# Internal carbon recycling by heterotrophic prokaryotes compensates for mismatches between phytoplankton production and heterotrophic consumption

**DOI:** 10.1093/ismejo/wrae103

**Published:** 2024-06-11

**Authors:** Falk Eigemann, Karen Tait, Ben Temperton, Ferdi L Hellweger

**Affiliations:** Water Quality Engineering, Technical University of Berlin, 10623 Berlin, Germany; Plymouth Marine Laboratory, PL1 Plymouth, United Kingdom; Faculty of Health and Life Sciences, University of Exeter, EX2 Exeter, United Kingdom; Water Quality Engineering, Technical University of Berlin, 10623 Berlin, Germany

**Keywords:** carbon cycling, phytoplankton–bacteria interactions, dissolved organic carbon, mechanistic inference

## Abstract

Molecular observational tools are useful for characterizing the composition and genetic endowment of microbial communities but cannot measure fluxes, which are critical for the understanding of ecosystems. To overcome these limitations, we used a mechanistic inference approach to estimate dissolved organic carbon (DOC) production and consumption by phytoplankton operational taxonomic units and heterotrophic prokaryotic amplicon sequence variants and inferred carbon fluxes between members of this microbial community from Western English Channel time-series data. Our analyses focused on phytoplankton spring and summer blooms, as well as bacteria summer blooms. In spring blooms, phytoplankton DOC production exceeds heterotrophic prokaryotic consumption, but in bacterial summer blooms heterotrophic prokaryotes consume three times more DOC than produced by the phytoplankton. This mismatch is compensated by heterotrophic prokaryotic DOC release by death, presumably from viral lysis. In both types of summer blooms, large amounts of the DOC liberated by heterotrophic prokaryotes are reused through internal recycling, with fluxes between different heterotrophic prokaryotes being at the same level as those between phytoplankton and heterotrophic prokaryotes. In context, internal recycling accounts for approximately 75% and 30% of the estimated net primary production (0.16 vs 0.22 and 0.08 vs 0.29 μmol l^−1^ d^−1^) in bacteria and phytoplankton summer blooms, respectively, and thus represents a major component of the Western English Channel carbon cycle. We have concluded that internal recycling compensates for mismatches between phytoplankton DOC production and heterotrophic prokaryotic consumption, and we encourage future analyses on aquatic carbon cycles to investigate fluxes between heterotrophic prokaryotes, specifically internal recycling.

## Introduction

Phytoplankton–bacteria interactions have global consequences for carbon and nutrient cycling [1–3]. Briefly, inorganic carbon is photosynthetically fixed by phytoplankton, and a substantial fraction is subsequently released in the form of dissolved organic matter (DOM), which is recycled/consumed by heterotrophic bacteria [3]. The microbial recycling of photosynthates, in turn, drives the microbial loop [4] as well as the microbial carbon pump [5]. High-throughput sequencing enables the incorporation of numerous taxa in analyses and advances understanding of microbial ecosystems. For example, high taxonomical resolution enabled the identification of pronounced seasonal differences in composition and richness of microbial communities in the North Atlantic [6] and temporal distributions of closely related bacteria in the Mediterranean [7]. However, understanding these ecosystems also requires quantification of interactions between individual components, which cannot be so readily observed. Most interaction analyses are based on empirical measures such as species co-occurrences or expression of specific genes [8, 9], but co-occurrence of species does not necessarily imply interaction [10], and the expression of genes for the degradation of specific polysaccharides by a defined bacterium may indeed target different producers/phytoplankton species [11]. Mechanistic inference, based on mass balances, offers the potential to overcome these limitations. In the present study, this approach was applied to a 7-year time series (2012–2018) at Station L4 in the Western English Channel to infer quantitative interactions in microbial communities.

Western English Channel Station L4 is a temperate coastal ocean site off Plymouth, UK, with well-mixed waters during the autumn and winter months, but weak stratification and declining nutrient concentrations from spring into summer (www.westernchannelobservatory.org.uk/data).

The phytoplankton community of Station L4 is dominated by phytoflagellates, diatoms, *Phaeocystis*, coccolithophorids, and dinoflagellates [12], and pronounced phytoplankton blooms occur in spring as well as in summer/autumn [13, 14], which vary in their timing, intensity, and key taxa present [12]. The bacterial community is dominated by *Alphaproteobacteria* and *Bacteroidetes* [15], with occasional peaks of *Gammaproteobacteria* [16] and reveals strong seasonal patterns [15, 16]. However, despite a few single-year analyses on seasonal primary and/or bacterial production performed in the English Channel [17, 18], to our knowledge an analysis involving the transformation of phytoplankton and bacteria seasonality into season-specific estimates of carbon production, consumption, and fluxes has not been undertaken. Furthermore, microbial association network analyses showed that abundance correlations between bacteria and phytoplankton were markedly low [15, 16], a finding that raises the question of the bacterial carbon source during periods with high bacterial but low phytoplankton concentrations. Different bacterial taxa revealed the highest correlations in these network association analyses [16], but the mechanisms underlying these putative bacteria–bacteria interactions were not explored.

In the present study we aimed to answer the following questions: (i) Which periods/seasons of the year have the highest carbon fluxes at Station L4? (ii) How do heterotrophic prokaryotes (i.e., heterotrophic archaea and heterotrophic bacteria) meet their carbon demands in periods with high heterotrophic prokaryote but low phytoplankton abundances? (iii) How do fluxes between phytoplankton and heterotrophic prokaryotes, as well as between different heterotrophic prokaryotes, develop during the course of microbial blooms? (iv) Which resources limit phytoplankton and heterotrophic prokaryotes at different seasons of the year?

In order to answer these questions, we enhanced the mass-balancing, mechanistically constrained inference approach FluxNet [19] for the investigation of 158 heterotrophic, prokaryotic amplicon sequence variants (ASVs; derived from 16S rRNA genes), 135 phototrophic, eukaryotic operational taxonomic units (OTUs; derived from 18S rRNA genes), *Synechococcus* (derived from flow-cytometric counts), and 162 hypothetical dissolved organic matter (DOM) species. The model consists of a set of differential mass balance equations and a customized optimization/calibration routine. Organic carbon is represented in phytoplankton, DOM, particulate organic matter (POM), and heterotrophic prokaryote compartments, each with many “species”. This high-resolution biogeochemical model was automatically calibrated to time-series data. The resulting flux network included quantitative carbon fluxes between all members in microbial communities for any time point, enabling insights in the functioning of the respective ecosystem.

## Materials and methods

### Data acquisition and selection

All data, except where noted otherwise, came from Western English Channel Station L4 and were obtained from the Western Channel Observatory (westernchannelobservatory.org.uk), with sampling performed by the Plymouth Marine Laboratory. Station L4 is a northern temperate coastal site approximately 50 km off Plymouth, UK, with occasional pulses of increased nitrate concentrations due to riverine input [14]. Data were collected from the beginning of 2012 until the end of 2018 with a weekly sampling period. Total phytoplankton carbon concentrations were derived from chlorophyll measurements, with a fixed chlorophyll/carbon conversion factor of 40 (1 g chlorophyll = 40 g phytoplankton carbon). Carbon concentrations of individual eukaryotic phytoplankton OTUs were derived from 18S rRNA gene sequence data (full ASV table provided as Supplementary Data 1). This unit derivation was done by converting the read fraction to the carbon fraction and multiplying it by the total phytoplankton concentration. Carbon concentrations of prokaryotic phytoplankton (only *Synechococcus*) were determined from flow cytometer cell counts (Supplementary Table 1), assuming a carbon content of 1.4E-14 mol cell^−1^ (for details, see Supplemental Information [SI] S4.3). Total phytoplankton as well as individual phytoplankton groups and OTU carbon concentrations derived from chlorophyll/18S rRNA genes were compared with microscopic data (Supplementary Table 2) and yielded highly significant correlations (see SI S4.4 for detailed analyses and possible limitations). The full 18S rRNA gene table contained 69 207 ASVs, but in order to establish manageable data loads, only the 10 000 most abundant ASVs were considered for further analyses. Heterotrophic as well as unassigned ASVs were filtered out, and the remaining phytoplankton ASVs were combined into 135 OTUs (for details see SI). Heterotrophic prokaryote concentrations were derived from 16S rRNA gene sequence data after conversion of relative read abundances into carbon concentrations (for details see SI S4.5-S4.6). The dataset of 16S rRNA genes contained the 200 most abundant ASVs (Supplementary Table 3), and after chloroplast, unassigned, cyanobacterial, and ammonia-oxidizing archaeal ASVs were filtered out, 157 ASVs were used for elaborated analyses. Additional data that were incorporated into the model were photosynthetic active radiation (obtained from the MODIS satellite, https://modis.gsfc.nasa.gov/data/), daylength (obtained from https://timeanddate.com for Plymouth), temperature, NO_2_ + NO_3_, NH_4_, PO_4_, silicate, salinity, oxygen concentrations, particulate organic carbon (POC), and chlorophyll (Supplementary Data 2).

### FluxNet method—mechanistic microbial ecosystem model inference

A complete description and explanation of the inference approach is available in [19] and details on the English Channel application are provided in the SI. Major differences (compared to [19]) are the upscaling of components and separate model runs for separate years. The latter were established in order to improve the fit between data and model. Briefly, modeling concepts and equations are based on past models of phytoplankton and bacteria [20–24], in which fluxes between ecological compartments, such as phytoplankton to DOM to heterotrophic prokaryotes, are explicitly simulated. For example, phytoplankton growth rates are a function of light, temperature, and inorganic nutrients, and heterotrophic prokaryotes grow by heterotrophy with different affinities for each hypothetical DOM species. The model accounts for intrusions of different water masses by variable loadings of nutrients (i.e., if total nitrogen measurements increase over time these changes were balanced with loadings of nitrate and equivalent for phosphorus). However, the model assumes all organic matter (including microbes and DOM) is produced internally and allochthonous sources are ignored. Key features of the approach are an optimization routine customized for microbial ecosystems, inclusion of dormancy to avoid extinction and support co-existence, and gradual delumping/diversification to increase the resolution (i.e., number of species).

### DOM production by phytoplankton and heterotrophic prokaryotes

Phytoplankton and heterotrophic prokaryotes produce DOM by death (directly and via POM, which dissolves), and phytoplankton additionally by exudation. Phytoplankton exudation consists of a basal exudation rate (ke) and a fraction of photosynthesis (fe). Both exudation parameters are based on values reported in the literature for total phytoplankton but were individually optimized for each OTU (within realistic ranges) by the optimization routine. Similarly, DOM and POM production by death are based on literature values but were optimized for each heterotrophic prokaryotic ASV/phytoplankton OTU as well as POM/DOM species individually. For details on equations and optimization routines for DOM production of phytoplankton and heterotrophic prokaryotes see the SI of our previous publication [19].

### Definition and duration of blooms

Phytoplankton spring blooms were defined as the highest chlorophyll concentration between February and May and phytoplankton summer blooms as the highest chlorophyll concentration between July and August. In the year 2014, no pronounced phytoplankton summer bloom was observed, and consequently no bloom was assigned. Bacterial summer blooms were defined as the highest bacterial concentrations between June and August. To ensure model/data overlaps, bloom starting days were not defined according to strict parameters and were assigned manually. Bloom durations were set to 28 days, except when concentrations fell below the starting concentration during that period. In those cases blooms were identified as having ended at the day their concentrations fell below the bloom starting concentration. However, in the years 2012 and 2017, bacterial summer bloom duration was increased to 34 and 42 days, respectively, because concentrations remained high. An overview of bloom durations and start and end days is presented in Supplementary Table 4.

### Statistics

All applied statistical tests and statistical outcomes are provided in Supplementary Table 5. Statistical tests were executed with the free software R [25] and R studio [26]. All graphics, except those for Fig. 6 (created with BioRender.com), were executed with the ggplot2 package [27] and refined with the freeware Inkscape (https://inkscape.com).

## Results

### Model performance

**Figure 1:**
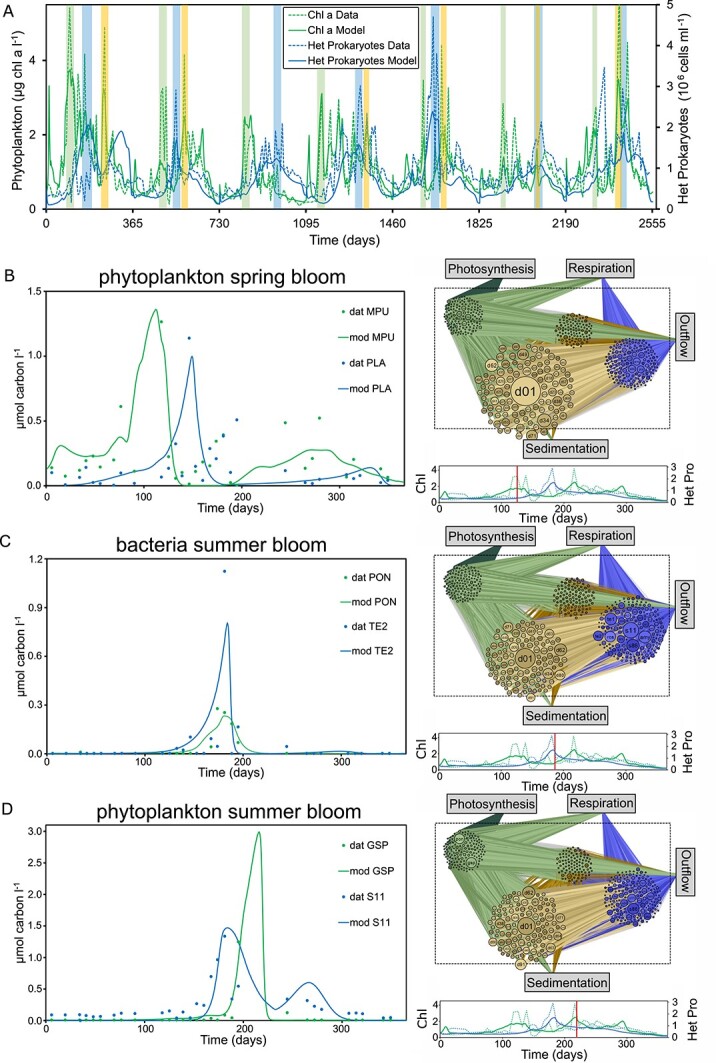
Model-data comparisons for the 7-year time series and illustration of the inferred flux network. (A) Model-data comparison for total phytoplankton and heterotrophic prokaryotes concentrations with t0 = 01.01.2012. Phytoplankton spring blooms are shaded green, bacteria summer blooms blue, and phytoplankton summer blooms yellow. (B–D) Model-data comparisons for phytoplankton OTUs (green) and heterotrophic prokaryotic ASVs (blue) with the highest carbon flux for each bloom type in the example year 2013 with t0 = 01.01.2013. MPU = *Micromonas pusila*, PON = *Prorocentrum donghaiense*, GSP = *Gonyaulax spinifera*, PLA = *Planktomarina*, TE2 = *Tenacibaculum*, S11 = SAR11. The right panel displays the network for the median day in each bloom period. Phytoplankton are depicted in green, DOM in light brown, POM in dark brown, and heterotrophic prokaryotes in blue. The size of the circles reflects the carbon outflow (phytoplankton), throughflow (average of inflow and outflow, DOM and POM) and inflow (heterotrophic prokaryotes), and the size of the lines between the compartments the magnitude of fluxes. Below each network a timeline with the corresponding chlorophyll a and heterotrophic prokaryotes concentrations is given (green: phytoplankton, blue: heterotrophic prokaryotes, solid lines: model, dashed lines: data). The red bar indicates the day for the network. (B) phytoplankton spring bloom, (C) bacteria summer bloom, (D) phytoplankton summer bloom.

The model presented here provides a continuous depiction of fluxes over the 7-year period, with distinct periods of high growth, known as blooms, and we focused our analyses on those periods. Specifically, we defined phytoplankton spring and summer blooms, as well as bacterial summer blooms for each year (Fig. 1A and Supplementary Table 4). The model reproduces the main patterns of the blooms (Fig. 1A), as well as concentration patterns of individual phytoplankton and heterotrophic prokaryotic taxa (Fig. 1B–D left panel and Supplementary Data 3).

The networks illustrate the direct flux of organic carbon from phytoplankton to heterotrophic prokaryotes via DOM, as well as the indirect transfer through POM (i.e., dead phytoplankton cells that partially dissolve) to DOM before being taken up by heterotrophic prokaryotes. Additionally, there is a significant flux from heterotrophic prokaryotes back to the DOM pool during bacterial summer blooms. Varying circle sizes of phytoplankton and heterotrophic prokaryotic taxa illustrate the changing carbon inflows and outflows for the different bloom types (Fig. 1B–D). For instance, *Micromonas pusila* played a major role in the 2013 phytoplankton spring bloom but vanishes towards the bacterial summer bloom, whereas SAR11 plays a minor role in the phytoplankton spring bloom but has the highest DOC influx in bacterial summer blooms. Detailed analyses of important phytoplankton producers, heterotrophic prokaryotic consumers, their recurrences, and possible limitations of converting 18S rRNA gene data into carbon concentrations and DOM fluxes are given in the SI (S4.4, S5.1-S5.3) and in Supplementary Table 6.

Besides the individual microbial concentration time series given in Supplementary Data 3, the model results can be compared to observations of bulk parameters. We calculated gross and net primary production, as well as gross and net heterotrophic prokaryotic production, as integrated values for each complete year and also for the selected bloom types of each year (Supplementary Table 7). Whole-year gross primary production ranged between 32 (year 2013) and 18 (year 2016) mmol carbon m^−2^ d^−1^, whereas net primary production was highest in 2012 and 2013 (each 23 mmol carbon m^−2^ d^−1^) and lowest in 2014 (13 mmol carbon m^−2^ d^−1^, Supplementary Table 7). For the different bloom types, phytoplankton spring blooms revealed the highest, and bacterial summer blooms the lowest, primary production, with the highest value for gross primary production achieved in the phytoplankton spring bloom in 2014 (91 mmol carbon m^−2^ d^−1^), and the lowest in bacterial summer blooms in the same year (8 mmol carbon m^−2^ d^−1^). These estimated primary production values are in the range of measured primary production rates in the English Channel (e.g. 8.3–217 mmol carbon m^−2^ d^−1^ ~20 km south of Plymouth [28], 0.42–2.4 mmol carbon m^−2^ d^−1^, measured at a transect between Portsmouth and Ouistreham [18], or 6–60 μmol carbon l^−1^ d^−1^ at Southampton surface waters [29]). The whole year gross heterotrophic prokaryotic production inferred with the model revealed contrasting patterns to the primary production with the highest value obtained in 2018 (0.24 μmol l^−1^ d^−1^), and the lowest in 2017 (0.13 μmol l^−1^ d^−1^), whereas net heterotrophic prokaryotic production ranged between 0.12 μmol l^−1^ d^−1^ (2012) and 0.07 μmol l^−1^ d^−1^ (2017, Supplementary Table 7). Heterotrophic prokaryotic production was highest in bacterial summer blooms (maximum value 0.73 μmol carbon l^−1^ d^−1^ in 2013), and mostly lowest in phytoplankton spring blooms (minimum value 0.04 μmol carbon l^−1^ d^−1^ in 2015). Similarly to the phytoplankton, the estimated values for heterotrophic prokaryotic production were in the range of measurements from the English Channel, with 0.5 μmol carbon l^−1^ d^−1^ [29] or 0.01–0.16 μmol carbon l^−1^ d^−1^ after a *Phaeocystis* bloom [17].

### Heterotrophic prokaryotes dominate DOC production in summer blooms

**Figure 2:**
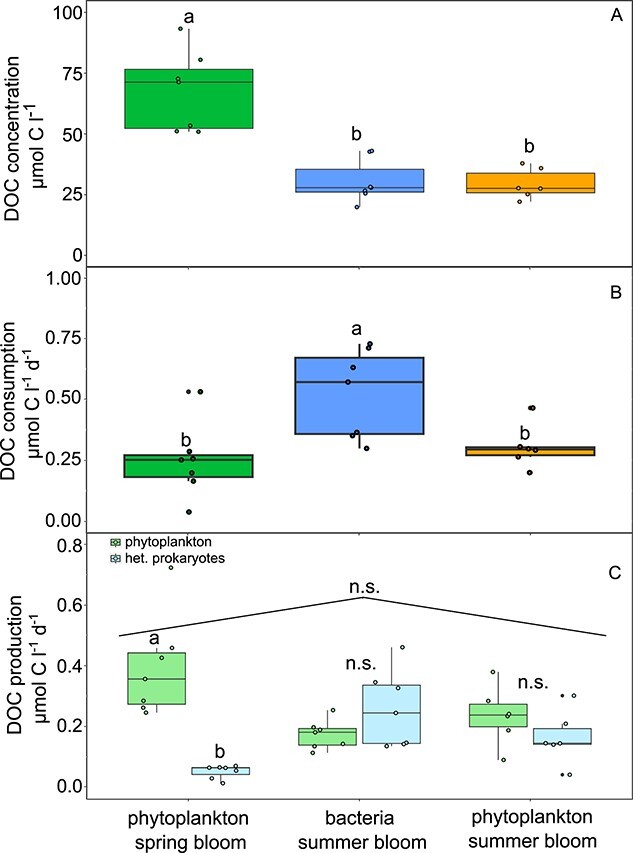
DOC concentration (A), heterotrophic prokaryotes DOC consumption (B), and DOC production of phytoplankton and heterotrophic prokaryotes (C) for the three different bloom types. Letters on the box-plots refer to Tukey post-hoc tests between the different bloom types (combined production of heterotrophic prokaryotes and phytoplankton for panel C), and to *t*-tests for differences in DOC production of phytoplankton and heterotrophic prokaryotes in the same bloom type (panel C). n.s. = not significant.

In order to explore the importance of the different bloom types for the Western English Channel carbon cycle, we estimated DOC concentration, consumption, and production (Fig. 2). The DOC in the model is solely produced by the microbial community present in the system (i.e., phytoplankton and heterotrophic prokaryotes), meaning it does not include allochthonous input or recalcitrant background DOC.

The DOC concentrations were highest during phytoplankton spring blooms, whereas DOC uptake by heterotrophic prokaryotes exhibited the opposite pattern, with the highest uptake rates occurring during bacterial summer blooms and the lowest uptake rates during phytoplankton spring blooms (Fig. 2A-B). We next asked how much DOC is produced and which organisms (phytoplankton or heterotrophic prokaryotes) are the producers. We hypothesized that during periods of high DOC consumption (bacterial summer blooms) the mostly low phytoplankton abundances (Fig. 1A) were balanced by high phytoplankton DOC production, indicating a disconnect between phytoplankton abundance and productivity. However, this was not the case, with lowest production in bacterial summer blooms, and highest production in phytoplankton spring blooms (Fig. 2C). Despite higher DOC production by phytoplankton compared to heterotrophic prokaryotes during spring blooms, we found that DOC production by heterotrophic prokaryotes exceeded that of phytoplankton during bacterial summer blooms. This resulted in overall similar combined production rates across different bloom types (Fig. 2C). Taking together the outcomes for phytoplankton DOC production and heterotrophic prokaryote consumption, two points become obvious: first, summer blooms are important to the Western English Channel carbon cycle, with higher carbon fluxes compared to spring blooms (Fig. 2B and C). Second, carbon consumption by heterotrophic prokaryotes during bacterial summer blooms cannot be satisfied by the DOC production from phytoplankton, as three times the amount of DOC produced by phytoplankton is taken up (Fig. 2B and C).

### Fluxes between heterotrophic prokaryotes substantially contribute to the Western English Channel carbon cycle

**Figure 3:**
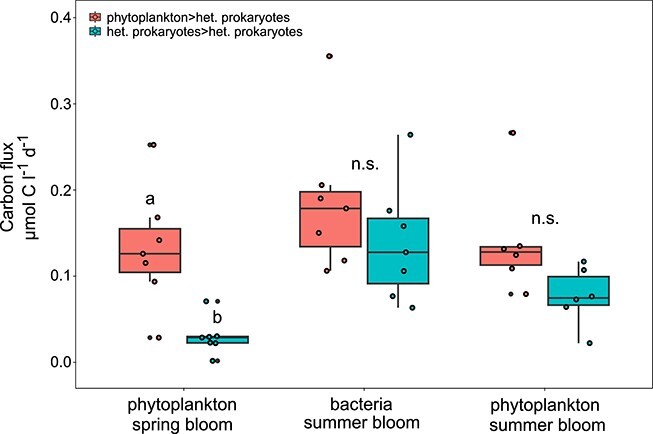
Quantitative bloom averaged carbon fluxes from phytoplankton to heterotrophic prokaryotes and heterotrophic prokaryotes to heterotrophic prokaryotes for the three bloom types for Western English Channel Station L4. Letters on the box-plots refer to *t*-tests for differences between fluxes from phytoplankton to heterotrophic prokaryotes and heterotrophic prokaryotes to heterotrophic prokaryotes. n.s. = not significant.

The high proportion of DOC produced by heterotrophic prokaryotes, coupled with high uptake rates during bacterial summer blooms, raises the question of whether heterotrophic prokaryotes may consume significant amounts of DOC derived from other heterotrophic prokaryotes. To address this question, we quantified carbon fluxes between heterotrophic prokaryotes, as well as between phytoplankton and heterotrophic prokaryotes (Fig. 3).

In phytoplankton spring blooms, fluxes from phytoplankton to heterotrophic prokaryotes dominated, whereas fluxes between heterotrophic prokaryotes were negligible. However, in bacterial summer blooms fluxes from heterotrophic prokaryotes to heterotrophic prokaryotes were in the same range as fluxes from phytoplankton to heterotrophic prokaryotes, and also in phytoplankton summer blooms both flux types did not differ significantly (Fig. 3). These results imply that the mostly neglected fluxes between heterotrophic prokaryotes are of major importance for summer blooms in the Western English Channel and should be considered in ecosystem analyses. Nevertheless, our model assumes that all DOM is produced internally (see model description), and it is possible that allochthonous sources supply some of the carbon and that estimated fluxes between heterotrophic prokaryotes are thus overestimated. However, we tested for different scenarios that may add external DOM (Tamar river inflow [the major source of allochthonous input for L4], resuspension due to extreme weather events, rapid exchanges of water masses) and found that they are highly unlikely to affect our main findings (detailed analyses and Figures in SI S5.5).

### Bacteria summer blooms are initiated by the recycling of photosynthetically fixed carbon and transition into internal recycling between heterotrophic prokaryotes

**Figure 4:**
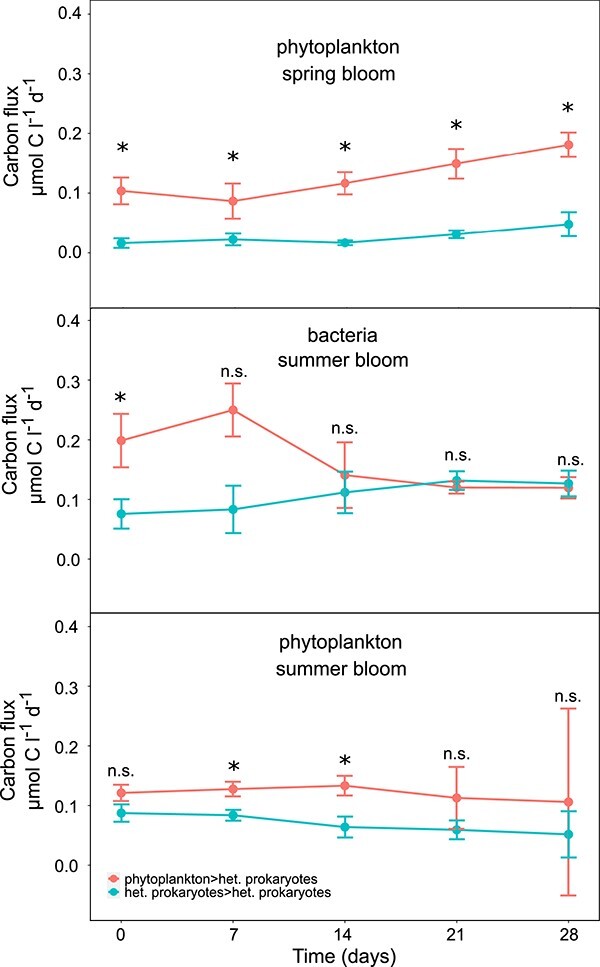
Dynamic carbon fluxes ± standard errors of fluxes from phytoplankton to heterotrophic prokaryotes and fluxes from heterotrophic prokaryotes to heterotrophic prokaryotes from bloom day 0 until 28, at weekly intervals for the three bloom types phytoplankton spring blooms, bacteria summer blooms and phytoplankton summer blooms. Asterix indicate significant differences between the flux types, n.s. = not significant.

In order to get insights into temporal developments of fluxes from phytoplankton to heterotrophic prokaryotes as well as fluxes between heterotrophic prokaryotes, we examined the evolution of fluxes over time. Fluxes from phytoplankton to heterotrophic prokaryotes were consistently higher than fluxes from heterotrophic prokaryotes to heterotrophic prokaryotes in spring blooms, whereas in bacterial summer blooms fluxes from phytoplankton to heterotrophic prokaryotes decreased and fluxes between heterotrophic prokaryotes significantly increased during the course of the blooms (Fig. 4, Supplementary Table 5). These opposed trends suggest that bacterial summer blooms are primarily initiated by the recycling of photosynthetically fixed carbon and then transition into a DOC re-recycling between different heterotrophic prokaryotes, a process for which the term “internal recycling” may be most fitting (to distinguish from “recycling” for consumption of phytoplankton-derived DOM, sensu [30]).

### Phages may cause fluxes between heterotrophic prokaryotes

In our analyses carbon fluxes from phytoplankton to heterotrophic prokaryotes are based on a combination of phytoplankton DOM exudation and DOM release due to phytoplankton death (directly or via POM). However, for heterotrophic prokaryotes, we did not include DOM exudation in the model. Consequently, all carbon fluxes between heterotrophic prokaryotes are based on DOM liberation upon death, specifically from viral predation or protist grazing. Fast and specific loss processes as drivers for the internal carbon recycling between heterotrophic prokaryotes are corroborated by high fluxes in bacterial summer blooms in years with pronounced successions of abundant taxa in periods of days (SI Fig. 4, Supplementary Fig. 1, Supplementary Table 8). However, our model does not explicitly distinguish among different death mechanisms. Instead, it simulates a general death process. Nonetheless, the model adjusts for the proportions of DOM and POM released, with viral lysis primarily releasing DOM and protist grazing releasing POM. The estimated DOM/POM ratio of 1.14 ± 0.12 (Supplementary Table 9) supports viral lysis as the predominant cause of death, as dominance of protist grazing would result in a ratio well below 1.

### Bacteria summer blooms are initiated by increasing temperatures and may recycle nitrogen for subsequent phytoplankton summer blooms

**Figure 5:**
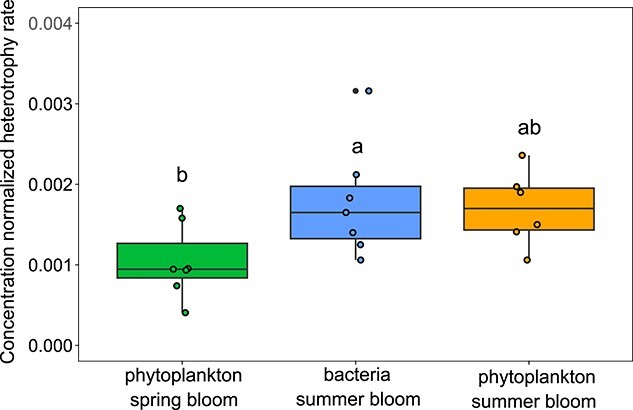
Concentration normalized heterotrophy rates (mol carbon consumed d^−1^/mol carbon bacteria l^−1^/mol DOC l^−1^). Letters on top of the box plots refer to Tukey post hoc tests between the different bloom types.

Bacteria summer blooms occur at periods with relatively low phytoplankton DOC production (Fig. 2C) and decreasing estimated DOC concentrations (Supplementary Data 3), raising the question of why they occur in summer rather than after phytoplankton spring blooms, which are periods with high phytoplankton DOC production and concentrations. The DOC concentration normalized heterotrophy rates (consumed DOC [mol carbon consumed d^−1^]/heterotrophic prokaryotes concentration [mol carbon l^−1^]/DOC concentration [mol carbon l^−1^]) revealed significantly lower rates in spring blooms compared to summer blooms, suggesting inhibition in the former (Fig. 5). We analyzed the relative strength of substrate and temperature inhibition for the most abundant heterotrophic prokaryotic taxa for each year and found that the onsets of bacterial summer blooms are caused by increases in water temperature (i.e., the temperature limitation decreases considerably, Supplementary Fig. 2).

Our data series depicts several phytoplankton summer blooms following bacterial summer blooms with a delay of ~15–30 days (Fig. 1A). To test whether we can mechanistically explain summer bloom sequences, we examined the growth inhibition due to PO_4_ and dissolved inorganic nitrogen concentrations, as well as due to light and temperature for the most abundant phytoplankton taxa for each year. Phytoplankton was predominantly limited by nitrogen in summer, and several onsets of phytoplankton summer blooms followed a decrease of nitrogen limitation accompanying bacteria summer blooms (e.g. in 2012, Supplementary Fig. 2). Thus, reductions of nitrogen limitation for the phytoplankton might be partly caused by previously occurring bacterial blooms. Ultimately, the mechanistic inhibition analyses suggest that the sequences of phytoplankton and bacteria blooms at Station L4 are interdependent not only due to the liberation of organic carbon by the phytoplankton, but also due to the liberation of nitrogen during bacterial blooms.

## Discussion

**Figure 6:**
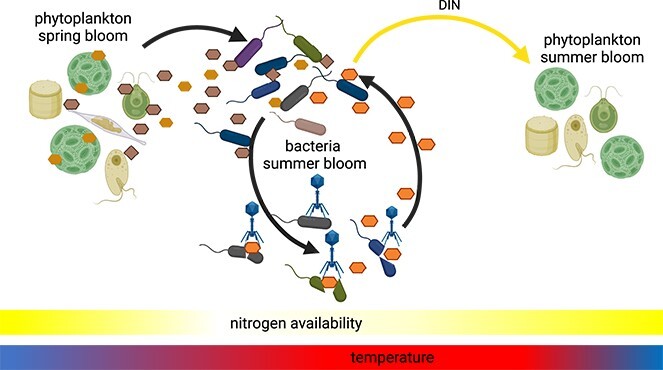
Summary figure for the major outcomes of the present study, created with BioRender.com. Phytoplankton produces large amounts of dissolved organic matter (DOM, brownish hexagons and squares) in spring blooms, whose instantaneous utilization is partly inhibited by low water temperatures. With increasing water temperatures, heterotrophic prokaryotes consume higher amounts of DOM, leading to bacteria summer blooms. Viruses infect abundant heterotrophic prokaryotes, and the lysed cells liberate heterotrophic prokaryotic DOM (orange hexagons) that is internally recycled. Bacteria summer blooms also liberate nitrogen, which may foster phytoplankton summer booms.

Through analysis of a 7-year time series from the Western English Channel Station L4 using a mechanistically constrained inference approach, we pinpointed carbon fluxes between heterotrophic prokaryotes as significant components of the L4 carbon cycle (Fig. 3). These fluxes are likely mediated by viruses, and we propose temperature and nitrogen limitations as drivers for the sequences of microbial summer blooms (Fig. 6).

Previous studies have shown that heterotrophs can use organic carbon released by other heterotrophs [31–34]. However, this process is not considered in most studies of aquatic carbon cycles, because organic carbon released by heterotrophic prokaryotes is often assumed to be mostly refractory [35–37] and because many studies focus exclusively on net fluxes (e.g. [38]). Nevertheless, our findings are supported by studies with different scopes. First, microbial association network analysis at Station L4 showed that abundance correlations were more pronounced within bacterial taxa than between bacteria and phytoplankton or between bacteria and environmental variables [16]. The rationale for the strong correlations within the bacterial compartment, however, were not addressed in that study. Second, several studies showed that heterotrophic bacteria exude large fractions/quantities of DOM, with 14%–31% of the previously ingested DOM being extracellularly released by an ocean beachside bacterial community [32] or exudation efficiencies by bacterial communities of Scottish coastal waters of 11% for DOC, 18% for dissolved organic nitrogen, and 17% for DOP [33]. Third, despite the assumption that heterotrophic bacteria are responsible for a high share of recalcitrant oceanic DOM [35, 37], the released DOM indeed consists of many bioavailable compounds such as amino acids, amino sugars [32], vitamins, polysaccharides [39], peptides, and saturated fatty acids [40]. Fourth, a recent study from the Helgoland time series showed that bacteria not only expressed polysaccharide utilization loci for utilization of beta-glucans (produced by phytoplankton), but also for alpha-glucans (produced by bacteria), and the authors hypothesized that a recycling of bacterial glucans occurs [41]. This hypothesis was corroborated by follow-up results in the same time series showing that the phytoplankton storage polysaccharide laminarin fuels the synthesis of alpha-glucans in bacteria during phytoplankton blooms, and that these are used as a carbon source by other bacteria if liberated by cell lysis ([42], also see the SI for overlaps in the suggested taxonomy of important taxa between this and our study). The authors of that study conclude that large amounts of carbon may get redirected via an intrapopulation loop, which corroborates the outcomes of our mechanistic inference approach by metatranscriptomic analyses. Fifth, incubation experiments confirmed that bacteria can grow on DOM exclusively derived from other heterotrophic bacteria [31, 43], yet yield the same cell numbers as if grown on glucose [34]. Thus, fluxes between heterotrophic prokaryotes indeed reflect well-known processes, whose importance for marine carbon cycles, however, may have been drastically underestimated. A short classification of fluxes between heterotrophic prokaryotes in relation to primary production for bacterial summer blooms (different blooms treated pari passu, then means were calculated), revealed that overall fluxes between heterotrophic prokaryotes accounted for 50% of gross primary production (0.16 vs 0.32 μmol carbon l^−1^ d^−1^), and were in the same range as net primary production (0.22 μmol l^−1^ d^−1^), as well as the flux from phytoplankton to heterotrophic prokaryotes (0.2 μmol l^−1^ d^−1^, Supplementary Table 7).

The starting points for the quantitative analyses of fluxes between heterotrophic prokaryotes, however, were temporal mismatches between phytoplankton DOC production and DOC consumption by heterotrophic prokaryotes (Fig. 2). Observations that heterotrophic prokaryotes consume more DOC than is allocated by the phytoplankton also exist from other environments [44–46]. Fouilland and Mostajir [45] reviewed numerous studies on aquatic primary vs bacterial production (among them 20 studies from marine environments) and concluded that the bacterial carbon utilization significantly exceeds the corresponding total primary production, raising the question whether fluxes between heterotrophic prokaryotes are important components of the carbon cycle as they are also in other systems. Indeed, the study by Beidler *et. al.* [42] suggests that the proposed internal recycling also takes place at other places in significant amounts, and we encourage future analyses of aquatic microbial communities to also resolve fluxes between heterotrophic prokaryotes. The assumption applied thus far that heterotrophic prokaryotes only consume DOM and do not also produce it for further consumption by others may be a gross oversimplification.

All fluxes between heterotrophic prokaryotes are a result of DOM liberation upon death (see above), and viral predation and grazing by protists are the major death causes for heterotrophic prokaryotes [47]. Both processes (due to cell lysis and sloppy feeding, respectively) liberate bioavailable DOC, but although sloppy feeding liberates significant amounts of DOC for copepod grazing on phytoplankton (up to 17% of the prey’s carbon content, [48]), protist grazing on bacteria liberates only minor amounts of DOC (because of phagotrophy and whole-prey engulfment [49]). Thus, the major part of DOC liberation can be attributed to viral lysis. Estimated DOM/POM release ratios >1 furthermore indicate viral lysis as the predominant death mechanism during bacterial blooms. As a consequence, the inferred fluxes between heterotrophic prokaryotes may be largely attributable to viral infections and considered a component of the “viral shunt” [50], i.e., the retainment of carbon for higher trophic levels due to the recycling of DOM derived from viral lysis of fish, zooplankton, phytoplankton, archaea, and bacteria by heterotrophic prokaryotes.

The growth of marine heterotrophic prokaryotes predominantly depends on the availability of organic carbon [51–53], and the question arises why bacteria blooms occur at times with comparably low DOC concentration and production. Analyses of growth-limiting factors in the model, however, suggest that the onsets of bacteria summer blooms are caused by increased water temperatures (Supplementary Fig. 2). Bacteria can metabolize less easily degradable microalgal polysaccharides long after phytoplankton blooms [54], and higher temperatures indeed facilitate the utilization of less bioavailable DOM [55], which includes more complex and less bioavailable compounds of phytoplankton exudates [56]. The occurrence of the latter is indicated by the highest phytoplankton to heterotrophic prokaryotes fluxes despite the lowest phytoplankton DOC production in bacteria summer blooms (compared to phytoplankton spring and summer blooms, Figs. 2 and 3), and may partly fill the gap between phytoplankton production and heterotrophic prokaryotic consumption in periods with high water temperatures. However, increased temperatures may also enable the internal recycling occurring at later phases in bacterial summer blooms, namely the utilization of less bioavailable DOM derived from heterotrophic prokaryotes [35–37]. On the phytoplankton side, mechanistic analyses suggested nitrogen deficiency as major inhibitor in summer (Supplementary Fig. 2), and that limitation decreases following bacterial blooms led to phytoplankton summer blooms in several years. We speculate that the bacteria blooms recycled nitrogen and thus partly initialized phytoplankton summer blooms. Our speculation that bacterial blooms foster phytoplankton summer blooms is supported by studies showing that a net production of dissolved inorganic nitrogen took place during bacterial utilization of glucose [33], and that the exploitation of organic material by coastal bacterioplankton communities led to increased NH_4_ levels [32]. Bloom sequences with distinct phytoplankton spring blooms and bacterial summer blooms preceding additional phytoplankton blooms also occured in other than the used years at Station L4 [15], as well as at other places (Helgoland roads time series, [57]). Yet, due to lacking analyses and availability of data, we cannot speculate on the underlying processes. Nevertheless, modeling efforts and/or experimental approaches with different time series at different places may help to increase understanding of the sequences of phytoplankton and bacteria blooms and would help to support or refute the suggested mechanisms.

Decreasing costs and efforts required for modern molecular tools (e.g. proteomics, high-throughput sequencing) enable high resolutions of observations and increase e.g. the number of heterotrophic prokaryotic taxa or DOM species in ecosystem analyses. Increased numbers of analyzed components in microbial communities, however, are accompanied by exponential increases of their interactions, which makes quantitative measurements of multi-species interactions impossible. As a consequence, the deployment of models is necessary for such analyses. In the present study, we used a mechanistic inference approach with hundreds of phytoplankton and heterotrophic prokaryotic species and were able to quantitatively estimate interactions (i.e., carbon fluxes) between phytoplankton and heterotrophic prokaryotic taxa, as well as between the latter. The code for the inference approach is open source and readily extendable in terms of dimensions (e.g. zooplankton and viruses) and model agents/species as well as constraints (e.g. the implementation of transcriptomic data) and thus may allow mechanistic analyses to keep track with progressions/outcomes made by modern molecular tools.

## Supplementary Material

Suppementary_wrae103

## Data Availability

The open-source model code is provided at https://github.com/fhellweger, and all input data for the model (except data for 16S and 18S rRNA gene sequences) are given in Supplementary Data 2. Raw data for 16S and 18S rRNA genes have been uploaded to NCBI with the submission ID of SUB13994125 and BioProject ID of PRJNA1045854. The top 200 16S rRNA gene ASVs are given in Supplementary Table 3, and the full 18S rRNA gene ASV table is given in Supplementary Data 1.
