# Correction to: Internal carbon recycling by heterotrophic prokaryotes compensates for mismatches between phytoplankton production and heterotrophic consumption

**DOI:** 10.1093/ismejo/wrae190

**Published:** 2024-10-11

This is a correction to: Falk Eigemann, Karen Tait, Ben Temperton, Ferdi L Hellweger, Internal carbon recycling by heterotrophic prokaryotes compensates for mismatches between phytoplankton production and heterotrophic consumption, *The ISME Journal*, Volume 18, Issue 1, January 2024, wrae103, https://doi.org/10.1093/ismejo/wrae103

The following corrections have been made:

1. In the originally published version of Figure 4, the captions were missing for the upper and middle panels. A number of `n.s.' labels were also missing in the middle panel. The figure has been updated to correct these errors.

2. In the section, `FluxNet method – mechanistic microbial ecosystem model inference', the sentence that originally read `The model accounts for intrusions of different water masses by variable loadings of nutrients (i.e., if total nitrate measurements increase over time these changes were balanced with loadings of nitrate and equivalent for phosphorus)' has been corrected to read `The model accounts for intrusions of different water masses by variable loadings of nutrients (i.e., if total nitrogen measurements increase over time these changes were balanced with loadings of nitrate and equivalent for phosphorus).'

3. In the section `Bacteria summer blooms are initiated by the recycling of photosynthetically fixed carbon and transition into internal recycling between heterotrophic prokaryotes', the sentence that originally read `Fluxes from phytoplankton to heterotrophic prokaryotes were consistently higher than fluxes from heterotrophic prokaryotes to heterotrophic prokaryotes in spring blooms, whereas in bacterial summer bloom fluxes from phytoplankton to heterotrophic prokaryotes decreased and fluxes between heterotrophic prokaryotes significantly increased during the course of the bloom' has been corrected grammatically to read `Fluxes from phytoplankton to heterotrophic prokaryotes were consistently higher than fluxes from heterotrophic prokaryotes to heterotrophic prokaryotes in spring blooms, whereas in bacterial summer blooms fluxes from phytoplankton to heterotrophic prokaryotes decreased and fluxes between heterotrophic prokaryotes significantly increased during the course of the blooms.' The publisher apologizes for this and the above error.

4. A sentence has been added to the `Acknowledgements' section in recognition of the open access funding provided by TU Berlin.

