# Supplementary material for: Internal carbon recycling by heterotrophic prokaryotes compensates for mismatches between phytoplankton production and heterotrophic consumption
Source: ISME J. 2024 Jun 11;18(1):wrae103. doi: 10.1093/ismejo/wrae103 (PMC11217553; doi:10.1093/ismejo/wrae103)
Supplement: Suppementary_wrae103 [file suppementary_wrae103.zip › SI_revised_clean.docx]

Supplementary Information for:

Internal carbon recycling by heterotrophic prokaryotes compensates for mismatches between phytoplankton production and heterotrophic consumption

Eigemann, et al. 2024

correspondence to: [eigemann@tu-berlin.de](mailto:eigemann@tu-berlin.de)

**This file includes:**

S1. Introduction

S2. Details of the mechanistic microbial ecosystem model FluxNet

S3. Optimization routine

S4. Data acquisition, processing and validation

S5. Additional results and discussion

S6. References

## S1: Introduction

The purpose of this section is twofold: (1) document the modeling framework with examples for the English Channel application, and (2) present additional analyses for the English Channel application. General information on model details can be found in the SI in [Mayerhofer et al [1]](#_ENREF_1) from the original Helgoland-roads application. In the paragraphs S1-S2 the most important model features are summarized.

# S2: Details of the mechanistic microbial ecosystem model

## S2.1: Concepts, components and processes

The modeling concepts and equations are generally based on past models of phytoplankton, bacteria, zooplankton and viruses (e.g. [Chapra [2]](#_ENREF_2), [Weitz et al [3]](#_ENREF_3)). Novel aspects (compared to [Mayerhofer et al [1]](#_ENREF_1)) include up-scaling of components, and separate model runs for separate years.

The model consists of a number of components (concentrations, state variables) that interact via a number of processes (functions) (see Supplementary Table 10 for components used in the English Channel application). The model structure is flexible and puts no constraints on the number and types of processes that connect components, so the model can, for example, simulate mixotrophy. However, it helps to think in terms of and organize components into traditional ecological compartments. In that case, the processes corresponding to each component depend on the ecological compartment it is in.

Model components are generally selected based on observations. However, for a mechanistic model, it is important to close the mass balance and that puts some constraints on the definition of components. First, state variables cannot overlap. Second, the state variables have to cover the trophic levels. To cover the trophic level, “hypothetical” species are added (conceptually similar to “others” in [Hellweger et al [4]](#_ENREF_4)).

For processes, the model includes loading [w], flow [q], settling [s], first-order [f], respiration [r], photosynthesis [p], grazing [z], heterotrophy [h], viral [v], exudation [e], death [y], loss [u] and inhibition [i].

To illustrate the approach, we present mass balance equations for a reduced system consisting of one phytoplankton OTU (mpu), two DOM species (d01, d02) and one heterotrophic prokaryotic ASV (s11).

Processes affecting phytoplankton concentration include photosynthesis, respiration, exudation, inhibition, death, settling and outflow. The mass balance equation for mpu is:

$\frac{d}{dt}C_{mpu}={kp}_{mpu} {Lp}_{mpu} C_{mpu}-{kr}_{mpu} {Lr}_{mpu} C_{mpu}-\left( {ke}_{mpu}+{ef}_{mpu} {kp}_{mpu} {Lp}_{mpu} \right) C_{mpu}-{ku}_{mpu} {Lu}_{mpu} C_{mpu}-\frac{{vs}_{mpu}}{H}C_{mpu}-\frac{Q}{V}C_{mpu}$

(1)

t (d) = time, C (mmolC/L) = concentration, kp (1/d) = max. photosynthesis rate constant, Lp = limitation/modification factor for photosynthesis (light, nutrients, temperature, salinity), kr (1/d) = max. respiration rate constant, Lr = limitation/modification factor for respiration (temperature), ke (1/d) = basal exudation rate constant, ef (1/d) = exudation/photosynthesis fraction, ku (1/d) = maximum death rate, Lu = limitation/modification factor for death (time-of-year, salinity), vs (m/d) = settling velocity, H (m) = water column depth, Q (m3/d) = flow rate, V (m3) = volume.

Processes affecting DOM include microbial exudation and death, POM dissolution, heterotrophy and outflow. The mass balance equation for d01, considering only mpu and s11 as source, s11 as sink, and p01 as corresponding POM species, is:

$$\frac{d}{dt}C_{d01}={Fe}_{mpu,d01} \left( {ke}_{mpu}+{ef}_{mpu} {kp}_{mpu} {Lp}_{mpu} \right) C_{mpu}+{Fx}_{mpu,d01} {ku}_{mpu} {Lu}_{mpu} C_{mpu}+{Fx}_{s11,d01} {ku}_{s11} {Lu}_{s11}C_{s11}+{kf}_{p01} {Lf}_{p01} C_{p01}-{kh}_{s11} \frac{{C_{d01}}/{{Ksh}_{s11,d01}}}{1+{C_{d01}}/{{Ksh}_{s11,d01}}+{C_{d02}}/{{Ksh}_{s11,d02}}} {Lh}_{s11} C_{s11}-\frac{Q}{V}C_{d01}$$

(2)

Fe = exudation fraction, Fx = composition fraction, kf (1/d) = dissolution rate constant, Lf = limitation/modification factor for dissolution (temperature), kh (1/d) = max. heterotrophy rate, Ksh (mmolC/L) = half-saturation constant, Lh = limitation/modification factor for heterotrophy (temperature, salinity, light).

Processes affecting heterotrophic prokaryotes concentration include heterotrophy, death and outflow. The mass balance equation for s11, considering only growth on d01 and d02, is:

$$\frac{d}{dt}C_{s11}={Yh}_{s11} {kh}_{s11} \frac{{C_{d01}}/{{Ksh}_{s11,d01}}+{C_{d02}}/{{Ksh}_{s11,d02}}}{1+{C_{d01}}/{{Ksh}_{s11,d01}}+{C_{d02}}/{{Ksh}_{s11,d02}}} {Lh}_{s11} C_{s11}-{ku}_{s11} {Lu}_{s11} C_{s11}$$

$$-\frac{Q}{V}C_{s11}$$

(3)

Yh = yield coefficient.

## S2.2: Dormancy

Dormancy is modeled by specifying a floor concentration (Cflr) and reducing any loss rate that would result in concentration below this value, an approach similar to previous models [[5](#_ENREF_5)]. Specifically, a floor concentration (*Cflr*) of ¼ of the lowest, measured concentration is given, and any loss process that would reduce the concentration below this value is blocked.

## S2.3: Environment and boundary conditions

The environment is a completely-mixed reactor with specified area (*A*) and depth (*H*). Boundary conditions include light intensity (*IT*), photoperiod (*f*), temperature (*T*) and nutrient loadings (*WNOX*, *WNH4*, *WPO4*, *WSIL*).

For the English Channel application, the dilution rate was set to 0.066 1/d, based on water residence times of ~2 weeks [[6](#_ENREF_6)].

External nutrient inputs (loadings) were calculated from observations *a priori* using a simplified approach. For nitrogen, it was assumed total nitrogen (TN) is made up of NOX, NH4 and phytoplankton nitrogen (PN), which was calculated from Chlorophyll *a* using conversions (see SI in [Mayerhofer et al [1]](#_ENREF_1)). It was further assumed the only loss processes are settling of PN and outflow of TN. These assumptions result in the following mass balance equation.

$$V \frac{dTN}{dt}=W_{NOX}-vs A PN-Q TN$$

(4)

This equation can be discretized and solved for the nitrogen input.

$$W_{NOX}=V\frac{\Delta TN}{\Delta t}+vs A \bar{PN}+Q \bar{TN}$$

(5)

When this calculation is applied to a data set with high variability, it can lead to negative values, which does not agree with the concept of external input. Therefore, the load is kept positive, but a deficit is tracked so that the resulting cumulative input is consistent with the mass balance. Input of PO4 and SIL are handled equivalently.

# S3: Optimization routine

The optimization method adjusts parameter values within literature ranges to minimize the disagreement i.e. error between model and data. The method generally follows previous numerical optimization approaches for microbial ecosystem models [[3](#_ENREF_3), [7](#_ENREF_7), [8](#_ENREF_8)]. Novel aspects include a two-dimensional (concentration and time) quantification of model-data disagreement, numerical optimization methods customized for microbial ecosystems and gradual increase in model complexity (de-lumping).

## S3.1: De-lumping

Species are introduced at specified de-lump levels, by de-lumping from an existing species. The new species generally inherits the parameter values (i.e. the genome, sensu [Daines et al [5]](#_ENREF_5)) from the old species. Subsequent optimization then diversifies the population. This is illustrated in Fig. 1C of [Mayerhofer et al [1]](#_ENREF_1), which shows the affinity of bacteria species for *mpu*. However, if parameter values are specified for the new species, they are adopted and overwrite those inherited from the old species. This is used, for example, to assign species-specific cell sizes (*MC*).

# S4. Data acquisition, processing and validation

## S4.1: 18S Illumina sequencing and phototroph/heterotroph factor

18S rRNA sequences were derived from Illumina sequencing and analyzed with the dada2 pipeline [[9](#_ENREF_9)]. The full 18S dataset comprised 69,207 amplicon sequence variants (ASVs, Supplementary Data 2). Absolute read counts were converted into relative abundances, and the 10,000 most abundant ASVs used for elaborated analyses (all dates/samples contained ~20,000 reads, and thus no subsampling was necessary, Supplementary Data 2), which covered 61-98% of all reads. In order to account for 18S reads that do not derive from phototrophic organisms, and are as such not in the scope of our study, all ASVs (10,000 most abundant) were manually classified into phototroph/not phototroph, whereby mixotrophic ASVs were treated as phototrophs. From these phototroph/not phototroph ratios, a factor for each sampling date was calculated, and each phototrophic ASV multiplied with the factor at the respective sampling point. This factor ensured that we received the true share of each ASV in the phototrophic community.

## S4.2: Assignment of OTUs

Because most 18S ASVs only occurred at one or few sampling dates, ASVs were pooled to OTUs. For all OTUs, we set the minimum occurrence to 4 sampling dates in the entire time-series. If less than 4 sampling dates were obtained, ASVs were pooled to the next lower taxonomic level until 4 or more occurrences in the data set were reached. This means, at first different ASVs of the same species were combined to OTUs, then all species with less than 4 occurrences were pooled to genera a.s.o. This was done successively for each taxonomic level (species/genera/family/order/class/phyla). If several ASVs of the same OTU occurred at the same sampling date(s), relative abundances of these ASVs were totalized. If ASVs were originally assigned to taxonomic levels lower than species, the highest resolved taxonomic level was taken and treated as described above. Altogether, we obtained 138 OTUs, consisting of 120 species, 7 genera (undefined family), 1 family (undefined species and family), 2 orders, 5 classes, and 3 phyla. In order to account for the share of the phytoplankton not covered by the 10,000 most abundant OTUs (see above), we also added 24 hypothetical phytoplankton OTUs.

## S4.3: Transformation of chlorophyll and 18S data in carbon concentrations

Chlorophyll measurements were transformed into total phytoplankton carbon concentrations with a fixed chl/carbon conversion factor of 40 (1 g chl = 40 g phytoplankton C). Carbon concentrations of individual eukaryotic phytoplankton OTUs were estimated by multiplying this total phytoplankton carbon concentration with the OTU’s 18S read count fraction (example calculation given at the end of the paragraph), and prokaryotic phytoplankton OTU (only Synechococcus) carbon concentrations were derived from flow-cytometer cell counts (Supplementary Table 7), with an assumed carbon content of 1.4E-14 mol cell^-1^. Because eukaryotic phytoplankton OTUs read count fractions did not sum up to 100% (only the 10,000 most abundant ASVs of the 18S dataset were considered, see above), and Synechococcus did not dominate the phytoplankton community (Supplementary Table 2), we did not subtract Synechococcus from the total phytoplankton biomass before calculating the individual concentrations of eukaryotic OTUs (i.e. Synechococcus and hypothetical OTUs were used by the model to achieve the total carbon concentration).

Thus, carbon concentrations of individual eukaryotic phytoplankton OTUs were estimated with the equation

individual OTU µg carbon l^-1^ = OTU’s 18S relative share of reads * total chl. a µg l^-1^ * 40 (6)

In general, for an individual ASV/OTU, the carbon concentration is the cell concentration multiplied by the carbon content per cell. We estimate it by multiplying the 18S read fraction with total phytoplankton carbon concentration, ignoring variability of gene copy numbers and cell size among OTUs. The justification of this approach is as follows:

The carbon content per cell depends on the cell volume, and can be calculated with

*pg carbon cell^-1^ = 0.76 cell volume ^0.819^ for dinoflagellates*

*pg carbon cell^-1^ = 0.288 cell volume ^0.811^ for diatoms, and*

*pg carbon cell^-1^ = 0.216 cell volume ^0.939^ for other protists (7)*

*with cell volume given in µm^3^* [[10](#_ENREF_10)].

Analogous, the share of 18S read counts of individual ASVs/OTUs depends on the ASV’s/OTU’s cell numbers per volume, as well as the ASV’s/OTU’s 18S gene copy number (GCN) per cell. Phytoplankton 18S GCN per cell, for its part, depend on the cell volume of the individual ASV/OTU, and can be estimated as follows:

*log (cell volume µm^3^) = -0.61 + 1.22 log GCN (8)*

[[11](#_ENREF_11)].

By opposing the share of 18S read counts and carbon concentrations of individual OTUs, the cell numbers per volume cancel out, and consequently GCN per cell can be compared with carbon content per cell. By combining the equations for GCN and carbon content per cell, and solving it for log(carbon cell^-1^), one gets:

*-0.61*0.819 + log(0.76) + 1.22*0.819 * log(GCN) = log(carbon cell^-1^)* *for dinoflagellates*

*-0.61*0.811 + log(0.288) + 1.22*0.811 * log(GCN) = log(carbon cell^-1^) for diatoms*

*-0.61*0.939 + log(0.216) + 1.22*0.939 * log(GCN) = log(carbon cell^-1^) for other protists (9)*

Thus, almost linear relations between GCN and carbon contents exist (0.999 log (GCN) = log (carbon cell^-1^) for dinoflagellates, (0.989 log (GCN) = log (carbon cell^-1^) for diatoms, and 1.14 log (GCN) = log (carbon cell^-1^) for diverse protists).

In other words, the relative share of read abundances of a specific phytoplankton OTU indeed corresponds to its relative share of carbon concentration. Example calculation: 20% of the phototrophic 18S reads (cleaned and filtered, see above) come from OTU A at 01.01.2012, and the chlorophyll concentration at this date is 5 µg l^-1^. Thus, the carbon concentration of OTU A at 01.01.2012 is 0.2*5 µg l^-1^ * 40 = 40 µg carbon l^-1^.

## S4.4: Comparison of phytoplankton carbon concentrations derived from microscopy and chlorophyll/18S sequencing

We compared total phytoplankton carbon concentrations based on chlorophyll measurements to those based on microscopic counting. Over the 7-year time-series, both estimates exhibited similar magnitudes and patterns (SI Fig. 1). Specifically, both showed phytoplankton carbon maxima concentrations between spring and autumn, and minima in winter, and also most peaks were reflected by both methods (e.g. narrow spring and autumn peaks in 2013, narrow summer peak in 2016). However, concentrations of individual phytoplankton groups/OTUs were calculated by multiplying the total phytoplankton carbon concentration with the relative share of 18S sequence reads (see above). Although 18S sequencing may reflect diversity of phytoplankton communities better than microscopy or flow-cytometry, it lacks robustness at higher taxonomic resolutions due to sequence length [[12](#_ENREF_12)], and adds uncertainties for community compositions, because primer pairs discriminate certain taxa [[13](#_ENREF_13)]. Furthermore, 18S gene copy numbers (GCN) vary by orders of magnitude between taxa [[14](#_ENREF_14)], and read abundances of individual OTUs do not account for actual cell sizes (see above for analyses on GCN and cell size impacts on carbon estimates), which may lead to limitations in inferring carbon concentrations from 18S data.

We tested for this by correlating estimated carbon concentrations inferred from relative shares of 18S sequences vs that inferred from microscopy for the two most abundant phytoplankton groups (i.e. diatoms and dinoflagellates), for a single diatom genus (Chaetoceros, multiple species/OTUs present in microscopic and 18S data), and a single taxonomic OTU (Phaeocystis)(SI Fig. 2). Indeed, all correlations between microscopic and 18S derived carbon concentrations were highly significant (SI Fig. 2A-D), validating the applied methods and values.

[[12](#_ENREF_12), [13](#_ENREF_13)]

SI Fig. 1: Comparison of estimated overall phytoplankton carbon concentrations derived from chlorophyll and microscopy. Note that not all measurements have matching sampling dates.

Si Fig. 2: Correlations between carbon estimates inferred from microscopic counts and shares of 18S sequence data. (A) Diatoms and (B) dinoflagellates estimates were calculated as sums of all species (microscope) or OTUs (18S) of the respective group. (C) Chaetoceros concentrations were calculated as sums of all Chaetoceros species (microscope, 31 species) or OTUs (18S, 10 OTUs). (D) Phaeocystis has a single taxonomic classification in microscopic data, and is correlated with a single Phaeocystis OTU (Phaeocystis pouchetii, ppo). For all correlations only matching sampling dates of microscopy and 18S were considered.

## S4.5: 16S rRNA Illumina sequencing and factor for relative abundances

16S sequencing was performed with primers 515F–806R targeting the V4 region of the 16S SSU rRNA), and analyzed with the dada2 pipeline [[9](#_ENREF_9)]. The current primers have been modified from the original 515F–806R primer pair [[15](#_ENREF_15)] in the following ways: Barcodes are now on the forward primer 515F (Parada et al., 2016). This enables the usage of various reverse primer constructs to obtain longer amplicons, for example the V4–V5 region using reverse primer 926R (Quince et al., 2011; Parada et al., 2016). Degeneracy was added to both the forward and reverse primers to remove known biases against Crenarachaeota/Thaumarchaeota (515F, also called 515F-Y, Parada et al., 2016) and the marine and freshwater Alphaproteobacterial clade SAR11 (806R, Apprill et al., 2015).

In order to reduce the full 16S dataset into manageable amounts of data, the 200 most abundant ASVs were used for elaborated analyses. After filtering out chloroplast, unassigned, cyanobacterial and ammonia-oxidizing archaeal ASVs, 157 ASVs were used for elaborated analyses, which covered between 18 and 94% of the reads at different sampling dates (average 82%). For each sampling date, a factor for heterotrophic 16S ASVs was introduced, considering the share of non-heterotrophic (i.e. cyanobacteria) and non-bacterial cell ASVs (i.e. chloroplasts).

## S4.6: Transformation of 16S data in carbon concentrations

Transformations of relative read abundances into carbon concentrations were based on flow cytometer counts of heterotrophic prokaryotic cells, and individual cell volumes.

The corrected relative abundance of each heterotrophic 16S ASV was multiplied with the overall heterotrophic prokaryotic cell number (heterotrophic prokaryotic cell numbers derived from flow cytometry, Supplementary Table 7), yielding cell numbers per volume for each ASV. Next, the carbon content of each 16S ASV cell was calculated with the formula

*fgC cell^–1^ = 133.754 × V ^0.438^  (10)*

*[*[*16*](#_ENREF_16)*]*

Cell volumes were looked up from the literature [[17-34](#_ENREF_17)]. If cell volumes were not known, Gammaproteobacterial ASVs were set as 13 fmol carbon cell^-1^, and all other ASVs with unknown cell volume as 5 fmol carbon cell^-1^ (all given carbon contents and sources are given in Supplementary Table 11).

# S5: Additional results and discussion

**S5.1: Important phytoplankton producer OTUs and heterotrophic prokaryotic consumer**

**ASVs**

In order to identify phytoplankton OTUs and heterotrophic prokaryotic ASVs that are important for the carbon production and consumption at station L4, we calculated for each year and bloom type: the ten primary phytoplankton DOM producers; the ten primary heterotrophic prokaryotic DOM consumers along with their top phytoplankton donor and top DOM species used; and the ten phytoplankton>heterotrophic prokaryotes as well as heterotrophic prokaryote>heterotrophic prokaryote pairs with the highest carbon fluxes (Supplementary Table 2). Primary phytoplankton DOM producers mostly differed between years as well as bloom types, with few exceptions: In phytoplankton spring blooms *Bathycoccus prasinos* (BPR), in bacteria summer blooms *Gonyaulax spinifera* (GSP), and in phytoplankton summer blooms *Pterocystis* sp. (PTE) were found in the top 10 of DOM producers, in 71%, 57% and 83% of the years, respectively (Supplementary Table 2). On the consumer side, phytoplankton spring blooms revealed frequent occurrences of SAR86 (S86) and *Amylibacter* (AMY), as primary heterotrophic prokaryotes (top 10 rank in 100% and 86% of the years), whereas SAR11 (S11) showed top 10 occurrences in phytoplankton and bacteria summer blooms in 86% and 57% of the years, respectively (Supplementary Table 2). The highest obtained carbon flux for phytoplankton>heterotrophic prokaryote pairs was in the 2018 bacteria summer bloom with 0.082 µmol C l^-1^ d^-1^ for *Dinophysis acuminata*>Marine group II archaea (DAC>M22). In contrast to the paradigm of carbon flux being dominated by flow from phototrophs to heterotrophs, fluxes between heterotrophic prokaryotic pairs were in the same range as fluxes for phytoplankton heterotrophic prokaryote pairs, with a maximum of 0.035 C l^-1^ d^-1^ for *Amylibacte*r>SAR86 (AMY>S86) in the bacteria summer bloom of 2016 (Supplementary Table 2).

**S5.2: Taxonomy of important heterotrophic prokaryotes DOM consumers:**

In all bloom events, FLUXNET predicted that most primary DOC consumers belong to Gammaproteobacteria, the Rhodobacterales clade of Alphaproteobacteria or the Flavobacteriales clade of Bacteroidetes (Supplementary Table 2). This outcome is supported by numerous studies, that defined these three bacterial groups as principle utilizers of DOM [[29](#_ENREF_29), [35-43](#_ENREF_35)]. However, in bacterial summer blooms, we found especially many Flavobacteriales such as Aquibacter (AQU), Cryomorphaceae (CR1), Flavicella (FAC)), Tenacibaculum (TE2), NS5 marine group (N51), and Fluviicola (FU2) as major carbon consumers (Supplementary Table 2). The (predicted) pivotal role of Flavobacteriales in periods with high heterotrophic prokaryotes>heterotrophic prokaryotes fluxes is supported by their genetic capacity for using manifold different sources of high-molecular-weight (HMW) DOM [[36](#_ENREF_36), [44](#_ENREF_44)], as well as experiments: Flavobacteriales were the principle degraders of bacterial derived polysaccharides [[45](#_ENREF_45)], and also dominated bacterial communities enriched with exudates of the copiotrophic Gammaproteobacterium Photobacterium angustum [[46](#_ENREF_46)]. A recent study additionally showed that 75% from 53 sequenced Bacteroidetes/Flavobacteriia strains had specific polysaccharide utilization loci (PULs) for α-glucans (which is the bacterial storage polysaccharide), and that the Bacteroidetes strain *Polaribacter* was able to grew solely on bacterial lysates [[47](#_ENREF_47)].

**S5.3: Lower recurrences of producers compared to consumers:**

In order to quantify the recurrences of producers, consumers and phytoplankton>heterotrophic prokaryotes and heterotrophic prokaryote>heterotrophic prokaryote interactions (fluxes), we calculated for each bloom type Bray-Curtis similarities of phytoplankton producer, heterotrophic prokaryotic consumers, phytoplankton>heterotrophic prokaryote and heterotrophic prokaryote>heterotrophic prokaryote pairs between all years. The highest recurrences were obtained for heterotrophic prokaryotic consumers with Bray-Curtis similarities of ~0.32 in spring blooms, whereas phytoplankton DOM producer revealed similarities between ~0.1 (phytoplankton summer blooms) and ~0.17 (phytoplankton spring and bacteria summer blooms). This decoupling of producers and consumers (same consumers supplied from different producers) is presumably driven by the production of similar DOM species by different phytoplankton species (Supplementary Table 2), which was also found at Helgoland Island [[1](#_ENREF_1), [35](#_ENREF_35)], and is further supported by low recurrences of phytoplankton>heterotrophic prokaryote and heterotrophic prokaryote>heterotrophic prokaryote pairs (SI Figure 3).

SI Figure 3: Recurrence of phytoplankton DOM producers (phy>dom), heterotrophic prokaryotic DOM consumers (hpr<dom), phytoplankton>heterotrophic prokaryote pairs (phy>hpr), and heterotrophic prokaryote>heterotrophic prokaryote pairs (hpr>hpr) in the years 2012-2018. Letters on top of the box plots refer to outcomes of Tukey post-hoc tests between the different bloom types, letters on top of the roofs to differences between variables (combined bloom types, Supplementary Table 4). n.s. = not significant.

**S5.4: Succession patterns of abundant phytoplankton OTUs and heterotrophic prokaryotic**

**ASVs differ between years and bloom types:**

To get a deeper insight into the dynamics of abundant phytoplankton OTUs and heterotrophic prokaryotic ASVs, we chose the OTUs/ASVs with the highest concentrations in each of the three different bloom types, and plotted their concentrations throughout bloom events for the example year 2013 (SI Fig. 4), and all other years (Supplementary Fig. 1). 2013’s phytoplankton spring bloom revealed a succession of phytoplankton OTUs to consecutive heterotrophic prokaryotic ASVs (SI Fig. 4). Accordingly, carbon concentrations of the phytoplankton OTUs *Bathycoccus prasinos* (bpr) and *Micromonas pusilla* (mpu) exceeded that of SAR86 (s86) and *Planktomarina* (pla) at the bloom start, but decreased below that of the heterotrophic prokaryotes at later phases. In contrast, the bacterial summer bloom in 2013 showed distinct successions of the most abundant heterotrophic prokaryotic ASVs without preceding abundant phytoplankton OTUs (SI Fig. 4). The increase of the most abundant phytoplankton OTU *Gonyaulax spinifera* (gsp) towards the end of the bacterial bloom ultimately led to the onset of the phytoplankton summer bloom with consecutive phytoplankton OTUs *Gonyaulax spinifera* (gsp) to Dinophyceae (din), in which, however, no successions of the three most abundant heterotrophic prokaryotic ASVs were obvious (SI Fig. 4). Successions from phytoplankton to consecutive heterotrophic prokaryotic ASVs in phytoplankton spring blooms also occurred in other years (Supplementary Fig. 1), and display a well-known process for marine phytoplankton blooms [[48](#_ENREF_48), [49](#_ENREF_49)], representing an initial DOM liberation by the phytoplankton and a subsequent exploitation of highly towards less bioavailable phytoplankton DOM by different heterotrophic prokaryotes [[29](#_ENREF_29), [35](#_ENREF_35), [36](#_ENREF_36), [43](#_ENREF_43)]. Phytoplankton summer blooms also revealed consistent patterns with consecutive abundant phytoplankton OTUs in all years, but without subsequent successions of the abundant heterotrophic prokaryotic ASVs (SI Fig. 4 and Supplementary Fig. 1). However, the latter may have been masked by high concentrations of heterotrophic prokaryotic ASVs remaining from forgoing bacteria summer blooms (SI Fig. 4 and Supplementary Fig. 1). In contrast, bacterial summer blooms did not show consistent patterns, with some years revealing successions of abundant heterotrophic prokaryotes (2013, 2014, 2018), whereas others did not (2015, 2017, SI Fig. 4, Supplementary Fig. 1, Supplementary Table 5). If successions occurred, succession periods in the range of days (i.e. DOM liberation by one ASV followed by the uptake/growth of another ASV, SI Fig. 4 and Supplementary Fig. 1) indicate the occurrence of fast and specific loss processes, such as protist grazing or infections with phages, where the latter may cause up to 61% of bacterial mortality after 24 h [[50](#_ENREF_50)]. Interestingly, bacterial summer blooms in years displaying successions of abundant heterotrophic prokaryotes showed higher fluxes between heterotrophic prokaryotes than those in years without successions (Supplementary Table 5), highlighting the importance of specific loss processes (and the pivotal role of phages, see main paper) as drivers of fluxes between heterotrophic prokaryotes.

SI Fig. 4: Temporal development of phytoplankton OTUs and heterotrophic prokaryotic ASVs with the highest concentrations in the three different bloom types in the example year 2013. Model outputs are indicated by lines (phytoplankton: lines including triangles, heterotrophic prokaryotes: smooth lines), data by dots. The day of the year 2013 is assigned to the x-axis, and the concentrations on the y-axis. The shaded areas correspond to the periods that were defined as bloom periods. mpu = Micromonas pusilla, bpr = Bathycoccus prasinos, s86 = SAR86, pla = Planktomarina, din = Dinophyceae, gsp = Gonyaulax spinifera, s11 = SAR11, n04 = NS4 marine group, pon = Prorocentrum donghaiense, te1 = Tenacibaculum, amy = Amylibacter, ros = Roseobacter.

## S5.5: Impact of allochthonous DOM sources on estimates of fluxes between heterotrophic prokaryotes

Our study outlines mismatches between phytoplankton DOM production and heterotrophic prokaryotic DOM consumption in bacteria summer blooms, that are partly balanced with heterotrophic prokaryotic DOM production (Fig. 2). However, in other systems allochthonous carbon sources were suggested to partly compensate for mismatches between phytoplankton DOC production and heterotrophic prokaryotic consumption [[51](#_ENREF_51)]. Our model assumes a closed system of DOM production and consumption, and thus allochthonous sources may have led to overestimations of heterotrophic prokaryotic DOM production (and the resulting fluxes between heterotrophic prokaryotes).

However, the average water residence time at L4 is approximately 2 weeks ([Lewis and Allen, 2009](https://www.ncbi.nlm.nih.gov/pmc/articles/PMC3260500/#bib16)), and most dynamics in carbon production and fluxes in our study happen at smaller time-scales (Fig. 4). Thus, it is unlikely that allochthonous sources have a big impact on our results. Nevertheless, we tested if allochthonous carbon sources may impact our estimated heterotrophic prokaryotic DOM production and the resulting fluxes between heterotrophic prokaryotes: For WEC station L4, the major source of allochthonous material is the Tamar River, whose outlet is ca. 40 km off L4. We plotted Tamar river flow rates together with bloom starts of bacterial summer blooms, which, however, did not reveal a relation (i.e. we did not find indications that allochthonous DOM fuels bacteria summer blooms, SI Fig. 5A). We also plotted heterotrophic prokaryotic DOC production against the forgoing weekly average Tamar river flow rates, again without finding indications for correlations (SI Fig. 5B).

SI Fig. 5: Tests for allochthonous DOM sources. A) 7-year time series of maximum daily (orange) and weekly average (grey) Tamar river run-off. The starting dates of bacterial summer blooms are indicated by blue arrows. B) Heterotrophic prokaryotic DOC production plotted against the foregoing river discharge. C) Heterotrophic prokaryotic DOC production plotted against the maximum wave height in the foregoing week. Color legends are given directly in the Fig.

Another possible source of allochthonous DOM input might be resuspension. Albeit this seems unlikely for L4 (water depth is ~52 m), we plotted the heterotrophic prokaryotic DOC production against the maximum wave height (which may also indicate extreme weather events), but did not find any positive correlations (SI Fig. 5C). Indeed, the slope revealed a slightly negative trend, indicating that stable weather conditions favor heterotrophic prokaryotic DOC production (SI Fig. 5C).

We then tested if sudden water mass exchanges may have occurred (which may introduce allochthonous DOC), and tested their potential impact on heterotrophic prokaryotic DOC production by plotting time-series data of nitrate, phosphate and water density as well as estimates of heterotrophic prokaryotic DOC production for all bacterial summer blooms (i.e. periods with high heterotrophic prokaryotic DOC production, Supplementary Fig. 3, all data for tests on possible allochthonous DOM inputs are given in Supplementary Data 3). Water density remained in narrow ranges in all years (between 1025.75 and 1026.75 kg m^-3^), and thus did not indicate major exchanges of water masses. Nevertheless, nitrate/phosphate concentrations did show rapid decreases (2014) or increases (2017), which, however, were not accompanied by increases of heterotrophic prokaryotic DOC production (and thus are probably related to internal processes). In conclusion, we could neither find indications that WEC station L4 receives extensive allochthonous input, nor did we find any correlations of the tested variables on the estimated heterotrophic prokaryotic DOC production, and thus on the major importance of the internal recycling of DOM.

# S6: References

1. Mayerhofer MM, Eigemann F, Lackner C *et al.* Dynamic carbon flux network of a diverse marine microbial community. *ISME Comm*. 2021;**1**:50 <https://doi.org/10.1038/s43705-021-00055-7>

2. Chapra SC. *Surface water-quality modeling*, Boston: McGraw-Hill, 1997.

3. Weitz JS, Stock CA, Wilhelm SW *et al.* A multitrophic model to quantify the effects of marine viruses on microbial food webs and ecosystem processes. *ISME J*. 2015;**9**:1352 <https://doi.org/10.1038/ismej.2014.220>

4. Hellweger FL, Kravchuk ES, Novotny V *et al.* Agent-based modeling of the complex life cycle of a cyanobacterium (anabaena) in a shallow reservoir. *Limnol Oceanogr*. 2008;**53**:1227-41 <https://doi.org/10.2307/40058247>

5. Daines SJ, Clark JR, Lenton TM. Multiple environmental controls on phytoplankton growth strategies determine adaptive responses of the n : p ratio. *Ecol Lett*. 2014;**17**:414-25 <https://doi.org/10.1111/ele.12239>

6. Lewis K, Allen JI. Validation of a hydrodynamic-ecosystem model simulation with time-series data collected in the western english channel. *J Mar Syst*. 2009;**77**:296-311 <https://doi.org/10.1016/j.jmarsys.2007.12.013>

7. Mieleitner J, Reichert P. Modelling functional groups of phytoplankton in three lakes of different trophic state. *Ecol Modell*. 2008;**211**:279-91 <https://doi.org/https://doi.org/10.1016/j.ecolmodel.2007.09.010>

8. Pinto F, Medina DA, Pérez-Correa JR *et al.* Modeling metabolic interactions in a consortium of the infant gut microbiome. *Front Microbiol*. 2017;**8** <https://doi.org/10.3389/fmicb.2017.02507>

9. Callahan BJ, McMurdie PJ, Rosen MJ *et al.* Dada2: High-resolution sample inference from illumina amplicon data. *Nat Methods*. 2016;**13**:581-83 <https://doi.org/10.1038/nmeth.3869>

10. Menden-Deuer S, Lessard EJ. Carbon to volume relationships for dinoflagellates, diatoms, and other protist plankton. *Limnol Oceanogr*. 2000;**45**:569-79 <https://doi.org/https://doi.org/10.4319/lo.2000.45.3.0569>

11. Godhe A, Asplund ME, Härnström K *et al.* Quantification of diatom and dinoflagellate biomasses in coastal marine seawater samples by real-time pcr. *Appl Environ Microbiol*. 2008;**74**:7174-82 <https://doi.org/10.1128/aem.01298-08>

12. Xiao X, Sogge H, Lagesen K *et al.* Use of high throughput sequencing and light microscopy show contrasting results in a study of phytoplankton occurrence in a freshwater environment. *PLOS ONE*. 2014;**9**:e106510 <https://doi.org/10.1371/journal.pone.0106510>

13. Bradley IM, Pinto AJ, Guest JS. Design and evaluation of illumina miseq-compatible, 18s rrna gene-specific primers for improved characterization of mixed phototrophic communities. *Appli Environ Microbiol.* 2016;**82**:5878-91 <https://doi.org/doi:10.1128/AEM.01630-16>

14. Gong W, Marchetti A. Estimation of 18s gene copy number in marine eukaryotic plankton using a next-generation sequencing approach. *Front Mar Sci*. 2019;**6** <https://doi.org/10.3389/fmars.2019.00219>

15. Caporaso JG, Lauber CL, Walters WA *et al.* Ultra-high-throughput microbial community analysis on the illumina hiseq and miseq platforms. *ISME J*. 2012;**6**:1621-4 <https://doi.org/10.1038/ismej.2012.8>

16. Romanova ND, Sazhin AF. Relationships between the cell volume and the carbon content of bacteria. *Oceanology*. 2010;**50**:522-30 <https://doi.org/10.1134/s0001437010040089>

17. Cottrell MT, Kirchman DL. Transcriptional control in marine copiotrophic and oligotrophic bacteria with streamlined genomes. *Appl Environ Microbiol*. 2016;**82**:6010-8 <https://doi.org/10.1128/AEM.01299-16>

18. Ho A, Di Lonardo DP, Bodelier PL. Revisiting life strategy concepts in environmental microbial ecology. *FEMS Microbiol Ecol*. 2017;**93** <https://doi.org/10.1093/femsec/fix006>

19. Kirchman DL. Growth rates of microbes in the oceans. *Ann Rev Mar Sci*. 2016;**8**:285-309 <https://doi.org/10.1146/annurev-marine-122414-033938>

20. Voget S, Wemheuer B, Brinkhoff T *et al.* Adaptation of an abundant roseobacter rca organism to pelagic systems revealed by genomic and transcriptomic analyses. *ISME J*. 2015;**9**:371-84 <https://doi.org/10.1038/ismej.2014.134>

21. Yao F, Yang S, Wang Z *et al.* Microbial taxa distribution is associated with ecological trophic cascades along an elevation gradient. *Front Microbiol*. 2017;**8**:2071 <https://doi.org/10.3389/fmicb.2017.02071>

22. Avci B, Hahnke RL, Chafee M *et al.* Genomic and physiological analyses of 'reinekea forsetii' reveal a versatile opportunistic lifestyle during spring algae blooms. *Environ Microbiol*. 2017;**19**:1209-21 <https://doi.org/10.1111/1462-2920.13646>

23. Pelve EA, Fontanez KM, DeLong EF. Bacterial succession on sinking particles in the ocean's interior. *Front Microbiol*. 2017;**8**:2269 <https://doi.org/10.3389/fmicb.2017.02269>

24. Cho JC, Giovannoni SJ. Cultivation and growth characteristics of a diverse group of oligotrophic marine gammaproteobacteria. *Appl Environ Microbiol*. 2004;**70**:432-40 <https://doi.org/10.1128/aem.70.1.432-440.2004>

25. Yan S, Fuchs BM, Lenk S *et al.* Biogeography and phylogeny of the nor5/om60 clade of gammaproteobacteria. *Syst Appl Microbiol*. 2009;**32**:124-39 <https://doi.org/10.1016/j.syapm.2008.12.001>

26. Duhaime MB, Wichels A, Sullivan MB. Six pseudoalteromonas strains isolated from surface waters of kabeltonne, offshore helgoland, north sea. *Genome Announc*. 2016;**4** <https://doi.org/10.1128/genomeA.01697-15>

27. Sheik AR. Viral regulation of nutrient assimilation by algae and prokaryotes. Max Planck Institut für Marine Mikrobiologie, University of Bremen, 2012

28. Mann AJ, Hahnke RL, Huang S *et al.* The genome of the alga-associated marine flavobacterium

Formosa agariphila KMM 3901t reveals a broad potential for degradation of algal polysaccharides. *Appl Environ Microbiol*. 2013;**79**:6813-22 <https://doi.org/10.1128/aem.01937-13>

29. Buchan A, LeCleir GR, Gulvik CA *et al.* Master recyclers: Features and functions of bacteria associated with phytoplankton blooms. *Nat Rev Microbiol*. 2014;**12**:686-98 <https://doi.org/10.1038/nrmicro3326>

30. Kruger K, Chafee M, Ben Francis T *et al.* In marine bacteroidetes the bulk of glycan degradation during algae blooms is mediated by few clades using a restricted set of genes. *ISME J*. 2019;**13**:2800-16 <https://doi.org/10.1038/s41396-019-0476-y>

31. Seo HS, Kwon KK, Yang SH *et al.* Marinoscillum gen. nov., a member of the family 'Flexibacteraceae', with Marinoscillum pacificum sp. nov. from a marine sponge and Marinoscillum furvescens nom. rev., comb. nov. *Int J Syst Evol Microbiol*. 2009;**59**:1204-8 <https://doi.org/10.1099/ijs.0.004317-0>

32. Lauro FM, McDougald D, Thomas T *et al.* The genomic basis of trophic strategy in marine bacteria. *Proc Natl Acad Sci U S A*. 2009;**106**:15527-33 <https://doi.org/10.1073/pnas.0903507106>

33. Morris RM, Longnecker K, Giovannoni SJ. Pirellula and om43 are among the dominant lineages identified in an oregon coast diatom bloom. *Environ Microbiol*. 2006;**8**:1361-70 <https://doi.org/10.1111/j.1462-2920.2006.01029.x>

34. Sheik CS, Jain S, Dick GJ. Metabolic flexibility of enigmatic sar324 revealed through metagenomics and metatranscriptomics. *Environ Microbiol*. 2014;**16**:304-17 <https://doi.org/10.1111/1462-2920.12165>

35. Teeling H, Fuchs BM, Bennke CM *et al.* Recurring patterns in bacterioplankton dynamics during coastal spring algae blooms. *Elife*. 2016;**5**:e11888 <https://doi.org/10.7554/eLife.11888>

36. Teeling H, Fuchs BM, Becher D *et al.* Substrate-controlled succession of marine bacterioplankton populations induced by a phytoplankton bloom. *Science*. 2012;**336**:608-11 <https://doi.org/10.1126/science.1218344>

37. Becker JW, Hogle SL, Rosendo K *et al.* Co-culture and biogeography of prochlorococcus and sar11. *ISME J*. 2019;**13**:1506-19 <https://doi.org/10.1038/s41396-019-0365-4>

38. Riemann L, Steward GF, Azam F. Dynamics of bacterial community composition and activity during a mesocosm diatom bloom. *Appl Environ Microbiol*. 2000;**66**:578-87 <https://doi.org/10.1128/aem.66.2.578-587.2000>

39. Pinhassi J, Sala MM, Havskum H *et al.* Changes in bacterioplankton composition under different phytoplankton regimens. *Appl Environ Microbiol*. 2004;**70**:6753-66 <https://doi.org/10.1128/AEM.70.11.6753-6766.2004>

40. Kieft B, Li Z, Bryson S *et al.* Phytoplankton exudates and lysates support distinct microbial consortia with specialized metabolic and ecophysiological traits. *Proc Natl Acad Sci*. 2021;**118**:e2101178118 <https://doi.org/doi:10.1073/pnas.2101178118>

41. Sarmento H, Gasol JM. Use of phytoplankton-derived dissolved organic carbon by different types of bacterioplankton. *Environ Microbiol*. 2012;**14**:2348-60 <https://doi.org/10.1111/j.1462-2920.2012.02787.x>

42. Eigemann F, Rahav E, Grossart H-P *et al.* Phytoplankton exudates provide full nutrition to a subset of accompanying heterotrophic bacteria via carbon, nitrogen and phosphorus allocation. *Environ Microbiol*. 2022;**24**:2467-83 <https://doi.org/https://doi.org/10.1111/1462-2920.15933>

43. Eigemann F, Rahav E, Grossart H-P *et al.* Phytoplankton producer species and transformation of released compounds over time define bacterial communities following phytoplankton dissolved organic matter pulses. *Appl Environ Microbiol*. 2023;**89**:e00539-23 <https://doi.org/doi:10.1128/aem.00539-23>

44. Lombard V, Golaconda Ramulu H, Drula E *et al.* The carbohydrate-active enzymes database (cazy) in 2013. *Nucleic Acids Res*. 2014;**42**:D490-5 <https://doi.org/10.1093/nar/gkt1178>

45. Zhang Z, Chen Y, Wang R *et al.* The fate of marine bacterial exopolysaccharide in natural marine microbial communities. *PLoS One*. 2015;**10**:e0142690 <https://doi.org/10.1371/journal.pone.0142690>

46. Ortega-Retuerta E, Devresse Q, Caparros J *et al.* Dissolved organic matter released by two marine heterotrophic bacterial strains and its bioavailability for natural prokaryotic communities. *Environ Microbiol*. 2021;**23**:1363-78 <https://doi.org/https://doi.org/10.1111/1462-2920.15306>

47. Beidler I, Steinke N, Schulze T *et al.* Alpha-glucans from bacterial necromass indicate an intra-population loop within the marine carbon cycle. *PREPRINT Research Square*. 2023;**Version 1** <https://doi.org/https://doi.org/10.21203/rs.3.rs-3205445/v1>

48. Luria CM, Amaral-Zettler LA, Ducklow HW *et al.* Seasonal shifts in bacterial community responses to phytoplankton-derived dissolved organic matter in the western antarctic peninsula. *Front Microbiol*. 2017;**8** <https://doi.org/10.3389/fmicb.2017.02117>

49. Sison-Mangus MP, Jiang S, Kudela RM *et al.* Phytoplankton-associated bacterial community composition and succession during toxic diatom bloom and non-bloom events. *Front Microbiol*. 2016;**7**:1433 <https://doi.org/10.3389/fmicb.2016.01433>

50. Fouilland E, Tolosa I, Bonnet D *et al.* Bacterial carbon dependence on freshly produced phytoplankton exudates under different nutrient availability and grazing pressure conditions in coastal marine waters. *FEMS Microbiol Ecol*. 2014;**87**:757-69 <https://doi.org/10.1111/1574-6941.12262>

51. Morán XA, G. , Josep MG, Pedros-Alio C *et al.* Partitioning of phytoplanktonic organic carbon production and bacterial production along a coastal-offshore gradient in the ne atlantic during different hydrographic regimes. *Aquat Microb Ecol*. 2002;**29**:239-52
