# Supplementary material for: Internal carbon recycling by heterotrophic prokaryotes compensates for mismatches between phytoplankton production and heterotrophic consumption
Source: ISME J. 2024 Jun 11;18(1):wrae103. doi: 10.1093/ismejo/wrae103 (PMC11217553; doi:10.1093/ismejo/wrae103)
Supplement: Suppementary_wrae103 [file suppementary_wrae103.zip › Suppementary Table 10.pdf]

Supplementary Table 10: Components with associated names, groups and compartments used in the English Chanel application.

icom	input	name	genus/group	compartment
1	nox	reactive nitrogen oxide	$NO_2 + NO_3$	nutrients
2	nh4	ammonium	-	nutrients
3	po4	phosphate	-	nutrients
4	sil	silicate	-	nutrients
11	p01	-	hypothetical phytoplankton	18S/phytoplankton
12	syn	Synechococcus	phototrophic cyanobacteria	18S/phytoplankton
13	pte	Prorocentrum texanum	Dinophyceae	18S/phytoplankton
14	mpu	Micromonas pusilla	Chlorophyta	18S/phytoplankton
15	bpr	Bathycoccus prasinos	Chlorophyta	18S/phytoplankton
16	lch	Lepidodinium chlorophorum	Dinophyceae	18S/phytoplankton
17	ppo	Phaeocystis pouchetii	haptophyceae	18S/phytoplankton
18	tam	Teleaulax amphioxeia	cryptophyta	18S/phytoplankton
19	kve	Karlodinium veneficum	Dinophyceae	18S/phytoplankton
20	kse	Karenia selliformis	Dinophyceae	18S/phytoplankton
21	gcr	Geminigera cryophila	cryptophyta	18S/phytoplankton
22	str	Scrippsiella trochoidea	Dinophyceae	18S/phytoplankton
23	tal	Thalassiosira allenii	Bacillariophyta	18S/phytoplankton
24	rse	Rhizosolenia setigera	Bacillariophyta	18S/phytoplankton
25	toc	Thalassiosira oceanica	Bacillariophyta	18S/phytoplankton
26	pin	Parvodinium inconspicuum	Dinophyceae	18S/phytoplankton
27	aan	Aureococcus anophagefferens	Pelagophyceae	18S/phytoplankton
28	ael	Adenoides eludens	Dinophyceae	18S/phytoplankton
29	pha	Pheopolykrikos hartmannii	Dinophyceae	18S/phytoplankton
30	che	Chaetoceros tenuissimus	Bacillariophyta	18S/phytoplankton
31	tcf	Takayama cf. Pulchellum	Dinophyceae	18S/phytoplankton
32	gsp	Gonyaulax spinifera	Dinophyceae	18S/phytoplankton
33	yye	Yihiella yeosuensis	Dinophyceae	18S/phytoplankton
34	pon	Prorocentrum donghaiense	Dinophyceae	18S/phytoplankton
35	ezo	Eucampia zodiacus	Bacillariophyta	18S/phytoplankton
36	lmi	Leptocylindrus minimus	Bacillariophyta	18S/phytoplankton
37	dac	Dinophysis acuminata	Dinophyceae	18S/phytoplankton
38	tte	Thalassiosira tenera	Bacillariophyta	18S/phytoplankton

39	pdi	Pyramimonas disomata	Chlorophyta	18S/phytoplankton
40	cbe	Cymatosira belgica	Bacillariophyta	18S/phytoplankton
41	tfu	Tripos fusus	Dinophyceae	18S/phytoplankton
42	pve	Pseudochattonella verruculosa	Dictyochophyceae	18S/phytoplankton
43	dia	Dinophysis acuta	Dinophyceae	18S/phytoplankton
44	cca	Chrysochromulina campanulifera	Haptophyceae	18S/phytoplankton
45	lda	Leptocylindrus danicus	Bacillariophyta	18S/phytoplankton
46	lvi	Lepidodinium viride	Dinophyceae	18S/phytoplankton
47	fna	Fragilaria nanana	Bacillariophyta	18S/phytoplankton
48	isp	Imantonia_cf. Imantonia sp. CCMP1404	Haptophyceae	18S/phytoplankton
49	nar	Navicula arenaria	Bacillariophyta	18S/phytoplankton
50	sgr	Skeletonema grevillei	Bacillariophyta	18S/phytoplankton
51	csp	Chrysoxys sp. CCMP591	Chrysophyceae	18S/phytoplankton
52	gde	Guinardia delicatula	Bacillariophyta	18S/phytoplankton
53	csi	Chrysochromulina simplex	Haptophyceae	18S/phytoplankton
54	ppa	Pyramimonas parkeae	Chlorophyta	18S/phytoplankton
55	tco	Thalassiosira concaviuscula	Bacillariophyta	18S/phytoplankton
56	tpa	Triparma pacifica	Bolidophyceae	18S/phytoplankton
57	pcu	Pseudo-nitzschia cuspidata	Bacillariophyta	18S/phytoplankton
58	gos	Goniomonas sp. SH-8	Cryptophyta	18S/phytoplankton
59	pel	Pseudopedinella elastica	Dictyochophyceae	18S/phytoplankton
60	trt	Tripos tenuis	Dinophyceae	18S/phytoplankton
61	psp	Proboscia sp.	Bacillariophyta	18S/phytoplankton
62	stu	Stephanopyxis turris	Bacillariophyta	18S/phytoplankton
63	lbo	Lauderia borealis	Bacillariophyta	18S/phytoplankton
64	pol	Pyramimonas olivacea	Chlorophyta	18S/phytoplankton
65	cle	Chrysochromulina leadbeateri	Haptophyceae	18S/phytoplankton
66	gsm	Gymnodinium smaydae	Dinophyceae	18S/phytoplankton
67	dsp	Dictyocha speculum	Dictyochophyceae	18S/phytoplankton
68	ehu	Emiliania huxleyi	Haptophyceae	18S/phytoplankton
69	chs	Chaetoceros sp. UNC1415	Bacillariophyta	18S/phytoplankton
70	pau	Pseudo-nitzschia australis	Bacillariophyta	18S/phytoplankton
71	pls	Pleurosigma sp. 102	Bacillariophyta	18S/phytoplankton
72	cry	Cryptophyta sp. CCMP2293	Cryptophyta	18S/phytoplankton
73	esp	Esoptrodinium sp. RP	Dinophyceae	18S/phytoplankton
74	fdo	Fragilariopsis doliolus	Bacillariophyta	18S/phytoplankton
75	tbe	Tenuicylindrus belgicus	Bacillariophyta	18S/phytoplankton

76	tro	Thalassiosira rotula	Bacillariophyta	18S/phytoplankton
77	cdi	Chaetoceros diadema	Bacillariophyta	18S/phytoplankton
78	ufl	Ulva flexuosa	Chlorophyta	18S/phytoplankton
79	php	Phaeomonas parva	Pinguiophyceae	18S/phytoplankton
80	pre	Protoceratium reticulatum	Dinophyceae	18S/phytoplankton
81	hak	Heterosigma akashiwo	Raphidophyceae	18S/phytoplankton
82	lpo	Lingulodinium polyedra	Dinophyceae	18S/phytoplankton
83	cs1	Chaetoceros sp. CCAP 1010/16	Bacillariophyta	18S/phytoplankton
84	tac	Teleaulax acuta	Cryptophyta	18S/phytoplankton
85	cro	Chaetoceros rostratus	Bacillariophyta	18S/phytoplankton
86	ssp	Spumella sp. GOT220	Chrysophyceae	18S/phytoplankton
87	cde	Chaetoceros debilis	Bacillariophyta	18S/phytoplankton
88	phe	Pseliodinium helix	Dinophyceae	18S/phytoplankton
89	lco	Leptocylindrus convexus	Bacillariophyta	18S/phytoplankton
90	psi	Protodinium simplex	Dinophyceae	18S/phytoplankton
91	pne	Pseudo-nitzschia heimii	Bacillariophyta	18S/phytoplankton
92	lgr	Leonella granifera	Dinophyceae	18S/phytoplankton
93	ctt	Chlorellidium tetrabotrys	Xanthophyceae	18S/phytoplankton
94	mco	Micromonas commoda	Chlorophyta	18S/phytoplankton
95	gca	Gymnodinium catenatum	Dinophyceae	18S/phytoplankton
96	sha	Scrippsiella hangoei	Dinophyceae	18S/phytoplankton
97	mpo	Margalefidinium polykrikoides	Dinophyceae	18S/phytoplankton
98	vac	Vacuolaria	Raphidophyceae	18S/phytoplankton
99	tcu	Thalassiosira curviseriata	Bacillariophyta	18S/phytoplankton
100	cwi	Chaetoceros cf. Wighamii	Bacillariophyta	18S/phytoplankton
101	pge	Polykrikos geminatum	Dinophyceae	18S/phytoplankton
102	ha2	Haptophyceae sp. EE-2014	Haptophyceae	18S/phytoplankton
103	han	Hemiselmis andersenii	Cryptophyta	18S/phytoplankton
104	cs2	Coscinodiscus sp. GGM-2004	Bacillariophyta	18S/phytoplankton
105	acu	Azadinium cuneatum	Dinophyceae	18S/phytoplankton
106	rsh	Rhizosolenia shrubsolei	Bacillariophyta	18S/phytoplankton
107	ps2	Plagiolemma sp. NC-2018a	Bacillariophyta	18S/phytoplankton
108	cc2	Crypthecodinium sp. CAAE-CL2	Dinophyceae	18S/phytoplankton
109	pya	Prymnesium parvum	Haptophyceae	18S/phytoplankton
110	cte	Cymbomonas tetramitiformis	Chlorophyta	18S/phytoplankton
111	mgi	Mamiella gilva	Chlorophyta	18S/phytoplankton

112	cci	Chaetoceros cinctus	Bacillariophyta	18S/phytoplankton
113	tch	Trieres chinensis	Bacillariophyta	18S/phytoplankton
114	pan	Phaeocystis antarctica	Haptophyceae	18S/phytoplankton
115	rat	Rhodomonas atrorosea	Cryptophyta	18S/phytoplankton
116	ns1	Nannochloris sp. MBIC10055	Chlorophyta	18S/phytoplankton
117	dno	Dinophysis norvegica	Dinophyceae	18S/phytoplankton
118	lfi	Levanderina fissa	Dinophyceae	18S/phytoplankton
119	cra	Chaetoceros radicans	Bacillariophyta	18S/phytoplankton
120	cwe	Conticribra weissflogii	Bacillariophyta	18S/phytoplankton
121	chy	Corethron hystrix	Bacillariophyta	18S/phytoplankton
122	mlo	Madanidinium loirii	Dinophyceae	18S/phytoplankton
123	rsi	Rhizosolenia similoides	Bacillariophyta	18S/phytoplankton
124	ppi	Prymnesium pigrum	Haptophyceae	18S/phytoplankton
125	psh	Paragymnodinium shiwhaense	Dinophyceae	18S/phytoplankton
126	pts	Pterocystis sp.	Acanthocystidae	18S/phytoplankton
127	cpu	Ceratodon purpureus	Streptophyta	18S/phytoplankton
128	pbl	Prototheca blaschkeae	Chlorophyta	18S/phytoplankton
129	asp	Amphora sp. 38	Bacillariophyta	18S/phytoplankton
130	cpa	Cyanophora paradoxa	Glaucocystophyceae	18S/phytoplankton
131	pio	Prototheca eriobotryae	Chlorophyta	18S/phytoplankton
132	ale	Alexandrium	Dinophyceae	18S/phytoplankton
133	och	Ochromonas	Chrysophyceae	18S/phytoplankton
134	ost	Ostreococcus	Chlorophyta	18S/phytoplankton
135	da2	Dactylodinium	Dinophyceae	18S/phytoplankton
136	tre	Trebouxiophyceae	Chlorophyta	18S/phytoplankton
137	pry	Prymnesiales	Haptophyceae	18S/phytoplankton
138	pep	Pelagophyceae	Pelagophyceae	18S/phytoplankton
139	cym	Cymatosiraceae	Bacillariophyta	18S/phytoplankton
140	dit	Ditylum	Bacillariophyta	18S/phytoplankton
141	cha	Chaetoceros	Bacillariophyta	18S/phytoplankton
142	tha	Thalassiosirales	Bacillariophyta	18S/phytoplankton
143	ske	Skeletonema	Bacillariophyta	18S/phytoplankton
144	dio	Bacillariophyta	Bacillariophyta	18S/phytoplankton
145	cph	Chlorophyta	Chlorophyta	18S/phytoplankton
146	cy2	Cryptophyta	Cryptophyta	18S/phytoplankton
147	crs	Chrysophyceae	Chrysophyceae	18S/phytoplankton
148	din	Dinophyceae	Dinophyceae	18S/phytoplankton
149	hap	Haptophyceae	Haptophyceae	18S/phytoplankton
150-172	h35-h57	-	hypothetical phytoplankton species	18S/phytoplankton
174	b01	-	hypothetical 16S ASV	16S/heterotrophic prokaryotes

175,178,180,181,206,211,250,257,269,279,282,305,322,325,329	s11-s23	SAR11	Alphaproteo-bacteriaa	16S/heterotrophic prokaryotes
176,182,215,231,232,245,255,272	s86-s93	SAR86	Gammaproteo-bacteria	16S/heterotrophic prokaryotes
177	amy	Amylibacter	Alphaproteo-bacteriaa	16S/heterotrophic prokaryotes
179	act	Actinomarina	Actinobacteria	16S/heterotrophic prokaryotes
183,284,288,289	n04-n07	NS4 marine group	Bacteroidetes	16S/heterotrophic prokaryotes
184	pla	Planktomarina	Alphaproteo-bacteriaa	16S/heterotrophic prokaryotes
185,233,236,237,256,286,298,301,336	n51-n59	NS5 marine group	Bacteroidetes	16S/heterotrophic prokaryotes
186	are	Arenicellaceae	Gammaproteo-bacteria	16S/heterotrophic prokaryotes
187,190,203,235,238,247,251,264,281,294,297,317,326	m21-m33	Marine group II archaea	Euryarchaeota	16S/heterotrophic prokaryotes
188,202,290	pu1-pu3	SAR116 clade	Alphaproteo-bacteriaa	16S/heterotrophic prokaryotes
189,222,310	cr1-cr3	Cryomorphoceae	Bacteroidetes	16S/heterotrophic prokaryotes
191,266,306	o43-o45	OM43 clade	Betaproteobacteriales	16S/heterotrophic prokaryotes
192	ros	Roseobacter	Alphaproteo-bacteriaa	16S/heterotrophic prokaryotes
193	ns2	NS2b marine group	Bacteroidetes	16S/heterotrophic prokaryotes
194,260	te1, te2	Tenacibaculum	Bacteroidetes	16S/heterotrophic prokaryotes
195,242,263,278,291,299,304,318	ma1-ma8	Marinimicrobia	SAR406 clade	16S/heterotrophic prokaryotes
196,204	th1, th2	Thioglobaceae	Gammaproteo-bacteria	16S/heterotrophic prokaryotes
197	sa2	SAR92 clade	Gammaproteo-bacteria	16S/heterotrophic prokaryotes
198	pav	Parvibaculales	Alphaproteo-bacteriaa	16S/heterotrophic prokaryotes
199,225,270	m03-m05	Marine group III archaea	Euryarchaeota	16S/heterotrophic prokaryotes
200,221	pl1, pl2	Polaribacter	Bacteroidetes	16S/heterotrophic prokaryotes
201	fla	Flavobacteriaceae	Bacteroidetes	16S/heterotrophic prokaryotes
205	ulv	Ulvibacter	Bacteroidetes	16S/heterotrophic prokaryotes
207,218,311,315	om6-om9	OM60(NOR5) clade	Gammaproteo-bacteria	16S/heterotrophic prokaryotes
208	psa	Pseudoalteromonas	Gammaproteo-bacteria	16S/heterotrophic prokaryotes

209	lum	Luminiphilus	Gammaproteo-bacteria	16S/heterotrophic prokaryotes
210,234	fo1, fo2	Formosa	Bacteroidetes	16S/heterotrophic prokaryotes
212,273	fu1, fu2	Fluviicola	Bacteroidetes	16S/heterotrophic prokaryotes
213,216,220,312	ae1-ae4	AEGEAN-169	Alphaproteo-bacteria	16S/heterotrophic prokaryotes
214	sa3	SAR92 clade	Gammaproteo-bacteria	16S/heterotrophic prokaryotes
217	dad	Dadabacteriales	Dadabacteria	16S/heterotrophic prokaryotes
219	pmy	Pla3 lineage	Planctomycetes	16S/heterotrophic prokaryotes
223,226,229,244	ro1-ro4	Rhodobacteraceae	Alphaproteo-bacteria	16S/heterotrophic prokaryotes
224	o75	OM75 clade	Alphaproteo-bacteria	16S/heterotrophic prokaryotes
227	rub	Rubritaleaceae	Verrucomicrobia	16S/heterotrophic prokaryotes
228	hoc	HOC36	Gammaproteo-bacteria	16S/heterotrophic prokaryotes
230	leb	Lentibacter	Alphaproteo-bacteria	16S/heterotrophic prokaryotes
239	n3a	NS3a marine group	Bacteroidetes	16S/heterotrophic prokaryotes
240,287,293,296	n91-n94	NS9 marine group	Bacteroidetes	16S/heterotrophic prokaryotes
241,246,248,259,327	po1-po3, po9, po5	Pseudohongiella	Gammaproteo-bacteria	16S/heterotrophic prokaryotes
243	aqu	Aquibacter	Bacteroidetes	16S/heterotrophic prokaryotes
249	per	Persicirhabdus	Verrucomicrobia	16S/heterotrophic prokaryotes
252	oce	Nitrincolaceae	Gammaproteo-bacteria	16S/heterotrophic prokaryotes
253	flc	Flavicella	Bacteroidetes	16S/heterotrophic prokaryotes
254	hyp	Hellea	Alphaproteo-bacteria	16S/heterotrophic prokaryotes
258	n7m	NS7 marine group	Bacteroidetes	16S/heterotrophic prokaryotes
261,328	ub1, ub2	UBA10353 marine group	Gammaproteo-bacteria	16S/heterotrophic prokaryotes
262	mas	Marinoscillum	Bacteroidetes	16S/heterotrophic prokaryotes
265,276	o18, o19	OM182 clade	Gammaproteo-bacteria	16S/heterotrophic prokaryotes
267	vib	Vibrio sp. hMe3-9	Gammaproteo-bacteria	16S/heterotrophic prokaryotes
268,316	s32, del	SAR324	Deltaproteobacteria	16S/heterotrophic prokaryotes
271	ect	Ectothiorhodospiraceae	Gammaproteo-bacteria	16S/heterotrophic prokaryotes
274,277,331	pi1-pi3	Pirrelulaceae	Planctomycetes	16S/heterotrophic prokaryotes
275	sul	Sulfitobacter sp.	Alphaproteo-bacteria	16S/heterotrophic prokaryotes
280,308,313	al1-al3	Alphaproteo-bacteria	Alphaproteo-bacteria	16S/heterotrophic prokaryotes

283	nmo	Nitrosomonadaceae	Gammaproteo-bacteria	16S/heterotrophic prokaryotes
285	pga	Paraglaciecola	Gammaproteo-bacteria	16S/heterotrophic prokaryotes
292,314	at1, at2	Alteromonas sp.	Gammaproteo-bacteria	16S/heterotrophic prokaryotes
295	ki1	KI89A clade	Gammaproteo-bacteria	16S/heterotrophic prokaryotes
300	fac	Flavicella	Bacteroidetes	16S/heterotrophic prokaryotes
302	wos	Woeseia	Gammaproteo-bacteria	16S/heterotrophic prokaryotes
303	ps1	PS1 clade	Alphaproteo-bacteria	16S/heterotrophic prokaryotes
307	ga1	Gammaproteo-bacteria	Gammaproteo-bacteria	16S/heterotrophic prokaryotes
309	clo	SAR202 clade	Chloroflexi	16S/heterotrophic prokaryotes
319	lem	Lentimonas	Verrucomicrobia	16S/heterotrophic prokaryotes
320	sap	Saprospiraceae	Bacteroidetes	16S/heterotrophic prokaryotes
321	psm	Pseudomonas	Gammaproteo-bacteria	16S/heterotrophic prokaryotes
323	slp	Salinisphaera	Gammaproteo-bacteria	16S/heterotrophic prokaryotes
324	ths	Thalassospira sp.	Alphaproteo-bacteria	16S/heterotrophic prokaryotes
332	b02-b05	-	hypothetical 16S ASV	16S/heterotrophic prokaryotes
336-497	a01-a99, c01-c63	-	hypothetical POM species	POM
503-664	d01-d99, f01-f63	-	hypothetical DOM species	DOM
