# Supplementary material for: Internal carbon recycling by heterotrophic prokaryotes compensates for mismatches between phytoplankton production and heterotrophic consumption
Source: ISME J. 2024 Jun 11;18(1):wrae103. doi: 10.1093/ismejo/wrae103 (PMC11217553; doi:10.1093/ismejo/wrae103)
Supplement: Suppementary_wrae103 [file suppementary_wrae103.zip › Supplementary Fig. 1.pdf]

Supplementary Fig. 1

succession of phytoplankton and heterotrophic prokaryotes

2012

phytoplankton spring bloom

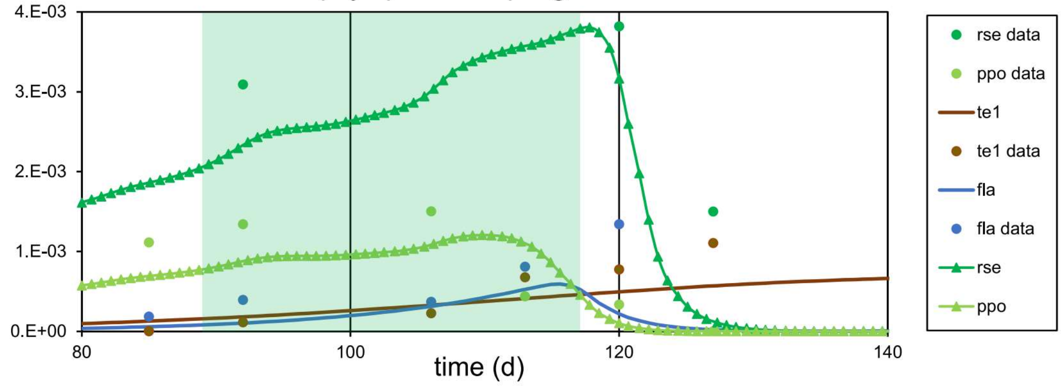

bacteria summer bloom

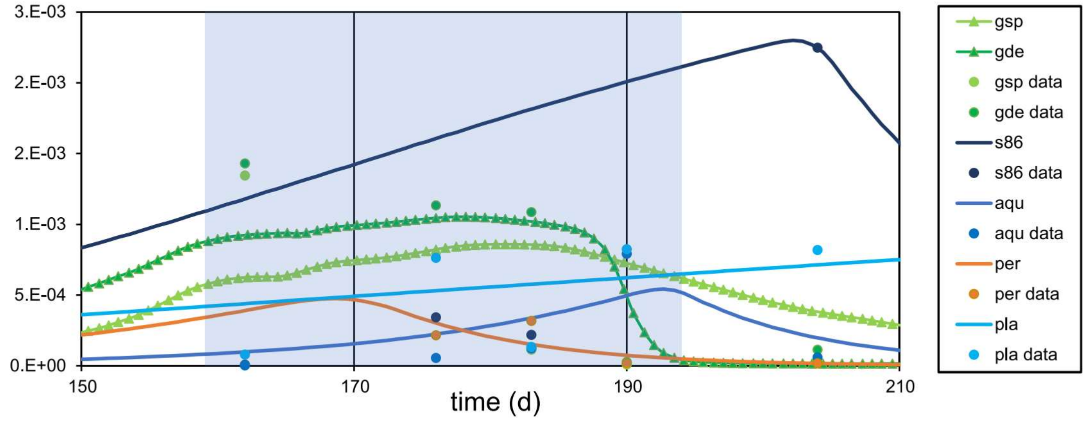

phytoplankton summer bloom

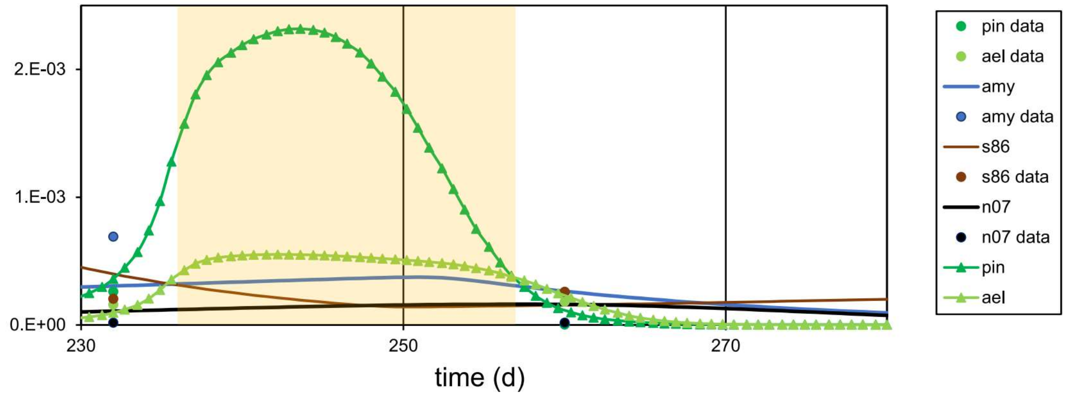

2014

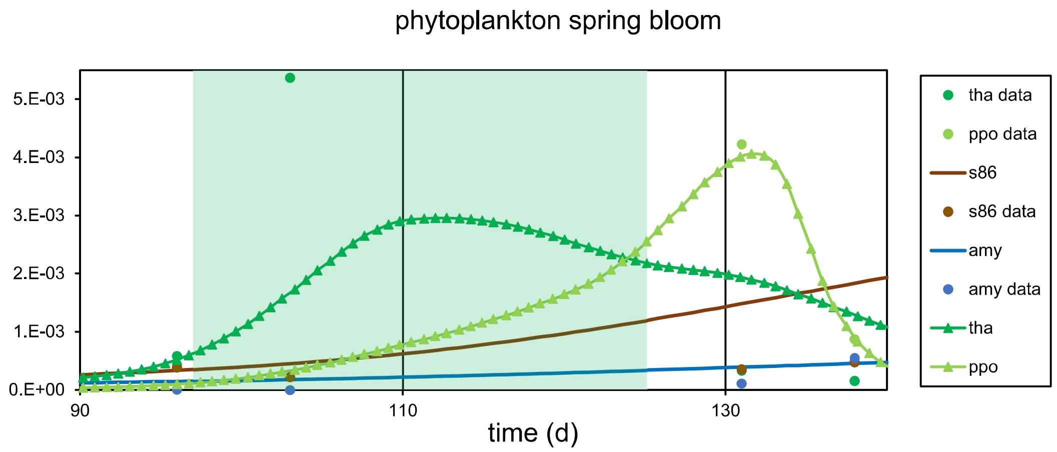
phytoplankton spring bloom
5.E-03
4.E-03
3.E-03
2.E-03
1.E-03
0.E+00
90
110
130
time (d)
tha data
ppo data
s86
s86 data
amy
amy data
tha
ppo

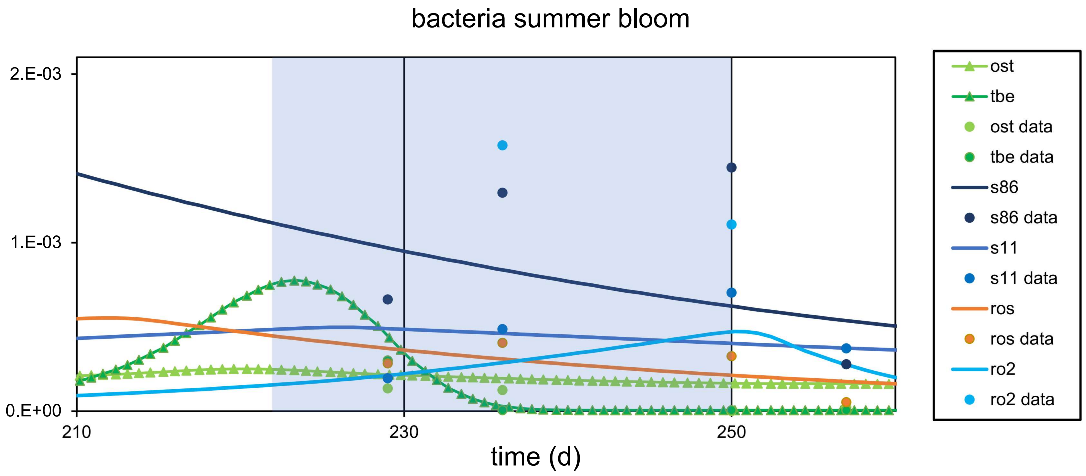
bacteria summer bloom
2.E-03
1.E-03
0.E+00
210
230
250
time (d)
ost
tbe
ost data
tbe data
s86
s86 data
s11
s11 data
ros
ros data
ro2
ro2 data

2015

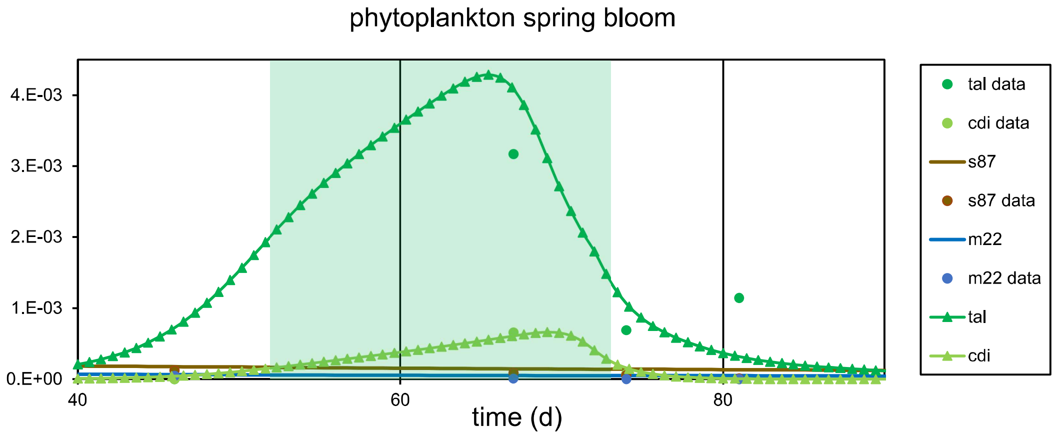
phytoplankton spring bloom
4.E-03
3.E-03
2.E-03
1.E-03
0.E+00
40
60
80
time (d)
tal data
cdi data
s87
s87 data
m22
m22 data
tal
cdi

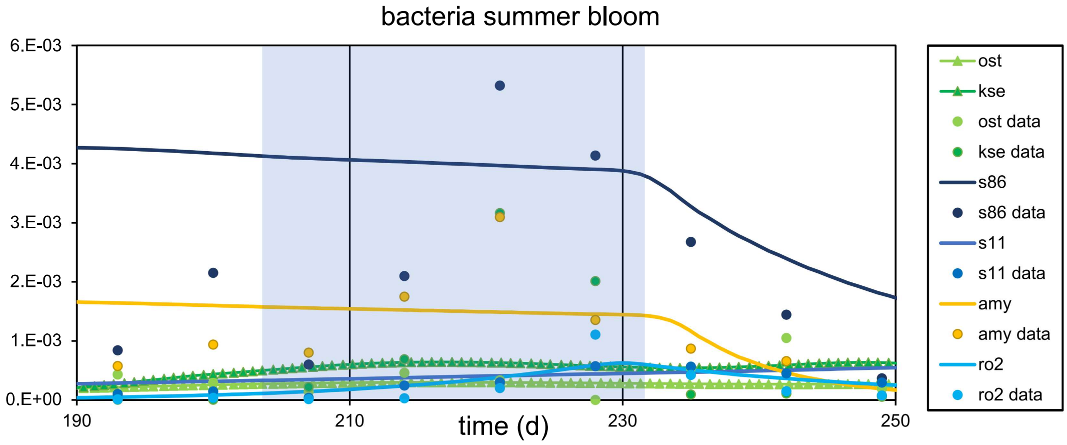
bacteria summer bloom
6.E-03
5.E-03
4.E-03
3.E-03
2.E-03
1.E-03
0.E+00
190
210
230
250
time (d)
ost
kse
ost data
kse data
s86
s86 data
s11
s11 data
amy
amy data
ro2
ro2 data

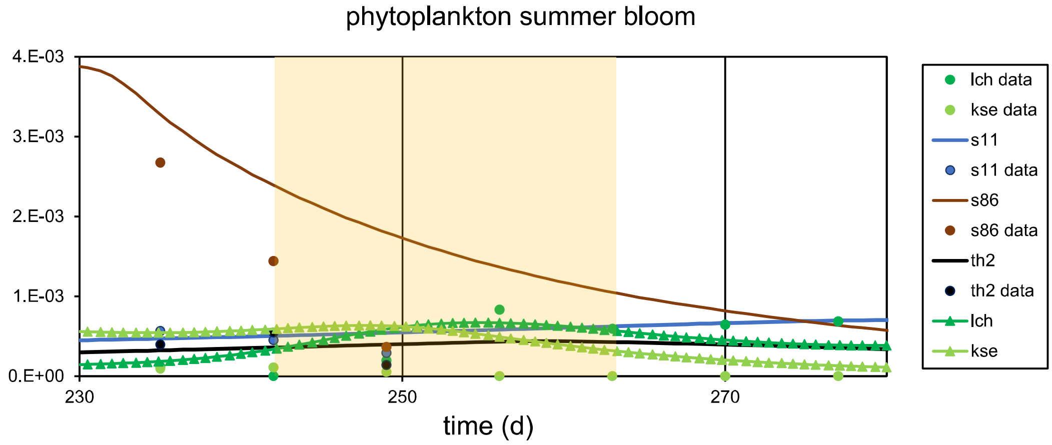
phytoplankton summer bloom
4.E-03
3.E-03
2.E-03
1.E-03
0.E+00
230
250
270
time (d)
lch data
kse data
s11
s11 data
s86
s86 data
th2
th2 data
lch
kse

2016

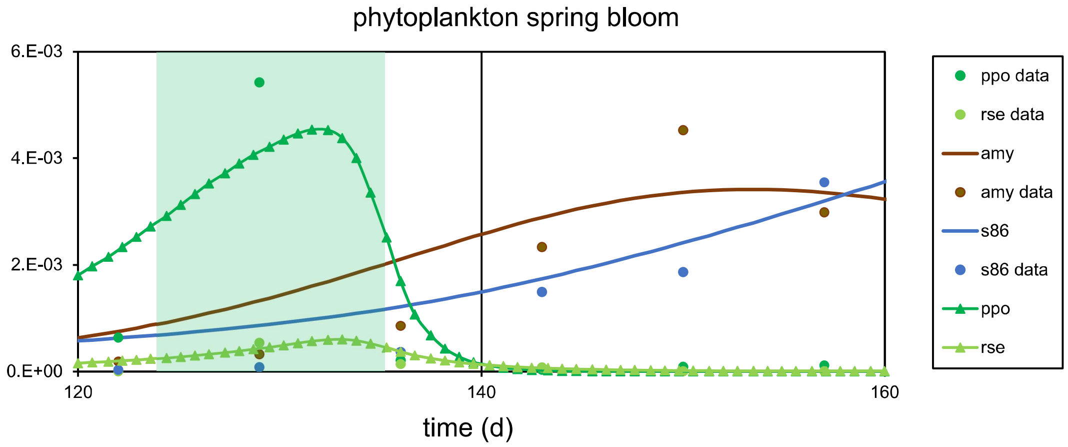
phytoplankton spring bloom
6.E-03
4.E-03
2.E-03
0.E+00
120
140
160
time (d)
ppo data
rse data
amy
amy data
s86
s86 data
ppo
rse

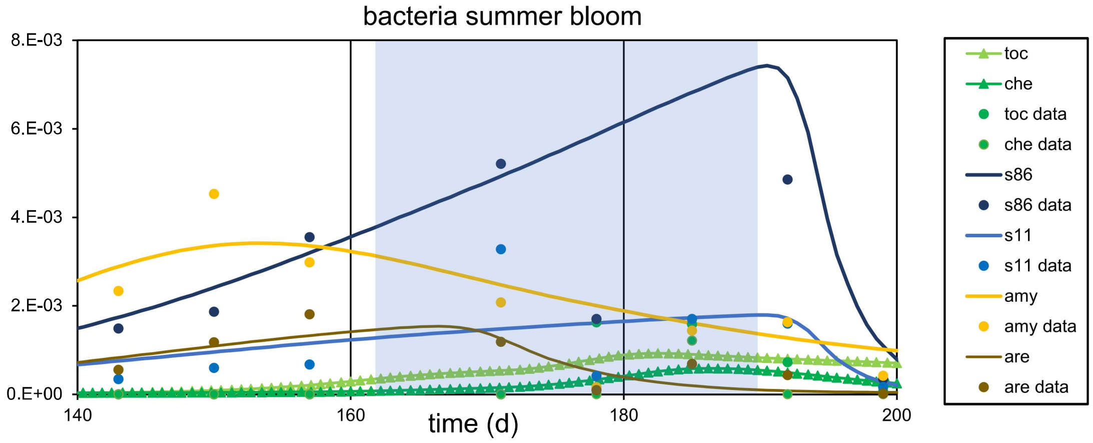
bacteria summer bloom
8.E-03
6.E-03
4.E-03
2.E-03
0.E+00
140
160
180
200
time (d)
toc
che
toc data
che data
s86
s86 data
s11
s11 data
amy
amy data
are
are data

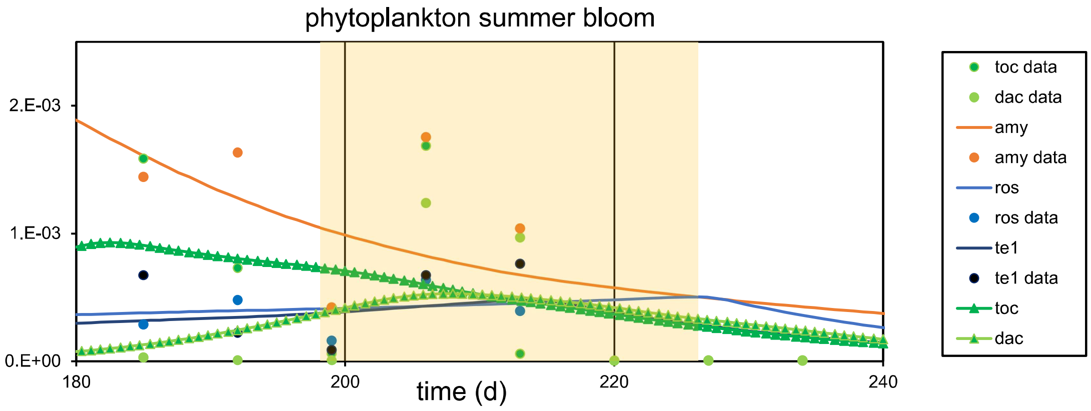
phytoplankton summer bloom
2.E-03
1.E-03
0.E+00
180
200
220
240
time (d)
toc data
dac data
amy
amy data
ros
ros data
te1
te1 data
toc
dac

2017

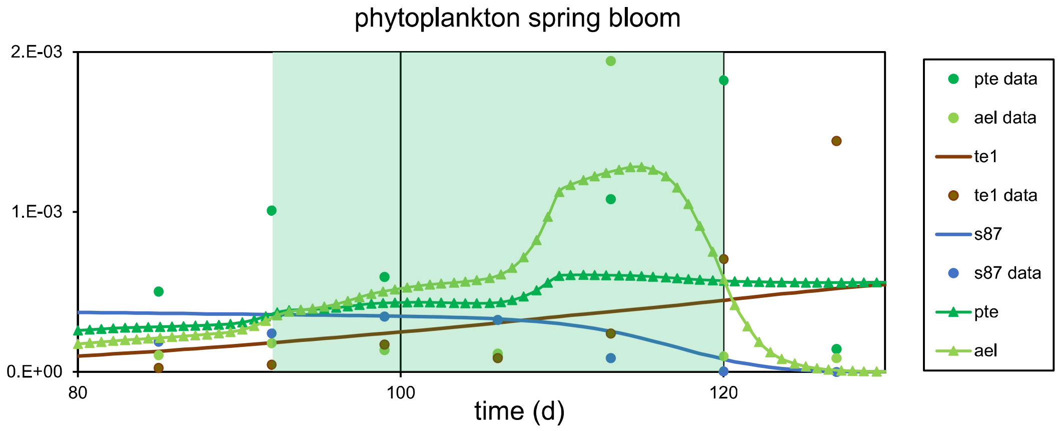

phytoplankton spring bloom
2.E-03
1.E-03
0.E+00
80
100
120
time (d)
pte data
ael data
te1
te1 data
s87
s87 data
pte
ael

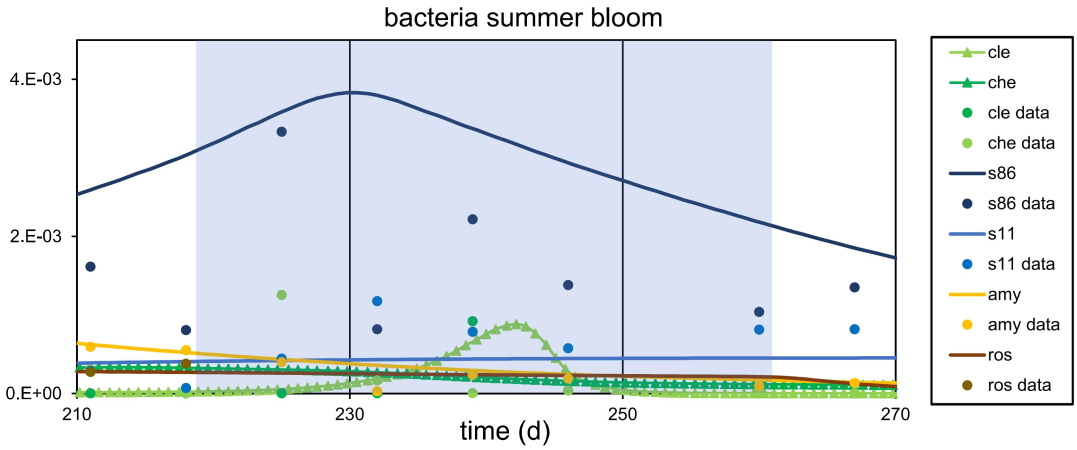

bacteria summer bloom
4.E-03
2.E-03
0.E+00
210
230
250
270
time (d)
cle
che
cle data
che data
s86
s86 data
s11
s11 data
amy
amy data
ros
ros data

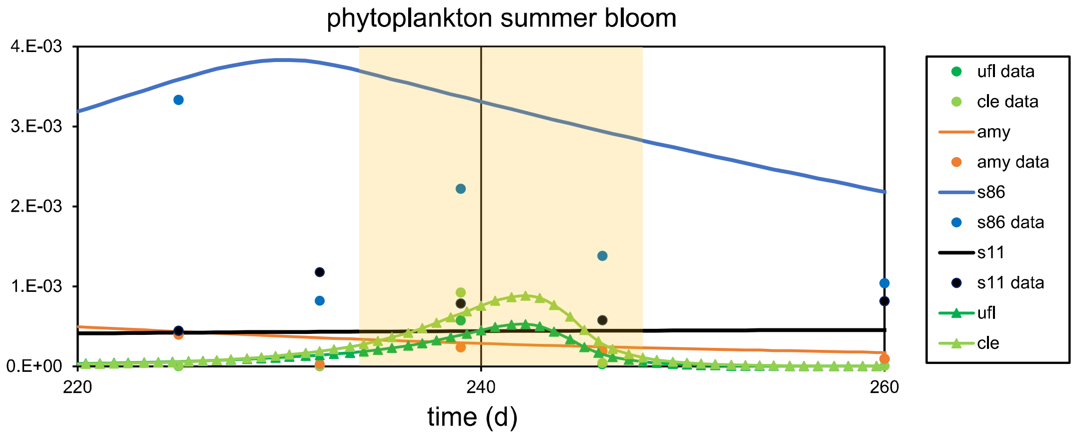

phytoplankton summer bloom
4.E-03
3.E-03
2.E-03
1.E-03
0.E+00
220
240
260
time (d)
ufl data
cle data
amy
amy data
s86
s86 data
s11
s11 data
ufl
cle

2018

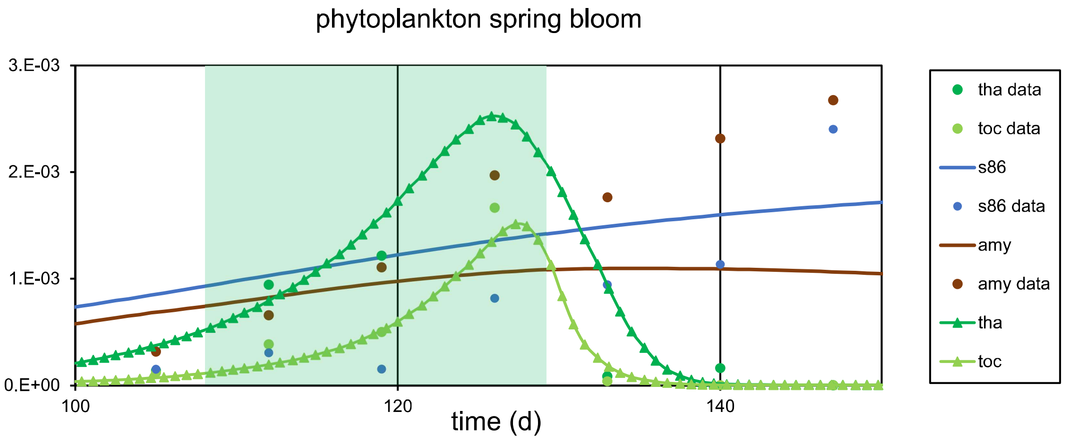
phytoplankton spring bloom
3.E-03
2.E-03
1.E-03
0.E+00
100
120
140
time (d)
tha data
toc data
s86
s86 data
amy
amy data
tha
toc

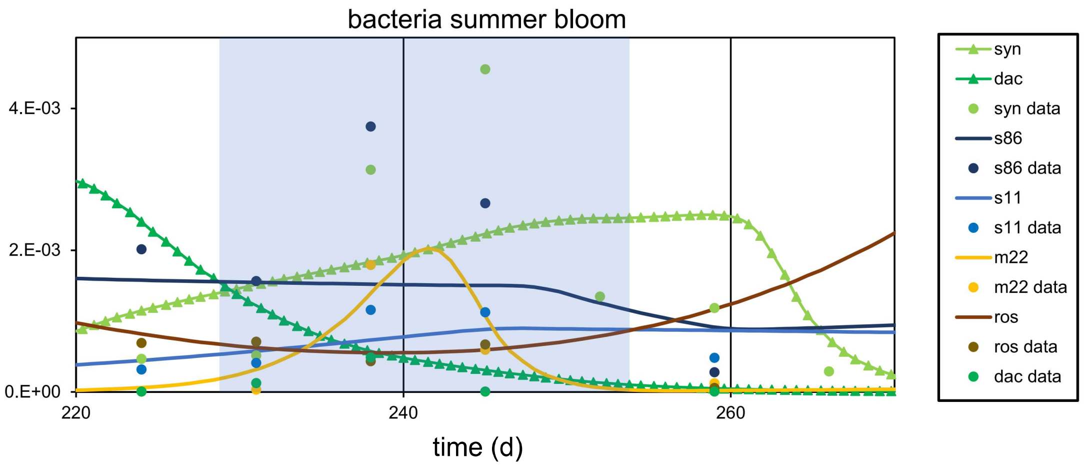
bacteria summer bloom
4.E-03
2.E-03
0.E+00
220
240
260
time (d)
syn
dac
syn data
s86
s86 data
s11
s11 data
m22
m22 data
ros
ros data
dac data

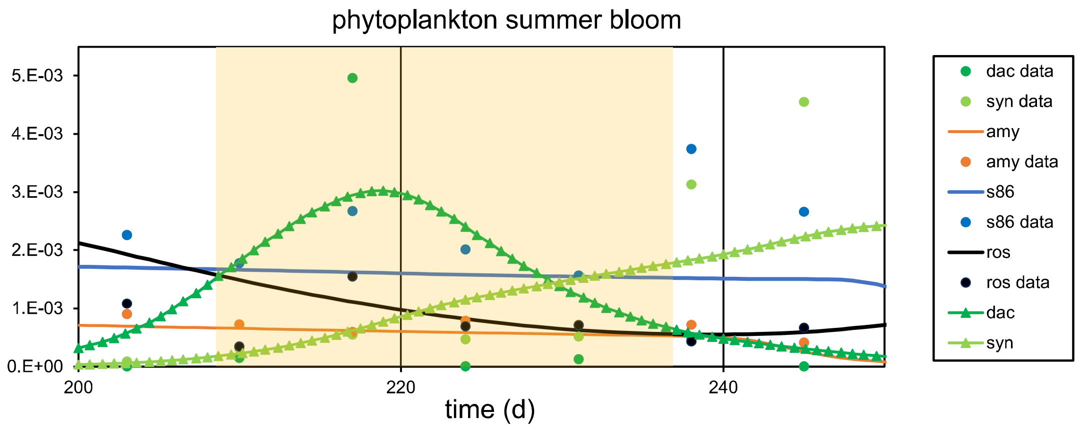
phytoplankton summer bloom
5.E-03
4.E-03
3.E-03
2.E-03
1.E-03
0.E+00
200
220
240
time (d)
dac data
syn data
amy
amy data
s86
s86 data
ros
ros data
dac
syn
