# Supplementary material for: Internal carbon recycling by heterotrophic prokaryotes compensates for mismatches between phytoplankton production and heterotrophic consumption
Source: ISME J. 2024 Jun 11;18(1):wrae103. doi: 10.1093/ismejo/wrae103 (PMC11217553; doi:10.1093/ismejo/wrae103)
Supplement: Suppementary_wrae103 [file suppementary_wrae103.zip › Supplementary Fig. 2.pdf]

Supplementary Fig. 2: Inhibition analyses

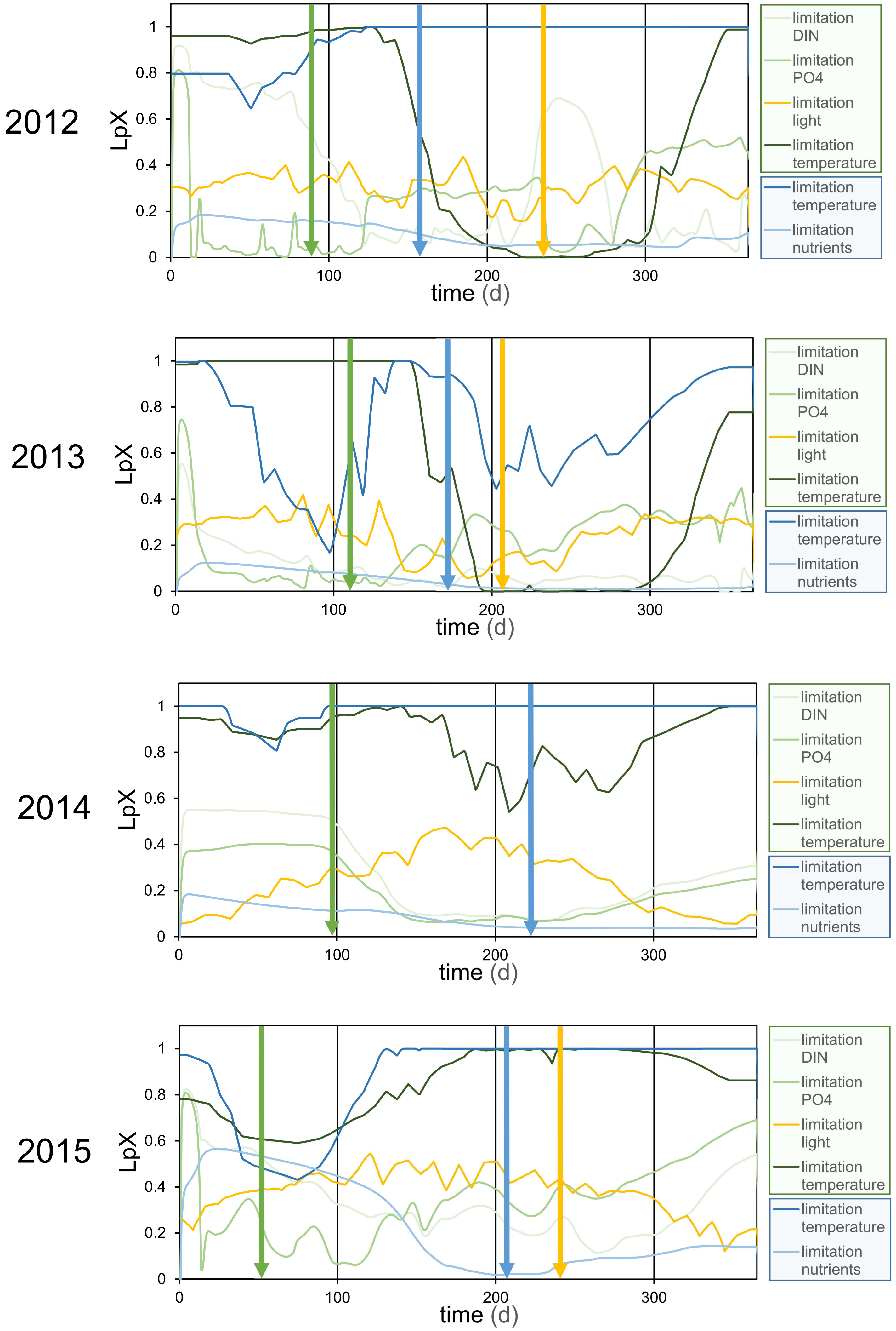

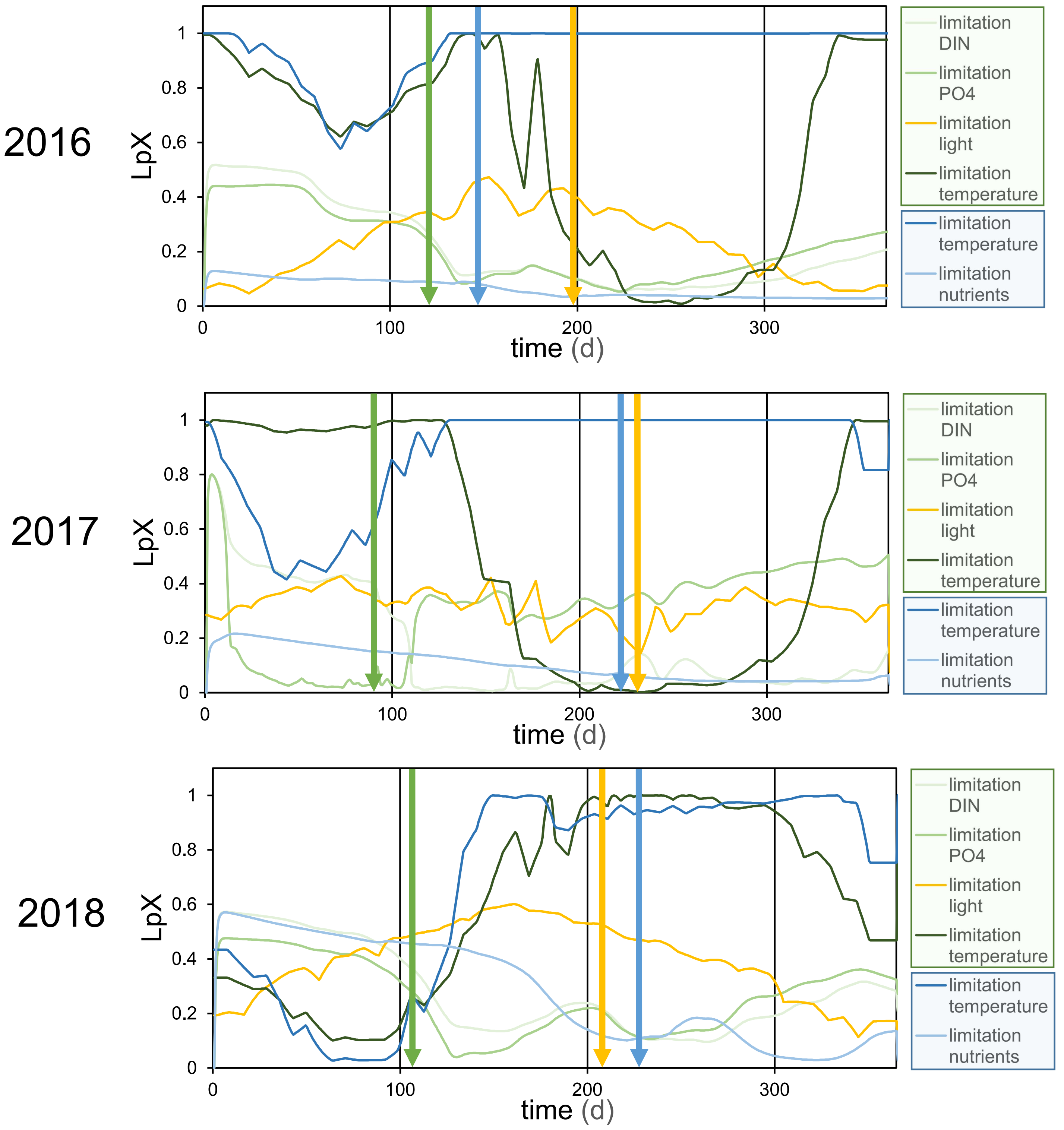

The y-axis refers to the relative inhibition, where a higher value stands for lower inhibition. The green shaded legend refers to phytoplankton inhibition, the blue shaded legend to heterotrophic prokaryotic inhibition. DIN = dissolved inoranic nitrogen, PO4 = phosphate. The arrows indicate the start of the blooms: green: phytoplankton spring bloom, blue: bacteria summer bloom, orange: phytoplankton summer bloom.
