# Supplementary material for: Internal carbon recycling by heterotrophic prokaryotes compensates for mismatches between phytoplankton production and heterotrophic consumption
Source: ISME J. 2024 Jun 11;18(1):wrae103. doi: 10.1093/ismejo/wrae103 (PMC11217553; doi:10.1093/ismejo/wrae103)
Supplement: Suppementary_wrae103 [file suppementary_wrae103.zip › Supplementary Fig. 3.pdf]

Supplementary Fig. 3: density, nitrate, phosphate and het. prokaryotic DOC production in bacterial summer blooms

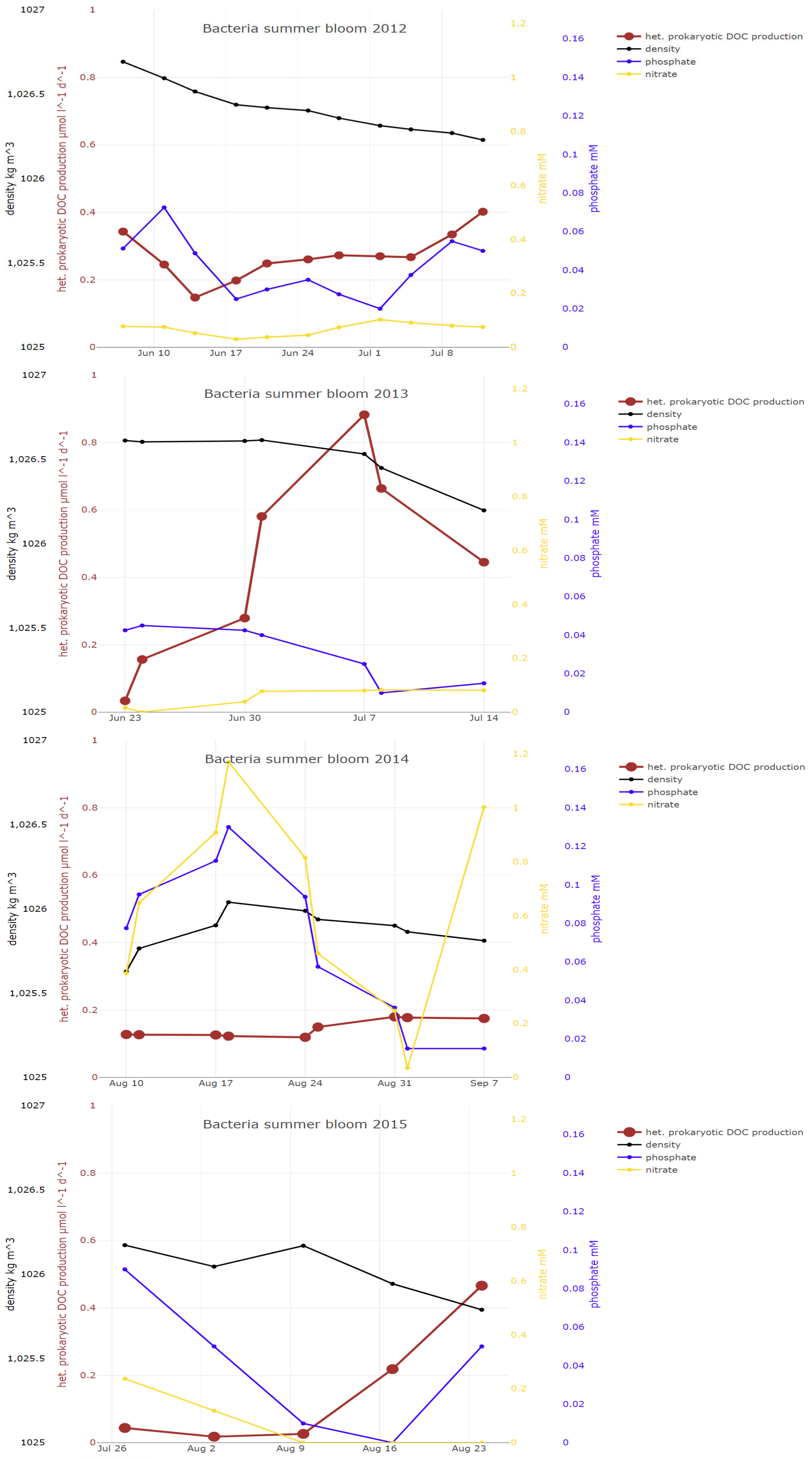

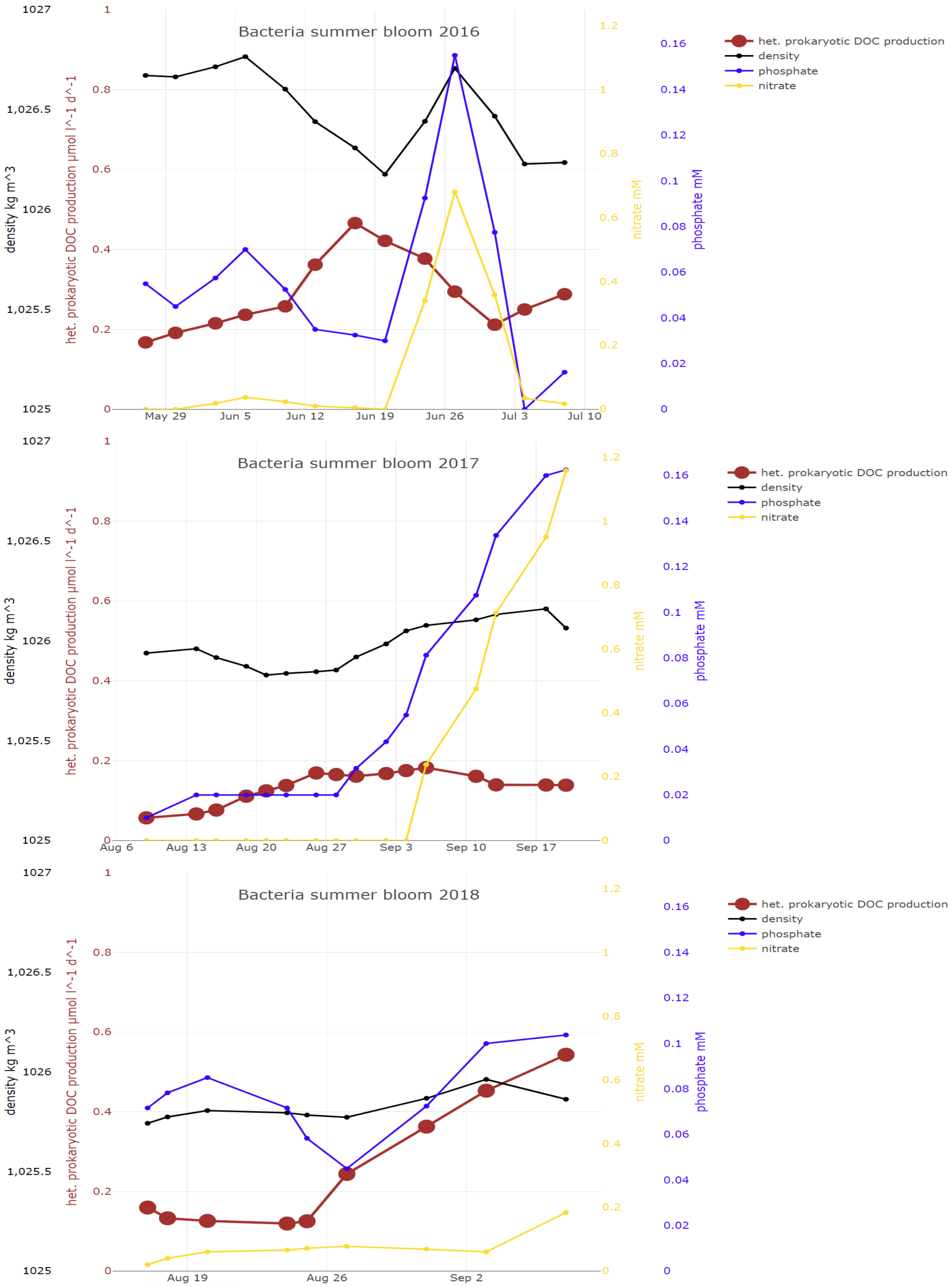

Time-series for measurements of phosphate, nitrate and water density as well as estimates of DOC production by heterotrophic prokaryotes for bacterial summer blooms at WEC station L4.
