# Supplementary material for: Internal carbon recycling by heterotrophic prokaryotes compensates for mismatches between phytoplankton production and heterotrophic consumption
Source: ISME J. 2024 Jun 11;18(1):wrae103. doi: 10.1093/ismejo/wrae103 (PMC11217553; doi:10.1093/ismejo/wrae103)
Supplement: Suppementary_wrae103 [file suppementary_wrae103.zip › Supplementary Table 4.docx]

Supplemental Table 4:

Definition of bloom periods that were chosen for elaborated analyses. Phytoplankton spring blooms were defined as the highest chlorophyll concentration between February and May, phytoplankton summer blooms as the highest chlorophyll concentration between July and August. Bacterial summer blooms were defined as the highest bacterial concentrations between June and August. Model-data overlaps, estimated peak chl. a or bacterial cell number concentrations, and estimated growth rates (positive/negative) were considered for the assignments of bloom periods without applying strict cut-offs to ensure reasonable definitions (for general rules see main text).

| Year | bloom | Days of the year | Comments | Duration (d) |
| --- | --- | --- | --- | --- |
| 2012 | Phytoplankton spring bloom | 89-117 | Start: 3.1 µg Chl. a l^-1^, end: 3.32 µg Chl. a l^-1^.  First overlap between model and data with positive growth rate. | 28 |
| 2013 | Phytoplankton spring bloom | 112-140 | First day above 2.5 µg Chl. a l^-1^. | 28 |
| 2014 | Phytoplankton spring bloom | 97-125 | First day above 1.5 µg Chl. a l^-1^ that fits the data. | 28 |
| 2015 | Phytoplankton spring bloom | 52-73 | First bloom of the year. First day above 1.7 µg Chl. a l^-1,^ last day above 1.3 µg Chl. a l^-1^. Only 21-day period applied. | 21 |
| 2016 | Phytoplankton spring bloom | 124-135 | Short, small spring bloom. Starting day: first day above 1.4 µg Chl. a l^-1^, last day: last day above 1.4 µg Chl. a l^-1^. | 11 |
| 2017 | Phytoplankton spring bloom | 92-120 | Double peak, first day above 2.8 µg Chl. a l^-1^ with data match. Full period, last day above 2 µg µg Chl. a l^-1^. | 28 |
| 2018 | Phytoplankton spring bloom | 108-129 | First day that fits the data and is above 2.5 µg Chl. a l^-1^. Last day above 2 µg Chl. a l^-1^. Sharp decrease. | 21 |
| 2012 | Phytoplankton summer bloom | 234-255 | First day 1.34 µg Chl. a l^-1^, last day 1.87 µg Chl. a l^-1^. | 21 |
| 2013 | Phytoplankton summer bloom | 206-234 | Fist day above 1.8 µg Chl. a l^-1^. | 28 |
| 2015 | Phytoplankton summer bloom | 242-265 | First day above 1.5 µg Chl. a l^-1^, last day with 1.5 µg Chl. a l^-1^. | 23 |
| 2016 | Phytoplankton summer bloom | 198-226 | Pronounced long bloom. First day that fits the data, and above 1.6 µg Chl. a l^-1^. | 28 |
| 2017 | Phytoplankton summer bloom | 231-246 | Small peak that overlaps with the data. First day above 0.9 µg Chl. a l^-1^ | 15 |
| 2018 | Phytoplankton summer bloom | 209-237 | Strong double peak. First day above 2 µg Chl. a l^-1^. | 28 |
| 2012 | Bacteria summer bloom | 159-193 | First day above 1.8 million cells ml^-1^ that matches the data, afterwards a long plateau. Last day above 1.8 million cells ml^-1^. | 34 |
| 2013 | Bacteria summer bloom | 174-200 | First day above 1.3 million cells ml^-1^ with positive growth rate that fits the data, last day above 1 million cells ml^-1^. | 26 |
| 2014 | Bacteria summer bloom | 222-250 | Second increase of a long plateau that reflects the data. | 28 |
| 2015 | Bacteria summer bloom | 208-236 | Chosen period reflects the data. Start: first day above 1.4 million cells ml^-1^. | 28 |
| 2016 | Bacteria summer bloom | 148-194 | Long, pronounced plateau. First day above 1.7 million cells ml^-1^ until last day above 1.7 million cells ml^-1^. | 46 |
| 2017 | Bacteria summer bloom | 221-263 | Long plateau. First day: positive growth rate that matches the data. Last day: first day with negative growth rates, i.e. decline. | 42 |
| 2018 | Bacteria summer bloom | 229-254 | Peak is reflected by the data (other peaks not). First day above 1.2 million cells ml^-1^. | 25 |
