# Supplementary material for: Internal carbon recycling by heterotrophic prokaryotes compensates for mismatches between phytoplankton production and heterotrophic consumption
Source: ISME J. 2024 Jun 11;18(1):wrae103. doi: 10.1093/ismejo/wrae103 (PMC11217553; doi:10.1093/ismejo/wrae103)
Supplement: Suppementary_wrae103 [file suppementary_wrae103.zip › Supplementary Table 5.docx]

Supplementary Table 5

Statistical tests and statistical outcomes.

1. Tests for differences of recurrences between different variables, and test for differences of recurrences in the tested variables between the different bloom types.
2. Tests for differences in DOM concentration, DOM consumption and DOM production between the three different bloom types
3. Differences in averaged fluxes of phytoplankton>heterotrophic prokaryotes and heterotrophic prokaryotes>heterotrophic prokaryotes
4. Differences in instantaneous fluxes between phytoplankton>heterotrophic prokaryotes and heterotrophic prokaryotes>heterotrophic prokaryotes between bloom types and trends over time
5. Differences in concentration normalized heterotrophy rates between bloom types
6. **Tests for differences of recurrences between different variables, and test for differences of recurrences in the tested variables between the different bloom types.**

Test for homogeneity of variances, Levene test

| Tested variables | F | *P* |
| --- | --- | --- |
| Variables (phyto>dom, pro. het<dom, phyto>pro. het, pro. het>pro. het) | 7.5 | <0.01 |
| Phyto>dom (different bloom types) | 0.6 | 0.53 |
| Pro. het<dom (different bloom types) | 1.6 | 0.22 |
| Phyto>pro. het (different bloom types) | 2.7 | 0.07 |
| pro. het>pro. het (different bloom types) | 3 | 0.06 |

Kruskal-Wallis and Tukey posthoc test for differences between variables

| Tested variables | Chi² | *P* | Tukey |
| --- | --- | --- | --- |
| Variables (phyto>dom, pro. het<dom, phyto>pro. het, pro. het>pro. het) | 104.7 | <0.01 | Phyto>dom = b, pro. het<dom = a, phyto>pro. het = c, pro. het>pro. het = c |

ANOVA and posthoc test for differences in the recurrence in the different variables between the three bloom types

| Tested variables | F | *P* | TukeyHSD |
| --- | --- | --- | --- |
| Phyto>dom | 2.4 | 0.1 | - |
| Pro. het<dom | 3.3 | 0.04 | Phytoplankton spring bloom = a, bacteria summer bloom = ab, phytoplankton summer bloom = b |
| Phyto>pro. het | 7.4 | <0.01 | Phytoplankton spring bloom = a, bacteria summer bloom = ab, phytoplankton summer bloom = b |
| Pro. het>pro. het | 1.1 | 0.35 | - |

1. **Tests for differences in DOM concentration, DOM consumption and DOM production between the three different bloom types**

Test for homogeneity of variances, Levene test

| Tested variables | F | *P* |
| --- | --- | --- |
| concentration~  bloom types | 2.7 | 0.1 |
| production combined~ bloom types | 0.6 | 0.56 |
| consumption ~ bloom types | 1.8 | 0.19 |

ANOVA and Tukey posthoc to test for differences between blooms

| Tested variables | F | *P* | TukeyHSD |
| --- | --- | --- | --- |
| DOC concentration | 23.9 | <0.01 | Phytoplankton spring bloom = a, bacteria summer bloom and phytoplankton summer bloom = b |
| DOC production combined | 0.2 | 0.84 | - |
| DOC consumption | 6.7 | 0.01 | bacteria summer bloom = a, phytoplankton spring and summer bloom = b |

Test for differences of phytoplankton and heterotrophic prokaryotic DOC production

Test for homogeneity of variances, Levene test

| Tested variables | F | *P* |
| --- | --- | --- |
| Phytoplankton spring bloom: Phytoplankton production~  Heterotrophic prokaryotes production | 5.2 | 0.04 |
| Bacteria summer bloom: Phytoplankton production~  Heterotrophic prokaryotes production | 6.3 | 0.03 |
| Phytoplankton summer bloom: Phytoplankton production~  Heterotrophic prokaryotes production | 0.1 | 0.78 |

Mann-Whitney-U and T-Tests for differences between phytoplankton and pheterotrophic prokaryotes production

| Tested variables | W or T value | *P* |
| --- | --- | --- |
| Phytoplankton spring bloom | W: 49 | <0.01 |
| Bacteria summer bloom | W: 15 | 0.26 |
| Phytoplankton summer bloom | T: 1.4 | 0.2 |

1. **Differences in averaged fluxes of phytoplankton>pro. hetteria and pro. hetteria>pro. hetteria**

Test for homogeneity of variances, Levene test

| Tested variables | F | *P* |
| --- | --- | --- |
| Phytoplankton spring bloom | 3.3 | 0.1 |
| Bacteria summer bloom | 0.02 | 0.9 |
| Phytoplankton summer bloom | 0.3 | 0.58 |

T-tests for differences between phytoplankton>heterotrophic prokaryotes and heterotrophic prokaryotes>heterotrophic prokaryotes fluxes for bloom averaged fluxes

| Tested variables | t | *P* | average µmol C l^-1^ d^-1^ |
| --- | --- | --- | --- |
| Phytoplankton spring bloom | 3.8 | <0.01 | phytoplankton> heterotrophic prokaryotes  0.13, heterotrophic prokaryotes > heterotrophic prokaryotes 0.03 |
| Bacteria summer bloom | 1.2 | 0.27 | phytoplankton> heterotrophic prokaryotes  0.18, heterotrophic prokaryotes>heterotrophic prokaryotes 0.15 |
| Phytoplankton summer bloom | 2.2 | 0.06 | phytoplankton>heterotrophic prokaryotes  0.14, heterotrophic prokaryotes>heterotrophic prokaryotes 0.08 |

1. **Differences in instantaneous fluxes between phytoplankton>heterotrophic prokaryotes and heterotrophic prokaryotes>heterotrophic prokaryotes**

Test for homogeneity of variances, Levene test

| Tested variables | F | *P* |
| --- | --- | --- |
| Phytoplankton spring bloom day 0 | 3.3 | 0.09 |
| Phytoplankton spring bloom day 7 | 2.2 | 0.16 |
| Phytoplankton spring bloom day 14 | 2.8 | 0.13 |
| Phytoplankton spring bloom day 21 | 3.1 | 0.11 |
| Phytoplankton spring bloom day 28 | 0 | 0.99 |
| Bacteria summer bloom day 0 | 1.6 | 0.22 |
| Bacteria summer bloom day 7 | 0.1 | 0.79 |
| Bacteria summer bloom day 14 | 0.3 | 0.59 |
| Bacteria summer bloom day 21 | 0.6 | 0.45 |
| Bacteria summer bloom day 28 | 0.3 | 0.6 |
| Phytoplankton summer bloom day 0 | 0.2 | 0.64 |
| Phytoplankton summer bloom day 7 | 0.8 | 0.4 |
| Phytoplankton summer bloom day 14 | 0.1 | 0.75 |
| Phytoplankton summer bloom day 21 | 0.6 | 0.45 |
| Phytoplankton summer bloom day 28 | 0.7 | 0.44 |

T-tests for differences between phytoplankton>heterotrophic prokaryotes and heterotrophic prokaryotes>heterotrophic prokaryotes fluxes for bloom averaged fluxes

| Tested variables | t | *P* |
| --- | --- | --- |
| Phytoplankton spring bloom day 0 | 3.5 | <0.01 |
| Phytoplankton spring bloom day 7 | 2.9 | 0.01 |
| Phytoplankton spring bloom day 14 | 5 | <0.01 |
| Phytoplankton spring bloom day 21 | 3.9 | <0.01 |
| Phytoplankton spring bloom day 28 | 4.2 | <0.01 |
| Bacteria summer bloom day 0 | 2.9 | 0.01 |
| Bacteria summer bloom day 7 | 1.9 | 0.08 |
| Bacteria summer bloom day 14 | 0.9 | 0.36 |
| Bacteria summer bloom day 21 | 0.03 | 0.97 |
| Bacteria summer bloom day 28 | 0.8 | 0.45 |
| Phytoplankton summer bloom day 0 | 1.7 | 0.13 |
| Phytoplankton summer bloom day 7 | 4 | <0.01 |
| Phytoplankton summer bloom day 14 | 2.7 | 0.02 |
| Phytoplankton summer bloom day 21 | 1.6 | 0.15 |
| Phytoplankton summer bloom day 28 | 0.9 | 0.39 |

Differences in trends of instantaneous fluxes between bloom types

Linear models to test for increases/decreases of phytoplankton>heterotrophic prokaryotes or heterotrophic prokaryotes>heterotrophic prokaryotes fluxes over time

| Tested variables | Formula | *R*² | *P* |
| --- | --- | --- | --- |
| Bacteria summer bloom heterotrophic prokaryotes>heterotrophic prokaryotes | Y=0.11+0.00081x | 0.01 | 0.56 |
| Bacteria summer bloom phytoplankton>heterotrophic prokaryotes | Y=0.25-0.005x | 0.15 | 0.02 |
| Phytoplankton summer bloom heterotrophic prokaryotes>heterotrophic prokaryotes | Y=0.07-0.00016x | <0.01 | 0.84 |
| Phytoplankton summer bloom phytoplankton>heterotrophic prokaryotes | Y=0.1+0.003x | 0.09 | 0.13 |
| Phytoplankton spring bloom heterotrophic prokaryotes>heterotrophic prokaryotes | Y=0.02+0.0005x | 0.05 | 0.23 |
| Phytoplankton spring bloom phytoplankton>heterotrophic prokaryotes | Y=0.1+0.0017x | 0.08 | 0.13 |

1. **Differences in concentration normalized heterotrophy rates:**

Test for homogeneity of variances of the concentration normalized heterotrophy rates: Levene test

| Tested variables | F | *P* |
| --- | --- | --- |
| Concentration normalized heterotrophy rate~  bloom types | 0.4 | 0.67 |

ANOVA and posthoc test for differences in the concentration normalized heterotrophy rate (heterotrophy rate: mol C consumed per day by mol C of pro. hetteria)

| Tested variables | F | *P* | TukeyHSD |
| --- | --- | --- | --- |
| concentration | 3.7 | <0.05 | Phytoplankton spring bloom = b, bacteria summer bloom = a, phytoplankton summer bloom = ab |
