# Supplementary material for: Internal carbon recycling by heterotrophic prokaryotes compensates for mismatches between phytoplankton production and heterotrophic consumption
Source: ISME J. 2024 Jun 11;18(1):wrae103. doi: 10.1093/ismejo/wrae103 (PMC11217553; doi:10.1093/ismejo/wrae103)
Supplement: Suppementary_wrae103 [file suppementary_wrae103.zip › Supplementary Table 6.docx]

Supplementary Table 6: (A) Top ten phytoplankton DOM provider, (B) top ten heterotrophic prokaryotic DOM consumers with the corresponding largest phytoplankton OTU provider, and consumed DOM species, (C) top ten phytoplankton>heterotrophic prokaryotes and heterotrophic prokaryotes>heterotrophic prokaryotes pairs with the fluxes between them (µmol C l^-1^ d^-1^). Analyses were done for separate years and the three different bloom types. For taxonomies/names of phytoplankton OTUs and heterotrophic prokaryotic ASVs see Supplementary Table 10. Bloom types: Psb = phytoplankton spring bloom, Bsu = bacteria summer bloom, Psu = phytoplankton summer bloom.

1. Top ten phytoplankton DOM provider

| year | 2012 | | | 2013 | | | 2014 | | 2015 | | | 2016 | | | 2017 | | | 2018 | | |
| --- | --- | --- | --- | --- | --- | --- | --- | --- | --- | --- | --- | --- | --- | --- | --- | --- | --- | --- | --- | --- |
| Bloom/rank | Psb | Bsu | Psu | Psb | Bsu | Psu | Psb | Bsu | Psb | Bsu | Psu | Psb | Bsu | Psu | Psb | Bsu | Psu | Psb | Bsu | Psu |
| 1 | ppo | lmi | pin | mpu | h37 | gsp | tha | tbe | tal | kse | kse | rse | che | toc | ael | cle | cle | tha | dac | dac |
| 2 | toc | ezo | dio | tro | pon | din | h39 | ost | bpr | syn | syn | ppo | ns1 | pte | tam | h44 | pcu | fna | gsp | gsp |
| 3 | rse | gde | ael | bpr | dsp | pha | pcu | dia | syn | dac | lch | cro | pte | dia | h50 | pcu | ufl | tam | syn | syn |
| 4 | kve | hap | acu | h37 | gsp | pin | bpr | che | cdi | pte | tte | csi | cwe | che | h44 | ufl | cwi | toc | ale | ale |
| 5 | lbo | lgr | tac | tam | stu | pte | h36 | pha | lch | che | pte | csp | cs1 | dac | bpr | cwi | che | csi | ael | ael |
| 6 | gcr | csi | ske | cra | pve | mpu | ppo | dno | ezo | gsm | rse | kse | toc | pre | mpu | che | lco | cca | chs | chs |
| 7 | tam | tre | lvi | tha | pha | gcr | h40 | rat | csp | lch | pon | stu | cro | ezo | pte | lco | gos | isp | rsh | ns1 |
| 8 | mpu | och | lda | ha2 | psp | ael | h35 | gsp | toc | lgr | yye | ns1 | pha | dno | aan | pte | pon | mpu | pre | pre |
| 9 | da2 | gsp | pon | cs1 | psh | str | tco | hap | tco | lco | gsm | kve | gde | gsm | cca | pve | pte | bpr | cci | lmi |
| 10 | tro | gsm | pte | h56 | pte | che | syn | gca | pin | str | ost | pte | pve | cle | h39 | toc | pve | sha | cwe | cwe |

(B): Top ten bacterial carbon consumers (con), corresponding top phytoplankton provider (prov) and corresponding top DOM species consumed for (B1) phytoplankton spring blooms, (B2) bacteria summer blooms, and (B3) phytoplankton summer blooms.

(B1) phytoplankton spring blooms

| year | 2012 | | | 2013 | | | 2014 | | | 2015 | | | 2016 | | | 2017 | | | 2018 | | |
| --- | --- | --- | --- | --- | --- | --- | --- | --- | --- | --- | --- | --- | --- | --- | --- | --- | --- | --- | --- | --- | --- |
| rank | con | prov | DOM | con | prov | DOM | con | prov | DOM | con | prov | DOM | con | prov | DOM | con | prov | DOM | con | prov | DOM |
| 1 | b03 | ppo | d01 | pla | mpu | d01 | s86 | h39 | d01 | s86 | tal | d60 | amy | rse | d01 | amy | ael | d01 | s86 | fna | d01 |
| 2 | fla | ppo | d01 | amy | bpr | d01 | s88 | tha | d69 | pla | tal | d01 | s86 | rse | d01 | te2 | ael | d01 | fo1 | tha | d46 |
| 3 | ulv | ppo | d05 | s86 | mpu | f31 | s87 | h39 | d01 | ro4 | tal | d05 | are | csi | d34 | pla | ael | d01 | amy | fna | d01 |
| 4 | b02 | ppo | d01 | cr1 | mpu | d45 | amy | h39 | d01 | n04 | tal | d01 | s11 | rse | d01 | te1 | h50 | d12 | m27 | tam | d44 |
| 5 | s86 | ppo | f14 | pl1 | mpu | d49 | s89 | tha | d01 | pmy | tal | d15 | pu1 | rse | d01 | s86 | ael | d01 | cr1 | fna | d01 |
| 6 | te1 | ppo | d06 | te1 | tro | d01 | b05 | tha | d01 | te1 | tal | d01 | ro1 | rse | d01 | n04 | ael | d01 | pla | fna | d01 |
| 7 | b05 | ppo | d01 | sul | mpu | d62 | m21 | h39 | d31 | cr1 | tal | d01 | n55 | ppo | d21 | hyp | ael | d01 | pl1 | fna | d01 |
| 8 | psm | da2 | d01 | hyp | mpu | d01 | are | pcu | d01 | amy | tal | d47 | sul | ppo | d34 | s87 | h44 | d76 | pl2 | tha | d01 |
| 9 | sa2 | ppo | d01 | n55 | mpu | d01 | ro4 | tha | d73 | te2 | tal | d01 | m21 | rse | d34 | ros | ael | d01 | cr3 | tam | d97 |
| 10 | pla | ppo | d01 | te2 | h37 | d69 | at2 | tha | d01 | n94 | tal | d01 | nmo | ppo | d01 | pl1 | tam | d01 | te1 | tha | d01 |

(B2) bacteria summer blooms

| year | 2012 | | | 2013 | | | 2014 | | | 2015 | | | 2016 | | | 2017 | | | 2018 | | |
| --- | --- | --- | --- | --- | --- | --- | --- | --- | --- | --- | --- | --- | --- | --- | --- | --- | --- | --- | --- | --- | --- |
| rank | con | prov | DOM | con | prov | DOM | con | prov | DOM | con | prov | DOM | con | prov | DOM | con | prov | DOM | con | prov | DOM |
| 1 | b02 | lmi | d01 | s11 | h37 | d01 | ro1 | ost | d61 | s86 | syn | d01 | s86 | che | d21 | s86 | cle | d01 | m22 | dac | d42 |
| 2 | s86 | lmi | d13 | te2 | h37 | d63 | ro2 | ost | d71 | ro2 | kse | d01 | amy | che | d01 | n91 | cle | d72 | th2 | dac | d42 |
| 3 | b05 | lmi | d01 | ros | h37 | d80 | b05 | tbe | d01 | ro3 | kse | d84 | pu1 | che | d01 | ma1 | cle | d01 | ro2 | dac | d42 |
| 4 | b03 | lmi | d01 | te1 | h37 | d01 | s86 | tbe | d01 | amy | syn | d01 | s11 | che | d01 | ect | cle | d34 | s11 | syn | d94 |
| 5 | aqu | gde | d82 | m21 | h37 | d01 | s89 | tbe | d01 | aqu | kse | d74 | are | ns1 | d34 | s11 | cle | d01 | s90 | dac | d42 |
| 6 | per | ezo | d01 | s86 | h37 | d01 | n51 | ost | d07 | n91 | kse | d74 | nmo | ns1 | d21 | s13 | cle | d08 | ro4 | dac | d42 |
| 7 | cr1 | gde | d01 | sa2 | h37 | d62 | pi2 | ost | d68 | fu2 | kse | d84 | fo1 | che | d21 | ro2 | cle | d81 | ros | syn | d01 |
| 8 | pla | gde | d01 | are | h37 | d01 | s11 | tbe | d01 | th2 | kse | d39 | po9 | che | d21 | m22 | cle | d01 | are | dac | d42 |
| 9 | om7 | gde | d01 | rub | h37 | d34 | ro3 | ost | d62 | s11 | kse | d01 | ro3 | che | d21 | amy | cle | d01 | oce | dac | d42 |
| 10 | fac | ezo | d01 | pi1 | gsp | d63 | s88 | tbe | d69 | pu2 | kse | d01 | ros | che | d01 | s14 | cle | d01 | ae2 | dac | d42 |

(B3) phytoplankton summer blooms

| year | 2012 | | | 2013 | | | 2015 | | | 2016 | | | 2017 | | | 2018 | | |
| --- | --- | --- | --- | --- | --- | --- | --- | --- | --- | --- | --- | --- | --- | --- | --- | --- | --- | --- |
| rank | con | prov | DOM | con | prov | DOM | con | prov | DOM | con | prov | DOM | con | prov | DOM | con | prov | DOM |
| 1 | b02 | pin | d01 | s11 | din | d01 | s86 | syn | d47 | aqu | toc | d75 | s86 | cle | d01 | m22 | dac | d42 |
| 2 | b05 | pin | d01 | aqu | gsp | d62 | ro2 | kse | d01 | amy | pte | d01 | ect | cle | d34 | ros | syn | d01 |
| 3 | s86 | pin | d13 | n05 | gsp | d62 | th2 | kse | d39 | pu1 | toc | d75 | n91 | cle | d72 | th2 | dac | d42 |
| 4 | amy | pin | f12 | lum | gsp | d34 | s11 | kse | d01 | pl1 | toc | d75 | ma1 | cle | d01 | ro2 | dac | d42 |
| 5 | n51 | pin | d79 | fo1 | din | d62 | ro3 | kse | d01 | cr1 | toc | d75 | s13 | cle | d08 | s90 | dac | d42 |
| 6 | n07 | pin | f28 | pi1 | gsp | d63 | s12 | kse | d70 | lem | toc | d79 | s11 | cle | d01 | are | dac | d42 |
| 7 | cr1 | pin | d68 | om7 | gsp | d62 | ect | kse | d74 | te1 | pte | d76 | ro2 | cle | d81 | s11 | syn | d94 |
| 8 | fu1 | pin | f17 | m22 | gsp | d38 | pu2 | kse | d01 | fu1 | toc | d79 | m22 | cle | d01 | n91 | dac | d42 |
| 9 | ub1 | pin | d01 | n04 | din | d01 | n91 | kse | d74 | ns2 | toc | d75 | fo2 | cle | d03 | ro3 | dac | d42 |
| 10 | s87 | pin | d01 | fla | gsp | d63 | ae1 | kse | d01 | te2 | toc | d97 | amy | cle | d01 | ro4 | dac | d42 |

(C) Top ten phytoplankton>heterotrophic prokaryotes and heterotrophic prokaryotes>heterotrophic prokaryotes carbon flux pairs for the respective bloom types and years. Fluxes are given in µmol C l^-1^ d^-1^.

(C1) Phytoplankton spring blooms

| year | 2012 | | 2013 | | 2014 | | 2015 | | 2016 | | 2017 | | 2018 | |
| --- | --- | --- | --- | --- | --- | --- | --- | --- | --- | --- | --- | --- | --- | --- |
|  | pair | flux | pair | flux | pair | flux | pair | flux | pair | flux | pair | flux | pair | flux |
| rank | phytoplankton>heterotrophic prokaryotes | | | | | | | | | | | | | |
| 1 | ppo>b03 | 0.006083 | mpu>pla | 0.00186 | tha>s88 | 0.006609 | tal>s86 | 0.001373 | rse>amy | 0.01566 | ael>te2 | 0.002117 | tha>fo1 | 0.004712 |
| 2 | ppo>fla | 0.003763 | bpr>amy | 0.001819 | h39>s86 | 0.005289 | tal>pla | 0.00082 | ppo>amy | 0.006817 | ael>amy | 0.00165 | fna>s86 | 0.002567 |
| 3 | toc>b03 | 0.00308 | tro>pla | 0.001654 | tha>s86 | 0.004394 | tal>ro4 | 0.000705 | cro>amy | 0.006605 | tam>amy | 0.001104 | tam>m27 | 0.002345 |
| 4 | ppo>b02 | 0.002745 | mpu>amy | 0.00144 | h39>s88 | 0.004054 | tal>n04 | 0.000526 | rse>s86 | 0.006353 | h50>amy | 0.000883 | tam>fo1 | 0.002099 |
| 5 | rse>b03 | 0.002305 | tro>amy | 0.001123 | tha>s89 | 0.002134 | bpr>s86 | 0.000515 | ppo>s86 | 0.005866 | tam>te2 | 0.000852 | tam>s86 | 0.001851 |
| 6 | ppo>s86 | 0.002138 | mpu>cr1 | 0.001113 | pcu>s86 | 0.002006 | tal>pmy | 0.000479 | csi>amy | 0.004483 | h44>te2 | 0.00083 | csi>s86 | 0.00178 |
| 7 | rse>fla | 0.002123 | mpu>sul | 0.001013 | tha>ro4 | 0.001235 | syn>s86 | 0.000411 | csp>amy | 0.003363 | ael>pla | 0.000771 | toc>s86 | 0.00176 |
| 8 | kve>b03 | 0.001789 | h37>sul | 0.000996 | h39>s87 | 0.001222 | tal>te1 | 0.000396 | cro>s86 | 0.003359 | h44>amy | 0.000707 | tha>s86 | 0.001754 |
| 9 | toc>fla | 0.001765 | tam>amy | 0.000985 | tha>s87 | 0.001179 | tal>amy | 0.000357 | kse>amy | 0.003248 | h50>te1 | 0.000702 | fna>fo1 | 0.001452 |
| 10 | ppo>ulv | 0.001699 | bpr>pla | 0.00085 | bpr>s86 | 0.001128 | syn>pmy | 0.000351 | stu>amy | 0.00283 | ael>s86 | 0.000687 | tha>n56 | 0.001388 |
| rank | heterotrophic prokaryotes>heterotrophic prokaryotes | | | | | | | | | | | | | |
| 1 | psm>psm | 0.001712 | s12>pla | 0.001134 | s87>s86 | 0.002023 | pmy>s86 | 0.0000277 | sul>amy | 0.01206 | s87>amy | 0.0005632 | m27>s86 | 0.001153 |
| 2 | fla>b03 | 0.001352 | s12>amy | 0.0007742 | s88>s86 | 0.001943 | pmy>pla | 0.00001928 | n55>amy | 0.009694 | s88>amy | 0.0004422 | at1>s86 | 0.001124 |
| 3 | psm>b03 | 0.001108 | s92>amy | 0.000735 | ma1>s86 | 0.001044 | pmy>ro4 | 0.00001554 | sul>s86 | 0.002871 | s87>pla | 0.0003301 | m27>amy | 0.0006487 |
| 4 | ths>psm | 0.0009146 | s87>pla | 0.000733 | pmy>s86 | 0.0008458 | pmy>te1 | 0.00001279 | s88>amy | 0.002831 | s87>te1 | 0.0002871 | at1>amy | 0.0005768 |
| 5 | fla>ulv | 0.0008168 | s12>s86 | 0.0005451 | s89>s86 | 0.0005814 | m23>s86 | 0.00001139 | n55>s86 | 0.00215 | s87>te2 | 0.00025 | m25>s86 | 0.0005463 |
| 6 | fla>fla | 0.0007099 | s92>pla | 0.0004877 | n51>s86 | 0.0005613 | pmy>n04 | 0.00001127 | m25>amy | 0.001728 | s92>amy | 0.0002439 | psa>s86 | 0.0005103 |
| 7 | fla>b02 | 0.0006251 | s87>amy | 0.0004872 | s87>amy | 0.0005267 | pmy>cr1 | 0.00001083 | s93>amy | 0.001226 | s13>amy | 0.0002424 | cr3>s86 | 0.0004862 |
| 8 | psm>ths | 0.0006246 | s92>s86 | 0.0003866 | sul>s86 | 0.0005157 | m04>s86 | 0.00001082 | amy>s86 | 0.001177 | s13>te1 | 0.0002195 | m27>te1 | 0.000384 |
| 9 | psm>b02 | 0.0005851 | s12>cr1 | 0.000352 | s92>s86 | 0.0003756 | n51>s86 | 0.00001074 | mas>amy | 0.00114 | s12>amy | 0.0002121 | m27>pl1 | 0.0003507 |
| 10 | psm>ulv | 0.0005444 | pav>amy | 0.000352 | s88>s89 | 0.0003654 | hoc>s86 | 0.00001023 | pav>amy | 0.00113 | leb>amy | 0.0001996 | m27>pla | 0.0003396 |

(C2) Bacteria summer bloom

| year | 2012 | | 2013 | | 2014 | | 2015 | | 2016 | | 2017 | | 2018 | |
| --- | --- | --- | --- | --- | --- | --- | --- | --- | --- | --- | --- | --- | --- | --- |
|  | pair | flux | pair | flux | pair | flux | pair | flux | pair | flux | pair | flux | pair | flux |
| rank | phytoplankton>heterotrophic prokaryotes | | | | | | | | | | | | | |
| 1 | lmi>b02 | 0.006751 | h37>s11 | 0.01043 | ost>ro1 | 0.004782 | syn>s86 | 0.02814 | che>s86 | 0.008755 | cle>s86 | 0.01206 | dac>m22 | 0.082 |
| 2 | ezo>b02 | 0.005079 | pon>s11 | 0.007398 | ost>ro2 | 0.002827 | kse>ro2 | 0.01262 | che>pu1 | 0.002197 | h44>s86 | 0.006327 | gsp>m22 | 0.02737 |
| 3 | gde>b02 | 0.005037 | dsp>s11 | 0.006269 | tbe>s86 | 0.002444 | kse>ro3 | 0.004241 | che>amy | 0.002173 | pcu>s86 | 0.005288 | dac>th2 | 0.02214 |
| 4 | lmi>s86 | 0.004873 | h37>te2 | 0.005367 | tbe>b05 | 0.002239 | kse>n91 | 0.004199 | pte>s86 | 0.002033 | cwi>s86 | 0.003994 | dac>ro2 | 0.01755 |
| 5 | hap>b02 | 0.004579 | pve>s11 | 0.003244 | dia>ro1 | 0.001661 | kse>aqu | 0.004049 | ns1>s86 | 0.001963 | ufl>s86 | 0.003522 | syn>m22 | 0.01507 |
| 6 | hap>s86 | 0.003998 | pha>s11 | 0.002965 | tbe>ro2 | 0.001424 | dac>ro2 | 0.0038 | pte>amy | 0.00145 | che>s86 | 0.00328 | syn>s11 | 0.01248 |
| 7 | lmi>b03 | 0.003626 | stu>s11 | 0.002917 | tbe>s11 | 0.001273 | syn>amy | 0.003321 | cwe>s86 | 0.001443 | lco>s86 | 0.001633 | dac>s90 | 0.01153 |
| 8 | lmi>b05 | 0.003417 | psp>s11 | 0.002572 | tbe>s89 | 0.001208 | kse>fu2 | 0.003224 | pha>s86 | 0.001334 | pte>s86 | 0.001436 | dac>ro4 | 0.01036 |
| 9 | gde>s86 | 0.003038 | h37>te1 | 0.002435 | tbe>ro1 | 0.001166 | dac>s86 | 0.003145 | gde>s86 | 0.001298 | toc>s86 | 0.001252 | dac>are | 0.008082 |
| 10 | csi>b02 | 0.002989 | h37>ros | 0.002346 | dia>ro2 | 0.001113 | kse>th2 | 0.002925 | ns1>amy | 0.001296 | pve>s86 | 0.000959 | gsp>th2 | 0.007297 |
| rank | heterotrophic prokaryotes>heterotrophic prokaryotes | | | | | | | | | | | | | |
| 1 | b03>b02 | 0.02053 | ros>s11 | 0.01702 | s86>ro1 | 0.005957 | s86>s86 | 0.003809 | ros>s11 | 0.02721 | s86>s86 | 0.01766 | ros>s11 | 0.01158 |
| 2 | b02>b02 | 0.01296 | rub>s11 | 0.01532 | s86>ro2 | 0.004152 | amy>s86 | 0.002326 | ros>th2 | 0.01303 | amy>s86 | 0.006449 | ros>m22 | 0.0103 |
| 3 | b03>s86 | 0.01254 | lem>s11 | 0.01313 | s86>n51 | 0.001168 | aqu>s86 | 0.002164 | ros>n07 | 0.009061 | te1>s86 | 0.005461 | m22>ros | 0.008451 |
| 4 | b05>b02 | 0.01099 | te1>s11 | 0.01234 | s86>om8 | 0.001105 | aqu>ro2 | 0.002156 | ros>n91 | 0.006328 | ect>s86 | 0.002377 | m22>s11 | 0.006105 |
| 5 | b03>b03 | 0.01034 | te2>s11 | 0.01181 | pla>s86 | 0.001021 | ro2>s86 | 0.002097 | ros>are | 0.005759 | s86>n91 | 0.002315 | ros>th2 | 0.004587 |
| 6 | b03>b05 | 0.00984 | amy>s11 | 0.008619 | n51>s86 | 0.001014 | fu2>s86 | 0.001688 | ros>ro2 | 0.005041 | s86>ma1 | 0.001538 | m22>m22 | 0.004123 |
| 7 | b02>s86 | 0.007497 | m21>s11 | 0.007703 | s86>s89 | 0.0009698 | fu2>ro2 | 0.001569 | n07>s11 | 0.004266 | s19>s86 | 0.001134 | pla>ros | 0.00301 |
| 8 | b05>s86 | 0.006265 | s11>s11 | 0.007483 | s86>ro3 | 0.0009172 | te1>s86 | 0.001403 | n07>ros | 0.004087 | s86>ect | 0.001014 | ros>ro2 | 0.002951 |
| 9 | b02>b05 | 0.00475 | s11>te2 | 0.005414 | s86>b05 | 0.0008774 | cr1>s86 | 0.001347 | ros>ros | 0.003938 | ae2>s86 | 0.0009376 | m22>s86 | 0.002536 |
| 10 | per>b02 | 0.00465 | ro1>s11 | 0.005295 | amy>s86 | 0.0008767 | s86>ro2 | 0.001273 | n91>s11 | 0.003178 | fu2>s86 | 0.0009006 | ros>ros | 0.002396 |

(C3) Phytoplankton summer blooms

| year | 2012 | | 2013 | | 2015 | | 2016 | | 2017 | | 2018 | |
| --- | --- | --- | --- | --- | --- | --- | --- | --- | --- | --- | --- | --- |
|  | pair | flux | pair | flux | pair | flux | pair | flux | Pair | Flux | pair |  |
| rank | phytoplankton>heterotrophic prokaryotes | | | | | | | | | | | |
| 1 | pin>b02 | 0.01612 | din>s11 | 0.007575 | syn>s86 | 0.02362 | toc>aqu | 0.004074 | cle>s86 | 0.02606 | dac>m22 | 0.03109 |
| 2 | pin>b05 | 0.009112 | gsp>s11 | 0.005128 | lch>s86 | 0.005699 | toc>lem | 0.001895 | pcu>s86 | 0.01484 | dac>th2 | 0.01448 |
| 3 | dio>b02 | 0.003694 | pha>s11 | 0.003383 | kse>ro2 | 0.005616 | pte>amy | 0.001858 | ufl>s86 | 0.007476 | dac>ro2 | 0.01378 |
| 4 | ael>b02 | 0.003689 | pin>s11 | 0.002761 | tte>s86 | 0.003823 | toc>pl1 | 0.001734 | cwi>s86 | 0.00408 | gsp>m22 | 0.00978 |
| 5 | acu>b02 | 0.003469 | gsp>aqu | 0.00256 | kse>th2 | 0.003379 | che>aqu | 0.001497 | che>s86 | 0.0033 | dac>s90 | 0.008944 |
| 6 | pin>amy | 0.002478 | gsp>lum | 0.002326 | kse>ro3 | 0.002091 | toc>cr1 | 0.001414 | lco>s86 | 0.001947 | dac>are | 0.008385 |
| 7 | ael>b05 | 0.002176 | gsp>pi1 | 0.002319 | kse>s11 | 0.002057 | toc>pu1 | 0.00117 | cle>ect | 0.001572 | dac>n91 | 0.006132 |
| 8 | pin>s86 | 0.00205 | gsp>om7 | 0.00225 | kse>s12 | 0.002028 | pte>aqu | 0.001032 | pte>s86 | 0.001507 | dac>ro3 | 0.00611 |
| 9 | acu>b05 | 0.002027 | gsp>n05 | 0.002216 | lch>ro2 | 0.001797 | toc>fu1 | 0.00103 | cle>n91 | 0.001498 | dac>ro4 | 0.00573 |
| 10 | dio>b05 | 0.002025 | pte>s11 | 0.001793 | rse>s86 | 0.001788 | toc>n05 | 0.001007 | cle>ma1 | 0.001395 | syn>s11 | 0.0053 |
| rank | heterotrophic prokaryotes>heterotrophic prokaryotes | | | | | | | | | | | |
| 1 | s86>b02 | 0.001864 | pi1>s11 | 0.003938 | s86>s86 | 0.01585 | s86>aqu | 0.005872 | s86>s86 | 0.0255 | ros>s11 | 0.01522 |
| 2 | cr1>b02 | 0.0009606 | s11>n05 | 0.002879 | ro2>s86 | 0.004515 | amy>aqu | 0.004685 | amy>s86 | 0.006559 | ros>ros | 0.008367 |
| 3 | s86>b05 | 0.0009451 | aqu>s11 | 0.002877 | amy>s86 | 0.004429 | s86>pl1 | 0.004224 | ect>s86 | 0.004169 | pla>ros | 0.004953 |
| 4 | amy>b02 | 0.0007938 | s11>pi1 | 0.002838 | s86>ro2 | 0.002252 | s86>lem | 0.002324 | s19>s86 | 0.001947 | te1>ros | 0.003863 |
| 5 | cr1>b05 | 0.000556 | s11>aqu | 0.002229 | ro3>s86 | 0.001624 | aqu>amy | 0.001823 | s86>n91 | 0.00187 | ros>m22 | 0.003848 |
| 6 | n07>b02 | 0.000554 | s11>lum | 0.001868 | m21>s86 | 0.001375 | s86>ns2 | 0.001652 | s86>ect | 0.001555 | ros>th2 | 0.003116 |
| 7 | s86>amy | 0.0004857 | n58>s11 | 0.001835 | ro2>ro2 | 0.001164 | s86>n05 | 0.00163 | ae2>s86 | 0.001524 | n05>ros | 0.002609 |
| 8 | amy>b05 | 0.0004601 | lum>s11 | 0.001706 | s86>s11 | 0.0009686 | amy>pl1 | 0.001575 | fo2>s86 | 0.001342 | ros>ro2 | 0.002472 |
| 9 | ps1>b02 | 0.0003854 | om7>s11 | 0.001706 | n51>s86 | 0.0008687 | amy>lem | 0.001481 | fu2>s86 | 0.00109 | n07>ros | 0.002432 |
| 10 | s11>b02 | 0.0003813 | om7>s11 | 0.001639 | pu2>s86 | 0.0007934 | amy>cr1 | 0.001406 | s86>ma1 | 0.001066 | ros>fu2 | 0.002402 |
