# Supplementary material for: Internal carbon recycling by heterotrophic prokaryotes compensates for mismatches between phytoplankton production and heterotrophic consumption
Source: ISME J. 2024 Jun 11;18(1):wrae103. doi: 10.1093/ismejo/wrae103 (PMC11217553; doi:10.1093/ismejo/wrae103)
Supplement: Suppementary_wrae103 [file suppementary_wrae103.zip › Supplementary Table 7.docx]

Supplementary Table 7:

Primary and heterotrophic prokaryotic production.

Net primary production was calculated as gross primary production – respiration. Gross heterotrophic prokaryotic production: all dissolved organic carbon (DOC) fluxes into heterotrophic prokaryotes, net heterotrophic prokaryotic production: gross production – heterotrophic respiration. Bloom types: Psp: phytoplankton spring bloom, Bsu: bacteria summer bloom, Psu: phytoplankton summer bloom.

| year | 2012 | | | 2013 | | | 2014 | | 2015 | | | 2016 | | | 2017 | | | 2018 | | |
| --- | --- | --- | --- | --- | --- | --- | --- | --- | --- | --- | --- | --- | --- | --- | --- | --- | --- | --- | --- | --- |
| Primary production gross mmol C m^-2^ d^-1^ | 25 | | | 32 | | | 20 | | 22 | | | 18 | | | 22 | | | 25 | | |
| Primary production net mmol C m^-2^ d^-1^ | 23 | | | 23 | | | 13 | | 20 | | | 13 | | | 19 | | | 15 | | |
| Heterotrophic prokaryotic production gross µmol C l^-1^ d^-1^ | 0.2 | | | 0.14 | | | 0.18 | | 0.21 | | | 0.2 | | | 0.13 | | | 0.24 | | |
| Heterotrophic prokaryotic production net µmol C l^-1^ d^-1^ | 0.12 | | | 0.07 | | | 0.08 | | 0.08 | | | 0.11 | | | 0.07 | | | 0.1 | | |
| Bloom type | Psp | Bsu | Psu | Psp | Bsu | Psu | Psp | Bsu | Psp | Bsu | Psu | Psp | Bsu | Psu | Psp | Bsu | Psu | Psp | Bsu | Psu |
| Primary production gross mmol C m^-2^ d^-1^ | 16 | 17 | 19 | 25 | 16 | 18 | 91 | 8 | 22 | 14 | 19 | 55 | 19 | 19 | 21 | 11 | 13 | 71 | 31 | 52 |
| Primary production net mmol C m^-2^ d^-1^ | 15 | 12 | 16 | 12 | 13 | 14 | 61 | 5 | 19 | 12 | 16 | 47 | 13 | 11 | 16 | 10 | 12 | 39 | 14 | 24 |
| Heterotrophic prokaryotic production gross µmol C l^-1^ d^-1^ | 0.26 | 0.57 | 0.19 | 0.2 | 0.73 | 0.26 | 0.25 | 0.35 | 0.04 | 0.37 | 0.29 | 0.53 | 0.65 | 0.3 | 0.17 | 0.3 | 0.31 | 0.29 | 0.62 | 0.47 |
| Heterotrophic prokaryotic production net µmol C l^-1^ d^-1^ | 0.25 | 0.55 | 0.19 | 0.19 | 0.4 | 0.15 | 0.13 | 0.19 | 0.02 | 0.18 | 0.13 | 0.36 | 0.45 | 0.22 | 0.12 | 0.18 | 0.18 | 0.2 | 0.42 | 0.3 |
