# Supplementary material for: Internal carbon recycling by heterotrophic prokaryotes compensates for mismatches between phytoplankton production and heterotrophic consumption
Source: ISME J. 2024 Jun 11;18(1):wrae103. doi: 10.1093/ismejo/wrae103 (PMC11217553; doi:10.1093/ismejo/wrae103)
Supplement: Suppementary_wrae103 [file suppementary_wrae103.zip › Supplementary Table 8.docx]

| Year | Flux het. prokaryotes>het. prokaryotes µmol C l-1 d-1 | Successions of abundant 16S ASVs |
| --- | --- | --- |
| 2012 | 0.176007 | partly |
| 2013 | 0.264127 | yes |
| 2014 | 0.105966 | partly |
| 2015 | 0.063408 | no |
| 2016 | 0.127775 | partly/yes |
| 2017 | 0.076782 | no/partly |
| 2018 | 0.158104 | yes/partly |

Supplementary Table 8: Correlations between heterotrophic prokaryotes>heterotrophic prokaryotes fluxes and occurrence of successions of abundant 16S ASVs in bacterial summer blooms.
