# Supplementary material for: Internal carbon recycling by heterotrophic prokaryotes compensates for mismatches between phytoplankton production and heterotrophic consumption
Source: ISME J. 2024 Jun 11;18(1):wrae103. doi: 10.1093/ismejo/wrae103 (PMC11217553; doi:10.1093/ismejo/wrae103)
Supplement: Suppementary_wrae103 [file Supplementary Data 3.pdf › Supplementary Data 3.pdf]

Supplementary Data 3: Model-data comparsions for nutrients, sum-parameters (phytoplankton, heterotrophic prokaryotes, etc), and each individual phytoplankton OTU and heterotrophic prokaryotic ASV for the years 2012-2018. Each year has 303 pages, i.e. year 2012: page 2-304, year 2013: page 305-607, year 2014: page 608-910, year 2015: page 911-1213, year 2016: page 1214-1516, year 2017: page 1517-1819, and year 2018: page 1820-2122. The first page for each year is an overview of the most important parameters (nutrients, chlorophyll, heterotrophic prokaryotes, POM, DOM), pages 2-5 are nutrients, page 6: summed up phytoplankton OTUs, pages 7-144: single phytoplankton OTUs, page 145: summed up Bacillariophyceae (BAP), pages 146-302: single heterotrophic prokaryotic ASVs, page 303: summed up heterotrophic prokaryotic ASVs.

324_Calib_S_A

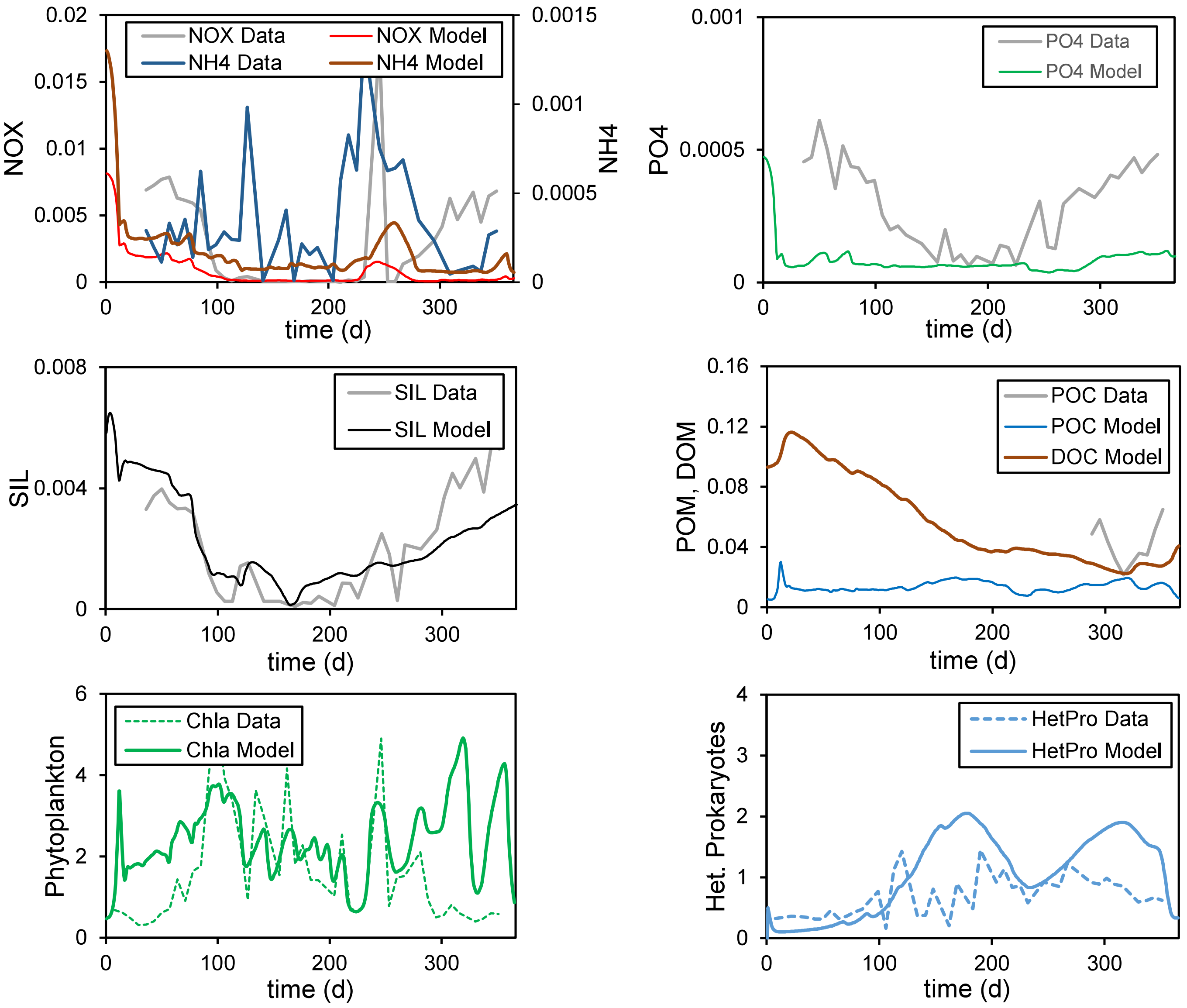
NOX Data
NOX Model
NH4 Data
NH4 Model
NOX
NH4
time (d)
PO4 Data
PO4 Model
PO4
SIL Data
SIL Model
SIL
POC Data
POC Model
DOC Model
POM, DOM
Chla Data
Chla Model
Phytoplankton
HetPro Data
HetPro Model
Het. Prokaryotes

324_Calib_S_A

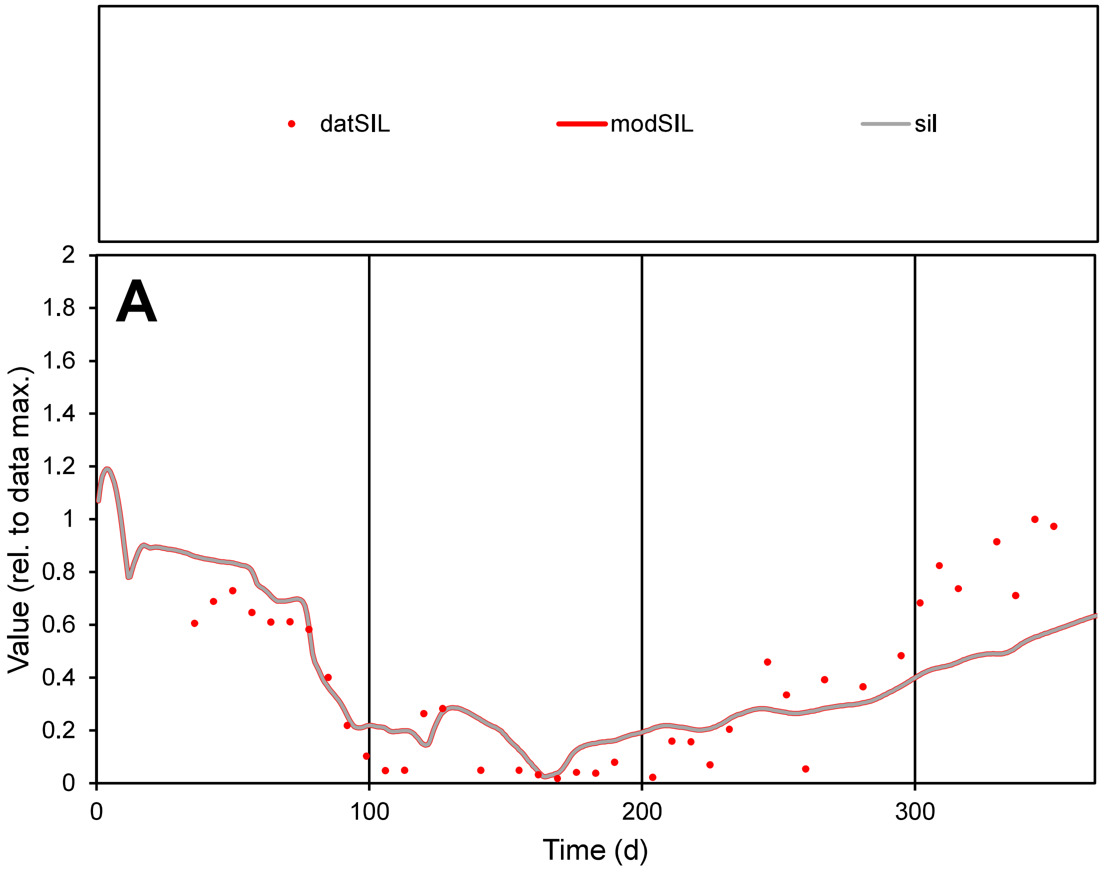

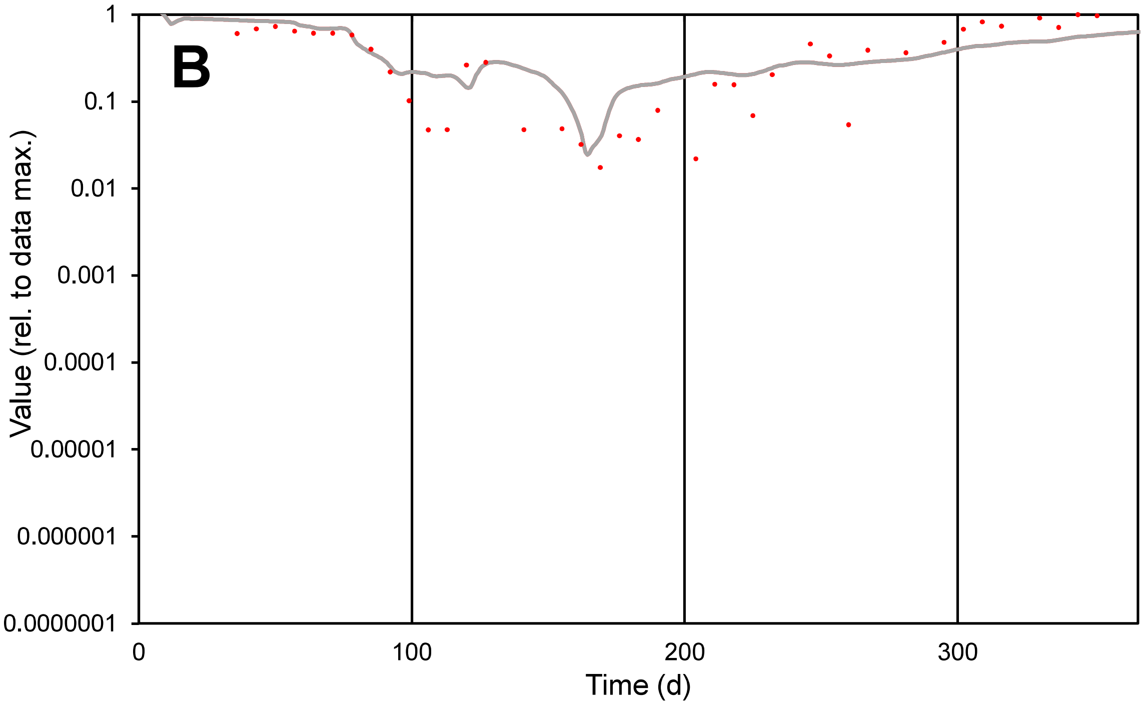

C1

Variables
SIL Provide jtd or Dst
CalibOnly?
2 1 to reproduce of
oNormalize?
2 1=no, 2=yes

ofxn calc*
1.45675
ofxn mod
1.4996

sRun
RunA1
zout
1

Ave
Data
0.00205
Model
0.00195

data/vMax
0.00545

ofxng
321.551

Min d/m
0.66387

ave/min d
21.5731
ave/min m
13.643

324_Calib_S_A

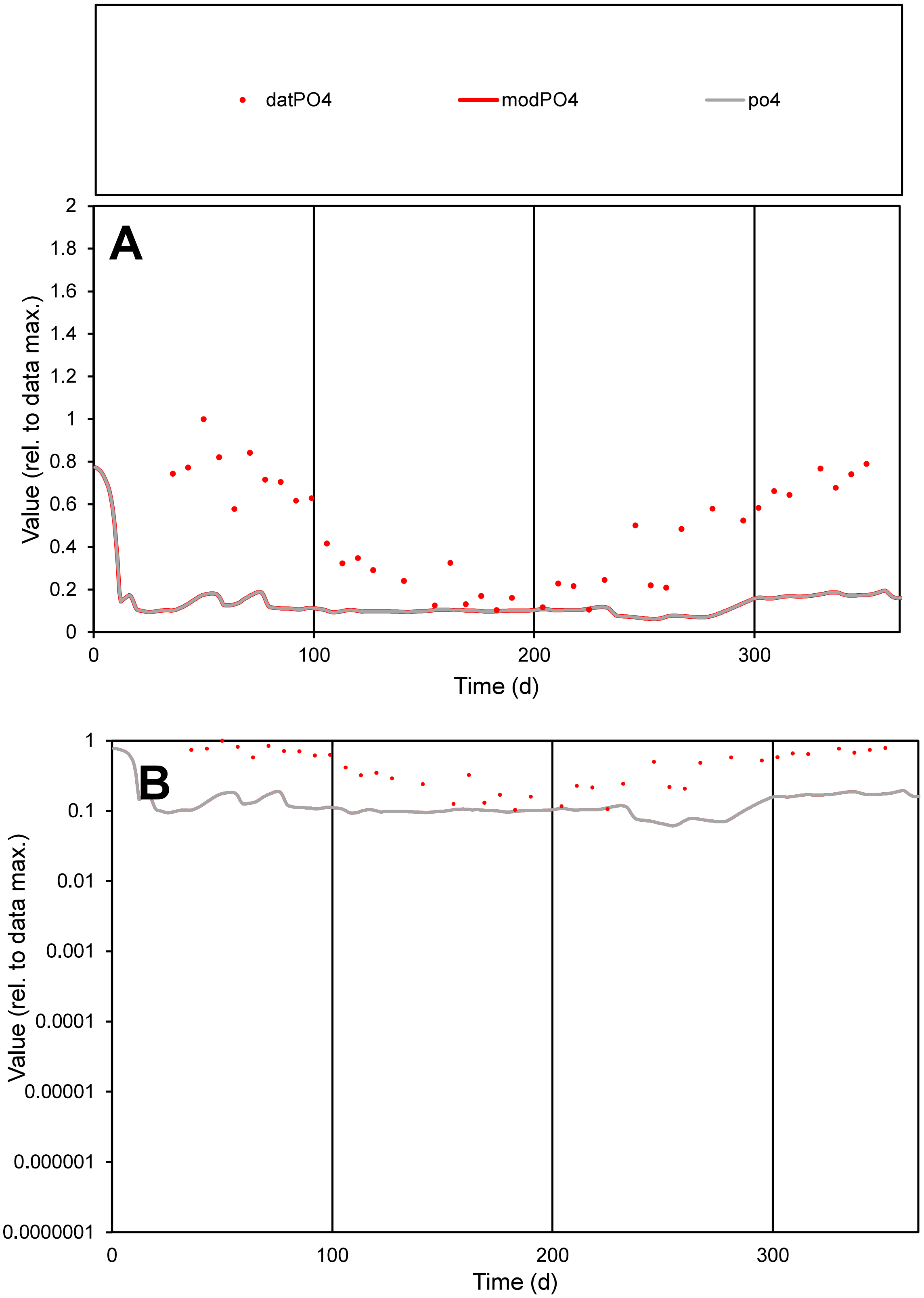

C1

Variables
PO4 Provide jtd or Dst
CalibOnly?
2 1 to reproduce of
oNormalize?
2 1=no, 2=yes

ofxn calc*
2.67649
ofxn mod
2.68748

sRun
RunA1
zout
1

Ave
Data
0.00029
Model
7.4E-05

data/vMax
0.00061

ofxng
321.551

Min d/m
1.68738

ave/min d
4.54642
ave/min m
1.97555

324_Calib_S_A

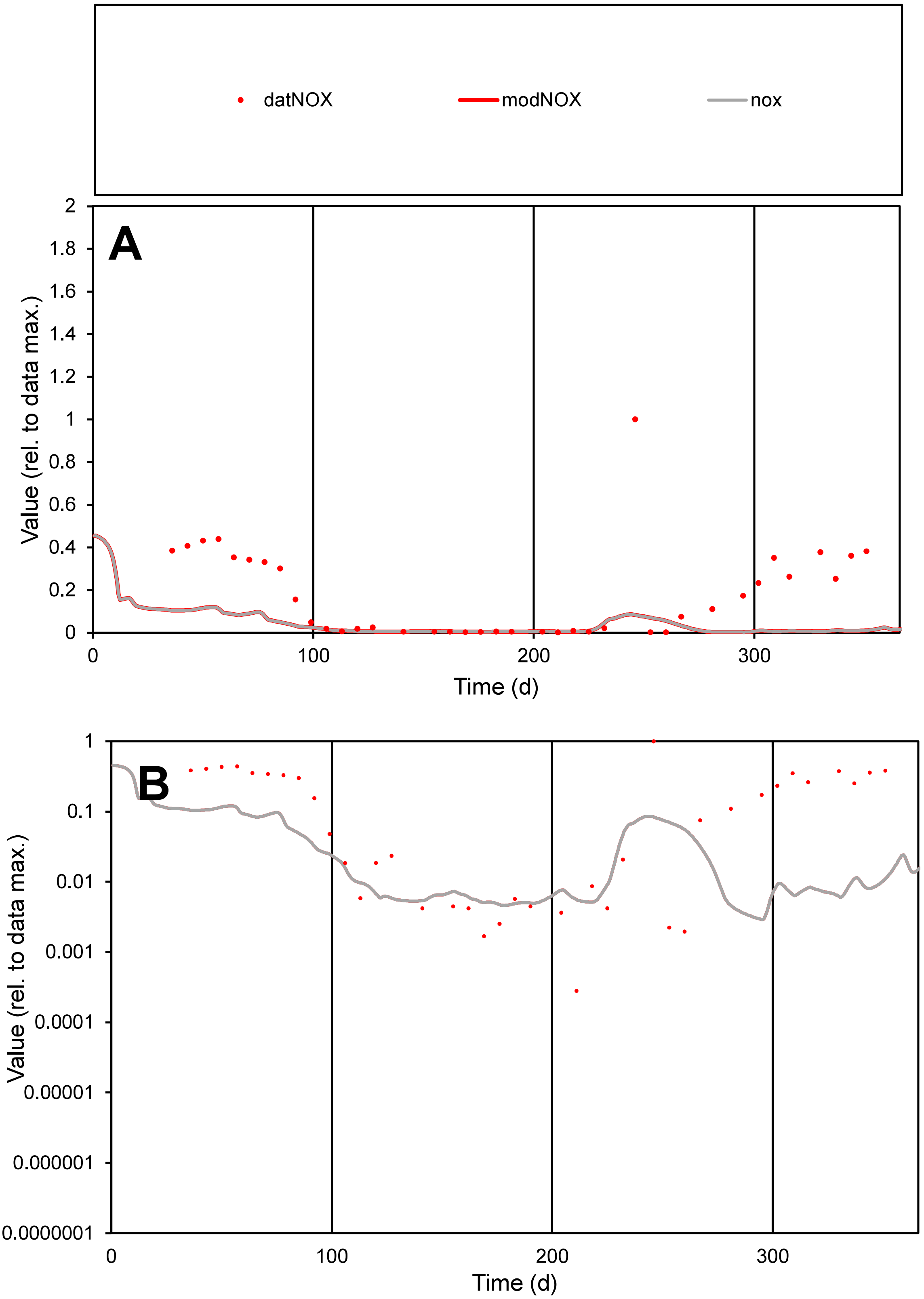

C1

Variables
NOX Provide jtd or Dst
CalibOnly?
2 1 to reproduce of
oNormalize?
2 1=no, 2=yes

ofxn calc*
4.06308
ofxn mod
4.07732

sRun
RunA1
zout
1

Ave
Data
0.00316
Model
0.00056

data/vMax
0.01789

ofxng
321.551

Min d/m
0.09463

ave/min d
632.862
ave/min m
10.6686

324_Calib_S_A

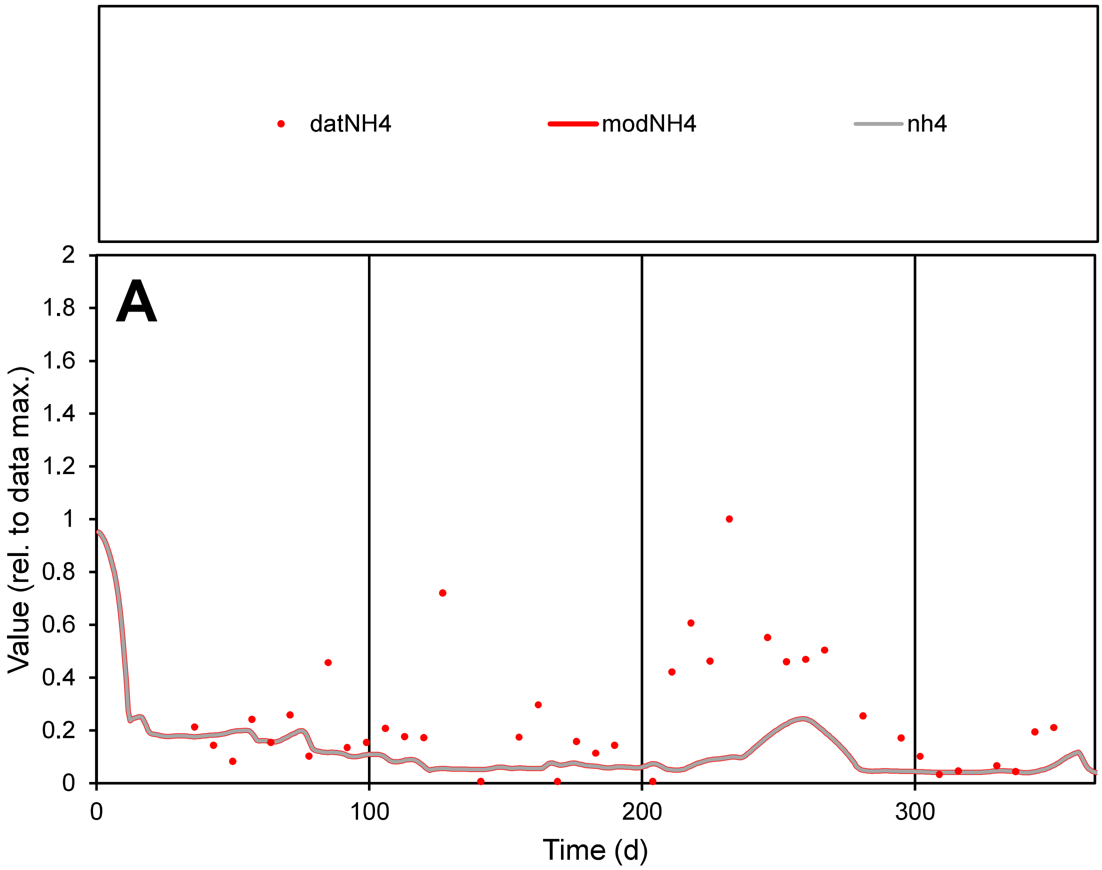

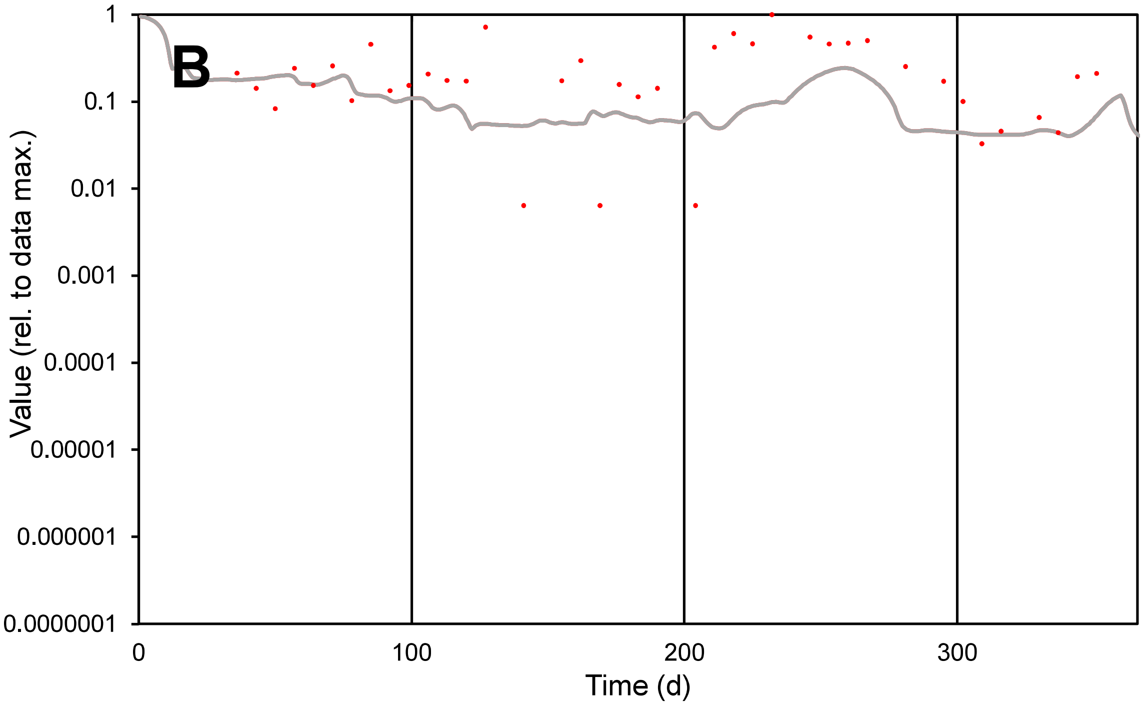

C1

Variables
NH4 Provide jtd or Dst
CalibOnly?
2 1 to reproduce of
oNormalize?
2 1=no, 2=yes

ofxn calc*
3.06601
ofxn mod
3.07966

sRun
RunA1
zout
1

Ave
Data
0.00034
Model
0.00014

data/vMax
0.00137

ofxng
321.551

Min d/m
0.15443

ave/min d
38.8368
ave/min m
2.43053

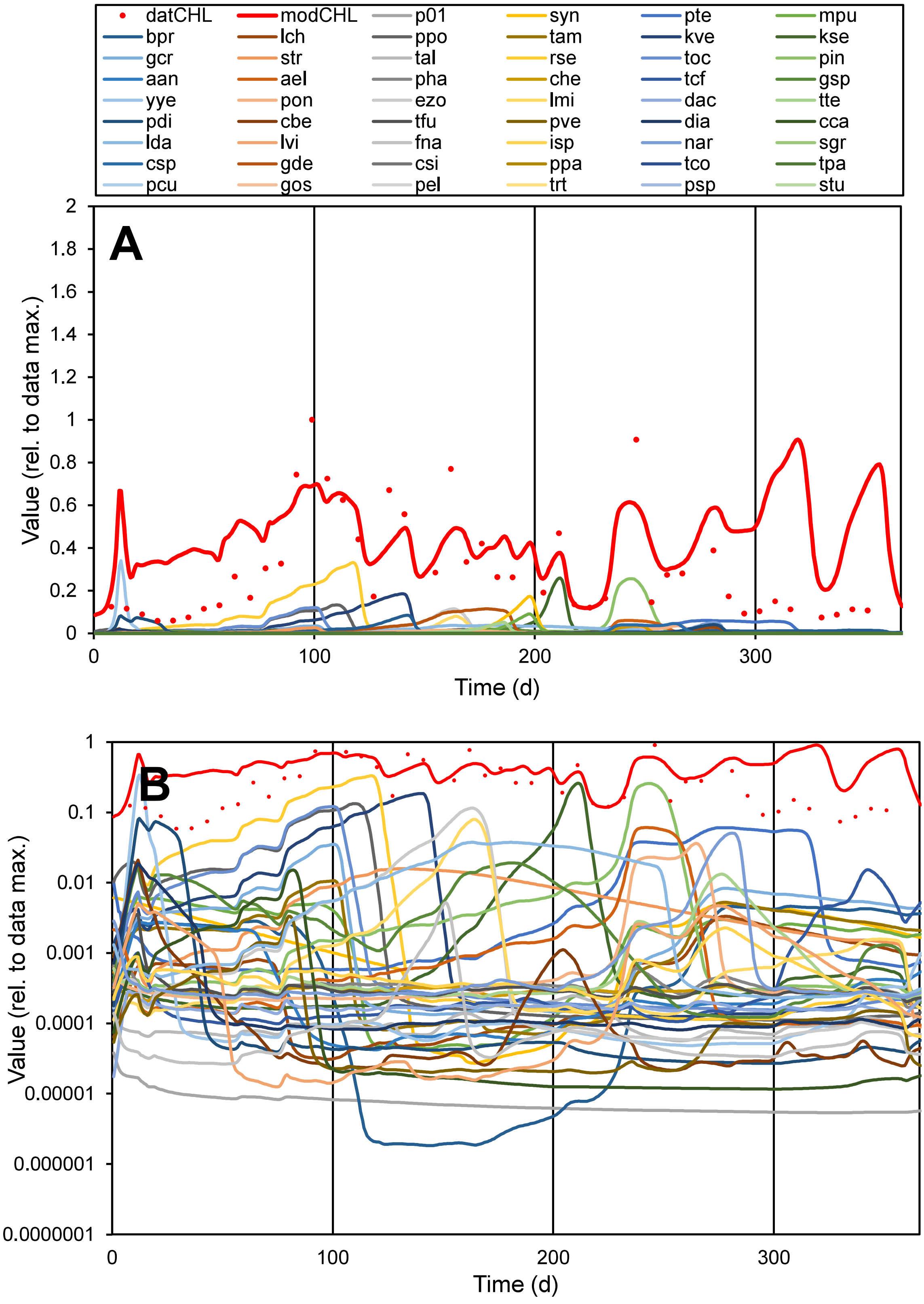

C1

Variables

CHL Provide jtd or Dst

CalibOnly?

2 1 to reproduce of

oNormalize?

2 1=no, 2=yes

ofxn calc*

4.05998

ofxn mod

4.31784

sRun

RunA1

zout

1

Ave

Data

1.58547

Model

1.59705

data/vMax

5.413

ofxng

321.551

Min d/m

0.795

ave/min d

4.98575

ave/min m

3.99263

324_Calib_S_A

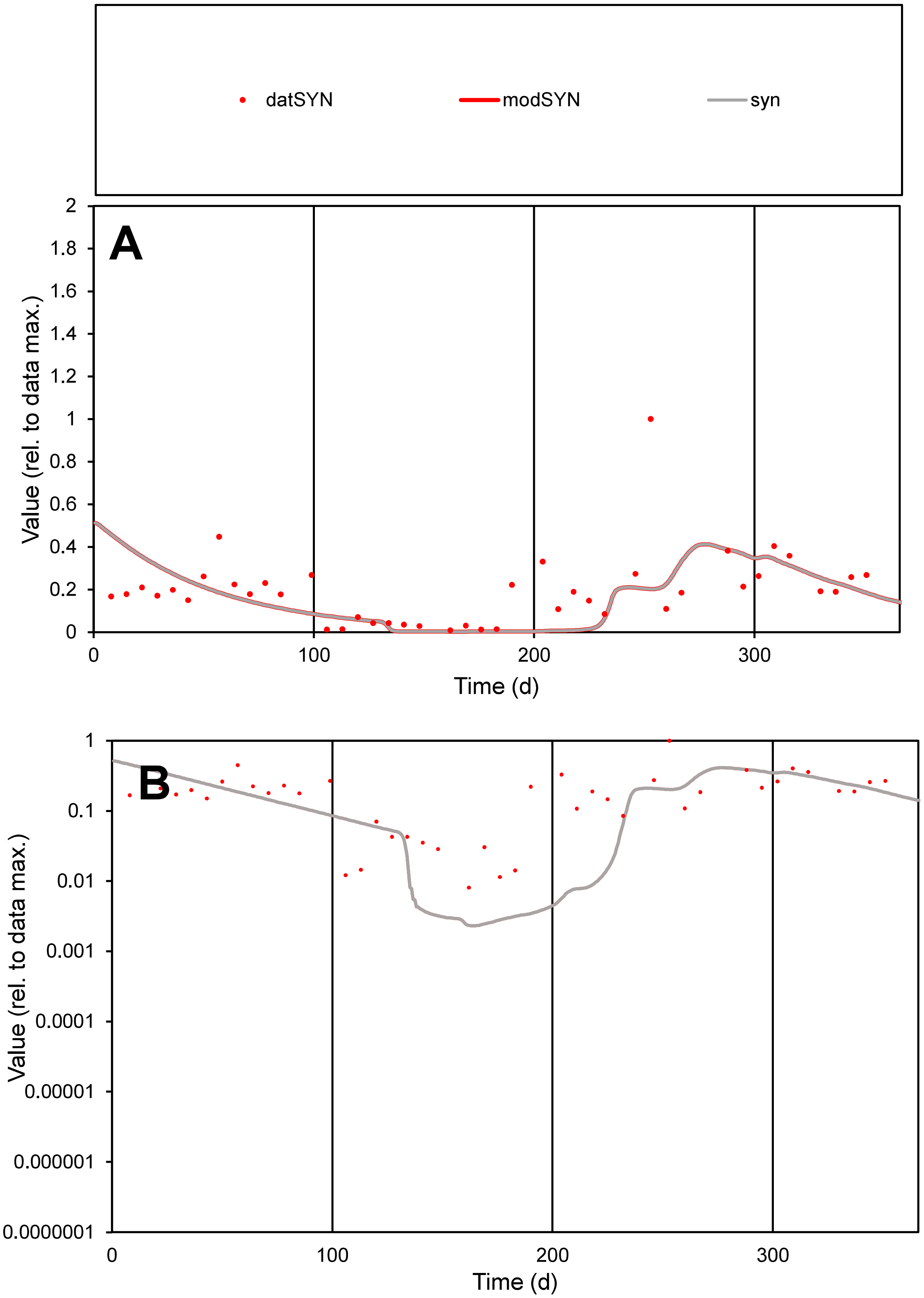

C1

Variables	
SYN	Provide jtd or Dst
CalibOnly?	
2	1 to reproduce of
oNormalize?	
2	1=no, 2=yes
ofxn calc*	
0.87922	
ofxn mod	
0.88836	
sRun	
RunA1	
zout	
1	
Ave	
Data	
7.37757	
Model	
6.20448	
data/vMax	
38	
ofxng	
321.551	
Min d/m	
3.48877	
ave/min d	
24.1097	
ave/min m	
70.7386	

324_Calib_S_A

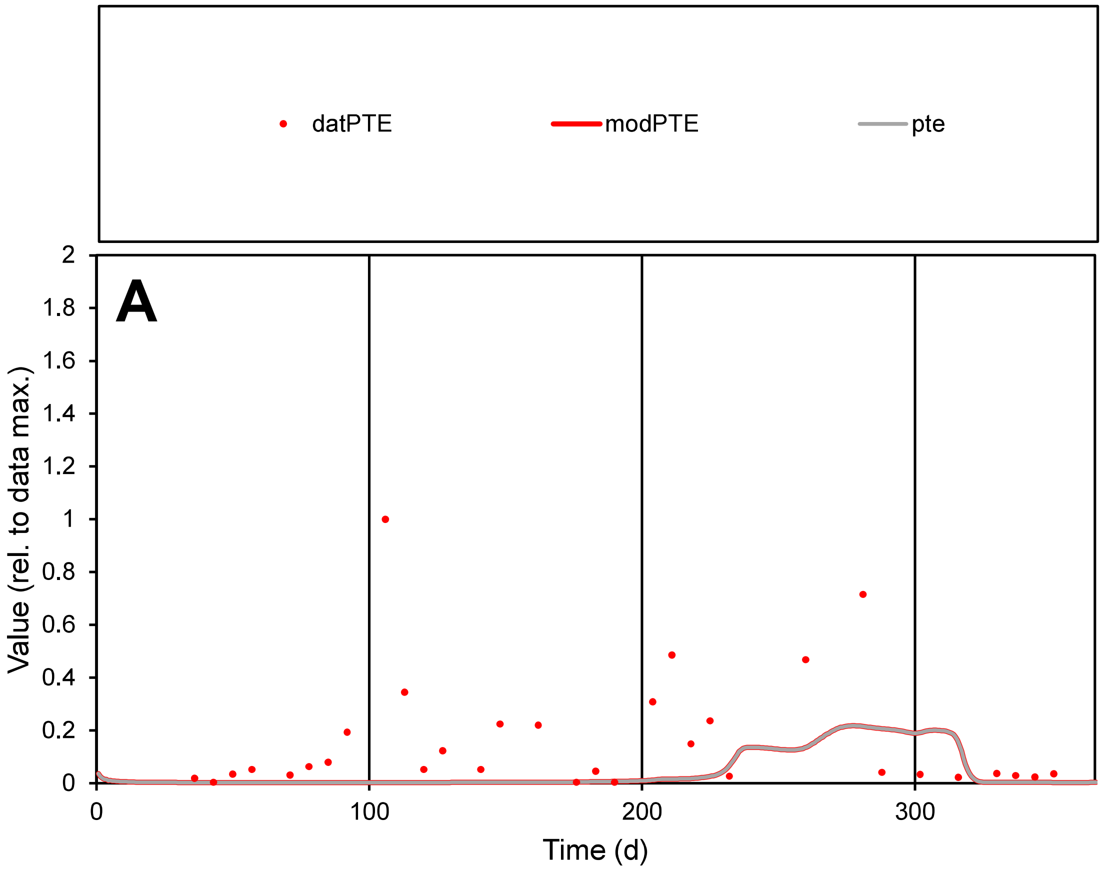

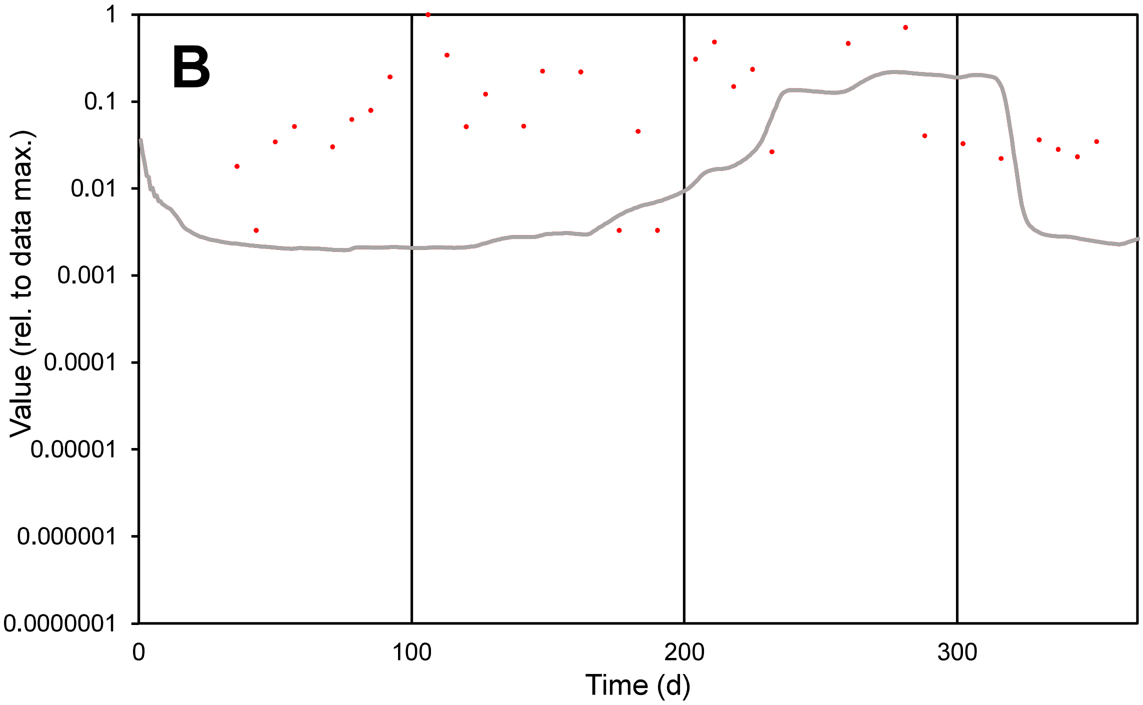

C1

Variables

PTE Provide jtd or Dst

CalibOnly?

2 1 to reproduce of

oNormalize?

2 1=no, 2=yes

ofxn calc*

1.58023

ofxn mod

1.58419

sRun

RunA1

zout

1

Ave

Data

0.00041

Model

8.1E-05

data/vMax

0.00252

ofxng

321.551

Min d/m

1.67602

ave/min d

48.4738

ave/min m

16.1986

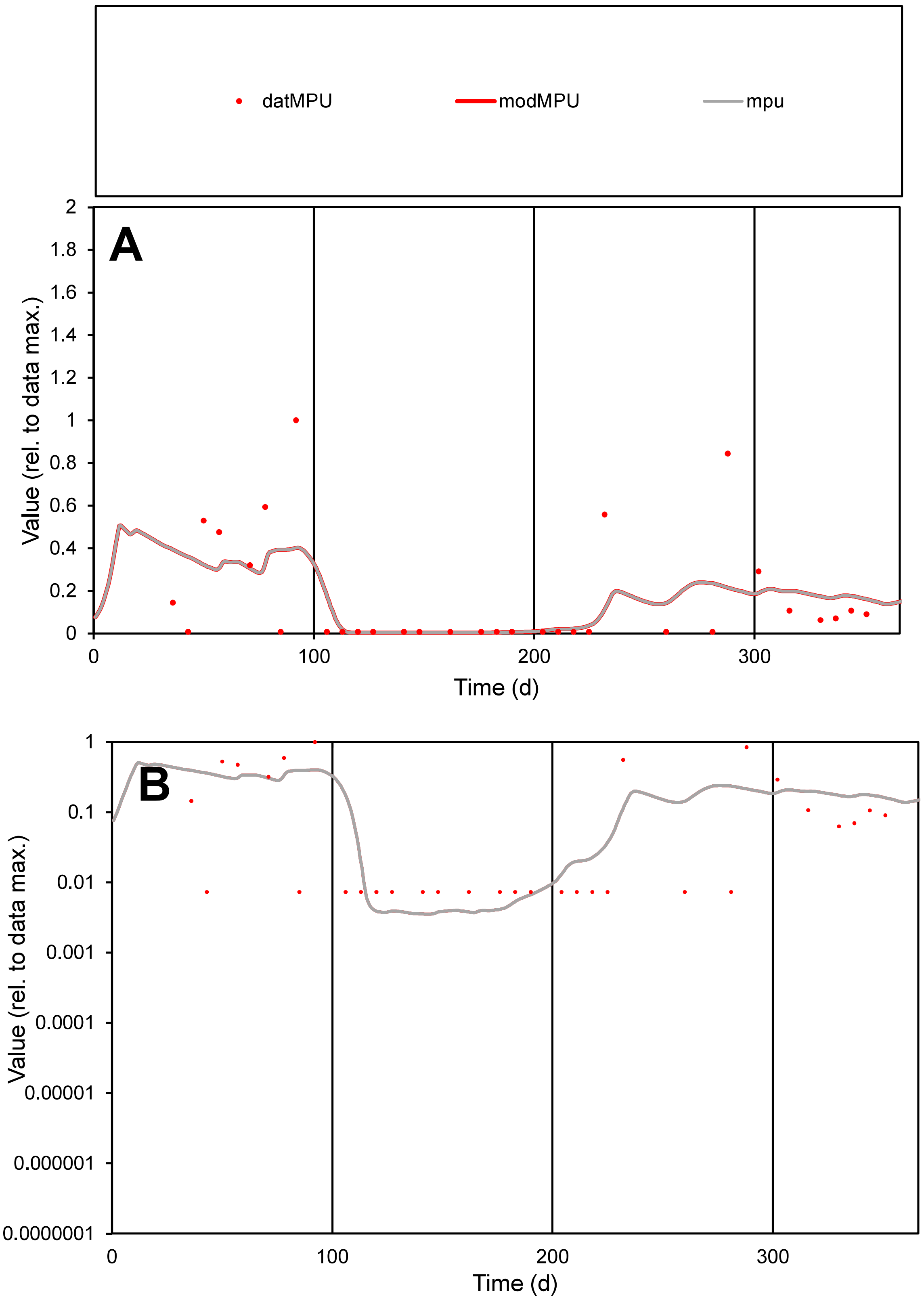

C1

Variables
MPU Provide jtd or Dst
CalibOnly?
2 1 to reproduce of
oNormalize?
2 1=no, 2=yes

ofxn calc*
1.30223
ofxn mod
1.31043

sRun
RunA1
zout
1

Ave
Data
9.3E-05
Model
8.5E-05

data/vMax
0.00056

ofxng
321.551

Min d/m
2.07306

ave/min d
22.7615
ave/min m
43.1917

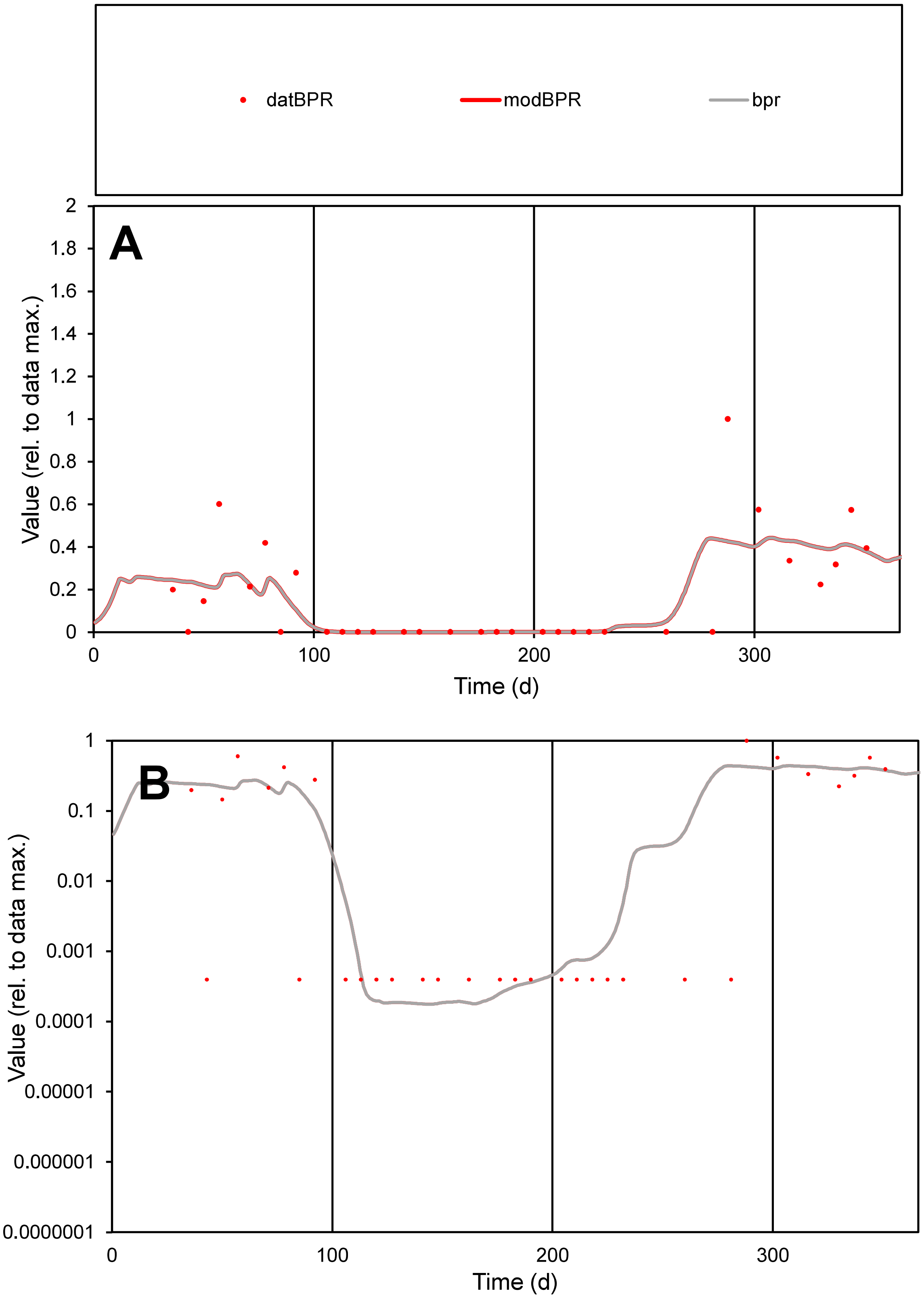

C1

Variables
BPR Provide jtd or Dst
CalibOnly?
2 1 to reproduce of
oNormalize?
2 1=no, 2=yes

ofxn calc*
1.00944
ofxn mod
1.02476

sRun
RunA1
zout
1

Ave
Data
7.7E-05
Model
7.3E-05

data/vMax
0.00047

ofxng
321.551

Min d/m
2.24497

ave/min d
417.634
ave/min m
883.266

324_Calib_S_A

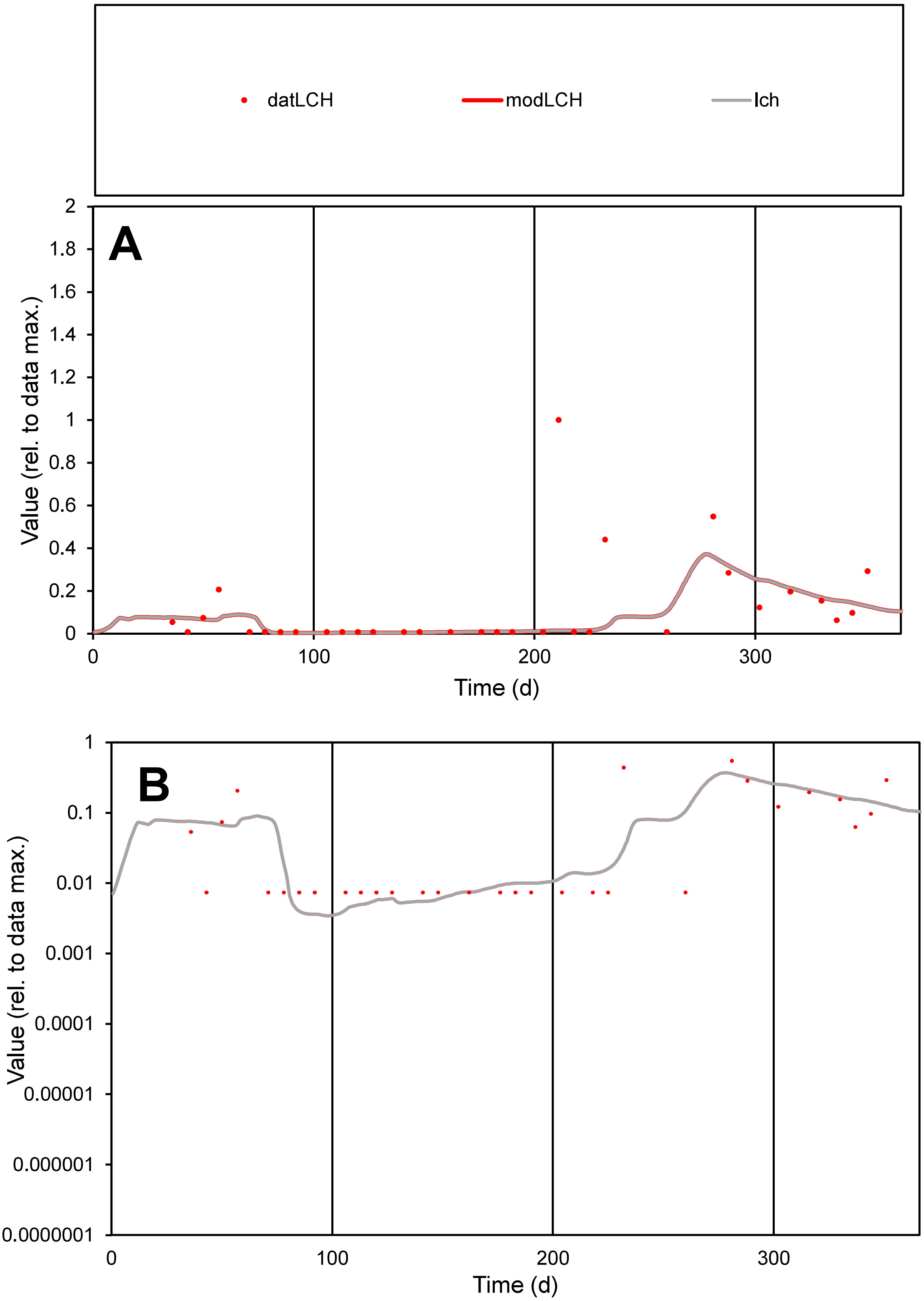

C1

Variables
LCH Provide jtd or Dst
CalibOnly?
2 1 to reproduce of
oNormalize?
2 1=no, 2=yes

ofxn calc*
1.72811
ofxn mod
1.7369

sRun
RunA1
zout
1

Ave
Data
3.8E-05
Model
2.4E-05

data/vMax
0.00033

ofxng
321.551

Min d/m
2.03833

ave/min d
15.5889
ave/min m
20.105

324_Calib_S_A

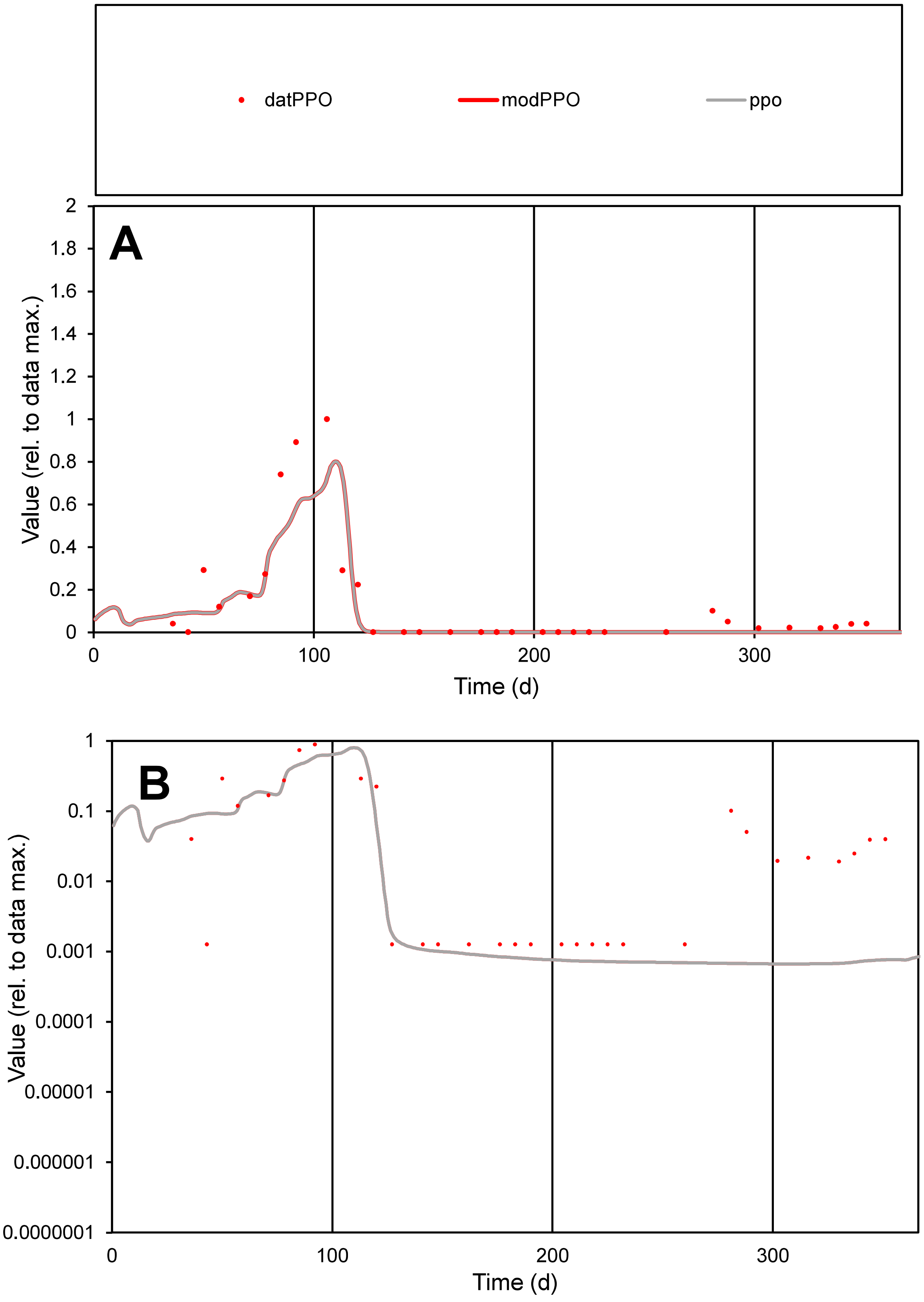

C1

Variables
PPO Provide jtd or Dst
CalibOnly?
2 1 to reproduce of
oNormalize?
2 1=no, 2=yes

ofxn calc*
0.72354
ofxn mod
0.82074

sRun
RunA1
zout
1

Ave
Data
0.00021
Model
0.00016

data/vMax
0.0015

ofxng
321.551

Min d/m
1.90348

ave/min d
107.521
ave/min m
158.968

324_Calib_S_A

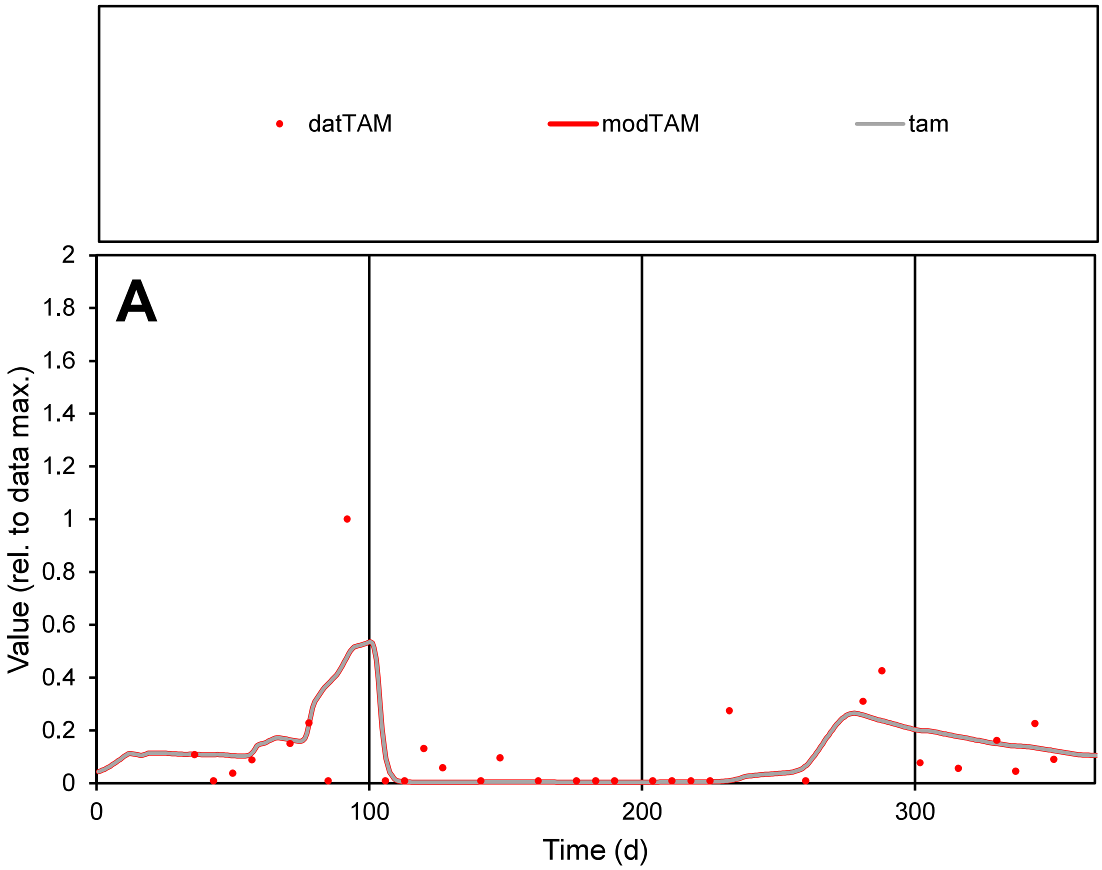

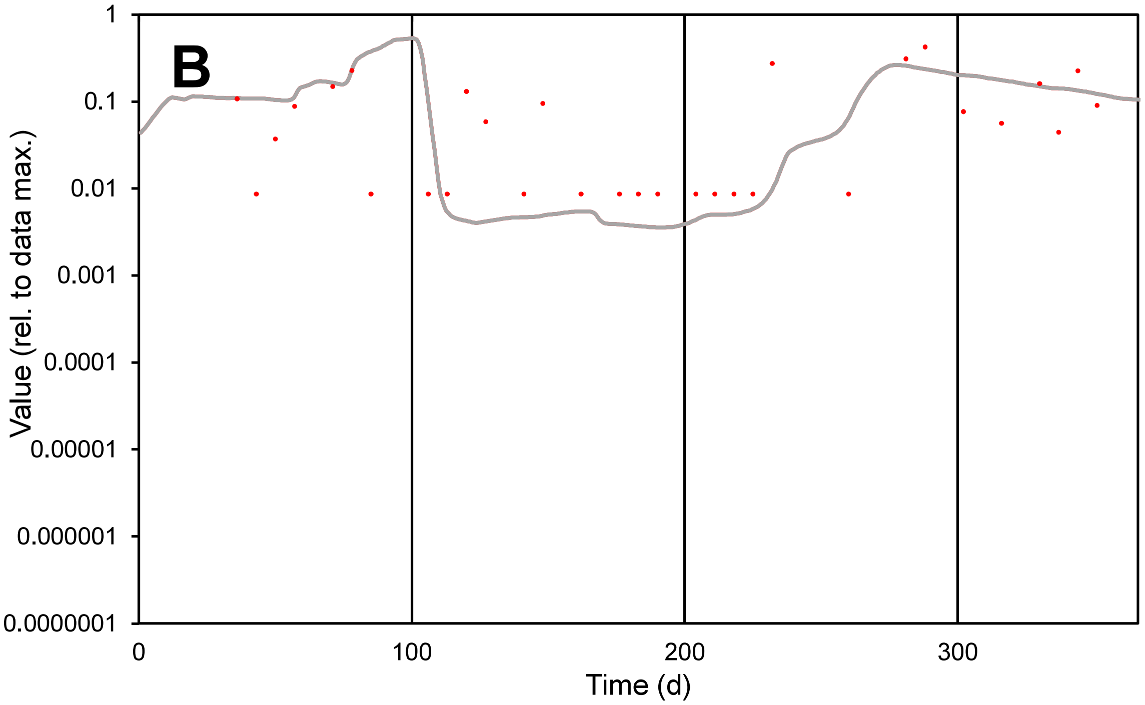

C1

Variables
TAM Provide jtd or Dst
CalibOnly?
2 1 to reproduce of
oNormalize?
2 1=no, 2=yes

ofxn calc*
1.17895
ofxn mod
1.19103

sRun
RunA1
zout
1

Ave
Data
7.7E-05
Model
6.8E-05

data/vMax
0.00066

ofxng
321.551

Min d/m
2.43407

ave/min d
13.2841
ave/min m
28.8877

324_Calib_S_A

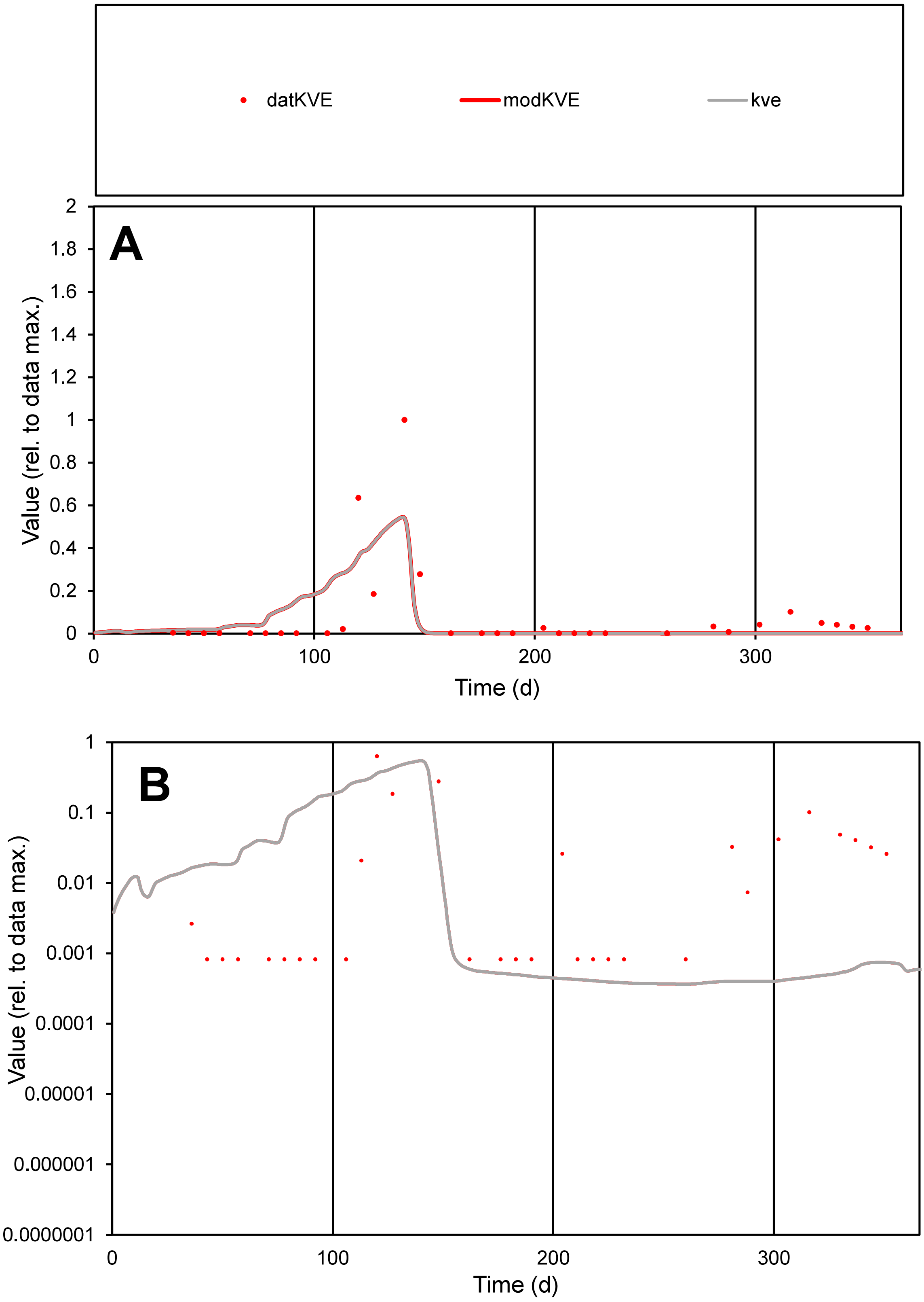

C1

Variables
KVE Provide jtd or Dst
CalibOnly?
2 1 to reproduce of
oNormalize?
2 1=no, 2=yes

ofxn calc*
1.60784
ofxn mod
1.76006

sRun
RunA1
zout
1

Ave
Data
0.0003
Model
0.0003

data/vMax
0.00381

ofxng
321.551

Min d/m
2.24284

ave/min d
94.7286
ave/min m
216.604

324_Calib_S_A

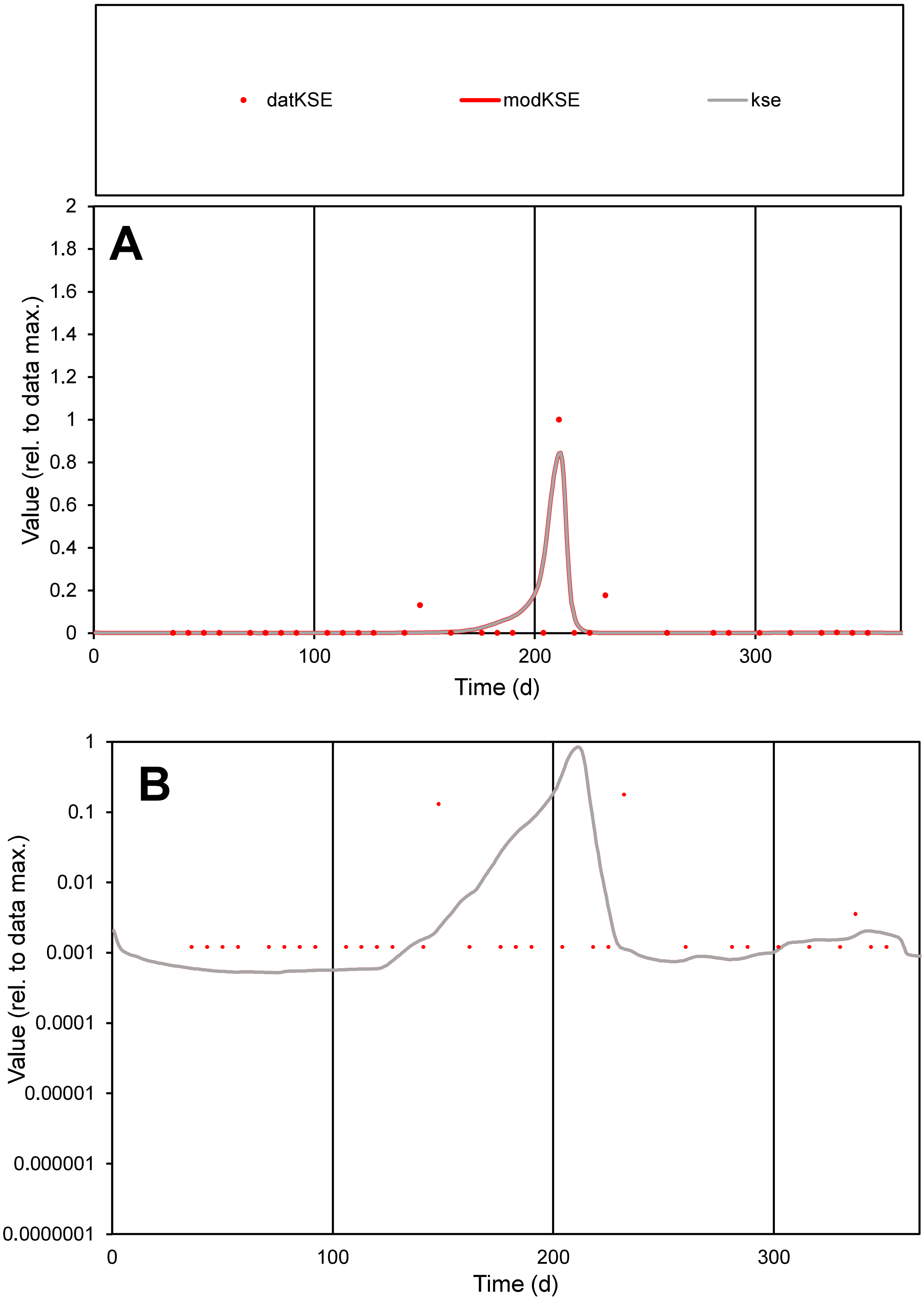

C1

Variables
KSE Provide jtd or Dst
CalibOnly?
2 1 to reproduce of
oNormalize?
2 1=no, 2=yes

ofxn calc*
1.57806
ofxn mod
1.83255

sRun
RunA1
zout
1

Ave
Data
0.00013
Model
0.00012

data/vMax
0.00309

ofxng
321.551

Min d/m
2.31215

ave/min d
34.7001
ave/min m
76.682

324_Calib_S_A

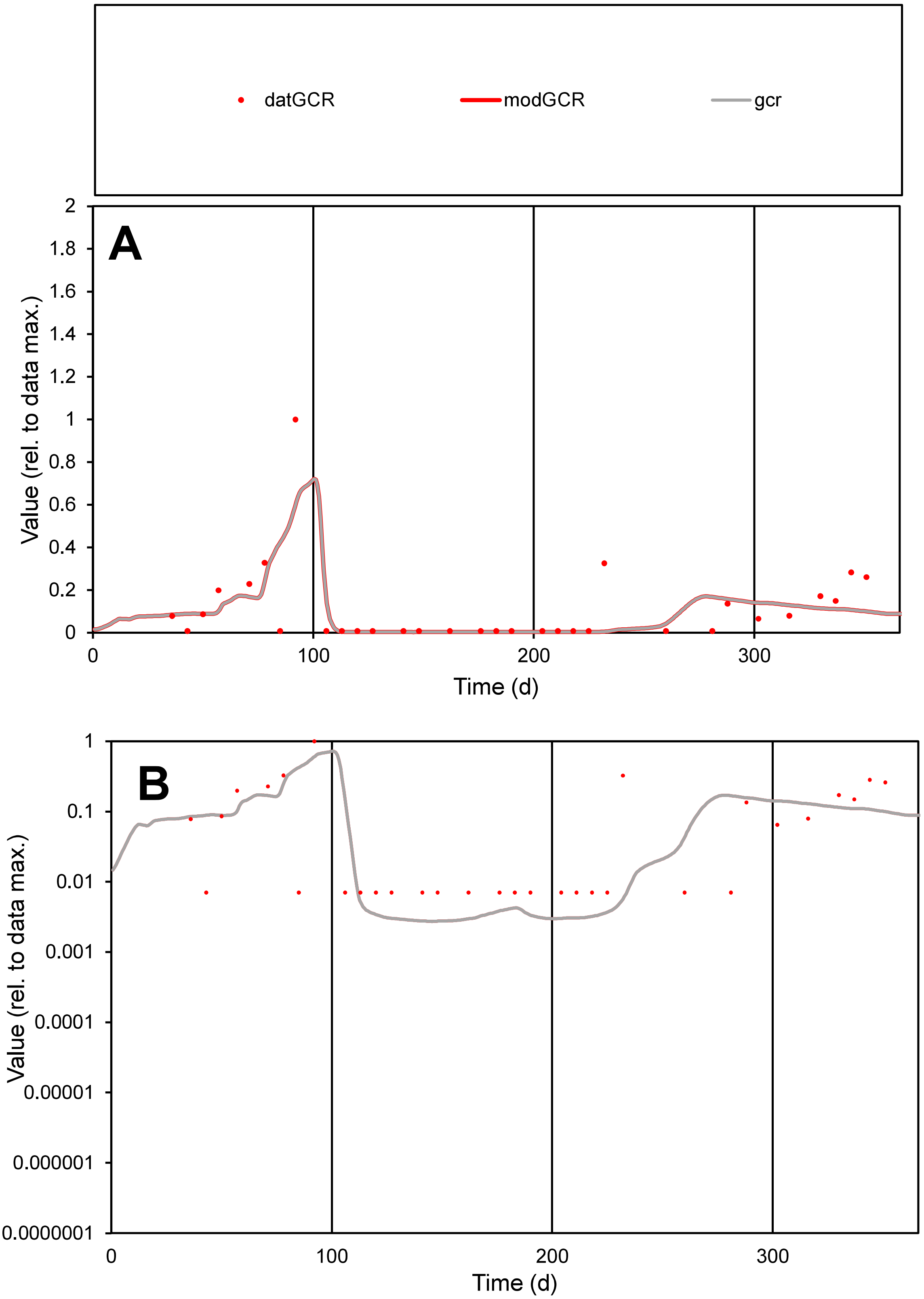

C1

Variables

GCR Provide jtd or Dst

CalibOnly?

2 1 to reproduce of

oNormalize?

2 1=no, 2=yes

ofxn calc*

1.15427

ofxn mod

1.17513

sRun

RunA1

zout

1

Ave

Data

4.9E-05

Model

4.2E-05

data/vMax

0.00044

ofxng

321.551

Min d/m

2.56255

ave/min d

15.5658

ave/min m

34.6041

324_Calib_S_A

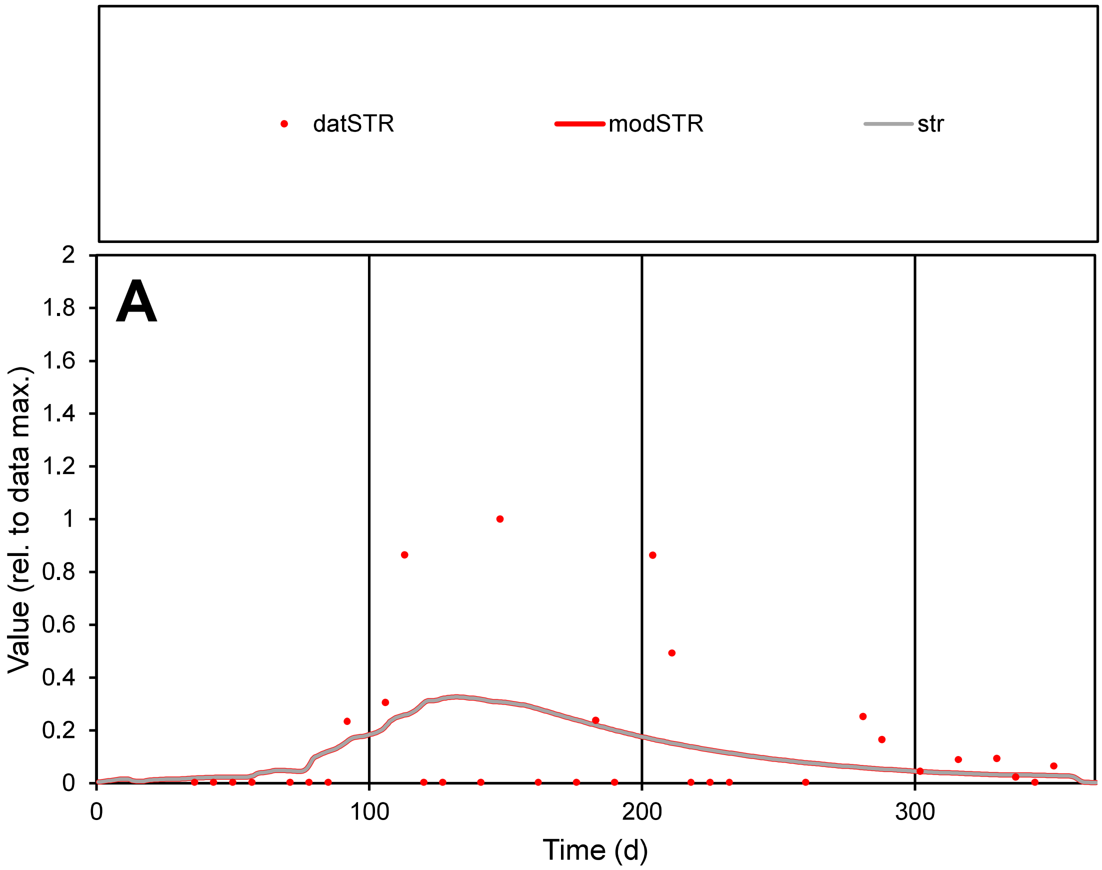

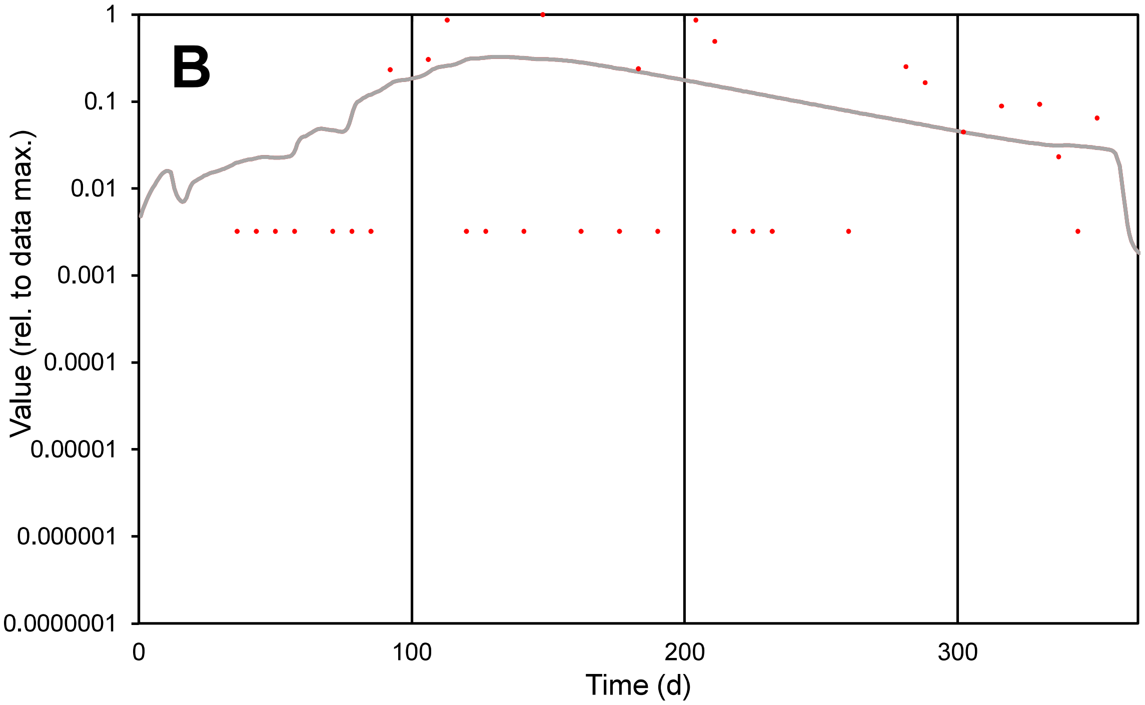

C1

Variables

STR Provide jtd or Dst

CalibOnly?

2 1 to reproduce of

oNormalize?

2 1=no, 2=yes

ofxn calc*

1.68925

ofxn mod

1.69541

sRun

RunA1

zout

1

Ave

Data

7.6E-05

Model

6.7E-05

data/vMax

0.00051

ofxng

321.551

Min d/m

0.16049

ave/min d

46.4835

ave/min m

6.57411

324_Calib_S_A

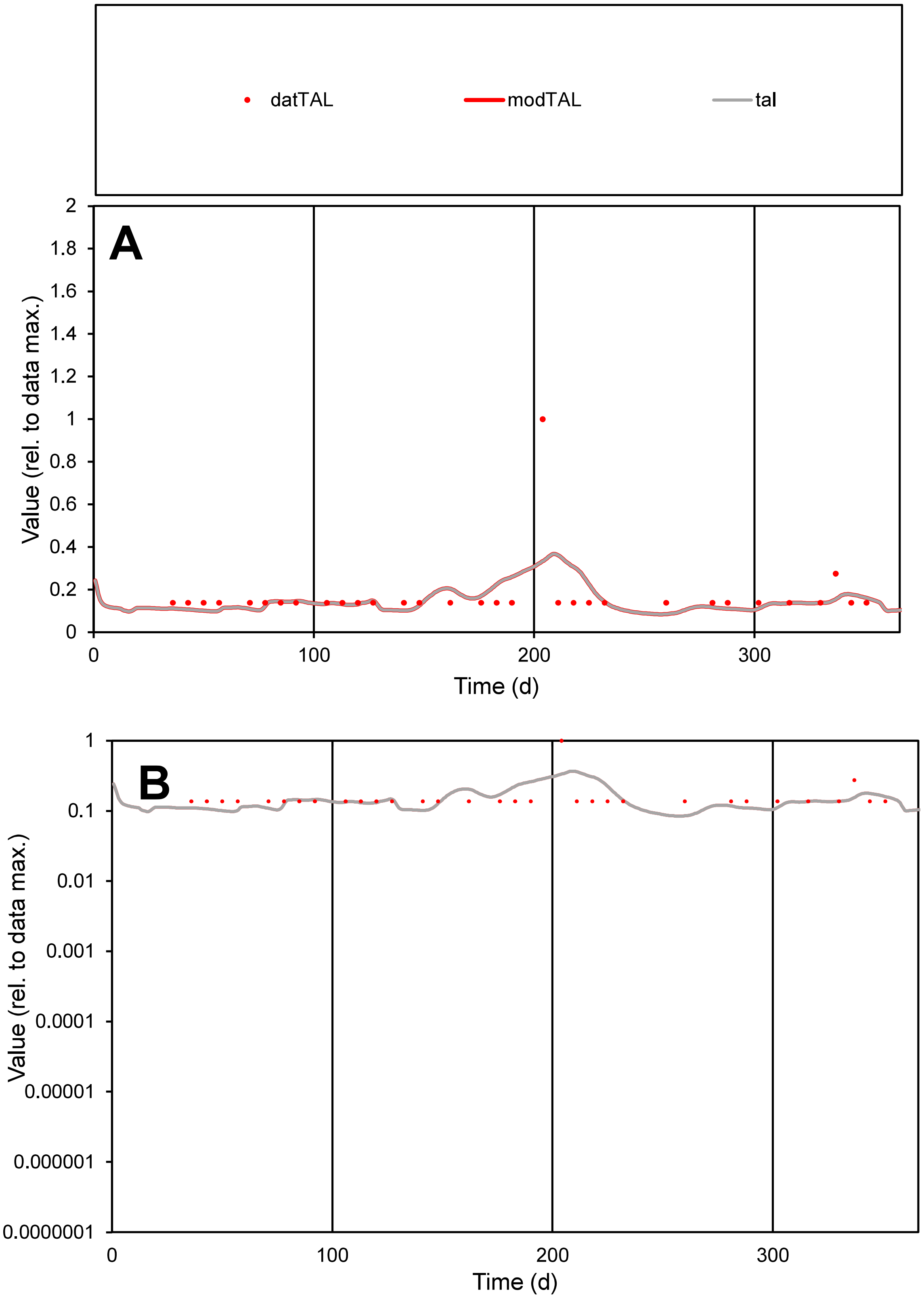

C1

Variables

TAL	Provide jtd or Dst

CalibOnly?

2	1 to reproduce of

oNormalize?

2	1=no, 2=yes

ofxn calc*
0.78646
ofxn mod
0.79419

sRun
RunA1
zout
1

Ave
Data
5.1E-06
Model
4.8E-06

data/vMax
3E-05

ofxng
321.551

Min d/m
1.60309

ave/min d
1.22743
ave/min m
1.87513

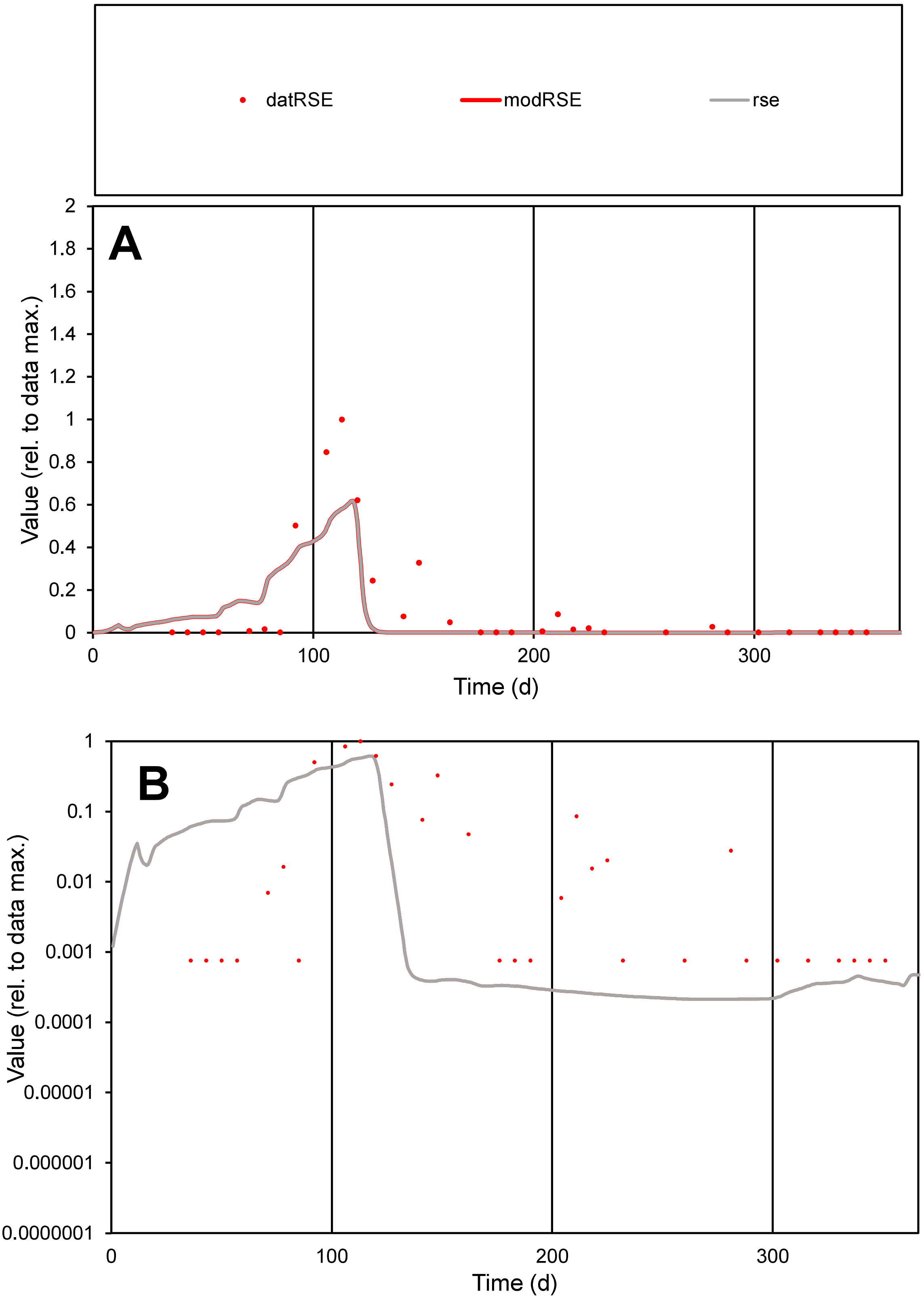

C1

Variables
RSE Provide jtd or Dst
CalibOnly?
2 1 to reproduce of
oNormalize?
2 1=no, 2=yes

ofxn calc*
1.14398
ofxn mod
1.18813

sRun
RunA1
zout
1

Ave
Data
0.00074
Model
0.00057

data/vMax
0.00616

ofxng
321.551

Min d/m
3.54062
ave/min d
159.036
ave/min m
434.881

324_Calib_S_A

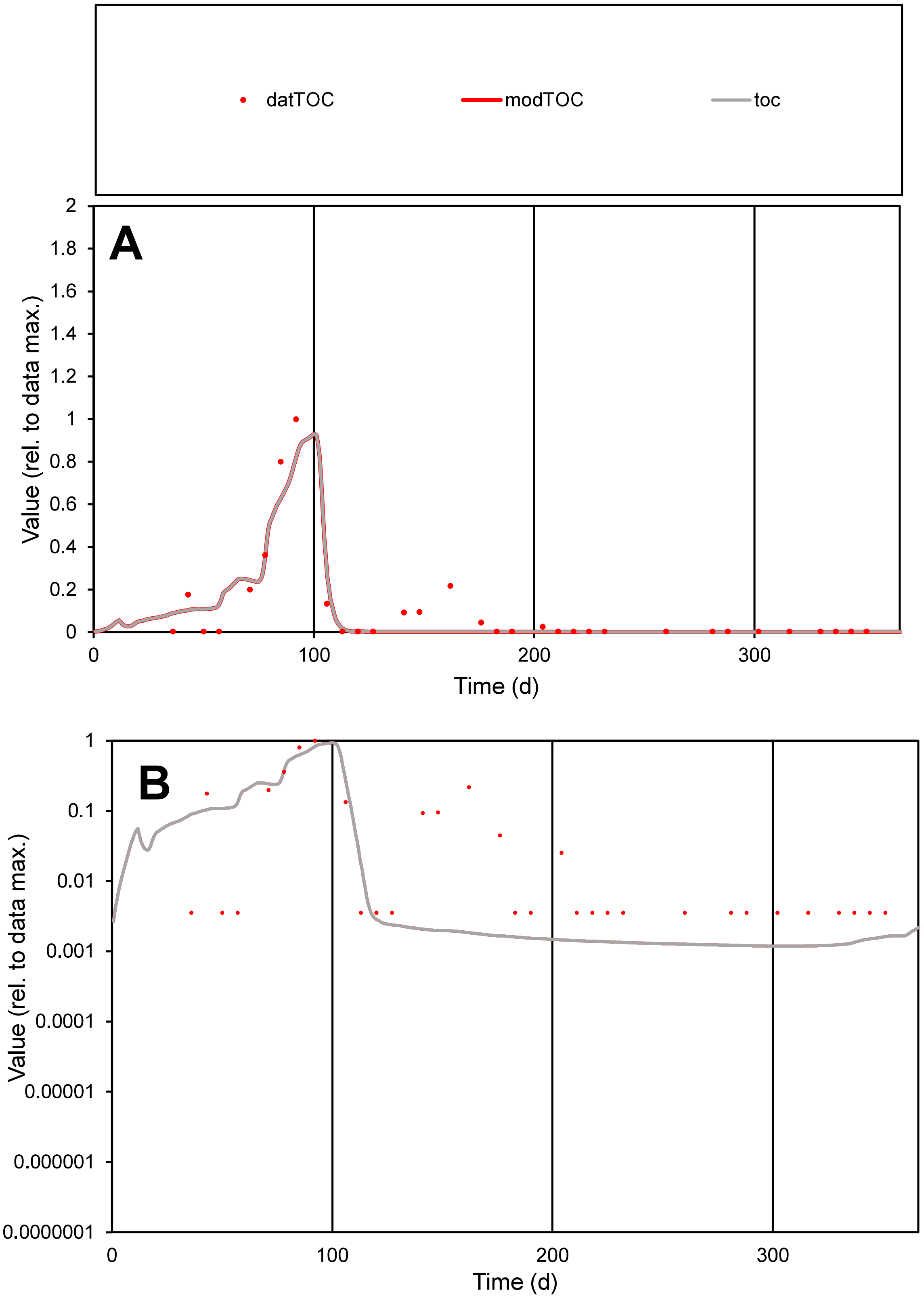

C1

Variables

TOC Provide jtd or Dst

CalibOnly?

2 1 to reproduce of

oNormalize?

2 1=no, 2=yes

ofxn calc*

0.6728

ofxn mod

0.71936

sRun

RunA1

zout

1

Ave

Data

0.00012

Model

0.00011

data/vMax

0.00122

ofxng

321.551

Min d/m

2.97658

ave/min d

28.4892

ave/min m

73.3878

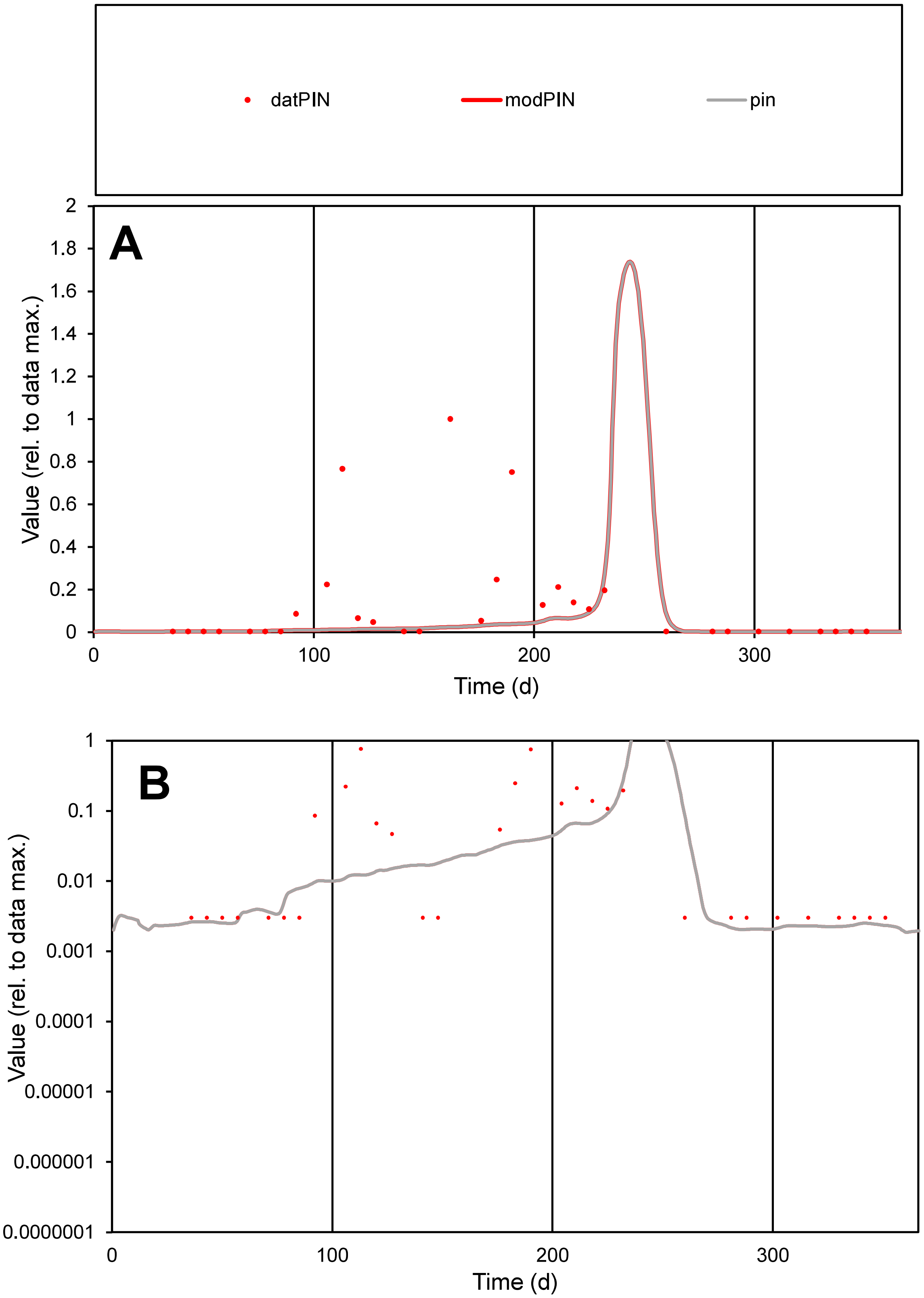

C1

Variables
PIN Provide jtd or Dst
CalibOnly?
2 1 to reproduce of
oNormalize?
2 1=no, 2=yes

ofxn calc*
2.04013
ofxn mod
2.04817

sRun
RunA1
zout
1

Ave
Data
0.00017
Model
3.4E-05

data/vMax
0.00133

ofxng
321.551

Min d/m
1.45924

ave/min d
42.3529
ave/min m
12.5292

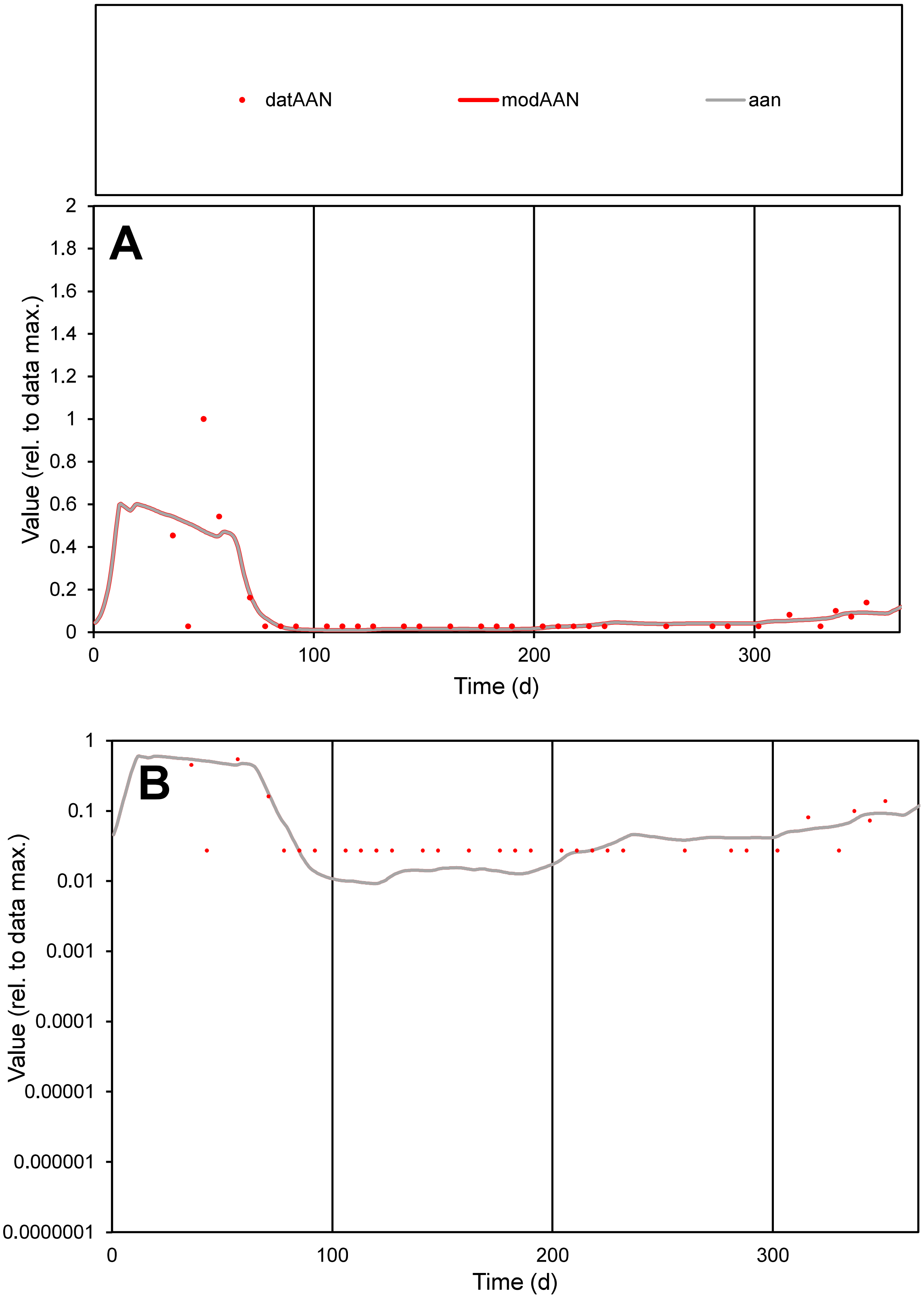

C1

Variables
AAN Provide jtd or Dst
CalibOnly?
2 1 to reproduce of
oNormalize?
2 1=no, 2=yes

ofxn calc*
1.29237
ofxn mod
1.3096

sRun
RunA1
zout
1

Ave
Data
2.1E-05
Model
1.9E-05

data/vMax
0.0002

ofxng
321.551

Min d/m
2.88269

ave/min d
3.67539
ave/min m
10.0694

324_Calib_S_A

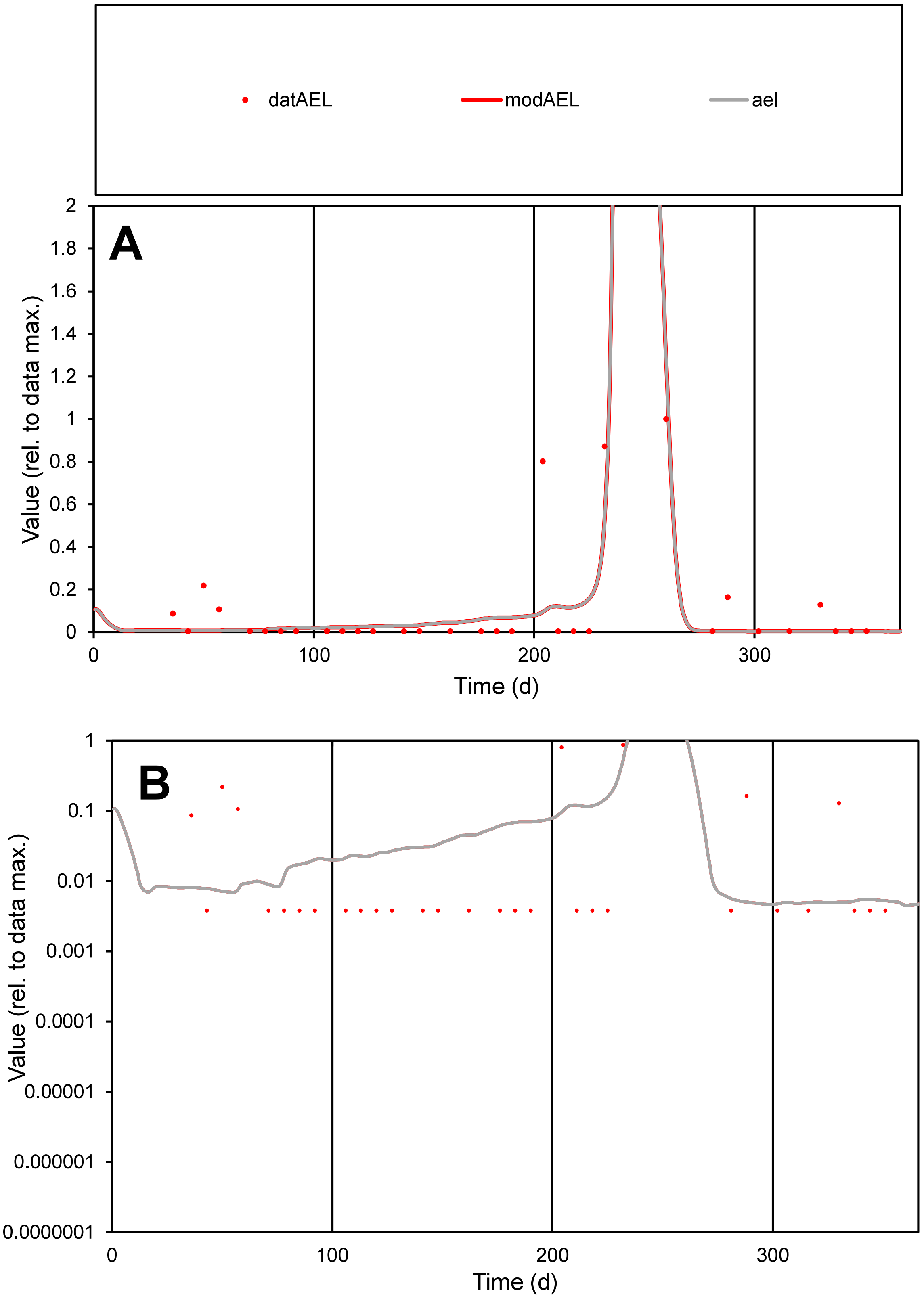

C1

Variables
AEL Provide jtd or Dst
CalibOnly?
2 1 to reproduce of
oNormalize?
2 1=no, 2=yes

ofxn calc*
1.31735
ofxn mod
1.33467

sRun
RunA1
zout
1

Ave
Data
2E-05
Model
1.6E-05

data/vMax
0.00018

ofxng
321.551

Min d/m
0.79621

ave/min d
28.2983
ave/min m
18.6393

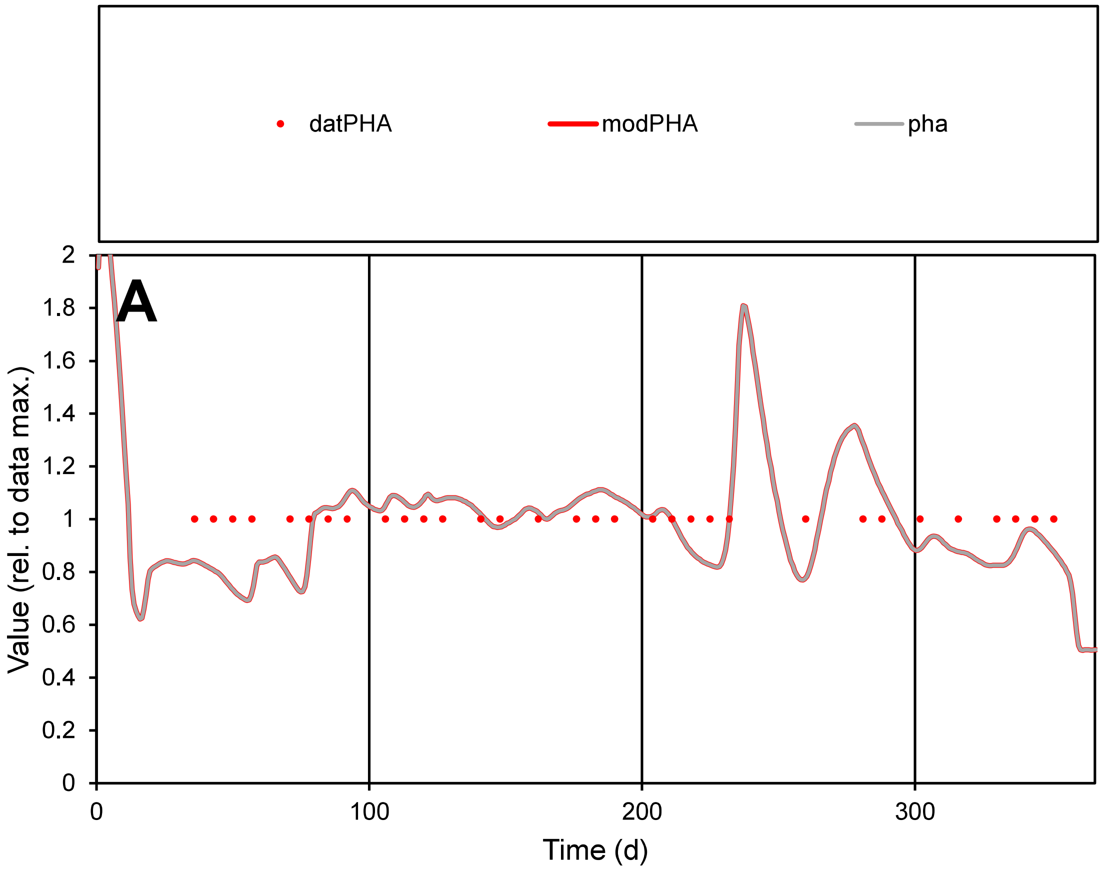

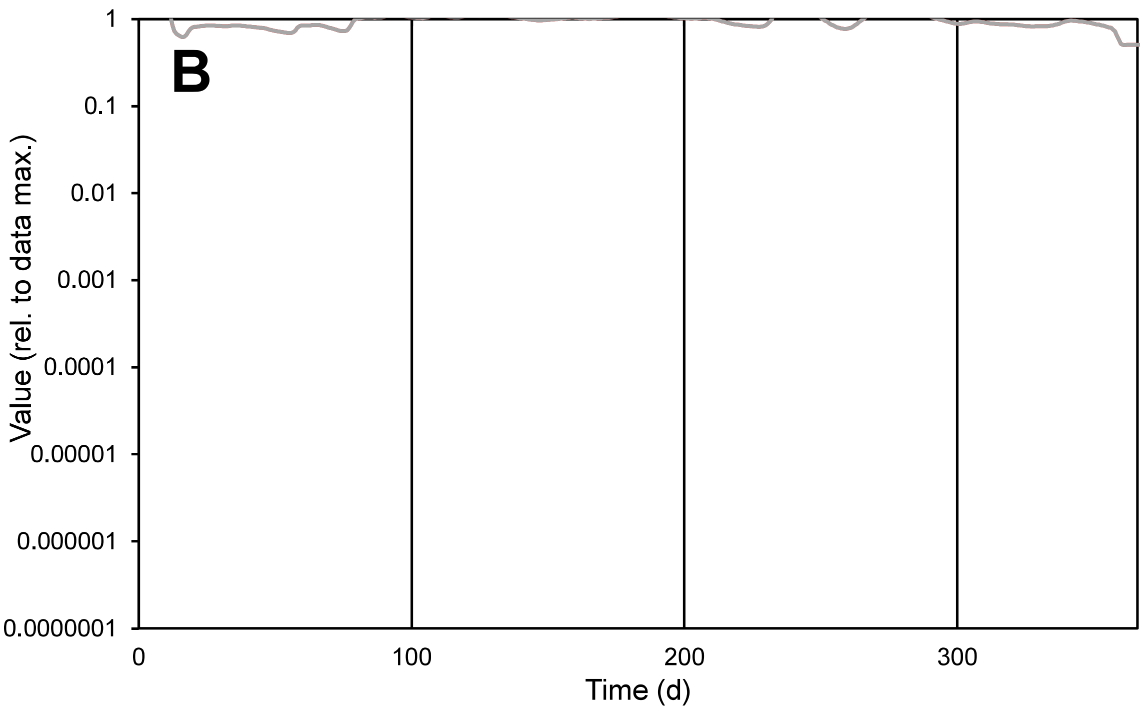

C1

Variables
PHA Provide jtd or Dst
CalibOnly?
2 1 to reproduce of
oNormalize?
2 1=no, 2=yes

ofxn calc*
0.13312
ofxn mod
0.16657

sRun
RunA1
zout
1

Ave
Data
1E-05
Model
1E-05

data/vMax
1E-05

ofxng
321.551

Min d/m
1.39488

ave/min d
1
ave/min m
1.34369

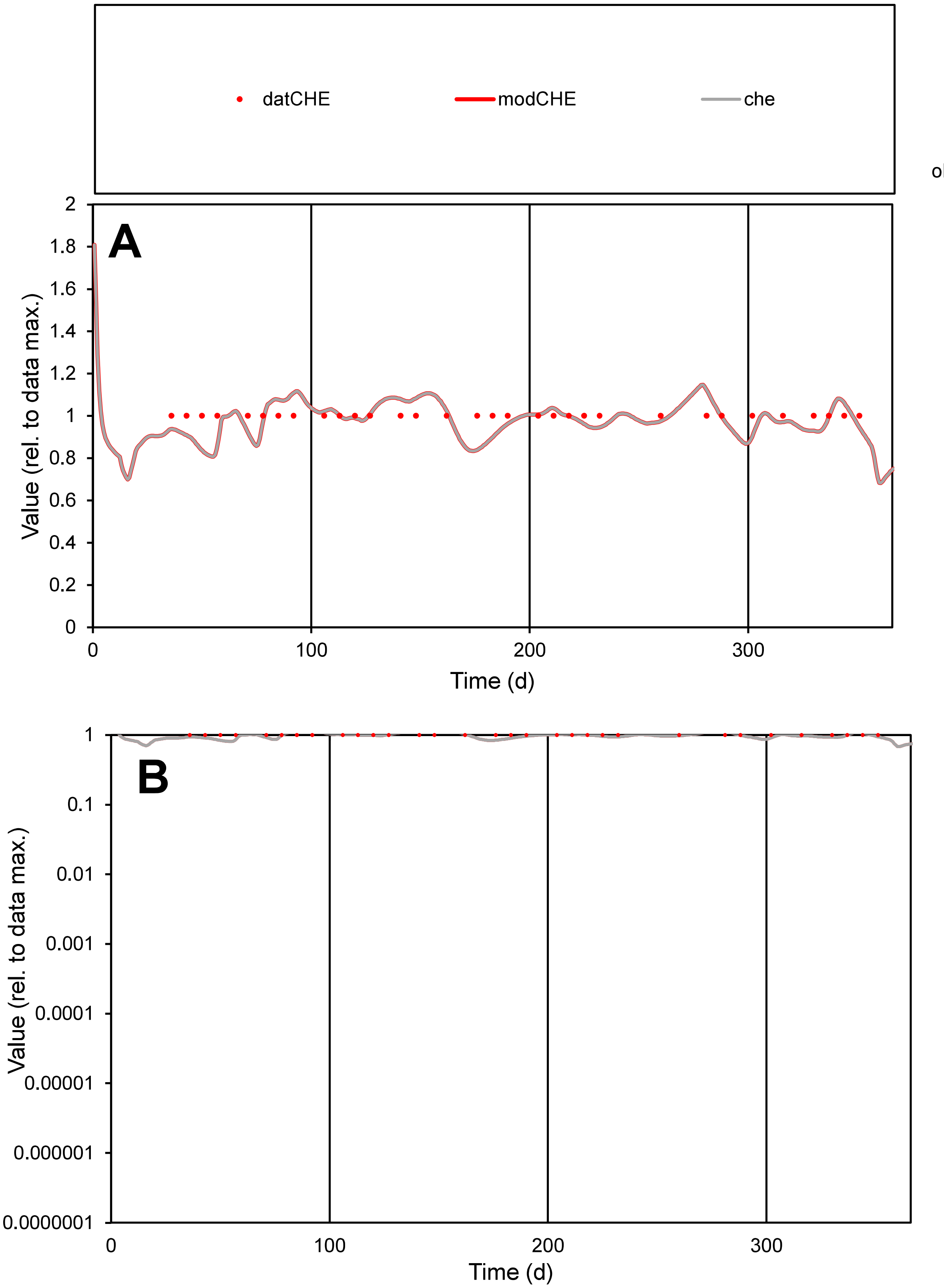

C1

Variables

CHE Provide jtd or Dst

CalibOnly?

2 1 to reproduce of

oNormalize?

2 1=no, 2=yes

ofxn calc*

0.07053

ofxn mod

0.11943

sRun

RunA1

zout

1

Ave

Data

7E-06

Model

6.9E-06

data/vMax

7E-06

ofxng

321.551

Min d/m

1.21579

ave/min d

1

ave/min m

1.19499

324_Calib_S_A

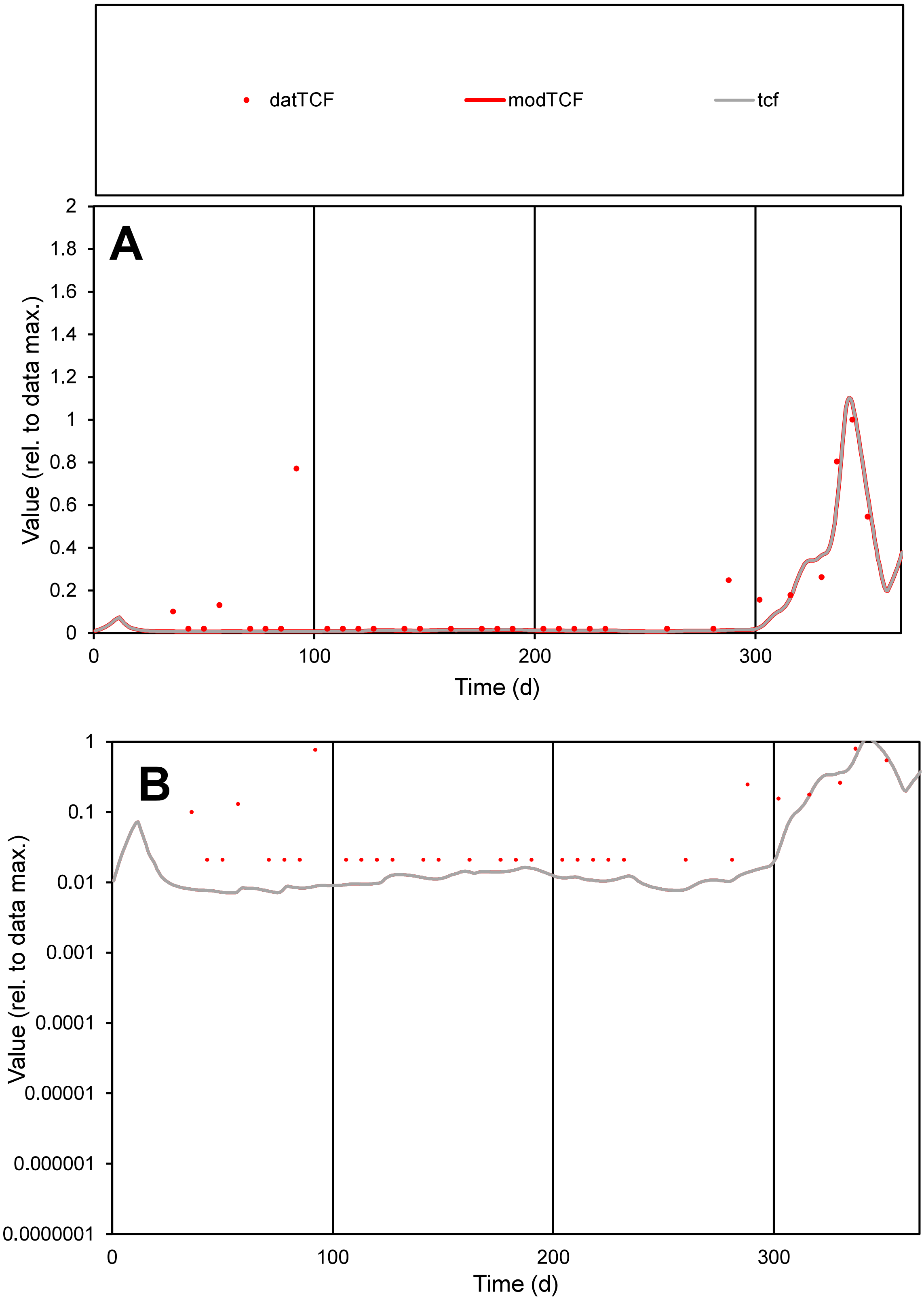

C1

Variables
TCF Provide jtd or Dst
CalibOnly?
2 1 to reproduce of
oNormalize?
2 1=no, 2=yes

ofxn calc*
1.00885
ofxn mod
1.02452

sRun
RunA1
zout
1

Ave
Data
2.2E-05
Model
1.5E-05

data/vMax
0.00015

ofxng
321.551

Min d/m
2.92864

ave/min d
6.91606
ave/min m
13.9276

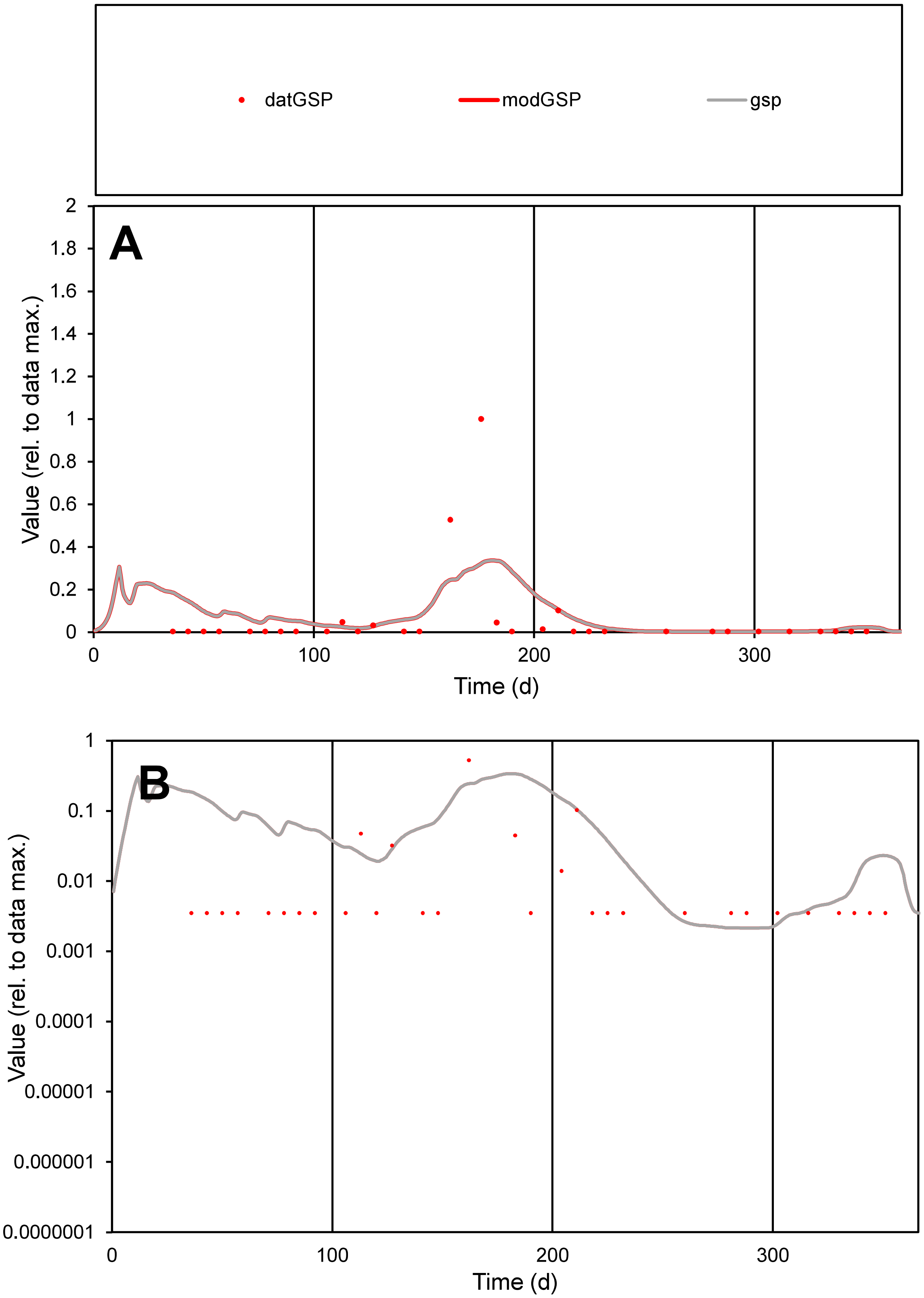

C1

Variables
GSP Provide jtd or Dst
CalibOnly?
2 1 to reproduce of
oNormalize?
2 1=no, 2=yes

ofxn calc*
2.66445
ofxn mod
2.70518

sRun
RunA1
zout
1

Ave
Data
0.00015
Model
0.0002

data/vMax
0.00255

ofxng
321.551

Min d/m
1.63641

ave/min d
16.4528
ave/min m
35.7555

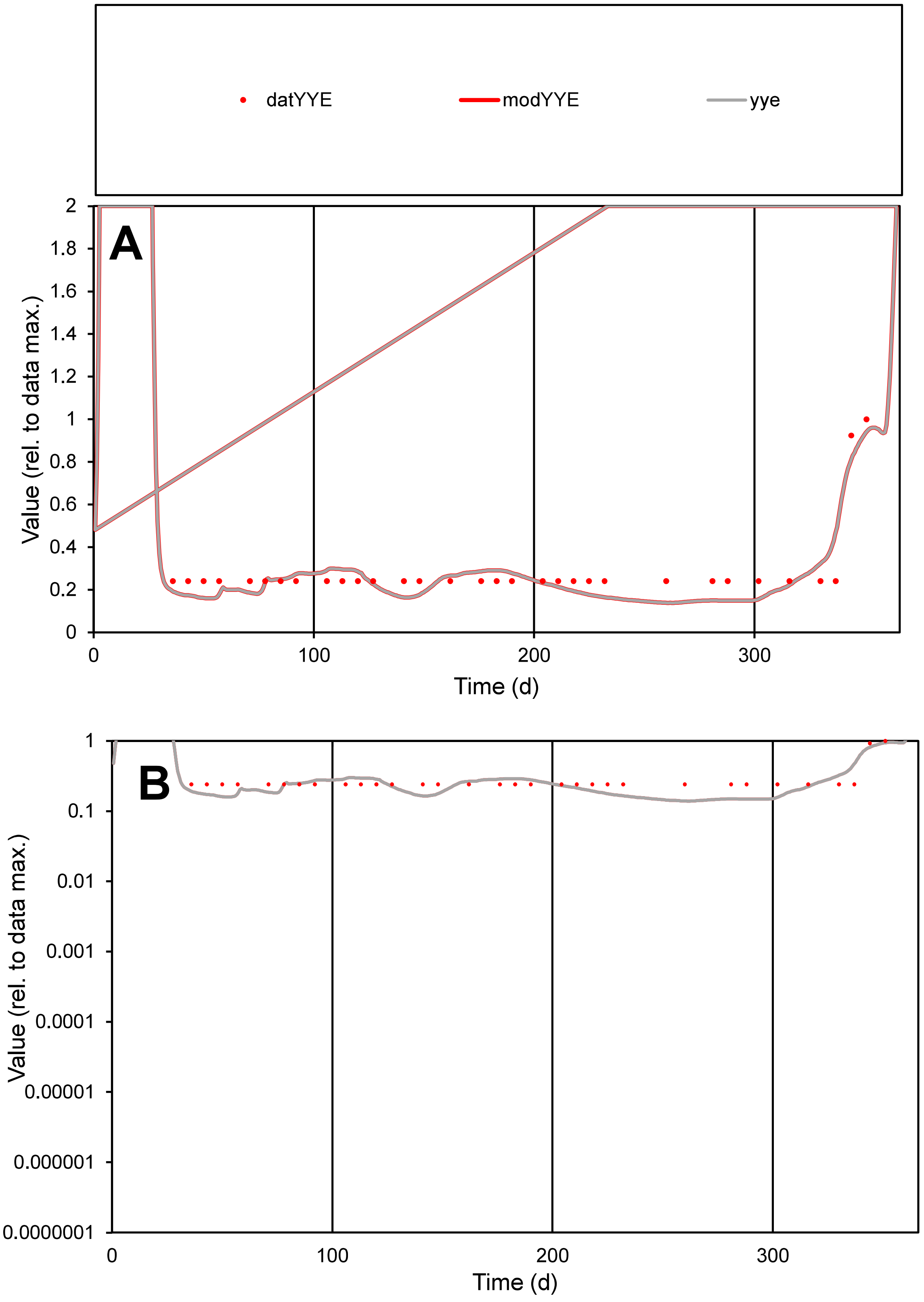

C1

Variables
YYE Provide jtd or Dst
CalibOnly?
2 1 to reproduce of
oNormalize?
2 1=no, 2=yes

ofxn calc*
0.25828
ofxn mod
0.28045

sRun
RunA1
zout
1

Ave
Data
4.5E-06
Model
4.2E-06

data/vMax
1.6E-05

ofxng
321.551

Min d/m
1.73011

ave/min d
1.18738
ave/min m
1.94948

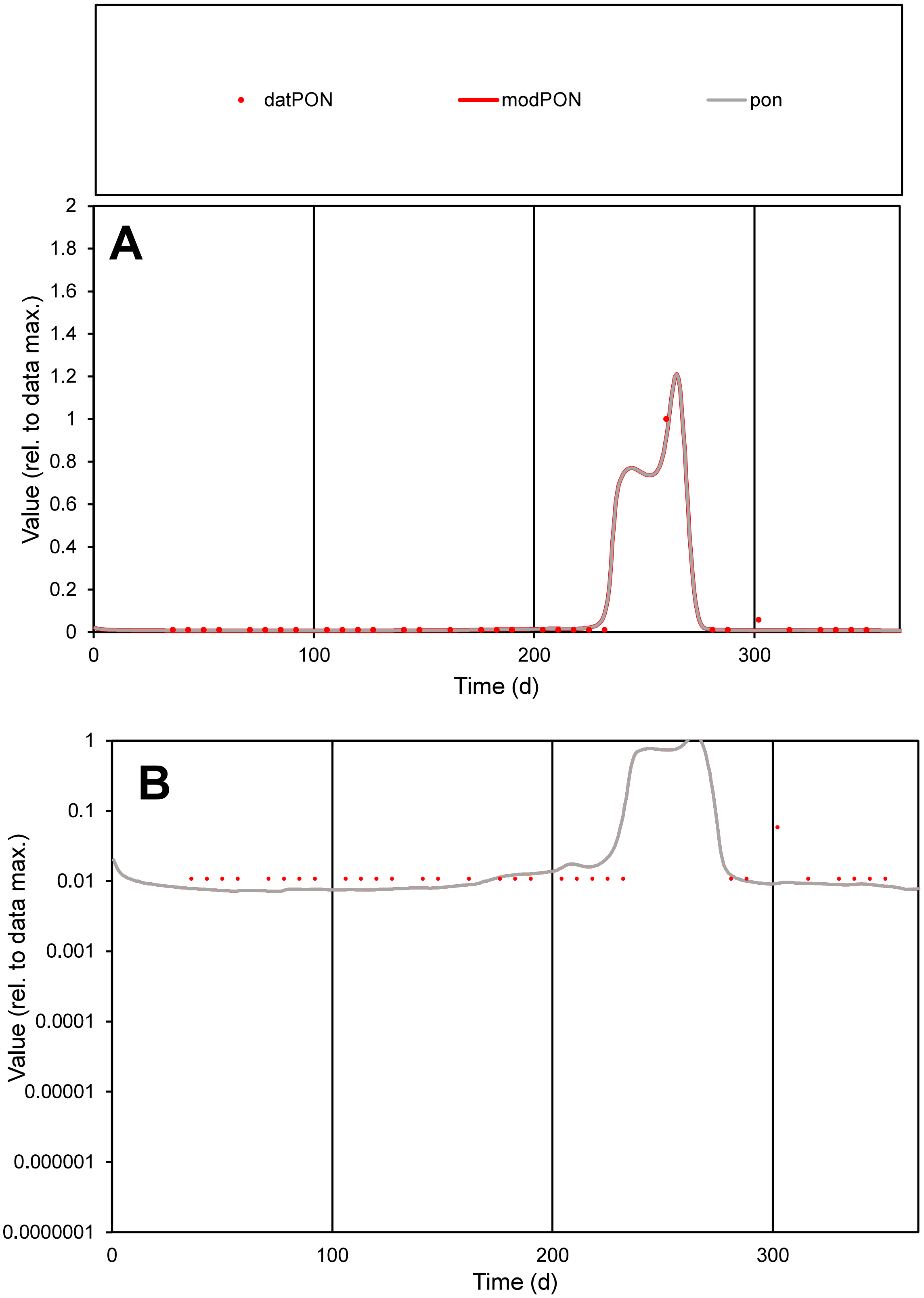

C1

Variables
PON Provide jtd or Dst
CalibOnly?
2 1 to reproduce of
oNormalize?
2 1=no, 2=yes

ofxn calc*
0.31705
ofxn mod
0.44233

sRun
RunA1
zout
1

Ave
Data
1.2E-05
Model
1.1E-05

data/vMax
0.00027

ofxng
321.551

Min d/m
1.51004

ave/min d
3.98209
ave/min m
5.89705

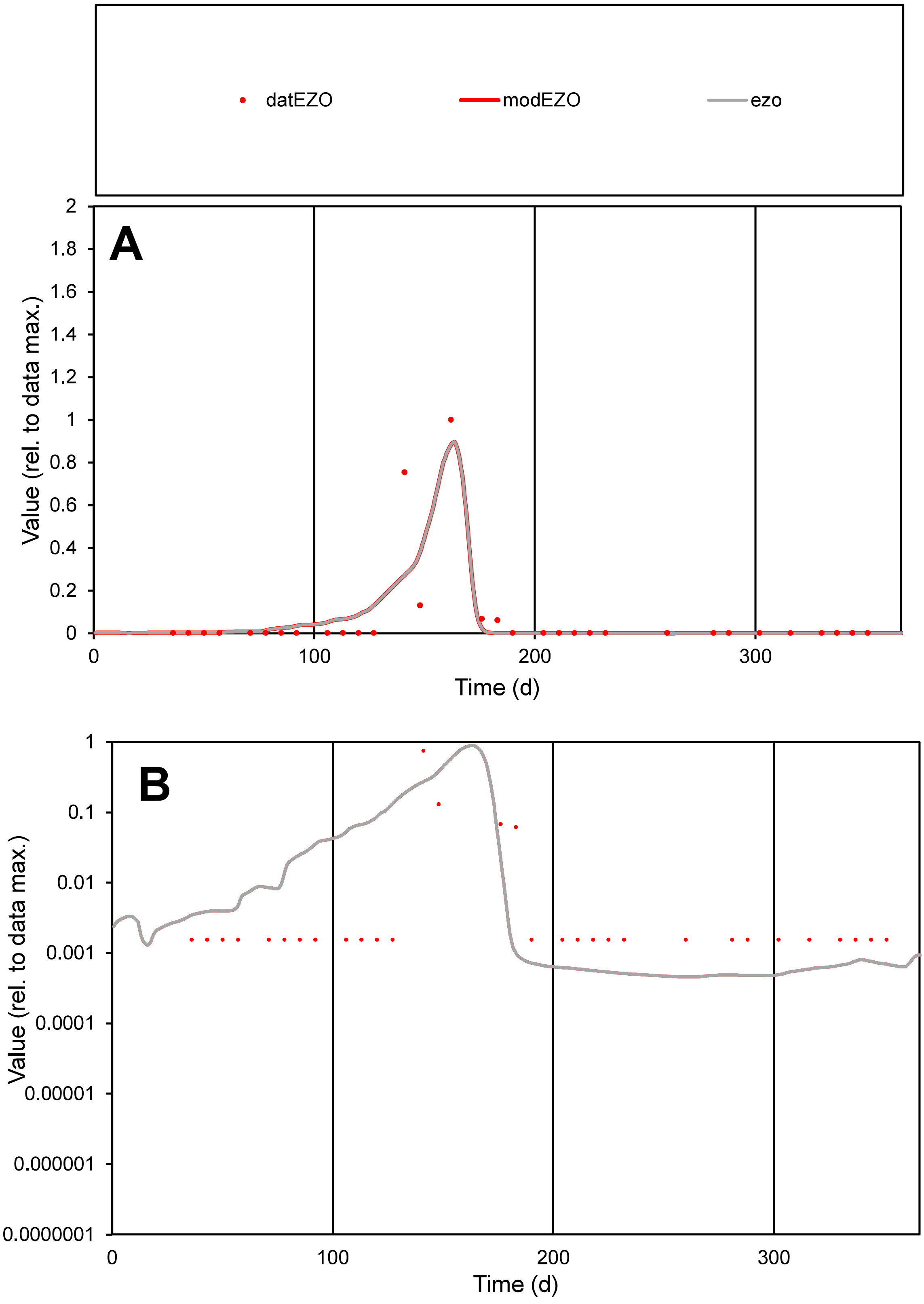

C1

Variables
EZO Provide jtd or Dst
CalibOnly?
2 1 to reproduce of
oNormalize?
2 1=no, 2=yes

ofxn calc*
1.5755
ofxn mod
1.63906

sRun
RunA1
zout
1

Ave
Data
0.00016
Model
0.00016

data/vMax
0.00254

ofxng
321.551

Min d/m
3.39914

ave/min d
41.3618
ave/min m
140.117

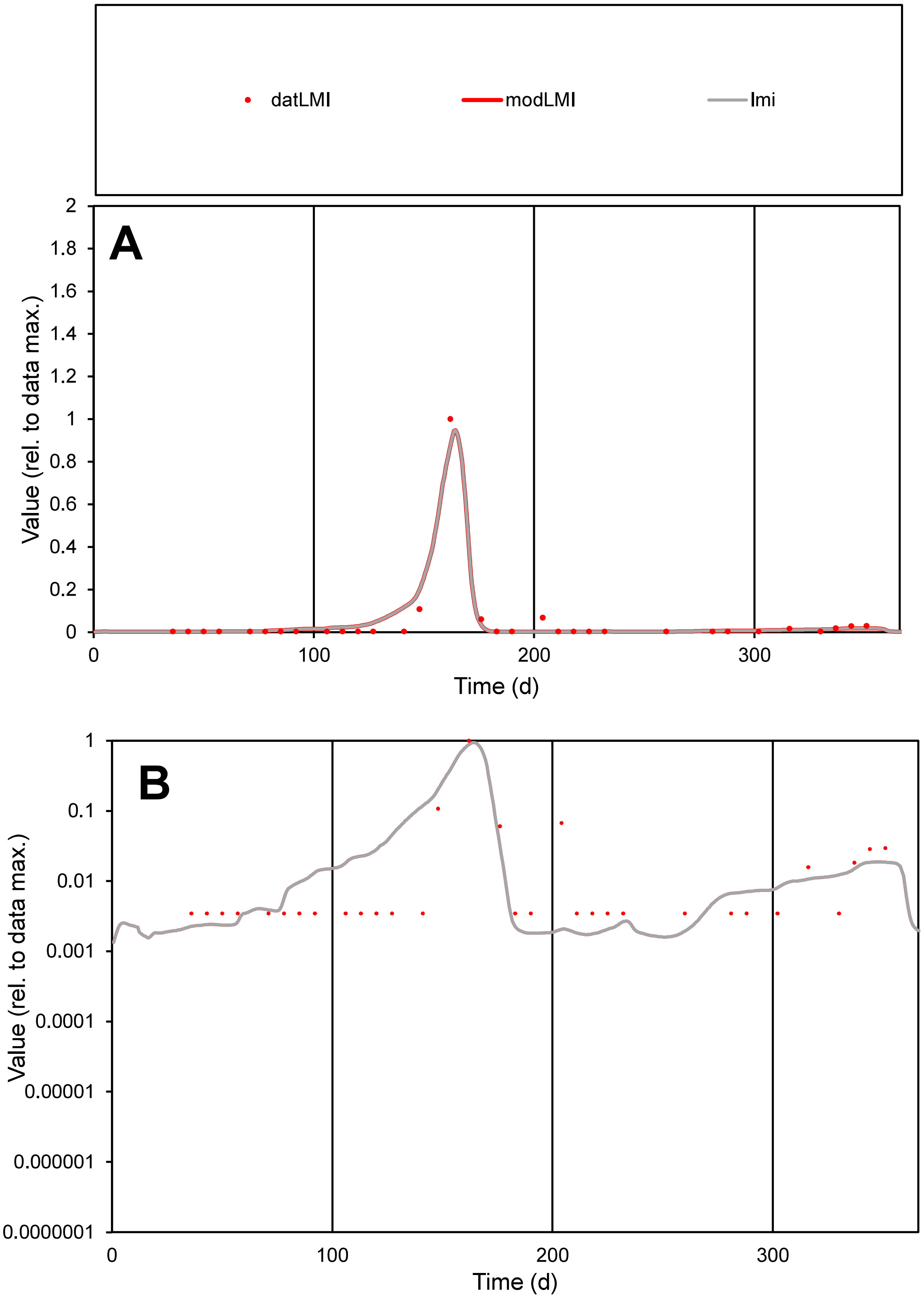

C1

Variables
LMI Provide jtd or Dst
CalibOnly?
2 1 to reproduce of
oNormalize?
2 1=no, 2=yes

ofxn calc*
0.72893
ofxn mod
0.9116

sRun
RunA1
zout
1

Ave
Data
3.8E-05
Model
4.3E-05

data/vMax
0.00087

ofxng
321.551

Min d/m
1.92401

ave/min d
12.7161
ave/min m
27.3531

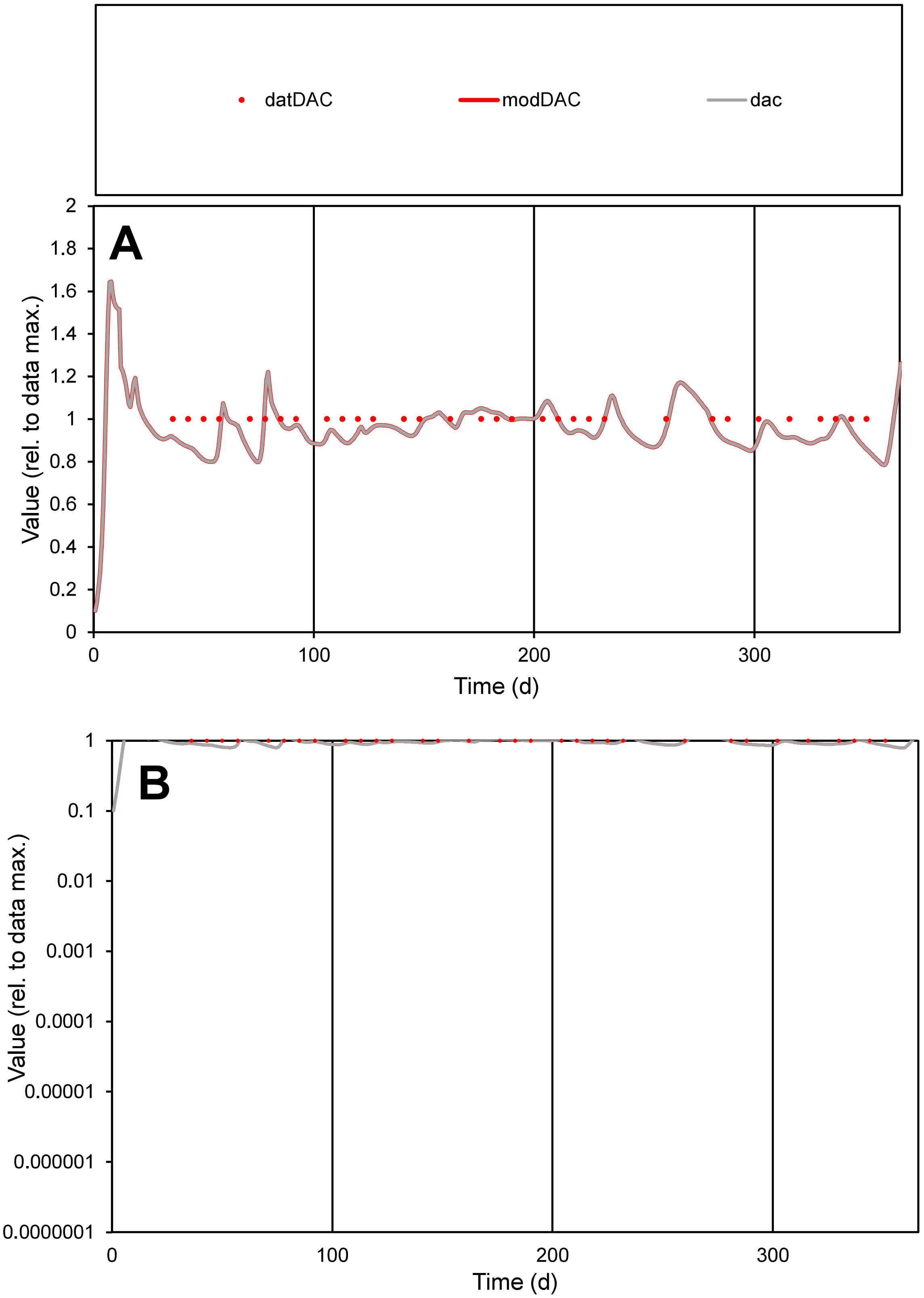

C1

Variables
DAC Provide jtd or Dst
CalibOnly?
2 1 to reproduce of
oNormalize?
2 1=no, 2=yes

ofxn calc*
0.08921
ofxn mod
0.13038

sRun
RunA1
zout
1

Ave
Data
7.7E-06
Model
7.4E-06

data/vMax
7.7E-06

ofxng
321.551

Min d/m
1.24612

ave/min d
1
ave/min m
1.19275

324_Calib_S_A

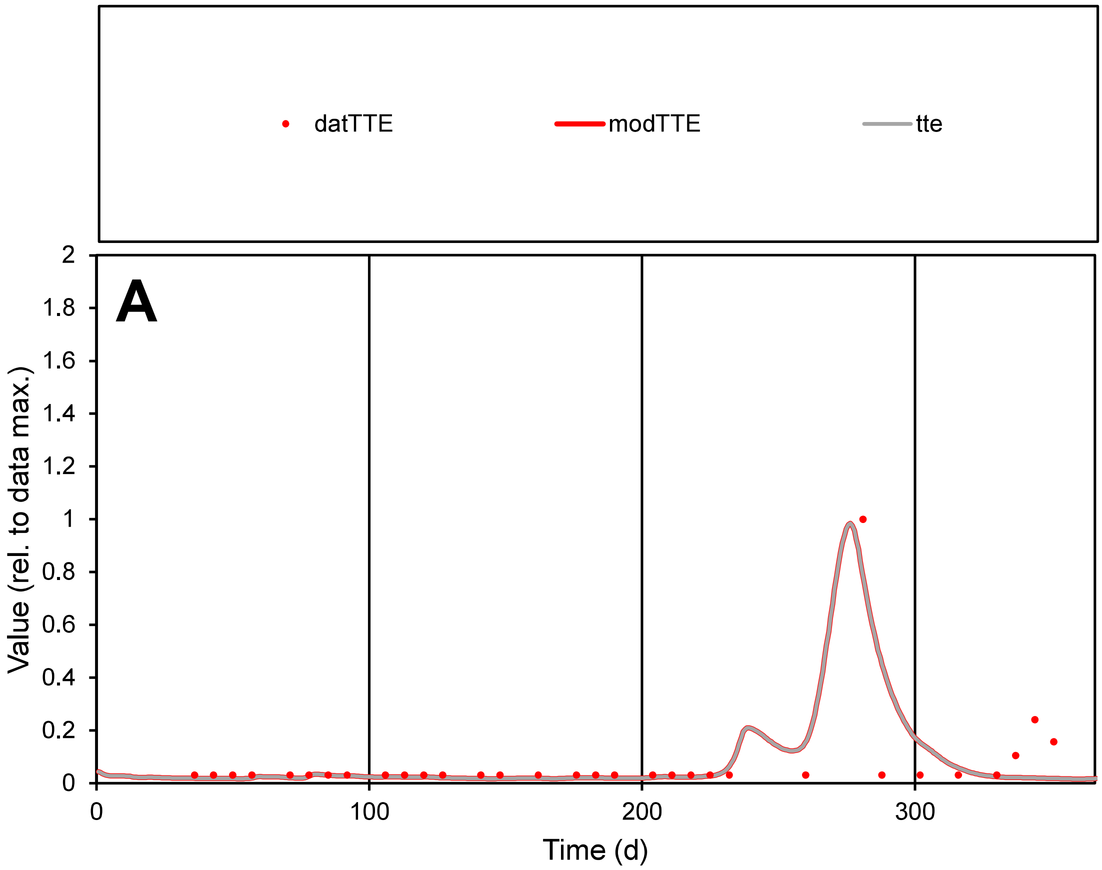

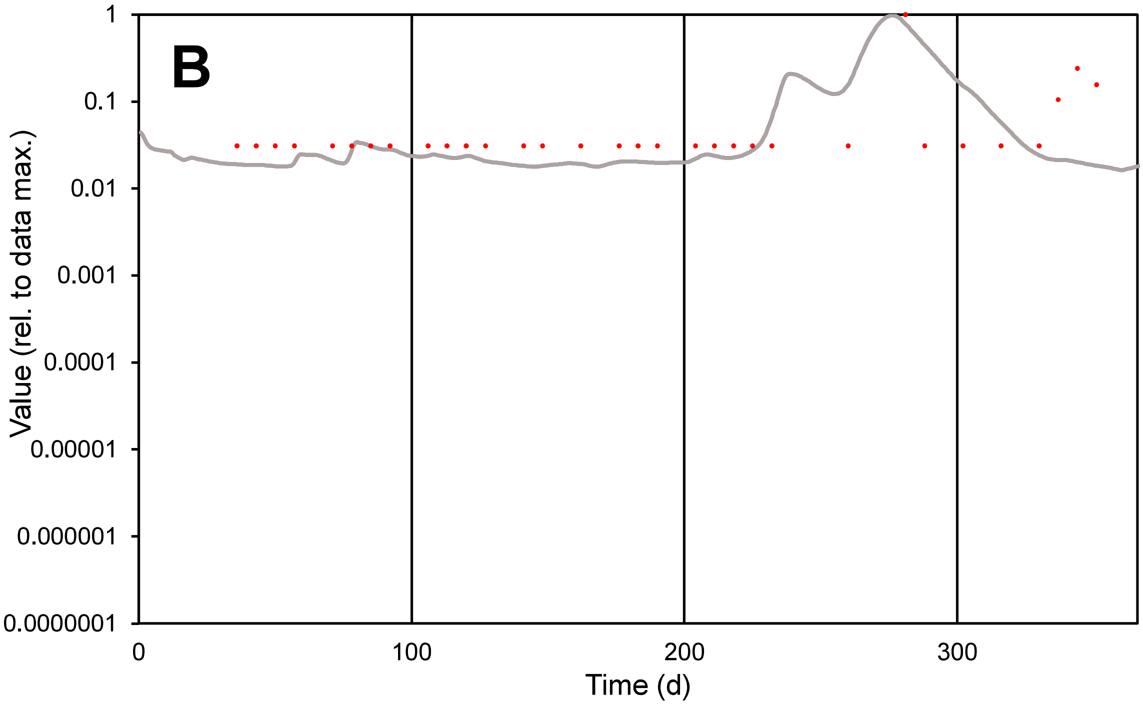

C1

Variables	
TTE	Provide jtd or Dst
CalibOnly?	
2	1 to reproduce of
oNormalize?	
2	1=no, 2=yes
ofxn calc*	
1.20965	
ofxn mod	
1.34693	
sRun	
RunA1	
zout	
1	
Ave	
Data	
9E-06	
Model	
7.8E-06	
data/vMax	
0.00012	
ofxng	
321.551	
Min d/m	
1.72761	
ave/min d	
2.3923	
ave/min m	
3.60523	

324_Calib_S_A

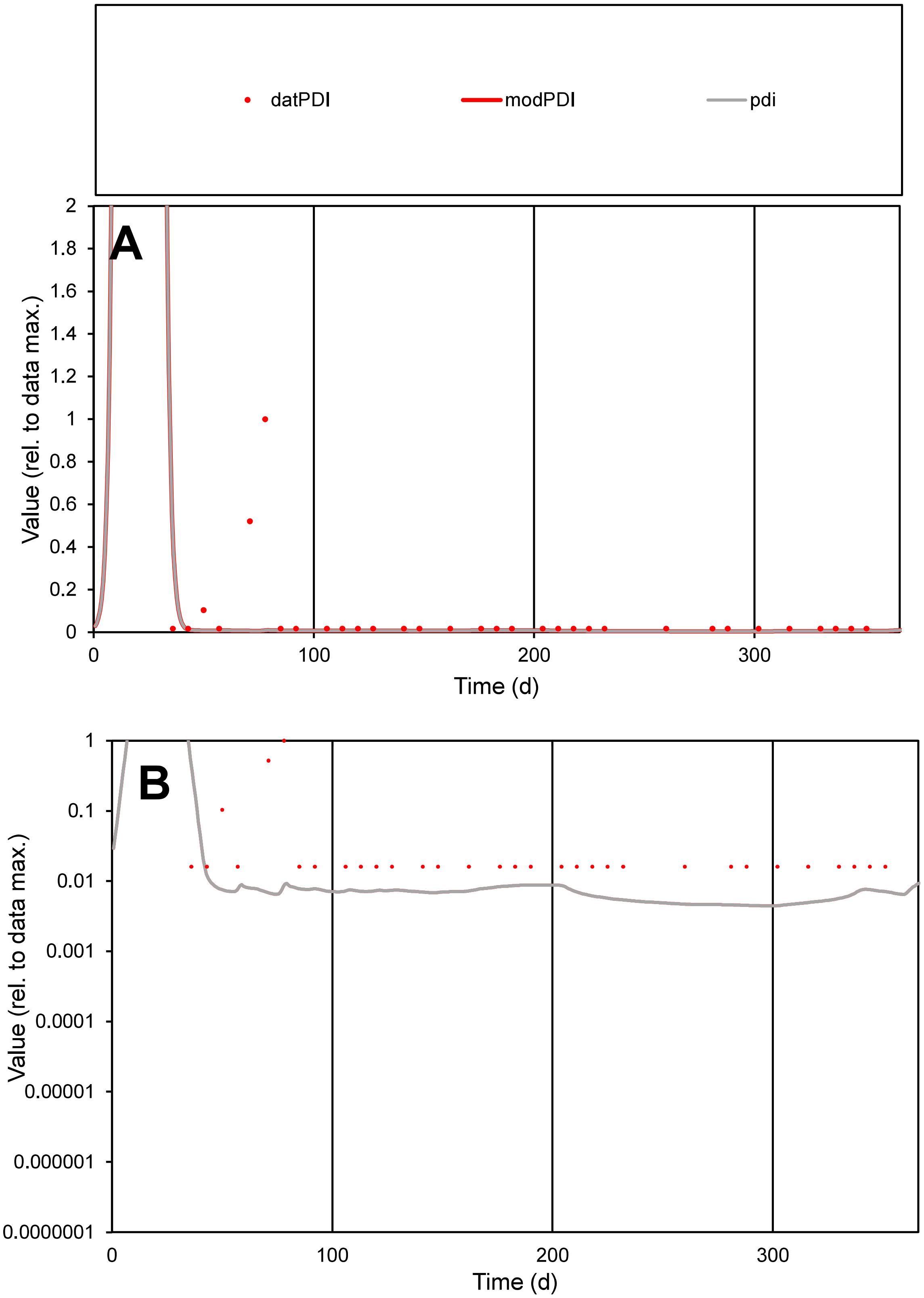

C1

Variables
PDI Provide jtd or Dst
CalibOnly?
2 1 to reproduce of
oNormalize?
2 1=no, 2=yes

ofxn calc*
3.04485
ofxn mod
3.05582

sRun
RunA1
zout
1

Ave
Data
1.8E-05
Model
2.8E-06

data/vMax
0.00027

ofxng
321.551

Min d/m
3.5798

ave/min d
4.06551
ave/min m
2.25662

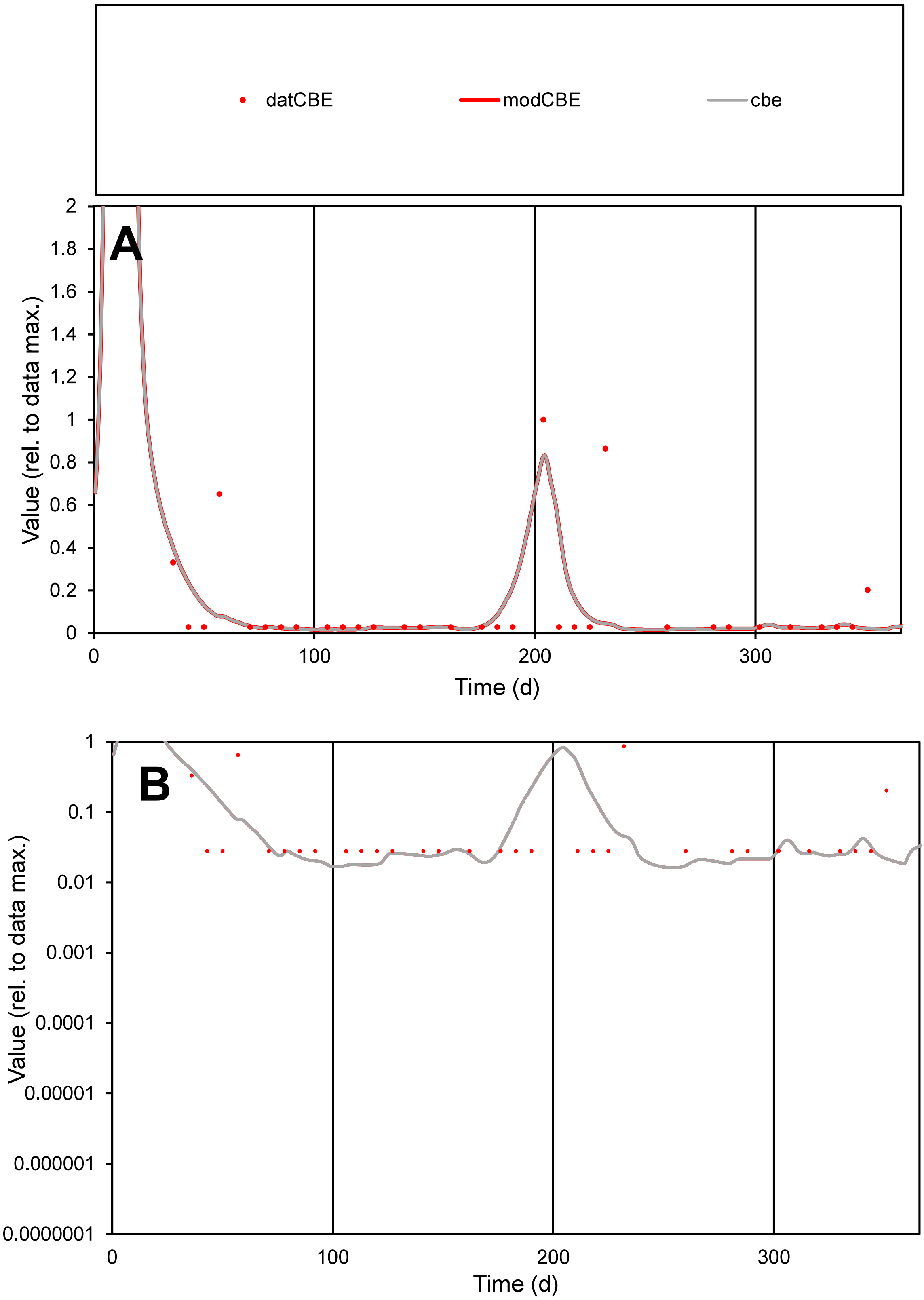

C1

Variables

CBE Provide jtd or Dst

CalibOnly?

2 1 to reproduce of

oNormalize?

2 1=no, 2=yes

ofxn calc*

1.62501

ofxn mod

1.66294

sRun

RunA1

zout

1

Ave

Data

7.1E-06

Model

5.3E-06

data/vMax

6E-05

ofxng

321.551

Min d/m

1.58254

ave/min d

4.25689

ave/min m

5.03128

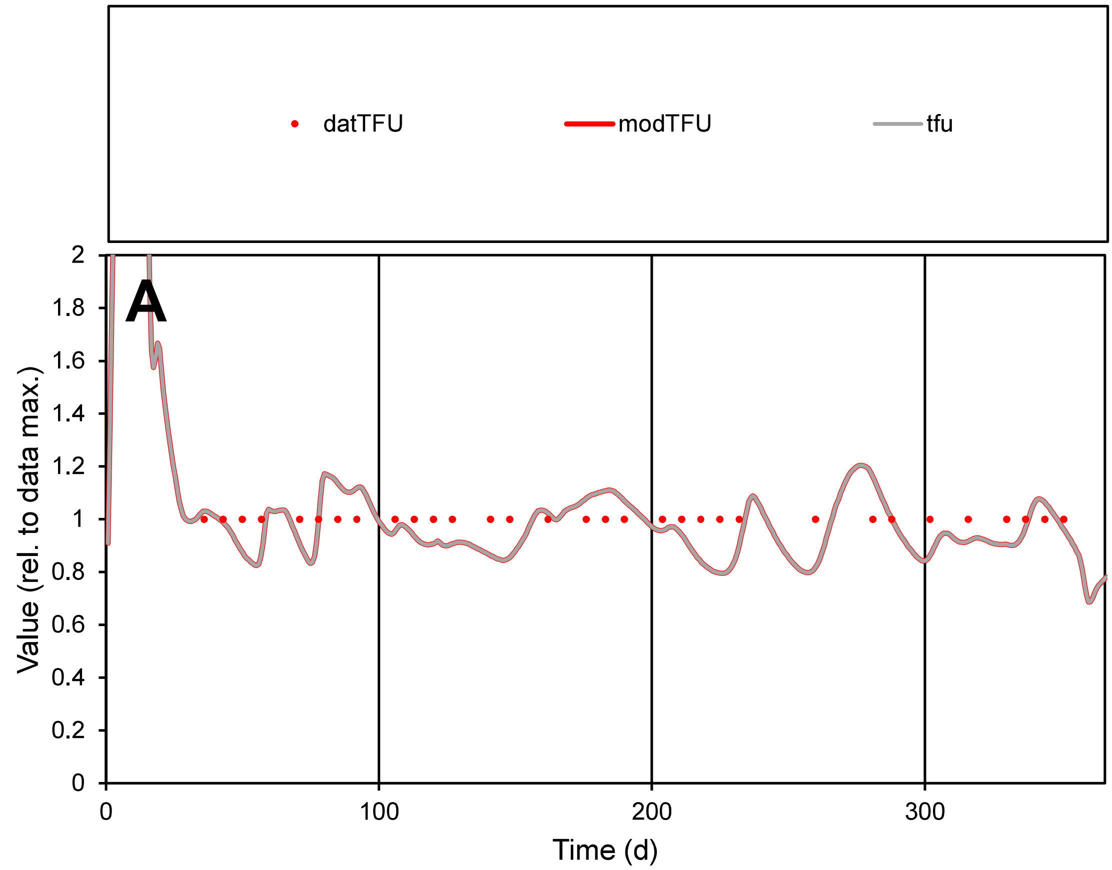

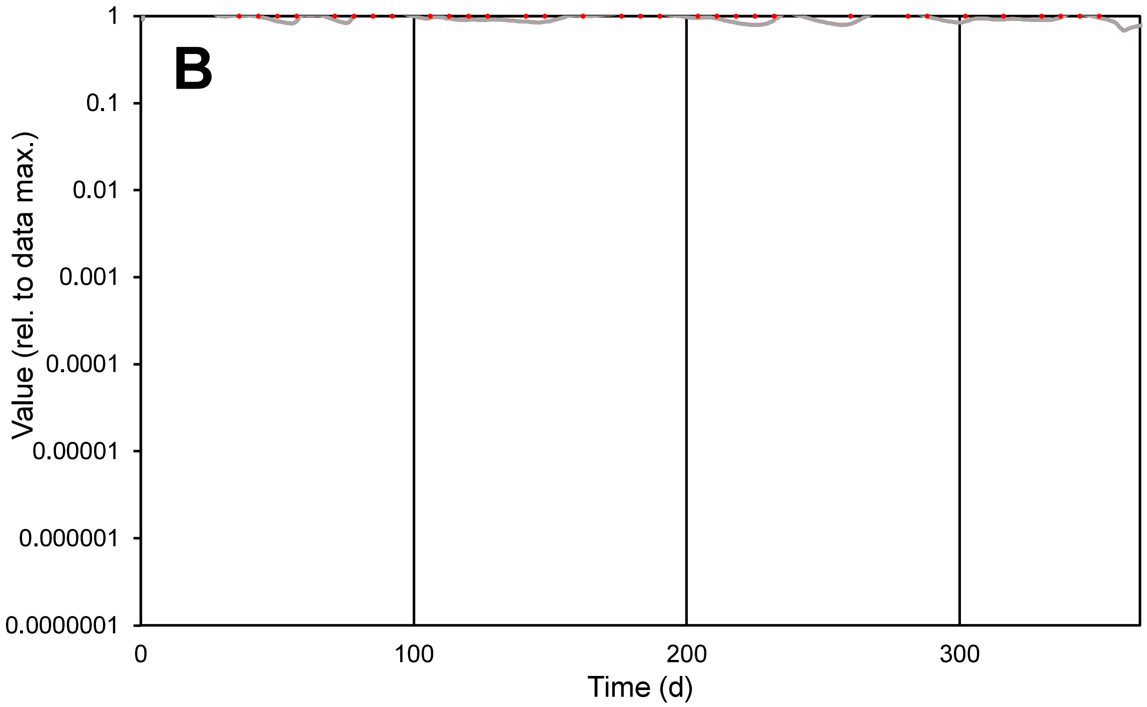

C1

Variables

TFU Provide jtd or Dst

CalibOnly?

2 1 to reproduce of

oNormalize?

2 1=no, 2=yes

ofxn calc*

0.10174

ofxn mod

0.13933

sRun

RunA1

zout

1

Ave

Data

1.3E-05

Model

1.2E-05

data/vMax

1.3E-05

ofxng

321.551

Min d/m

1.2574

ave/min d

1

ave/min m

1.21521

324_Calib_S_A

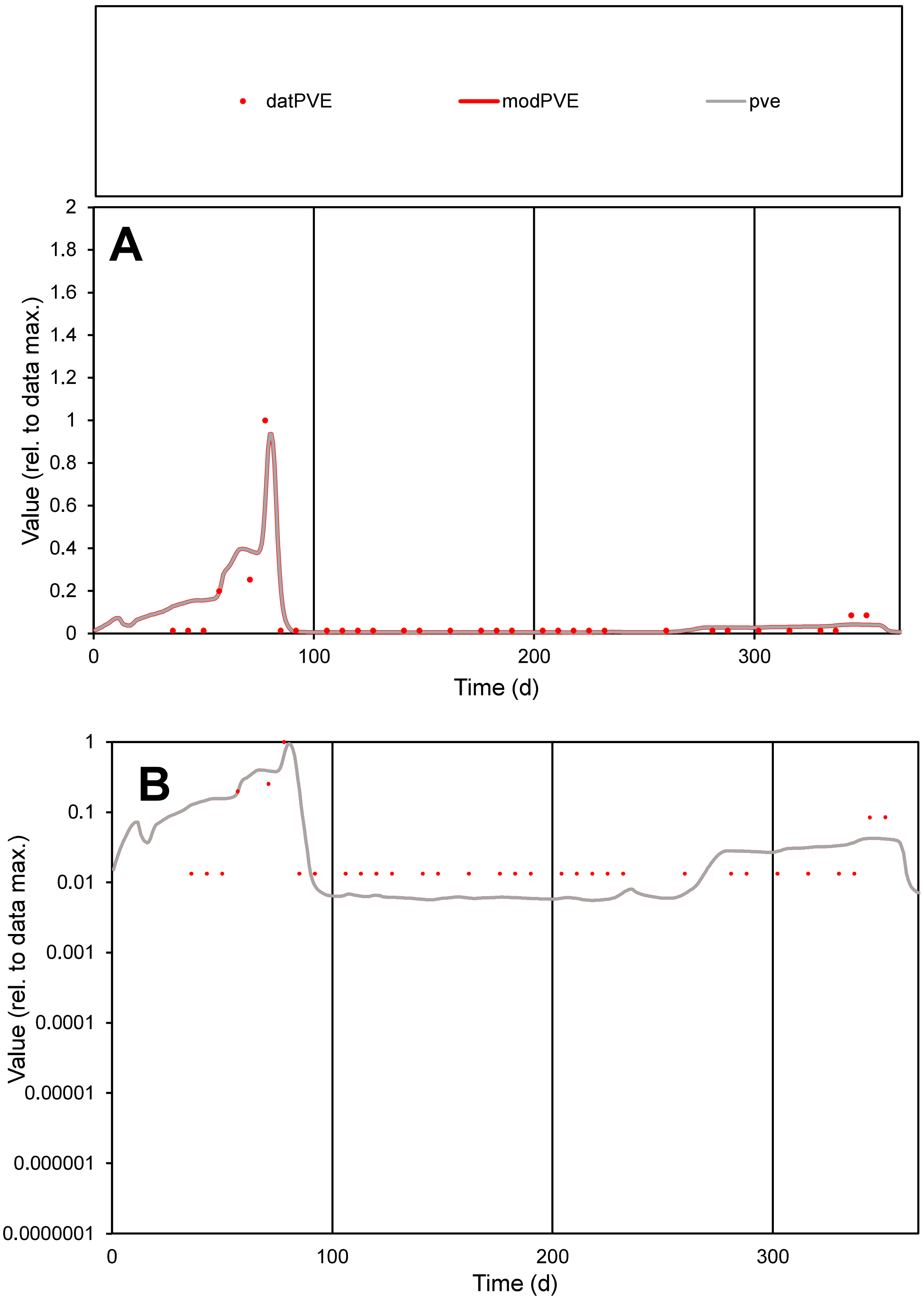

C1

Variables

PVE	Provide jtd or Dst
CalibOnly?	
2	1 to reproduce of
oNormalize?	
2	1=no, 2=yes

ofxn calc*
0.88725
ofxn mod
0.94148

sRun
RunA1
zout
1

Ave
Data
9.7E-06
Model
1.1E-05

data/vMax
0.00016

ofxng
321.551

Min d/m
2.38052

ave/min d
4.65051
ave/min m
13.0562

324_Calib_S_A

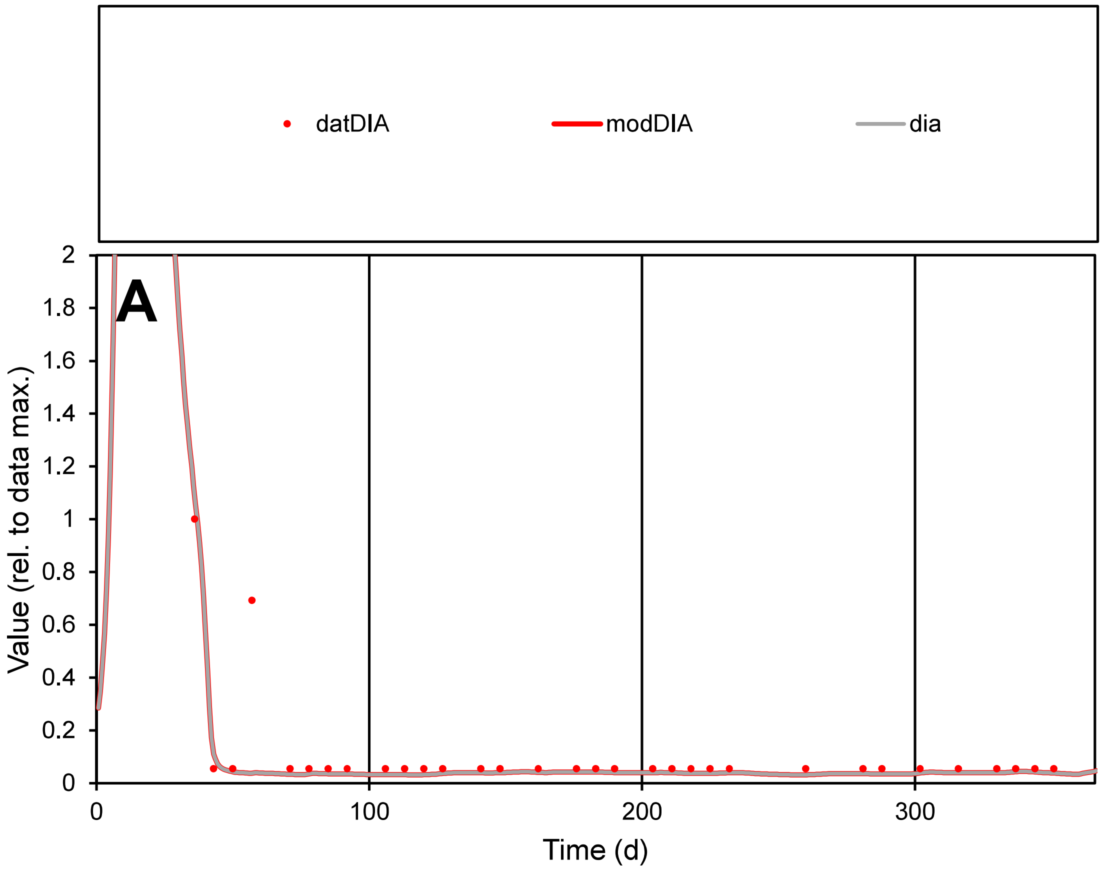

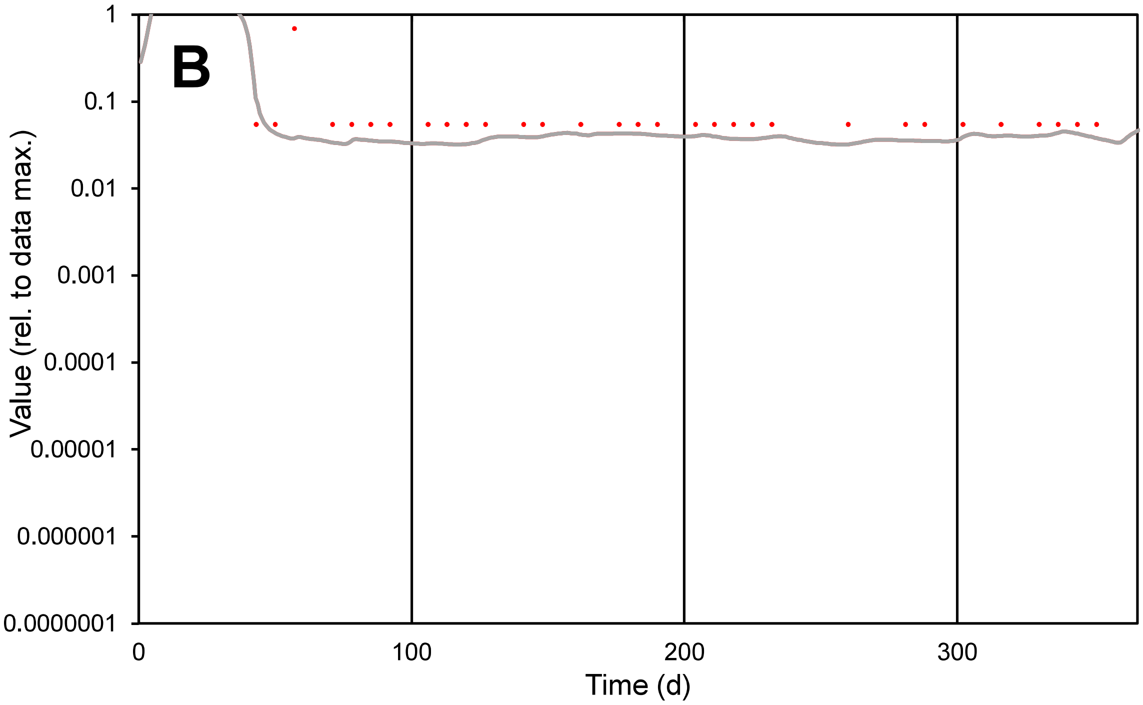

C1

Variables

DIA Provide jtd or Dst

CalibOnly?

2 1 to reproduce of

oNormalize?

2 1=no, 2=yes

ofxn calc*

1.11884

ofxn mod

1.12821

sRun

RunA1

zout

1

Ave

Data

1.2E-05

Model

7.8E-06

data/vMax

0.00011

ofxng

321.551

Min d/m

1.69997

ave/min d

1.90215

ave/min m

2.13966

324_Calib_S_A

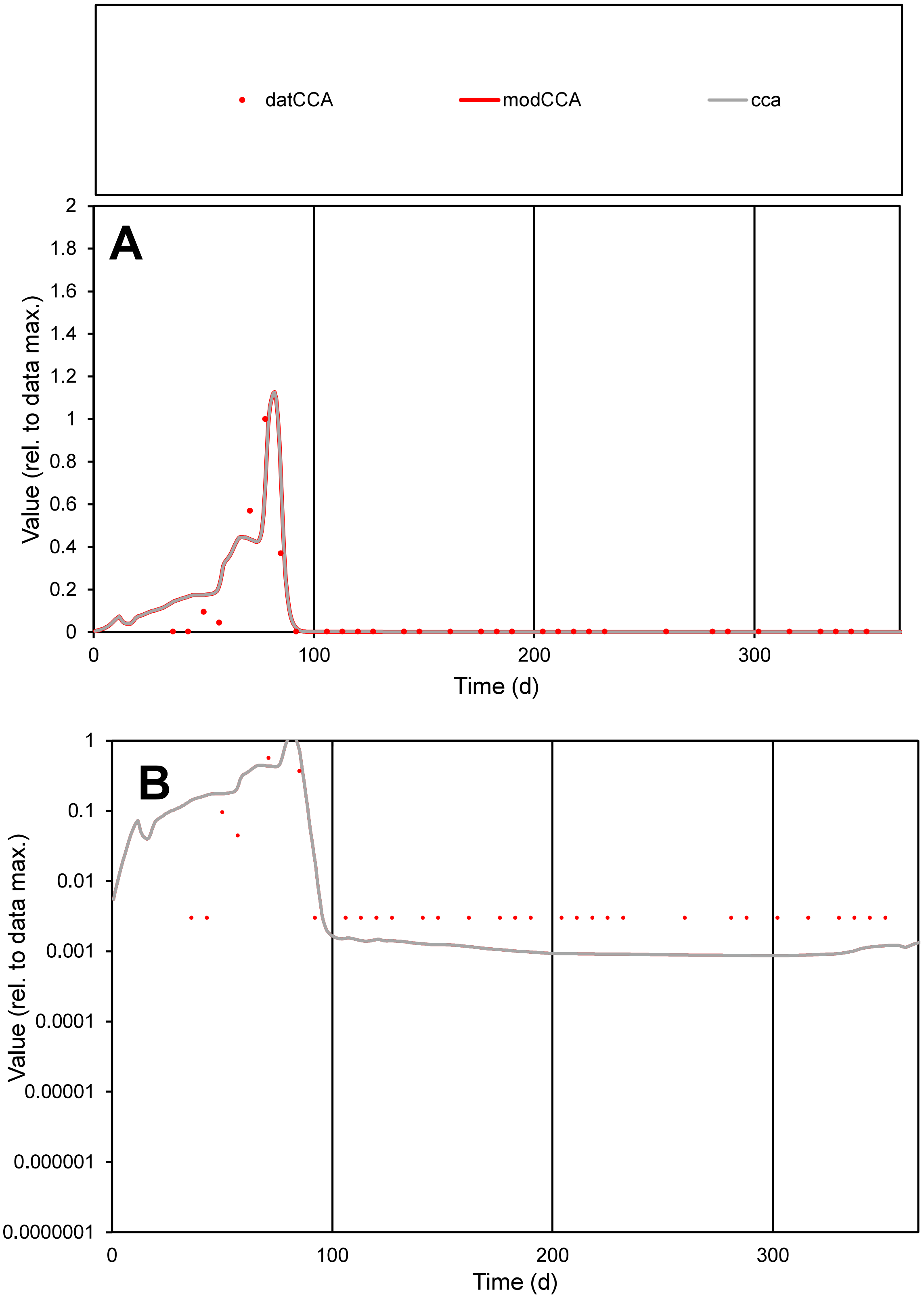

C1

Variables

CCA Provide jtd or Dst

CalibOnly?

2 1 to reproduce of

oNormalize?

2 1=no, 2=yes

ofxn calc*

0.81691

ofxn mod

0.89189

sRun

RunA1

zout

1

Ave

Data

4.1E-05

Model

4.7E-05

data/vMax

0.00061

ofxng

321.551

Min d/m

3.47067

ave/min d

22.482

ave/min m

89.8714

324_Calib_S_A

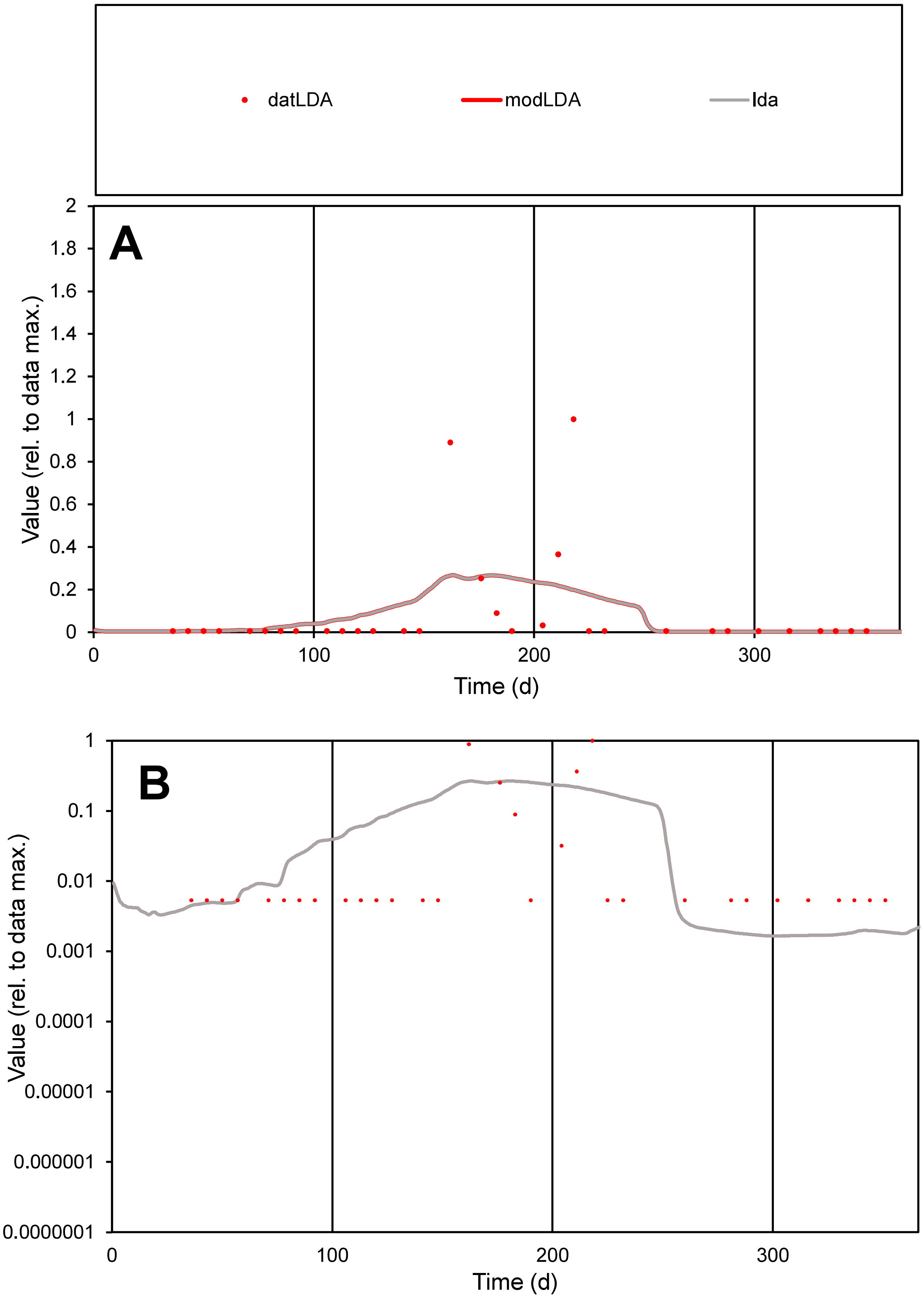

C1

Variables
LDA Provide jtd or Dst
CalibOnly?
2 1 to reproduce of
oNormalize?
2 1=no, 2=yes

ofxn calc*
2.32637
ofxn mod
2.34841

sRun
RunA1
zout
1

Ave
Data
0.00011
Model
0.00011

data/vMax
0.0013

ofxng
321.551

Min d/m
3.22041

ave/min d
16.2699
ave/min m
51.1145

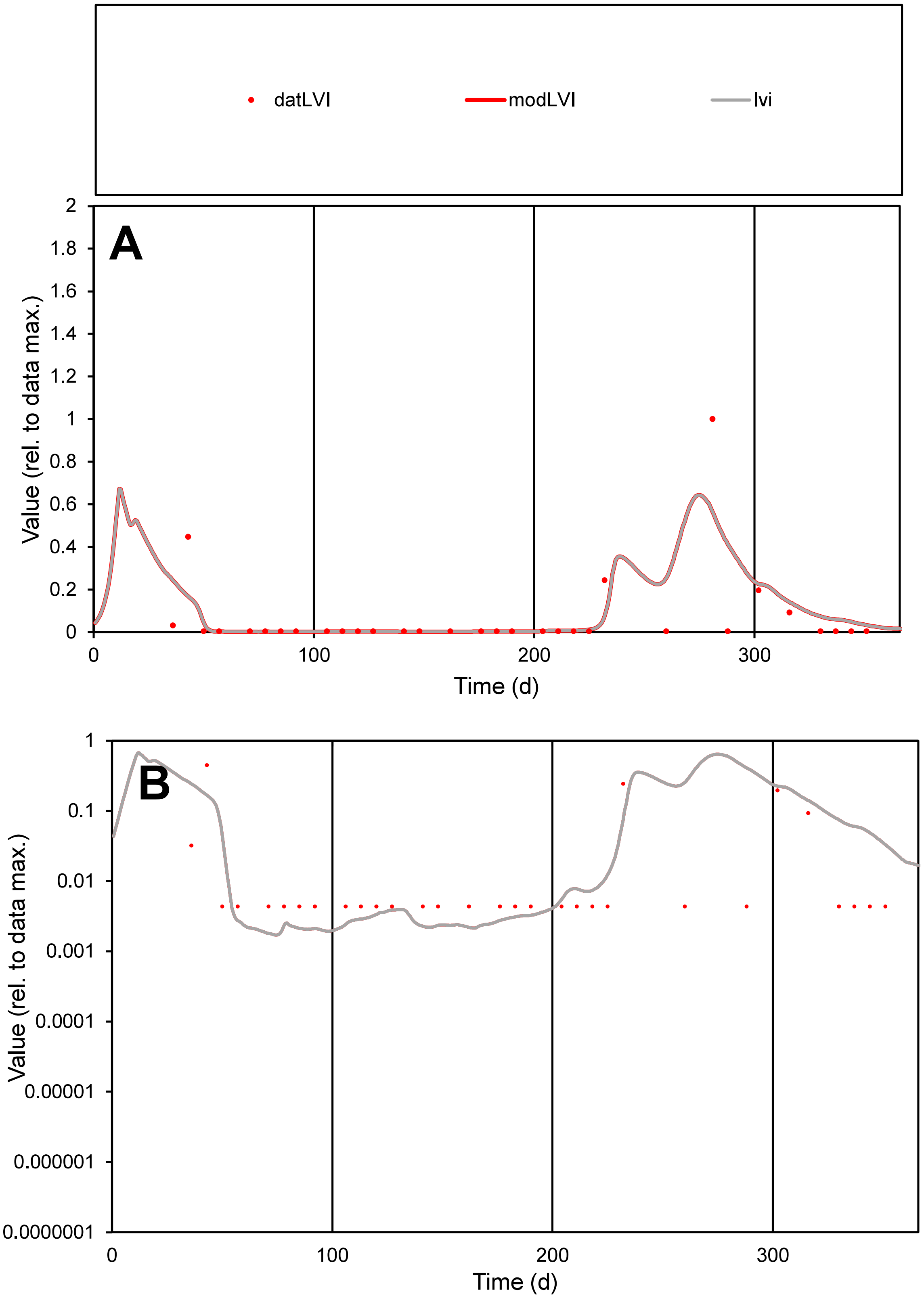

C1

Variables
LVI Provide jtd or Dst
CalibOnly?
2 1 to reproduce of
oNormalize?
2 1=no, 2=yes

ofxn calc*
1.93438
ofxn mod
2.00786

sRun
RunA1
zout
1

Ave
Data
2.2E-05
Model
2.4E-05

data/vMax
0.00033

ofxng
321.551

Min d/m
2.46021

ave/min d
15.2416
ave/min m
41.8323

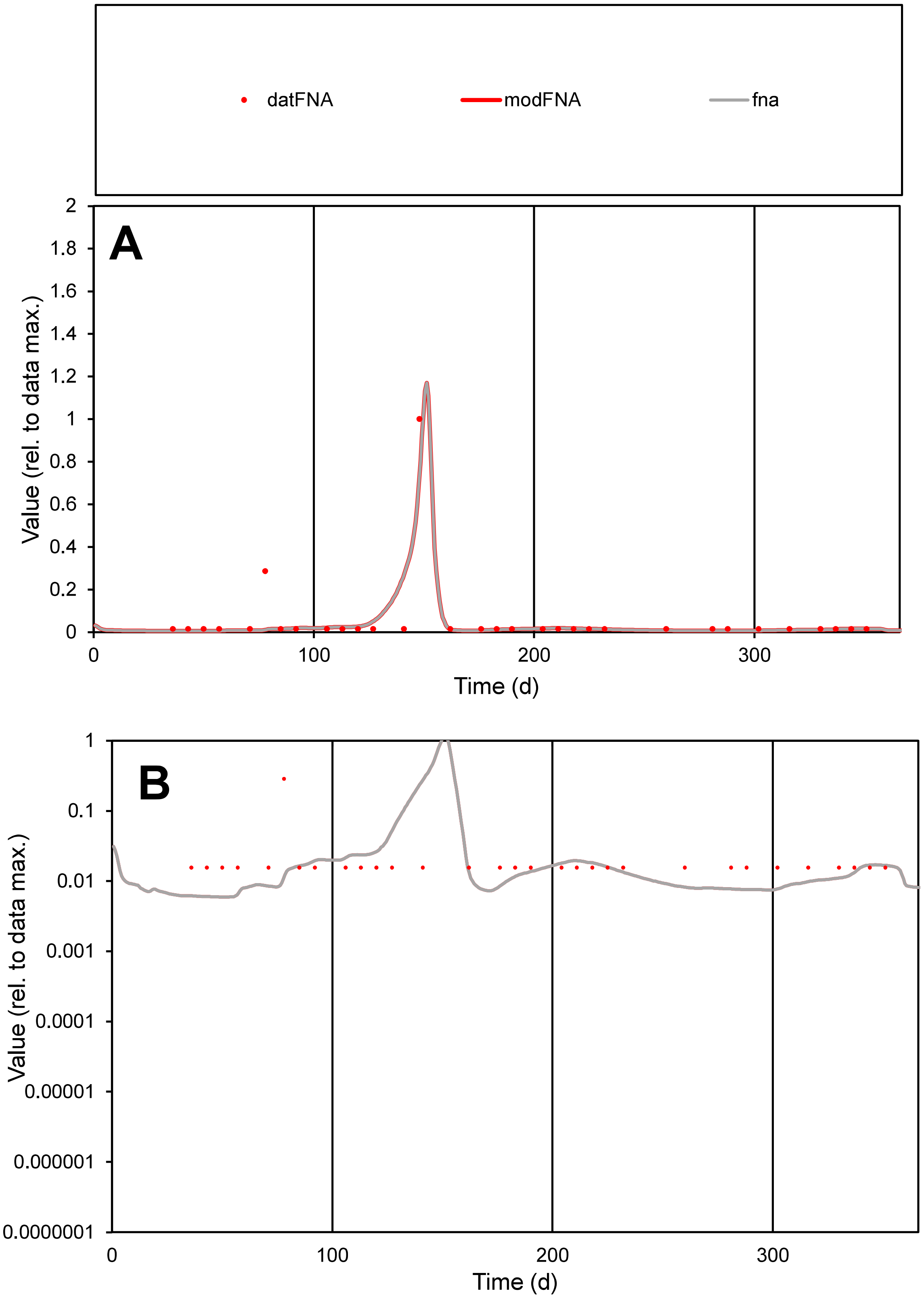

C1

Variables
FNA Provide jtd or Dst
CalibOnly?
2 1 to reproduce of
oNormalize?
2 1=no, 2=yes

ofxn calc*
1.16804
ofxn mod
1.24352

sRun
RunA1
zout
1

Ave
Data
1.1E-05
Model
9.9E-06

data/vMax
0.0002

ofxng
321.551

Min d/m
2.64461

ave/min d
3.51307
ave/min m
8.42364

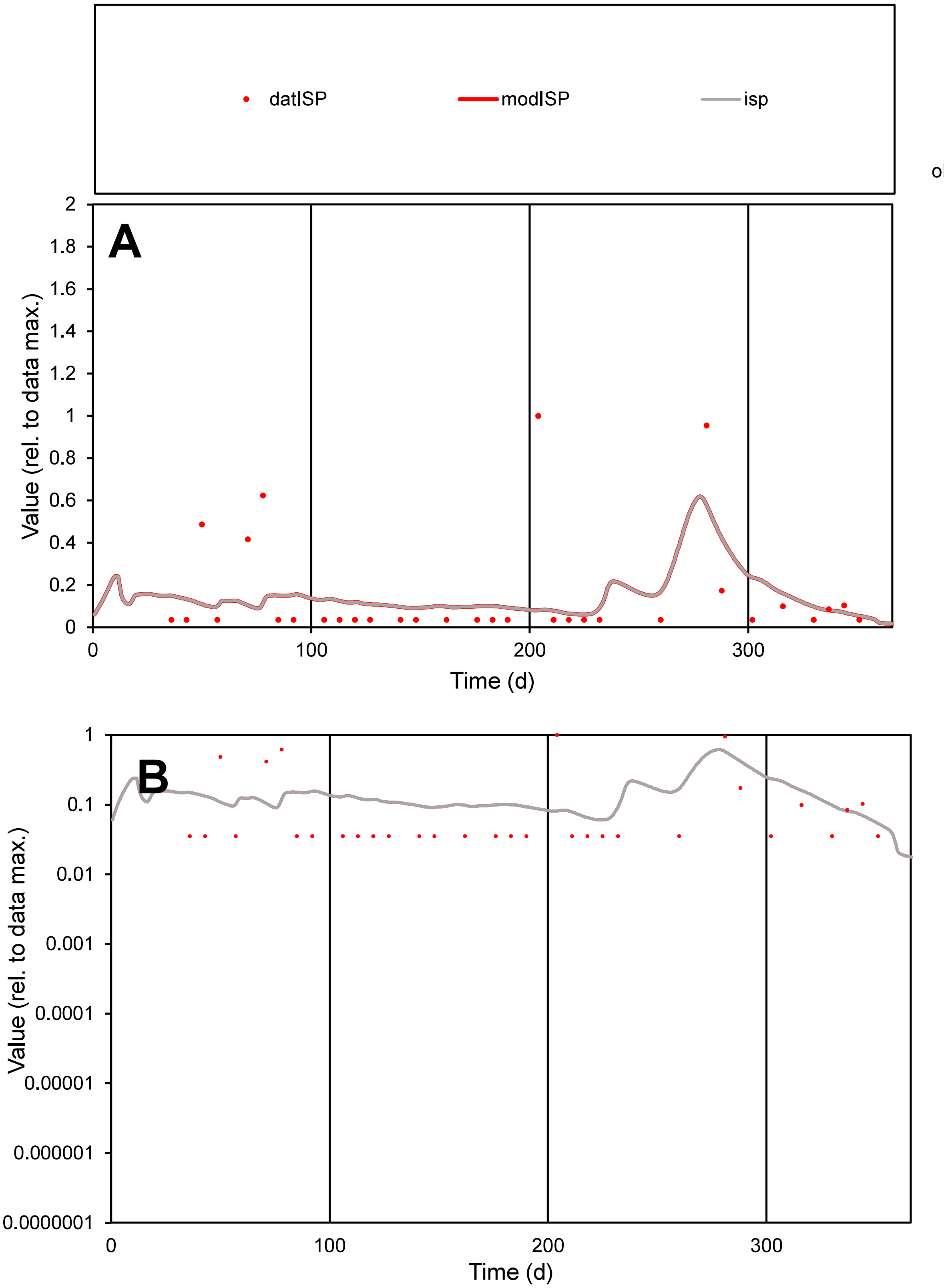

C1

Variables
ISP Provide jtd or Dst
CalibOnly?
2 1 to reproduce of
oNormalize?
2 1=no, 2=yes

ofxn calc*
1.54635
ofxn mod
1.56045

sRun
RunA1
zout
1

Ave
Data
2.1E-05
Model
1.9E-05

data/vMax
0.00014

ofxng
321.551

Min d/m
0.6786

ave/min d
4.21023
ave/min m
2.55958

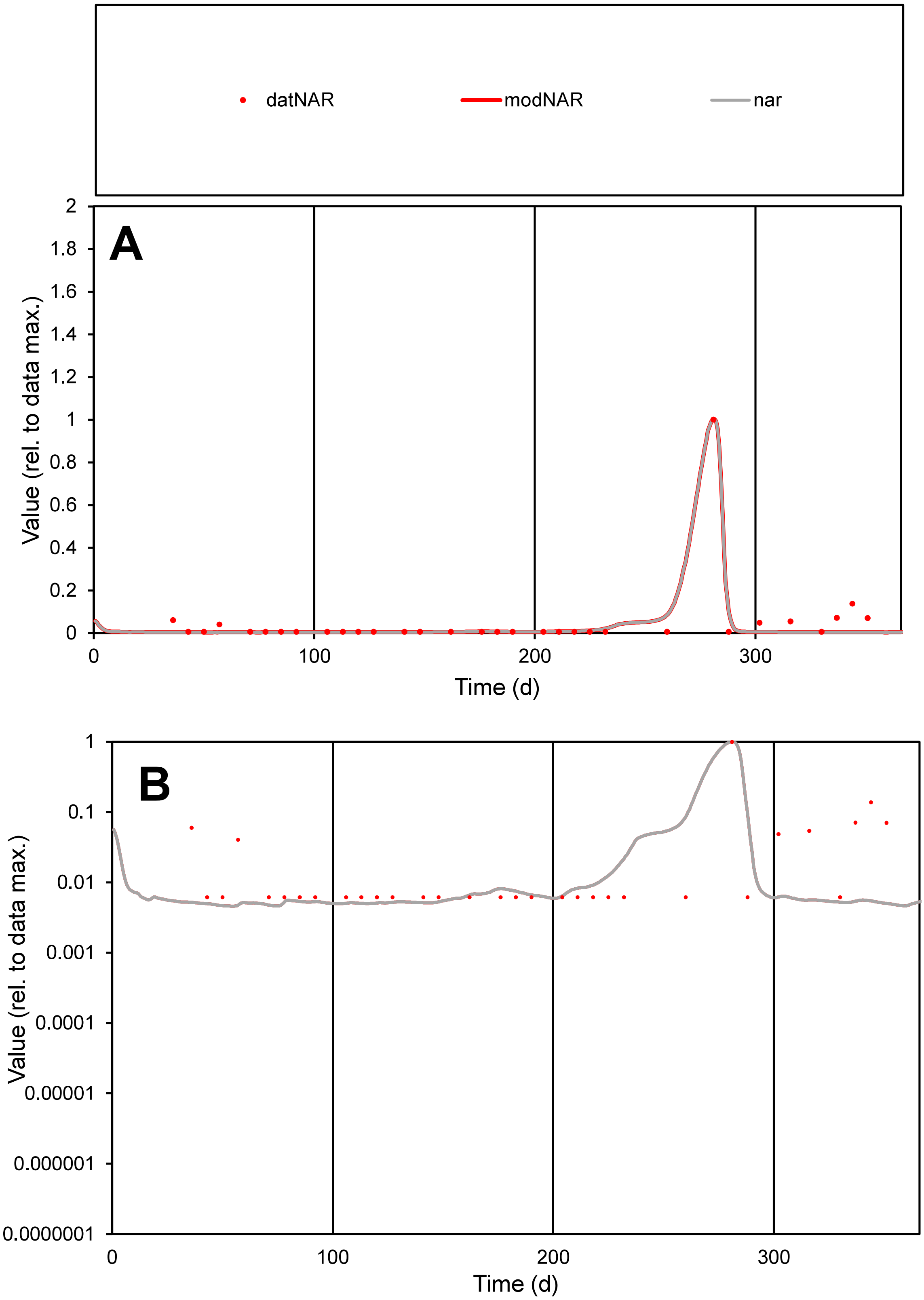

C1

Variables
NAR Provide jtd or Dst
CalibOnly?
2 1 to reproduce of
oNormalize?
2 1=no, 2=yes

ofxn calc*
0.68982
ofxn mod
0.75715

sRun
RunA1
zout
1

Ave
Data
2.3E-05
Model
1.8E-05

data/vMax
0.00046

ofxng
321.551

Min d/m
1.33318
ave/min d
8.24347
ave/min m
8.62367

324_Calib_S_A

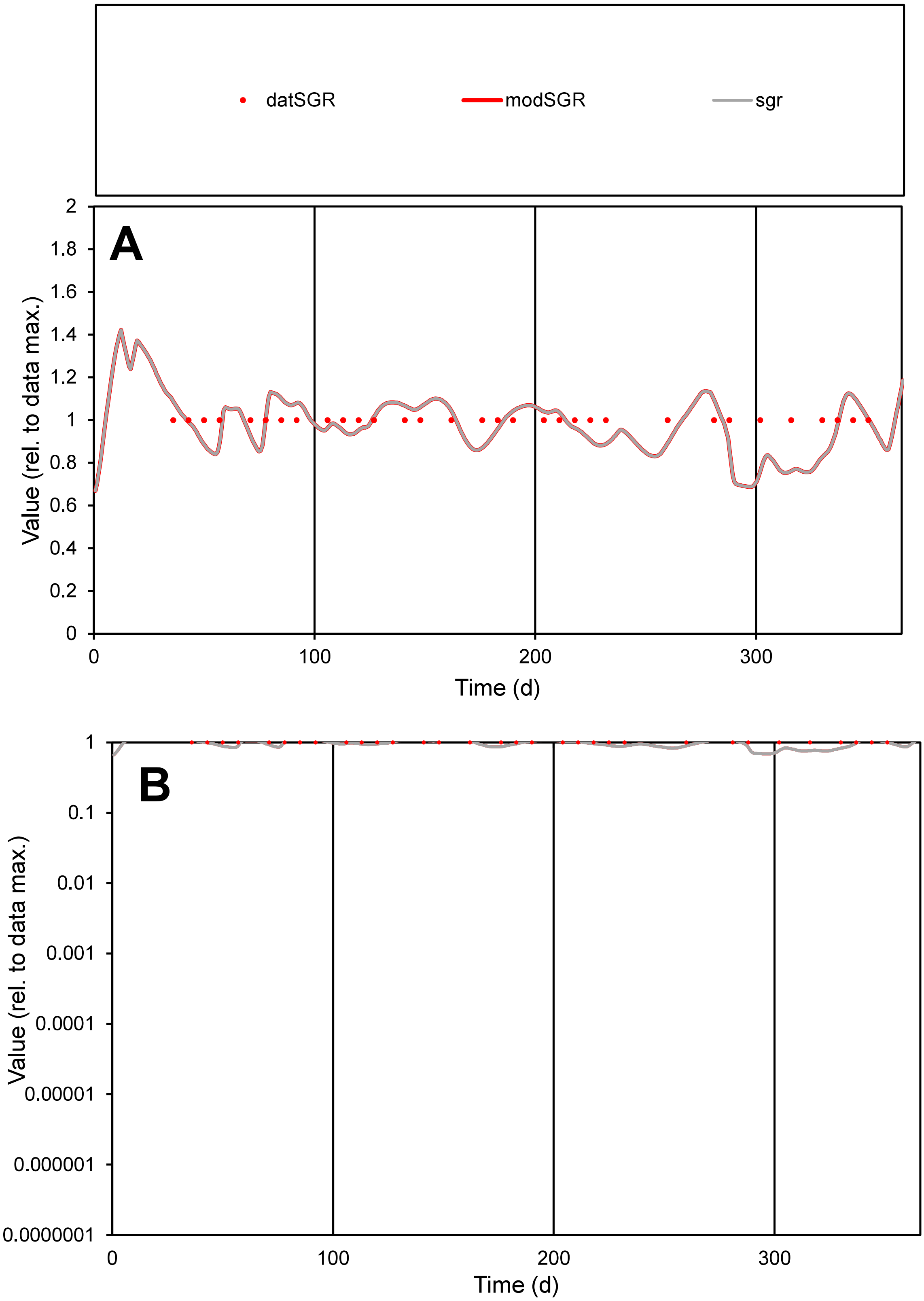

C1

Variables
SGR Provide jtd or Dst
CalibOnly?
2 1 to reproduce of
oNormalize?
2 1=no, 2=yes

ofxn calc*
0.09217
ofxn mod
0.13797

sRun
RunA1
zout
1

Ave
Data
4.5E-06
Model
4.4E-06

data/vMax
4.5E-06

ofxng
321.551

Min d/m
1.30166

ave/min d
1
ave/min m
1.2702

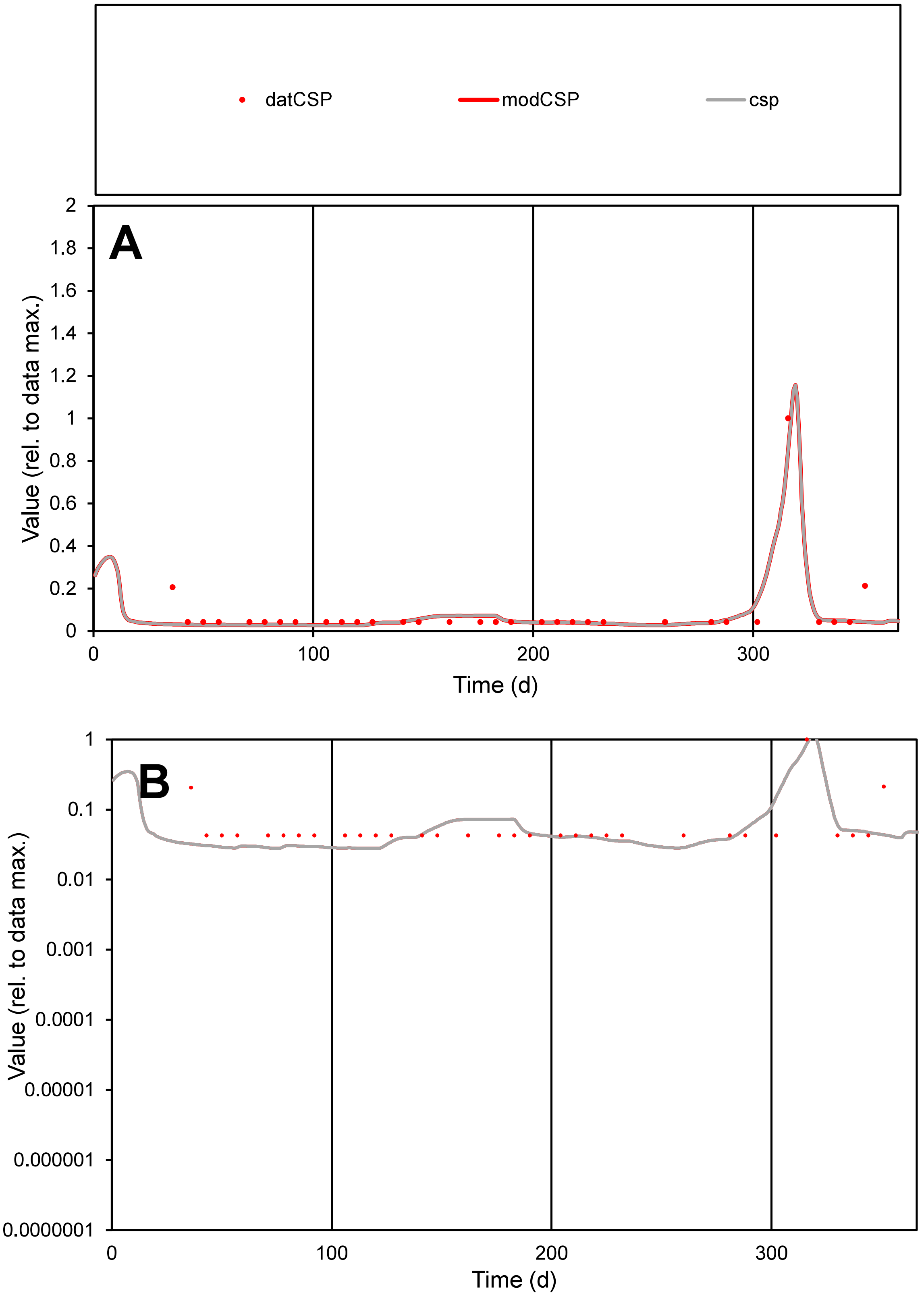

C1

Variables
CSP Provide jtd or Dst
CalibOnly?
2 1 to reproduce of
oNormalize?
2 1=no, 2=yes

ofxn calc*
0.56932
ofxn mod
0.60432

sRun
RunA1
zout
1

Ave
Data
4.6E-06
Model
4.1E-06

data/vMax
5.6E-05

ofxng
321.551

Min d/m
1.53013

ave/min d
1.94416
ave/min m
2.62316

324_Calib_S_A

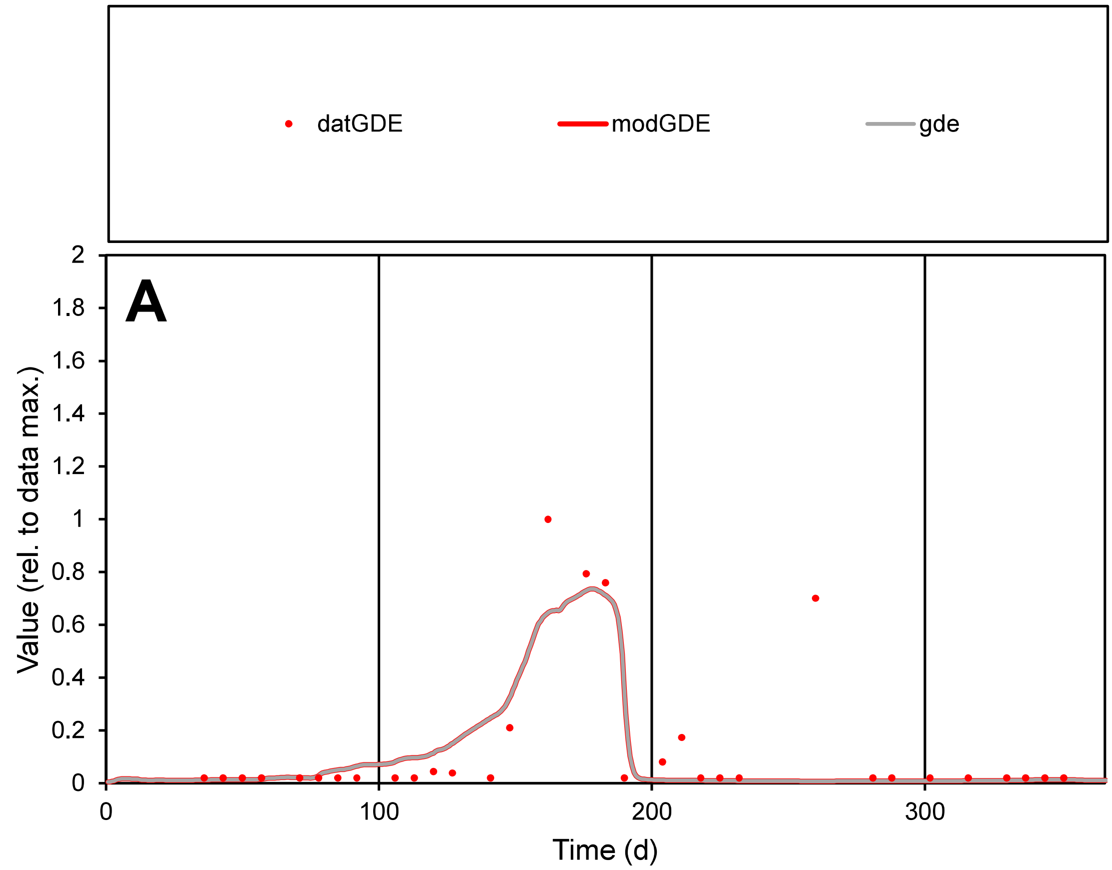

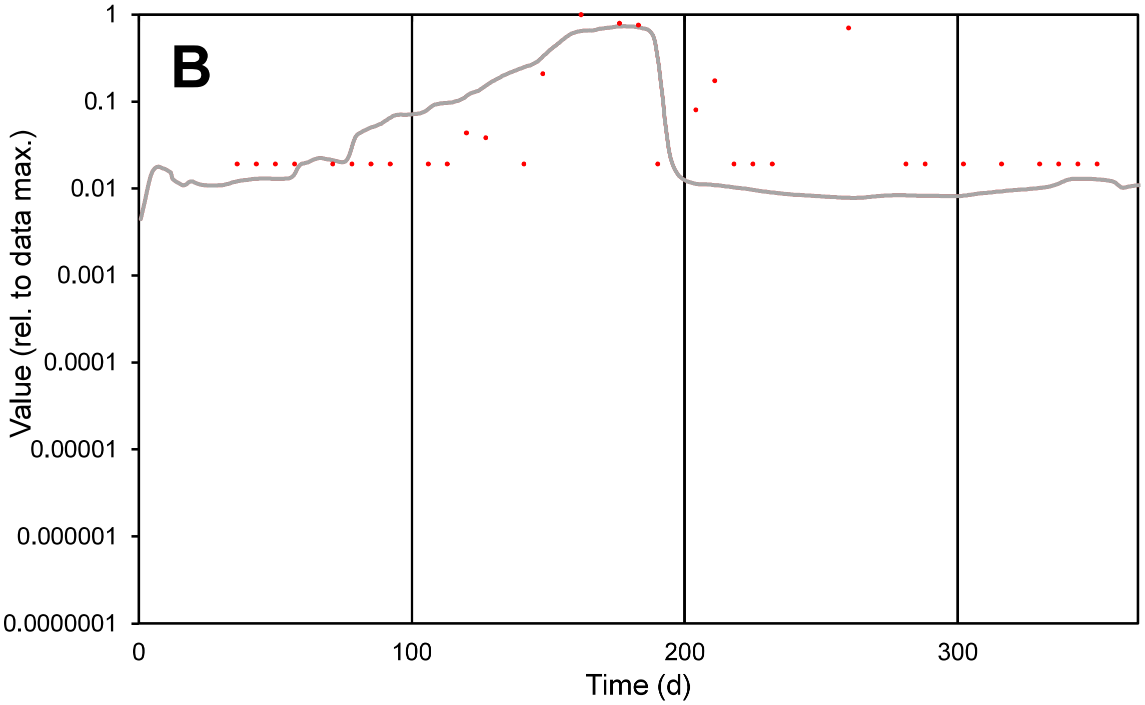

C1

Variables
GDE Provide jtd or Dst
CalibOnly?
2 1 to reproduce of
oNormalize?
2 1=no, 2=yes

ofxn calc*
1.14895
ofxn mod
1.17258

sRun
RunA1
zout
1

Ave
Data
0.00019
Model
0.00016

data/vMax
0.00143

ofxng
321.551

Min d/m
2.45138

ave/min d
6.8984
ave/min m
14.1142

324_Calib_S_A

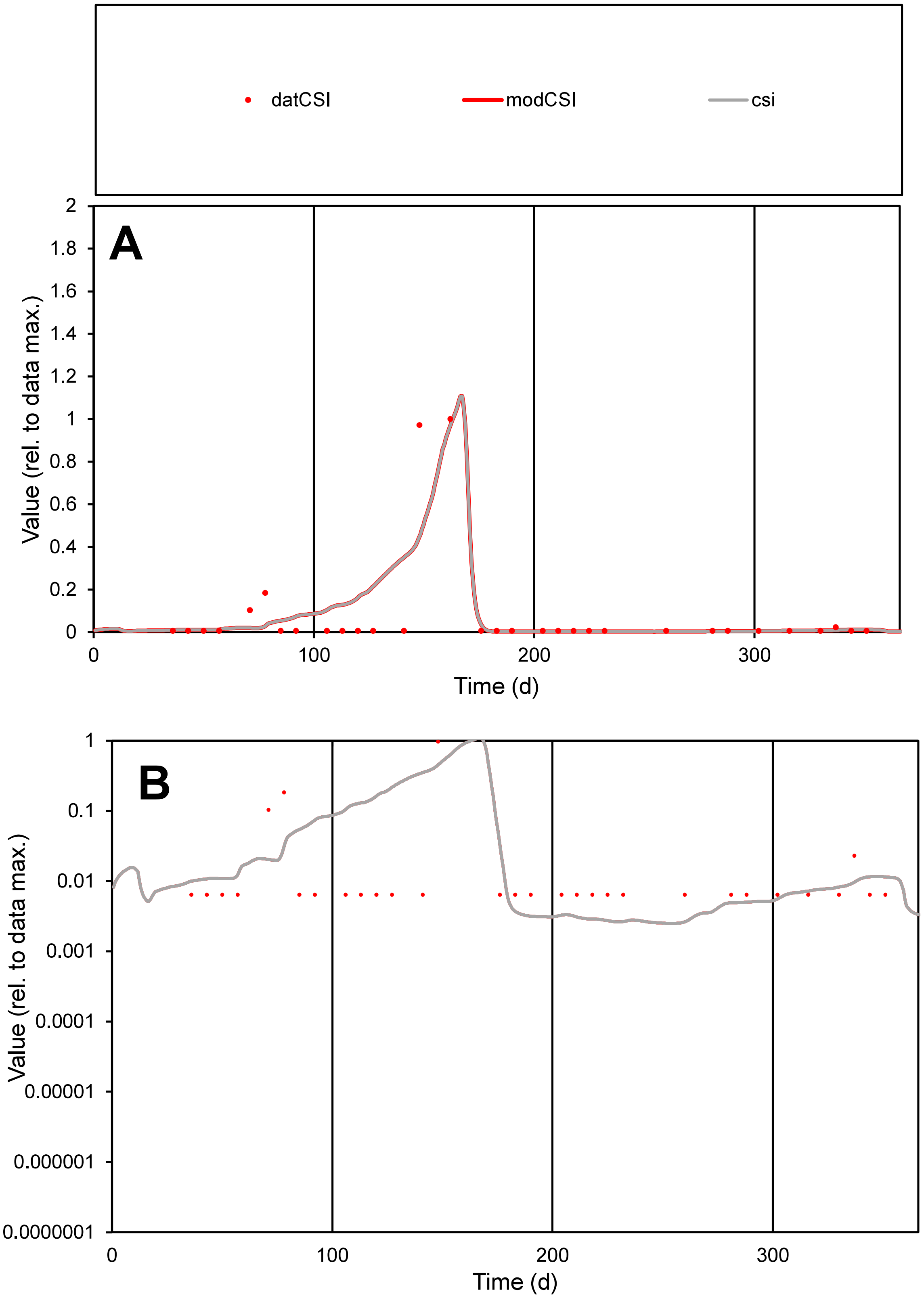

C1

Variables

CSI Provide jtd or Dst

CalibOnly?

2 1 to reproduce of

oNormalize?

2 1=no, 2=yes

ofxn calc*

1.59901

ofxn mod

1.6453

sRun

RunA1

zout

1

Ave

Data

2.8E-05

Model

3.1E-05

data/vMax

0.00036

ofxng

321.551

Min d/m

2.38046

ave/min d

11.9807

ave/min m

32.2635

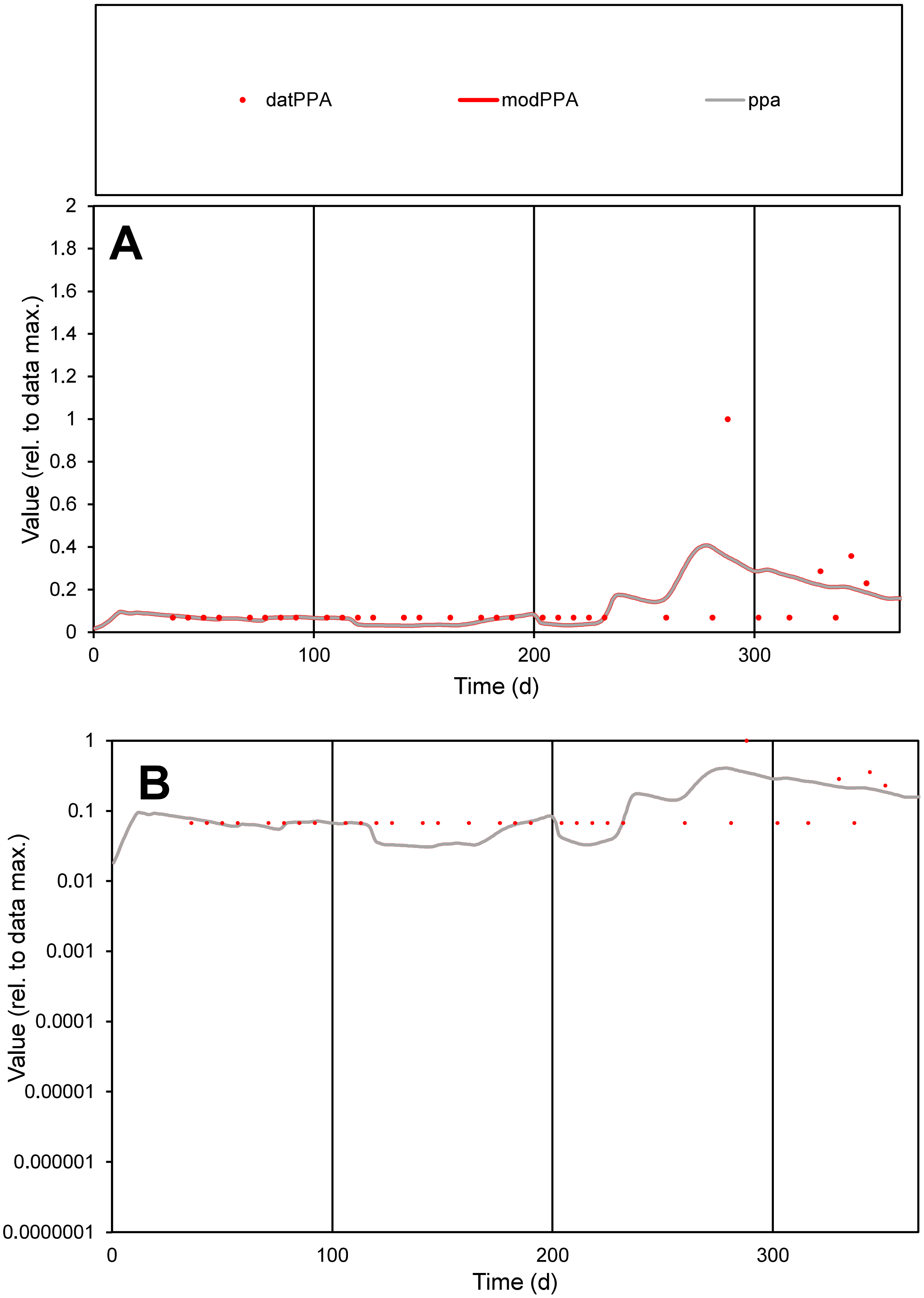

C1

Variables
PPA Provide jtd or Dst
CalibOnly?
2 1 to reproduce of
oNormalize?
2 1=no, 2=yes

ofxn calc*
1.2489
ofxn mod
1.25728

sRun
RunA1
zout
1

Ave
Data
5.6E-06
Model
5.2E-06

data/vMax
4.8E-05

ofxng
321.551

Min d/m
2.19631

ave/min d
1.74029
ave/min m
3.57644

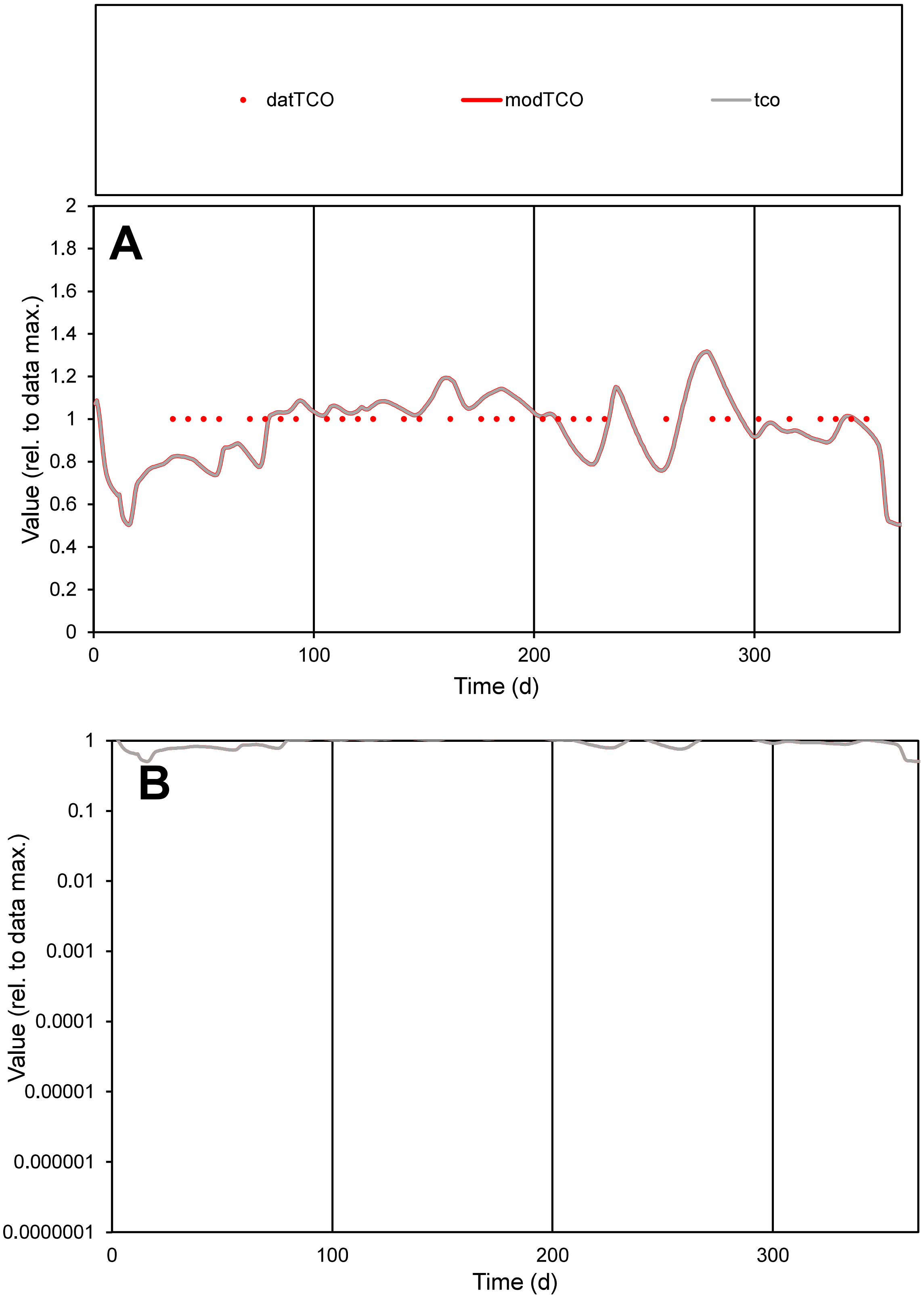

C1

Variables
TCO Provide jtd or Dst
CalibOnly?
2 1 to reproduce of
oNormalize?
2 1=no, 2=yes

ofxn calc*
0.12467
ofxn mod
0.15715

sRun
RunA1
zout
1

Ave
Data
8.6E-06
Model
8.5E-06

data/vMax
8.6E-06

ofxng
321.551

Min d/m
1.32376

ave/min d
1
ave/min m
1.29799

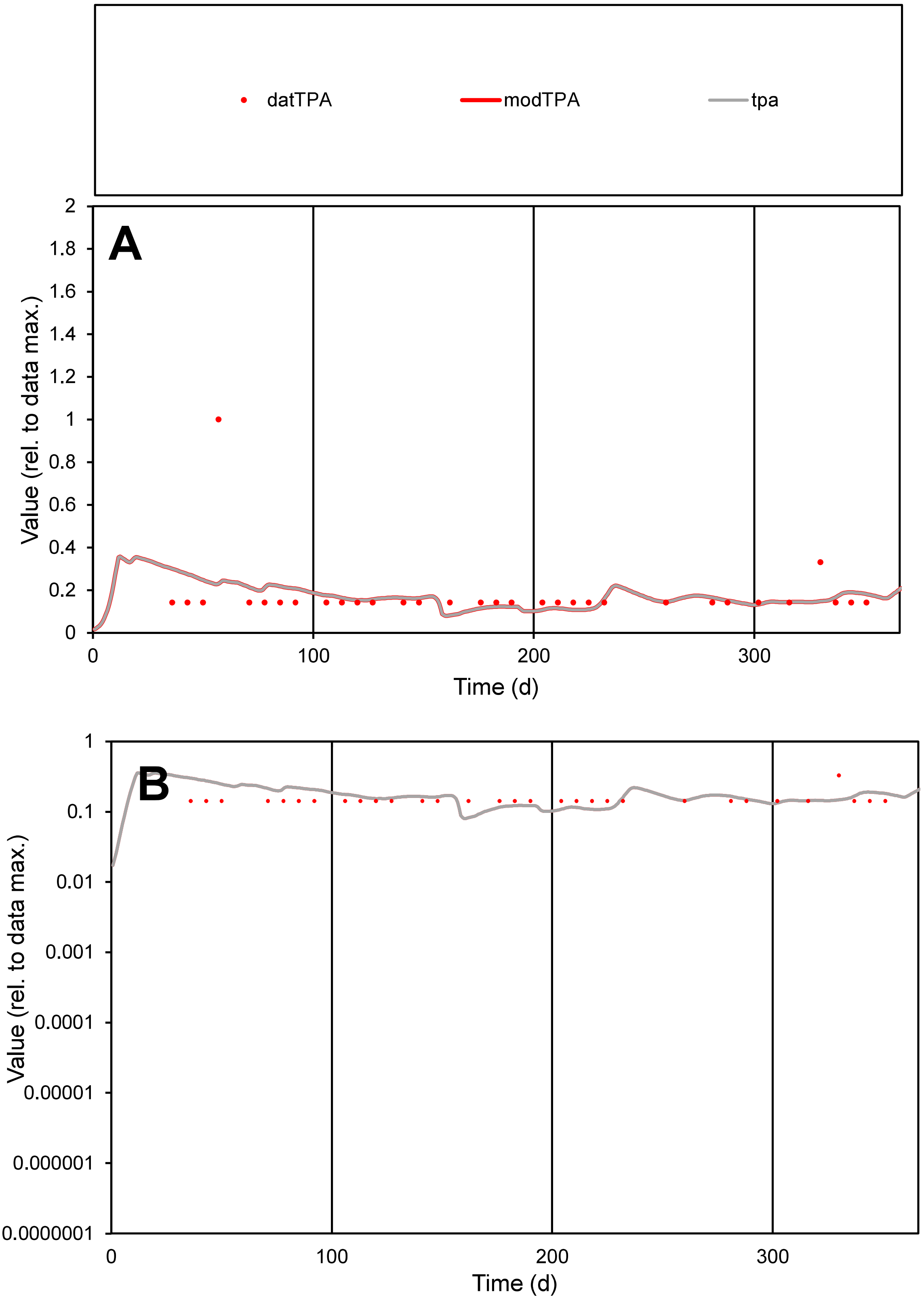

C1

Variables

TPA Provide jtd or Dst

CalibOnly?

2 1 to reproduce of

oNormalize?

2 1=no, 2=yes

ofxn calc*

0.84314

ofxn mod

0.85017

sRun

RunA1

zout

1

Ave

Data

5.8E-06

Model

5.6E-06

data/vMax

3.3E-05

ofxng

321.551

Min d/m

1.68534

ave/min d

1.22943

ave/min m

1.98561

324_Calib_S_A

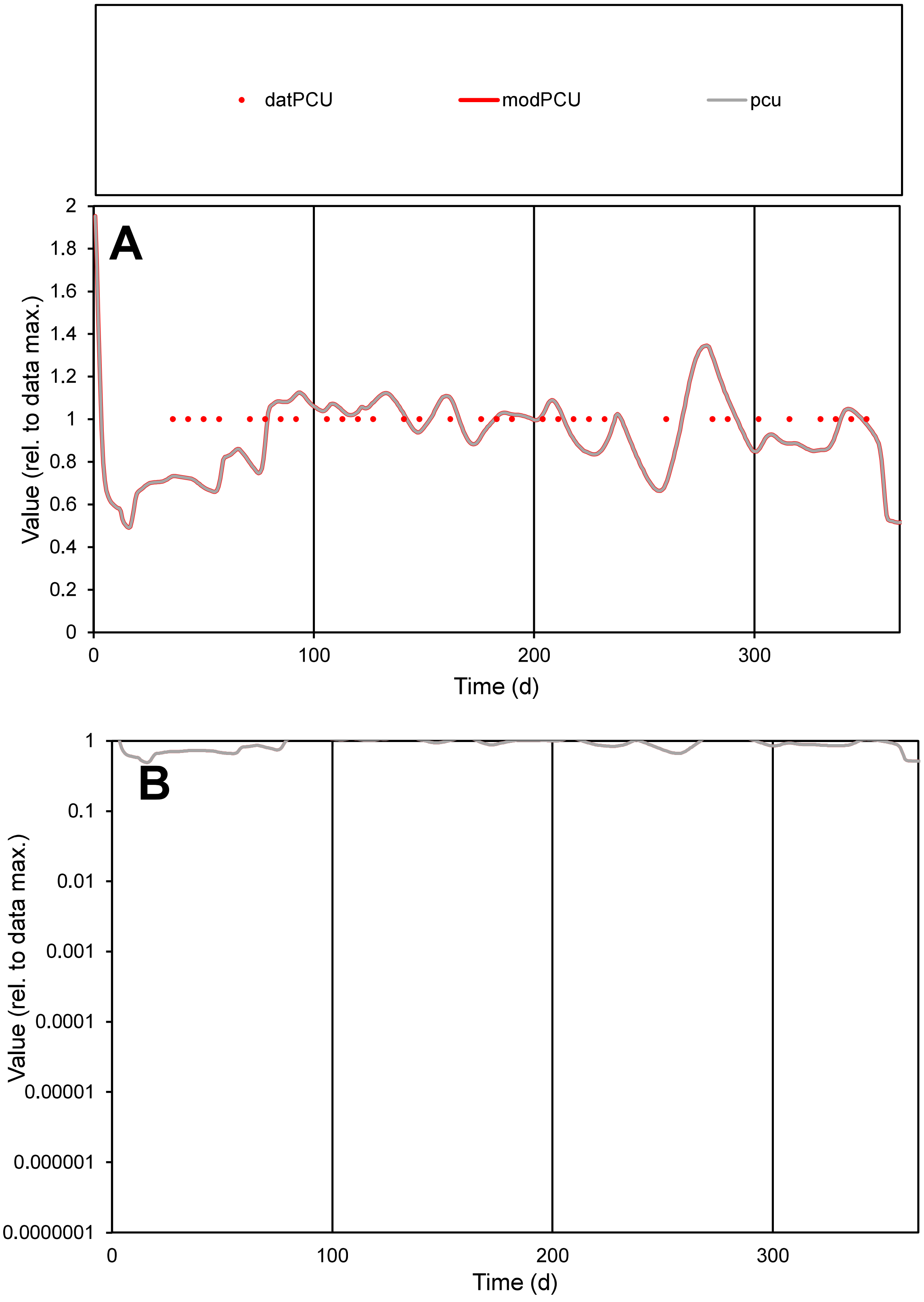

C1

Variables
PCU Provide jtd or Dst
CalibOnly?
2 1 to reproduce of
oNormalize?
2 1=no, 2=yes

ofxn calc*
0.14311
ofxn mod
0.17156

sRun
RunA1
zout
1

Ave
Data
6.2E-06
Model
5.9E-06

data/vMax
6.2E-06

ofxng
321.551

Min d/m
1.48966

ave/min d
1
ave/min m
1.41997

324_Calib_S_A

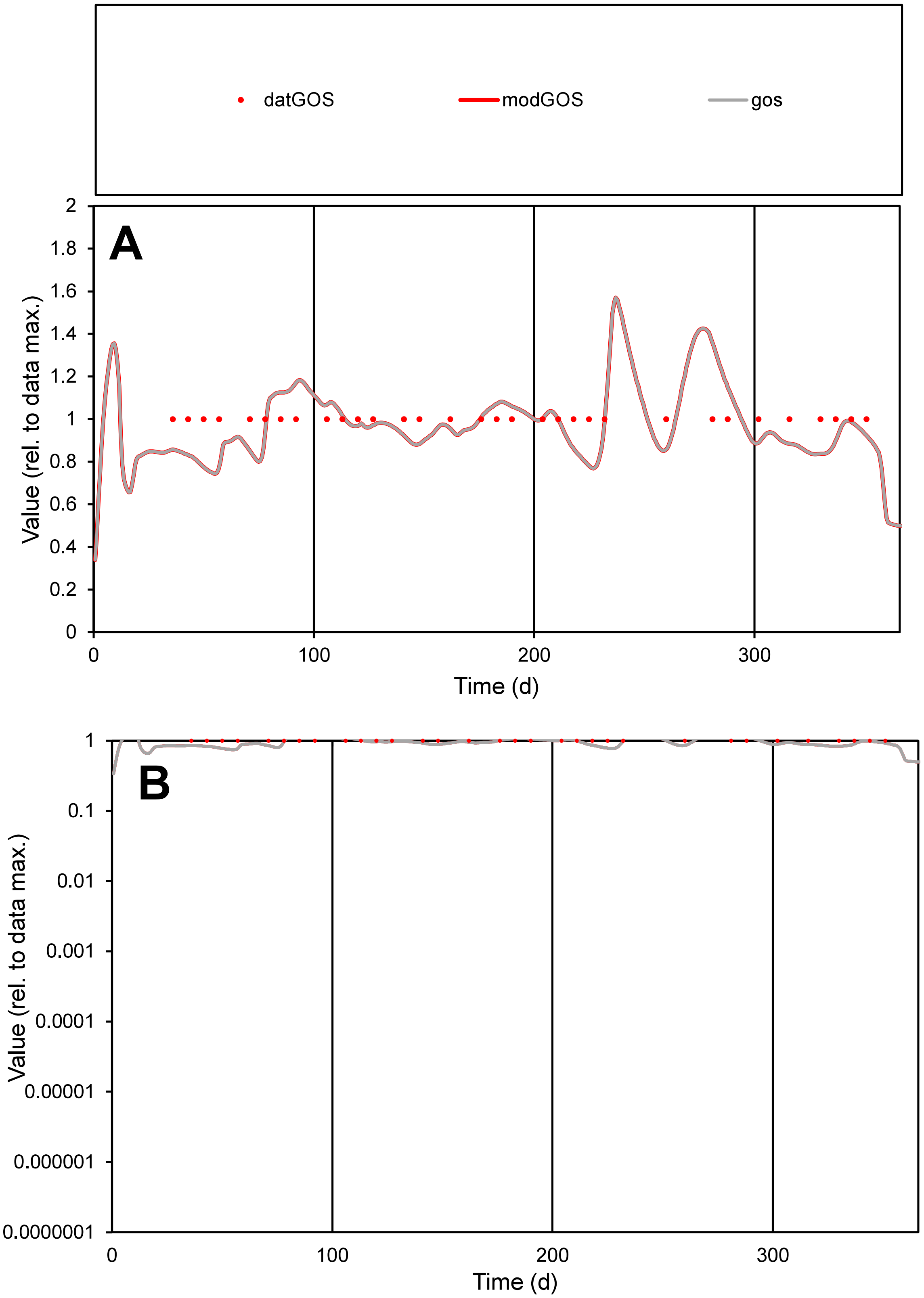

C1

Variables
GOS Provide jtd or Dst
CalibOnly?
2 1 to reproduce of
oNormalize?
2 1=no, 2=yes

ofxn calc*
0.12915
ofxn mod
0.16307

sRun
RunA1
zout
1

Ave
Data
1.8E-06
Model
1.7E-06

data/vMax
1.8E-06

ofxng
321.551

Min d/m
1.31209

ave/min d
1
ave/min m
1.26521

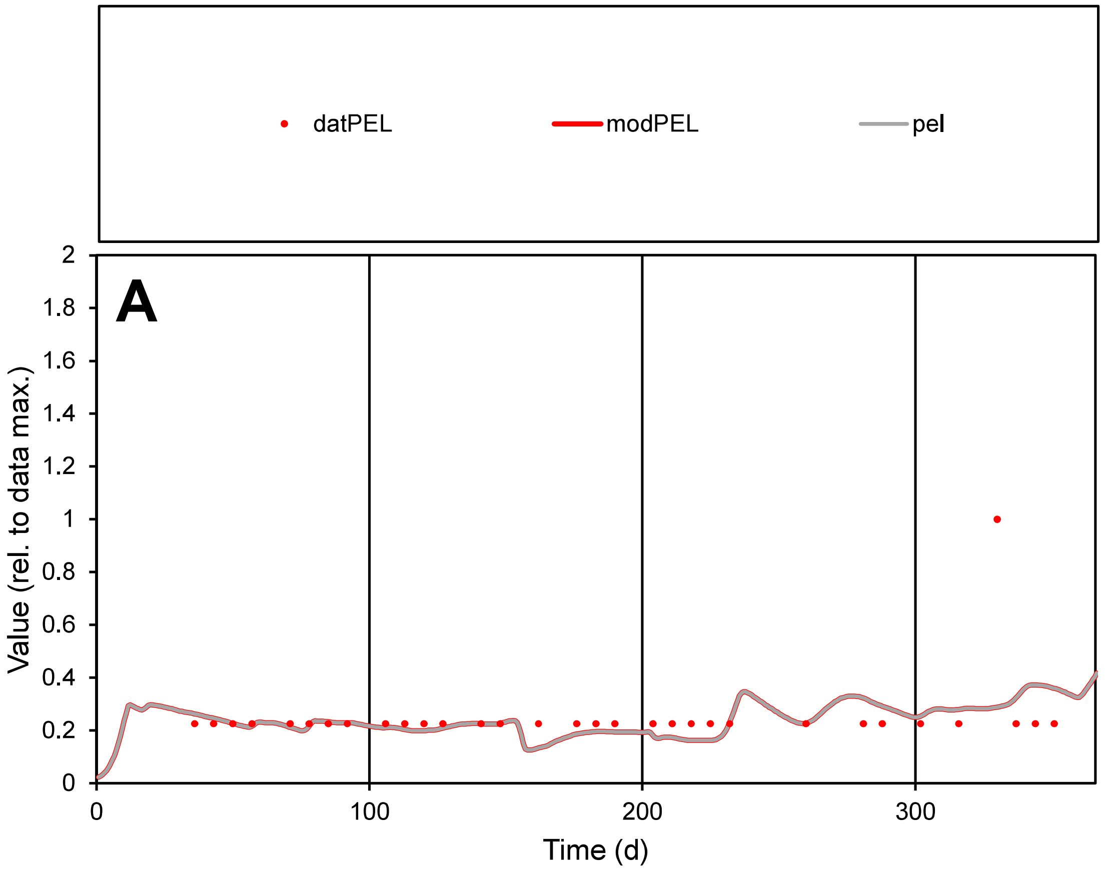

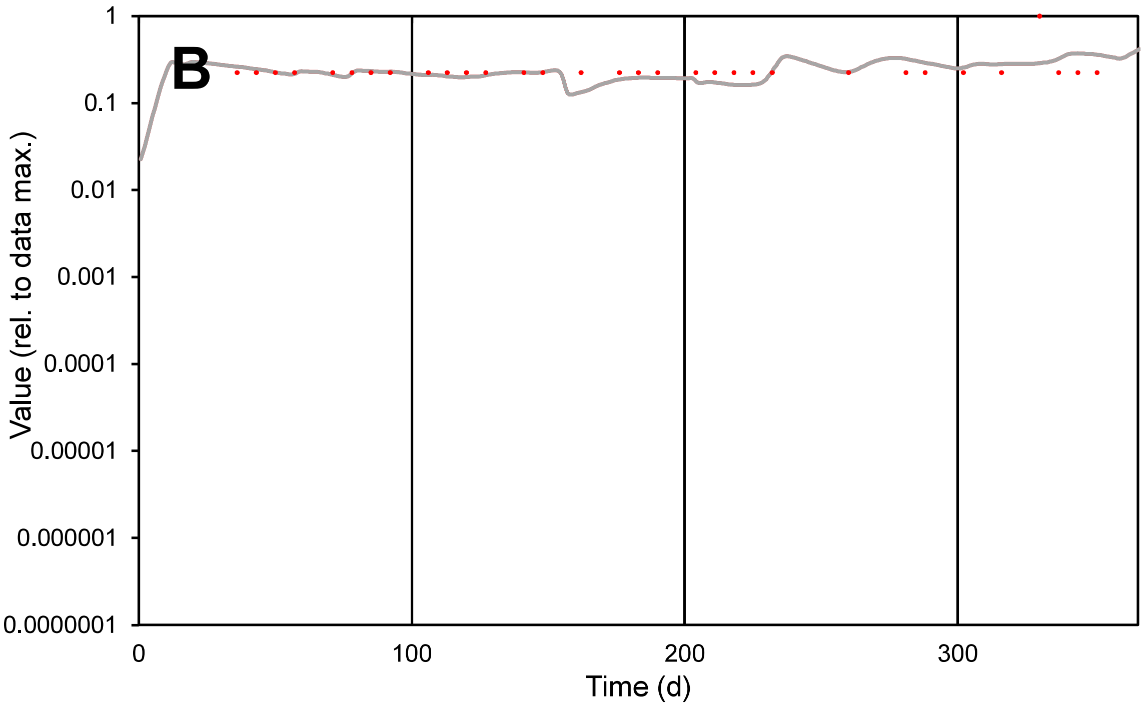

C1

Variables
PEL Provide jtd or Dst
CalibOnly?
2 1 to reproduce of
oNormalize?
2 1=no, 2=yes

ofxn calc*
0.55041
ofxn mod
0.56131

sRun
RunA1
zout
1

Ave
Data
3.6E-06
Model
3.4E-06

data/vMax
1.5E-05

ofxng
321.551

Min d/m
1.62959

ave/min d
1.10805
ave/min m
1.70294

324_Calib_S_A

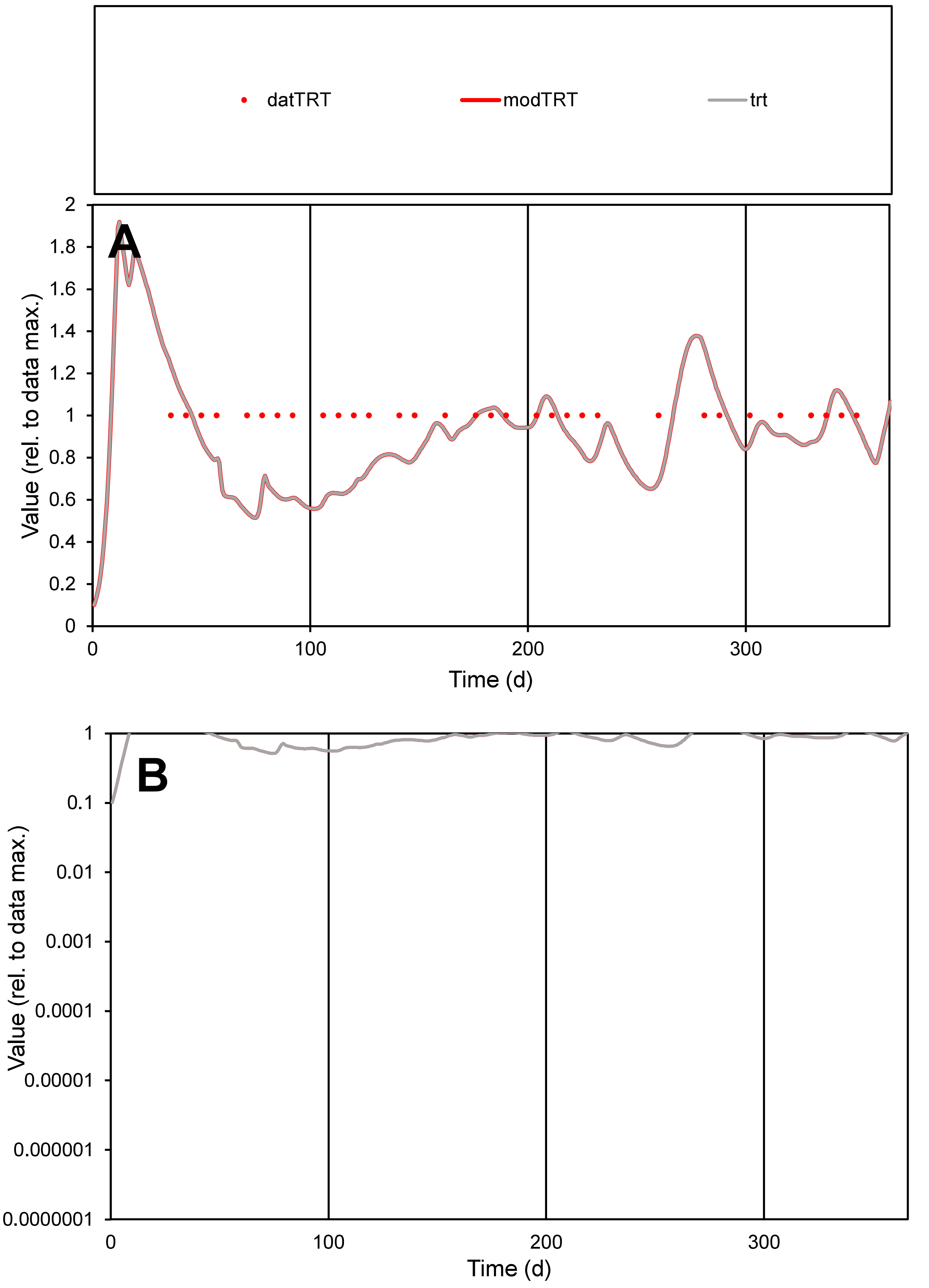

C1

Variables
TRT Provide jtd or Dst
CalibOnly?
2 1 to reproduce of
oNormalize?
2 1=no, 2=yes

ofxn calc*
0.21838
ofxn mod
0.23666

sRun
RunA1
zout
1

Ave
Data
2.3E-05
Model
2E-05

data/vMax
2.3E-05

ofxng
321.551

Min d/m
1.88374

ave/min d
1
ave/min m
1.64499

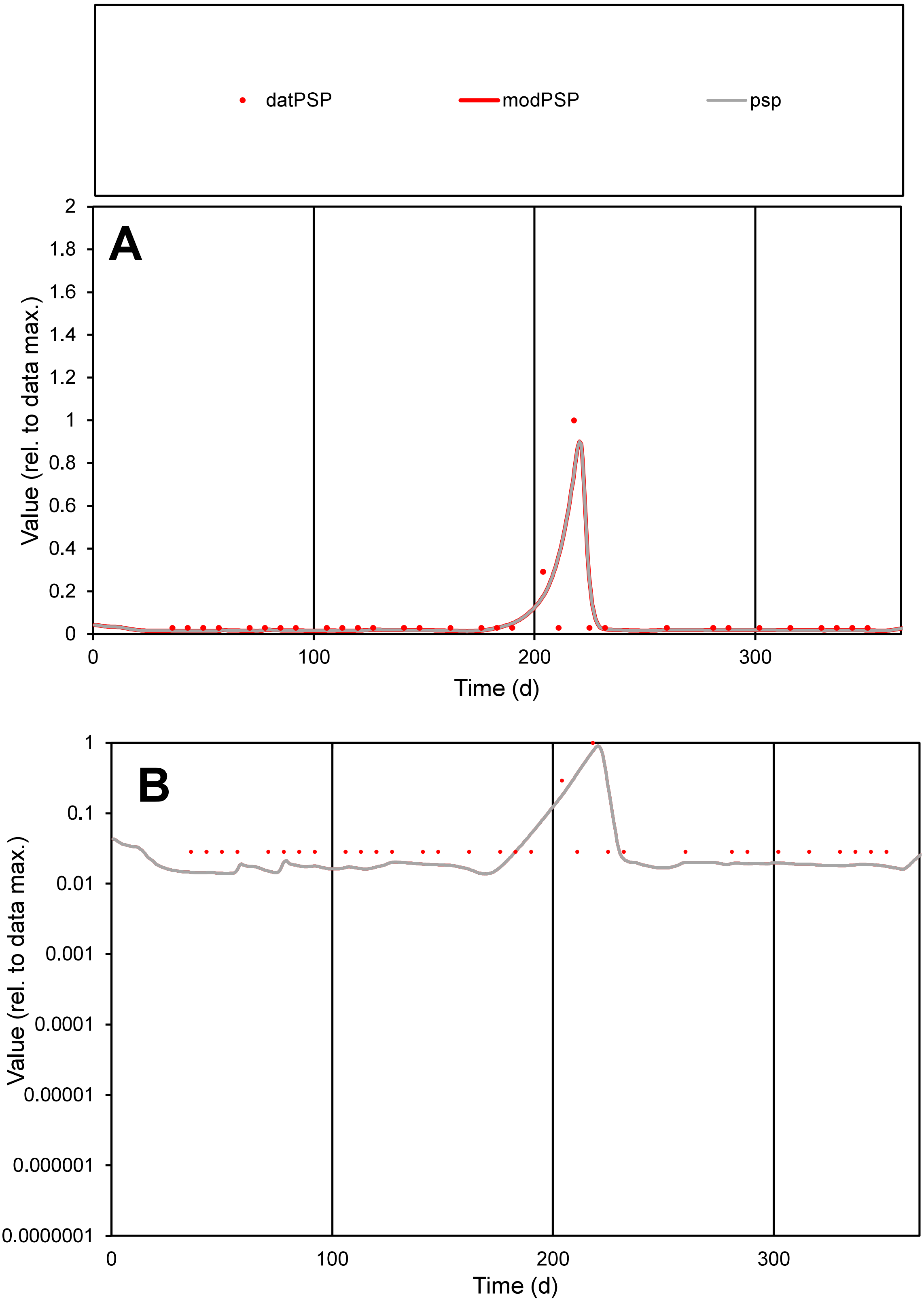

C1

Variables
PSP Provide jtd or Dst
CalibOnly?
2 1 to reproduce of
oNormalize?
2 1=no, 2=yes

ofxn calc*
0.87984
ofxn mod
1.04027

sRun
RunA1
zout
1

Ave
Data
1E-05
Model
9.5E-06

data/vMax
0.00015

ofxng
321.551

Min d/m
2.03151

ave/min d
2.36173
ave/min m
4.48881

324_Calib_S_A

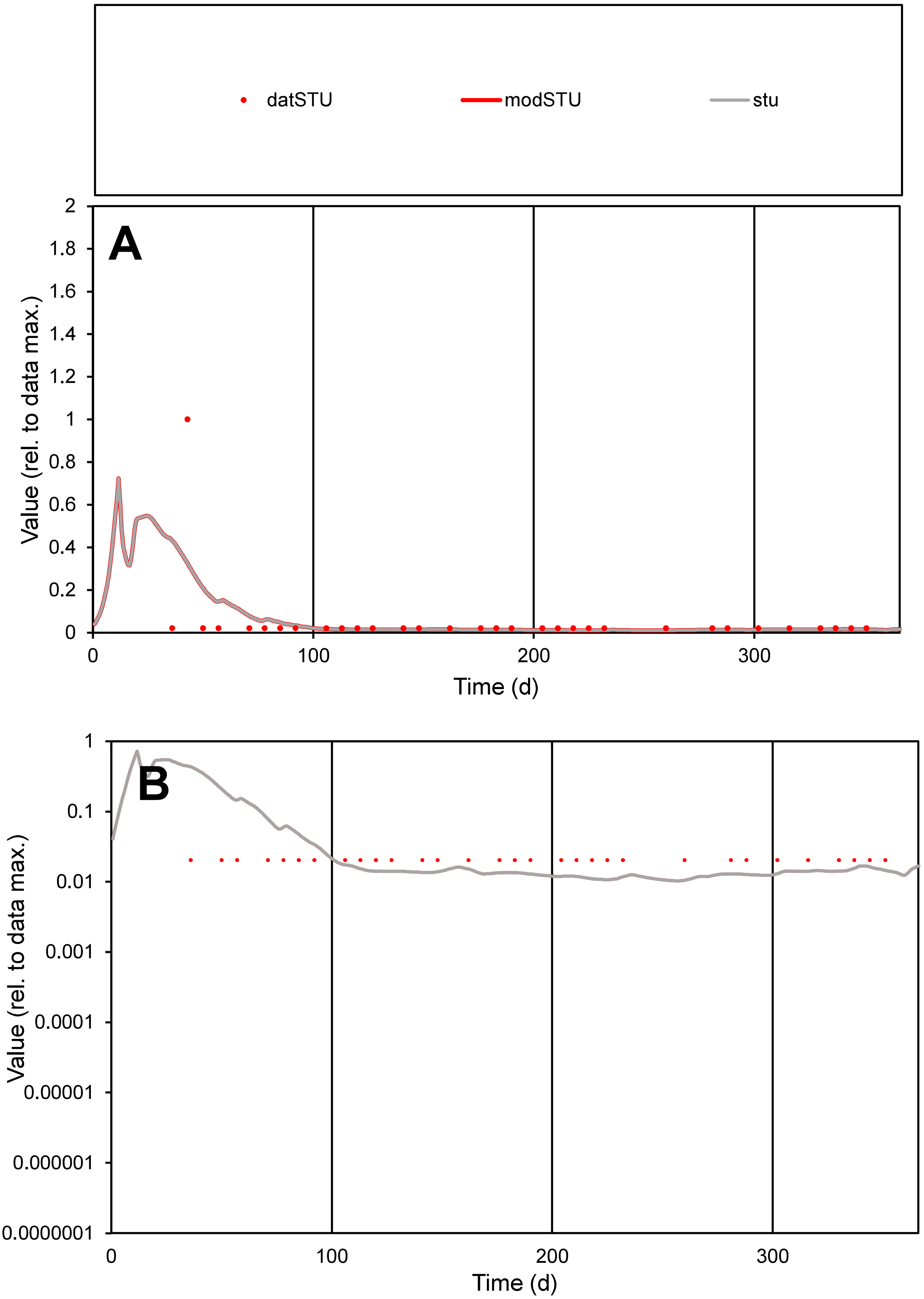

C1

Variables
STU Provide jtd or Dst
CalibOnly?
2 1 to reproduce of
oNormalize?
2 1=no, 2=yes

ofxn calc*
2.81773
ofxn mod
2.86889

sRun
RunA1
zout
1

Ave
Data
6.2E-06
Model
6E-06

data/vMax
0.00012

ofxng
321.551

Min d/m
1.91181

ave/min d
2.50054
ave/min m
4.58675

324_Calib_S_A

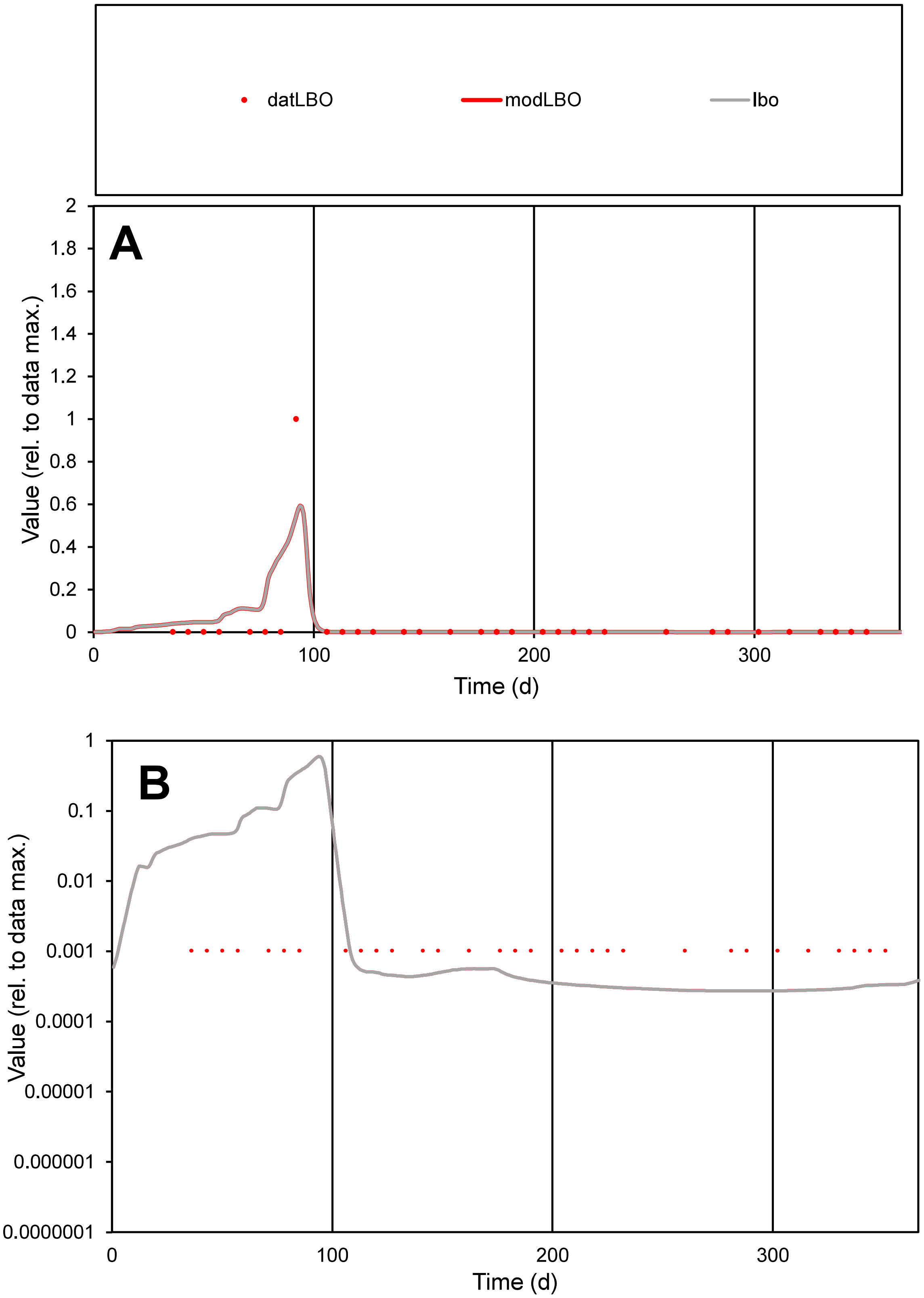

C1

Variables
LBO Provide jtd or Dst
CalibOnly?
2 1 to reproduce of
oNormalize?
2 1=no, 2=yes

ofxn calc*
3.16426
ofxn mod
3.24972

sRun
RunA1
zout
1

Ave
Data
4.4E-05
Model
5.7E-05

data/vMax
0.00135

ofxng
321.551

Min d/m
3.74258

ave/min d
31.3851
ave/min m
154.296

324_Calib_S_A

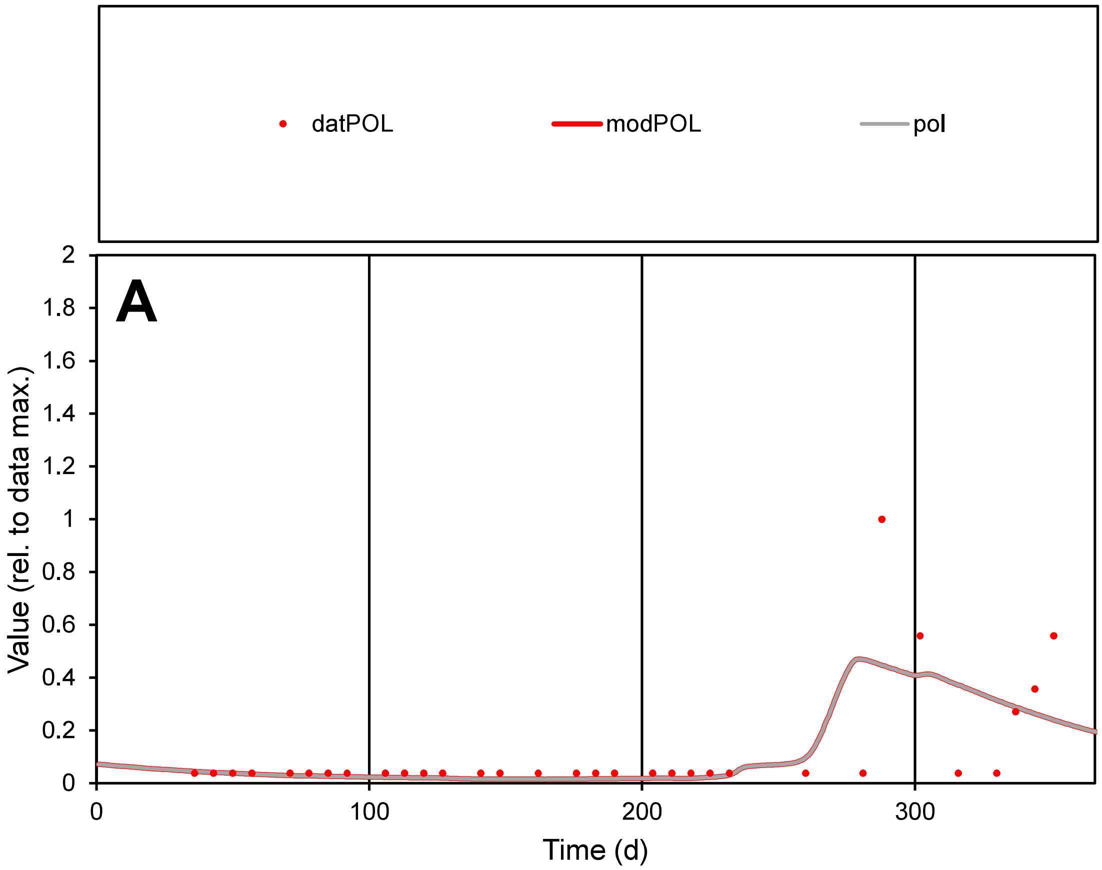

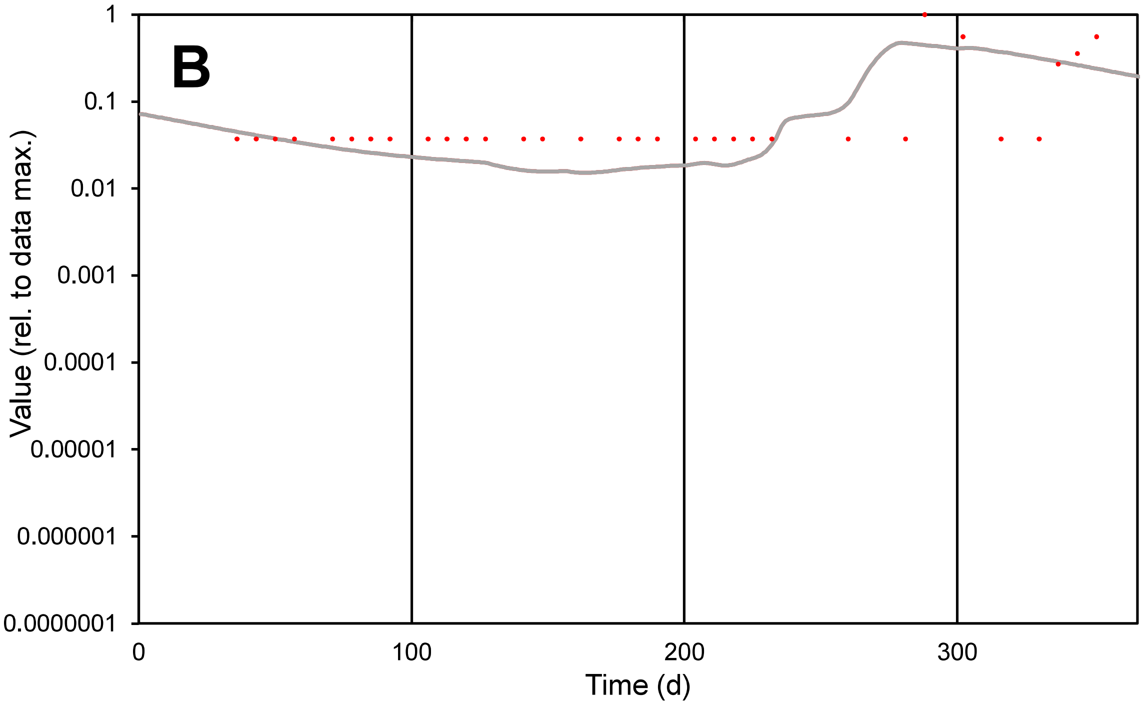

C1

Variables
POL Provide jtd or Dst
CalibOnly?
2 1 to reproduce of
oNormalize?
2 1=no, 2=yes

ofxn calc*
1.36996
ofxn mod
1.37903

sRun
RunA1
zout
1

Ave
Data
5E-06
Model
4.6E-06

data/vMax
4.3E-05

ofxng
321.551

Min d/m
2.45199

ave/min d
3.1548
ave/min m
7.09757

324_Calib_S_A

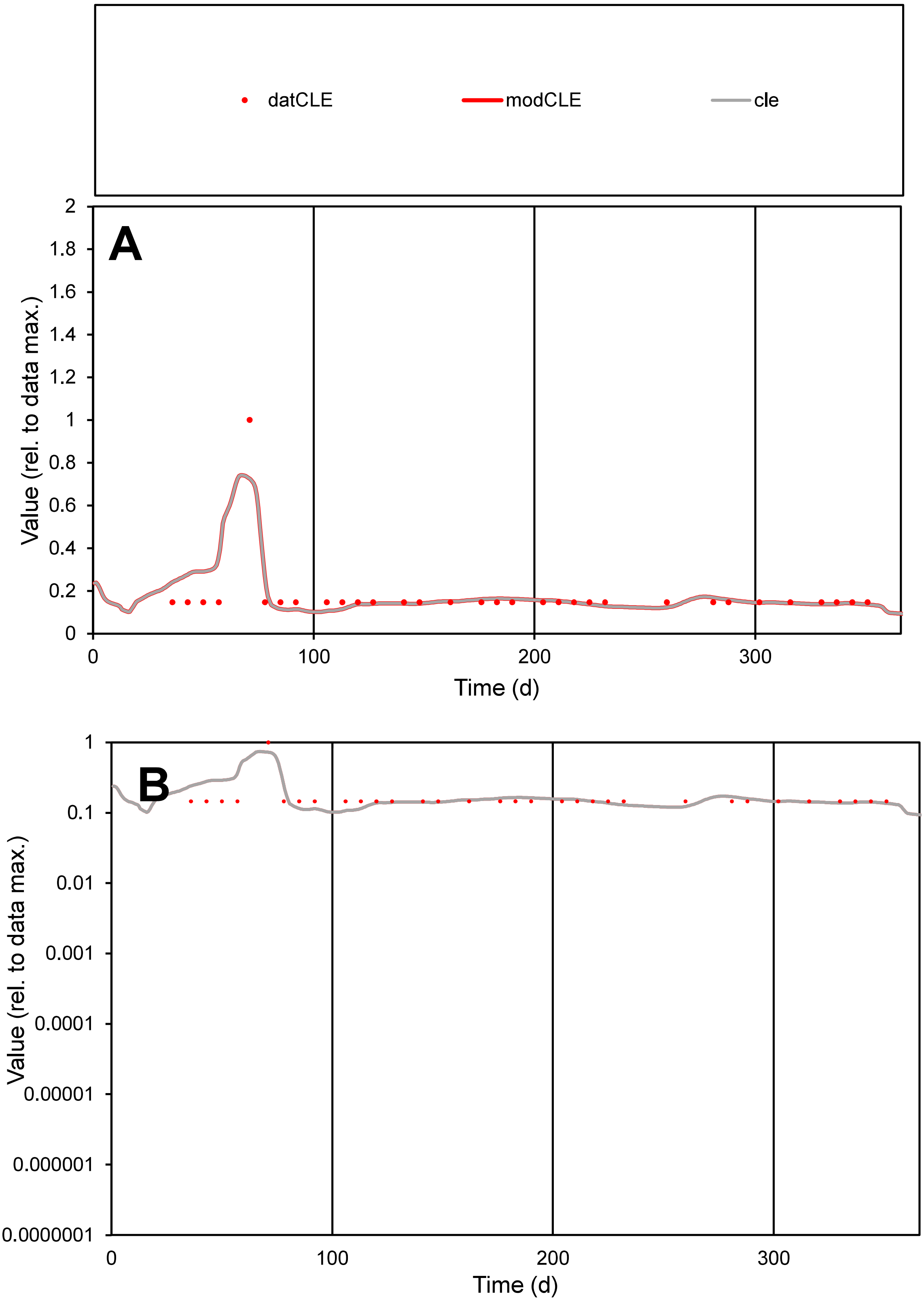

C1

Variables
CLE Provide jtd or Dst
CalibOnly?
2 1 to reproduce of
oNormalize?
2 1=no, 2=yes

ofxn calc*
0.43238
ofxn mod
0.45831

sRun
RunA1
zout
1

Ave
Data
5.6E-06
Model
5.7E-06

data/vMax
3.2E-05

ofxng
321.551

Min d/m
1.35137

ave/min d
1.1825
ave/min m
1.65397

324_Calib_S_A

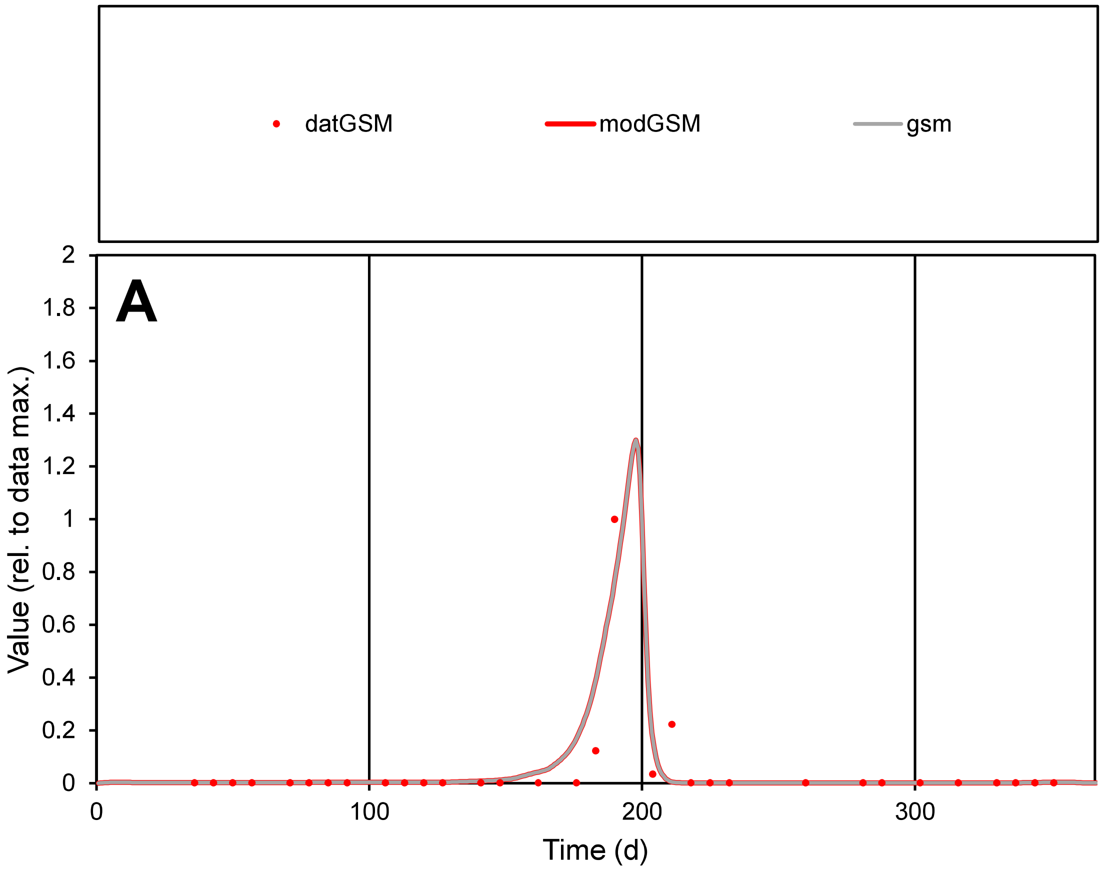

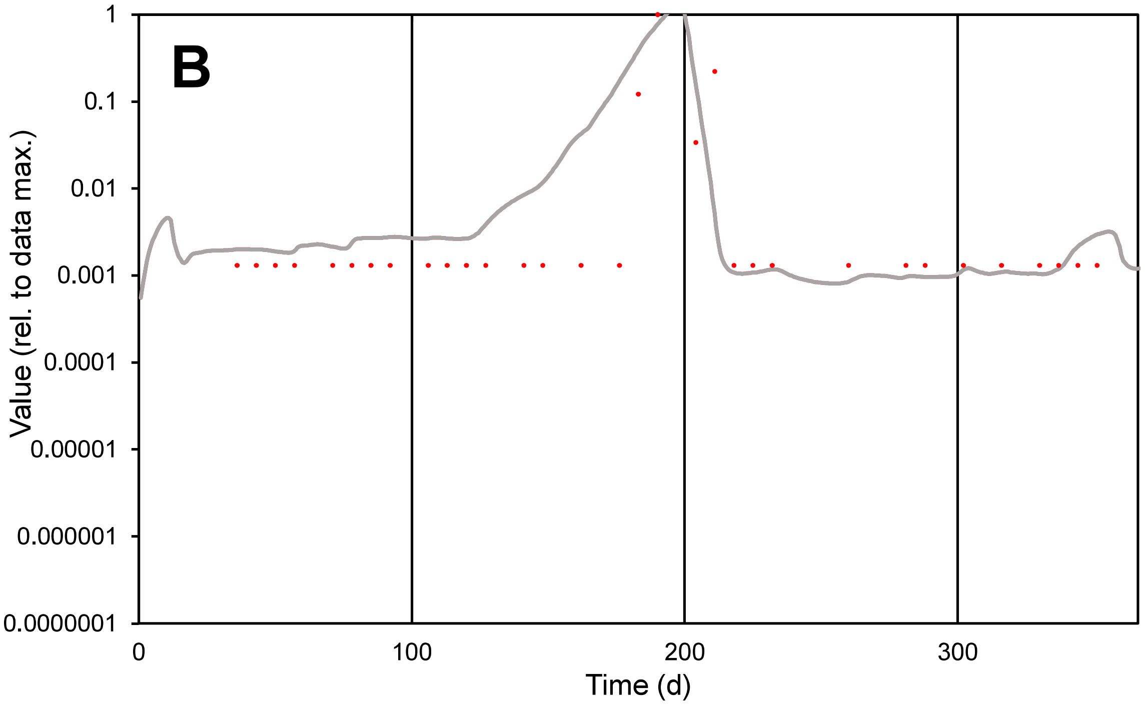

C1

Variables

GSM Provide jtd or Dst

CalibOnly?

2 1 to reproduce of

oNormalize?

2 1=no, 2=yes

ofxn calc*

1.52797

ofxn mod

1.70759

sRun

RunA1

zout

1

Ave

Data

5.3E-05

Model

5.7E-05

data/vMax

0.00121

ofxng

321.551

Min d/m

1.48682

ave/min d

33.8575

ave/min m

53.2512

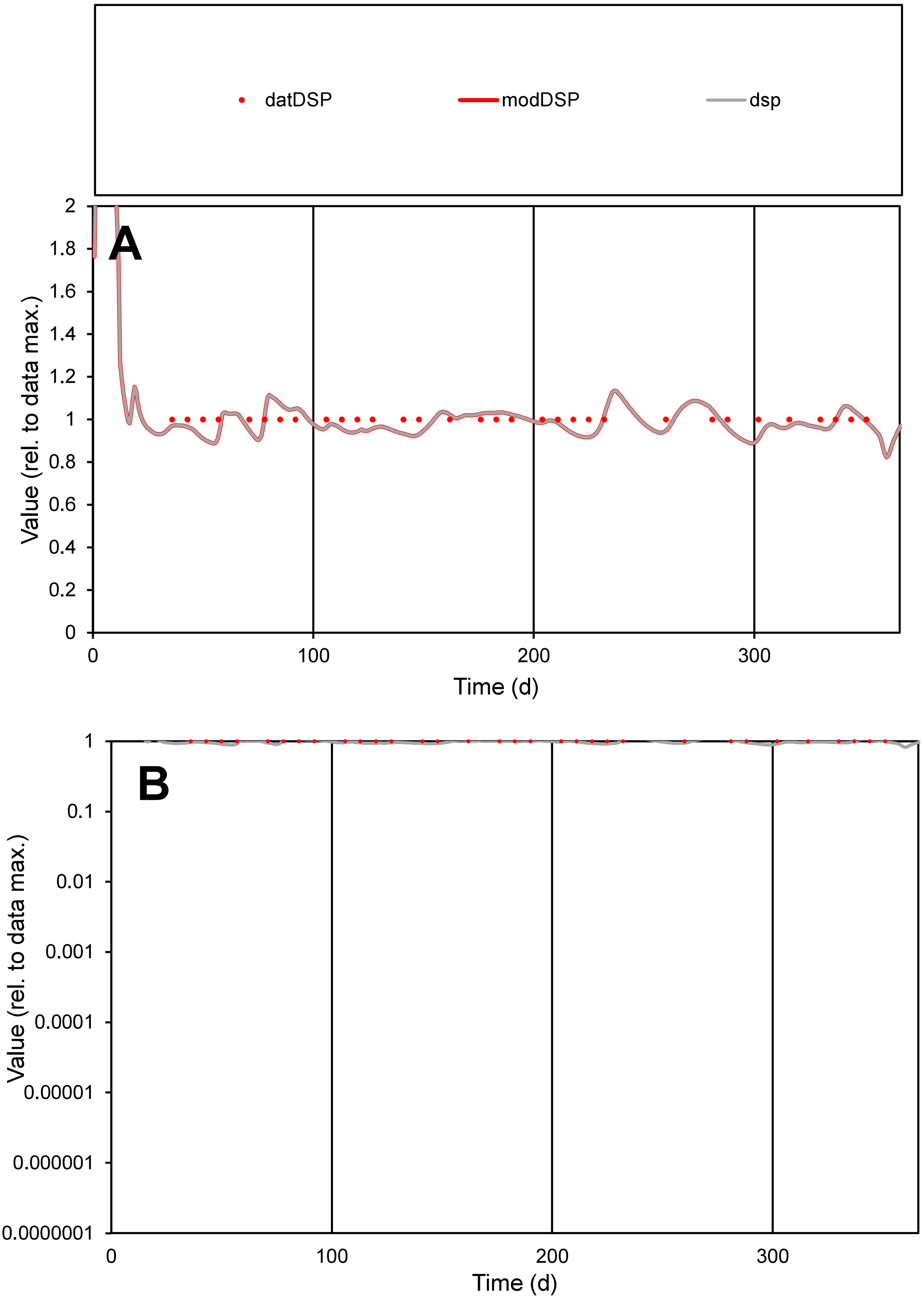

C1

Variables
DSP Provide jtd or Dst
CalibOnly?
2 1 to reproduce of
oNormalize?
2 1=no, 2=yes

ofxn calc*
0.05055
ofxn mod
0.10892

sRun
RunA1
zout
1

Ave
Data
5E-06
Model
4.9E-06

data/vMax
5E-06

ofxng
321.551

Min d/m
1.11123

ave/min d
1
ave/min m
1.09244

324_Calib_S_A

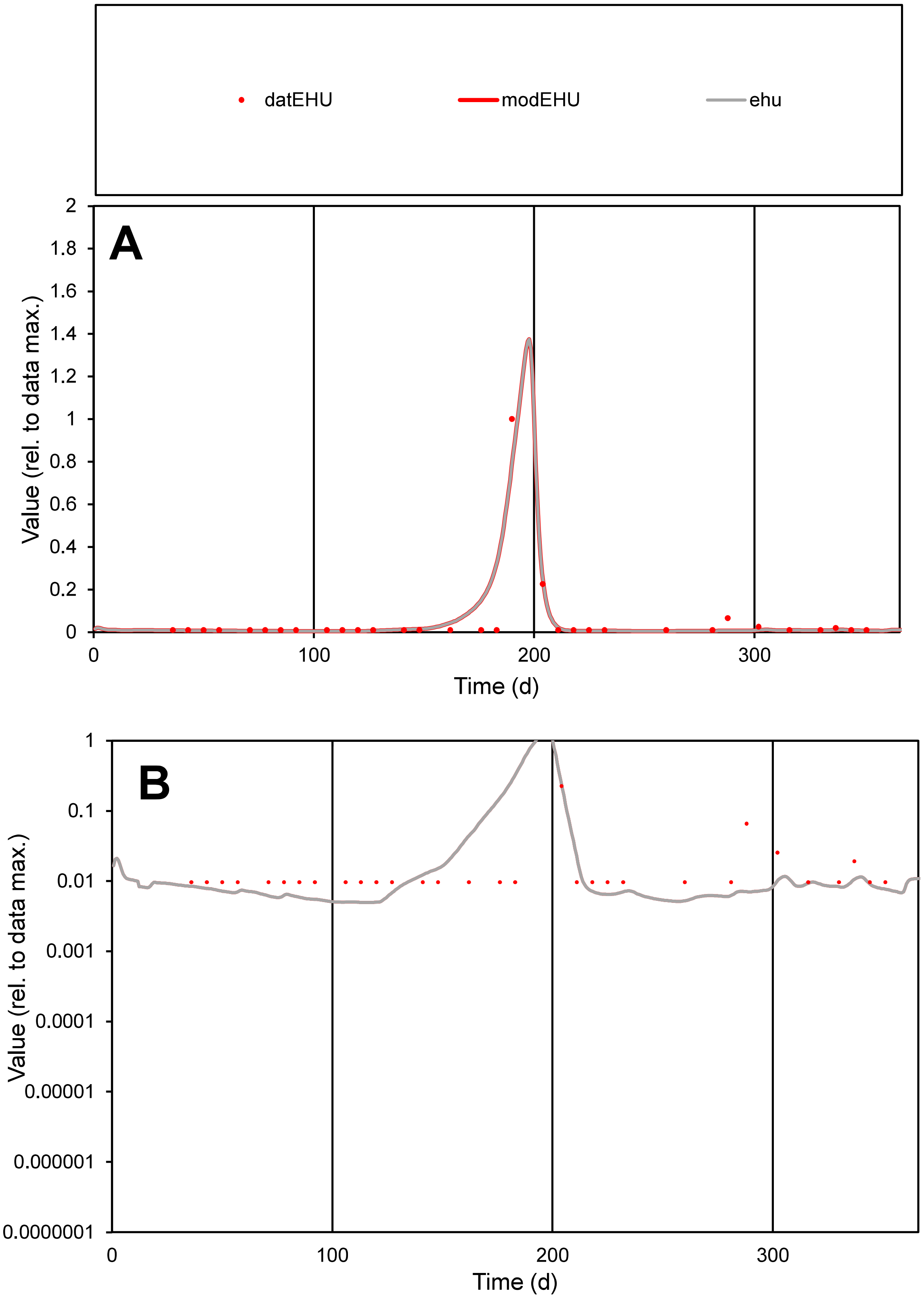

C1

Variables

EHU Provide jtd or Dst

CalibOnly?

2 1 to reproduce of

oNormalize?

2 1=no, 2=yes

ofxn calc*

1.08225

ofxn mod

1.25522

sRun

RunA1

zout

1

Ave

Data

3E-05

Model

3.3E-05

data/vMax

0.0006

ofxng

321.551

Min d/m

1.9436

ave/min d

5.18704

ave/min m

11.1308

324_Calib_S_A

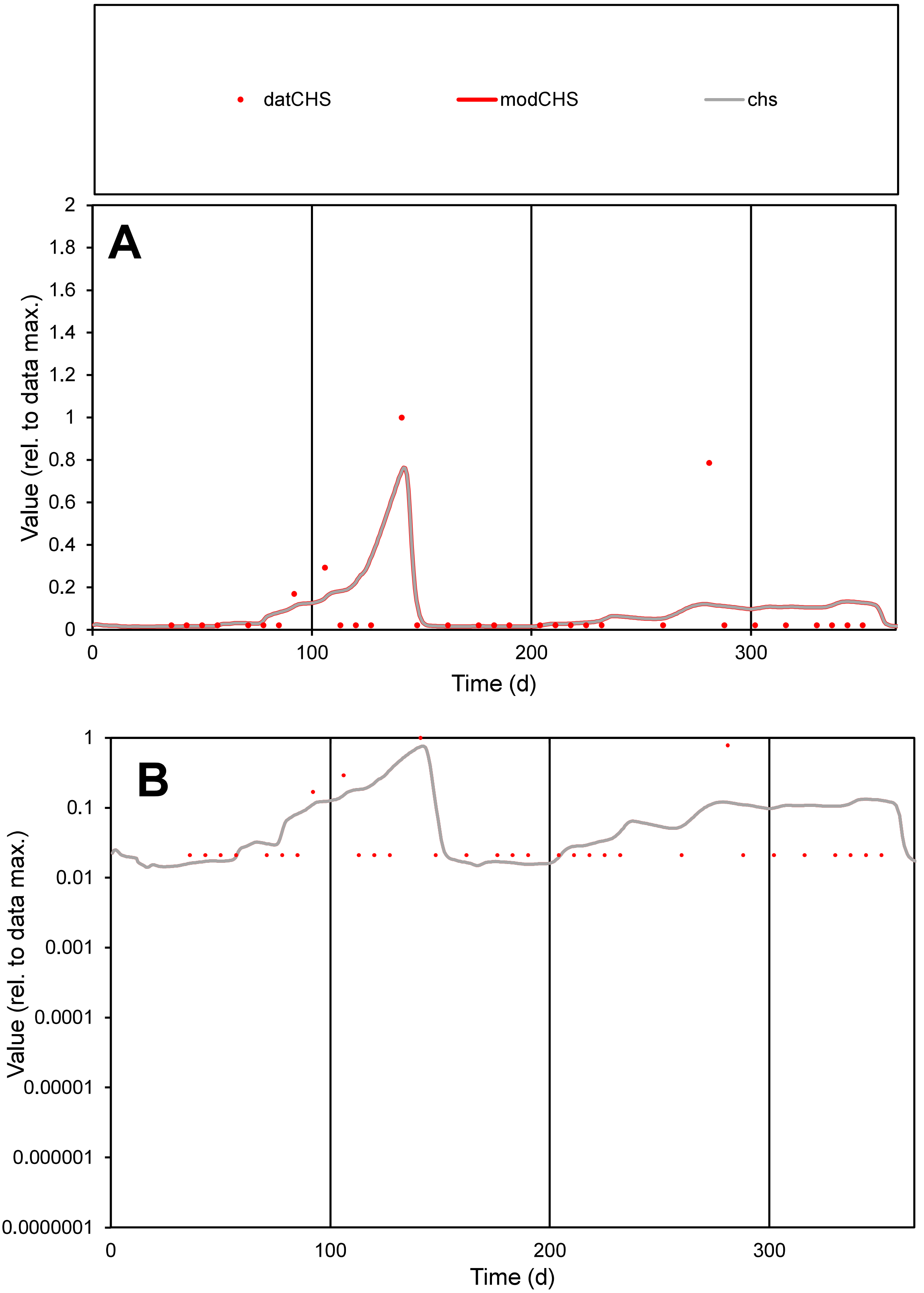

C1

Variables
CHS Provide jtd or Dst
CalibOnly?
2 1 to reproduce of
oNormalize?
2 1=no, 2=yes

ofxn calc*
1.70163
ofxn mod
1.7502

sRun
RunA1
zout
1

Ave
Data
9E-05
Model
0.0001

data/vMax
0.00101

ofxng
321.551

Min d/m
1.35123

ave/min d
4.20322
ave/min m
6.38884

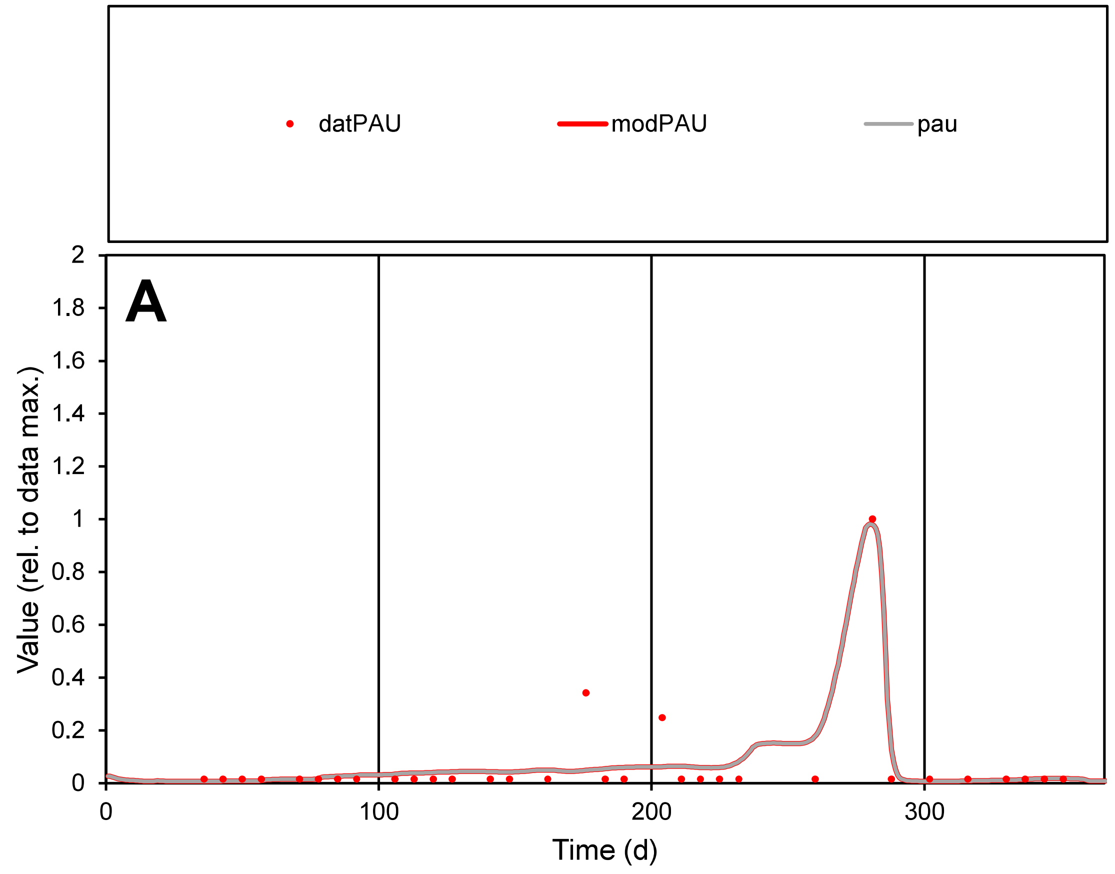

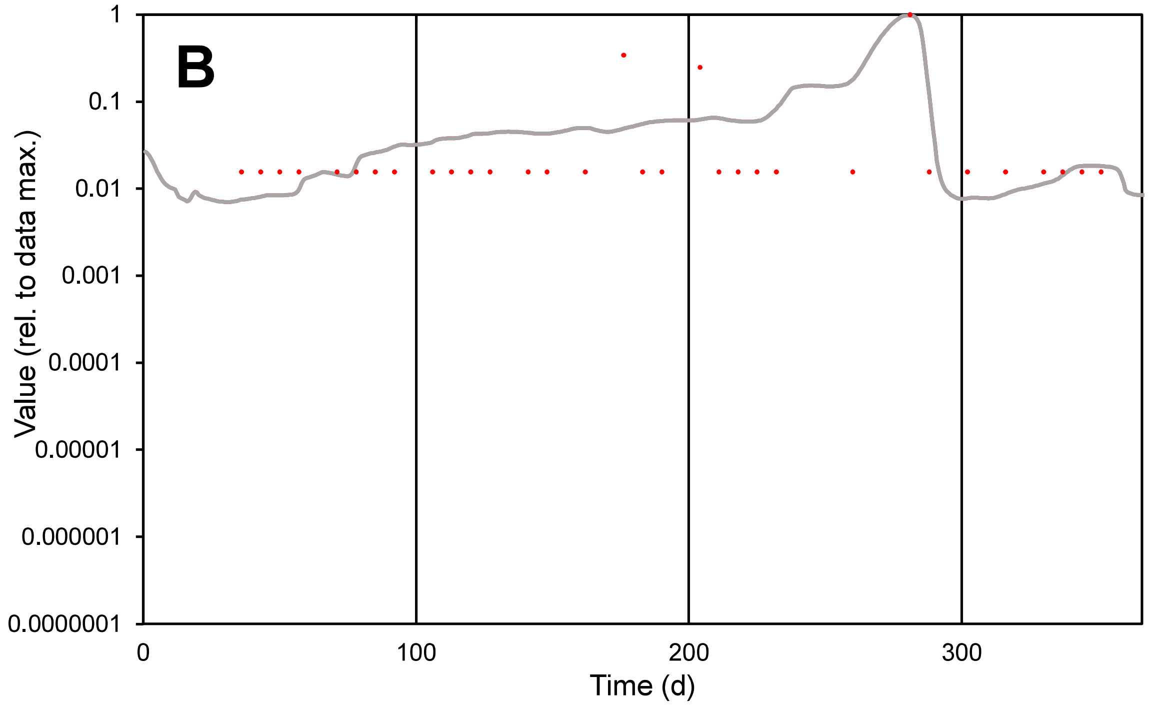

C1

Variables
PAU Provide jtd or Dst
CalibOnly?
2 1 to reproduce of
oNormalize?
2 1=no, 2=yes

ofxn calc*
1.12671
ofxn mod
1.16106

sRun
RunA1
zout
1

Ave
Data
1.6E-05
Model
1.7E-05

data/vMax
0.00025

ofxng
321.551

Min d/m
2.06332

ave/min d
4.10036
ave/min m
8.97115

324_Calib_S_A

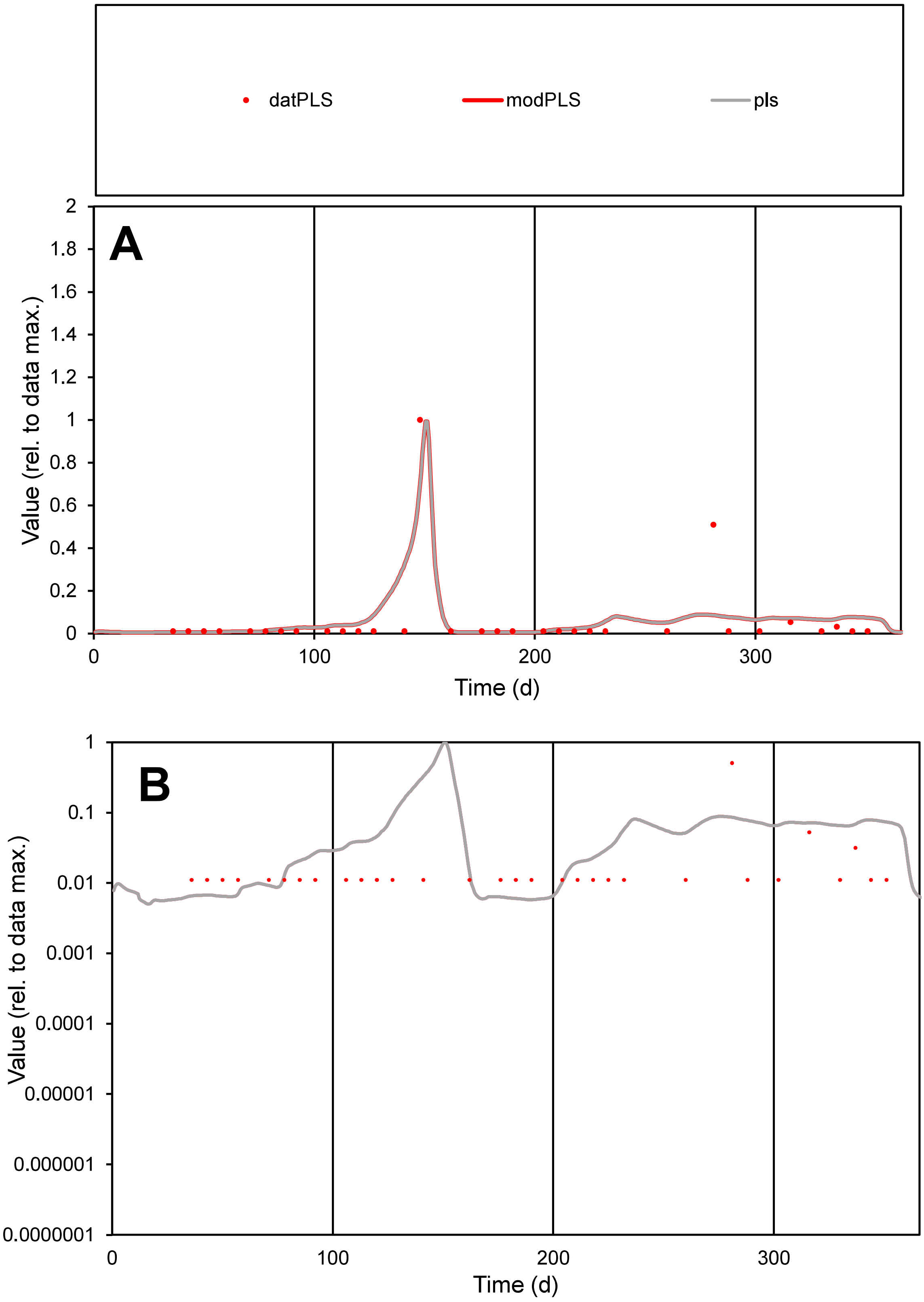

C1

Variables

PLS Provide jtd or Dst

CalibOnly?

2 1 to reproduce of

oNormalize?

2 1=no, 2=yes

ofxn calc*

1.69337

ofxn mod

1.72935

sRun

RunA1

zout

1

Ave

Data

2.1E-05

Model

2.4E-05

data/vMax

0.00035

ofxng

321.551

Min d/m

1.90715

ave/min d

5.3661

ave/min m

12.0676

324_Calib_S_A

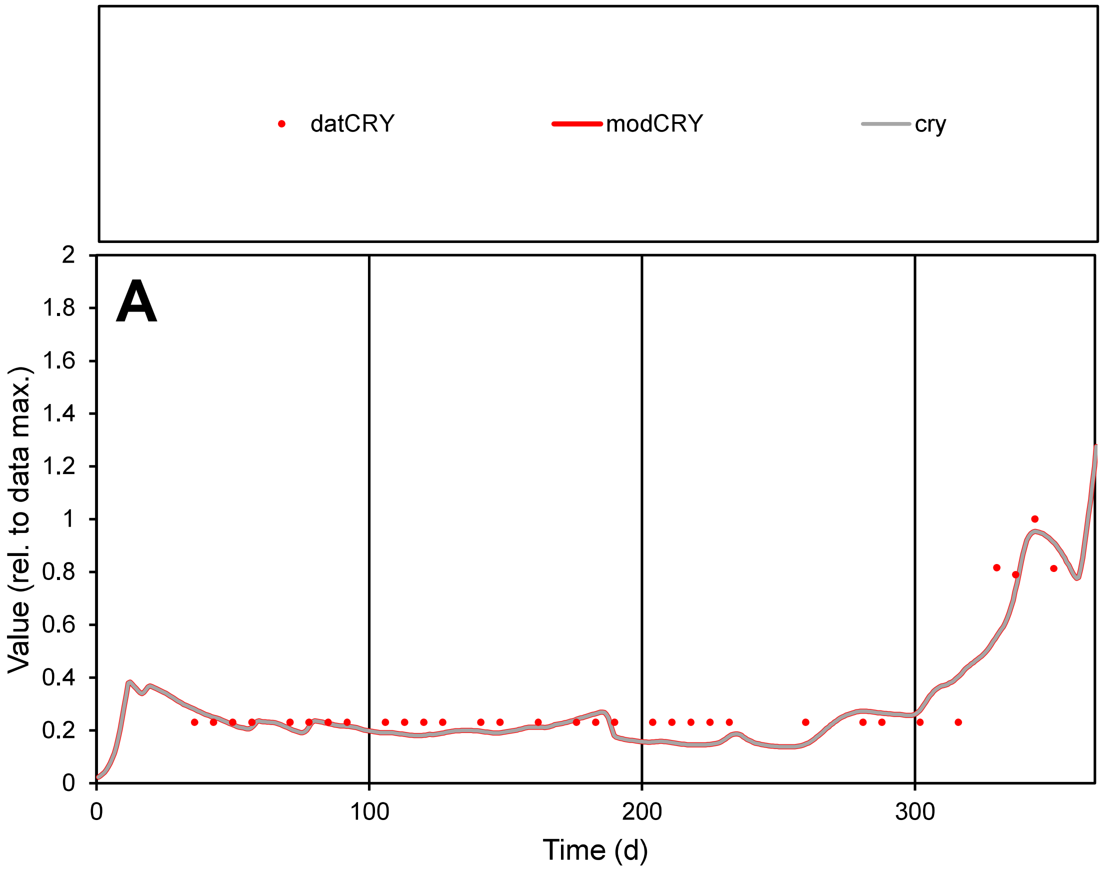

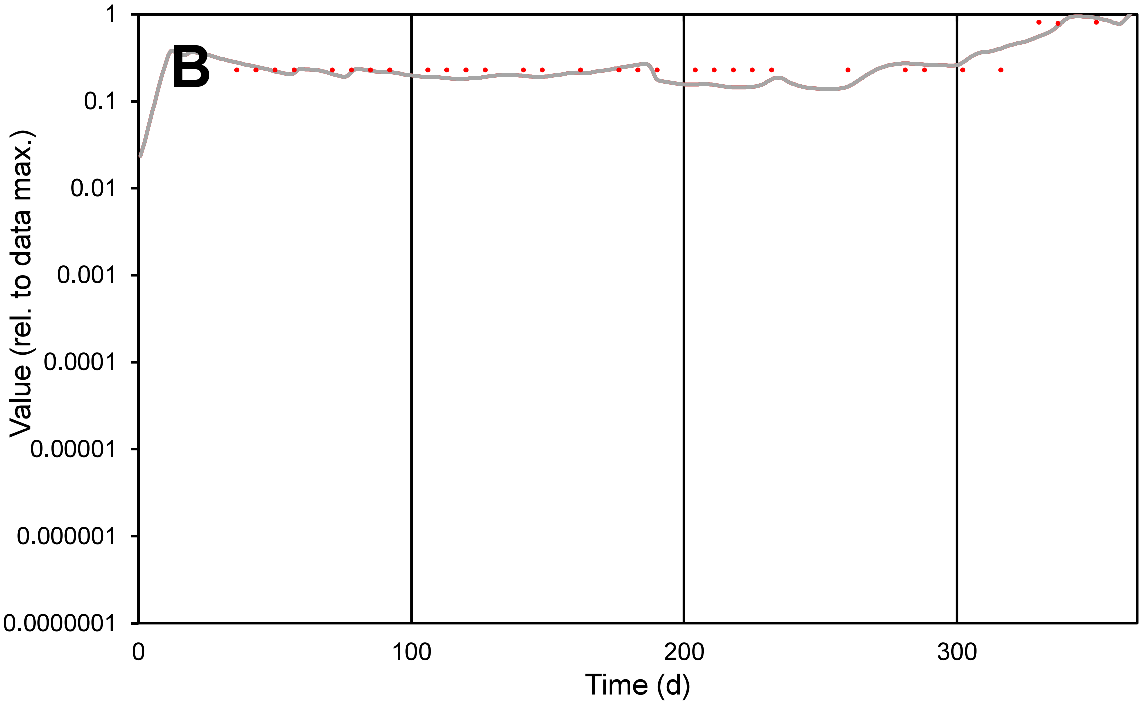

C1

Variables
CRY Provide jtd or Dst
CalibOnly?
2 1 to reproduce of
oNormalize?
2 1=no, 2=yes

ofxn calc*
0.22648
ofxn mod
0.25324

sRun
RunA1
zout
1

Ave
Data
4E-06
Model
3.8E-06

data/vMax
1.3E-05

ofxng
321.551

Min d/m
1.58914

ave/min d
1.33894
ave/min m
2.00138

324_Calib_S_A

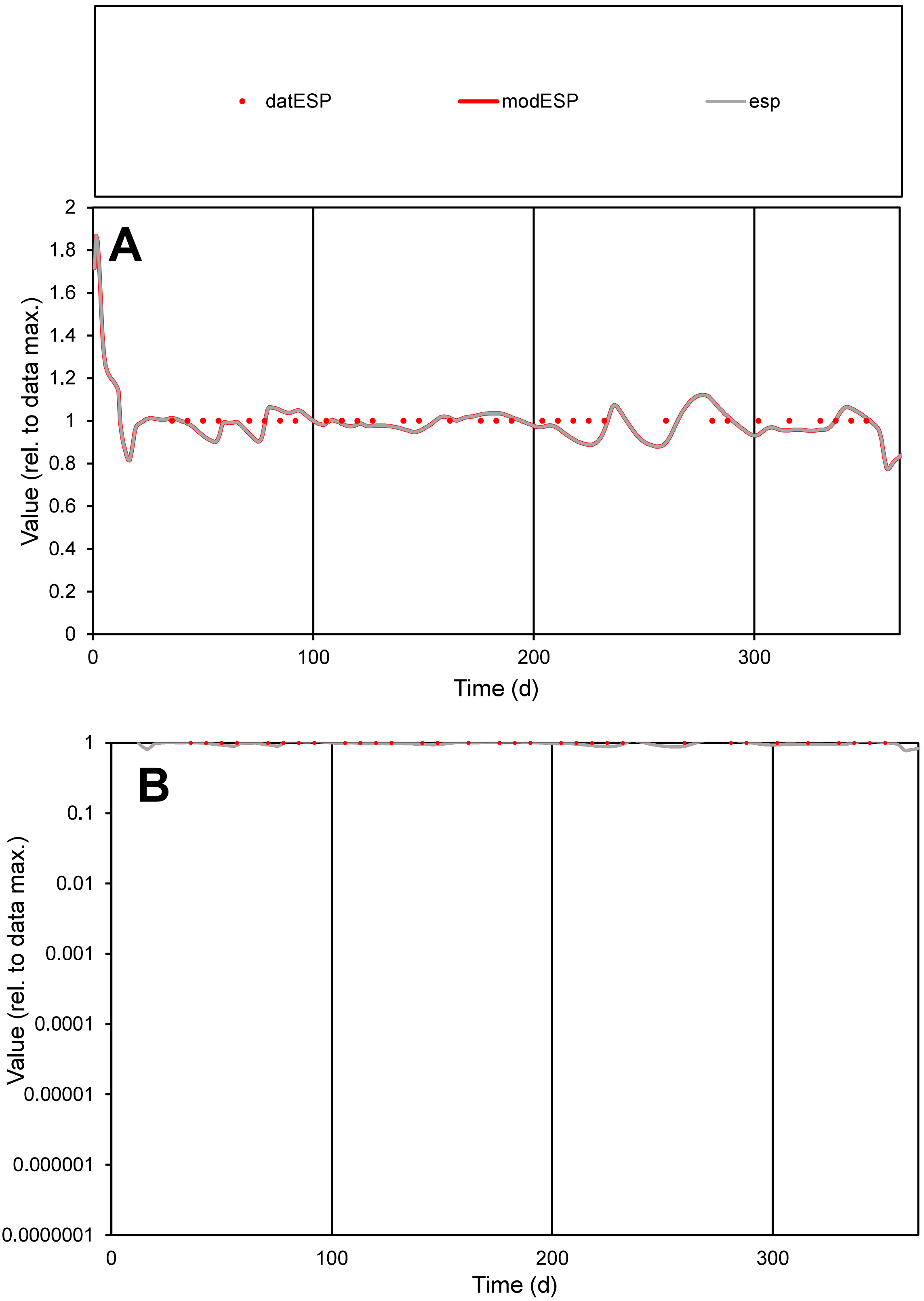

C1

Variables
ESP Provide jtd or Dst
CalibOnly?
2 1 to reproduce of
oNormalize?
2 1=no, 2=yes

ofxn calc*
0.04888
ofxn mod
0.10782

sRun
RunA1
zout
1

Ave
Data
8E-06
Model
7.9E-06

data/vMax
8E-06

ofxng
321.551

Min d/m
1.12507

ave/min d
1
ave/min m
1.10926

324_Calib_S_A

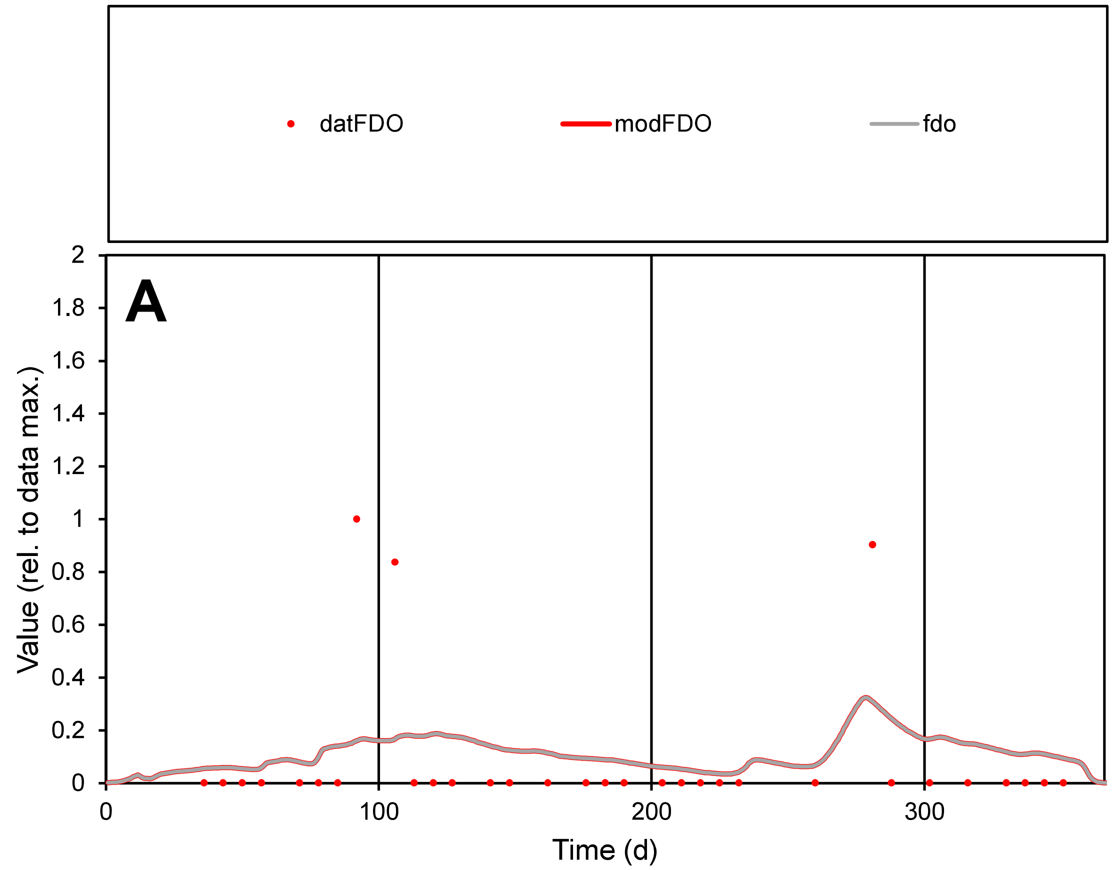

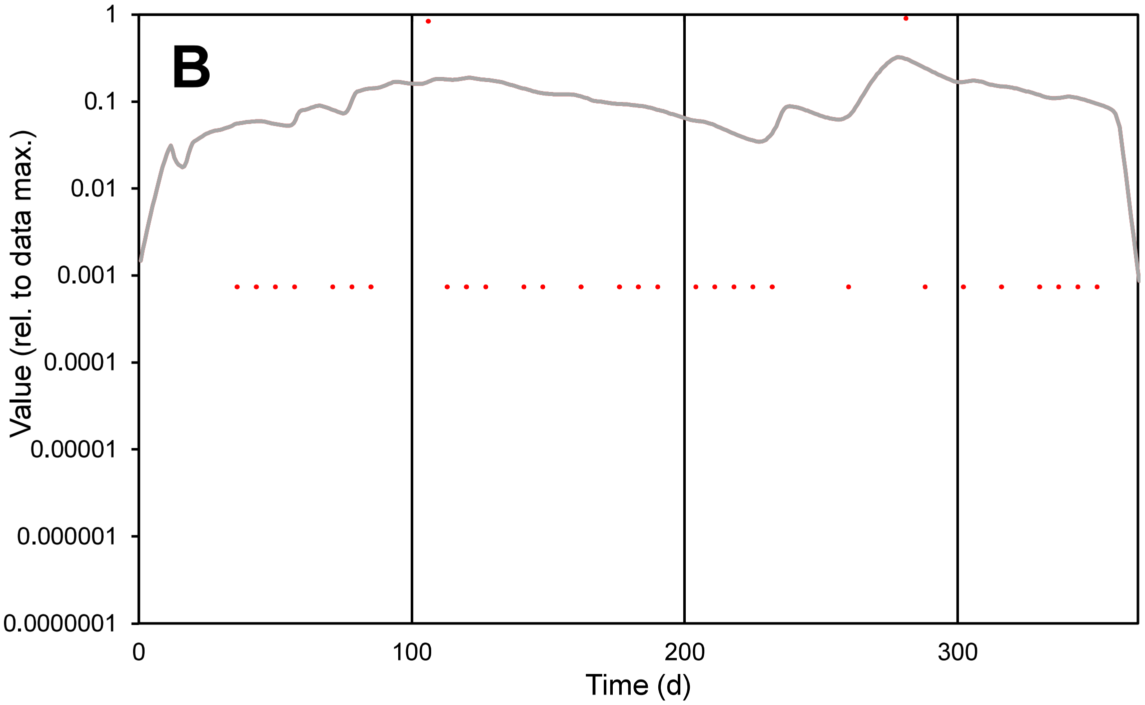

C1

Variables

FDO Provide jtd or Dst

CalibOnly?

2 1 to reproduce of

oNormalize?

2 1=no, 2=yes

ofxn calc*

2.78999

ofxn mod

2.80797

sRun

RunA1

zout

1

Ave

Data

1.2E-05

Model

1.5E-05

data/vMax

0.00013

ofxng

321.551

Min d/m

0.02118

ave/min d

116.584

ave/min m

3.21851

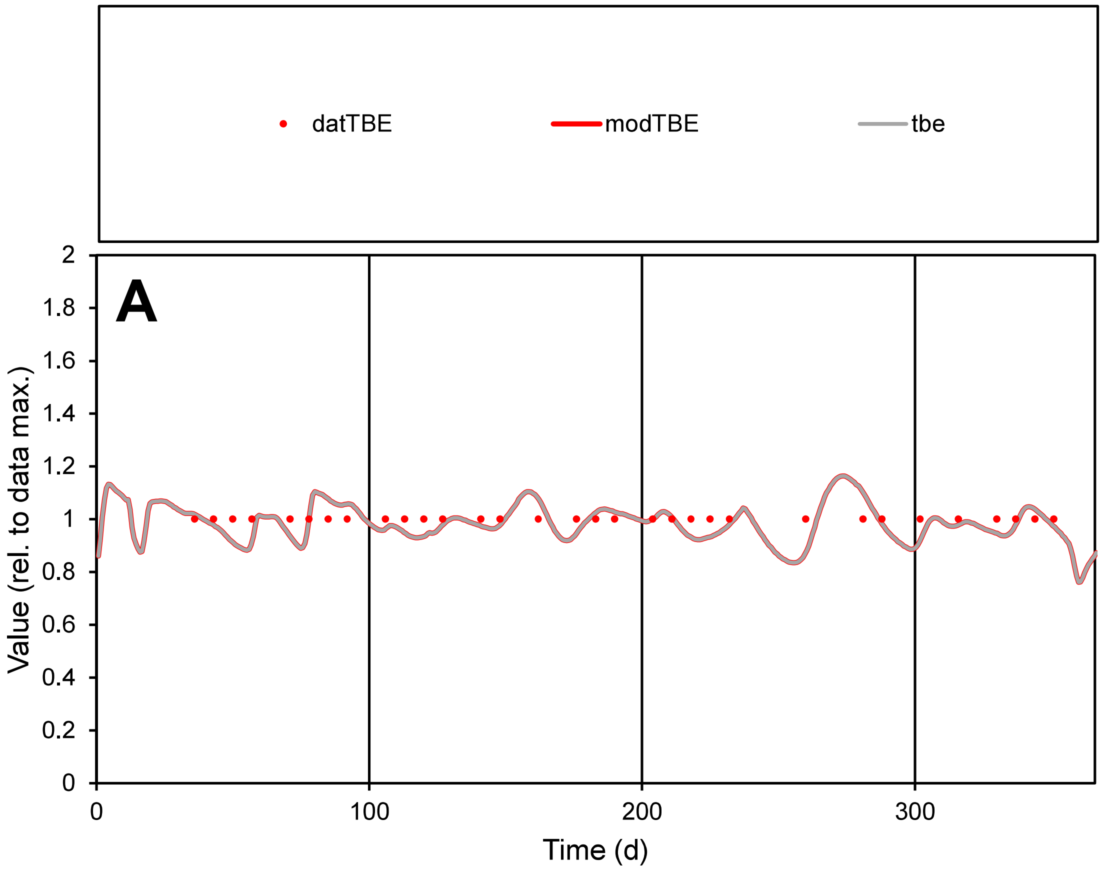

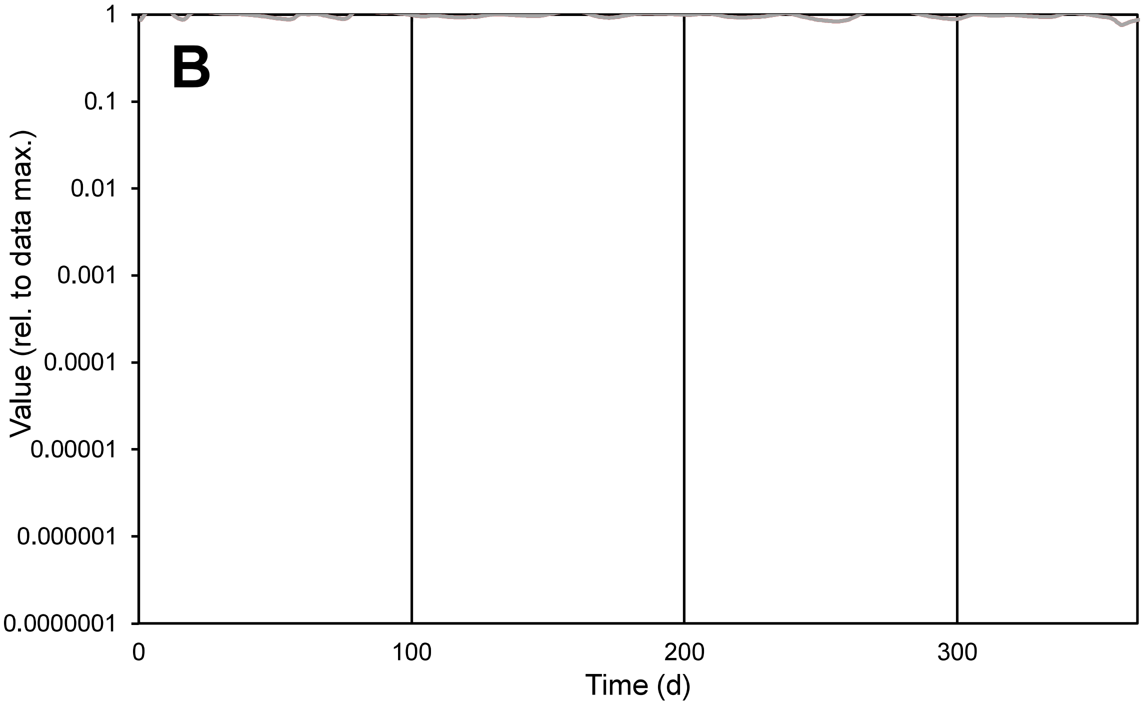

C1

Variables
TBE Provide jtd or Dst
CalibOnly?
2 1 to reproduce of
oNormalize?
2 1=no, 2=yes

ofxn calc*
0.05111
ofxn mod
0.10887

sRun
RunA1
zout
1

Ave
Data
6.7E-06
Model
6.6E-06

data/vMax
6.7E-06

ofxng
321.551

Min d/m
1.11176

ave/min d
1
ave/min m
1.09584

324_Calib_S_A

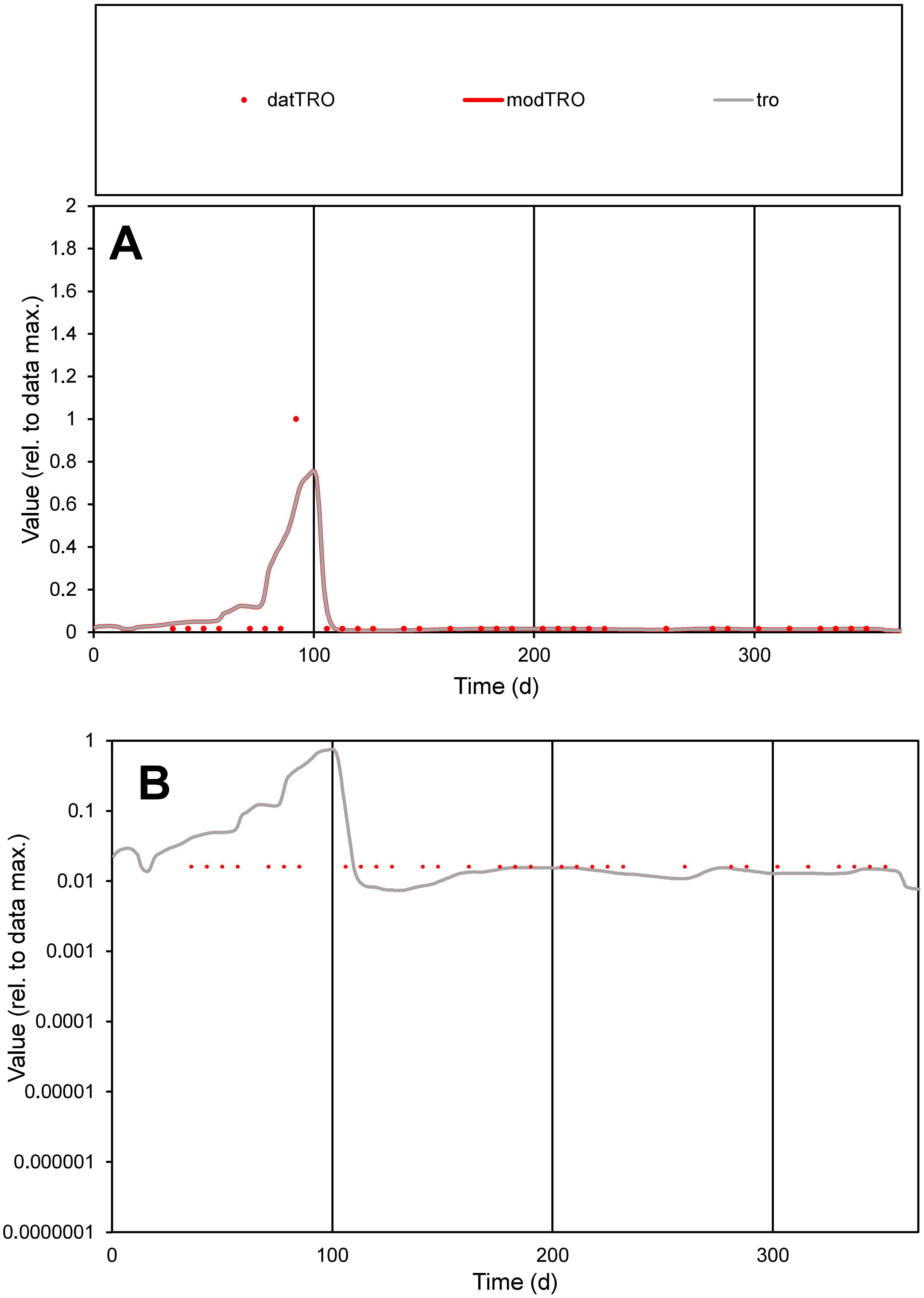

C1

Variables

TRO Provide jtd or Dst

CalibOnly?

2 1 to reproduce of

oNormalize?

2 1=no, 2=yes

ofxn calc*

2.03579

ofxn mod

2.11912

sRun

RunA1

zout

1

Ave

Data

1.5E-05

Model

1.8E-05

data/vMax

0.00031

ofxng

321.551

Min d/m

2.15979

ave/min d

2.92094

ave/min m

7.72715

324_Calib_S_A

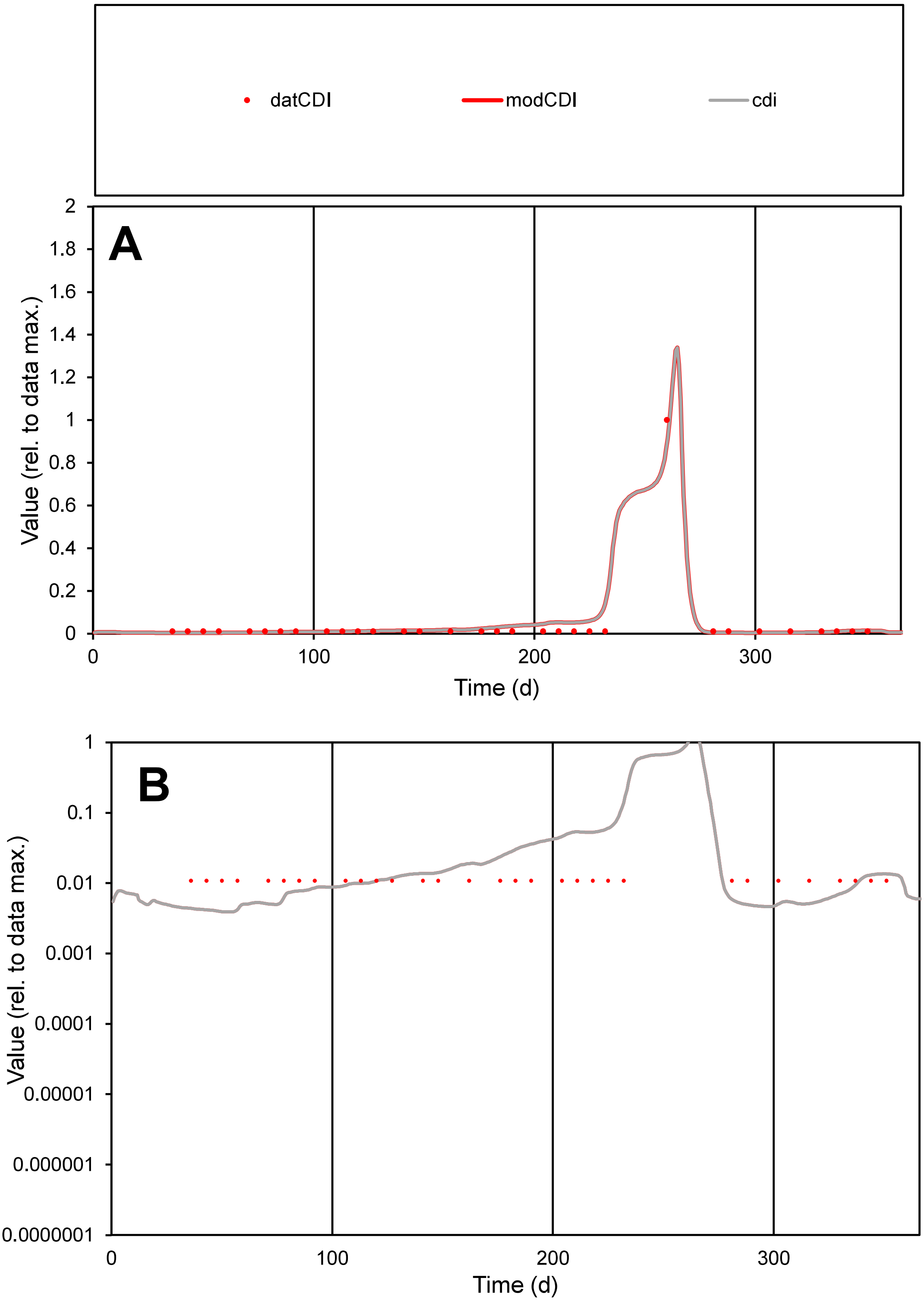

C1

Variables

CDI Provide jtd or Dst

CalibOnly?

2 1 to reproduce of

oNormalize?

2 1=no, 2=yes

ofxn calc*

0.55836

ofxn mod

0.68649

sRun

RunA1

zout

1

Ave

Data

6.7E-07

Model

8E-07

data/vMax

1.6E-05

ofxng

321.551

Min d/m

2.76324

ave/min d

3.85559

ave/min m

12.6881

324_Calib_S_A

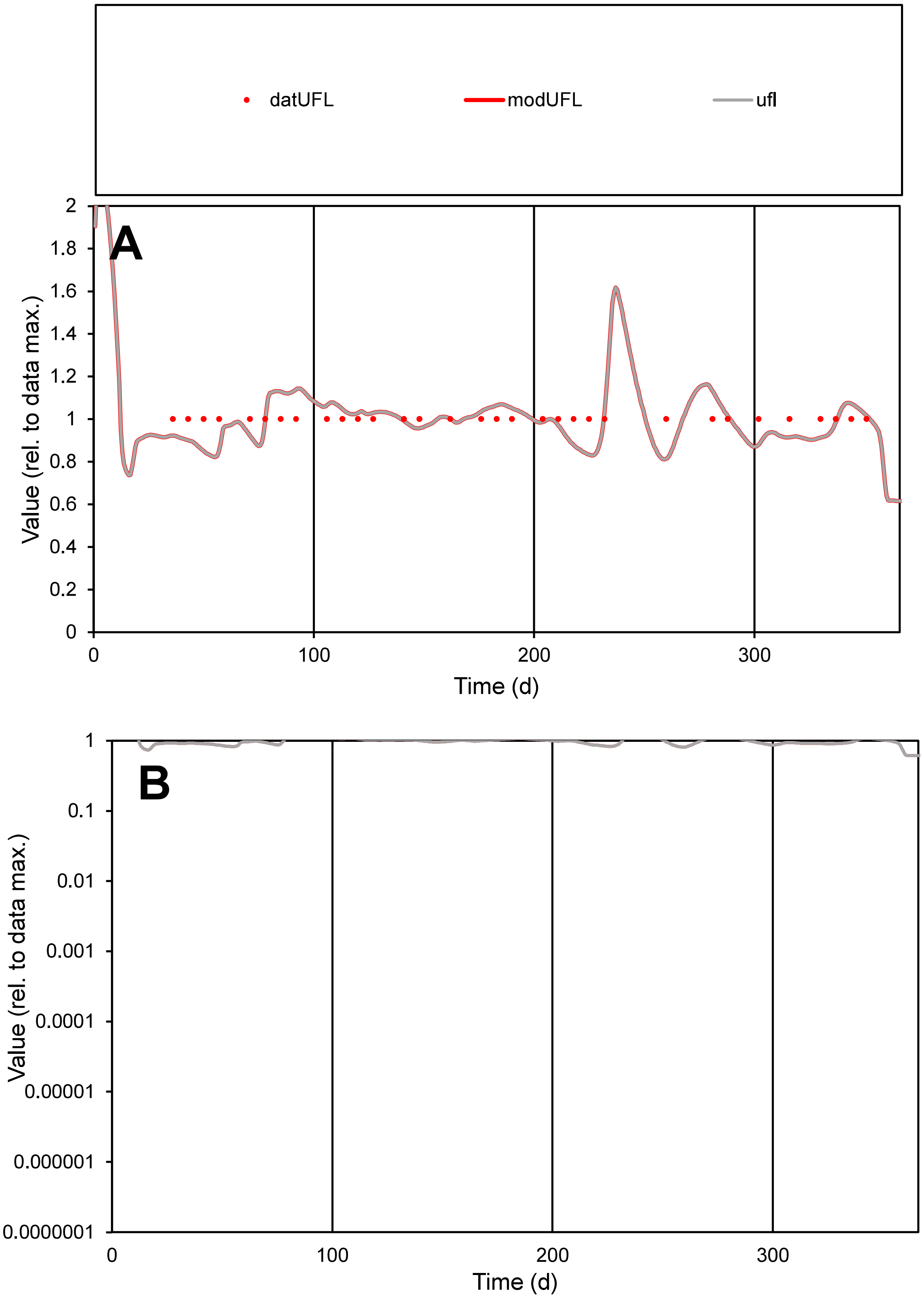

C1

Variables
UFL Provide jtd or Dst
CalibOnly?
2 1 to reproduce of
oNormalize?
2 1=no, 2=yes

ofxn calc*
0.09045
ofxn mod
0.13663

sRun
RunA1
zout
1

Ave
Data
3.1E-06
Model
3E-06

data/vMax
3.1E-06

ofxng
321.551

Min d/m
1.21171

ave/min d
1
ave/min m
1.19797

324_Calib_S_A

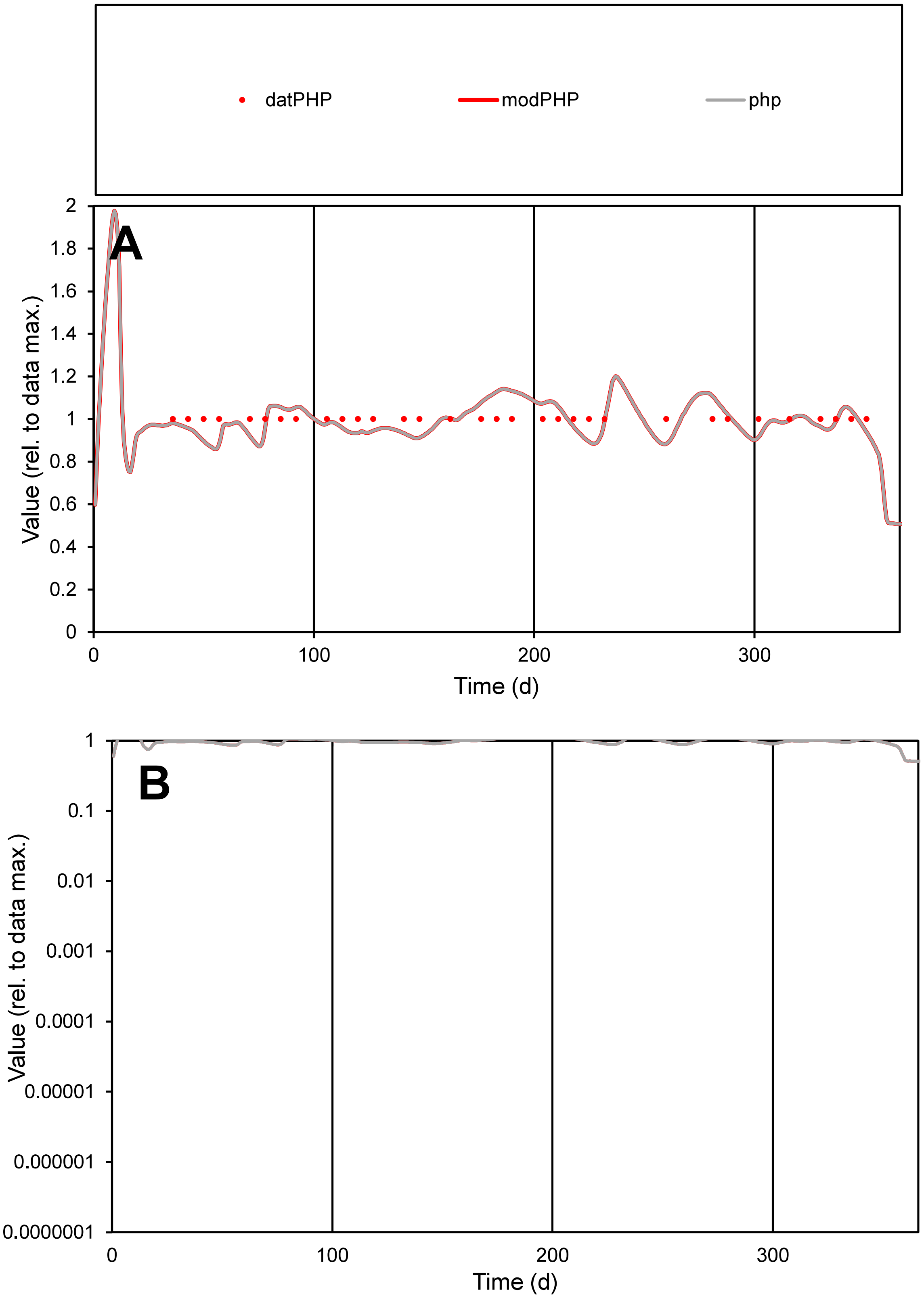

C1

Variables
PHP Provide jtd or Dst
CalibOnly?
2 1 to reproduce of
oNormalize?
2 1=no, 2=yes

ofxn calc*
0.07065
ofxn mod
0.11998

sRun
RunA1
zout
1

Ave
Data
6.9E-06
Model
6.9E-06

data/vMax
6.9E-06

ofxng
321.551

Min d/m
1.13304

ave/min d
1
ave/min m
1.12104

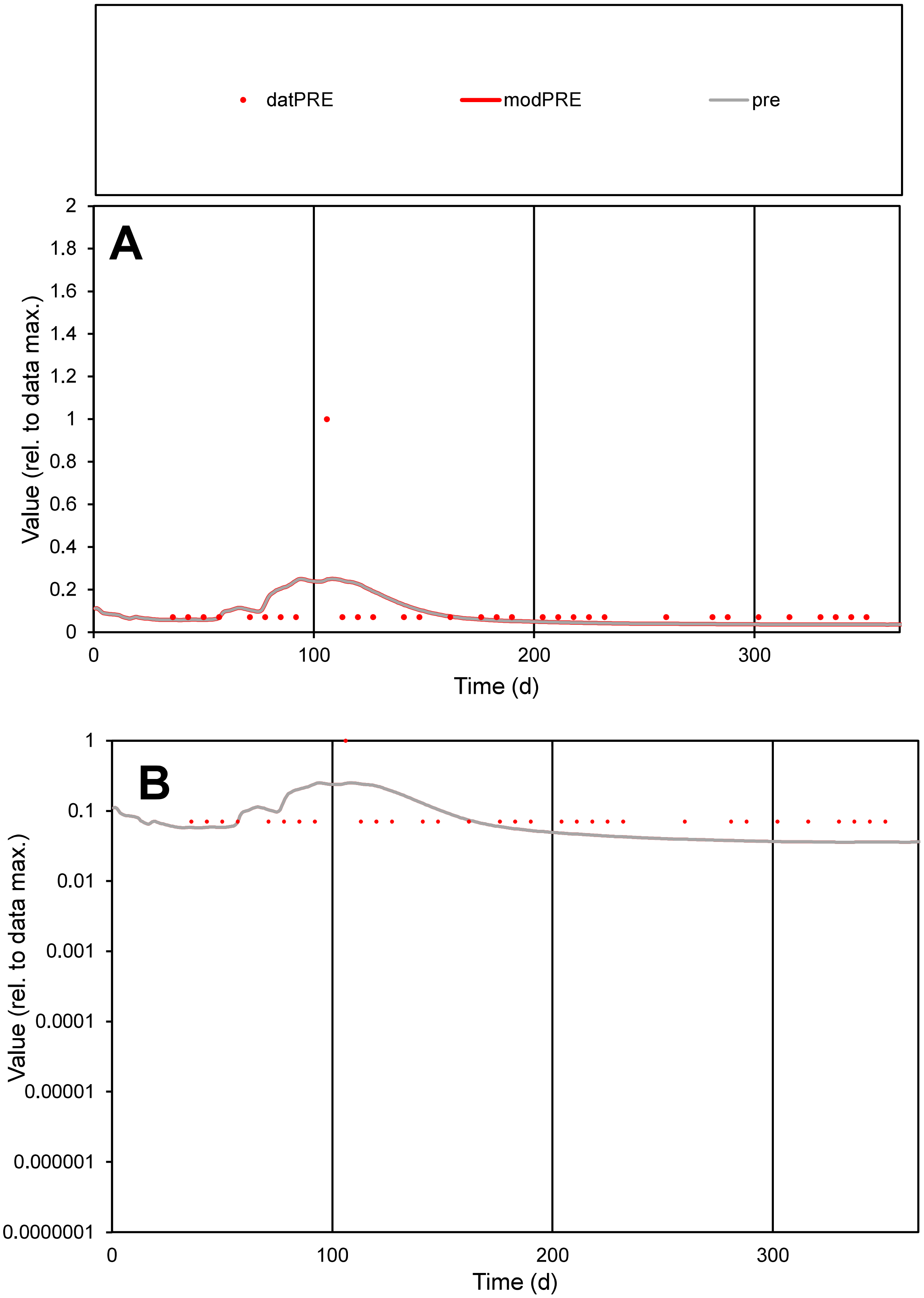

C1

Variables
PRE Provide jtd or Dst
CalibOnly?
2 1 to reproduce of
oNormalize?
2 1=no, 2=yes

ofxn calc*
1.47965
ofxn mod
1.49411

sRun
RunA1
zout
1

Ave
Data
1.9E-05
Model
1.7E-05

data/vMax
0.00019

ofxng
321.551

Min d/m
1.97733

ave/min d
1.41086
ave/min m
2.47894

324_Calib_S_A

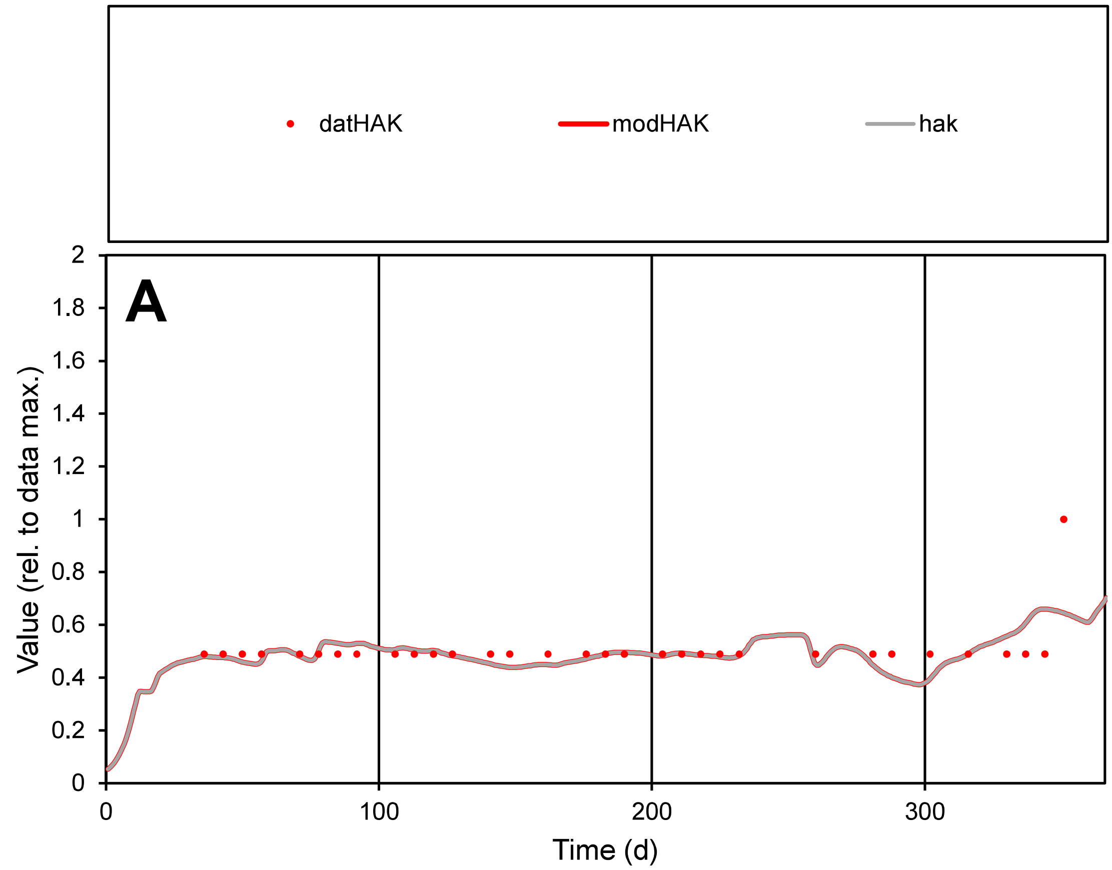

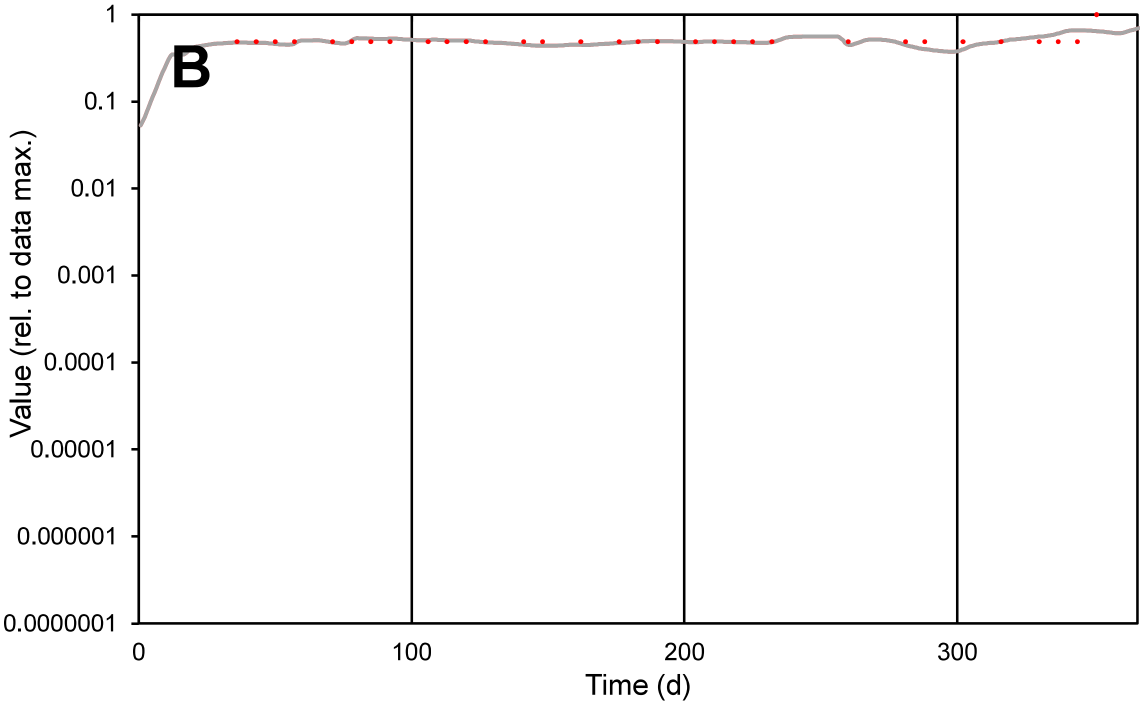

C1

Variables
HAK Provide jtd or Dst
CalibOnly?
2 1 to reproduce of
oNormalize?
2 1=no, 2=yes

ofxn calc*
0.16197
ofxn mod
0.18767

sRun
RunA1
zout
1

Ave
Data
5.3E-06
Model
5.2E-06

data/vMax
1E-05

ofxng
321.551

Min d/m
1.23901

ave/min d
1.03265
ave/min m
1.25539

324_Calib_S_A

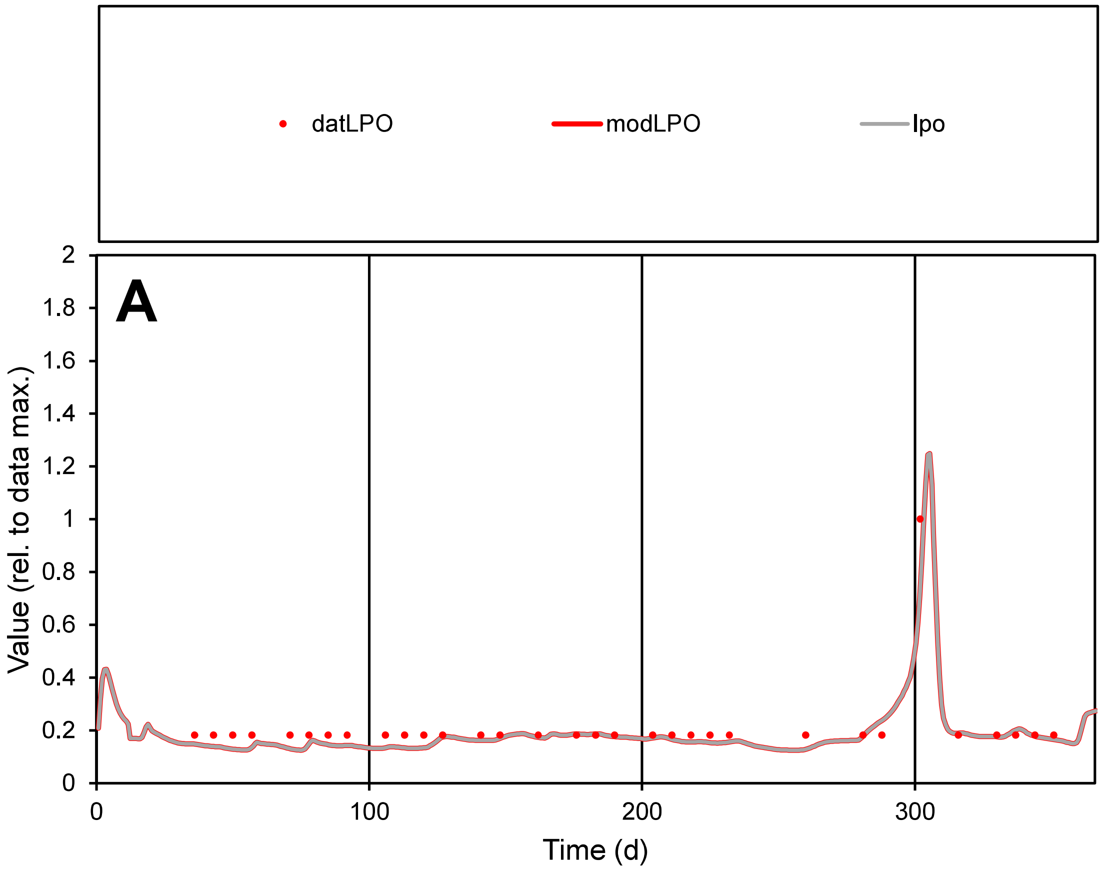

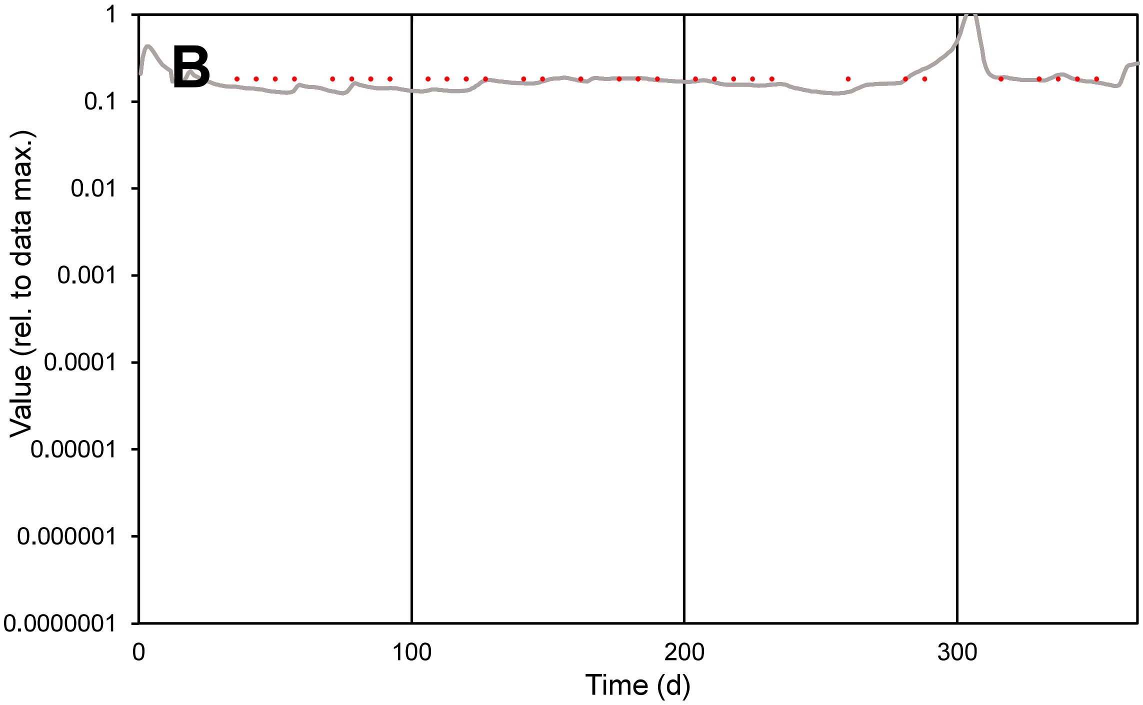

C1

Variables
LPO Provide jtd or Dst
CalibOnly?
2 1 to reproduce of
oNormalize?
2 1=no, 2=yes

ofxn calc*
0.15064
ofxn mod
0.18836

sRun
RunA1
zout
1

Ave
Data
6.7E-06
Model
6.1E-06

data/vMax
3.2E-05

ofxng
321.551

Min d/m
1.42274

ave/min d
1.14058
ave/min m
1.48056

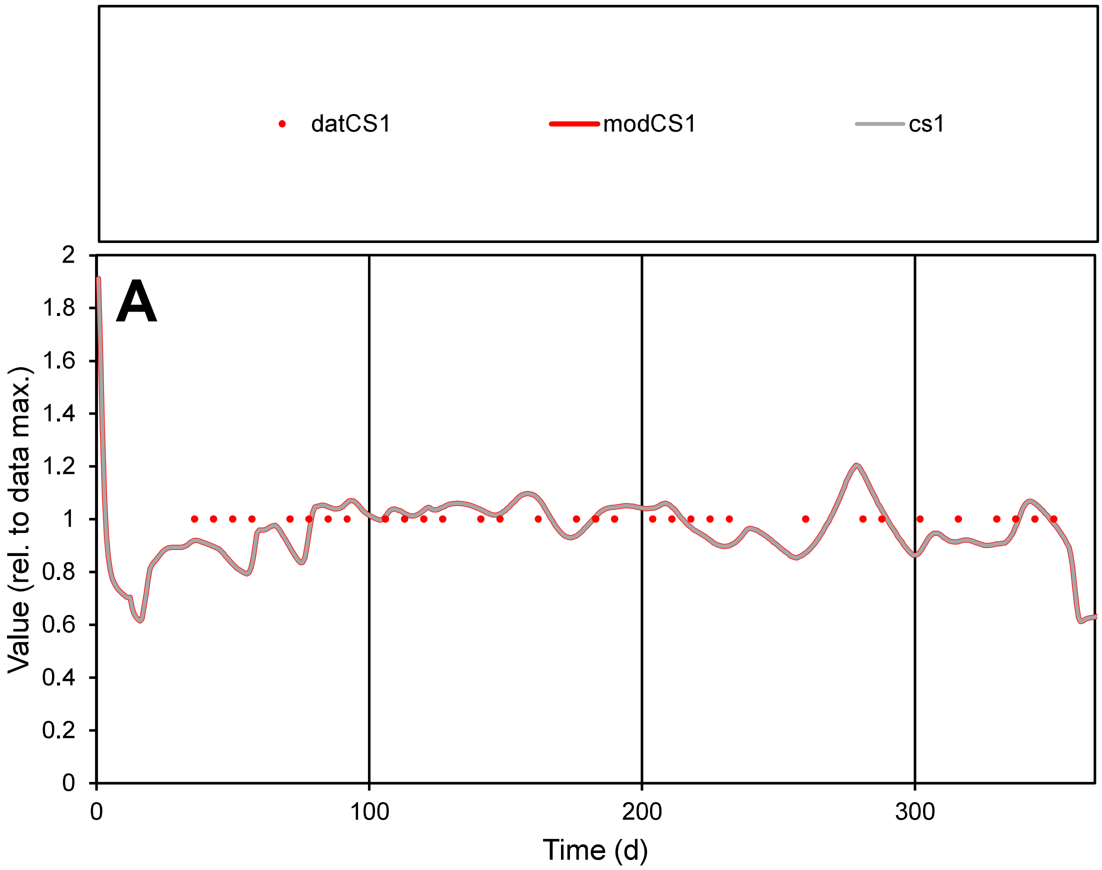

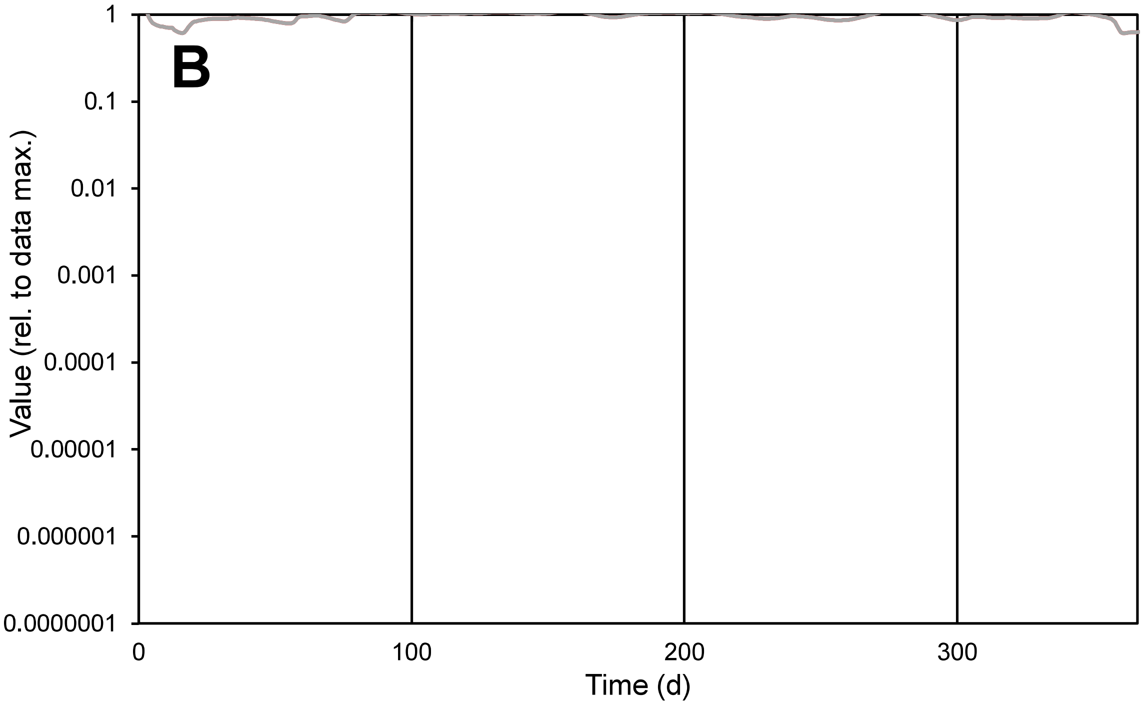

C1

Variables	
CS1	Provide jtd or Dst
CalibOnly?	
2	1 to reproduce of
oNormalize?	
2	1=no, 2=yes
ofxn calc*	
0.07668	
ofxn mod	
0.12281	
sRun	
RunA1	
zout	
1	
Ave	
Data	
1.8E-05	
Model	
1.7E-05	
data/vMax	
1.8E-05	
ofxng	
321.551	
Min d/m	
1.22775	
ave/min d	
1	
ave/min m	
1.20692	

324_Calib_S_A

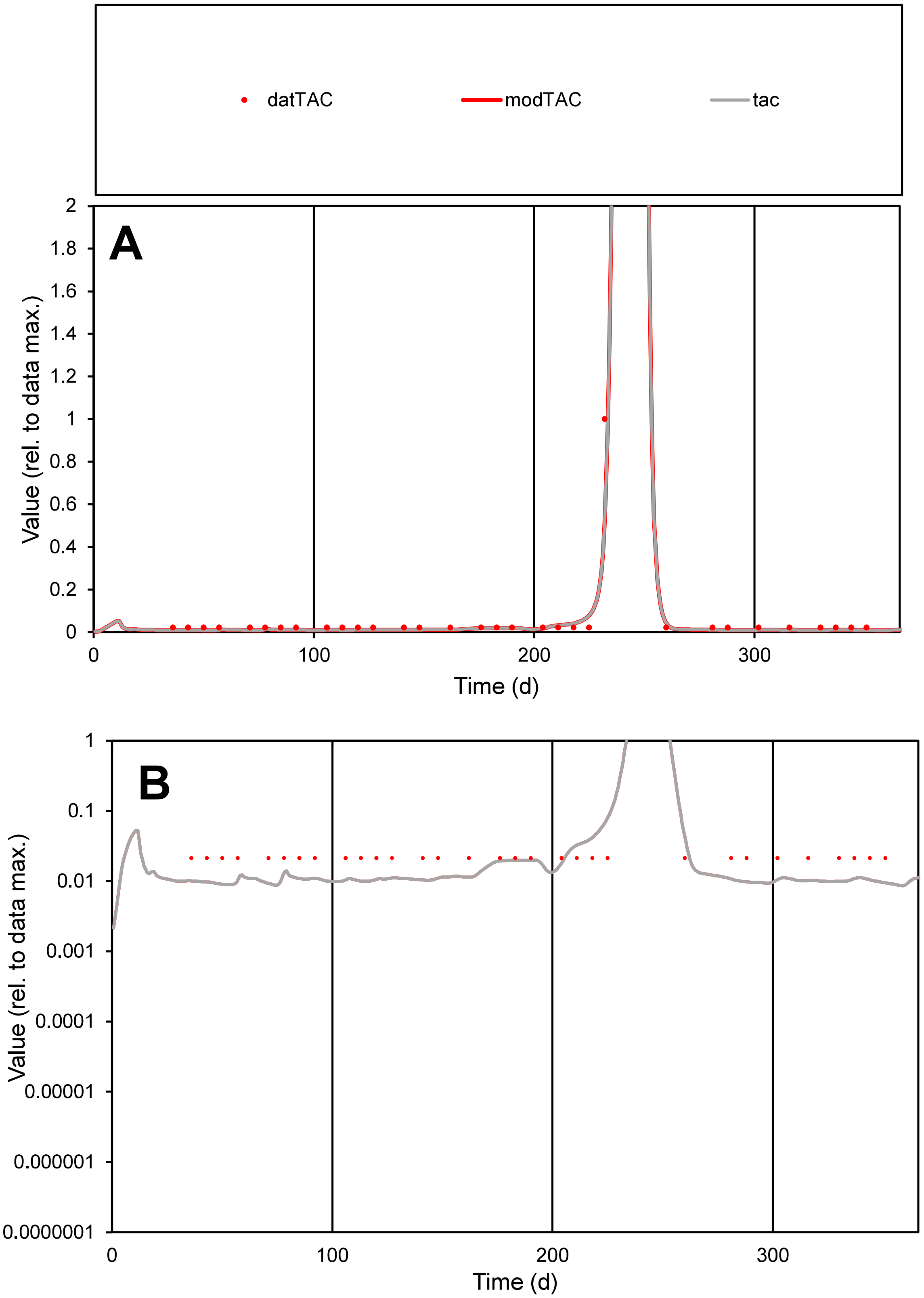

C1

Variables
TAC Provide jtd or Dst
CalibOnly?
2 1 to reproduce of
oNormalize?
2 1=no, 2=yes

ofxn calc*
0.27286
ofxn mod
0.37123

sRun
RunA1
zout
1

Ave
Data
3.2E-06
Model
2.8E-06

data/vMax
6.1E-05

ofxng
321.551

Min d/m
2.38666

ave/min d
2.43424
ave/min m
5.24003

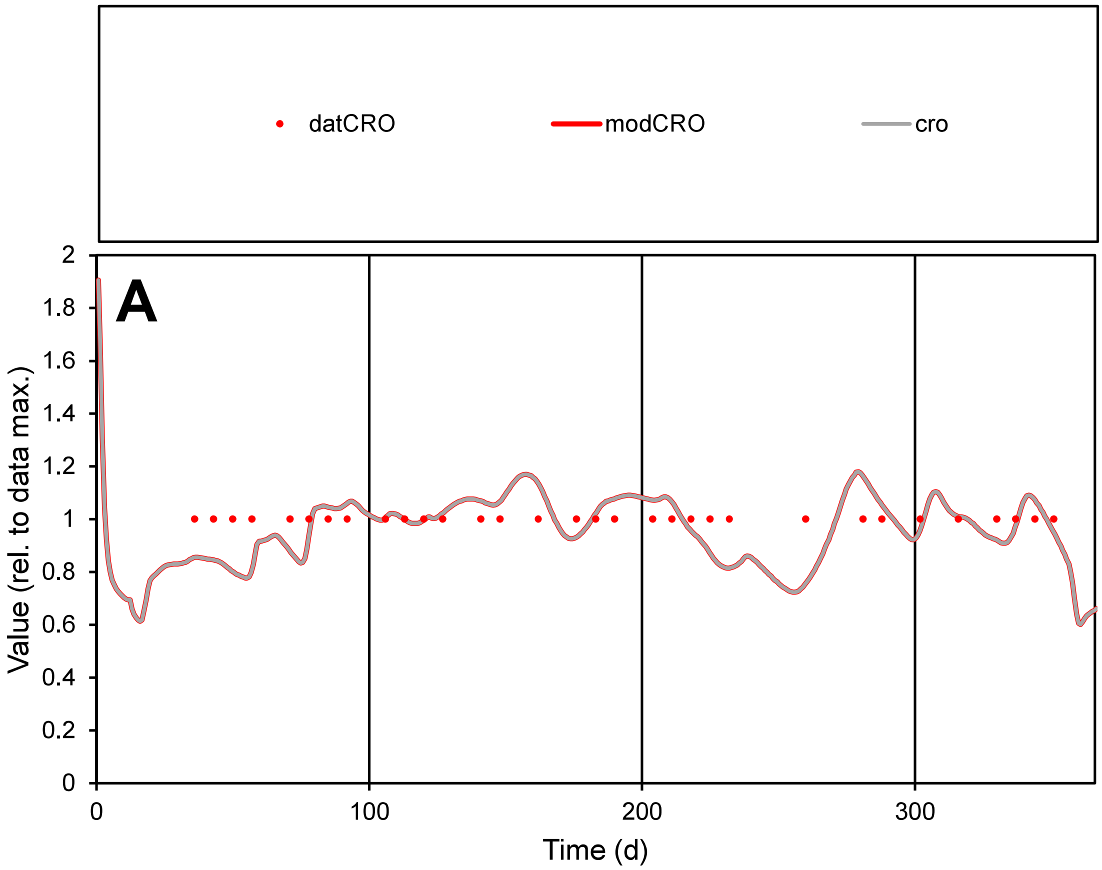

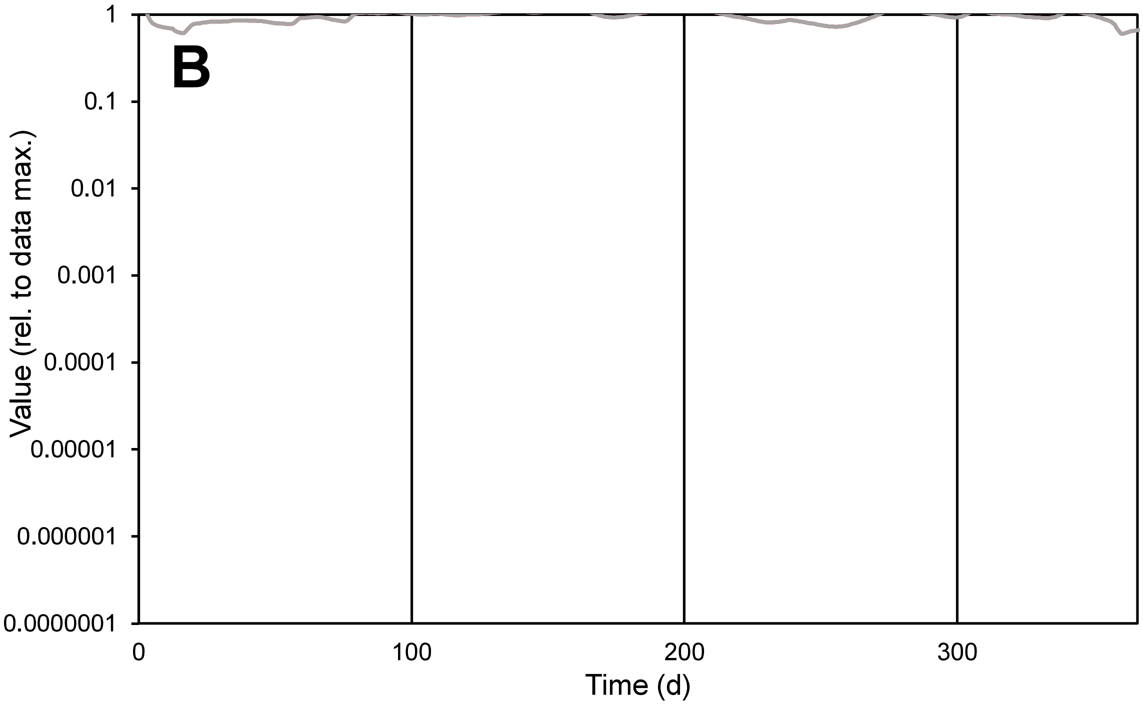

C1

Variables
CRO Provide jtd or Dst
CalibOnly?
2 1 to reproduce of
oNormalize?
2 1=no, 2=yes

ofxn calc*
0.10042
ofxn mod
0.13856

sRun
RunA1
zout
1

Ave
Data
1E-05
Model
1E-05

data/vMax
1E-05

ofxng
321.551

Min d/m
1.29555

ave/min d
1
ave/min m
1.26811

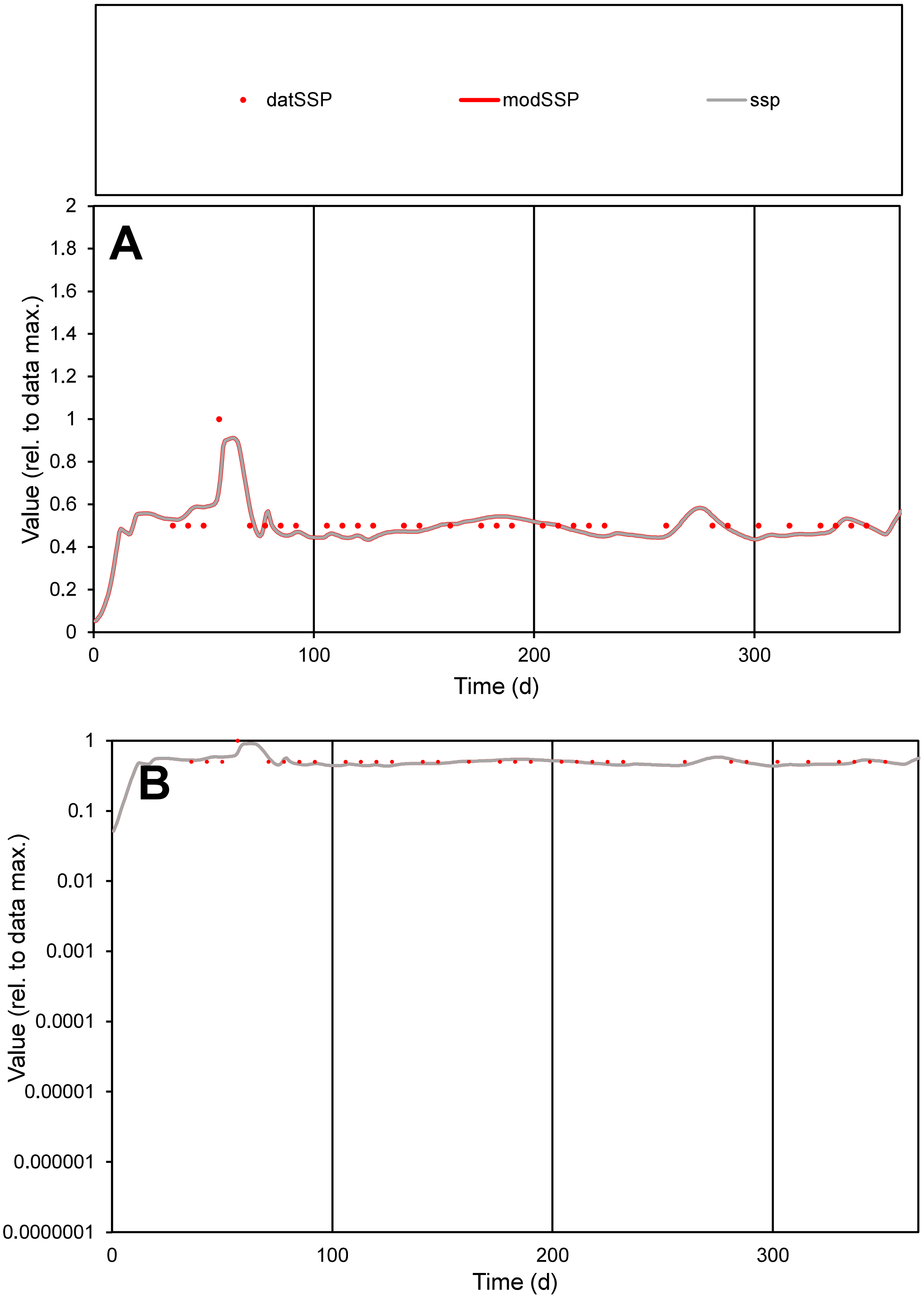

C1

Variables
SSP Provide jtd or Dst
CalibOnly?
2 1 to reproduce of
oNormalize?
2 1=no, 2=yes

ofxn calc*
0.10609
ofxn mod
0.14259

sRun
RunA1
zout
1

Ave
Data
1.4E-05
Model
1.3E-05

data/vMax
2.6E-05

ofxng
321.551

Min d/m
1.12884

ave/min d
1.03125
ave/min m
1.13929

324_Calib_S_A

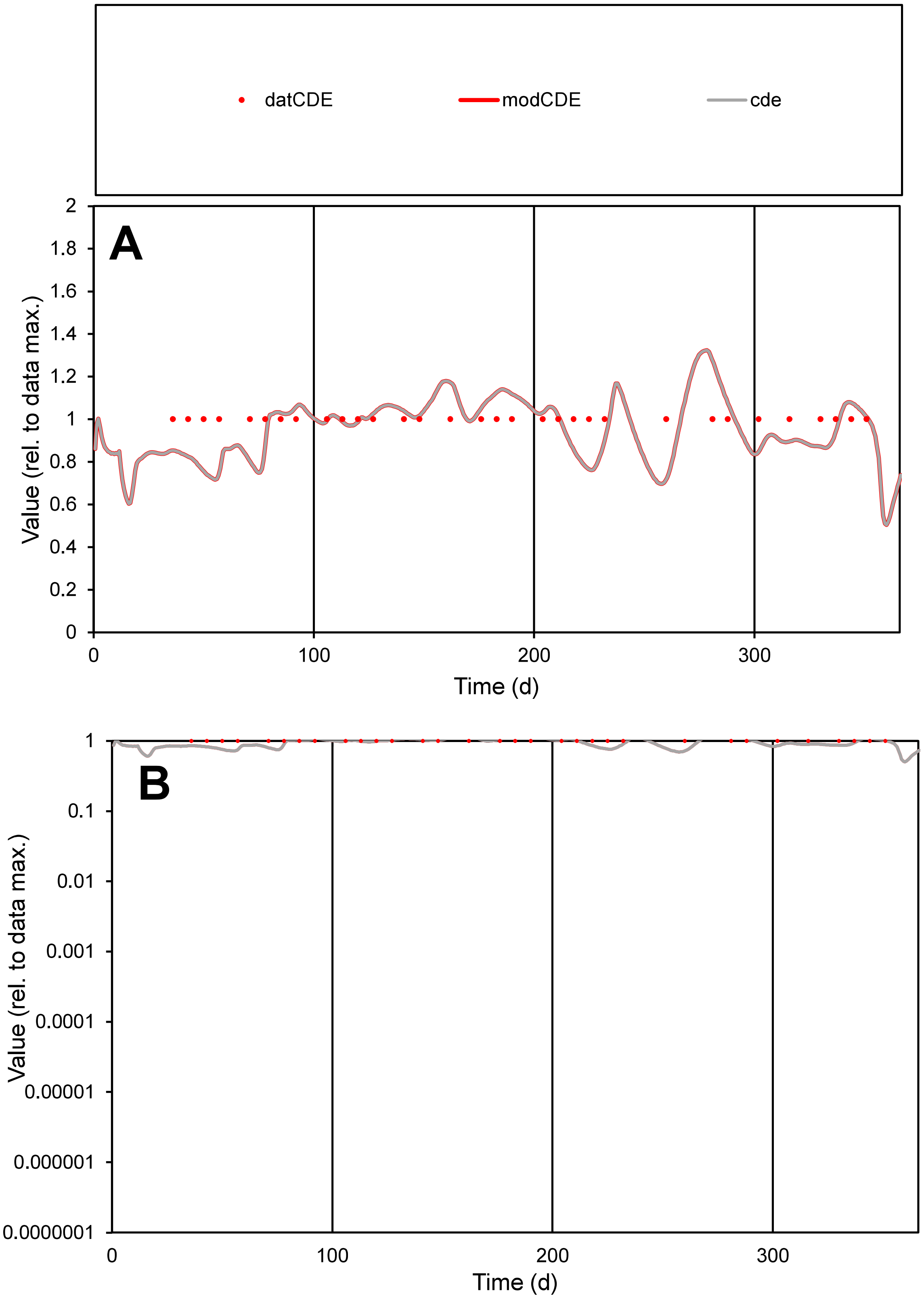

C1

Variables
CDE Provide jtd or Dst
CalibOnly?
2 1 to reproduce of
oNormalize?
2 1=no, 2=yes

ofxn calc*
0.12952
ofxn mod
0.16086

sRun
RunA1
zout
1

Ave
Data
1E-05
Model
9.7E-06

data/vMax
1E-05

ofxng
321.551

Min d/m
1.35069

ave/min d
1
ave/min m
1.30671

324_Calib_S_A

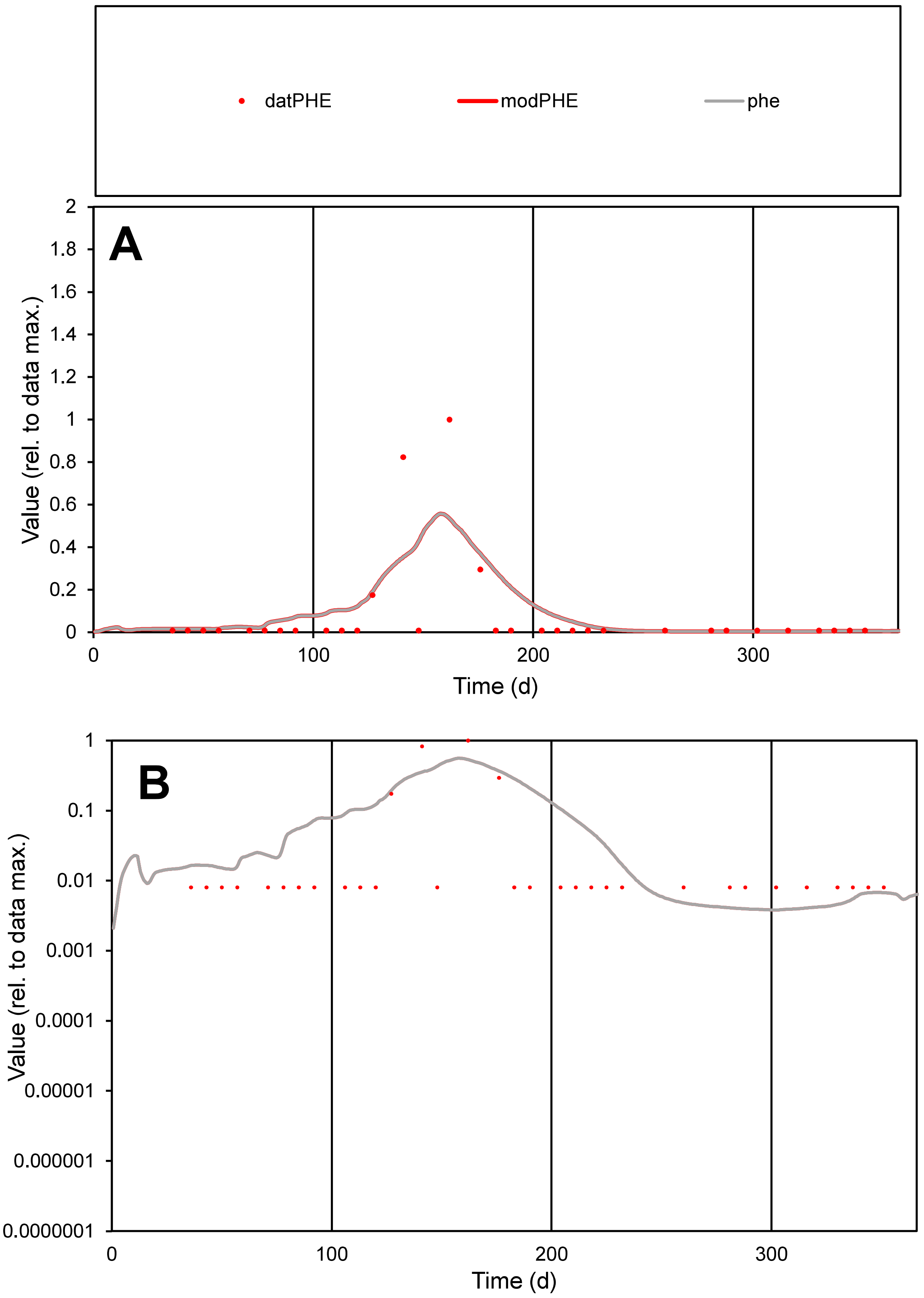

C1

Variables

PHE Provide jtd or Dst

CalibOnly?

2 1 to reproduce of

oNormalize?

2 1=no, 2=yes

ofxn calc*

1.96282

ofxn mod

2.0103

sRun

RunA1

zout

1

Ave

Data

3.9E-05

Model

4.9E-05

data/vMax

0.0005

ofxng

321.551

Min d/m

2.0923

ave/min d

9.82722

ave/min m

25.6765

324_Calib_S_A

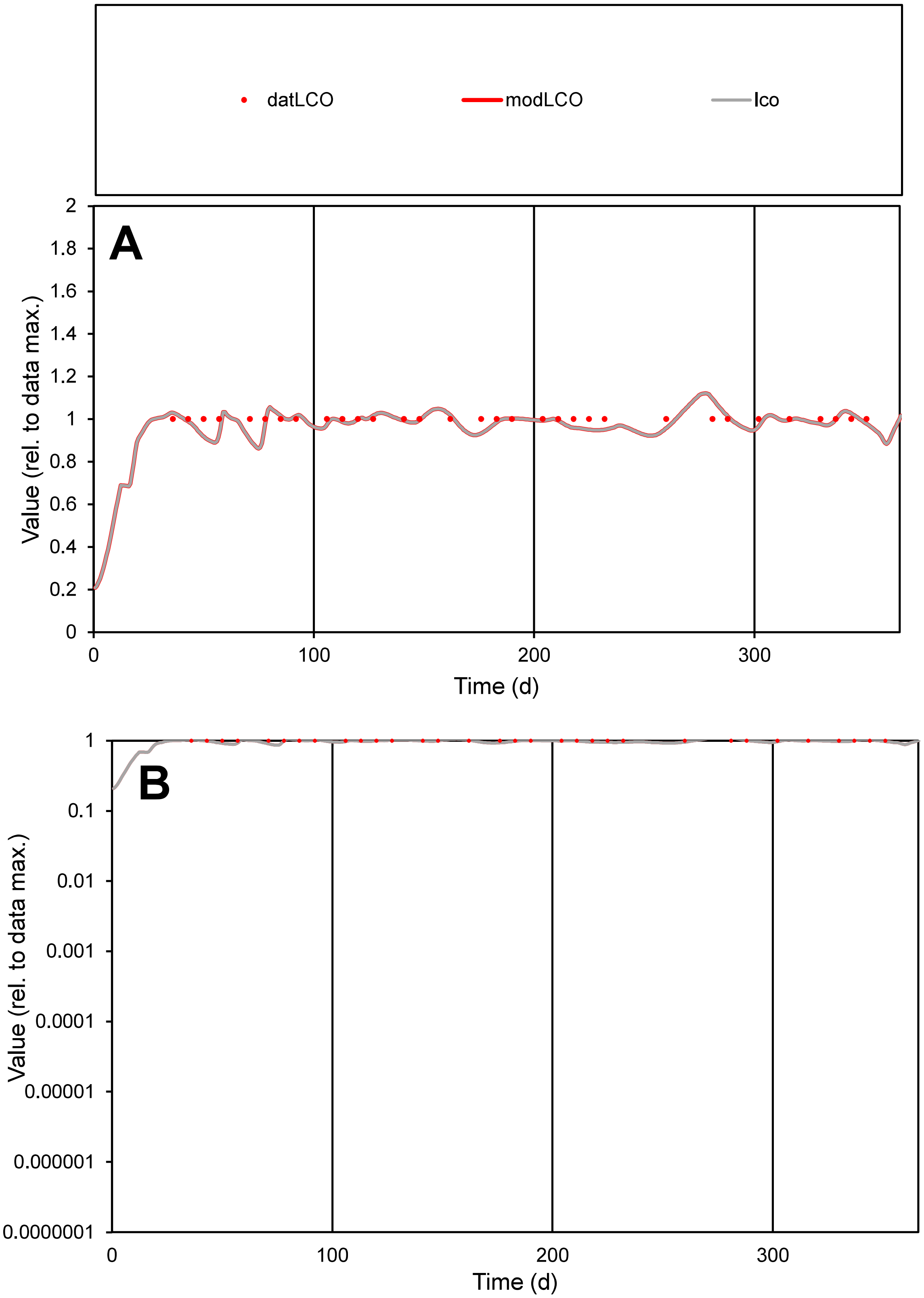

C1

Variables
LCO Provide jtd or Dst
CalibOnly?
2 1 to reproduce of
oNormalize?
2 1=no, 2=yes

ofxn calc*
0.03775
ofxn mod
0.10344

sRun
RunA1
zout
1

Ave
Data
7.4E-06
Model
7.3E-06

data/vMax
7.4E-06

ofxng
321.551

Min d/m
1.12329

ave/min d
1
ave/min m
1.11117

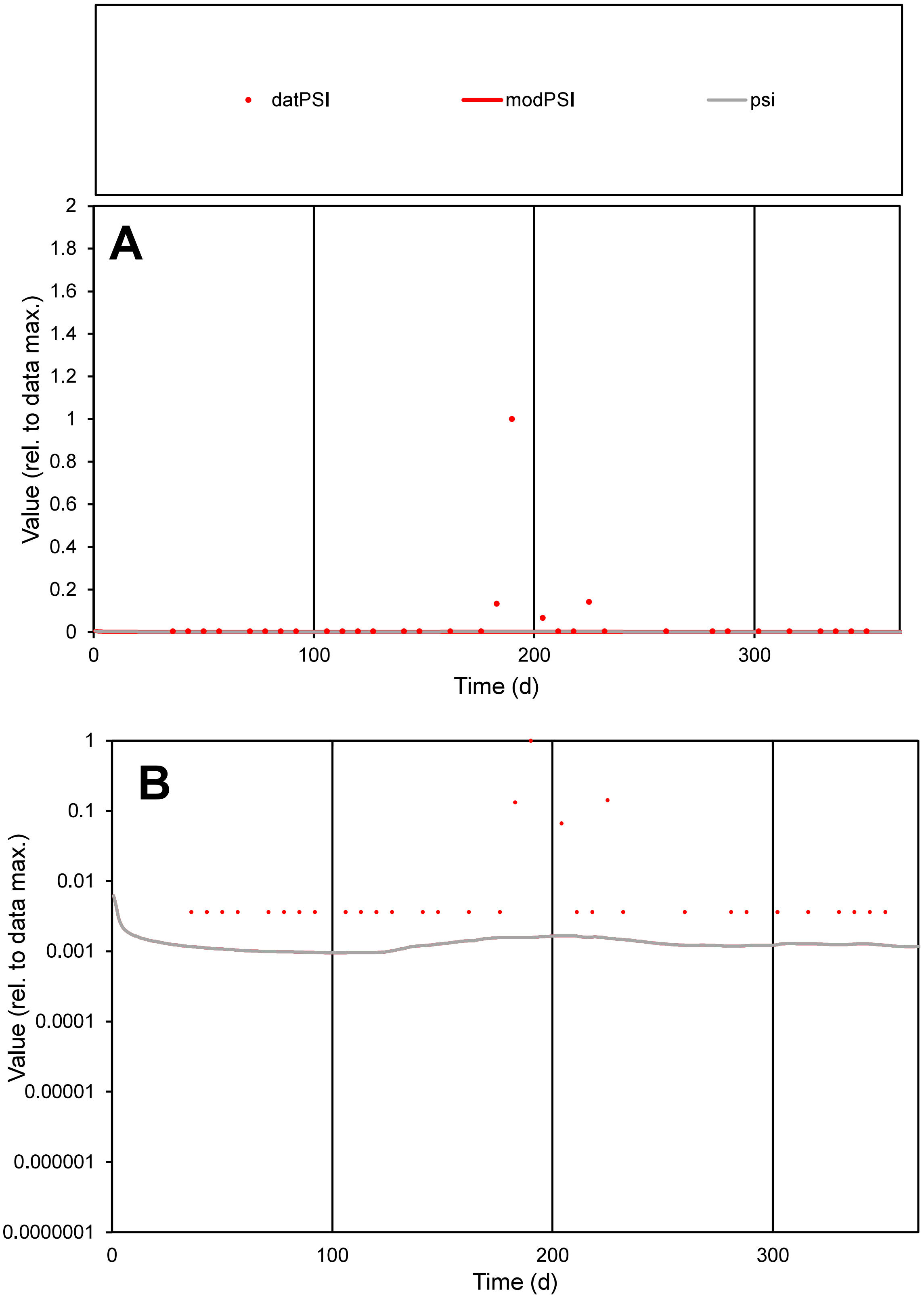

C1

Variables
PSI Provide jtd or Dst
CalibOnly?
2 1 to reproduce of
oNormalize?
2 1=no, 2=yes

ofxn calc*
3.9932
ofxn mod
3.99829

sRun
RunA1
zout
1

Ave
Data
4.4E-05
Model
1.2E-06

data/vMax
0.00098

ofxng
321.551

Min d/m
3.80428

ave/min d
12.4077
ave/min m
1.30324

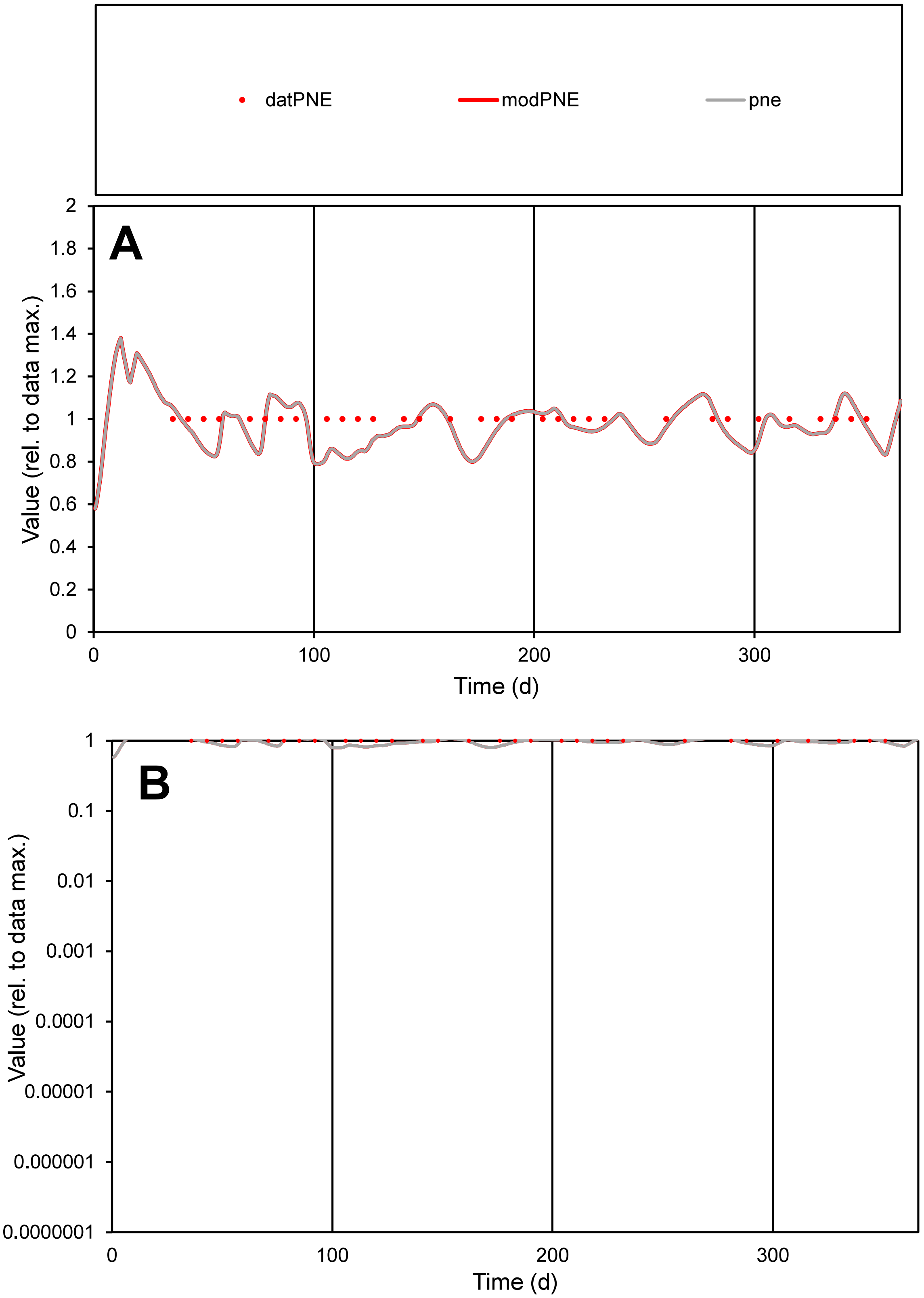

C1

Variables
PNE Provide jtd or Dst
CalibOnly?
2 1 to reproduce of
oNormalize?
2 1=no, 2=yes

ofxn calc*
0.08092
ofxn mod
0.12499

sRun
RunA1
zout
1

Ave
Data
1.5E-05
Model
1.4E-05

data/vMax
1.5E-05

ofxng
321.551

Min d/m
1.22532

ave/min d
1
ave/min m
1.18241

324_Calib_S_A

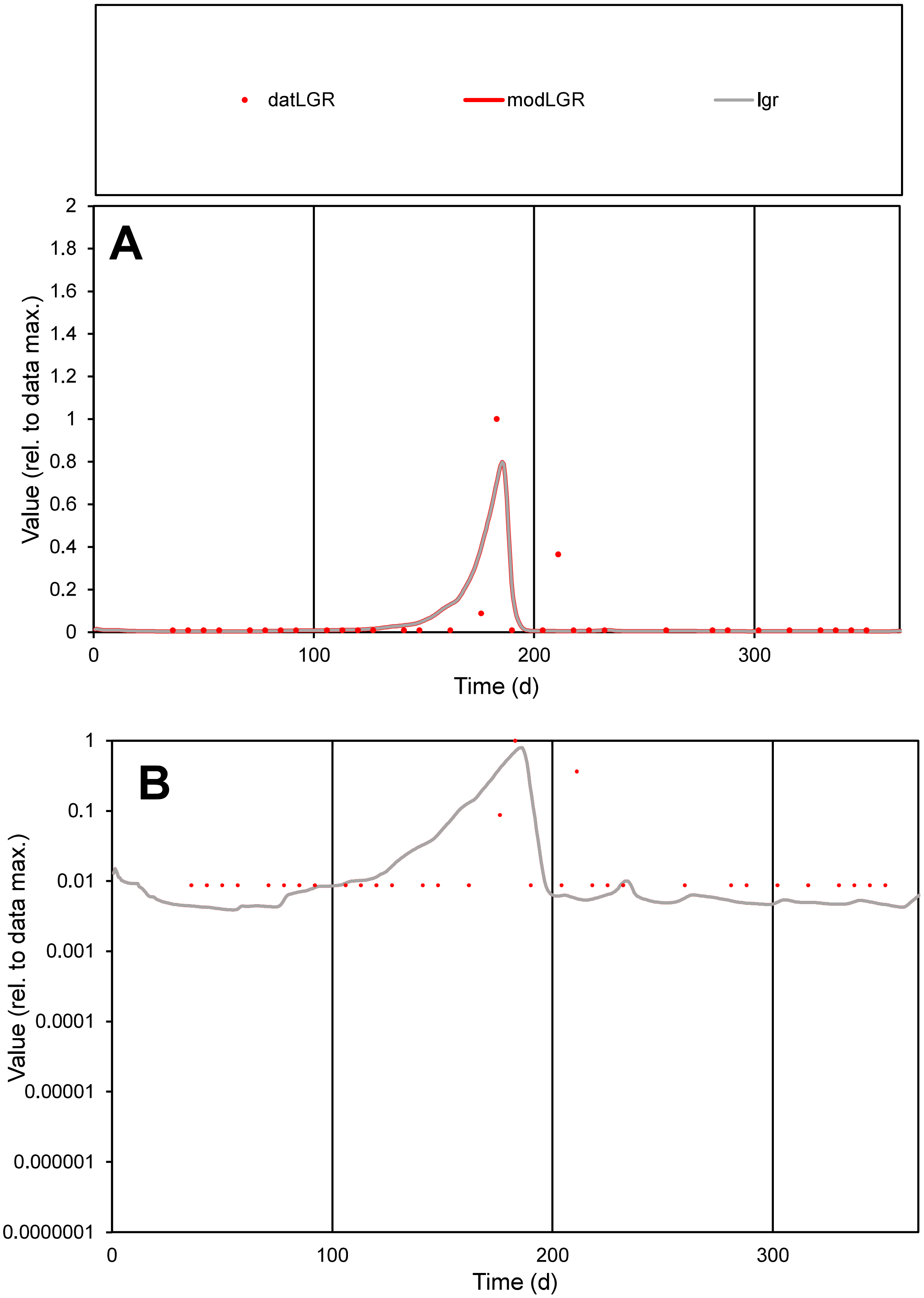

C1

Variables
LGR Provide jtd or Dst
CalibOnly?
2 1 to reproduce of
oNormalize?
2 1=no, 2=yes

ofxn calc*
1.74974
ofxn mod
1.85605

sRun
RunA1
zout
1

Ave
Data
3.2E-05
Model
2.9E-05

data/vMax
0.00059

ofxng
321.551

Min d/m
2.20787

ave/min d
6.10911
ave/min m
12.5738

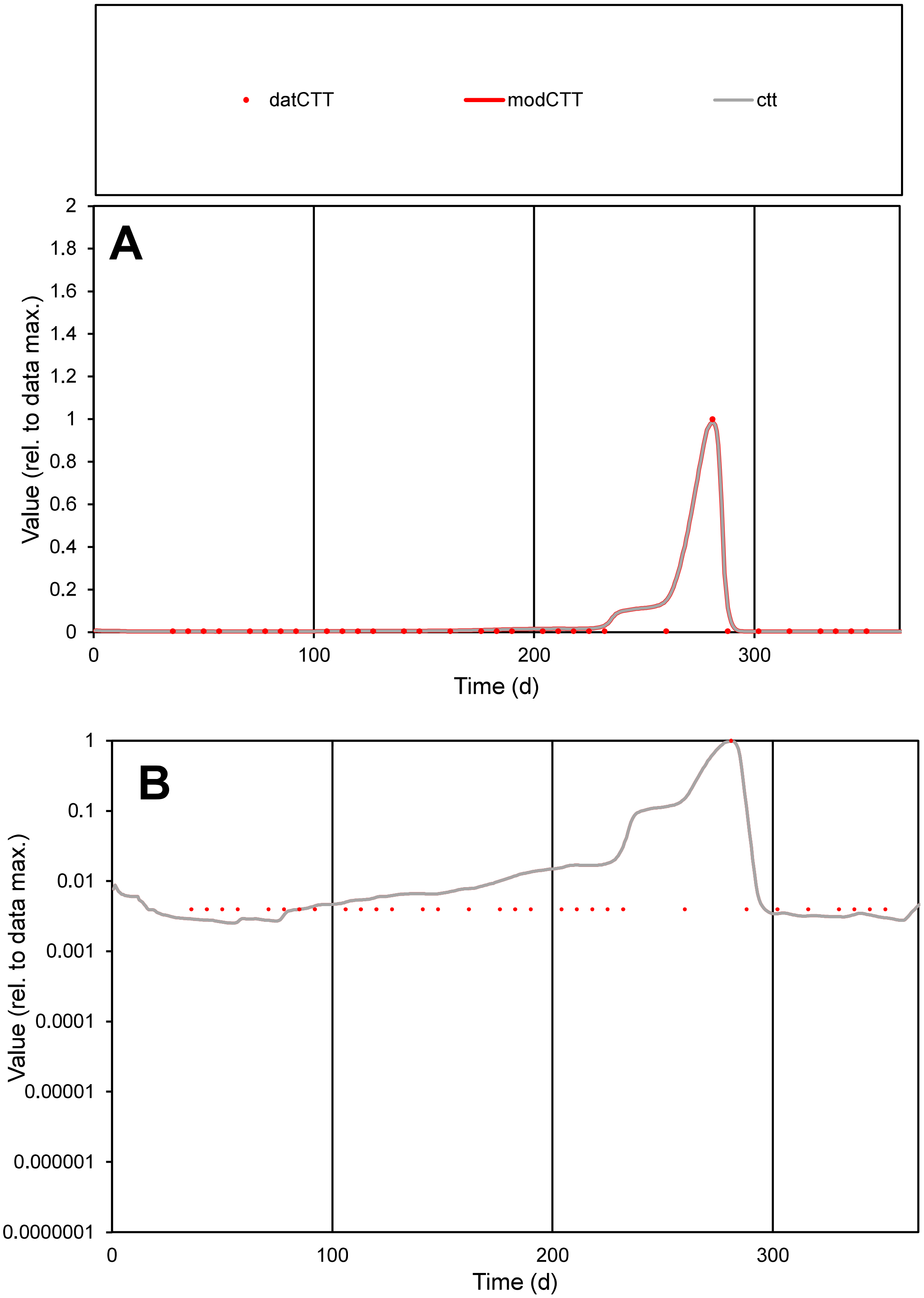

C1

Variables
CTT Provide jtd or Dst
CalibOnly?
2 1 to reproduce of
oNormalize?
2 1=no, 2=yes

ofxn calc*
0.70902
ofxn mod
0.88118

sRun
RunA1
zout
1

Ave
Data
1.3E-05
Model
1.6E-05

data/vMax
0.00037

ofxng
321.551

Min d/m
1.54705

ave/min d
8.84131
ave/min m
16.5407

324_Calib_S_A

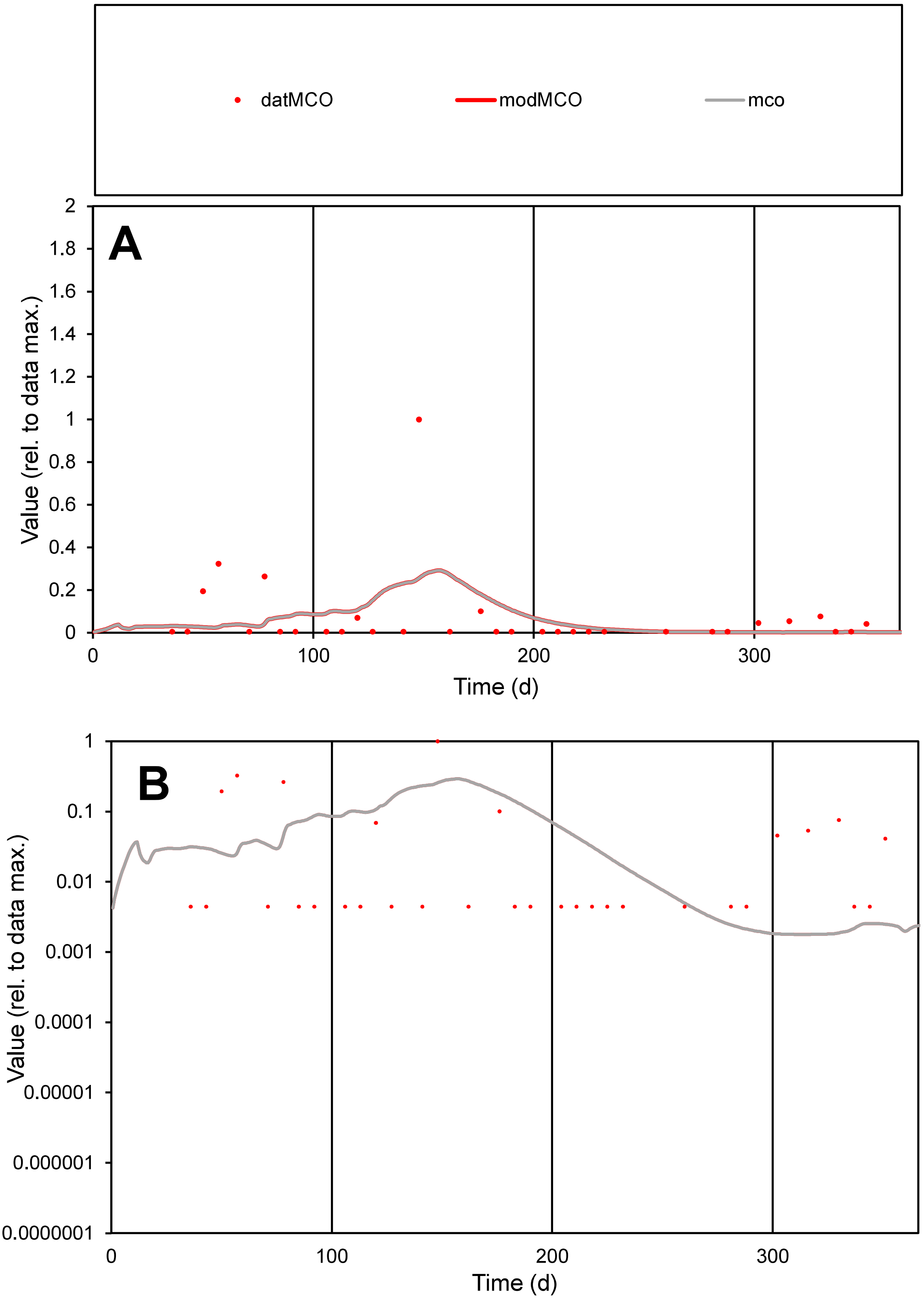

C1

Variables
MCO Provide jtd or Dst
CalibOnly?
2 1 to reproduce of
oNormalize?
2 1=no, 2=yes

ofxn calc*
2.37978
ofxn mod
2.39527

sRun
RunA1
zout
1

Ave
Data
9.8E-07
Model
9.2E-07

data/vMax
1.4E-05

ofxng
321.551

Min d/m
2.48274

ave/min d
15.9851
ave/min m
37.2463

324_Calib_S_A

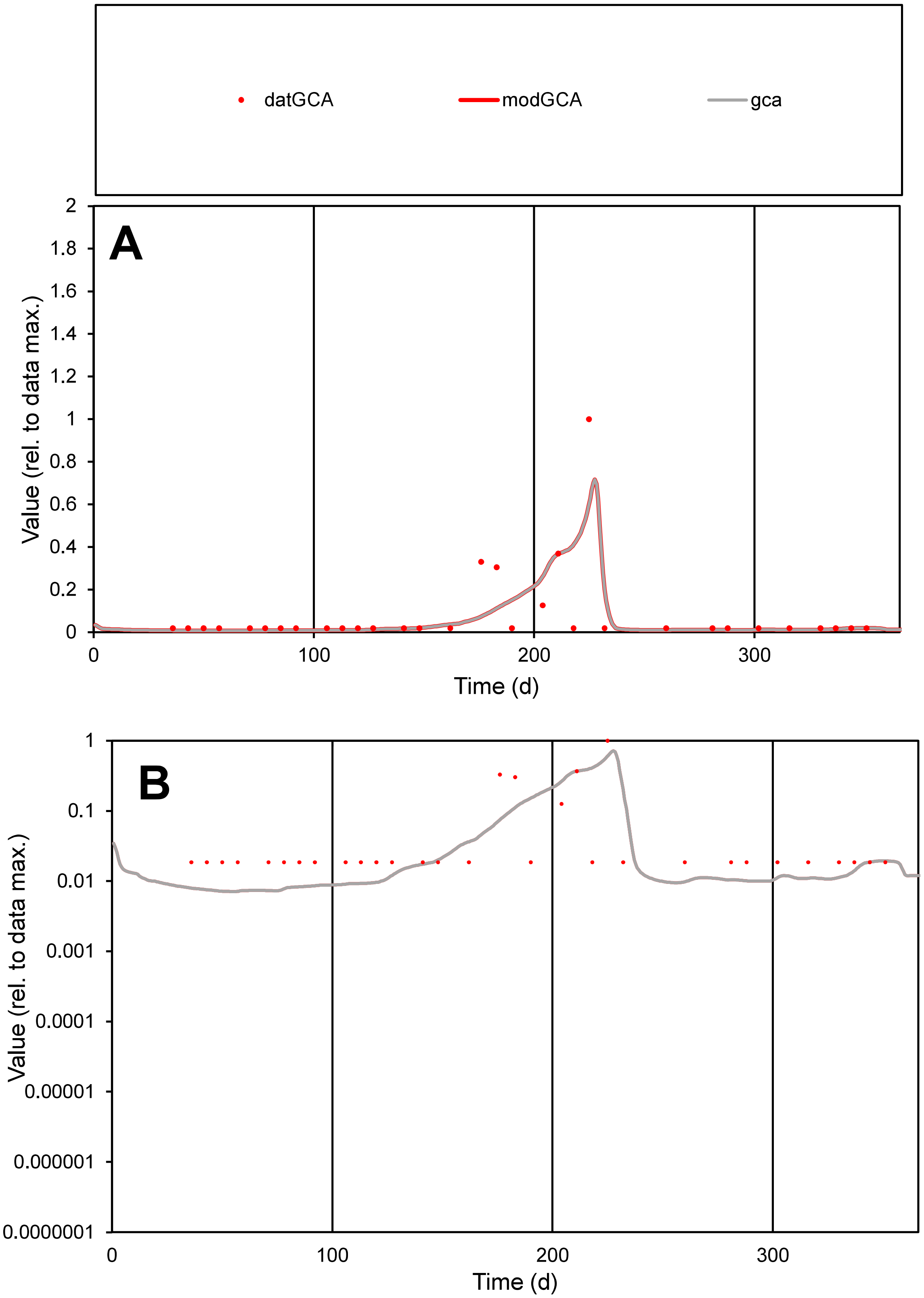

C1

Variables
GCA Provide jtd or Dst
CalibOnly?
2 1 to reproduce of
oNormalize?
2 1=no, 2=yes

ofxn calc*
1.36205
ofxn mod
1.39329

sRun
RunA1
zout
1

Ave
Data
3.7E-05
Model
3.4E-05

data/vMax
0.00045

ofxng
321.551

Min d/m
2.60174

ave/min d
4.42368
ave/min m
10.548

324_Calib_S_A

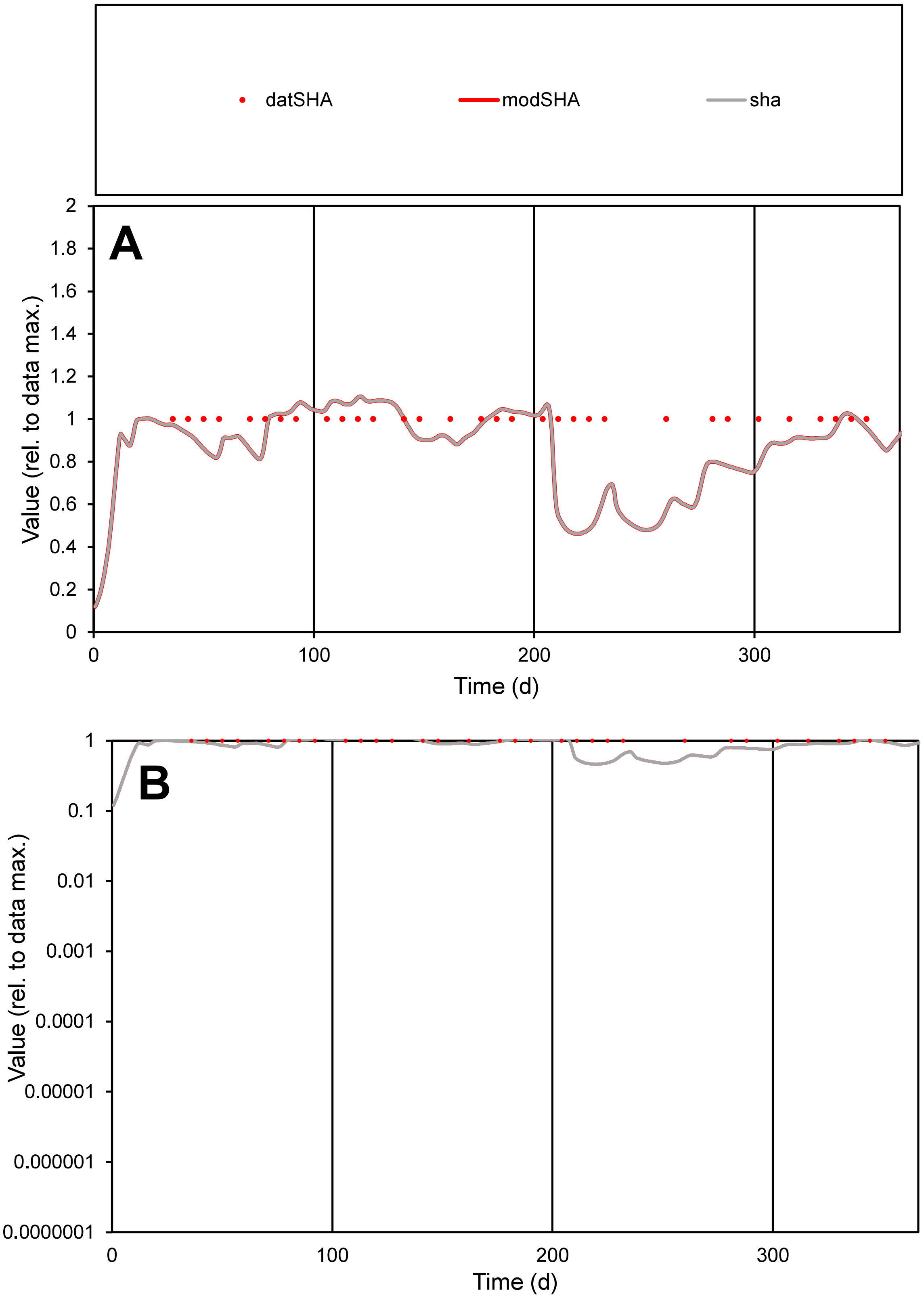

C1

Variables

SHA Provide jtd or Dst

CalibOnly?

2 1 to reproduce of

oNormalize?

2 1=no, 2=yes

ofxn calc*

0.20225

ofxn mod

0.22248

sRun

RunA1

zout

1

Ave

Data

7E-07

Model

6.3E-07

data/vMax

7E-07

ofxng

321.551

Min d/m

2.16517

ave/min d

1

ave/min m

1.94224

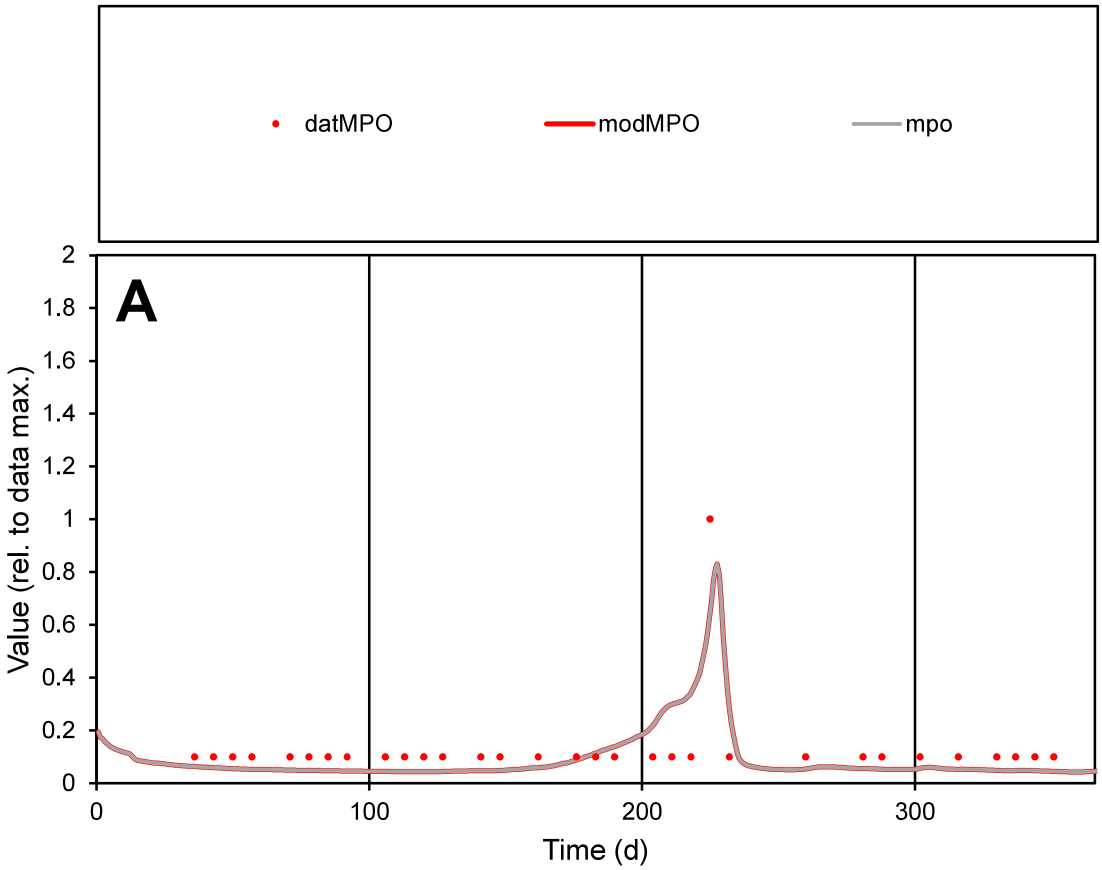

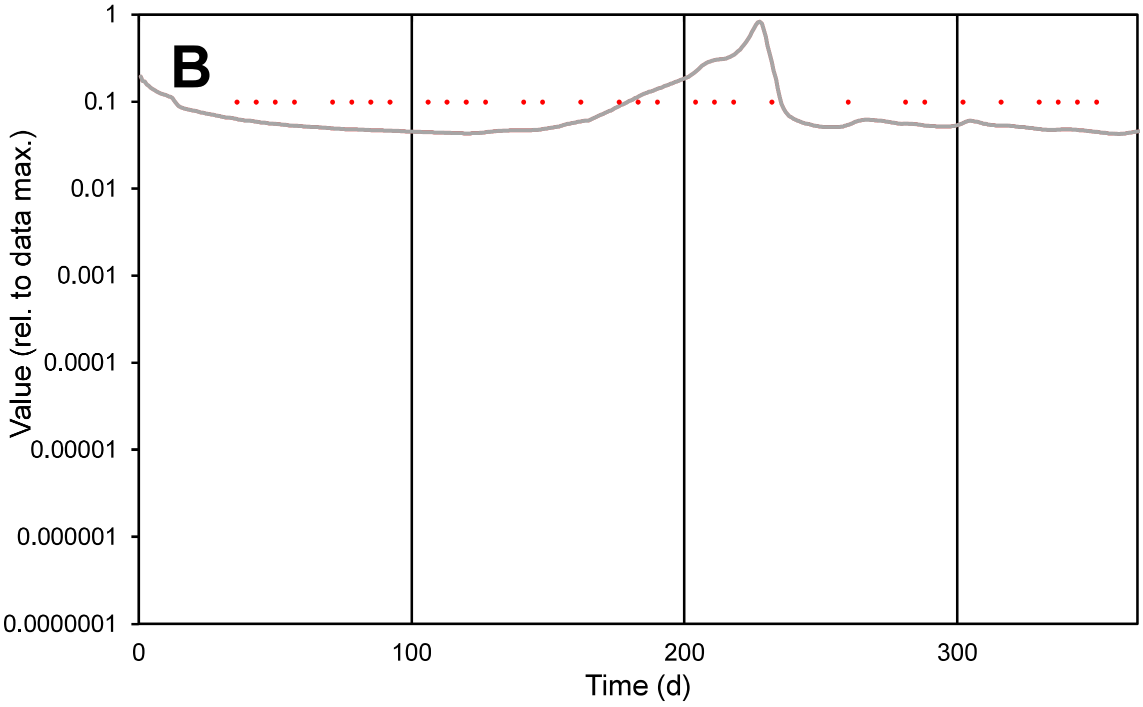

C1

Variables
MPO Provide jtd or Dst
CalibOnly?
2 1 to reproduce of
oNormalize?
2 1=no, 2=yes

ofxn calc*
0.67401
ofxn mod
0.6962

sRun
RunA1
zout
1

Ave
Data
1.6E-05
Model
1.3E-05

data/vMax
0.00012

ofxng
321.551

Min d/m
2.29305

ave/min d
1.28326
ave/min m
2.41483

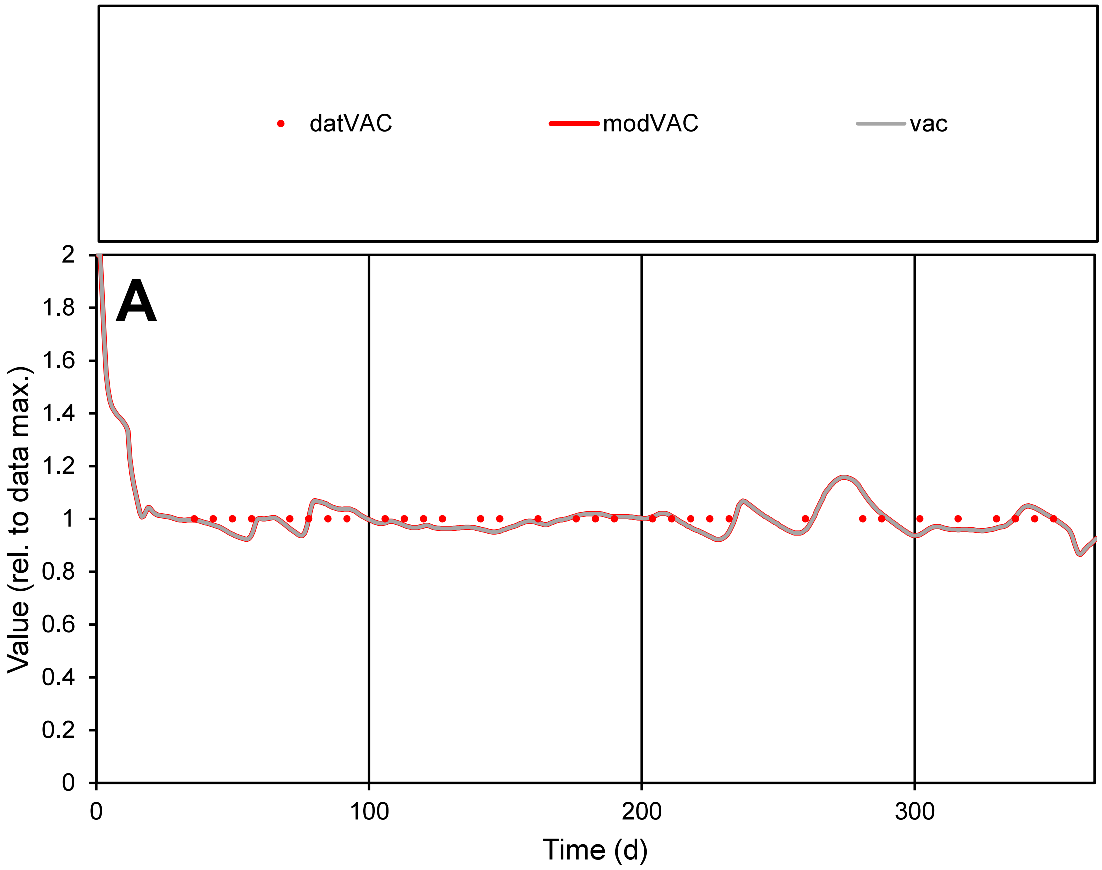

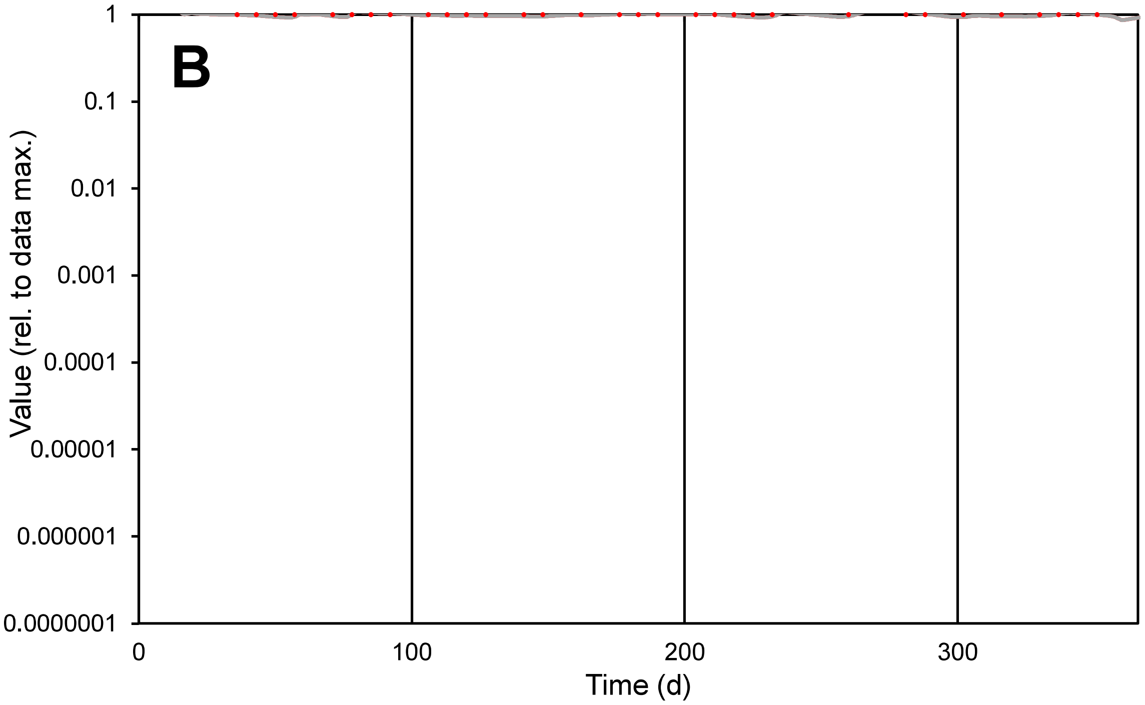

C1

Variables
VAC Provide jtd or Dst
CalibOnly?
2 1 to reproduce of
oNormalize?
2 1=no, 2=yes

ofxn calc*
0.03758
ofxn mod
0.10334

sRun
RunA1
zout
1

Ave
Data
9.5E-06
Model
9.4E-06

data/vMax
9.5E-06

ofxng
321.551

Min d/m
1.07832

ave/min d
1
ave/min m
1.06772

324_Calib_S_A

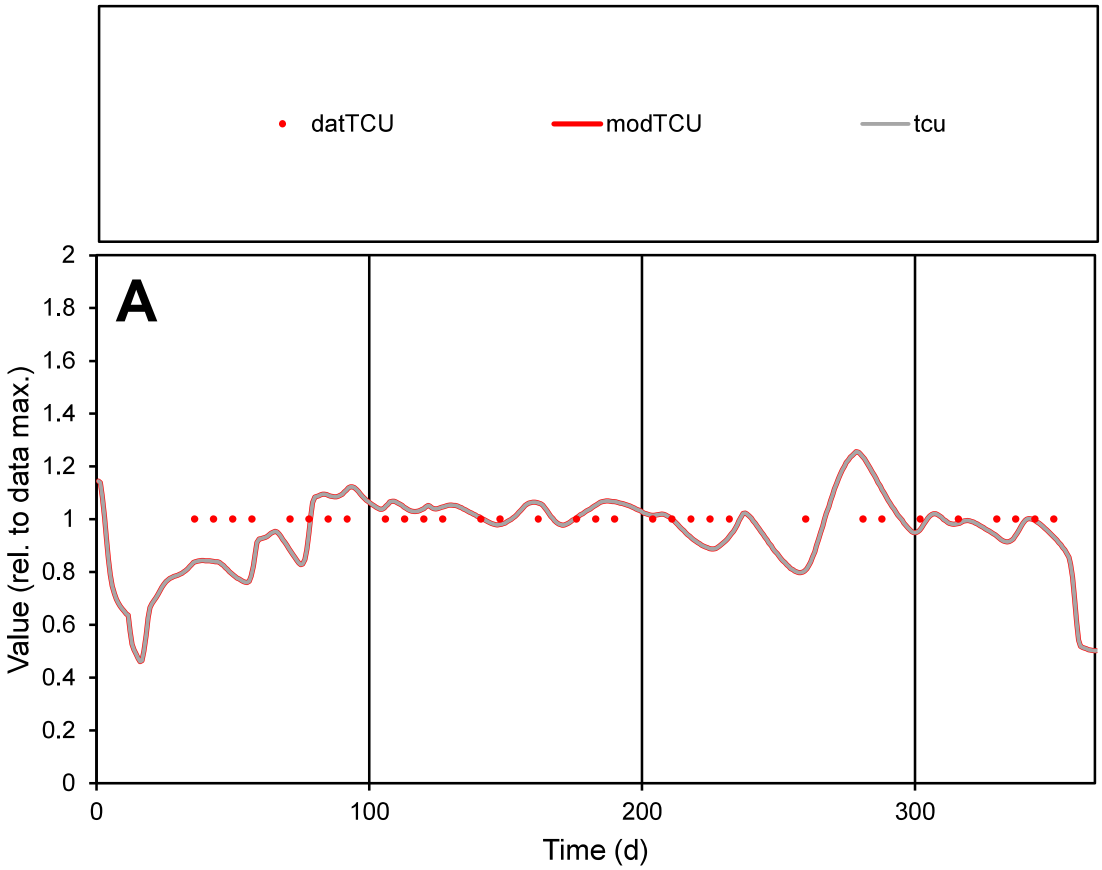

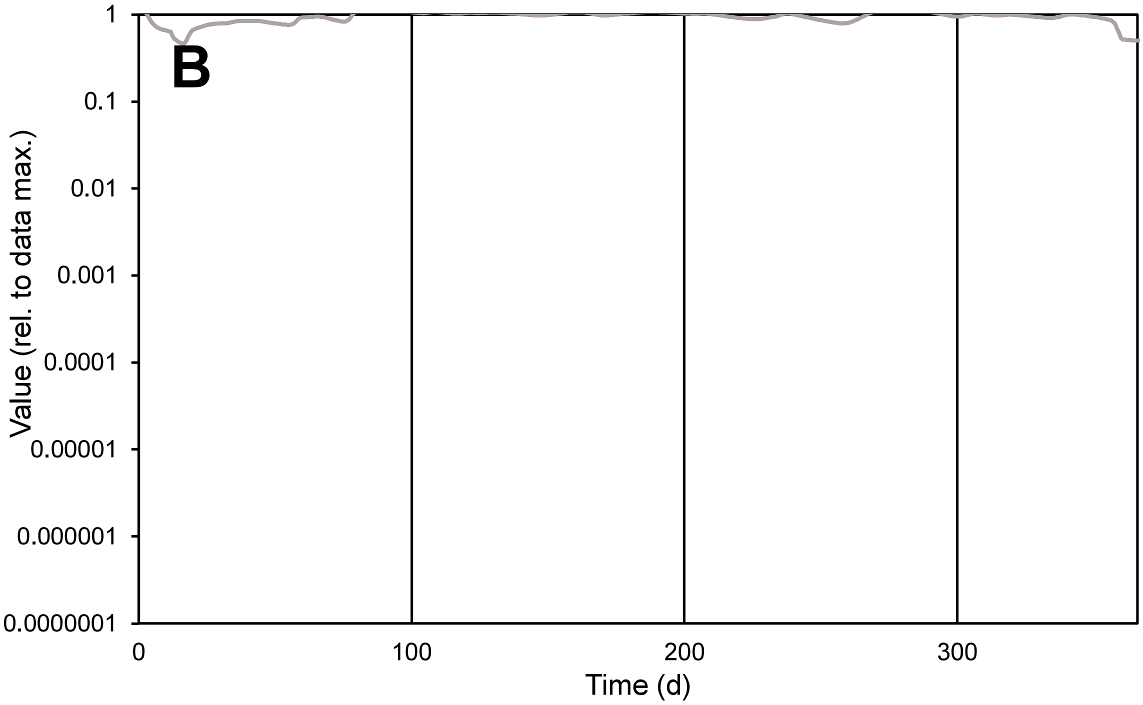

C1

Variables
TCU Provide jtd or Dst
CalibOnly?
2 1 to reproduce of
oNormalize?
2 1=no, 2=yes

ofxn calc*
0.09801
ofxn mod
0.13703

sRun
RunA1
zout
1

Ave
Data
9.8E-06
Model
9.6E-06

data/vMax
9.8E-06

ofxng
321.551

Min d/m
1.28609

ave/min d
1
ave/min m
1.26542

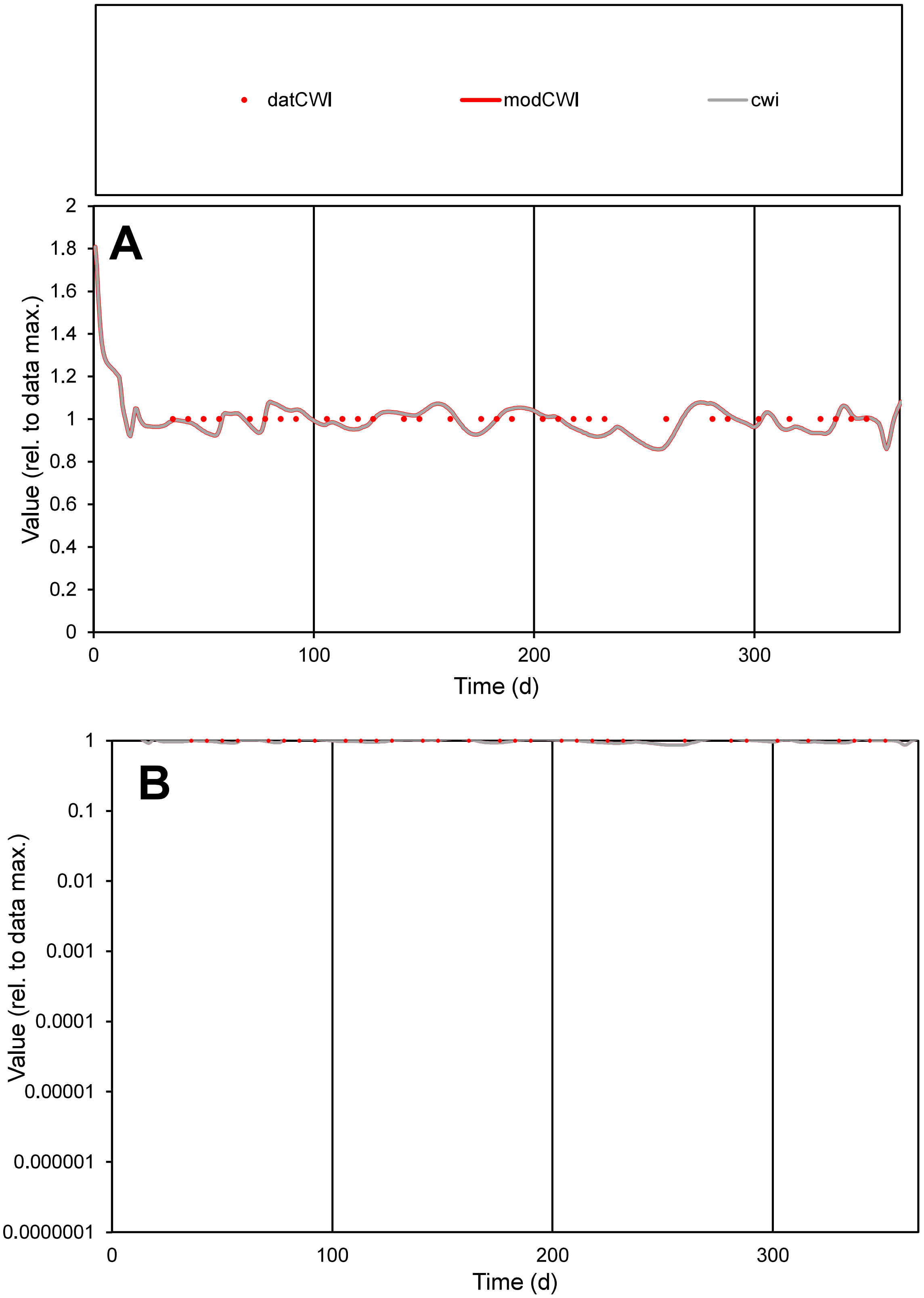

C1

Variables

CWI Provide jtd or Dst

CalibOnly?

2 1 to reproduce of

oNormalize?

2 1=no, 2=yes

ofxn calc*

0.04501

ofxn mod

0.1064

sRun

RunA1

zout

1

Ave

Data

1.3E-05

Model

1.3E-05

data/vMax

1.3E-05

ofxng

321.551

Min d/m

1.12247

ave/min d

1

ave/min m

1.11386

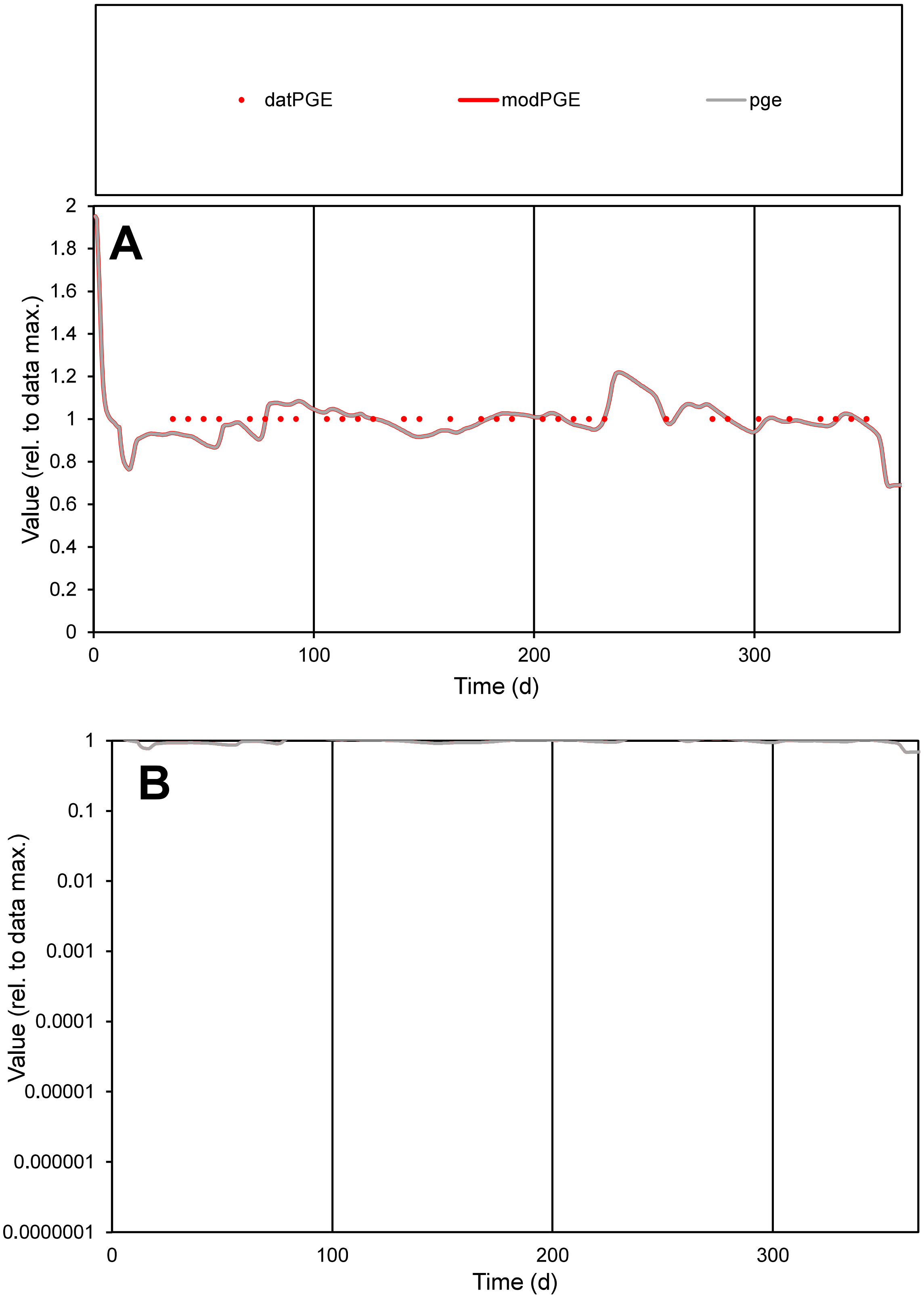

C1

Variables
PGE Provide jtd or Dst
CalibOnly?
2 1 to reproduce of
oNormalize?
2 1=no, 2=yes

ofxn calc*
0.0501
ofxn mod
0.10887

sRun
RunA1
zout
1

Ave
Data
5.9E-06
Model
5.8E-06

data/vMax
5.9E-06

ofxng
321.551

Min d/m
1.14006

ave/min d
1
ave/min m
1.12738

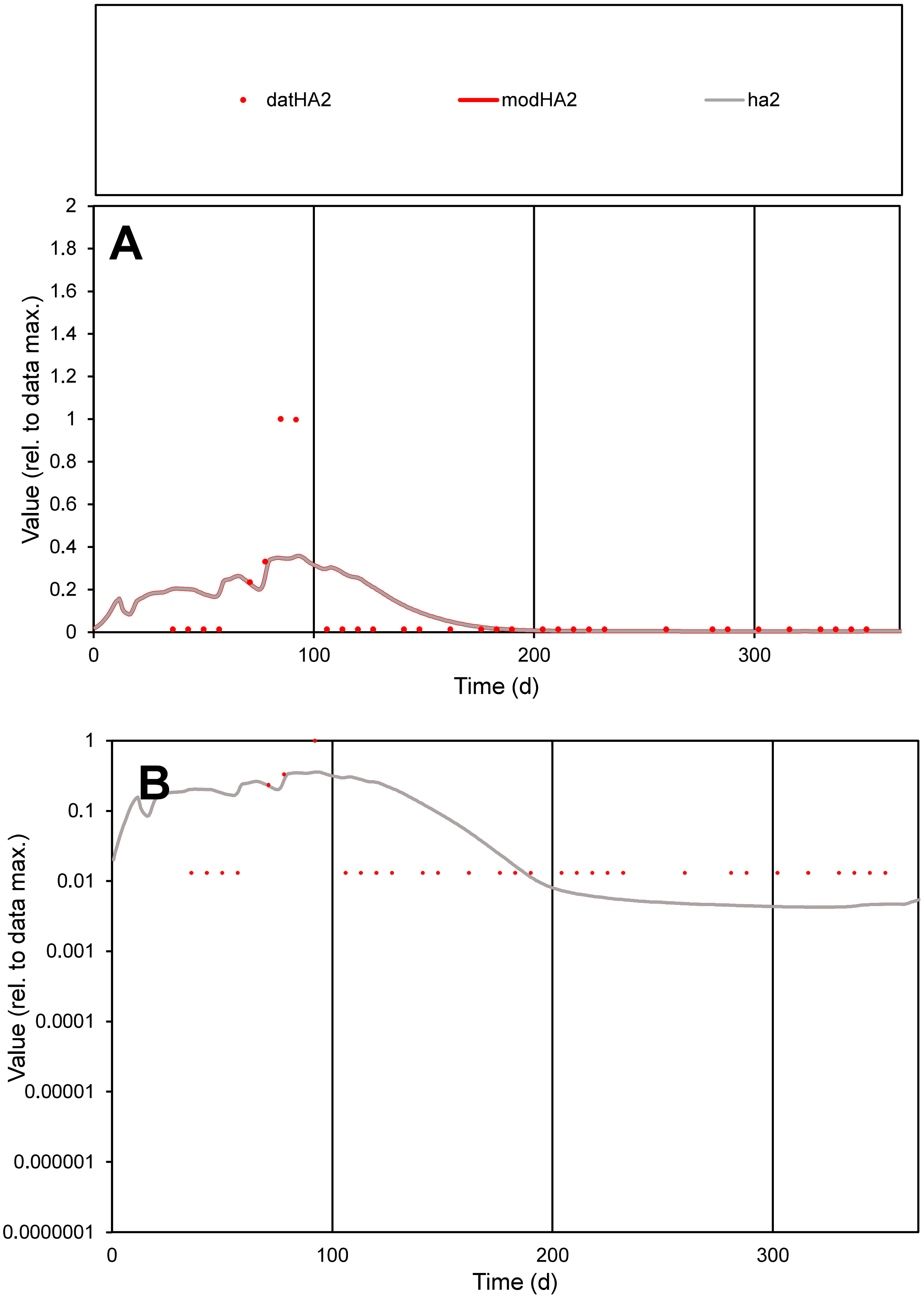

C1

Variables
HA2 Provide jtd or Dst
CalibOnly?
2 1 to reproduce of
oNormalize?
2 1=no, 2=yes

ofxn calc*
2.12622
ofxn mod
2.14191

sRun
RunA1
zout
1

Ave
Data
1.8E-05
Model
2.1E-05

data/vMax
0.0002

ofxng
321.551

Min d/m
3.07317

ave/min d
6.95053
ave/min m
24.6361

324_Calib_S_A

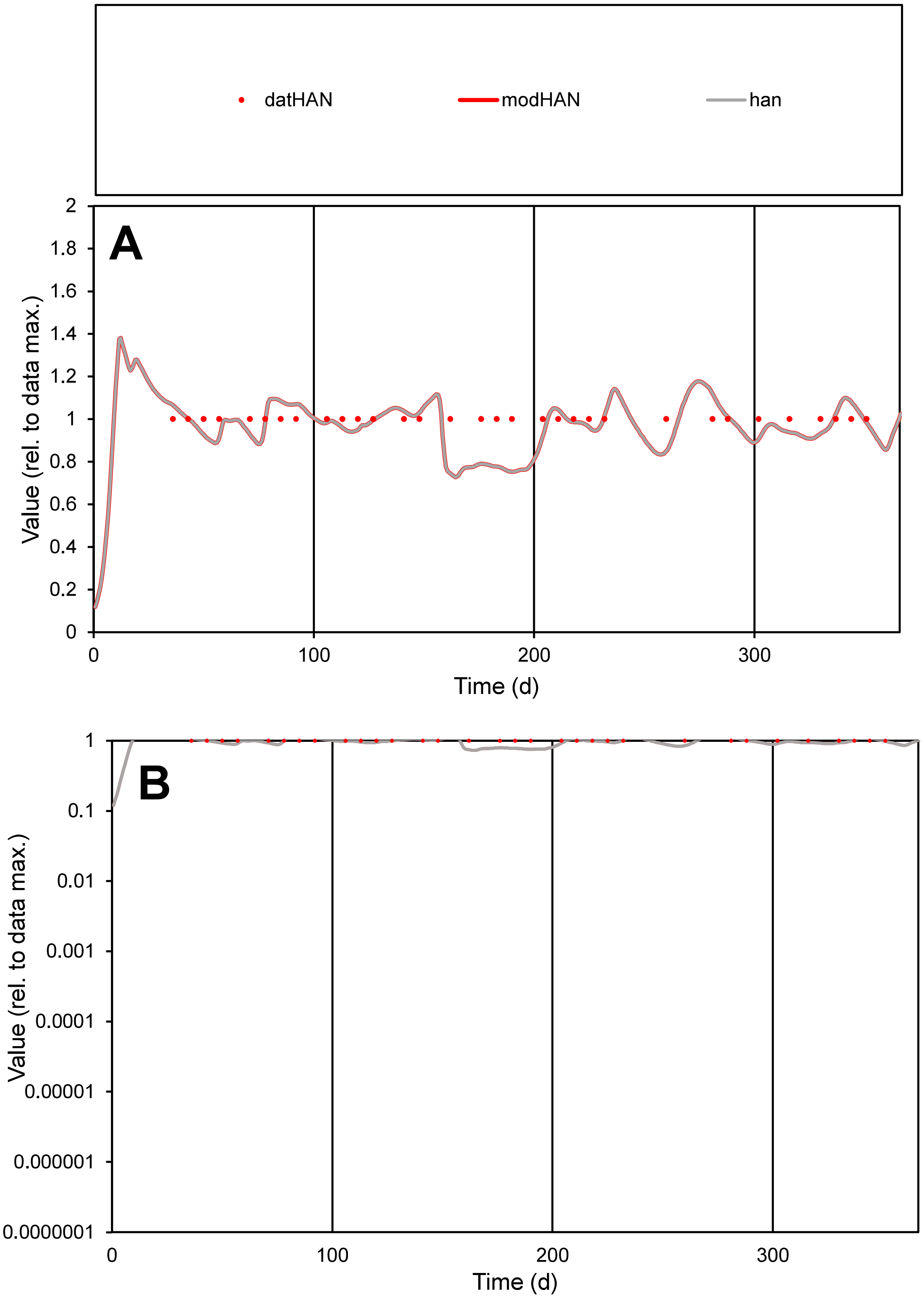

C1	
Variables	
HAN	Provide jtd or Dst
CalibOnly?	
2	1 to reproduce of
oNormalize?	
2	1=no, 2=yes
ofxn calc*	
0.10068	
ofxn mod	
0.13889	
sRun	
RunA1	
zout	
1	
Ave	
Data	
5.1E-06	
Model	
5E-06	
data/vMax	
5.1E-06	
ofxng	
321.551	
Min d/m	
1.36604	
ave/min d	
1	
ave/min m	
1.31968	

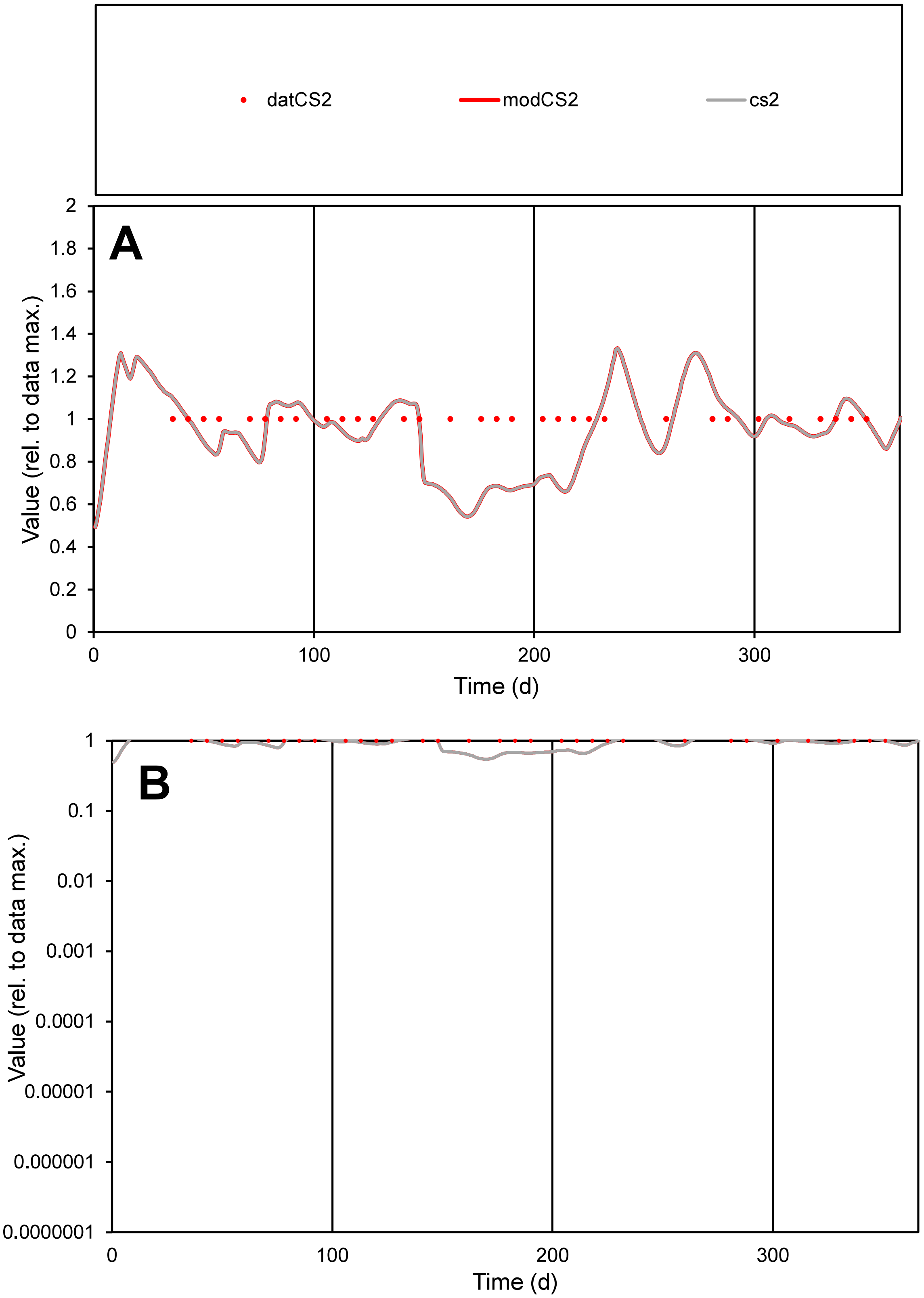

C1

Variables
CS2 Provide jtd or Dst
CalibOnly?
2 1 to reproduce of
oNormalize?
2 1=no, 2=yes

ofxn calc*
0.16523
ofxn mod
0.19211

sRun
RunA1
zout
1

Ave
Data
7.6E-06
Model
7E-06

data/vMax
7.6E-06

ofxng
321.551

Min d/m
1.64525

ave/min d
1
ave/min m
1.52056

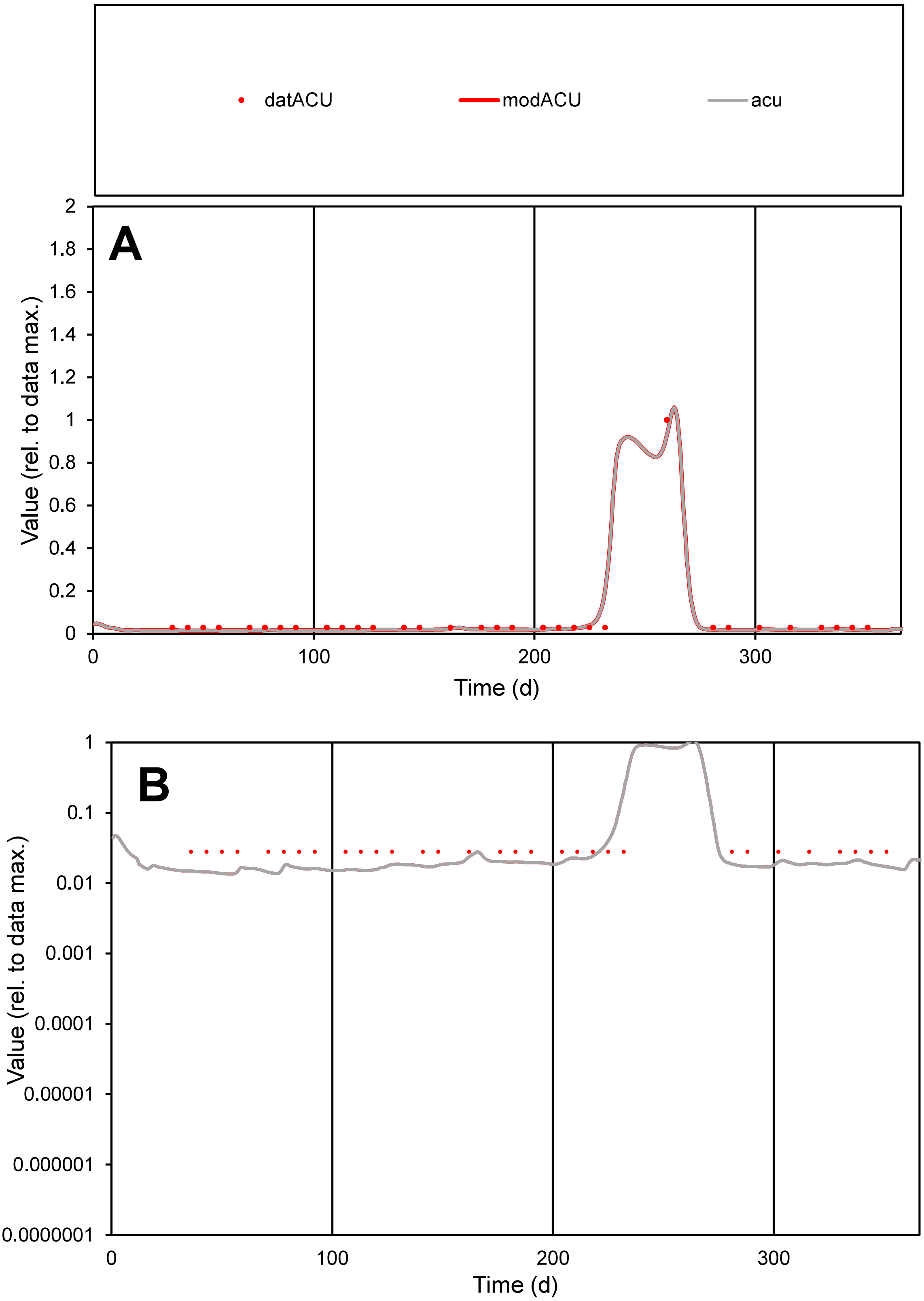

C1

Variables
ACU Provide jtd or Dst
CalibOnly?
2 1 to reproduce of
oNormalize?
2 1=no, 2=yes

ofxn calc*
0.34741
ofxn mod
0.49726

sRun
RunA1
zout
1

Ave
Data
2.3E-05
Model
2.1E-05

data/vMax
0.0004

ofxng
321.551

Min d/m
2.05817

ave/min d
2.08824
ave/min m
3.87083

324_Calib_S_A

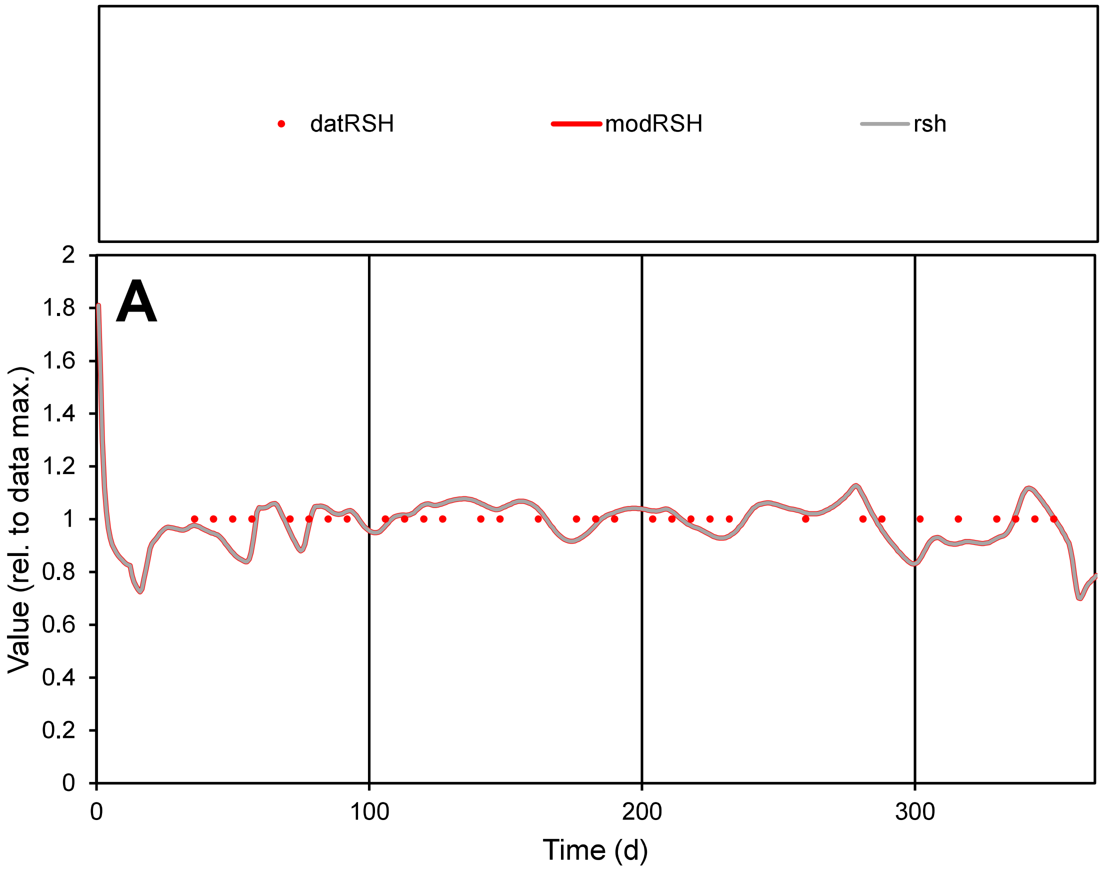

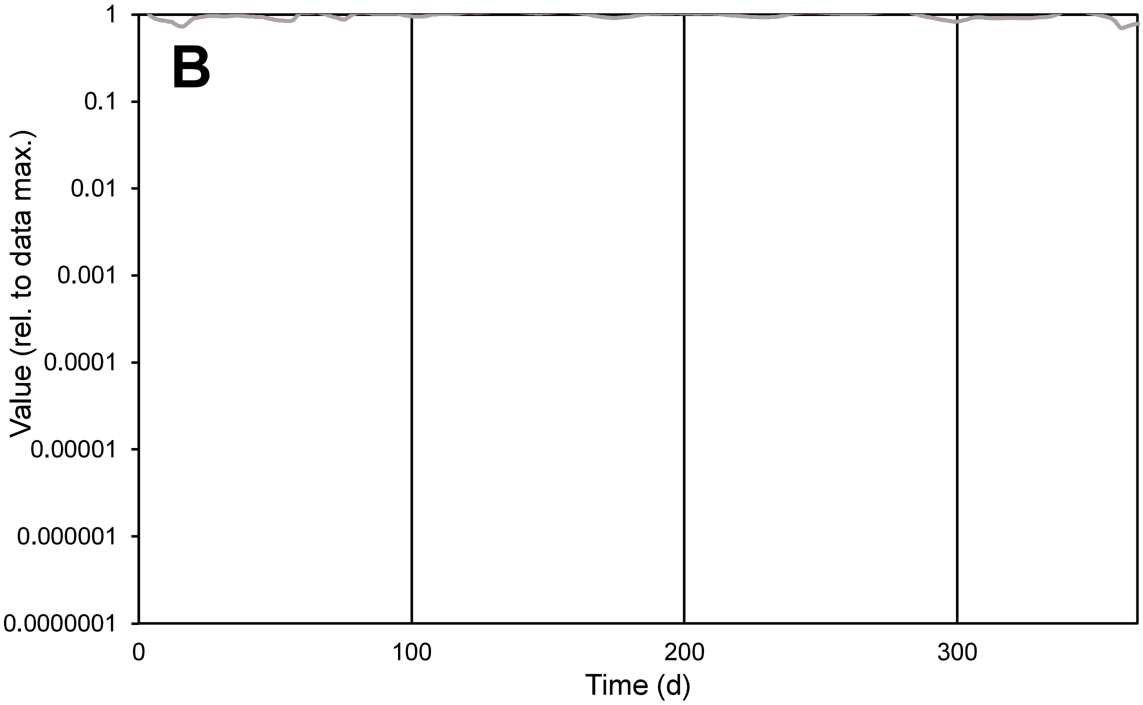

C1

Variables
RSH Provide jtd or Dst
CalibOnly?
2 1 to reproduce of
oNormalize?
2 1=no, 2=yes

ofxn calc*
0.05832
ofxn mod
0.11254

sRun
RunA1
zout
1

Ave
Data
1.8E-05
Model
1.8E-05

data/vMax
1.8E-05

ofxng
321.551

Min d/m
1.16656

ave/min d
1
ave/min m
1.15678

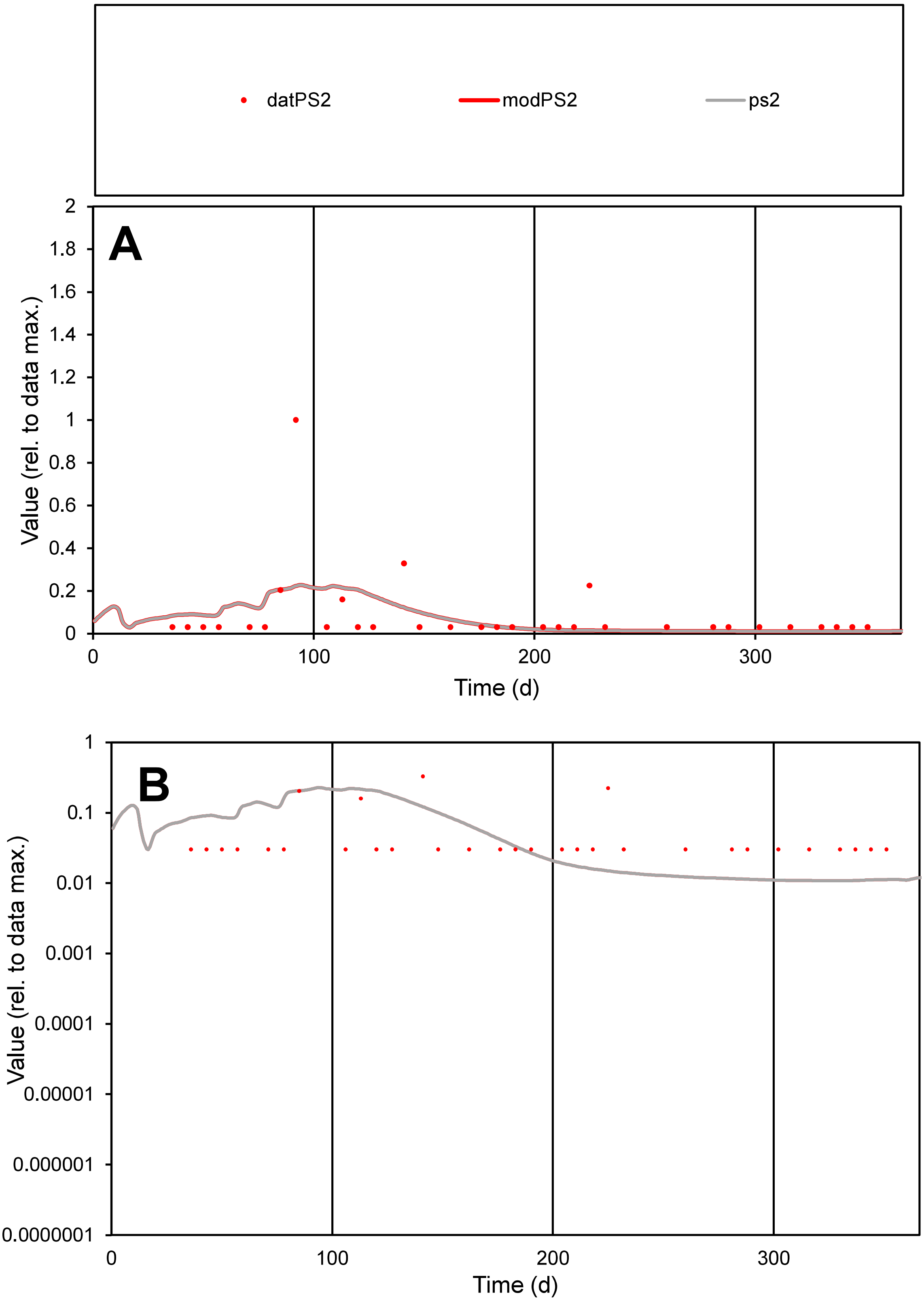

C1

Variables
PS2 Provide jtd or Dst
CalibOnly?
2 1 to reproduce of
oNormalize?
2 1=no, 2=yes

ofxn calc*
1.86721
ofxn mod
1.88123

sRun
RunA1
zout
1

Ave
Data
5.4E-05
Model
4.8E-05

data/vMax
0.00063

ofxng
321.551

Min d/m
2.79736

ave/min d
2.8326
ave/min m
6.97819

324_Calib_S_A

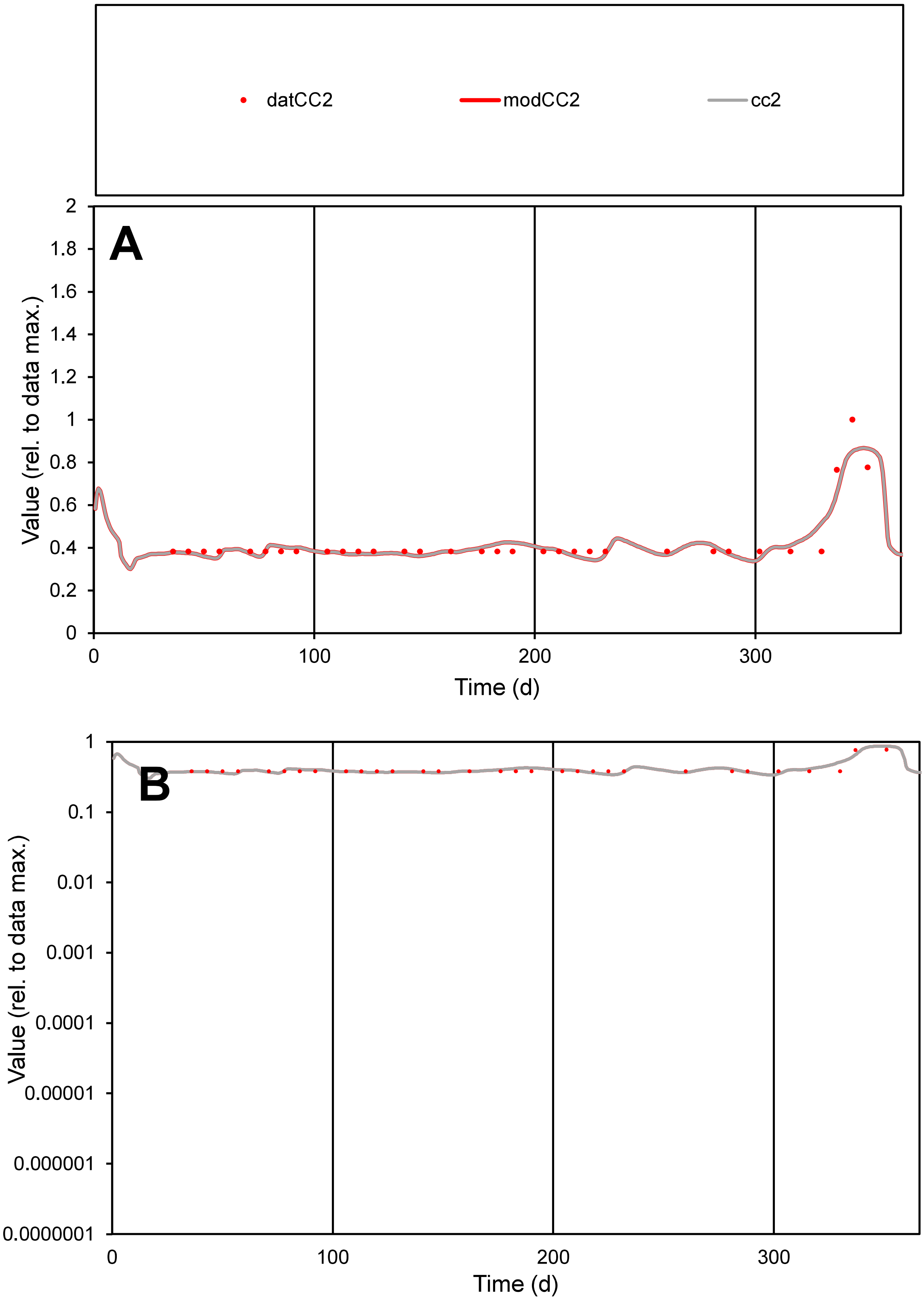

C1

Variables
CC2 Provide jtd or Dst
CalibOnly?
2 1 to reproduce of
oNormalize?
2 1=no, 2=yes

ofxn calc*
0.10713
ofxn mod
0.14481

sRun
RunA1
zout
1

Ave
Data
6.5E-06
Model
6.5E-06

data/vMax
1.5E-05

ofxng
321.551

Min d/m
1.11767

ave/min d
1.11371
ave/min m
1.24695

324_Calib_S_A

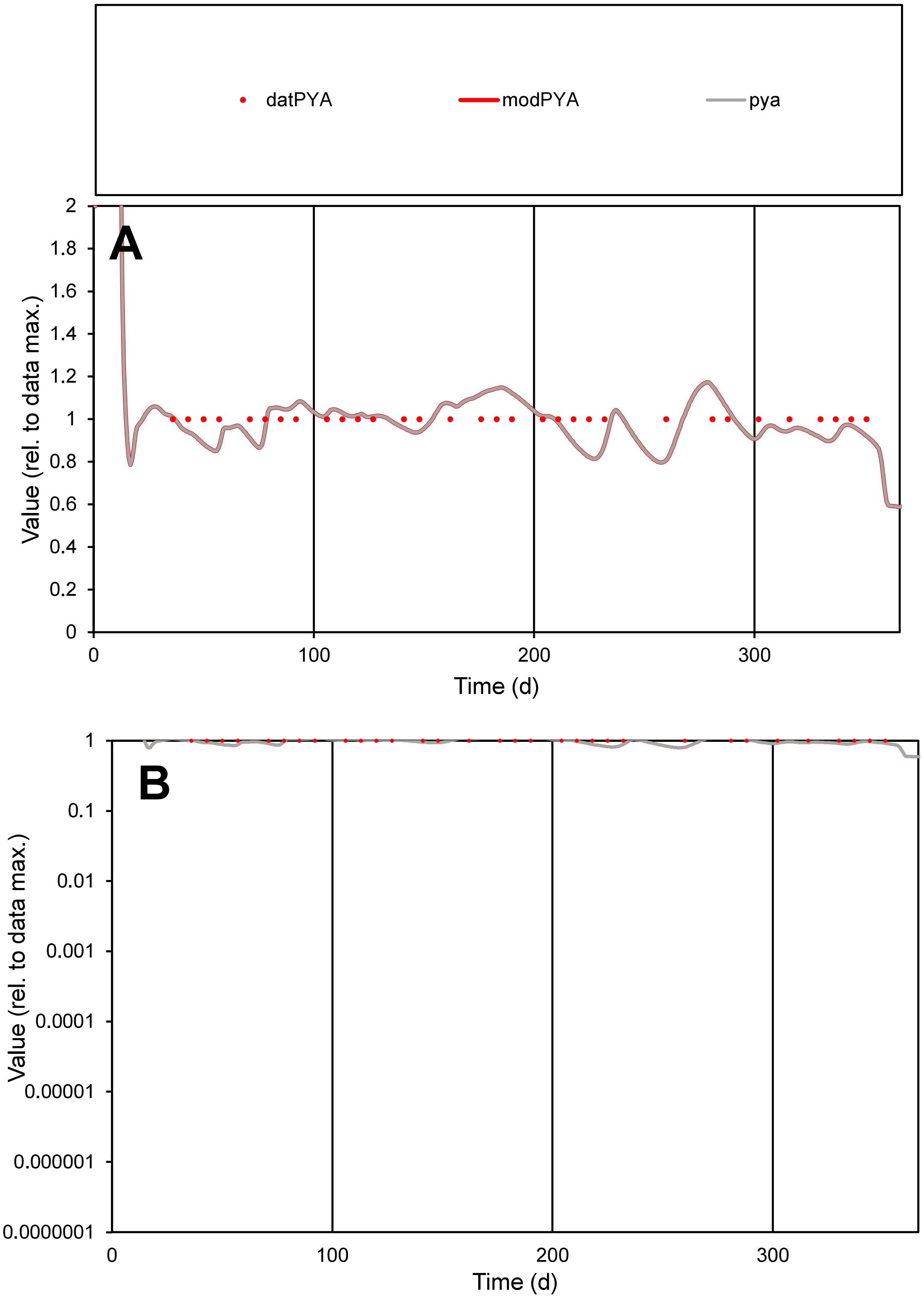

C1

Variables
PYA Provide jtd or Dst
CalibOnly?
2 1 to reproduce of
oNormalize?
2 1=no, 2=yes

ofxn calc*
0.08781
ofxn mod
0.13018

sRun
RunA1
zout
1

Ave
Data
1.8E-06
Model
1.8E-06

data/vMax
1.8E-06

ofxng
321.551

Min d/m
1.22515

ave/min d
1
ave/min m
1.20305

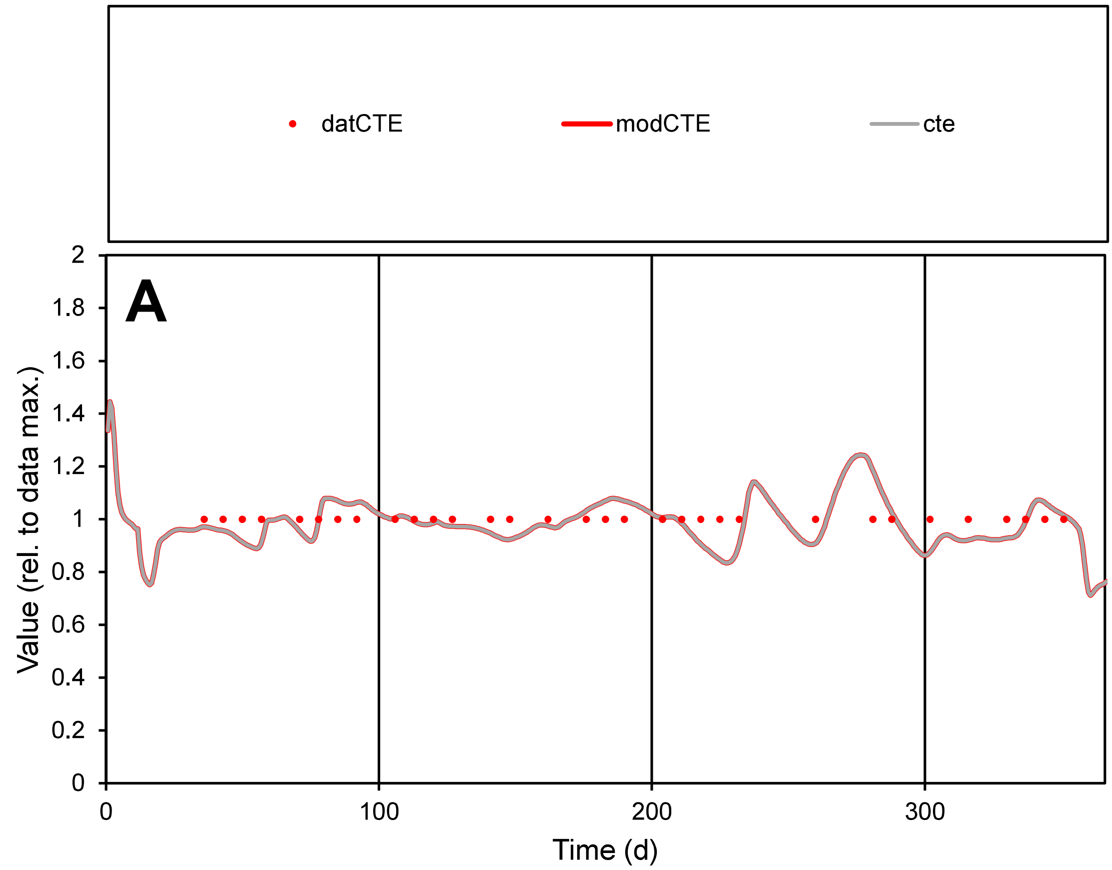

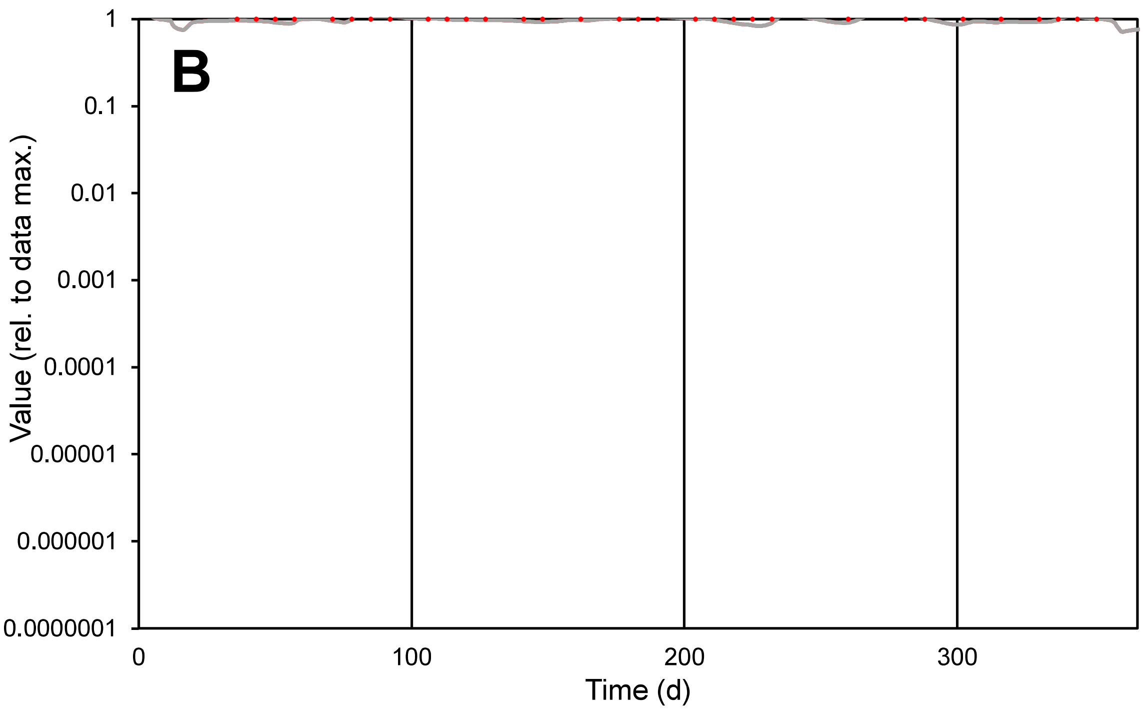

C1

Variables
CTE Provide jtd or Dst
CalibOnly?
2 1 to reproduce of
oNormalize?
2 1=no, 2=yes

ofxn calc*
0.068
ofxn mod
0.11752

sRun
RunA1
zout
1

Ave
Data
6.1E-06
Model
6E-06

data/vMax
6.1E-06

ofxng
321.551

Min d/m
1.19299

ave/min d
1
ave/min m
1.17406

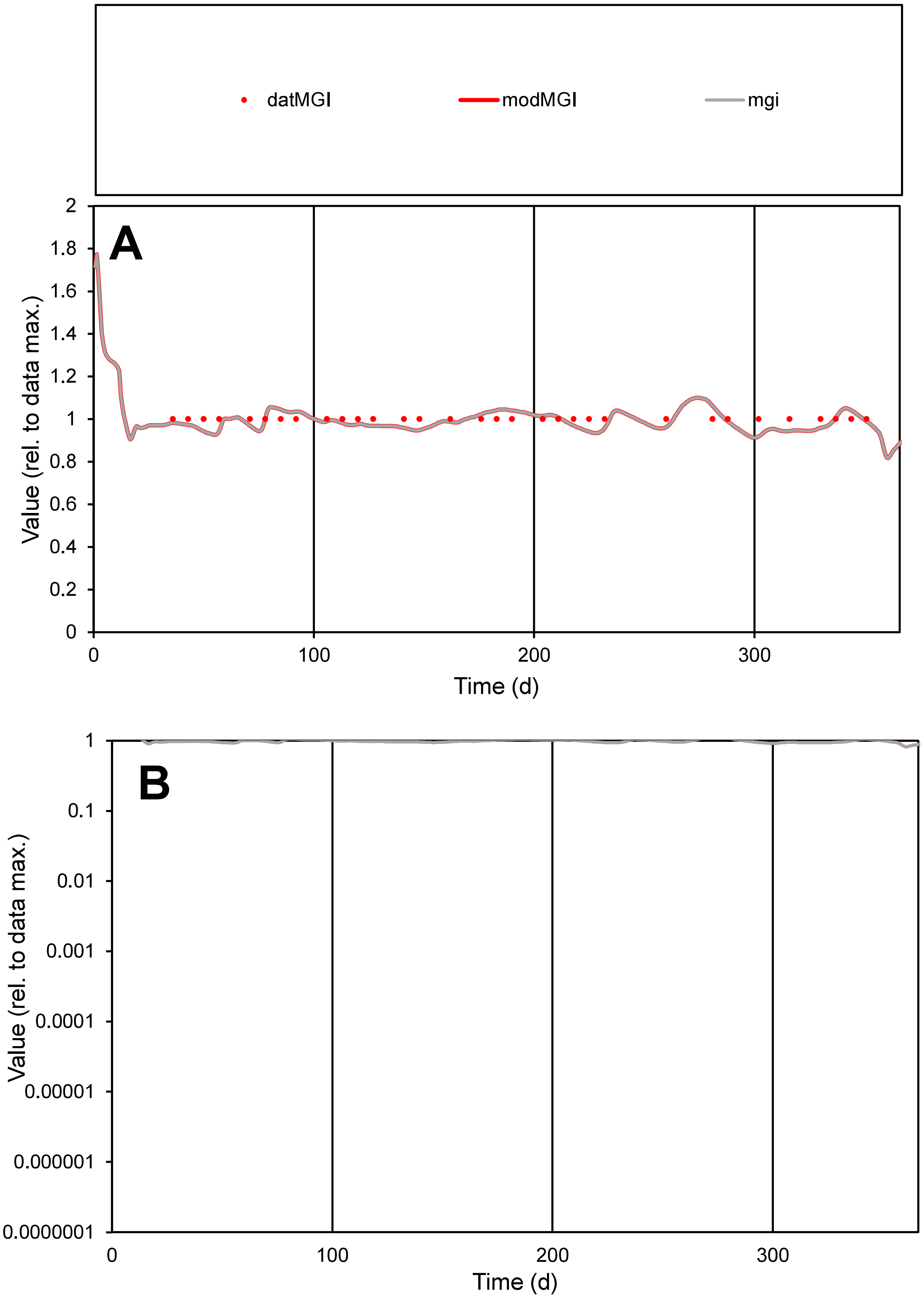

C1

Variables
MGI Provide jtd or Dst
CalibOnly?
2 1 to reproduce of
oNormalize?
2 1=no, 2=yes

ofxn calc*
0.03757
ofxn mod
0.10331

sRun
RunA1
zout
1

Ave
Data
1E-05
Model
1E-05

data/vMax
1E-05

ofxng
321.551

Min d/m
1.07792

ave/min d
1
ave/min m
1.06557

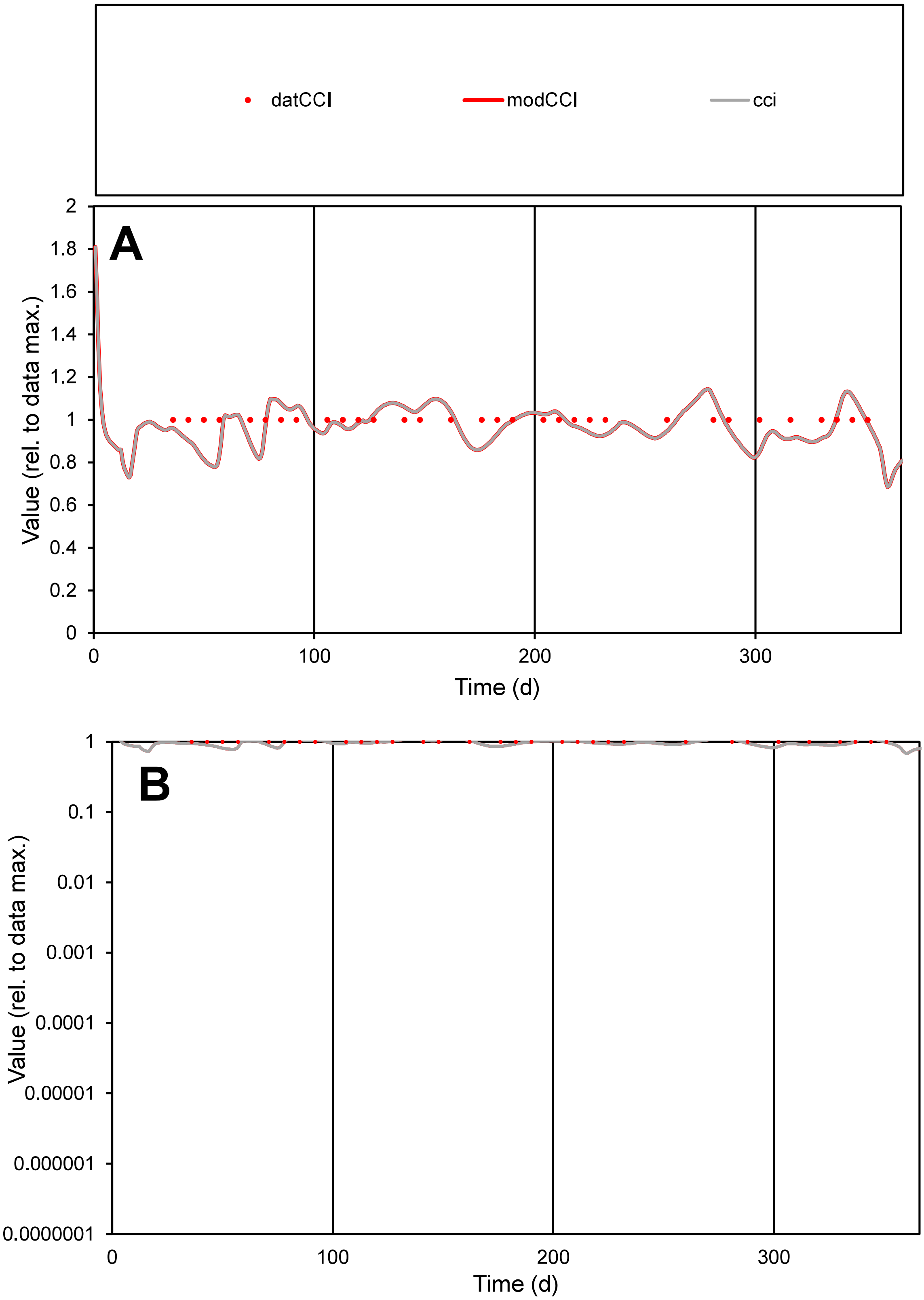

C1

Variables
CCI Provide jtd or Dst
CalibOnly?
2 1 to reproduce of
oNormalize?
2 1=no, 2=yes

ofxn calc*
0.0749
ofxn mod
0.12156

sRun
RunA1
zout
1

Ave
Data
1.1E-05
Model
1.1E-05

data/vMax
1.1E-05

ofxng
321.551

Min d/m
1.25602

ave/min d
1
ave/min m
1.226

324_Calib_S_A

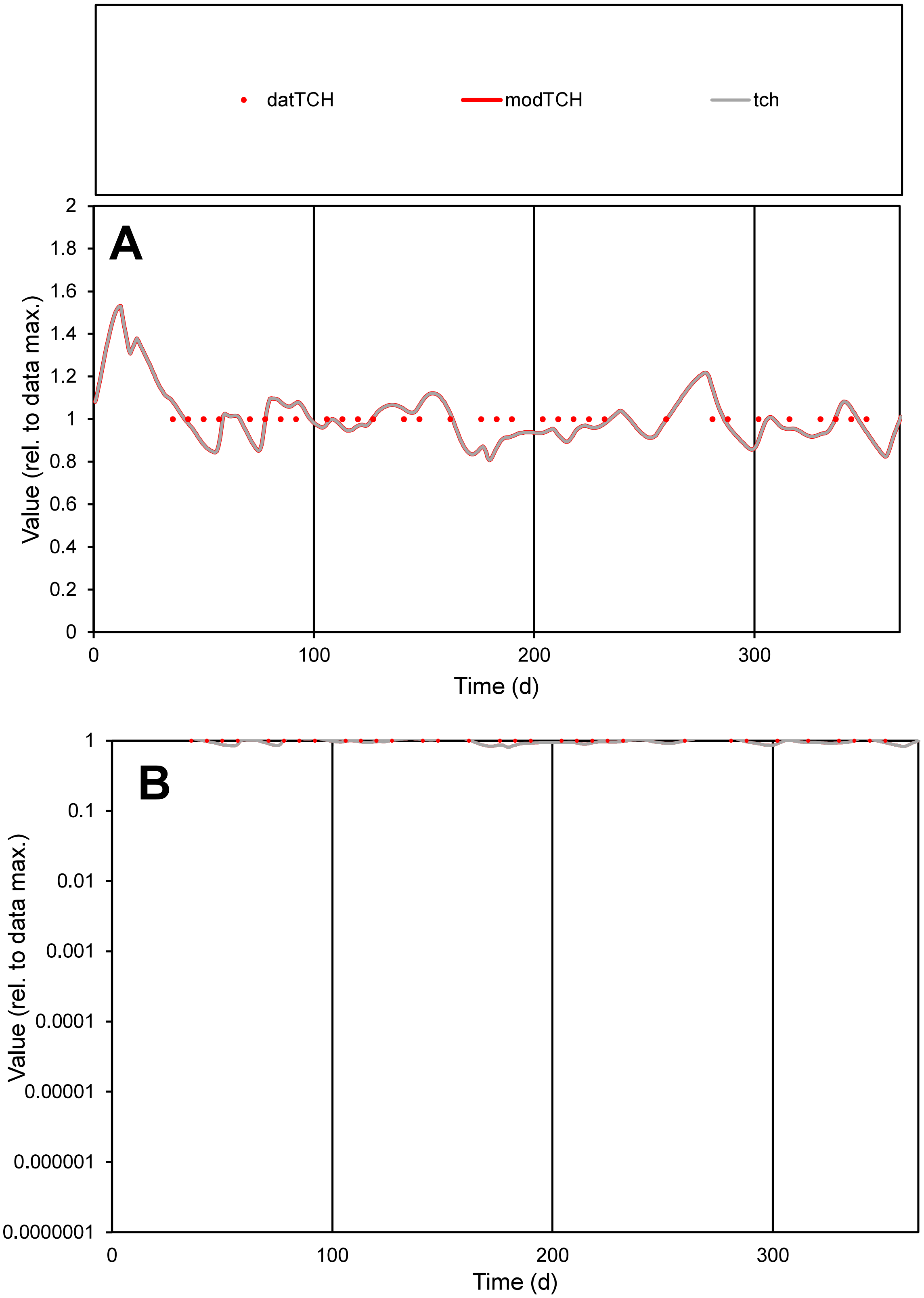

C1

Variables
TCH Provide jtd or Dst
CalibOnly?
2 1 to reproduce of
oNormalize?
2 1=no, 2=yes

ofxn calc*
0.06787
ofxn mod
0.1178

sRun
RunA1
zout
1

Ave
Data
1.7E-06
Model
1.6E-06

data/vMax
1.7E-06

ofxng
321.551

Min d/m
1.1576

ave/min d
1
ave/min m
1.13367

324_Calib_S_A

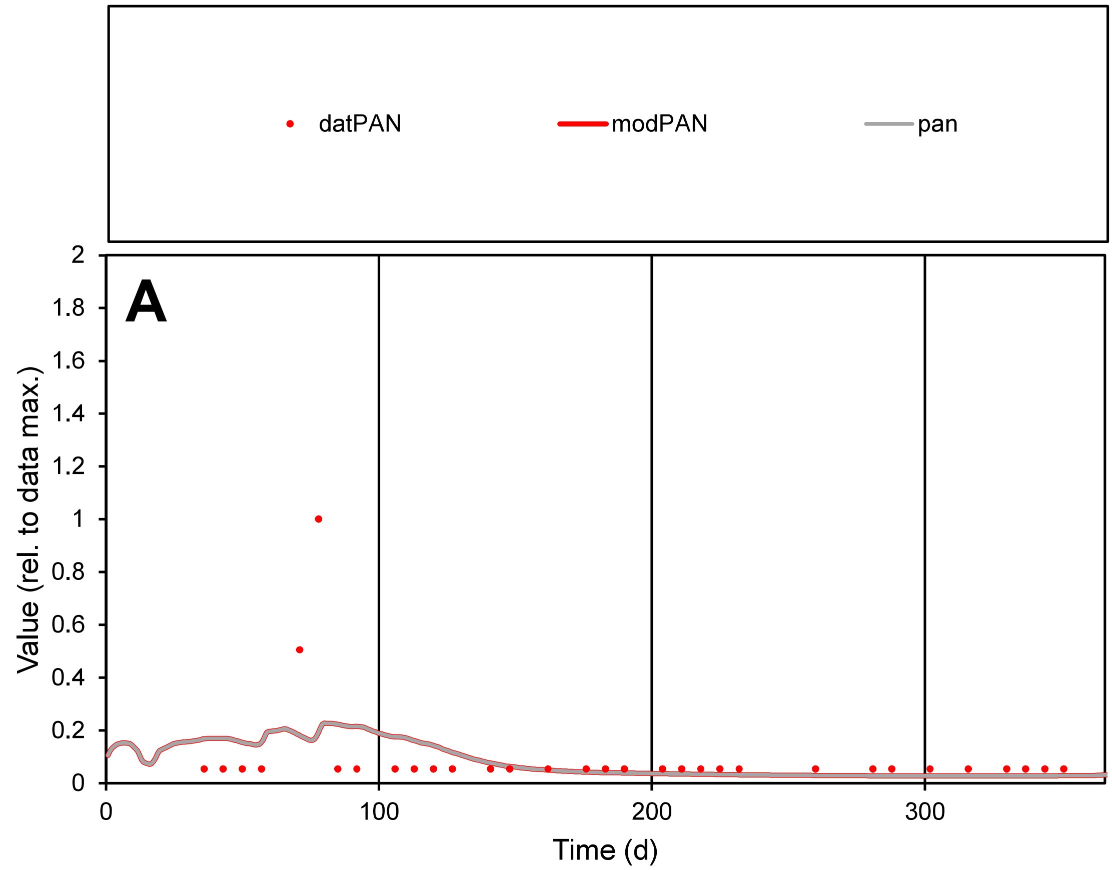

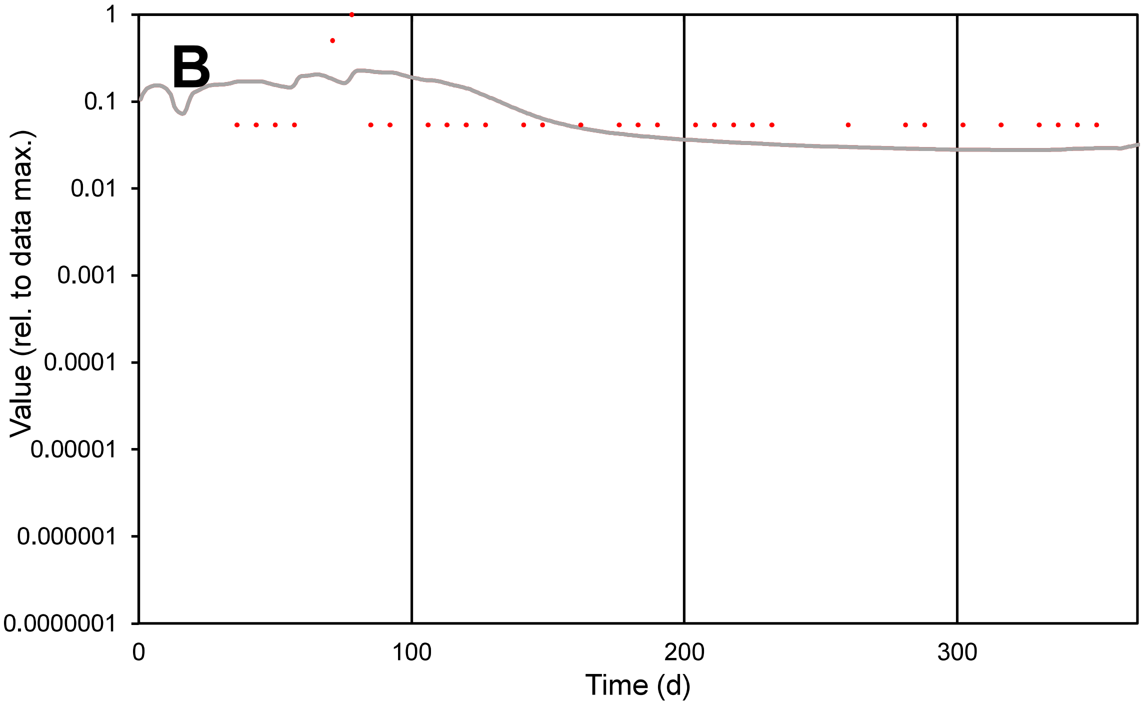

C1

Variables

PAN Provide jtd or Dst

CalibOnly?

2 1 to reproduce of

oNormalize?

2 1=no, 2=yes

ofxn calc*

1.67748

ofxn mod

1.68538

sRun

RunA1

zout

1

Ave

Data

1.7E-05

Model

1.5E-05

data/vMax

0.00017

ofxng

321.551

Min d/m

1.94872

ave/min d

1.80931

ave/min m

3.15023

324_Calib_S_A

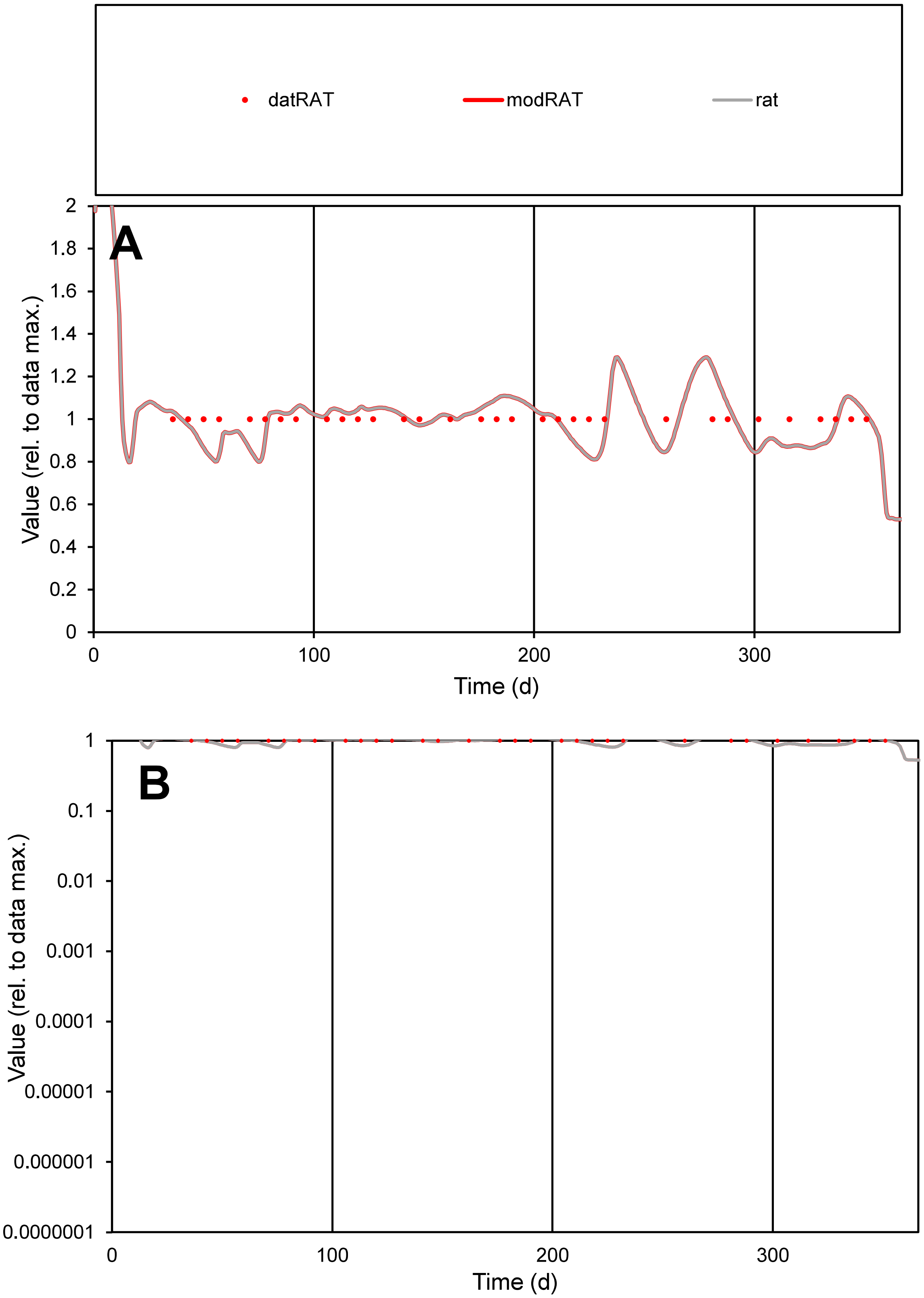

C1

Variables

RAT Provide jtd or Dst

CalibOnly?

2 1 to reproduce of

oNormalize?

2 1=no, 2=yes

ofxn calc*

0.09314

ofxn mod

0.13359

sRun

RunA1

zout

1

Ave

Data

1.1E-05

Model

1.1E-05

data/vMax

1.1E-05

ofxng

321.551

Min d/m

1.22919

ave/min d

1

ave/min m

1.21336

324_Calib_S_A

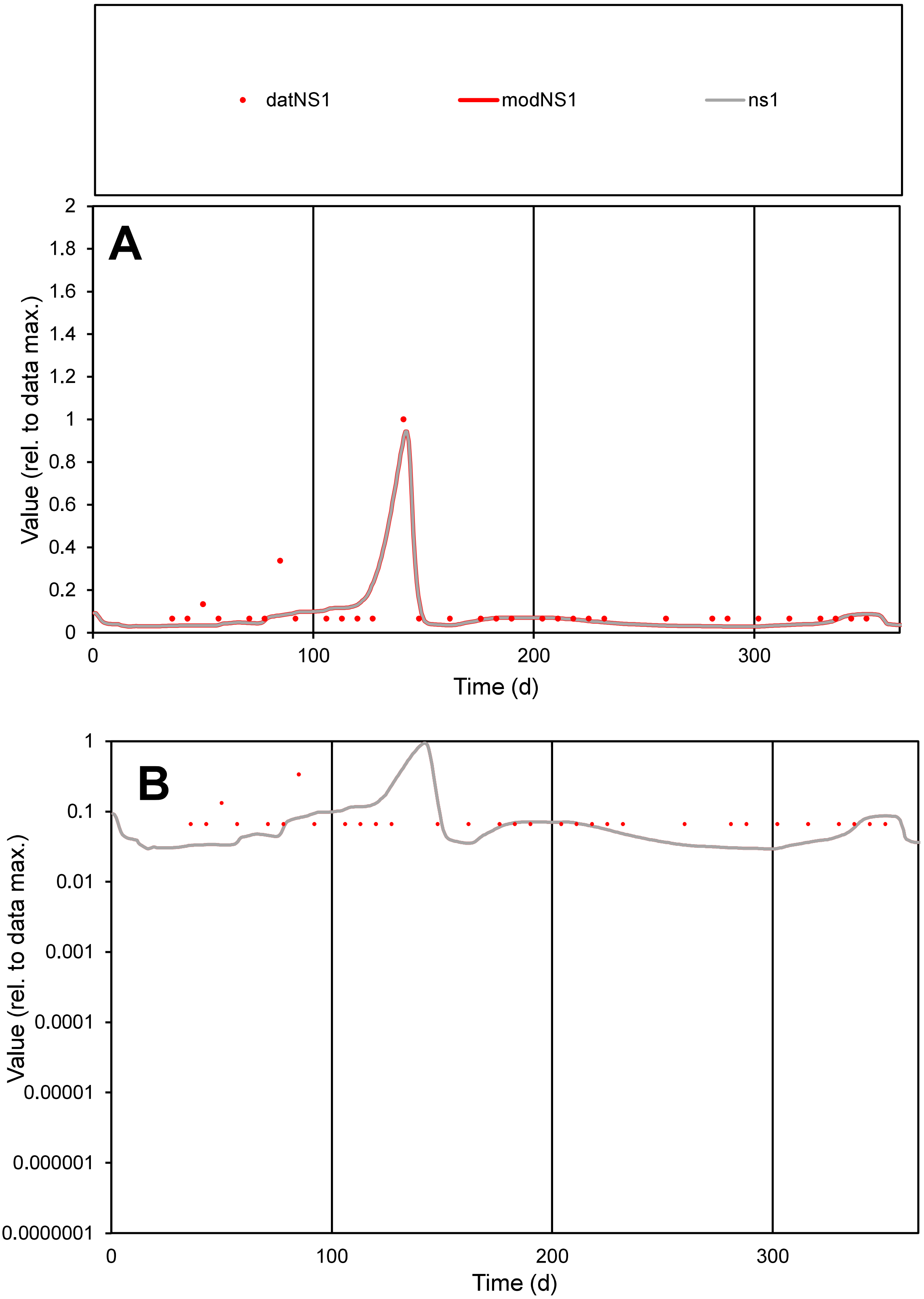

C1

Variables

NS1 Provide jtd or Dst

CalibOnly?

2 1 to reproduce of

oNormalize?

2 1=no, 2=yes

ofxn calc*

0.58358

ofxn mod

0.63191

sRun

RunA1

zout

1

Ave

Data

4.4E-05

Model

3.9E-05

data/vMax

0.00041

ofxng

321.551

Min d/m

2.21989

ave/min d

1.59763

ave/min m

3.16241

324_Calib_S_A

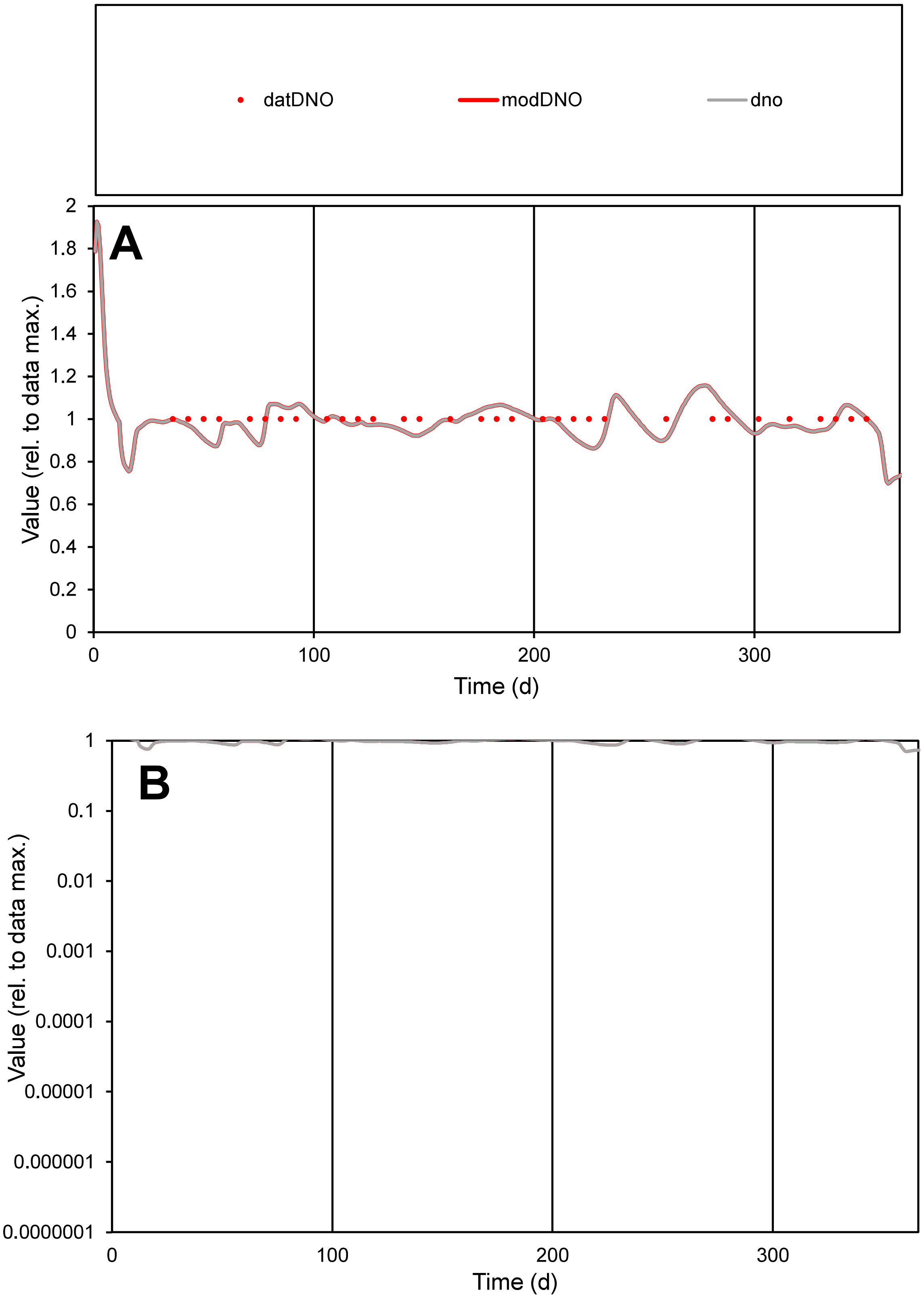

C1

Variables
DNO Provide jtd or Dst
CalibOnly?
2 1 to reproduce of
oNormalize?
2 1=no, 2=yes

ofxn calc*
0.06004
ofxn mod
0.11318

sRun
RunA1
zout
1

Ave
Data
1.8E-05
Model
1.8E-05

data/vMax
1.8E-05

ofxng
321.551

Min d/m
1.15803

ave/min d
1
ave/min m
1.14207

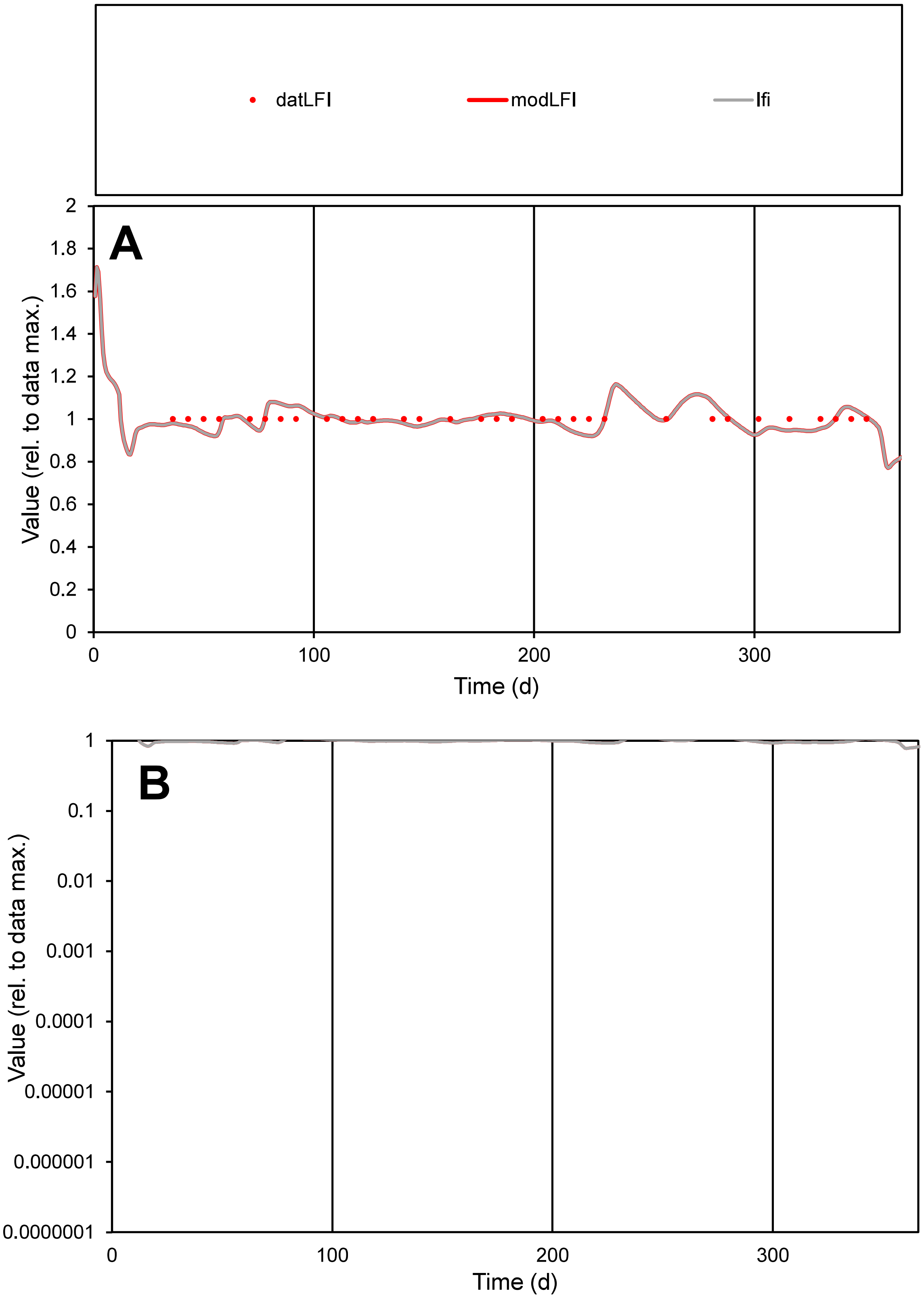

C1

Variables
LFI Provide jtd or Dst
CalibOnly?
2 1 to reproduce of
oNormalize?
2 1=no, 2=yes

ofxn calc*
0.04055
ofxn mod
0.10517

sRun
RunA1
zout
1

Ave
Data
8.3E-06
Model
8.3E-06

data/vMax
8.3E-06

ofxng
321.551

Min d/m
1.08643

ave/min d
1
ave/min m
1.08103

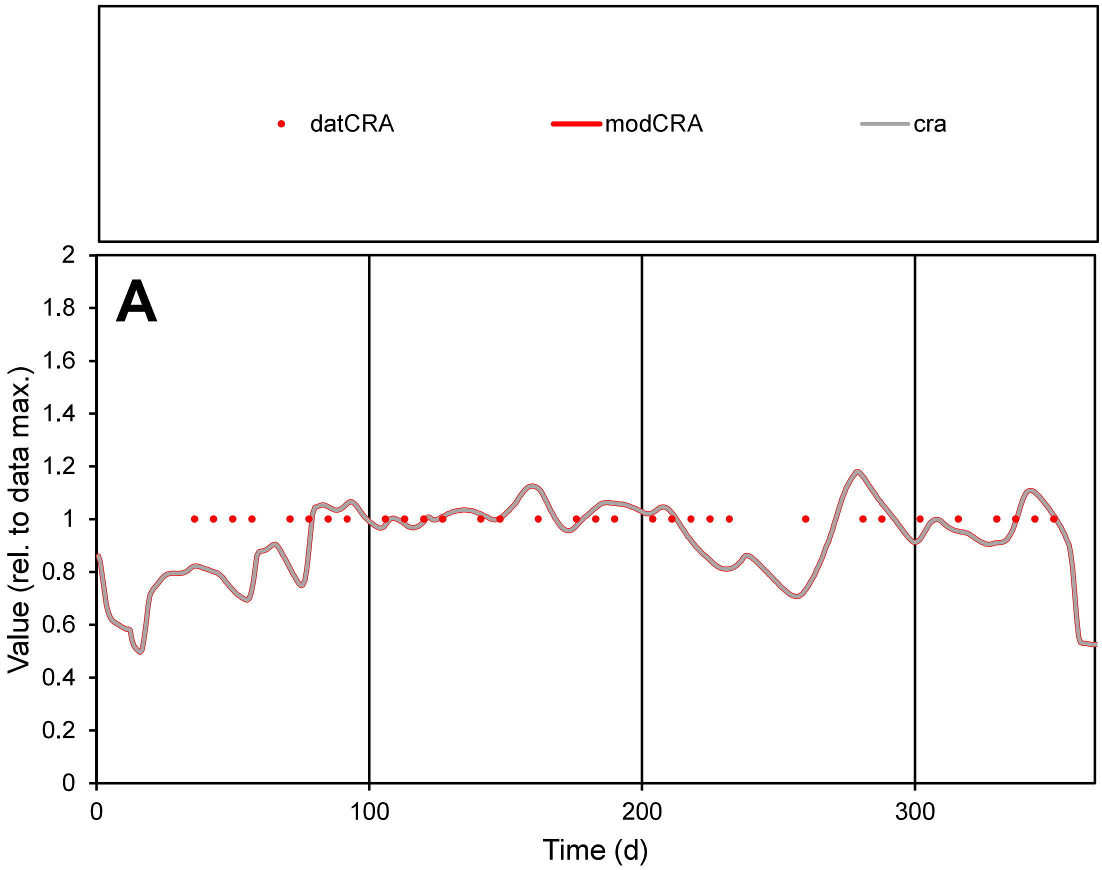

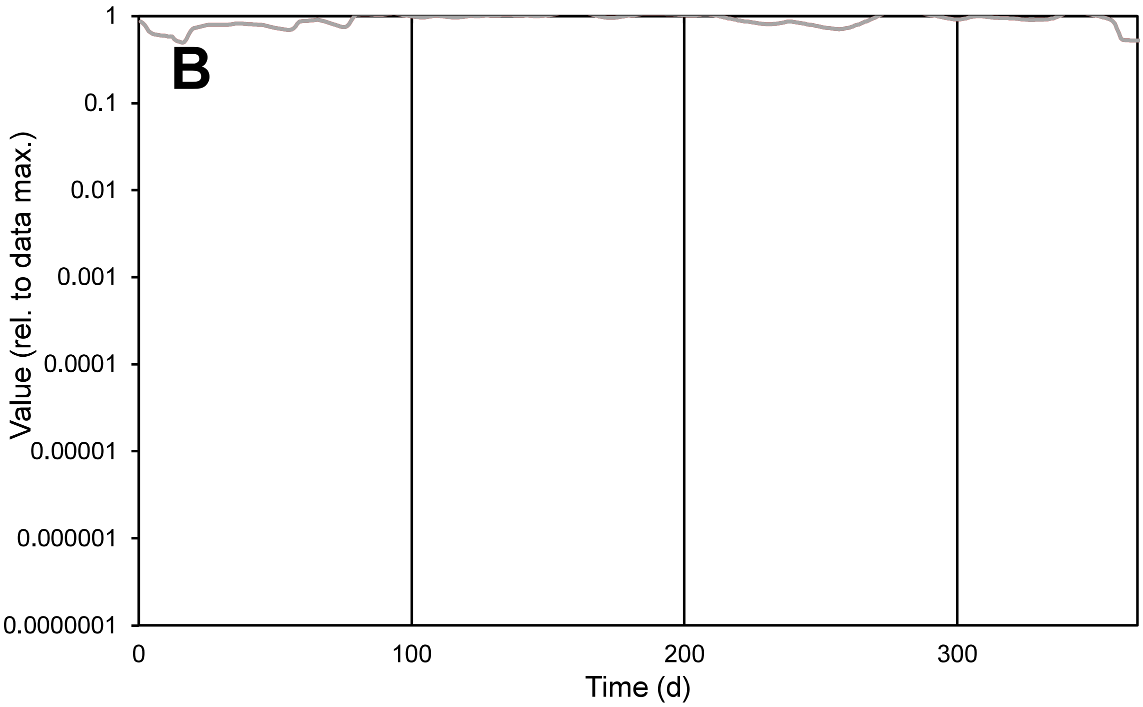

C1

Variables
CRA Provide jtd or Dst
CalibOnly?
2 1 to reproduce of
oNormalize?
2 1=no, 2=yes

ofxn calc*
0.11631
ofxn mod
0.15026

sRun
RunA1
zout
1

Ave
Data
4.9E-06
Model
4.7E-06

data/vMax
4.9E-06

ofxng
321.551

Min d/m
1.3969

ave/min d
1
ave/min m
1.34163

324_Calib_S_A

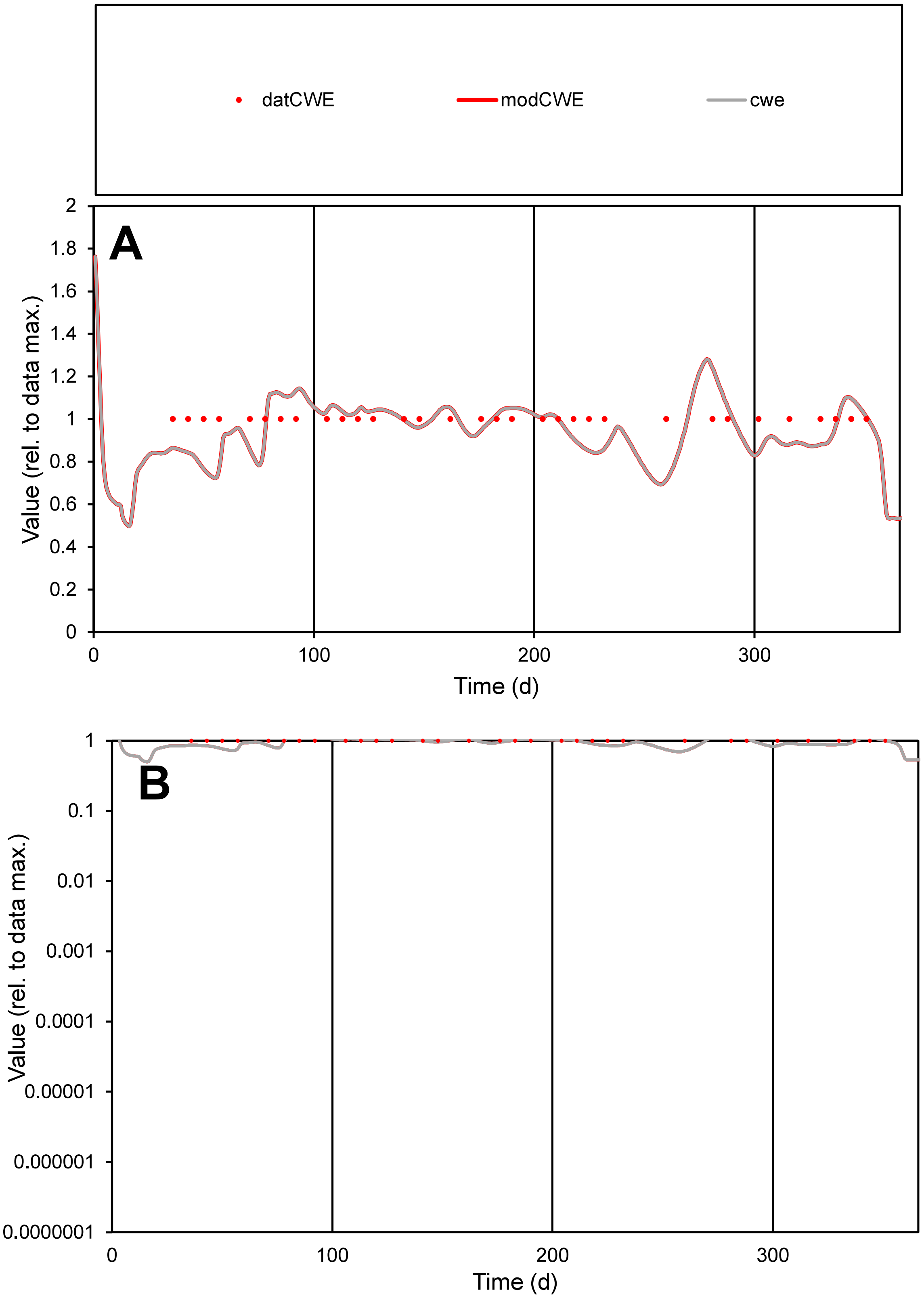

C1

Variables
CWE Provide jtd or Dst
CalibOnly?
2 1 to reproduce of
oNormalize?
2 1=no, 2=yes

ofxn calc*
0.1161
ofxn mod
0.15038

sRun
RunA1
zout
1

Ave
Data
2.6E-05
Model
2.5E-05

data/vMax
2.6E-05

ofxng
321.551

Min d/m
1.36656

ave/min d
1
ave/min m
1.32412

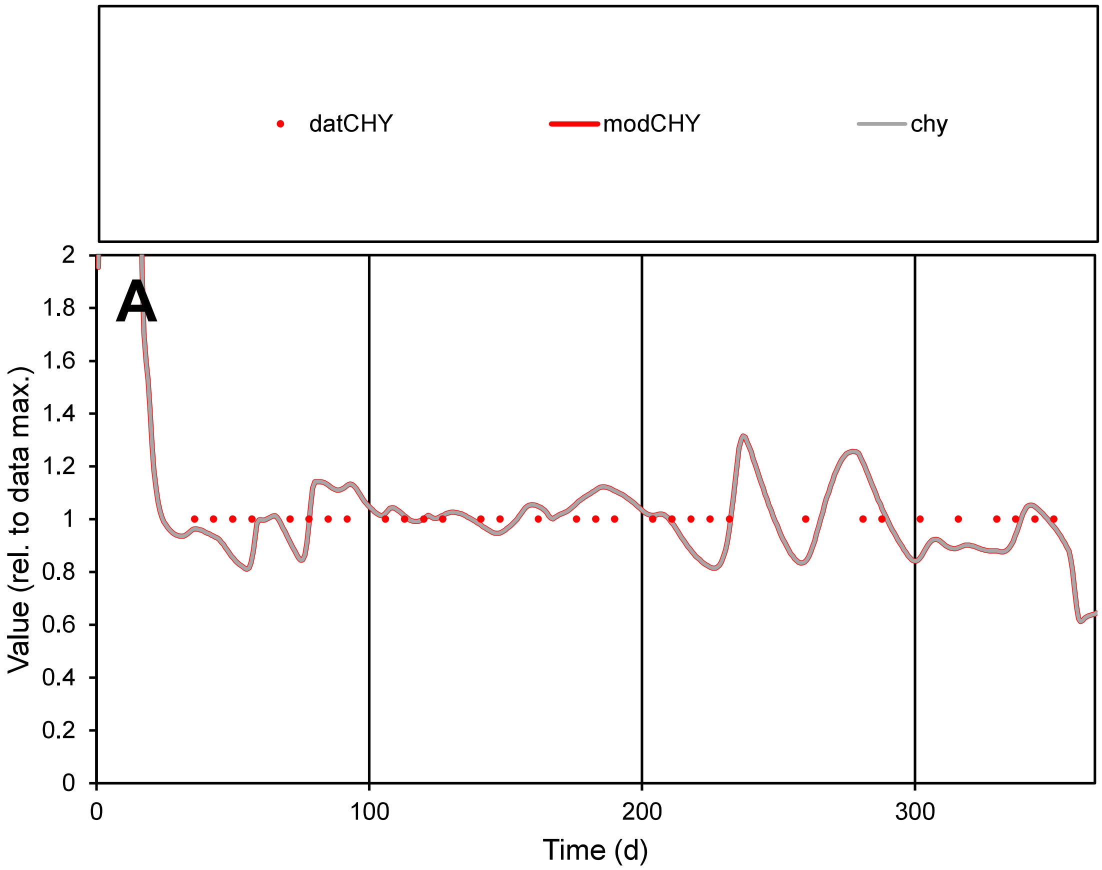

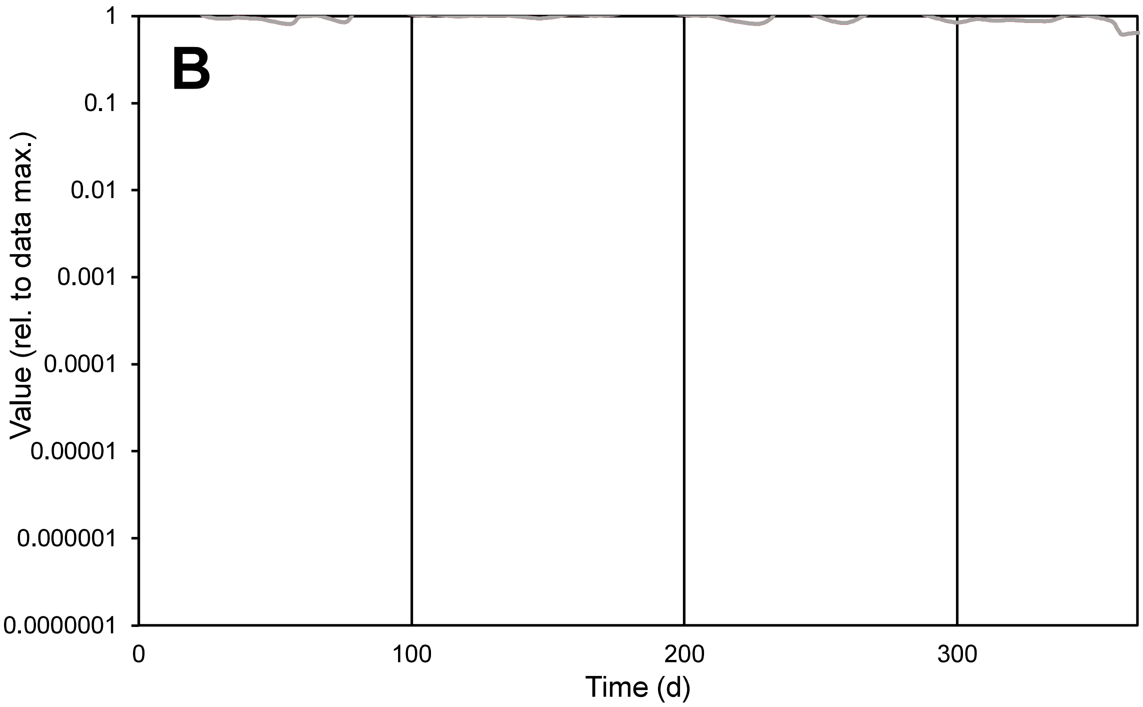

C1

Variables
CHY Provide jtd or Dst
CalibOnly?
2 1 to reproduce of
oNormalize?
2 1=no, 2=yes

ofxn calc*
0.09454
ofxn mod
0.13556

sRun
RunA1
zout
1

Ave
Data
6.7E-06
Model
6.6E-06

data/vMax
6.7E-06

ofxng
321.551

Min d/m
1.22723

ave/min d
1
ave/min m
1.21239

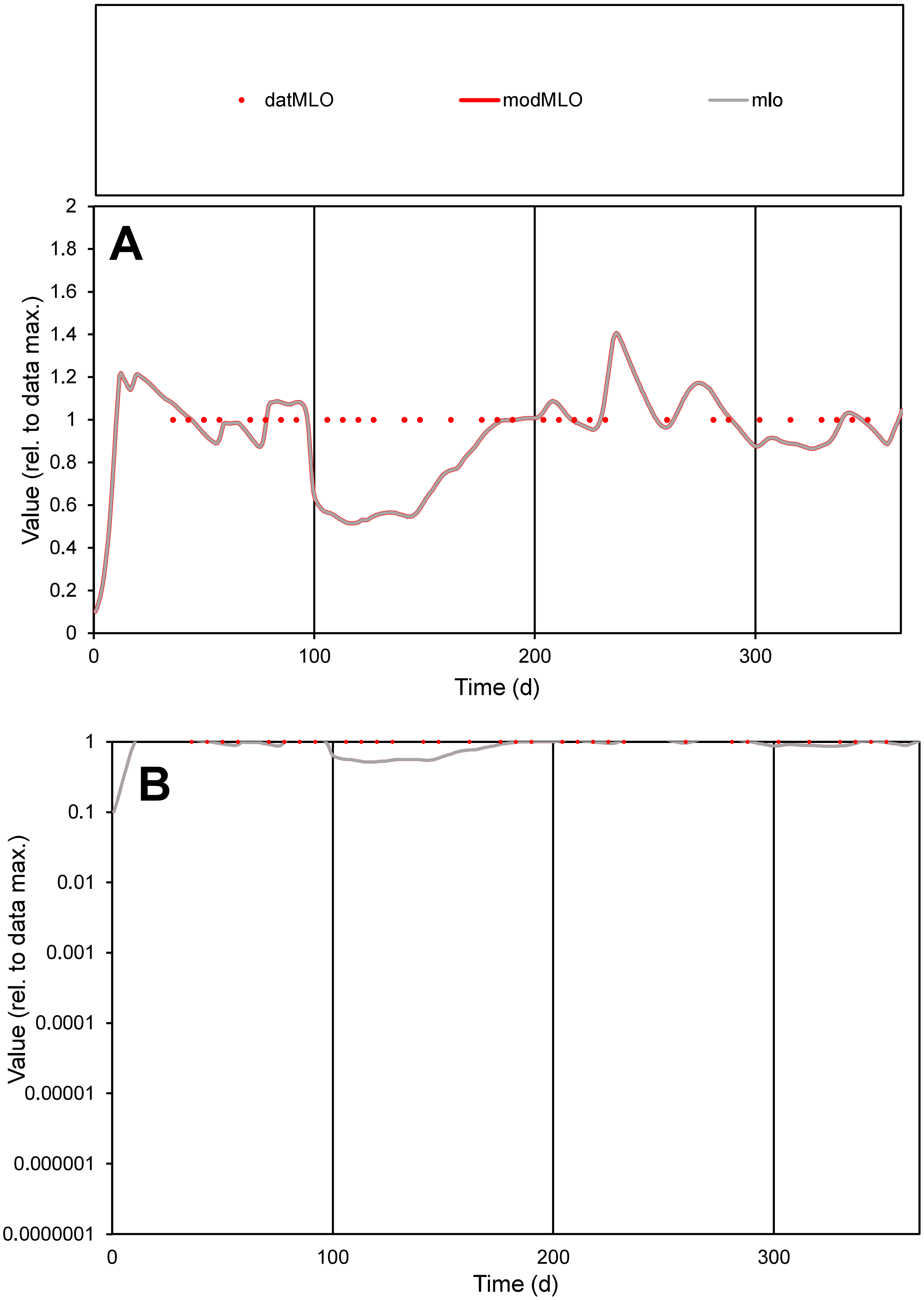

C1

Variables
MLO Provide jtd or Dst
CalibOnly?
2 1 to reproduce of
oNormalize?
2 1=no, 2=yes

ofxn calc*
0.20829
ofxn mod
0.22932

sRun
RunA1
zout
1

Ave
Data
1E-05
Model
9.5E-06

data/vMax
1E-05

ofxng
321.551

Min d/m
1.92081

ave/min d
1
ave/min m
1.74088

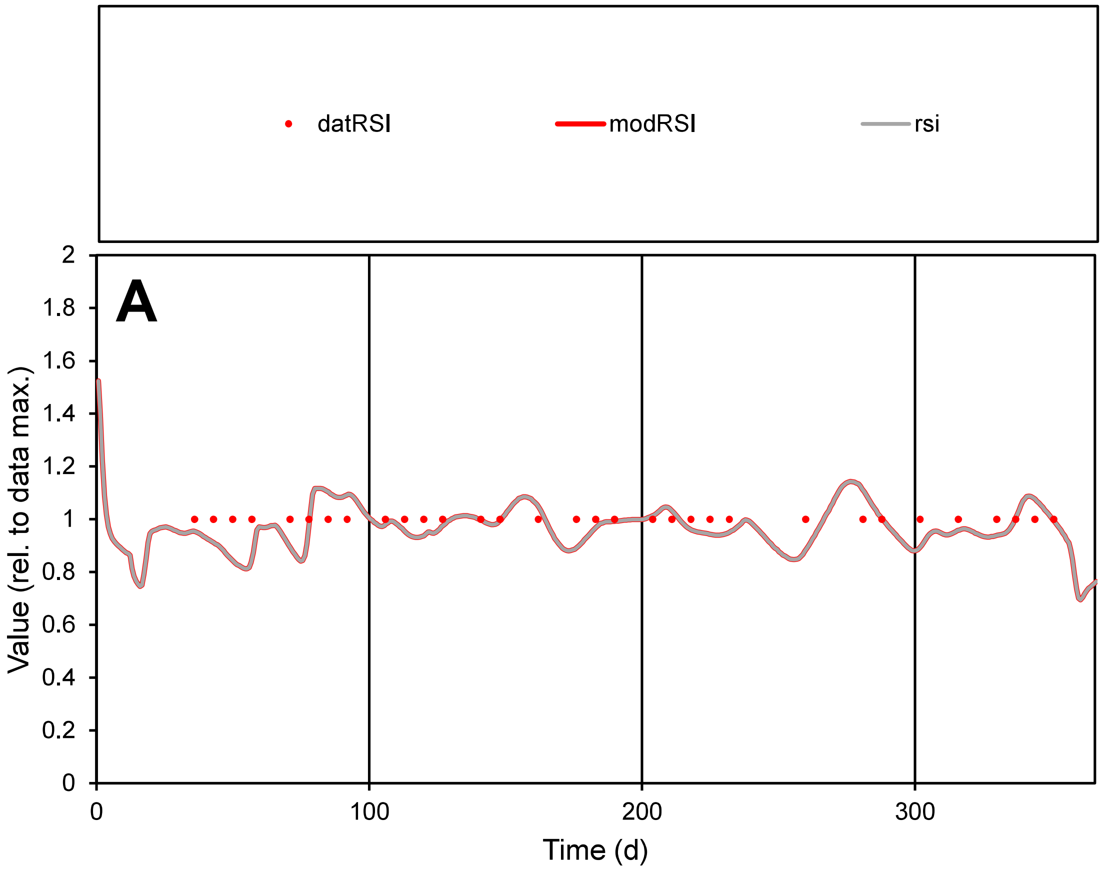

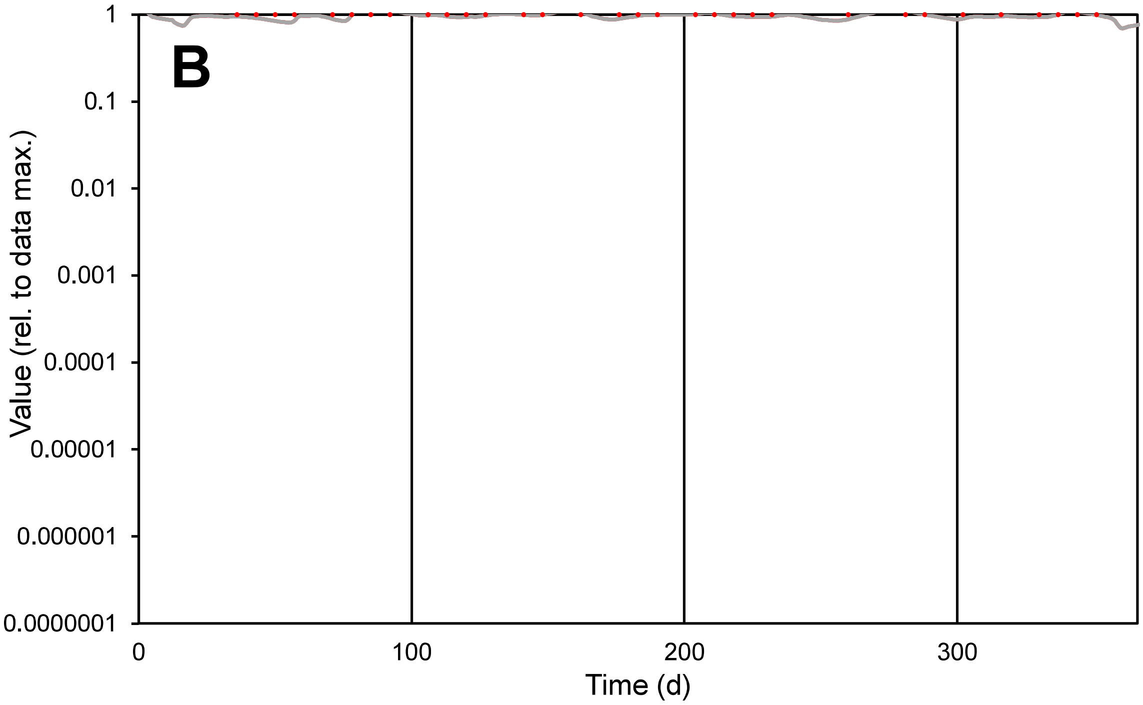

C1

Variables
RSI Provide jtd or Dst
CalibOnly?
2 1 to reproduce of
oNormalize?
2 1=no, 2=yes

ofxn calc*
0.06802
ofxn mod
0.11754

sRun
RunA1
zout
1

Ave
Data
1E-05
Model
1E-05

data/vMax
1E-05

ofxng
321.551

Min d/m
1.20649

ave/min d
1
ave/min m
1.17959

324_Calib_S_A

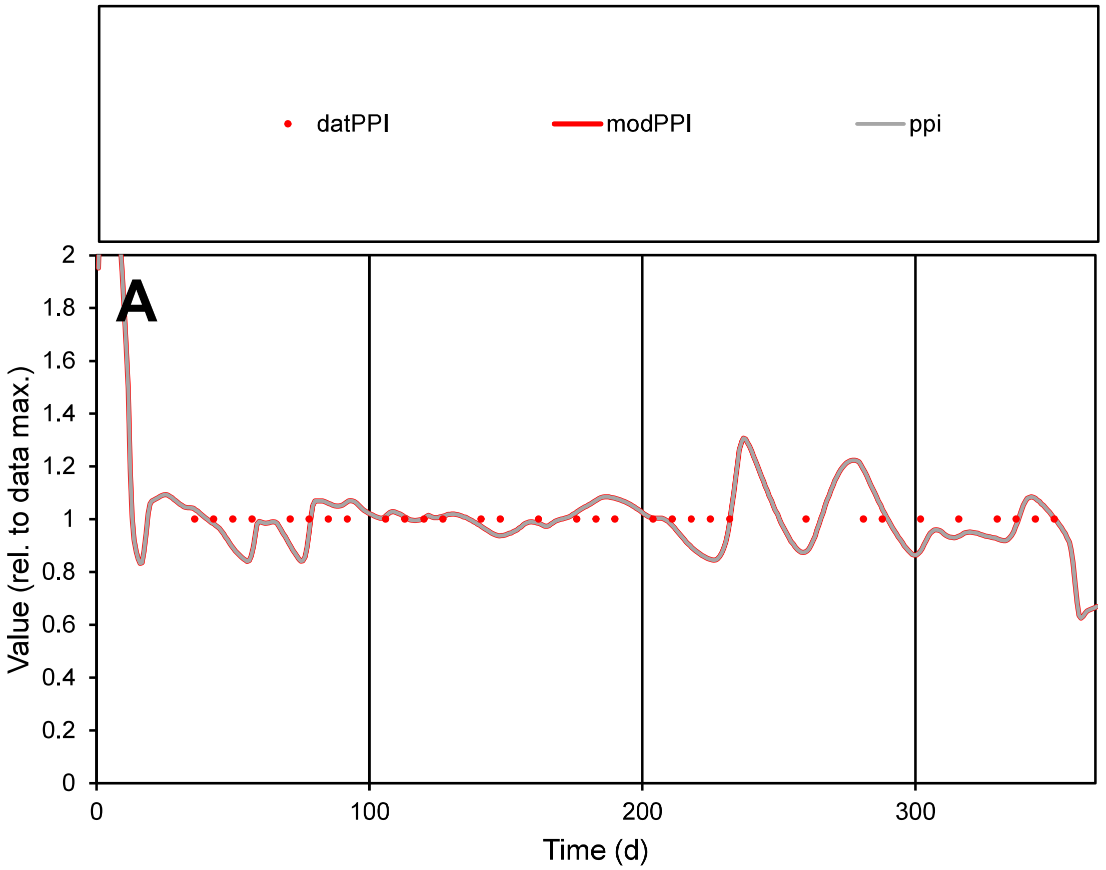

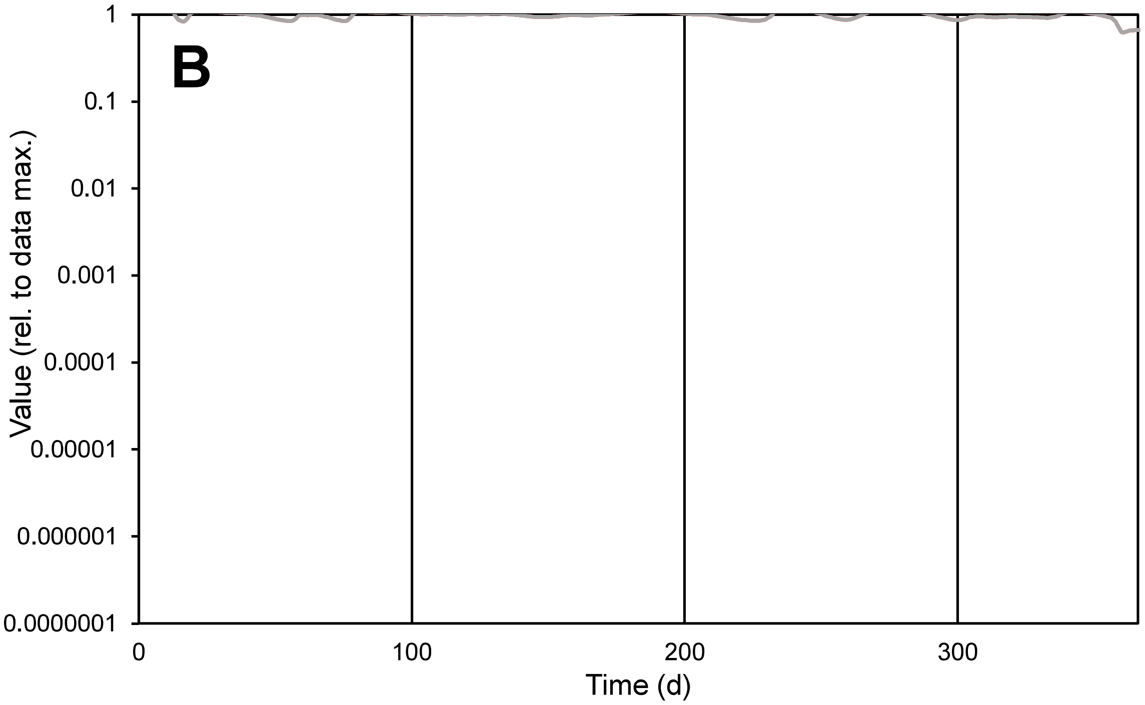

C1

Variables
PPI Provide jtd or Dst
CalibOnly?
2 1 to reproduce of
oNormalize?
2 1=no, 2=yes

ofxn calc*
0.07309
ofxn mod
0.12179

sRun
RunA1
zout
1

Ave
Data
1.4E-06
Model
1.4E-06

data/vMax
1.4E-06

ofxng
321.551

Min d/m
1.18331

ave/min d
1
ave/min m
1.16991

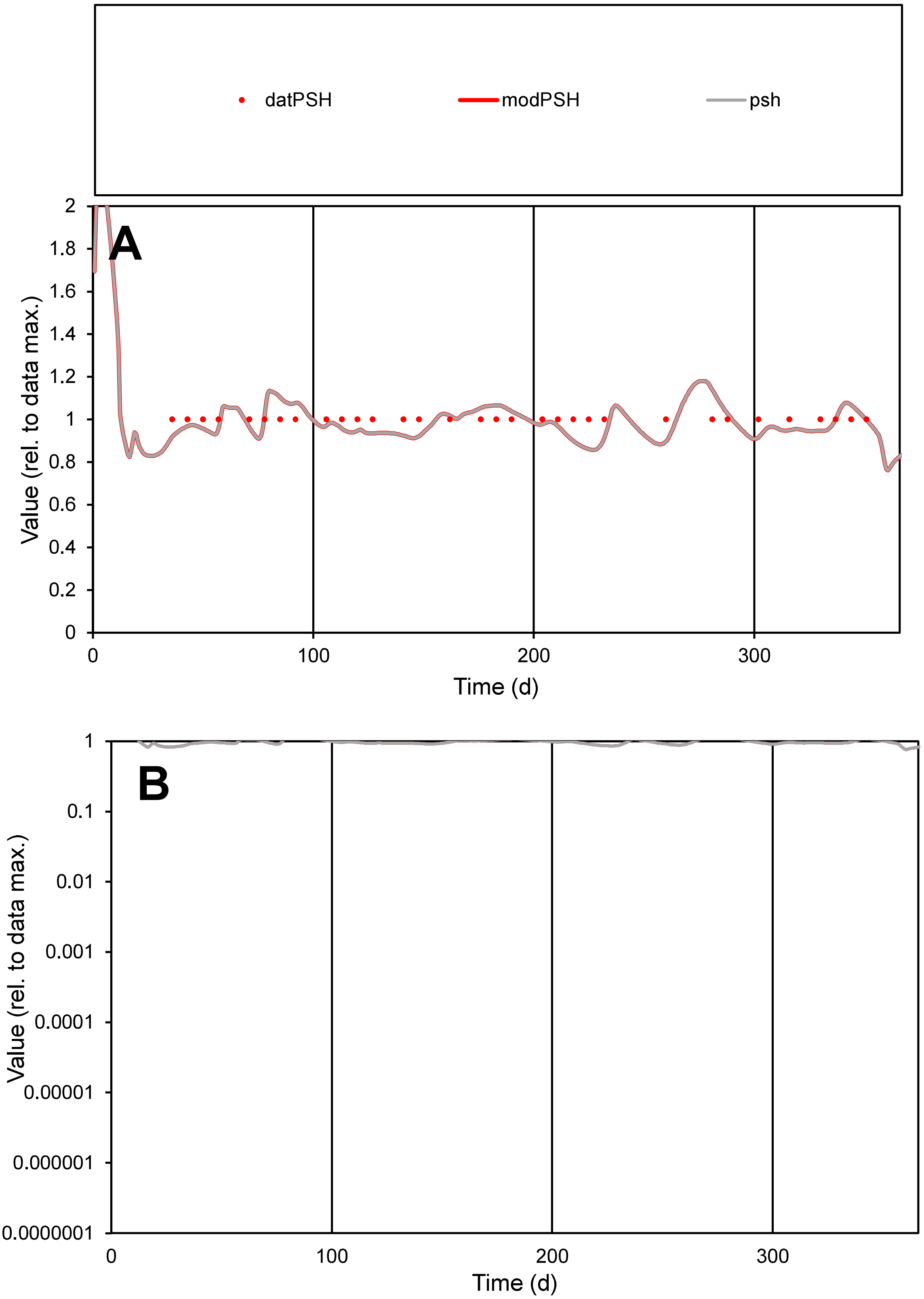

C1

Variables
PSH Provide jtd or Dst
CalibOnly?
2 1 to reproduce of
oNormalize?
2 1=no, 2=yes

ofxn calc*
0.0665
ofxn mod
0.11696

sRun
RunA1
zout
1

Ave
Data
1.3E-05
Model
1.3E-05

data/vMax
1.3E-05

ofxng
321.551

Min d/m
1.16637

ave/min d
1
ave/min m
1.14913

324_Calib_S_A

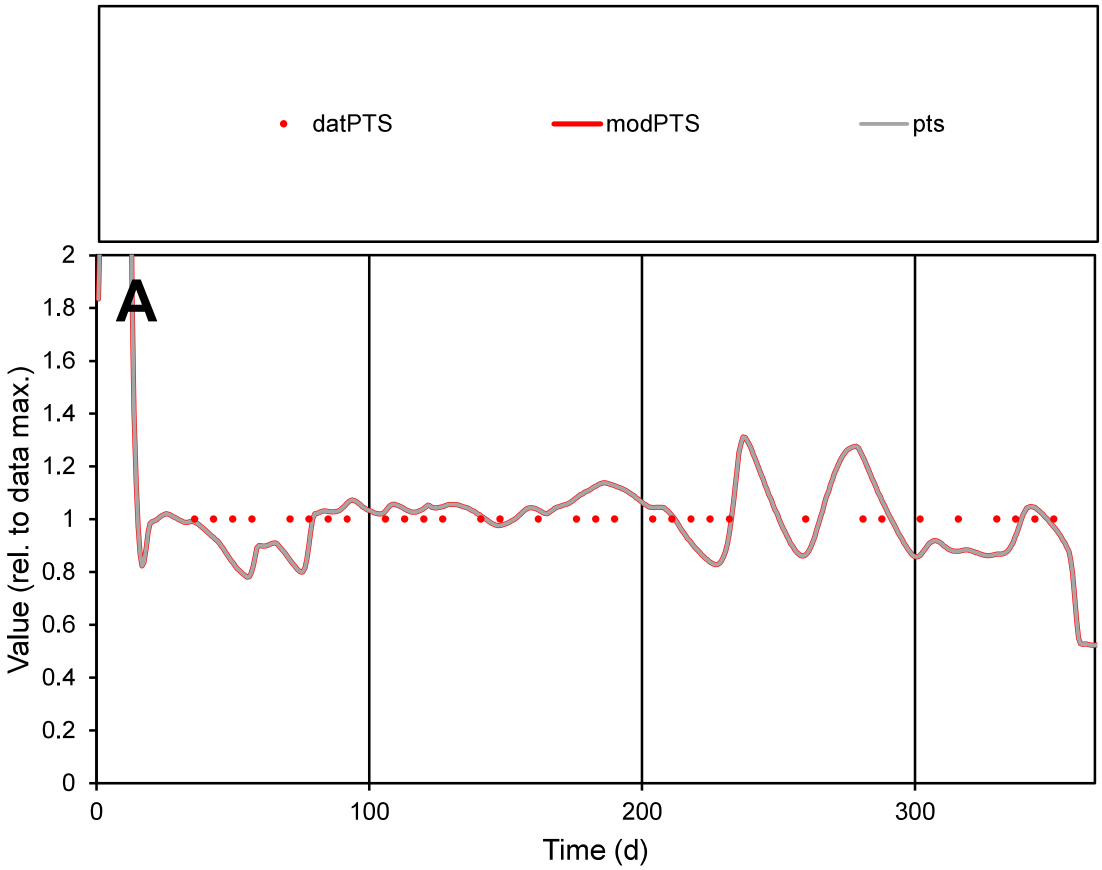

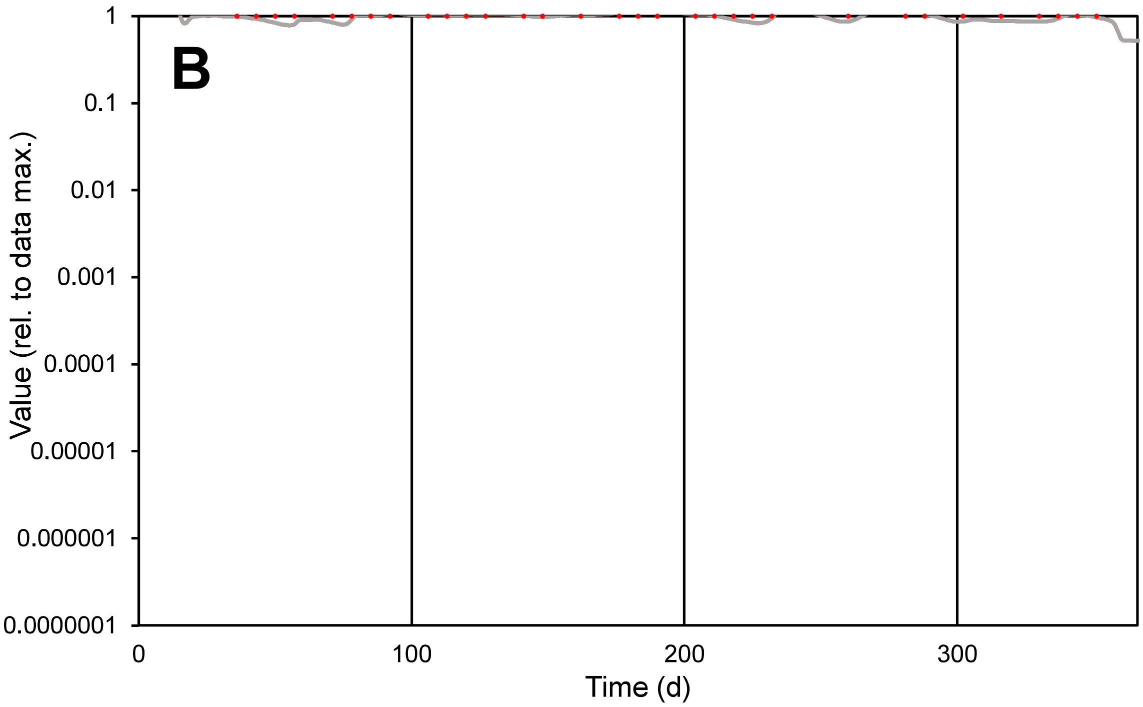

C1

Variables

PTS Provide jtd or Dst

CalibOnly?

2 1 to reproduce of

oNormalize?

2 1=no, 2=yes

ofxn calc*

0.09652

ofxn mod

0.13674

sRun

RunA1

zout

1

Ave

Data

9.7E-06

Model

9.5E-06

data/vMax

9.7E-06

ofxng

321.551

Min d/m

1.22787

ave/min d

1

ave/min m

1.20972

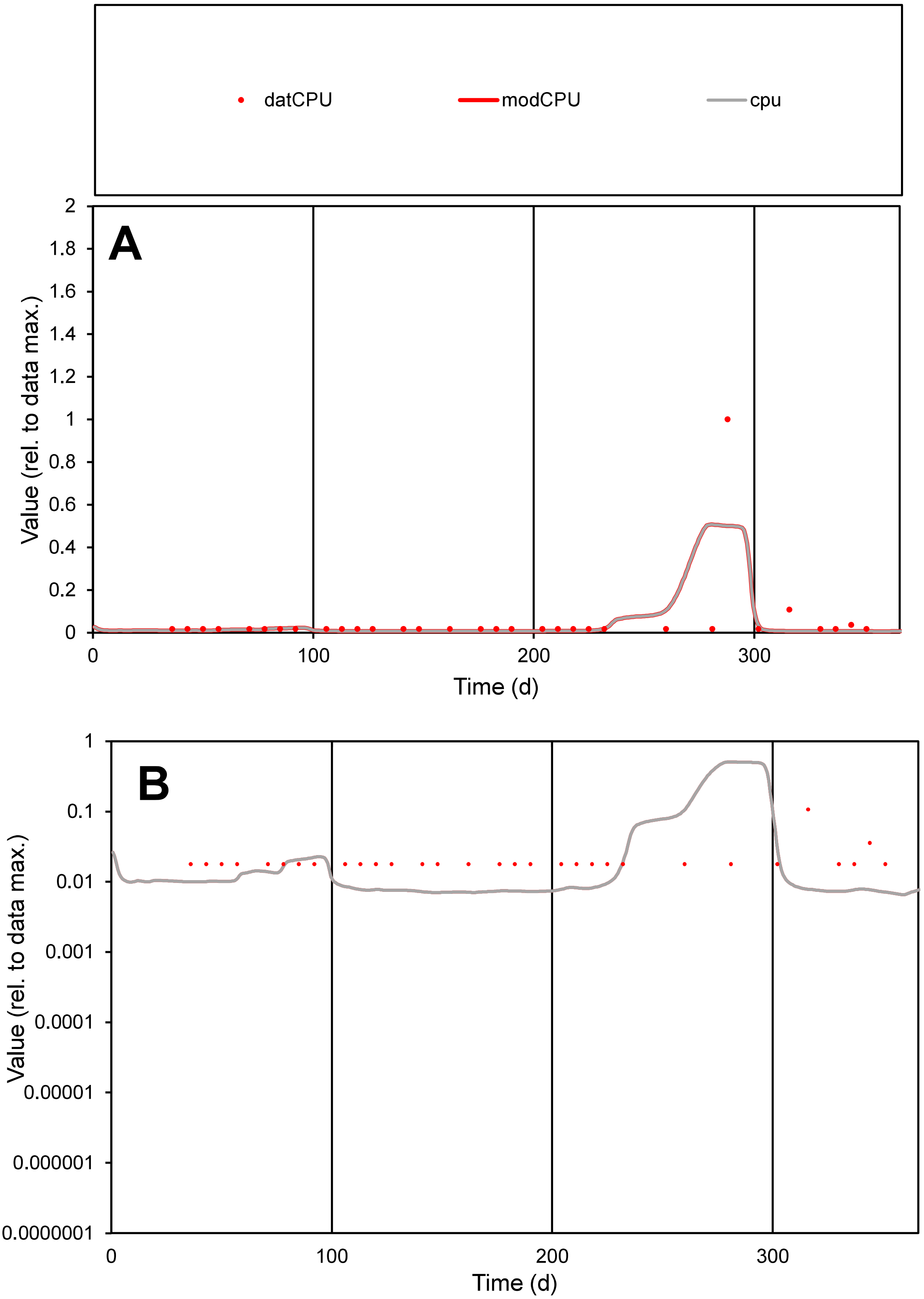

C1

Variables
CPU Provide jtd or Dst
CalibOnly?
2 1 to reproduce of
oNormalize?
2 1=no, 2=yes

ofxn calc*
2.42994
ofxn mod
2.44574

sRun
RunA1
zout
1

Ave
Data
1.3E-06
Model
1.1E-06

data/vMax
2.5E-05

ofxng
321.551

Min d/m
2.54284

ave/min d
2.9087
ave/min m
6.30267

324_Calib_S_A

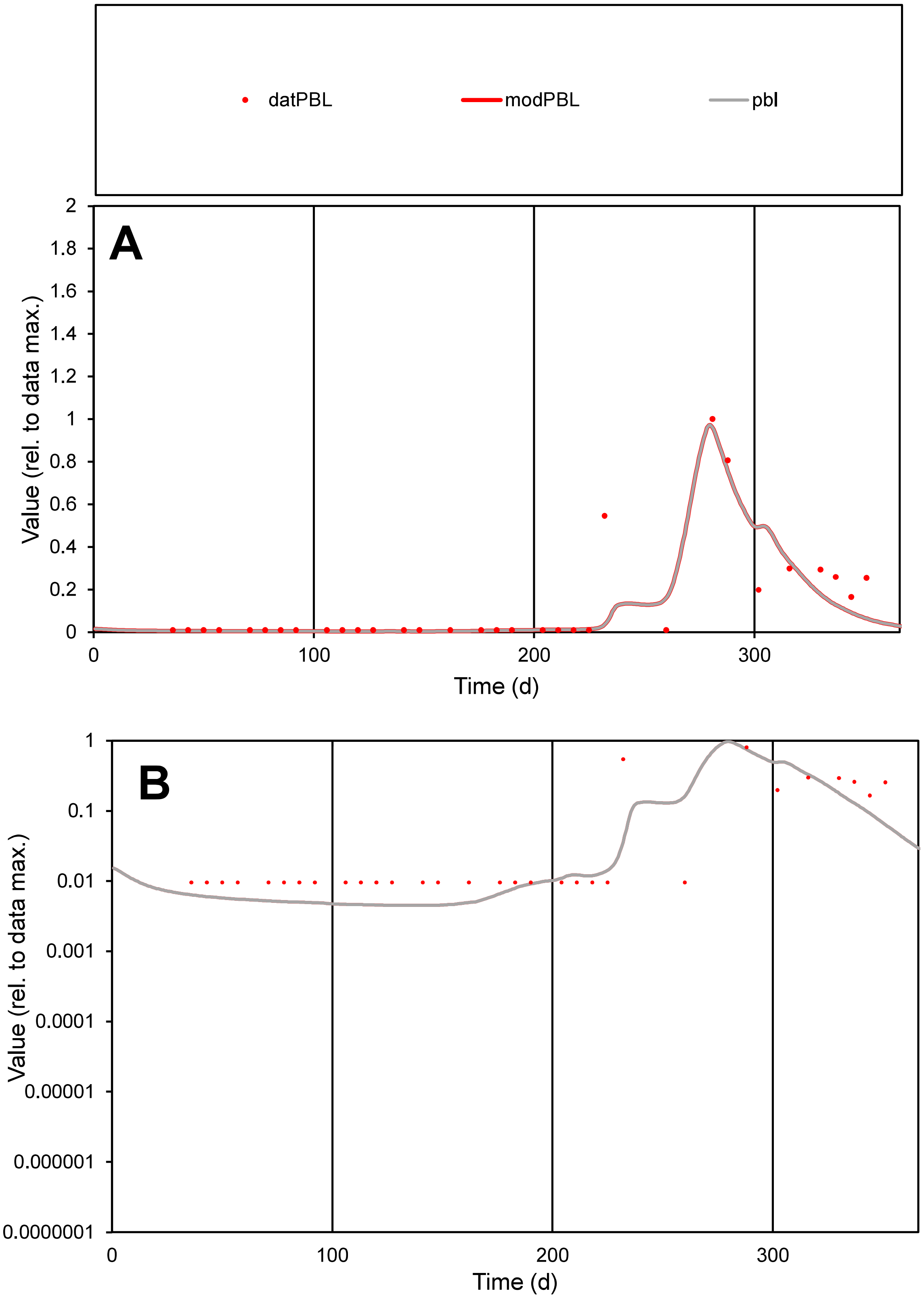

C1

Variables
PBL Provide jtd or Dst
CalibOnly?
2 1 to reproduce of
oNormalize?
2 1=no, 2=yes

ofxn calc*
0.9137
ofxn mod
0.95044

sRun
RunA1
zout
1

Ave
Data
5.6E-07
Model
4.6E-07

data/vMax
4.4E-06

ofxng
321.551

Min d/m
2.12481

ave/min d
13.2001
ave/min m
22.7212

324_Calib_S_A

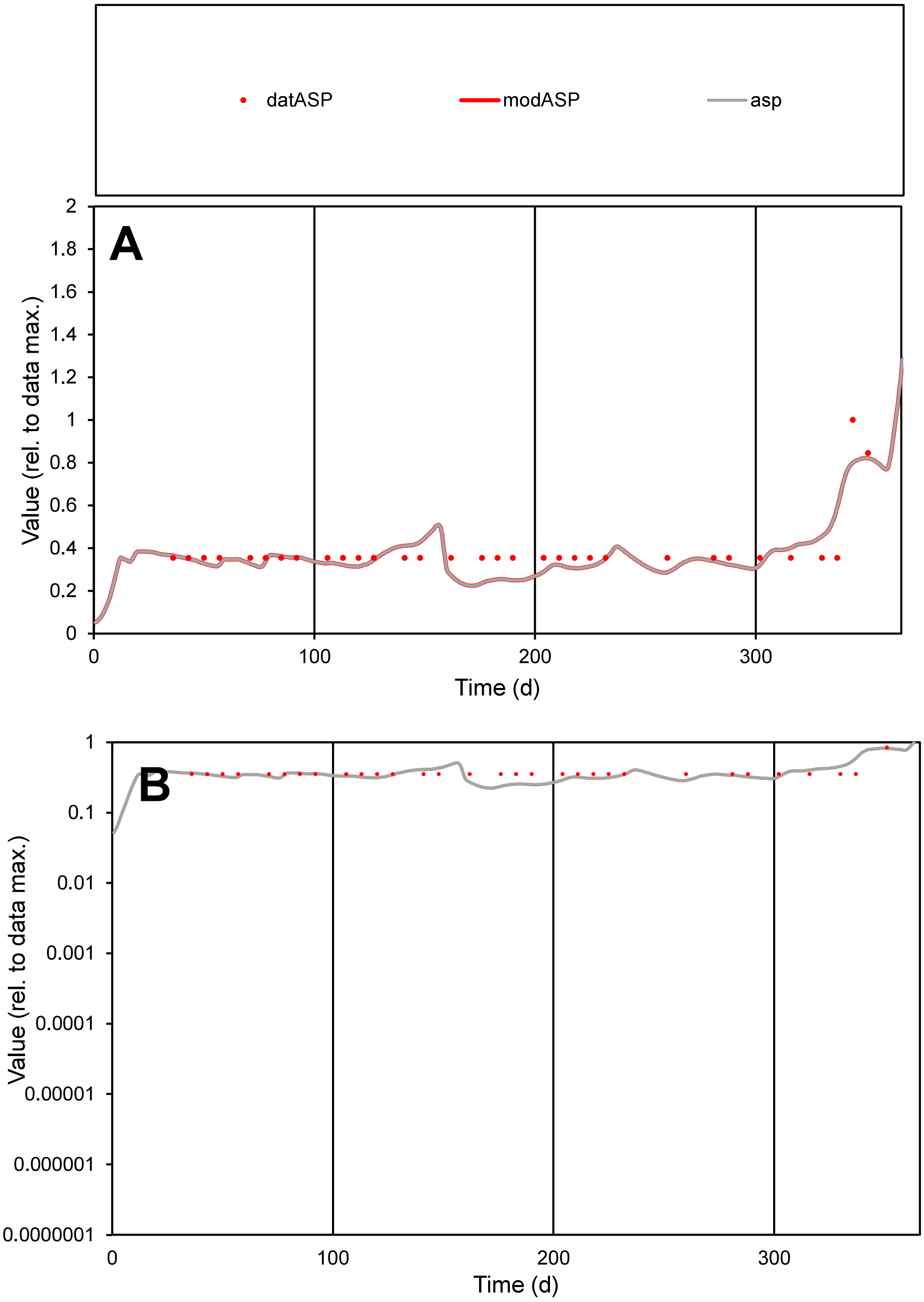

C1

Variables

ASP Provide jtd or Dst

CalibOnly?

2 1 to reproduce of

oNormalize?

2 1=no, 2=yes

ofxn calc*

0.19807

ofxn mod

0.22402

sRun

RunA1

zout

1

Ave

Data

6.4E-06

Model

6.1E-06

data/vMax

1.6E-05

ofxng

321.551

Min d/m

1.45972

ave/min d

1.10016

ave/min m

1.53802

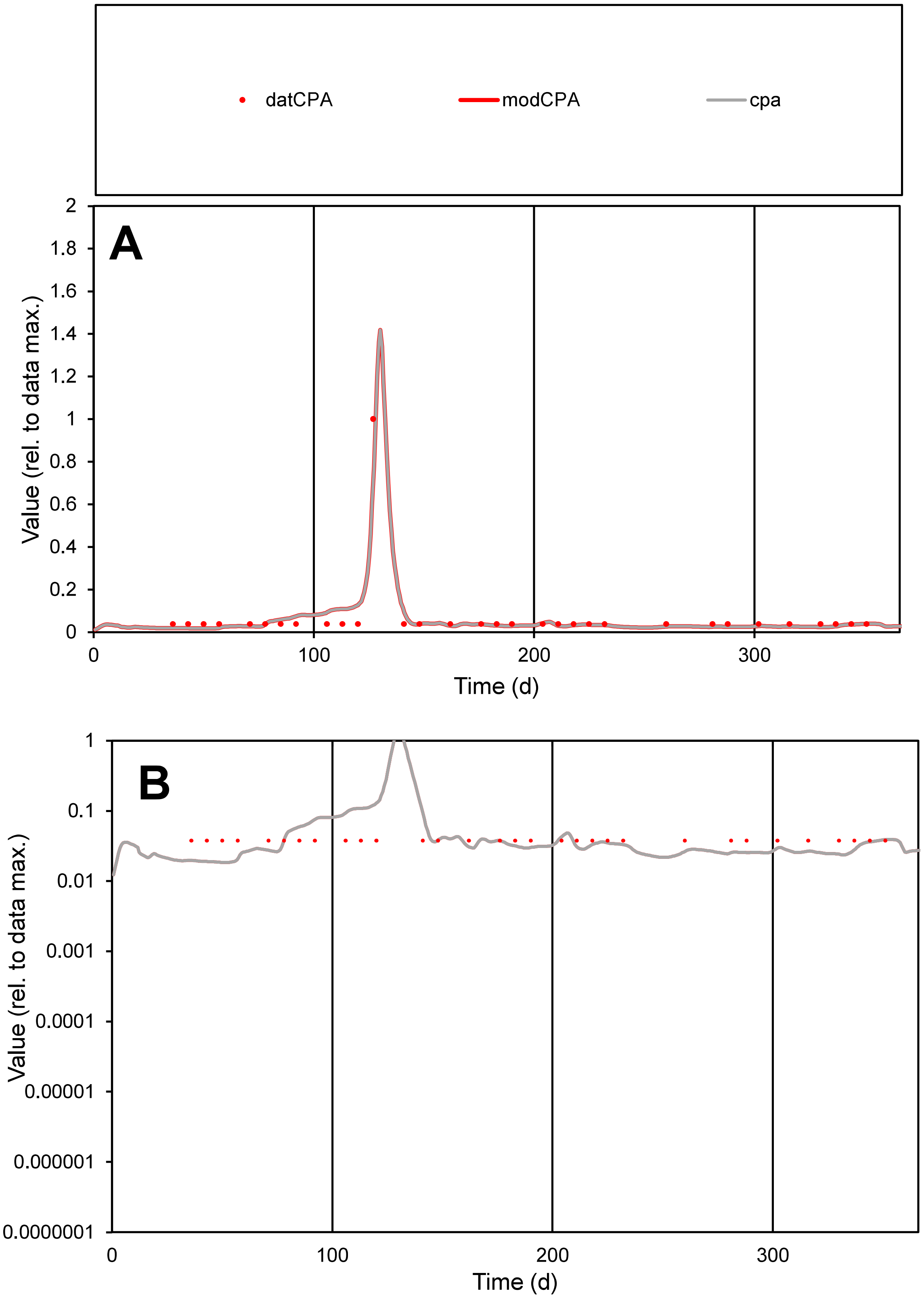

C1

Variables
CPA Provide jtd or Dst
CalibOnly?
2 1 to reproduce of
oNormalize?
2 1=no, 2=yes

ofxn calc*
0.38335
ofxn mod
0.41247

sRun
RunA1
zout
1

Ave
Data
1.2E-07
Model
1.2E-07

data/vMax
1.8E-06

ofxng
321.551

Min d/m
2.04621

ave/min d
1.79399
ave/min m
3.84579

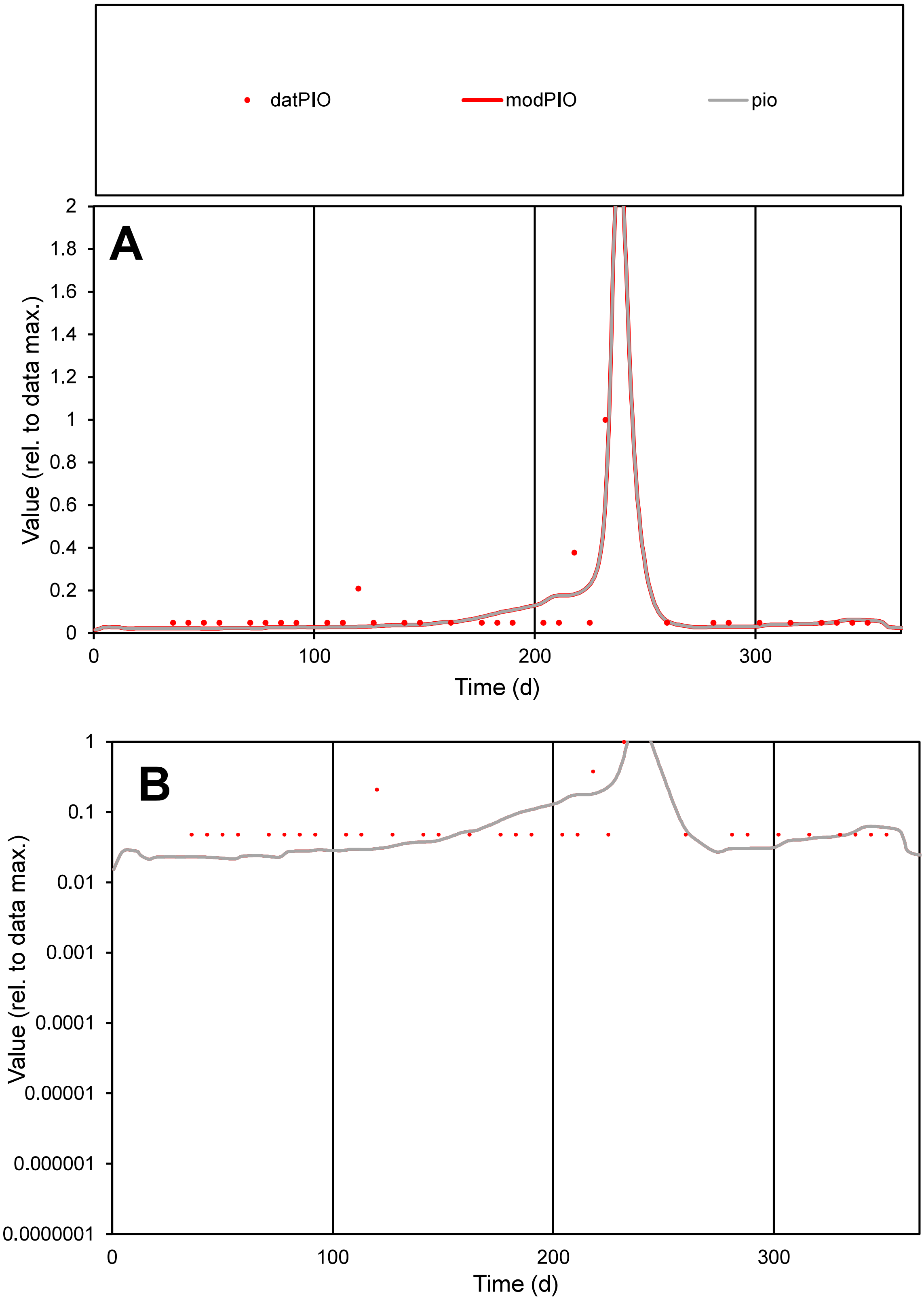

C1

Variables
PIO Provide jtd or Dst
CalibOnly?
2 1 to reproduce of
oNormalize?
2 1=no, 2=yes

ofxn calc*
0.71466
ofxn mod
0.74422

sRun
RunA1
zout
1

Ave
Data
2.8E-07
Model
2.7E-07

data/vMax
3E-06

ofxng
321.551

Min d/m
2.17659

ave/min d
1.94301
ave/min m
4.05151

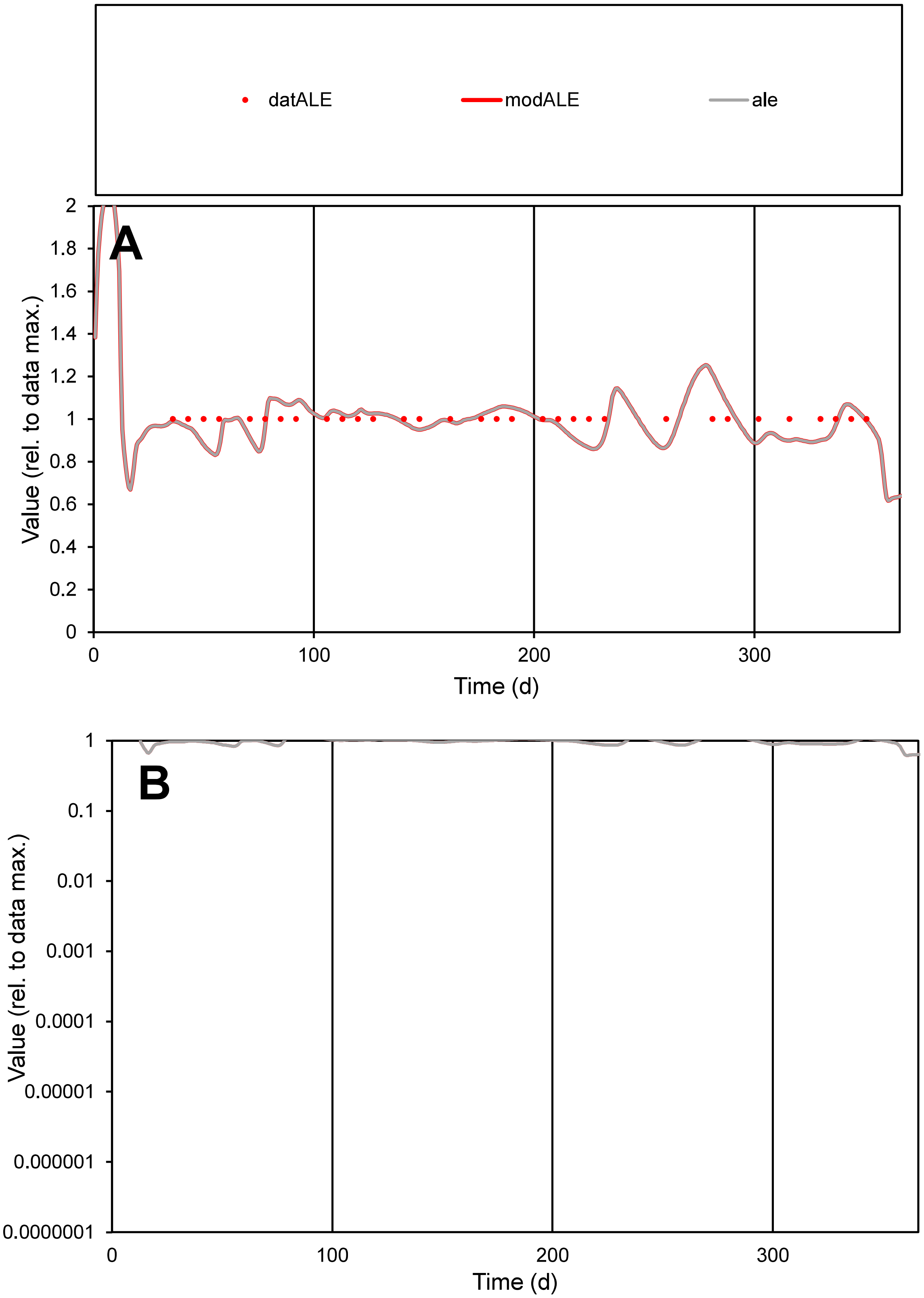

C1

Variables
ALE Provide jtd or Dst
CalibOnly?
2 1 to reproduce of
oNormalize?
2 1=no, 2=yes

ofxn calc*
0.0762
ofxn mod
0.12233

sRun
RunA1
zout
1

Ave
Data
1.1E-05
Model
1.1E-05

data/vMax
1.1E-05

ofxng
321.551

Min d/m
1.16201

ave/min d
1
ave/min m
1.14666

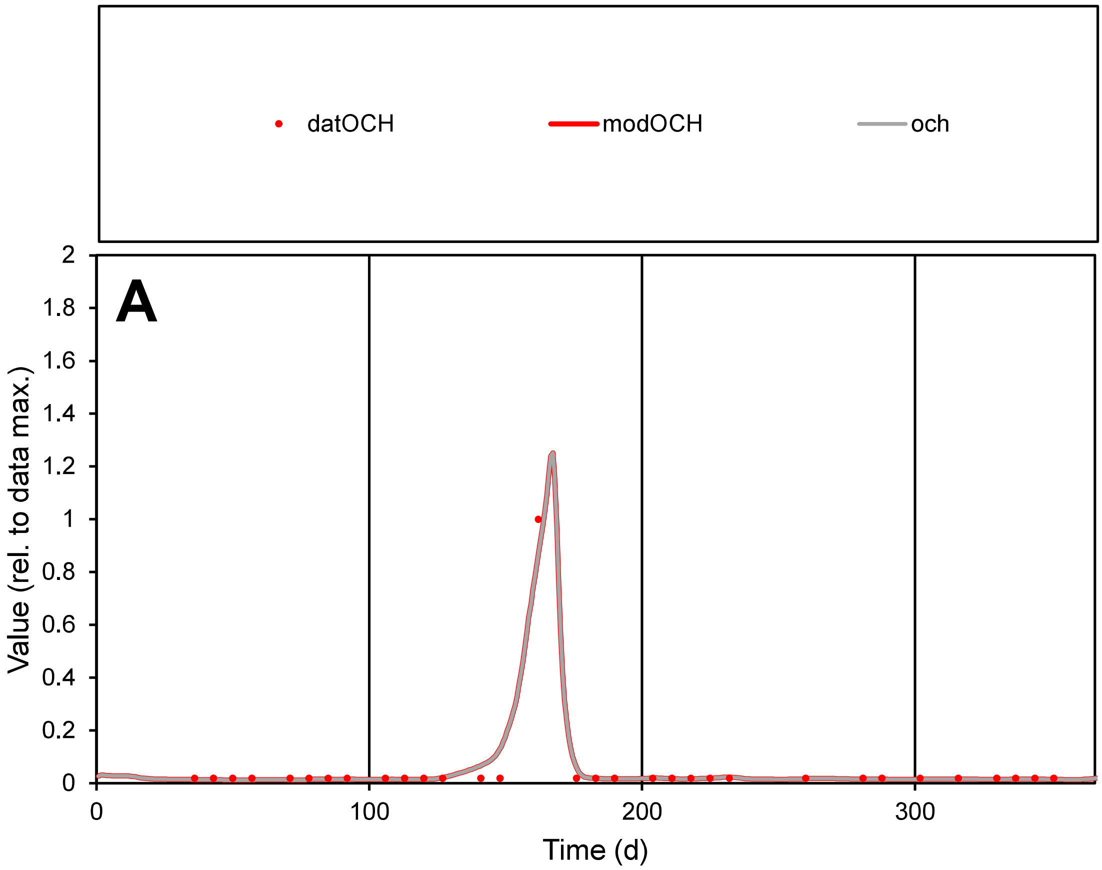

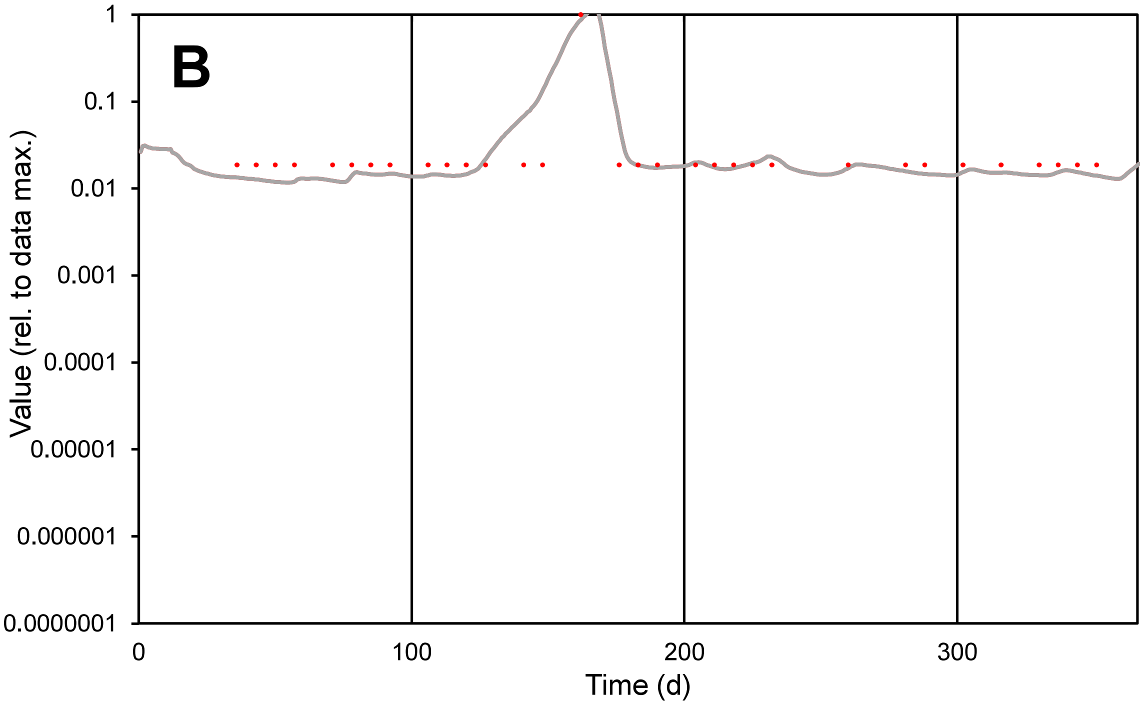

C1

Variables
OCH Provide jtd or Dst
CalibOnly?
2 1 to reproduce of
oNormalize?
2 1=no, 2=yes

ofxn calc*
0.37428
ofxn mod
0.63279

sRun
RunA1
zout
1

Ave
Data
1.2E-05
Model
1.2E-05

data/vMax
0.00023

ofxng
321.551

Min d/m
1.58279

ave/min d
2.63821
ave/min m
4.29487

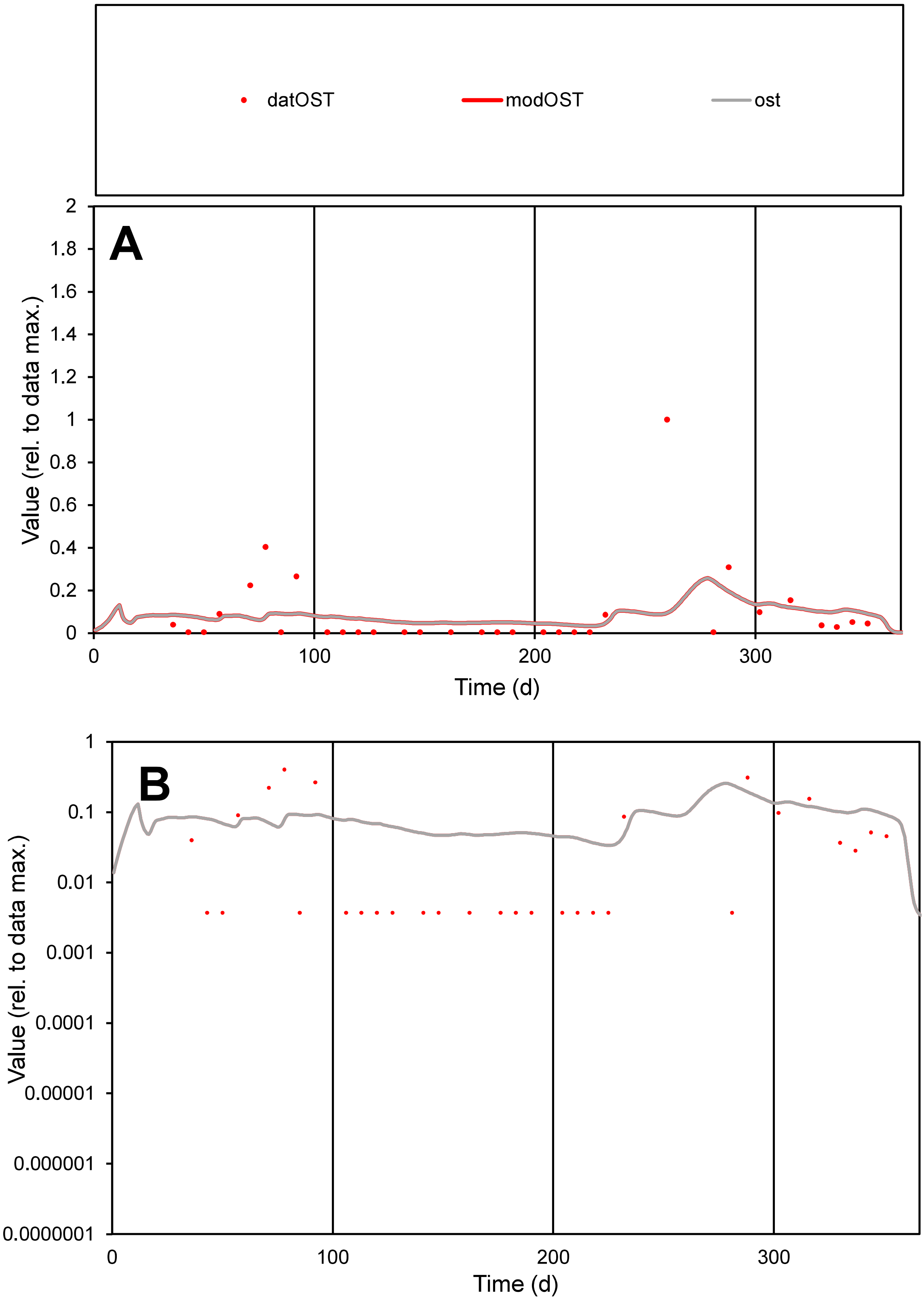

C1

Variables
OST Provide jtd or Dst
CalibOnly?
2 1 to reproduce of
oNormalize?
2 1=no, 2=yes

ofxn calc*
2.0452
ofxn mod
2.05117

sRun
RunA1
zout
1

Ave
Data
5.3E-05
Model
4.7E-05

data/vMax
0.00058

ofxng
321.551

Min d/m
0.11

ave/min d
24.4059
ave/min m
2.40376

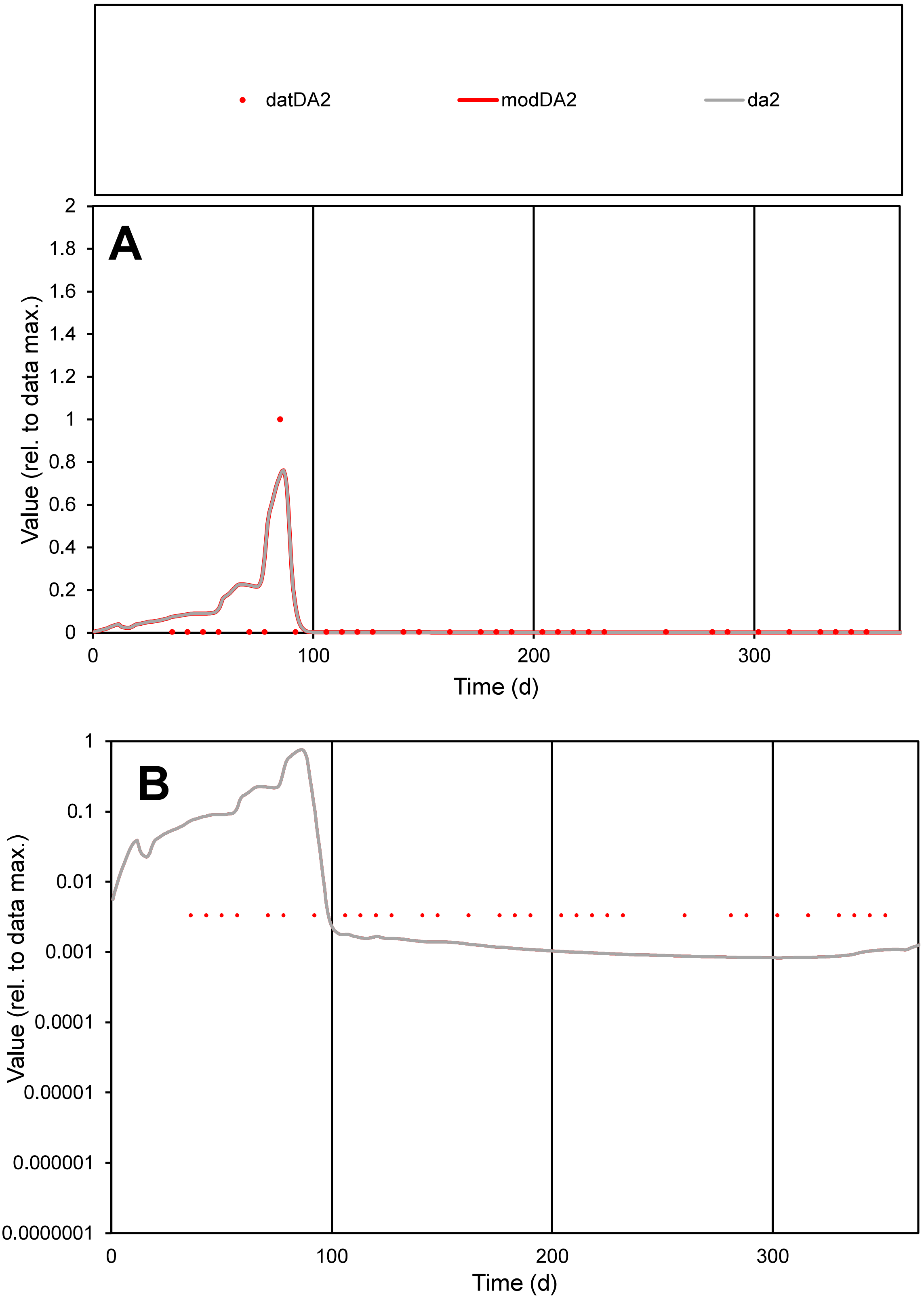

C1

Variables
DA2 Provide jtd or Dst
CalibOnly?
2 1 to reproduce of
oNormalize?
2 1=no, 2=yes

ofxn calc*
2.23639
ofxn mod
2.46729

sRun
RunA1
zout
1

Ave
Data
3.2E-05
Model
4.8E-05

data/vMax
0.00093

ofxng
321.551

Min d/m
4.05037

ave/min d
10.3257
ave/min m
61.7059

324_Calib_S_A

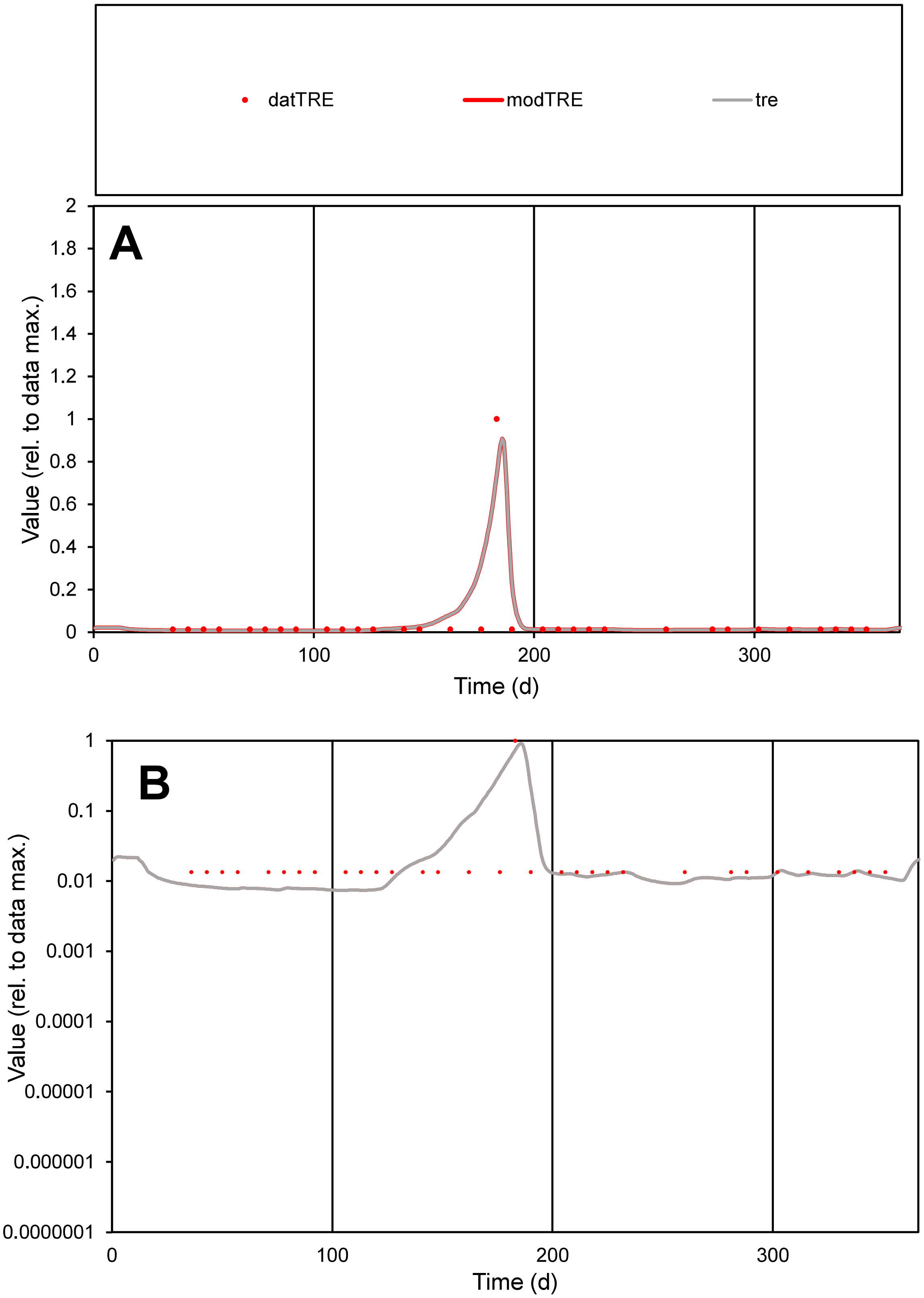

C1

Variables
TRE Provide jtd or Dst
CalibOnly?
2 1 to reproduce of
oNormalize?
2 1=no, 2=yes

ofxn calc*
1.06229
ofxn mod
1.43589

sRun
RunA1
zout
1

Ave
Data
2.3E-05
Model
2.5E-05

data/vMax
0.00052

ofxng
321.551

Min d/m
1.80946

ave/min d
3.29839
ave/min m
6.62061

324_Calib_S_A

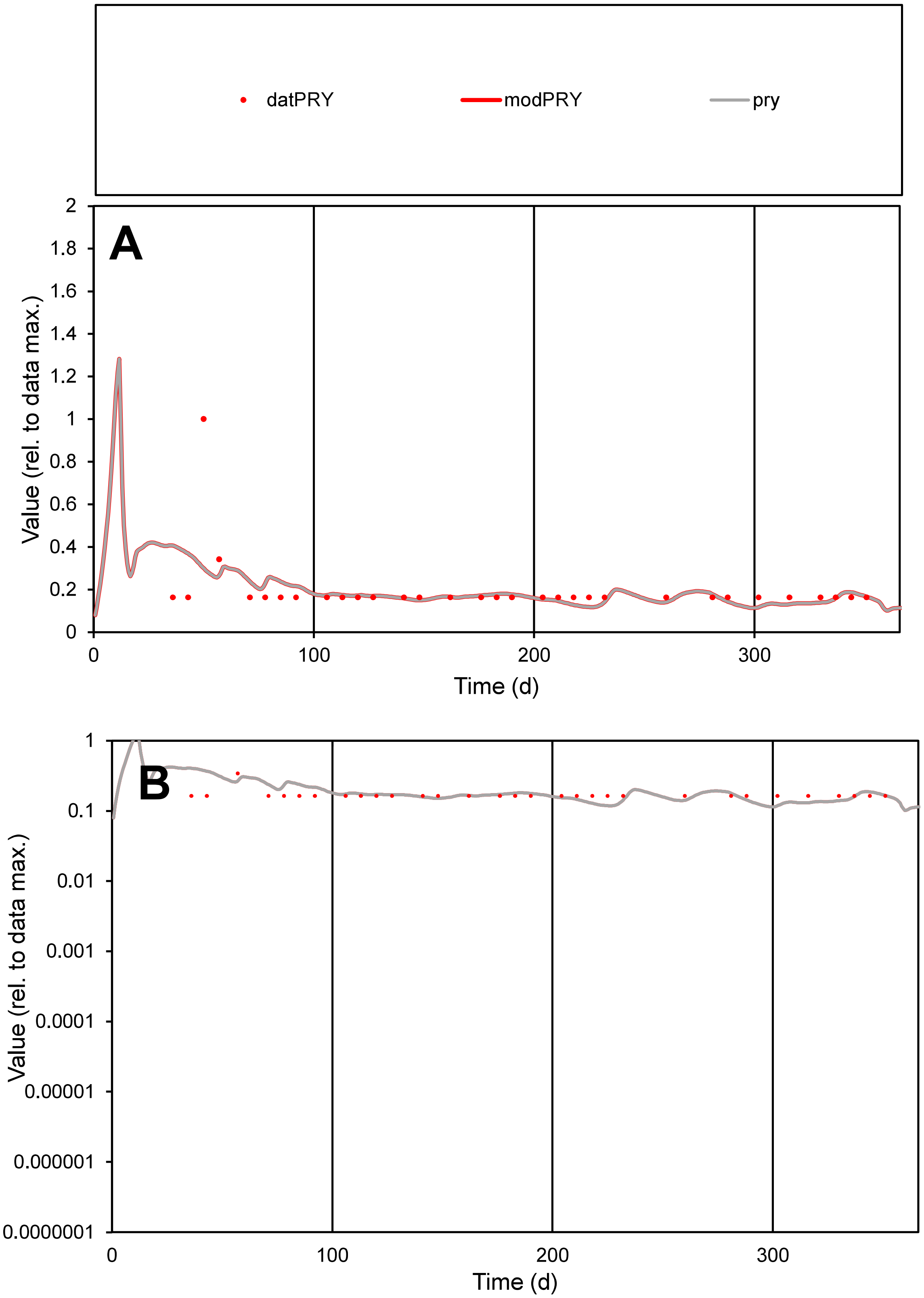

C1

Variables
PRY Provide jtd or Dst
CalibOnly?
2 1 to reproduce of
oNormalize?
2 1=no, 2=yes

ofxn calc*
0.7138
ofxn mod
0.73175

sRun
RunA1
zout
1

Ave
Data
1.1E-05
Model
1E-05

data/vMax
5.4E-05

ofxng
321.551

Min d/m
1.38172

ave/min d
1.19472
ave/min m
1.59737

324_Calib_S_A

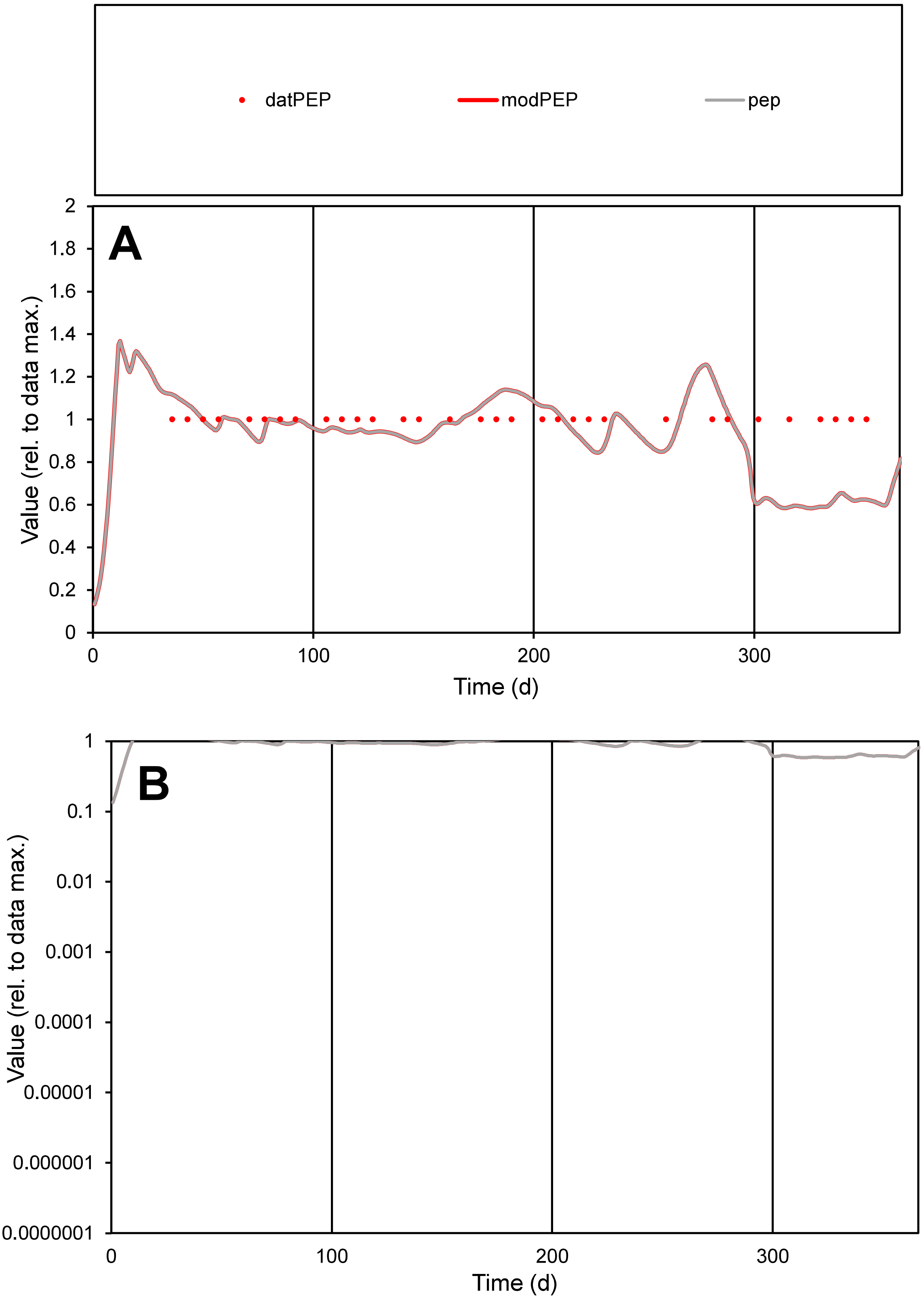

C1

Variables
PEP Provide jtd or Dst
CalibOnly?
2 1 to reproduce of
oNormalize?
2 1=no, 2=yes

ofxn calc*
0.18285
ofxn mod
0.20521

sRun
RunA1
zout
1

Ave
Data
4.9E-06
Model
4.5E-06

data/vMax
4.9E-06

ofxng
321.551

Min d/m
1.69553

ave/min d
1
ave/min m
1.56226

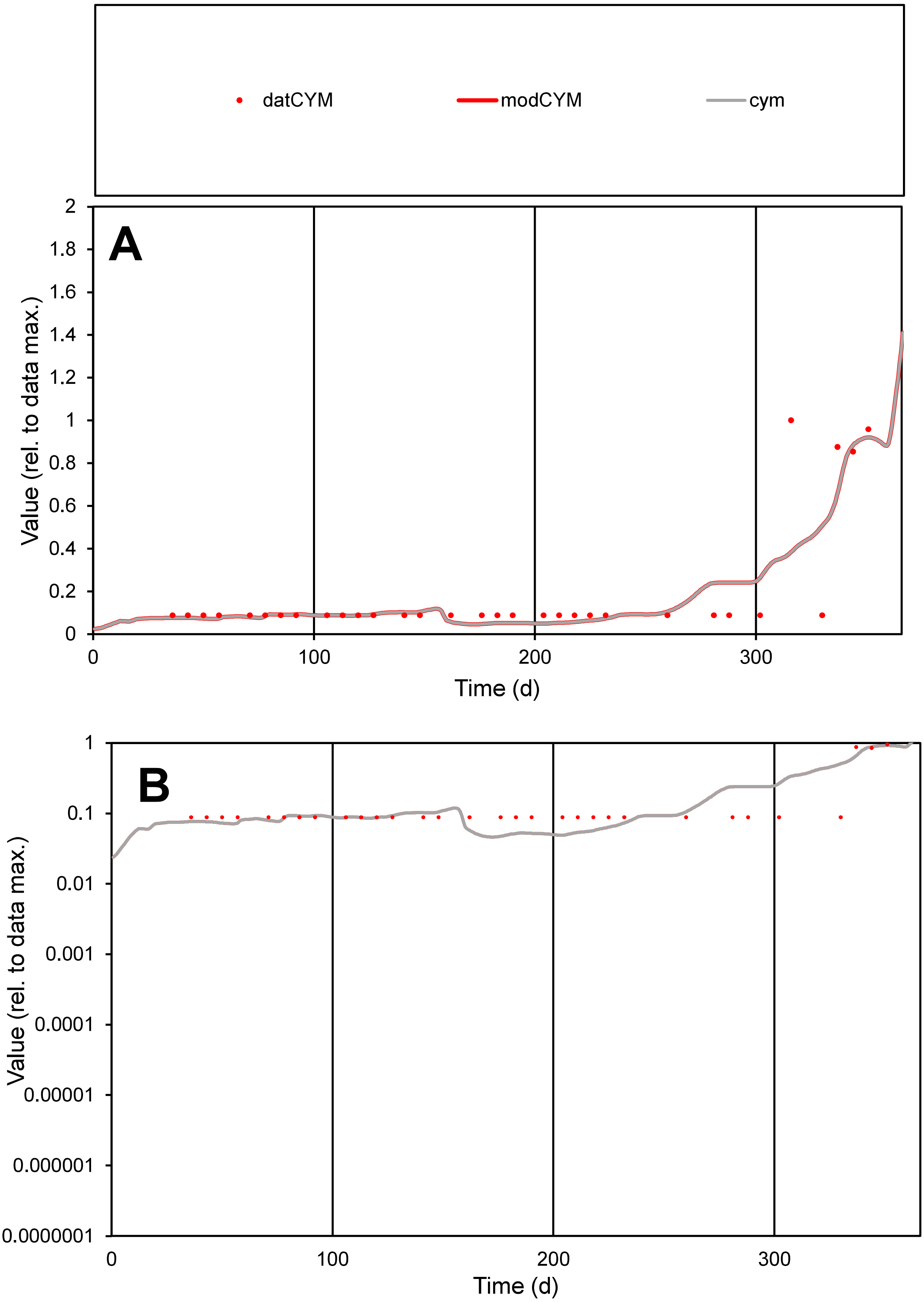

C1

Variables
CYM Provide jtd or Dst
CalibOnly?
2 1 to reproduce of
oNormalize?
2 1=no, 2=yes

ofxn calc*
0.74811
ofxn mod
0.76144

sRun
RunA1
zout
1

Ave
Data
3E-06
Model
2.9E-06

data/vMax
1.5E-05

ofxng
321.551

Min d/m
1.83374

ave/min d
2.17666
ave/min m
3.90541

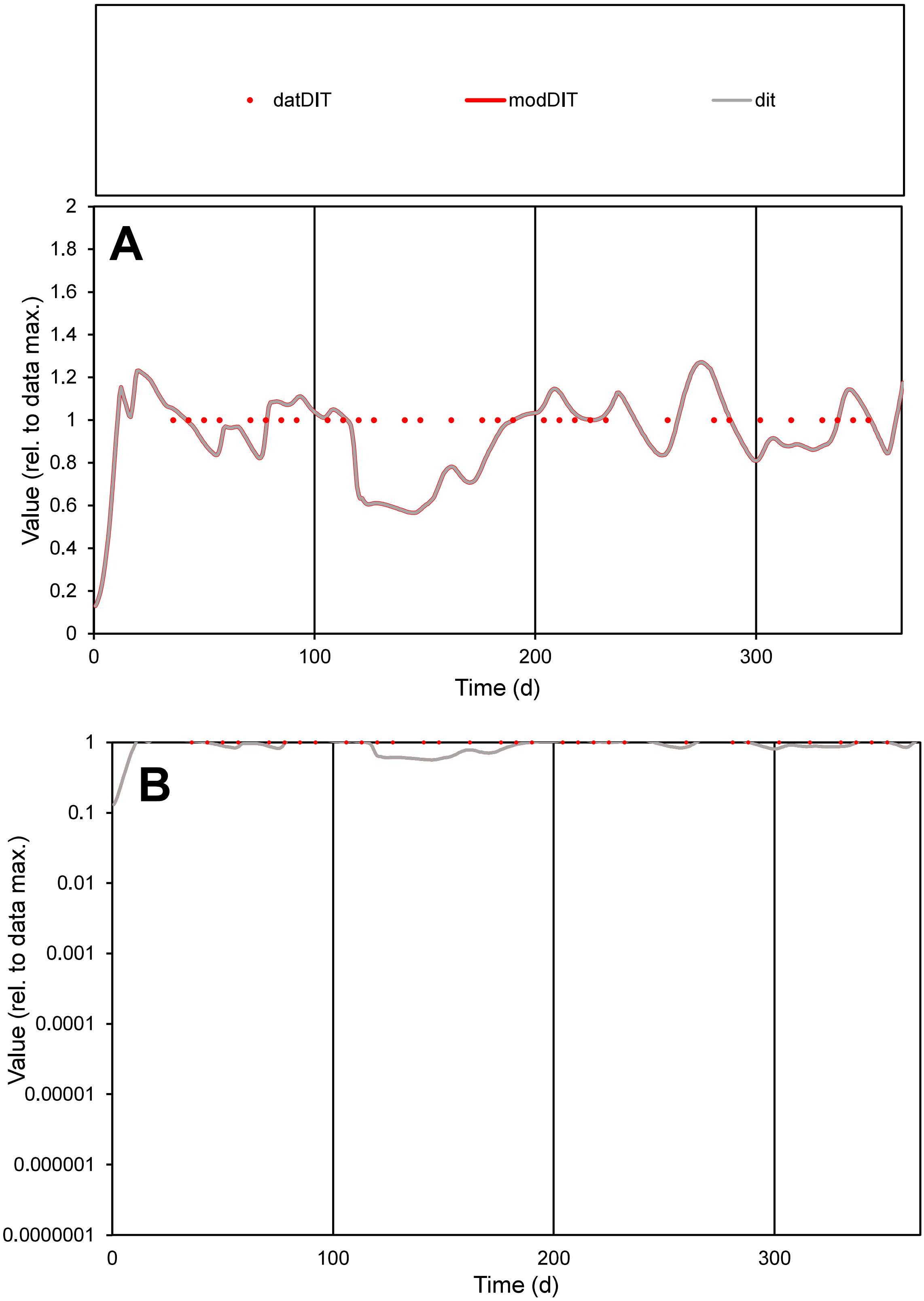

C1

Variables
DIT Provide jtd or Dst
CalibOnly?
2 1 to reproduce of
oNormalize?
2 1=no, 2=yes

ofxn calc*
0.16955
ofxn mod
0.19402

sRun
RunA1
zout
1

Ave
Data
5.1E-06
Model
4.8E-06

data/vMax
5.1E-06

ofxng
321.551

Min d/m
1.7567

ave/min d
1
ave/min m
1.64732

324_Calib_S_A

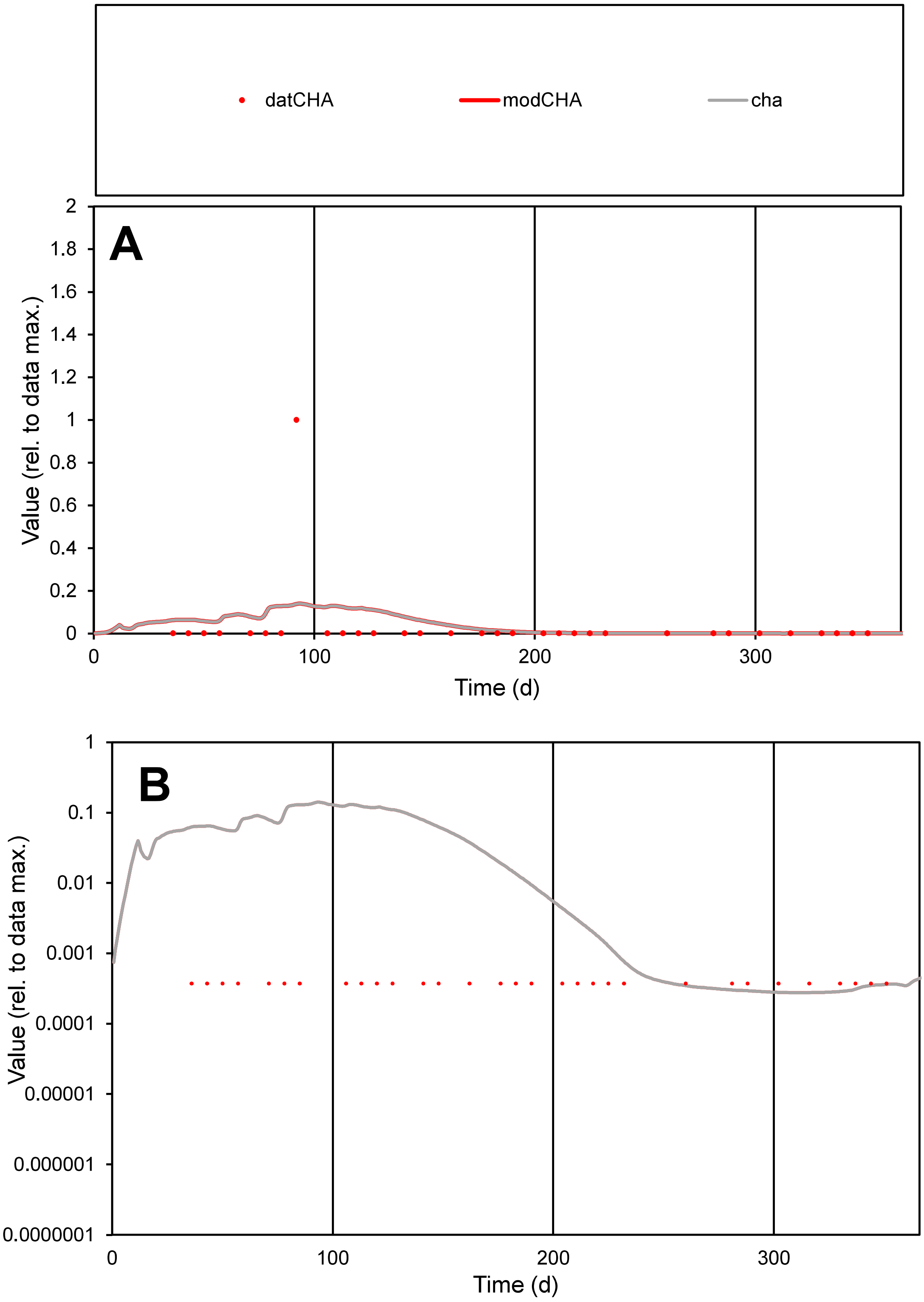

C1

Variables

CHA Provide jtd or Dst

CalibOnly?

2 1 to reproduce of

oNormalize?

2 1=no, 2=yes

ofxn calc*

5.14584

ofxn mod

5.16177

sRun

RunA1

zout

1

Ave

Data

7.3E-06

Model

9.8E-06

data/vMax

0.00023

ofxng

321.551

Min d/m

1.33792

ave/min d

84.9159

ave/min m

151.347

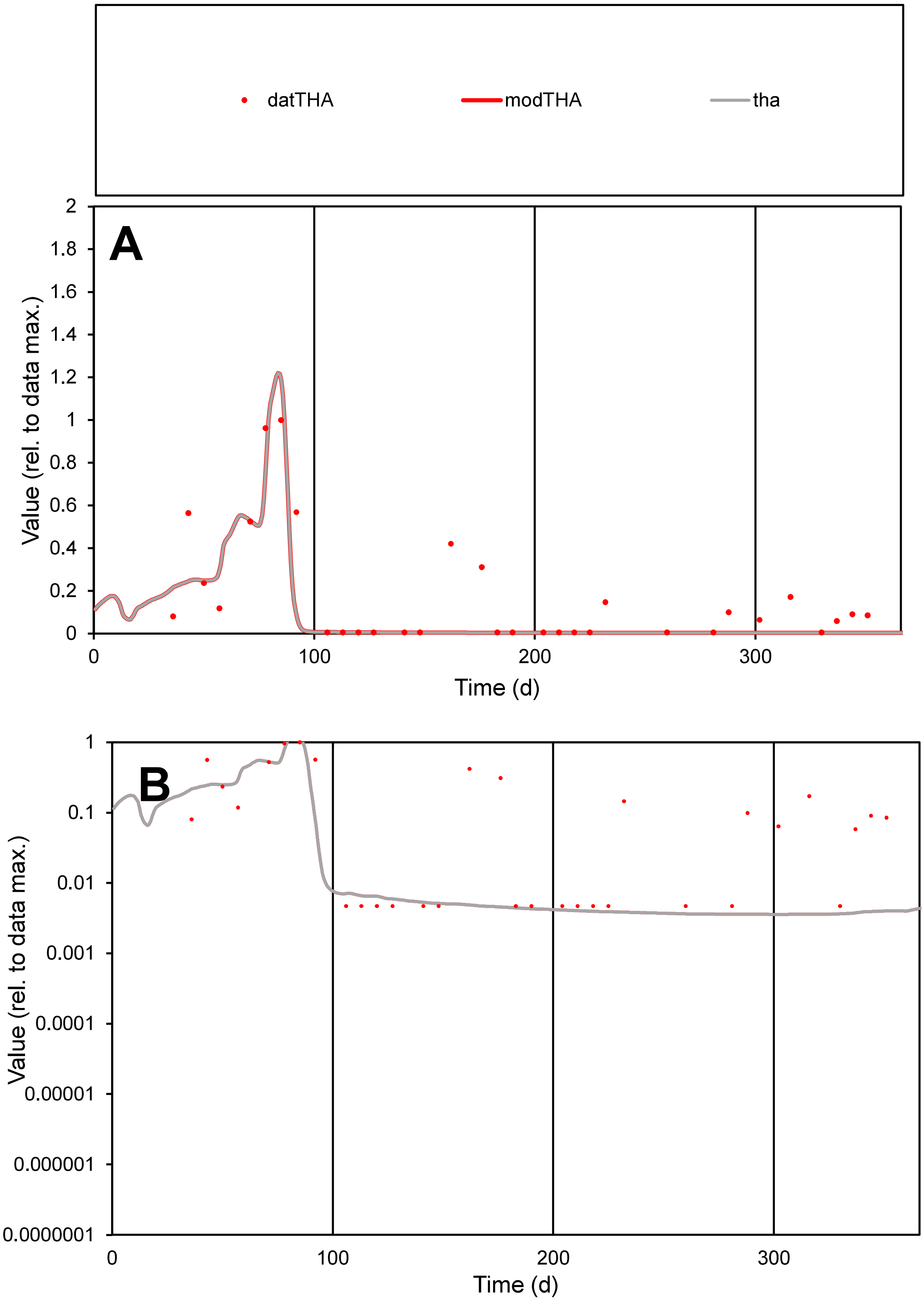

C1

Variables
THA Provide jtd or Dst
CalibOnly?
2 1 to reproduce of
oNormalize?
2 1=no, 2=yes

ofxn calc*
0.75122
ofxn mod
0.85652

sRun
RunA1
zout
1

Ave
Data
0.0001
Model
7.2E-05

data/vMax
0.00058

ofxng
321.551

Min d/m
1.3269

ave/min d
36.7898
ave/min m
34.6756

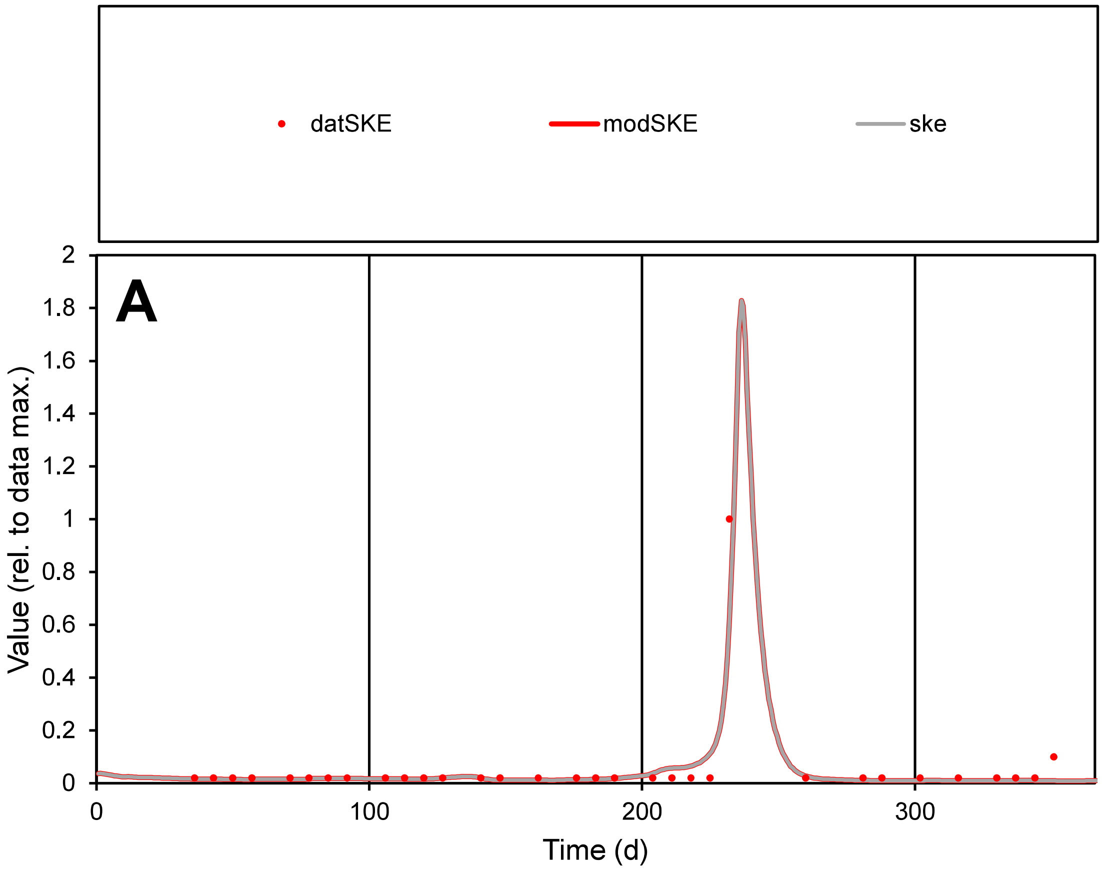

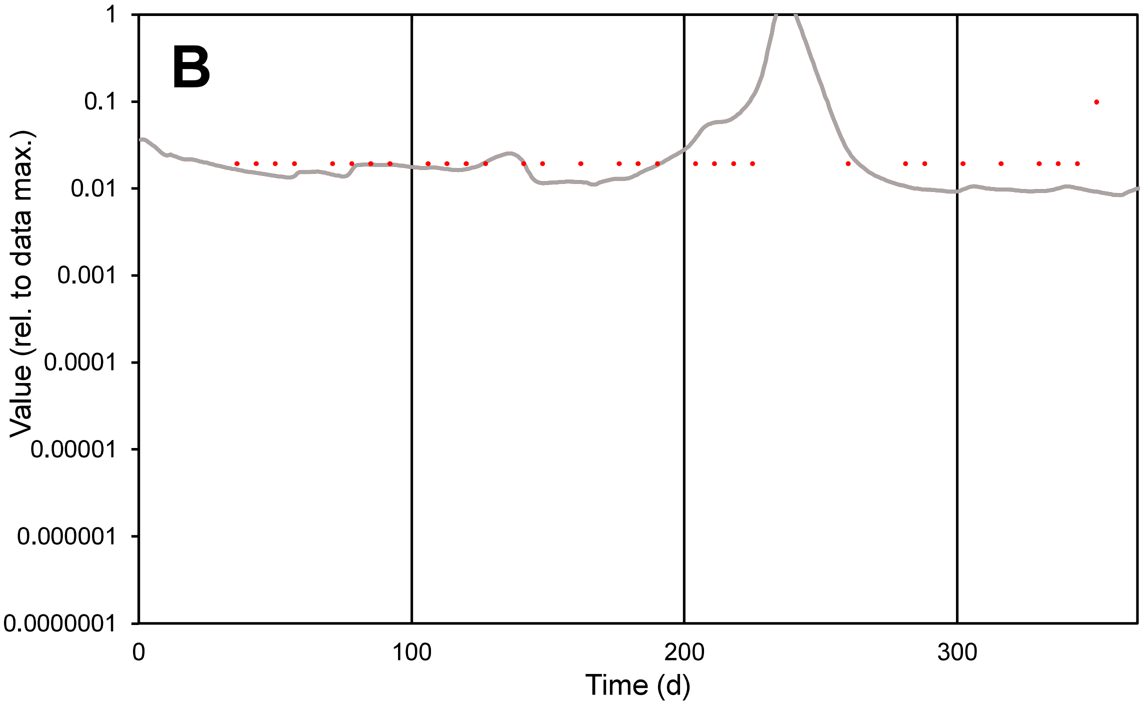

C1

Variables
SKE Provide jtd or Dst
CalibOnly?
2 1 to reproduce of
oNormalize?
2 1=no, 2=yes

ofxn calc*
0.4618
ofxn mod
0.55787

sRun
RunA1
zout
1

Ave
Data
6.2E-06
Model
6.1E-06

data/vMax
0.00012

ofxng
321.551

Min d/m
2.13056

ave/min d
2.71377
ave/min m
5.6364

324_Calib_S_A

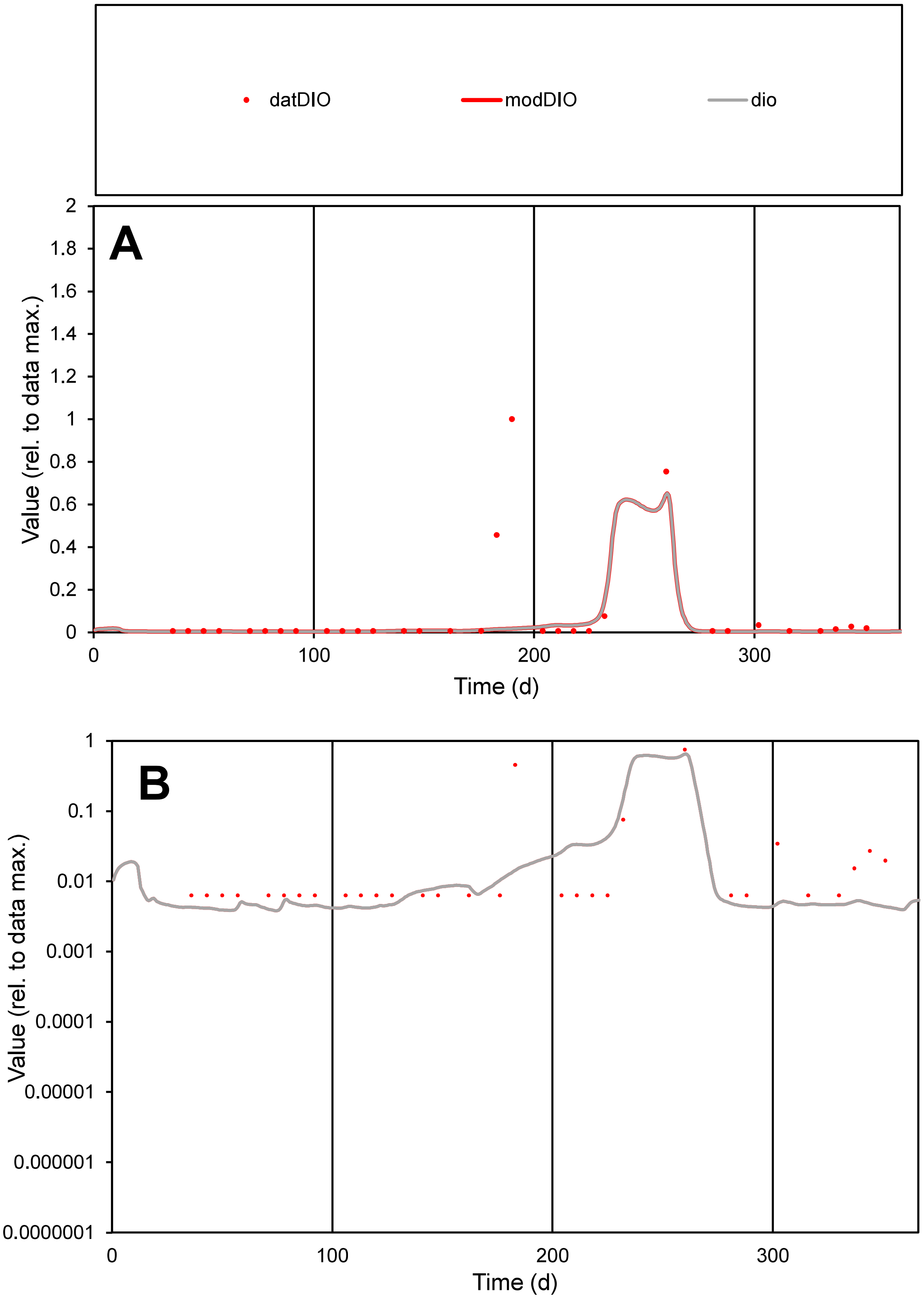

C1

Variables
DIO Provide jtd or Dst
CalibOnly?
2 1 to reproduce of
oNormalize?
2 1=no, 2=yes

ofxn calc*
2.41988
ofxn mod
2.43078

sRun
RunA1
zout
1

Ave
Data
5.8E-05
Model
2.4E-05

data/vMax
0.00074

ofxng
321.551

Min d/m
1.64055

ave/min d
12.5052
ave/min m
8.53849

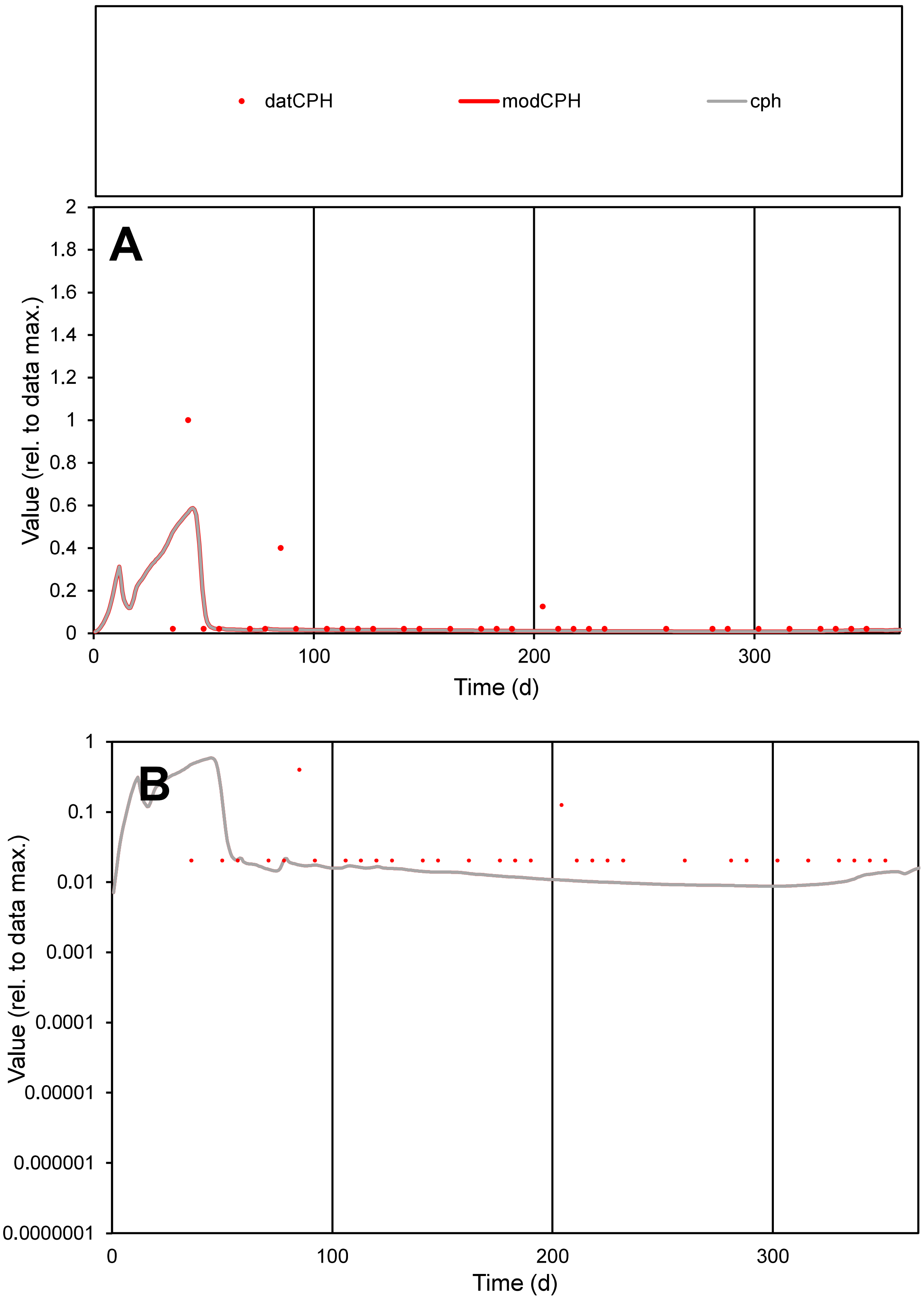

C1

Variables
CPH Provide jtd or Dst
CalibOnly?
2 1 to reproduce of
oNormalize?
2 1=no, 2=yes

ofxn calc*
1.90414
ofxn mod
1.99904

sRun
RunA1
zout
1

Ave
Data
3.1E-05
Model
2.1E-05

data/vMax
0.00047

ofxng
321.551

Min d/m
2.33423

ave/min d
3.24139
ave/min m
5.20484

324_Calib_S_A

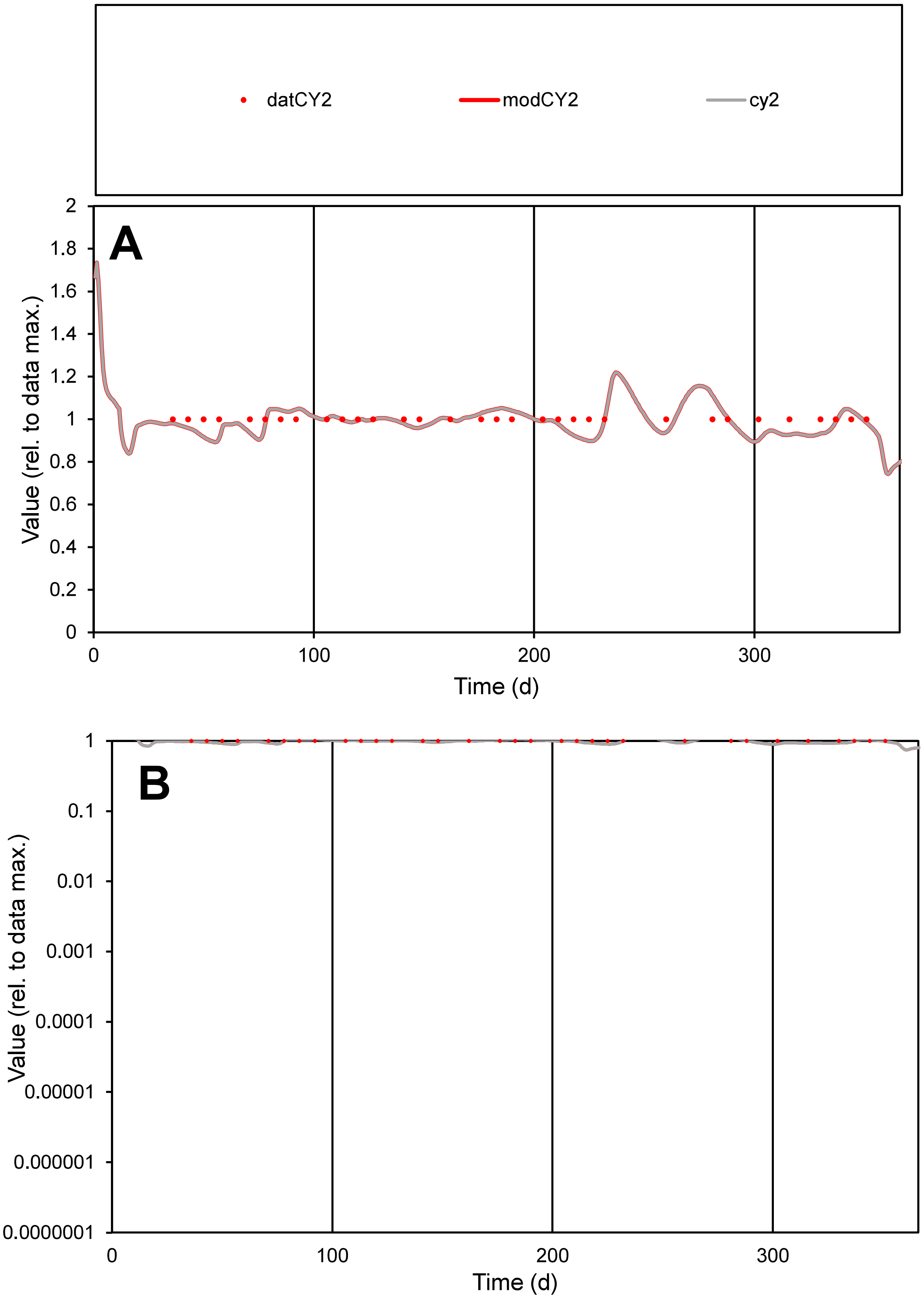

C1

Variables
CY2 Provide jtd or Dst
CalibOnly?
2 1 to reproduce of
oNormalize?
2 1=no, 2=yes

ofxn calc*
0.05029
ofxn mod
0.10911

sRun
RunA1
zout
1

Ave
Data
1.1E-05
Model
1.1E-05

data/vMax
1.1E-05

ofxng
321.551

Min d/m
1.11289

ave/min d
1
ave/min m
1.09672

324_Calib_S_A

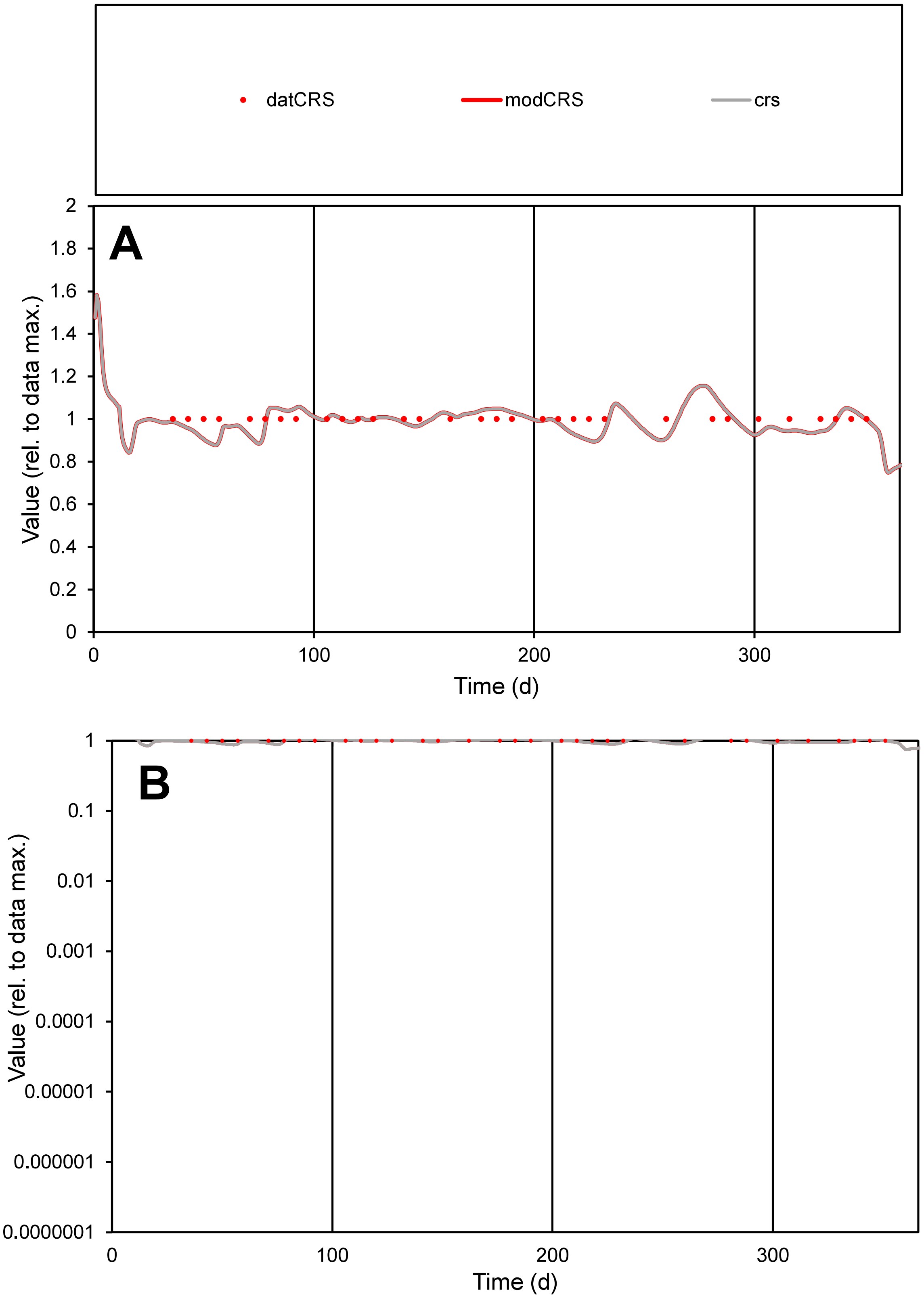

C1

Variables
CRS Provide jtd or Dst
CalibOnly?
2 1 to reproduce of
oNormalize?
2 1=no, 2=yes

ofxn calc*
0.05233
ofxn mod
0.10937

sRun
RunA1
zout
1

Ave
Data
5.4E-06
Model
5.3E-06

data/vMax
5.4E-06

ofxng
321.551

Min d/m
1.11823

ave/min d
1
ave/min m
1.10383

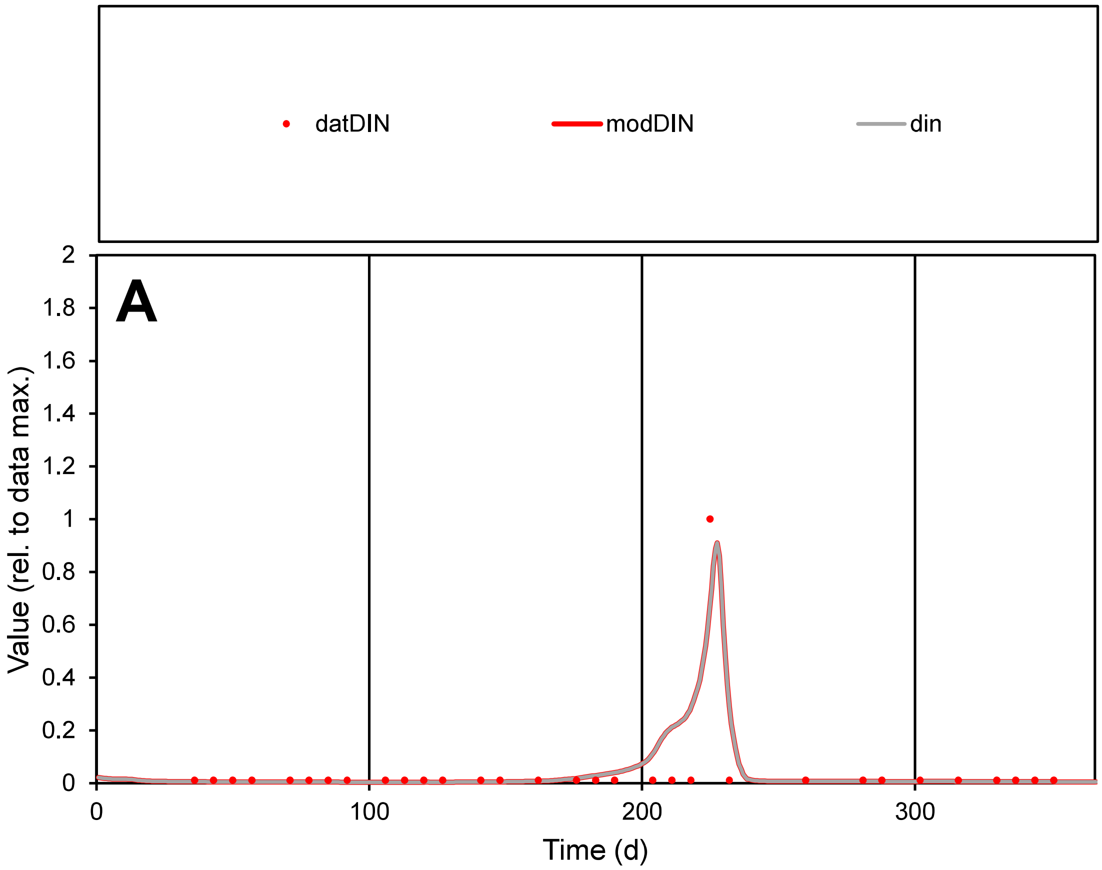

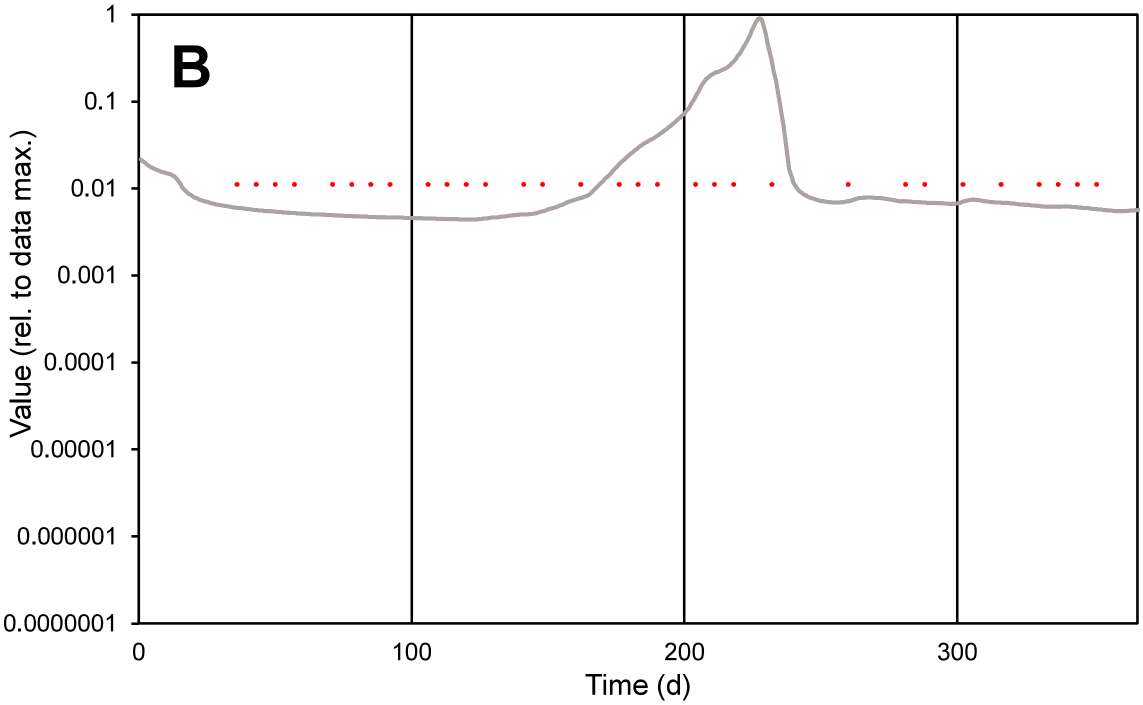

C1

Variables
DIN Provide jtd or Dst
CalibOnly?
2 1 to reproduce of
oNormalize?
2 1=no, 2=yes

ofxn calc*
1.6368
ofxn mod
1.7763

sRun
RunA1
zout
1

Ave
Data
1.1E-05
Model
1.4E-05

data/vMax
0.00026

ofxng
321.551

Min d/m
2.52761

ave/min d
3.76976
ave/min m
11.994

324_Calib_S_A

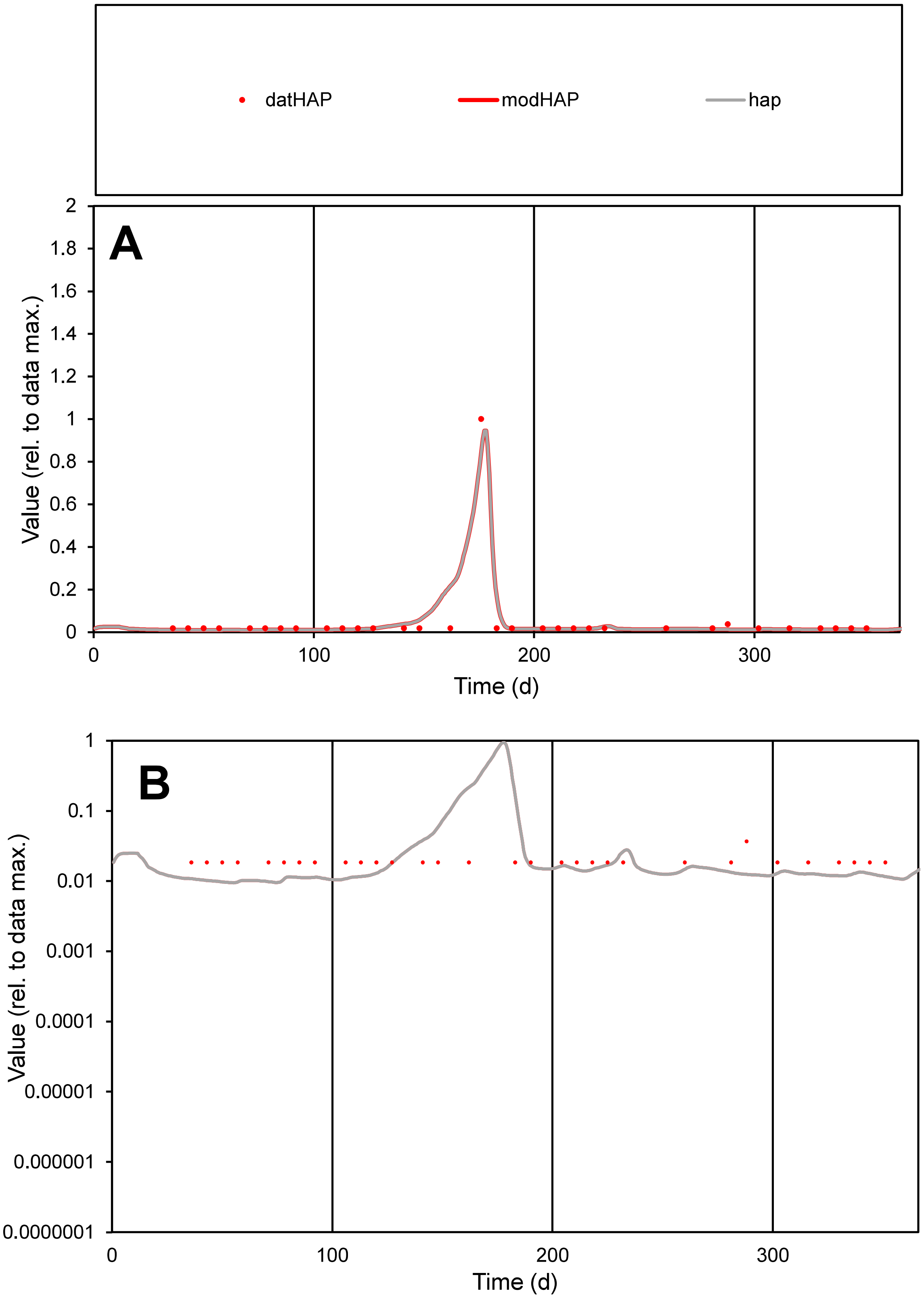

C1

Variables
HAP Provide jtd or Dst
CalibOnly?
2 1 to reproduce of
oNormalize?
2 1=no, 2=yes

ofxn calc*
0.69123
ofxn mod
0.84944

sRun
RunA1
zout
1

Ave
Data
3.1E-05
Model
3.2E-05

data/vMax
0.00062

ofxng
321.551

Min d/m
1.91432

ave/min d
2.69411
ave/min m
5.34485

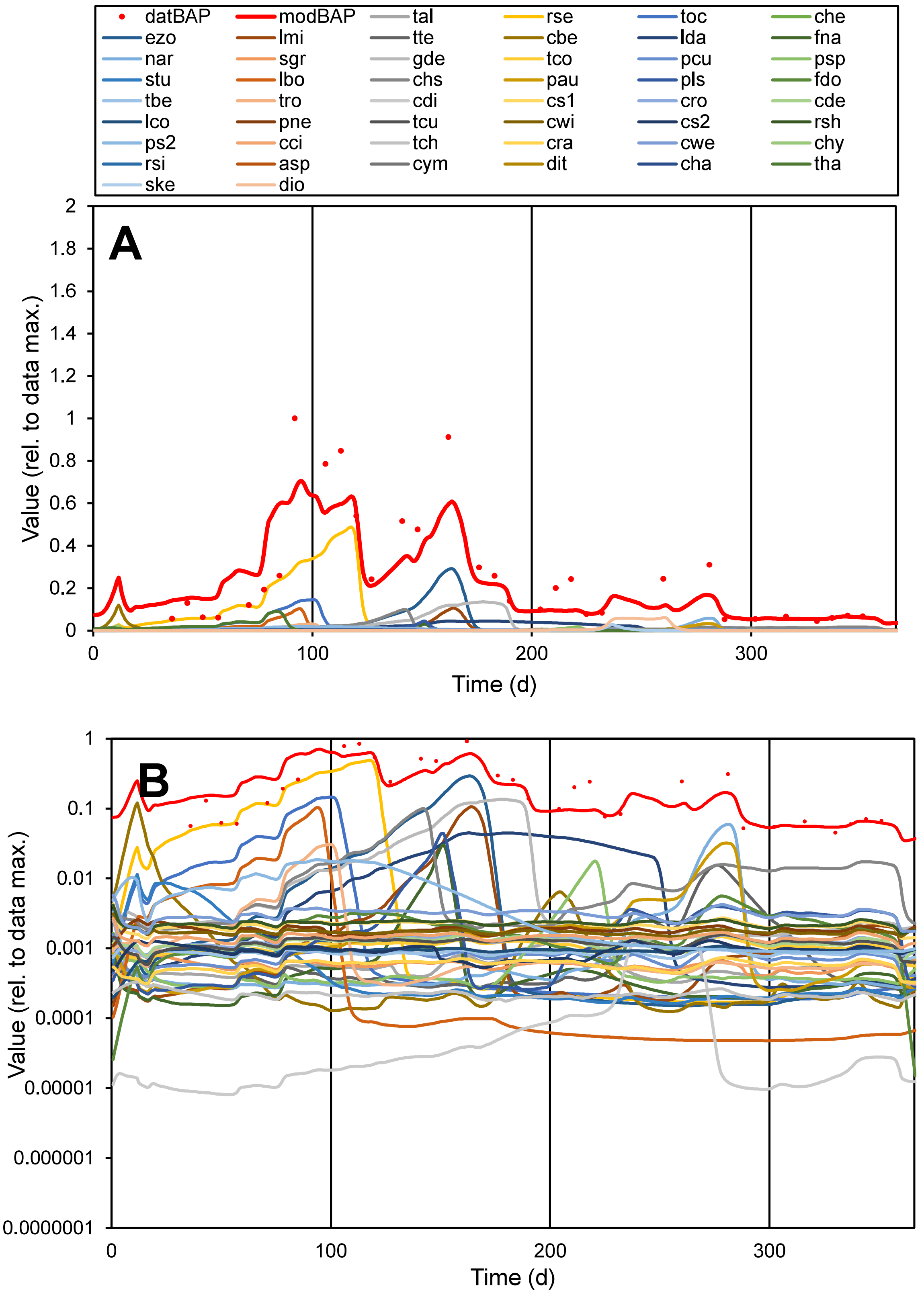

C1

Variables

BAP Provide jtd or Dst

CalibOnly?

2 1 to reproduce of

oNormalize?

2 1=no, 2=yes

ofxn calc*

0.51612

ofxn mod

0.54321

sRun

RunA1

zout

1

Ave

Data

0.00208

Model

0.00179

data/vMax

0.00777

ofxng

321.551

Min d/m

0.8331

ave/min d

5.92081

ave/min m

4.24395

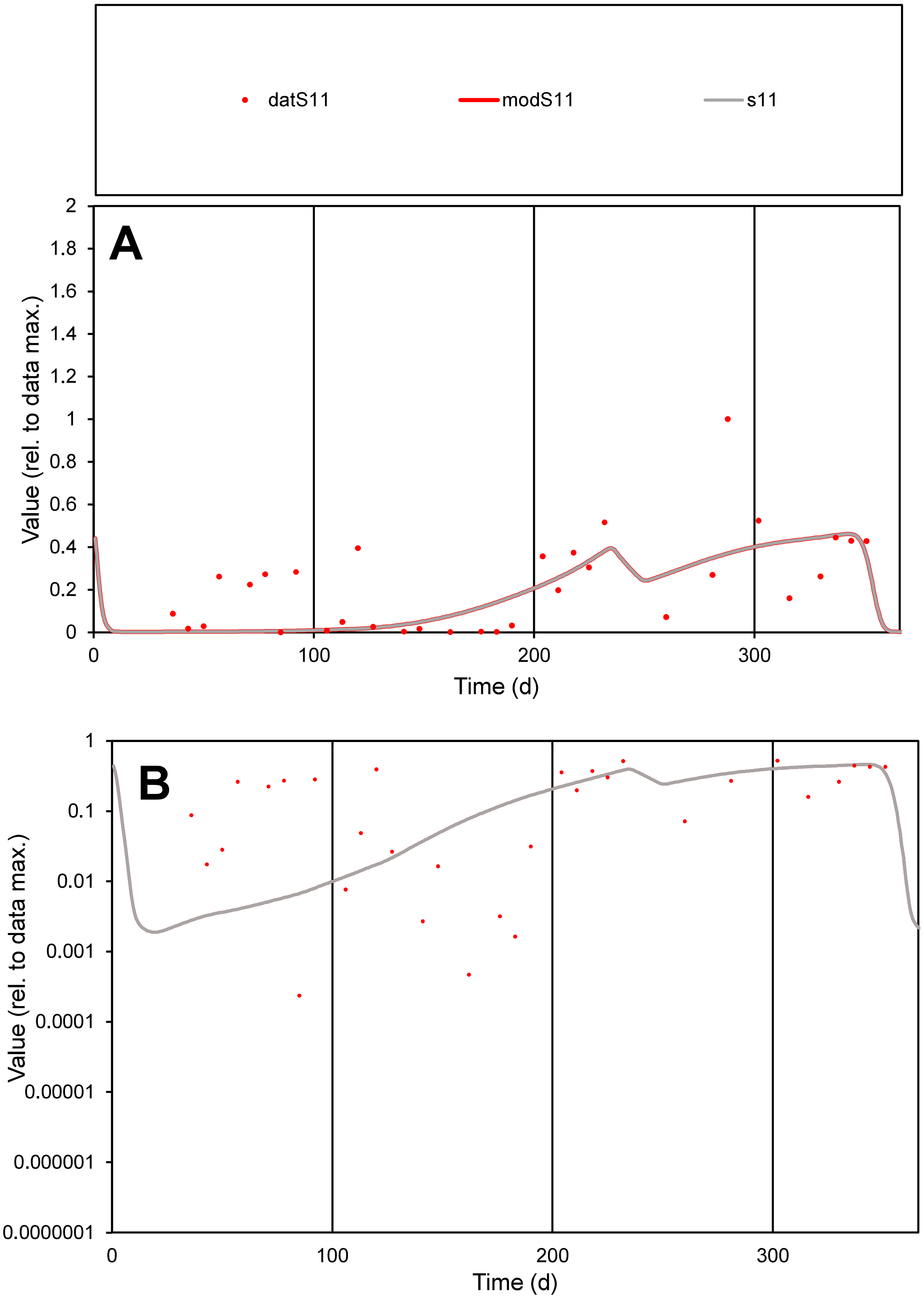

C1

Variables
S11 Provide jtd or Dst
CalibOnly?
2 1 to reproduce of
oNormalize?
2 1=no, 2=yes

ofxn calc*
0.83583
ofxn mod
0.84652

sRun
RunA1
zout
1

Ave
Data
0.01629
Model
0.01335

data/vMax
0.07402

ofxng
321.551

Min d/m
0.08224

ave/min d
933.164
ave/min m
62.8988

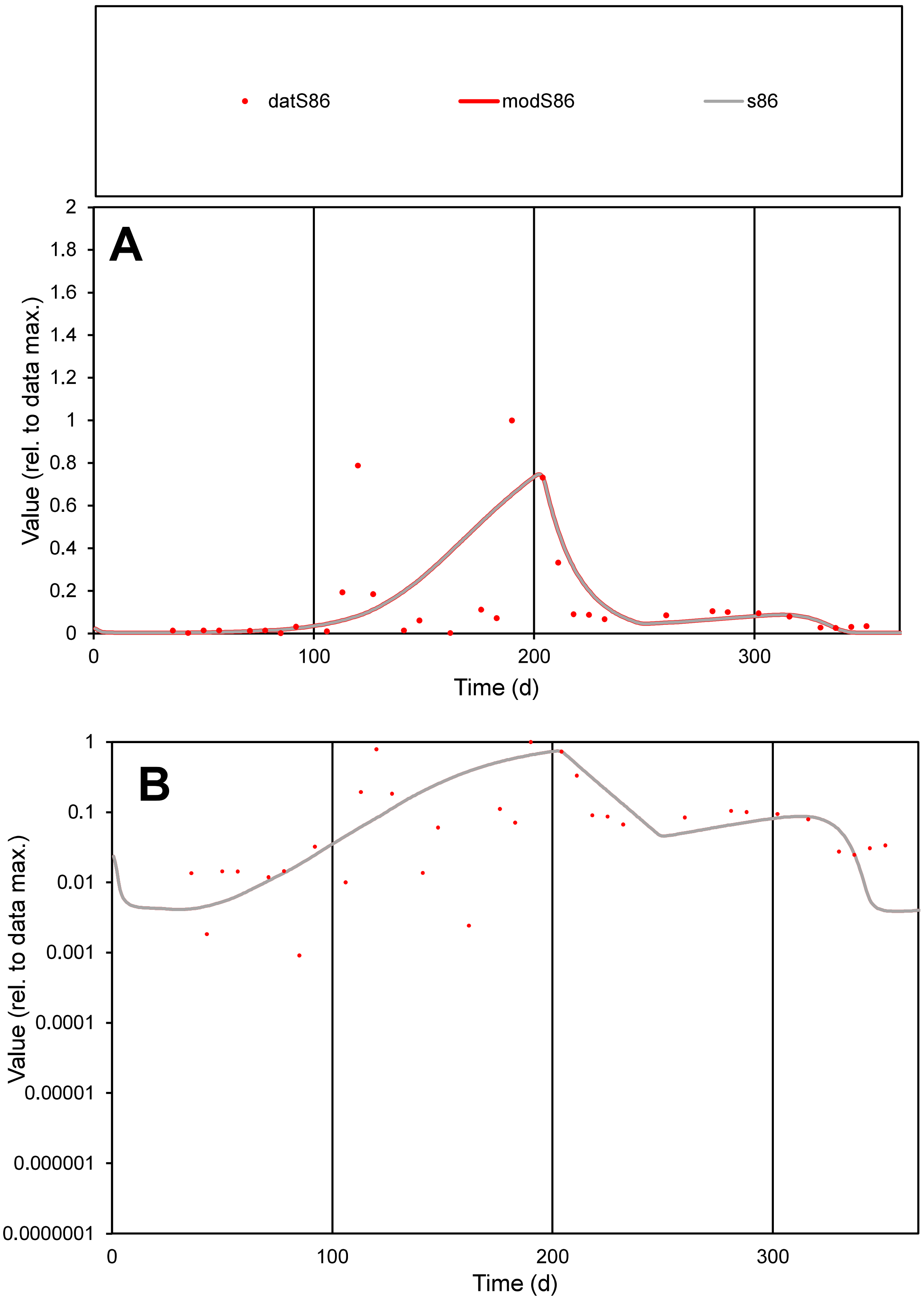

C1

Variables
S86 Provide jtd or Dst
CalibOnly?
2 1 to reproduce of
oNormalize?
2 1=no, 2=yes

ofxn calc*
1.48706
ofxn mod
1.50293

sRun
RunA1
zout
1

Ave
Data
0.0327
Model
0.03833

data/vMax
0.2366

ofxng
321.551

Min d/m
0.23211

ave/min d
151.616
ave/min m
41.2502

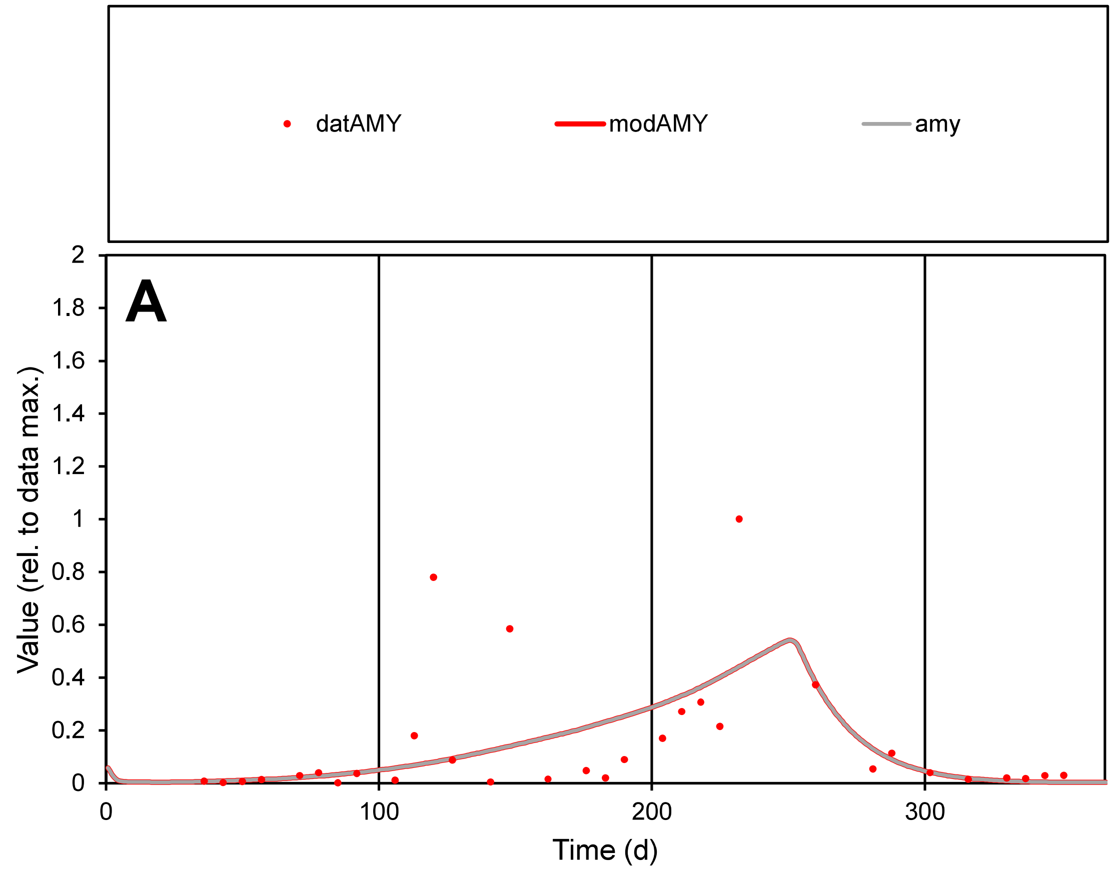

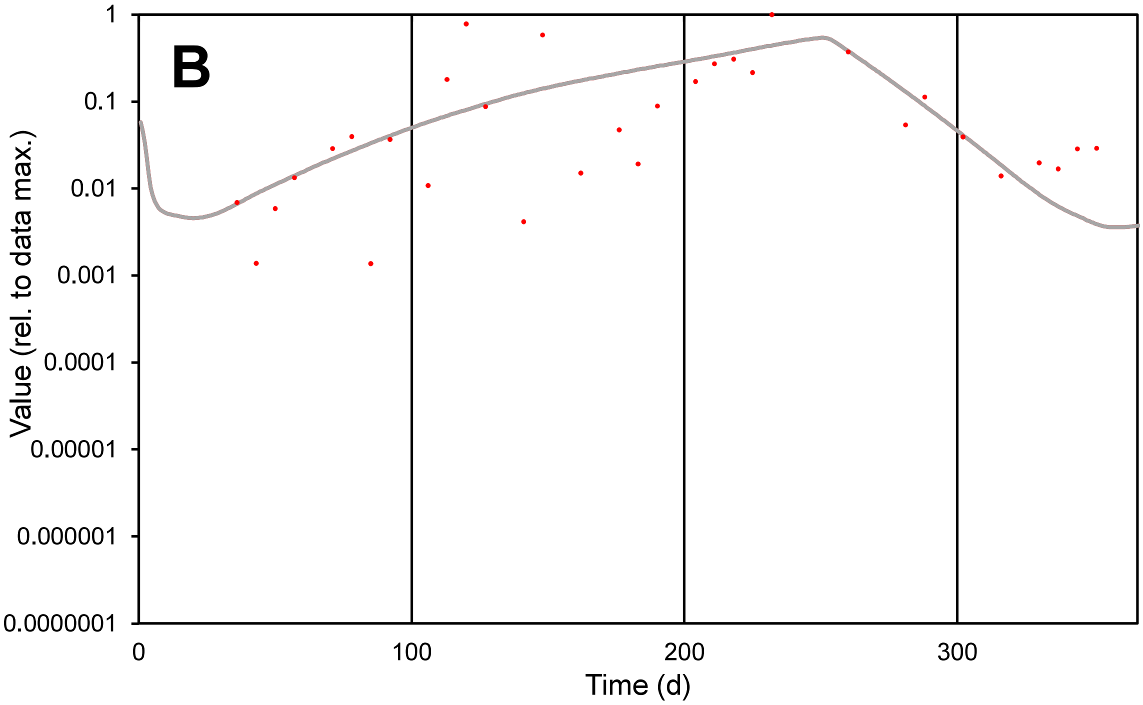

C1

Variables
AMY Provide jtd or Dst
CalibOnly?
2 1 to reproduce of
oNormalize?
2 1=no, 2=yes

ofxn calc*
1.35268
ofxn mod
1.3624

sRun
RunA1
zout
1

Ave
Data
0.01057
Model
0.00951

data/vMax
0.07351

ofxng
321.551

Min d/m
0.35913

ave/min d
104.953
ave/min m
33.9029

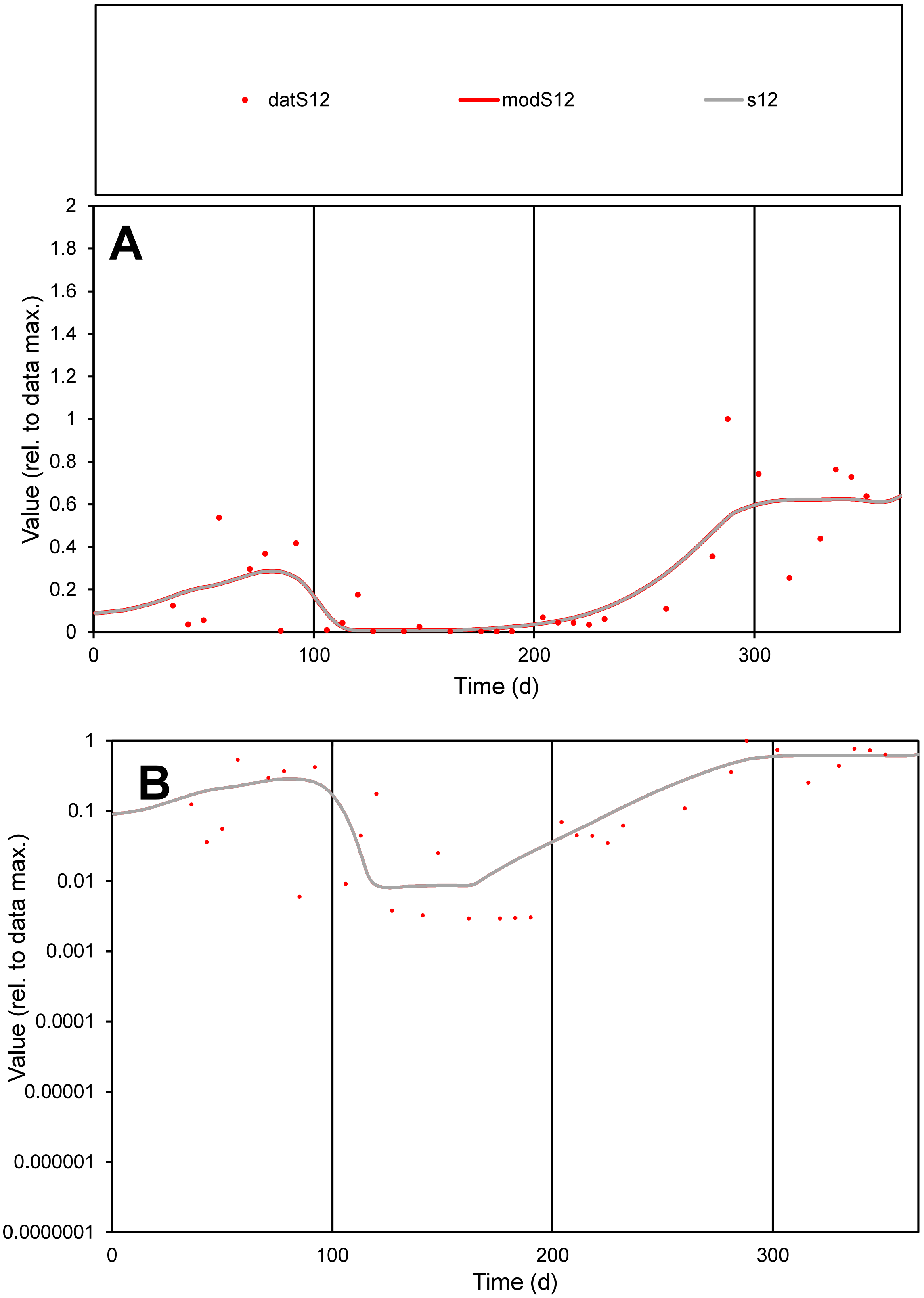

C1

Variables
S12 Provide jtd or Dst
CalibOnly?
2 1 to reproduce of
oNormalize?
2 1=no, 2=yes

ofxn calc*
0.66723
ofxn mod
0.68189

sRun
RunA1
zout
1

Ave
Data
0.0108
Model
0.01091

data/vMax
0.04671

ofxng
321.551

Min d/m
0.36229

ave/min d
78.6873
ave/min m
28.8172

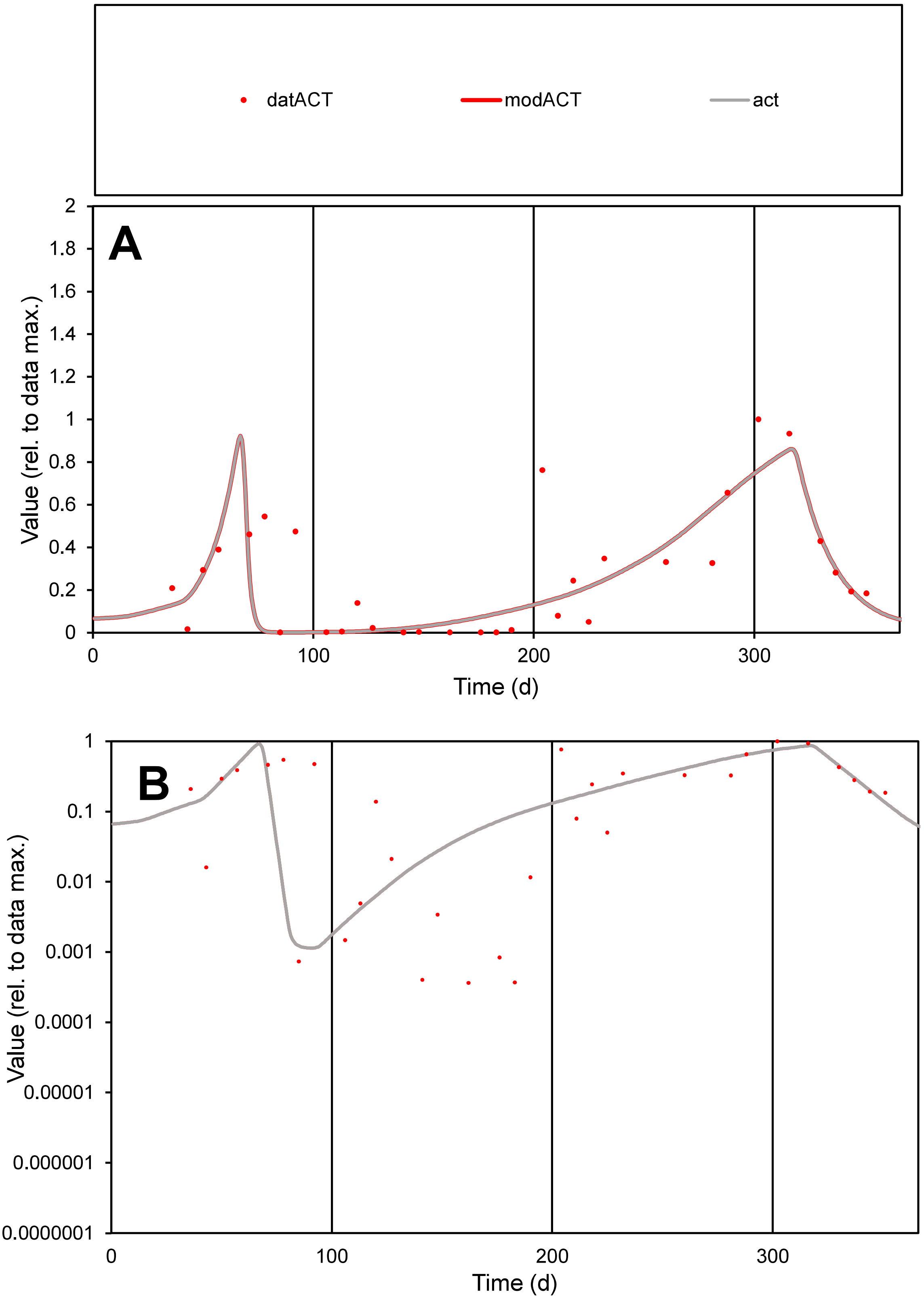

C1

Variables
ACT Provide jtd or Dst
CalibOnly?
2 1 to reproduce of
oNormalize?
2 1=no, 2=yes

ofxn calc*
0.72847
ofxn mod
0.75112

sRun
RunA1
zout
1

Ave
Data
0.01836
Model
0.01556

data/vMax
0.07003

ofxng
321.551

Min d/m
0.31294

ave/min d
722.16
ave/min m
191.604

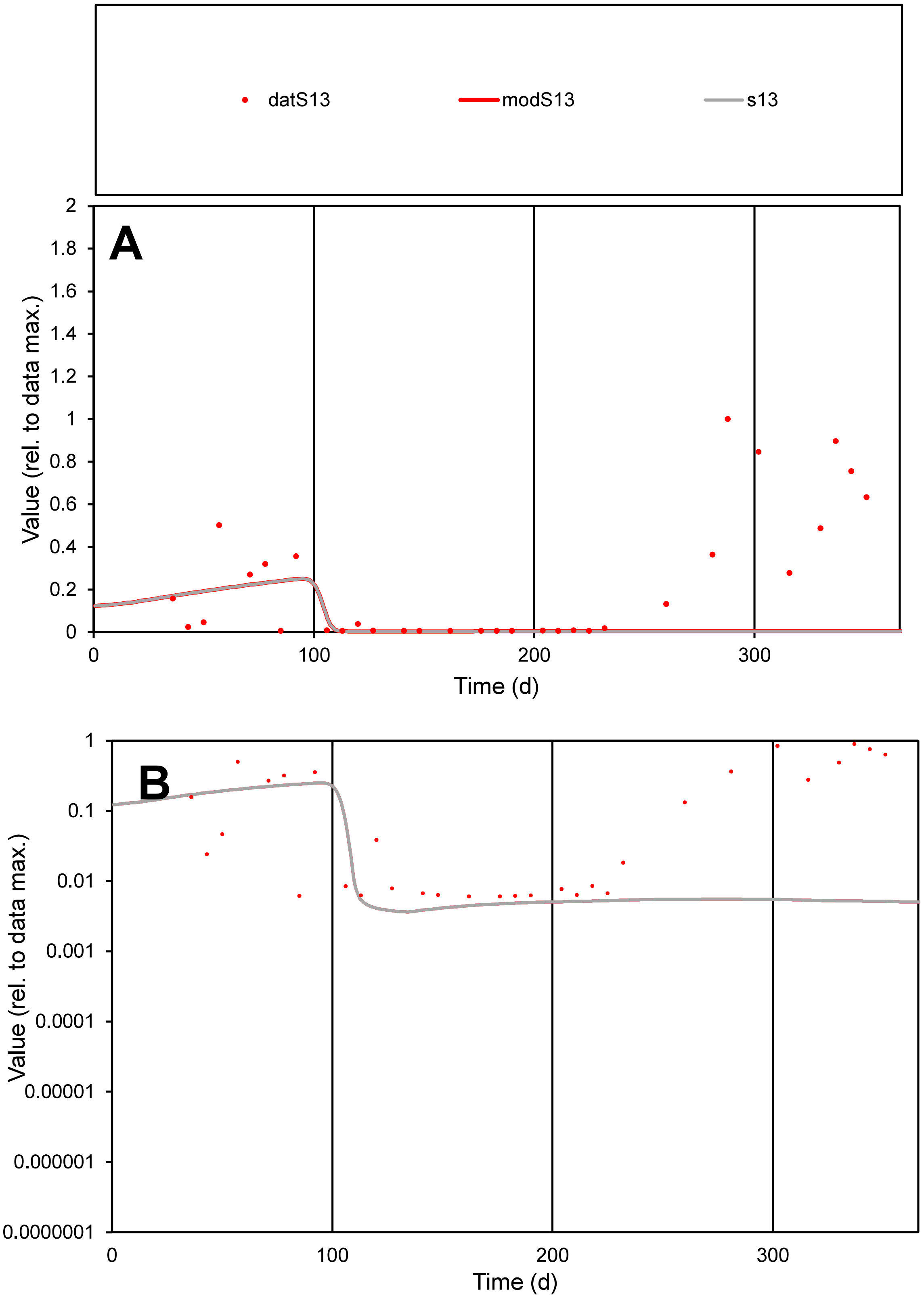

C1

Variables
S13 Provide jtd or Dst
CalibOnly?
2 1 to reproduce of
oNormalize?
2 1=no, 2=yes

ofxn calc*
1.58842
ofxn mod
1.59094

sRun
RunA1
zout
1

Ave
Data
0.01016
Model
0.00259

data/vMax
0.04502

ofxng
321.551

Min d/m
1.61775

ave/min d
37.1655
ave/min m
15.3113

324_Calib_S_A

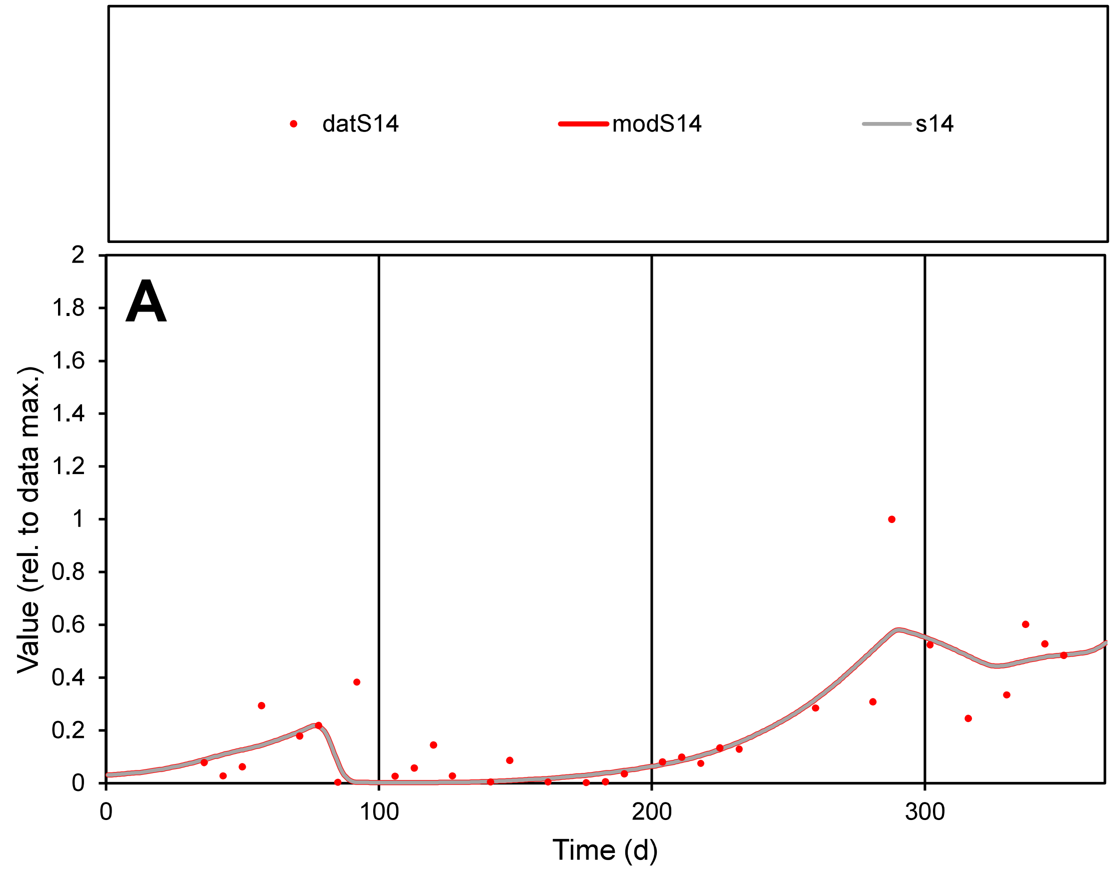

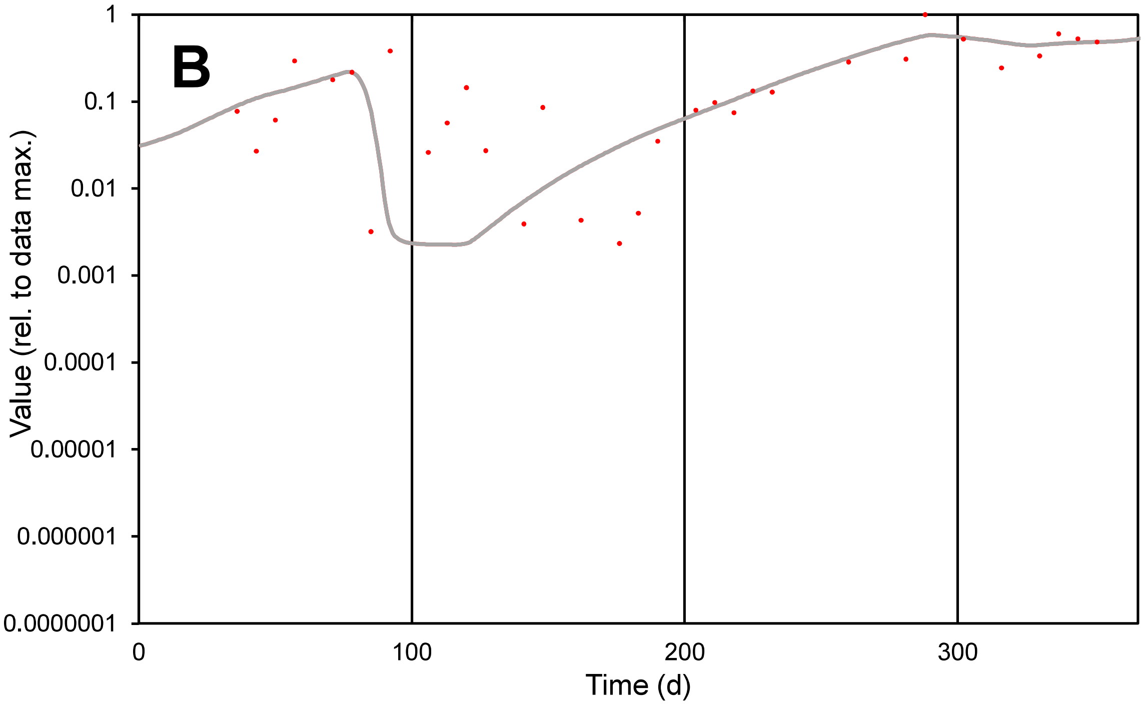

C1

Variables
S14 Provide jtd or Dst
CalibOnly?
2 1 to reproduce of
oNormalize?
2 1=no, 2=yes

ofxn calc*
0.63145
ofxn mod
0.64642

sRun
RunA1
zout
1

Ave
Data
0.00847
Model
0.00781

data/vMax
0.04201

ofxng
321.551

Min d/m
1.03068

ave/min d
86.3407
ave/min m
82.0862

324_Calib_S_A

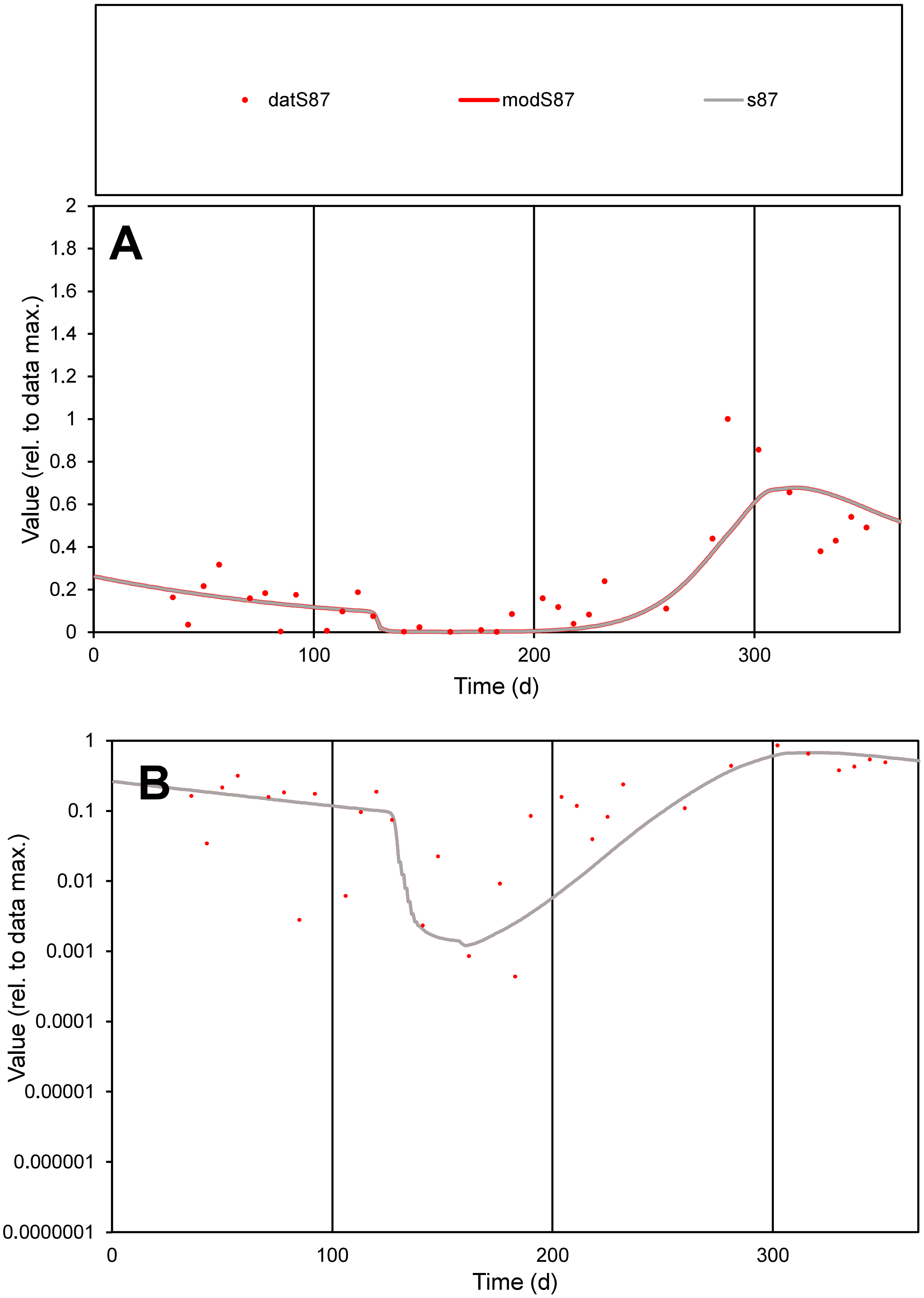

C1

Variables
S87 Provide jtd or Dst
CalibOnly?
2 1 to reproduce of
oNormalize?
2 1=no, 2=yes

ofxn calc*
0.6161
ofxn mod
0.62986

sRun
RunA1
zout
1

Ave
Data
0.00917
Model
0.0083

data/vMax
0.04035

ofxng
321.551

Min d/m
0.34521

ave/min d
519.757
ave/min m
162.462

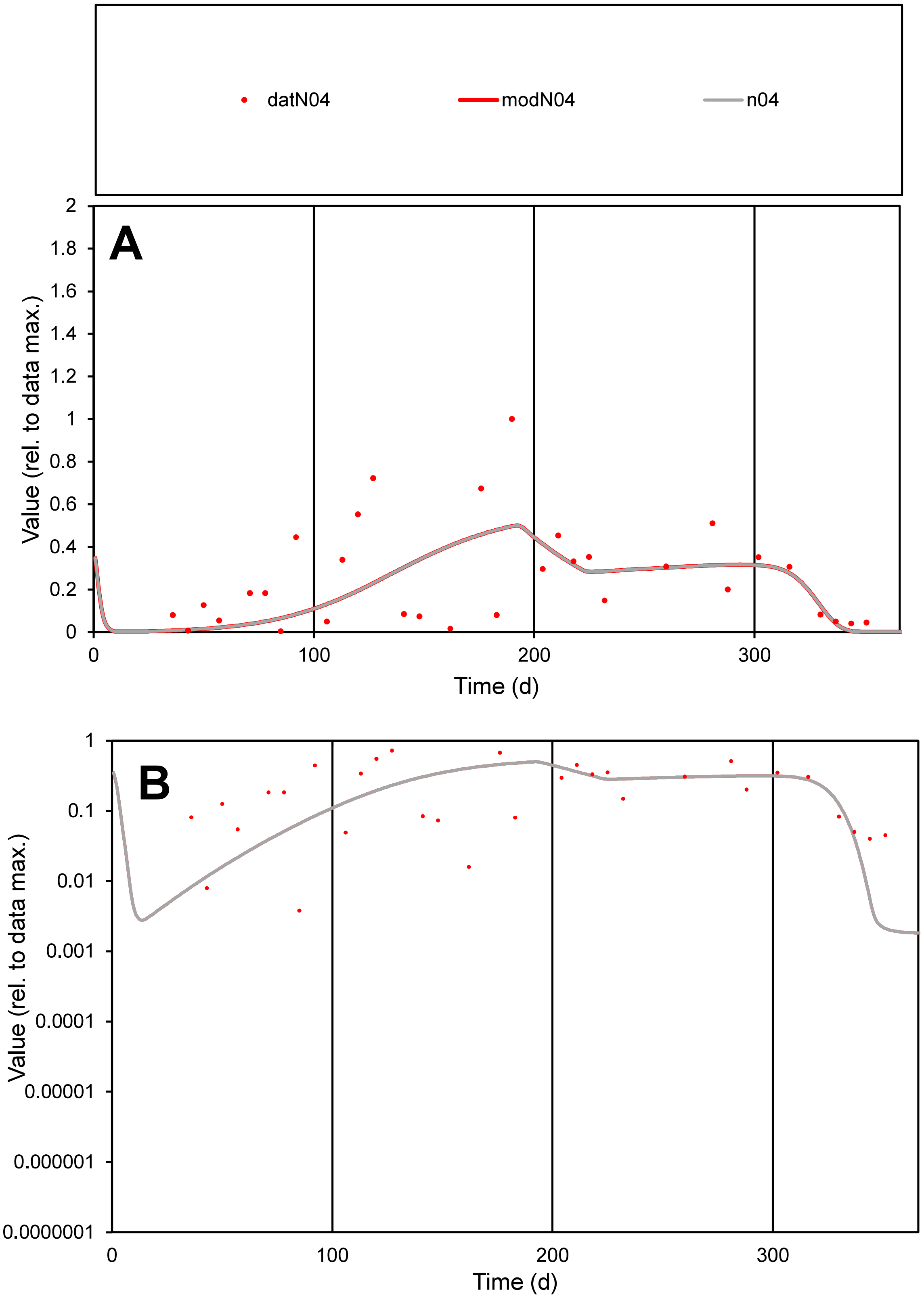

C1

Variables
N04 Provide jtd or Dst
CalibOnly?
2 1 to reproduce of
oNormalize?
2 1=no, 2=yes

ofxn calc*
0.82597
ofxn mod
0.83471

sRun
RunA1
zout
1

Ave
Data
0.01589
Model
0.01335

data/vMax
0.06235

ofxng
321.551

Min d/m
1.84825

ave/min d
66.9171
ave/min m
103.859

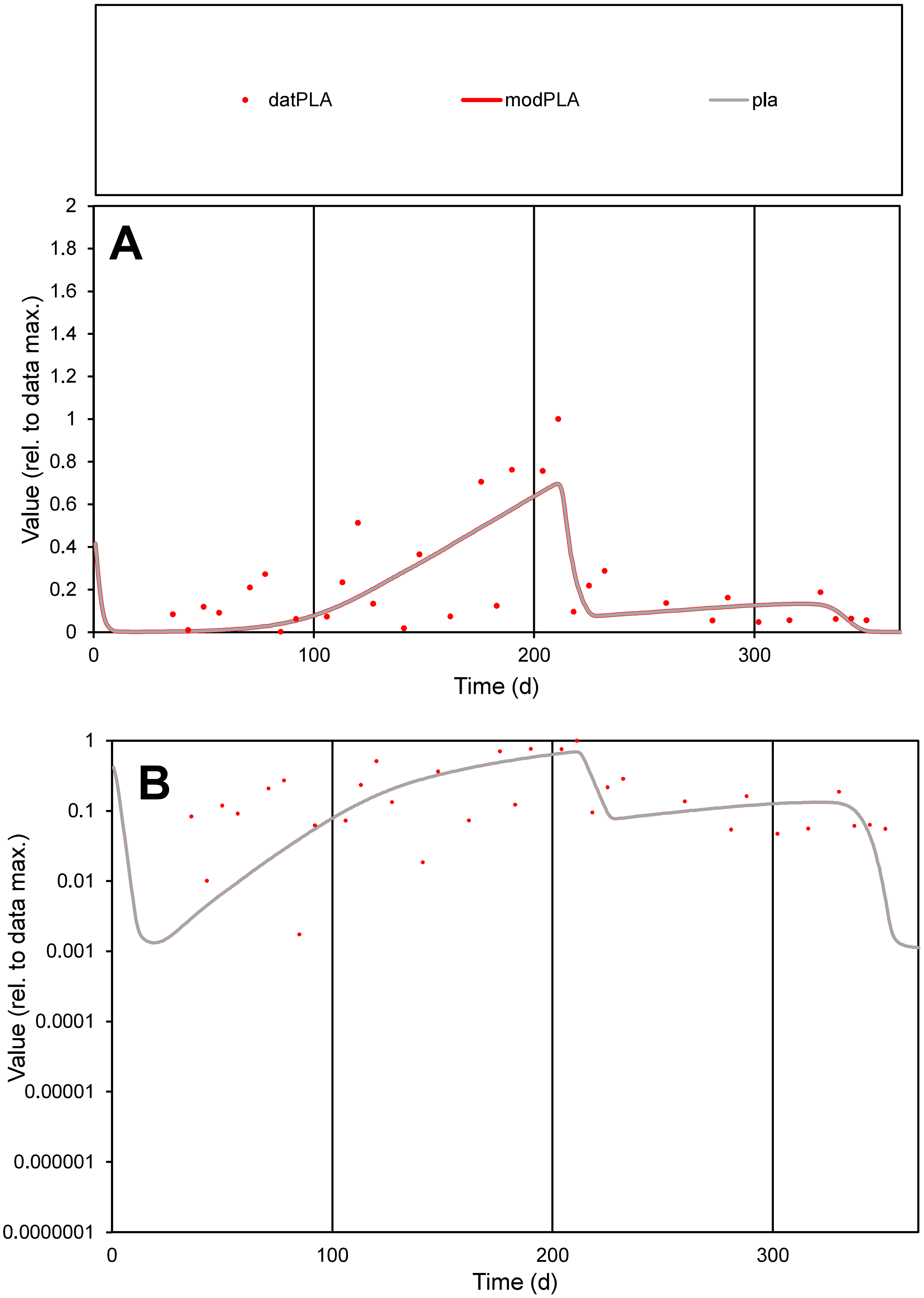

C1

Variables

PLA Provide jtd or Dst

CalibOnly?

2 1 to reproduce of

oNormalize?

2 1=no, 2=yes

ofxn calc*

0.75531

ofxn mod

0.77674

sRun

RunA1

zout

1

Ave

Data

0.01949

Model

0.01673

data/vMax

0.08867

ofxng

321.551

Min d/m

0.55396

ave/min d

126.128

ave/min m

59.971

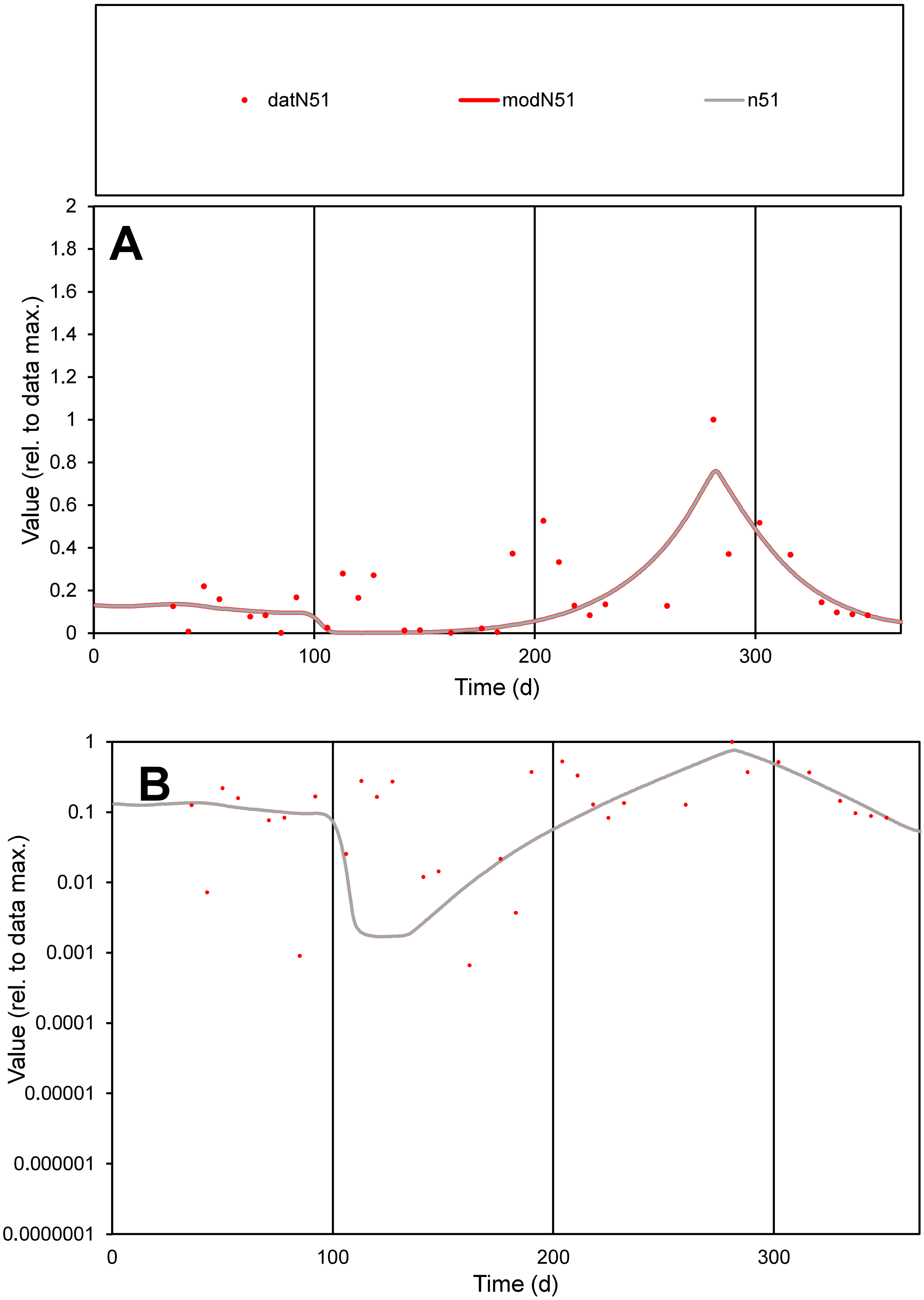

C1

Variables
N51 Provide jtd or Dst
CalibOnly?
2 1 to reproduce of
oNormalize?
2 1=no, 2=yes

ofxn calc*
0.86421
ofxn mod
0.87322

sRun
RunA1
zout
1

Ave
Data
0.01715
Model
0.01338

data/vMax
0.09136

ofxng
321.551

Min d/m
0.39187

ave/min d
282.544
ave/min m
86.3488

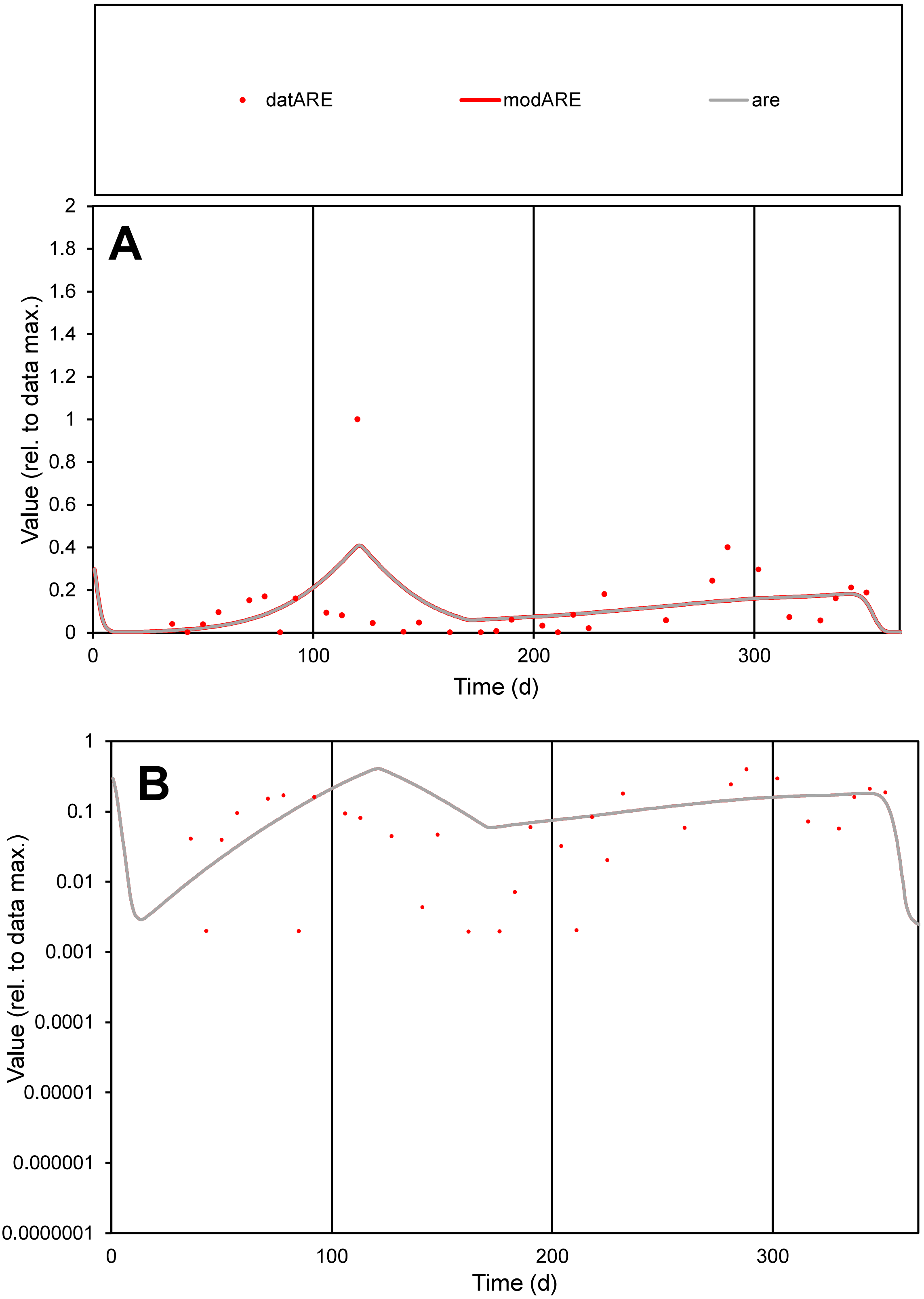

C1

Variables
ARE Provide jtd or Dst
CalibOnly?
2 1 to reproduce of
oNormalize?
2 1=no, 2=yes

ofxn calc*
1.22132
ofxn mod
1.24007

sRun
RunA1
zout
1

Ave
Data
0.0034
Model
0.00366

data/vMax
0.02708

ofxng
321.551

Min d/m
0.17782

ave/min d
63.9911
ave/min m
12.2537

324_Calib_S_A

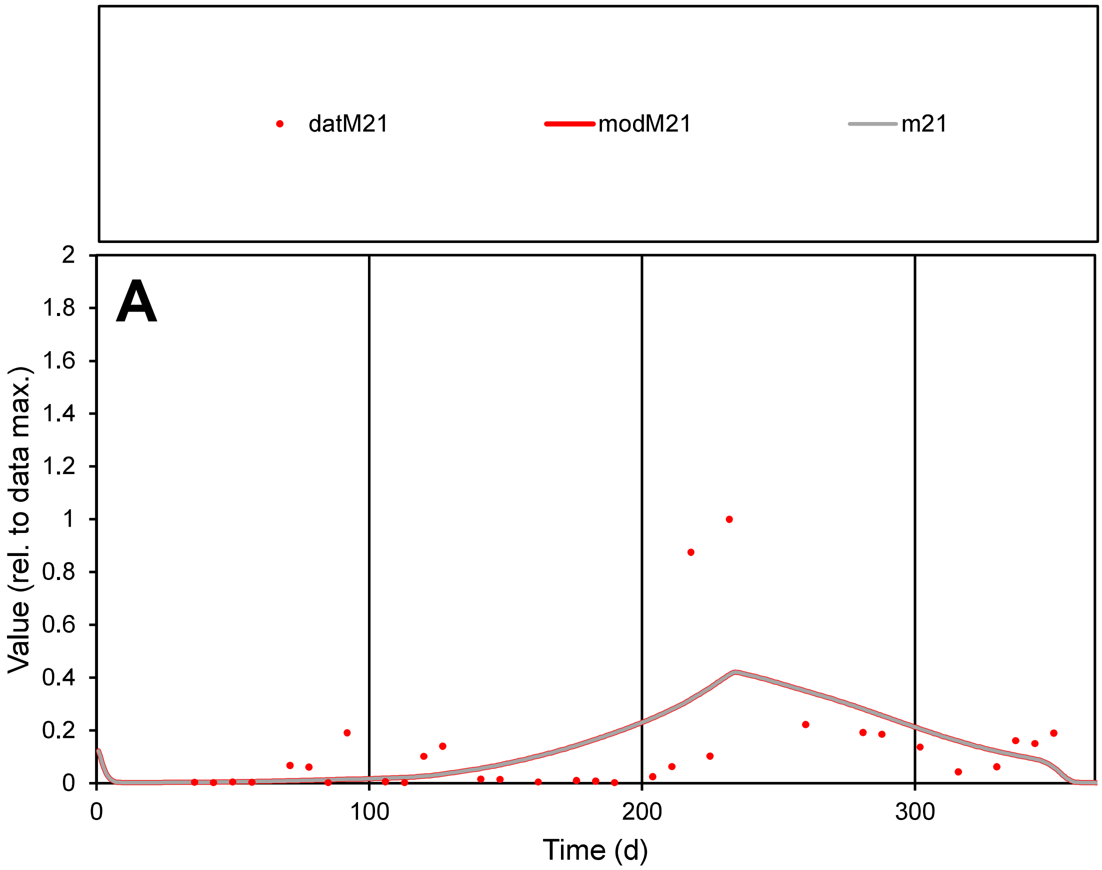

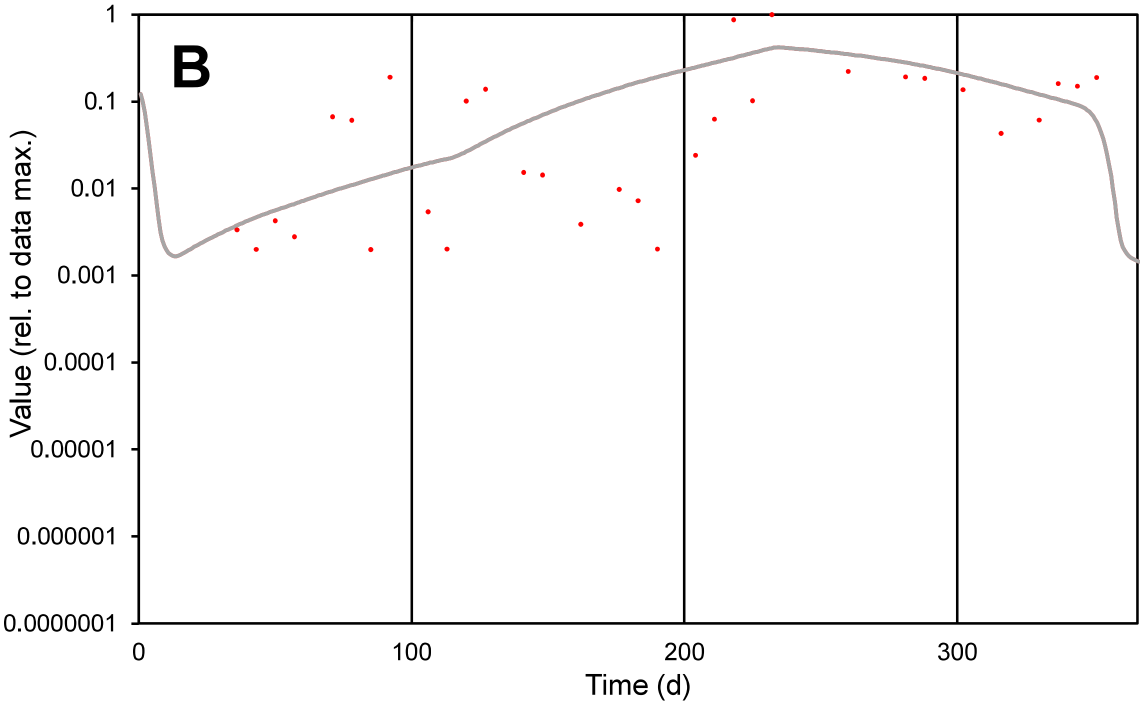

C1

Variables
M21 Provide jtd or Dst
CalibOnly?
2 1 to reproduce of
oNormalize?
2 1=no, 2=yes

ofxn calc*
1.41845
ofxn mod
1.43123

sRun
RunA1
zout
1

Ave
Data
0.00126
Model
0.0013

data/vMax
0.01001

ofxng
321.551

Min d/m
0.50873

ave/min d
63.7315
ave/min m
33.3484

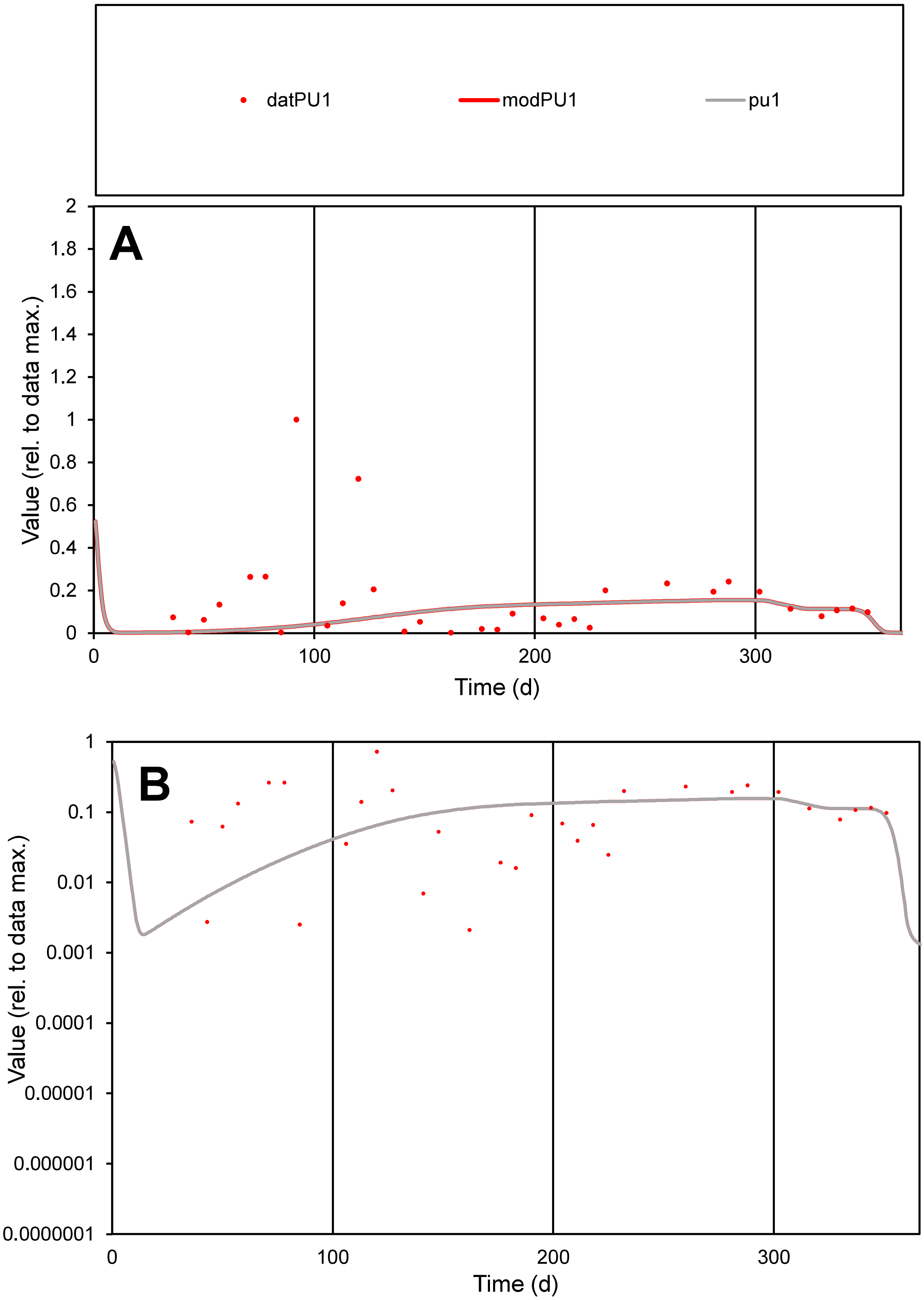

C1

Variables

PU1 Provide jtd or Dst

CalibOnly?

2 1 to reproduce of

oNormalize?

2 1=no, 2=yes

ofxn calc*

1.48493

ofxn mod

1.48794

sRun

RunA1

zout

1

Ave

Data

0.00435

Model

0.00262

data/vMax

0.02863

ofxng

321.551

Min d/m

0.43388

ave/min d

71.694

ave/min m

18.7336

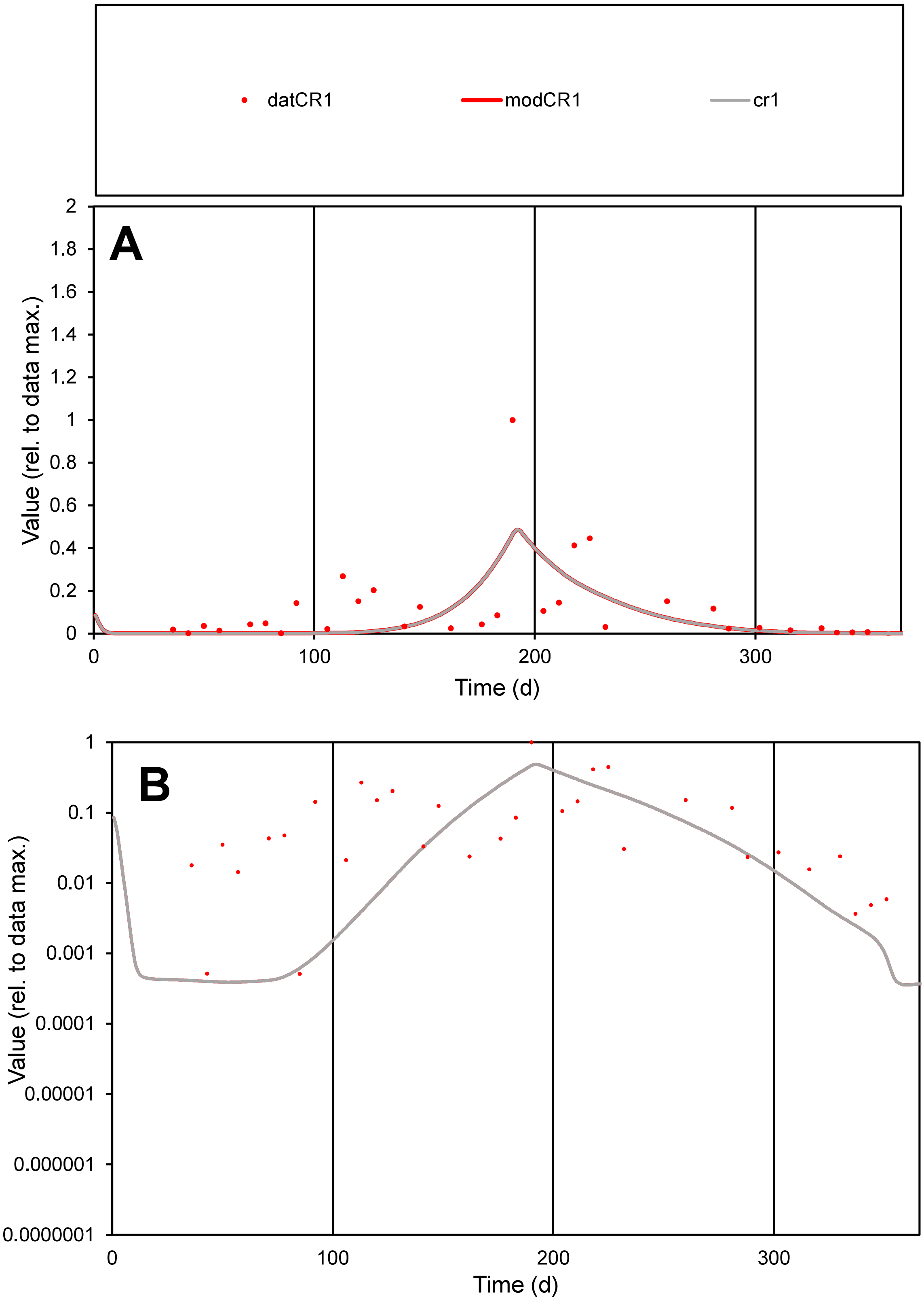

C1

Variables
CR1 Provide jtd or Dst
CalibOnly?
2 1 to reproduce of
oNormalize?
2 1=no, 2=yes

ofxn calc*
1.2958
ofxn mod
1.31119

sRun
RunA1
zout
1

Ave
Data
0.01501
Model
0.01084

data/vMax
0.1276

ofxng
321.551

Min d/m
1.30584

ave/min d
230.64
ave/min m
217.624

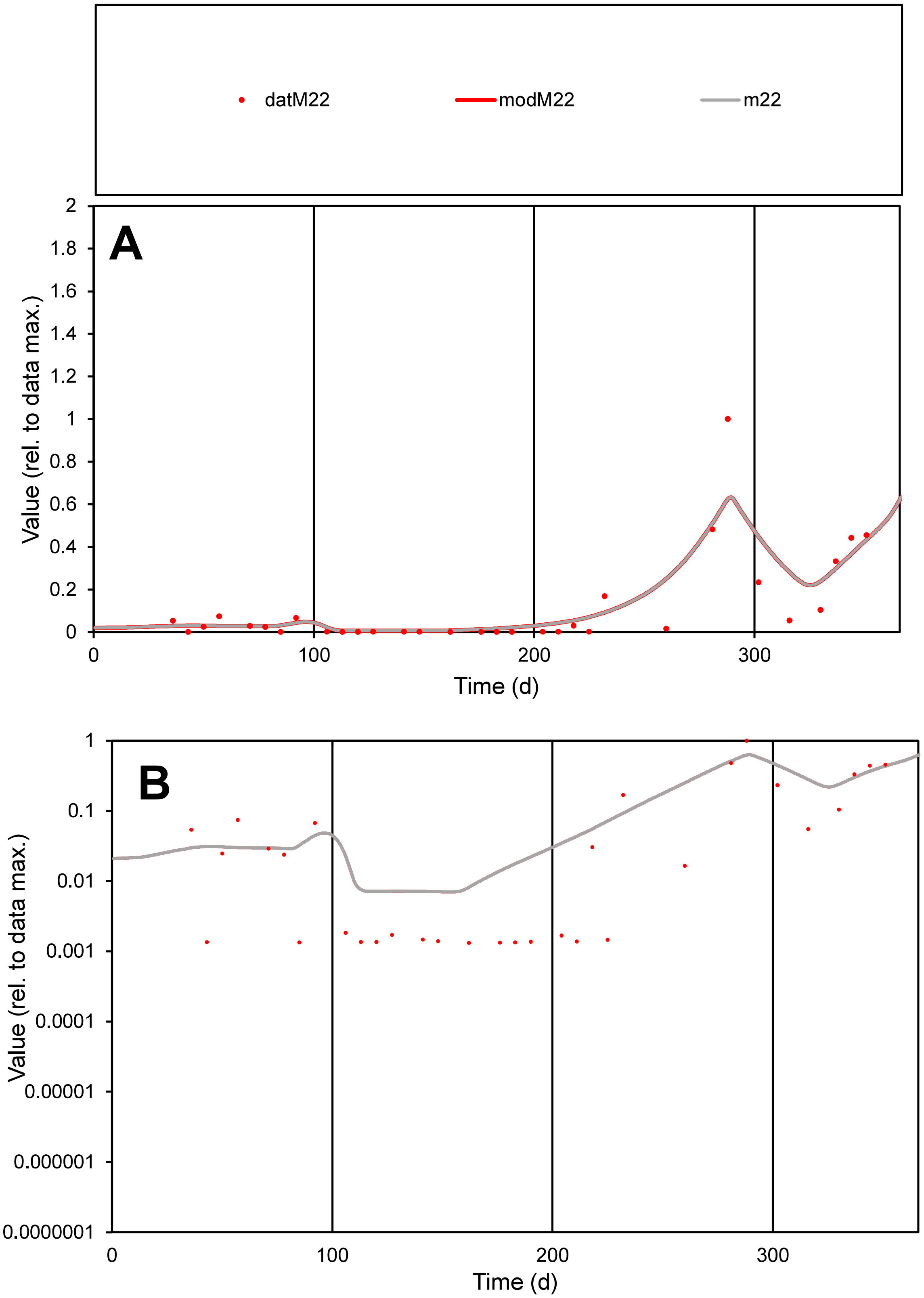

C1

Variables
M22 Provide jtd or Dst
CalibOnly?
2 1 to reproduce of
oNormalize?
2 1=no, 2=yes

ofxn calc*
0.87817
ofxn mod
0.90441

sRun
RunA1
zout
1

Ave
Data
0.0018
Model
0.00207

data/vMax
0.01596

ofxng
321.551

Min d/m
0.18798

ave/min d
85.4301
ave/min m
18.4327

324_Calib_S_A

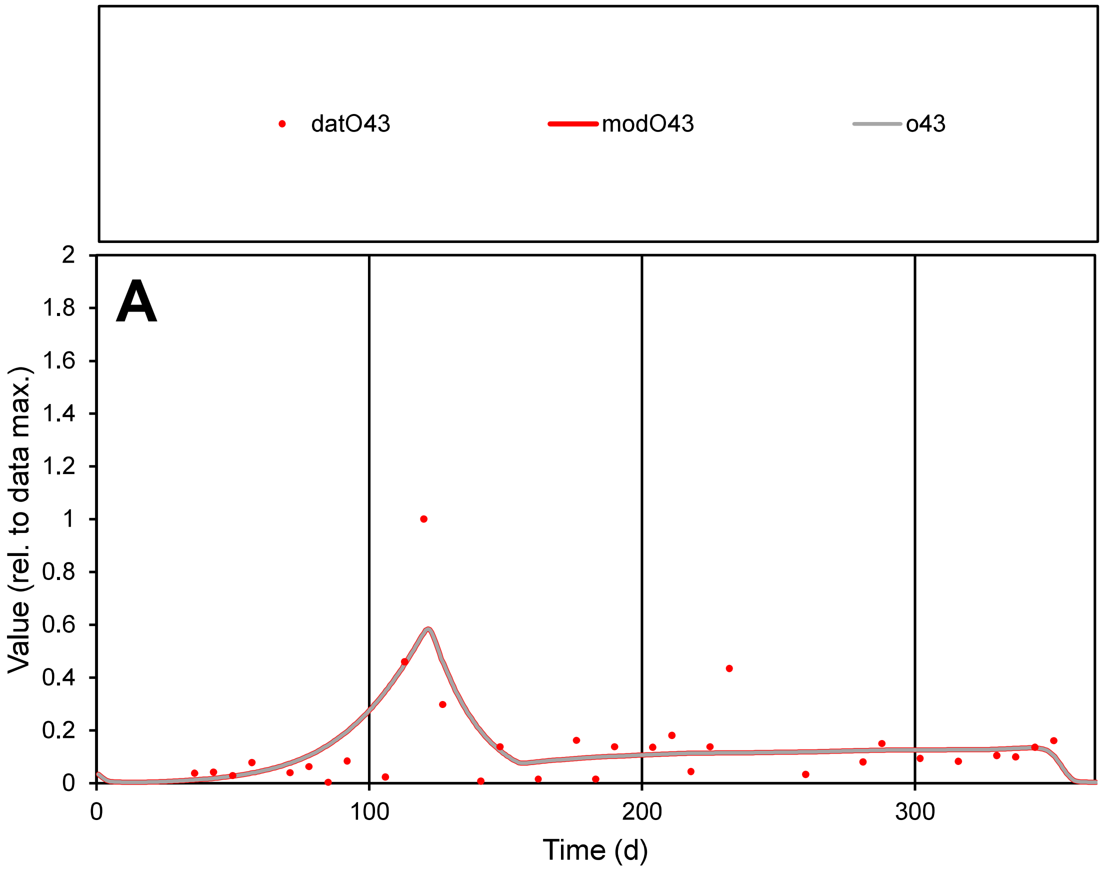

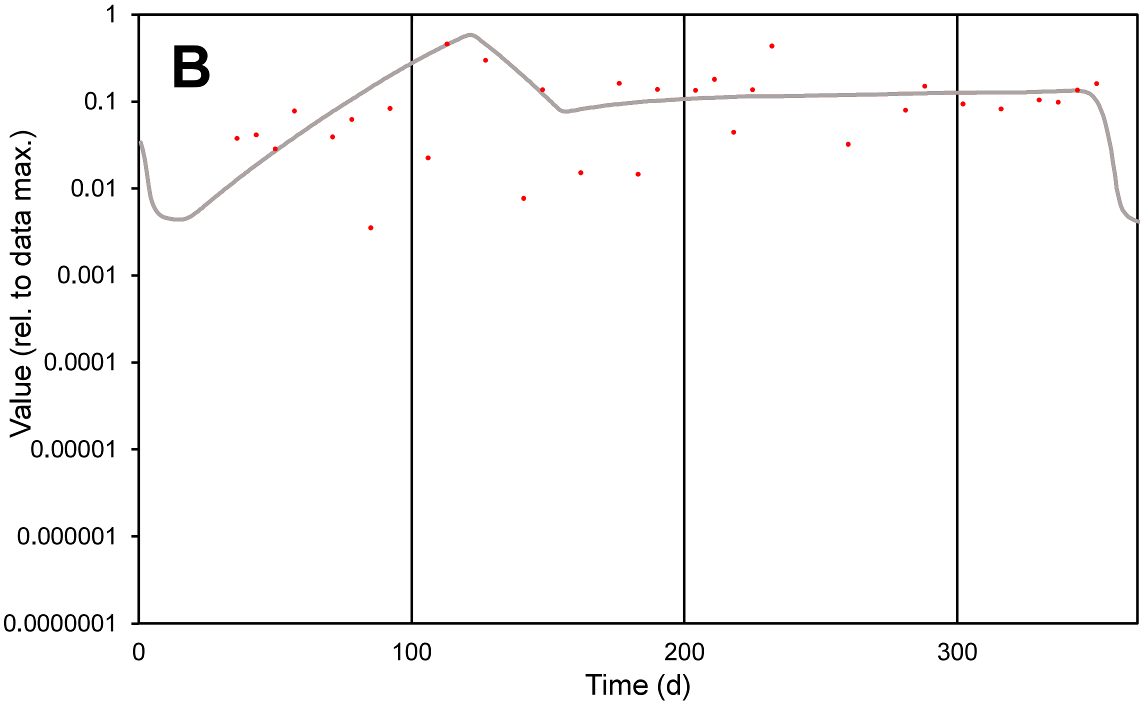

C1

Variables
O43 Provide jtd or Dst
CalibOnly?
2 1 to reproduce of
oNormalize?
2 1=no, 2=yes

ofxn calc*
0.901
ofxn mod
0.91521

sRun
RunA1
zout
1

Ave
Data
0.00557
Model
0.00597

data/vMax
0.03966

ofxng
321.551

Min d/m
0.26289

ave/min d
39.7632
ave/min m
11.1859

324_Calib_S_A

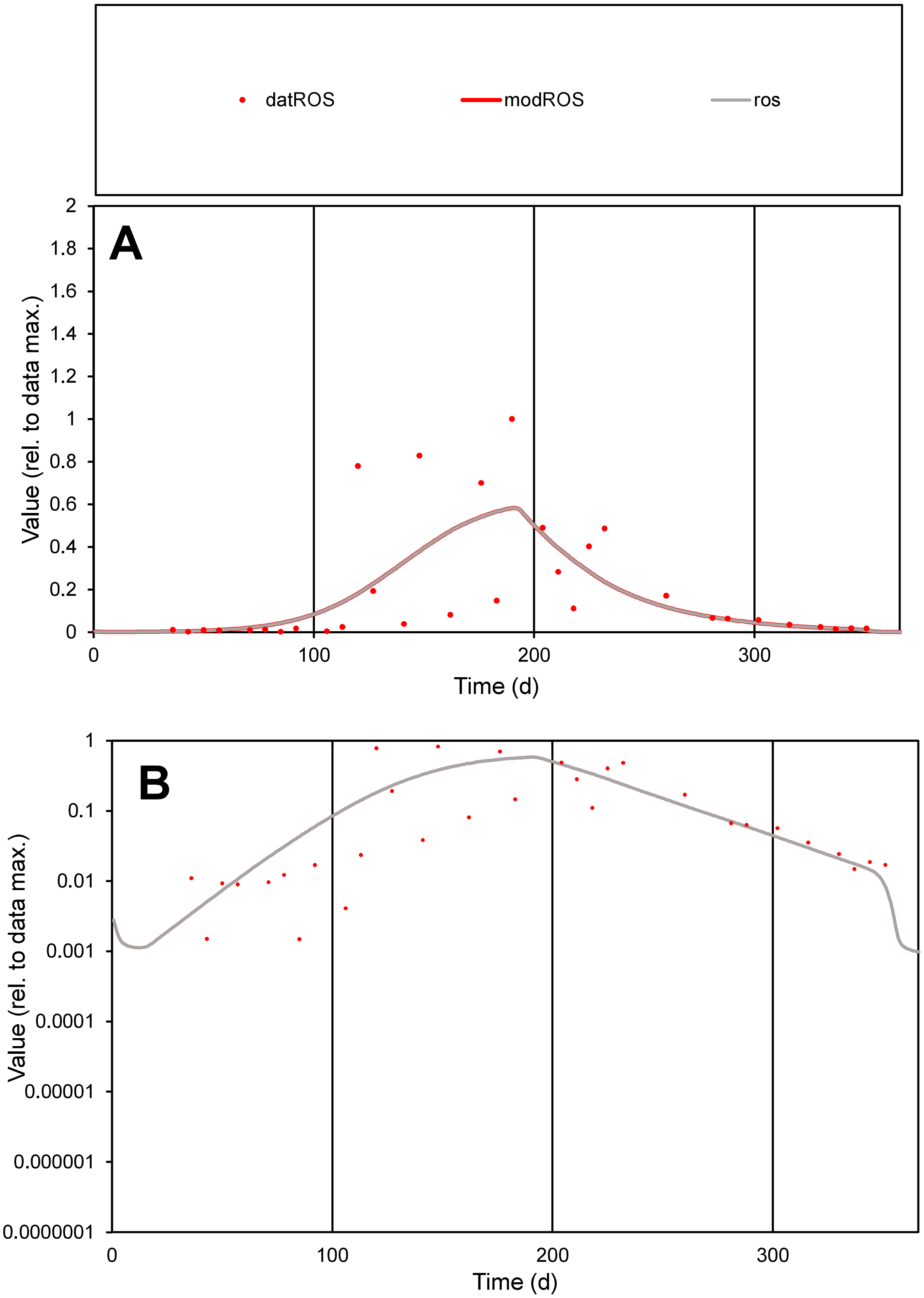

C1

Variables
ROS Provide jtd or Dst
CalibOnly?
2 1 to reproduce of
oNormalize?
2 1=no, 2=yes

ofxn calc*
1.06655
ofxn mod
1.07898

sRun
RunA1
zout
1

Ave
Data
0.00633
Model
0.00601

data/vMax
0.03317

ofxng
321.551

Min d/m
0.40923

ave/min d
127.557
ave/min m
49.5101

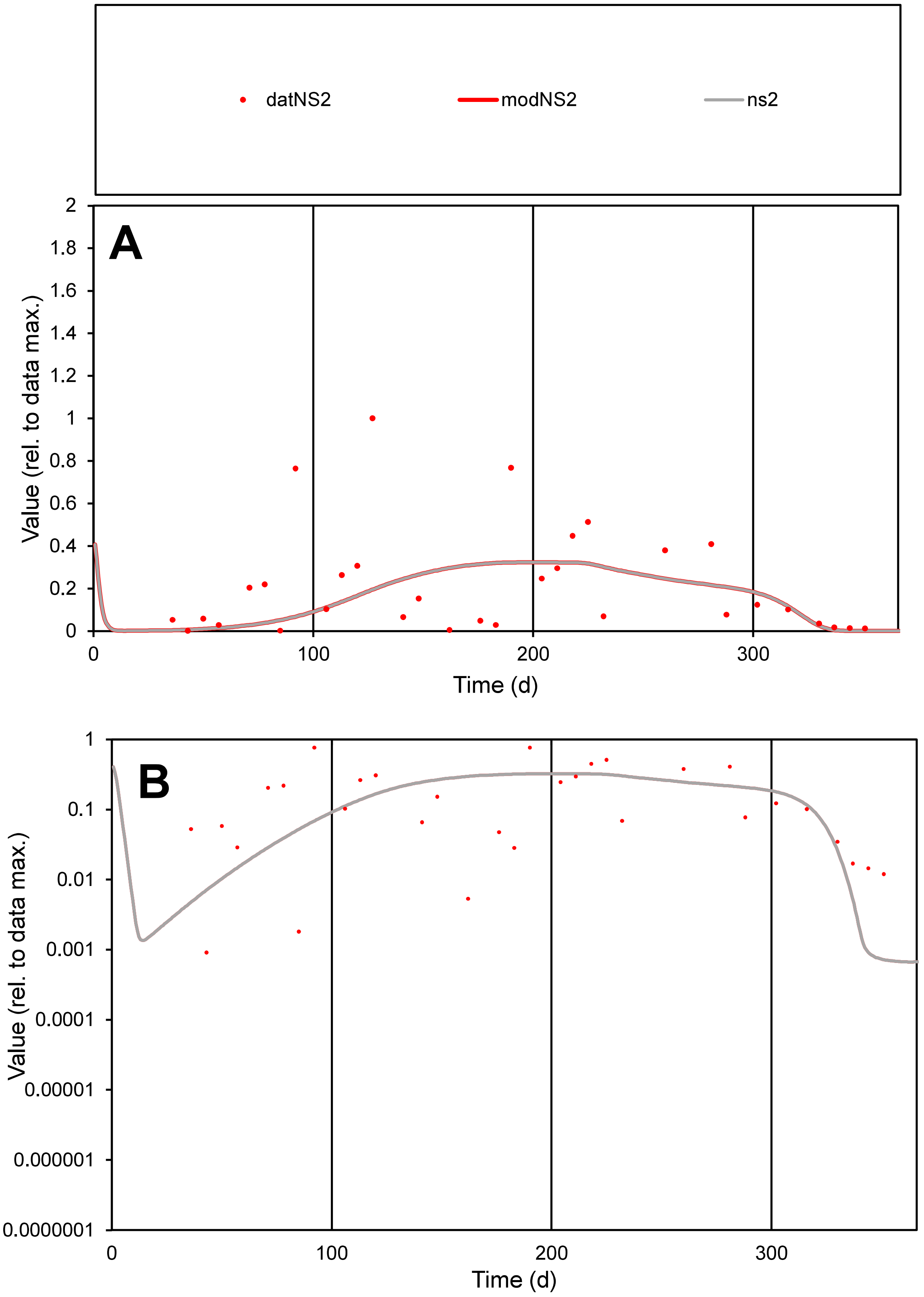

C1

Variables
NS2 Provide jtd or Dst
CalibOnly?
2 1 to reproduce of
oNormalize?
2 1=no, 2=yes

ofxn calc*
1.14439
ofxn mod
1.14947

sRun
RunA1
zout
1

Ave
Data
0.01338
Model
0.01022

data/vMax
0.06286

ofxng
321.551

Min d/m
1.27904
ave/min d
233.709
ave/min m
228.41

324_Calib_S_A

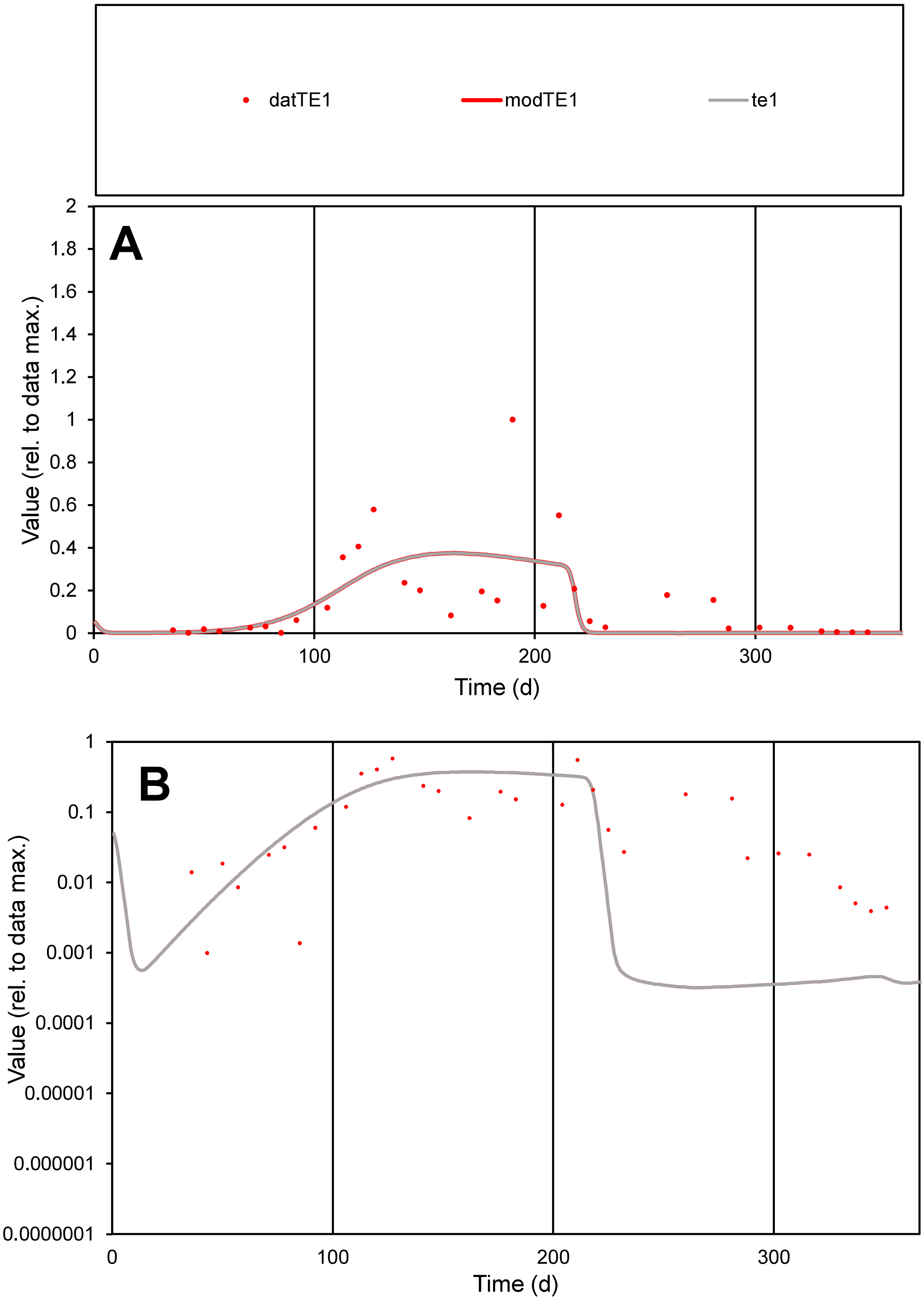

C1

Variables

TE1 Provide jtd or Dst

CalibOnly?

2 1 to reproduce of

oNormalize?

2 1=no, 2=yes

ofxn calc*

1.09705

ofxn mod

1.10877

sRun

RunA1

zout

1

Ave

Data

0.01612

Model

0.01413

data/vMax

0.1056

ofxng

321.551

Min d/m

3.07919

ave/min d

154.111

ave/min m

416.086

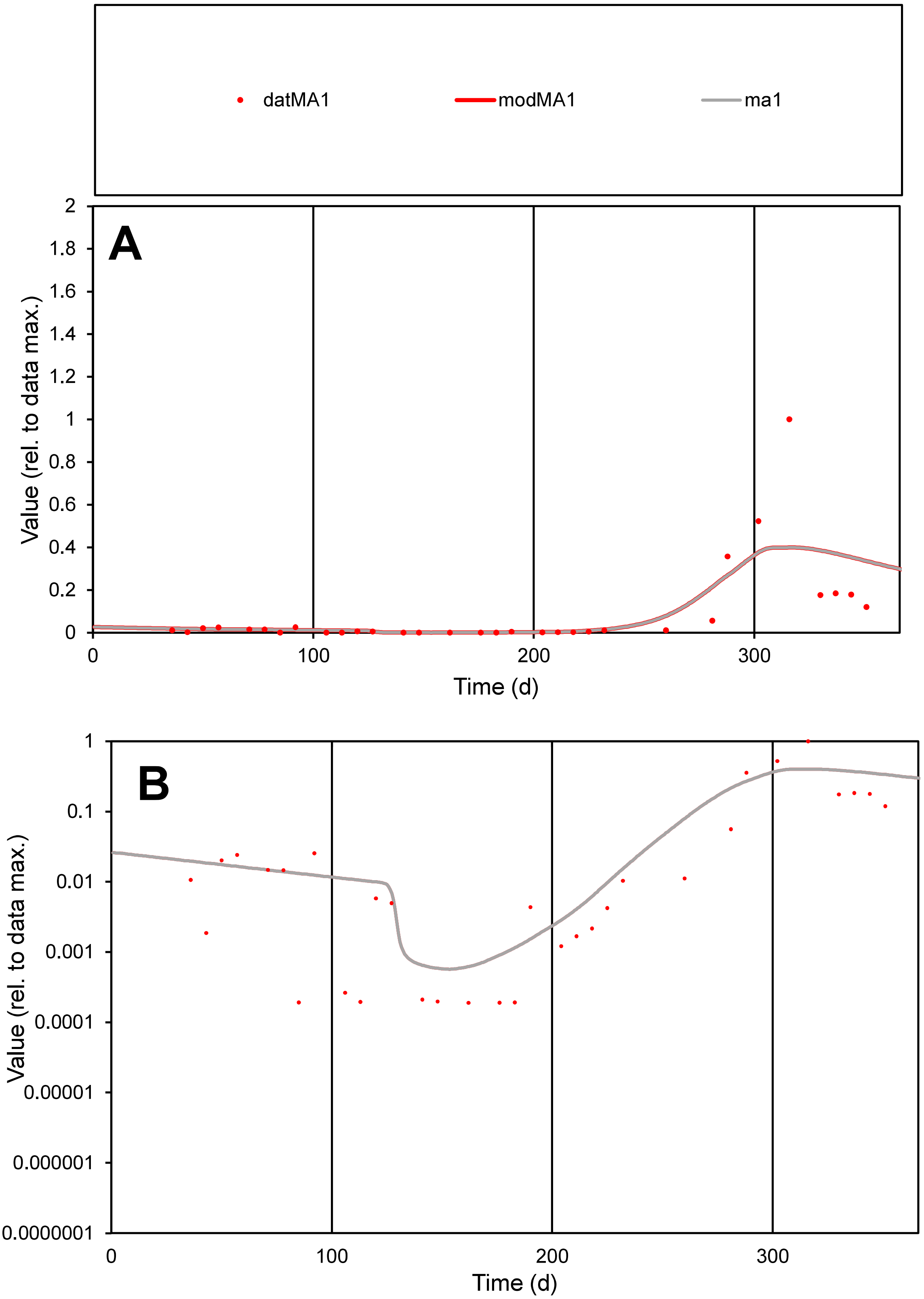

C1

Variables
MA1 Provide jtd or Dst
CalibOnly?
2 1 to reproduce of
oNormalize?
2 1=no, 2=yes

ofxn calc*
1.55026
ofxn mod
1.56452

sRun
RunA1
zout
1

Ave
Data
0.00643
Model
0.00701

data/vMax
0.07472

ofxng
321.551

Min d/m
0.32588

ave/min d
454.56
ave/min m
161.554

324_Calib_S_A

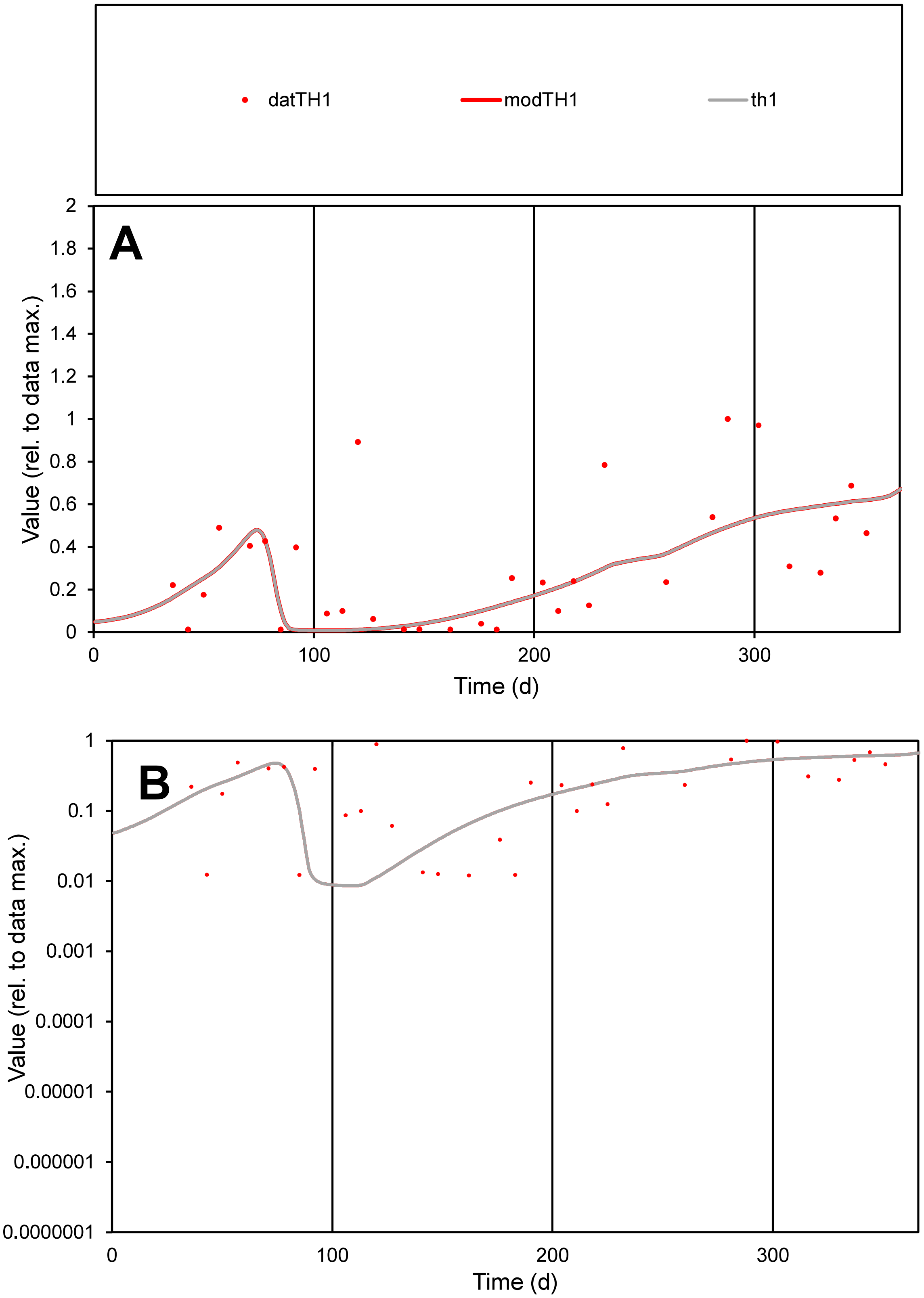

C1

Variables
TH1 Provide jtd or Dst
CalibOnly?
2 1 to reproduce of
oNormalize?
2 1=no, 2=yes

ofxn calc*
0.78604
ofxn mod
0.79468

sRun
RunA1
zout
1

Ave
Data
0.00239
Model
0.00201

data/vMax
0.00756

ofxng
321.551

Min d/m
1.39755

ave/min d
26.233
ave/min m
30.8464

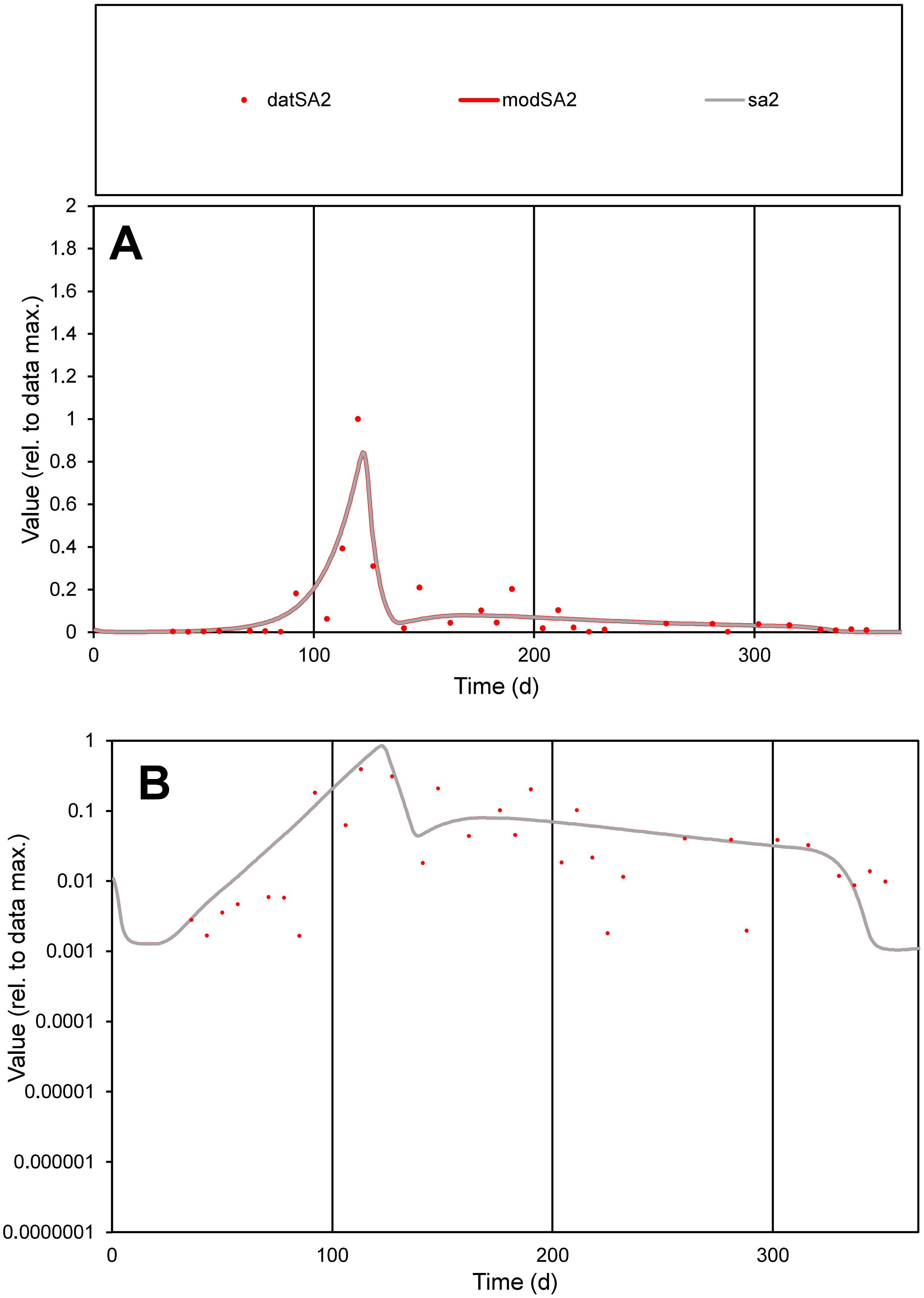

C1

Variables
SA2 Provide jtd or Dst
CalibOnly?
2 1 to reproduce of
oNormalize?
2 1=no, 2=yes

ofxn calc*
0.79051
ofxn mod
0.83309

sRun
RunA1
zout
1

Ave
Data
0.00561
Model
0.00612

data/vMax
0.06091

ofxng
321.551

Min d/m
1.55682

ave/min d
55.5519
ave/min m
94.3152

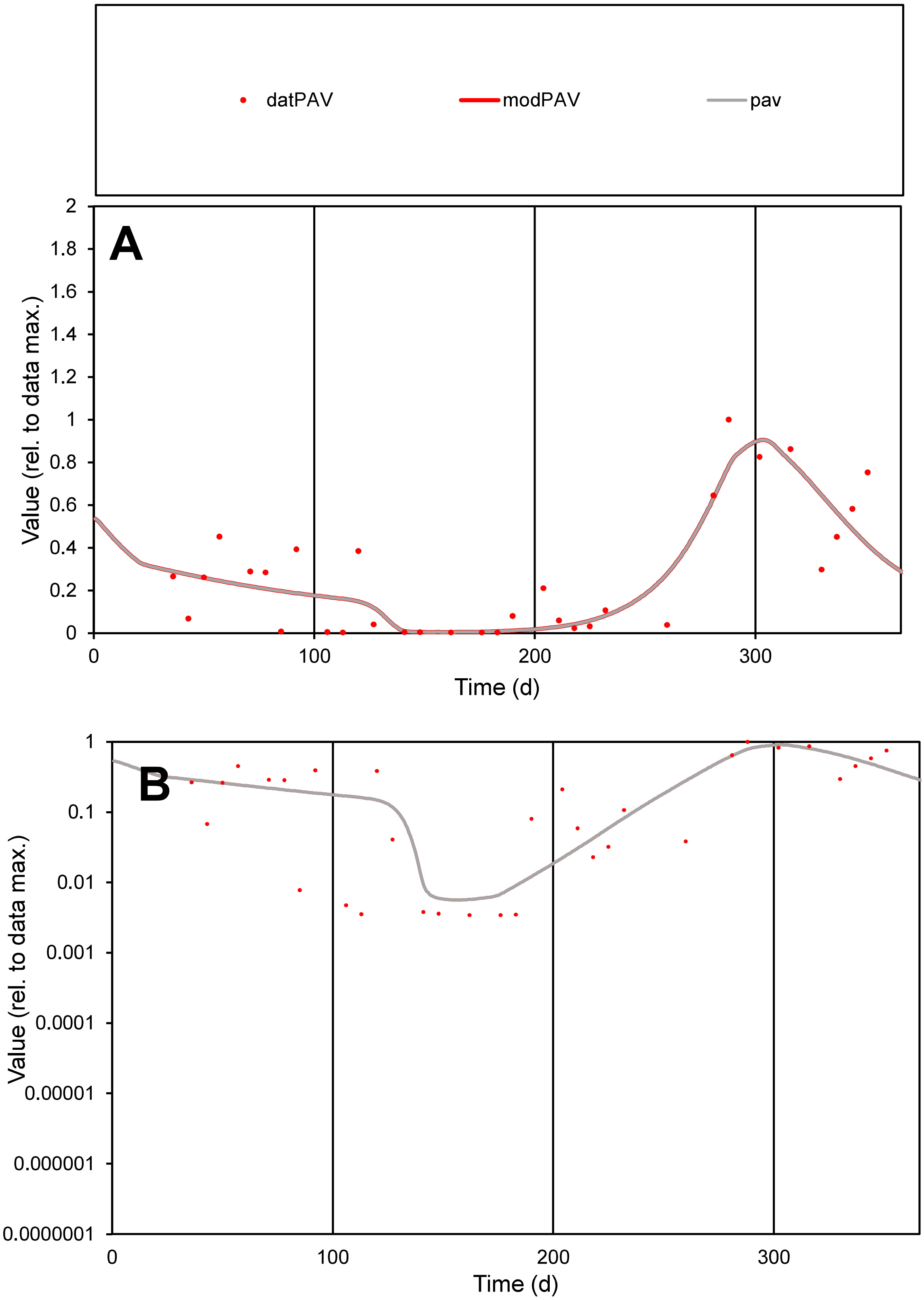

C1

Variables
PAV Provide jtd or Dst
CalibOnly?
2 1 to reproduce of
oNormalize?
2 1=no, 2=yes

ofxn calc*
0.55687
ofxn mod
0.57221

sRun
RunA1
zout
1

Ave
Data
0.00281
Model
0.00274

data/vMax
0.01064

ofxng
321.551

Min d/m
0.59374

ave/min d
76.9879
ave/min m
44.6126

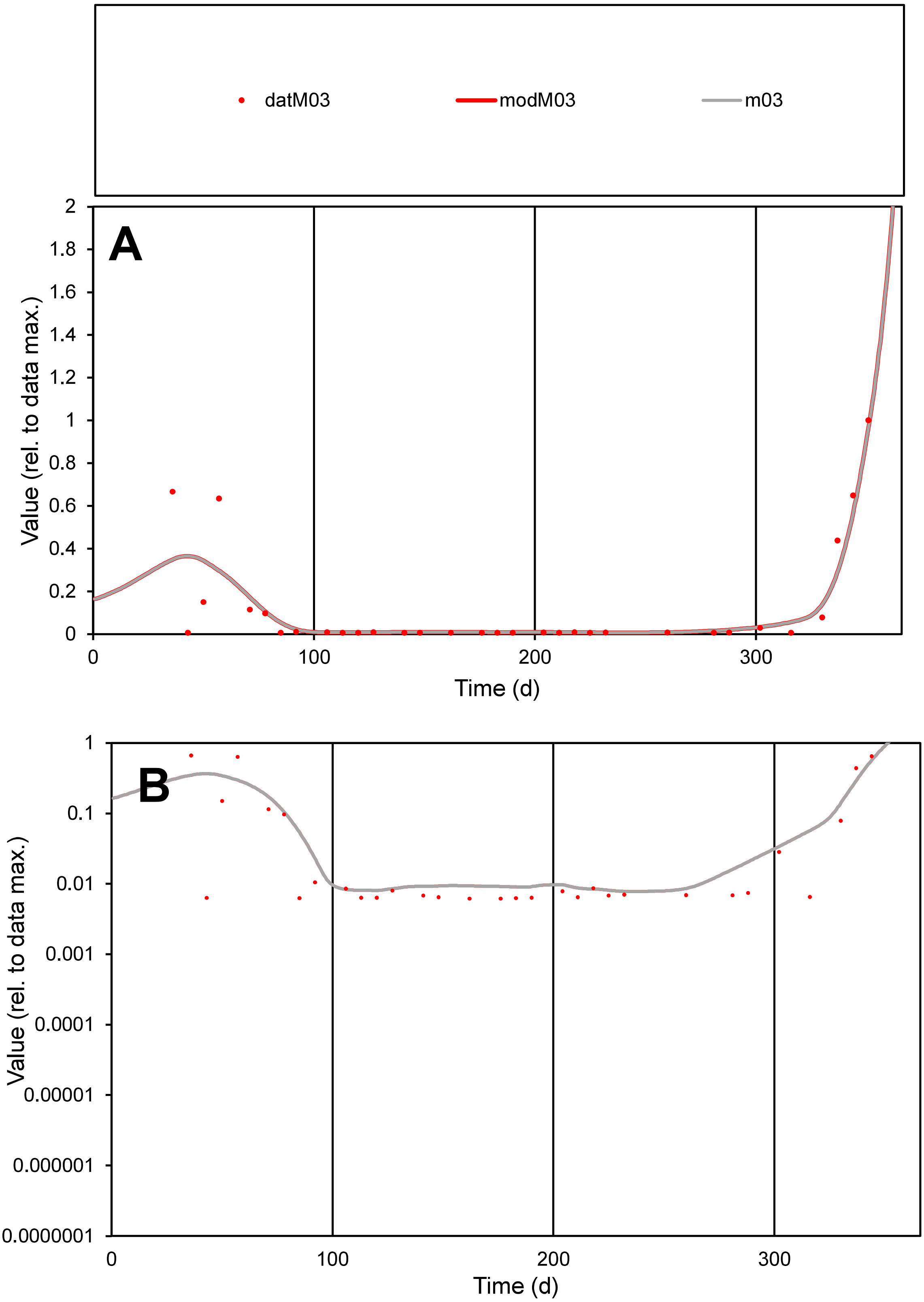

C1

Variables
M03 Provide jtd or Dst
CalibOnly?
2 1 to reproduce of
oNormalize?
2 1=no, 2=yes

ofxn calc*
0.89015
ofxn mod
0.90805

sRun
RunA1
zout
1

Ave
Data
0.00035
Model
0.00036

data/vMax
0.00279

ofxng
321.551

Min d/m
0.78836

ave/min d
20.3356
ave/min m
16.2996

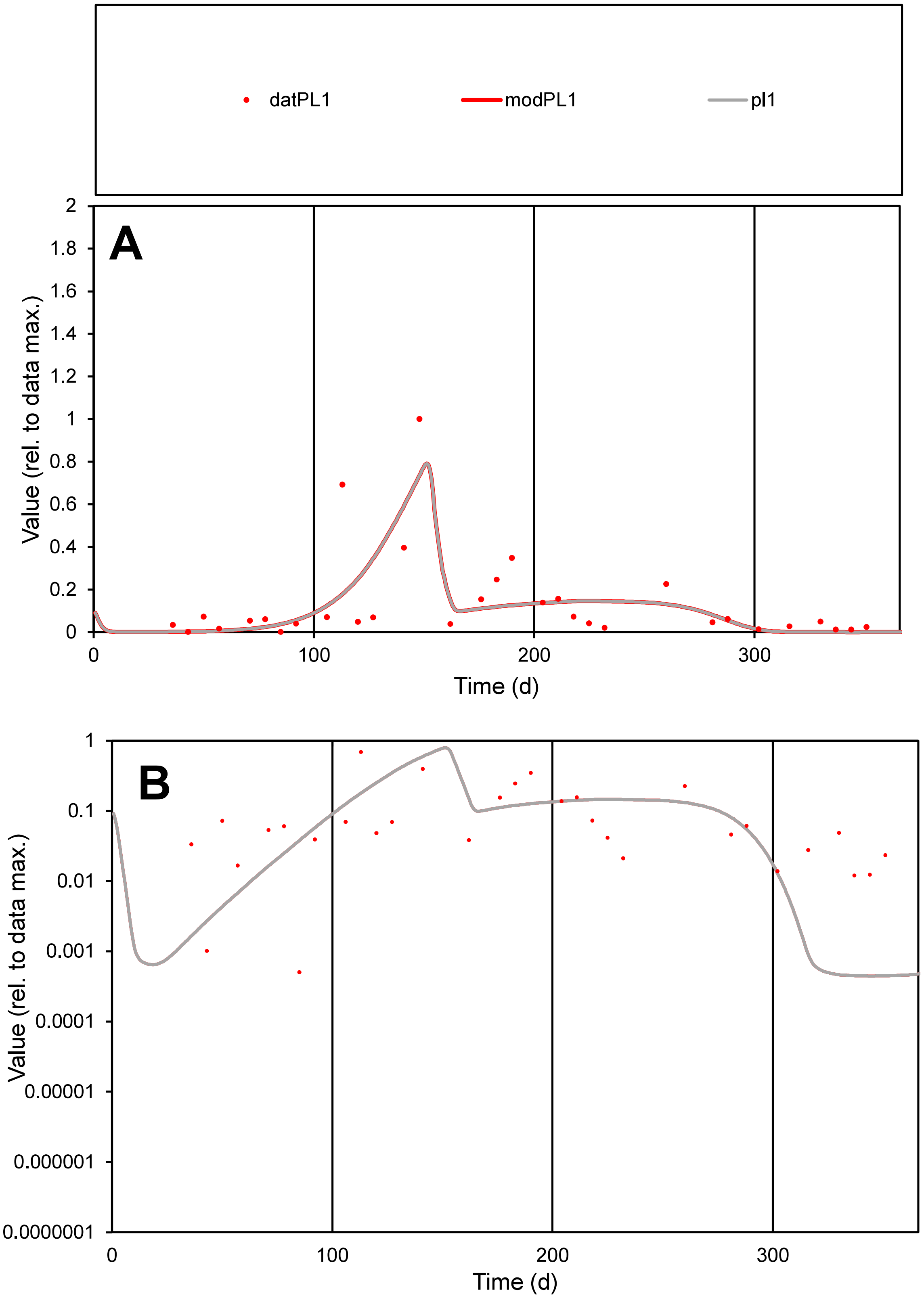

C1

Variables
PL1 Provide jtd or Dst
CalibOnly?
2 1 to reproduce of
oNormalize?
2 1=no, 2=yes

ofxn calc*
1.0344
ofxn mod
1.05488

sRun
RunA1
zout
1

Ave
Data
0.00909
Model
0.00834

data/vMax
0.06855

ofxng
321.551

Min d/m
1.13377

ave/min d
262.93
ave/min m
273.538

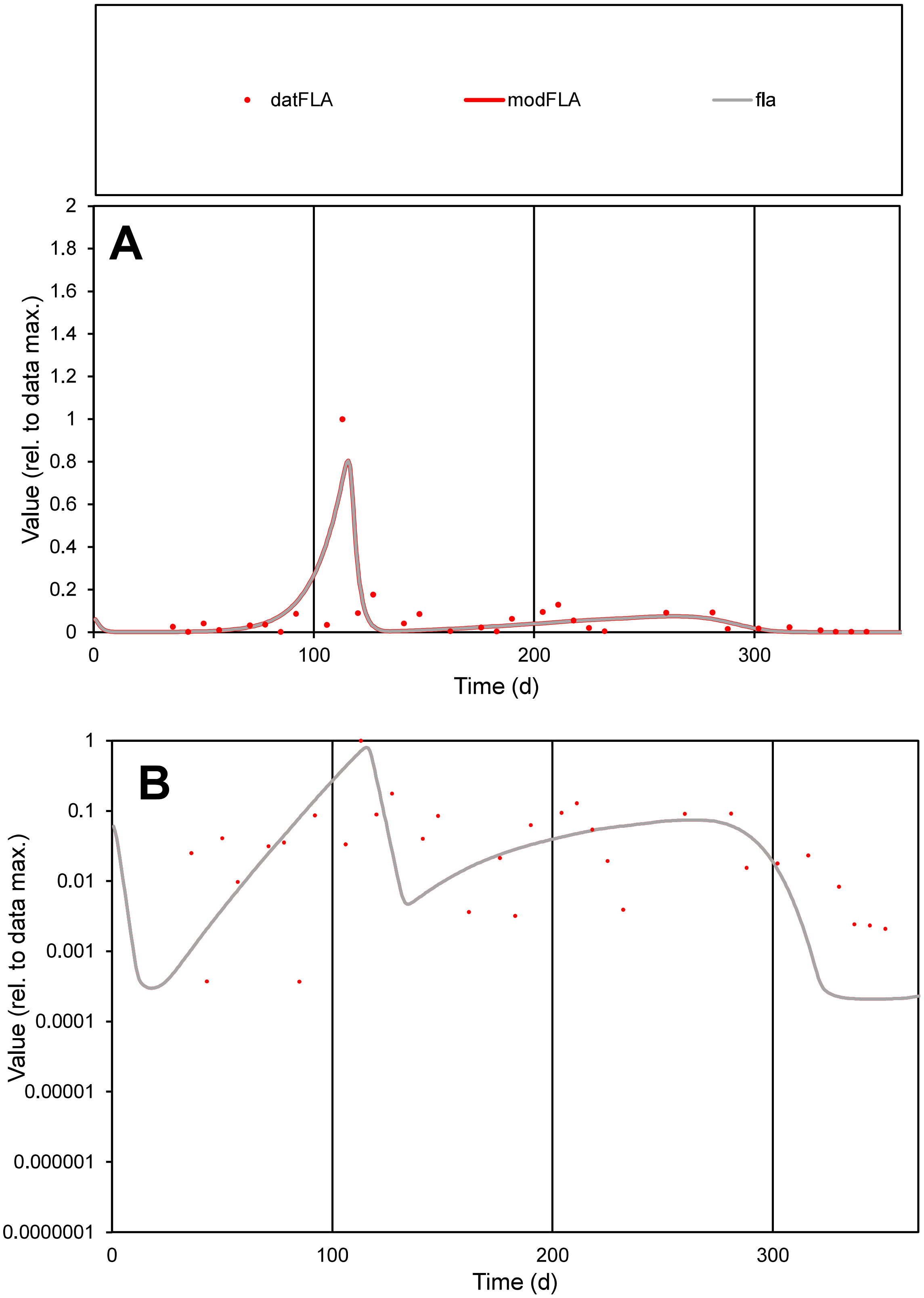

C1

Variables
FLA Provide jtd or Dst
CalibOnly?
2 1 to reproduce of
oNormalize?
2 1=no, 2=yes

ofxn calc*
1.14451
ofxn mod
1.28731

sRun
RunA1
zout
1

Ave
Data
0.0106
Model
0.01022

data/vMax
0.1475

ofxng
321.551

Min d/m
1.7846

ave/min d
193.835
ave/min m
333.422

324_Calib_S_A

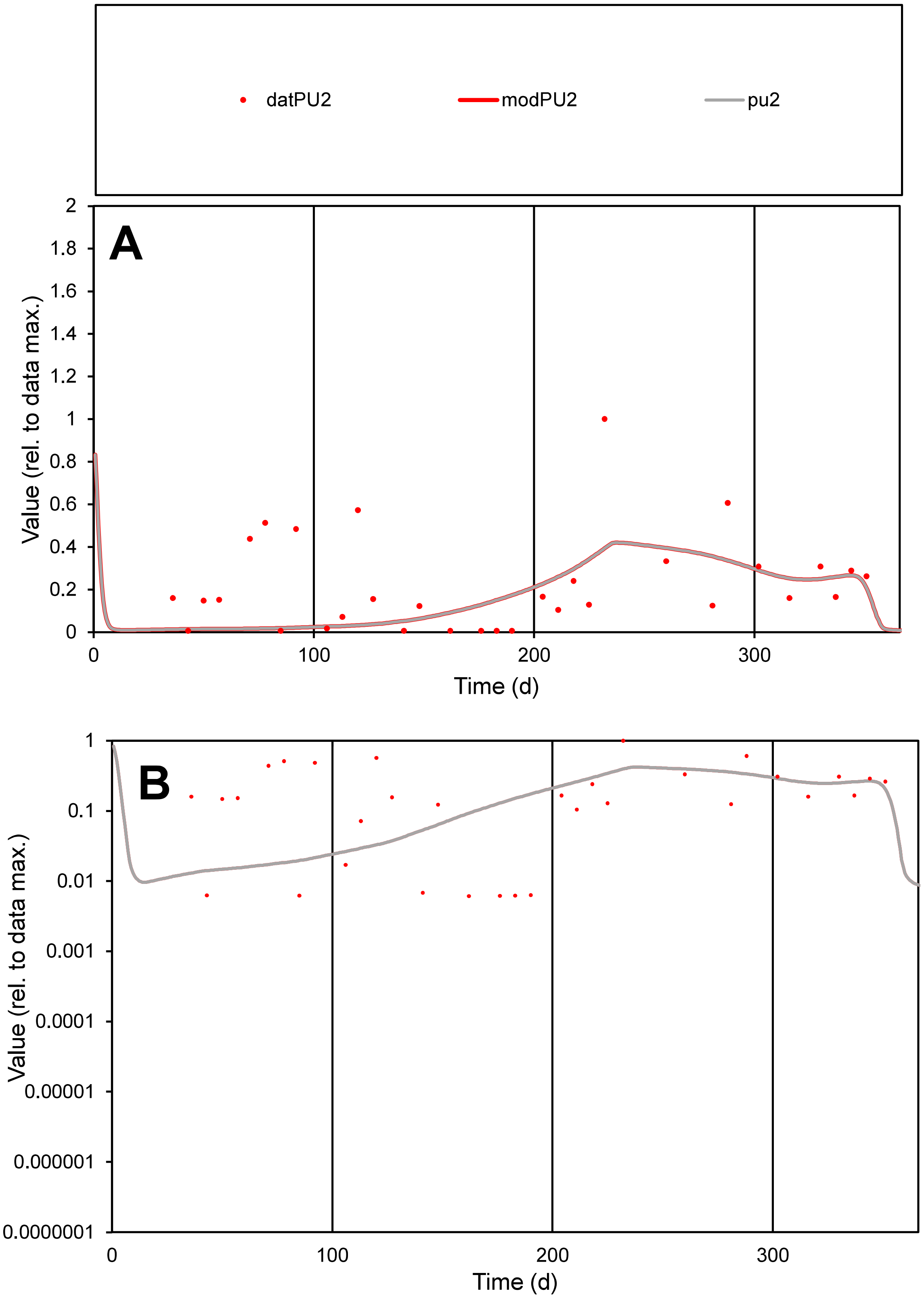

C1

Variables	
PU2	Provide jtd or Dst
CalibOnly?	
2	1 to reproduce of
oNormalize?	
2	1=no, 2=yes
ofxn calc*	
1.03295	
ofxn mod	
1.03953	
sRun	
RunA1	
zout	
1	
Ave	
Data	
0.00194	
Model	
0.00139	
data/vMax	
0.00876	
ofxng	
321.551	
Min d/m	
0.46385	
ave/min d	
36.0381	
ave/min m	
12.0117	

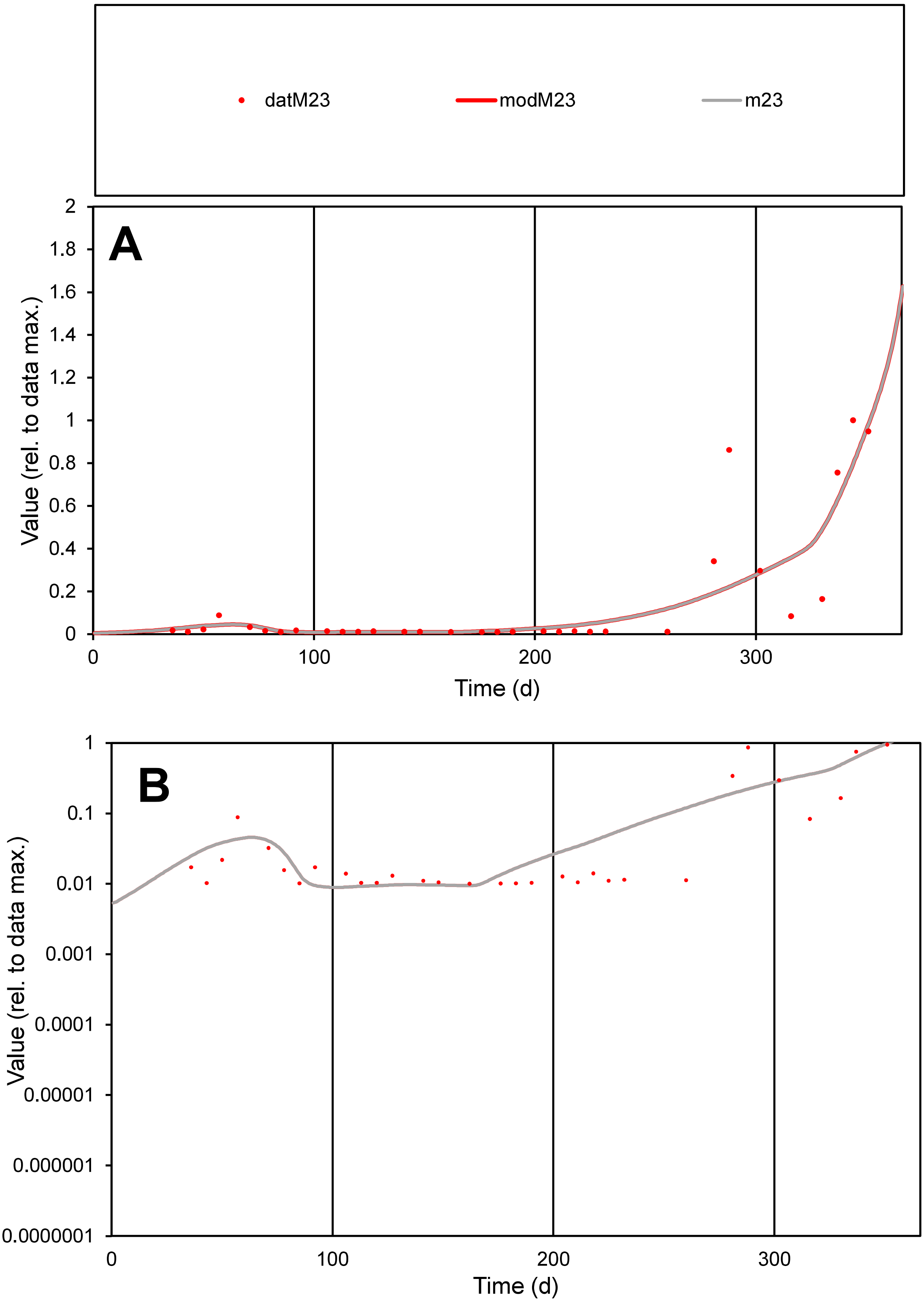

C1

Variables
M23 Provide jtd or Dst
CalibOnly?
2 1 to reproduce of
oNormalize?
2 1=no, 2=yes

ofxn calc*
0.95533
ofxn mod
0.97269

sRun
RunA1
zout
1

Ave
Data
0.00159
Model
0.00156

data/vMax
0.0105

ofxng
321.551

Min d/m
1.11381

ave/min d
15.0836
ave/min m
16.4779

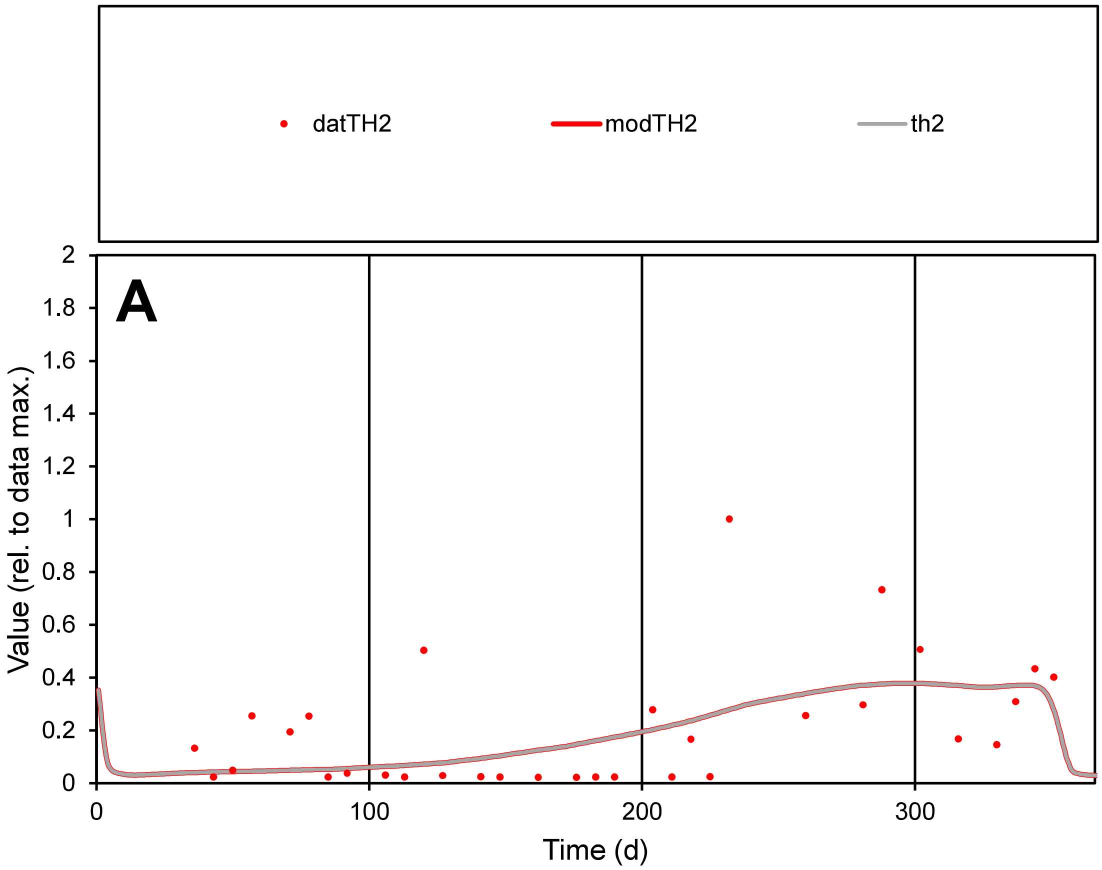

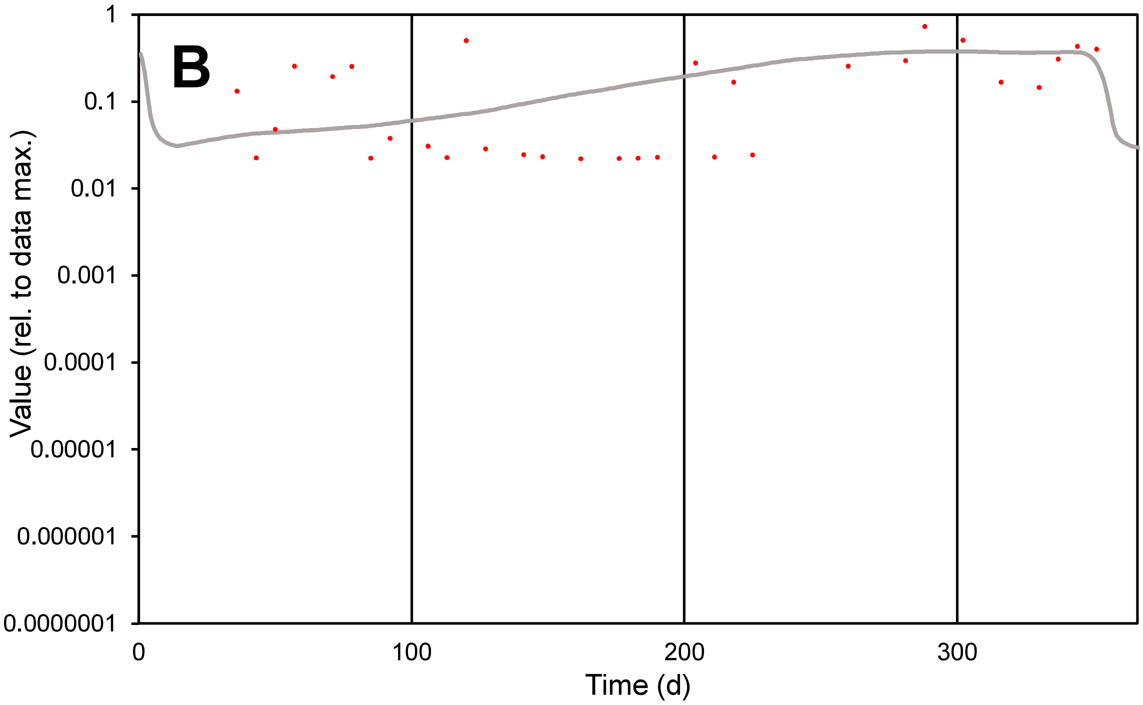

C1

Variables
TH2 Provide jtd or Dst
CalibOnly?
2 1 to reproduce of
oNormalize?
2 1=no, 2=yes

ofxn calc*
0.99476
ofxn mod
1.00382

sRun
RunA1
zout
1

Ave
Data
0.00094
Model
0.00086

data/vMax
0.0047

ofxng
321.551

Min d/m
0.53982

ave/min d
9.09682
ave/min m
4.50171

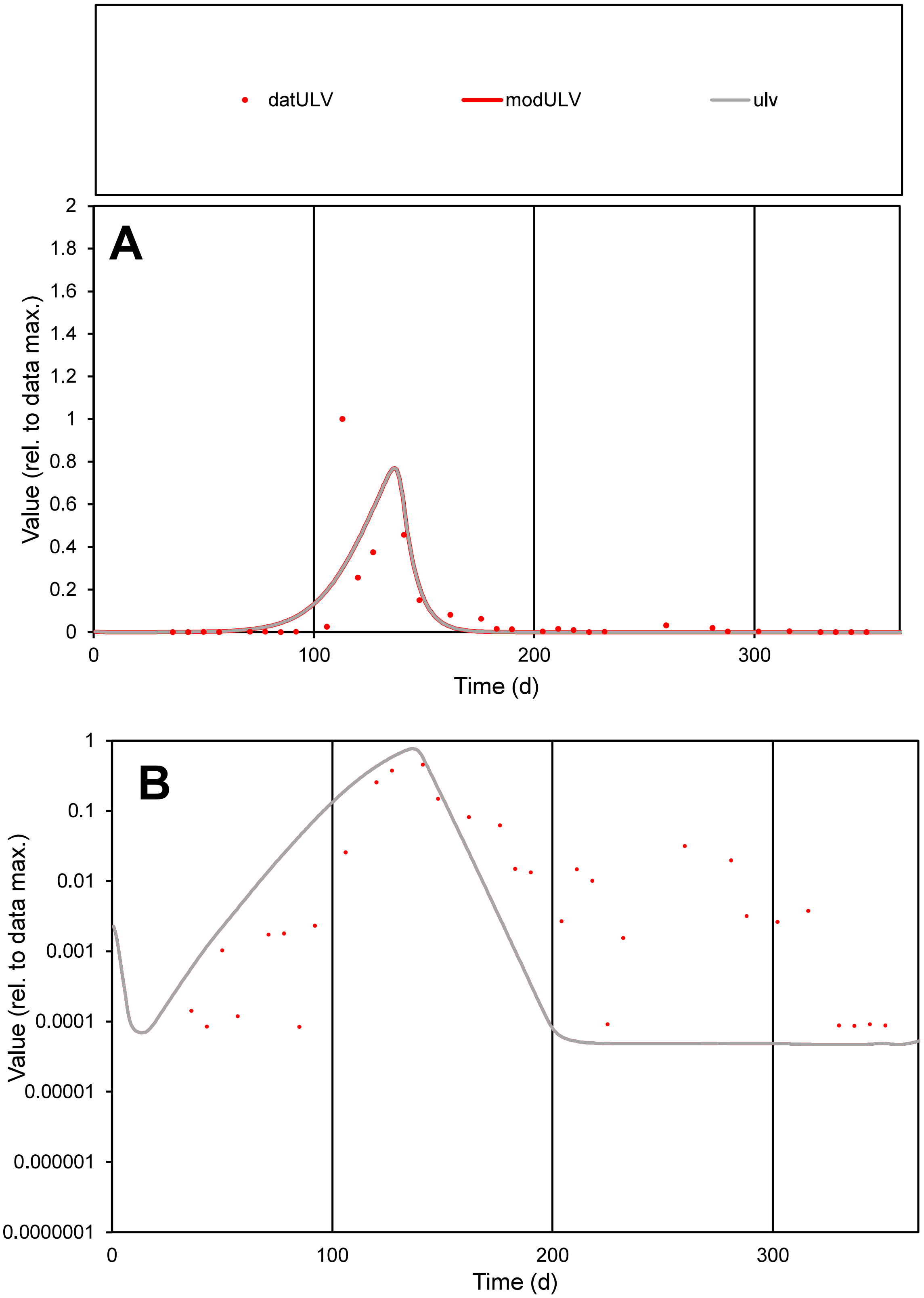

C1

Variables

ULV Provide jtd or Dst

CalibOnly?

2 1 to reproduce of

oNormalize?

2 1=no, 2=yes

ofxn calc*

1.71419

ofxn mod

1.74207

sRun

RunA1

zout

1

Ave

Data

0.01612

Model

0.01508

data/vMax

0.2037

ofxng

321.551

Min d/m

1.79005

ave/min d

941.729

ave/min m

1576.99

324_Calib_S_A

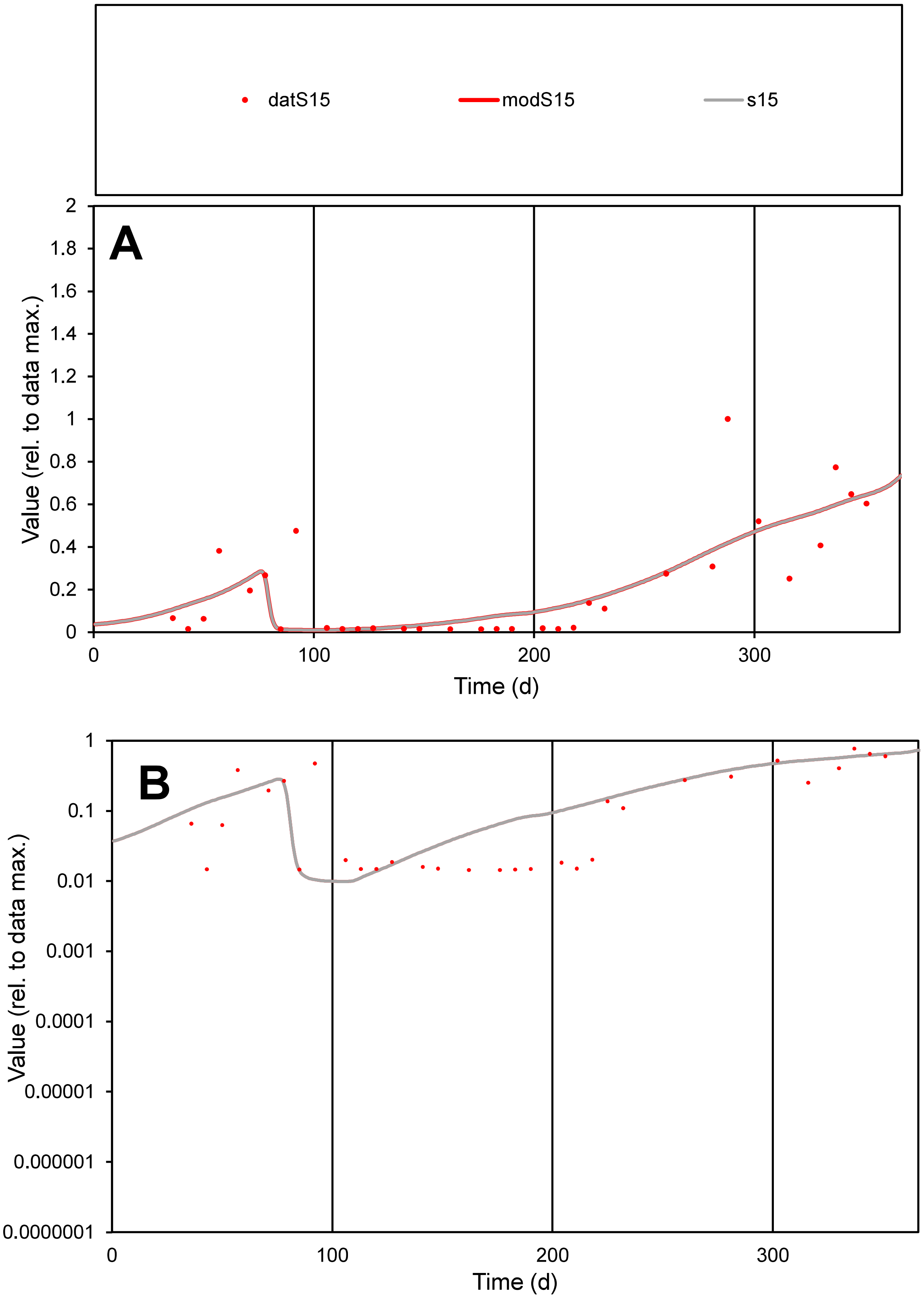

C1

Variables
S15 Provide jtd or Dst
CalibOnly?
2 1 to reproduce of
oNormalize?
2 1=no, 2=yes

ofxn calc*
0.75716
ofxn mod
0.76975

sRun
RunA1
zout
1

Ave
Data
0.00178
Model
0.00178

data/vMax
0.00847

ofxng
321.551

Min d/m
1.454

ave/min d
14.6064
ave/min m
21.2892

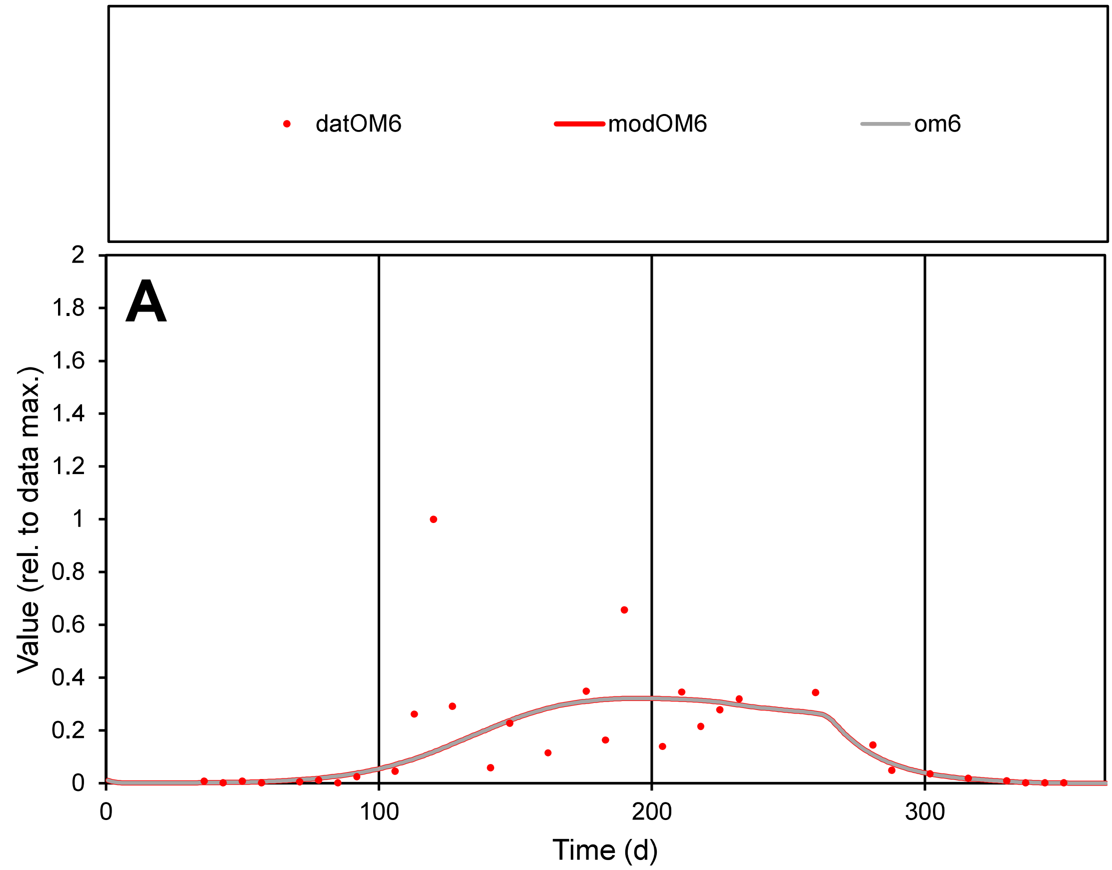

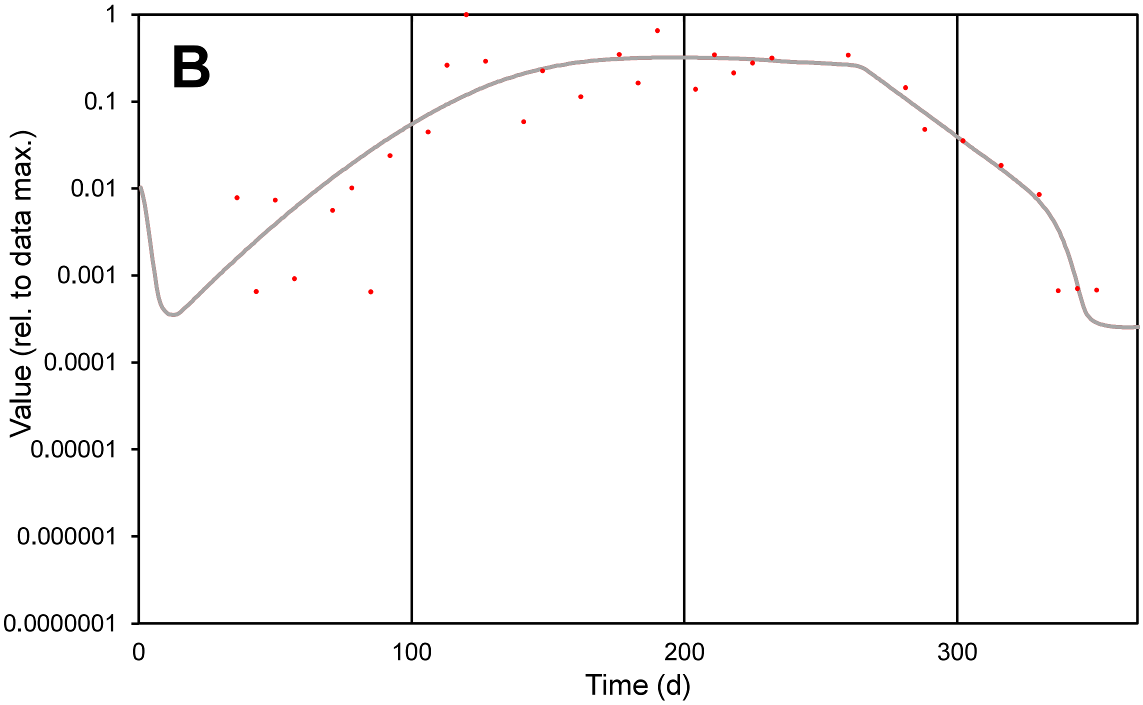

C1

Variables
OM6 Provide jtd or Dst
CalibOnly?
2 1 to reproduce of
oNormalize?
2 1=no, 2=yes

ofxn calc*
1.13717
ofxn mod
1.1448

sRun
RunA1
zout
1

Ave
Data
0.00615
Model
0.00517

data/vMax
0.03846

ofxng
321.551

Min d/m
2.31628

ave/min d
246.963
ave/min m
480.612

324_Calib_S_A

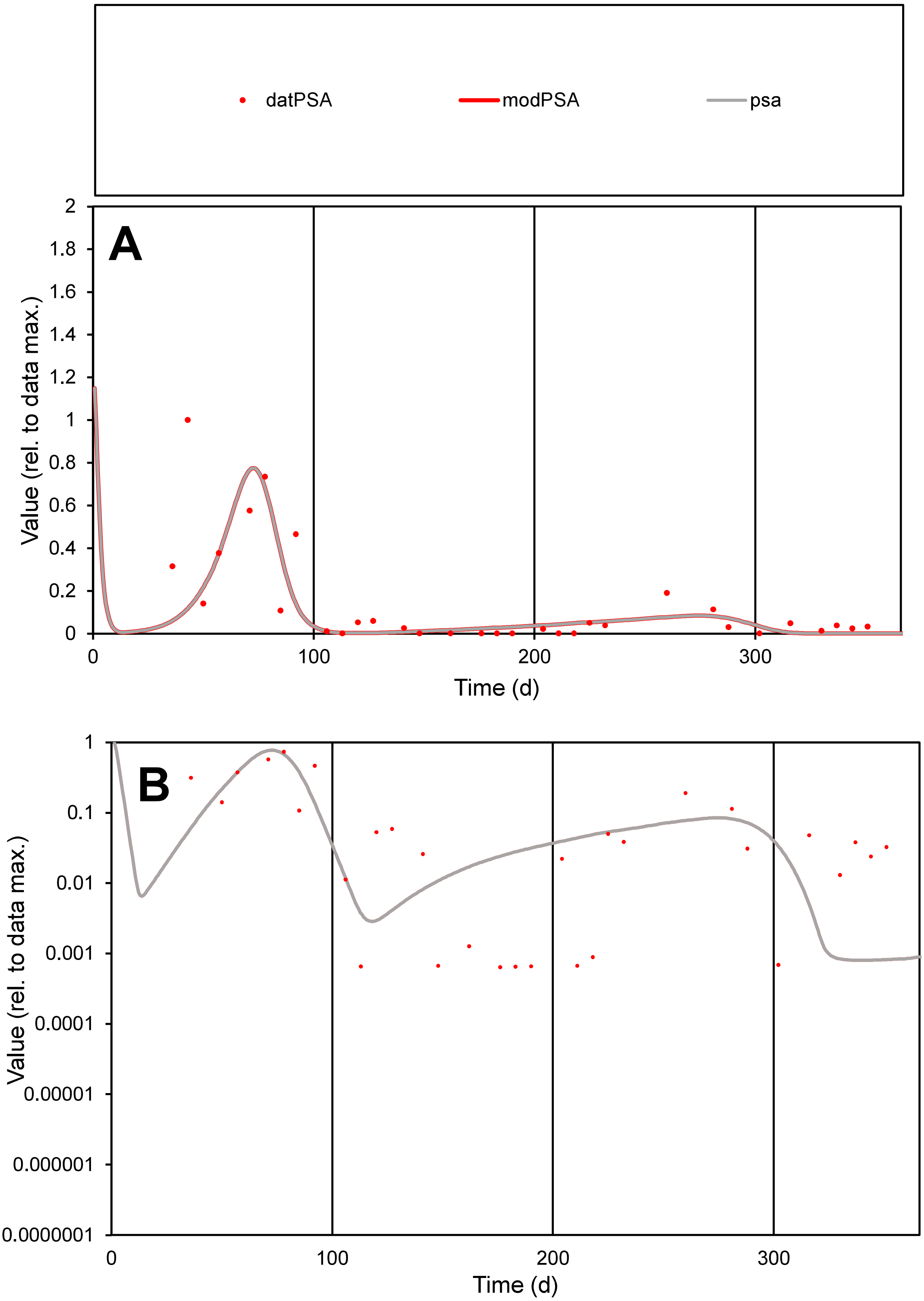

C1

Variables
PSA Provide jtd or Dst
CalibOnly?
2 1 to reproduce of
oNormalize?
2 1=no, 2=yes

ofxn calc*
1.29206
ofxn mod
1.30818

sRun
RunA1
zout
1

Ave
Data
0.0019
Model
0.00141

data/vMax
0.01362

ofxng
321.551

Min d/m
0.79817

ave/min d
218.654
ave/min m
128.847

324_Calib_S_A

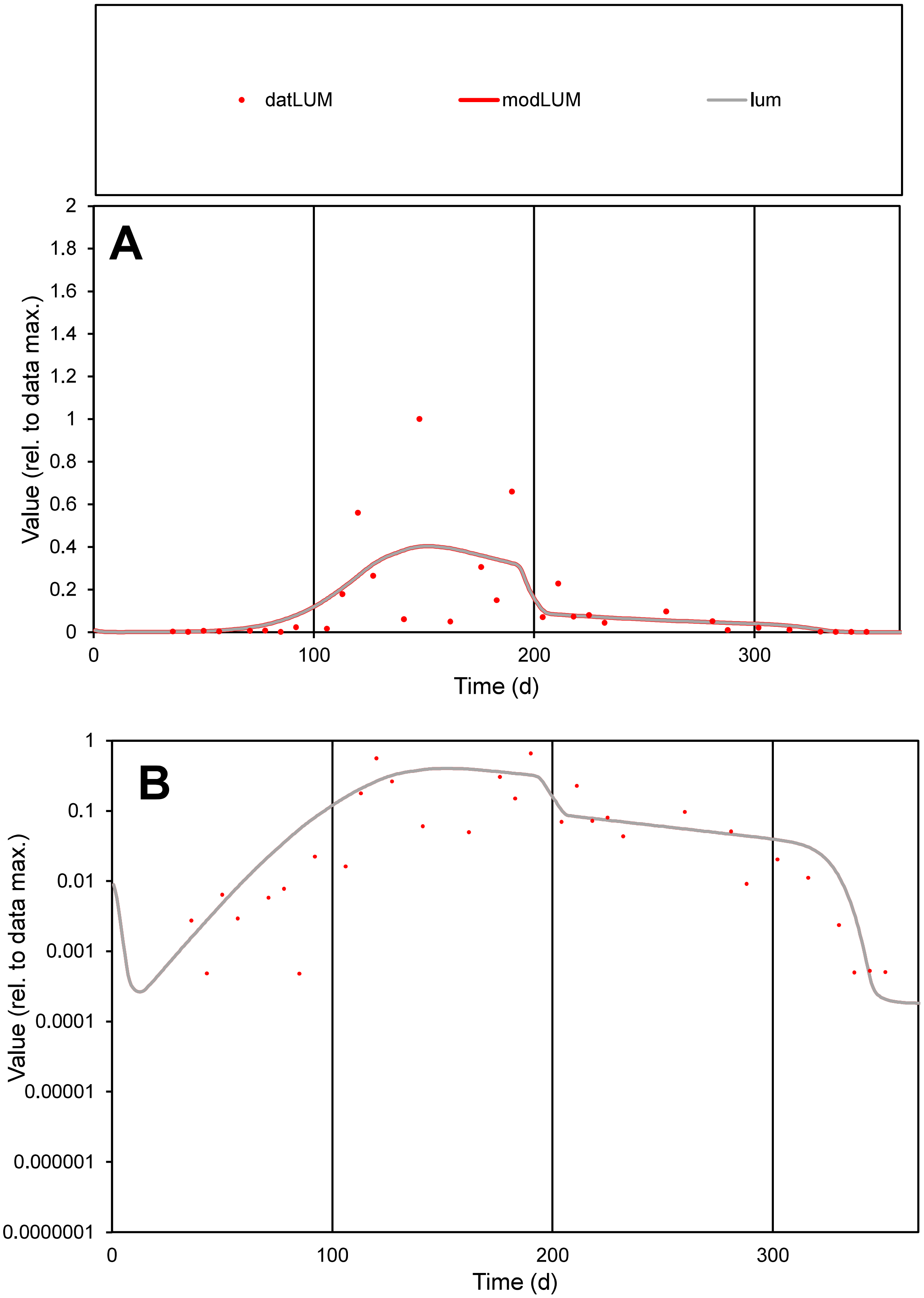

C1

Variables
LUM Provide jtd or Dst
CalibOnly?
2 1 to reproduce of
oNormalize?
2 1=no, 2=yes

ofxn calc*
1.32592
ofxn mod
1.33658

sRun
RunA1
zout
1

Ave
Data
0.01148
Model
0.01153

data/vMax
0.09231

ofxng
321.551

Min d/m
2.37567

ave/min d
257.957
ave/min m
615.358

324_Calib_S_A

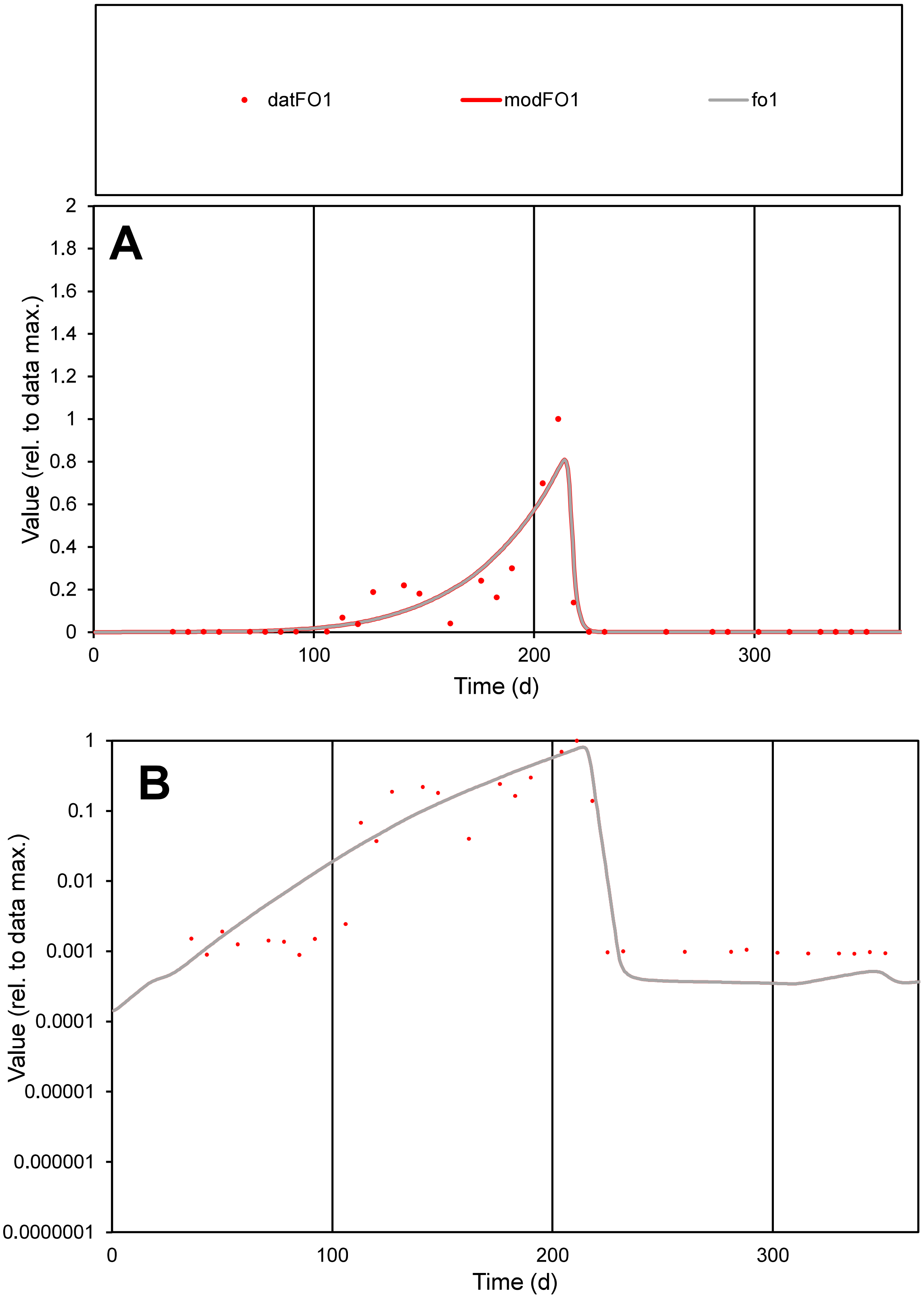

C1

Variables	
FO1	Provide jtd or Dst
CalibOnly?	
2	1 to reproduce of
oNormalize?	
2	1=no, 2=yes
ofxn calc*	
0.71603	
ofxn mod	
0.75912	
sRun	
RunA1	
zout	
1	
Ave	
Data	
0.00669	
Model	
0.00684	
data/vMax	
0.06485	
ofxng	
321.551	
Min d/m	
2.57035	
ave/min d	
115.439	
ave/min m	
303.625	

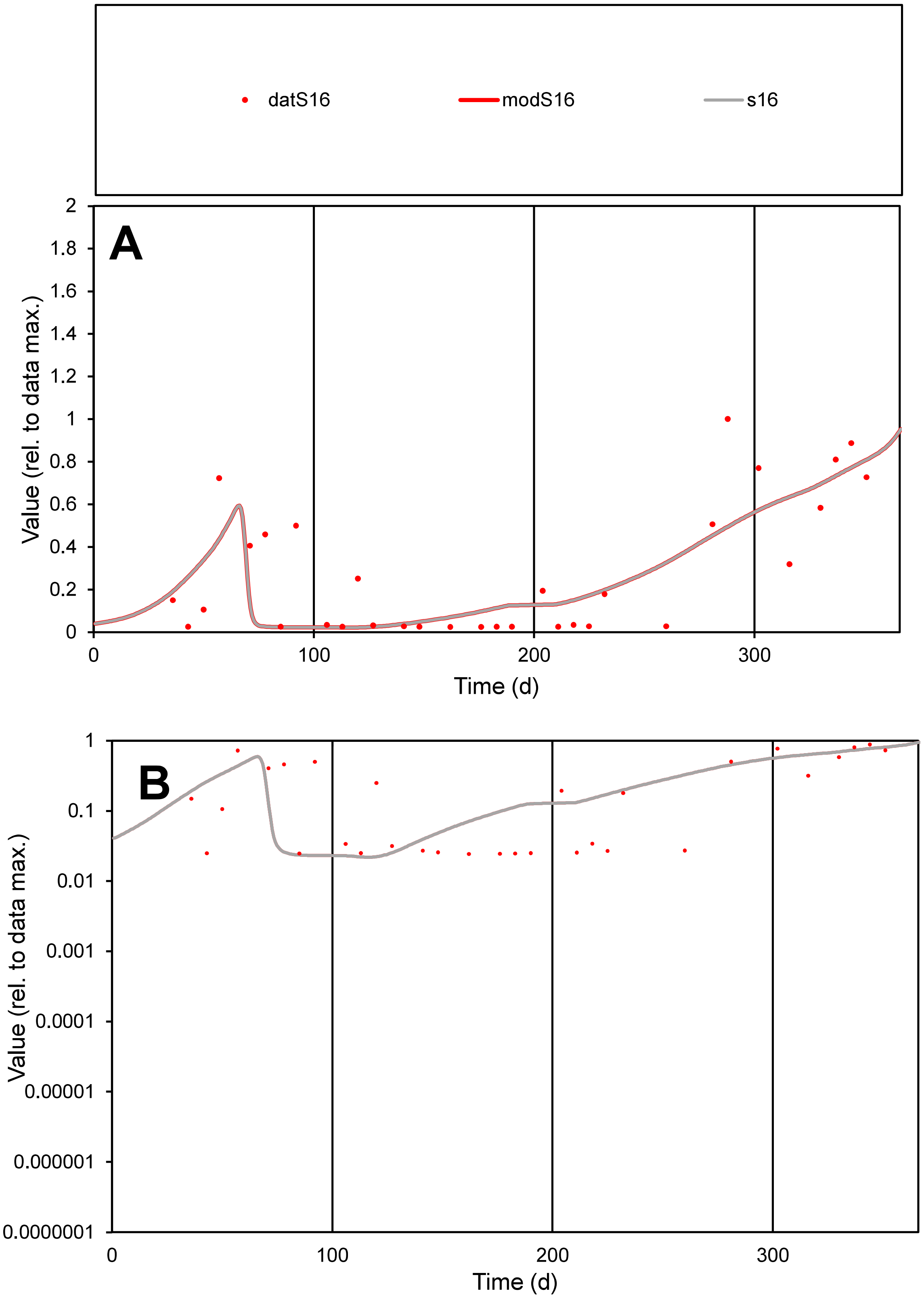

C1

Variables
S16 Provide jtd or Dst
CalibOnly?
2 1 to reproduce of
oNormalize?
2 1=no, 2=yes

ofxn calc*
0.70324
ofxn mod
0.72923

sRun
RunA1
zout
1

Ave
Data
0.0014
Model
0.00135

data/vMax
0.005

ofxng
321.551

Min d/m
1.10536

ave/min d
11.5091
ave/min m
12.2305

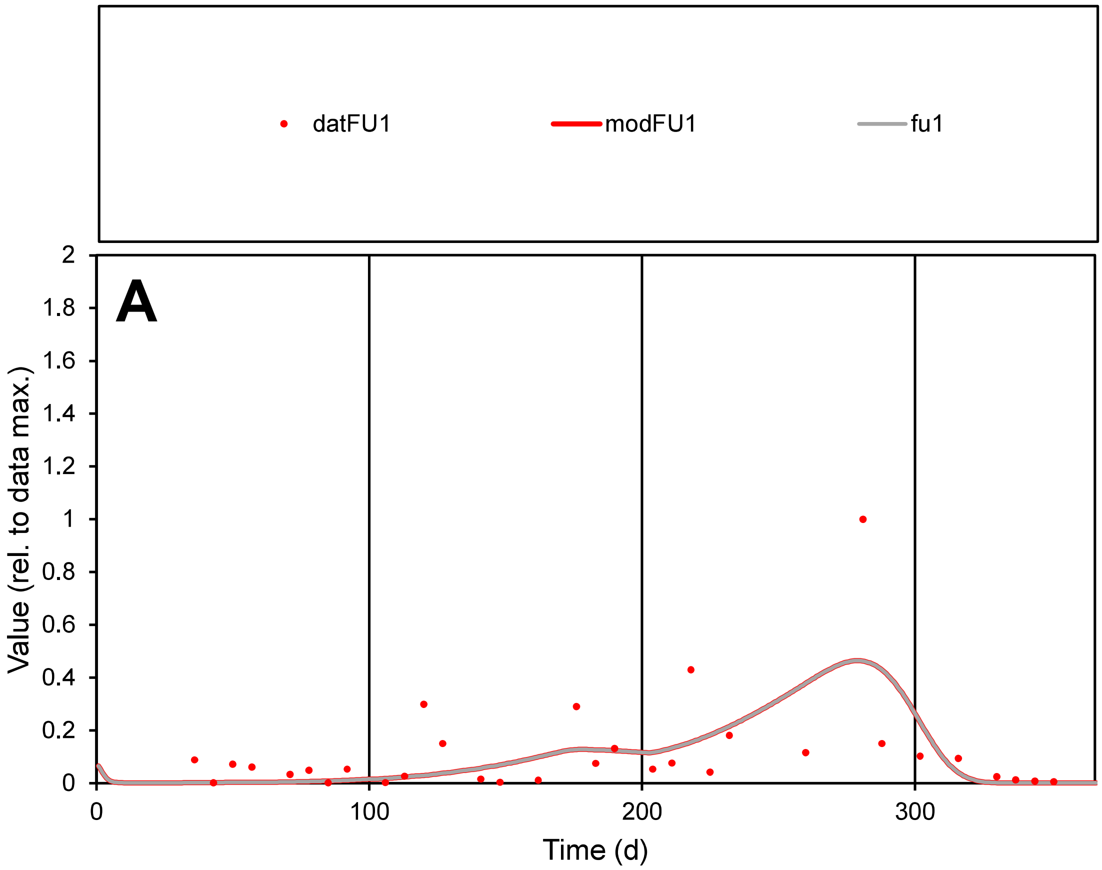

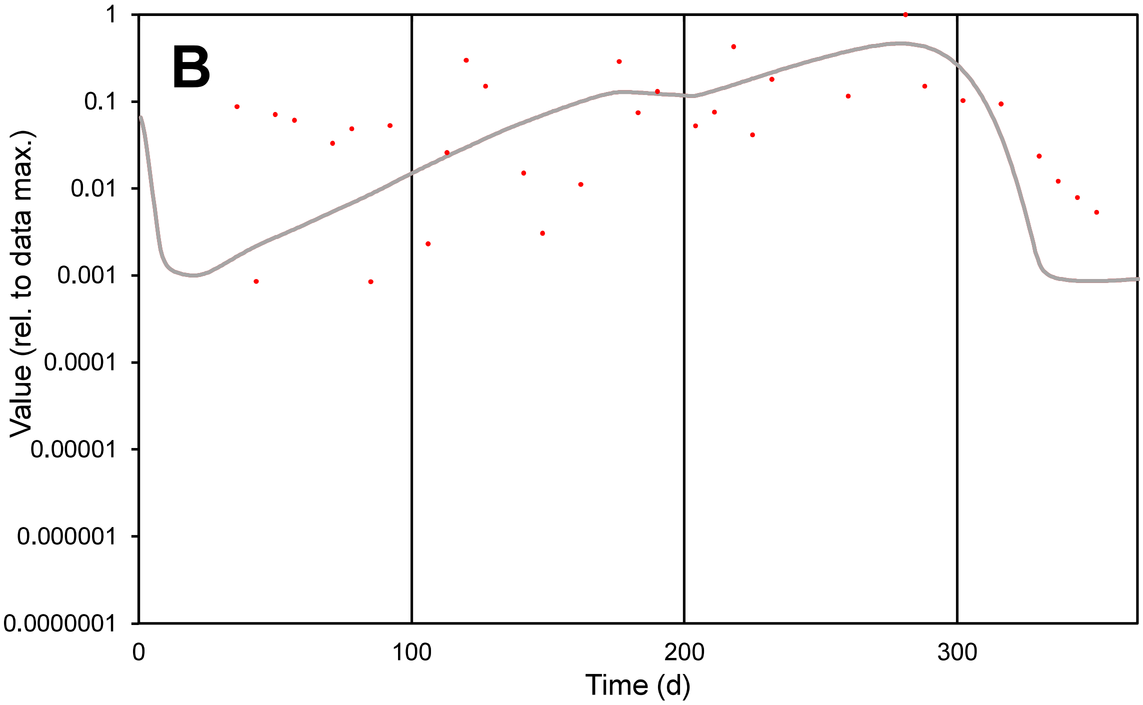

C1

Variables
FU1 Provide jtd or Dst
CalibOnly?
2 1 to reproduce of
oNormalize?
2 1=no, 2=yes

ofxn calc*
1.29296
ofxn mod
1.30261

sRun
RunA1
zout
1

Ave
Data
0.00622
Model
0.00527

data/vMax
0.0546

ofxng
321.551

Min d/m
0.98011

ave/min d
134.333
ave/min m
111.434

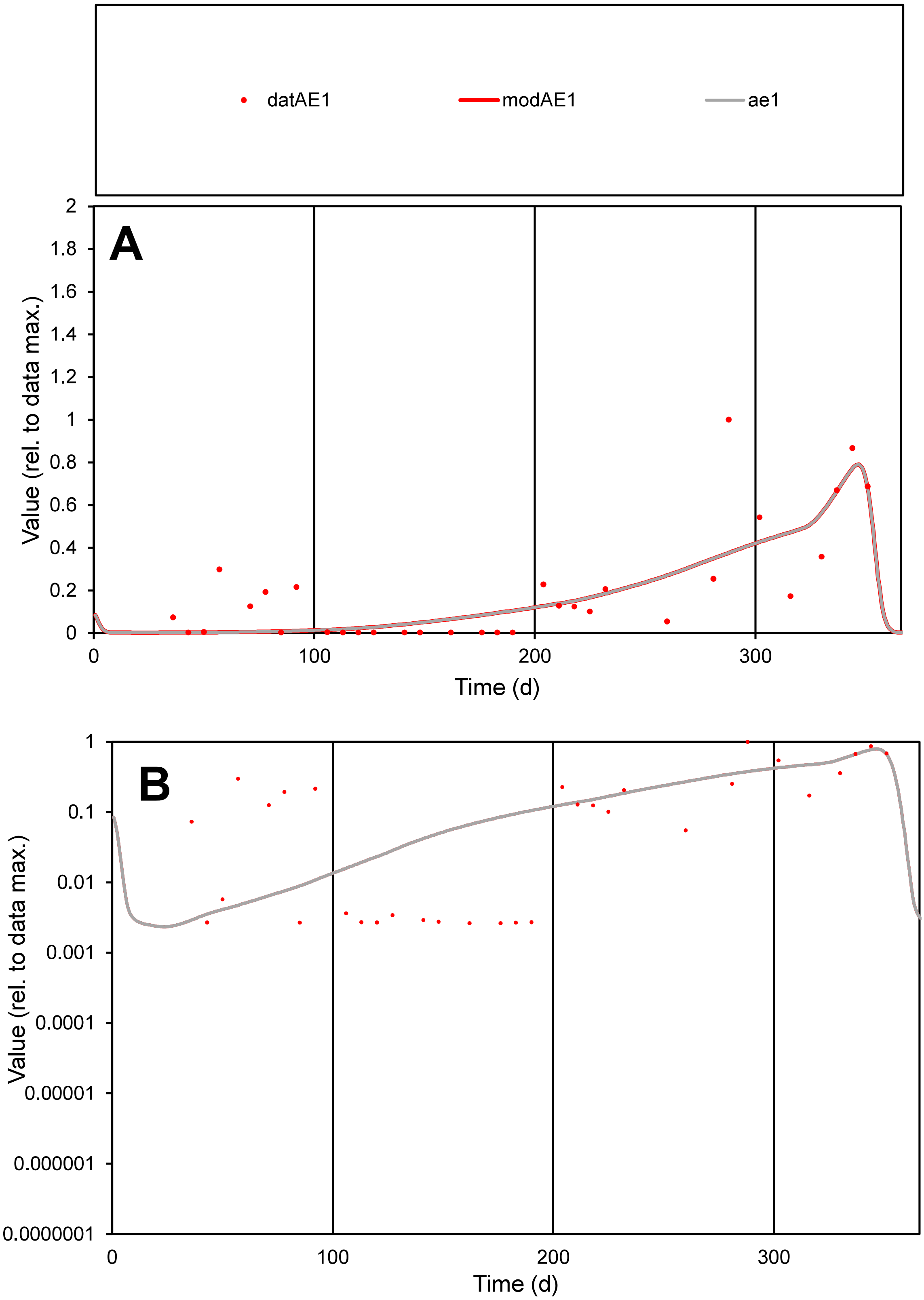

C1

Variables
AE1 Provide jtd or Dst
CalibOnly?
2 1 to reproduce of
oNormalize?
2 1=no, 2=yes

ofxn calc*
0.81403
ofxn mod
0.82961

sRun
RunA1
zout
1

Ave
Data
0.00103
Model
0.00096

data/vMax
0.00519

ofxng
321.551

Min d/m
0.87173

ave/min d
75.307
ave/min m
61.3139

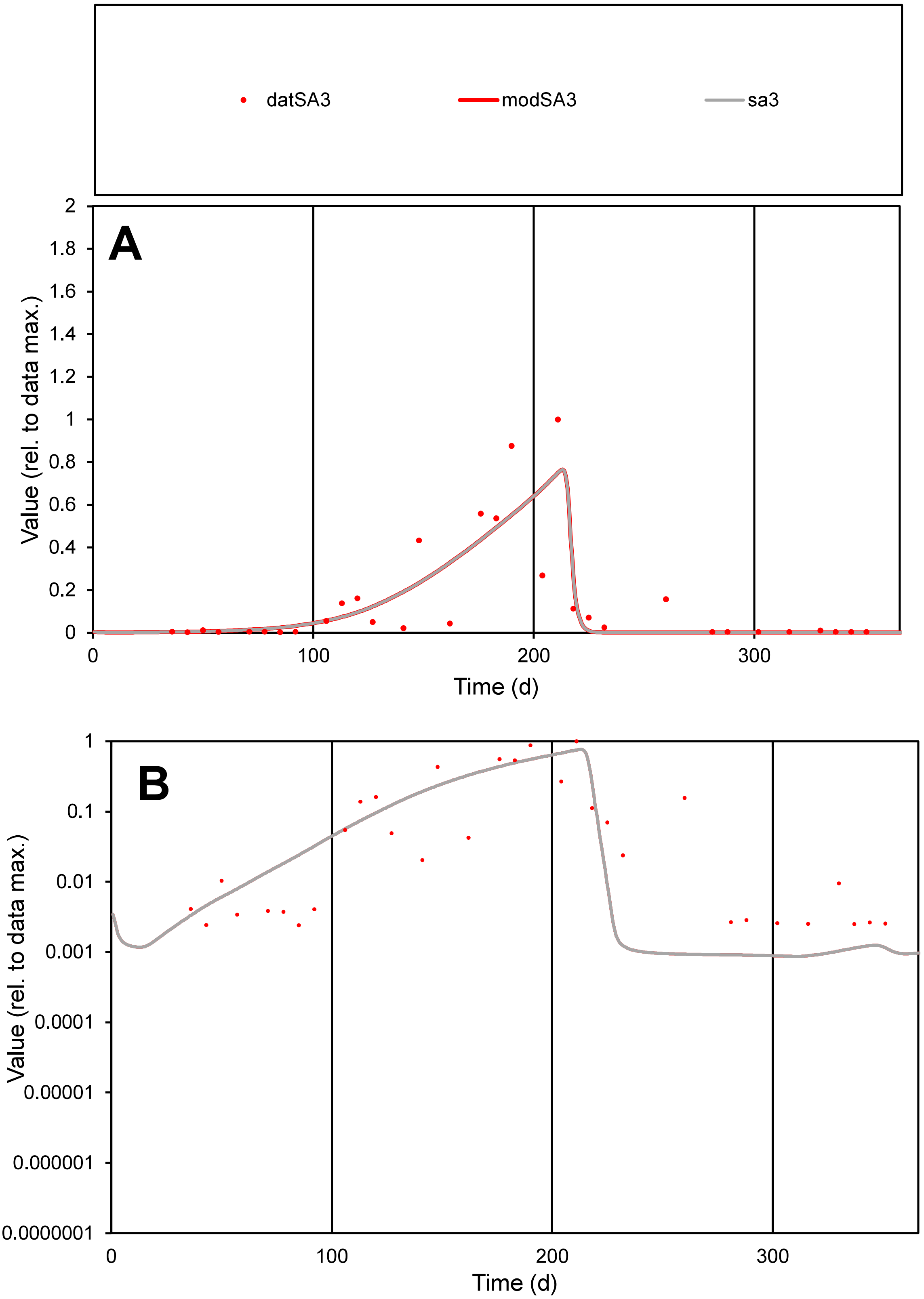

C1

Variables

SA3 Provide jtd or Dst

CalibOnly?

2 1 to reproduce of

oNormalize?

2 1=no, 2=yes

ofxn calc*

0.90053

ofxn mod

0.92959

sRun

RunA1

zout

1

Ave

Data

0.00576

Model

0.00555

data/vMax

0.04045

ofxng

321.551

Min d/m

2.74866

ave/min d

58.9588

ave/min m

155.977

324_Calib_S_A

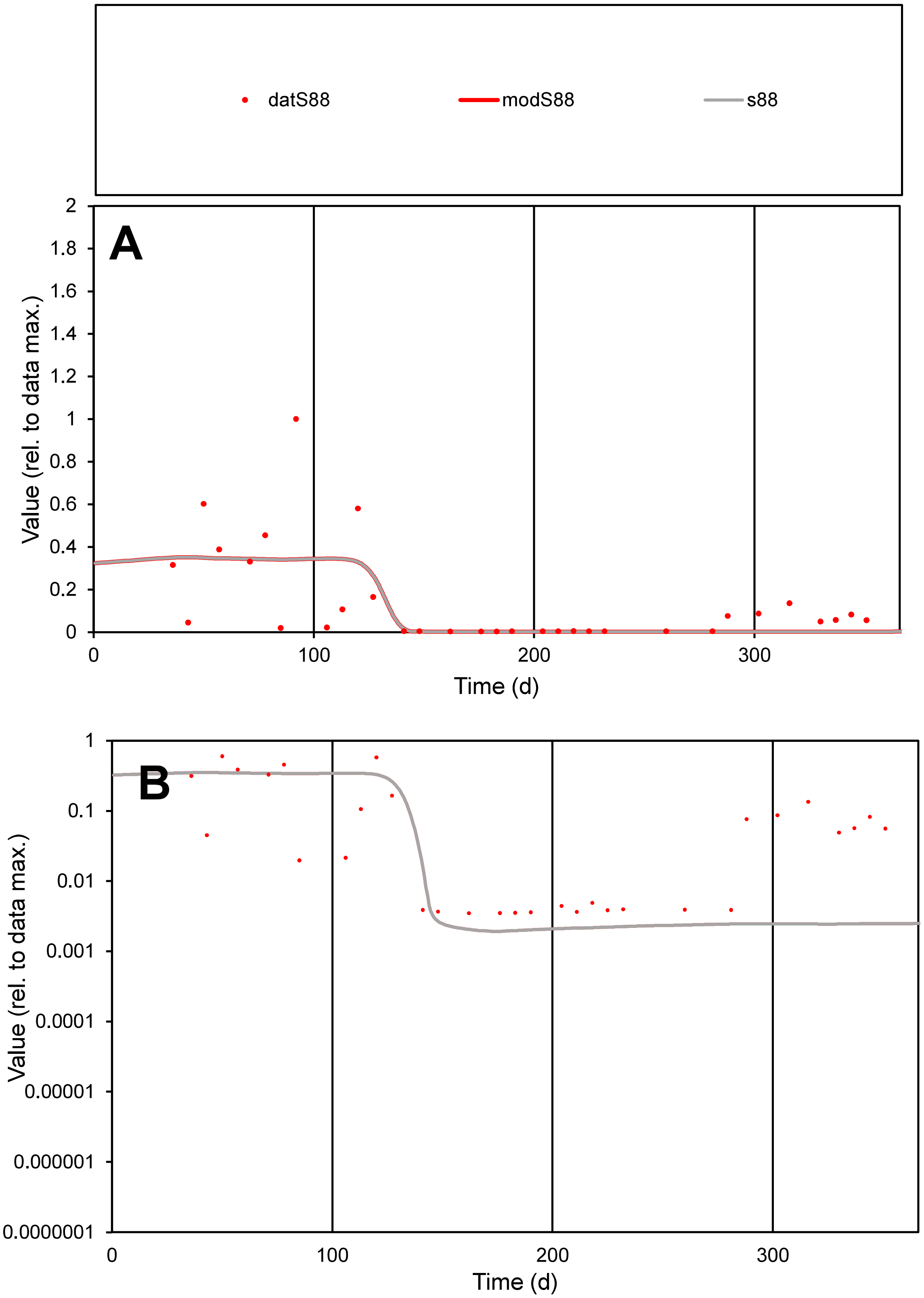

C1

Variables
S88 Provide jtd or Dst
CalibOnly?
2 1 to reproduce of
oNormalize?
2 1=no, 2=yes

ofxn calc*
1.21683
ofxn mod
1.22536

sRun
RunA1
zout
1

Ave
Data
0.00234
Model
0.00206

data/vMax
0.01619

ofxng
321.551

Min d/m
1.81166

ave/min d
41.3364
ave/min m
66.0209

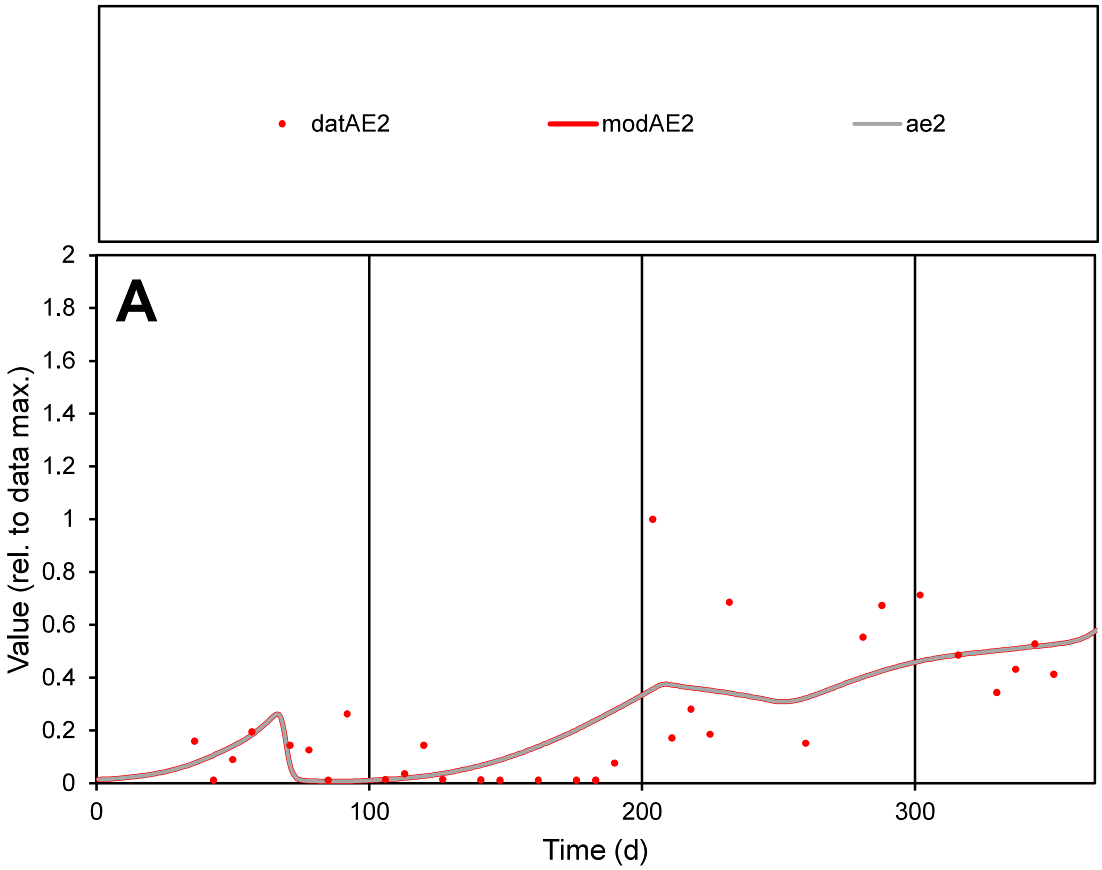

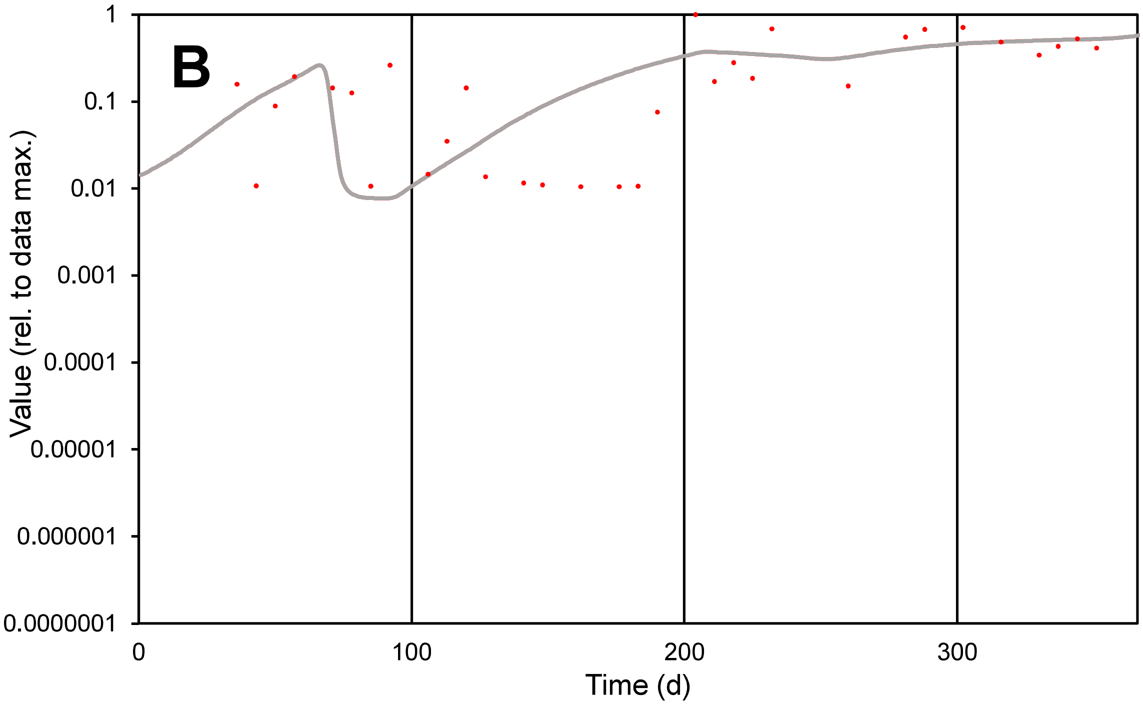

C1

Variables
AE2 Provide jtd or Dst
CalibOnly?
2 1 to reproduce of
oNormalize?
2 1=no, 2=yes

ofxn calc*
0.74254
ofxn mod
0.75404

sRun
RunA1
zout
1

Ave
Data
0.00134
Model
0.0013

data/vMax
0.0054

ofxng
321.551

Min d/m
1.36276

ave/min d
23.637
ave/min m
31.2867

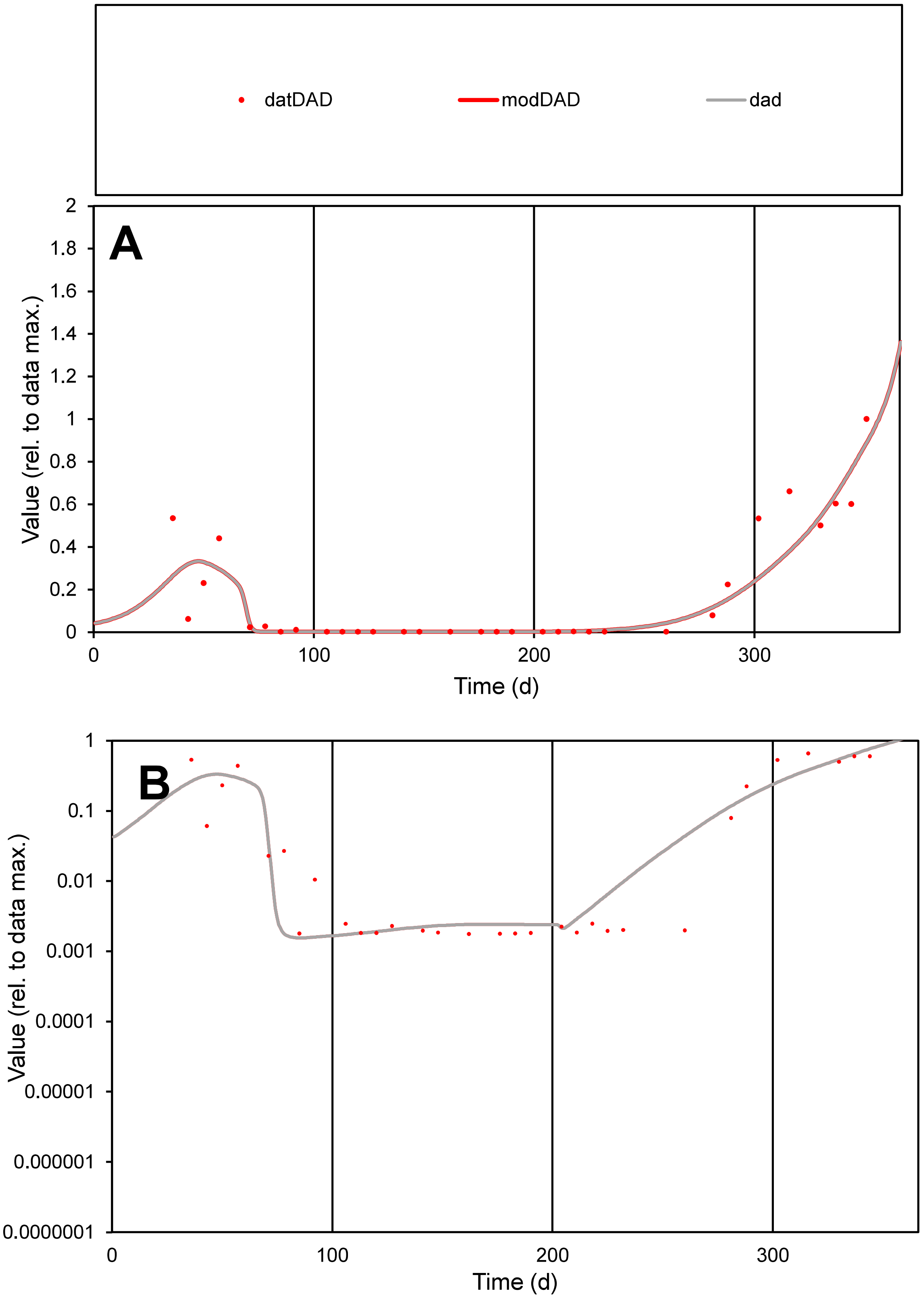

C1

Variables
DAD Provide jtd or Dst
CalibOnly?
2 1 to reproduce of
oNormalize?
2 1=no, 2=yes

ofxn calc*
0.61992
ofxn mod
0.63865

sRun
RunA1
zout
1

Ave
Data
0.00149
Model
0.00139

data/vMax
0.00855

ofxng
321.551

Min d/m
1.13835

ave/min d
98.1937
ave/min m
104.328

324_Calib_S_A

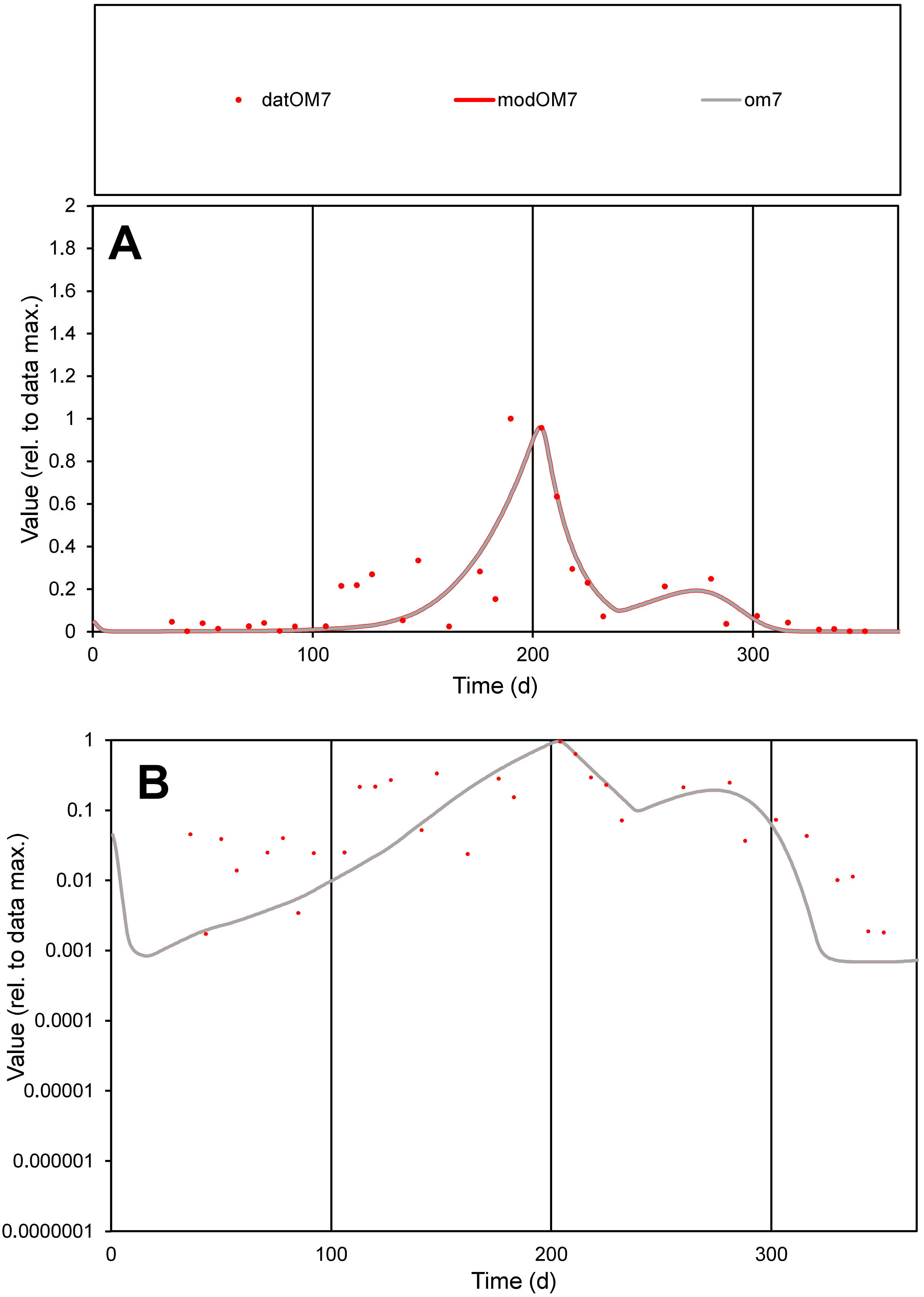

C1

Variables
OM7 Provide jtd or Dst
CalibOnly?
2 1 to reproduce of
oNormalize?
2 1=no, 2=yes

ofxn calc*
0.71317
ofxn mod
0.733

sRun
RunA1
zout
1

Ave
Data
0.00945
Model
0.00814

data/vMax
0.05409

ofxng
321.551

Min d/m
2.52616

ave/min d
100.91
ave/min m
219.453

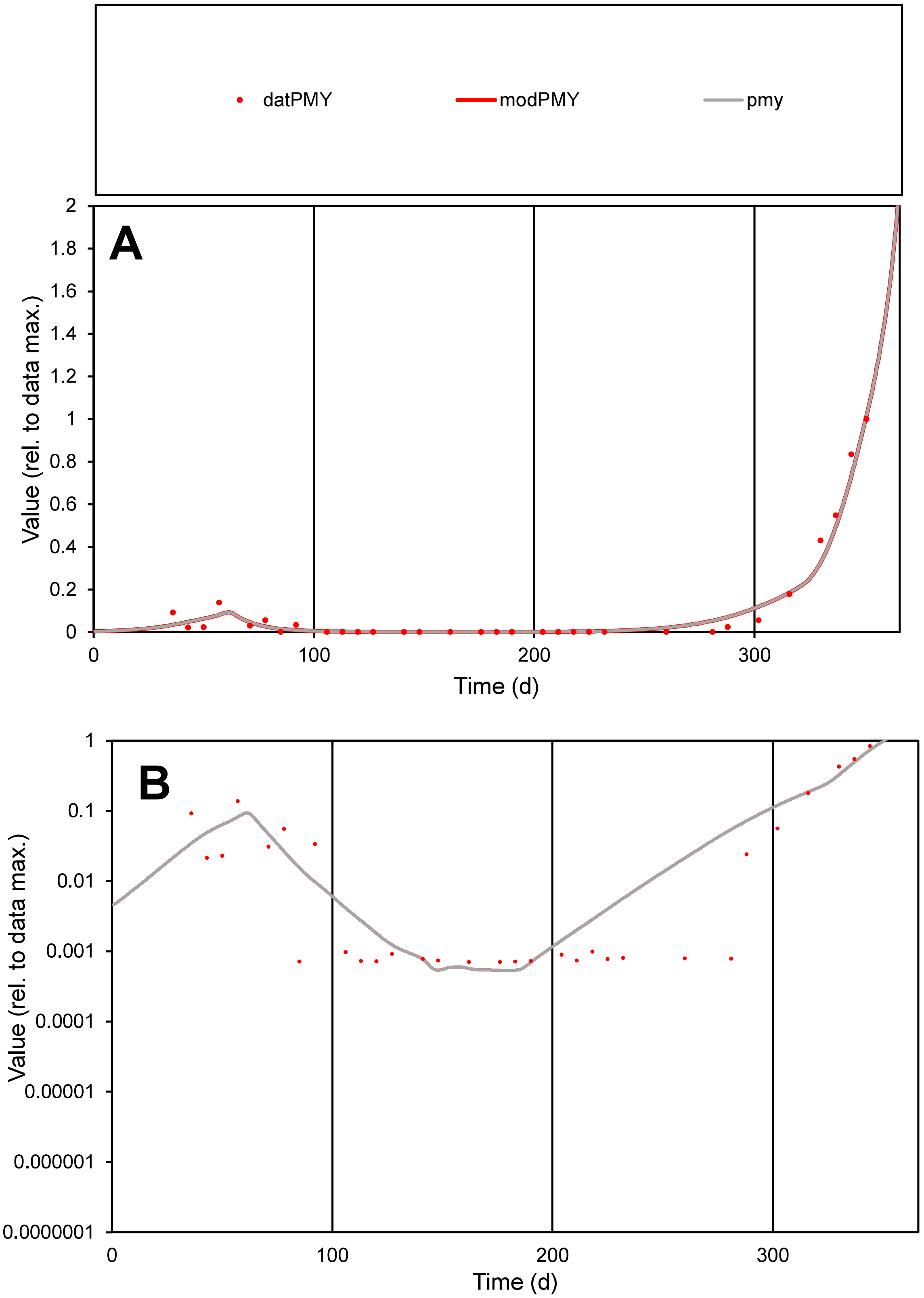

C1

Variables
PMY Provide jtd or Dst
CalibOnly?
2 1 to reproduce of
oNormalize?
2 1=no, 2=yes

ofxn calc*
0.29002
ofxn mod
0.34859

sRun
RunA1
zout
1

Ave
Data
0.00146
Model
0.00149

data/vMax
0.01338

ofxng
321.551

Min d/m
1.31699

ave/min d
154.456
ave/min m
208.027

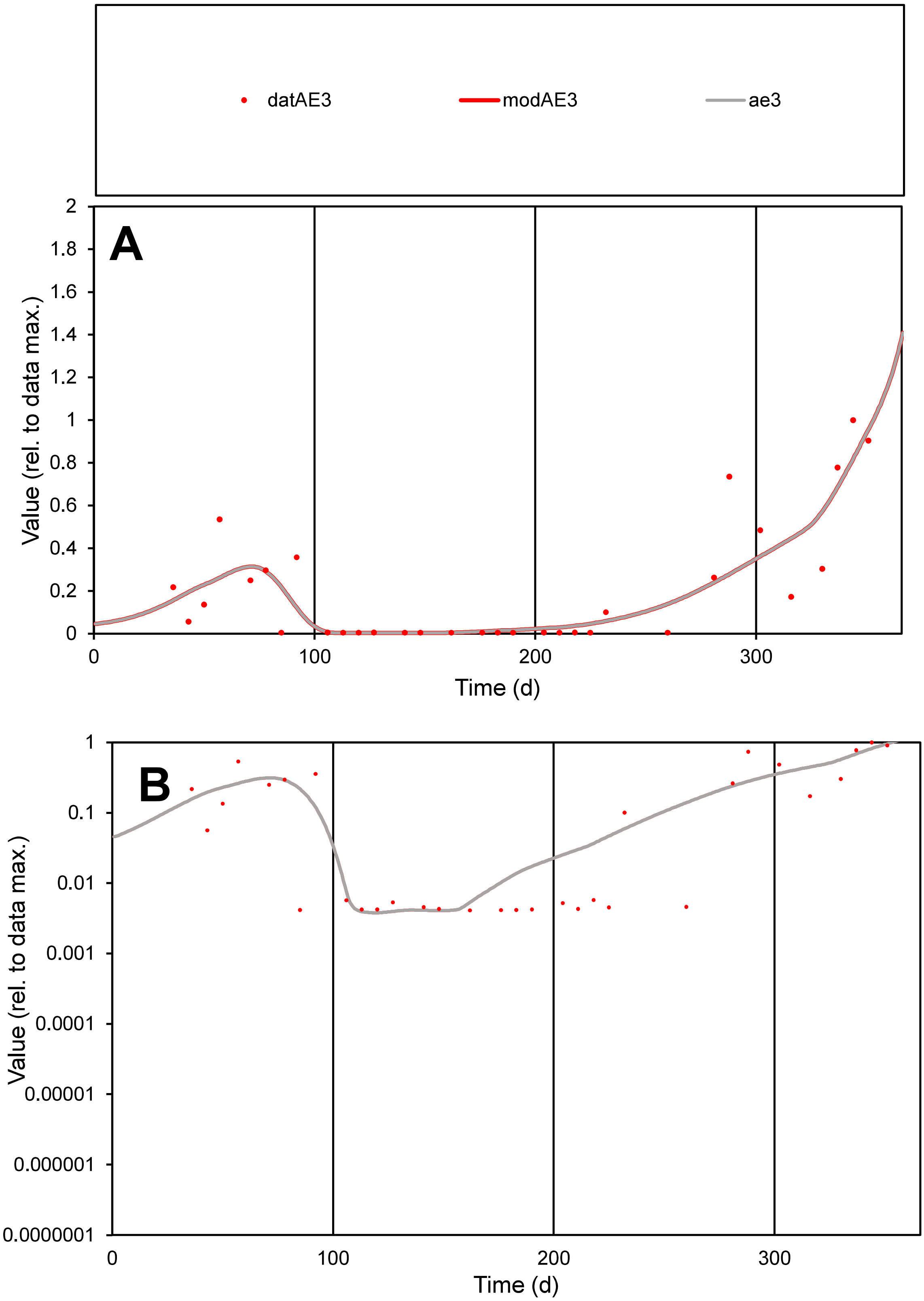

C1

Variables

AE3 Provide jtd or Dst

CalibOnly?

2 1 to reproduce of

oNormalize?

2 1=no, 2=yes

ofxn calc*

0.66888

ofxn mod

0.69003

sRun

RunA1

zout

1

Ave

Data

0.00084

Model

0.00083

data/vMax

0.00402

ofxng

321.551

Min d/m

1.08055

ave/min d

50.6894

ave/min m

54.5212

324_Calib_S_A

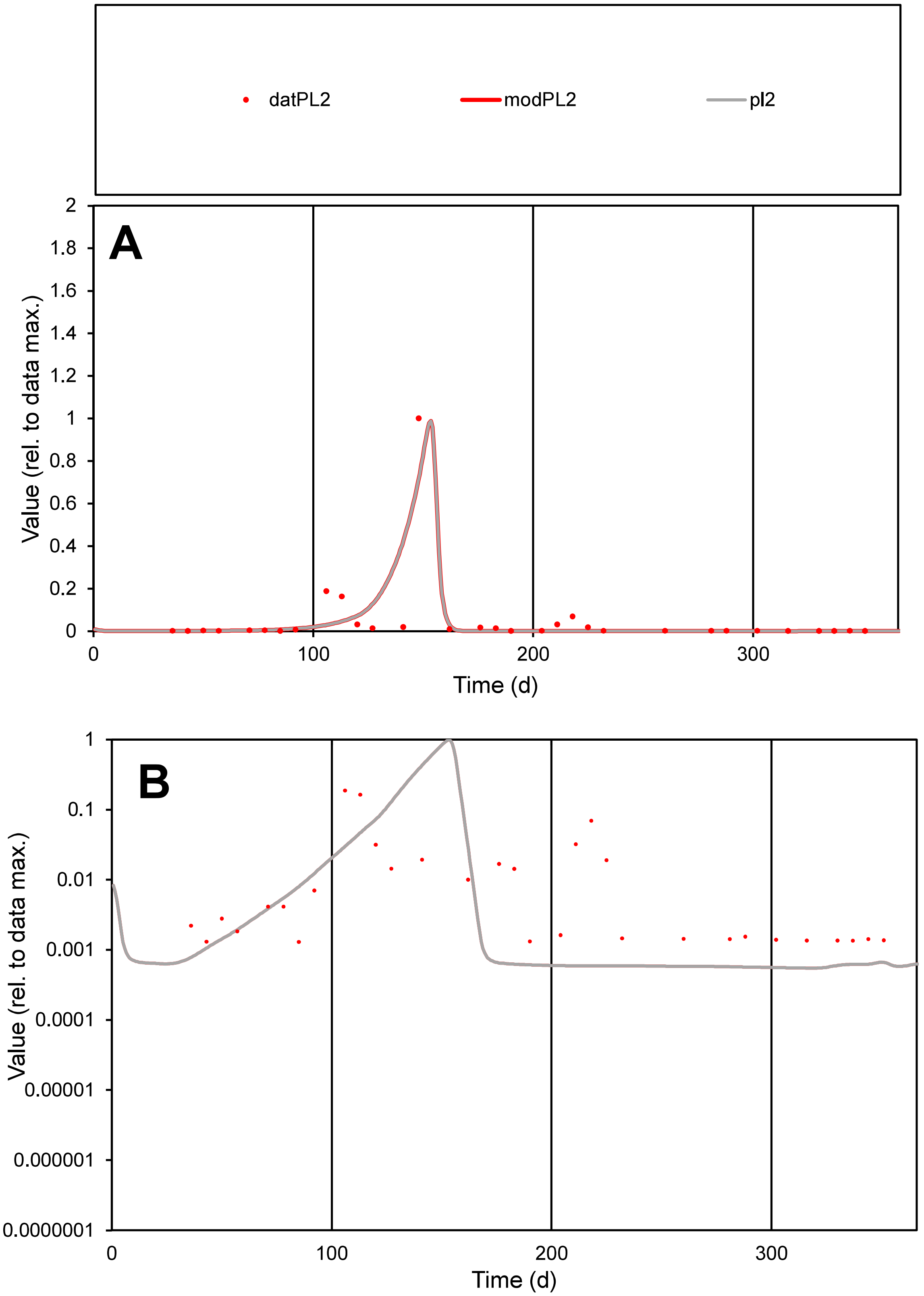

C1

Variables
PL2 Provide jtd or Dst
CalibOnly?
2 1 to reproduce of
oNormalize?
2 1=no, 2=yes

ofxn calc*
1.75122
ofxn mod
1.8192

sRun
RunA1
zout
1

Ave
Data
0.0063
Model
0.00587

data/vMax
0.1245

ofxng
321.551

Min d/m
2.35953

ave/min d
38.8893
ave/min m
85.3875

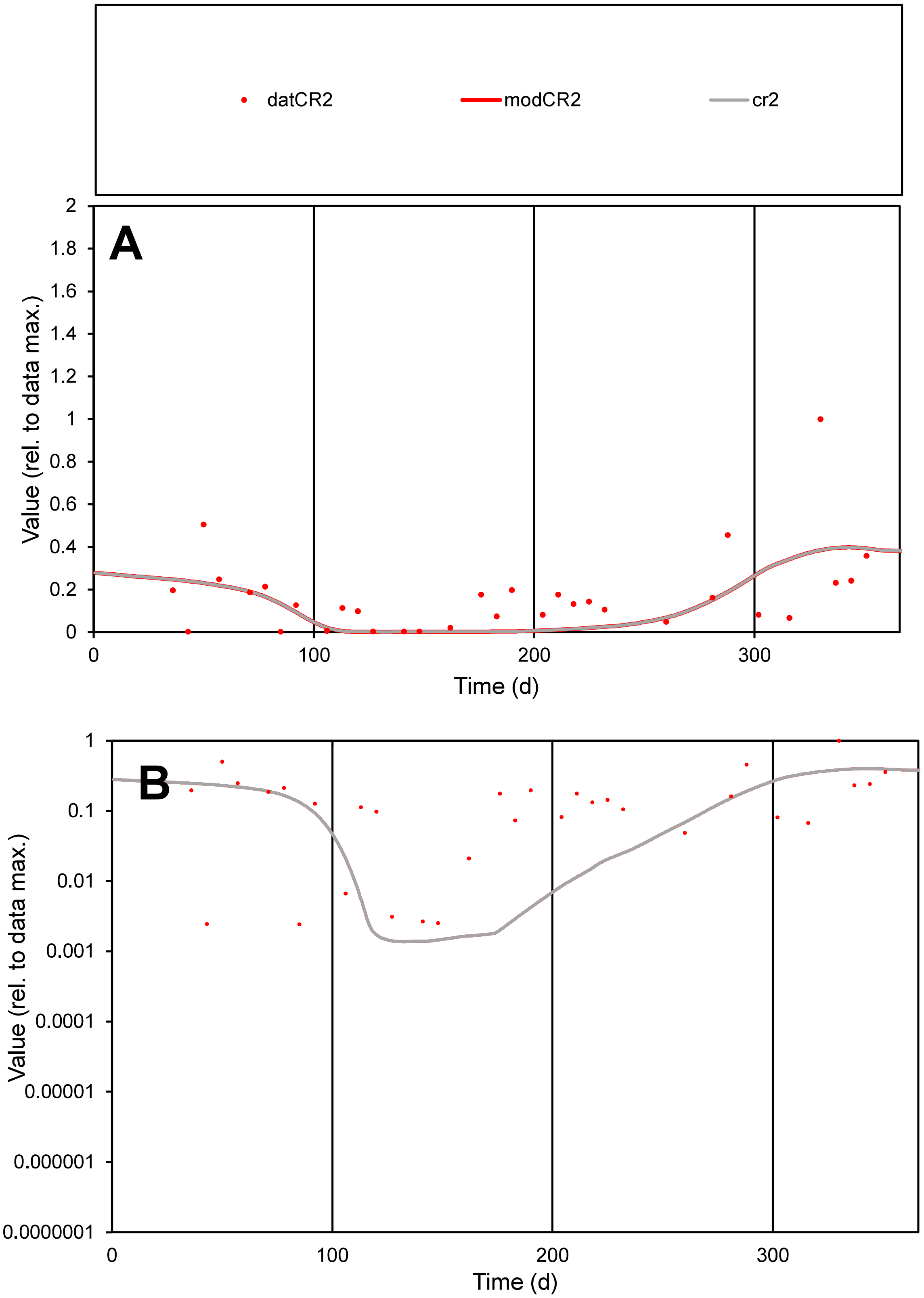

C1

Variables
CR2 Provide jtd or Dst
CalibOnly?
2 1 to reproduce of
oNormalize?
2 1=no, 2=yes

ofxn calc*
0.99631
ofxn mod
1.00682

sRun
RunA1
zout
1

Ave
Data
0.00279
Model
0.00216

data/vMax
0.01633

ofxng
321.551

Min d/m
1.7409

ave/min d
70.1737
ave/min m
94.8661

324_Calib_S_A

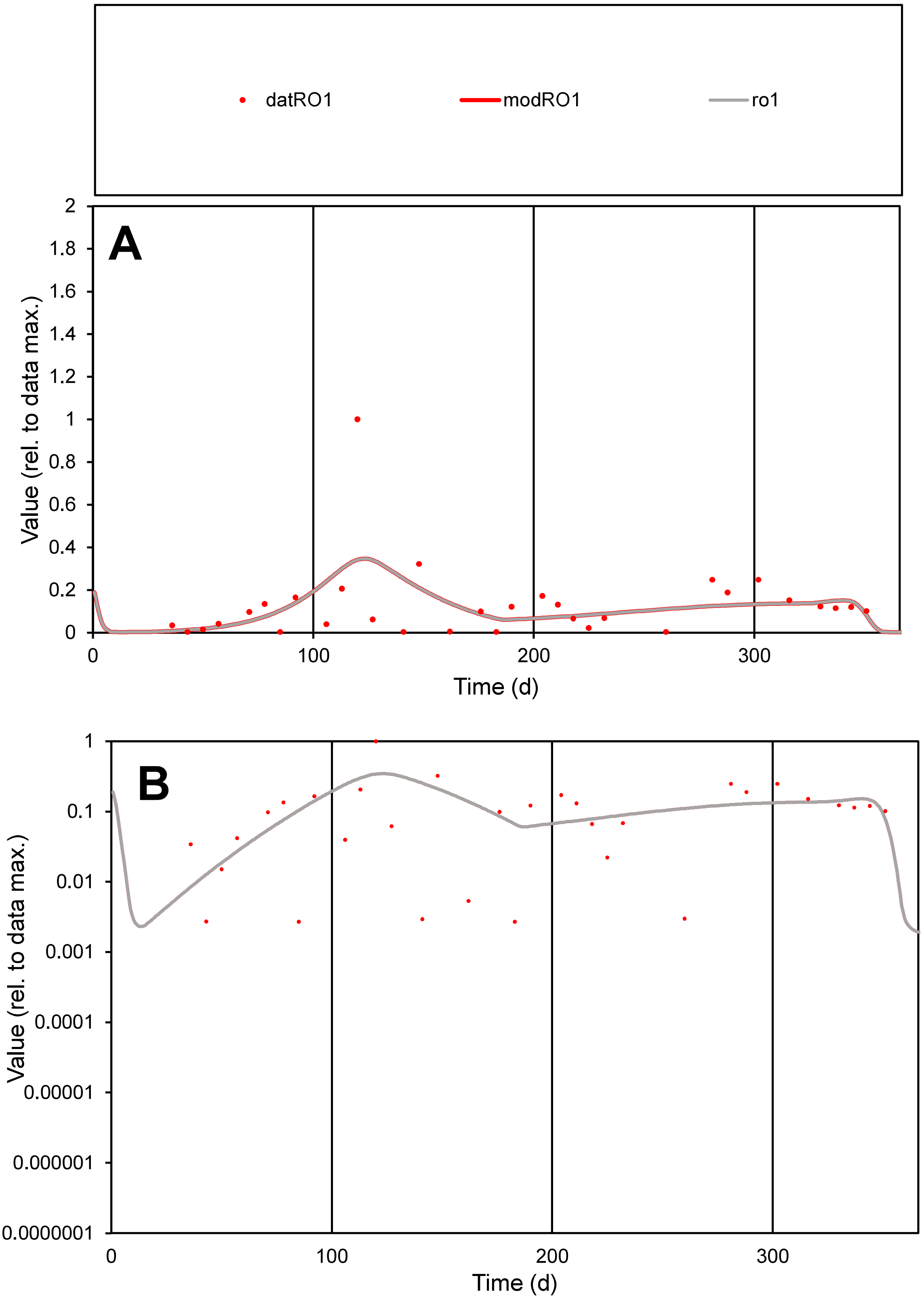

C1

Variables

RO1 Provide jtd or Dst

CalibOnly?

2 1 to reproduce of

oNormalize?

2 1=no, 2=yes

ofxn calc*

1.17585

ofxn mod

1.18367

sRun

RunA1

zout

1

Ave

Data

0.00168

Model

0.00164

data/vMax

0.01306

ofxng

321.551

Min d/m

0.30009

ave/min d

47.6311

ave/min m

13.9746

324_Calib_S_A

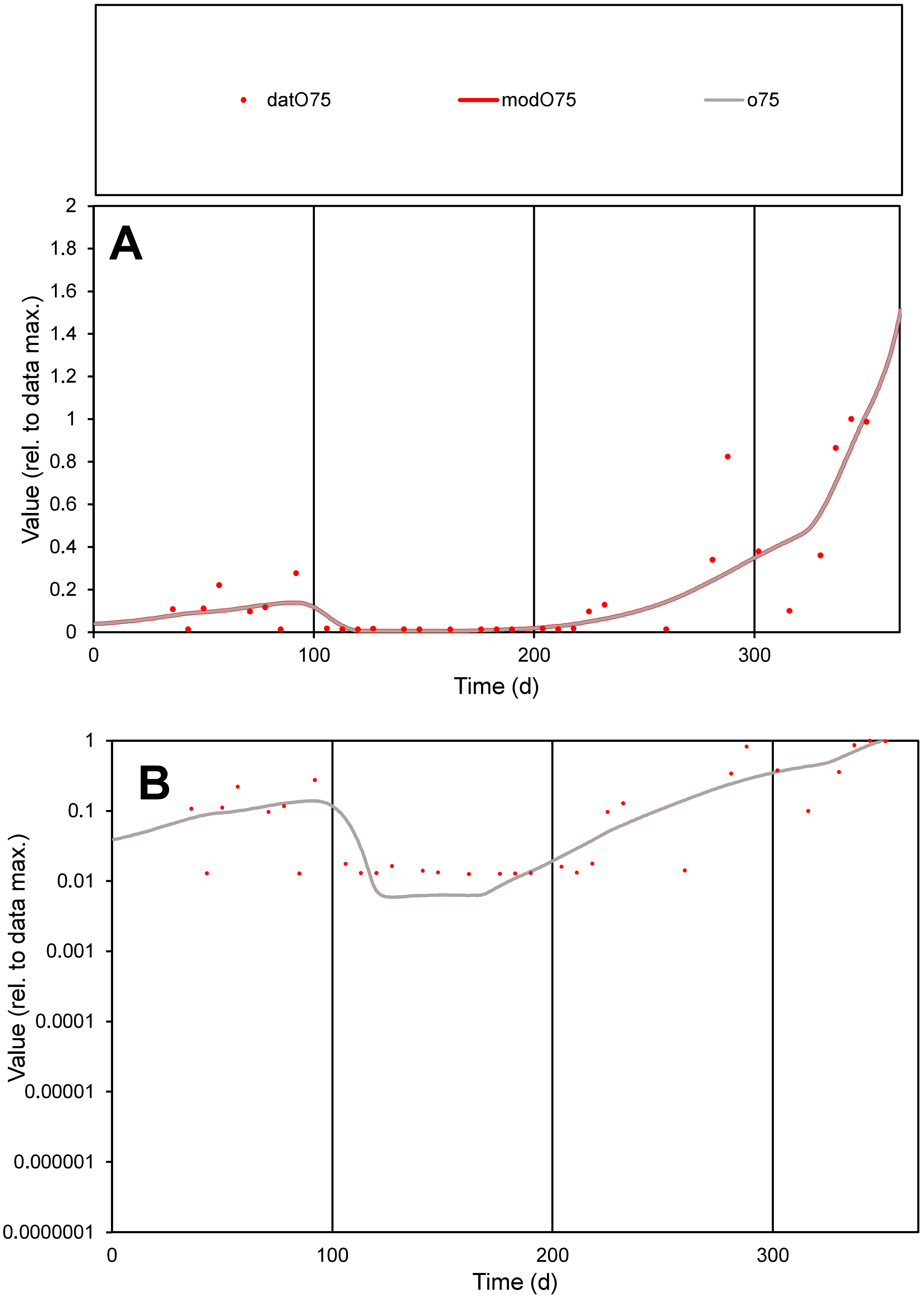

C1

Variables
O75 Provide jtd or Dst
CalibOnly?
2 1 to reproduce of
oNormalize?
2 1=no, 2=yes

ofxn calc*
0.67809
ofxn mod
0.69878

sRun
RunA1
zout
1

Ave
Data
0.00106
Model
0.00101

data/vMax
0.00543

ofxng
321.551

Min d/m
2.15

ave/min d
15.3945
ave/min m
31.6589

324_Calib_S_A

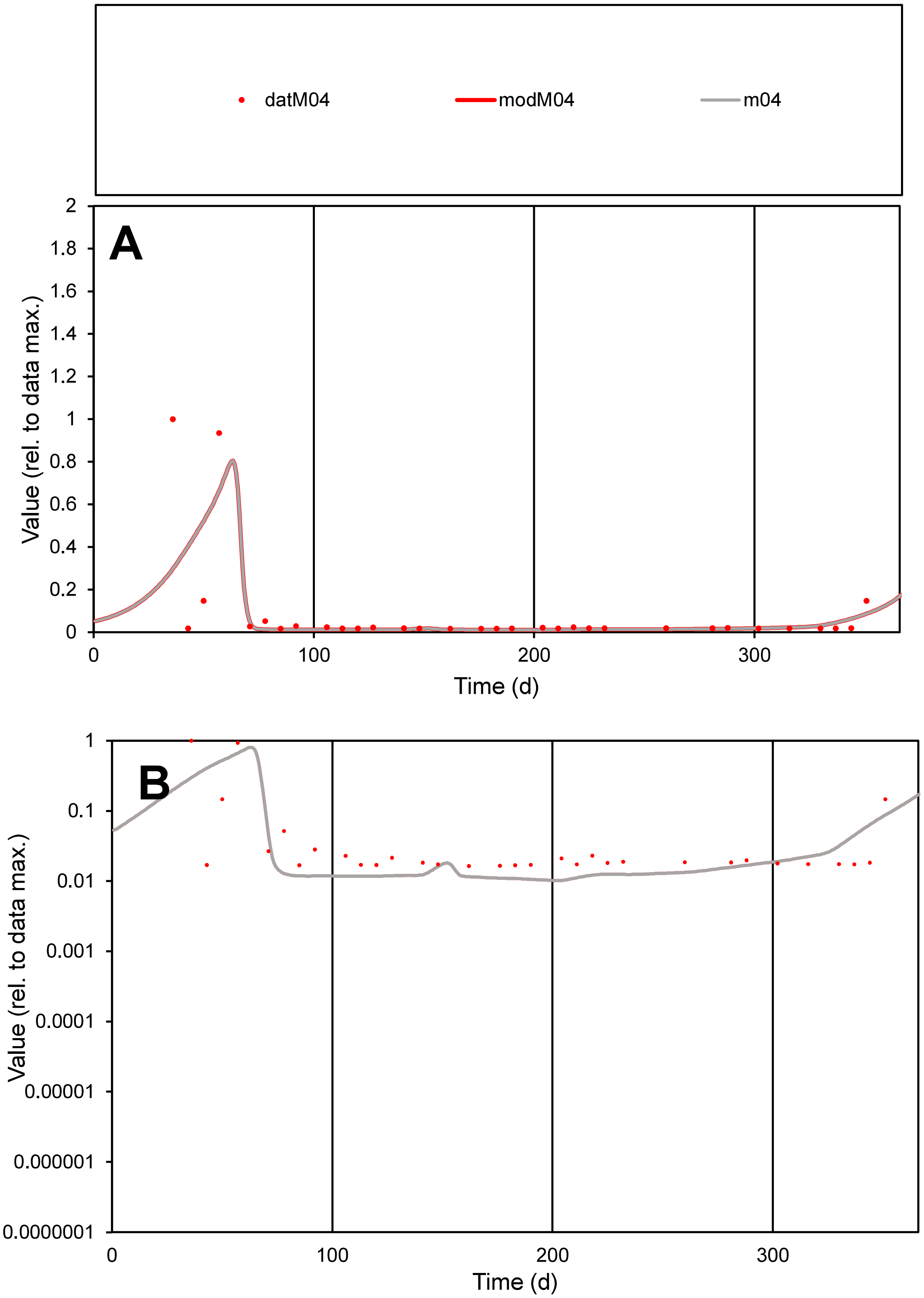

C1

Variables
M04 Provide jtd or Dst
CalibOnly?
2 1 to reproduce of
oNormalize?
2 1=no, 2=yes

ofxn calc*
1.8358
ofxn mod
1.86333

sRun
RunA1
zout
1

Ave
Data
0.00018
Model
0.00016

data/vMax
0.0021

ofxng
321.551

Min d/m
1.60259

ave/min d
5.29342
ave/min m
7.55823

324_Calib_S_A

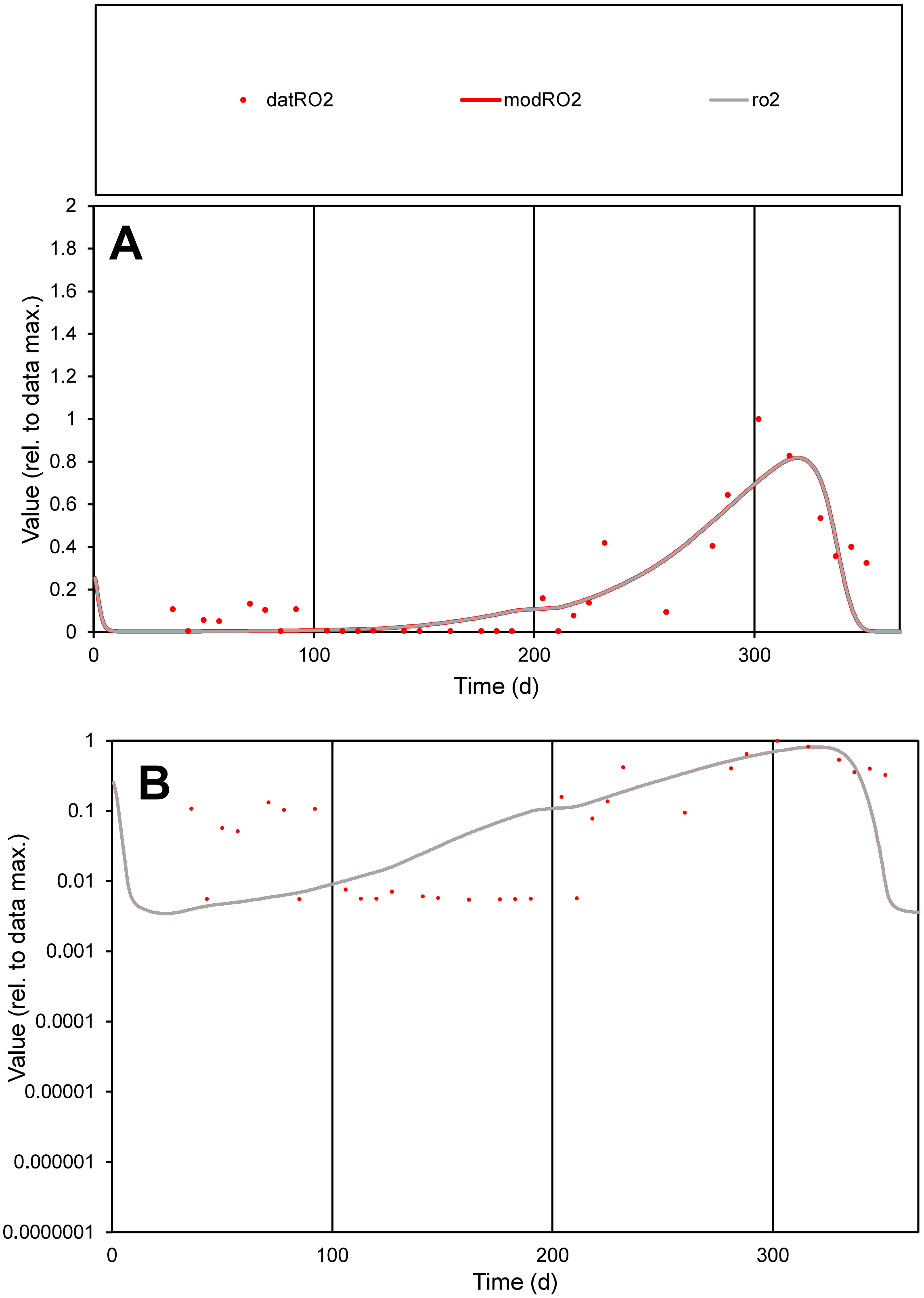

C1

Variables
RO2 Provide jtd or Dst
CalibOnly?
2 1 to reproduce of
oNormalize?
2 1=no, 2=yes

ofxn calc*
0.65504
ofxn mod
0.68549

sRun
RunA1
zout
1

Ave
Data
0.0013
Model
0.00116

data/vMax
0.0069

ofxng
321.551

Min d/m
1.35113

ave/min d
34.4269
ave/min m
41.5831

324_Calib_S_A

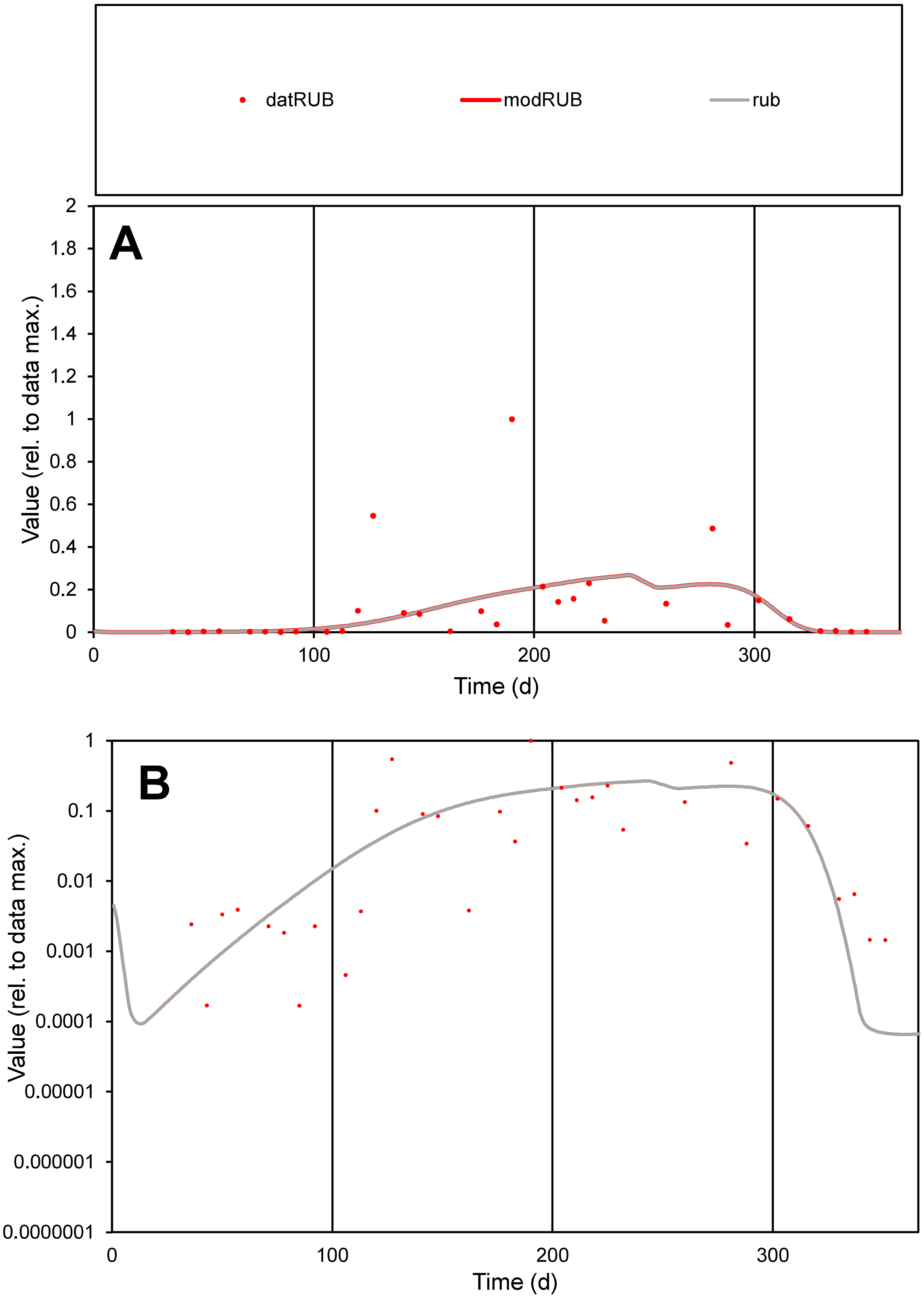

C1

Variables
RUB Provide jtd or Dst
CalibOnly?
2 1 to reproduce of
oNormalize?
2 1=no, 2=yes

ofxn calc*
1.62803
ofxn mod
1.63447

sRun
RunA1
zout
1

Ave
Data
0.00674
Model
0.00567

data/vMax
0.05902

ofxng
321.551

Min d/m
2.48597

ave/min d
679.131
ave/min m
1419.78

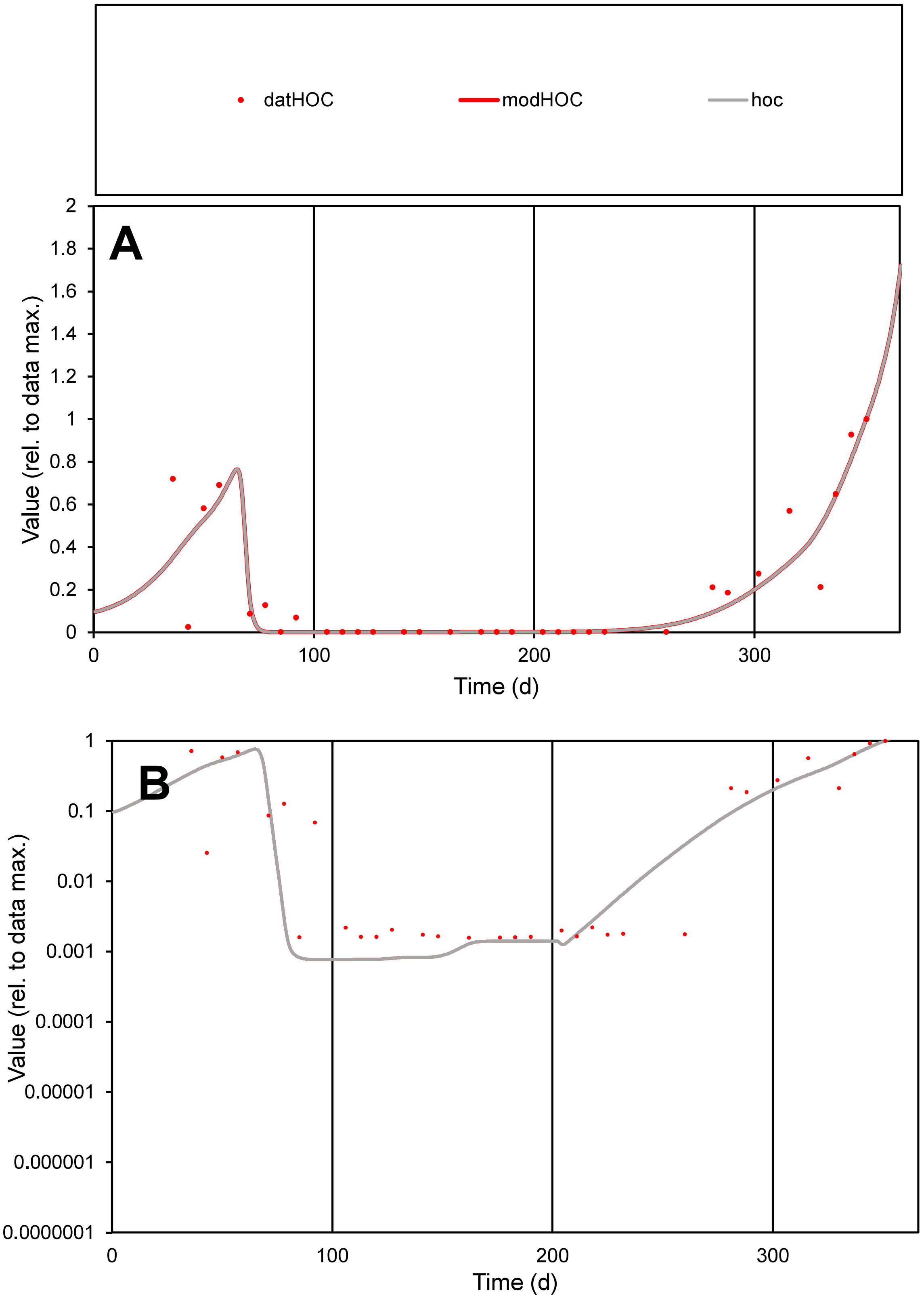

C1

Variables
HOC Provide jtd or Dst
CalibOnly?
2 1 to reproduce of
oNormalize?
2 1=no, 2=yes

ofxn calc*
0.61514
ofxn mod
0.64457

sRun
RunA1
zout
1

Ave
Data
0.00156
Model
0.00147

data/vMax
0.00783

ofxng
321.551

Min d/m
2.03967

ave/min d
126.306
ave/min m
242.483

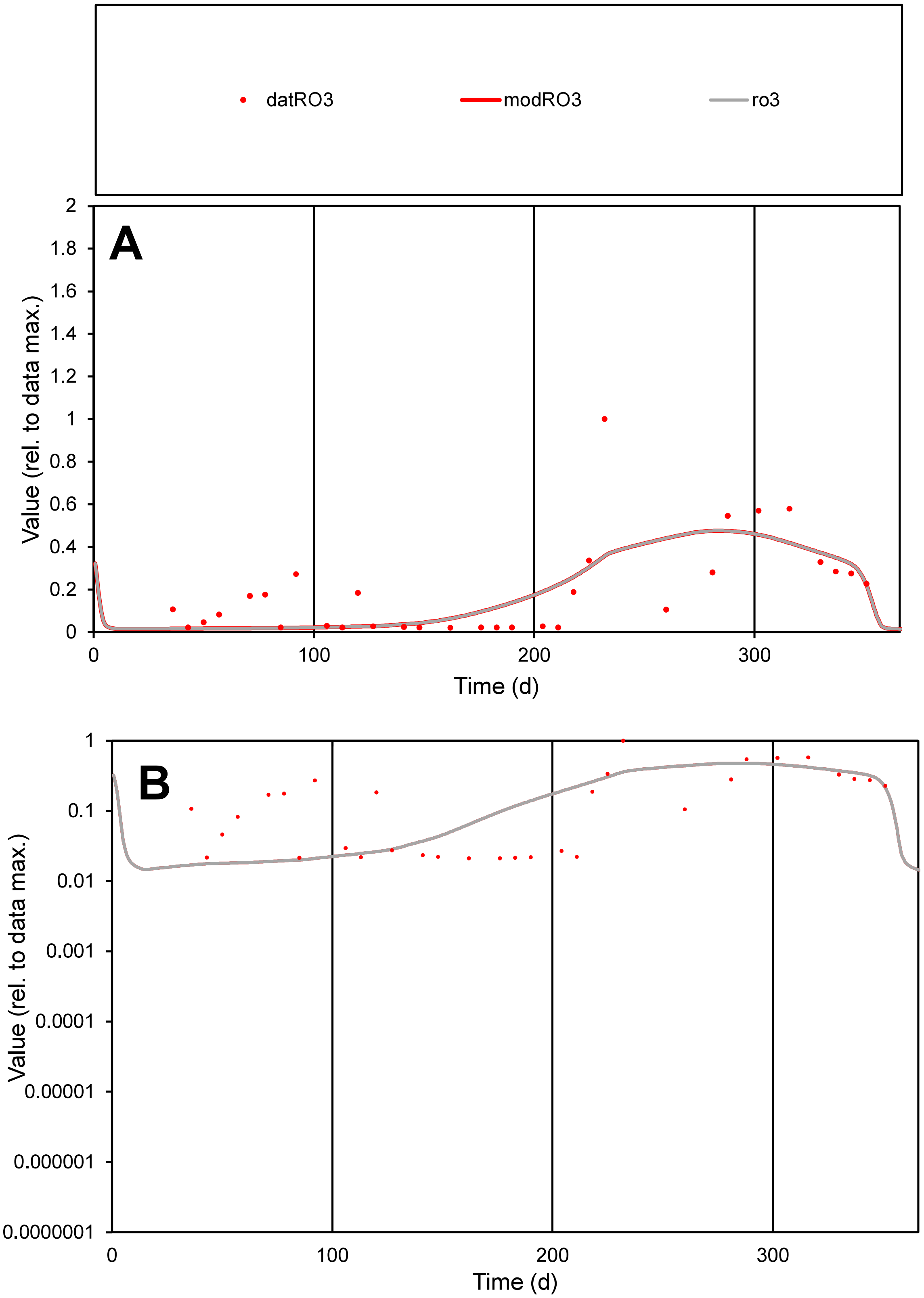

C1

Variables
RO3 Provide jtd or Dst
CalibOnly?
2 1 to reproduce of
oNormalize?
2 1=no, 2=yes

ofxn calc*
0.86622
ofxn mod
0.87671

sRun
RunA1
zout
1

Ave
Data
0.00084
Model
0.00078

data/vMax
0.00445

ofxng
321.551

Min d/m
1.23581

ave/min d
8.9451
ave/min m
10.2029

324_Calib_S_A

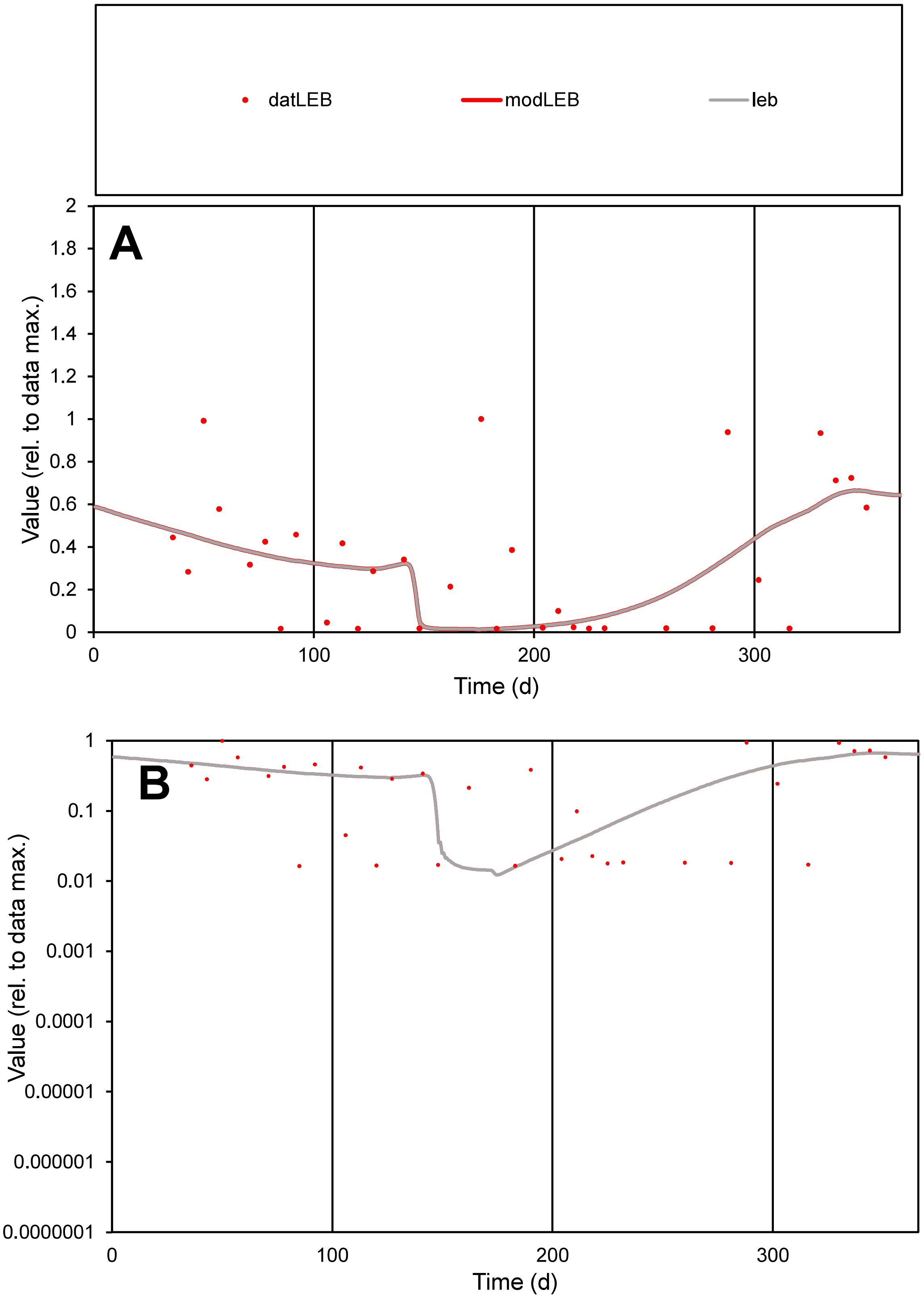

C1

Variables

LEB Provide jtd or Dst

CalibOnly?

2 1 to reproduce of

oNormalize?

2 1=no, 2=yes

ofxn calc*

0.87421

ofxn mod

0.88256

sRun

RunA1

zout

1

Ave

Data

0.0014

Model

0.00125

data/vMax

0.00422

ofxng

321.551

Min d/m

1.26318

ave/min d

20.1429

ave/min m

22.7786

324_Calib_S_A

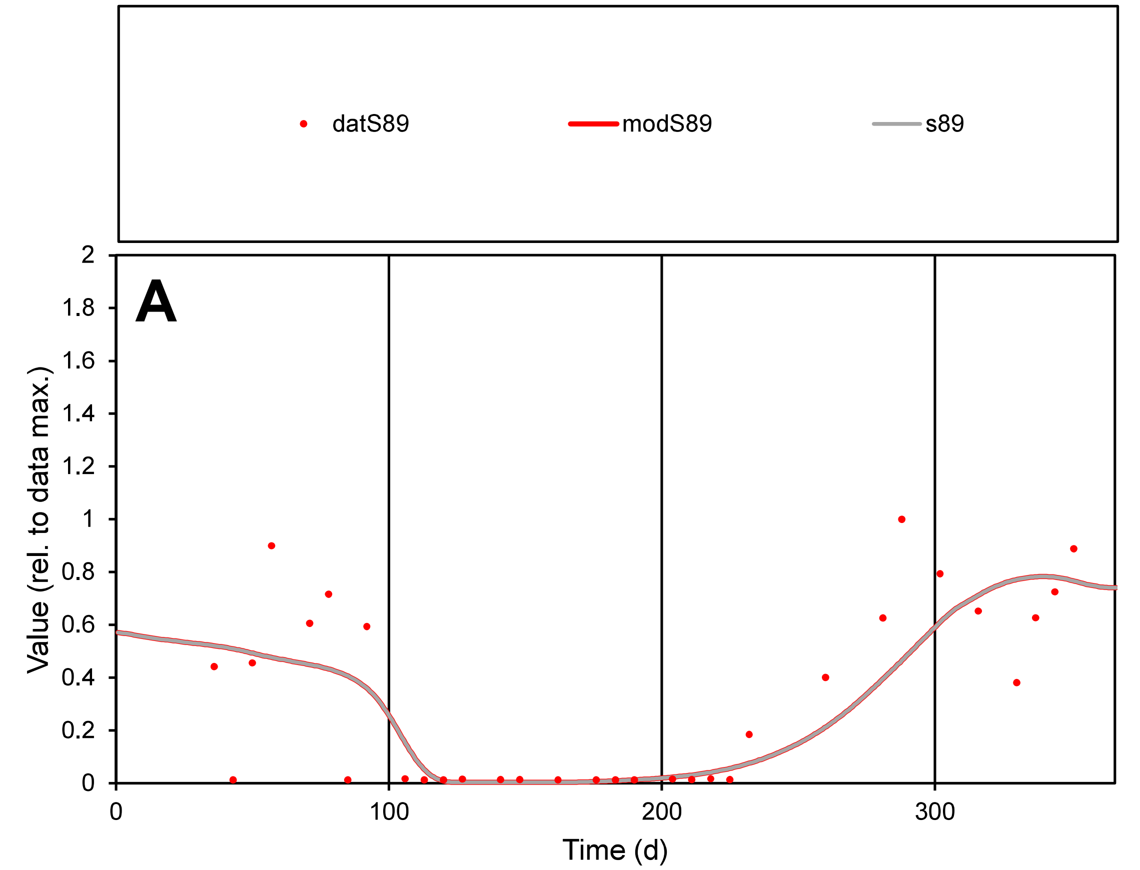

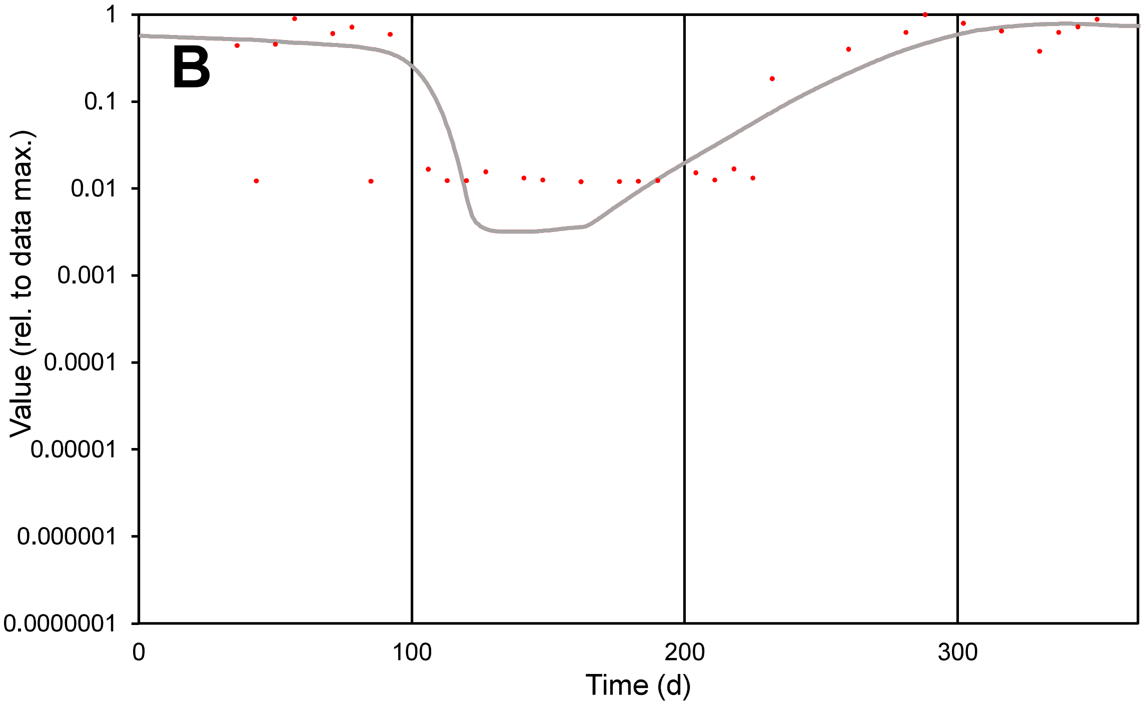

C1

Variables
S89 Provide jtd or Dst
CalibOnly?
2 1 to reproduce of
oNormalize?
2 1=no, 2=yes

ofxn calc*
0.64501
ofxn mod
0.65686

sRun
RunA1
zout
1

Ave
Data
0.00094
Model
0.00088

data/vMax
0.00295

ofxng
321.551

Min d/m
3.75704

ave/min d
26.6158
ave/min m
93.9281

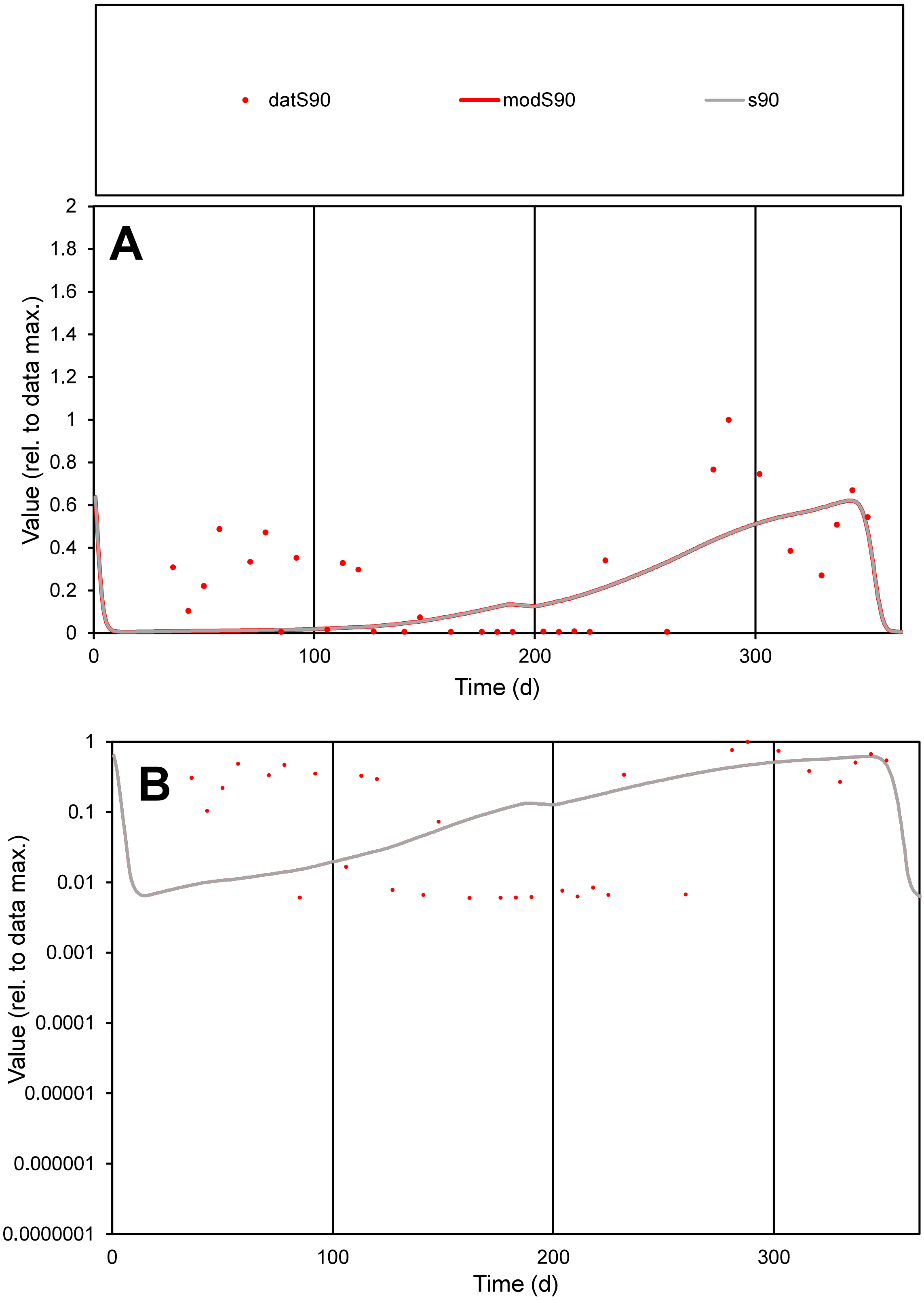

C1

Variables
S90 Provide jtd or Dst
CalibOnly?
2 1 to reproduce of
oNormalize?
2 1=no, 2=yes

ofxn calc*
0.91971
ofxn mod
0.92672

sRun
RunA1
zout
1

Ave
Data
0.00084
Model
0.00063

data/vMax
0.00324

ofxng
321.551

Min d/m
0.64585

ave/min d
43.0877
ave/min m
20.7562

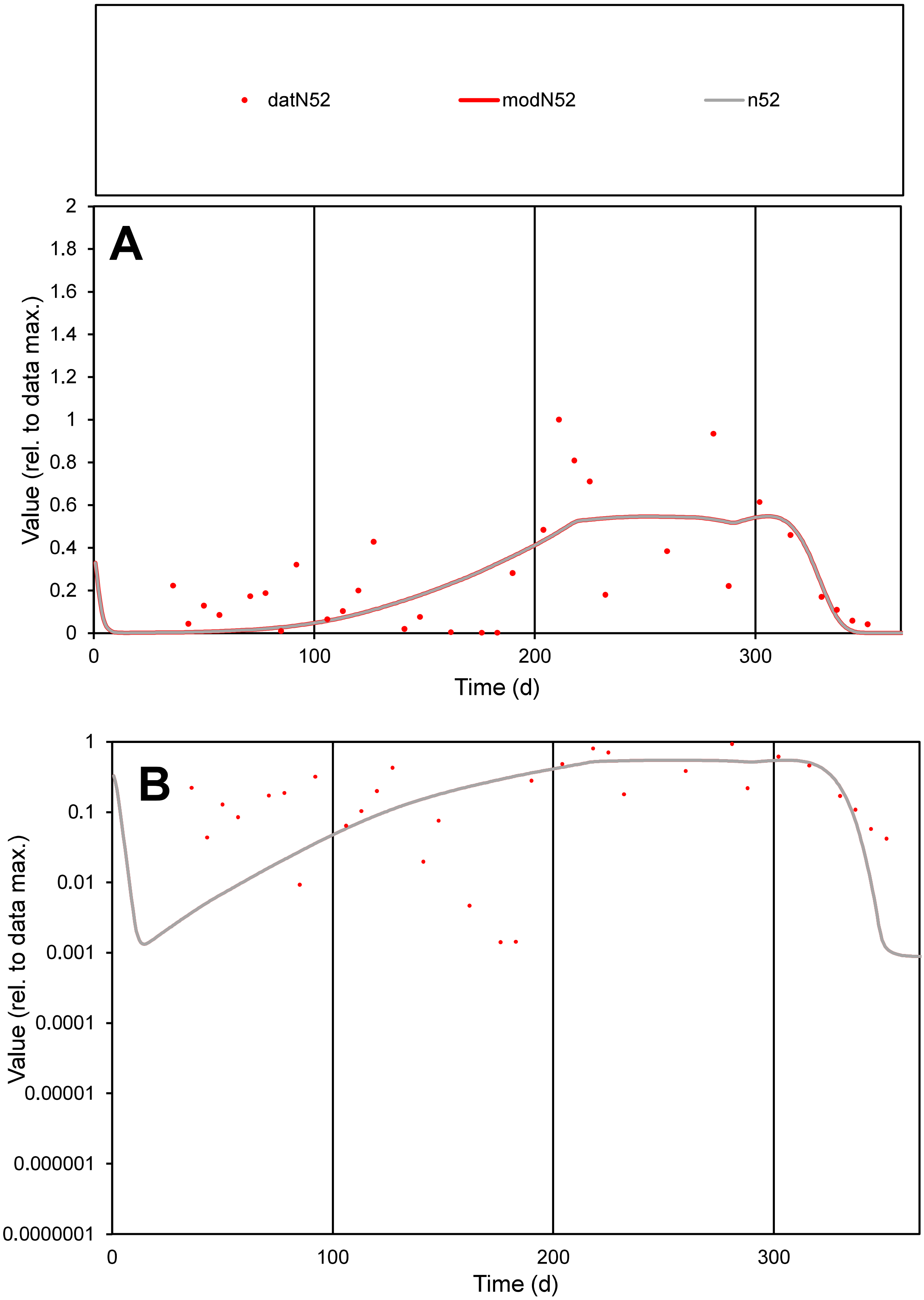

C1

Variables
N52 Provide jtd or Dst
CalibOnly?
2 1 to reproduce of
oNormalize?
2 1=no, 2=yes

ofxn calc*
0.78216
ofxn mod
0.79324

sRun
RunA1
zout
1

Ave
Data
0.00444
Model
0.00389

data/vMax
0.01667

ofxng
321.551

Min d/m
1.29841

ave/min d
187.552
ave/min m
213.153

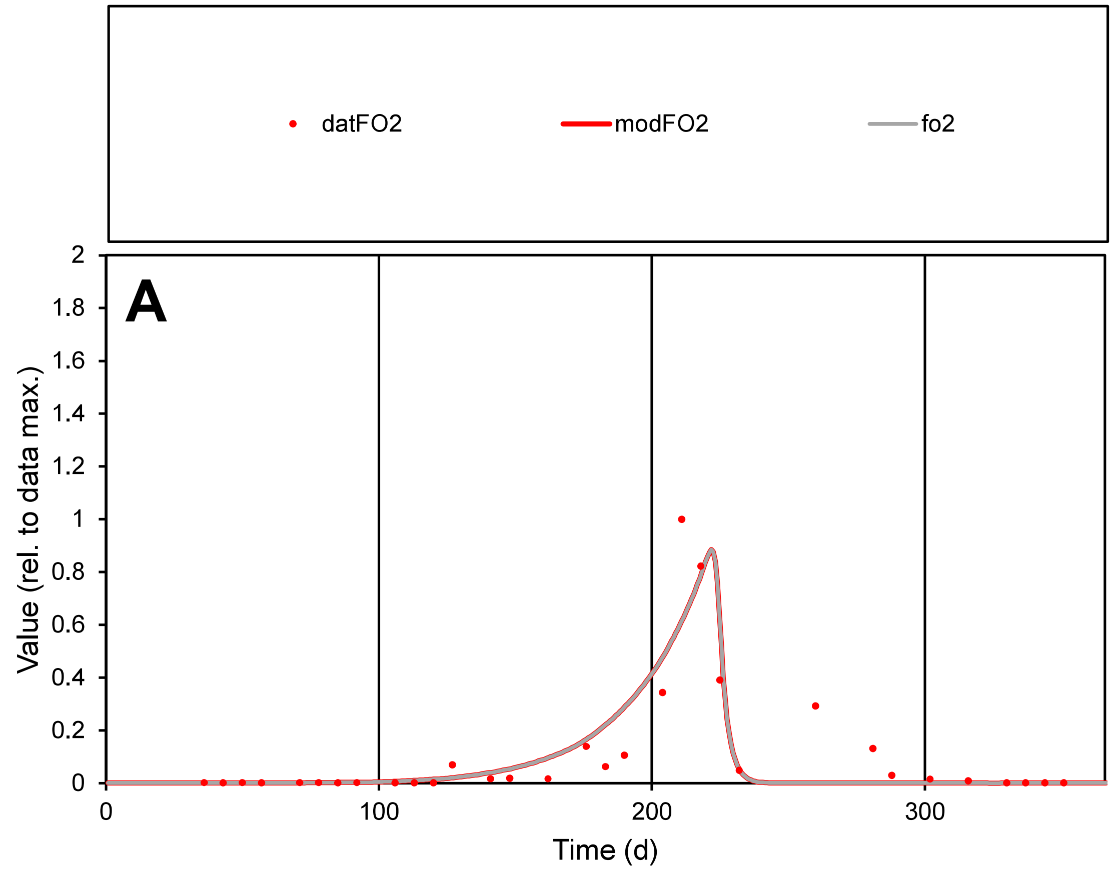

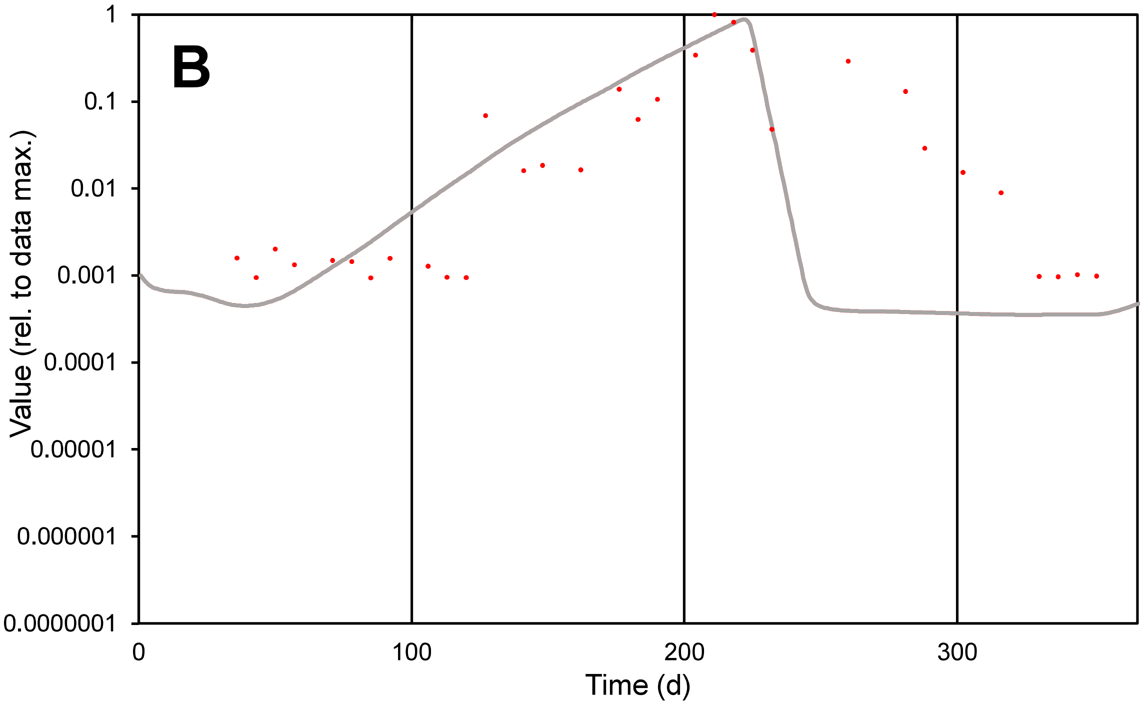

C1

Variables
FO2 Provide jtd or Dst
CalibOnly?
2 1 to reproduce of
oNormalize?
2 1=no, 2=yes

ofxn calc*
0.91725
ofxn mod
0.95431

sRun
RunA1
zout
1

Ave
Data
0.00786
Model
0.00753

data/vMax
0.07133

ofxng
321.551

Min d/m
2.65317

ave/min d
117.545
ave/min m
298.795

324_Calib_S_A

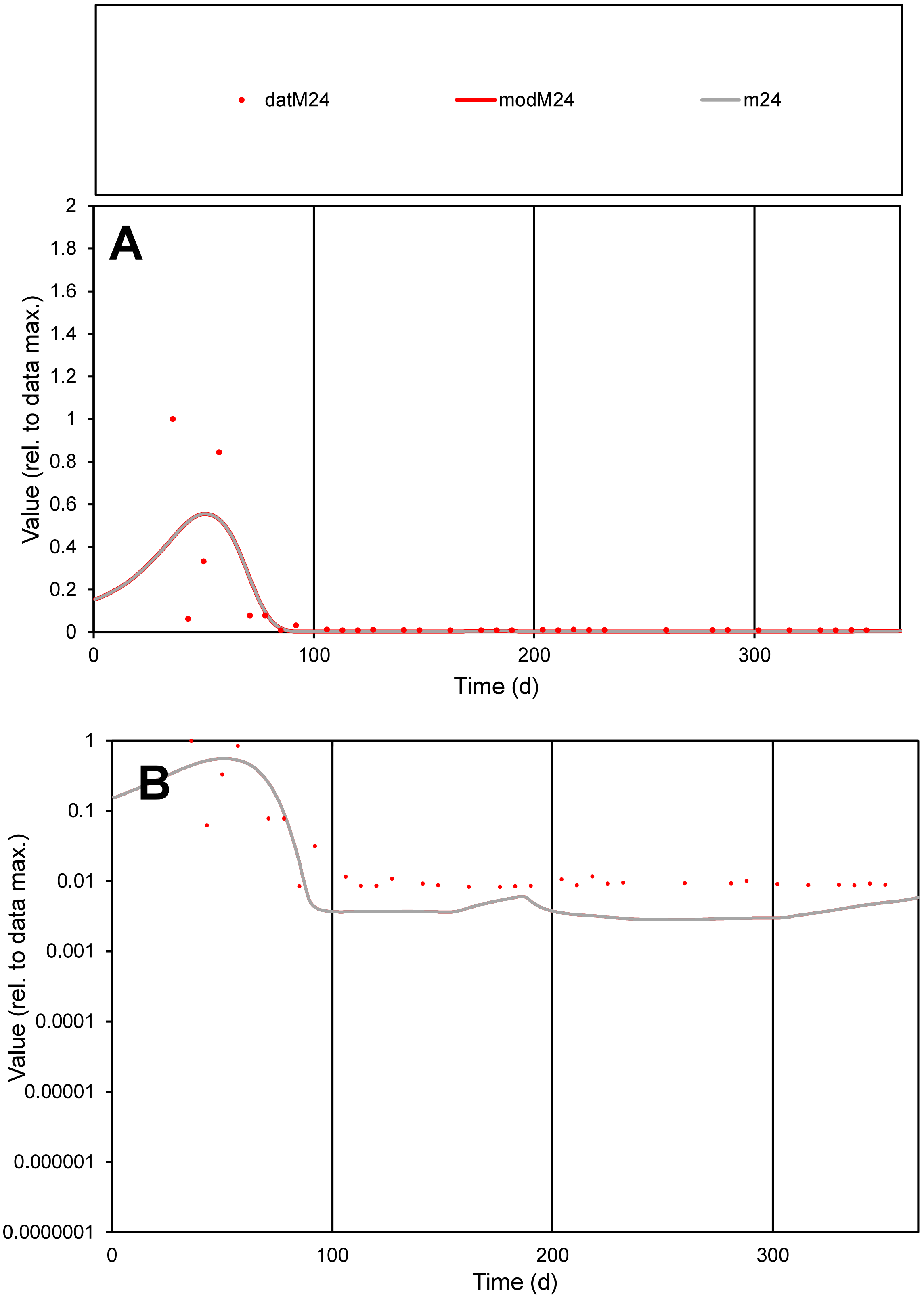

C1

Variables
M24 Provide jtd or Dst
CalibOnly?
2 1 to reproduce of
oNormalize?
2 1=no, 2=yes

ofxn calc*
1.75155
ofxn mod
1.77155

sRun
RunA1
zout
1

Ave
Data
0.00071
Model
0.00064

data/vMax
0.0085

ofxng
321.551

Min d/m
2.95411

ave/min d
9.97386
ave/min m
26.8905

324_Calib_S_A

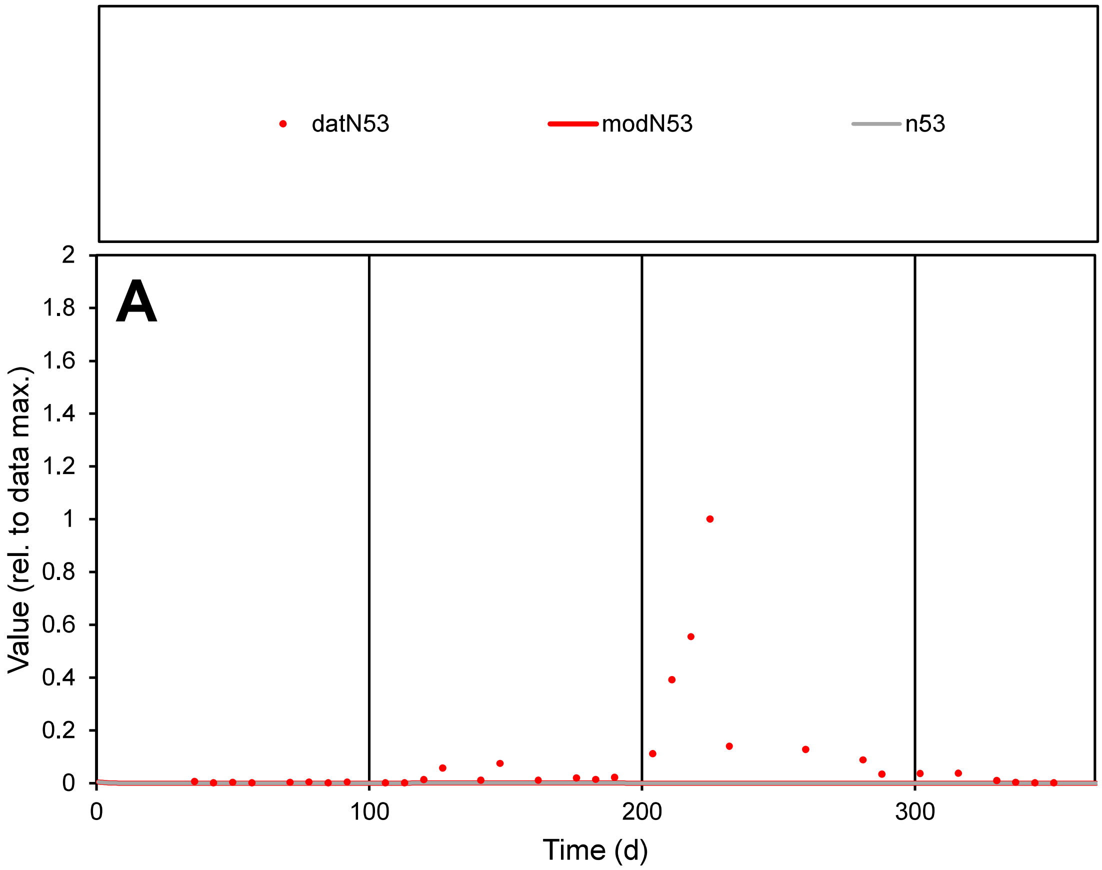

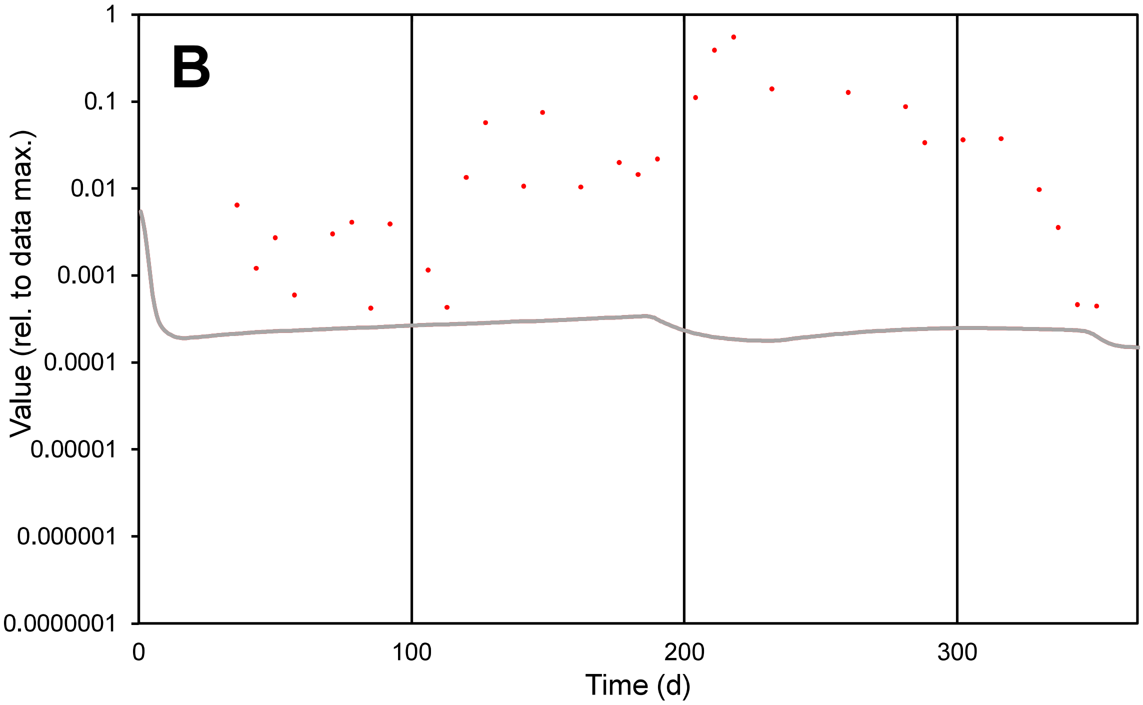

C1

Variables
N53 Provide jtd or Dst
CalibOnly?
2 1 to reproduce of
oNormalize?
2 1=no, 2=yes

ofxn calc*
2.5159
ofxn mod
2.51886

sRun
RunA1
zout
1

Ave
Data
0.00747
Model
2.1E-05

data/vMax
0.08598

ofxng
321.551

Min d/m
2.38376

ave/min d
205.257
ave/min m
1.4014

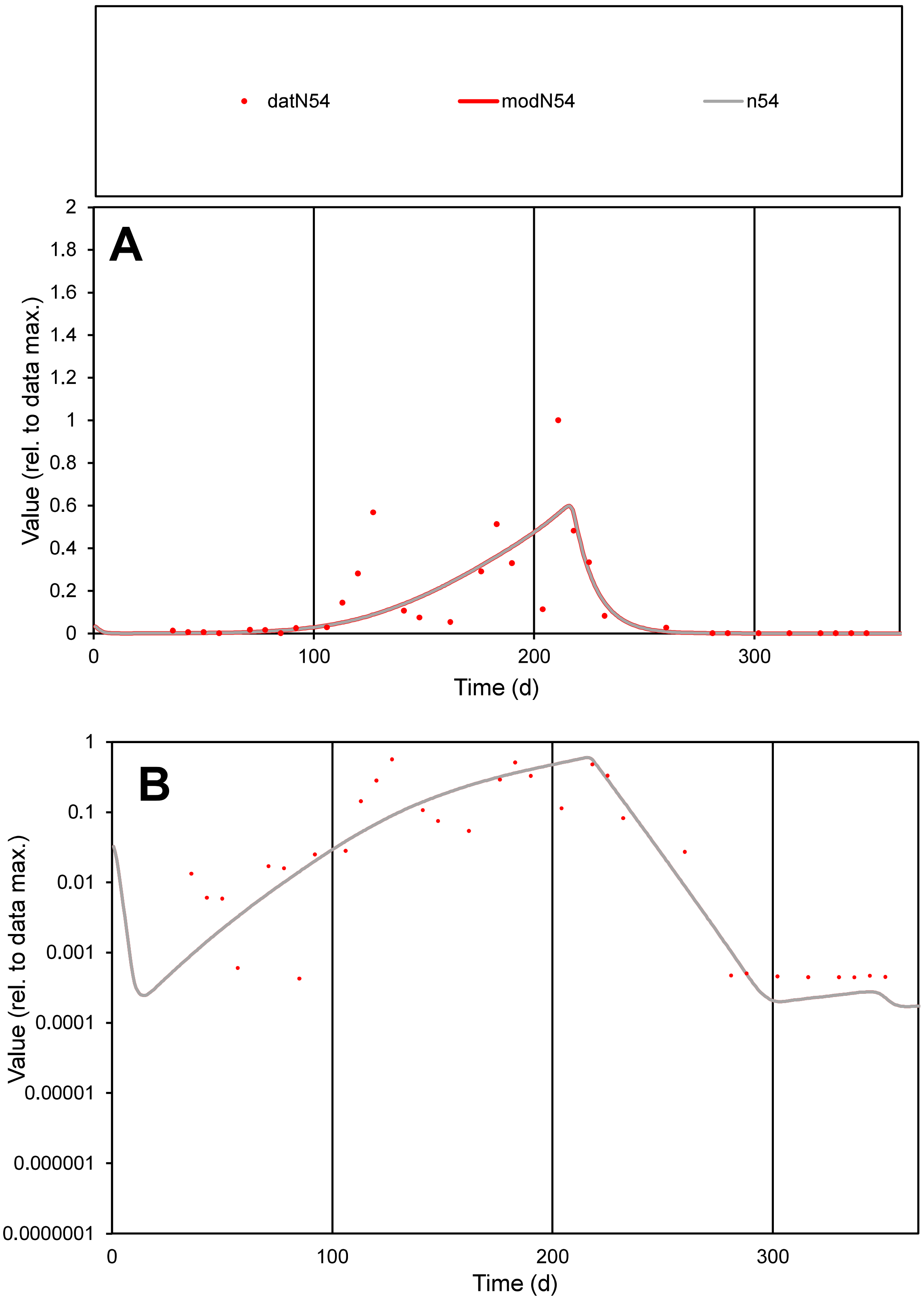

C1

Variables
N54 Provide jtd or Dst
CalibOnly?
2 1 to reproduce of
oNormalize?
2 1=no, 2=yes

ofxn calc*
1.04284
ofxn mod
1.06303

sRun
RunA1
zout
1

Ave
Data
0.00708
Model
0.00623

data/vMax
0.05014

ofxng
321.551

Min d/m
2.14756

ave/min d
328.705
ave/min m
621.6

324_Calib_S_A

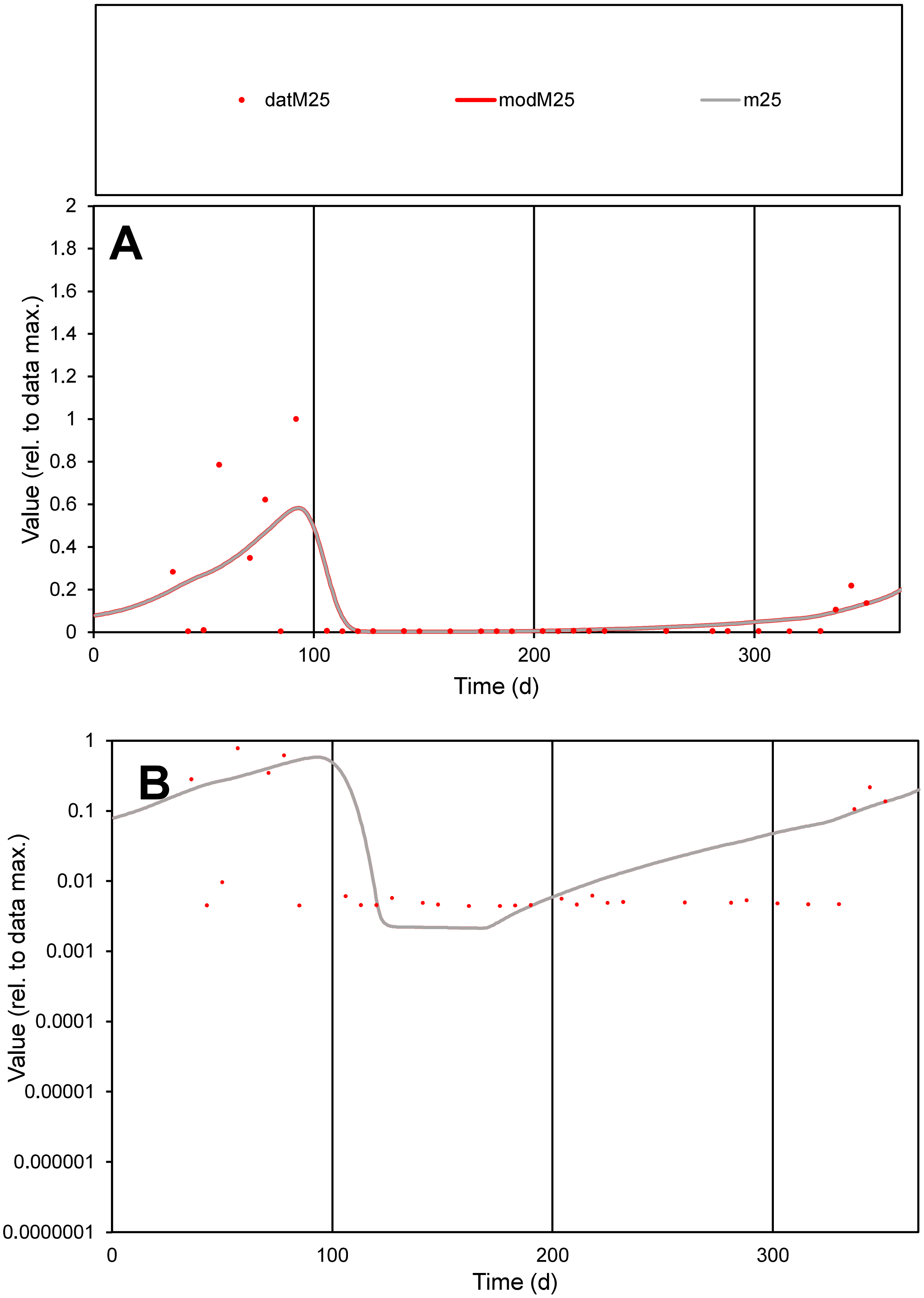

C1	
Variables	
M25	Provide jtd or Dst
CalibOnly?	
2	1 to reproduce of
oNormalize?	
2	1=no, 2=yes
ofxn calc*	
1.45607	
ofxn mod	
1.48844	
sRun	
RunA1	
zout	
1	
Ave	
Data	
0.00024	
Model	
0.00026	
data/vMax	
0.00213	
ofxng	
321.551	
Min d/m	
2.0574	
ave/min d	
25.6214	
ave/min m	
56.8642	

324_Calib_S_A

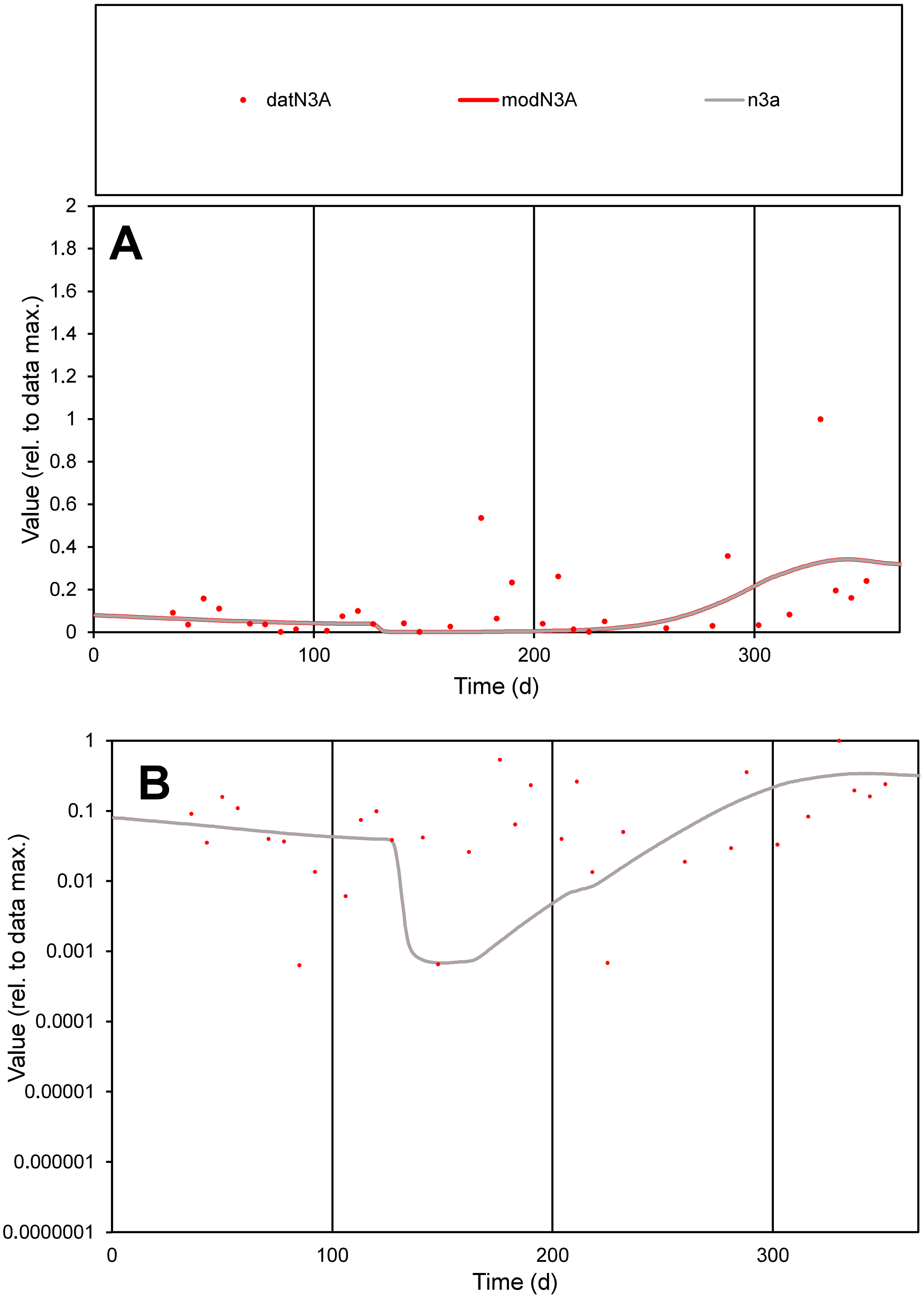

C1

Variables

N3A Provide jtd or Dst

CalibOnly?

2 1 to reproduce of

oNormalize?

2 1=no, 2=yes

ofxn calc*

1.43433

ofxn mod

1.44323

sRun

RunA1

zout

1

Ave

Data

0.0036

Model

0.00251

data/vMax

0.02818

ofxng

321.551

Min d/m

0.92909

ave/min d

201.995

ave/min m

130.697

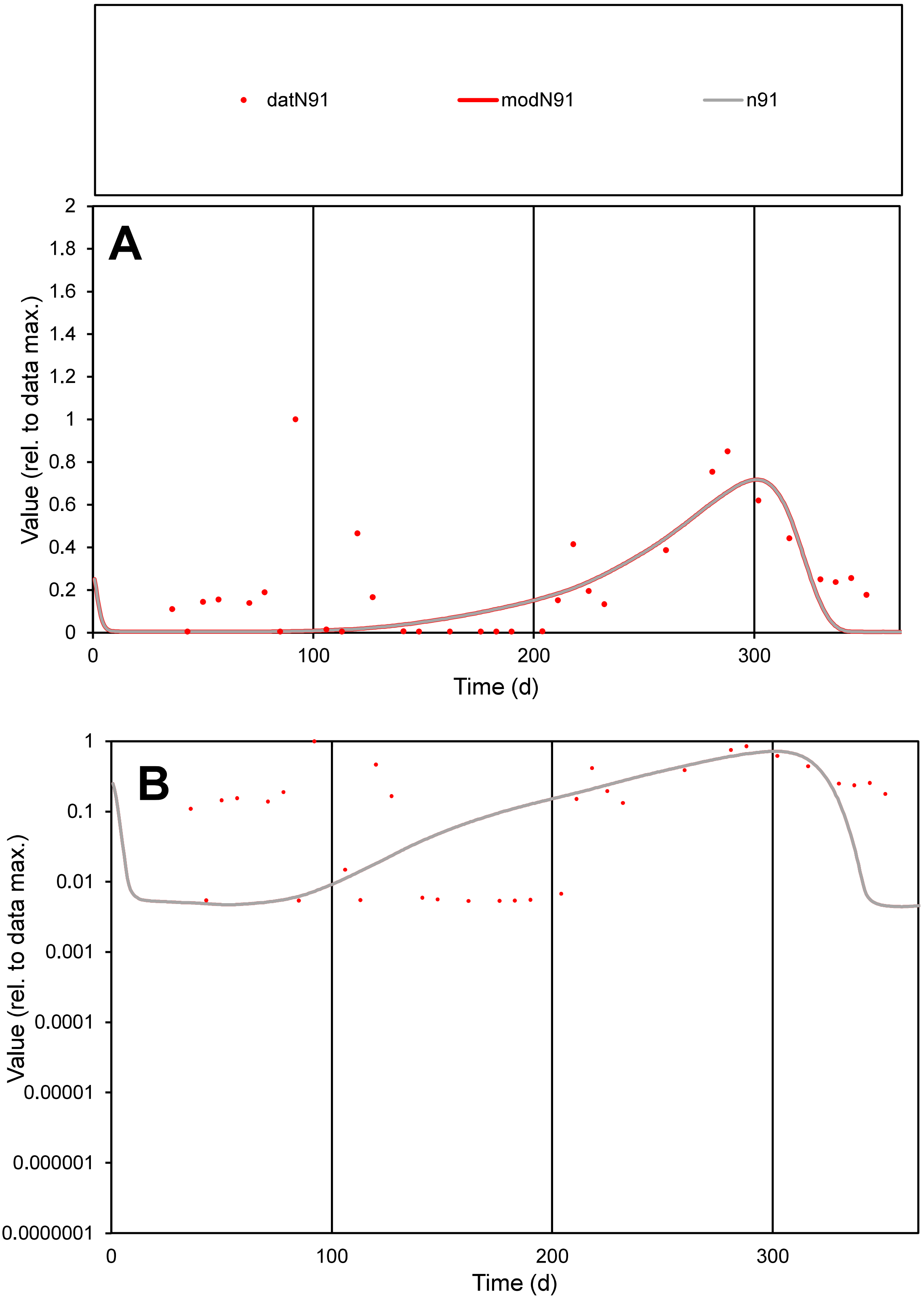

C1

Variables

N91 Provide jtd or Dst

CalibOnly?

2 1 to reproduce of

oNormalize?

2 1=no, 2=yes

ofxn calc*

0.9955

ofxn mod

1.00435

sRun

RunA1

zout

1

Ave

Data

0.00132

Model

0.00088

data/vMax

0.00579

ofxng

321.551

Min d/m

1.1804

ave/min d

42.7666

ave/min m

33.5727

324_Calib_S_A

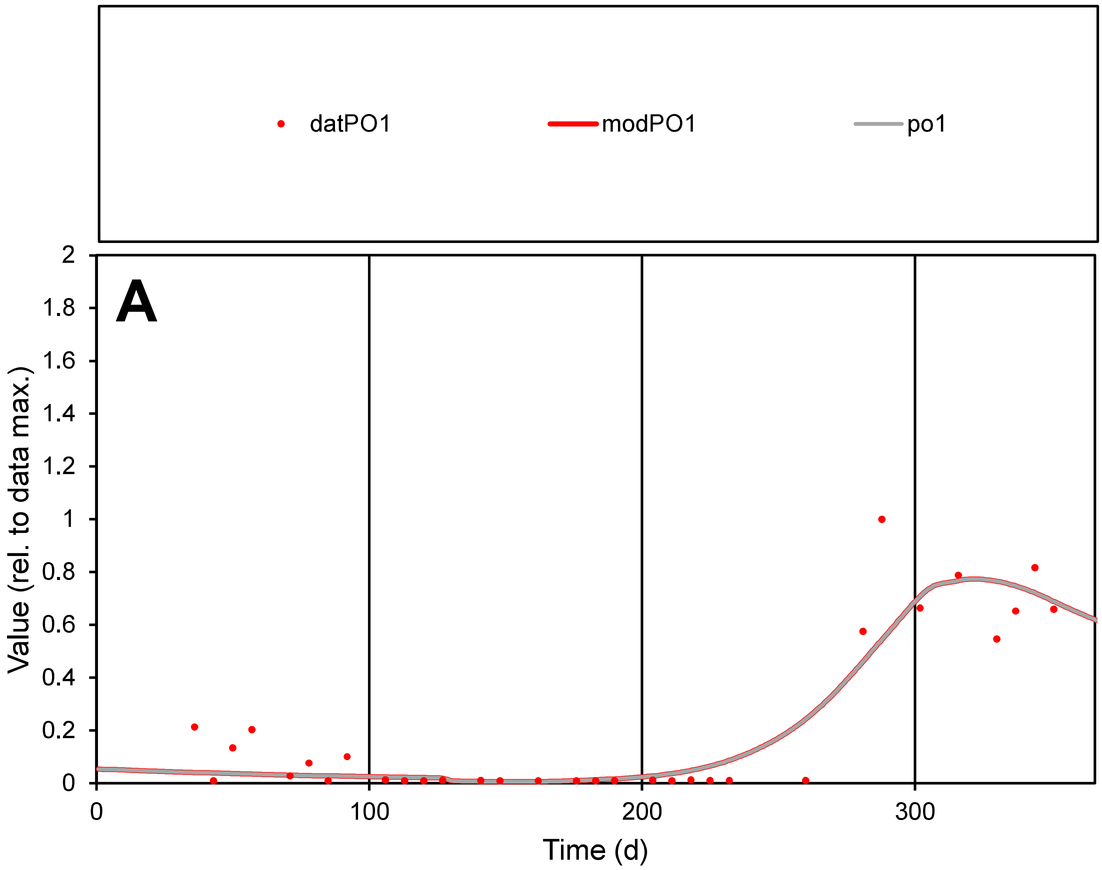

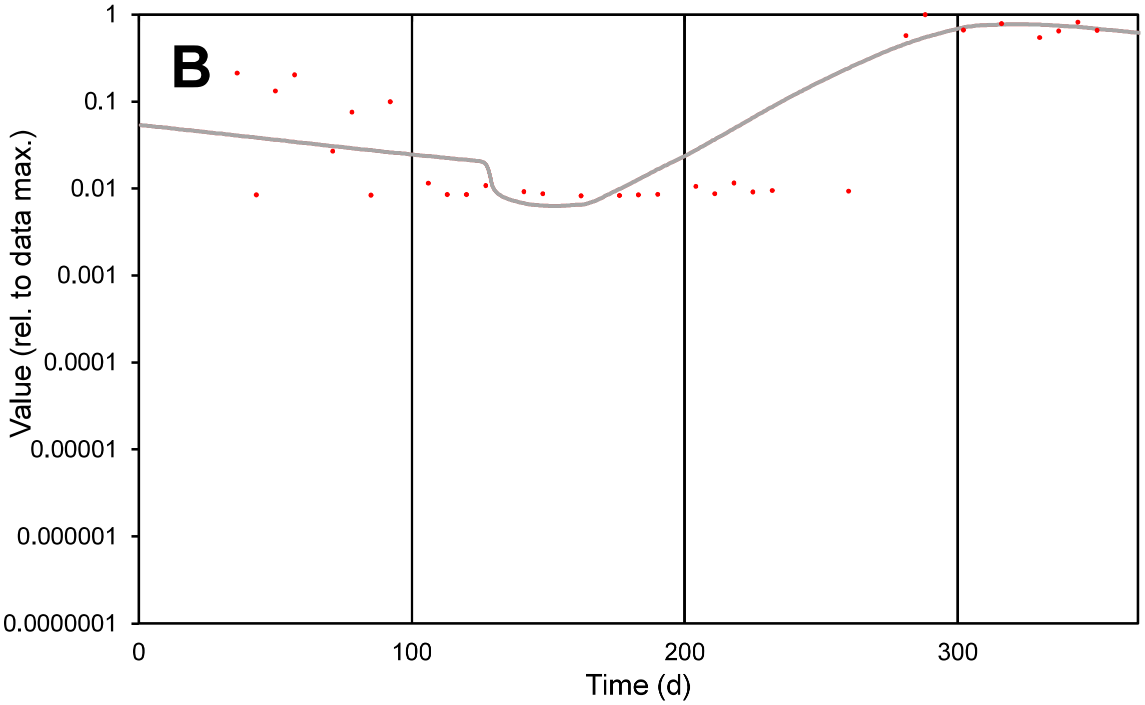

C1

Variables
PO1 Provide jtd or Dst
CalibOnly?
2 1 to reproduce of
oNormalize?
2 1=no, 2=yes

ofxn calc*
0.551
ofxn mod
0.57368

sRun
RunA1
zout
1

Ave
Data
0.00111
Model
0.00107

data/vMax
0.00539

ofxng
321.551

Min d/m
1.31085

ave/min d
24.907
ave/min m
31.303

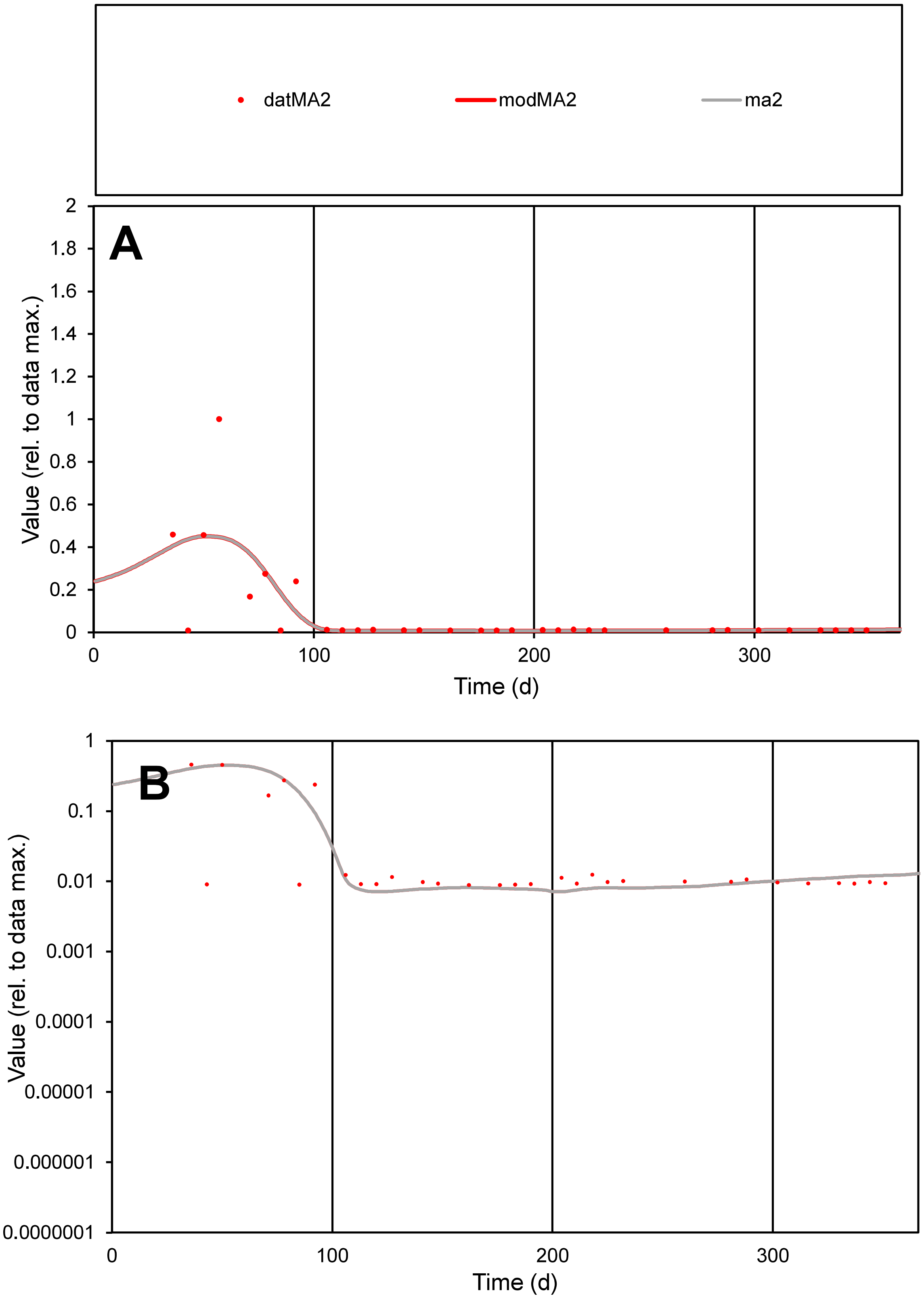

C1

Variables
MA2 Provide jtd or Dst
CalibOnly?
2 1 to reproduce of
oNormalize?
2 1=no, 2=yes

ofxn calc*
1.50081
ofxn mod
1.52029

sRun
RunA1
zout
1

Ave
Data
0.00018
Model
0.00017

data/vMax
0.00197

ofxng
321.551

Min d/m
1.23955

ave/min d
10.0576
ave/min m
12.3286

324_Calib_S_A

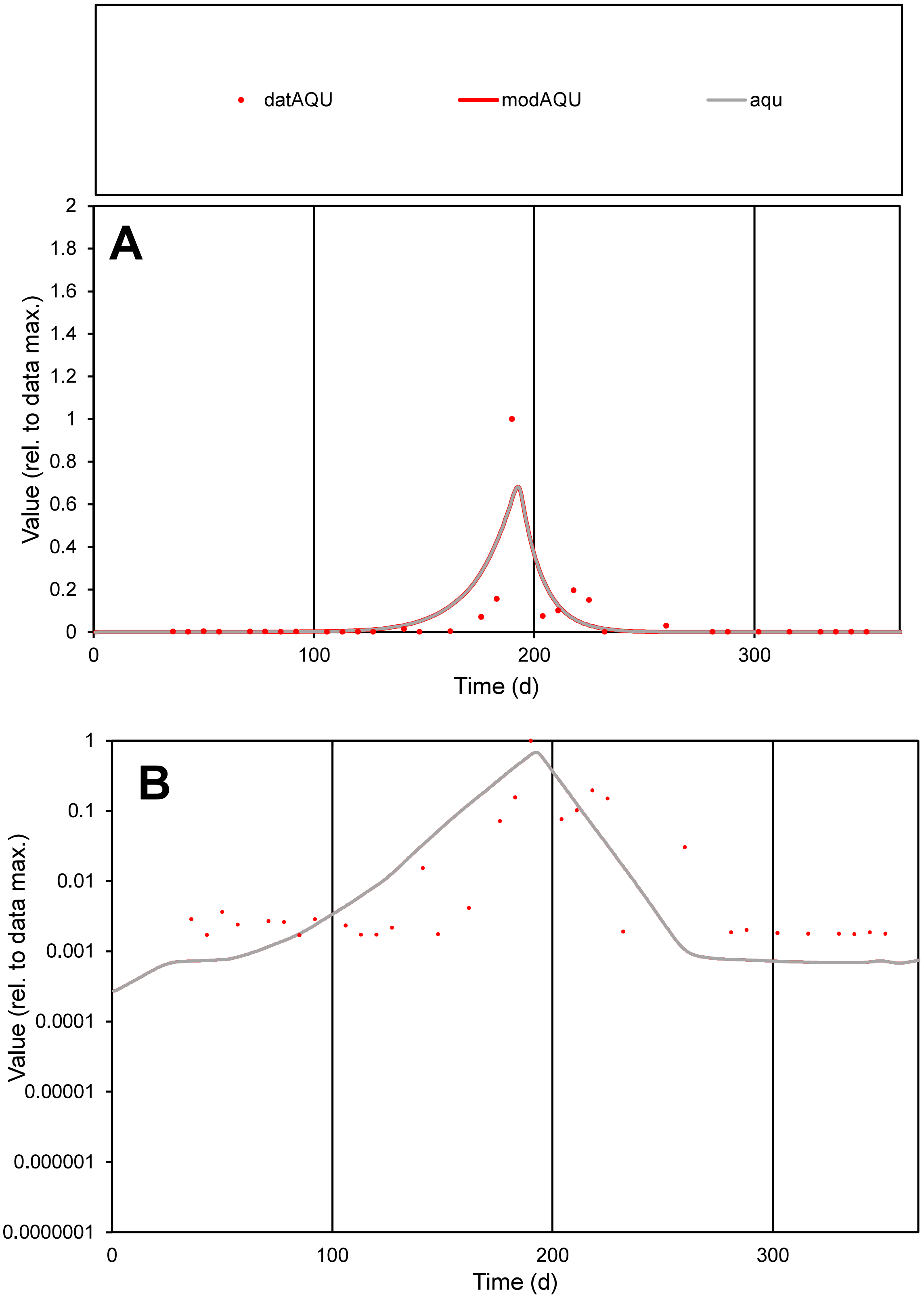

C1

Variables
AQU Provide jtd or Dst
CalibOnly?
2 1 to reproduce of
oNormalize?
2 1=no, 2=yes

ofxn calc*
1.65995
ofxn mod
1.72021

sRun
RunA1
zout
1

Ave
Data
0.0044
Model
0.00481

data/vMax
0.07607

ofxng
321.551

Min d/m
2.47801

ave/min d
33.939
ave/min m
91.8741

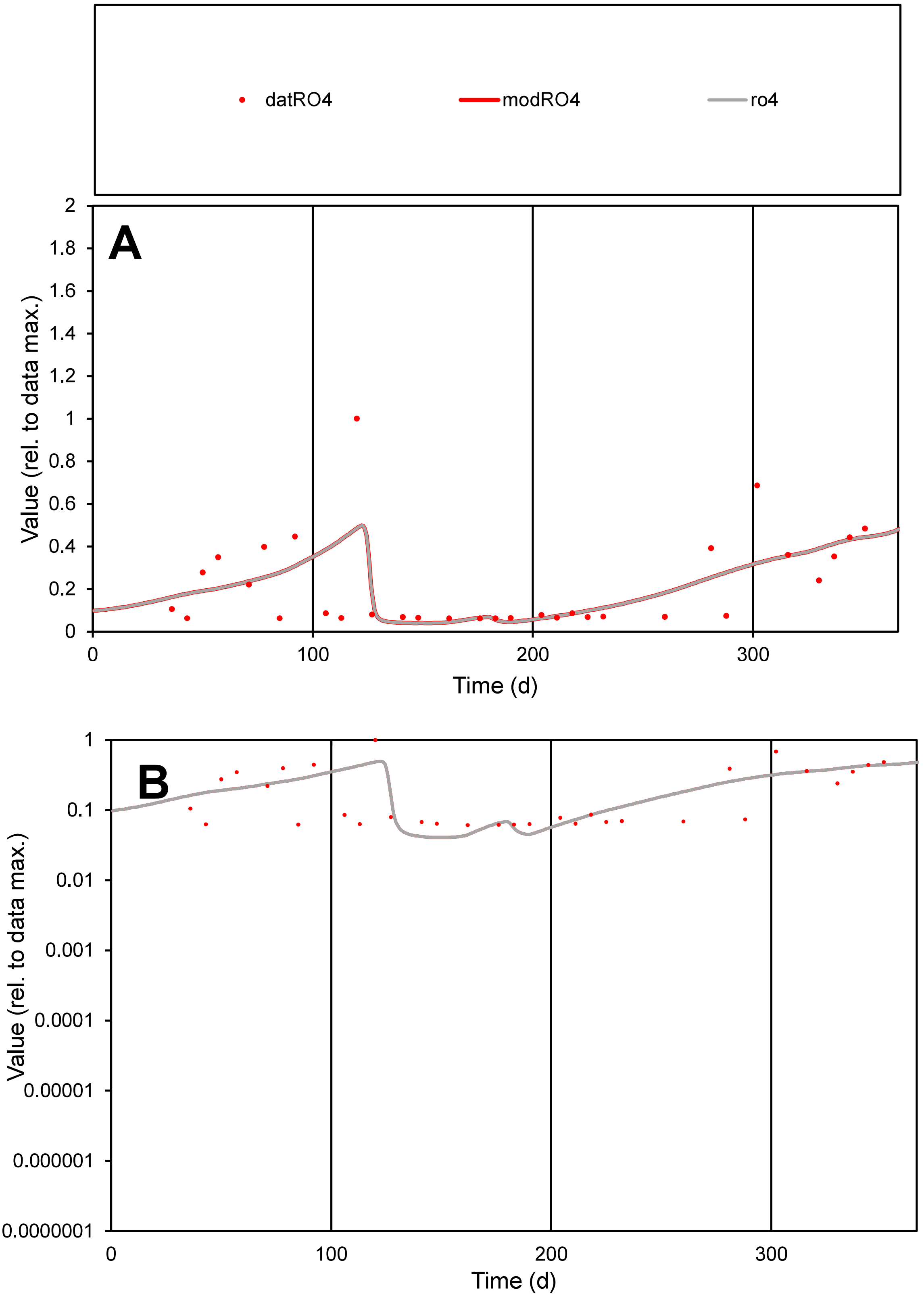

C1

Variables

RO4 Provide jtd or Dst

CalibOnly?

2 1 to reproduce of

oNormalize?

2 1=no, 2=yes

ofxn calc*

0.74542

ofxn mod

0.75777

sRun

RunA1

zout

1

Ave

Data

0.00076

Model

0.00077

data/vMax

0.00347

ofxng

321.551

Min d/m

1.5007

ave/min d

3.56181

ave/min m

5.41761

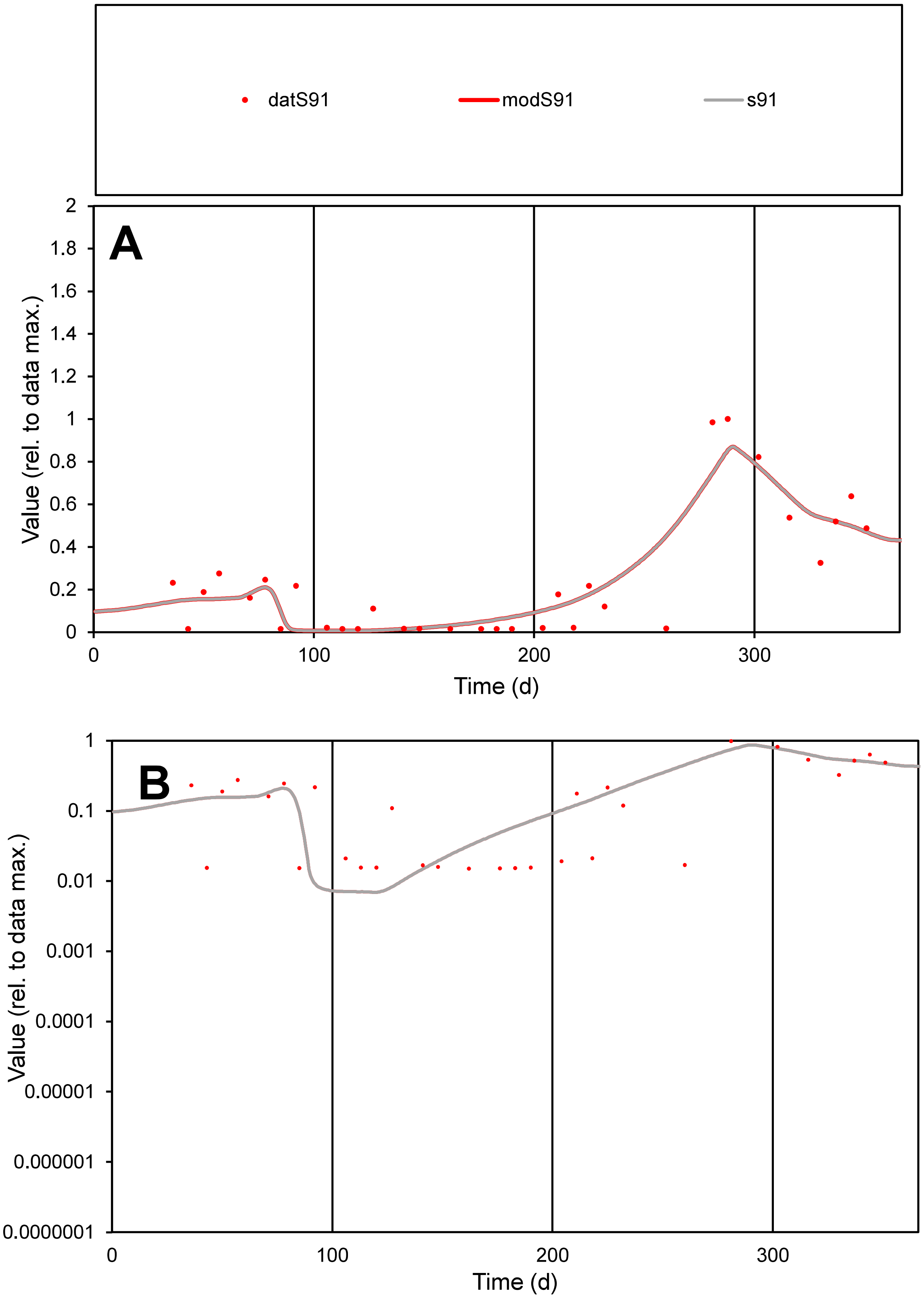

C1

Variables

S91 Provide jtd or Dst

CalibOnly?

2 1 to reproduce of

oNormalize?

2 1=no, 2=yes

ofxn calc*

0.52135

ofxn mod

0.5409

sRun

RunA1

zout

1

Ave

Data

0.00099

Model

0.00101

data/vMax

0.00425

ofxng

321.551

Min d/m

2.15542

ave/min d

15.4874

ave/min m

33.8976

324_Calib_S_A

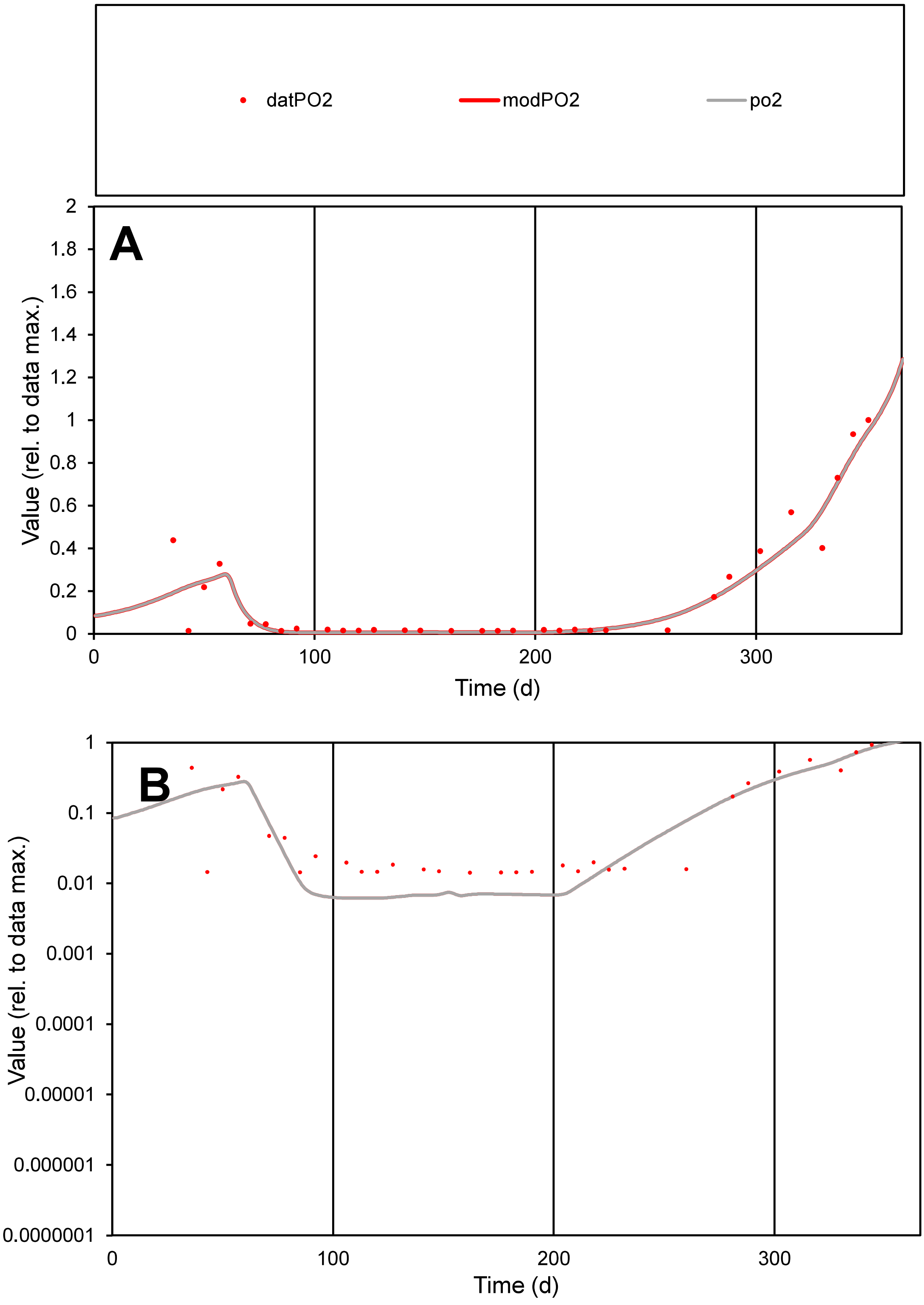

C1

Variables

PO2 Provide jtd or Dst

CalibOnly?

2 1 to reproduce of

oNormalize?

2 1=no, 2=yes

ofxn calc*

0.40633

ofxn mod

0.44106

sRun

RunA1

zout

1

Ave

Data

0.001

Model

0.00095

data/vMax

0.00548

ofxng

321.551

Min d/m

2.29743

ave/min d

12.8647

ave/min m

28.1401

324_Calib_S_A

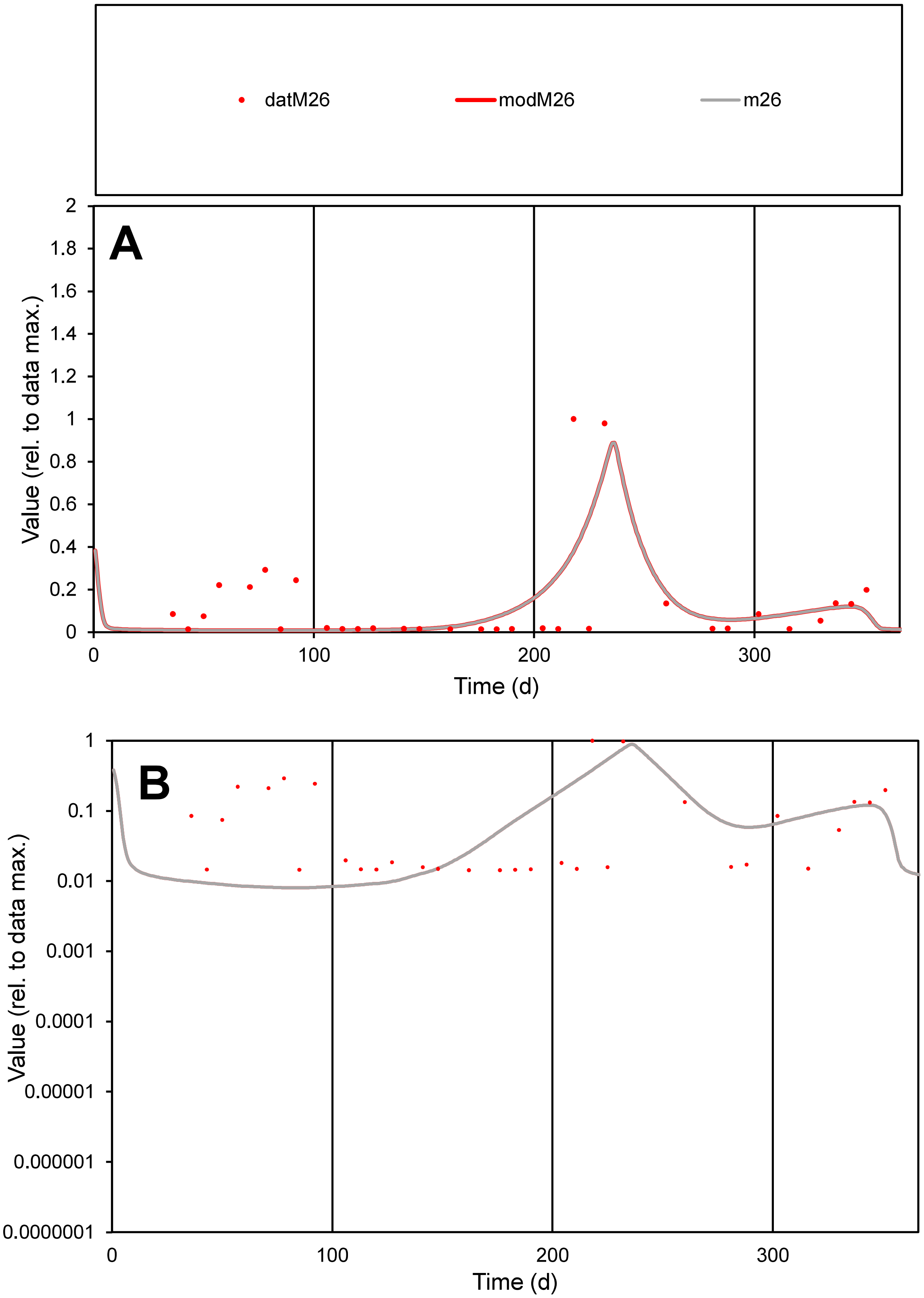

C1

Variables
M26 Provide jtd or Dst
CalibOnly?
2 1 to reproduce of
oNormalize?
2 1=no, 2=yes

ofxn calc*
1.34362
ofxn mod
1.39181

sRun
RunA1
zout
1

Ave
Data
0.00072
Model
0.00061

data/vMax
0.00562

ofxng
321.551

Min d/m
1.77458

ave/min d
9.04196
ave/min m
13.4113

324_Calib_S_A

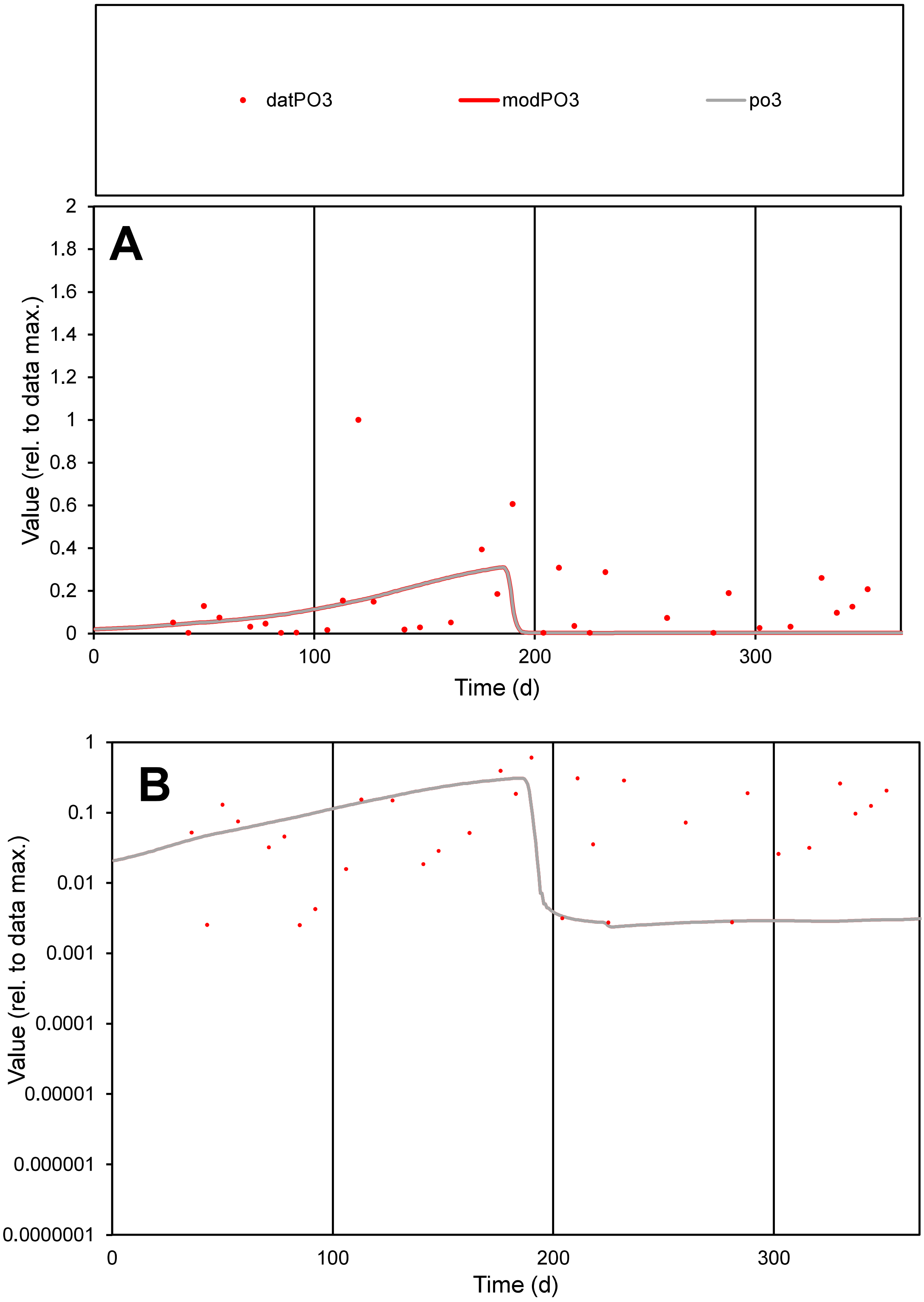

C1

Variables
PO3 Provide jtd or Dst
CalibOnly?
2 1 to reproduce of
oNormalize?
2 1=no, 2=yes

ofxn calc*
1.46035
ofxn mod
1.48243

sRun
RunA1
zout
1

Ave
Data
0.00263
Model
0.00155

data/vMax
0.01831

ofxng
321.551

Min d/m
1.05784

ave/min d
56.8299
ave/min m
35.3242

324_Calib_S_A

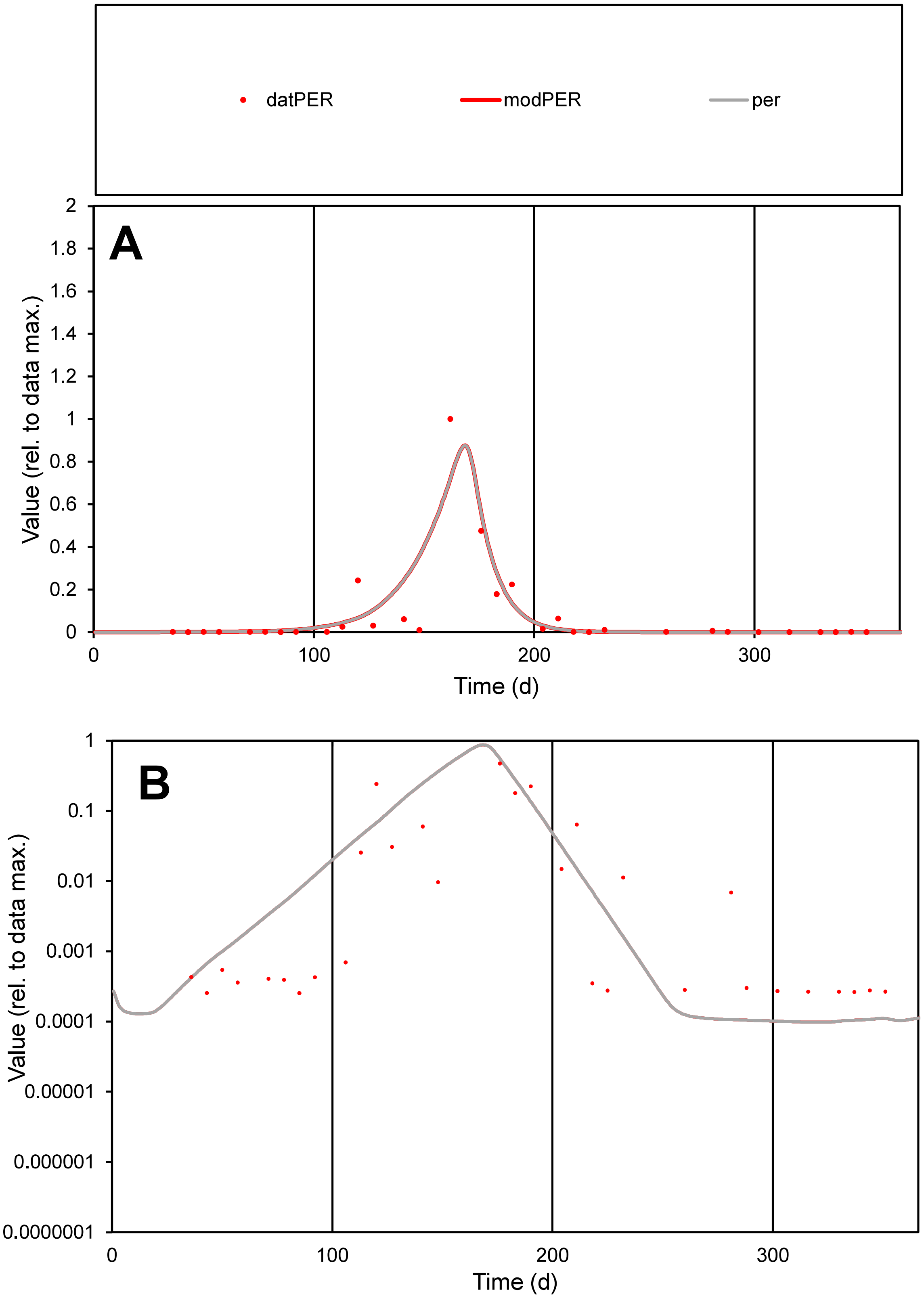

C1

Variables
PER Provide jtd or Dst
CalibOnly?
2 1 to reproduce of
oNormalize?
2 1=no, 2=yes

ofxn calc*
1.17676
ofxn mod
1.28516

sRun
RunA1
zout
1

Ave
Data
0.00525
Model
0.00566

data/vMax
0.07156

ofxng
321.551

Min d/m
2.59444

ave/min d
288.729
ave/min m
806.607

324_Calib_S_A

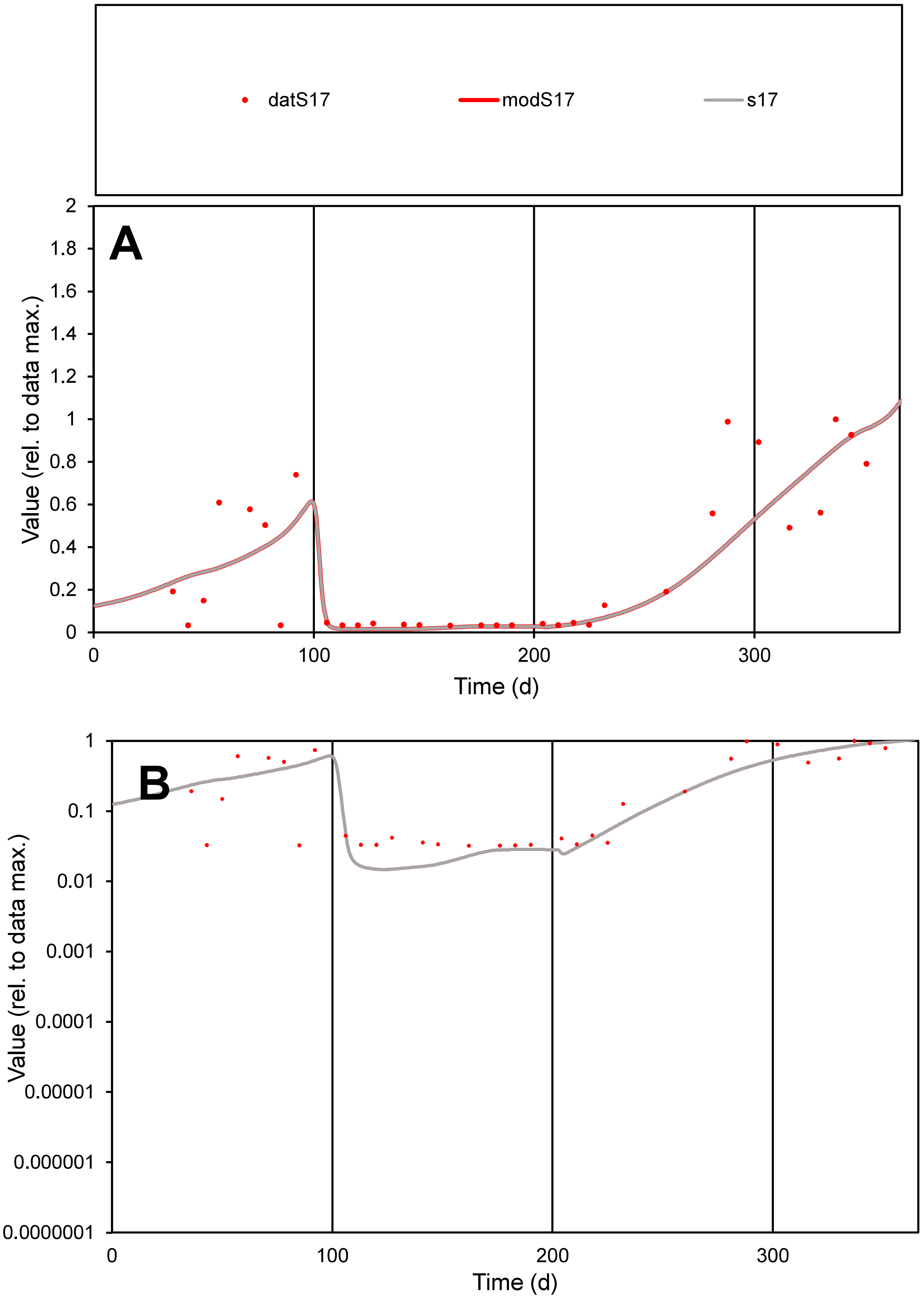

C1

Variables

S17 Provide jtd or Dst

CalibOnly?

2 1 to reproduce of

oNormalize?

2 1=no, 2=yes

ofxn calc*

0.58566

ofxn mod

0.59947

sRun

RunA1

zout

1

Ave

Data

0.00102

Model

0.00095

data/vMax

0.00331

ofxng

321.551

Min d/m

2.17702

ave/min d

9.59316

ave/min m

19.3758

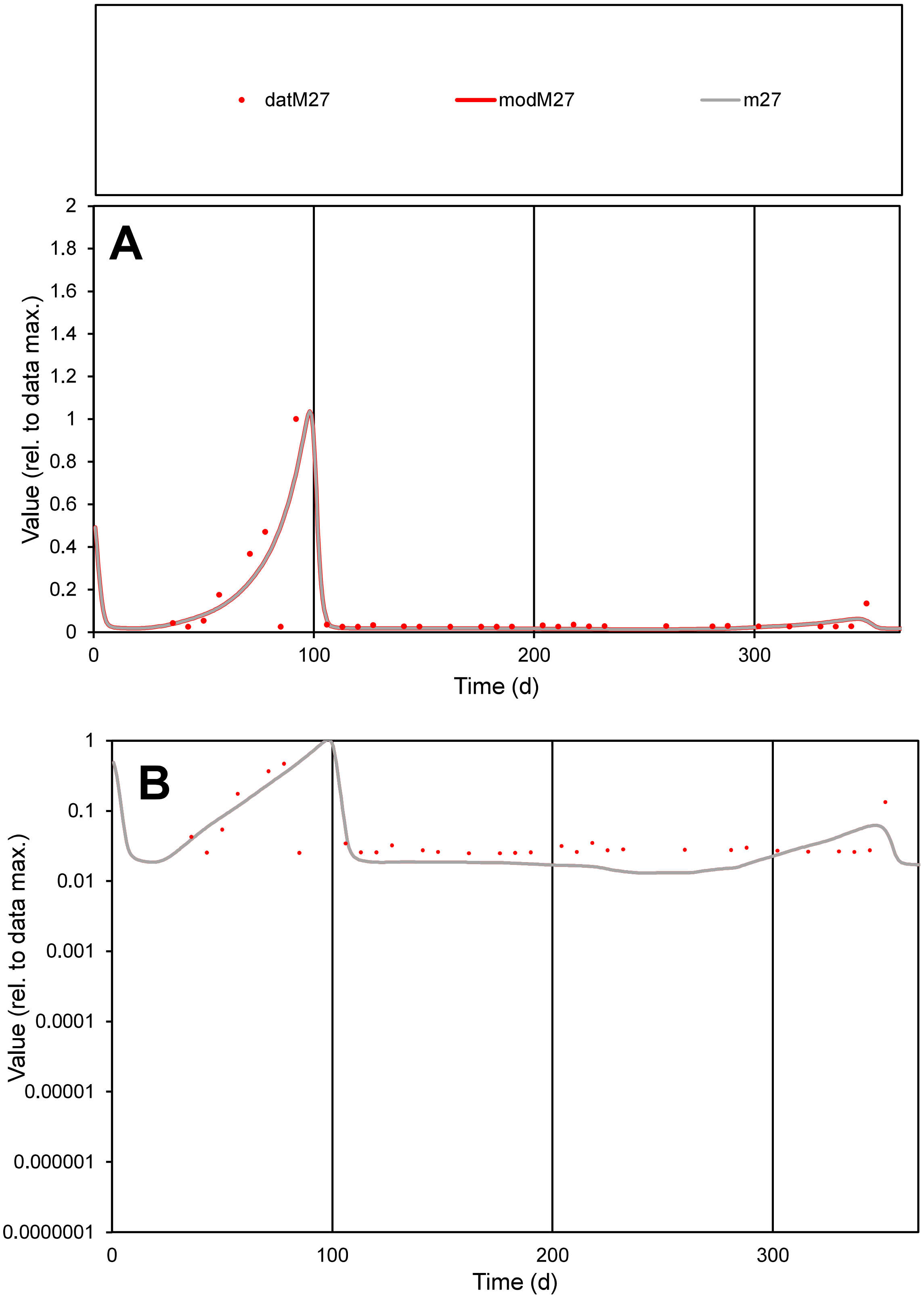

C1

Variables
M27 Provide jtd or Dst
CalibOnly?
2 1 to reproduce of
oNormalize?
2 1=no, 2=yes

ofxn calc*
1.03311
ofxn mod
1.07427

sRun
RunA1
zout
1

Ave
Data
0.00023
Model
0.00022

data/vMax
0.00256

ofxng
321.551

Min d/m
1.88619

ave/min d
3.69002
ave/min m
6.61932

324_Calib_S_A

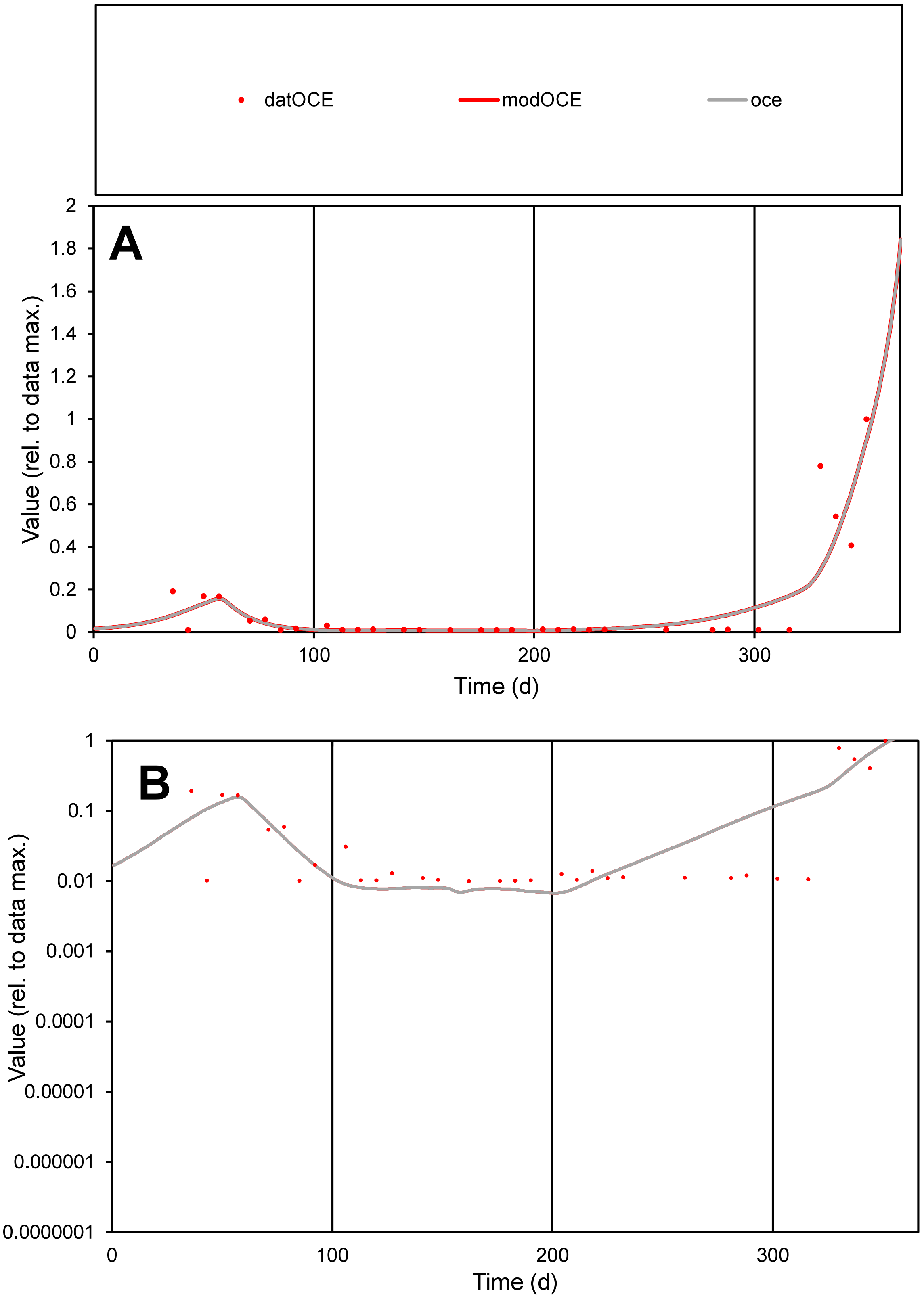

C1

Variables
OCE Provide jtd or Dst
CalibOnly?
2 1 to reproduce of
oNormalize?
2 1=no, 2=yes

ofxn calc*
0.91499
ofxn mod
0.95208

sRun
RunA1
zout
1

Ave
Data
0.00042
Model
0.00041

data/vMax
0.00365

ofxng
321.551

Min d/m
1.42885

ave/min d
11.4516
ave/min m
16.3075

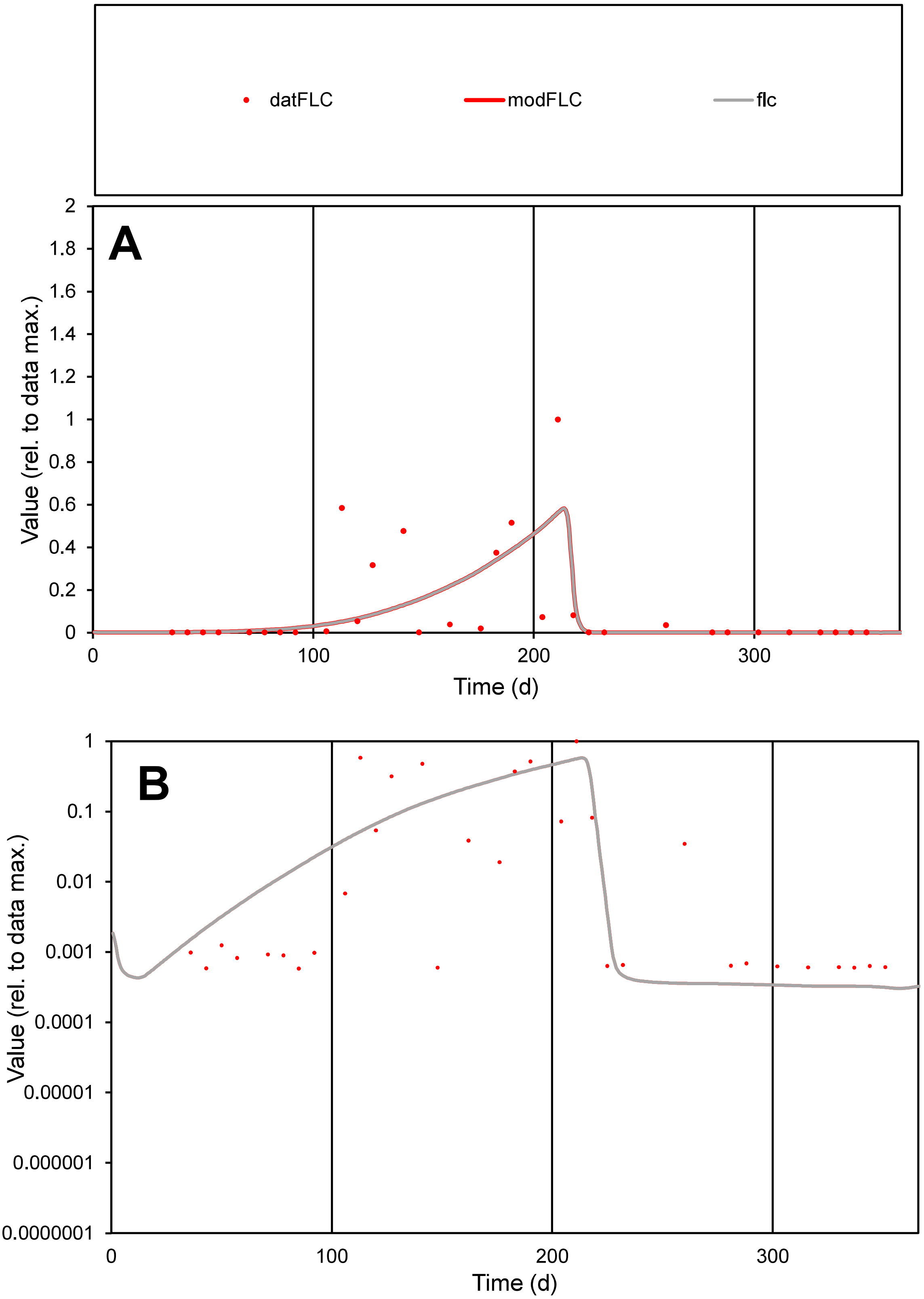

C1

Variables
FLC Provide jtd or Dst
CalibOnly?
2 1 to reproduce of
oNormalize?
2 1=no, 2=yes

ofxn calc*
1.54884
ofxn mod
1.56435

sRun
RunA1
zout
1

Ave
Data
0.0023
Model
0.00196

data/vMax
0.02056

ofxng
321.551

Min d/m
1.8642

ave/min d
192.527
ave/min m
304.919

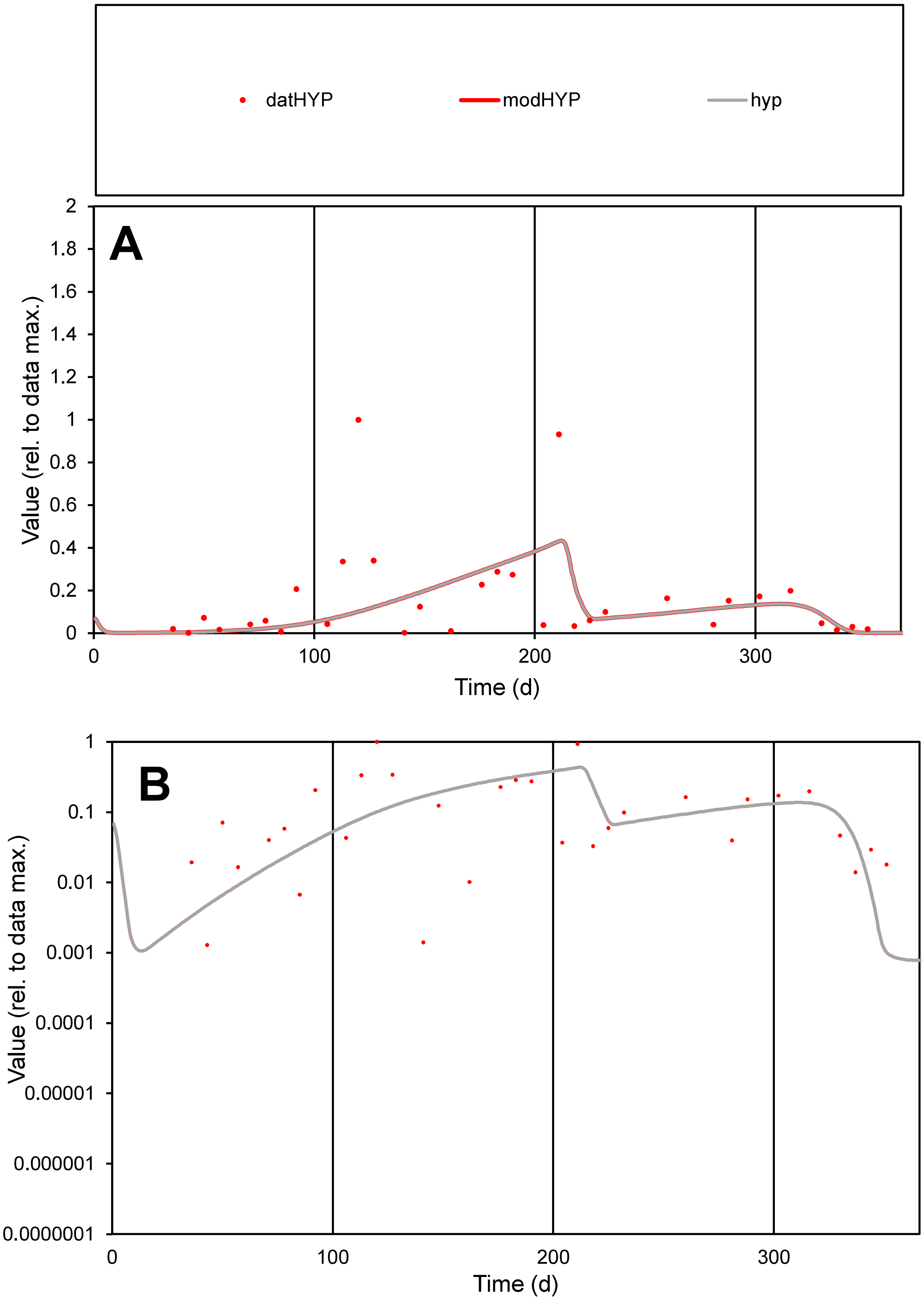

C1

Variables
HYP Provide jtd or Dst
CalibOnly?
2 1 to reproduce of
oNormalize?
2 1=no, 2=yes

ofxn calc*
1.35197
ofxn mod
1.36067

sRun
RunA1
zout
1

Ave
Data
0.00195
Model
0.00152

data/vMax
0.01234

ofxng
321.551

Min d/m
1.34599

ave/min d
122.204
ave/min m
128.298

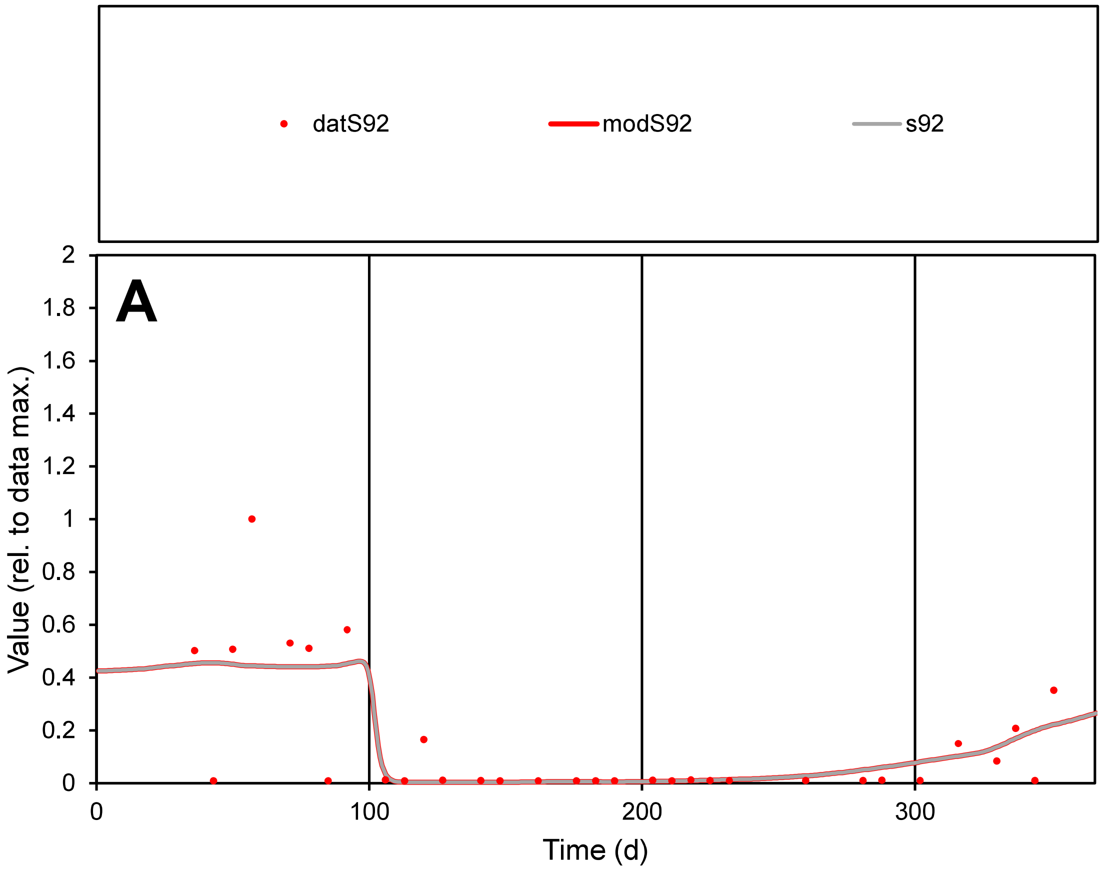

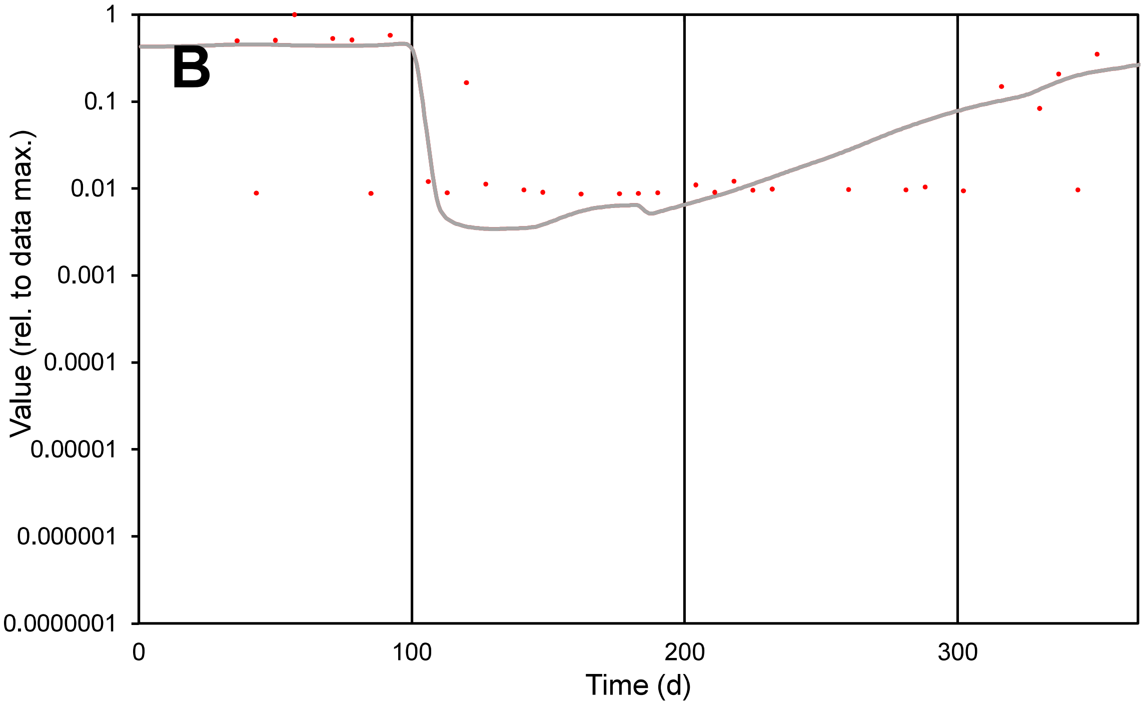

C1

Variables
S92 Provide jtd or Dst
CalibOnly?
2 1 to reproduce of
oNormalize?
2 1=no, 2=yes

ofxn calc*
1.07338
ofxn mod
1.0839

sRun
RunA1
zout
1

Ave
Data
0.00039
Model
0.00039

data/vMax
0.00263

ofxng
321.551

Min d/m
2.52186

ave/min d
17.2784
ave/min m
43.2474

324_Calib_S_A

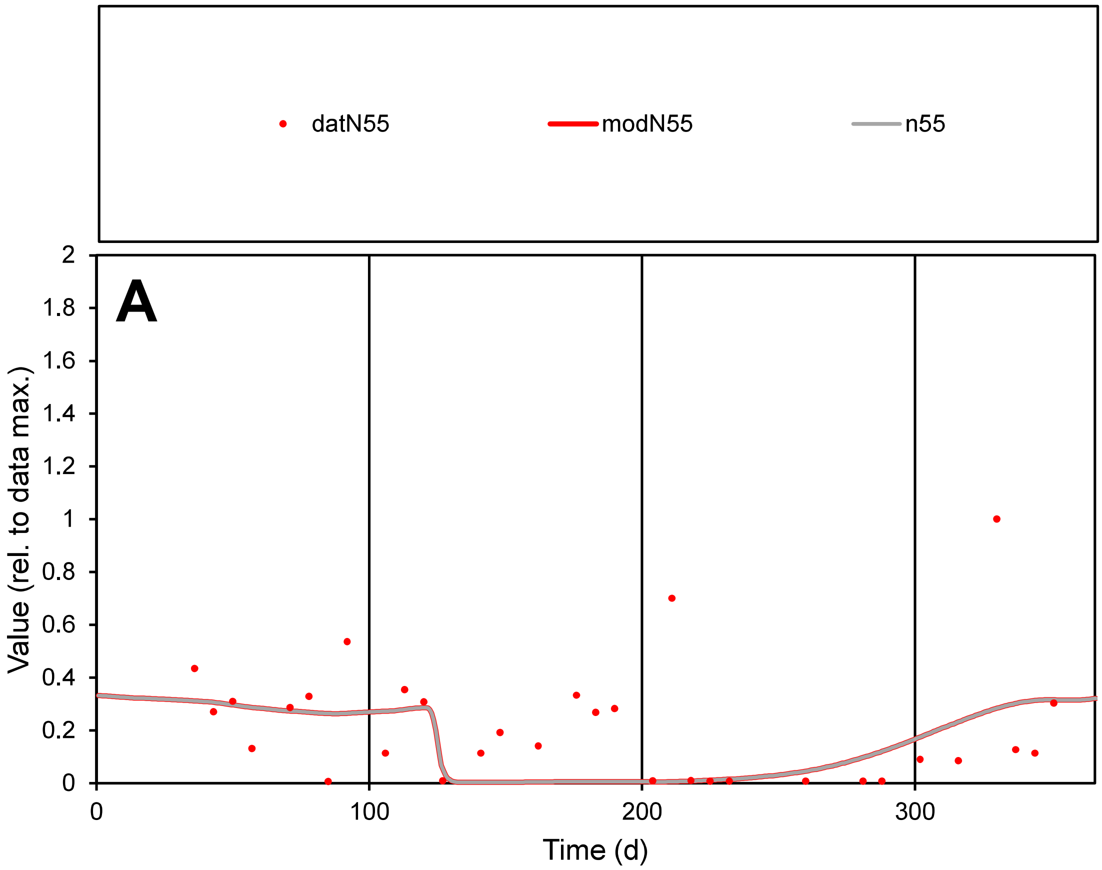

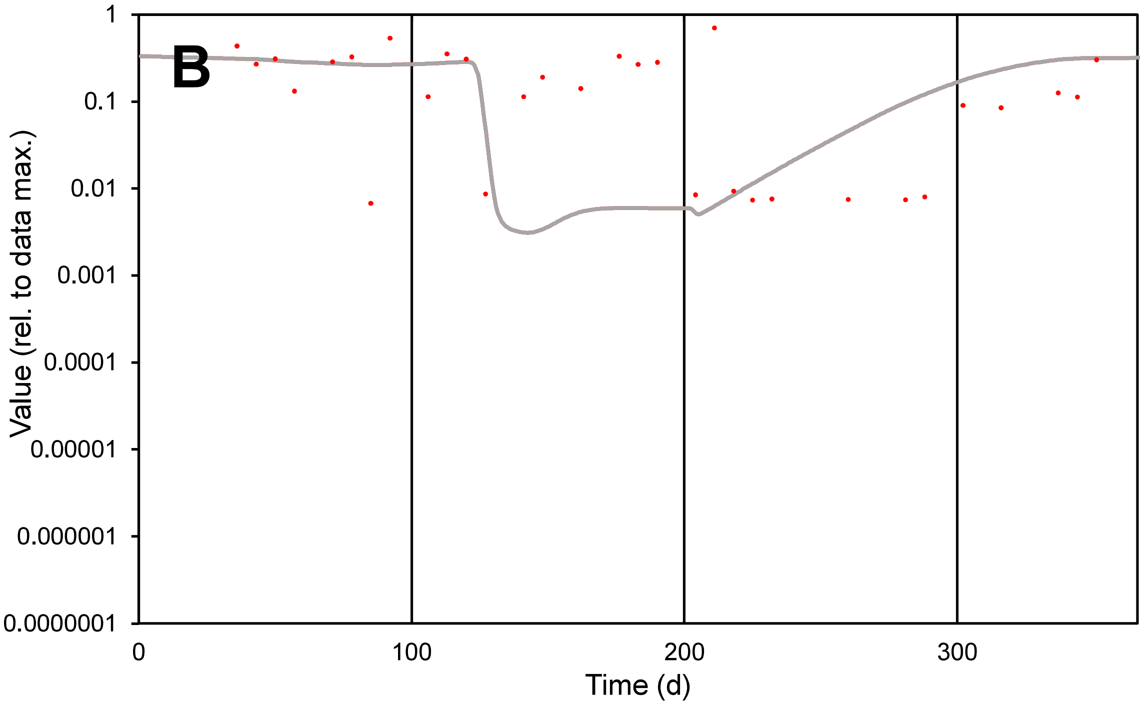

C1

Variables

N55	Provide jtd or Dst

CalibOnly?

2	1 to reproduce of

oNormalize?

2	1=no, 2=yes

ofxn calc*
1.05301
ofxn mod
1.05983

sRun
RunA1
zout
1

Ave
Data
0.00118
Model
0.00087

data/vMax
0.00549

ofxng
321.551

Min d/m
2.17616

ave/min d
31.8827
ave/min m
51.341

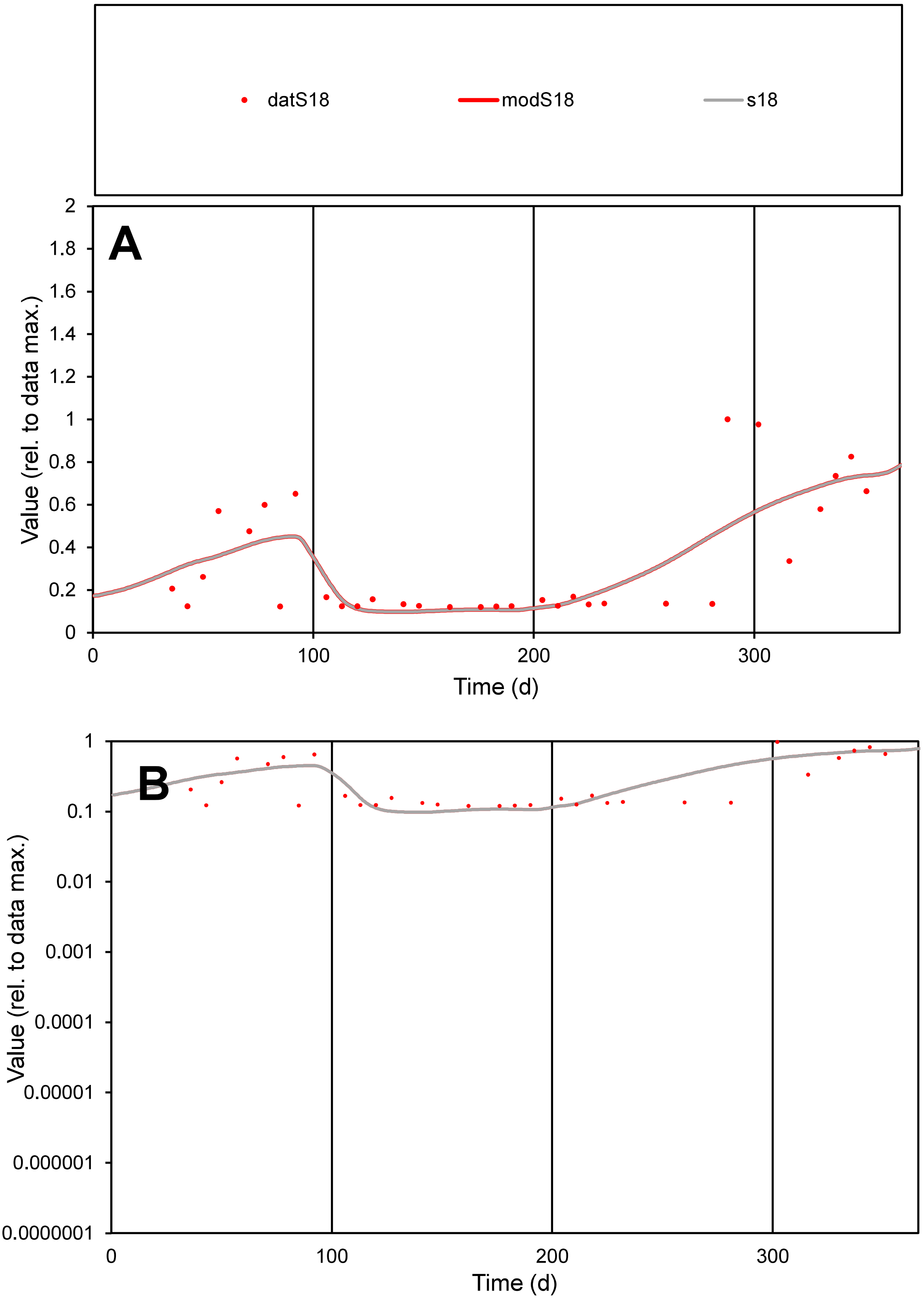

C1

Variables
S18 Provide jtd or Dst
CalibOnly?
2 1 to reproduce of
oNormalize?
2 1=no, 2=yes

ofxn calc*
0.53128
ofxn mod
0.54504

sRun
RunA1
zout
1

Ave
Data
0.00071
Model
0.00071

data/vMax
0.00217

ofxng
321.551

Min d/m
1.22787

ave/min d
2.71389
ave/min m
3.34618

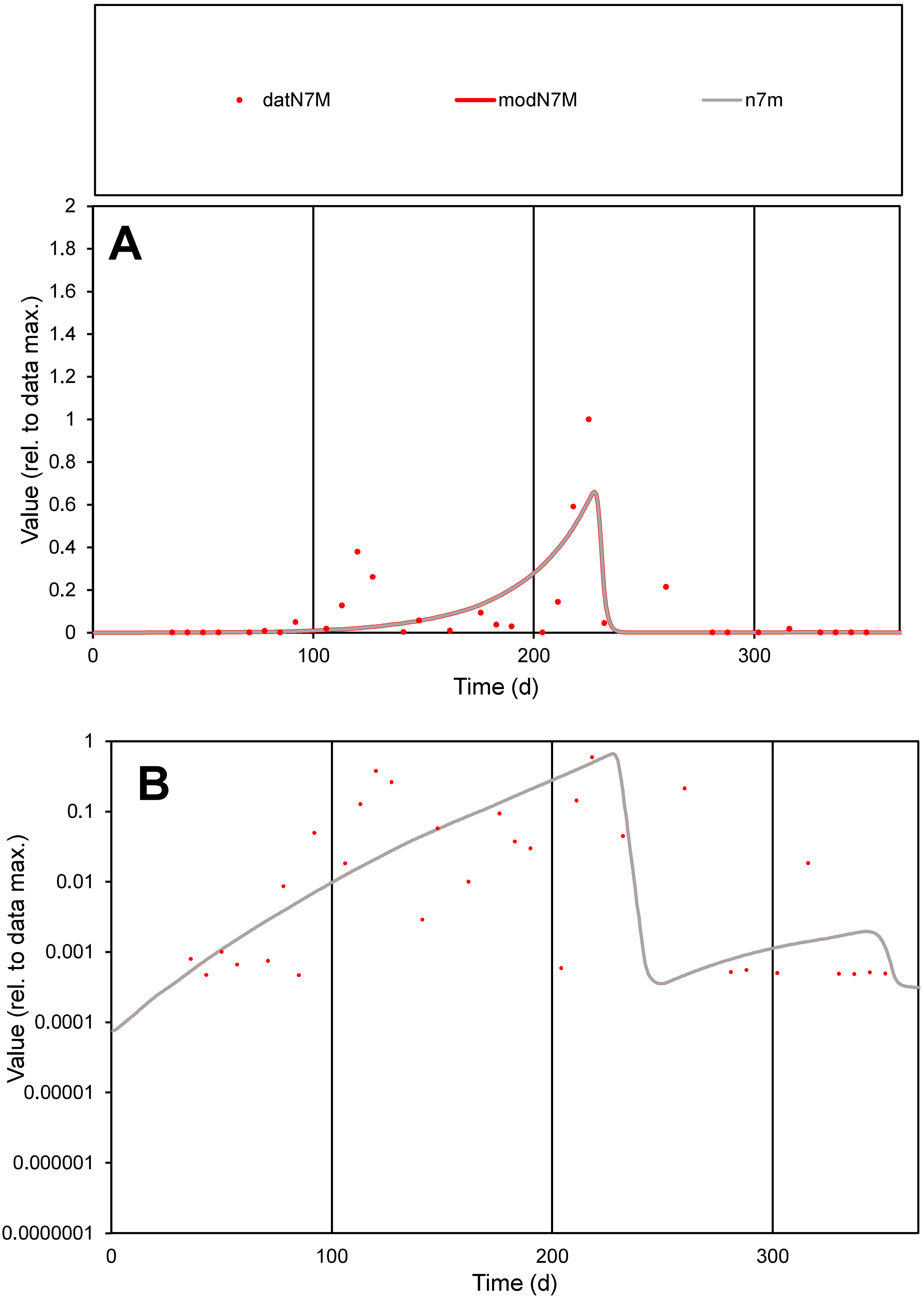

C1

Variables
N7M Provide jtd or Dst
CalibOnly?
2 1 to reproduce of
oNormalize?
2 1=no, 2=yes

ofxn calc*
1.40954
ofxn mod
1.43285

sRun
RunA1
zout
1

Ave
Data
0.00197
Model
0.00175

data/vMax
0.02033

ofxng
321.551

Min d/m
0.97784

ave/min d
205.57
ave/min m
178.973

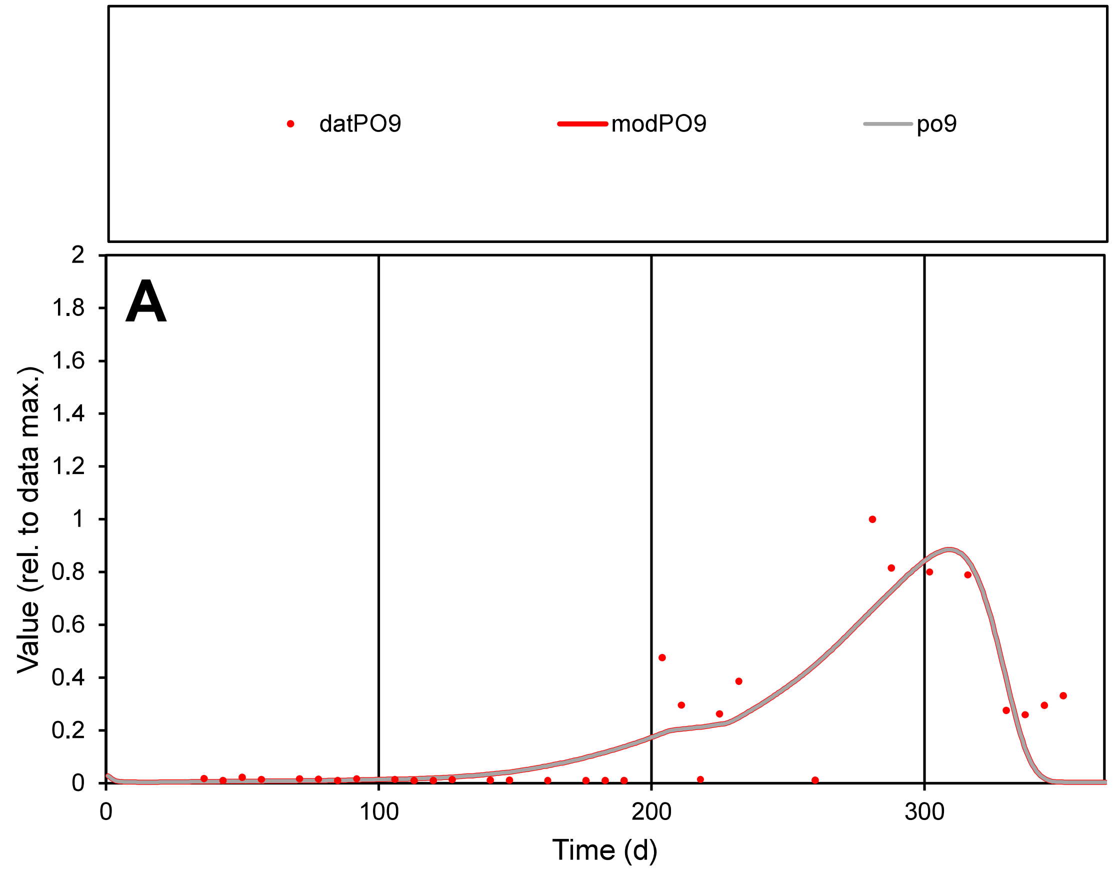

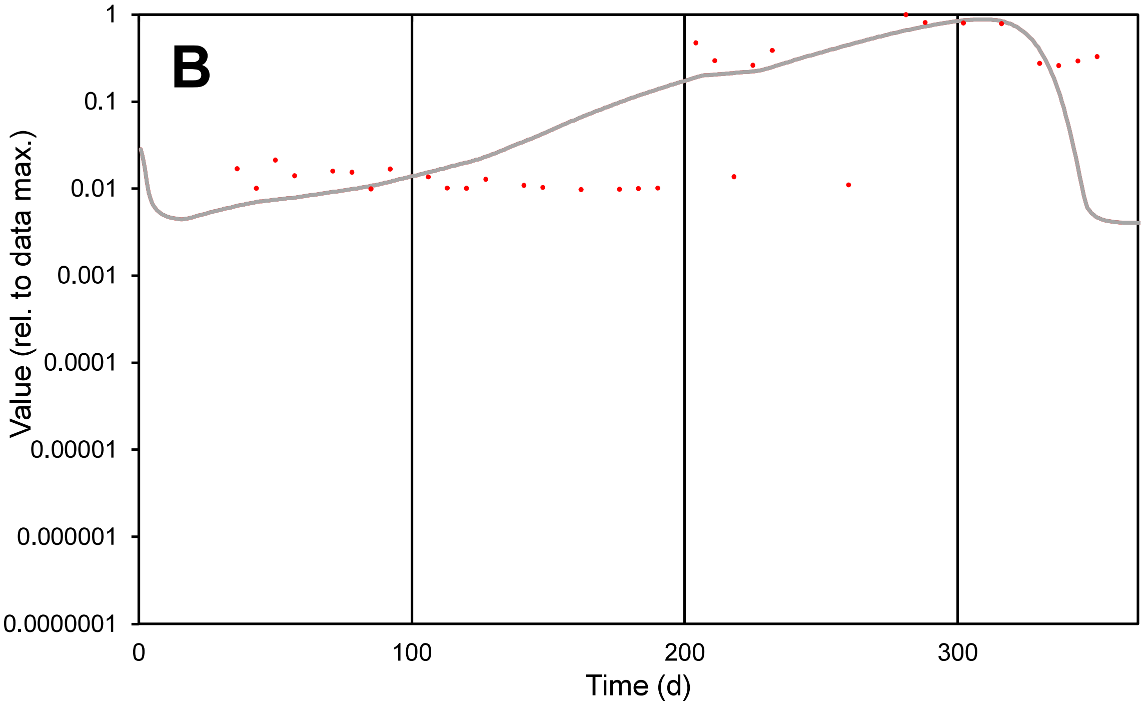

C1

Variables
PO9 Provide jtd or Dst
CalibOnly?
2 1 to reproduce of
oNormalize?
2 1=no, 2=yes

ofxn calc*
0.75644
ofxn mod
0.77849

sRun
RunA1
zout
1

Ave
Data
0.00113
Model
0.00105

data/vMax
0.00581

ofxng
321.551

Min d/m
2.16484

ave/min d
19.8044
ave/min m
39.966

324_Calib_S_A

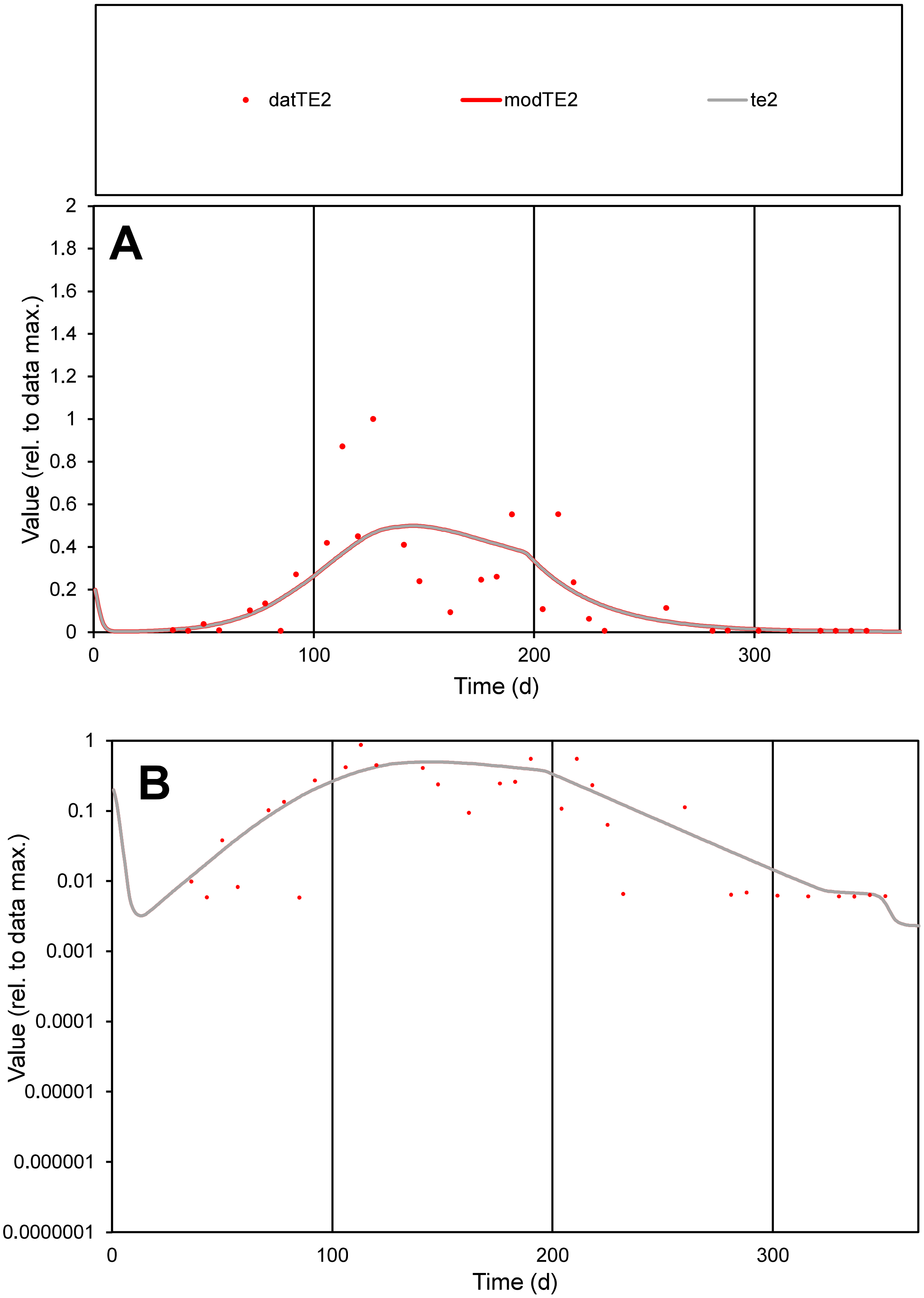

C1

Variables

TE2 Provide jtd or Dst

CalibOnly?

2 1 to reproduce of

oNormalize?

2 1=no, 2=yes

ofxn calc*

0.9137

ofxn mod

0.92537

sRun

RunA1

zout

1

Ave

Data

0.00285

Model

0.00278

data/vMax

0.01458

ofxng

321.551

Min d/m

1.21978

ave/min d

33.4876

ave/min m

39.8375

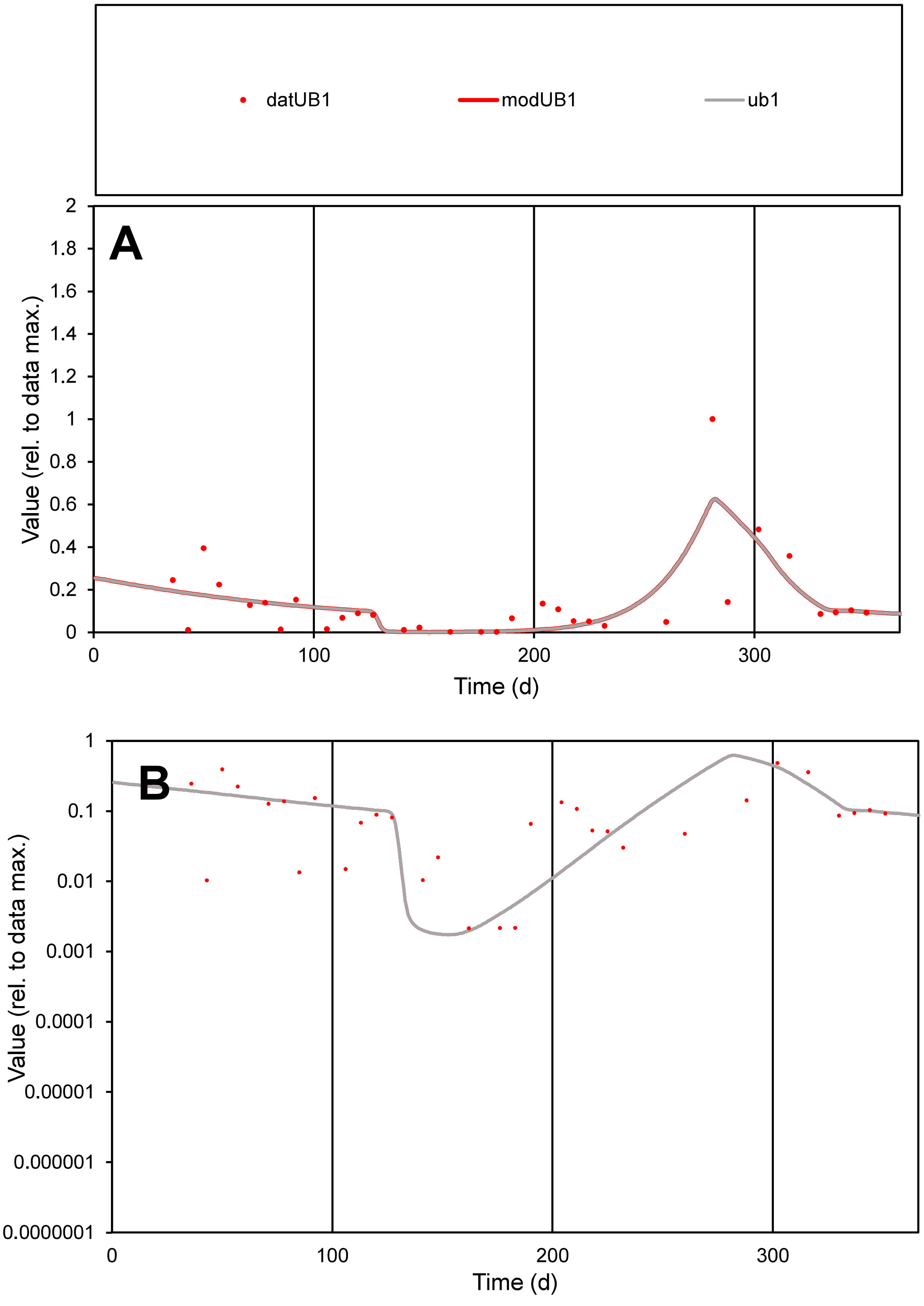

C1

Variables

UB1 Provide jtd or Dst

CalibOnly?

2 1 to reproduce of

oNormalize?

2 1=no, 2=yes

ofxn calc*

0.91795

ofxn mod

0.92817

sRun

RunA1

zout

1

Ave

Data

0.00362

Model

0.00354

data/vMax

0.02605

ofxng

321.551

Min d/m

1.21813

ave/min d

64.7792

ave/min m

77.0325

324_Calib_S_A

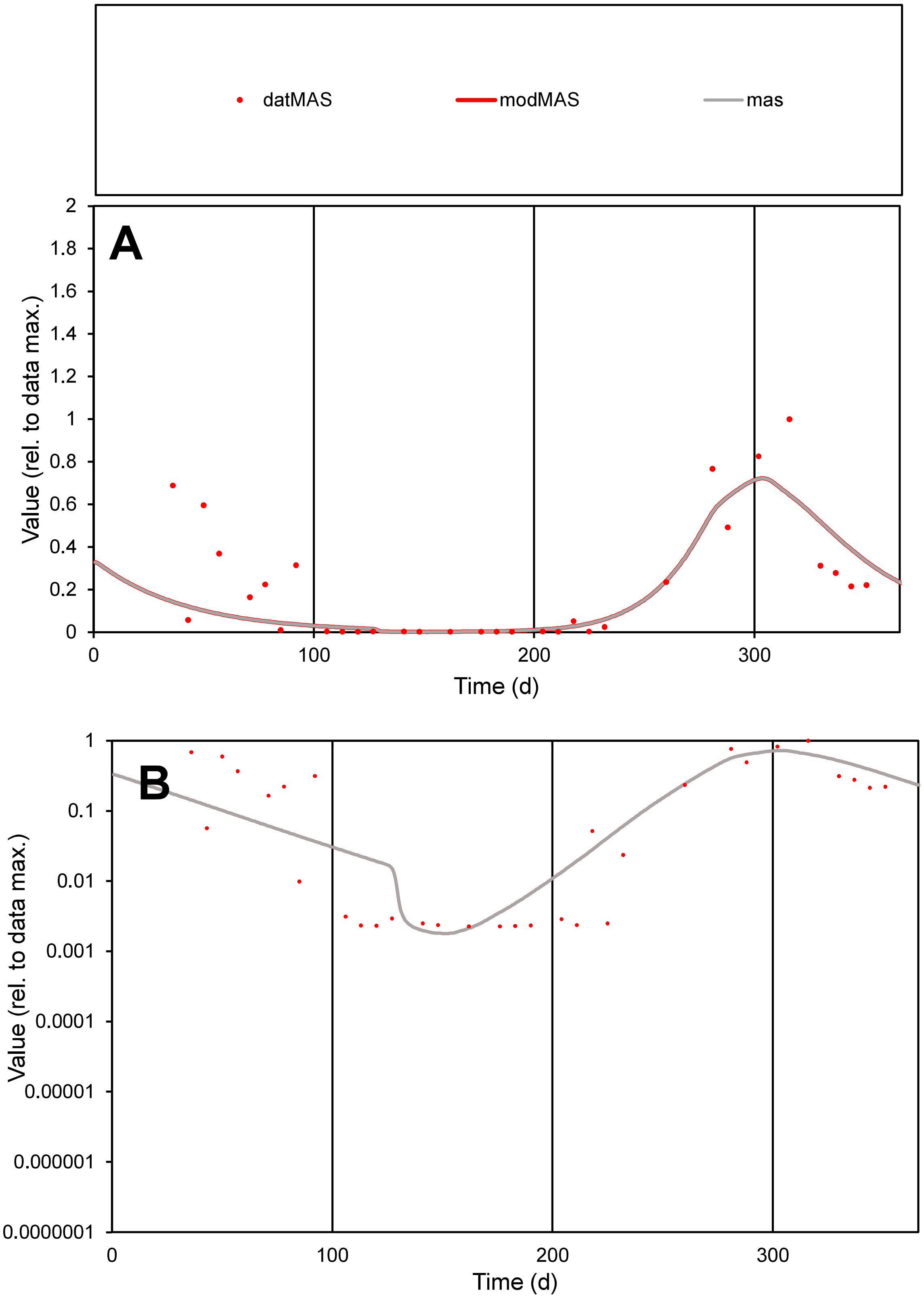

C1

Variables
MAS Provide jtd or Dst
CalibOnly?
2 1 to reproduce of
oNormalize?
2 1=no, 2=yes

ofxn calc*
0.84629
ofxn mod
0.85823

sRun
RunA1
zout
1

Ave
Data
0.00112
Model
0.00088

data/vMax
0.00522

ofxng
321.551

Min d/m
1.25386

ave/min d
95.0783
ave/min m
93.2118

324_Calib_S_A

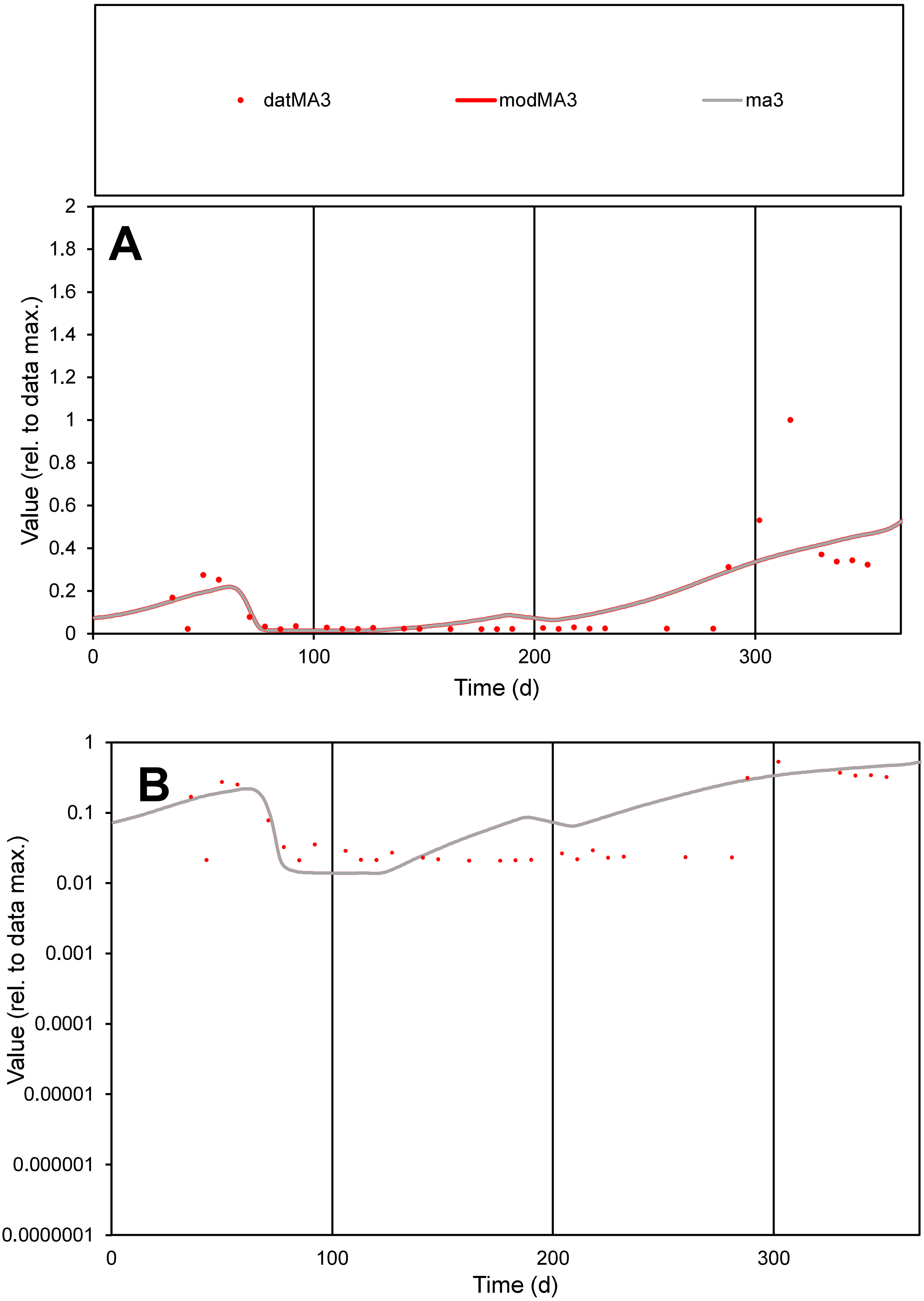

C1

Variables
MA3 Provide jtd or Dst
CalibOnly?
2 1 to reproduce of
oNormalize?
2 1=no, 2=yes

ofxn calc*
0.97792
ofxn mod
0.99001

sRun
RunA1
zout
1

Ave
Data
0.00041
Model
0.00045

data/vMax
0.00292

ofxng
321.551

Min d/m
1.51145

ave/min d
6.75587
ave/min m
11.2275

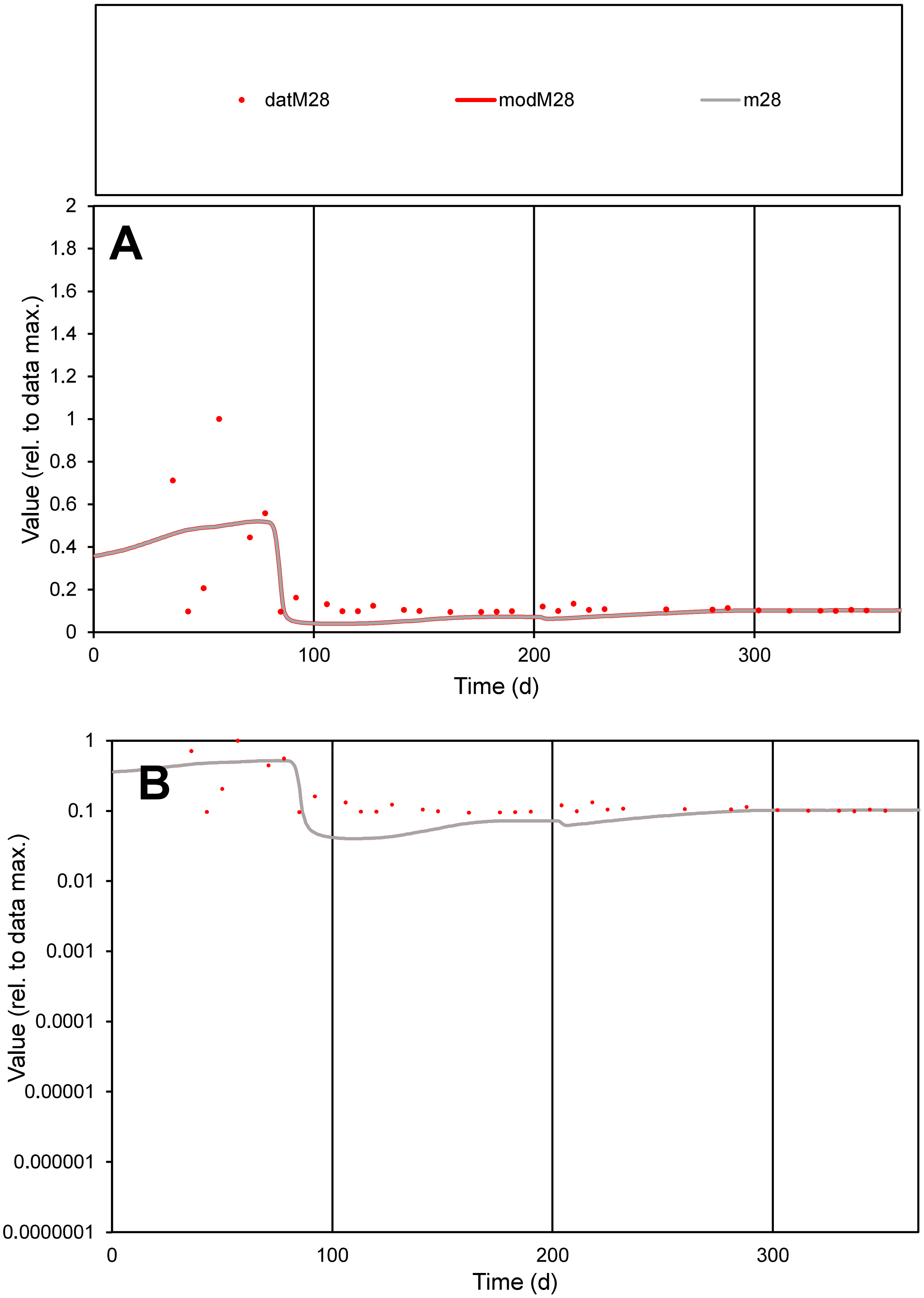

C1

Variables
M28 Provide jtd or Dst
CalibOnly?
2 1 to reproduce of
oNormalize?
2 1=no, 2=yes

ofxn calc*
0.75463
ofxn mod
0.7643

sRun
RunA1
zout
1

Ave
Data
0.0001
Model
8.6E-05

data/vMax
0.00056

ofxng
321.551

Min d/m
2.3431

ave/min d
1.92008
ave/min m
3.81661

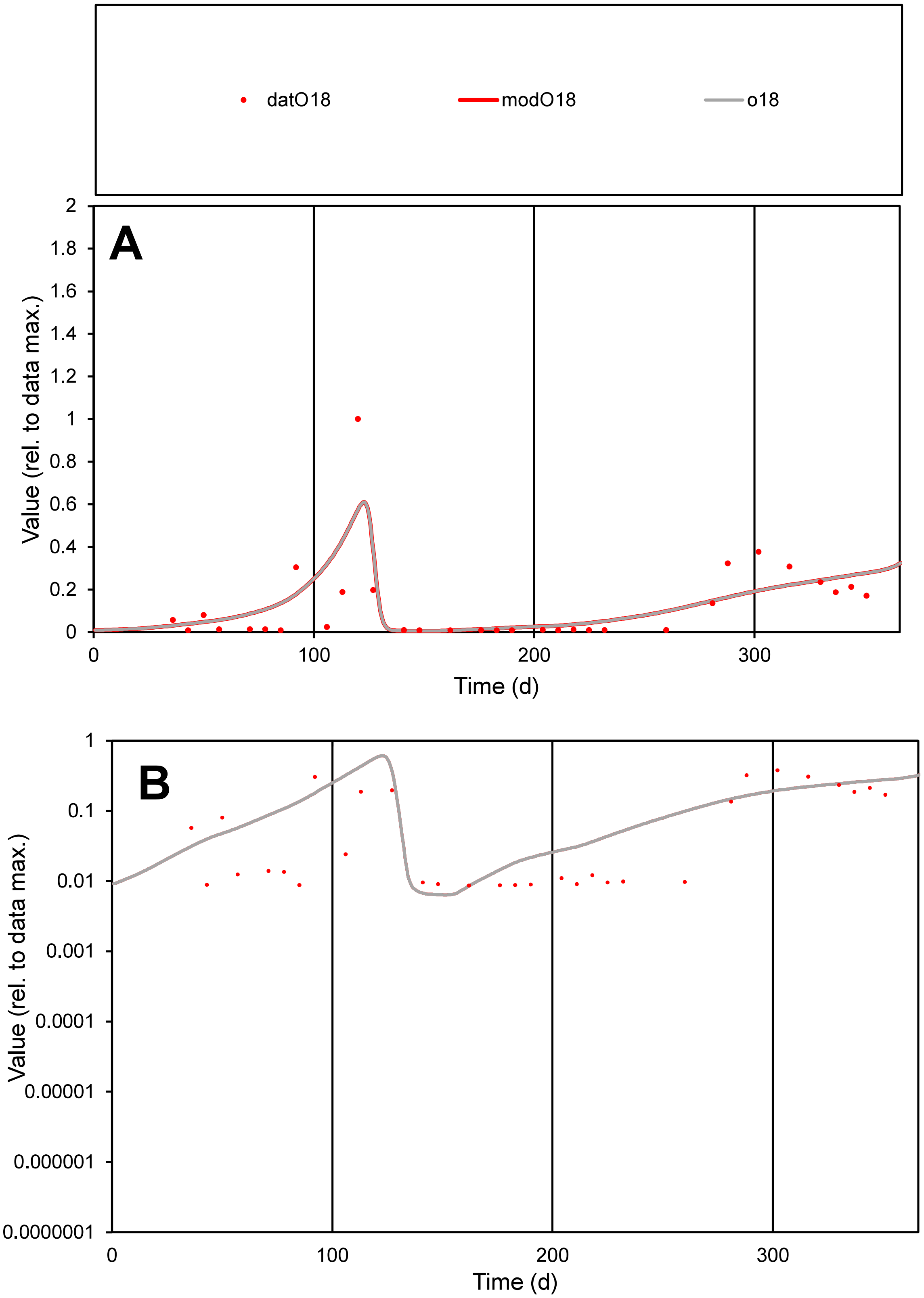

C1

Variables
O18 Provide jtd or Dst
CalibOnly?
2 1 to reproduce of
oNormalize?
2 1=no, 2=yes

ofxn calc*
0.97069
ofxn mod
0.98787

sRun
RunA1
zout
1

Ave
Data
0.00072
Model
0.00082

data/vMax
0.00583

ofxng
321.551

Min d/m
1.35643

ave/min d
14.3347
ave/min m
22.0606

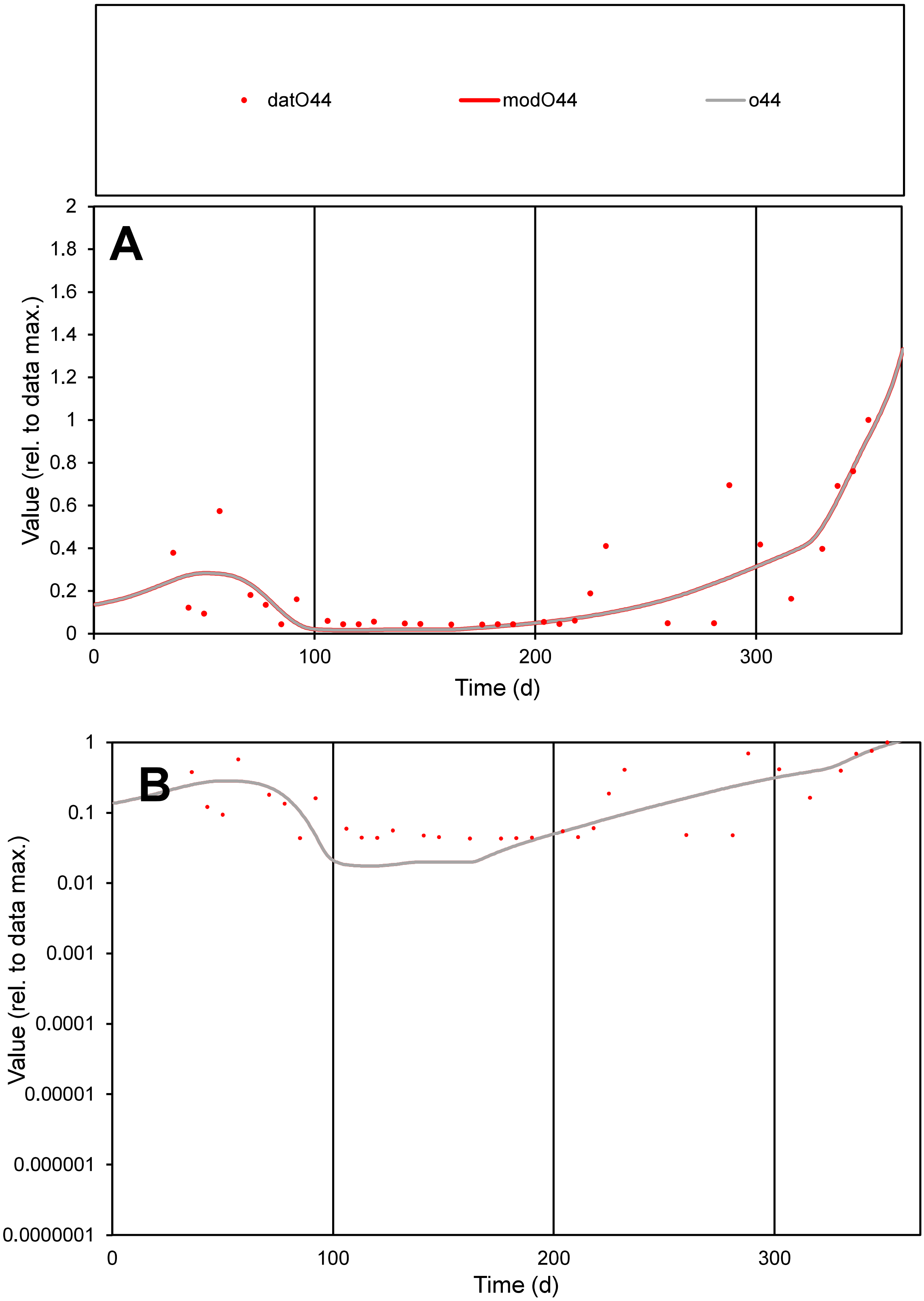

C1

Variables

O44 Provide jtd or Dst

CalibOnly?

2 1 to reproduce of

oNormalize?

2 1=no, 2=yes

ofxn calc*

0.61811

ofxn mod

0.63066

sRun

RunA1

zout

1

Ave

Data

0.00056

Model

0.00051

data/vMax

0.00252

ofxng

321.551

Min d/m

2.44128

ave/min d

5.19536

ave/min m

11.6251

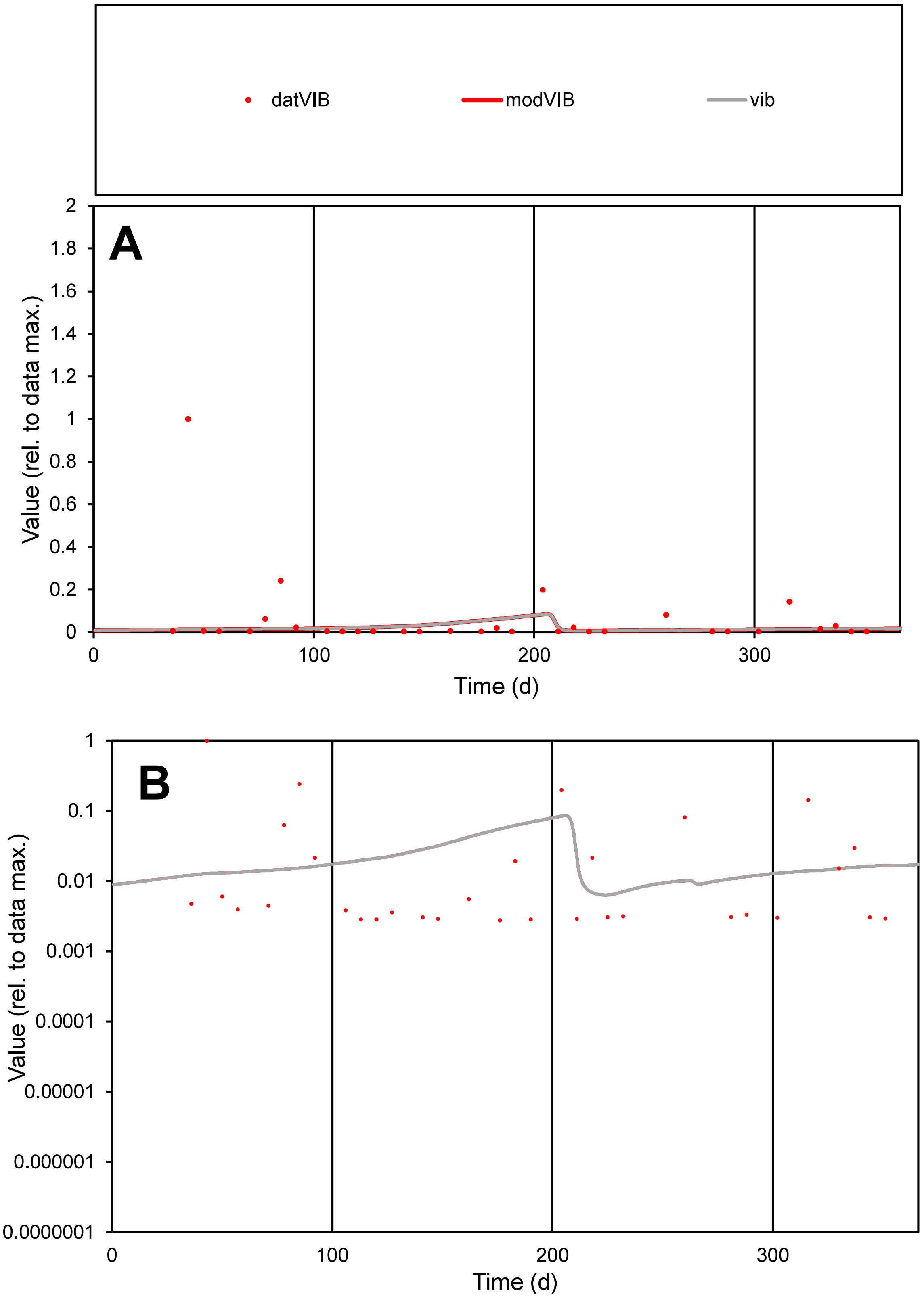

C1

Variables
VIB Provide jtd or Dst
CalibOnly?
2 1 to reproduce of
oNormalize?
2 1=no, 2=yes

ofxn calc*
3.07657
ofxn mod
3.08298

sRun
RunA1
zout
1

Ave
Data
0.00037
Model
0.00014

data/vMax
0.00628

ofxng
321.551

Min d/m
0.43488
ave/min d
21.4673
ave/min m
3.54932

324_Calib_S_A

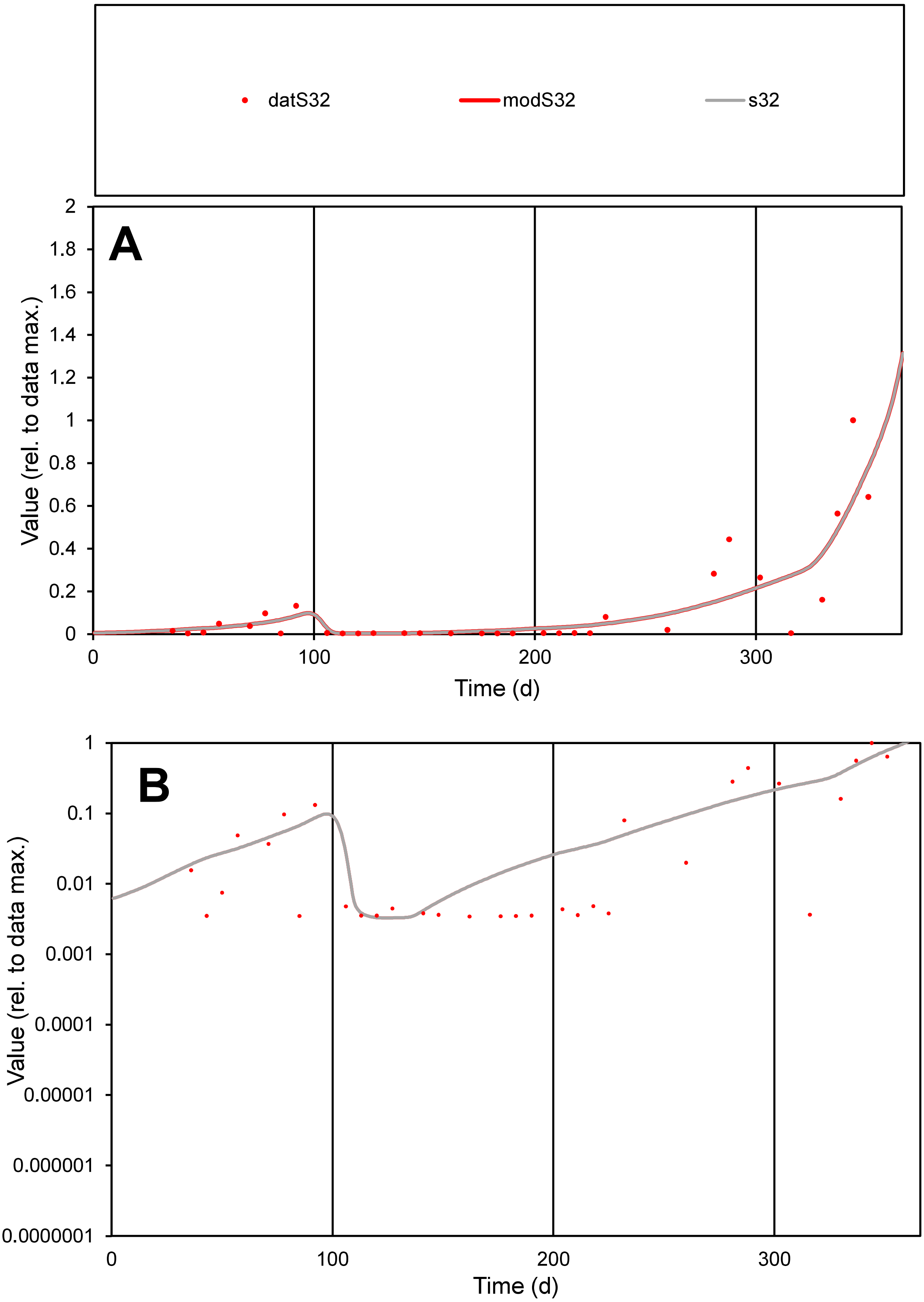

C1

Variables

S32 Provide jtd or Dst

CalibOnly?

2 1 to reproduce of

oNormalize?

2 1=no, 2=yes

ofxn calc*

0.89922

ofxn mod

0.9181

sRun

RunA1

zout

1

Ave

Data

0.00037

Model

0.00038

data/vMax

0.00307

ofxng

321.551

Min d/m

1.04866

ave/min d

35.0626

ave/min m

37.4801

324_Calib_S_A

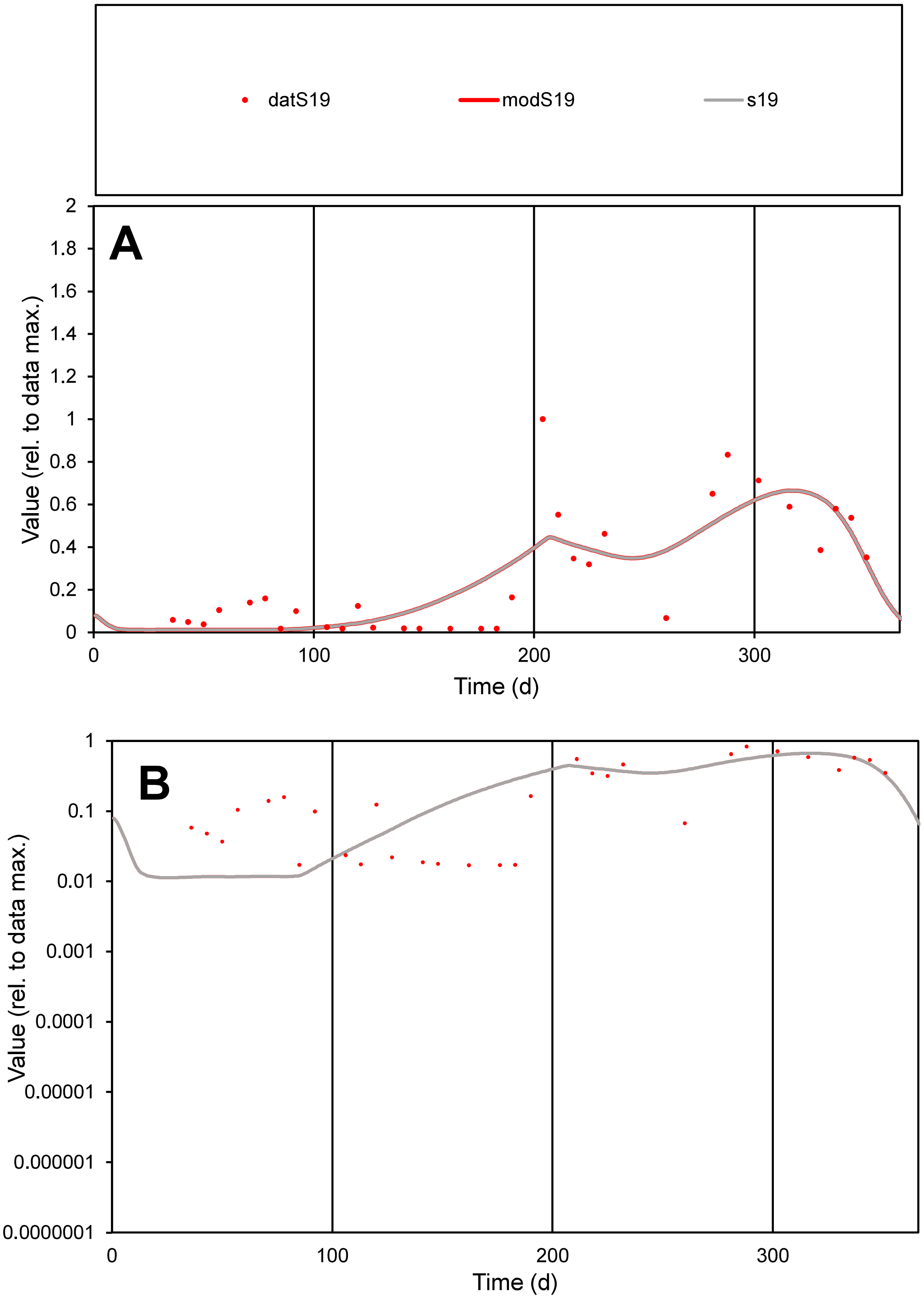

C1

Variables

S19 Provide jtd or Dst

CalibOnly?

2 1 to reproduce of

oNormalize?

2 1=no, 2=yes

ofxn calc*

0.62375

ofxn mod

0.63845

sRun

RunA1

zout

1

Ave

Data

0.00072

Model

0.0007

data/vMax

0.00273

ofxng

321.551

Min d/m

1.46713

ave/min d

15.661

ave/min m

22.2377

324_Calib_S_A

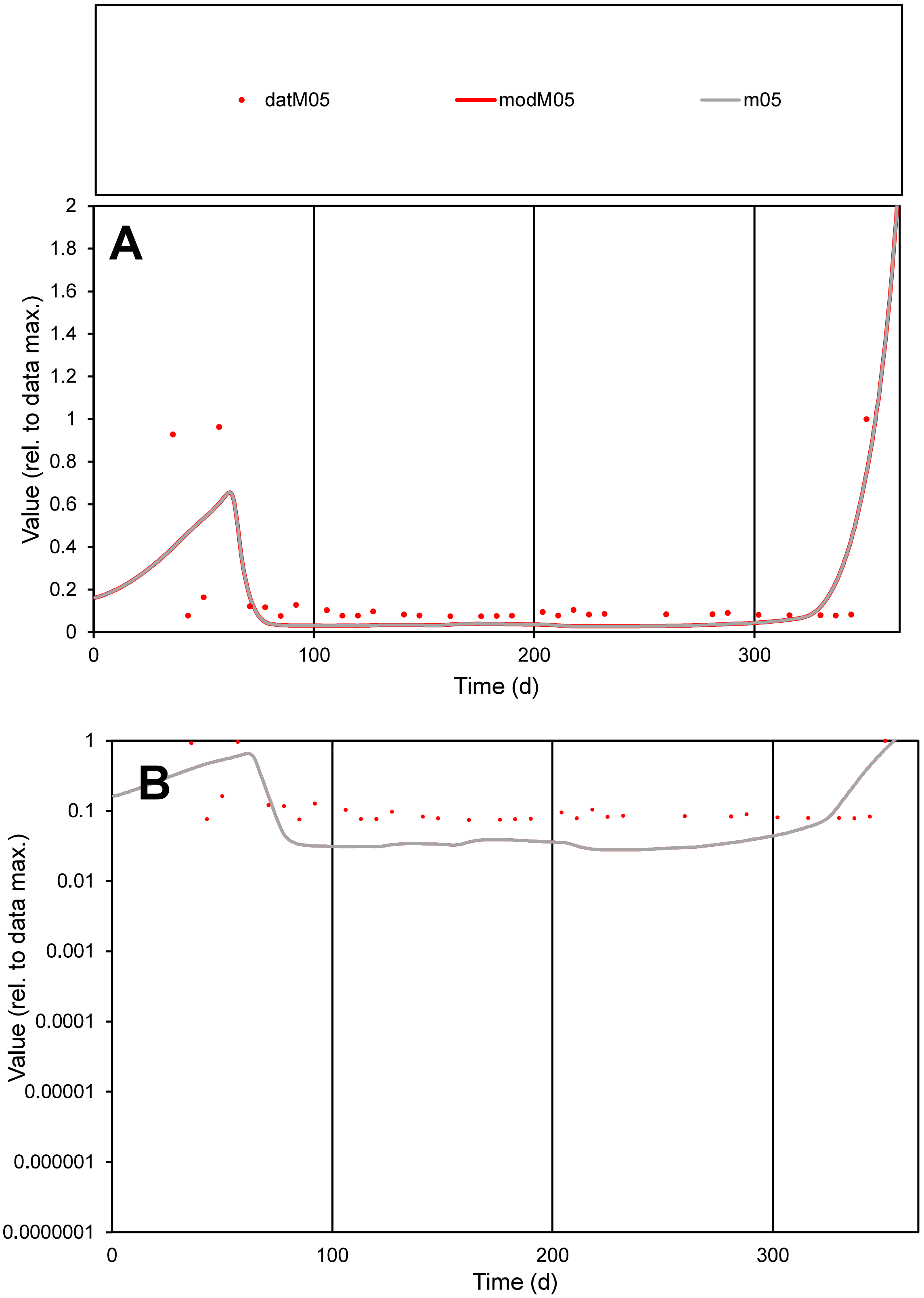

C1

Variables
M05 Provide jtd or Dst
CalibOnly?
2 1 to reproduce of
oNormalize?
2 1=no, 2=yes

ofxn calc*
0.98523
ofxn mod
1.00766

sRun
RunA1
zout
1

Ave
Data
9.7E-05
Model
8E-05

data/vMax
0.00057

ofxng
321.551

Min d/m
2.67914

ave/min d
2.29837
ave/min m
5.08935

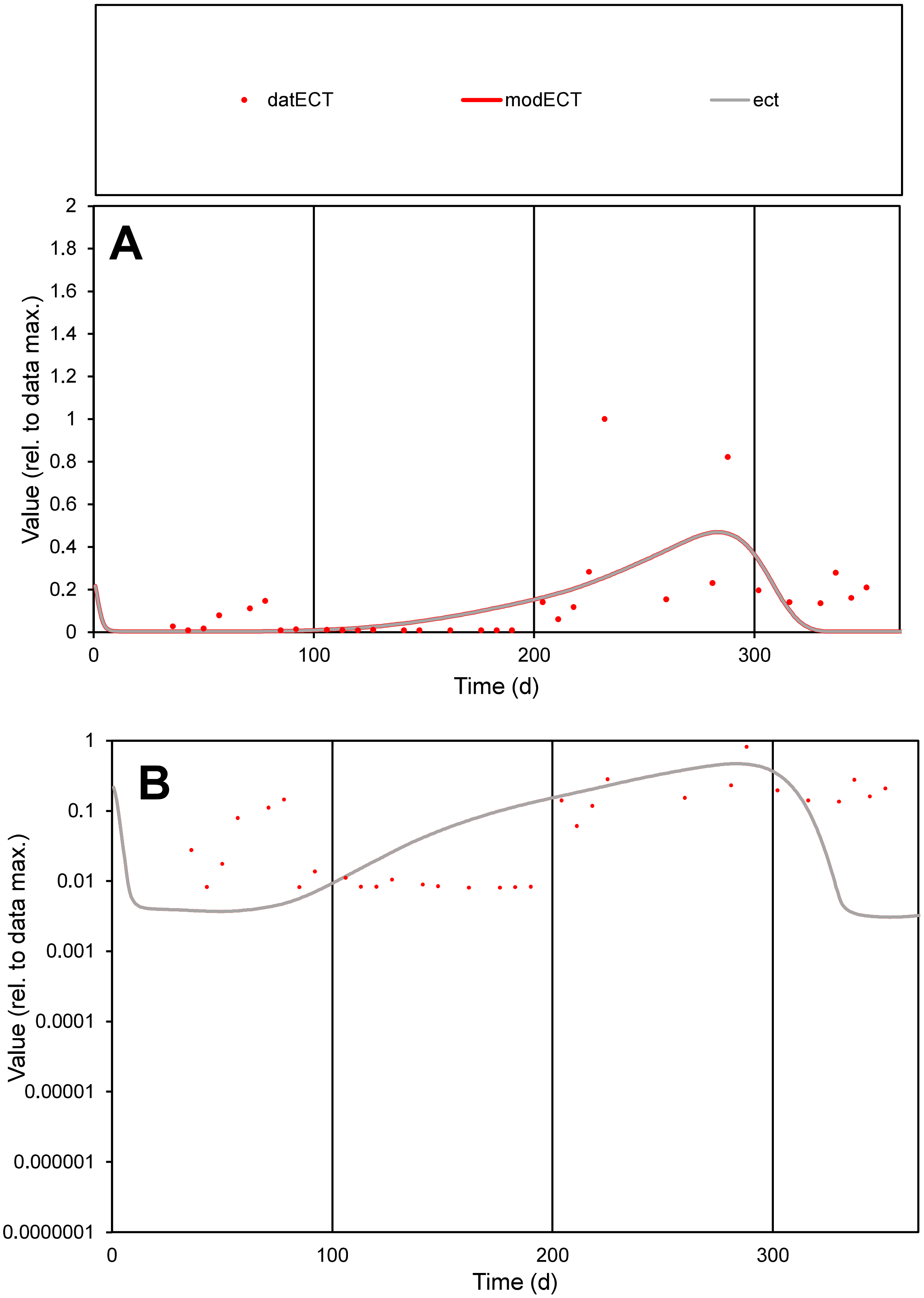

C1

Variables
ECT Provide jtd or Dst
CalibOnly?
2 1 to reproduce of
oNormalize?
2 1=no, 2=yes

ofxn calc*
1.32029
ofxn mod
1.32845

sRun
RunA1
zout
1

Ave
Data
0.00048
Model
0.00037

data/vMax
0.00344

ofxng
321.551

Min d/m
2.61509

ave/min d
17.1646
ave/min m
34.6573

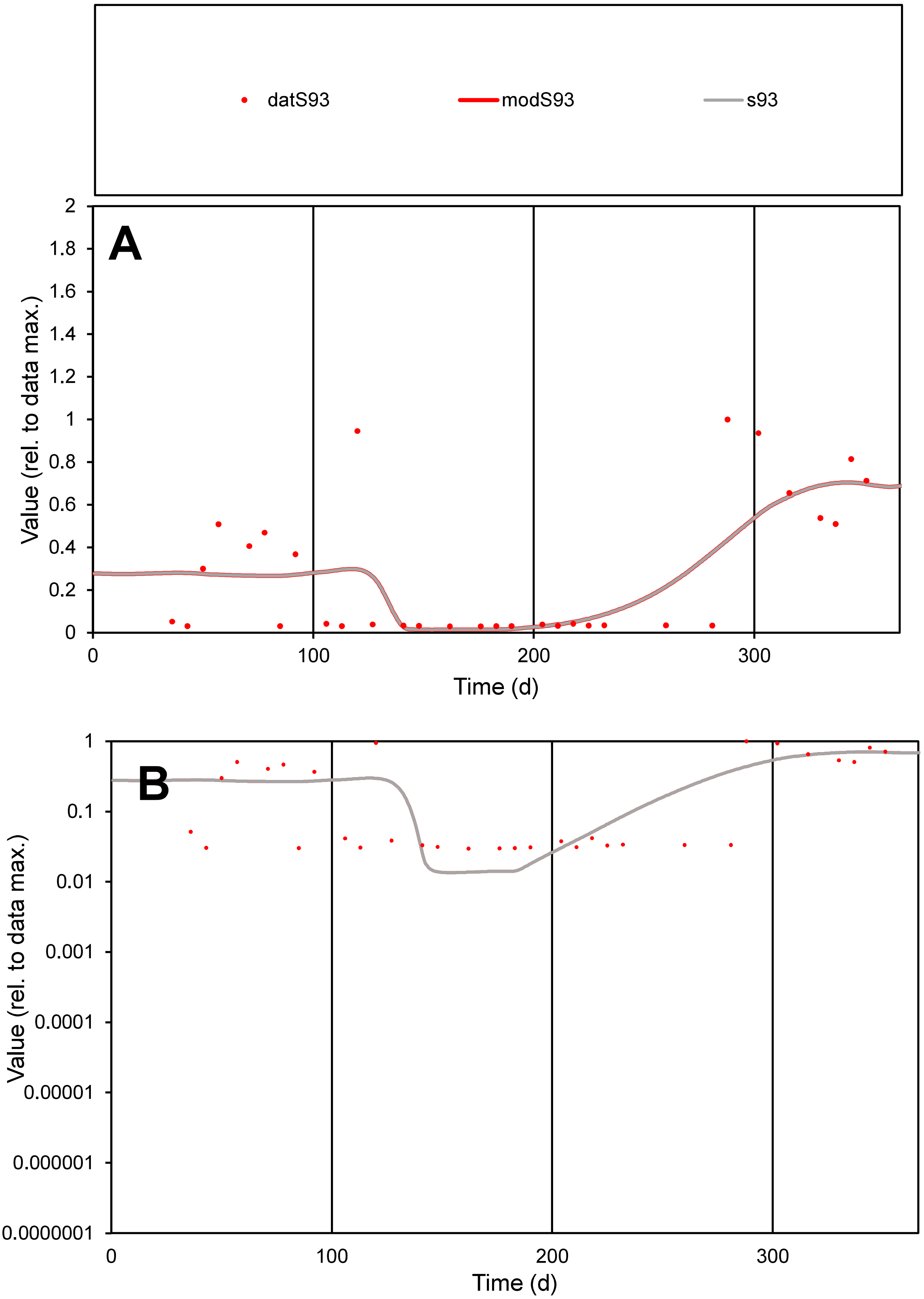

C1

Variables
S93 Provide jtd or Dst
CalibOnly?
2 1 to reproduce of
oNormalize?
2 1=no, 2=yes

ofxn calc*
0.80365
ofxn mod
0.81503

sRun
RunA1
zout
1

Ave
Data
0.00059
Model
0.00058

data/vMax
0.00214

ofxng
321.551

Min d/m
2.17337

ave/min d
9.23739
ave/min m
19.8746

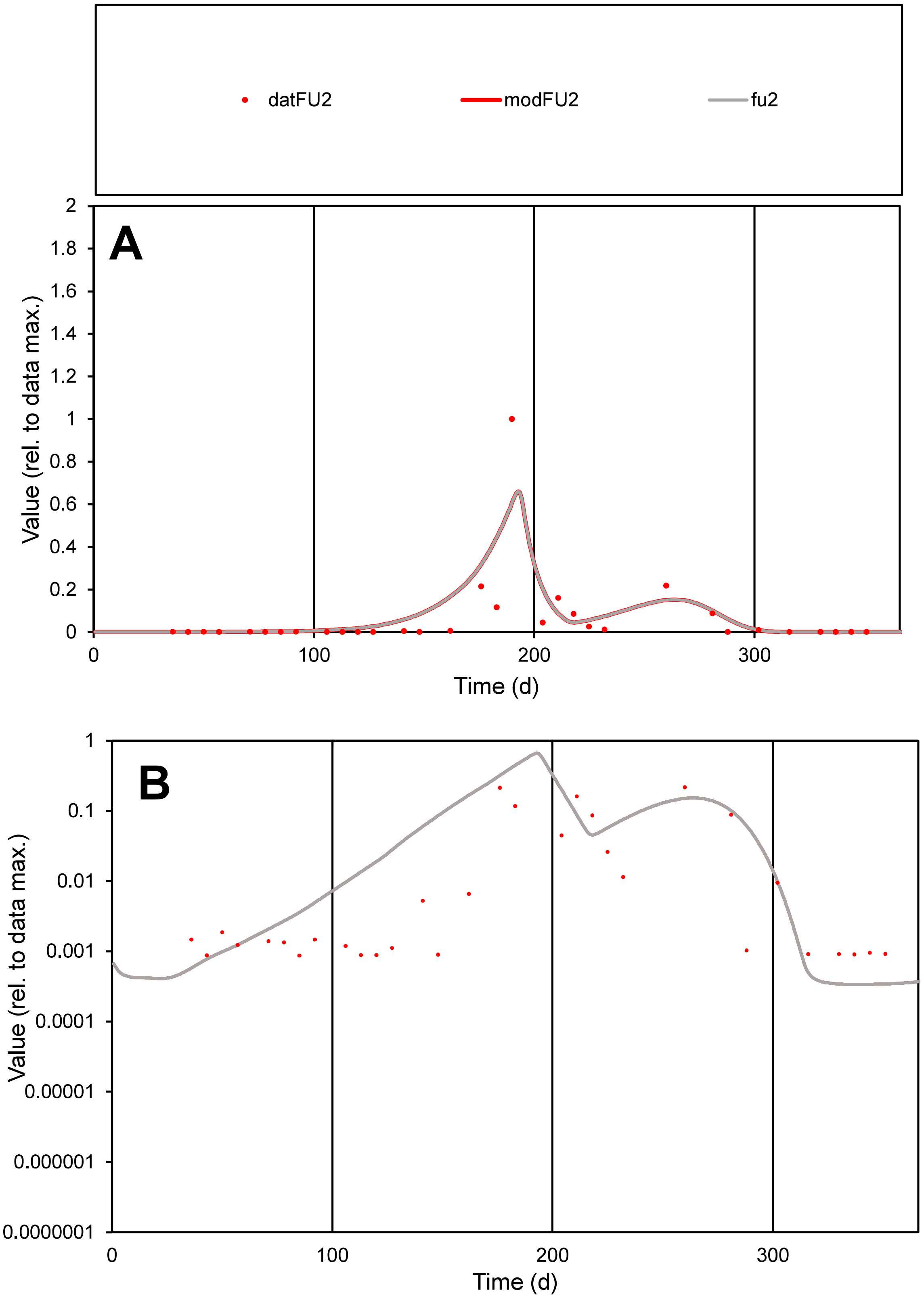

C1

Variables
FU2 Provide jtd or Dst
CalibOnly?
2 1 to reproduce of
oNormalize?
2 1=no, 2=yes

ofxn calc*
1.58268
ofxn mod
1.6381

sRun
RunA1
zout
1

Ave
Data
0.00183
Model
0.00228

data/vMax
0.02914

ofxng
321.551

Min d/m
2.58055

ave/min d
72.0769
ave/min m
231.443

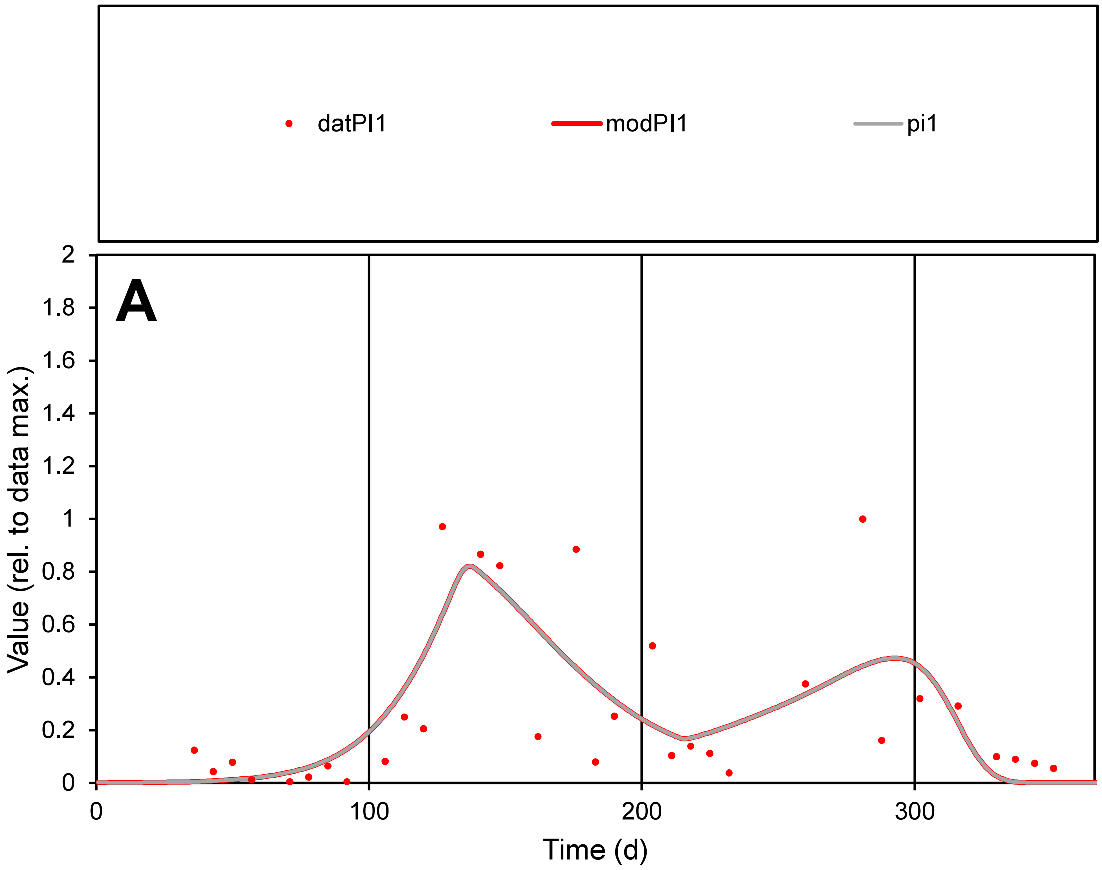

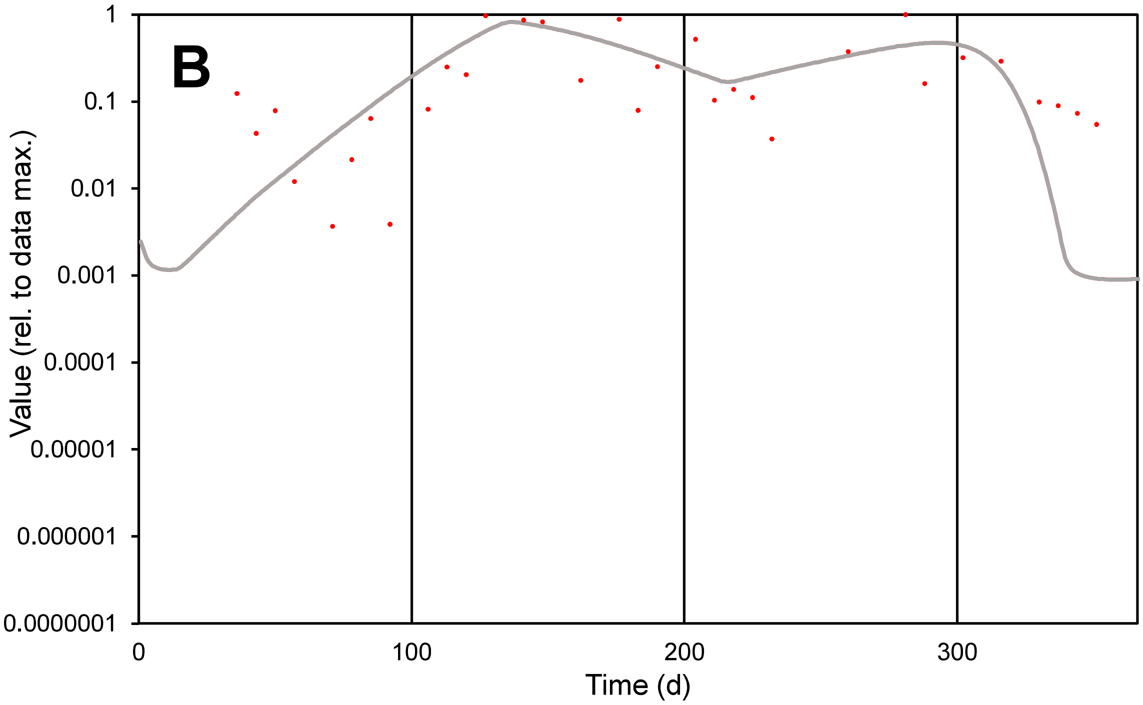

C1

Variables
PI1 Provide jtd or Dst
CalibOnly?
2 1 to reproduce of
oNormalize?
2 1=no, 2=yes

ofxn calc*
0.77625
ofxn mod
0.791

sRun
RunA1
zout
1

Ave
Data
0.00147
Model
0.00145

data/vMax
0.00566

ofxng
321.551

Min d/m
4.02203

ave/min d
70.581
ave/min m
280.227

324_Calib_S_A

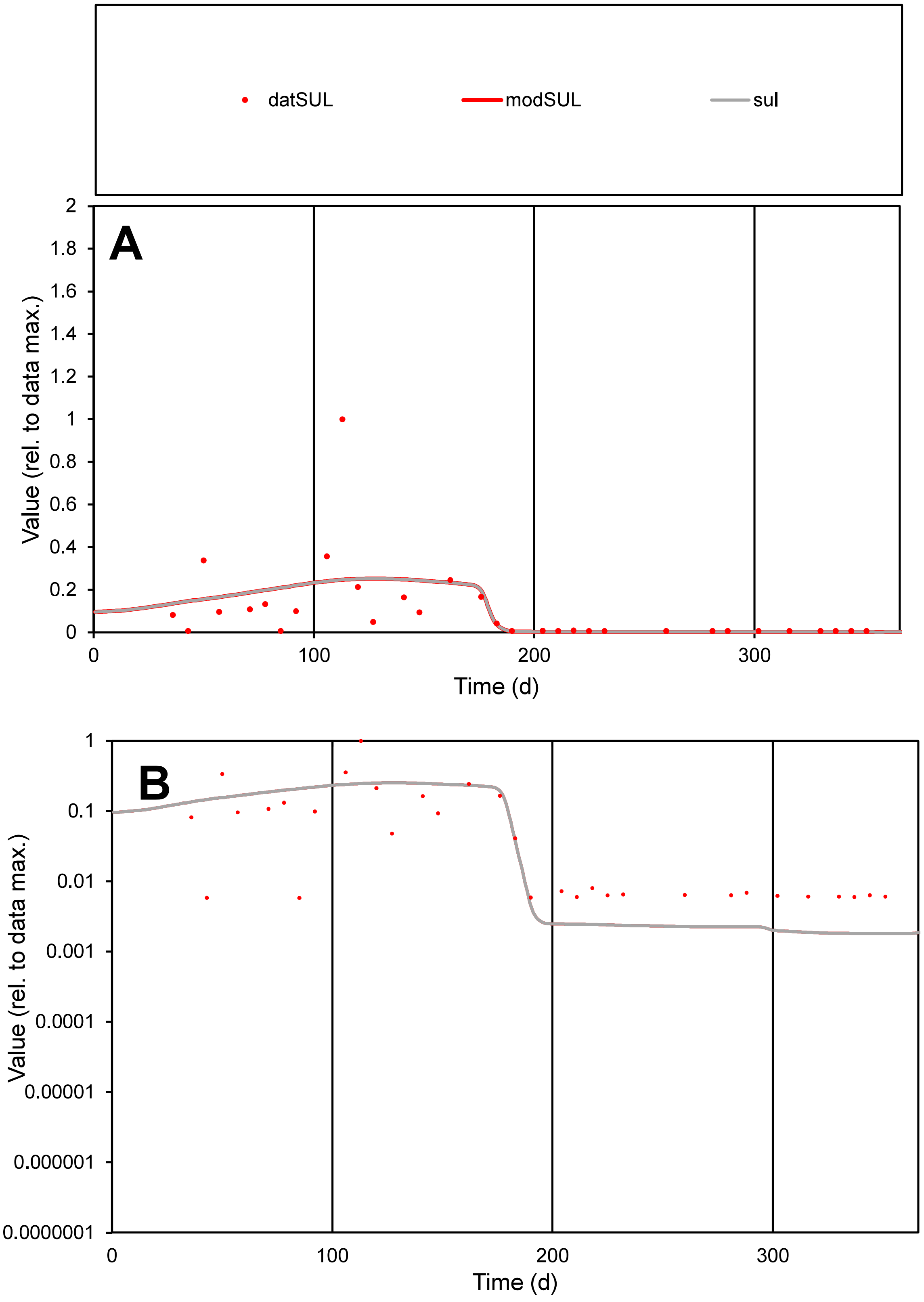

C1

Variables
SUL Provide jtd or Dst
CalibOnly?
2 1 to reproduce of
oNormalize?
2 1=no, 2=yes

ofxn calc*
1.51902
ofxn mod
1.52747

sRun
RunA1
zout
1

Ave
Data
0.0014
Model
0.00144

data/vMax
0.01358

ofxng
321.551

Min d/m
3.20838

ave/min d
17.703
ave/min m
58.4516

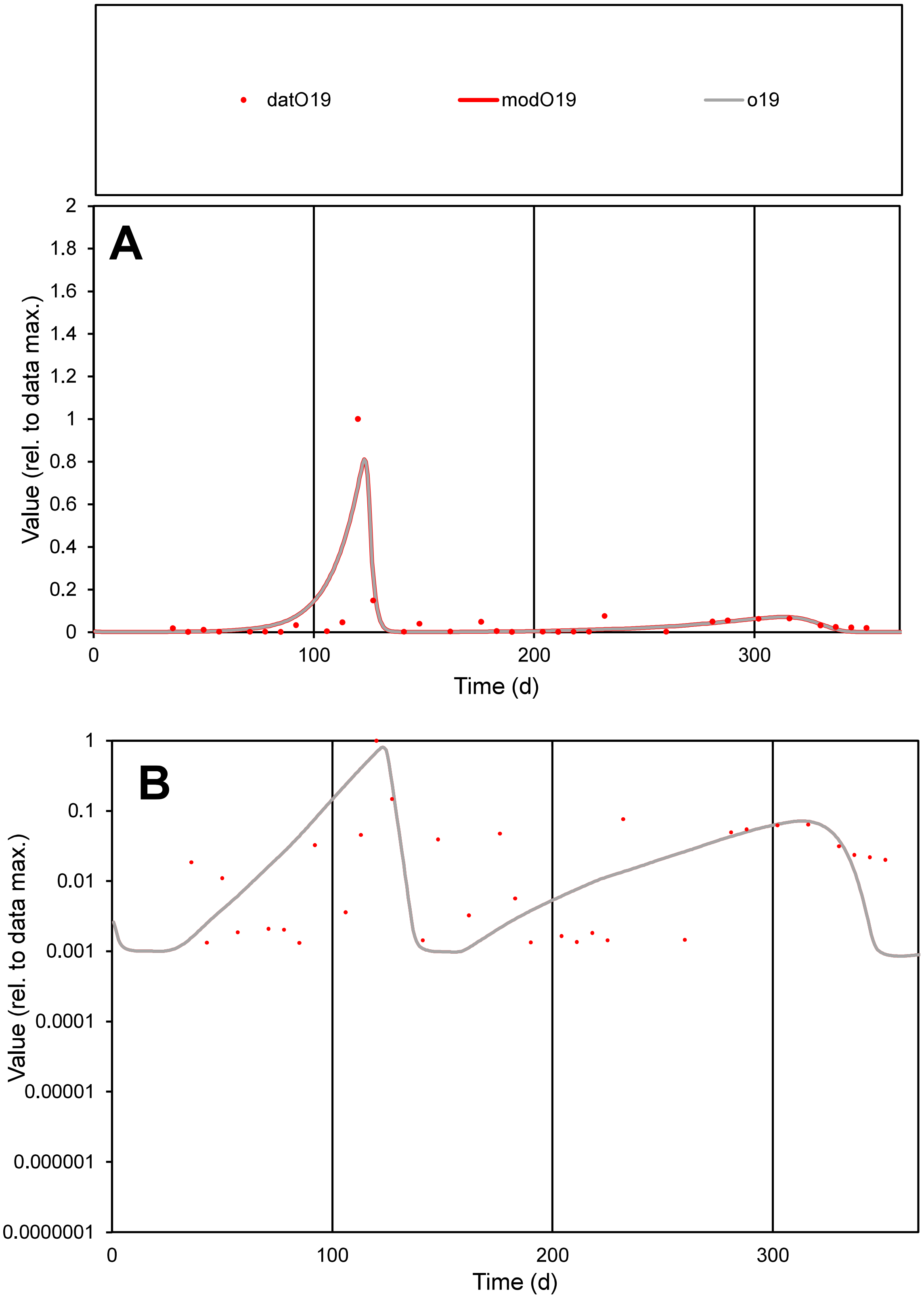

C1

Variables
O19 Provide jtd or Dst
CalibOnly?
2 1 to reproduce of
oNormalize?
2 1=no, 2=yes

ofxn calc*
1.49104
ofxn mod
1.60151

sRun
RunA1
zout
1

Ave
Data
0.00119
Model
0.00136

data/vMax
0.02135

ofxng
321.551

Min d/m
1.47897

ave/min d
42.176
ave/min m
71.3825

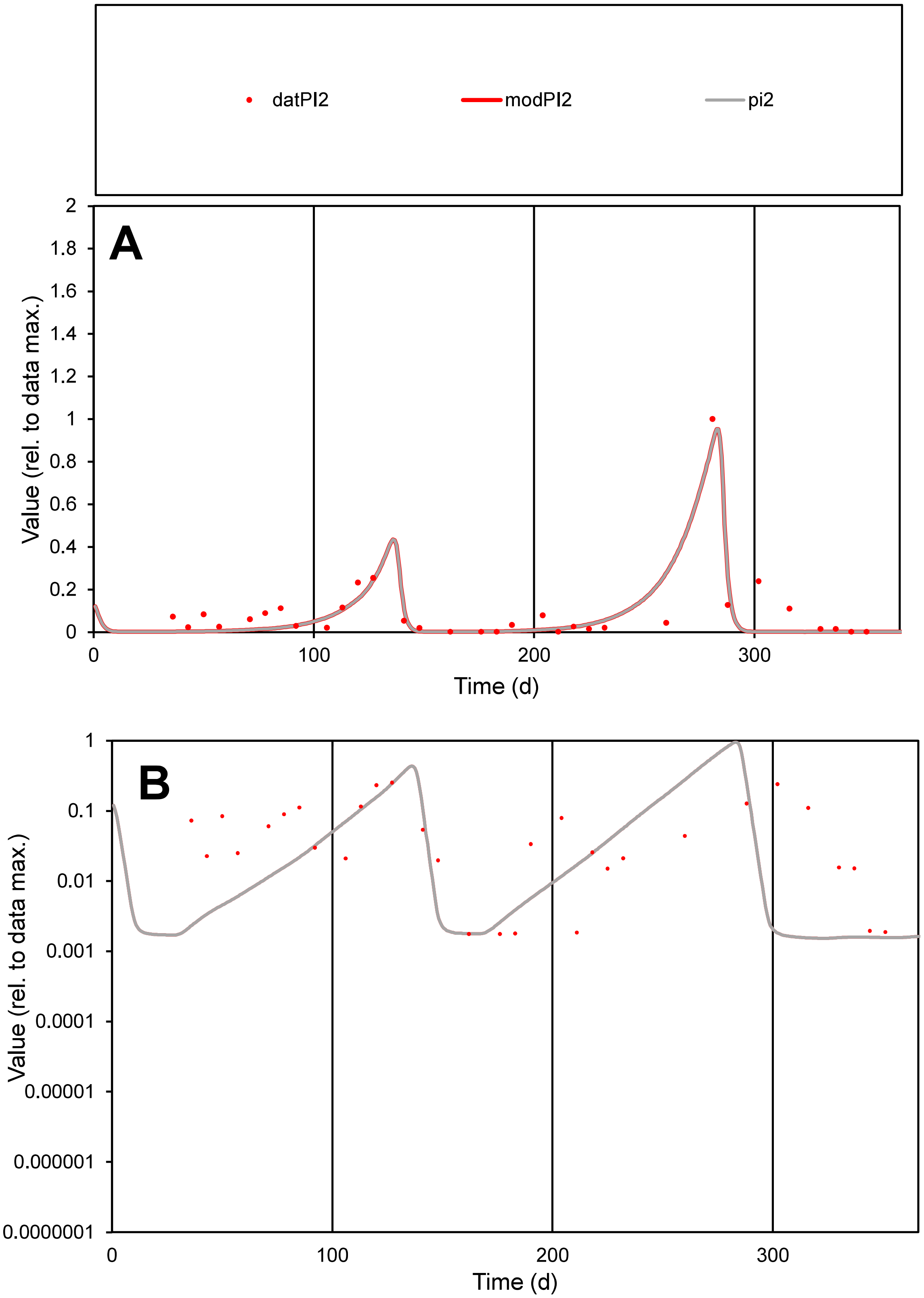

C1

Variables
PI2 Provide jtd or Dst
CalibOnly?
2 1 to reproduce of
oNormalize?
2 1=no, 2=yes

ofxn calc*
0.78687
ofxn mod
0.8167

sRun
RunA1
zout
1

Ave
Data
0.00144
Model
0.00113

data/vMax
0.01572

ofxng
321.551

Min d/m
1.13519

ave/min d
52.0151
ave/min m
46.1126

324_Calib_S_A

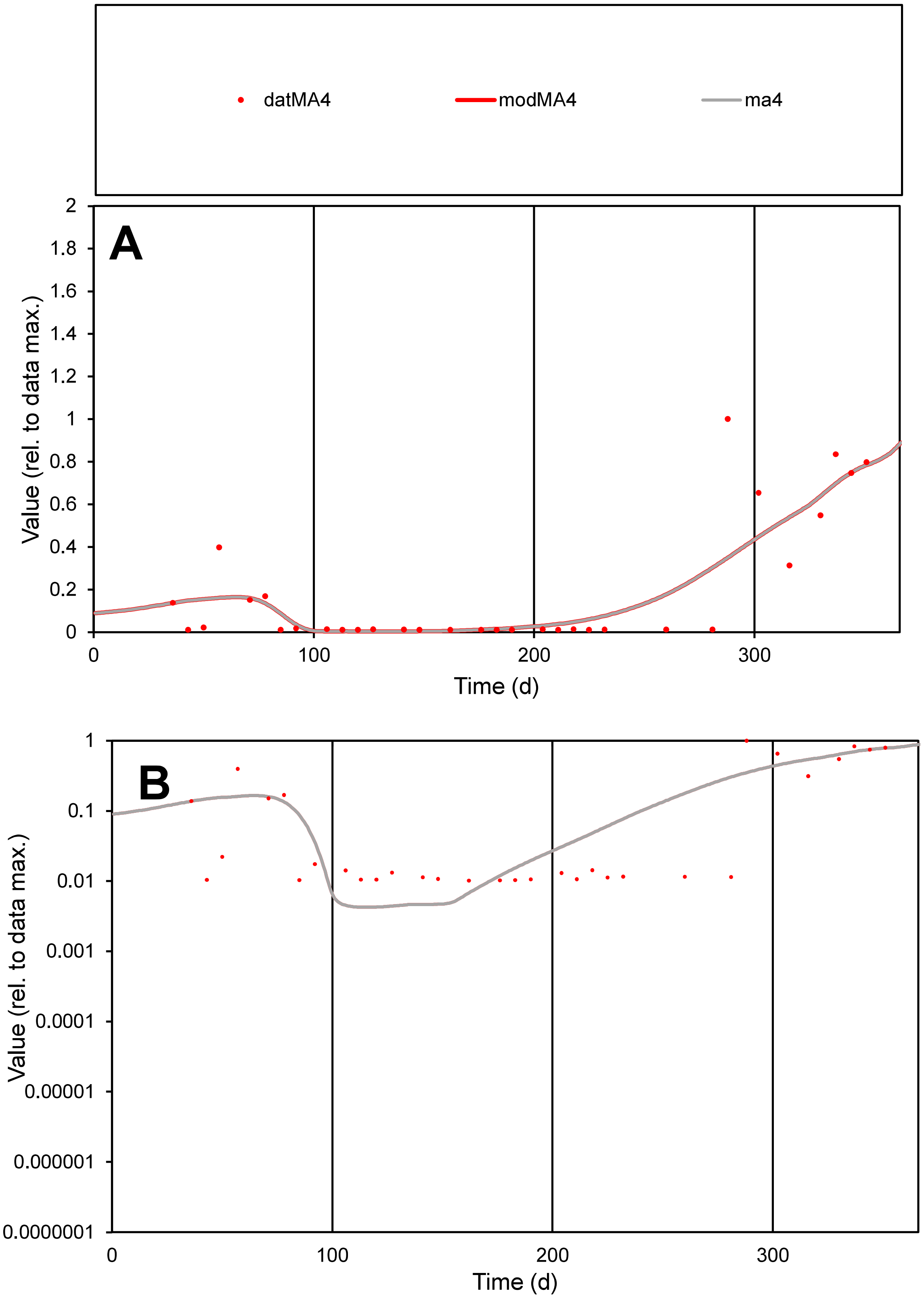

C1

Variables

MA4	Provide jtd or Dst
CalibOnly?	
2	1 to reproduce of
oNormalize?	
2	1=no, 2=yes

ofxn calc*
0.81644
ofxn mod
0.83273

sRun
RunA1
zout
1

Ave
Data
0.00035
Model
0.00035

data/vMax
0.00185

ofxng
321.551

Min d/m
2.38746

ave/min d
18.377
ave/min m
44.3696

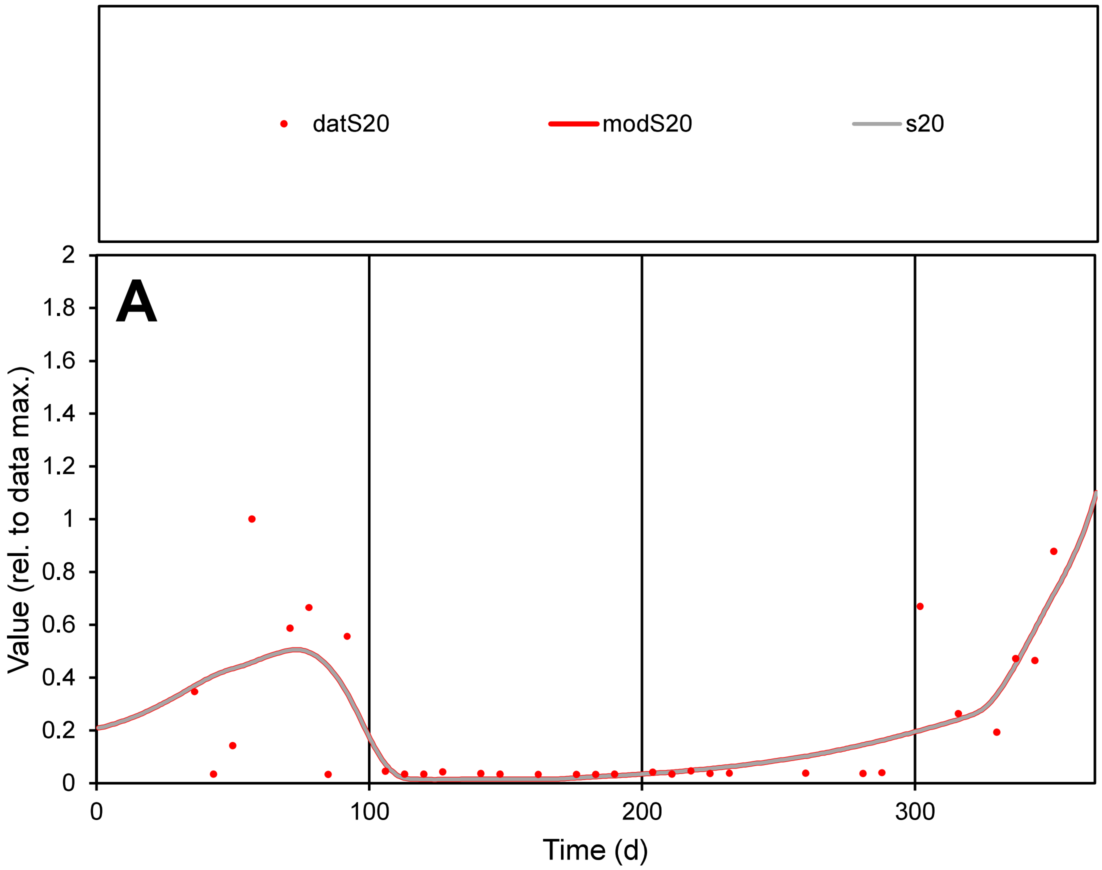

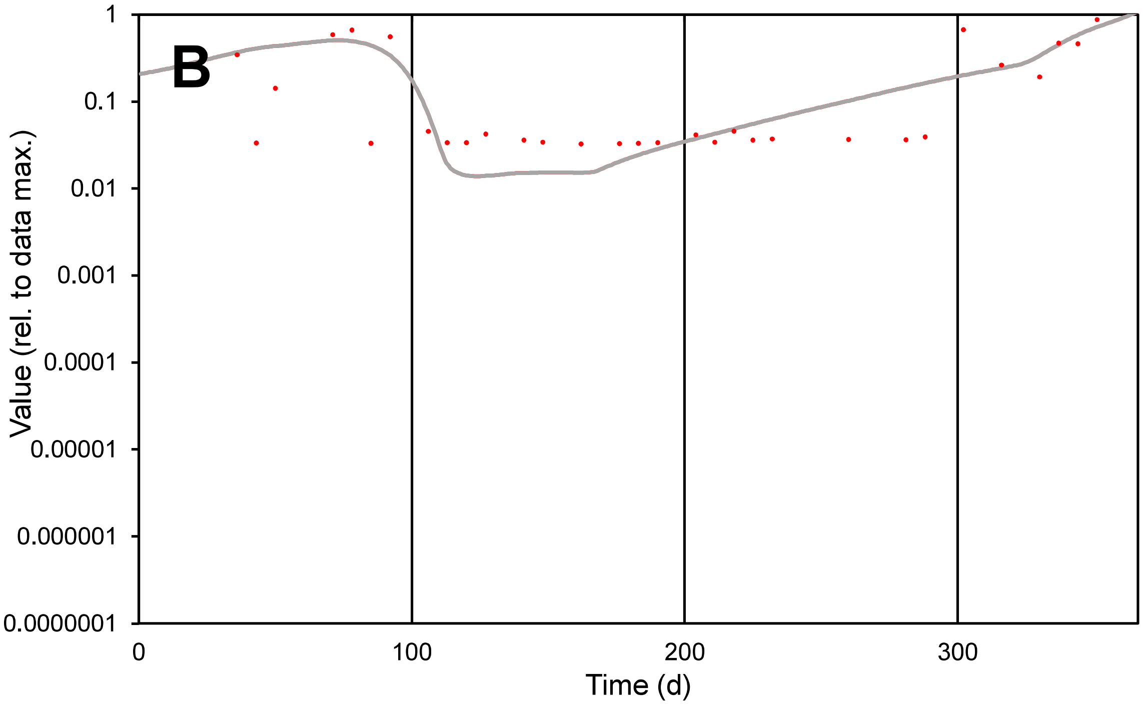

C1

Variables

S20 Provide jtd or Dst

CalibOnly?

2 1 to reproduce of

oNormalize?

2 1=no, 2=yes

ofxn calc*

0.84124

ofxn mod

0.85412

sRun

RunA1

zout

1

Ave

Data

0.00051

Model

0.00051

data/vMax

0.00234

ofxng

321.551

Min d/m

2.33761

ave/min d

6.67049

ave/min m

15.4998

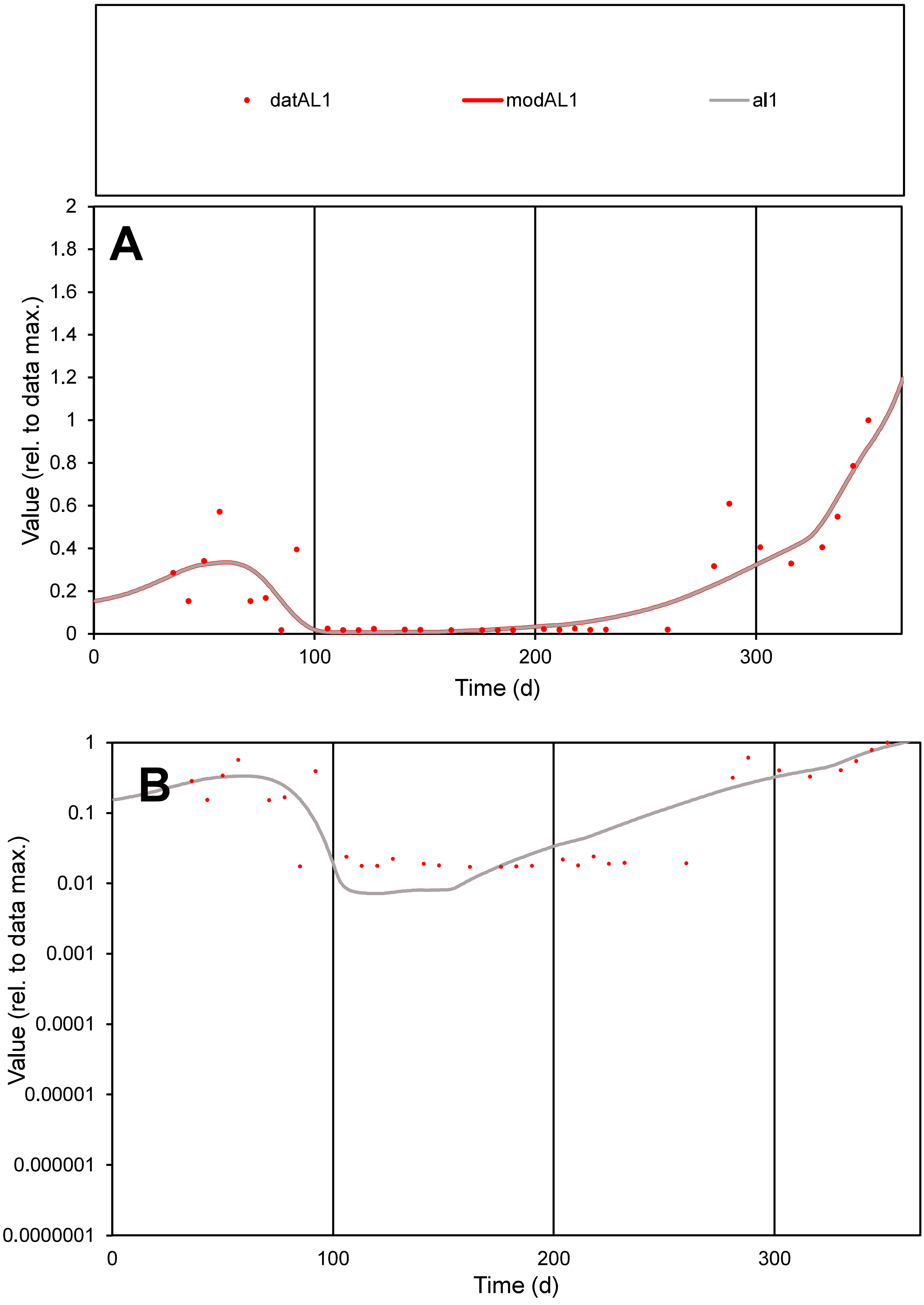

C1

Variables
AL1 Provide jtd or Dst
CalibOnly?
2 1 to reproduce of
oNormalize?
2 1=no, 2=yes

ofxn calc*
0.54067
ofxn mod
0.55814

sRun
RunA1
zout
1

Ave
Data
0.00053
Model
0.00052

data/vMax
0.0025

ofxng
321.551

Min d/m
2.38227

ave/min d
12.3485
ave/min m
28.7864

324_Calib_S_A

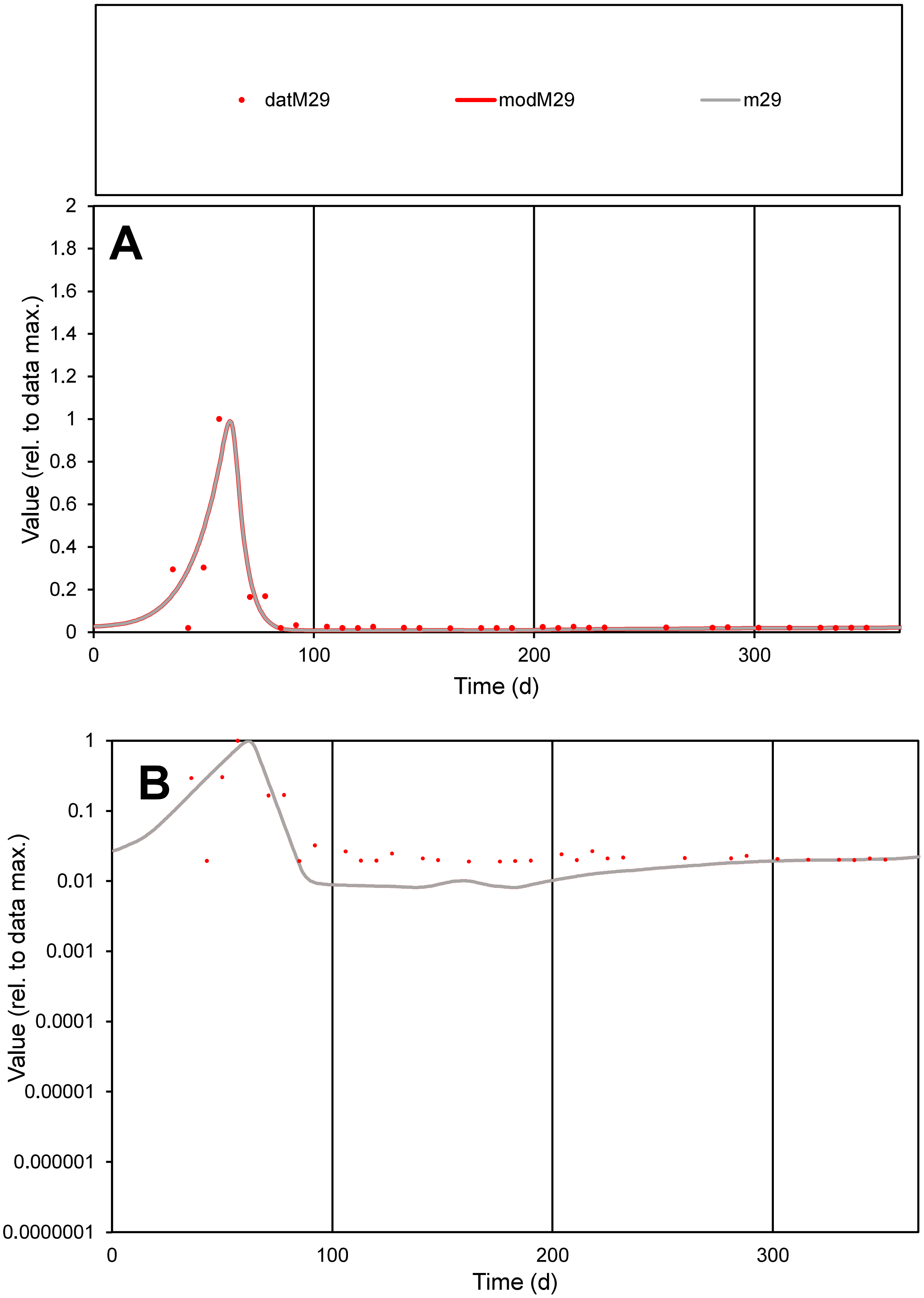

C1

Variables
M29 Provide jtd or Dst
CalibOnly?
2 1 to reproduce of
oNormalize?
2 1=no, 2=yes

ofxn calc*
0.873
ofxn mod
0.94052

sRun
RunA1
zout
1

Ave
Data
0.00022
Model
0.00021

data/vMax
0.00281

ofxng
321.551

Min d/m
2.32737

ave/min d
4.13262
ave/min m
9.35388

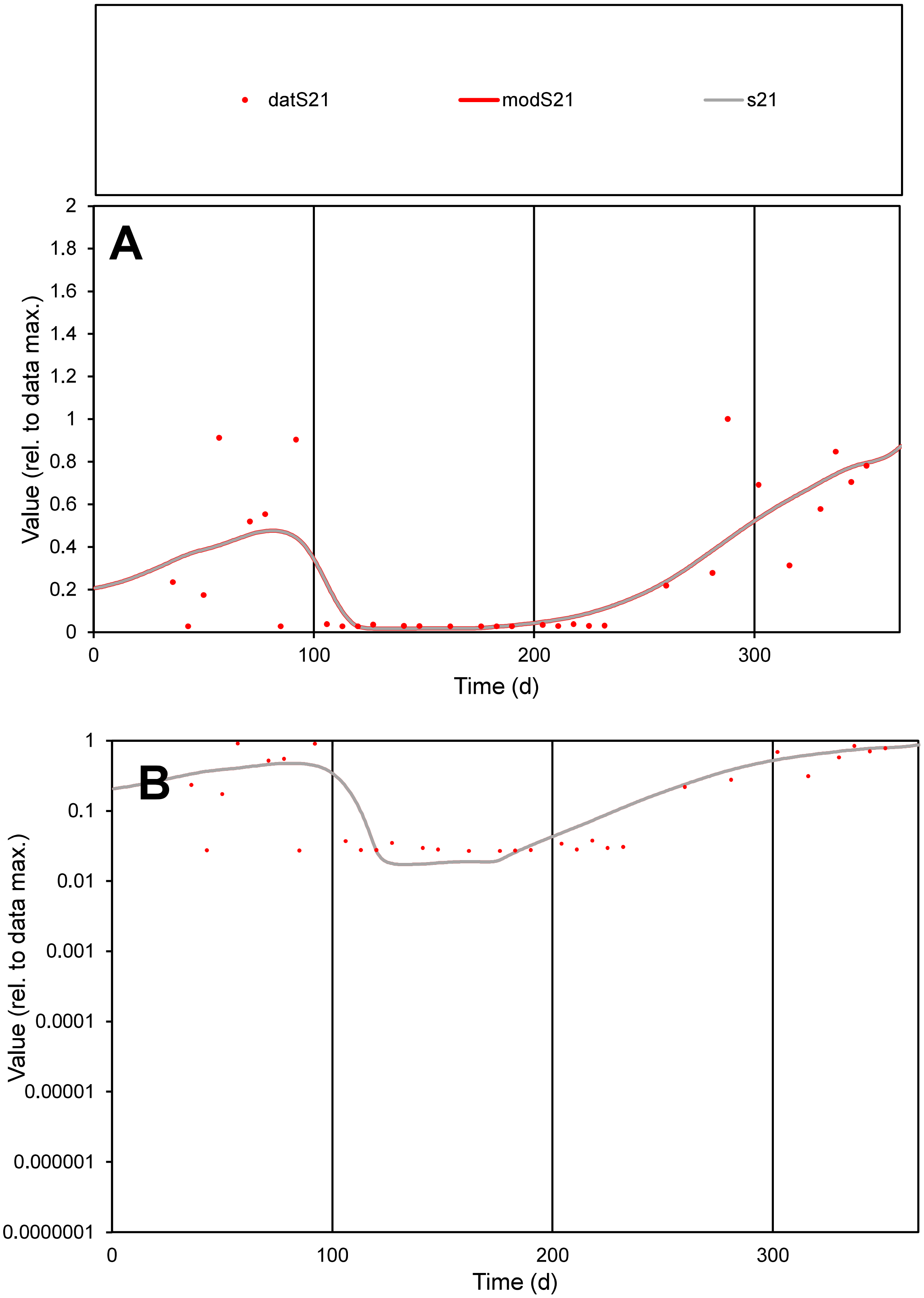

C1

Variables

S21 Provide jtd or Dst

CalibOnly?

2 1 to reproduce of

oNormalize?

2 1=no, 2=yes

ofxn calc*

0.71545

ofxn mod

0.72779

sRun

RunA1

zout

1

Ave

Data

0.00043

Model

0.00044

data/vMax

0.00149

ofxng

321.551

Min d/m

1.5261

ave/min d

10.7288

ave/min m

16.7928

324_Calib_S_A

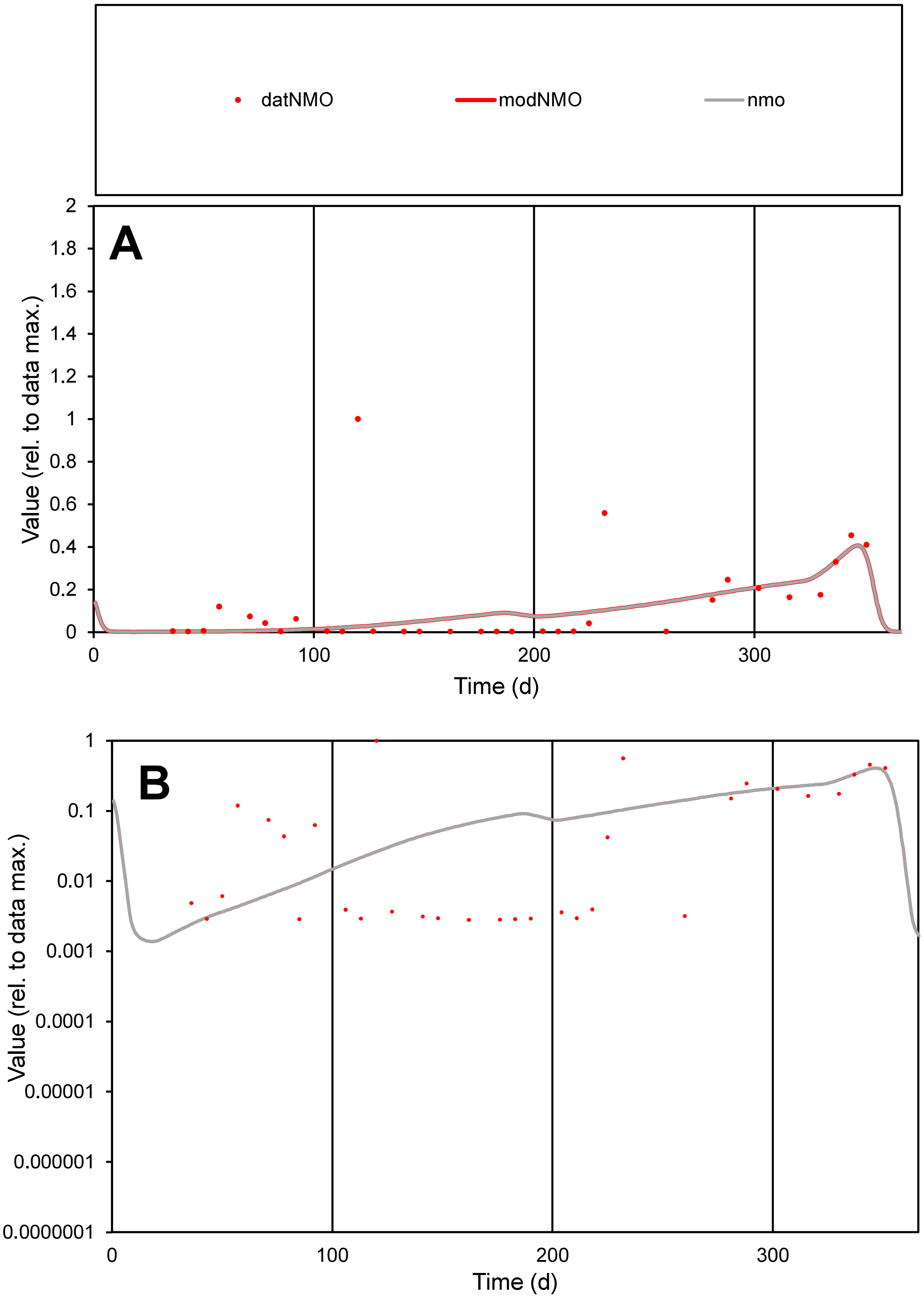

C1

Variables

NMO Provide jtd or Dst

CalibOnly?

2 1 to reproduce of

oNormalize?

2 1=no, 2=yes

ofxn calc*

1.56046

ofxn mod

1.56711

sRun

RunA1

zout

1

Ave

Data

0.00032

Model

0.00026

data/vMax

0.00251

ofxng

321.551

Min d/m

1.11761

ave/min d

45.3497

ave/min m

41.0456

324_Calib_S_A

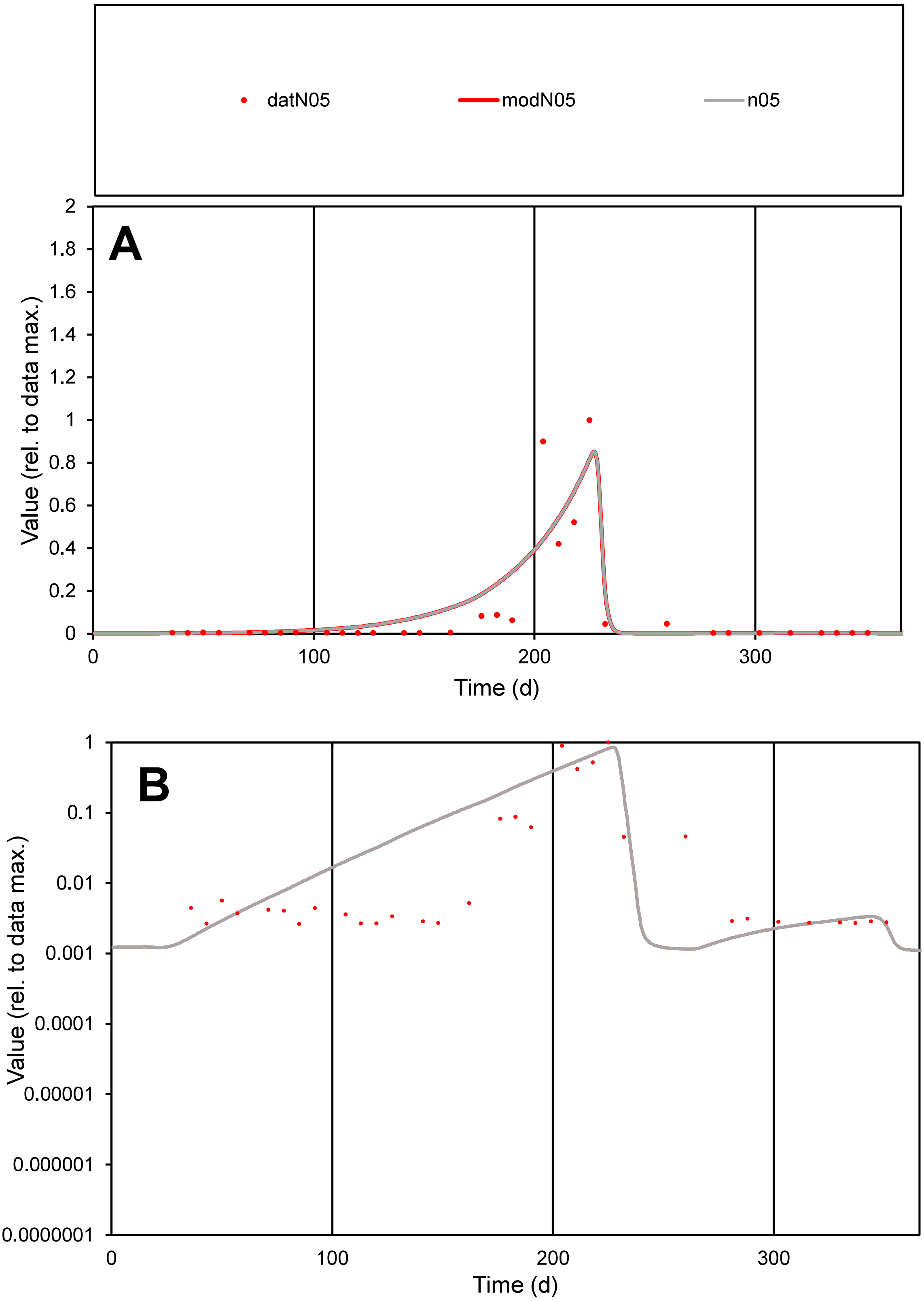

C1

Variables
N05 Provide jtd or Dst
CalibOnly?
2 1 to reproduce of
oNormalize?
2 1=no, 2=yes

ofxn calc*
1.07118
ofxn mod
1.12005

sRun
RunA1
zout
1

Ave
Data
0.00152
Model
0.00176

data/vMax
0.01502

ofxng
321.551

Min d/m
2.29075

ave/min d
38.4106
ave/min m
101.723

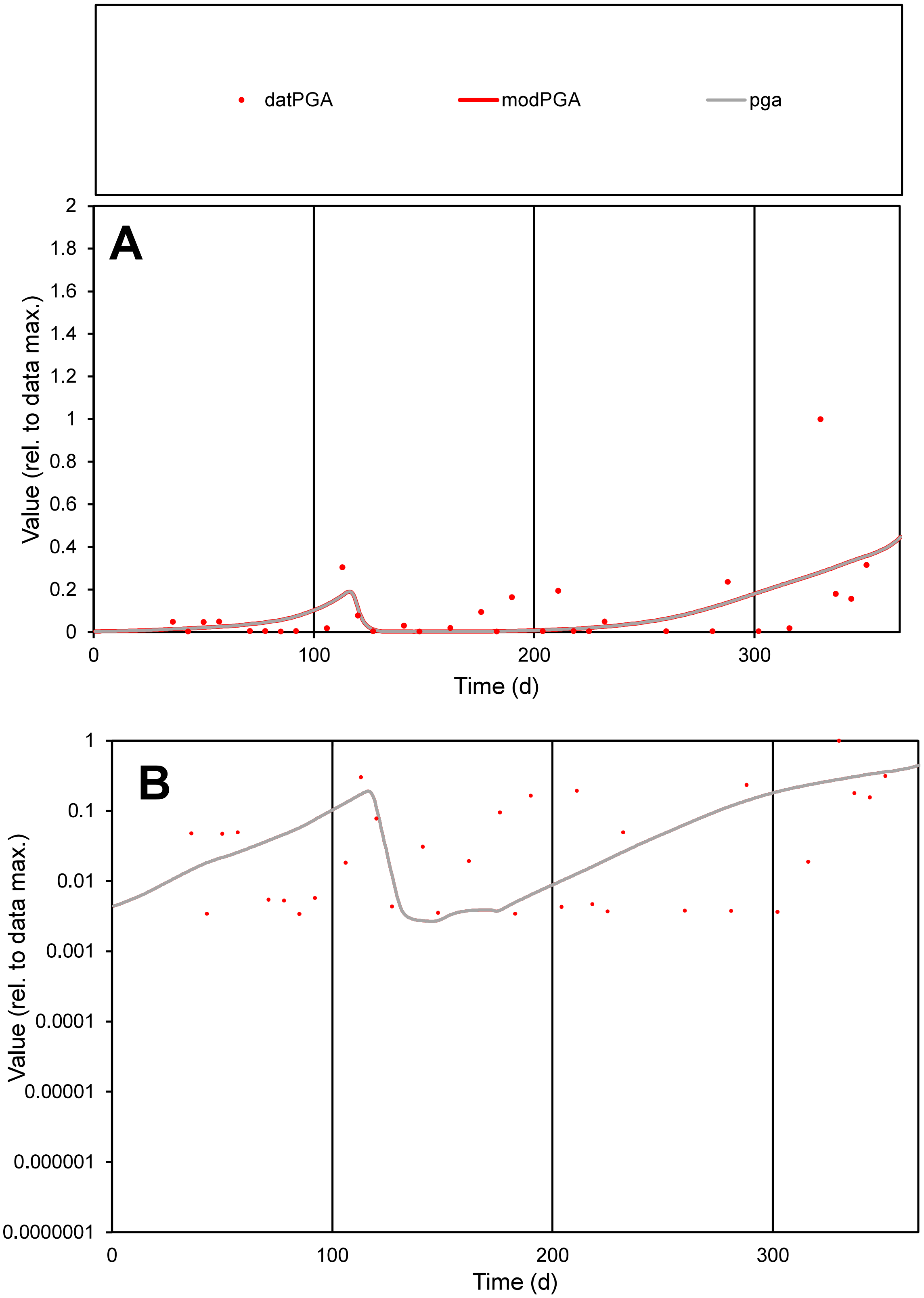

C1

Variables

PGA Provide jtd or Dst

CalibOnly?

2 1 to reproduce of

oNormalize?

2 1=no, 2=yes

ofxn calc*

1.62714

ofxn mod

1.63912

sRun

RunA1

zout

1

Ave

Data

0.00047

Model

0.00044

data/vMax

0.00495

ofxng

321.551

Min d/m

1.26816

ave/min d

28.003

ave/min m

33.0875

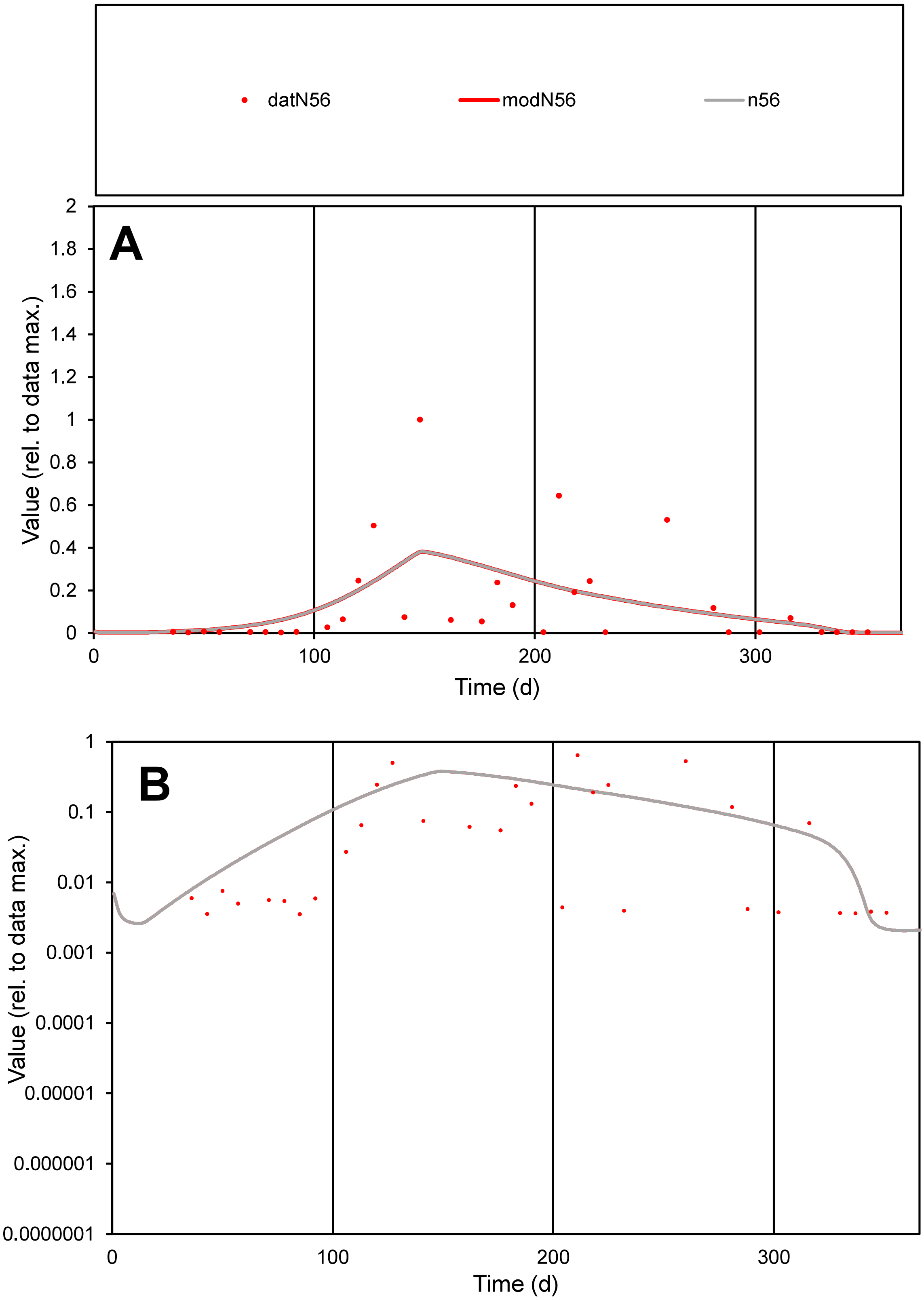

C1

Variables
N56 Provide jtd or Dst
CalibOnly?
2 1 to reproduce of
oNormalize?
2 1=no, 2=yes

ofxn calc*
1.43131
ofxn mod
1.44066

sRun
RunA1
zout
1

Ave
Data
0.00128
Model
0.00132

data/vMax
0.0096

ofxng
321.551

Min d/m
1.65205

ave/min d
37.8275
ave/min m
64.2839

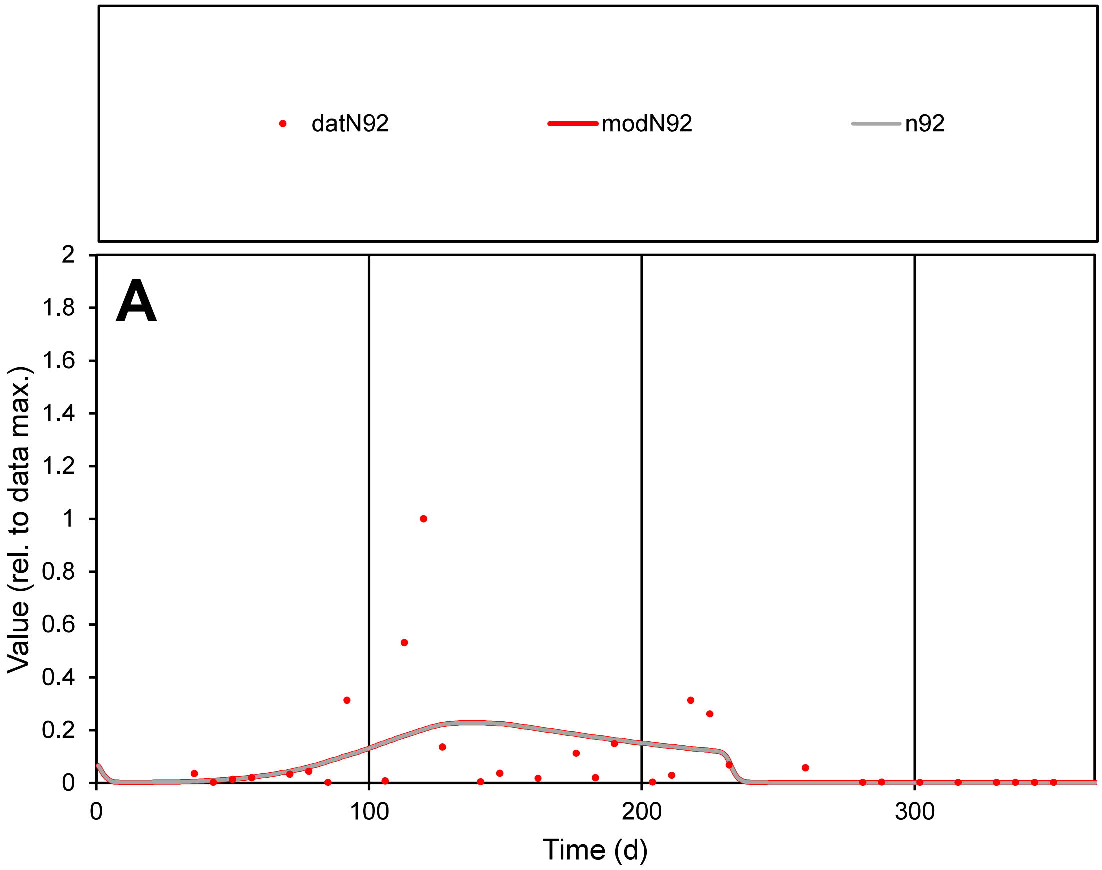

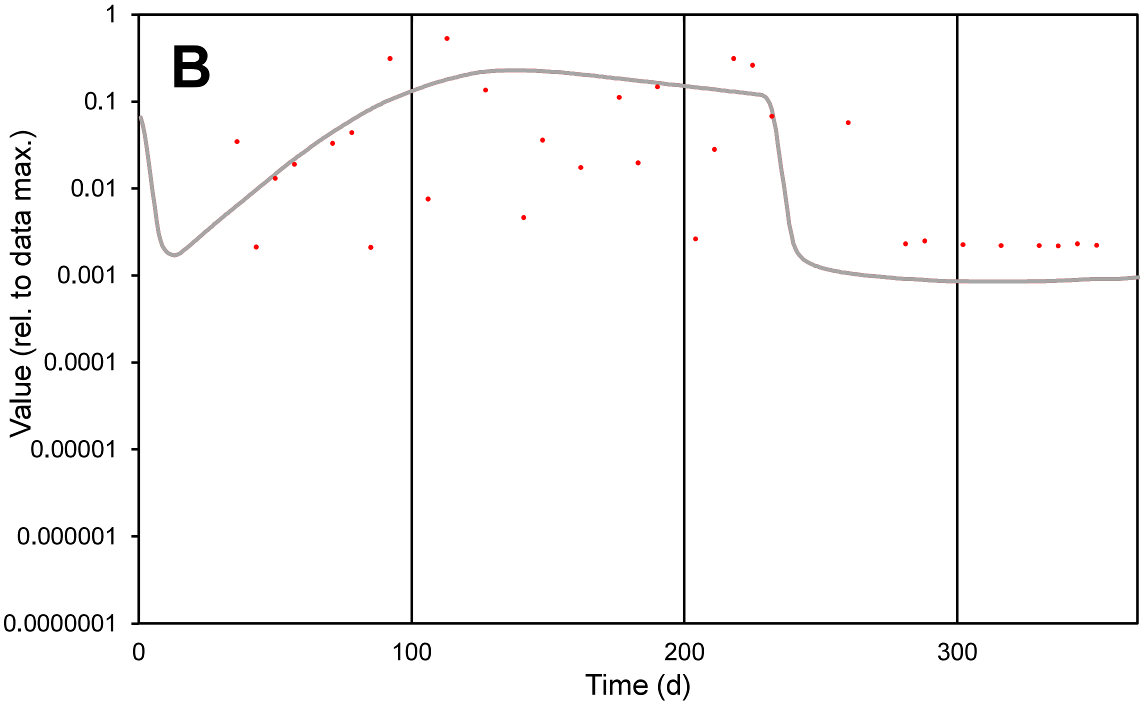

C1

Variables
N92 Provide jtd or Dst
CalibOnly?
2 1 to reproduce of
oNormalize?
2 1=no, 2=yes

ofxn calc*
1.80999
ofxn mod
1.81687

sRun
RunA1
zout
1

Ave
Data
0.0018
Model
0.00161

data/vMax
0.01783

ofxng
321.551

Min d/m
2.47248

ave/min d
47.6071
ave/min m
105.553

324_Calib_S_A

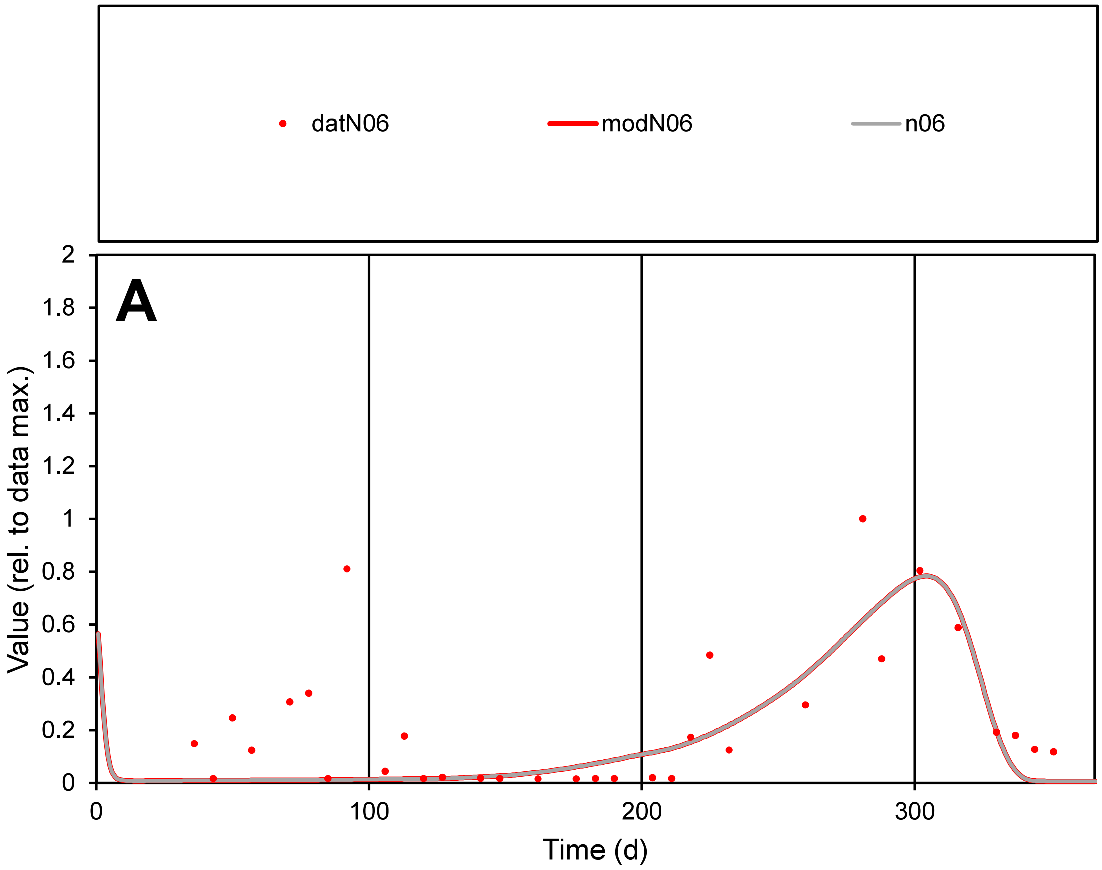

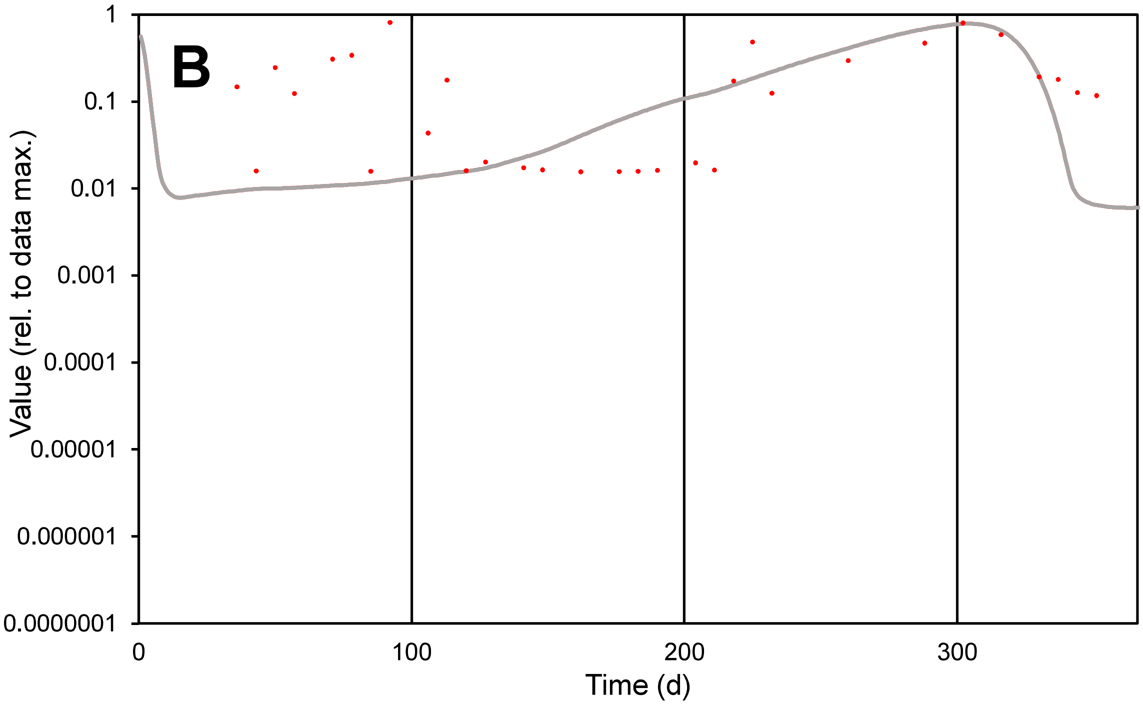

C1

Variables
N06 Provide jtd or Dst
CalibOnly?
2 1 to reproduce of
oNormalize?
2 1=no, 2=yes

ofxn calc*
0.9376
ofxn mod
0.94713

sRun
RunA1
zout
1

Ave
Data
0.00088
Model
0.0006

data/vMax
0.00407

ofxng
321.551

Min d/m
2.44534

ave/min d
13.9226
ave/min m
23.0837

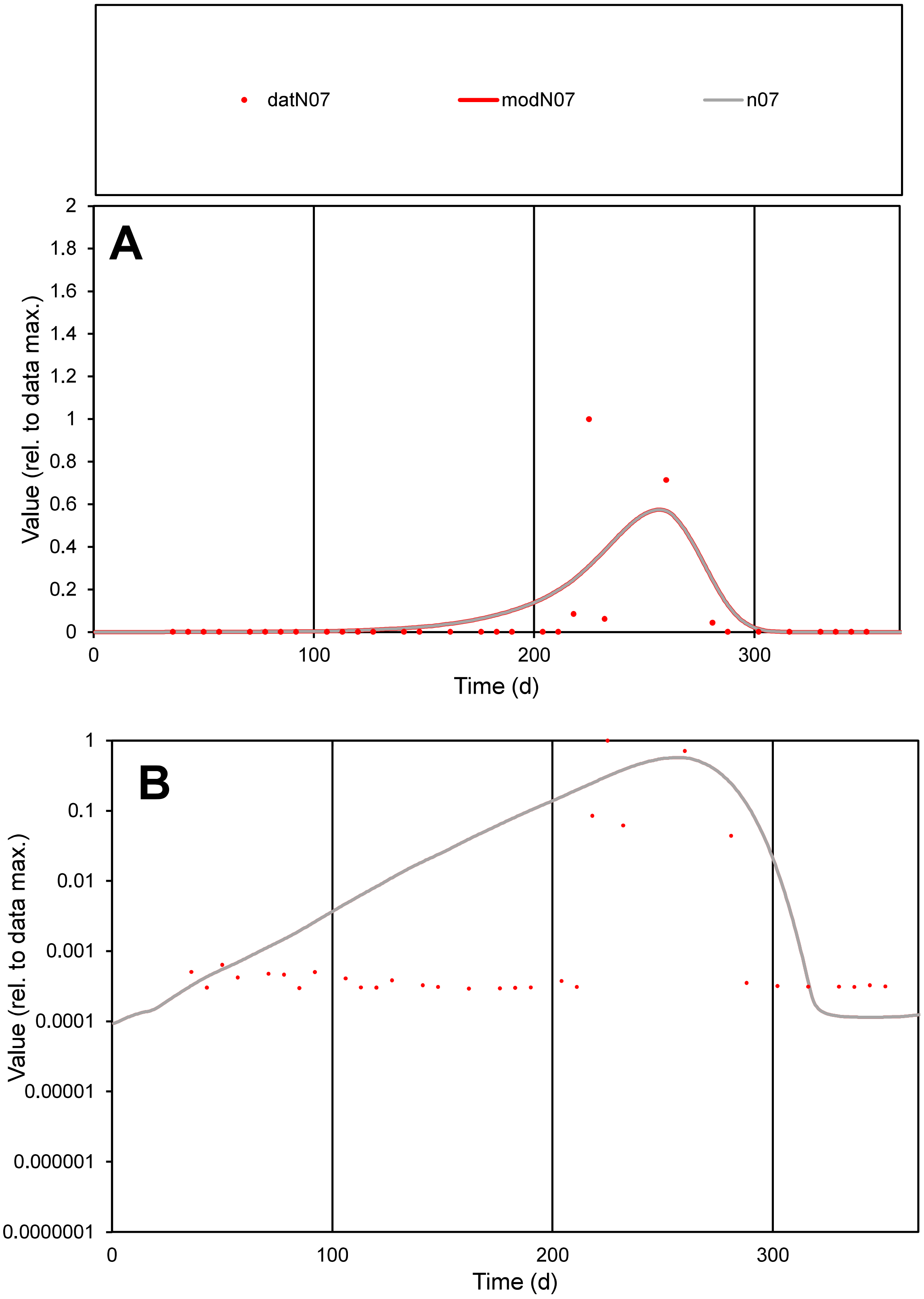

C1

Variables
N07 Provide jtd or Dst
CalibOnly?
2 1 to reproduce of
oNormalize?
2 1=no, 2=yes

ofxn calc*
2.52478
ofxn mod
2.55134

sRun
RunA1
zout
1

Ave
Data
0.00338
Model
0.00446

data/vMax
0.05658

ofxng
321.551

Min d/m
2.5797

ave/min d
203.052
ave/min m
690.409

324_Calib_S_A

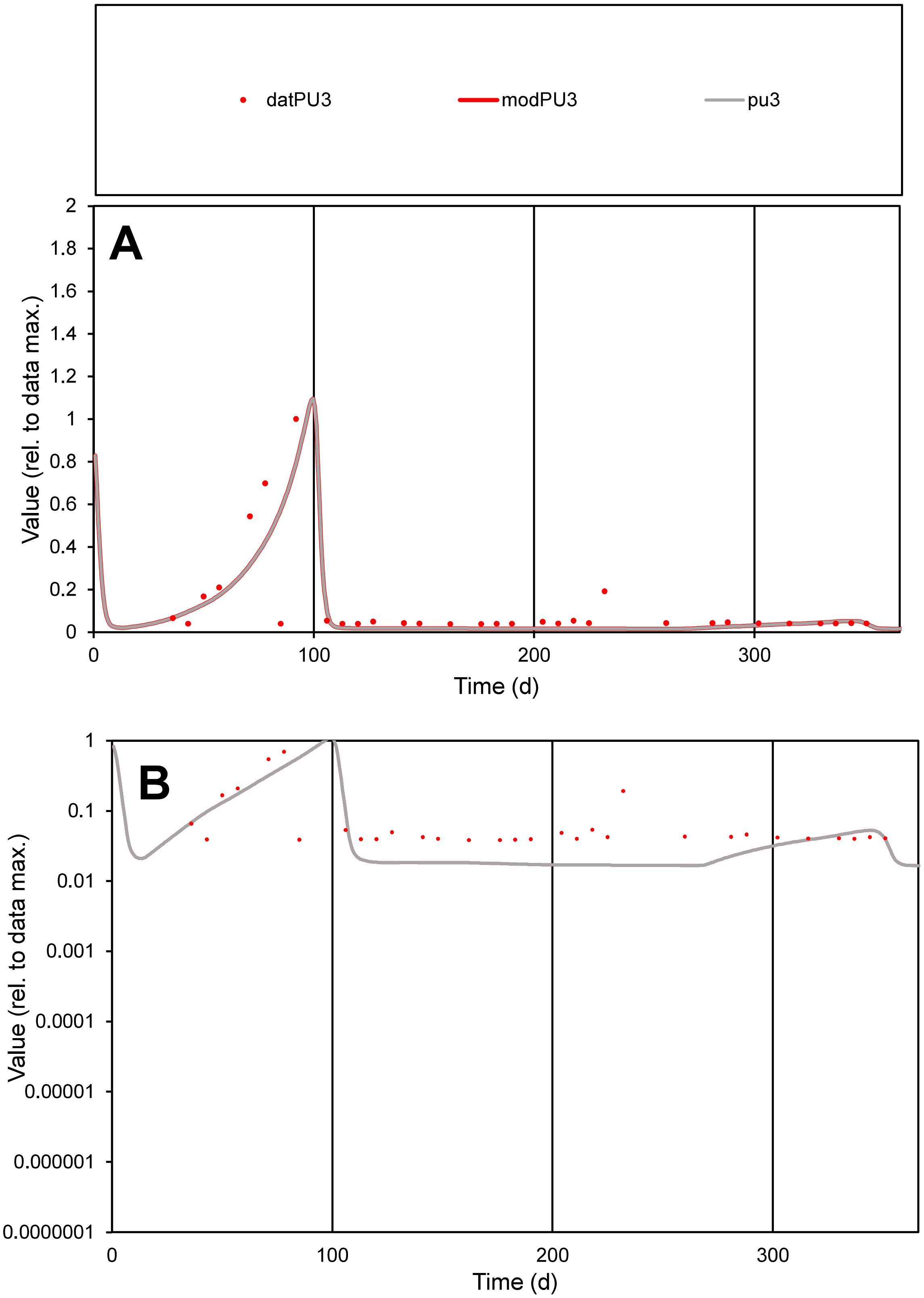

C1

Variables
PU3 Provide jtd or Dst
CalibOnly?
2 1 to reproduce of
oNormalize?
2 1=no, 2=yes

ofxn calc*
0.96284
ofxn mod
0.99587

sRun
RunA1
zout
1

Ave
Data
0.00047
Model
0.00039

data/vMax
0.00384

ofxng
321.551

Min d/m
2.30072

ave/min d
3.21081
ave/min m
6.17192

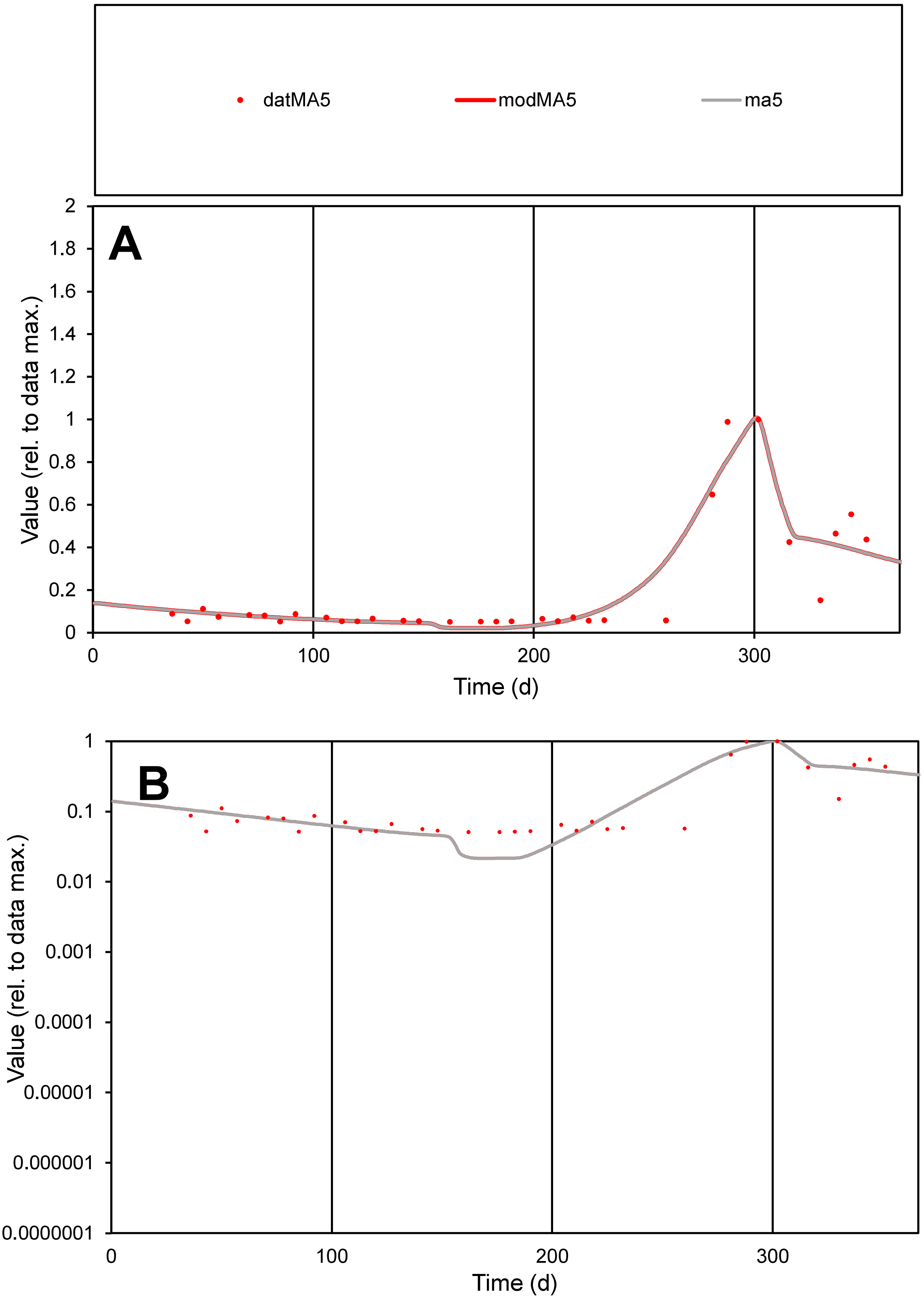

C1

Variables
MA5 Provide jtd or Dst
CalibOnly?
2 1 to reproduce of
oNormalize?
2 1=no, 2=yes

ofxn calc*
0.44088
ofxn mod
0.46843

sRun
RunA1
zout
1

Ave
Data
0.00035
Model
0.00036

data/vMax
0.00181

ofxng
321.551

Min d/m
2.35356

ave/min d
3.81158
ave/min m
9.20905

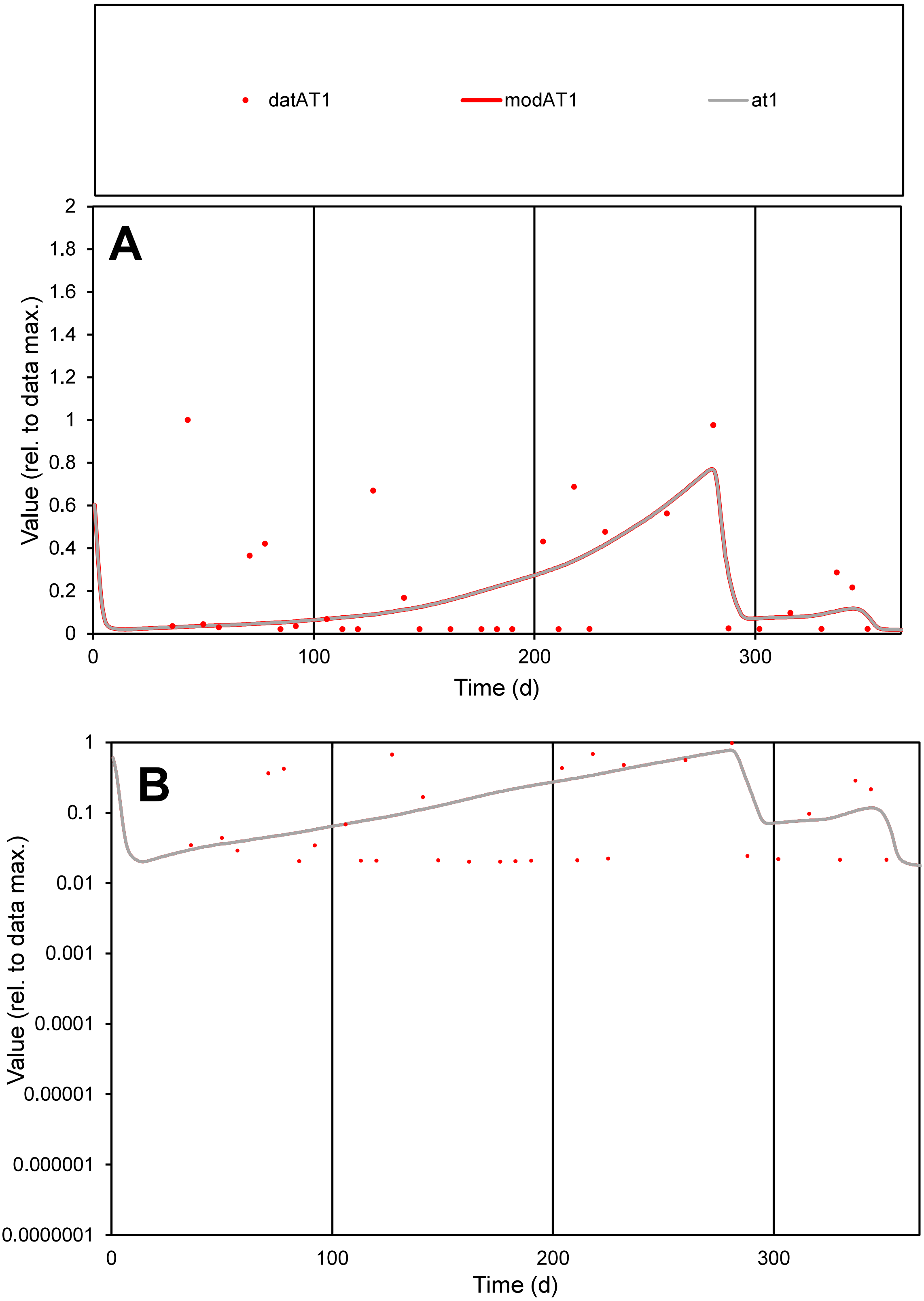

C1

Variables
AT1 Provide jtd or Dst
CalibOnly?
2 1 to reproduce of
oNormalize?
2 1=no, 2=yes

ofxn calc*
1.20345
ofxn mod
1.2171

sRun
RunA1
zout
1

Ave
Data
0.0005
Model
0.0004

data/vMax
0.00232

ofxng
321.551

Min d/m
0.66139

ave/min d
10.6218
ave/min m
5.6539

324_Calib_S_A

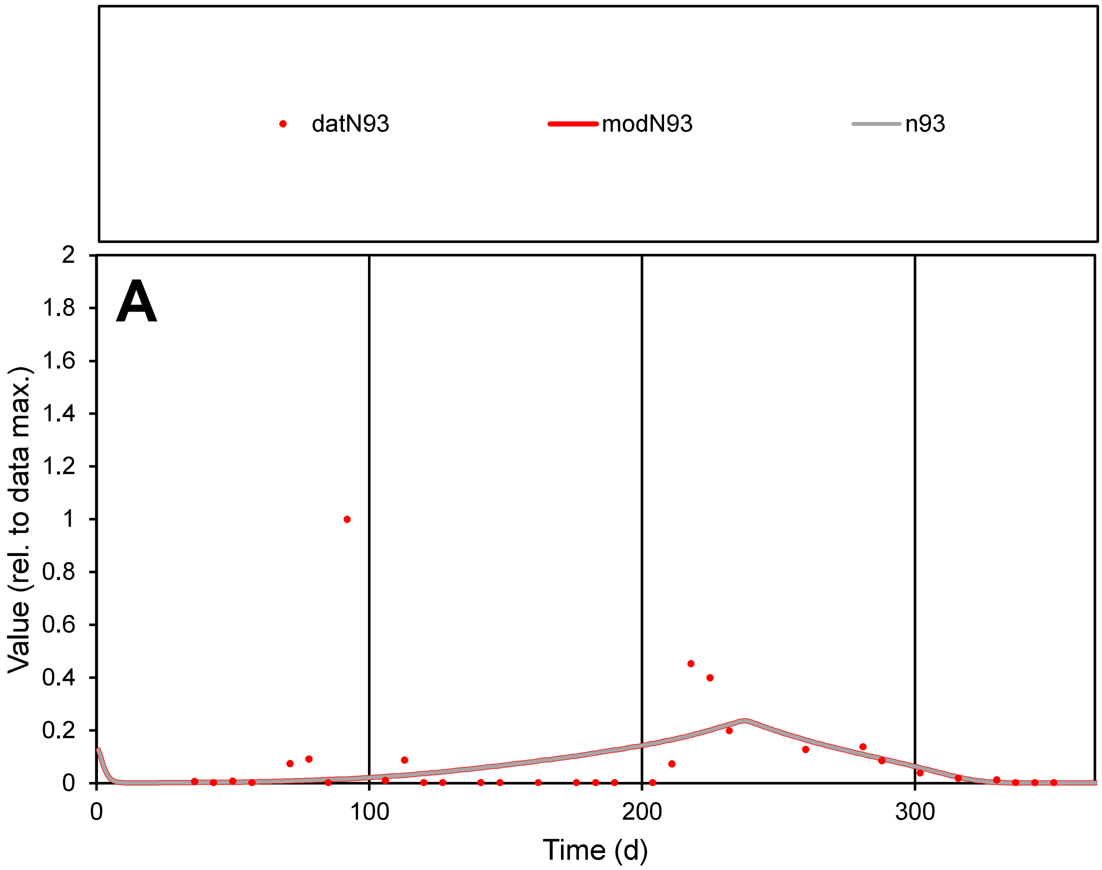

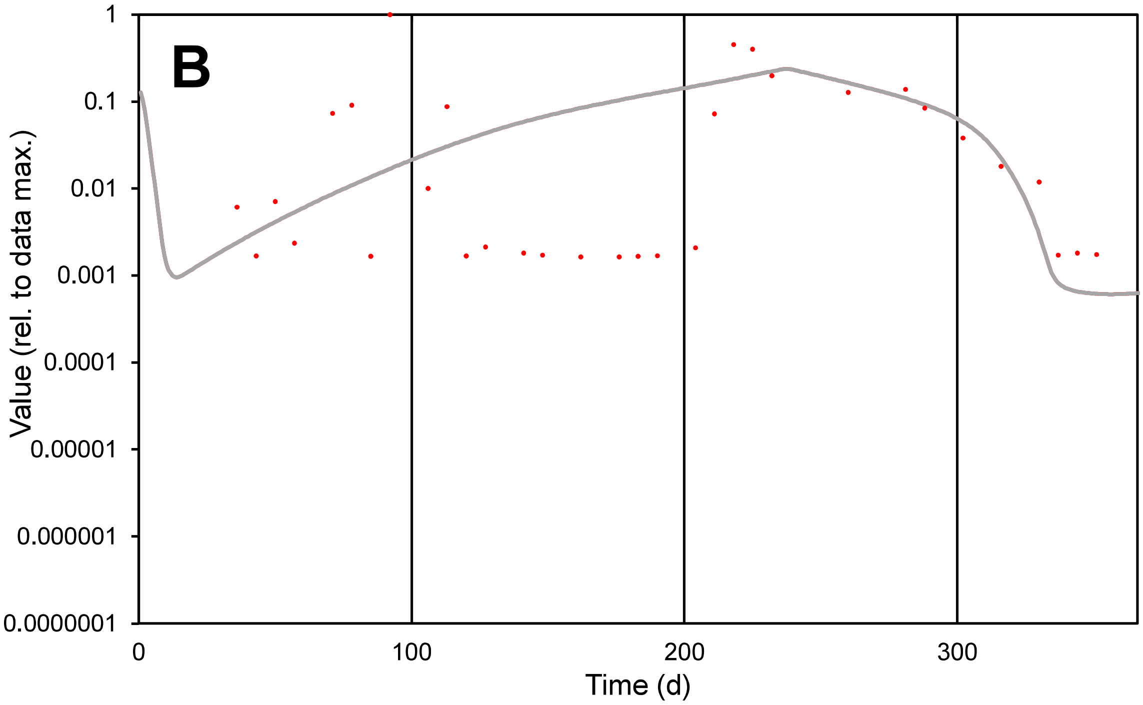

C1

Variables

N93 Provide jtd or Dst

CalibOnly?

2 1 to reproduce of

oNormalize?

2 1=no, 2=yes

ofxn calc*

2.16057

ofxn mod

2.16577

sRun

RunA1

zout

1

Ave

Data

0.00092

Model

0.00069

data/vMax

0.0104

ofxng

321.551

Min d/m

2.65686

ave/min d

54.4042

ave/min m

108.244

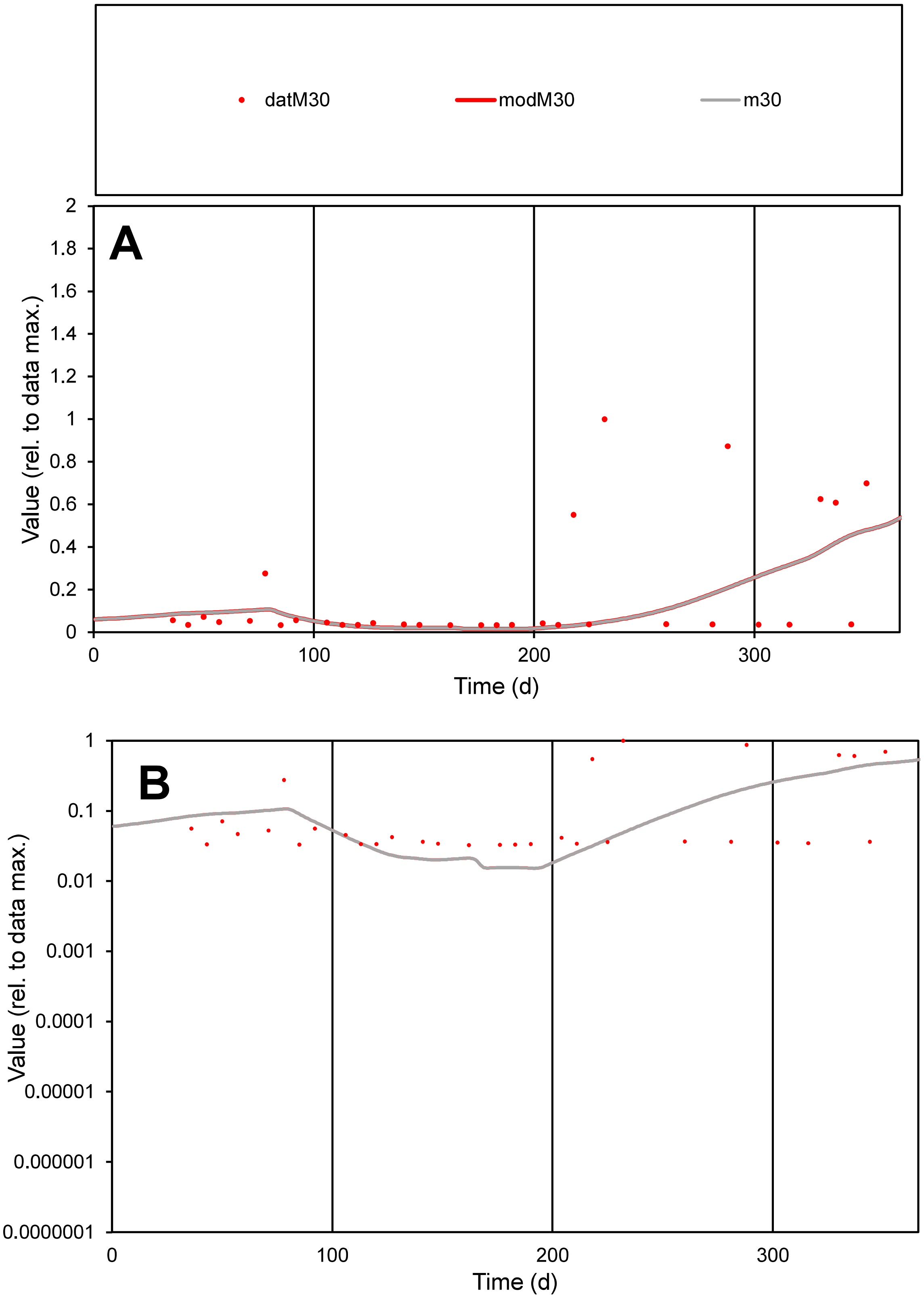

C1

Variables
M30 Provide jtd or Dst
CalibOnly?
2 1 to reproduce of
oNormalize?
2 1=no, 2=yes

ofxn calc*
1.46188
ofxn mod
1.46911

sRun
RunA1
zout
1

Ave
Data
0.00022
Model
0.00015

data/vMax
0.00123

ofxng
321.551

Min d/m
2.15145

ave/min d
5.38627
ave/min m
8.18287

324_Calib_S_A

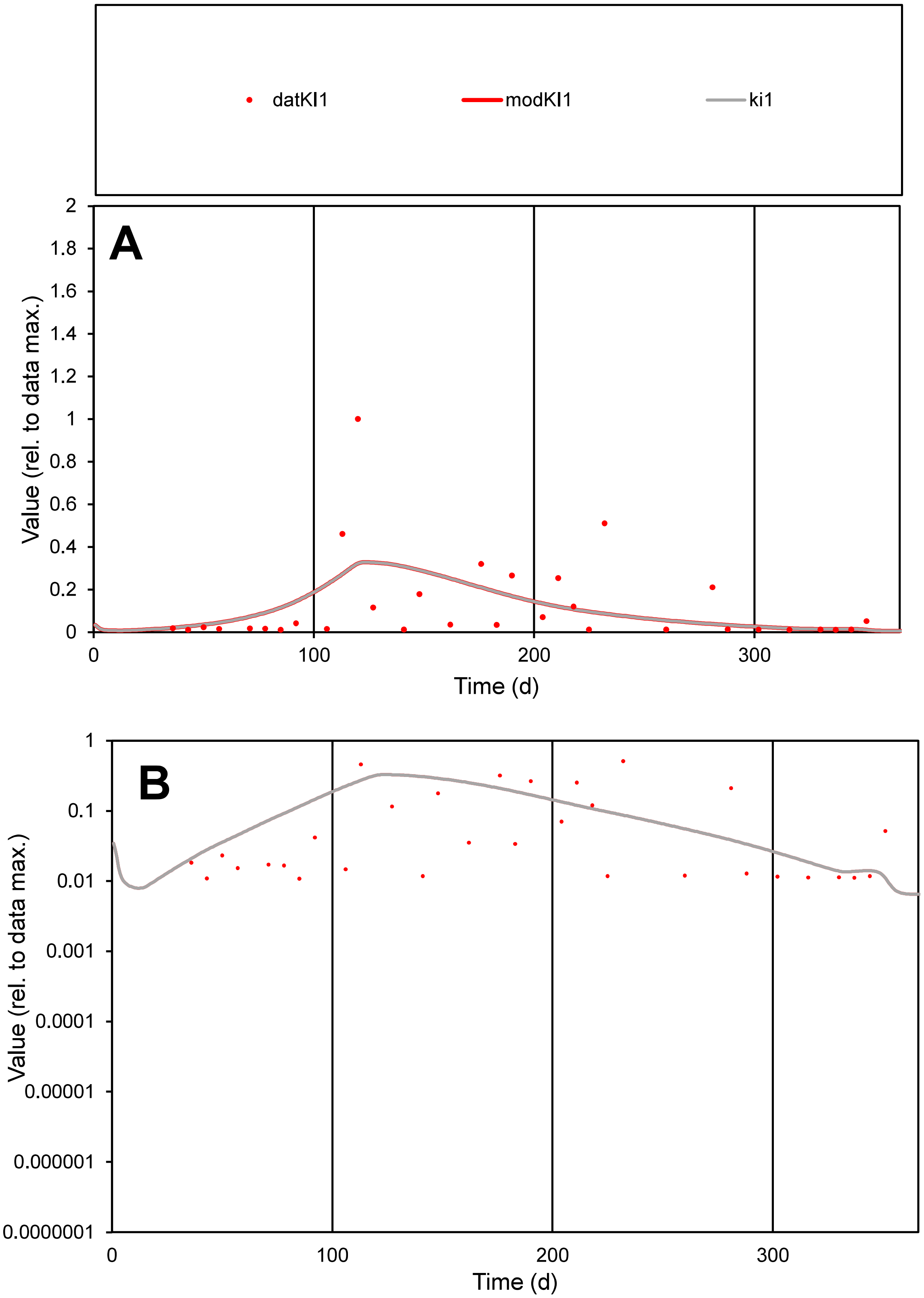

C1

Variables

KI1 Provide jtd or Dst

CalibOnly?

2 1 to reproduce of

oNormalize?

2 1=no, 2=yes

ofxn calc*

1.47719

ofxn mod

1.48573

sRun

RunA1

zout

1

Ave

Data

0.00052

Model

0.00052

data/vMax

0.00429

ofxng

321.551

Min d/m

0.98934

ave/min d

11.2727

ave/min m

11.0871

324_Calib_S_A

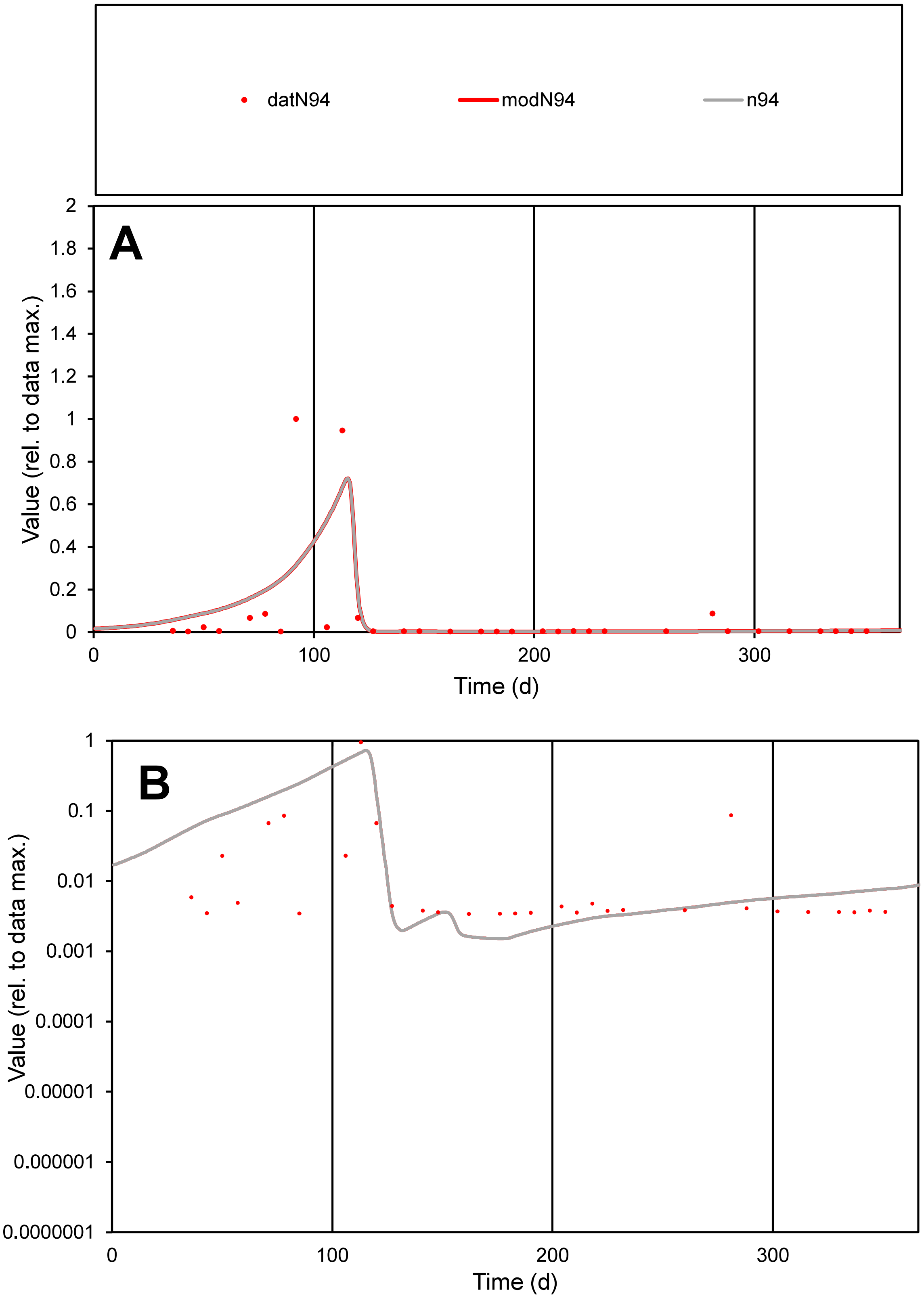

C1

Variables

N94 Provide jtd or Dst

CalibOnly?

2 1 to reproduce of

oNormalize?

2 1=no, 2=yes

ofxn calc*

2.20812

ofxn mod

2.22799

sRun

RunA1

zout

1

Ave

Data

0.00097

Model

0.00108

data/vMax

0.01298

ofxng

321.551

Min d/m

2.257

ave/min d

21.885

ave/min m

54.9631

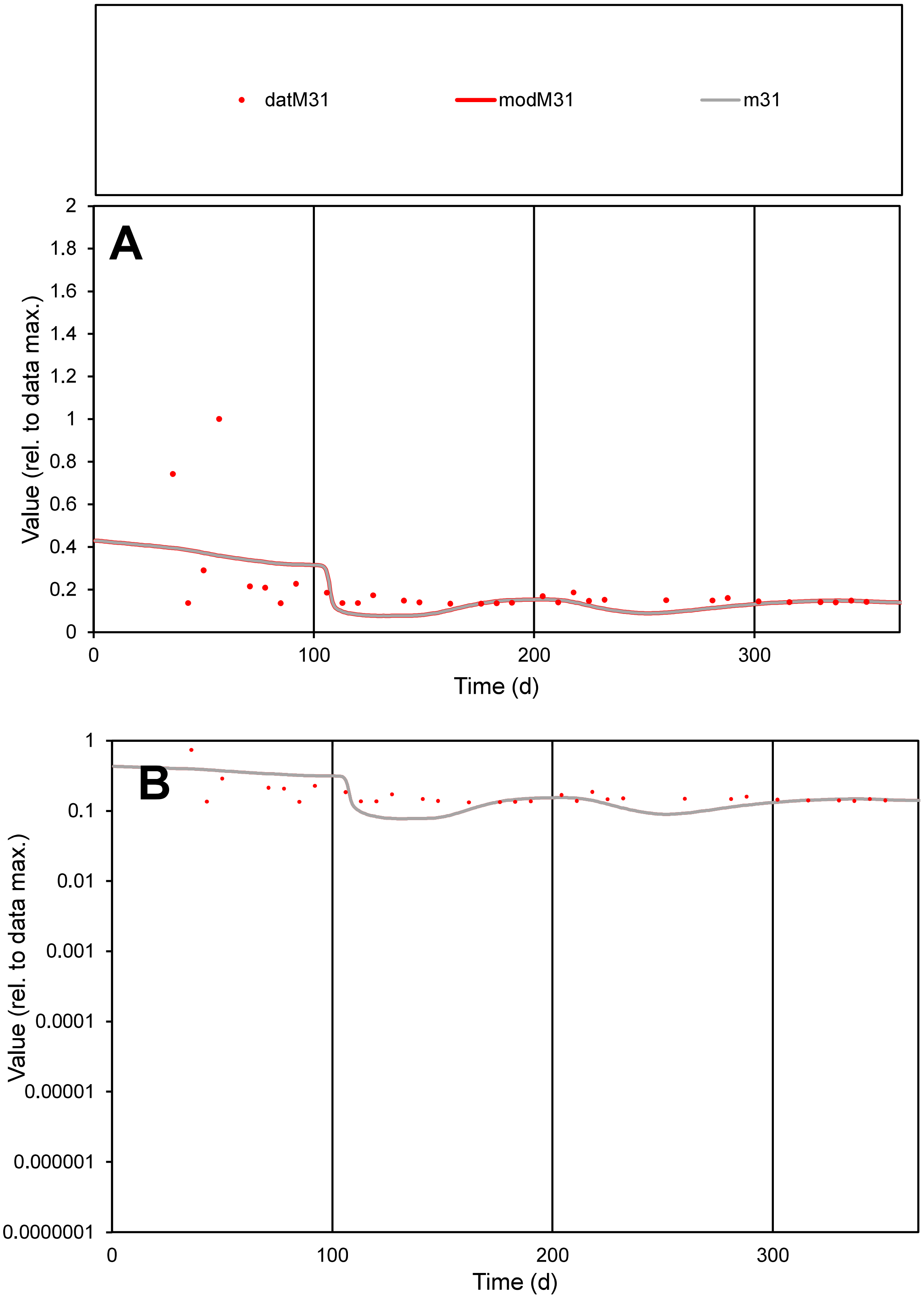

C1

Variables
M31 Provide jtd or Dst
CalibOnly?
2 1 to reproduce of
oNormalize?
2 1=no, 2=yes

ofxn calc*
0.73047
ofxn mod
0.73793

sRun
RunA1
zout
1

Ave
Data
0.00012
Model
0.00011

data/vMax
0.0006

ofxng
321.551

Min d/m
1.71198

ave/min d
1.53269
ave/min m
2.34189

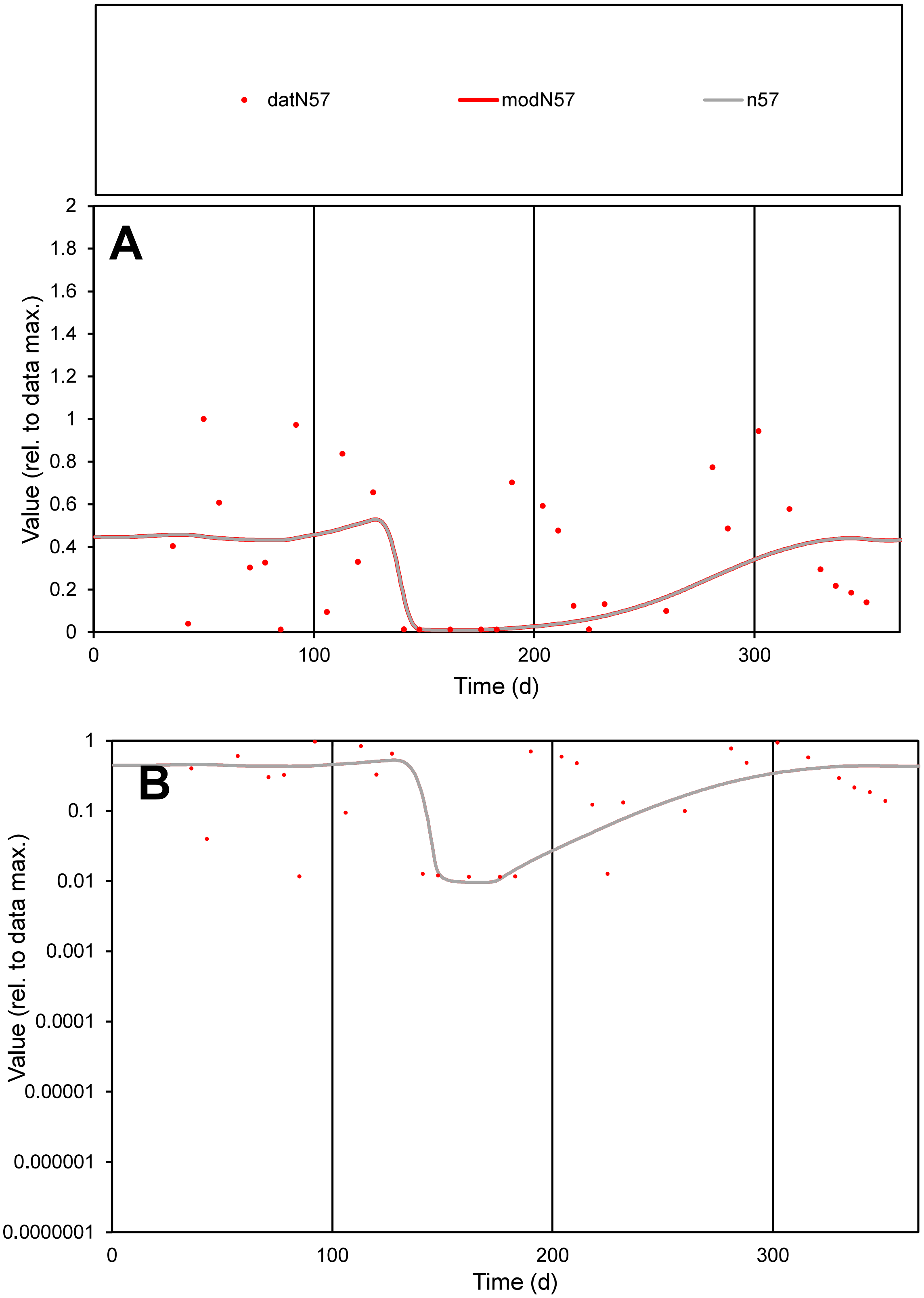

C1

Variables
N57 Provide jtd or Dst
CalibOnly?
2 1 to reproduce of
oNormalize?
2 1=no, 2=yes

ofxn calc*
0.8866
ofxn mod
0.89274

sRun
RunA1
zout
1

Ave
Data
0.00109
Model
0.00088

data/vMax
0.00307

ofxng
321.551

Min d/m
1.19871

ave/min d
30.9358
ave/min m
29.8447

324_Calib_S_A

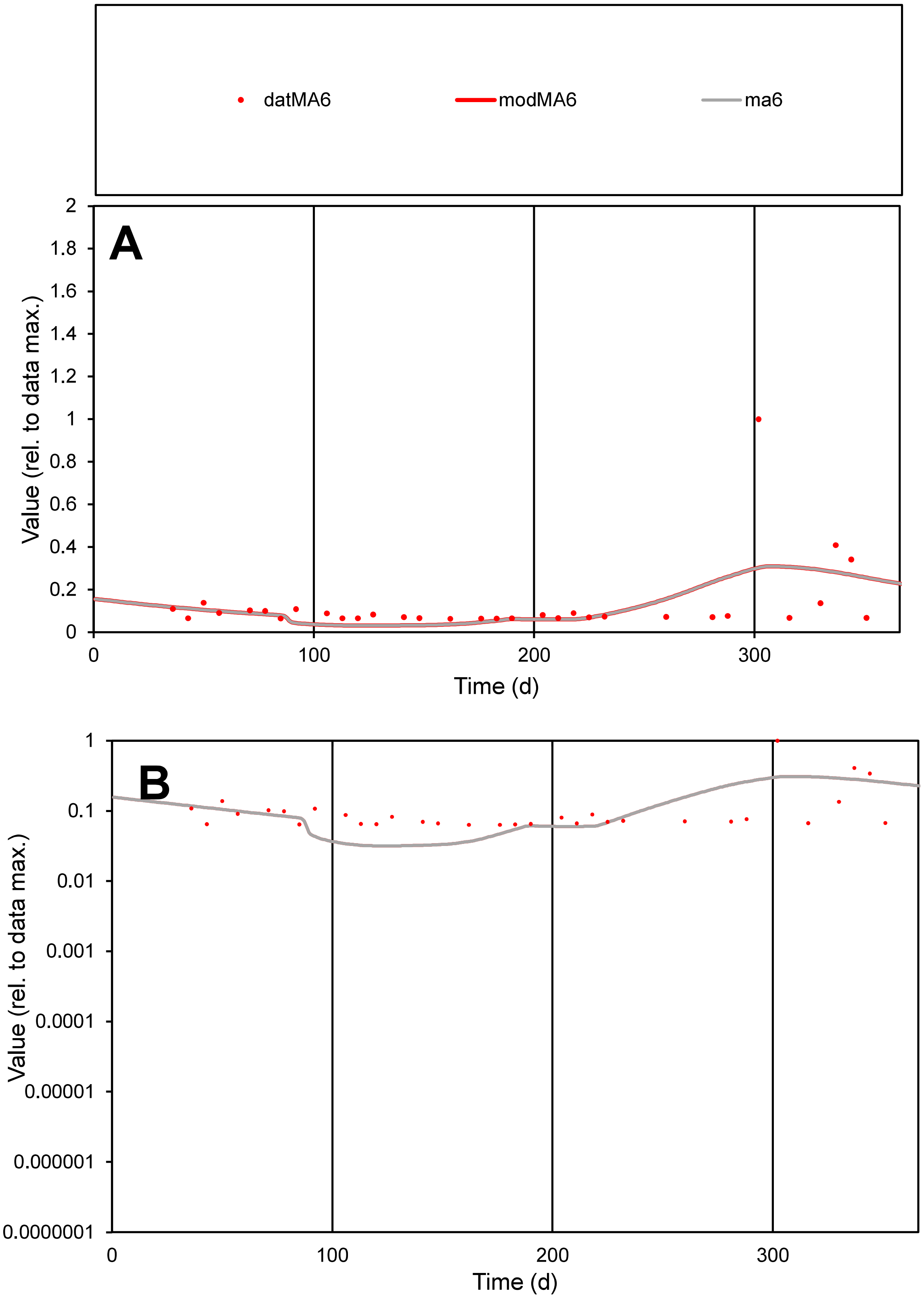

C1

Variables
MA6 Provide jtd or Dst
CalibOnly?
2 1 to reproduce of
oNormalize?
2 1=no, 2=yes

ofxn calc*
1.1685
ofxn mod
1.17792

sRun
RunA1
zout
1

Ave
Data
0.00023
Model
0.00022

data/vMax
0.00183

ofxng
321.551

Min d/m
1.98347

ave/min d
2.01953
ave/min m
3.74089

324_Calib_S_A

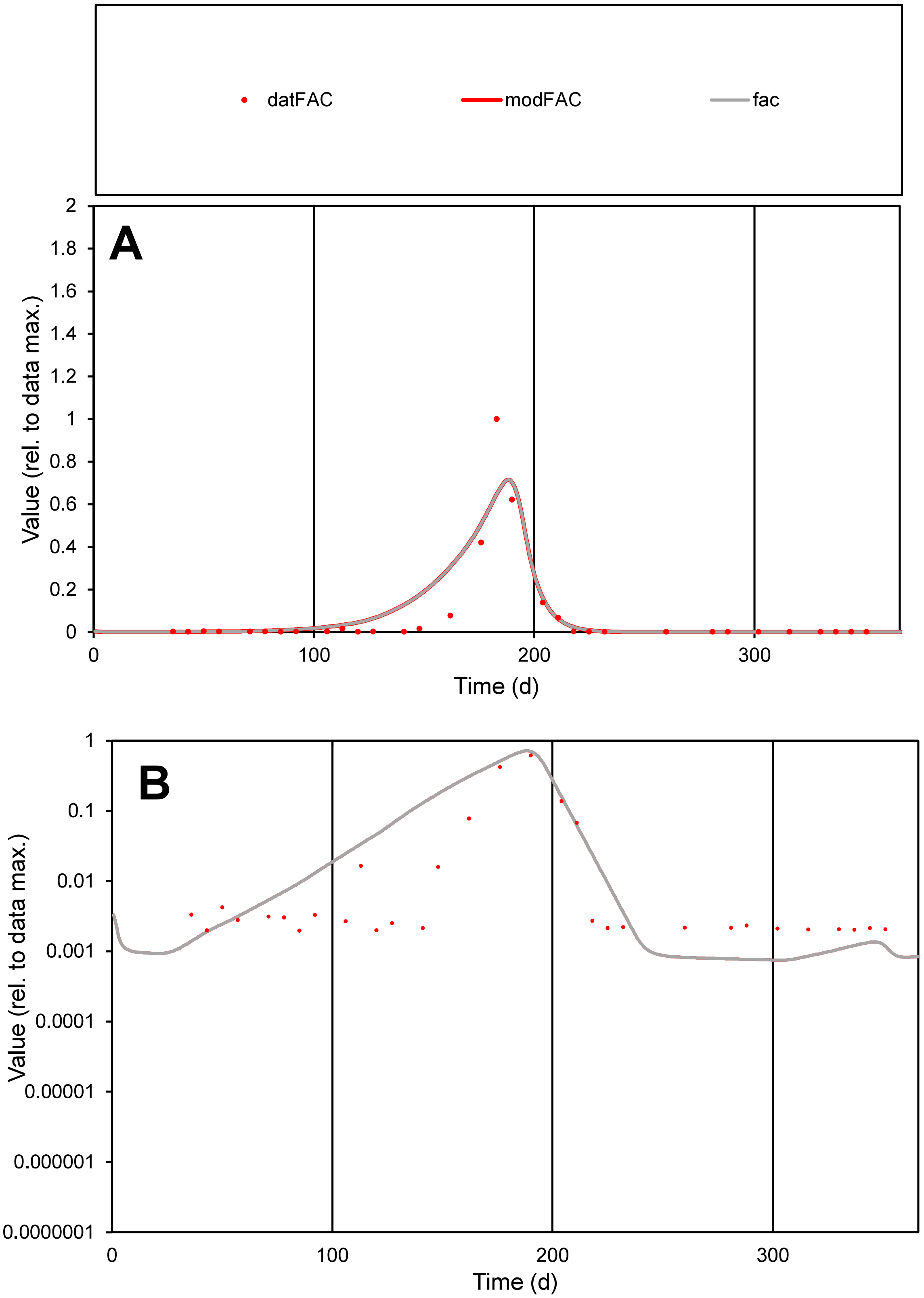

C1

Variables

FAC Provide jtd or Dst

CalibOnly?

2 1 to reproduce of

oNormalize?

2 1=no, 2=yes

ofxn calc*

1.0971

ofxn mod

1.15282

sRun

RunA1

zout

1

Ave

Data

0.0033

Model

0.00401

data/vMax

0.04359

ofxng

321.551

Min d/m

2.63583

ave/min d

38.4265

ave/min m

123.224

324_Calib_S_A

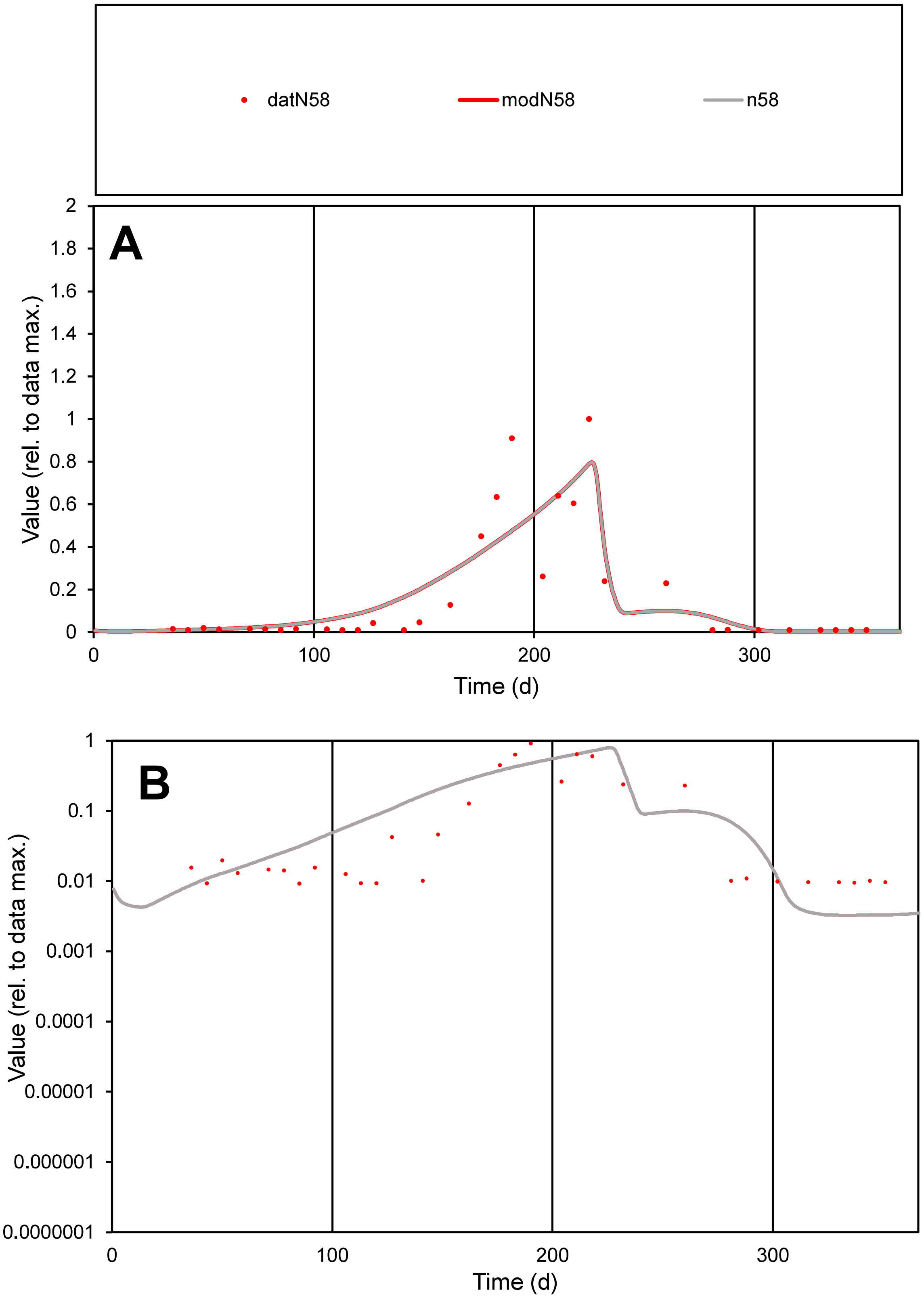

C1

Variables

N58 Provide jtd or Dst

CalibOnly?

2 1 to reproduce of

oNormalize?

2 1=no, 2=yes

ofxn calc*

0.7435

ofxn mod

0.76841

sRun

RunA1

zout

1

Ave

Data

0.00149

Model

0.00159

data/vMax

0.00881

ofxng

321.551

Min d/m

2.83659

ave/min d

18.3529

ave/min m

55.4445

324_Calib_S_A

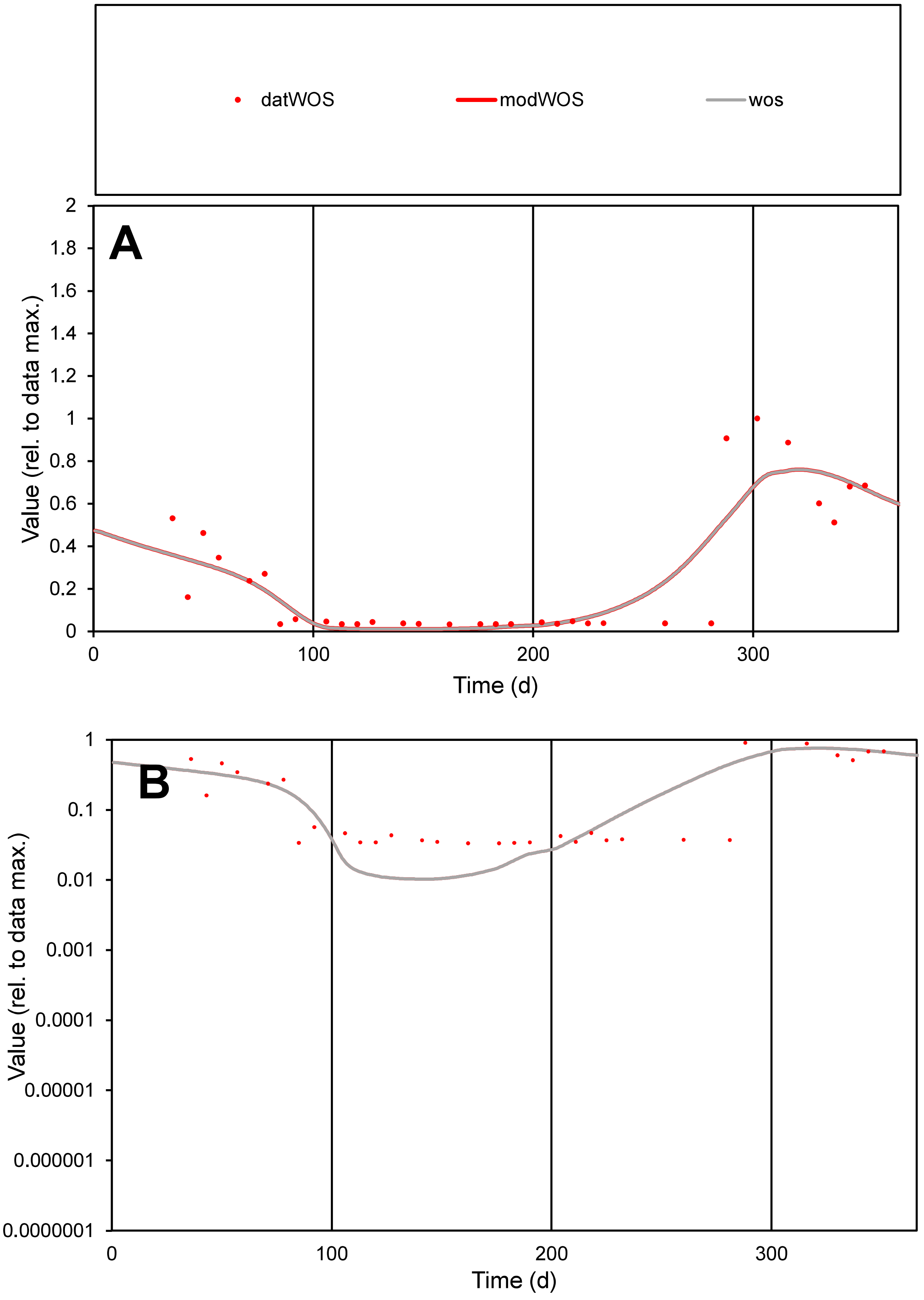

C1

Variables

WOS Provide jtd or Dst

CalibOnly?

2 1 to reproduce of

oNormalize?

2 1=no, 2=yes

ofxn calc*

0.55658

ofxn mod

0.57532

sRun

RunA1

zout

1

Ave

Data

0.0005

Model

0.00049

data/vMax

0.002

ofxng

321.551

Min d/m

3.2374

ave/min d

7.50367

ave/min m

23.9106

324_Calib_S_A

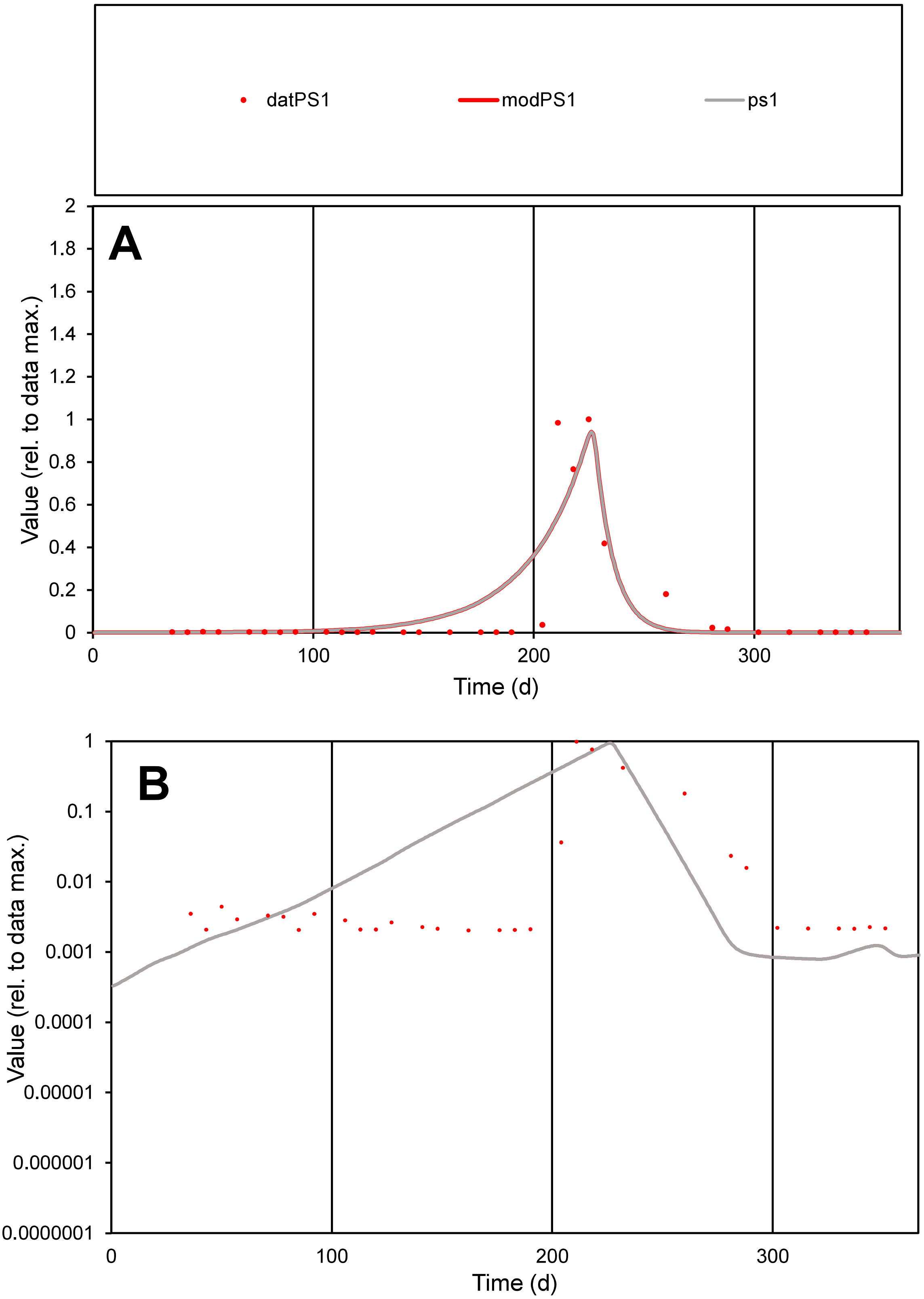

C1

Variables
PS1 Provide jtd or Dst
CalibOnly?
2 1 to reproduce of
oNormalize?
2 1=no, 2=yes

ofxn calc*
1.12211
ofxn mod
1.16503

sRun
RunA1
zout
1

Ave
Data
0.00246
Model
0.00285

data/vMax
0.02259

ofxng
321.551

Min d/m
2.55438

ave/min d
53.4522
ave/min m
157.946

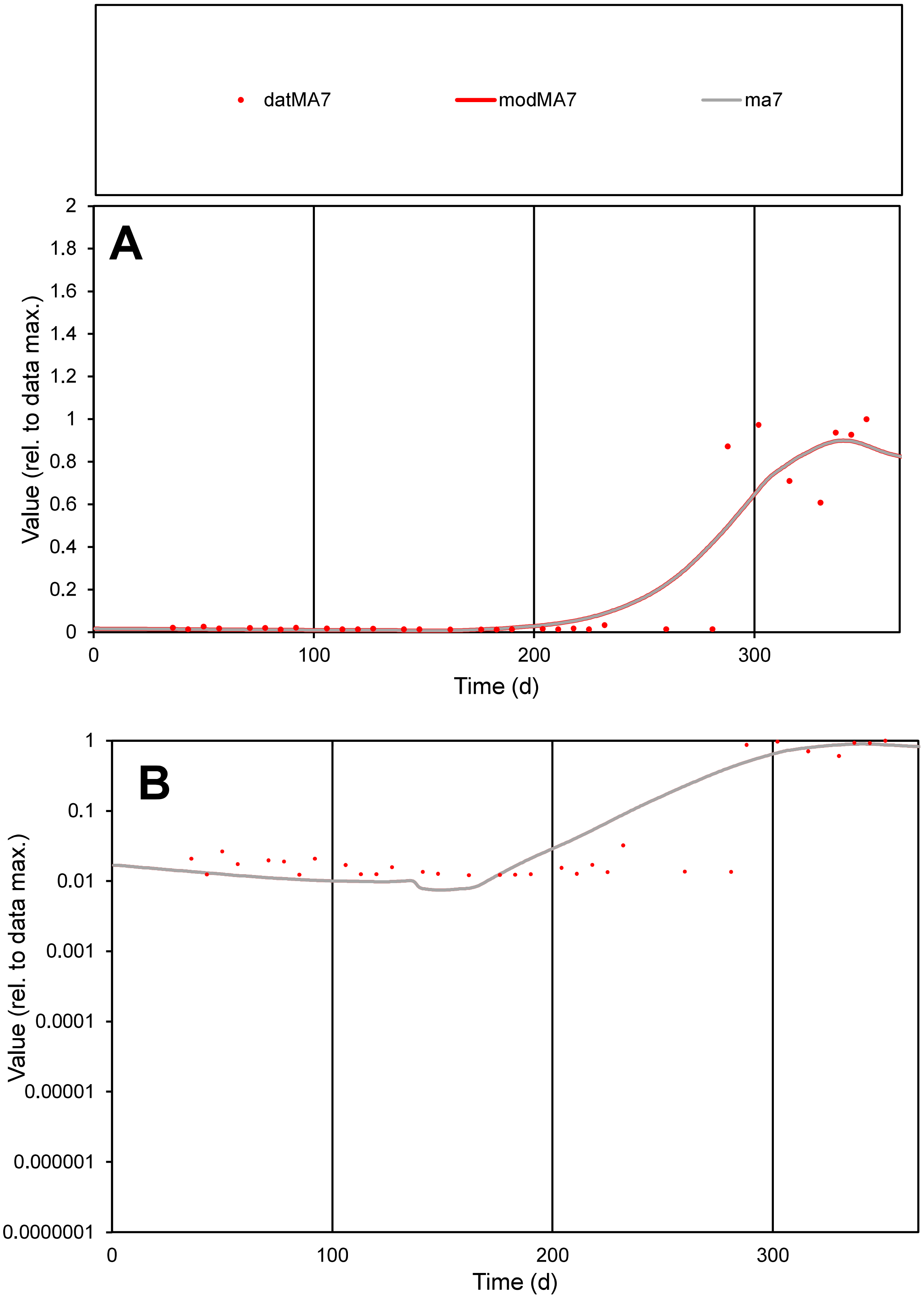

C1

Variables
MA7 Provide jtd or Dst
CalibOnly?
2 1 to reproduce of
oNormalize?
2 1=no, 2=yes

ofxn calc*
0.64382
ofxn mod
0.66922

sRun
RunA1
zout
1

Ave
Data
0.00022
Model
0.00023

data/vMax
0.00108

ofxng
321.551

Min d/m
1.63293

ave/min d
16.4912
ave/min m
28.0767

324_Calib_S_A

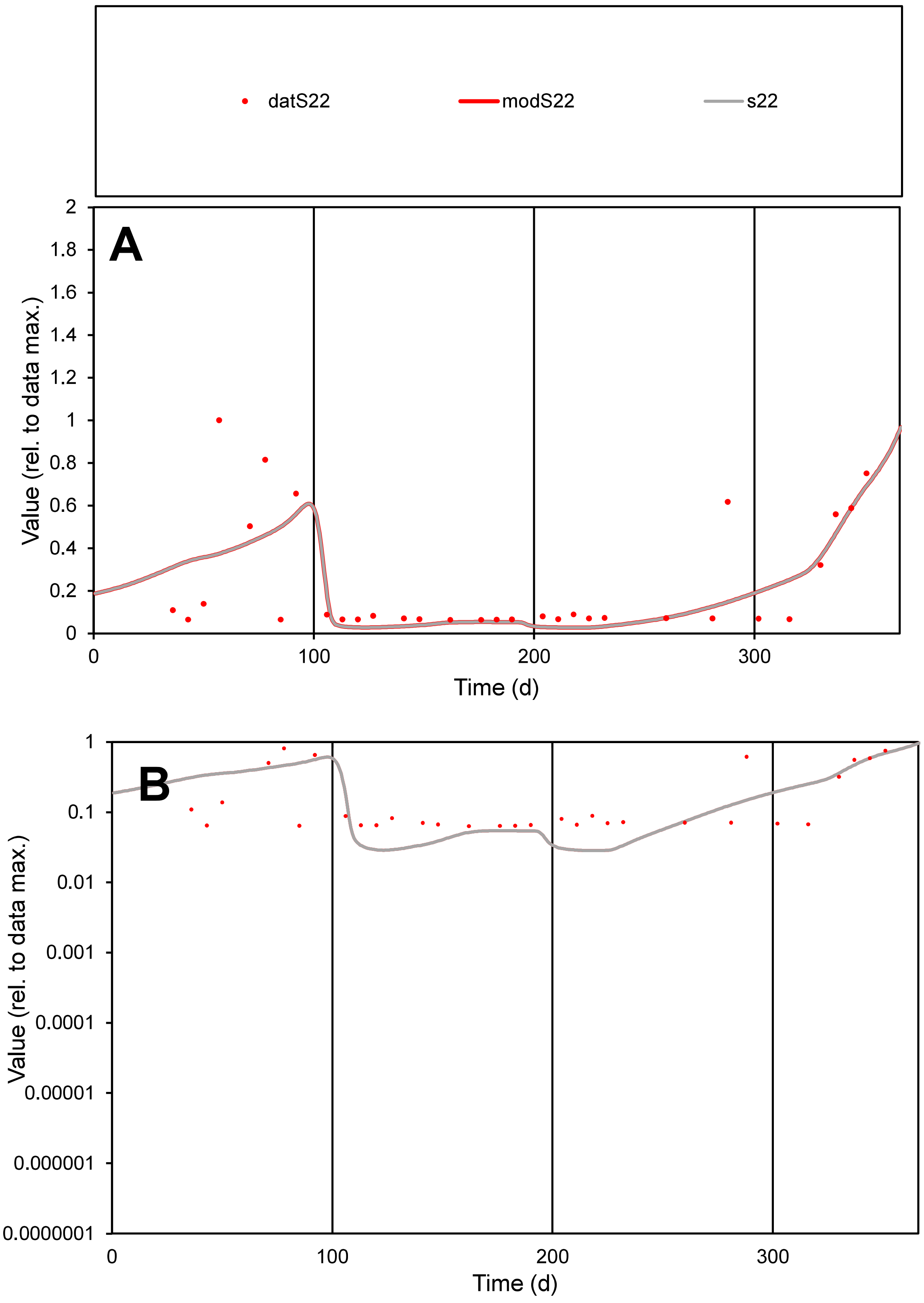

C1

Variables
S22 Provide jtd or Dst
CalibOnly?
2 1 to reproduce of
oNormalize?
2 1=no, 2=yes

ofxn calc*
0.81383
ofxn mod
0.82444

sRun
RunA1
zout
1

Ave
Data
0.00053
Model
0.00049

data/vMax
0.00226

ofxng
321.551

Min d/m
2.2293

ave/min d
3.71095
ave/min m
7.663

324_Calib_S_A

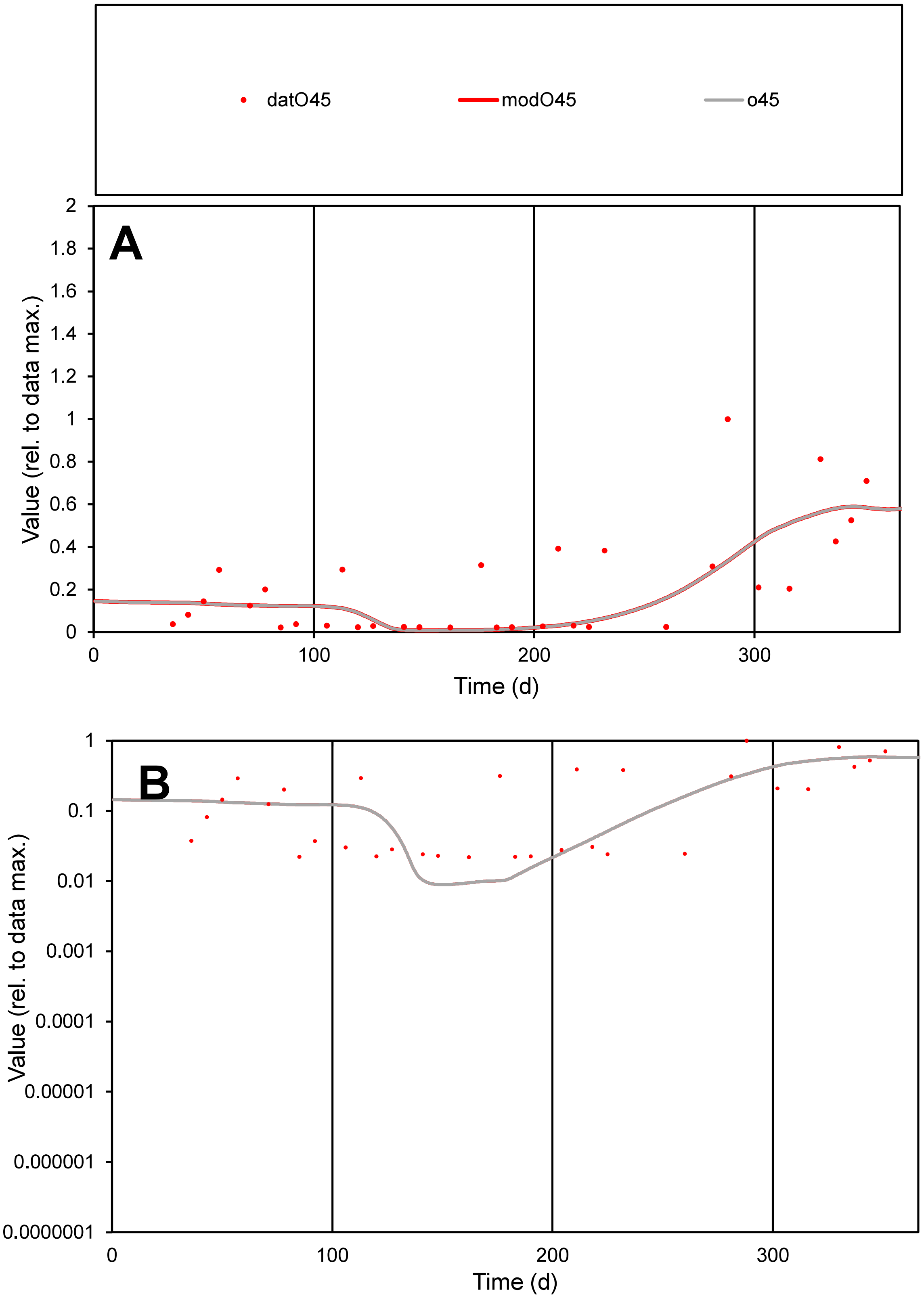

C1

Variables
O45 Provide jtd or Dst
CalibOnly?
2 1 to reproduce of
oNormalize?
2 1=no, 2=yes

ofxn calc*
0.88362
ofxn mod
0.8939

sRun
RunA1
zout
1

Ave
Data
0.00055
Model
0.00046

data/vMax
0.00256

ofxng
321.551

Min d/m
2.45391

ave/min d
9.76754
ave/min m
20.274

324_Calib_S_A

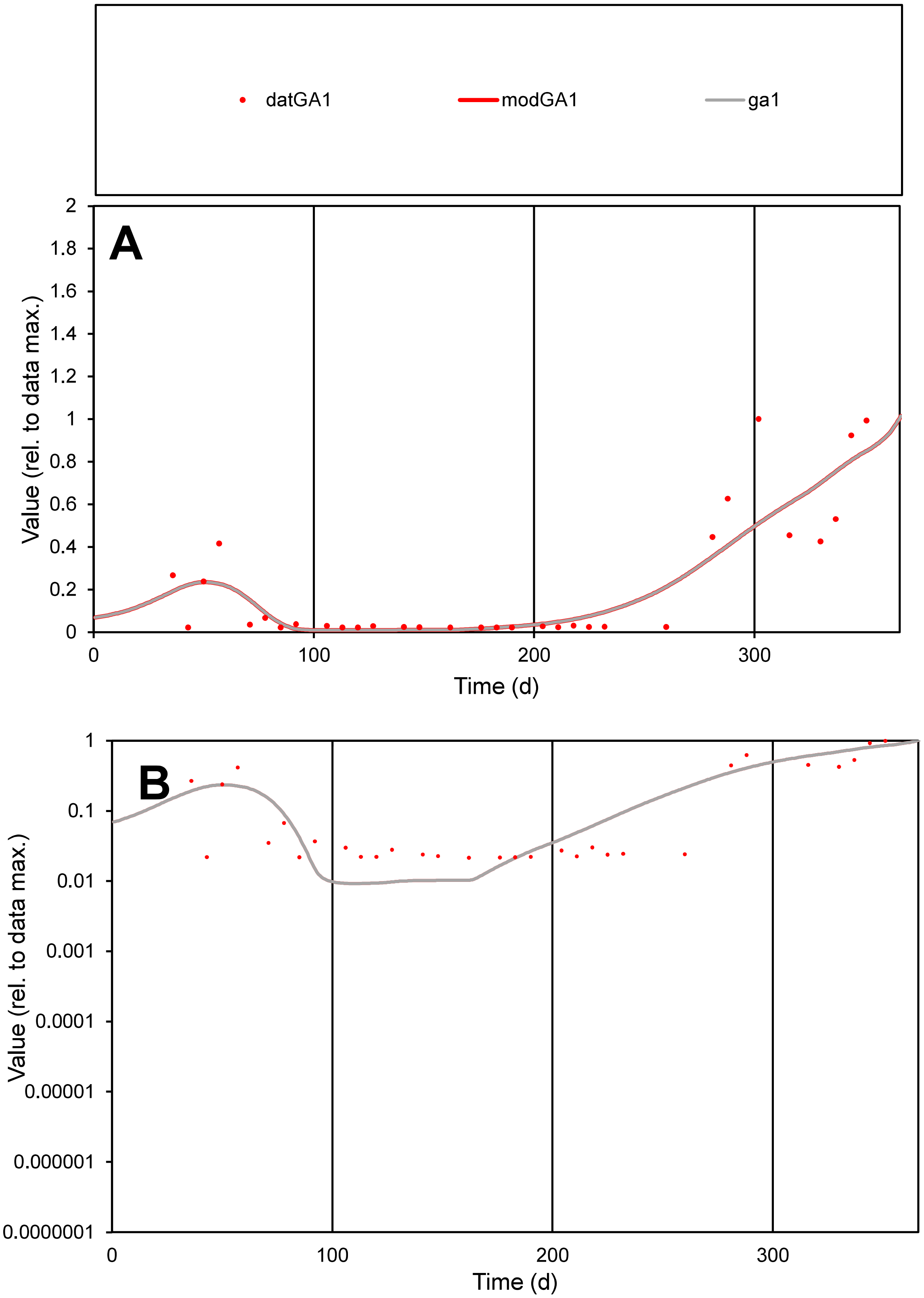

C1

Variables
GA1 Provide jtd or Dst
CalibOnly?
2 1 to reproduce of
oNormalize?
2 1=no, 2=yes

ofxn calc*
0.64227
ofxn mod
0.65961

sRun
RunA1
zout
1

Ave
Data
0.00035
Model
0.00035

data/vMax
0.00161

ofxng
321.551

Min d/m
2.32215

ave/min d
9.99632
ave/min m
23.1841

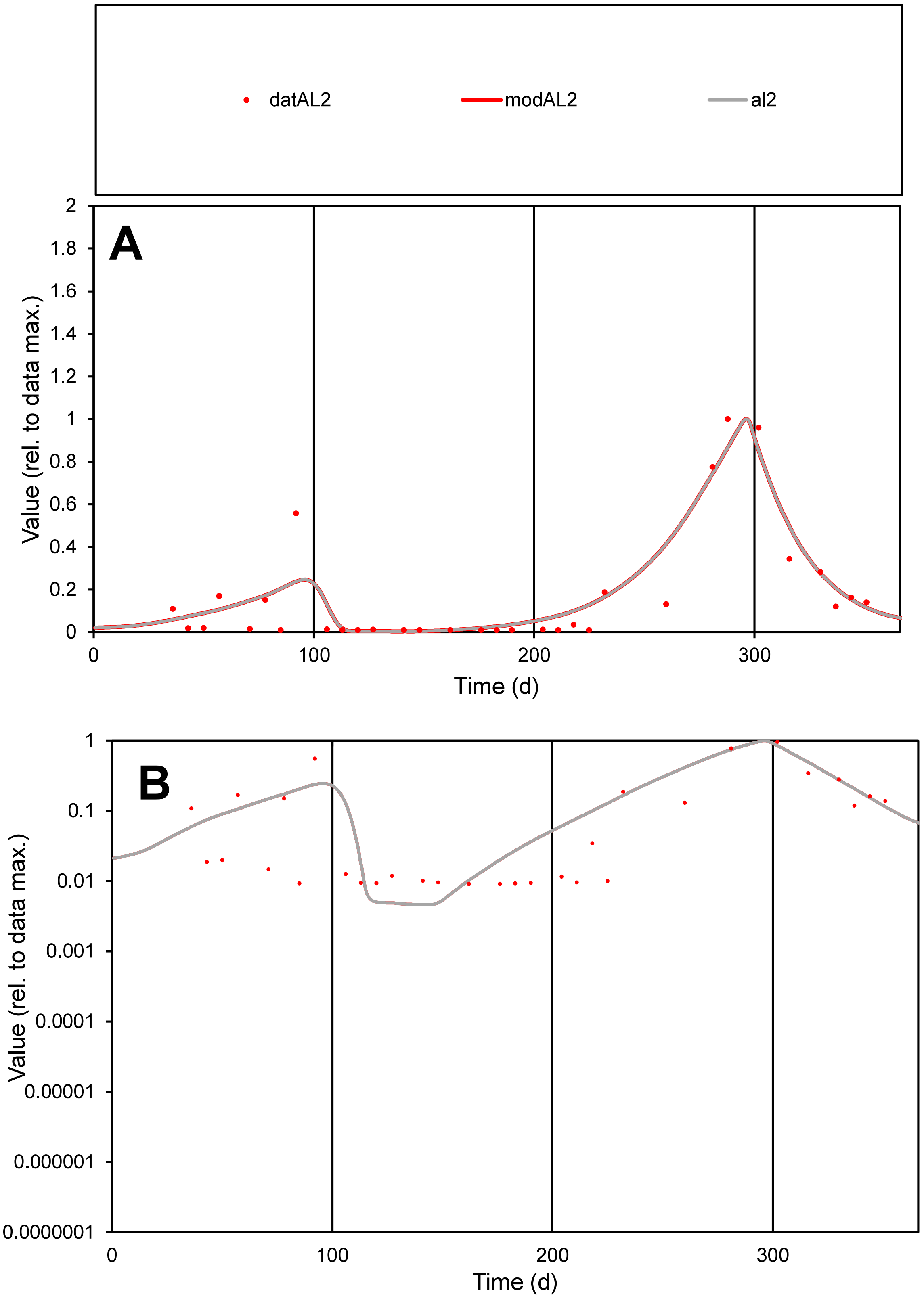

C1

Variables
AL2 Provide jtd or Dst
CalibOnly?
2 1 to reproduce of
oNormalize?
2 1=no, 2=yes

ofxn calc*
0.62282
ofxn mod
0.65048

sRun
RunA1
zout
1

Ave
Data
0.00039
Model
0.00043

data/vMax
0.00234

ofxng
321.551

Min d/m
1.96491

ave/min d
18.2282
ave/min m
39.9228

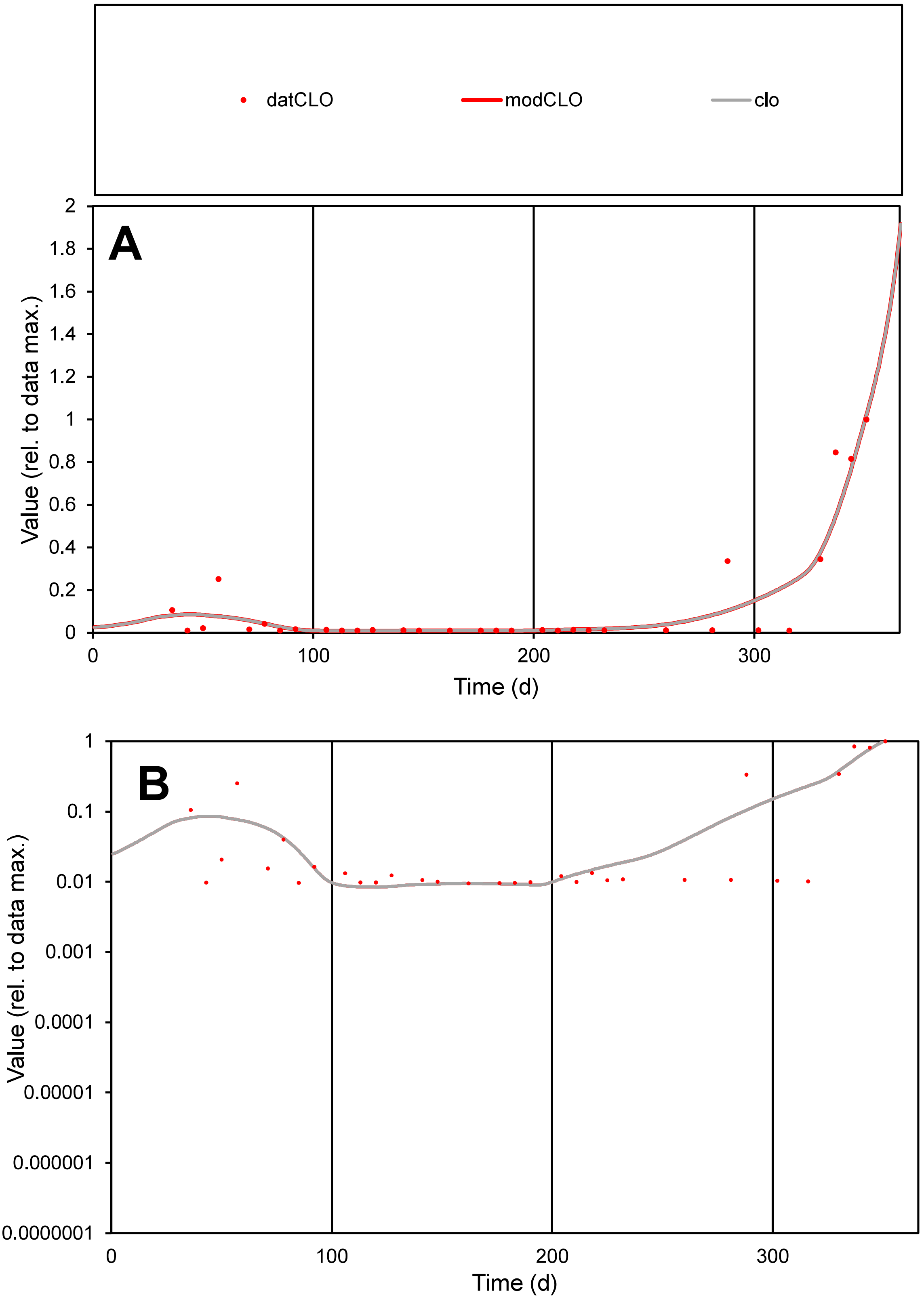

C1

Variables

CLO Provide jtd or Dst

CalibOnly?

2 1 to reproduce of

oNormalize?

2 1=no, 2=yes

ofxn calc*

0.70344

ofxn mod

0.72847

sRun

RunA1

zout

1

Ave

Data

0.00017

Model

0.00017

data/vMax

0.00135

ofxng

321.551

Min d/m

1.13556

ave/min d

13.1572

ave/min m

15.3354

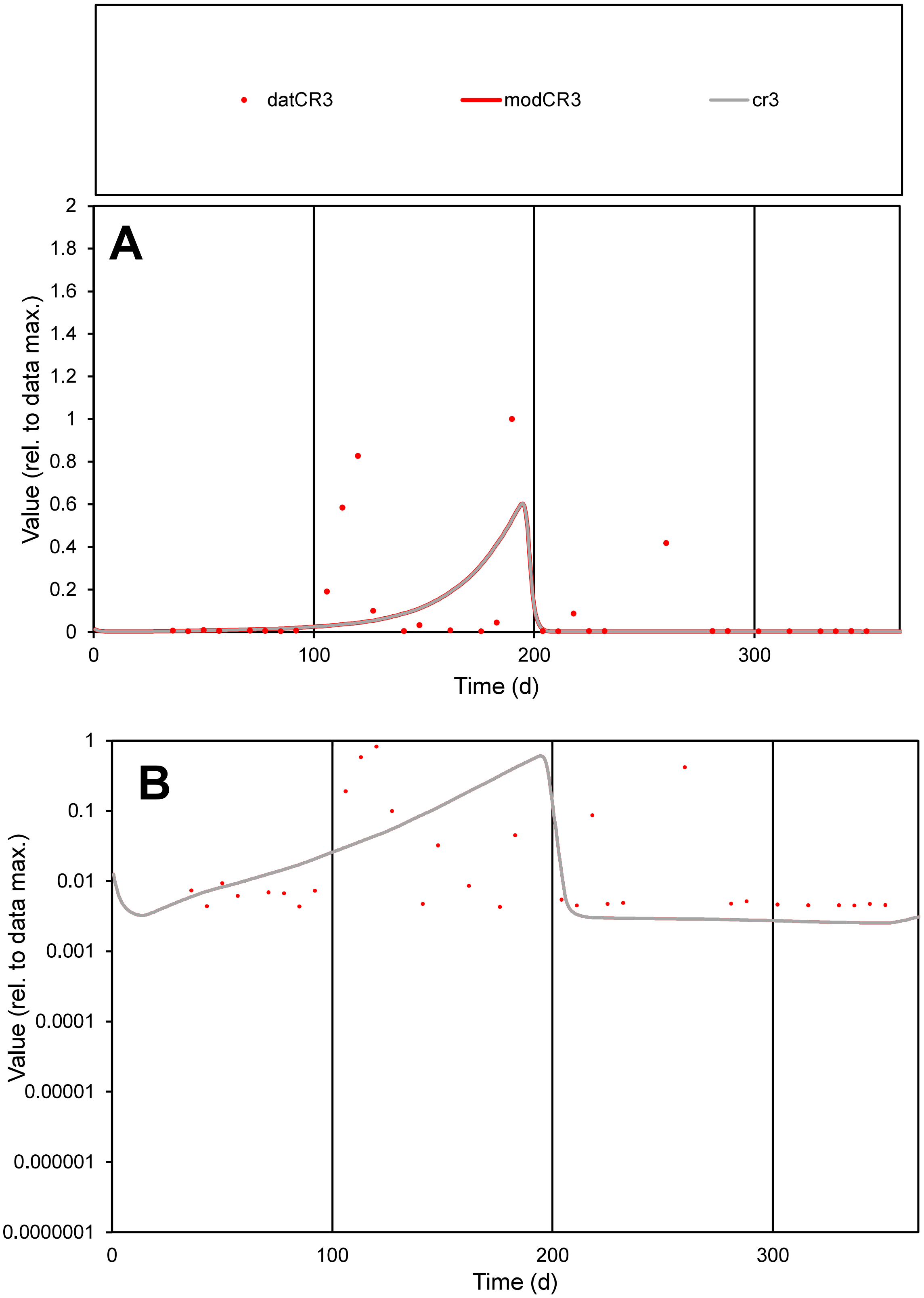

C1

Variables
CR3 Provide jtd or Dst
CalibOnly?
2 1 to reproduce of
oNormalize?
2 1=no, 2=yes

ofxn calc*
2.09294
ofxn mod
2.10479

sRun
RunA1
zout
1

Ave
Data
0.00086
Model
0.0005

data/vMax
0.0081

ofxng
321.551

Min d/m
1.69455

ave/min d
24.7795
ave/min m
24.4165

324_Calib_S_A

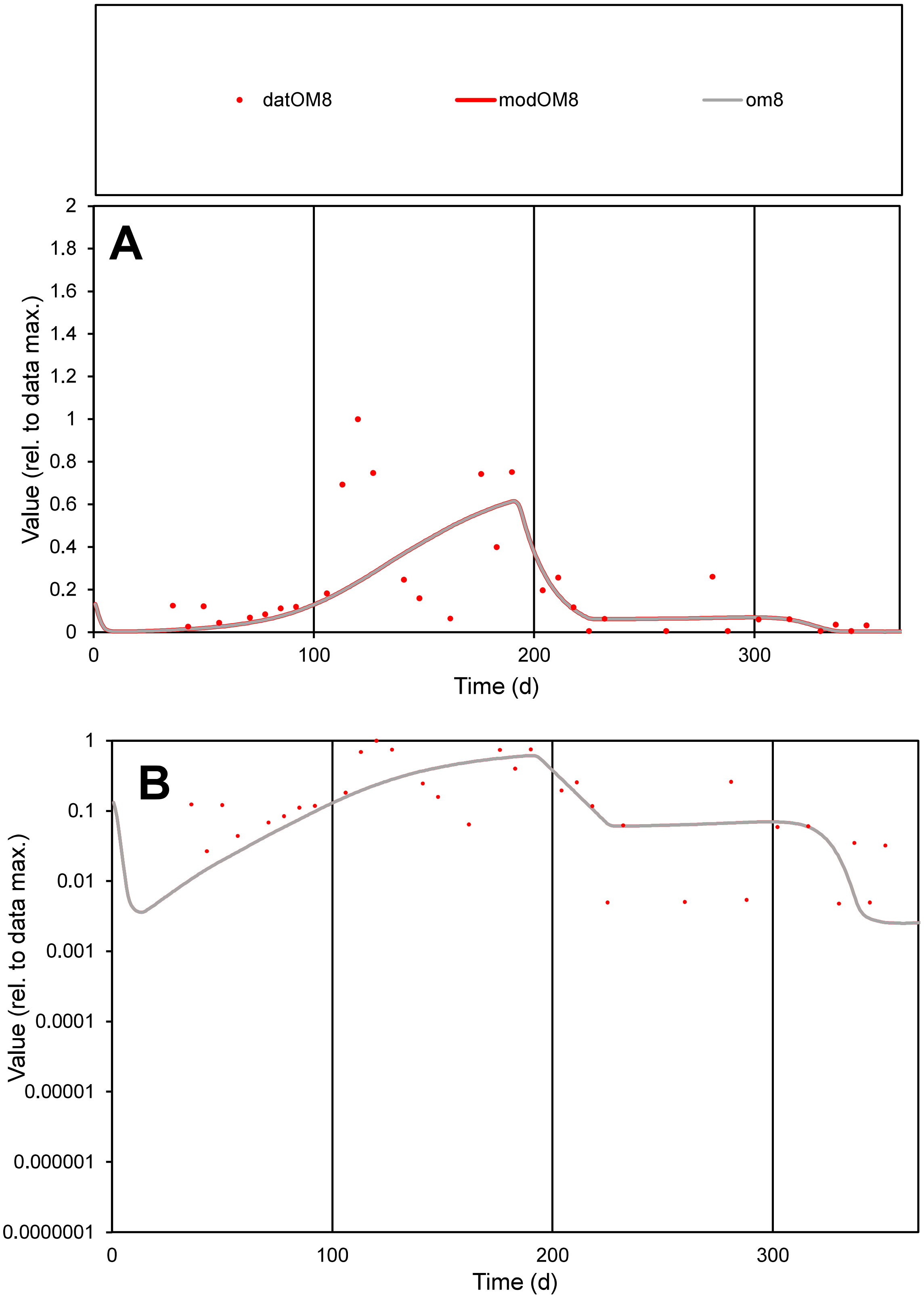

C1

Variables
OM8 Provide jtd or Dst
CalibOnly?
2 1 to reproduce of
oNormalize?
2 1=no, 2=yes

ofxn calc*
1.01111
ofxn mod
1.01939

sRun
RunA1
zout
1

Ave
Data
0.00249
Model
0.00197

data/vMax
0.01176

ofxng
321.551

Min d/m
1.85937

ave/min d
44.4775
ave/min m
65.2142

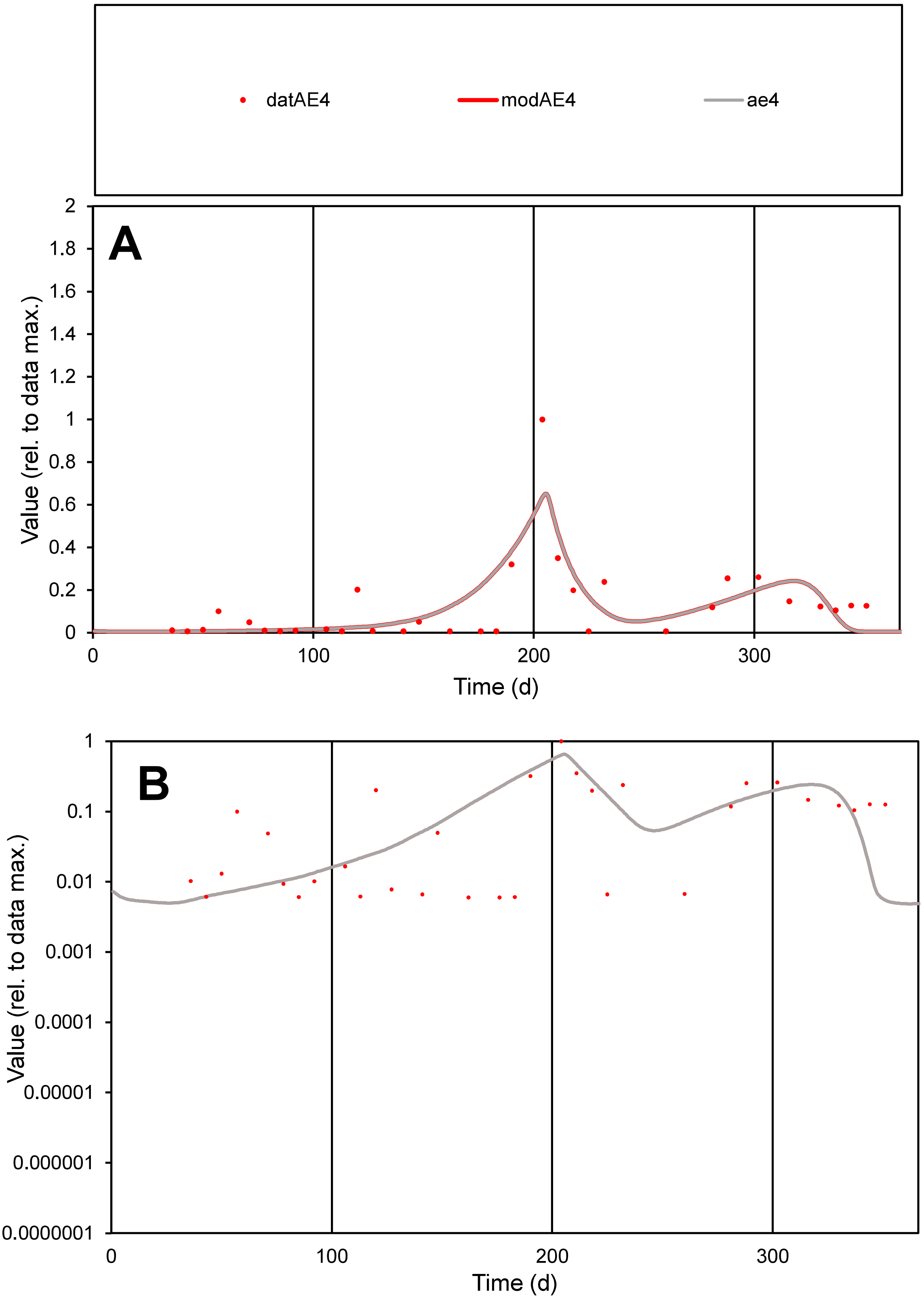

C1

Variables
AE4 Provide jtd or Dst
CalibOnly?
2 1 to reproduce of
oNormalize?
2 1=no, 2=yes

ofxn calc*
0.93064
ofxn mod
0.9562

sRun
RunA1
zout
1

Ave
Data
0.00023
Model
0.00024

data/vMax
0.00191

ofxng
321.551

Min d/m
1.11984

ave/min d
20.356
ave/min m
23.1273

324_Calib_S_A

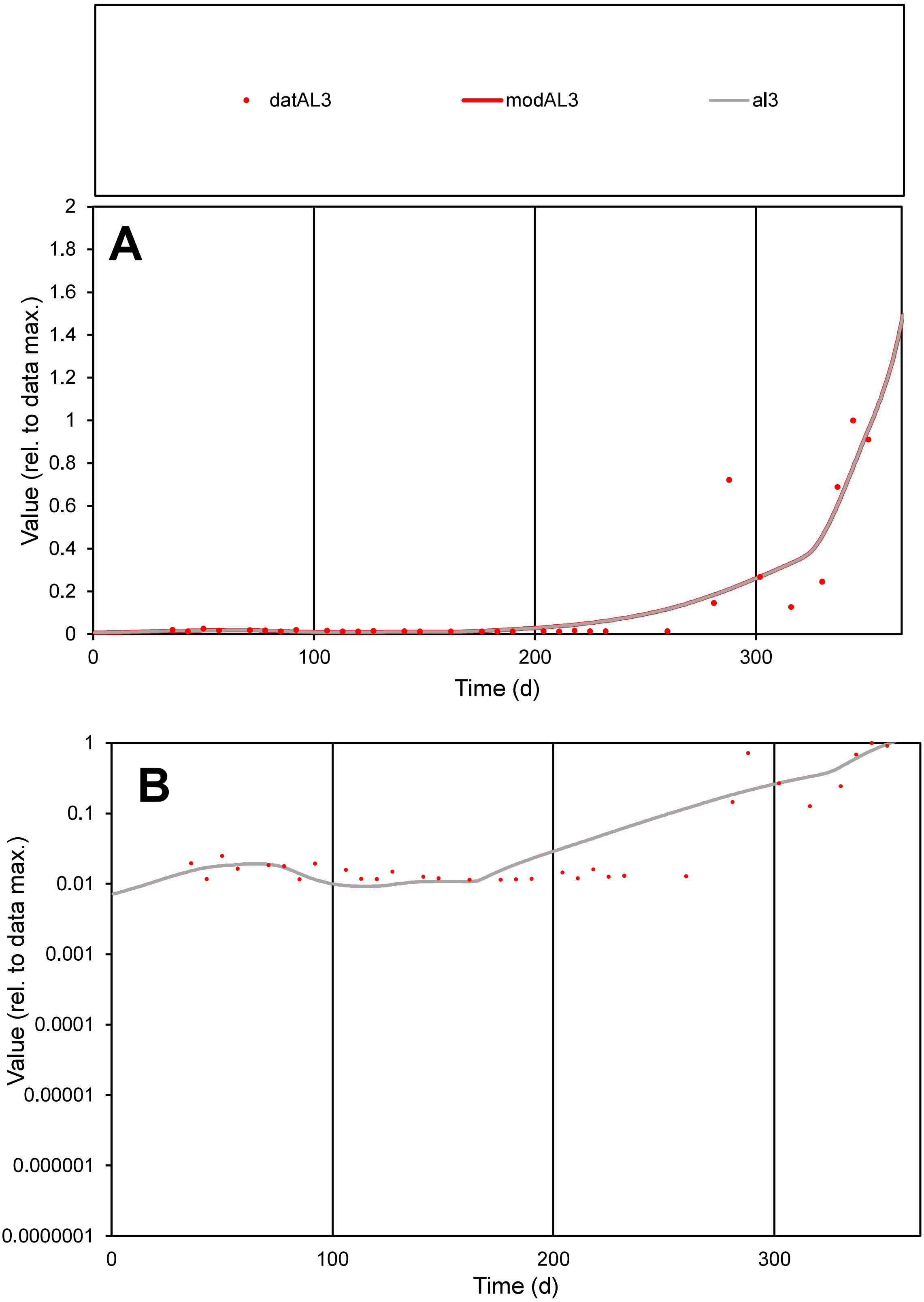

C1

Variables
AL3 Provide jtd or Dst
CalibOnly?
2 1 to reproduce of
oNormalize?
2 1=no, 2=yes

ofxn calc*
0.80891
ofxn mod
0.83225

sRun
RunA1
zout
1

Ave
Data
0.00029
Model
0.00029

data/vMax
0.00207

ofxng
321.551

Min d/m
1.22905

ave/min d
12.1928
ave/min m
15.2231

324_Calib_S_A

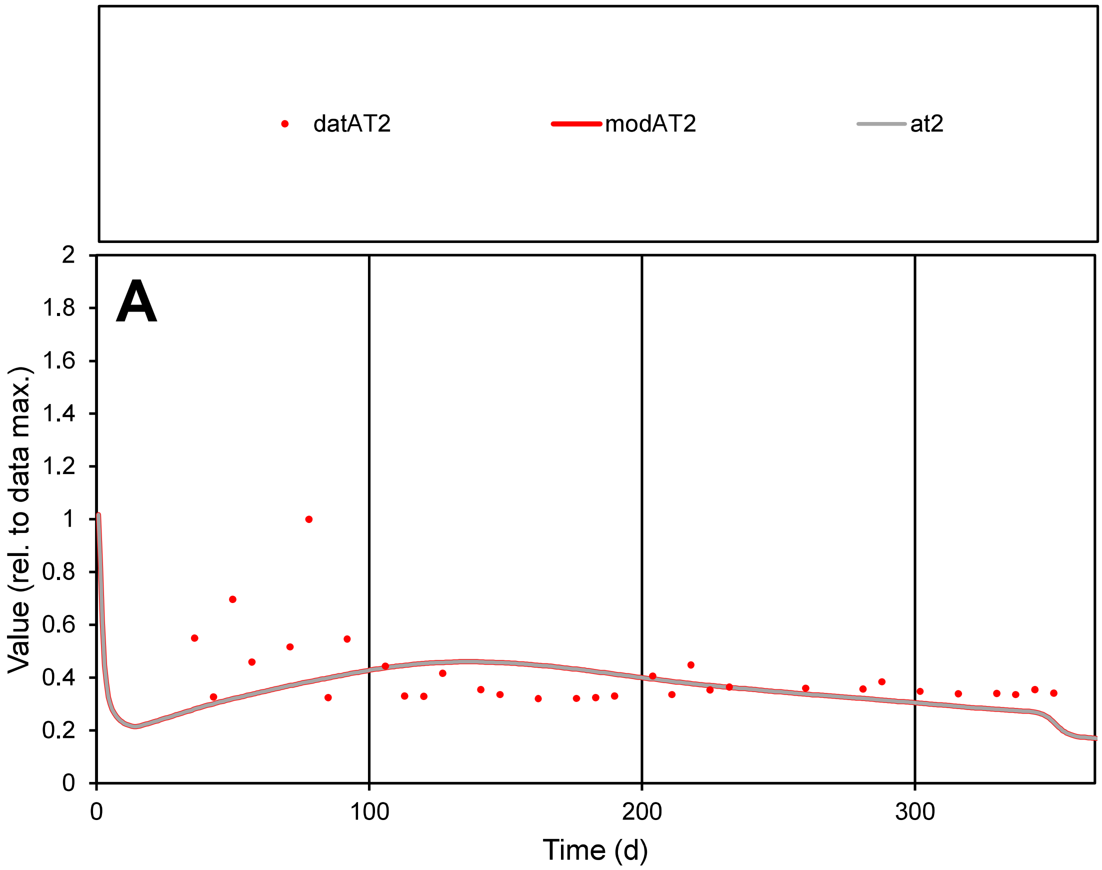

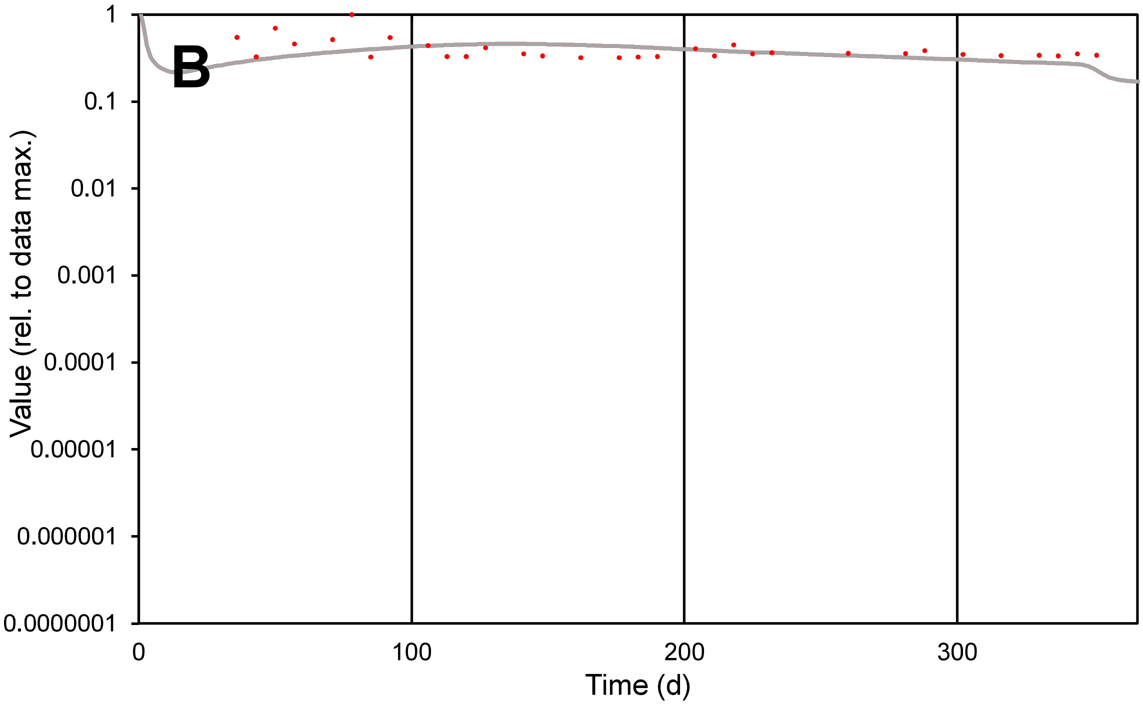

C1

Variables
AT2 Provide jtd or Dst
CalibOnly?
2 1 to reproduce of
oNormalize?
2 1=no, 2=yes

ofxn calc*
0.38801
ofxn mod
0.39953

sRun
RunA1
zout
1

Ave
Data
9.9E-05
Model
8.9E-05

data/vMax
0.00024

ofxng
321.551

Min d/m
1.45261

ave/min d
1.2703
ave/min m
1.66964

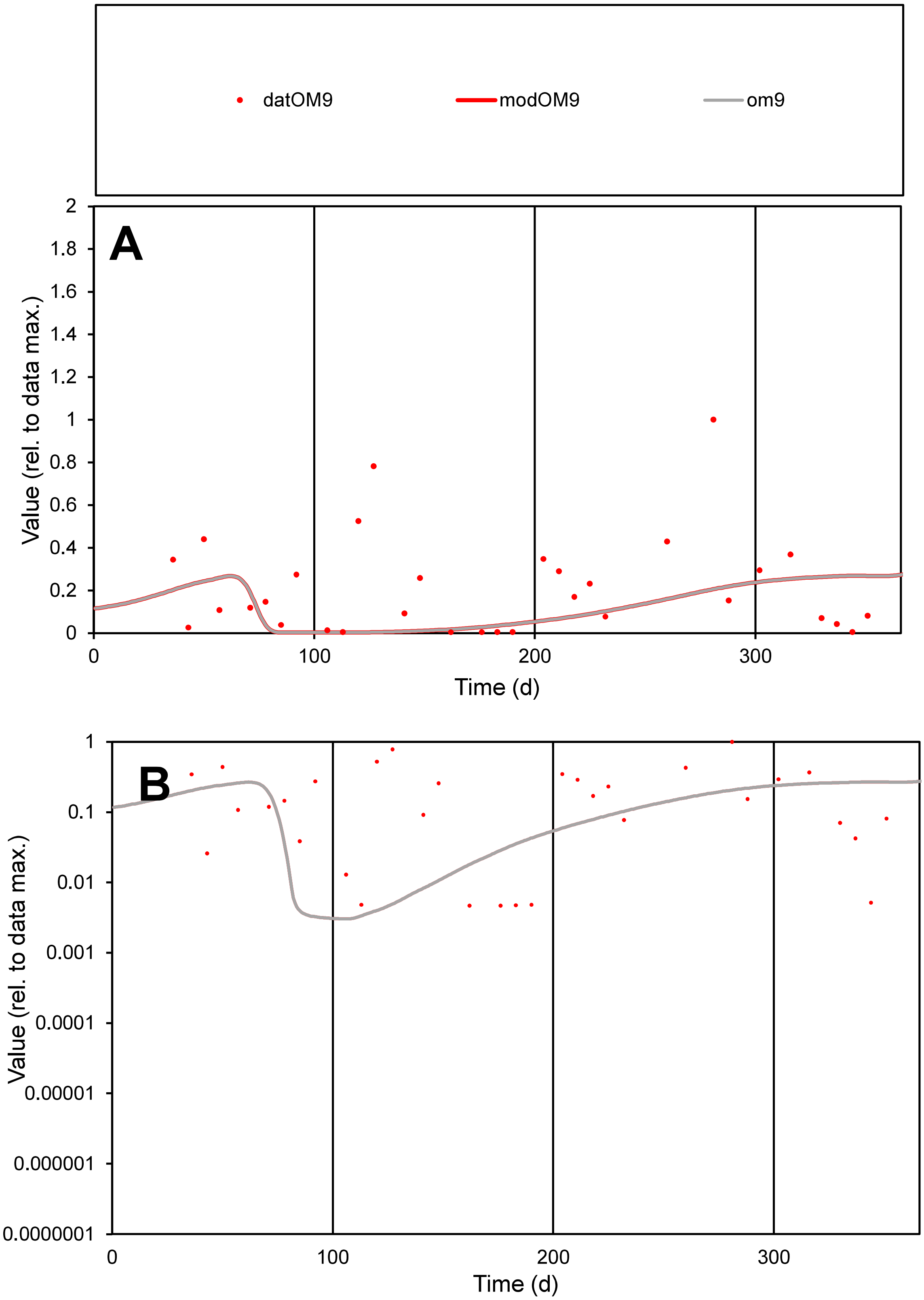

C1

Variables

OM9 Provide jtd or Dst

CalibOnly?

2 1 to reproduce of

oNormalize?

2 1=no, 2=yes

ofxn calc*

1.24869

ofxn mod

1.25257

sRun

RunA1

zout

1

Ave

Data

0.00192

Model

0.00111

data/vMax

0.00909

ofxng

321.551

Min d/m

1.52429

ave/min d

45.2401

ave/min m

39.8169

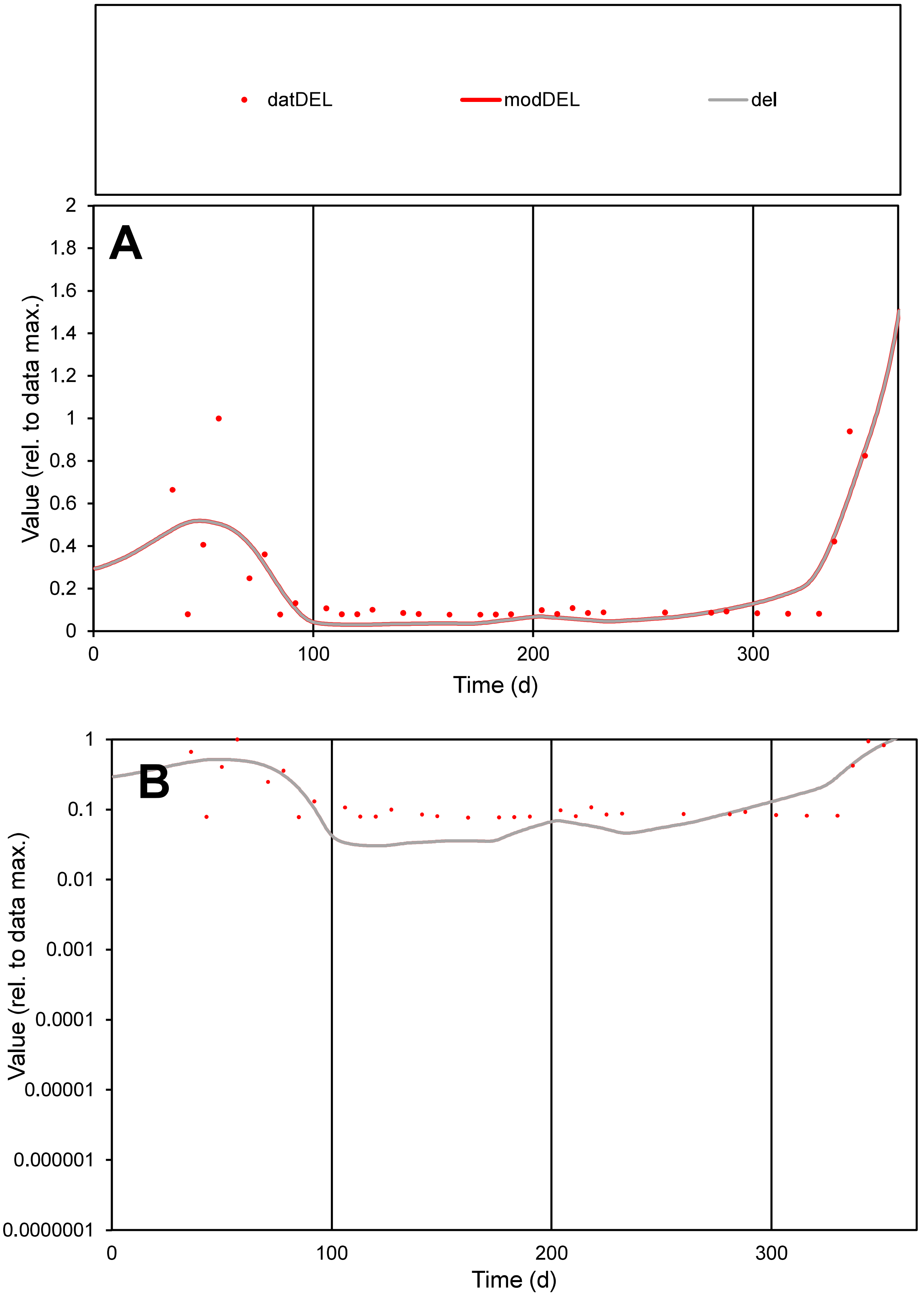

C1

Variables
DEL Provide jtd or Dst
CalibOnly?
2 1 to reproduce of
oNormalize?
2 1=no, 2=yes

ofxn calc*
0.66952
ofxn mod
0.68204

sRun
RunA1
zout
1

Ave
Data
0.00017
Model
0.00016

data/vMax
0.00079

ofxng
321.551

Min d/m
2.52961

ave/min d
2.83301
ave/min m
6.75571

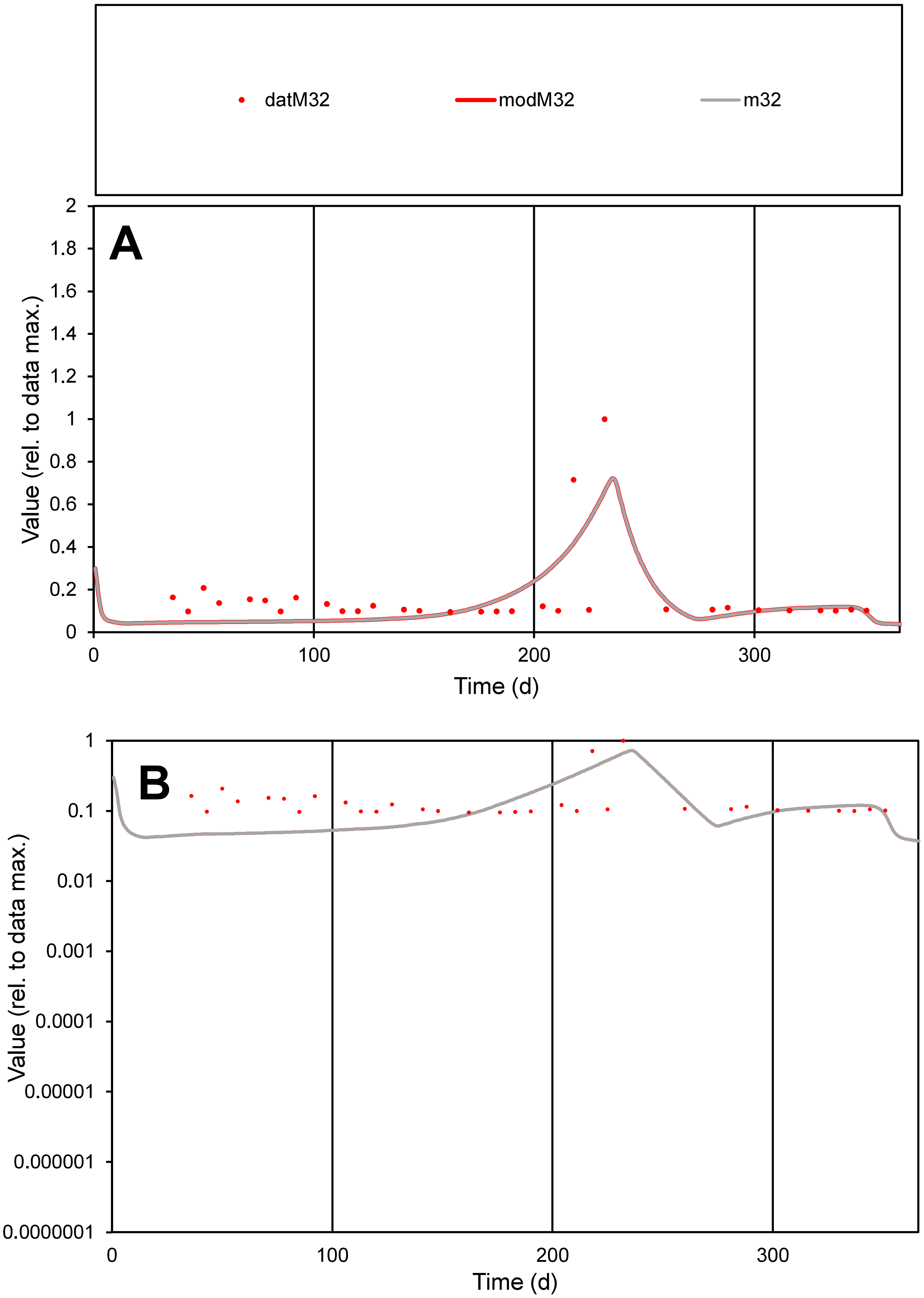

C1

Variables
M32 Provide jtd or Dst
CalibOnly?
2 1 to reproduce of
oNormalize?
2 1=no, 2=yes

ofxn calc*
0.81124
ofxn mod
0.8273

sRun
RunA1
zout
1

Ave
Data
0.00035
Model
0.0003

data/vMax
0.00216

ofxng
321.551

Min d/m
2.06365

ave/min d
1.70522
ave/min m
3.07008

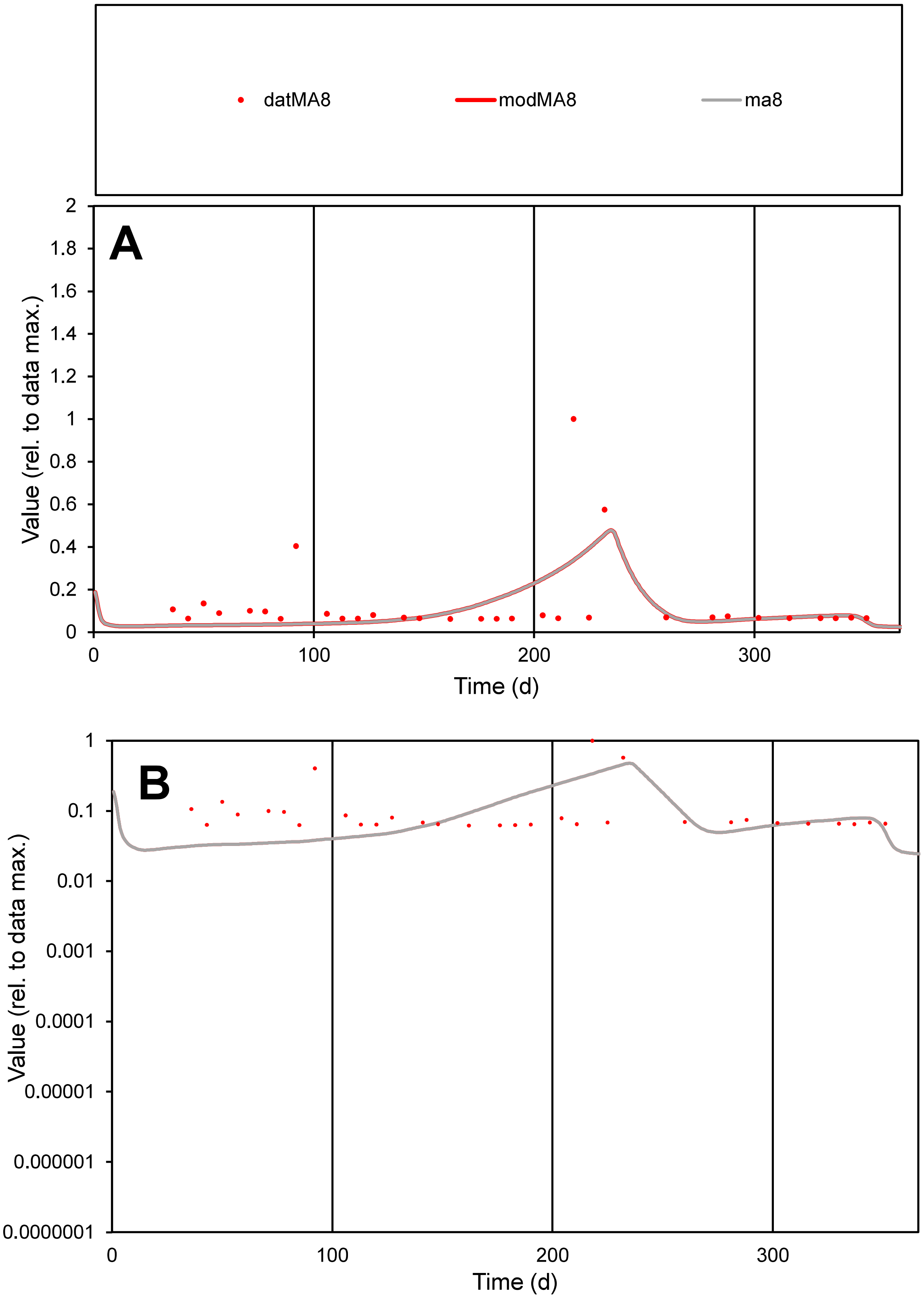

C1

Variables
MA8 Provide jtd or Dst
CalibOnly?
2 1 to reproduce of
oNormalize?
2 1=no, 2=yes

ofxn calc*
1.24054
ofxn mod
1.25391

sRun
RunA1
zout
1

Ave
Data
0.00012
Model
0.0001

data/vMax
0.00095

ofxng
321.551

Min d/m
1.94578

ave/min d
2.0879
ave/min m
3.47373

324_Calib_S_A

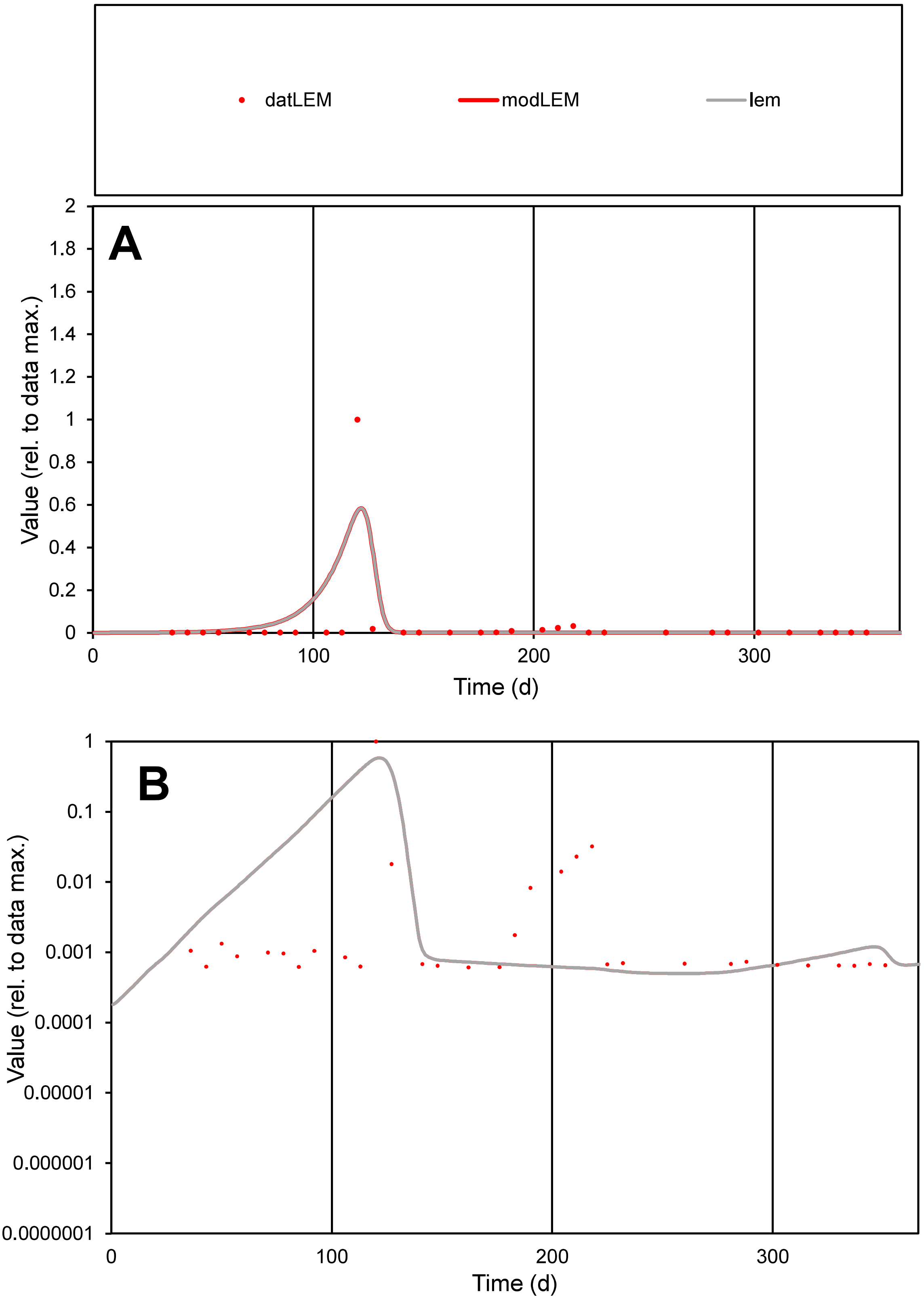

C1

Variables
LEM Provide jtd or Dst
CalibOnly?
2 1 to reproduce of
oNormalize?
2 1=no, 2=yes

ofxn calc*
3.1334
ofxn mod
3.32198

sRun
RunA1
zout
1

Ave
Data
0.0012
Model
0.00166

data/vMax
0.03451

ofxng
321.551

Min d/m
1.23122

ave/min d
56.9388
ave/min m
96.4306

324_Calib_S_A

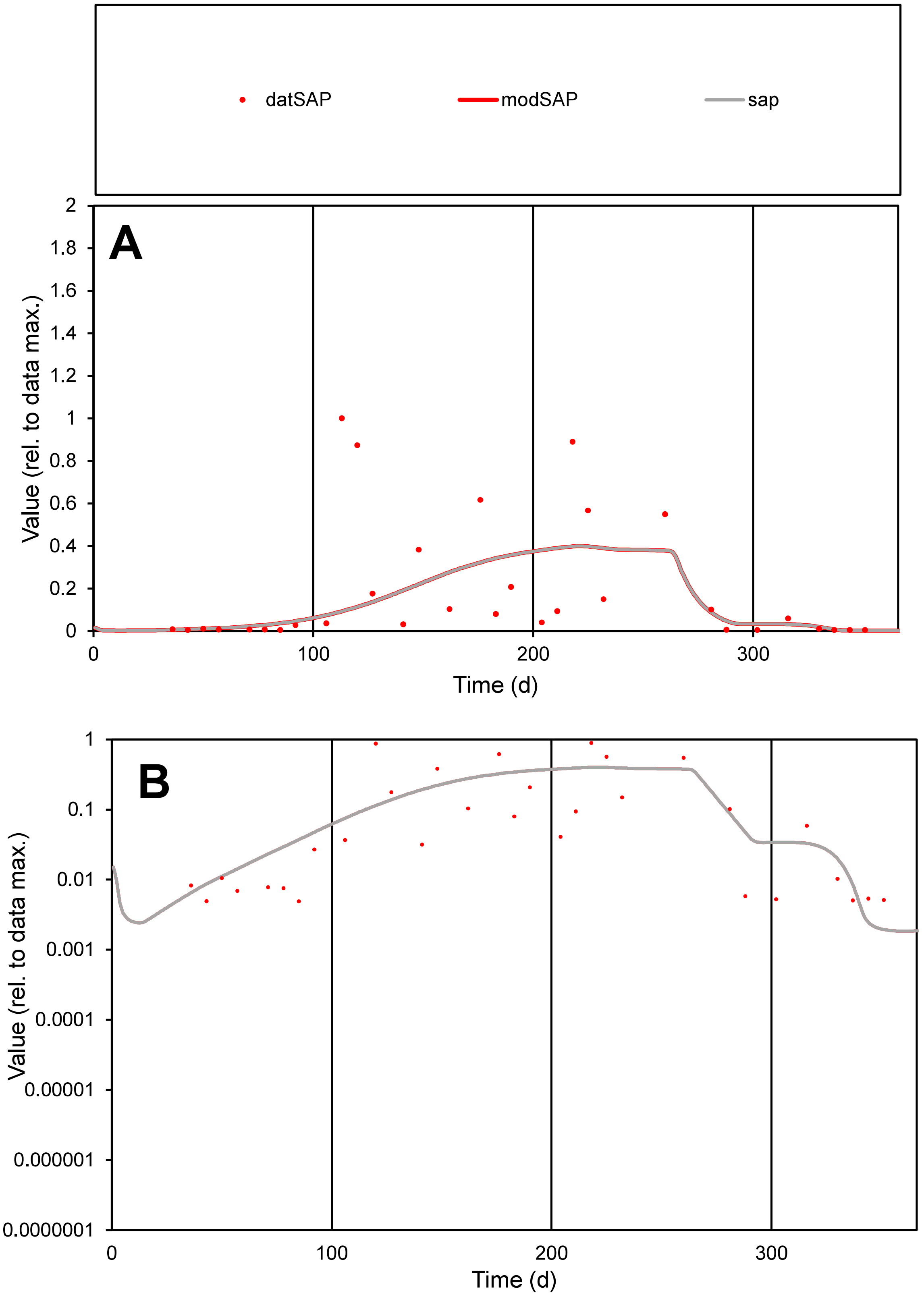

C1

Variables
SAP Provide jtd or Dst
CalibOnly?
2 1 to reproduce of
oNormalize?
2 1=no, 2=yes

ofxn calc*
1.38494
ofxn mod
1.39259

sRun
RunA1
zout
1

Ave
Data
0.00127
Model
0.00102

data/vMax
0.00668

ofxng
321.551

Min d/m
2.55747

ave/min d
38.7563
ave/min m
79.8779

324_Calib_S_A

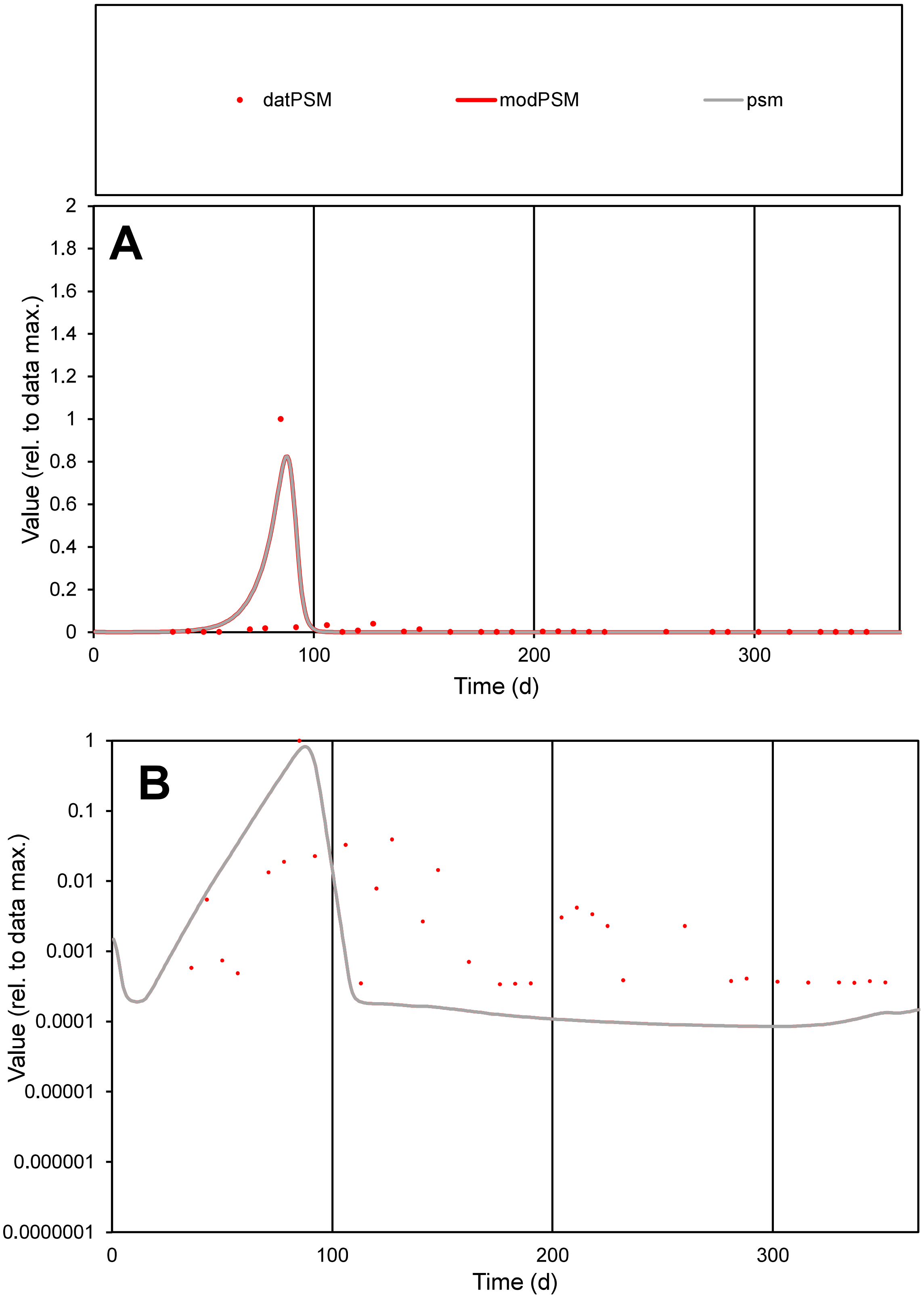

C1

Variables
PSM Provide jtd or Dst
CalibOnly?
2 1 to reproduce of
oNormalize?
2 1=no, 2=yes

ofxn calc*
1.92607
ofxn mod
2.20262

sRun
RunA1
zout
1

Ave
Data
0.00294
Model
0.00368

data/vMax
0.07978

ofxng
321.551

Min d/m
4.01624

ave/min d
108.125
ave/min m
543.024

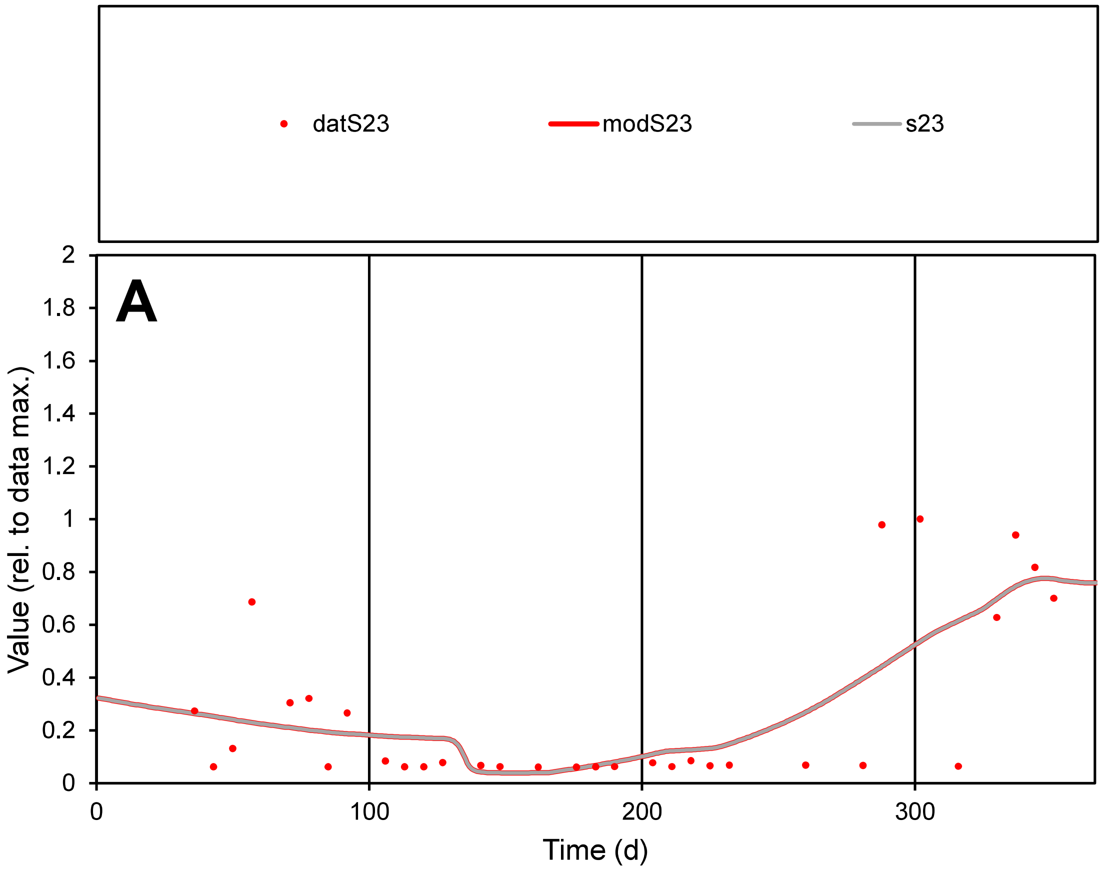

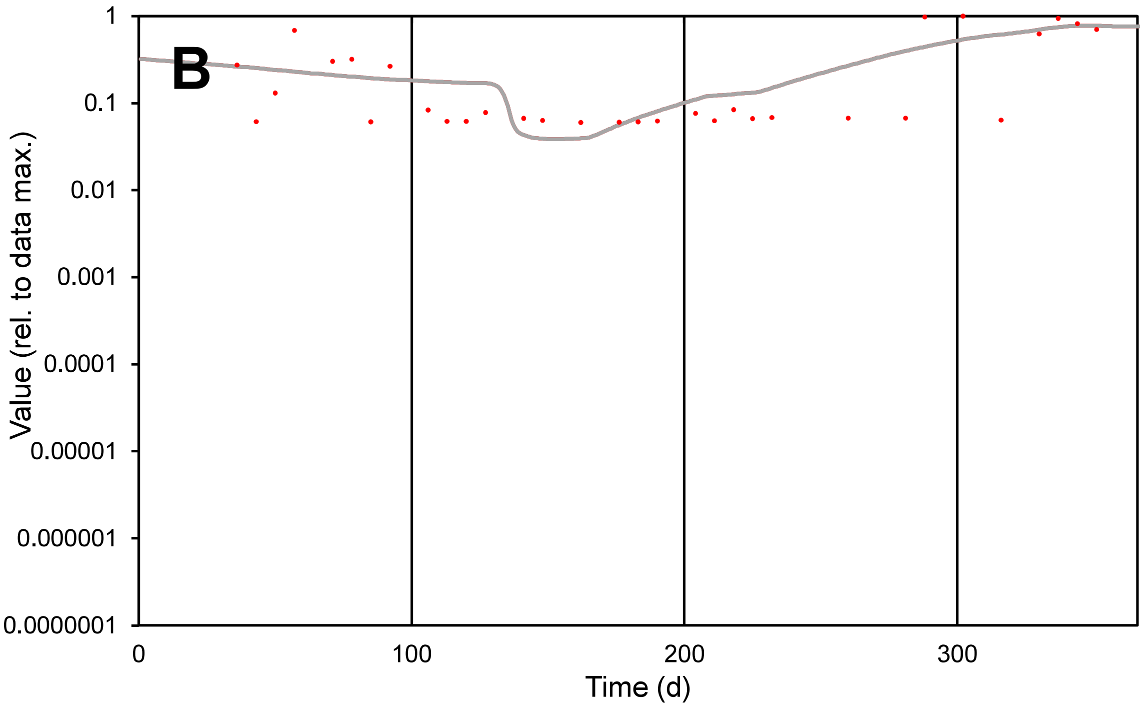

C1

Variables
S23 Provide jtd or Dst
CalibOnly?
2 1 to reproduce of
oNormalize?
2 1=no, 2=yes

ofxn calc*
0.78585
ofxn mod
0.79837

sRun
RunA1
zout
1

Ave
Data
0.00069
Model
0.00071

data/vMax
0.00262

ofxng
321.551

Min d/m
1.55227

ave/min d
4.35777
ave/min m
7.04888

324_Calib_S_A

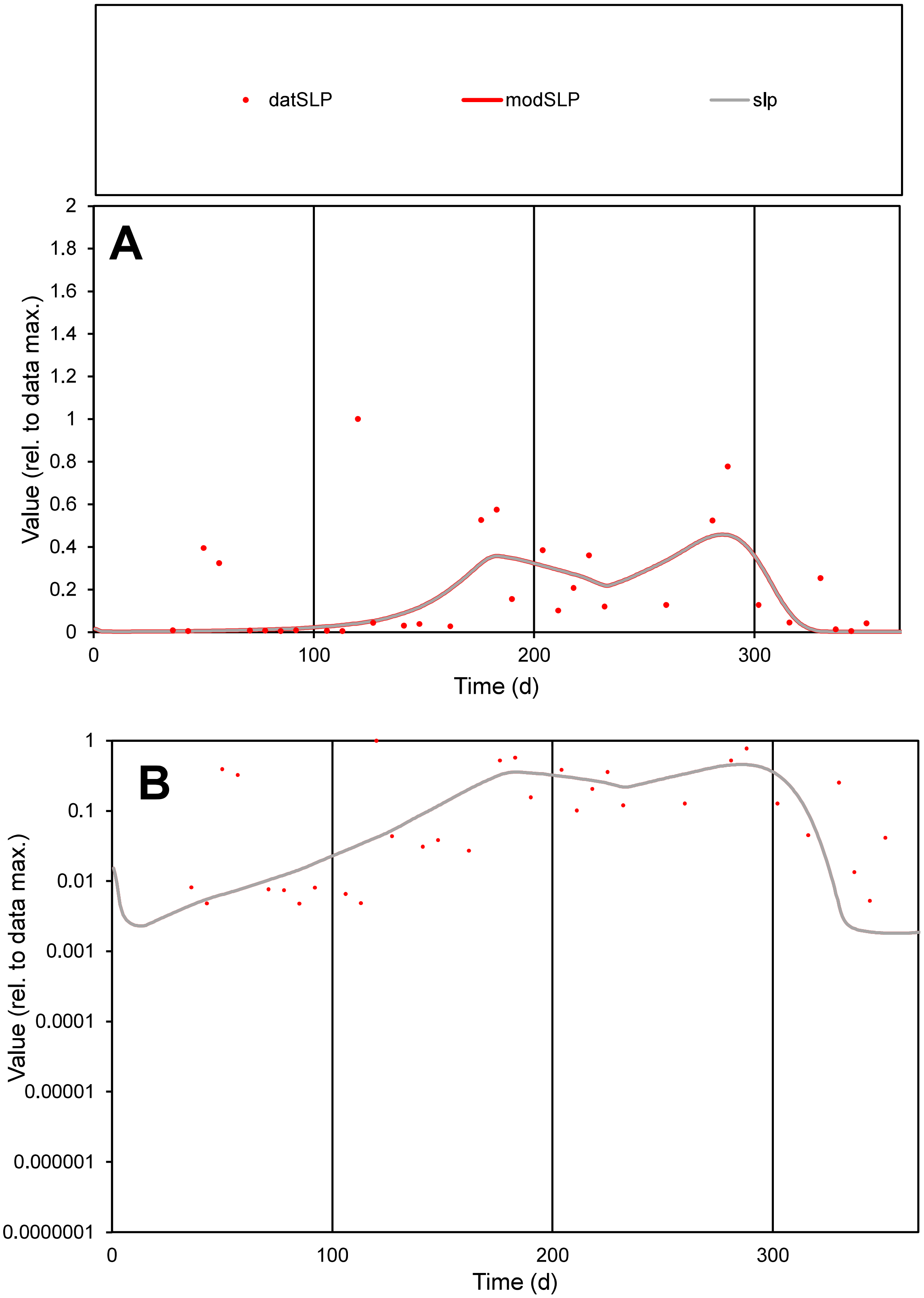

C1

Variables
SLP Provide jtd or Dst
CalibOnly?
2 1 to reproduce of
oNormalize?
2 1=no, 2=yes

ofxn calc*
1.16081
ofxn mod
1.16754

sRun
RunA1
zout
1

Ave
Data
0.00097
Model
0.00072

data/vMax
0.00496

ofxng
321.551

Min d/m
2.64138

ave/min d
40.7431
ave/min m
79.8586

324_Calib_S_A

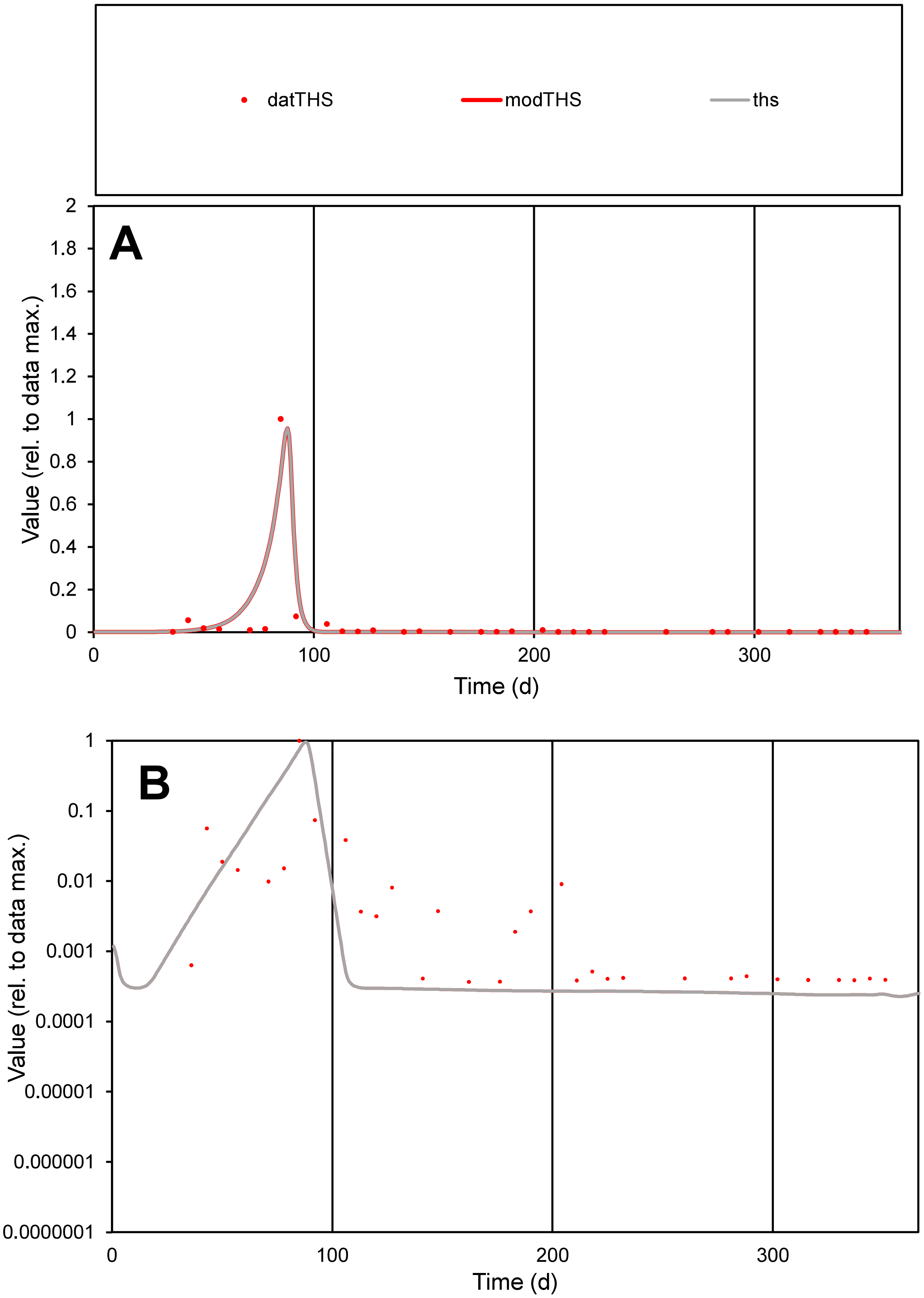

C1

Variables

THS Provide jtd or Dst

CalibOnly?

2 1 to reproduce of

oNormalize?

2 1=no, 2=yes

ofxn calc*

1.42946

ofxn mod

1.69417

sRun

RunA1

zout

1

Ave

Data

0.00303

Model

0.00348

data/vMax

0.07656

ofxng

321.551

Min d/m

1.53377

ave/min d

107.646

ave/min m

189.734

324_Calib_S_A

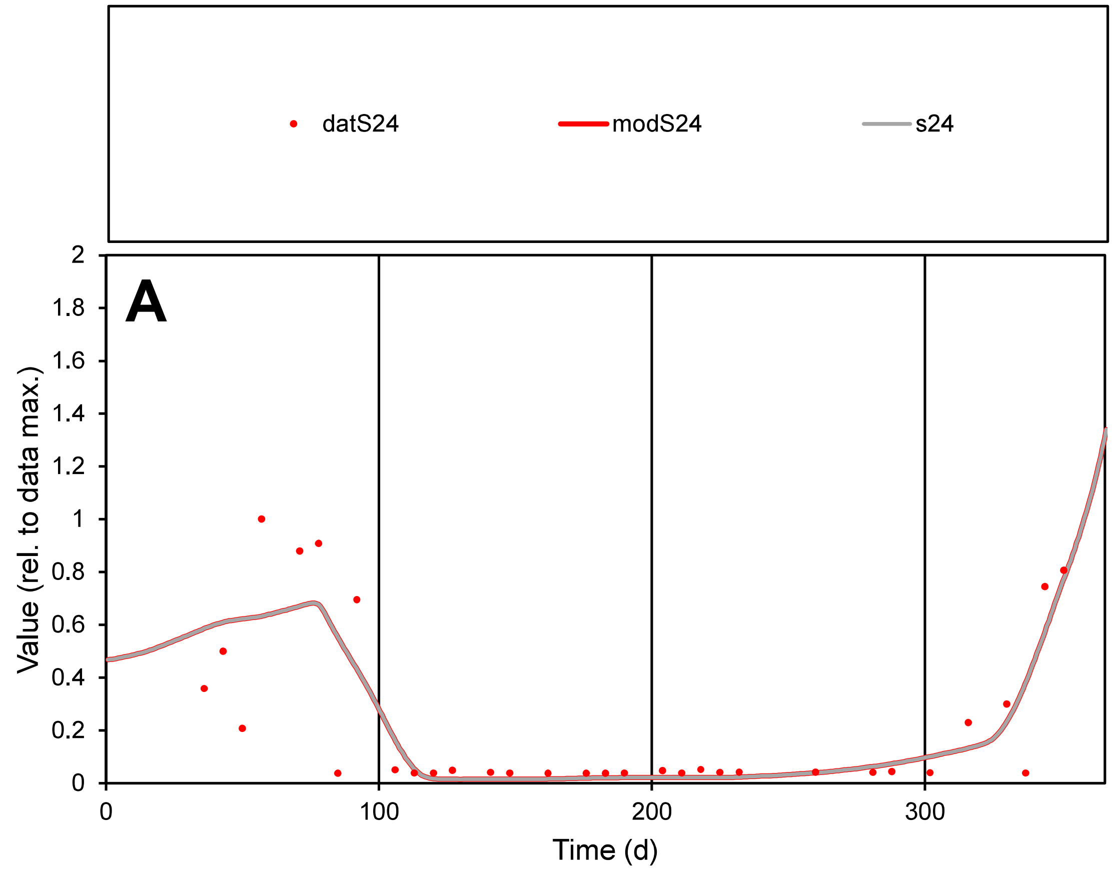

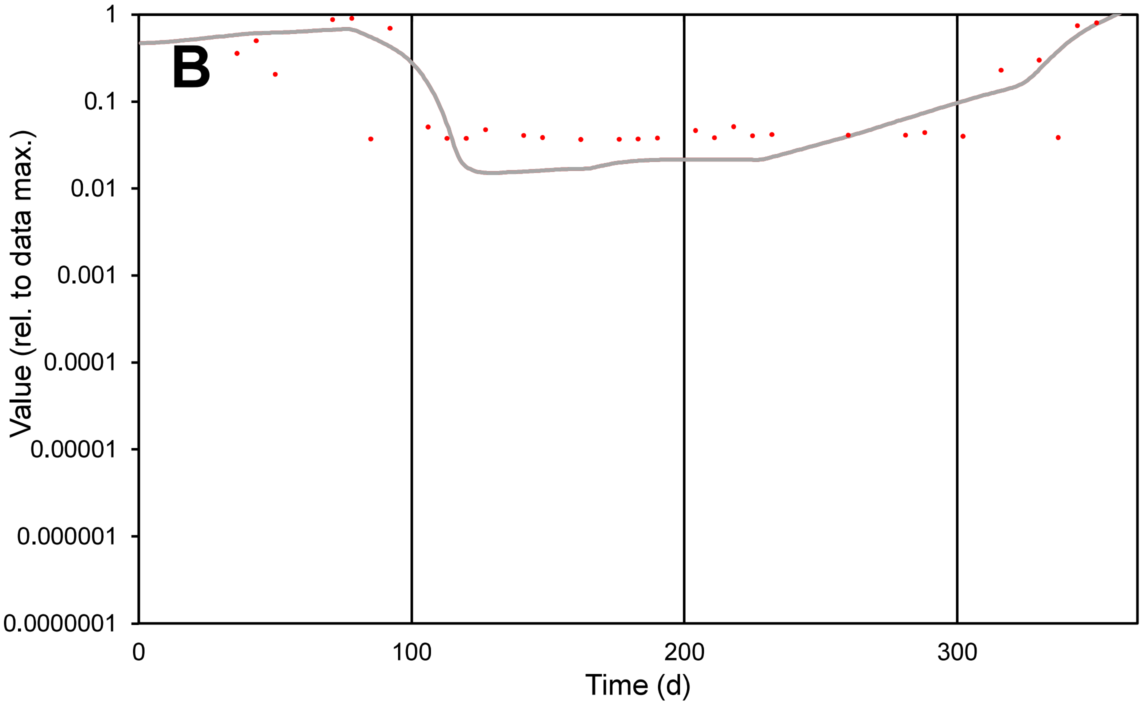

C1

Variables
S24 Provide jtd or Dst
CalibOnly?
2 1 to reproduce of
oNormalize?
2 1=no, 2=yes

ofxn calc*
0.73186
ofxn mod
0.75014

sRun
RunA1
zout
1

Ave
Data
0.00036
Model
0.00037

data/vMax
0.00155

ofxng
321.551

Min d/m
2.43566

ave/min d
6.37593
ave/min m
15.7759

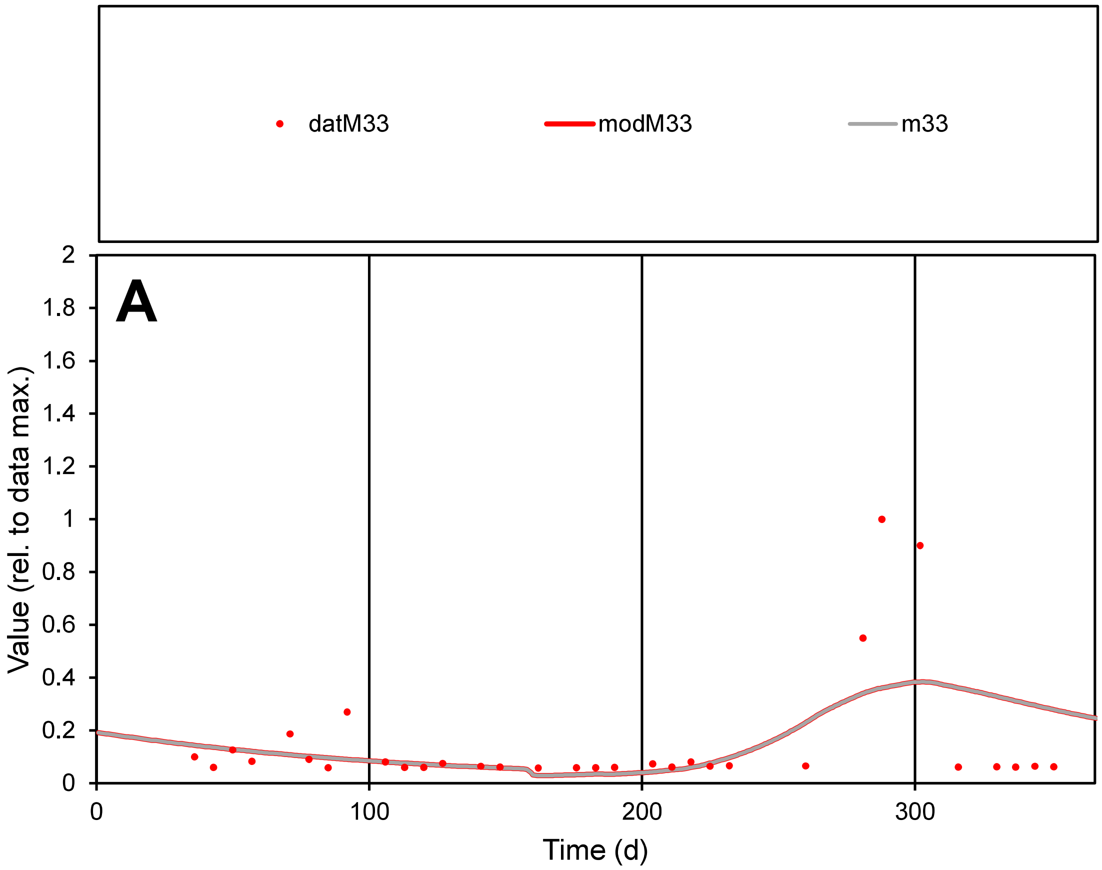

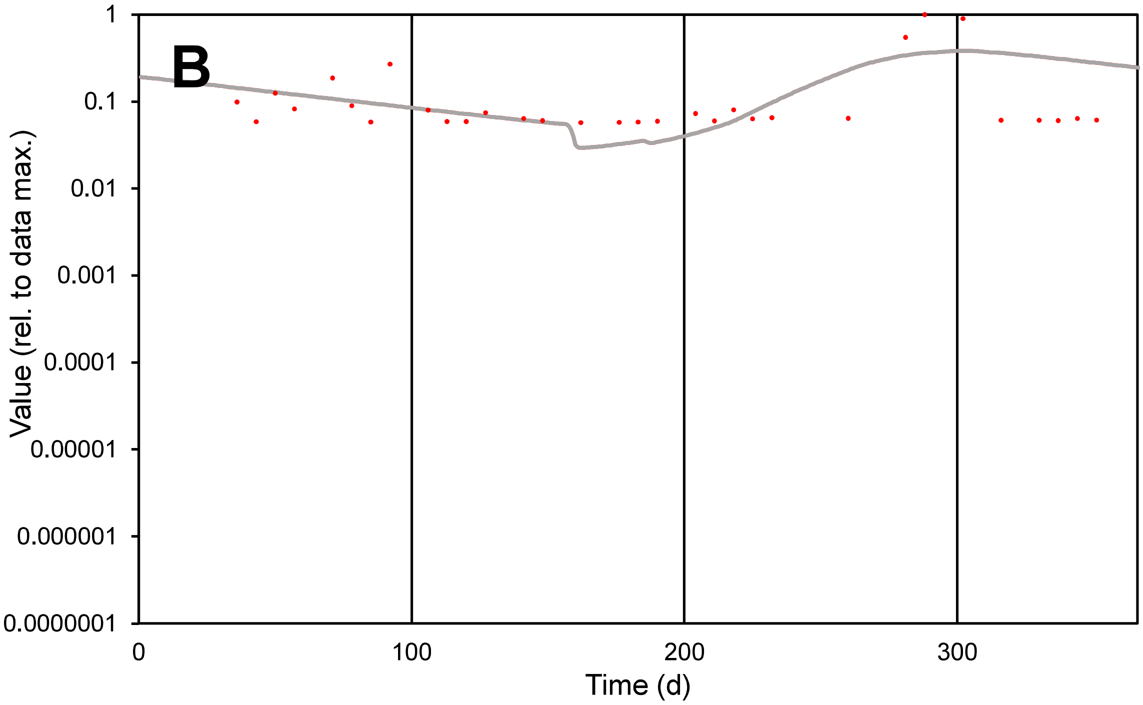

C1

Variables
M33 Provide jtd or Dst
CalibOnly?
2 1 to reproduce of
oNormalize?
2 1=no, 2=yes

ofxn calc*
1.25378
ofxn mod
1.26146

sRun
RunA1
zout
1

Ave
Data
0.00017
Model
0.00017

data/vMax
0.00117

ofxng
321.551

Min d/m
1.94686

ave/min d
2.59155
ave/min m
4.96204

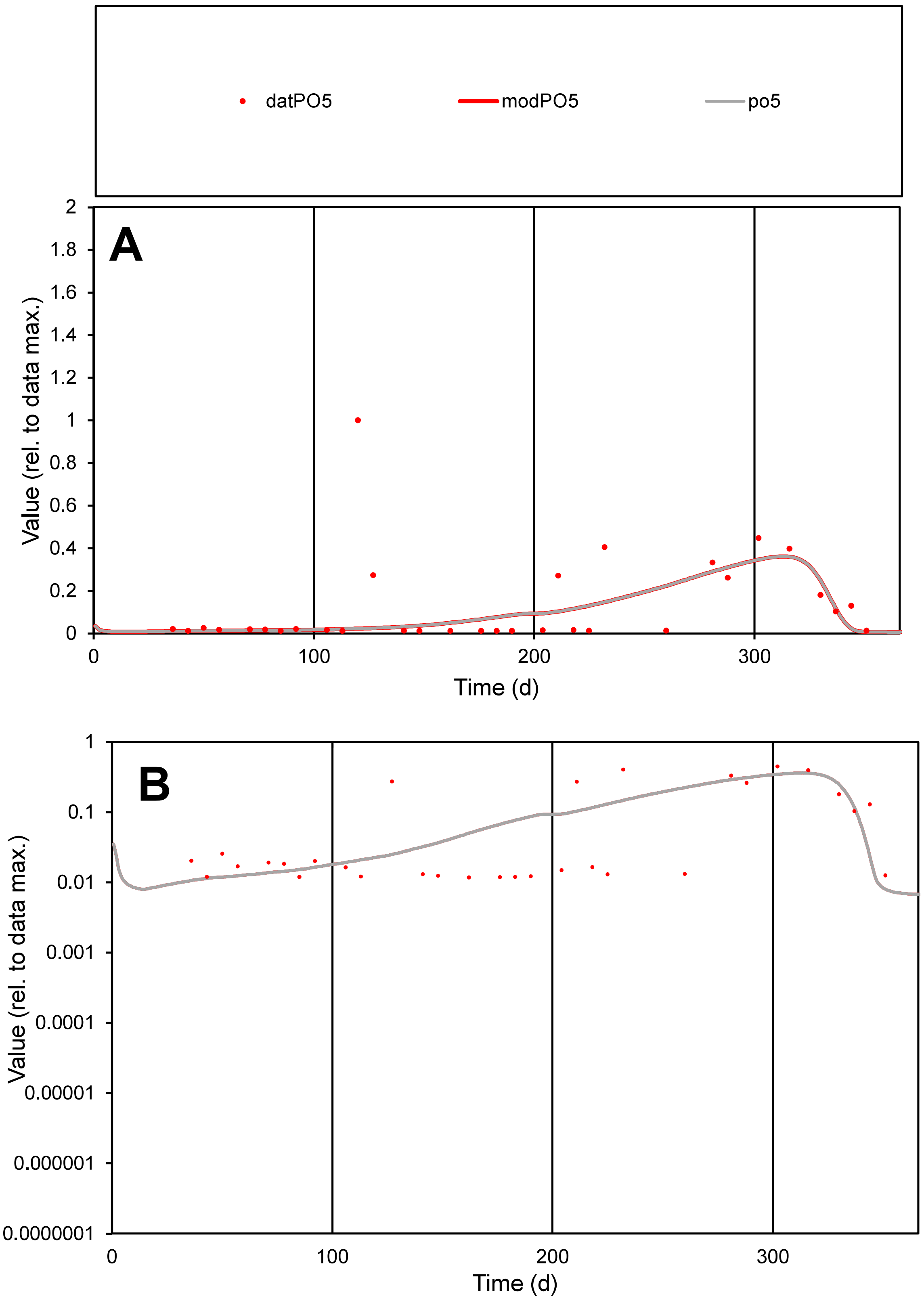

C1

Variables
PO5 Provide jtd or Dst
CalibOnly?
2 1 to reproduce of
oNormalize?
2 1=no, 2=yes

ofxn calc*
1.52037
ofxn mod
1.52741

sRun
RunA1
zout
1

Ave
Data
0.0005
Model
0.00037

data/vMax
0.00386

ofxng
321.551

Min d/m
1.51968

ave/min d
10.8939
ave/min m
12.2333

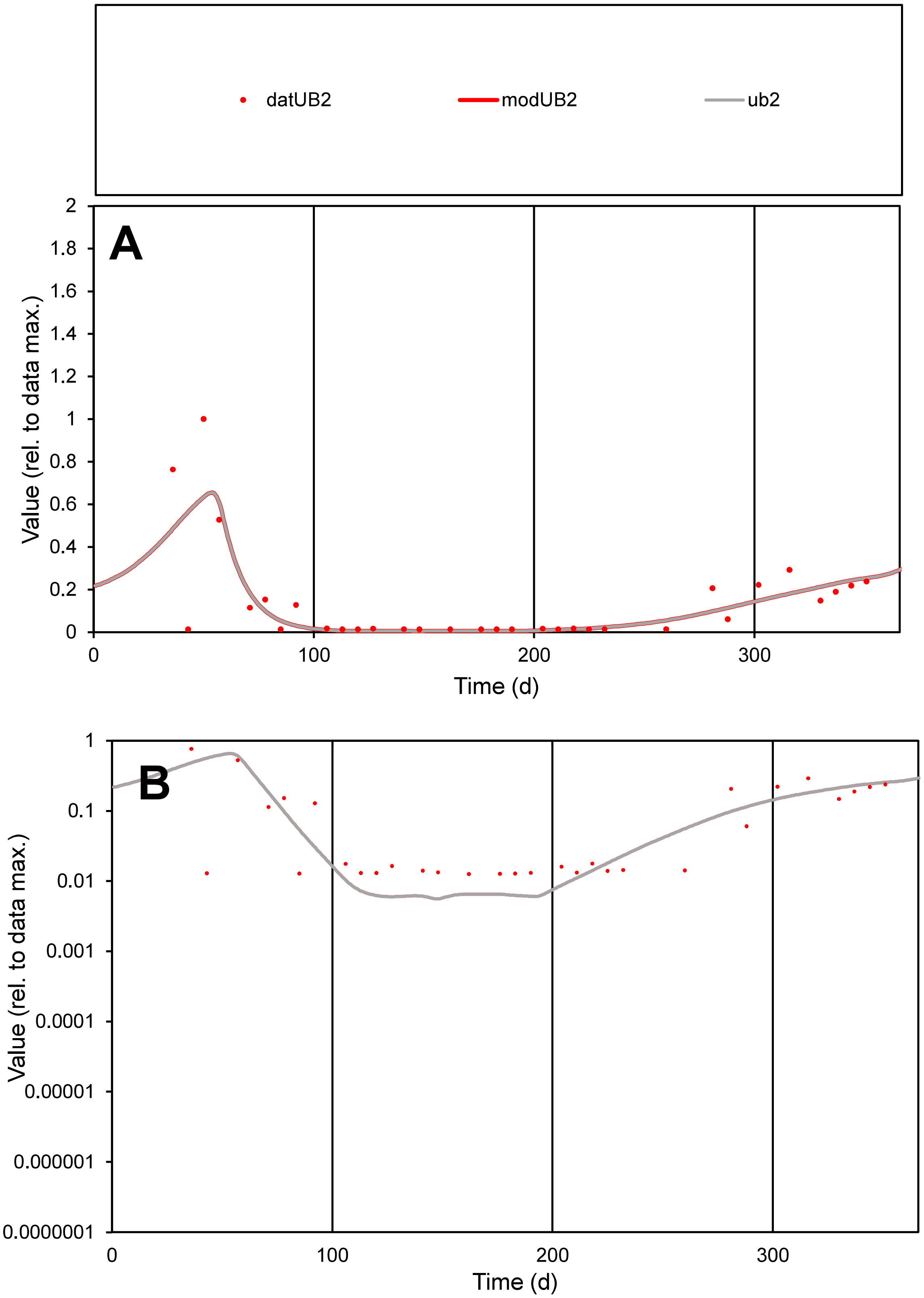

C1

Variables
UB2 Provide jtd or Dst
CalibOnly?
2 1 to reproduce of
oNormalize?
2 1=no, 2=yes

ofxn calc*
0.94359
ofxn mod
0.96145

sRun
RunA1
zout
1

Ave
Data
0.00114
Model
0.00109

data/vMax
0.00805

ofxng
321.551

Min d/m
2.23635

ave/min d
11.1436
ave/min m
23.8679

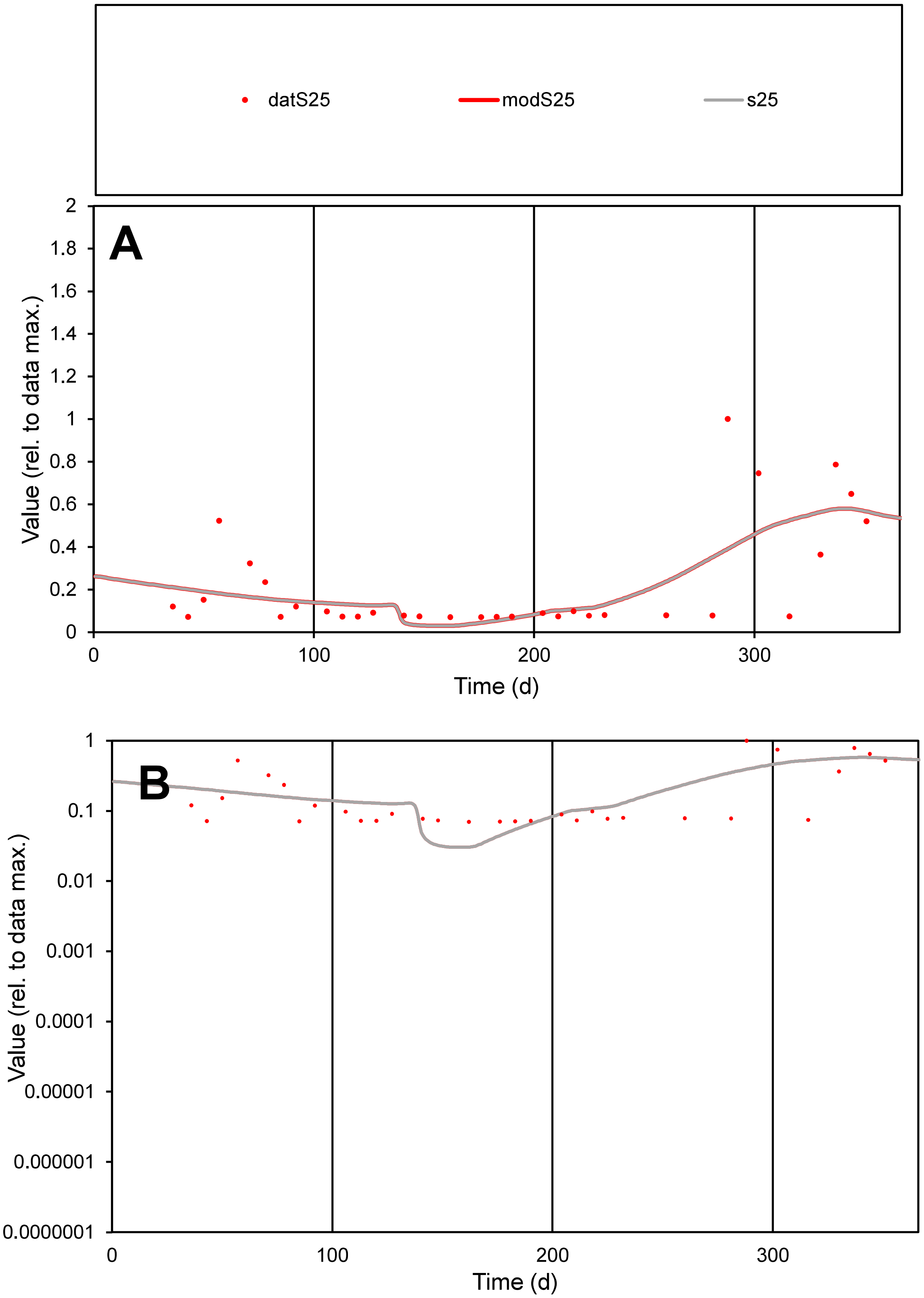

C1

Variables
S25 Provide jtd or Dst
CalibOnly?
2 1 to reproduce of
oNormalize?
2 1=no, 2=yes

ofxn calc*
0.8104
ofxn mod
0.82093

sRun
RunA1
zout
1

Ave
Data
0.00053
Model
0.00052

data/vMax
0.00237

ofxng
321.551

Min d/m
2.26273

ave/min d
3.1696
ave/min m
7.10553

324_Calib_S_A

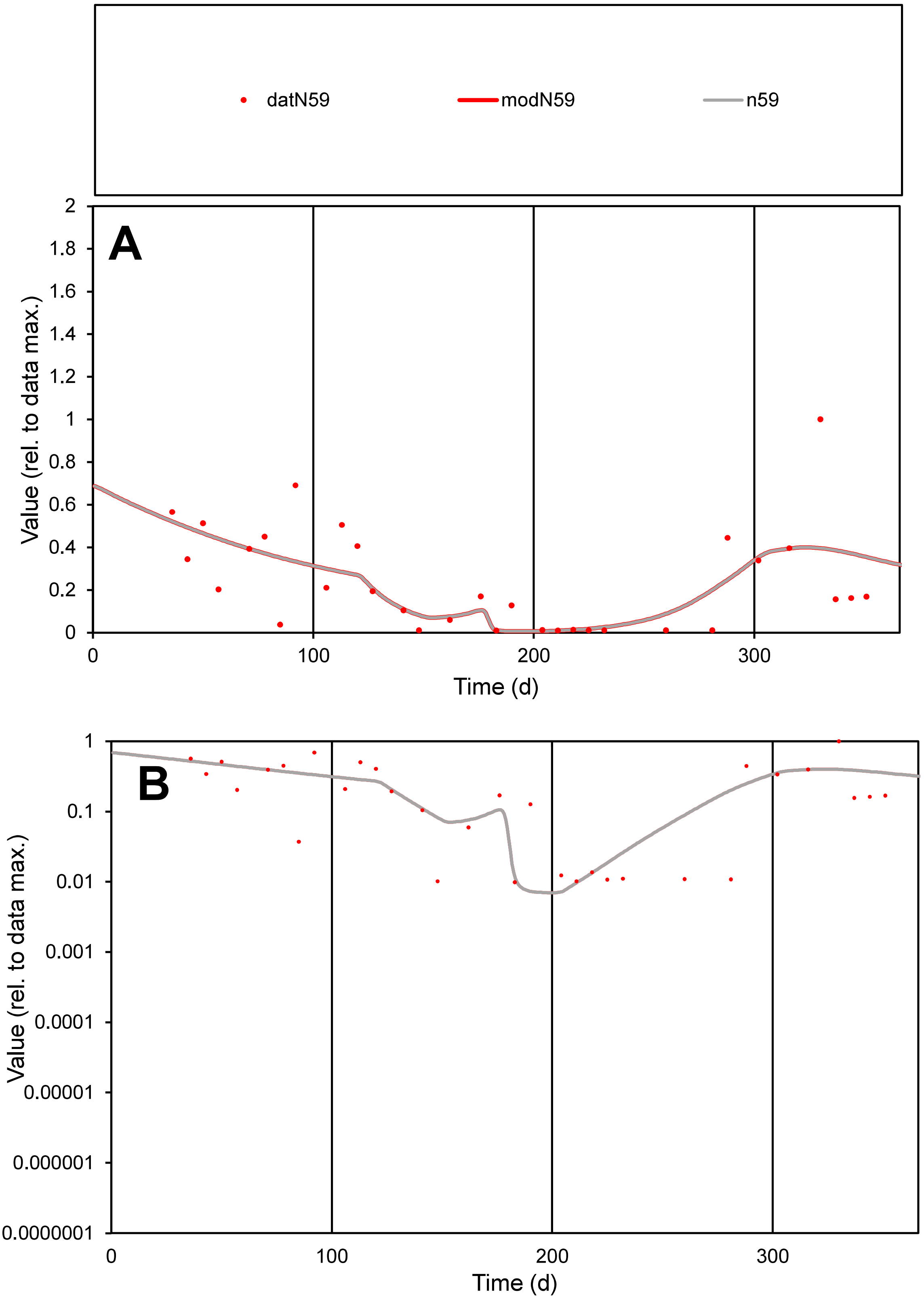

C1

Variables
N59 Provide jtd or Dst
CalibOnly?
2 1 to reproduce of
oNormalize?
2 1=no, 2=yes

ofxn calc*
0.7265
ofxn mod
0.7377

sRun
RunA1
zout
1

Ave
Data
0.00101
Model
0.00099

data/vMax
0.00416

ofxng
321.551

Min d/m
1.37212

ave/min d
24.4394
ave/min m
33.1222

324_Calib_S_A

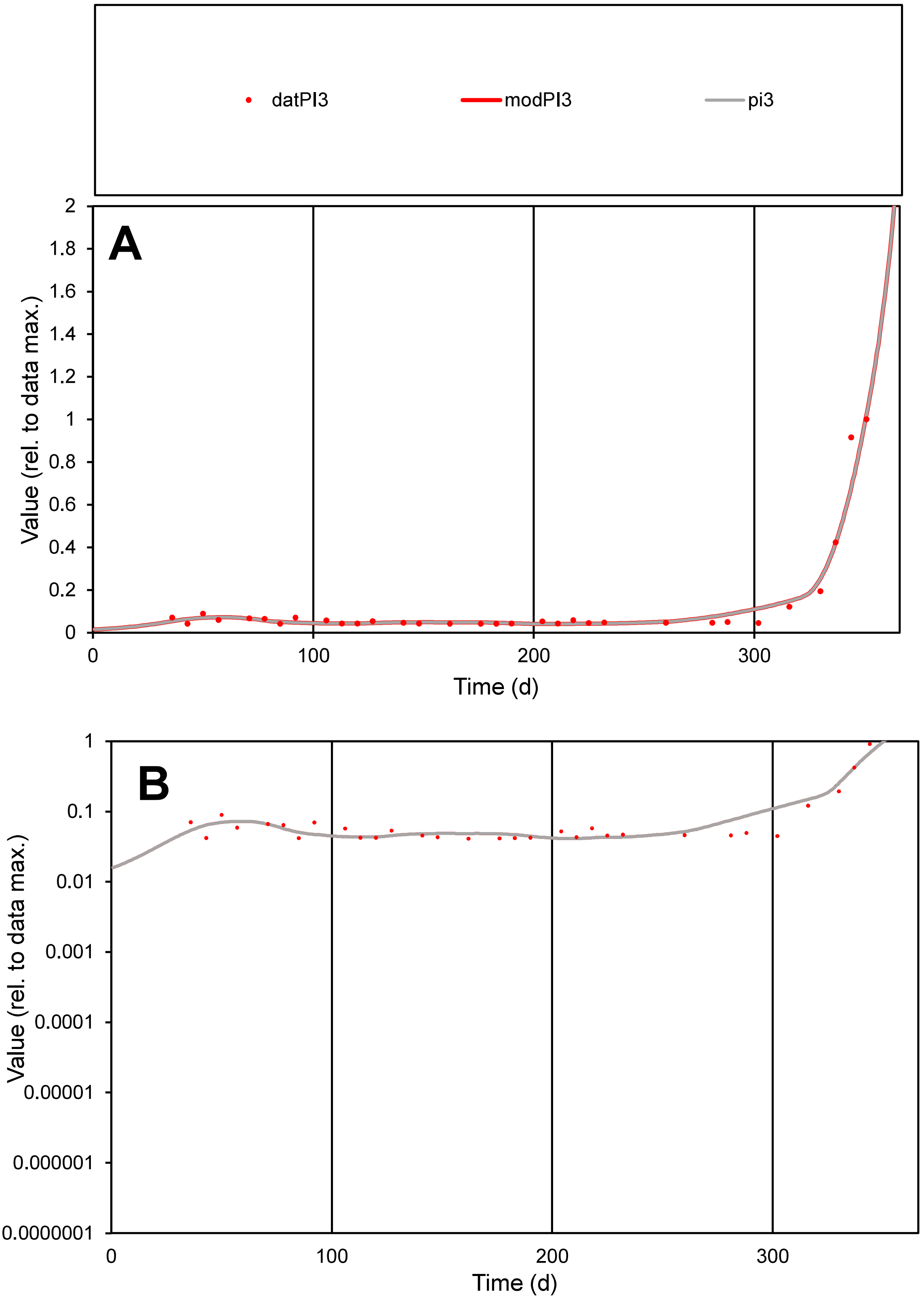

C1

Variables
PI3 Provide jtd or Dst
CalibOnly?
2 1 to reproduce of
oNormalize?
2 1=no, 2=yes

ofxn calc*
0.31817
ofxn mod
0.35792

sRun
RunA1
zout
1

Ave
Data
0.00021
Model
0.00022

data/vMax
0.0017

ofxng
321.551

Min d/m
0.99671

ave/min d
3.07047
ave/min m
3.167

324_Calib_S_A

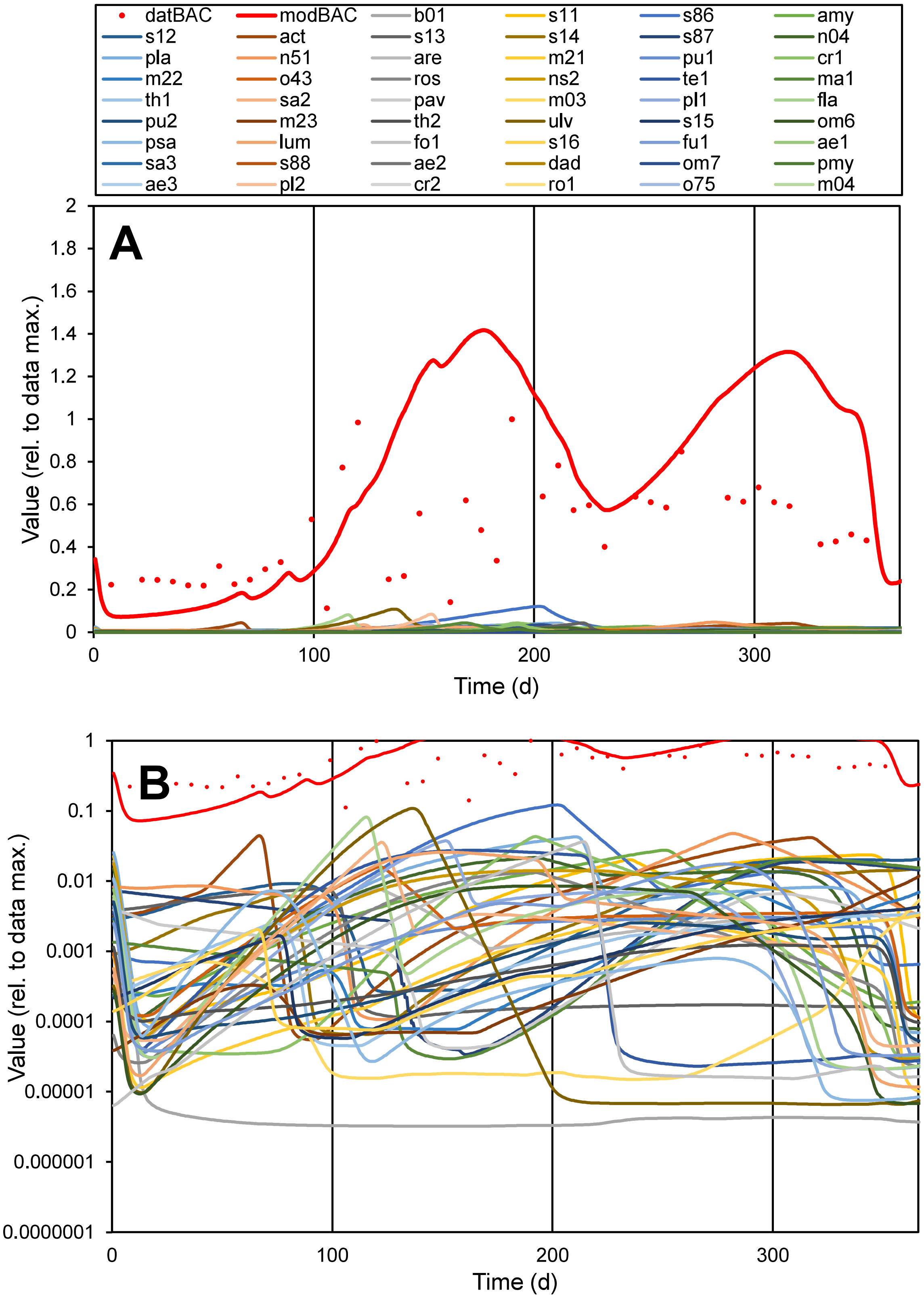

C1

Variables

BAC Provide jtd or Dst

CalibOnly?

2 1 to reproduce of

oNormalize?

2 1=no, 2=yes

ofxn calc*

1.01337

ofxn mod

1.02928

sRun

RunA1

zout

1

Ave

Data

0.68283

Model

1.06607

data/vMax

1.447

ofxng

321.551

Min d/m

1.51115

ave/min d

4.19944

ave/min m

9.90774

325_Calib_S_A

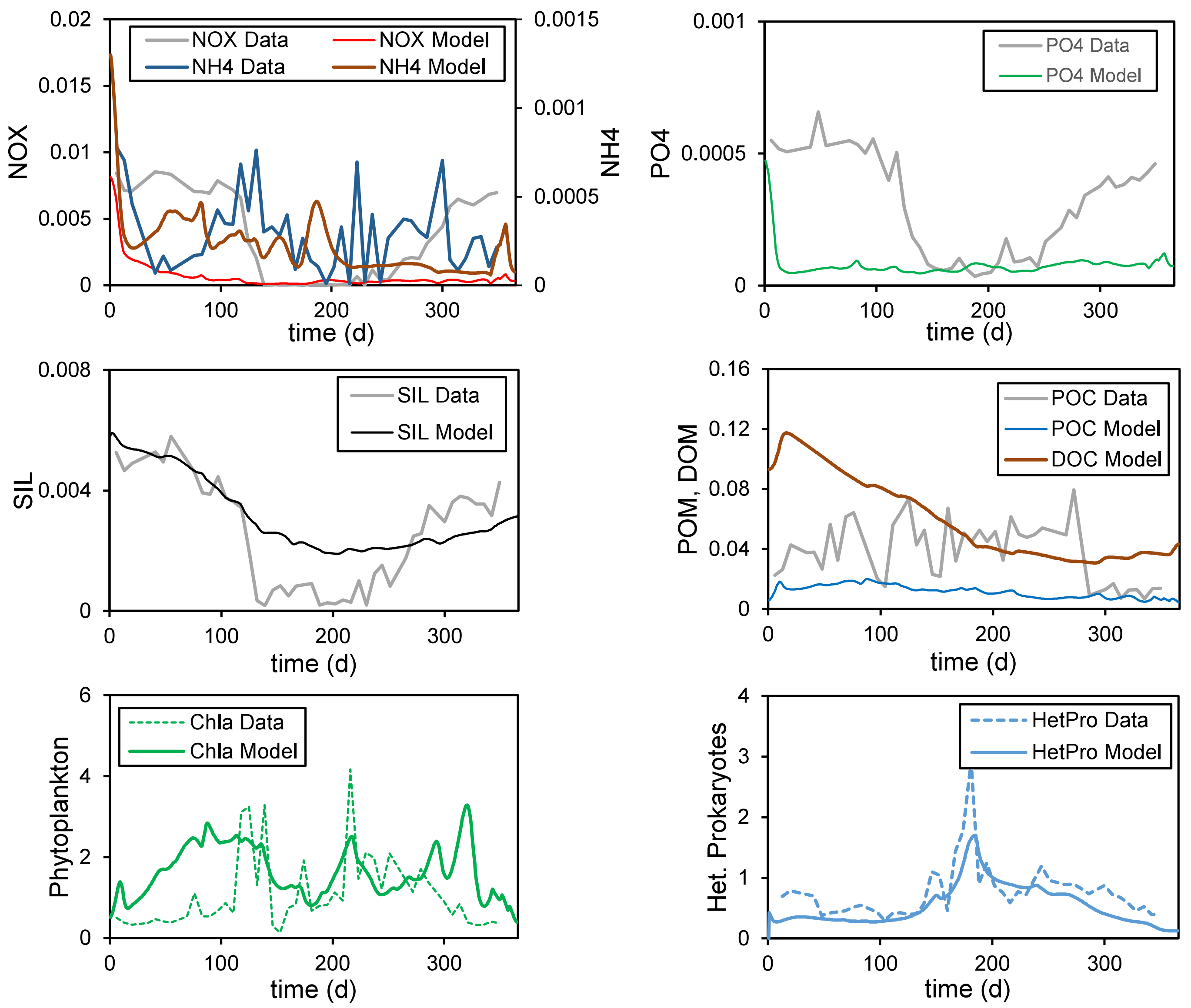
NOX Data
NOX Model
NH4 Data
NH4 Model
NOX
NH4
time (d)
PO4 Data
PO4 Model
PO4
time (d)
SIL Data
SIL Model
SIL
time (d)
POC Data
POC Model
DOC Model
POM, DOM
time (d)
Chla Data
Chla Model
Phytoplankton
time (d)
HetPro Data
HetPro Model
Het. Prokaryotes
time (d)

325_Calib_S_A

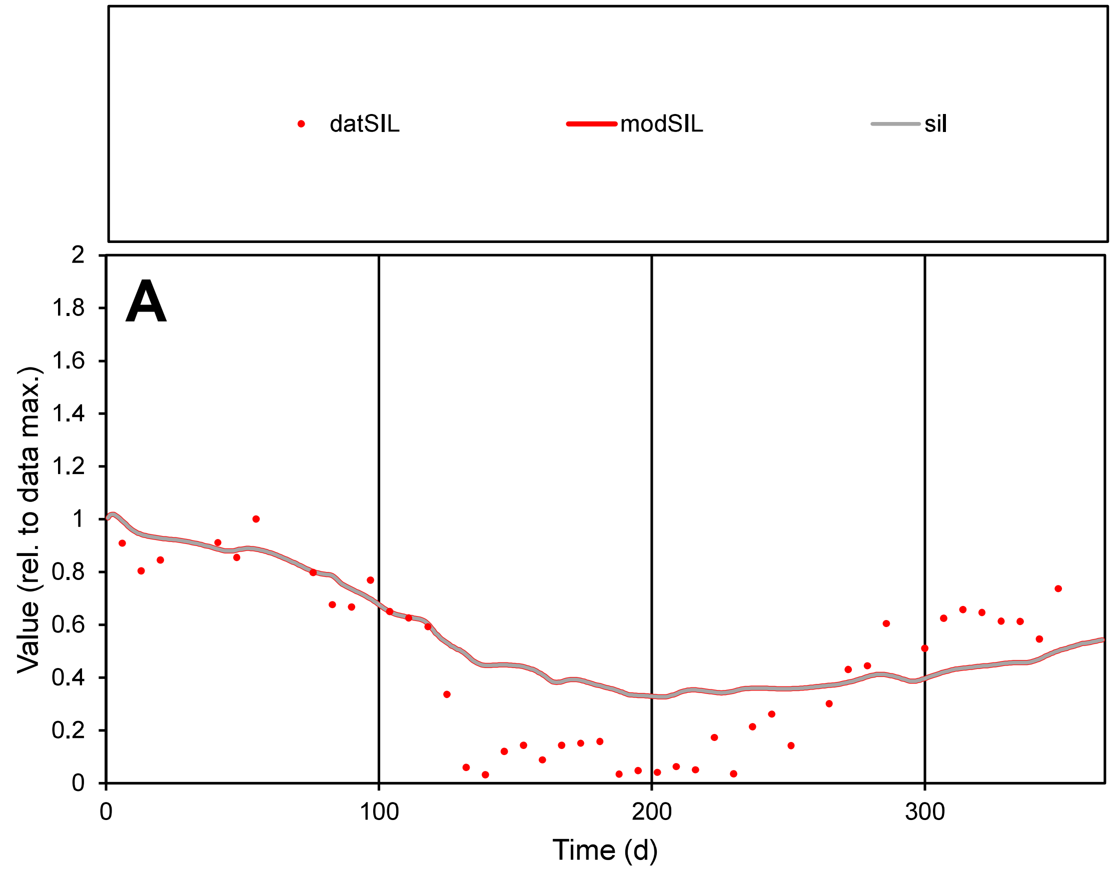

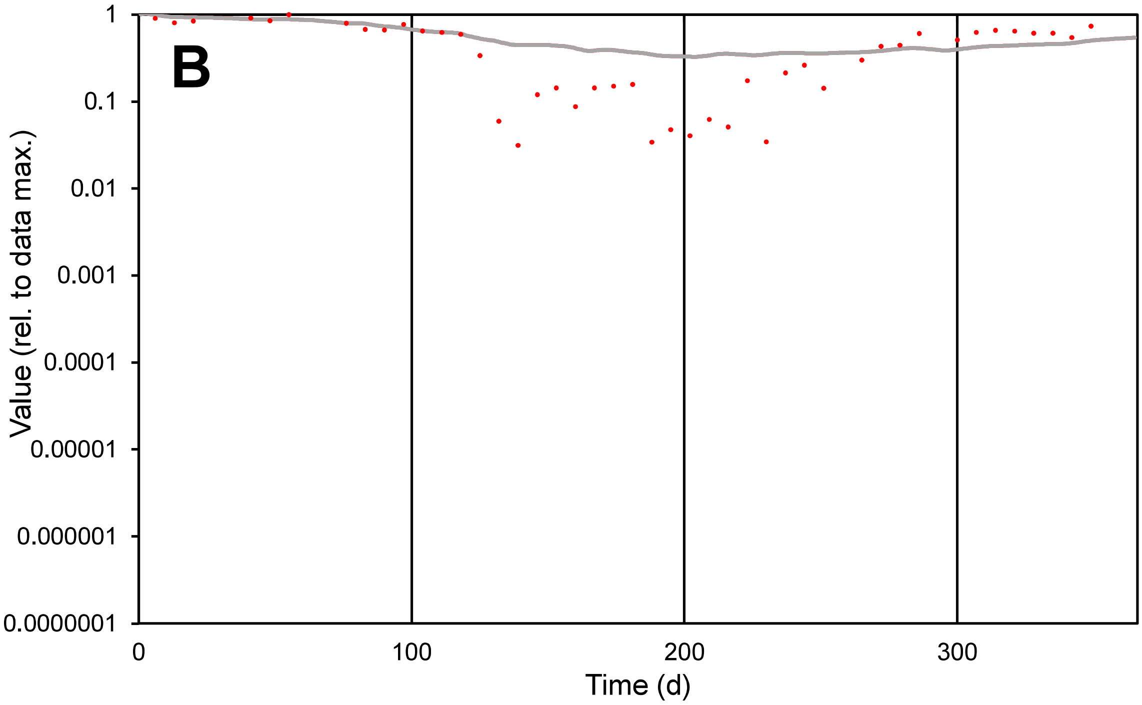

C1

Variables
SIL Provide jtd or Dst
CalibOnly?
2 1 to reproduce of
oNormalize?
2 1=no, 2=yes

ofxn calc*
1.40634
ofxn mod
1.46444

sRun
RunA1
zout
1

Ave
Data
0.00252
Model
0.00301

data/vMax
0.0058

ofxng
275.646

Min d/m
0.09636

ave/min d
13.7979
ave/min m
1.58969

325_Calib_S_A

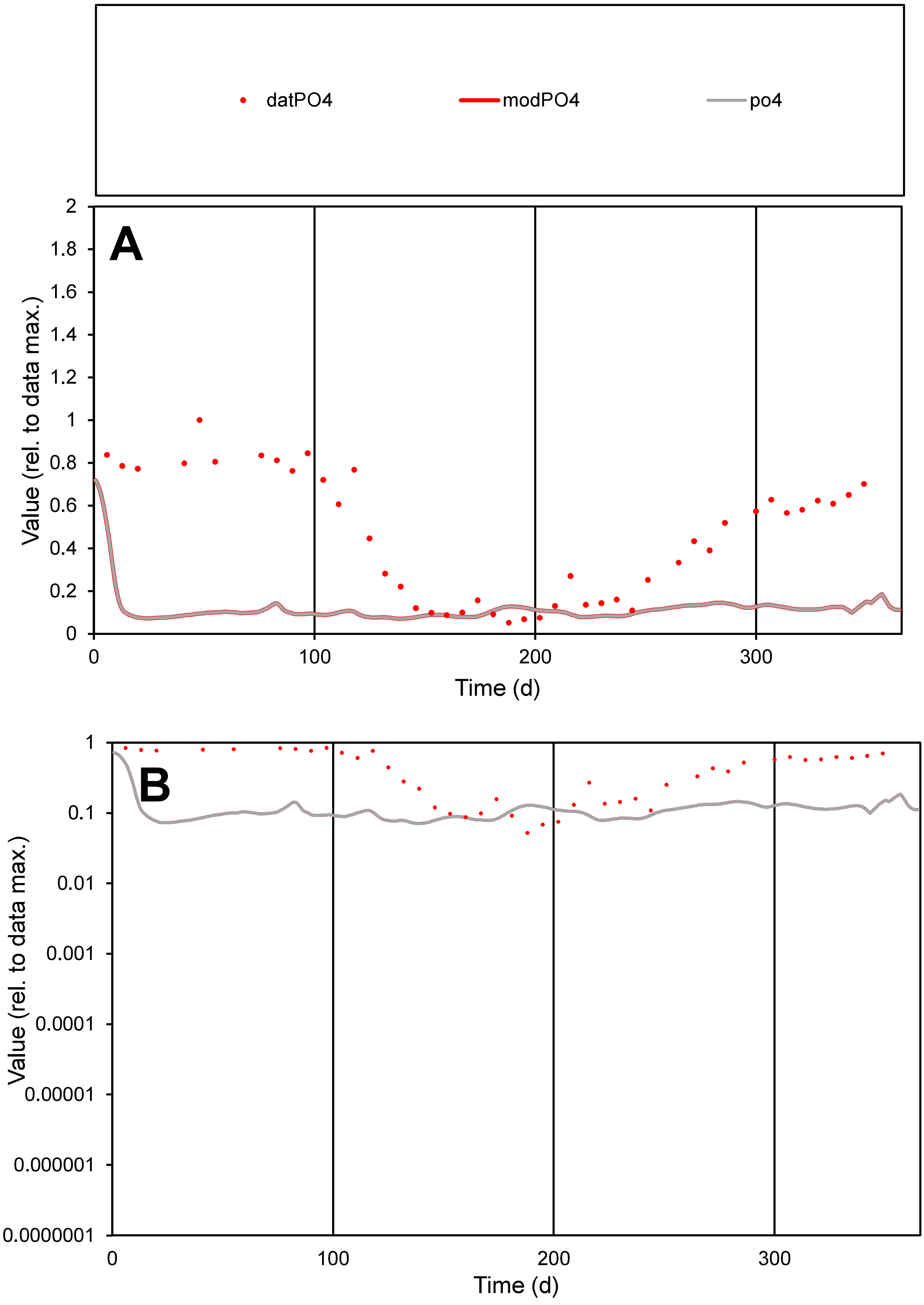

C1

Variables
PO4 Provide jtd or Dst
CalibOnly?
2 1 to reproduce of
oNormalize?
2 1=no, 2=yes

ofxn calc*
2.91573
ofxn mod
2.92973

sRun
RunA1
zout
1

Ave
Data
0.0003
Model
7.4E-05

data/vMax
0.00066

ofxng
275.646

Min d/m
0.73204

ave/min d
8.70723
ave/min m
1.59127

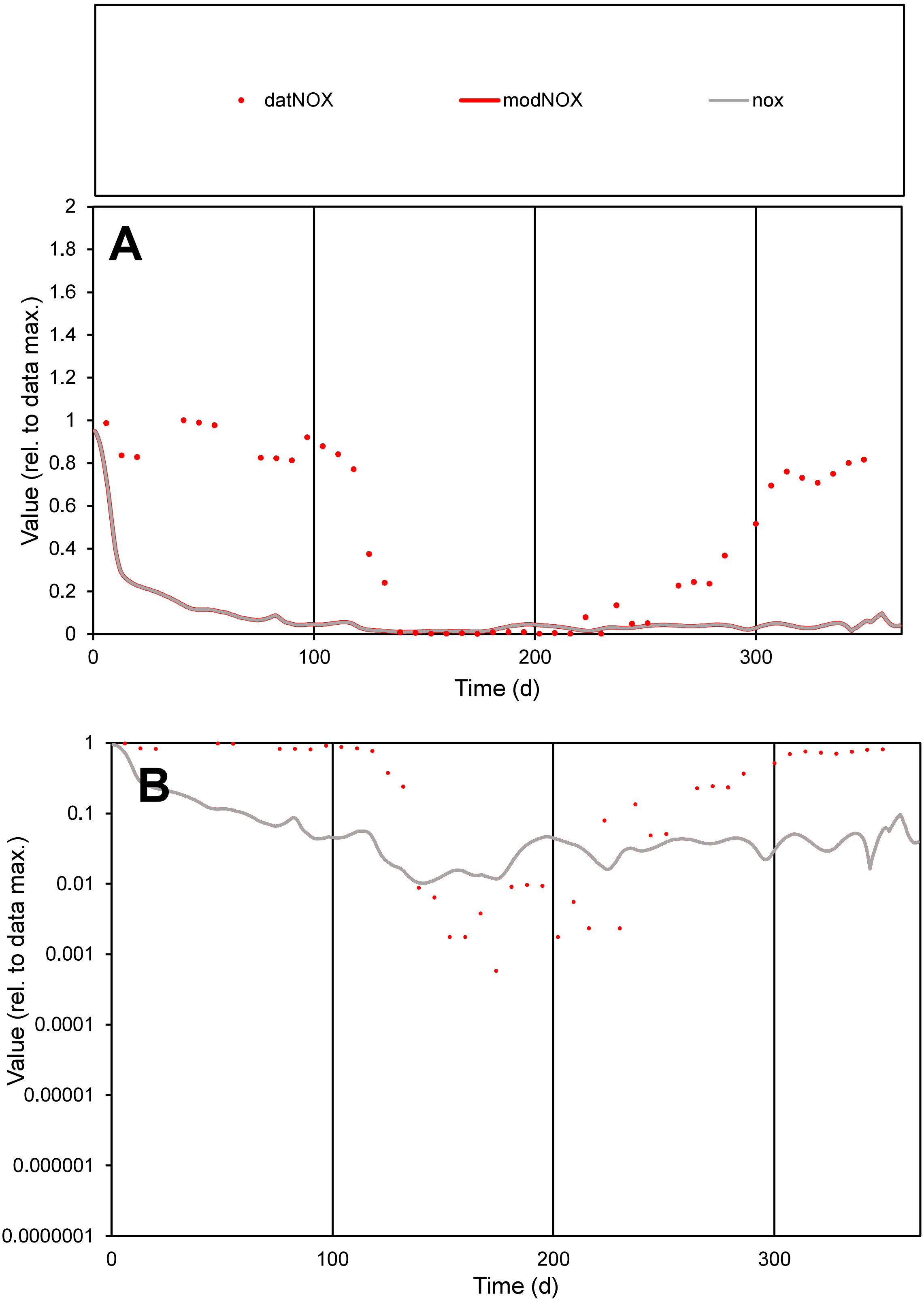

C1

Variables
NOX Provide jtd or Dst
CalibOnly?
2 1 to reproduce of
oNormalize?
2 1=no, 2=yes

ofxn calc*
3.49493
ofxn mod
3.51061

sRun
RunA1
zout
1

Ave
Data
0.00375
Model
0.00056

data/vMax
0.00855

ofxng
275.646

Min d/m
0.05711

ave/min d
750.993
ave/min m
6.39278

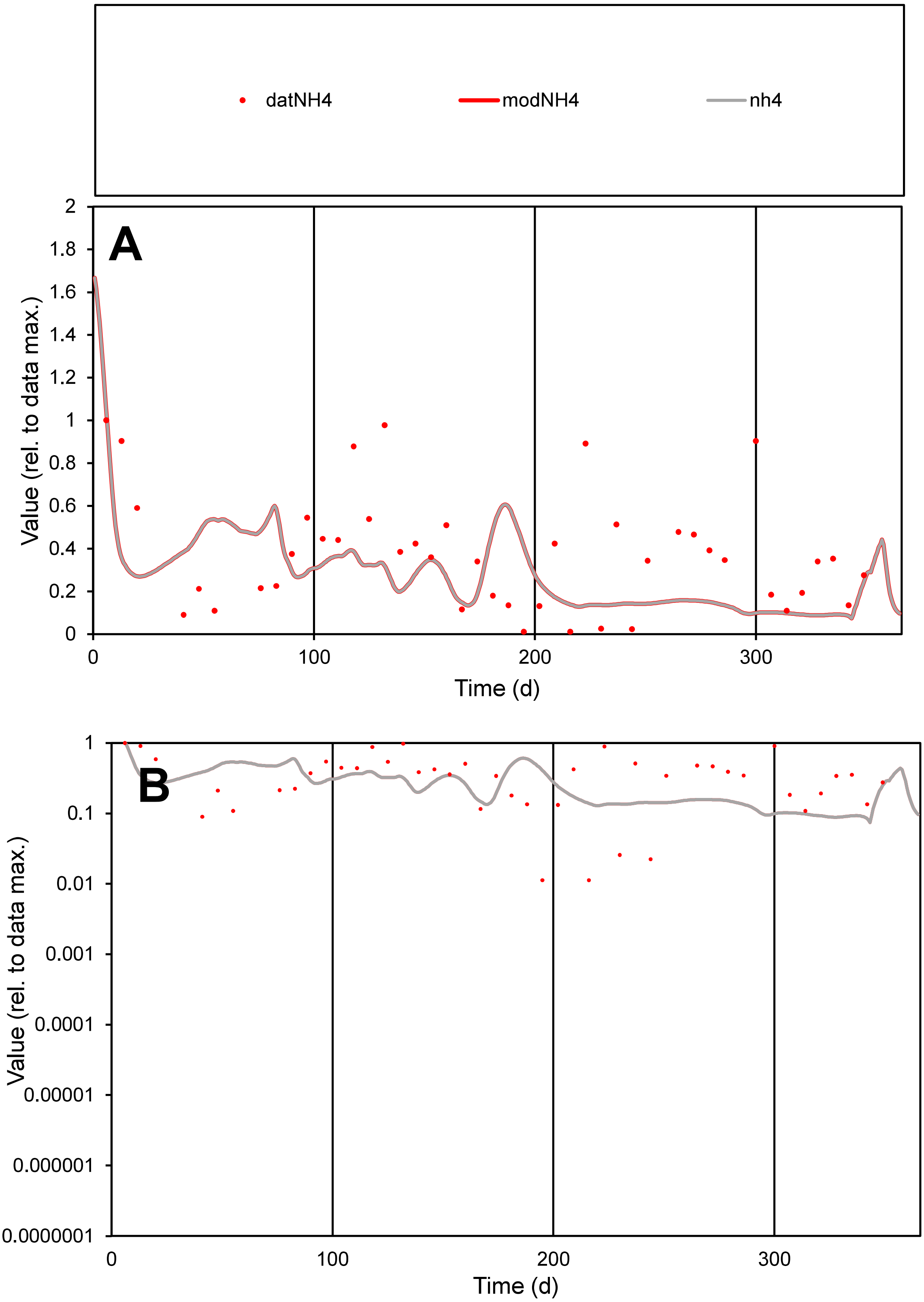

C1

Variables
NH4 Provide jtd or Dst
CalibOnly?
2 1 to reproduce of
oNormalize?
2 1=no, 2=yes

ofxn calc*
2.45165
ofxn mod
2.50635

sRun
RunA1
zout
1

Ave
Data
0.00029
Model
0.00021

data/vMax
0.00078

ofxng
275.646

Min d/m
0.13732

ave/min d
33.4921
ave/min m
3.2728

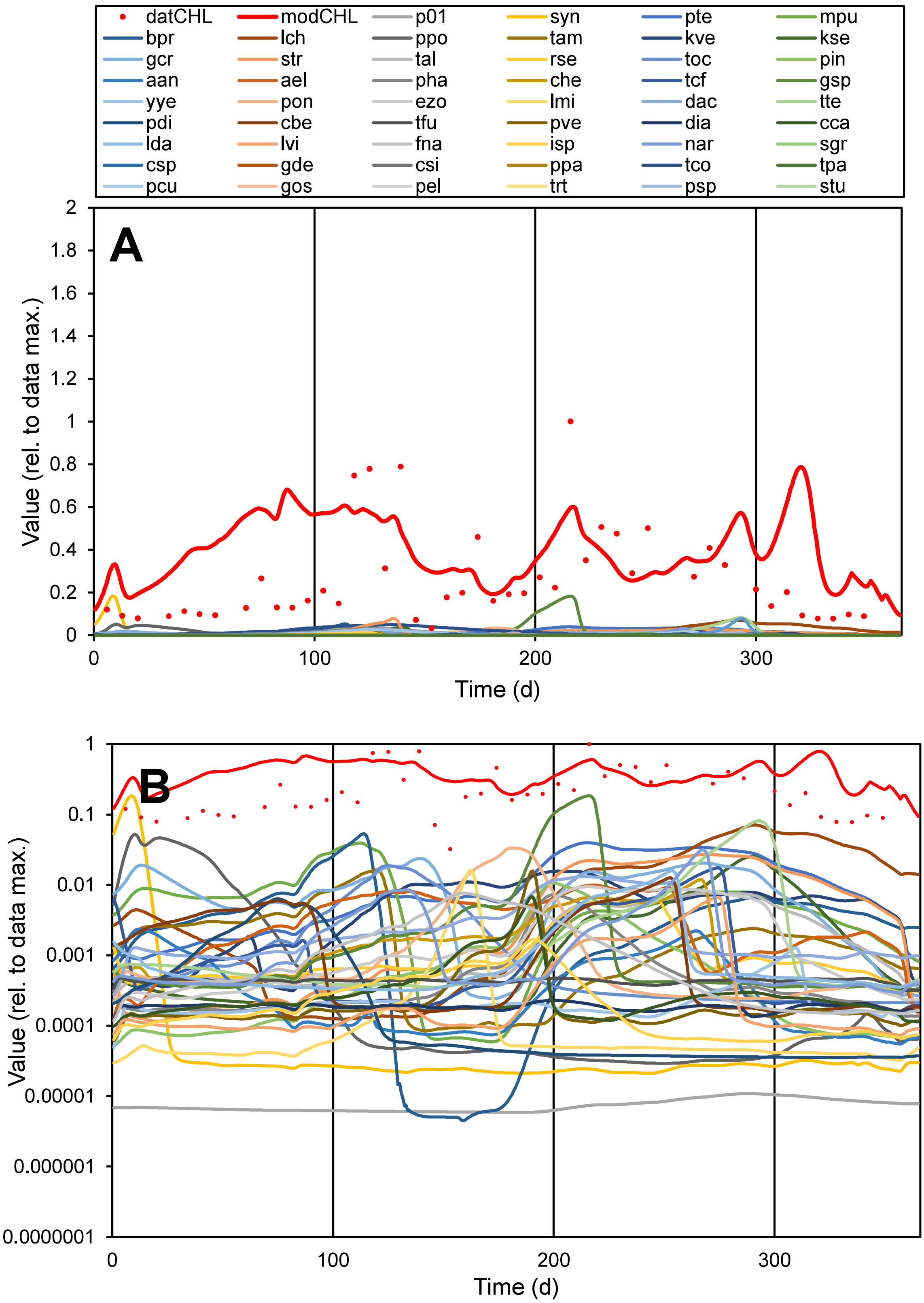

C1

Variables

CHL Provide jtd or Dst

CalibOnly?

2 1 to reproduce of

oNormalize?

2 1=no, 2=yes

ofxn calc*

6.05275

ofxn mod

6.27912

sRun

RunA1

zout

1

Ave

Data

1.07345

Model

1.03876

data/vMax

4.17

ofxng

275.646

Min d/m

0.42006

ave/min d

7.93388

ave/min m

3.22497

325_Calib_S_A

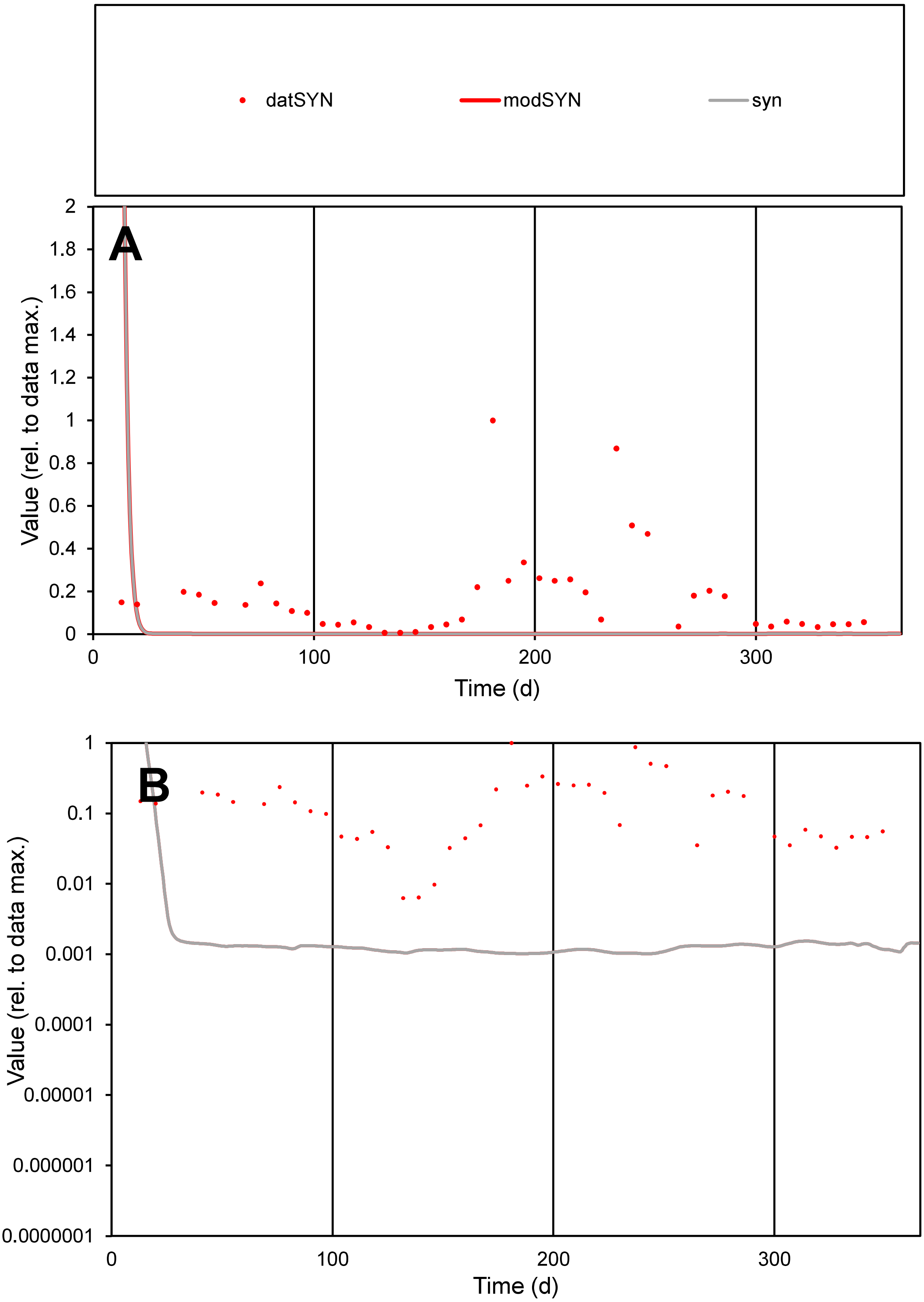

C1

Variables

SYN Provide jtd or Dst

CalibOnly?

2 1 to reproduce of

oNormalize?

2 1=no, 2=yes

ofxn calc*

1.78988

ofxn mod

1.89714

sRun

RunA1

zout

1

Ave

Data

8.84665

Model

1.59559

data/vMax

51.72

ofxng

275.646

Min d/m

6.11331

ave/min d

27.3298

ave/min m

30.134

325_Calib_S_A

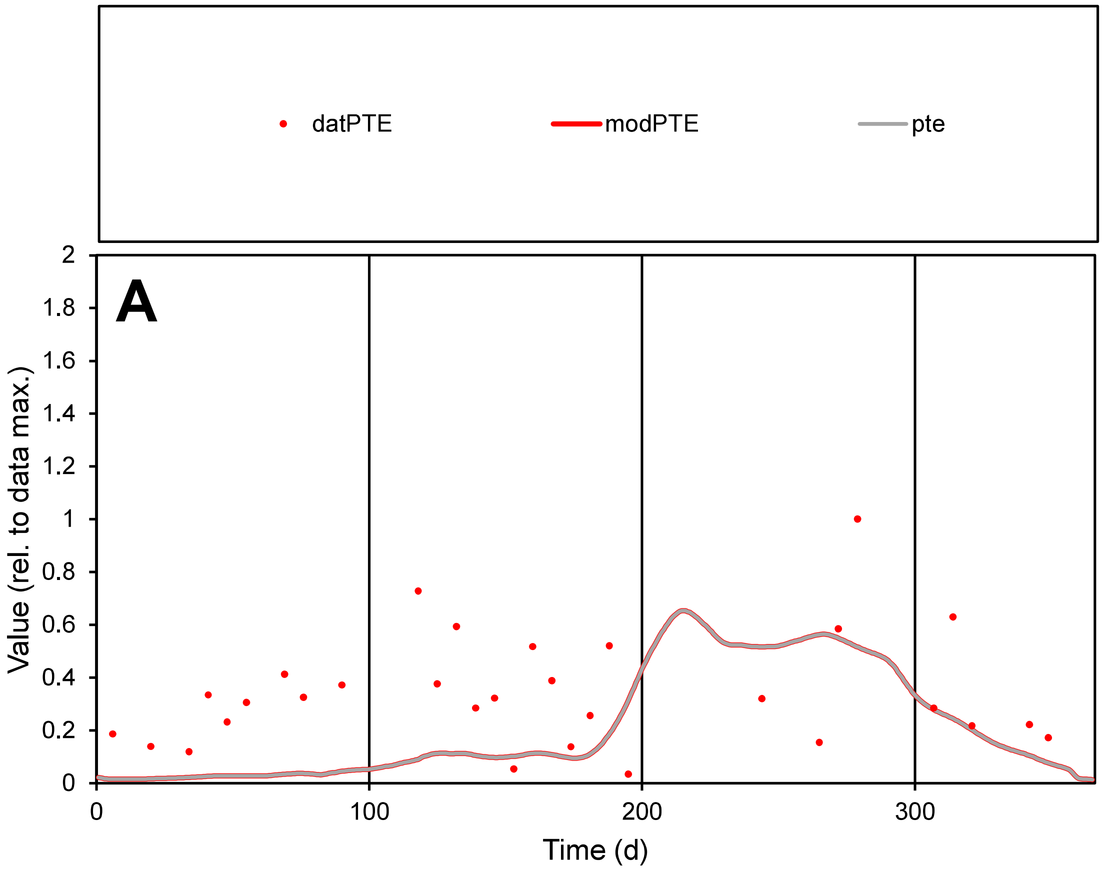

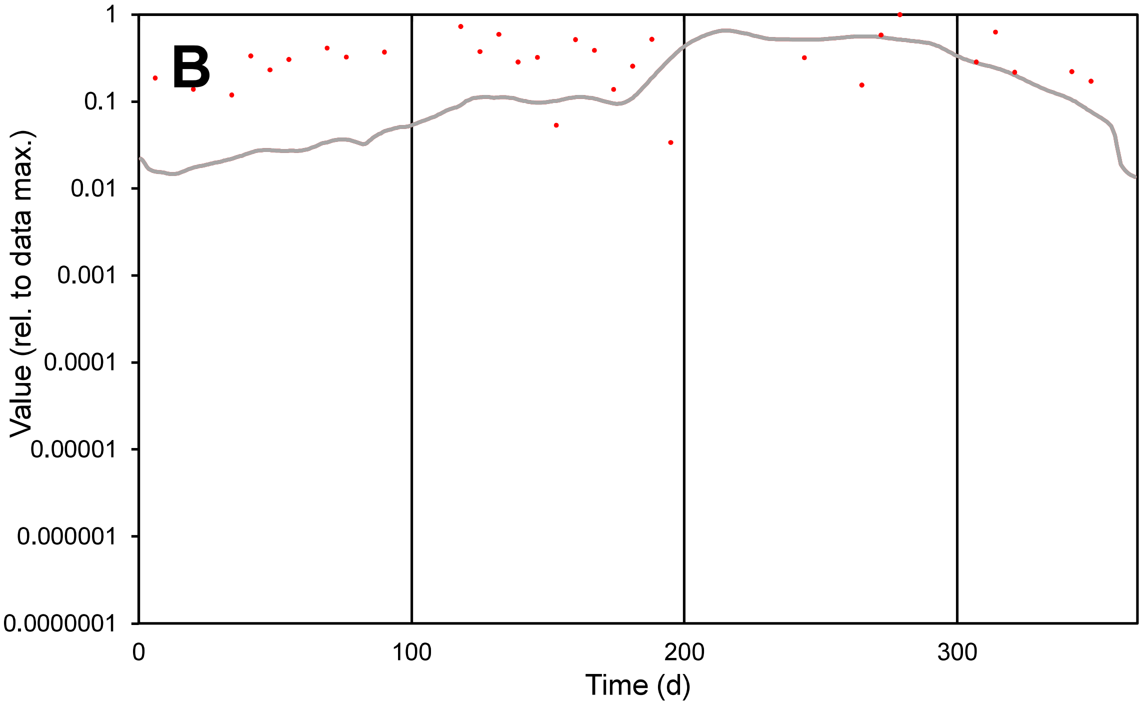

C1

Variables
PTE Provide jtd or Dst
CalibOnly?
2 1 to reproduce of
oNormalize?
2 1=no, 2=yes

ofxn calc*
0.83794
ofxn mod
0.84617

sRun
RunA1
zout
1

Ave
Data
0.00022
Model
0.0001

data/vMax
0.00063

ofxng
275.646

Min d/m
2.19739

ave/min d
10.0057
ave/min m
10.4027

325_Calib_S_A

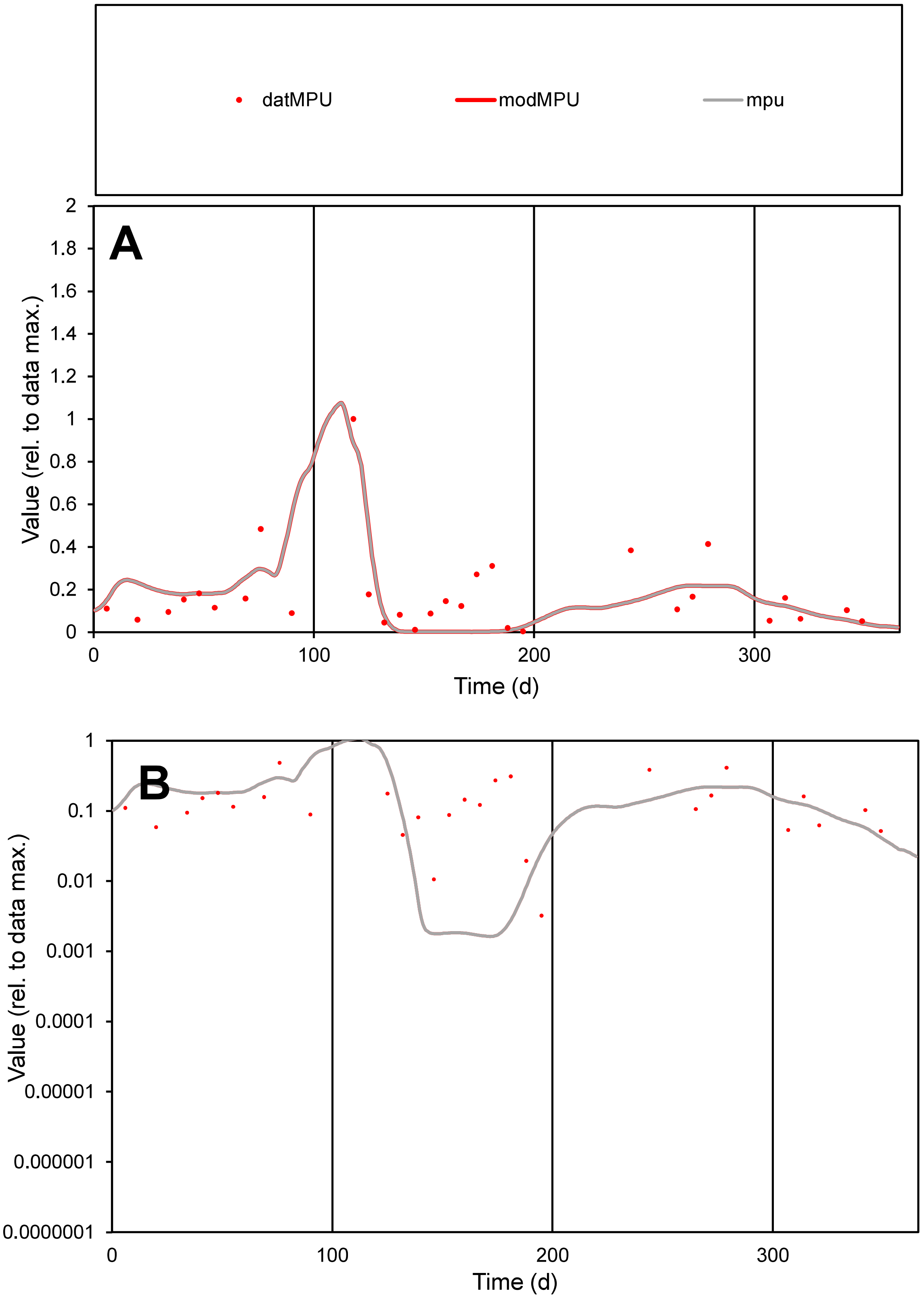

C1

Variables
MPU Provide jtd or Dst
CalibOnly?
2 1 to reproduce of
oNormalize?
2 1=no, 2=yes

ofxn calc*
0.81064
ofxn mod
0.88469

sRun
RunA1
zout
1

Ave
Data
0.00022
Model
0.0002

data/vMax
0.00127

ofxng
275.646

Min d/m
1.93191

ave/min d
53.8371
ave/min m
92.437

325_Calib_S_A

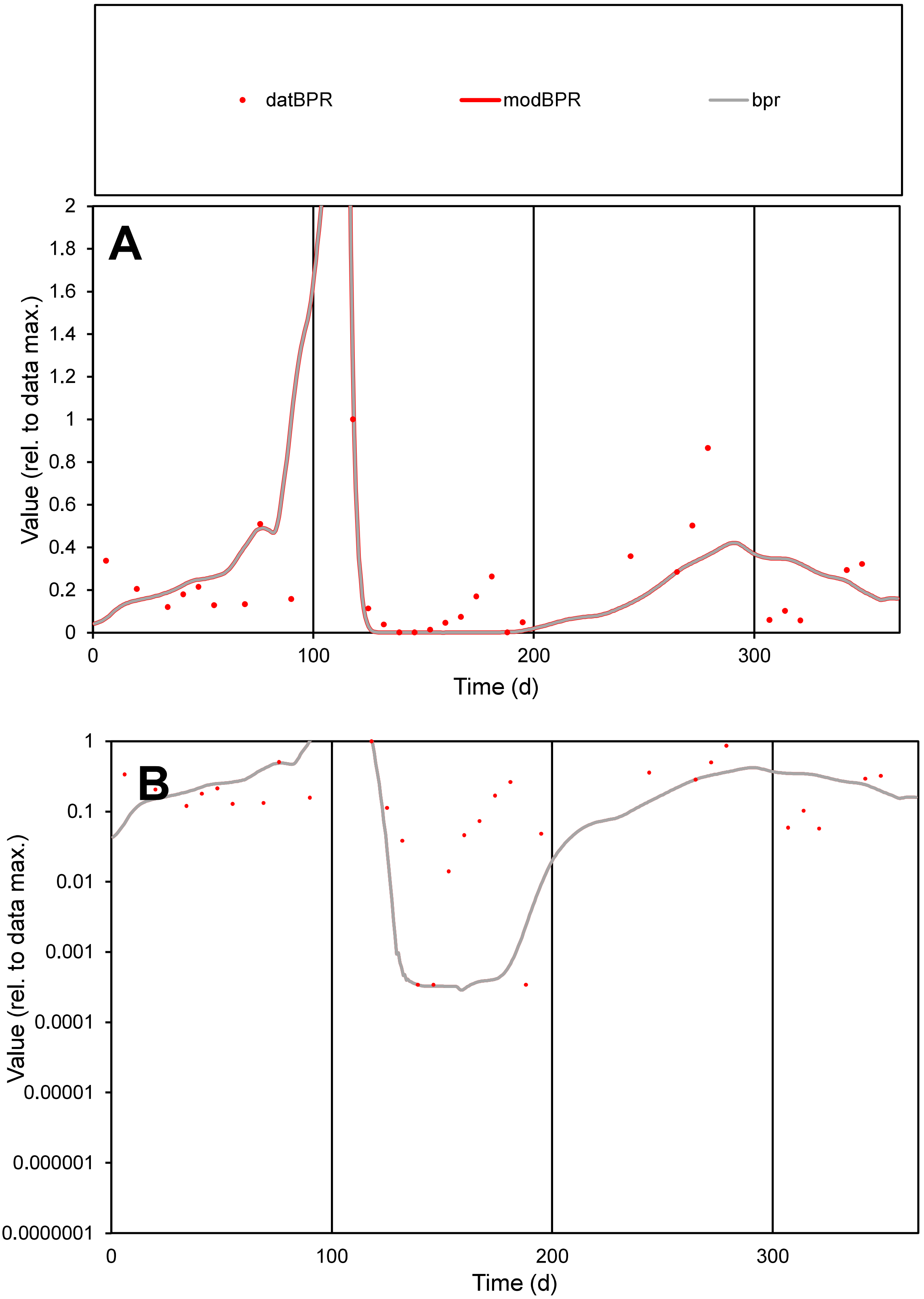

C1

Variables

BPR Provide jtd or Dst

CalibOnly?

2 1 to reproduce of

oNormalize?

2 1=no, 2=yes

ofxn calc*

0.9313

ofxn mod

1.03933

sRun

RunA1

zout

1

Ave

Data

0.00012

Model

0.00012

data/vMax

0.00054

ofxng

275.646

Min d/m

1.07483

ave/min d

640.405

ave/min m

675.893

325_Calib_S_A

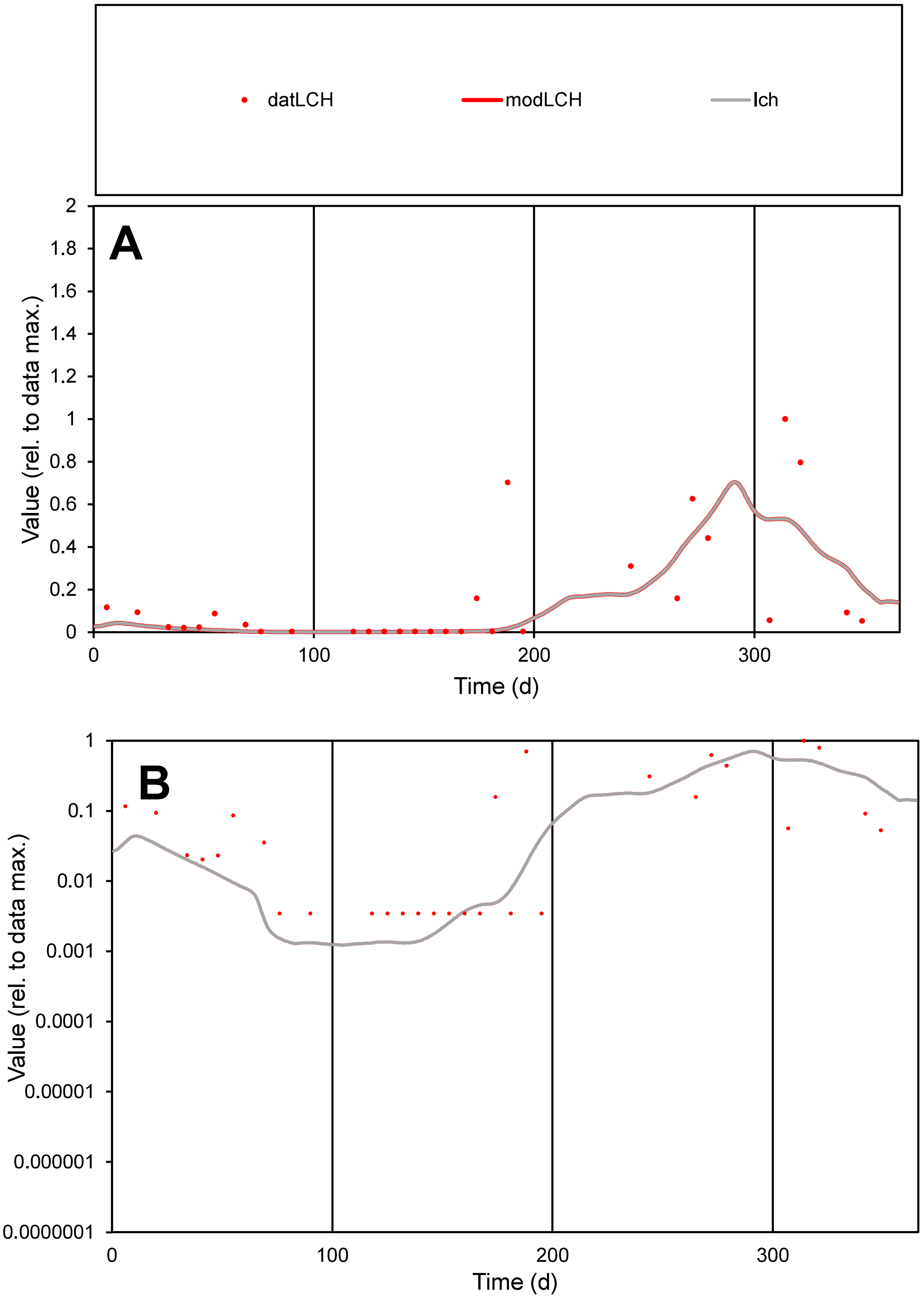

C1

Variables
LCH Provide jtd or Dst
CalibOnly?
2 1 to reproduce of
oNormalize?
2 1=no, 2=yes

ofxn calc*
1.24903
ofxn mod
1.2663

sRun
RunA1
zout
1

Ave
Data
0.00011
Model
8.9E-05

data/vMax
0.00071

ofxng
275.646

Min d/m
2.62503

ave/min d
46.5353
ave/min m
95.9419

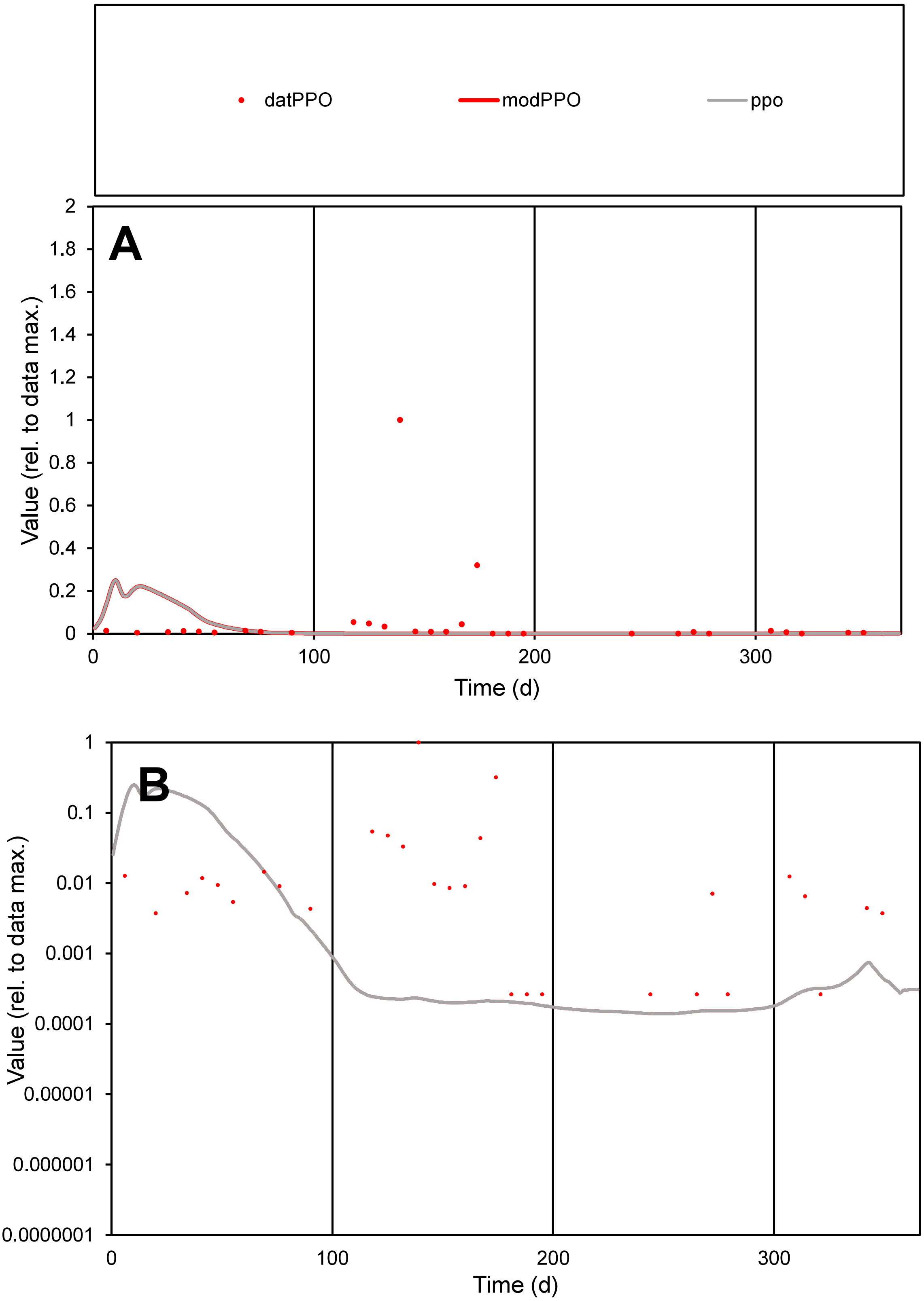

C1

Variables
PPO Provide jtd or Dst
CalibOnly?
2 1 to reproduce of
oNormalize?
2 1=no, 2=yes

ofxn calc*
3.65785
ofxn mod
3.67409

sRun
RunA1
zout
1

Ave
Data
0.0004
Model
0.00017

data/vMax
0.00728

ofxng
275.646

Min d/m
1.87549

ave/min d
207.953
ave/min m
167.288

325_Calib_S_A

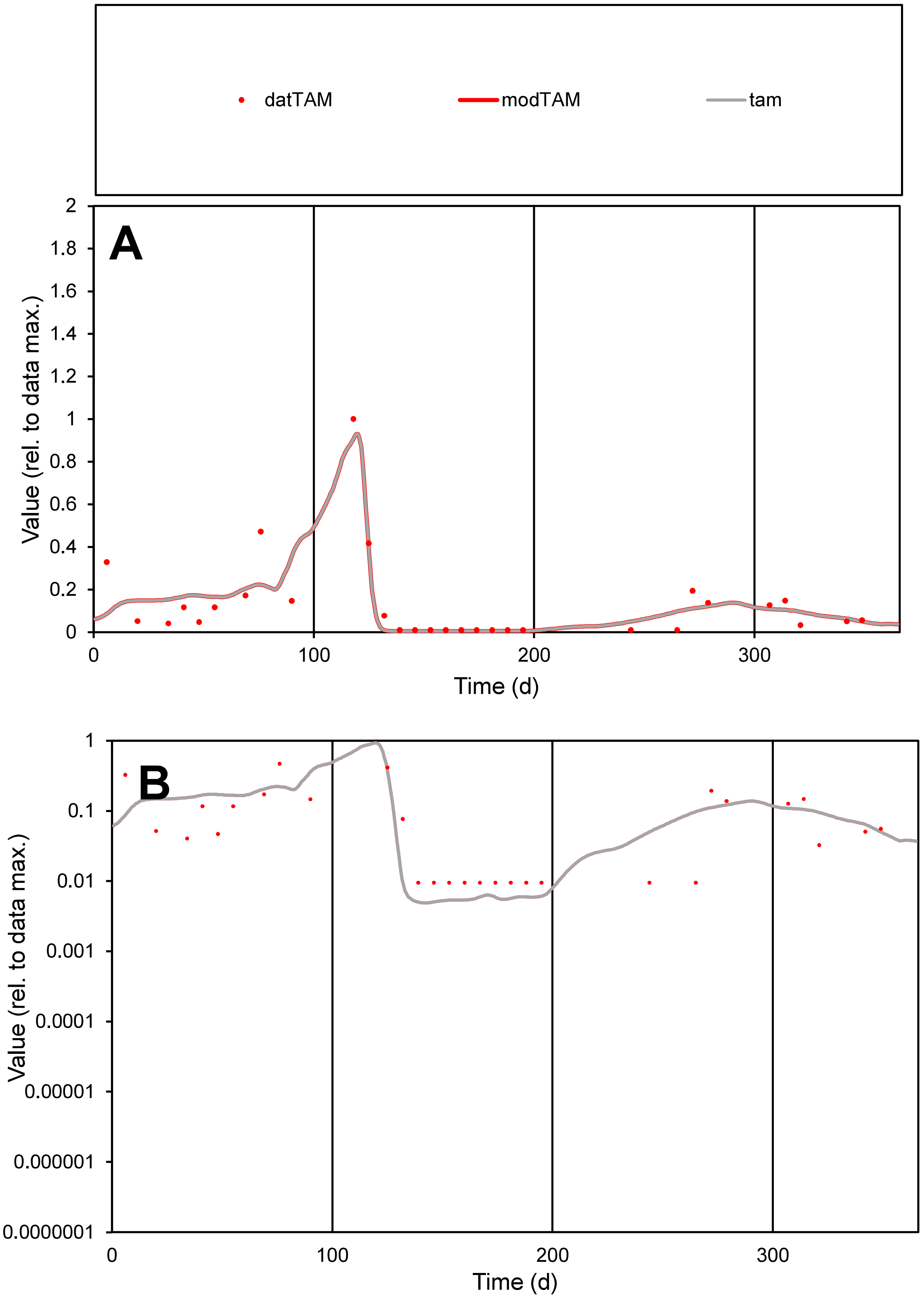

C1

Variables
TAM Provide jtd or Dst
CalibOnly?
2 1 to reproduce of
oNormalize?
2 1=no, 2=yes

ofxn calc*
0.69746
ofxn mod
0.73643

sRun
RunA1
zout
1

Ave
Data
7.7E-05
Model
7.8E-05

data/vMax
0.0006

ofxng
275.646

Min d/m
1.9384

ave/min d
13.3954
ave/min m
26.3242

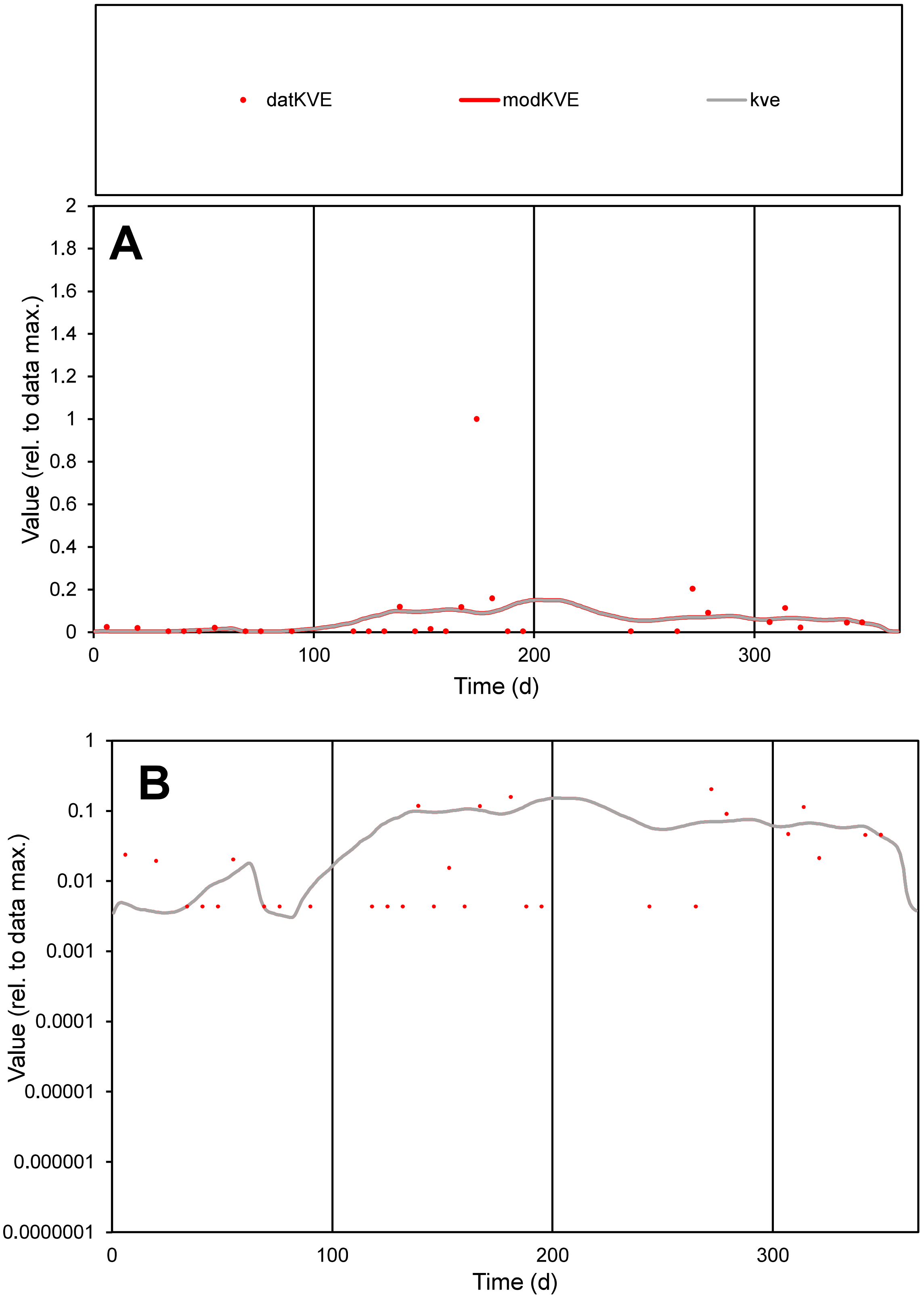

C1

Variables
KVE Provide jtd or Dst
CalibOnly?
2 1 to reproduce of
oNormalize?
2 1=no, 2=yes

ofxn calc*
2.50893
ofxn mod
2.51755

sRun
RunA1
zout
1

Ave
Data
5E-05
Model
4.2E-05

data/vMax
0.00072

ofxng
275.646

Min d/m
1.34841

ave/min d
16.1194
ave/min m
18.2245

325_Calib_S_A

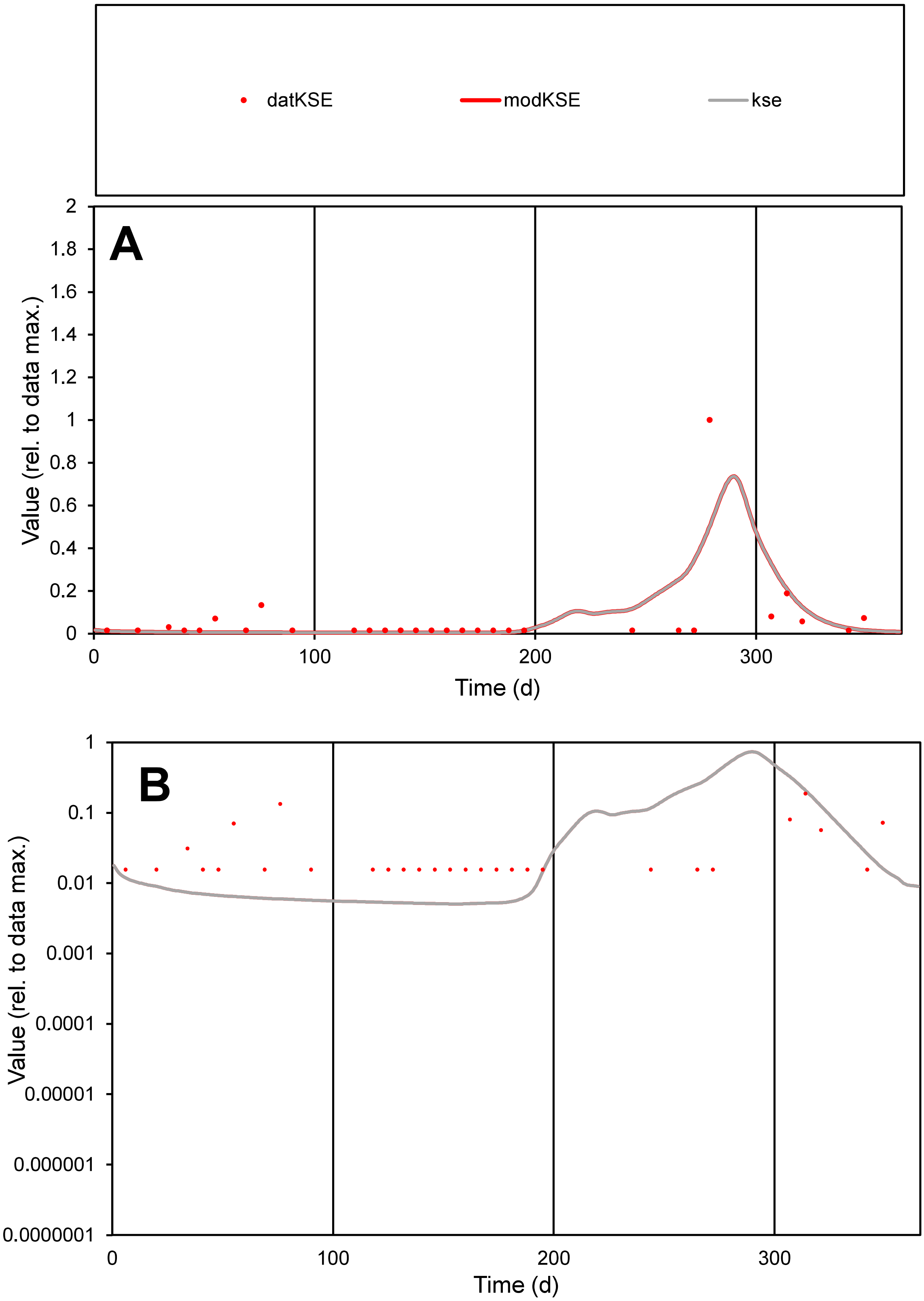

C1

Variables
KSE Provide jtd or Dst
CalibOnly?
2 1 to reproduce of
oNormalize?
2 1=no, 2=yes

ofxn calc*
1.84488
ofxn mod
1.90638

sRun
RunA1
zout
1

Ave
Data
1.6E-05
Model
1.6E-05

data/vMax
0.00024

ofxng
275.646

Min d/m
3.0571

ave/min d
4.23445
ave/min m
13.2685

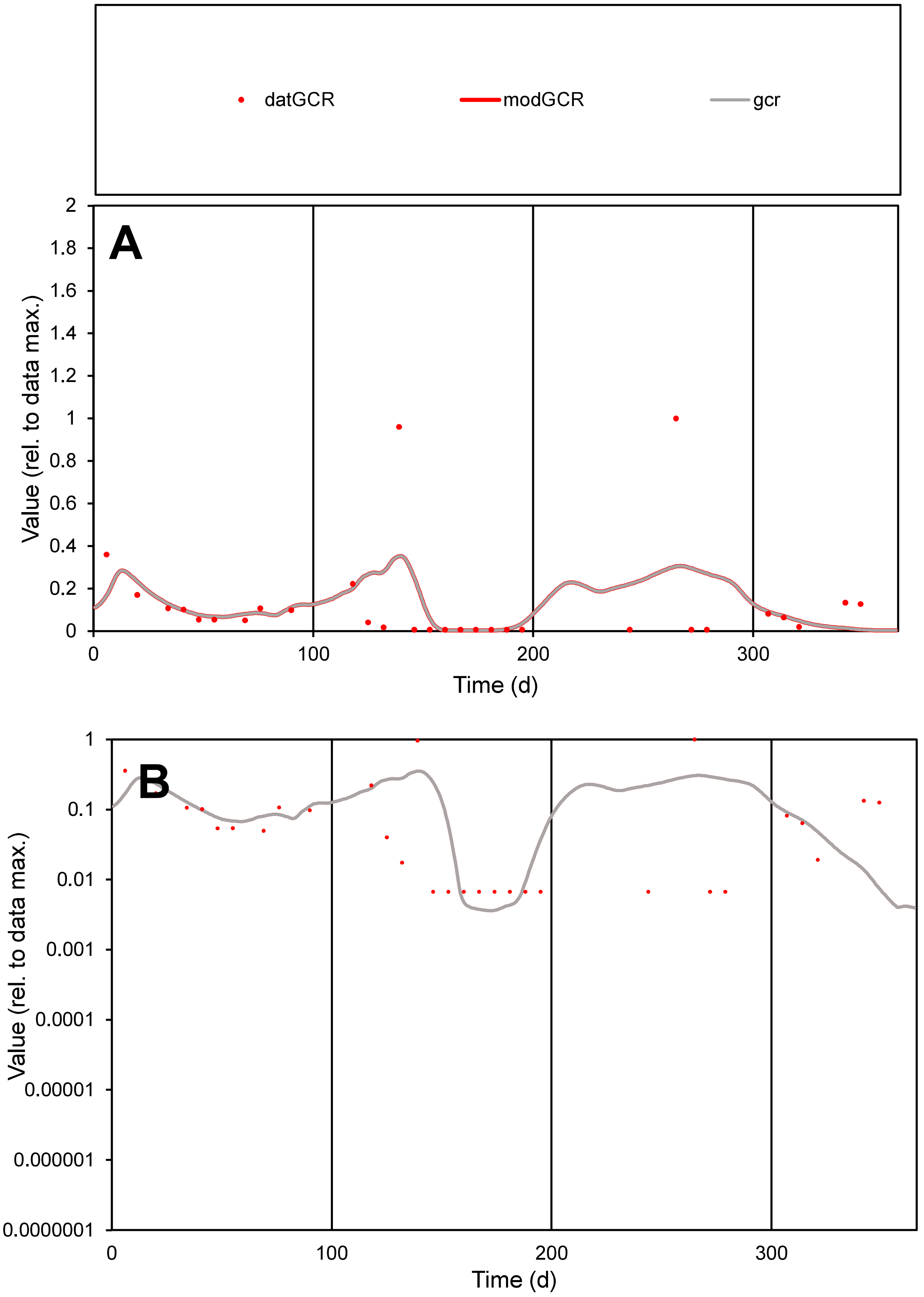

C1

Variables

GCR Provide jtd or Dst

CalibOnly?

2 1 to reproduce of

oNormalize?

2 1=no, 2=yes

ofxn calc*

1.59285

ofxn mod

1.61409

sRun

RunA1

zout

1

Ave

Data

6E-05

Model

5.7E-05

data/vMax

0.00047

ofxng

275.646

Min d/m

1.80634

ave/min d

19.0111

ave/min m

32.8563

325_Calib_S_A

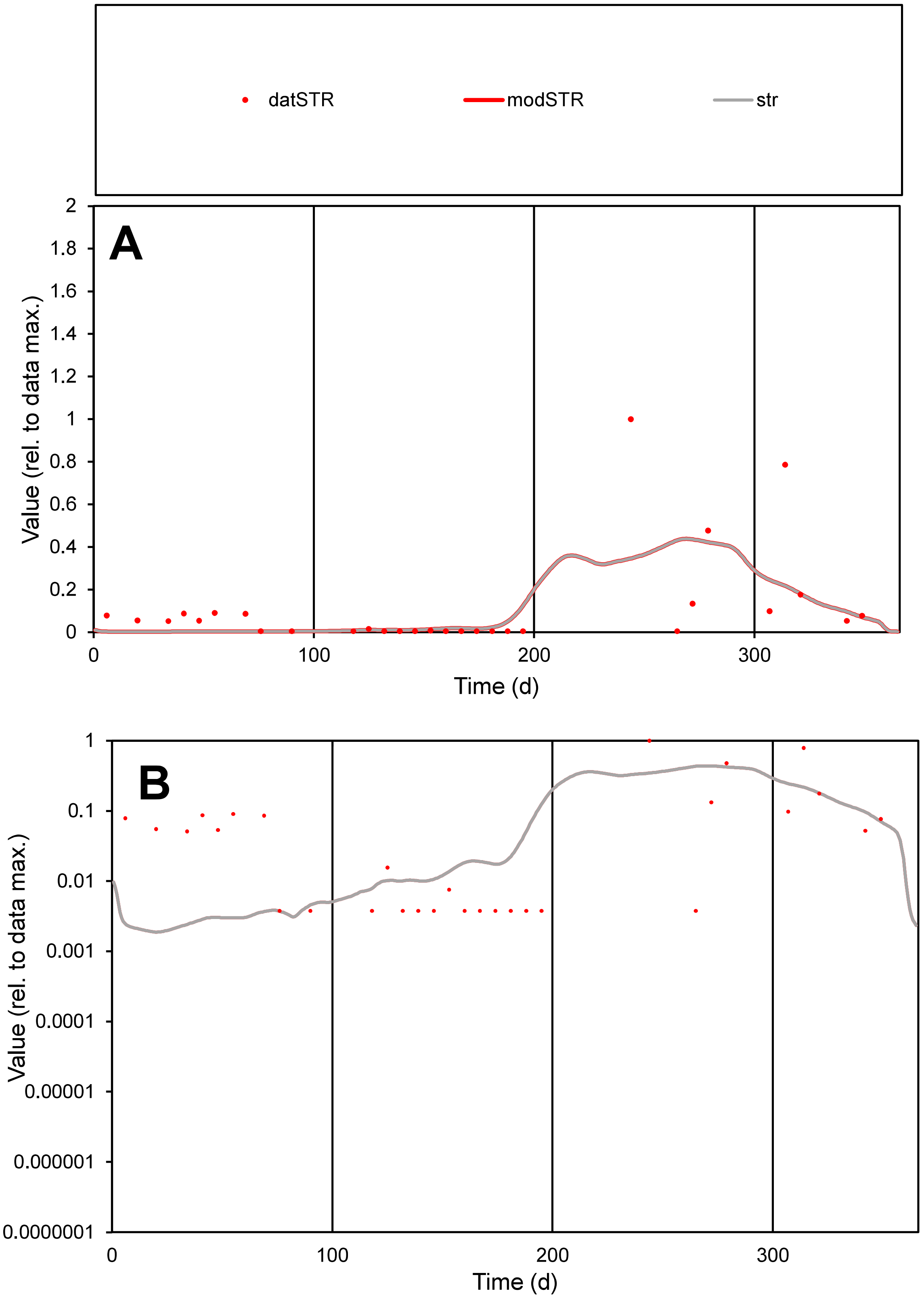

C1

Variables
STR Provide jtd or Dst
CalibOnly?
2 1 to reproduce of
oNormalize?
2 1=no, 2=yes

ofxn calc*
1.70596
ofxn mod
1.72061

sRun
RunA1
zout
1

Ave
Data
4.9E-05
Model
3.9E-05

data/vMax
0.00043

ofxng
275.646

Min d/m
2.00443

ave/min d
29.7746
ave/min m
48.2752

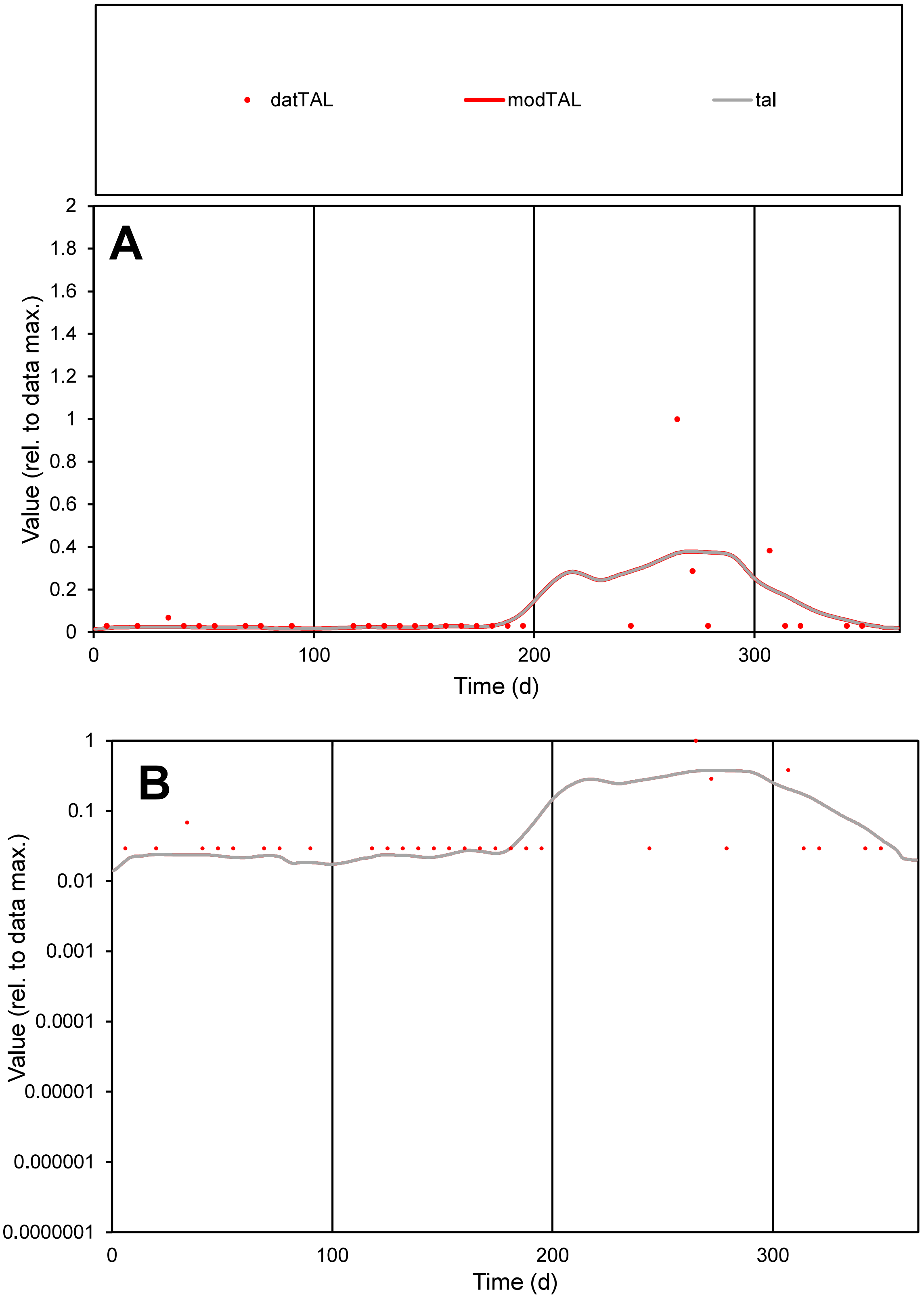

C1

Variables
TAL Provide jtd or Dst
CalibOnly?
2 1 to reproduce of
oNormalize?
2 1=no, 2=yes

ofxn calc*
1.76604
ofxn mod
1.78468

sRun
RunA1
zout
1

Ave
Data
1.2E-05
Model
1.2E-05

data/vMax
0.00014

ofxng
275.646

Min d/m
1.60682

ave/min d
2.83606
ave/min m
4.69131

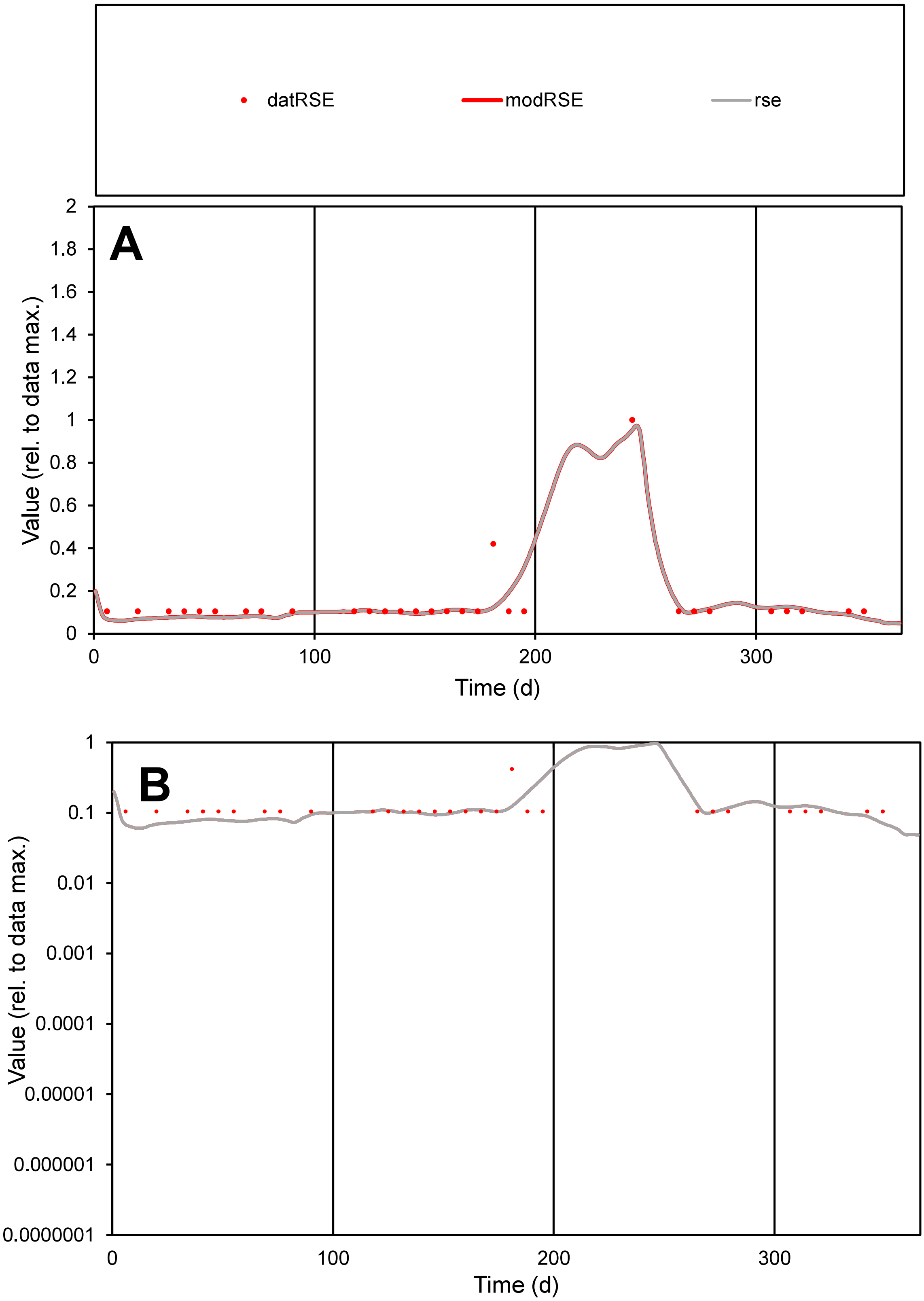

C1

Variables
RSE Provide jtd or Dst
CalibOnly?
2 1 to reproduce of
oNormalize?
2 1=no, 2=yes

ofxn calc*
0.46758
ofxn mod
0.50424

sRun
RunA1
zout
1

Ave
Data
6.5E-06
Model
6.1E-06

data/vMax
4.5E-05

ofxng
275.646

Min d/m
1.60904

ave/min d
1.38712
ave/min m
2.10023

325_Calib_S_A

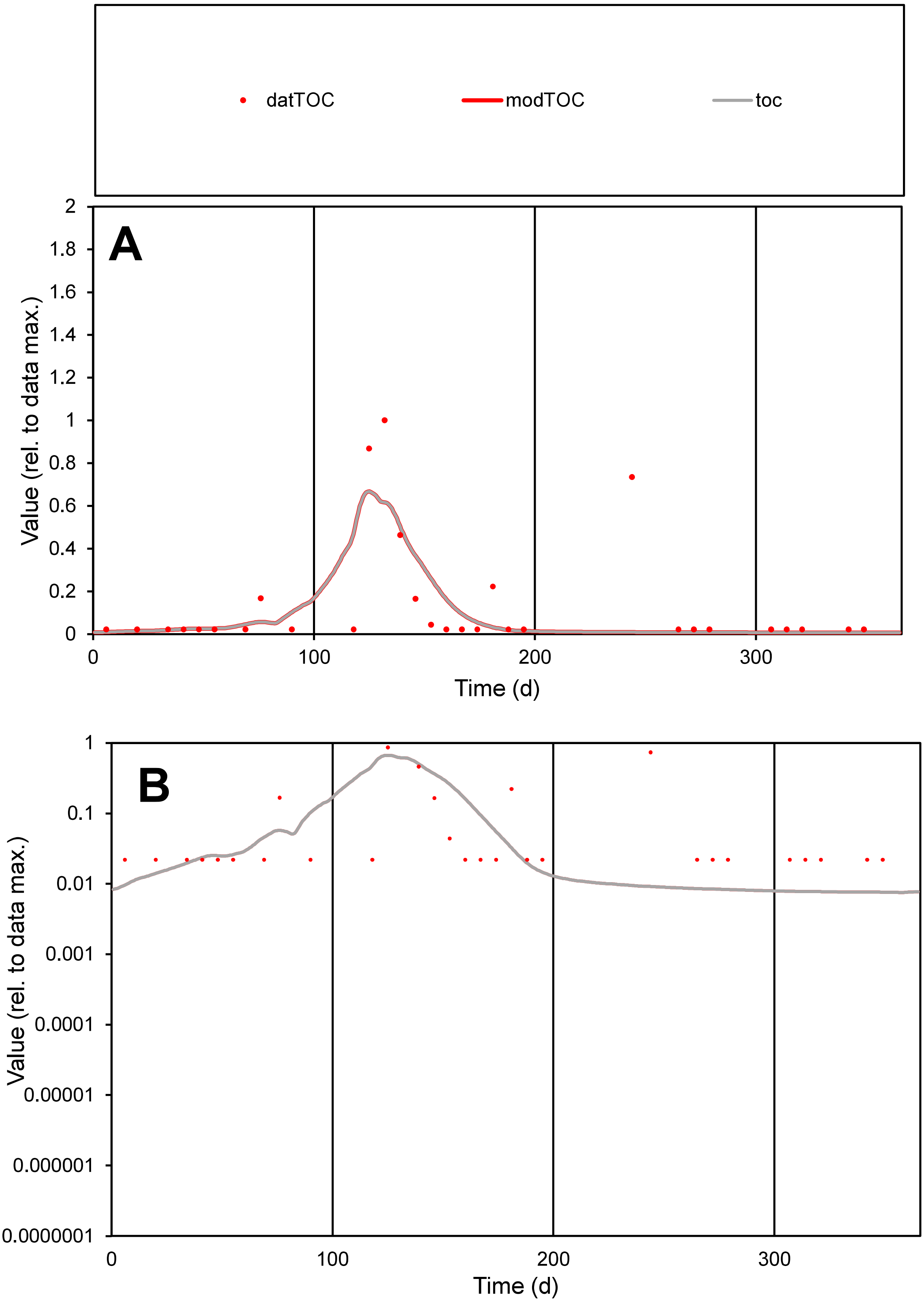

C1

Variables
TOC Provide jtd or Dst
CalibOnly?
2 1 to reproduce of
oNormalize?
2 1=no, 2=yes

ofxn calc*
1.34145
ofxn mod
1.36939

sRun
RunA1
zout
1

Ave
Data
2.7E-05
Model
2.3E-05

data/vMax
0.0002

ofxng
275.646

Min d/m
2.90262

ave/min d
6.28357
ave/min m
15.3168

325_Calib_S_A

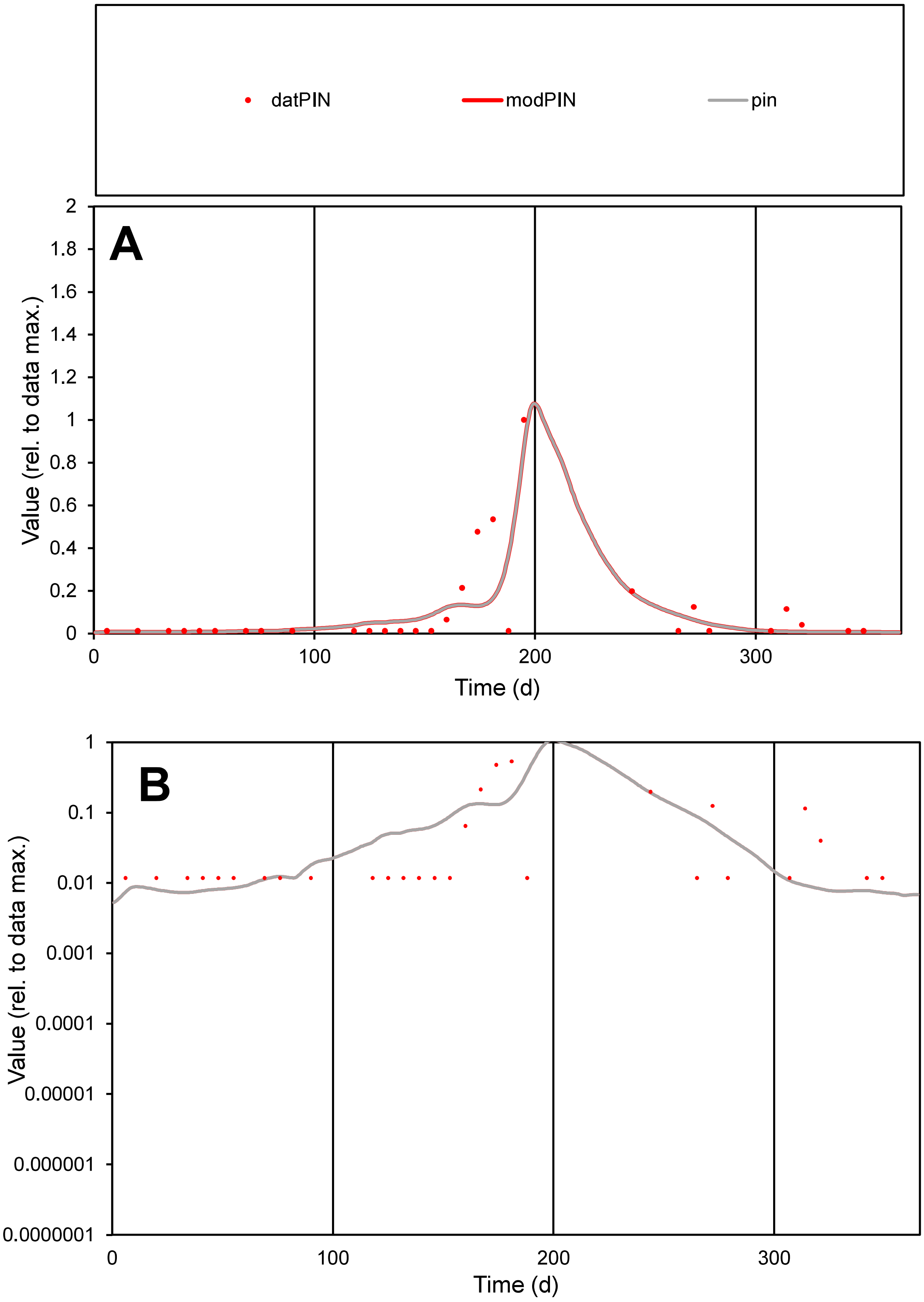

C1

Variables

PIN Provide jtd or Dst

CalibOnly?

2 1 to reproduce of

oNormalize?

2 1=no, 2=yes

ofxn calc*

1.0934

ofxn mod

1.16036

sRun

RunA1

zout

1

Ave

Data

3.4E-05

Model

3E-05

data/vMax

0.00034

ofxng

275.646

Min d/m

1.61955

ave/min d

8.52934

ave/min m

12.2901

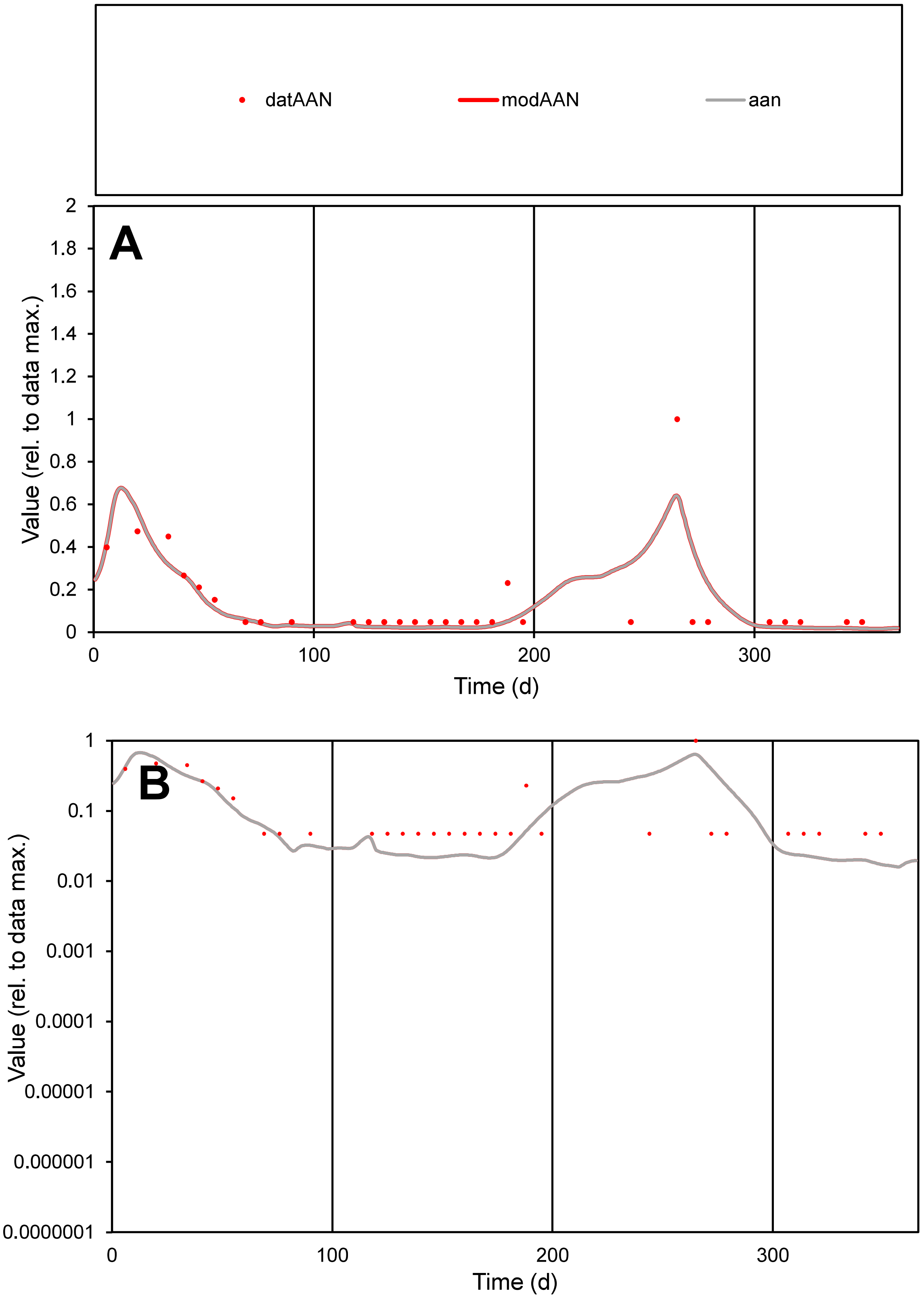

C1

Variables
AAN Provide jtd or Dst
CalibOnly?
2 1 to reproduce of
oNormalize?
2 1=no, 2=yes

ofxn calc*
0.78978
ofxn mod
0.84897

sRun
RunA1
zout
1

Ave
Data
1.7E-05
Model
1.6E-05

data/vMax
0.00012

ofxng
275.646

Min d/m
2.76824

ave/min d
2.98312
ave/min m
7.72073

325_Calib_S_A

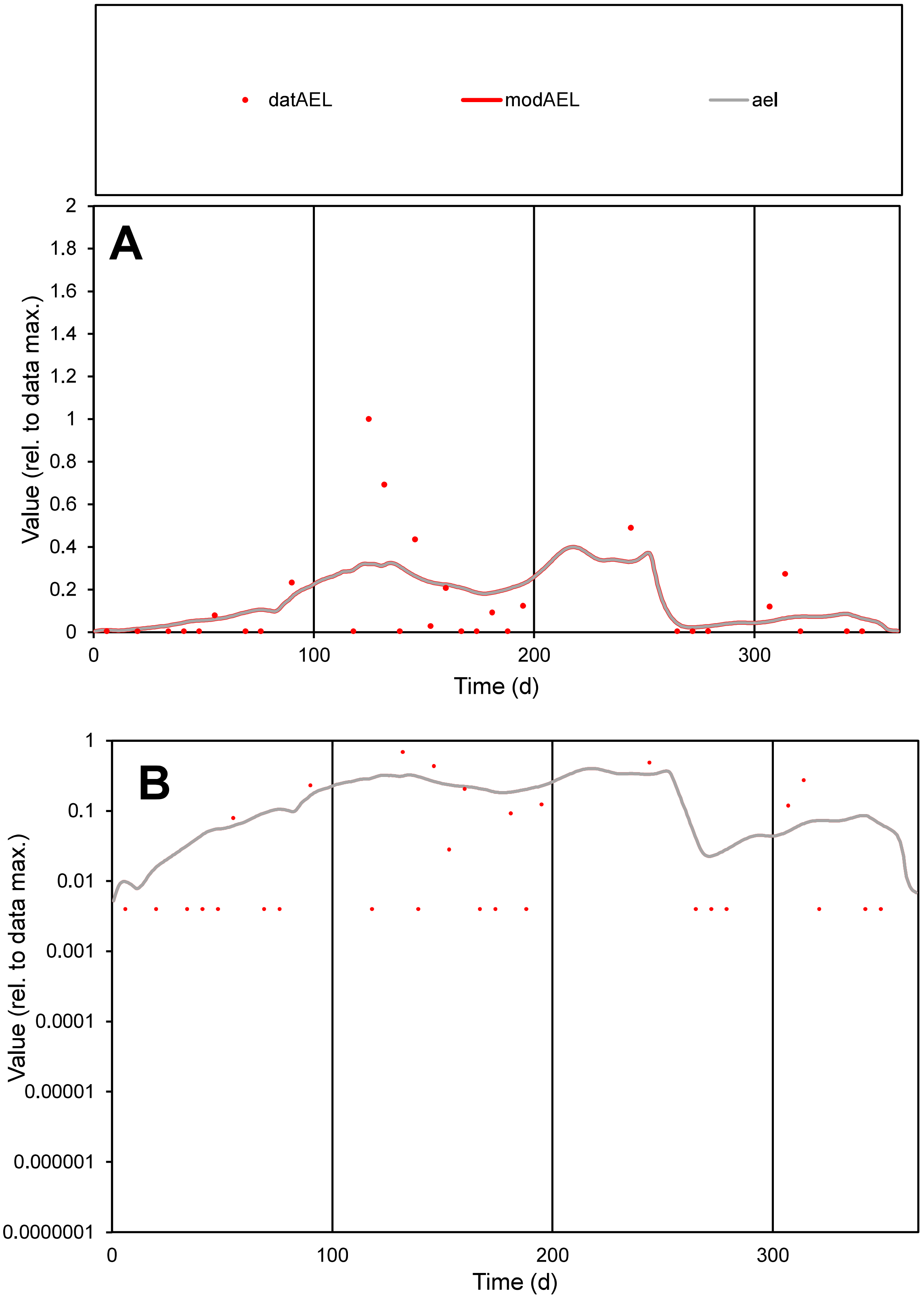

C1

Variables
AEL Provide jtd or Dst
CalibOnly?
2 1 to reproduce of
oNormalize?
2 1=no, 2=yes

ofxn calc*
1.48923
ofxn mod
1.50066

sRun
RunA1
zout
1

Ave
Data
2.2E-05
Model
2.5E-05

data/vMax
0.00017

ofxng
275.646

Min d/m
0.41987

ave/min d
32.0439
ave/min m
15.0475

325_Calib_S_A

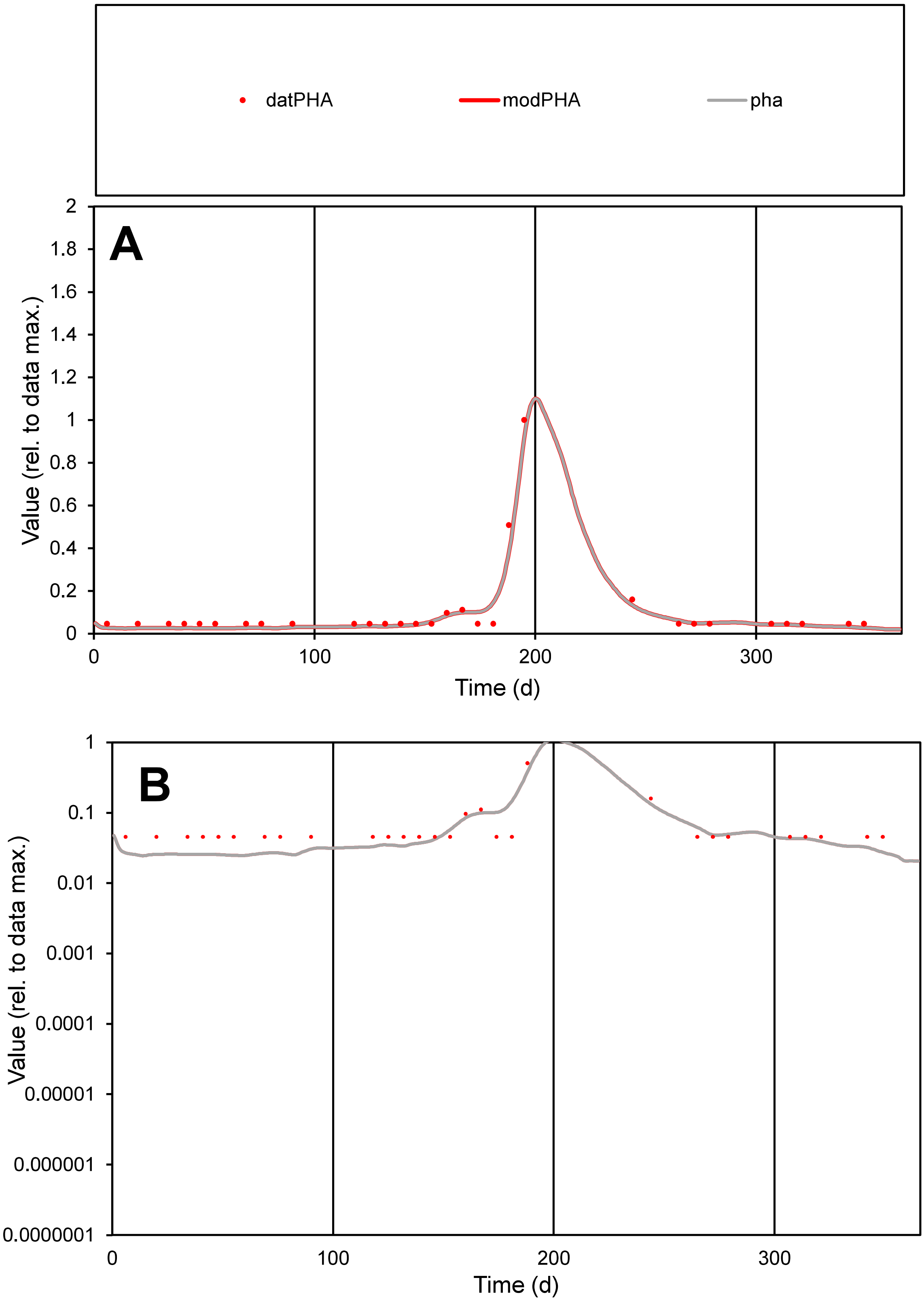

C1

Variables
PHA Provide jtd or Dst
CalibOnly?
2 1 to reproduce of
oNormalize?
2 1=no, 2=yes

ofxn calc*
0.26914
ofxn mod
0.37184

sRun
RunA1
zout
1

Ave
Data
2.3E-05
Model
2.2E-05

data/vMax
0.00023

ofxng
275.646

Min d/m
1.83987

ave/min d
2.2074
ave/min m
3.80596

325_Calib_S_A

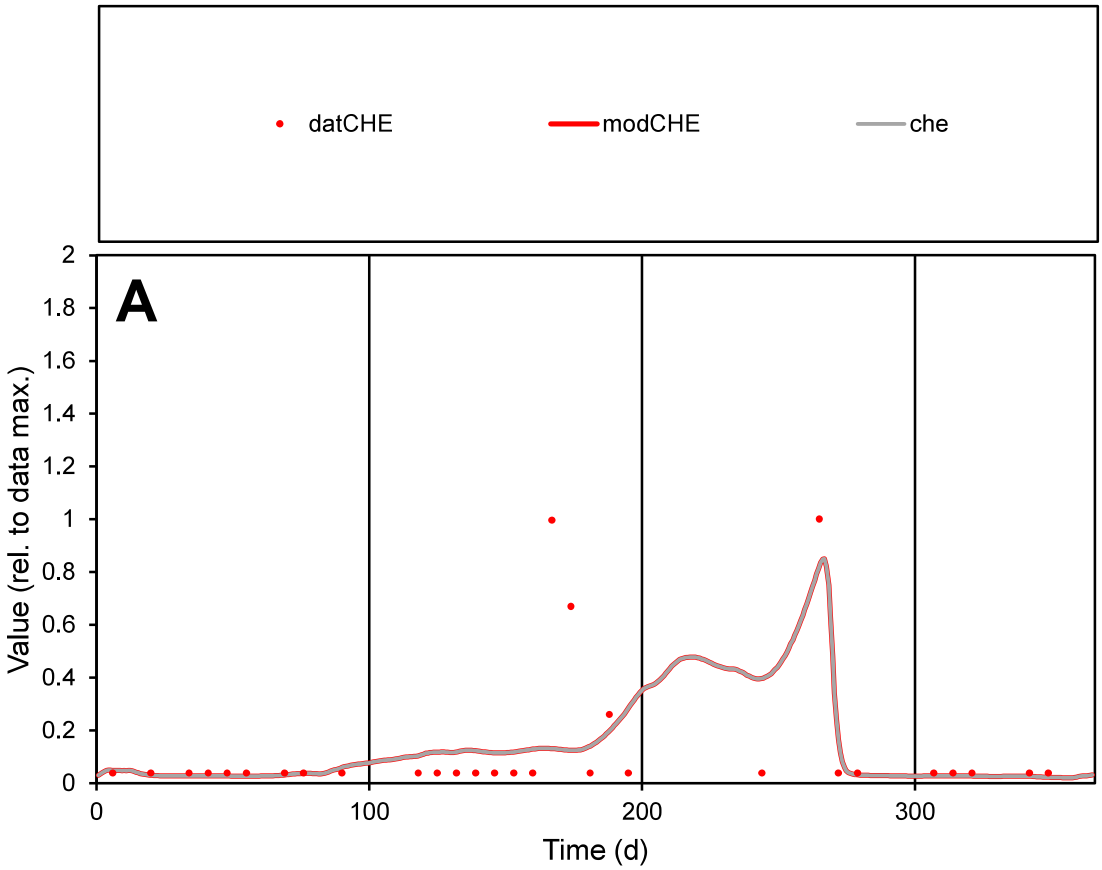

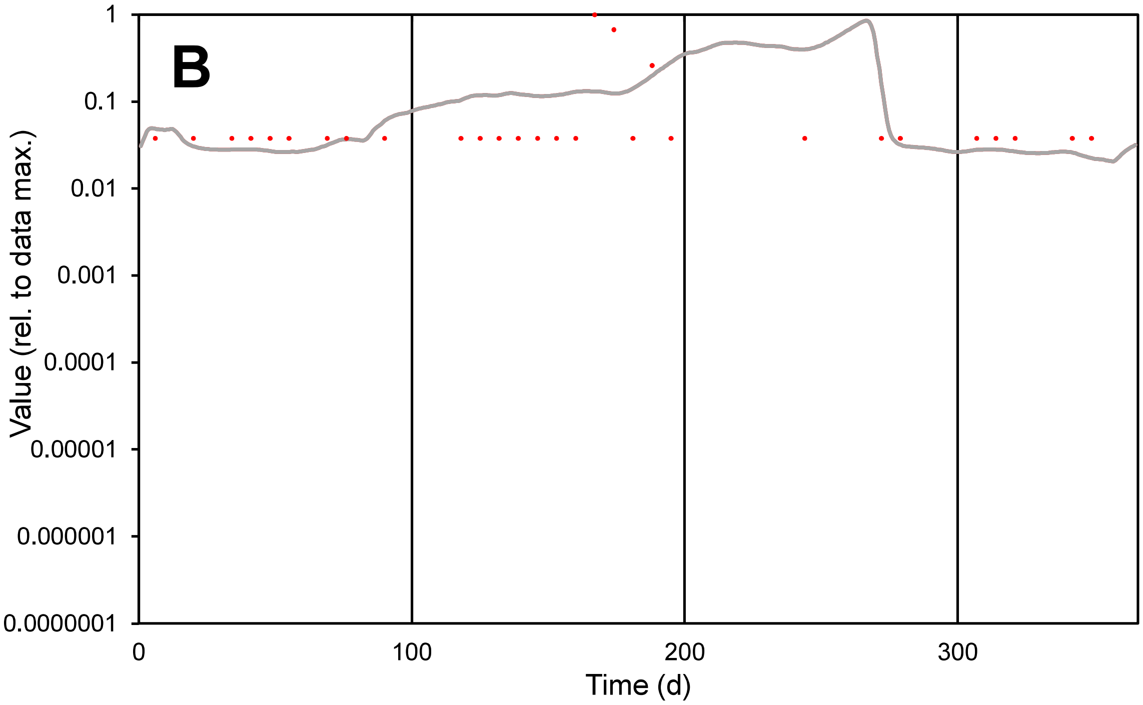

C1

Variables
CHE Provide jtd or Dst
CalibOnly?
2 1 to reproduce of
oNormalize?
2 1=no, 2=yes

ofxn calc*
1.60793
ofxn mod
1.62126

sRun
RunA1
zout
1

Ave
Data
2.4E-05
Model
2.2E-05

data/vMax
0.00019

ofxng
275.646

Min d/m
1.71932

ave/min d
3.44606
ave/min m
5.29625

325_Calib_S_A

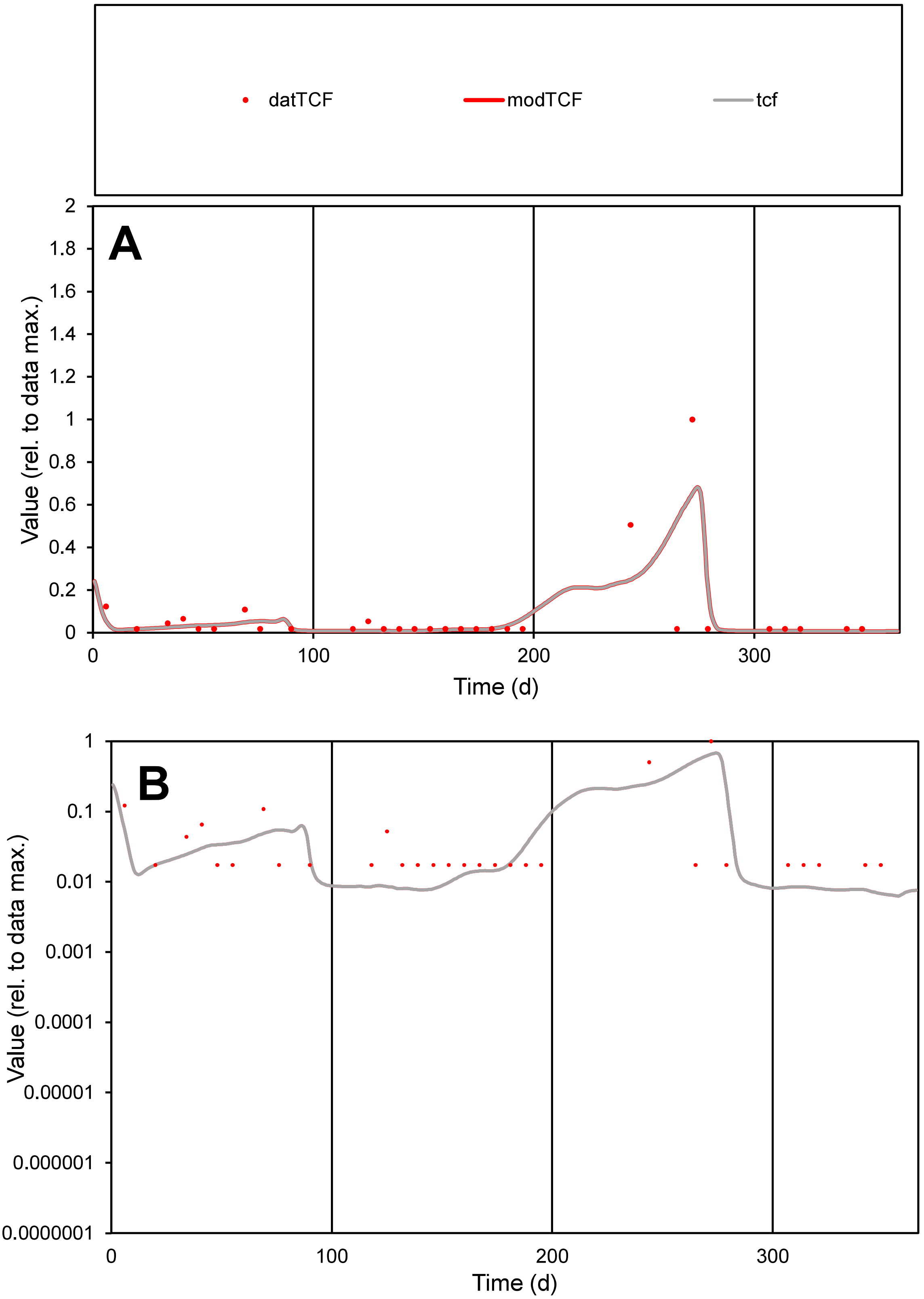

C1

Variables

TCF Provide jtd or Dst

CalibOnly?

2 1 to reproduce of

oNormalize?

2 1=no, 2=yes

ofxn calc*

1.58362

ofxn mod

1.64615

sRun

RunA1

zout

1

Ave

Data

1.4E-05

Model

1.3E-05

data/vMax

0.00018

ofxng

275.646

Min d/m

2.59917

ave/min d

4.41291

ave/min m

10.4548

325_Calib_S_A

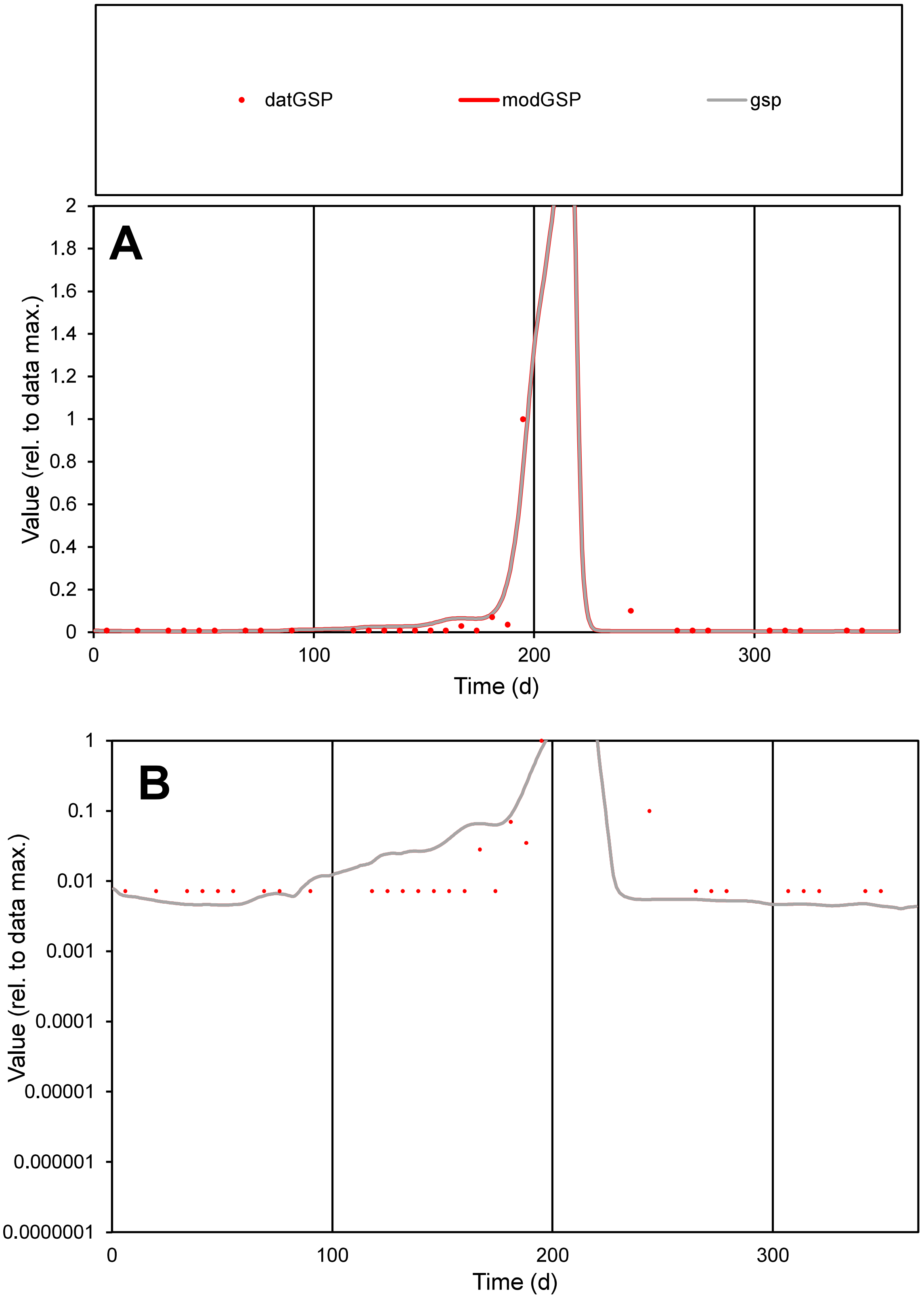

C1

Variables
GSP Provide jtd or Dst
CalibOnly?
2 1 to reproduce of
oNormalize?
2 1=no, 2=yes

ofxn calc*
0.81977
ofxn mod
1.04652

sRun
RunA1
zout
1

Ave
Data
5.9E-05
Model
6.8E-05

data/vMax
0.00125

ofxng
275.646

Min d/m
1.64961
ave/min d
6.53853
ave/min m
12.5244

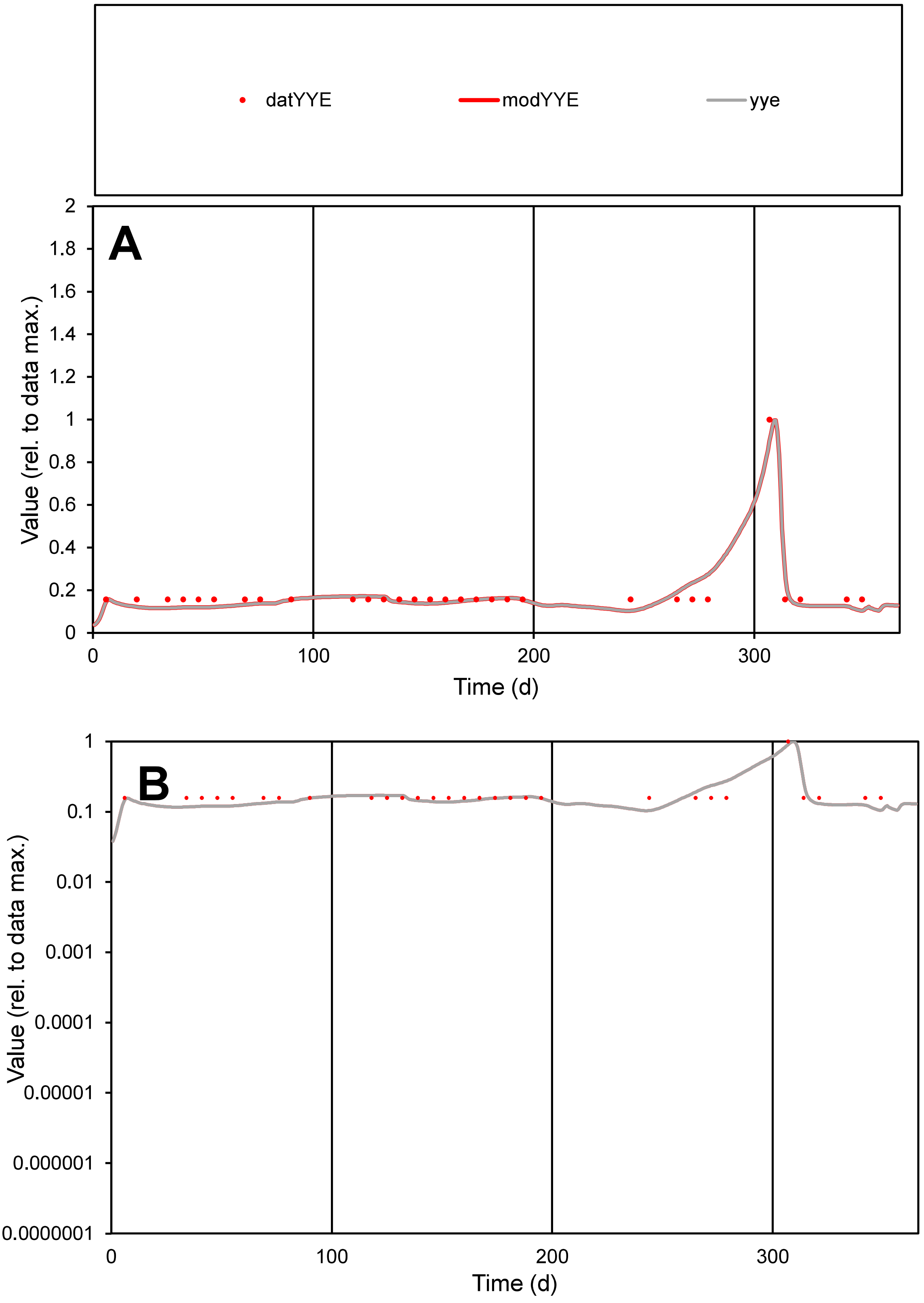

C1

Variables
YYE Provide jtd or Dst
CalibOnly?
2 1 to reproduce of
oNormalize?
2 1=no, 2=yes

ofxn calc*
0.19548
ofxn mod
0.24834

sRun
RunA1
zout
1

Ave
Data
4.4E-06
Model
4.3E-06

data/vMax
2.4E-05

ofxng
275.646

Min d/m
1.49325

ave/min d
1.17909
ave/min m
1.70087

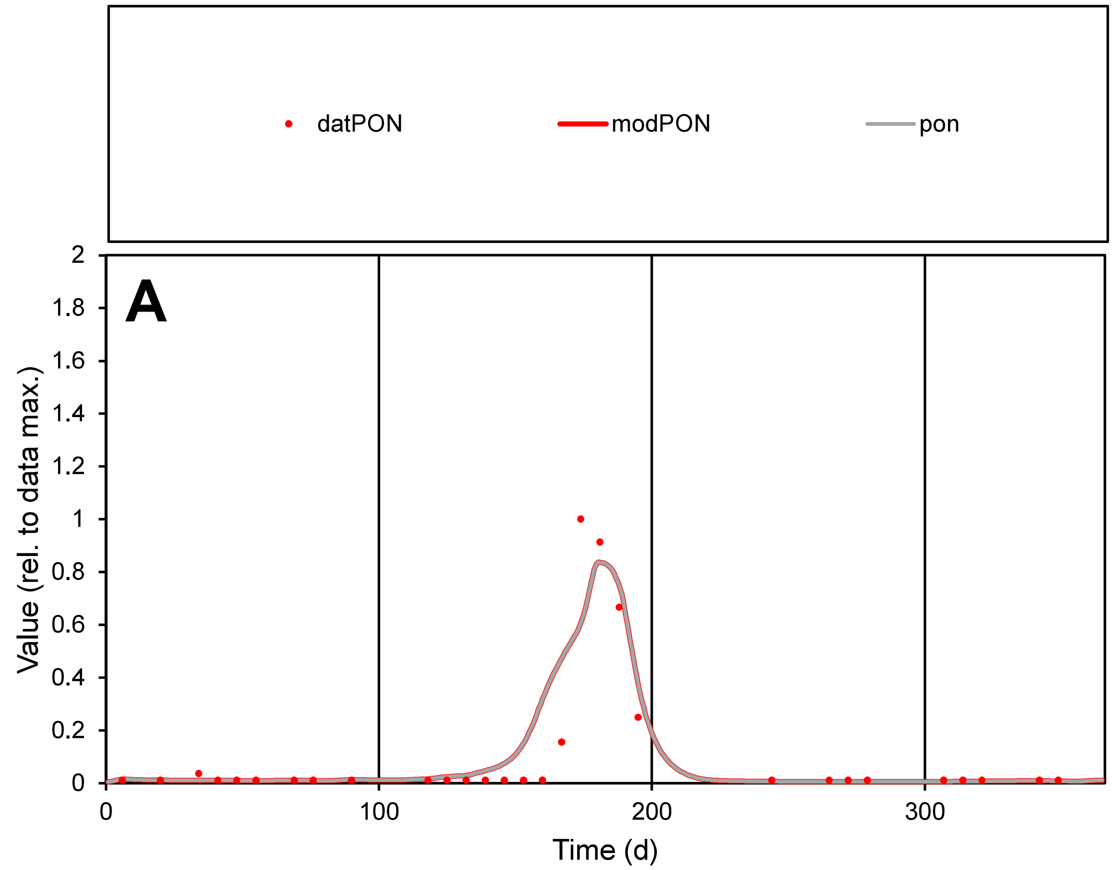

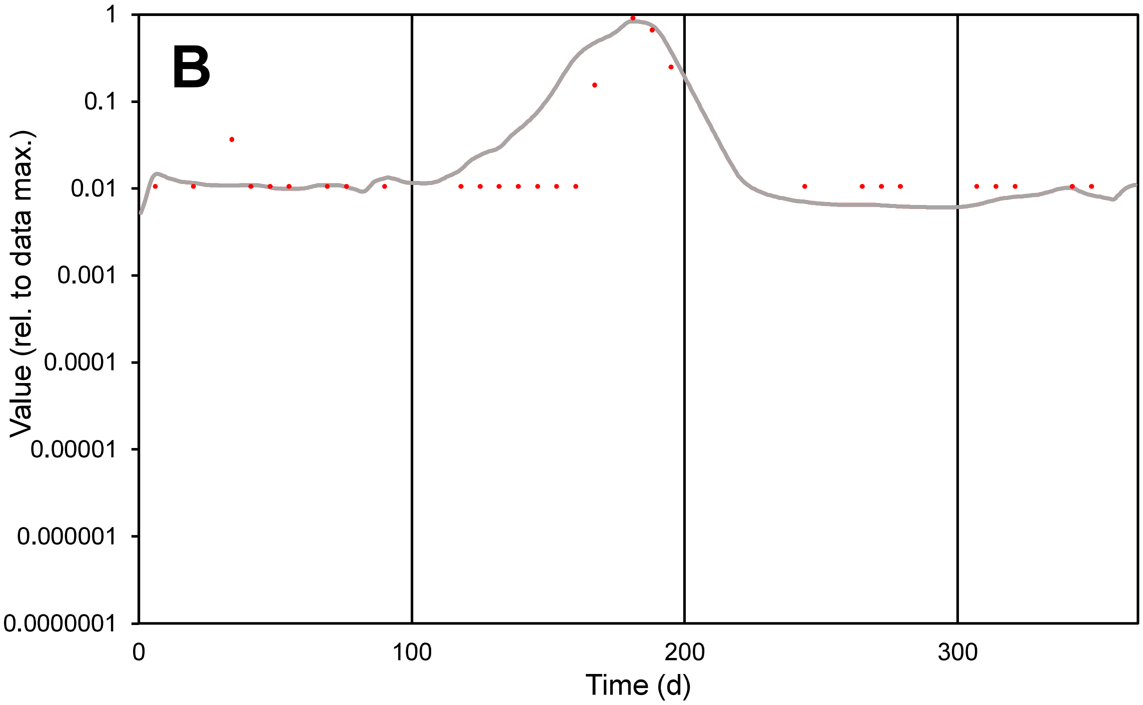

C1

Variables
PON Provide jtd or Dst
CalibOnly?
2 1 to reproduce of
oNormalize?
2 1=no, 2=yes

ofxn calc*
0.93724
ofxn mod
1.03099

sRun
RunA1
zout
1

Ave
Data
3E-05
Model
3.4E-05

data/vMax
0.00028

ofxng
275.646

Min d/m
1.70087

ave/min d
10.3424
ave/min m
19.9324

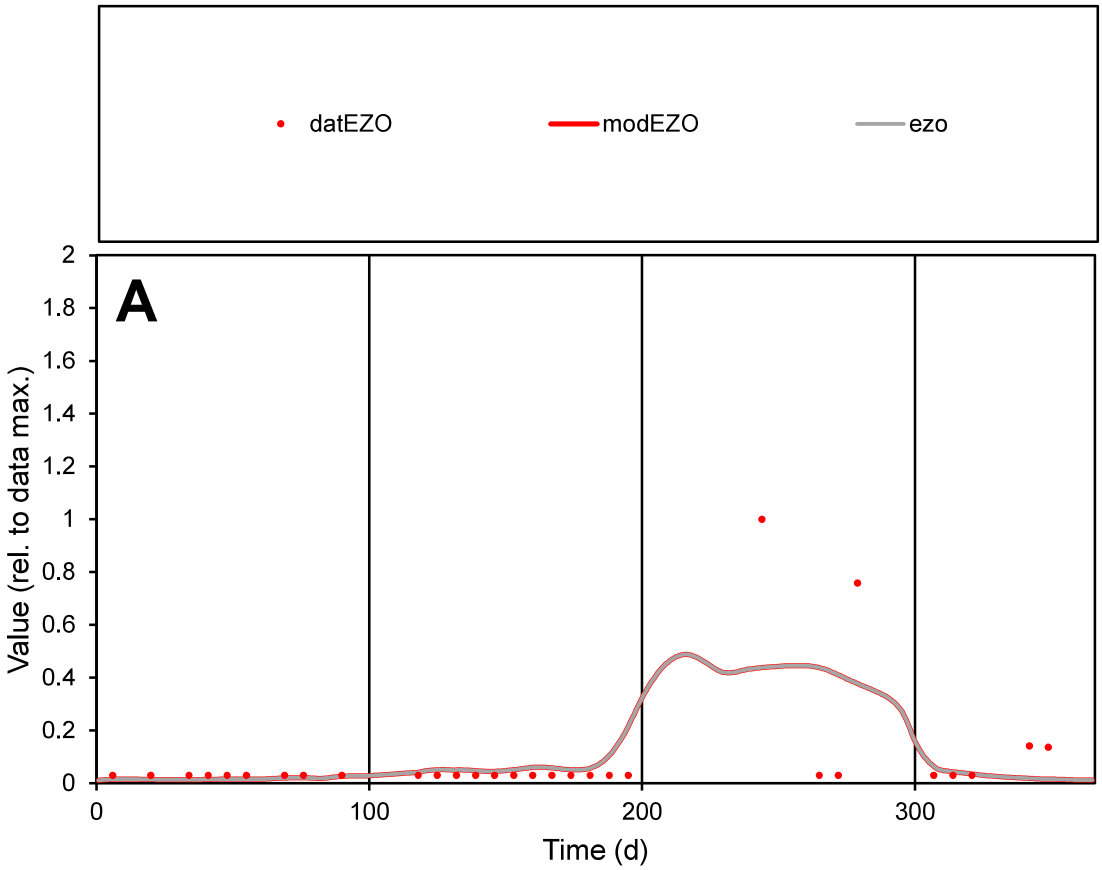

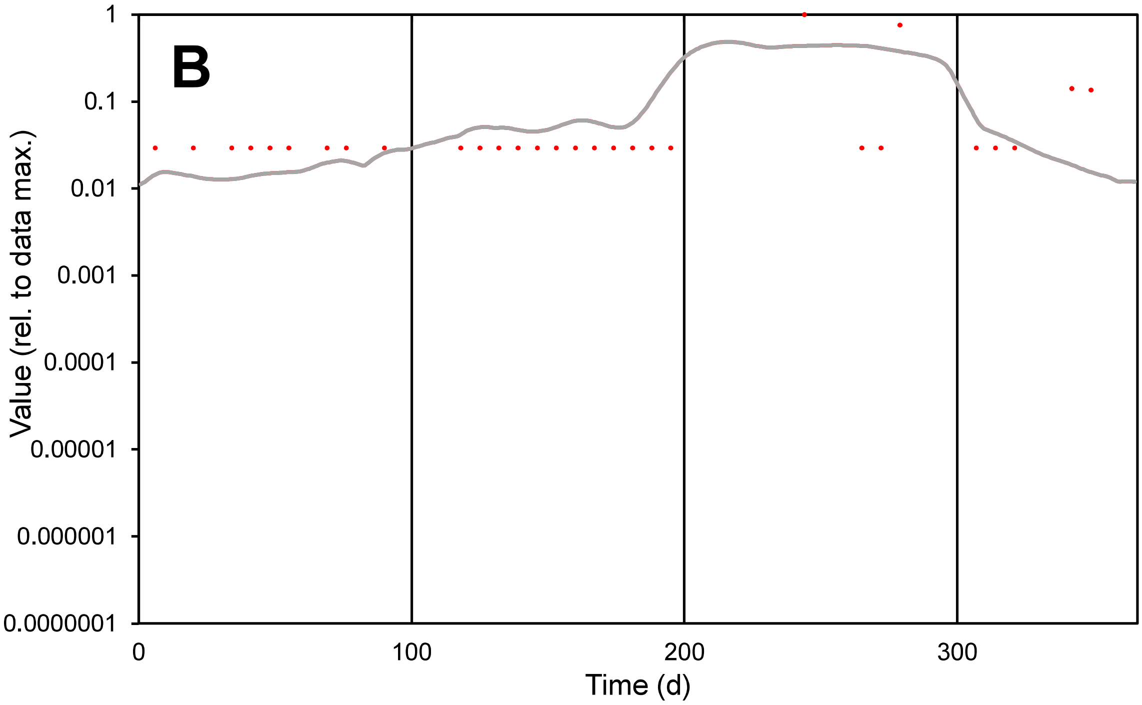

C1

Variables
EZO Provide jtd or Dst
CalibOnly?
2 1 to reproduce of
oNormalize?
2 1=no, 2=yes

ofxn calc*
1.79345
ofxn mod
1.81787

sRun
RunA1
zout
1

Ave
Data
1.3E-05
Model
1.3E-05

data/vMax
0.00014

ofxng
275.646

Min d/m
2.25057

ave/min d
3.19045
ave/min m
7.15732

325_Calib_S_A

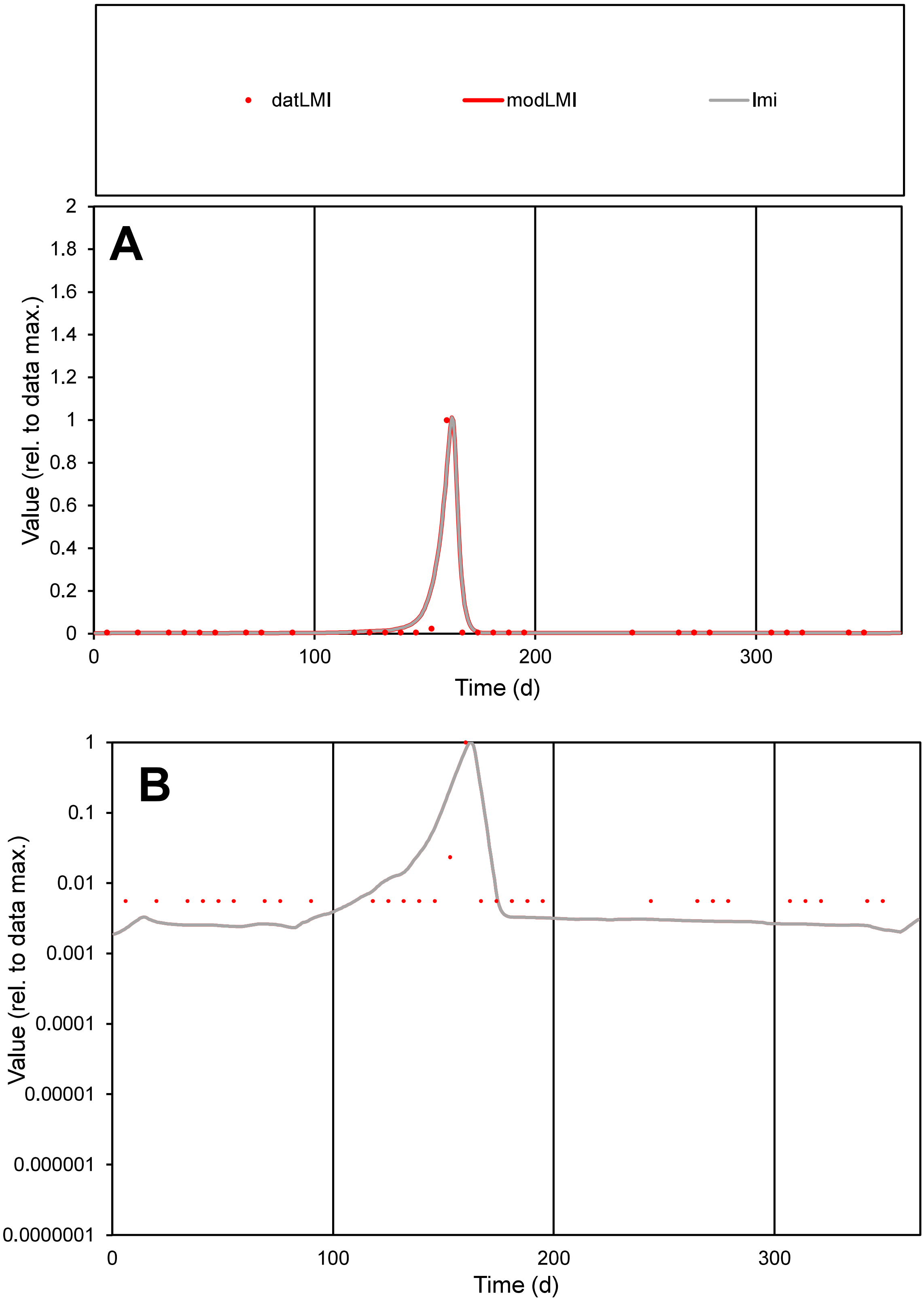

C1

Variables
LMI Provide jtd or Dst
CalibOnly?
2 1 to reproduce of
oNormalize?
2 1=no, 2=yes

ofxn calc*
0.73561
ofxn mod
0.98737

sRun
RunA1
zout
1

Ave
Data
2.1E-05
Model
2.4E-05

data/vMax
0.00054

ofxng
275.646

Min d/m
2.59071

ave/min d
7.035
ave/min m
20.8853

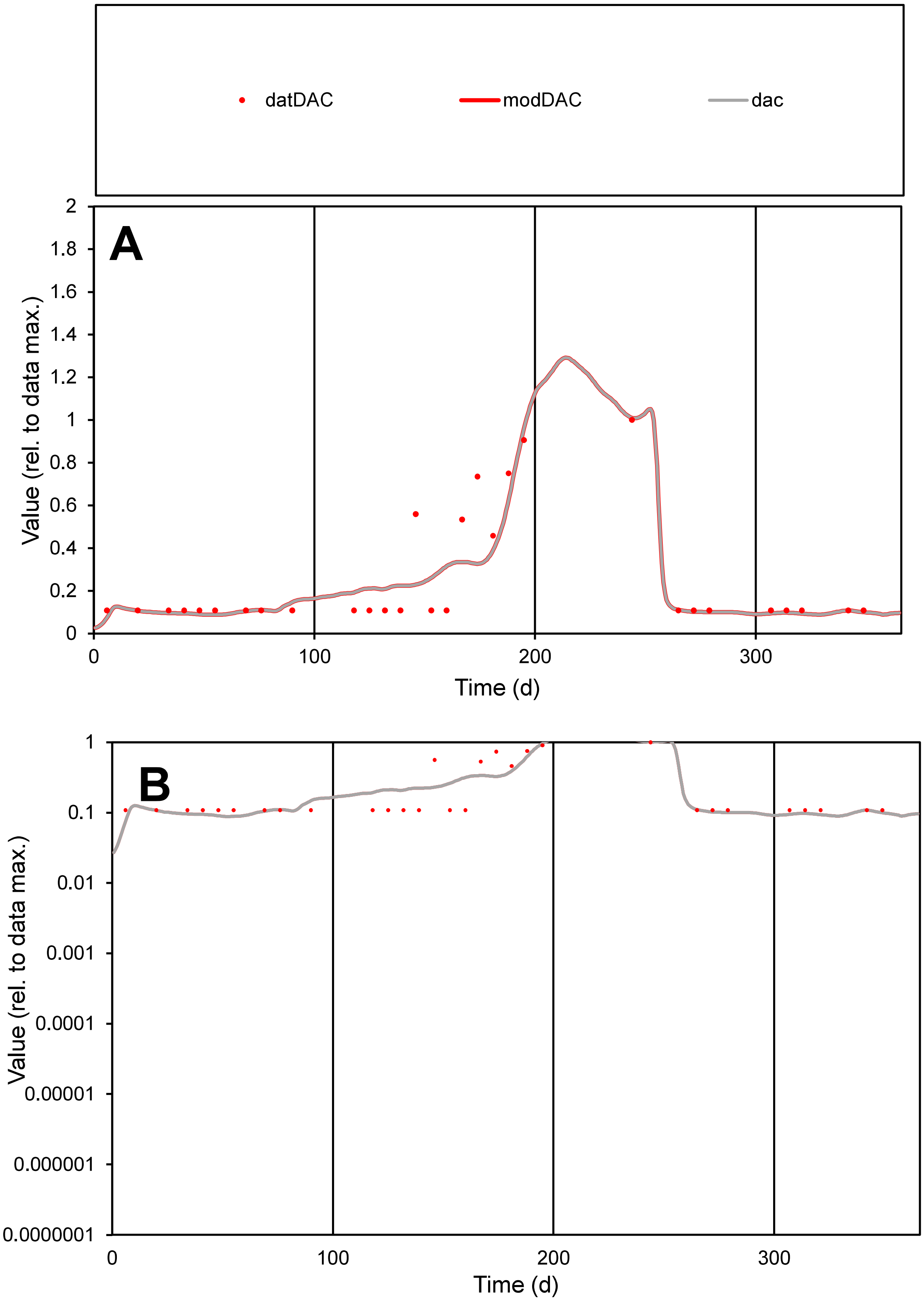

C1

Variables
DAC Provide jtd or Dst
CalibOnly?
2 1 to reproduce of
oNormalize?
2 1=no, 2=yes

ofxn calc*
0.48986
ofxn mod
0.51197

sRun
RunA1
zout
1

Ave
Data
1.8E-05
Model
1.7E-05

data/vMax
7.1E-05

ofxng
275.646

Min d/m
1.21469

ave/min d
2.28648
ave/min m
2.68643

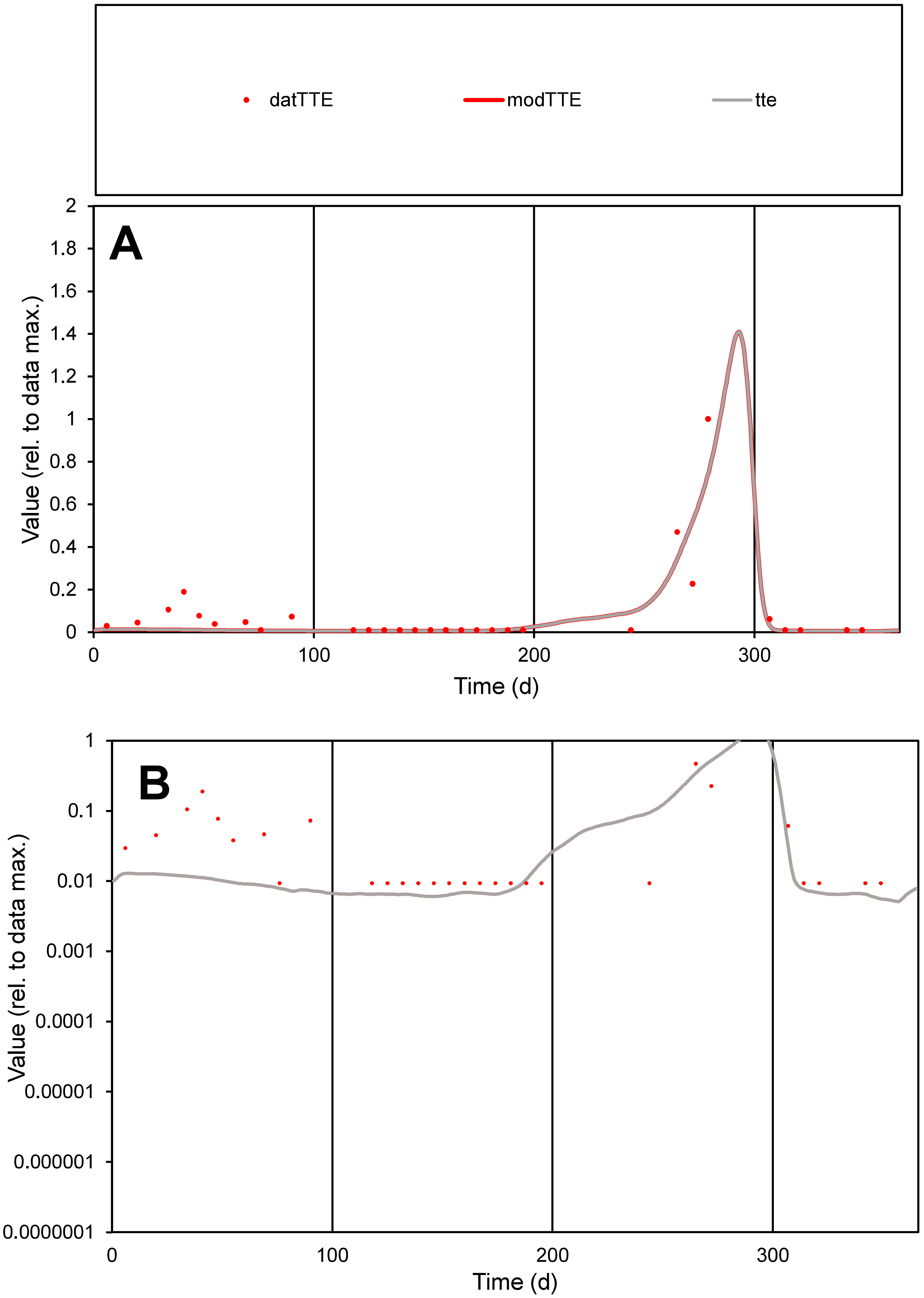

C1

Variables
TTE Provide jtd or Dst
CalibOnly?
2 1 to reproduce of
oNormalize?
2 1=no, 2=yes

ofxn calc*
0.97439
ofxn mod
1.03319

sRun
RunA1
zout
1

Ave
Data
3.4E-05
Model
2.8E-05

data/vMax
0.0004

ofxng
275.646

Min d/m
1.70258

ave/min d
9.01647
ave/min m
12.654

325_Calib_S_A

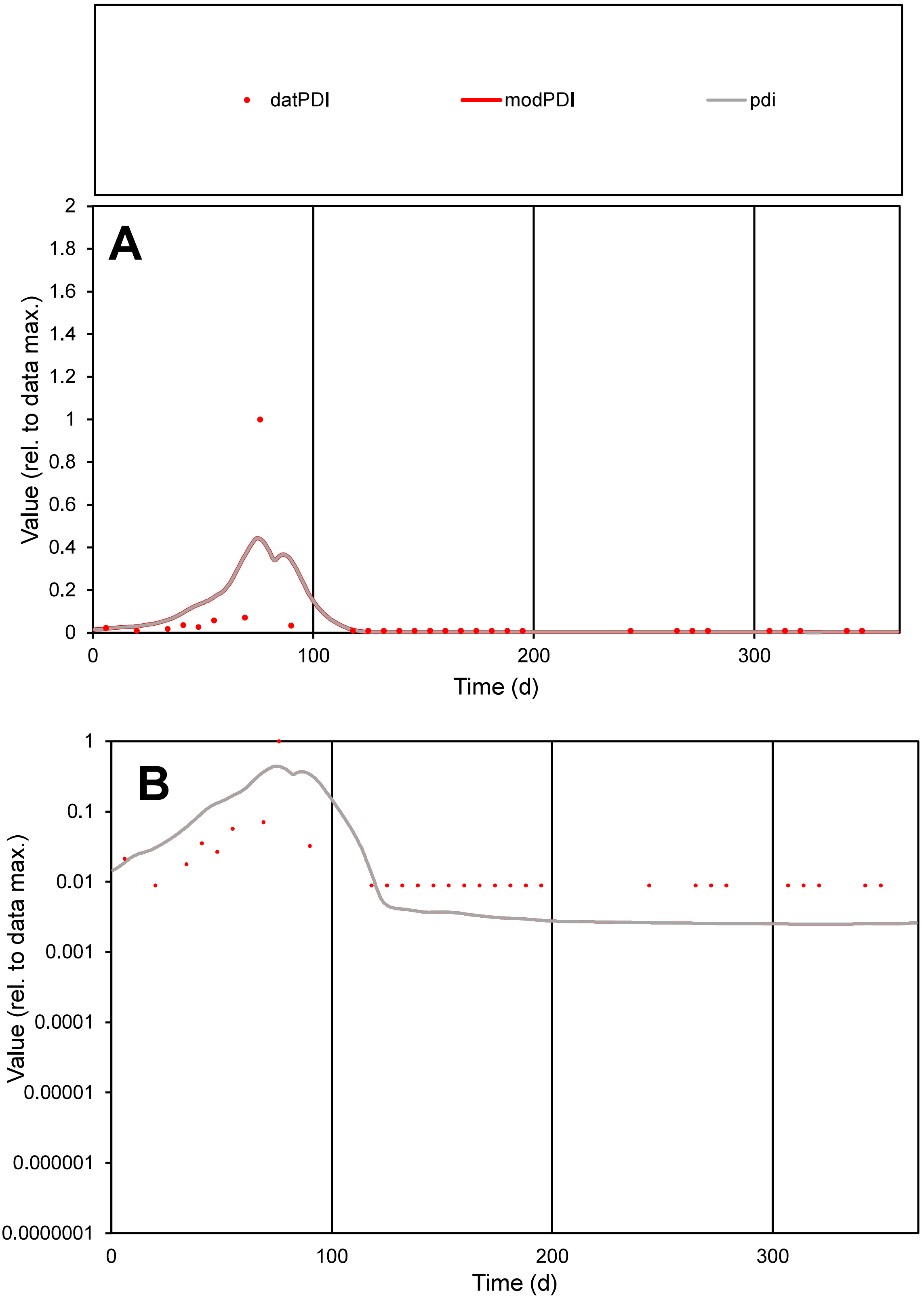

C1

Variables
PDI Provide jtd or Dst
CalibOnly?
2 1 to reproduce of
oNormalize?
2 1=no, 2=yes

ofxn calc*
2.69335
ofxn mod
2.72471

sRun
RunA1
zout
1

Ave
Data
2.4E-05
Model
2.8E-05

data/vMax
0.00049

ofxng
275.646

Min d/m
3.57398

ave/min d
5.45598
ave/min m
22.4614

325_Calib_S_A

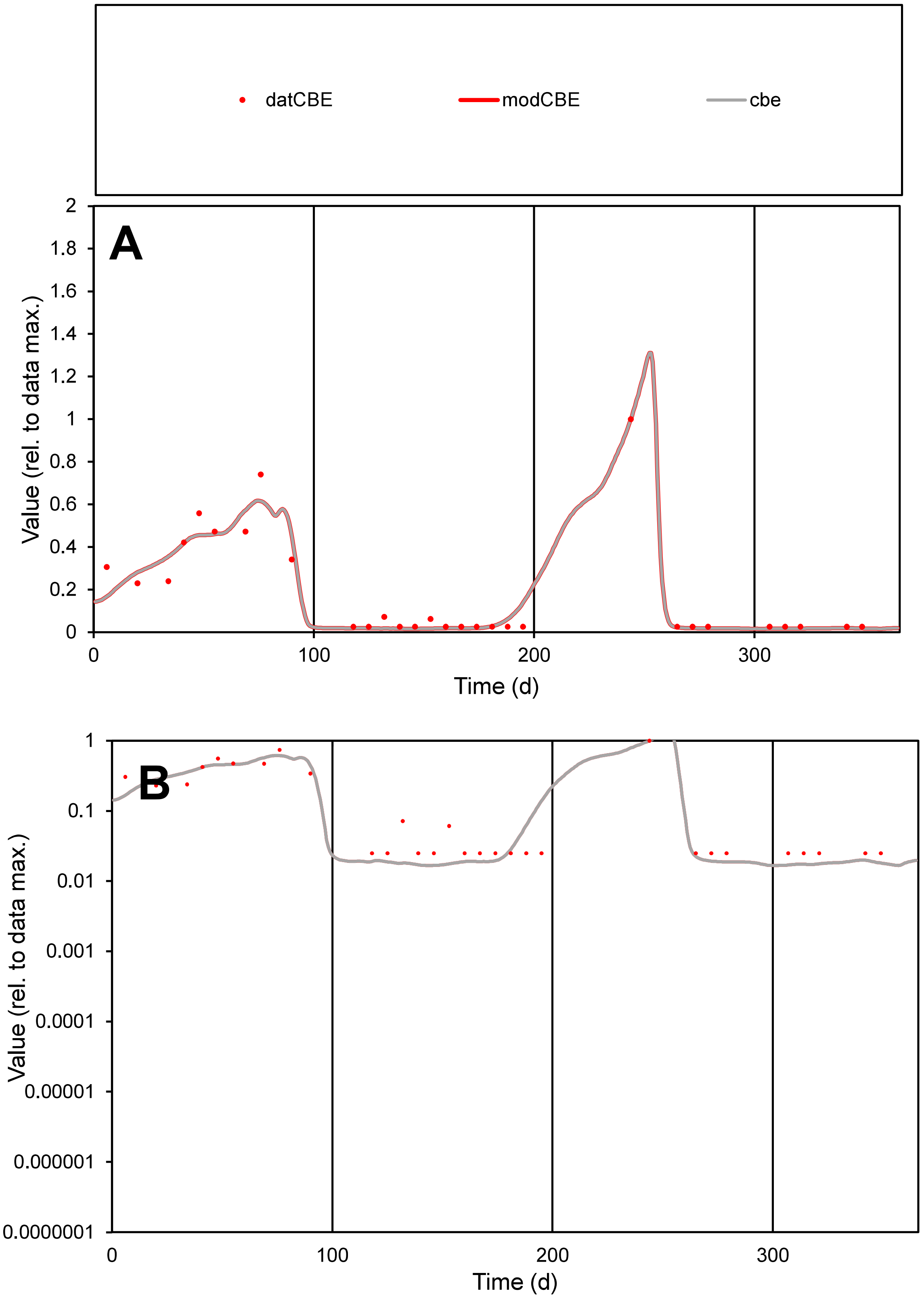

C1

Variables
CBE Provide jtd or Dst
CalibOnly?
2 1 to reproduce of
oNormalize?
2 1=no, 2=yes

ofxn calc*
0.31269
ofxn mod
0.36596

sRun
RunA1
zout
1

Ave
Data
1.2E-05
Model
1.2E-05

data/vMax
6.7E-05

ofxng
275.646

Min d/m
1.47872

ave/min d
7.17866
ave/min m
10.4539

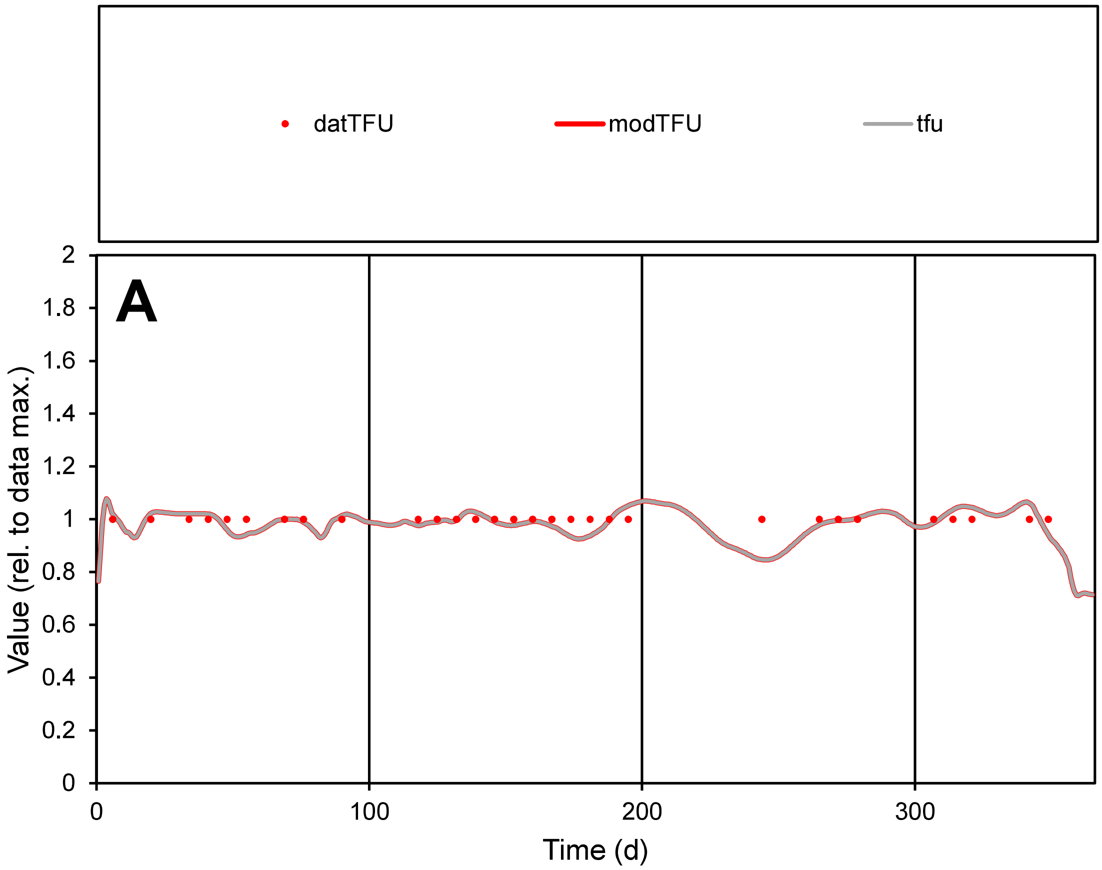

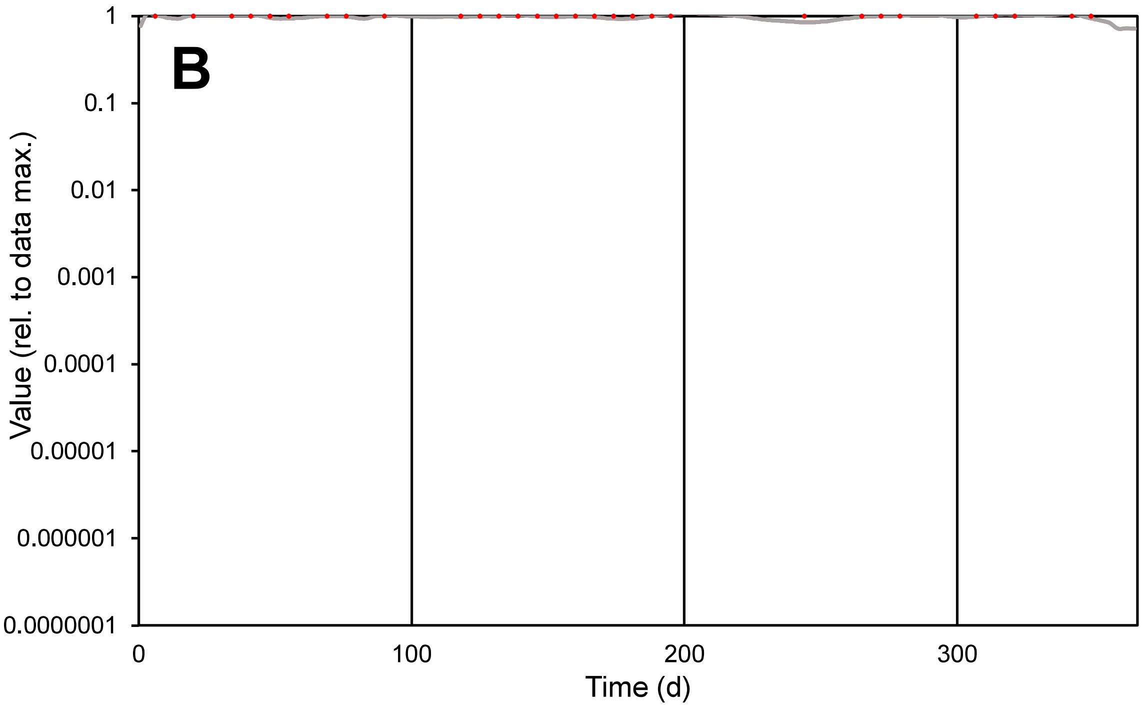

C1

Variables
TFU Provide jtd or Dst
CalibOnly?
2 1 to reproduce of
oNormalize?
2 1=no, 2=yes

ofxn calc*
0.04413
ofxn mod
0.12033

sRun
RunA1
zout
1

Ave
Data
1.3E-05
Model
1.3E-05

data/vMax
1.3E-05

ofxng
275.646

Min d/m
1.18275

ave/min d
1
ave/min m
1.17124

325_Calib_S_A

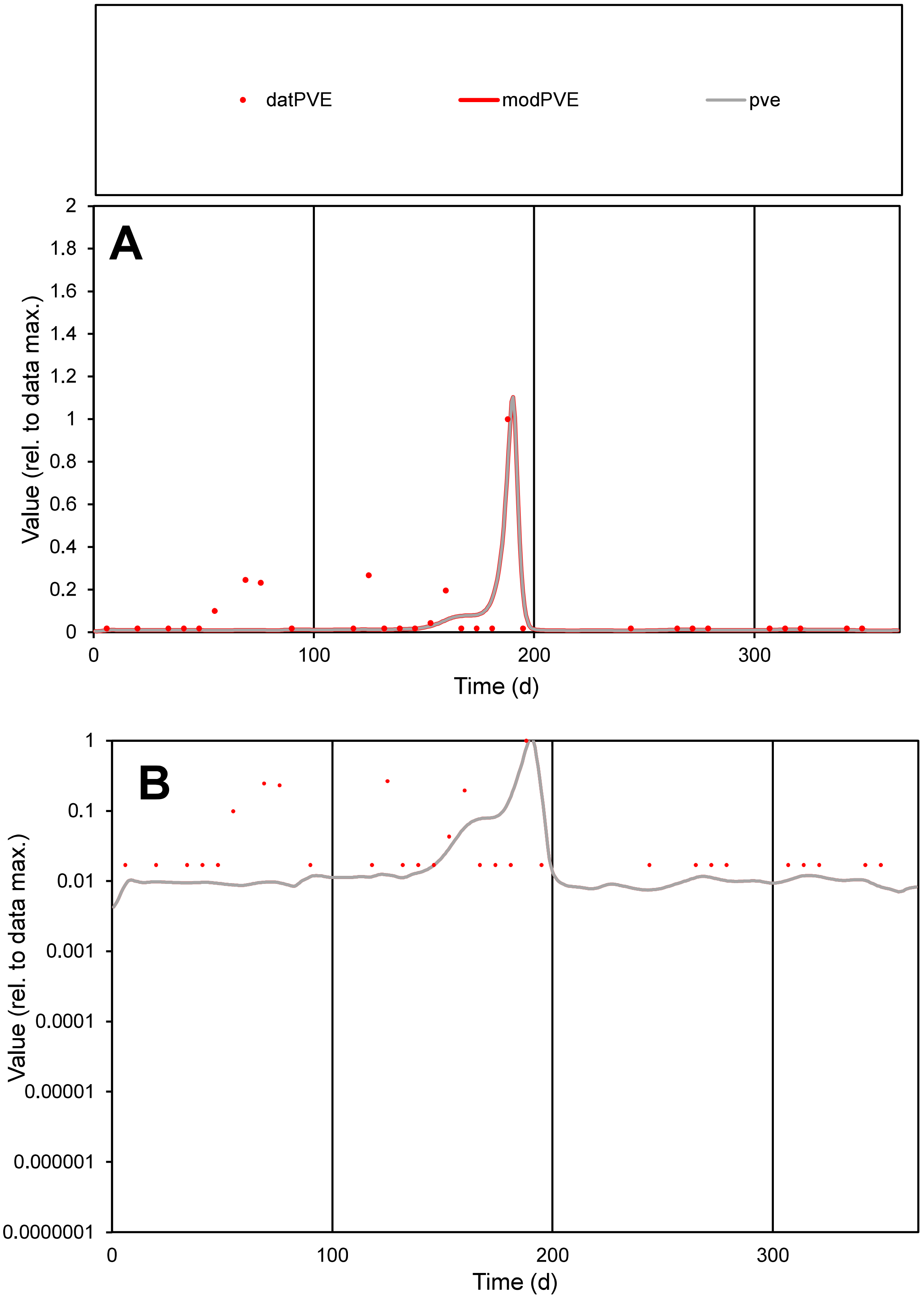

C1

Variables
PVE Provide jtd or Dst
CalibOnly?
2 1 to reproduce of
oNormalize?
2 1=no, 2=yes

ofxn calc*
1.03424
ofxn mod
1.06122

sRun
RunA1
zout
1

Ave
Data
1E-05
Model
6.8E-06

data/vMax
0.00012

ofxng
275.646

Min d/m
2.25577

ave/min d
4.85699
ave/min m
7.36408

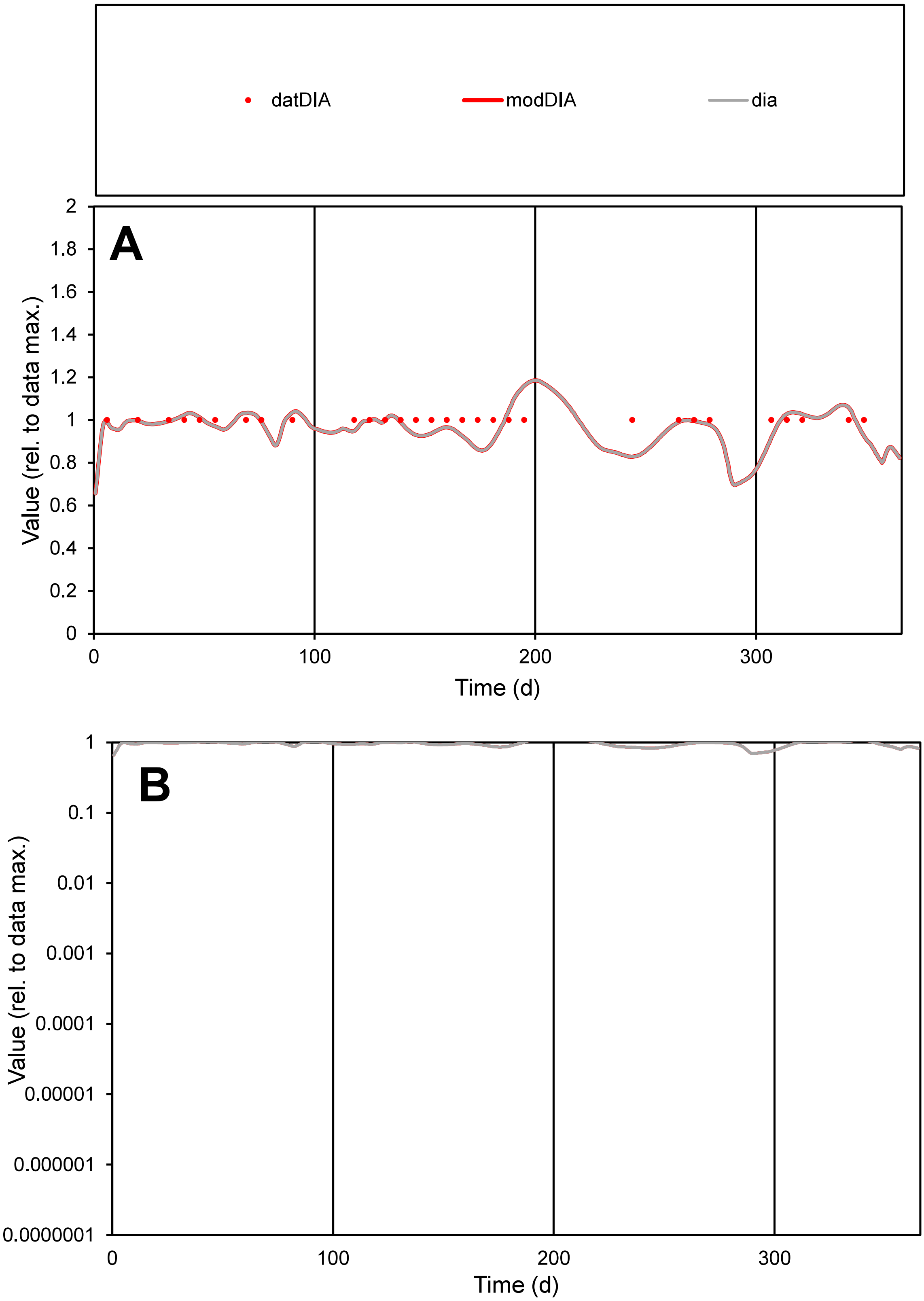

C1

Variables
DIA Provide jtd or Dst
CalibOnly?
2 1 to reproduce of
oNormalize?
2 1=no, 2=yes

ofxn calc*
0.06615
ofxn mod
0.12984

sRun
RunA1
zout
1

Ave
Data
6.2E-06
Model
6.1E-06

data/vMax
6.2E-06

ofxng
275.646

Min d/m
1.20445

ave/min d
1
ave/min m
1.18369

325_Calib_S_A

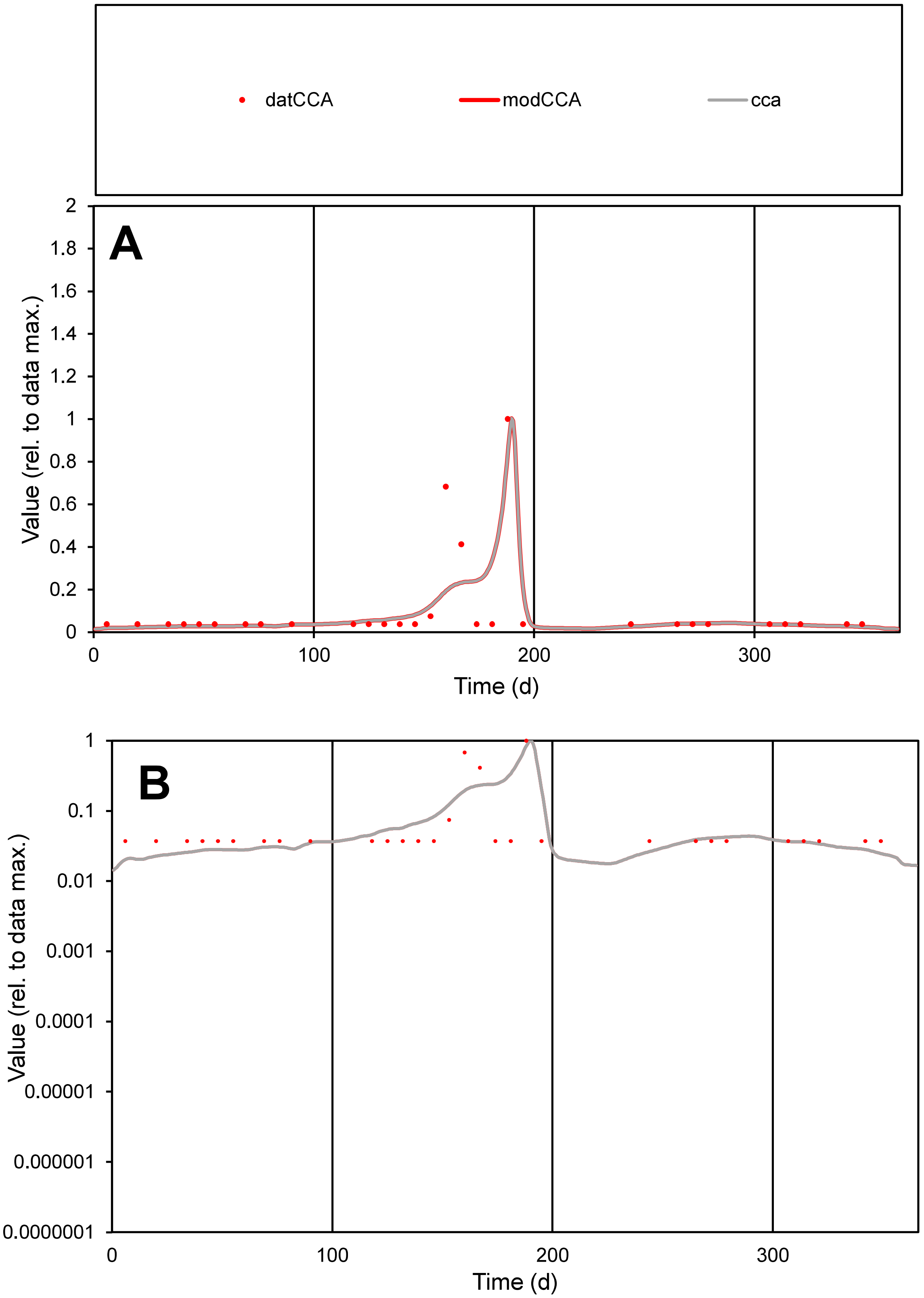

C1

Variables
CCA Provide jtd or Dst
CalibOnly?
2 1 to reproduce of
oNormalize?
2 1=no, 2=yes

ofxn calc*
1.09469
ofxn mod
1.11991

sRun
RunA1
zout
1

Ave
Data
5.1E-06
Model
5E-06

data/vMax
4.9E-05

ofxng
275.646

Min d/m
1.77994

ave/min d
2.80753
ave/min m
4.84106

325_Calib_S_A

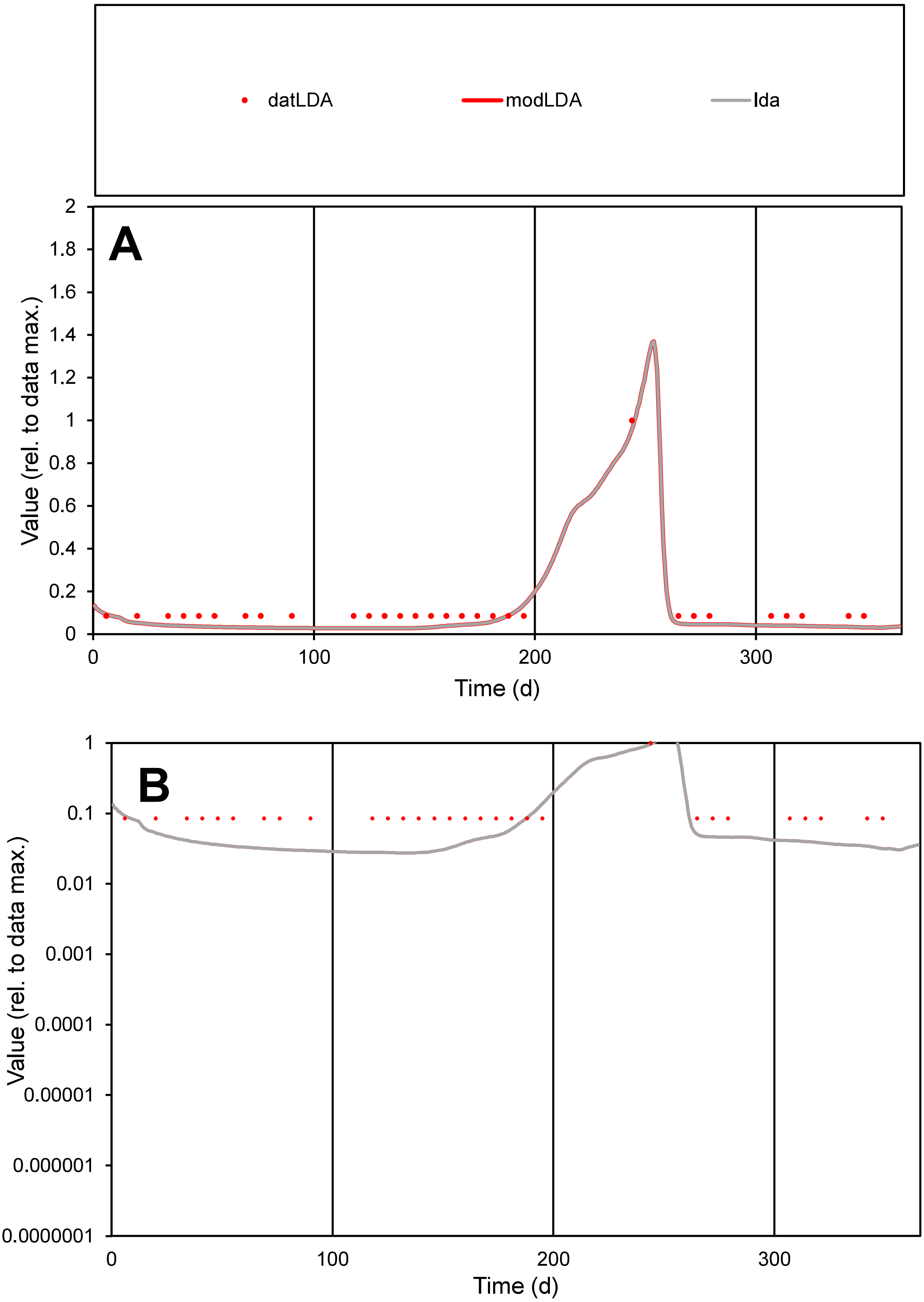

C1

Variables
LDA Provide jtd or Dst
CalibOnly?
2 1 to reproduce of
oNormalize?
2 1=no, 2=yes

ofxn calc*
0.39717
ofxn mod
0.42045

sRun
RunA1
zout
1

Ave
Data
9.4E-06
Model
6.3E-06

data/vMax
8.1E-05

ofxng
275.646

Min d/m
3.10467

ave/min d
1.3586
ave/min m
2.81294

325_Calib_S_A

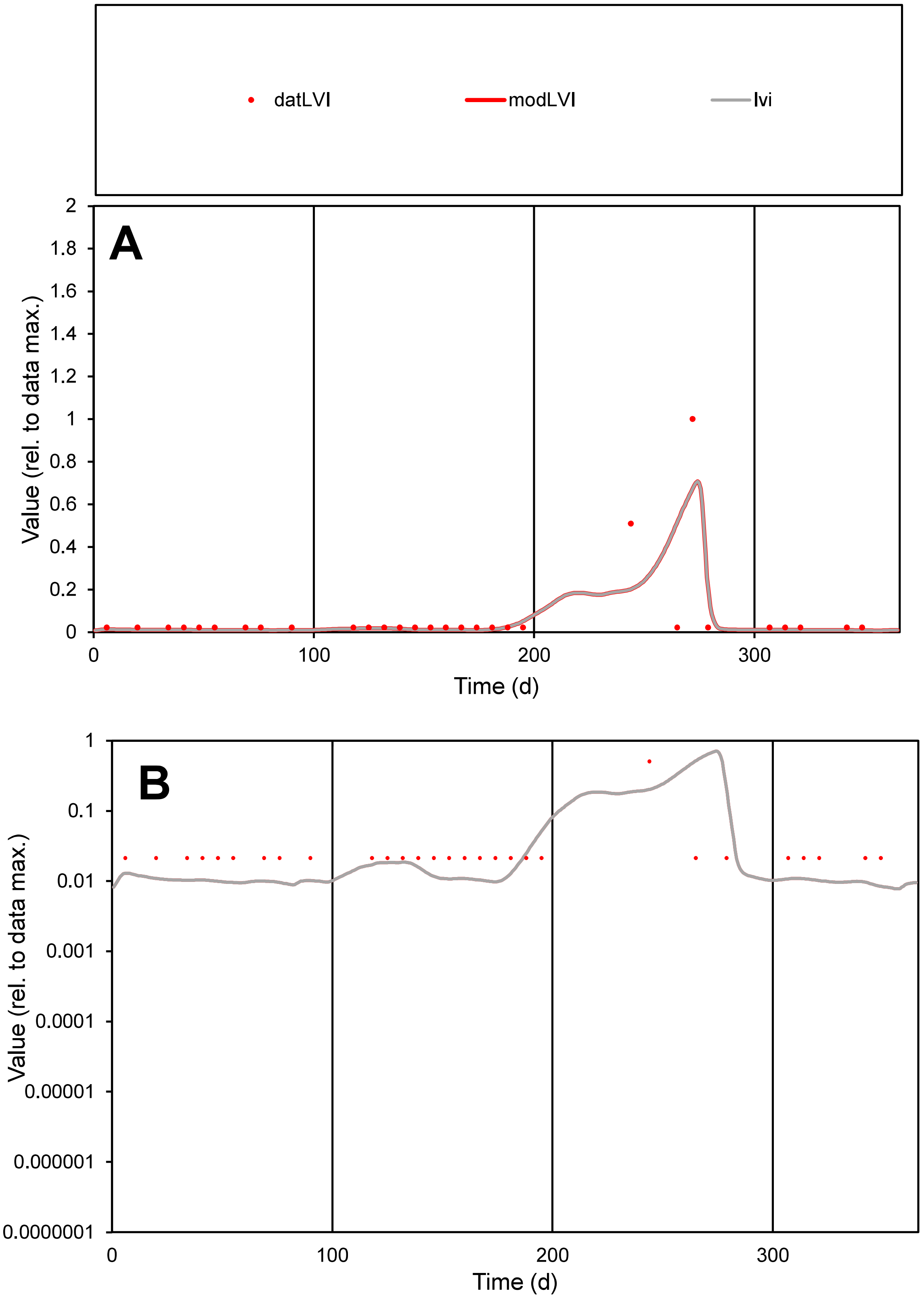

C1

Variables

LVI Provide jtd or Dst

CalibOnly?

2 1 to reproduce of

oNormalize?

2 1=no, 2=yes

ofxn calc*

1.69415

ofxn mod

1.7596

sRun

RunA1

zout

1

Ave

Data

4.7E-06

Model

4.1E-06

data/vMax

6.7E-05

ofxng

275.646

Min d/m

2.57515

ave/min d

3.29644

ave/min m

7.37031

325_Calib_S_A

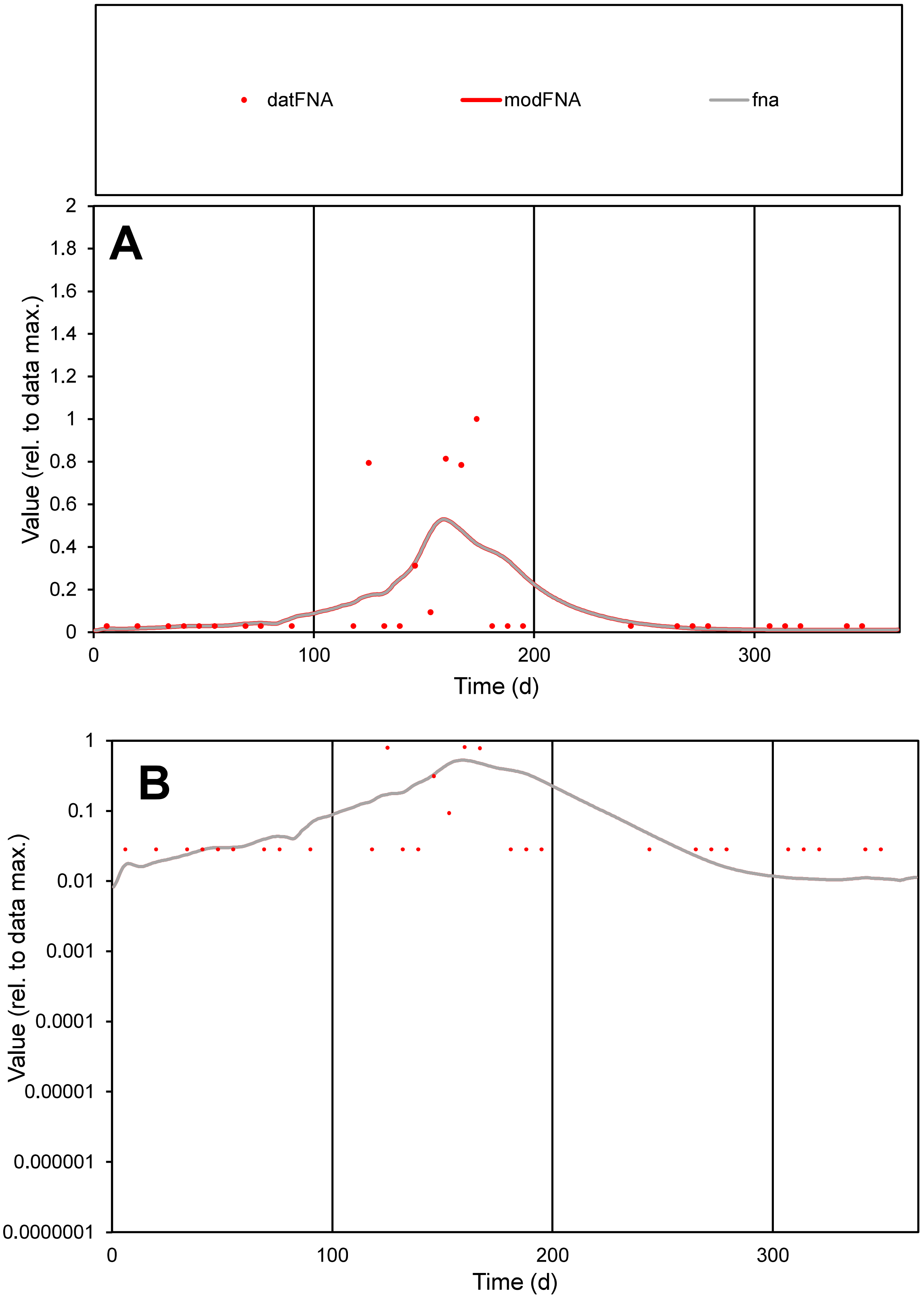

C1

Variables

FNA Provide jtd or Dst

CalibOnly?

2 1 to reproduce of

oNormalize?

2 1=no, 2=yes

ofxn calc*

1.45063

ofxn mod

1.46839

sRun

RunA1

zout

1

Ave

Data

1.6E-05

Model

1.6E-05

data/vMax

0.00011

ofxng

275.646

Min d/m

2.69025

ave/min d

5.26077

ave/min m

13.8244

325_Calib_S_A

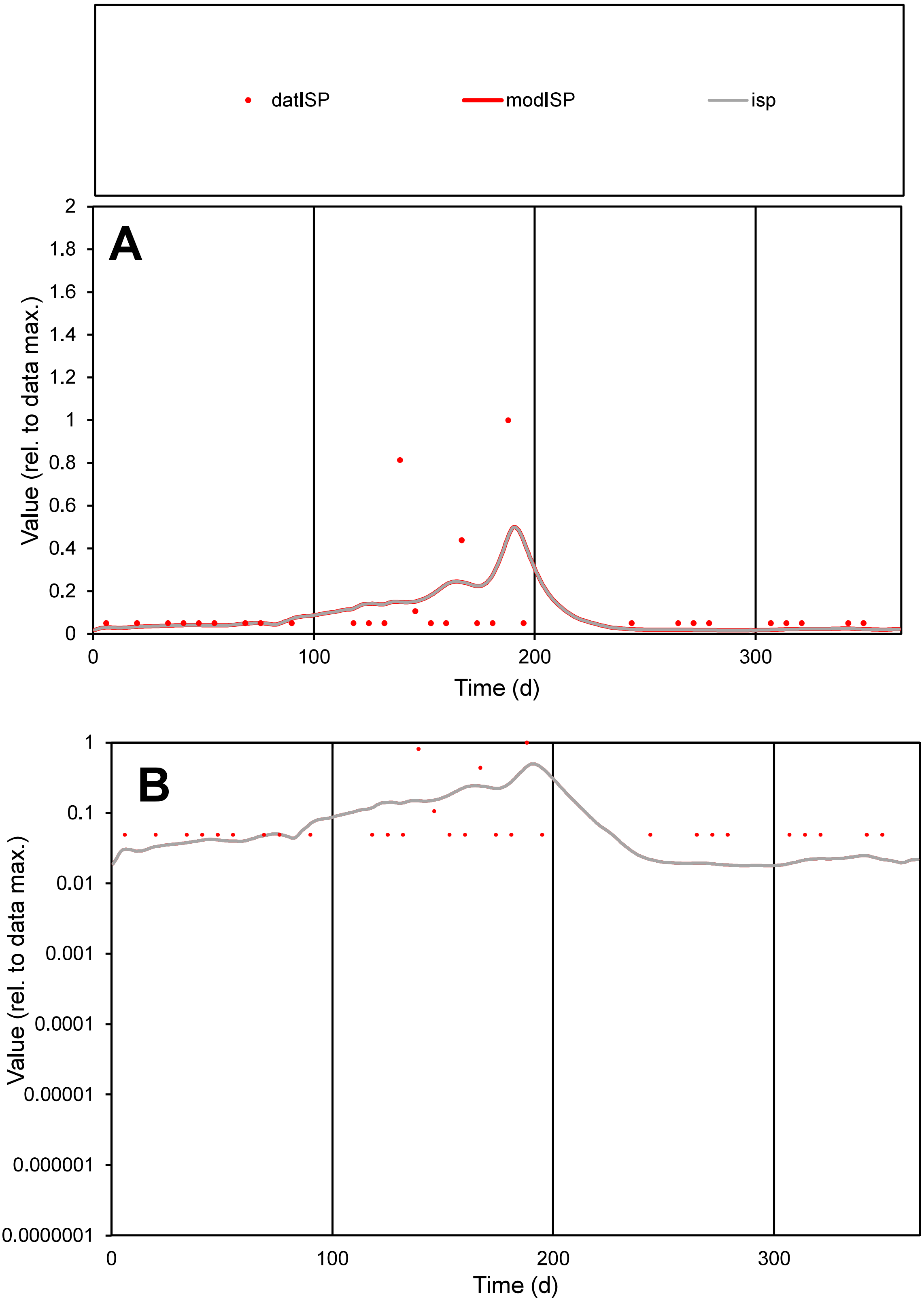

C1

Variables
ISP Provide jtd or Dst
CalibOnly?
2 1 to reproduce of
oNormalize?
2 1=no, 2=yes

ofxn calc*
1.50333
ofxn mod
1.53688

sRun
RunA1
zout
1

Ave
Data
1.2E-05
Model
1.1E-05

data/vMax
0.0001

ofxng
275.646

Min d/m
2.72688

ave/min d
2.46213
ave/min m
6.01741

325_Calib_S_A

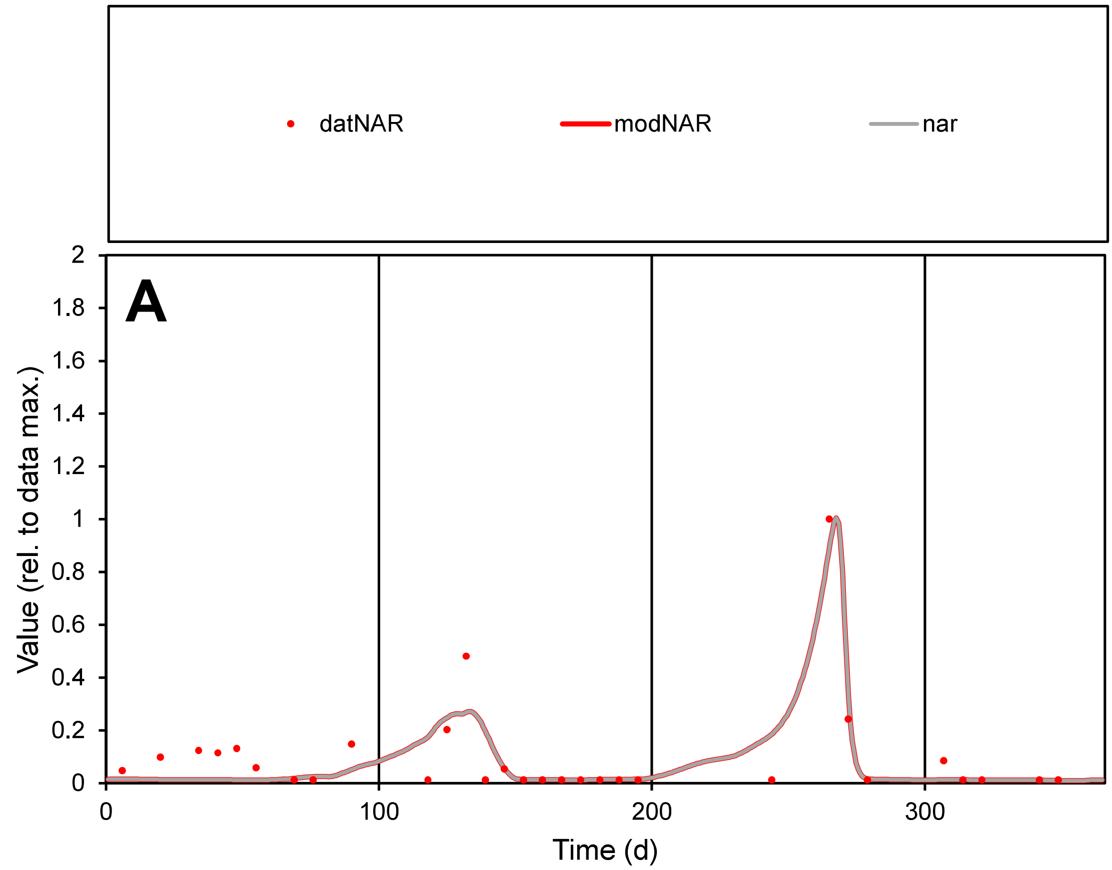

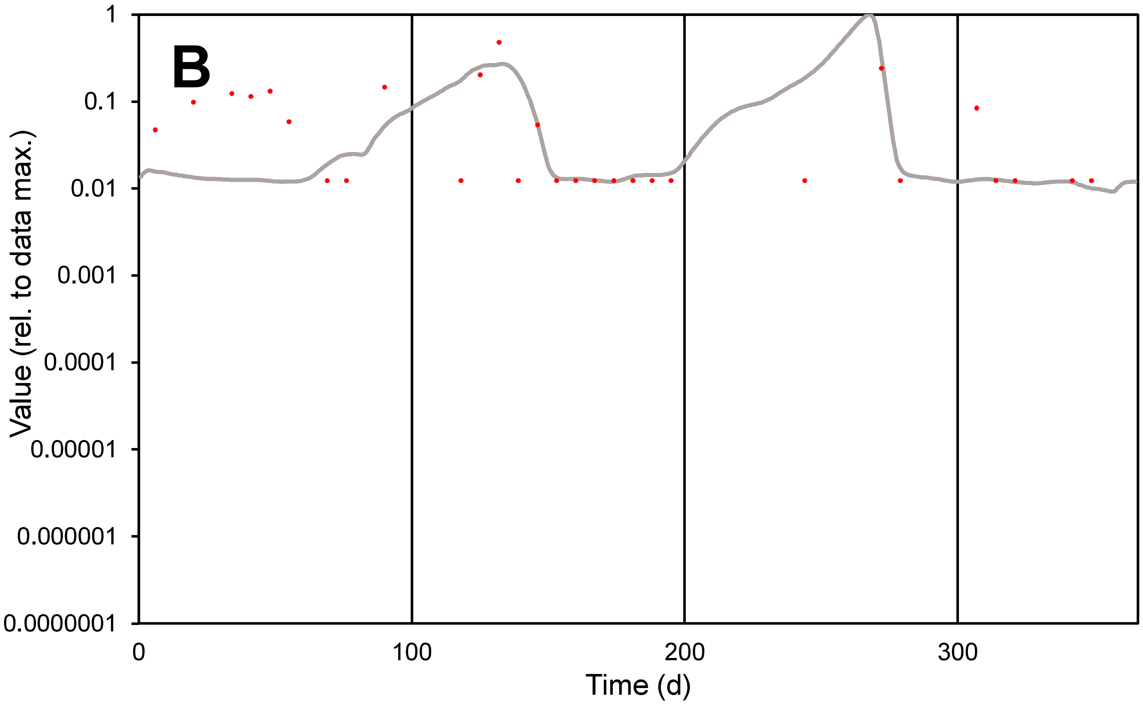

C1

Variables
NAR Provide jtd or Dst
CalibOnly?
2 1 to reproduce of
oNormalize?
2 1=no, 2=yes

ofxn calc*
0.76218
ofxn mod
0.80966

sRun
RunA1
zout
1

Ave
Data
2.3E-05
Model
2E-05

data/vMax
0.00023

ofxng
275.646

Min d/m
1.24669

ave/min d
8.08973
ave/min m
8.73236

325_Calib_S_A

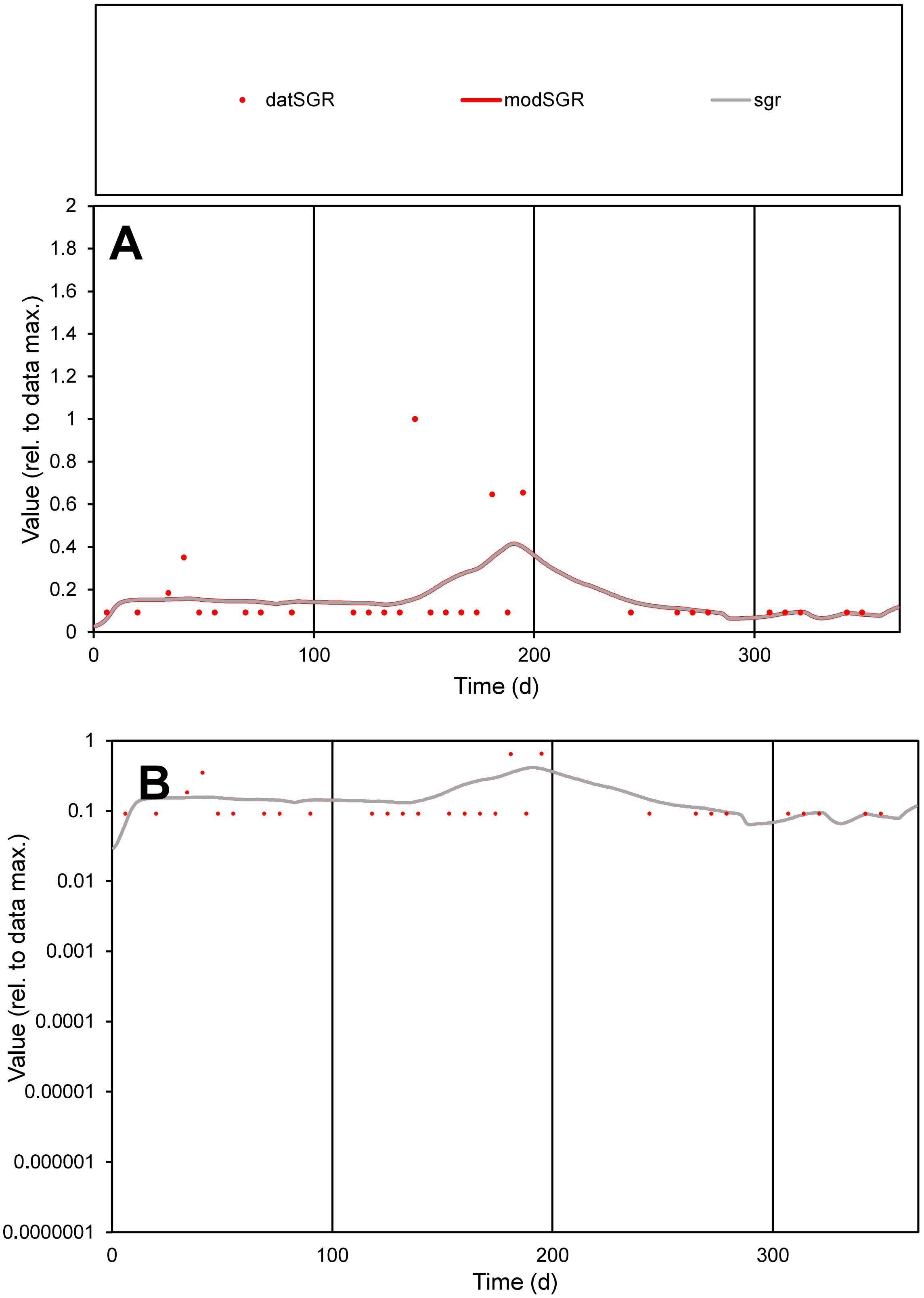

C1

Variables
SGR Provide jtd or Dst
CalibOnly?
2 1 to reproduce of
oNormalize?
2 1=no, 2=yes

ofxn calc*
1.13041
ofxn mod
1.14355

sRun
RunA1
zout
1

Ave
Data
8.4E-06
Model
8.3E-06

data/vMax
4.9E-05

ofxng
275.646

Min d/m
1.17123

ave/min d
1.86118
ave/min m
2.14281

325_Calib_S_A

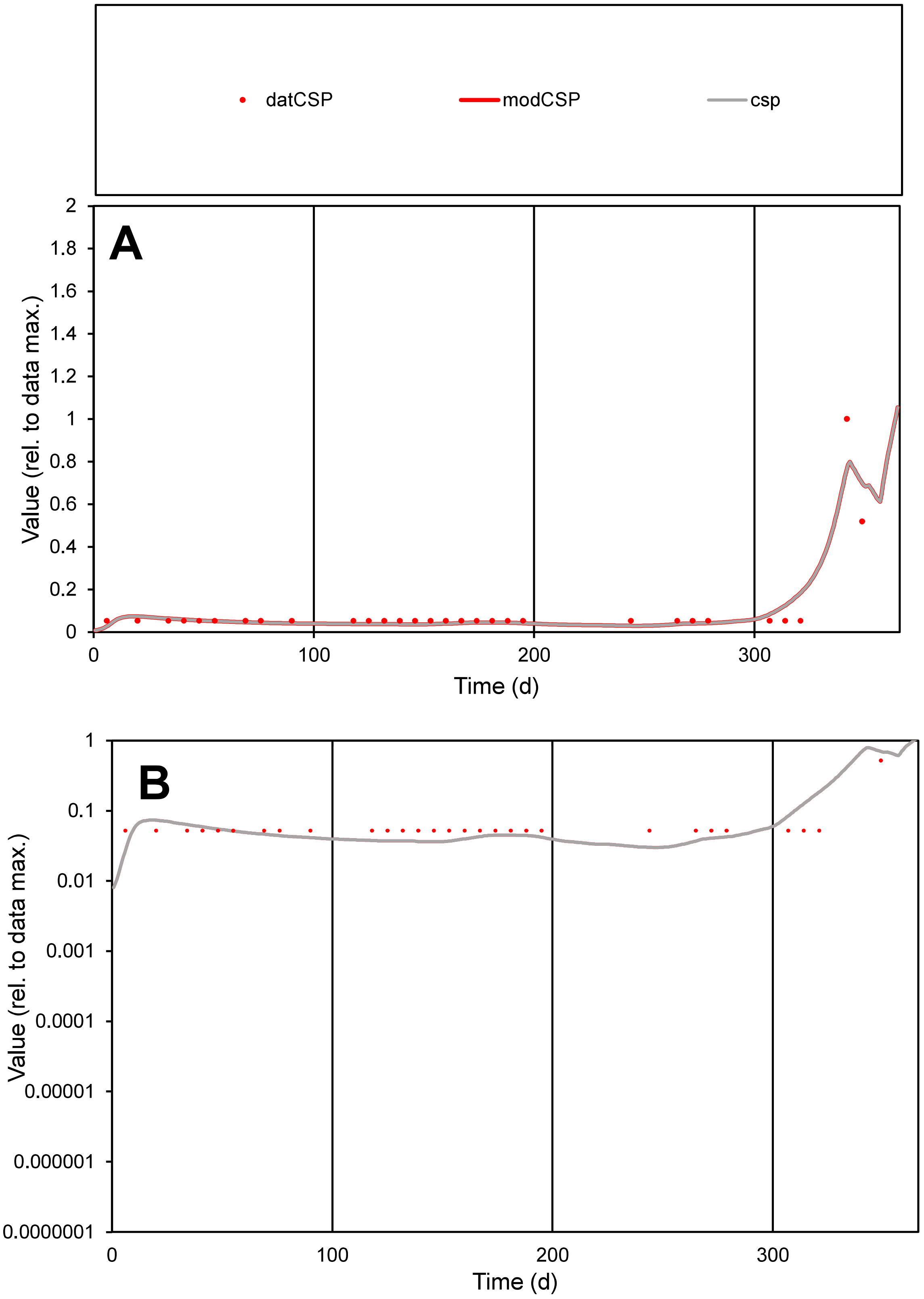

C1

Variables

CSP Provide jtd or Dst

CalibOnly?

2 1 to reproduce of

oNormalize?

2 1=no, 2=yes

ofxn calc*

0.57194

ofxn mod

0.61491

sRun

RunA1

zout

1

Ave

Data

4.5E-06

Model

4.5E-06

data/vMax

4.6E-05

ofxng

275.646

Min d/m

1.74488

ave/min d

1.90066

ave/min m

3.31674

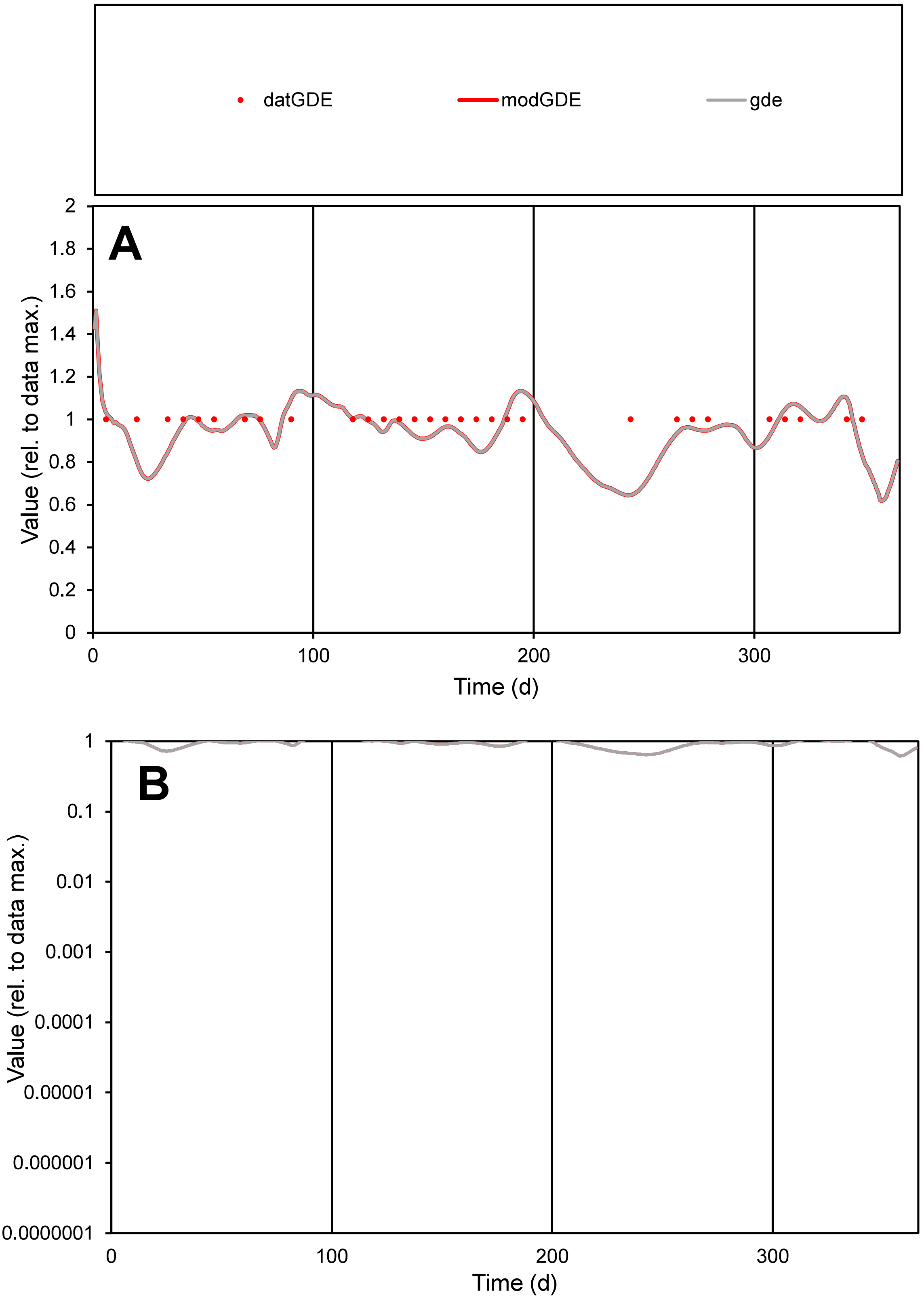

C1

Variables

GDE Provide jtd or Dst

CalibOnly?

2 1 to reproduce of

oNormalize?

2 1=no, 2=yes

ofxn calc*

0.10755

ofxn mod

0.15419

sRun

RunA1

zout

1

Ave

Data

2.7E-05

Model

2.6E-05

data/vMax

2.7E-05

ofxng

275.646

Min d/m

1.5395

ave/min d

1

ave/min m

1.47585

325_Calib_S_A

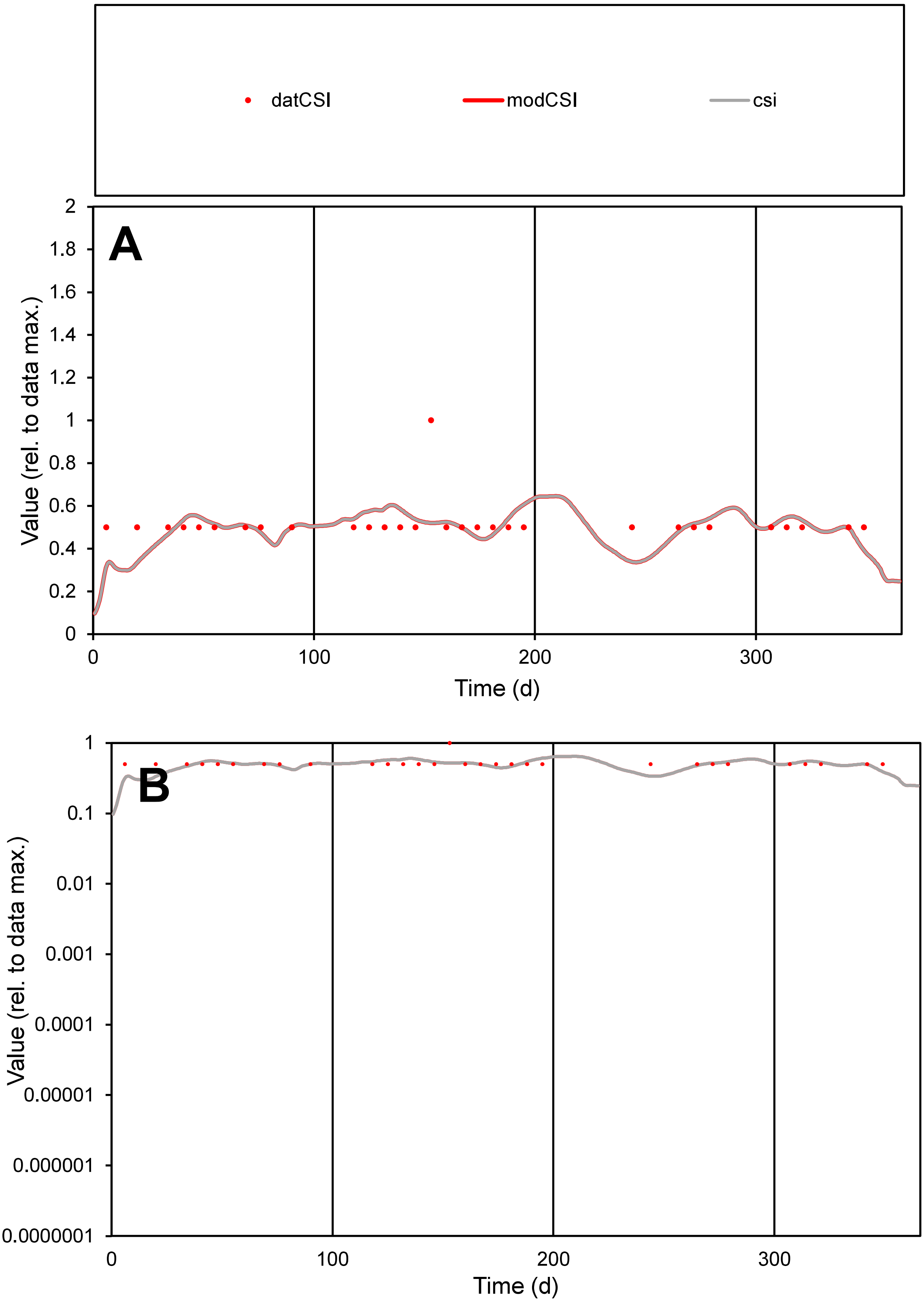

C1

Variables

CSI Provide jtd or Dst

CalibOnly?

2 1 to reproduce of

oNormalize?

2 1=no, 2=yes

ofxn calc*

0.21792

ofxn mod

0.24449

sRun

RunA1

zout

1

Ave

Data

2.4E-06

Model

2.3E-06

data/vMax

4.6E-06

ofxng

275.646

Min d/m

1.48362

ave/min d

1.03335

ave/min m

1.48917

325_Calib_S_A

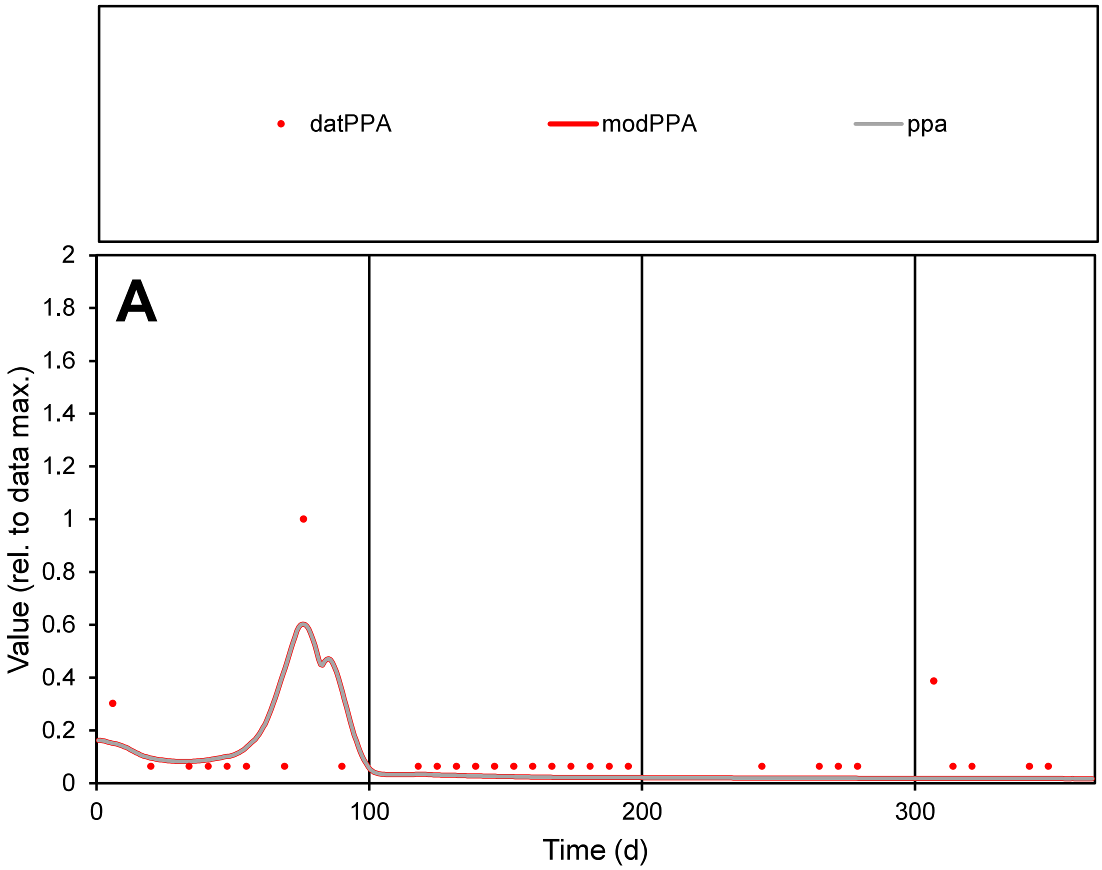

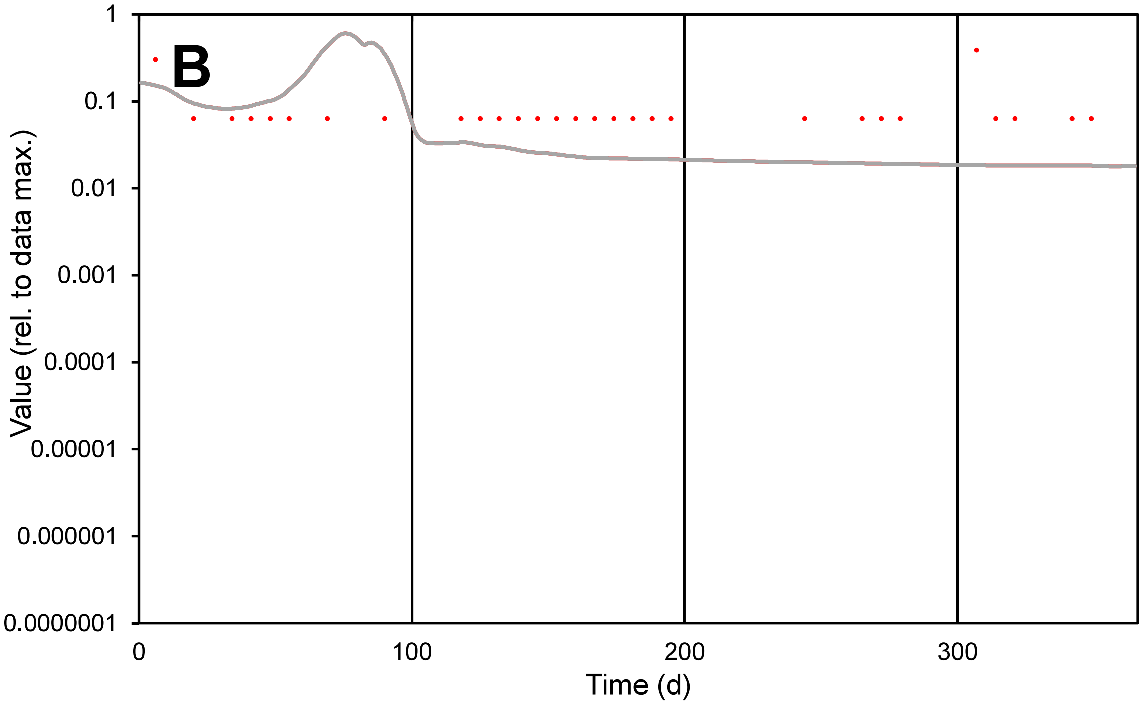

C1

Variables
PPA Provide jtd or Dst
CalibOnly?
2 1 to reproduce of
oNormalize?
2 1=no, 2=yes

ofxn calc*
1.16971
ofxn mod
1.19962

sRun
RunA1
zout
1

Ave
Data
5.7E-06
Model
4.1E-06

data/vMax
5.1E-05

ofxng
275.646

Min d/m
3.48454

ave/min d
1.79069
ave/min m
4.402

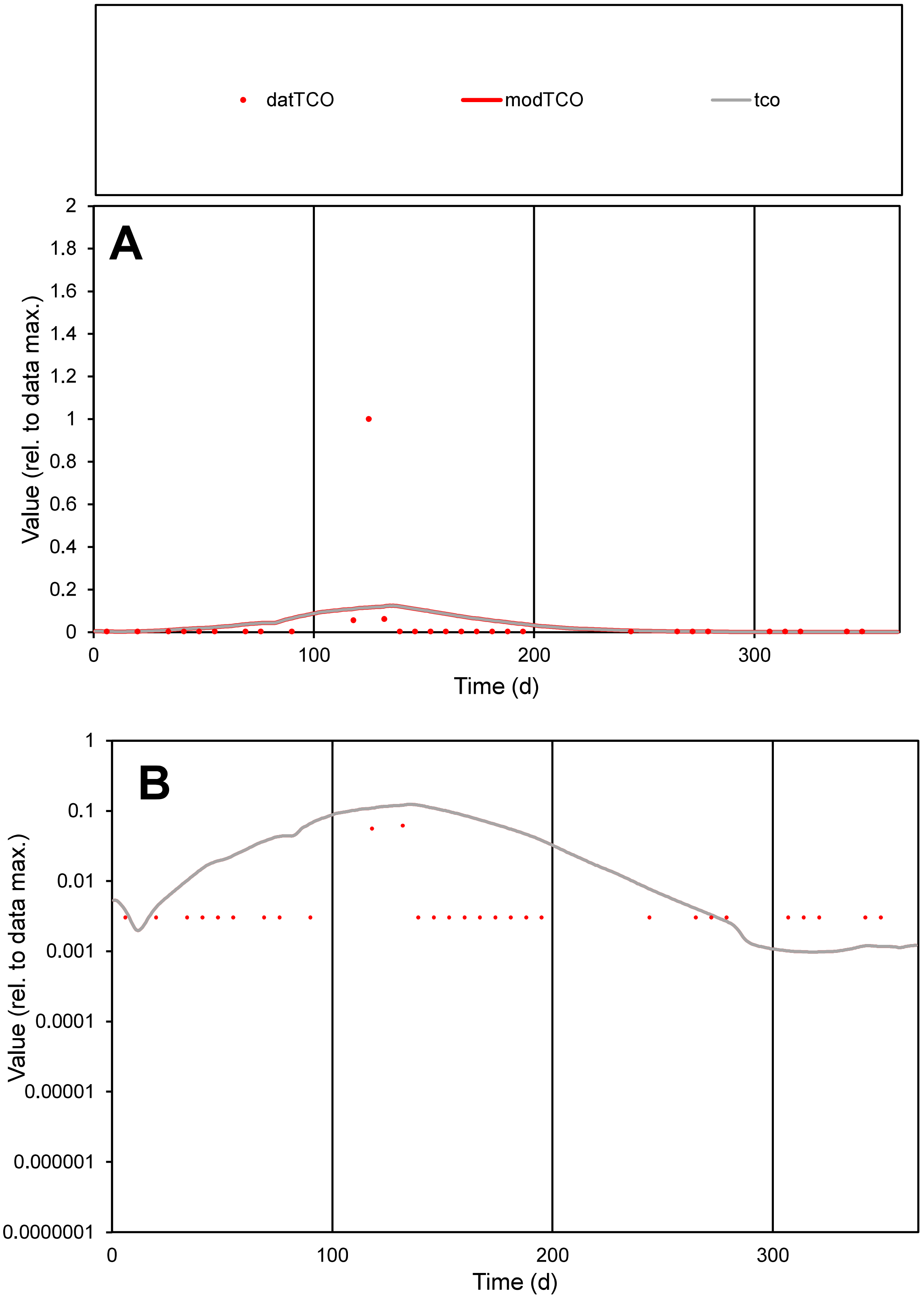

C1

Variables
TCO Provide jtd or Dst
CalibOnly?
2 1 to reproduce of
oNormalize?
2 1=no, 2=yes

ofxn calc*
4.19707
ofxn mod
4.20999

sRun
RunA1
zout
1

Ave
Data
0.00011
Model
0.00012

data/vMax
0.00283

ofxng
275.646

Min d/m
3.11525

ave/min d
13.1405
ave/min m
43.2451

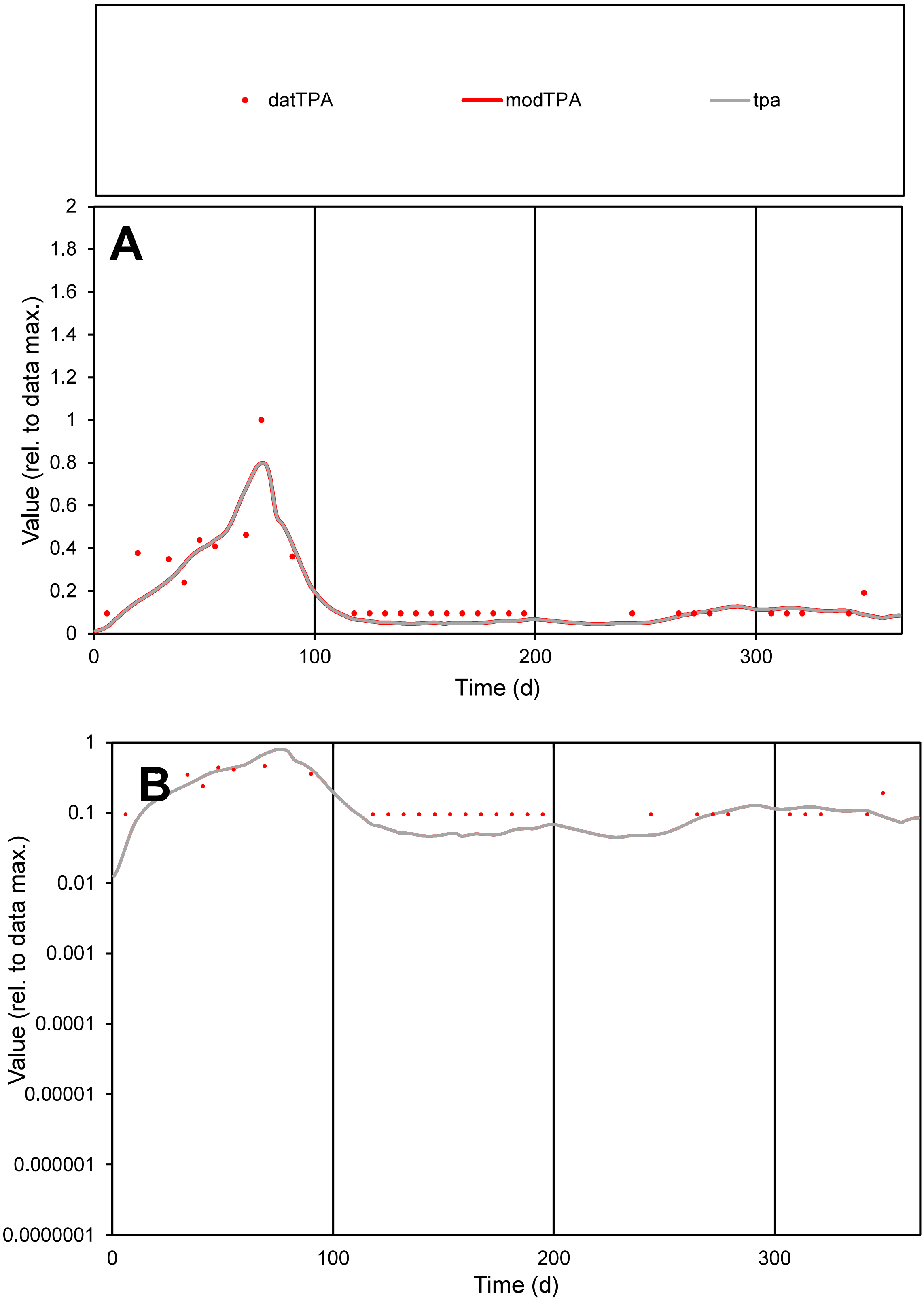

C1

Variables
TPA Provide jtd or Dst
CalibOnly?
2 1 to reproduce of
oNormalize?
2 1=no, 2=yes

ofxn calc*
0.4143
ofxn mod
0.45318

sRun
RunA1
zout
1

Ave
Data
9.6E-06
Model
8.4E-06

data/vMax
5E-05

ofxng
275.646

Min d/m
2.01967

ave/min d
2.03938
ave/min m
3.5713

325_Calib_S_A

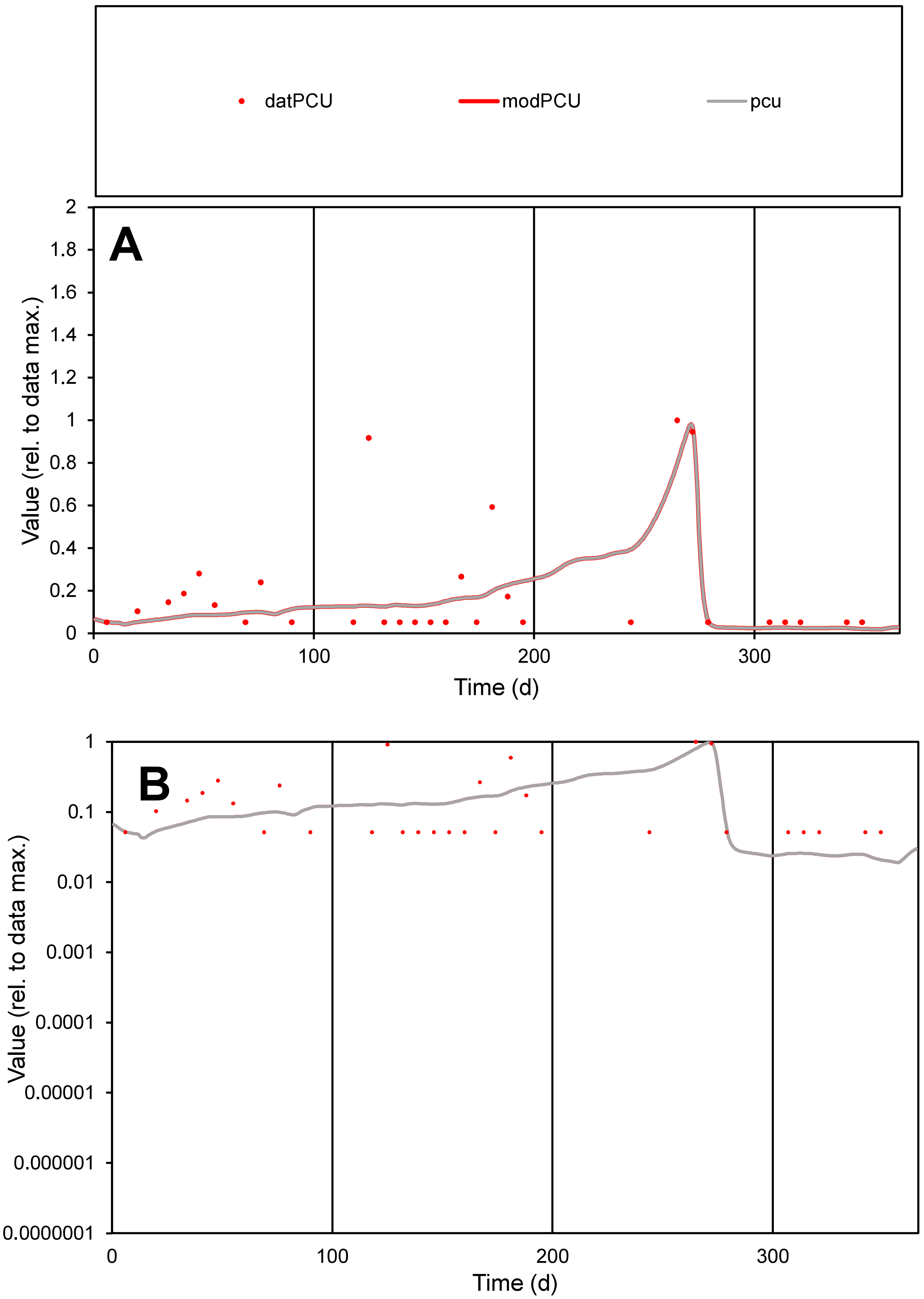

C1

Variables
PCU Provide jtd or Dst
CalibOnly?
2 1 to reproduce of
oNormalize?
2 1=no, 2=yes

ofxn calc*
0.9762
ofxn mod
0.99283

sRun
RunA1
zout
1

Ave
Data
2.4E-05
Model
2E-05

data/vMax
0.00012

ofxng
275.646

Min d/m
2.52816

ave/min d
3.81779
ave/min m
8.1977

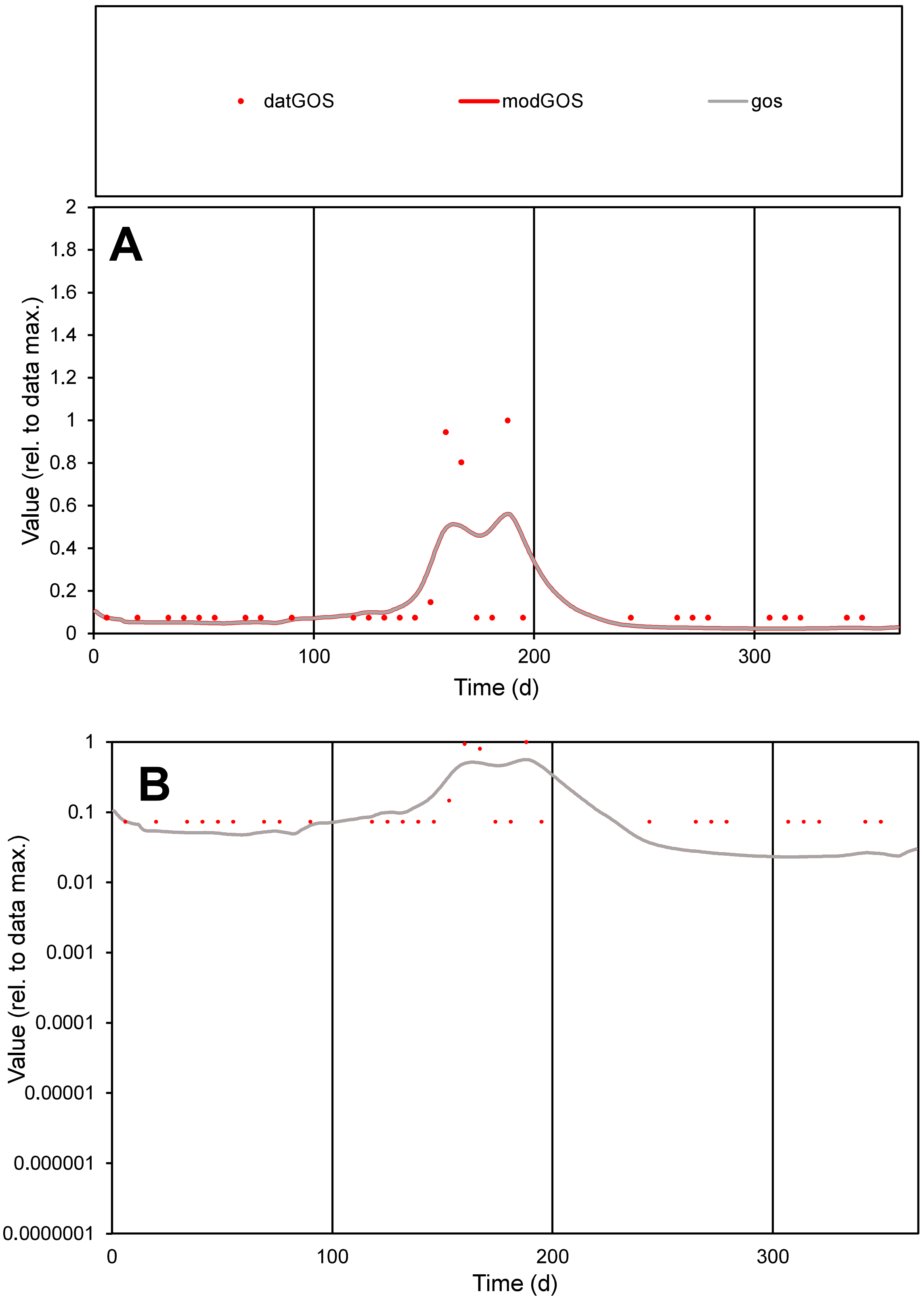

C1

Variables
GOS Provide jtd or Dst
CalibOnly?
2 1 to reproduce of
oNormalize?
2 1=no, 2=yes

ofxn calc*
1.13526
ofxn mod
1.15108

sRun
RunA1
zout
1

Ave
Data
4E-06
Model
3.8E-06

data/vMax
2.5E-05

ofxng
275.646

Min d/m
3.19126

ave/min d
2.18076
ave/min m
6.64833

325_Calib_S_A

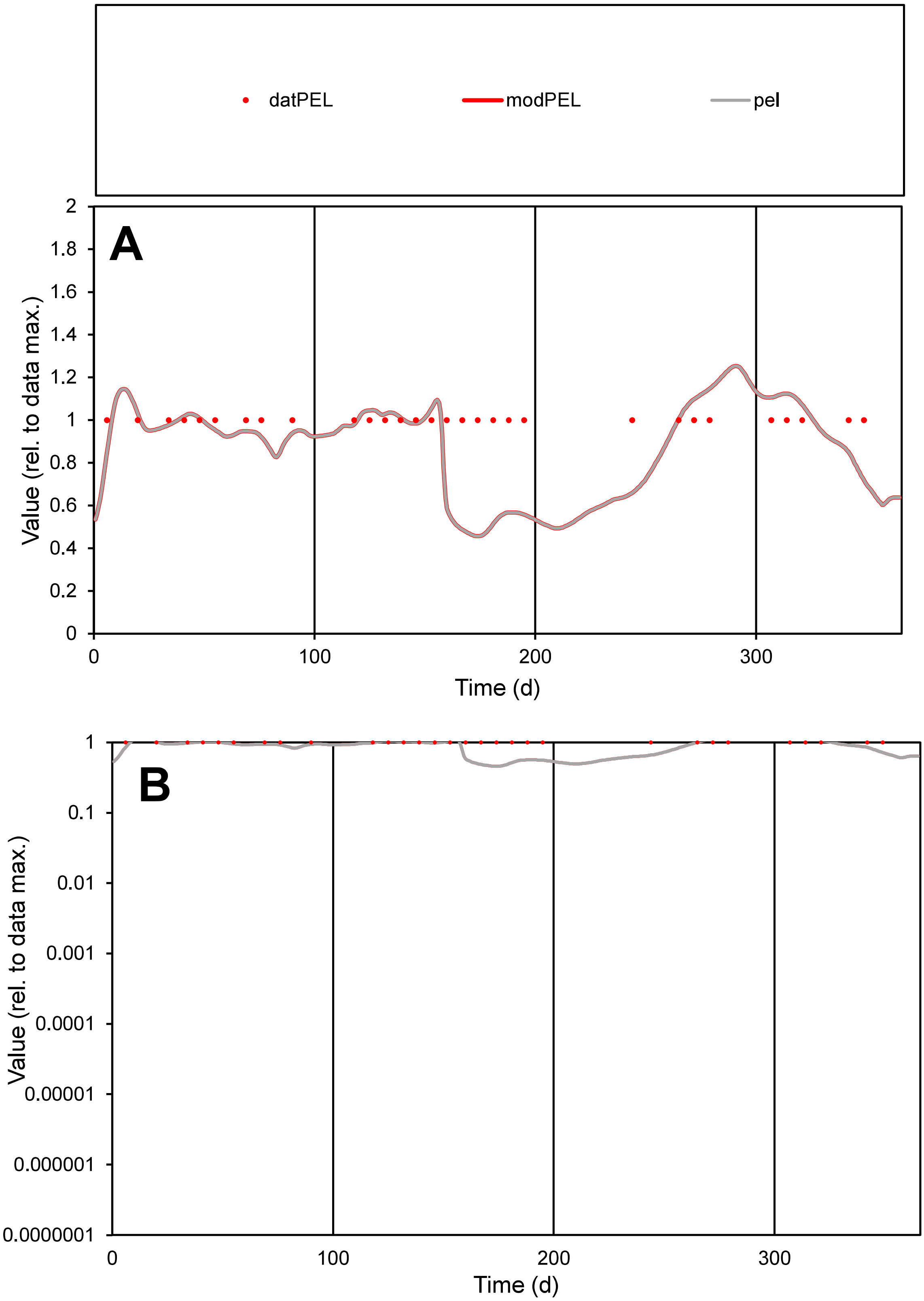

C1

Variables
PEL Provide jtd or Dst
CalibOnly?
2 1 to reproduce of
oNormalize?
2 1=no, 2=yes

ofxn calc*
0.23832
ofxn mod
0.26191

sRun
RunA1
zout
1

Ave
Data
3.3E-06
Model
2.9E-06

data/vMax
3.3E-06

ofxng
275.646

Min d/m
2.18218

ave/min d
1
ave/min m
1.94648

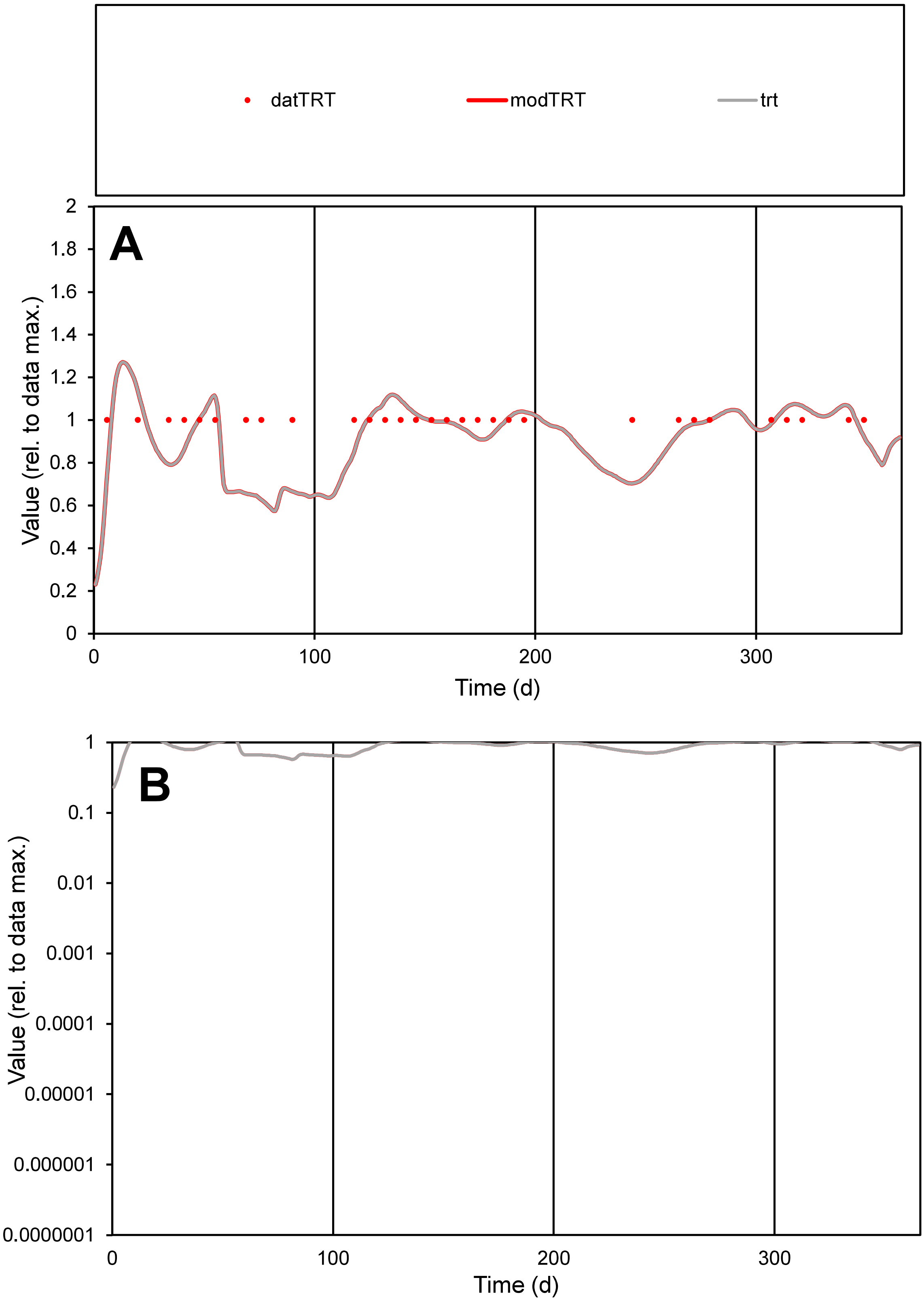

C1

Variables
TRT Provide jtd or Dst
CalibOnly?
2 1 to reproduce of
oNormalize?
2 1=no, 2=yes

ofxn calc*
0.1425
ofxn mod
0.17994

sRun
RunA1
zout
1

Ave
Data
2.3E-05
Model
2.2E-05

data/vMax
2.3E-05

ofxng
275.646

Min d/m
1.61915

ave/min d
1
ave/min m
1.52986

325_Calib_S_A

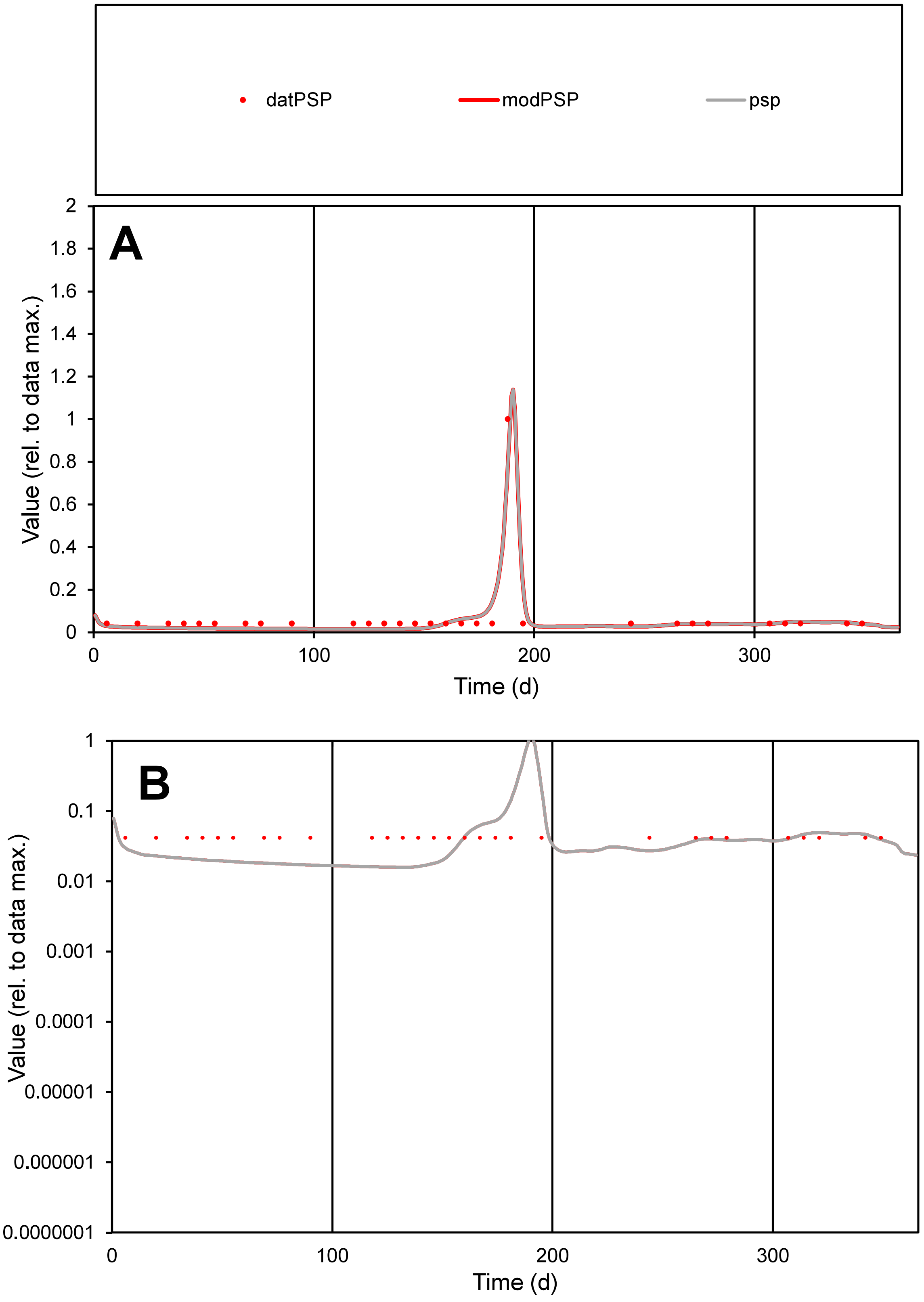

C1

Variables
PSP Provide jtd or Dst
CalibOnly?
2 1 to reproduce of
oNormalize?
2 1=no, 2=yes

ofxn calc*
0.32818
ofxn mod
0.40258

sRun
RunA1
zout
1

Ave
Data
7.6E-06
Model
7E-06

data/vMax
0.0001

ofxng
275.646

Min d/m
2.63354

ave/min d
1.76315
ave/min m
4.26321

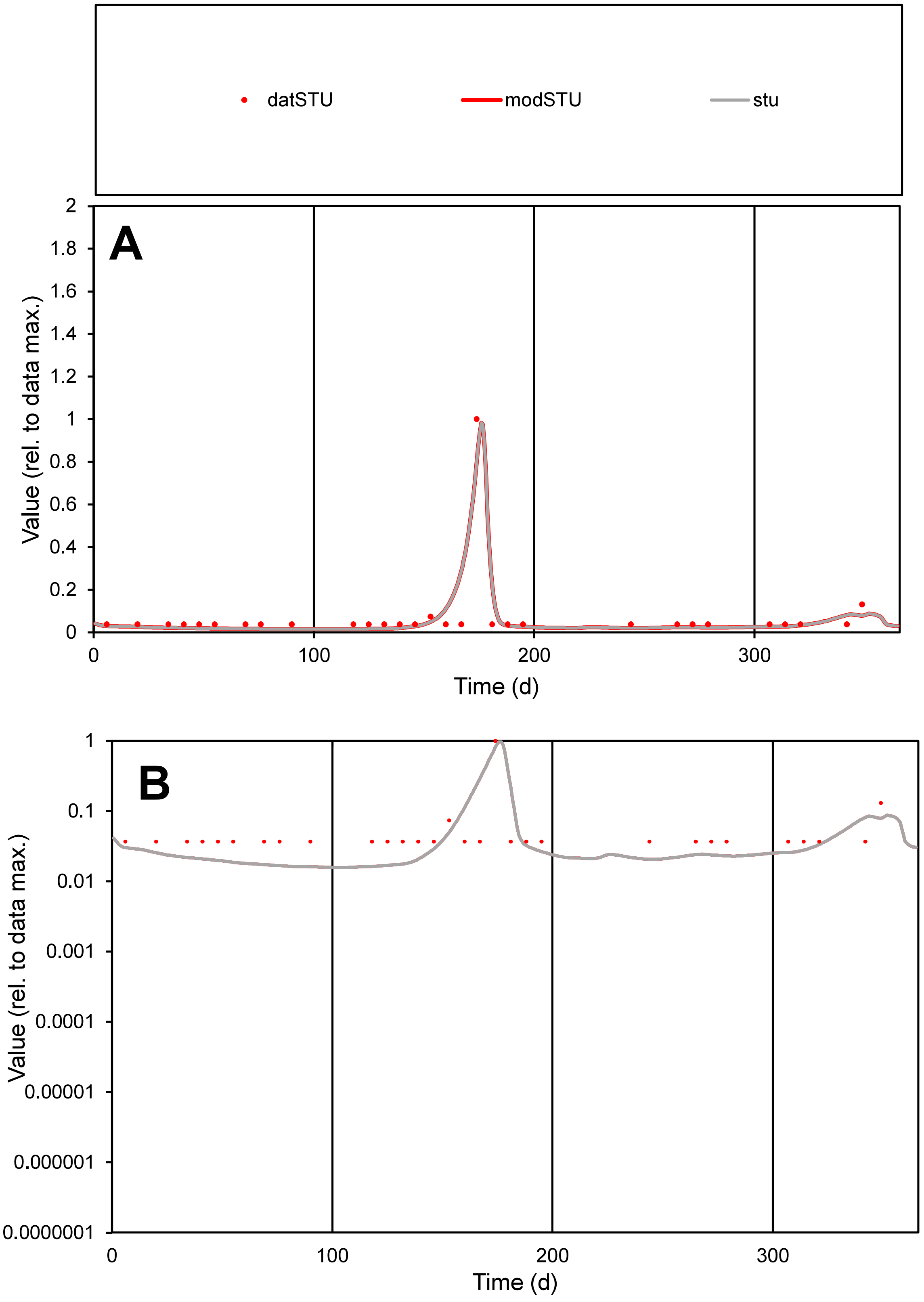

C1

Variables
STU Provide jtd or Dst
CalibOnly?
2 1 to reproduce of
oNormalize?
2 1=no, 2=yes

ofxn calc*
0.57746
ofxn mod
0.73179

sRun
RunA1
zout
1

Ave
Data
4.9E-06
Model
4.7E-06

data/vMax
6.7E-05

ofxng
275.646

Min d/m
2.2977

ave/min d
1.98387
ave/min m
4.32037

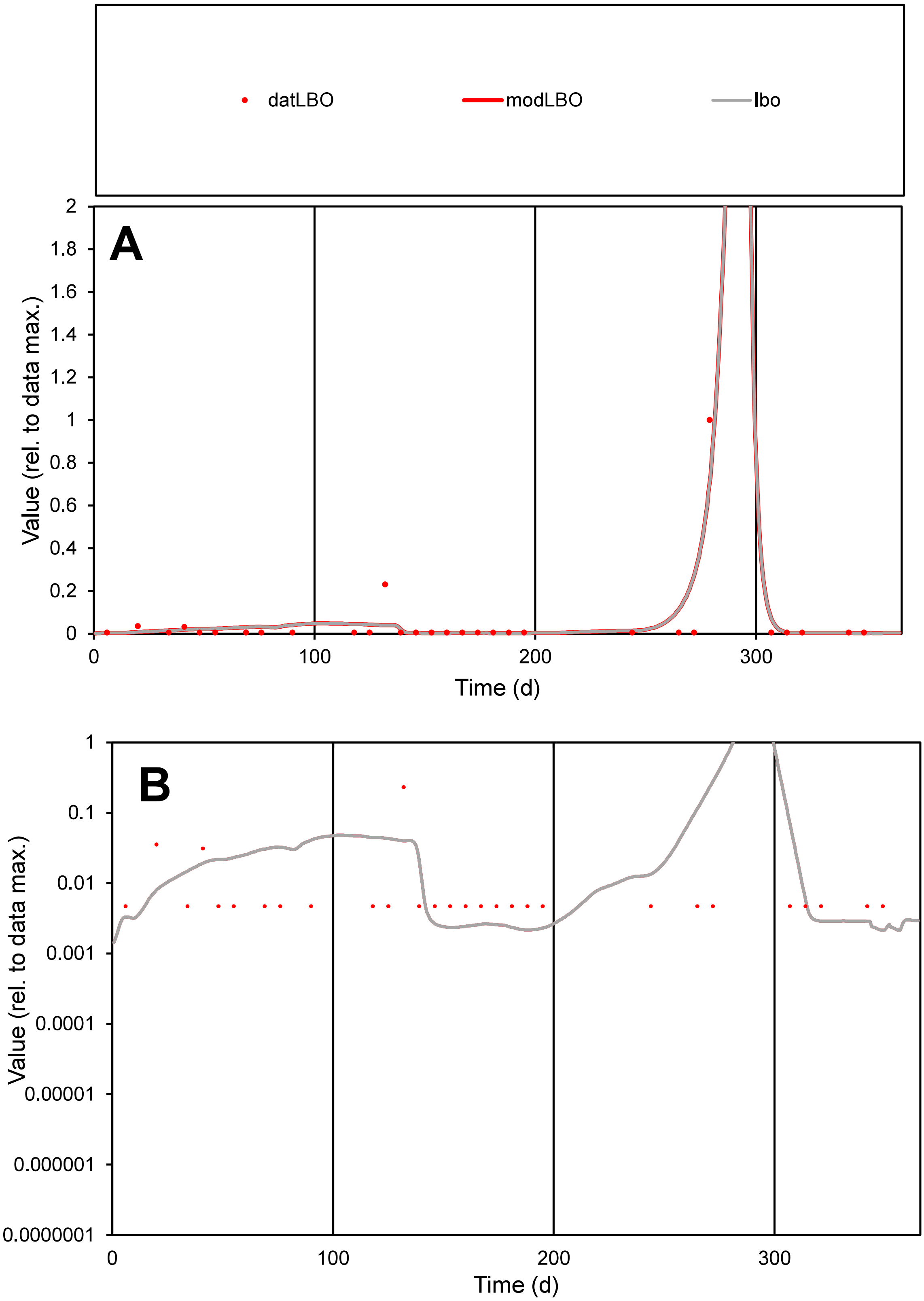

C1

Variables
LBO Provide jtd or Dst
CalibOnly?
2 1 to reproduce of
oNormalize?
2 1=no, 2=yes

ofxn calc*
1.17096
ofxn mod
1.46428

sRun
RunA1
zout
1

Ave
Data
1.4E-05
Model
1.6E-05

data/vMax
0.00029

ofxng
275.646

Min d/m
2.17671

ave/min d
10.0306
ave/min m
24.5555

325_Calib_S_A

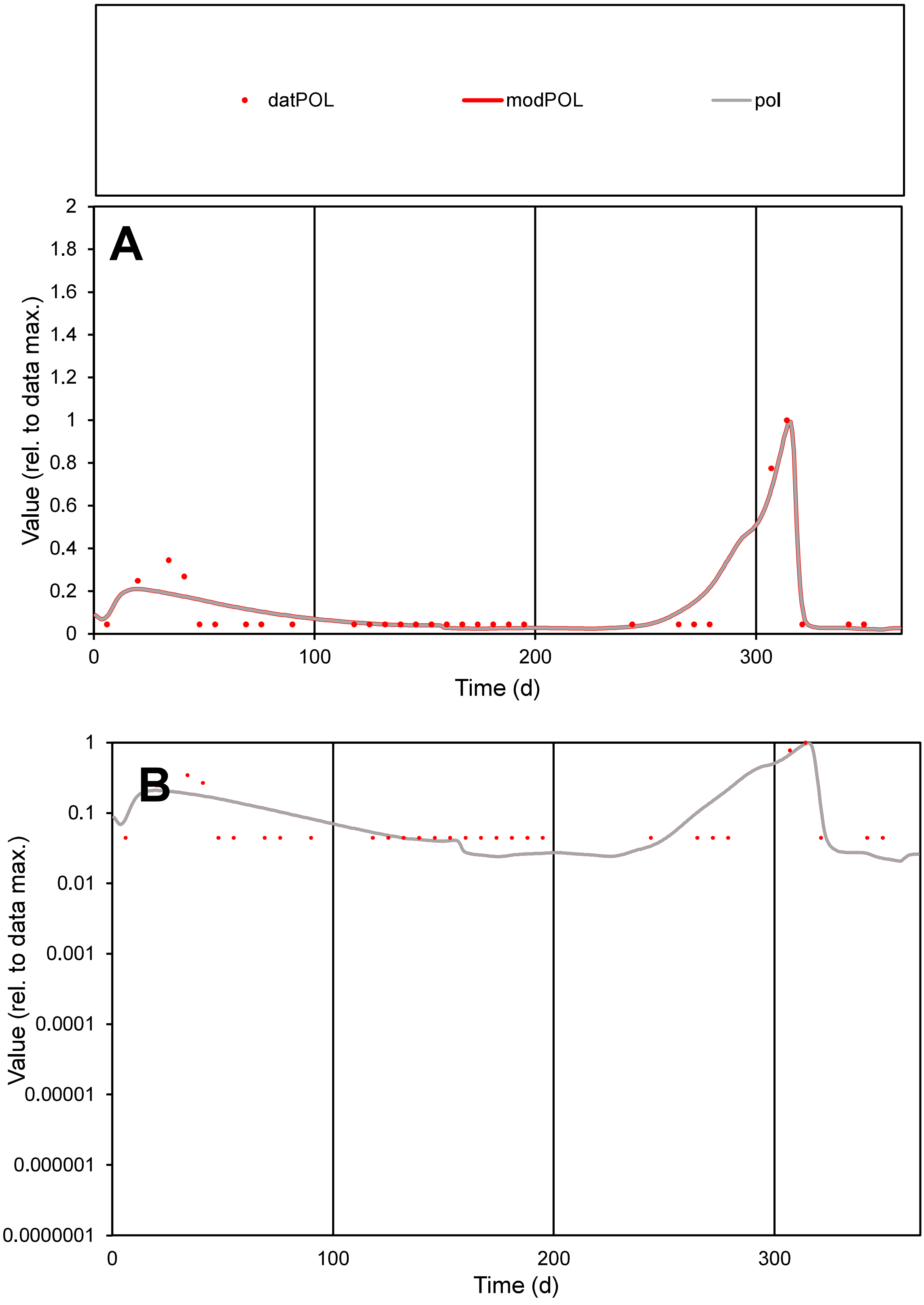

C1

Variables
POL Provide jtd or Dst
CalibOnly?
2 1 to reproduce of
oNormalize?
2 1=no, 2=yes

ofxn calc*
0.5024
ofxn mod
0.5679

sRun
RunA1
zout
1

Ave
Data
4.5E-06
Model
4.8E-06

data/vMax
3.6E-05

ofxng
275.646

Min d/m
1.99525

ave/min d
2.81571
ave/min m
5.9933

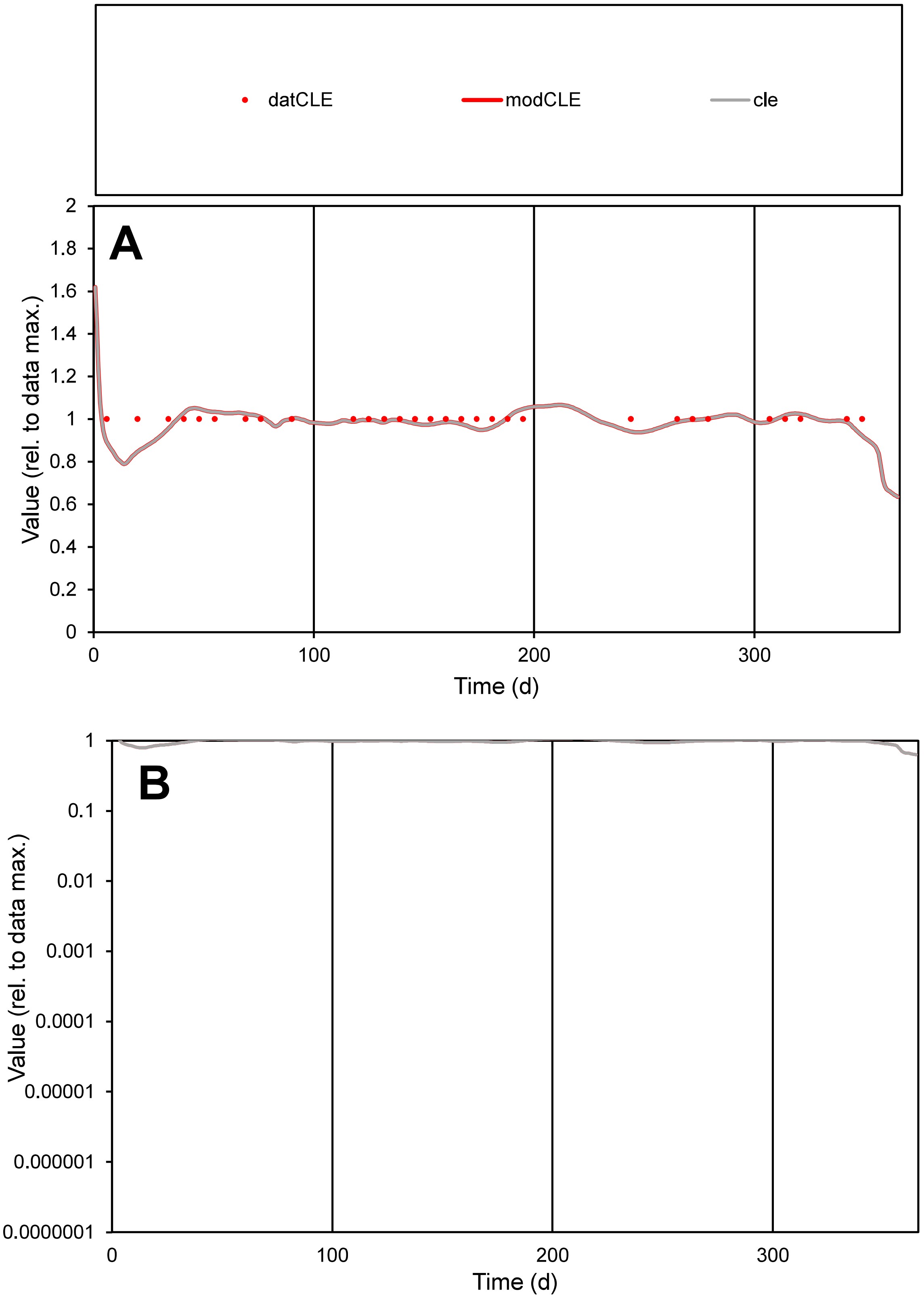

C1

Variables
CLE Provide jtd or Dst
CalibOnly?
2 1 to reproduce of
oNormalize?
2 1=no, 2=yes

ofxn calc*
0.04704
ofxn mod
0.12144

sRun
RunA1
zout
1

Ave
Data
4.7E-06
Model
4.6E-06

data/vMax
4.7E-06

ofxng
275.646

Min d/m
1.16845

ave/min d
1
ave/min m
1.15185

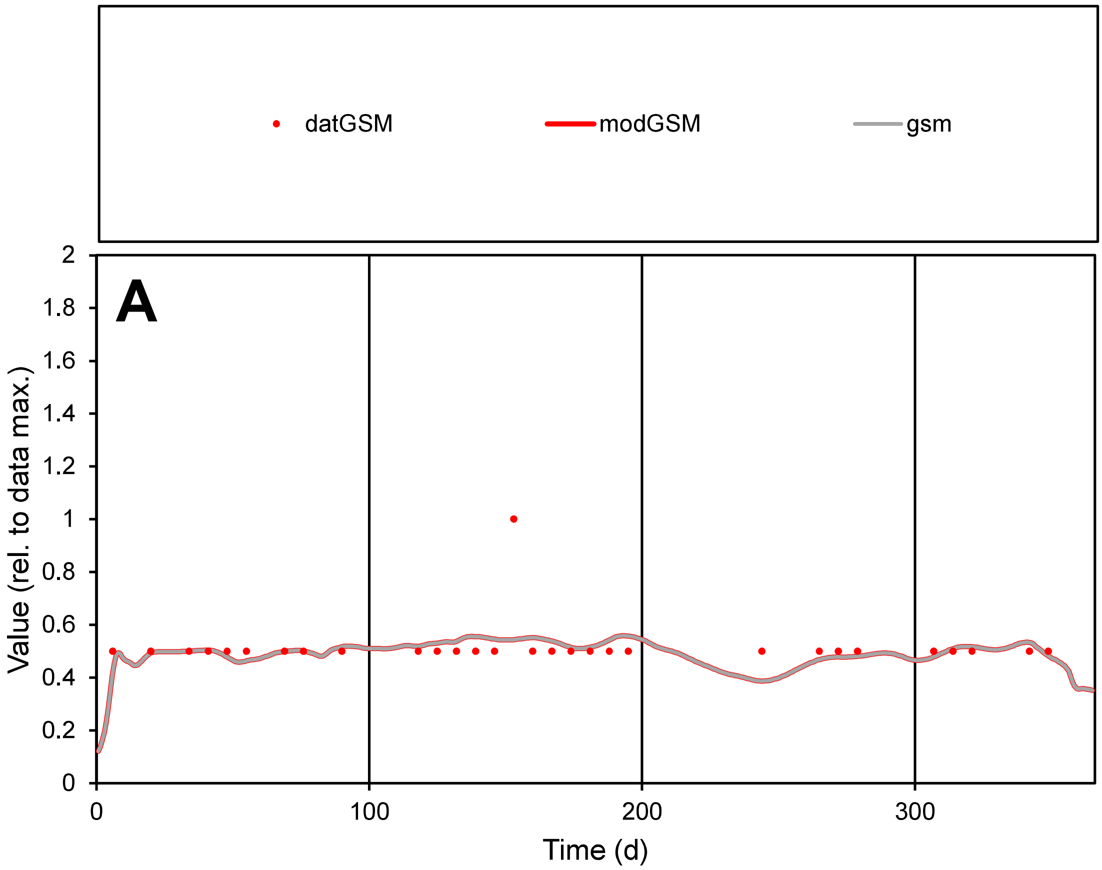

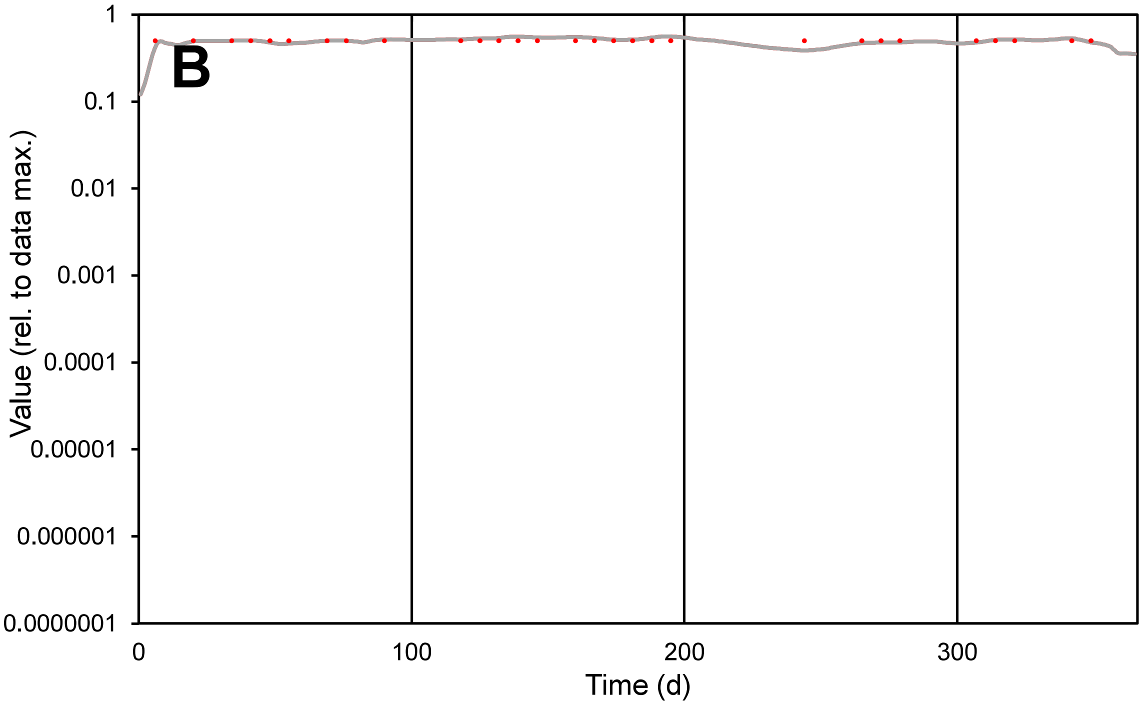

C1

Variables
GSM Provide jtd or Dst
CalibOnly?
2 1 to reproduce of
oNormalize?
2 1=no, 2=yes

ofxn calc*
0.17436
ofxn mod
0.20628

sRun
RunA1
zout
1

Ave
Data
1.6E-06
Model
1.6E-06

data/vMax
3.2E-06

ofxng
275.646

Min d/m
1.29003

ave/min d
1.03333
ave/min m
1.31122

325_Calib_S_A

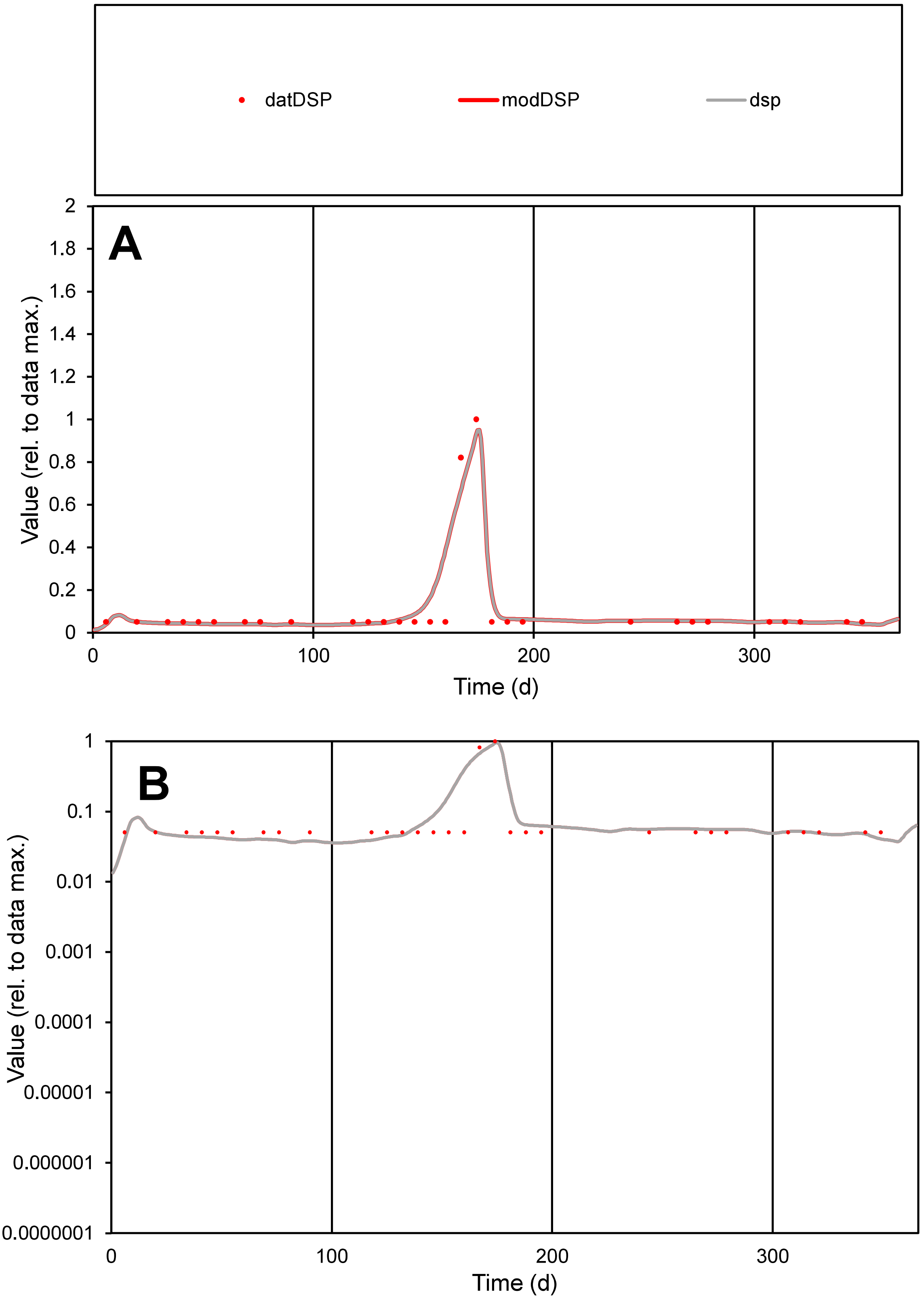

C1

Variables
DSP Provide jtd or Dst
CalibOnly?
2 1 to reproduce of
oNormalize?
2 1=no, 2=yes

ofxn calc*
0.59779
ofxn mod
0.66594

sRun
RunA1
zout
1

Ave
Data
1.1E-05
Model
1.2E-05

data/vMax
9.9E-05

ofxng
275.646

Min d/m
1.32198

ave/min d
2.13513
ave/min m
3.07126

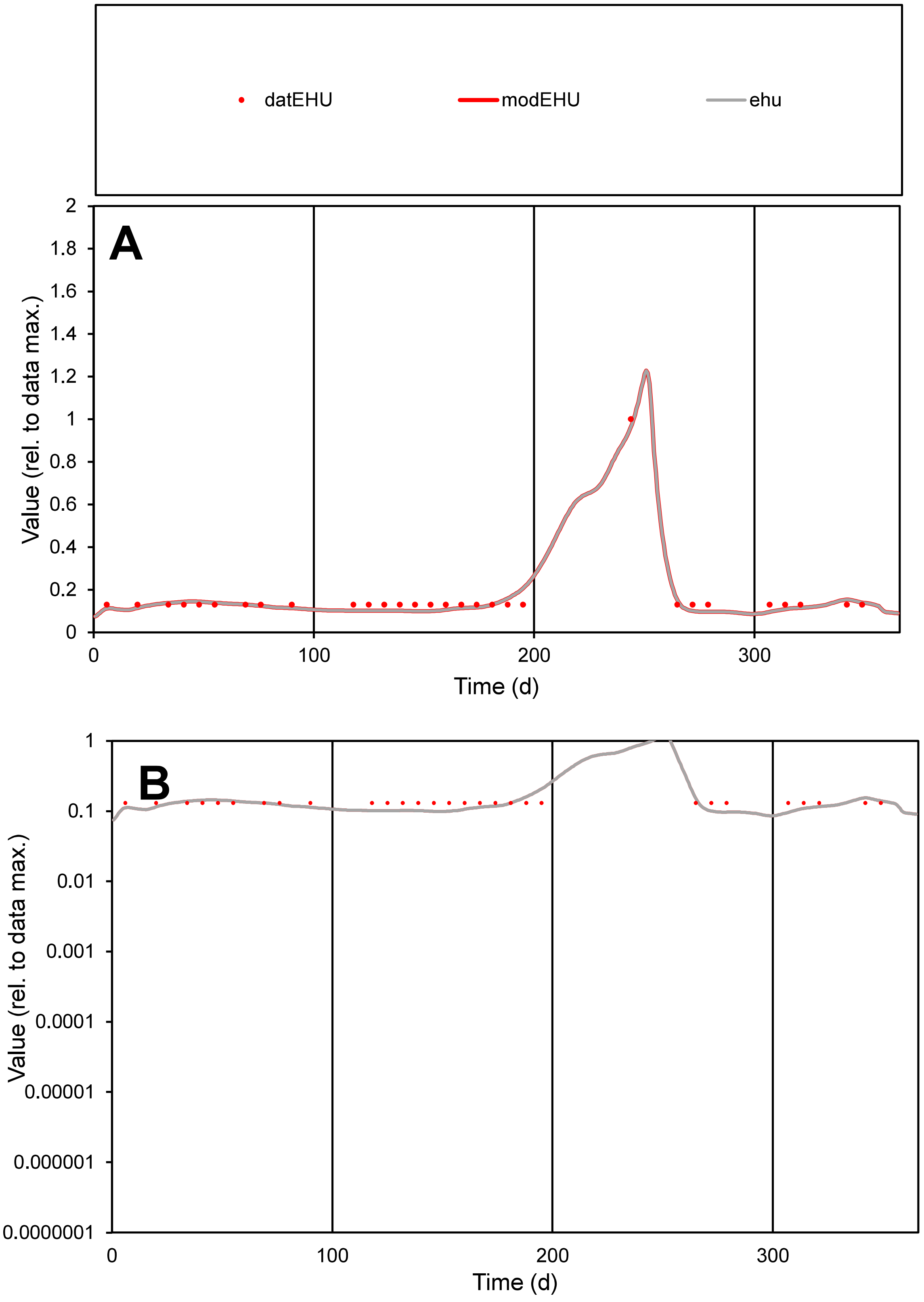

C1

Variables
EHU Provide jtd or Dst
CalibOnly?
2 1 to reproduce of
oNormalize?
2 1=no, 2=yes

ofxn calc*
0.16094
ofxn mod
0.20462

sRun
RunA1
zout
1

Ave
Data
7E-06
Model
6.7E-06

data/vMax
4.4E-05

ofxng
275.646

Min d/m
1.34118

ave/min d
1.22256
ave/min m
1.56747

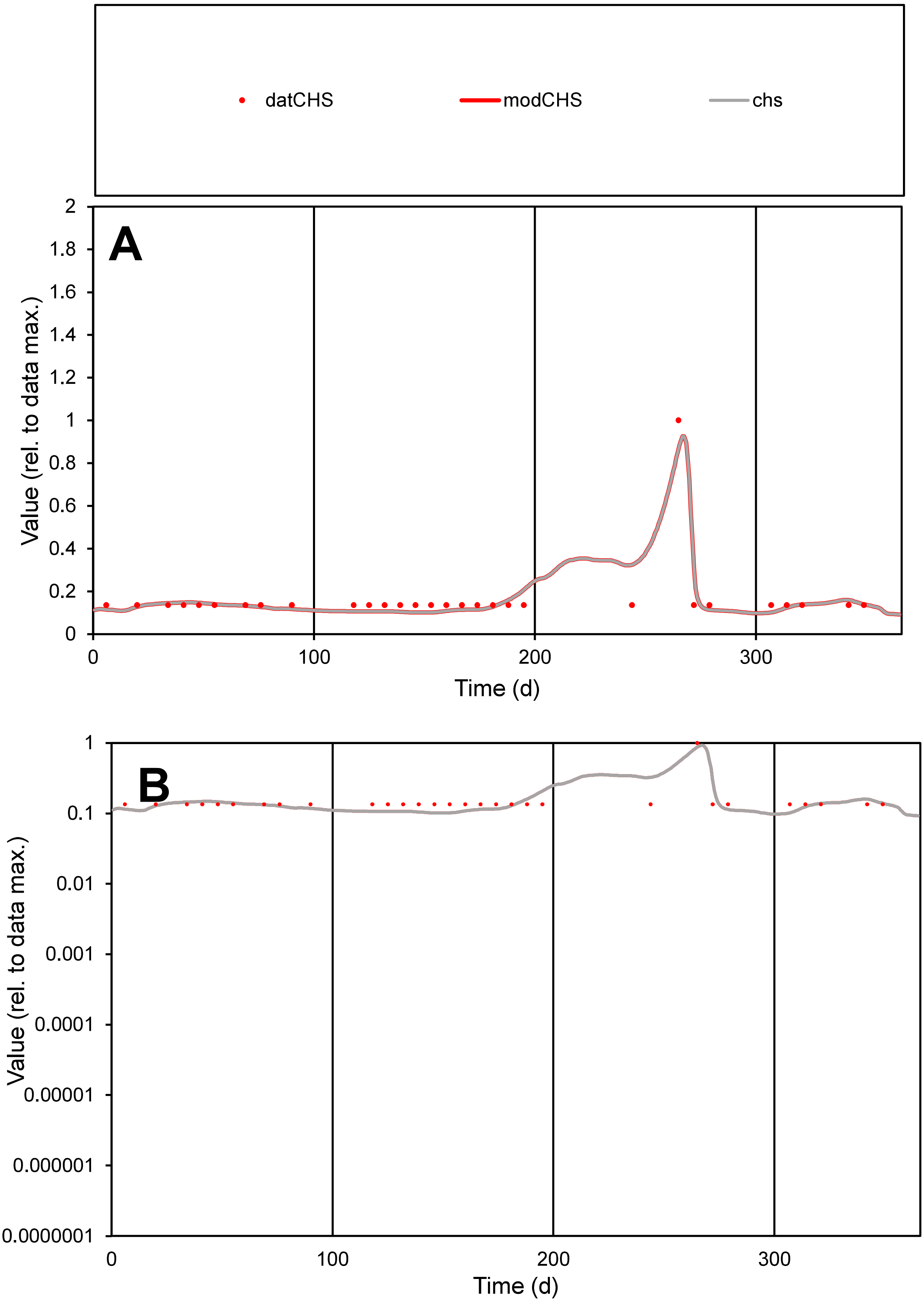

C1

Variables
CHS Provide jtd or Dst
CalibOnly?
2 1 to reproduce of
oNormalize?
2 1=no, 2=yes

ofxn calc*
0.28054
ofxn mod
0.3172

sRun
RunA1
zout
1

Ave
Data
2.6E-05
Model
2.6E-05

data/vMax
0.00016

ofxng
275.646

Min d/m
1.33271

ave/min d
1.21367
ave/min m
1.60399

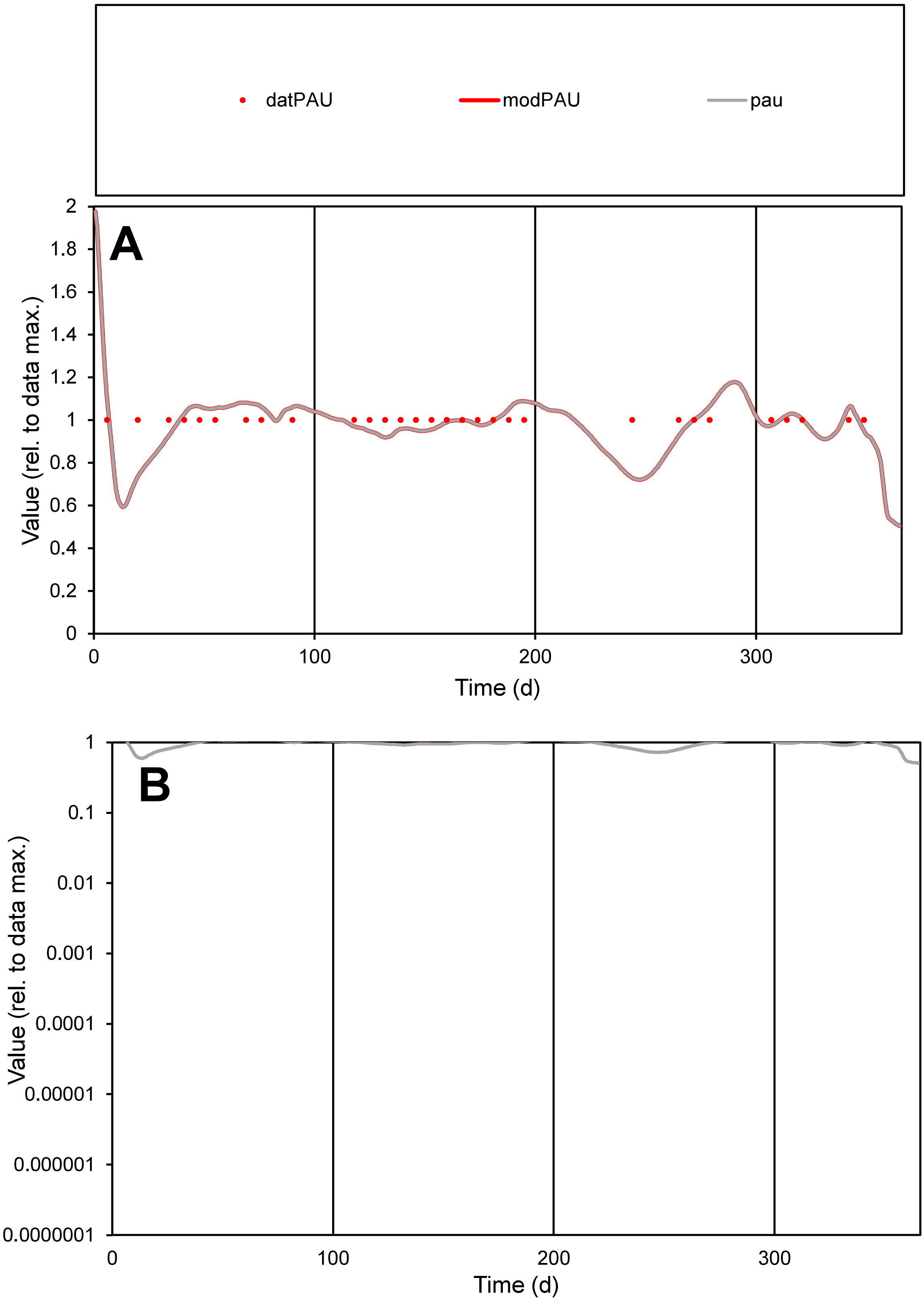

C1

Variables
PAU Provide jtd or Dst
CalibOnly?
2 1 to reproduce of
oNormalize?
2 1=no, 2=yes

ofxn calc*
0.08427
ofxn mod
0.1406

sRun
RunA1
zout
1

Ave
Data
3.9E-06
Model
3.9E-06

data/vMax
3.9E-06

ofxng
275.646

Min d/m
1.38012

ave/min d
1
ave/min m
1.36248

325_Calib_S_A

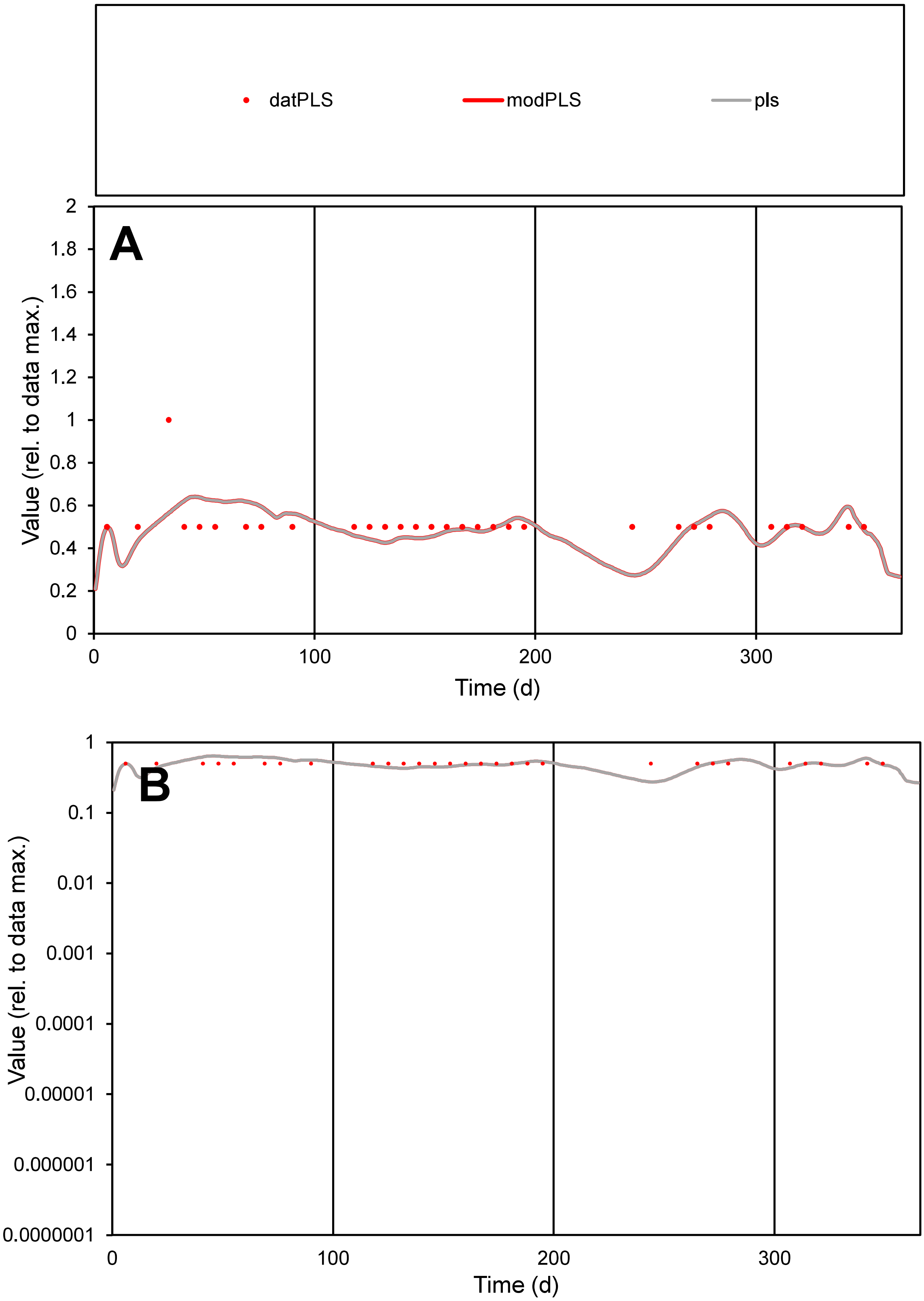

C1

Variables
PLS Provide jtd or Dst
CalibOnly?
2 1 to reproduce of
oNormalize?
2 1=no, 2=yes

ofxn calc*
0.20699
ofxn mod
0.23684

sRun
RunA1
zout
1

Ave
Data
4E-06
Model
3.9E-06

data/vMax
7.7E-06

ofxng
275.646

Min d/m
1.82644

ave/min d
1.03332
ave/min m
1.84603

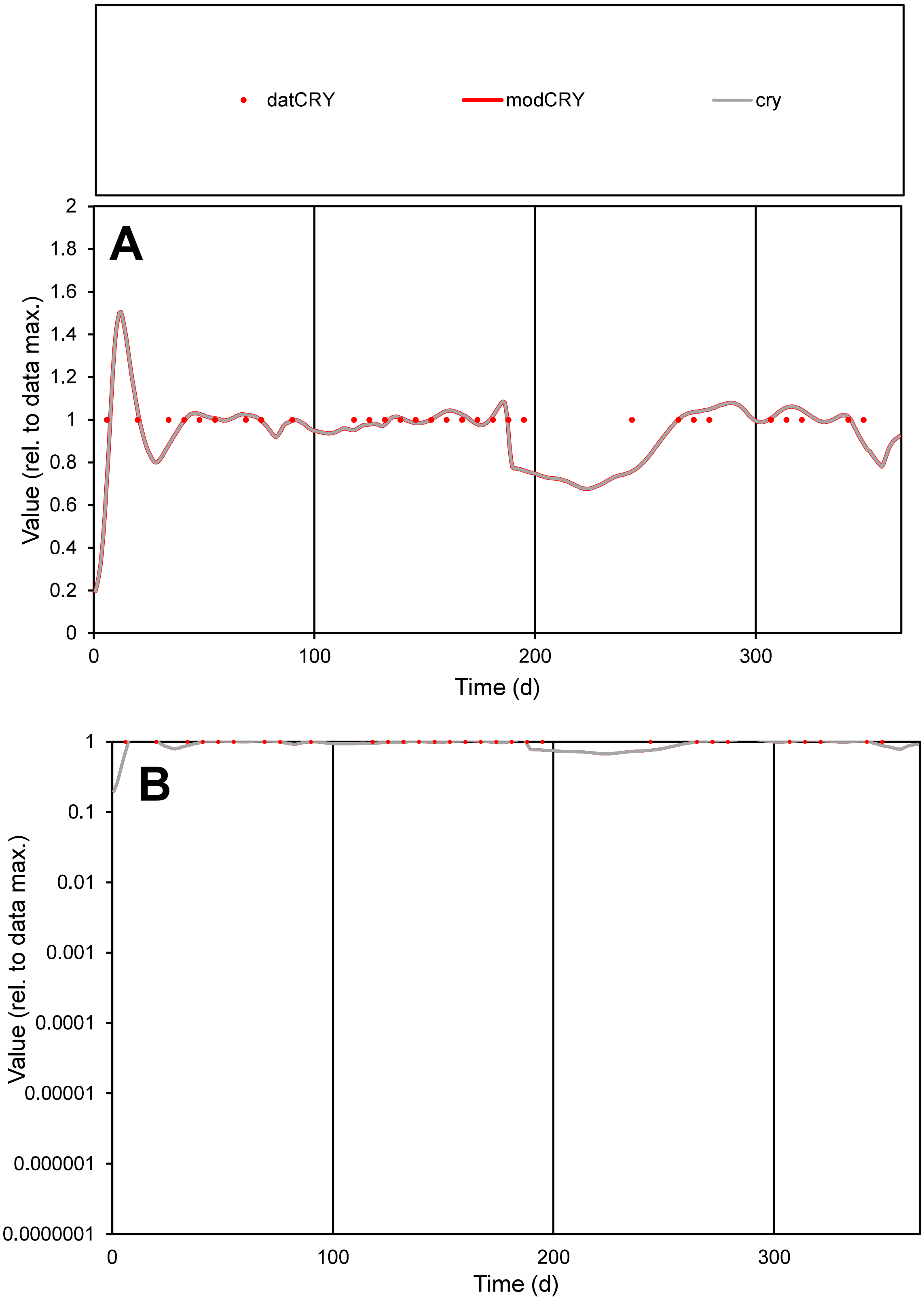

C1

Variables
CRY Provide jtd or Dst
CalibOnly?
2 1 to reproduce of
oNormalize?
2 1=no, 2=yes

ofxn calc*
0.07612
ofxn mod
0.13742

sRun
RunA1
zout
1

Ave
Data
3E-06
Model
2.9E-06

data/vMax
3E-06

ofxng
275.646

Min d/m
1.31949

ave/min d
1
ave/min m
1.29333

325_Calib_S_A

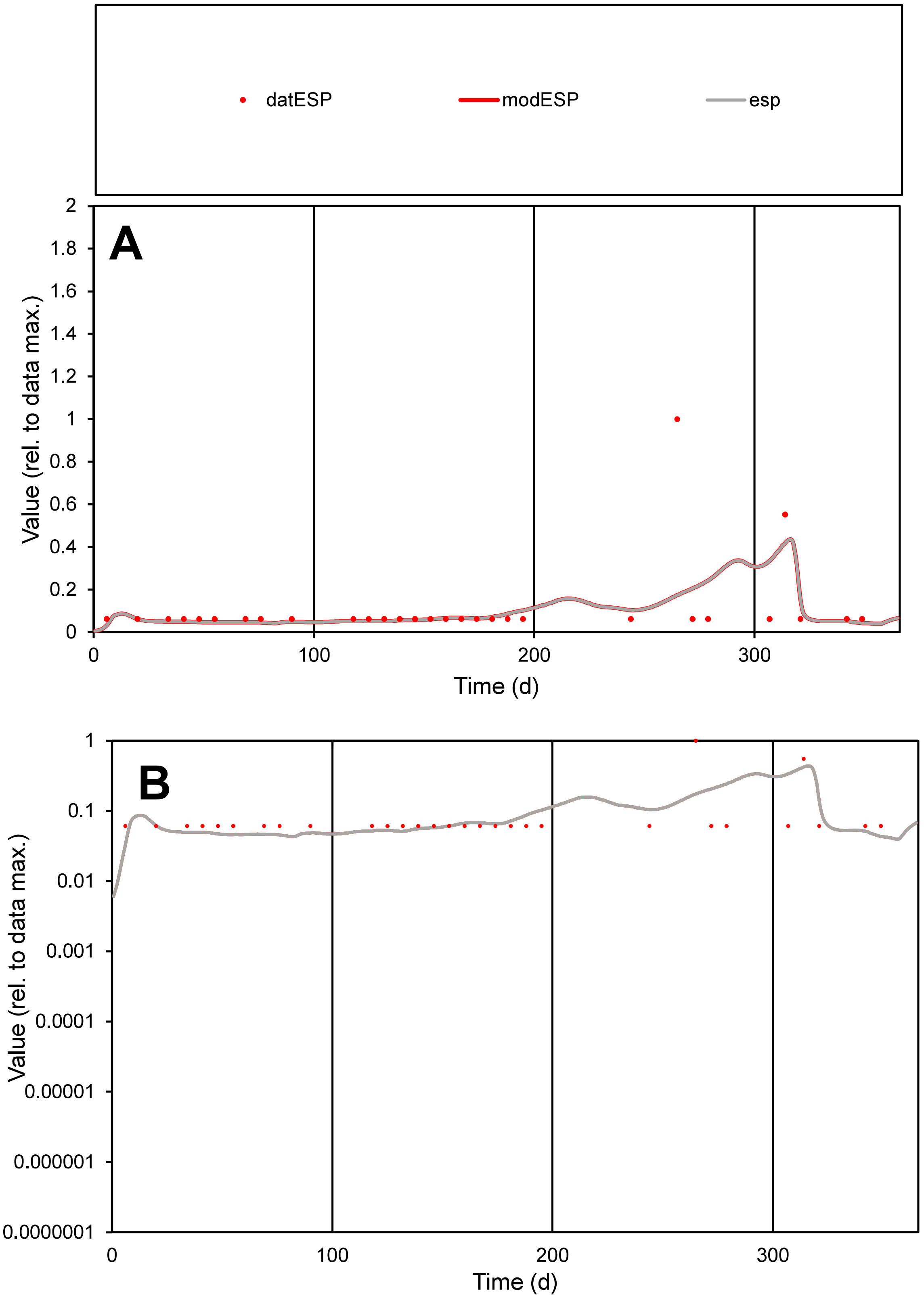

C1

Variables

ESP Provide jtd or Dst

CalibOnly?

2 1 to reproduce of

oNormalize?

2 1=no, 2=yes

ofxn calc*

1.5308

ofxn mod

1.54097

sRun

RunA1

zout

1

Ave

Data

1.4E-05

Model

1.3E-05

data/vMax

0.00013

ofxng

275.646

Min d/m

1.434

ave/min d

1.78153

ave/min m

2.30663

325_Calib_S_A

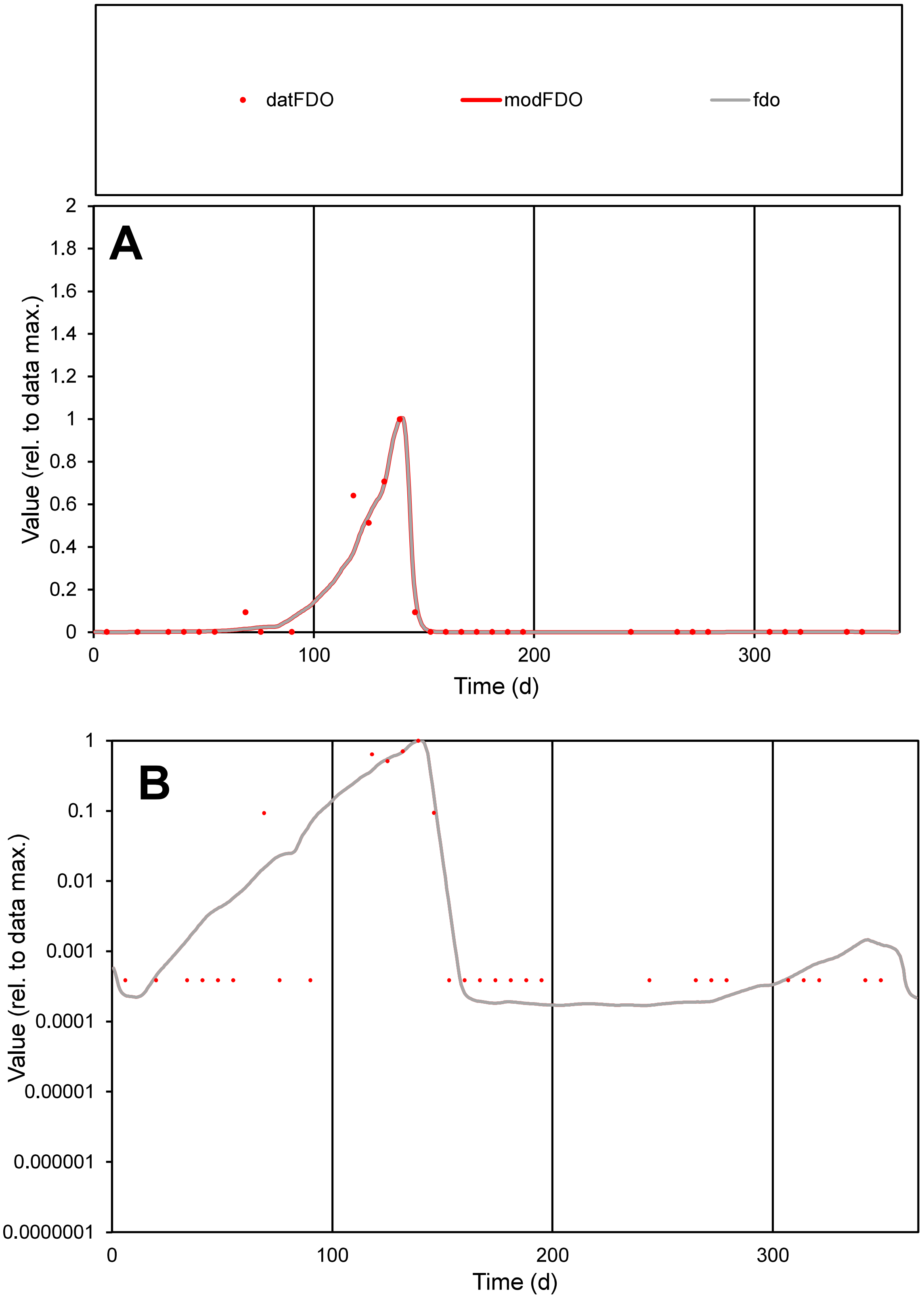

C1

Variables

FDO	Provide jtd or Dst
CalibOnly?	
2	1 to reproduce of
oNormalize?	
2	1=no, 2=yes

ofxn calc*
0.46984
ofxn mod
0.54964

sRun
RunA1
zout
1

Ave
Data
2.6E-05
Model
2.5E-05

data/vMax
0.00026

ofxng
275.646

Min d/m
2.25765

ave/min d
263.408
ave/min m
568.579

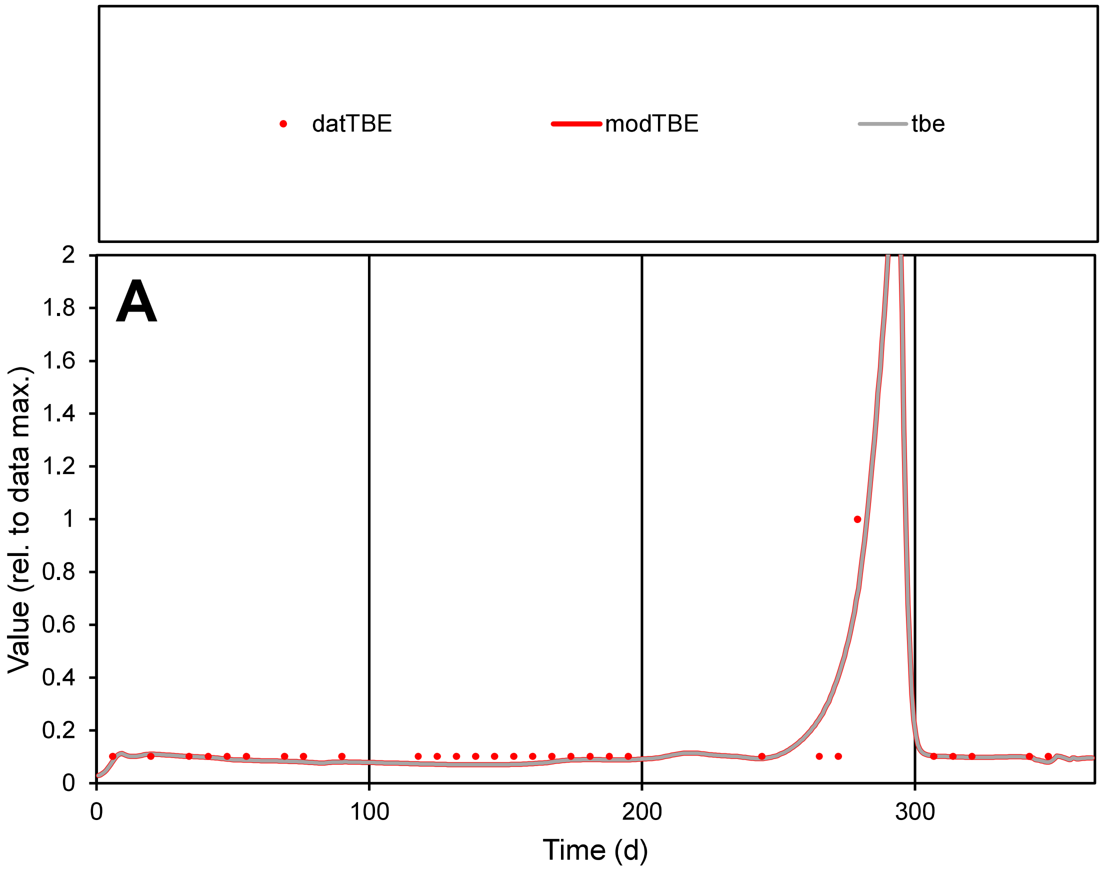

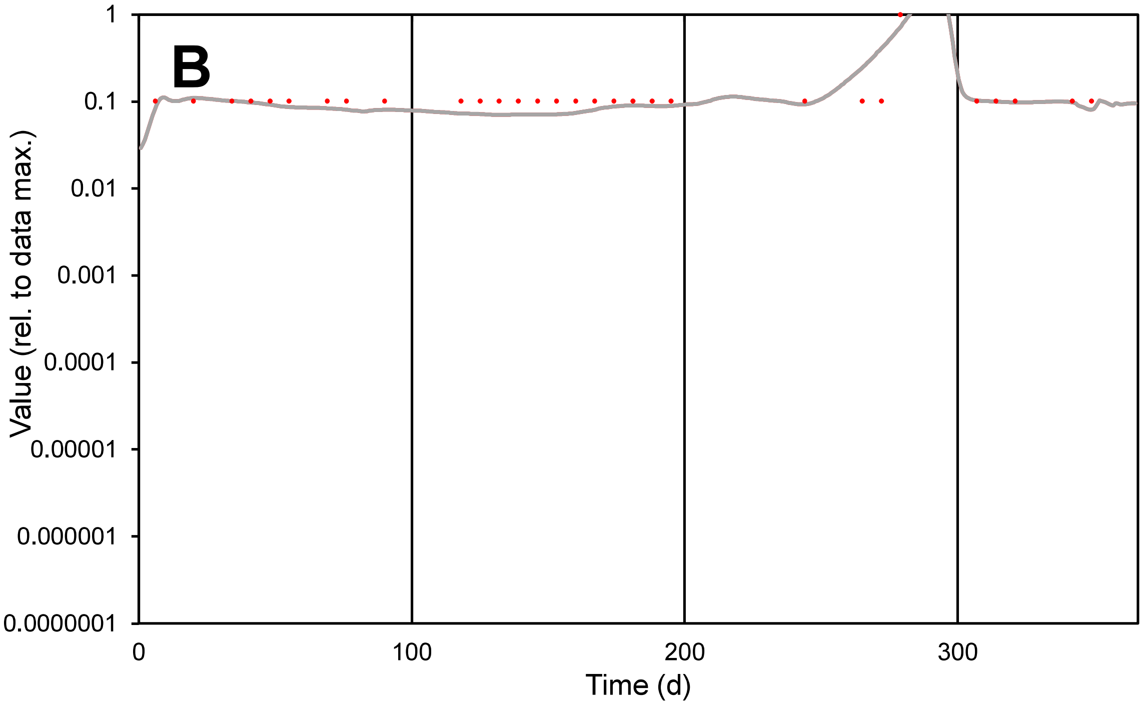

C1

Variables
TBE Provide jtd or Dst
CalibOnly?
2 1 to reproduce of
oNormalize?
2 1=no, 2=yes

ofxn calc*
0.51181
ofxn mod
0.5835

sRun
RunA1
zout
1

Ave
Data
8.7E-06
Model
8.4E-06

data/vMax
6.6E-05

ofxng
275.646

Min d/m
1.44013

ave/min d
1.29655
ave/min m
1.8224

325_Calib_S_A

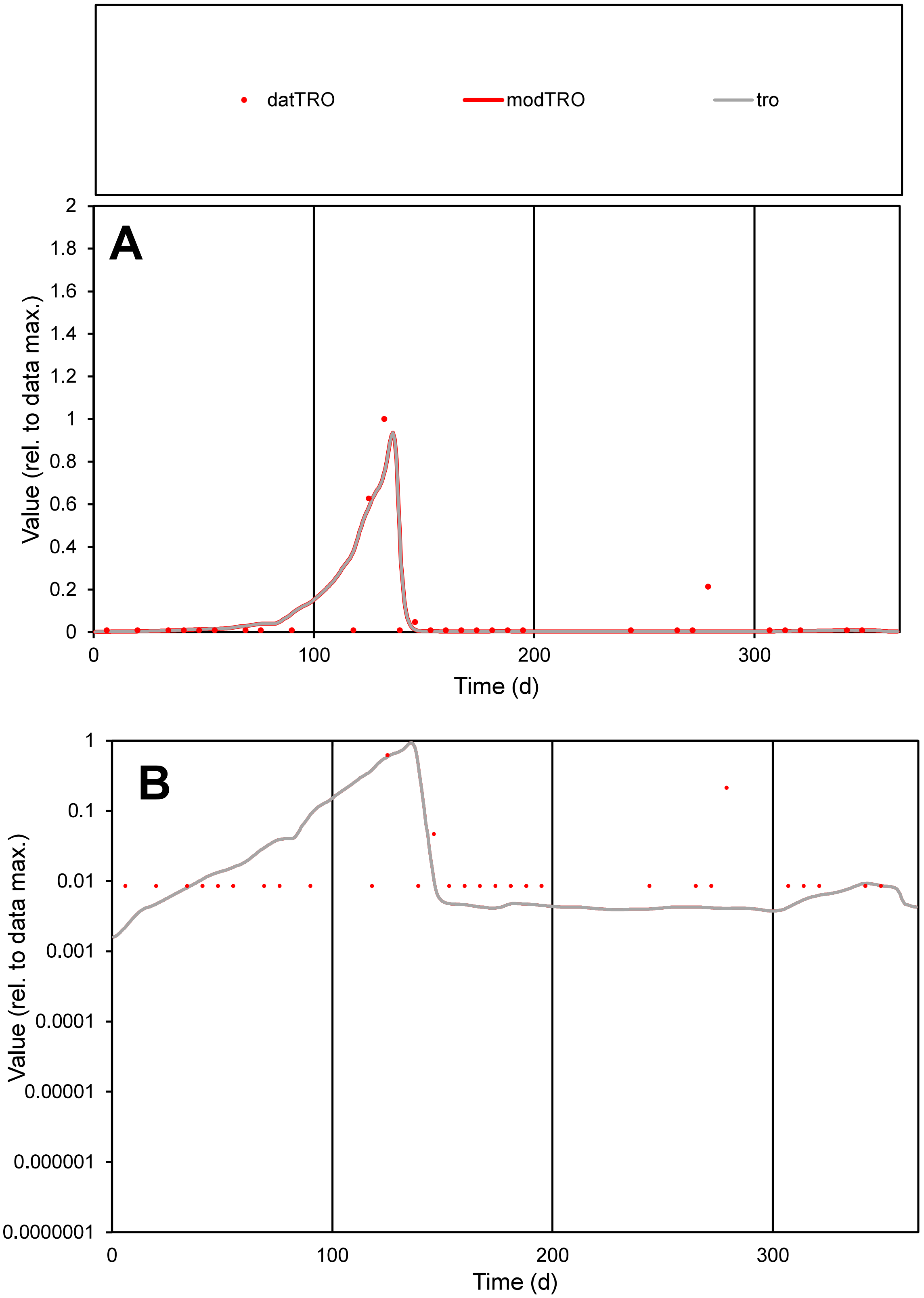

C1

Variables
TRO Provide jtd or Dst
CalibOnly?
2 1 to reproduce of
oNormalize?
2 1=no, 2=yes

ofxn calc*
1.20885
ofxn mod
1.31034

sRun
RunA1
zout
1

Ave
Data
4.1E-05
Model
4.2E-05

data/vMax
0.00059

ofxng
275.646

Min d/m
3.49409

ave/min d
8.2232
ave/min m
29.4583

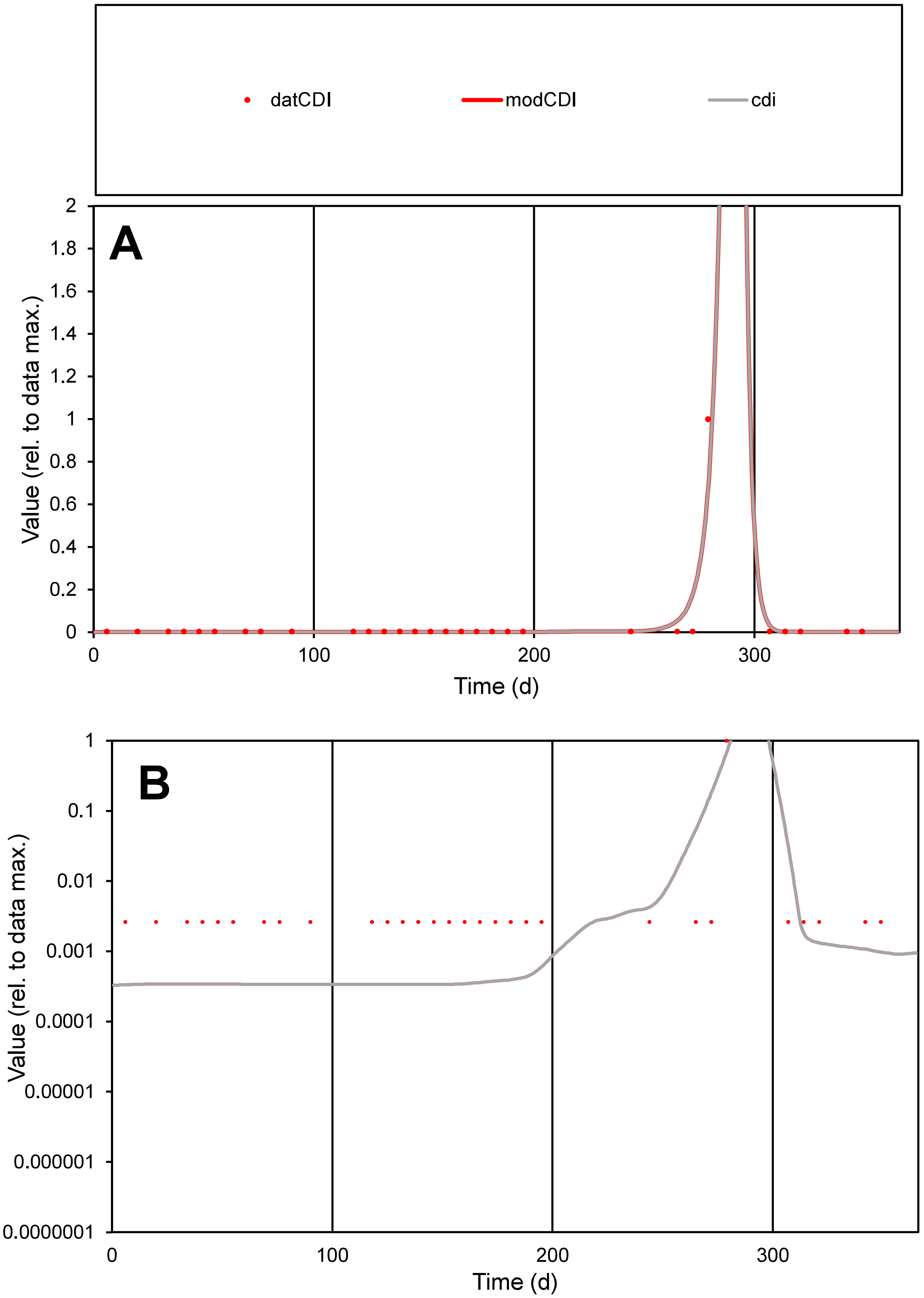

C1

Variables
CDI Provide jtd or Dst
CalibOnly?
2 1 to reproduce of
oNormalize?
2 1=no, 2=yes

ofxn calc*
0.58131
ofxn mod
1.08907

sRun
RunA1
zout
1

Ave
Data
2.4E-06
Model
2.5E-06

data/vMax
6.6E-05

ofxng
275.646

Min d/m
7.81588

ave/min d
13.6803
ave/min m
113.759

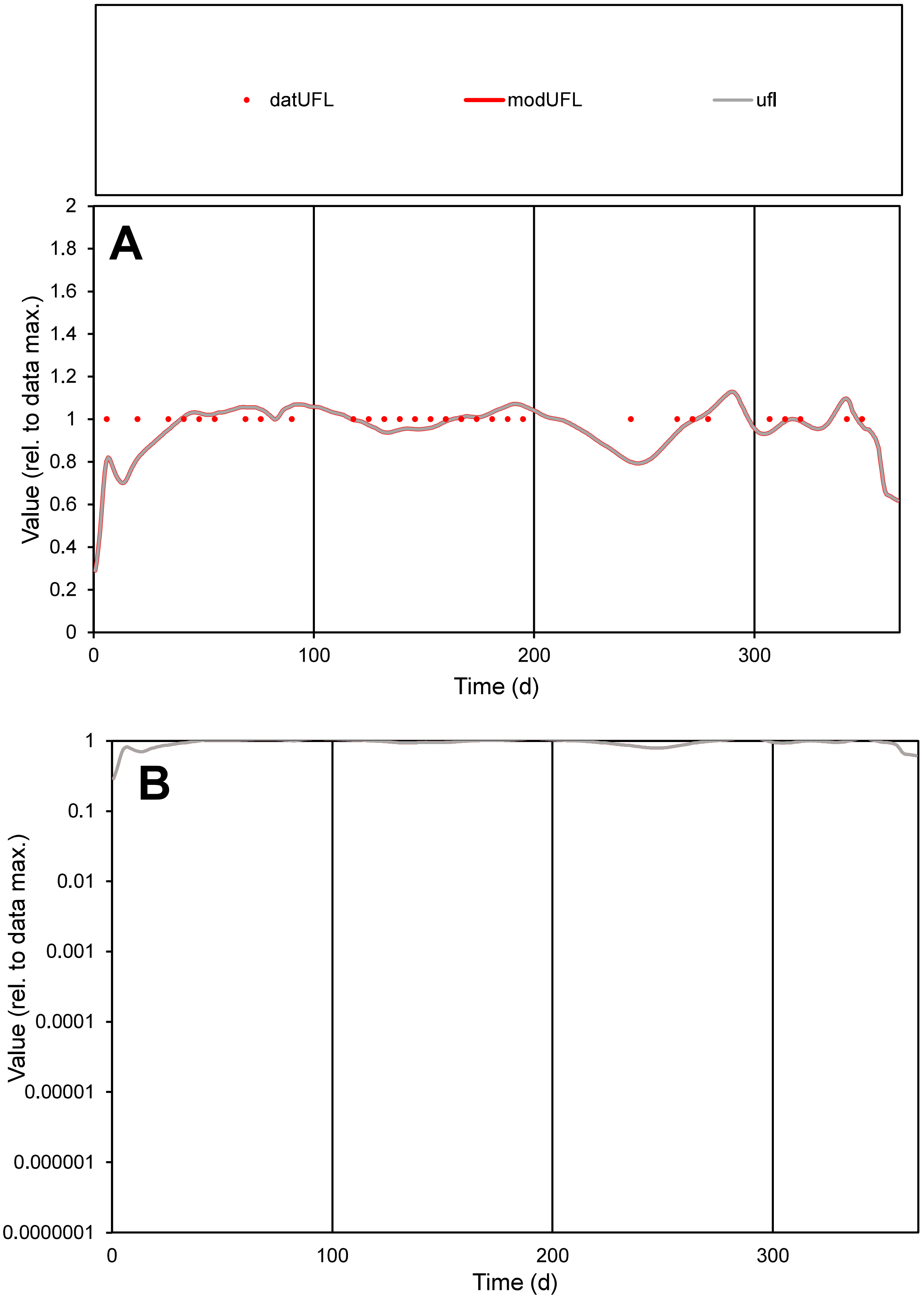

C1

Variables
UFL Provide jtd or Dst
CalibOnly?
2 1 to reproduce of
oNormalize?
2 1=no, 2=yes

ofxn calc*
0.07173
ofxn mod
0.13305

sRun
RunA1
zout
1

Ave
Data
3.1E-06
Model
3E-06

data/vMax
3.1E-06

ofxng
275.646

Min d/m
1.25646

ave/min d
1
ave/min m
1.23813

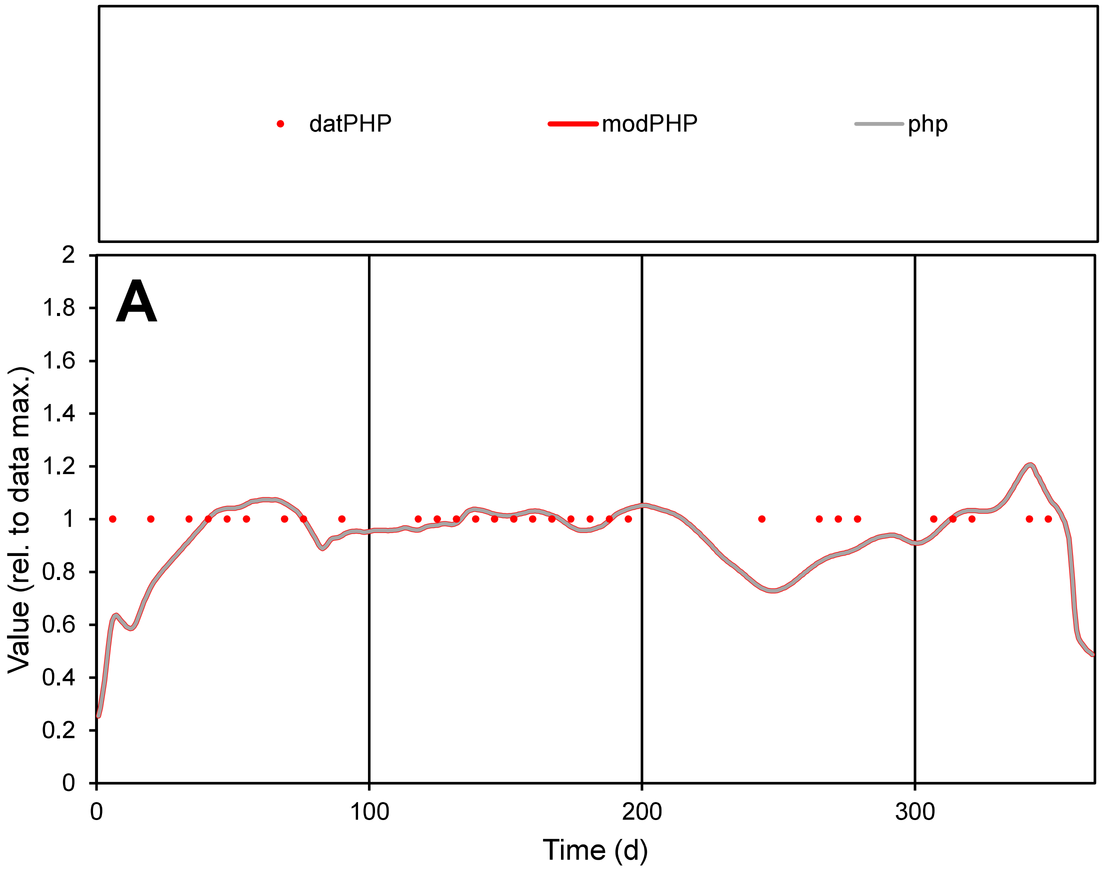

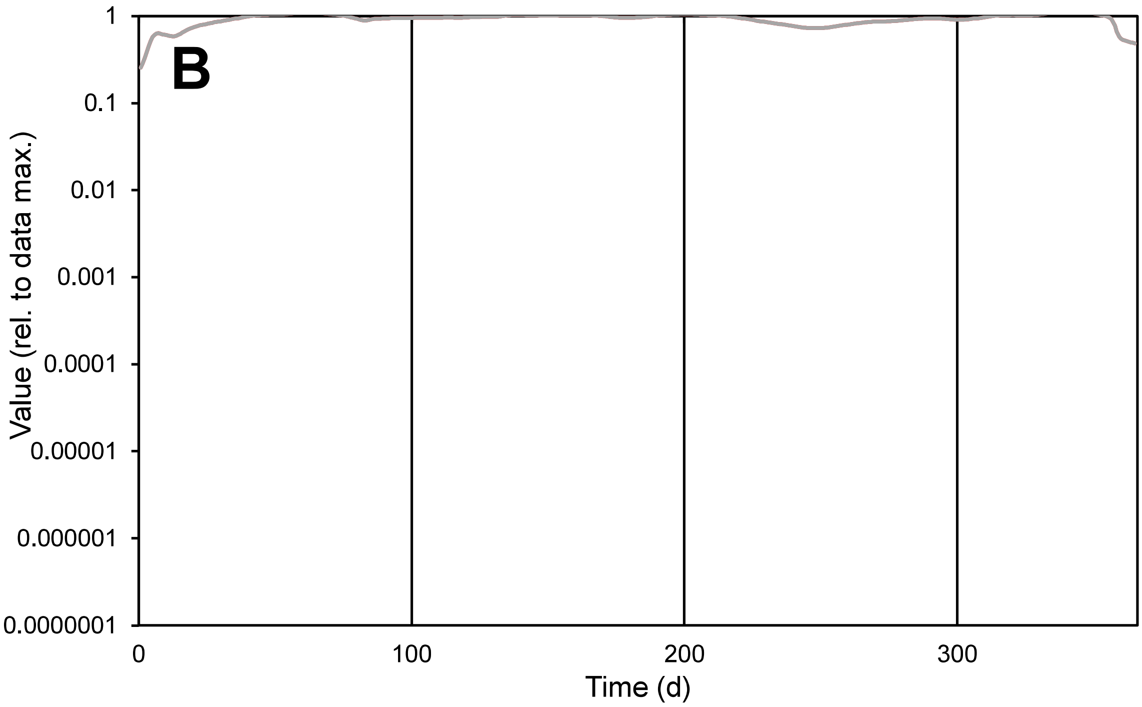

C1

Variables
PHP Provide jtd or Dst
CalibOnly?
2 1 to reproduce of
oNormalize?
2 1=no, 2=yes

ofxn calc*
0.11295
ofxn mod
0.15835

sRun
RunA1
zout
1

Ave
Data
6.9E-06
Model
6.7E-06

data/vMax
6.9E-06

ofxng
275.646

Min d/m
1.57143

ave/min d
1
ave/min m
1.52338

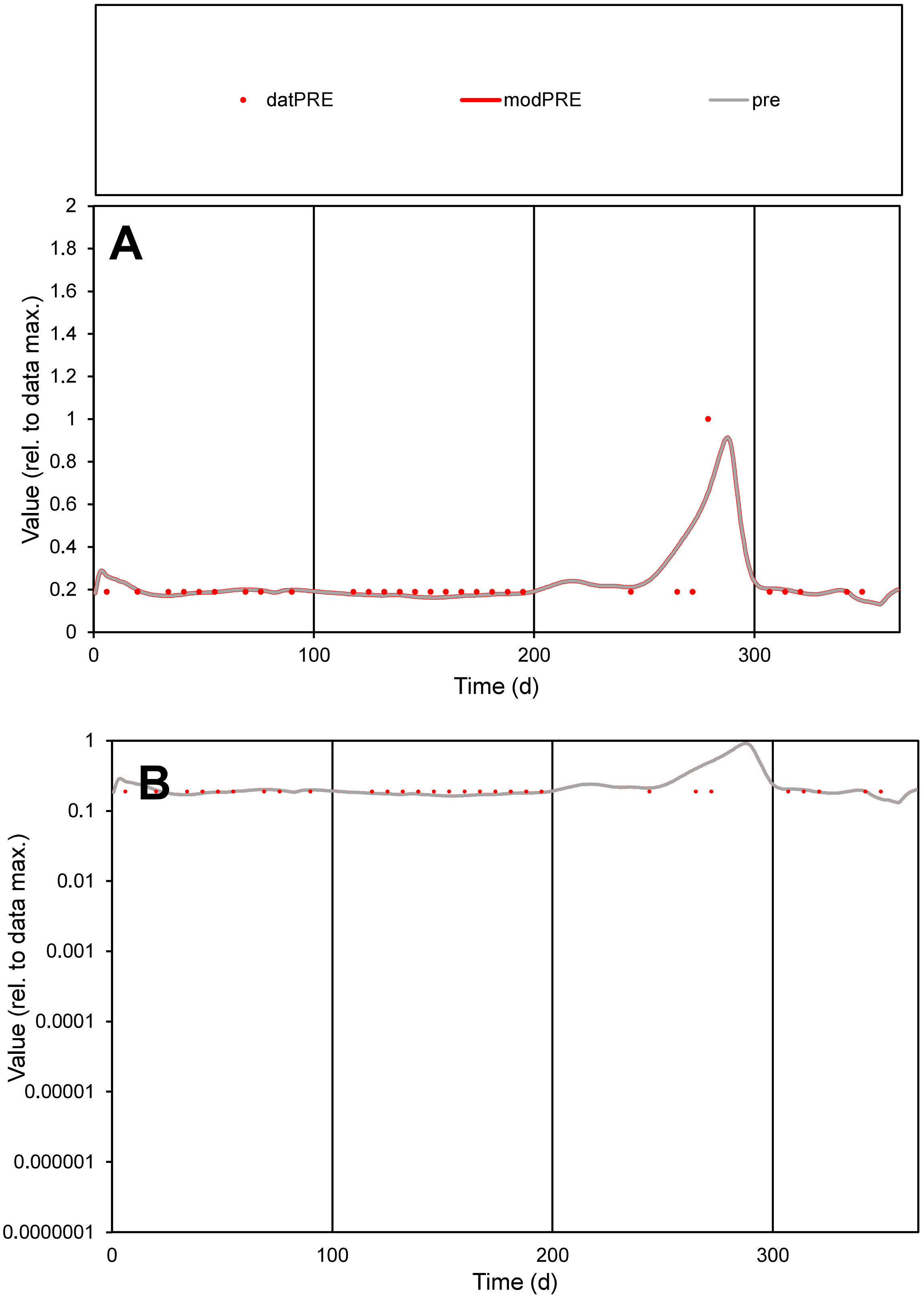

C1

Variables
PRE Provide jtd or Dst
CalibOnly?
2 1 to reproduce of
oNormalize?
2 1=no, 2=yes

ofxn calc*
0.42411
ofxn mod
0.45145

sRun
RunA1
zout
1

Ave
Data
1.5E-05
Model
1.6E-05

data/vMax
7.1E-05

ofxng
275.646

Min d/m
1.31024

ave/min d
1.14291
ave/min m
1.52608

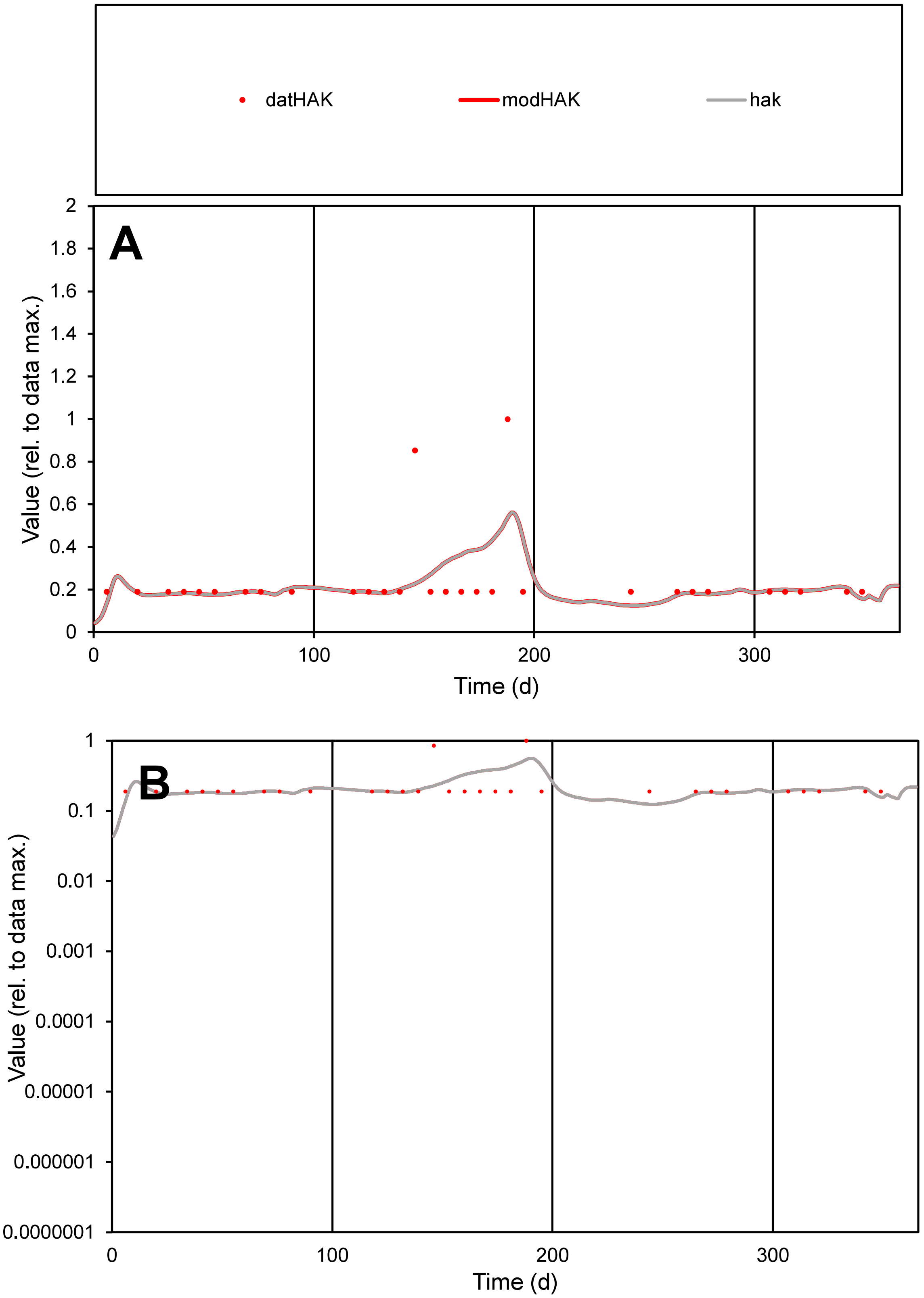

C1

Variables
HAK Provide jtd or Dst
CalibOnly?
2 1 to reproduce of
oNormalize?
2 1=no, 2=yes

ofxn calc*
0.67869
ofxn mod
0.69034

sRun
RunA1
zout
1

Ave
Data
6.4E-06
Model
6.3E-06

data/vMax
2.7E-05

ofxng
275.646

Min d/m
1.52269

ave/min d
1.25967
ave/min m
1.88818

325_Calib_S_A

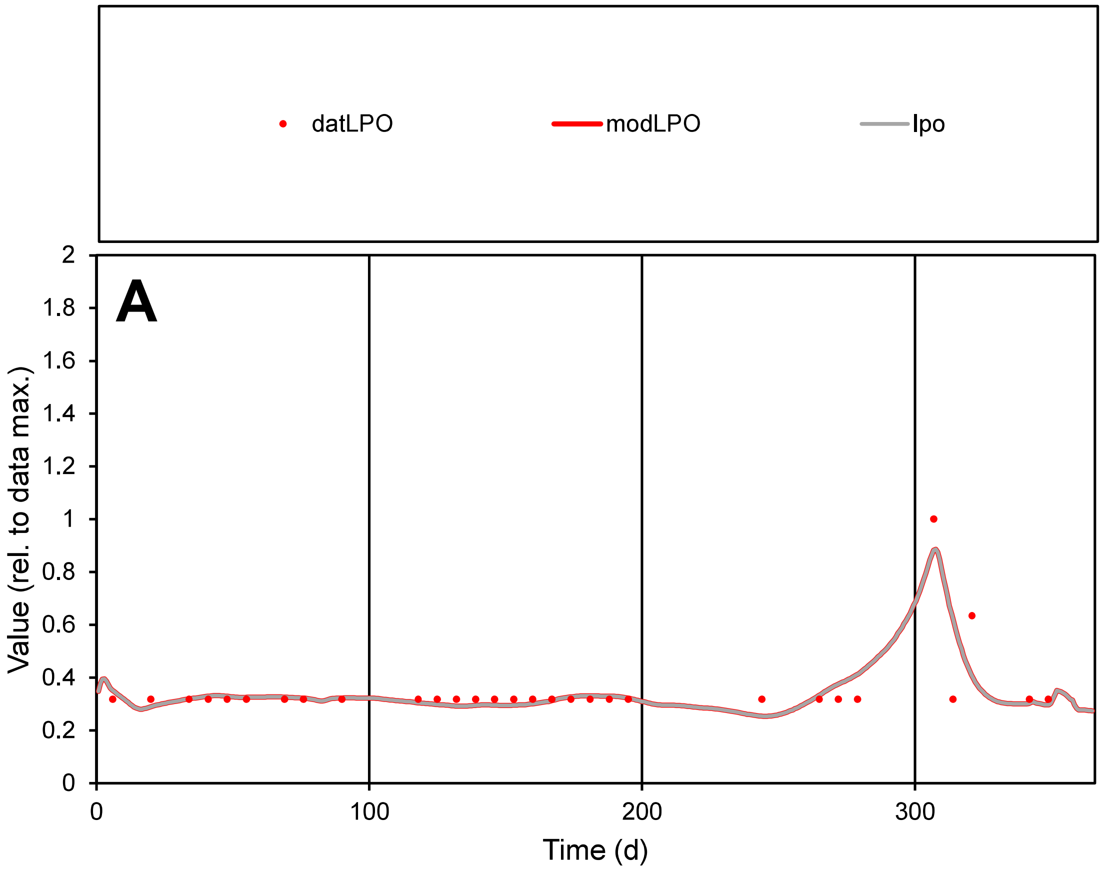

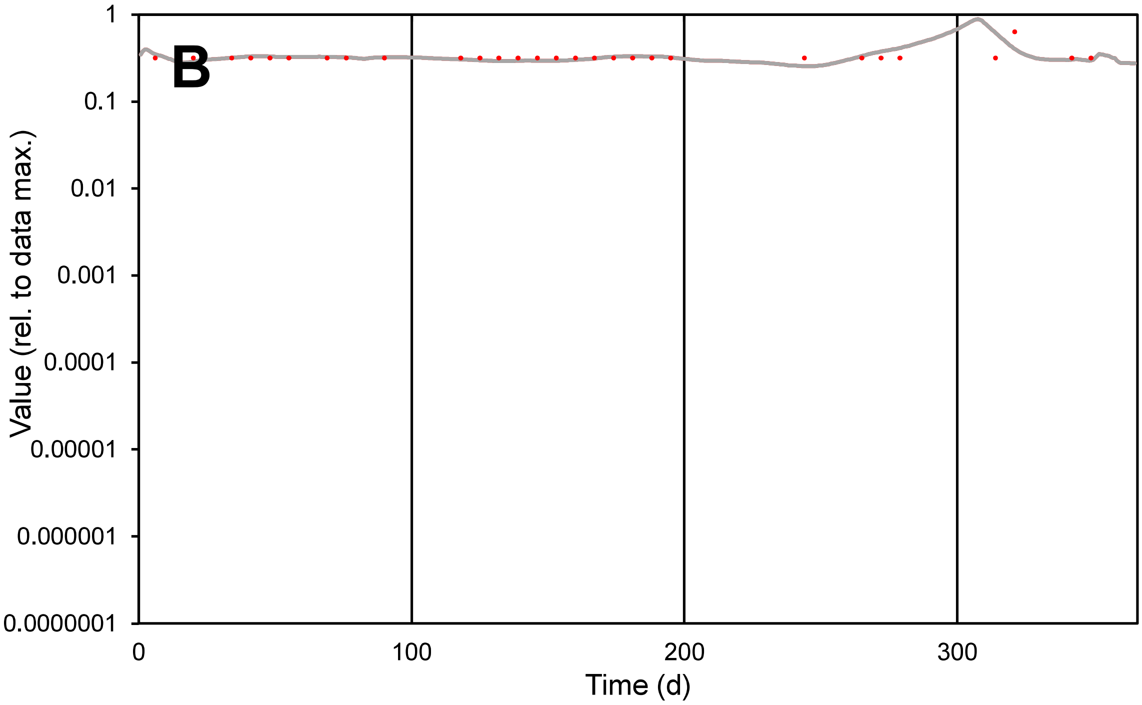

C1

Variables
LPO Provide jtd or Dst
CalibOnly?
2 1 to reproduce of
oNormalize?
2 1=no, 2=yes

ofxn calc*
0.20785
ofxn mod
0.24146

sRun
RunA1
zout
1

Ave
Data
6.5E-06
Model
6.4E-06

data/vMax
1.8E-05

ofxng
275.646

Min d/m
1.24915

ave/min d
1.10512
ave/min m
1.37213

325_Calib_S_A

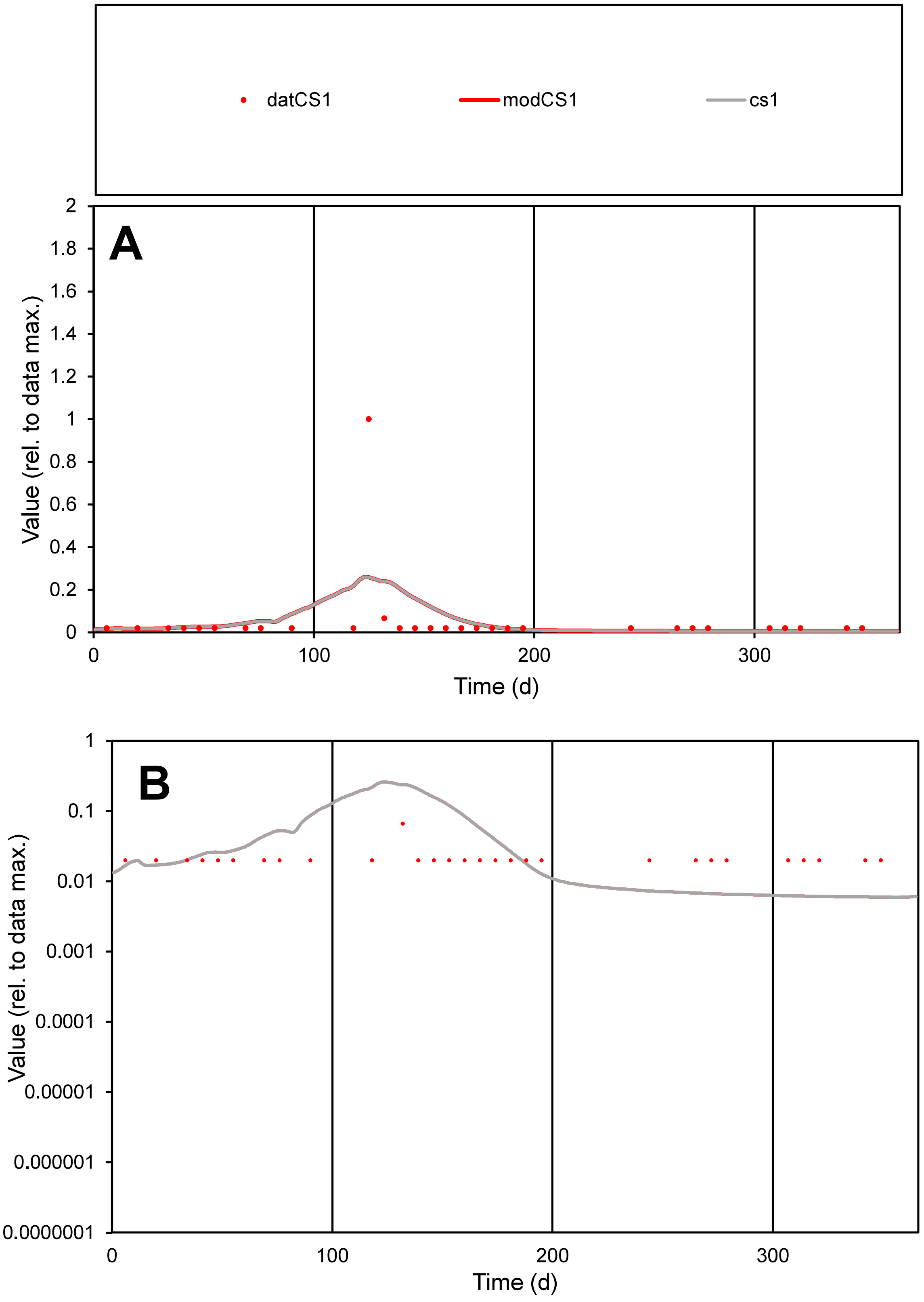

C1

Variables
CS1 Provide jtd or Dst
CalibOnly?
2 1 to reproduce of
oNormalize?
2 1=no, 2=yes

ofxn calc*
2.79929
ofxn mod
2.82573

sRun
RunA1
zout
1

Ave
Data
4.8E-05
Model
5.1E-05

data/vMax
0.00088

ofxng
275.646

Min d/m
3.34607

ave/min d
2.71621
ave/min m
9.77403

325_Calib_S_A

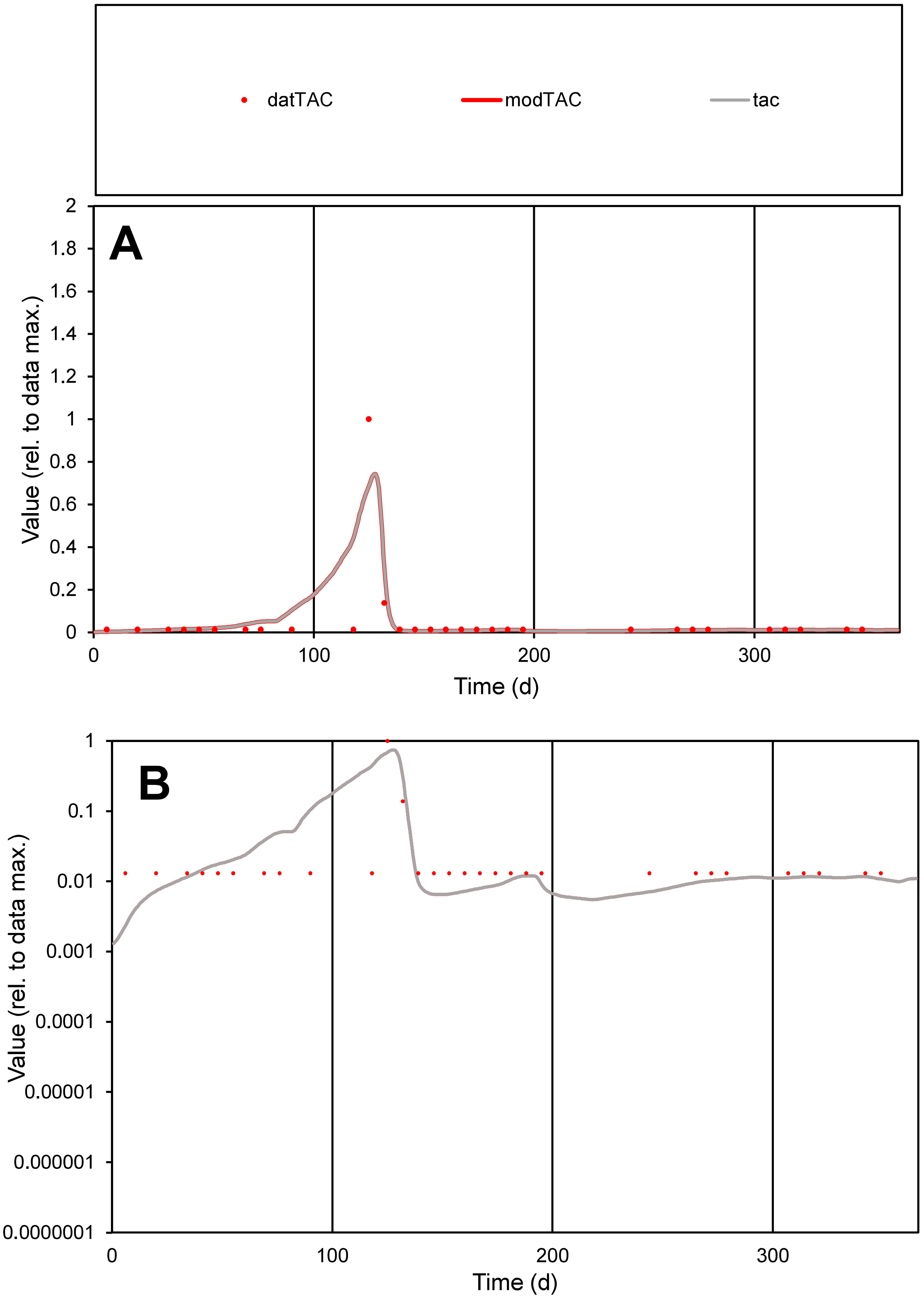

C1

Variables
TAC Provide jtd or Dst
CalibOnly?
2 1 to reproduce of
oNormalize?
2 1=no, 2=yes

ofxn calc*
1.81025
ofxn mod
1.91186

sRun
RunA1
zout
1

Ave
Data
5E-06
Model
5.8E-06

data/vMax
9.9E-05

ofxng
275.646

Min d/m
4.78213

ave/min d
3.84221
ave/min m
21.3338

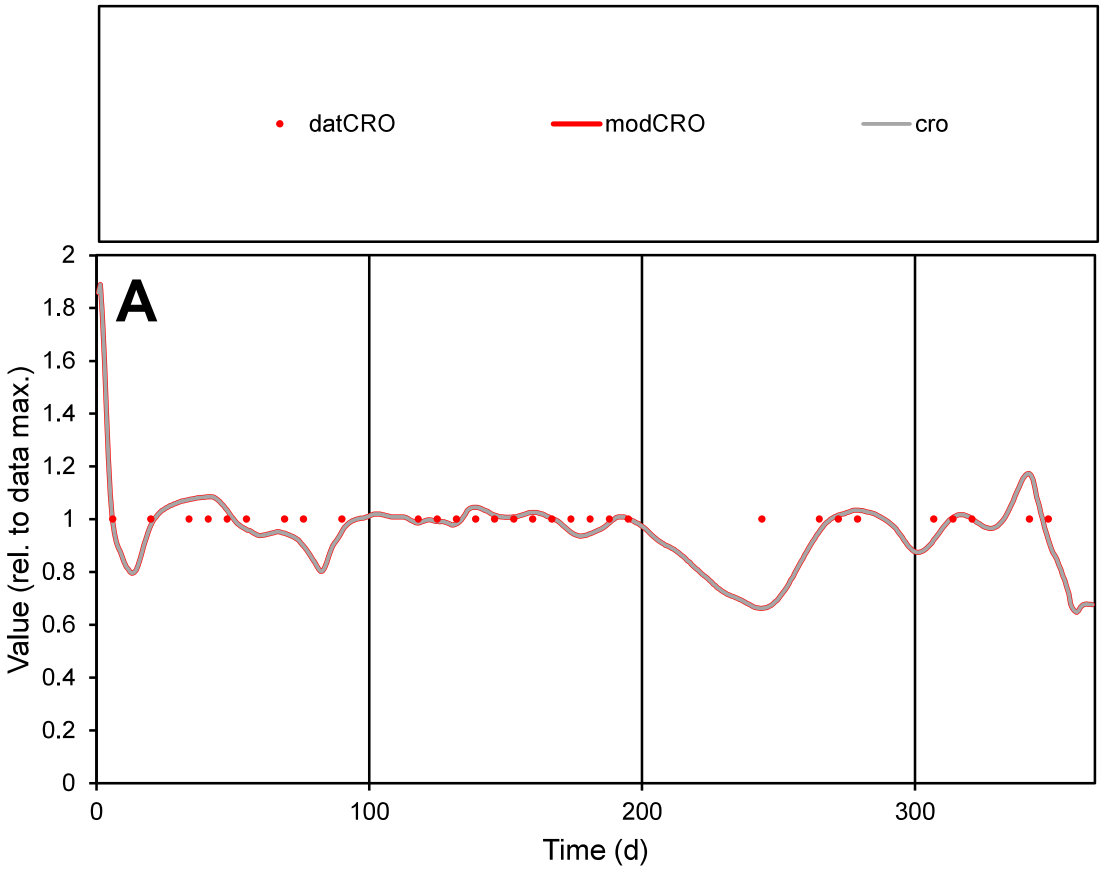

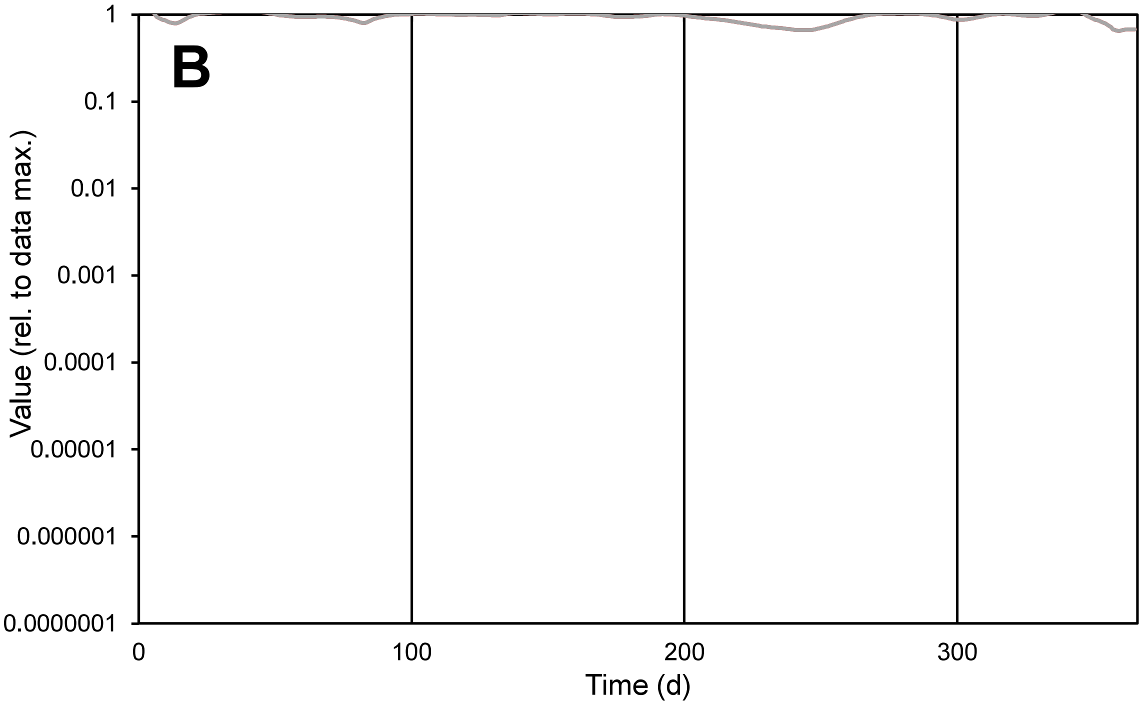

C1

Variables
CRO Provide jtd or Dst
CalibOnly?
2 1 to reproduce of
oNormalize?
2 1=no, 2=yes

ofxn calc*
0.08299
ofxn mod
0.13941

sRun
RunA1
zout
1

Ave
Data
1E-05
Model
1E-05

data/vMax
1E-05

ofxng
275.646

Min d/m
1.50633

ave/min d
1
ave/min m
1.47915

325_Calib_S_A

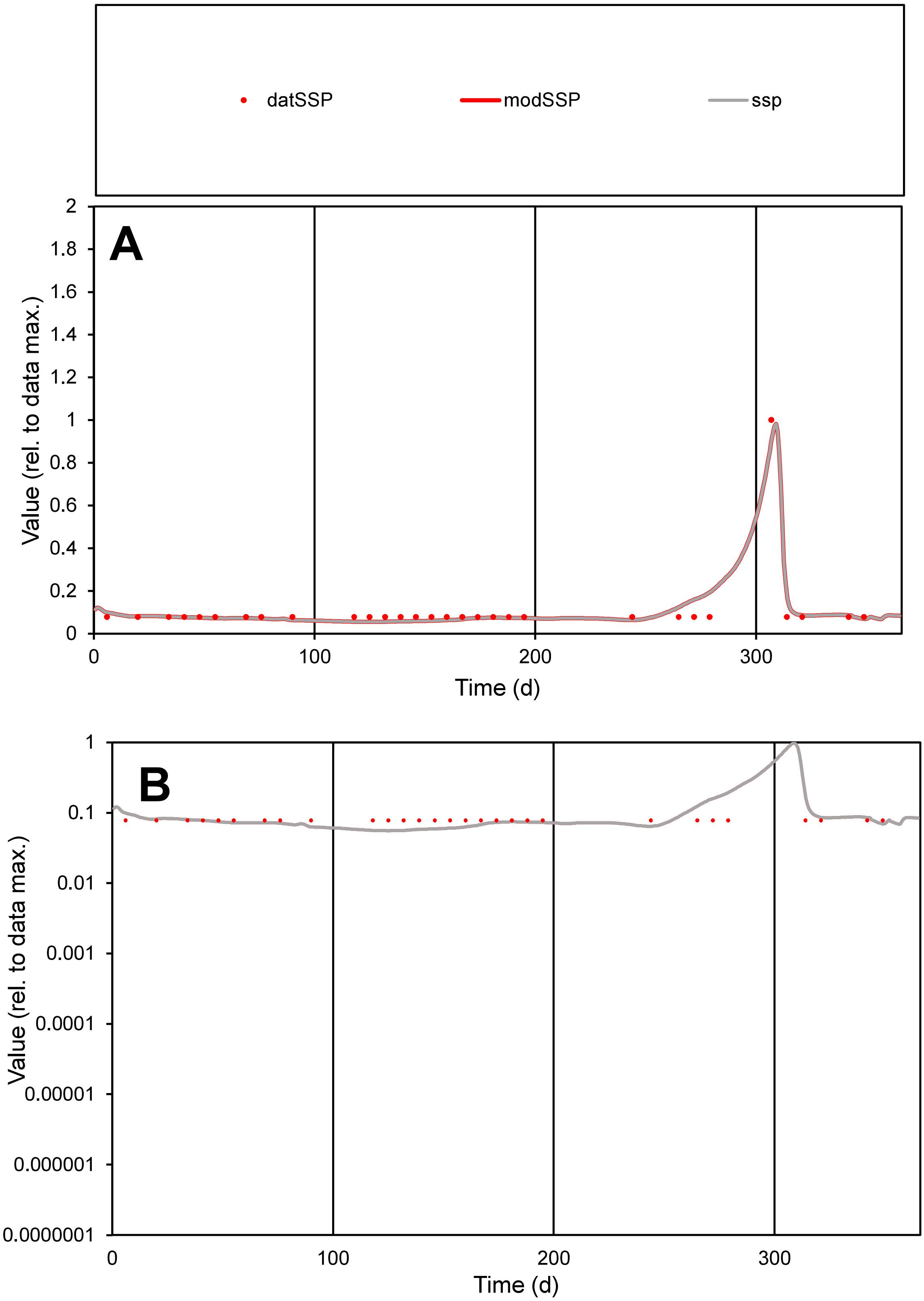

C1

Variables
SSP Provide jtd or Dst
CalibOnly?
2 1 to reproduce of
oNormalize?
2 1=no, 2=yes

ofxn calc*
0.28676
ofxn mod
0.36029

sRun
RunA1
zout
1

Ave
Data
1.8E-05
Model
1.9E-05

data/vMax
0.00017

ofxng
275.646

Min d/m
1.39117

ave/min d
1.39448
ave/min m
1.98691

325_Calib_S_A

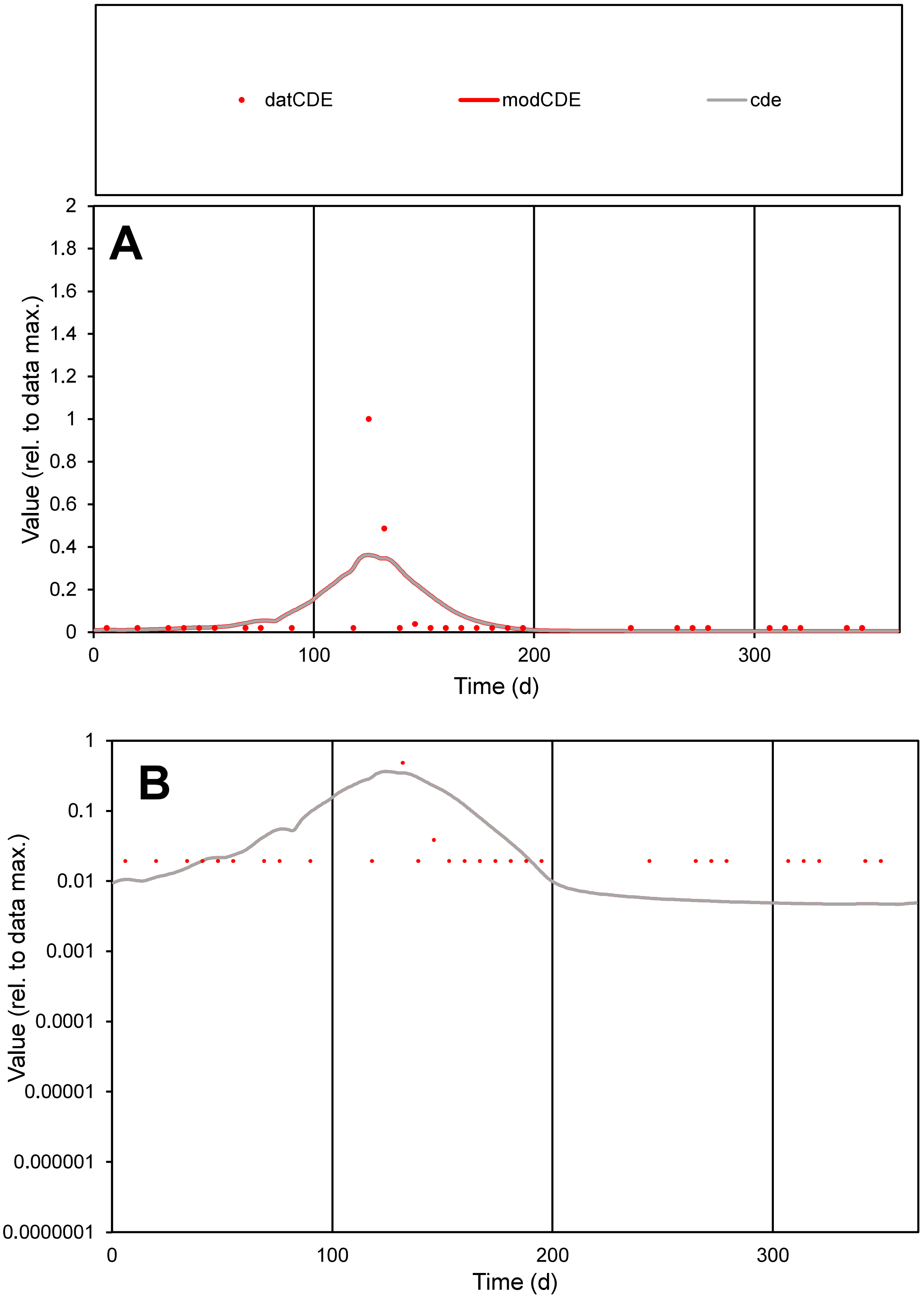

C1

Variables
CDE Provide jtd or Dst
CalibOnly?
2 1 to reproduce of
oNormalize?
2 1=no, 2=yes

ofxn calc*
2.13671
ofxn mod
2.17299

sRun
RunA1
zout
1

Ave
Data
3.5E-05
Model
3.9E-05

data/vMax
0.00052

ofxng
275.646

Min d/m
4.08299

ave/min d
3.52932
ave/min m
16.0003

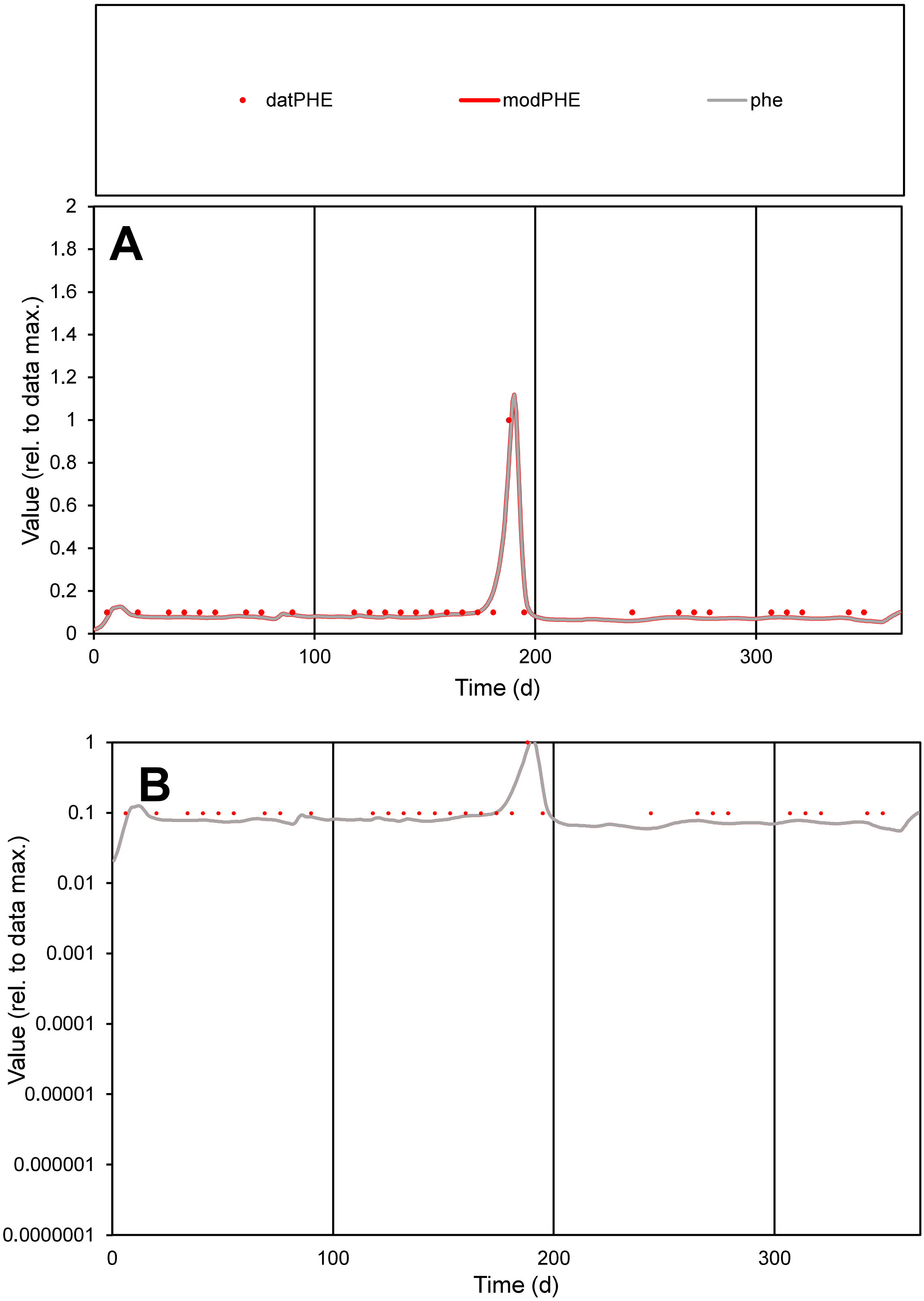

C1

Variables
PHE Provide jtd or Dst
CalibOnly?
2 1 to reproduce of
oNormalize?
2 1=no, 2=yes

ofxn calc*
0.22321
ofxn mod
0.26176

sRun
RunA1
zout
1

Ave
Data
5.2E-06
Model
4.6E-06

data/vMax
4E-05

ofxng
275.646

Min d/m
1.66331

ave/min d
1.30513
ave/min m
1.94261

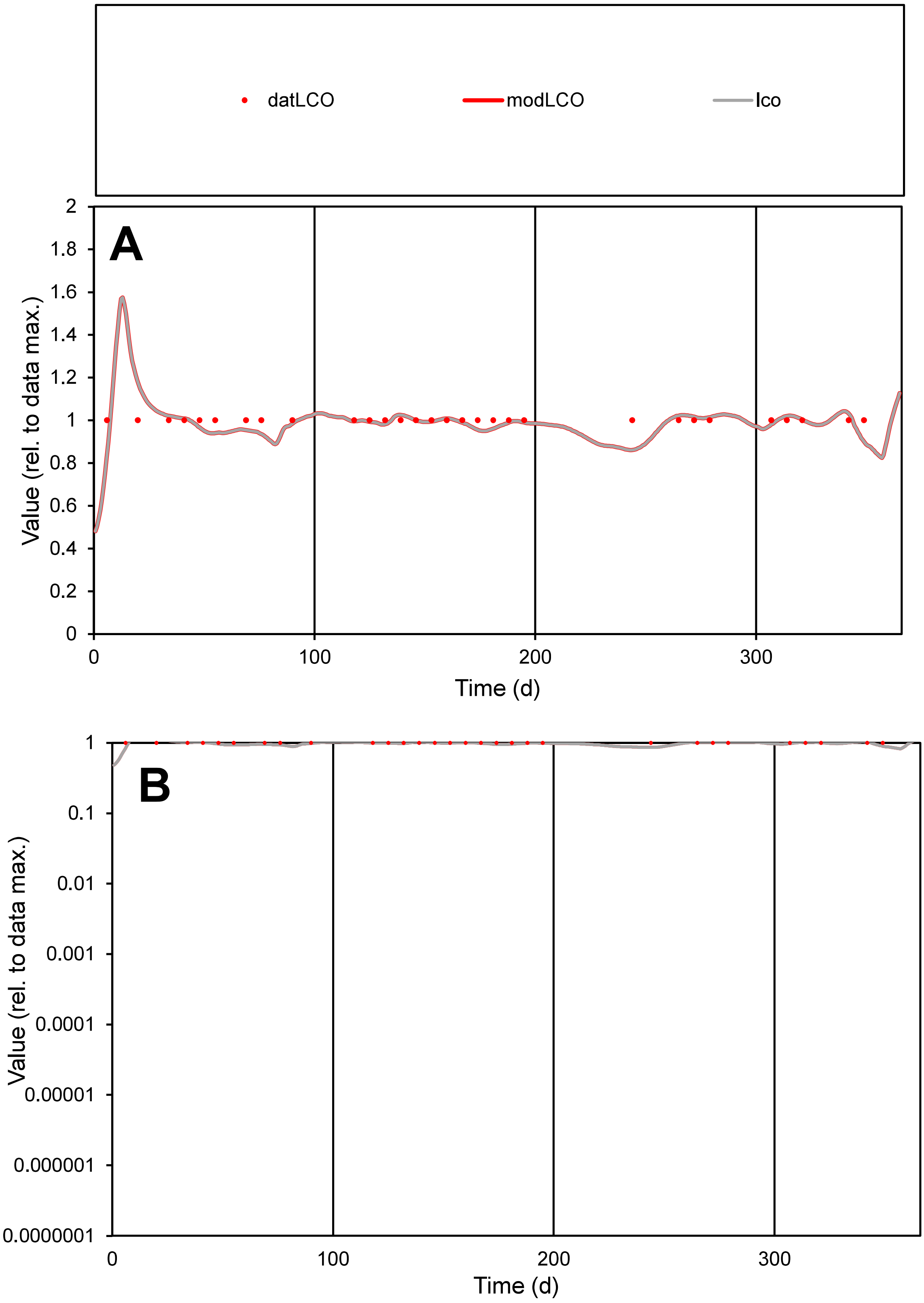

C1

Variables

LCO	Provide jtd or Dst

CalibOnly?

2	1 to reproduce of

oNormalize?

2	1=no, 2=yes

ofxn calc*
0.04949
ofxn mod
0.12213

sRun
RunA1
zout
1

Ave
Data
7.4E-06
Model
7.3E-06

data/vMax
7.4E-06

ofxng
275.646

Min d/m
1.15734

ave/min d
1
ave/min m
1.14377

325_Calib_S_A

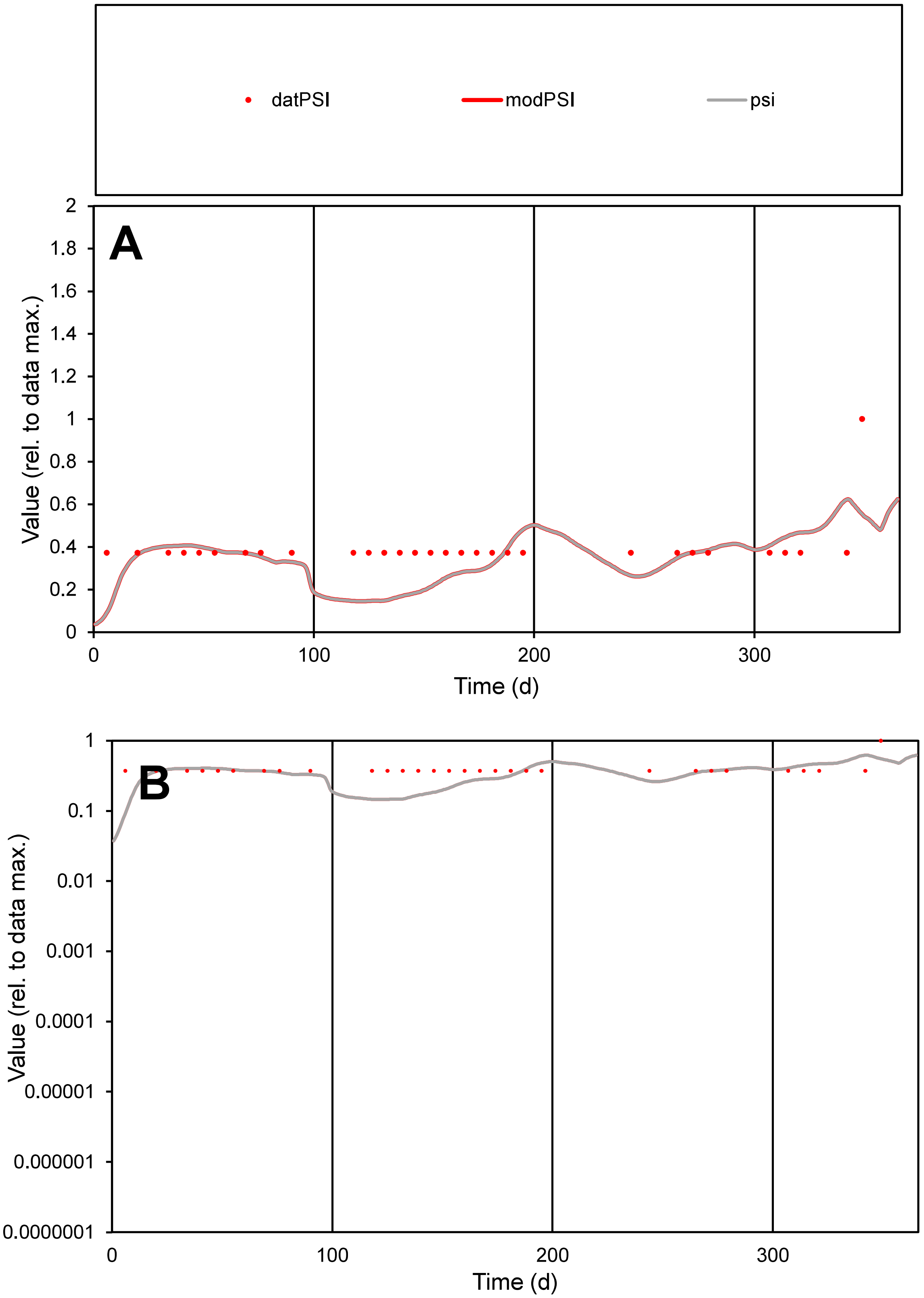

C1

Variables
PSI Provide jtd or Dst
CalibOnly?
2 1 to reproduce of
oNormalize?
2 1=no, 2=yes

ofxn calc*
0.37722
ofxn mod
0.39459

sRun
RunA1
zout
1

Ave
Data
3.8E-06
Model
3.2E-06

data/vMax
9.6E-06

ofxng
275.646

Min d/m
2.7898

ave/min d
1.05622
ave/min m
2.52847

325_Calib_S_A

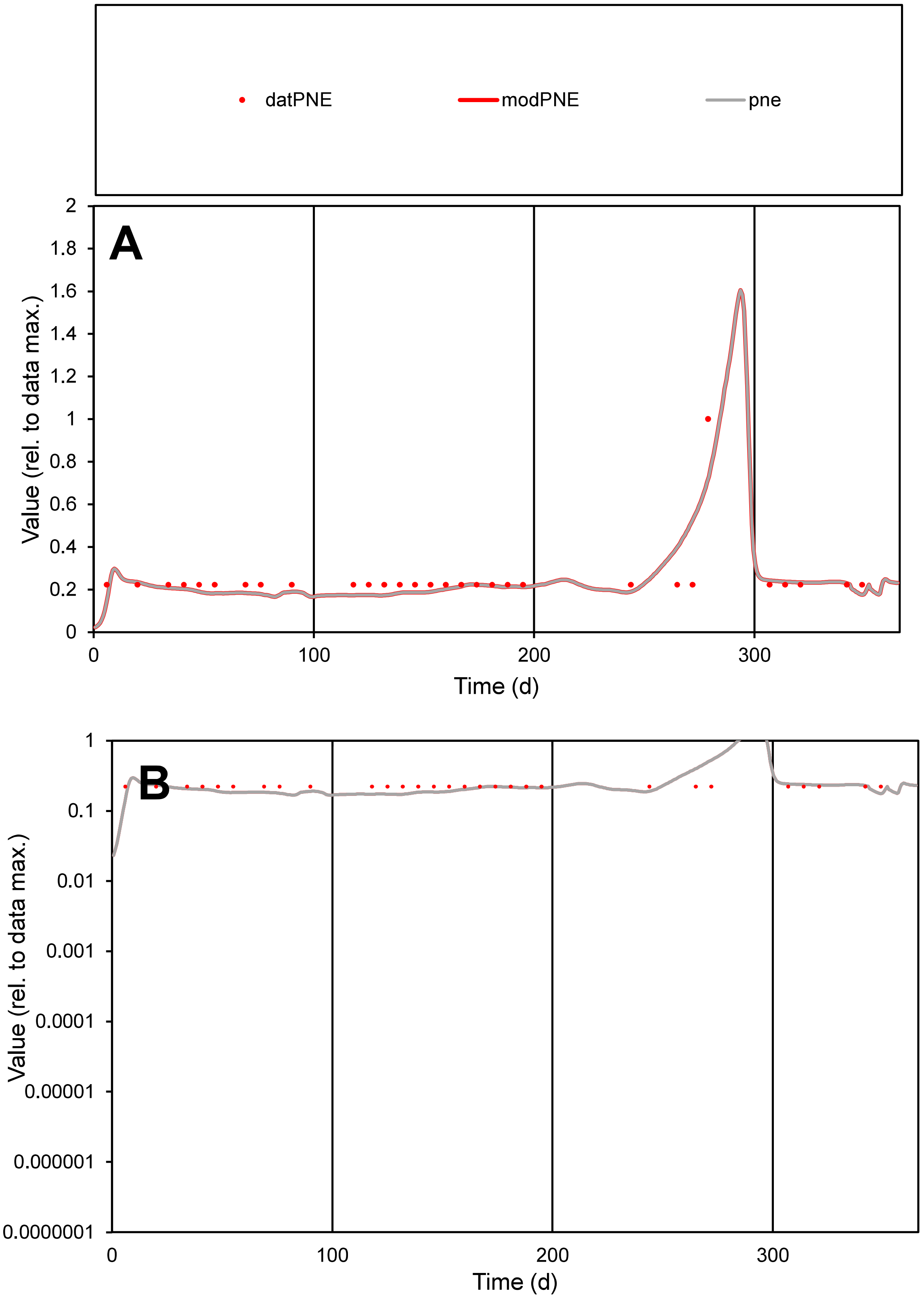

C1

Variables

PNE Provide jtd or Dst

CalibOnly?

2 1 to reproduce of

oNormalize?

2 1=no, 2=yes

ofxn calc*

0.32761

ofxn mod

0.35812

sRun

RunA1

zout

1

Ave

Data

1.6E-05

Model

1.6E-05

data/vMax

6.5E-05

ofxng

275.646

Min d/m

1.28155

ave/min d

1.11641

ave/min m

1.38676

325_Calib_S_A

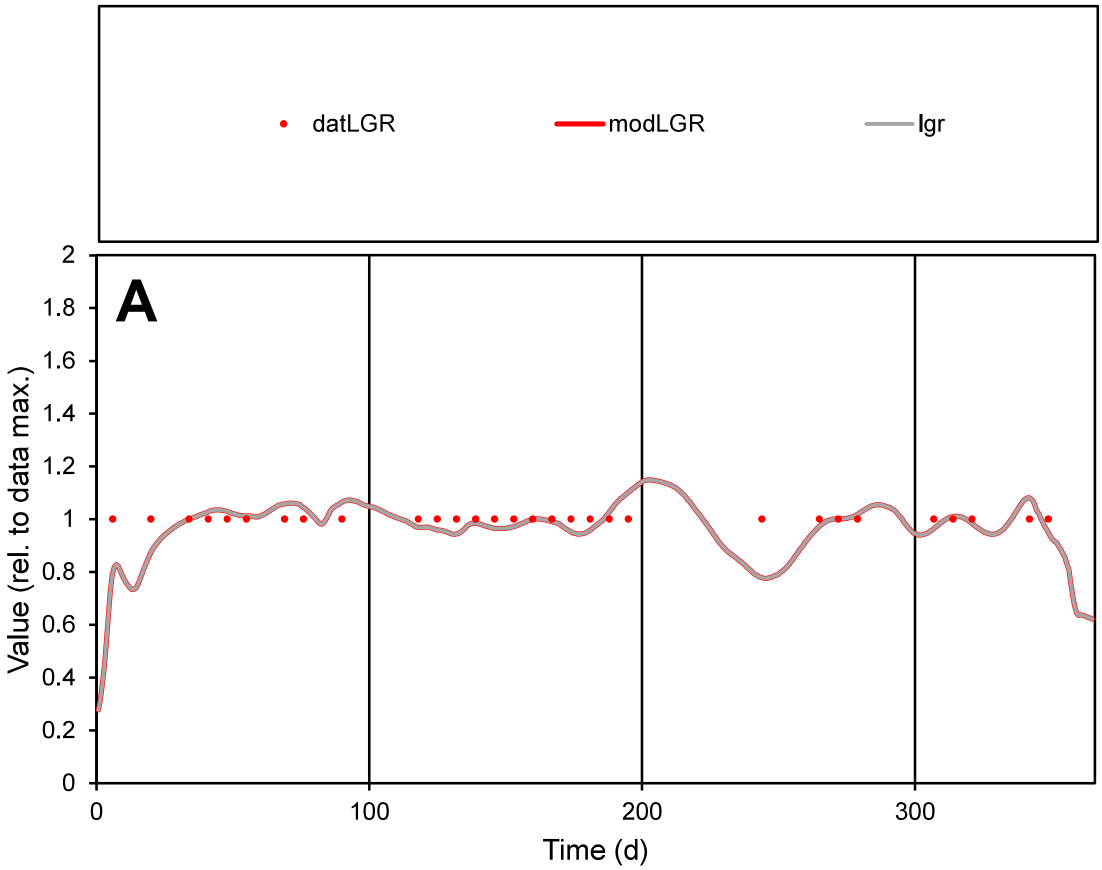

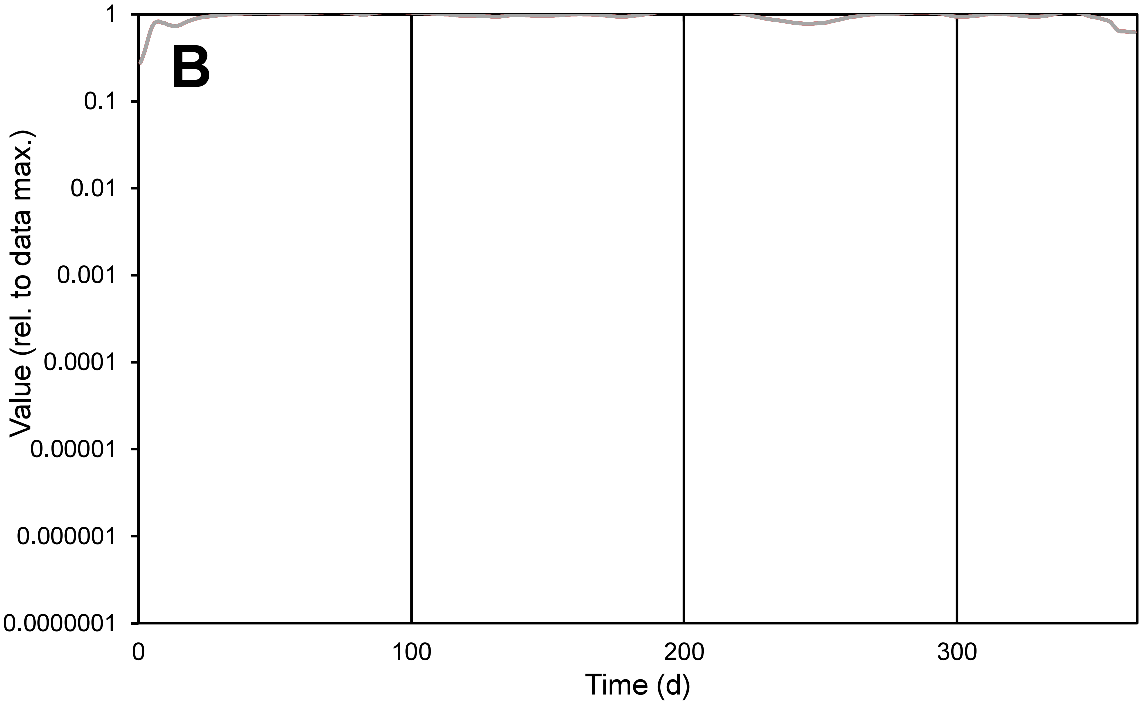

C1

Variables
LGR Provide jtd or Dst
CalibOnly?
2 1 to reproduce of
oNormalize?
2 1=no, 2=yes

ofxn calc*
0.06889
ofxn mod
0.13162

sRun
RunA1
zout
1

Ave
Data
5.2E-06
Model
5.1E-06

data/vMax
5.2E-06

ofxng
275.646

Min d/m
1.28696

ave/min d
1
ave/min m
1.2687

325_Calib_S_A

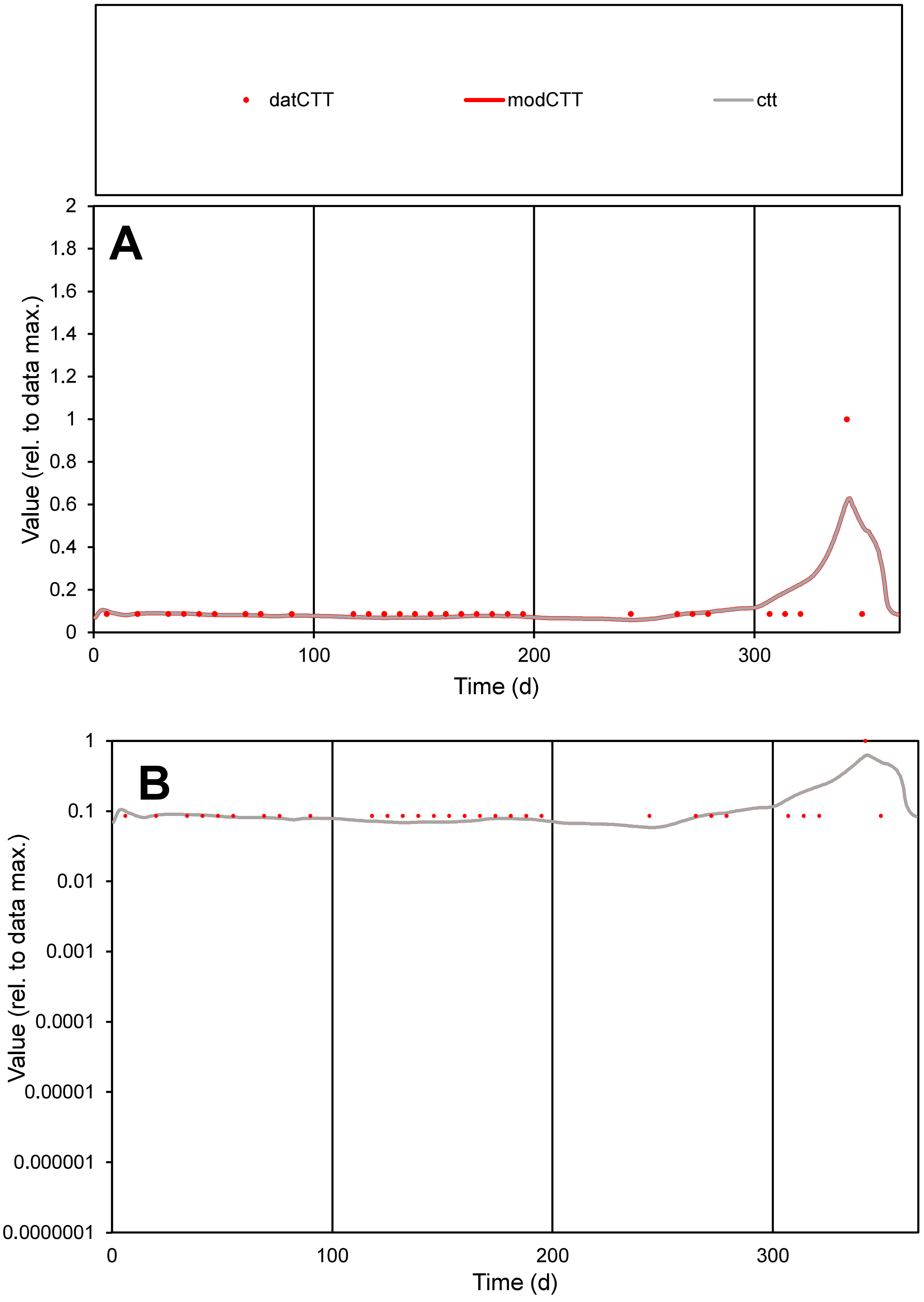

C1

Variables
CTT Provide jtd or Dst
CalibOnly?
2 1 to reproduce of
oNormalize?
2 1=no, 2=yes

ofxn calc*
0.9089
ofxn mod
0.92594

sRun
RunA1
zout
1

Ave
Data
2E-06
Model
2.1E-06

data/vMax
1.7E-05

ofxng
275.646

Min d/m
1.45965

ave/min d
1.35754
ave/min m
2.08163

325_Calib_S_A

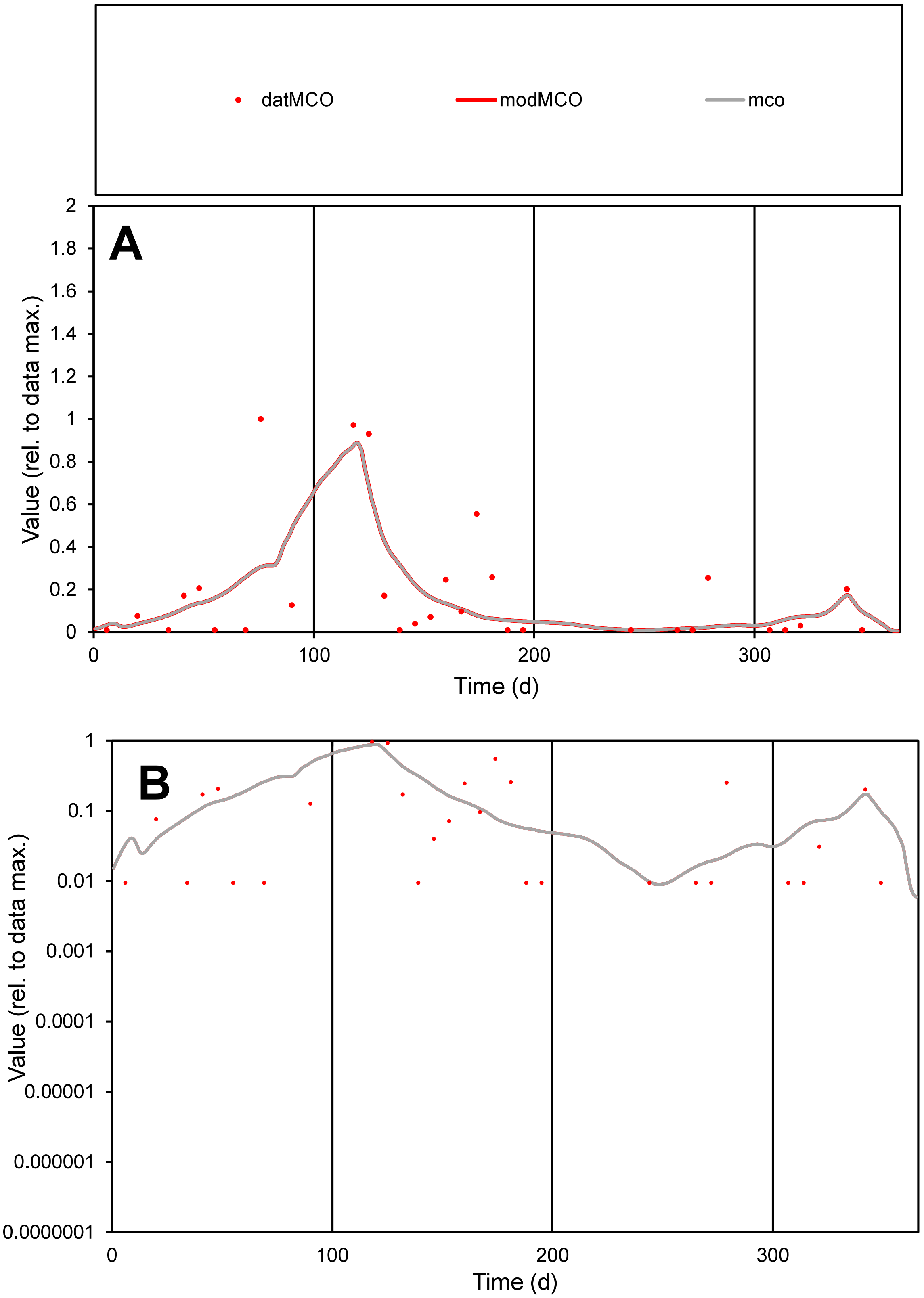

C1

Variables

MCO Provide jtd or Dst

CalibOnly?

2 1 to reproduce of

oNormalize?

2 1=no, 2=yes

ofxn calc*

1.1223

ofxn mod

1.15192

sRun

RunA1

zout

1

Ave

Data

1.2E-06

Model

1.1E-06

data/vMax

6.5E-06

ofxng

275.646

Min d/m

0.99512

ave/min d

19.5697

ave/min m

18.311

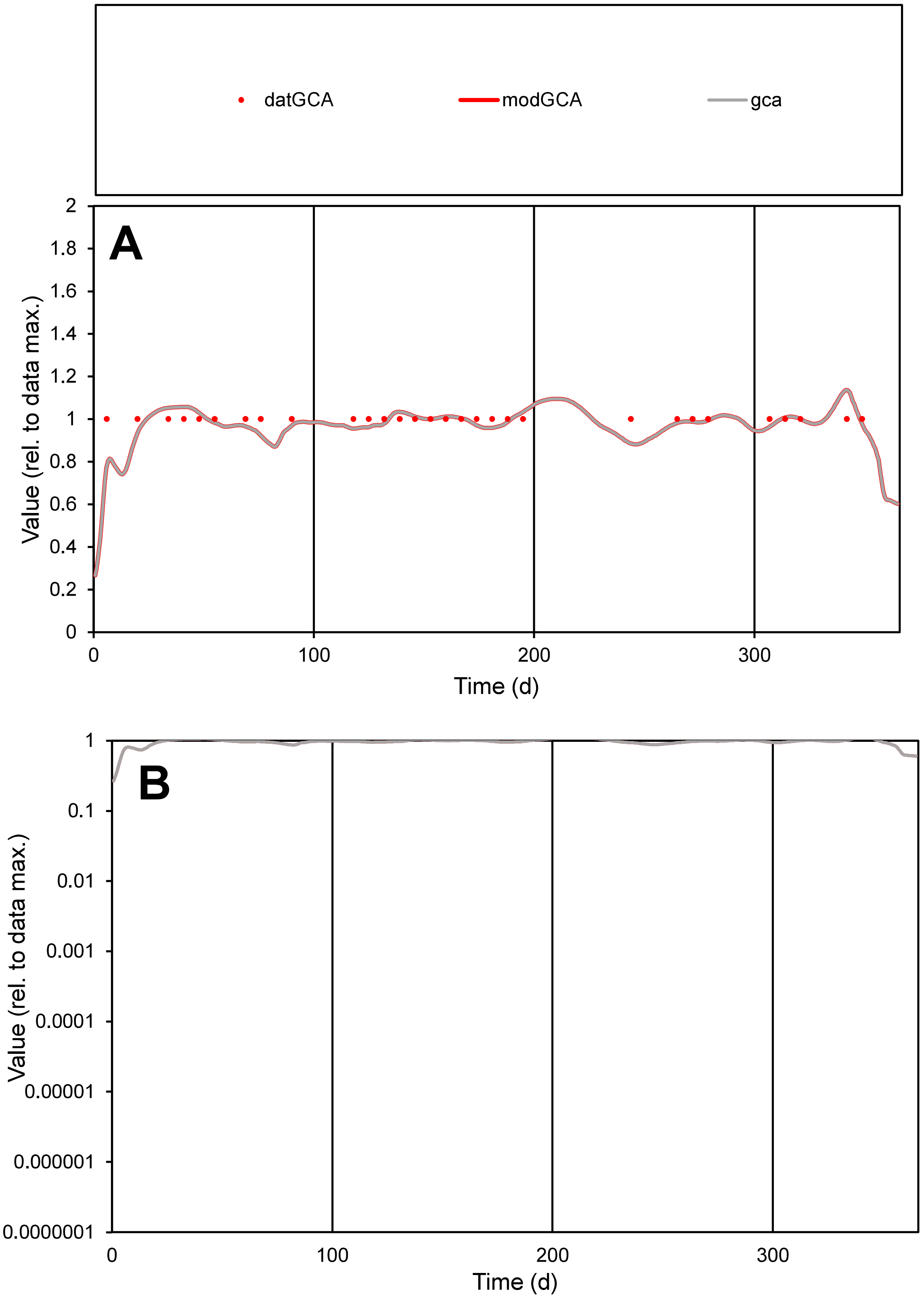

C1

Variables
GCA Provide jtd or Dst
CalibOnly?
2 1 to reproduce of
oNormalize?
2 1=no, 2=yes

ofxn calc*
0.05653
ofxn mod
0.1254

sRun
RunA1
zout
1

Ave
Data
8.4E-06
Model
8.3E-06

data/vMax
8.4E-06

ofxng
275.646

Min d/m
1.22693

ave/min d
1
ave/min m
1.20976

325_Calib_S_A

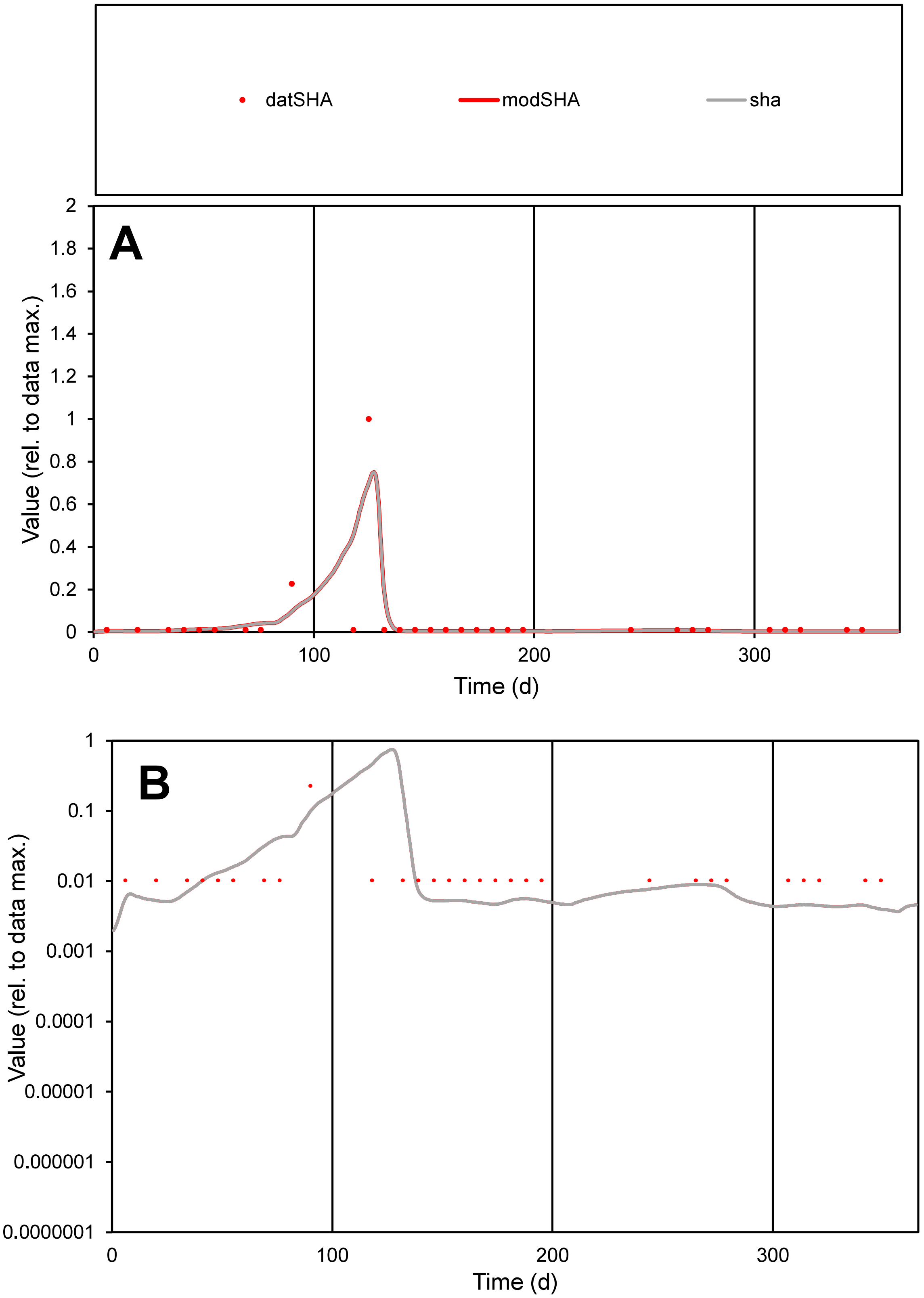

C1

Variables

SHA Provide jtd or Dst

CalibOnly?

2 1 to reproduce of

oNormalize?

2 1=no, 2=yes

ofxn calc*

1.84622

ofxn mod

1.94428

sRun

RunA1

zout

1

Ave

Data

3.4E-06

Model

3.6E-06

data/vMax

6.8E-05

ofxng

275.646

Min d/m

2.62575

ave/min d

4.90857

ave/min m

13.4191

325_Calib_S_A

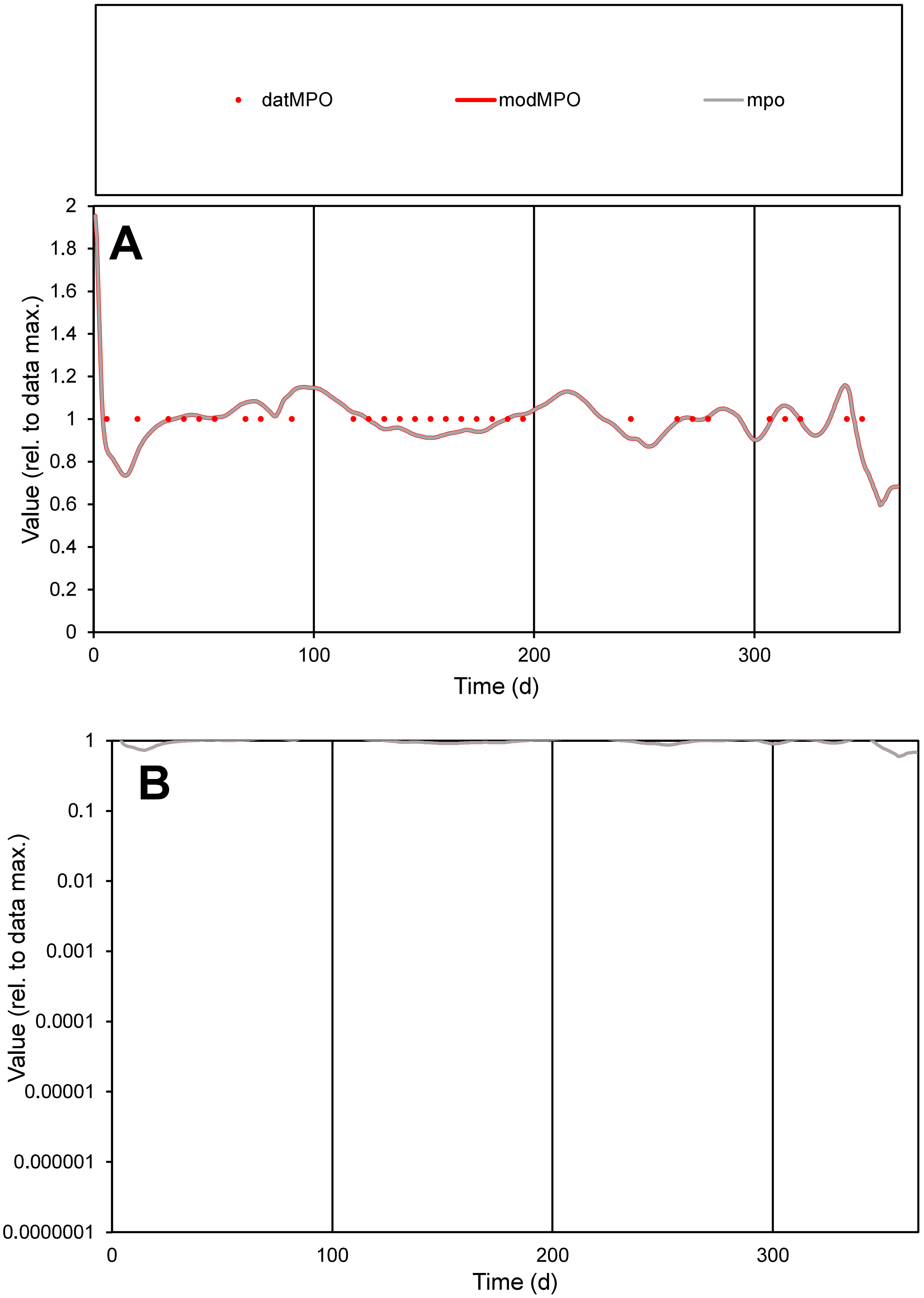

C1

Variables
MPO Provide jtd or Dst
CalibOnly?
2 1 to reproduce of
oNormalize?
2 1=no, 2=yes

ofxn calc*
0.07945
ofxn mod
0.13737

sRun
RunA1
zout
1

Ave
Data
1.2E-05
Model
1.2E-05

data/vMax
1.2E-05

ofxng
275.646

Min d/m
1.3033

ave/min d
1
ave/min m
1.27996

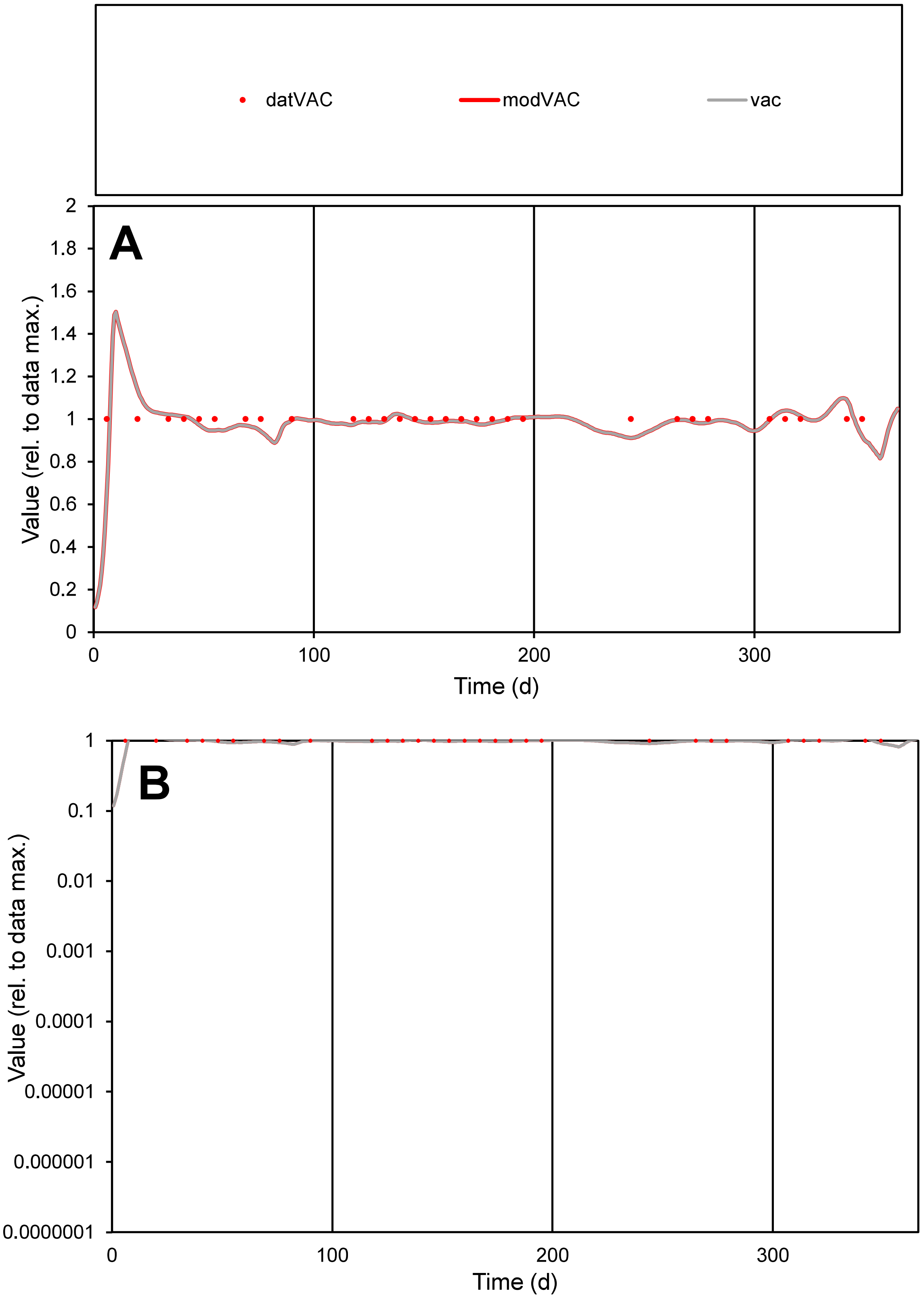

C1

Variables
VAC Provide jtd or Dst
CalibOnly?
2 1 to reproduce of
oNormalize?
2 1=no, 2=yes

ofxn calc*
0.04019
ofxn mod
0.11864

sRun
RunA1
zout
1

Ave
Data
9.5E-06
Model
9.4E-06

data/vMax
9.5E-06

ofxng
275.646

Min d/m
1.11529

ave/min d
1
ave/min m
1.10615

325_Calib_S_A

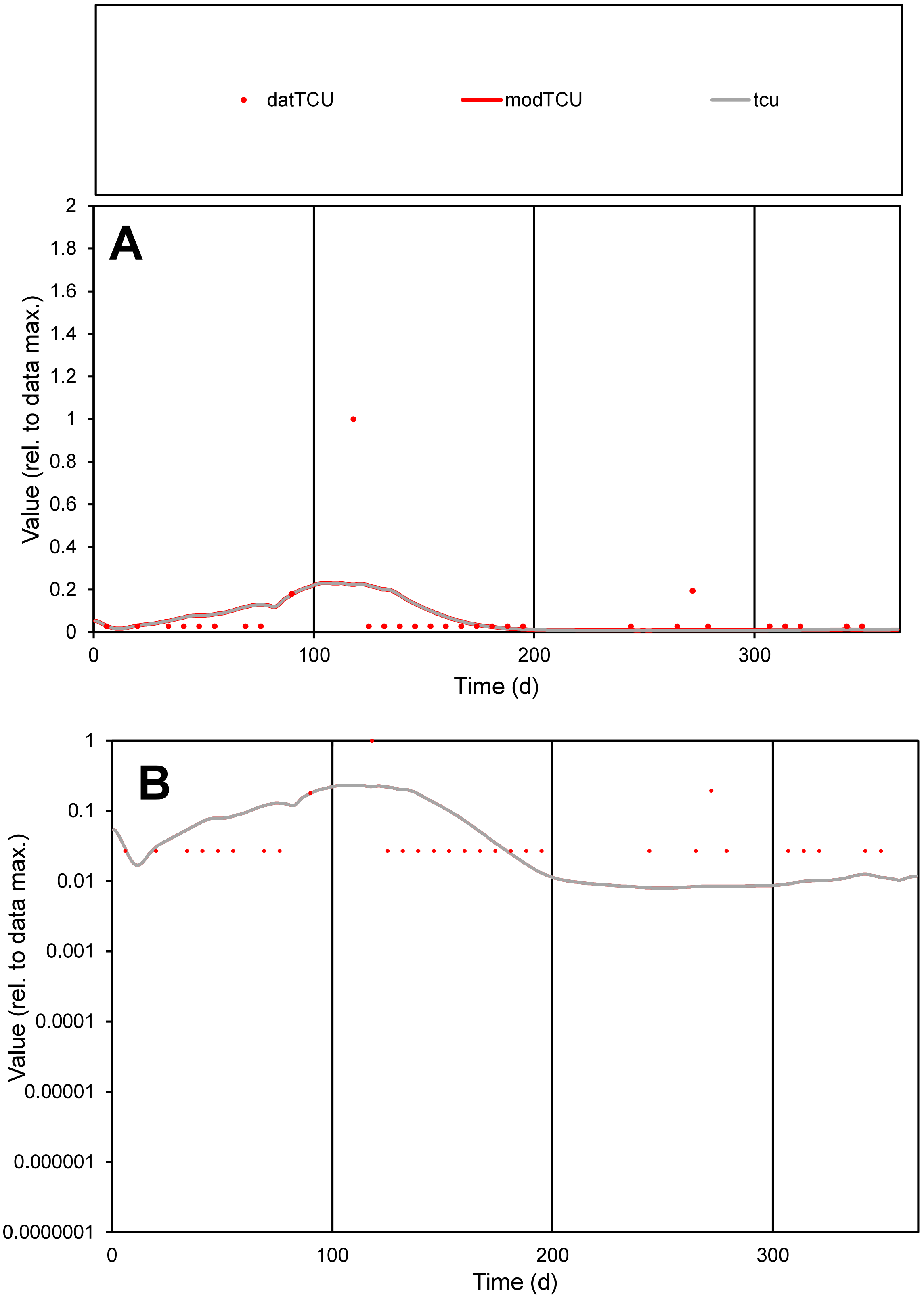

C1

Variables
TCU Provide jtd or Dst
CalibOnly?
2 1 to reproduce of
oNormalize?
2 1=no, 2=yes

ofxn calc*
2.27901
ofxn mod
2.29033

sRun
RunA1
zout
1

Ave
Data
2.5E-05
Model
2.5E-05

data/vMax
0.00036

ofxng
275.646

Min d/m
3.37038

ave/min d
2.59712
ave/min m
8.66882

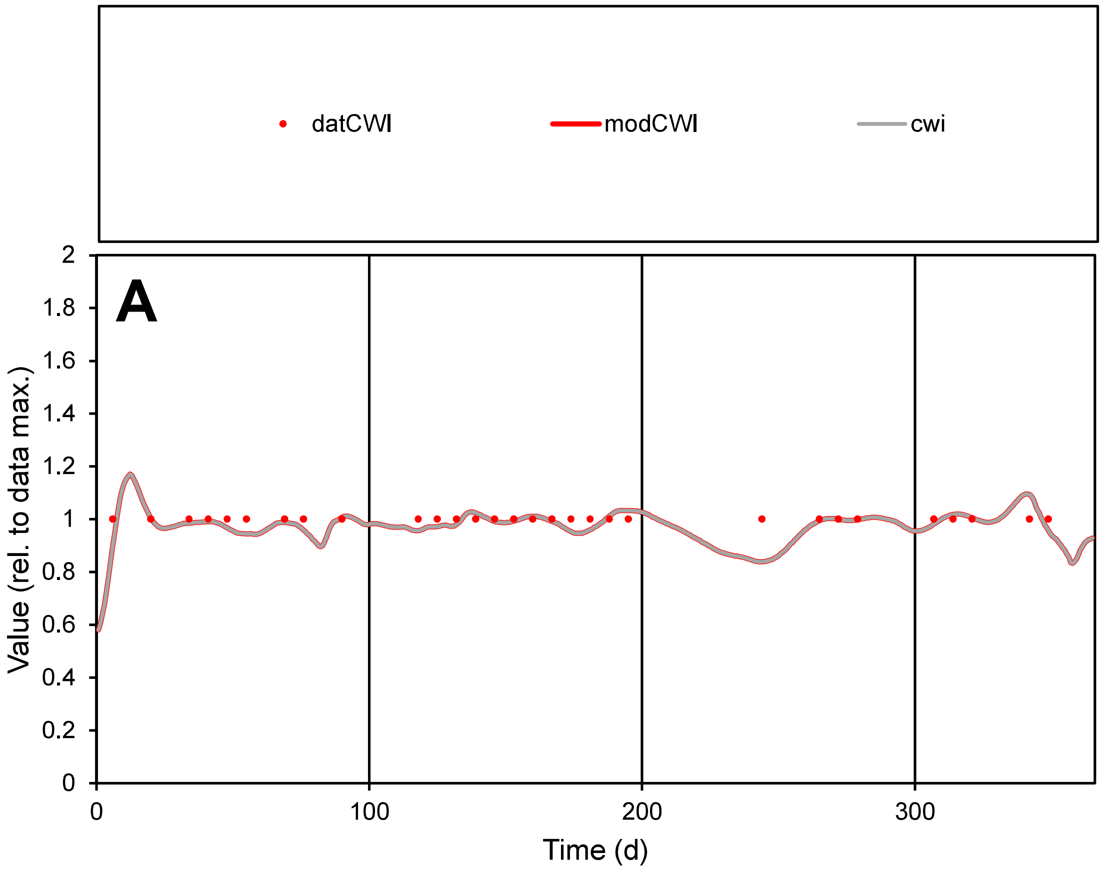

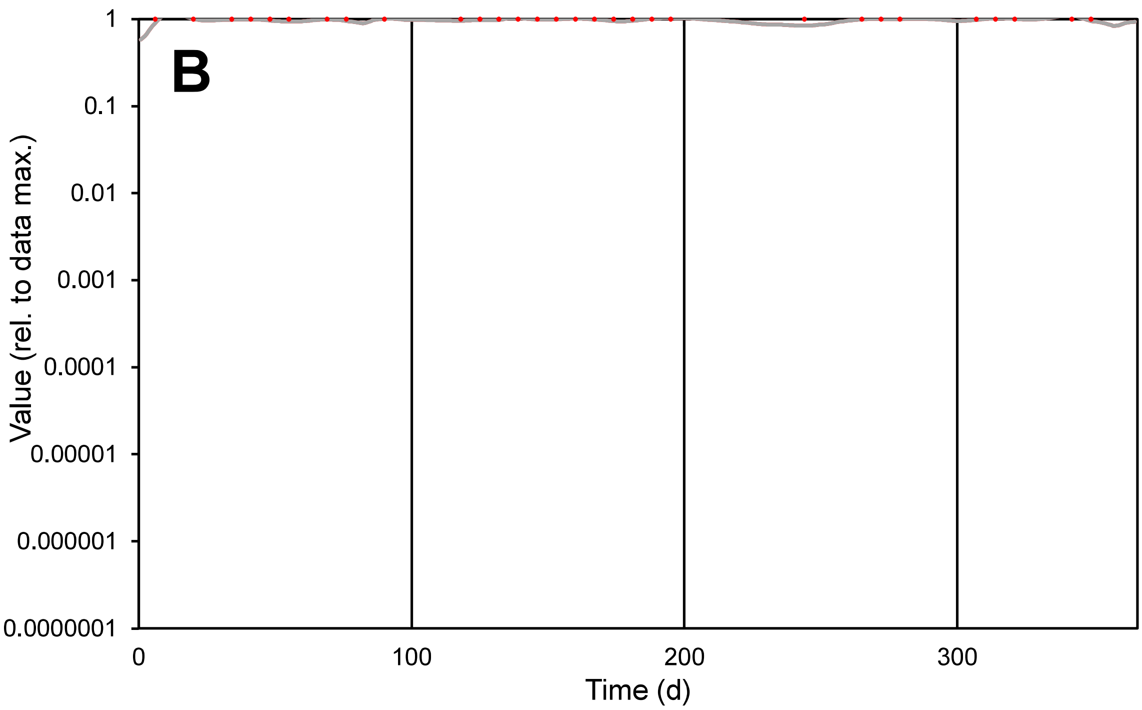

C1

Variables

CWI	Provide jtd or Dst
CalibOnly?	
2	1 to reproduce of
oNormalize?	
2	1=no, 2=yes
ofxn calc*	
0.04174	
ofxn mod	
0.11917	
sRun	
RunA1	
zout	
1	
Ave	
Data	
1.3E-05	
Model	
1.3E-05	
data/vMax	
1.3E-05	
ofxng	
275.646	
Min d/m	
1.18979	
ave/min d	
1	
ave/min m	
1.17302	

325_Calib_S_A

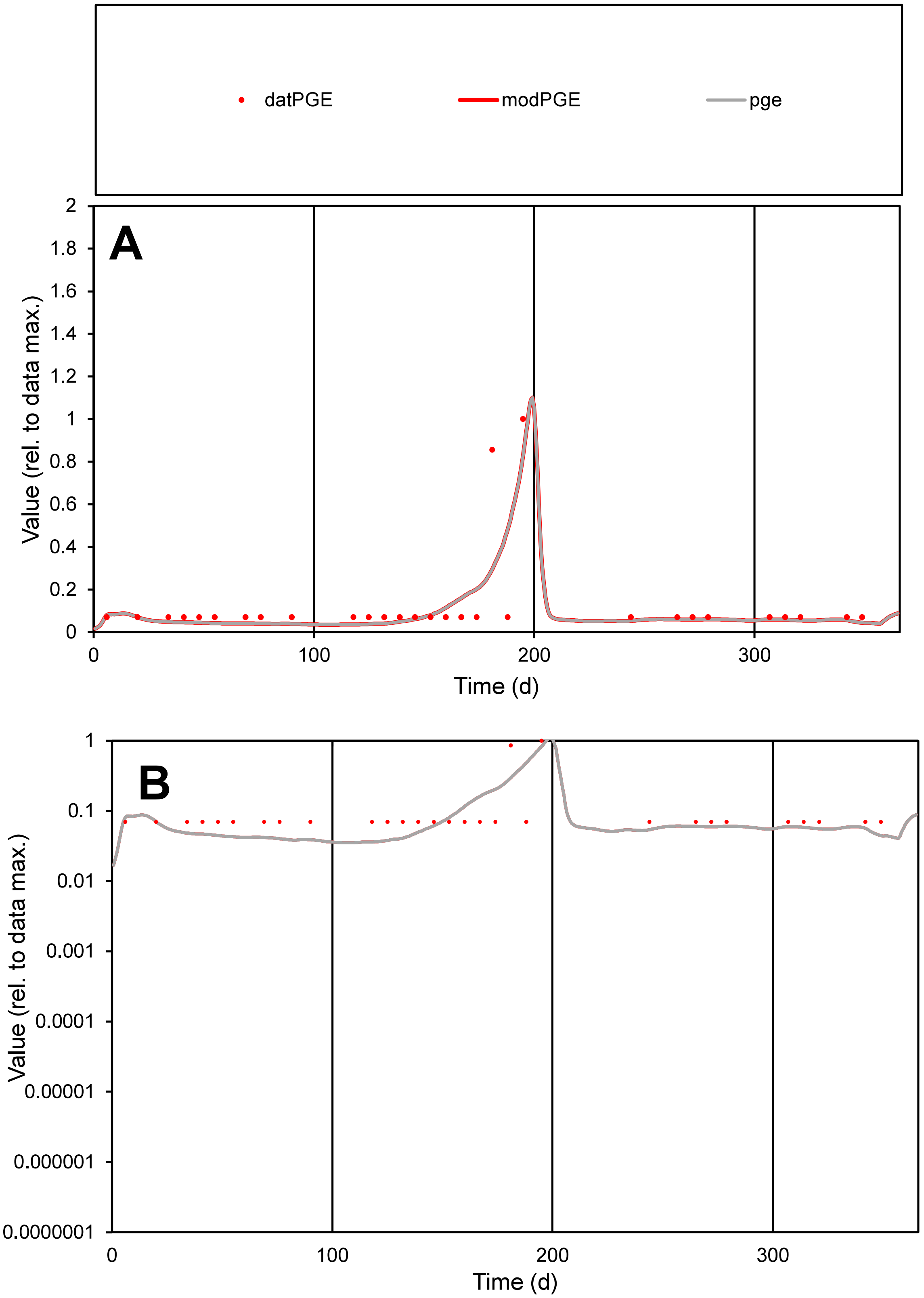

C1

Variables
PGE Provide jtd or Dst
CalibOnly?
2 1 to reproduce of
oNormalize?
2 1=no, 2=yes

ofxn calc*
0.95893
ofxn mod
1.03438

sRun
RunA1
zout
1

Ave
Data
1.1E-05
Model
9.7E-06

data/vMax
8.4E-05

ofxng
275.646

Min d/m
1.93204

ave/min d
1.81943
ave/min m
3.17737

325_Calib_S_A

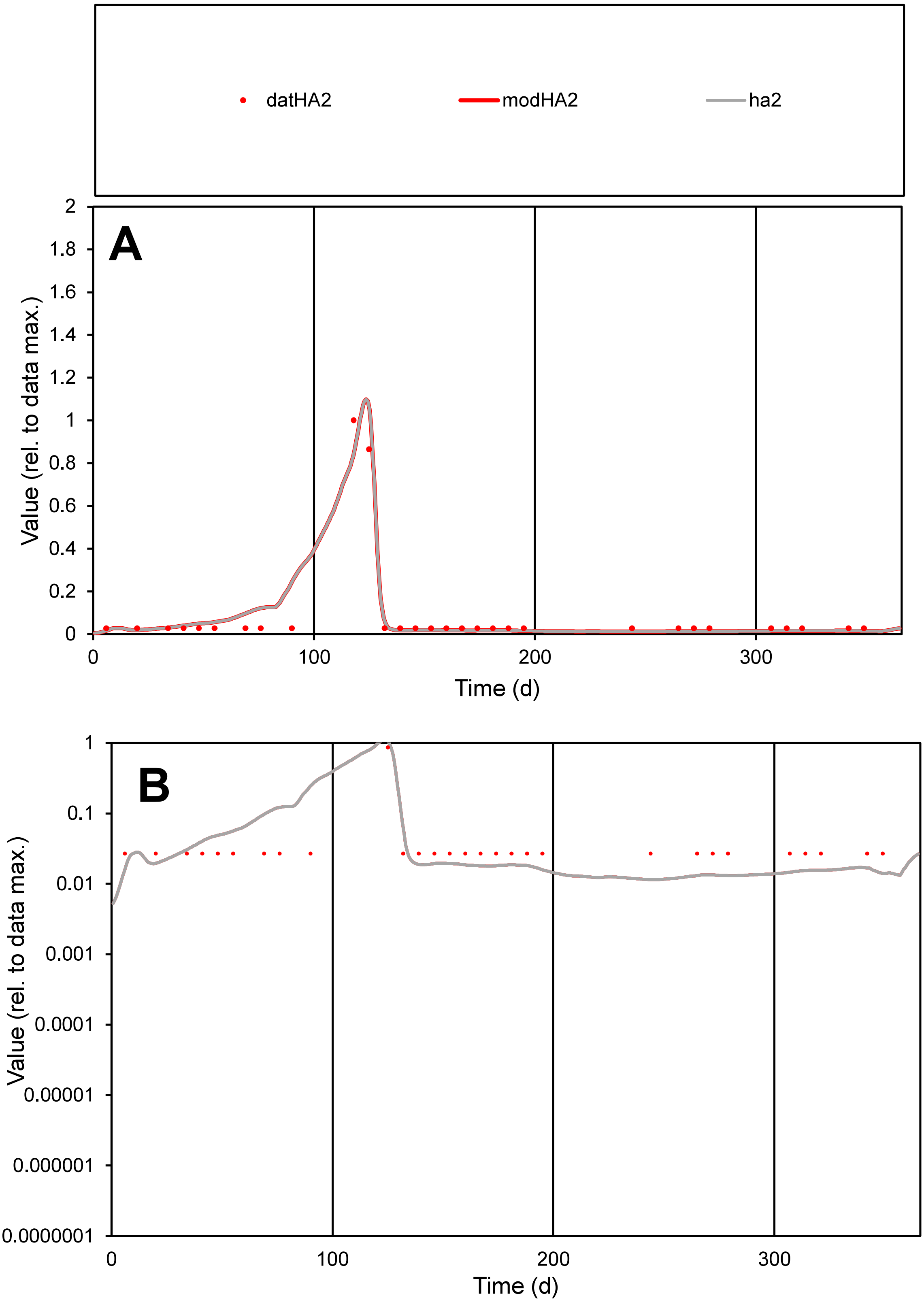

C1

Variables
HA2 Provide jtd or Dst
CalibOnly?
2 1 to reproduce of
oNormalize?
2 1=no, 2=yes

ofxn calc*
0.50272
ofxn mod
0.64009

sRun
RunA1
zout
1

Ave
Data
8.5E-06
Model
9E-06

data/vMax
9.8E-05

ofxng
275.646

Min d/m
2.35263

ave/min d
3.23386
ave/min m
7.9748

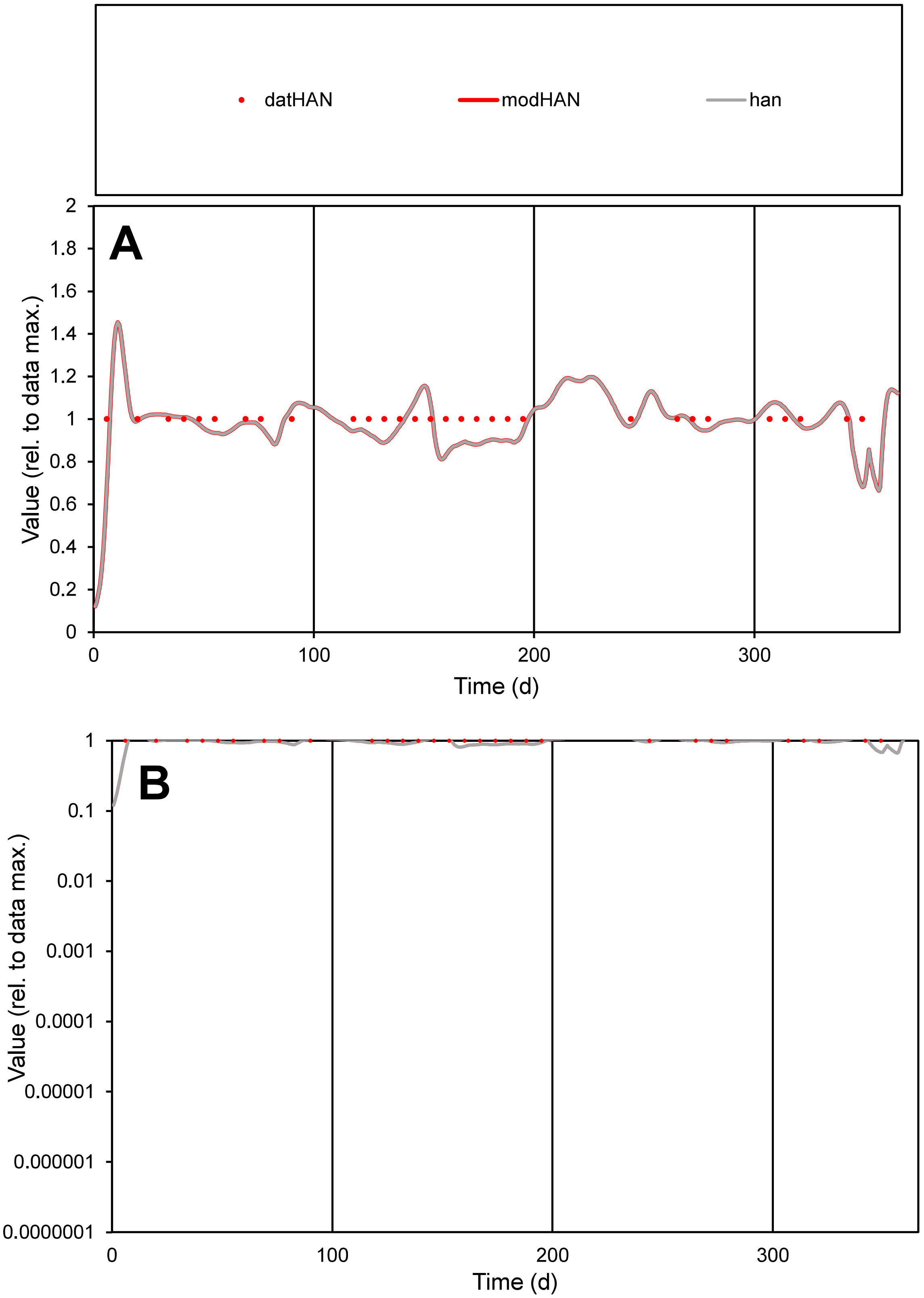

C1

Variables

HAN Provide jtd or Dst

CalibOnly?

2 1 to reproduce of

oNormalize?

2 1=no, 2=yes

ofxn calc*

0.08329

ofxn mod

0.13827

sRun

RunA1

zout

1

Ave

Data

5.1E-06

Model

4.9E-06

data/vMax

5.1E-06

ofxng

275.646

Min d/m

1.39088

ave/min d

1

ave/min m

1.33826

325_Calib_S_A

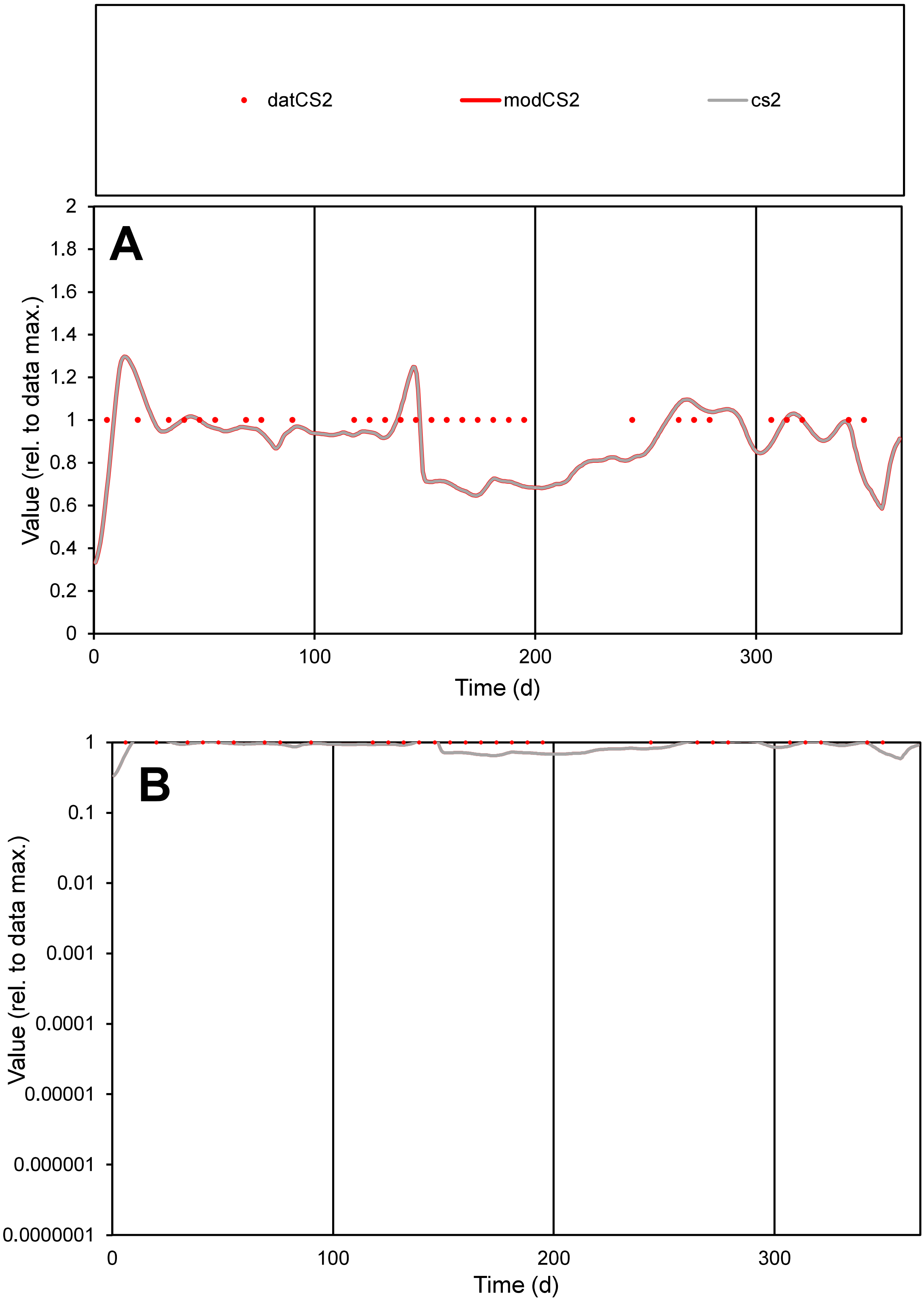

C1

Variables
CS2 Provide jtd or Dst
CalibOnly?
2 1 to reproduce of
oNormalize?
2 1=no, 2=yes

ofxn calc*
0.17395
ofxn mod
0.20529

sRun
RunA1
zout
1

Ave
Data
7.6E-06
Model
6.9E-06

data/vMax
7.6E-06

ofxng
275.646

Min d/m
1.52567

ave/min d
1
ave/min m
1.37948

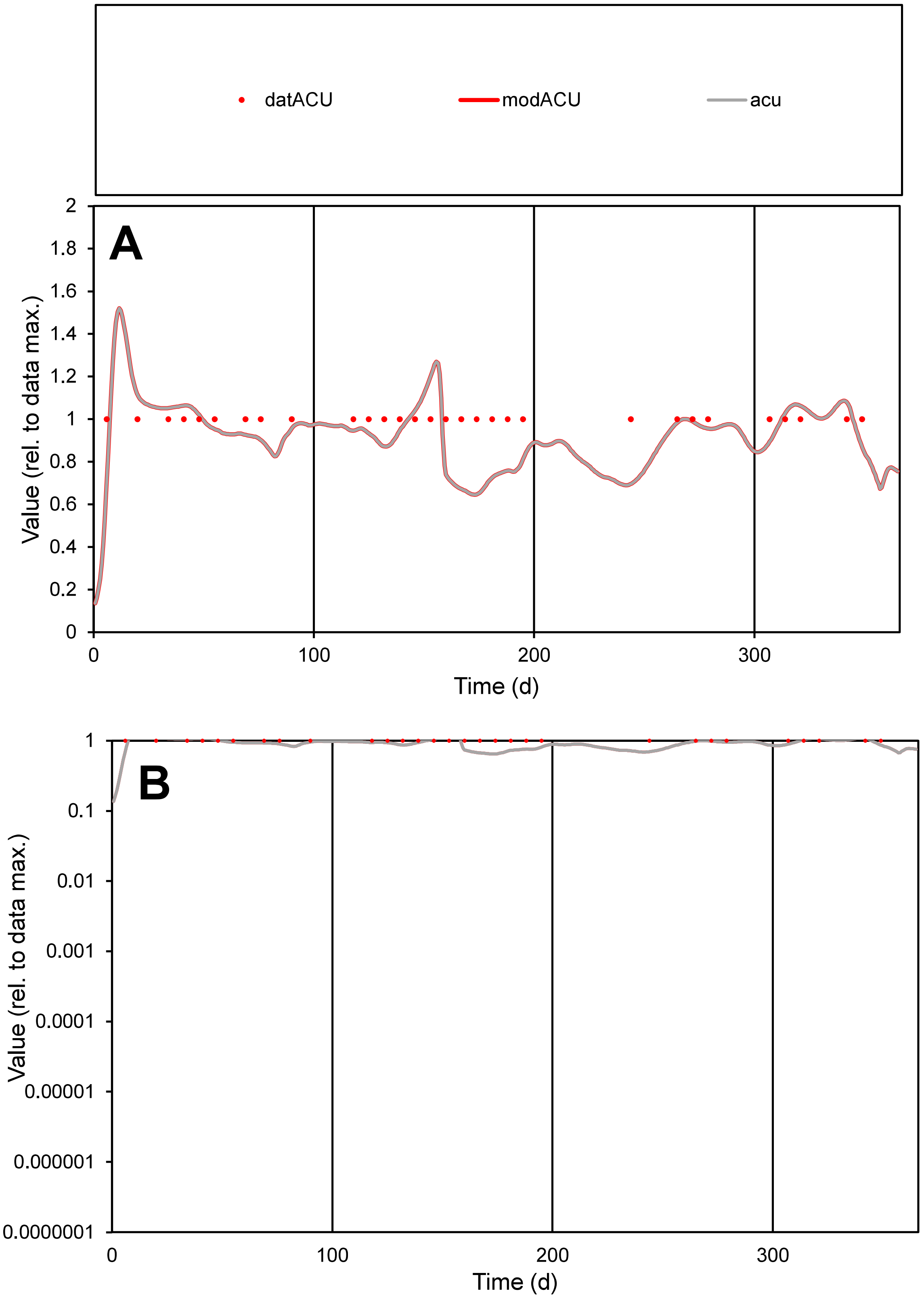

C1

Variables
ACU Provide jtd or Dst
CalibOnly?
2 1 to reproduce of
oNormalize?
2 1=no, 2=yes

ofxn calc*
0.155
ofxn mod
0.18948

sRun
RunA1
zout
1

Ave
Data
1.1E-05
Model
1E-05

data/vMax
1.1E-05

ofxng
275.646

Min d/m
1.52757

ave/min d
1
ave/min m
1.41972

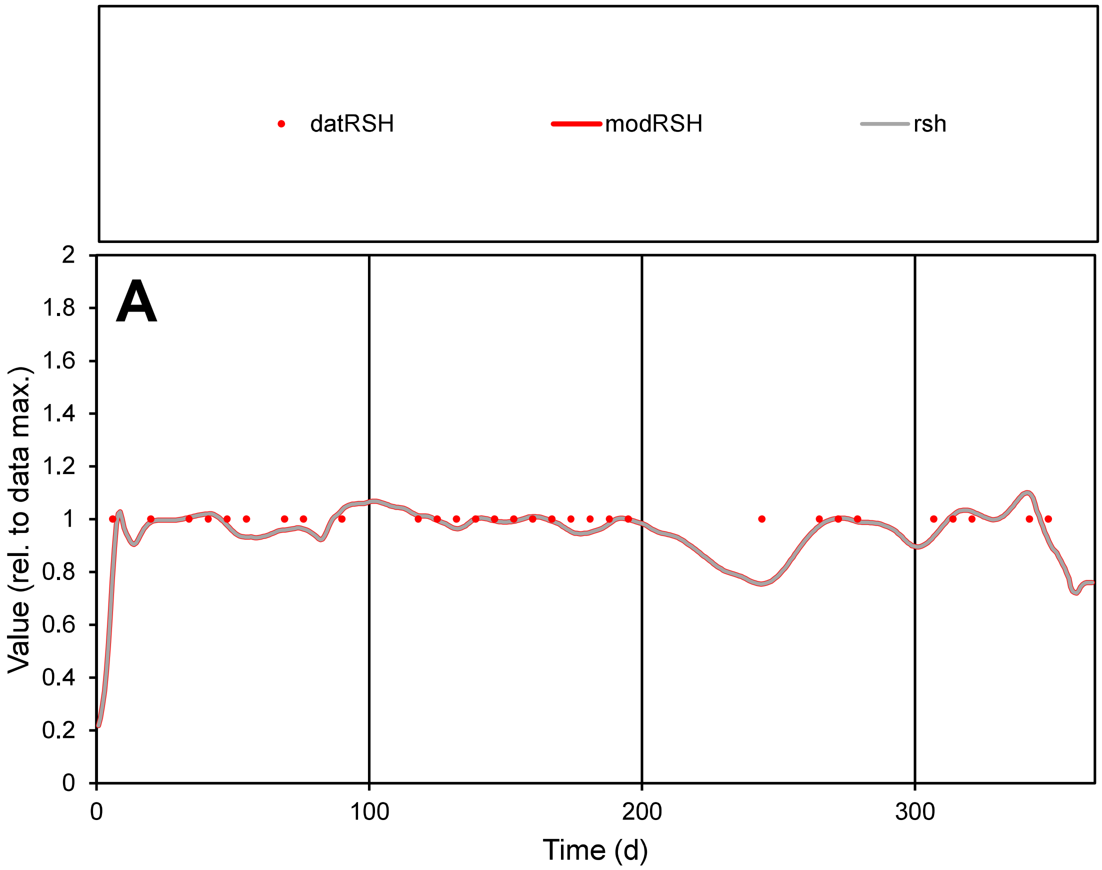

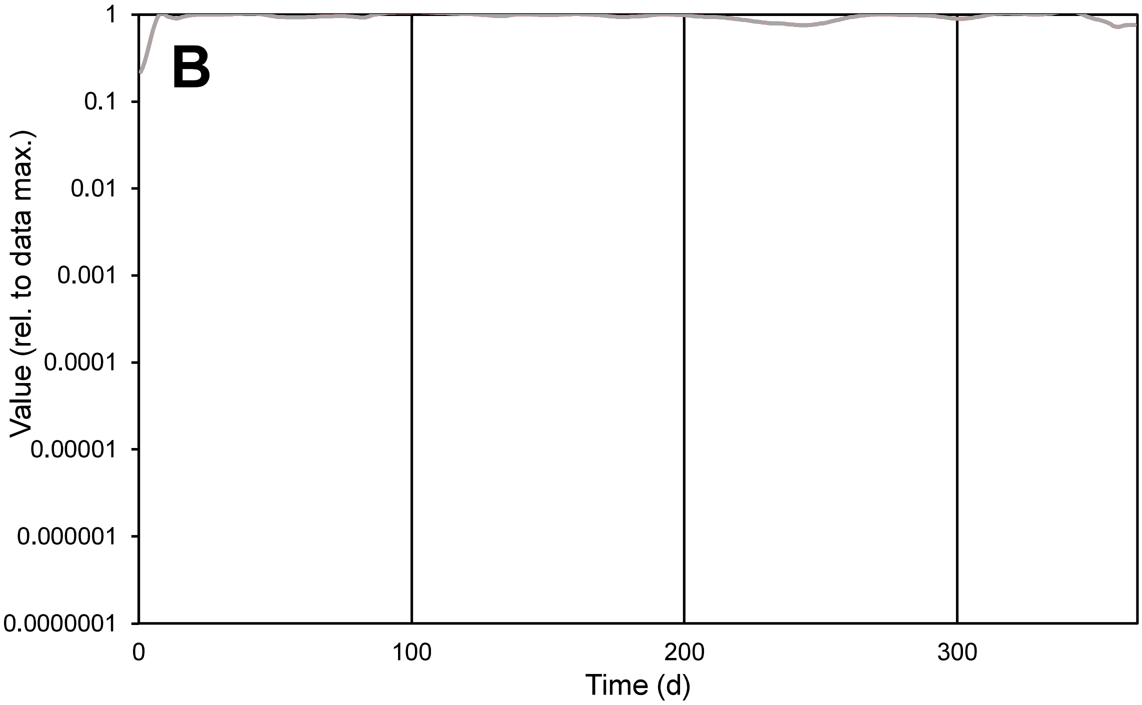

C1

Variables
RSH Provide jtd or Dst
CalibOnly?
2 1 to reproduce of
oNormalize?
2 1=no, 2=yes

ofxn calc*
0.05796
ofxn mod
0.12551

sRun
RunA1
zout
1

Ave
Data
1.8E-05
Model
1.8E-05

data/vMax
1.8E-05

ofxng
275.646

Min d/m
1.32198

ave/min d
1
ave/min m
1.29674

325_Calib_S_A

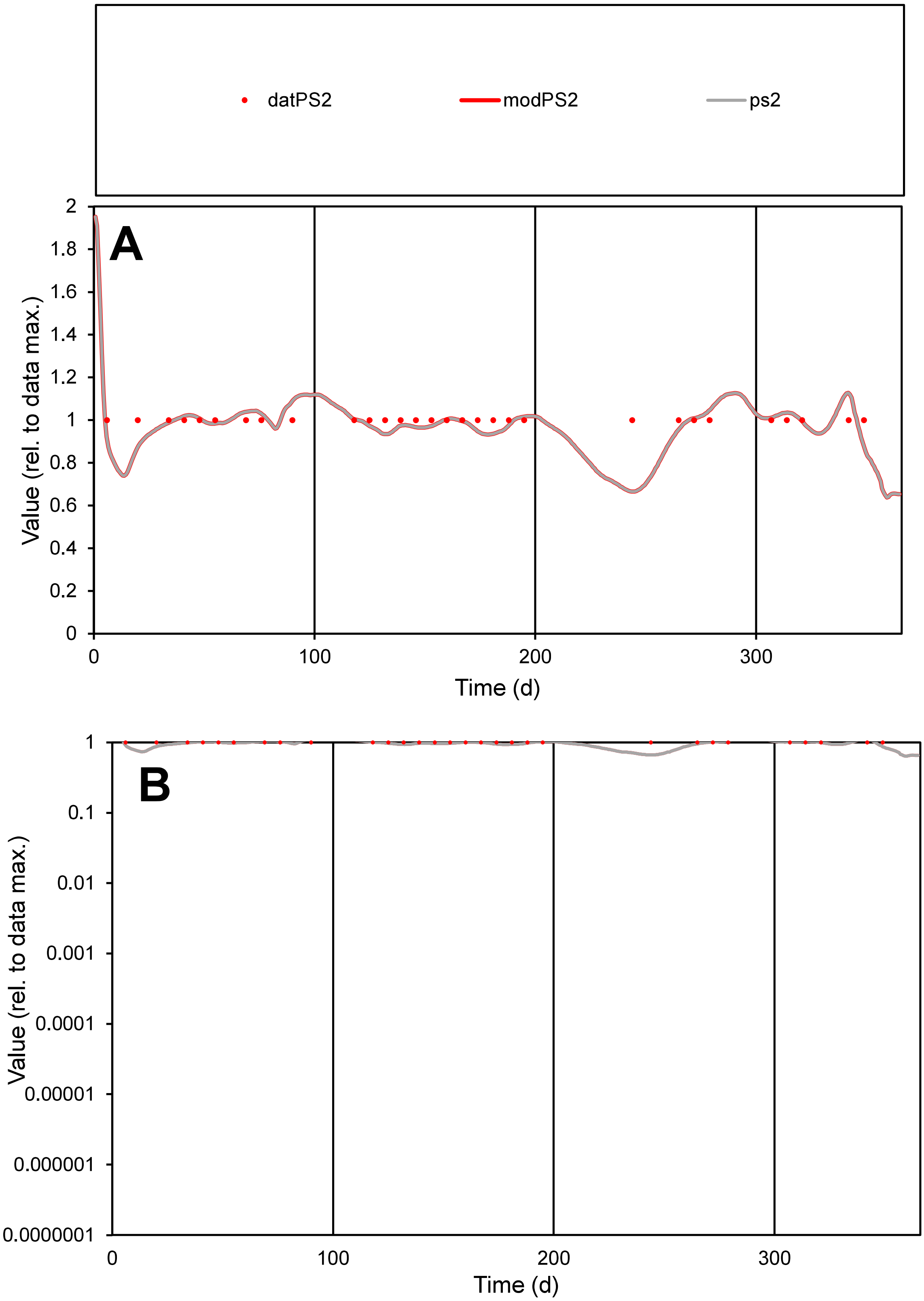

C1

Variables
PS2 Provide jtd or Dst
CalibOnly?
2 1 to reproduce of
oNormalize?
2 1=no, 2=yes

ofxn calc*
0.08608
ofxn mod
0.14115

sRun
RunA1
zout
1

Ave
Data
1.9E-05
Model
1.9E-05

data/vMax
1.9E-05

ofxng
275.646

Min d/m
1.5

ave/min d
1
ave/min m
1.46507

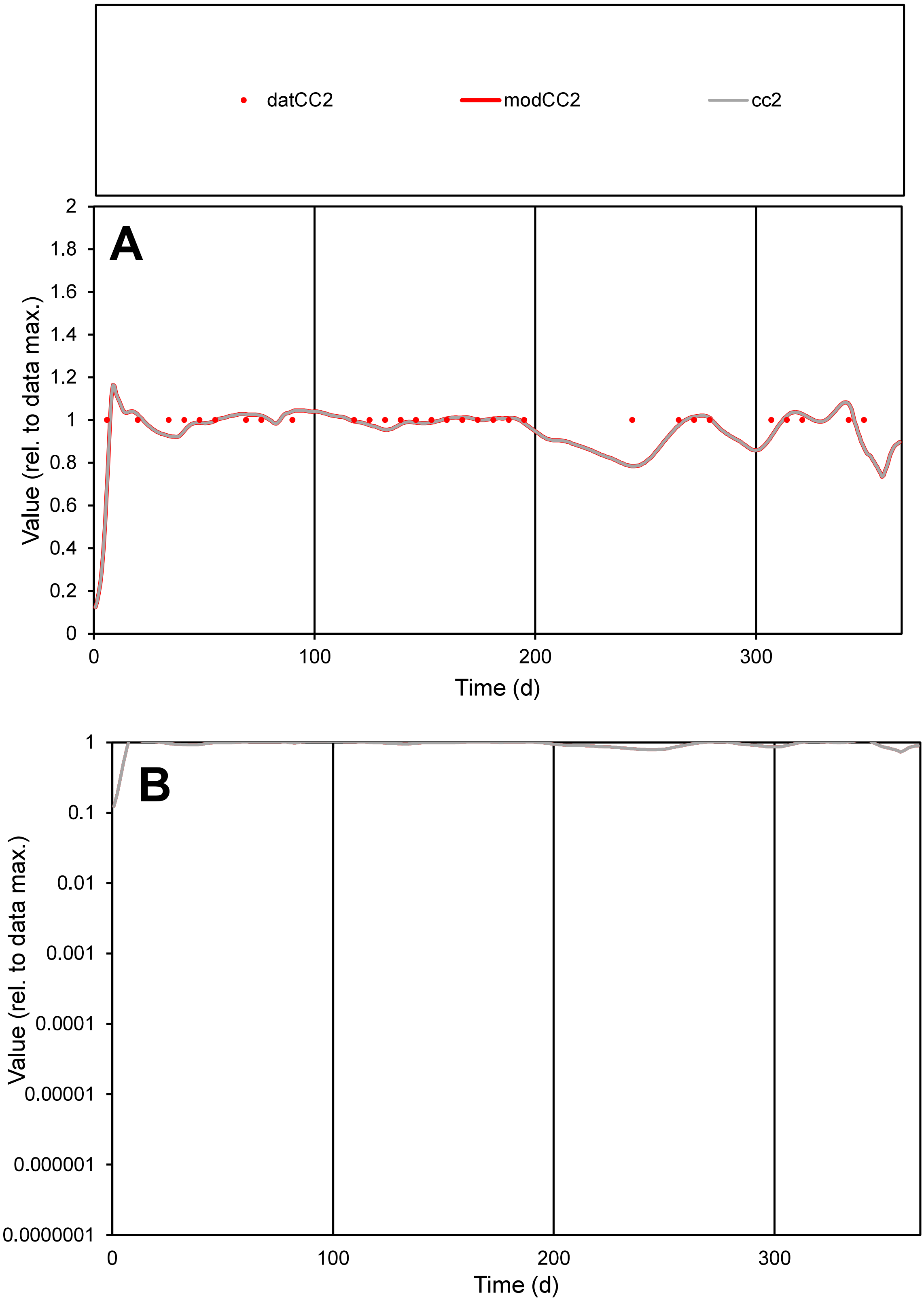

C1

Variables
CC2 Provide jtd or Dst
CalibOnly?
2 1 to reproduce of
oNormalize?
2 1=no, 2=yes

ofxn calc*
0.05603
ofxn mod
0.12473

sRun
RunA1
zout
1

Ave
Data
5.9E-06
Model
5.8E-06

data/vMax
5.9E-06

ofxng
275.646

Min d/m
1.27498

ave/min d
1
ave/min m
1.25573

325_Calib_S_A

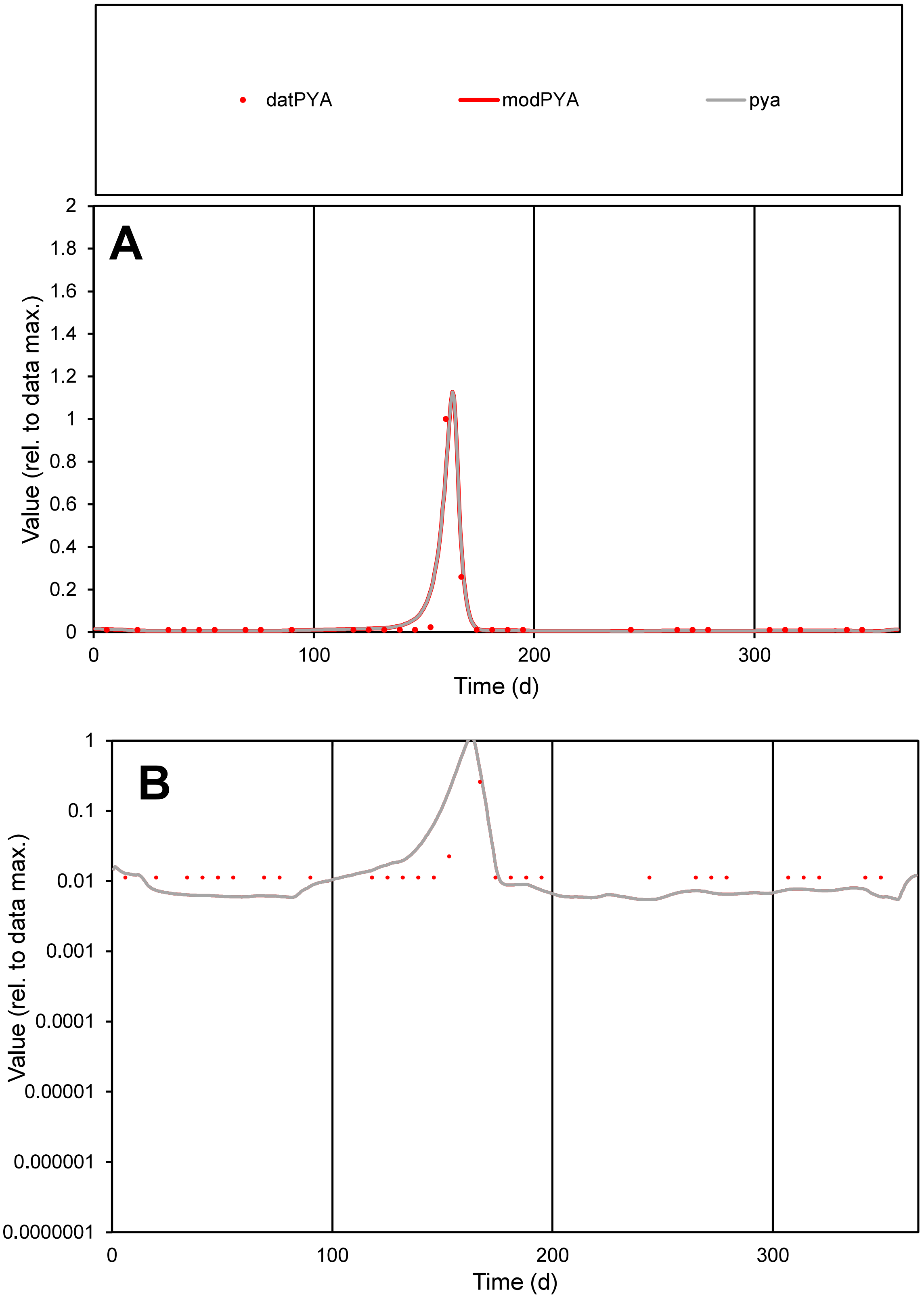

C1

Variables
PYA Provide jtd or Dst
CalibOnly?
2 1 to reproduce of
oNormalize?
2 1=no, 2=yes

ofxn calc*
0.4468
ofxn mod
0.63297

sRun
RunA1
zout
1

Ave
Data
8.5E-06
Model
9E-06

data/vMax
0.00016

ofxng
275.646

Min d/m
2.06543

ave/min d
4.69202
ave/min m
10.2634

325_Calib_S_A

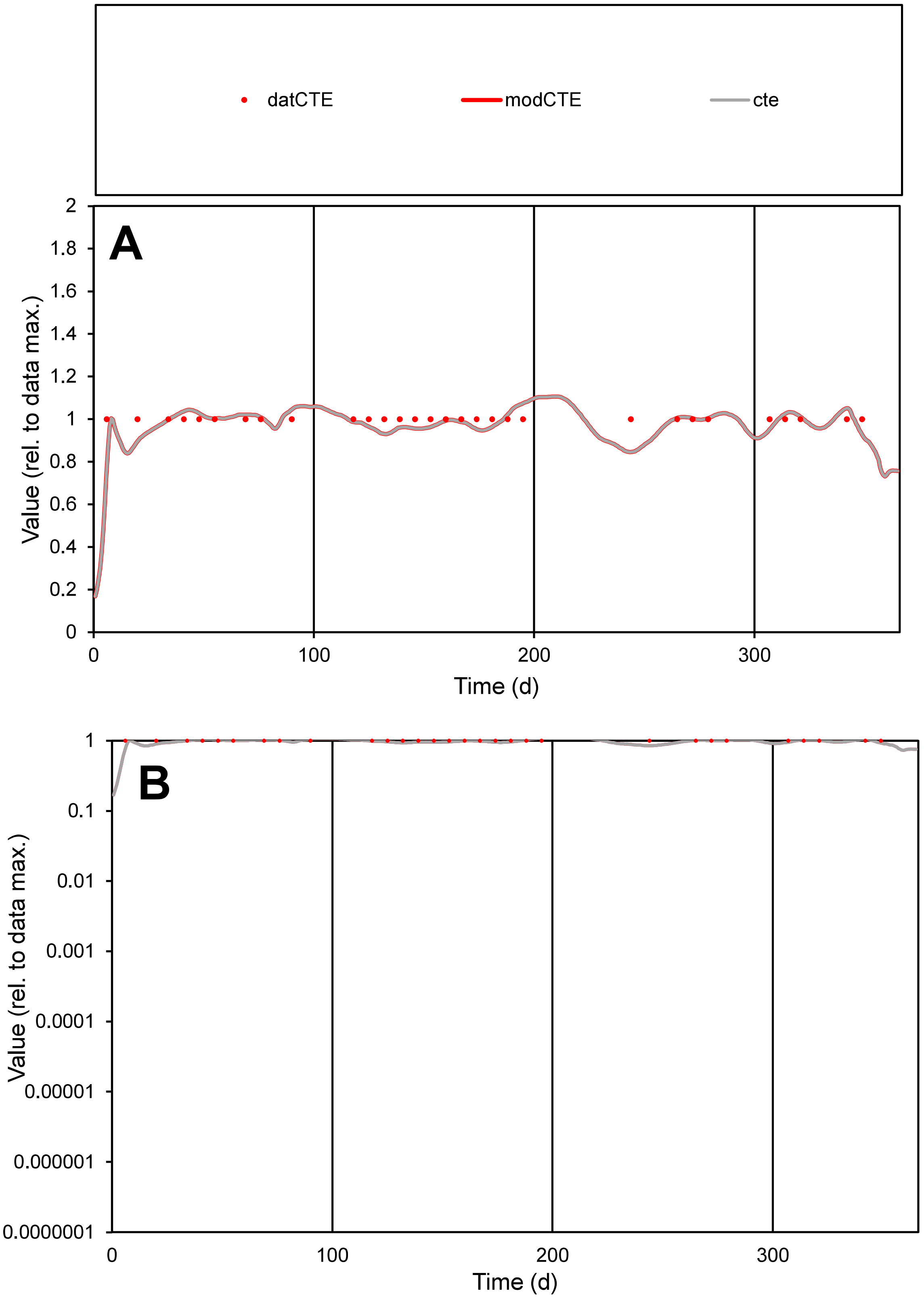

C1

Variables
CTE Provide jtd or Dst
CalibOnly?
2 1 to reproduce of
oNormalize?
2 1=no, 2=yes

ofxn calc*
0.04992
ofxn mod
0.12253

sRun
RunA1
zout
1

Ave
Data
6.1E-06
Model
6E-06

data/vMax
6.1E-06

ofxng
275.646

Min d/m
1.18057

ave/min d
1
ave/min m
1.16583

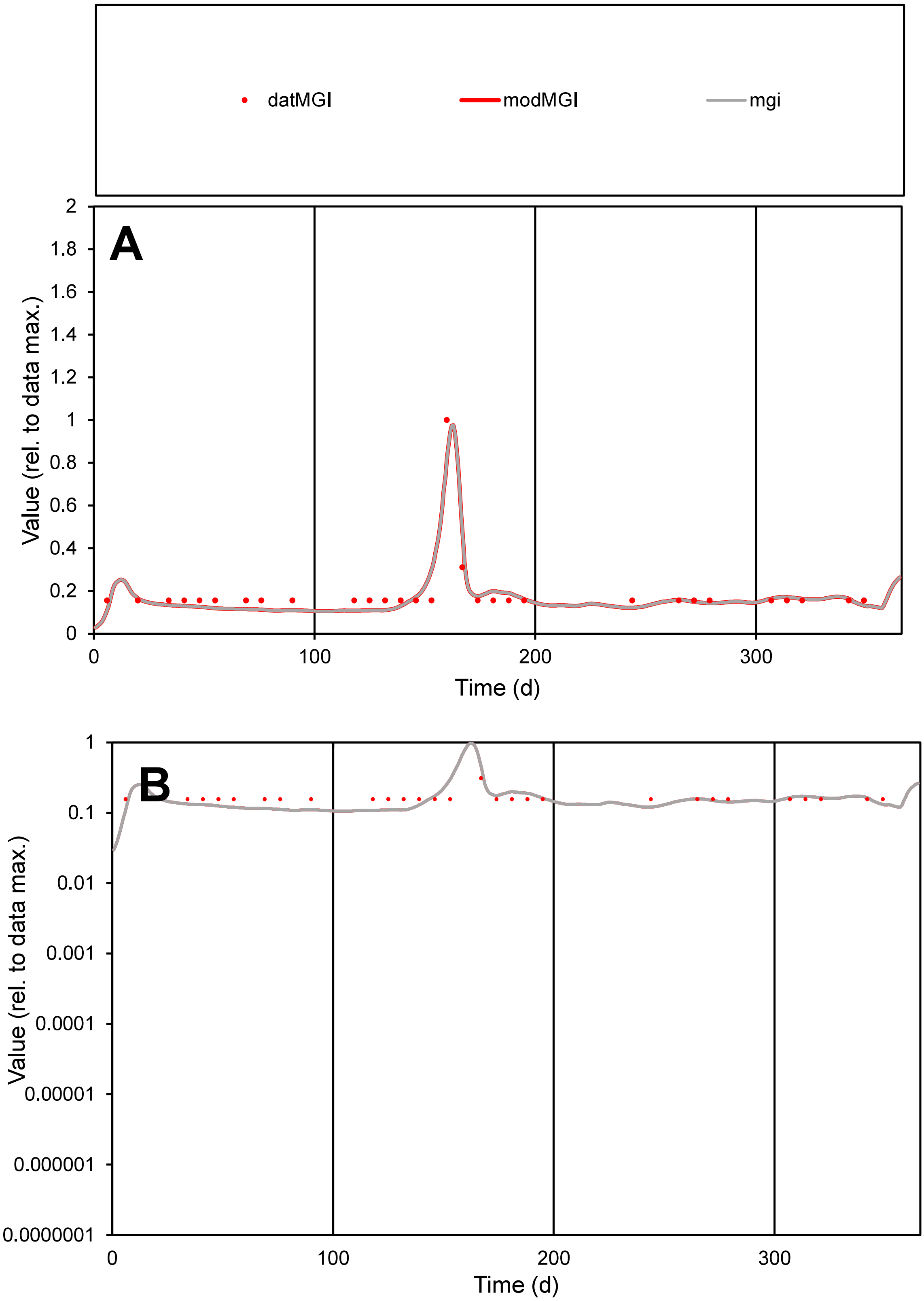

C1

Variables

MGI Provide jtd or Dst

CalibOnly?

2 1 to reproduce of

oNormalize?

2 1=no, 2=yes

ofxn calc*

0.21974

ofxn mod

0.25508

sRun

RunA1

zout

1

Ave

Data

1.3E-05

Model

1.2E-05

data/vMax

6.6E-05

ofxng

275.646

Min d/m

1.44255

ave/min d

1.21437

ave/min m

1.69176

325_Calib_S_A

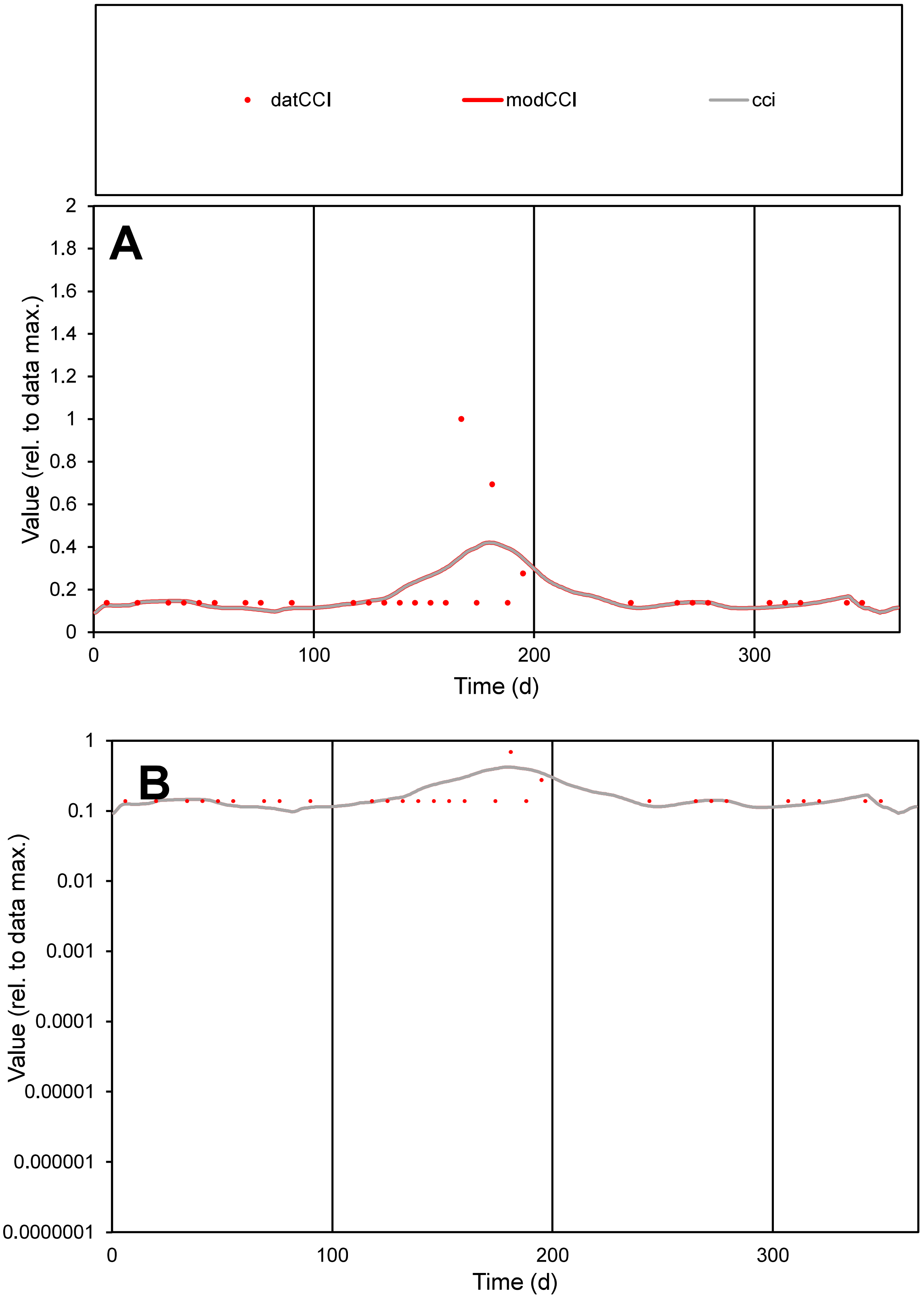

C1

Variables

CCI Provide jtd or Dst

CalibOnly?

2 1 to reproduce of

oNormalize?

2 1=no, 2=yes

ofxn calc*

0.79397

ofxn mod

0.8062

sRun

RunA1

zout

1

Ave

Data

1.6E-05

Model

1.6E-05

data/vMax

8.3E-05

ofxng

275.646

Min d/m

1.3355

ave/min d

1.37581

ave/min m

1.832

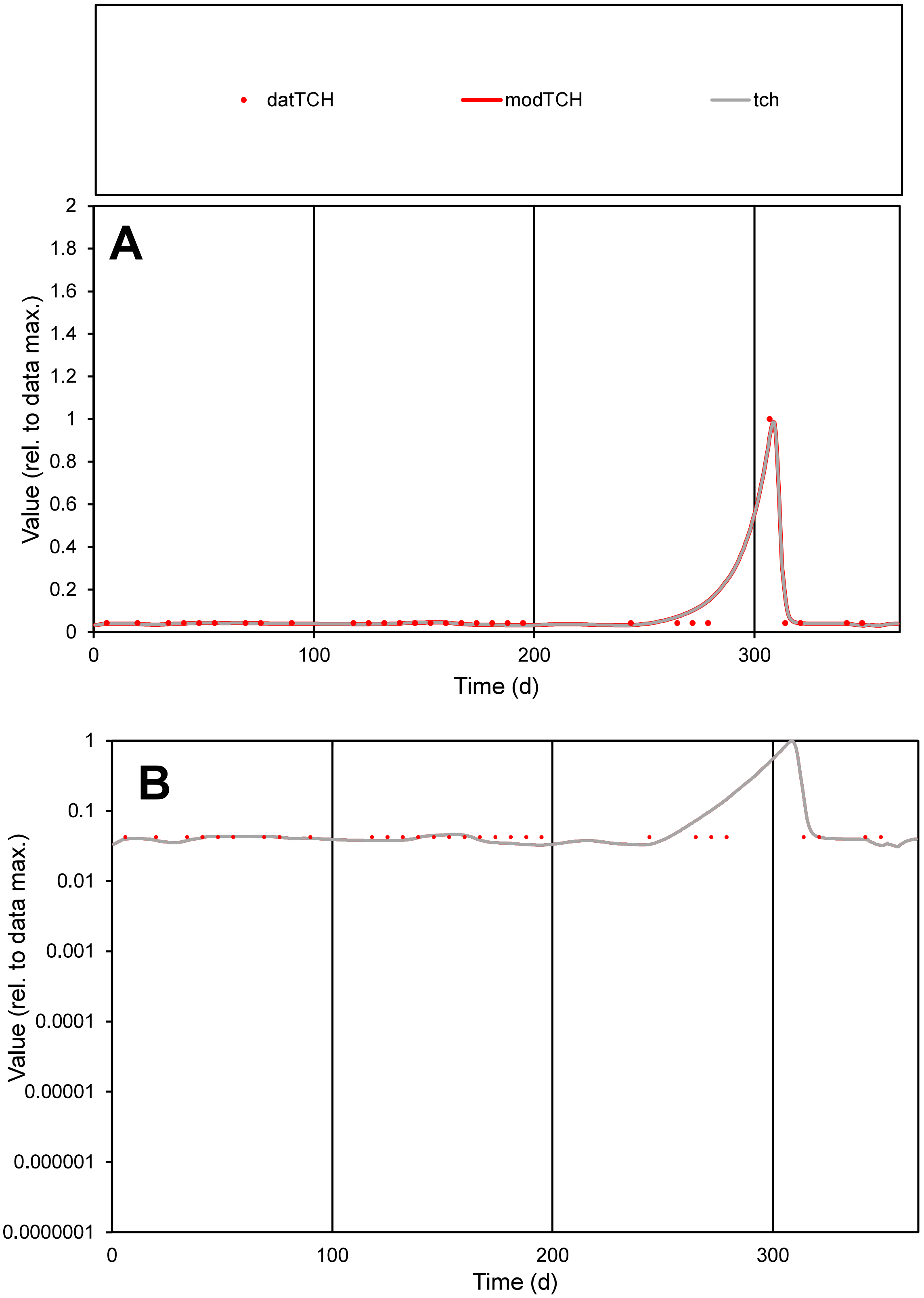

C1

Variables
TCH Provide jtd or Dst
CalibOnly?
2 1 to reproduce of
oNormalize?
2 1=no, 2=yes

ofxn calc*
0.32932
ofxn mod
0.44669

sRun
RunA1
zout
1

Ave
Data
2.9E-06
Model
3.1E-06

data/vMax
4E-05

ofxng
275.646

Min d/m
1.31201

ave/min d
1.74946
ave/min m
2.43593

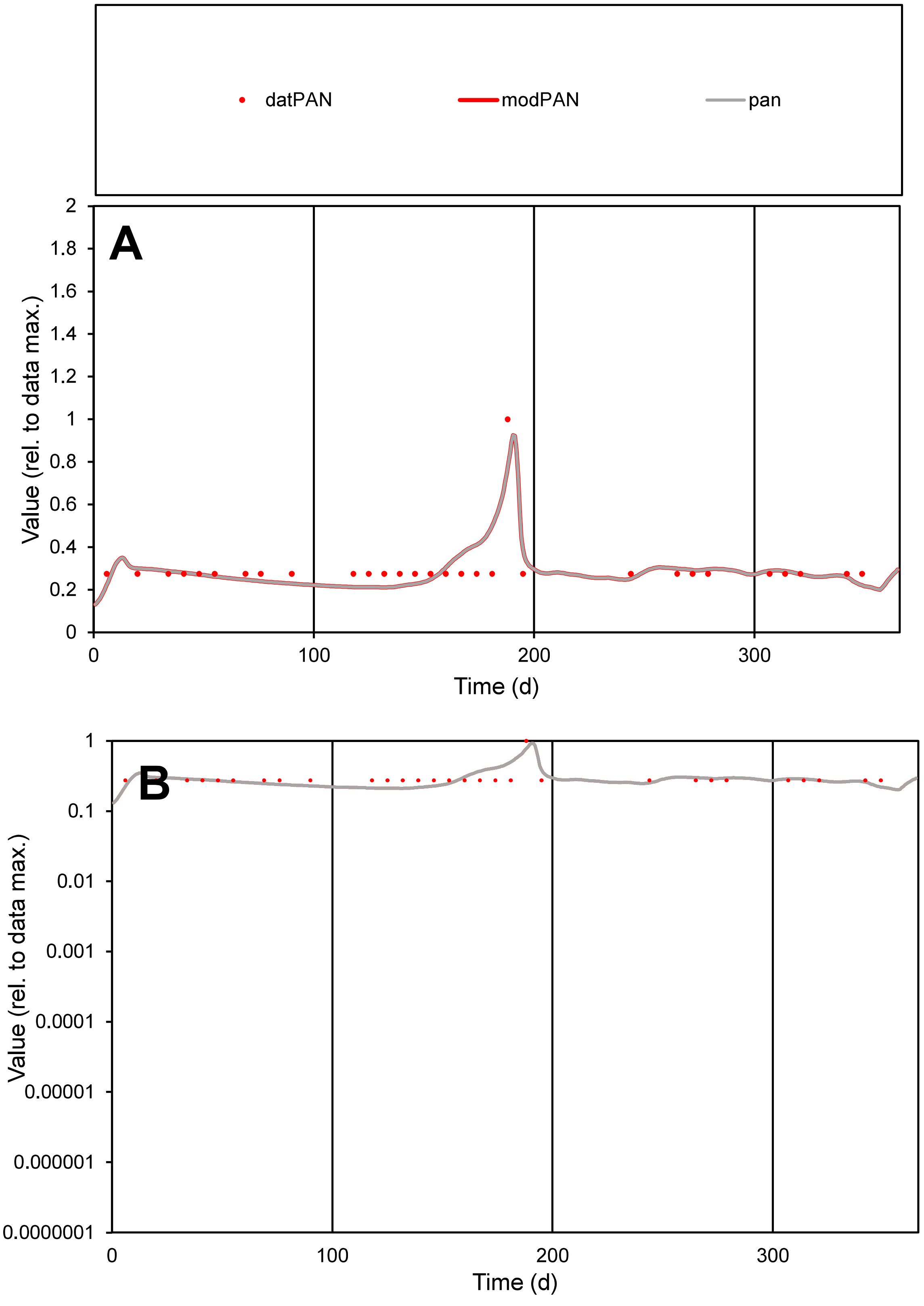

C1

Variables
PAN Provide jtd or Dst
CalibOnly?
2 1 to reproduce of
oNormalize?
2 1=no, 2=yes

ofxn calc*
0.23051
ofxn mod
0.26045

sRun
RunA1
zout
1

Ave
Data
9.9E-06
Model
9.9E-06

data/vMax
3.3E-05

ofxng
275.646

Min d/m
1.3023

ave/min d
1.08798
ave/min m
1.41573

325_Calib_S_A

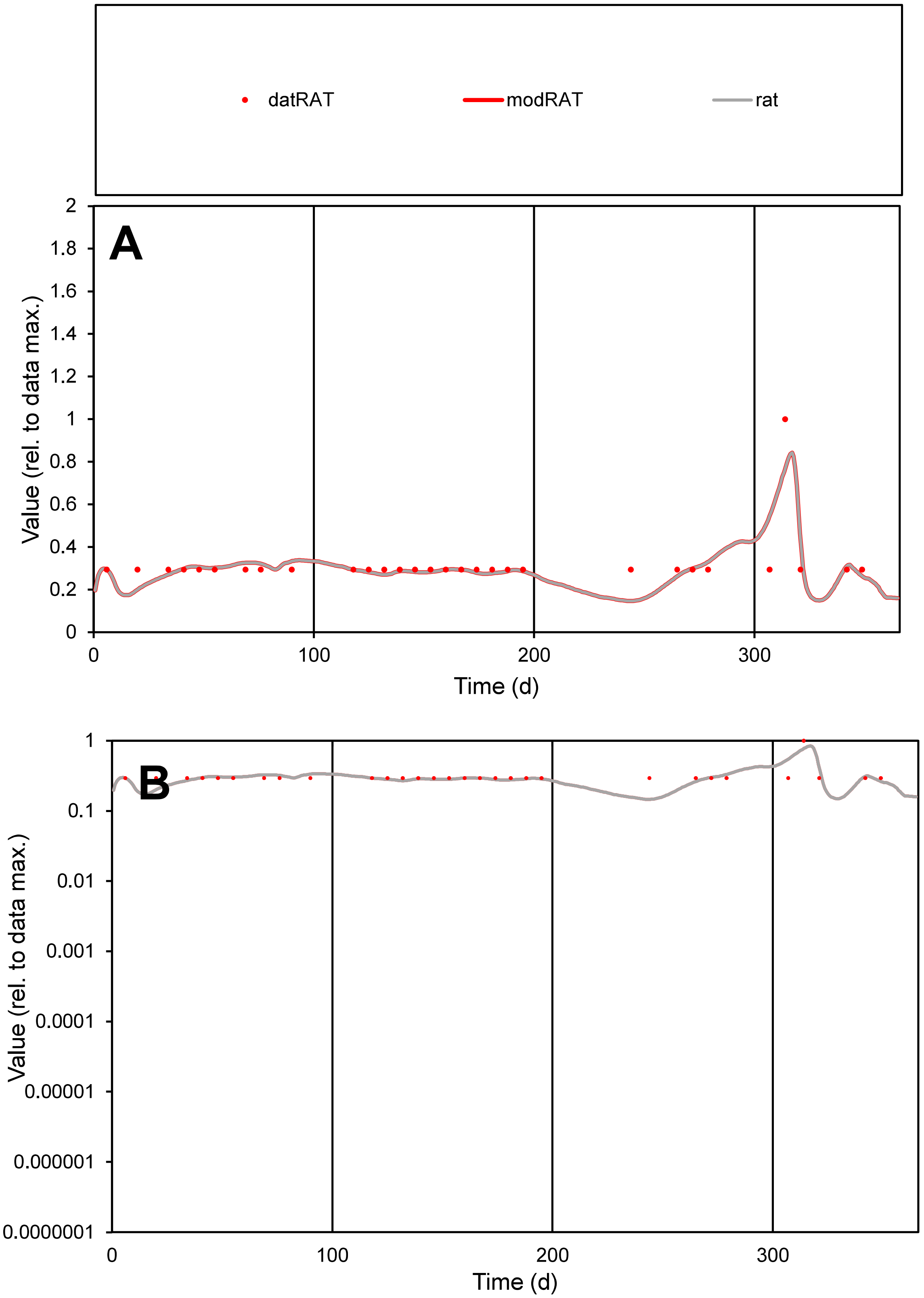

C1

Variables
RAT Provide jtd or Dst
CalibOnly?
2 1 to reproduce of
oNormalize?
2 1=no, 2=yes

ofxn calc*
0.21946
ofxn mod
0.25374

sRun
RunA1
zout
1

Ave
Data
1.2E-05
Model
1.2E-05

data/vMax
3.9E-05

ofxng
275.646

Min d/m
1.99754

ave/min d
1.08021
ave/min m
2.12815

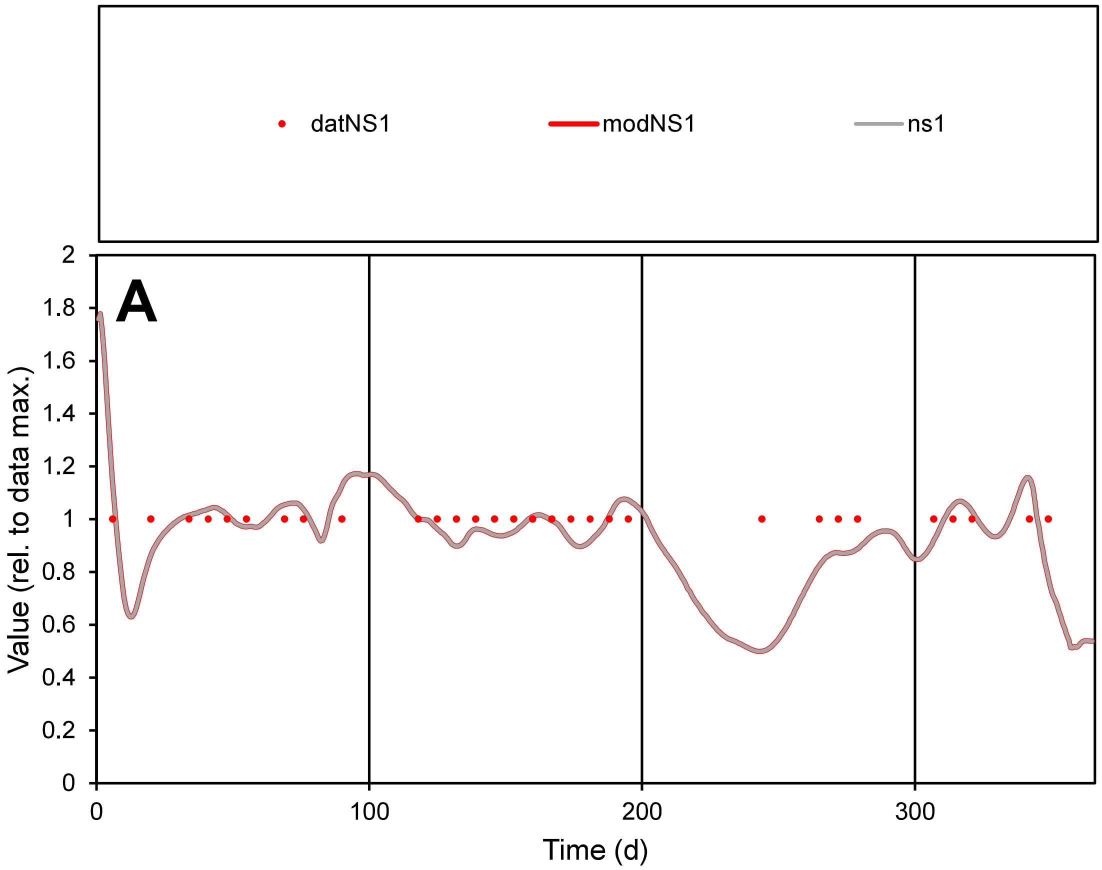

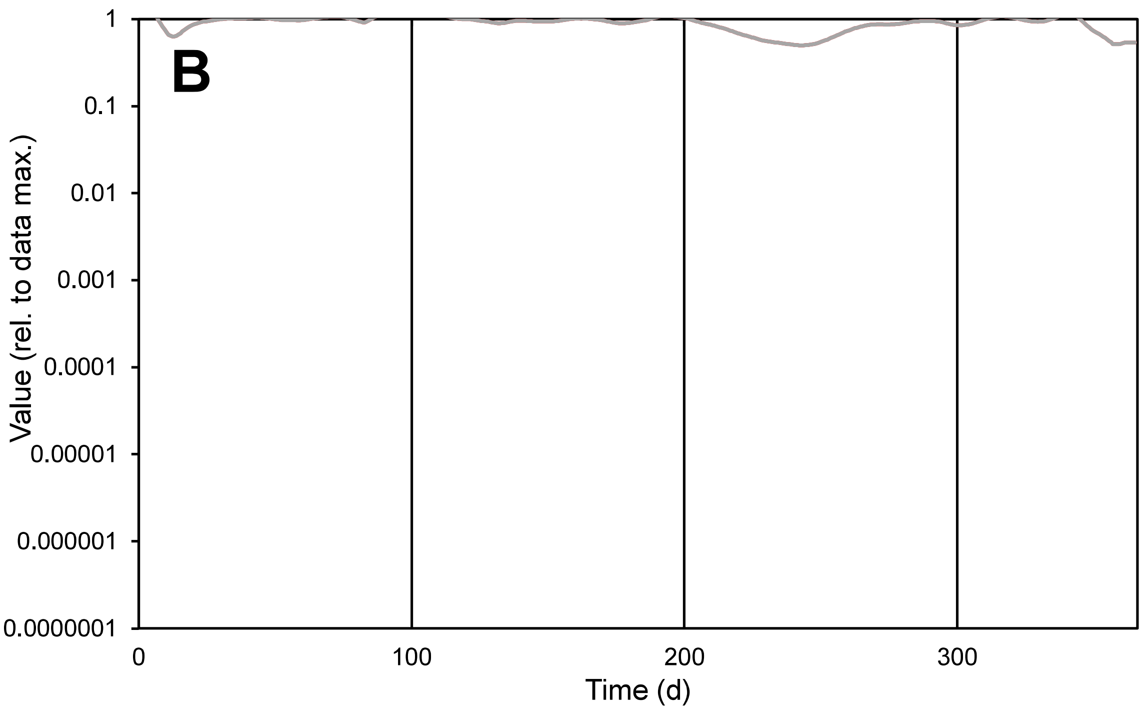

C1

Variables
NS1 Provide jtd or Dst
CalibOnly?
2 1 to reproduce of
oNormalize?
2 1=no, 2=yes

ofxn calc*
0.12693
ofxn mod
0.16846

sRun
RunA1
zout
1

Ave
Data
2.7E-05
Model
2.6E-05

data/vMax
2.7E-05

ofxng
275.646

Min d/m
1.98698

ave/min d
1
ave/min m
1.90523

325_Calib_S_A

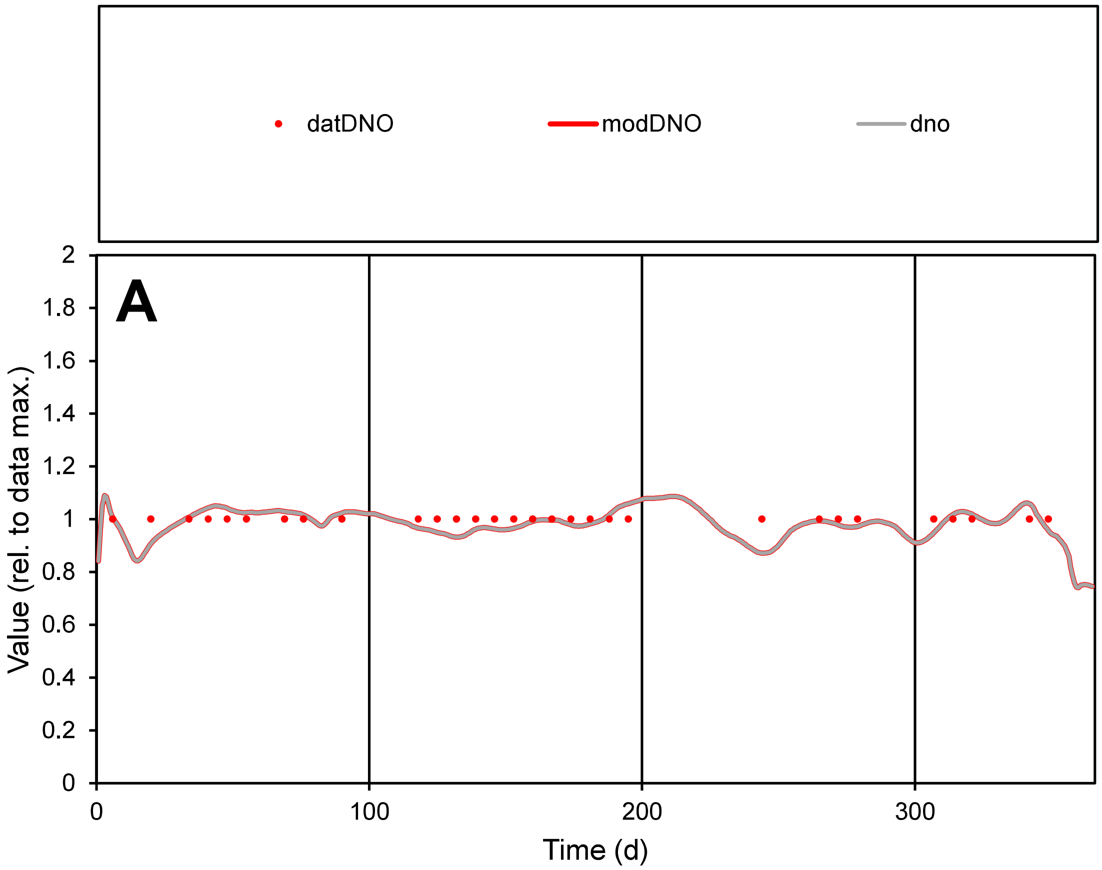

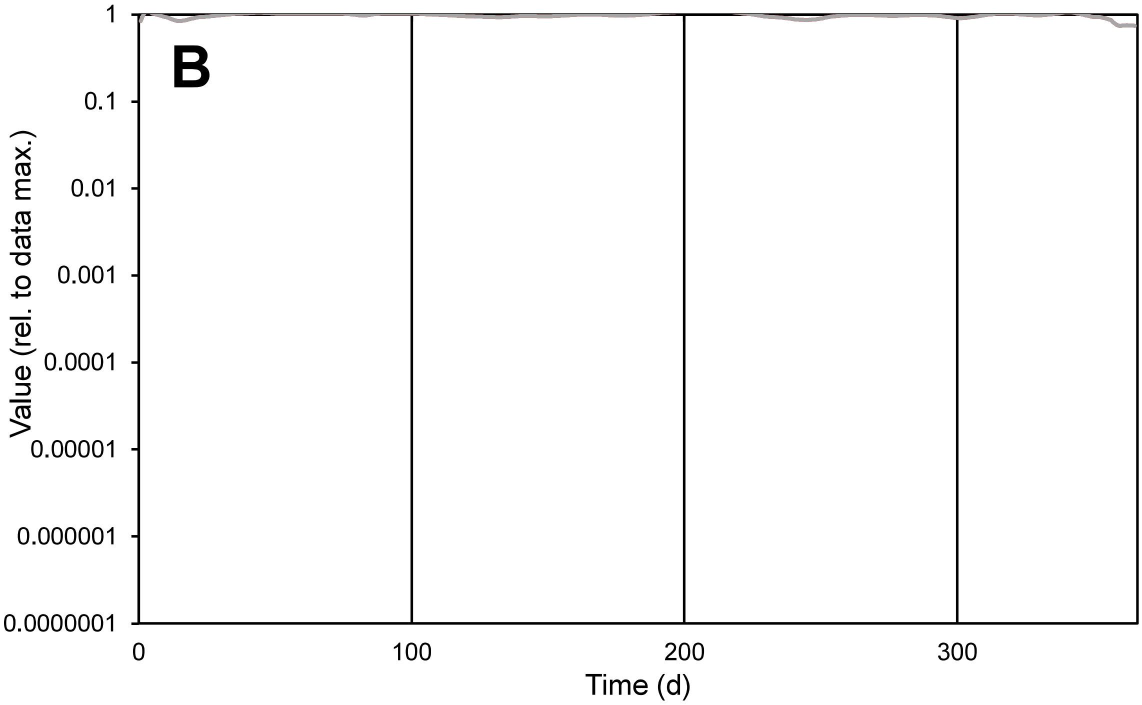

C1

Variables
DNO Provide jtd or Dst
CalibOnly?
2 1 to reproduce of
oNormalize?
2 1=no, 2=yes

ofxn calc*
0.04318
ofxn mod
0.12014

sRun
RunA1
zout
1

Ave
Data
1.8E-05
Model
1.8E-05

data/vMax
1.8E-05

ofxng
275.646

Min d/m
1.14775

ave/min d
1
ave/min m
1.13619

325_Calib_S_A

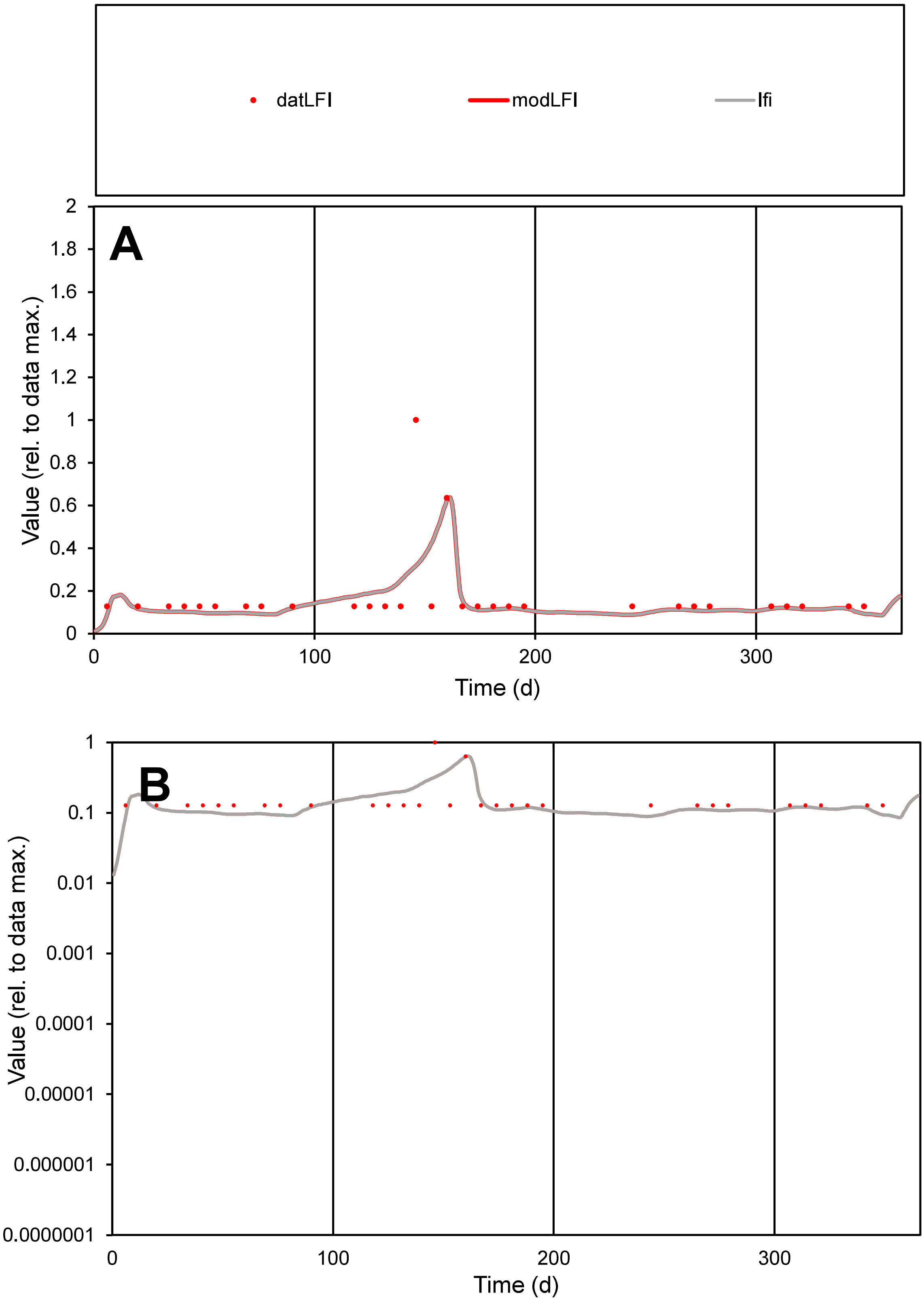

C1

Variables

LFI Provide jtd or Dst

CalibOnly?

2 1 to reproduce of

oNormalize?

2 1=no, 2=yes

ofxn calc*

0.79789

ofxn mod

0.81186

sRun

RunA1

zout

1

Ave

Data

1.1E-05

Model

1E-05

data/vMax

6.5E-05

ofxng

275.646

Min d/m

1.41816

ave/min d

1.36142

ave/min m

1.77051

325_Calib_S_A

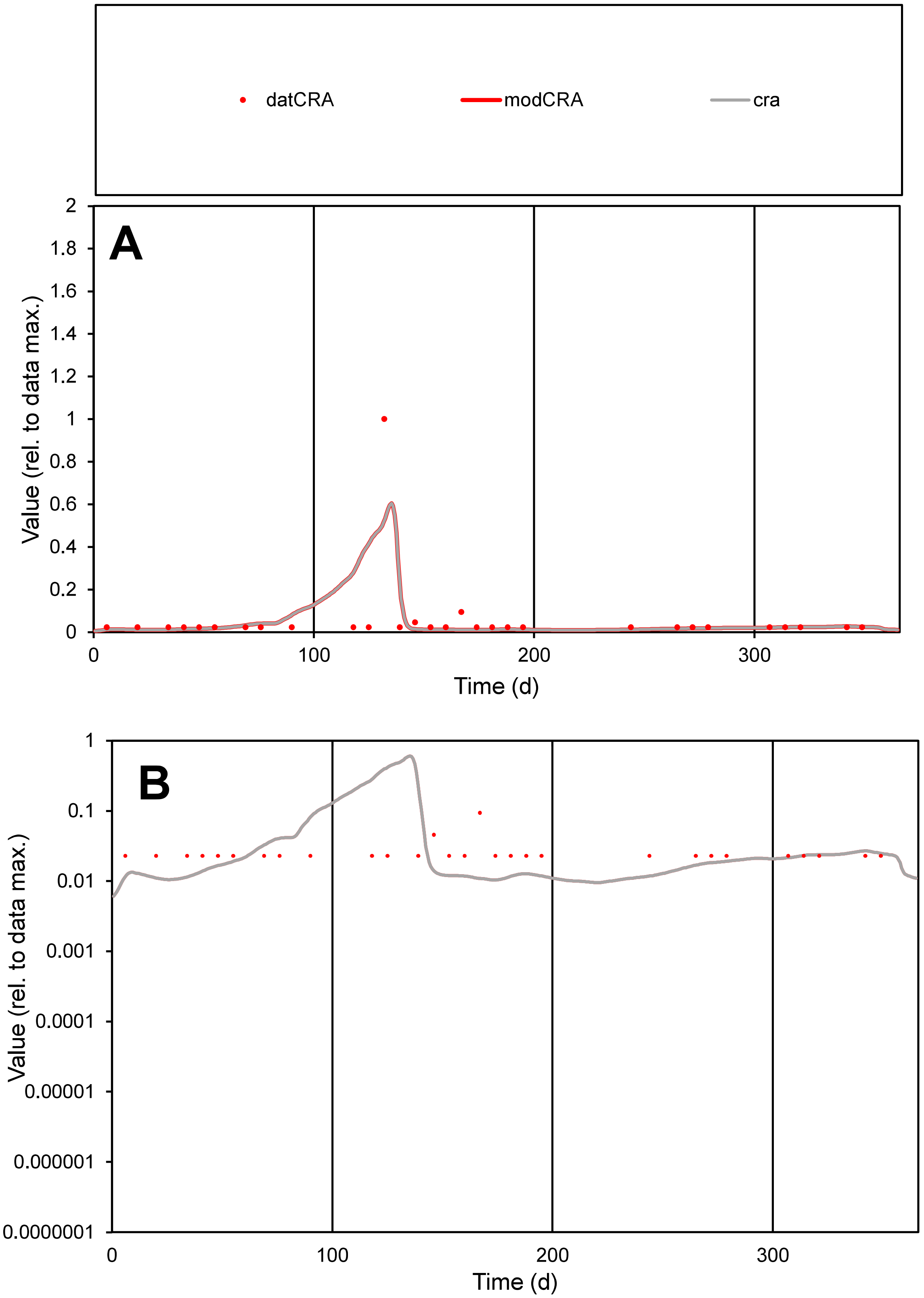

C1

Variables
CRA Provide jtd or Dst
CalibOnly?
2 1 to reproduce of
oNormalize?
2 1=no, 2=yes

ofxn calc*
2.01471
ofxn mod
2.06874

sRun
RunA1
zout
1

Ave
Data
1.2E-05
Model
1.3E-05

data/vMax
0.00021

ofxng
275.646

Min d/m
2.19198

ave/min d
2.56151
ave/min m
5.83484

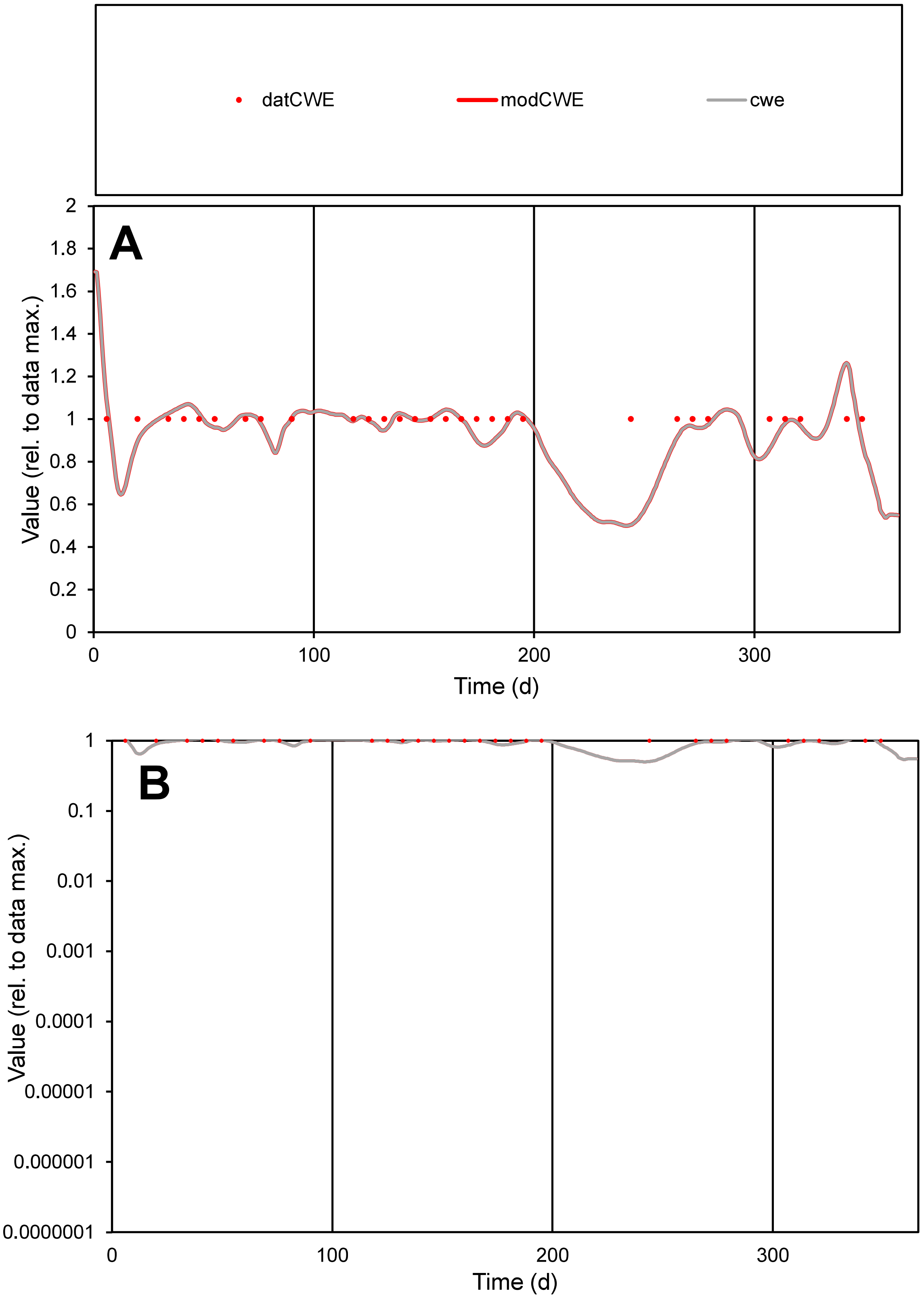

C1

Variables
CWE Provide jtd or Dst
CalibOnly?
2 1 to reproduce of
oNormalize?
2 1=no, 2=yes

ofxn calc*
0.11399
ofxn mod
0.15929

sRun
RunA1
zout
1

Ave
Data
2.6E-05
Model
2.5E-05

data/vMax
2.6E-05

ofxng
275.646

Min d/m
1.95853

ave/min d
1
ave/min m
1.89736

325_Calib_S_A

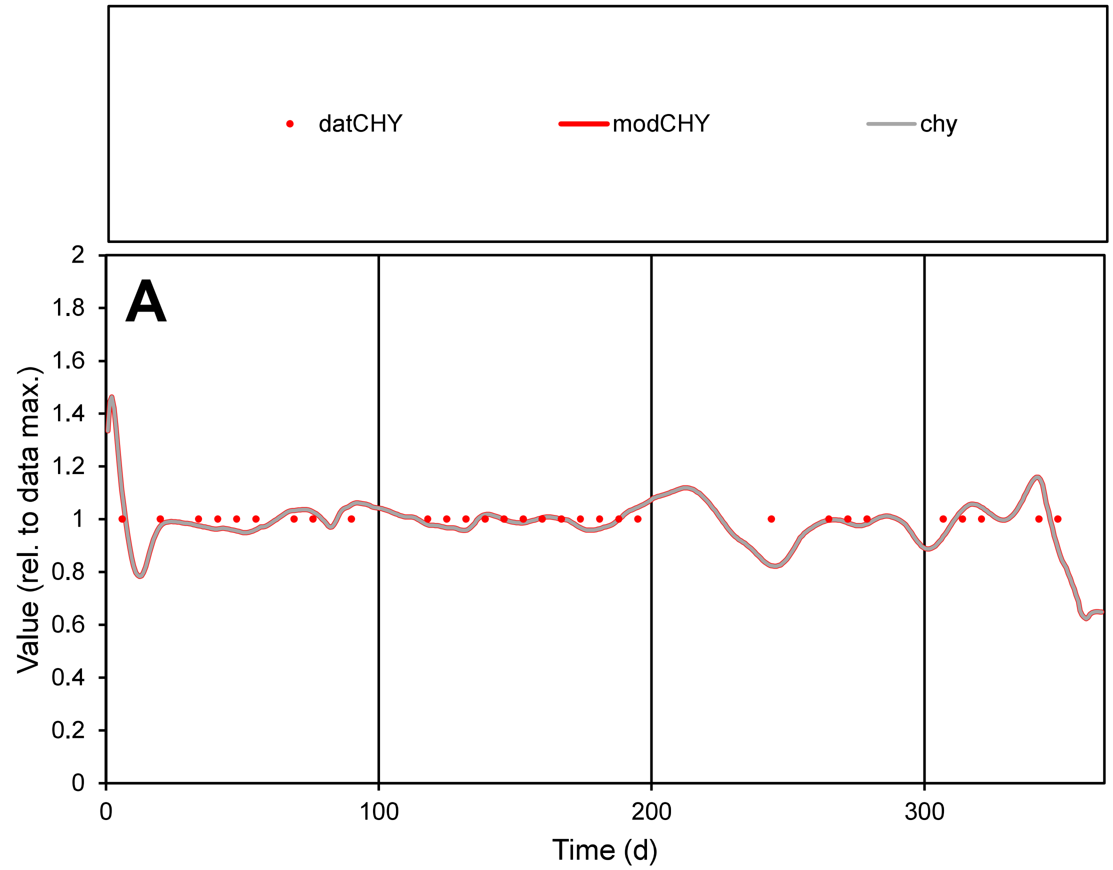

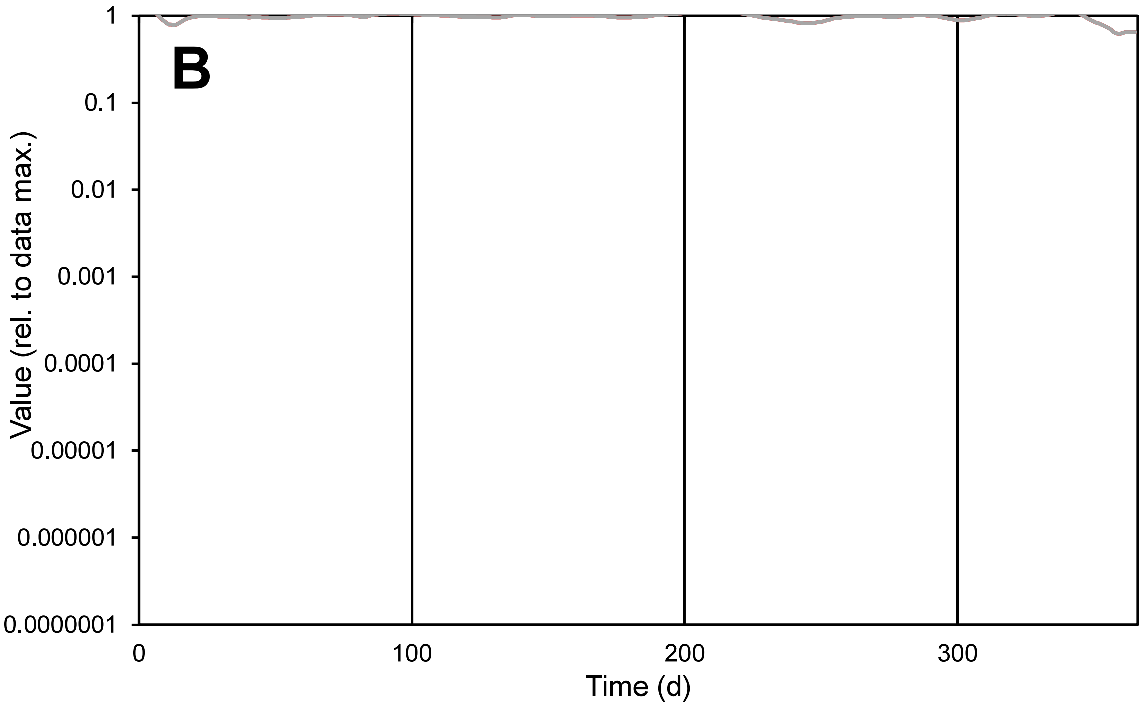

C1

Variables
CHY Provide jtd or Dst
CalibOnly?
2 1 to reproduce of
oNormalize?
2 1=no, 2=yes

ofxn calc*
0.05752
ofxn mod
0.12582

sRun
RunA1
zout
1

Ave
Data
6.7E-06
Model
6.6E-06

data/vMax
6.7E-06

ofxng
275.646

Min d/m
1.21719

ave/min d
1
ave/min m
1.20387

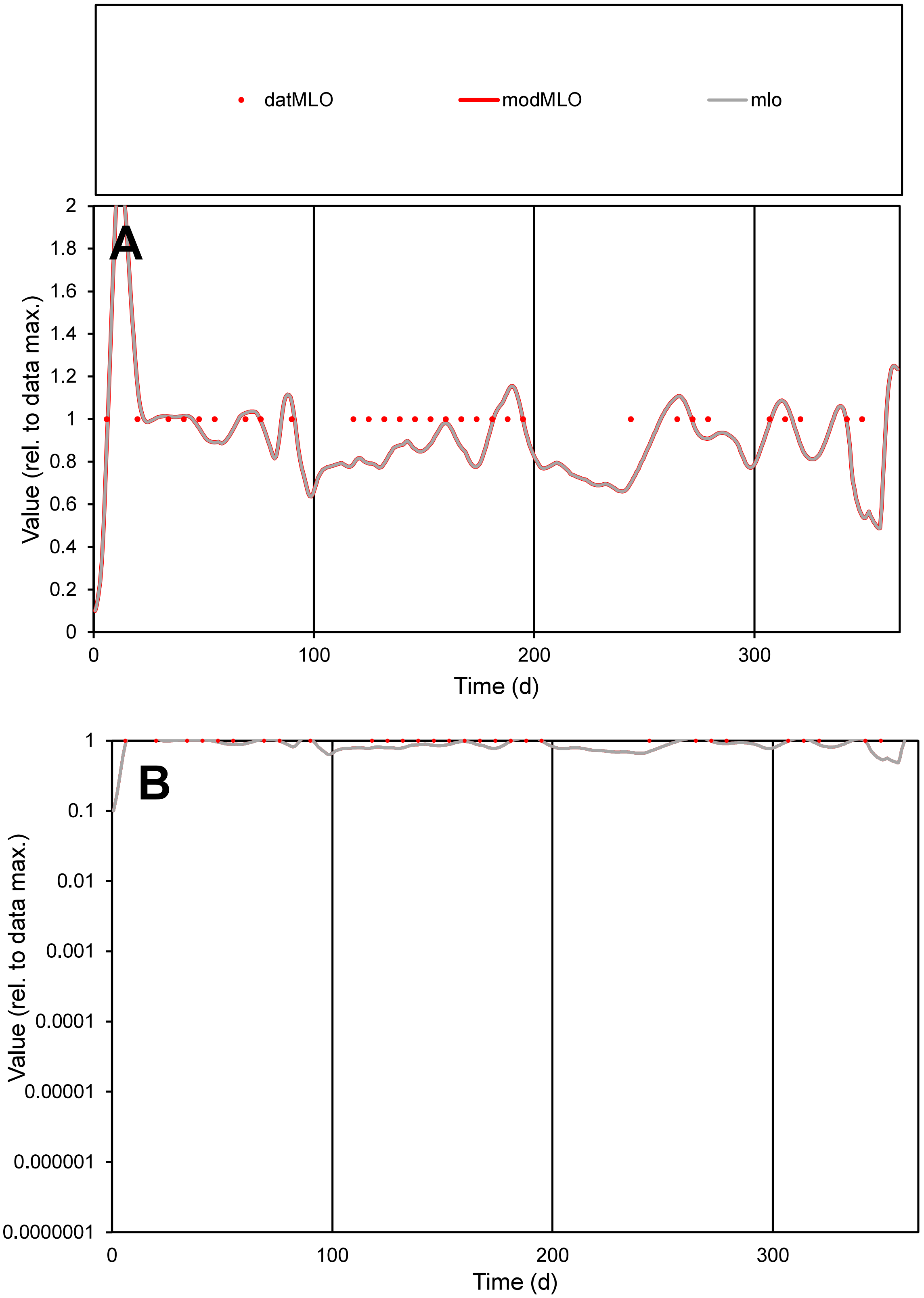

C1

Variables
MLO Provide jtd or Dst
CalibOnly?
2 1 to reproduce of
oNormalize?
2 1=no, 2=yes

ofxn calc*
0.14497
ofxn mod
0.18329

sRun
RunA1
zout
1

Ave
Data
1E-05
Model
9.6E-06

data/vMax
1E-05

ofxng
275.646

Min d/m
1.86017

ave/min d
1

ave/min m
1.72098

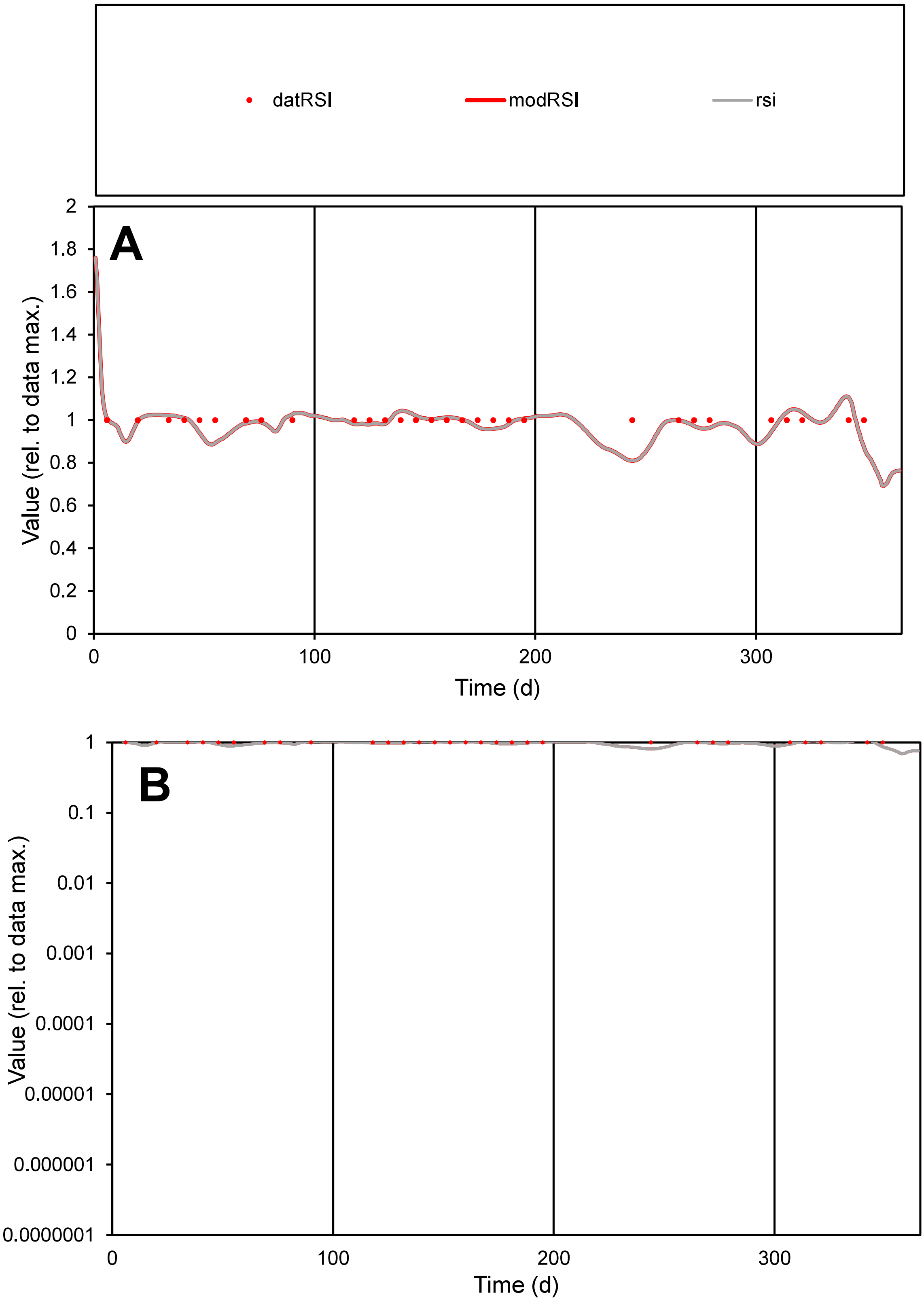

C1

Variables
RSI Provide jtd or Dst
CalibOnly?
2 1 to reproduce of
oNormalize?
2 1=no, 2=yes

ofxn calc*
0.05696
ofxn mod
0.12507

sRun
RunA1
zout
1

Ave
Data
1E-05
Model
1E-05

data/vMax
1E-05

ofxng
275.646

Min d/m
1.23358

ave/min d
1
ave/min m
1.2139

325_Calib_S_A

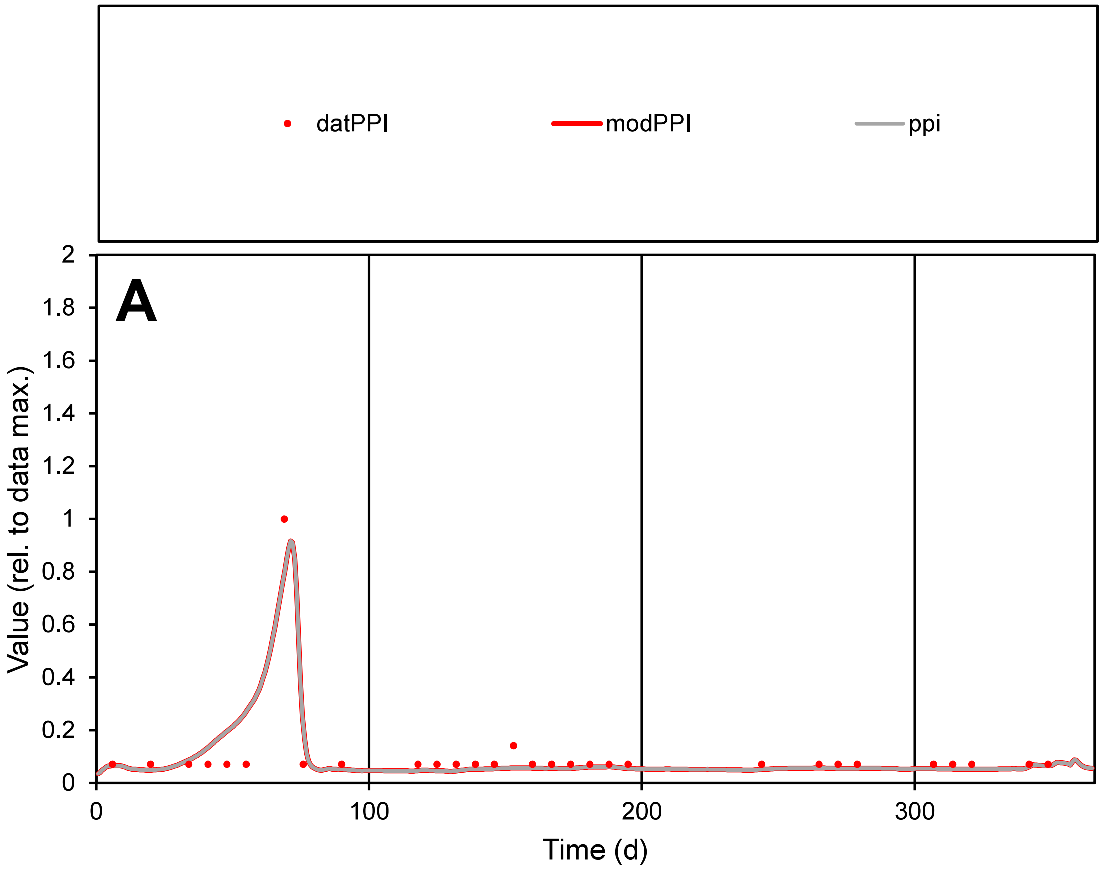

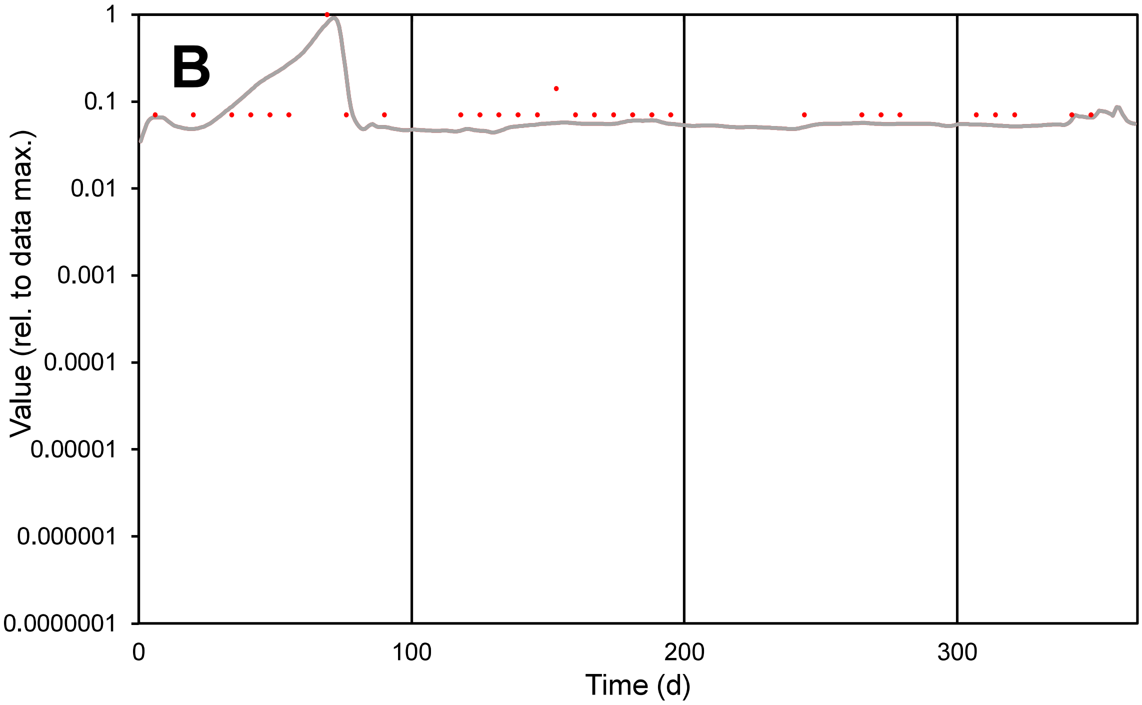

C1

Variables

PPI Provide jtd or Dst

CalibOnly?

2 1 to reproduce of

oNormalize?

2 1=no, 2=yes

ofxn calc*

0.53165

ofxn mod

0.57367

sRun

RunA1

zout

1

Ave

Data

2.1E-06

Model

2.1E-06

data/vMax

2.1E-05

ofxng

275.646

Min d/m

1.53797

ave/min d

1.47303

ave/min m

2.2088

325_Calib_S_A

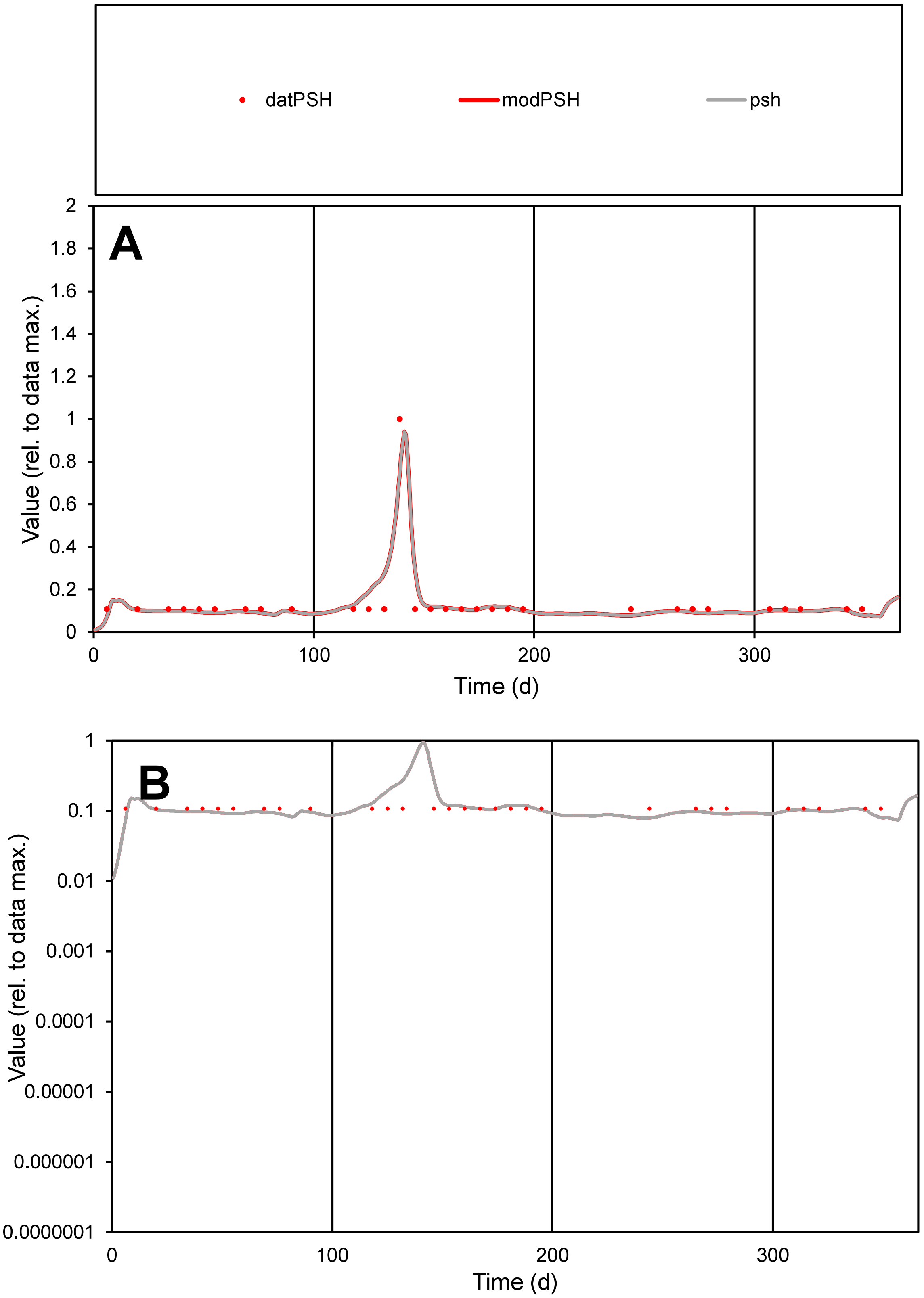

C1

Variables
PSH Provide jtd or Dst
CalibOnly?
2 1 to reproduce of
oNormalize?
2 1=no, 2=yes

ofxn calc*
0.32568
ofxn mod
0.37276

sRun
RunA1
zout
1

Ave
Data
1.6E-05
Model
1.7E-05

data/vMax
0.00012

ofxng
275.646

Min d/m
1.35704

ave/min d
1.27674
ave/min m
1.76729

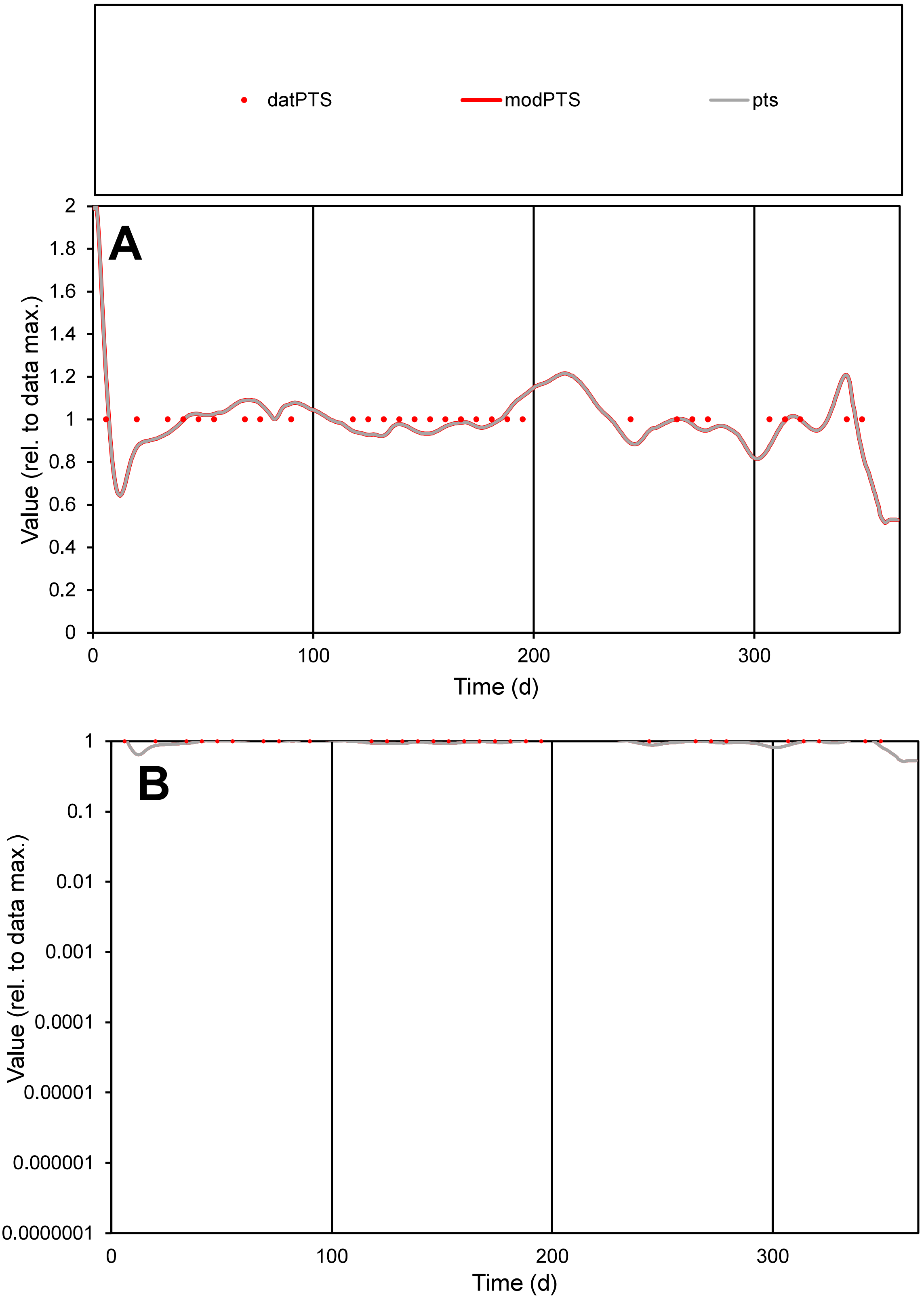

C1

Variables
PTS Provide jtd or Dst
CalibOnly?
2 1 to reproduce of
oNormalize?
2 1=no, 2=yes

ofxn calc*
0.07724
ofxn mod
0.136

sRun
RunA1
zout
1

Ave
Data
9.7E-06
Model
9.5E-06

data/vMax
9.7E-06

ofxng
275.646

Min d/m
1.2714

ave/min d
1
ave/min m
1.25165

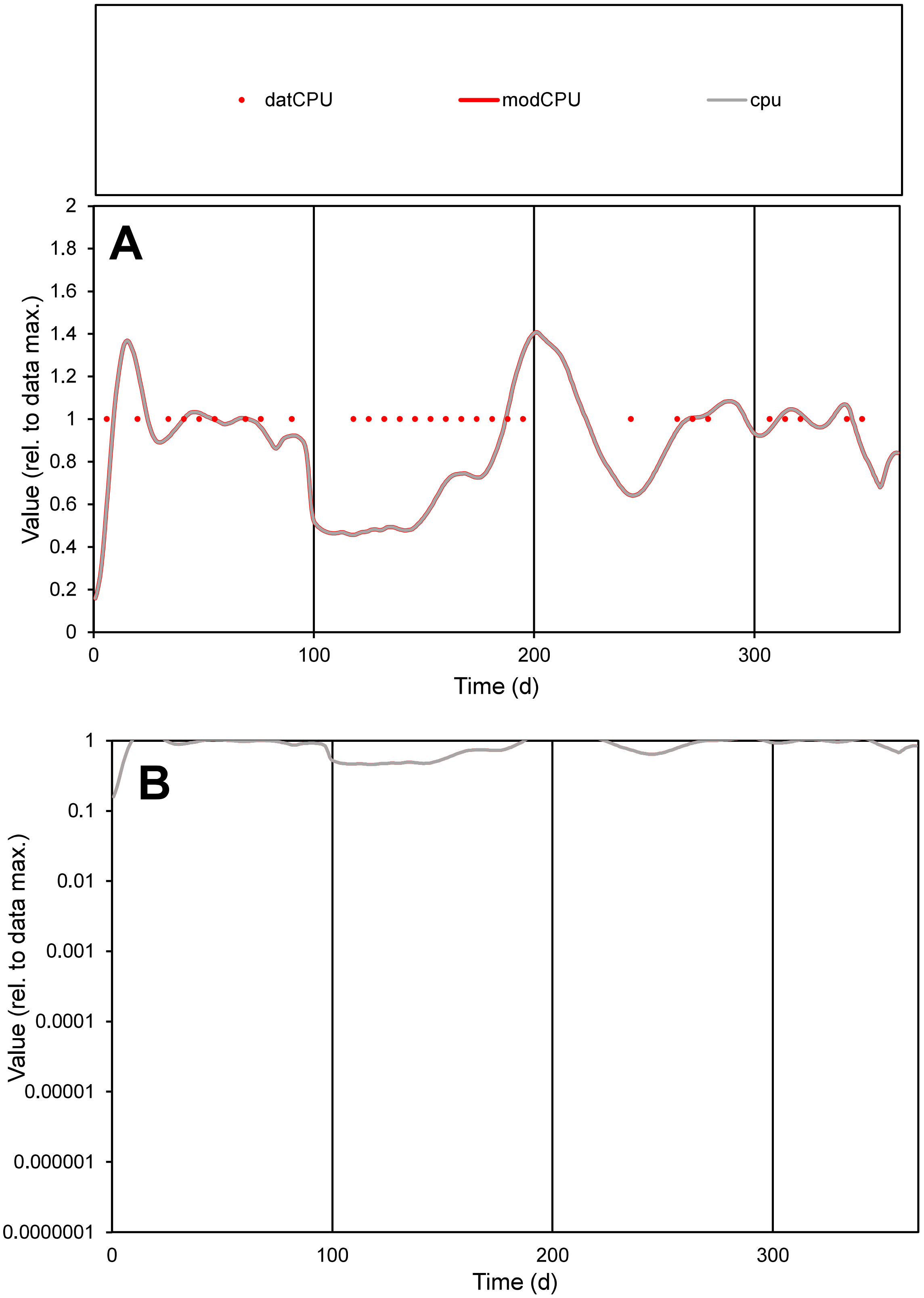

C1

Variables
CPU Provide jtd or Dst
CalibOnly?
2 1 to reproduce of
oNormalize?
2 1=no, 2=yes

ofxn calc*
0.26508
ofxn mod
0.28622

sRun
RunA1
zout
1

Ave
Data
4.4E-07
Model
3.8E-07

data/vMax
4.4E-07

ofxng
275.646

Min d/m
2.17511

ave/min d
1
ave/min m
1.87167

325_Calib_S_A

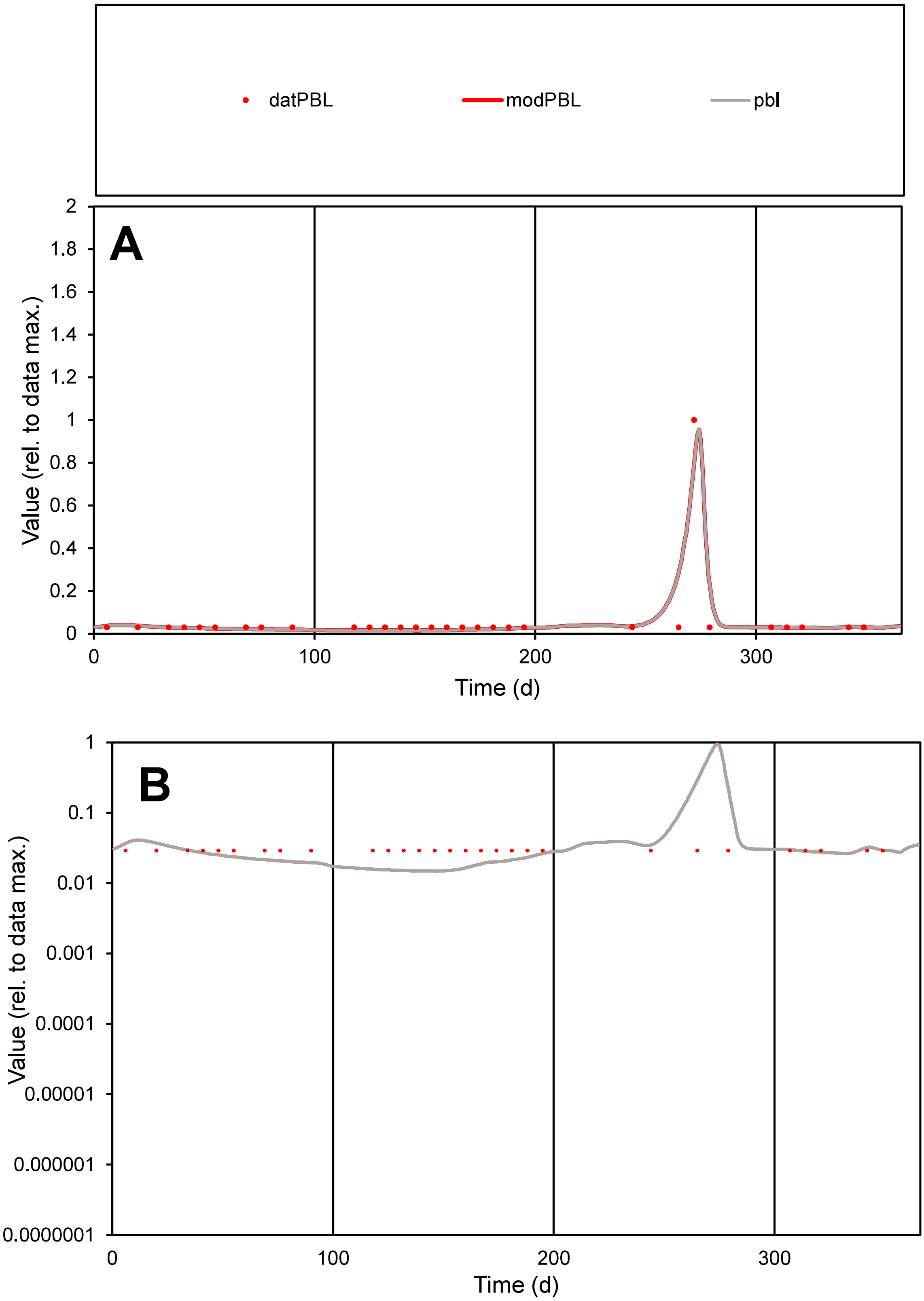

C1

Variables
PBL Provide jtd or Dst
CalibOnly?
2 1 to reproduce of
oNormalize?
2 1=no, 2=yes

ofxn calc*
0.6562
ofxn mod
0.88652

sRun
RunA1
zout
1

Ave
Data
9E-08
Model
9.2E-08

data/vMax
1.5E-06

ofxng
275.646

Min d/m
1.95858

ave/min d
2.11172
ave/min m
4.23214

325_Calib_S_A

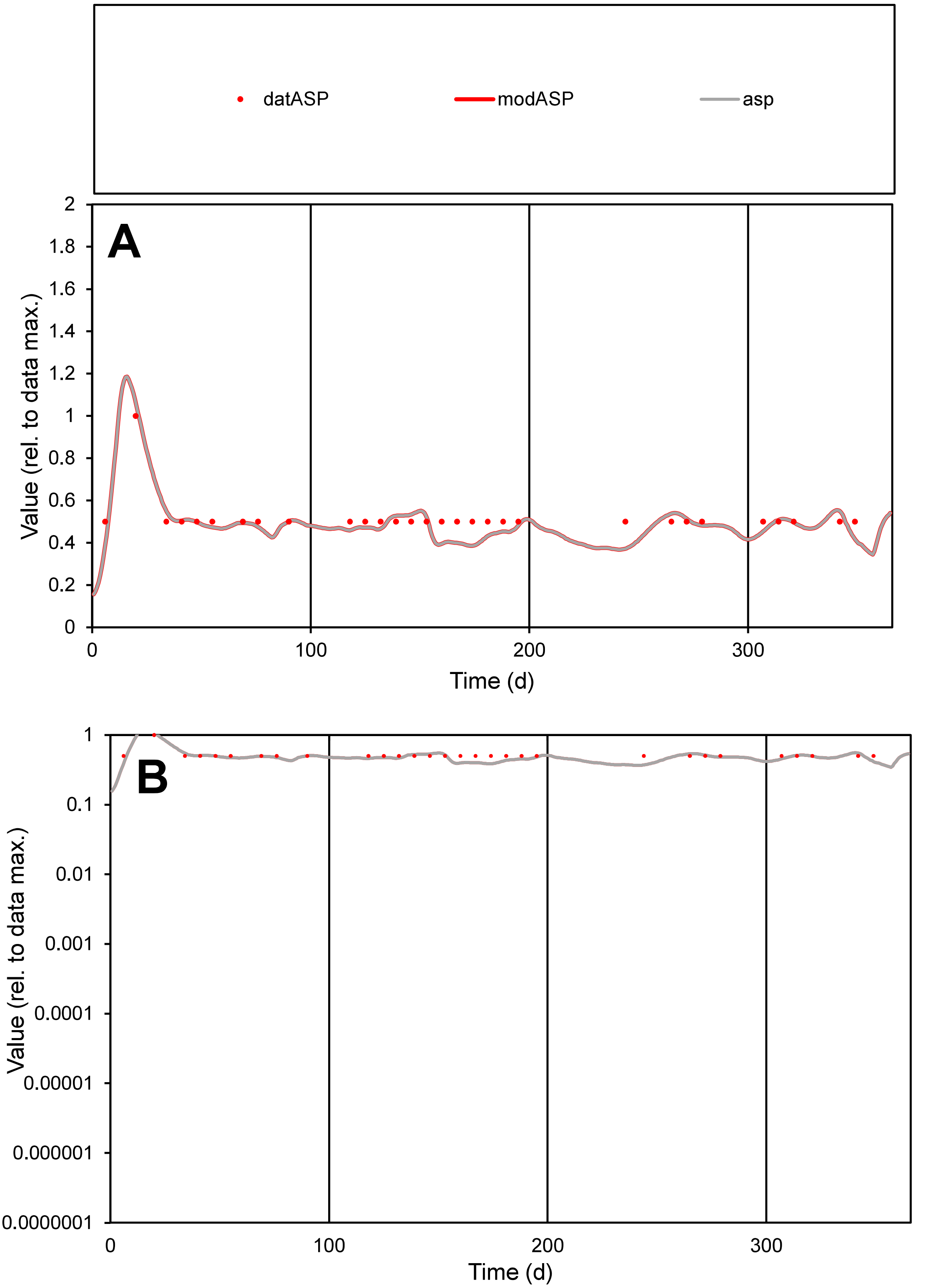

C1

Variables
ASP Provide jtd or Dst
CalibOnly?
2 1 to reproduce of
oNormalize?
2 1=no, 2=yes

ofxn calc*
0.09753
ofxn mod
0.14691

sRun
RunA1
zout
1

Ave
Data
6E-06
Model
5.7E-06

data/vMax
1.2E-05

ofxng
275.646

Min d/m
1.32556

ave/min d
1.03336
ave/min m
1.3098

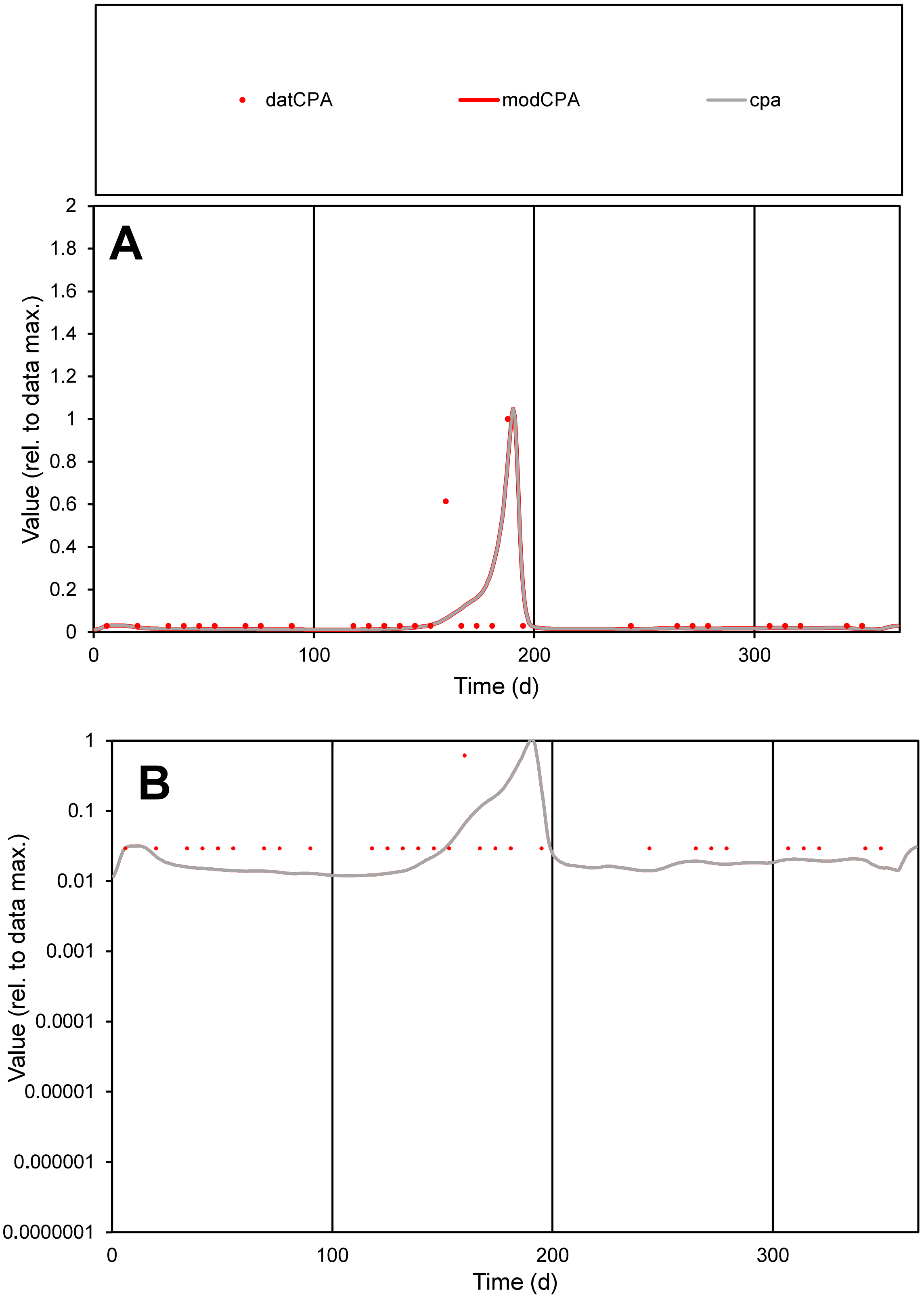

C1

Variables
CPA Provide jtd or Dst
CalibOnly?
2 1 to reproduce of
oNormalize?
2 1=no, 2=yes

ofxn calc*
1.37844
ofxn mod
1.42582

sRun
RunA1
zout
1

Ave
Data
1.8E-07
Model
1.5E-07

data/vMax
2.3E-06

ofxng
275.646

Min d/m
2.38406

ave/min d
2.77214
ave/min m
5.50526

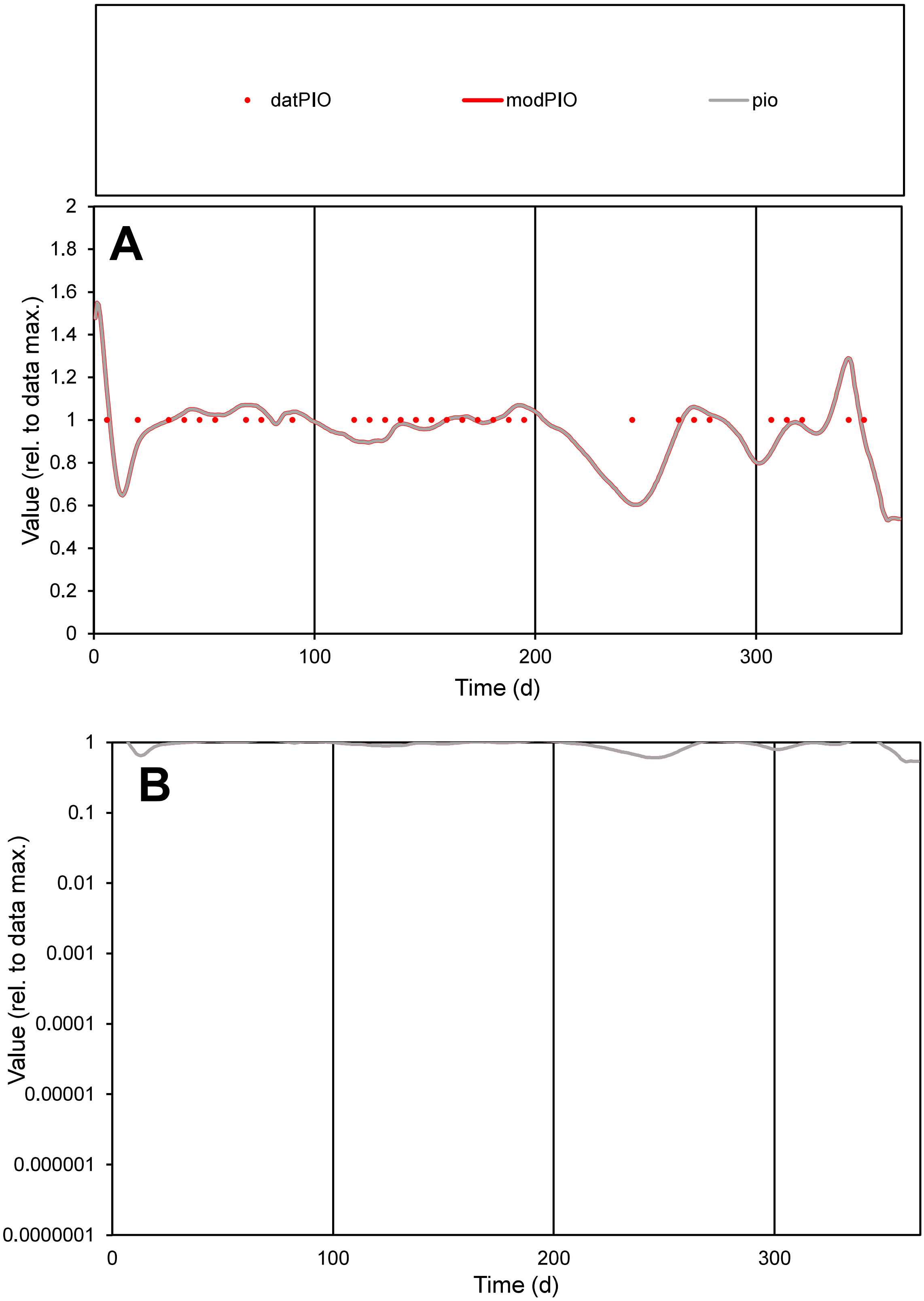

C1

Variables
PIO Provide jtd or Dst
CalibOnly?
2 1 to reproduce of
oNormalize?
2 1=no, 2=yes

ofxn calc*
0.10403
ofxn mod
0.15315

sRun
RunA1
zout
1

Ave
Data
1.5E-07
Model
1.4E-07

data/vMax
1.5E-07

ofxng
275.646

Min d/m
1.65848

ave/min d
1
ave/min m
1.63387

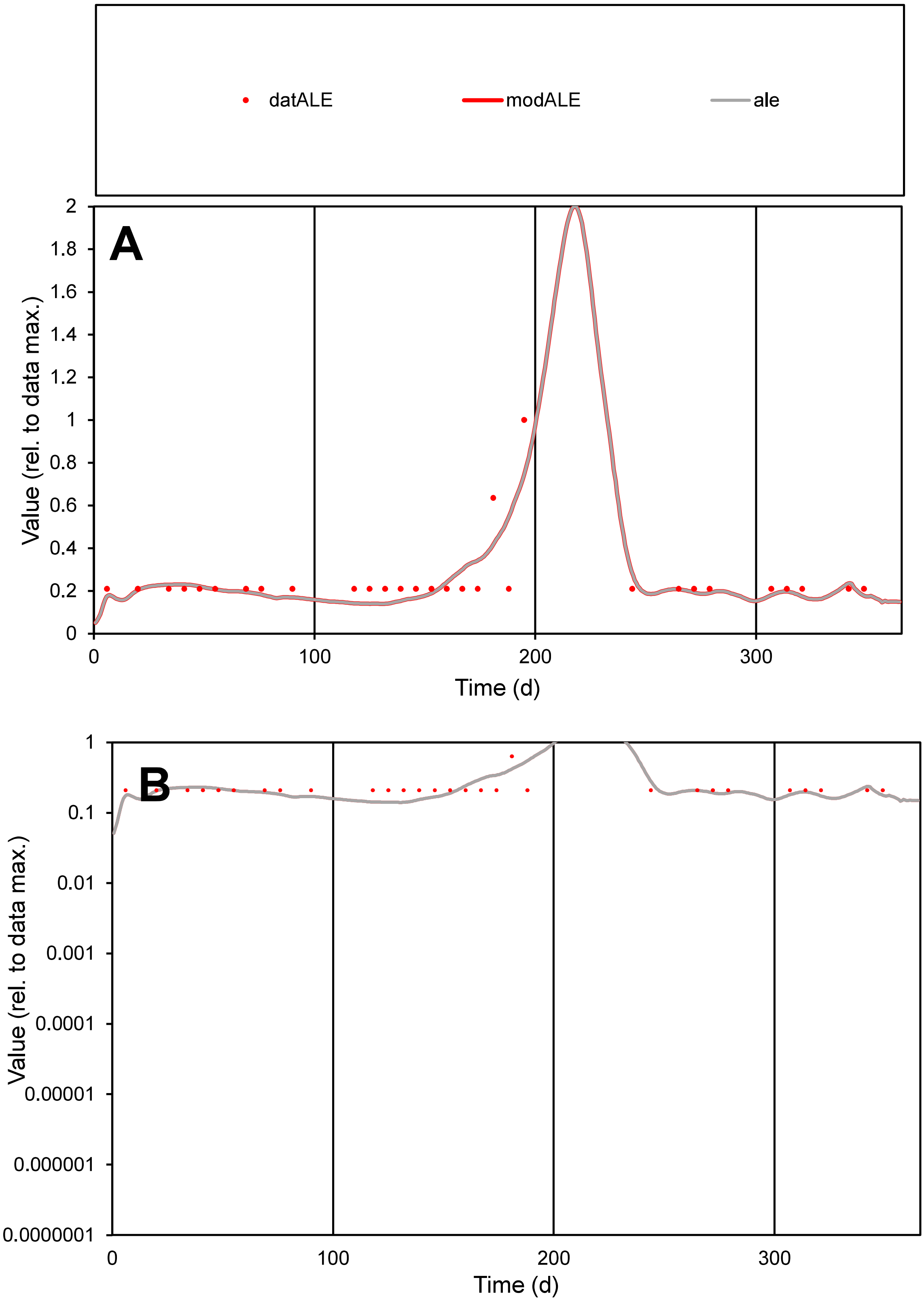

C1

Variables
ALE Provide jtd or Dst
CalibOnly?
2 1 to reproduce of
oNormalize?
2 1=no, 2=yes

ofxn calc*
0.37209
ofxn mod
0.39654

sRun
RunA1
zout
1

Ave
Data
1.3E-05
Model
1.3E-05

data/vMax
5.3E-05

ofxng
275.646

Min d/m
1.48956

ave/min d
1.19361
ave/min m
1.72519

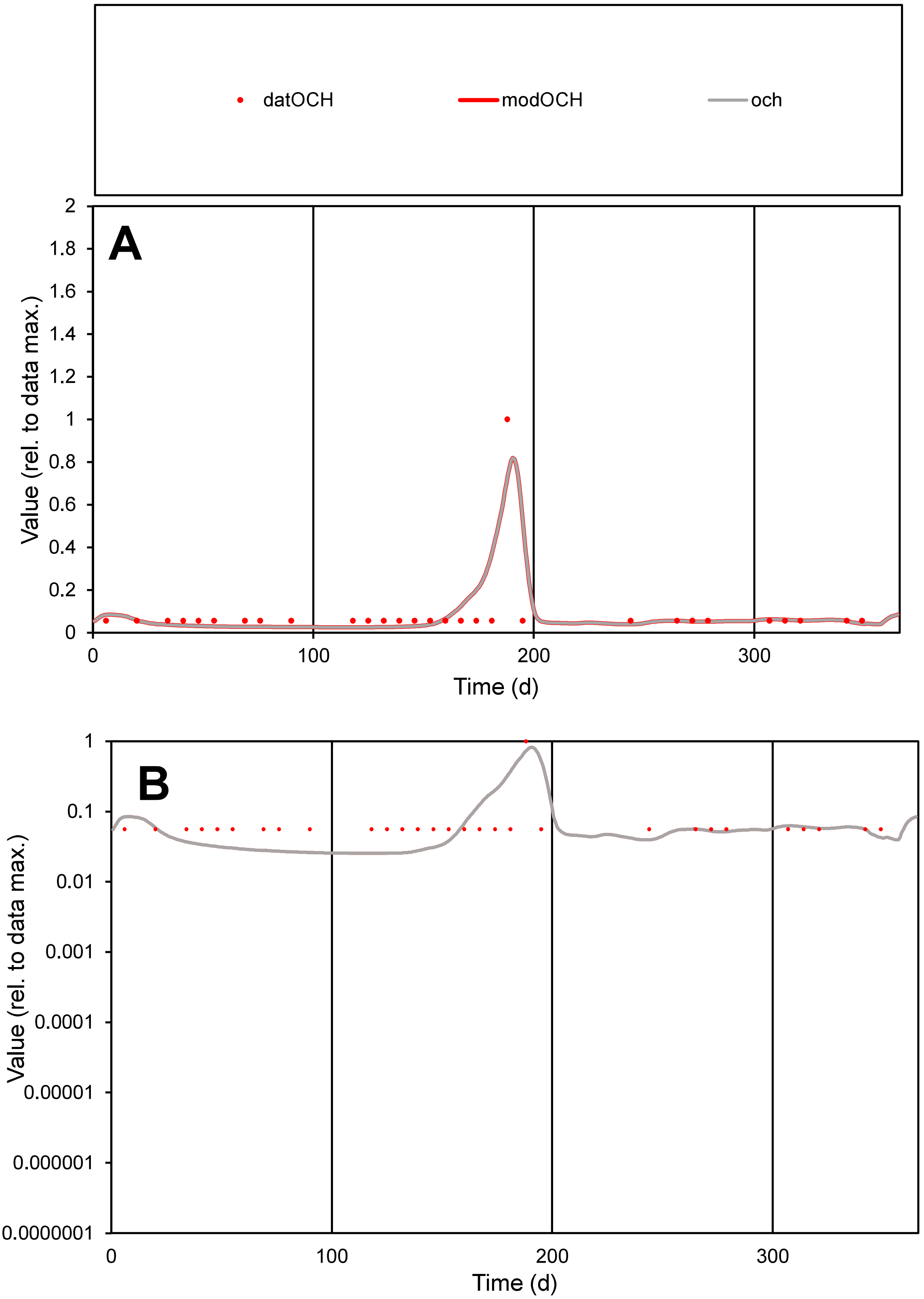

C1

Variables
OCH Provide jtd or Dst
CalibOnly?
2 1 to reproduce of
oNormalize?
2 1=no, 2=yes

ofxn calc*
0.89752
ofxn mod
1.02325

sRun
RunA1
zout
1

Ave
Data
6.9E-06
Model
7.1E-06

data/vMax
7.9E-05

ofxng
275.646

Min d/m
2.20844

ave/min d
1.5623
ave/min m
3.56456

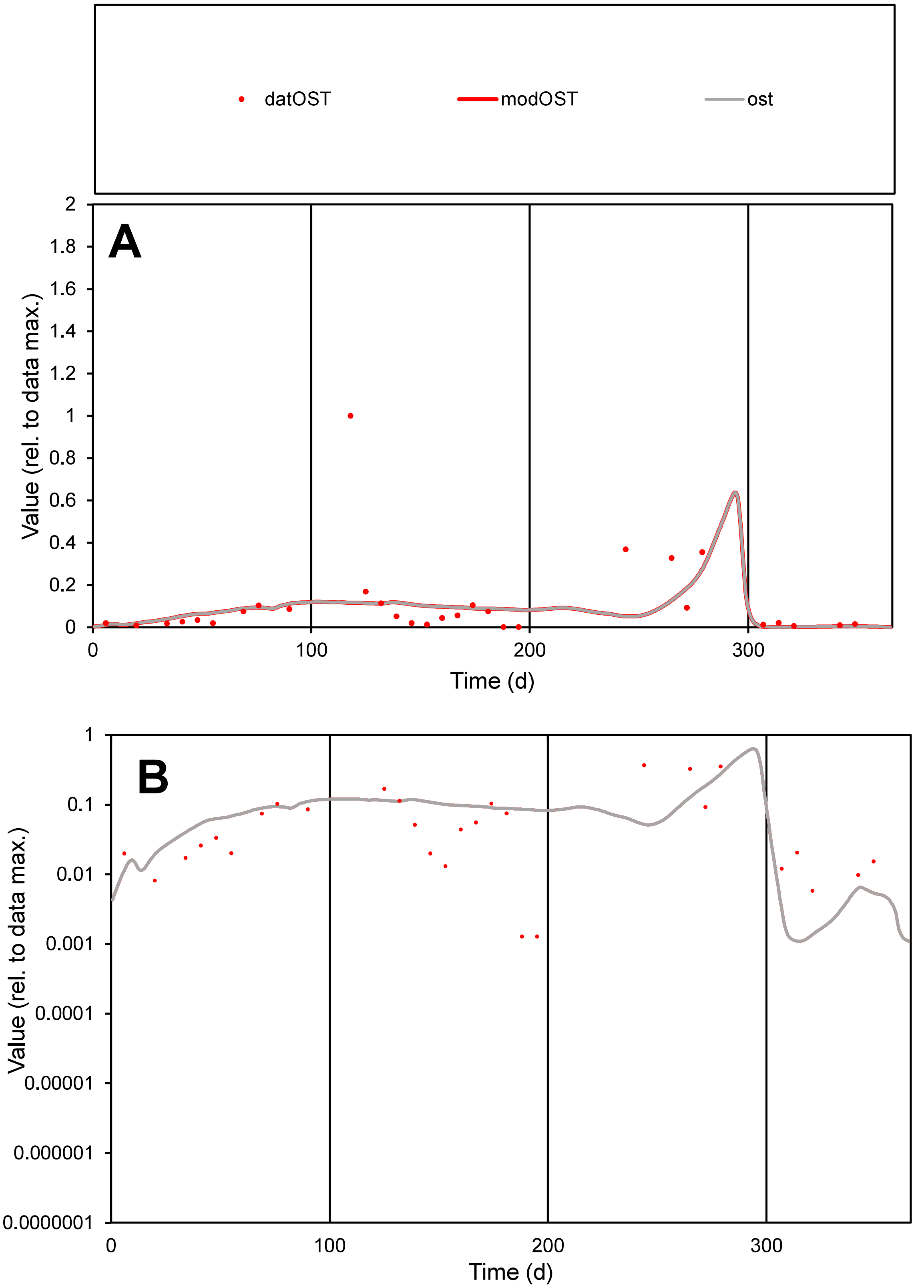

C1

Variables
OST Provide jtd or Dst
CalibOnly?
2 1 to reproduce of
oNormalize?
2 1=no, 2=yes

ofxn calc*
1.66859
ofxn mod
1.67564

sRun
RunA1
zout
1

Ave
Data
0.00018
Model
0.00014

data/vMax
0.00169

ofxng
275.646

Min d/m
1.16568

ave/min d
84.3763
ave/min m
75.0903

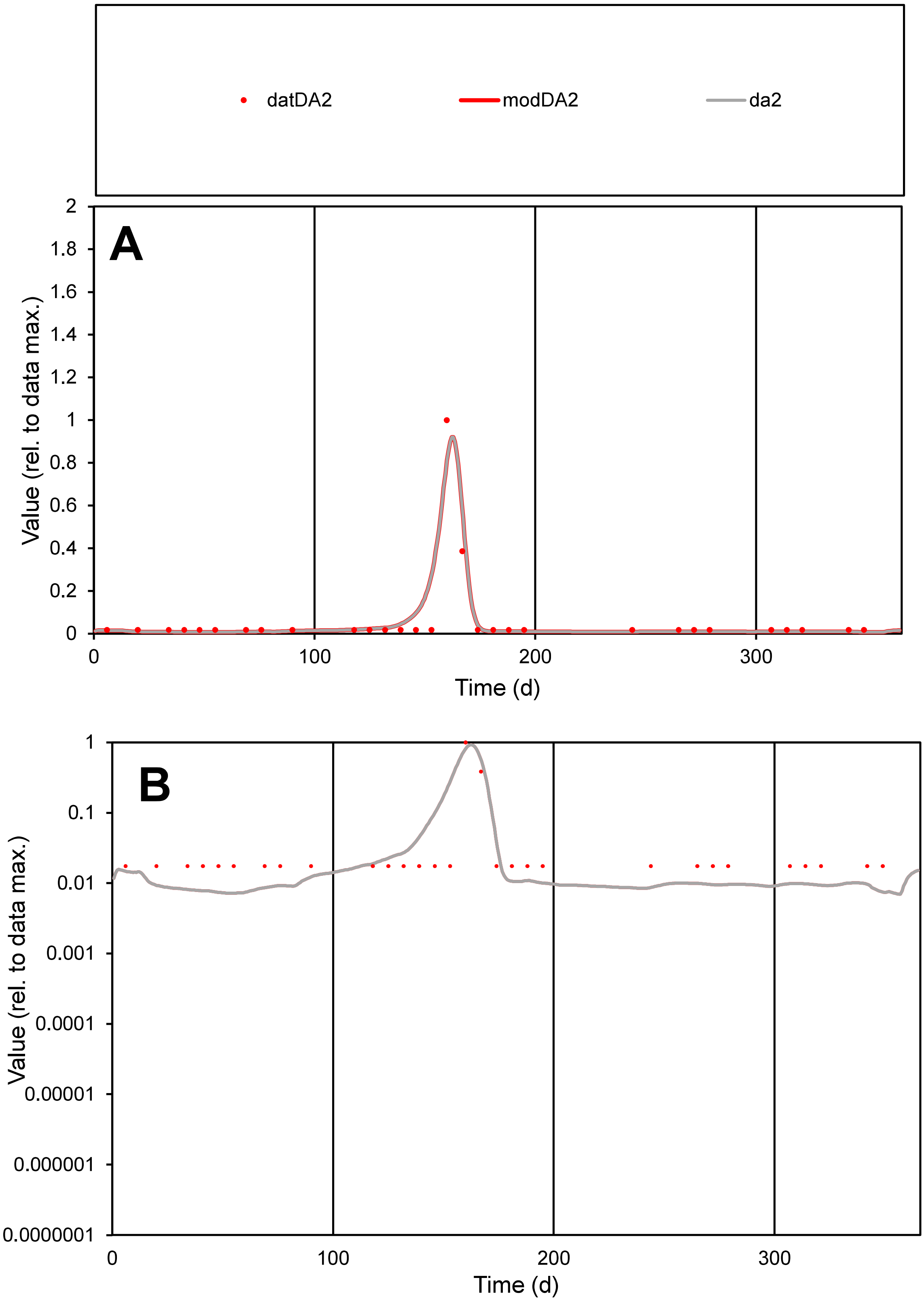

C1

Variables
DA2 Provide jtd or Dst
CalibOnly?
2 1 to reproduce of
oNormalize?
2 1=no, 2=yes

ofxn calc*
0.69207
ofxn mod
0.83872

sRun
RunA1
zout
1

Ave
Data
1.1E-05
Model
1.2E-05

data/vMax
0.00018

ofxng
275.646

Min d/m
2.40185

ave/min d
3.58323
ave/min m
9.05607

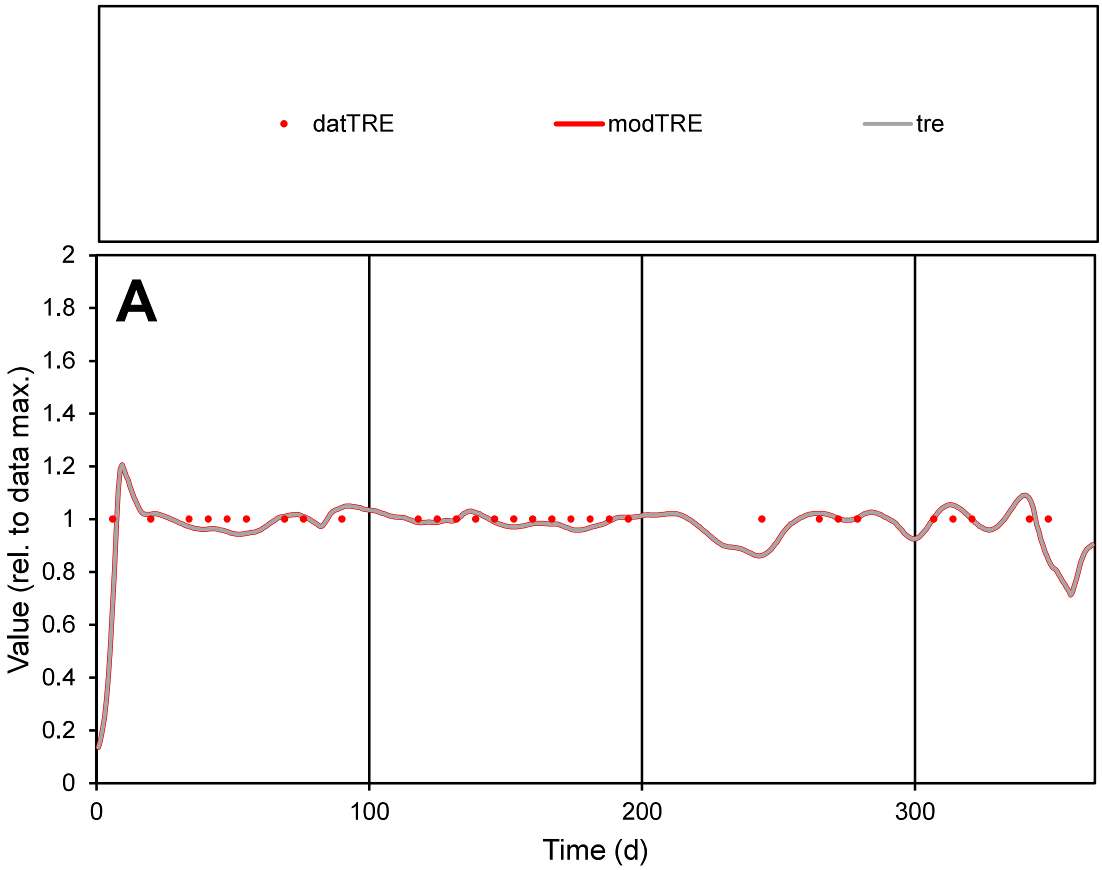

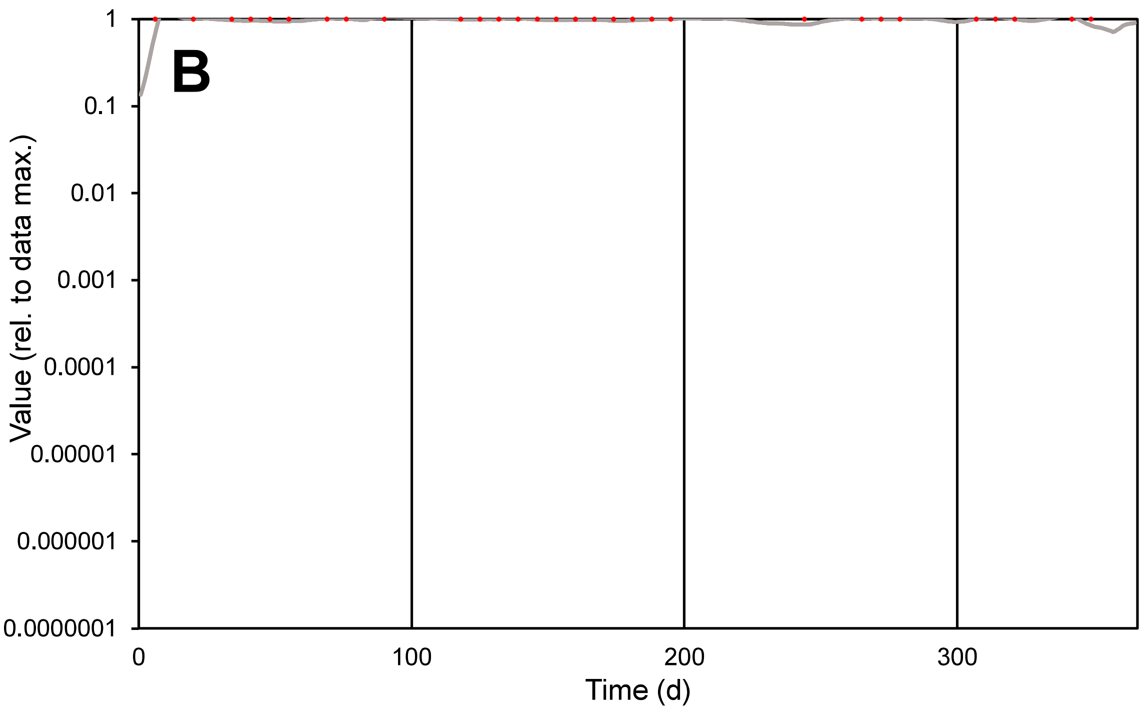

C1

Variables
TRE Provide jtd or Dst
CalibOnly?
2 1 to reproduce of
oNormalize?
2 1=no, 2=yes

ofxn calc*
0.04923
ofxn mod
0.12188

sRun
RunA1
zout
1

Ave
Data
6.9E-06
Model
6.8E-06

data/vMax
6.9E-06

ofxng
275.646

Min d/m
1.22379

ave/min d
1
ave/min m
1.20687

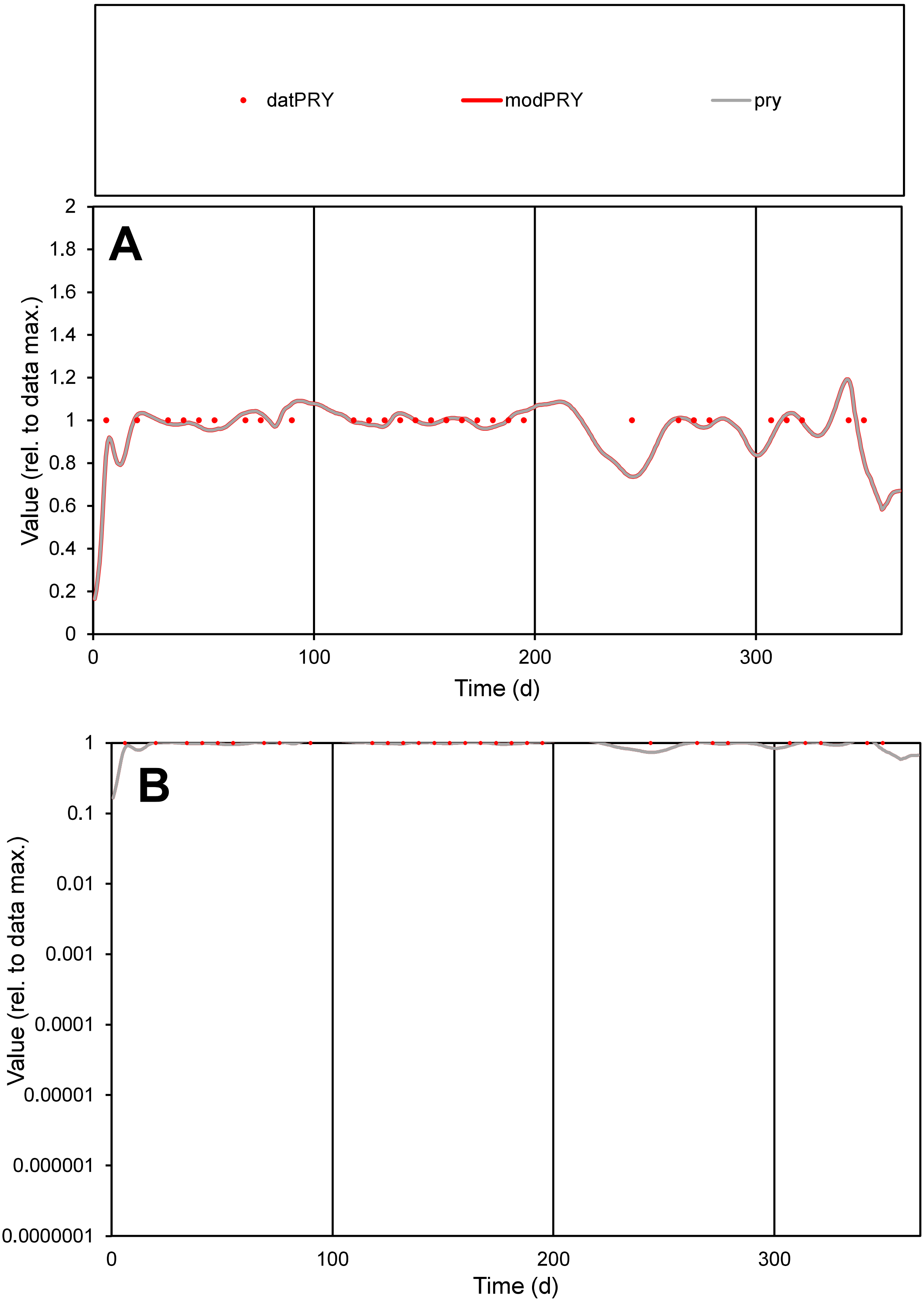

C1

Variables
PRY Provide jtd or Dst
CalibOnly?
2 1 to reproduce of
oNormalize?
2 1=no, 2=yes

ofxn calc*
0.07827
ofxn mod
0.13638

sRun
RunA1
zout
1

Ave
Data
8.8E-06
Model
8.7E-06

data/vMax
8.8E-06

ofxng
275.646

Min d/m
1.35804

ave/min d
1
ave/min m
1.33812

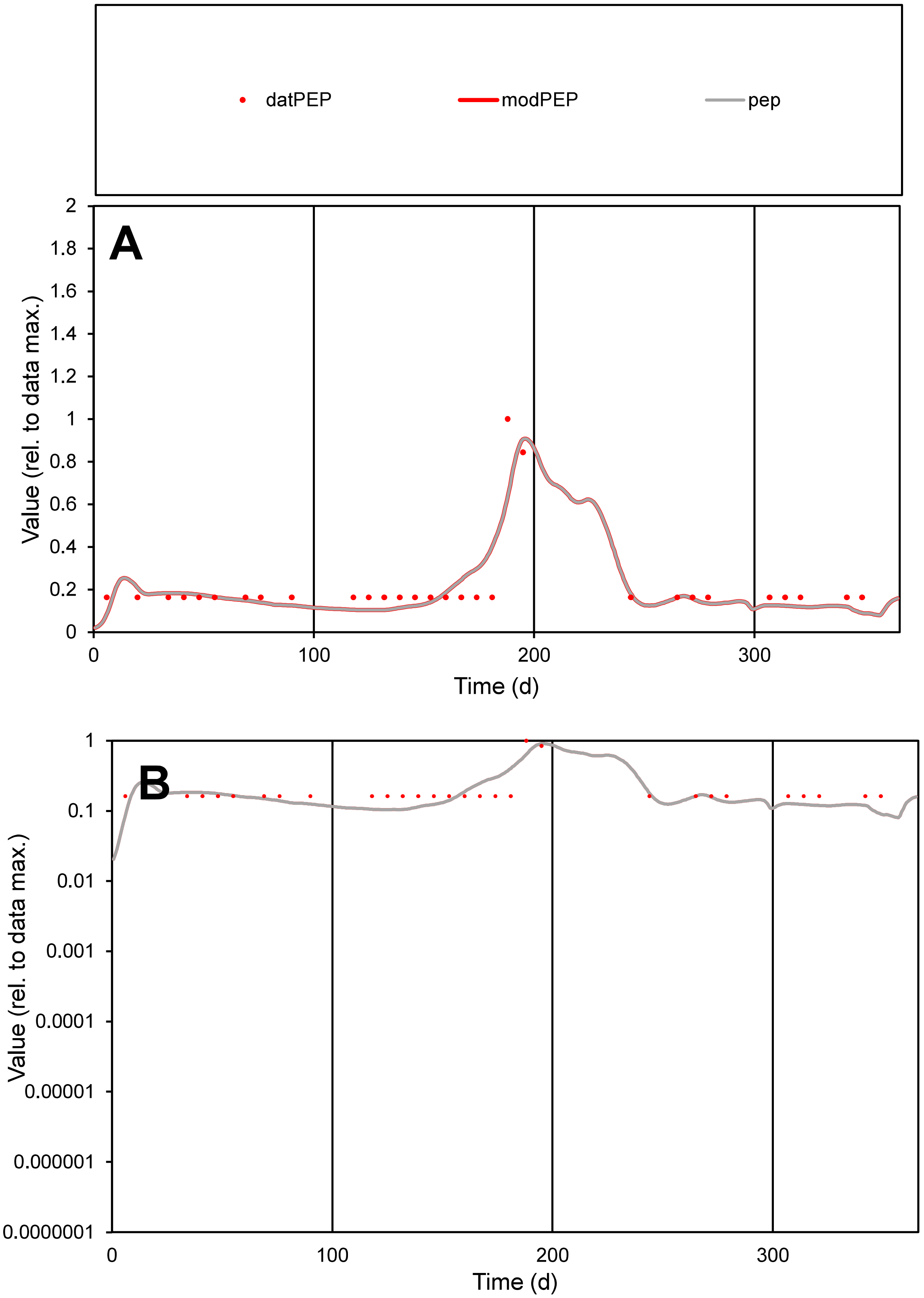

C1

Variables
PEP Provide jtd or Dst
CalibOnly?
2 1 to reproduce of
oNormalize?
2 1=no, 2=yes

ofxn calc*
0.40405
ofxn mod
0.43072

sRun
RunA1
zout
1

Ave
Data
6.5E-06
Model
6.2E-06

data/vMax
3E-05

ofxng
275.646

Min d/m
1.84449

ave/min d
1.31126
ave/min m
2.30041

325_Calib_S_A

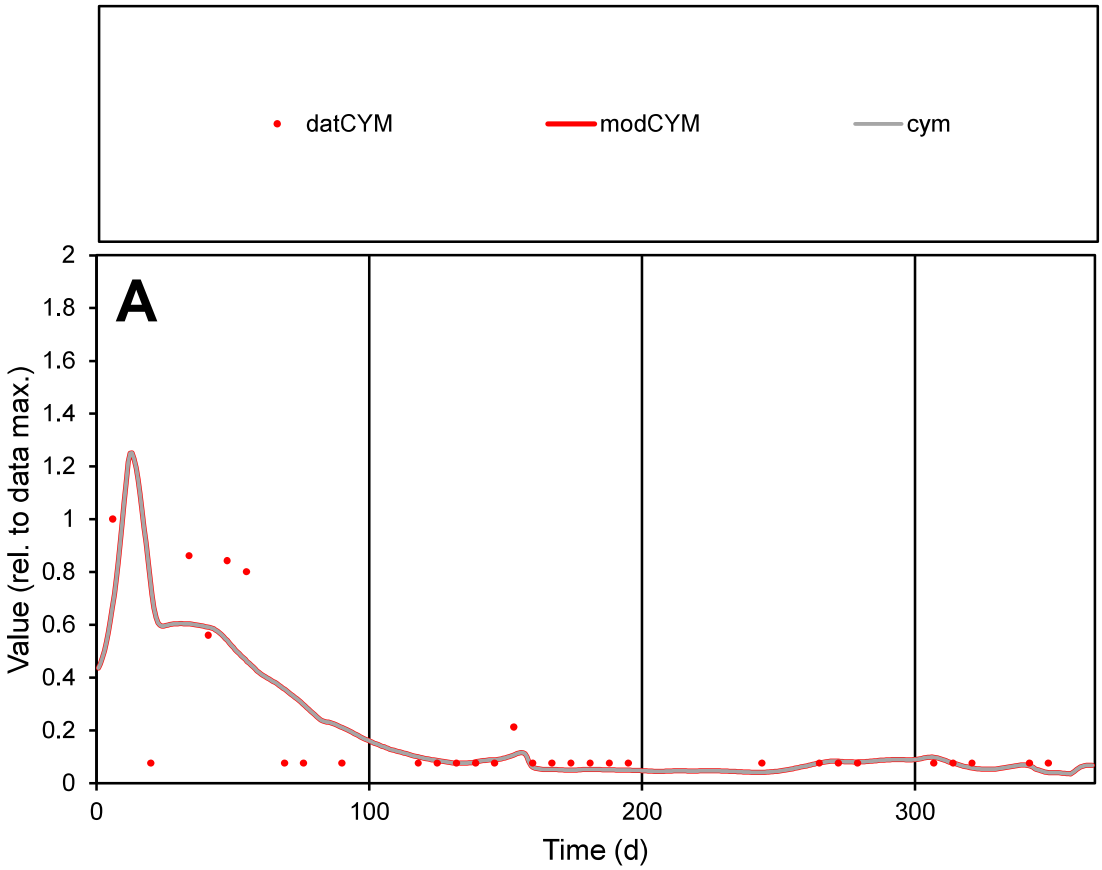

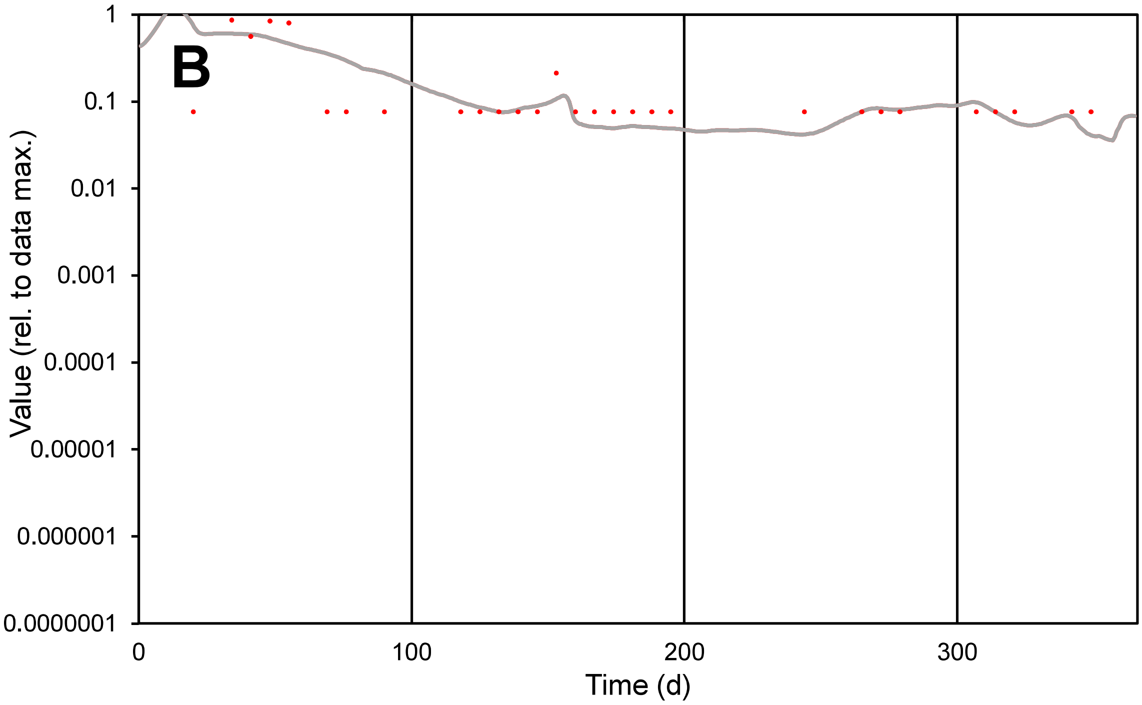

C1

Variables
CYM Provide jtd or Dst
CalibOnly?
2 1 to reproduce of
oNormalize?
2 1=no, 2=yes

ofxn calc*
0.7839
ofxn mod
0.82316

sRun
RunA1
zout
1

Ave
Data
3.6E-06
Model
3.5E-06

data/vMax
1.8E-05

ofxng
275.646

Min d/m
1.91367

ave/min d
2.66806
ave/min m
4.89547

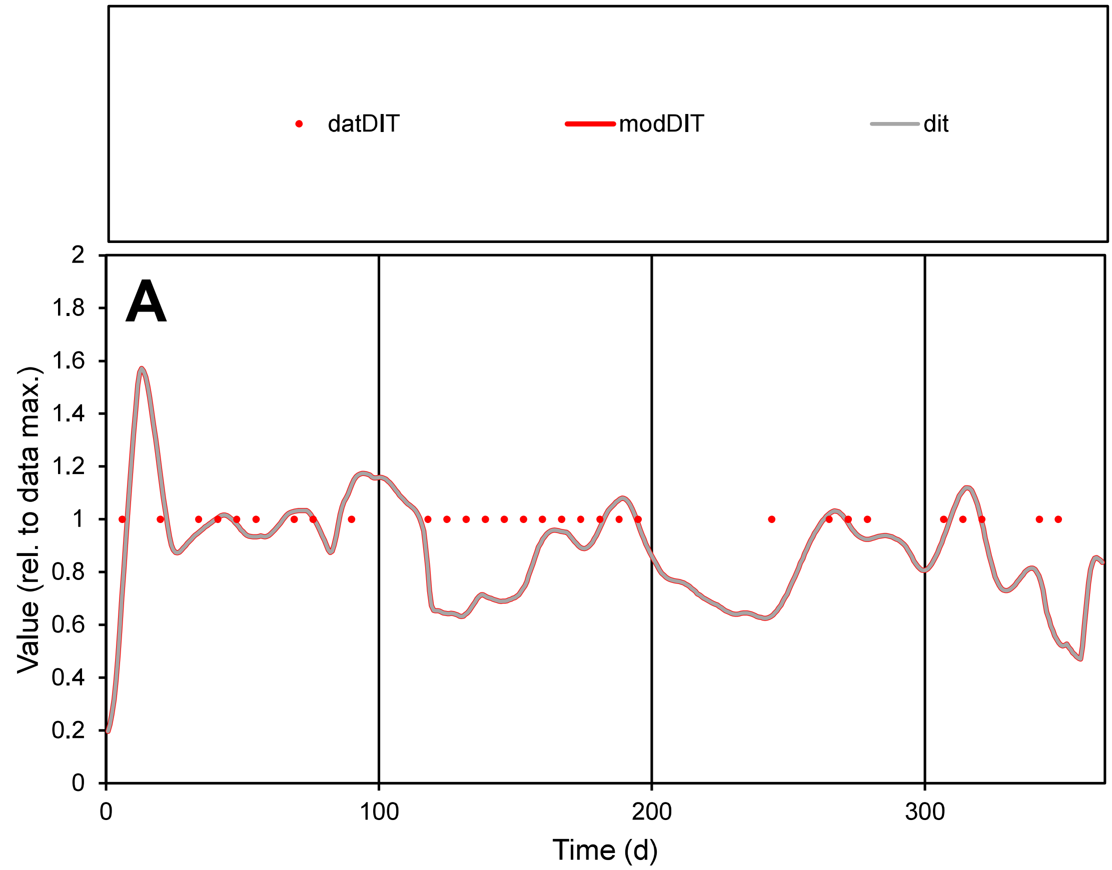

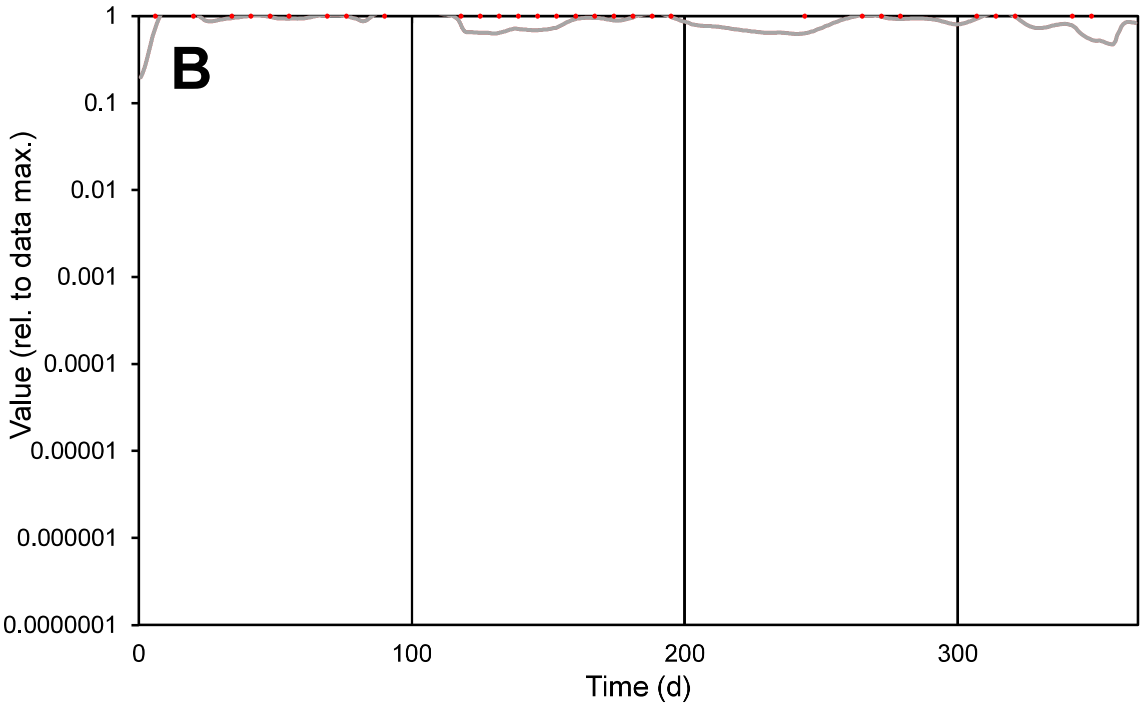

C1

Variables

DIT Provide jtd or Dst

CalibOnly?

2 1 to reproduce of

oNormalize?

2 1=no, 2=yes

ofxn calc*

0.19019

ofxn mod

0.21962

sRun

RunA1

zout

1

Ave

Data

5.1E-06

Model

4.6E-06

data/vMax

5.1E-06

ofxng

275.646

Min d/m

1.9218

ave/min d

1

ave/min m

1.7245

325_Calib_S_A

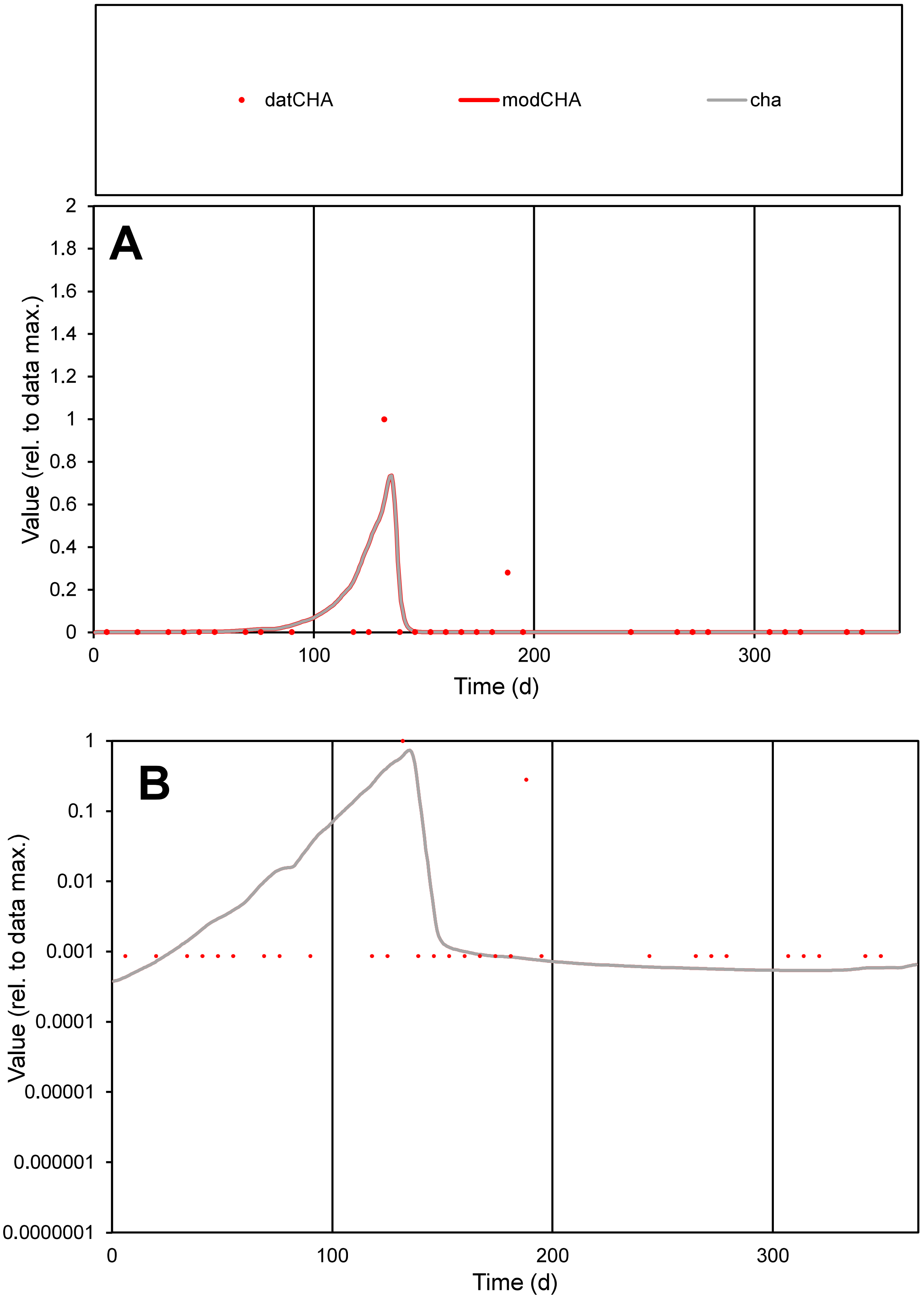

C1

Variables
CHA Provide jtd or Dst
CalibOnly?
2 1 to reproduce of
oNormalize?
2 1=no, 2=yes

ofxn calc*
2.6494
ofxn mod
2.71964

sRun
RunA1
zout
1

Ave
Data
4.4E-06
Model
4.7E-06

data/vMax
0.0001

ofxng
275.646

Min d/m
1.91313

ave/min d
50.2998
ave/min m
103.277

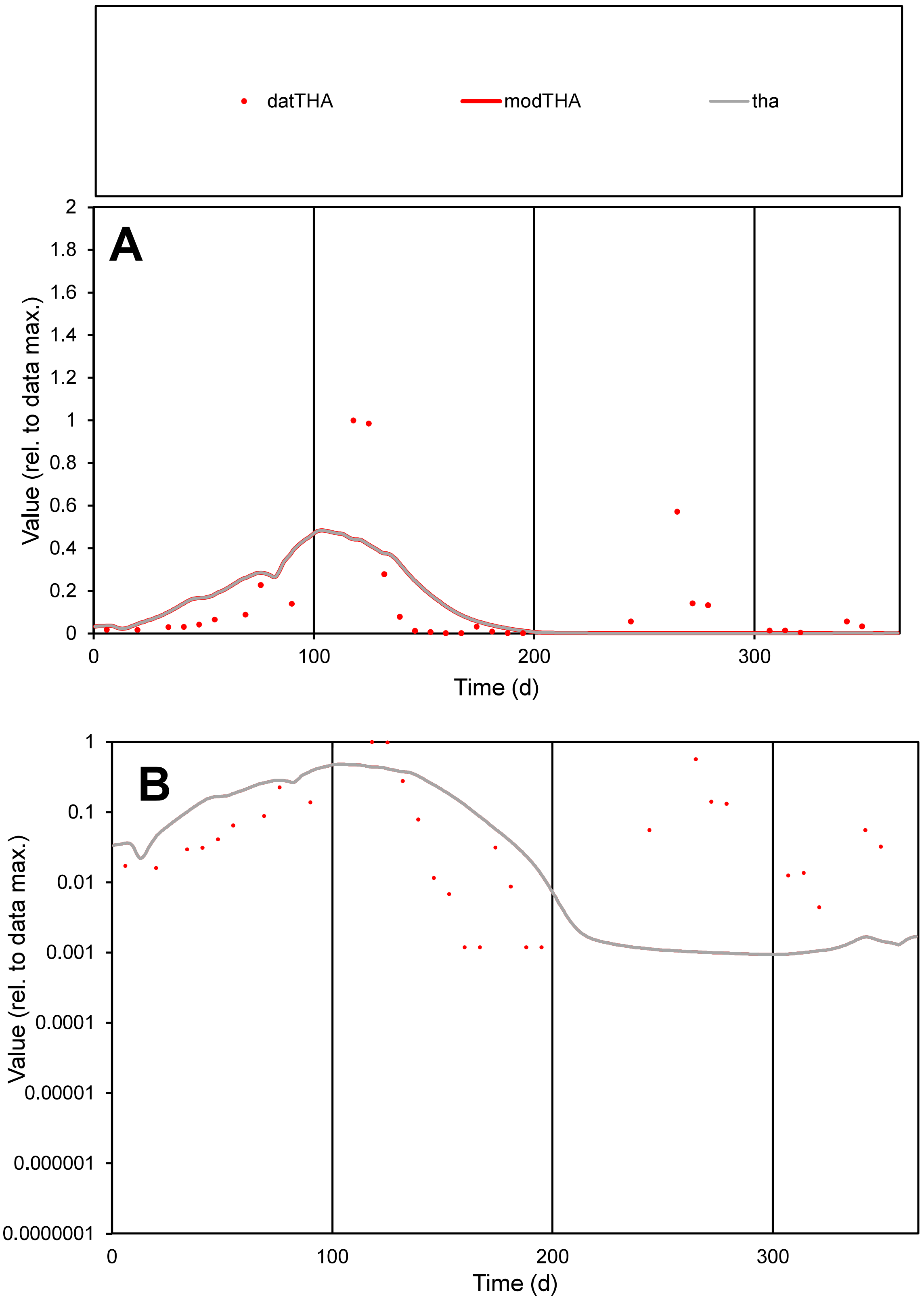

C1

Variables

THA Provide jtd or Dst

CalibOnly?

2 1 to reproduce of

oNormalize?

2 1=no, 2=yes

ofxn calc*

1.53644

ofxn mod

1.55279

sRun

RunA1

zout

1

Ave

Data

0.00031

Model

0.00029

data/vMax

0.0023

ofxng

275.646

Min d/m

1.24751

ave/min d

113.513

ave/min m

133.134

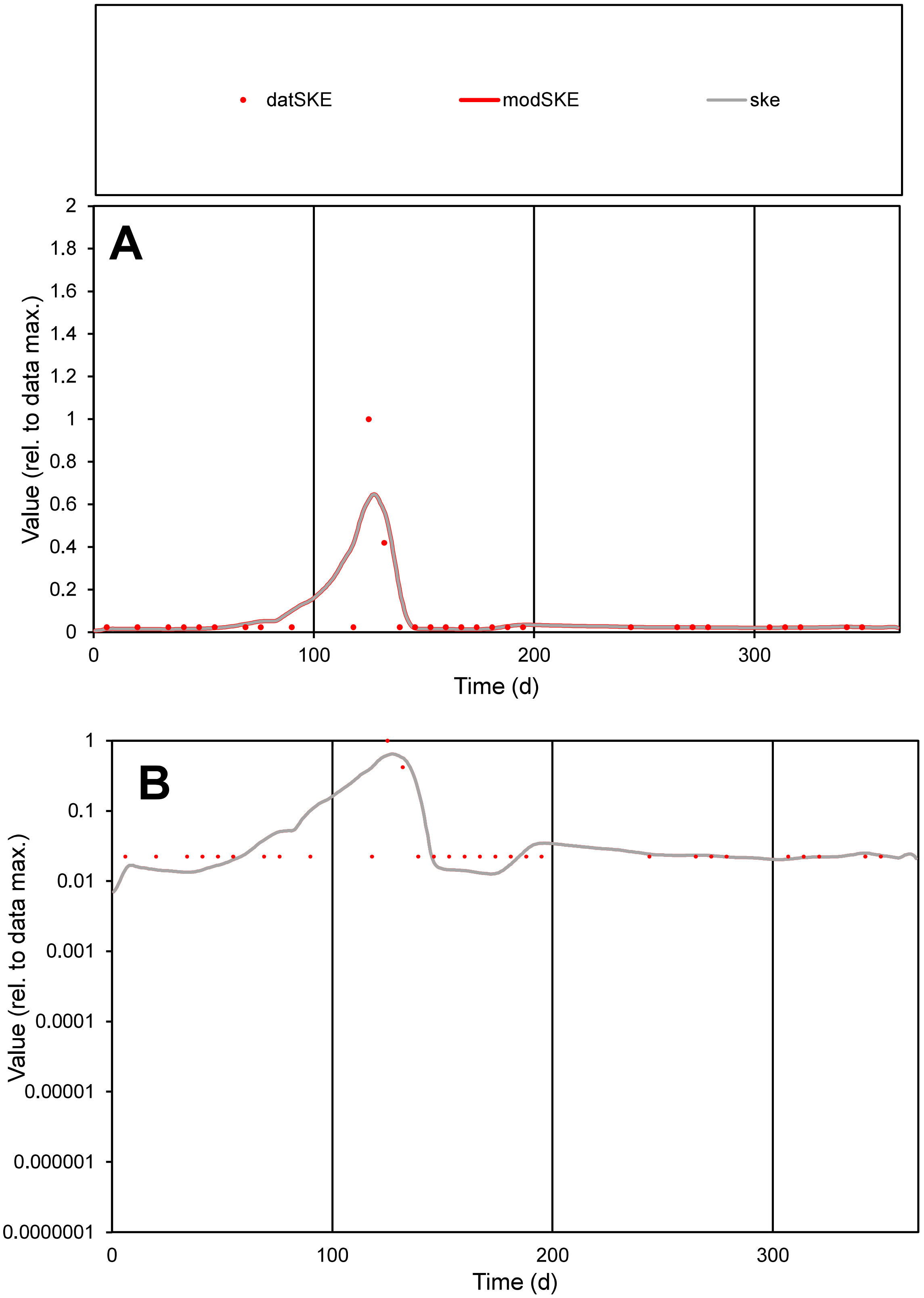

C1

Variables
SKE Provide jtd or Dst
CalibOnly?
2 1 to reproduce of
oNormalize?
2 1=no, 2=yes

ofxn calc*
1.43863
ofxn mod
1.51105

sRun
RunA1
zout
1

Ave
Data
7E-06
Model
7.8E-06

data/vMax
0.0001

ofxng
275.646

Min d/m
1.72102

ave/min d
3.04386
ave/min m
5.84341

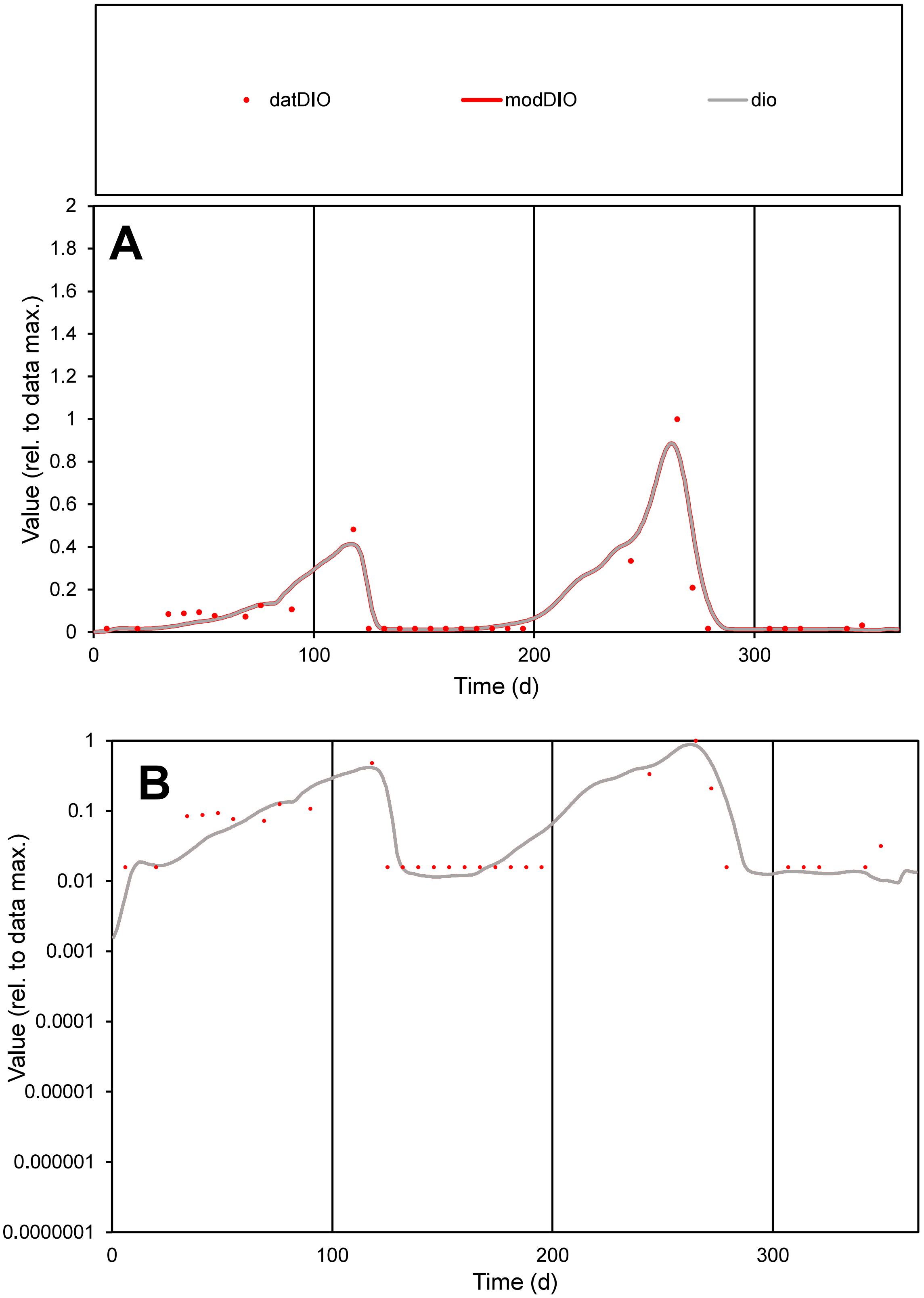

C1

Variables
DIO Provide jtd or Dst
CalibOnly?
2 1 to reproduce of
oNormalize?
2 1=no, 2=yes

ofxn calc*
0.62228
ofxn mod
0.67056

sRun
RunA1
zout
1

Ave
Data
2.9E-05
Model
3.1E-05

data/vMax
0.00029

ofxng
275.646

Min d/m
1.56573

ave/min d
6.28265
ave/min m
10.3259

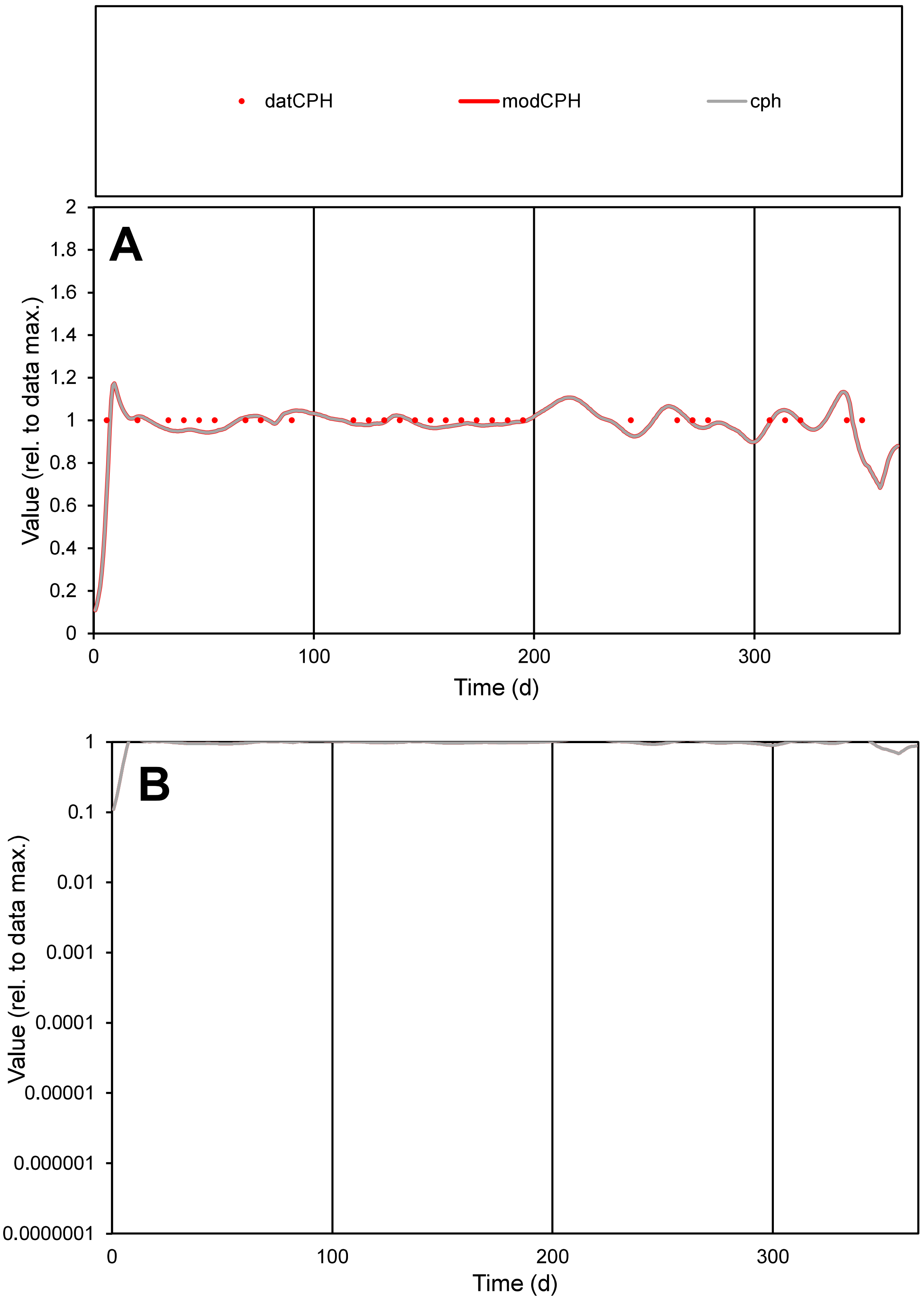

C1

Variables
CPH Provide jtd or Dst
CalibOnly?
2 1 to reproduce of
oNormalize?
2 1=no, 2=yes

ofxn calc*
0.05089
ofxn mod
0.12232

sRun
RunA1
zout
1

Ave
Data
9.6E-06
Model
9.4E-06

data/vMax
9.6E-06

ofxng
275.646

Min d/m
1.26096

ave/min d
1

ave/min m
1.24094

325_Calib_S_A

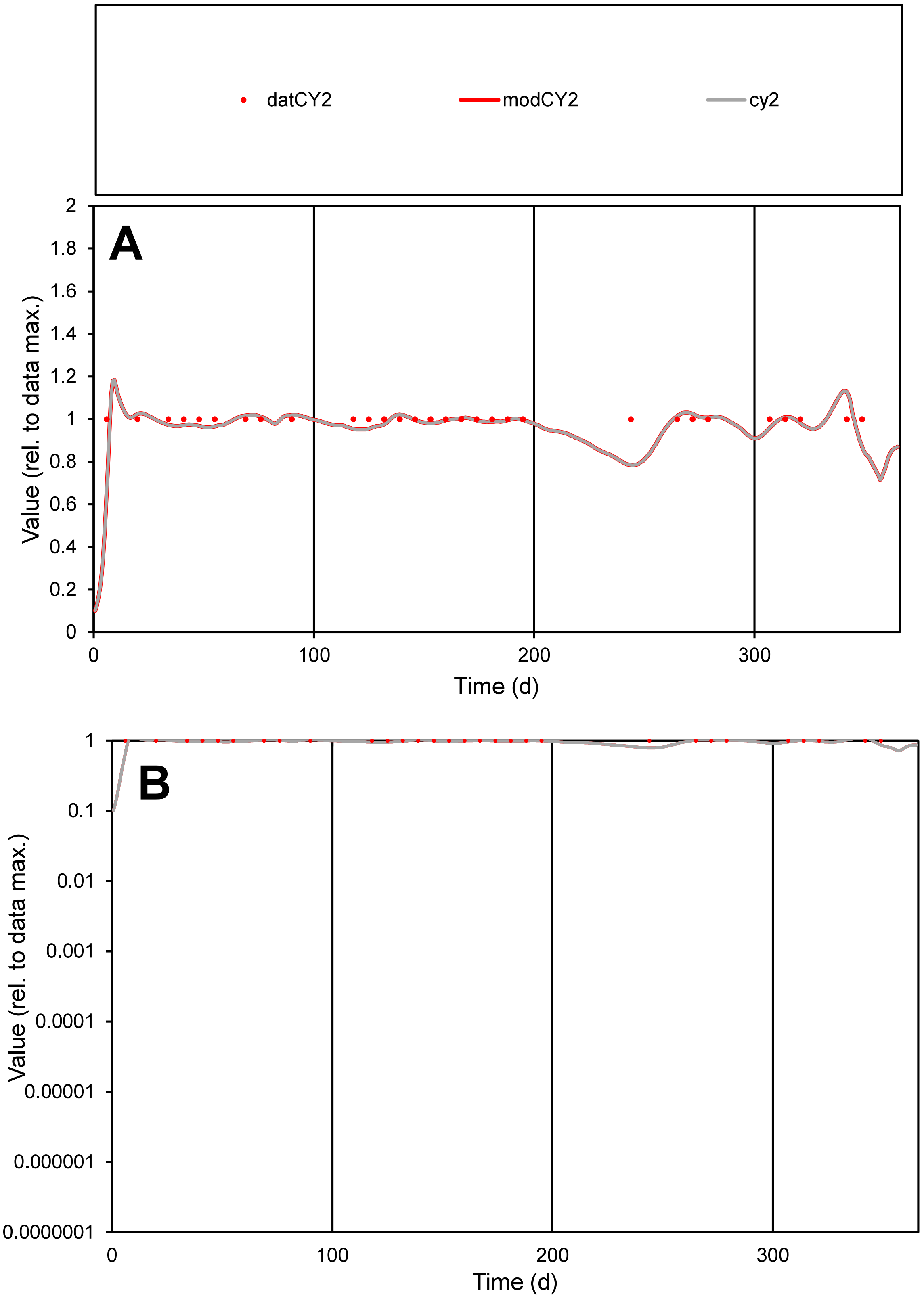

C1

Variables
CY2 Provide jtd or Dst
CalibOnly?
2 1 to reproduce of
oNormalize?
2 1=no, 2=yes

ofxn calc*
0.0566
ofxn mod
0.12494

sRun
RunA1
zout
1

Ave
Data
1.1E-05
Model
1.1E-05

data/vMax
1.1E-05

ofxng
275.646

Min d/m
1.27518

ave/min d
1

ave/min m
1.25437

325_Calib_S_A

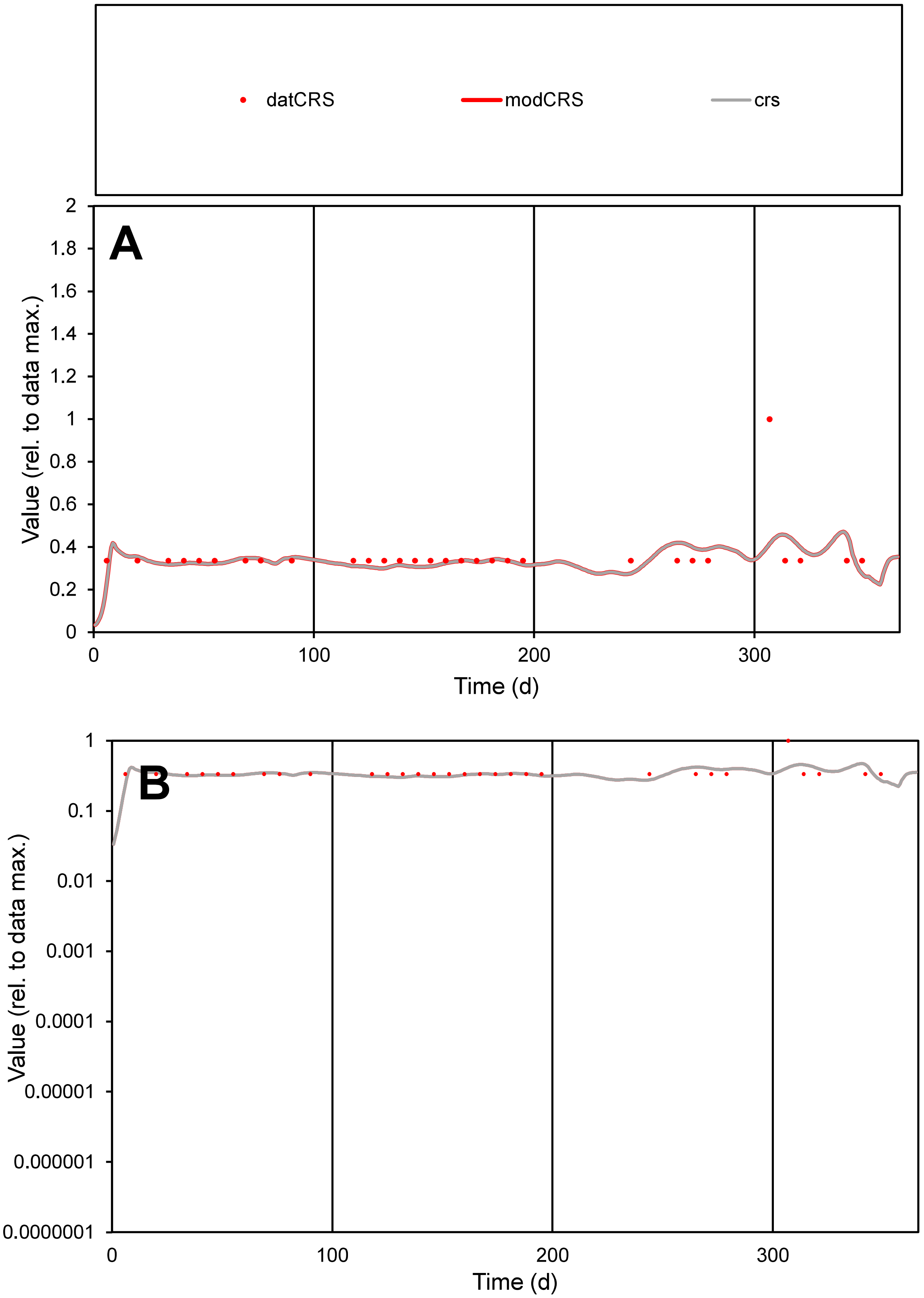

C1

Variables
CRS Provide jtd or Dst
CalibOnly?
2 1 to reproduce of
oNormalize?
2 1=no, 2=yes

ofxn calc*
0.31272
ofxn mod
0.33269

sRun
RunA1
zout
1

Ave
Data
5.8E-06
Model
5.5E-06

data/vMax
1.6E-05

ofxng
275.646

Min d/m
1.28382

ave/min d
1.06599
ave/min m
1.31366

325_Calib_S_A

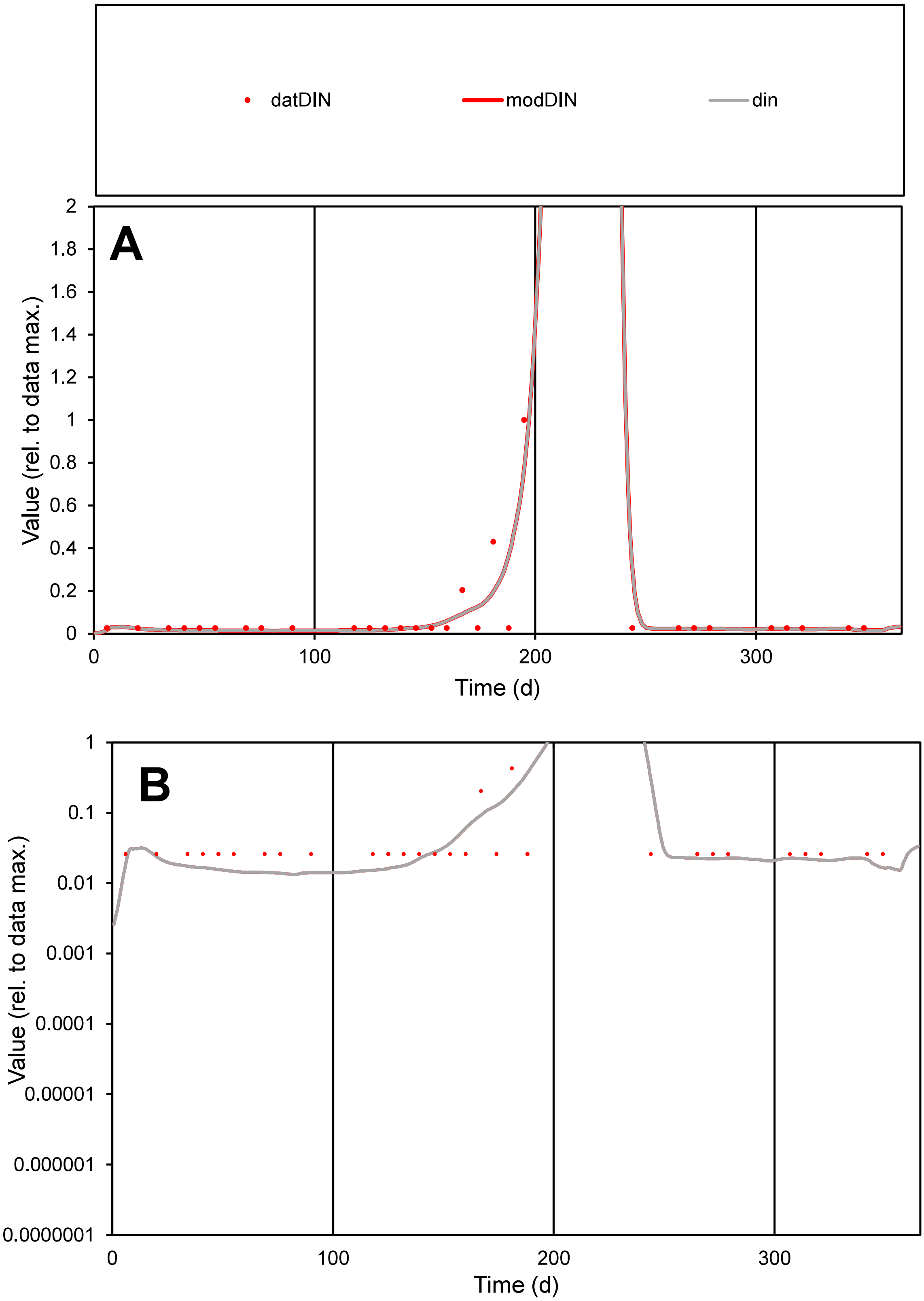

C1

Variables

DIN Provide jtd or Dst

CalibOnly?

2 1 to reproduce of

oNormalize?

2 1=no, 2=yes

ofxn calc*

0.931

ofxn mod

1.06159

sRun

RunA1

zout

1

Ave

Data

8.7E-06

Model

8.5E-06

data/vMax

0.00011

ofxng

275.646

Min d/m

1.88374

ave/min d

3.00599

ave/min m

5.54986

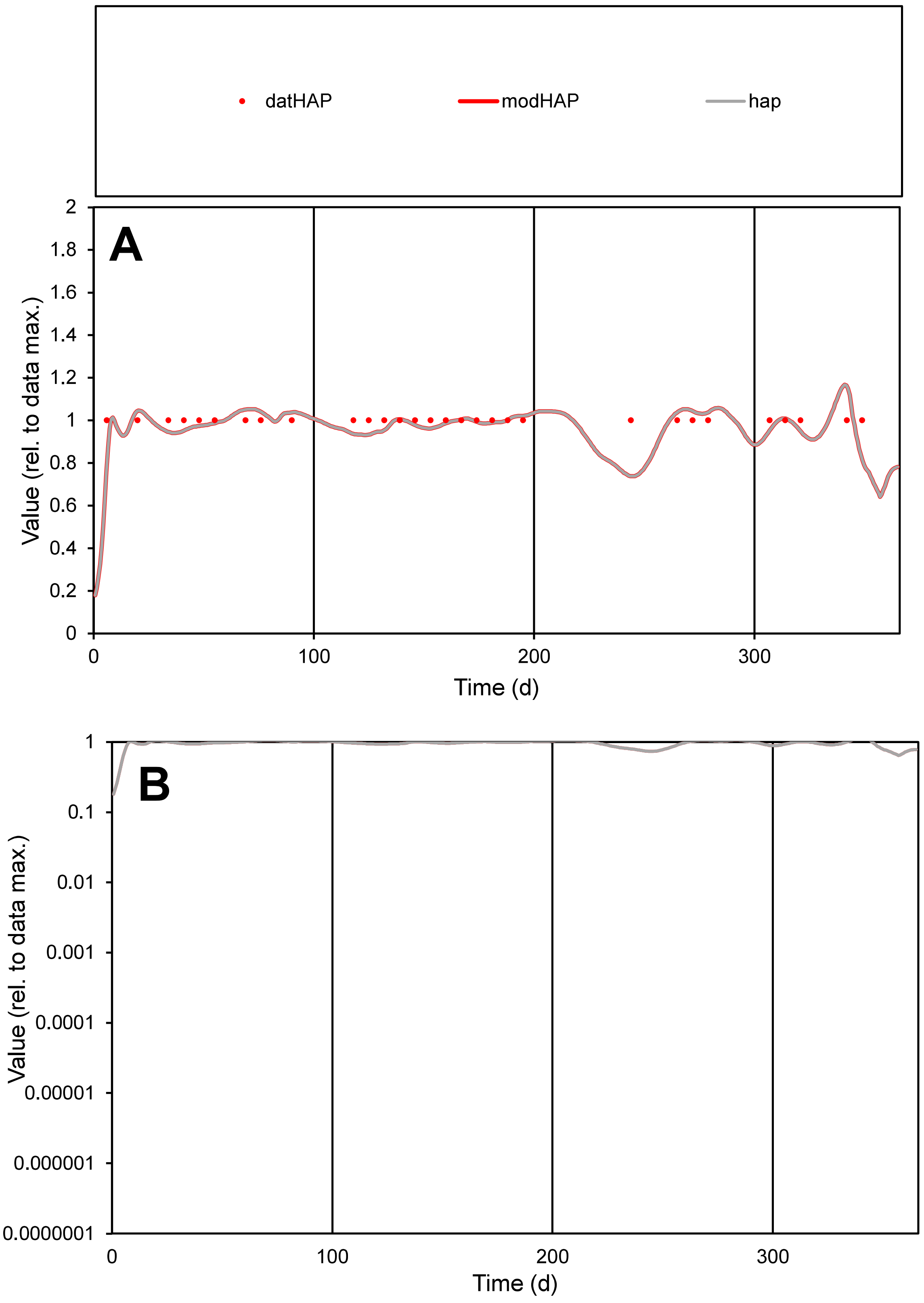

C1

Variables
HAP Provide jtd or Dst
CalibOnly?
2 1 to reproduce of
oNormalize?
2 1=no, 2=yes

ofxn calc*
0.07564
ofxn mod
0.13487

sRun
RunA1
zout
1

Ave
Data
1.1E-05
Model
1.1E-05

data/vMax
1.1E-05

ofxng
275.646

Min d/m
1.35474

ave/min d
1
ave/min m
1.33064

325_Calib_S_A

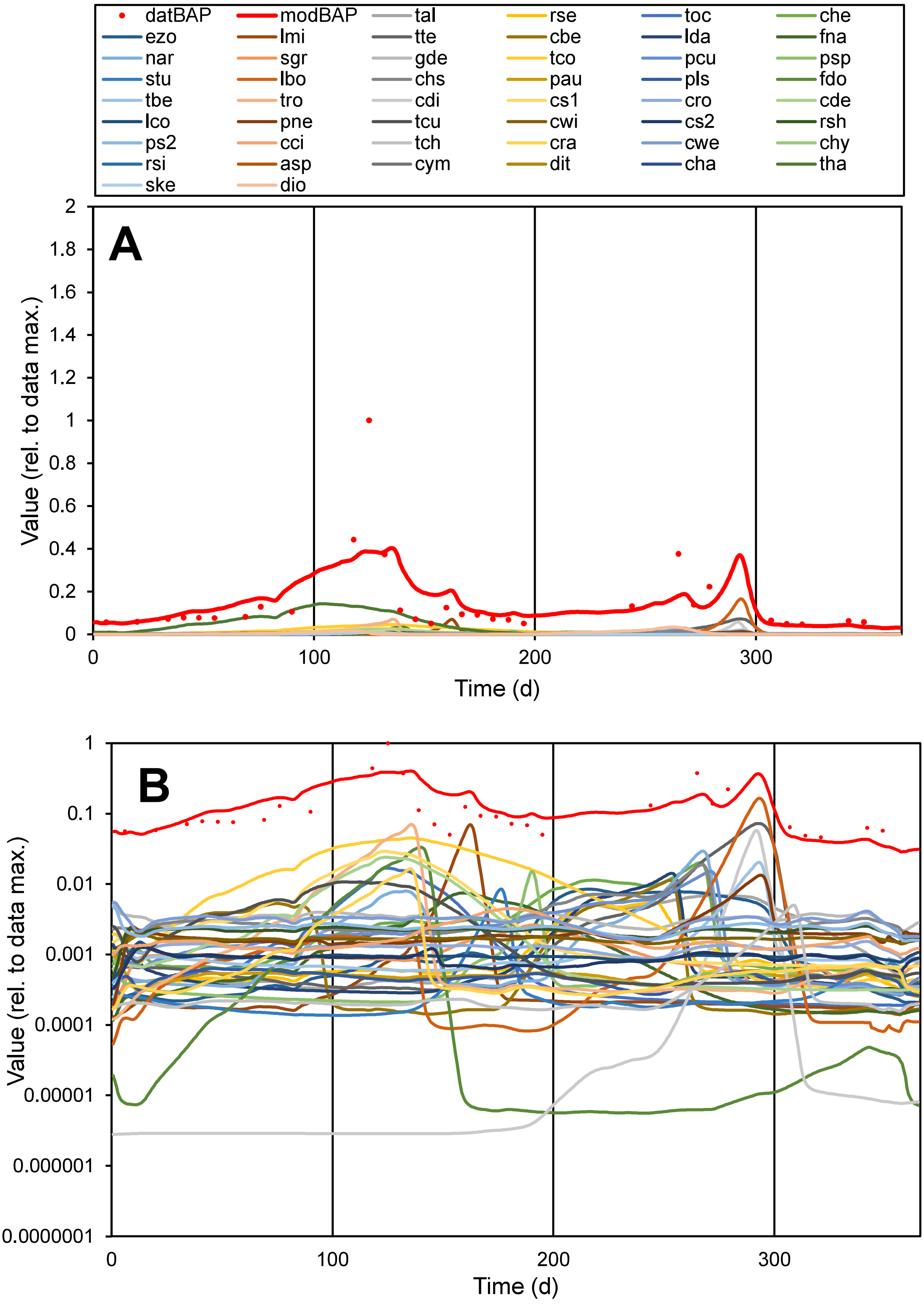

C1

Variables

BAP Provide jtd or Dst

CalibOnly?

2 1 to reproduce of

oNormalize?

2 1=no, 2=yes

ofxn calc*

0.90262

ofxn mod

0.91619

sRun

RunA1

zout

1

Ave

Data

0.00115

Model

0.00115

data/vMax

0.0078

ofxng

275.646

Min d/m

1.35639

ave/min d

3.20741

ave/min m

4.33886

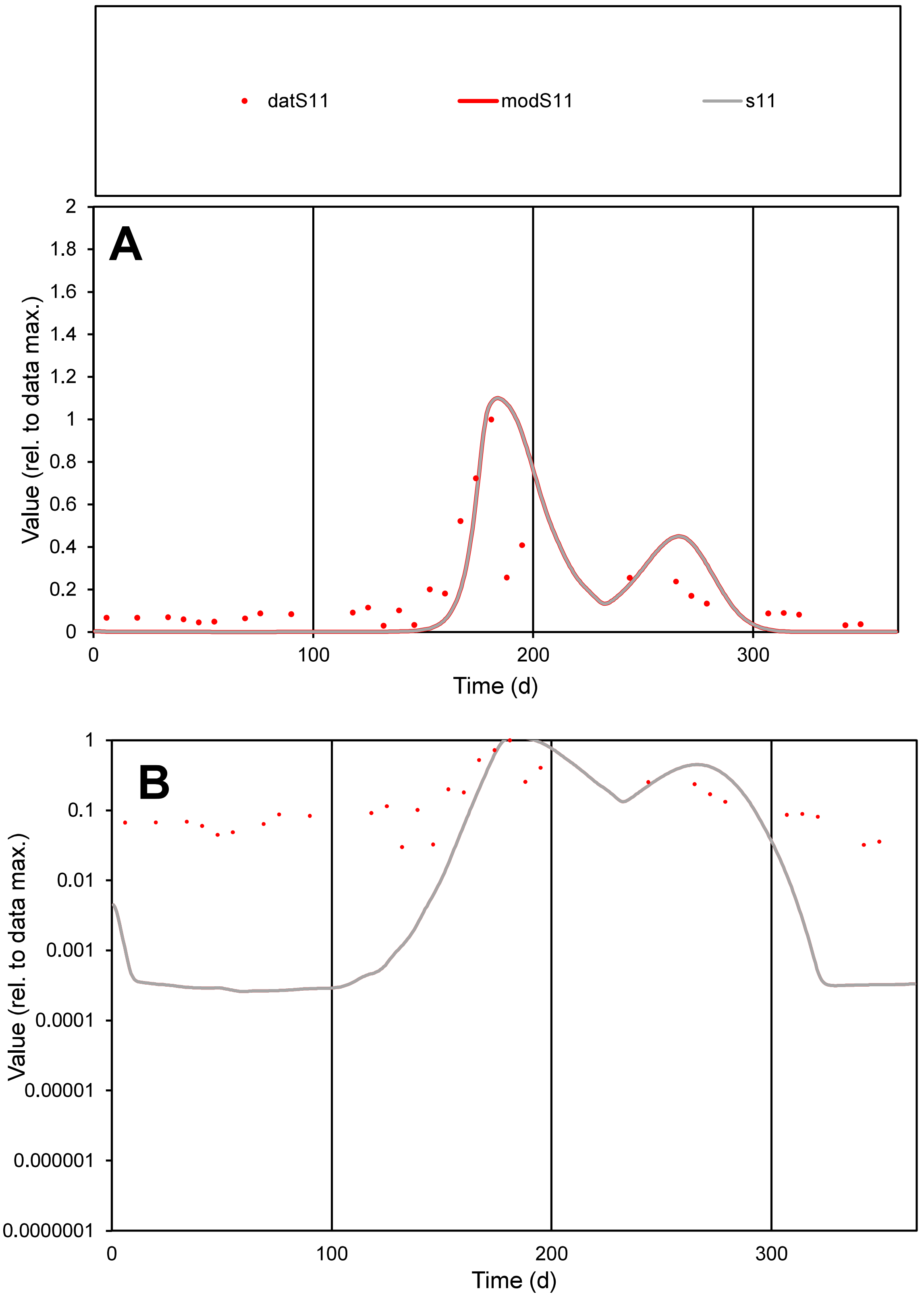

C1

Variables
S11 Provide jtd or Dst
CalibOnly?
2 1 to reproduce of
oNormalize?
2 1=no, 2=yes

ofxn calc*
1.11532
ofxn mod
1.14298

sRun
RunA1
zout
1

Ave
Data
0.09093
Model
0.09505

data/vMax
0.5081

ofxng
275.646

Min d/m
113.647

ave/min d
5.96636
ave/min m
708.823

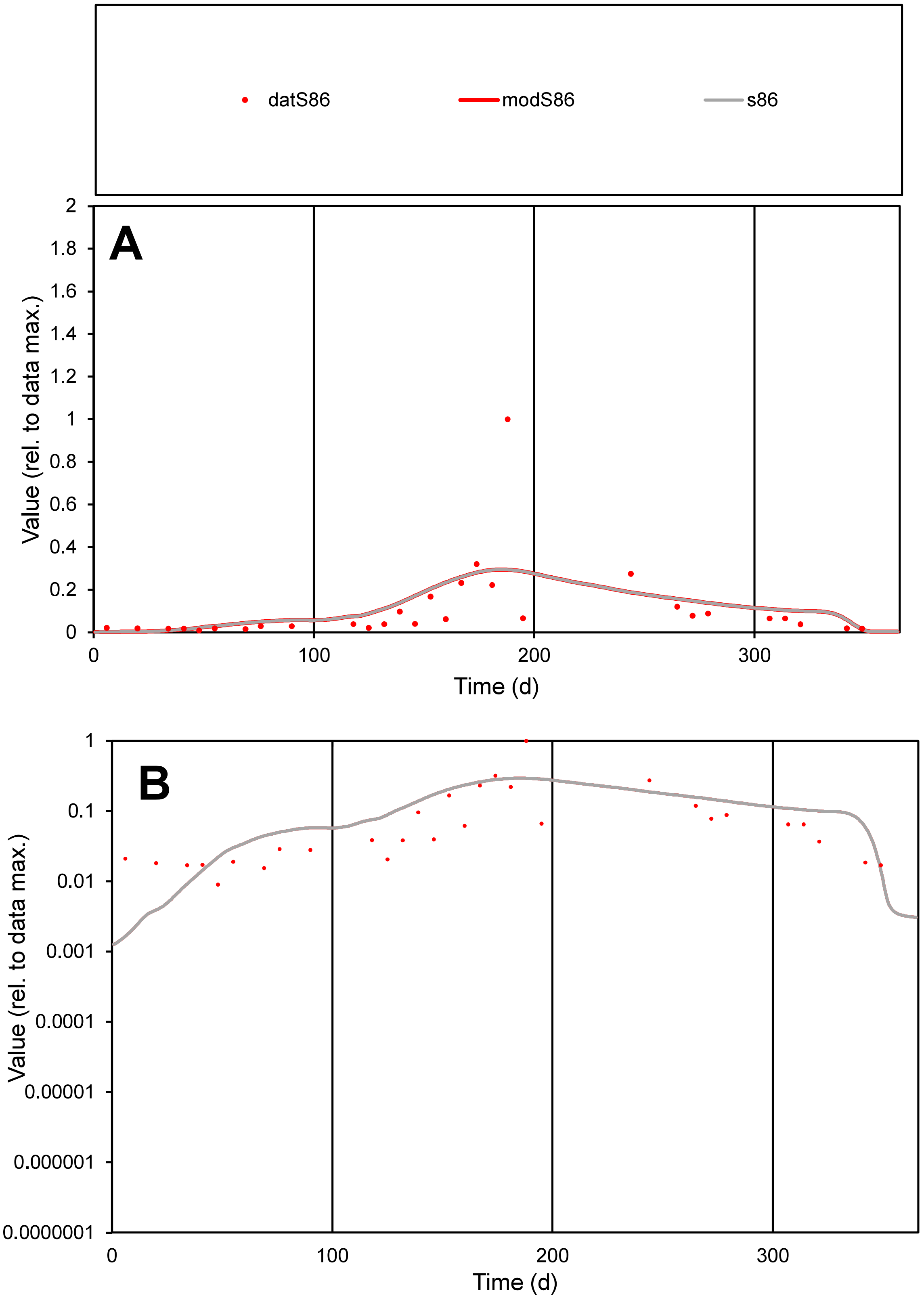

C1

Variables
S86 Provide jtd or Dst
CalibOnly?
2 1 to reproduce of
oNormalize?
2 1=no, 2=yes

ofxn calc*
1.35801
ofxn mod
1.36789

sRun
RunA1
zout
1

Ave
Data
0.03132
Model
0.03569

data/vMax
0.2902

ofxng
275.646

Min d/m
5.01155

ave/min d
12.0295
ave/min m
68.6913

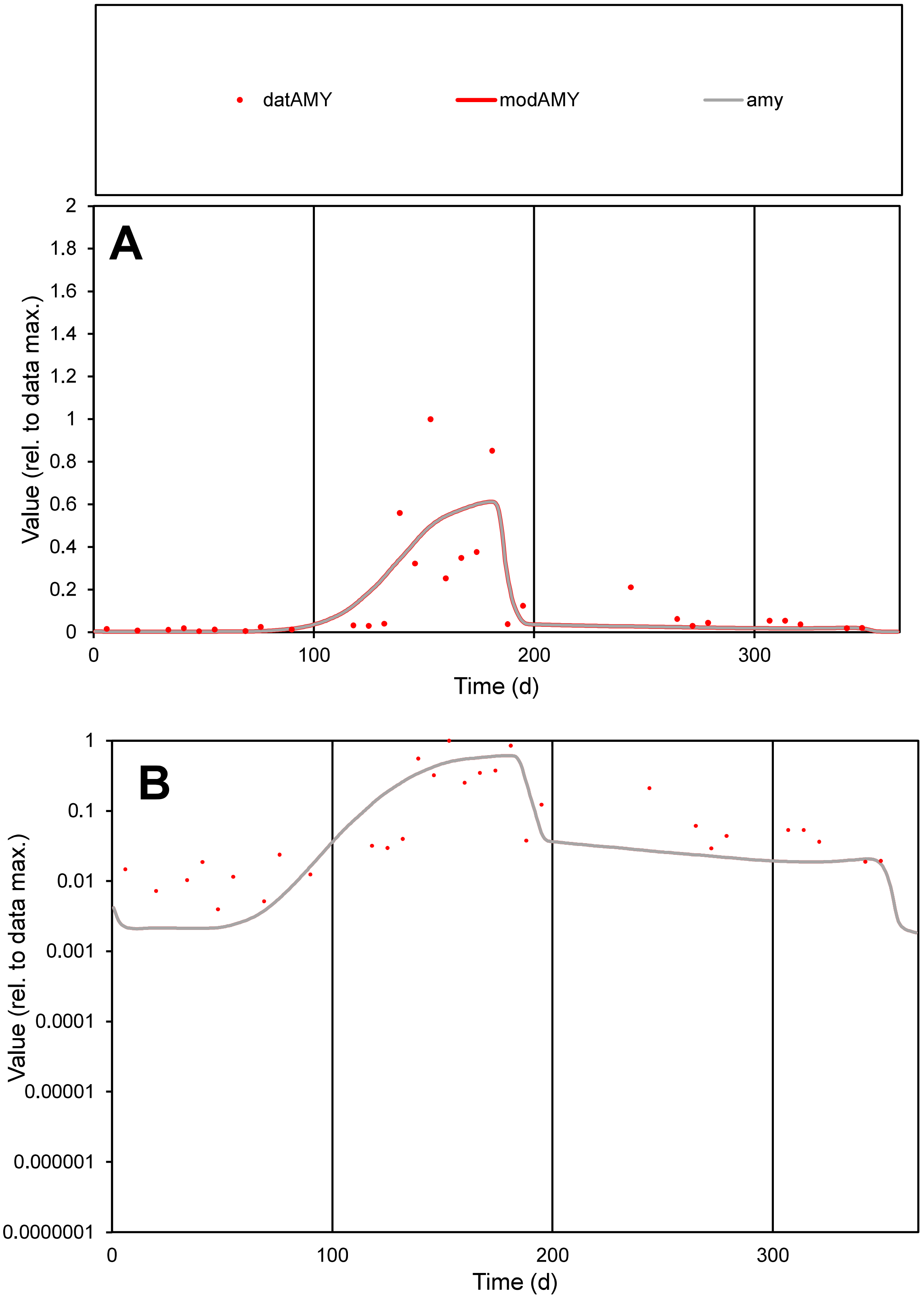

C1

Variables
AMY Provide jtd or Dst
CalibOnly?
2 1 to reproduce of
oNormalize?
2 1=no, 2=yes

ofxn calc*
0.98759
ofxn mod
1.00805

sRun
RunA1
zout
1

Ave
Data
0.02619
Model
0.02644

data/vMax
0.1706

ofxng
275.646

Min d/m
1.87514

ave/min d
38.6695
ave/min m
73.1898

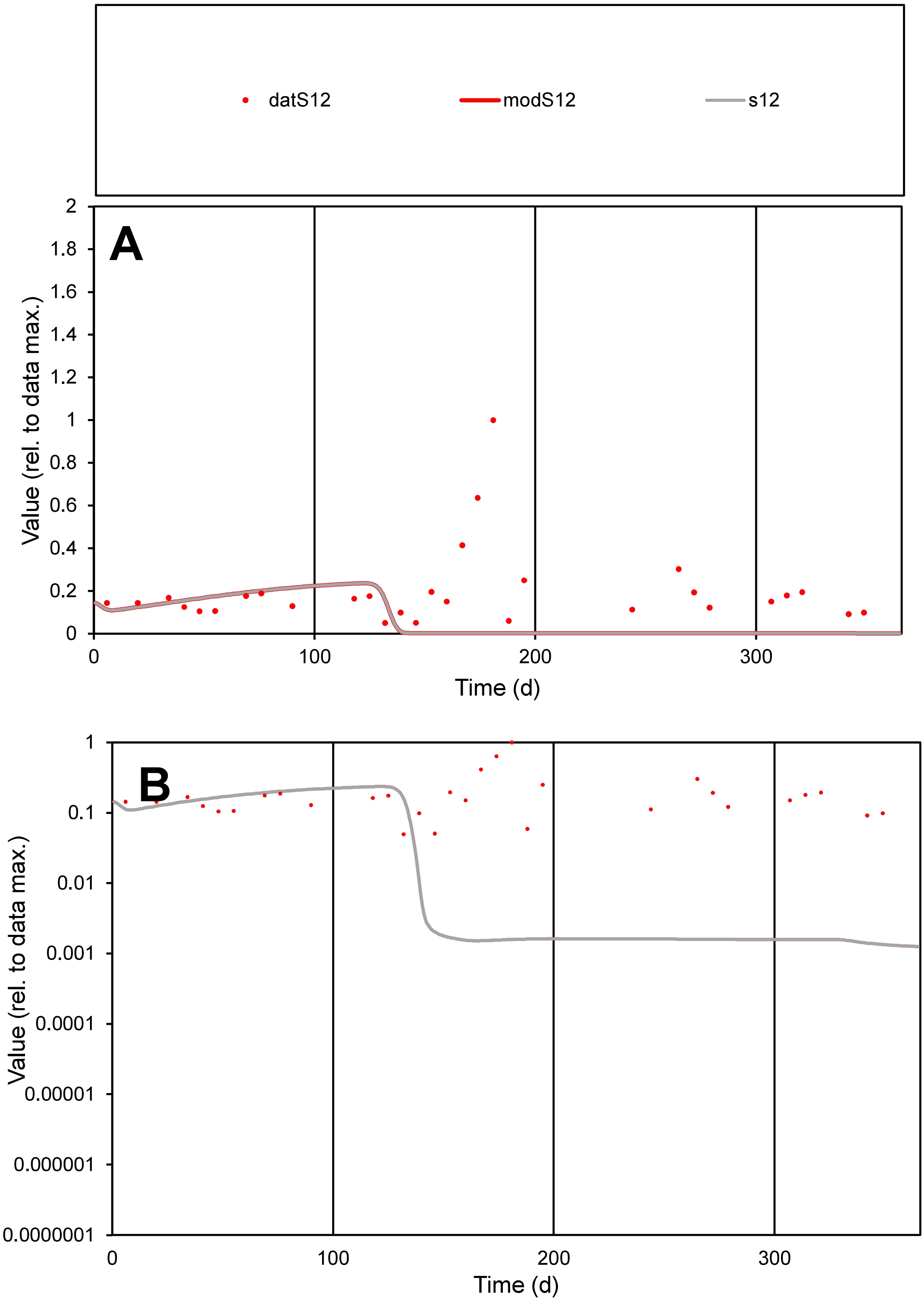

C1

Variables
S12 Provide jtd or Dst
CalibOnly?
2 1 to reproduce of
oNormalize?
2 1=no, 2=yes

ofxn calc*
1.29536
ofxn mod
1.30032

sRun
RunA1
zout
1

Ave
Data
0.04112
Model
0.0147

data/vMax
0.2069

ofxng
275.646

Min d/m
37.1511

ave/min d
4.01174
ave/min m
53.2806

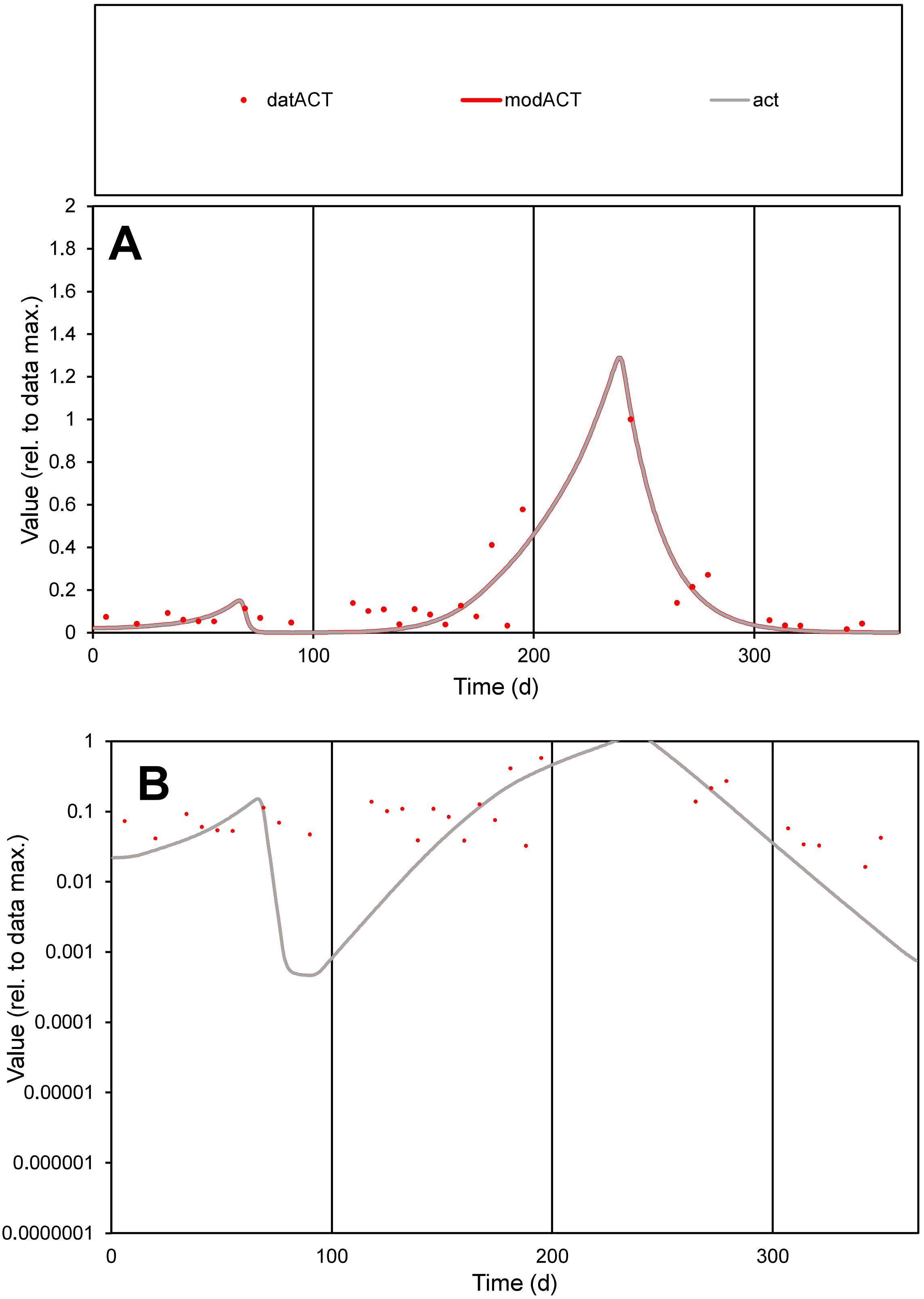

C1

Variables
ACT Provide jtd or Dst
CalibOnly?
2 1 to reproduce of
oNormalize?
2 1=no, 2=yes

ofxn calc*
0.63974
ofxn mod
0.66182

sRun
RunA1
zout
1

Ave
Data
0.0246
Model
0.02032

data/vMax
0.1733

ofxng
275.646

Min d/m
34.541

ave/min d
8.7186
ave/min m
248.682

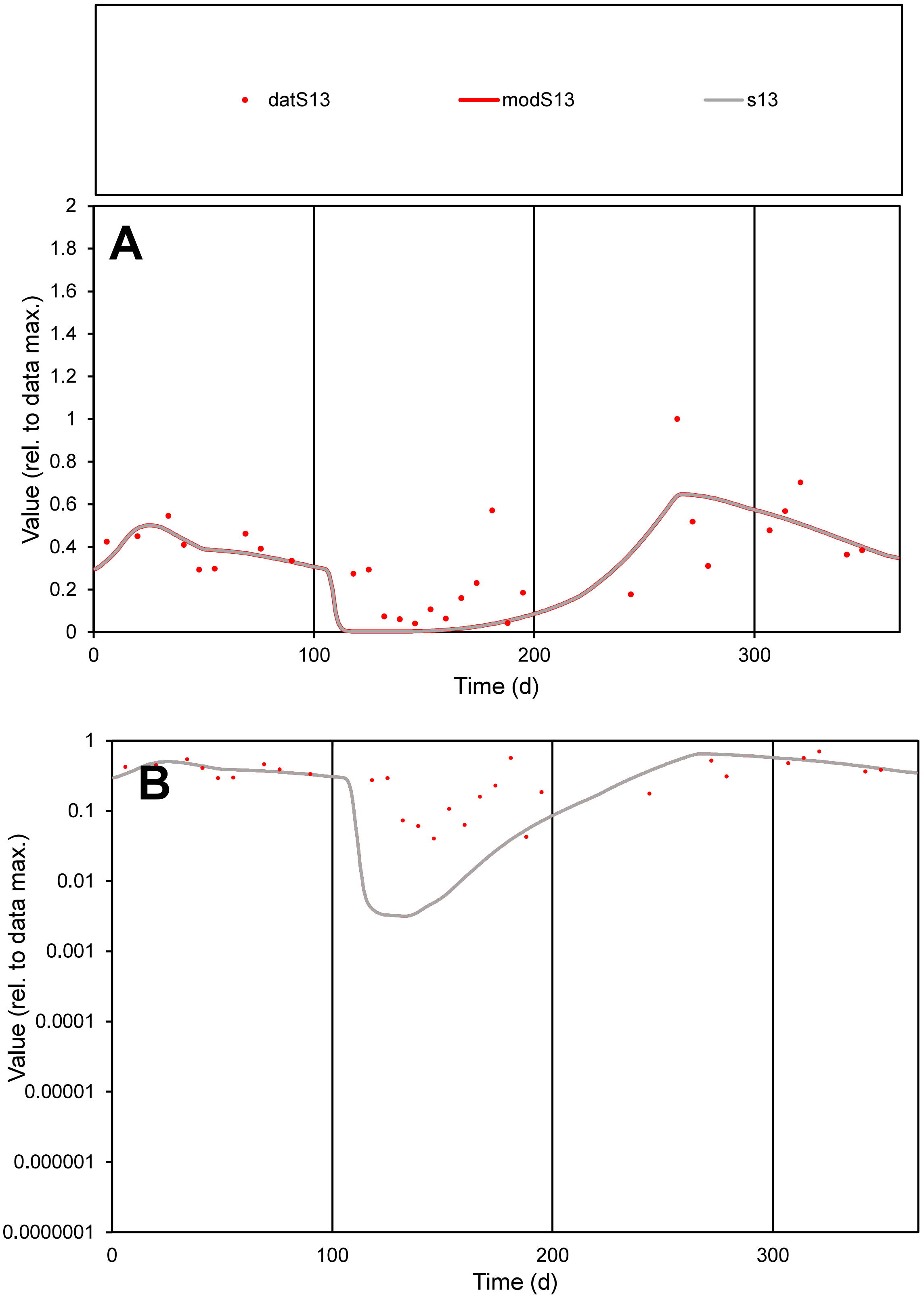

C1

Variables

S13	Provide jtd or Dst

CalibOnly?

2	1 to reproduce of

oNormalize?

2	1=no, 2=yes

ofxn calc*
0.51078

ofxn mod
0.52738

sRun
RunA1

zout
1

Ave
Data
0.01909

Model
0.01592

data/vMax
0.05605

ofxng
275.646

Min d/m
12.7872

ave/min d
8.4038

ave/min m
89.6293

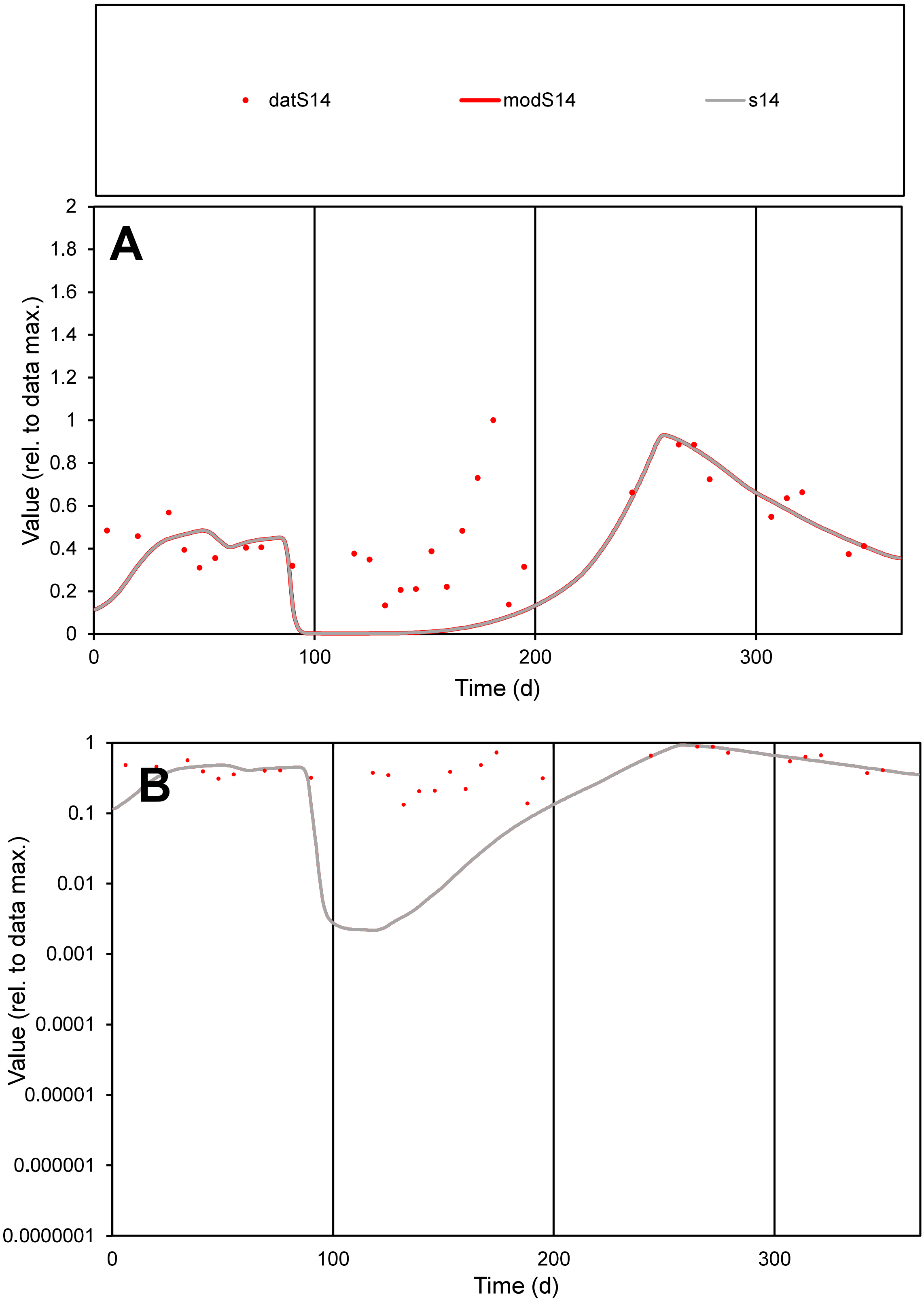

C1

Variables
S14 Provide jtd or Dst
CalibOnly?
2 1 to reproduce of
oNormalize?
2 1=no, 2=yes

ofxn calc*
0.59999
ofxn mod
0.61251

sRun
RunA1
zout
1

Ave
Data
0.01746
Model
0.01196

data/vMax
0.03734

ofxng
275.646

Min d/m
61.1179

ave/min d
3.50986
ave/min m
146.88

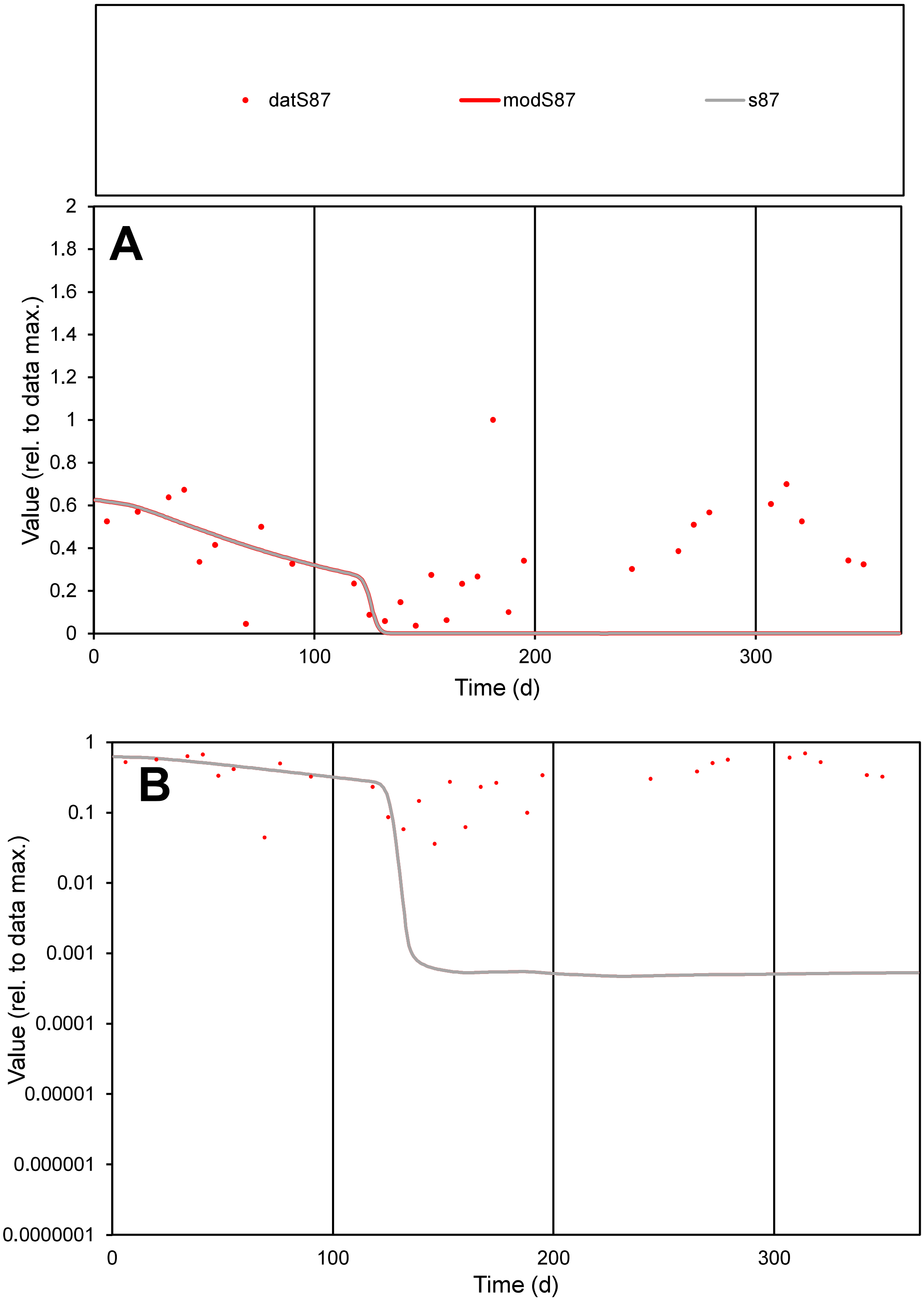

C1

Variables
S87 Provide jtd or Dst
CalibOnly?
2 1 to reproduce of
oNormalize?
2 1=no, 2=yes

ofxn calc*
0.95194
ofxn mod
0.95762

sRun
RunA1
zout
1

Ave
Data
0.01745
Model
0.00747

data/vMax
0.04707

ofxng
275.646

Min d/m
75.2983

ave/min d
10.2425
ave/min m
329.898

325_Calib_S_A

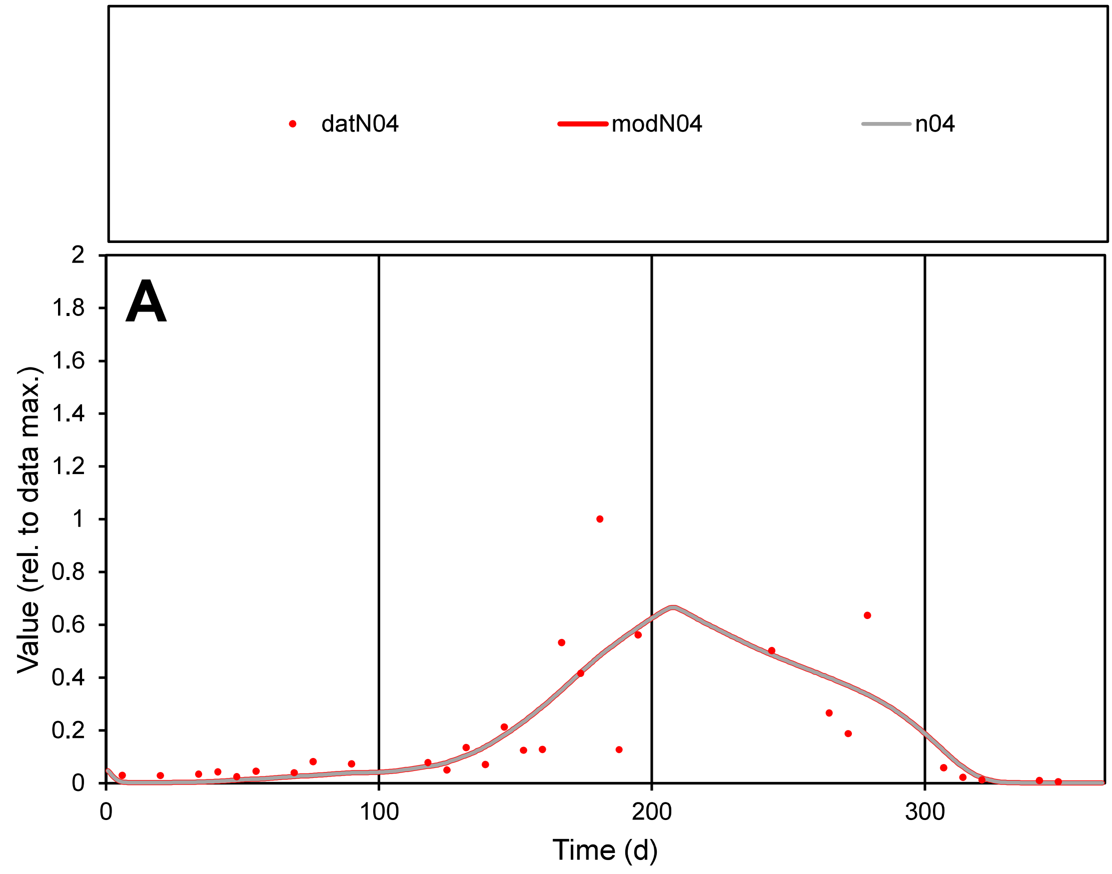

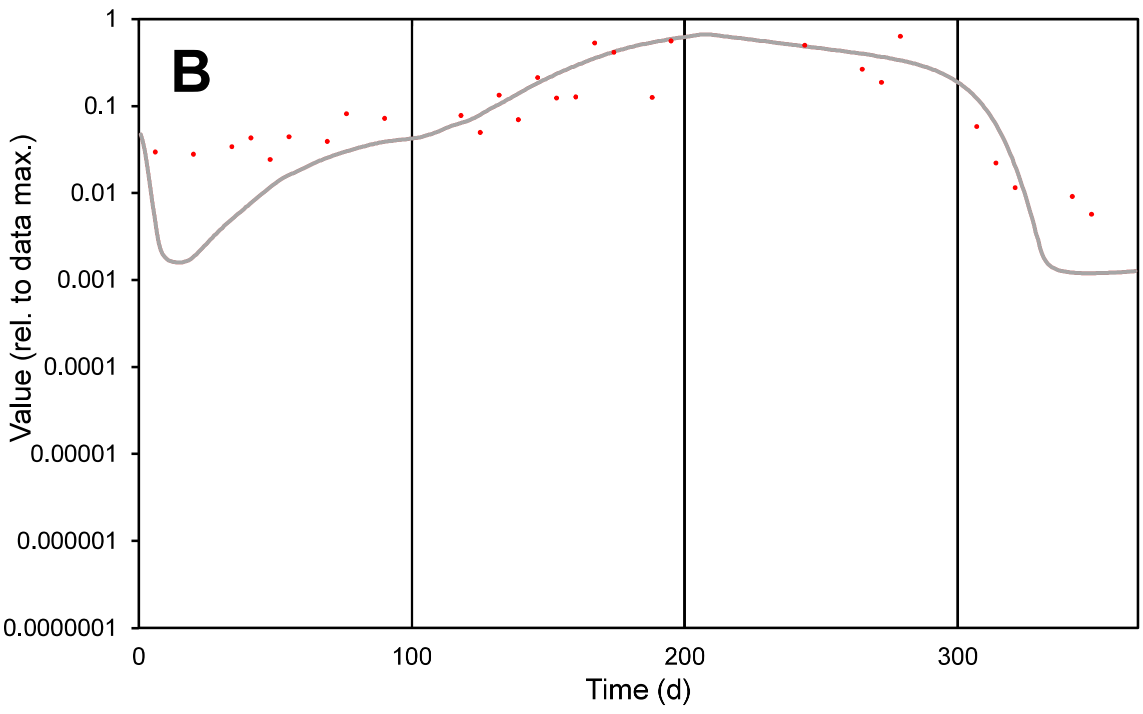

C1

Variables
N04 Provide jtd or Dst
CalibOnly?
2 1 to reproduce of
oNormalize?
2 1=no, 2=yes

ofxn calc*
0.80039
ofxn mod
0.82373

sRun
RunA1
zout
1

Ave
Data
0.01862
Model
0.01841

data/vMax
0.1011

ofxng
275.646

Min d/m
4.74589

ave/min d
32.2651
ave/min m
151.379

325_Calib_S_A

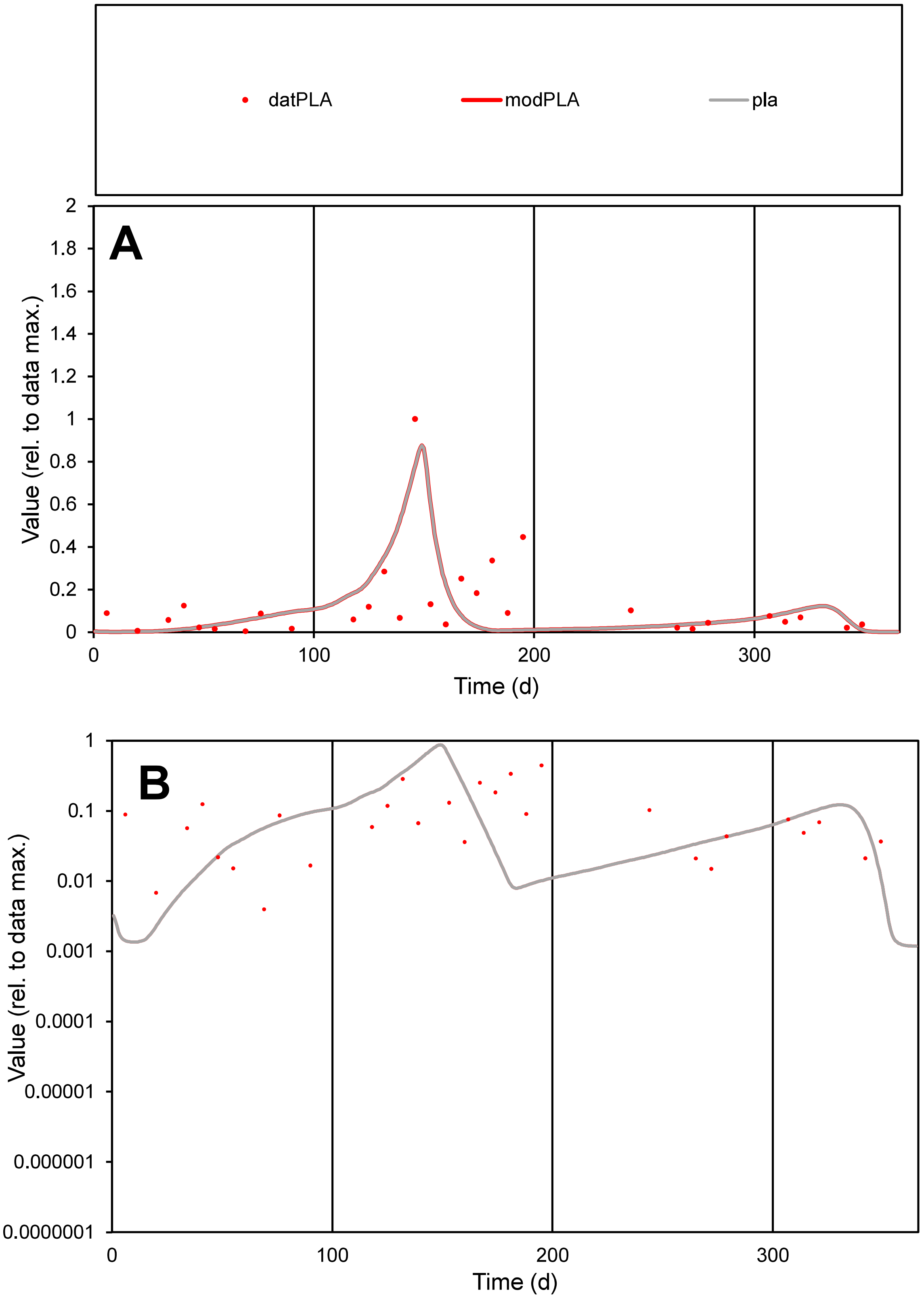

C1

Variables
PLA Provide jtd or Dst
CalibOnly?
2 1 to reproduce of
oNormalize?
2 1=no, 2=yes

ofxn calc*
1.22314
ofxn mod
1.28584

sRun
RunA1
zout
1

Ave
Data
0.01205
Model
0.01125

data/vMax
0.09369

ofxng
275.646

Min d/m
2.87693

ave/min d
32.421
ave/min m
87.0882

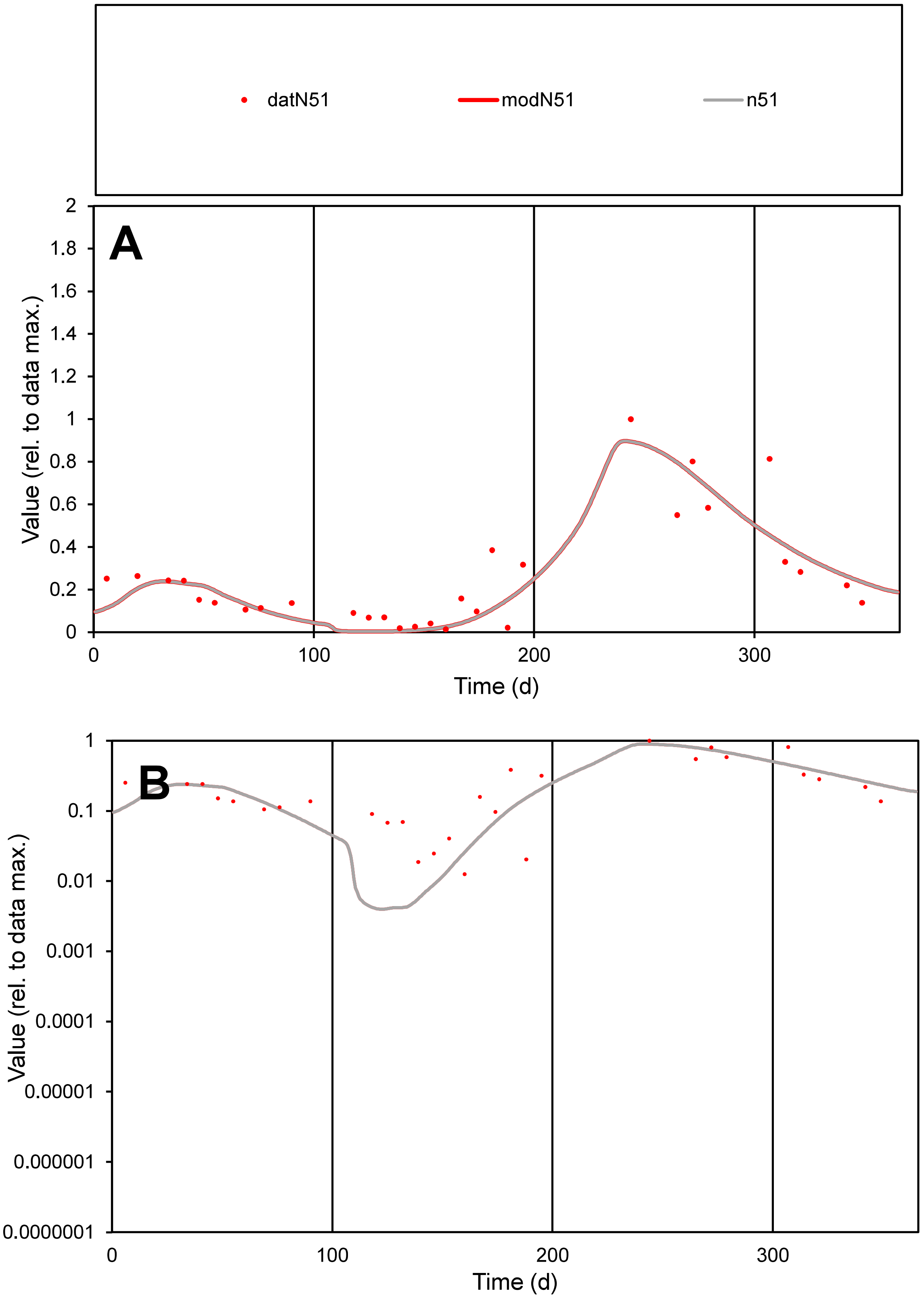

C1

Variables
N51 Provide jtd or Dst
CalibOnly?
2 1 to reproduce of
oNormalize?
2 1=no, 2=yes

ofxn calc*
0.45041
ofxn mod
0.47709

sRun
RunA1
zout
1

Ave
Data
0.00881
Model
0.00799

data/vMax
0.03451

ofxng
275.646

Min d/m
3.04645

ave/min d
20.3493
ave/min m
56.214

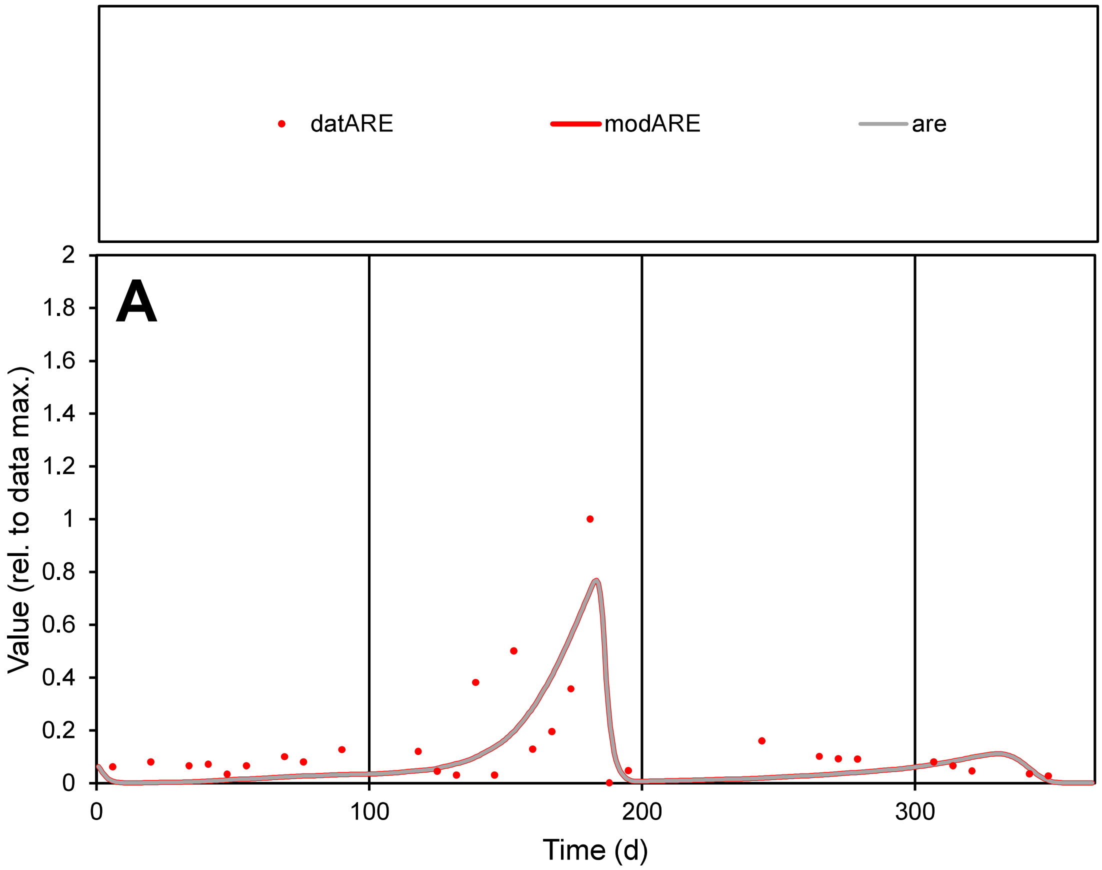

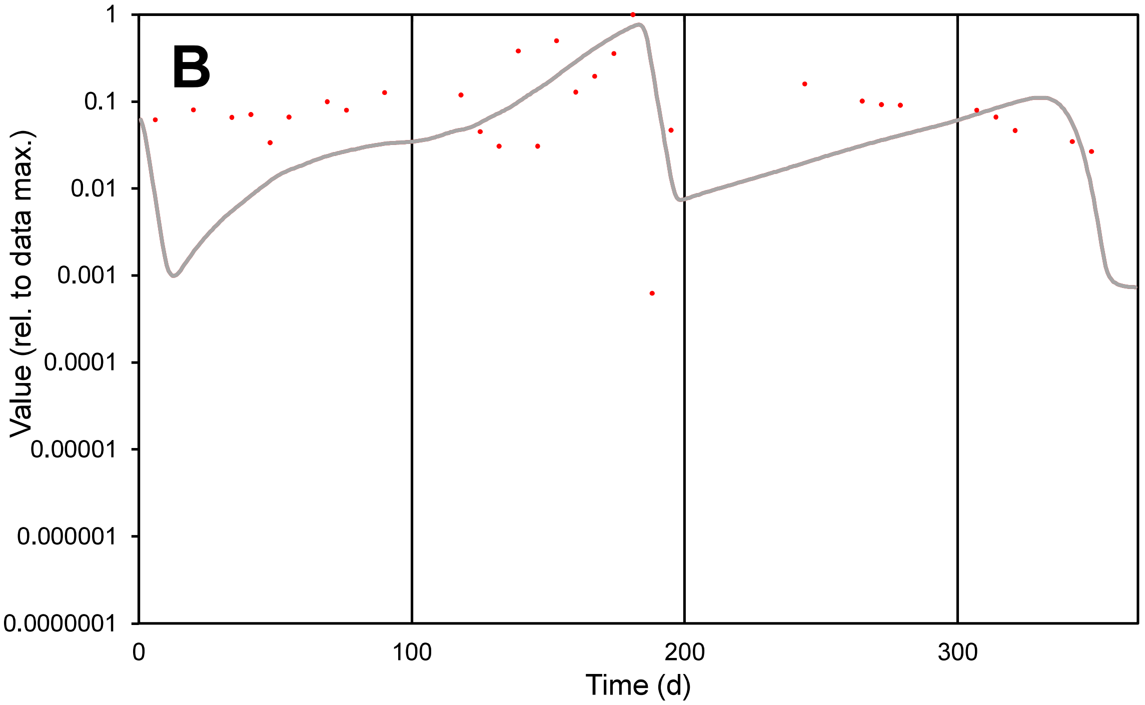

C1

Variables

ARE Provide jtd or Dst

CalibOnly?

2 1 to reproduce of

oNormalize?

2 1=no, 2=yes

ofxn calc*

0.85191

ofxn mod

0.88287

sRun

RunA1

zout

1

Ave

Data

0.01225

Model

0.00989

data/vMax

0.08707

ofxng

275.646

Min d/m

0.30334

ave/min d

224.498

ave/min m

54.975

325_Calib_S_A

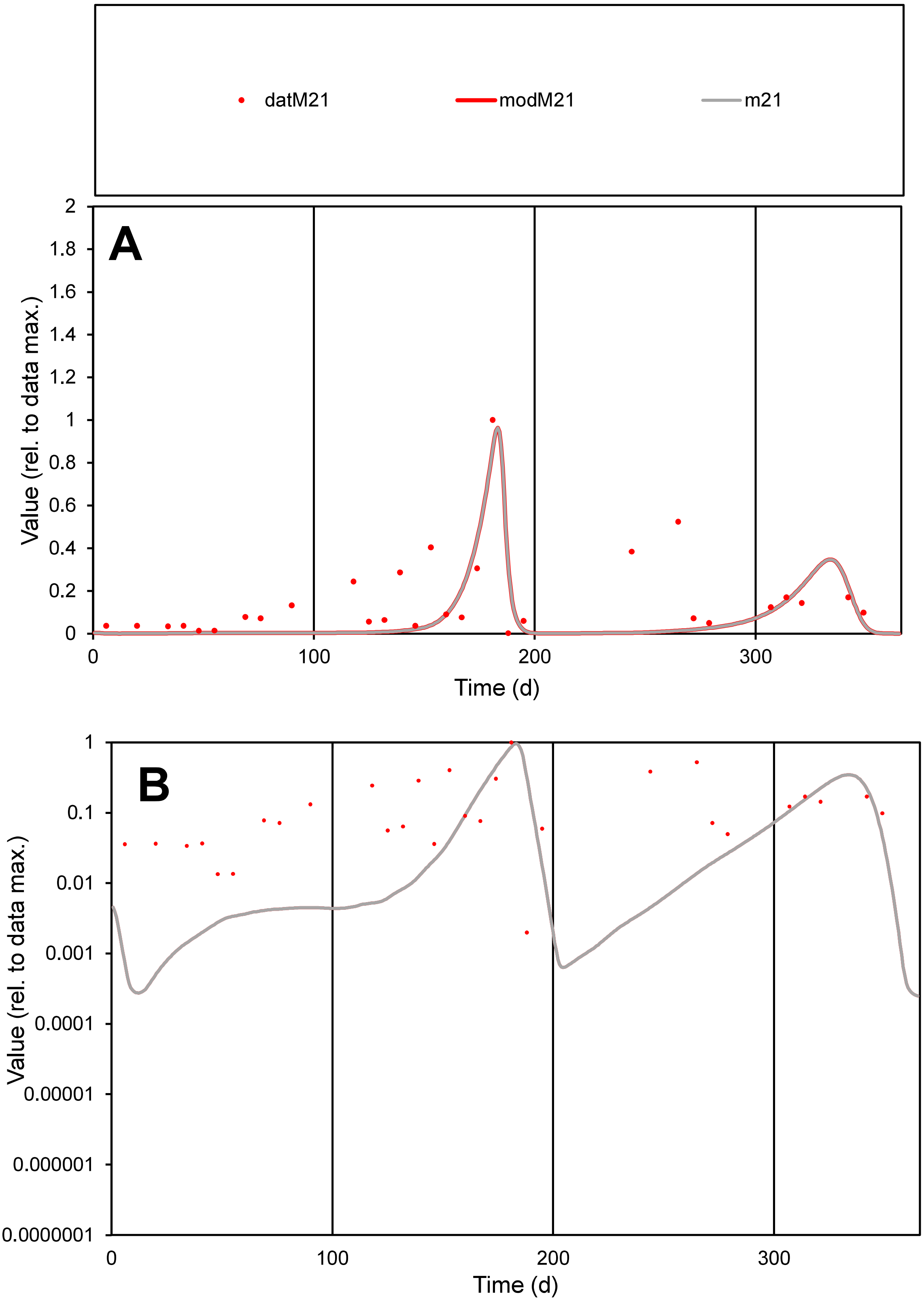

C1

Variables
M21 Provide jtd or Dst
CalibOnly?
2 1 to reproduce of
oNormalize?
2 1=no, 2=yes

ofxn calc*
0.99993
ofxn mod
1.02064

sRun
RunA1
zout
1

Ave
Data
0.01013
Model
0.00595

data/vMax
0.06319

ofxng
275.646

Min d/m
3.54674

ave/min d
80.9014
ave/min m
168.53

325_Calib_S_A

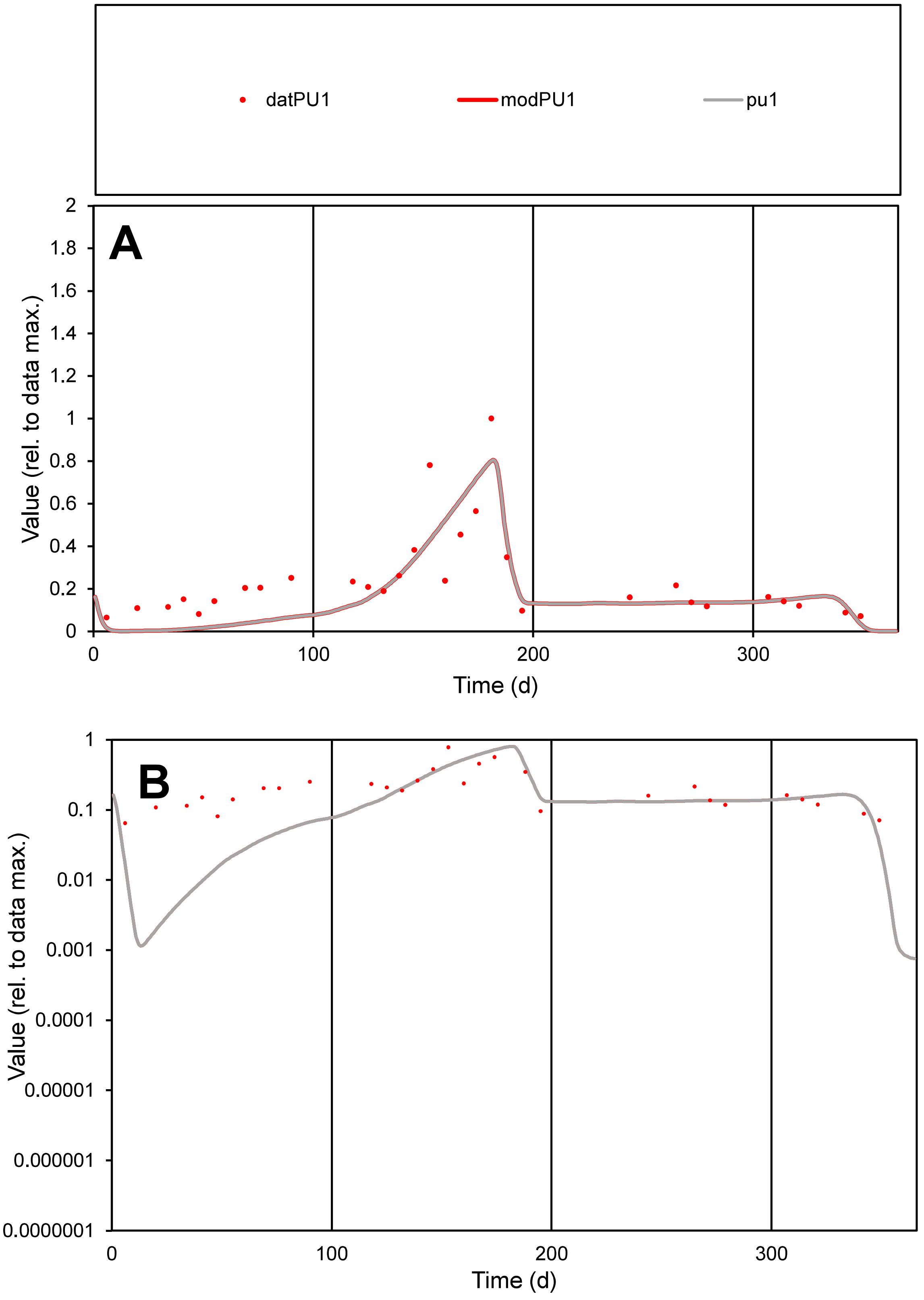

C1

Variables
PU1 Provide jtd or Dst
CalibOnly?
2 1 to reproduce of
oNormalize?
2 1=no, 2=yes

ofxn calc*
0.51264
ofxn mod
0.53281

sRun
RunA1
zout
1

Ave
Data
0.01157
Model
0.00978

data/vMax
0.04756

ofxng
275.646

Min d/m
30.3846

ave/min d
3.75419
ave/min m
96.4702

325_Calib_S_A

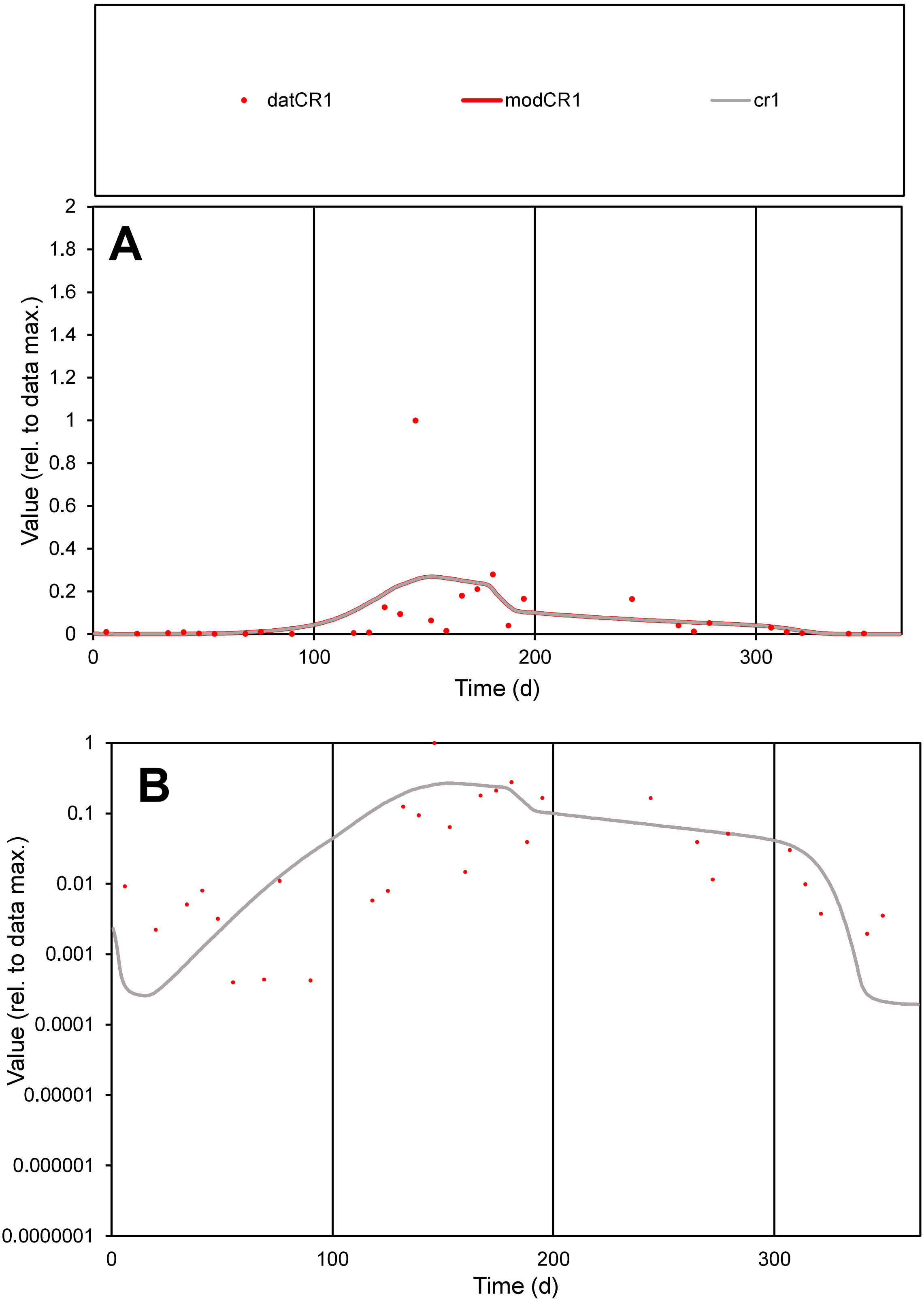

C1

Variables

CR1 Provide jtd or Dst

CalibOnly?

2 1 to reproduce of

oNormalize?

2 1=no, 2=yes

ofxn calc*

1.84449

ofxn mod

1.86527

sRun

RunA1

zout

1

Ave

Data

0.01516

Model

0.01629

data/vMax

0.179

ofxng

275.646

Min d/m

1.9118

ave/min d

211.313

ave/min m

434.182

325_Calib_S_A

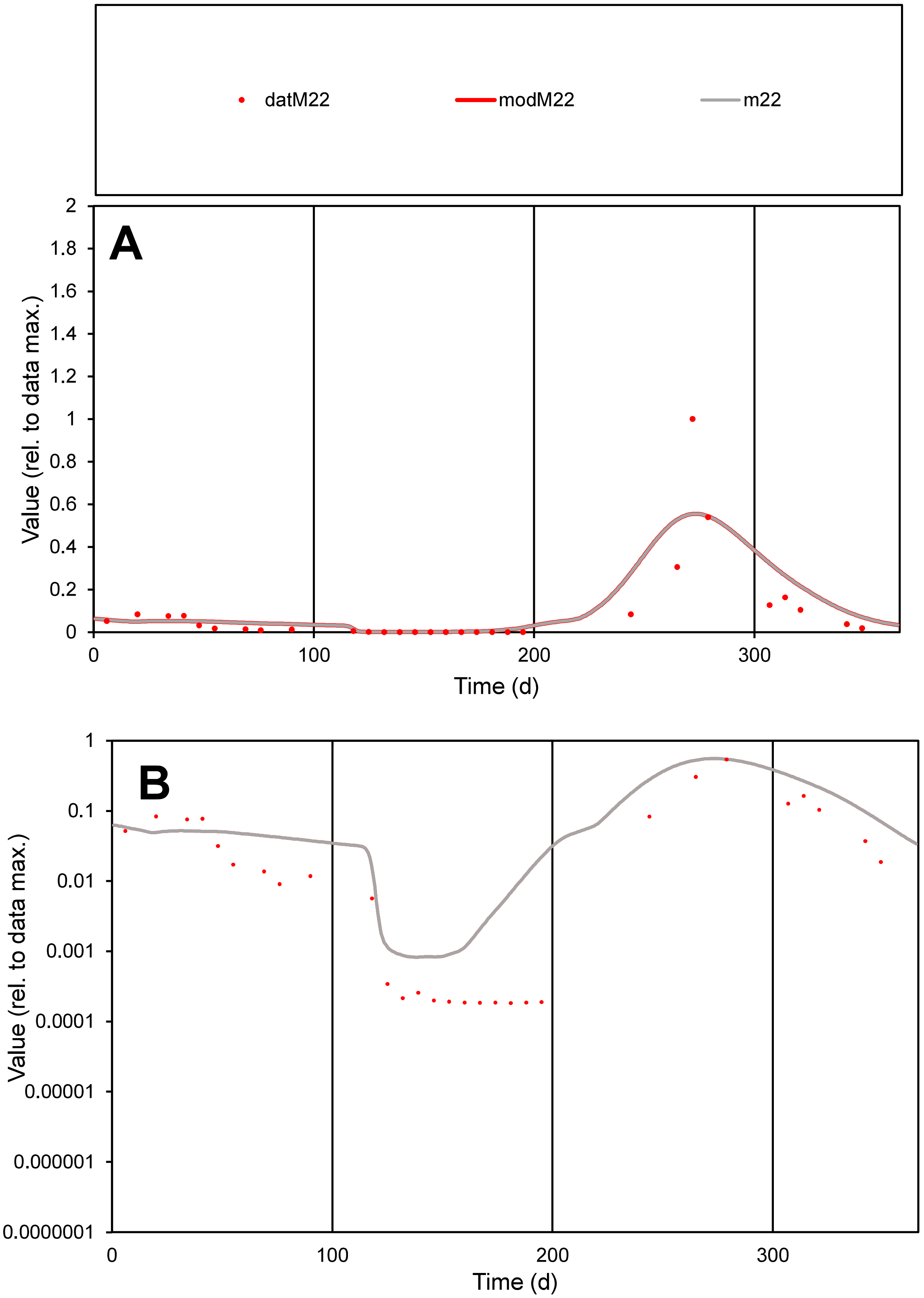

C1

Variables

M22 Provide jtd or Dst

CalibOnly?

2 1 to reproduce of

oNormalize?

2 1=no, 2=yes

ofxn calc*

1.18546

ofxn mod

1.22549

sRun

RunA1

zout

1

Ave

Data

0.01075

Model

0.01298

data/vMax

0.1169

ofxng

275.646

Min d/m

0.22094

ave/min d

502.18

ave/min m

134.002

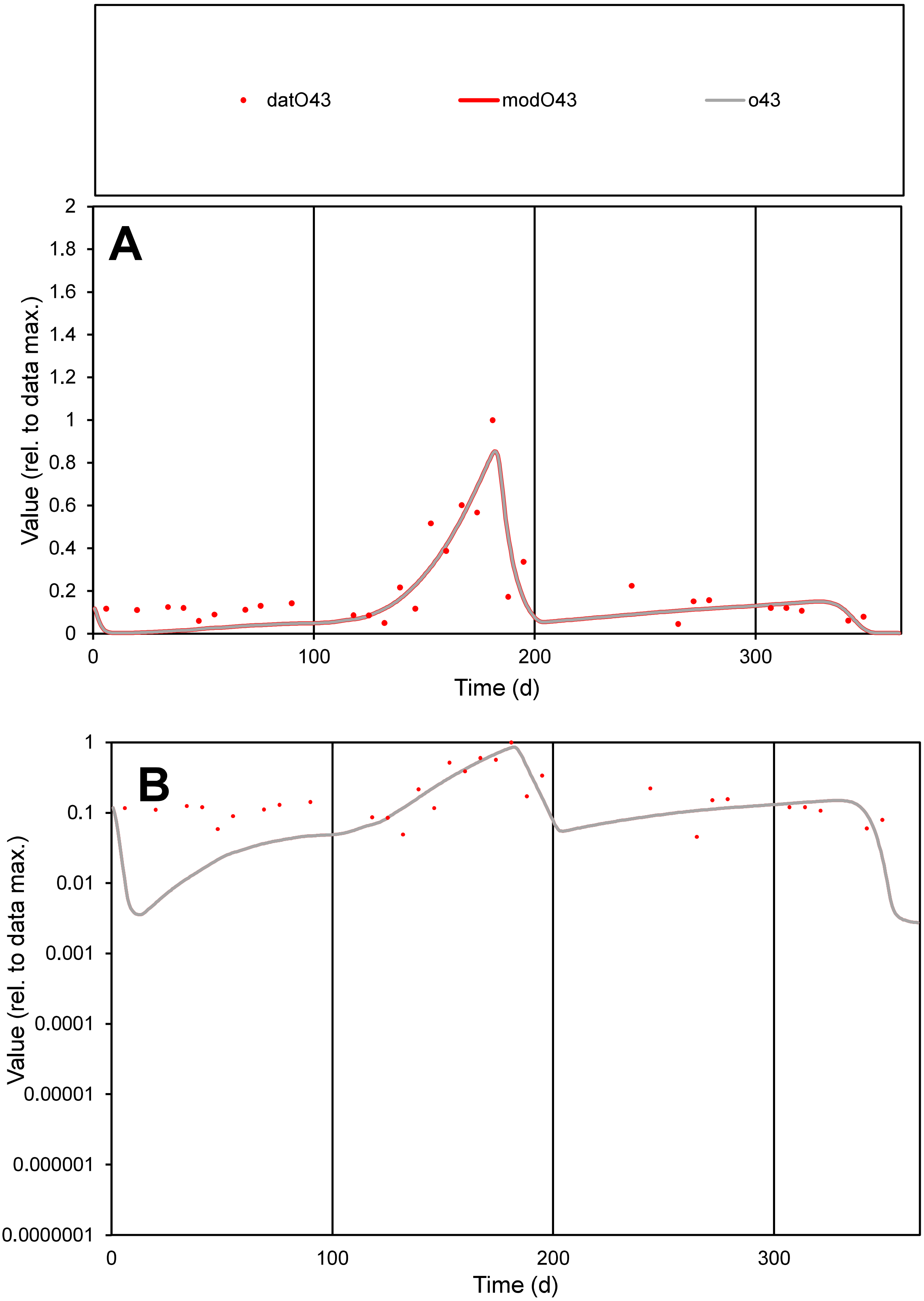

C1

Variables
O43 Provide jtd or Dst
CalibOnly?
2 1 to reproduce of
oNormalize?
2 1=no, 2=yes

ofxn calc*
0.44474
ofxn mod
0.48625

sRun
RunA1
zout
1

Ave
Data
0.01121
Model
0.00958

data/vMax
0.05425

ofxng
275.646

Min d/m
8.08134

ave/min d
4.54786
ave/min m
31.4306

325_Calib_S_A

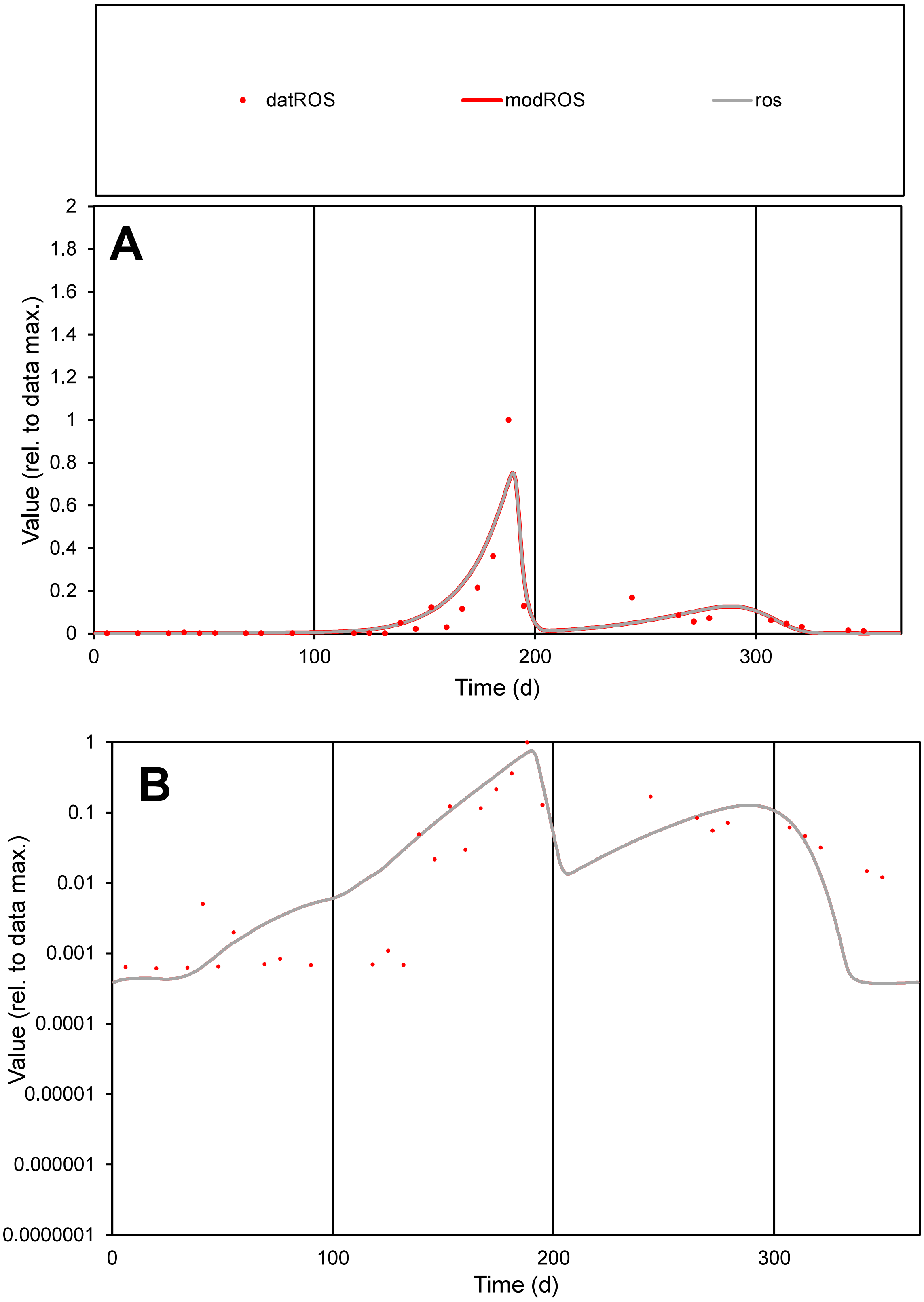

C1

Variables

ROS Provide jtd or Dst

CalibOnly?

2 1 to reproduce of

oNormalize?

2 1=no, 2=yes

ofxn calc*

0.83717

ofxn mod

0.88875

sRun

RunA1

zout

1

Ave

Data

0.0074

Model

0.00834

data/vMax

0.08513

ofxng

275.646

Min d/m

1.6485

ave/min d

141.311

ave/min m

262.788

325_Calib_S_A

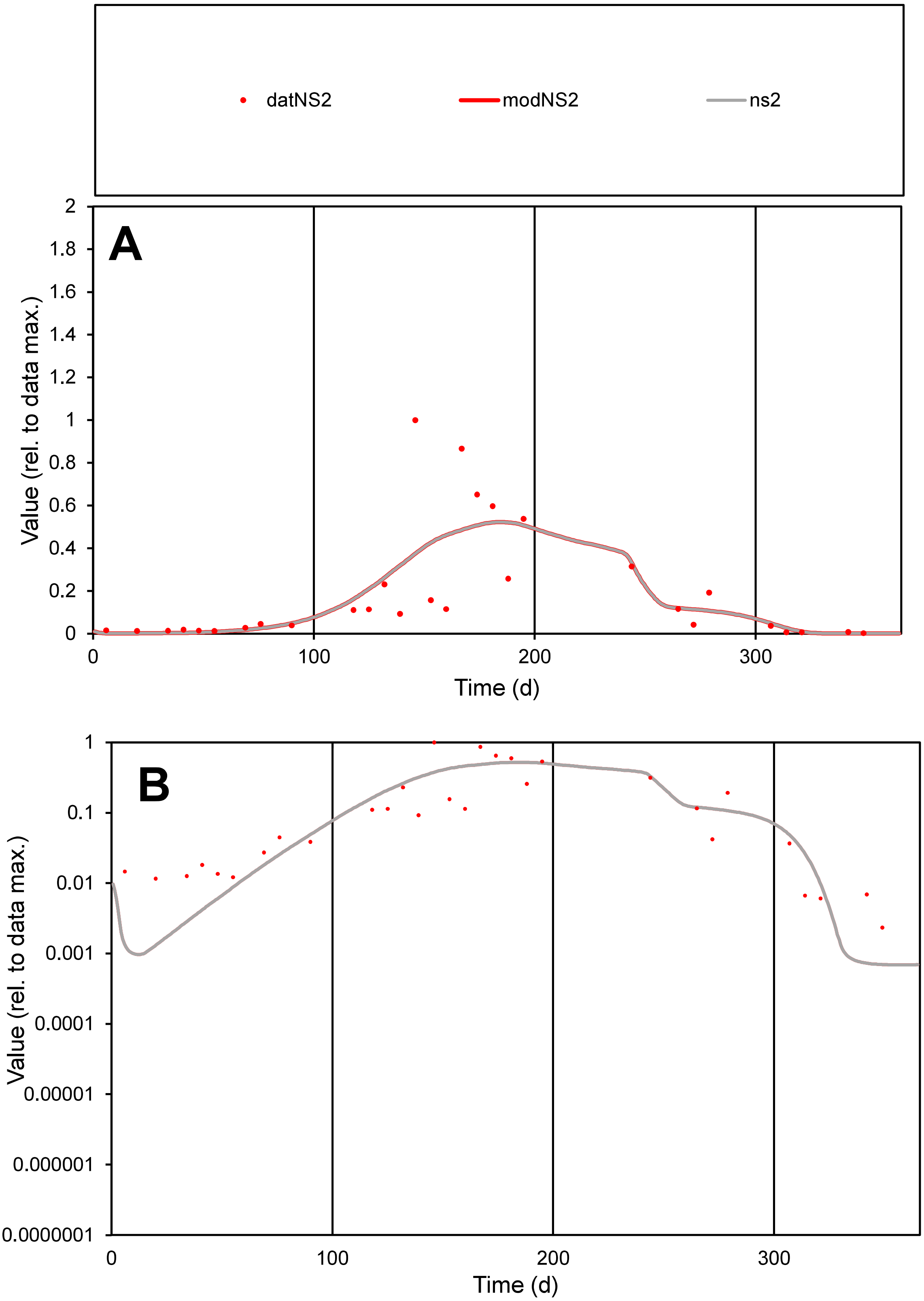

C1

Variables
NS2 Provide jtd or Dst
CalibOnly?
2 1 to reproduce of
oNormalize?
2 1=no, 2=yes

ofxn calc*
0.91864
ofxn mod
0.93339

sRun
RunA1
zout
1

Ave
Data
0.01118
Model
0.01119

data/vMax
0.05945

ofxng
275.646

Min d/m
3.36247

ave/min d
80.7126
ave/min m
271.742

325_Calib_S_A

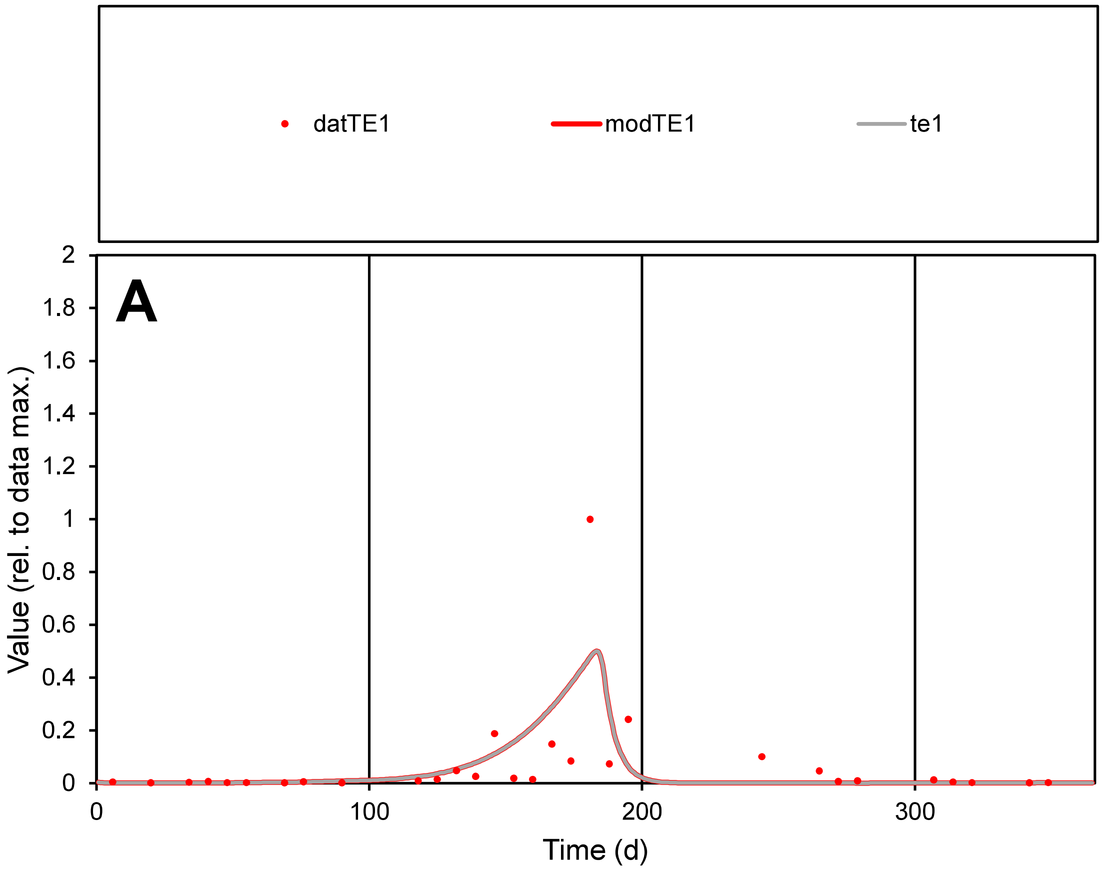

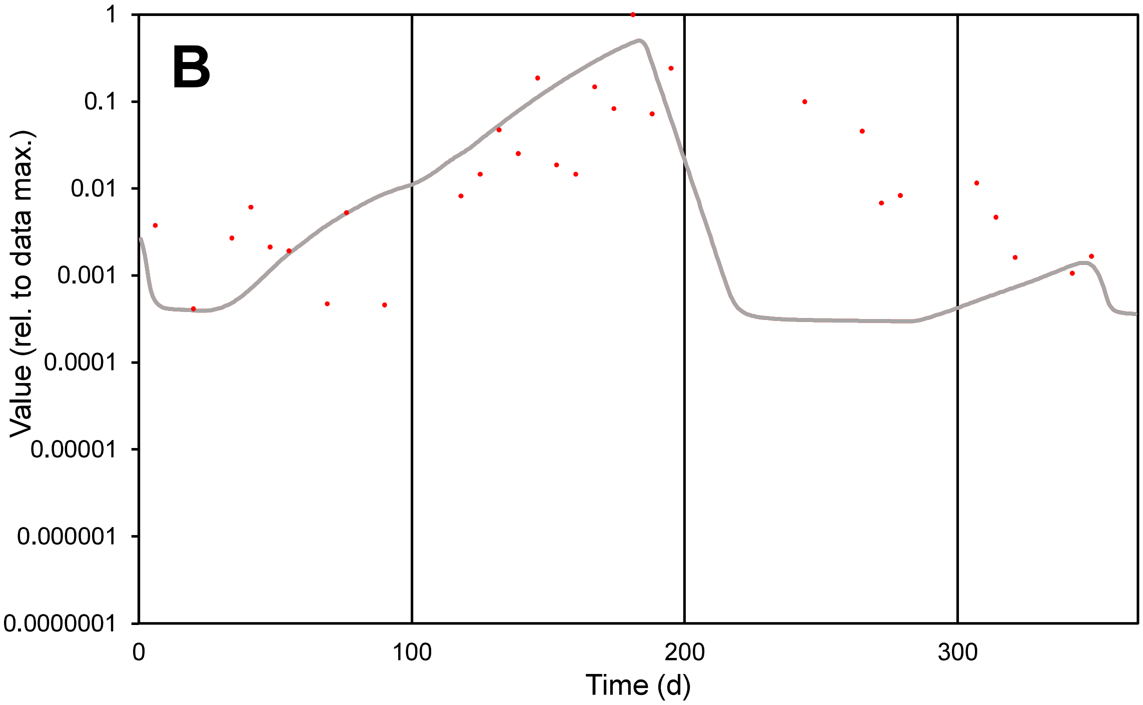

C1

Variables

TE1 Provide jtd or Dst

CalibOnly?

2 1 to reproduce of

oNormalize?

2 1=no, 2=yes

ofxn calc*

1.83217

ofxn mod

1.88488

sRun

RunA1

zout

1

Ave

Data

0.00908

Model

0.00931

data/vMax

0.1319

ofxng

275.646

Min d/m

1.38246

ave/min d

166.033

ave/min m

235.263

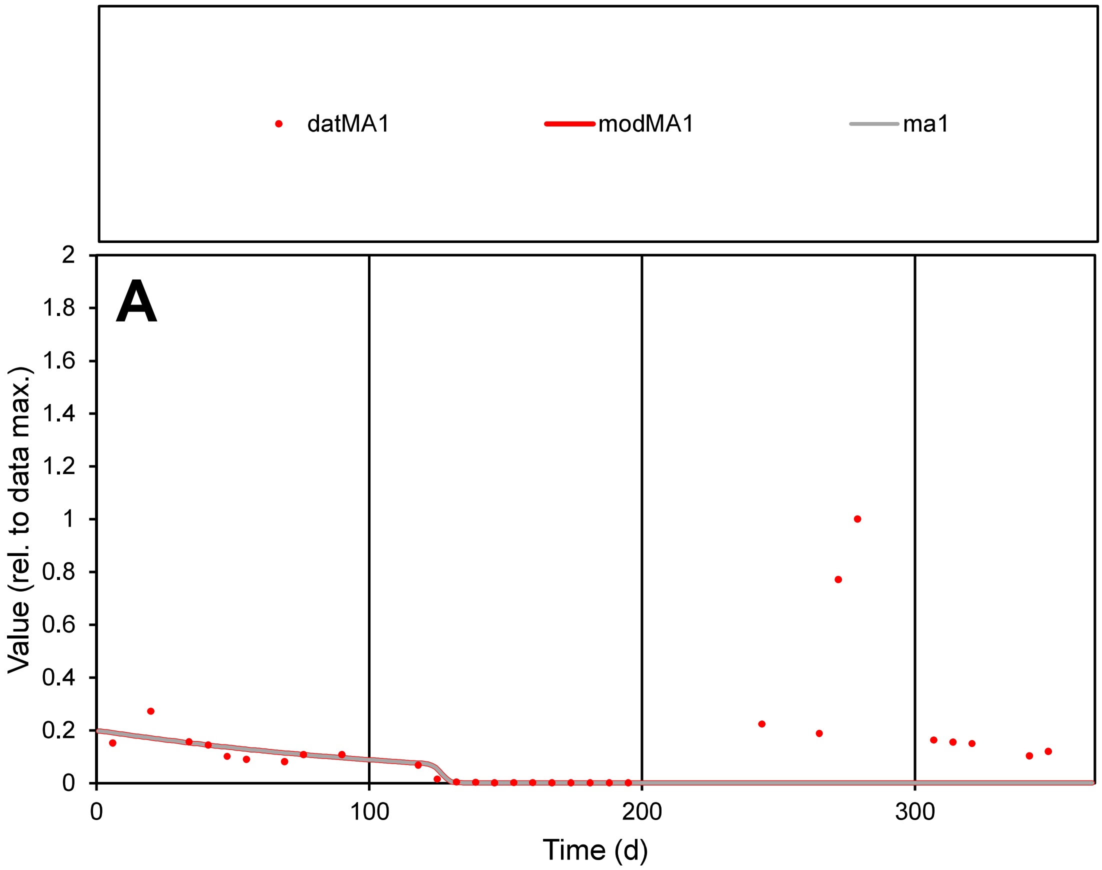

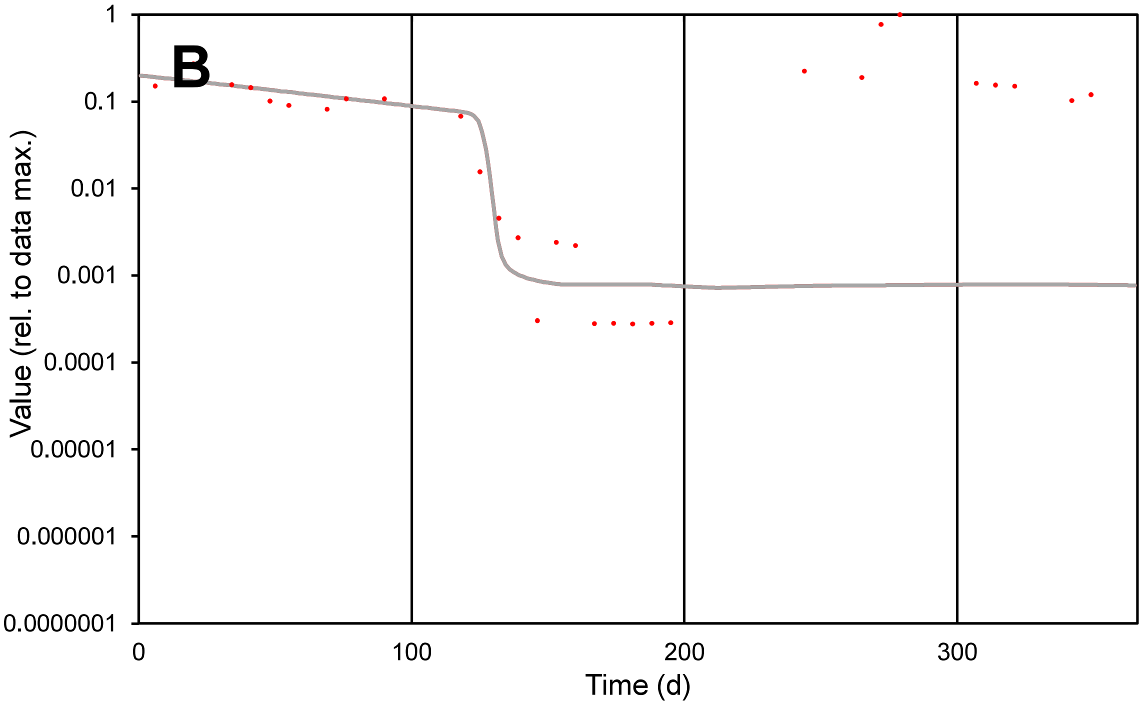

C1

Variables

MA1	Provide jtd or Dst
CalibOnly?	
2	1 to reproduce of
oNormalize?	
2	1=no, 2=yes
ofxn calc*	
1.75129	
ofxn mod	
1.75732	
sRun	
RunA1	
zout	
1	
Ave	
Data	
0.00718	
Model	
0.00232	
data/vMax	
0.05151	
ofxng	
275.646	
Min d/m	
0.36807	
ave/min d	
500.99	
ave/min m	
59.5934	

325_Calib_S_A

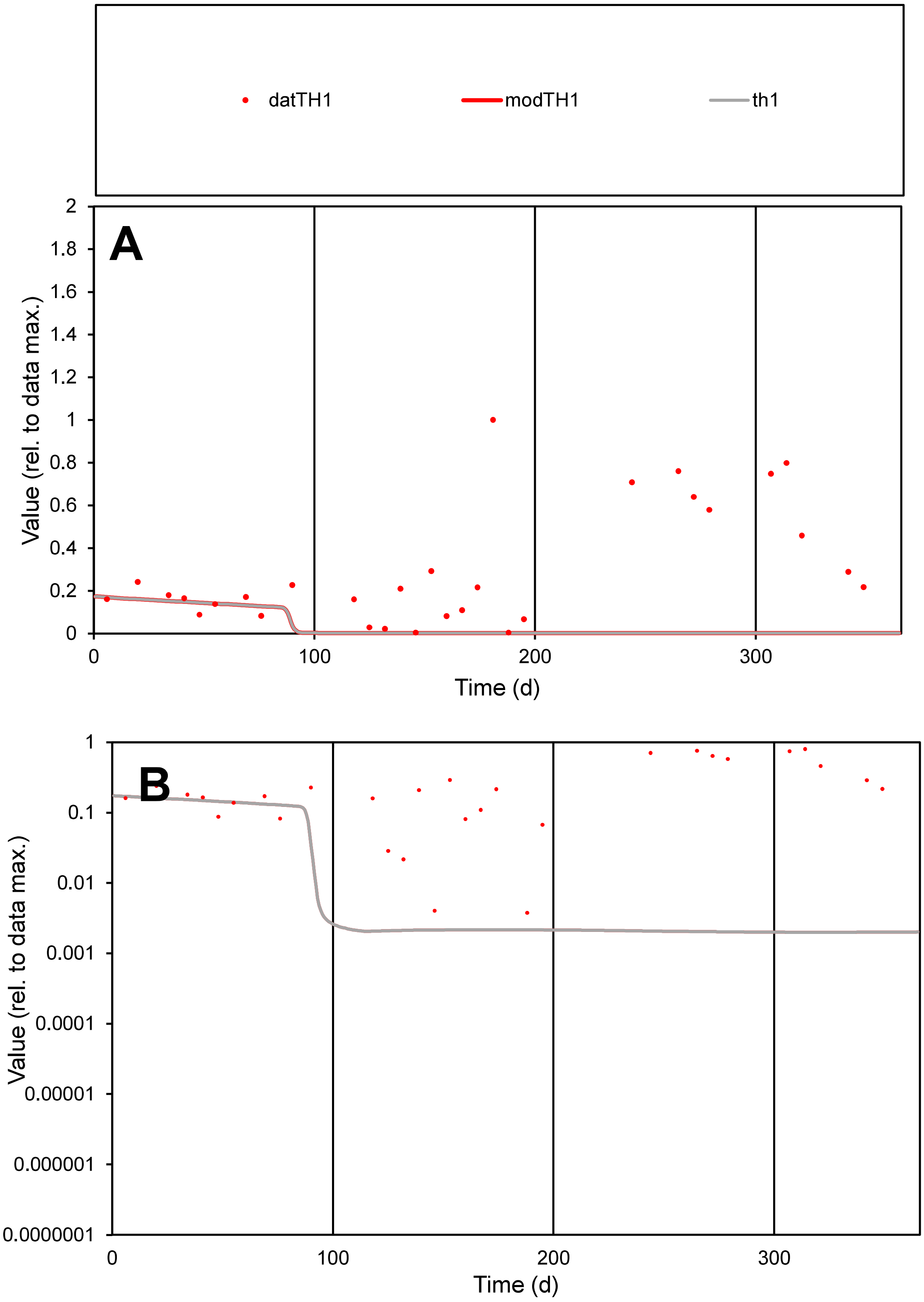

C1

Variables

TH1 Provide jtd or Dst

CalibOnly?

2 1 to reproduce of

oNormalize?

2 1=no, 2=yes

ofxn calc*

1.32664

ofxn mod

1.33108

sRun

RunA1

zout

1

Ave

Data

0.00736

Model

0.00109

data/vMax

0.02496

ofxng

275.646

Min d/m

1.87848

ave/min d

78.5634

ave/min m

21.8767

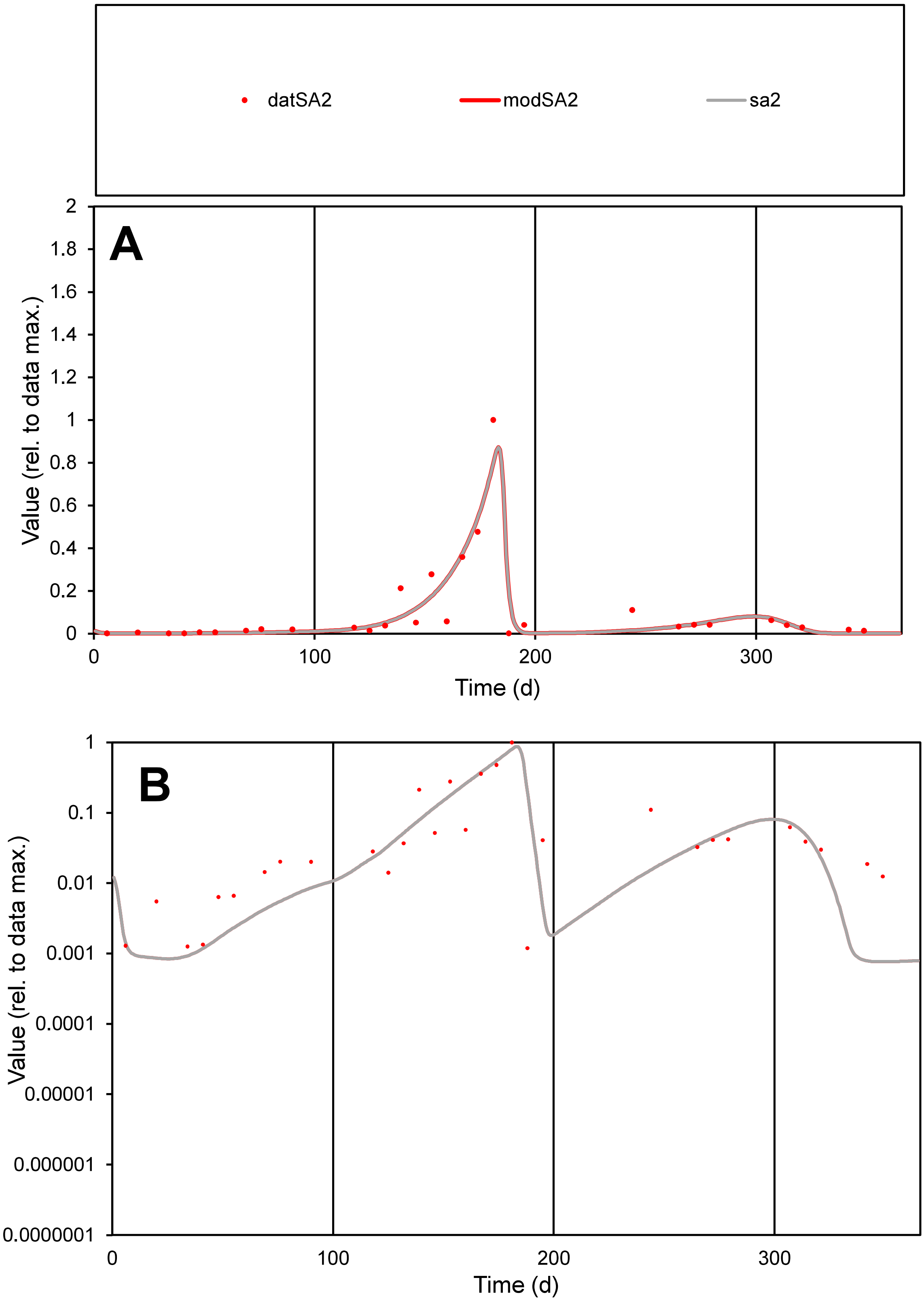

C1

Variables
SA2 Provide jtd or Dst
CalibOnly?
2 1 to reproduce of
oNormalize?
2 1=no, 2=yes

ofxn calc*
0.61662
ofxn mod
0.6669

sRun
RunA1
zout
1

Ave
Data
0.00866
Model
0.0086

data/vMax
0.08599

ofxng
275.646

Min d/m
1.55165

ave/min d
84.6655
ave/min m
130.45

325_Calib_S_A

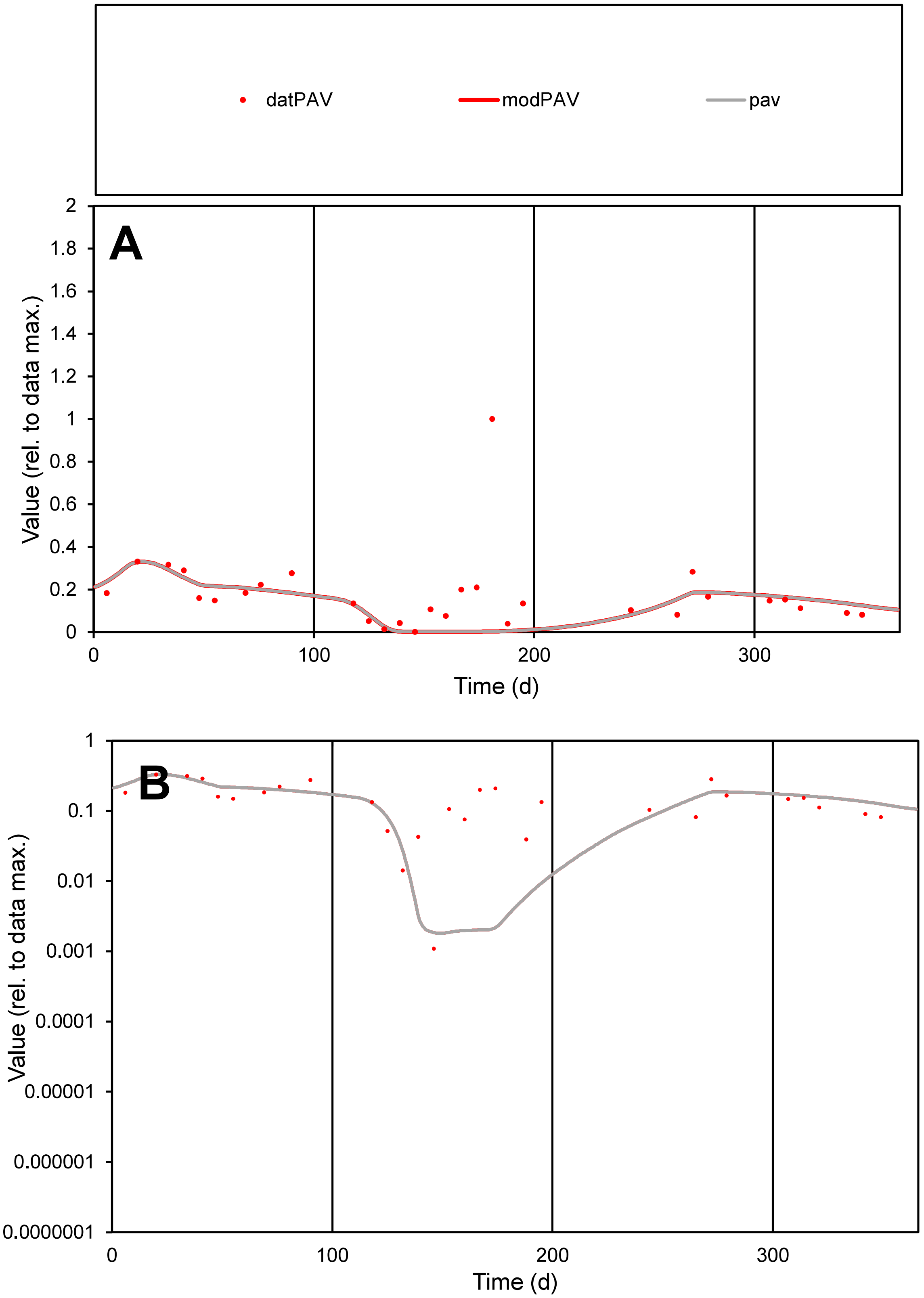

C1

Variables
PAV Provide jtd or Dst
CalibOnly?
2 1 to reproduce of
oNormalize?
2 1=no, 2=yes

ofxn calc*
1.10279
ofxn mod
1.10984

sRun
RunA1
zout
1

Ave
Data
0.00656
Model
0.0046

data/vMax
0.03685

ofxng
275.646

Min d/m
0.59857

ave/min d
163.088
ave/min m
68.4424

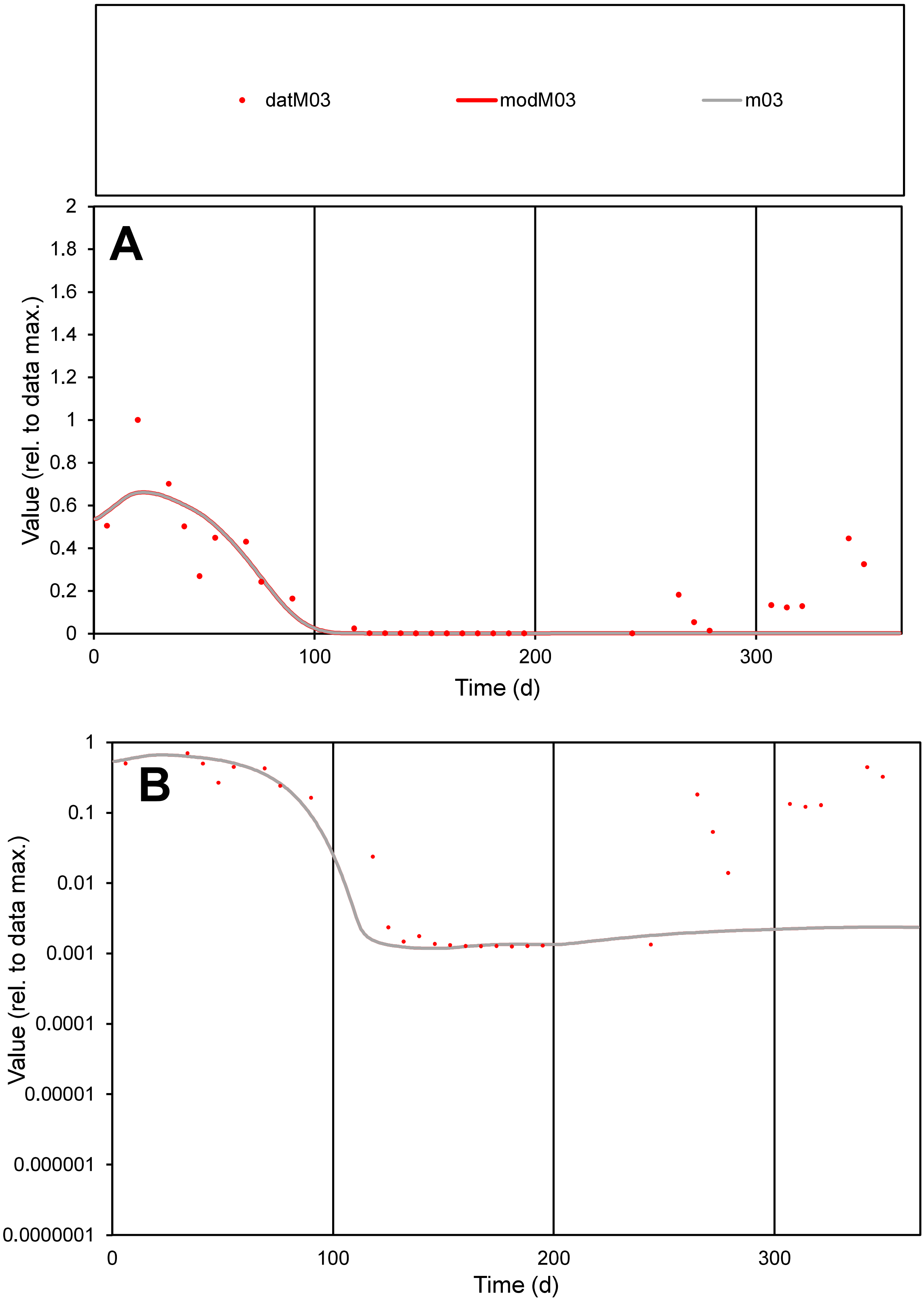

C1

Variables
M03 Provide jtd or Dst
CalibOnly?
2 1 to reproduce of
oNormalize?
2 1=no, 2=yes

ofxn calc*
0.7535
ofxn mod
0.77403

sRun
RunA1
zout
1

Ave
Data
0.00264
Model
0.00195

data/vMax
0.01386

ofxng
275.646

Min d/m
1.06147

ave/min d
151.262
ave/min m
118.65

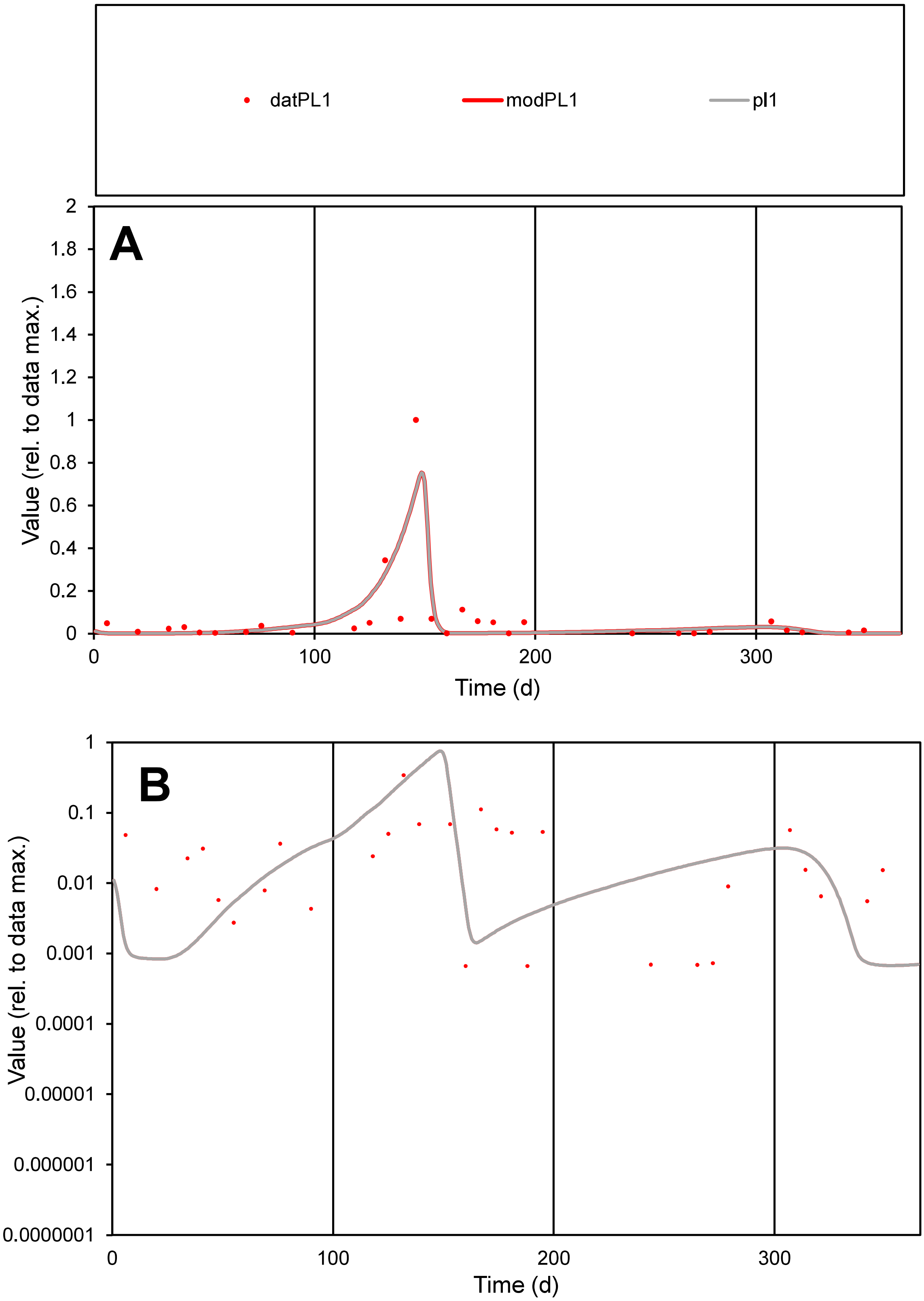

C1

Variables

PL1 Provide jtd or Dst

CalibOnly?

2 1 to reproduce of

oNormalize?

2 1=no, 2=yes

ofxn calc*

1.2881

ofxn mod

1.37554

sRun

RunA1

zout

1

Ave

Data

0.00371

Model

0.00369

data/vMax

0.05277

ofxng

275.646

Min d/m

0.98149

ave/min d

106.084

ave/min m

103.459

325_Calib_S_A

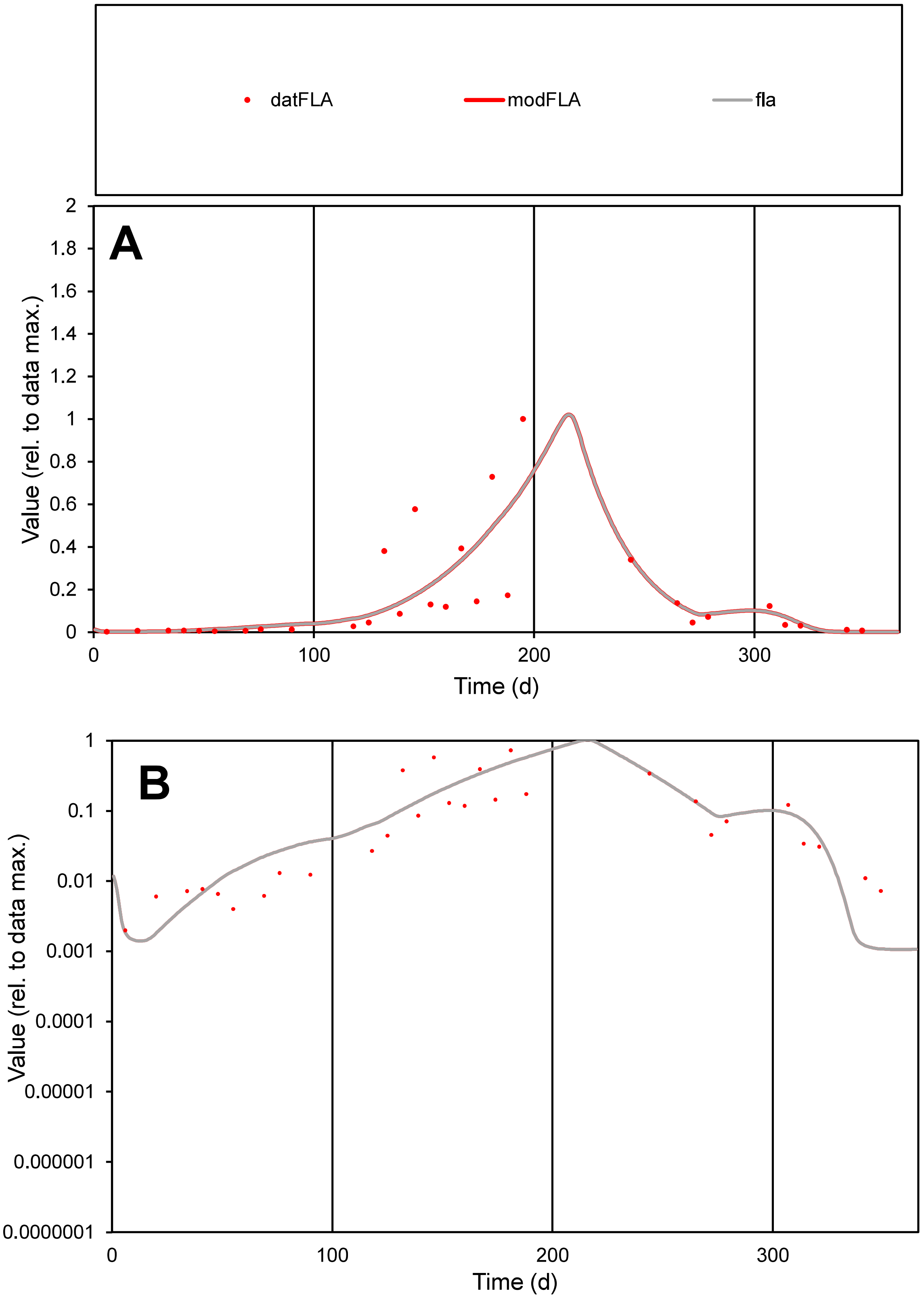

C1

Variables

FLA Provide jtd or Dst

CalibOnly?

2 1 to reproduce of

oNormalize?

2 1=no, 2=yes

ofxn calc*

0.9498

ofxn mod

0.97238

sRun

RunA1

zout

1

Ave

Data

0.0047

Model

0.00463

data/vMax

0.03019

ofxng

275.646

Min d/m

1.85185

ave/min d

78.2627

ave/min m

142.944

325_Calib_S_A

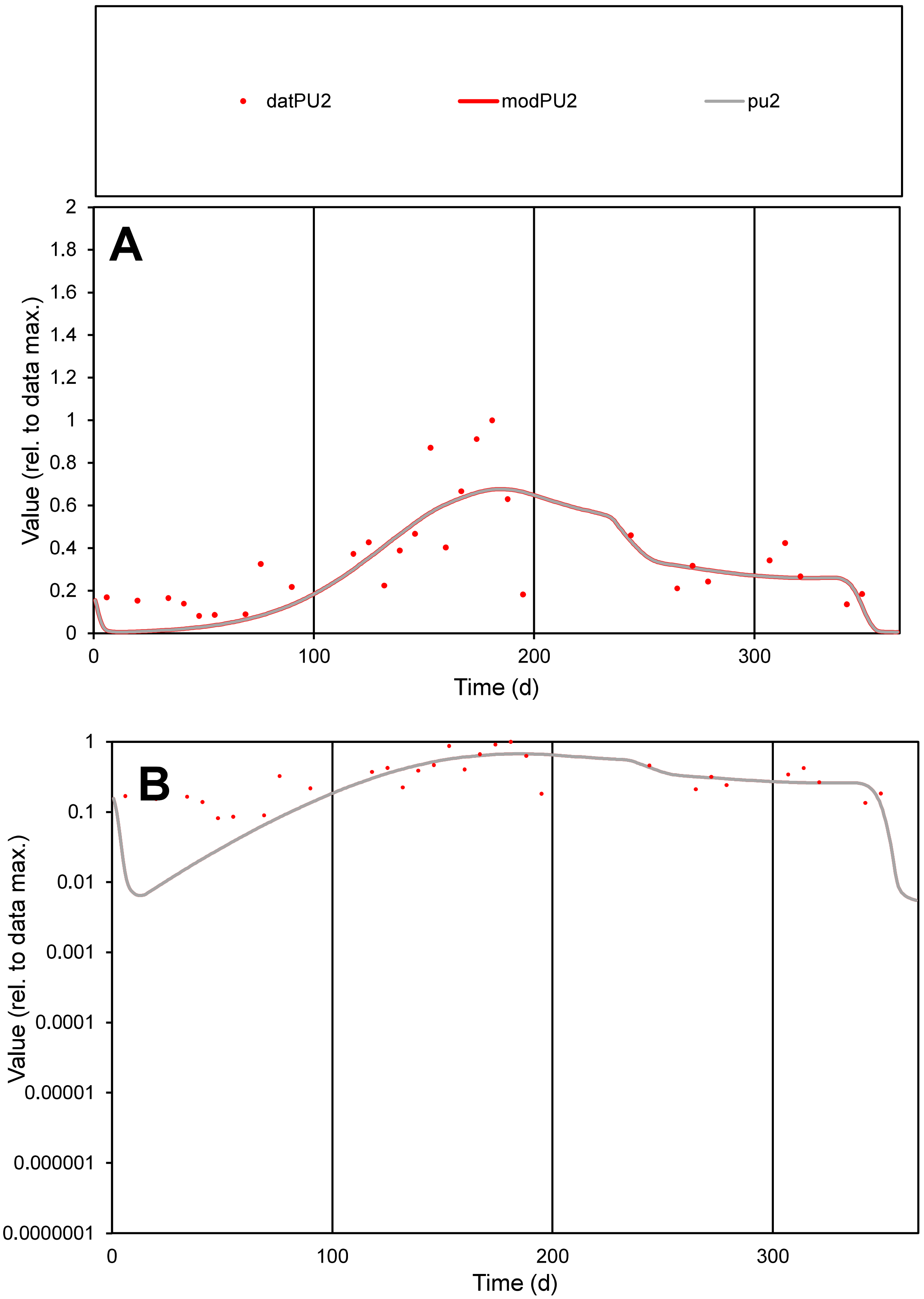

C1

Variables
PU2 Provide jtd or Dst
CalibOnly?
2 1 to reproduce of
oNormalize?
2 1=no, 2=yes

ofxn calc*
0.45735
ofxn mod
0.47637

sRun
RunA1
zout
1

Ave
Data
0.00463
Model
0.00421

data/vMax
0.01317

ofxng
275.646

Min d/m
9.25065

ave/min d
4.30999
ave/min m
36.2835

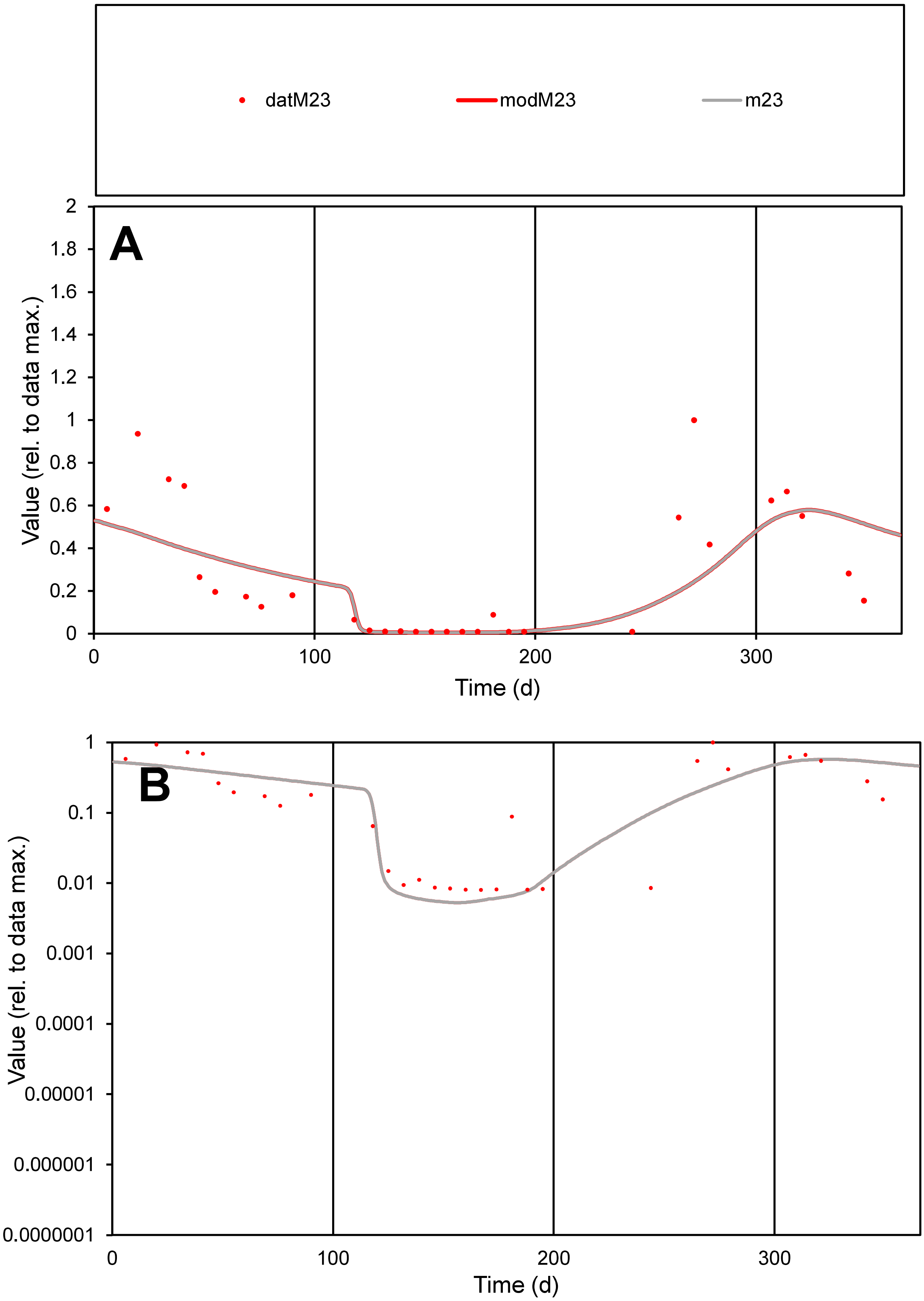

C1

Variables
M23 Provide jtd or Dst
CalibOnly?
2 1 to reproduce of
oNormalize?
2 1=no, 2=yes

ofxn calc*
0.77677
ofxn mod
0.79092

sRun
RunA1
zout
1

Ave
Data
0.00373
Model
0.00317

data/vMax
0.01338

ofxng
275.646

Min d/m
1.51849

ave/min d
34.6434
ave/min m
44.7632

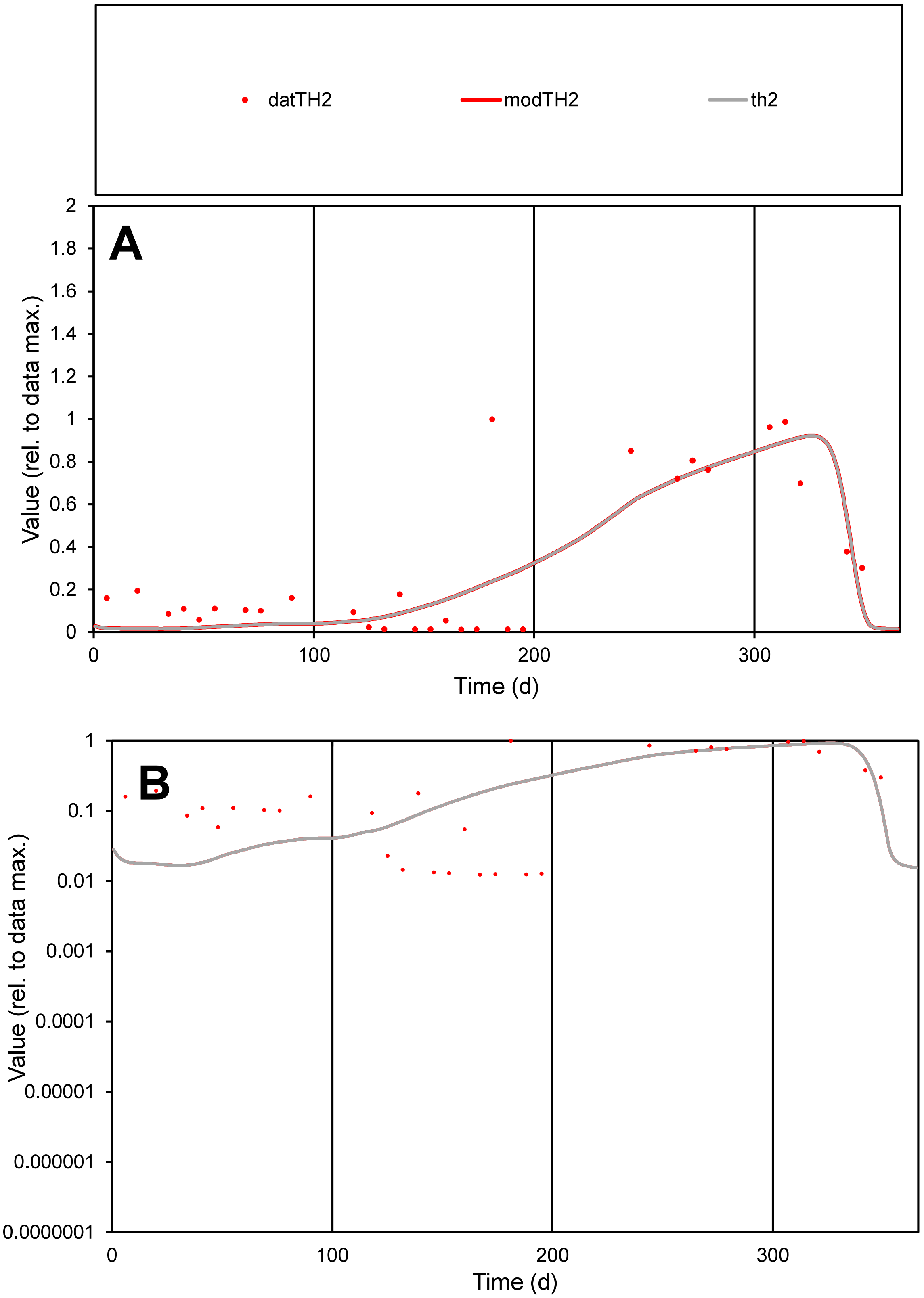

C1

Variables

TH2 Provide jtd or Dst

CalibOnly?

2 1 to reproduce of

oNormalize?

2 1=no, 2=yes

ofxn calc*

0.63331

ofxn mod

0.65883

sRun

RunA1

zout

1

Ave

Data

0.00256

Model

0.00236

data/vMax

0.00855

ofxng

275.646

Min d/m

0.72287

ave/min d

24.1806

ave/min m

16.1204

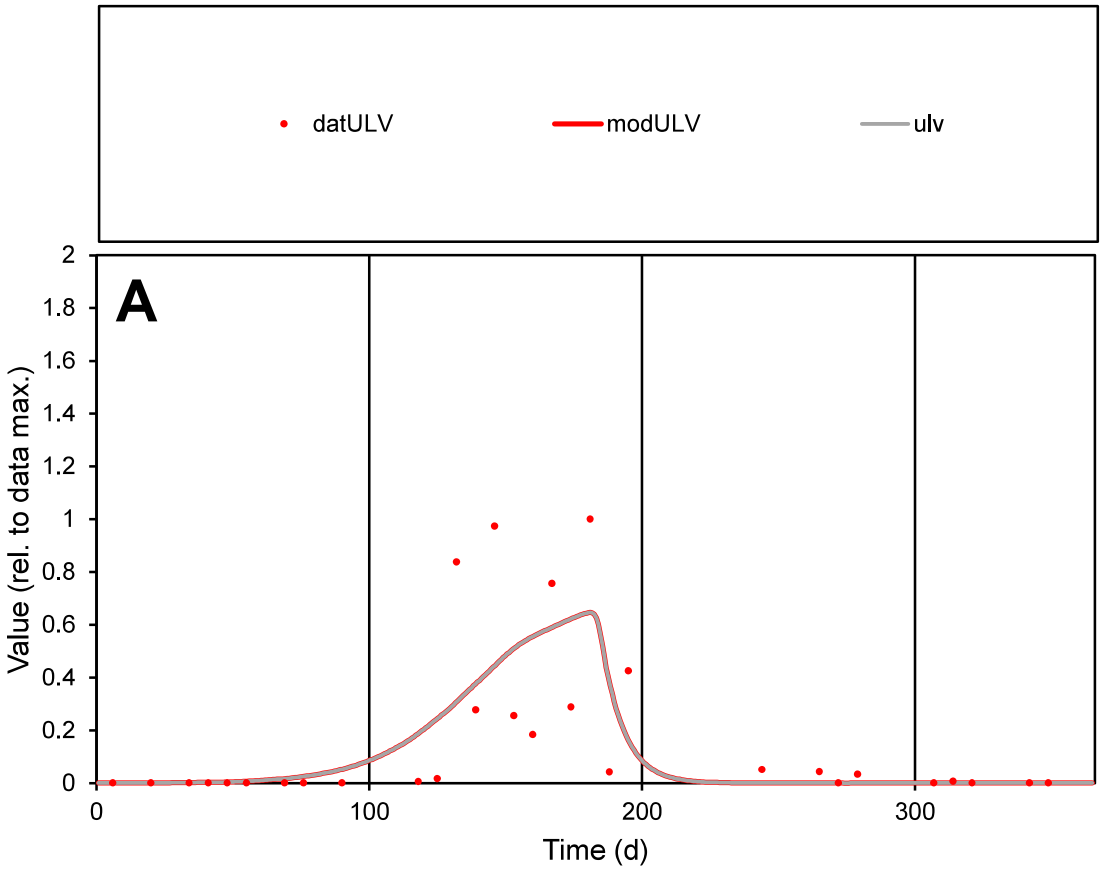

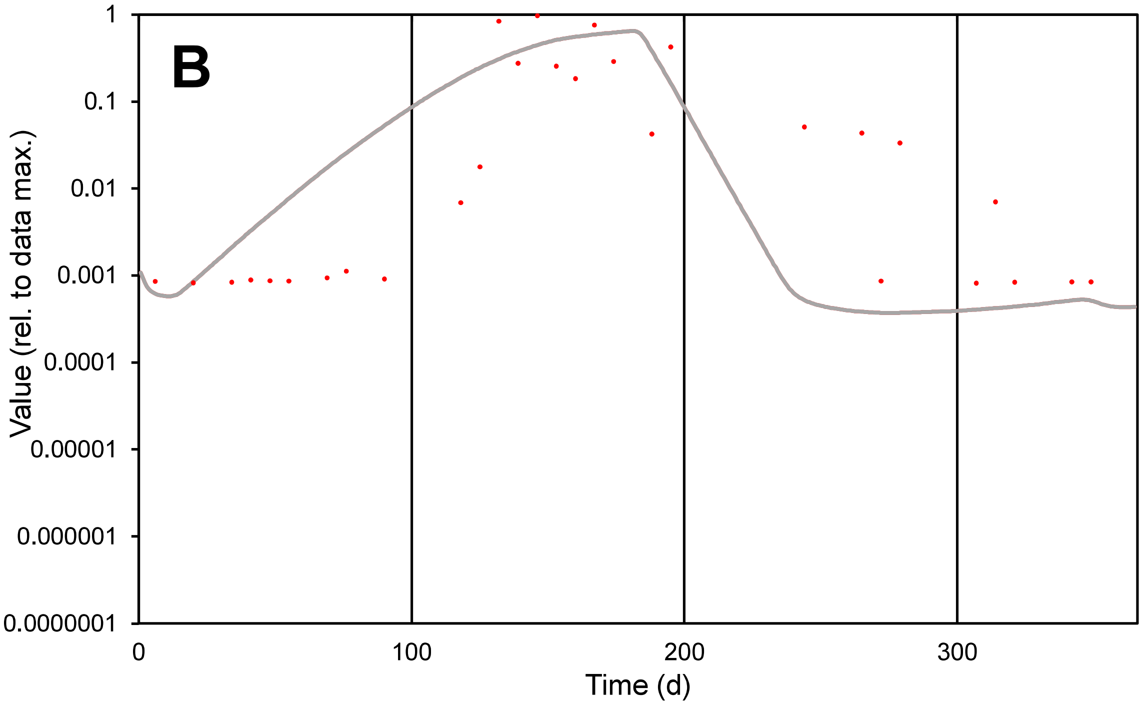

C1

Variables
ULV Provide jtd or Dst
CalibOnly?
2 1 to reproduce of
oNormalize?
2 1=no, 2=yes

ofxn calc*
1.17527
ofxn mod
1.19511

sRun
RunA1
zout
1

Ave
Data
0.00381
Model
0.00371

data/vMax
0.02191

ofxng
275.646

Min d/m
2.2041

ave/min d
211.96
ave/min m
454.905

325_Calib_S_A

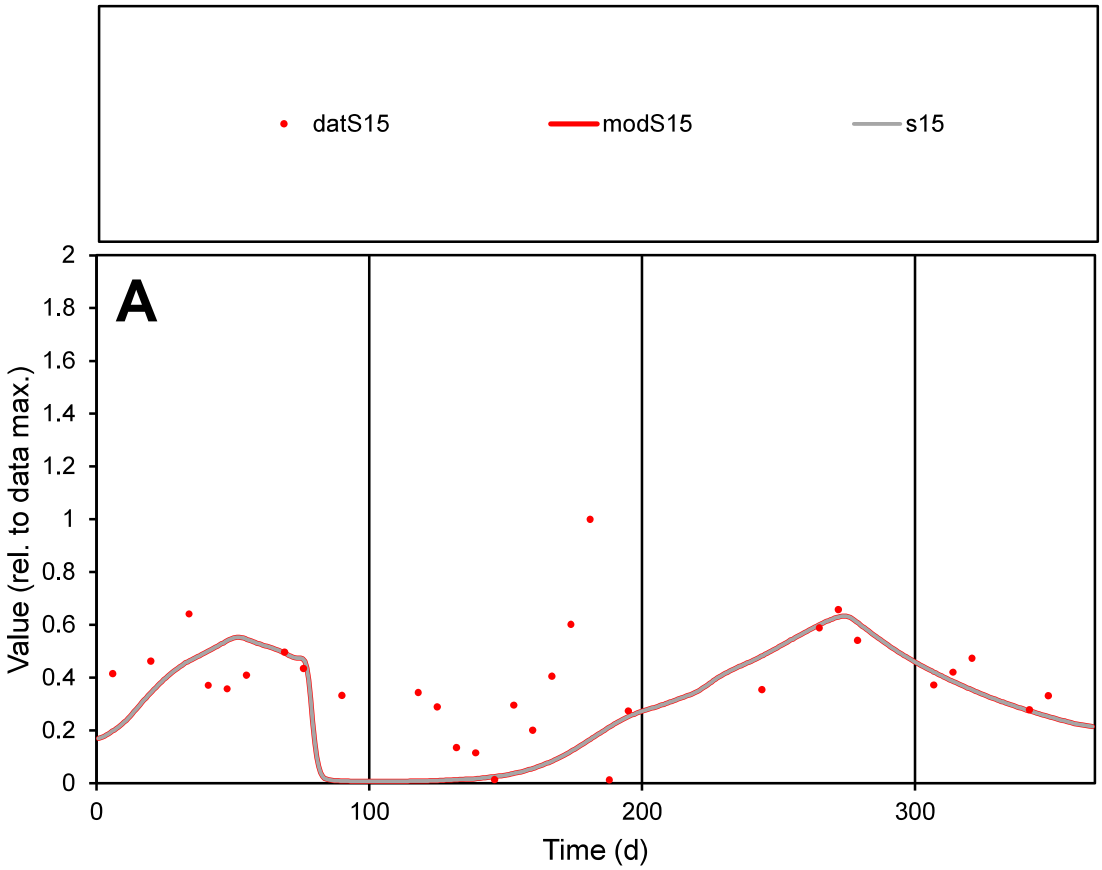

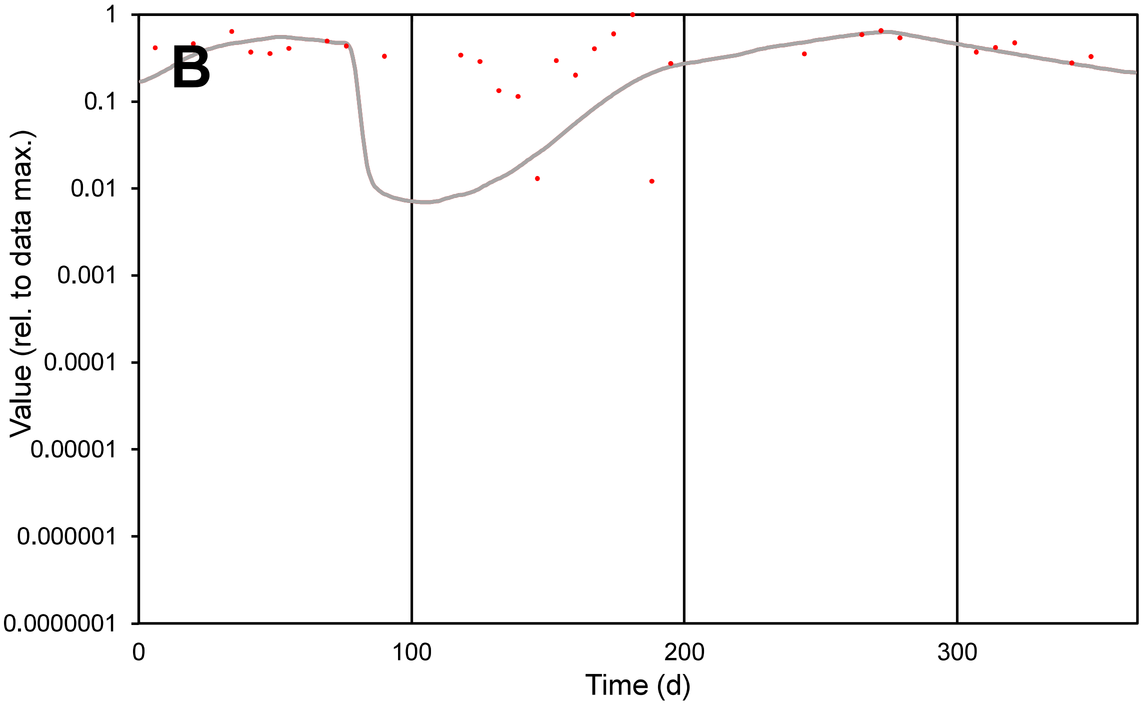

C1

Variables

S15	Provide jtd or Dst
CalibOnly?	
2	1 to reproduce of
oNormalize?	
2	1=no, 2=yes

ofxn calc*
0.60542
ofxn mod
0.61695

sRun
RunA1
zout
1

Ave
Data
0.00399
Model
0.00295

data/vMax
0.01032

ofxng
275.646

Min d/m
1.47485

ave/min d
31.9072
ave/min m
34.7637

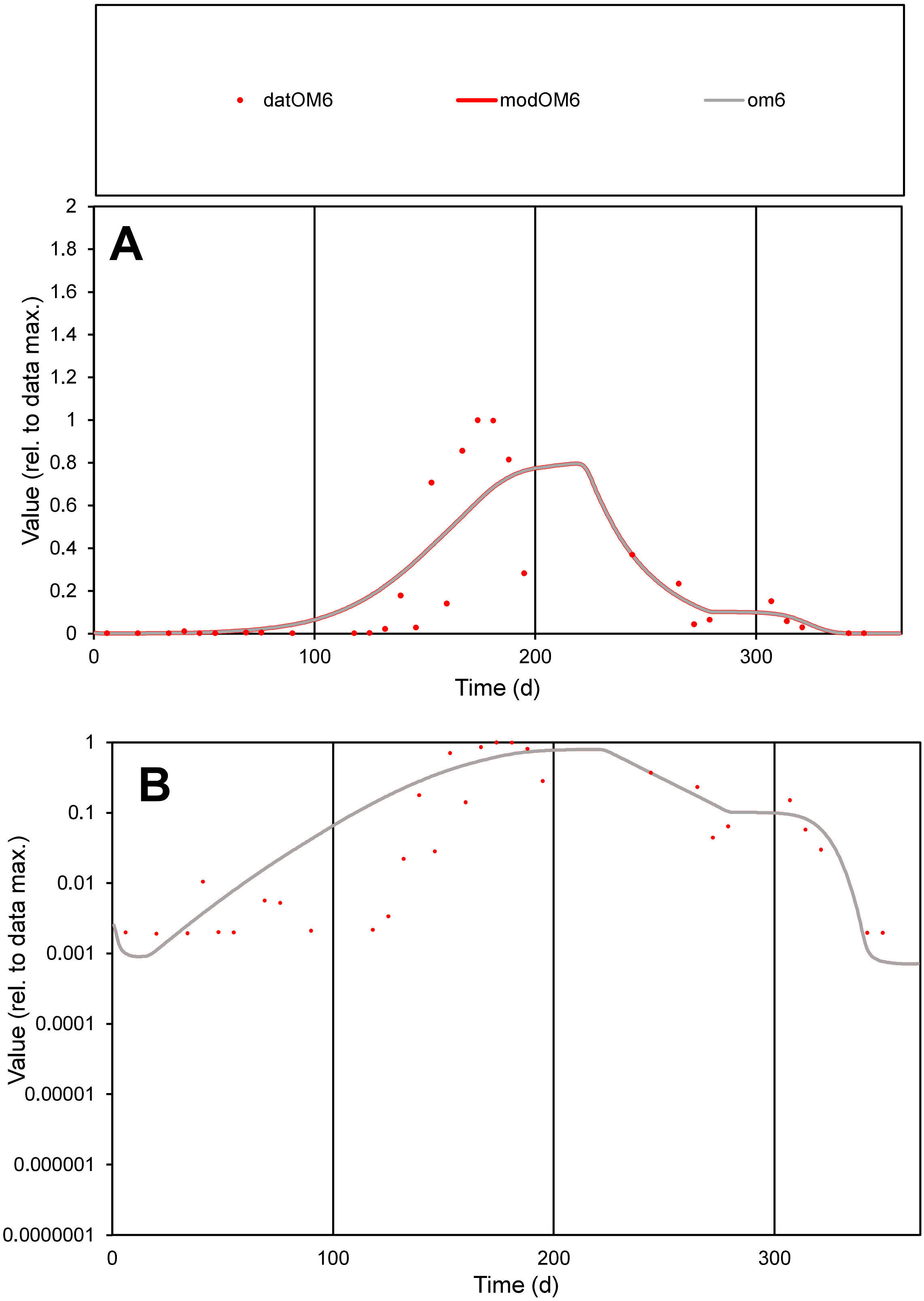

C1

Variables

OM6 Provide jtd or Dst

CalibOnly?

2 1 to reproduce of

oNormalize?

2 1=no, 2=yes

ofxn calc*

0.8937

ofxn mod

0.91337

sRun

RunA1

zout

1

Ave

Data

0.00275

Model

0.00298

data/vMax

0.0137

ofxng

275.646

Min d/m

2.54607

ave/min d

104.781

ave/min m

289.366

325_Calib_S_A

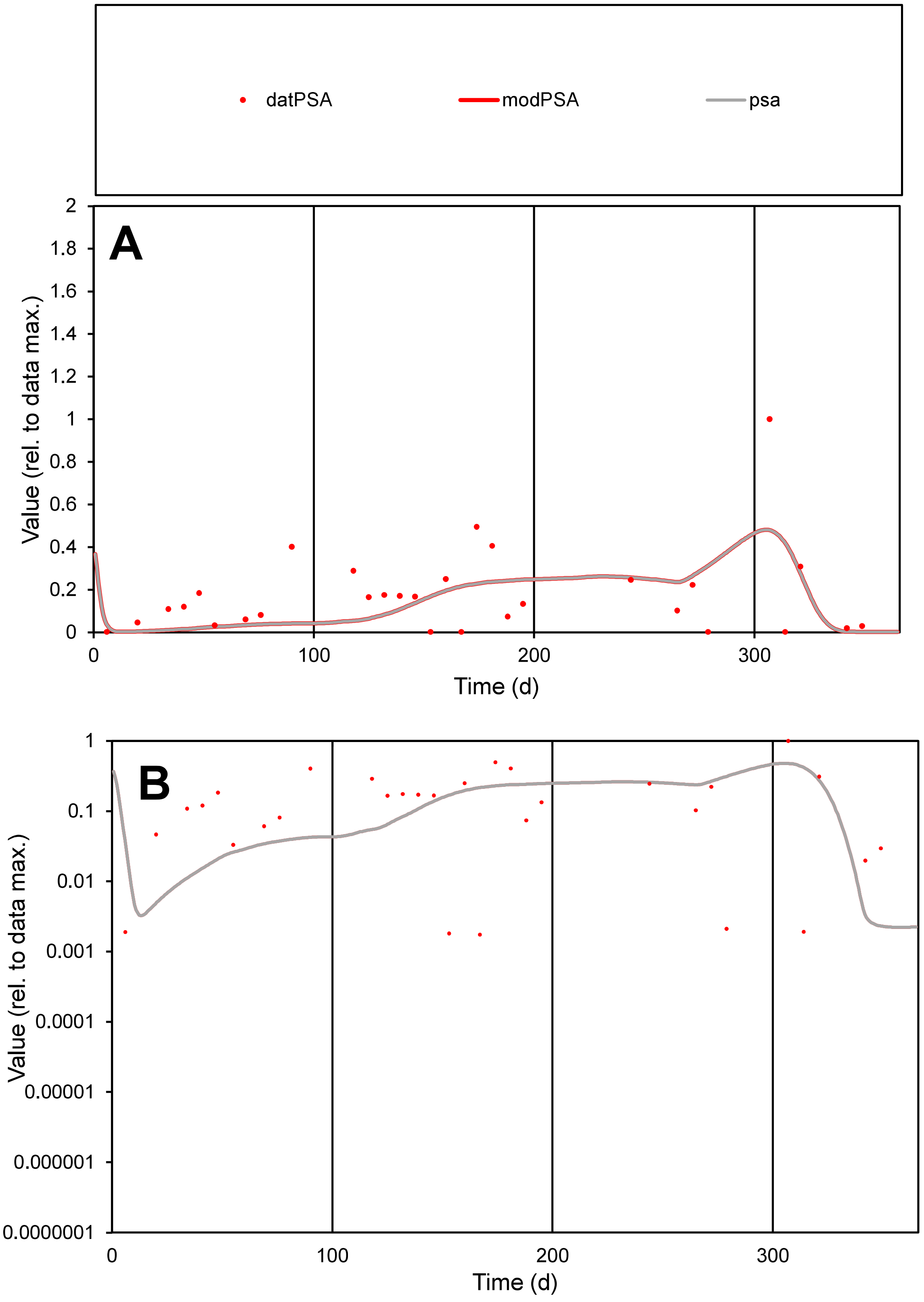

C1

Variables
PSA Provide jtd or Dst
CalibOnly?
2 1 to reproduce of
oNormalize?
2 1=no, 2=yes

ofxn calc*
1.03872
ofxn mod
1.05321

sRun
RunA1
zout
1

Ave
Data
0.0009
Model
0.00075

data/vMax
0.00509

ofxng
275.646

Min d/m
0.76534

ave/min d
101.611
ave/min m
64.5401

325_Calib_S_A

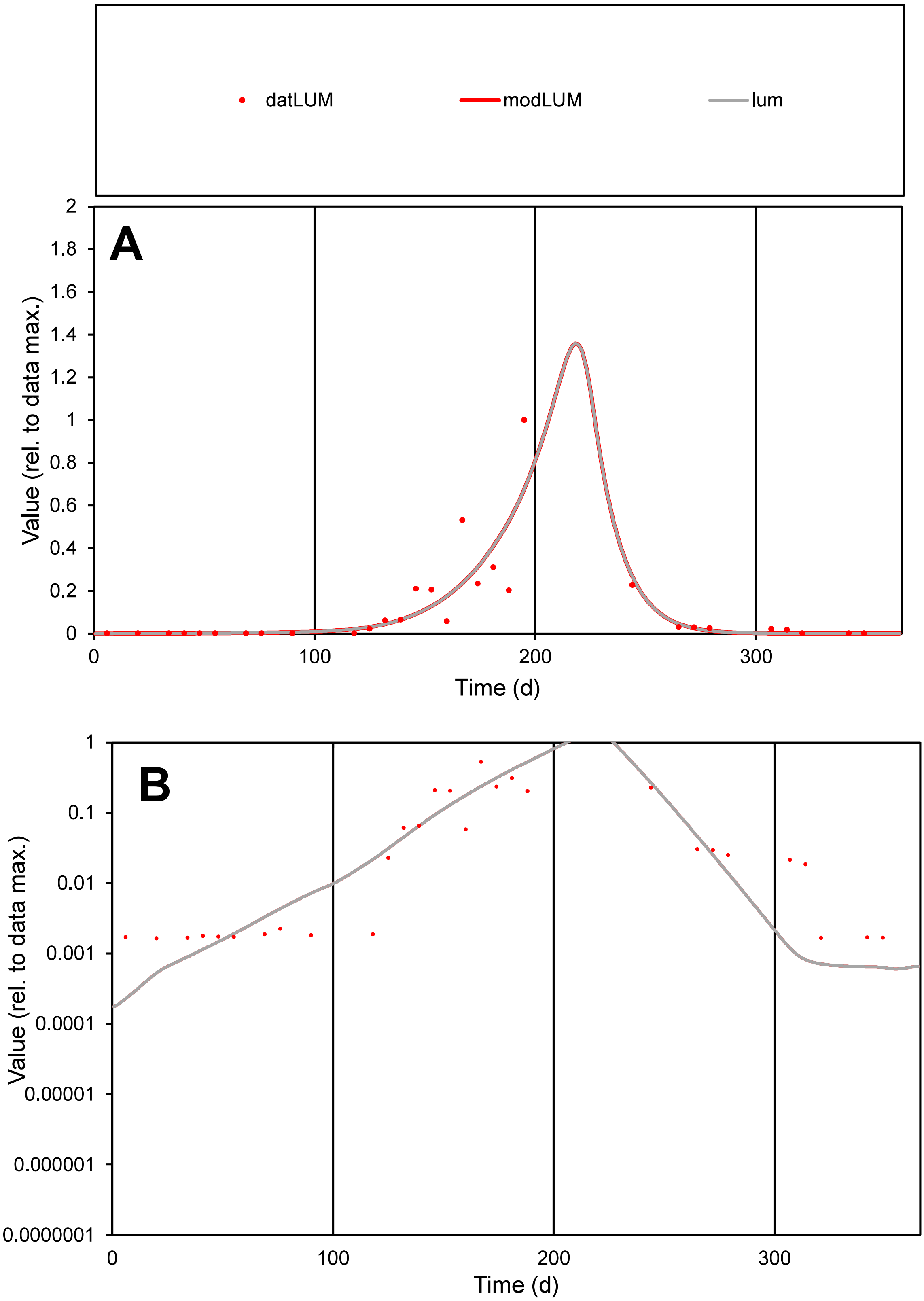

C1

Variables

LUM Provide jtd or Dst

CalibOnly?

2 1 to reproduce of

oNormalize?

2 1=no, 2=yes

ofxn calc*

0.95272

ofxn mod

0.98934

sRun

RunA1

zout

1

Ave

Data

0.00311

Model

0.003

data/vMax

0.0284

ofxng

275.646

Min d/m

6.74232

ave/min d

66.1651

ave/min m

430.271

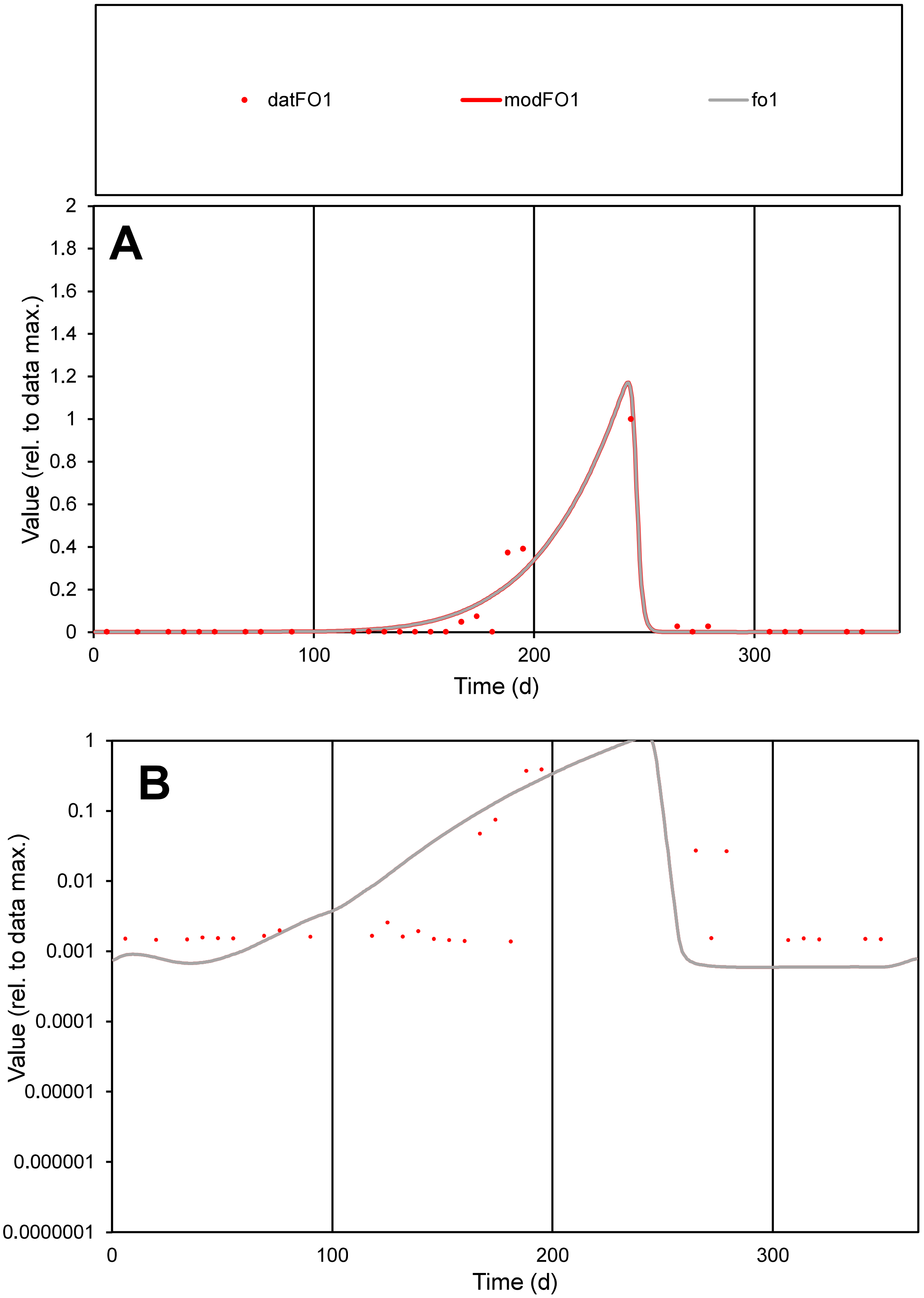

C1

Variables
FO1 Provide jtd or Dst
CalibOnly?
2 1 to reproduce of
oNormalize?
2 1=no, 2=yes

ofxn calc*
0.74716
ofxn mod
0.80683

sRun
RunA1
zout
1

Ave
Data
0.00276
Model
0.00302

data/vMax
0.04184

ofxng
275.646

Min d/m
2.30963

ave/min d
47.7364
ave/min m
120.632

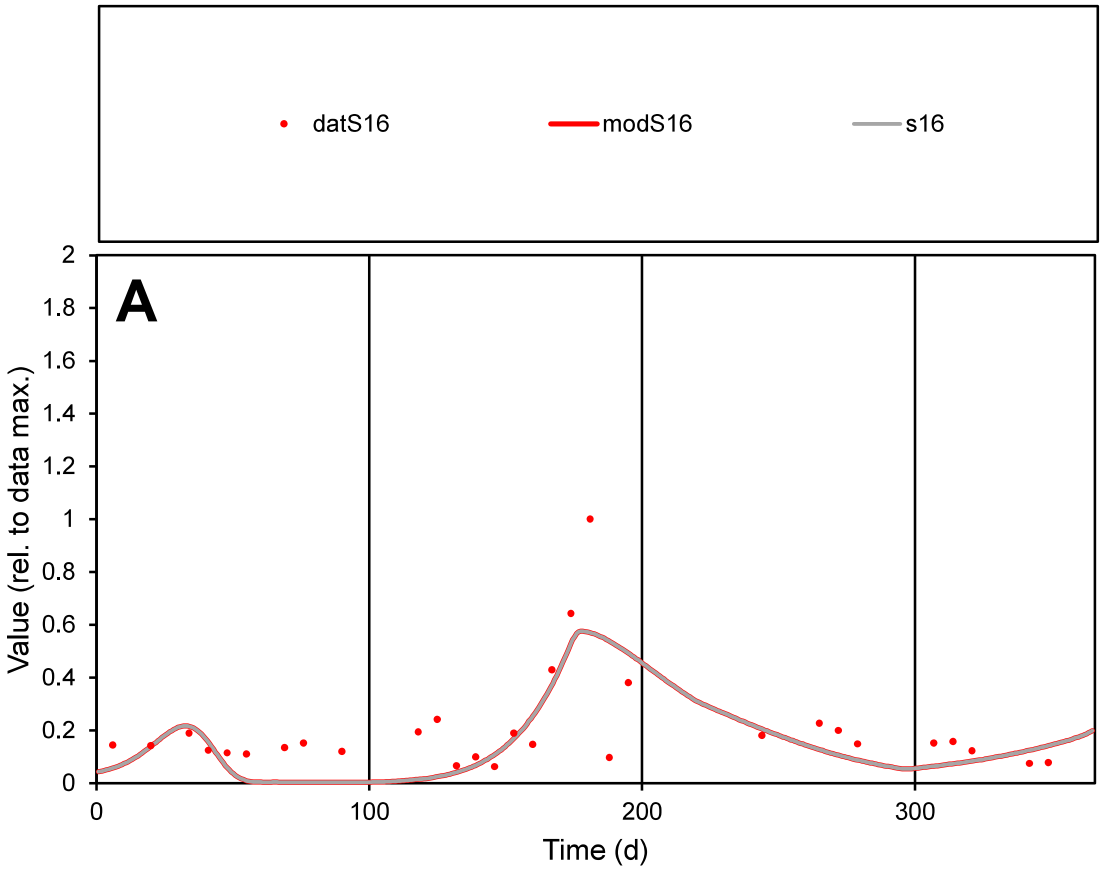

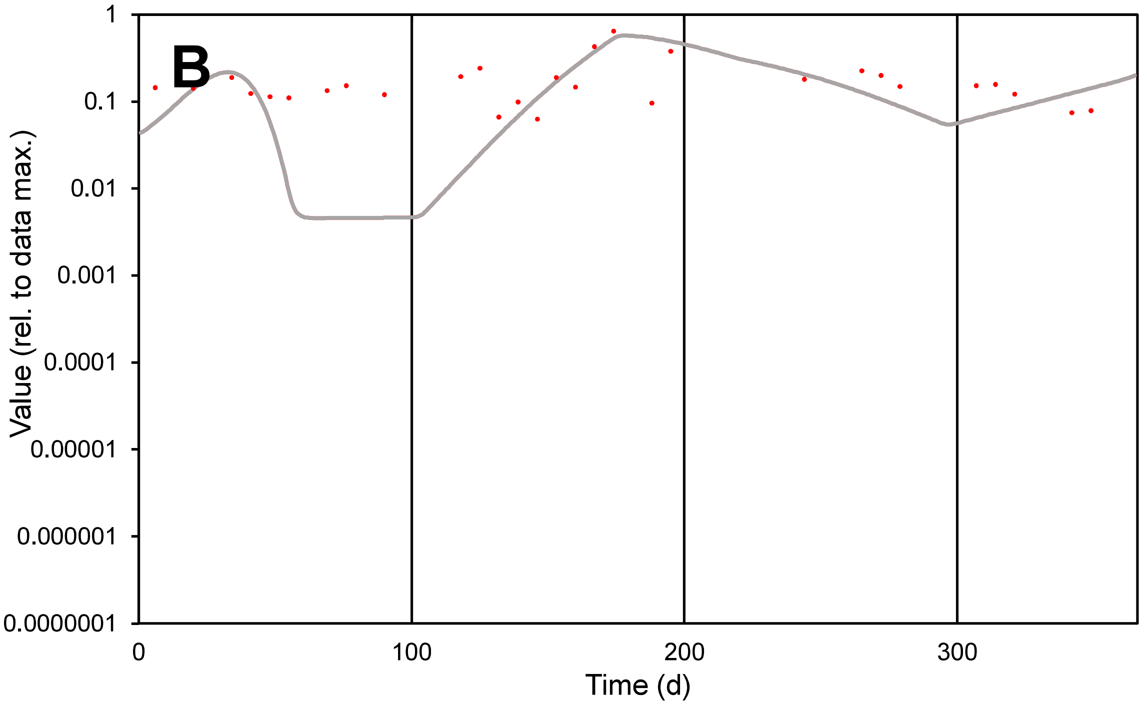

C1

Variables
S16 Provide jtd or Dst
CalibOnly?
2 1 to reproduce of
oNormalize?
2 1=no, 2=yes

ofxn calc*
0.69785
ofxn mod
0.71284

sRun
RunA1
zout
1

Ave
Data
0.00551
Model
0.00442

data/vMax
0.02702

ofxng
275.646

Min d/m
13.753

ave/min d
3.234
ave/min m
35.6592

325_Calib_S_A

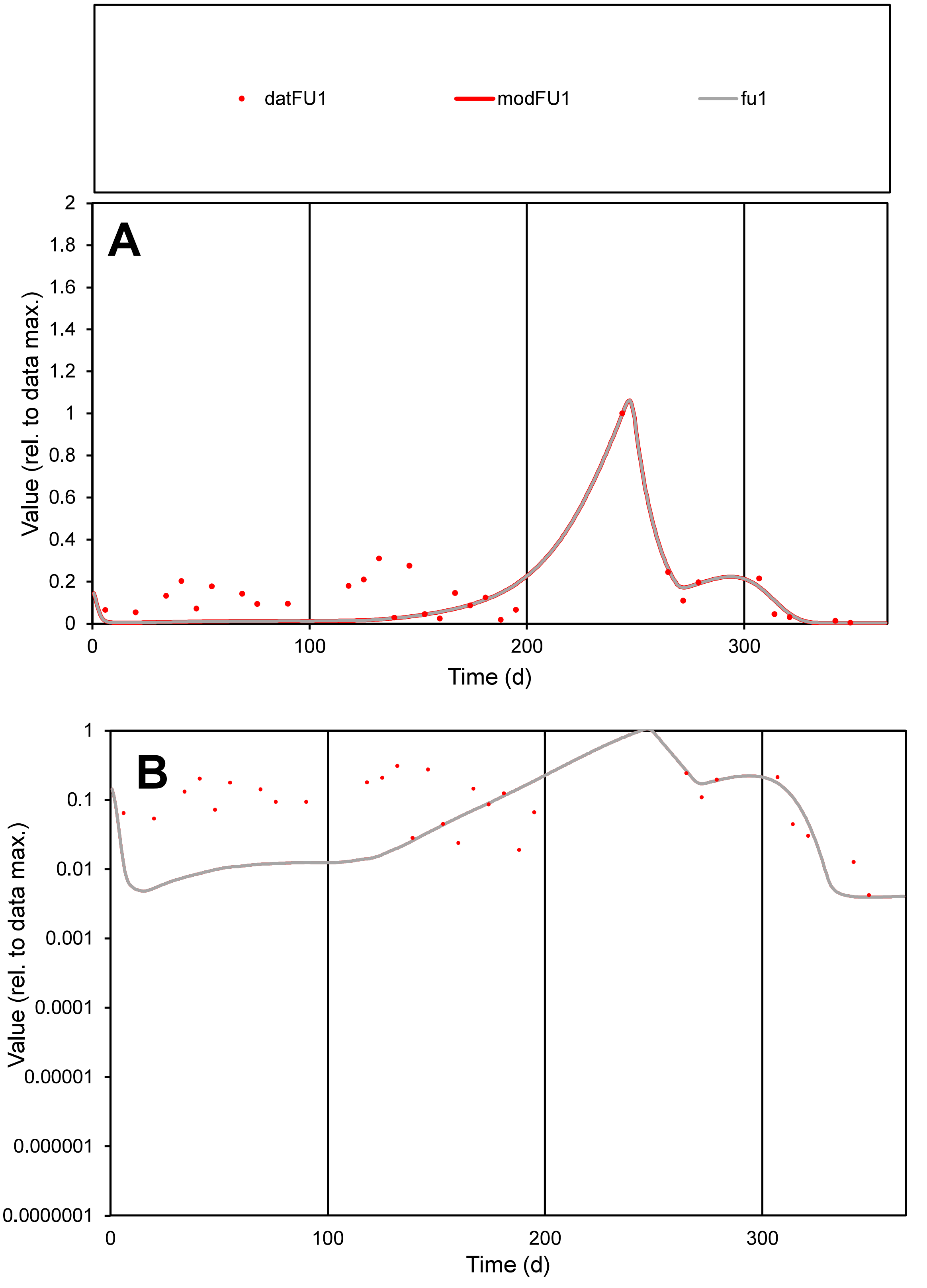

C1

Variables
FU1 Provide jtd or Dst
CalibOnly?
2 1 to reproduce of
oNormalize?
2 1=no, 2=yes

ofxn calc*
0.75841
ofxn mod
0.77168

sRun
RunA1
zout
1

Ave
Data
0.00174
Model
0.00115

data/vMax
0.01185

ofxng
275.646

Min d/m
1.07048

ave/min d
34.7189
ave/min m
24.5625

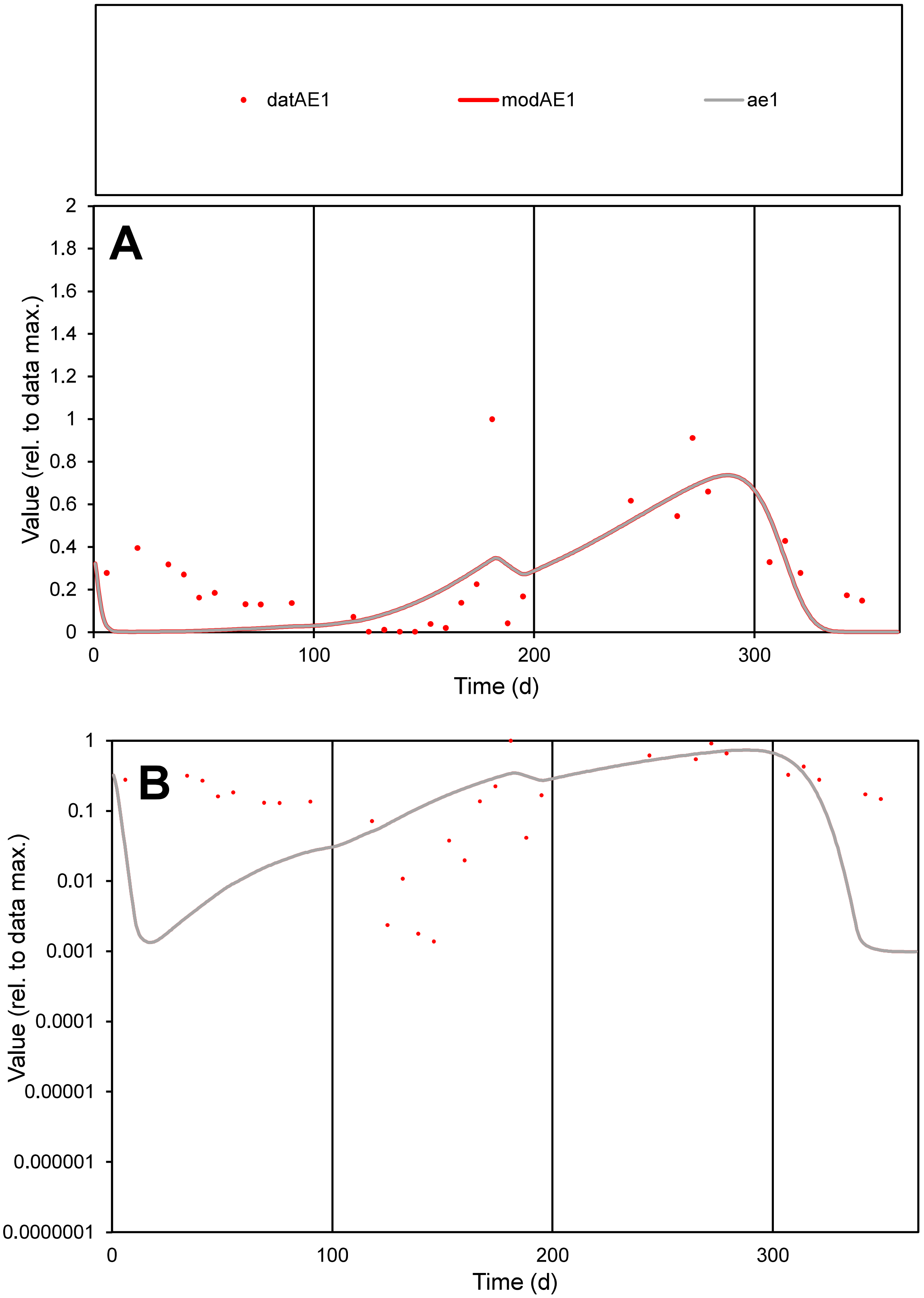

C1

Variables
AE1 Provide jtd or Dst
CalibOnly?
2 1 to reproduce of
oNormalize?
2 1=no, 2=yes

ofxn calc*
0.77346
ofxn mod
0.80567

sRun
RunA1
zout
1

Ave
Data
0.00284
Model
0.00225

data/vMax
0.01091

ofxng
275.646

Min d/m
1.35036

ave/min d
188.409
ave/min m
201.631

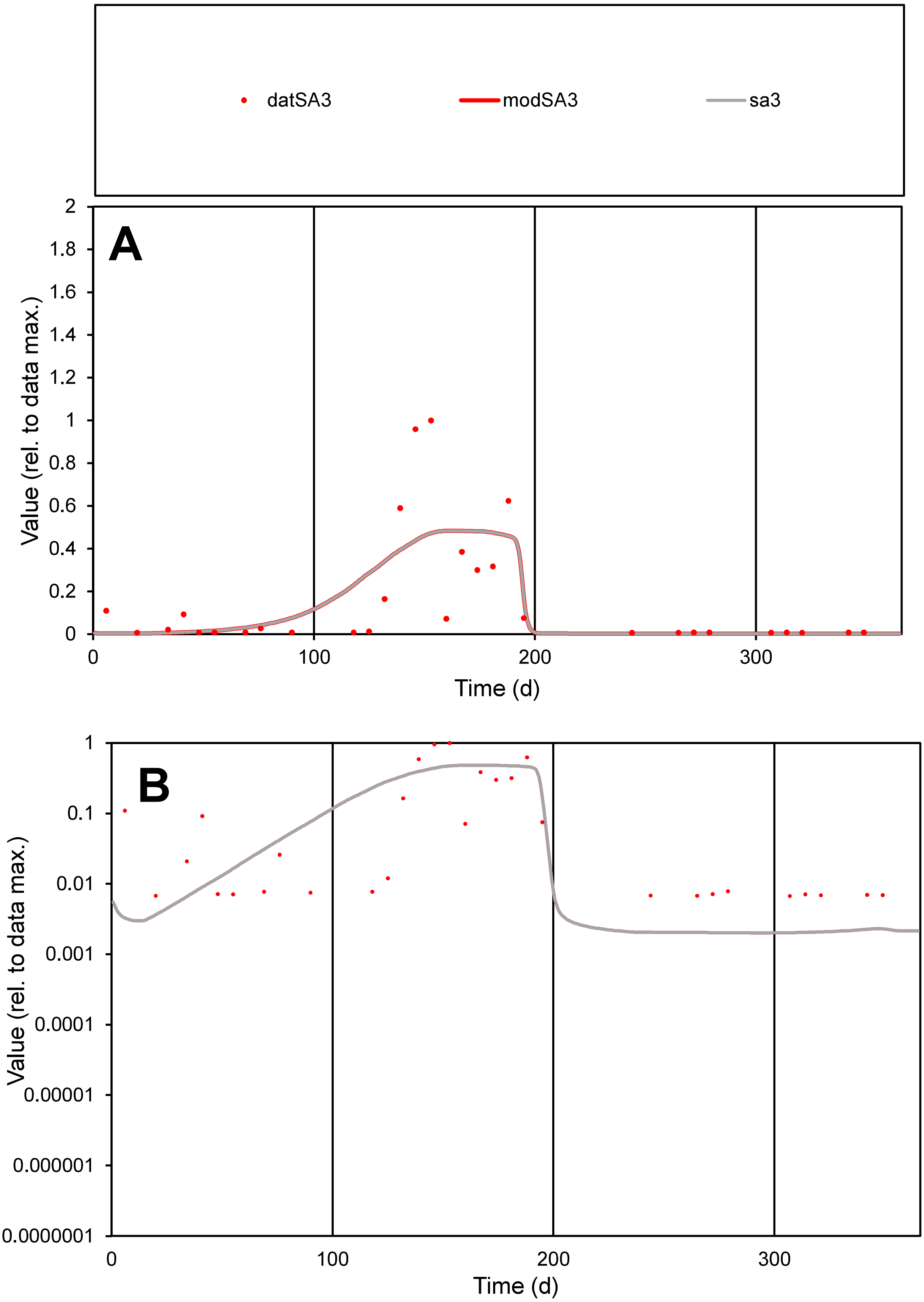

C1

Variables
SA3 Provide jtd or Dst
CalibOnly?
2 1 to reproduce of
oNormalize?
2 1=no, 2=yes

ofxn calc*
1.13692
ofxn mod
1.15314

sRun
RunA1
zout
1

Ave
Data
0.00246
Model
0.00246

data/vMax
0.01522

ofxng
275.646

Min d/m
3.33225

ave/min d
23.9845
ave/min m
79.954

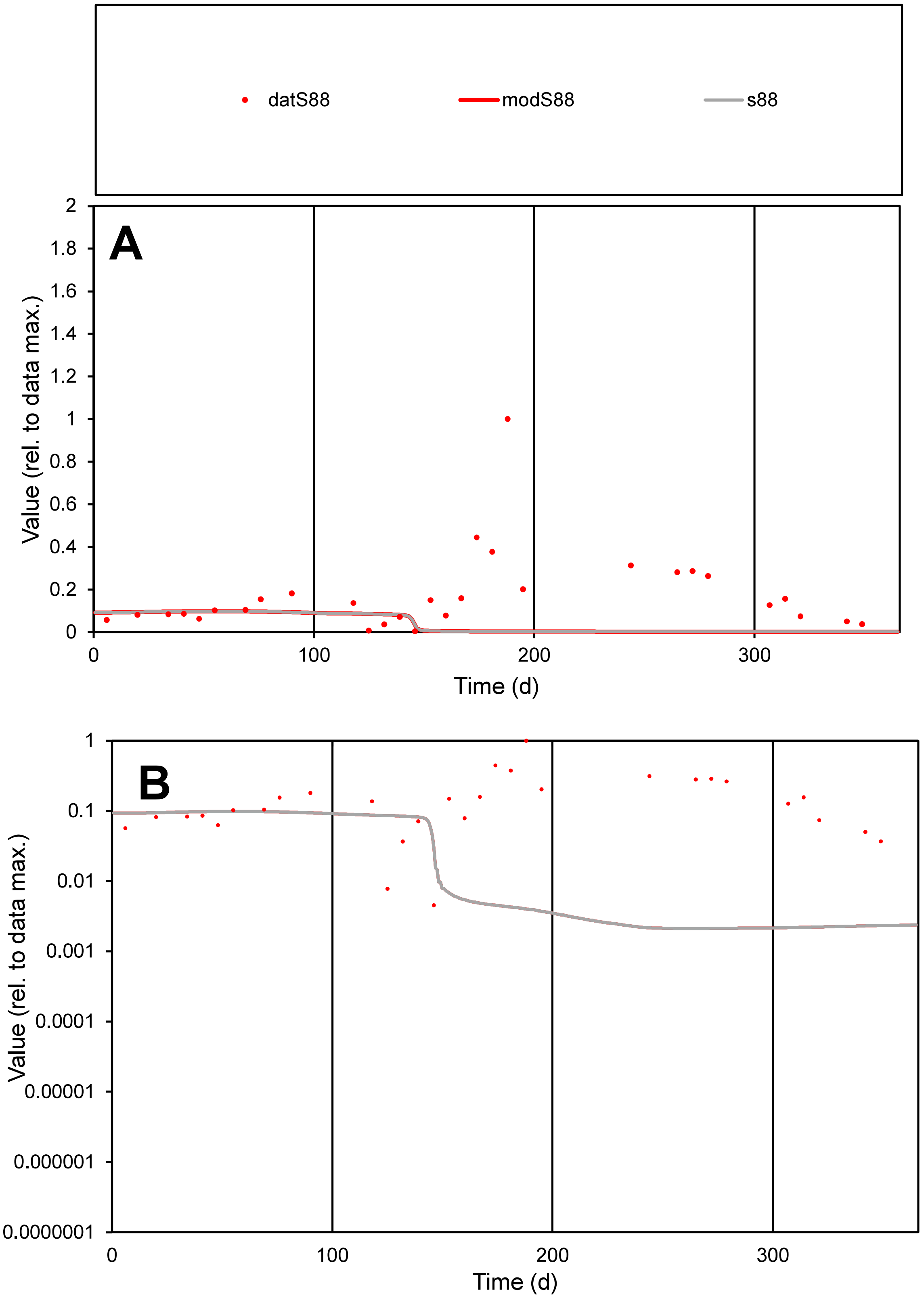

C1

Variables

S88 Provide jtd or Dst

CalibOnly?

2 1 to reproduce of

oNormalize?

2 1=no, 2=yes

ofxn calc*

1.42568

ofxn mod

1.42797

sRun

RunA1

zout

1

Ave

Data

0.00238

Model

0.00058

data/vMax

0.0138

ofxng

275.646

Min d/m

2.14364

ave/min d

38.1233

ave/min m

19.9979

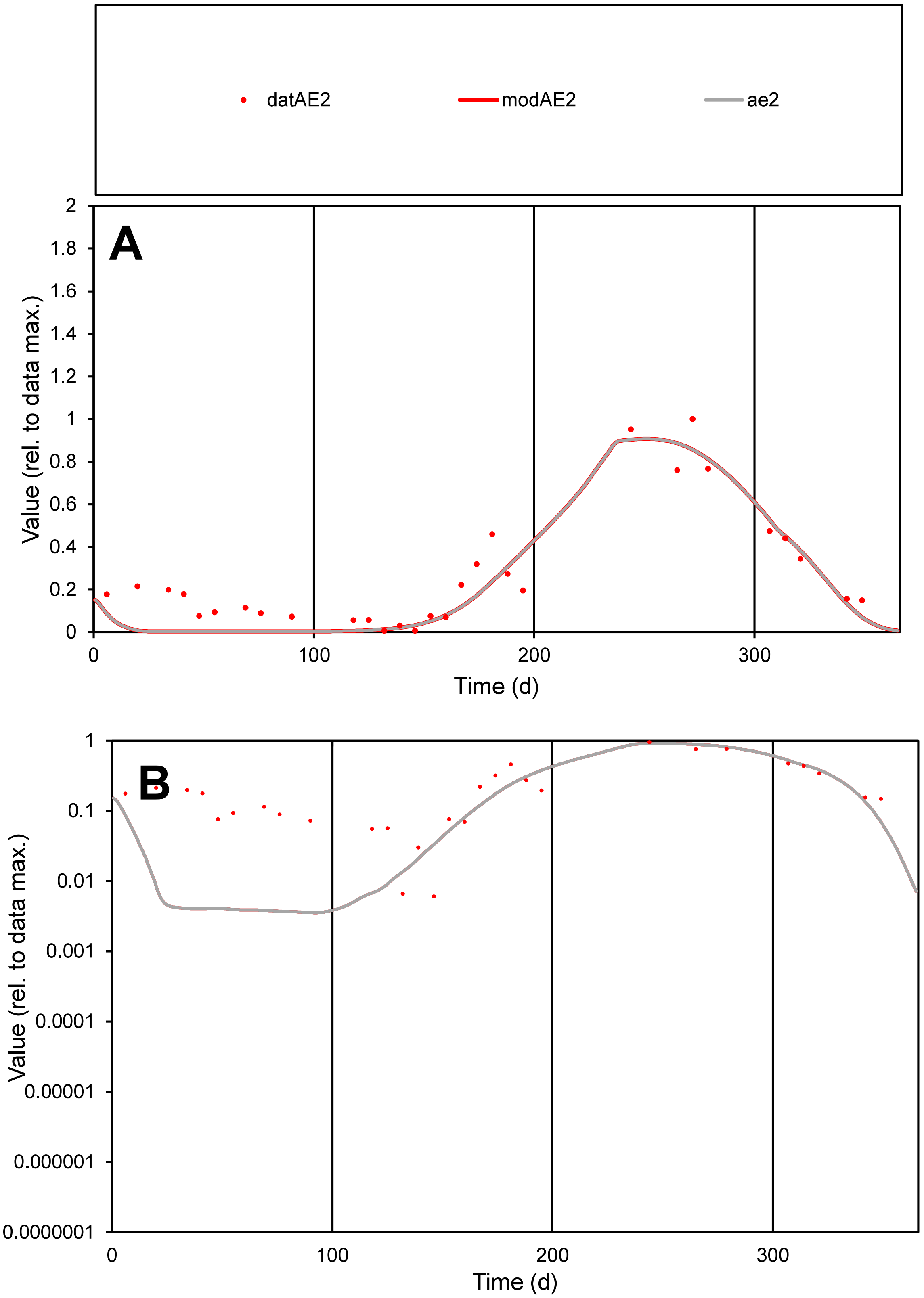

C1

Variables
AE2 Provide jtd or Dst
CalibOnly?
2 1 to reproduce of
oNormalize?
2 1=no, 2=yes

ofxn calc*
0.38653
ofxn mod
0.42132

sRun
RunA1
zout
1

Ave
Data
0.00275
Model
0.00225

data/vMax
0.01026

ofxng
275.646

Min d/m
1.71979

ave/min d
43.9394
ave/min m
61.854

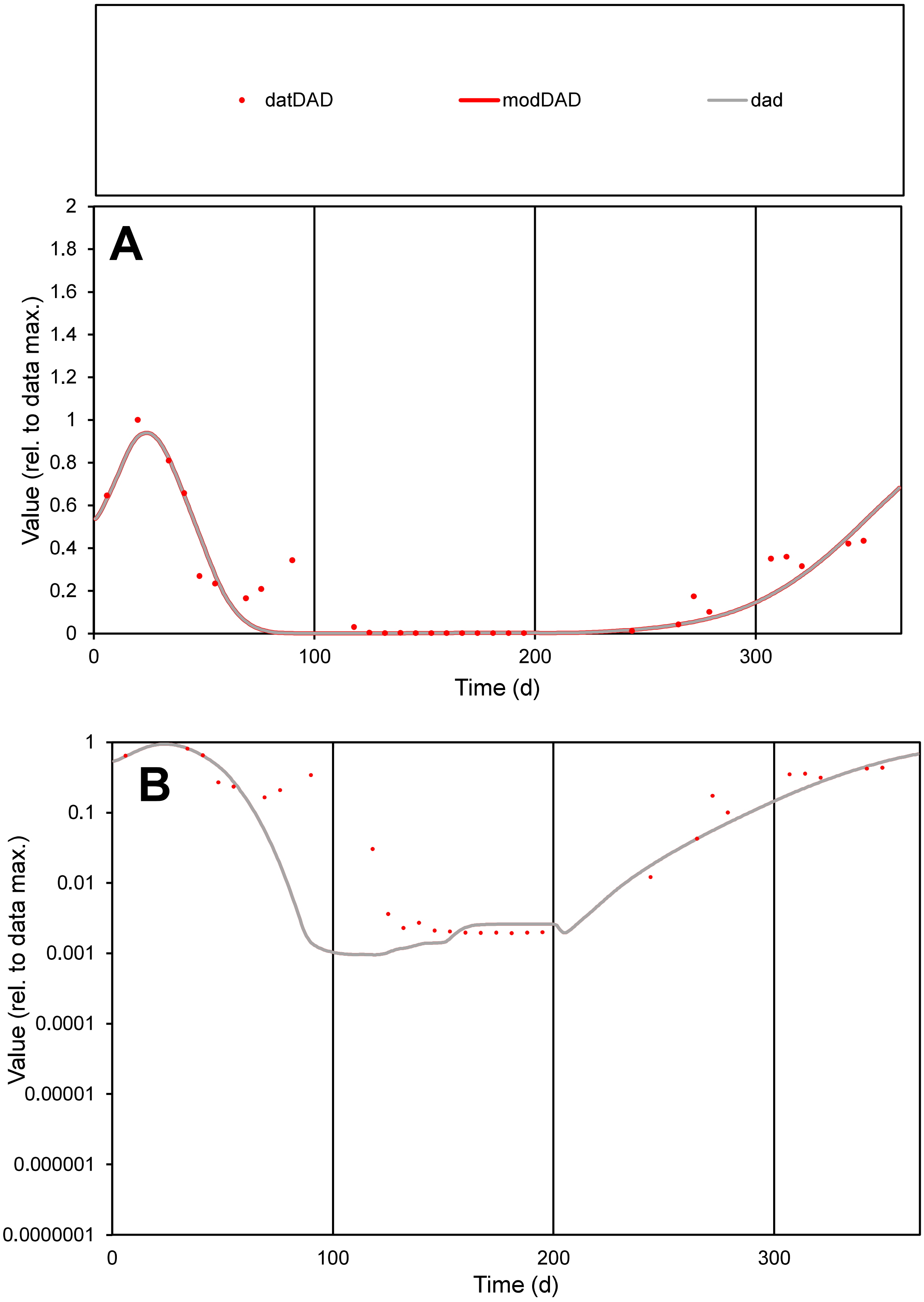

C1

Variables
DAD Provide jtd or Dst
CalibOnly?
2 1 to reproduce of
oNormalize?
2 1=no, 2=yes

ofxn calc*
0.42823
ofxn mod
0.46148

sRun
RunA1
zout
1

Ave
Data
0.00174
Model
0.00149

data/vMax
0.0079

ofxng
275.646

Min d/m
2.03797

ave/min d
113.144
ave/min m
197.549

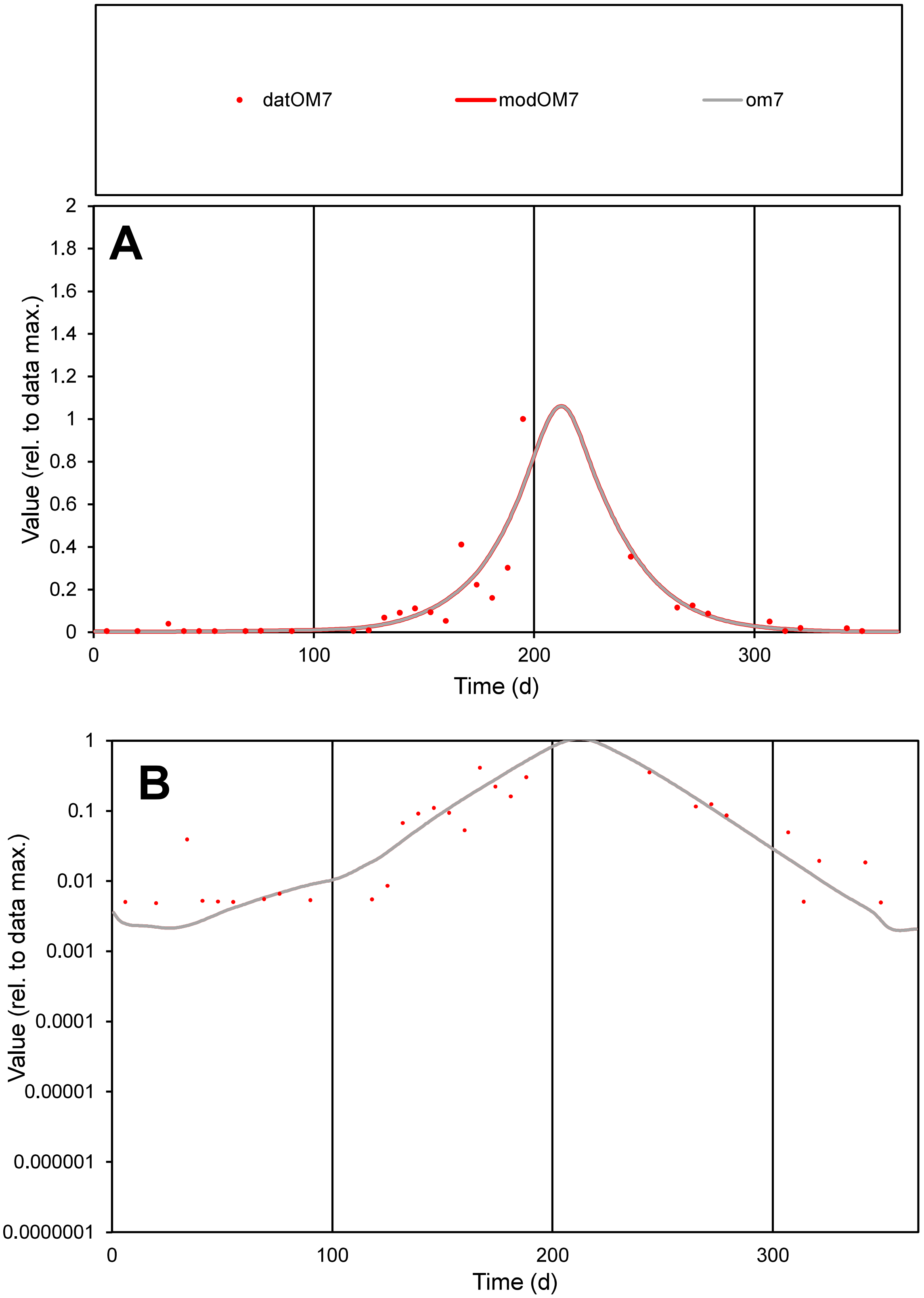

C1

Variables
OM7 Provide jtd or Dst
CalibOnly?
2 1 to reproduce of
oNormalize?
2 1=no, 2=yes

ofxn calc*
0.78481
ofxn mod
0.82372

sRun
RunA1
zout
1

Ave
Data
0.00228
Model
0.0023

data/vMax
0.02021

ofxng
275.646

Min d/m
2.20108

ave/min d
23.2988
ave/min m
51.7521

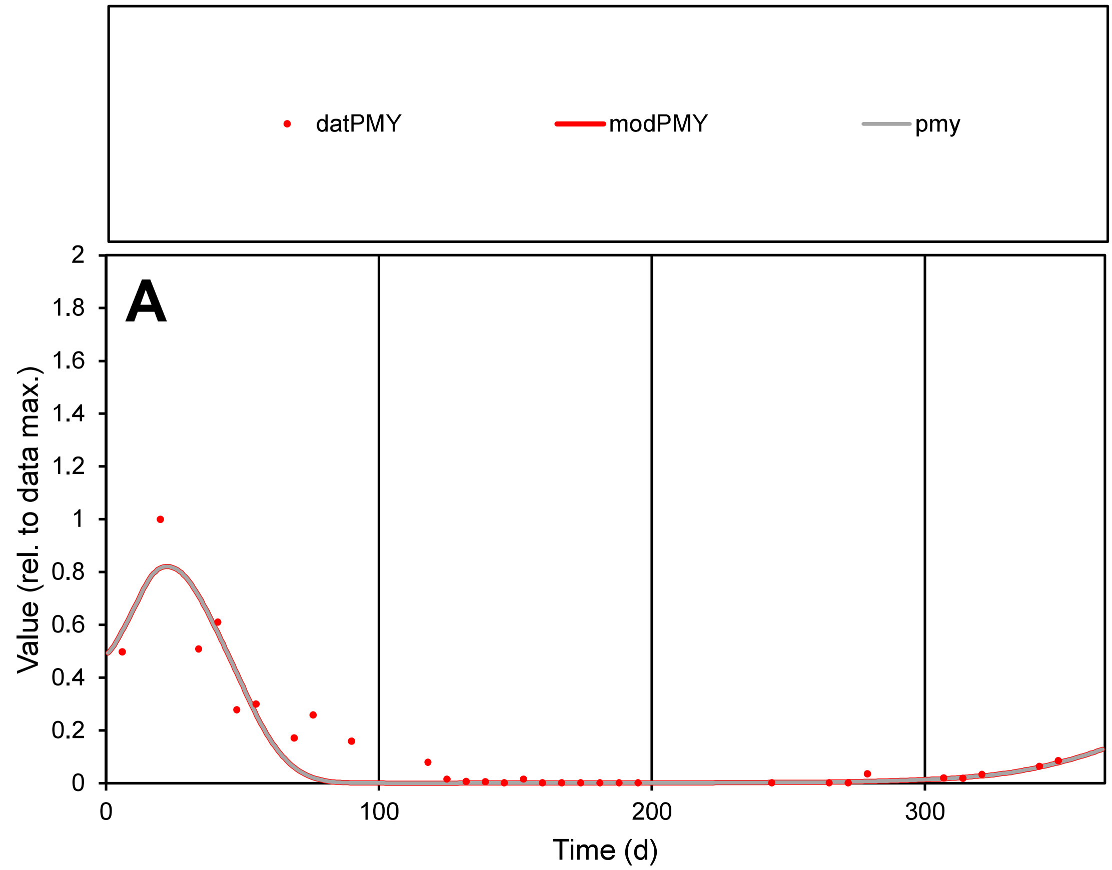

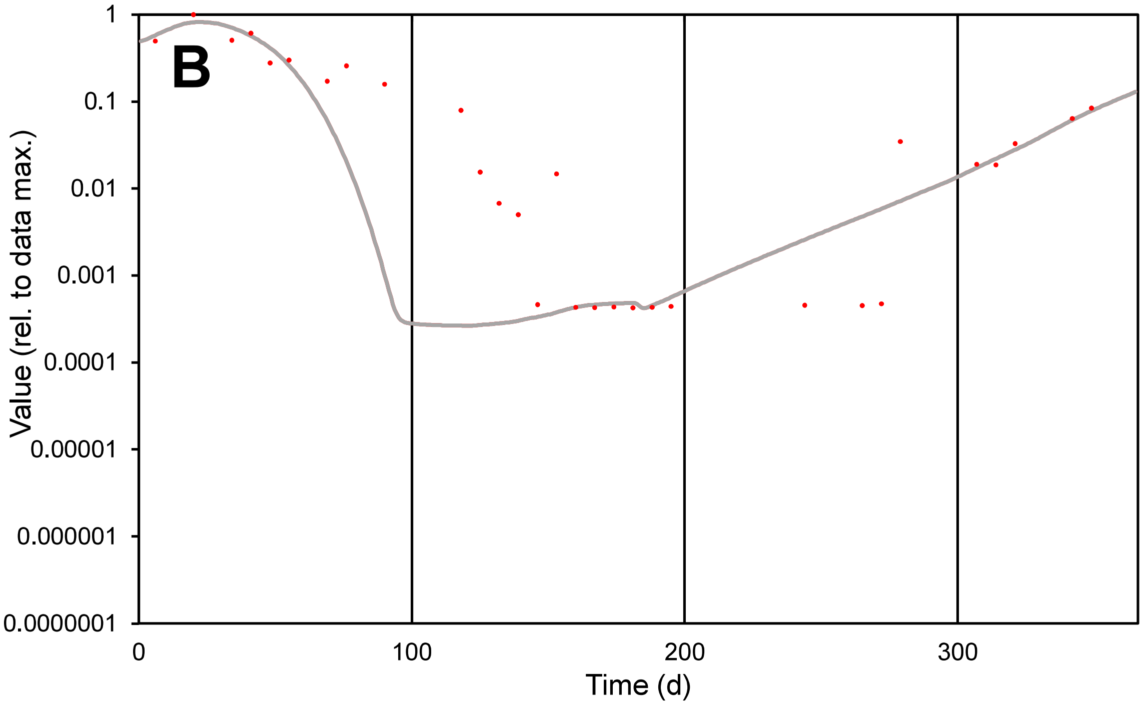

C1

Variables

PMY	Provide jtd or Dst
CalibOnly?	
2	1 to reproduce of
oNormalize?	
2	1=no, 2=yes

ofxn calc*
0.57677
ofxn mod
0.61536

sRun
RunA1
zout
1

Ave
Data
0.00311
Model
0.00269

data/vMax
0.02242

ofxng
275.646

Min d/m
1.60828

ave/min d
325.368
ave/min m
453.189

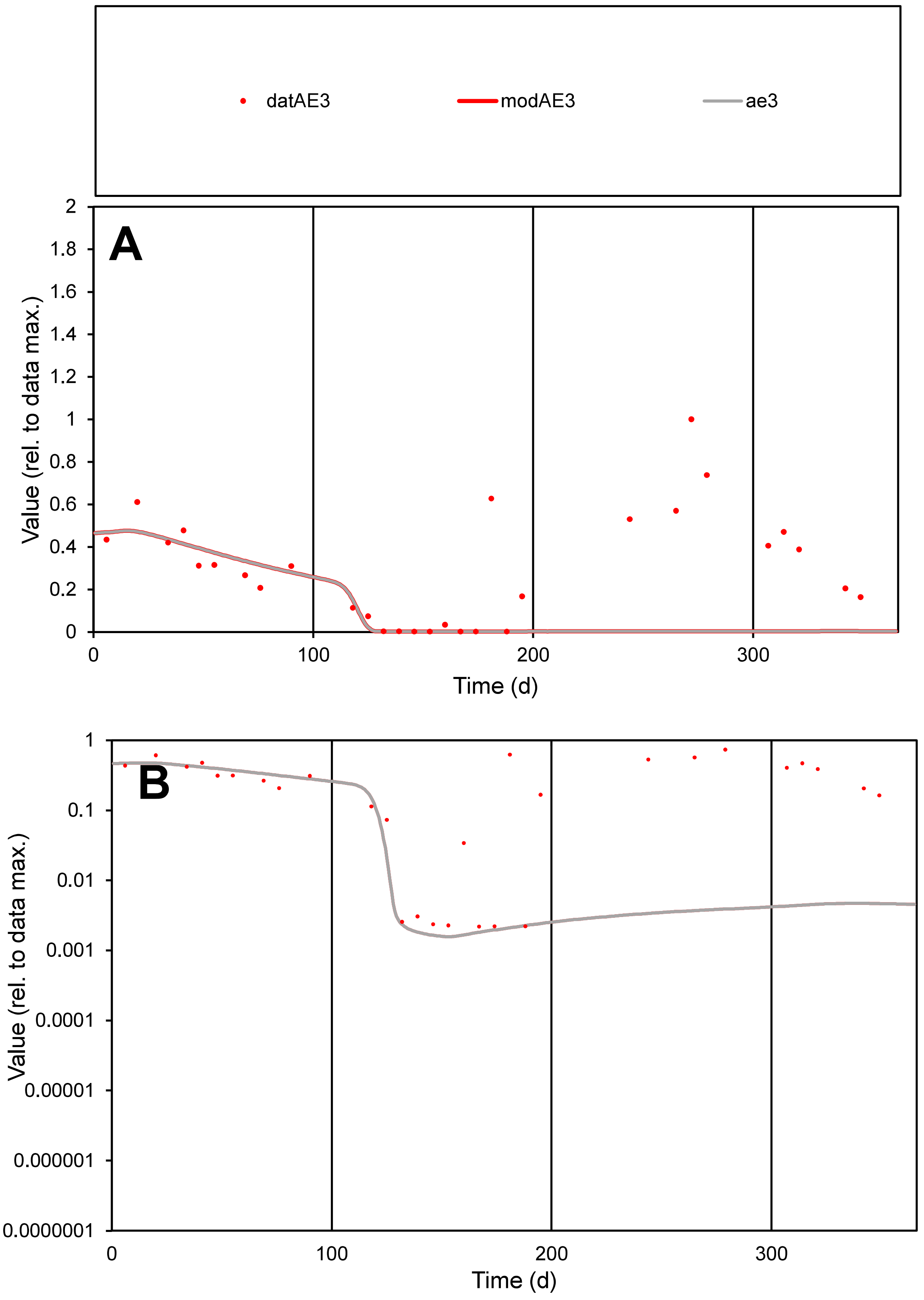

C1

Variables
AE3 Provide jtd or Dst
CalibOnly?
2 1 to reproduce of
oNormalize?
2 1=no, 2=yes

ofxn calc*
1.10474
ofxn mod
1.1104

sRun
RunA1
zout
1

Ave
Data
0.00227
Model
0.00094

data/vMax
0.00768

ofxng
275.646

Min d/m
1.39371

ave/min d
134.534
ave/min m
77.7127

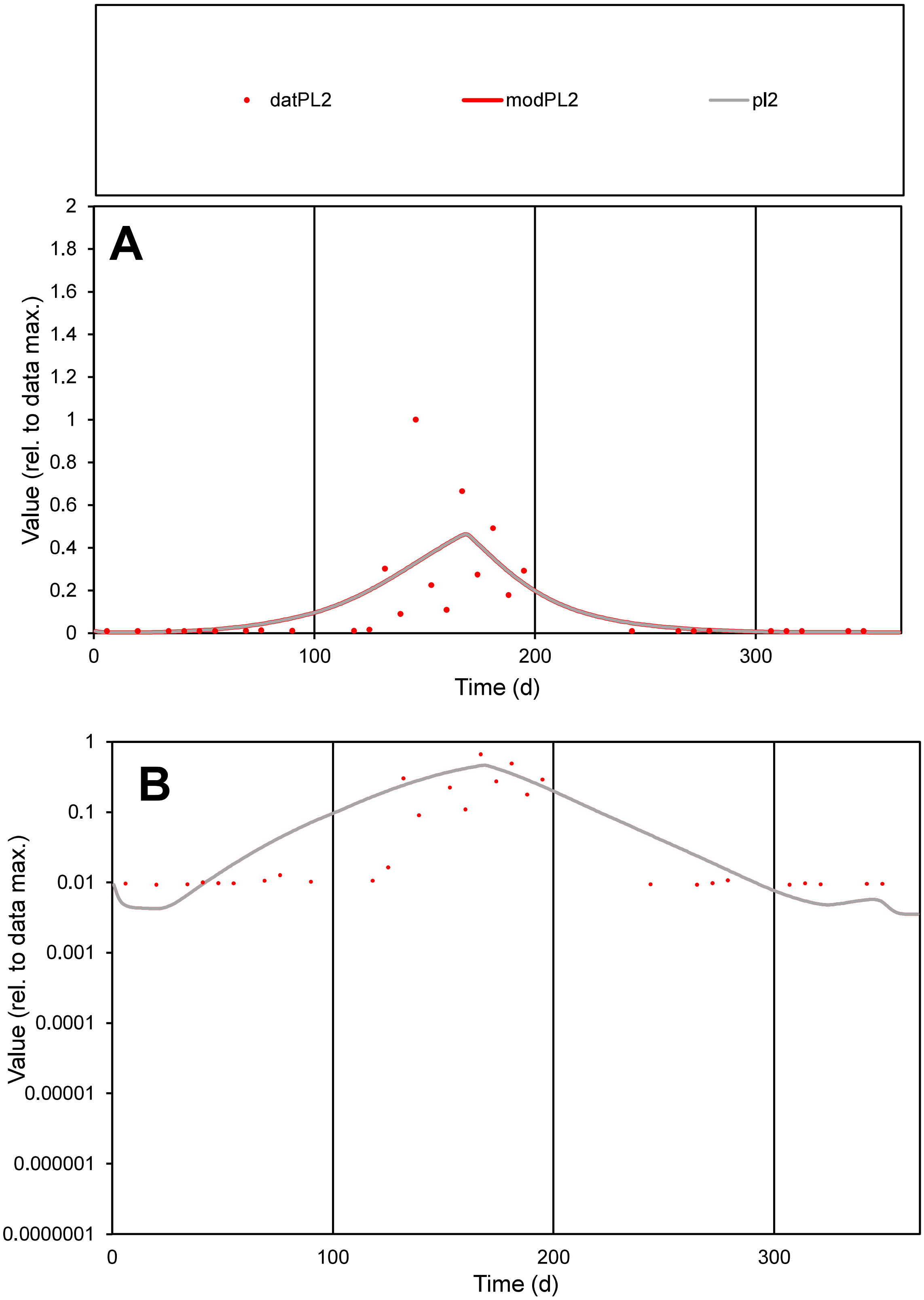

C1

Variables
PL2 Provide jtd or Dst
CalibOnly?
2 1 to reproduce of
oNormalize?
2 1=no, 2=yes

ofxn calc*
1.24492
ofxn mod
1.26424

sRun
RunA1
zout
1

Ave
Data
0.00235
Model
0.00251

data/vMax
0.01838

ofxng
275.646

Min d/m
2.17258

ave/min d
13.7978
ave/min m
31.9958

325_Calib_S_A

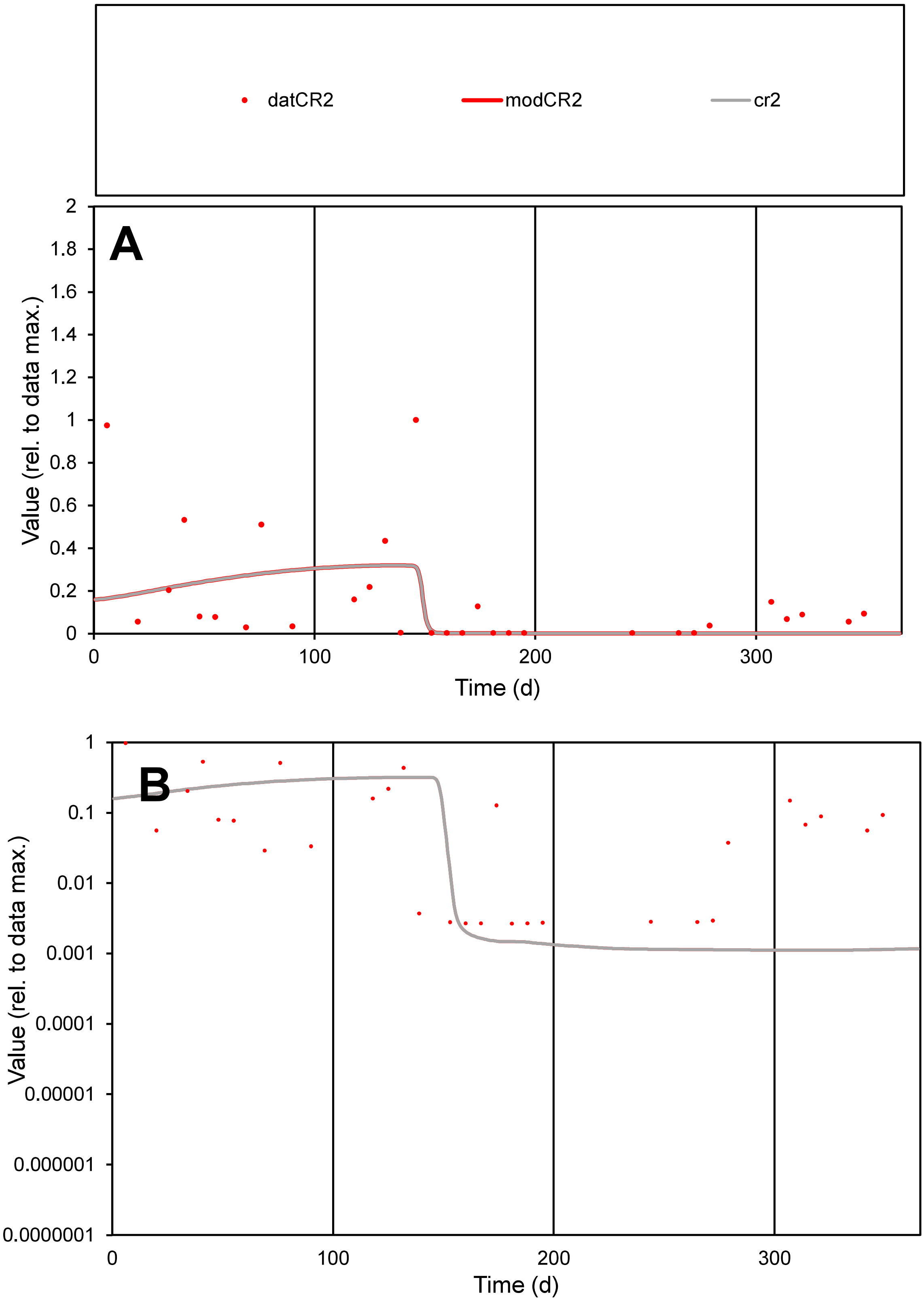

C1

Variables

CR2	Provide jtd or Dst
CalibOnly?	
2	1 to reproduce of
oNormalize?	
2	1=no, 2=yes

ofxn calc*
1.43438
ofxn mod
1.44922

sRun
RunA1
zout
1

Ave
Data
0.00247
Model
0.00187

data/vMax
0.01494

ofxng
275.646

Min d/m
2.38138

ave/min d
62.2938
ave/min m
112.478

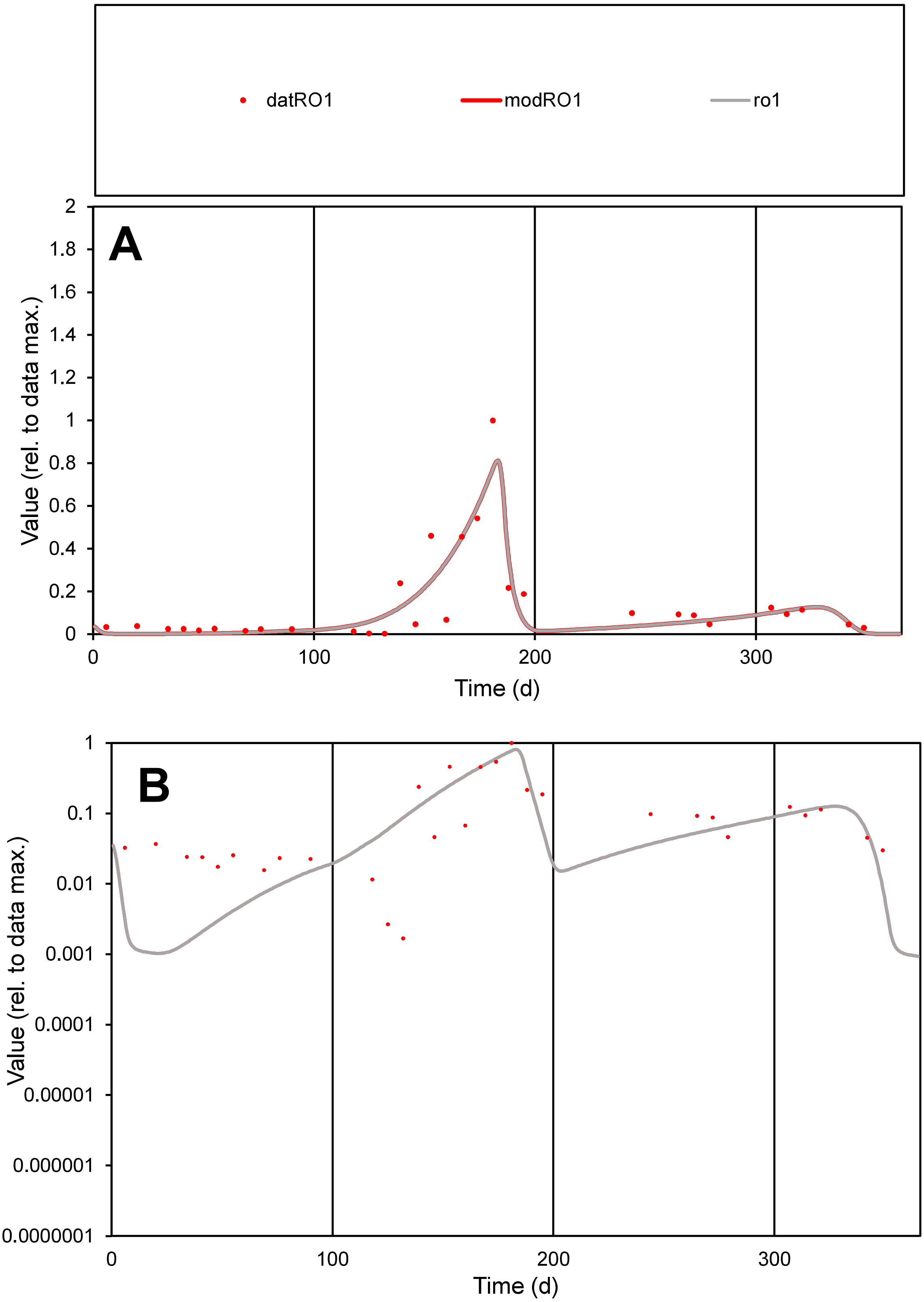

C1

Variables
RO1 Provide jtd or Dst
CalibOnly?
2 1 to reproduce of
oNormalize?
2 1=no, 2=yes

ofxn calc*
0.61974
ofxn mod
0.65546

sRun
RunA1
zout
1

Ave
Data
0.00344
Model
0.00332

data/vMax
0.02467

ofxng
275.646

Min d/m
1.63515

ave/min d
82.8191
ave/min m
130.796

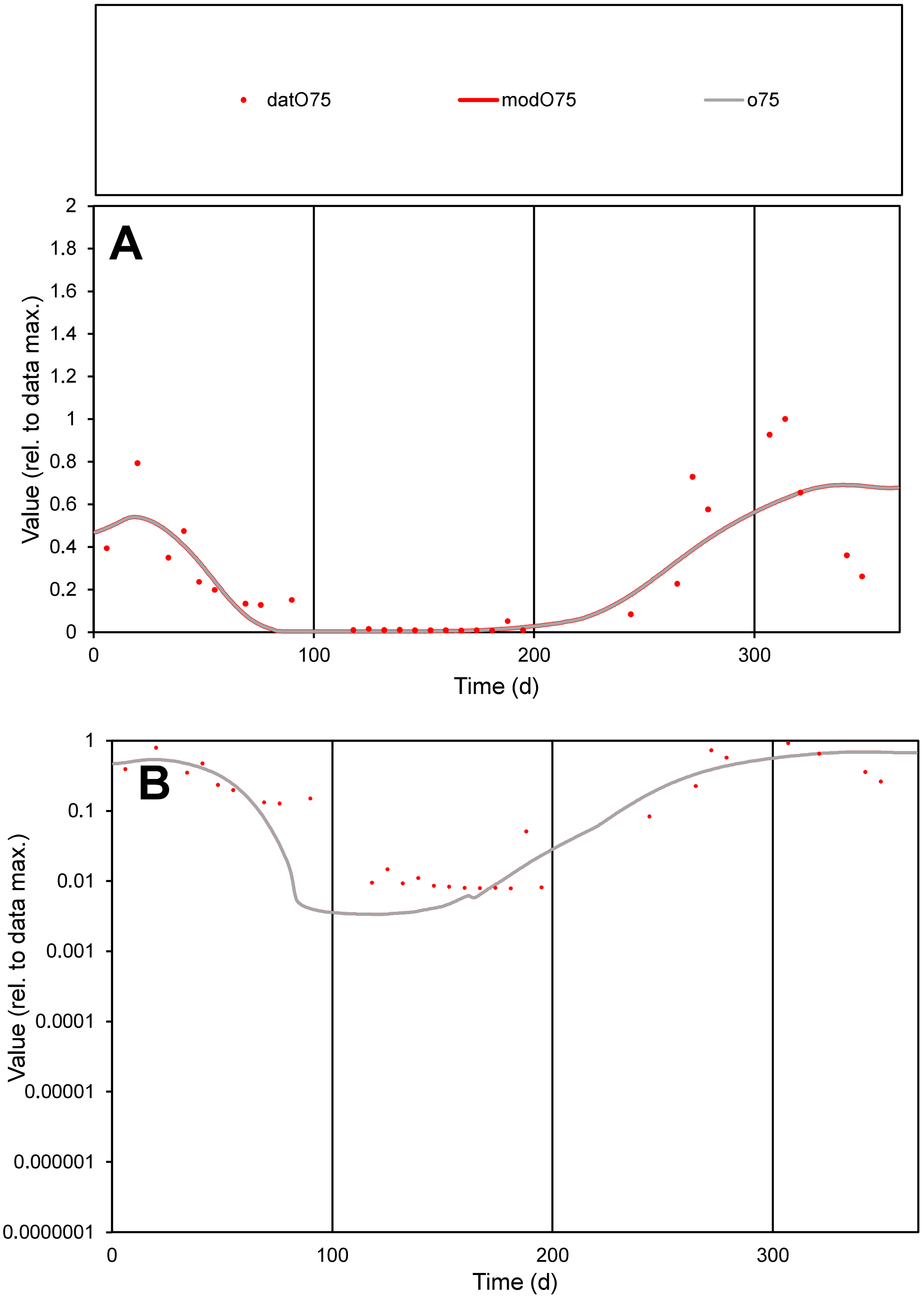

C1

Variables
O75 Provide jtd or Dst
CalibOnly?
2 1 to reproduce of
oNormalize?
2 1=no, 2=yes

ofxn calc*
0.62916
ofxn mod
0.65139

sRun
RunA1
zout
1

Ave
Data
0.0023
Model
0.00214

data/vMax
0.00883

ofxng
275.646

Min d/m
2.3406

ave/min d
33.0355
ave/min m
71.7486

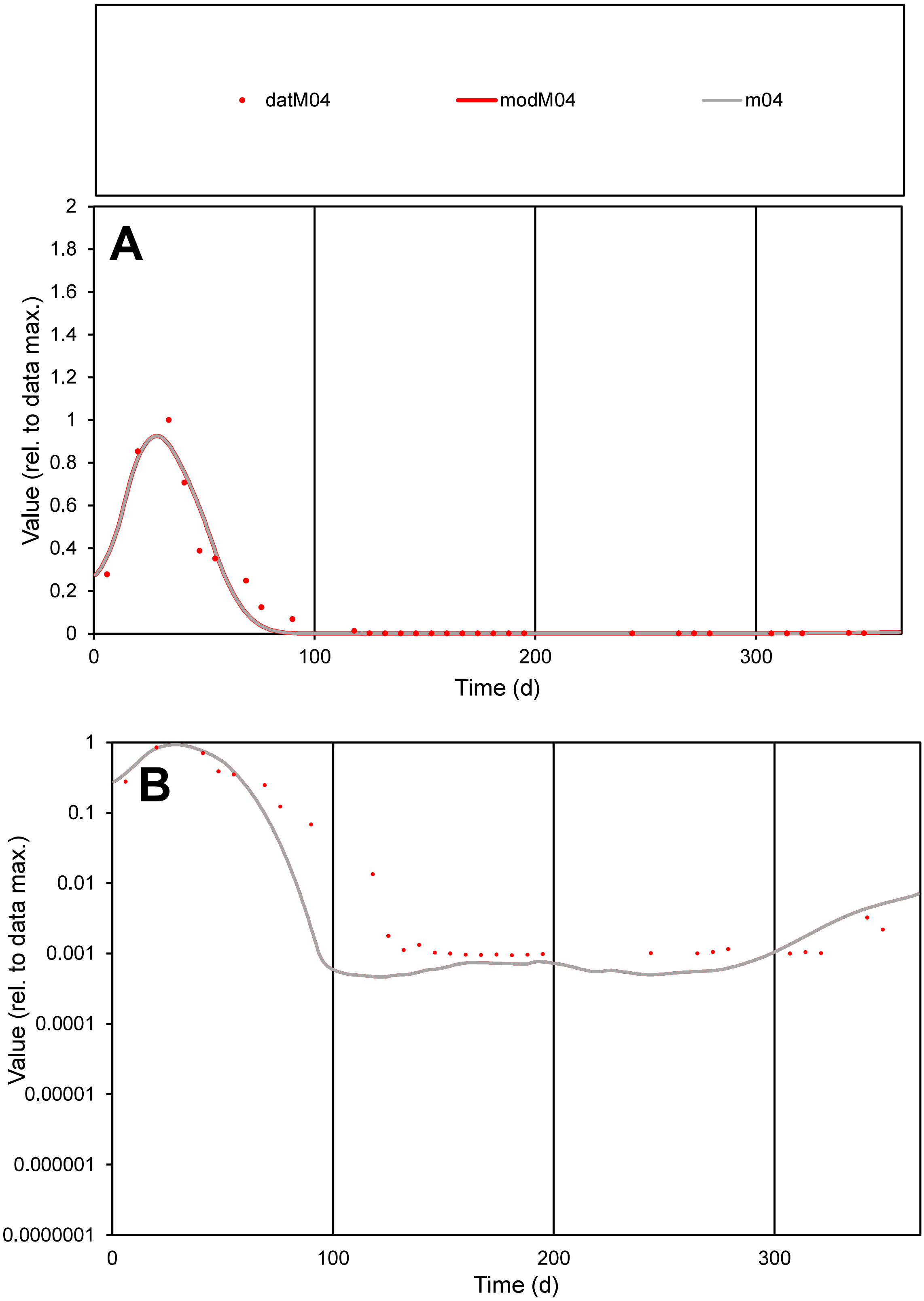

C1

Variables
M04 Provide jtd or Dst
CalibOnly?
2 1 to reproduce of
oNormalize?
2 1=no, 2=yes

ofxn calc*
0.40787
ofxn mod
0.49305

sRun
RunA1
zout
1

Ave
Data
0.00499
Model
0.00474

data/vMax
0.03689

ofxng
275.646

Min d/m
2.0348

ave/min d
142.168
ave/min m
274.953

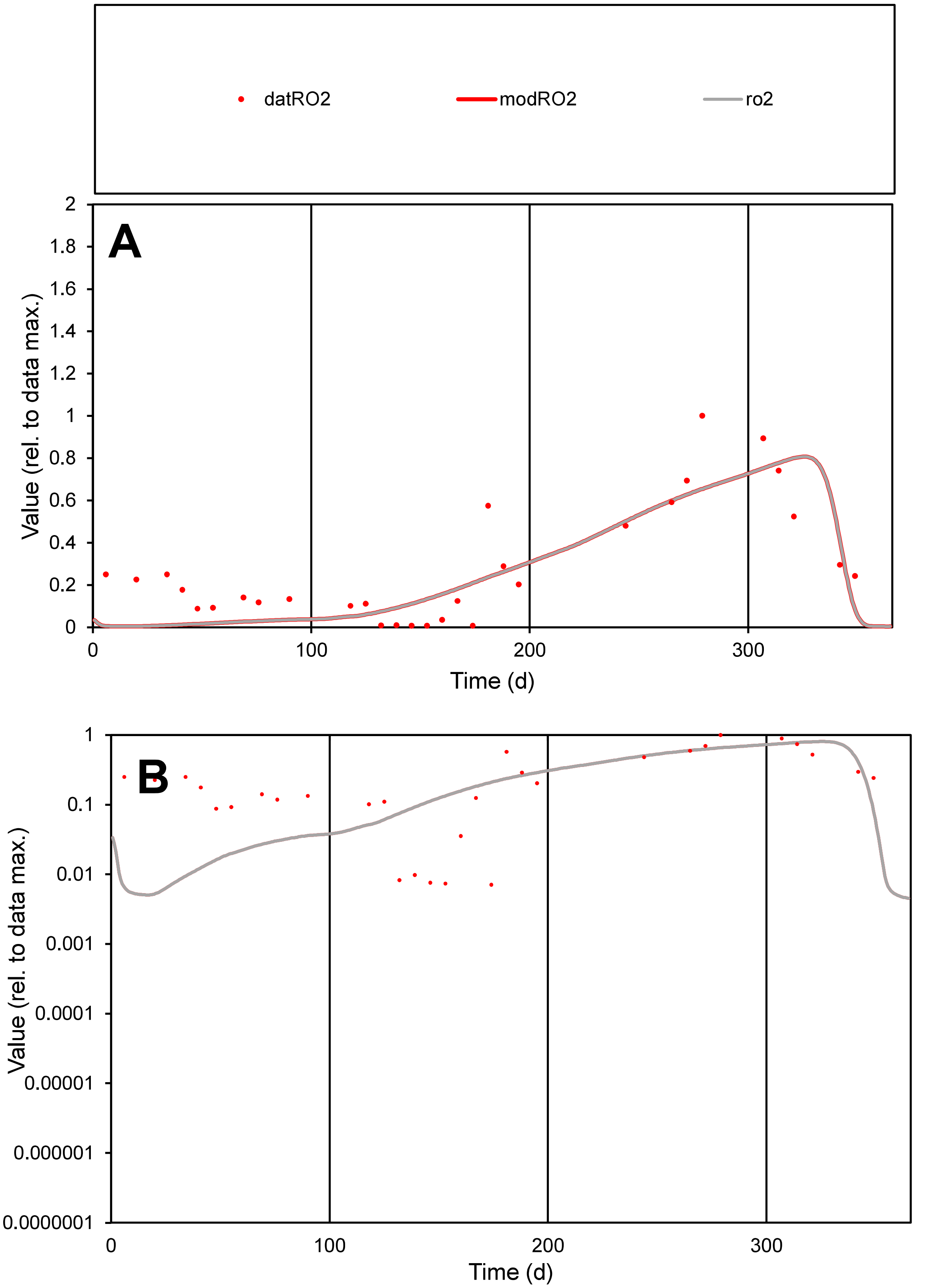

C1

Variables
RO2 Provide jtd or Dst
CalibOnly?
2 1 to reproduce of
oNormalize?
2 1=no, 2=yes

ofxn calc*
0.54267
ofxn mod
0.56856

sRun
RunA1
zout
1

Ave
Data
0.00153
Model
0.00132

data/vMax
0.00547

ofxng
275.646

Min d/m
1.31451

ave/min d
39.4904
ave/min m
44.7074

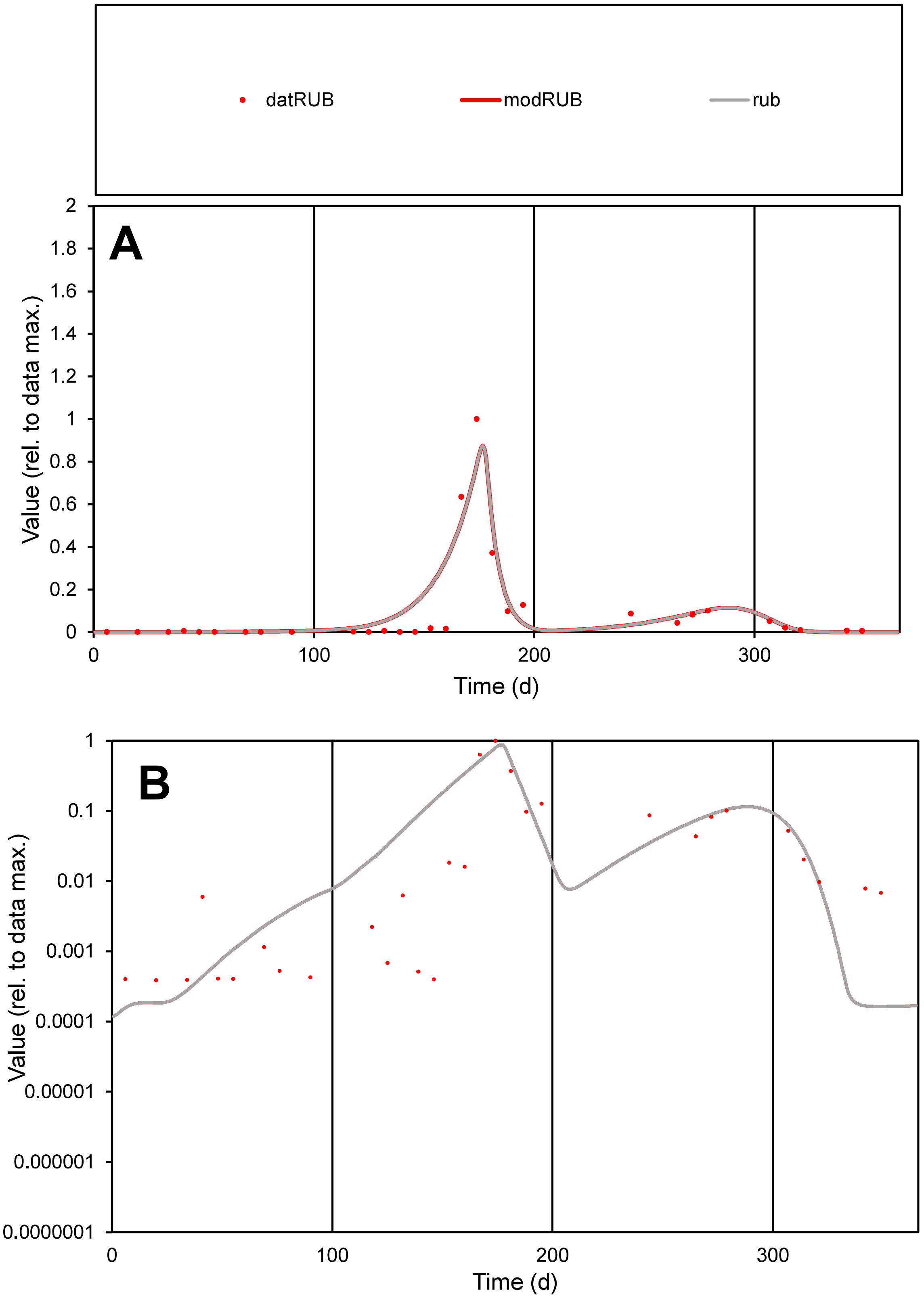

C1

Variables
RUB Provide jtd or Dst
CalibOnly?
2 1 to reproduce of
oNormalize?
2 1=no, 2=yes

ofxn calc*
0.902
ofxn mod
0.9714

sRun
RunA1
zout
1

Ave
Data
0.00243
Model
0.00288

data/vMax
0.02699

ofxng
275.646

Min d/m
2.37457

ave/min d
231.837
ave/min m
654.842

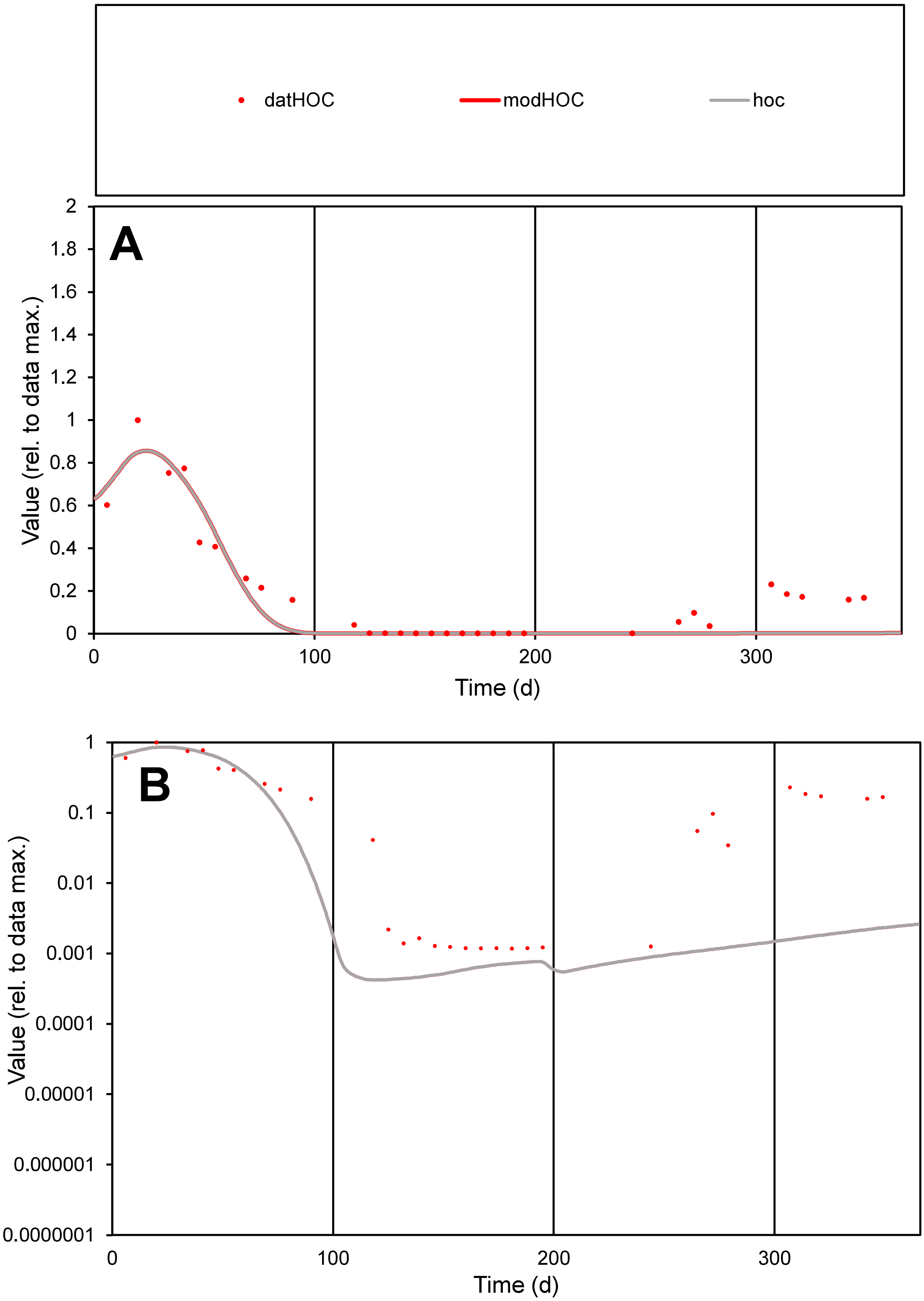

C1

Variables
HOC Provide jtd or Dst
CalibOnly?
2 1 to reproduce of
oNormalize?
2 1=no, 2=yes

ofxn calc*
0.50703
ofxn mod
0.54414

sRun
RunA1
zout
1

Ave
Data
0.00203
Model
0.00156

data/vMax
0.01061

ofxng
275.646

Min d/m
2.78495

ave/min d
162.512
ave/min m
346.239

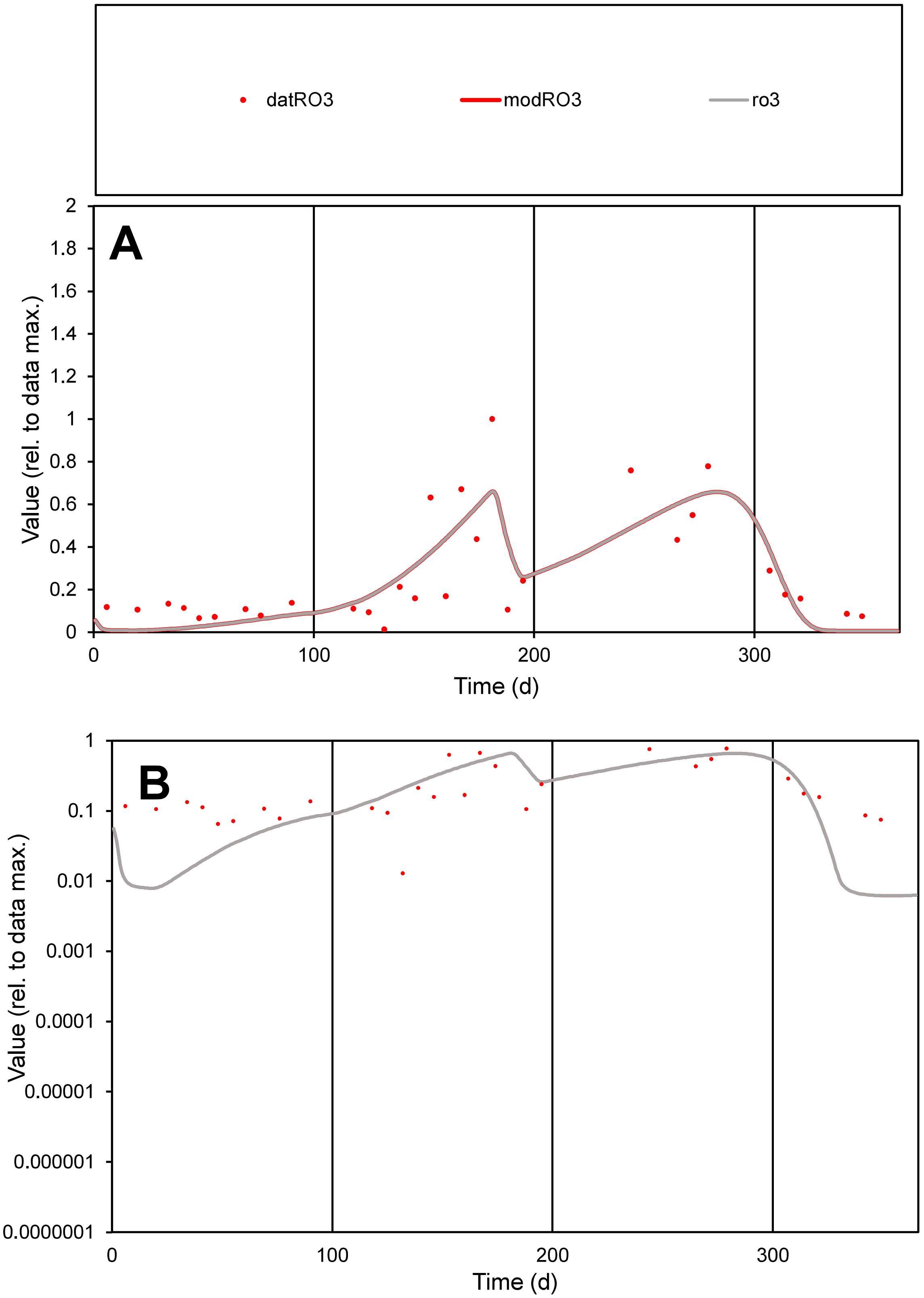

C1

Variables
RO3 Provide jtd or Dst
CalibOnly?
2 1 to reproduce of
oNormalize?
2 1=no, 2=yes

ofxn calc*
0.53955
ofxn mod
0.56746

sRun
RunA1
zout
1

Ave
Data
0.00235
Model
0.00223

data/vMax
0.00875

ofxng
275.646

Min d/m
2.07811

ave/min d
20.8684
ave/min m
40.9932

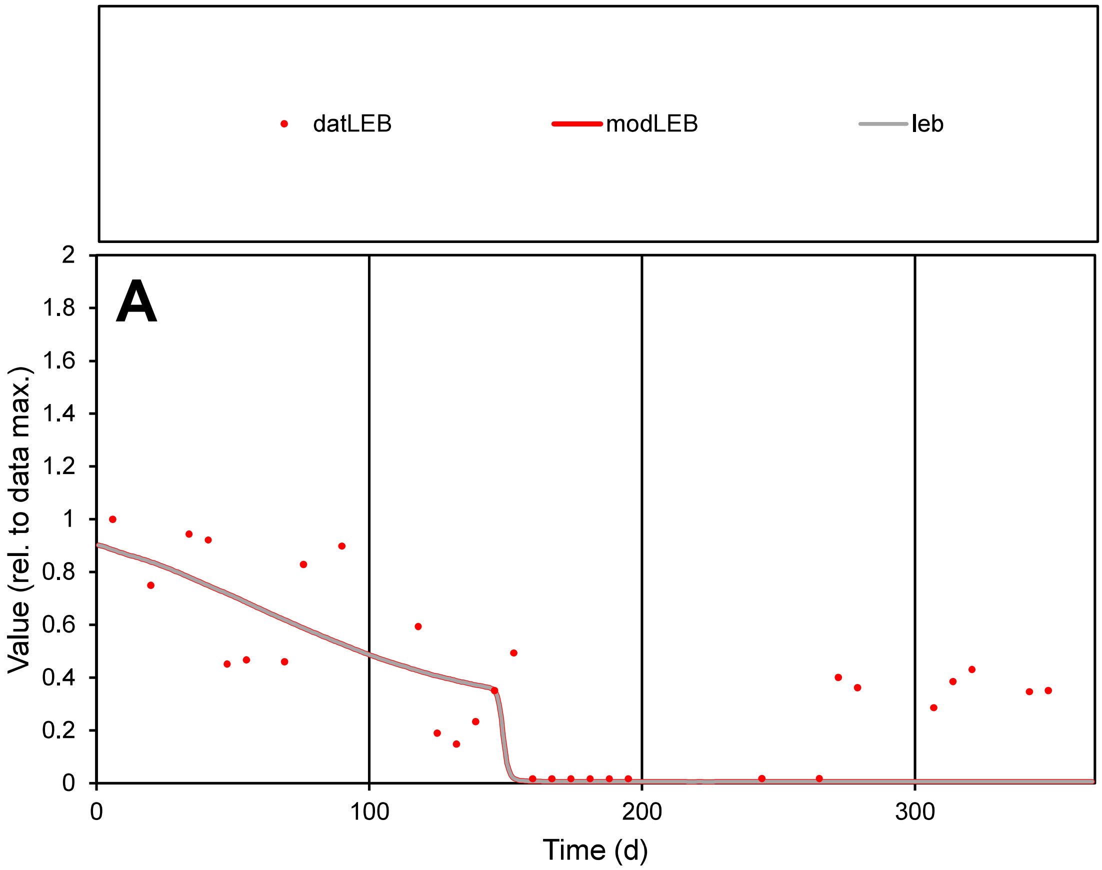

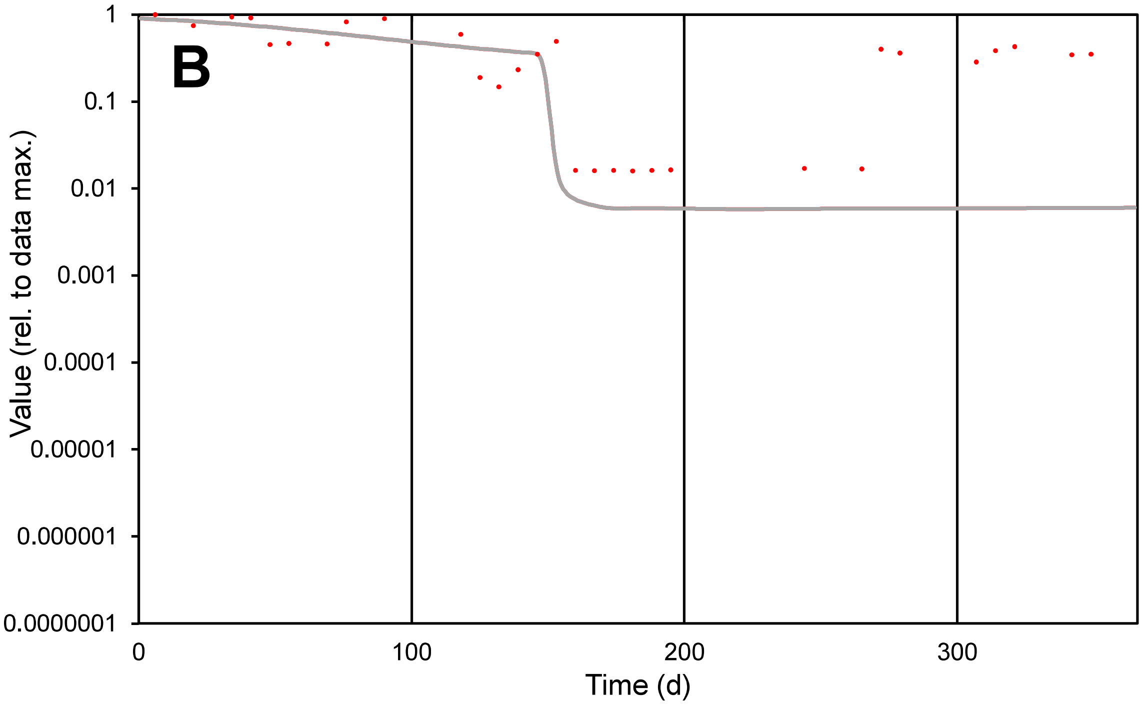

C1

Variables

LEB Provide jtd or Dst

CalibOnly?

2 1 to reproduce of

oNormalize?

2 1=no, 2=yes

ofxn calc*

0.60505

ofxn mod

0.6439

sRun

RunA1

zout

1

Ave

Data

0.00165

Model

0.00123

data/vMax

0.00435

ofxng

275.646

Min d/m

2.73218

ave/min d

23.8481

ave/min m

48.4188

325_Calib_S_A

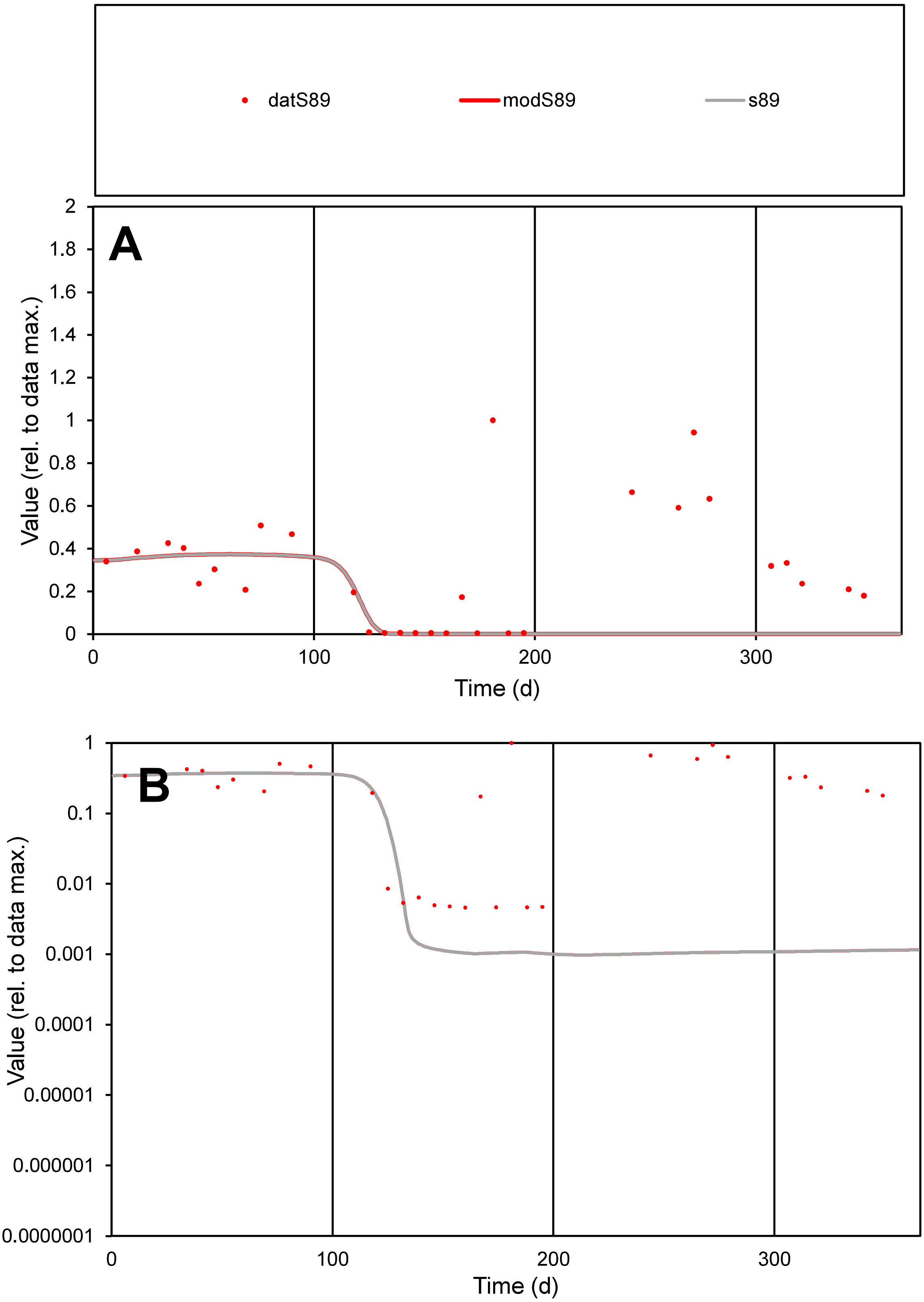

C1

Variables
S89 Provide jtd or Dst
CalibOnly?
2 1 to reproduce of
oNormalize?
2 1=no, 2=yes

ofxn calc*
1.16941
ofxn mod
1.17458

sRun
RunA1
zout
1

Ave
Data
0.00231
Model
0.00093

data/vMax
0.00786

ofxng
275.646

Min d/m
4.52316

ave/min d
63.4786
ave/min m
115.747

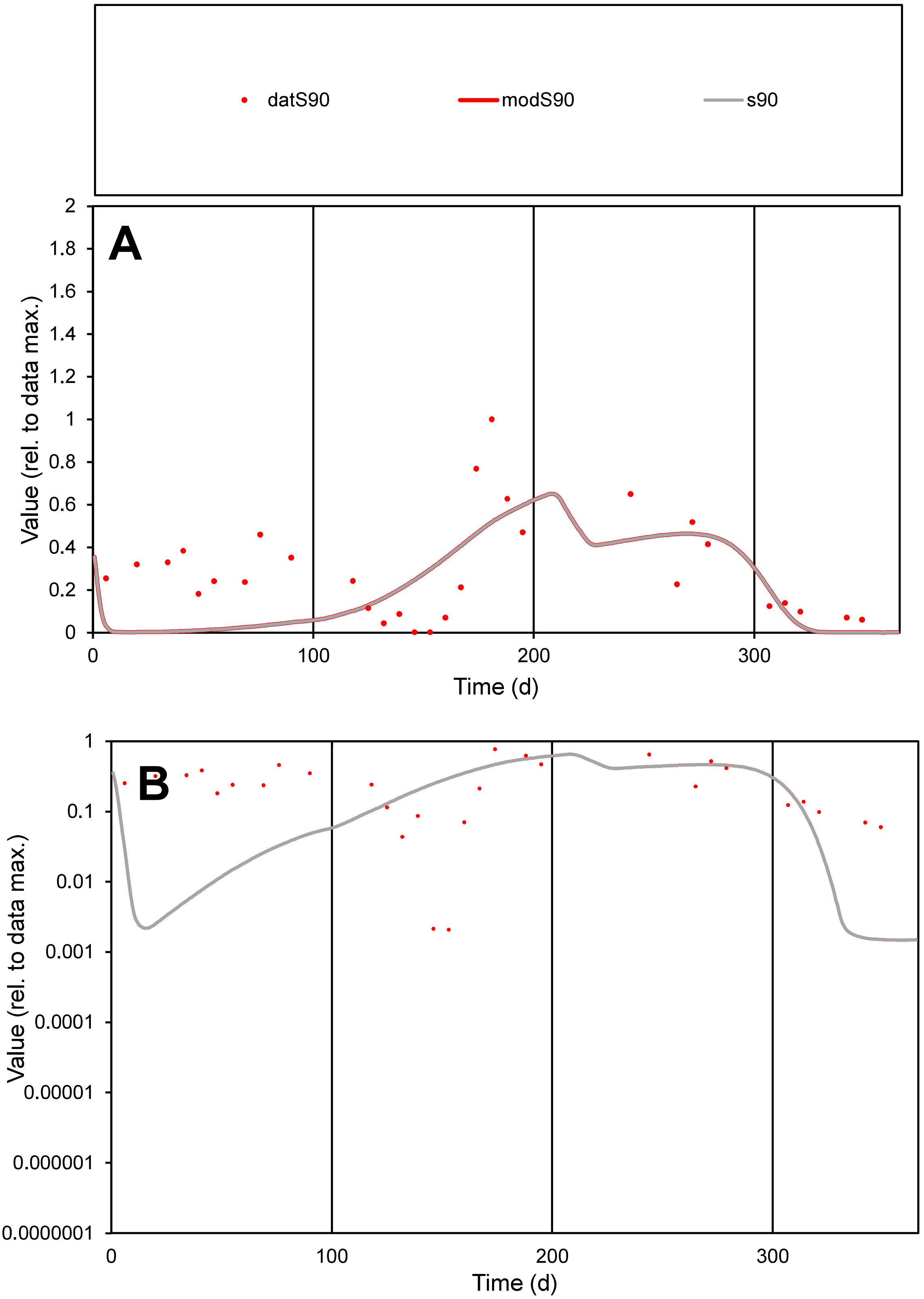

C1

Variables
S90 Provide jtd or Dst
CalibOnly?
2 1 to reproduce of
oNormalize?
2 1=no, 2=yes

ofxn calc*
0.77235
ofxn mod
0.79381

sRun
RunA1
zout
1

Ave
Data
0.00291
Model
0.00221

data/vMax
0.01002

ofxng
275.646

Min d/m
1.39093

ave/min d
139.393
ave/min m
147.479

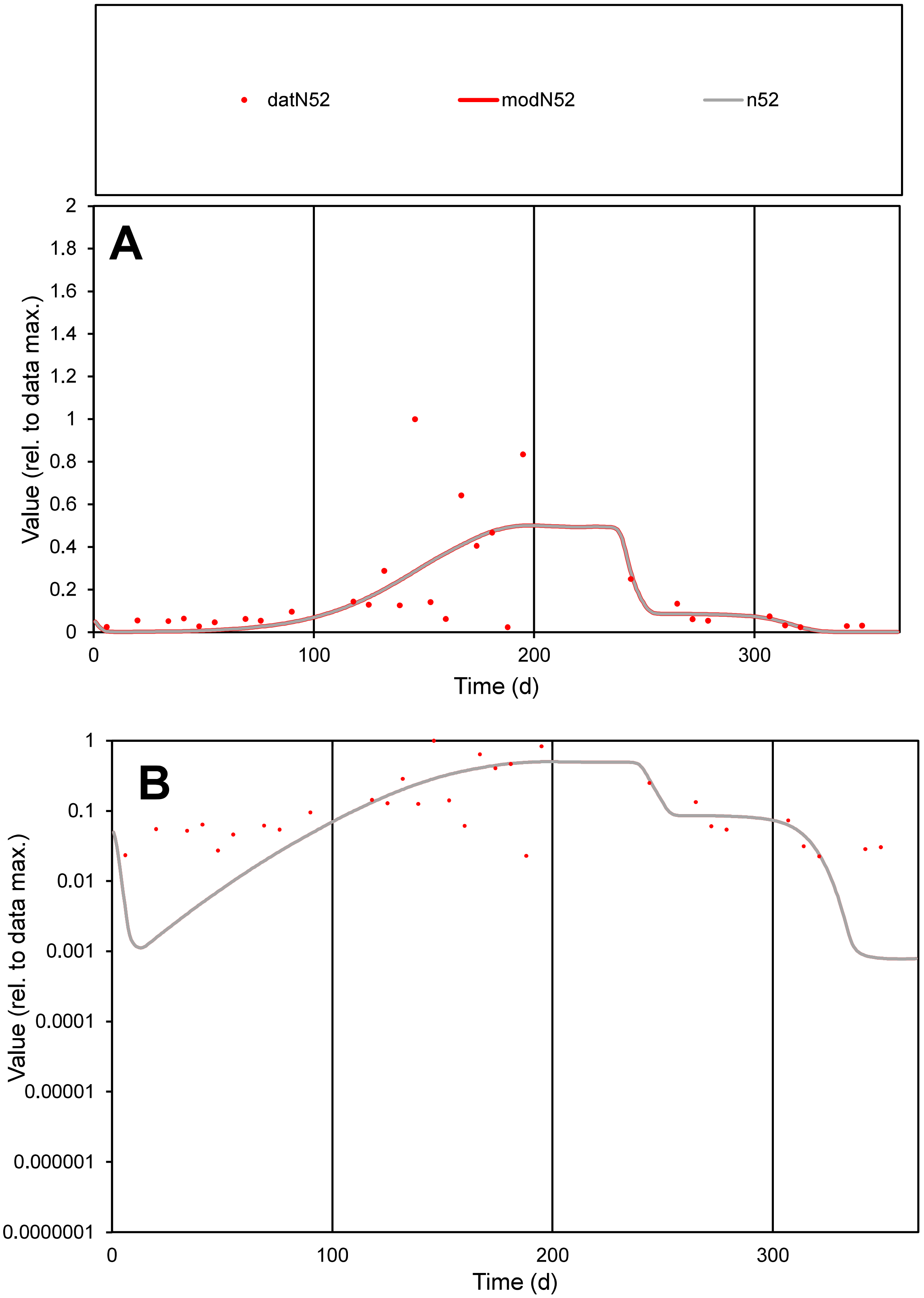

C1

Variables
N52 Provide jtd or Dst
CalibOnly?
2 1 to reproduce of
oNormalize?
2 1=no, 2=yes

ofxn calc*
1.03968
ofxn mod
1.0527

sRun
RunA1
zout
1

Ave
Data
0.00316
Model
0.00284

data/vMax
0.0175

ofxng
275.646

Min d/m
28.4043

ave/min d
8.03759
ave/min m
205.235

325_Calib_S_A

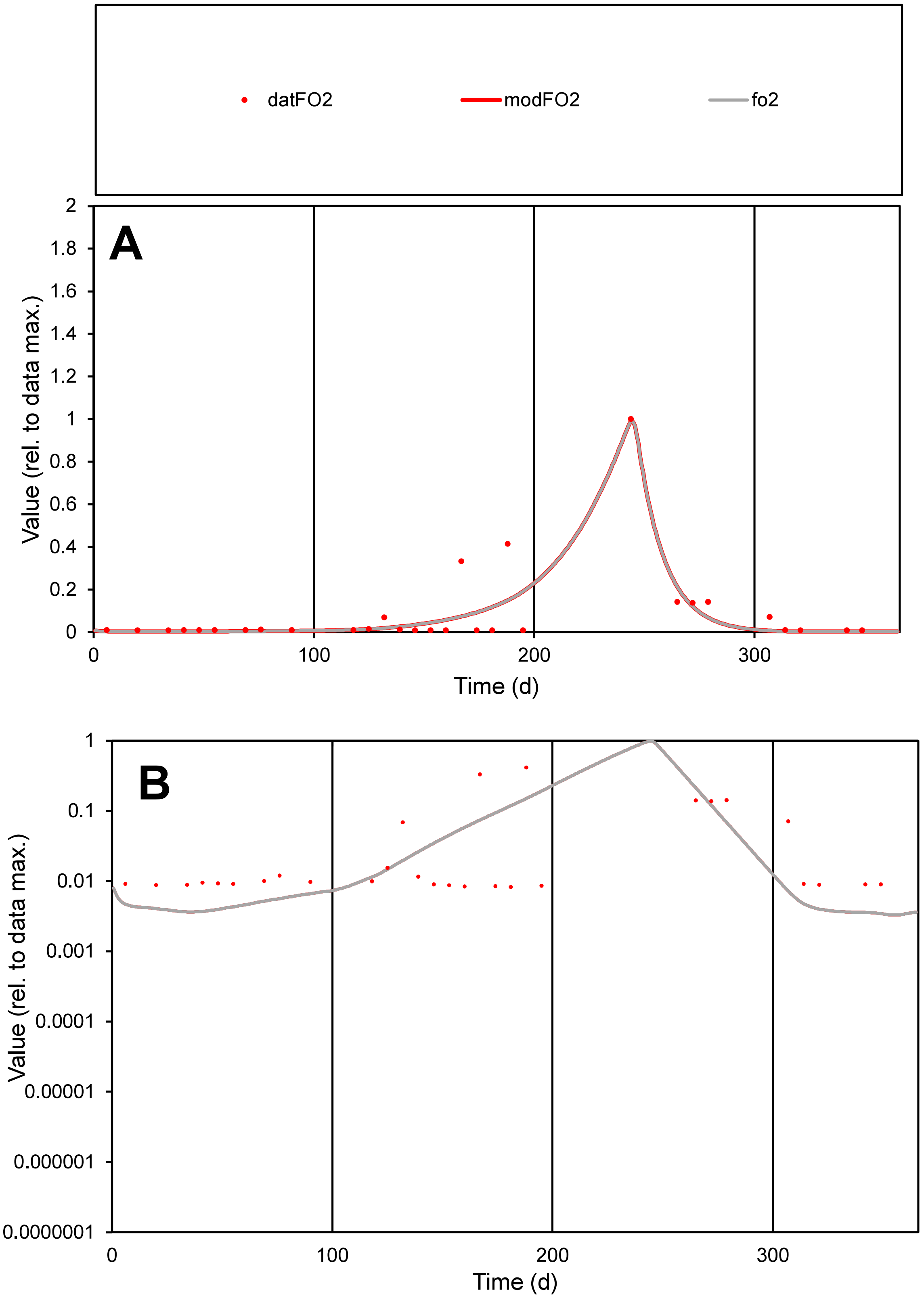

C1

Variables

FO2 Provide jtd or Dst

CalibOnly?

2 1 to reproduce of

oNormalize?

2 1=no, 2=yes

ofxn calc*

0.98187

ofxn mod

1.00876

sRun

RunA1

zout

1

Ave

Data

0.00068

Model

0.00061

data/vMax

0.00804

ofxng

275.646

Min d/m

2.45007

ave/min d

10.1226

ave/min m

22.3047

325_Calib_S_A

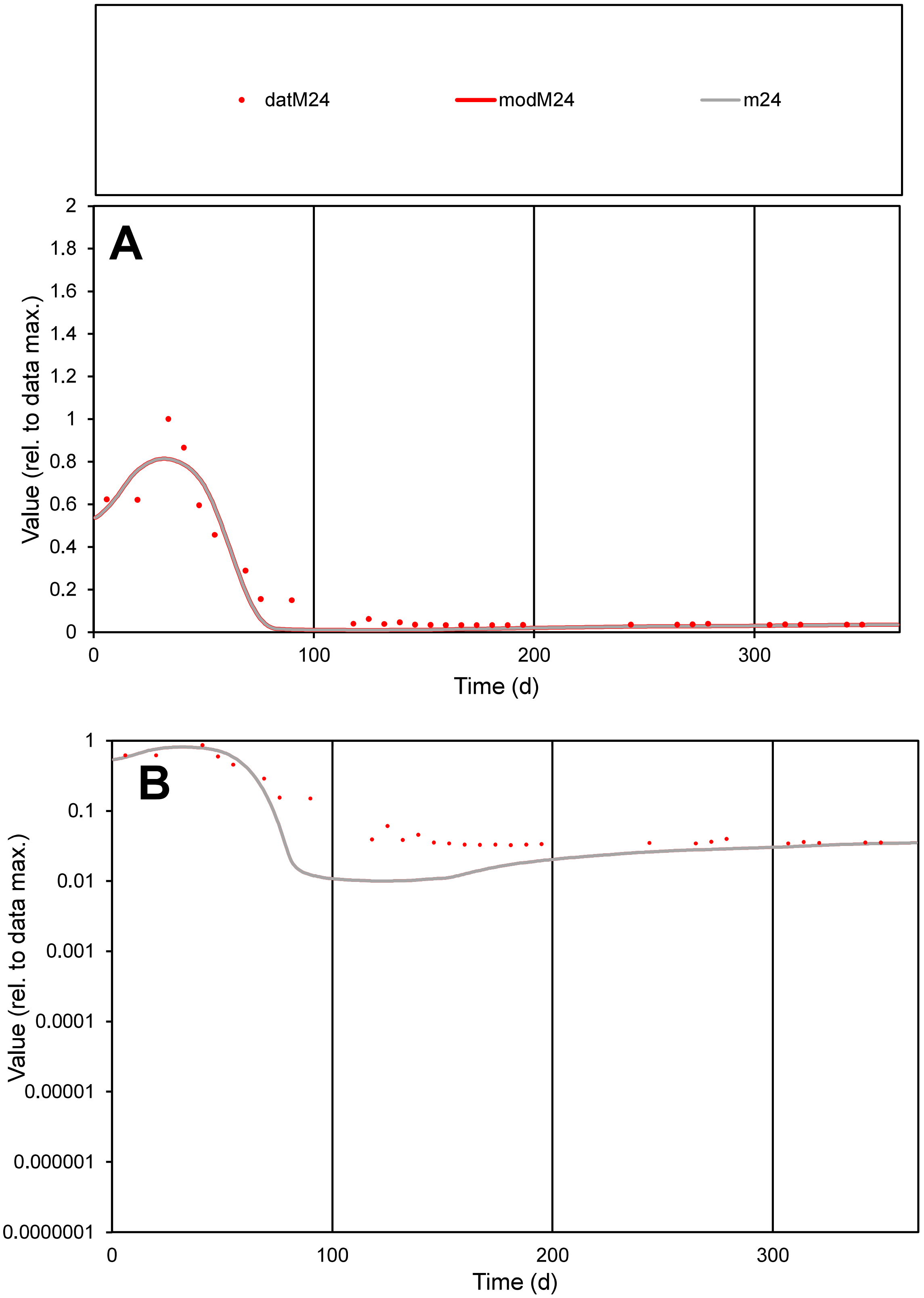

C1

Variables
M24 Provide jtd or Dst
CalibOnly?
2 1 to reproduce of
oNormalize?
2 1=no, 2=yes

ofxn calc*
0.35983
ofxn mod
0.42383

sRun
RunA1
zout
1

Ave
Data
0.00041
Model
0.00036

data/vMax
0.0022

ofxng
275.646

Min d/m
3.24695

ave/min d
5.64328
ave/min m
16.2067

325_Calib_S_A

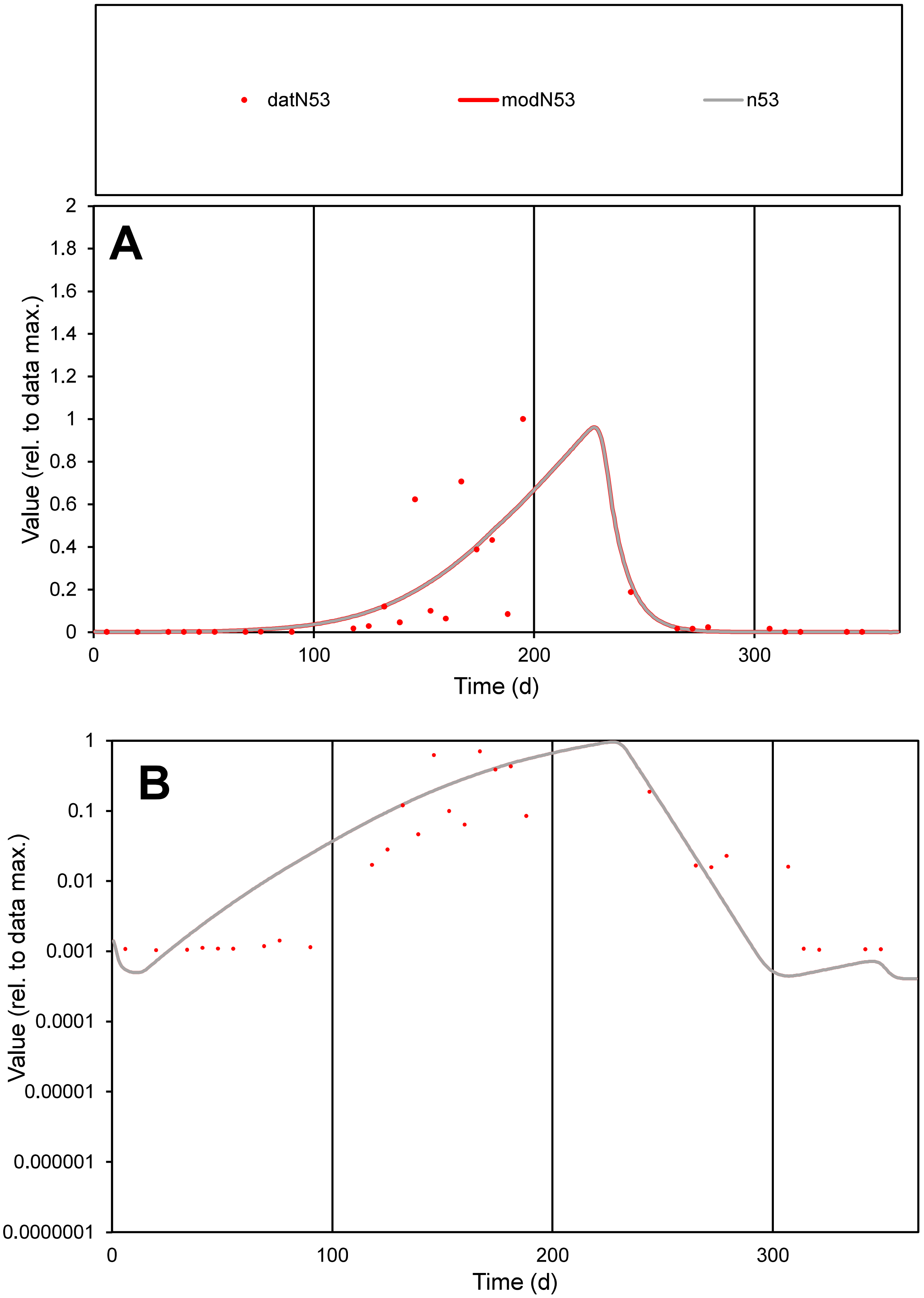

C1

Variables
N53 Provide jtd or Dst
CalibOnly?
2 1 to reproduce of
oNormalize?
2 1=no, 2=yes

ofxn calc*
1.22056
ofxn mod
1.24142

sRun
RunA1
zout
1

Ave
Data
0.00477
Model
0.00482

data/vMax
0.03684

ofxng
275.646

Min d/m
2.33536

ave/min d
124.344
ave/min m
293.502

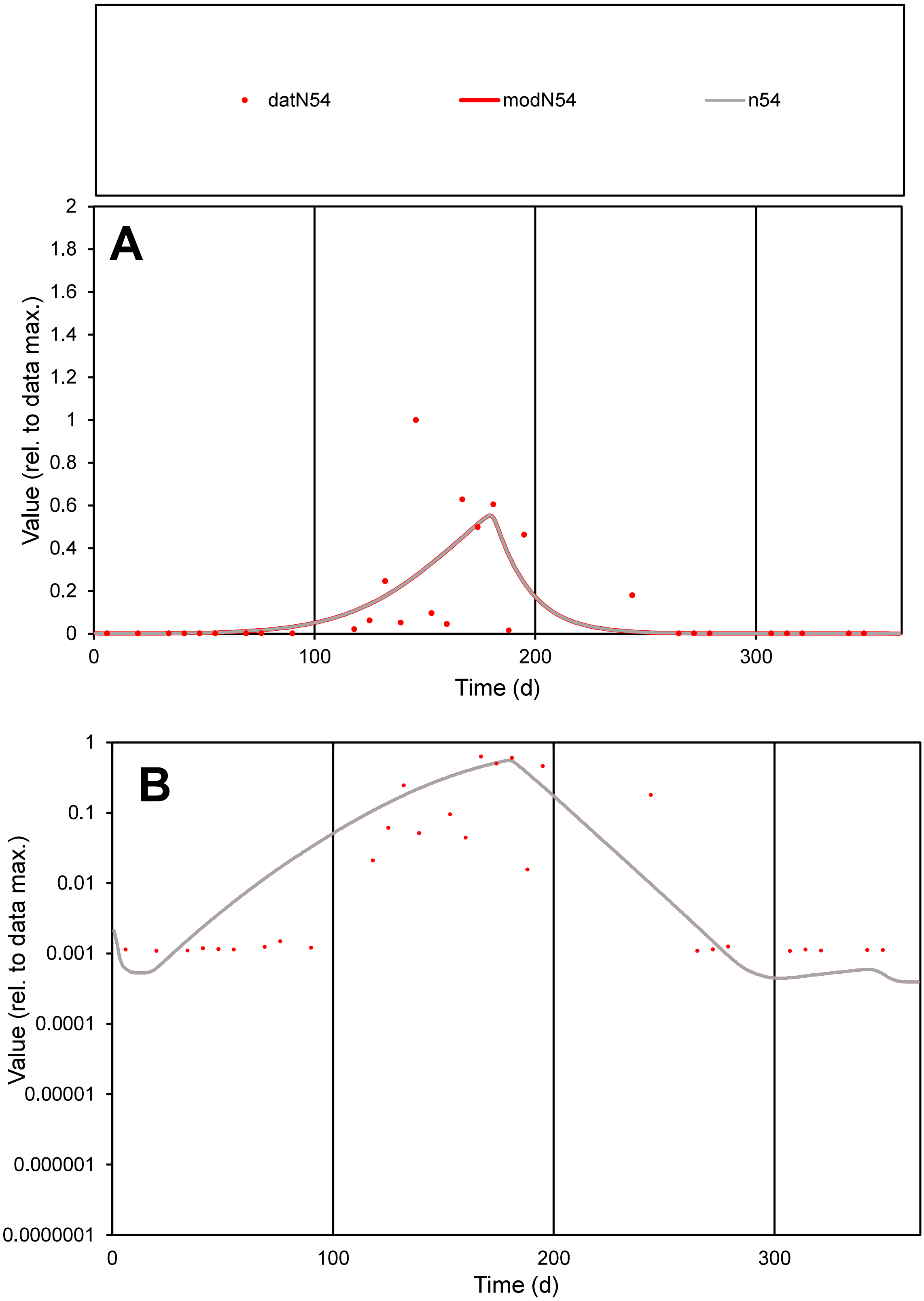

C1

Variables
N54 Provide jtd or Dst
CalibOnly?
2 1 to reproduce of
oNormalize?
2 1=no, 2=yes

ofxn calc*
1.35559
ofxn mod
1.37748

sRun
RunA1
zout
1

Ave
Data
0.00272
Model
0.00262

data/vMax
0.02076

ofxng
275.646

Min d/m
2.38578

ave/min d
120.291
ave/min m
276.966

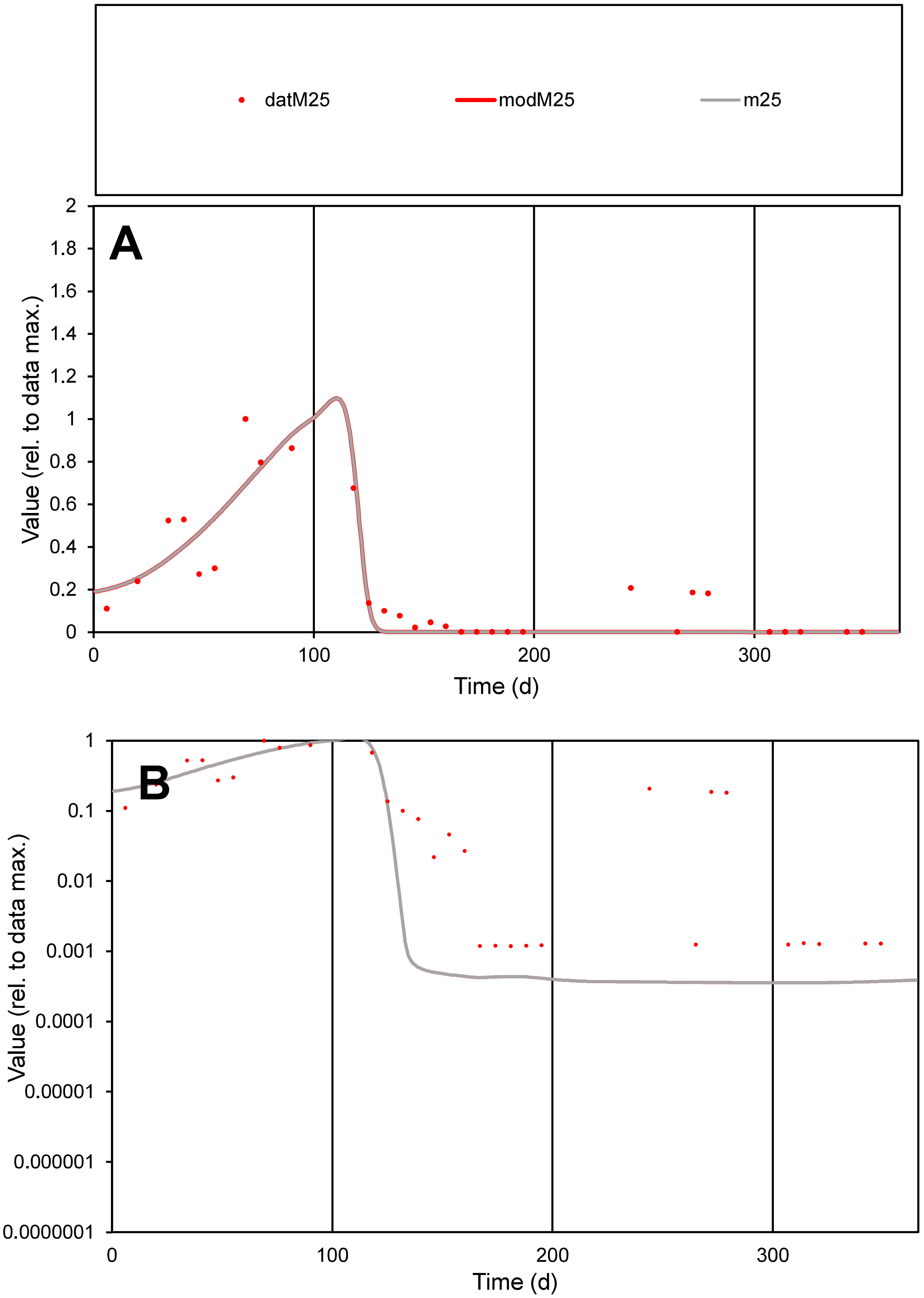

C1

Variables
M25 Provide jtd or Dst
CalibOnly?
2 1 to reproduce of
oNormalize?
2 1=no, 2=yes

ofxn calc*
0.52569
ofxn mod
0.56106

sRun
RunA1
zout
1

Ave
Data
0.00169
Model
0.00147

data/vMax
0.00805

ofxng
275.646

Min d/m
3.33147

ave/min d
177.215
ave/min m
513.902

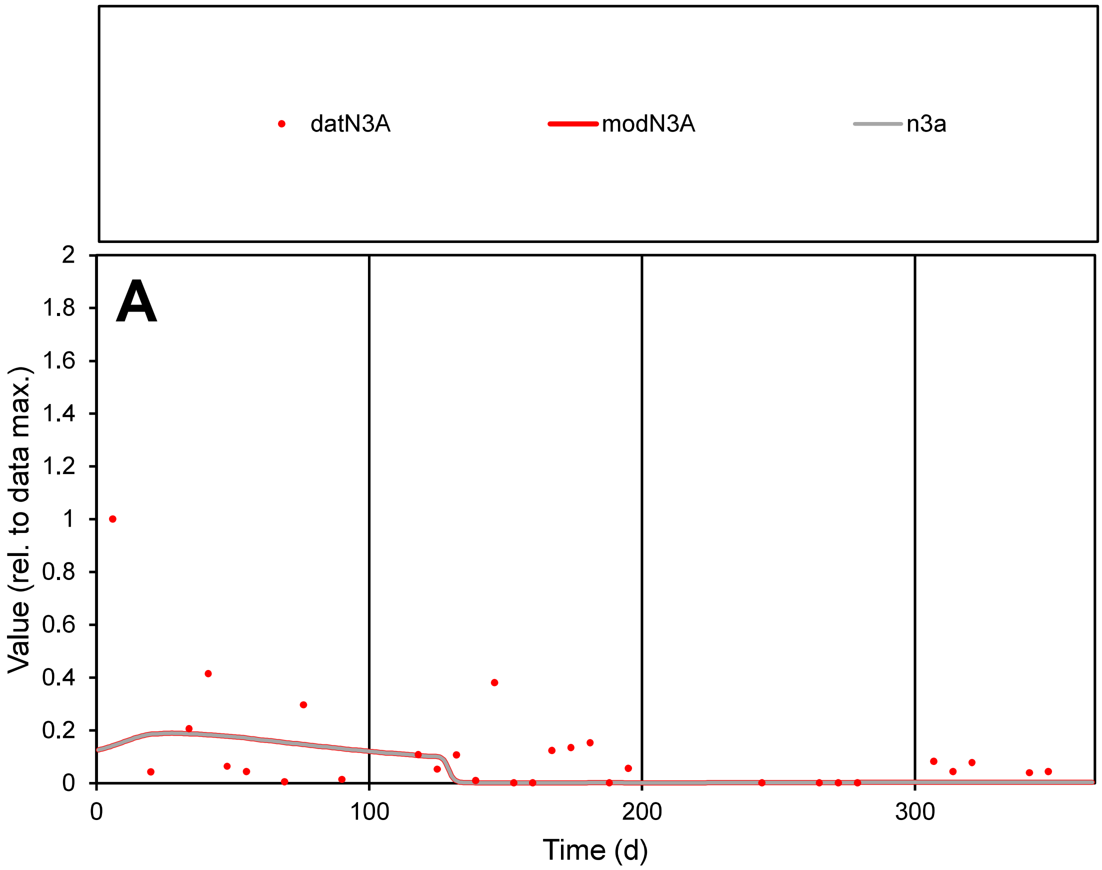

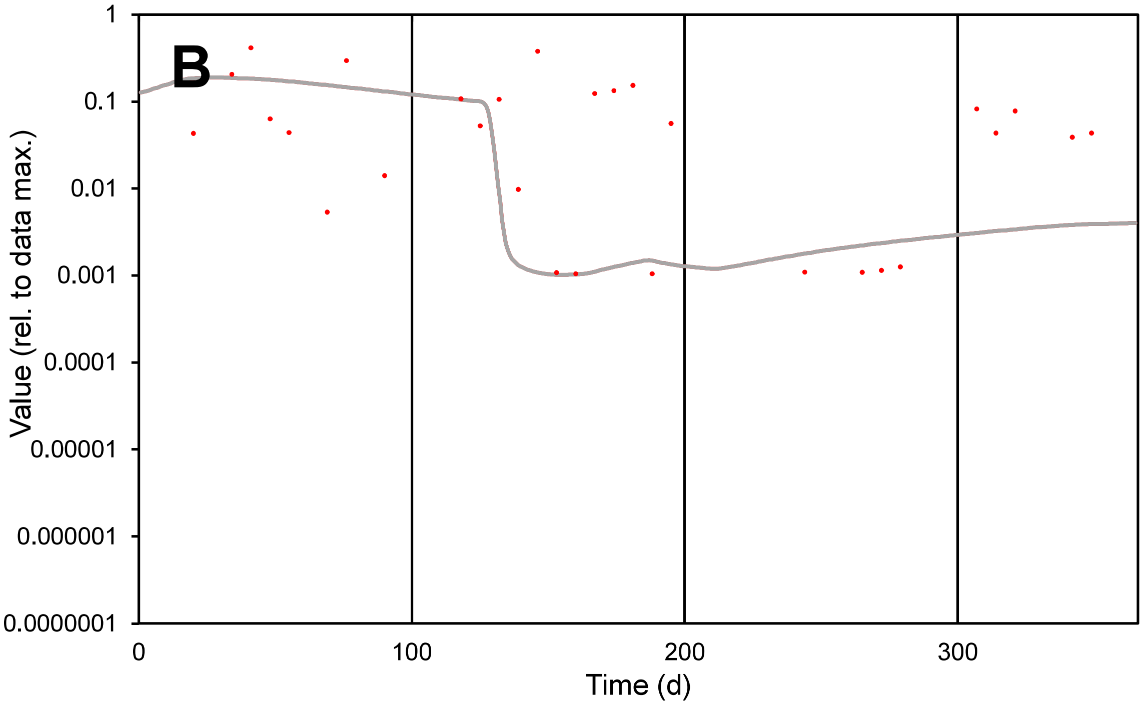

C1

Variables
N3A Provide jtd or Dst
CalibOnly?
2 1 to reproduce of
oNormalize?
2 1=no, 2=yes

ofxn calc*
1.64671
ofxn mod
1.65715

sRun
RunA1
zout
1

Ave
Data
0.00202
Model
0.00103

data/vMax
0.01727

ofxng
275.646

Min d/m
1.02269
ave/min d
111.948
ave/min m
58.4537

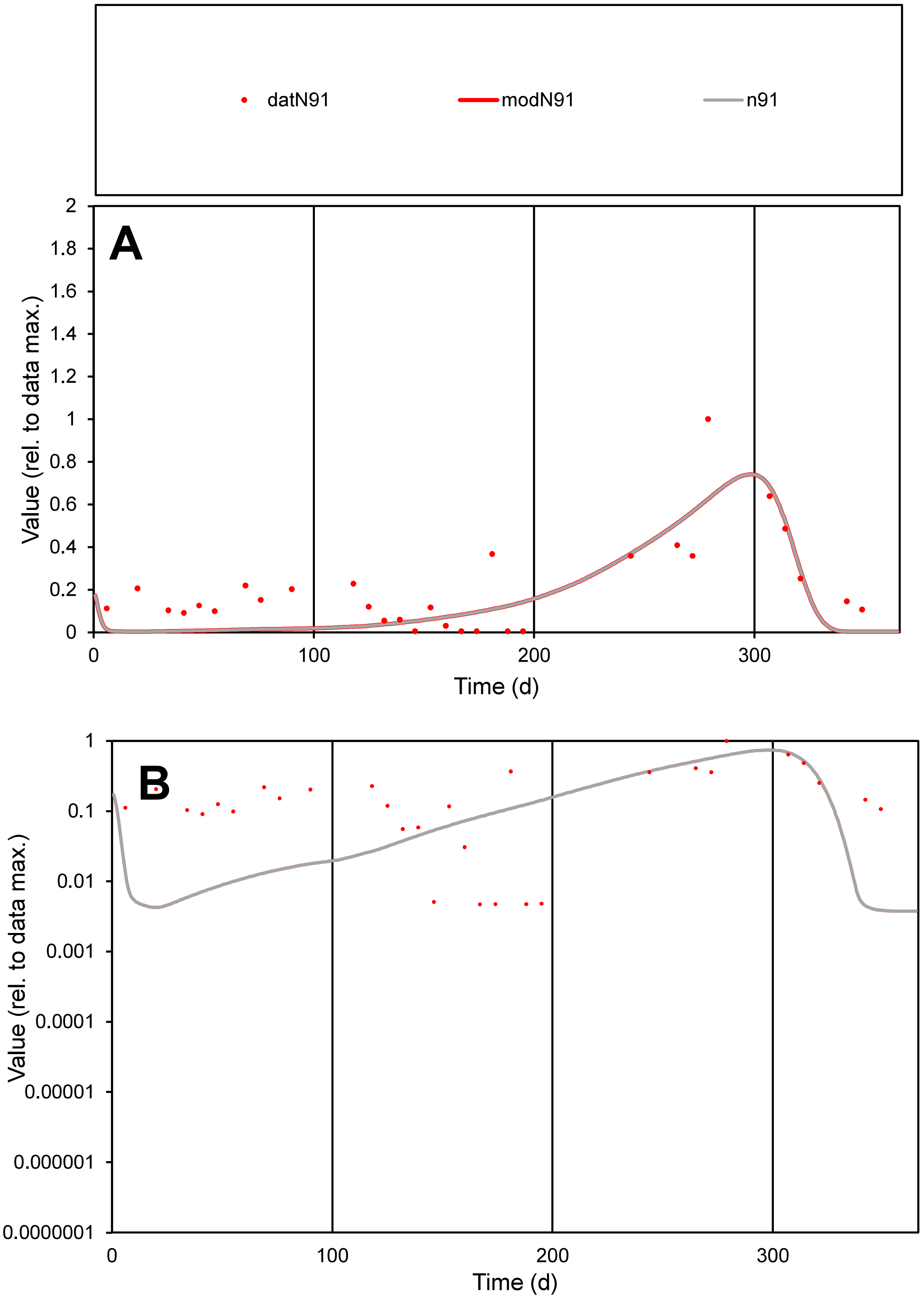

C1

Variables
N91 Provide jtd or Dst
CalibOnly?
2 1 to reproduce of
oNormalize?
2 1=no, 2=yes

ofxn calc*
0.67706
ofxn mod
0.70353

sRun
RunA1
zout
1

Ave
Data
0.00136
Model
0.00103

data/vMax
0.0067

ofxng
275.646

Min d/m
1.23083

ave/min d
42.8626
ave/min m
39.9224

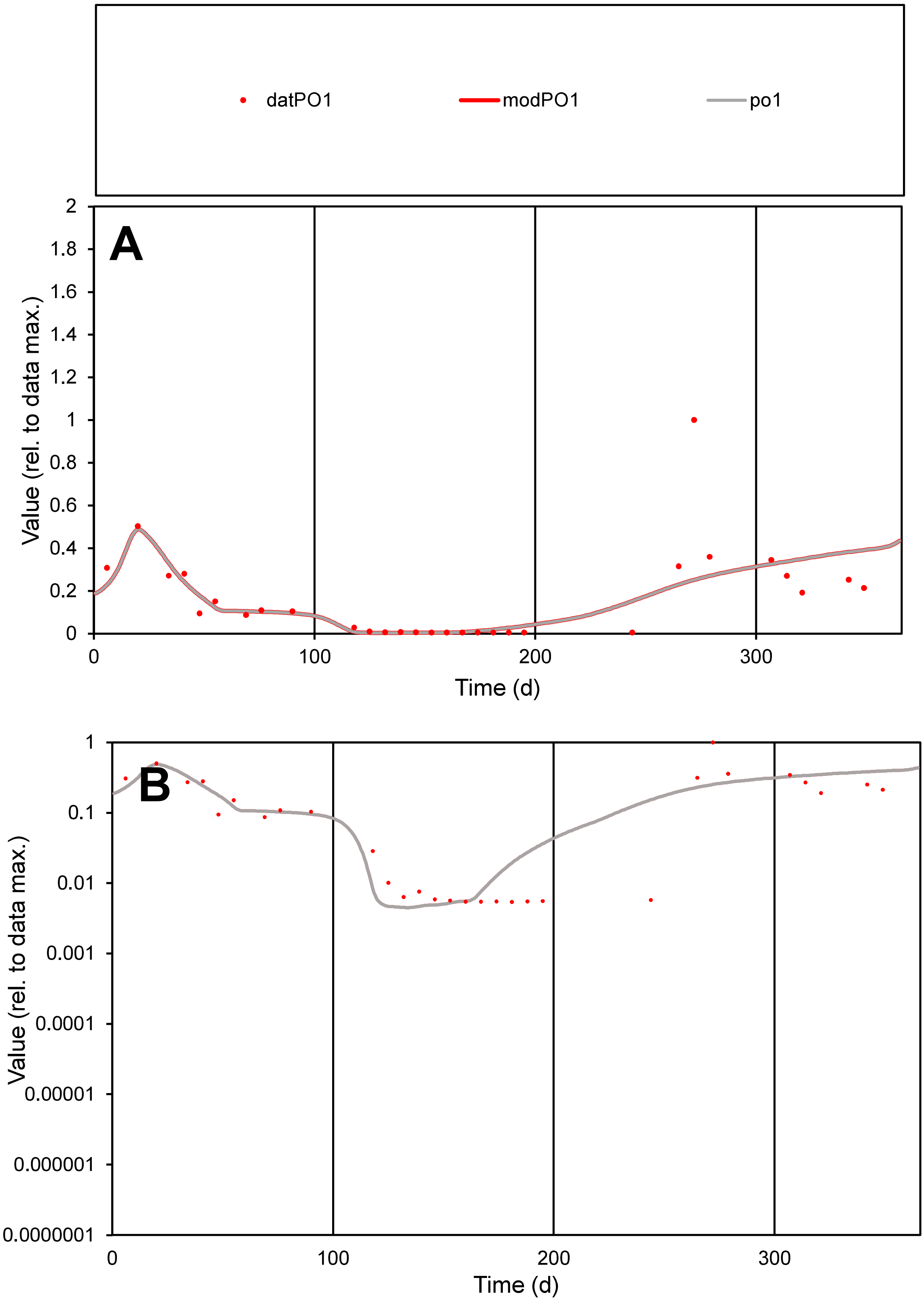

C1

Variables

PO1 Provide jtd or Dst

CalibOnly?

2 1 to reproduce of

oNormalize?

2 1=no, 2=yes

ofxn calc*

0.91541

ofxn mod

0.93162

sRun

RunA1

zout

1

Ave

Data

0.00139

Model

0.00132

data/vMax

0.00839

ofxng

275.646

Min d/m

1.20276

ave/min d

30.6011

ave/min m

35.0175

325_Calib_S_A

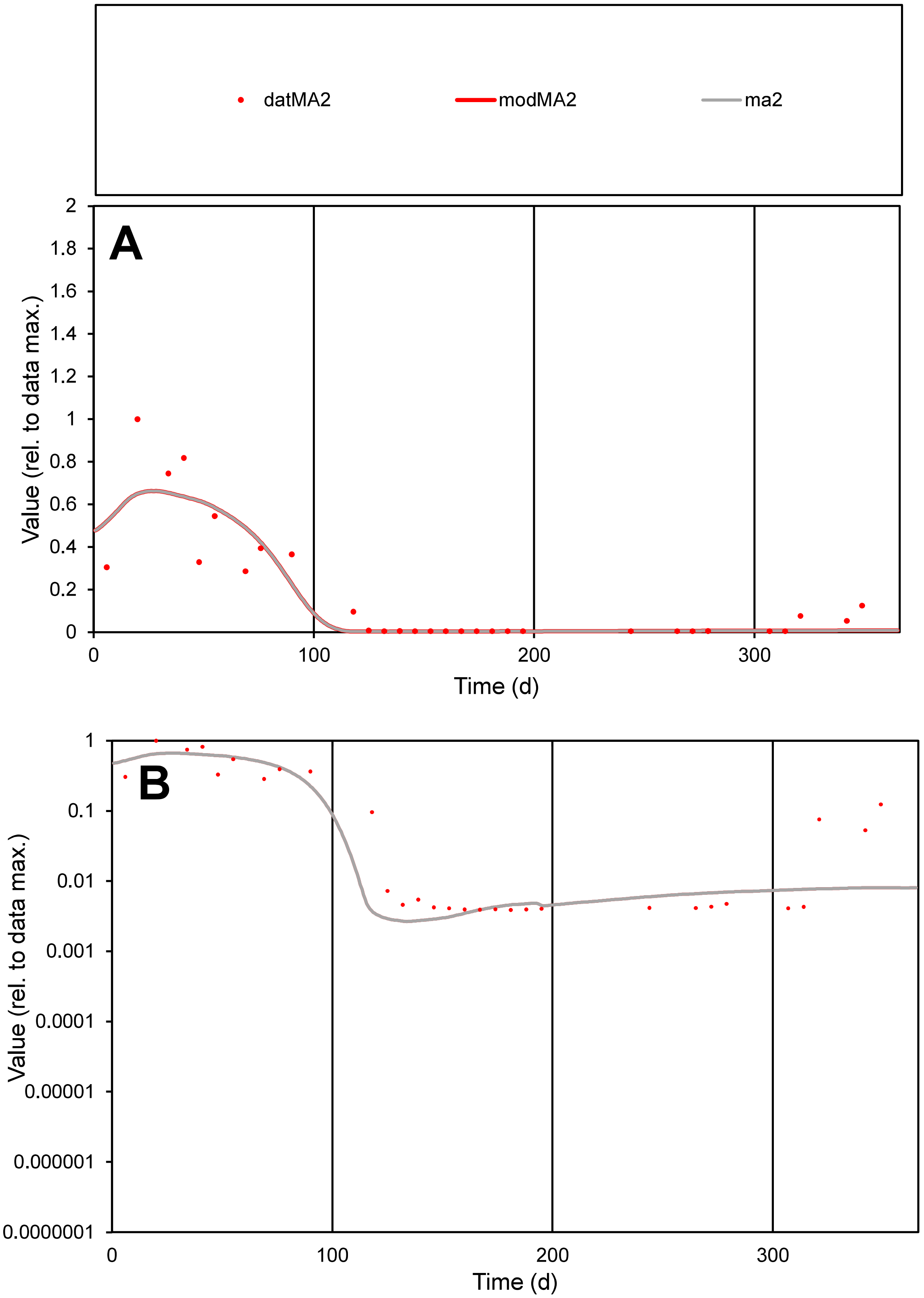

C1

Variables

MA2 Provide jtd or Dst

CalibOnly?

2 1 to reproduce of

oNormalize?

2 1=no, 2=yes

ofxn calc*

0.64926

ofxn mod

0.68421

sRun

RunA1

zout

1

Ave

Data

0.00079

Model

0.00074

data/vMax

0.00455

ofxng

275.646

Min d/m

1.46287

ave/min d

44.5386

ave/min m

60.9769

325_Calib_S_A

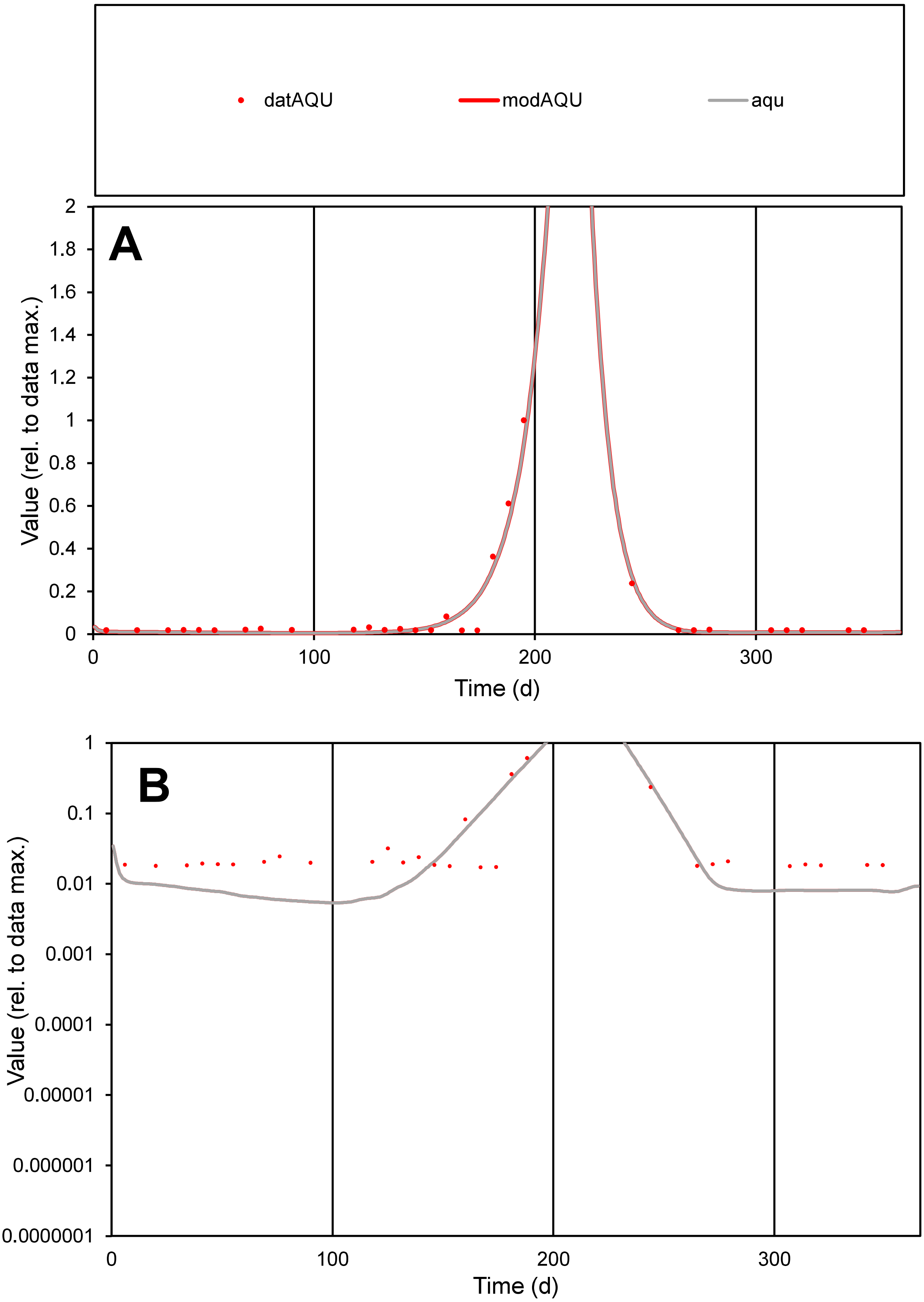

C1

Variables
AQU Provide jtd or Dst
CalibOnly?
2 1 to reproduce of
oNormalize?
2 1=no, 2=yes

ofxn calc*
0.35959
ofxn mod
0.46665

sRun
RunA1
zout
1

Ave
Data
0.0007
Model
0.00068

data/vMax
0.00758

ofxng
275.646

Min d/m
3.11291

ave/min d
5.40427
ave/min m
16.333

325_Calib_S_A

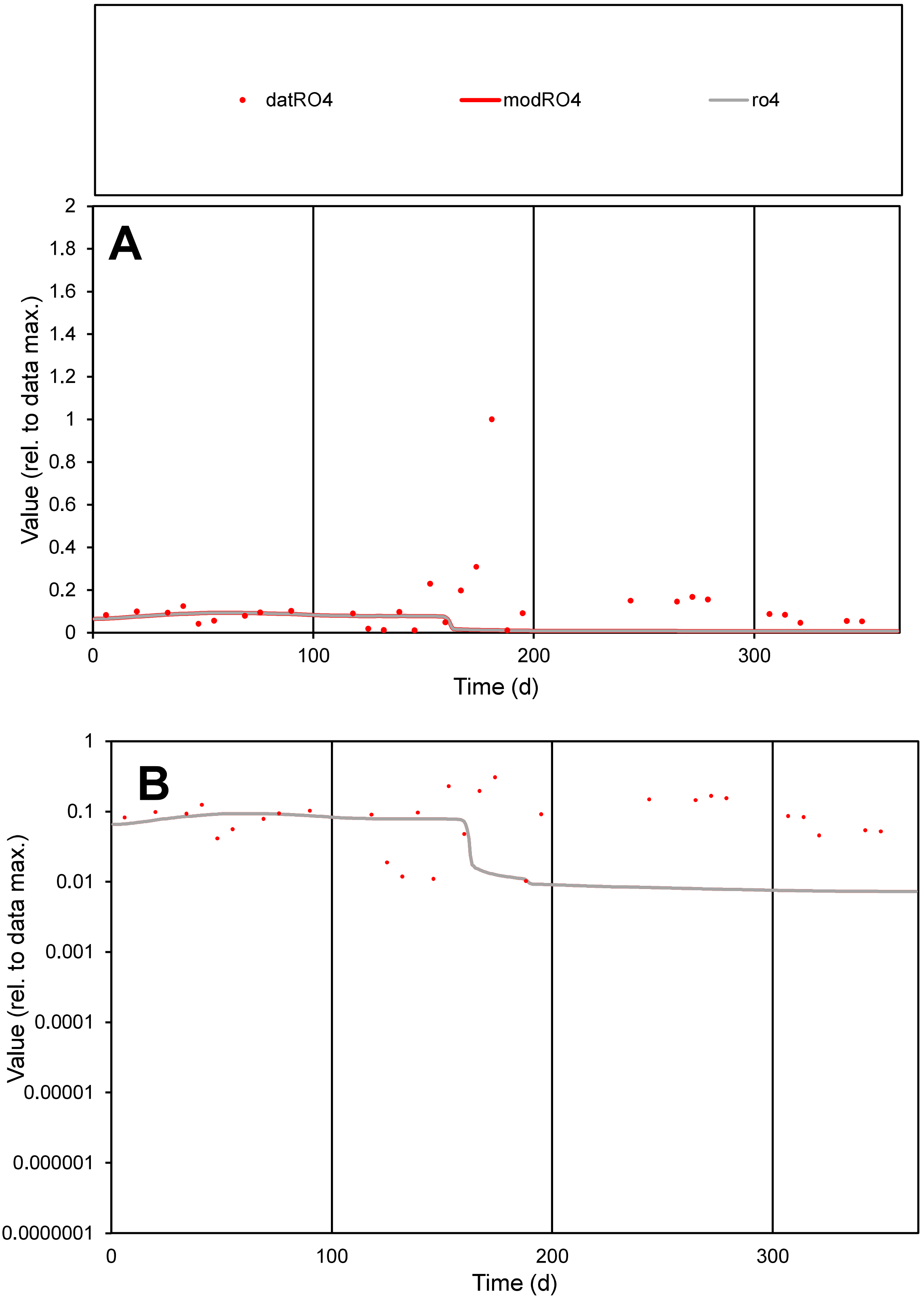

C1

Variables
RO4 Provide jtd or Dst
CalibOnly?
2 1 to reproduce of
oNormalize?
2 1=no, 2=yes

ofxn calc*
1.60127
ofxn mod
1.60506

sRun
RunA1
zout
1

Ave
Data
0.00273
Model
0.00102

data/vMax
0.02142

ofxng
275.646

Min d/m
1.4

ave/min d
12.4694
ave/min m
6.51042

325_Calib_S_A

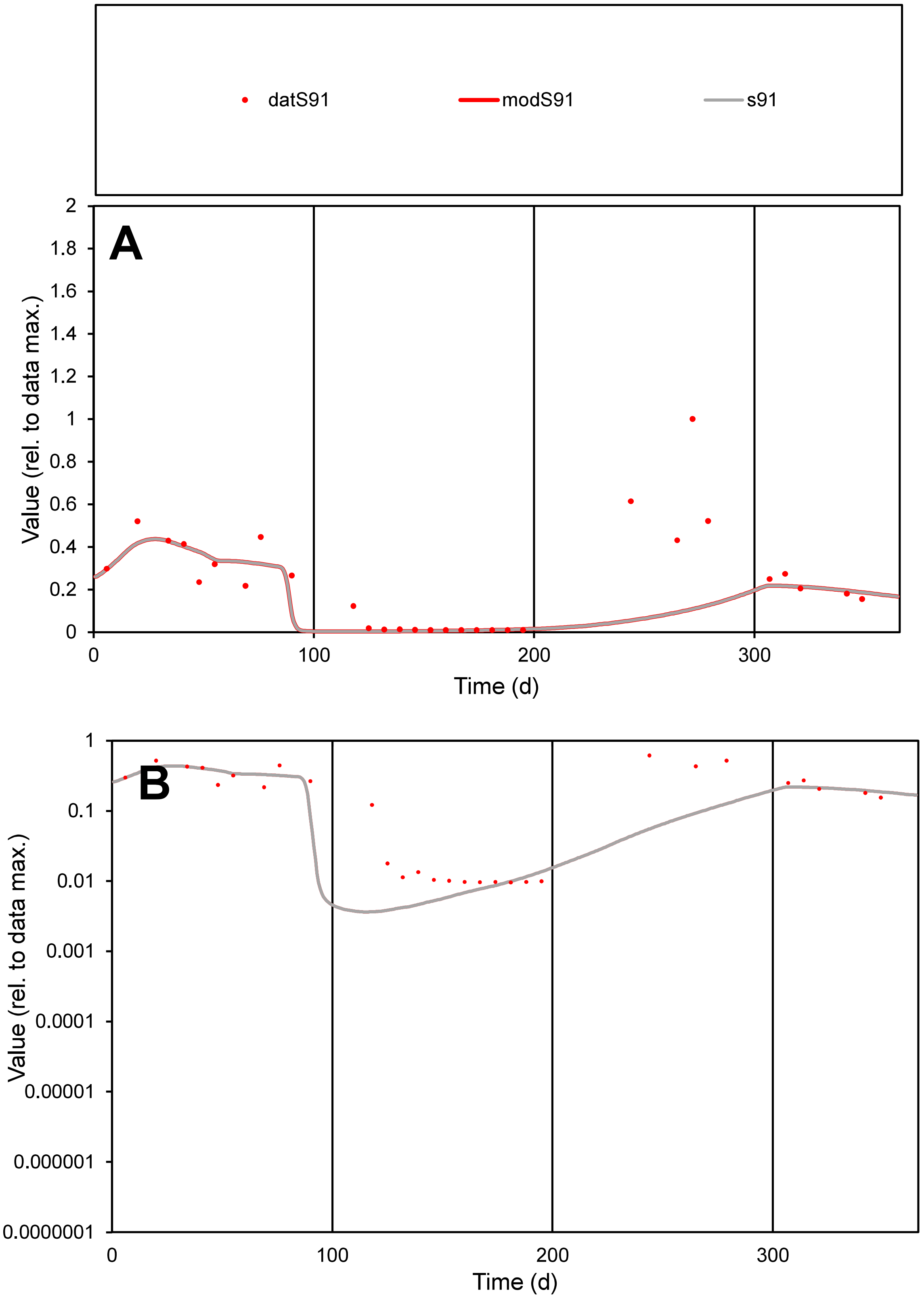

C1

Variables
S91 Provide jtd or Dst
CalibOnly?
2 1 to reproduce of
oNormalize?
2 1=no, 2=yes

ofxn calc*
0.93734
ofxn mod
0.95334

sRun
RunA1
zout
1

Ave
Data
0.00159
Model
0.00105

data/vMax
0.00678

ofxng
275.646

Min d/m
2.61236

ave/min d
24.3609
ave/min m
42.0682

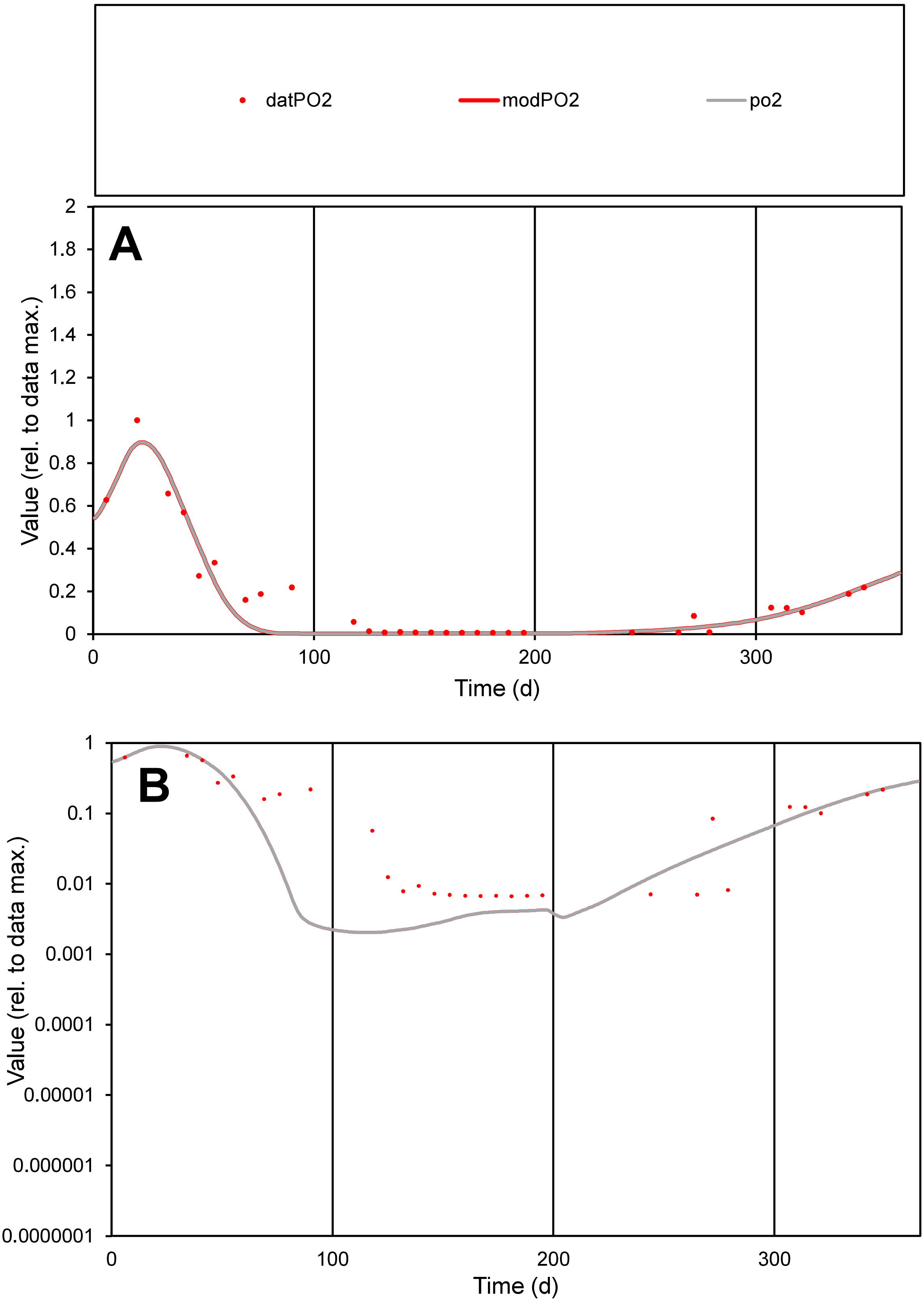

C1

Variables
PO2 Provide jtd or Dst
CalibOnly?
2 1 to reproduce of
oNormalize?
2 1=no, 2=yes

ofxn calc*
0.39505
ofxn mod
0.44042

sRun
RunA1
zout
1

Ave
Data
0.00199
Model
0.00173

data/vMax
0.01186

ofxng
275.646

Min d/m
3.2566
ave/min d
25.1707
ave/min m
71.5713

325_Calib_S_A

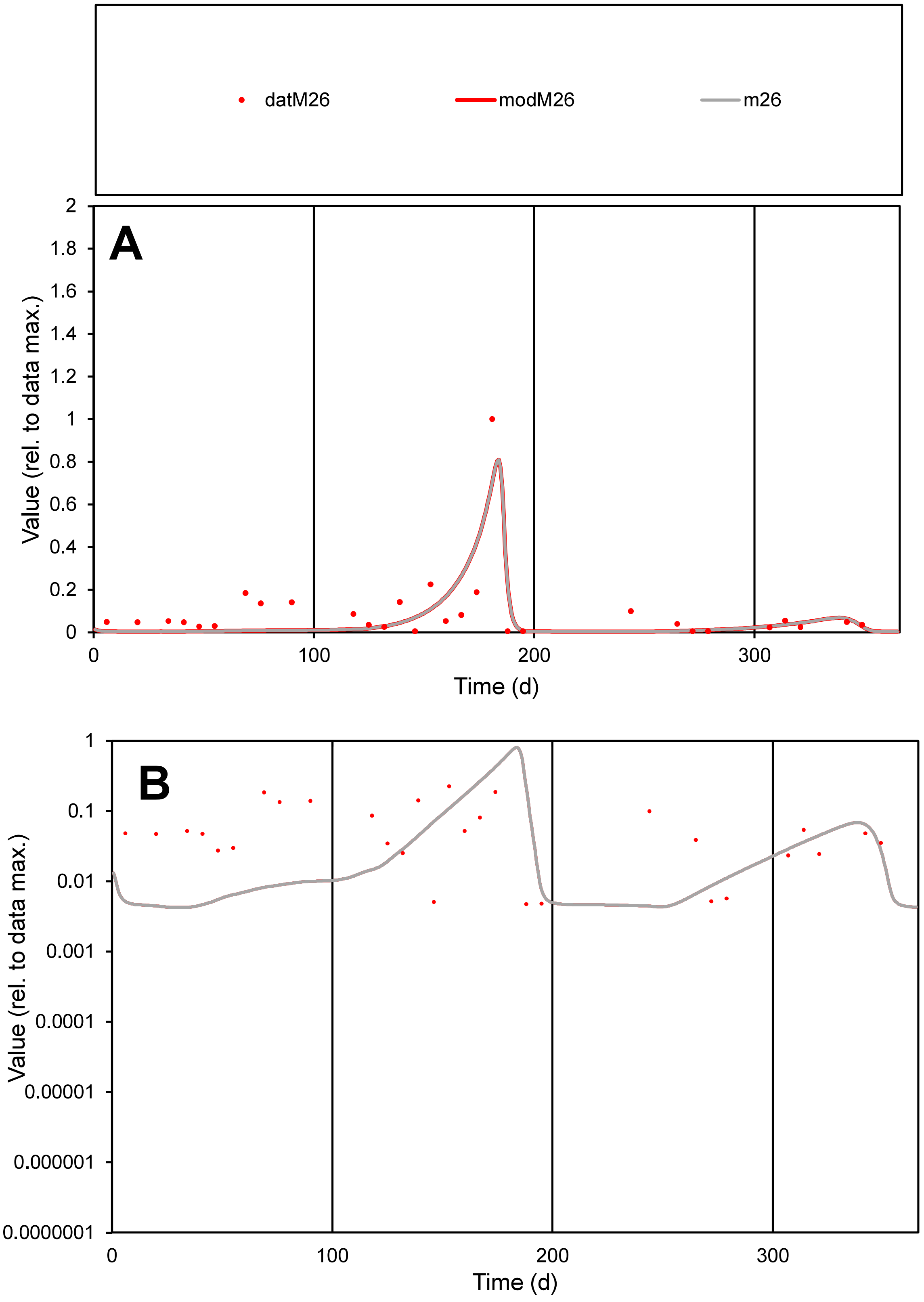

C1

Variables

M26 Provide jtd or Dst

CalibOnly?

2 1 to reproduce of

oNormalize?

2 1=no, 2=yes

ofxn calc*

0.99476

ofxn mod

1.02816

sRun

RunA1

zout

1

Ave

Data

0.00168

Model

0.00134

data/vMax

0.01739

ofxng

275.646

Min d/m

1.09912

ave/min d

20.3908

ave/min m

17.8783

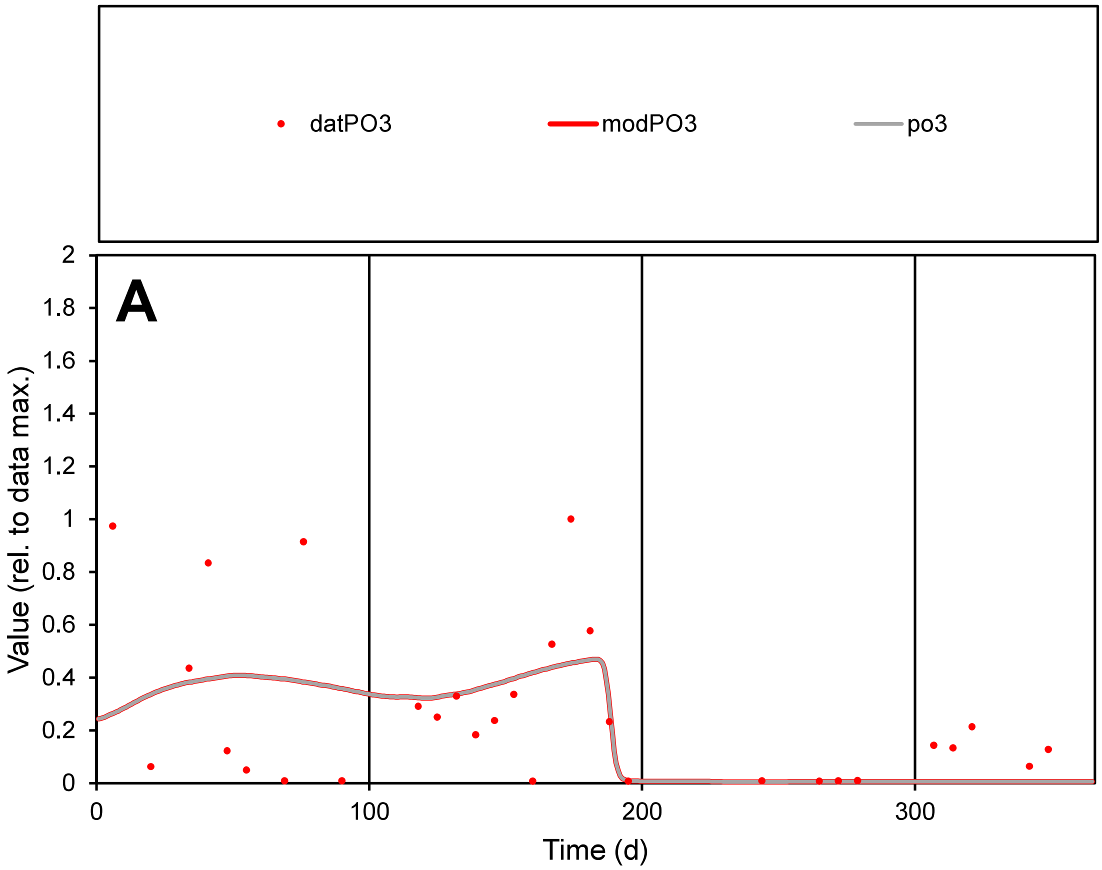

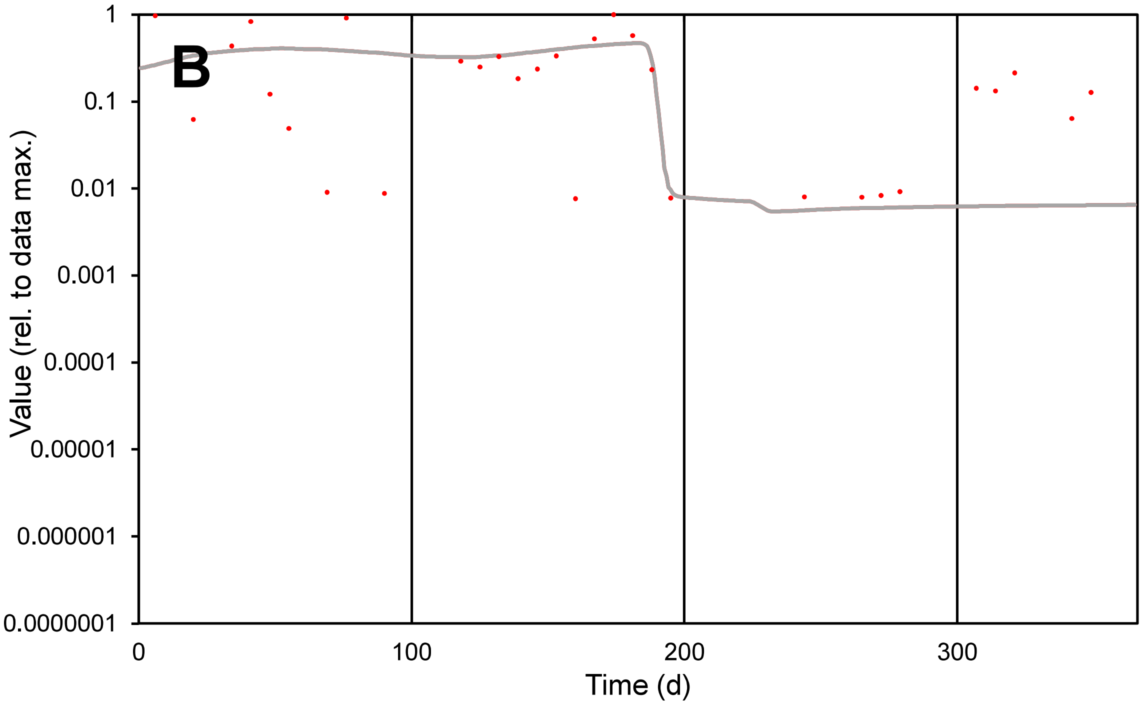

C1

Variables
PO3 Provide jtd or Dst
CalibOnly?
2 1 to reproduce of
oNormalize?
2 1=no, 2=yes

ofxn calc*
0.995
ofxn mod
1.00377

sRun
RunA1
zout
1

Ave
Data
0.00166
Model
0.00153

data/vMax
0.00613

ofxng
275.646

Min d/m
1.35123

ave/min d
35.3879
ave/min m
44.2909

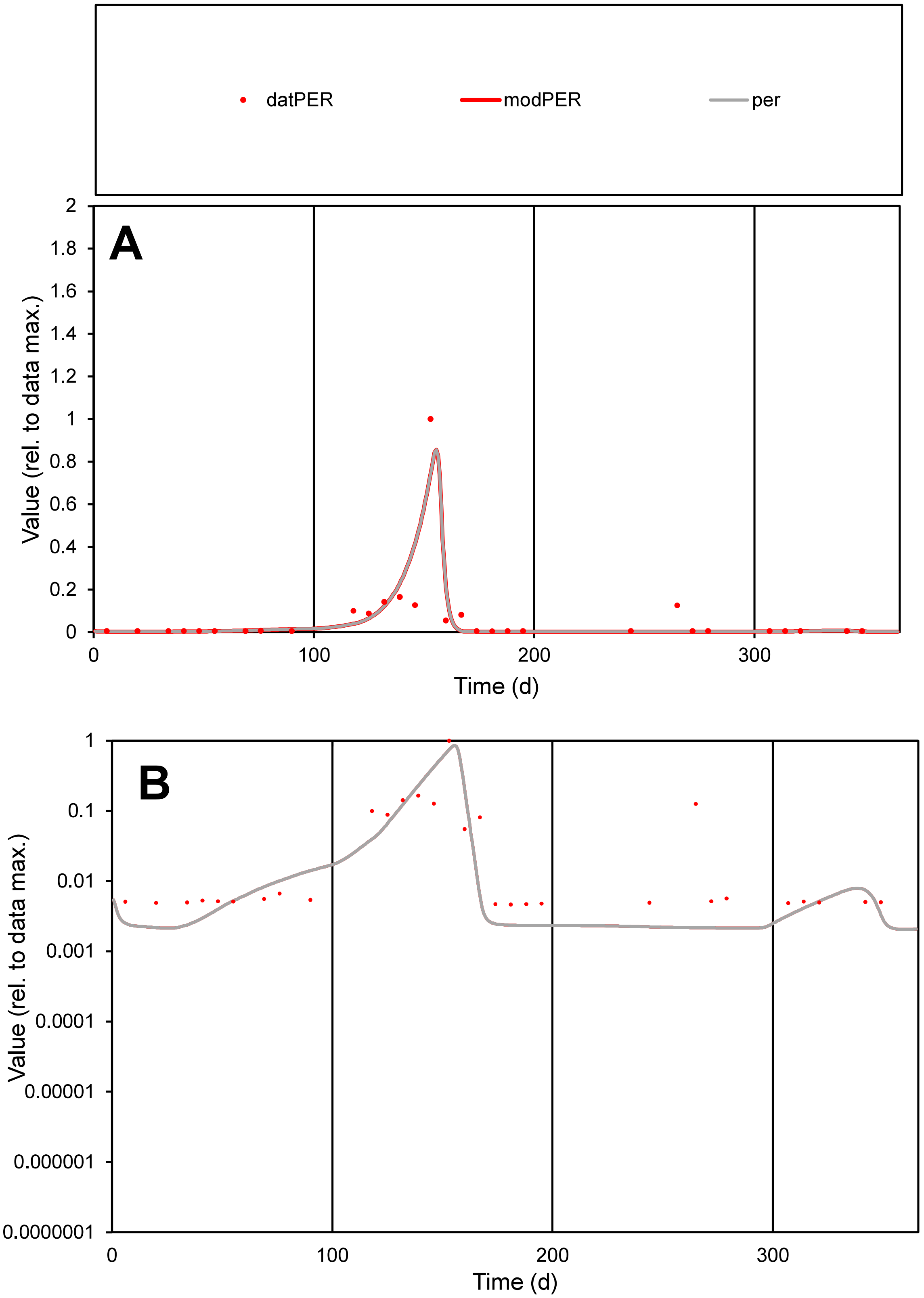

C1

Variables
PER Provide jtd or Dst
CalibOnly?
2 1 to reproduce of
oNormalize?
2 1=no, 2=yes

ofxn calc*
1.08695
ofxn mod
1.12492

sRun
RunA1
zout
1

Ave
Data
0.00026
Model
0.00025

data/vMax
0.00392

ofxng
275.646

Min d/m
2.15131

ave/min d
14.3179
ave/min m
30.0345

325_Calib_S_A

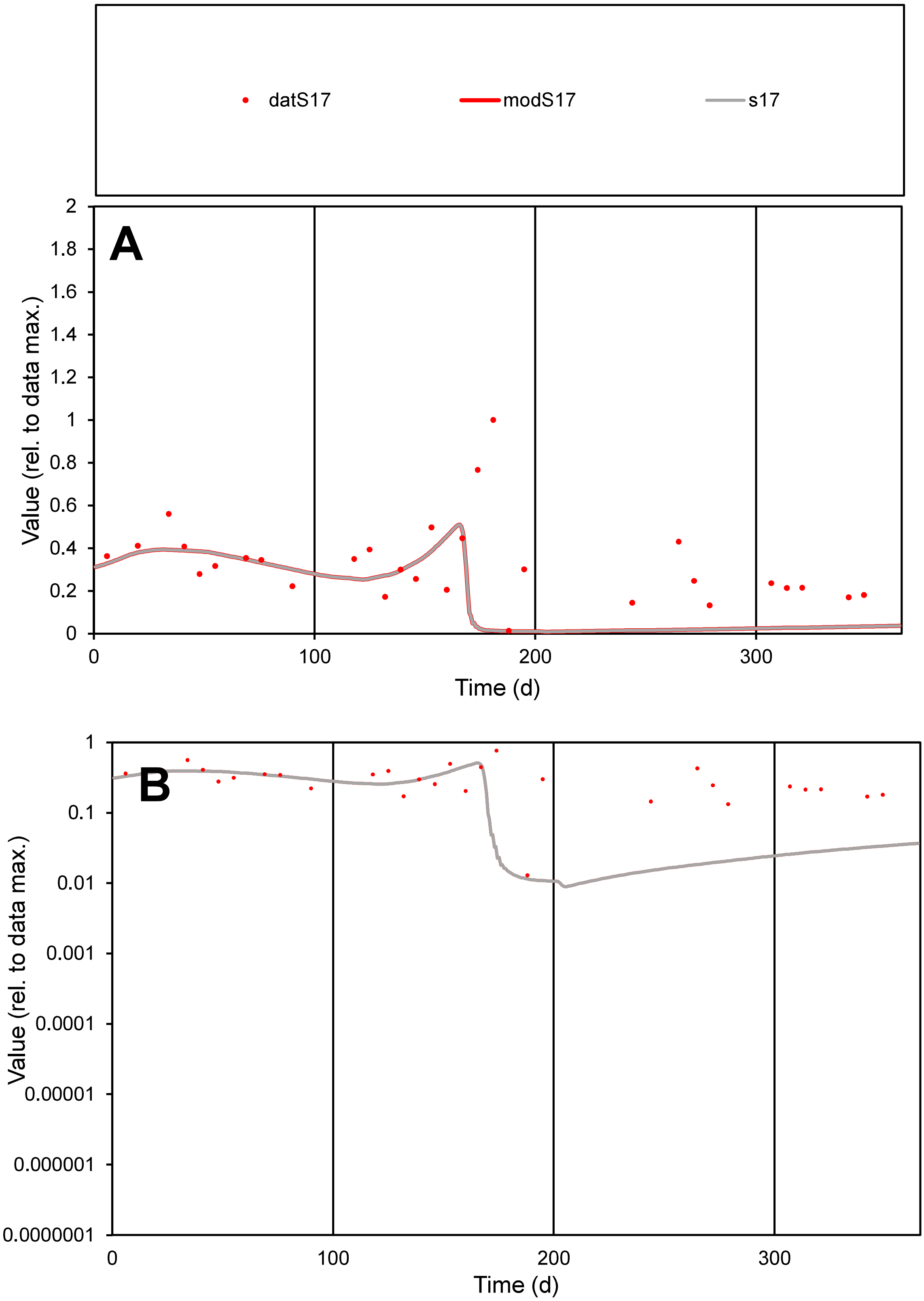

C1

Variables
S17 Provide jtd or Dst
CalibOnly?
2 1 to reproduce of
oNormalize?
2 1=no, 2=yes

ofxn calc*
0.81573
ofxn mod
0.823

sRun
RunA1
zout
1

Ave
Data
0.0028
Model
0.00175

data/vMax
0.00845

ofxng
275.646

Min d/m
1.20396

ave/min d
25.5374
ave/min m
19.22

325_Calib_S_A

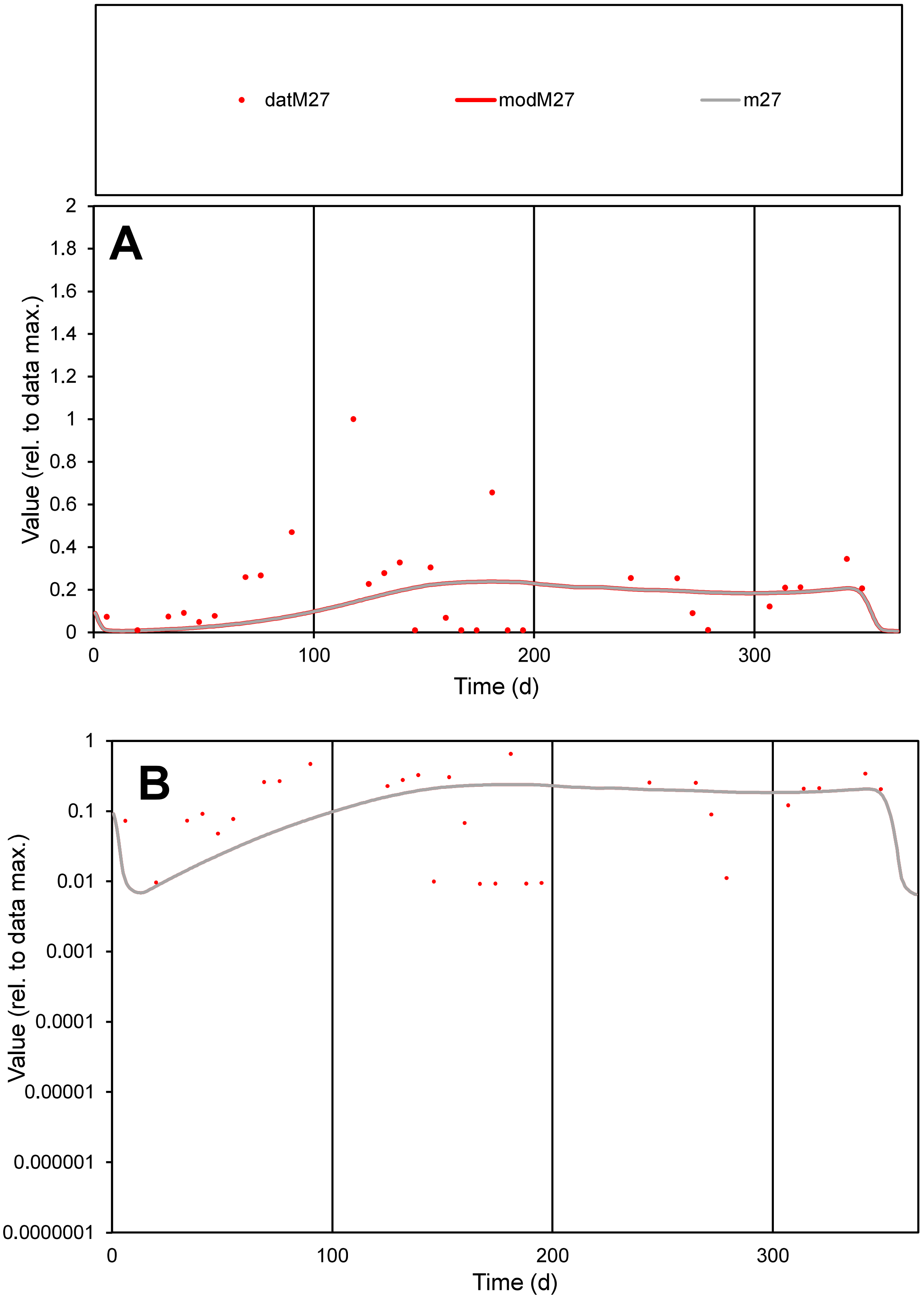

C1

Variables
M27 Provide jtd or Dst
CalibOnly?
2 1 to reproduce of
oNormalize?
2 1=no, 2=yes

ofxn calc*
1.13594
ofxn mod
1.14331

sRun
RunA1
zout
1

Ave
Data
0.0014
Model
0.00108

data/vMax
0.00704

ofxng
275.646

Min d/m
1.03784

ave/min d
21.5891
ave/min m
17.2209

325_Calib_S_A

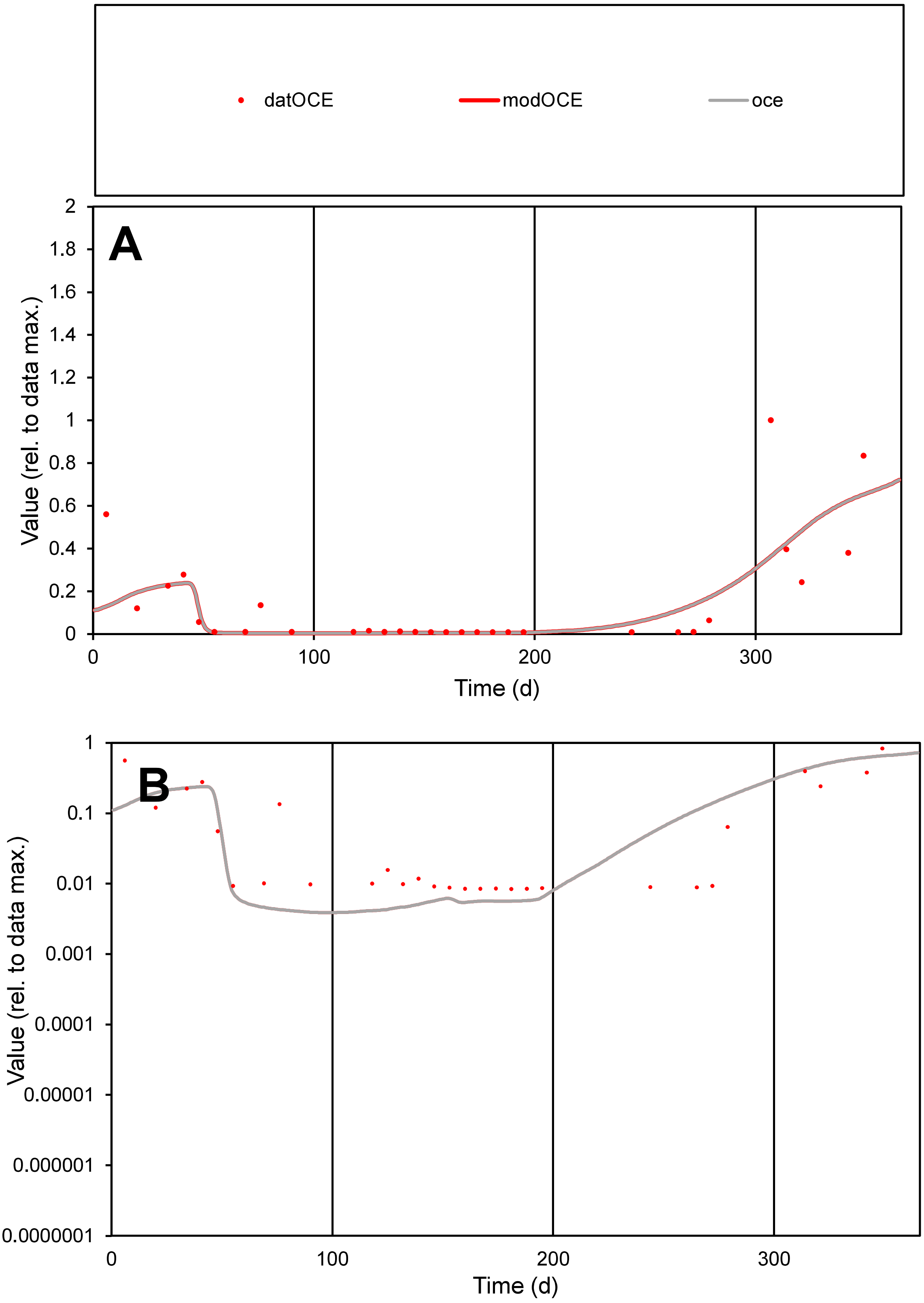

C1

Variables
OCE Provide jtd or Dst
CalibOnly?
2 1 to reproduce of
oNormalize?
2 1=no, 2=yes

ofxn calc*
1.09509
ofxn mod
1.11313

sRun
RunA1
zout
1

Ave
Data
0.00065
Model
0.00059

data/vMax
0.0044

ofxng
275.646

Min d/m
2.14302

ave/min d
17.7428
ave/min m
34.2867

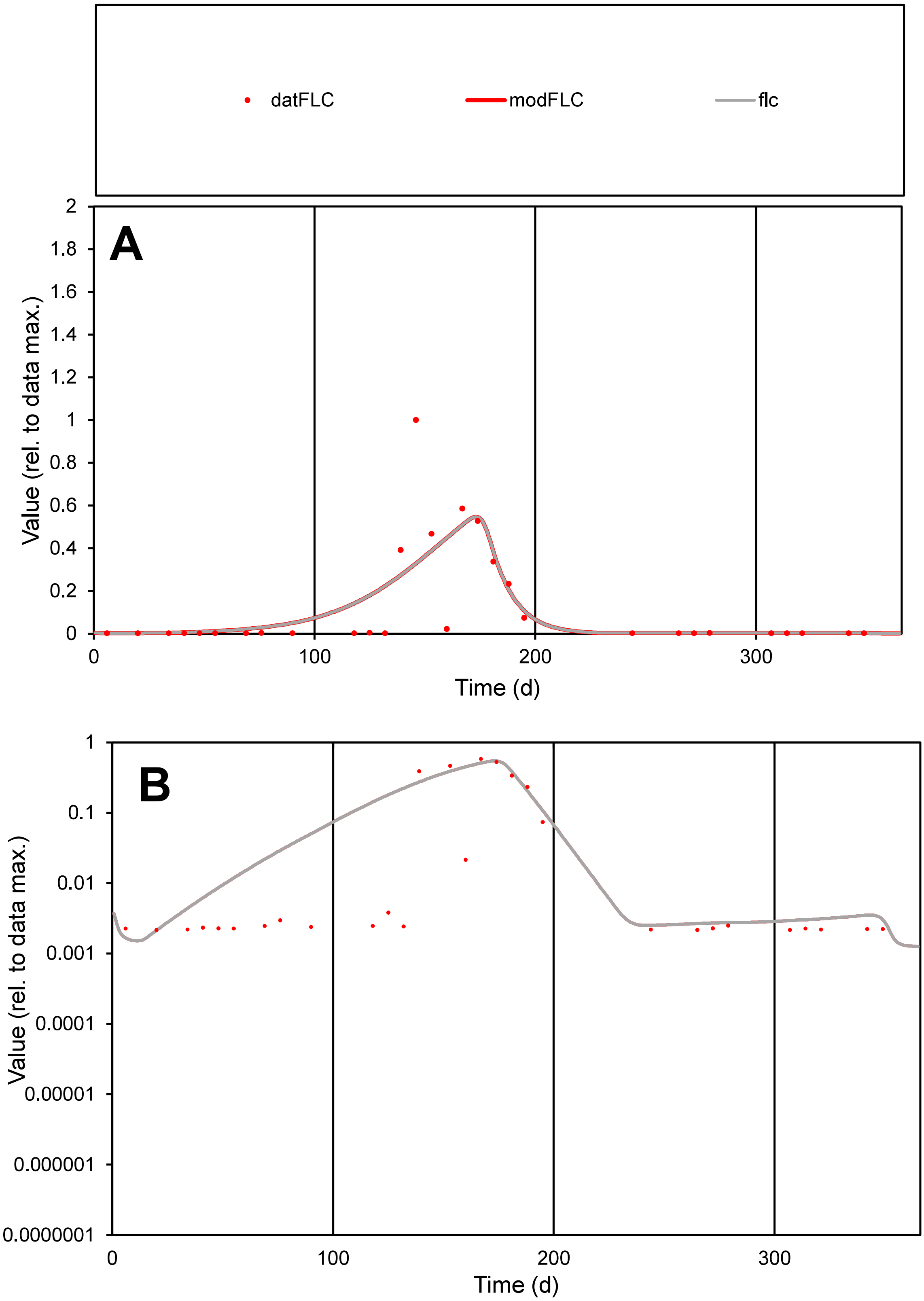

C1

Variables
FLC Provide jtd or Dst
CalibOnly?
2 1 to reproduce of
oNormalize?
2 1=no, 2=yes

ofxn calc*
1.27841
ofxn mod
1.29742

sRun
RunA1
zout
1

Ave
Data
0.00072
Model
0.00075

data/vMax
0.00582

ofxng
275.646

Min d/m
1.34923

ave/min d
56.9542
ave/min m
80.2935

325_Calib_S_A

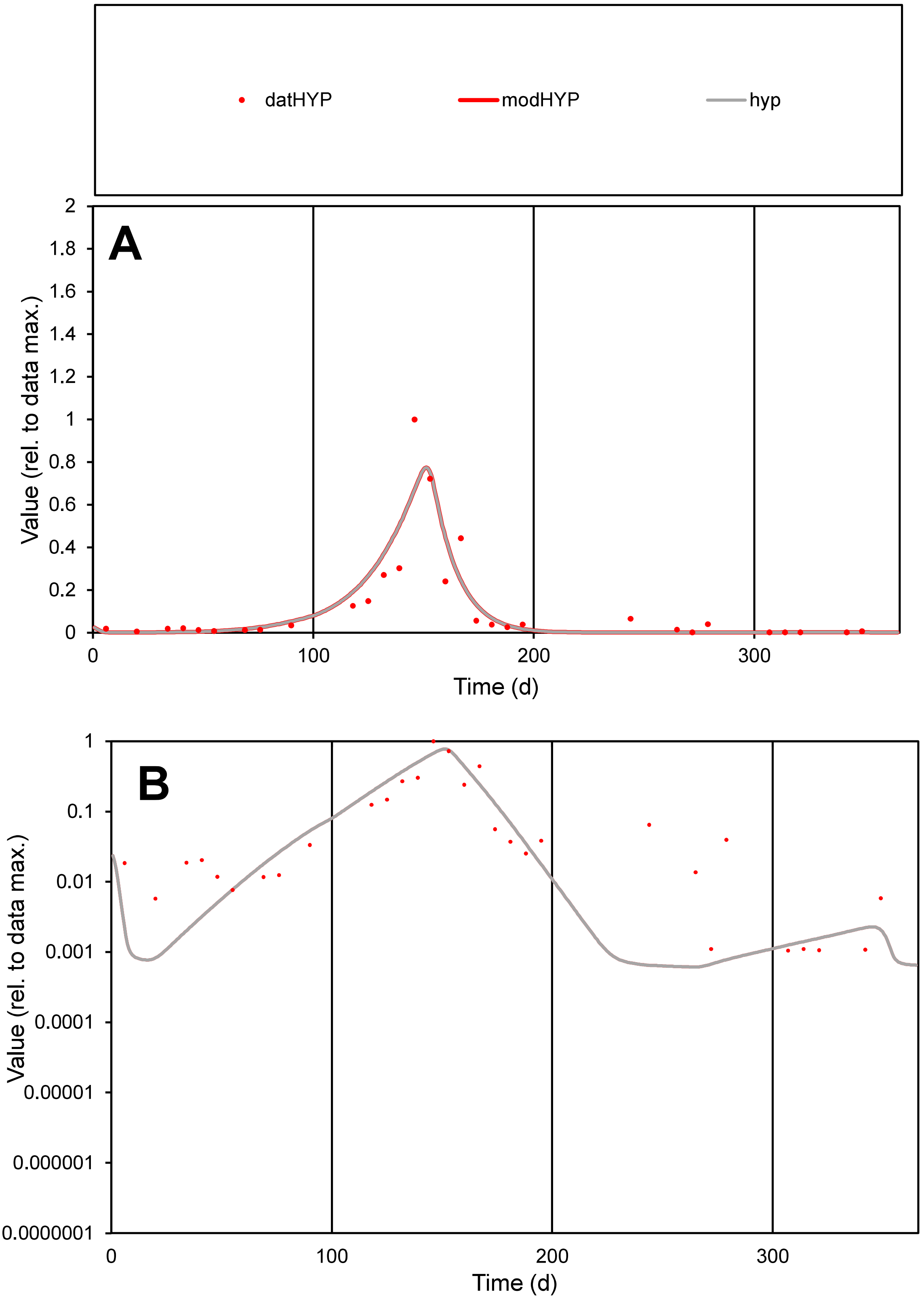

C1

Variables
HYP Provide jtd or Dst
CalibOnly?
2 1 to reproduce of
oNormalize?
2 1=no, 2=yes

ofxn calc*
0.72433
ofxn mod
0.77251

sRun
RunA1
zout
1

Ave
Data
0.00194
Model
0.002

data/vMax
0.01583

ofxng
275.646

Min d/m
1.70887

ave/min d
116.803
ave/min m
205.703

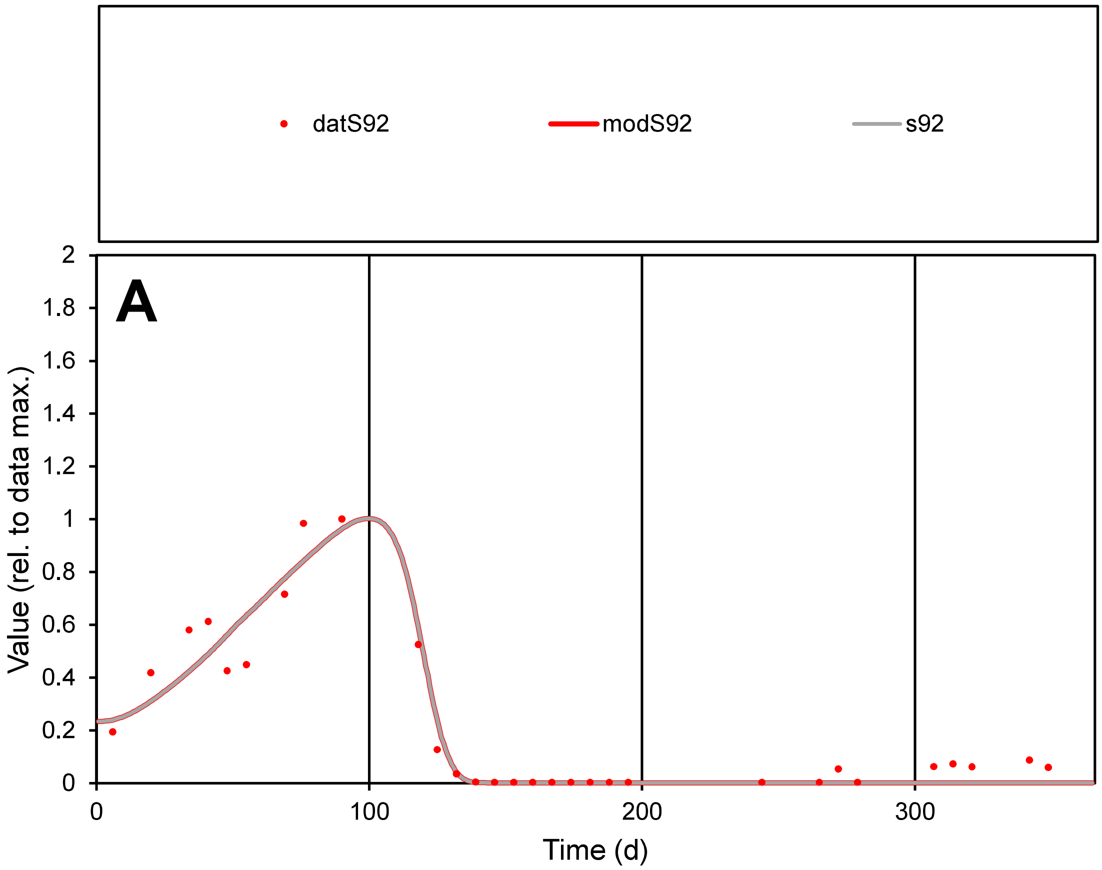

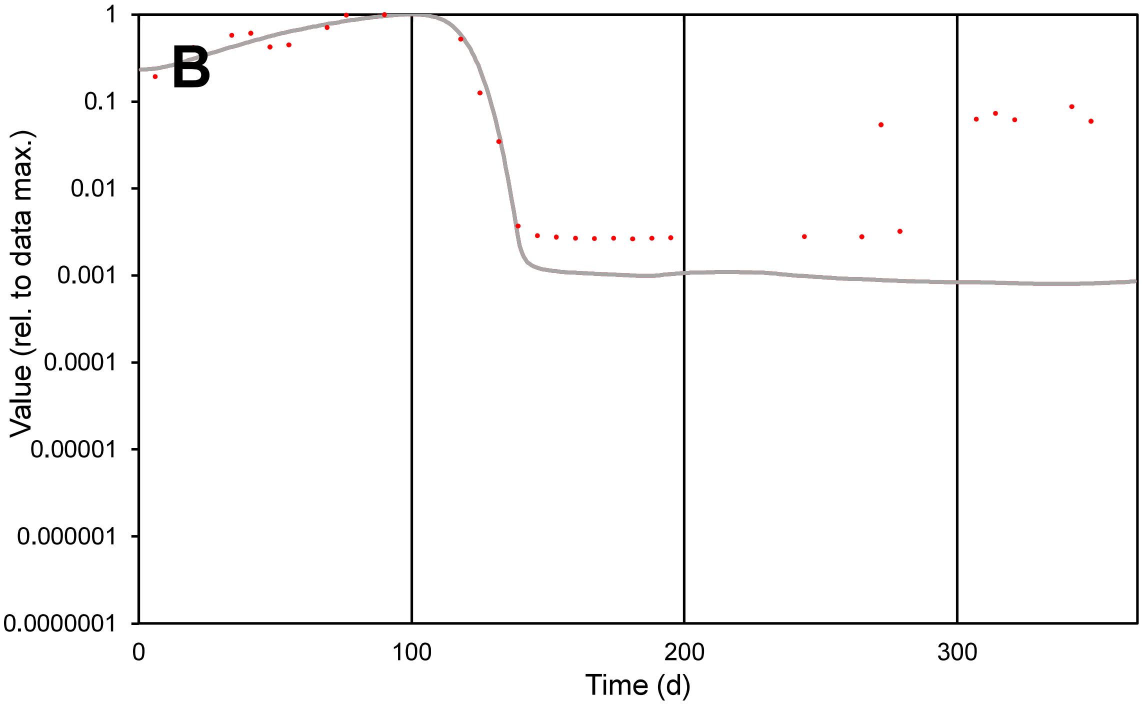

C1

Variables
S92 Provide jtd or Dst
CalibOnly?
2 1 to reproduce of
oNormalize?
2 1=no, 2=yes

ofxn calc*
0.33024
ofxn mod
0.38909

sRun
RunA1
zout
1

Ave
Data
0.00189
Model
0.00177

data/vMax
0.00874

ofxng
275.646

Min d/m
3.28543

ave/min d
82.0084
ave/min m
251.983

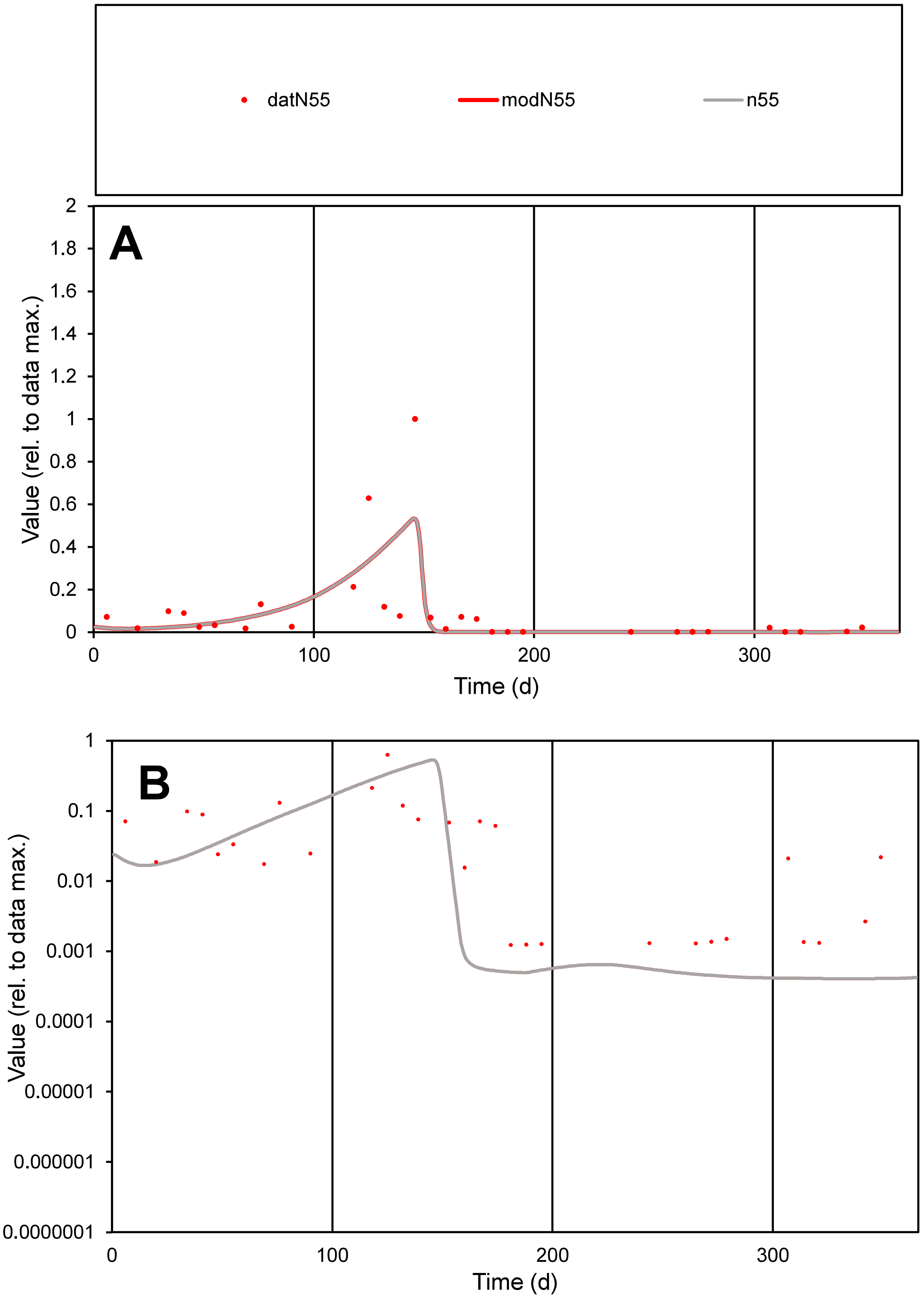

C1

Variables
N55 Provide jtd or Dst
CalibOnly?
2 1 to reproduce of
oNormalize?
2 1=no, 2=yes

ofxn calc*
1.48282
ofxn mod
1.53235

sRun
RunA1
zout
1

Ave
Data
0.00282
Model
0.00257

data/vMax
0.03006

ofxng
275.646

Min d/m
3.01058

ave/min d
76.294
ave/min m
208.886

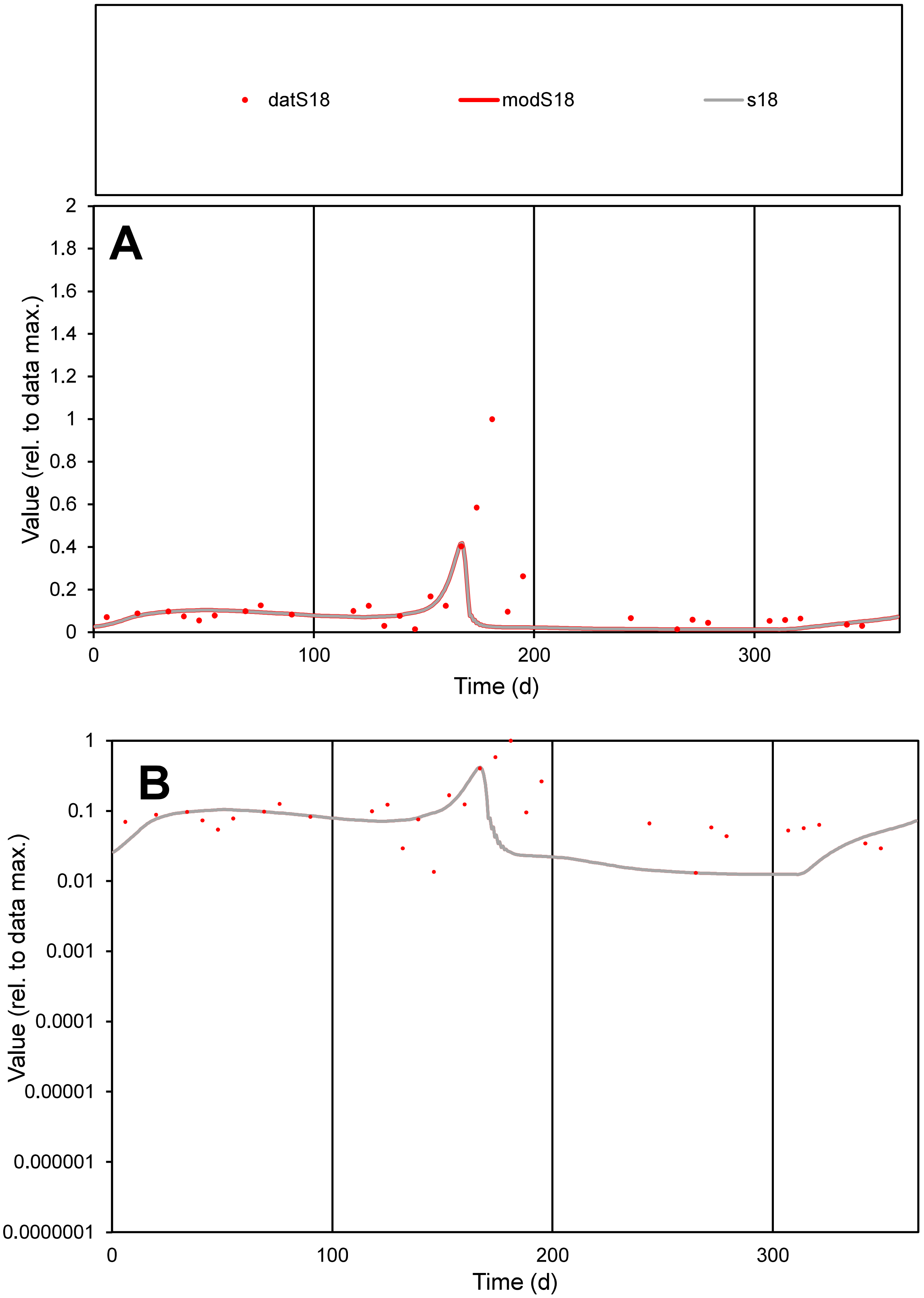

C1

Variables
S18 Provide jtd or Dst
CalibOnly?
2 1 to reproduce of
oNormalize?
2 1=no, 2=yes

ofxn calc*
1.52588
ofxn mod
1.53219

sRun
RunA1
zout
1

Ave
Data
0.00295
Model
0.00158

data/vMax
0.02123

ofxng
275.646

Min d/m
1.05356

ave/min d
10.5451
ave/min m
5.97211

325_Calib_S_A

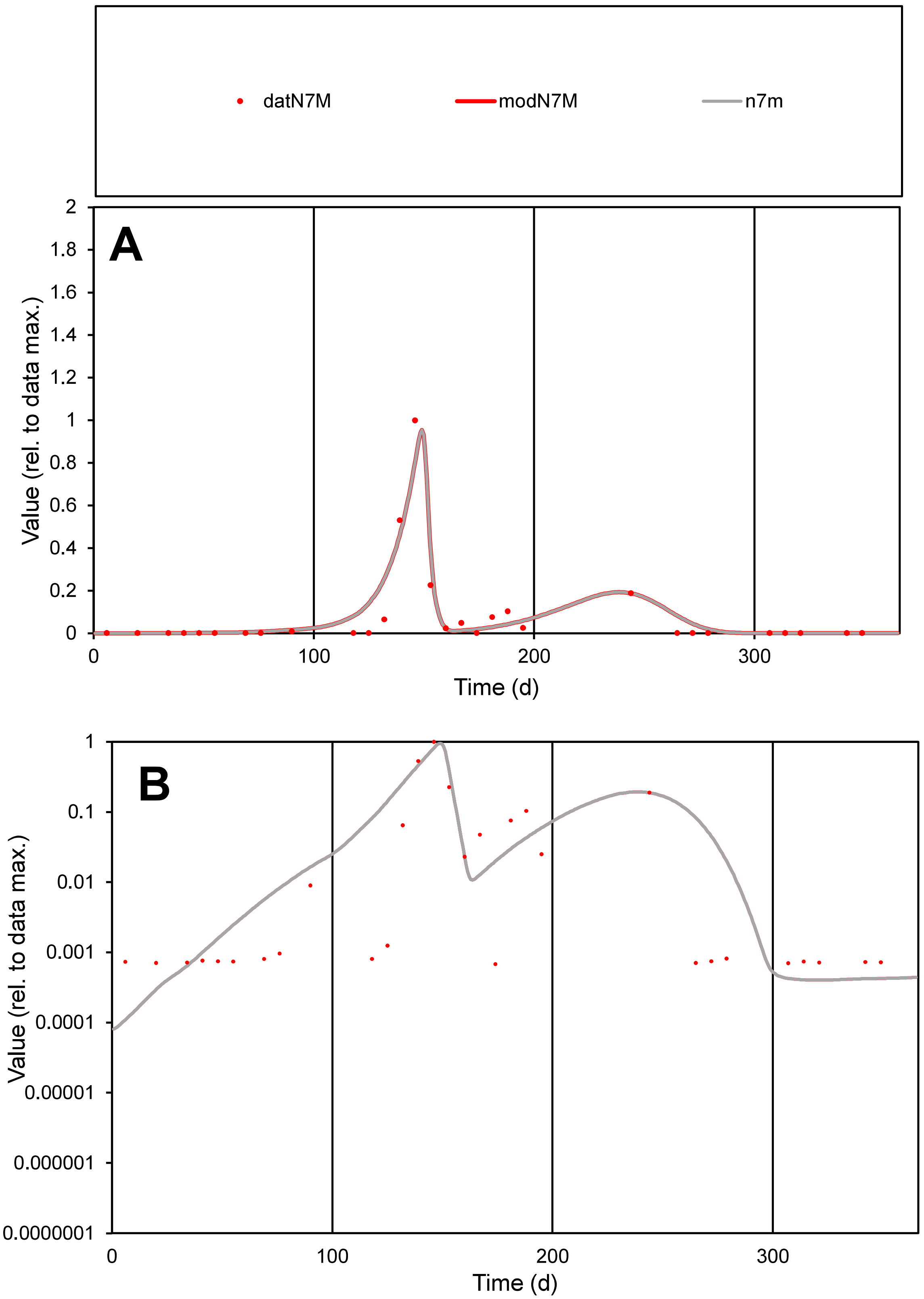

C1

Variables

N7M Provide jtd or Dst

CalibOnly?

2 1 to reproduce of

oNormalize?

2 1=no, 2=yes

ofxn calc*

0.66476

ofxn mod

0.77879

sRun

RunA1

zout

1

Ave

Data

0.0011

Model

0.00126

data/vMax

0.01427

ofxng

275.646

Min d/m

5.71252

ave/min d

112.996

ave/min m

740.646

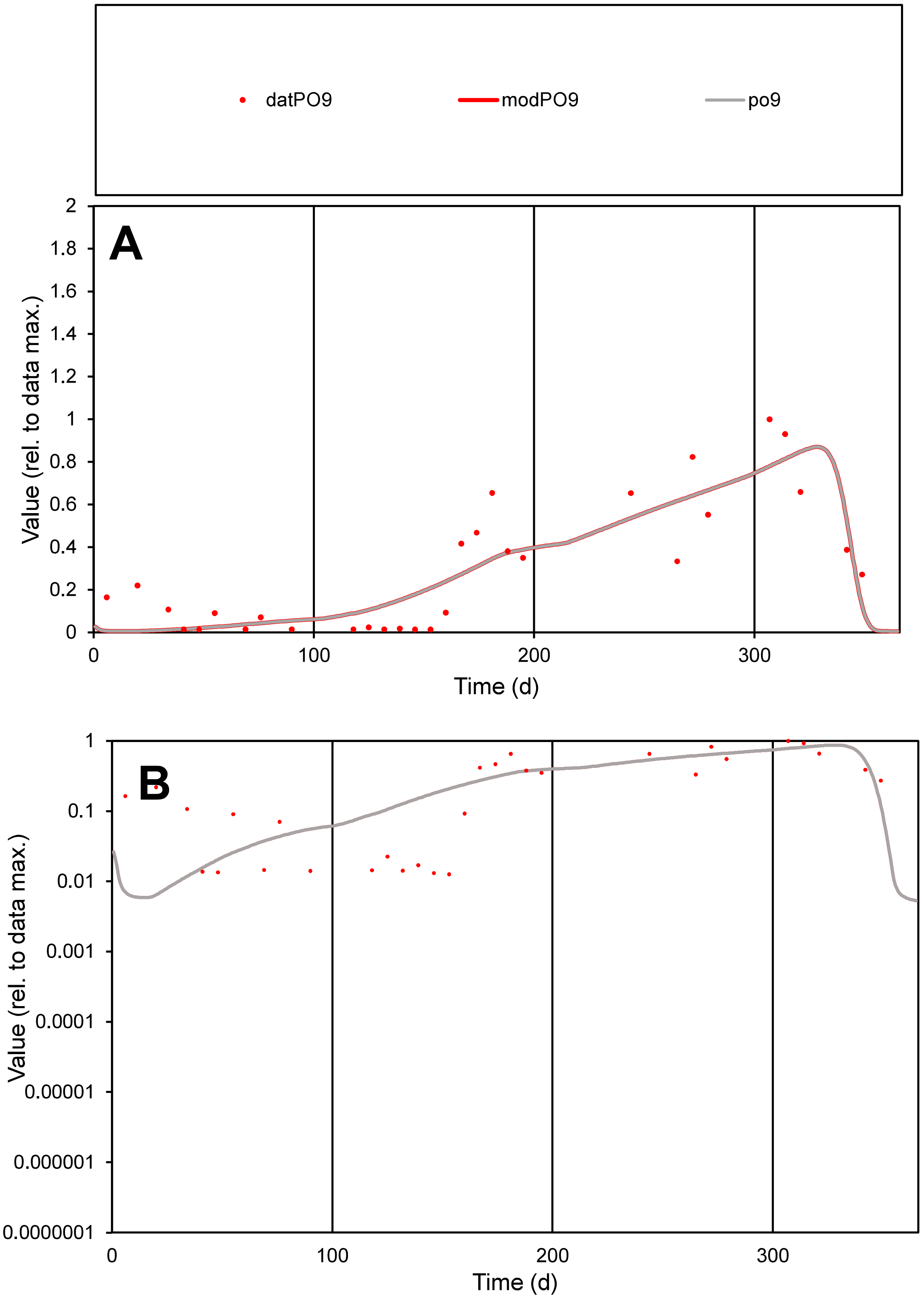

C1

Variables
PO9 Provide jtd or Dst
CalibOnly?
2 1 to reproduce of
oNormalize?
2 1=no, 2=yes

ofxn calc*
0.48765
ofxn mod
0.51696

sRun
RunA1
zout
1

Ave
Data
0.00141
Model
0.00138

data/vMax
0.00481

ofxng
275.646

Min d/m
1.93611

ave/min d
23.0681
ave/min m
43.7348

325_Calib_S_A

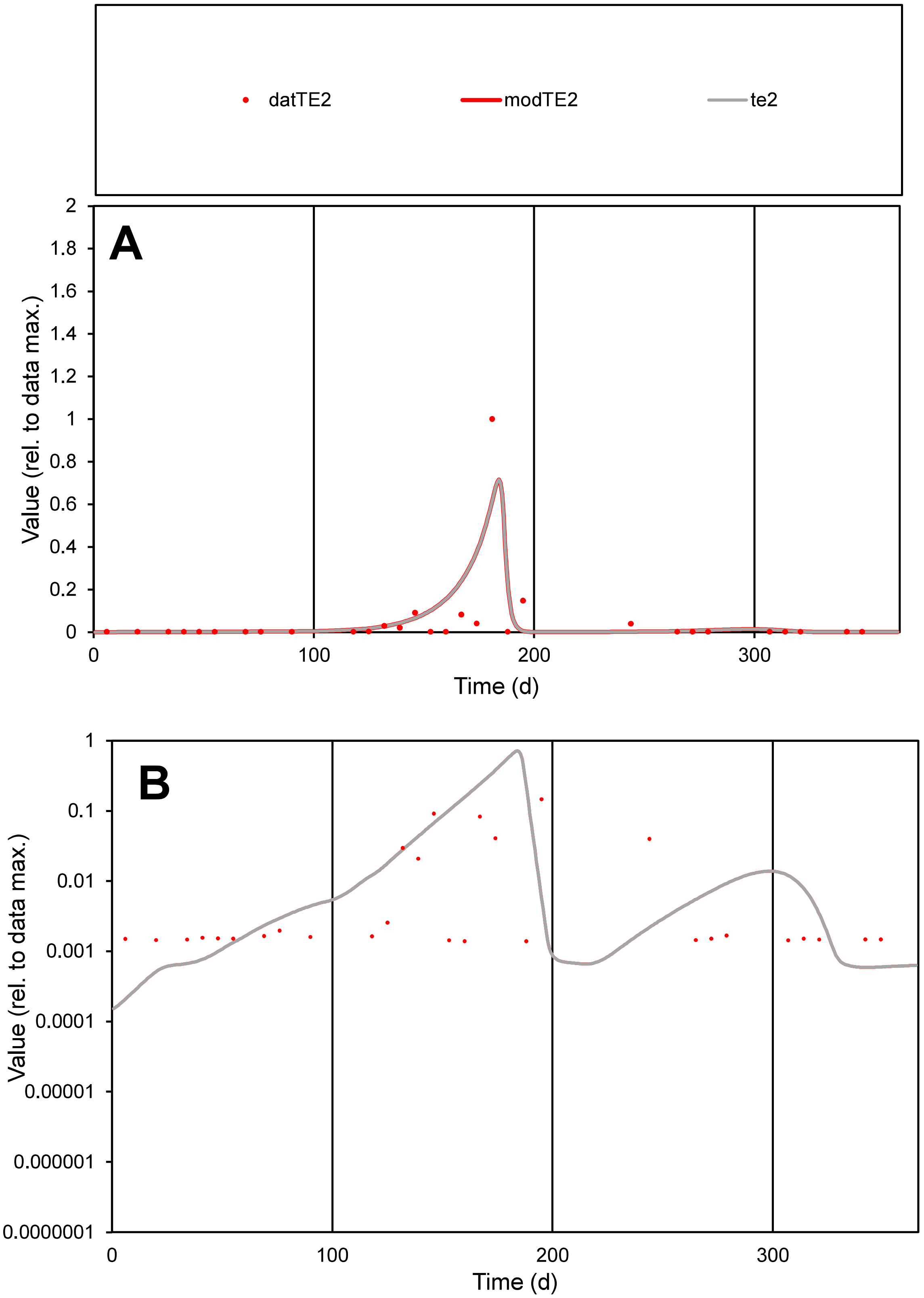

C1

Variables
TE2 Provide jtd or Dst
CalibOnly?
2 1 to reproduce of
oNormalize?
2 1=no, 2=yes

ofxn calc*
1.96538
ofxn mod
2.06282

sRun
RunA1
zout
1

Ave
Data
0.00307
Model
0.00378

data/vMax
0.06191

ofxng
275.646

Min d/m
6.13195

ave/min d
35.7163
ave/min m
269.853

325_Calib_S_A

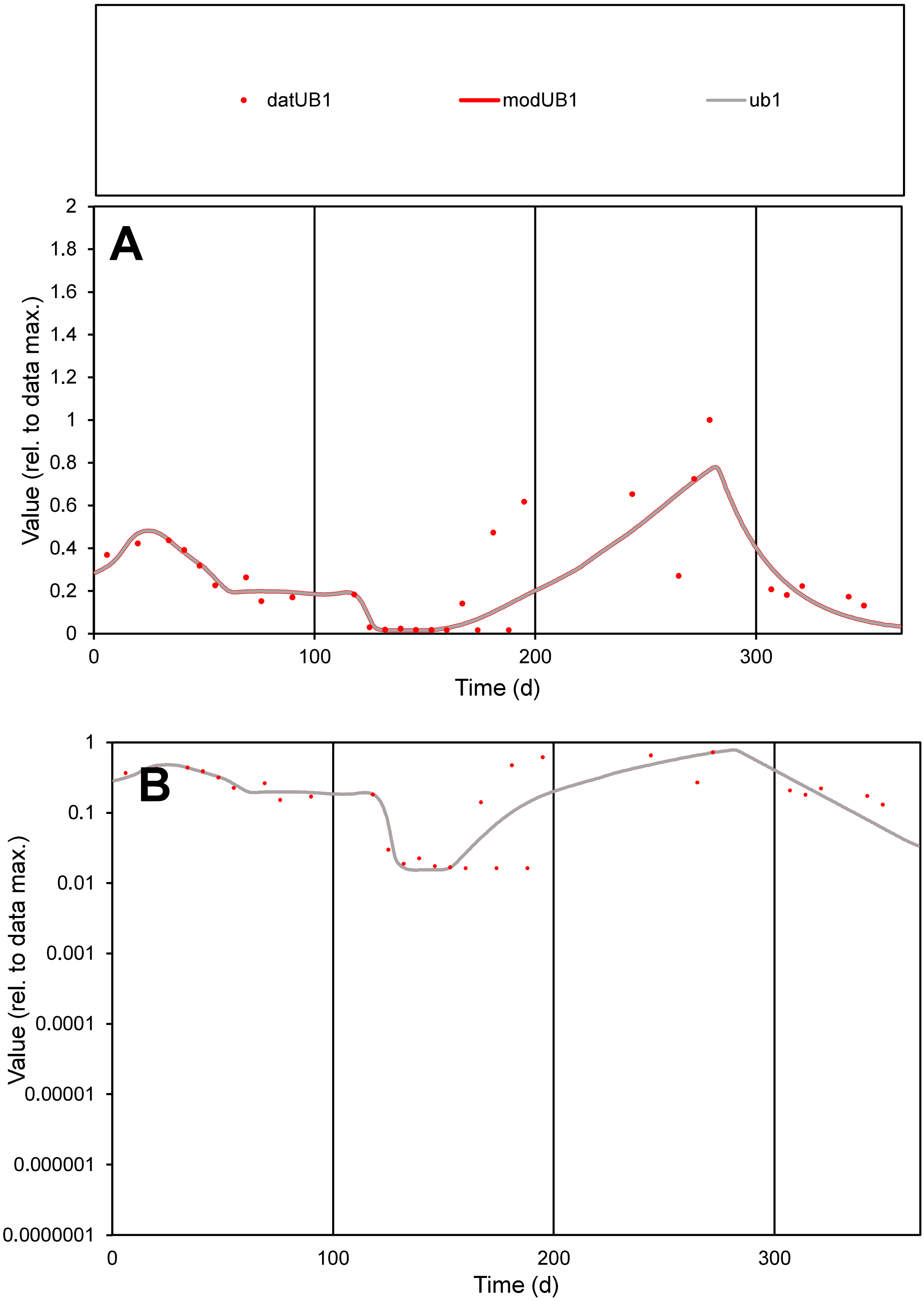

C1

Variables

UB1	Provide jtd or Dst

CalibOnly?

2	1 to reproduce of

oNormalize?

2	1=no, 2=yes

ofxn calc*
0.54464
ofxn mod
0.5697

sRun
RunA1
zout
1

Ave
Data
0.00093
Model
0.00083

data/vMax
0.00353

ofxng
275.646

Min d/m
1.05452

ave/min d
16.1523
ave/min m
15.2729

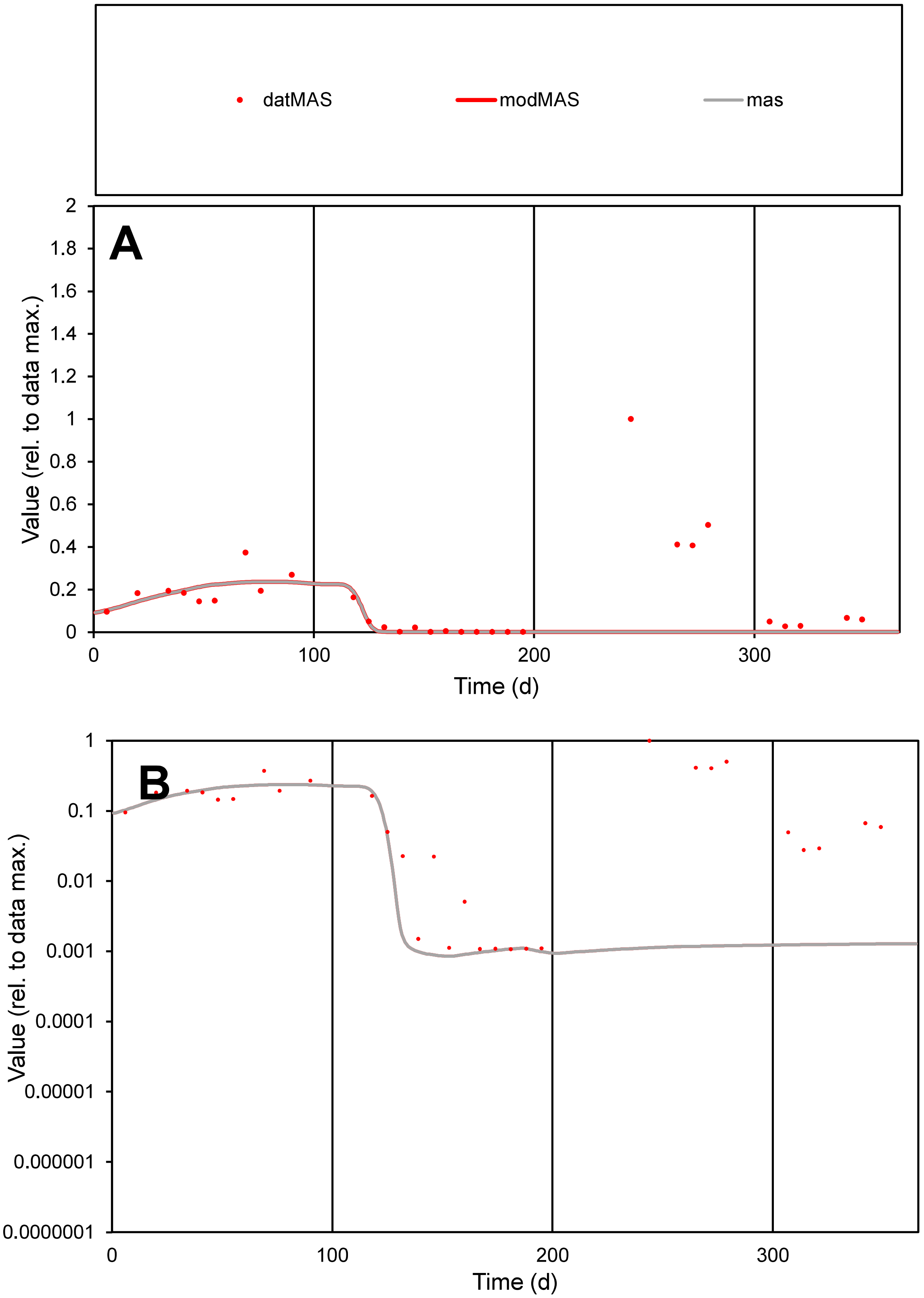

C1

Variables
MAS Provide jtd or Dst
CalibOnly?
2 1 to reproduce of
oNormalize?
2 1=no, 2=yes

ofxn calc*
1.51685
ofxn mod
1.52174

sRun
RunA1
zout
1

Ave
Data
0.00171
Model
0.00075

data/vMax
0.01115

ofxng
275.646

Min d/m
1.25829

ave/min d
143.349
ave/min m
78.9169

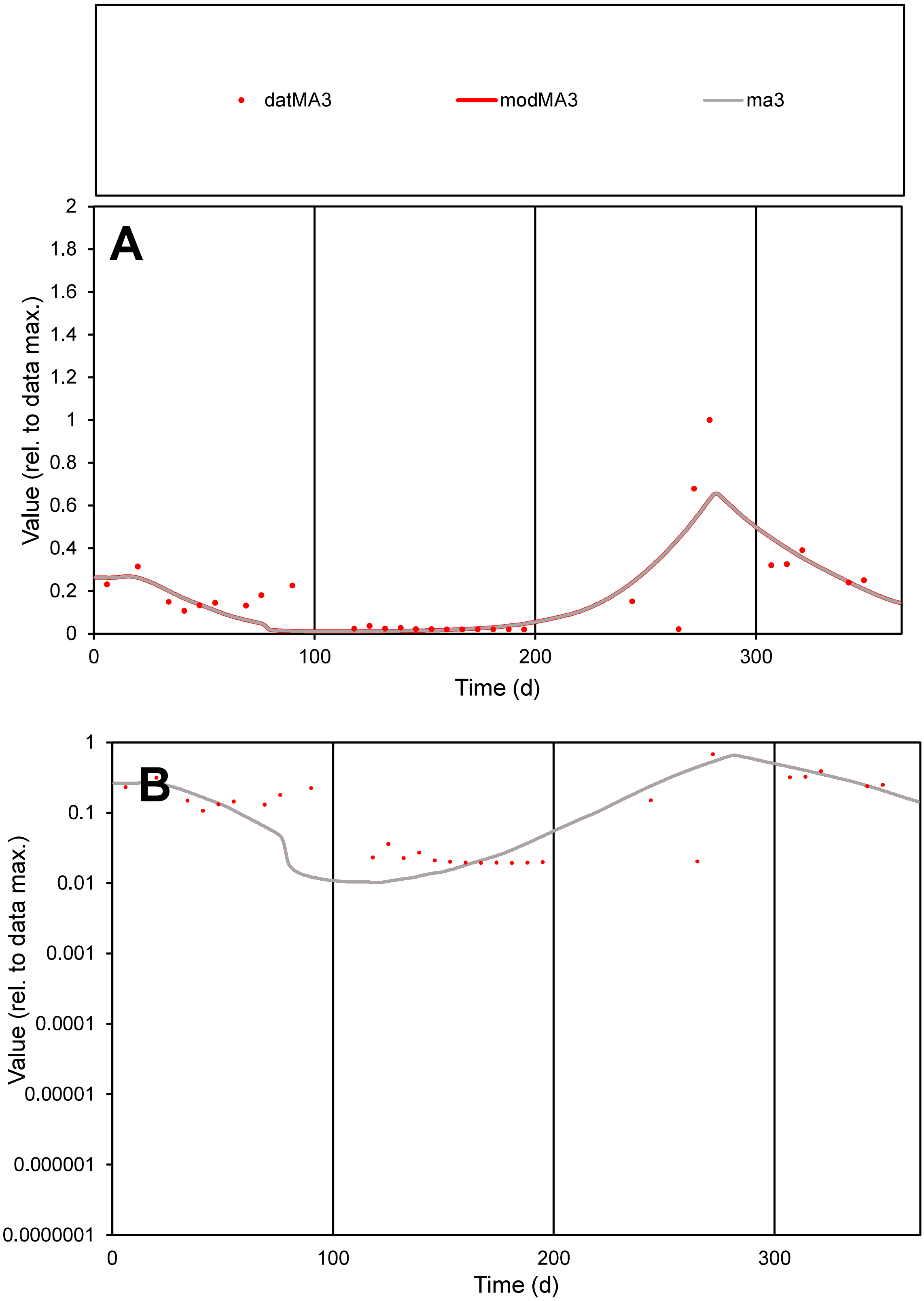

C1

Variables

MA3	Provide jtd or Dst

CalibOnly?

2	1 to reproduce of

oNormalize?

2	1=no, 2=yes

ofxn calc*
0.68798
ofxn mod
0.71757

sRun
RunA1
zout
1

Ave
Data
0.00056
Model
0.00053

data/vMax
0.00319

ofxng
275.646

Min d/m
1.88657

ave/min d
9.07213
ave/min m
16.2951

325_Calib_S_A

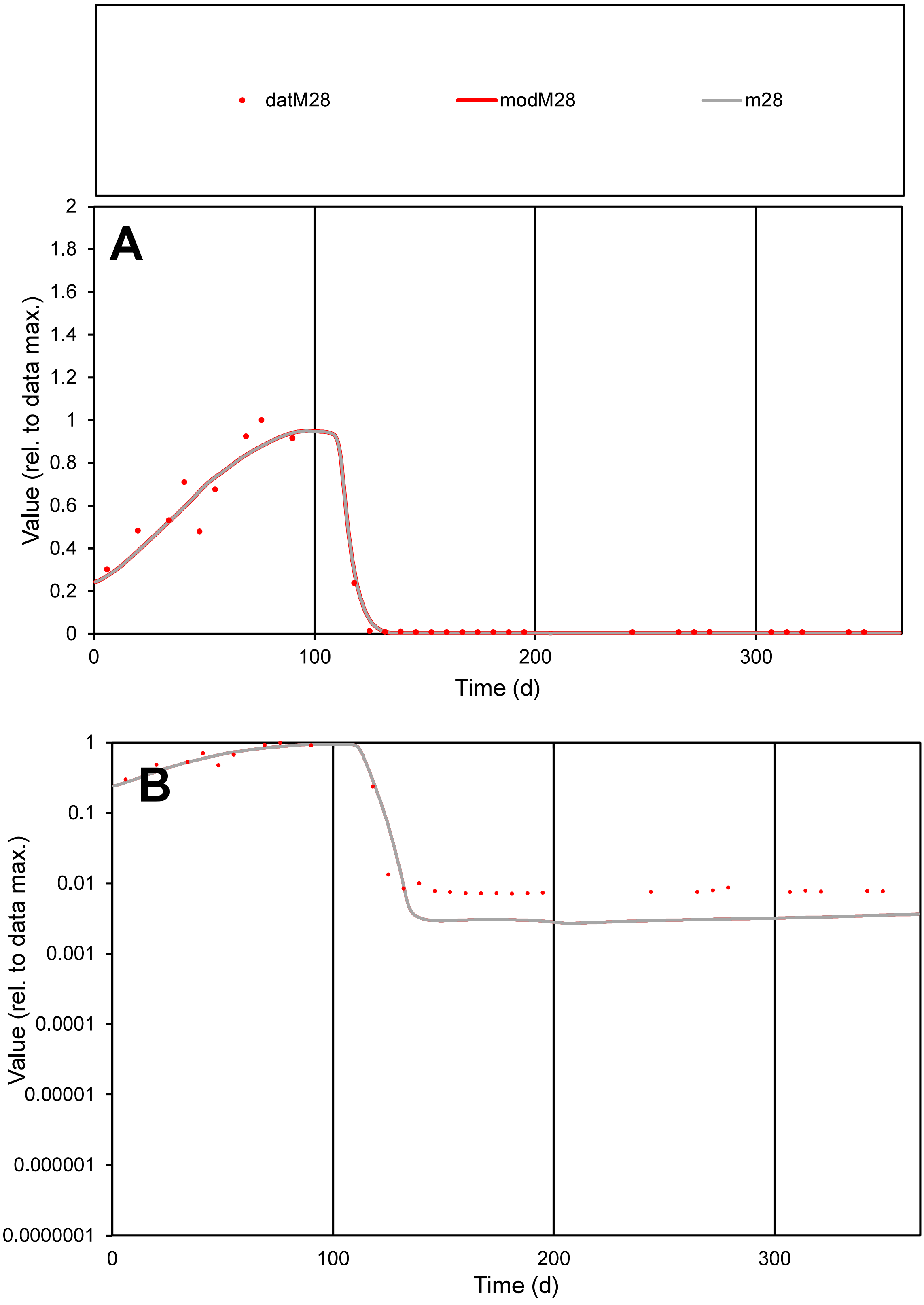

C1

Variables
M28 Provide jtd or Dst
CalibOnly?
2 1 to reproduce of
oNormalize?
2 1=no, 2=yes

ofxn calc*
0.24656
ofxn mod
0.32737

sRun
RunA1
zout
1

Ave
Data
0.0016
Model
0.00156

data/vMax
0.00749

ofxng
275.646

Min d/m
2.47091

ave/min d
29.9437
ave/min m
72.0714

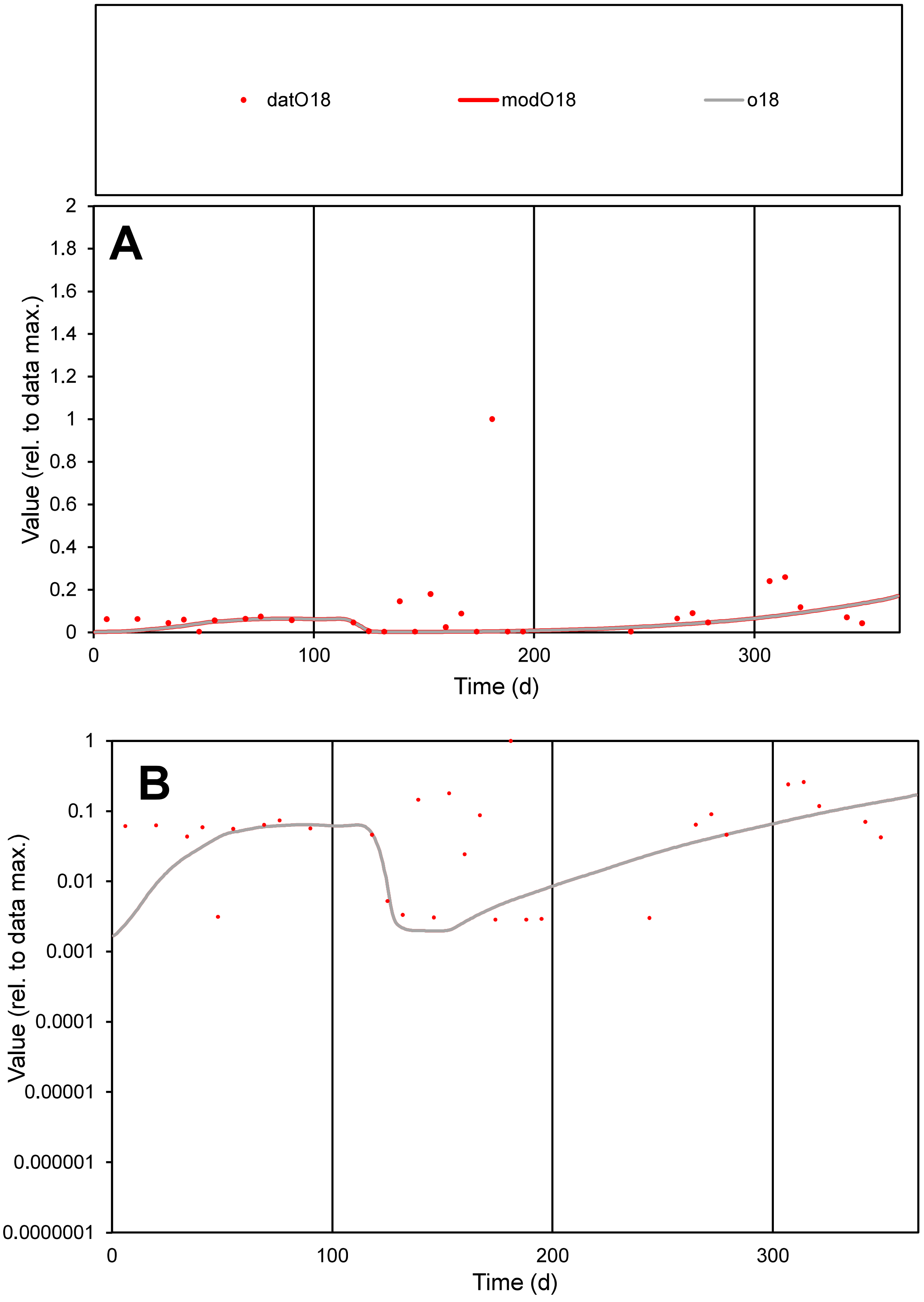

C1

Variables
O18 Provide jtd or Dst
CalibOnly?
2 1 to reproduce of
oNormalize?
2 1=no, 2=yes

ofxn calc*
1.99619
ofxn mod
2.00125

sRun
RunA1
zout
1

Ave
Data
0.00177
Model
0.00067

data/vMax
0.01818

ofxng
275.646

Min d/m
1.44866

ave/min d
34.053
ave/min m
18.6163

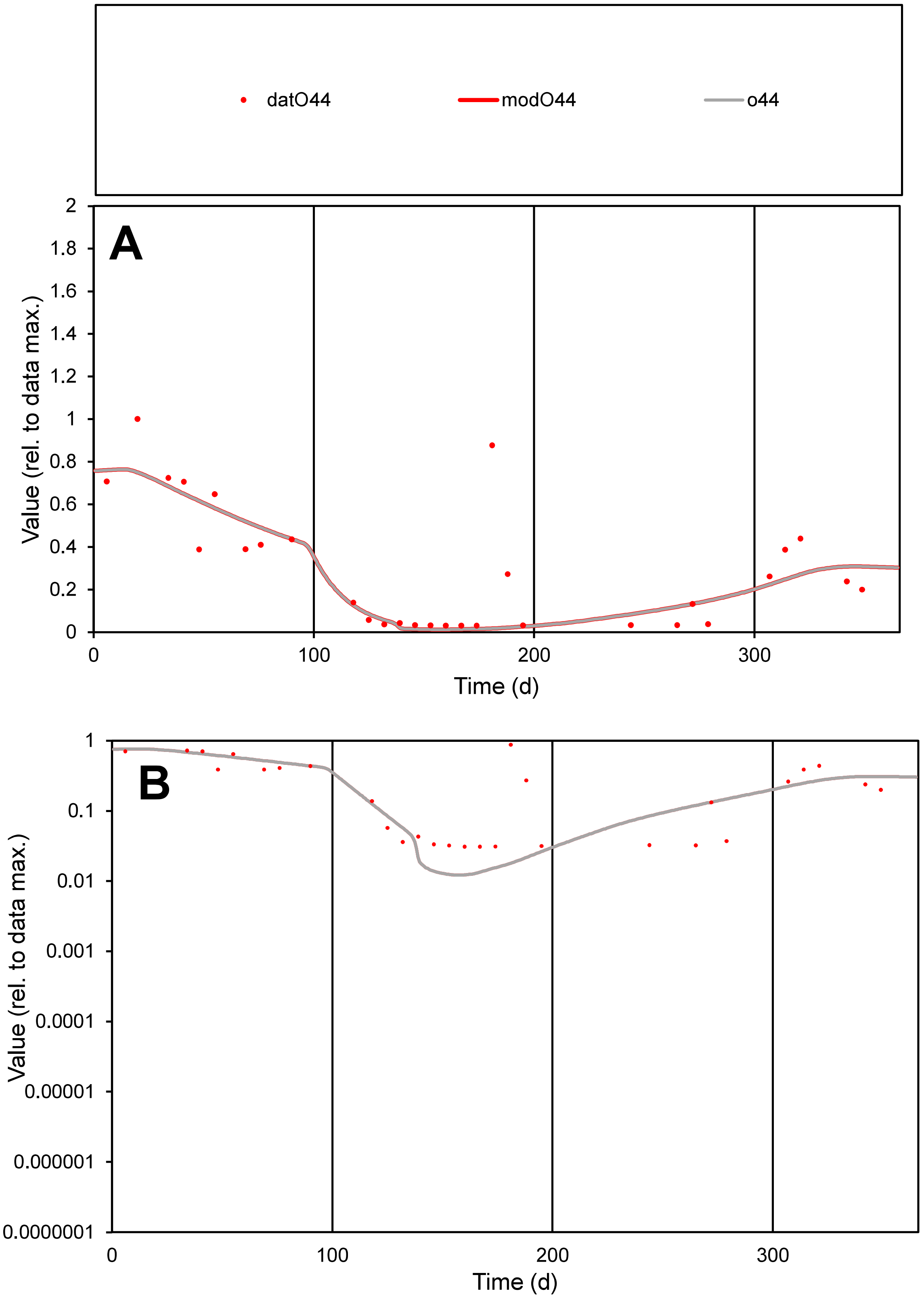

C1

Variables
O44 Provide jtd or Dst
CalibOnly?
2 1 to reproduce of
oNormalize?
2 1=no, 2=yes

ofxn calc*
0.63535
ofxn mod
0.65272

sRun
RunA1
zout
1

Ave
Data
0.00105
Model
0.00092

data/vMax
0.00358

ofxng
275.646

Min d/m
2.50283

ave/min d
9.49348
ave/min m
20.8861

325_Calib_S_A

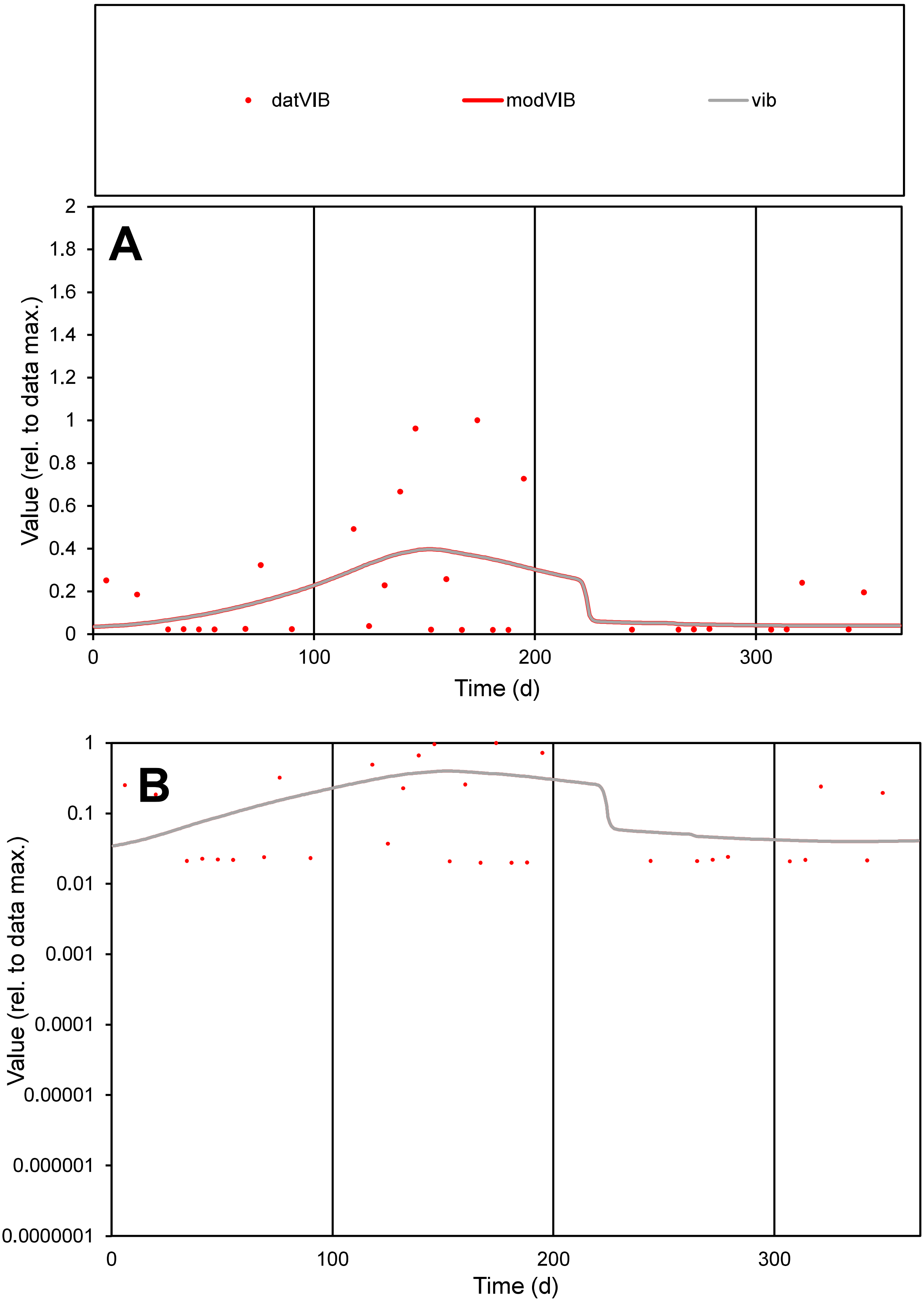

C1

Variables

VIB Provide jtd or Dst

CalibOnly?

2 1 to reproduce of

oNormalize?

2 1=no, 2=yes

ofxn calc*

1.24755

ofxn mod

1.25748

sRun

RunA1

zout

1

Ave

Data

0.00018

Model

0.00016

data/vMax

0.00089

ofxng

275.646

Min d/m

0.52429

ave/min d

9.96123

ave/min m

4.91536

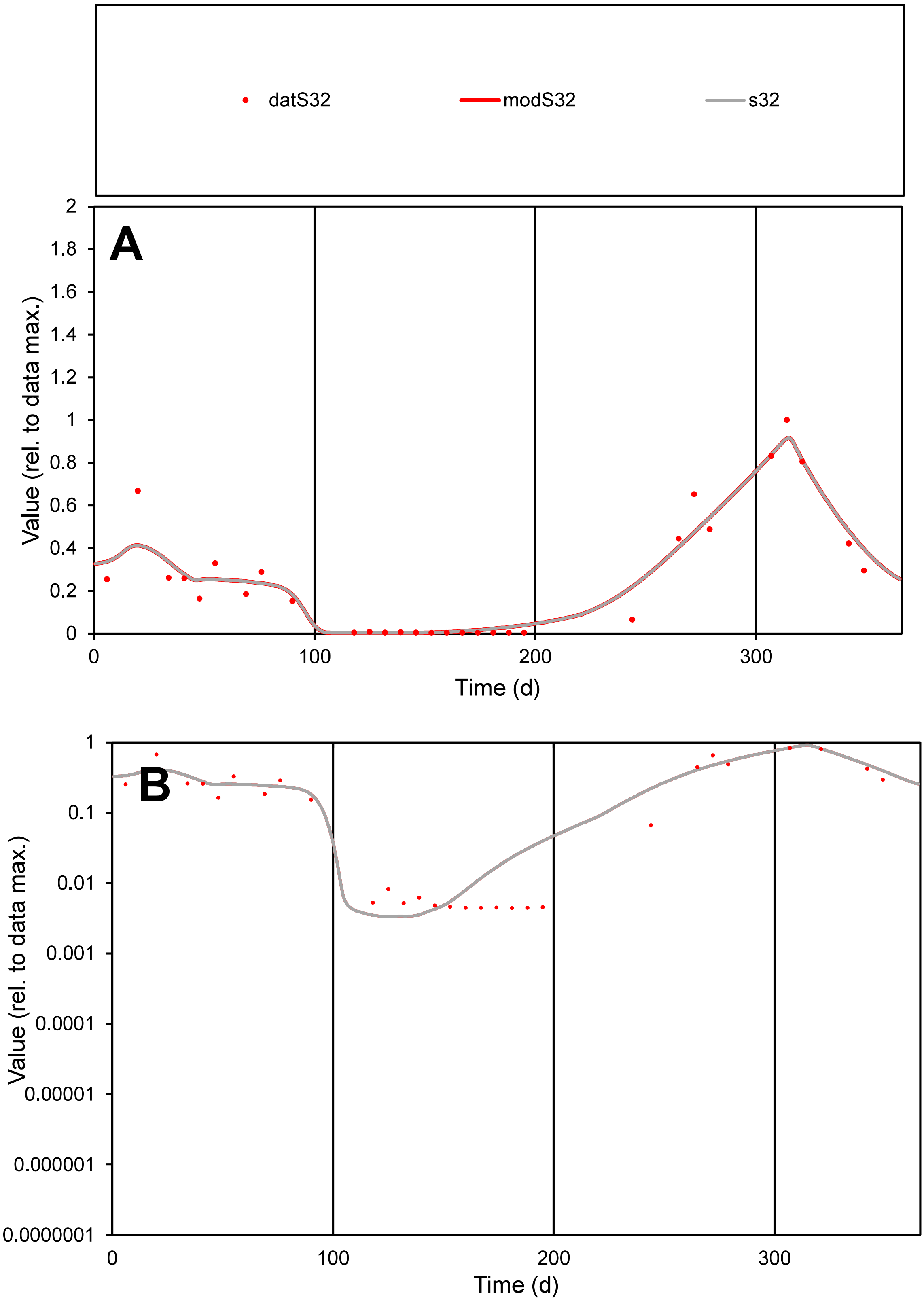

C1

Variables
S32 Provide jtd or Dst
CalibOnly?
2 1 to reproduce of
oNormalize?
2 1=no, 2=yes

ofxn calc*
0.29975
ofxn mod
0.34746

sRun
RunA1
zout
1

Ave
Data
0.00062
Model
0.00063

data/vMax
0.00242

ofxng
275.646

Min d/m
1.3133

ave/min d
57.5632
ave/min m
76.6899

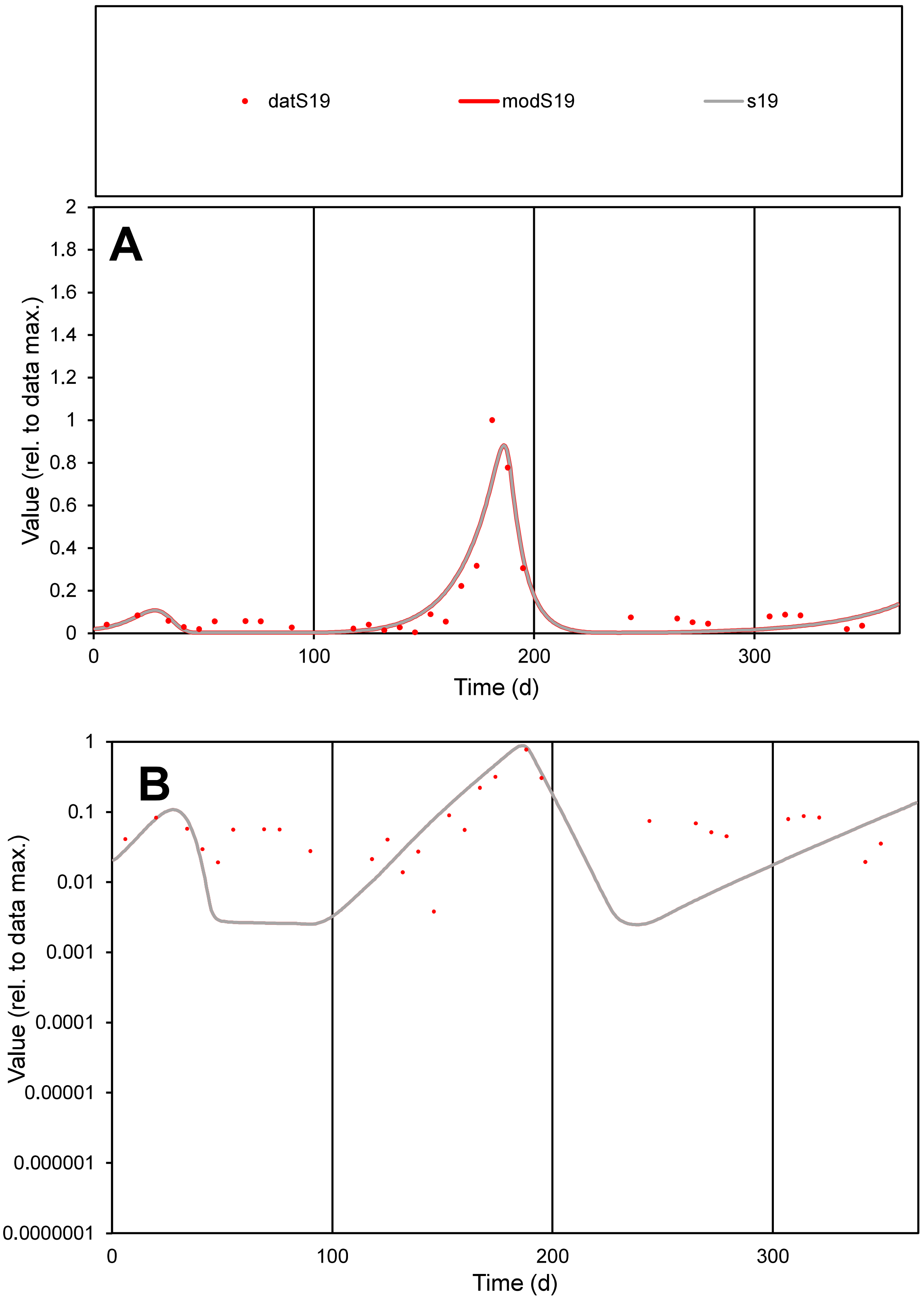

C1

Variables
S19 Provide jtd or Dst
CalibOnly?
2 1 to reproduce of
oNormalize?
2 1=no, 2=yes

ofxn calc*
0.54848
ofxn mod
0.59728

sRun
RunA1
zout
1

Ave
Data
0.00171
Model
0.00162

data/vMax
0.01336

ofxng
275.646

Min d/m
1.50473

ave/min d
33.6065
ave/min m
47.7043

325_Calib_S_A

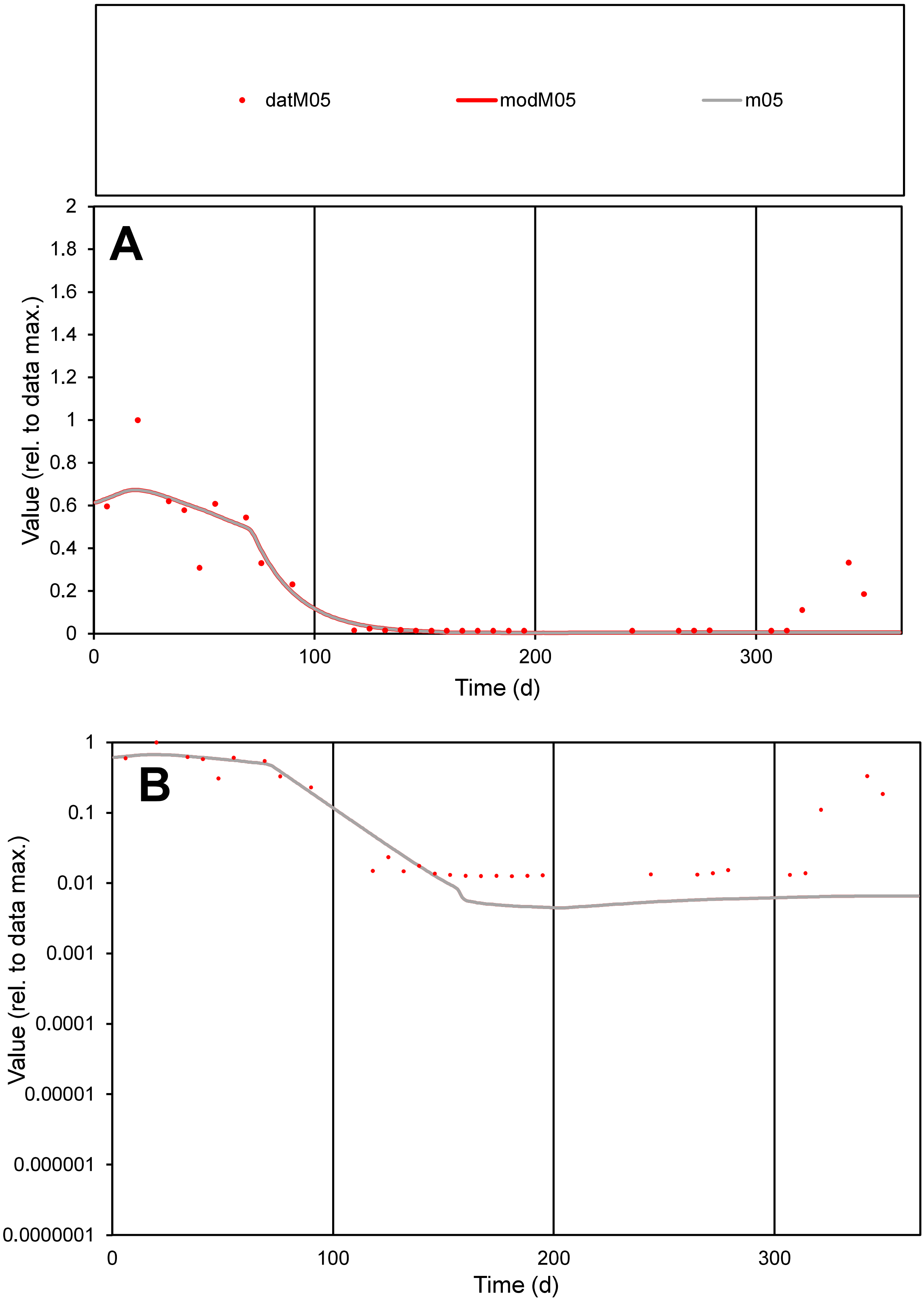

C1

Variables
M05 Provide jtd or Dst
CalibOnly?
2 1 to reproduce of
oNormalize?
2 1=no, 2=yes

ofxn calc*
0.56549
ofxn mod
0.59648

sRun
RunA1
zout
1

Ave
Data
0.00065
Model
0.00056

data/vMax
0.00342

ofxng
275.646

Min d/m
2.77038

ave/min d
15.1609
ave/min m
36.3731

325_Calib_S_A

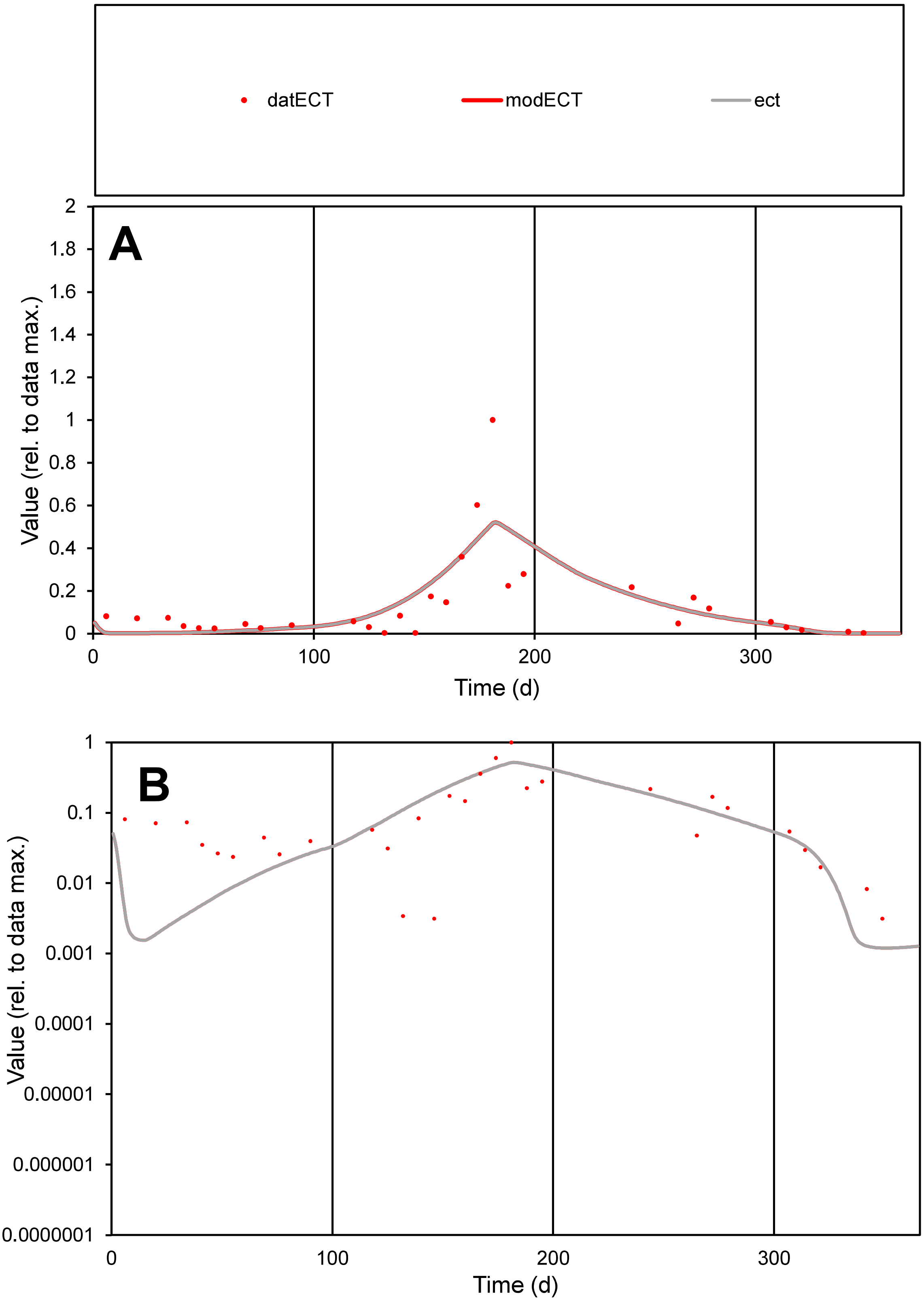

C1

Variables
ECT Provide jtd or Dst
CalibOnly?
2 1 to reproduce of
oNormalize?
2 1=no, 2=yes

ofxn calc*
0.90695
ofxn mod
0.93057

sRun
RunA1
zout
1

Ave
Data
0.00131
Model
0.00133

data/vMax
0.00973

ofxng
275.646

Min d/m
2.61235

ave/min d
43.0908
ave/min m
114.19

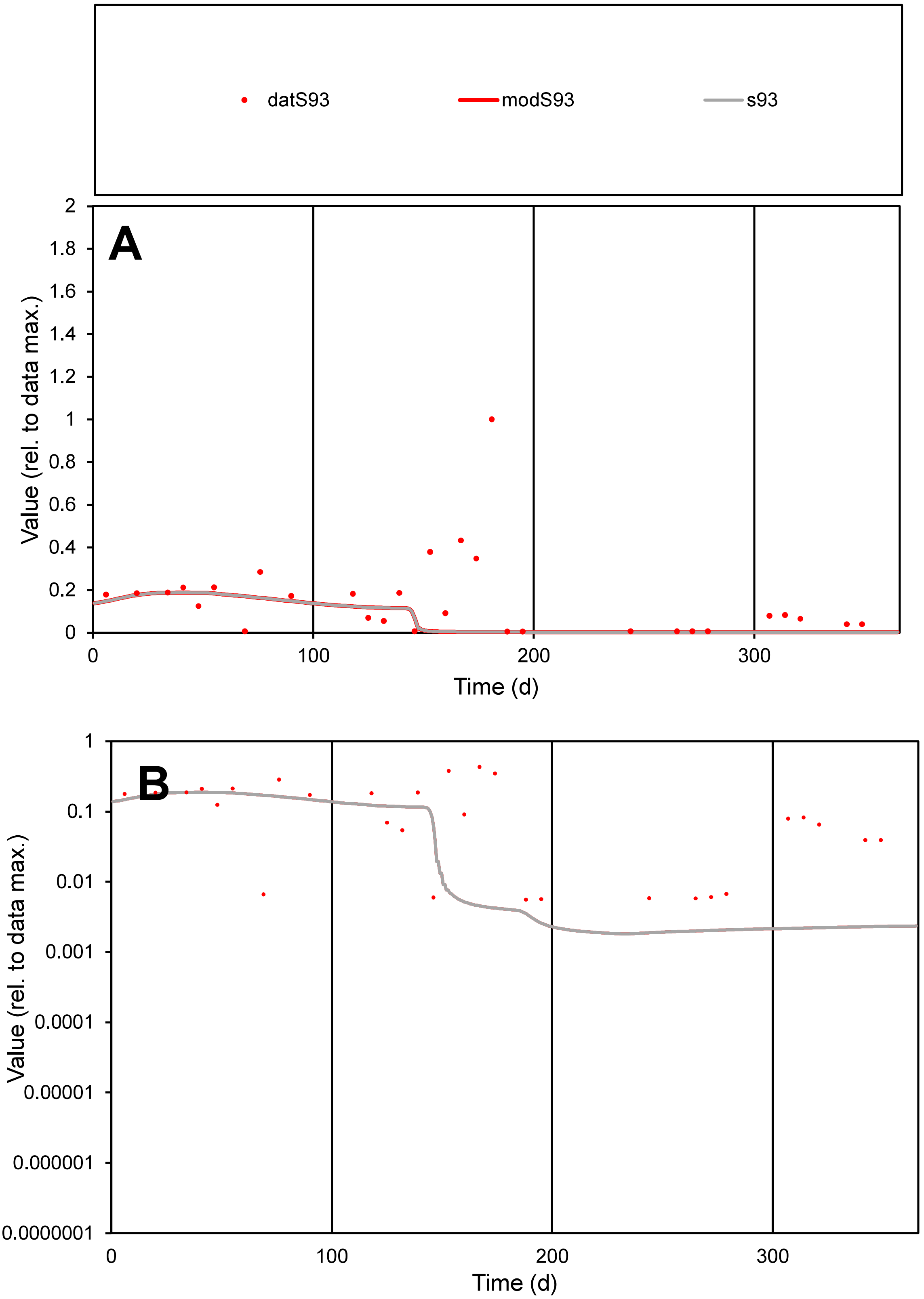

C1

Variables
S93 Provide jtd or Dst
CalibOnly?
2 1 to reproduce of
oNormalize?
2 1=no, 2=yes

ofxn calc*
1.45162
ofxn mod
1.45605

sRun
RunA1
zout
1

Ave
Data
0.00183
Model
0.00082

data/vMax
0.01179

ofxng
275.646

Min d/m
2.94831

ave/min d
27.8637
ave/min m
36.9832

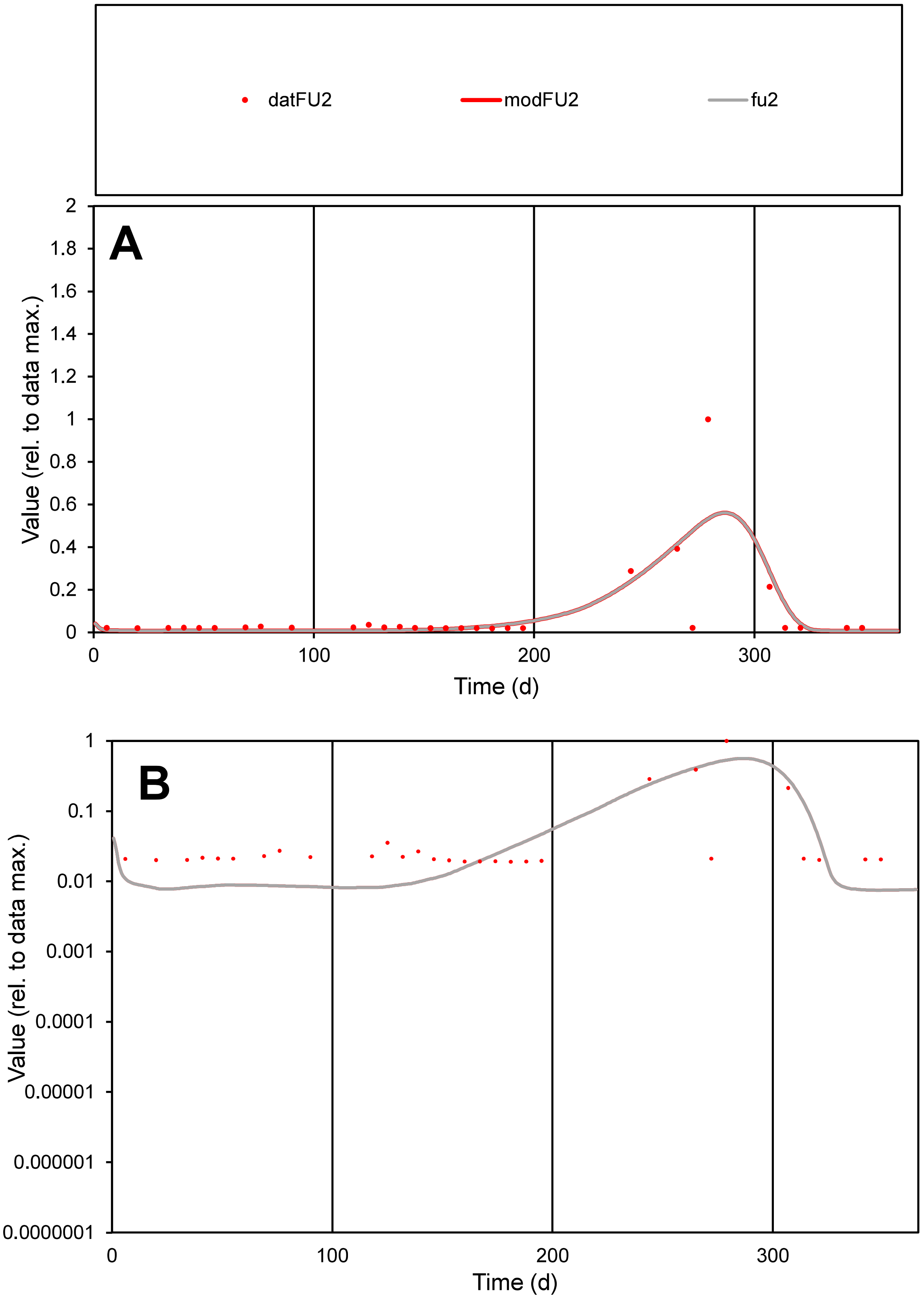

C1

Variables
FU2 Provide jtd or Dst
CalibOnly?
2 1 to reproduce of
oNormalize?
2 1=no, 2=yes

ofxn calc*
1.47343
ofxn mod
1.50716

sRun
RunA1
zout
1

Ave
Data
0.00011
Model
0.00011

data/vMax
0.00134

ofxng
275.646

Min d/m
2.535

ave/min d
4.31682
ave/min m
10.7374

325_Calib_S_A

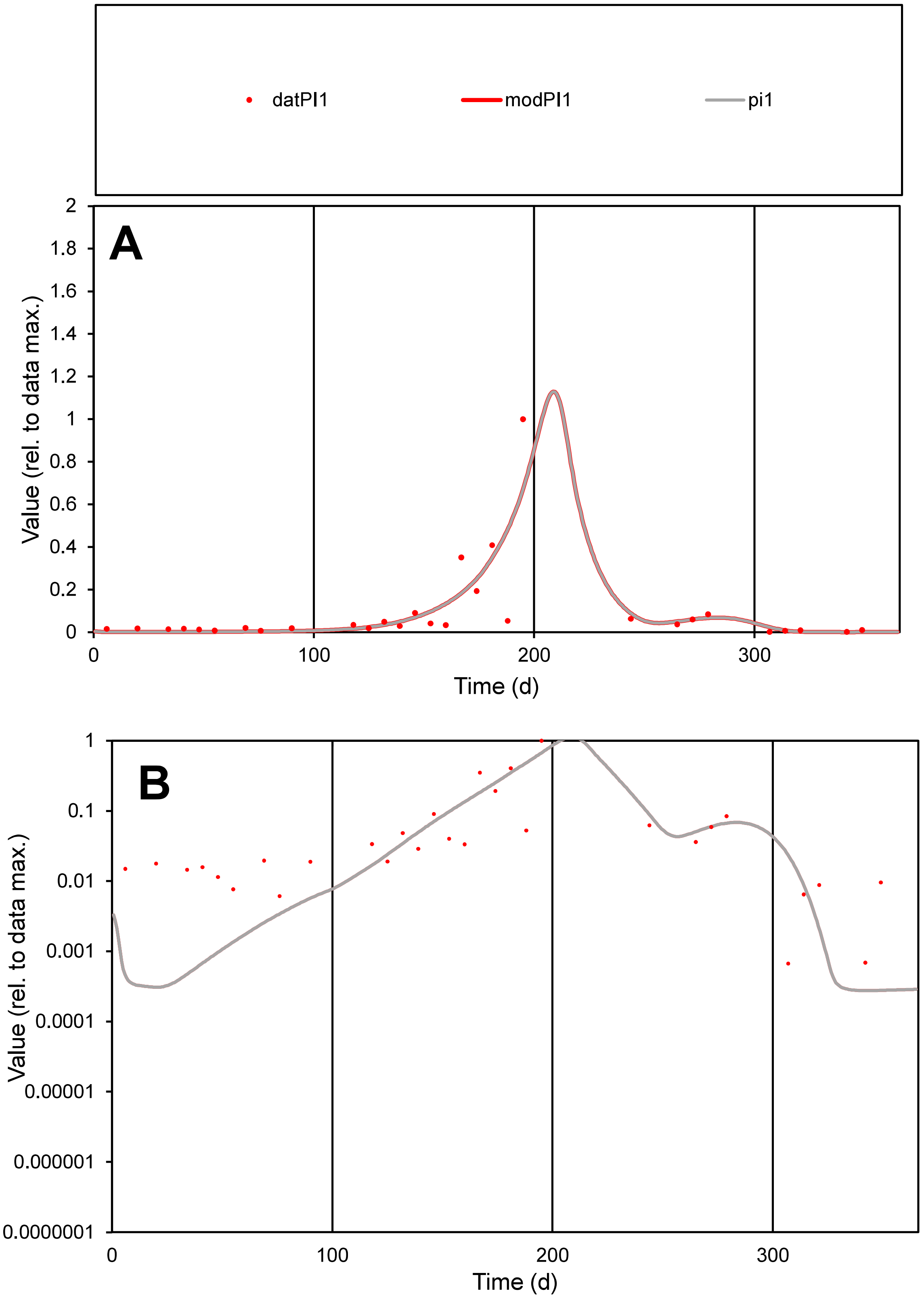

C1

Variables

PI1 Provide jtd or Dst

CalibOnly?

2 1 to reproduce of

oNormalize?

2 1=no, 2=yes

ofxn calc*

1.12367

ofxn mod

1.18133

sRun

RunA1

zout

1

Ave

Data

0.00184

Model

0.00189

data/vMax

0.0205

ofxng

275.646

Min d/m

2.42274

ave/min d

134.052

ave/min m

333.187

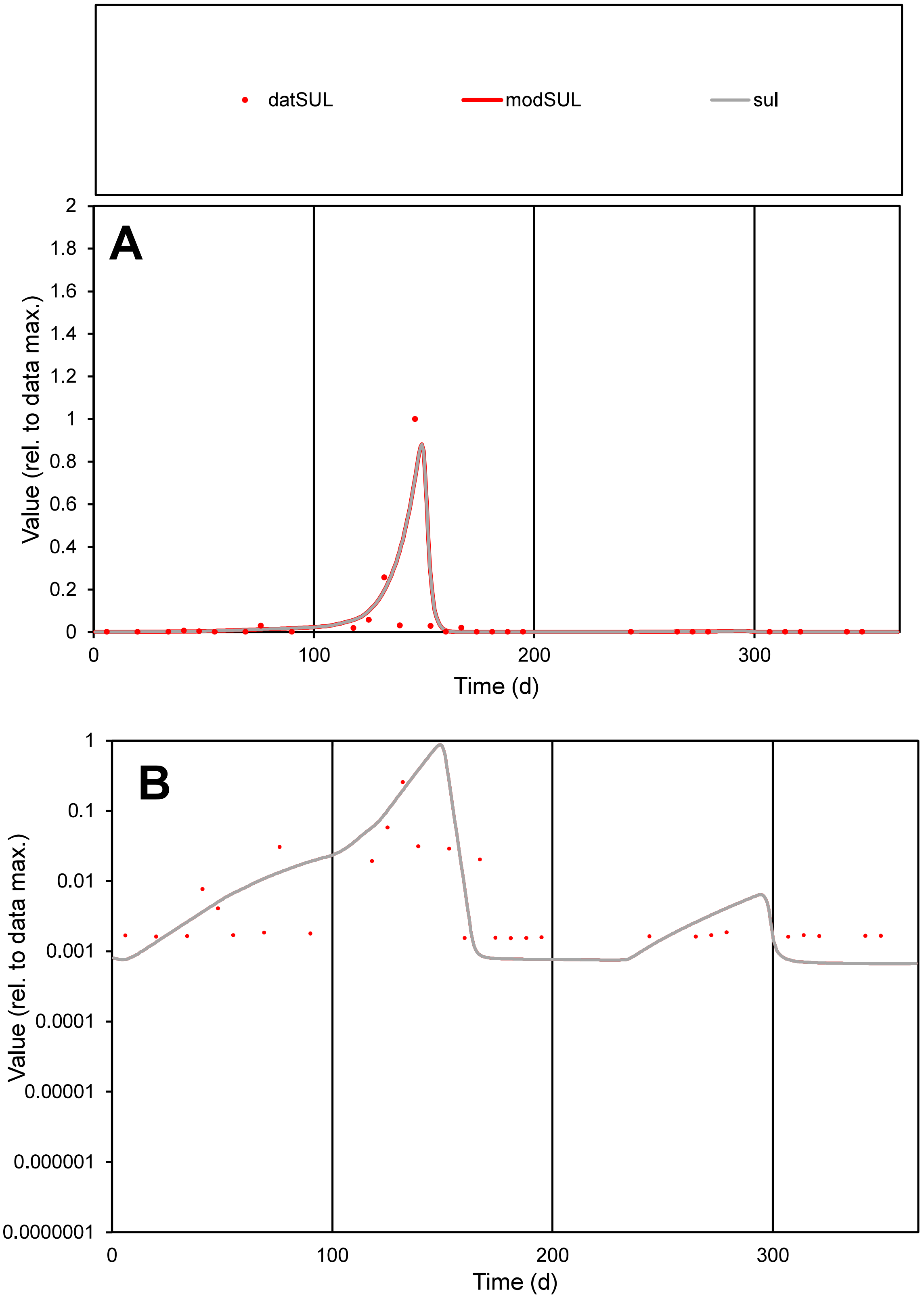

C1

Variables
SUL Provide jtd or Dst
CalibOnly?
2 1 to reproduce of
oNormalize?
2 1=no, 2=yes

ofxn calc*
1.37778
ofxn mod
1.51718

sRun
RunA1
zout
1

Ave
Data
0.00255
Model
0.00294

data/vMax
0.05121

ofxng
275.646

Min d/m
2.3005

ave/min d
32.3475
ave/min m
86.0317

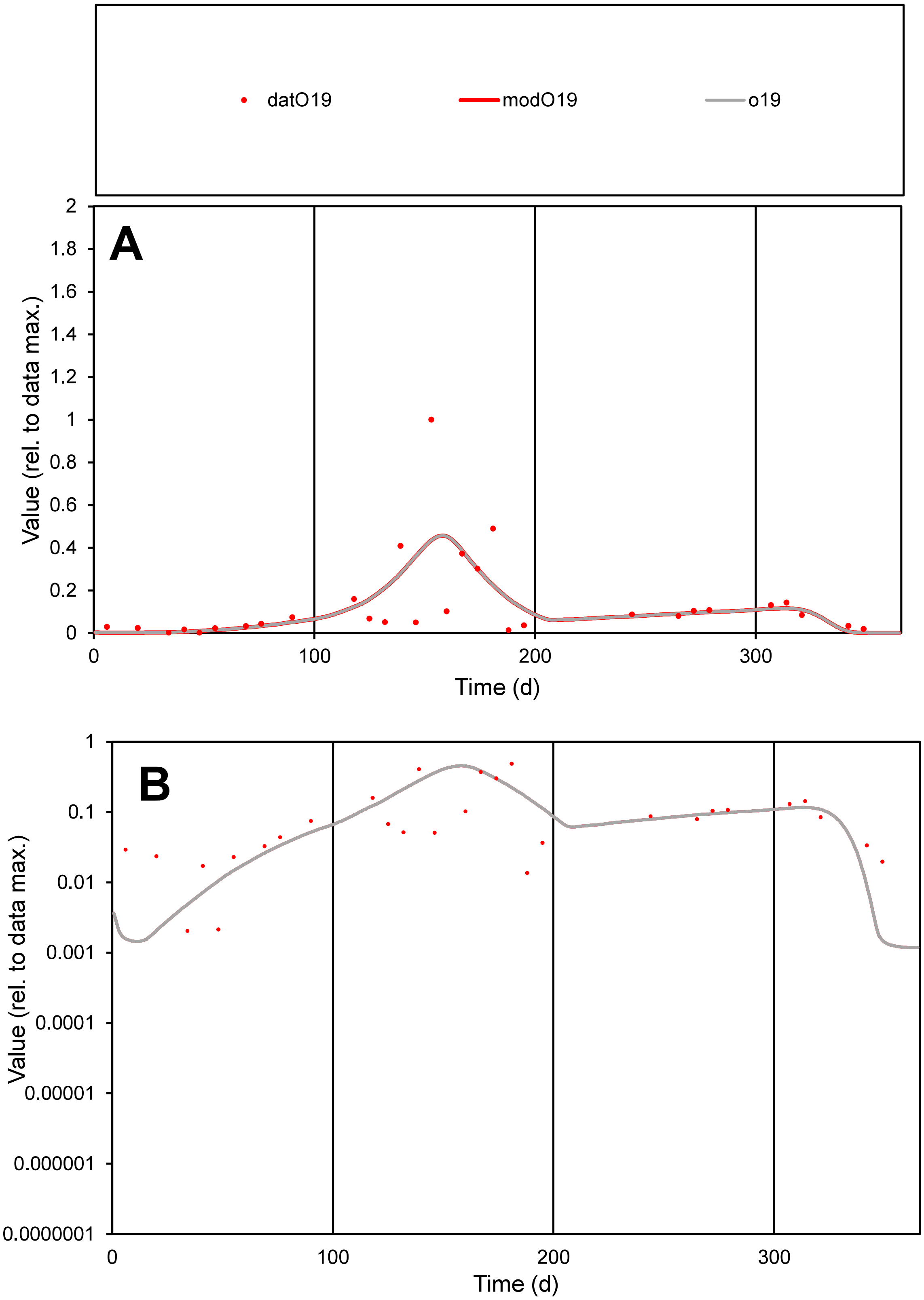

C1

Variables

O19 Provide jtd or Dst

CalibOnly?

2 1 to reproduce of

oNormalize?

2 1=no, 2=yes

ofxn calc*

1.09444

ofxn mod

1.11026

sRun

RunA1

zout

1

Ave

Data

0.002

Model

0.00198

data/vMax

0.01464

ofxng

275.646

Min d/m

1.36653

ave/min d

66.5687

ave/min m

90.1318

325_Calib_S_A

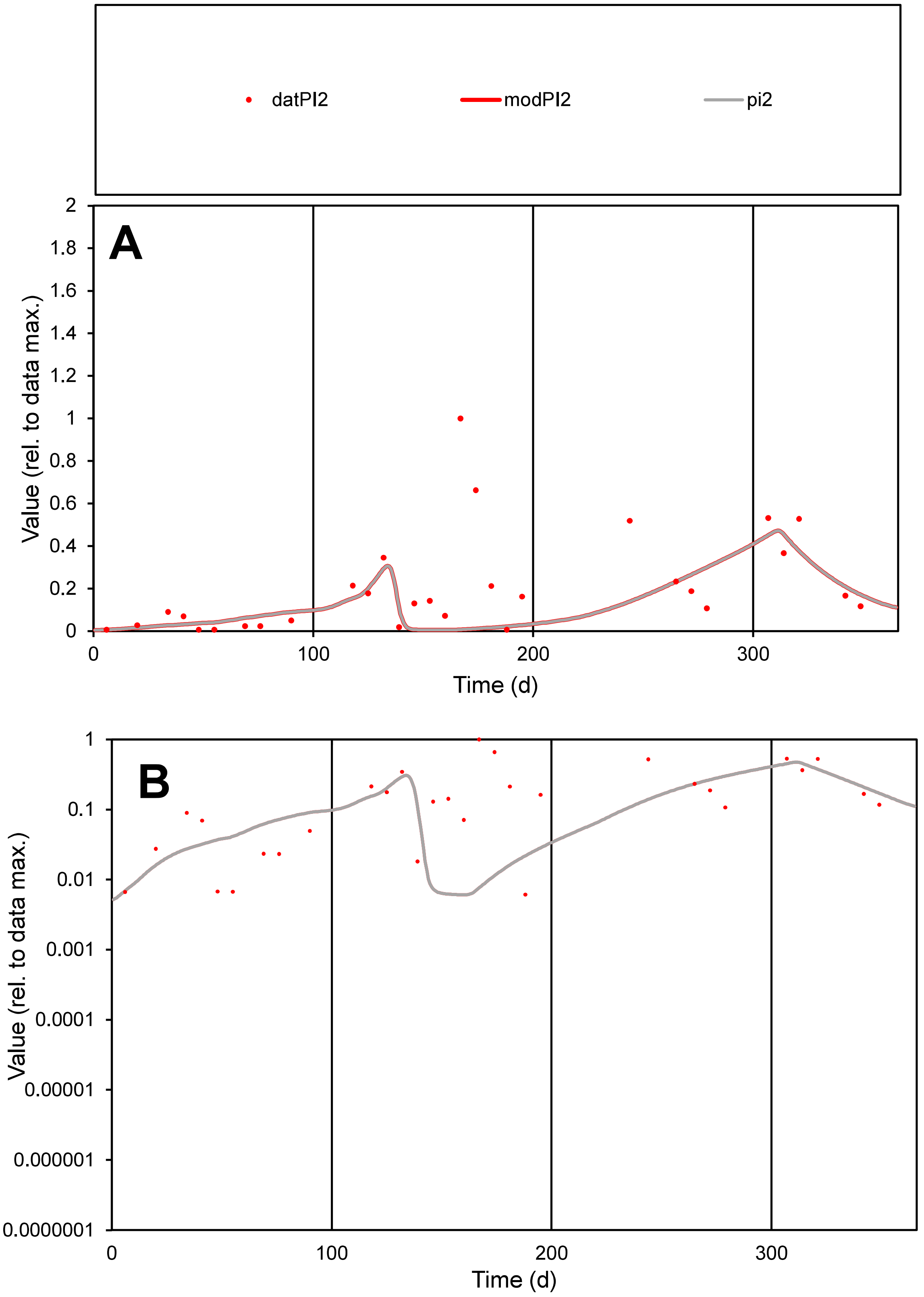

C1

Variables
PI2 Provide jtd or Dst
CalibOnly?
2 1 to reproduce of
oNormalize?
2 1=no, 2=yes

ofxn calc*
1.16726
ofxn mod
1.17275

sRun
RunA1
zout
1

Ave
Data
0.00096
Model
0.00059

data/vMax
0.00464

ofxng
275.646

Min d/m
1.00743

ave/min d
33.6113
ave/min m
20.7677

325_Calib_S_A

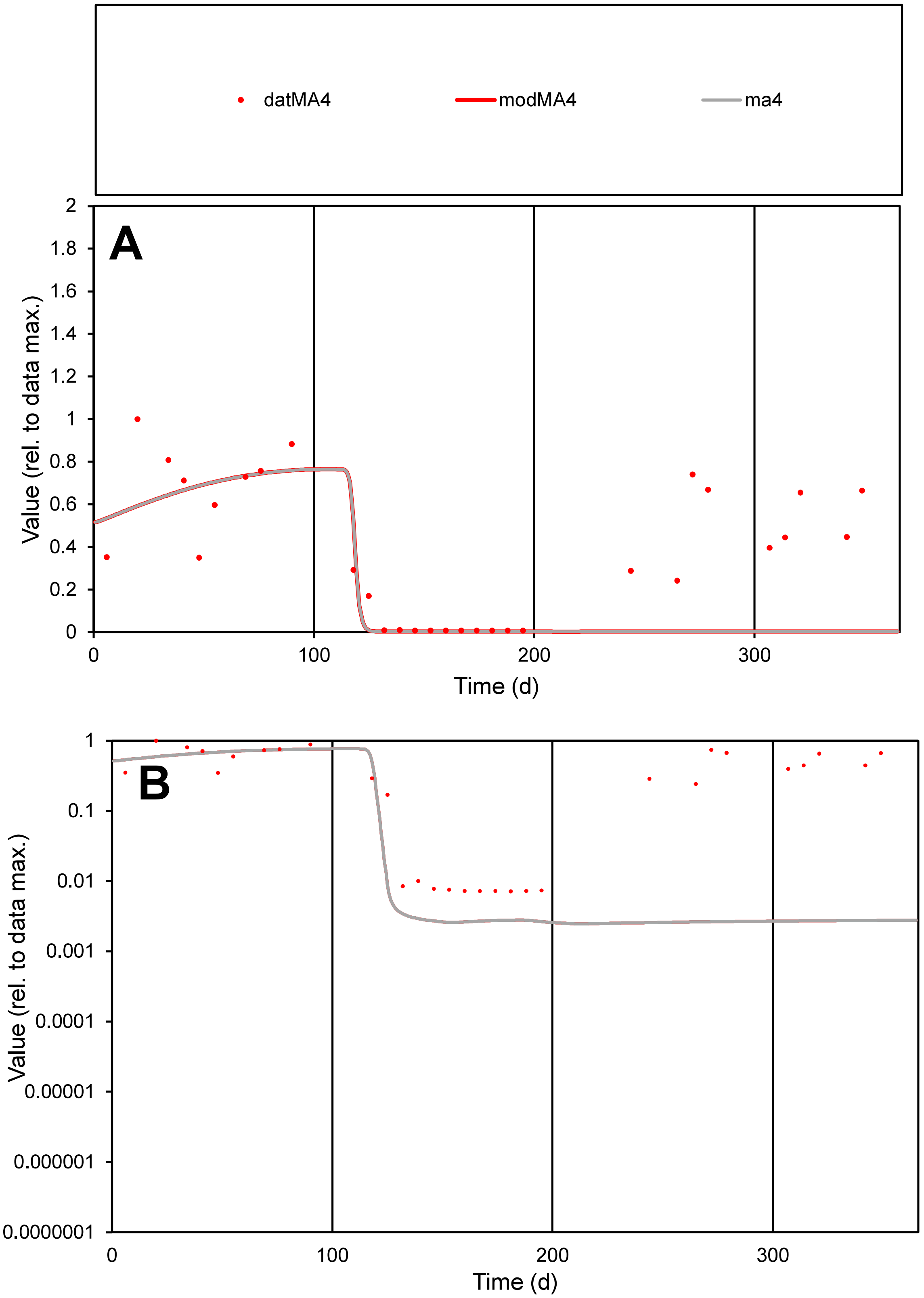

C1

Variables
MA4 Provide jtd or Dst
CalibOnly?
2 1 to reproduce of
oNormalize?
2 1=no, 2=yes

ofxn calc*
0.82959
ofxn mod
0.84199

sRun
RunA1
zout
1

Ave
Data
0.001
Model
0.00058

data/vMax
0.00268

ofxng
275.646

Min d/m
2.78181

ave/min d
52.4667
ave/min m
84.5546

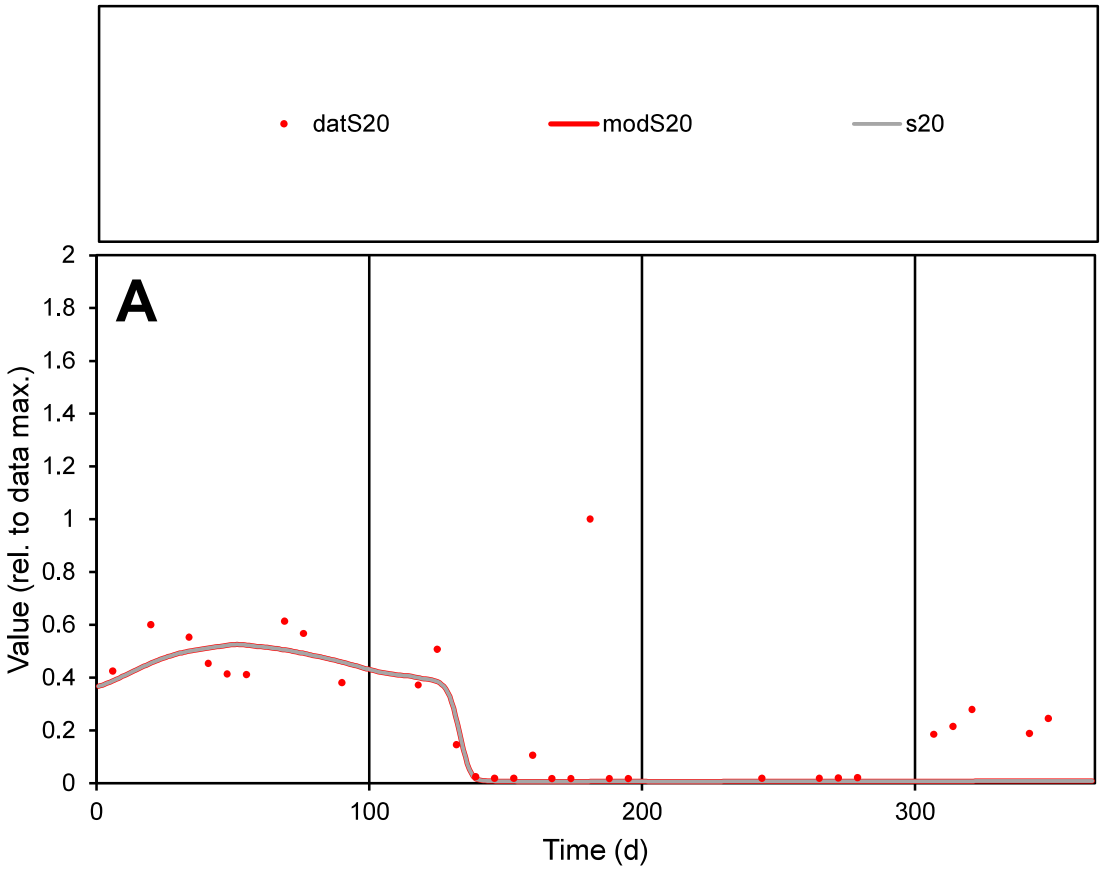

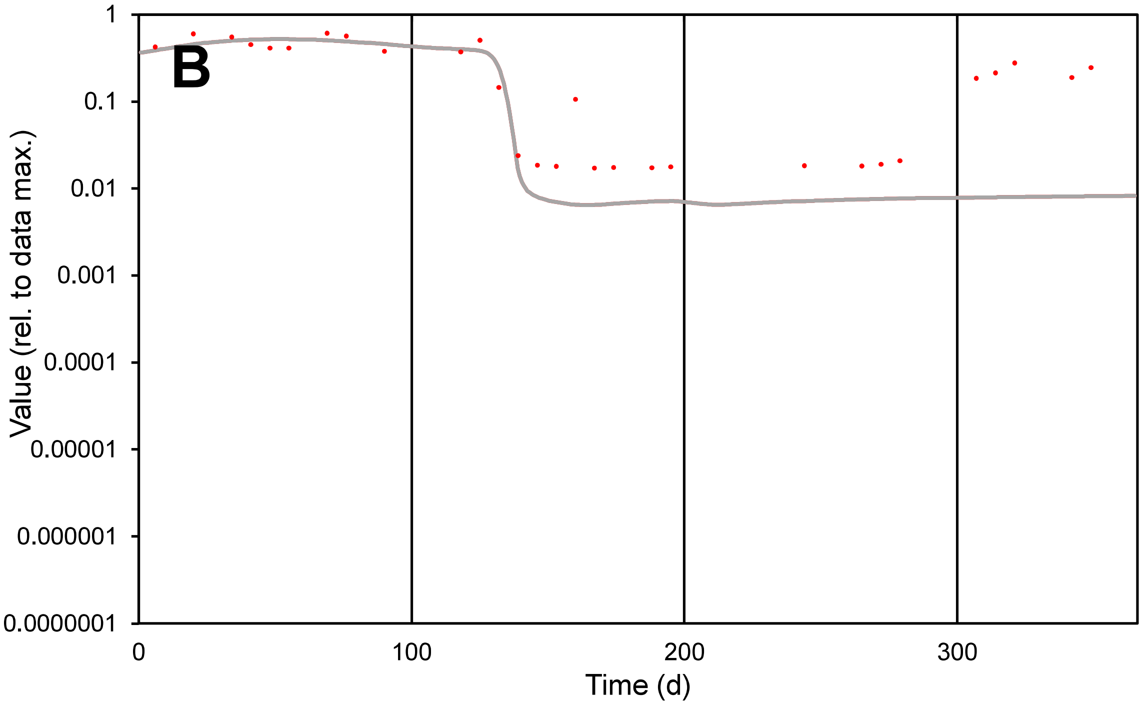

C1

Variables
S20 Provide jtd or Dst
CalibOnly?
2 1 to reproduce of
oNormalize?
2 1=no, 2=yes

ofxn calc*
0.80287
ofxn mod
0.81483

sRun
RunA1
zout
1

Ave
Data
0.00118
Model
0.00083

data/vMax
0.00452

ofxng
275.646

Min d/m
2.66849

ave/min d
15.174
ave/min m
28.2104

325_Calib_S_A

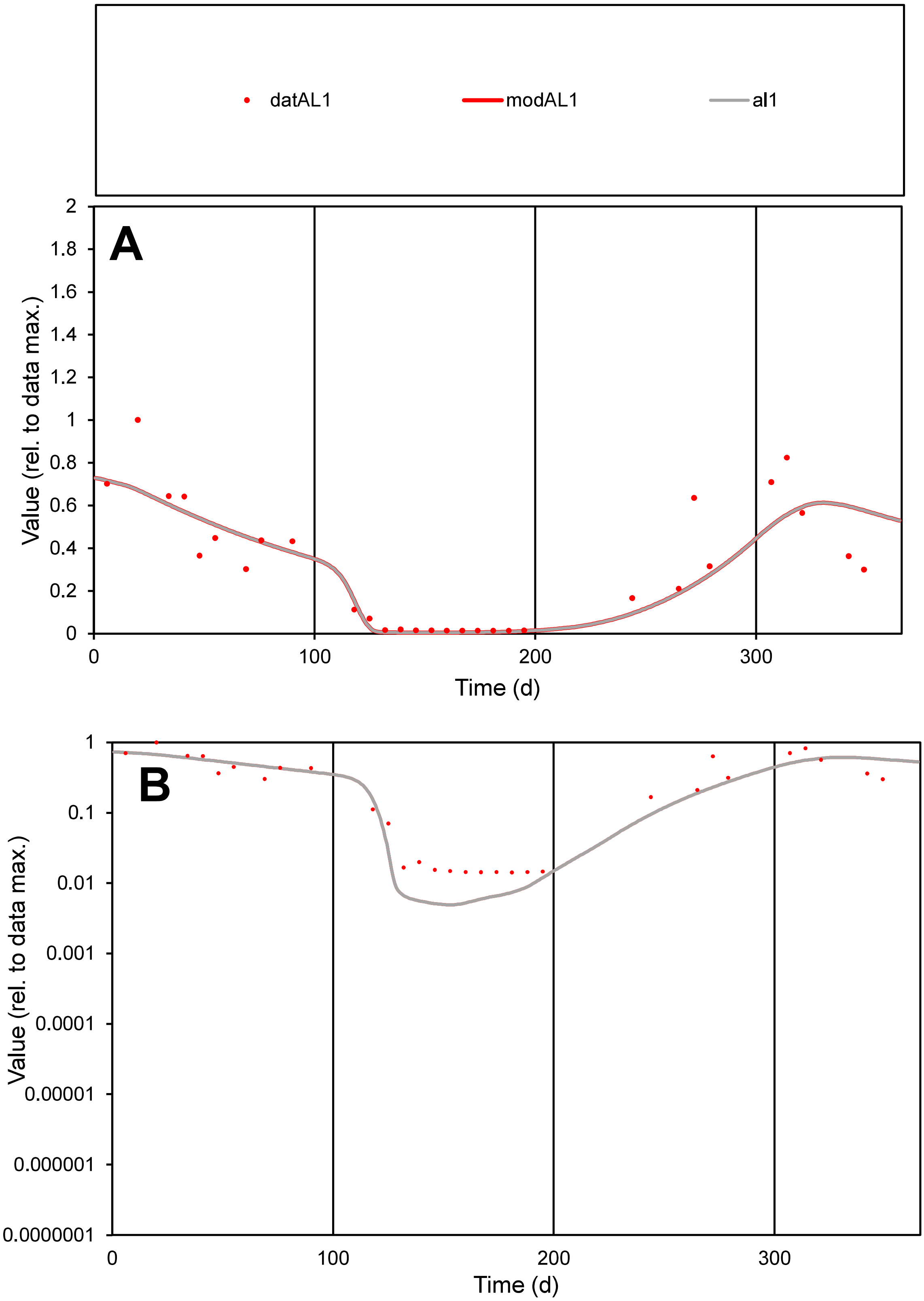

C1

Variables
AL1 Provide jtd or Dst
CalibOnly?
2 1 to reproduce of
oNormalize?
2 1=no, 2=yes

ofxn calc*
0.44015
ofxn mod
0.46496

sRun
RunA1
zout
1

Ave
Data
0.00096
Model
0.00089

data/vMax
0.00308

ofxng
275.646

Min d/m
2.88484

ave/min d
22.0931
ave/min m
59.2193

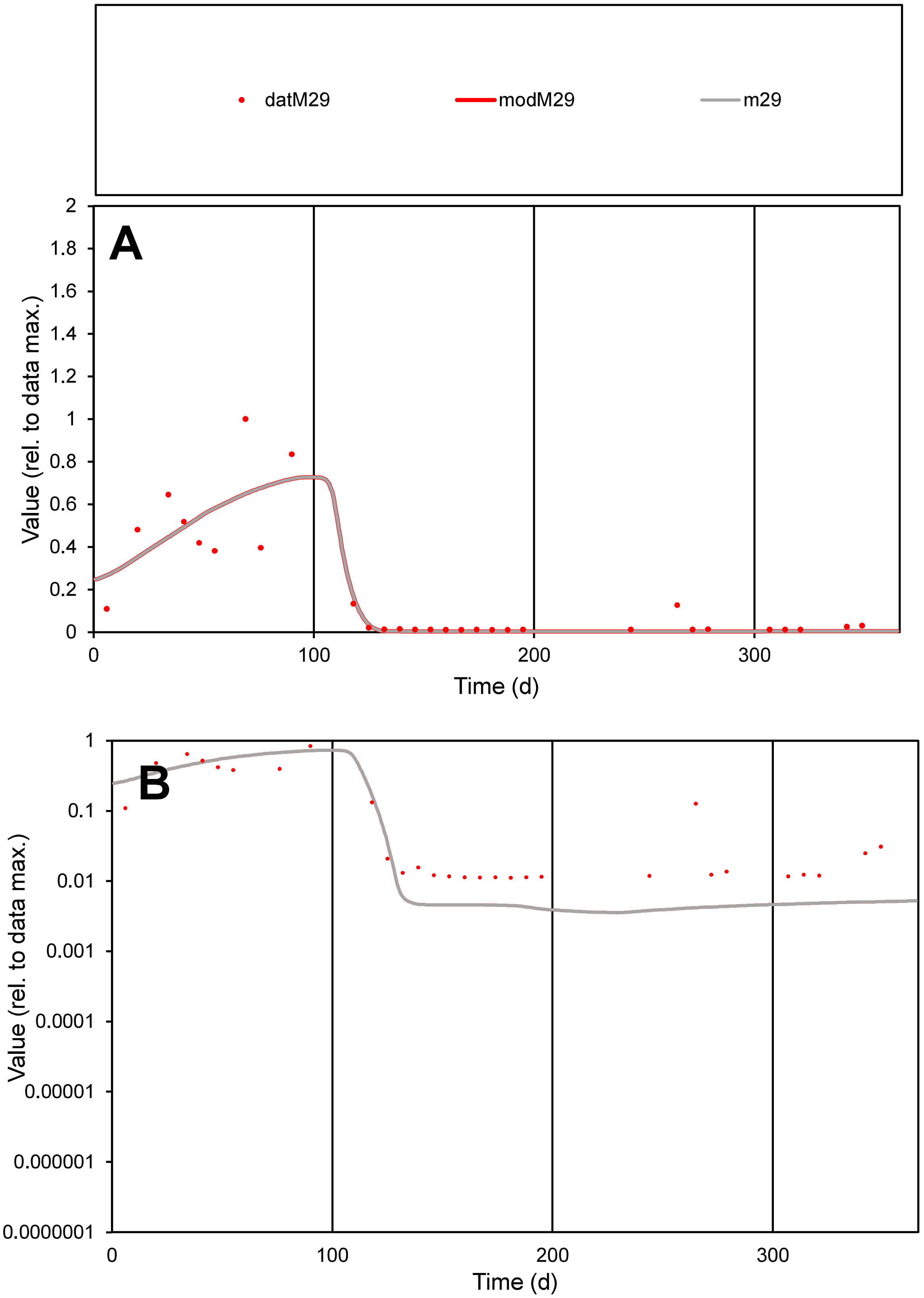

C1

Variables
M29 Provide jtd or Dst
CalibOnly?
2 1 to reproduce of
oNormalize?
2 1=no, 2=yes

ofxn calc*
0.62252
ofxn mod
0.6591

sRun
RunA1
zout
1

Ave
Data
0.00086
Model
0.00081

data/vMax
0.00485

ofxng
275.646

Min d/m
2.91272

ave/min d
15.891
ave/min m
43.7459

325_Calib_S_A

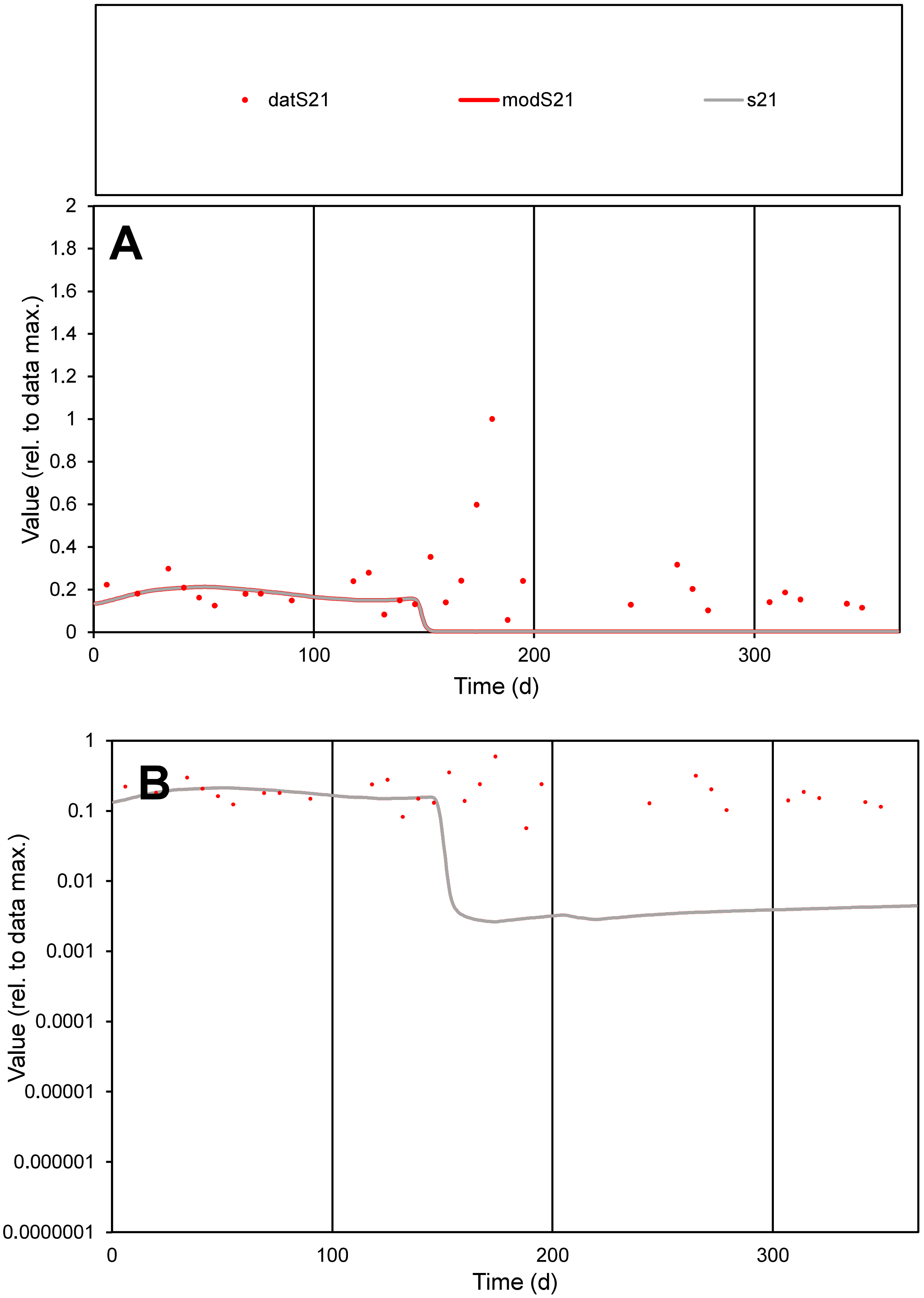

C1

Variables
S21 Provide jtd or Dst
CalibOnly?
2 1 to reproduce of
oNormalize?
2 1=no, 2=yes

ofxn calc*
1.13552
ofxn mod
1.1393

sRun
RunA1
zout
1

Ave
Data
0.00206
Model
0.00078

data/vMax
0.00924

ofxng
275.646

Min d/m
21.4869

ave/min d
3.9164
ave/min m
31.8597

325_Calib_S_A

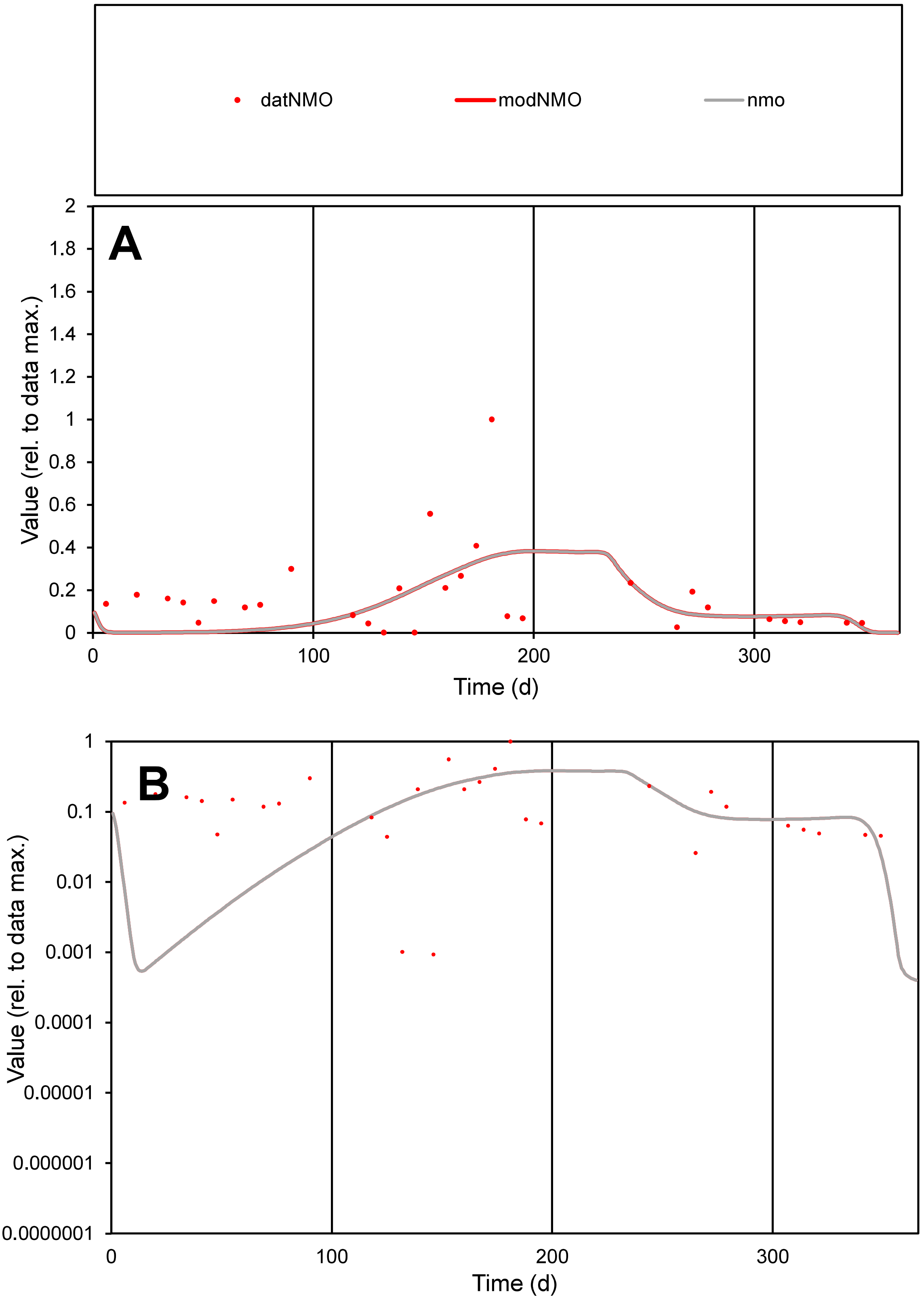

C1

Variables

NMO Provide jtd or Dst

CalibOnly?

2 1 to reproduce of

oNormalize?

2 1=no, 2=yes

ofxn calc*

1.06567

ofxn mod

1.07834

sRun

RunA1

zout

1

Ave

Data

0.00143

Model

0.00111

data/vMax

0.00835

ofxng

275.646

Min d/m

1.18748

ave/min d

182.857

ave/min m

168.347

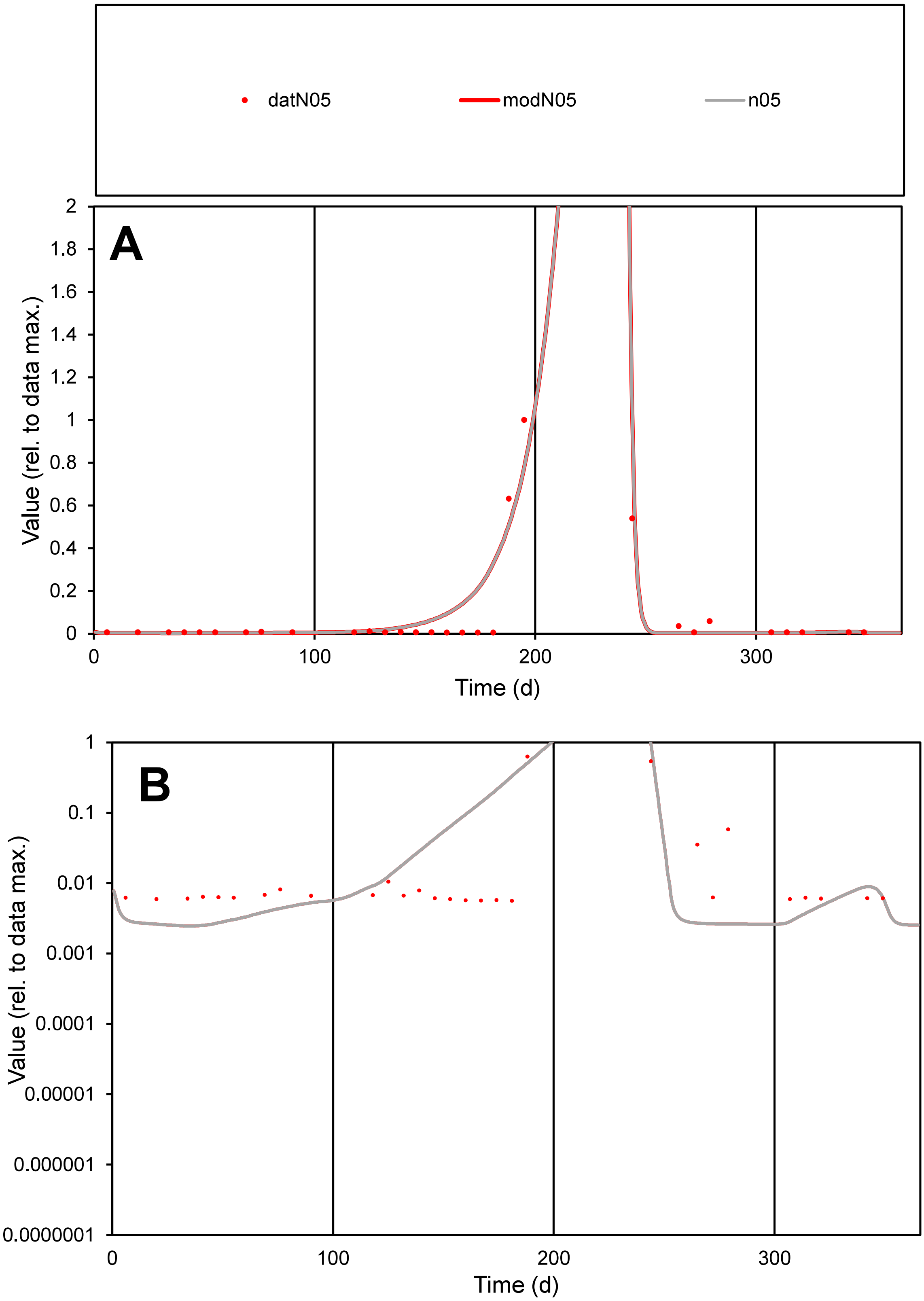

C1

Variables
N05 Provide jtd or Dst
CalibOnly?
2 1 to reproduce of
oNormalize?
2 1=no, 2=yes

ofxn calc*
0.99747
ofxn mod
1.07862

sRun
RunA1
zout
1

Ave
Data
0.00057
Model
0.00068

data/vMax
0.00701

ofxng
275.646

Min d/m
2.29866

ave/min d
14.3391
ave/min m
39.3017

325_Calib_S_A

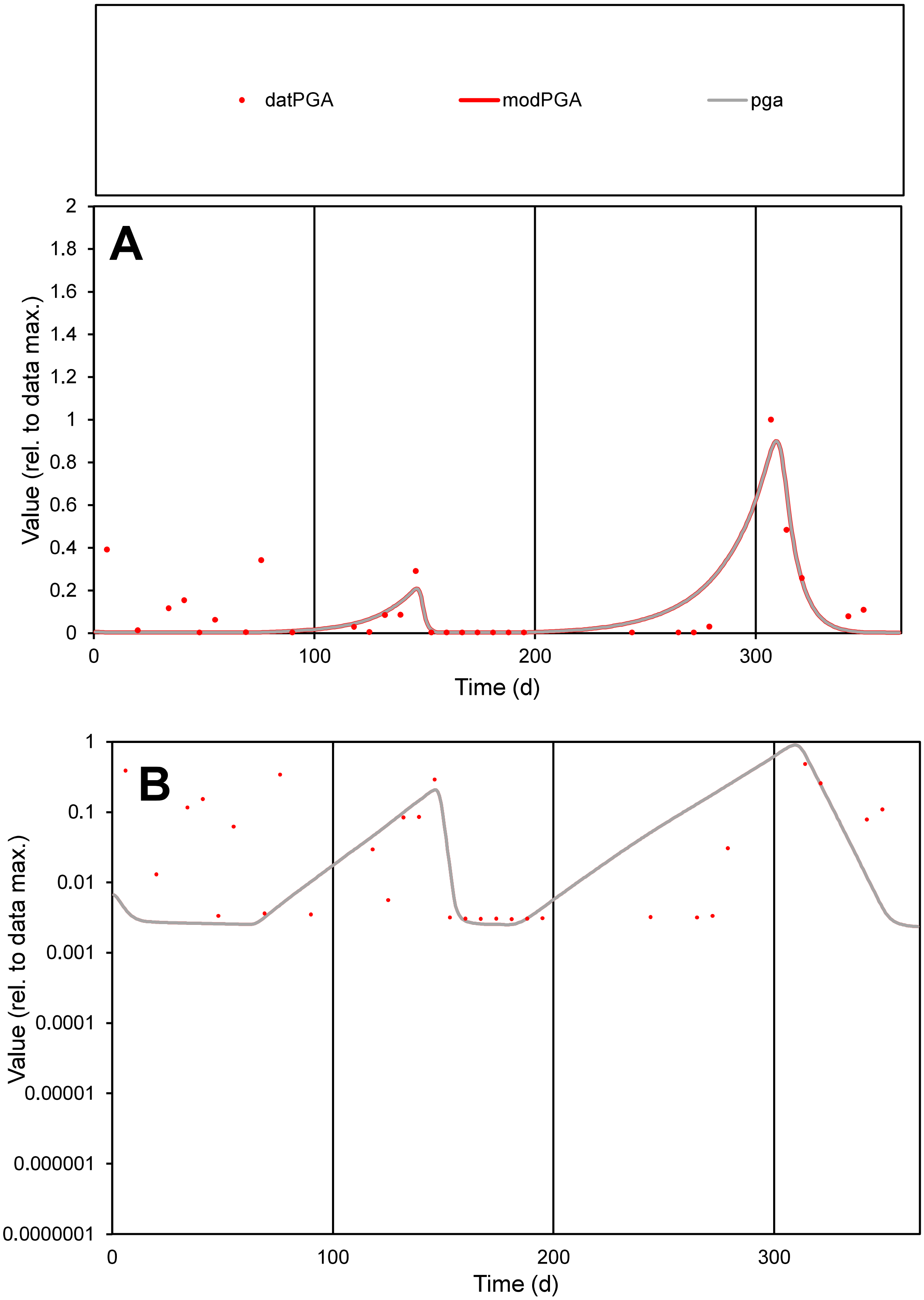

C1

Variables
PGA Provide jtd or Dst
CalibOnly?
2 1 to reproduce of
oNormalize?
2 1=no, 2=yes

ofxn calc*
1.01845
ofxn mod
1.04977

sRun
RunA1
zout
1

Ave
Data
0.00067
Model
0.00054

data/vMax
0.0056

ofxng
275.646

Min d/m
1.19182

ave/min d
39.5243
ave/min m
37.8345

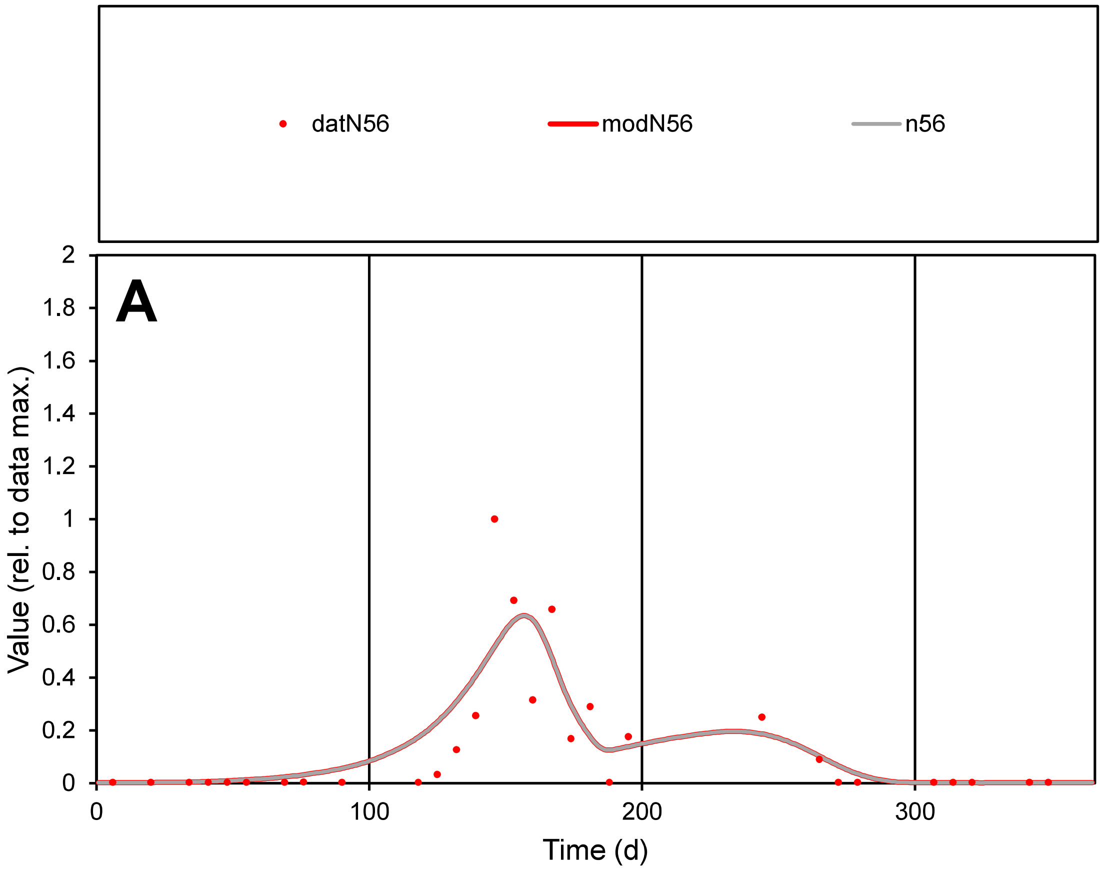

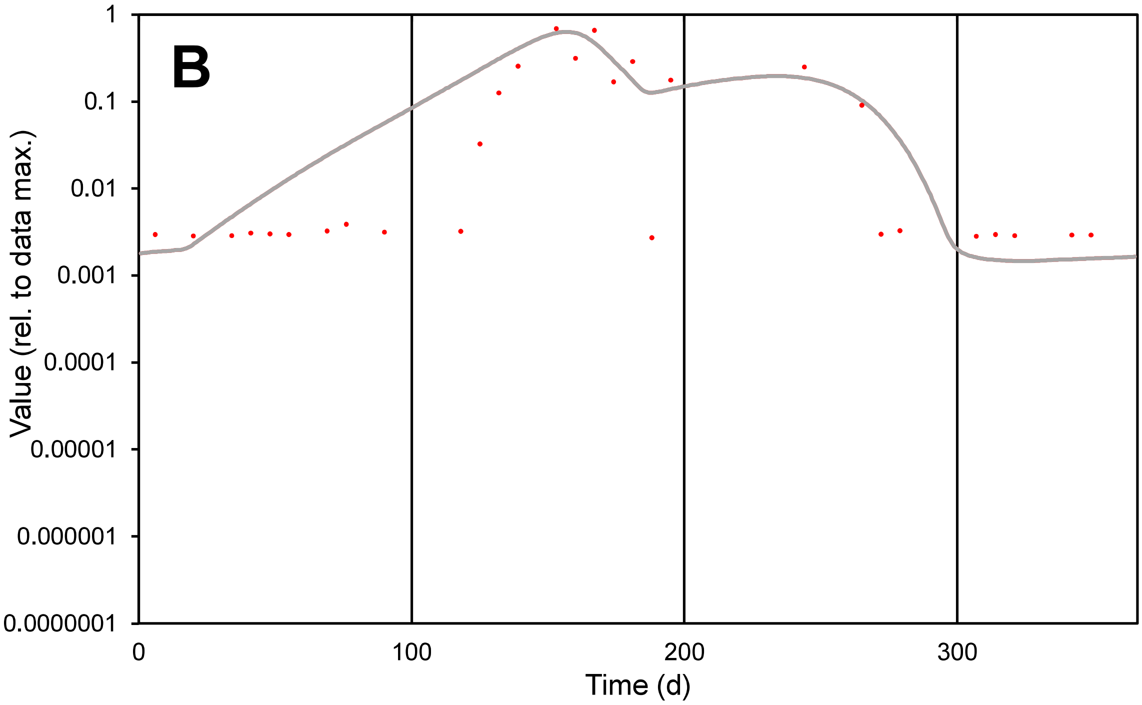

C1

Variables
N56 Provide jtd or Dst
CalibOnly?
2 1 to reproduce of
oNormalize?
2 1=no, 2=yes

ofxn calc*
0.9587
ofxn mod
0.98442

sRun
RunA1
zout
1

Ave
Data
0.00172
Model
0.00192

data/vMax
0.01256

ofxng
275.646

Min d/m
1.86366

ave/min d
50.1549
ave/min m
104.124

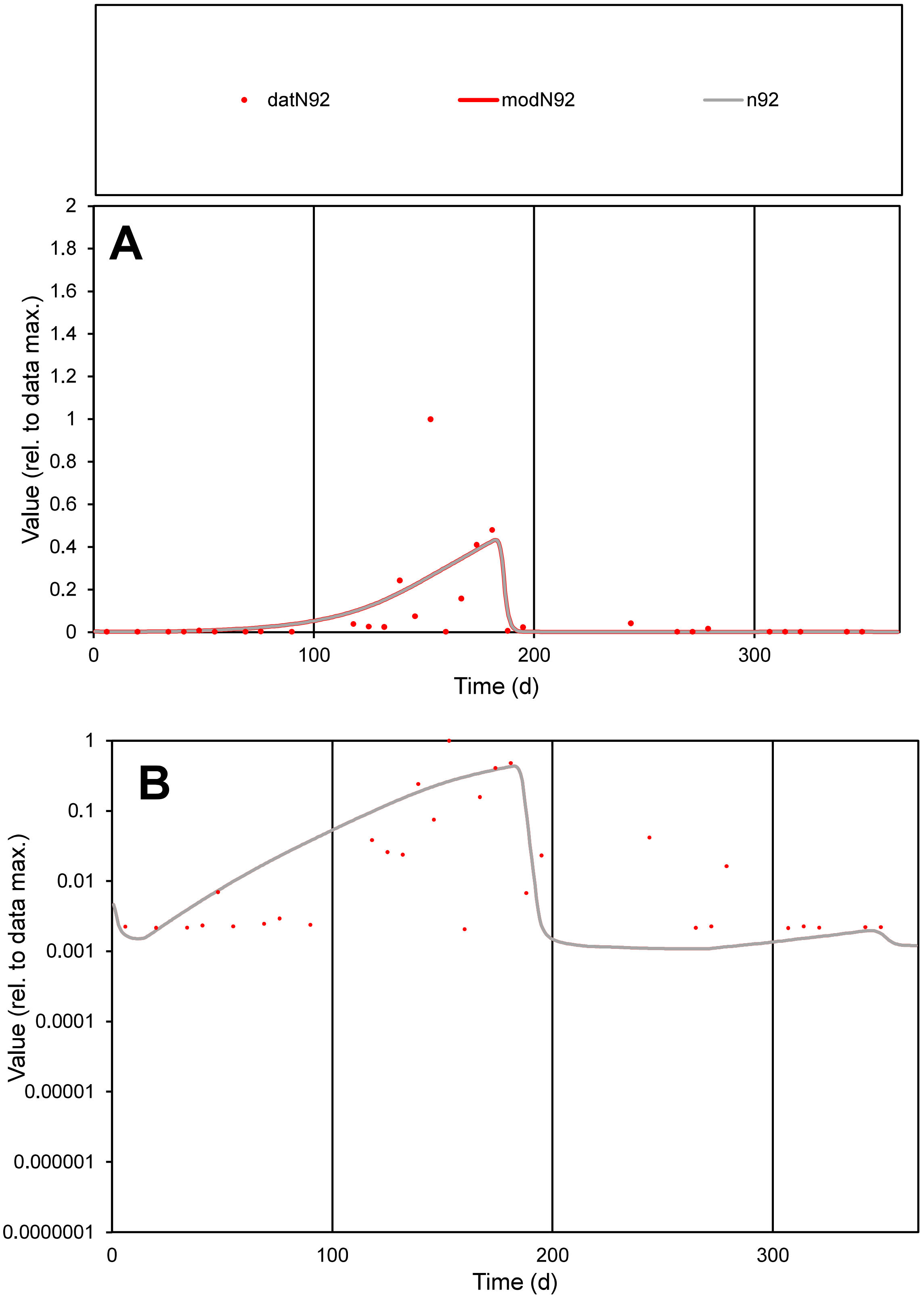

C1

Variables
N92 Provide jtd or Dst
CalibOnly?
2 1 to reproduce of
oNormalize?
2 1=no, 2=yes

ofxn calc*
1.80055
ofxn mod
1.81463

sRun
RunA1
zout
1

Ave
Data
0.00159
Model
0.00166

data/vMax
0.01844

ofxng
275.646

Min d/m
1.90045

ave/min d
41.6094
ave/min m
82.7084

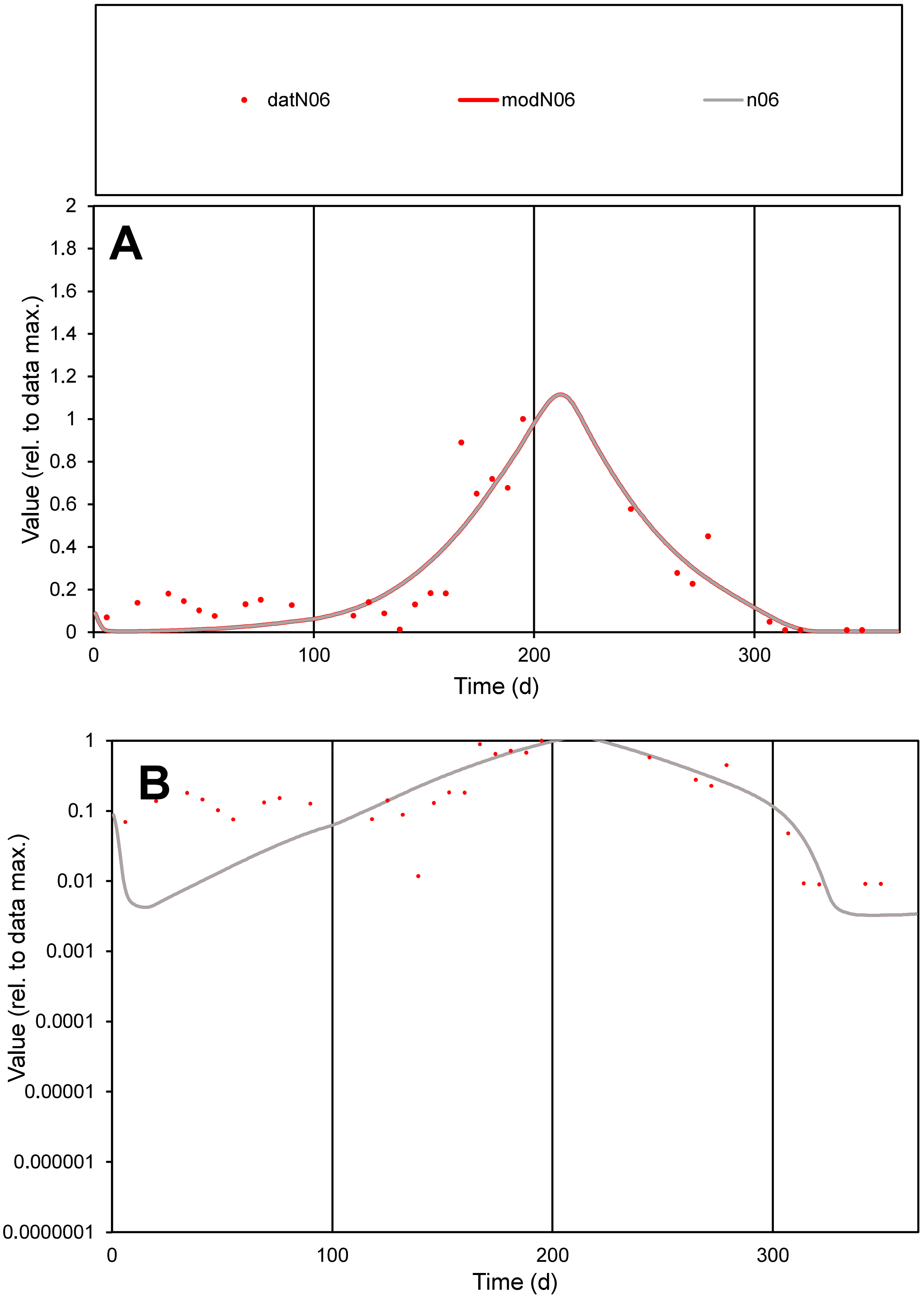

C1

Variables
N06 Provide jtd or Dst
CalibOnly?
2 1 to reproduce of
oNormalize?
2 1=no, 2=yes

ofxn calc*
0.5104
ofxn mod
0.5387

sRun
RunA1
zout
1

Ave
Data
0.00191
Model
0.00179

data/vMax
0.00766

ofxng
275.646

Min d/m
2.76796

ave/min d
27.713
ave/min m
71.8835

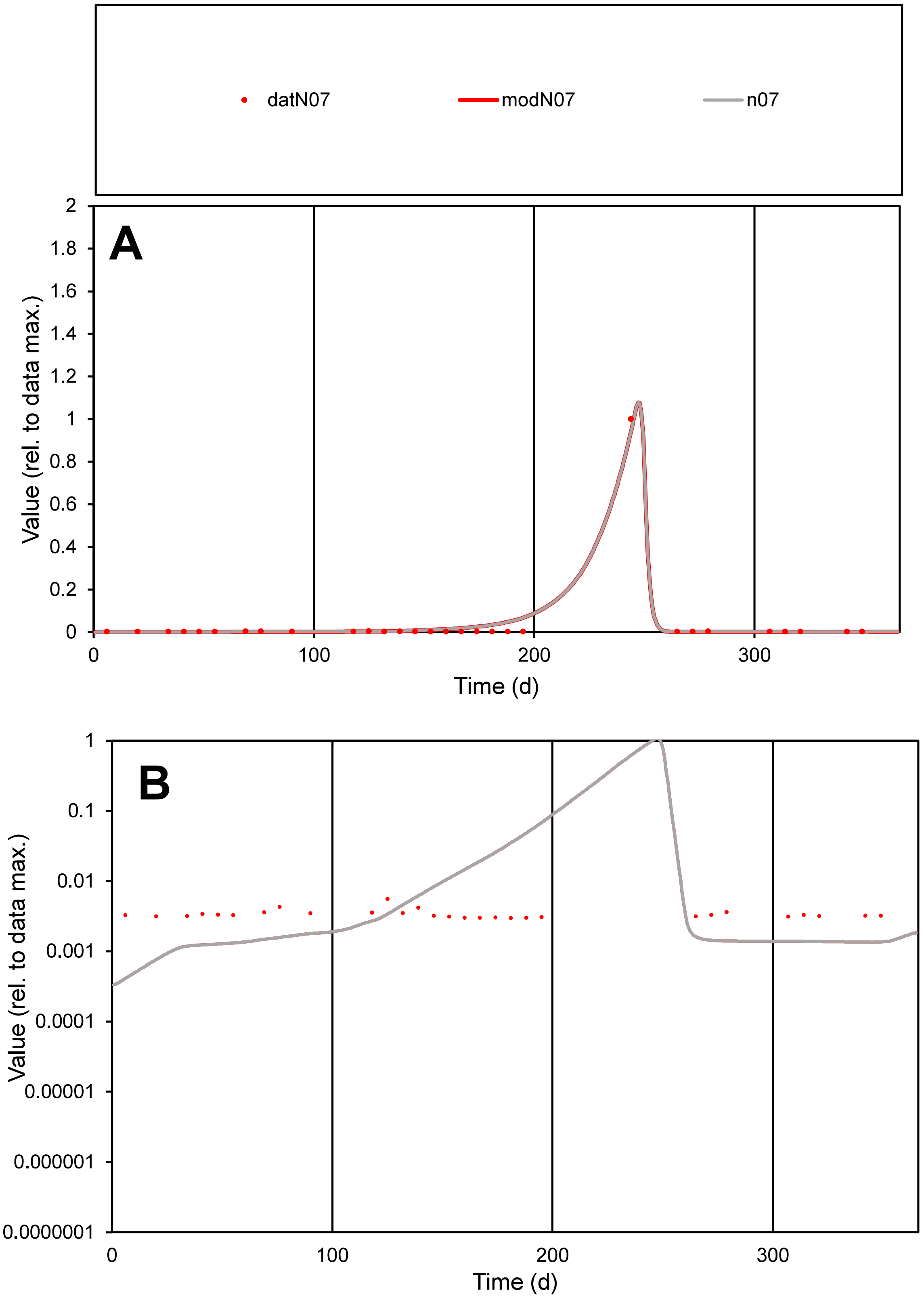

C1

Variables

N07 Provide jtd or Dst

CalibOnly?

2 1 to reproduce of

oNormalize?

2 1=no, 2=yes

ofxn calc*

0.44244

ofxn mod

0.59543

sRun

RunA1

zout

1

Ave

Data

0.00021

Model

0.00024

data/vMax

0.00567

ofxng

275.646

Min d/m

6.87551

ave/min d

12.2875

ave/min m

96.7448

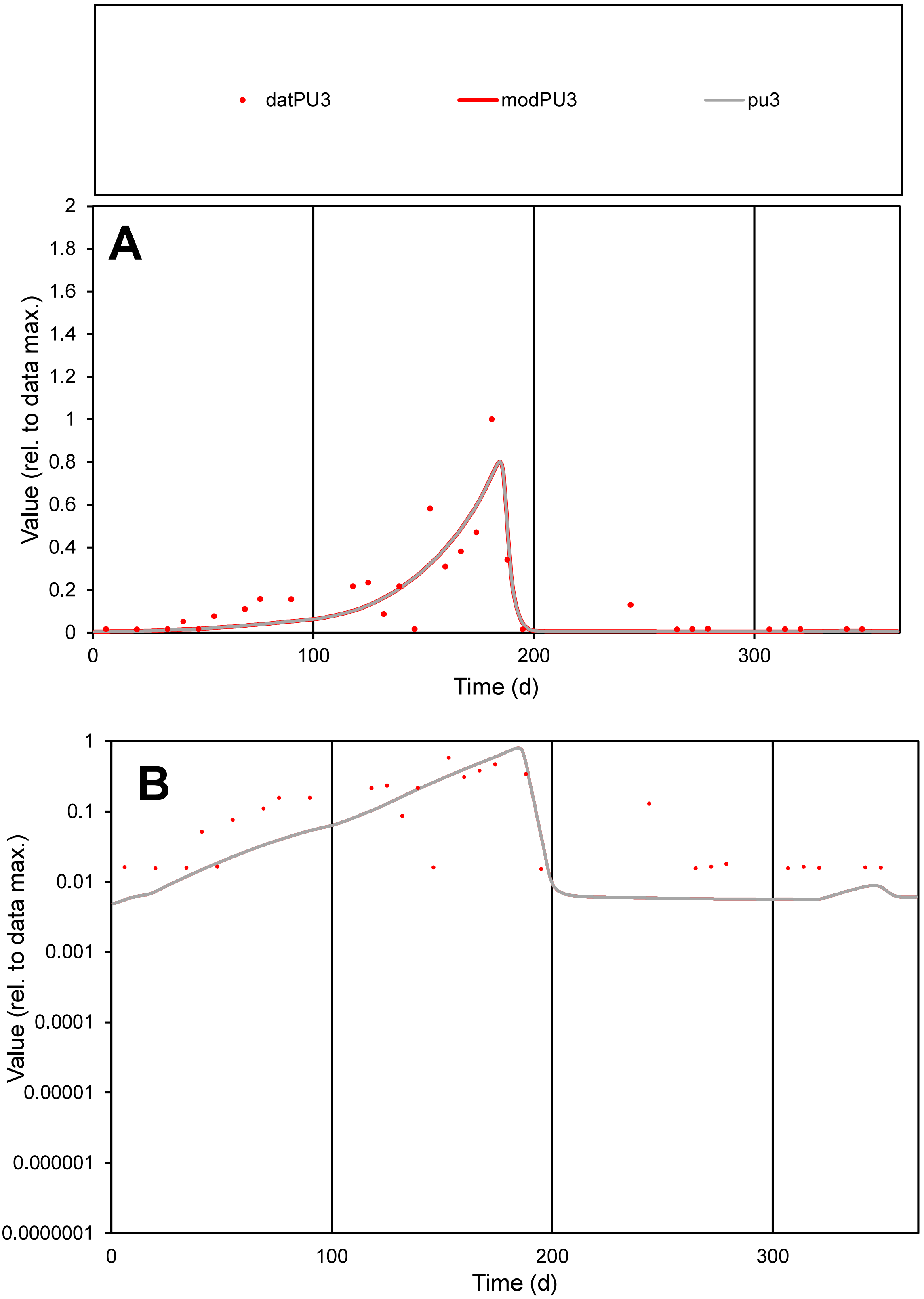

C1

Variables
PU3 Provide jtd or Dst
CalibOnly?
2 1 to reproduce of
oNormalize?
2 1=no, 2=yes

ofxn calc*
0.63255
ofxn mod
0.65395

sRun
RunA1
zout
1

Ave
Data
0.0016
Model
0.00141

data/vMax
0.01012

ofxng
275.646

Min d/m
2.69755
ave/min d
10.3832
ave/min m
24.5837

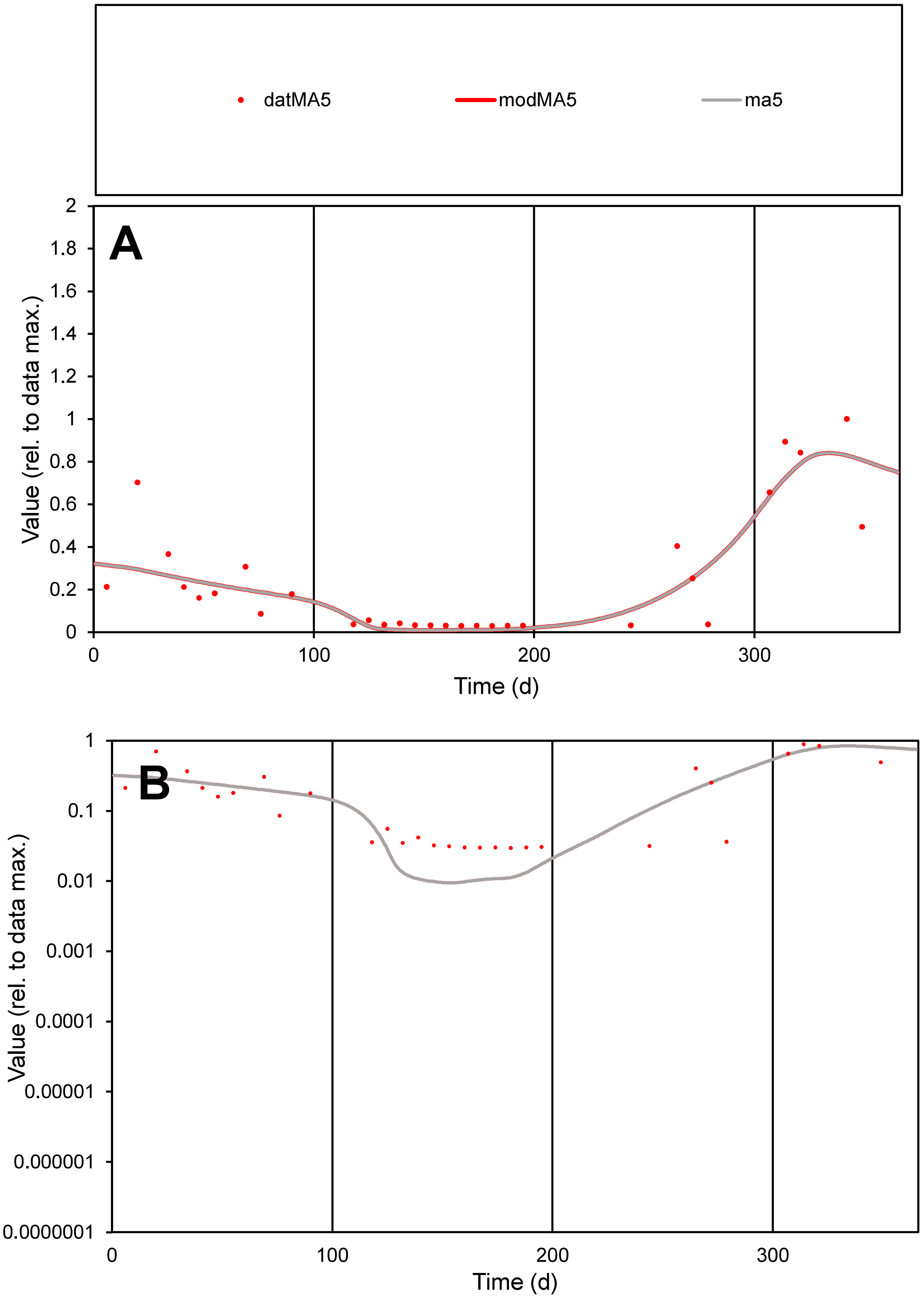

C1

Variables
MA5 Provide jtd or Dst
CalibOnly?
2 1 to reproduce of
oNormalize?
2 1=no, 2=yes

ofxn calc*
0.51768
ofxn mod
0.54218

sRun
RunA1
zout
1

Ave
Data
0.00078
Model
0.00074

data/vMax
0.00313

ofxng
275.646

Min d/m
3.1405

ave/min d
8.32342
ave/min m
24.7681

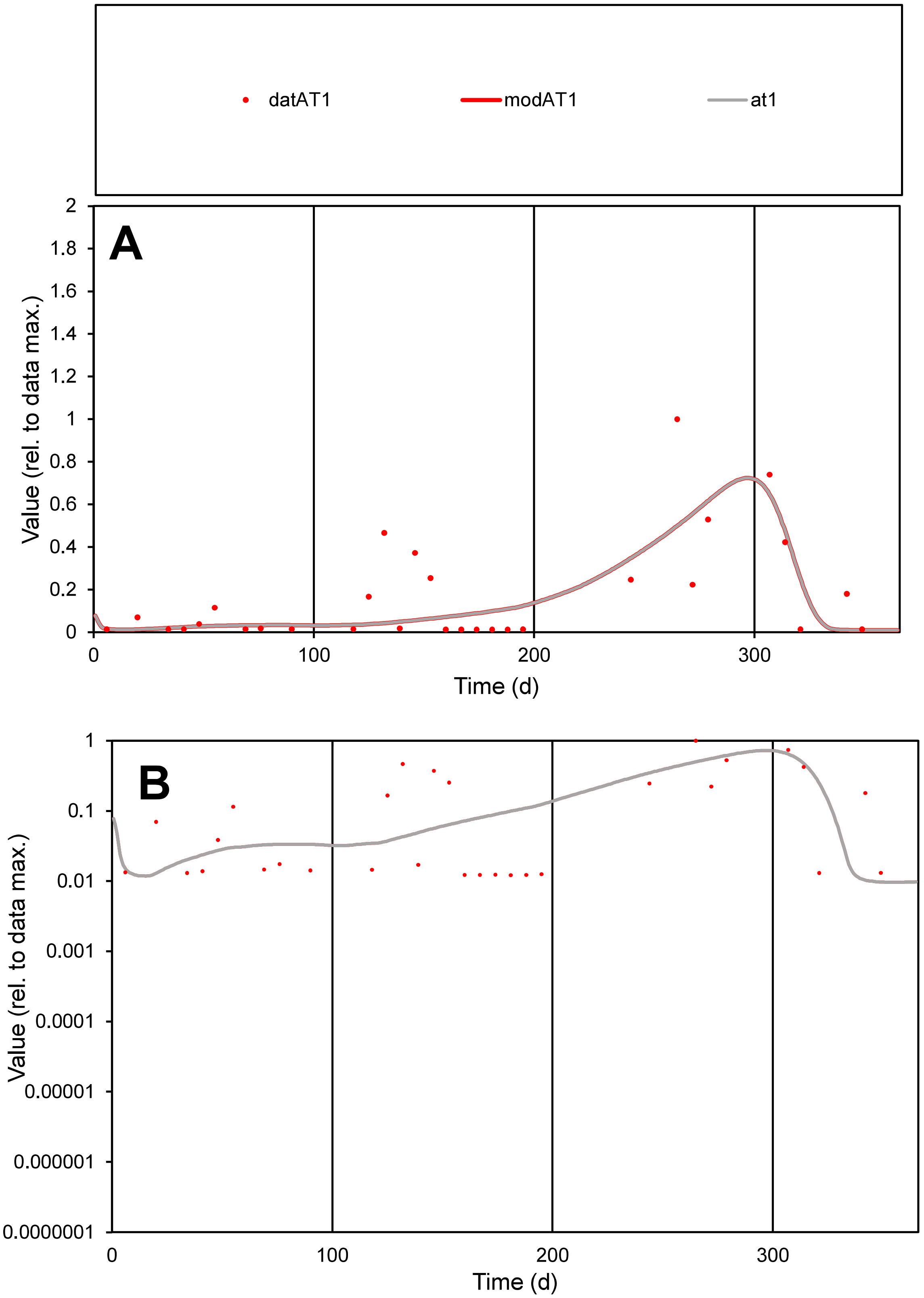

C1

Variables

AT1 Provide jtd or Dst

CalibOnly?

2 1 to reproduce of

oNormalize?

2 1=no, 2=yes

ofxn calc*

0.98989

ofxn mod

1.01264

sRun

RunA1

zout

1

Ave

Data

0.00066

Model

0.00057

data/vMax

0.00391

ofxng

275.646

Min d/m

1.25648

ave/min d

13.8321

ave/min m

15.0941

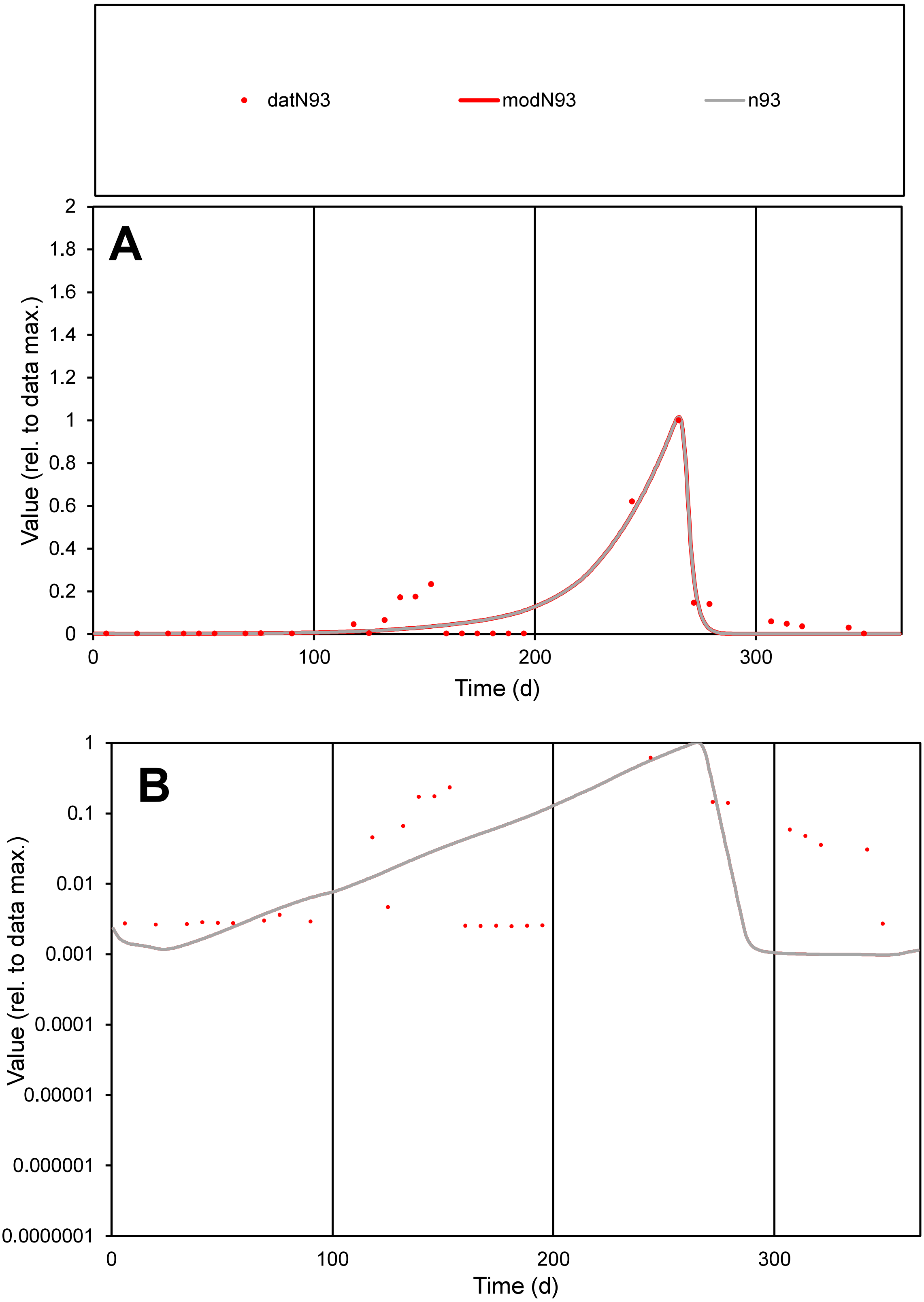

C1

Variables
N93 Provide jtd or Dst
CalibOnly?
2 1 to reproduce of
oNormalize?
2 1=no, 2=yes

ofxn calc*
0.7098
ofxn mod
0.75993

sRun
RunA1
zout
1

Ave
Data
0.00065
Model
0.00055

data/vMax
0.00687

ofxng
275.646

Min d/m
2.5763

ave/min d
37.5474
ave/min m
81.7758

325_Calib_S_A

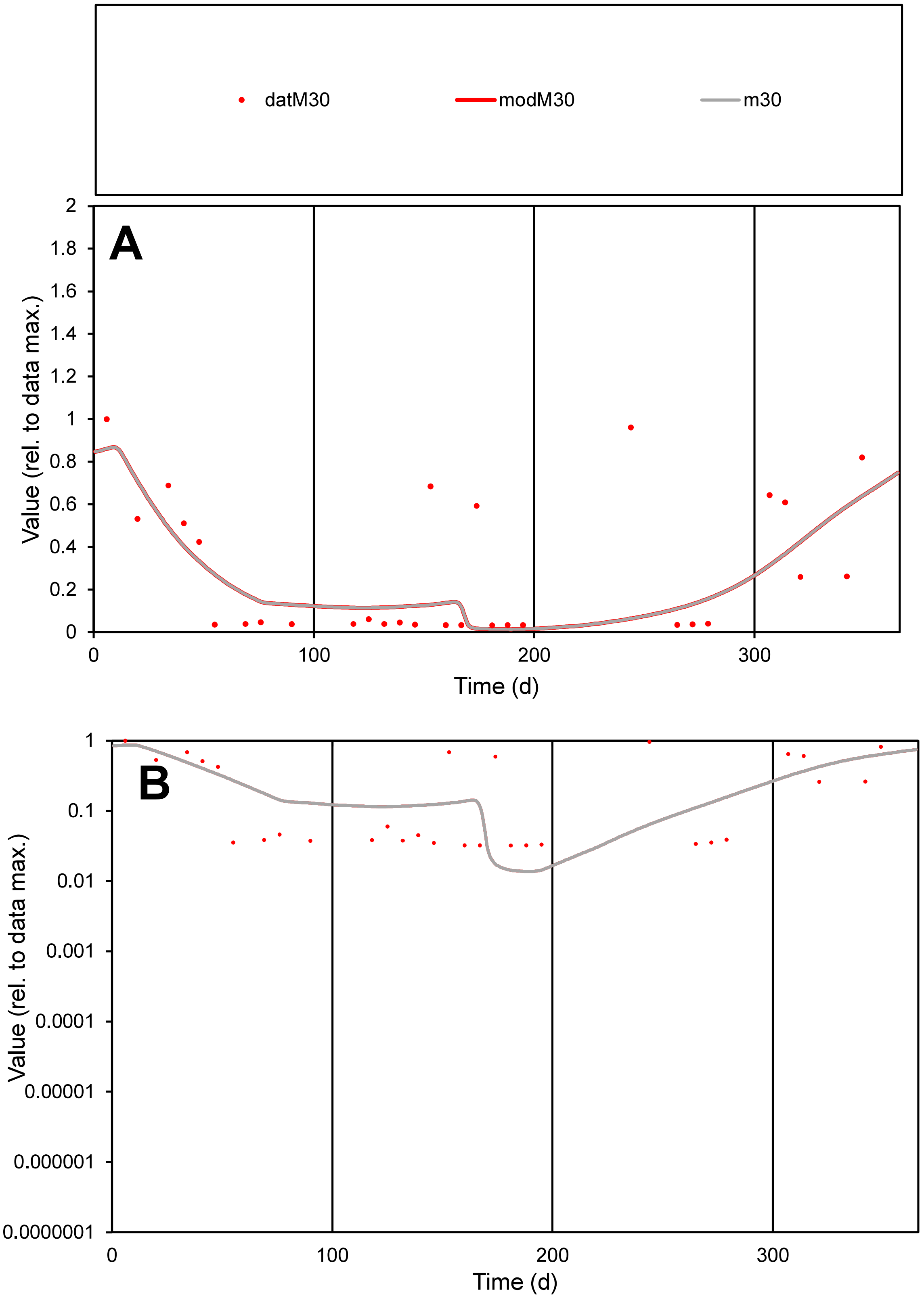

C1

Variables
M30 Provide jtd or Dst
CalibOnly?
2 1 to reproduce of
oNormalize?
2 1=no, 2=yes

ofxn calc*
0.90391
ofxn mod
0.91581

sRun
RunA1
zout
1

Ave
Data
0.00036
Model
0.00031

data/vMax
0.00127

ofxng
275.646

Min d/m
2.33851

ave/min d
8.96921
ave/min m
17.7067

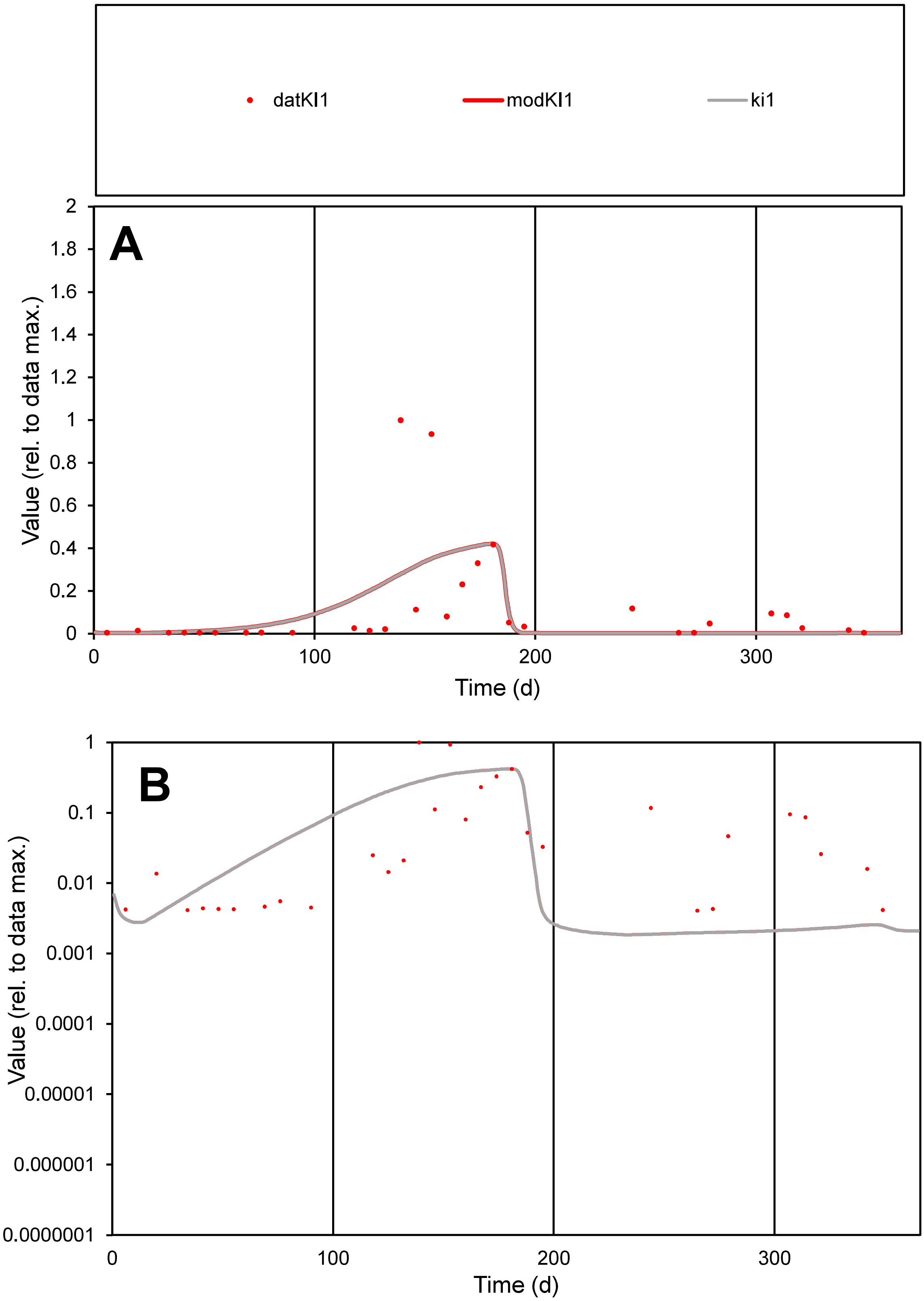

C1

Variables

Kl1	Provide jtd or Dst

CalibOnly?

2	1 to reproduce of

oNormalize?

2	1=no, 2=yes

ofxn calc*
1.59033
ofxn mod
1.60185

sRun
RunA1
zout
1

Ave
Data
0.00148
Model
0.00138

data/vMax
0.01201

ofxng
275.646

Min d/m
2.16313

ave/min d
30.2648
ave/min m
61.0384

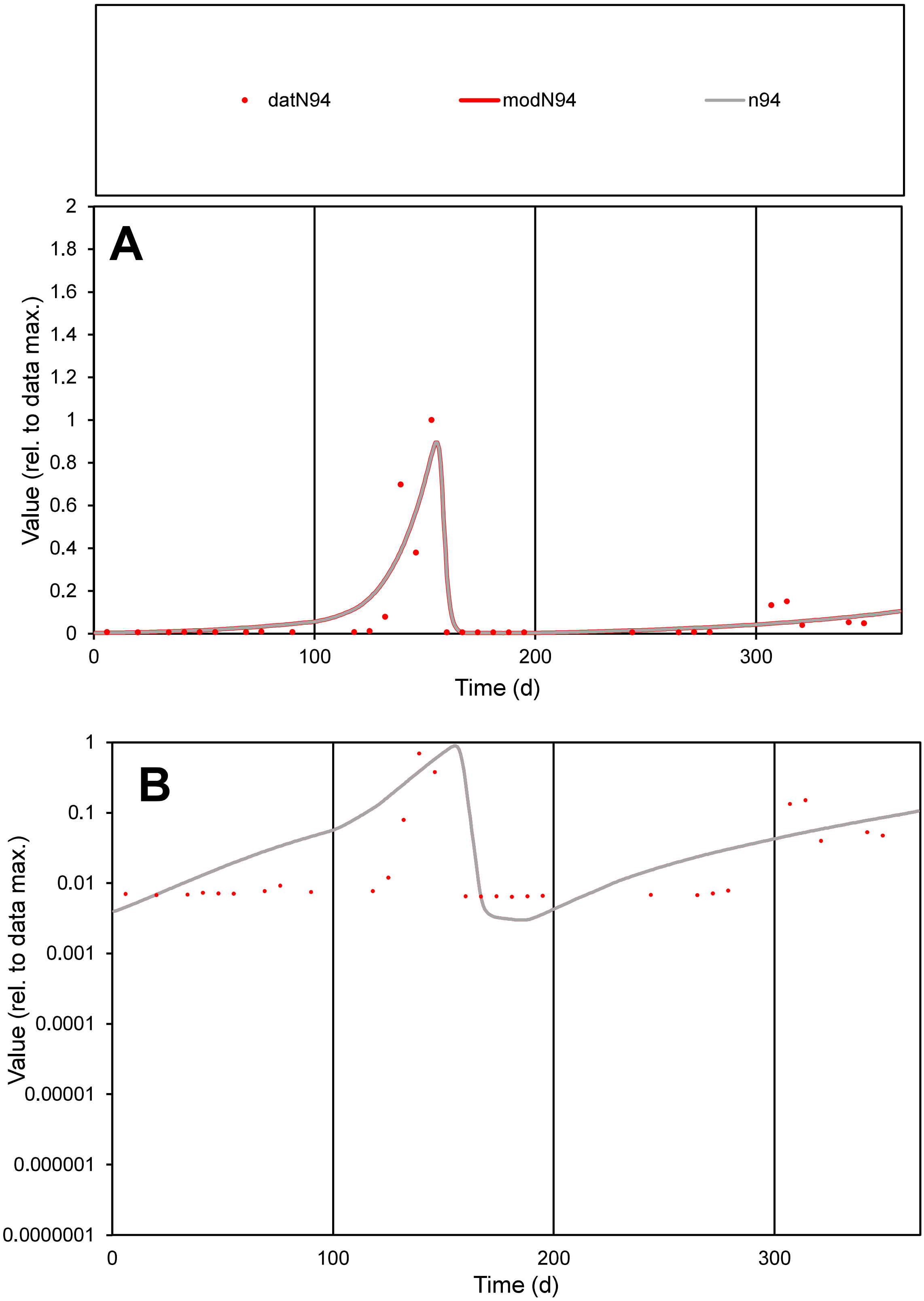

C1

Variables
N94 Provide jtd or Dst
CalibOnly?
2 1 to reproduce of
oNormalize?
2 1=no, 2=yes

ofxn calc*
0.95798
ofxn mod
1.01491

sRun
RunA1
zout
1

Ave
Data
0.00064
Model
0.00073

data/vMax
0.00701

ofxng
275.646

Min d/m
2.11325

ave/min d
14.2176
ave/min m
34.2187

325_Calib_S_A

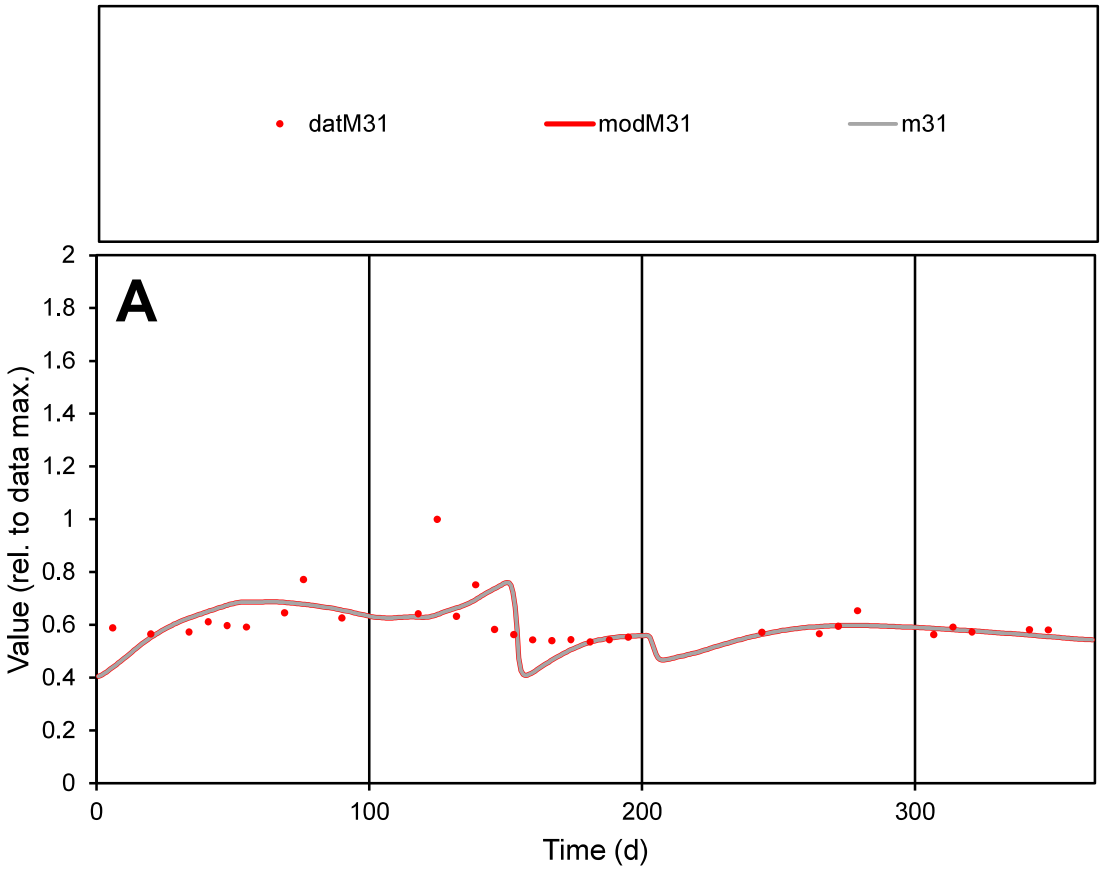

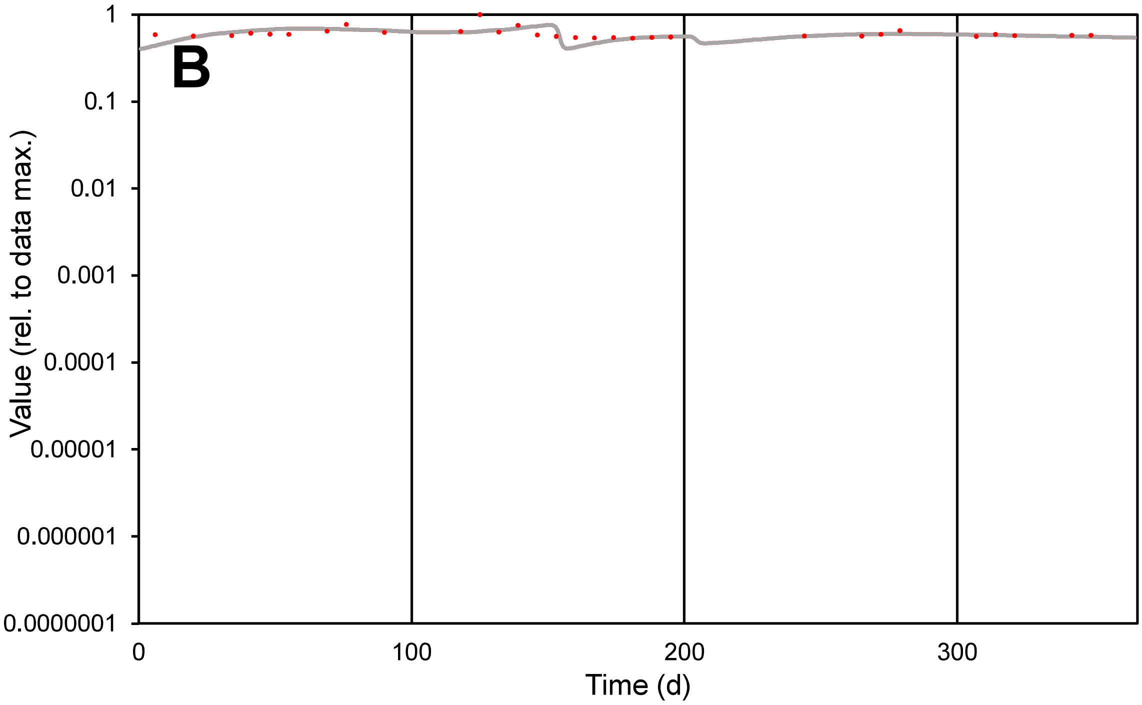

C1

Variables
M31 Provide jtd or Dst
CalibOnly?
2 1 to reproduce of
oNormalize?
2 1=no, 2=yes

ofxn calc*
0.14448
ofxn mod
0.18382

sRun
RunA1
zout
1

Ave
Data
9.2E-05
Model
9E-05

data/vMax
0.00015

ofxng
275.646

Min d/m
1.24422

ave/min d
1.1376
ave/min m
1.38866

325_Calib_S_A

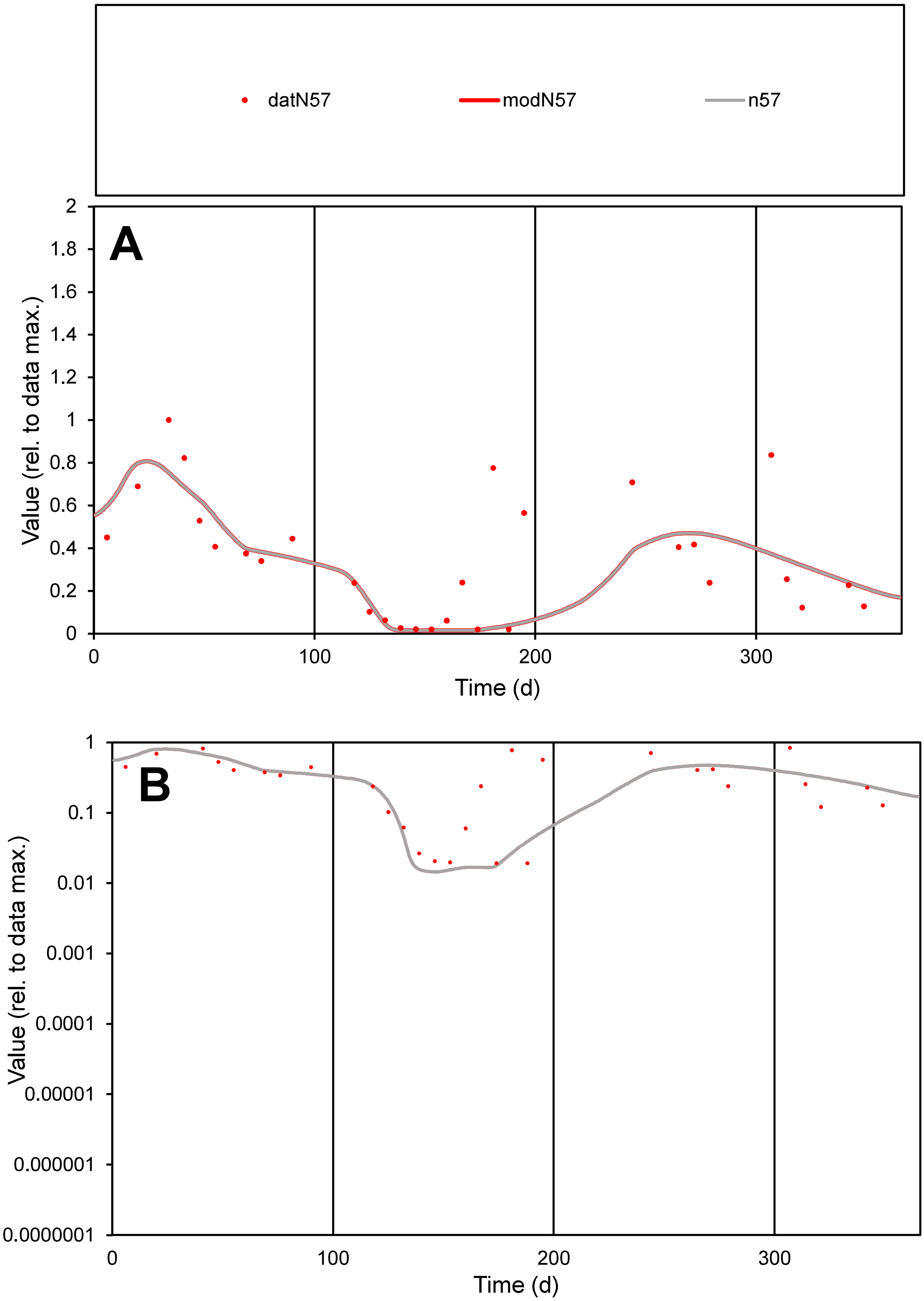

C1

Variables
N57 Provide jtd or Dst
CalibOnly?
2 1 to reproduce of
oNormalize?
2 1=no, 2=yes

ofxn calc*
0.62642
ofxn mod
0.64146

sRun
RunA1
zout
1

Ave
Data
0.00067
Model
0.00057

data/vMax
0.0019

ofxng
275.646

Min d/m
1.31858

ave/min d
18.3663
ave/min m
20.6275

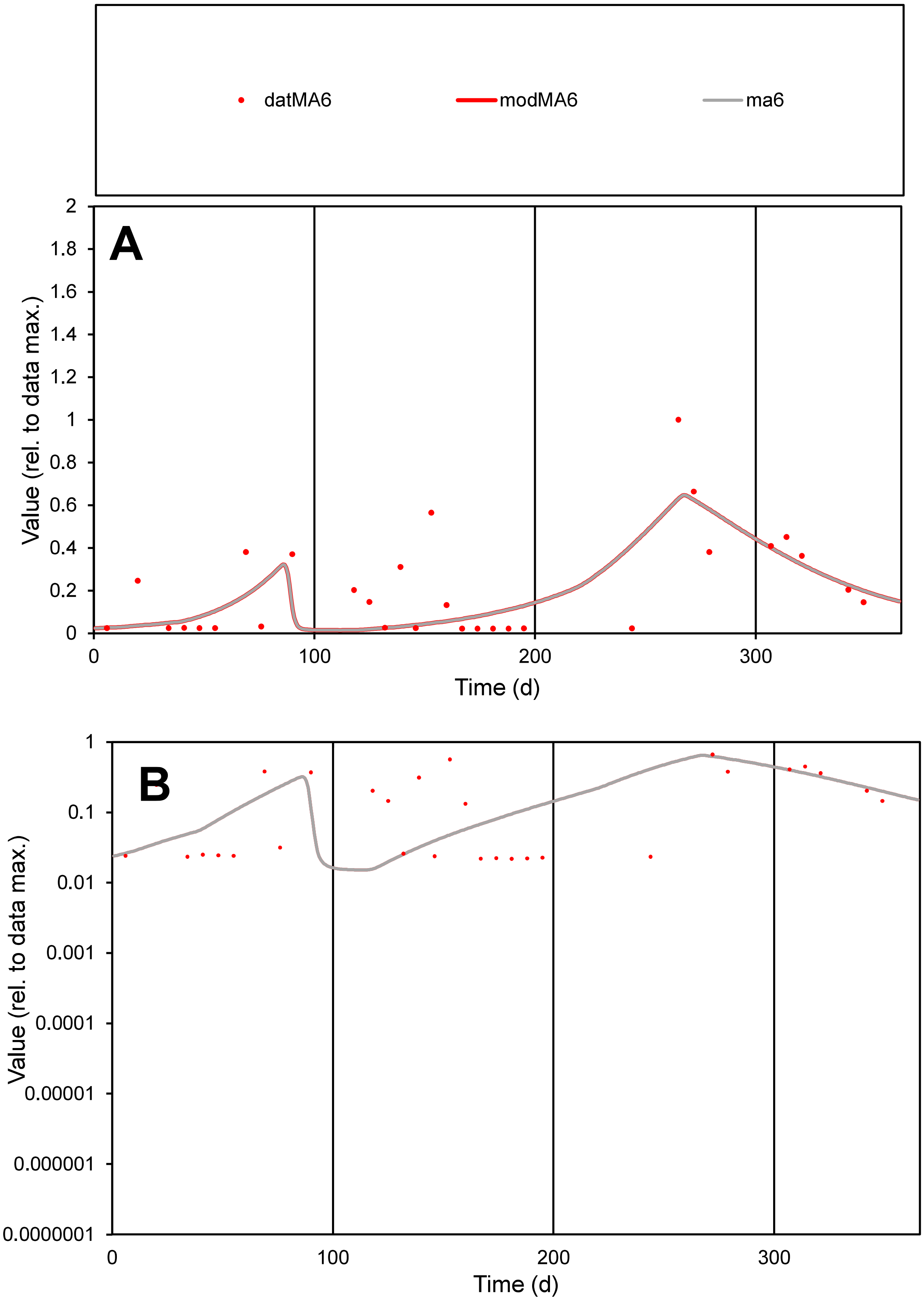

C1

Variables

MA6 Provide jtd or Dst

CalibOnly?

2 1 to reproduce of

oNormalize?

2 1=no, 2=yes

ofxn calc*

0.82765

ofxn mod

0.86112

sRun

RunA1

zout

1

Ave

Data

0.00112

Model

0.00098

data/vMax

0.00533

ofxng

275.646

Min d/m

1.3553

ave/min d

9.59084

ave/min m

11.375

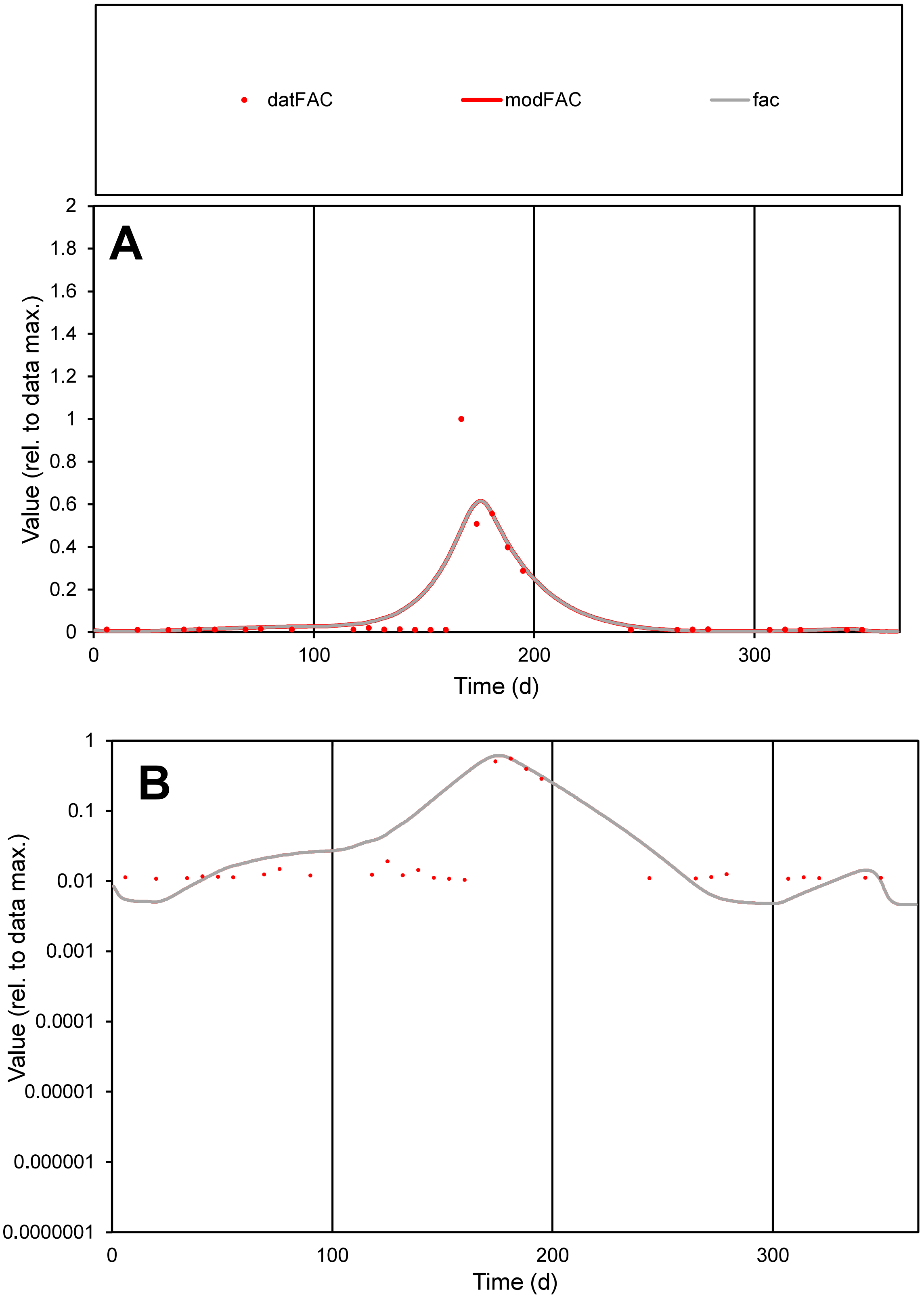

C1

Variables
FAC Provide jtd or Dst
CalibOnly?
2 1 to reproduce of
oNormalize?
2 1=no, 2=yes

ofxn calc*
1.16446
ofxn mod
1.1997

sRun
RunA1
zout
1

Ave
Data
0.00085
Model
0.00097

data/vMax
0.00832

ofxng
275.646

Min d/m
2.03805

ave/min d
9.738
ave/min m
22.6855

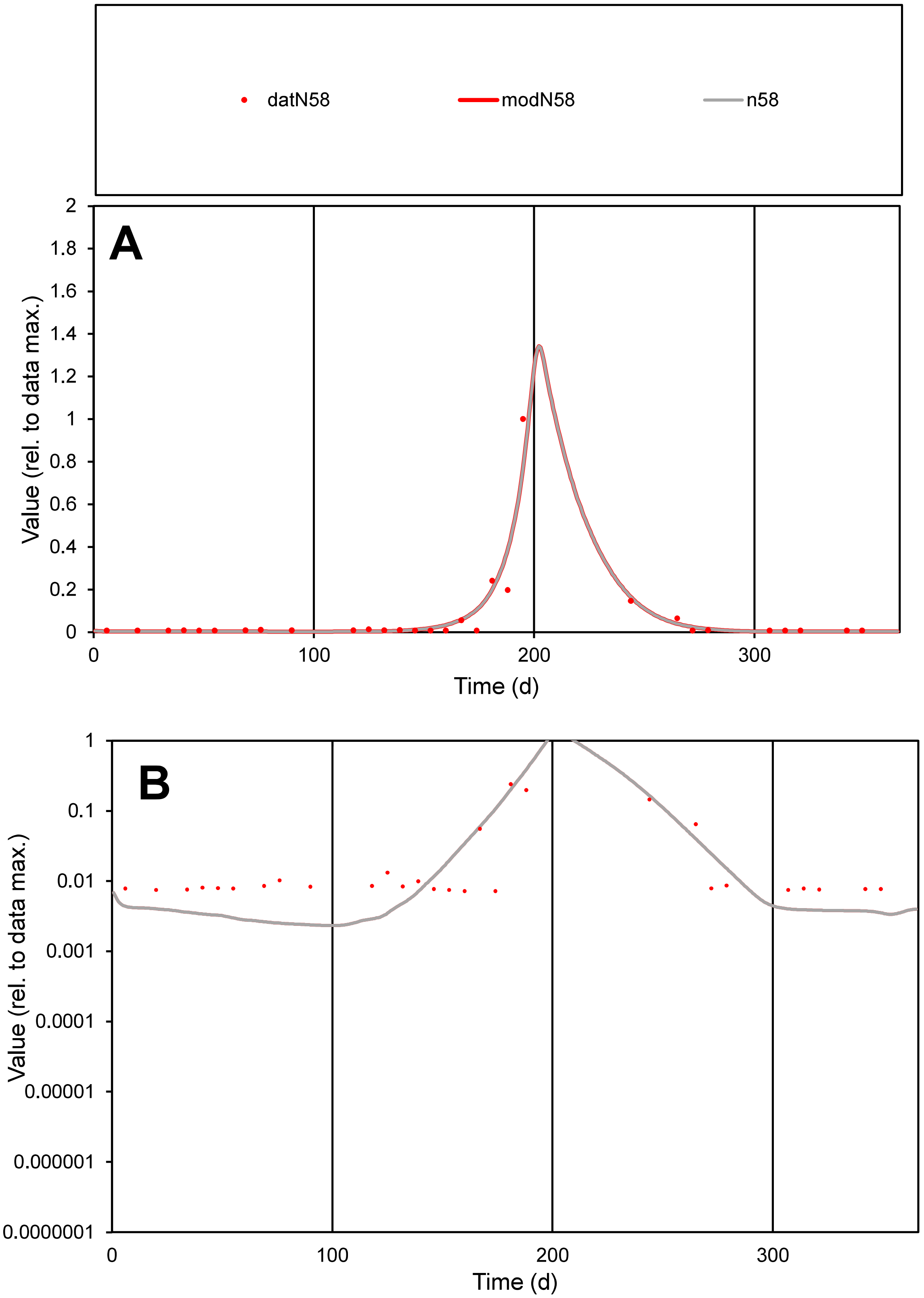

C1

Variables
N58 Provide jtd or Dst
CalibOnly?
2 1 to reproduce of
oNormalize?
2 1=no, 2=yes

ofxn calc*
0.72579
ofxn mod
0.82343

sRun
RunA1
zout
1

Ave
Data
0.00072
Model
0.00078

data/vMax
0.01141

ofxng
275.646

Min d/m
3.03209

ave/min d
8.80948
ave/min m
28.6504

325_Calib_S_A

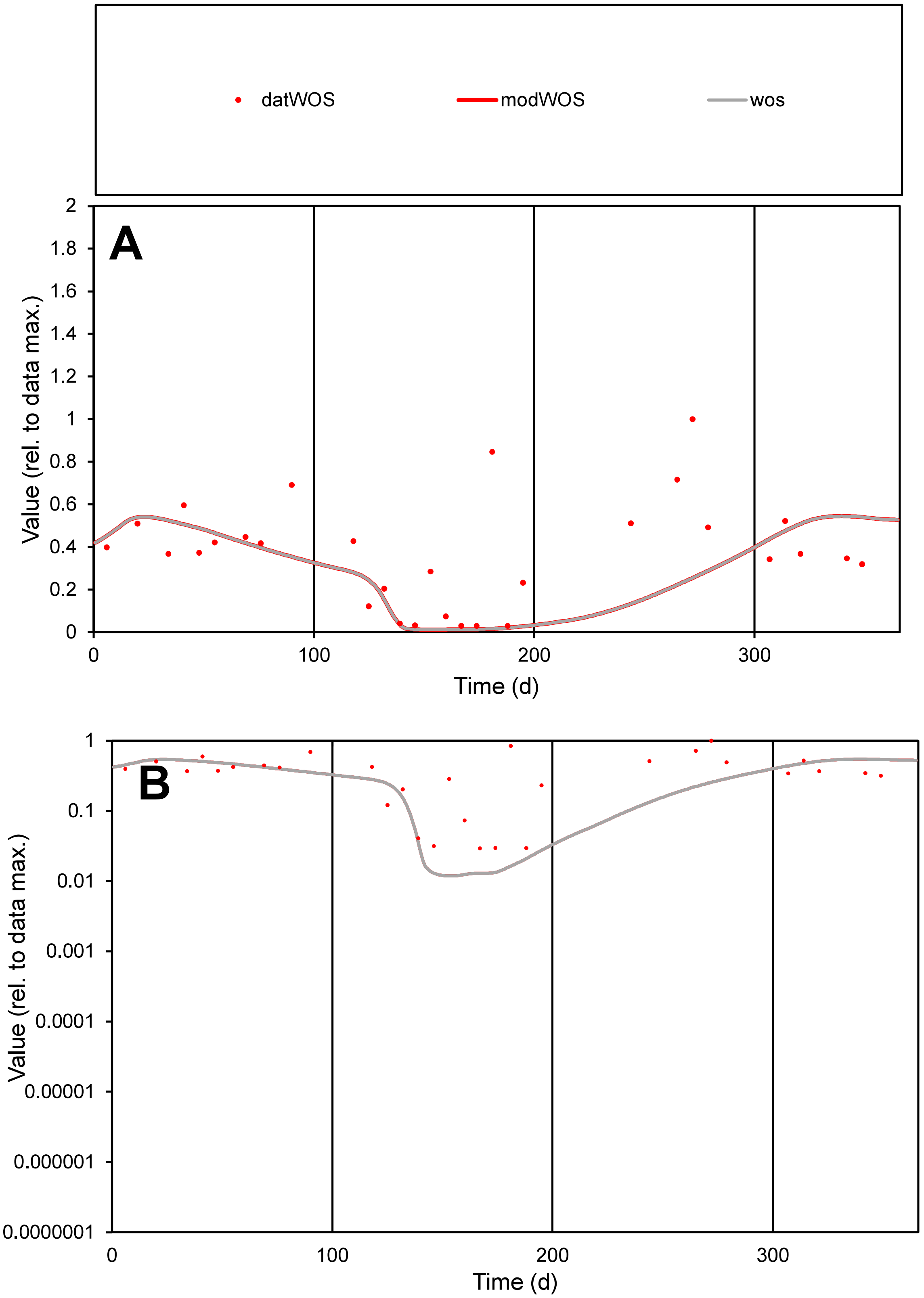

C1

Variables
WOS Provide jtd or Dst
CalibOnly?
2 1 to reproduce of
oNormalize?
2 1=no, 2=yes

ofxn calc*
0.70942
ofxn mod
0.7199

sRun
RunA1
zout
1

Ave
Data
0.00087
Model
0.00065

data/vMax
0.00232

ofxng
275.646

Min d/m
2.47588

ave/min d
12.675
ave/min m
23.5509

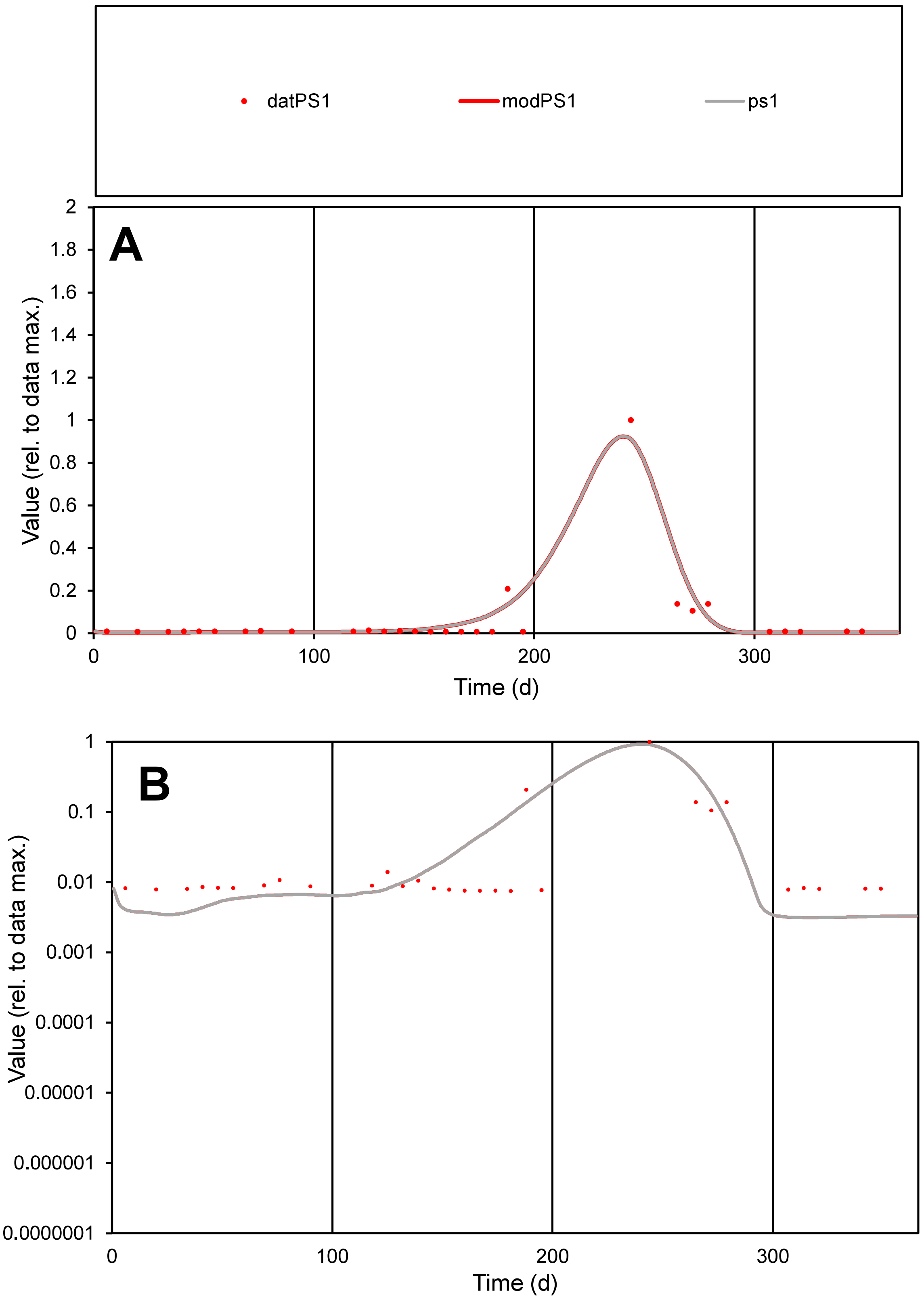

C1

Variables
PS1 Provide jtd or Dst
CalibOnly?
2 1 to reproduce of
oNormalize?
2 1=no, 2=yes

ofxn calc*
0.89975
ofxn mod
1.00597

sRun
RunA1
zout
1

Ave
Data
0.00038
Model
0.00044

data/vMax
0.00625

ofxng
275.646

Min d/m
2.38843

ave/min d
8.05032
ave/min m
22.5503

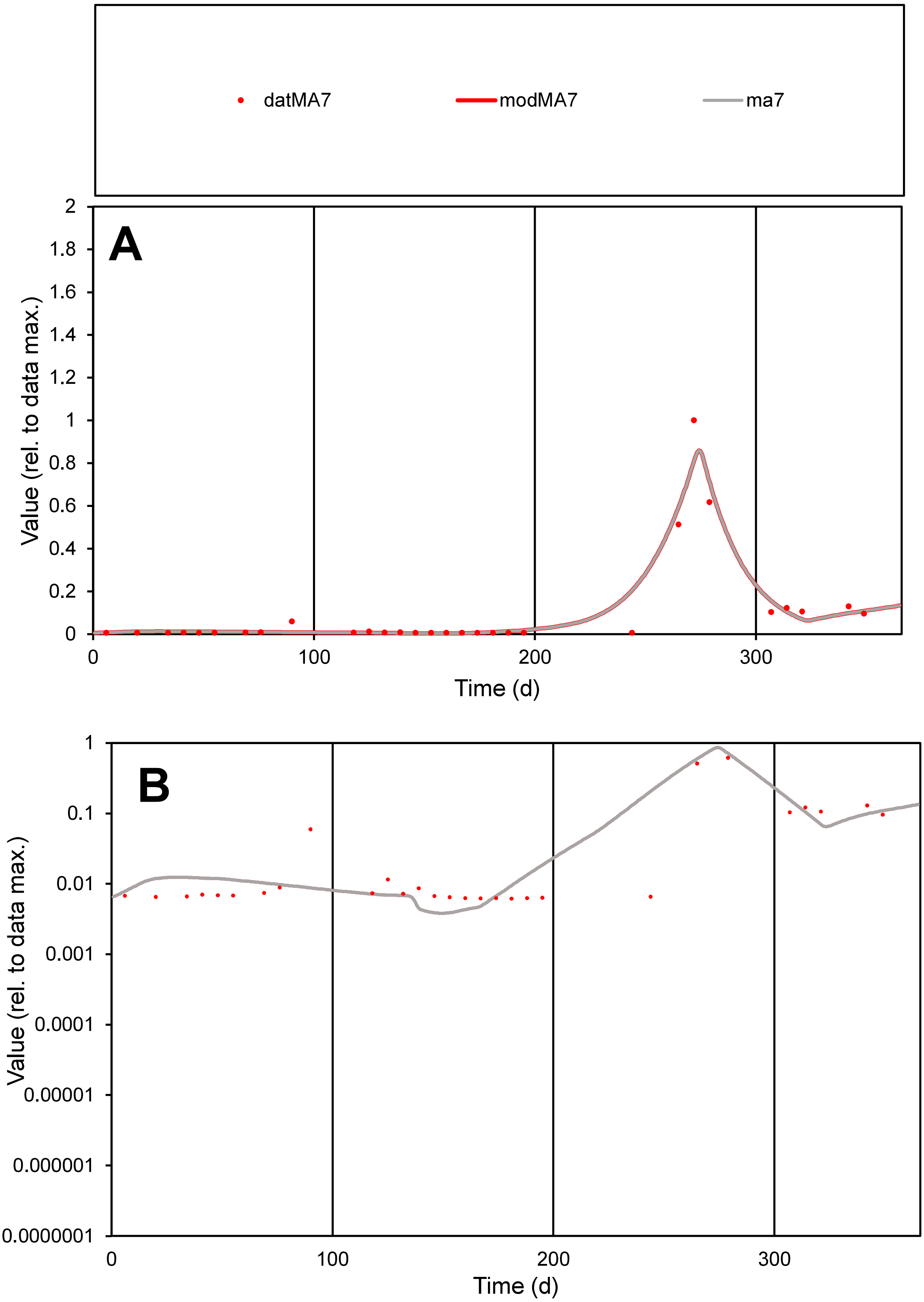

C1

Variables
MA7 Provide jtd or Dst
CalibOnly?
2 1 to reproduce of
oNormalize?
2 1=no, 2=yes

ofxn calc*
0.51083
ofxn mod
0.65018

sRun
RunA1
zout
1

Ave
Data
0.00021
Model
0.00022

data/vMax
0.00216

ofxng
275.646

Min d/m
1.60757

ave/min d
15.6687
ave/min m
26.0979

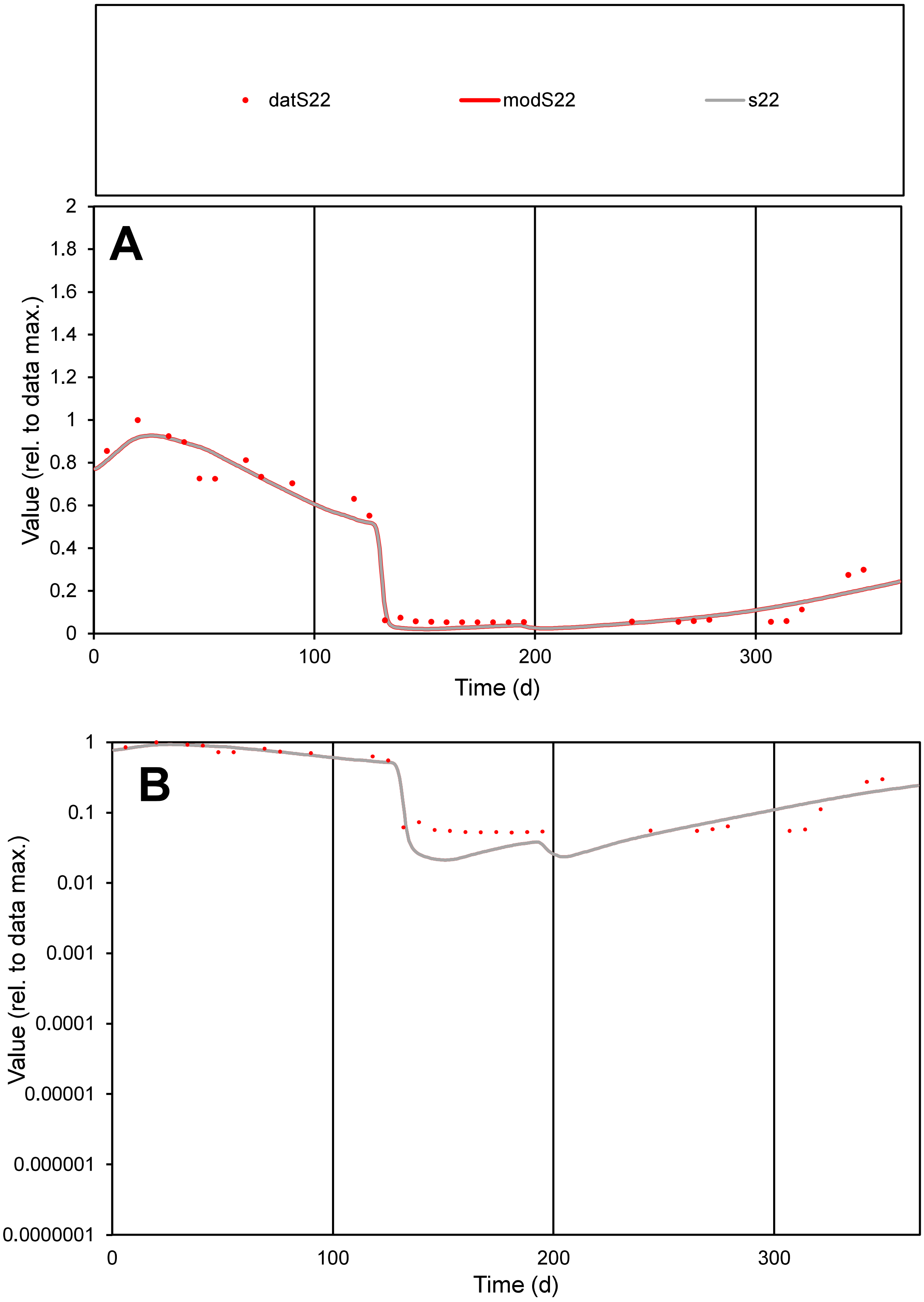

C1

Variables

S22 Provide jtd or Dst

CalibOnly?

2 1 to reproduce of

oNormalize?

2 1=no, 2=yes

ofxn calc*

0.16343

ofxn mod

0.23466

sRun

RunA1

zout

1

Ave

Data

0.00094

Model

0.00091

data/vMax

0.00278

ofxng

275.646

Min d/m

2.43027

ave/min d

6.47544

ave/min m

15.2408

325_Calib_S_A

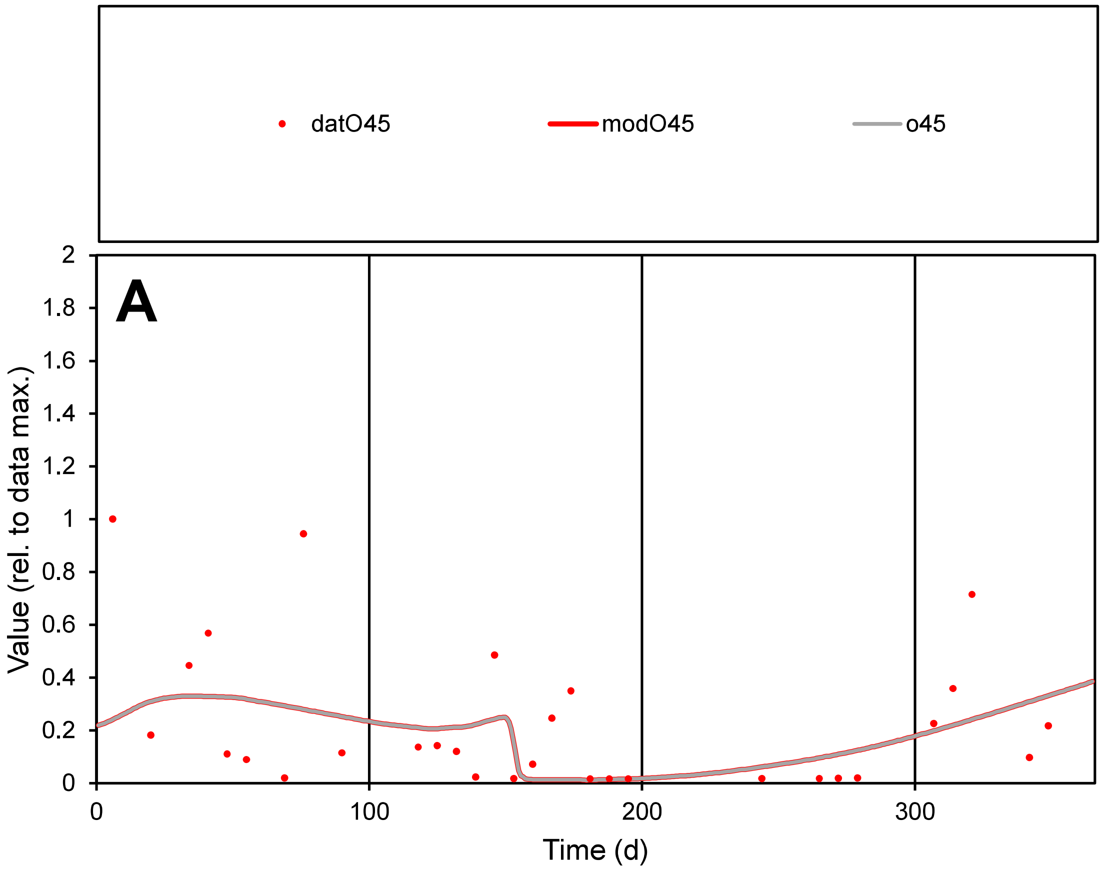

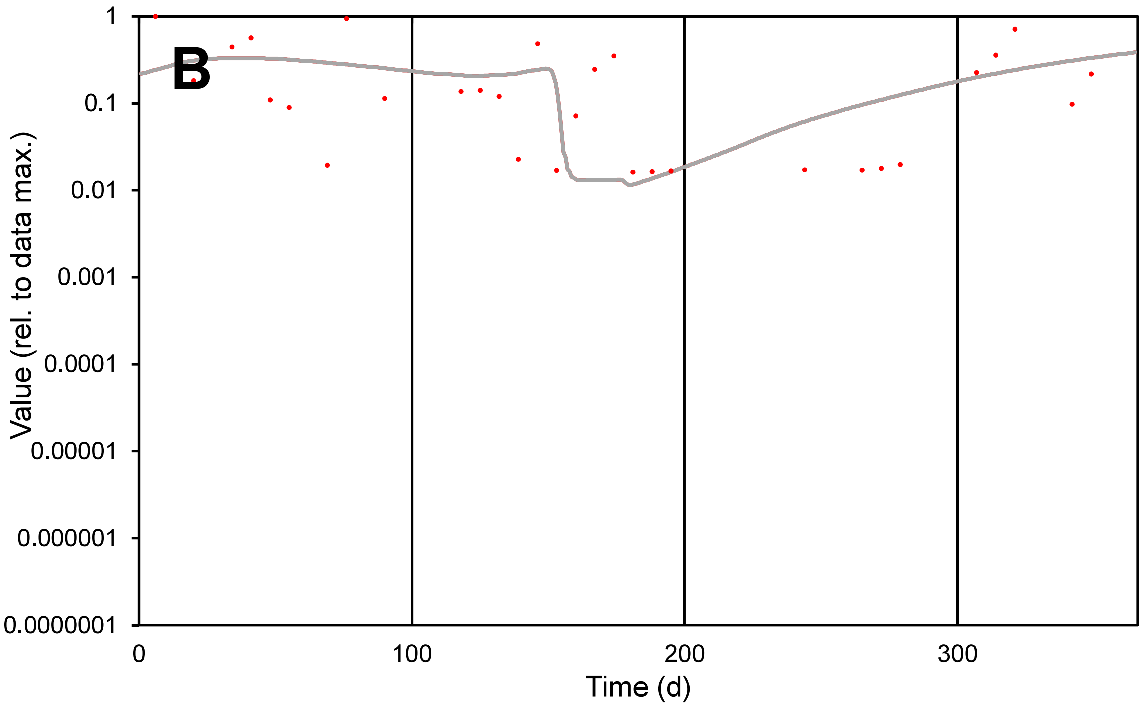

C1

Variables

O45 Provide jtd or Dst

CalibOnly?

2 1 to reproduce of

oNormalize?

2 1=no, 2=yes

ofxn calc*

1.11079

ofxn mod

1.11835

sRun

RunA1

zout

1

Ave

Data

0.0008

Model

0.00066

data/vMax

0.00351

ofxng

275.646

Min d/m

1.34568

ave/min d

14.0295

ave/min m

15.5649

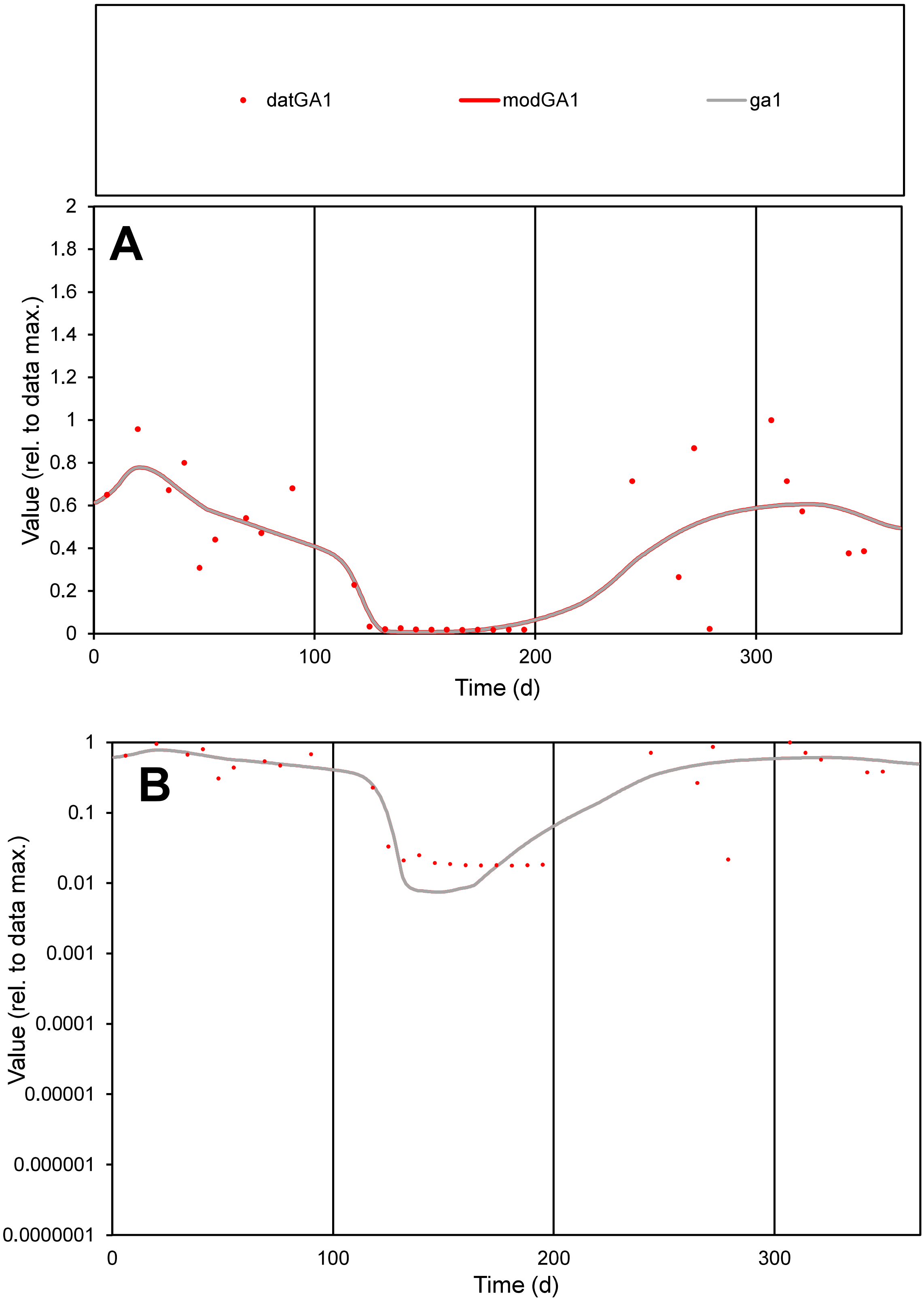

C1

Variables
GA1 Provide jtd or Dst
CalibOnly?
2 1 to reproduce of
oNormalize?
2 1=no, 2=yes

ofxn calc*
0.51048
ofxn mod
0.5318

sRun
RunA1
zout
1

Ave
Data
0.00072
Model
0.0007

data/vMax
0.00198

ofxng
275.646

Min d/m
2.38802

ave/min d
20.4429
ave/min m
47.7723

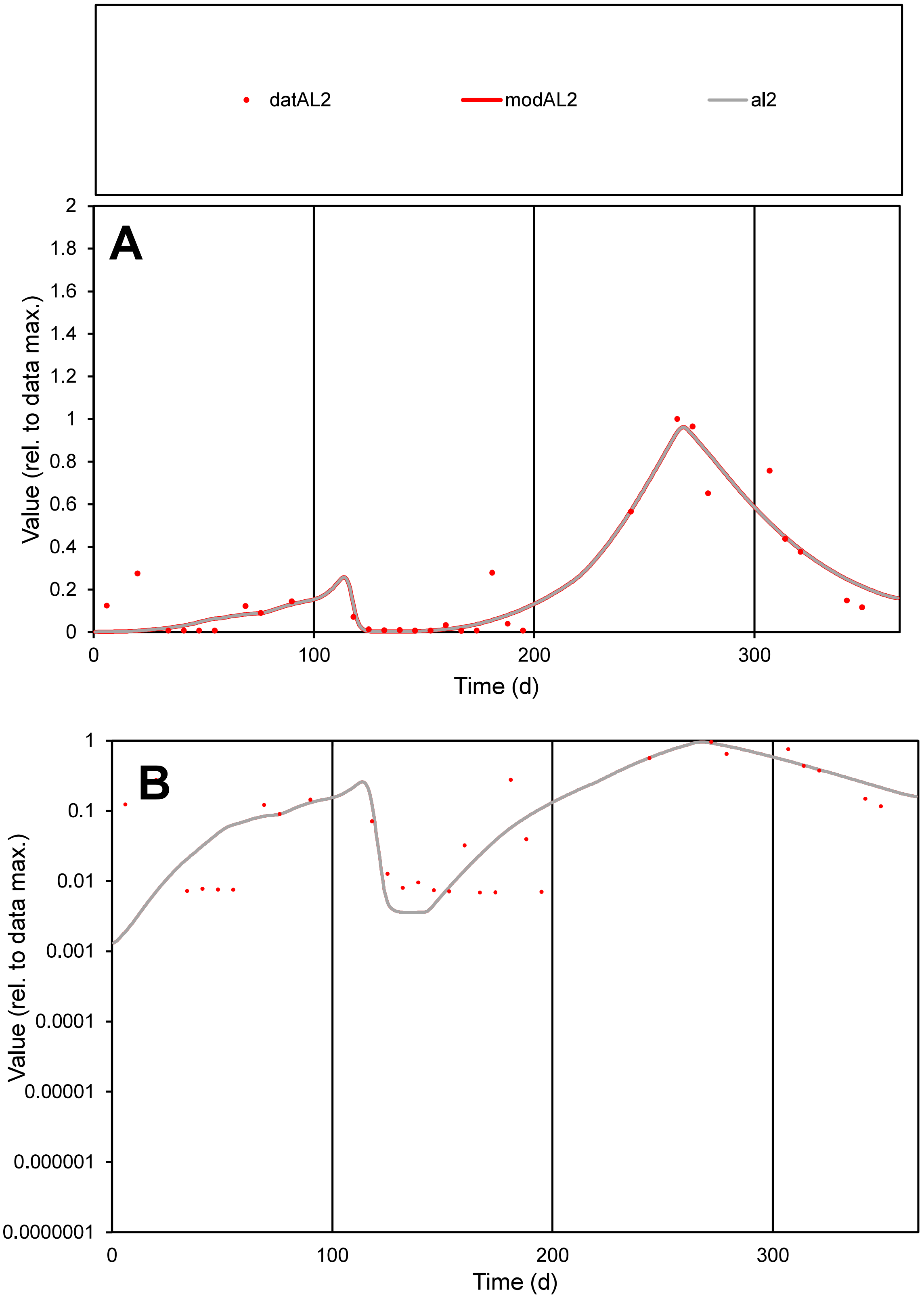

C1

Variables
AL2 Provide jtd or Dst
CalibOnly?
2 1 to reproduce of
oNormalize?
2 1=no, 2=yes

ofxn calc*
0.4518
ofxn mod
0.49704

sRun
RunA1
zout
1

Ave
Data
0.00067
Model
0.00063

data/vMax
0.00317

ofxng
275.646

Min d/m
3.25108

ave/min d
30.6072
ave/min m
94.0272

325_Calib_S_A

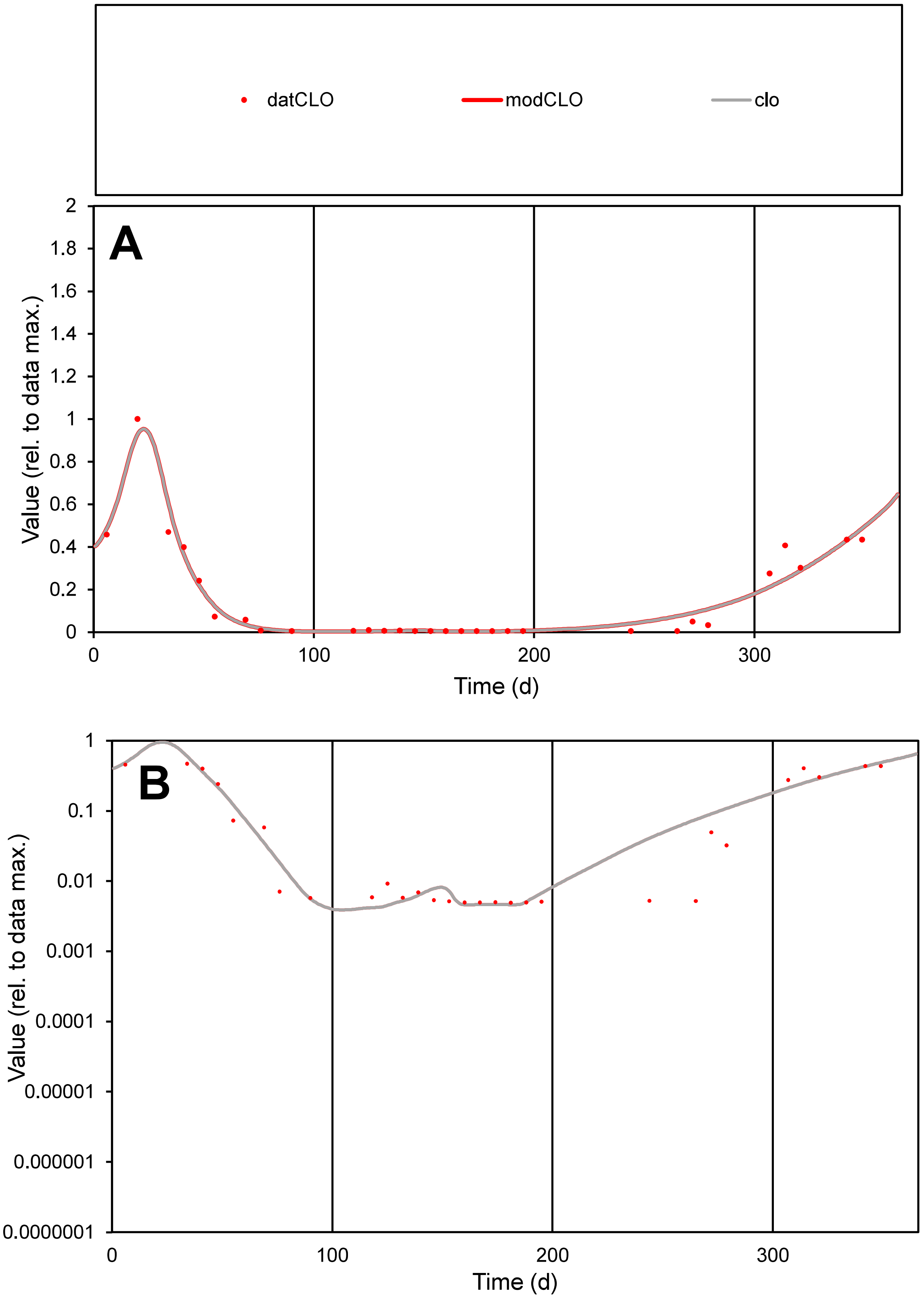

C1

Variables

CLO Provide jtd or Dst

CalibOnly?

2 1 to reproduce of

oNormalize?

2 1=no, 2=yes

ofxn calc*

0.28338

ofxn mod

0.34194

sRun

RunA1

zout

1

Ave

Data

0.00042

Model

0.00043

data/vMax

0.00266

ofxng

275.646

Min d/m

1.1752

ave/min d

31.9936

ave/min m

38.3191

325_Calib_S_A

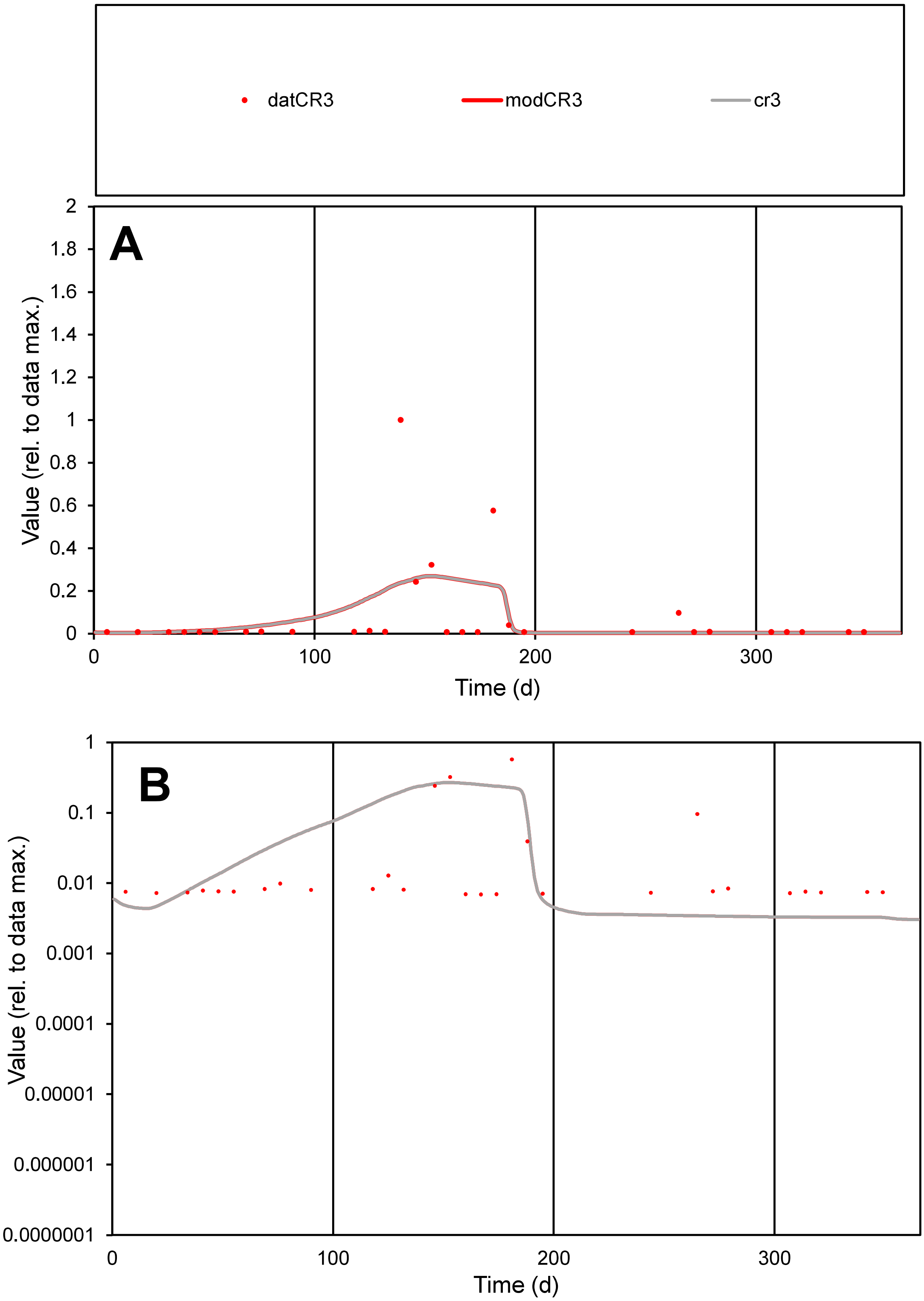

C1

Variables
CR3 Provide jtd or Dst
CalibOnly?
2 1 to reproduce of
oNormalize?
2 1=no, 2=yes

ofxn calc*
2.18683
ofxn mod
2.20062

sRun
RunA1
zout
1

Ave
Data
0.00042
Model
0.00043

data/vMax
0.00512

ofxng
275.646

Min d/m
2.14988

ave/min d
11.872
ave/min m
26.0837

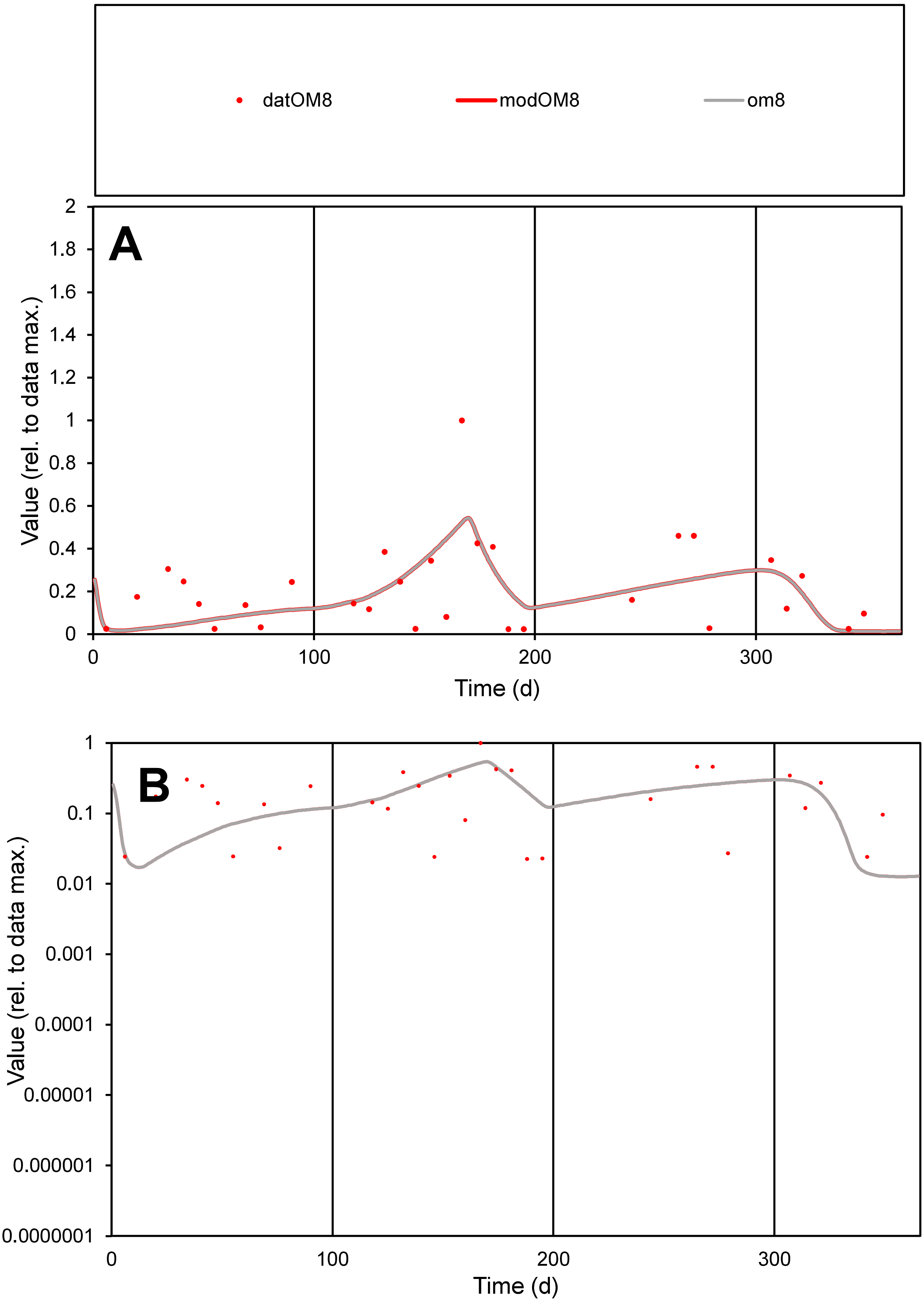

C1

Variables
OM8 Provide jtd or Dst
CalibOnly?
2 1 to reproduce of
oNormalize?
2 1=no, 2=yes

ofxn calc*
0.78049
ofxn mod
0.79448

sRun
RunA1
zout
1

Ave
Data
0.00052
Model
0.00047

data/vMax
0.00241

ofxng
275.646

Min d/m
1.76112

ave/min d
9.62441
ave/min m
15.2456

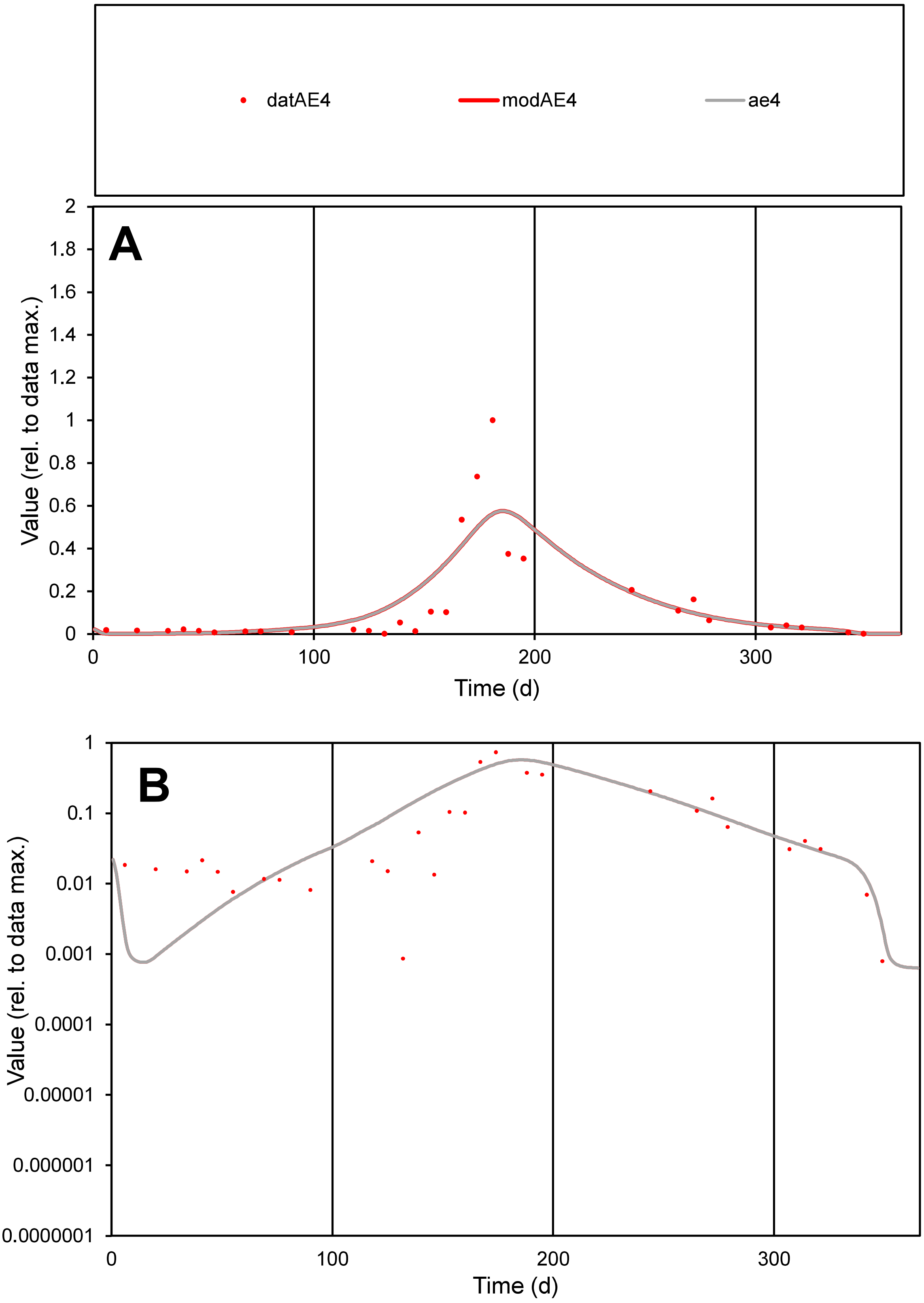

C1

Variables
AE4 Provide jtd or Dst
CalibOnly?
2 1 to reproduce of
oNormalize?
2 1=no, 2=yes

ofxn calc*
0.9174
ofxn mod
0.94175

sRun
RunA1
zout
1

Ave
Data
0.00215
Model
0.00238

data/vMax
0.01582

ofxng
275.646

Min d/m
0.80566

ave/min d
172.059
ave/min m
153.103

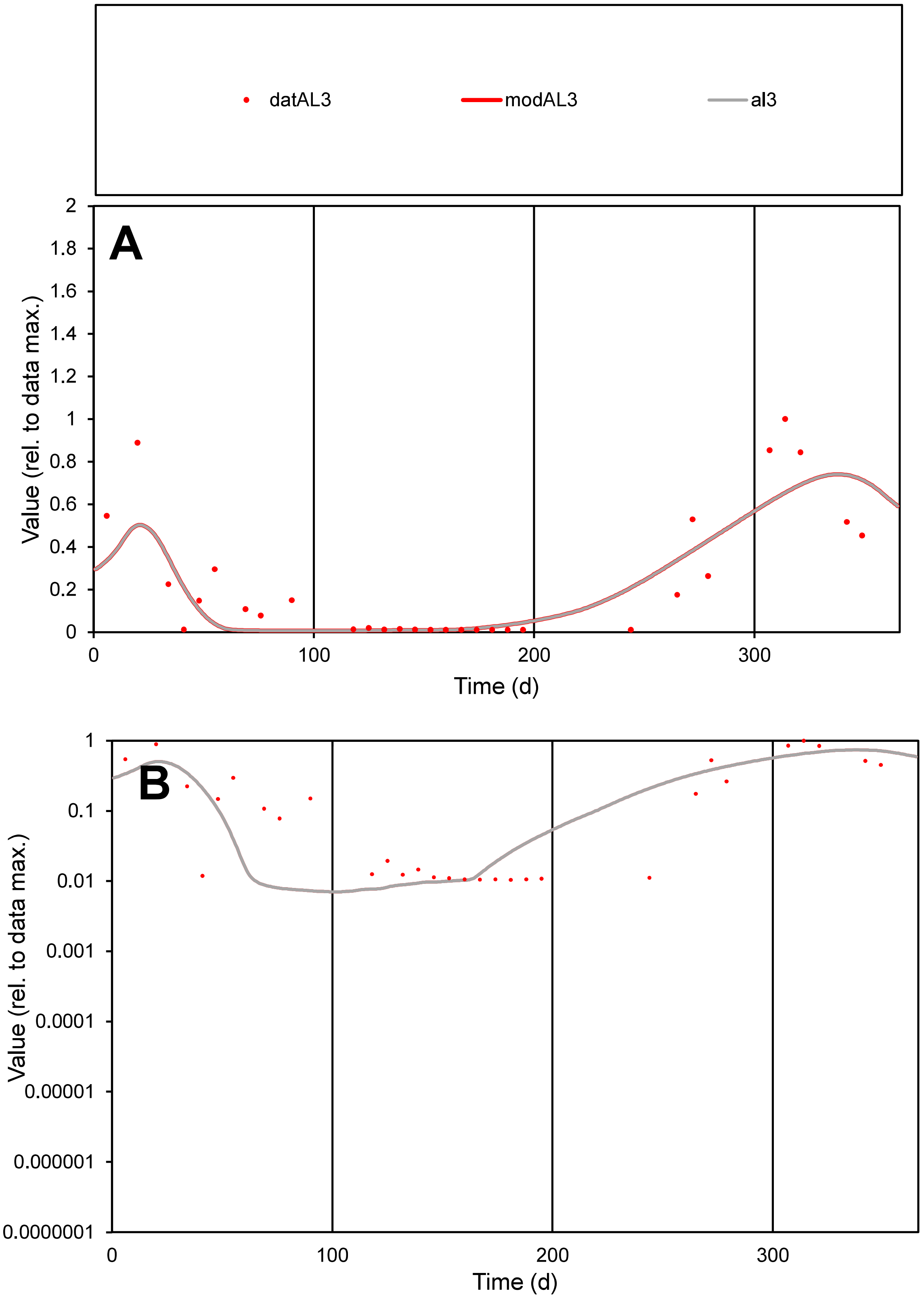

C1

Variables
AL3 Provide jtd or Dst
CalibOnly?
2 1 to reproduce of
oNormalize?
2 1=no, 2=yes

ofxn calc*
0.65067
ofxn mod
0.67392

sRun
RunA1
zout
1

Ave
Data
0.00055
Model
0.0005

data/vMax
0.00229

ofxng
275.646

Min d/m
1.4407

ave/min d
23.1163
ave/min m
29.898

325_Calib_S_A

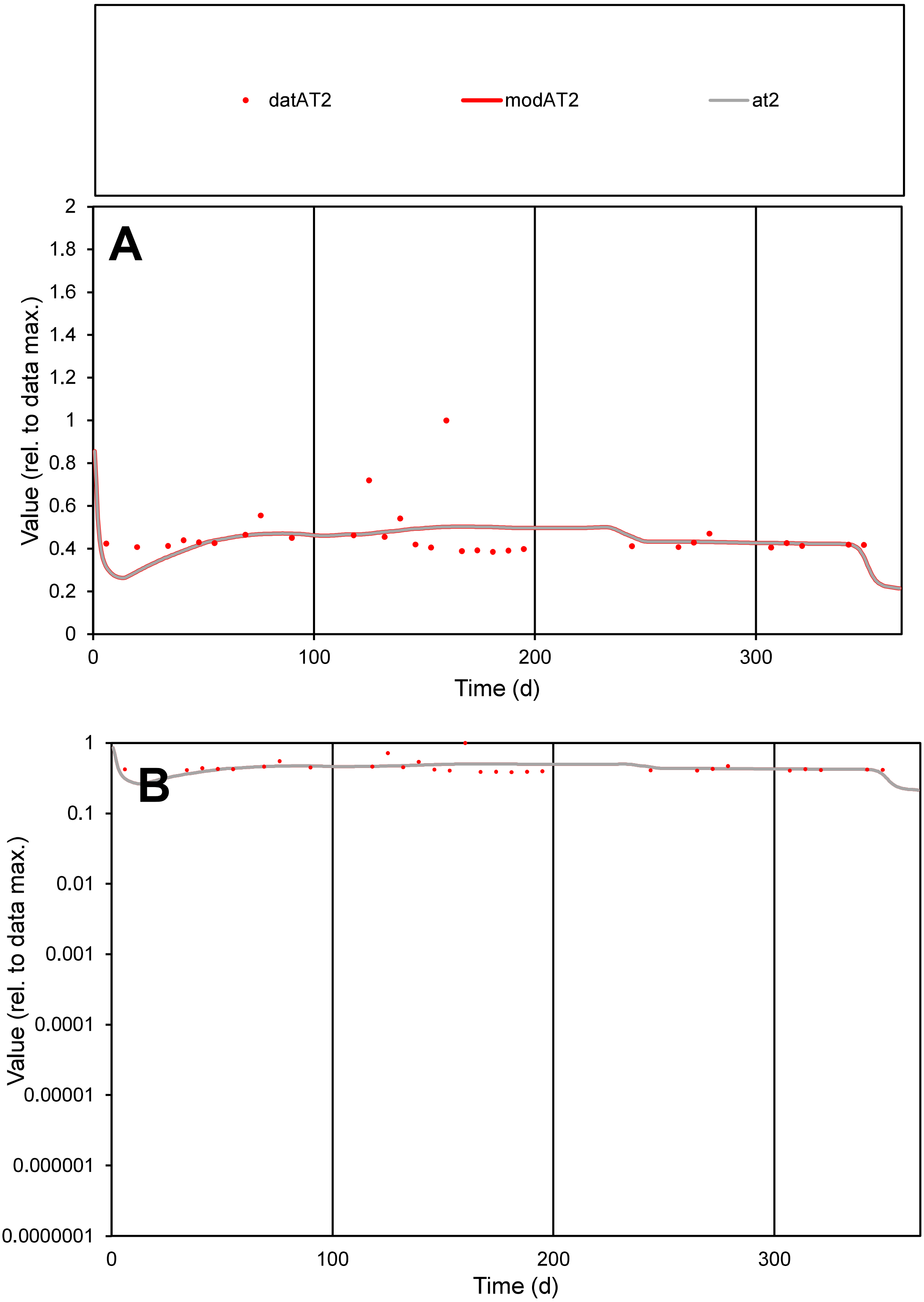

C1

Variables

AT2 Provide jtd or Dst

CalibOnly?

2 1 to reproduce of

oNormalize?

2 1=no, 2=yes

ofxn calc*

0.26439

ofxn mod

0.28663

sRun

RunA1

zout

1

Ave

Data

9.4E-05

Model

9.1E-05

data/vMax

0.00021

ofxng

275.646

Min d/m

1.30479

ave/min d

1.19052

ave/min m

1.50159

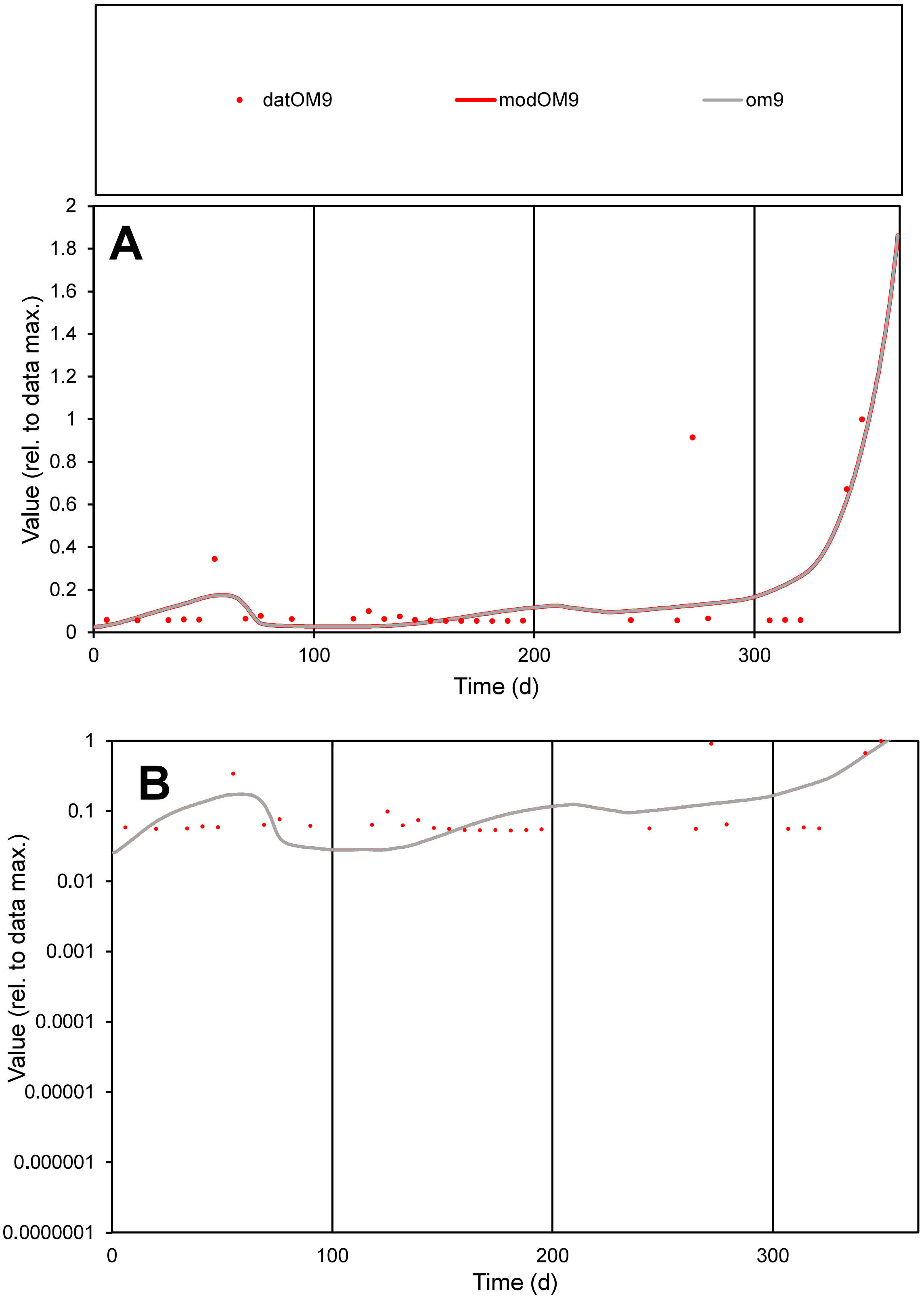

C1

Variables
OM9 Provide jtd or Dst
CalibOnly?
2 1 to reproduce of
oNormalize?
2 1=no, 2=yes

ofxn calc*
1.08191
ofxn mod
1.10156

sRun
RunA1
zout
1

Ave
Data
0.00012
Model
0.00012

data/vMax
0.00081

ofxng
275.646

Min d/m
1.89374

ave/min d
2.82836
ave/min m
5.20097

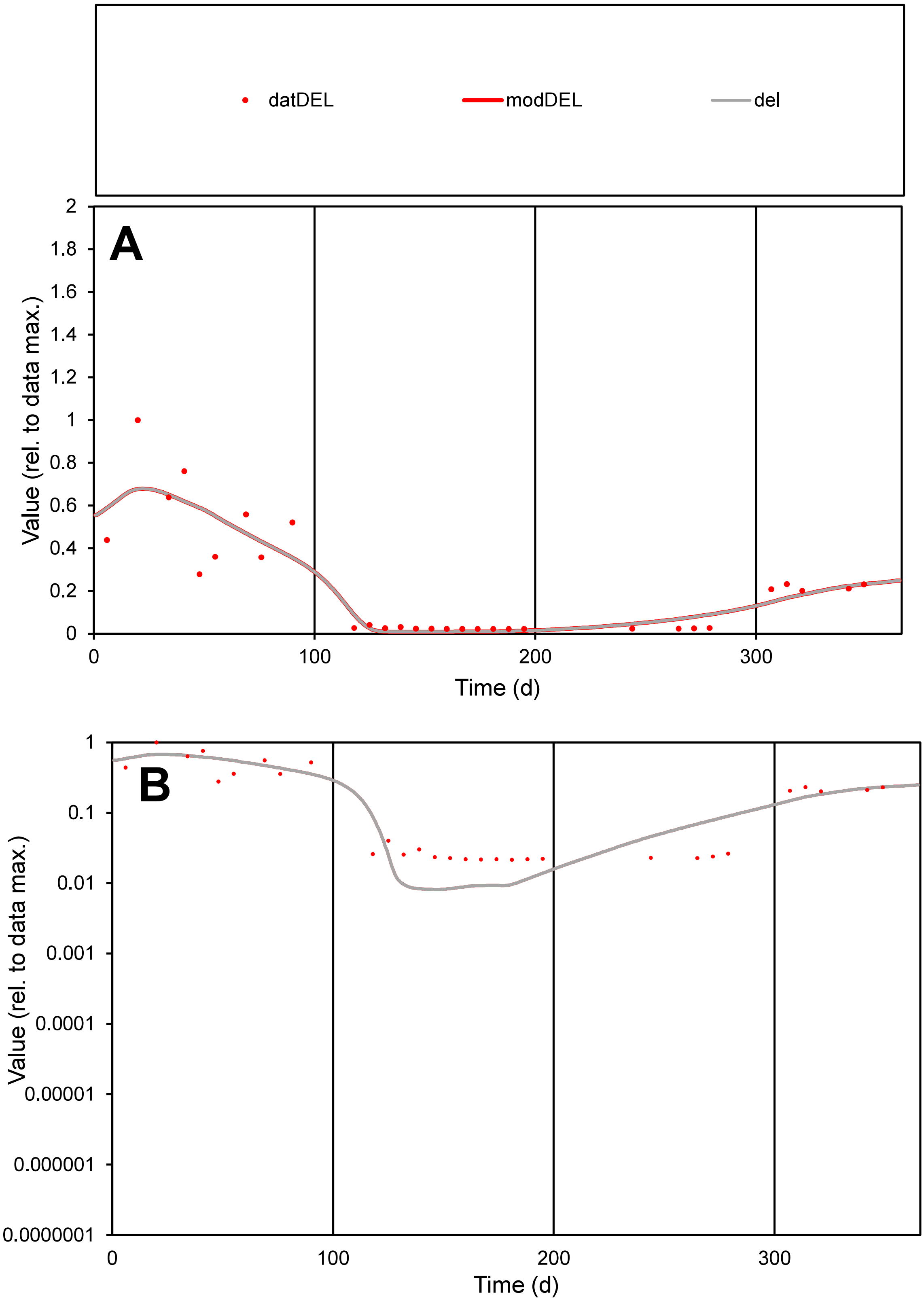

C1

Variables

DEL Provide jtd or Dst

CalibOnly?

2 1 to reproduce of

oNormalize?

2 1=no, 2=yes

ofxn calc*

0.50152

ofxn mod

0.53099

sRun

RunA1

zout

1

Ave

Data

0.00061

Model

0.0006

data/vMax

0.00286

ofxng

275.646

Min d/m

2.64858

ave/min d

9.88615

ave/min m

25.9798

325_Calib_S_A

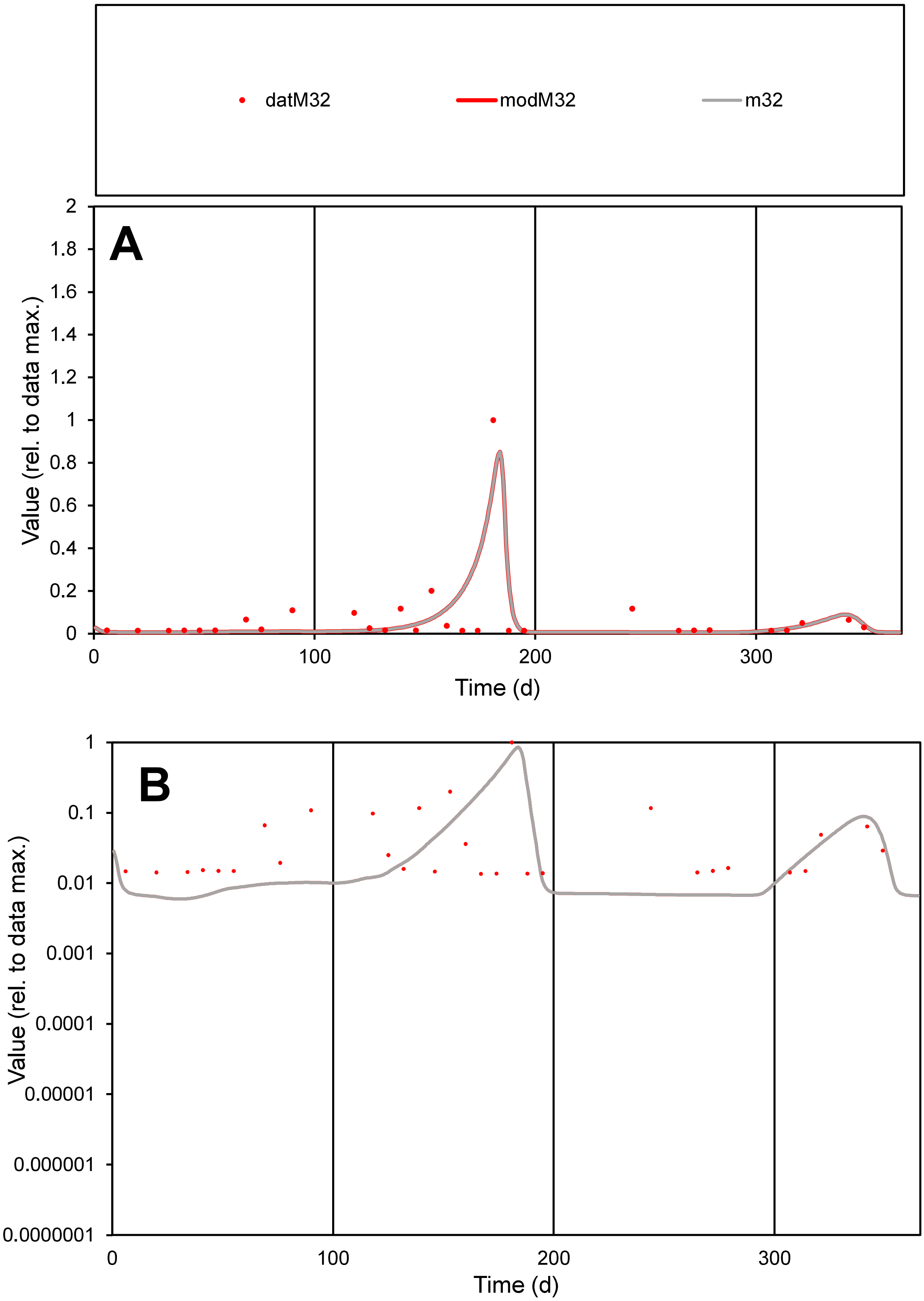

C1

Variables
M32 Provide jtd or Dst
CalibOnly?
2 1 to reproduce of
oNormalize?
2 1=no, 2=yes

ofxn calc*
1.21911
ofxn mod
1.30028

sRun
RunA1
zout
1

Ave
Data
0.00112
Model
0.00103

data/vMax
0.01543

ofxng
275.646

Min d/m
2.22376

ave/min d
5.34861
ave/min m
10.9669

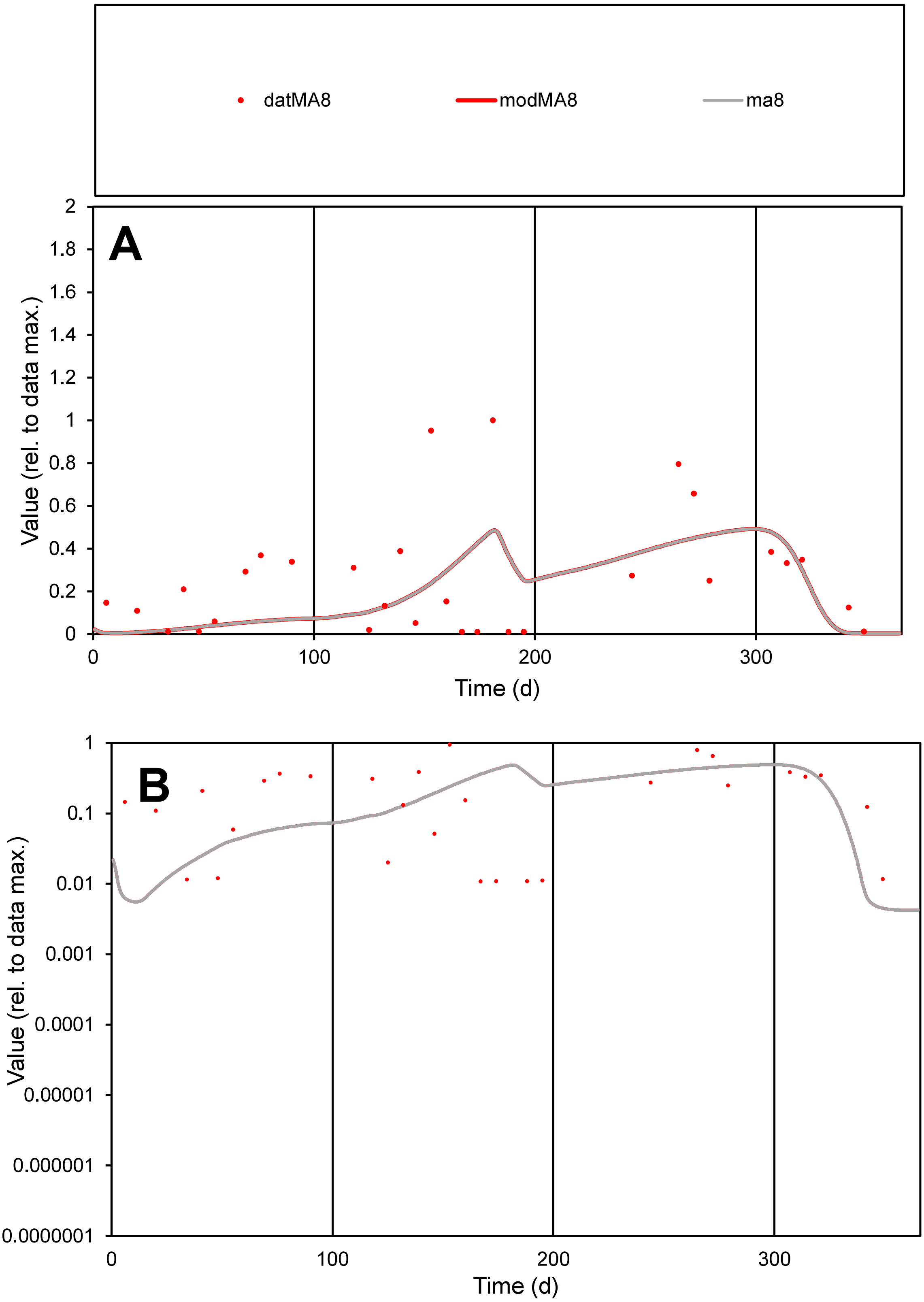

C1

Variables
MA8 Provide jtd or Dst
CalibOnly?
2 1 to reproduce of
oNormalize?
2 1=no, 2=yes

ofxn calc*
0.9718
ofxn mod
0.98356

sRun
RunA1
zout
1

Ave
Data
0.00143
Model
0.00115

data/vMax
0.0055

ofxng
275.646

Min d/m
2.45986

ave/min d
23.8669
ave/min m
47.472

325_Calib_S_A

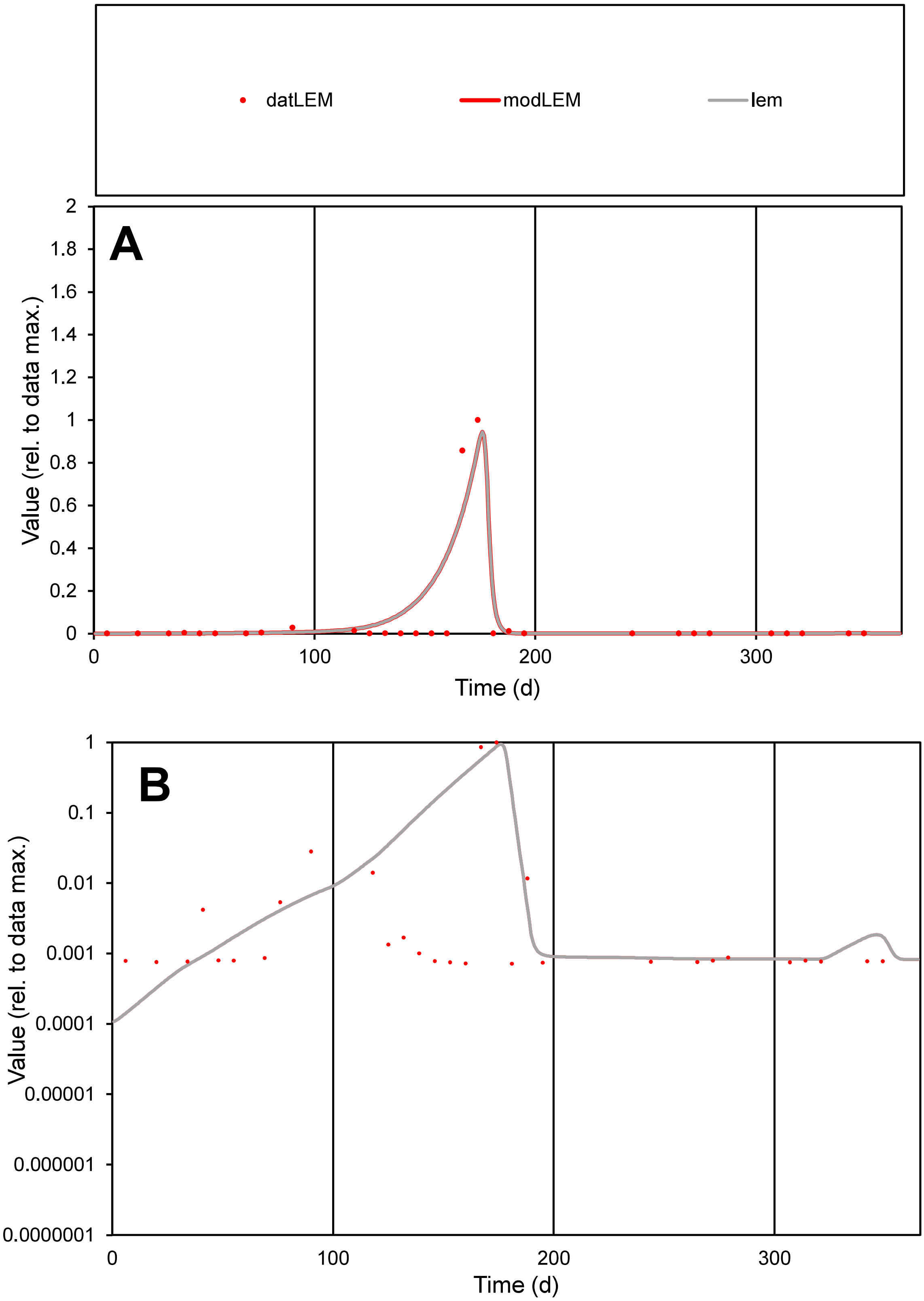

C1

Variables
LEM Provide jtd or Dst
CalibOnly?
2 1 to reproduce of
oNormalize?
2 1=no, 2=yes

ofxn calc*
1.47383
ofxn mod
1.55353

sRun
RunA1
zout
1

Ave
Data
0.00193
Model
0.00252

data/vMax
0.02992

ofxng
275.646

Min d/m
4.71819

ave/min d
90.2745
ave/min m
555.168

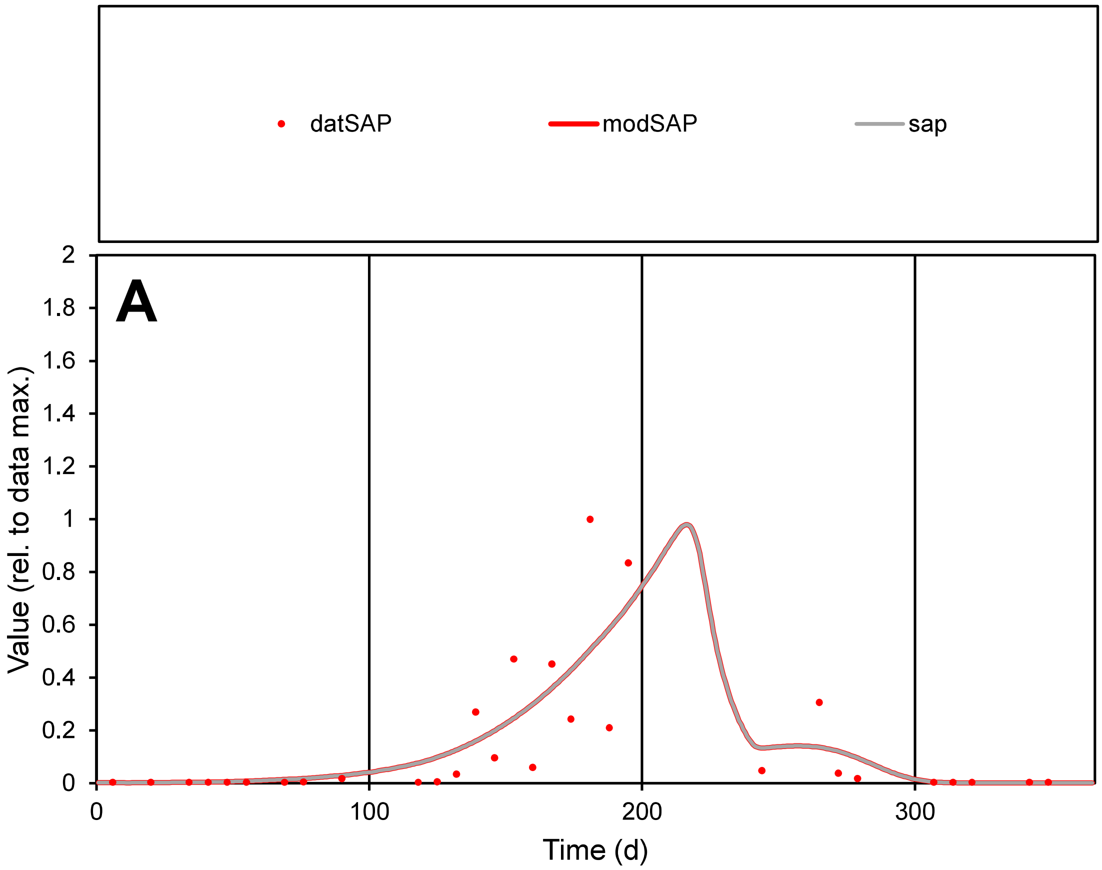

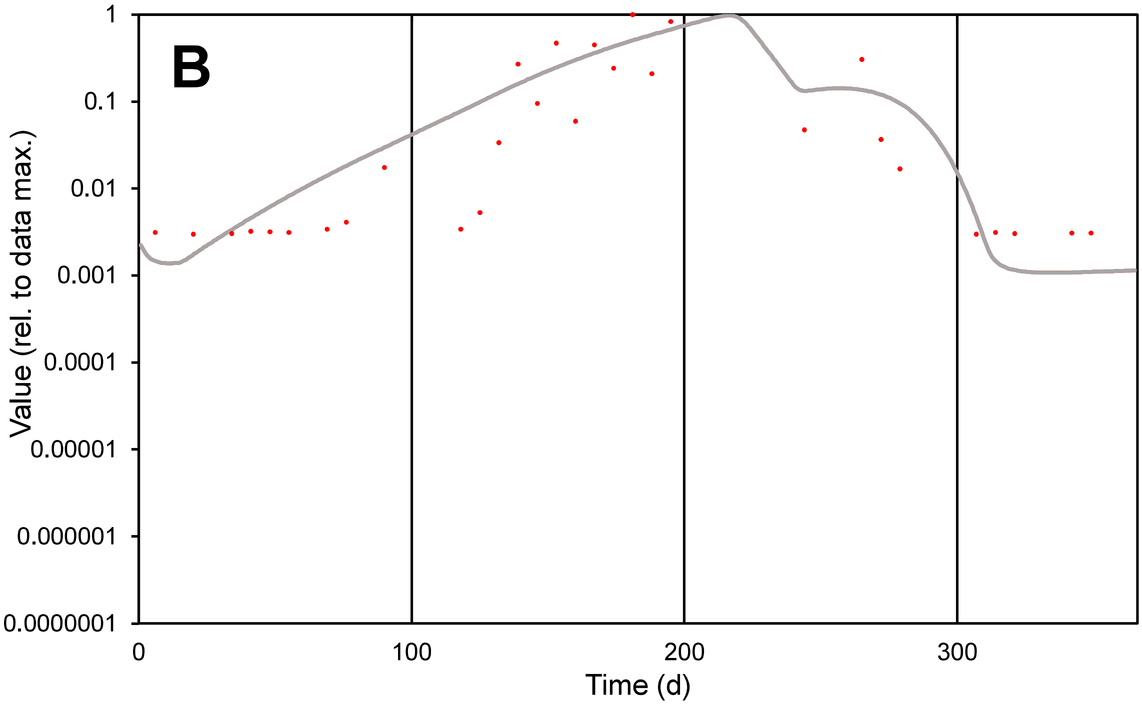

C1

Variables
SAP Provide jtd or Dst
CalibOnly?
2 1 to reproduce of
oNormalize?
2 1=no, 2=yes

ofxn calc*
1.05204
ofxn mod
1.07863

sRun
RunA1
zout
1

Ave
Data
0.00159
Model
0.00169

data/vMax
0.0115

ofxng
275.646

Min d/m
2.73328

ave/min d
46.2019
ave/min m
134.428

325_Calib_S_A

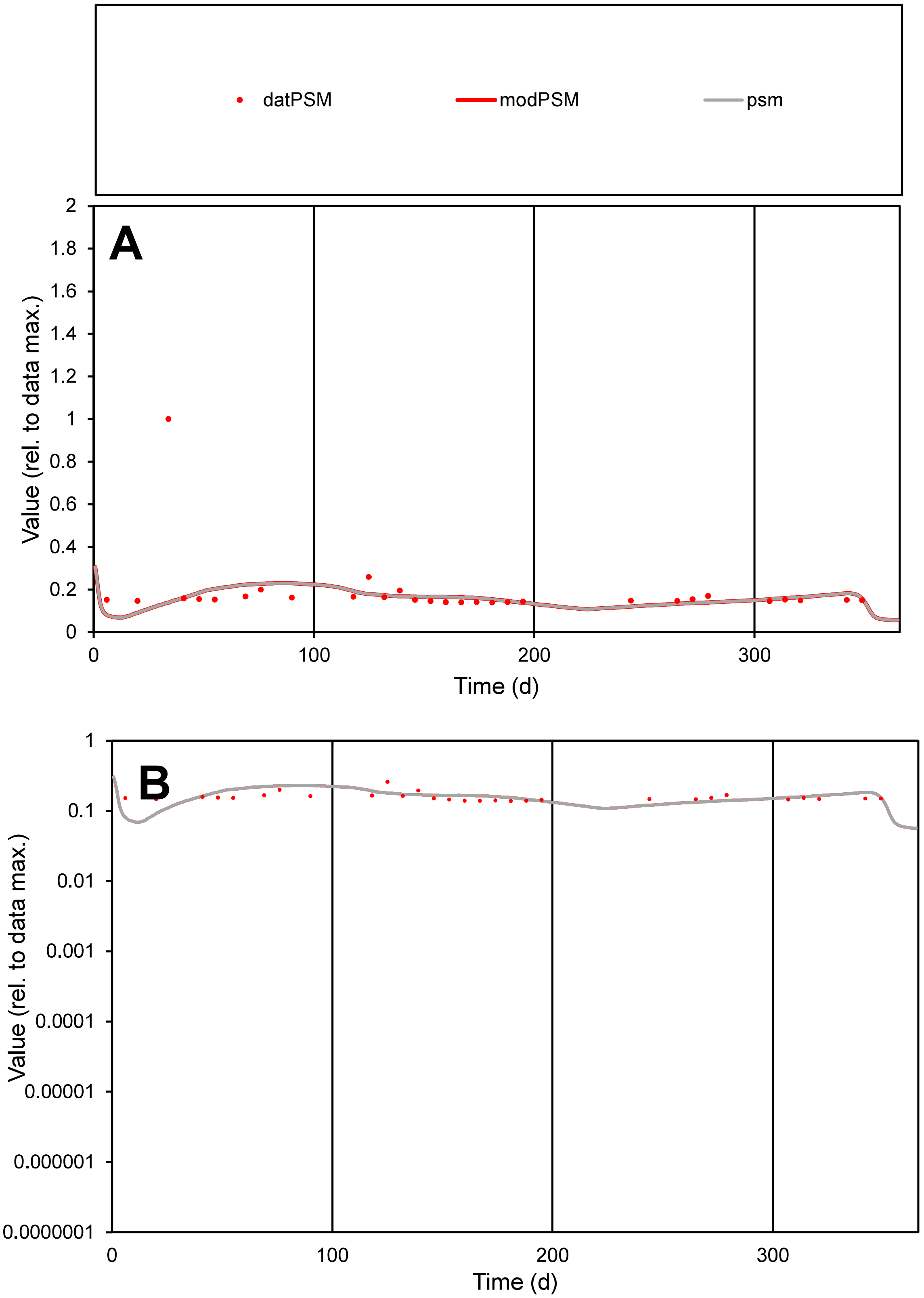

C1

Variables
PSM Provide jtd or Dst
CalibOnly?
2 1 to reproduce of
oNormalize?
2 1=no, 2=yes

ofxn calc*
0.85815
ofxn mod
0.86767

sRun
RunA1
zout
1

Ave
Data
3.7E-05
Model
3.2E-05

data/vMax
0.0002

ofxng
275.646

Min d/m
1.77161

ave/min d
1.34244
ave/min m
2.06923

325_Calib_S_A

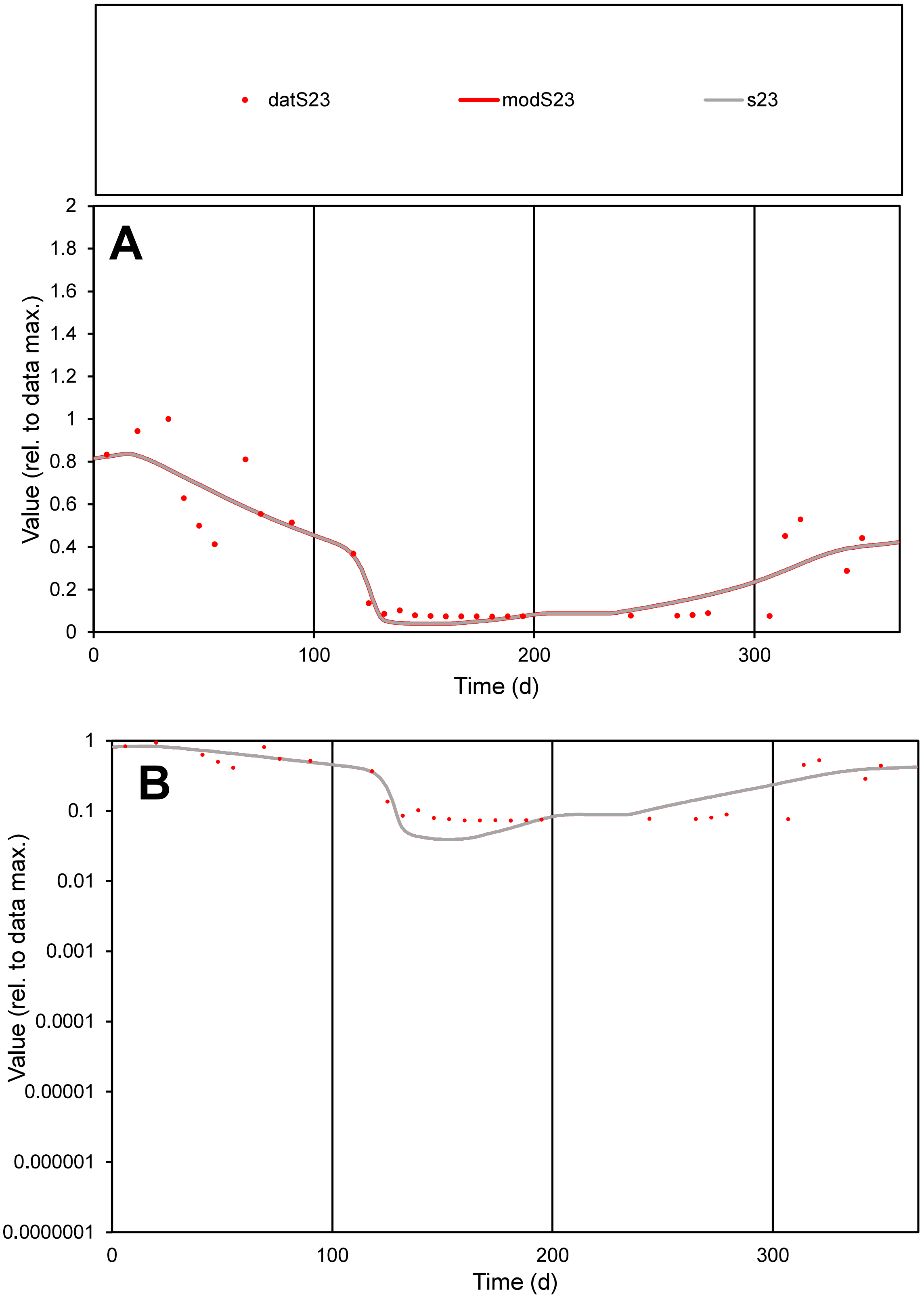

C1

Variables
S23 Provide jtd or Dst
CalibOnly?
2 1 to reproduce of
oNormalize?
2 1=no, 2=yes

ofxn calc*
0.3474
ofxn mod
0.38005

sRun
RunA1
zout
1

Ave
Data
0.0007
Model
0.00069

data/vMax
0.0022

ofxng
275.646

Min d/m
1.85884

ave/min d
4.40457
ave/min m
7.99294

325_Calib_S_A

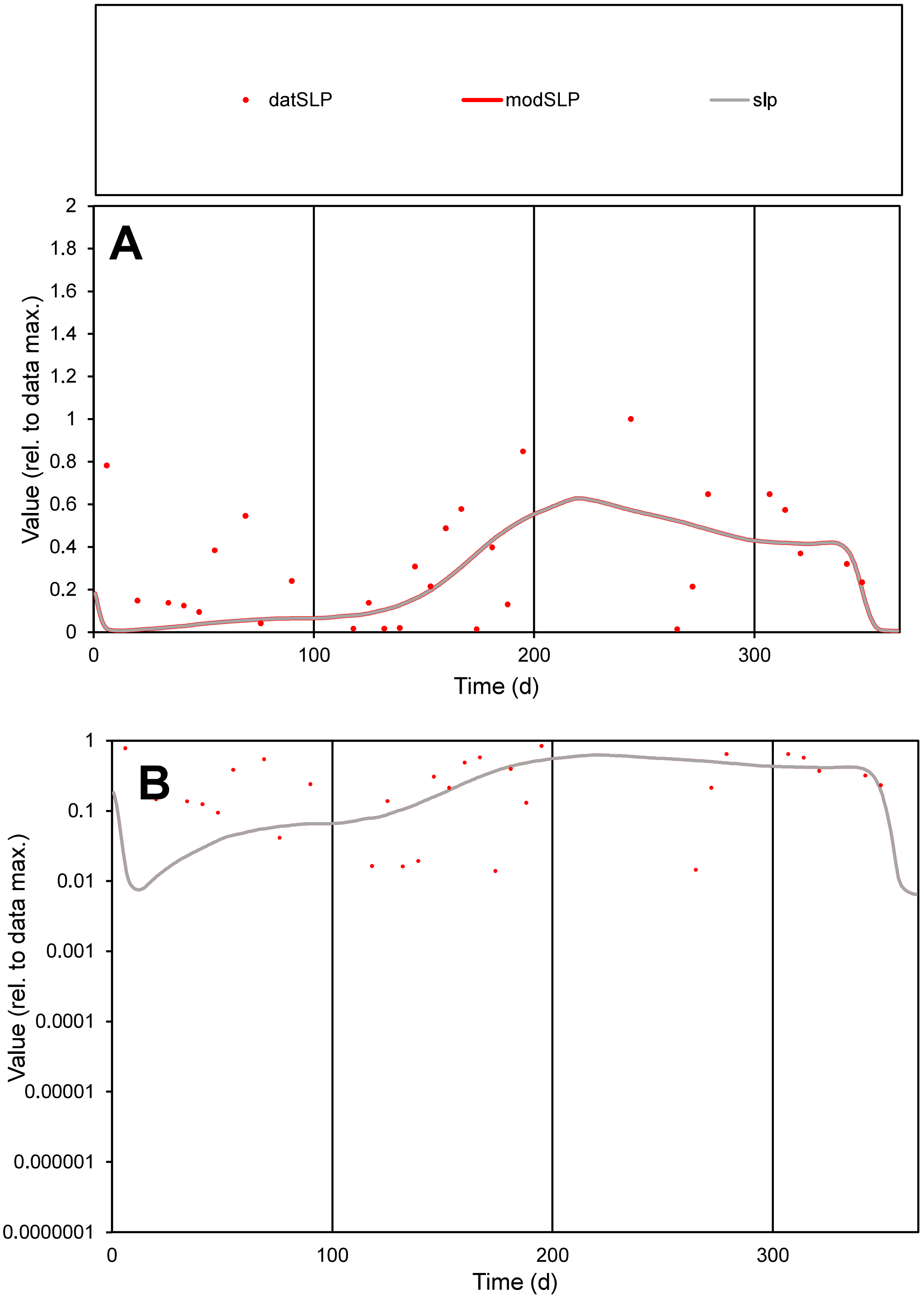

C1

Variables
SLP Provide jtd or Dst
CalibOnly?
2 1 to reproduce of
oNormalize?
2 1=no, 2=yes

ofxn calc*
0.83194
ofxn mod
0.84406

sRun
RunA1
zout
1

Ave
Data
0.00056
Model
0.00043

data/vMax
0.00173

ofxng
275.646

Min d/m
1.1438

ave/min d
23.09
ave/min m
20.2228

325_Calib_S_A

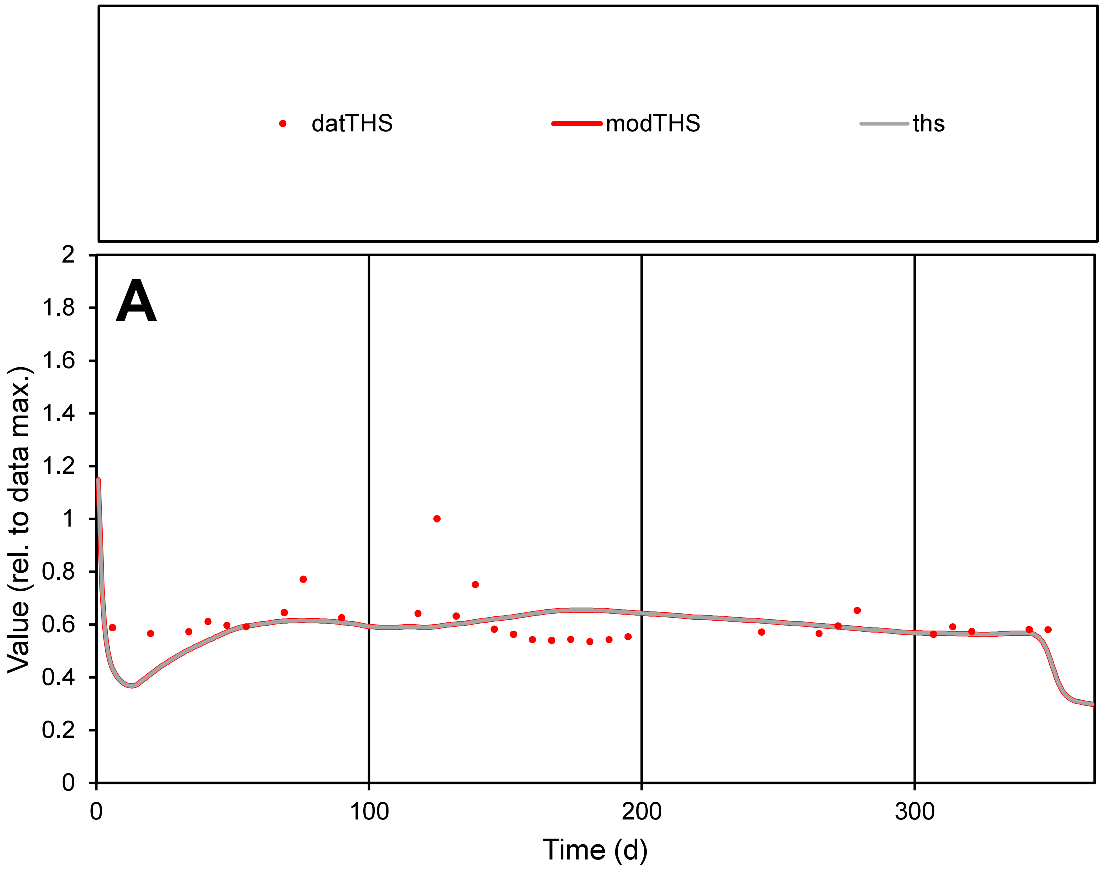

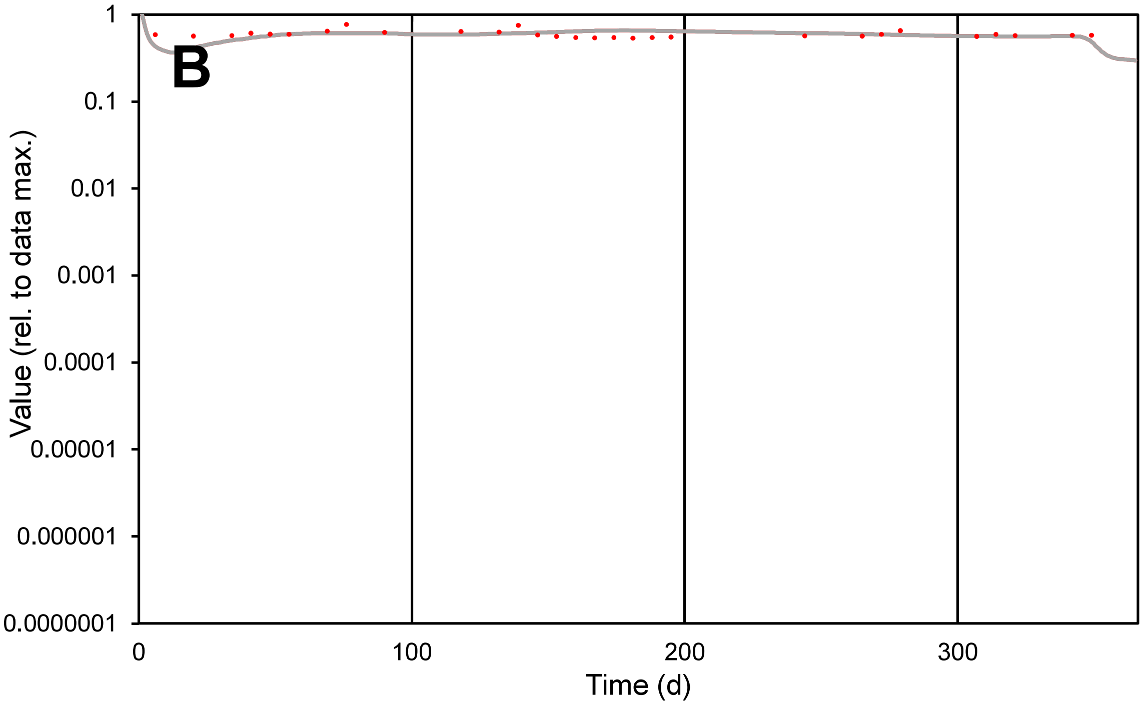

C1

Variables
THS Provide jtd or Dst
CalibOnly?
2 1 to reproduce of
oNormalize?
2 1=no, 2=yes

ofxn calc*
0.1841
ofxn mod
0.21493

sRun
RunA1
zout
1

Ave
Data
3.2E-05
Model
3.1E-05

data/vMax
5.3E-05

ofxng
275.646

Min d/m
1.31023

ave/min d
1.13763
ave/min m
1.4307

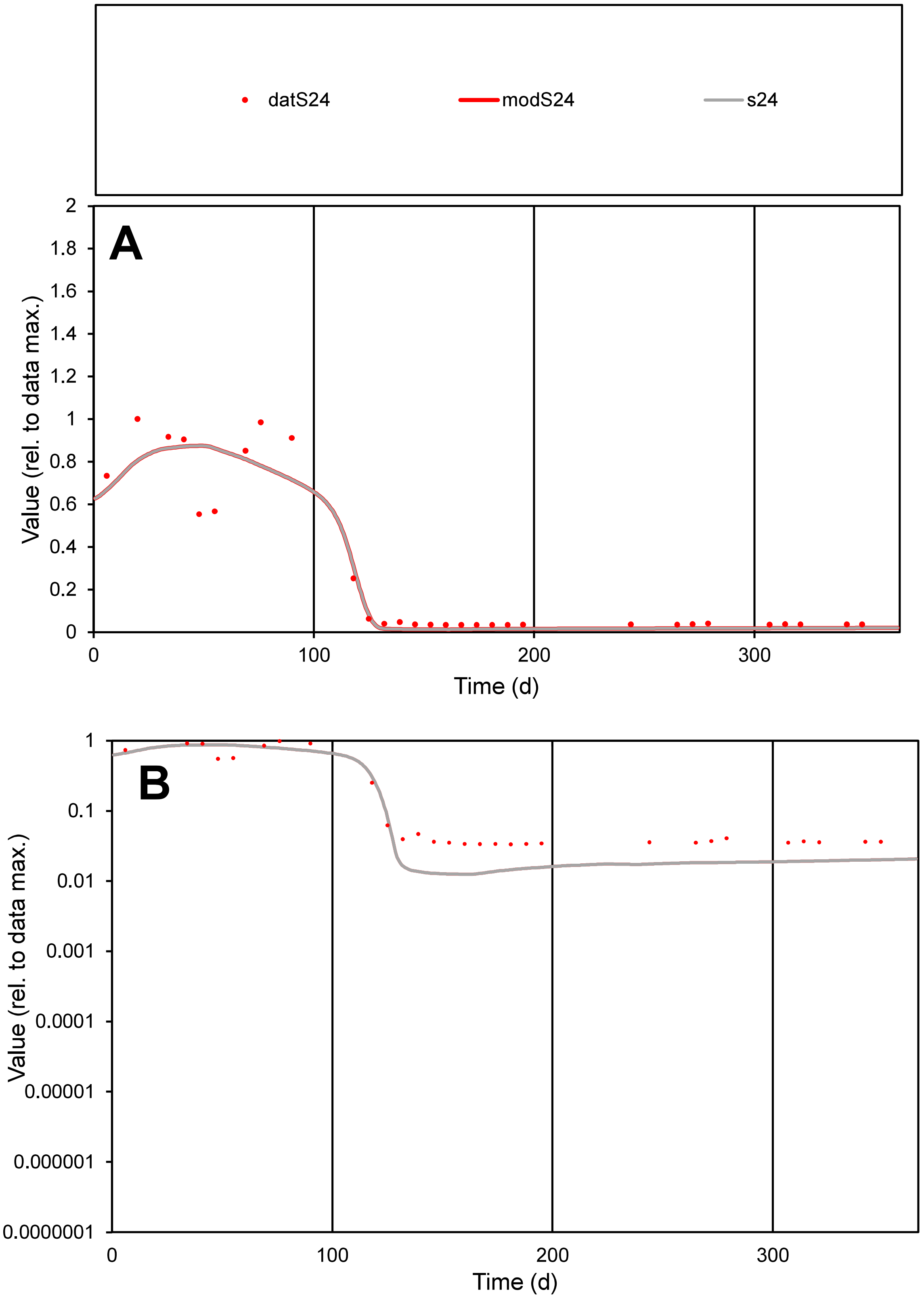

C1

Variables
S24 Provide jtd or Dst
CalibOnly?
2 1 to reproduce of
oNormalize?
2 1=no, 2=yes

ofxn calc*
0.37007
ofxn mod
0.41574

sRun
RunA1
zout
1

Ave
Data
0.00049
Model
0.00045

data/vMax
0.00173

ofxng
275.646

Min d/m
2.67531

ave/min d
8.4031
ave/min m
21.018

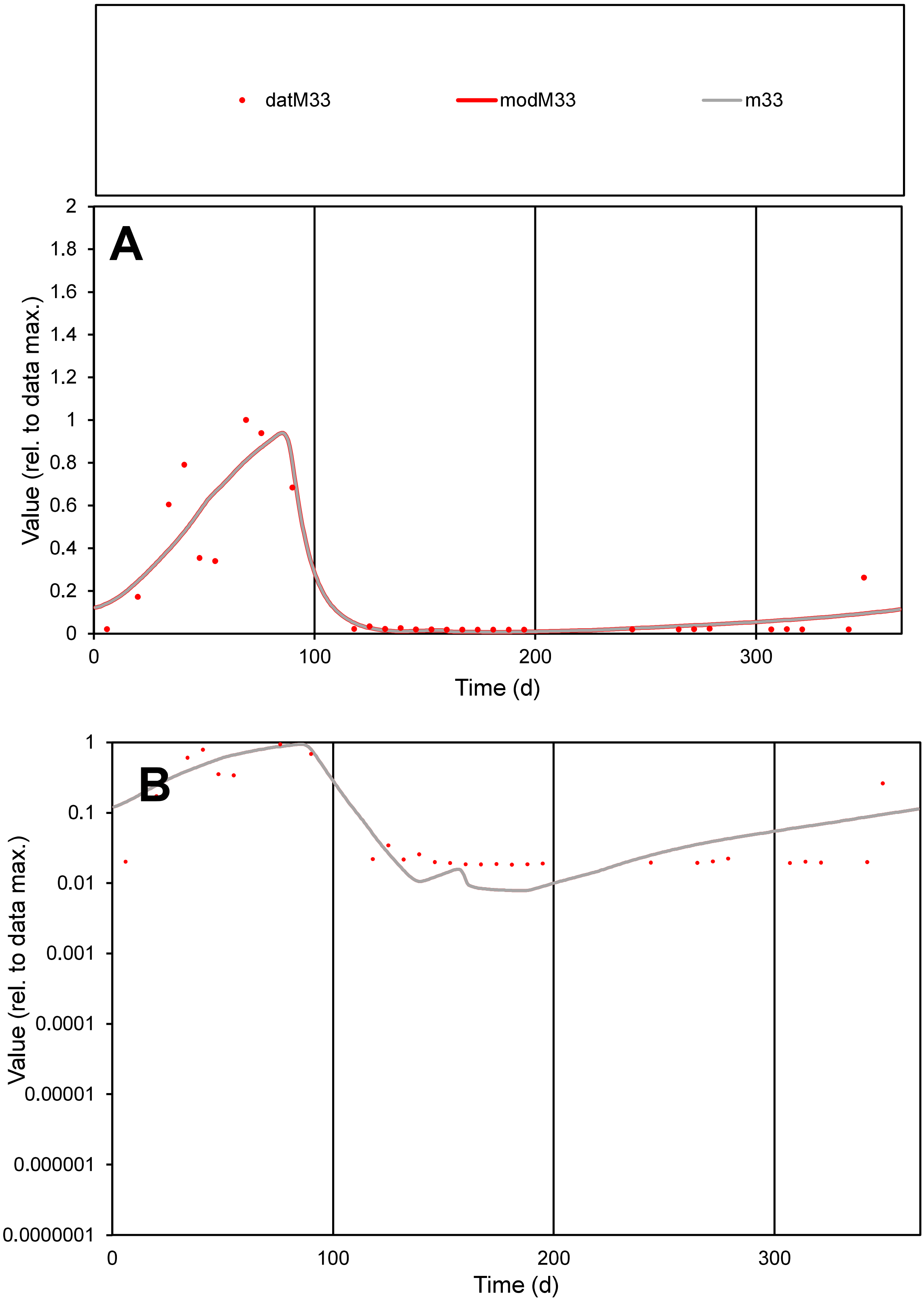

C1

Variables
M33 Provide jtd or Dst
CalibOnly?
2 1 to reproduce of
oNormalize?
2 1=no, 2=yes

ofxn calc*
0.60579
ofxn mod
0.64726

sRun
RunA1
zout
1

Ave
Data
0.00069
Model
0.0007

data/vMax
0.0037

ofxng
275.646

Min d/m
2.33608

ave/min d
10.1296
ave/min m
23.9021

325_Calib_S_A

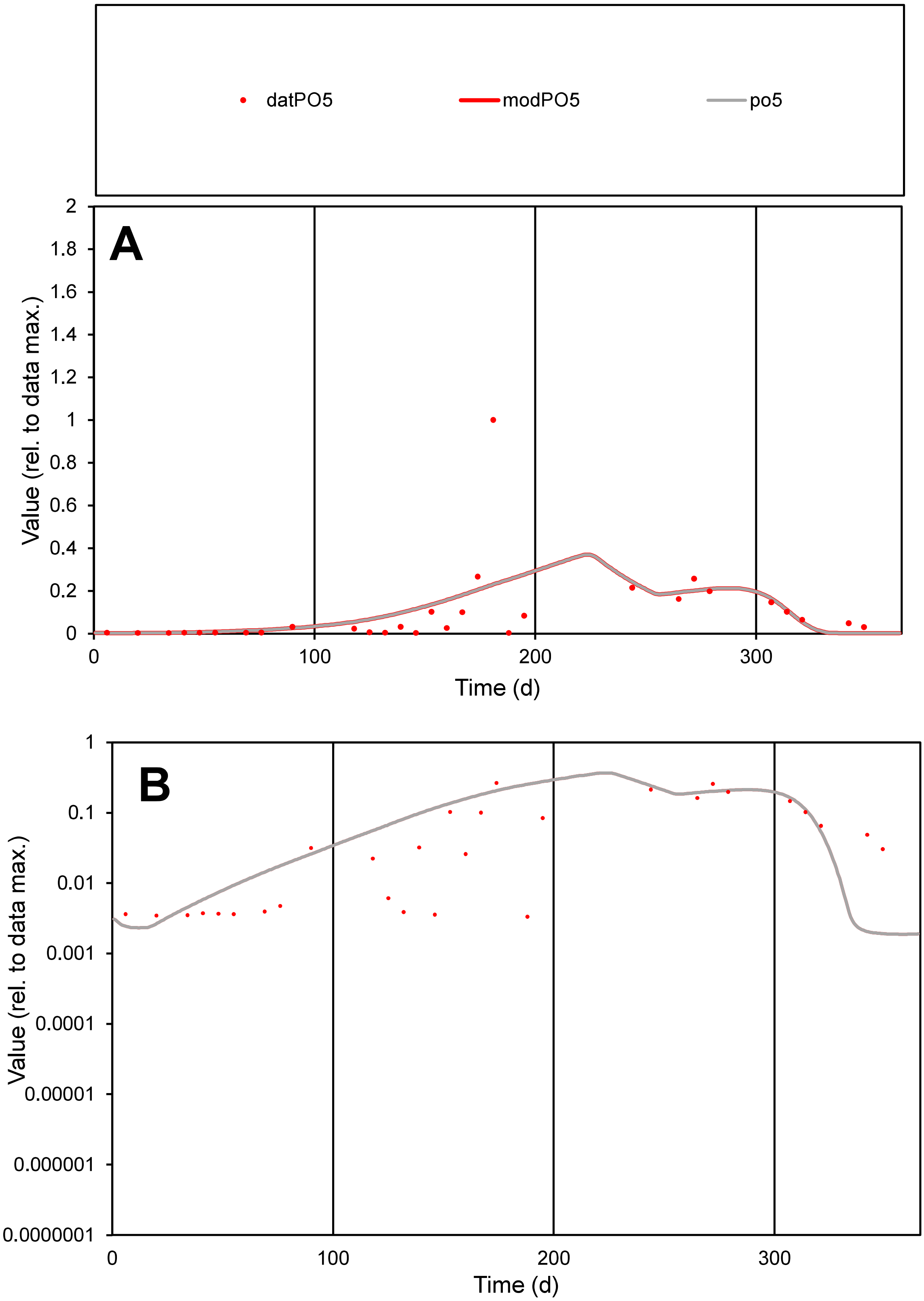

C1

Variables

PO5 Provide jtd or Dst

CalibOnly?

2 1 to reproduce of

oNormalize?

2 1=no, 2=yes

ofxn calc*

1.61613

ofxn mod

1.62893

sRun

RunA1

zout

1

Ave

Data

0.00138

Model

0.00147

data/vMax

0.01405

ofxng

275.646

Min d/m

1.75393

ave/min d

29.3747

ave/min m

54.8936

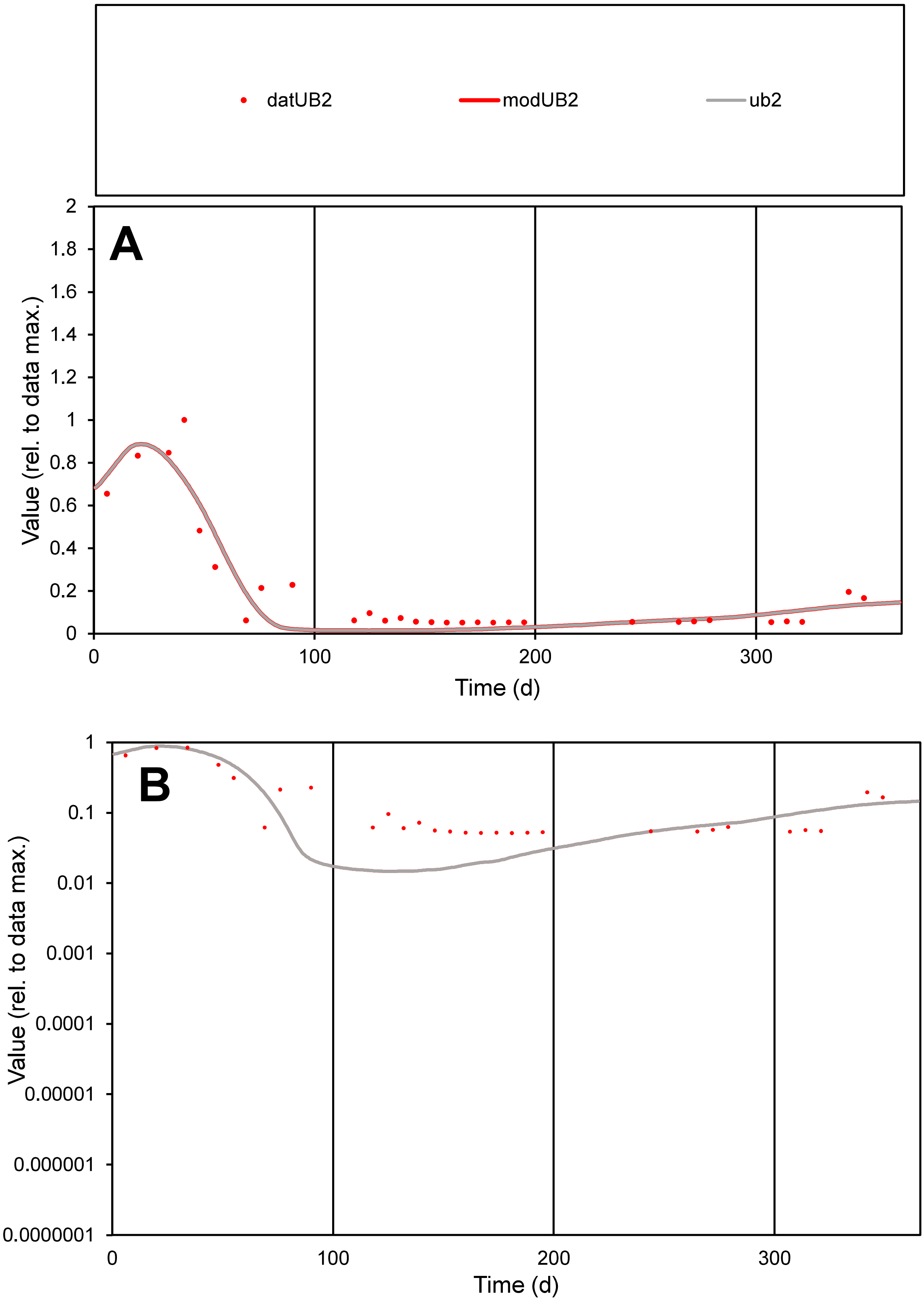

C1

Variables

UB2 Provide jtd or Dst

CalibOnly?

2 1 to reproduce of

oNormalize?

2 1=no, 2=yes

ofxn calc*

0.43181

ofxn mod

0.47132

sRun

RunA1

zout

1

Ave

Data

0.00041

Model

0.00037

data/vMax

0.00201

ofxng

275.646

Min d/m

3.48971

ave/min d

3.95616

ave/min m

12.4433

325_Calib_S_A

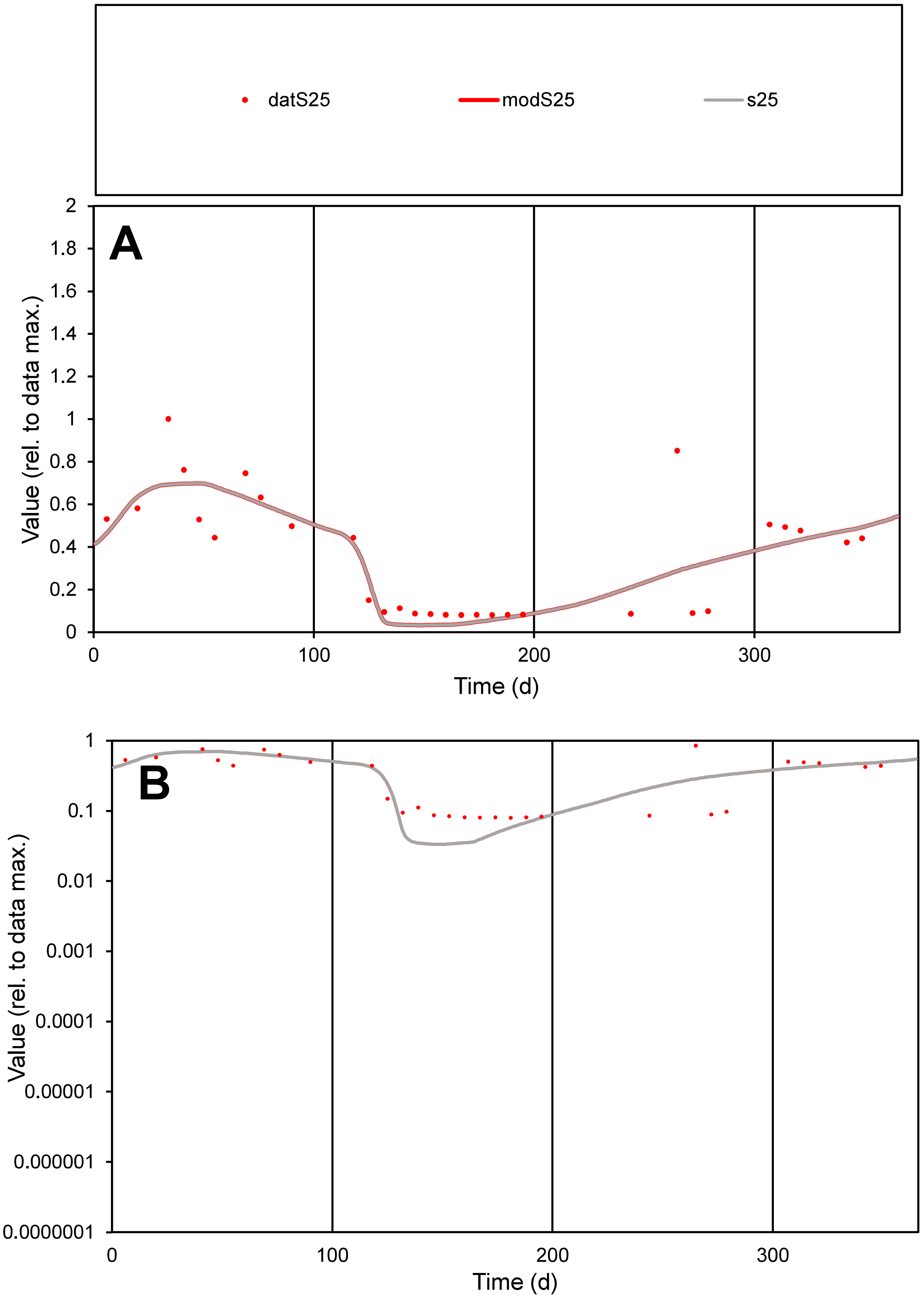

C1

Variables
S25 Provide jtd or Dst
CalibOnly?
2 1 to reproduce of
oNormalize?
2 1=no, 2=yes

ofxn calc*
0.4255
ofxn mod
0.44903

sRun
RunA1
zout
1

Ave
Data
0.00074
Model
0.00071

data/vMax
0.0021

ofxng
275.646

Min d/m
2.4024

ave/min d
4.42871
ave/min m
10.1158

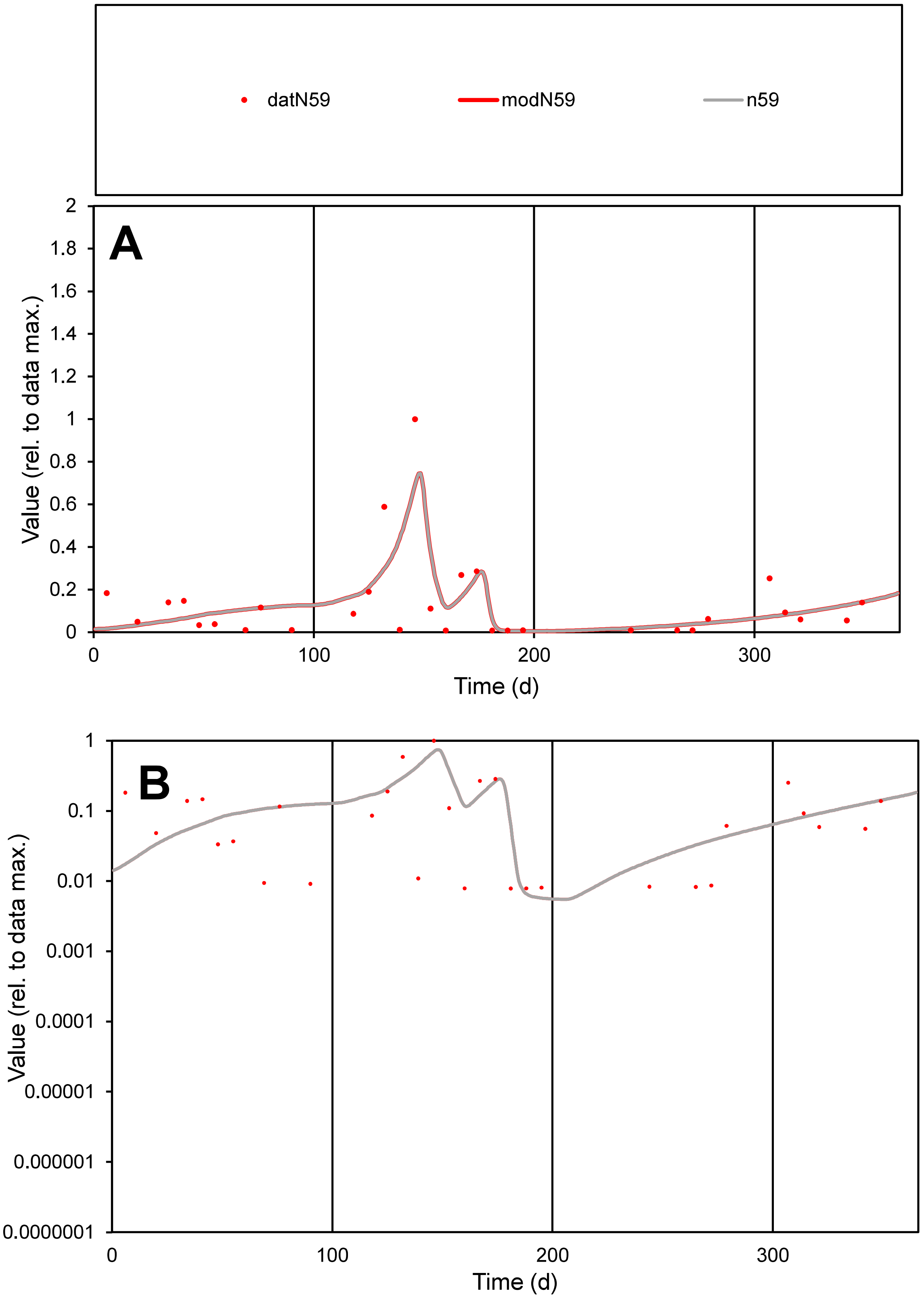

C1

Variables

N59 Provide jtd or Dst

CalibOnly?

2 1 to reproduce of

oNormalize?

2 1=no, 2=yes

ofxn calc*

0.93384

ofxn mod

0.97679

sRun

RunA1

zout

1

Ave

Data

0.0007

Model

0.00071

data/vMax

0.00525

ofxng

275.646

Min d/m

1.37638

ave/min d

16.9642

ave/min m

23.9445

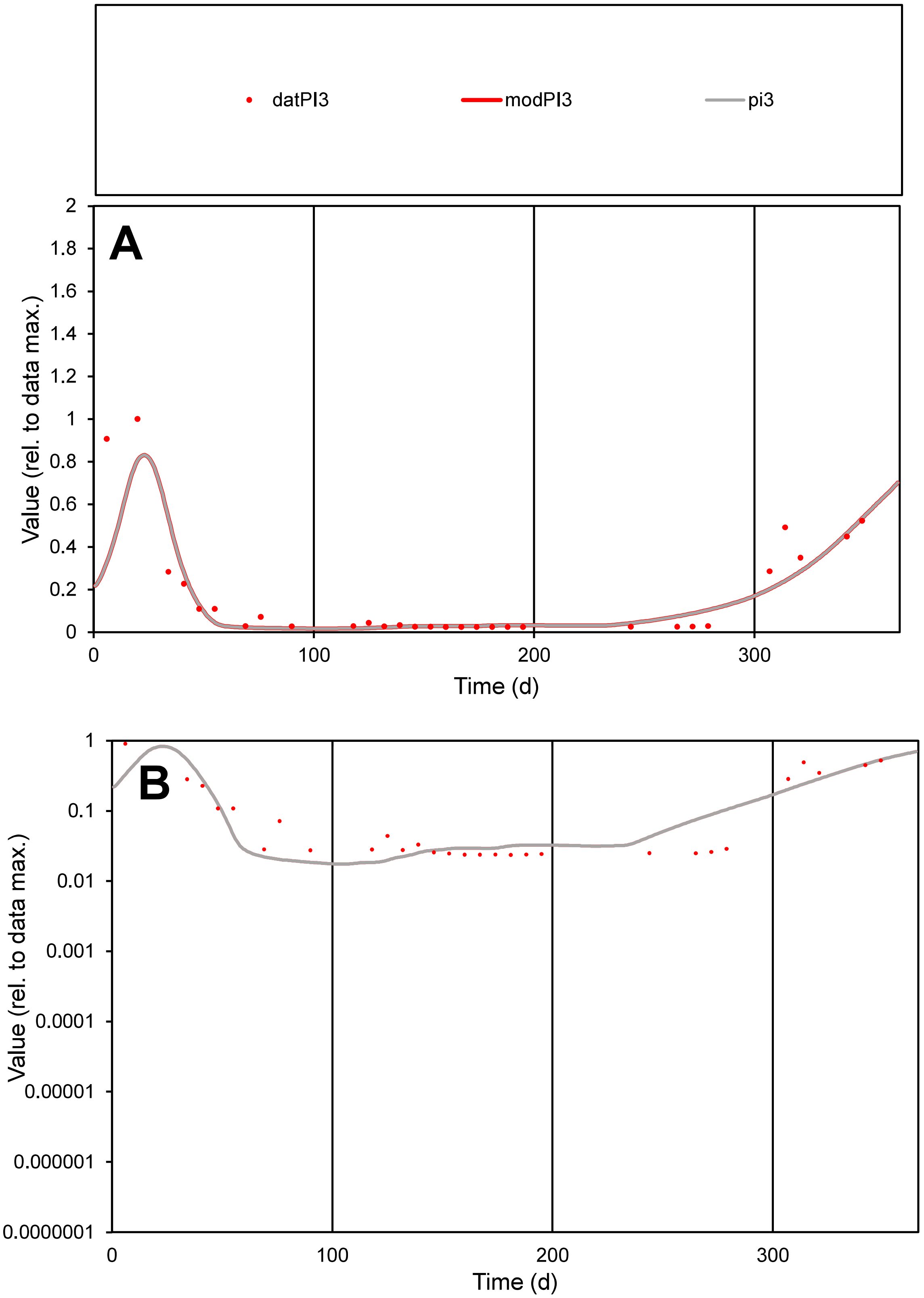

C1

Variables
PI3 Provide jtd or Dst
CalibOnly?
2 1 to reproduce of
oNormalize?
2 1=no, 2=yes

ofxn calc*
0.69344
ofxn mod
0.71165

sRun
RunA1
zout
1

Ave
Data
0.00053
Model
0.00045

data/vMax
0.003

ofxng
275.646

Min d/m
1.28186

ave/min d
7.48807
ave/min m
8.24096

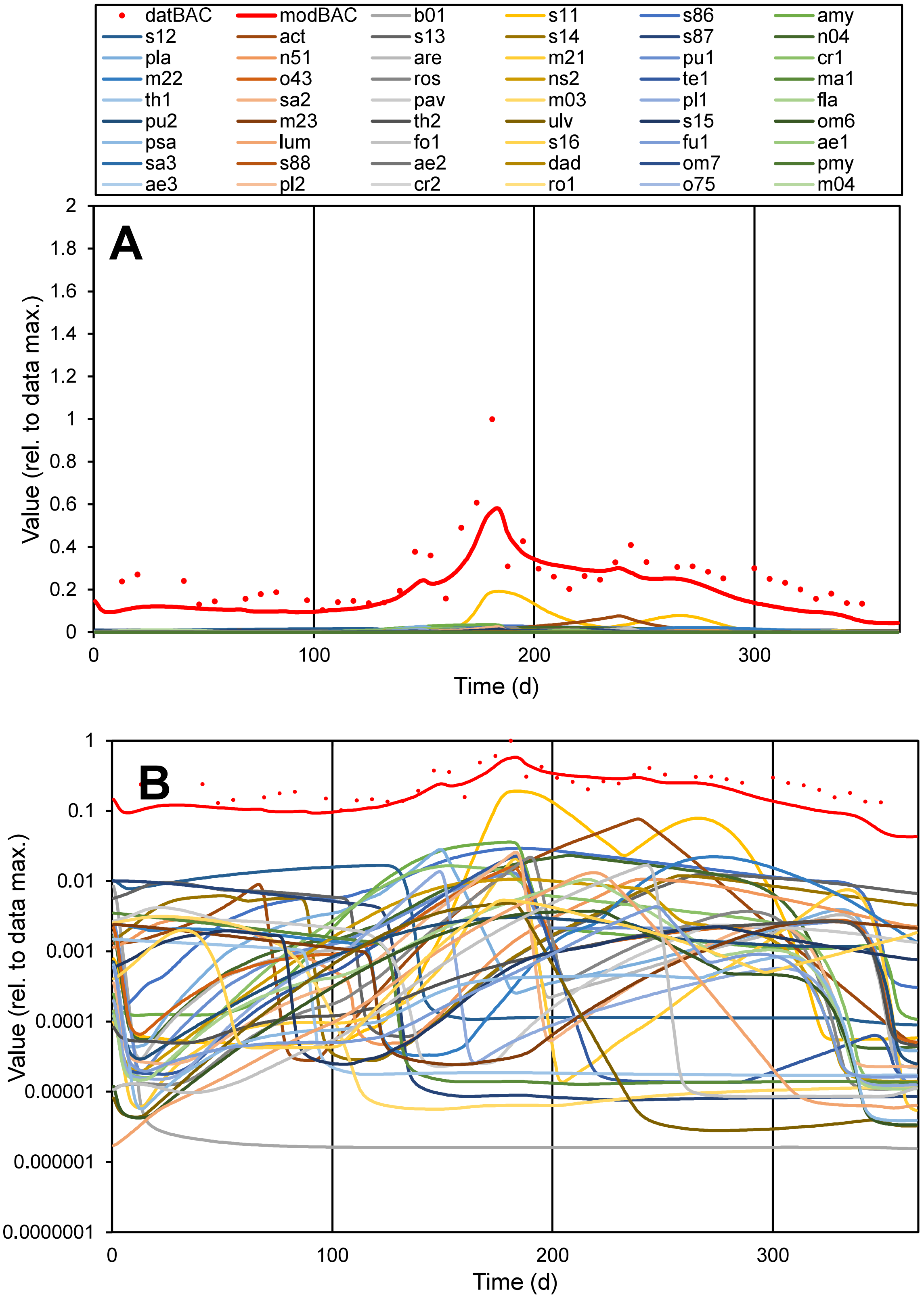

C1

Variables

BAC Provide jtd or Dst

CalibOnly?

2 1 to reproduce of

oNormalize?

2 1=no, 2=yes

ofxn calc*

0.41963

ofxn mod

0.43501

sRun

RunA1

zout

1

Ave

Data

0.76978

Model

0.58298

data/vMax

2.916

ofxng

275.646

Min d/m

2.00066

ave/min d

2.55657

ave/min m

3.87365

326_Calib_S_A

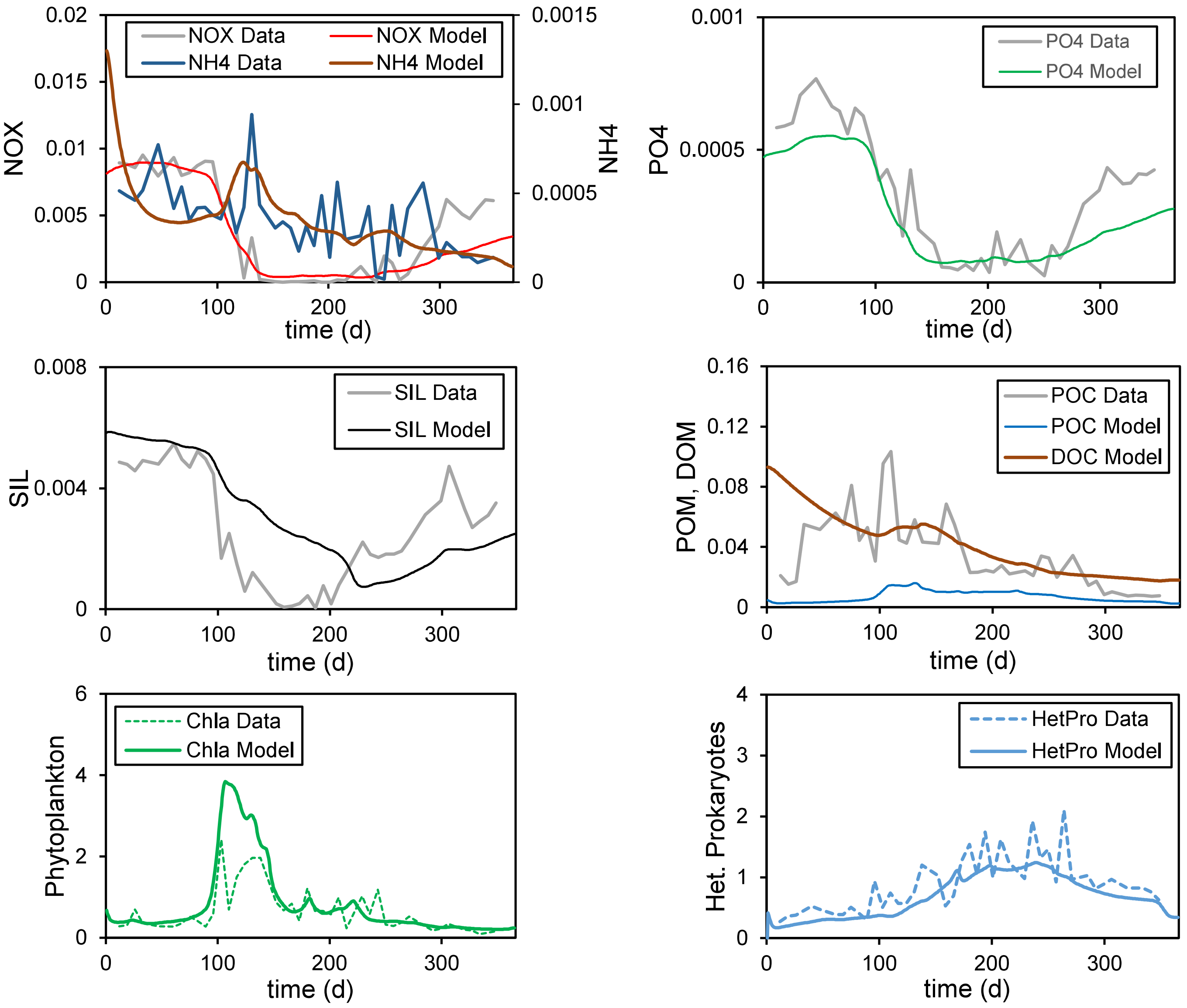

NOX Data
NOX Model
NH4 Data
NH4 Model
NOX
NH4
0.02
0.015
0.01
0.005
0
0.0015
0.001
0.0005
0
0
100
200
300
time (d)
PO4 Data
PO4 Model
PO4
0.001
0.0005
0
time (d)
SIL Data
SIL Model
SIL
0.008
0.004
0
time (d)
POC Data
POC Model
DOC Model
POM, DOM
0.16
0.12
0.08
0.04
0
time (d)
Chla Data
Chla Model
Phytoplankton
6
4
2
0
time (d)
HetPro Data
HetPro Model
Het. Prokaryotes
4
3
2
1
0
time (d)

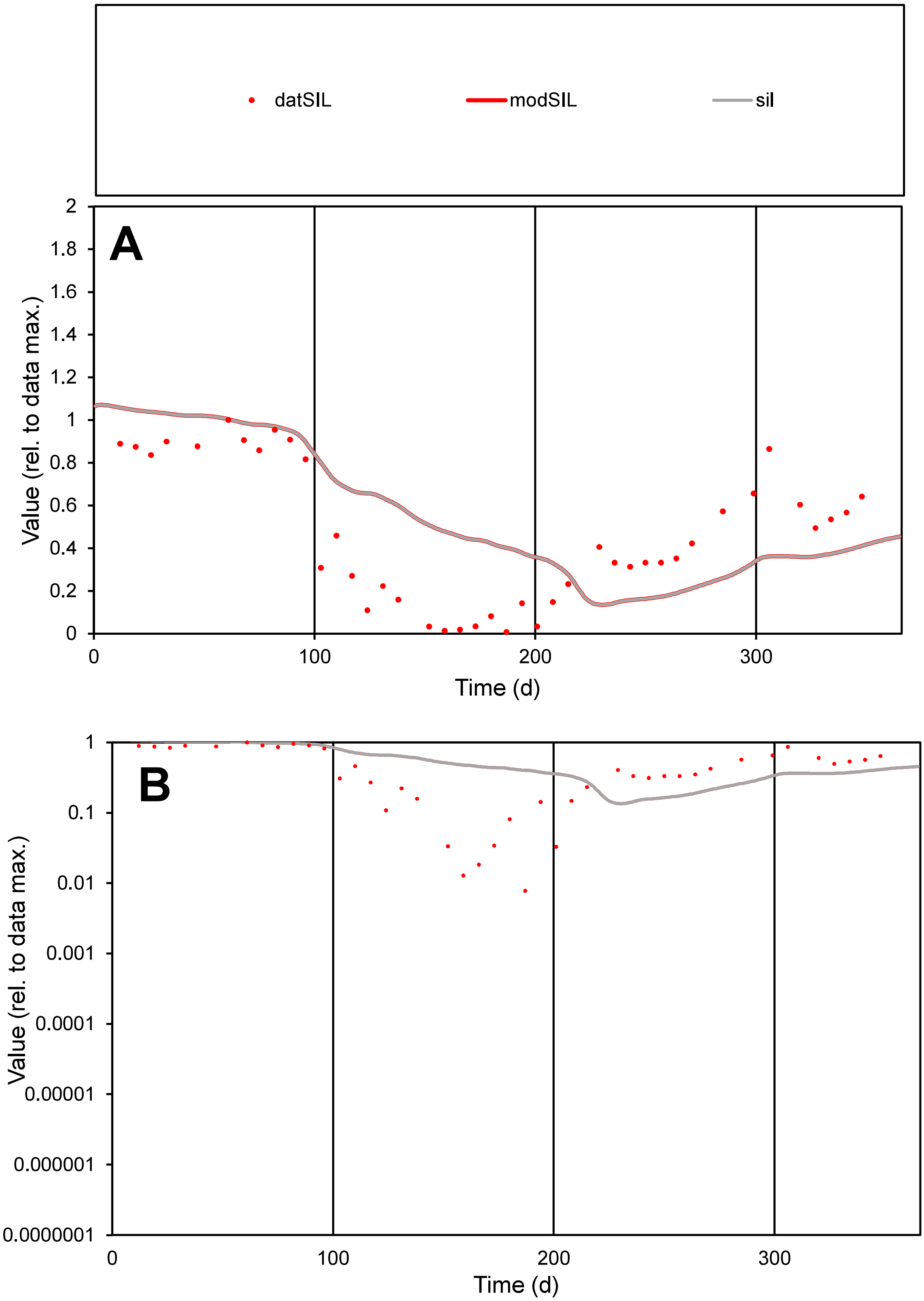

C1

Variables
SIL Provide jtd or Dst
CalibOnly?
2 1 to reproduce of
oNormalize?
2 1=no, 2=yes

ofxn calc*
1.82787
ofxn mod
1.8767

sRun
RunA1
zout
1

Ave
Data
0.00254
Model
0.003

data/vMax
0.00547

ofxng
263.459

Min d/m
0.05764

ave/min d
59.7881
ave/min m
4.06705

326_Calib_S_A

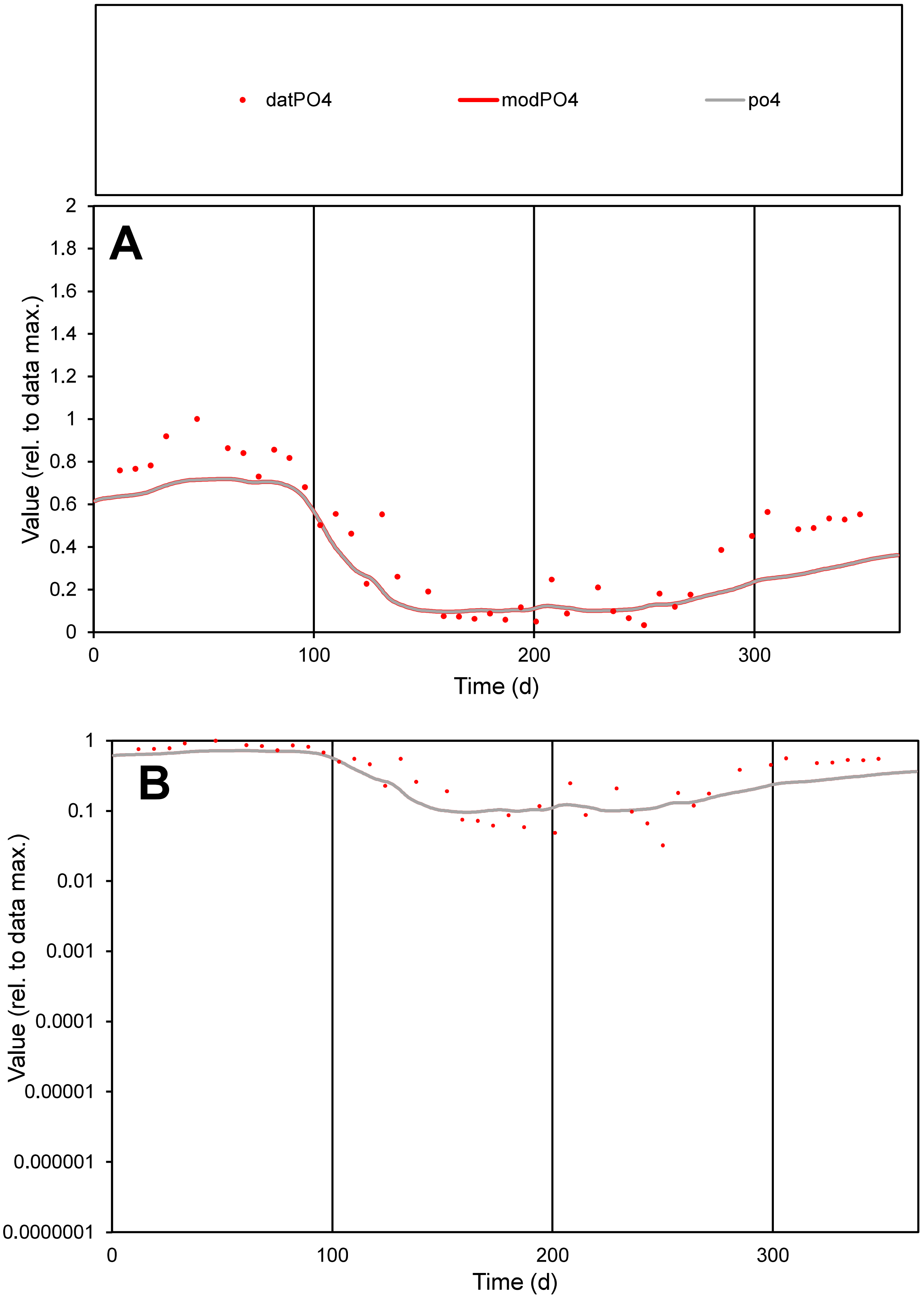

C1

Variables

PO4 Provide jtd or Dst

CalibOnly?

2 1 to reproduce of

oNormalize?

2 1=no, 2=yes

ofxn calc*

1.07645

ofxn mod

1.1394

sRun

RunA1

zout

1

Ave

Data

0.00032

Model

0.00024

data/vMax

0.00077

ofxng

263.459

Min d/m

0.33696

ave/min d

12.9022

ave/min m

3.31481

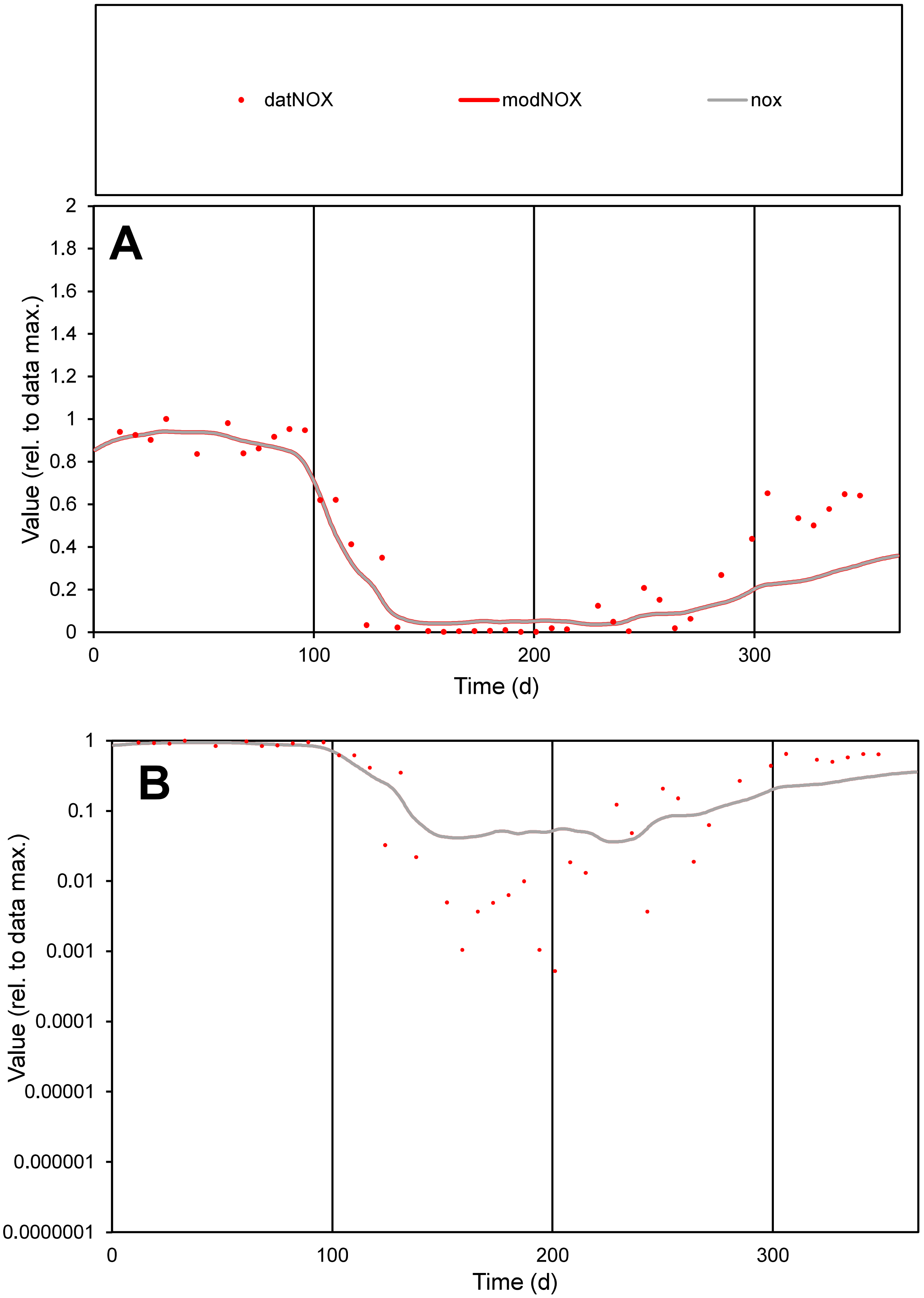

C1

Variables
NOX Provide jtd or Dst
CalibOnly?
2 1 to reproduce of
oNormalize?
2 1=no, 2=yes

ofxn calc*
1.11743
ofxn mod
1.19869

sRun
RunA1
zout
1

Ave
Data
0.00388
Model
0.00329

data/vMax
0.00952

ofxng
263.459

Min d/m
0.01433

ave/min d
775.146
ave/min m
9.43392

326_Calib_S_A

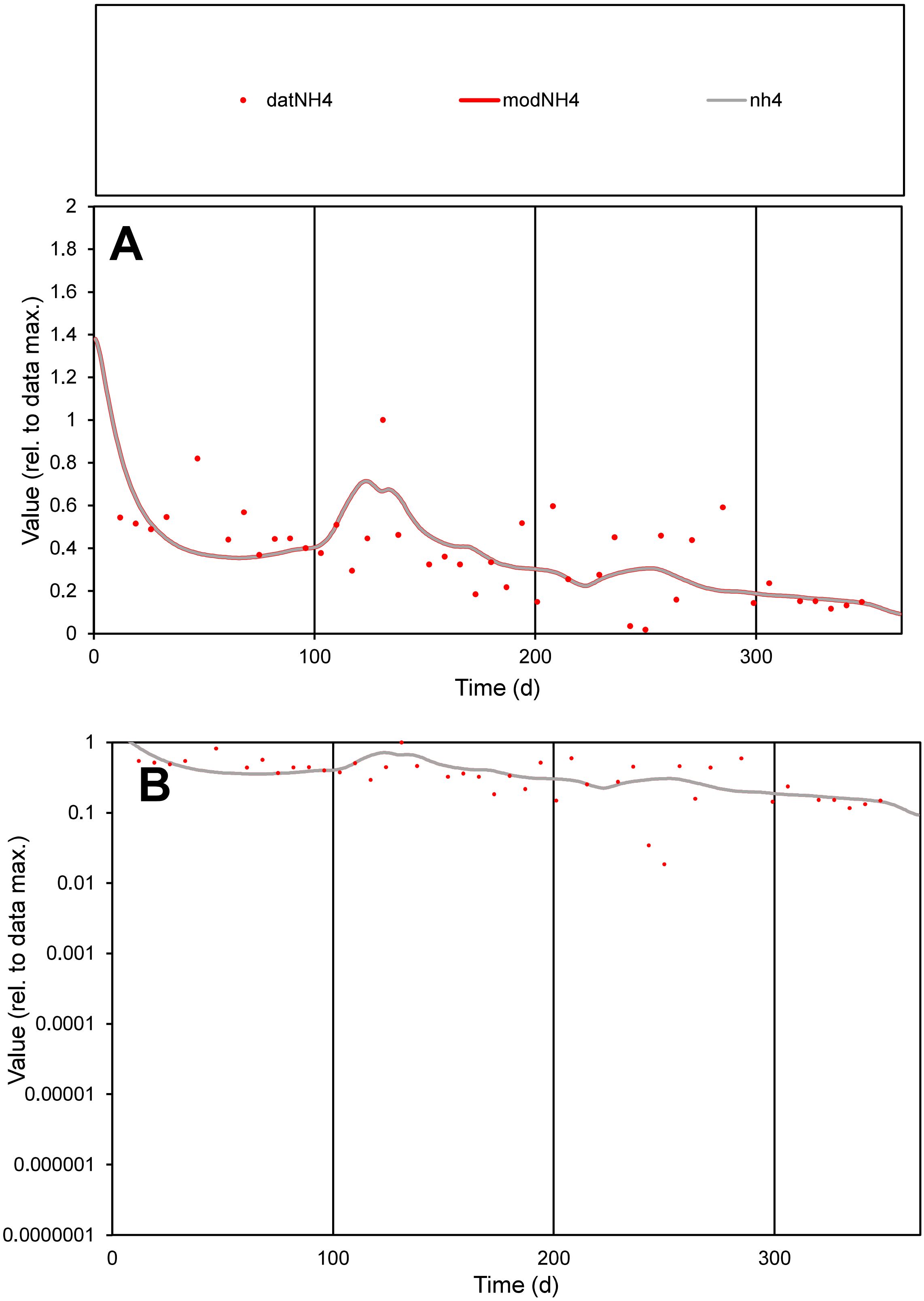

C1

Variables
NH4 Provide jtd or Dst
CalibOnly?
2 1 to reproduce of
oNormalize?
2 1=no, 2=yes

ofxn calc*
1.45721
ofxn mod
1.50925

sRun
RunA1
zout
1

Ave
Data
0.00035
Model
0.00034

data/vMax
0.00094

ofxng
263.459

Min d/m
0.1305

ave/min d
19.7924
ave/min m
2.56278

326_Calib_S_A

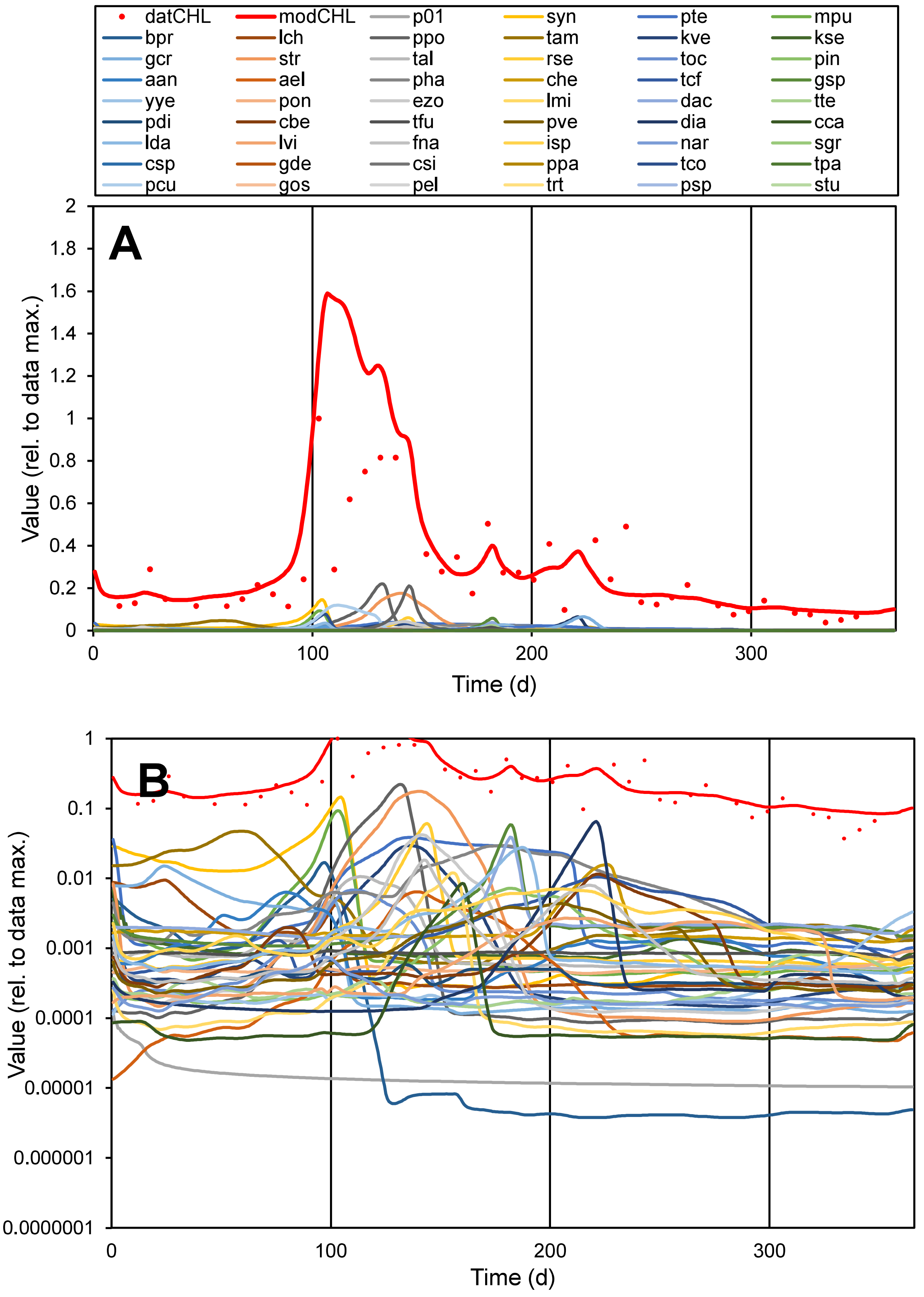

C1

Variables

CHL Provide jtd or Dst

CalibOnly?

2 1 to reproduce of

oNormalize?

2 1=no, 2=yes

ofxn calc*

3.8297

ofxn mod

4.1006

sRun

RunA1

zout

1

Ave

Data

0.64951

Model

0.63412

data/vMax

2.417

ofxng

263.459

Min d/m

0.45478

ave/min d

7.2168

ave/min m

3.20423

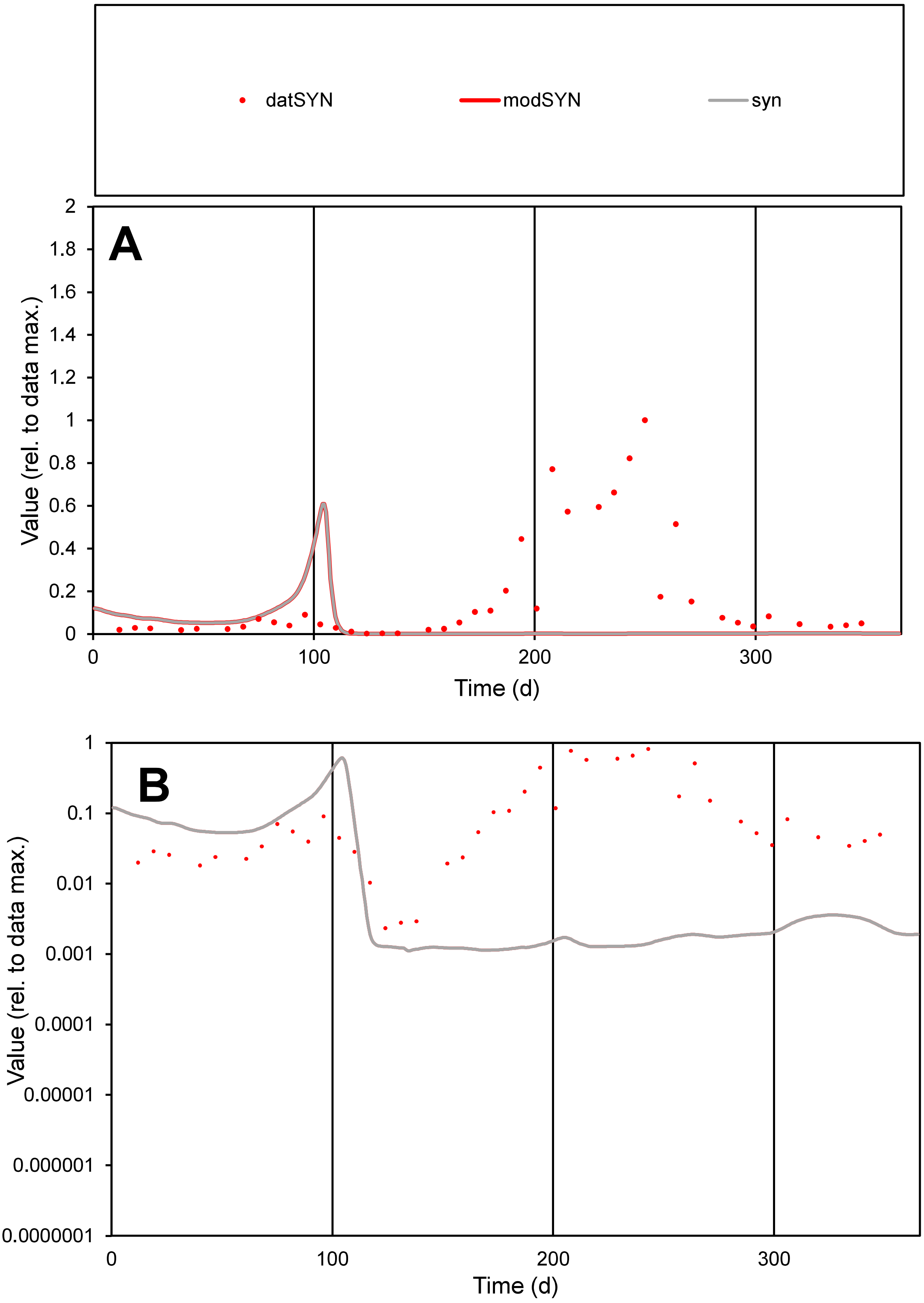

C1

Variables

SYN Provide jtd or Dst

CalibOnly?

2 1 to reproduce of

oNormalize?

2 1=no, 2=yes

ofxn calc*

1.80271

ofxn mod

1.83168

sRun

RunA1

zout

1

Ave

Data

11.9291

Model

2.34417

data/vMax

68.87

ofxng

263.459

Min d/m

2.05905

ave/min d

74.048

ave/min m

29.9613

326_Calib_S_A

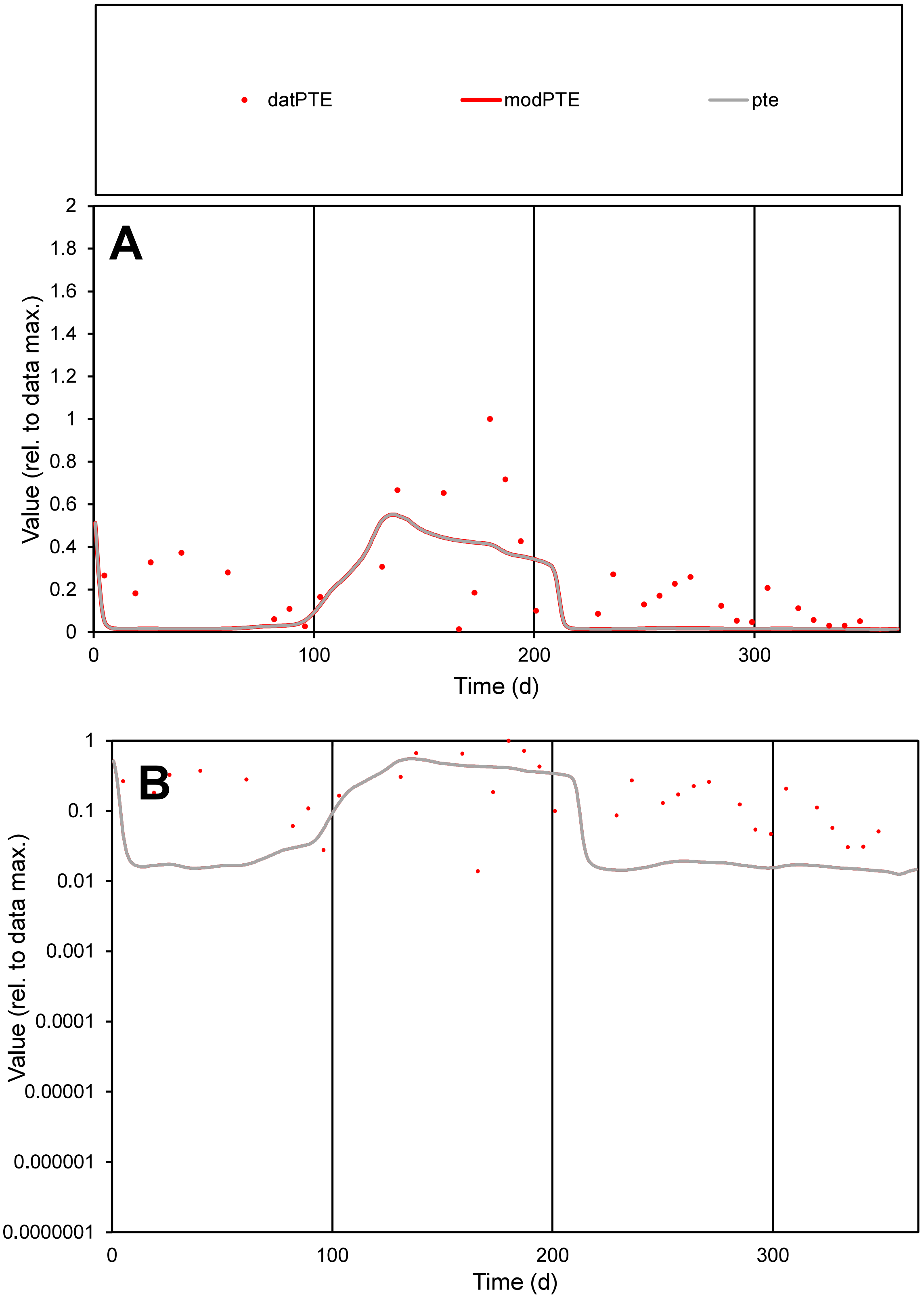

C1

Variables
PTE Provide jtd or Dst
CalibOnly?
2 1 to reproduce of
oNormalize?
2 1=no, 2=yes

ofxn calc*
0.90207
ofxn mod
0.91918

sRun
RunA1
zout
1

Ave
Data
0.00014
Model
8.6E-05

data/vMax
0.0006

ofxng
263.459

Min d/m
0.99749

ave/min d
16.8781
ave/min m
10.2483

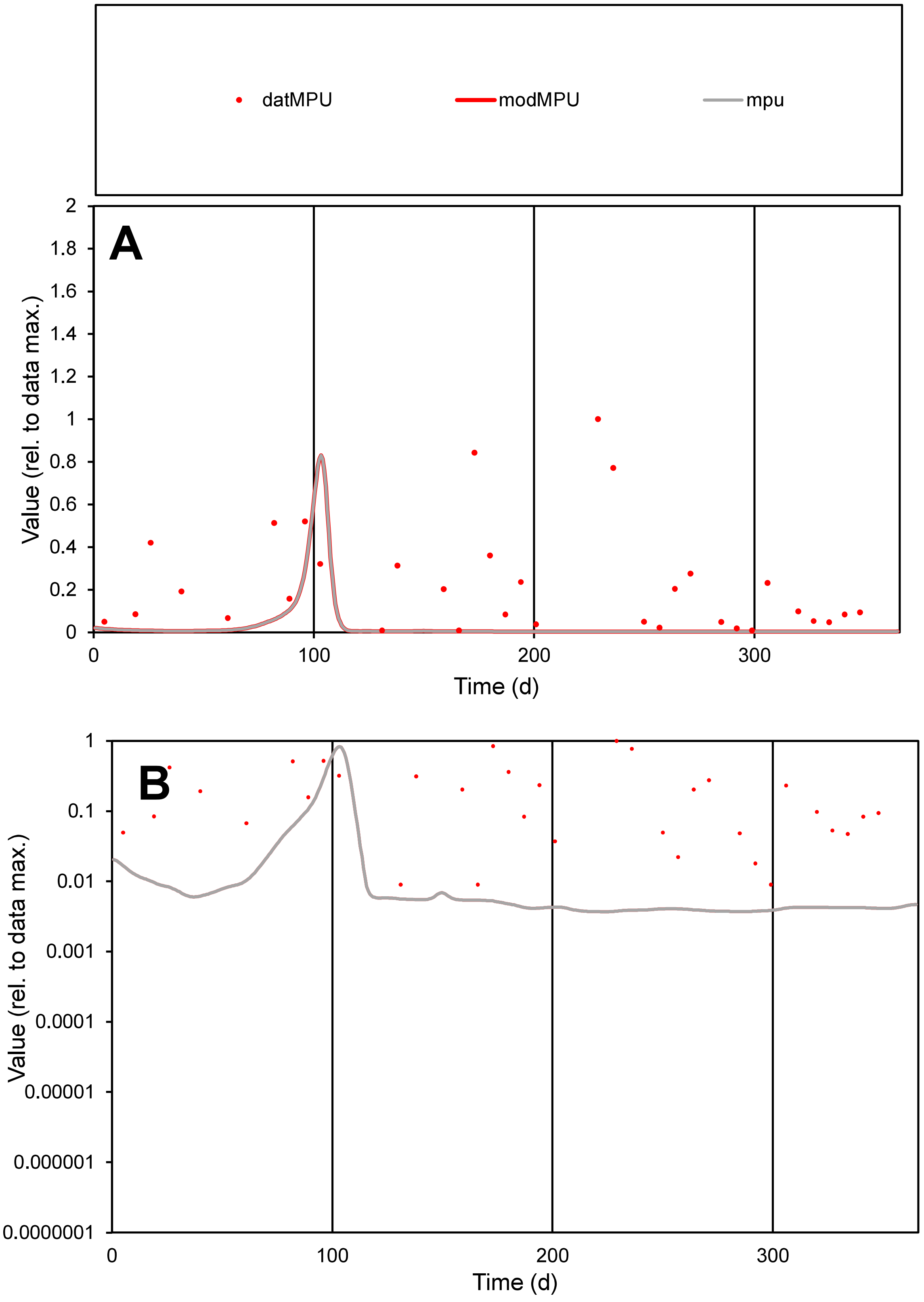

C1

Variables

MPU Provide jtd or Dst

CalibOnly?

2 1 to reproduce of

oNormalize?

2 1=no, 2=yes

ofxn calc*

1.41856

ofxn mod

1.43216

sRun

RunA1

zout

1

Ave

Data

0.0001

Model

1.9E-05

data/vMax

0.00045

ofxng

263.459

Min d/m

2.42349

ave/min d

24.9568

ave/min m

11.5431

326_Calib_S_A

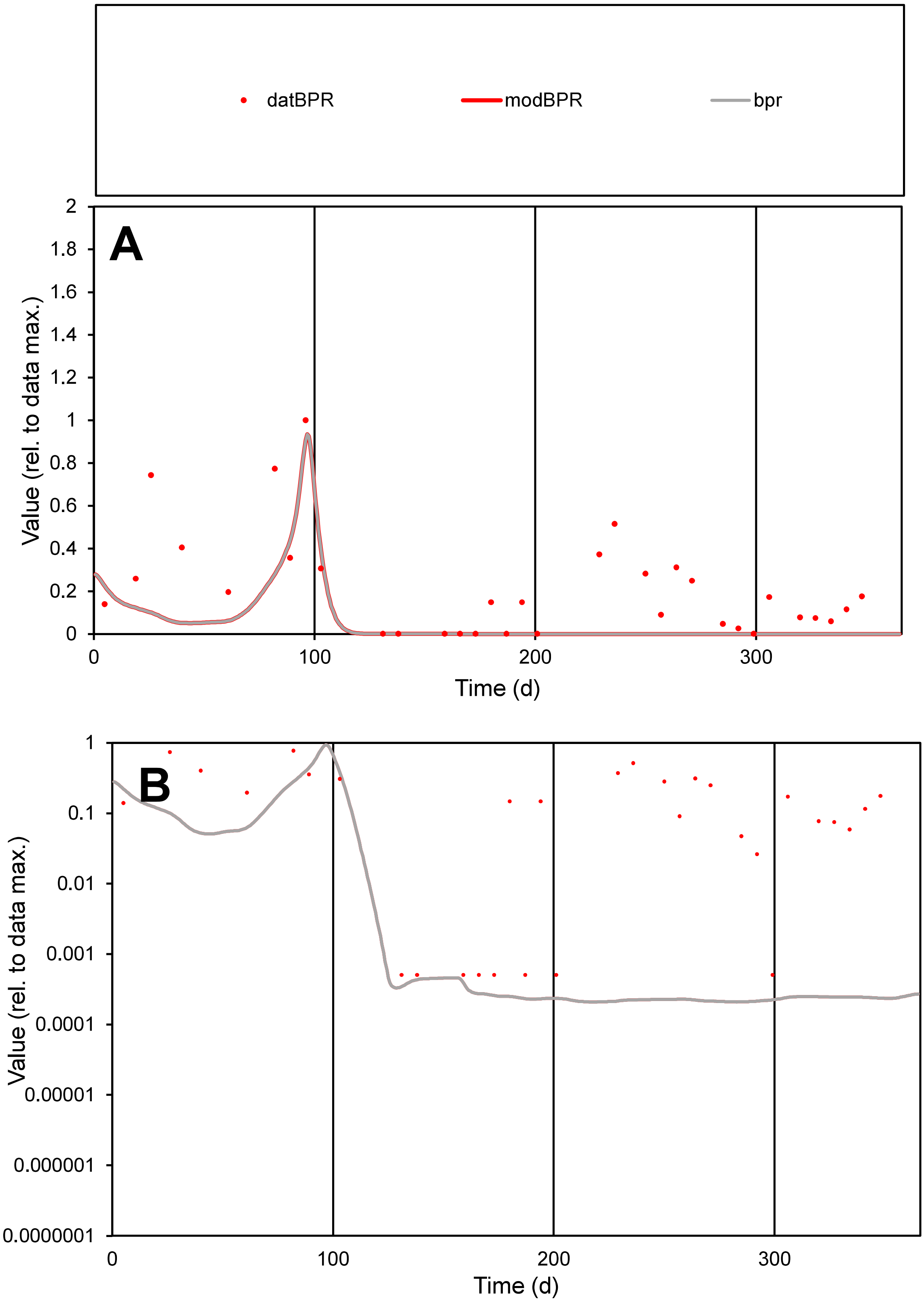

C1

Variables
BPR Provide jtd or Dst
CalibOnly?
2 1 to reproduce of
oNormalize?
2 1=no, 2=yes

ofxn calc*
1.02908
ofxn mod
1.03945

sRun
RunA1
zout
1

Ave
Data
7.8E-05
Model
2.8E-05

data/vMax
0.00036

ofxng
263.459

Min d/m
2.42857

ave/min d
419.475
ave/min m
368.701

326_Calib_S_A

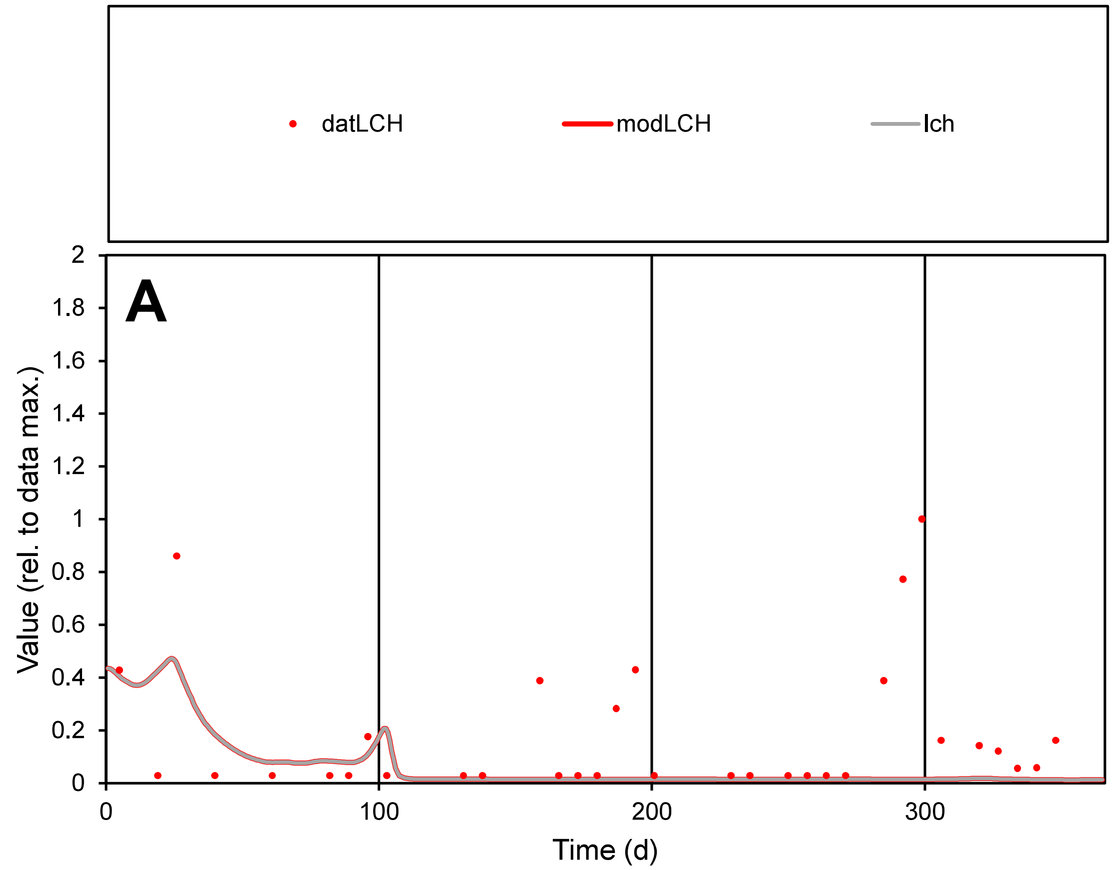

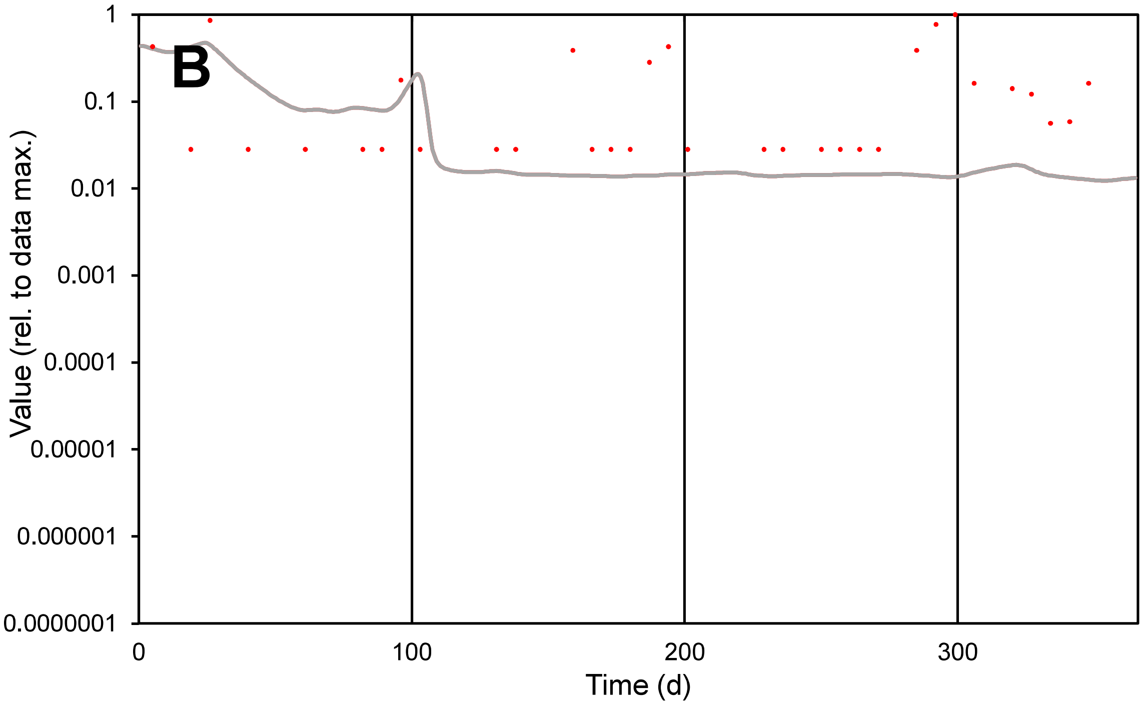

C1

Variables

LCH Provide jtd or Dst

CalibOnly?

2 1 to reproduce of

oNormalize?

2 1=no, 2=yes

ofxn calc*

1.53552

ofxn mod

1.54513

sRun

RunA1

zout

1

Ave

Data

1.6E-05

Model

5.9E-06

data/vMax

8.7E-05

ofxng

263.459

Min d/m

2.24404

ave/min d

6.39238

ave/min m

5.4109

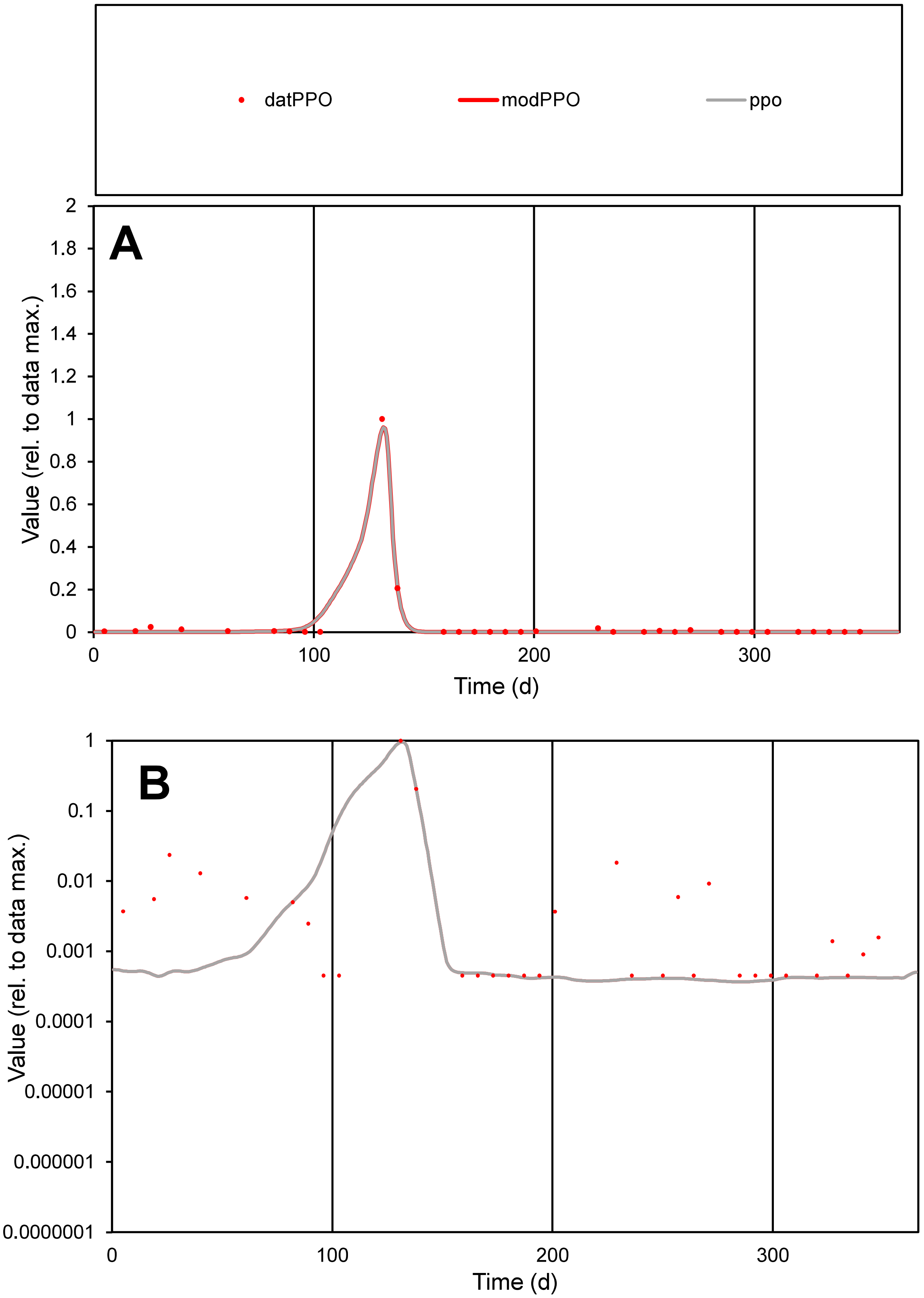

C1

Variables

PPO Provide jtd or Dst

CalibOnly?

2 1 to reproduce of

oNormalize?

2 1=no, 2=yes

ofxn calc*

0.36485

ofxn mod

0.6836

sRun

RunA1

zout

1

Ave

Data

0.00017

Model

0.00016

data/vMax

0.00422

ofxng

263.459

Min d/m

1.23181

ave/min d

87.9118

ave/min m

103.126

326_Calib_S_A

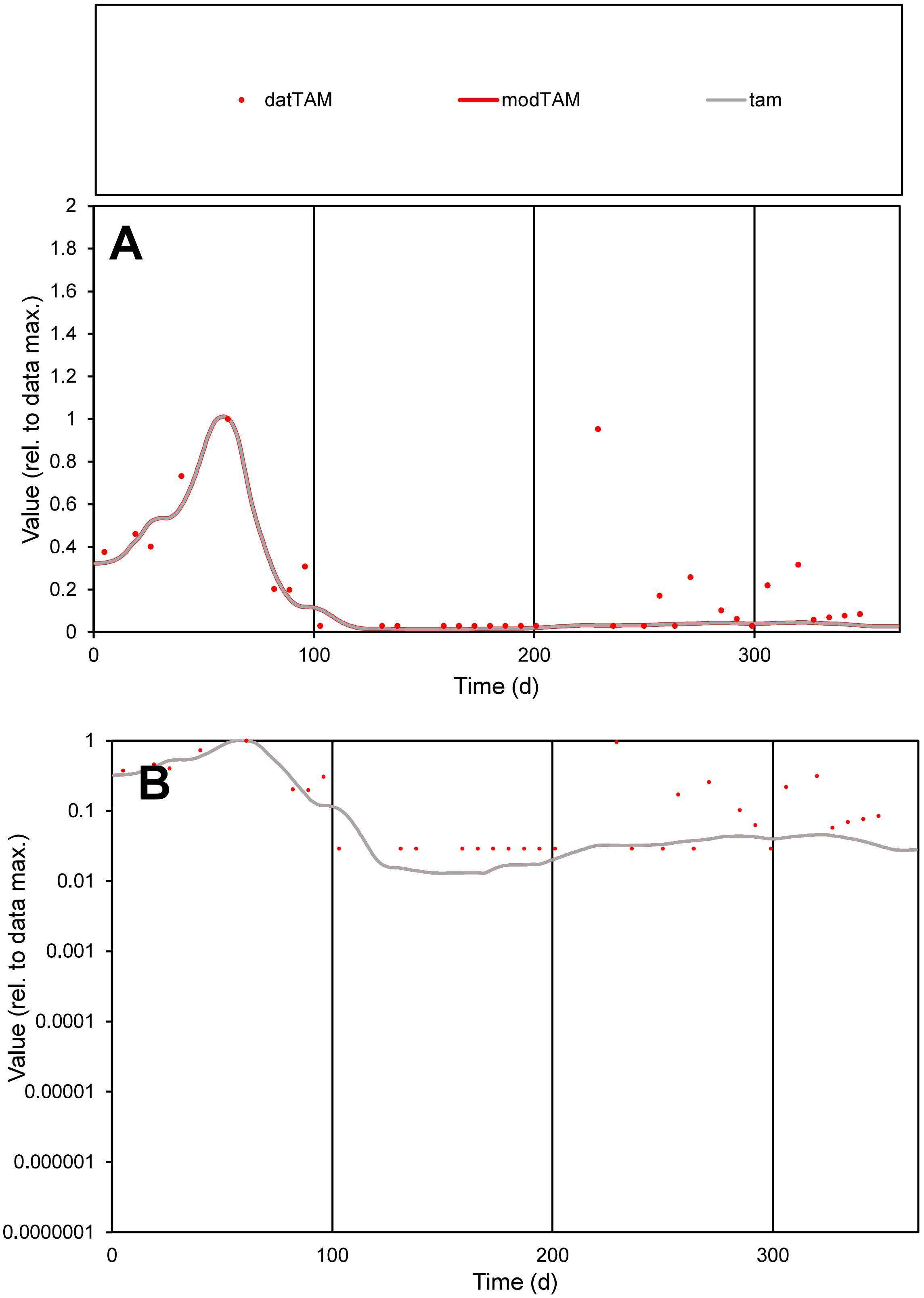

C1

Variables
TAM Provide jtd or Dst
CalibOnly?
2 1 to reproduce of
oNormalize?
2 1=no, 2=yes

ofxn calc*
0.93473
ofxn mod
0.95216

sRun
RunA1
zout
1

Ave
Data
3.9E-05
Model
2.5E-05

data/vMax
0.0002

ofxng
263.459

Min d/m
2.21585

ave/min d
6.75562
ave/min m
9.79765

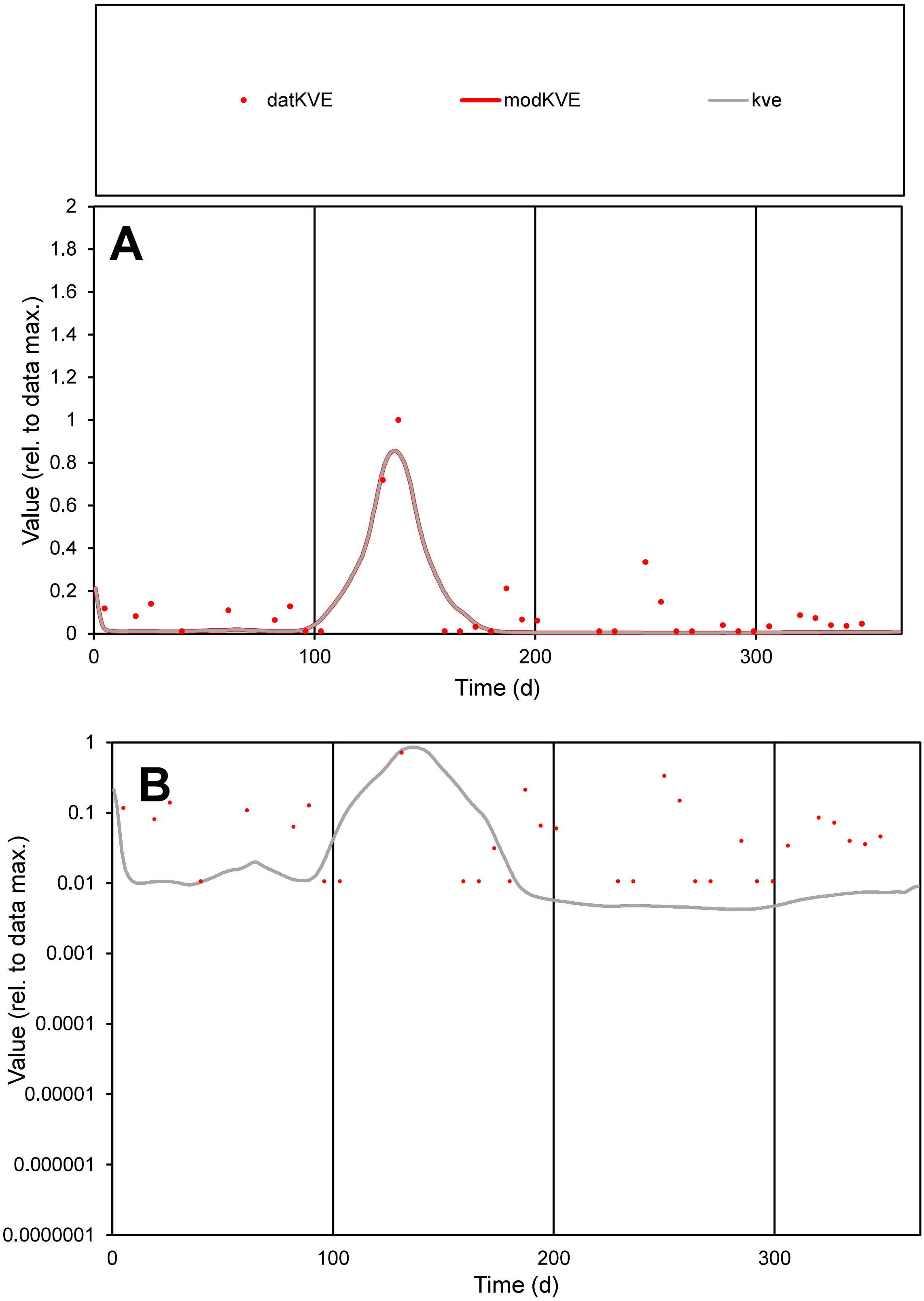

C1

Variables
KVE Provide jtd or Dst
CalibOnly?
2 1 to reproduce of
oNormalize?
2 1=no, 2=yes

ofxn calc*
0.8625
ofxn mod
0.9092

sRun
RunA1
zout
1

Ave
Data
3.3E-05
Model
2E-05

data/vMax
0.00029

ofxng
263.459

Min d/m
2.5008

ave/min d
10.5202
ave/min m
16.2034

326_Calib_S_A

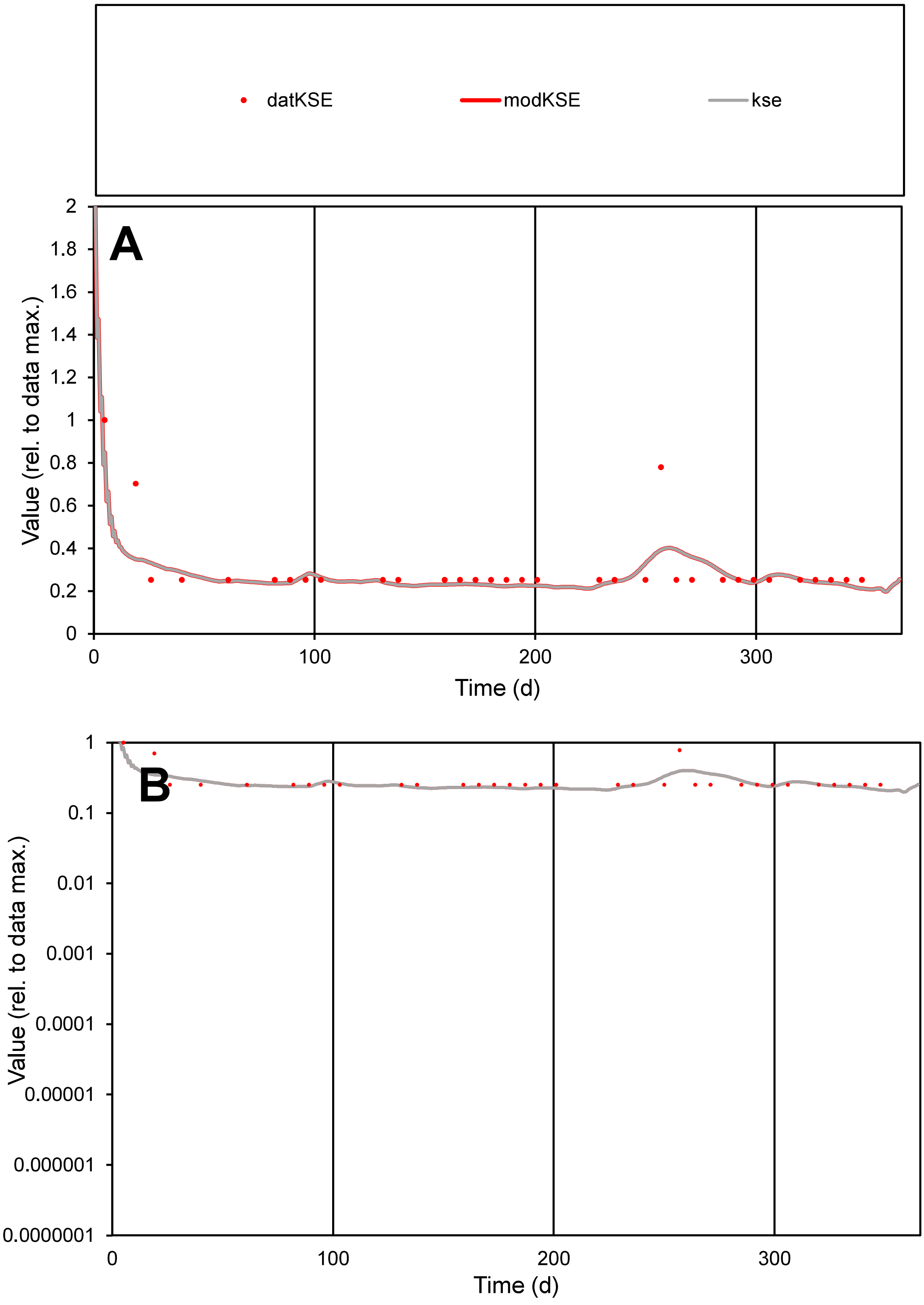

C1

Variables

KSE Provide jtd or Dst

CalibOnly?

2 1 to reproduce of

oNormalize?

2 1=no, 2=yes

ofxn calc*

0.33705

ofxn mod

0.37247

sRun

RunA1

zout

1

Ave

Data

4.5E-06

Model

4.2E-06

data/vMax

1.5E-05

ofxng

263.459

Min d/m

1.19706

ave/min d

1.20767

ave/min m

1.3387

326_Calib_S_A

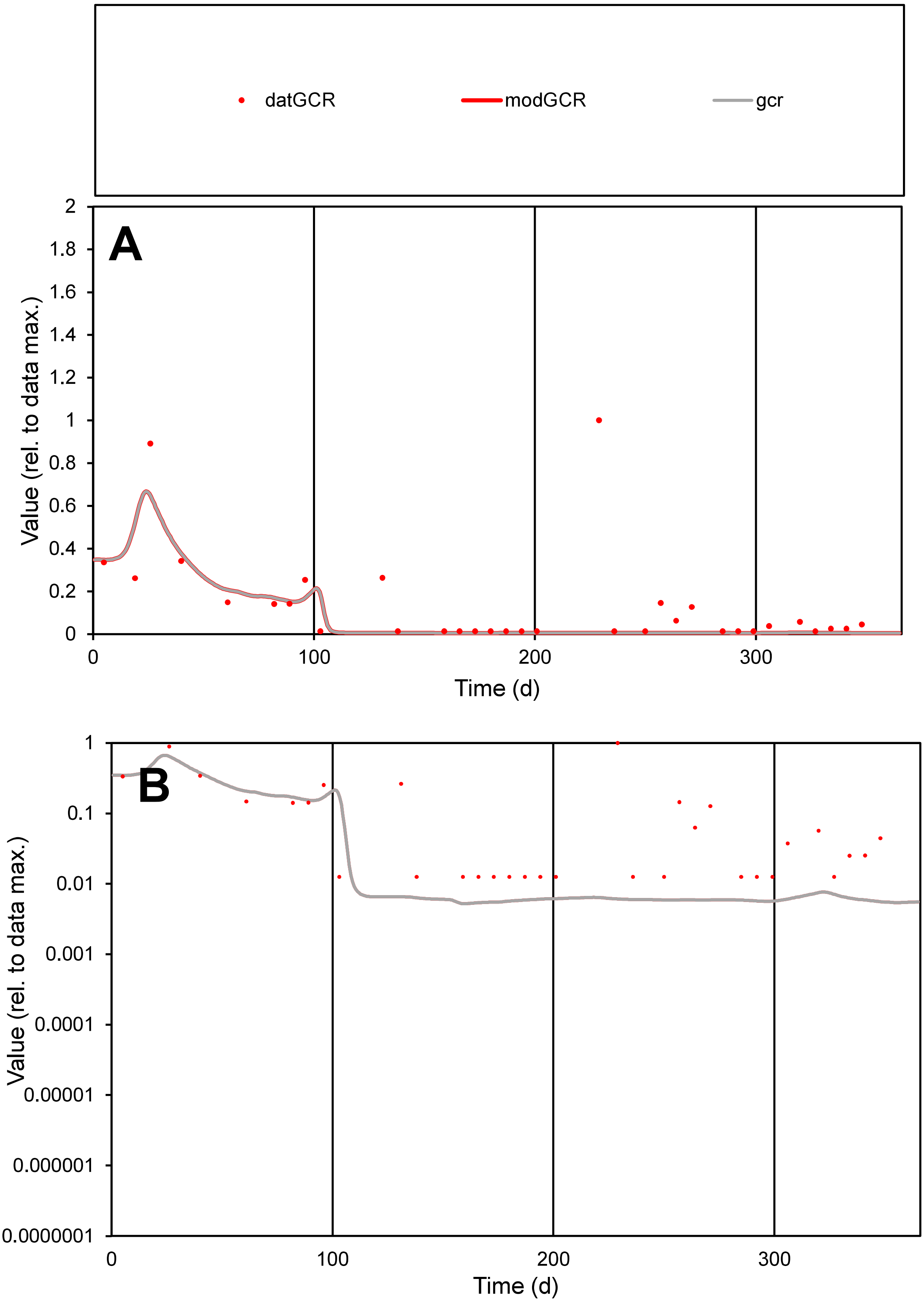

C1

Variables
GCR Provide jtd or Dst
CalibOnly?
2 1 to reproduce of
oNormalize?
2 1=no, 2=yes

ofxn calc*
1.43222
ofxn mod
1.45401

sRun
RunA1
zout
1

Ave
Data
3.4E-05
Model
2.1E-05

data/vMax
0.00025

ofxng
263.459

Min d/m
2.40707

ave/min d
10.821
ave/min m
16.3114

326_Calib_S_A

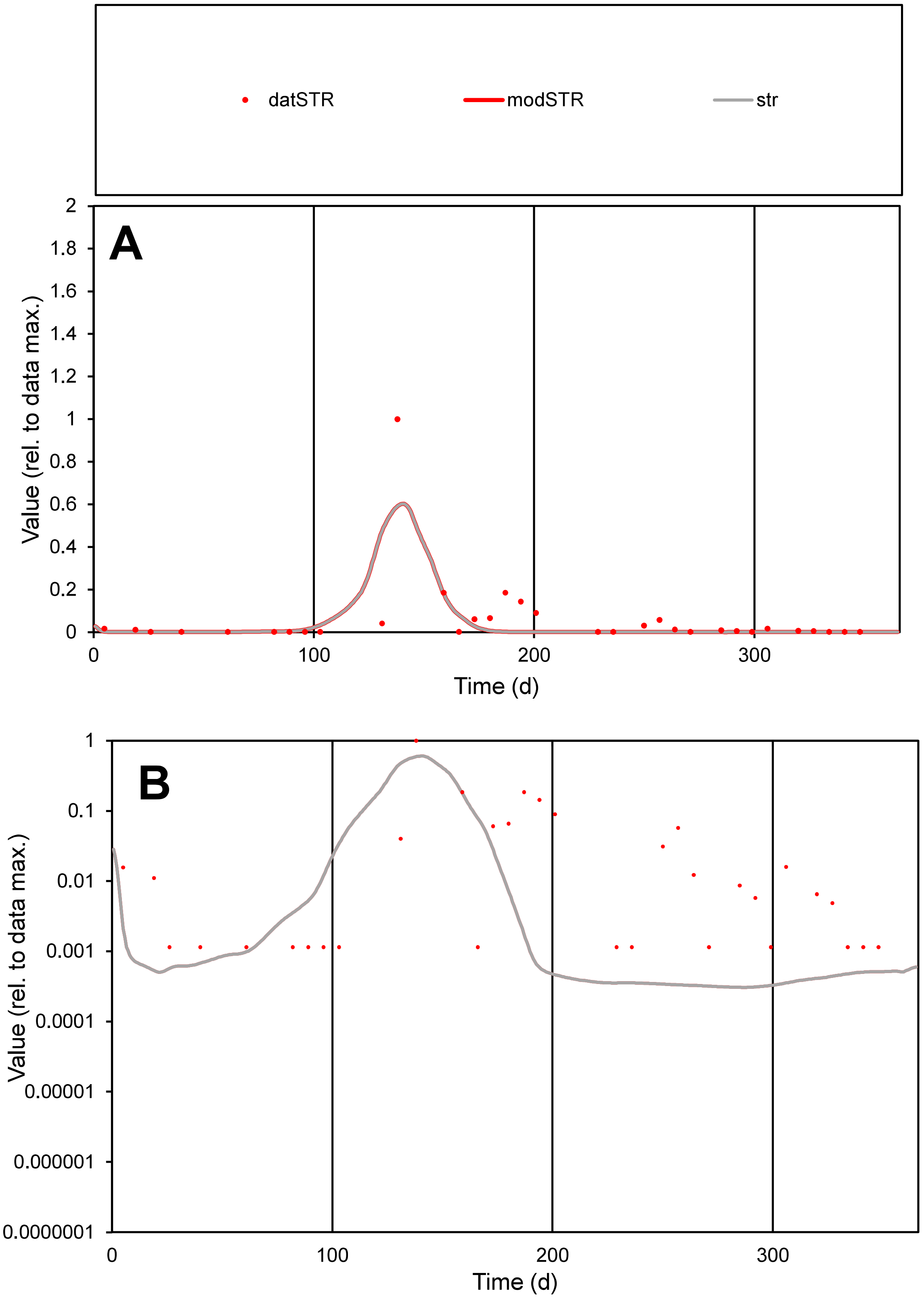

C1

Variables

STR	Provide jtd or Dst
CalibOnly?	
2	1 to reproduce of
oNormalize?	
2	1=no, 2=yes

ofxn calc*
1.87155
ofxn mod
1.94416

sRun
RunA1
zout
1

Ave
Data
8.4E-05
Model
5.9E-05

data/vMax
0.00142

ofxng
263.459

Min d/m
3.72769

ave/min d
51.7566
ave/min m
135.142

326_Calib_S_A

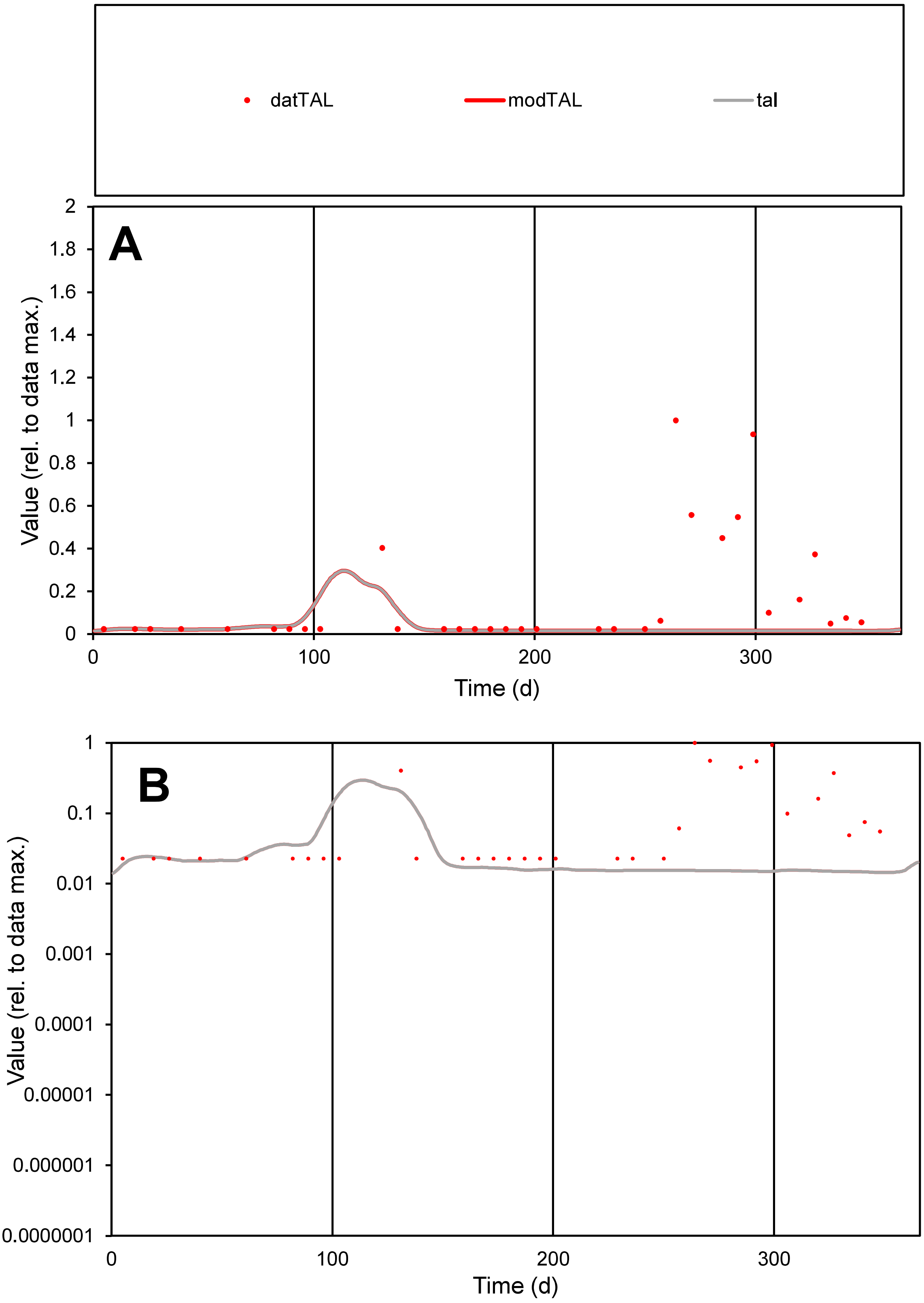

C1

Variables

TAL Provide jtd or Dst

CalibOnly?

2 1 to reproduce of

oNormalize?

2 1=no, 2=yes

ofxn calc*

1.84669

ofxn mod

1.85452

sRun

RunA1

zout

1

Ave

Data

2.9E-05

Model

5.7E-06

data/vMax

0.00018

ofxng

263.459

Min d/m

1.56614

ave/min d

6.98226

ave/min m

2.16961

326_Calib_S_A

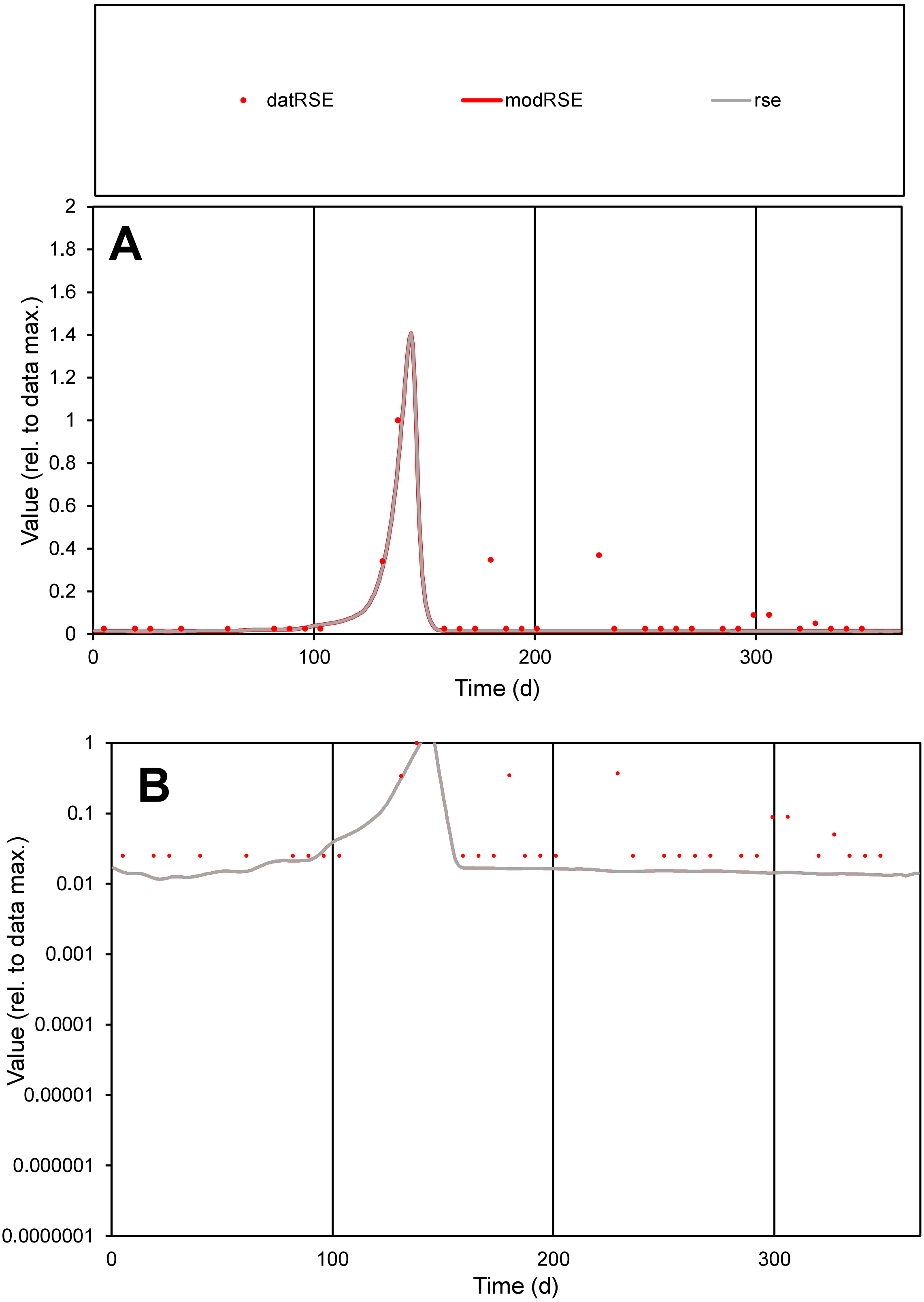

C1

Variables
RSE Provide jtd or Dst
CalibOnly?
2 1 to reproduce of
oNormalize?
2 1=no, 2=yes

ofxn calc*
0.98264
ofxn mod
1.01528

sRun
RunA1
zout
1

Ave
Data
1.7E-05
Model
1.1E-05

data/vMax
0.00019

ofxng
263.459

Min d/m
2.11378

ave/min d
3.55009
ave/min m
4.76236

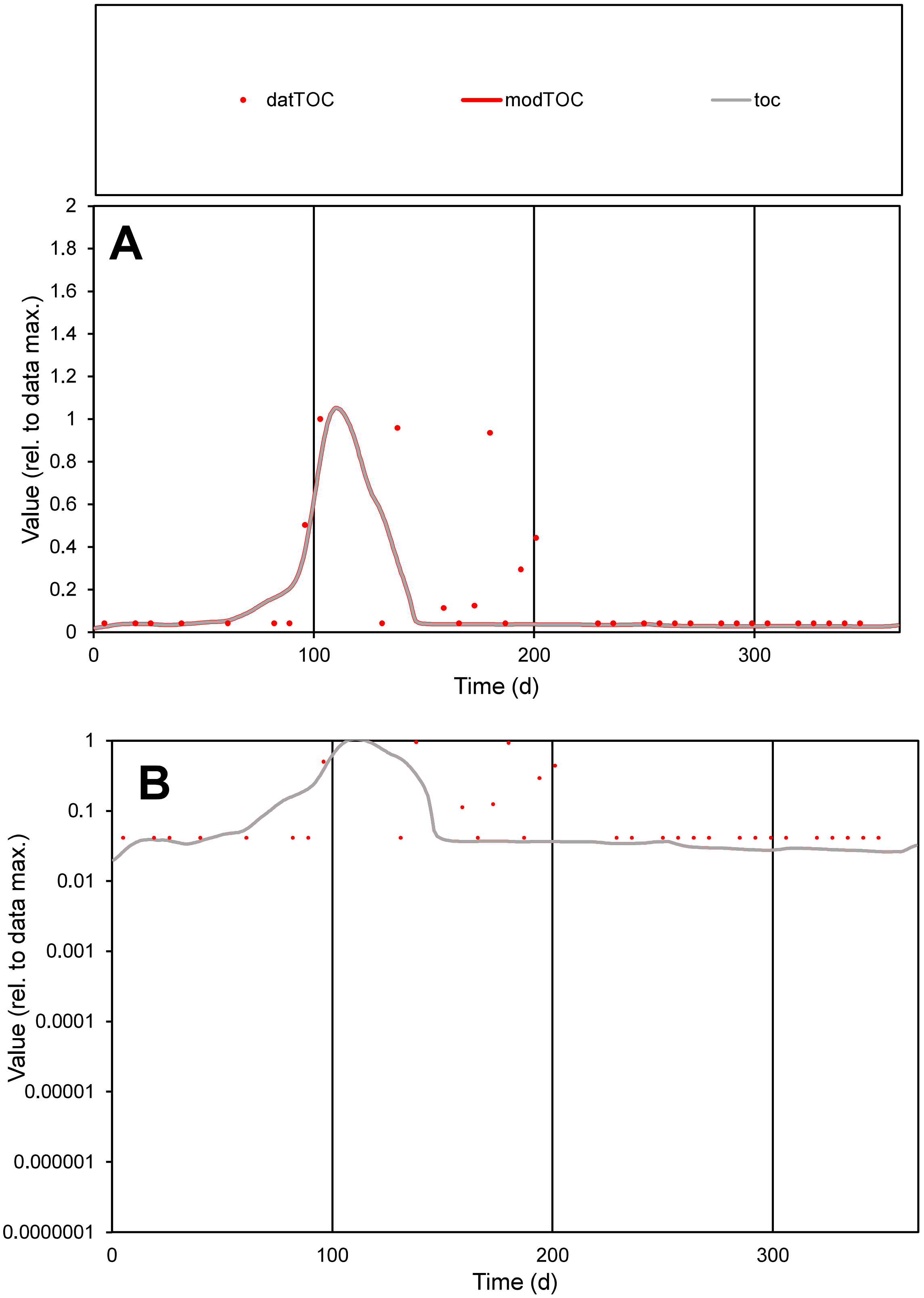

C1

Variables
TOC Provide jtd or Dst
CalibOnly?
2 1 to reproduce of
oNormalize?
2 1=no, 2=yes

ofxn calc*
1.37739
ofxn mod
1.40672

sRun
RunA1
zout
1

Ave
Data
1.7E-05
Model
1.1E-05

data/vMax
0.0001

ofxng
263.459

Min d/m
1.5745

ave/min d
3.95314
ave/min m
3.89494

326_Calib_S_A

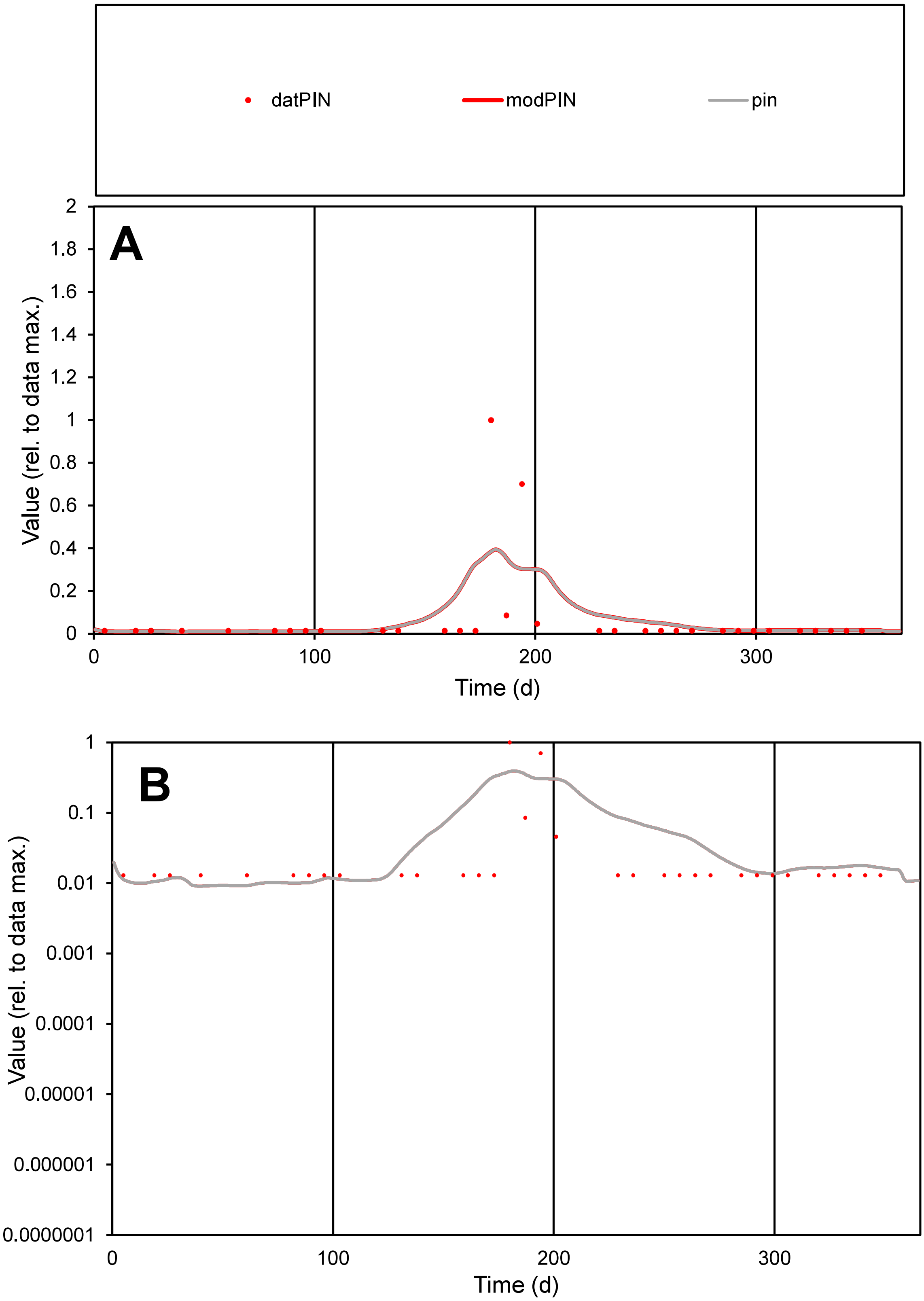

C1

Variables
PIN Provide jtd or Dst
CalibOnly?
2 1 to reproduce of
oNormalize?
2 1=no, 2=yes

ofxn calc*
2.34469
ofxn mod
2.37034

sRun
RunA1
zout
1

Ave
Data
2.1E-05
Model
2.4E-05

data/vMax
0.00031

ofxng
263.459

Min d/m
1.423

ave/min d
5.18288
ave/min m
8.64379

326_Calib_S_A

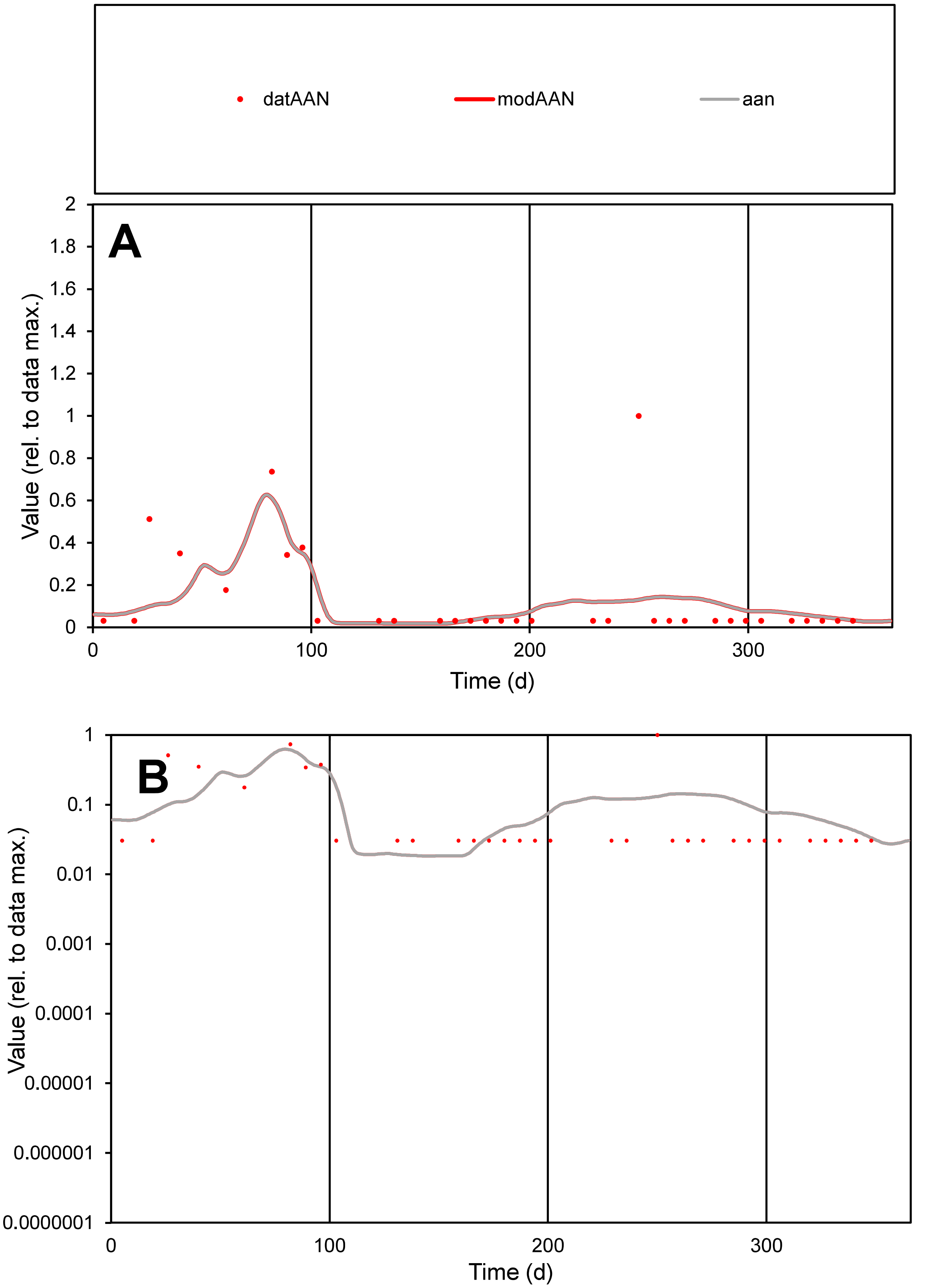

C1

Variables

AAN Provide jtd or Dst

CalibOnly?

2 1 to reproduce of

oNormalize?

2 1=no, 2=yes

ofxn calc*

1.39135

ofxn mod

1.40631

sRun

RunA1

zout

1

Ave

Data

2.4E-05

Model

2.2E-05

data/vMax

0.00018

ofxng

263.459

Min d/m

1.64107

ave/min d

4.27441

ave/min m

6.33898

326_Calib_S_A

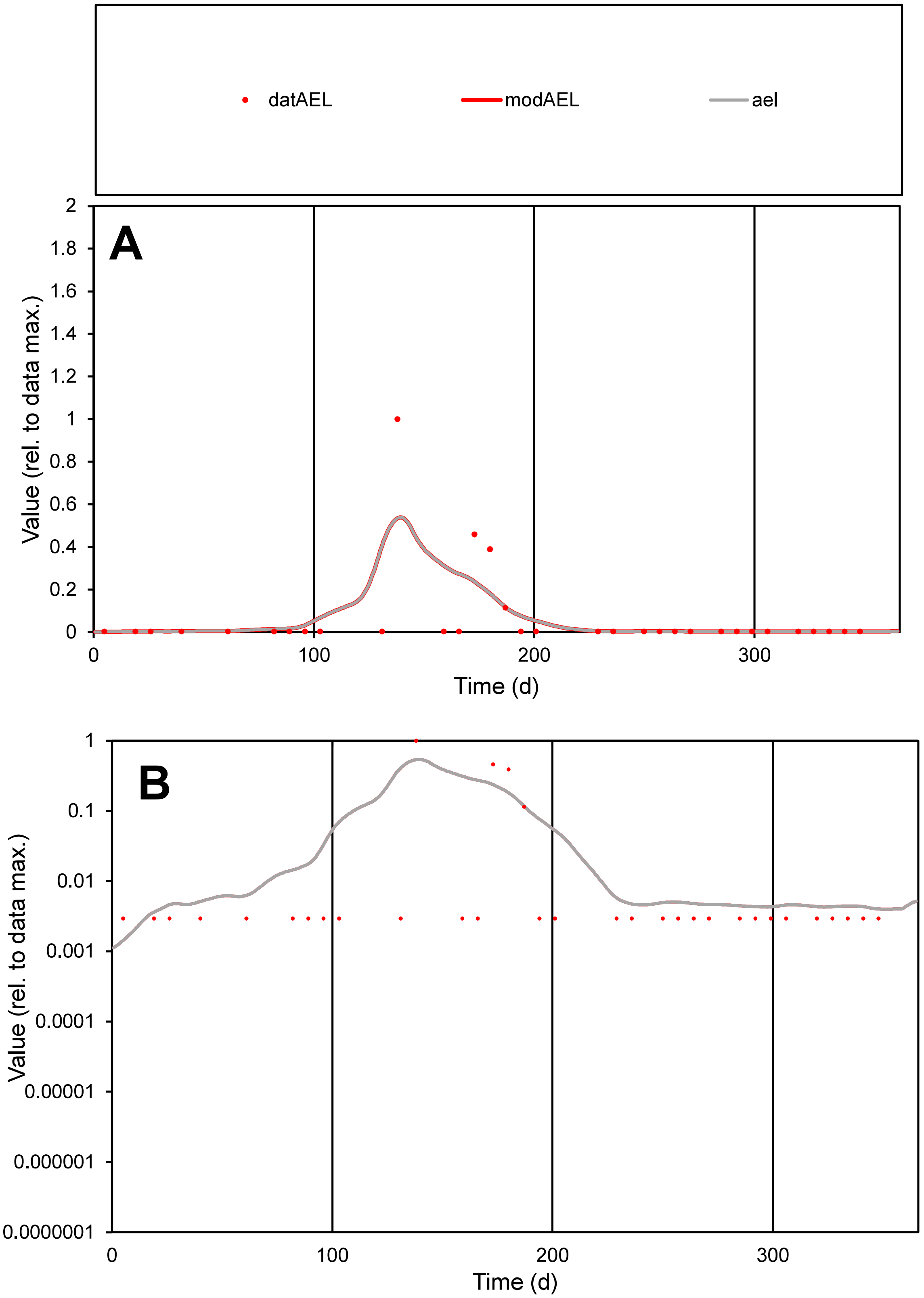

C1

Variables
AEL Provide jtd or Dst
CalibOnly?
2 1 to reproduce of
oNormalize?
2 1=no, 2=yes

ofxn calc*
2.11293
ofxn mod
2.26653

sRun
RunA1
zout
1

Ave
Data
1.5E-05
Model
1.6E-05

data/vMax
0.00024

ofxng
263.459

Min d/m
1.8657

ave/min d
21.1441
ave/min m
43.4787

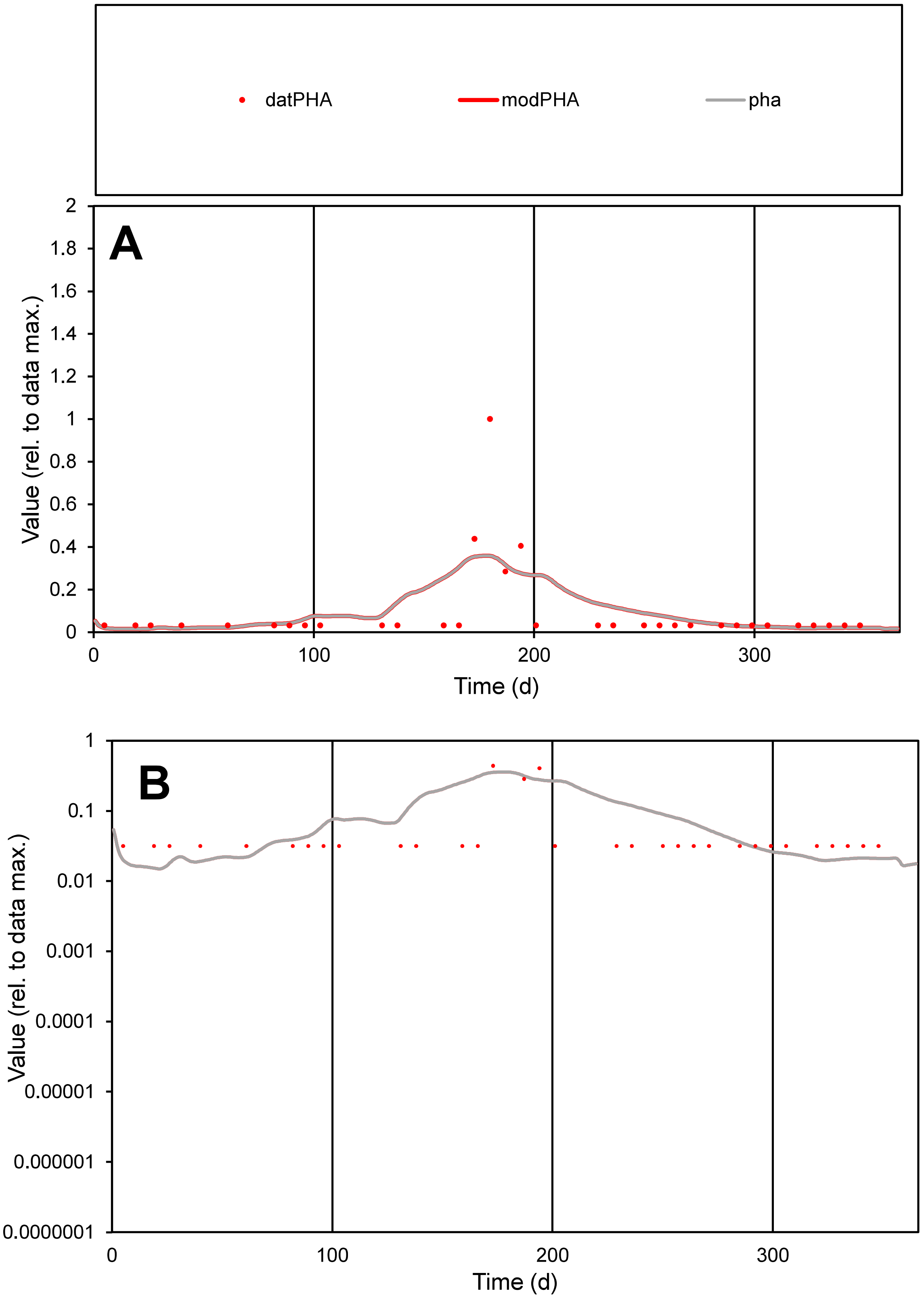

C1

Variables
PHA Provide jtd or Dst
CalibOnly?
2 1 to reproduce of
oNormalize?
2 1=no, 2=yes

ofxn calc*
1.53319
ofxn mod
1.55607

sRun
RunA1
zout
1

Ave
Data
3E-05
Model
3.4E-05

data/vMax
0.00033

ofxng
263.459

Min d/m
2.08743

ave/min d
2.91975
ave/min m
6.794

326_Calib_S_A

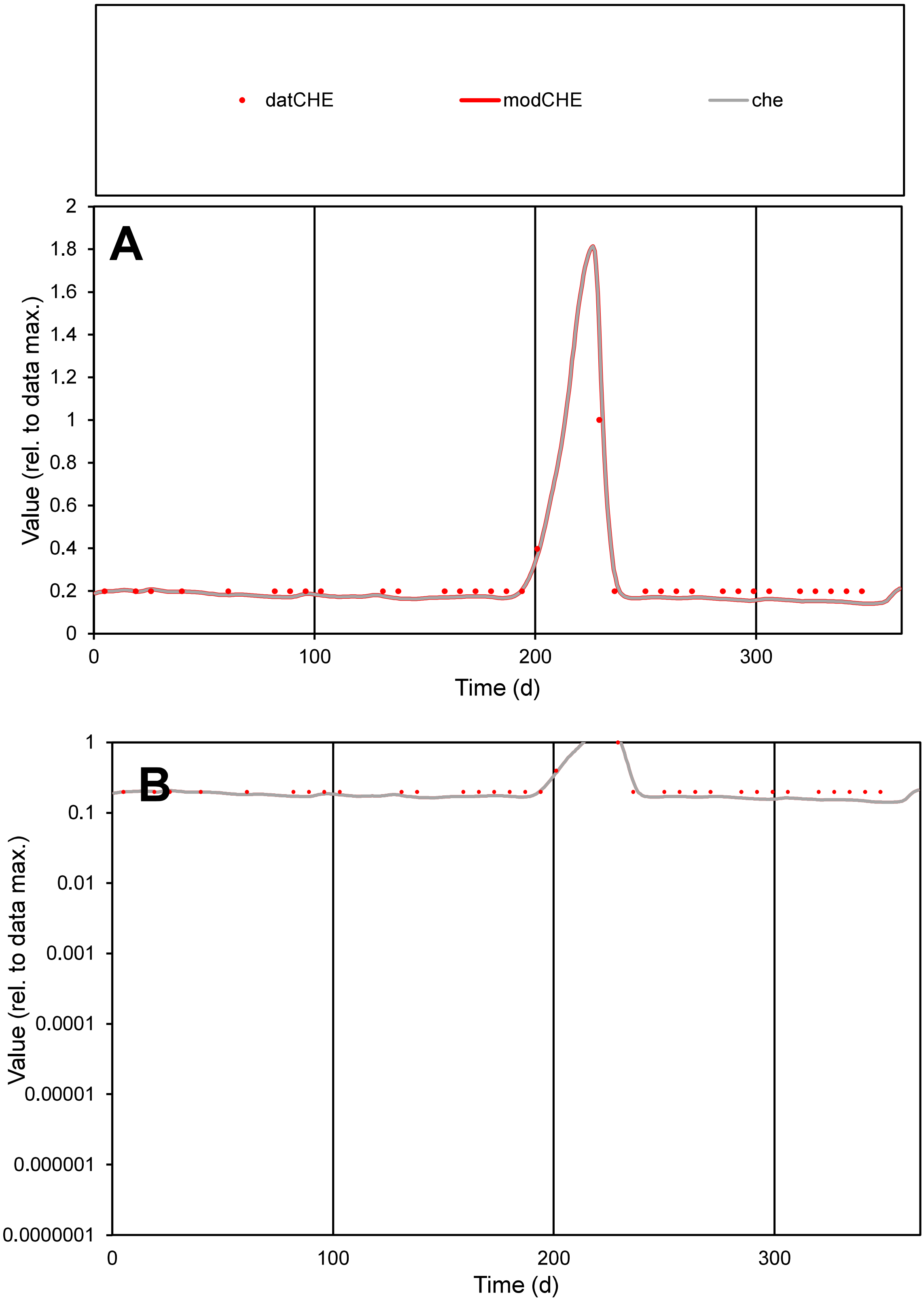

C1

Variables

CHE Provide jtd or Dst

CalibOnly?

2 1 to reproduce of

oNormalize?

2 1=no, 2=yes

ofxn calc*

0.13437

ofxn mod

0.1992

sRun

RunA1

zout

1

Ave

Data

8.1E-06

Model

7.3E-06

data/vMax

3.5E-05

ofxng

263.459

Min d/m

1.40681

ave/min d

1.15255

ave/min m

1.45649

326_Calib_S_A

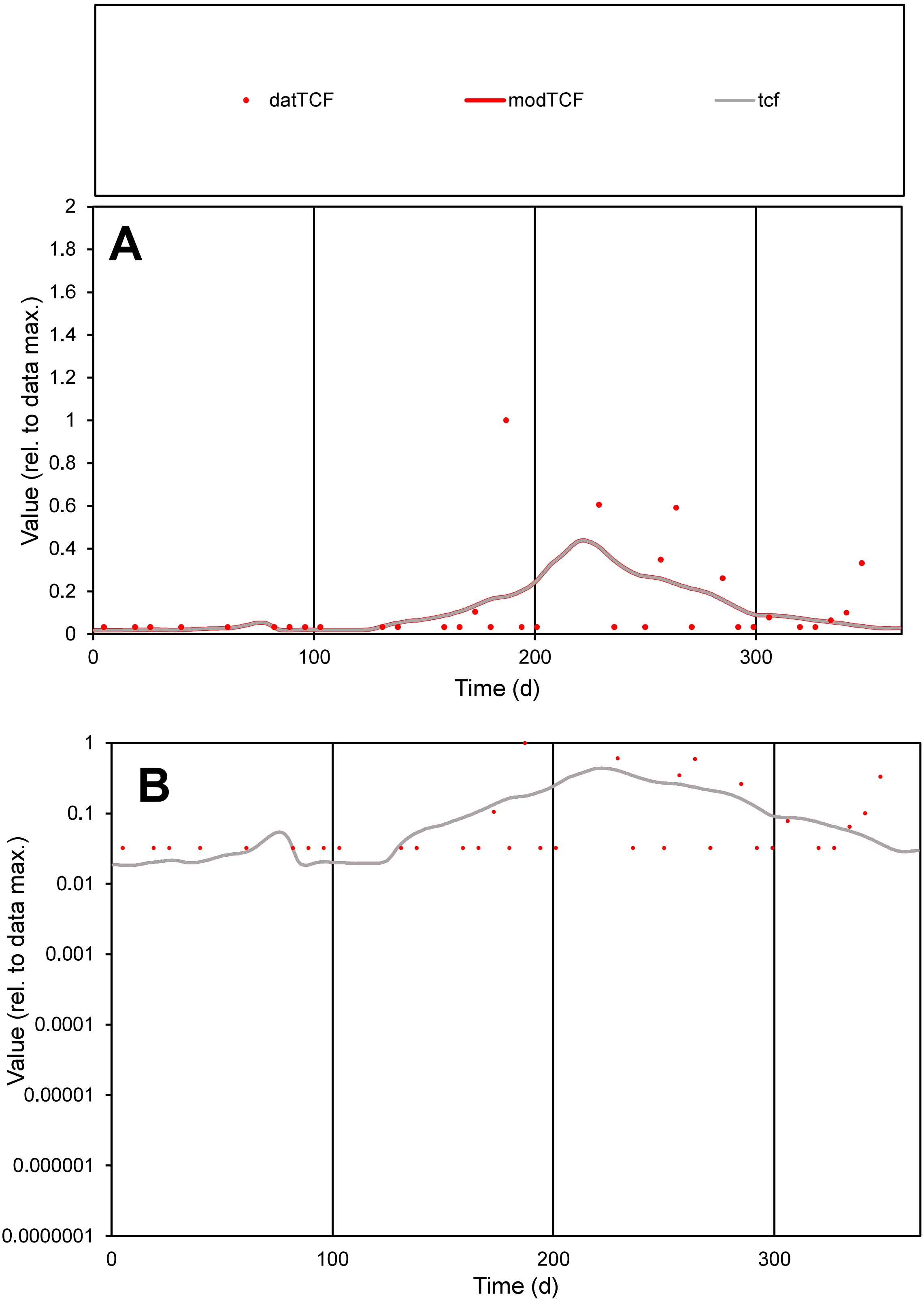

C1

Variables
TCF Provide jtd or Dst
CalibOnly?
2 1 to reproduce of
oNormalize?
2 1=no, 2=yes

ofxn calc*
1.51669
ofxn mod
1.53286

sRun
RunA1
zout
1

Ave
Data
1.2E-05
Model
1.1E-05

data/vMax
9.6E-05

ofxng
263.459

Min d/m
1.76514

ave/min d
3.95464
ave/min m
6.3315

326_Calib_S_A

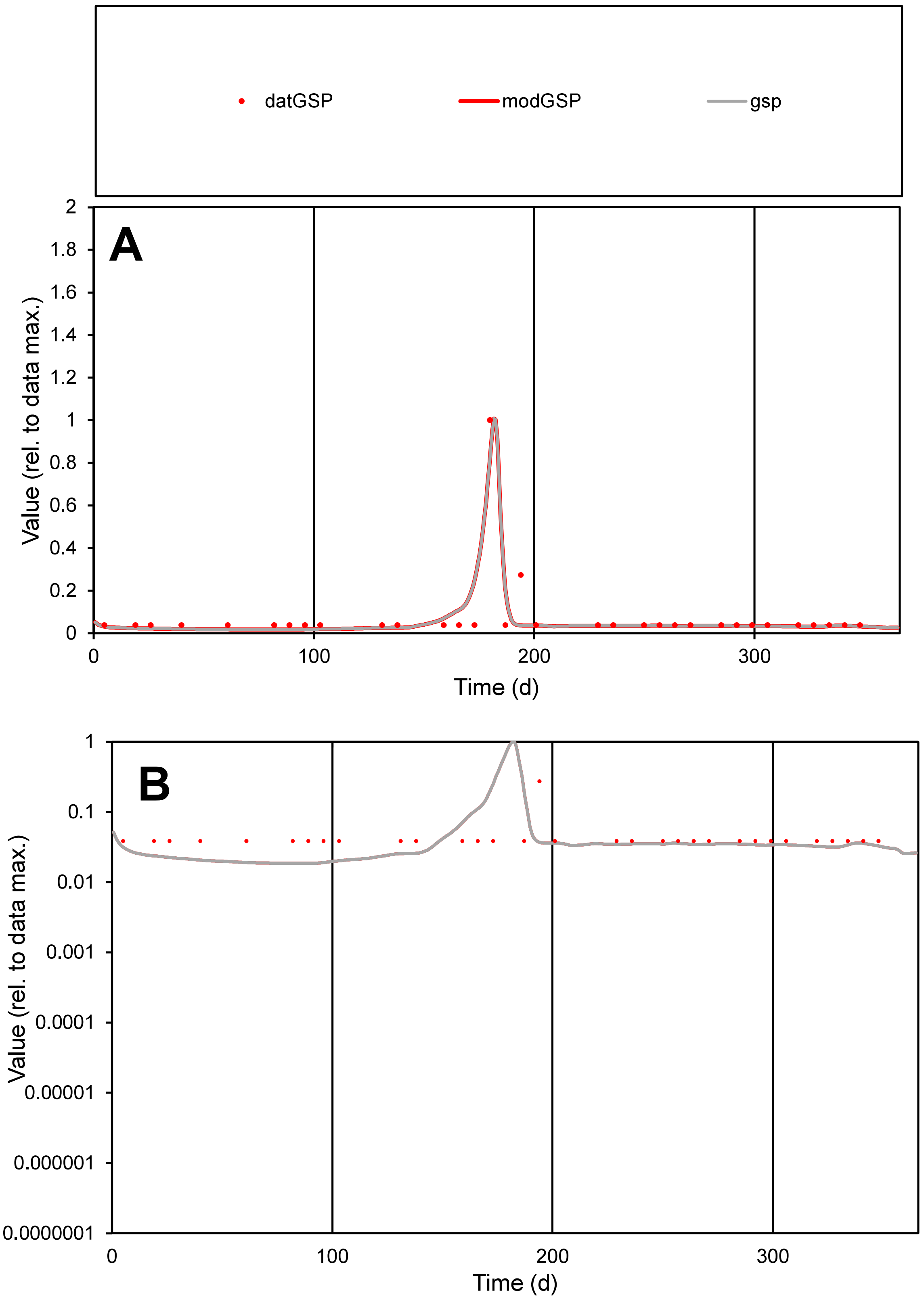

C1

Variables
GSP Provide jtd or Dst
CalibOnly?
2 1 to reproduce of
oNormalize?
2 1=no, 2=yes

ofxn calc*
0.72153
ofxn mod
0.79297

sRun
RunA1
zout
1

Ave
Data
1.7E-05
Model
1.6E-05

data/vMax
0.00023

ofxng
263.459

Min d/m
2.07352

ave/min d
1.94003
ave/min m
3.66244

326_Calib_S_A

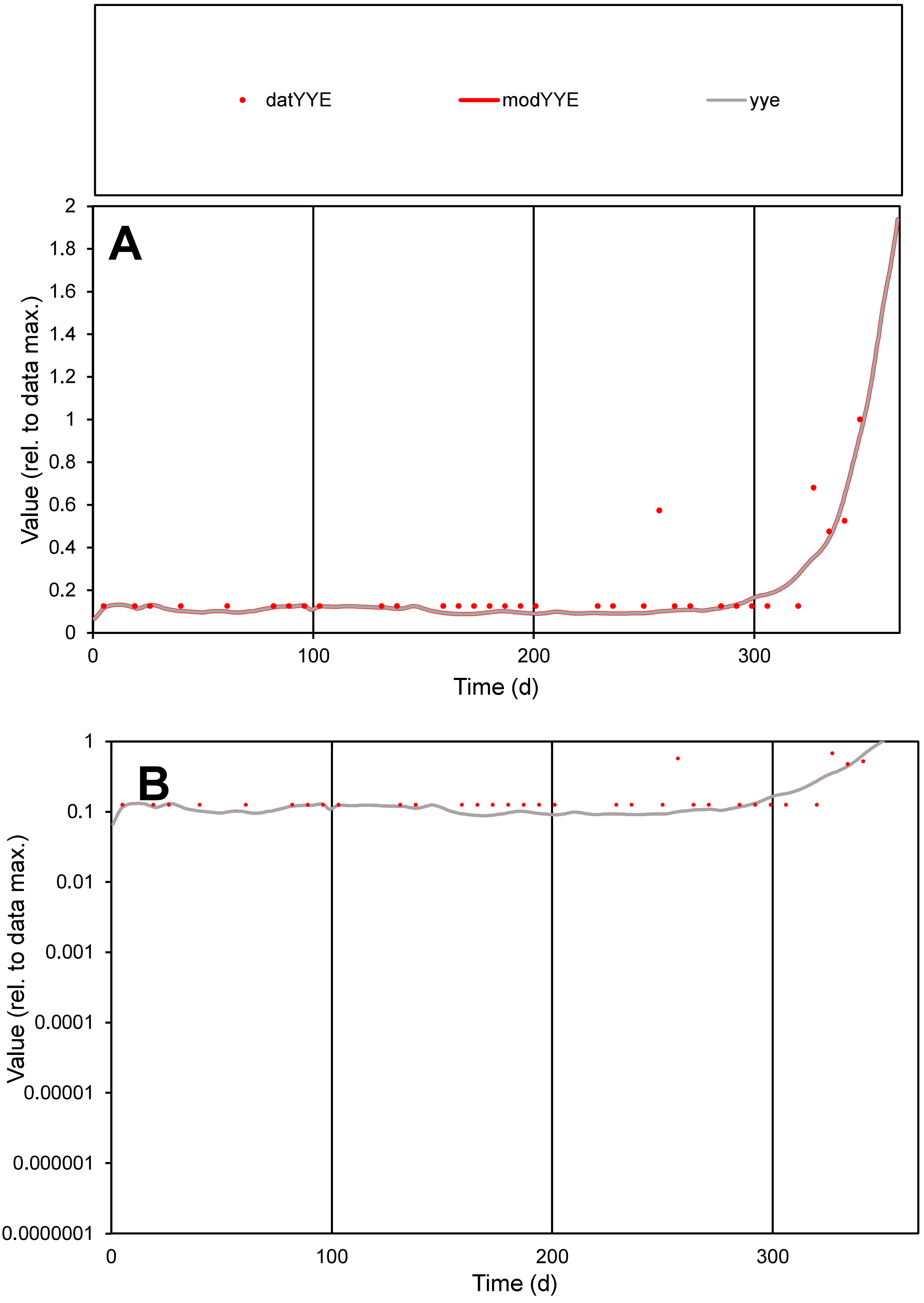

C1

Variables
YYE Provide jtd or Dst
CalibOnly?
2 1 to reproduce of
oNormalize?
2 1=no, 2=yes

ofxn calc*
0.52255
ofxn mod
0.5457

sRun
RunA1
zout
1

Ave
Data
6.1E-06
Model
5.3E-06

data/vMax
3E-05

ofxng
263.459

Min d/m
1.427

ave/min d
1.63155
ave/min m
2.01387

326_Calib_S_A

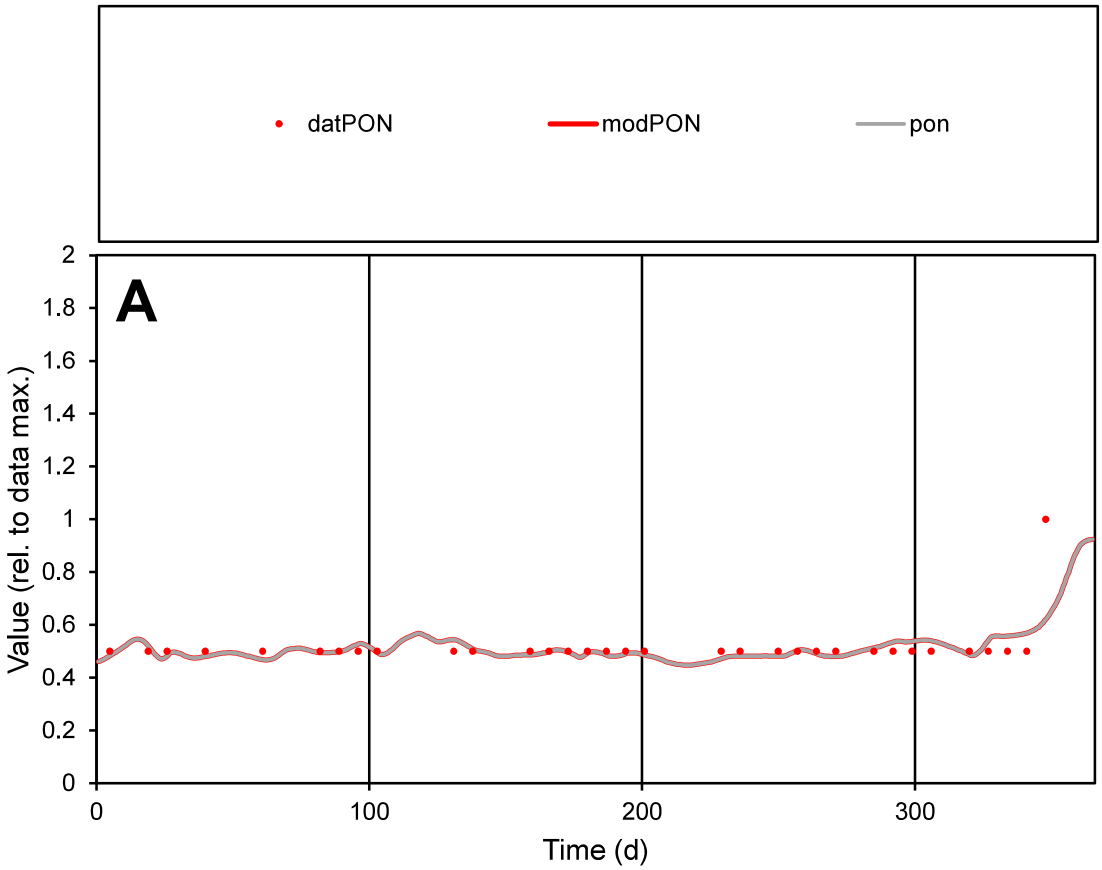

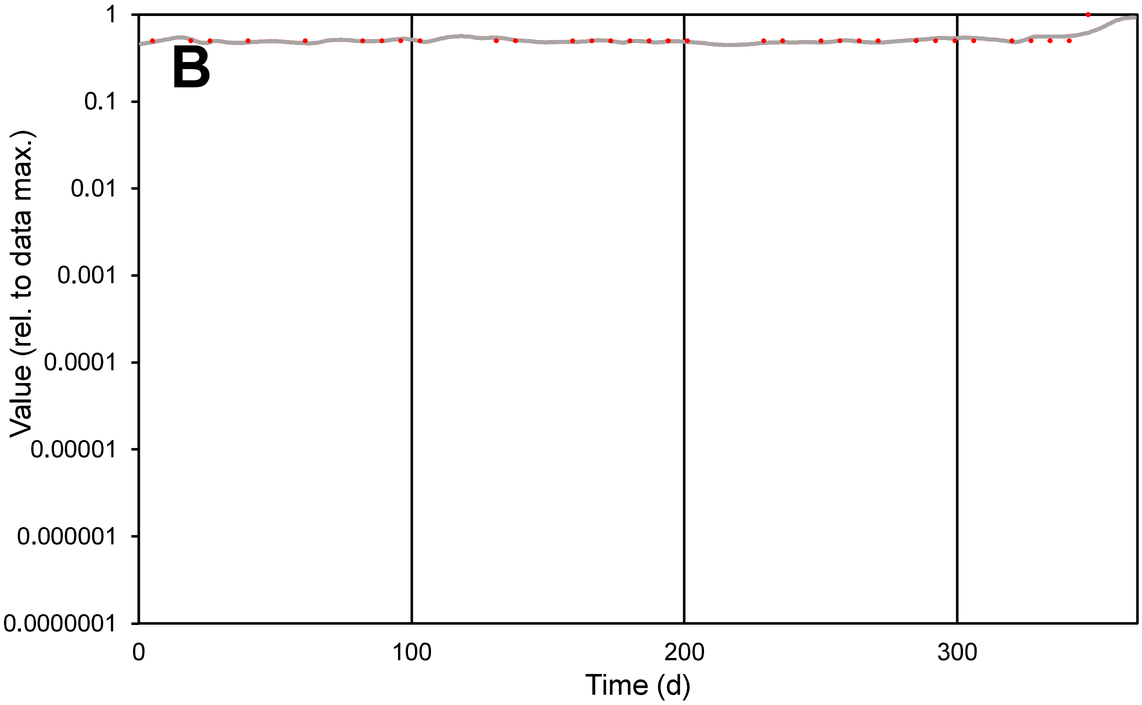

C1

Variables
PON Provide jtd or Dst
CalibOnly?
2 1 to reproduce of
oNormalize?
2 1=no, 2=yes

ofxn calc*
0.13352
ofxn mod
0.17383

sRun
RunA1
zout
1

Ave
Data
3E-06
Model
3E-06

data/vMax
5.9E-06

ofxng
263.459

Min d/m
1.07276

ave/min d
1.0303
ave/min m
1.09085

326_Calib_S_A

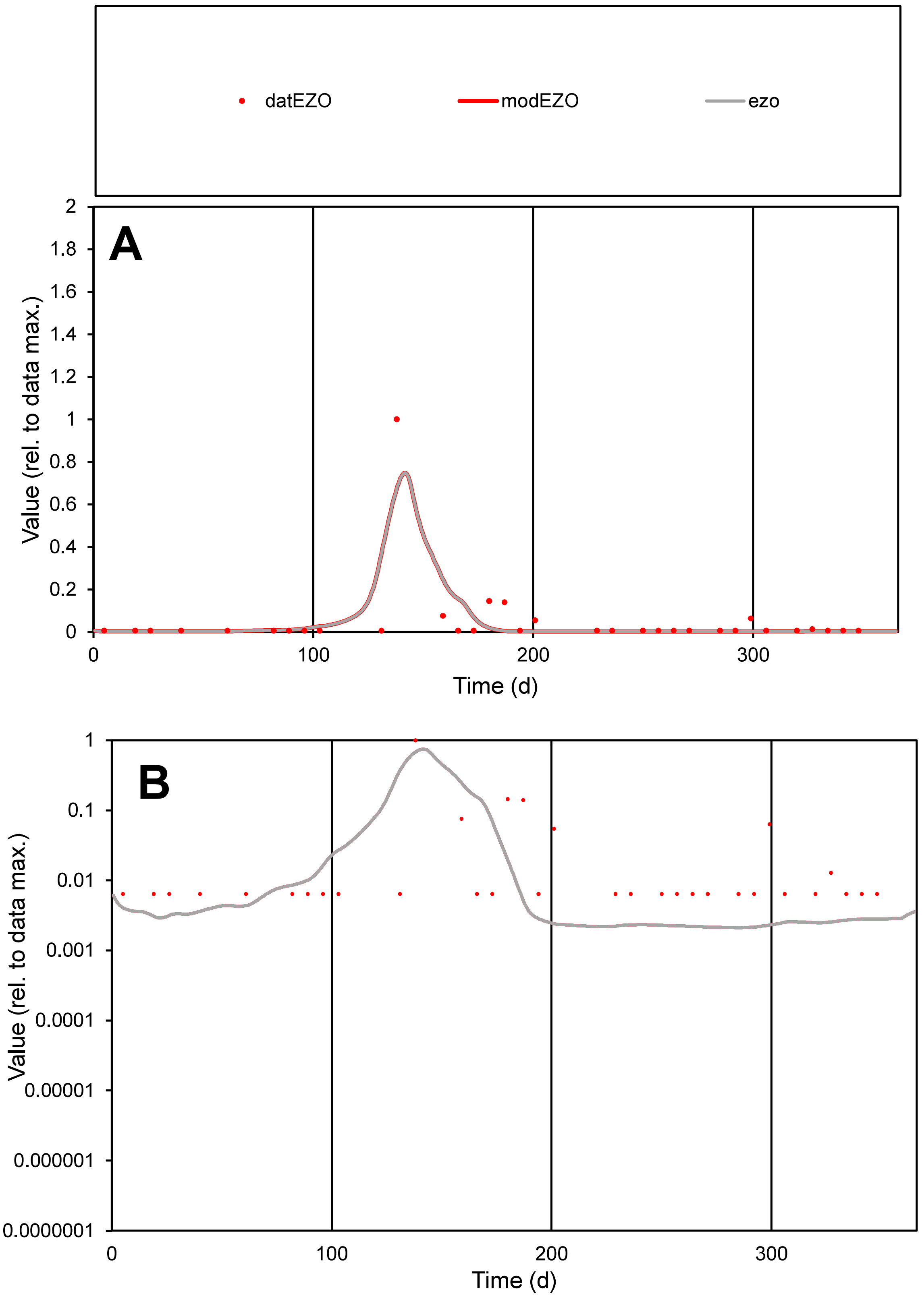

C1

Variables
EZO Provide jtd or Dst
CalibOnly?
2 1 to reproduce of
oNormalize?
2 1=no, 2=yes

ofxn calc*
1.5486
ofxn mod
1.816

sRun
RunA1
zout
1

Ave
Data
3.1E-05
Model
2.7E-05

data/vMax
0.00061

ofxng
263.459

Min d/m
3.04714

ave/min d
7.80666
ave/min m
21.0145

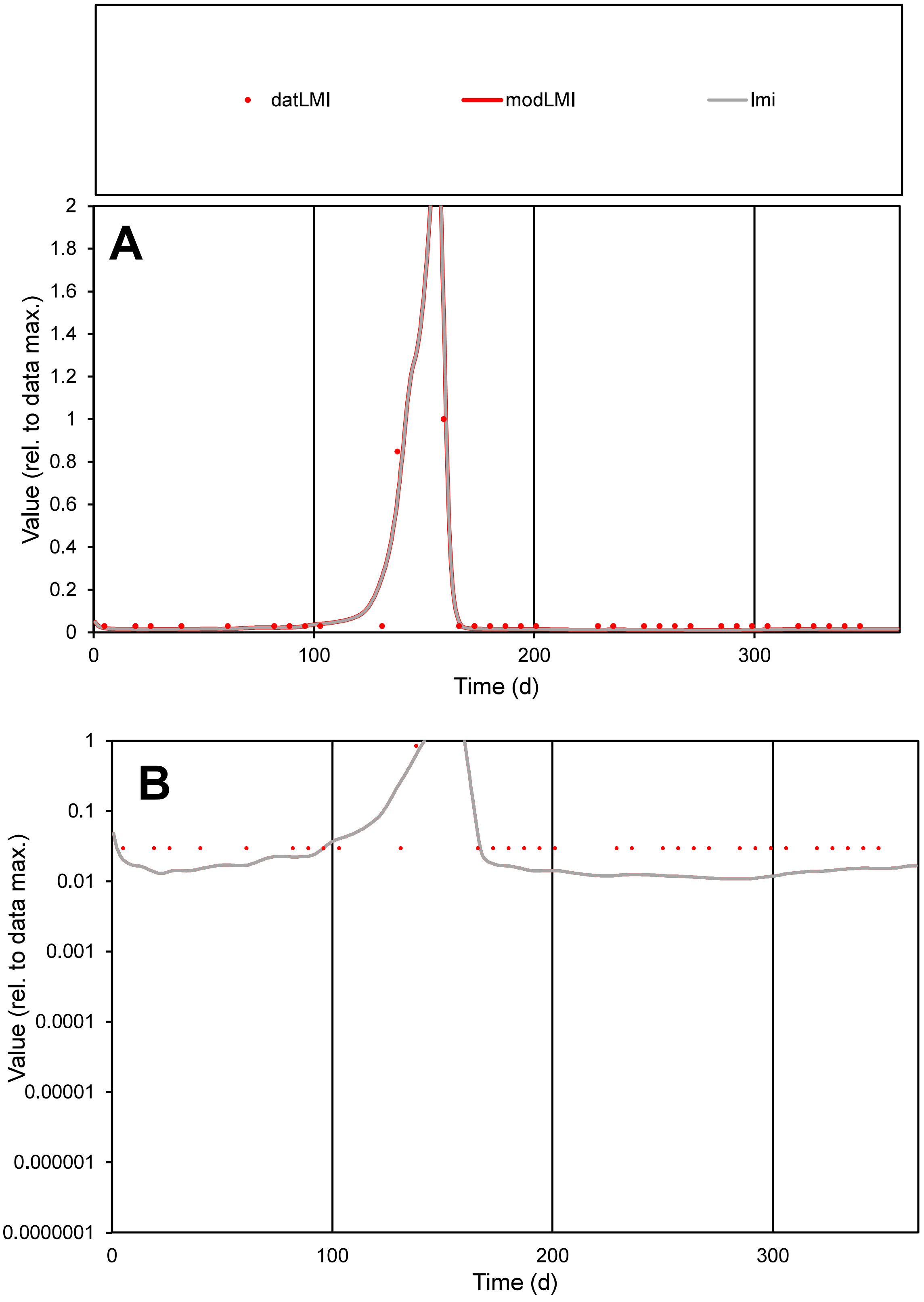

C1

Variables
LMI Provide jtd or Dst
CalibOnly?
2 1 to reproduce of
oNormalize?
2 1=no, 2=yes

ofxn calc*
0.48654
ofxn mod
0.56868

sRun
RunA1
zout
1

Ave
Data
8.5E-06
Model
7.7E-06

data/vMax
0.0001

ofxng
263.459

Min d/m
2.73909

ave/min d
2.82275
ave/min m
7.02444

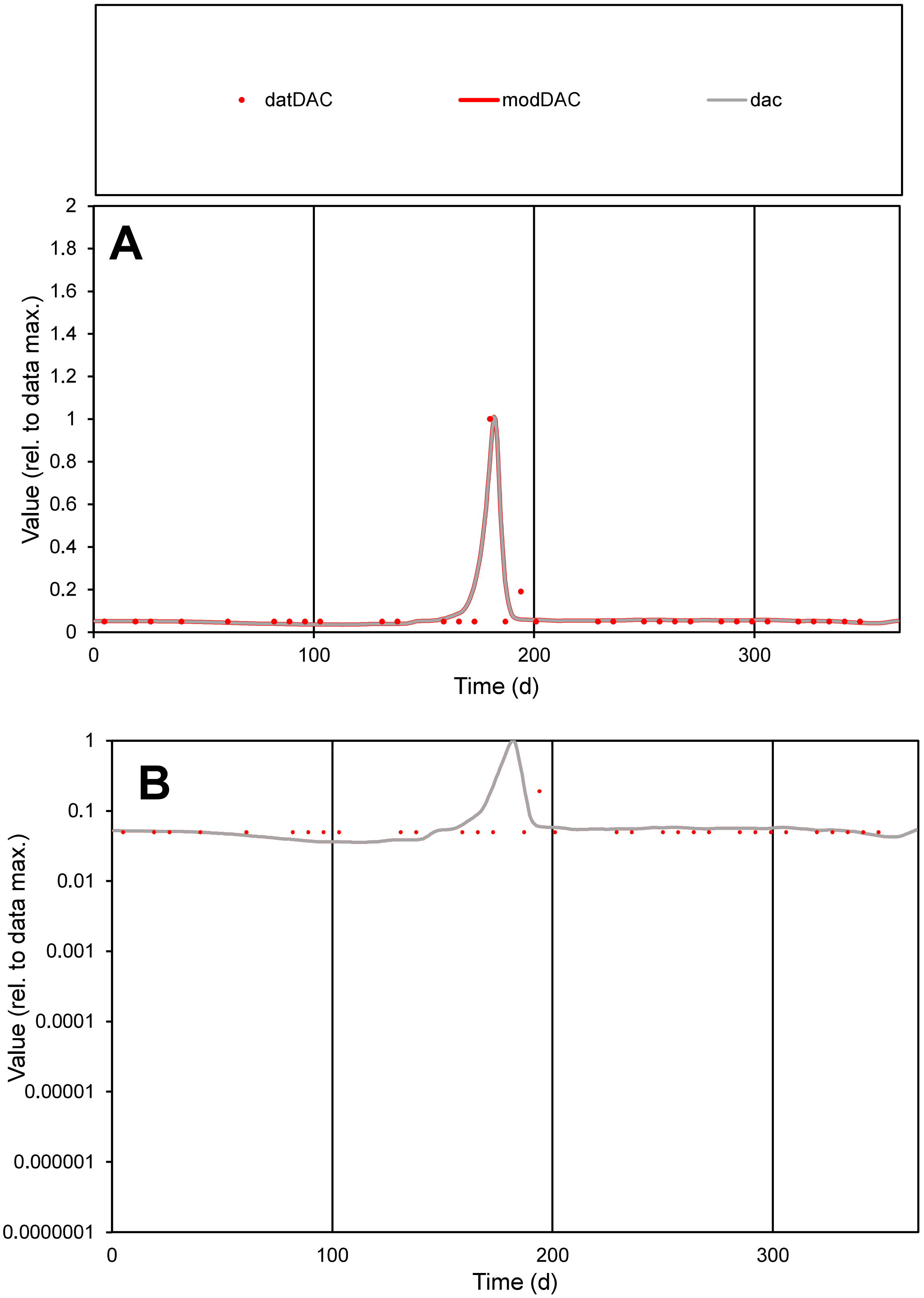

C1

Variables
DAC Provide jtd or Dst
CalibOnly?
2 1 to reproduce of
oNormalize?
2 1=no, 2=yes

ofxn calc*
0.46603
ofxn mod
0.52202

sRun
RunA1
zout
1

Ave
Data
1.3E-05
Model
1.3E-05

data/vMax
0.00015

ofxng
263.459

Min d/m
1.38398

ave/min d
1.66295
ave/min m
2.37849

326_Calib_S_A

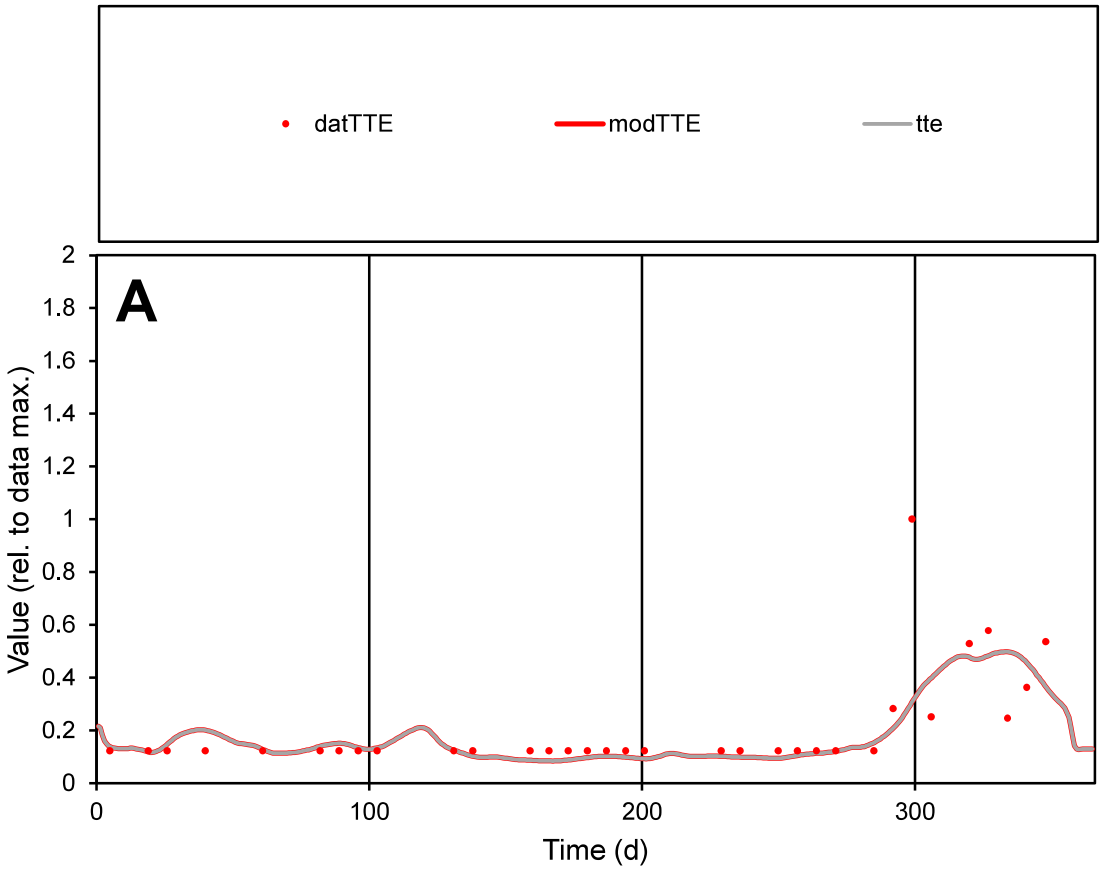

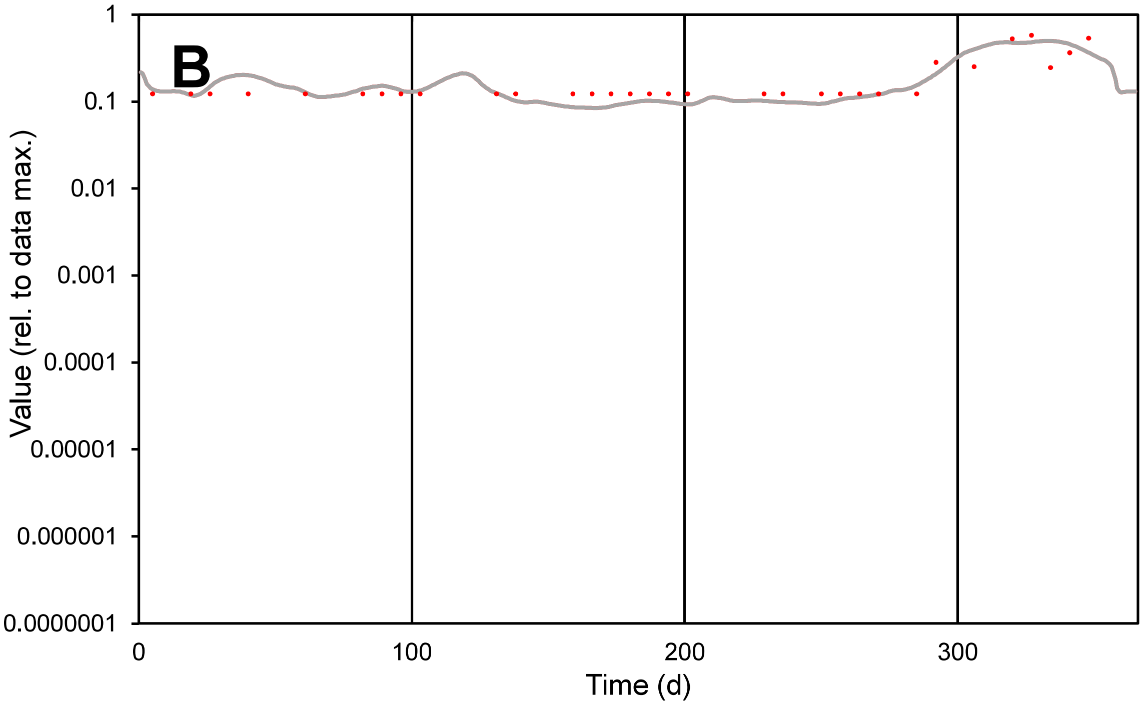

C1

Variables

TTE Provide jtd or Dst

CalibOnly?

2 1 to reproduce of

oNormalize?

2 1=no, 2=yes

ofxn calc*

0.65385

ofxn mod

0.66749

sRun

RunA1

zout

1

Ave

Data

6.4E-06

Model

5.7E-06

data/vMax

3.1E-05

ofxng

263.459

Min d/m

1.45945

ave/min d

1.69168

ave/min m

2.21507

326_Calib_S_A

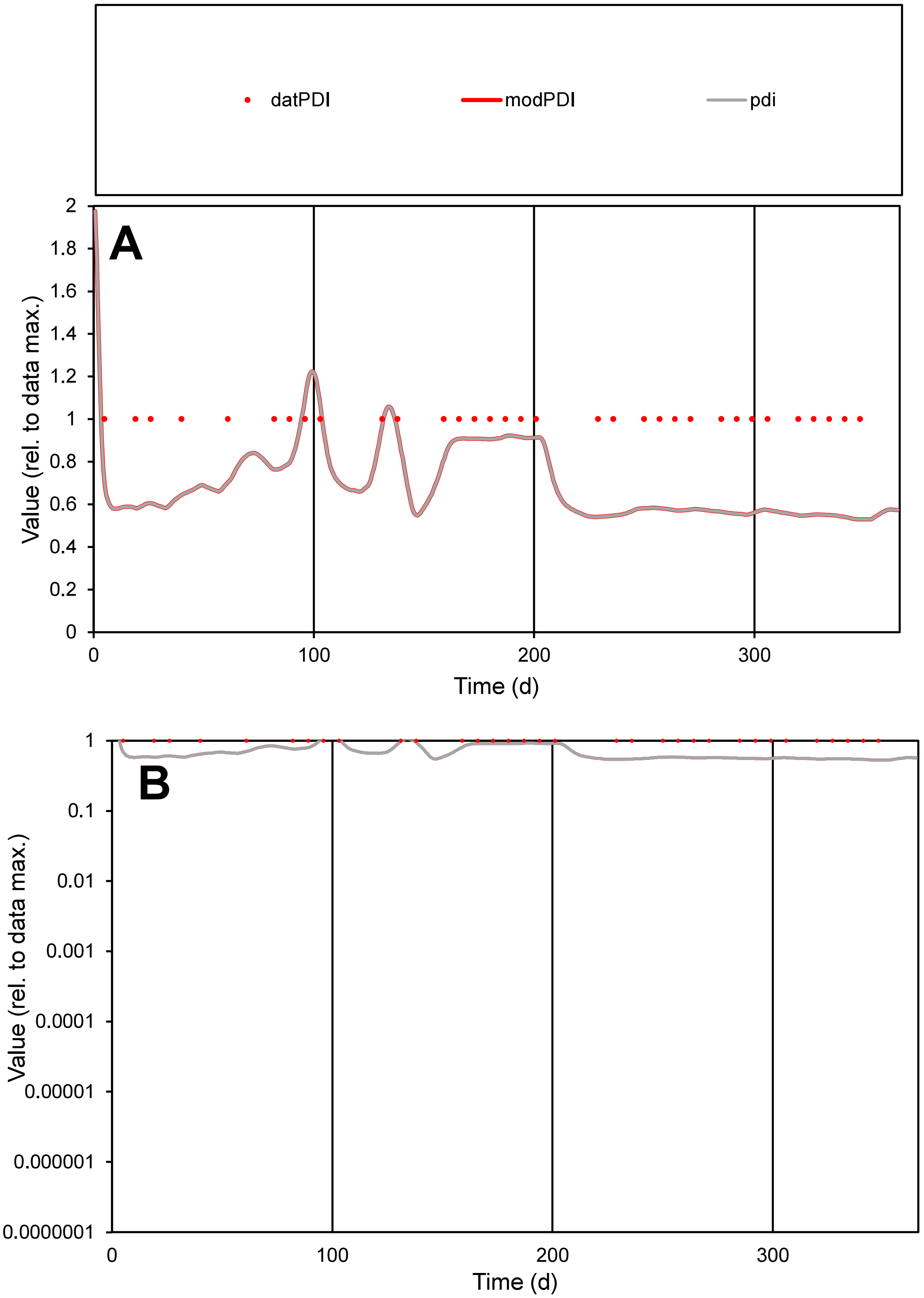

C1

Variables
PDI Provide jtd or Dst
CalibOnly?
2 1 to reproduce of
oNormalize?
2 1=no, 2=yes

ofxn calc*
0.33833
ofxn mod
0.35297

sRun
RunA1
zout
1

Ave
Data
4.4E-06
Model
3.1E-06

data/vMax
4.4E-06

ofxng
263.459

Min d/m
1.8867

ave/min d
1
ave/min m
1.3472

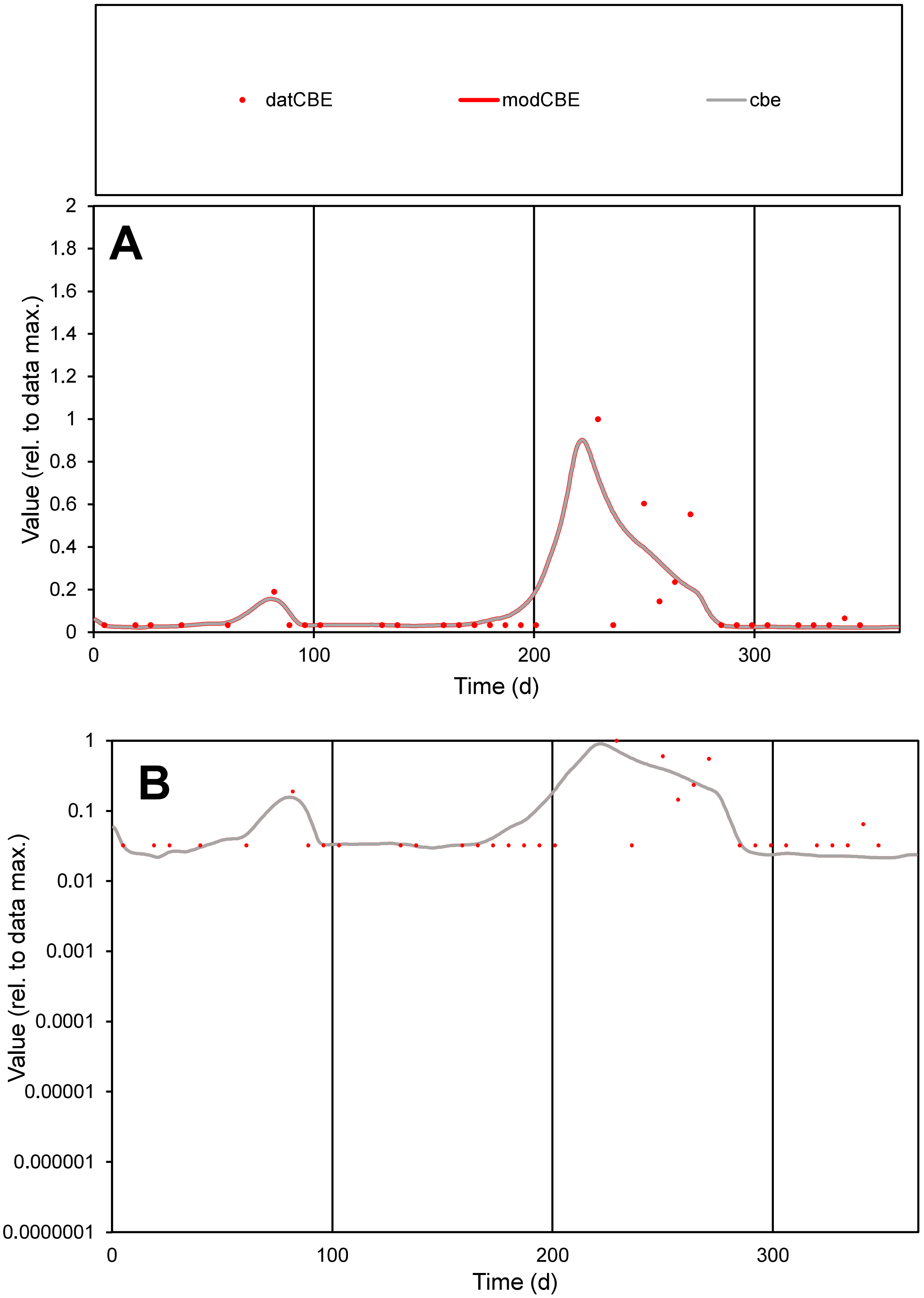

C1

Variables
CBE Provide jtd or Dst
CalibOnly?
2 1 to reproduce of
oNormalize?
2 1=no, 2=yes

ofxn calc*
1.18355
ofxn mod
1.24042

sRun
RunA1
zout
1

Ave
Data
5.7E-06
Model
5.6E-06

data/vMax
5.1E-05

ofxng
263.459

Min d/m
1.51499

ave/min d
3.38787
ave/min m
5.06961

326_Calib_S_A

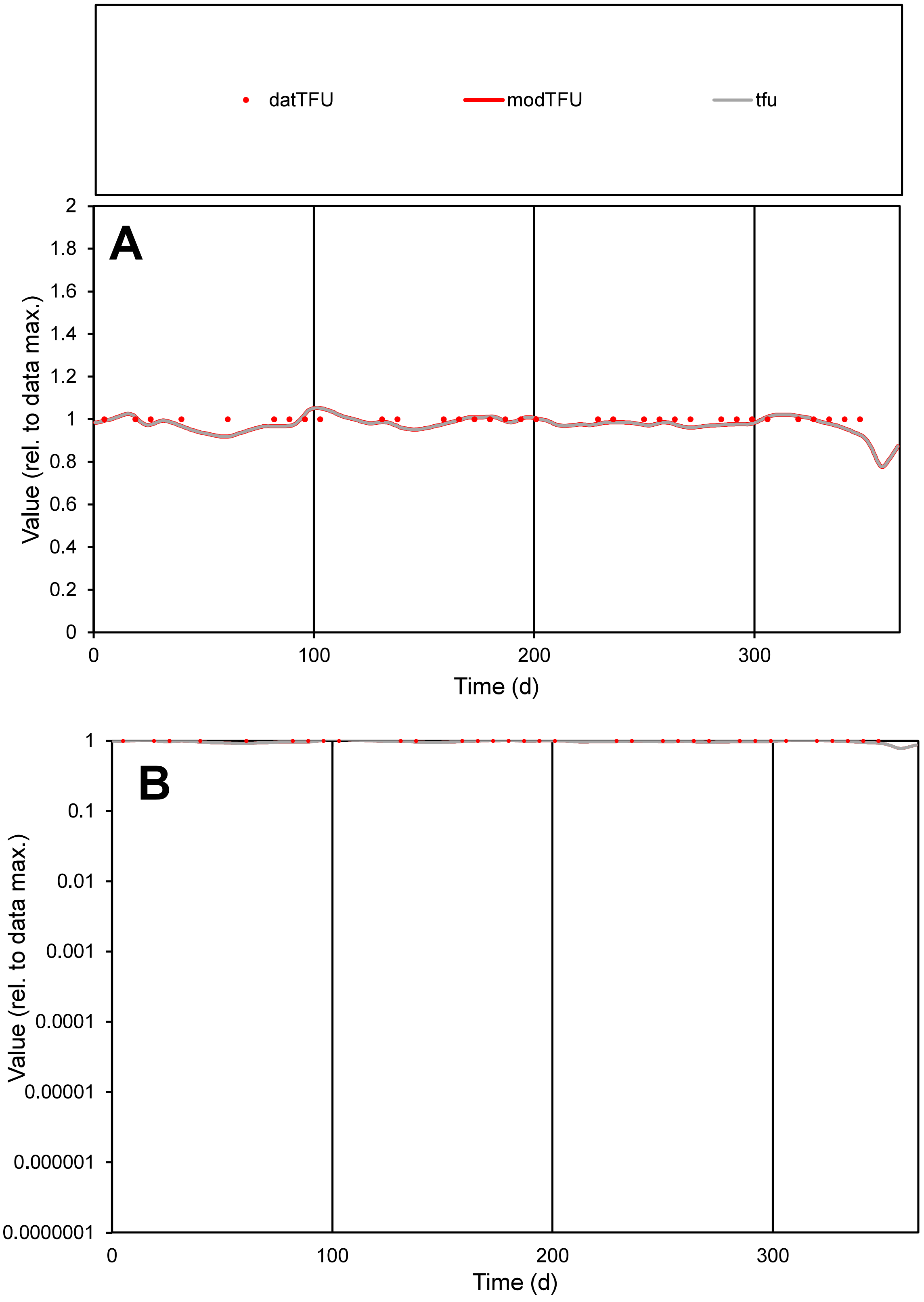

C1

Variables
TFU Provide jtd or Dst
CalibOnly?
2 1 to reproduce of
oNormalize?
2 1=no, 2=yes

ofxn calc*
0.03072
ofxn mod
0.11634

sRun
RunA1
zout
1

Ave
Data
1.3E-05
Model
1.3E-05

data/vMax
1.3E-05

ofxng
263.459

Min d/m
1.08603

ave/min d
1
ave/min m
1.06974

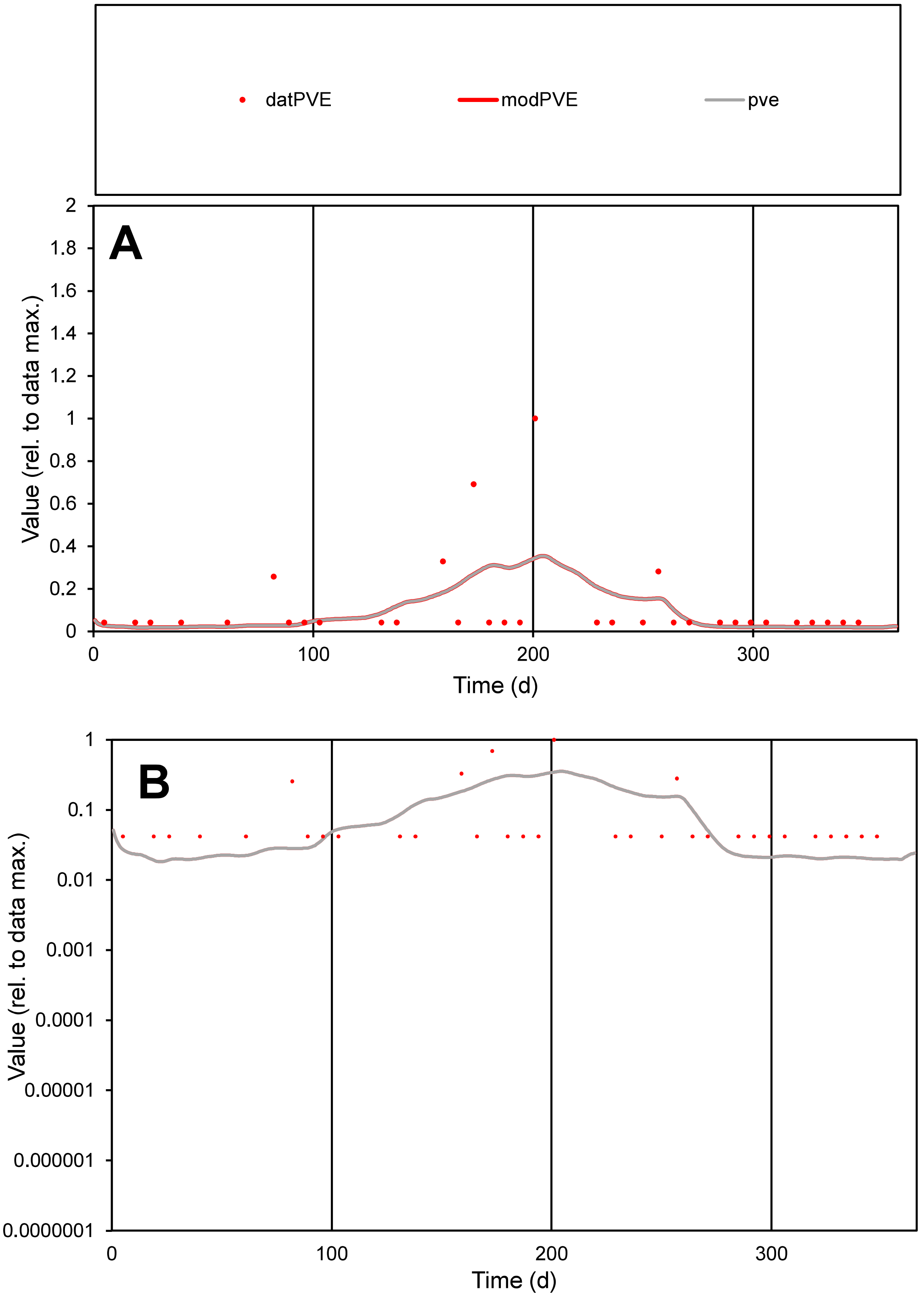

C1

Variables
PVE Provide jtd or Dst
CalibOnly?
2 1 to reproduce of
oNormalize?
2 1=no, 2=yes

ofxn calc*
1.54088
ofxn mod
1.55126

sRun
RunA1
zout
1

Ave
Data
5.7E-06
Model
5.2E-06

data/vMax
5E-05

ofxng
263.459

Min d/m
2.21775

ave/min d
2.70751
ave/min m
5.54221

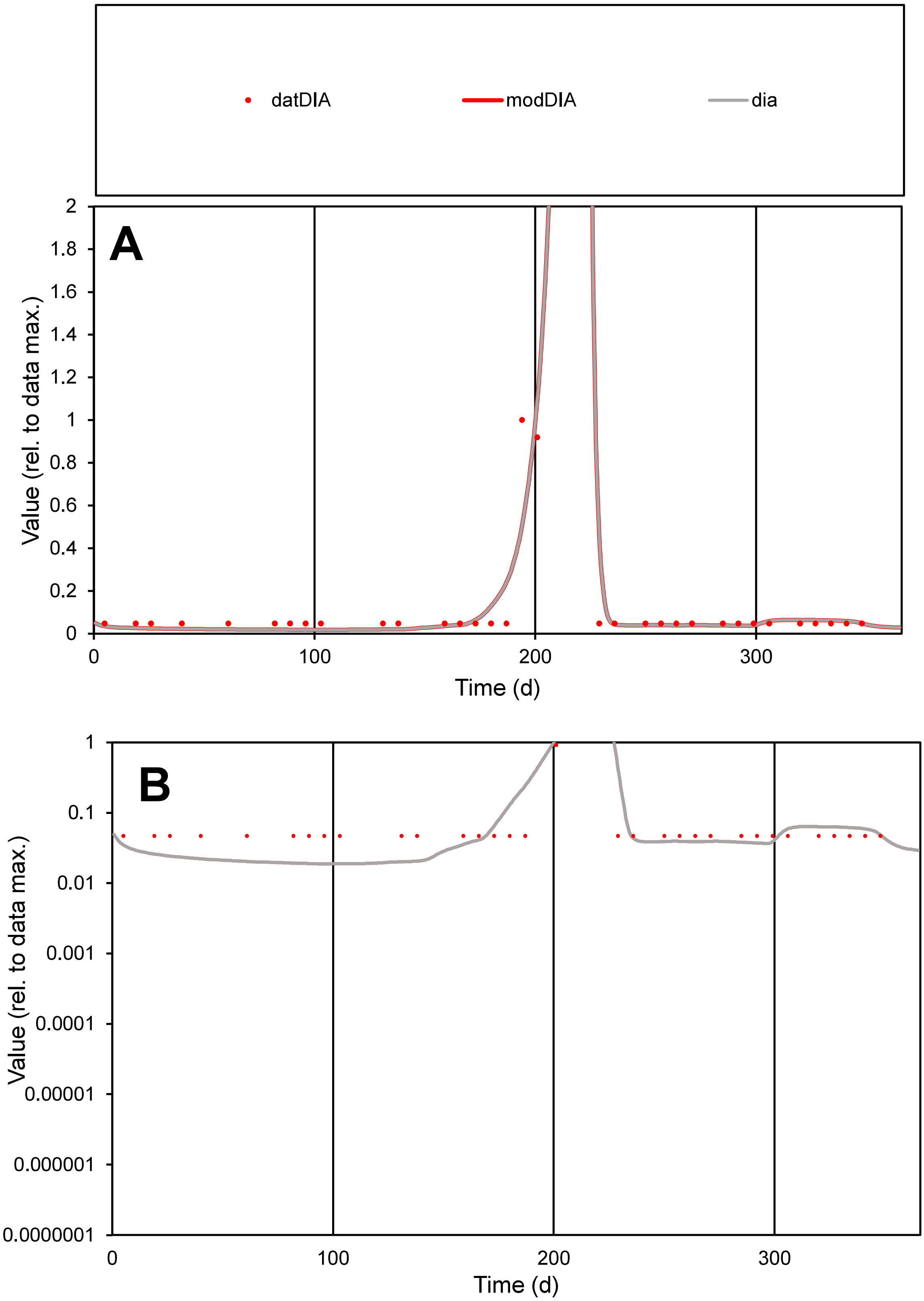

C1

Variables
DIA Provide jtd or Dst
CalibOnly?
2 1 to reproduce of
oNormalize?
2 1=no, 2=yes

ofxn calc*
0.88717
ofxn mod
0.97116

sRun
RunA1
zout
1

Ave
Data
1.4E-05
Model
1.3E-05

data/vMax
0.00013

ofxng
263.459

Min d/m
2.48088

ave/min d
2.18227
ave/min m
5.36667

326_Calib_S_A

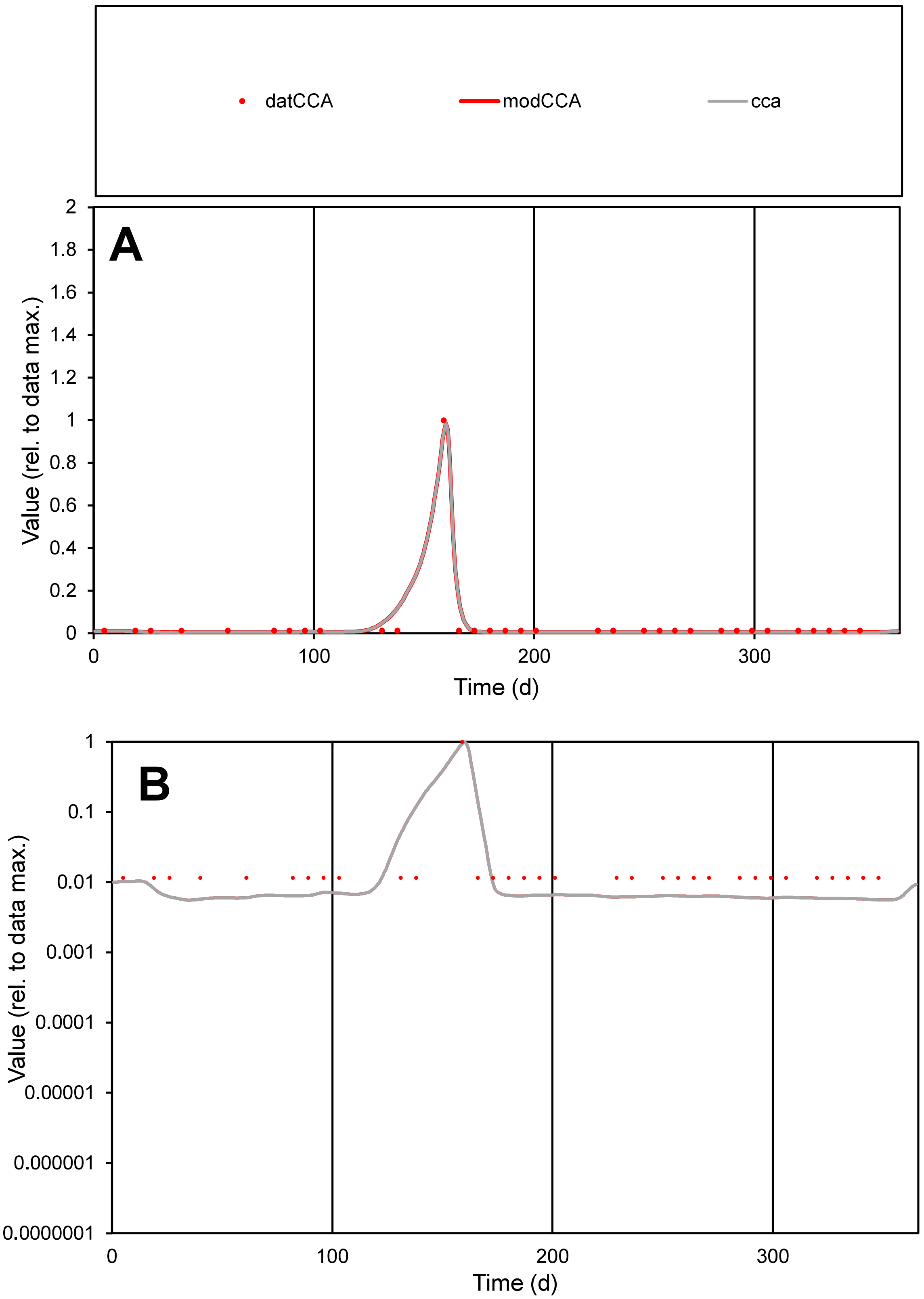

C1

Variables
CCA Provide jtd or Dst
CalibOnly?
2 1 to reproduce of
oNormalize?
2 1=no, 2=yes

ofxn calc*
0.40693
ofxn mod
0.55332

sRun
RunA1
zout
1

Ave
Data
6.6E-06
Model
6.5E-06

data/vMax
0.00016

ofxng
263.459

Min d/m
2.05232

ave/min d
3.60546
ave/min m
7.28636

326_Calib_S_A

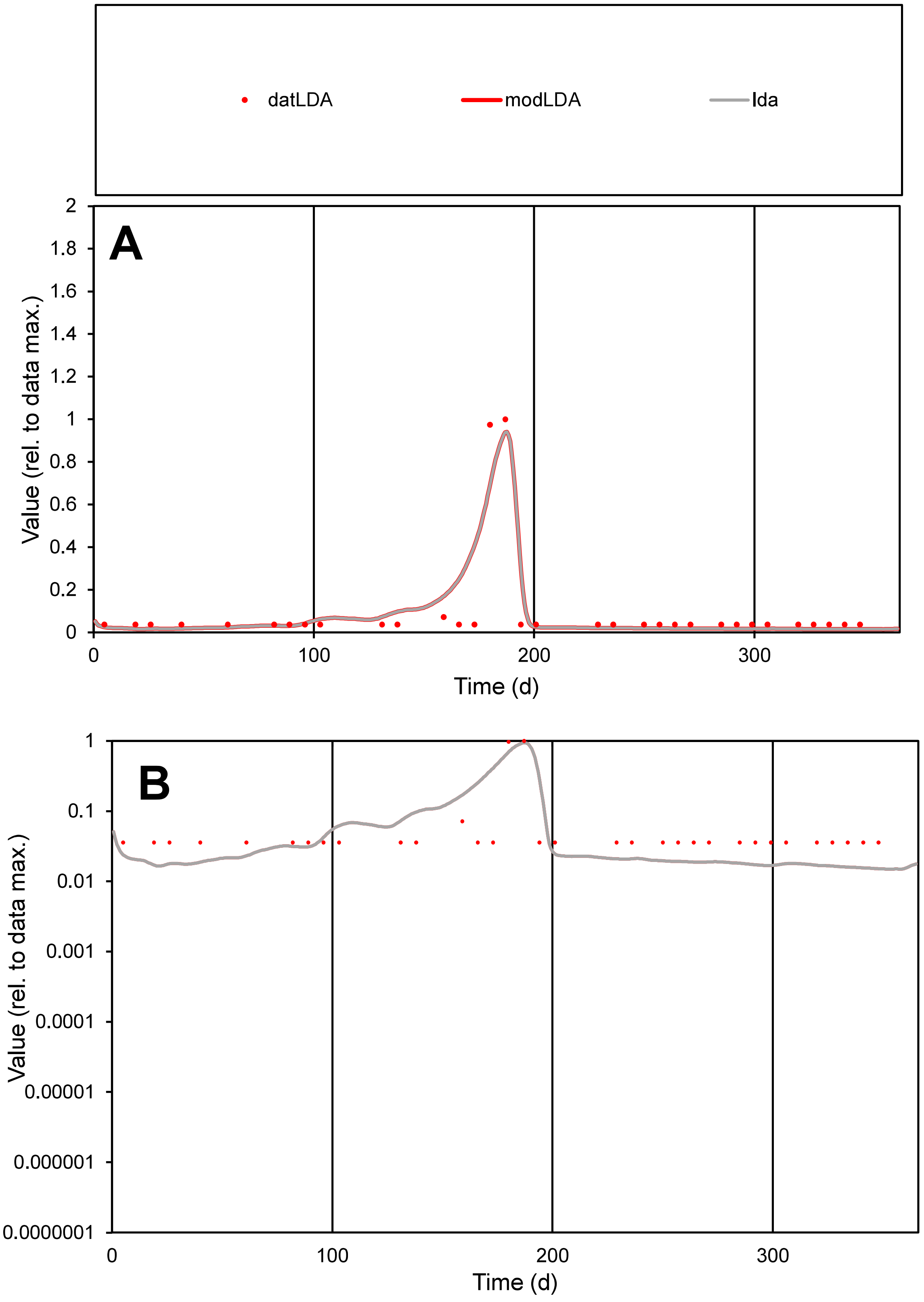

C1

Variables
LDA Provide jtd or Dst
CalibOnly?
2 1 to reproduce of
oNormalize?
2 1=no, 2=yes

ofxn calc*
0.92202
ofxn mod
0.99715

sRun
RunA1
zout
1

Ave
Data
1.8E-05
Model
1.9E-05

data/vMax
0.00019

ofxng
263.459

Min d/m
2.38393

ave/min d
2.63853
ave/min m
6.6581

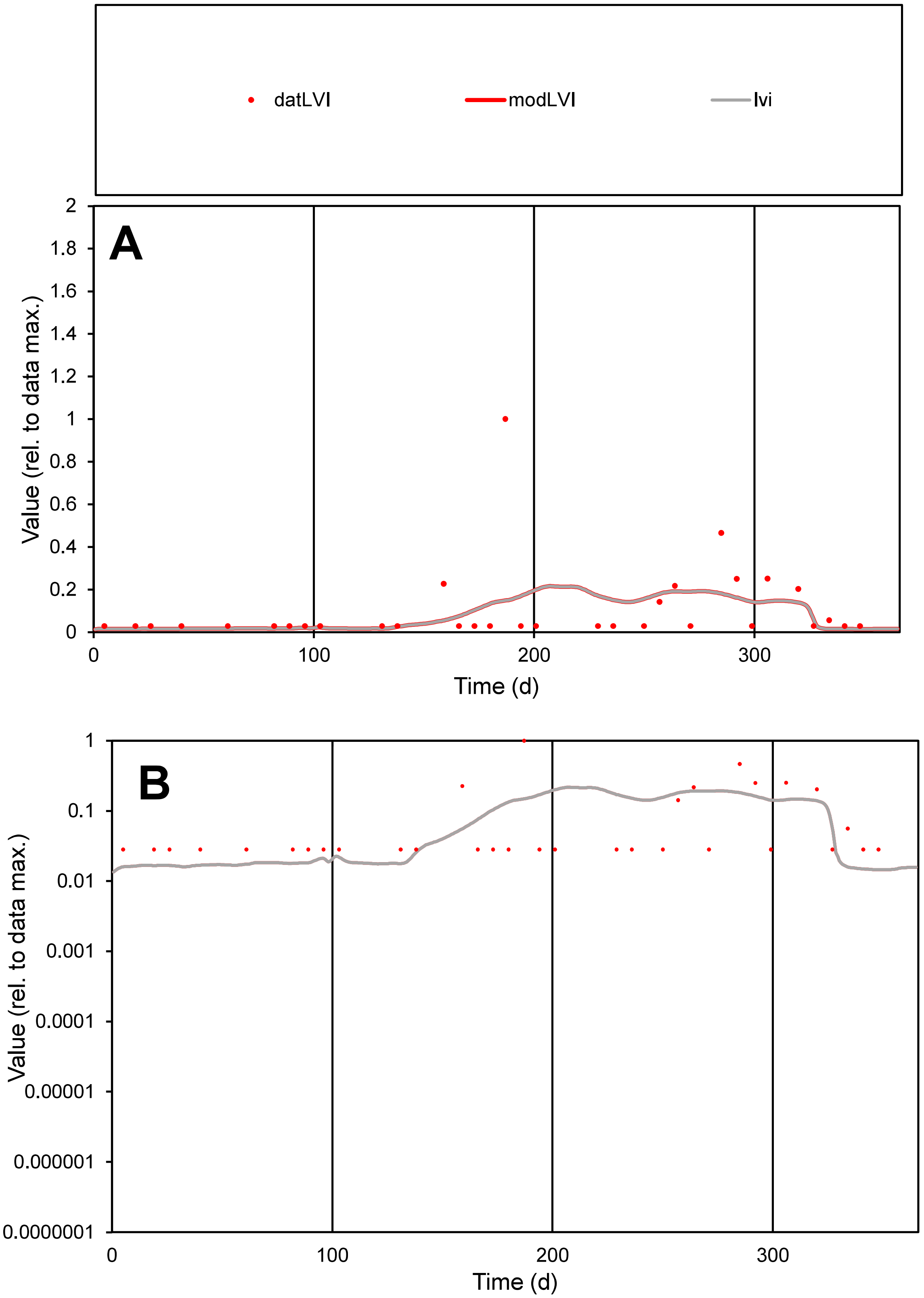

C1

Variables
LVI Provide jtd or Dst
CalibOnly?
2 1 to reproduce of
oNormalize?
2 1=no, 2=yes

ofxn calc*
1.66483
ofxn mod
1.67442

sRun
RunA1
zout
1

Ave
Data
5.3E-06
Model
4.6E-06

data/vMax
5.1E-05

ofxng
263.459

Min d/m
1.93285

ave/min d
3.76005
ave/min m
6.22202

326_Calib_S_A

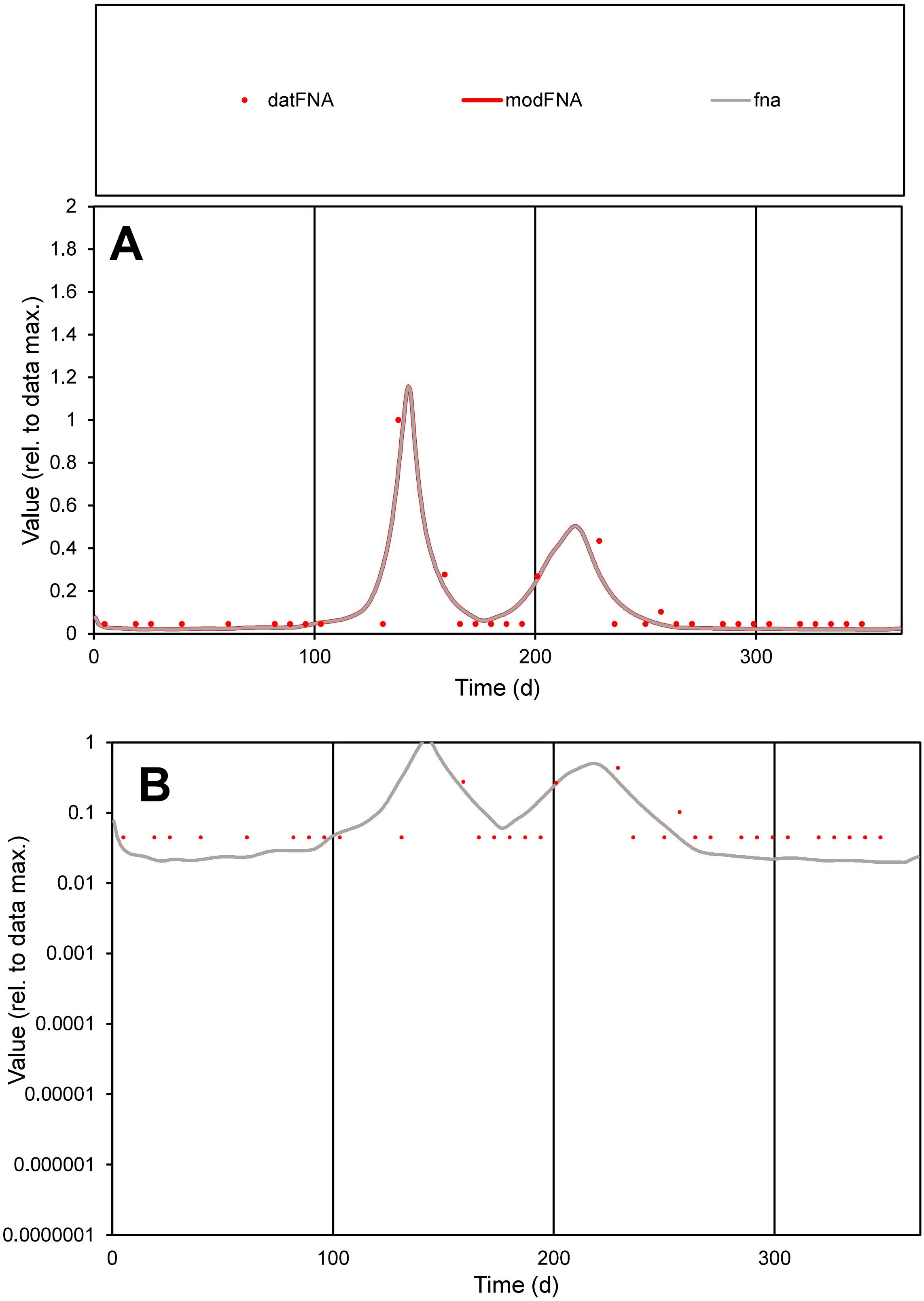

C1

Variables
FNA Provide jtd or Dst
CalibOnly?
2 1 to reproduce of
oNormalize?
2 1=no, 2=yes

ofxn calc*
0.6348
ofxn mod
0.71489

sRun
RunA1
zout
1

Ave
Data
7E-06
Model
6.8E-06

data/vMax
7E-05

ofxng
263.459

Min d/m
2.24317

ave/min d
2.26026
ave/min m
4.9104

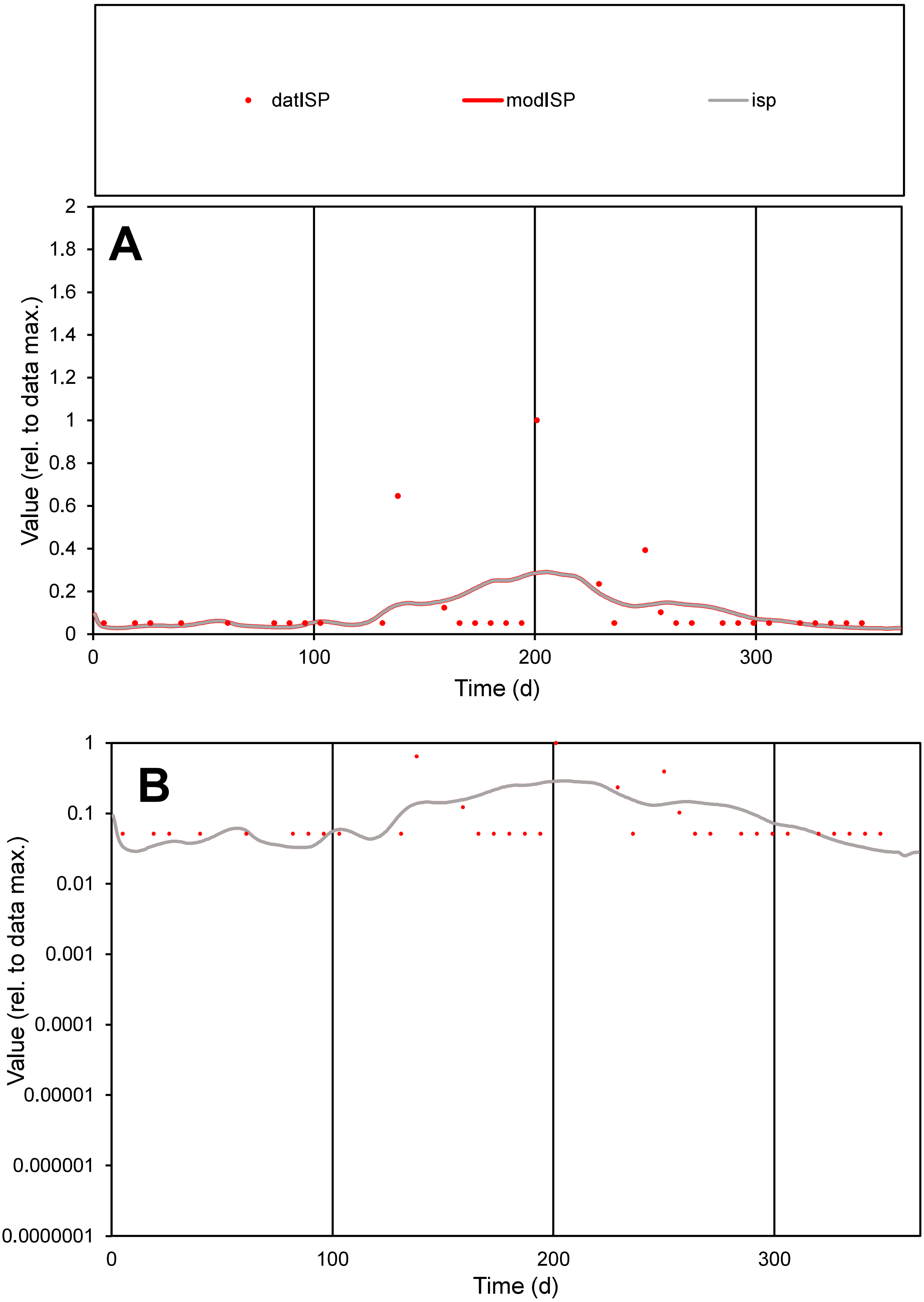

C1

Variables
ISP Provide jtd or Dst
CalibOnly?
2 1 to reproduce of
oNormalize?
2 1=no, 2=yes

ofxn calc*
1.50508
ofxn mod
1.51538

sRun
RunA1
zout
1

Ave
Data
1.2E-05
Model
1.1E-05

data/vMax
9.8E-05

ofxng
263.459

Min d/m
1.76232

ave/min d
2.29198
ave/min m
3.79449

326_Calib_S_A

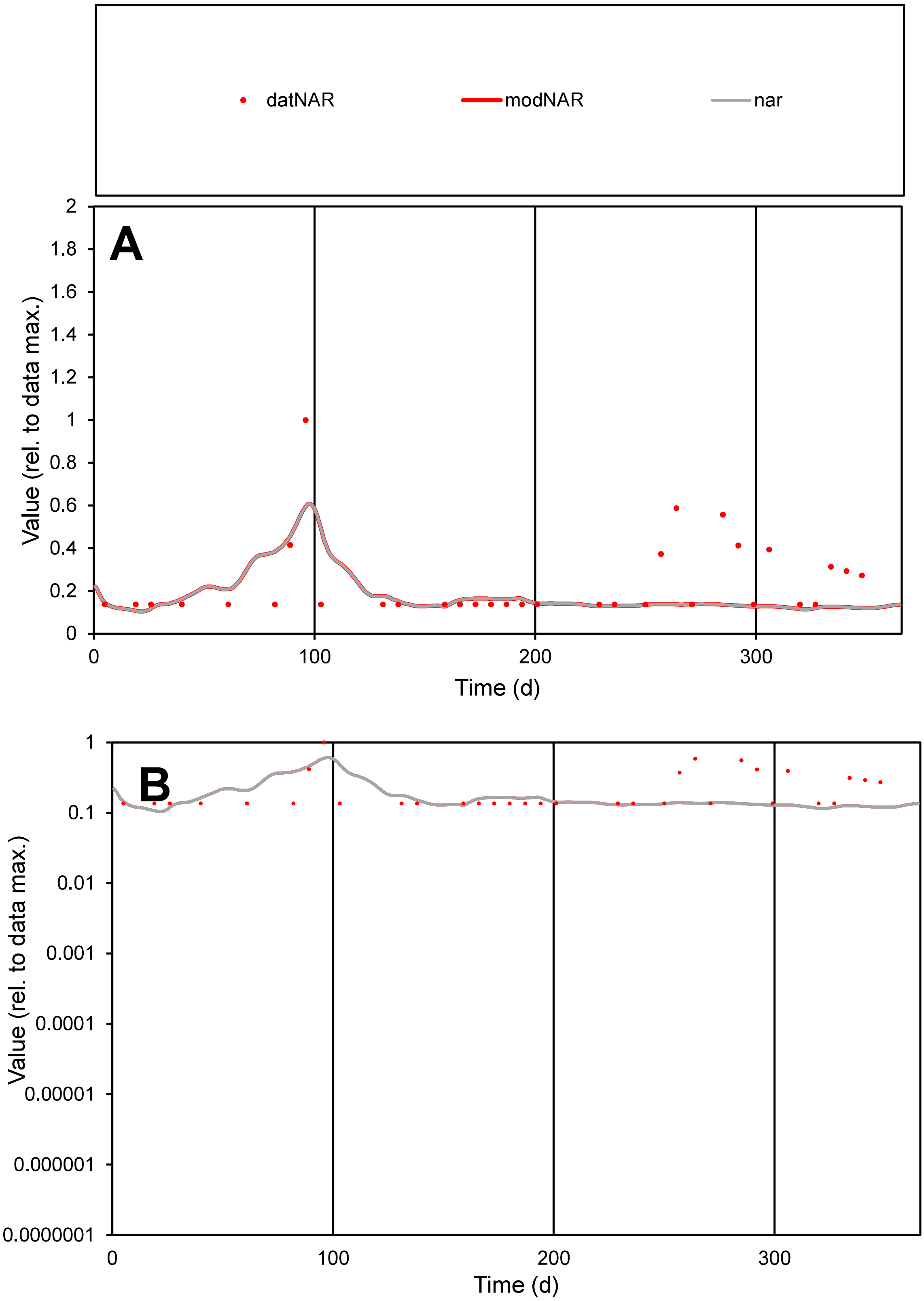

C1

Variables
NAR Provide jtd or Dst
CalibOnly?
2 1 to reproduce of
oNormalize?
2 1=no, 2=yes

ofxn calc*
0.73321
ofxn mod
0.74736

sRun
RunA1
zout
1

Ave
Data
4.9E-06
Model
3.7E-06

data/vMax
2.1E-05

ofxng
263.459

Min d/m
1.30005

ave/min d
1.72364
ave/min m
1.72518

326_Calib_S_A

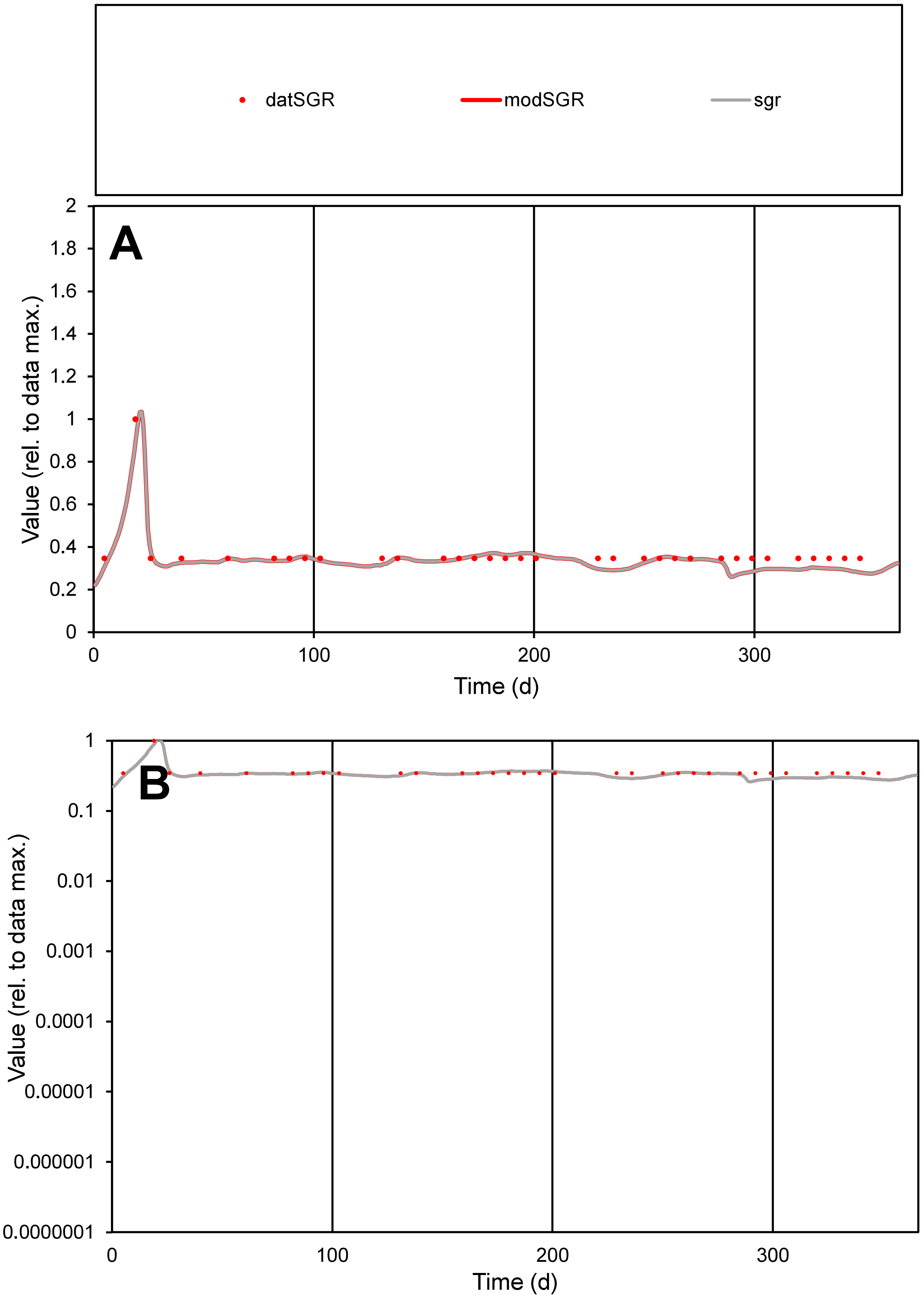

C1

Variables

SGR Provide jtd or Dst

CalibOnly?

2 1 to reproduce of

oNormalize?

2 1=no, 2=yes

ofxn calc*

0.09099

ofxn mod

0.14942

sRun

RunA1

zout

1

Ave

Data

4.8E-06

Model

4.5E-06

data/vMax

1.3E-05

ofxng

263.459

Min d/m

1.27173

ave/min d

1.0571

ave/min m

1.27592

326_Calib_S_A

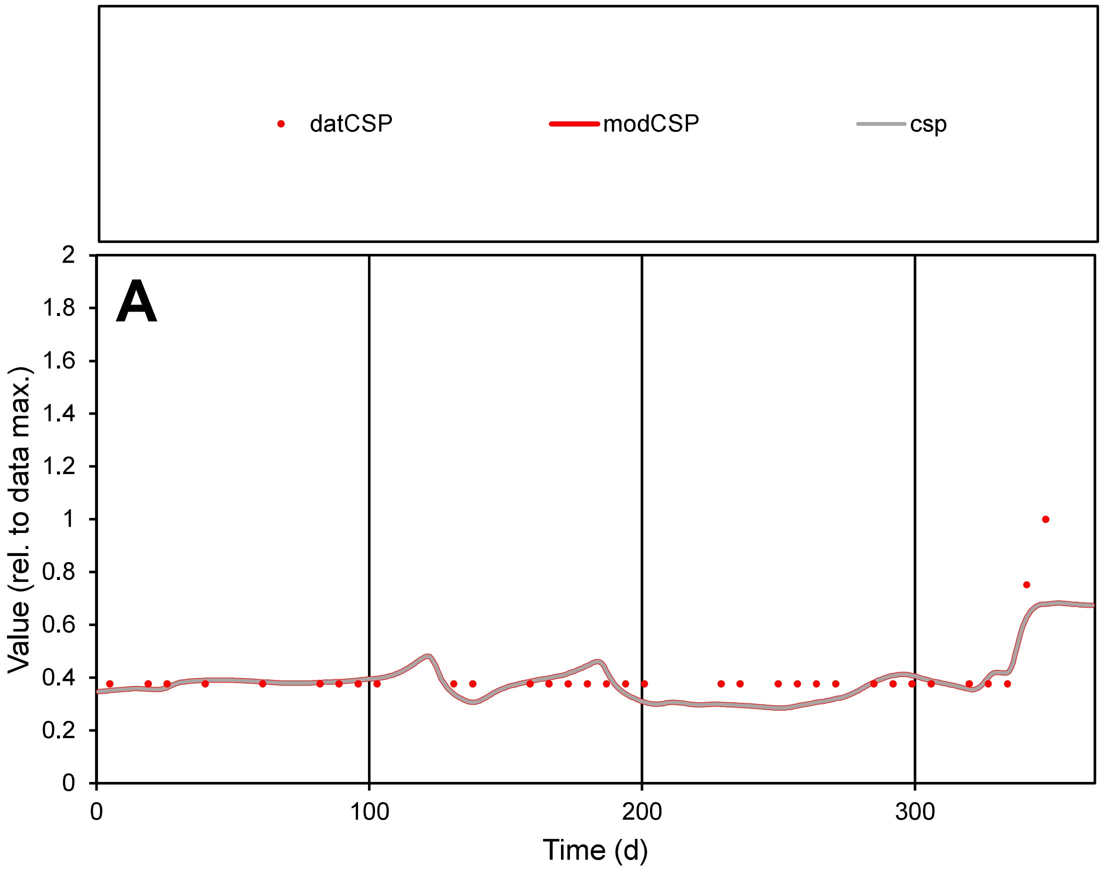

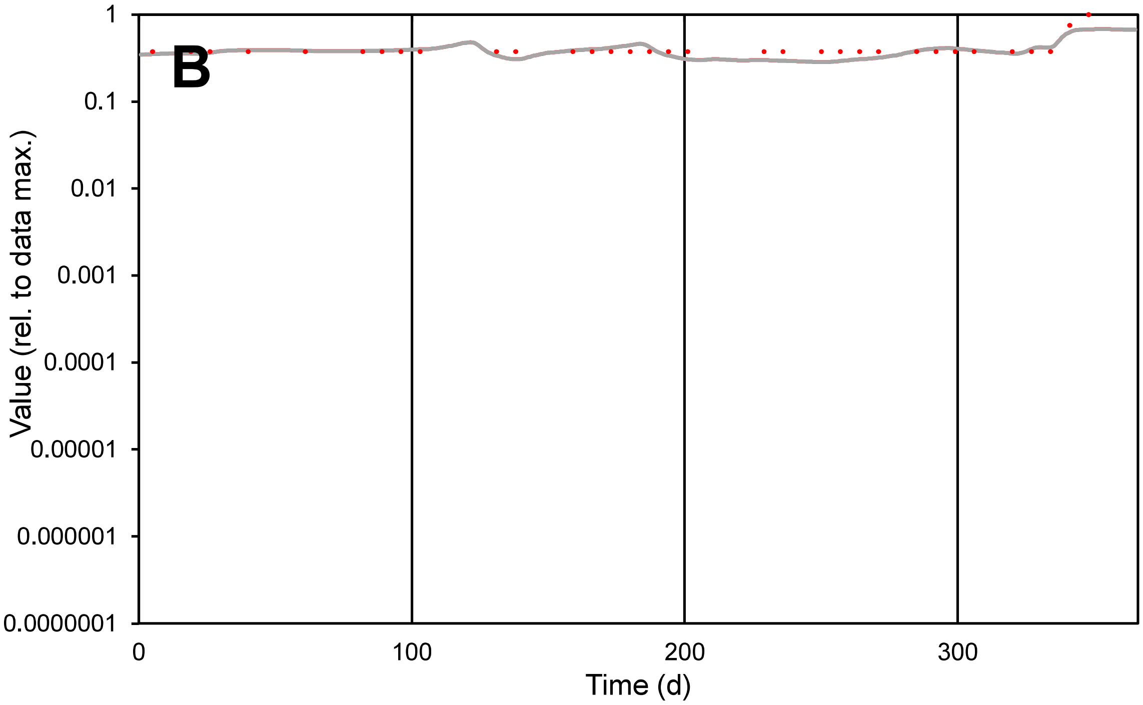

C1

Variables
CSP Provide jtd or Dst
CalibOnly?
2 1 to reproduce of
oNormalize?
2 1=no, 2=yes

ofxn calc*
0.18212
ofxn mod
0.21239

sRun
RunA1
zout
1

Ave
Data
2.6E-06
Model
2.4E-06

data/vMax
6.4E-06

ofxng
263.459

Min d/m
1.31588

ave/min d
1.0807
ave/min m
1.34556

326_Calib_S_A

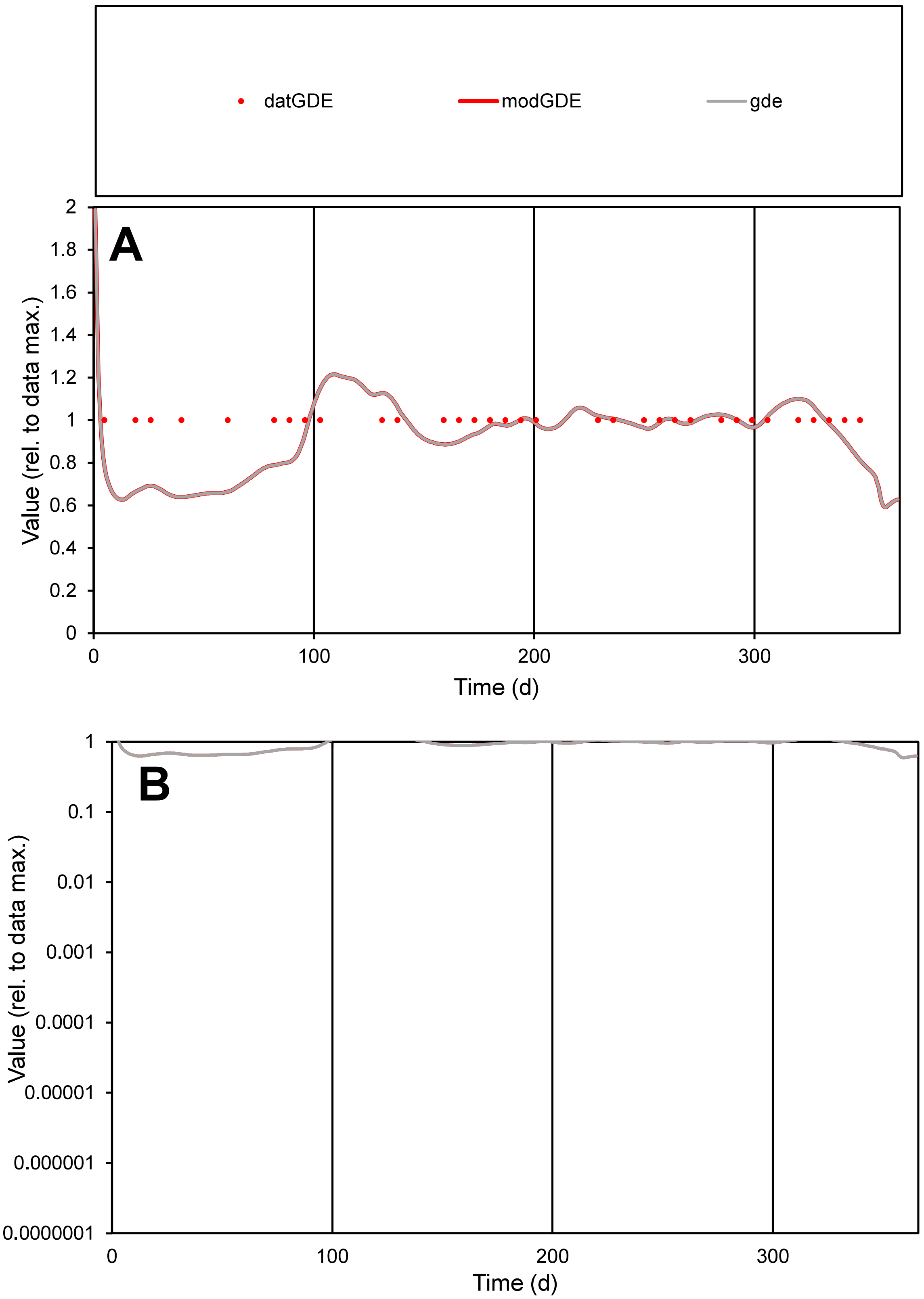

C1

Variables

GDE Provide jtd or Dst

CalibOnly?

2 1 to reproduce of

oNormalize?

2 1=no, 2=yes

ofxn calc*

0.14736

ofxn mod

0.18539

sRun

RunA1

zout

1

Ave

Data

2.7E-05

Model

2.6E-05

data/vMax

2.7E-05

ofxng

263.459

Min d/m

1.56048

ave/min d

1

ave/min m

1.45849

326_Calib_S_A

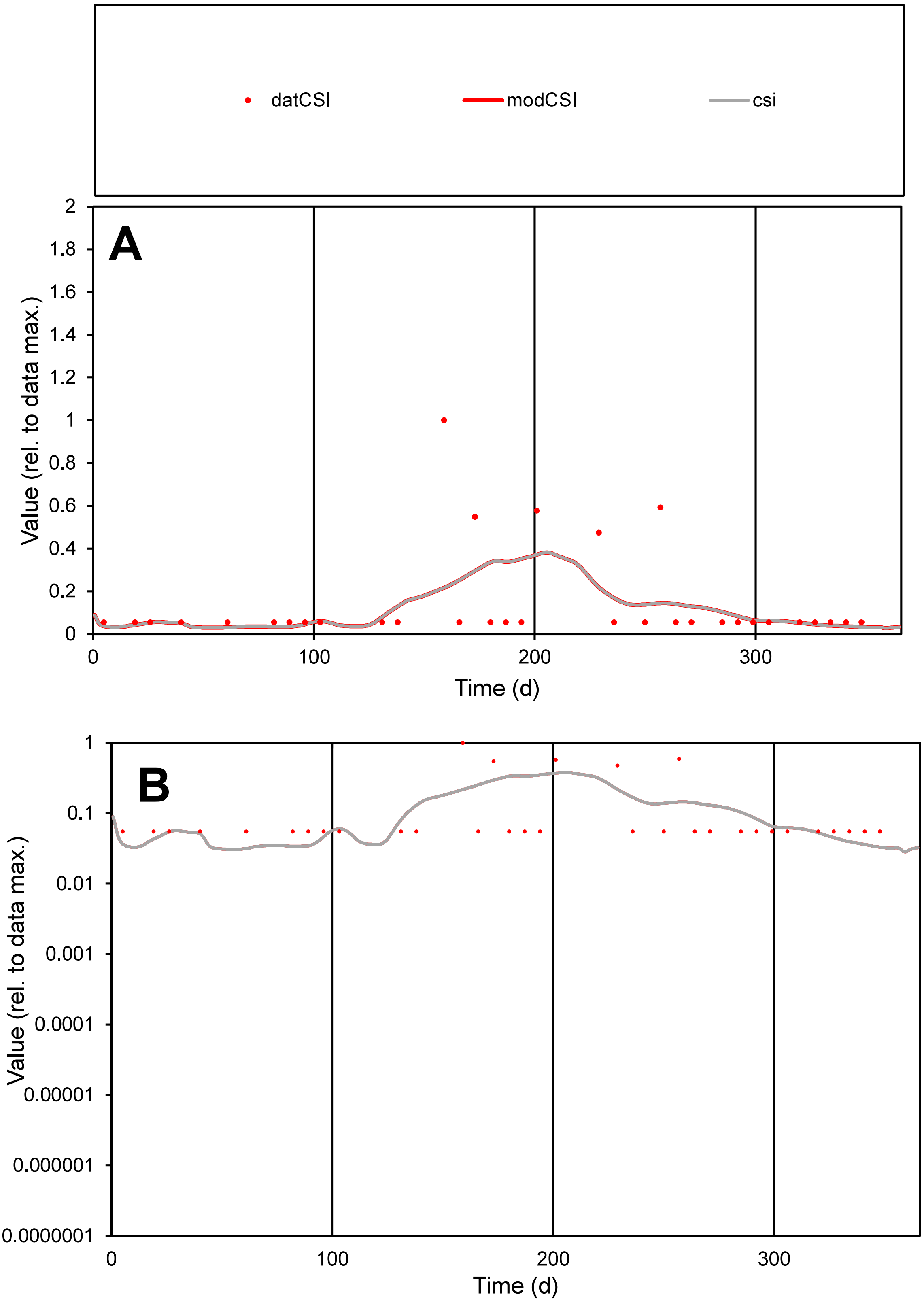

C1

Variables
CSI Provide jtd or Dst
CalibOnly?
2 1 to reproduce of
oNormalize?
2 1=no, 2=yes

ofxn calc*
1.38253
ofxn mod
1.39292

sRun
RunA1
zout
1

Ave
Data
6E-06
Model
5.4E-06

data/vMax
4.2E-05

ofxng
263.459

Min d/m
1.72131

ave/min d
2.59753
ave/min m
4.00029

326_Calib_S_A

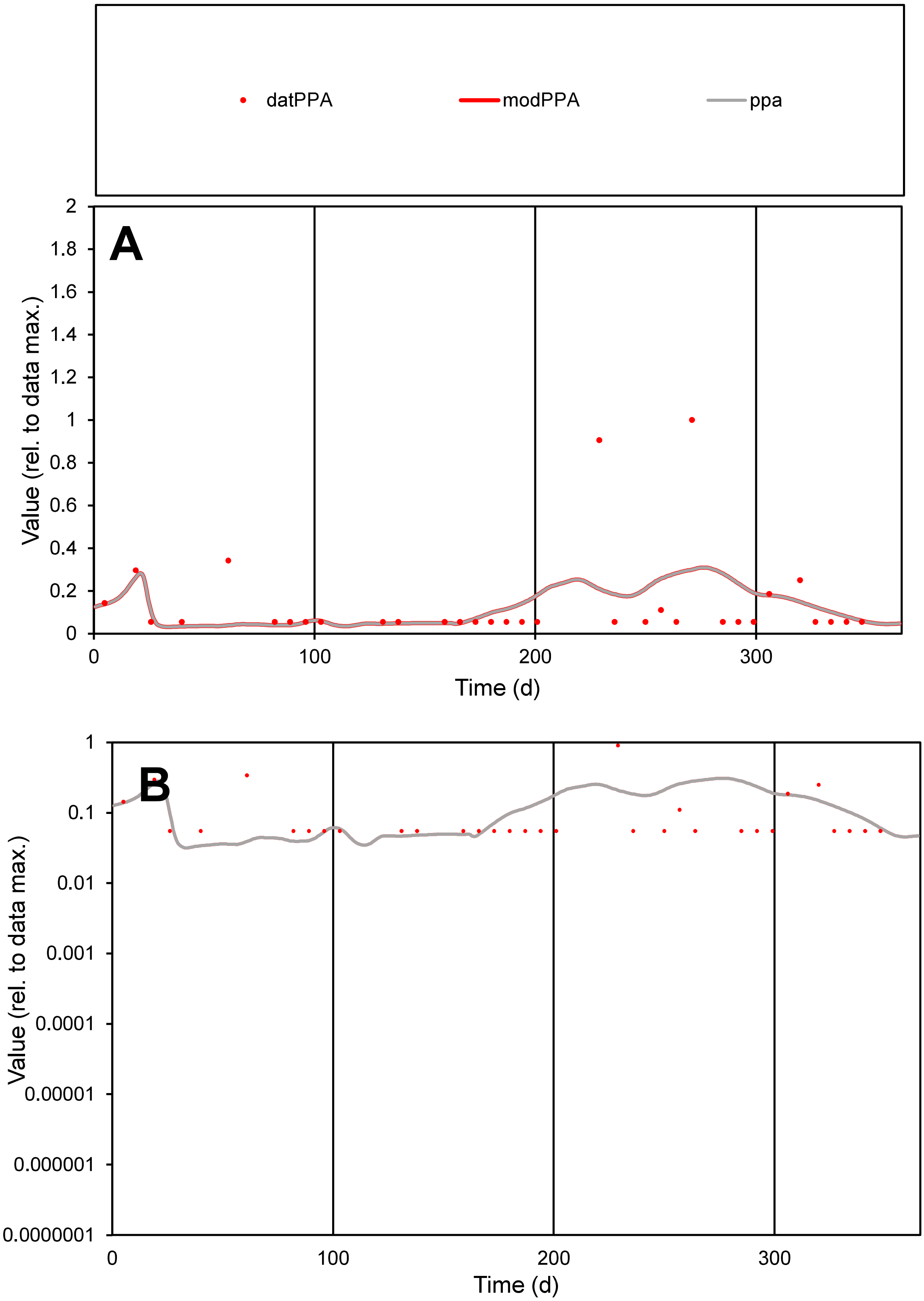

C1

Variables
PPA Provide jtd or Dst
CalibOnly?
2 1 to reproduce of
oNormalize?
2 1=no, 2=yes

ofxn calc*
1.43489
ofxn mod
1.44732

sRun
RunA1
zout
1

Ave
Data
8.1E-06
Model
7.7E-06

data/vMax
5.8E-05

ofxng
263.459

Min d/m
1.6055

ave/min d
2.53064
ave/min m
3.84979

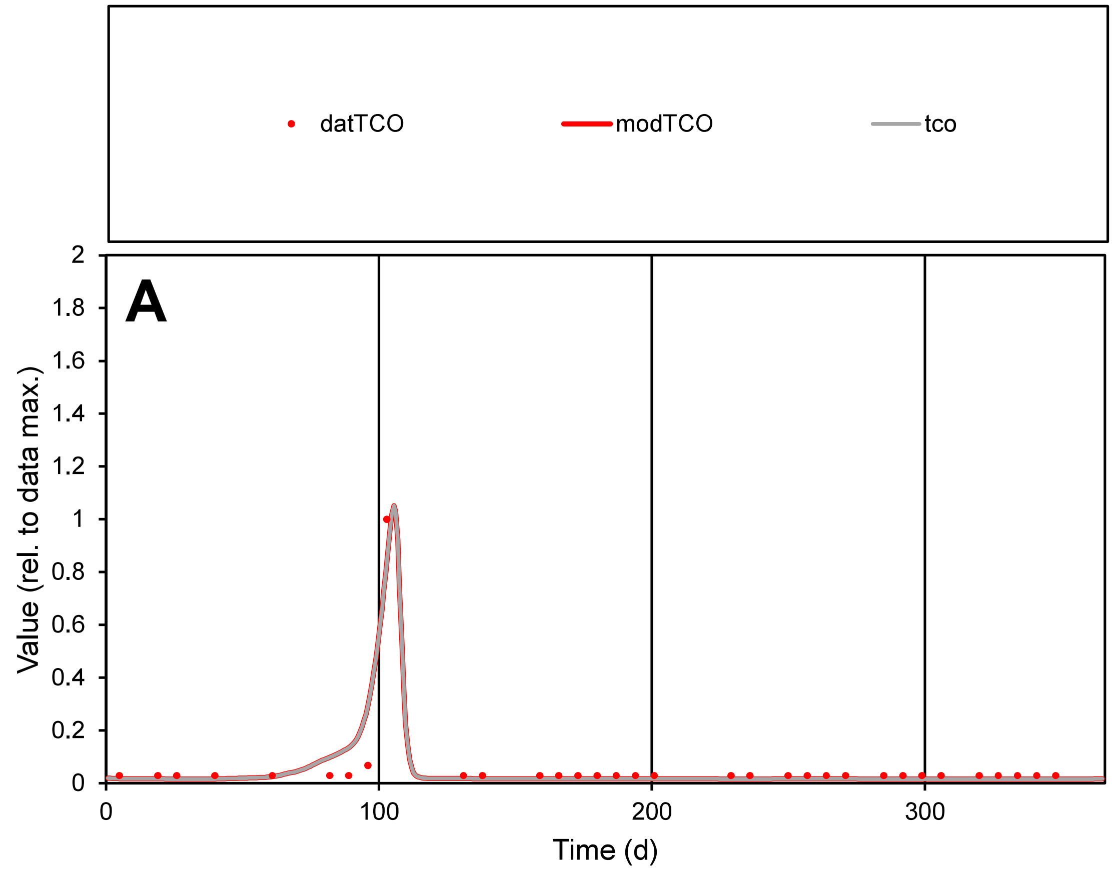

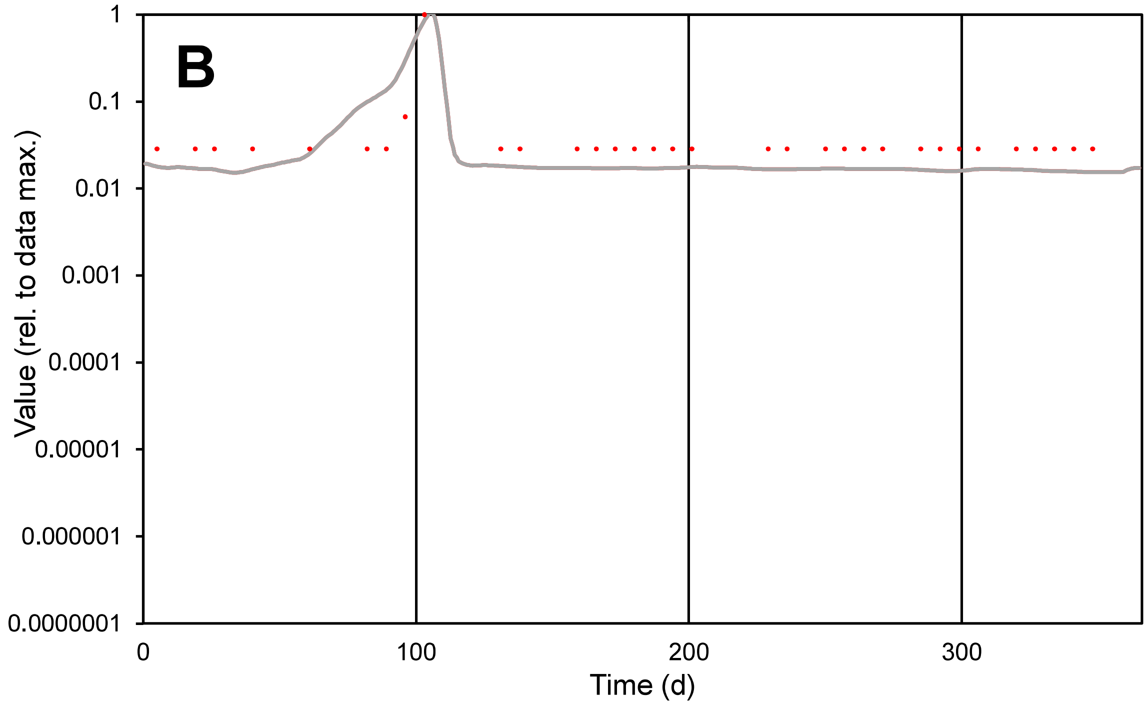

C1

Variables
TCO Provide jtd or Dst
CalibOnly?
2 1 to reproduce of
oNormalize?
2 1=no, 2=yes

ofxn calc*
0.71555
ofxn mod
0.7894

sRun
RunA1
zout
1

Ave
Data
1.8E-05
Model
1.8E-05

data/vMax
0.0003

ofxng
263.459

Min d/m
1.86484

ave/min d
2.06514
ave/min m
3.81896

326_Calib_S_A

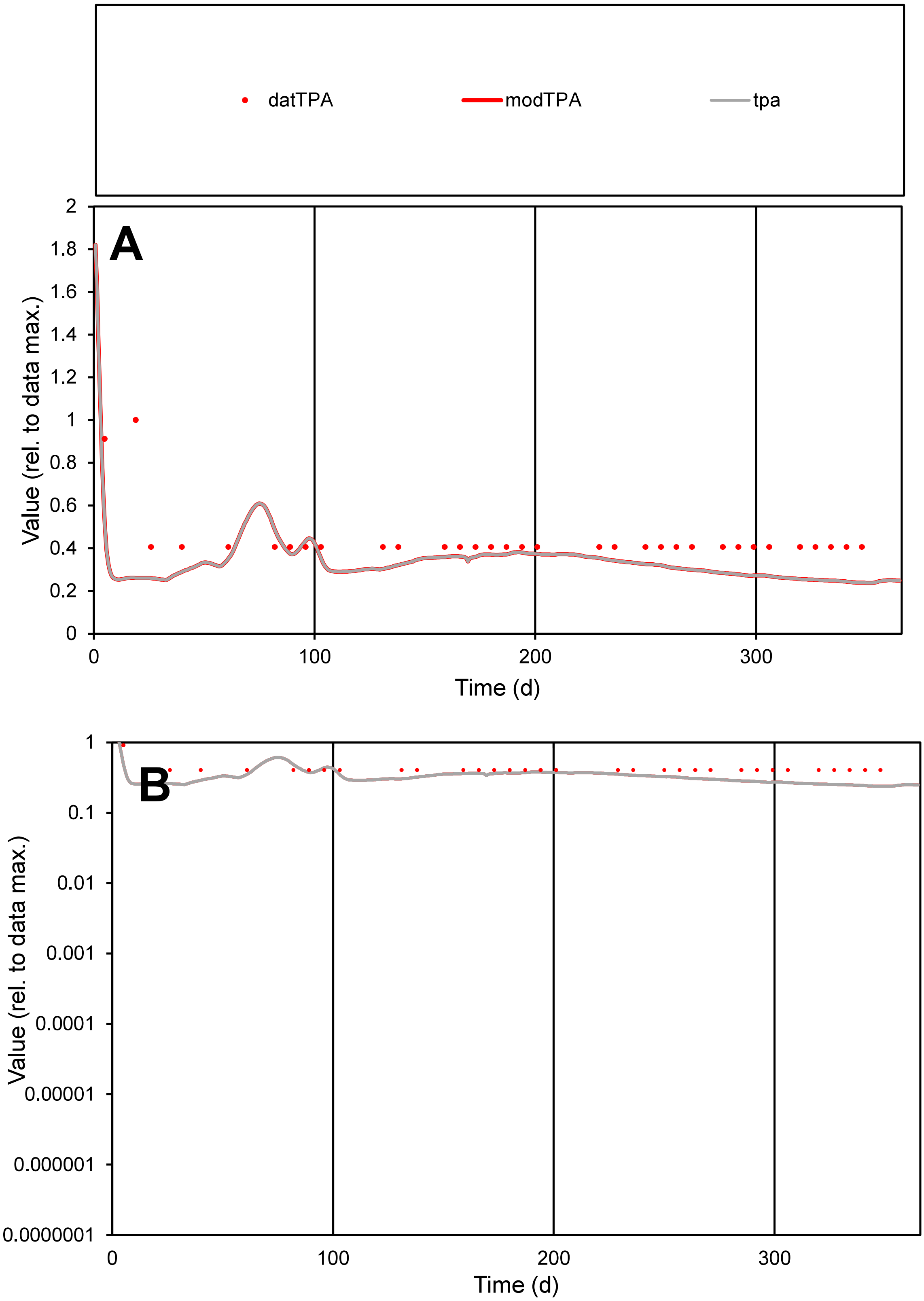

C1

Variables
TPA Provide jtd or Dst
CalibOnly?
2 1 to reproduce of
oNormalize?
2 1=no, 2=yes

ofxn calc*
0.38302
ofxn mod
0.41549

sRun
RunA1
zout
1

Ave
Data
5.1E-06
Model
3.9E-06

data/vMax
1.2E-05

ofxng
263.459

Min d/m
1.69928

ave/min d
1.08231
ave/min m
1.38531

326_Calib_S_A

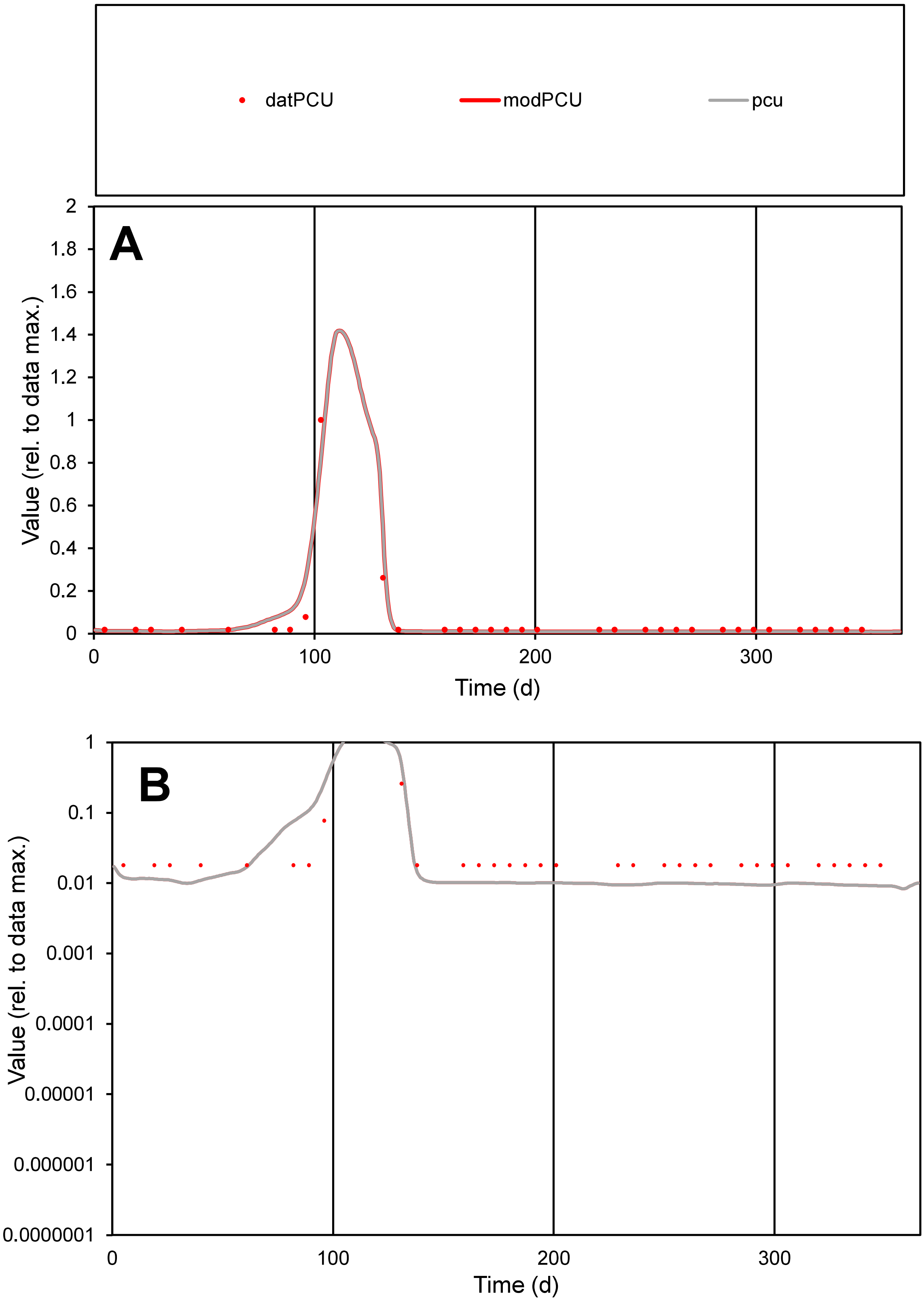

C1

Variables

PCU Provide jtd or Dst

CalibOnly?

2 1 to reproduce of

oNormalize?

2 1=no, 2=yes

ofxn calc*

0.61002

ofxn mod

0.69786

sRun

RunA1

zout

1

Ave

Data

2E-05

Model

2E-05

data/vMax

0.00034

ofxng

263.459

Min d/m

1.99036

ave/min d

3.15109

ave/min m

6.28007

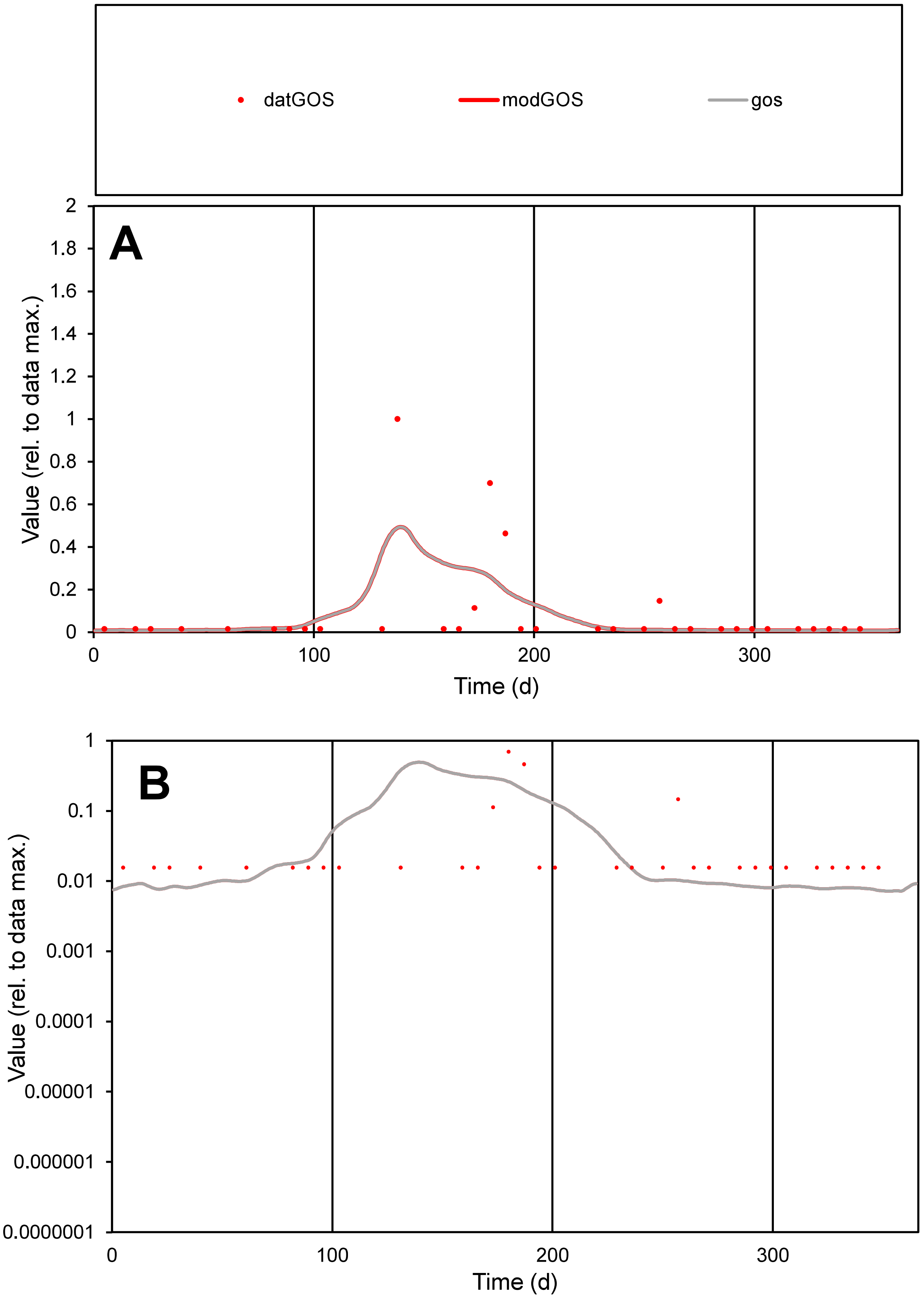

C1

Variables
GOS Provide jtd or Dst
CalibOnly?
2 1 to reproduce of
oNormalize?
2 1=no, 2=yes

ofxn calc*
1.90686
ofxn mod
1.9358

sRun
RunA1
zout
1

Ave
Data
1E-05
Model
9.8E-06

data/vMax
0.00012

ofxng
263.459

Min d/m
2.13252

ave/min d
5.54696
ave/min m
11.5738

326_Calib_S_A

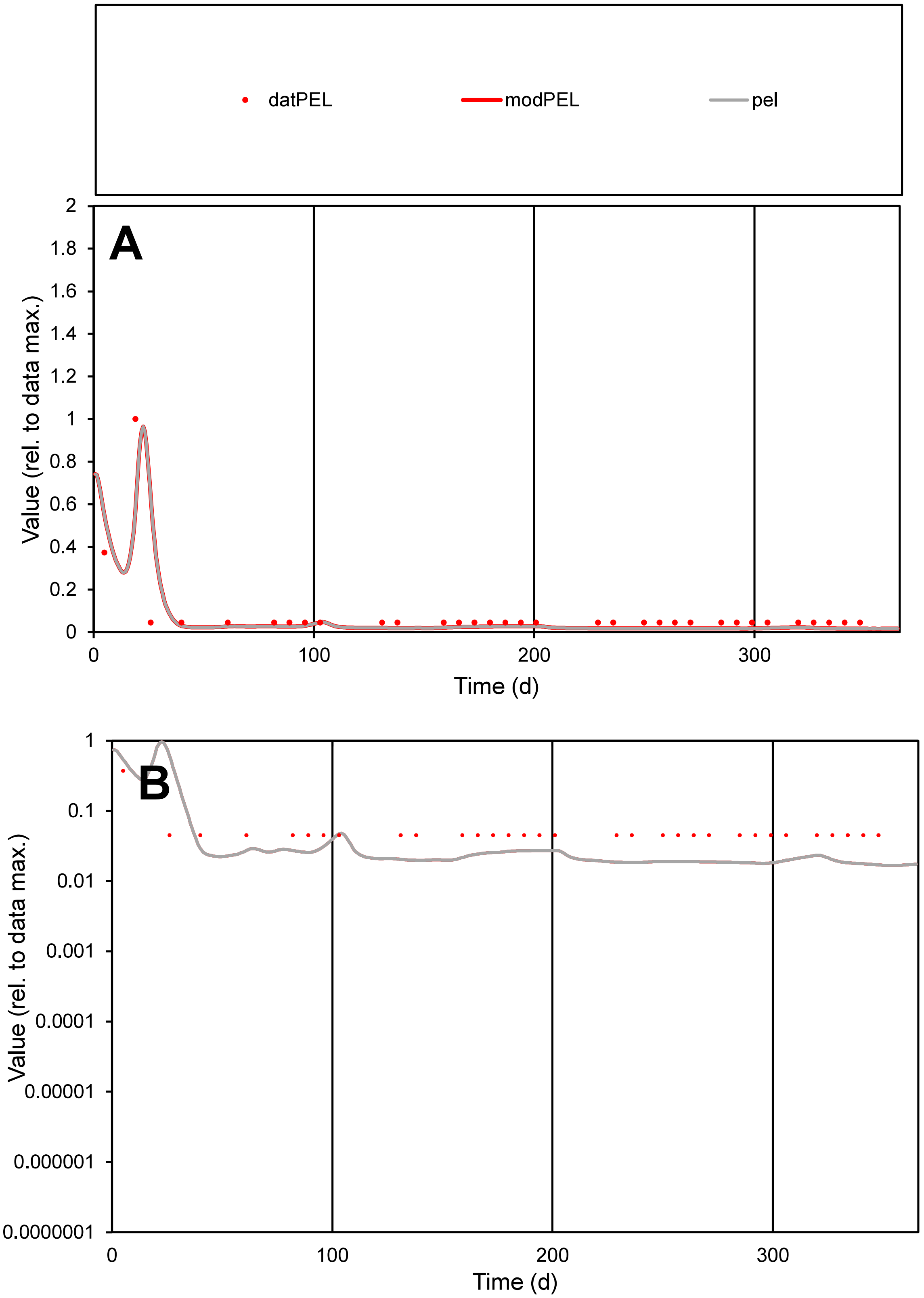

C1

Variables
PEL Provide jtd or Dst
CalibOnly?
2 1 to reproduce of
oNormalize?
2 1=no, 2=yes

ofxn calc*
0.76175
ofxn mod
0.92143

sRun
RunA1
zout
1

Ave
Data
6.1E-06
Model
5E-06

data/vMax
7.3E-05

ofxng
263.459

Min d/m
2.64677

ave/min d
1.8651
ave/min m
4.01618

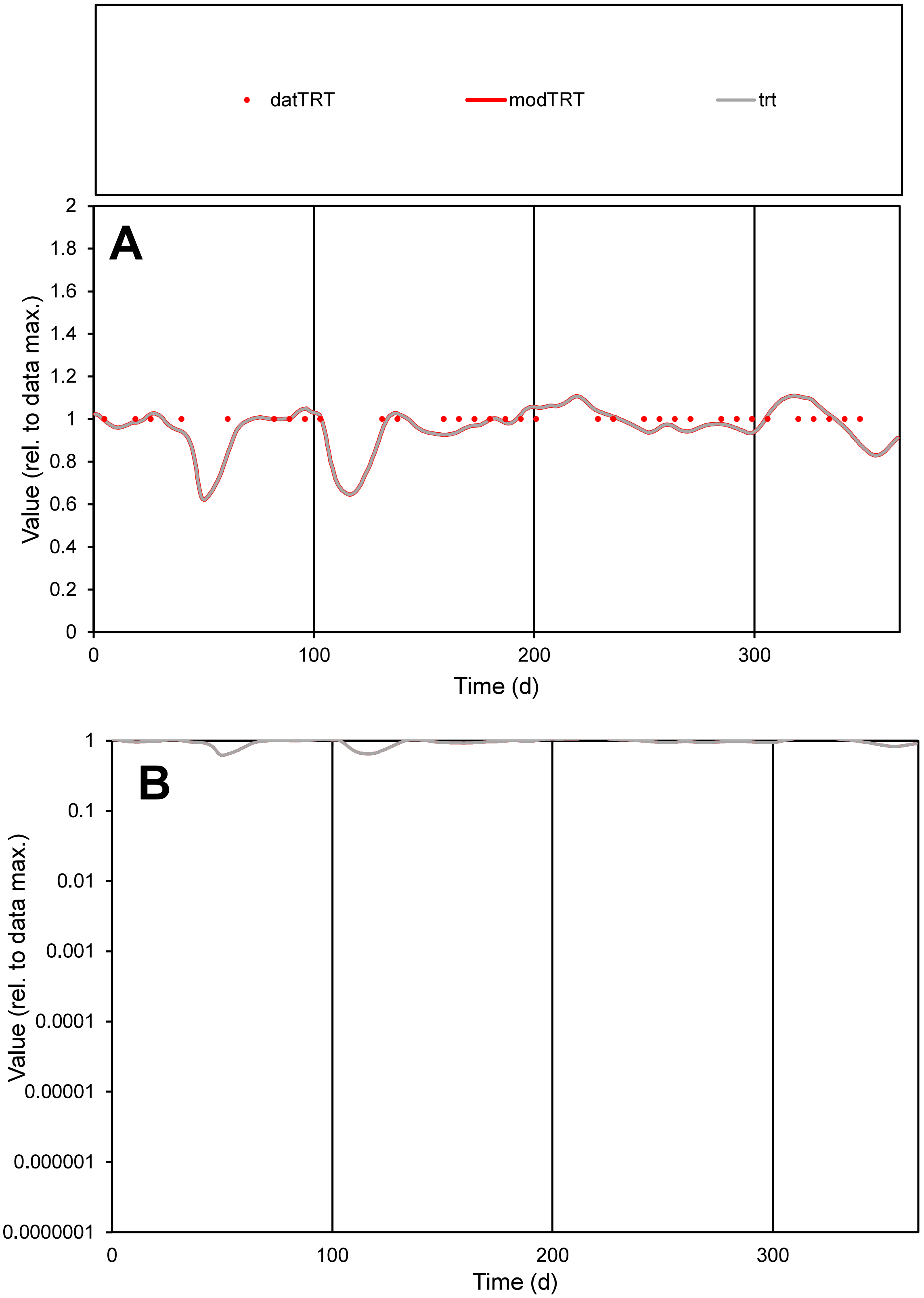

C1

Variables

TRT Provide jtd or Dst

CalibOnly?

2 1 to reproduce of

oNormalize?

2 1=no, 2=yes

ofxn calc*

0.05173

ofxn mod

0.12367

sRun

RunA1

zout

1

Ave

Data

2.3E-05

Model

2.3E-05

data/vMax

2.3E-05

ofxng

263.459

Min d/m

1.14817

ave/min d

1

ave/min m

1.13235

326_Calib_S_A

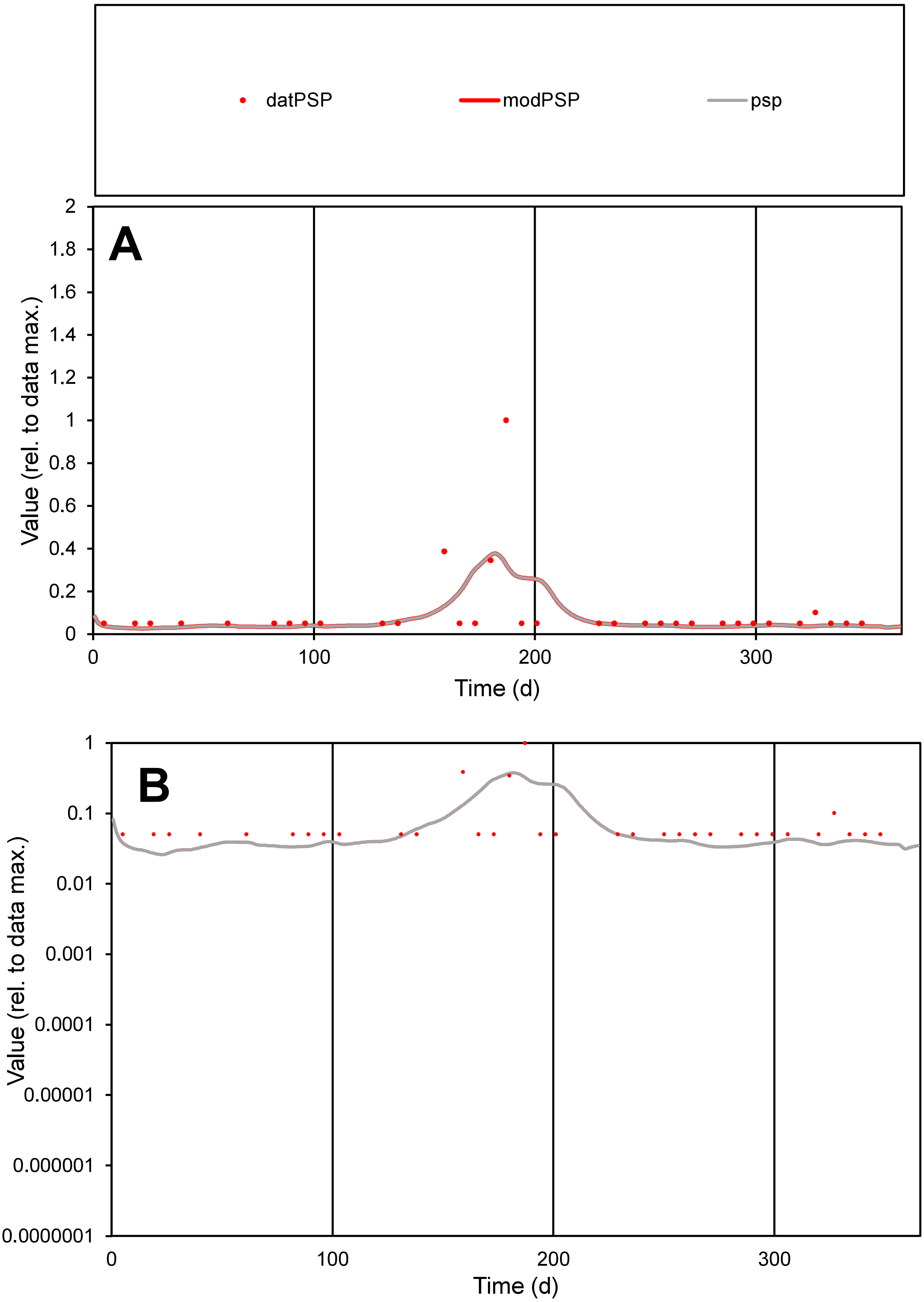

C1

Variables
PSP Provide jtd or Dst
CalibOnly?
2 1 to reproduce of
oNormalize?
2 1=no, 2=yes

ofxn calc*
1.46498
ofxn mod
1.48427

sRun
RunA1
zout
1

Ave
Data
8.5E-06
Model
7.4E-06

data/vMax
8.5E-05

ofxng
263.459

Min d/m
1.88274

ave/min d
1.9759
ave/min m
3.21809

326_Calib_S_A

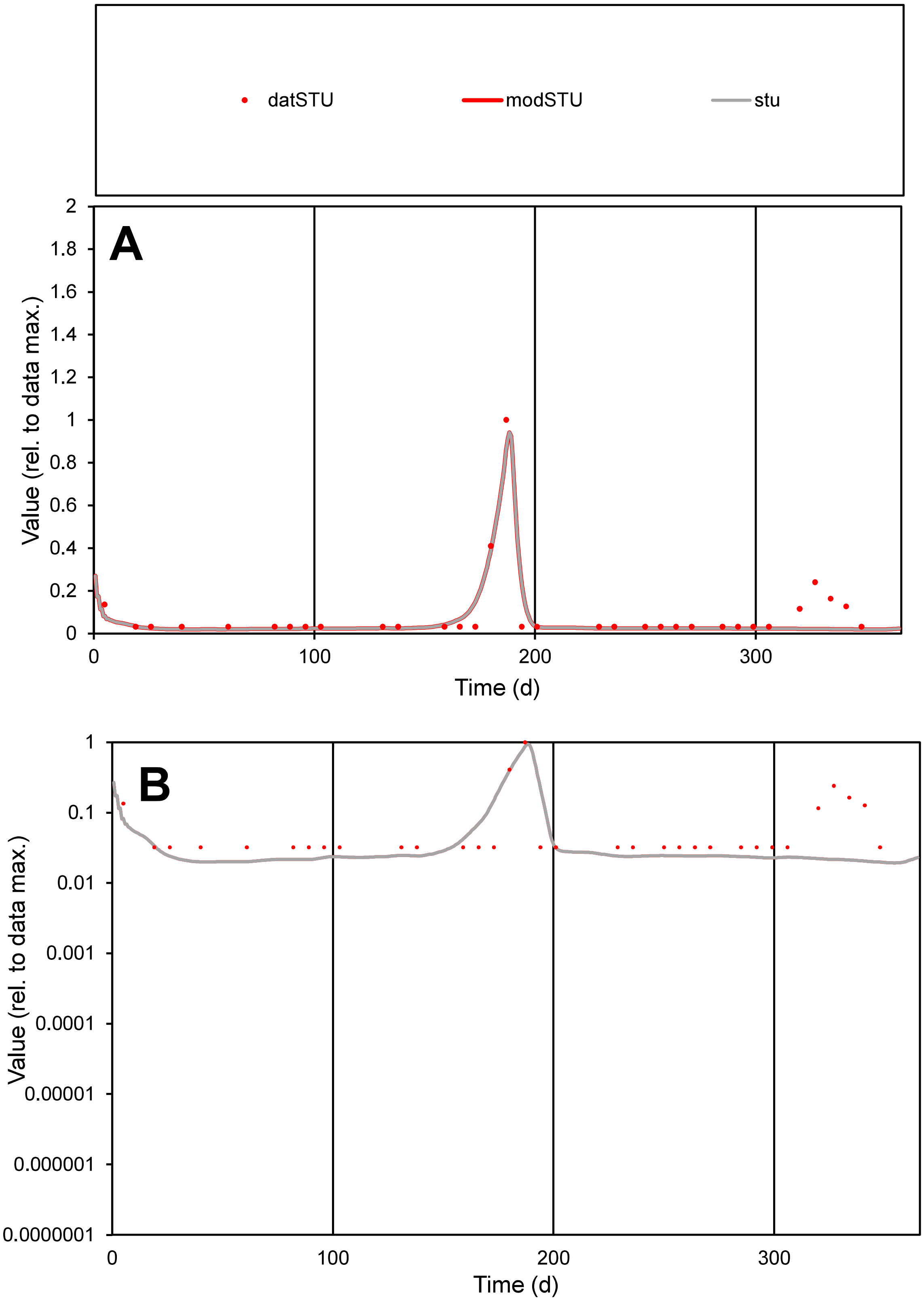

C1

Variables

STU Provide jtd or Dst

CalibOnly?

2 1 to reproduce of

oNormalize?

2 1=no, 2=yes

ofxn calc*

0.64422

ofxn mod

0.70216

sRun

RunA1

zout

1

Ave

Data

7.1E-06

Model

5.7E-06

data/vMax

7.7E-05

ofxng

263.459

Min d/m

1.64121

ave/min d

2.84852

ave/min m

3.76326

326_Calib_S_A

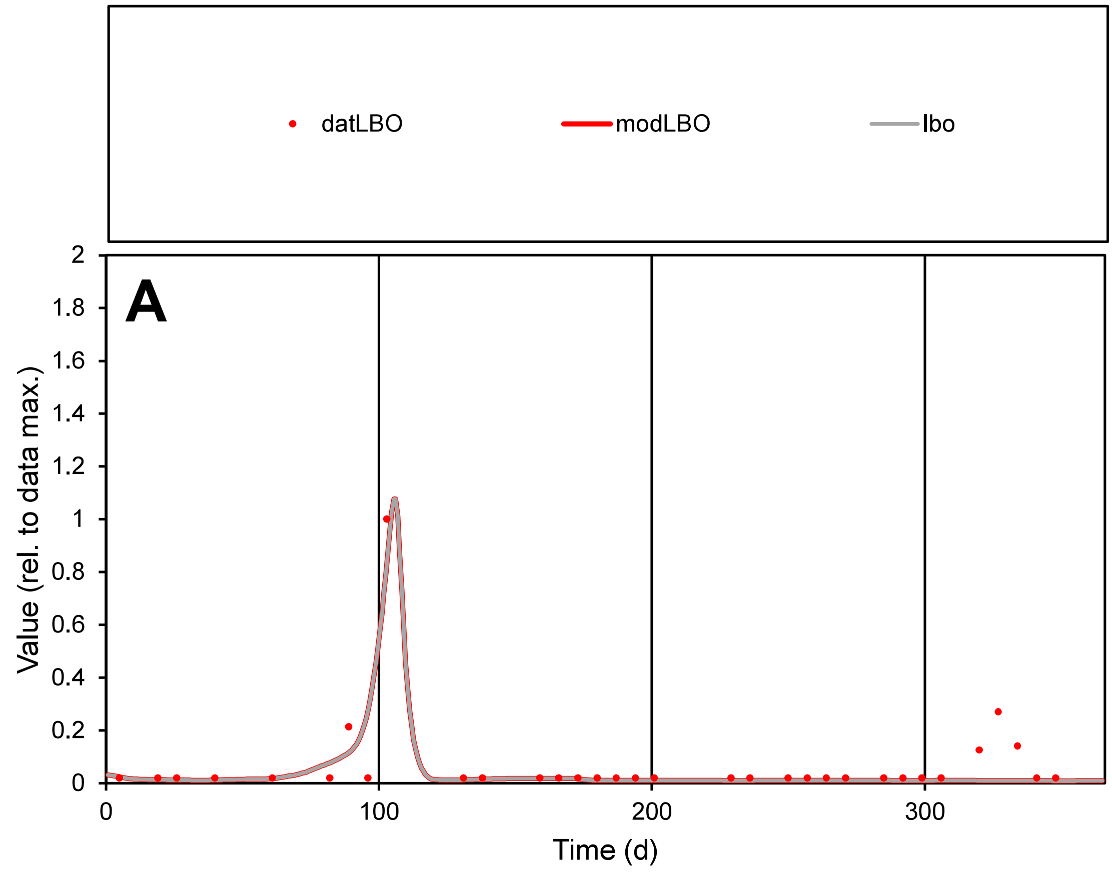

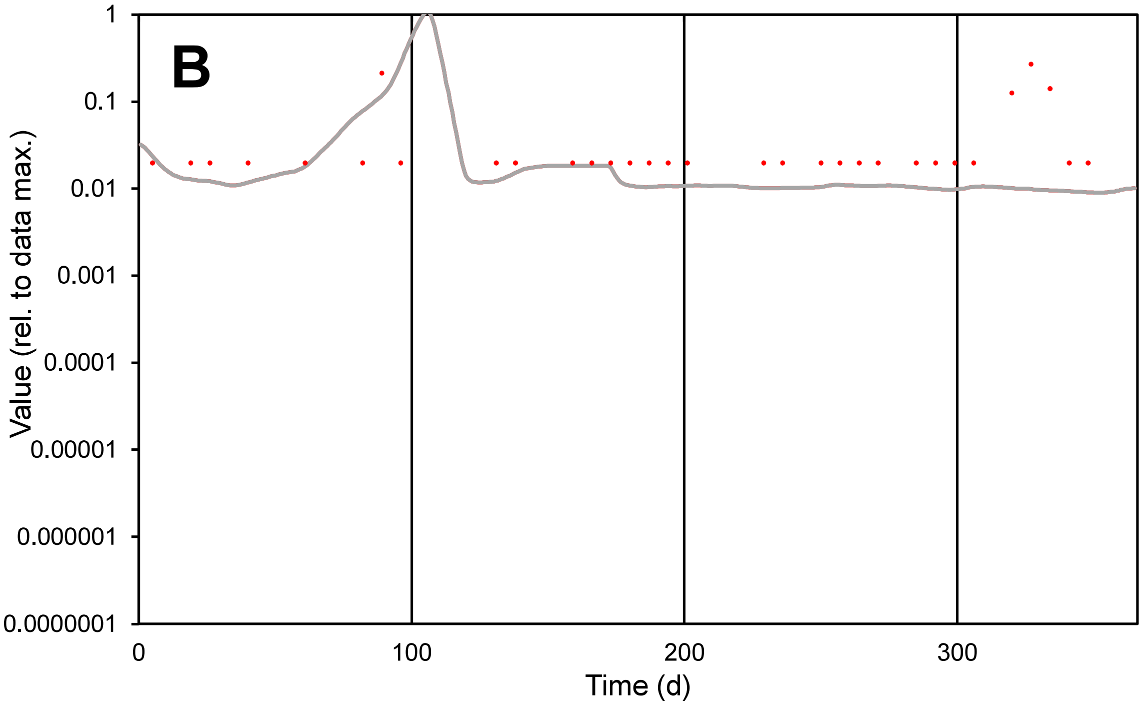

C1

Variables
LBO Provide jtd or Dst
CalibOnly?
2 1 to reproduce of
oNormalize?
2 1=no, 2=yes

ofxn calc*
0.92359
ofxn mod
1.02081

sRun
RunA1
zout
1

Ave
Data
4.9E-06
Model
3.6E-06

data/vMax
7E-05

ofxng
263.459

Min d/m
2.18391

ave/min d
3.53925
ave/min m
5.67503

326_Calib_S_A

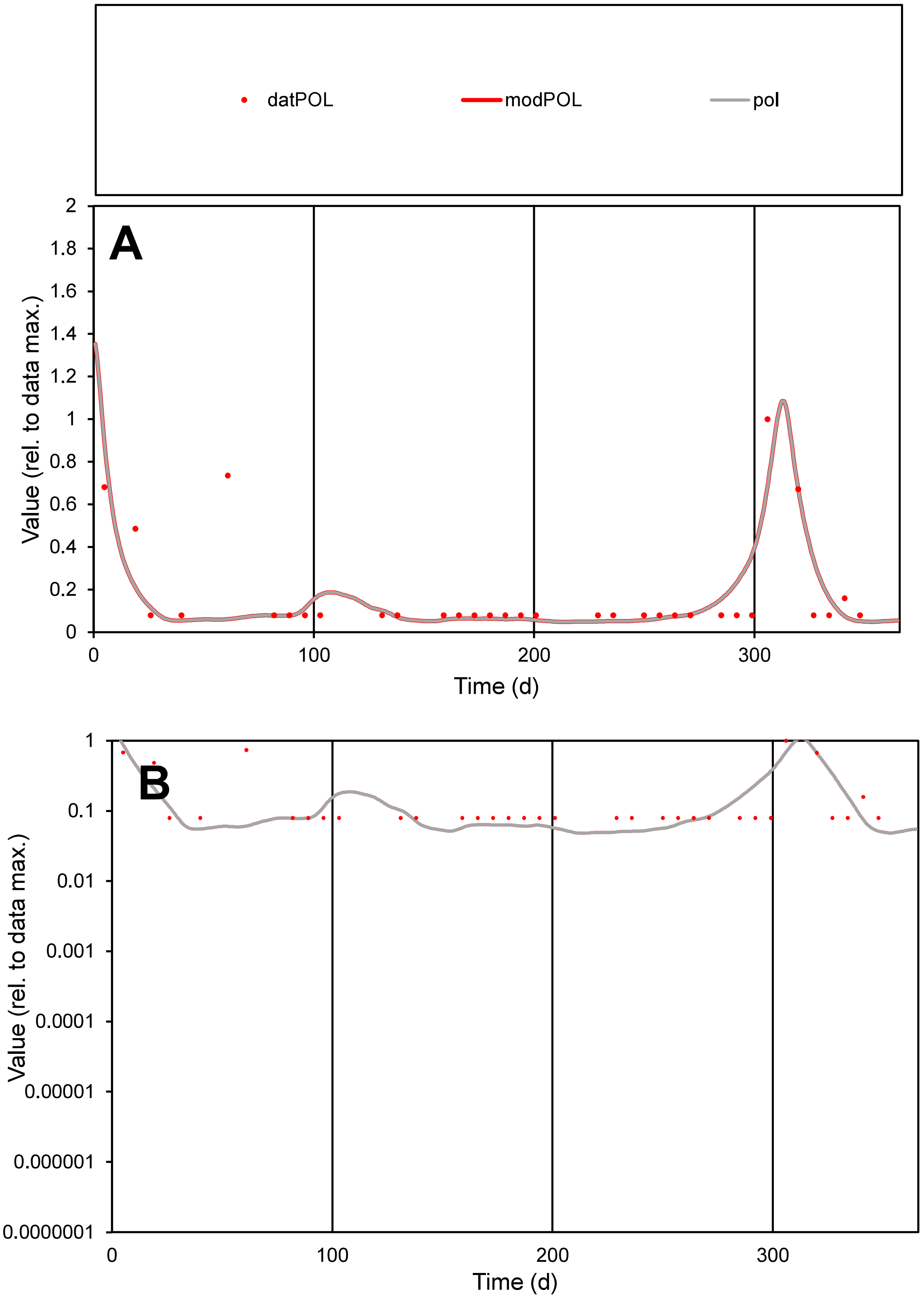

C1

Variables
POL Provide jtd or Dst
CalibOnly?
2 1 to reproduce of
oNormalize?
2 1=no, 2=yes

ofxn calc*
0.84626
ofxn mod
0.87768

sRun
RunA1
zout
1

Ave
Data
3.6E-06
Model
3.2E-06

data/vMax
2E-05

ofxng
263.459

Min d/m
1.57087

ave/min d
2.24358
ave/min m
3.13991

326_Calib_S_A

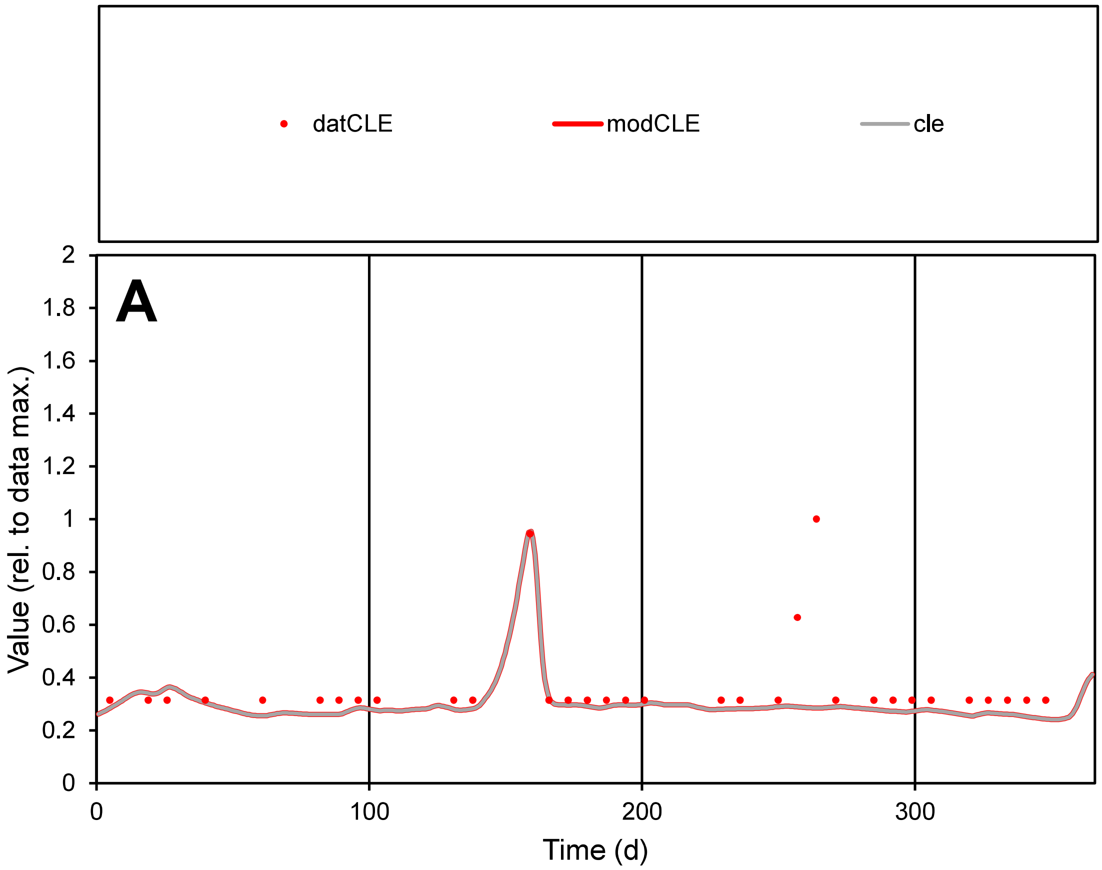

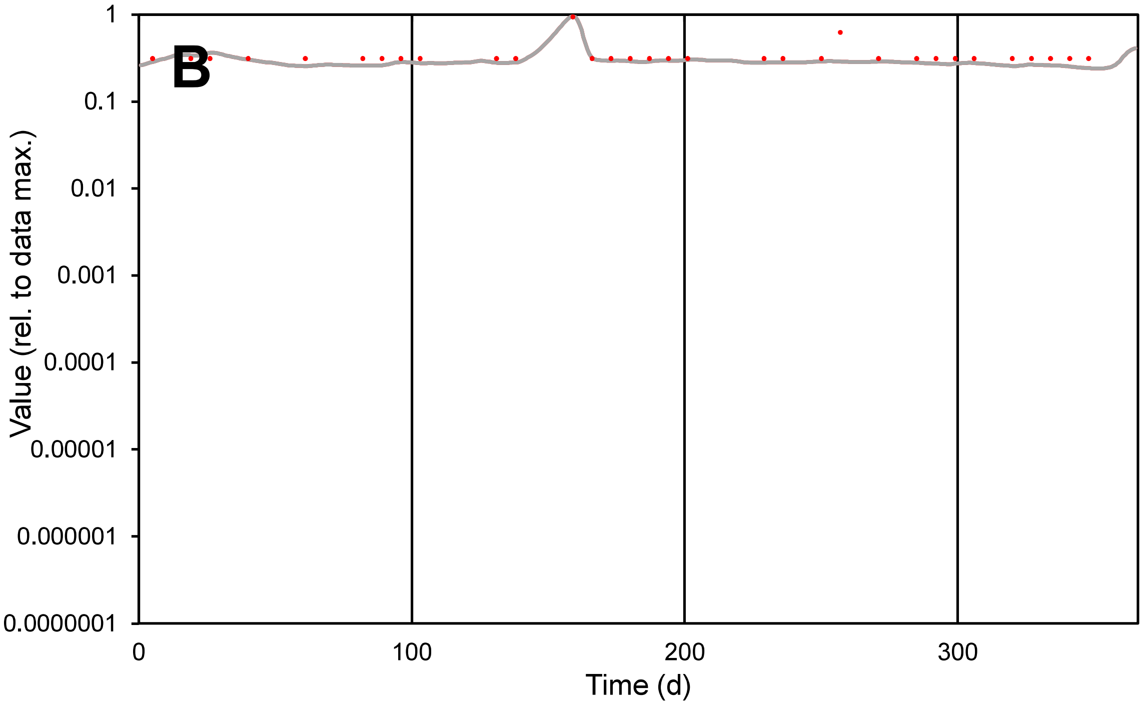

C1

Variables	
CLE	Provide jtd or Dst
CalibOnly?	
2	1 to reproduce of
oNormalize?	
2	1=no, 2=yes
ofxn calc*	
0.39277	
ofxn mod	
0.40668	
sRun	
RunA1	
zout	
1	
Ave	
Data	
5.4E-06	
Model	
4.5E-06	
data/vMax	
1.5E-05	
ofxng	
263.459	
Min d/m	
1.29473	
ave/min d	
1.15754	
ave/min m	
1.25131	

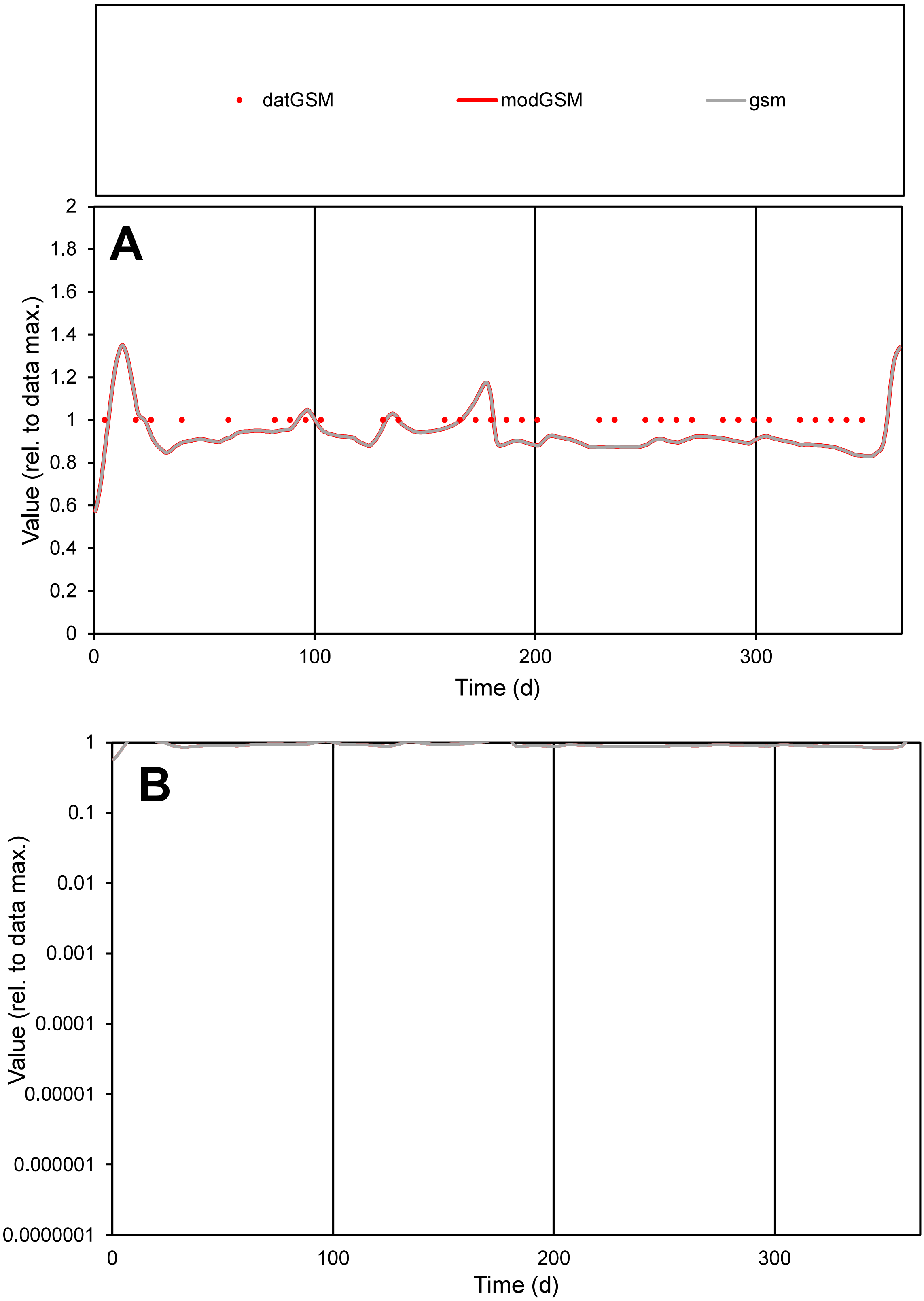

C1

Variables
GSM Provide jtd or Dst
CalibOnly?
2 1 to reproduce of
oNormalize?
2 1=no, 2=yes

ofxn calc*
0.09299
ofxn mod
0.14346

sRun
RunA1
zout
1

Ave
Data
1.6E-06
Model
1.5E-06

data/vMax
1.6E-06

ofxng
263.459

Min d/m
1.20167

ave/min d
1
ave/min m
1.11911

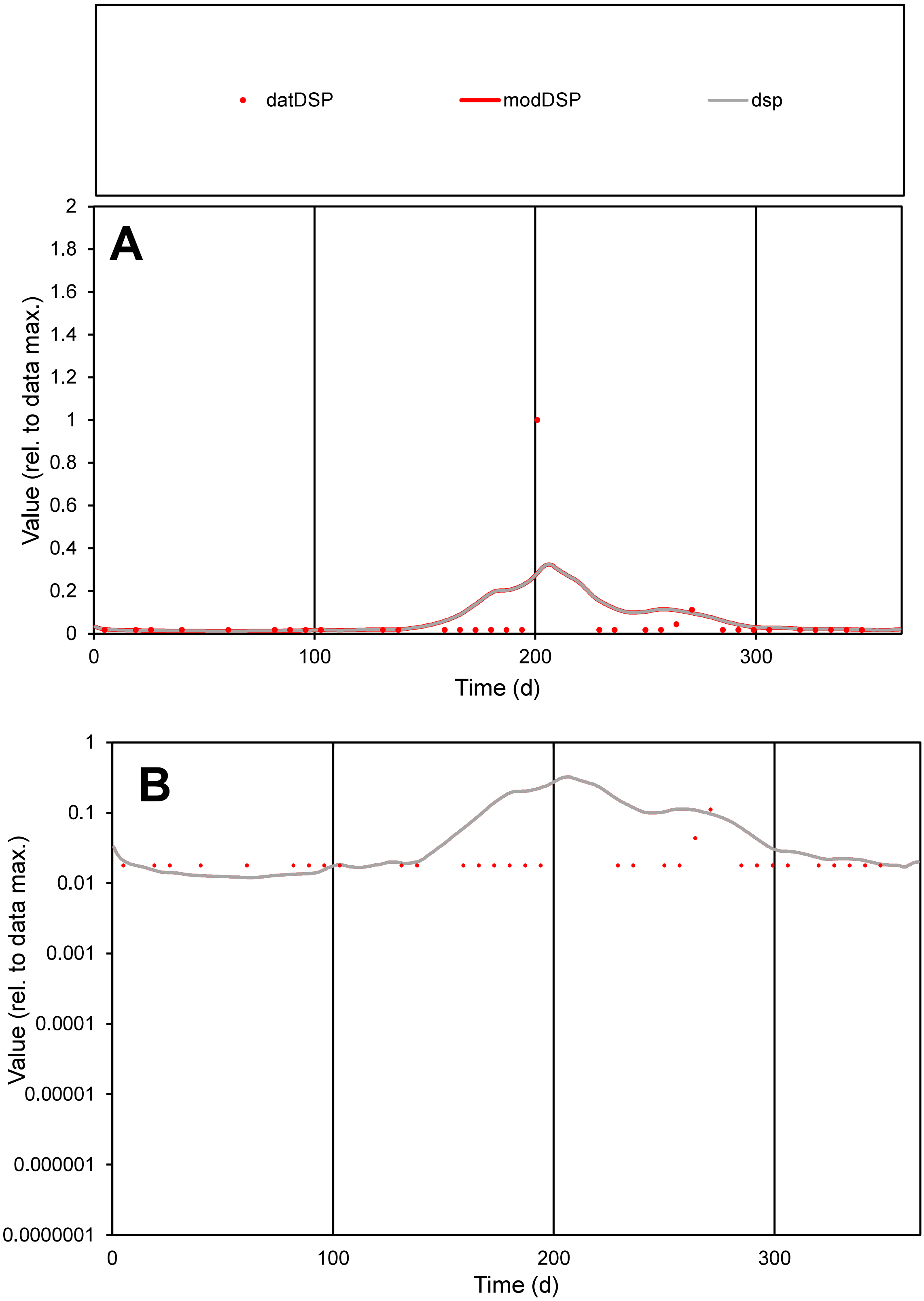

C1

Variables
DSP Provide jtd or Dst
CalibOnly?
2 1 to reproduce of
oNormalize?
2 1=no, 2=yes

ofxn calc*
2.77783
ofxn mod
2.79751

sRun
RunA1
zout
1

Ave
Data
1.4E-05
Model
2E-05

data/vMax
0.00028

ofxng
263.459

Min d/m
1.48384

ave/min d
2.86713
ave/min m
5.80938

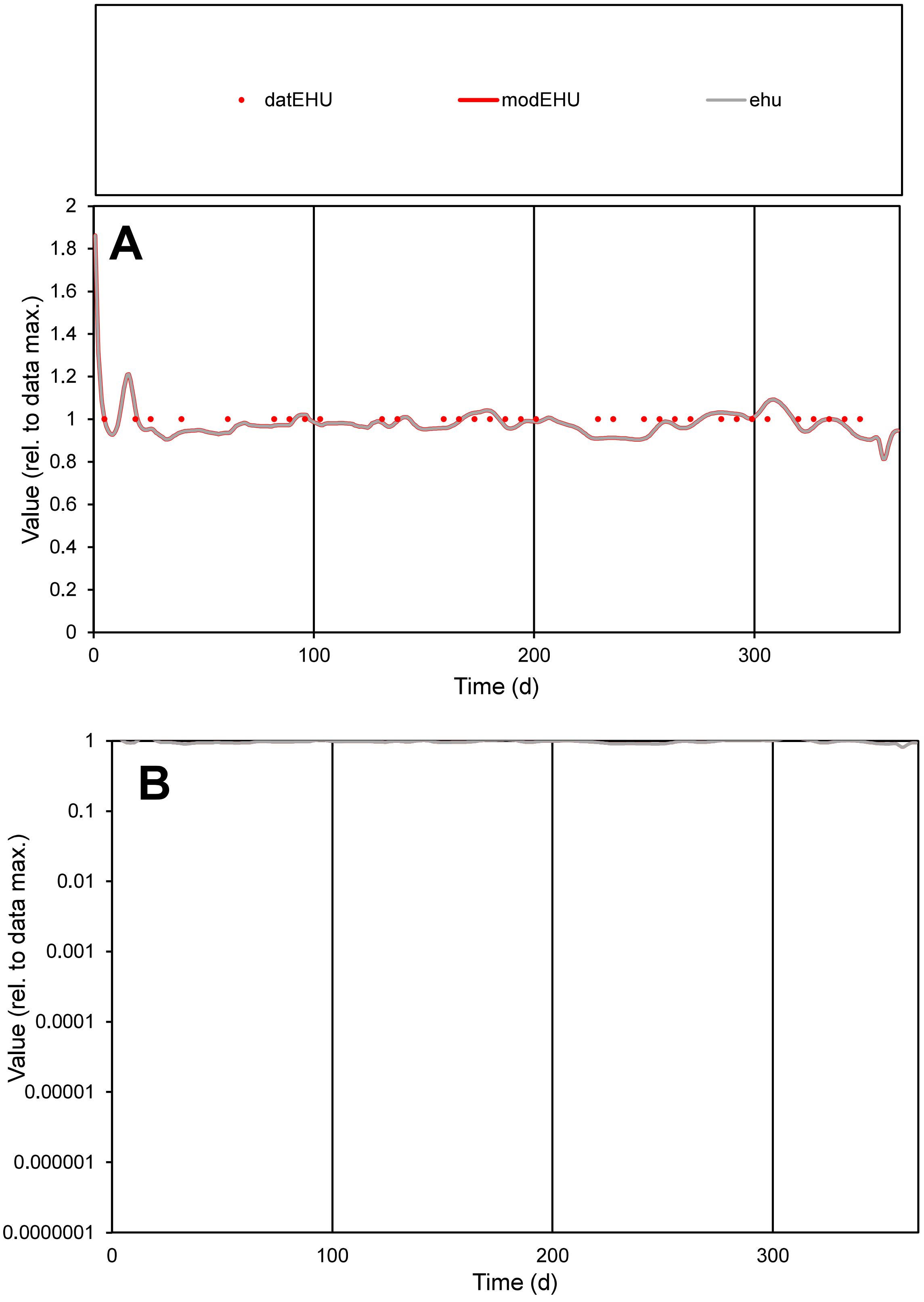

C1

Variables
EHU Provide jtd or Dst
CalibOnly?
2 1 to reproduce of
oNormalize?
2 1=no, 2=yes

ofxn calc*
0.04547
ofxn mod
0.12098

sRun
RunA1
zout
1

Ave
Data
5.8E-06
Model
5.6E-06

data/vMax
5.8E-06

ofxng
263.459

Min d/m
1.09912

ave/min d
1
ave/min m
1.07476

326_Calib_S_A

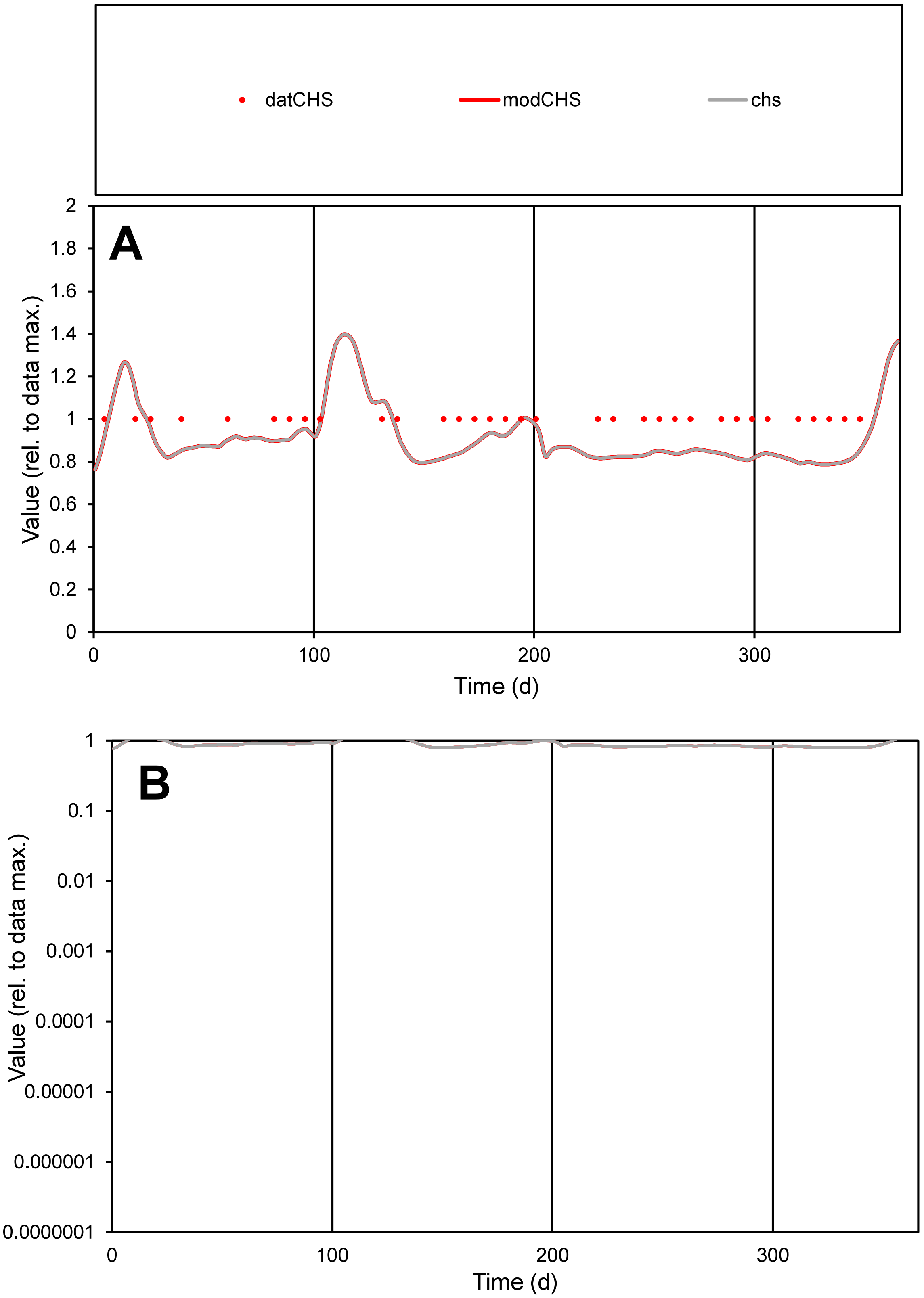

C1

Variables
CHS Provide jtd or Dst
CalibOnly?
2 1 to reproduce of
oNormalize?
2 1=no, 2=yes

ofxn calc*
0.13822
ofxn mod
0.17503

sRun
RunA1
zout
1

Ave
Data
2.1E-05
Model
1.9E-05

data/vMax
2.1E-05

ofxng
263.459

Min d/m
1.26493

ave/min d
1
ave/min m
1.12152

326_Calib_S_A

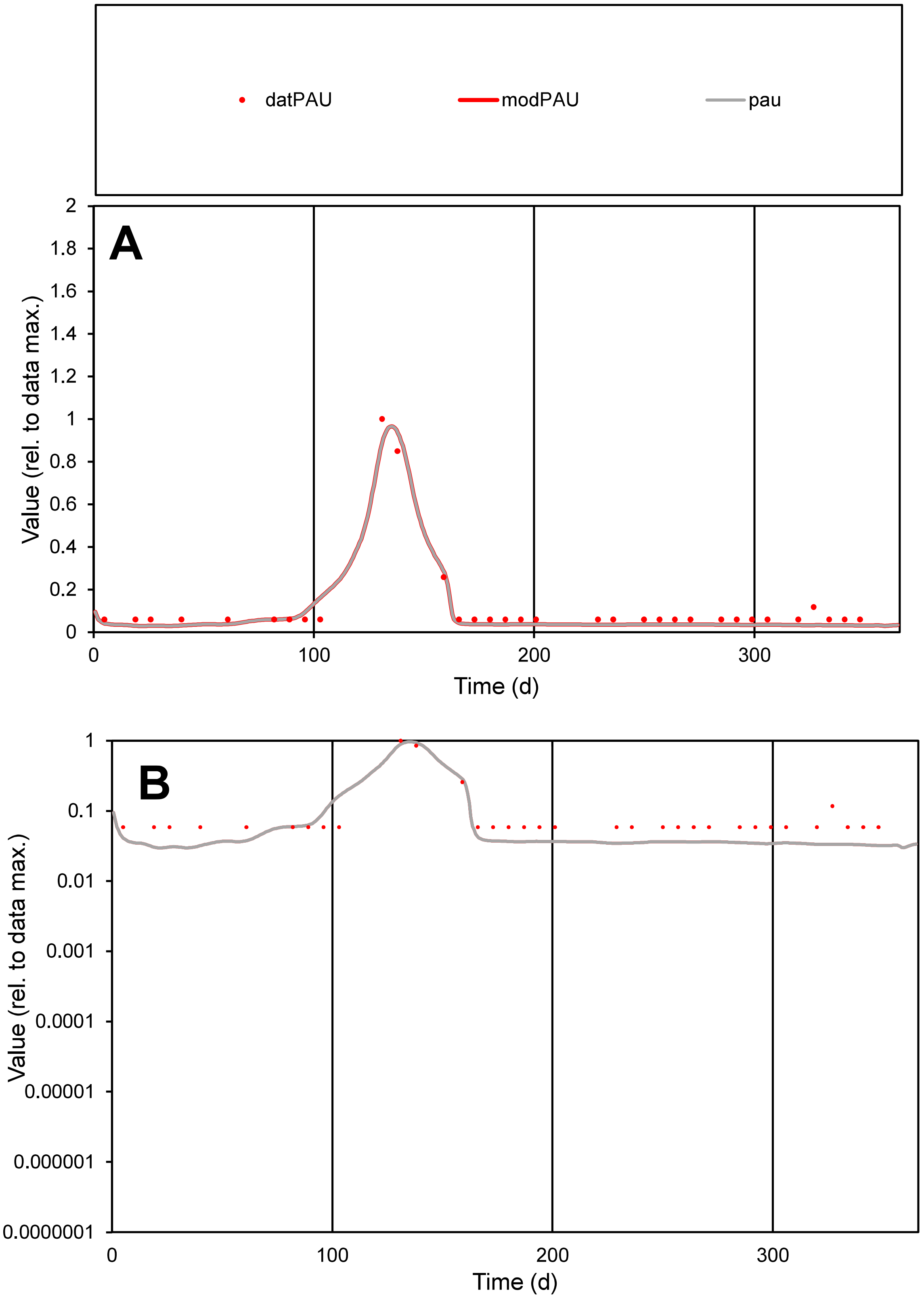

C1

Variables
PAU Provide jtd or Dst
CalibOnly?
2 1 to reproduce of
oNormalize?
2 1=no, 2=yes

ofxn calc*
0.29484
ofxn mod
0.38672

sRun
RunA1
zout
1

Ave
Data
8E-06
Model
6.9E-06

data/vMax
6.7E-05

ofxng
263.459

Min d/m
1.95198

ave/min d
2.02533
ave/min m
3.40575

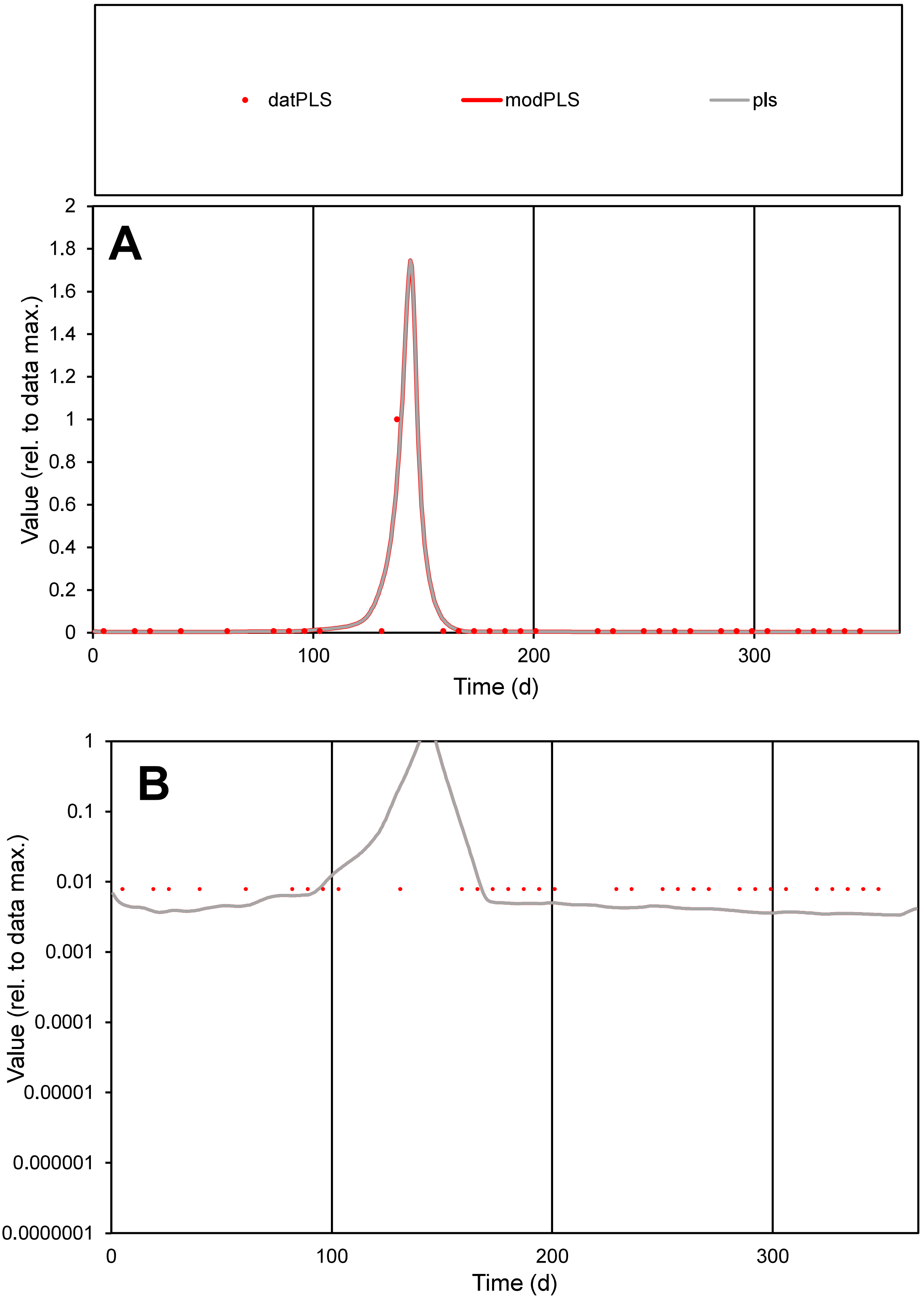

C1

Variables
PLS Provide jtd or Dst
CalibOnly?
2 1 to reproduce of
oNormalize?
2 1=no, 2=yes

ofxn calc*
0.65433
ofxn mod
1.08523

sRun
RunA1
zout
1

Ave
Data
1.9E-05
Model
1.9E-05

data/vMax
0.00049

ofxng
263.459

Min d/m
2.30829

ave/min d
4.81813
ave/min m
11.4589

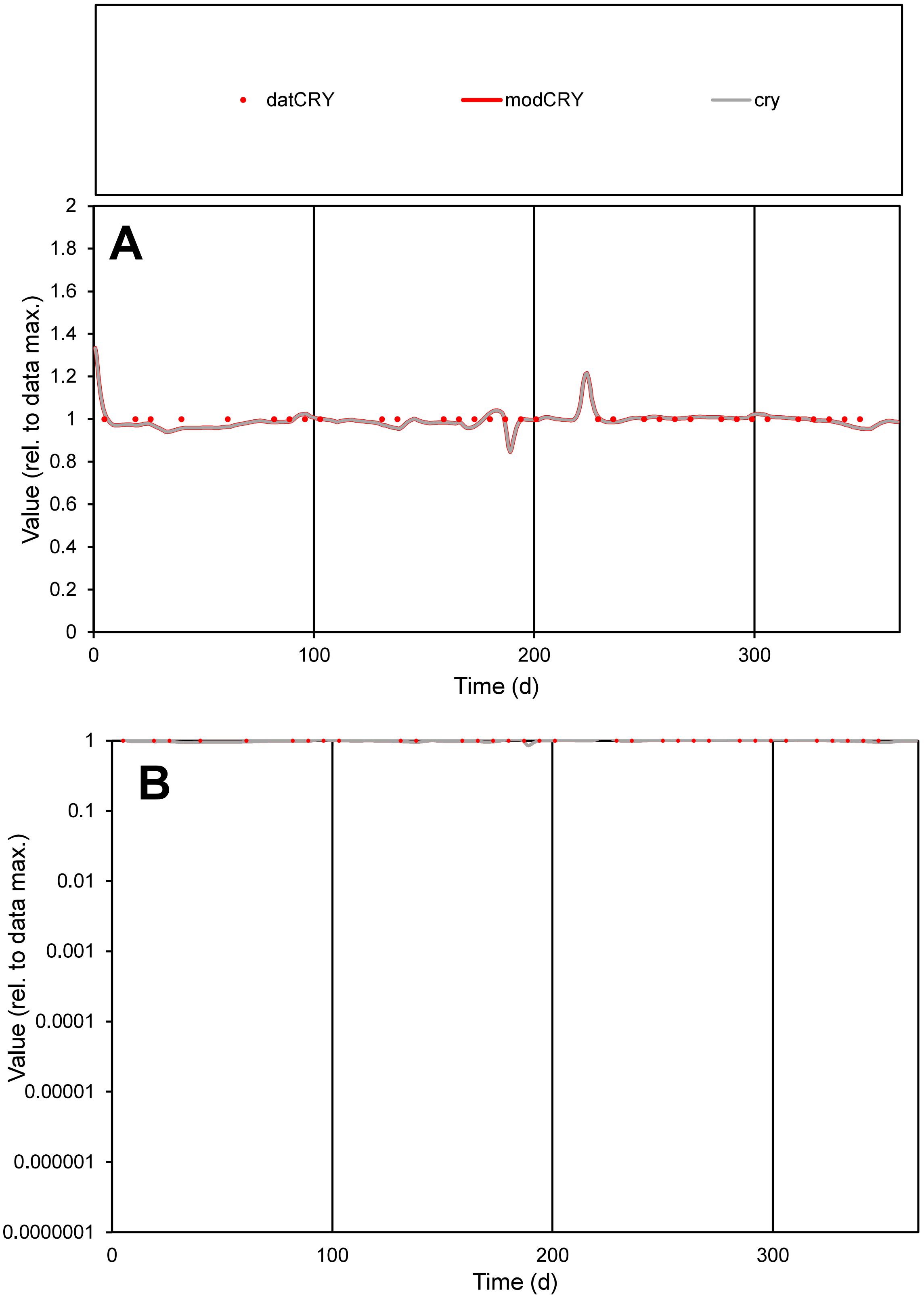

C1

Variables
CRY Provide jtd or Dst
CalibOnly?
2 1 to reproduce of
oNormalize?
2 1=no, 2=yes

ofxn calc*
0.02471
ofxn mod
0.11627

sRun
RunA1
zout
1

Ave
Data
3E-06
Model
3E-06

data/vMax
3E-06

ofxng
263.459

Min d/m
1.08032

ave/min d
1
ave/min m
1.06886

326_Calib_S_A

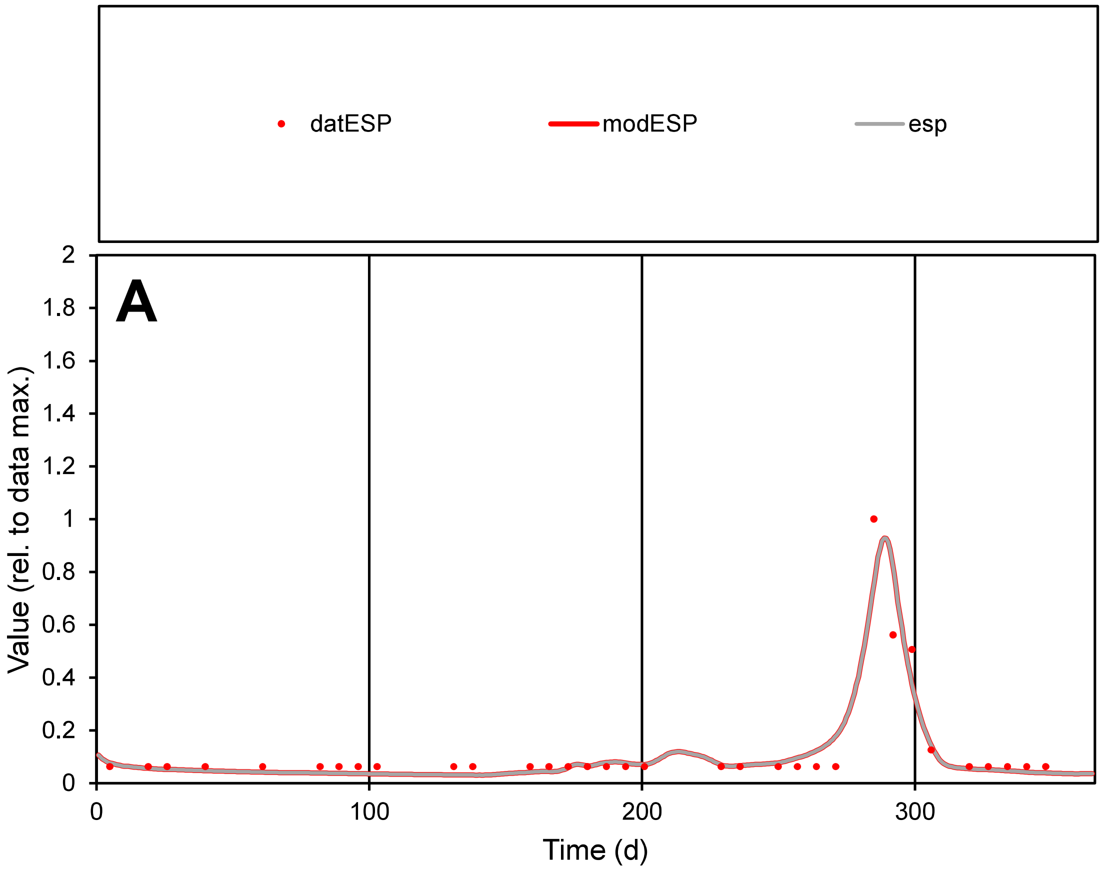

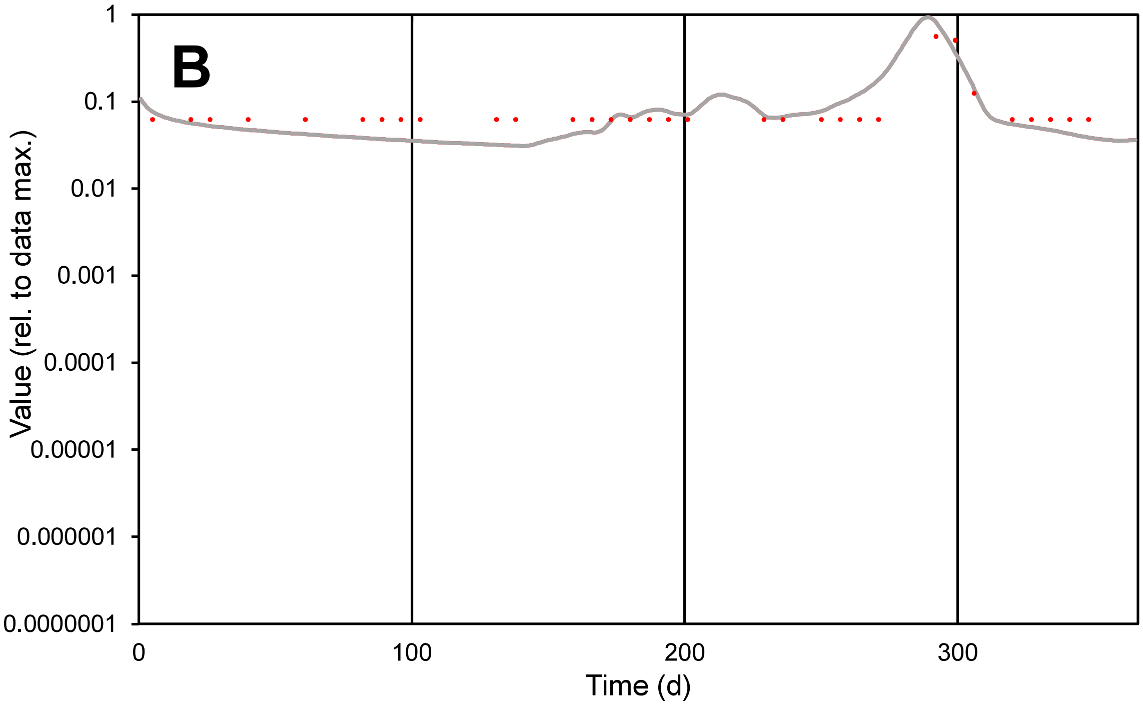

C1

Variables
ESP Provide jtd or Dst
CalibOnly?
2 1 to reproduce of
oNormalize?
2 1=no, 2=yes

ofxn calc*
0.4705
ofxn mod
0.51499

sRun
RunA1
zout
1

Ave
Data
1.6E-05
Model
1.5E-05

data/vMax
0.00013

ofxng
263.459

Min d/m
2.00853

ave/min d
1.94223
ave/min m
3.72635

326_Calib_S_A

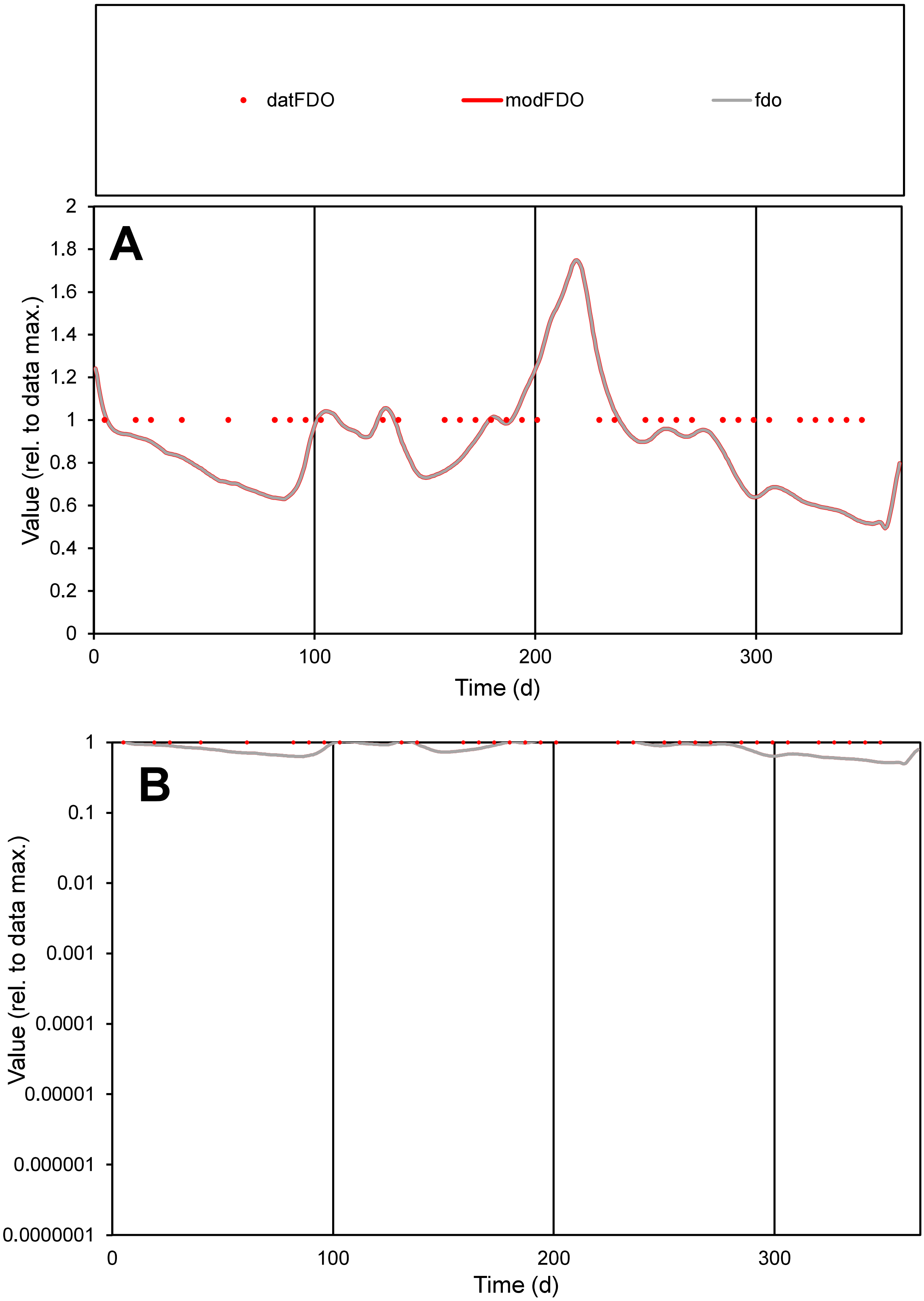

C1

Variables

FDO Provide jtd or Dst

CalibOnly?

2 1 to reproduce of

oNormalize?

2 1=no, 2=yes

ofxn calc*

0.24201

ofxn mod

0.26446

sRun

RunA1

zout

1

Ave

Data

1E-07

Model

8.5E-08

data/vMax

1E-07

ofxng

263.459

Min d/m

1.92188

ave/min d

1

ave/min m

1.63881

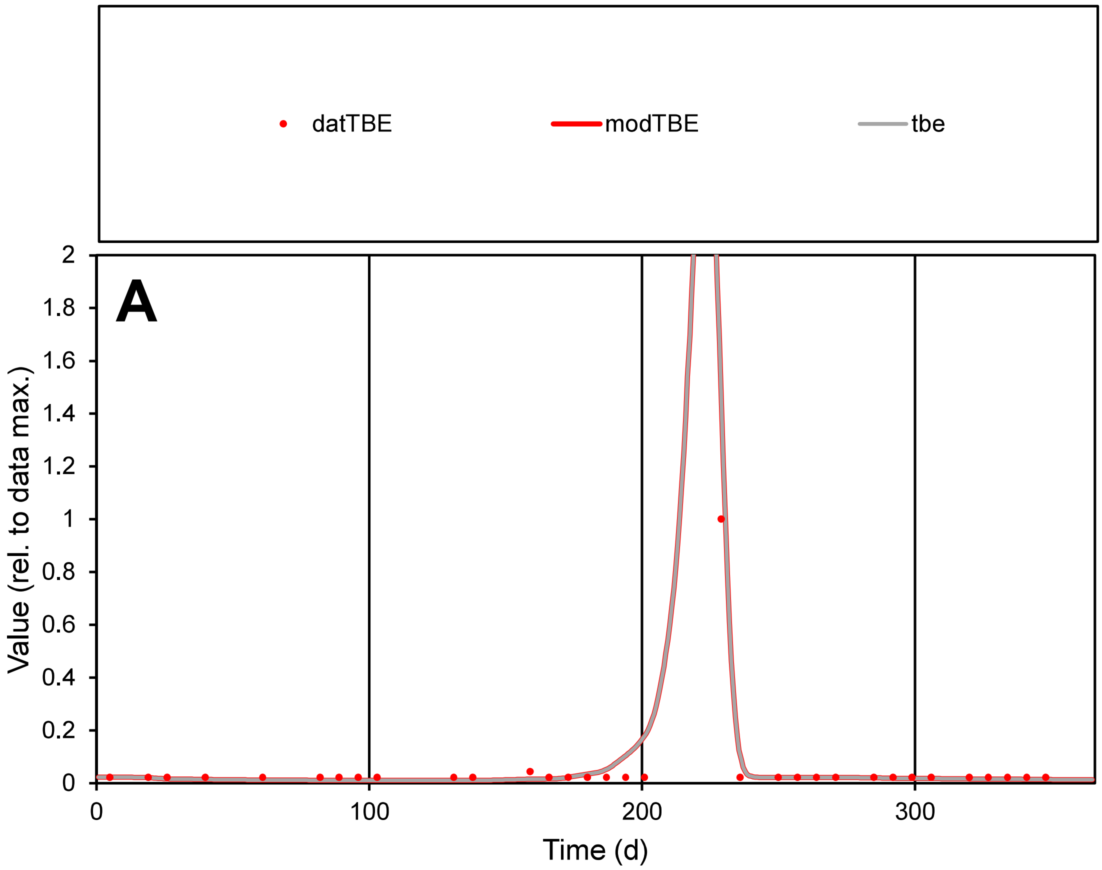

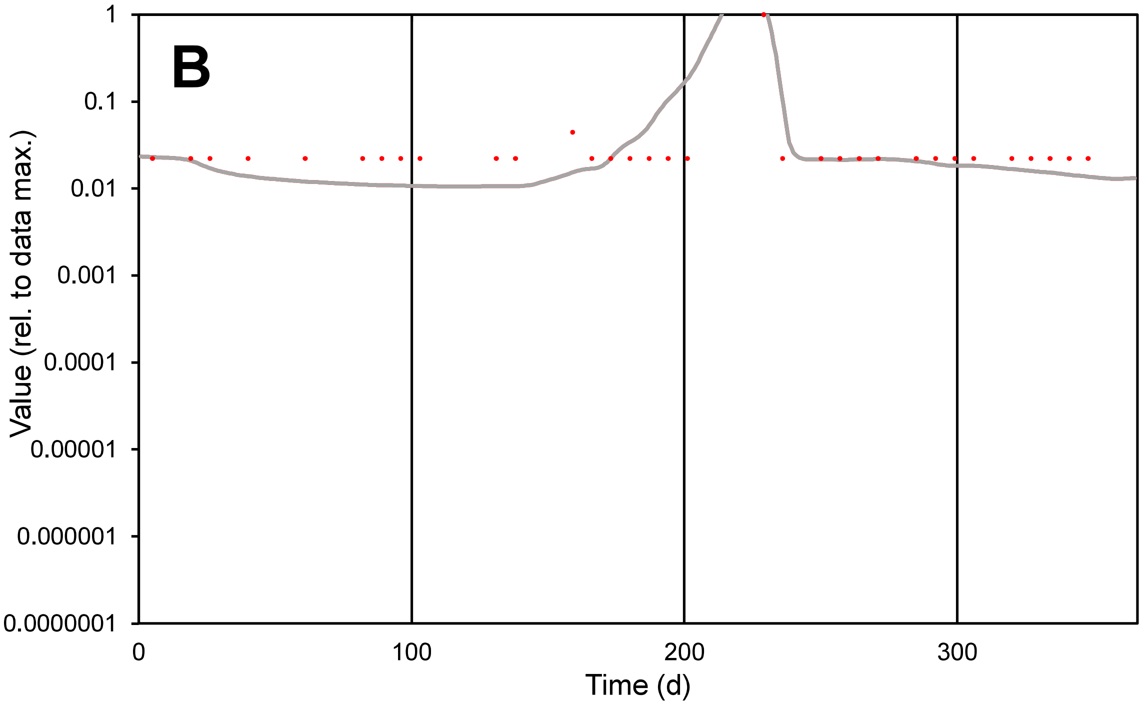

C1

Variables	
TBE	Provide jtd or Dst
CalibOnly?	
2	1 to reproduce of
oNormalize?	
2	1=no, 2=yes
ofxn calc*	
0.548	
ofxn mod	
0.79958	
sRun	
RunA1	
zout	
1	
Ave	
Data	
1.6E-05	
Model	
1.7E-05	
data/vMax	
0.0003	
ofxng	
263.459	
Min d/m	
2.08333	
ave/min d	
2.36874	
ave/min m	
5.17036	

326_Calib_S_A

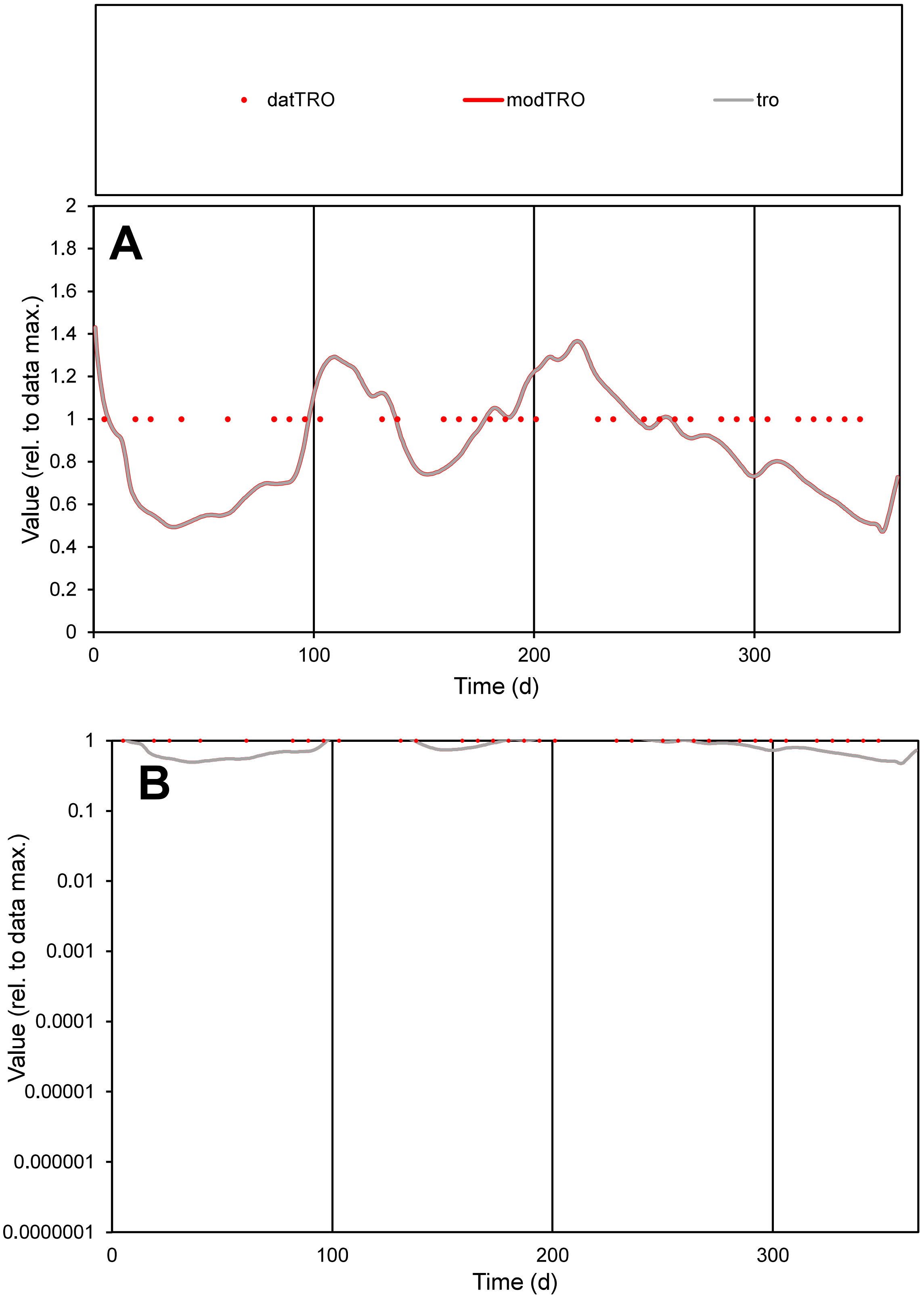

C1

Variables
TRO Provide jtd or Dst
CalibOnly?
2 1 to reproduce of
oNormalize?
2 1=no, 2=yes

ofxn calc*
0.25604
ofxn mod
0.27734

sRun
RunA1
zout
1

Ave
Data
5E-06
Model
4.3E-06

data/vMax
5E-06

ofxng
263.459

Min d/m
1.96637

ave/min d
1
ave/min m
1.68841

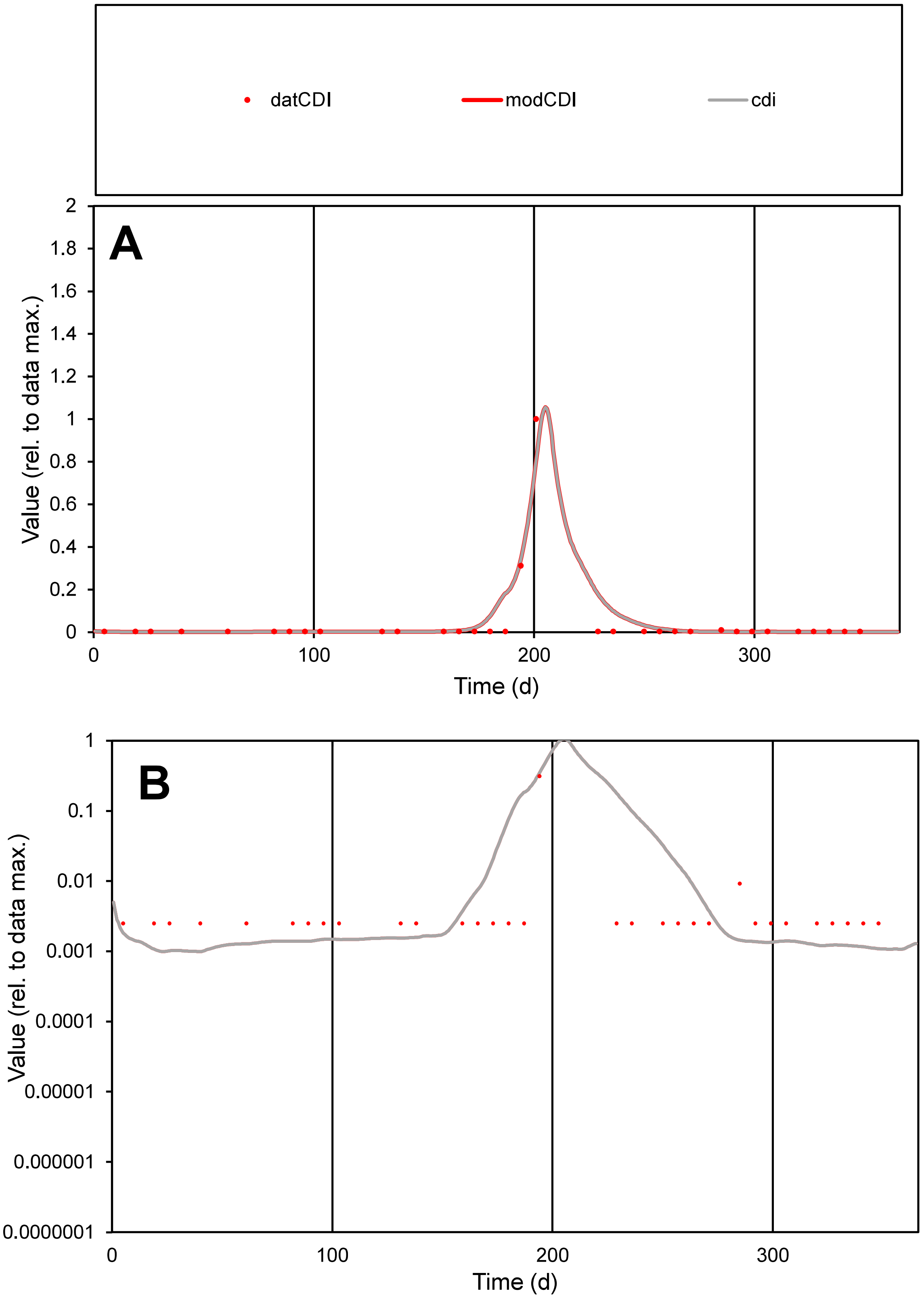

C1

Variables
CDI Provide jtd or Dst
CalibOnly?
2 1 to reproduce of
oNormalize?
2 1=no, 2=yes

ofxn calc*
1.02155
ofxn mod
1.20988

sRun
RunA1
zout
1

Ave
Data
2.9E-06
Model
3.8E-06

data/vMax
6.9E-05

ofxng
263.459

Min d/m
2.46408

ave/min d
16.9549

ave/min m
53.8661

326_Calib_S_A

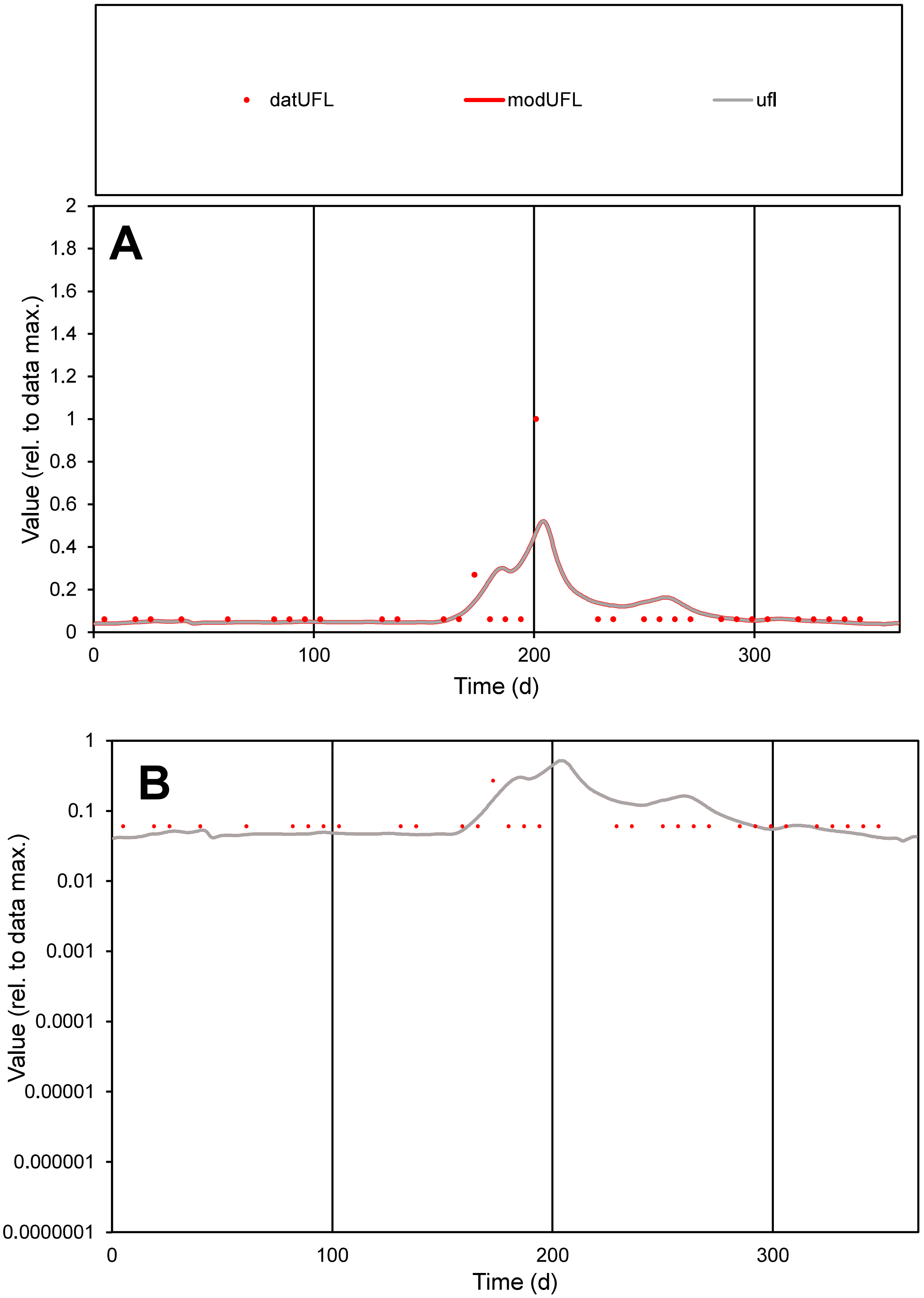

C1

Variables
UFL Provide jtd or Dst
CalibOnly?
2 1 to reproduce of
oNormalize?
2 1=no, 2=yes

ofxn calc*
1.23926
ofxn mod
1.25437

sRun
RunA1
zout
1

Ave
Data
4.8E-06
Model
5.3E-06

data/vMax
5.1E-05

ofxng
263.459

Min d/m
1.45671

ave/min d
1.57524
ave/min m
2.54234

326_Calib_S_A

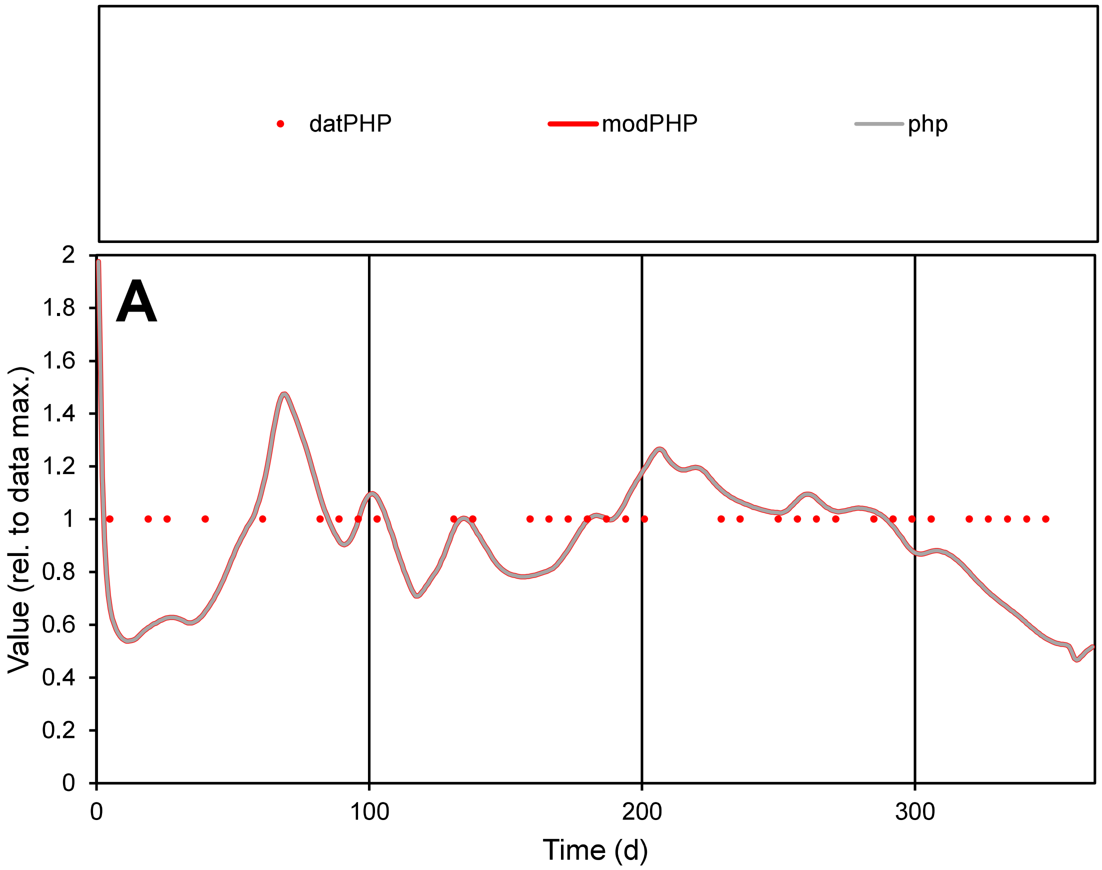

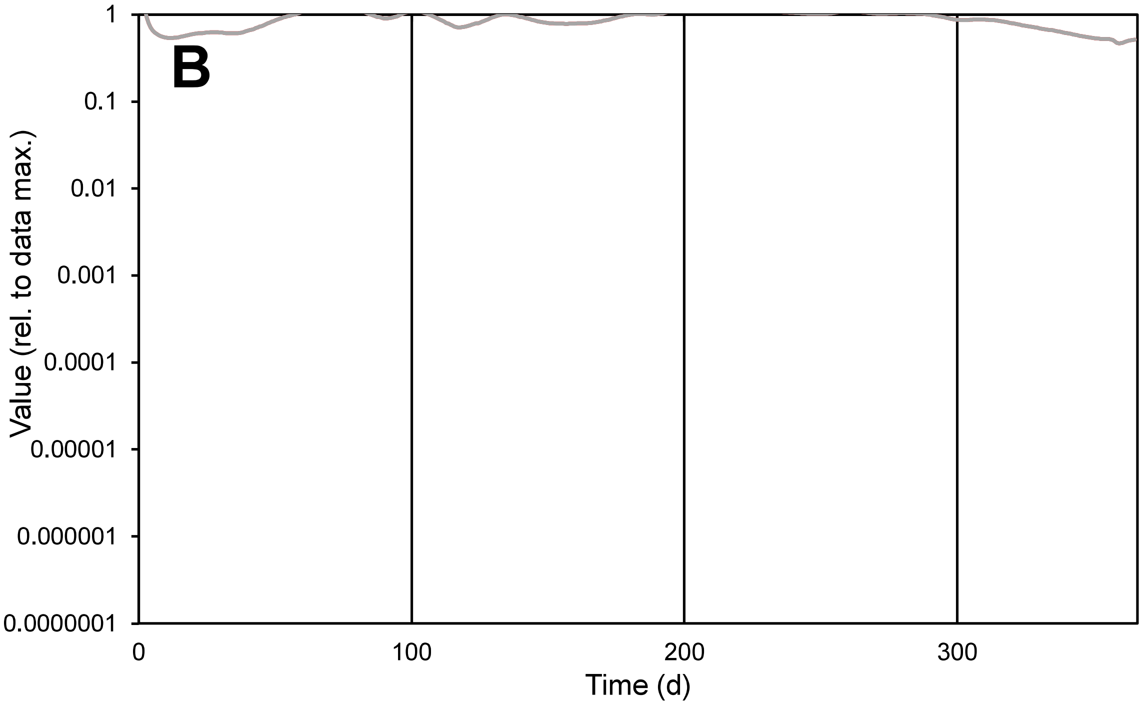

C1

Variables
PHP Provide jtd or Dst
CalibOnly?
2 1 to reproduce of
oNormalize?
2 1=no, 2=yes

ofxn calc*
0.20639
ofxn mod
0.2349

sRun
RunA1
zout
1

Ave
Data
6.9E-06
Model
6.3E-06

data/vMax
6.9E-06

ofxng
263.459

Min d/m
1.84111

ave/min d
1
ave/min m
1.6717

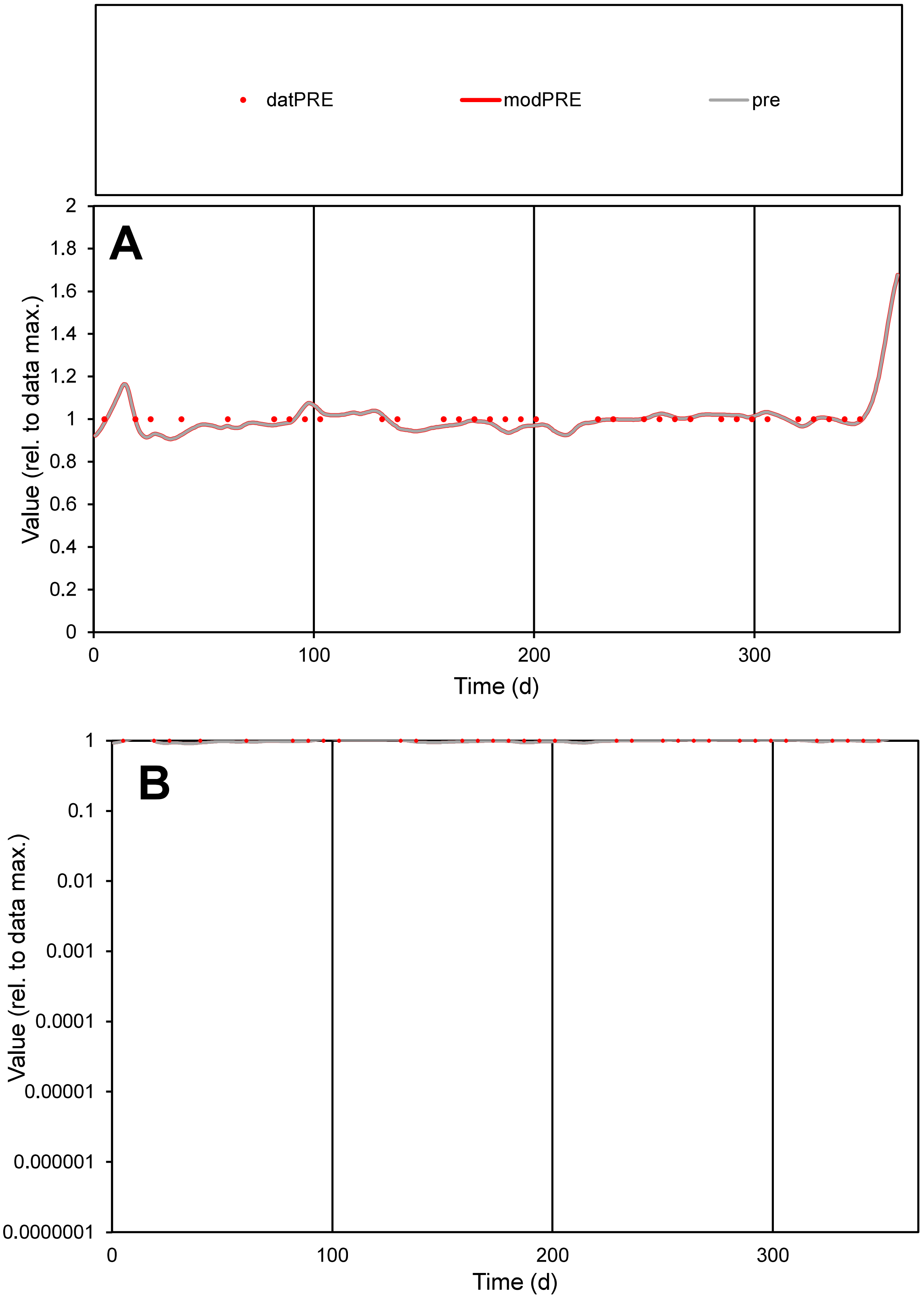

C1

Variables
PRE Provide jtd or Dst
CalibOnly?
2 1 to reproduce of
oNormalize?
2 1=no, 2=yes

ofxn calc*
0.03251
ofxn mod
0.11711

sRun
RunA1
zout
1

Ave
Data
1.3E-05
Model
1.3E-05

data/vMax
1.3E-05

ofxng
263.459

Min d/m
1.0744

ave/min d
1
ave/min m
1.06424

326_Calib_S_A

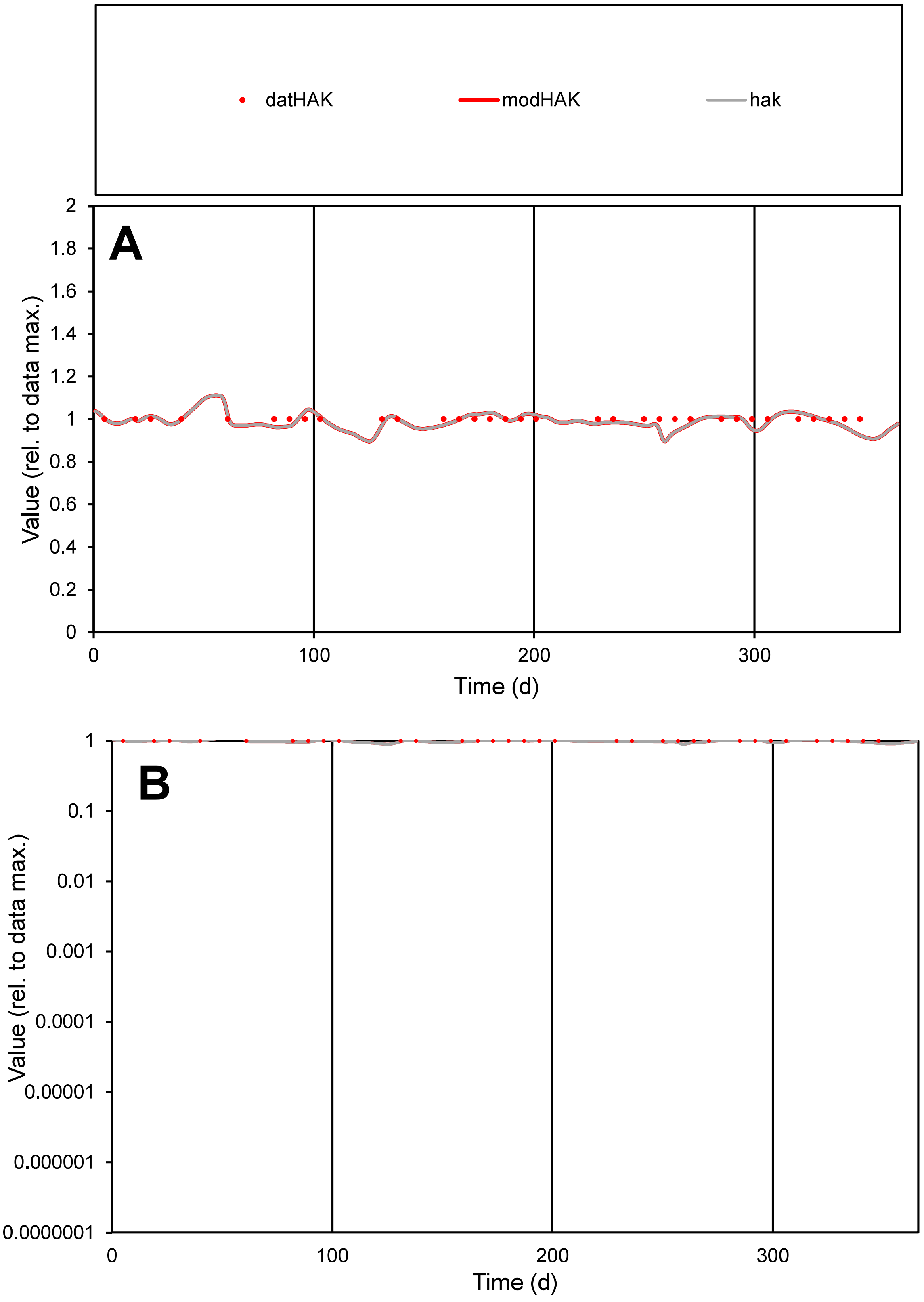

C1

Variables
HAK Provide jtd or Dst
CalibOnly?
2 1 to reproduce of
oNormalize?
2 1=no, 2=yes

ofxn calc*
0.03175
ofxn mod
0.11716

sRun
RunA1
zout
1

Ave
Data
5.1E-06
Model
5E-06

data/vMax
5.1E-06

ofxng
263.459

Min d/m
1.09912

ave/min d
1
ave/min m
1.08809

326_Calib_S_A

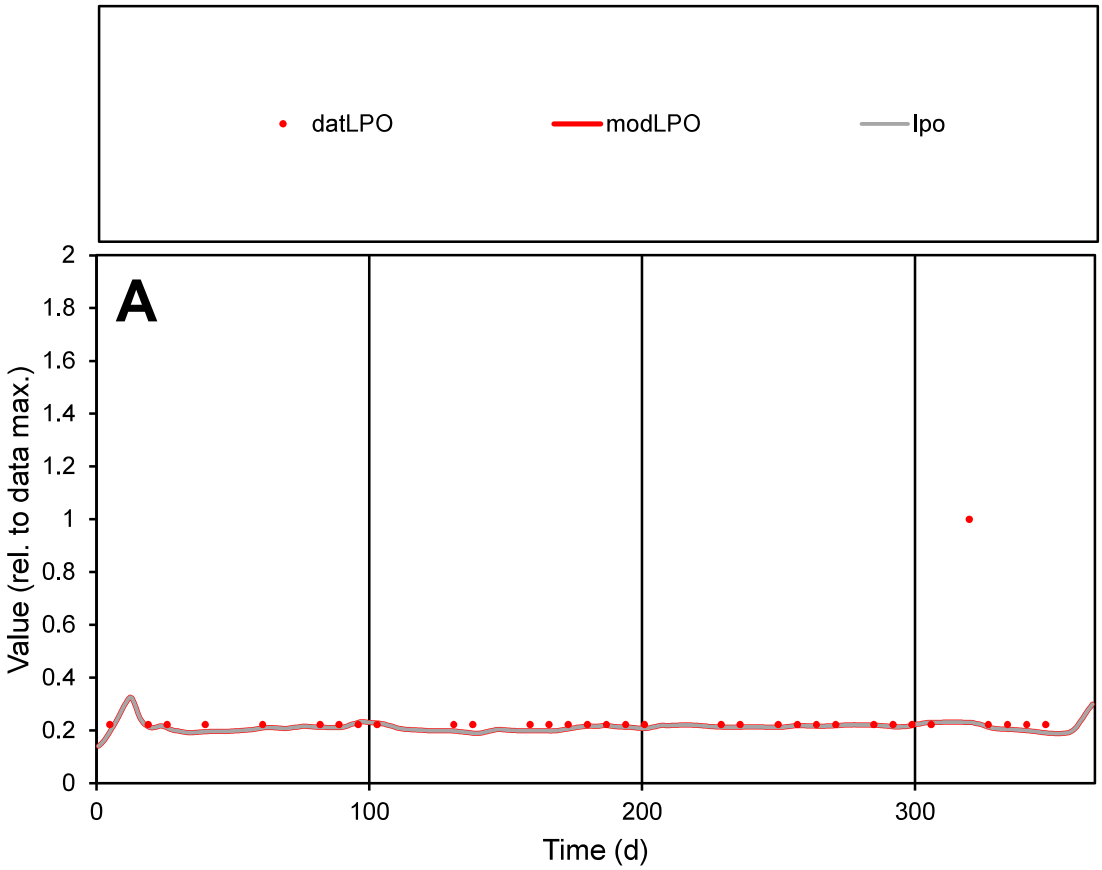

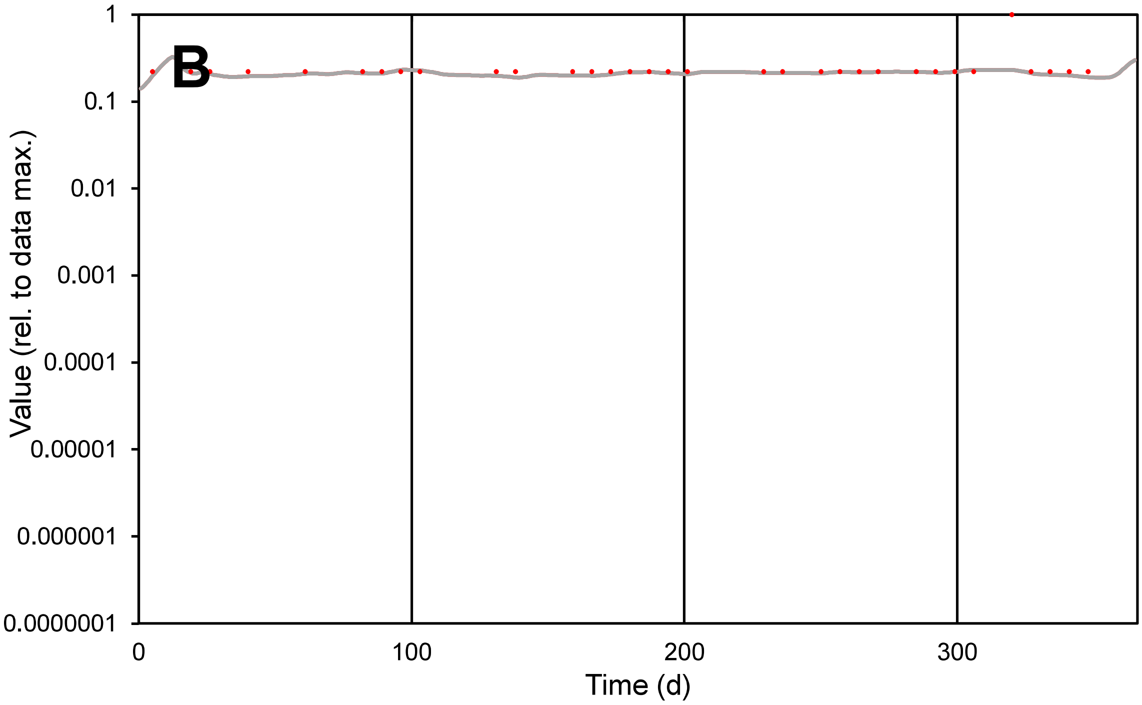

C1

Variables

LPO Provide jtd or Dst

CalibOnly?

2 1 to reproduce of

oNormalize?

2 1=no, 2=yes

ofxn calc*

0.55134

ofxn mod

0.56151

sRun

RunA1

zout

1

Ave

Data

6.5E-06

Model

5.6E-06

data/vMax

2.6E-05

ofxng

263.459

Min d/m

1.1698

ave/min d

1.10657

ave/min m

1.11674

326_Calib_S_A

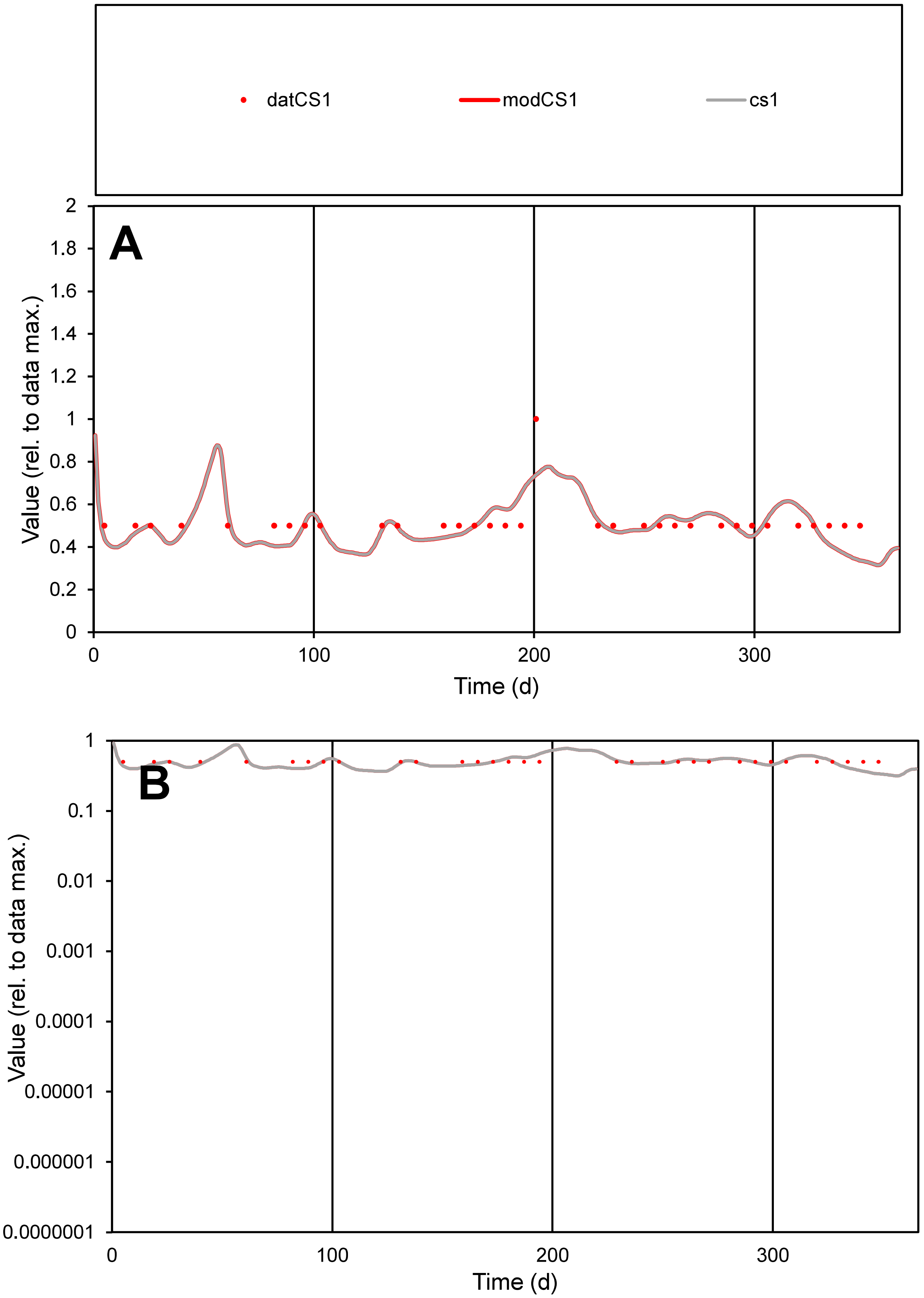

C1

Variables
CS1 Provide jtd or Dst
CalibOnly?
2 1 to reproduce of
oNormalize?
2 1=no, 2=yes

ofxn calc*
0.15363
ofxn mod
0.19241

sRun
RunA1
zout
1

Ave
Data
1.8E-05
Model
1.7E-05

data/vMax
3.5E-05

ofxng
263.459

Min d/m
1.49616

ave/min d
1.0303
ave/min m
1.48674

326_Calib_S_A

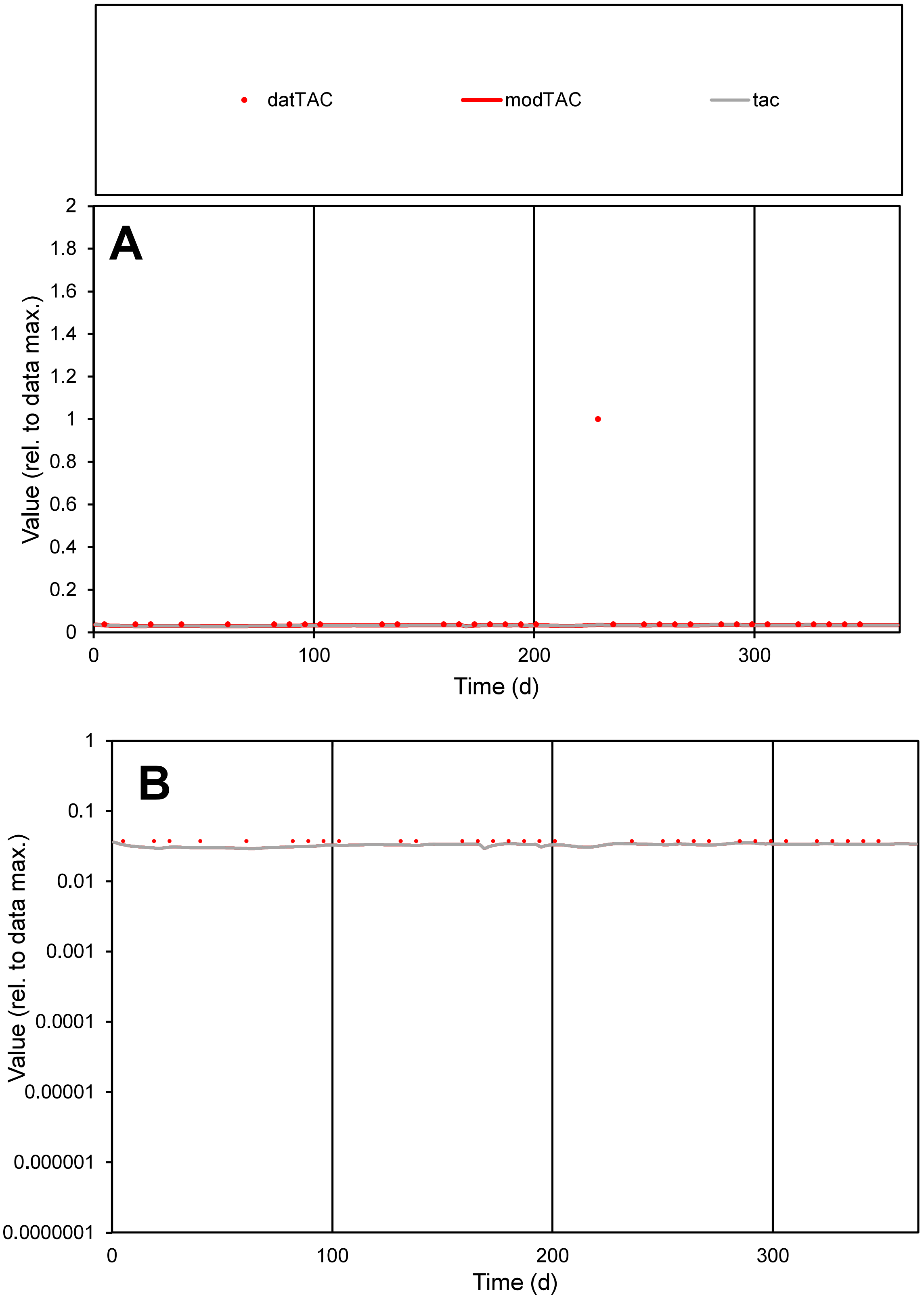

C1

Variables
TAC Provide jtd or Dst
CalibOnly?
2 1 to reproduce of
oNormalize?
2 1=no, 2=yes

ofxn calc*
2.51861
ofxn mod
2.5244

sRun
RunA1
zout
1

Ave
Data
2.3E-06
Model
1.1E-06

data/vMax
3.4E-05

ofxng
263.459

Min d/m
1.28472

ave/min d
1.77583
ave/min m
1.12626

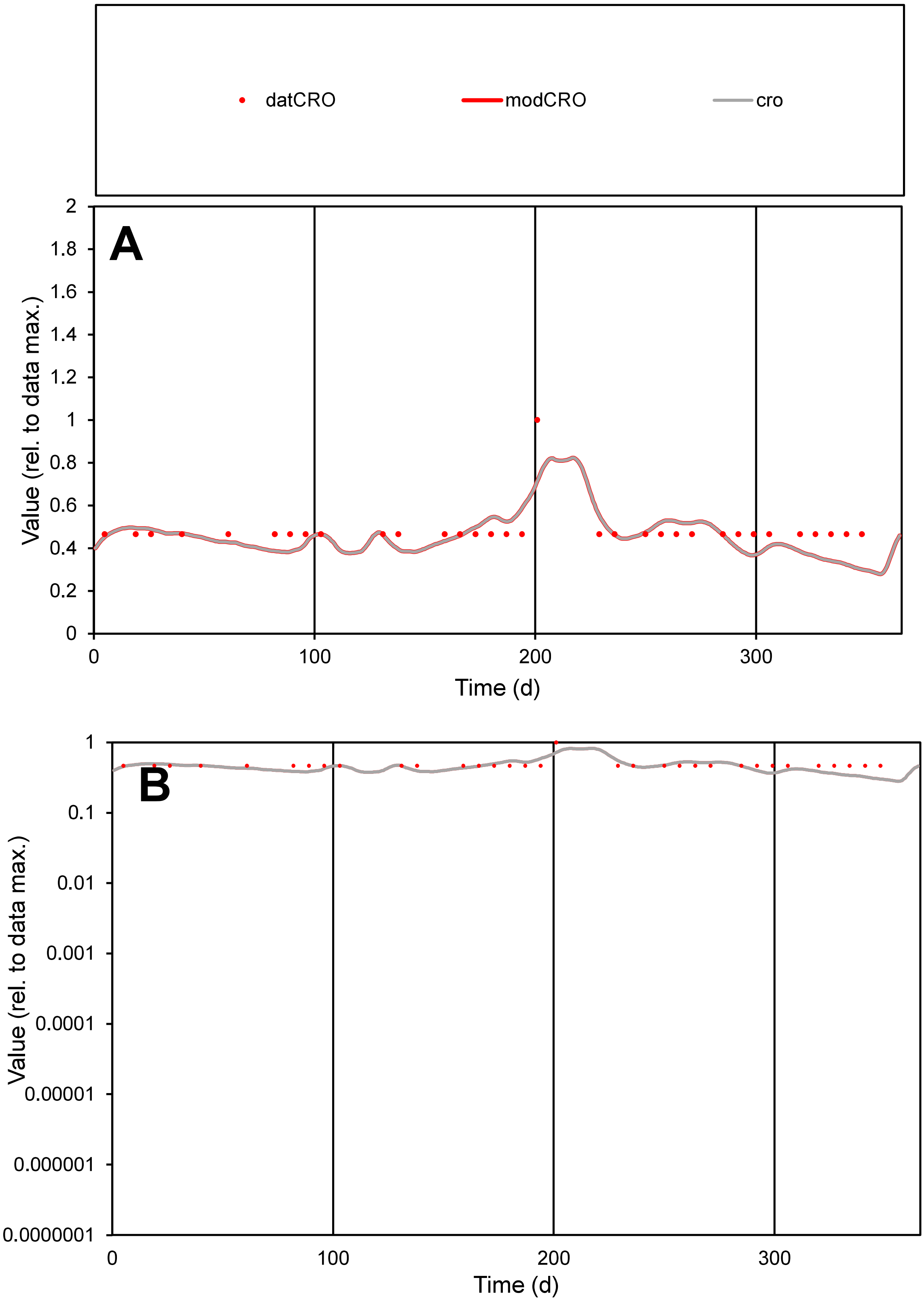

C1

Variables
CRO Provide jtd or Dst
CalibOnly?
2 1 to reproduce of
oNormalize?
2 1=no, 2=yes

ofxn calc*
0.1741
ofxn mod
0.20879

sRun
RunA1
zout
1

Ave
Data
1.1E-05
Model
1E-05

data/vMax
2.2E-05

ofxng
263.459

Min d/m
1.55931

ave/min d
1.0348
ave/min m
1.52565

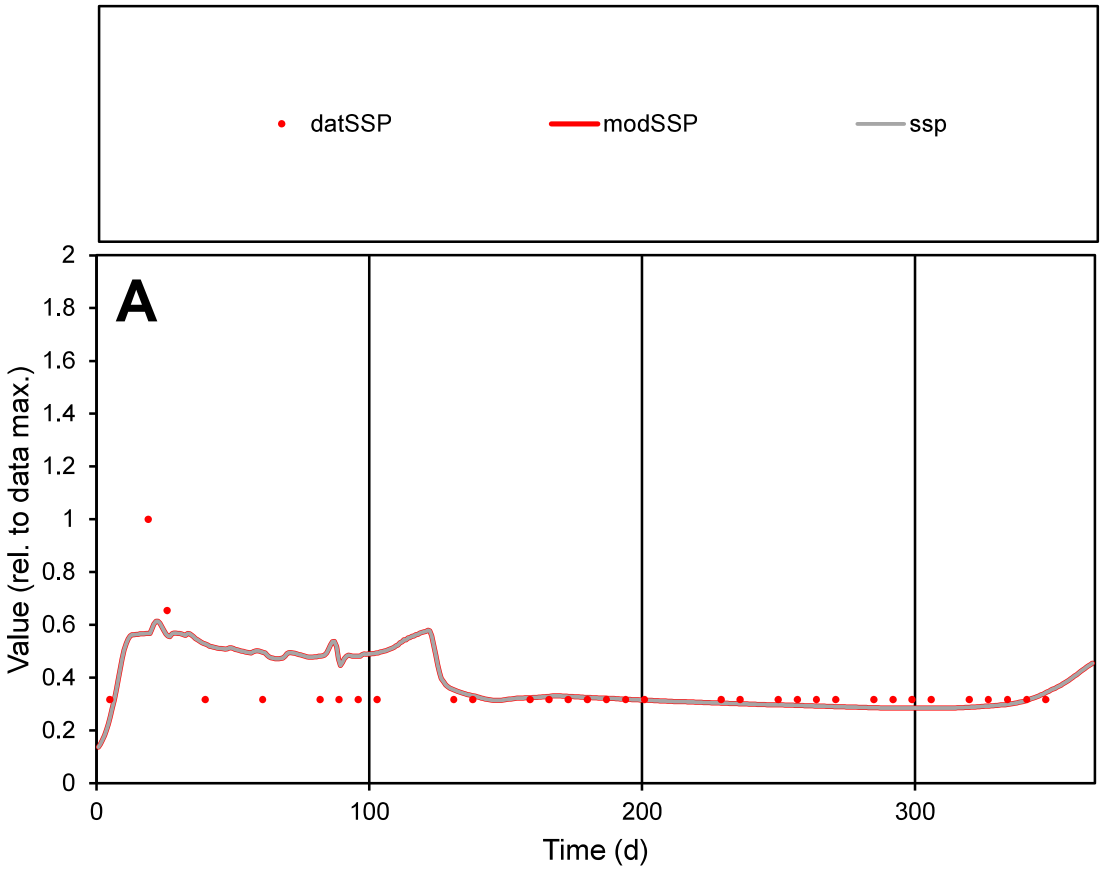

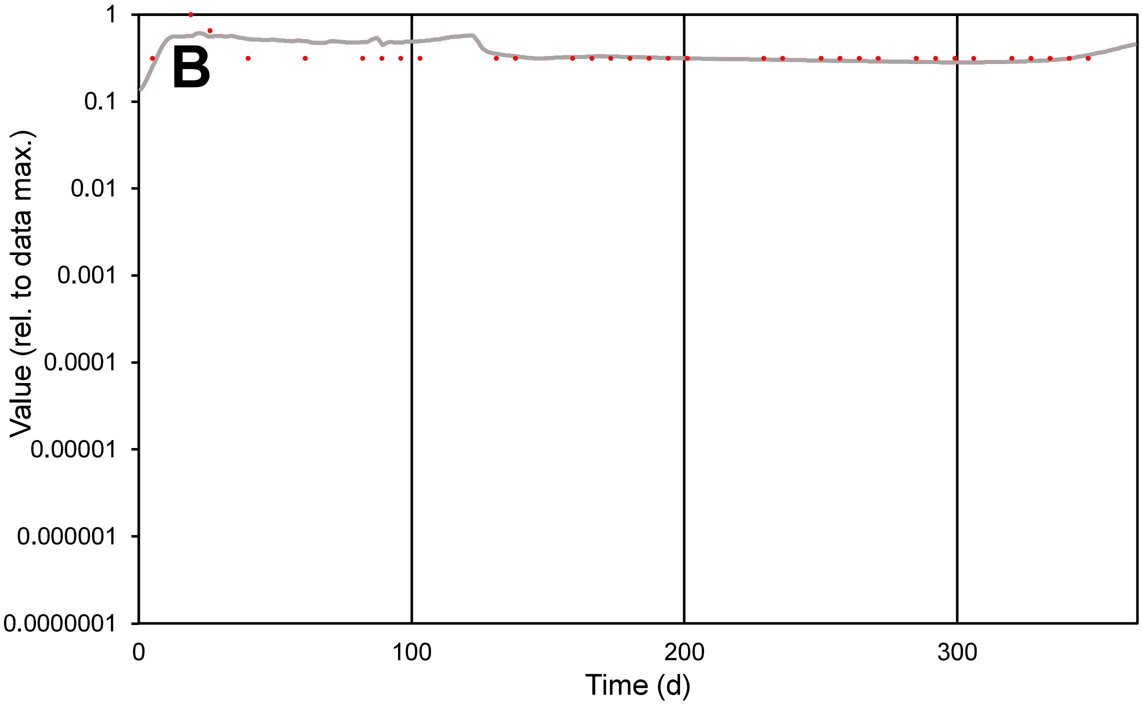

C1

Variables

SSP Provide jtd or Dst

CalibOnly?

2 1 to reproduce of

oNormalize?

2 1=no, 2=yes

ofxn calc*

0.30591

ofxn mod

0.32976

sRun

RunA1

zout

1

Ave

Data

1.5E-05

Model

1.5E-05

data/vMax

4.2E-05

ofxng

263.459

Min d/m

1.1127

ave/min d

1.09803

ave/min m

1.2595

326_Calib_S_A

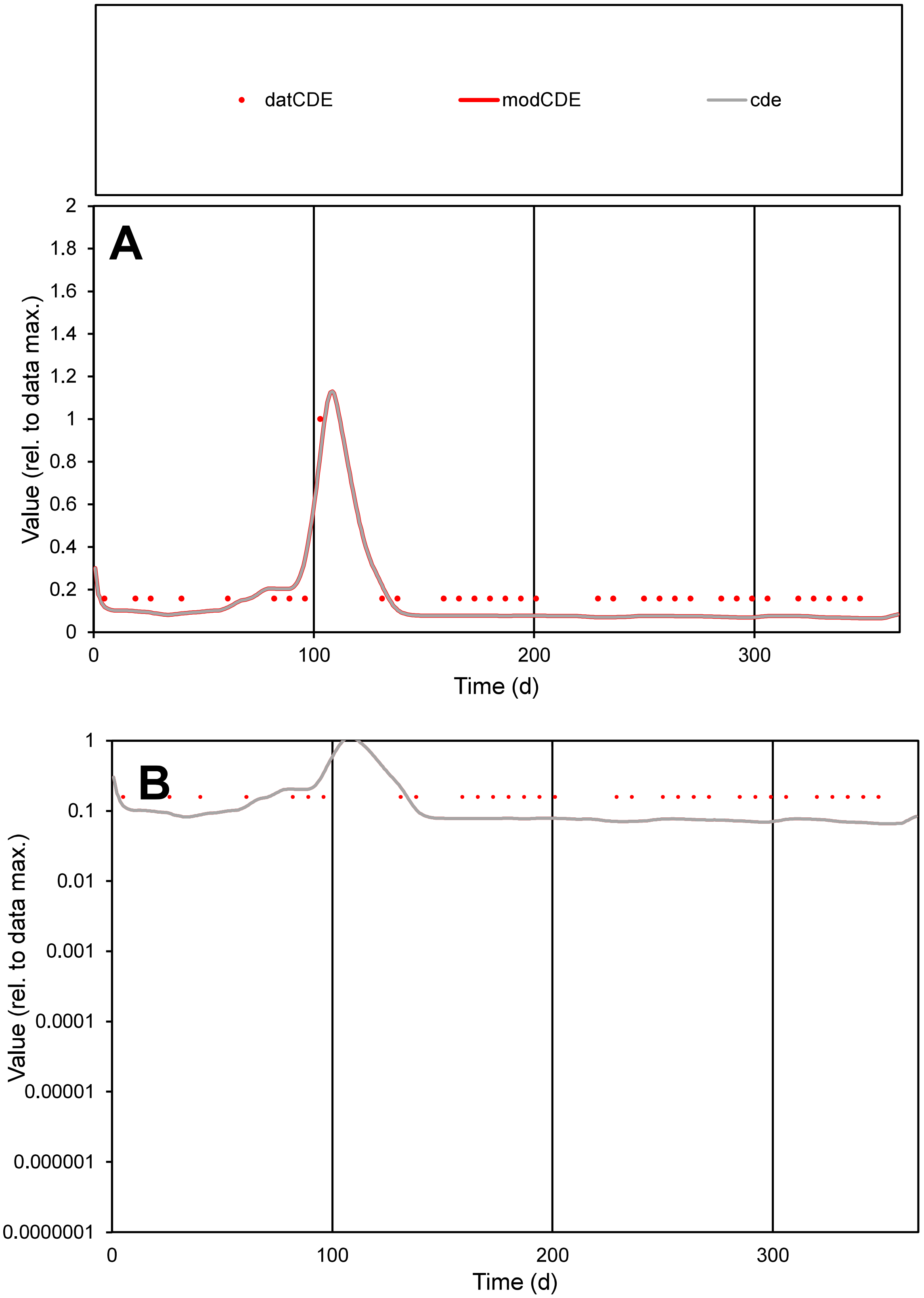

C1

Variables
CDE Provide jtd or Dst
CalibOnly?
2 1 to reproduce of
oNormalize?
2 1=no, 2=yes

ofxn calc*
0.44455
ofxn mod
0.45873

sRun
RunA1
zout
1

Ave
Data
1.2E-05
Model
7.9E-06

data/vMax
6.3E-05

ofxng
263.459

Min d/m
2.38923

ave/min d
1.16146
ave/min m
1.88973

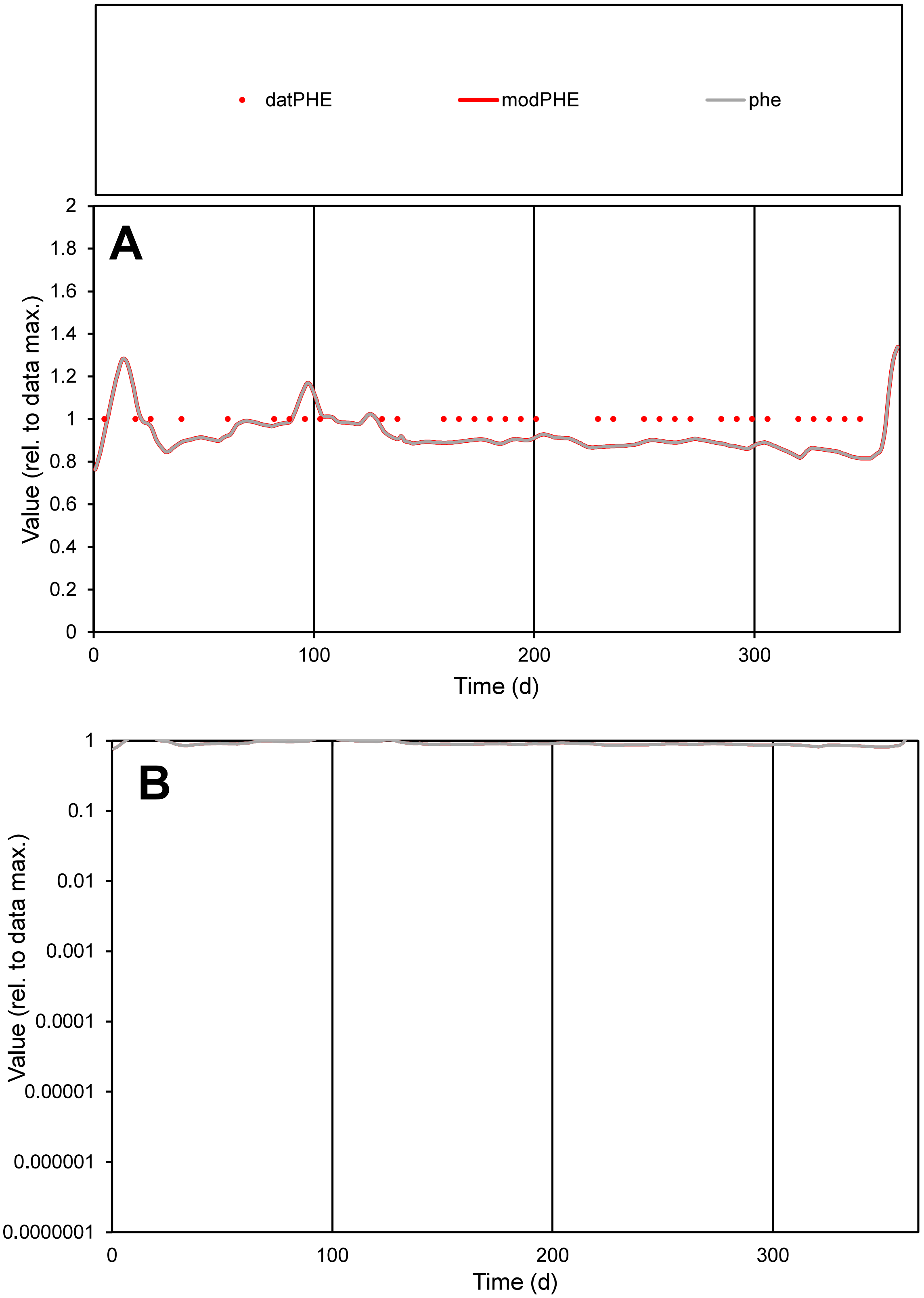

C1

Variables
PHE Provide jtd or Dst
CalibOnly?
2 1 to reproduce of
oNormalize?
2 1=no, 2=yes

ofxn calc*
0.11044
ofxn mod
0.15482

sRun
RunA1
zout
1

Ave
Data
4E-06
Model
3.6E-06

data/vMax
4E-06

ofxng
263.459

Min d/m
1.22552

ave/min d
1
ave/min m
1.11995

326_Calib_S_A

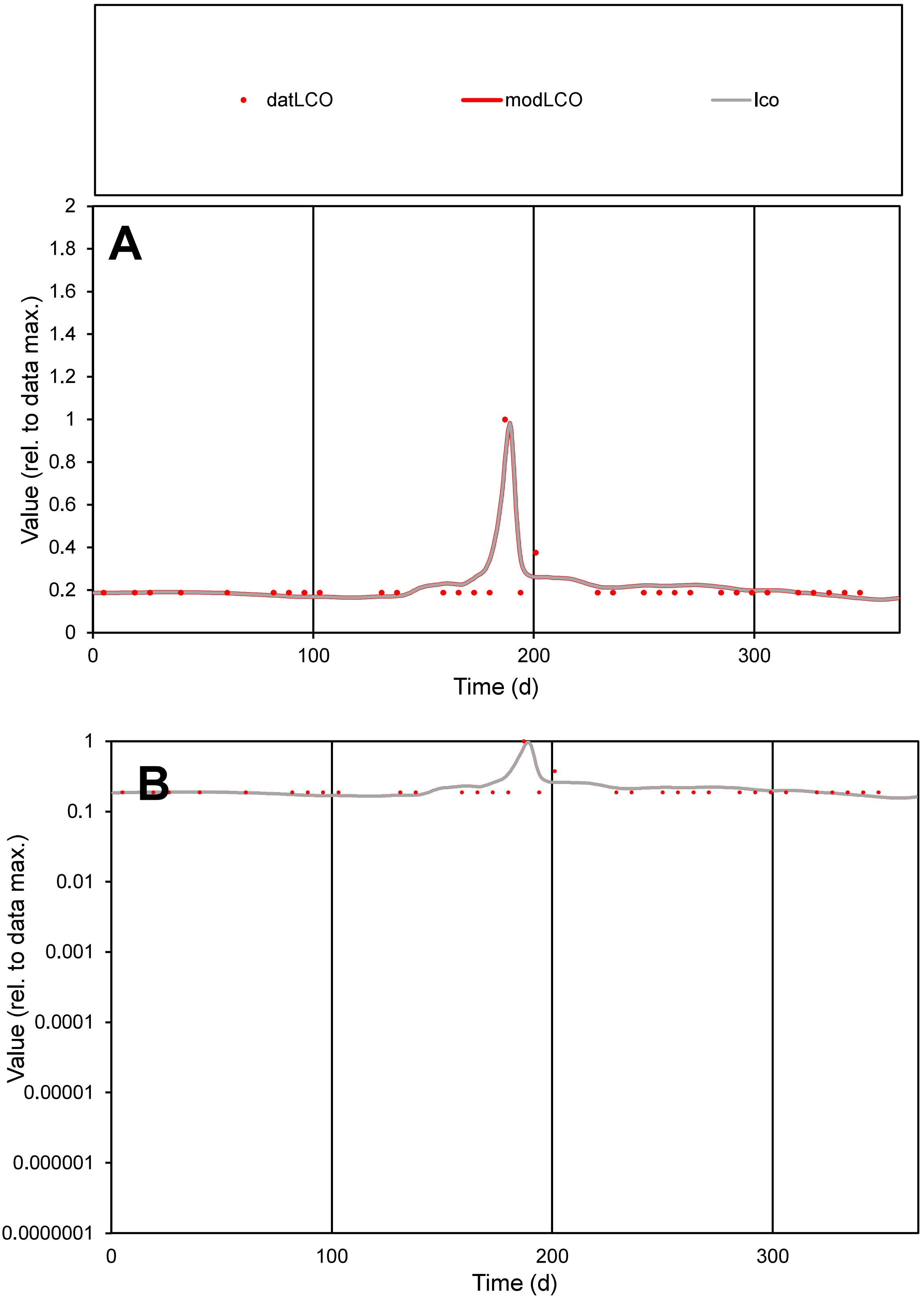

C1

Variables
LCO Provide jtd or Dst
CalibOnly?
2 1 to reproduce of
oNormalize?
2 1=no, 2=yes

ofxn calc*
0.21711
ofxn mod
0.26128

sRun
RunA1
zout
1

Ave
Data
8.6E-06
Model
9E-06

data/vMax
4E-05

ofxng
263.459

Min d/m
1.16774

ave/min d
1.16167
ave/min m
1.42251

326_Calib_S_A

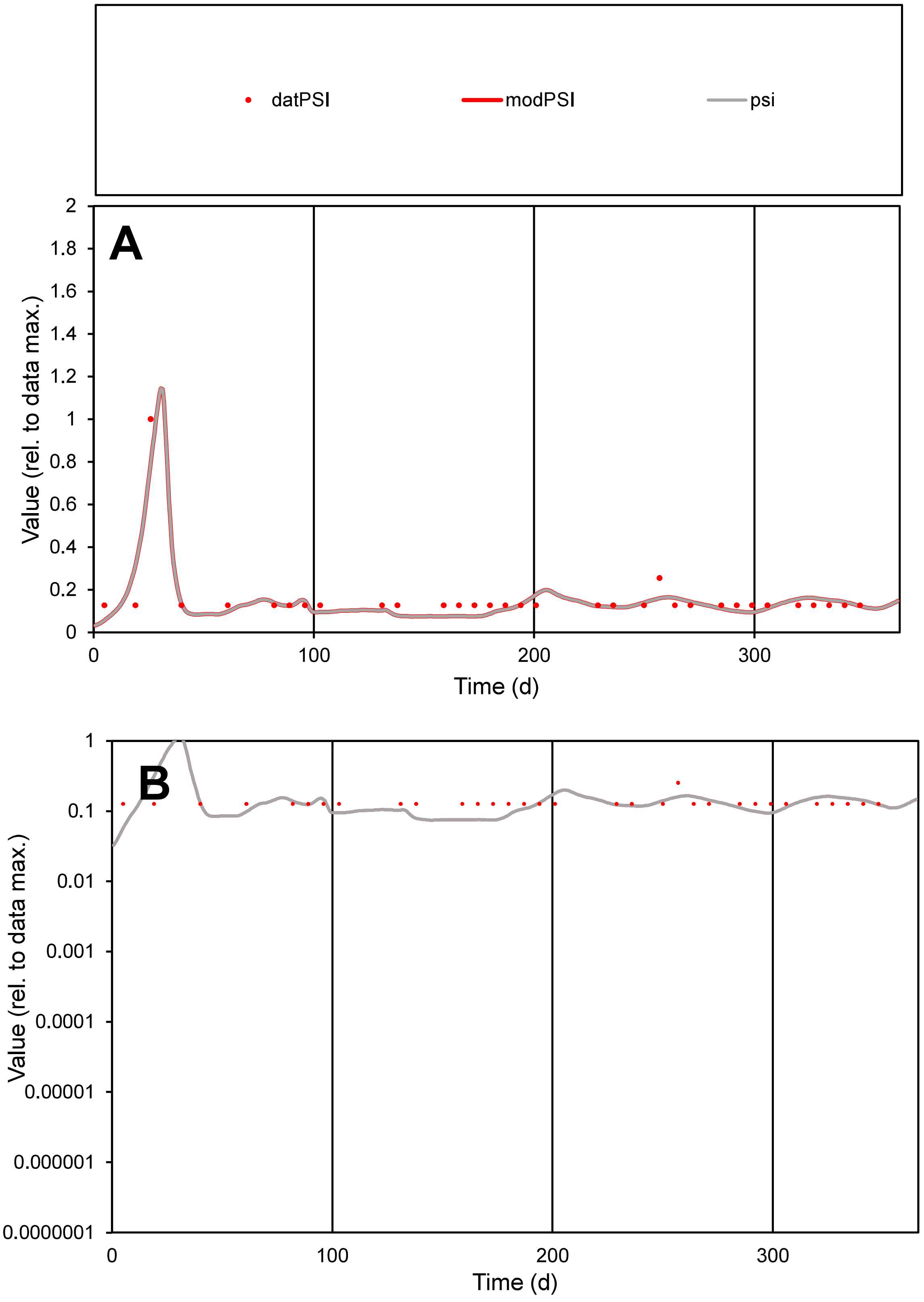

C1

Variables
PSI Provide jtd or Dst
CalibOnly?
2 1 to reproduce of
oNormalize?
2 1=no, 2=yes

ofxn calc*
0.27784
ofxn mod
0.3517

sRun
RunA1
zout
1

Ave
Data
4.4E-06
Model
4.2E-06

data/vMax
2.8E-05

ofxng
263.459

Min d/m
1.83162

ave/min d
1.23836
ave/min m
2.14927

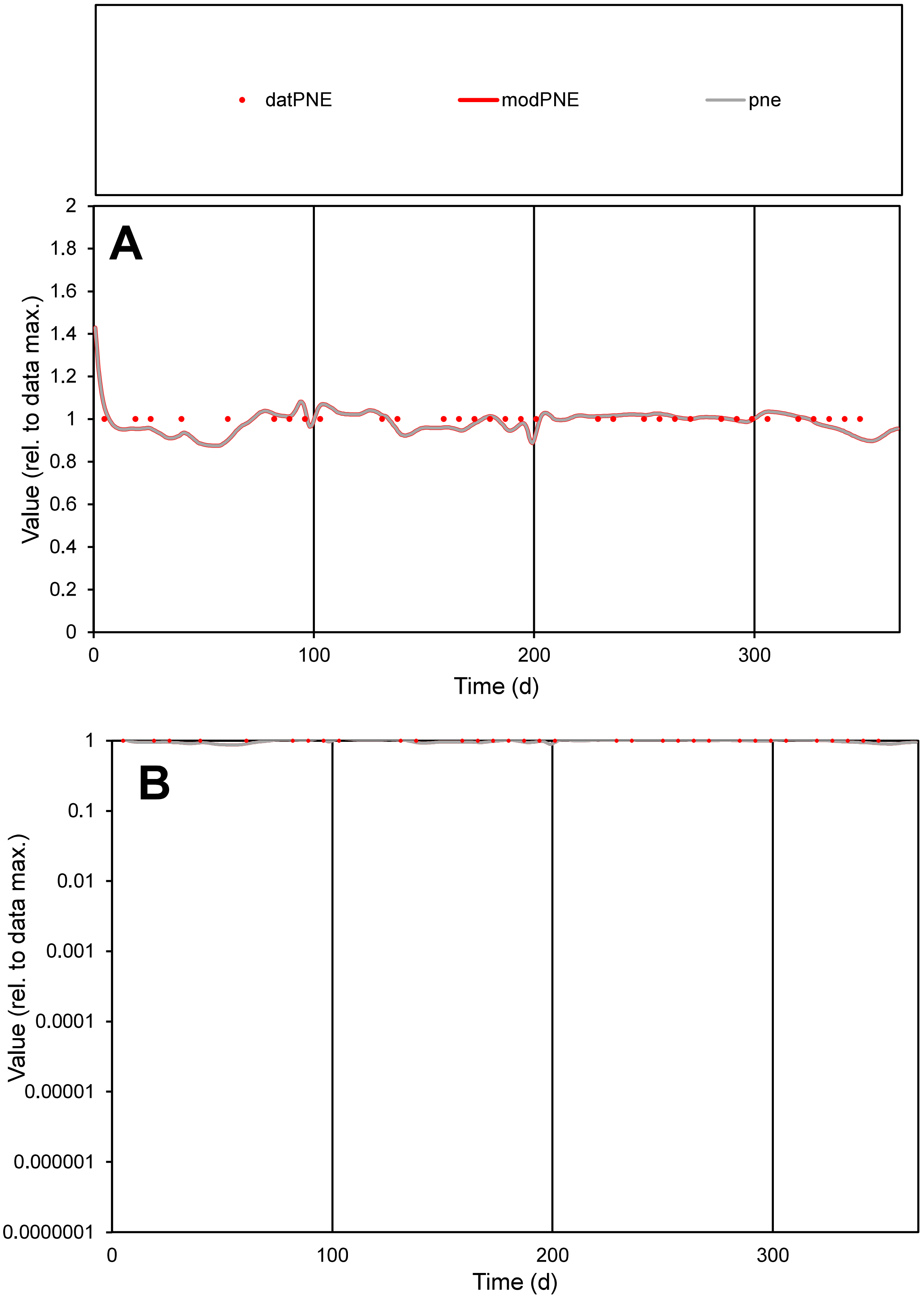

C1

Variables

PNE Provide jtd or Dst

CalibOnly?

2 1 to reproduce of

oNormalize?

2 1=no, 2=yes

ofxn calc*

0.0403

ofxn mod

0.11956

sRun

RunA1

zout

1

Ave

Data

1.5E-05

Model

1.4E-05

data/vMax

1.5E-05

ofxng

263.459

Min d/m

1.10502

ave/min d

1

ave/min m

1.08927

326_Calib_S_A

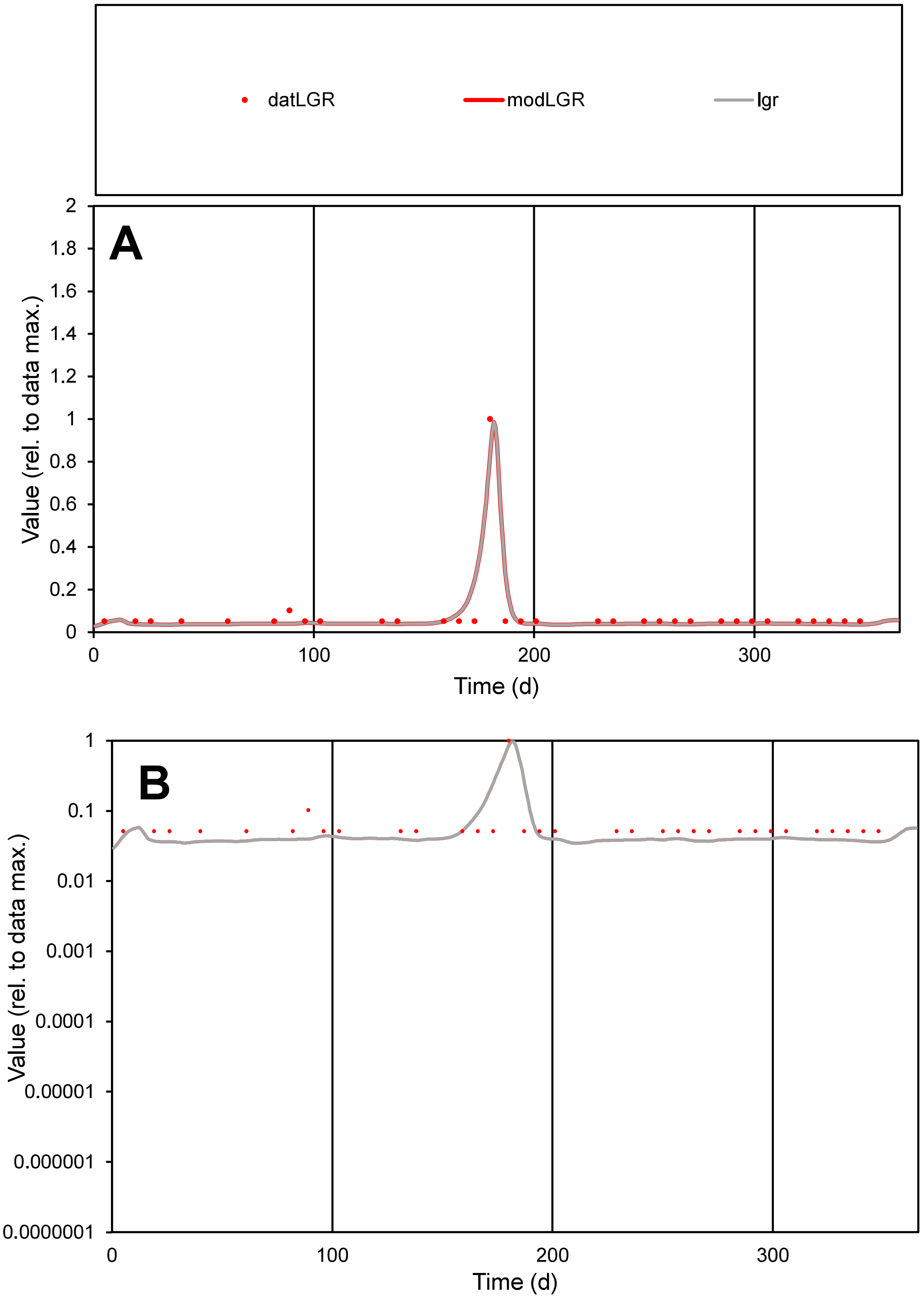

C1

Variables

LGR Provide jtd or Dst

CalibOnly?

2 1 to reproduce of

oNormalize?

2 1=no, 2=yes

ofxn calc*

0.46495

ofxn mod

0.52696

sRun

RunA1

zout

1

Ave

Data

8.2E-06

Model

7.8E-06

data/vMax

0.0001

ofxng

263.459

Min d/m

1.41309

ave/min d

1.59054

ave/min m

2.12911

326_Calib_S_A

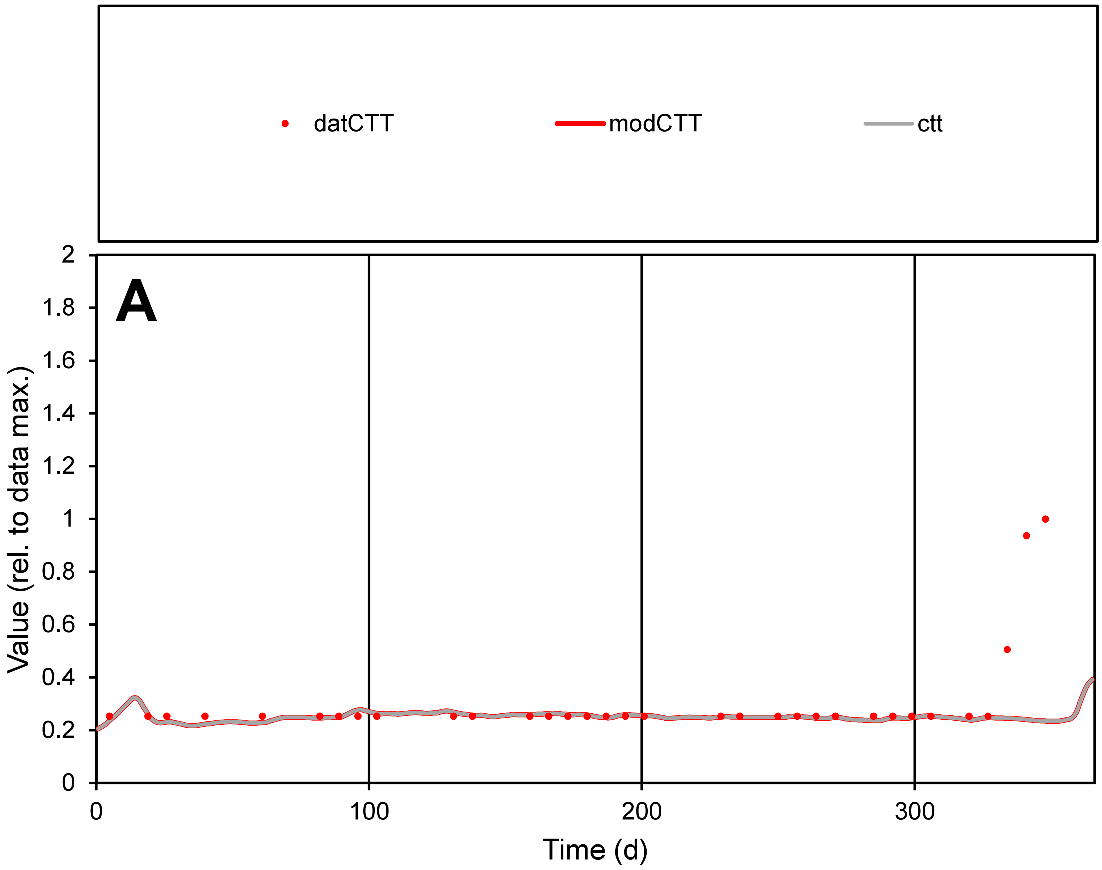

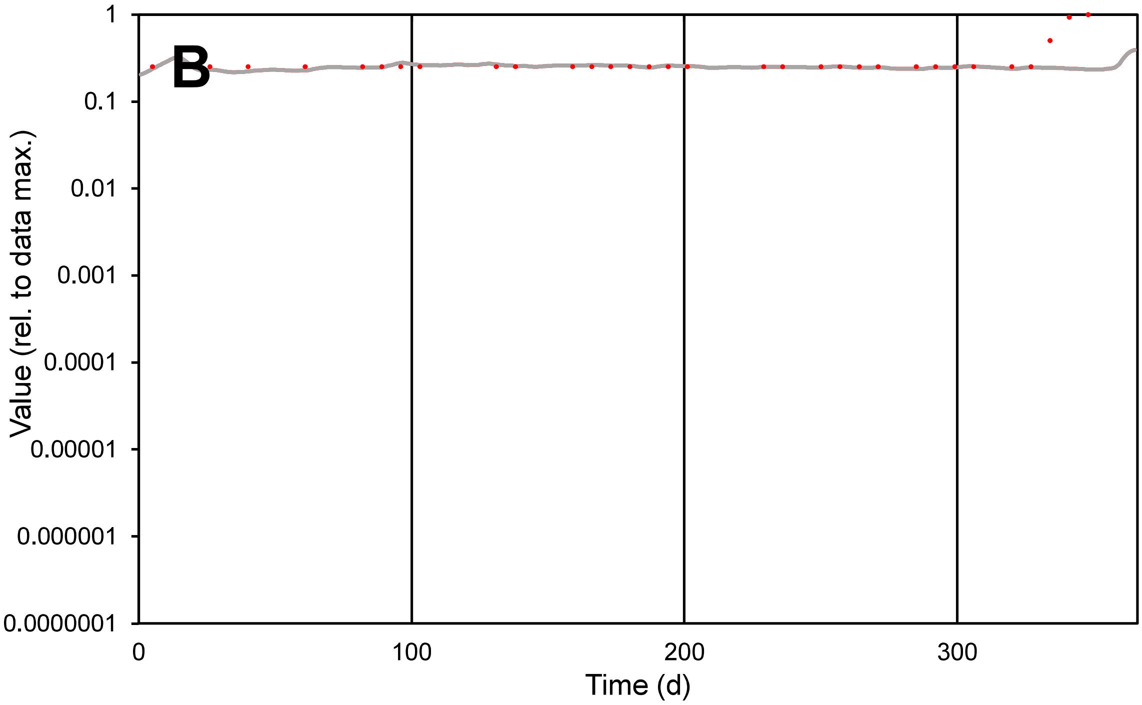

C1

Variables
CTT Provide jtd or Dst
CalibOnly?
2 1 to reproduce of
oNormalize?
2 1=no, 2=yes

ofxn calc*
0.61331
ofxn mod
0.62211

sRun
RunA1
zout
1

Ave
Data
1.8E-06
Model
1.5E-06

data/vMax
5.9E-06

ofxng
263.459

Min d/m
1.1269

ave/min d
1.20184
ave/min m
1.10979

326_Calib_S_A

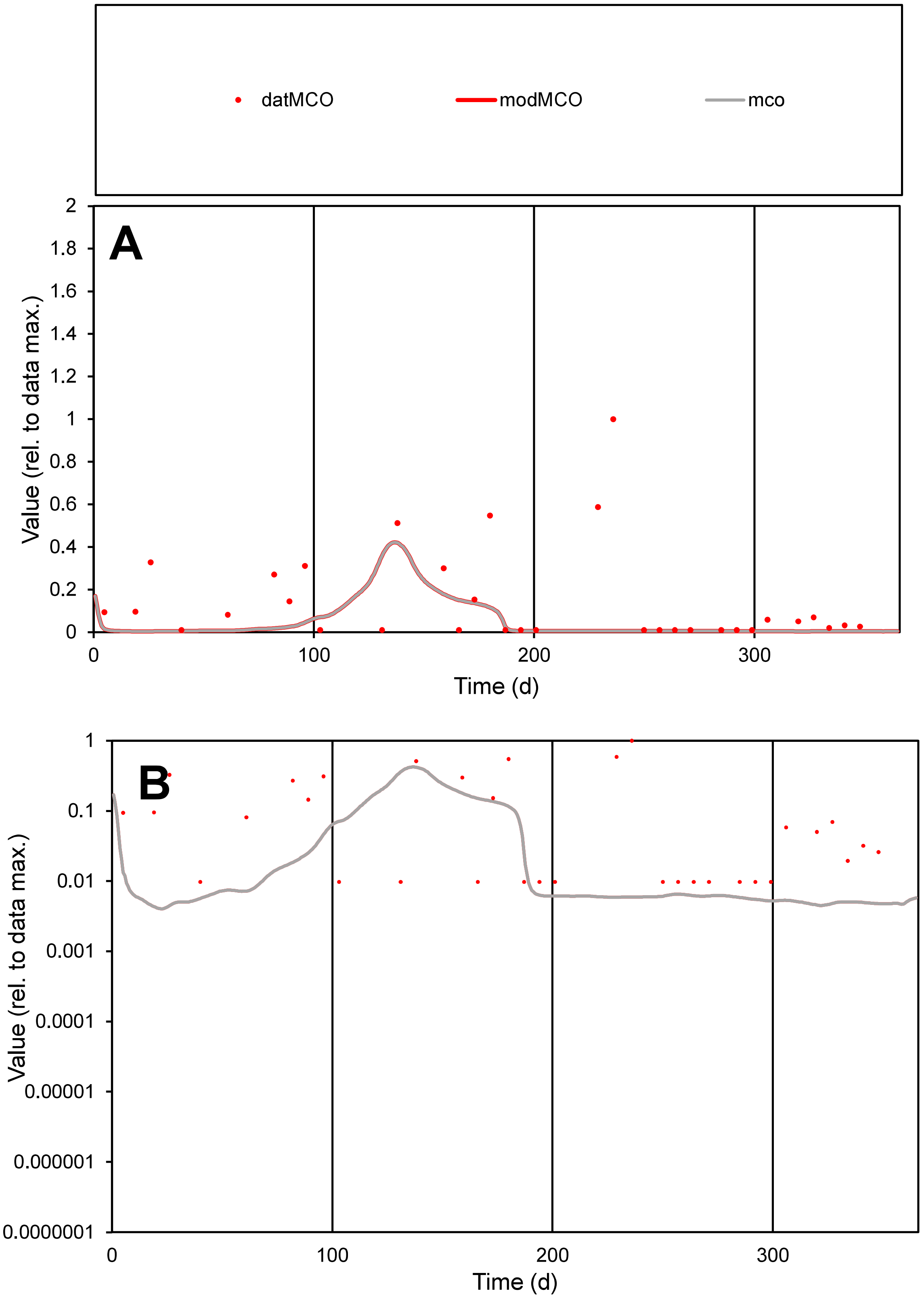

C1

Variables
MCO Provide jtd or Dst
CalibOnly?
2 1 to reproduce of
oNormalize?
2 1=no, 2=yes

ofxn calc*
1.66469
ofxn mod
1.67552

sRun
RunA1
zout
1

Ave
Data
9.2E-07
Model
3.3E-07

data/vMax
6.3E-06

ofxng
263.459

Min d/m
2.34201
ave/min d
15.0153
ave/min m
12.4603

326_Calib_S_A

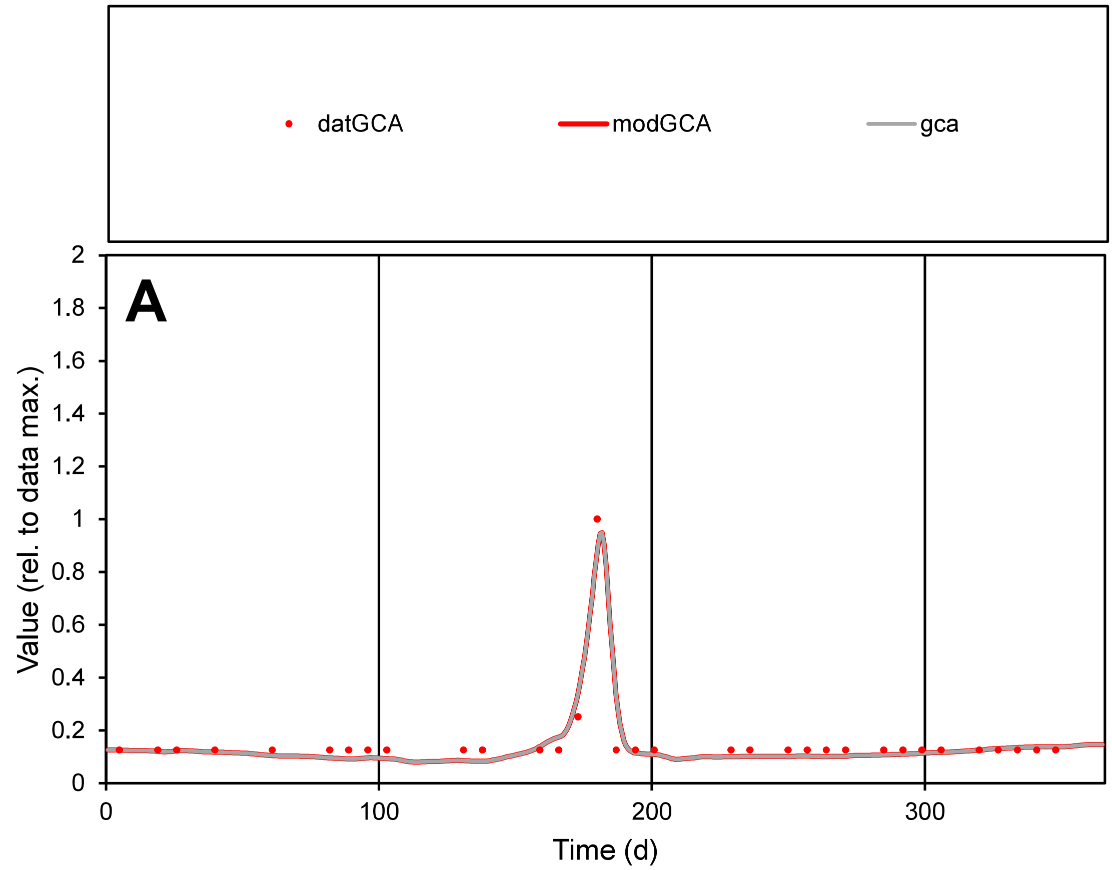

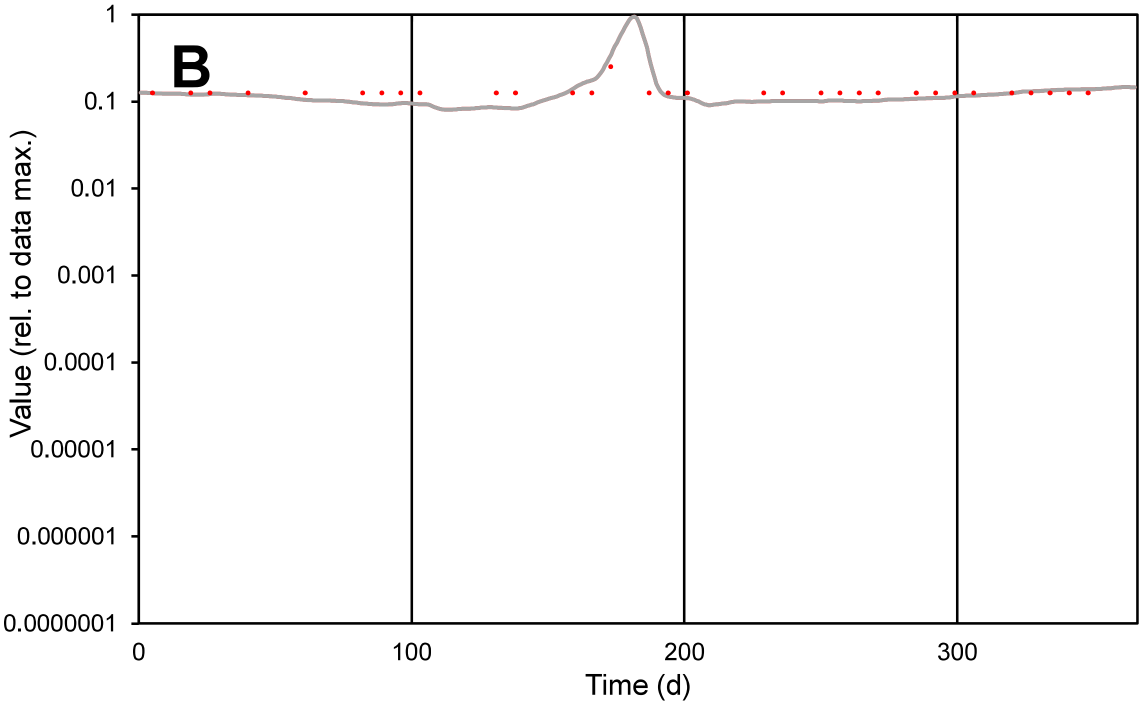

C1

Variables
GCA Provide jtd or Dst
CalibOnly?
2 1 to reproduce of
oNormalize?
2 1=no, 2=yes

ofxn calc*
0.20138
ofxn mod
0.25754

sRun
RunA1
zout
1

Ave
Data
1E-05
Model
9.9E-06

data/vMax
6.7E-05

ofxng
263.459

Min d/m
1.5017

ave/min d
1.2408
ave/min m
1.76937

326_Calib_S_A

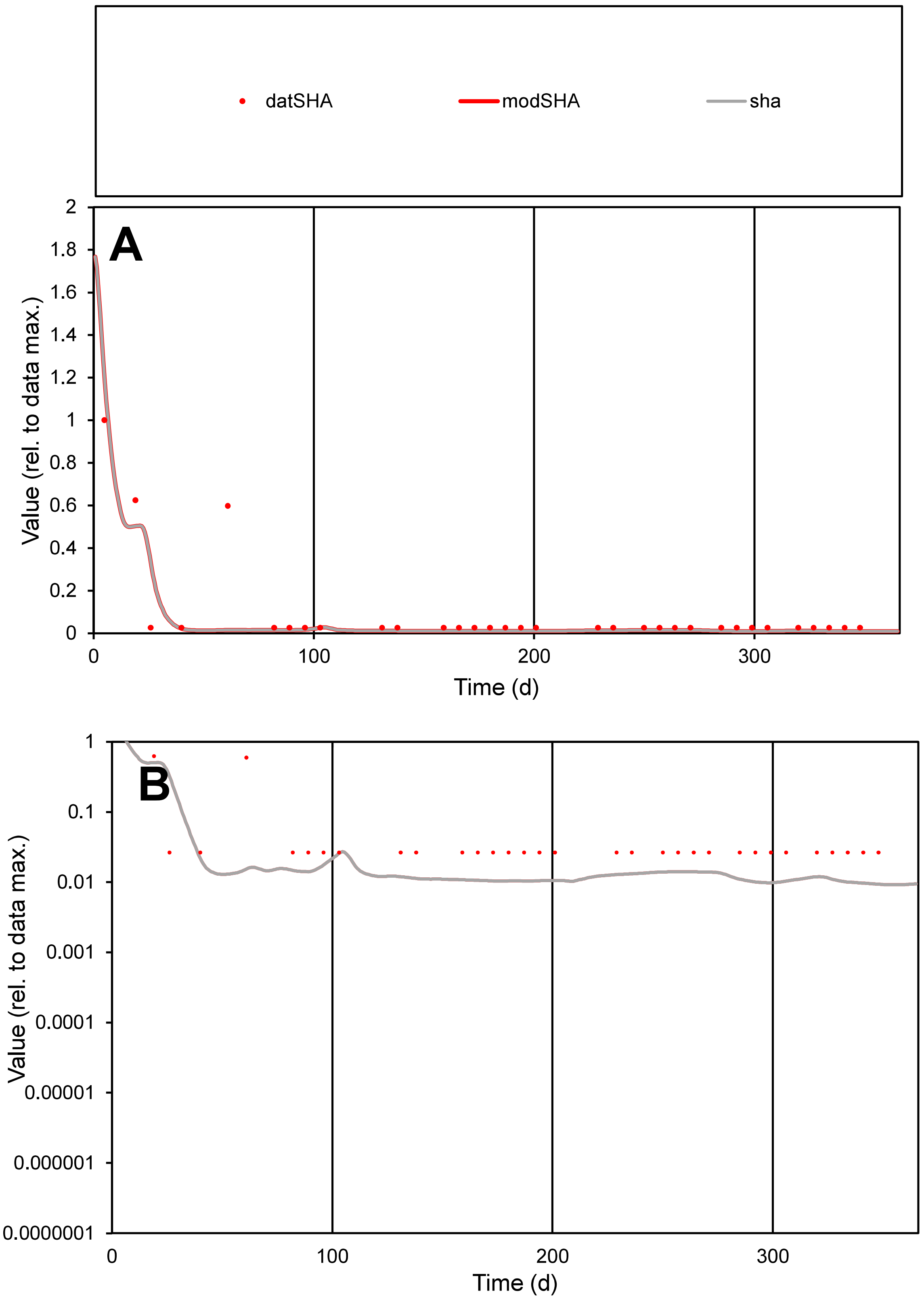

C1

Variables
SHA Provide jtd or Dst
CalibOnly?
2 1 to reproduce of
oNormalize?
2 1=no, 2=yes

ofxn calc*
1.17647
ofxn mod
1.23373

sRun
RunA1
zout
1

Ave
Data
2.4E-06
Model
1.7E-06

data/vMax
2.6E-05

ofxng
263.459

Min d/m
2.83266

ave/min d
3.45665
ave/min m
6.70763

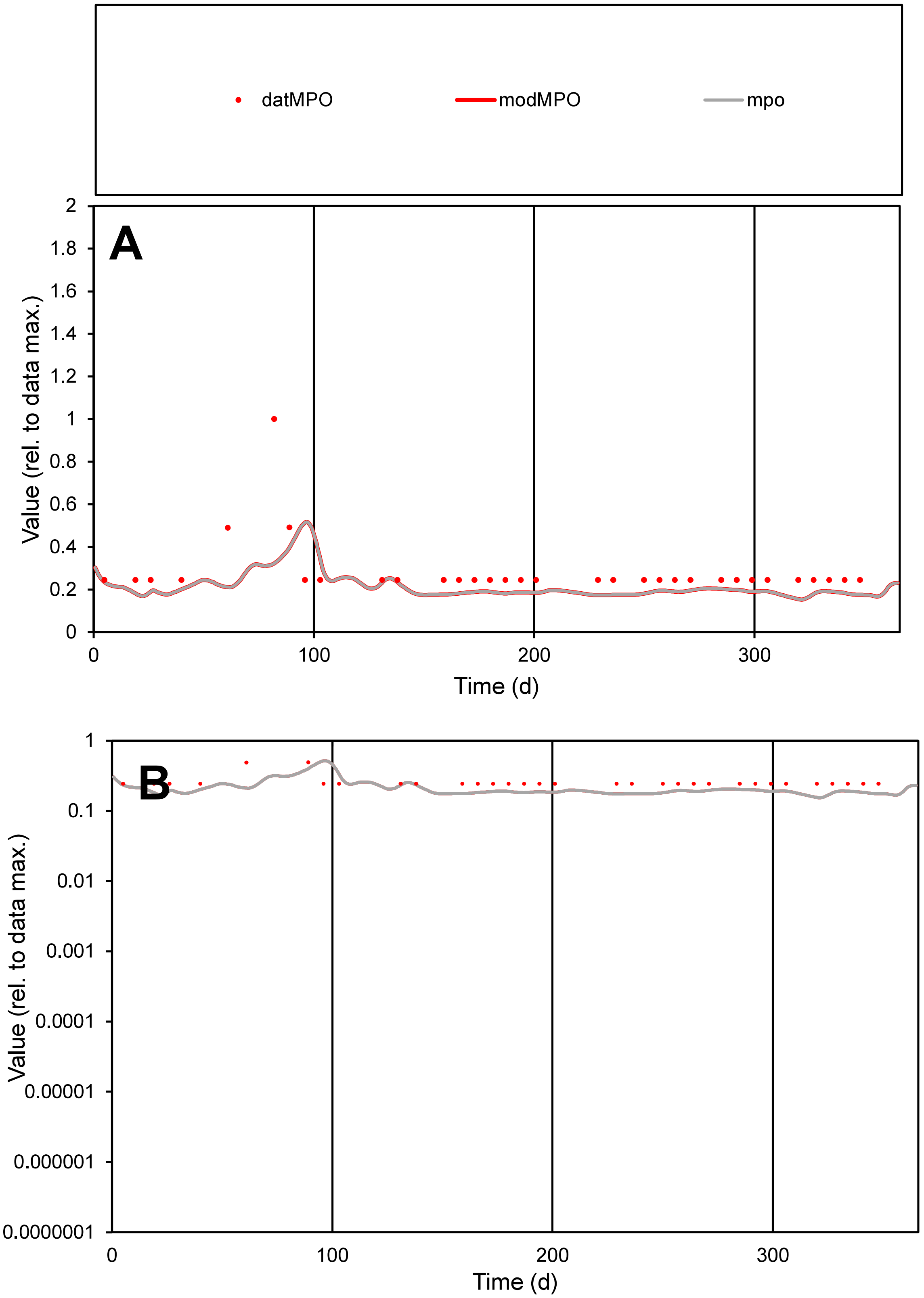

C1

Variables
MPO Provide jtd or Dst
CalibOnly?
2 1 to reproduce of
oNormalize?
2 1=no, 2=yes

ofxn calc*
0.51136
ofxn mod
0.52249

sRun
RunA1
zout
1

Ave
Data
1.4E-05
Model
1.1E-05

data/vMax
5.1E-05

ofxng
263.459

Min d/m
1.59389

ave/min d
1.15459
ave/min m
1.40877

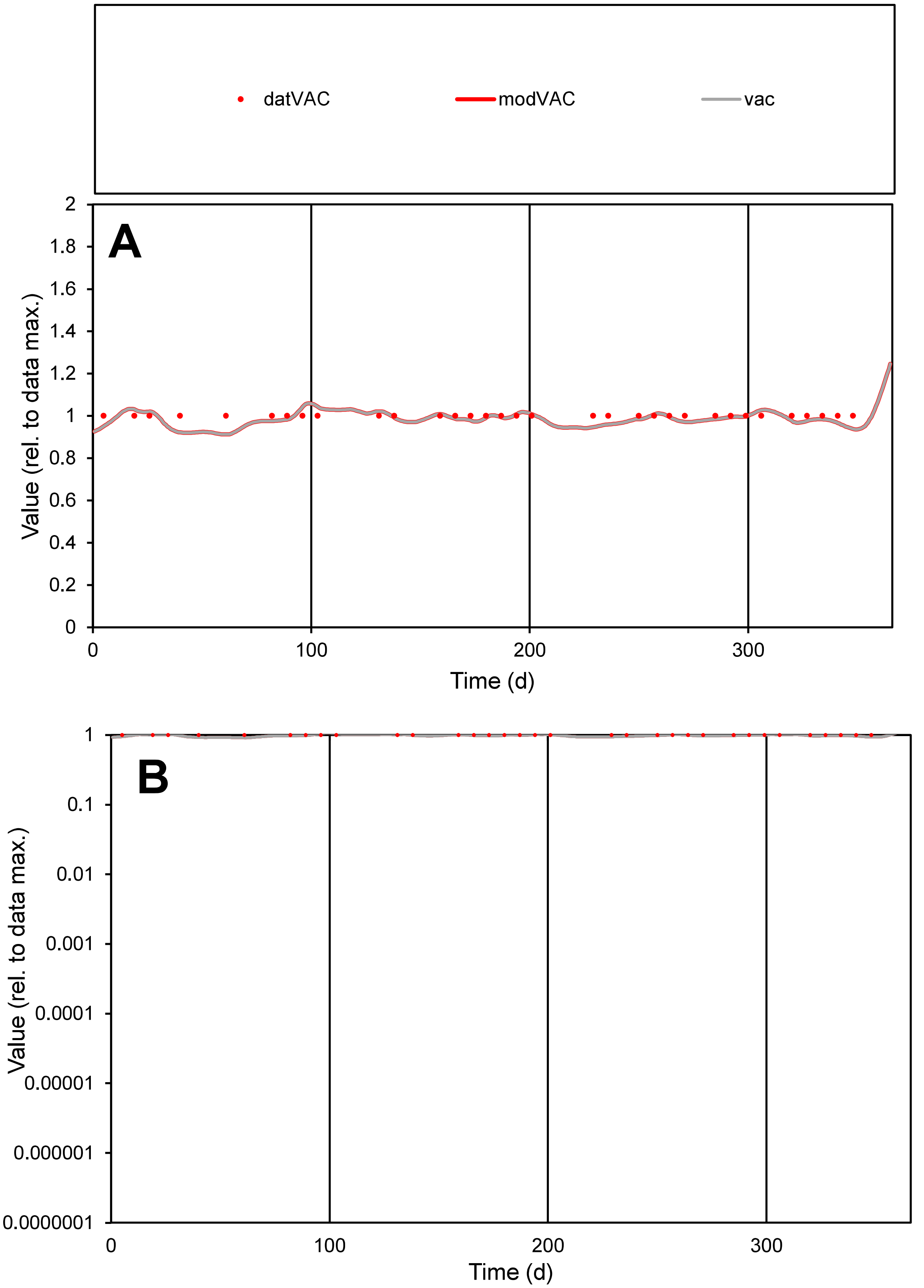

C1

Variables
VAC Provide jtd or Dst
CalibOnly?
2 1 to reproduce of
oNormalize?
2 1=no, 2=yes

ofxn calc*
0.03341
ofxn mod
0.11736

sRun
RunA1
zout
1

Ave
Data
9.5E-06
Model
9.4E-06

data/vMax
9.5E-06

ofxng
263.459

Min d/m
1.09447

ave/min d
1
ave/min m
1.08066

326_Calib_S_A

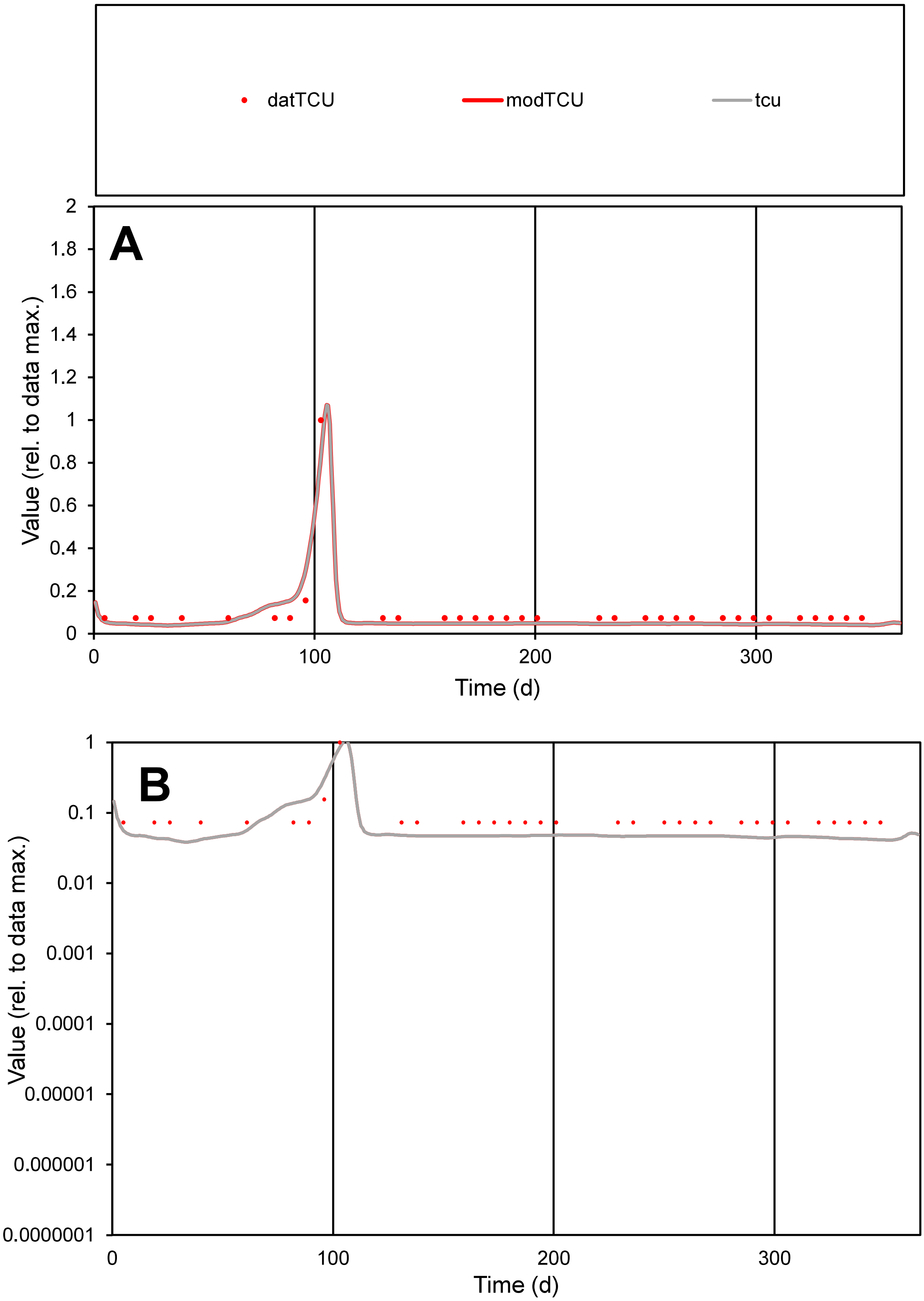

C1

Variables
TCU Provide jtd or Dst
CalibOnly?
2 1 to reproduce of
oNormalize?
2 1=no, 2=yes

ofxn calc*
0.395
ofxn mod
0.42306

sRun
RunA1
zout
1

Ave
Data
1.4E-05
Model
1.2E-05

data/vMax
0.00013

ofxng
263.459

Min d/m
1.77077

ave/min d
1.41902
ave/min m
2.1243

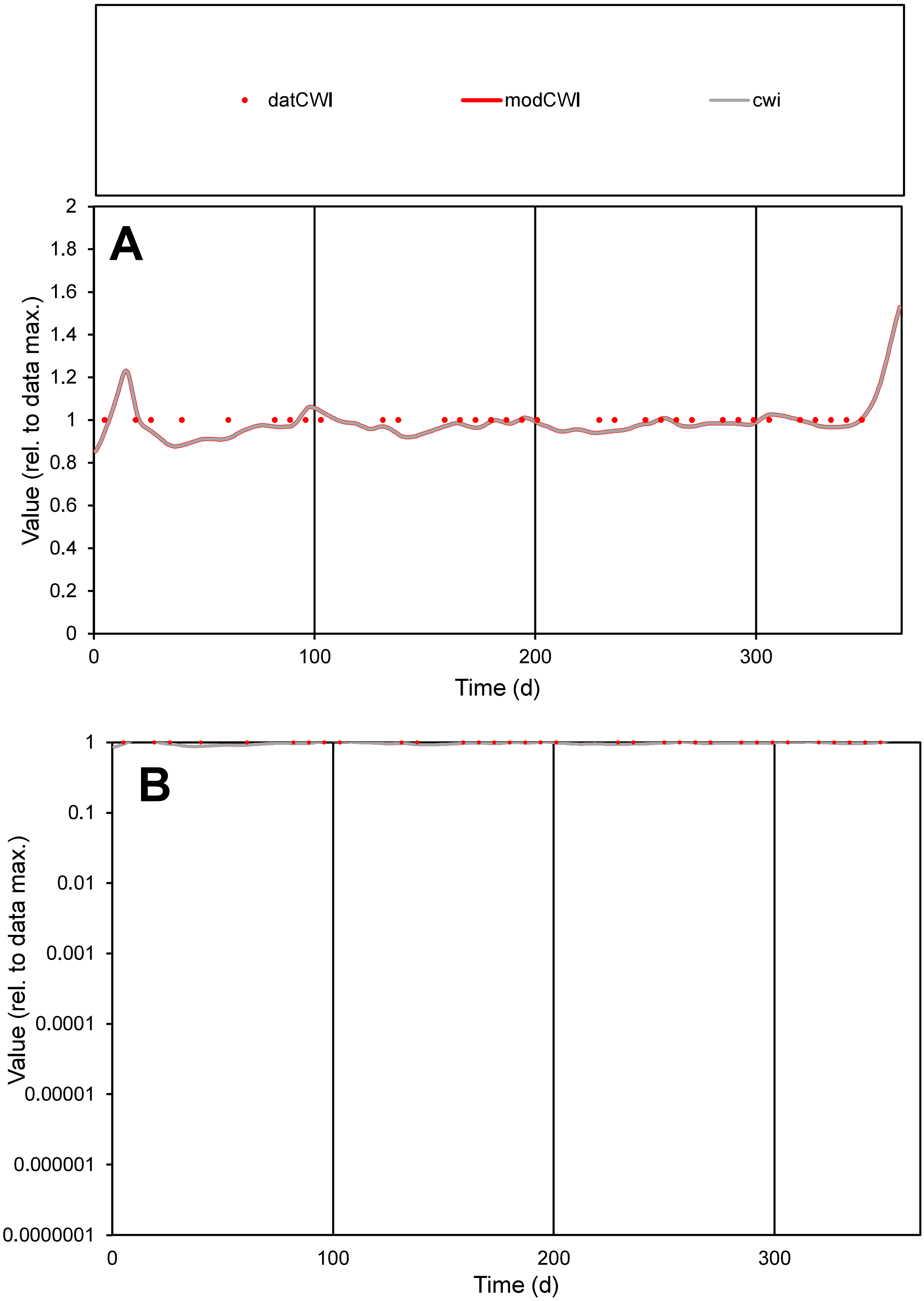

C1

Variables
CWI Provide jtd or Dst
CalibOnly?
2 1 to reproduce of
oNormalize?
2 1=no, 2=yes

ofxn calc*
0.03968
ofxn mod
0.11863

sRun
RunA1
zout
1

Ave
Data
1.3E-05
Model
1.3E-05

data/vMax
1.3E-05

ofxng
263.459

Min d/m
1.13106

ave/min d
1
ave/min m
1.10749

326_Calib_S_A

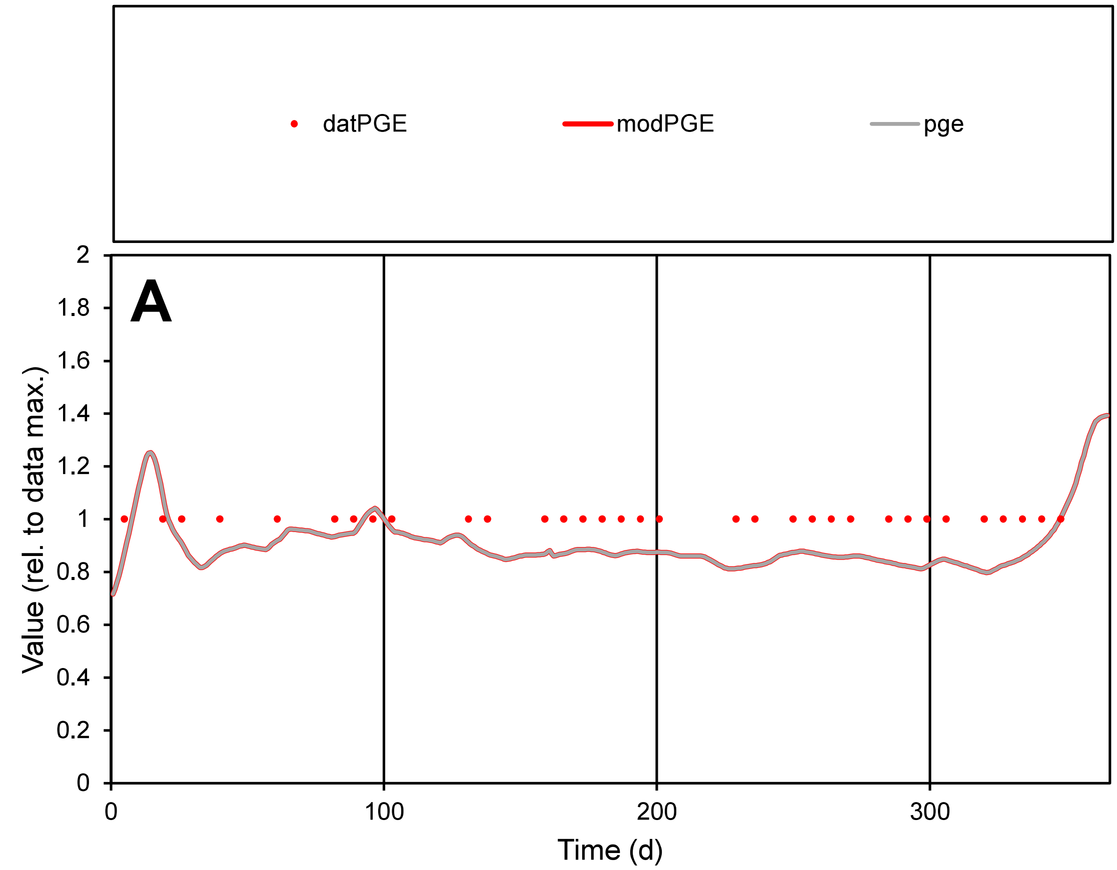

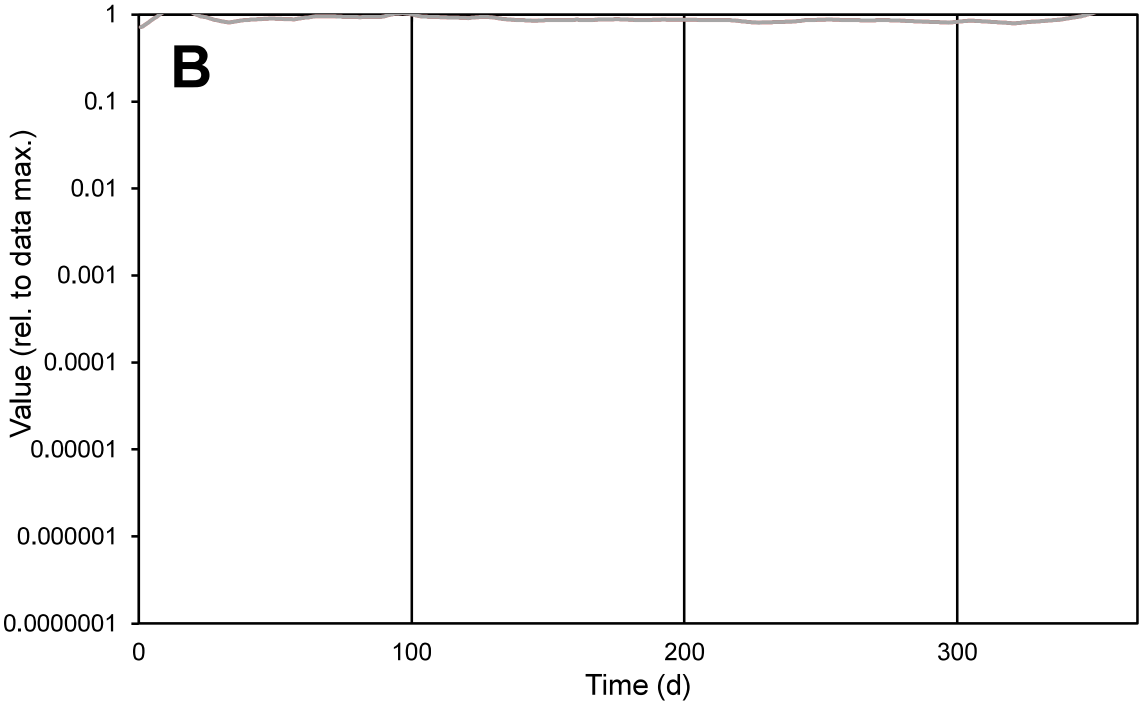

C1

Variables
PGE Provide jtd or Dst
CalibOnly?
2 1 to reproduce of
oNormalize?
2 1=no, 2=yes

ofxn calc*
0.12681
ofxn mod
0.16581

sRun
RunA1
zout
1

Ave
Data
5.9E-06
Model
5.2E-06

data/vMax
5.9E-06

ofxng
263.459

Min d/m
1.25266

ave/min d
1
ave/min m
1.11262

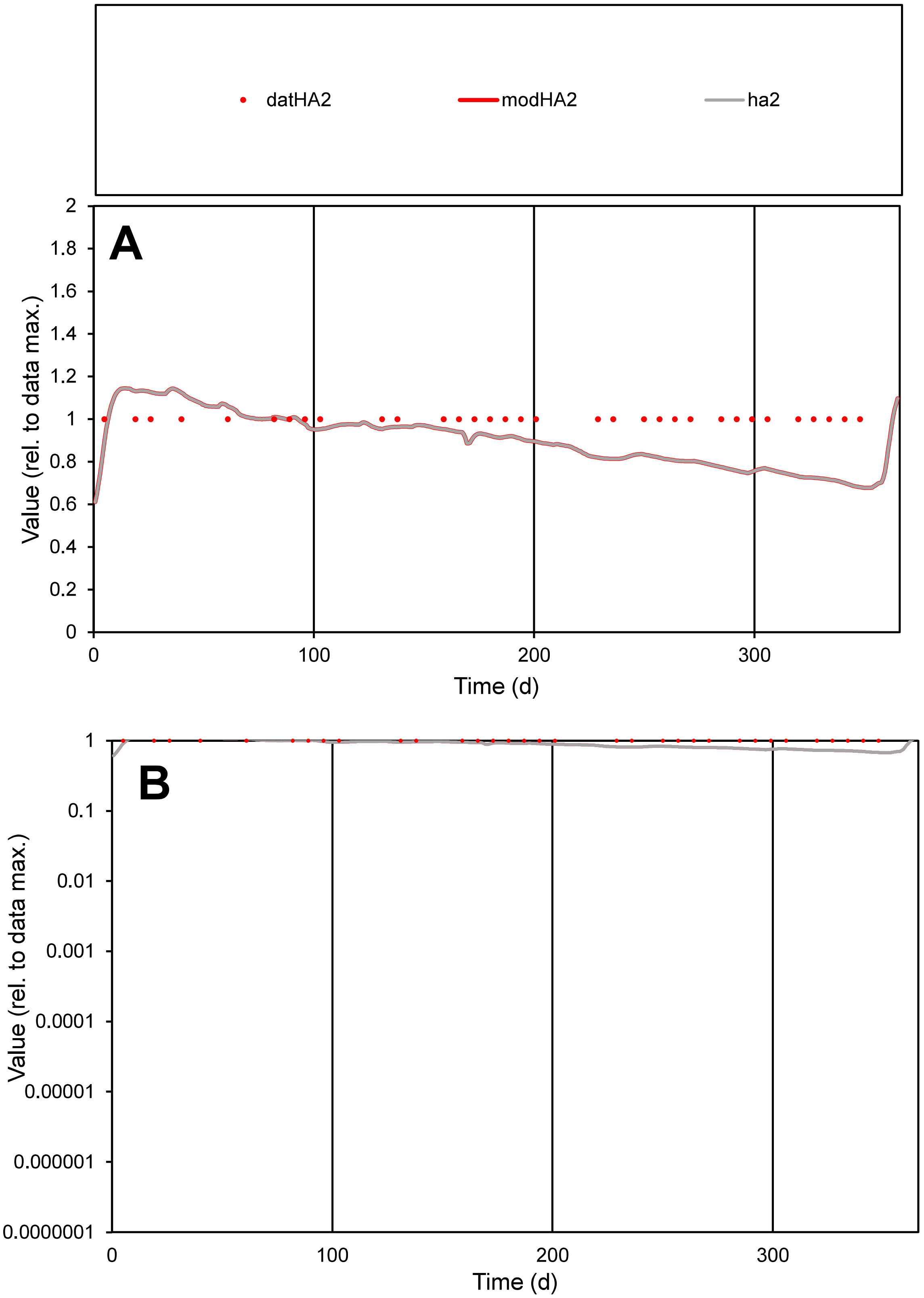

C1

Variables
HA2 Provide jtd or Dst
CalibOnly?
2 1 to reproduce of
oNormalize?
2 1=no, 2=yes

ofxn calc*
0.17086
ofxn mod
0.20195

sRun
RunA1
zout
1

Ave
Data
2.6E-06
Model
2.3E-06

data/vMax
2.6E-06

ofxng
263.459

Min d/m
1.47187

ave/min d
1

ave/min m
1.30274

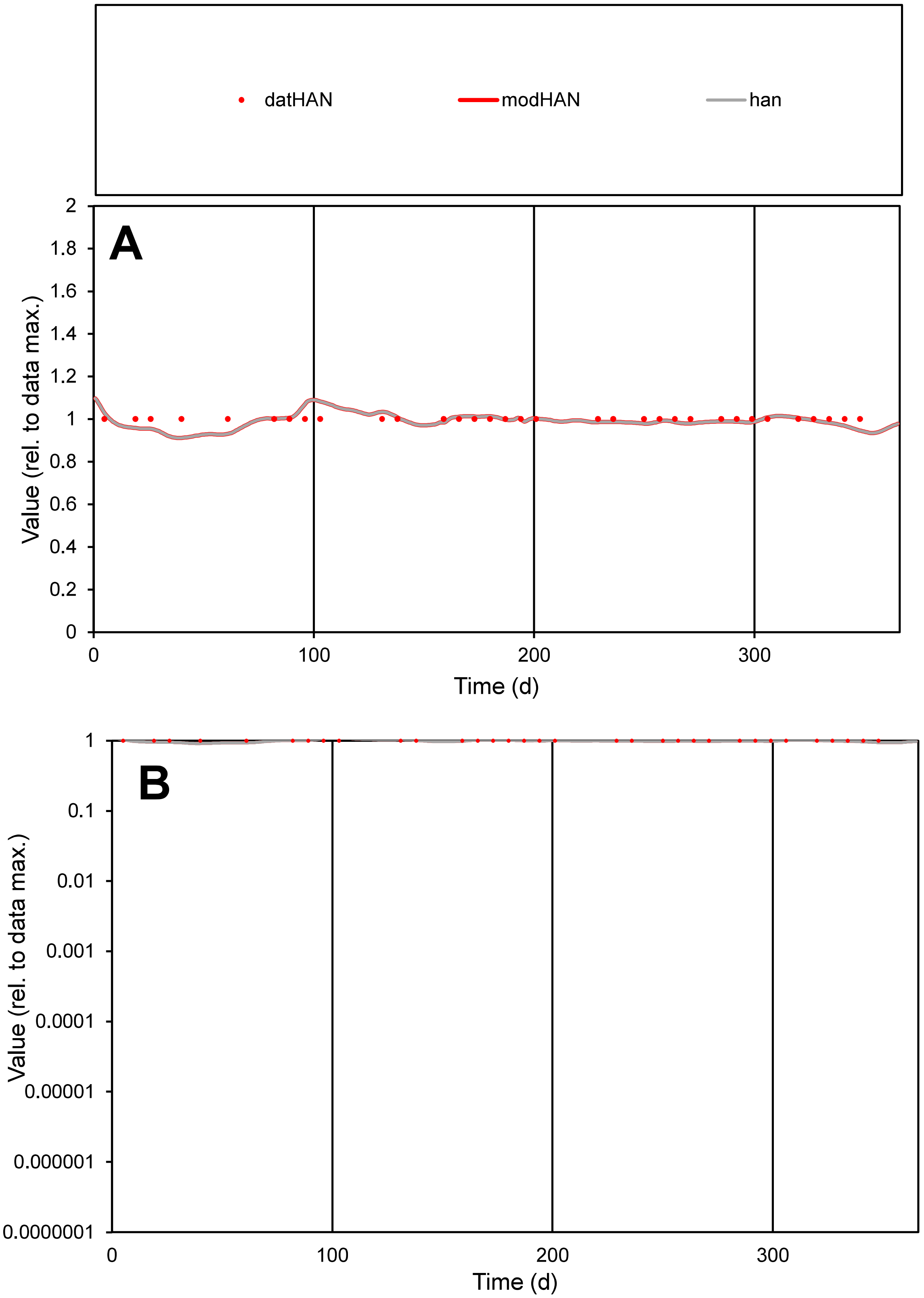

C1

Variables
HAN Provide jtd or Dst
CalibOnly?
2 1 to reproduce of
oNormalize?
2 1=no, 2=yes

ofxn calc*
0.03428
ofxn mod
0.11775

sRun
RunA1
zout
1

Ave
Data
5.1E-06
Model
5.1E-06

data/vMax
5.1E-06

ofxng
263.459

Min d/m
1.09511

ave/min d
1
ave/min m
1.08713

326_Calib_S_A

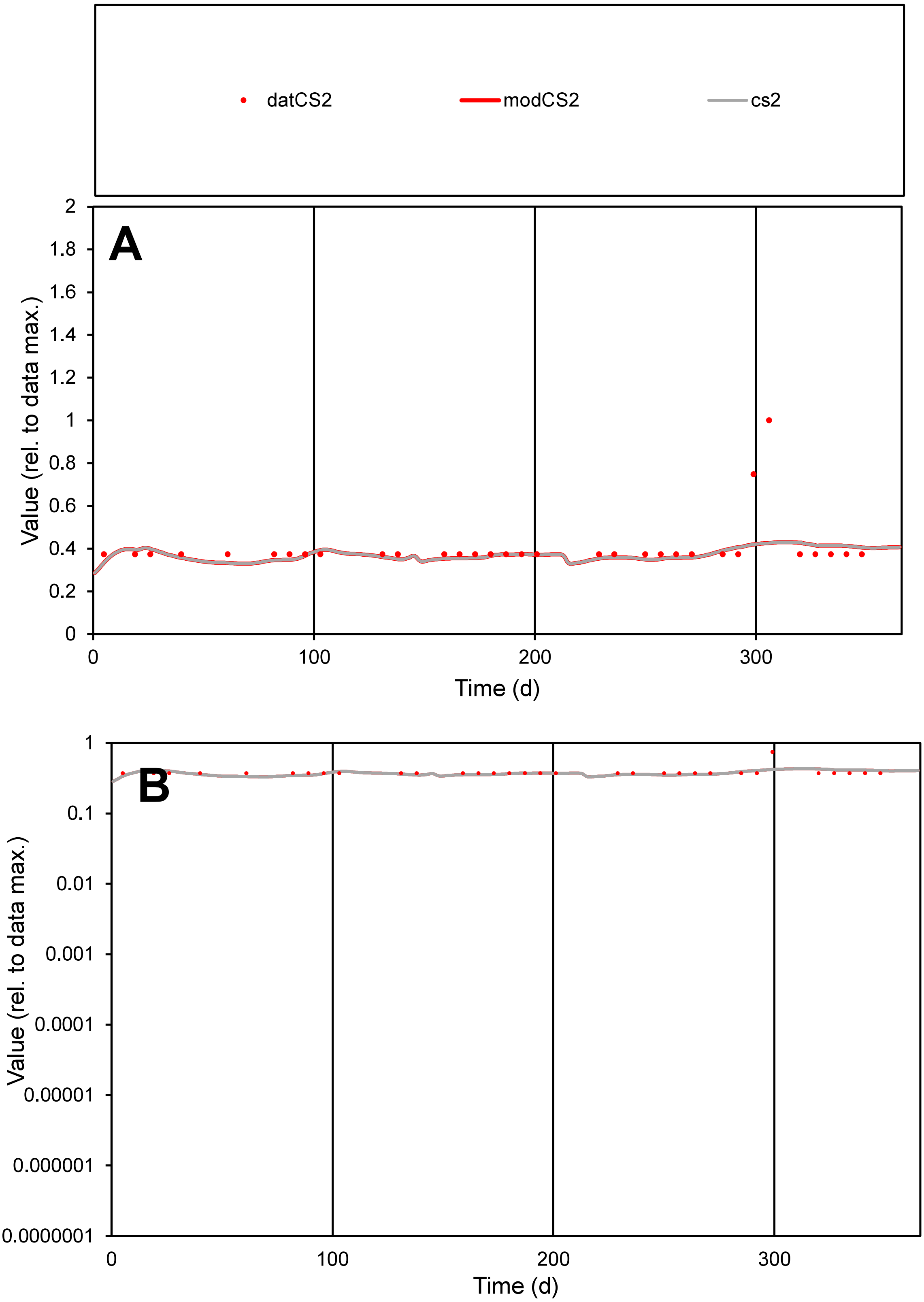

C1

Variables
CS2 Provide jtd or Dst
CalibOnly?
2 1 to reproduce of
oNormalize?
2 1=no, 2=yes

ofxn calc*
0.28972
ofxn mod
0.31069

sRun
RunA1
zout
1

Ave
Data
8.2E-06
Model
7.6E-06

data/vMax
2E-05

ofxng
263.459

Min d/m
1.12651

ave/min d
1.08107
ave/min m
1.13362

326_Calib_S_A

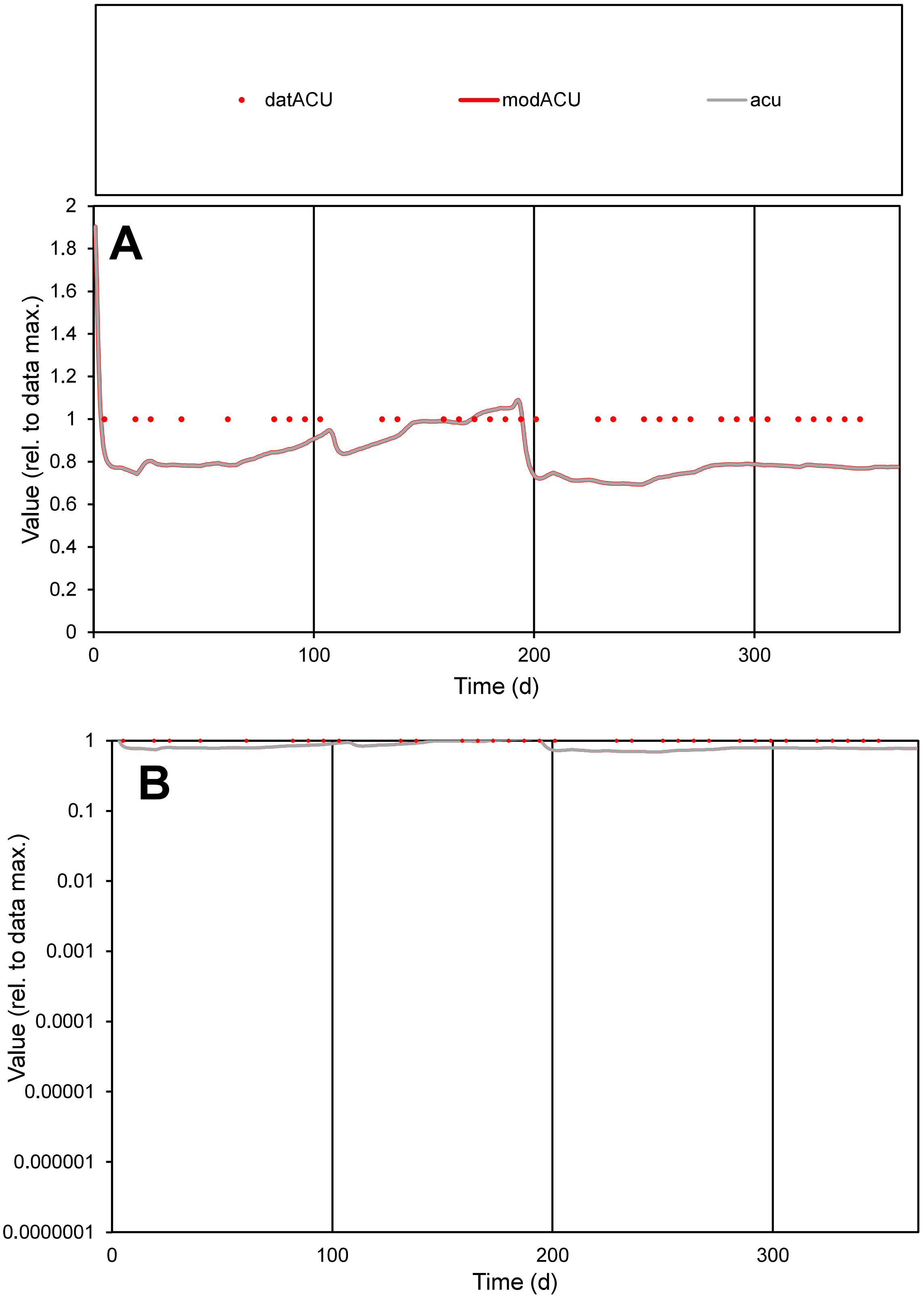

C1

Variables
ACU Provide jtd or Dst
CalibOnly?
2 1 to reproduce of
oNormalize?
2 1=no, 2=yes

ofxn calc*
0.19918
ofxn mod
0.22424

sRun
RunA1
zout
1

Ave
Data
1.1E-05
Model
9.2E-06

data/vMax
1.1E-05

ofxng
263.459

Min d/m
1.43392

ave/min d
1
ave/min m
1.18772

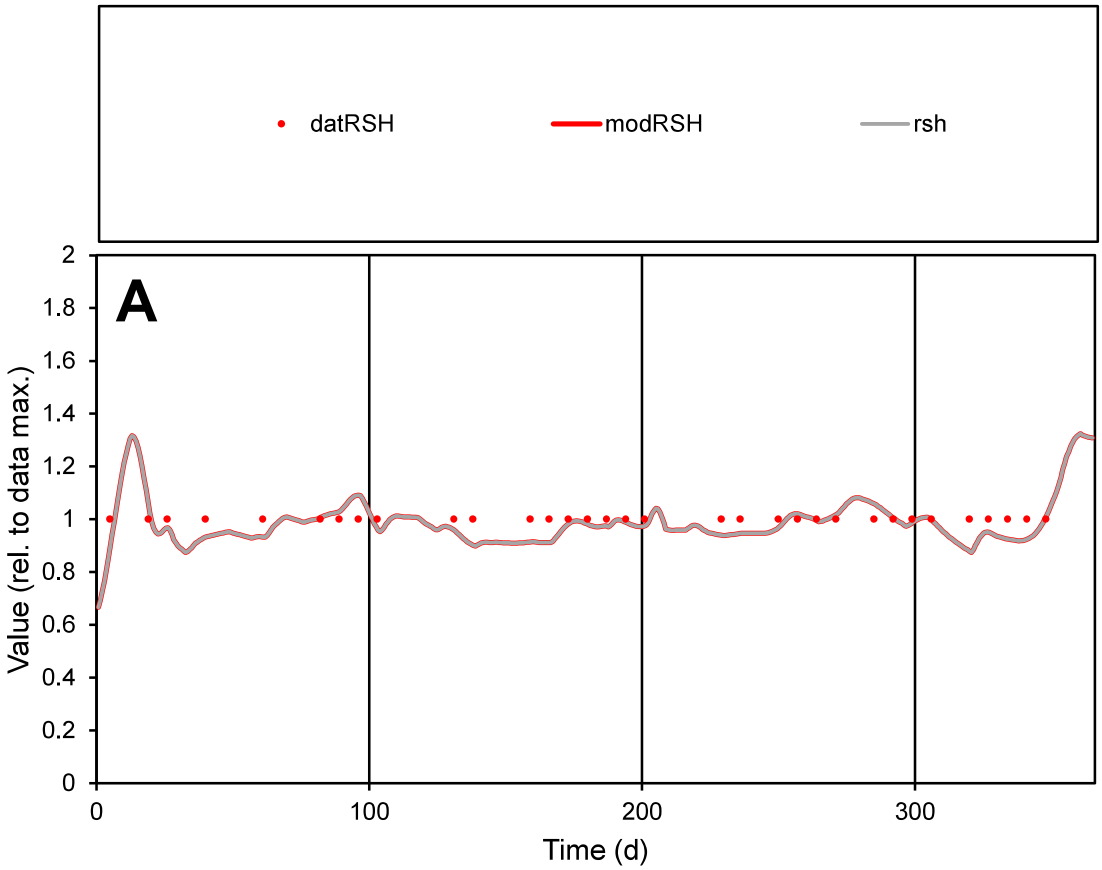

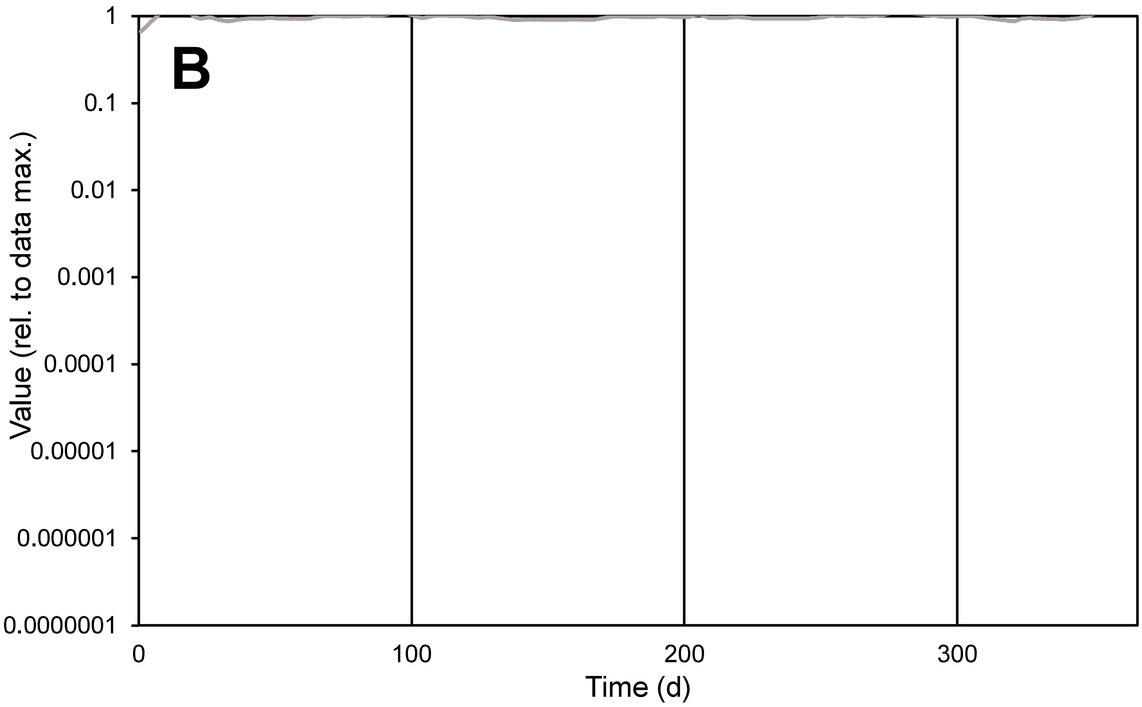

C1

Variables
RSH Provide jtd or Dst
CalibOnly?
2 1 to reproduce of
oNormalize?
2 1=no, 2=yes

ofxn calc*
0.0518
ofxn mod
0.1232

sRun
RunA1
zout
1

Ave
Data
1.8E-05
Model
1.7E-05

data/vMax
1.8E-05

ofxng
263.459

Min d/m
1.13038

ave/min d
1
ave/min m
1.10056

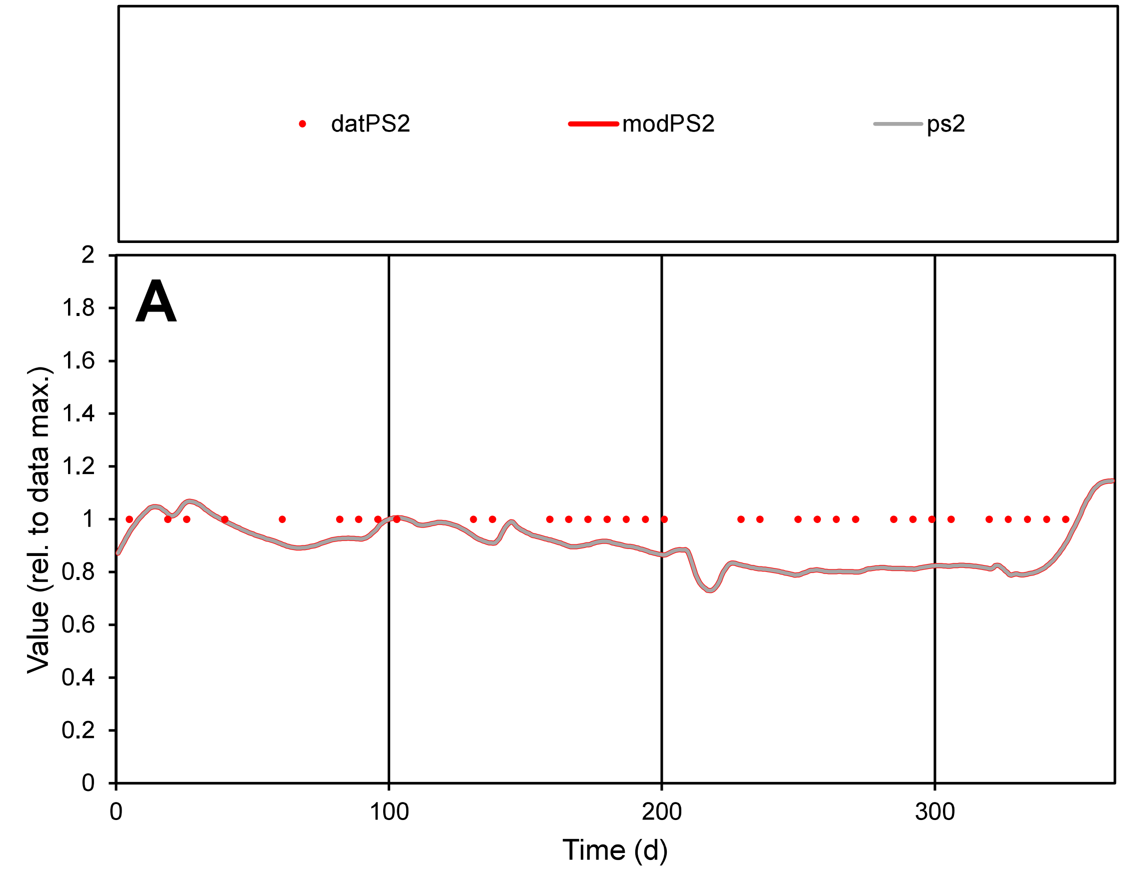

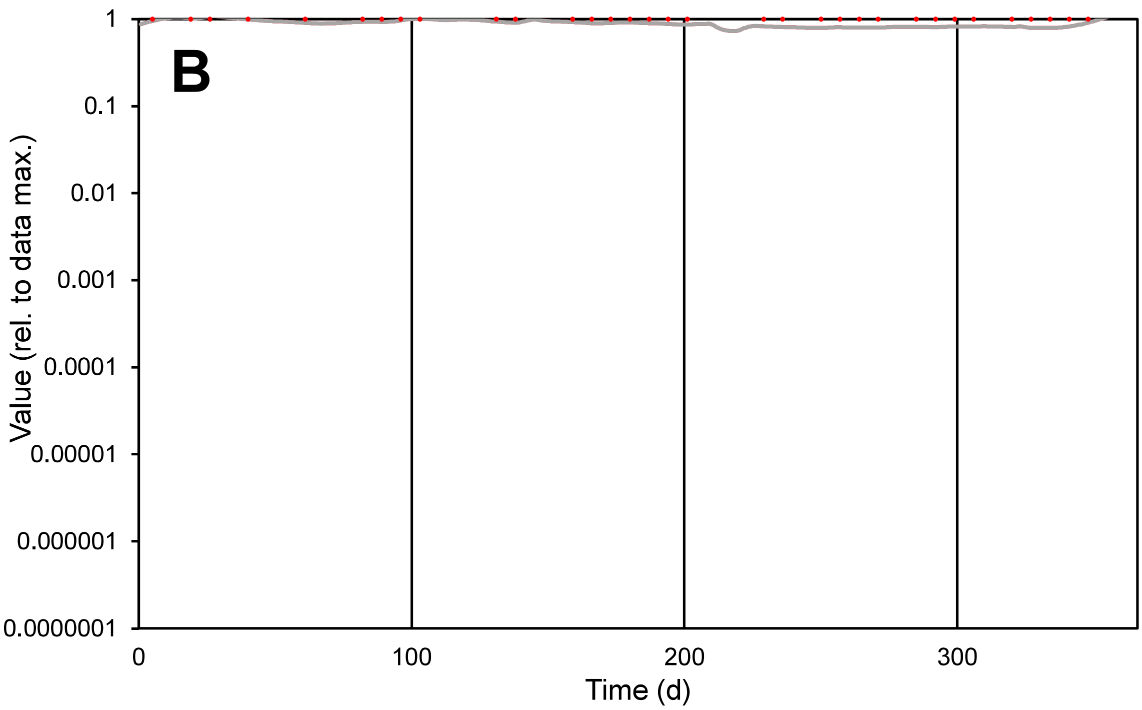

C1

Variables

PS2	Provide jtd or Dst
CalibOnly?	
2	1 to reproduce of
oNormalize?	
2	1=no, 2=yes

ofxn calc*
0.13745
ofxn mod
0.17397

sRun
RunA1
zout
1

Ave
Data
1.9E-05
Model
1.7E-05

data/vMax
1.9E-05

ofxng
263.459

Min d/m
1.26831

ave/min d
1
ave/min m
1.12194

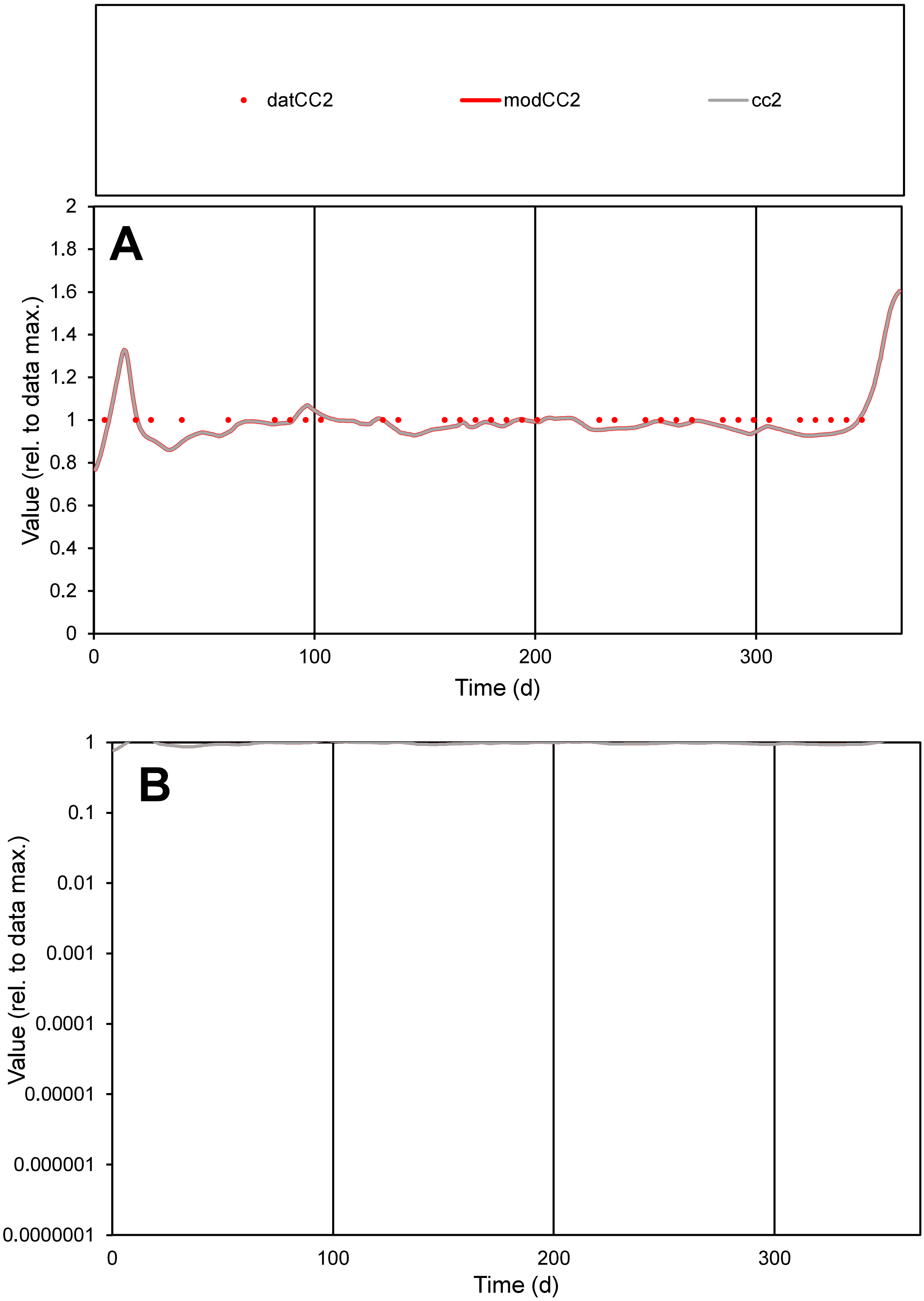

C1

Variables
CC2 Provide jtd or Dst
CalibOnly?
2 1 to reproduce of
oNormalize?
2 1=no, 2=yes

ofxn calc*
0.04357
ofxn mod
0.11971

sRun
RunA1
zout
1

Ave
Data
5.9E-06
Model
5.7E-06

data/vMax
5.9E-06

ofxng
263.459

Min d/m
1.10964

ave/min d
1
ave/min m
1.07982

326_Calib_S_A

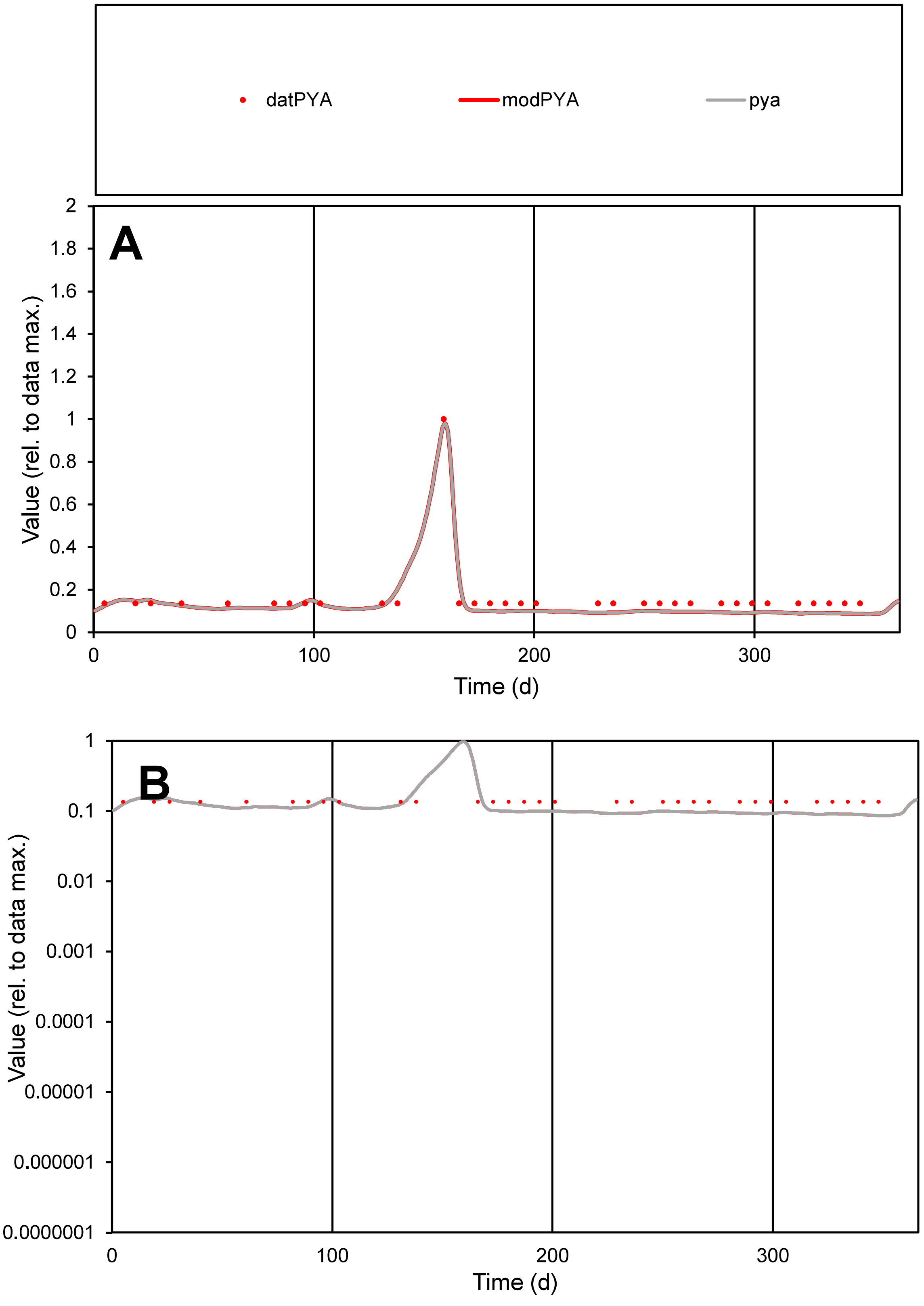

C1

Variables
PYA Provide jtd or Dst
CalibOnly?
2 1 to reproduce of
oNormalize?
2 1=no, 2=yes

ofxn calc*
0.22261
ofxn mod
0.24752

sRun
RunA1
zout
1

Ave
Data
2.2E-06
Model
1.8E-06

data/vMax
1.3E-05

ofxng
263.459

Min d/m
1.56883

ave/min d
1.19296
ave/min m
1.5936

326_Calib_S_A

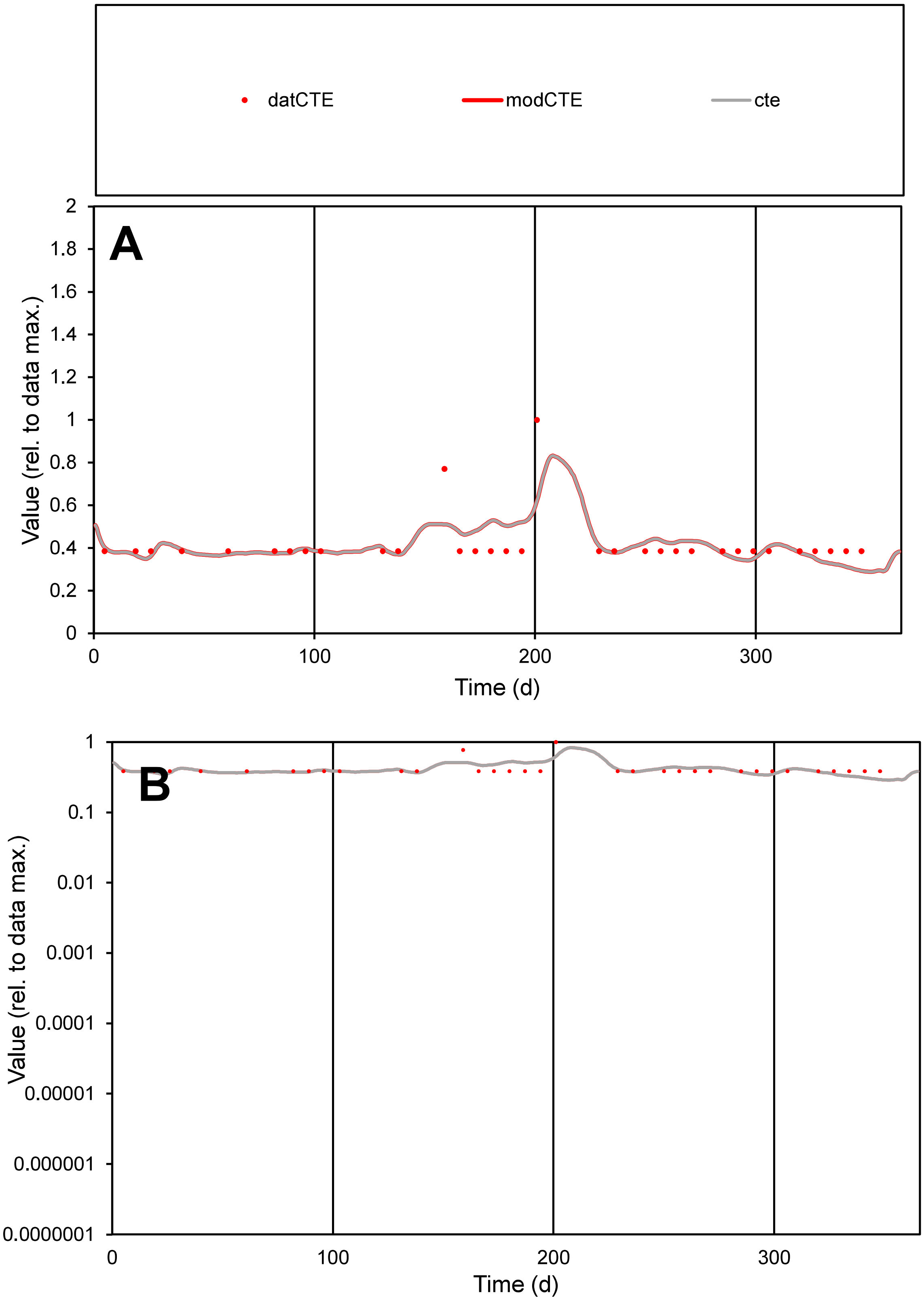

C1

Variables
CTE Provide jtd or Dst
CalibOnly?
2 1 to reproduce of
oNormalize?
2 1=no, 2=yes

ofxn calc*
0.22168
ofxn mod
0.24874

sRun
RunA1
zout
1

Ave
Data
6.6E-06
Model
6.5E-06

data/vMax
1.6E-05

ofxng
263.459

Min d/m
1.3214

ave/min d
1.07874
ave/min m
1.40581

326_Calib_S_A

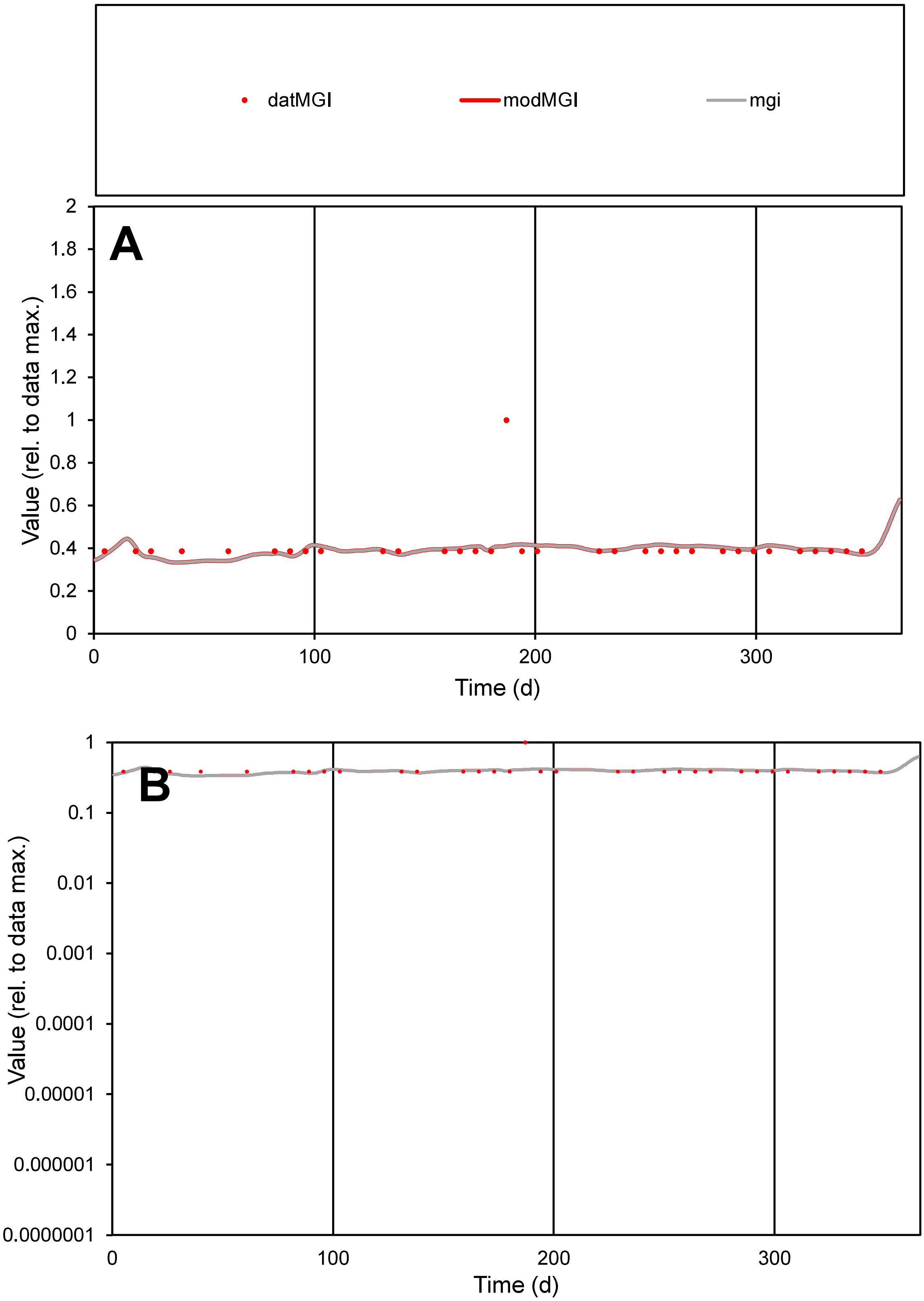

C1

Variables
MGI Provide jtd or Dst
CalibOnly?
2 1 to reproduce of
oNormalize?
2 1=no, 2=yes

ofxn calc*
0.25772
ofxn mod
0.28147

sRun
RunA1
zout
1

Ave
Data
1.1E-05
Model
1E-05

data/vMax
2.7E-05

ofxng
263.459

Min d/m
1.15488

ave/min d
1.04824
ave/min m
1.17309

326_Calib_S_A

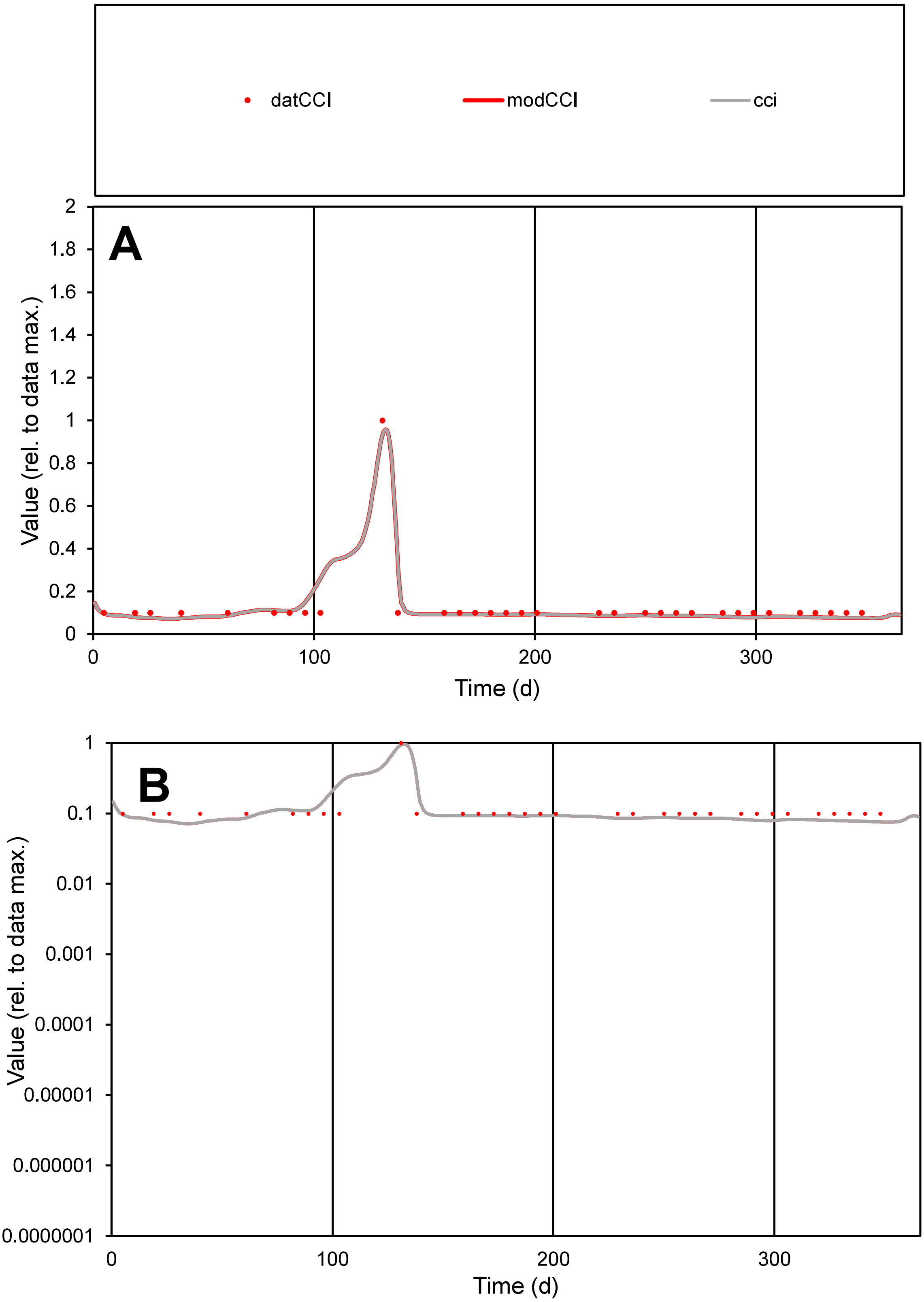

C1

Variables
CCI Provide jtd or Dst
CalibOnly?
2 1 to reproduce of
oNormalize?
2 1=no, 2=yes

ofxn calc*
0.28218
ofxn mod
0.34345

sRun
RunA1
zout
1

Ave
Data
1.5E-05
Model
1.4E-05

data/vMax
0.00012

ofxng
263.459

Min d/m
1.30886

ave/min d
1.27642
ave/min m
1.61794

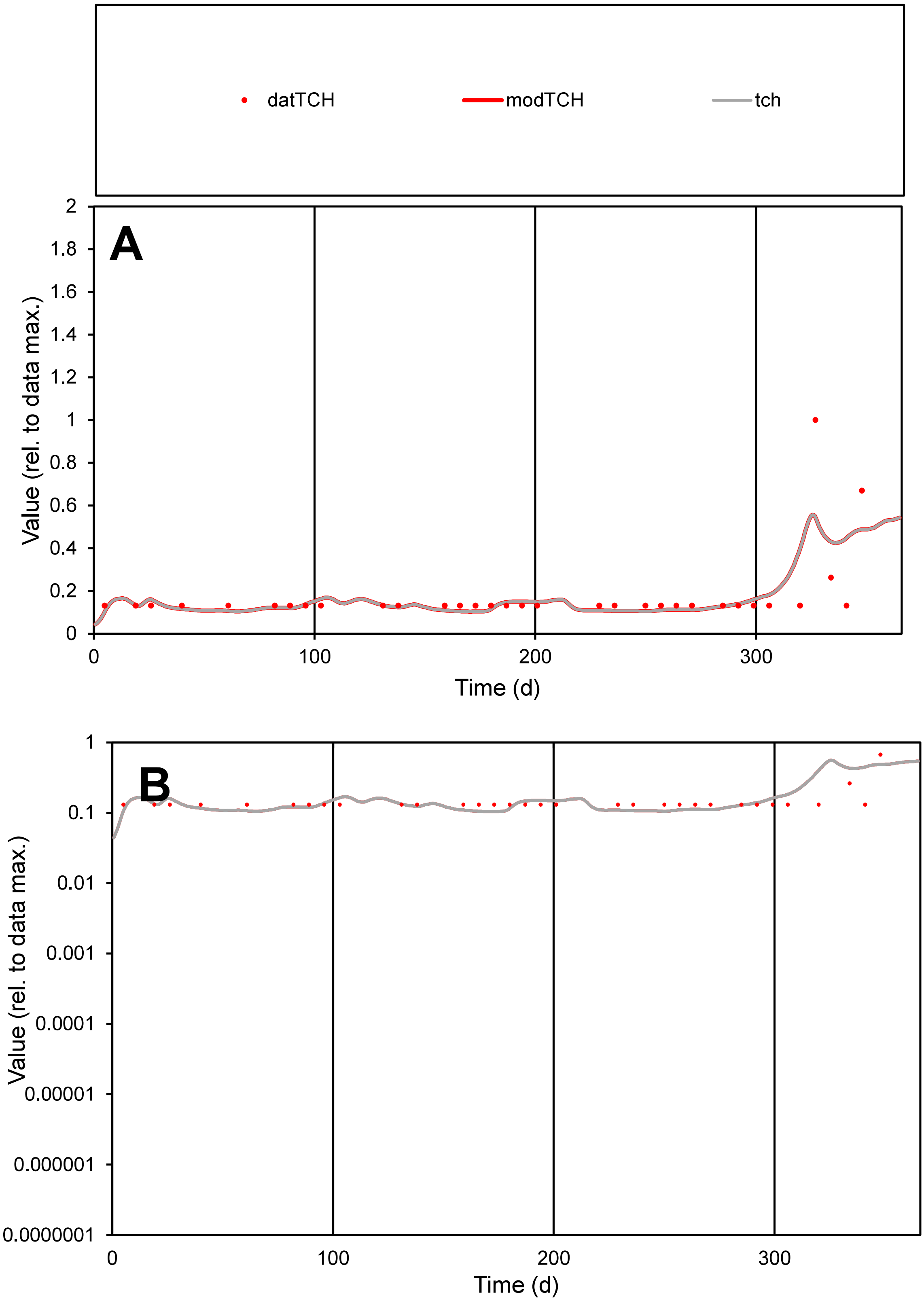

C1

Variables
TCH Provide jtd or Dst
CalibOnly?
2 1 to reproduce of
oNormalize?
2 1=no, 2=yes

ofxn calc*
0.67712
ofxn mod
0.69168

sRun
RunA1
zout
1

Ave
Data
2.3E-06
Model
2.3E-06

data/vMax
1.3E-05

ofxng
263.459

Min d/m
1.2571

ave/min d
1.35548
ave/min m
1.70823

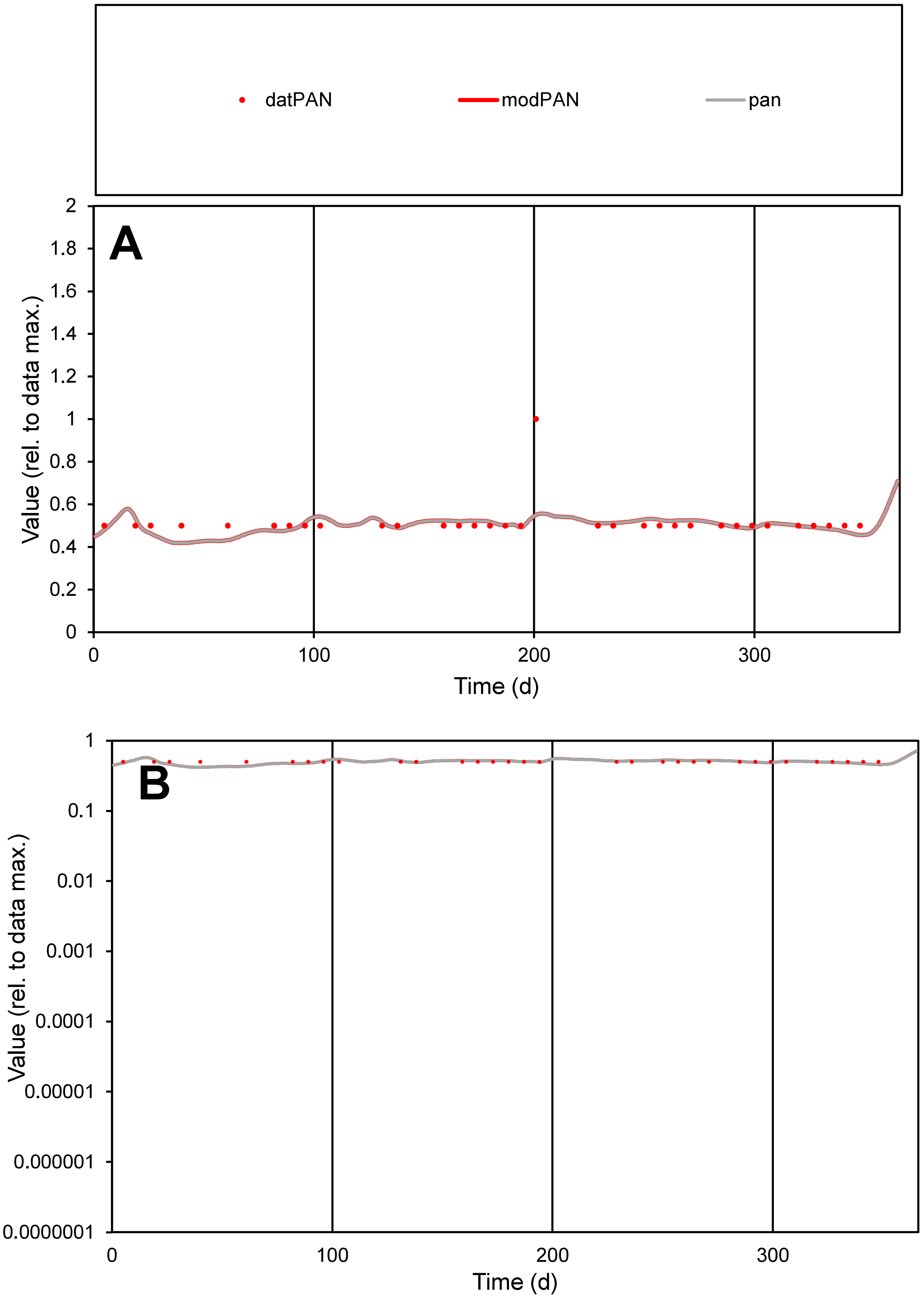

C1

Variables
PAN Provide jtd or Dst
CalibOnly?
2 1 to reproduce of
oNormalize?
2 1=no, 2=yes

ofxn calc*
0.15925
ofxn mod
0.19438

sRun
RunA1
zout
1

Ave
Data
9.4E-06
Model
9.1E-06

data/vMax
1.8E-05

ofxng
263.459

Min d/m
1.19294

ave/min d
1.0303
ave/min m
1.19317

326_Calib_S_A

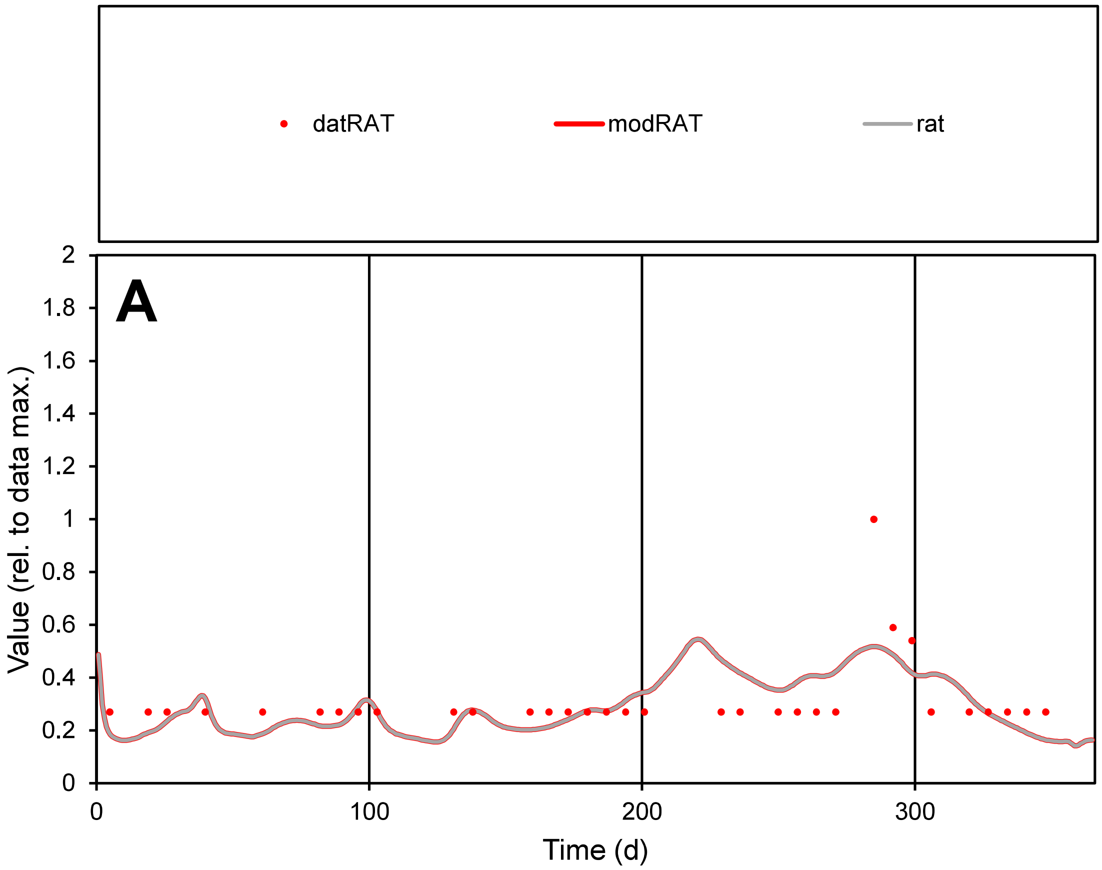

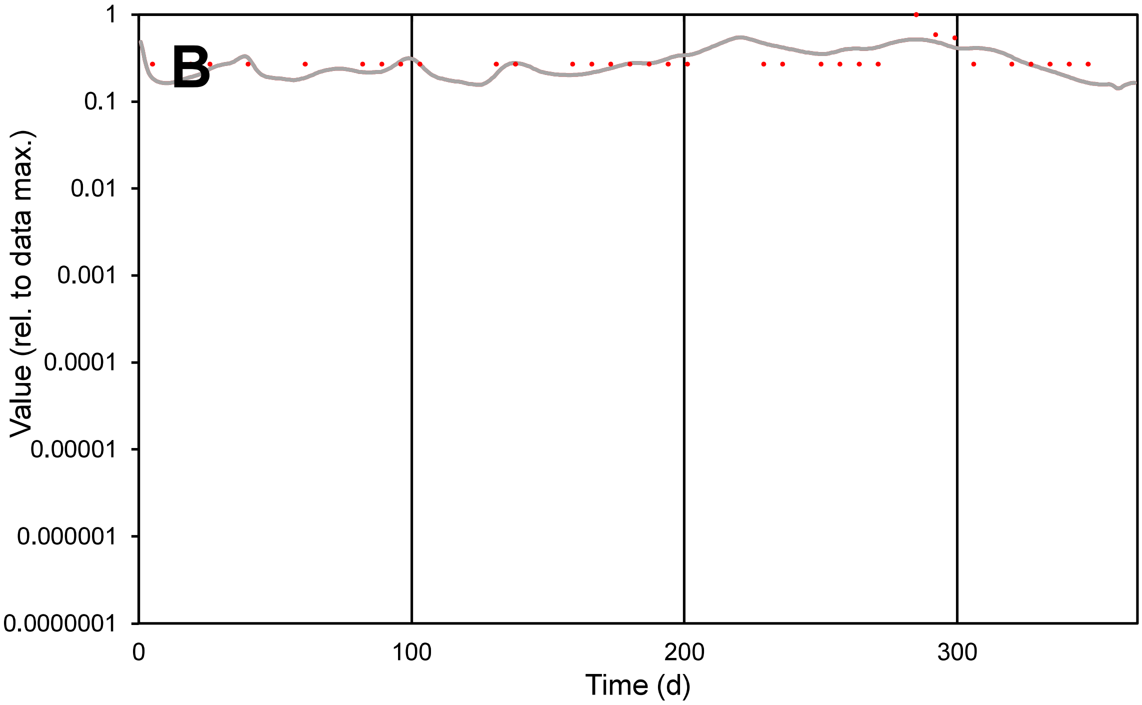

C1

Variables
RAT Provide jtd or Dst
CalibOnly?
2 1 to reproduce of
oNormalize?
2 1=no, 2=yes

ofxn calc*
0.3856
ofxn mod
0.40505

sRun
RunA1
zout
1

Ave
Data
1.3E-05
Model
1.3E-05

data/vMax
4.2E-05

ofxng
263.459

Min d/m
1.66325

ave/min d
1.14824
ave/min m
1.851

326_Calib_S_A

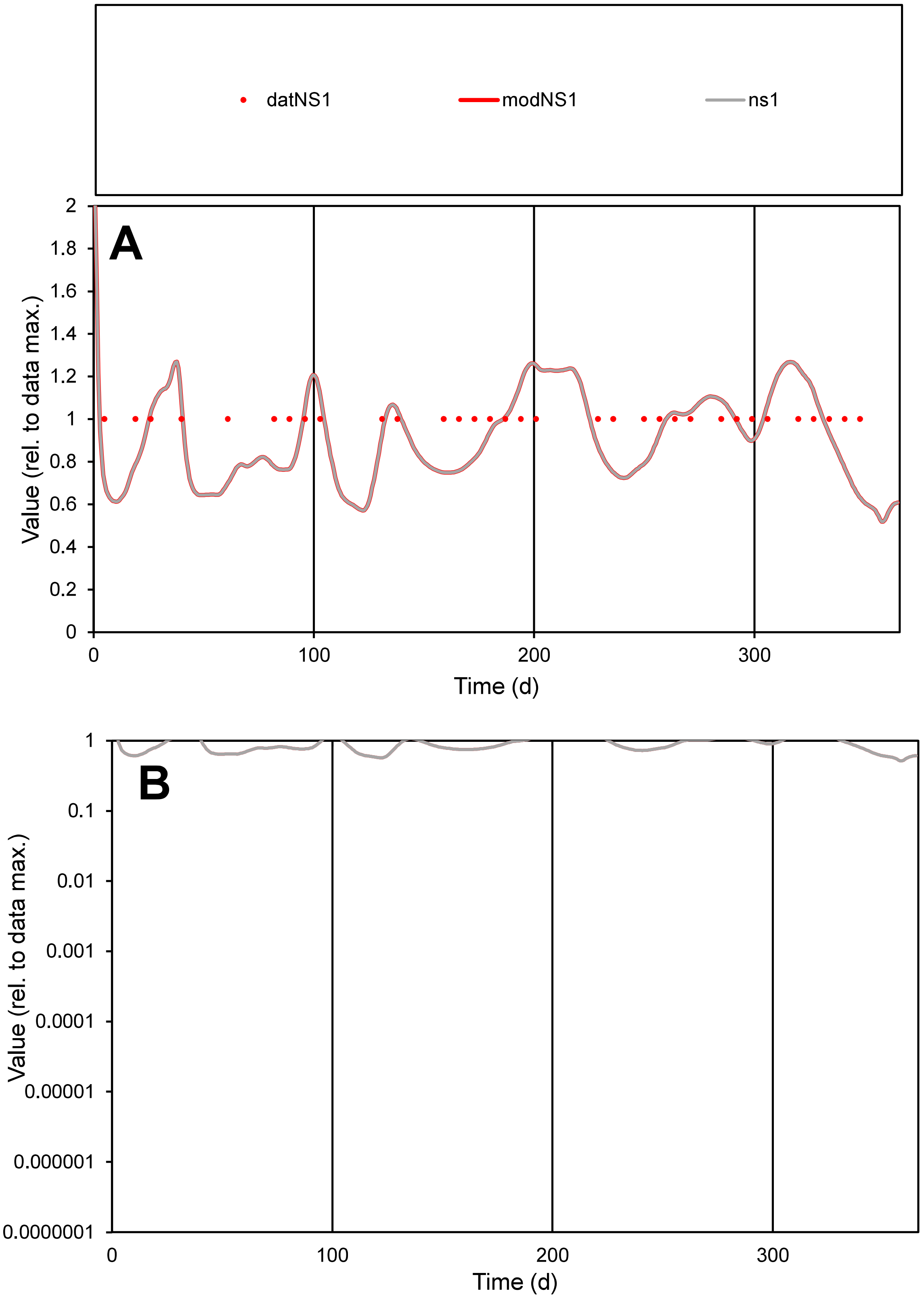

C1

Variables

NS1 Provide jtd or Dst

CalibOnly?

2 1 to reproduce of

oNormalize?

2 1=no, 2=yes

ofxn calc*

0.17669

ofxn mod

0.21002

sRun

RunA1

zout

1

Ave

Data

2.7E-05

Model

2.6E-05

data/vMax

2.7E-05

ofxng

263.459

Min d/m

1.61624

ave/min d

1

ave/min m

1.51383

326_Calib_S_A

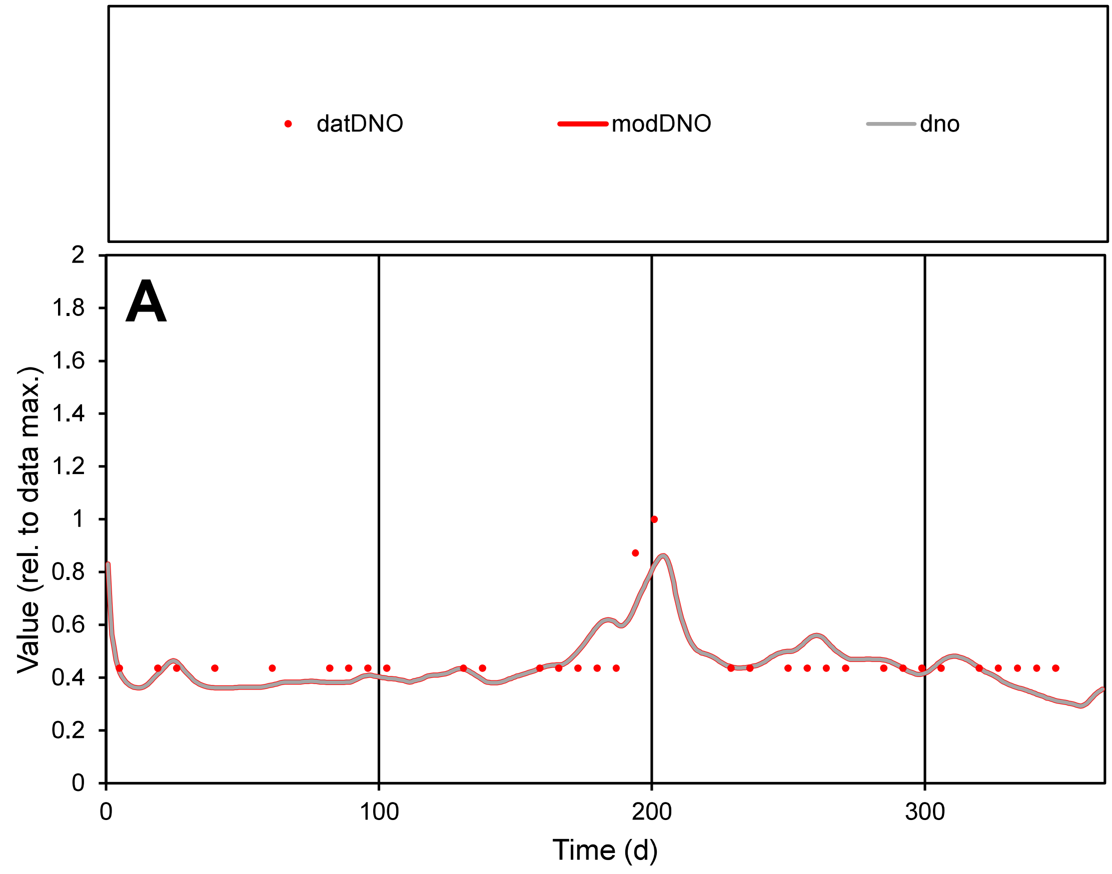

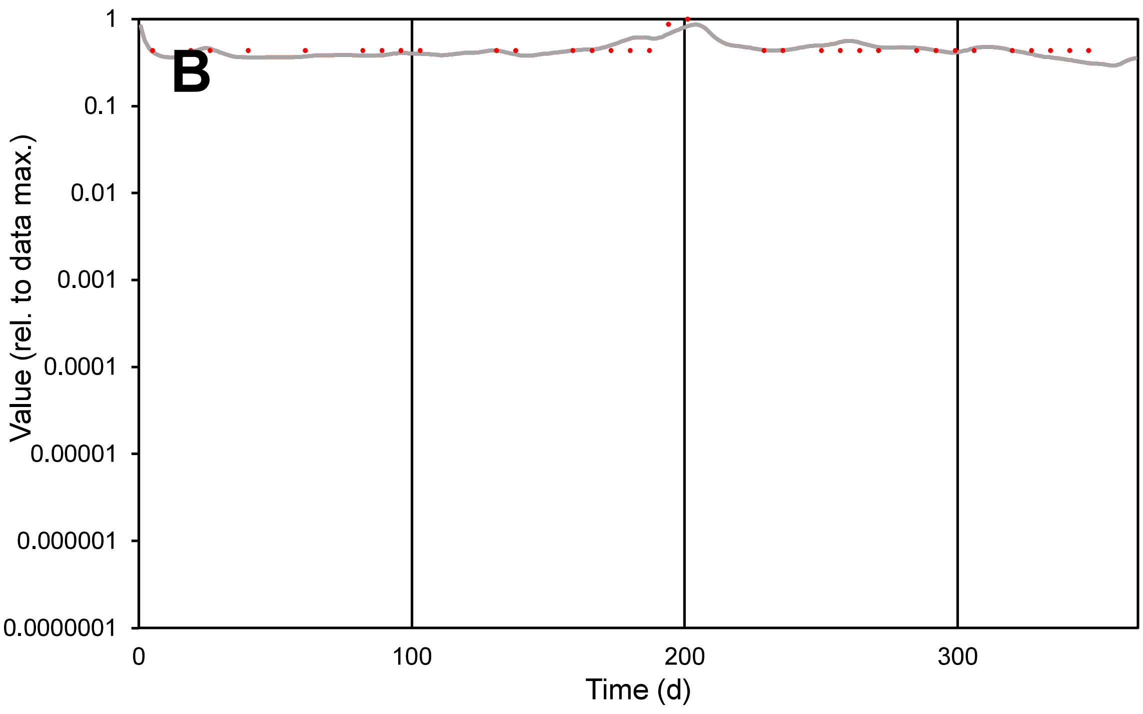

C1

Variables
DNO Provide jtd or Dst
CalibOnly?
2 1 to reproduce of
oNormalize?
2 1=no, 2=yes

ofxn calc*
0.16559
ofxn mod
0.20277

sRun
RunA1
zout
1

Ave
Data
1.9E-05
Model
1.9E-05

data/vMax
4.2E-05

ofxng
263.459

Min d/m
1.40201

ave/min d
1.0696
ave/min m
1.47119

326_Calib_S_A

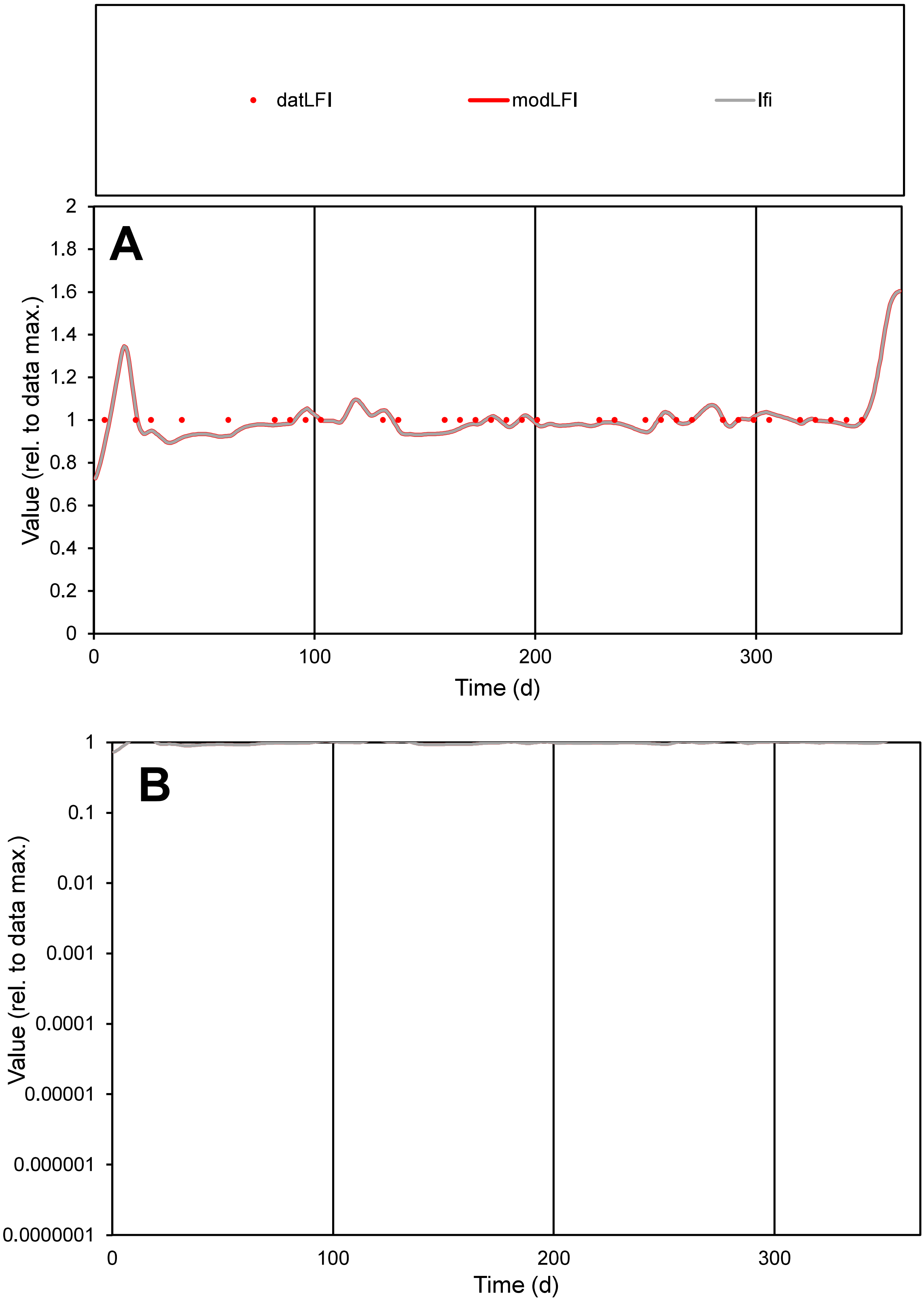

C1

Variables
LFI Provide jtd or Dst
CalibOnly?
2 1 to reproduce of
oNormalize?
2 1=no, 2=yes

ofxn calc*
0.03484
ofxn mod
0.1176

sRun
RunA1
zout
1

Ave
Data
8.3E-06
Model
8.2E-06

data/vMax
8.3E-06

ofxng
263.459

Min d/m
1.08558

ave/min d
1
ave/min m
1.07093

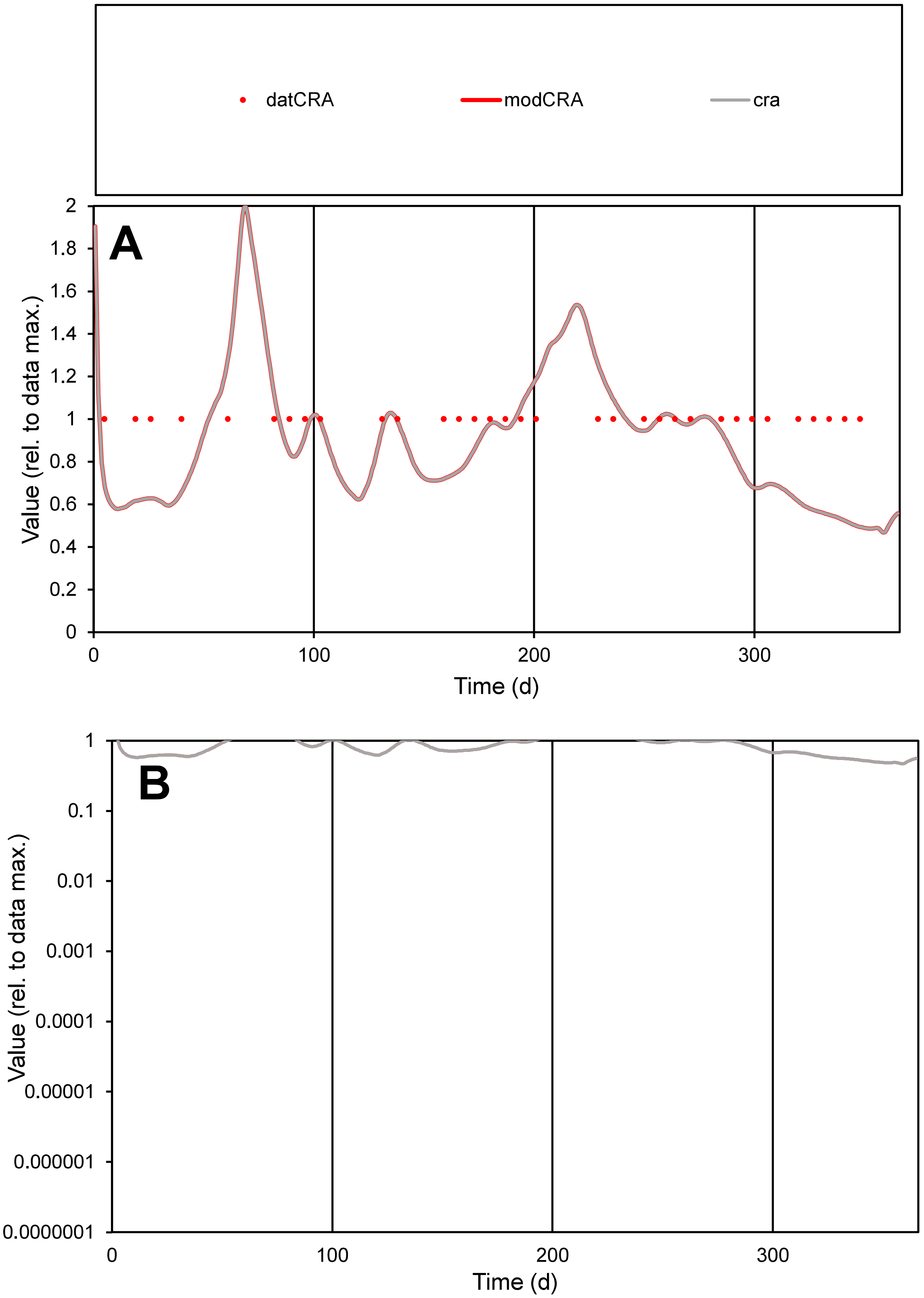

C1

Variables
CRA Provide jtd or Dst
CalibOnly?
2 1 to reproduce of
oNormalize?
2 1=no, 2=yes

ofxn calc*
0.25506
ofxn mod
0.28046

sRun
RunA1
zout
1

Ave
Data
4.9E-06
Model
4.2E-06

data/vMax
4.9E-06

ofxng
263.459

Min d/m
2.0343

ave/min d
1

ave/min m
1.74474

326_Calib_S_A

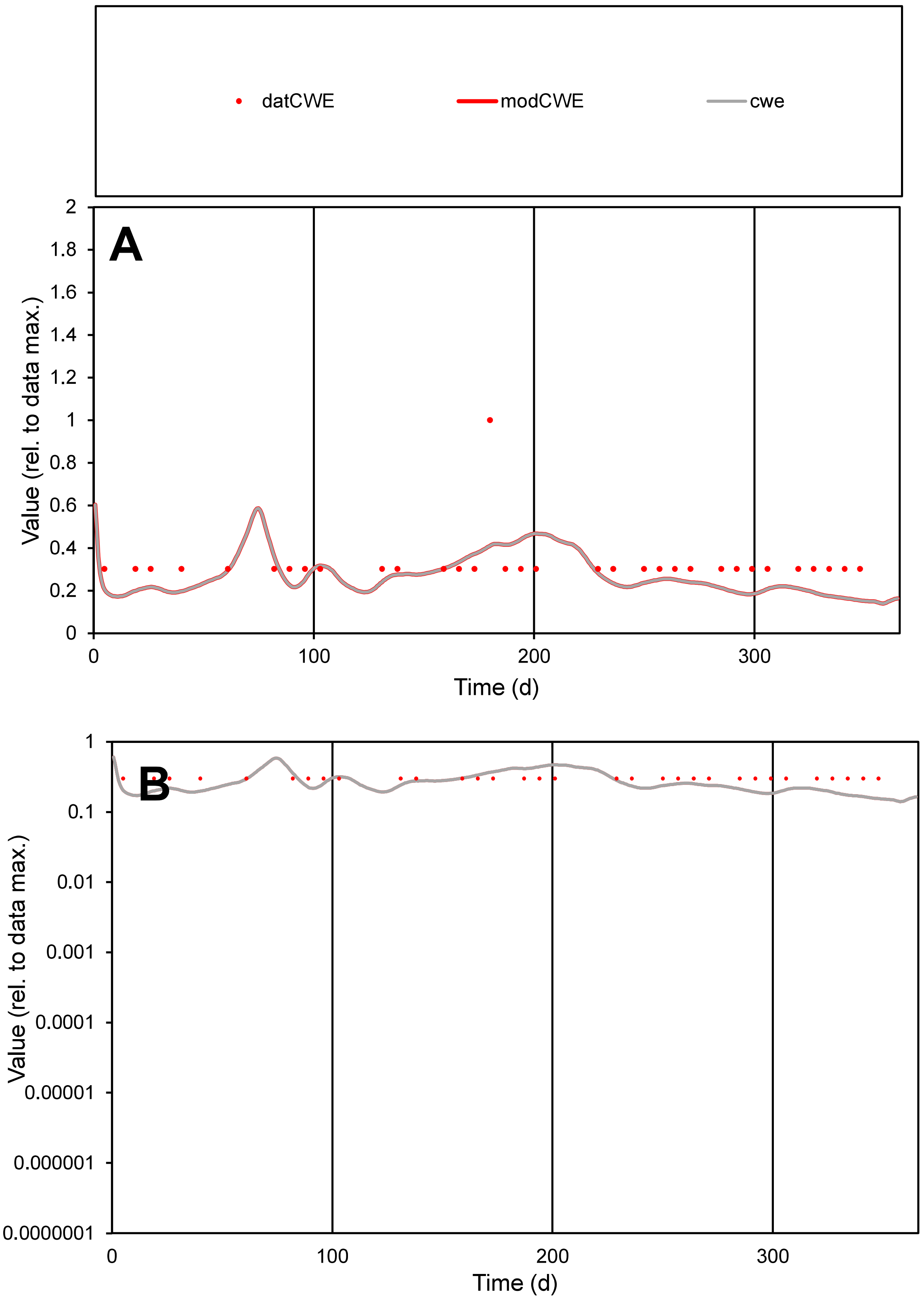

C1

Variables

CWE Provide jtd or Dst

CalibOnly?

2 1 to reproduce of

oNormalize?

2 1=no, 2=yes

ofxn calc*

0.41638

ofxn mod

0.43071

sRun

RunA1

zout

1

Ave

Data

2.7E-05

Model

2.2E-05

data/vMax

8.5E-05

ofxng

263.459

Min d/m

1.96456

ave/min d

1.07015

ave/min m

1.724

326_Calib_S_A

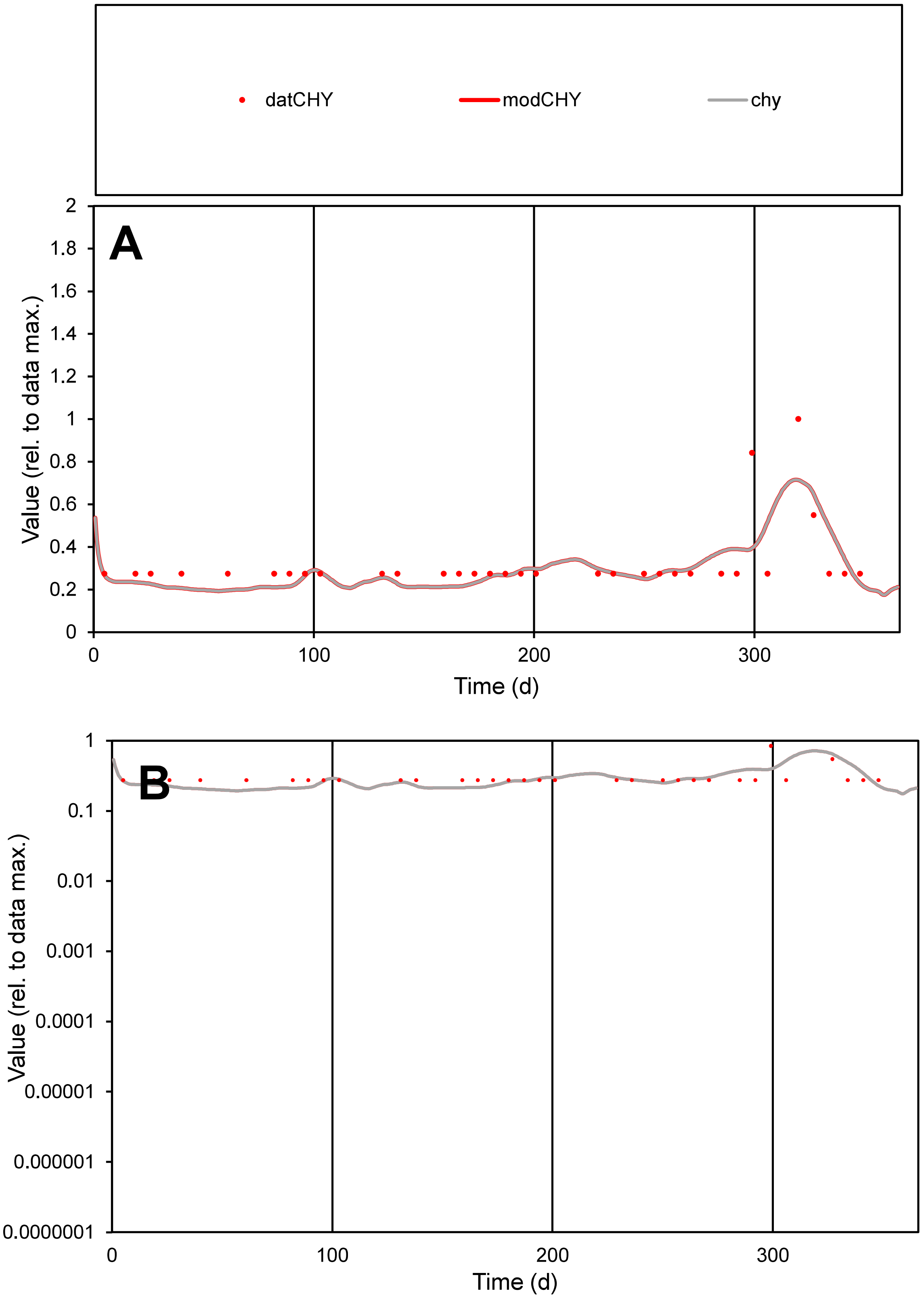

C1

Variables
CHY Provide jtd or Dst
CalibOnly?
2 1 to reproduce of
oNormalize?
2 1=no, 2=yes

ofxn calc*
0.36148
ofxn mod
0.3809

sRun
RunA1
zout
1

Ave
Data
7.9E-06
Model
7.5E-06

data/vMax
2.4E-05

ofxng
263.459

Min d/m
1.38913

ave/min d
1.17298
ave/min m
1.54672

326_Calib_S_A

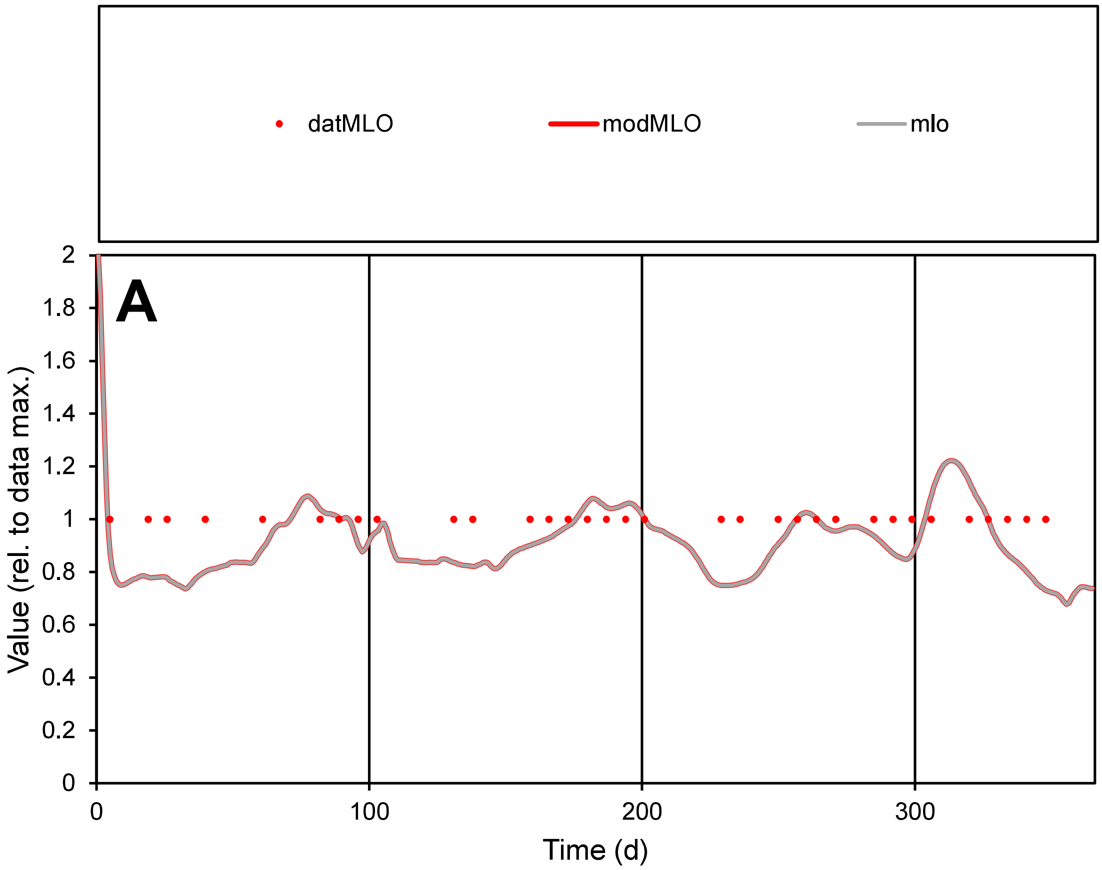

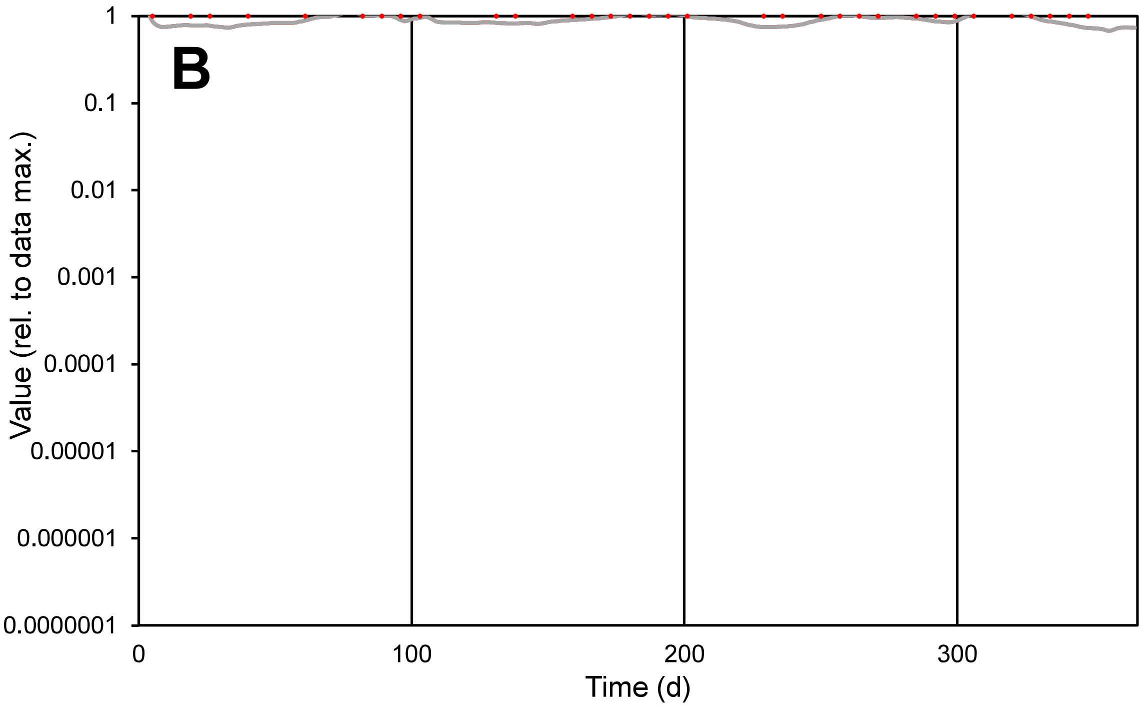

C1

Variables
MLO Provide jtd or Dst
CalibOnly?
2 1 to reproduce of
oNormalize?
2 1=no, 2=yes

ofxn calc*
0.13886
ofxn mod
0.17631

sRun
RunA1
zout
1

Ave
Data
1E-05
Model
9.6E-06

data/vMax
1E-05

ofxng
263.459

Min d/m
1.37763

ave/min d
1

ave/min m
1.26242

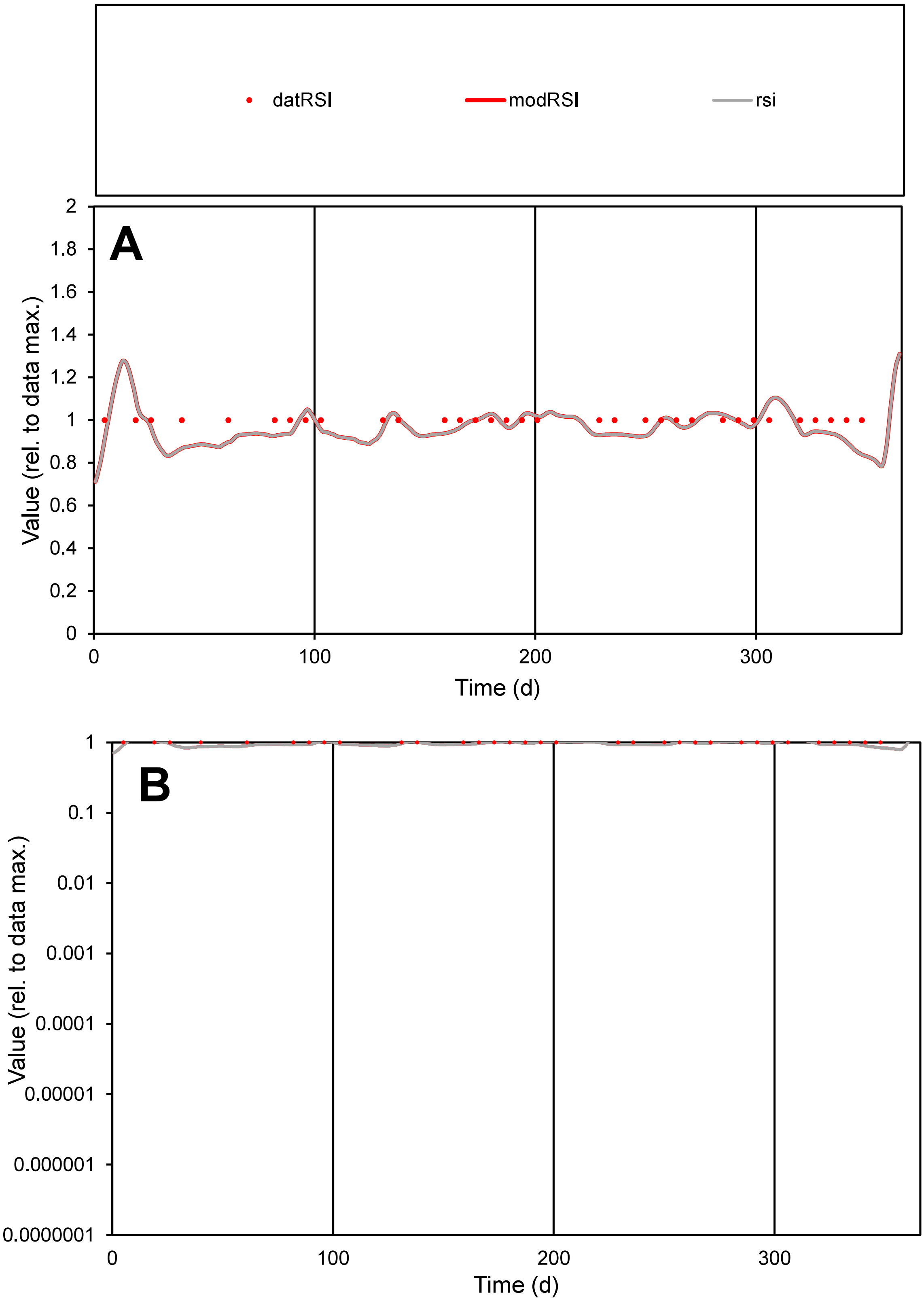

C1

Variables
RSI Provide jtd or Dst
CalibOnly?
2 1 to reproduce of
oNormalize?
2 1=no, 2=yes

ofxn calc*
0.06376
ofxn mod
0.12851

sRun
RunA1
zout
1

Ave
Data
1E-05
Model
1E-05

data/vMax
1E-05

ofxng
263.459

Min d/m
1.19907

ave/min d
1
ave/min m
1.16075

326_Calib_S_A

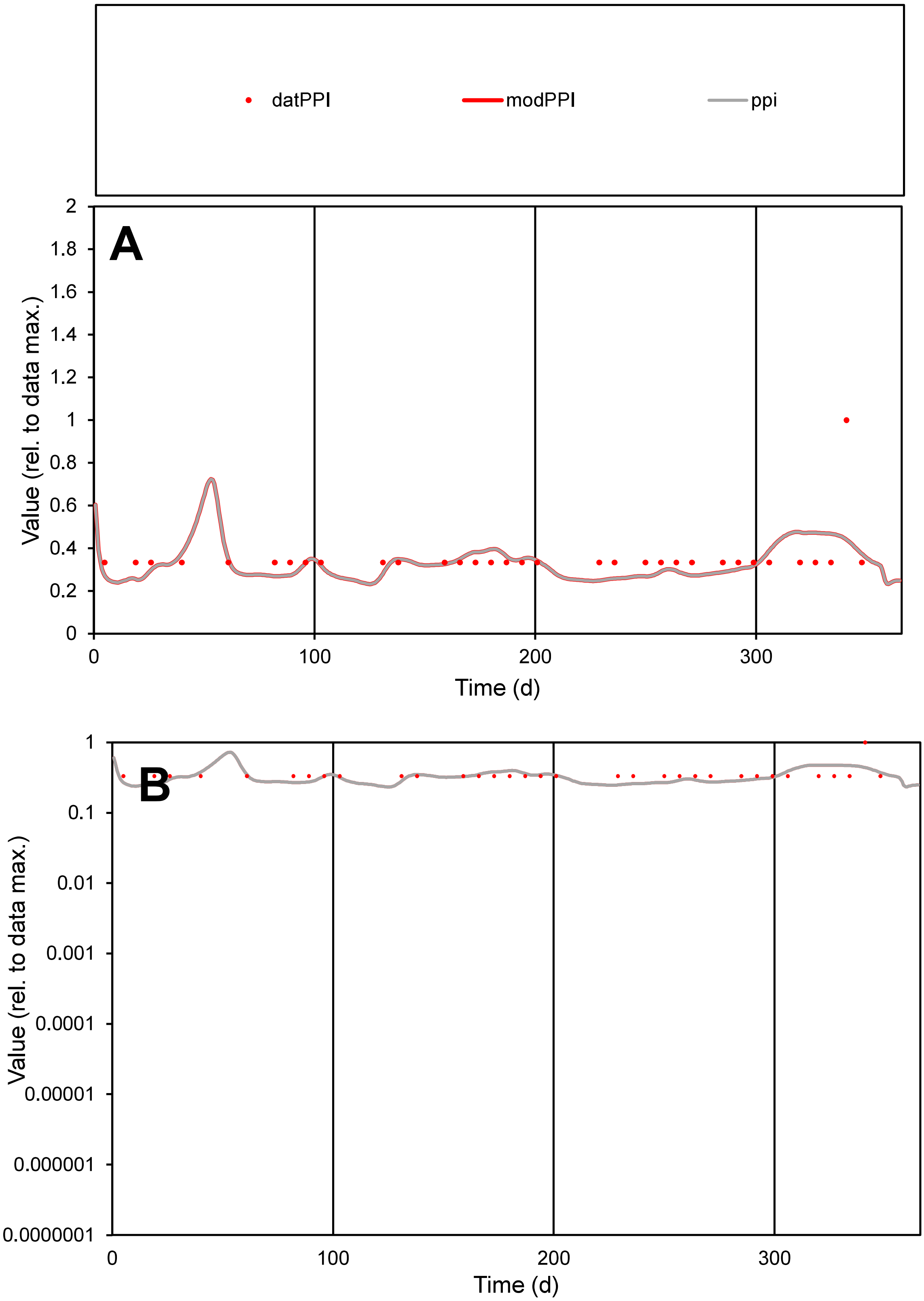

C1

Variables
PPI Provide jtd or Dst
CalibOnly?
2 1 to reproduce of
oNormalize?
2 1=no, 2=yes

ofxn calc*
0.33082
ofxn mod
0.34924

sRun
RunA1
zout
1

Ave
Data
1.5E-06
Model
1.5E-06

data/vMax
4.3E-06

ofxng
263.459

Min d/m
1.32418

ave/min d
1.06071
ave/min m
1.33164

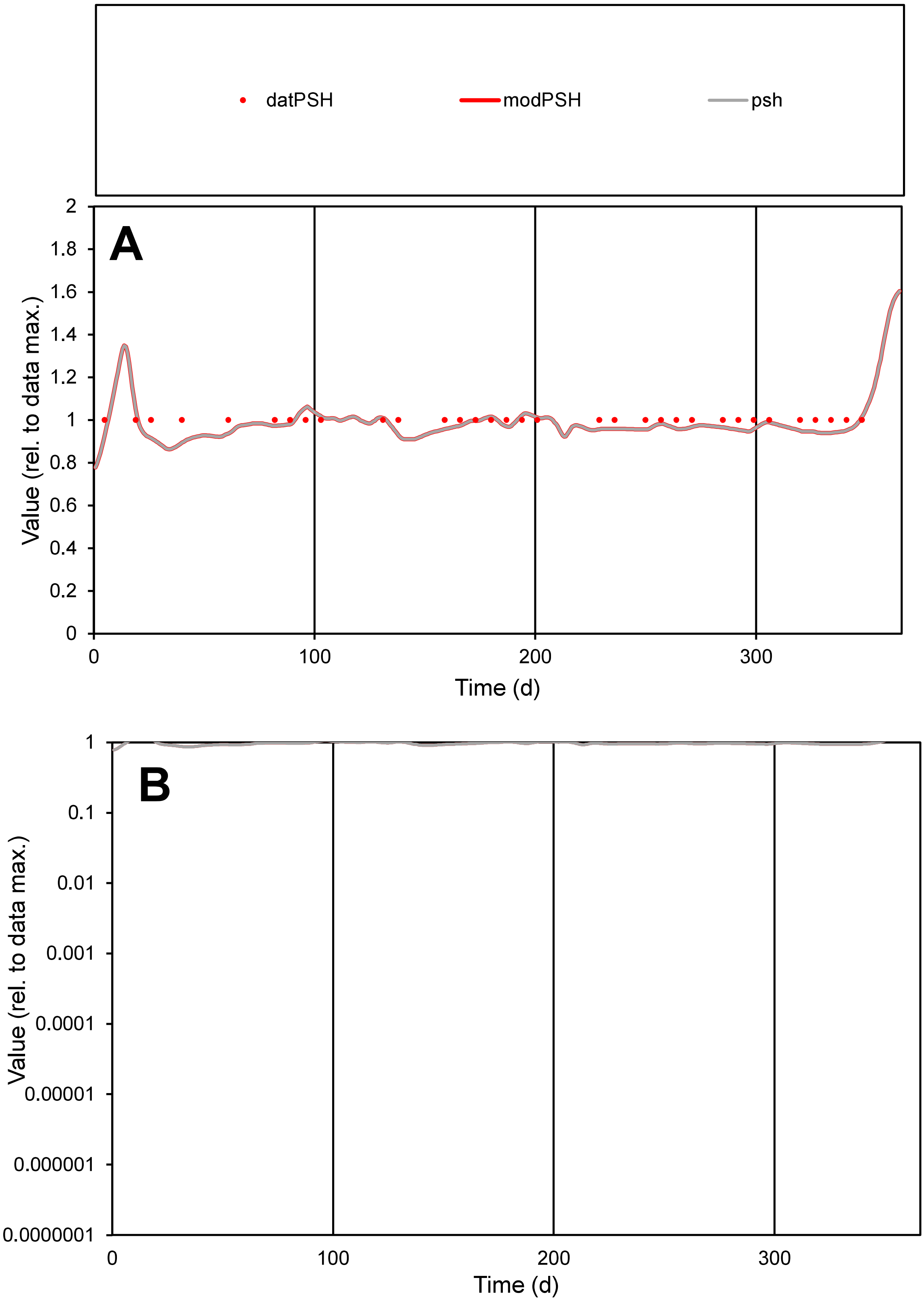

C1

Variables
PSH Provide jtd or Dst
CalibOnly?
2 1 to reproduce of
oNormalize?
2 1=no, 2=yes

ofxn calc*
0.04385
ofxn mod
0.11983

sRun
RunA1
zout
1

Ave
Data
1.3E-05
Model
1.3E-05

data/vMax
1.3E-05

ofxng
263.459

Min d/m
1.11207

ave/min d
1
ave/min m
1.08284

326_Calib_S_A

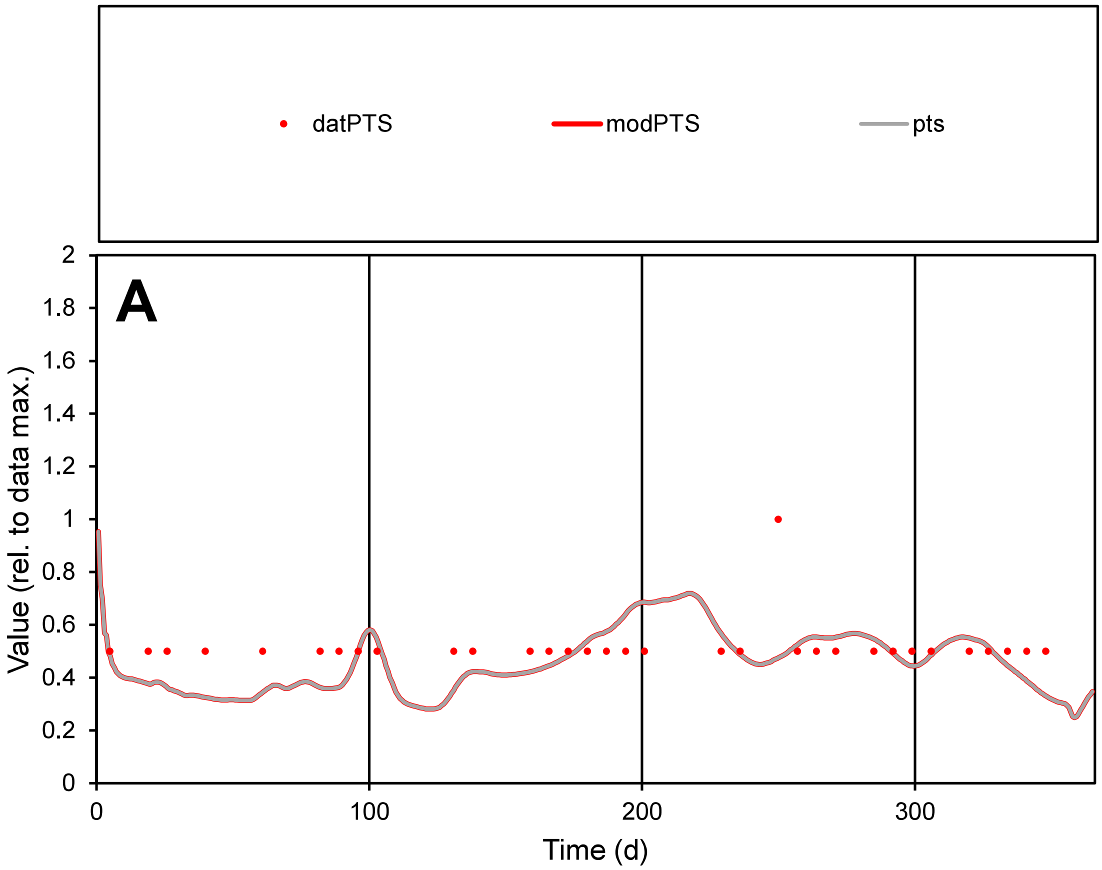

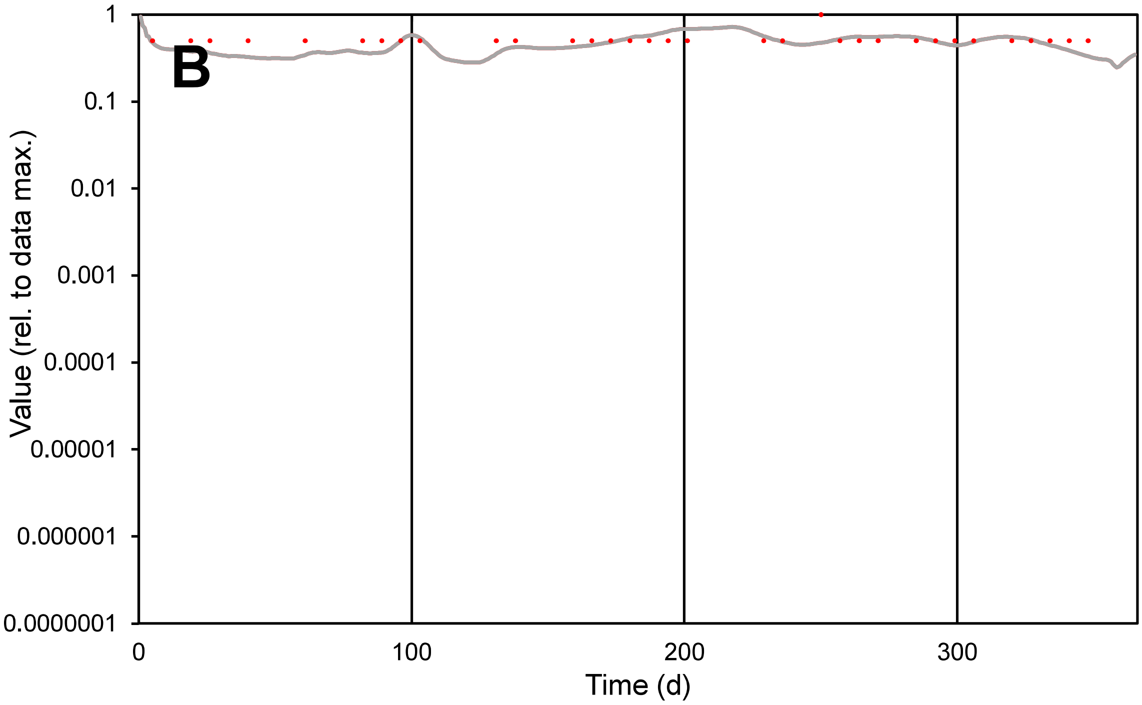

C1

Variables

PTS Provide jtd or Dst

CalibOnly?

2 1 to reproduce of

oNormalize?

2 1=no, 2=yes

ofxn calc*

0.25331

ofxn mod

0.27645

sRun

RunA1

zout

1

Ave

Data

1E-05

Model

9.1E-06

data/vMax

1.9E-05

ofxng

263.459

Min d/m

1.54409

ave/min d

1.03032

ave/min m

1.45833

326_Calib_S_A

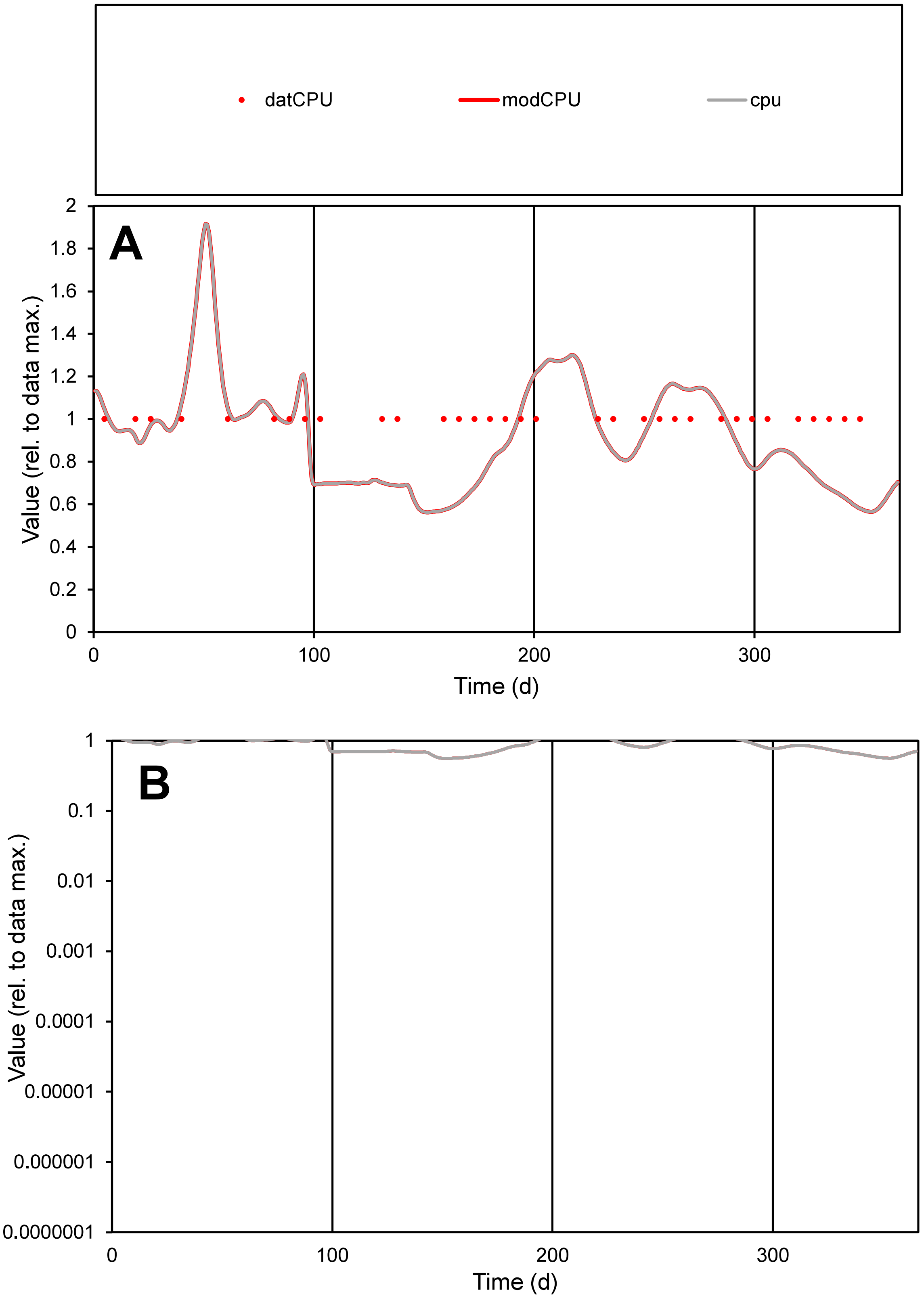

C1

Variables
CPU Provide jtd or Dst
CalibOnly?
2 1 to reproduce of
oNormalize?
2 1=no, 2=yes

ofxn calc*
0.21868
ofxn mod
0.2446

sRun
RunA1
zout
1

Ave
Data
4.4E-07
Model
3.9E-07

data/vMax
4.4E-07

ofxng
263.459

Min d/m
1.73821

ave/min d
1
ave/min m
1.52893

326_Calib_S_A

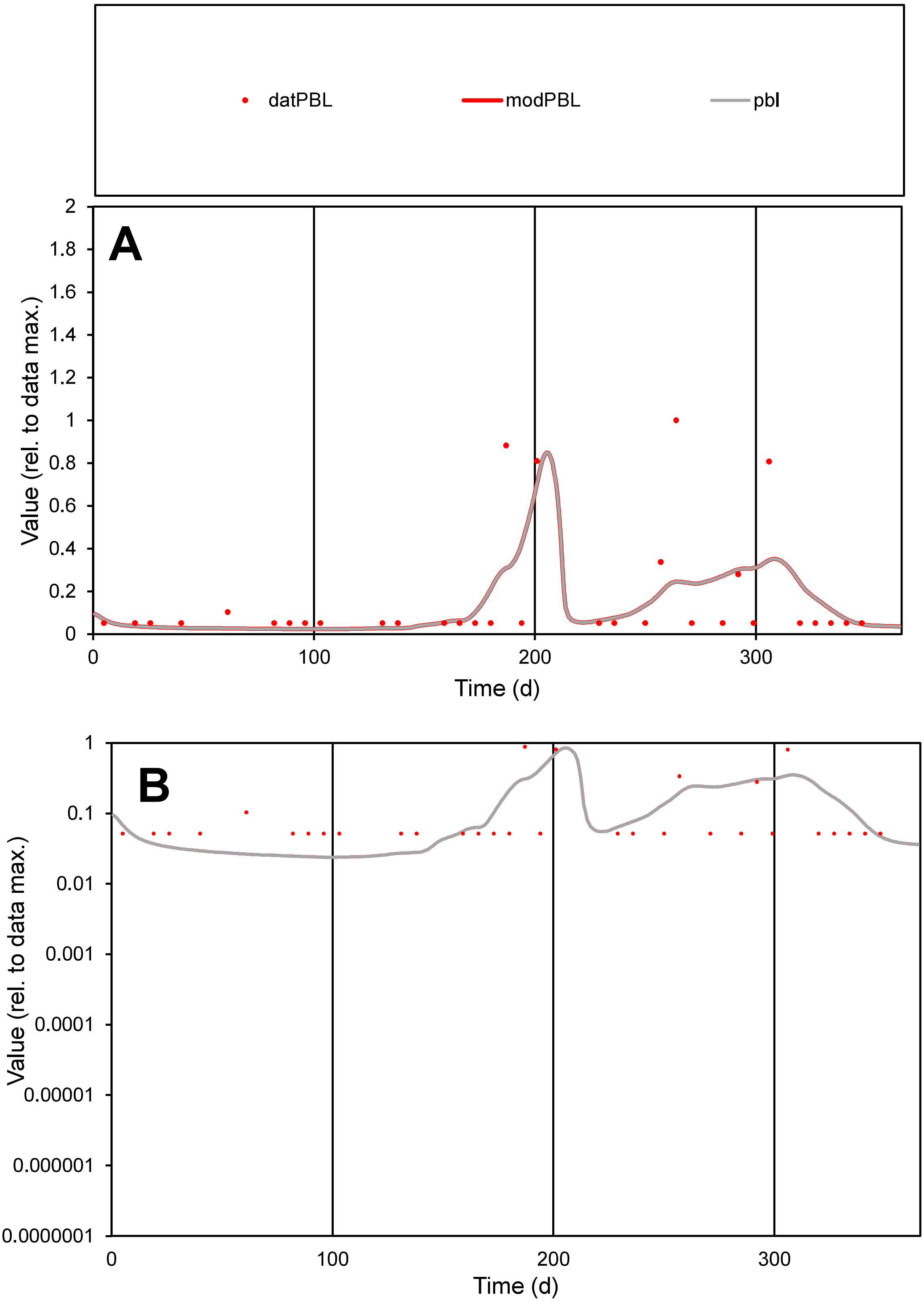

C1

Variables
PBL Provide jtd or Dst
CalibOnly?
2 1 to reproduce of
oNormalize?
2 1=no, 2=yes

ofxn calc*
1.25308
ofxn mod
1.2746

sRun
RunA1
zout
1

Ave
Data
1.4E-07
Model
1.3E-07

data/vMax
8.2E-07

ofxng
263.459

Min d/m
2.17476

ave/min d
3.24895
ave/min m
6.40074

326_Calib_S_A

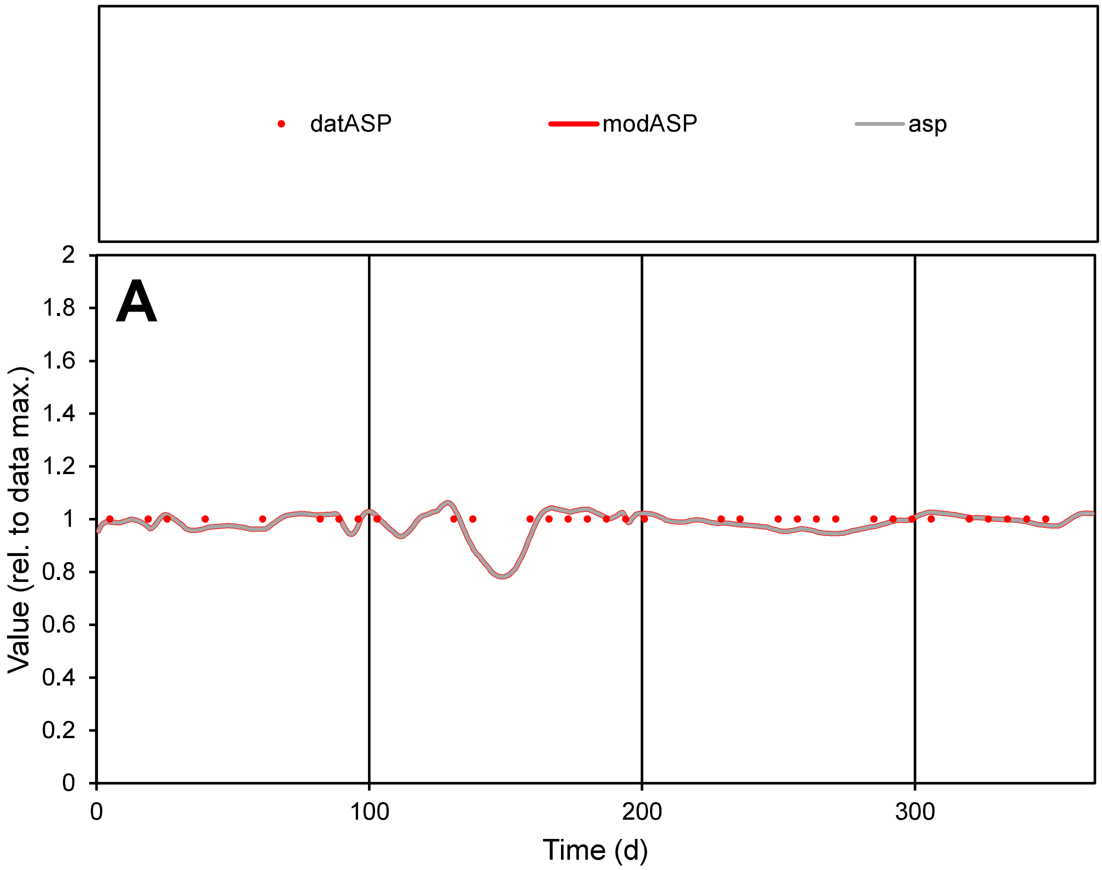

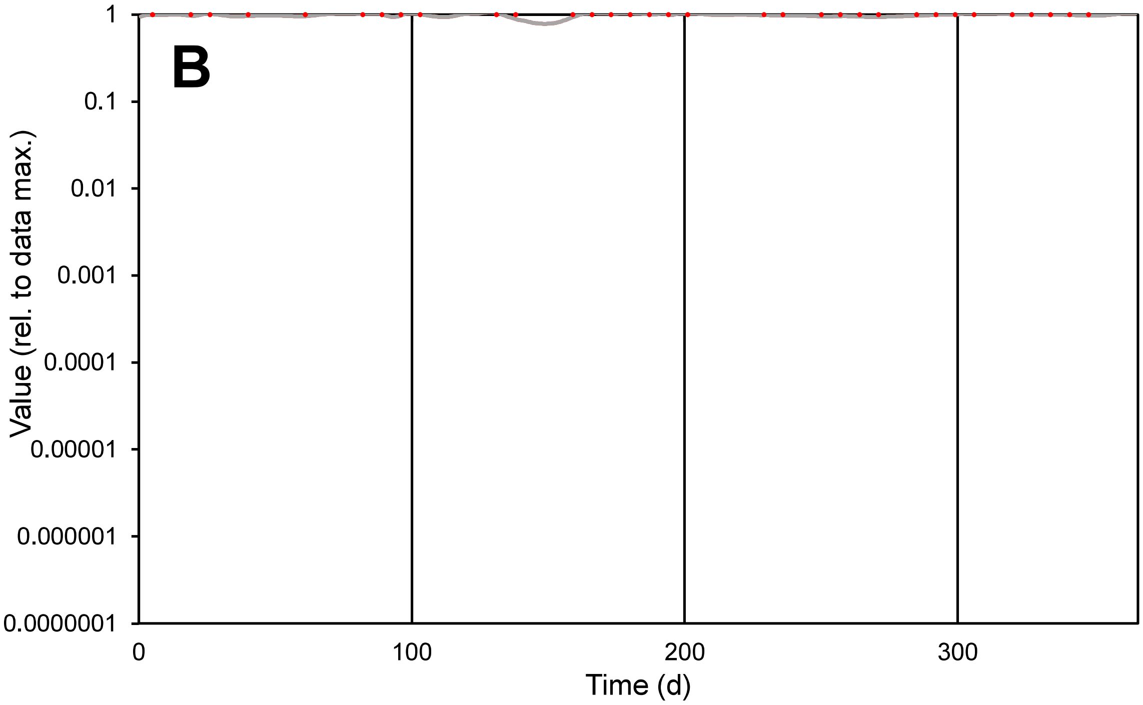

C1

Variables

ASP Provide jtd or Dst

CalibOnly?

2 1 to reproduce of

oNormalize?

2 1=no, 2=yes

ofxn calc*

0.03465

ofxn mod

0.11773

sRun

RunA1

zout

1

Ave

Data

5.8E-06

Model

5.7E-06

data/vMax

5.8E-06

ofxng

263.459

Min d/m

1.15383

ave/min d

1

ave/min m

1.14071

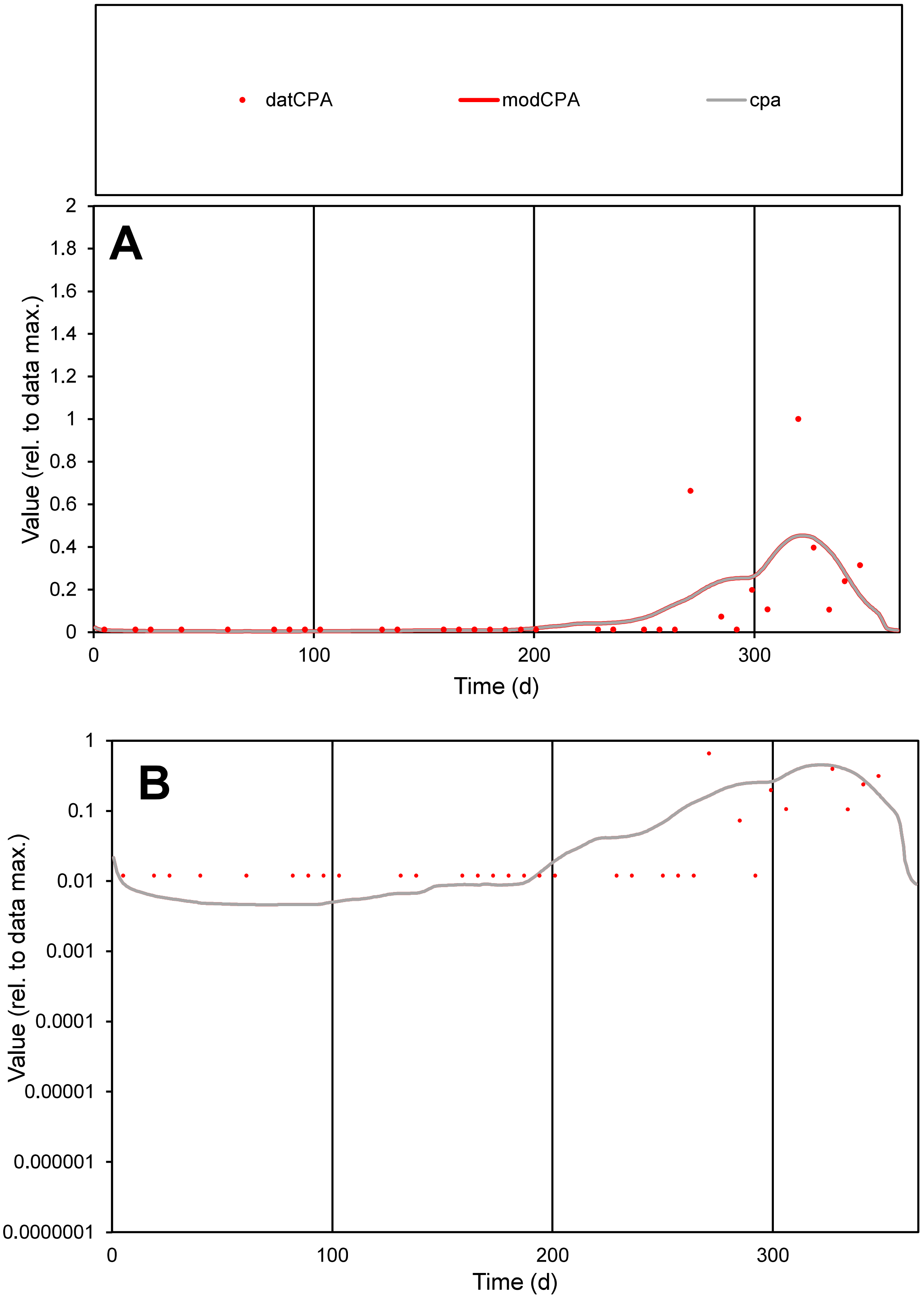

C1

Variables
CPA Provide jtd or Dst
CalibOnly?
2 1 to reproduce of
oNormalize?
2 1=no, 2=yes

ofxn calc*
1.51566
ofxn mod
1.53401

sRun
RunA1
zout
1

Ave
Data
5.7E-07
Model
5.8E-07

data/vMax
5.5E-06

ofxng
263.459

Min d/m
2.60471

ave/min d
8.53224
ave/min m
22.7593

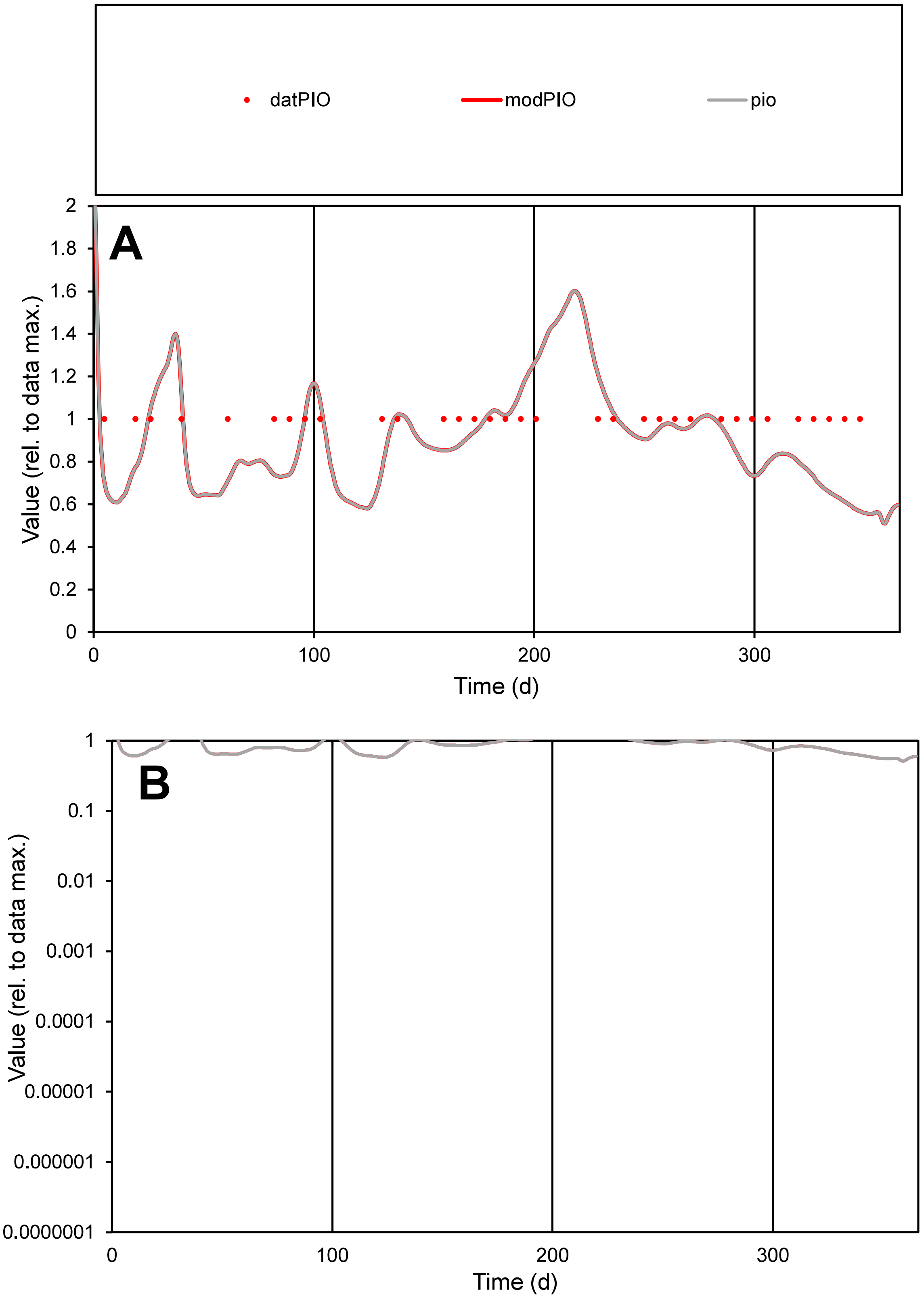

C1

Variables
PIO Provide jtd or Dst
CalibOnly?
2 1 to reproduce of
oNormalize?
2 1=no, 2=yes

ofxn calc*
0.19992
ofxn mod
0.22905

sRun
RunA1
zout
1

Ave
Data
1.5E-07
Model
1.3E-07

data/vMax
1.5E-07

ofxng
263.459

Min d/m
1.77658

ave/min d
1
ave/min m
1.59674

326_Calib_S_A

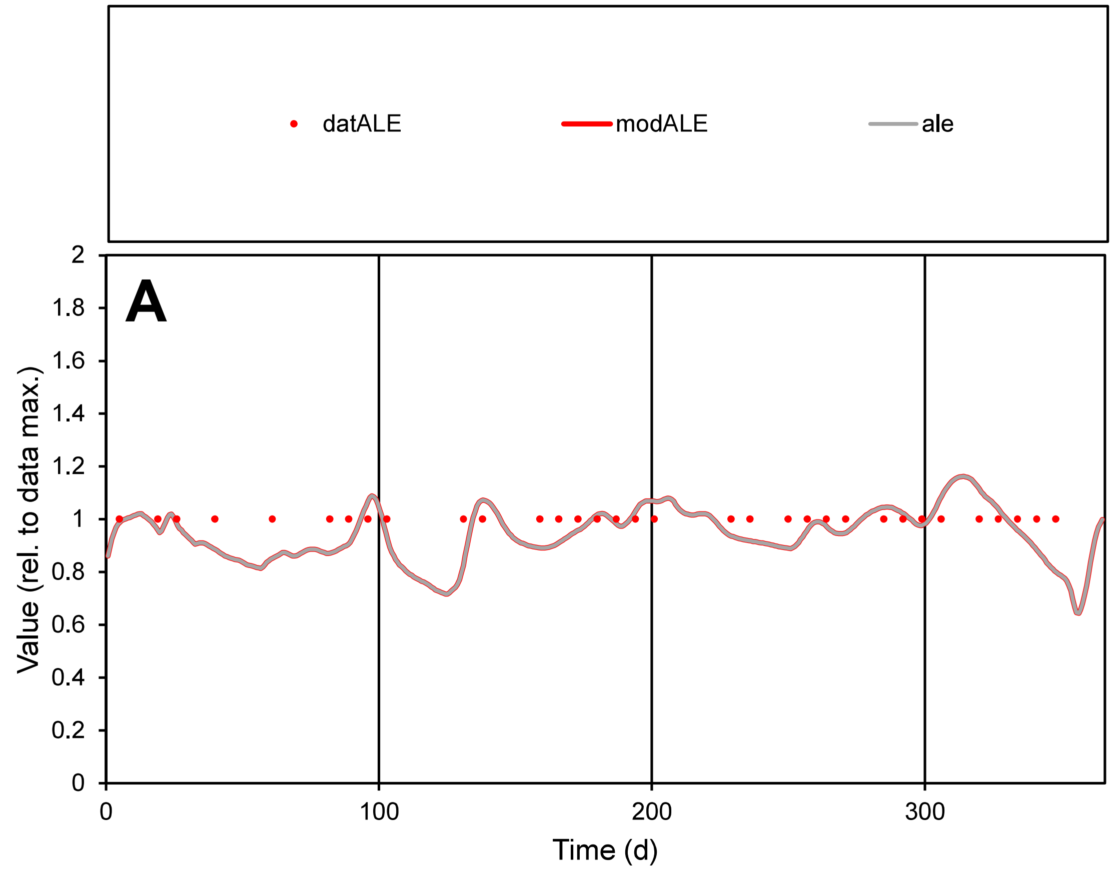

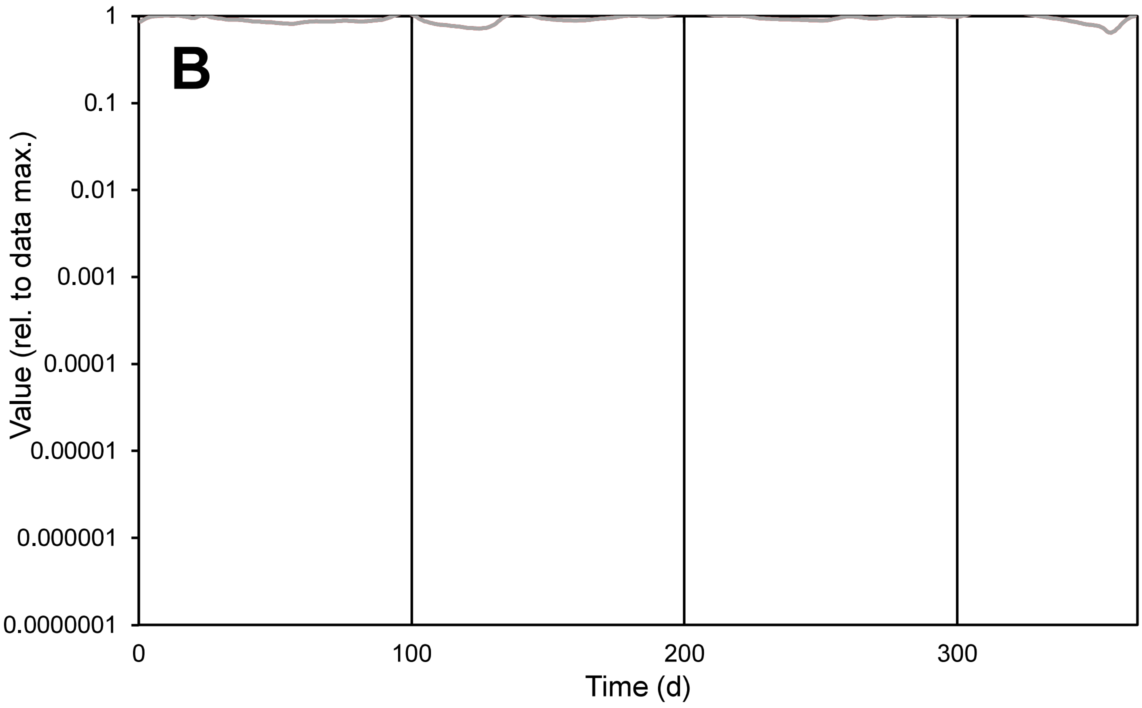

C1

Variables
ALE Provide jtd or Dst
CalibOnly?
2 1 to reproduce of
oNormalize?
2 1=no, 2=yes

ofxn calc*
0.08443
ofxn mod
0.13974

sRun
RunA1
zout
1

Ave
Data
1.1E-05
Model
1.1E-05

data/vMax
1.1E-05

ofxng
263.459

Min d/m
1.26198

ave/min d
1
ave/min m
1.21733

326_Calib_S_A

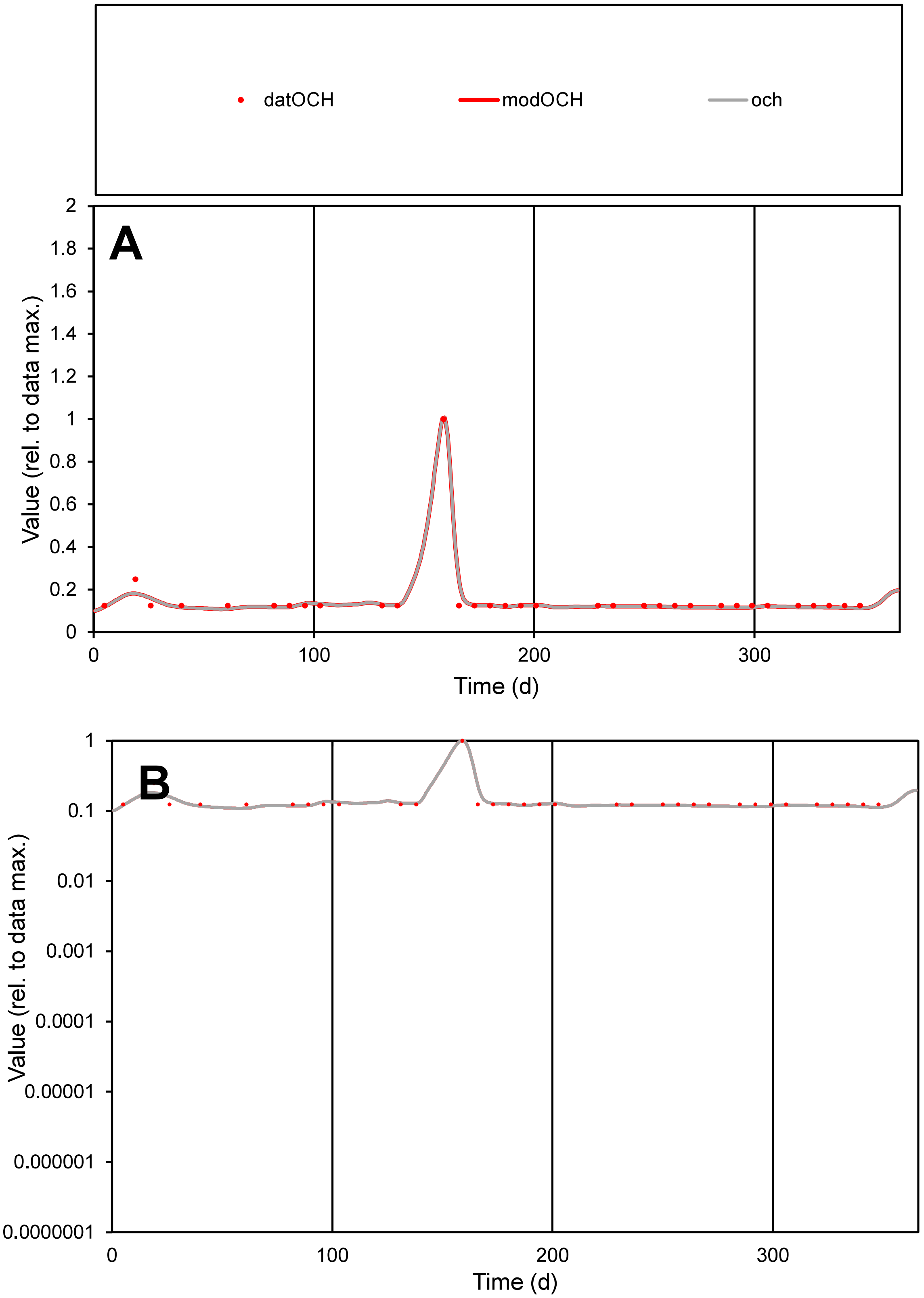

C1

Variables
OCH Provide jtd or Dst
CalibOnly?
2 1 to reproduce of
oNormalize?
2 1=no, 2=yes

ofxn calc*
0.11207
ofxn mod
0.15726

sRun
RunA1
zout
1

Ave
Data
5.5E-06
Model
5.4E-06

data/vMax
3.6E-05

ofxng
263.459

Min d/m
1.12369

ave/min d
1.24466
ave/min m
1.37567

326_Calib_S_A

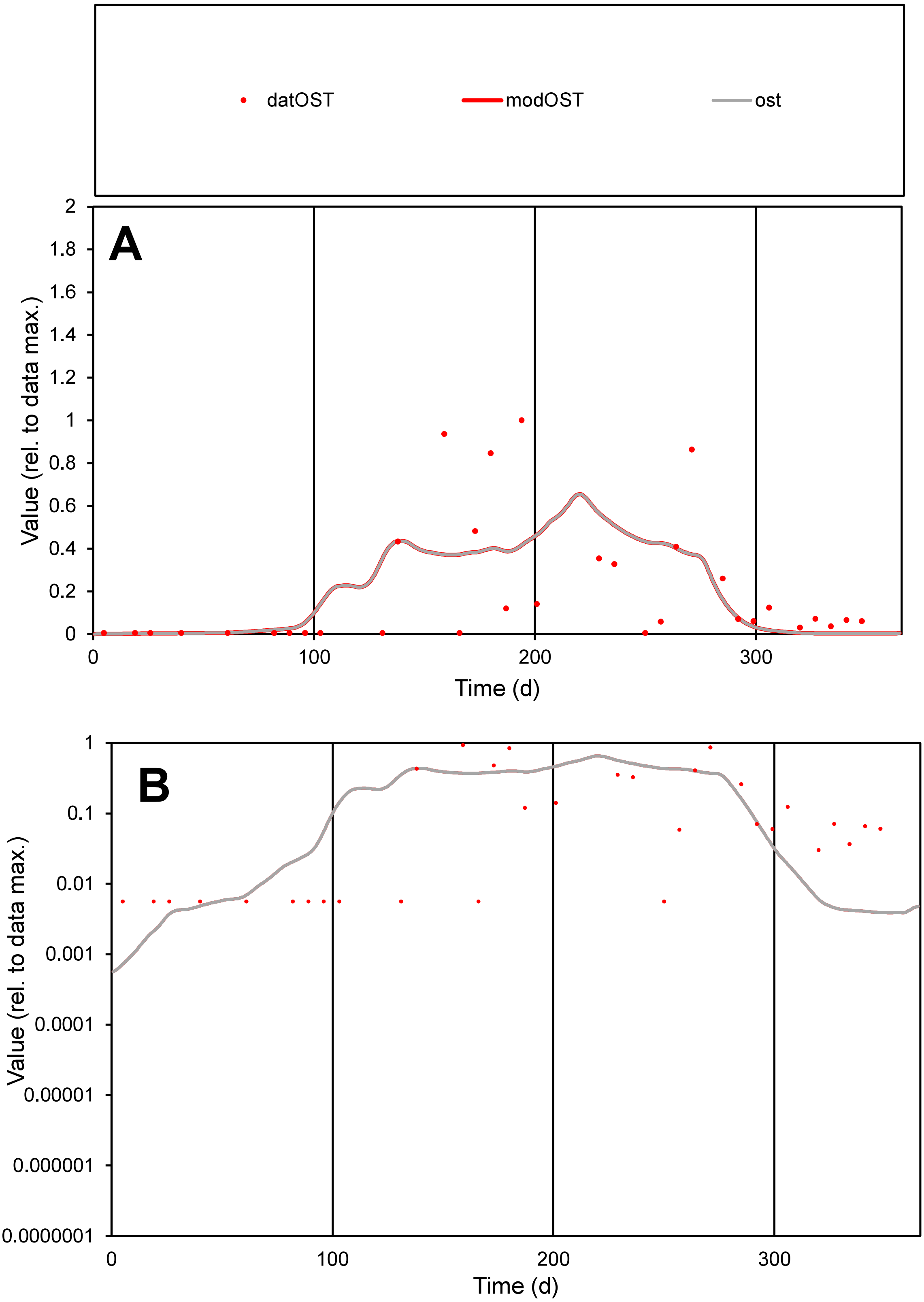

C1

Variables	
OST	Provide jtd or Dst
CalibOnly?	
2	1 to reproduce of
oNormalize?	
2	1=no, 2=yes
ofxn calc*	
1.17735	
ofxn mod	
1.19519	
sRun	
RunA1	
zout	
1	
Ave	
Data	
7.9E-05	
Model	
7.9E-05	
data/vMax	
0.00038	
ofxng	
263.459	
Min d/m	
7.038	
ave/min d	
36.6456	
ave/min m	
257.168	

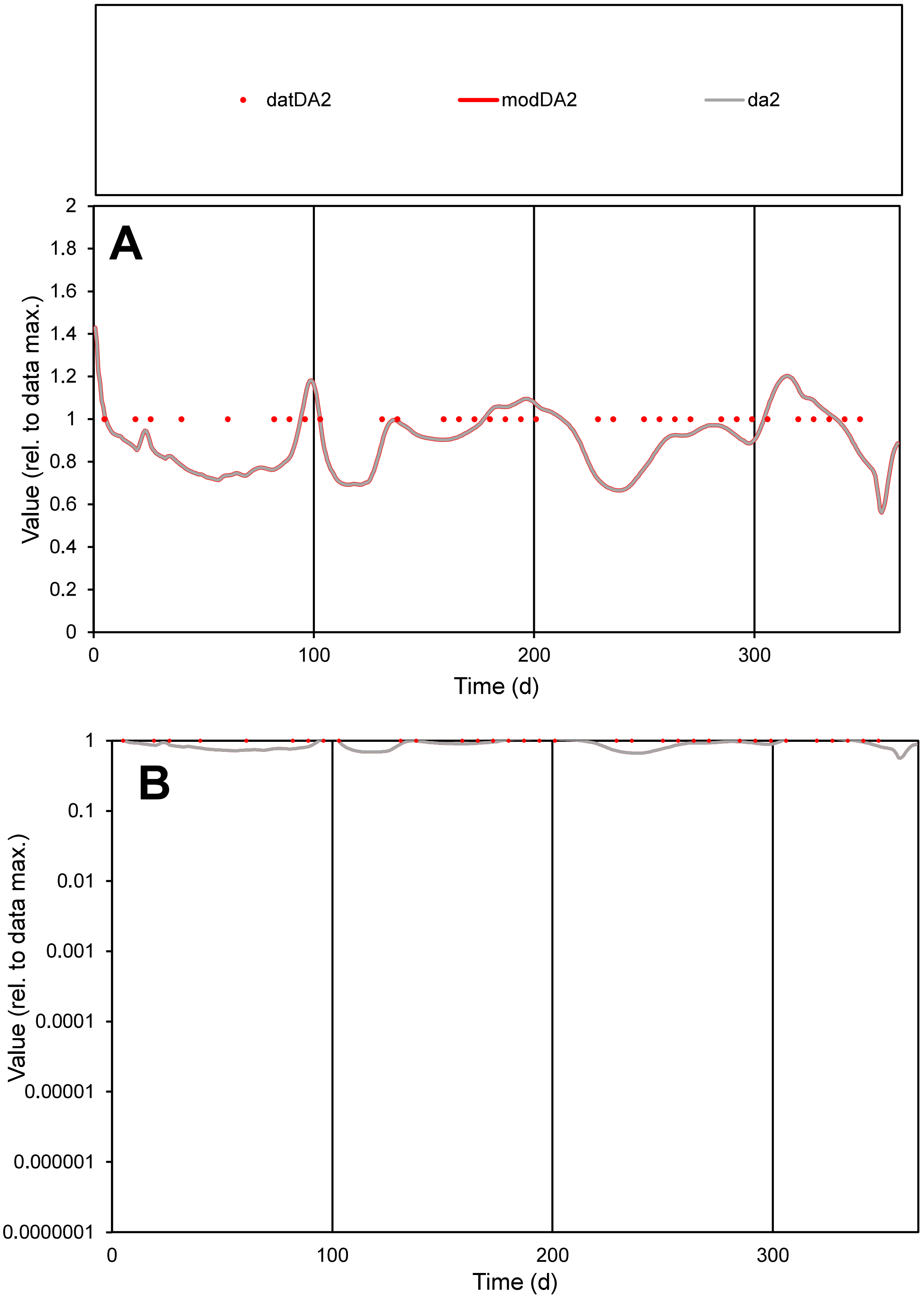

C1

Variables
DA2 Provide jtd or Dst
CalibOnly?
2 1 to reproduce of
oNormalize?
2 1=no, 2=yes

ofxn calc*
0.14138
ofxn mod
0.17851

sRun
RunA1
zout
1

Ave
Data
3.1E-06
Model
2.9E-06

data/vMax
3.1E-06

ofxng
263.459

Min d/m
1.49856

ave/min d
1
ave/min m
1.39004

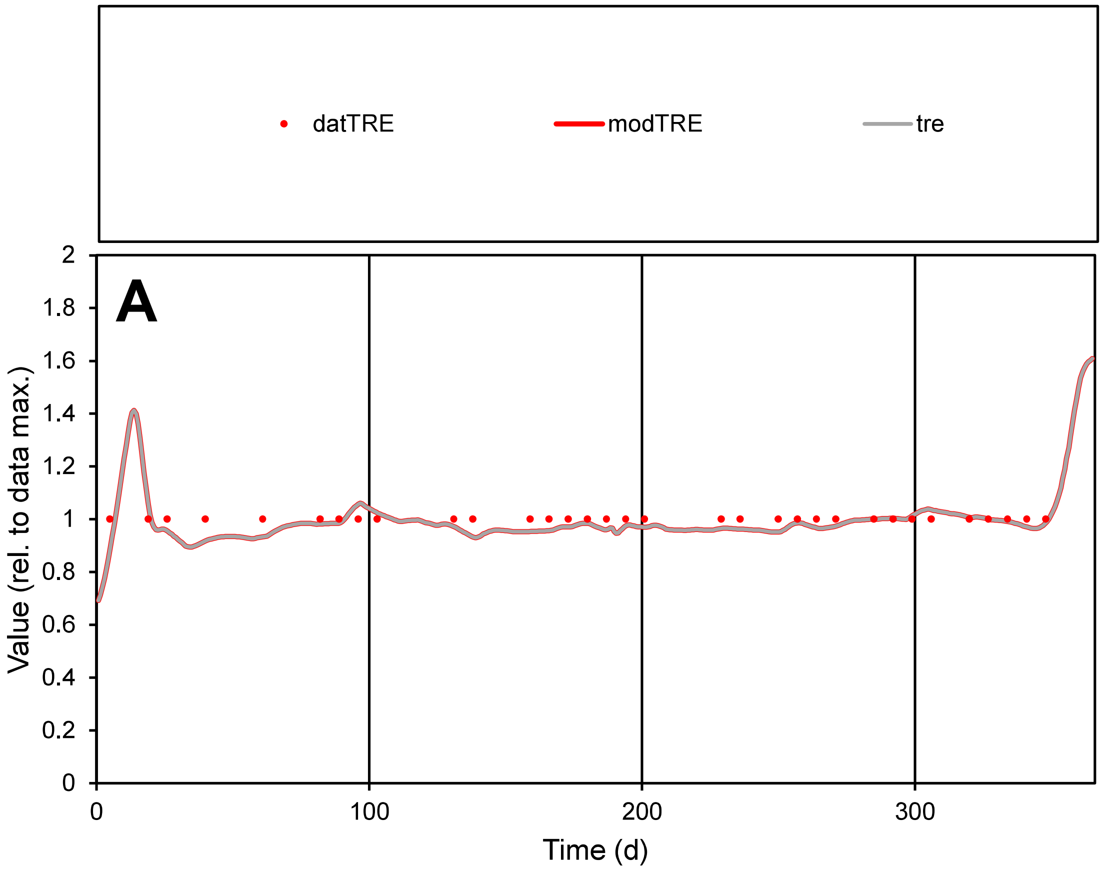

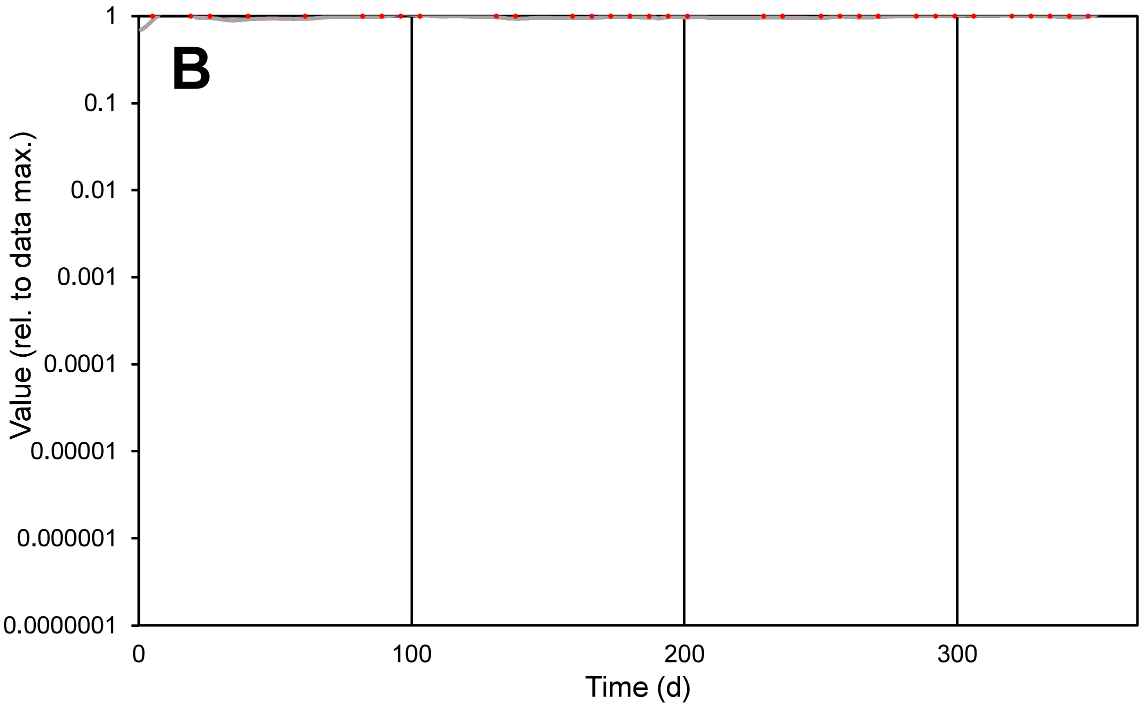

C1

Variables
TRE Provide jtd or Dst
CalibOnly?
2 1 to reproduce of
oNormalize?
2 1=no, 2=yes

ofxn calc*
0.03488
ofxn mod
0.11707

sRun
RunA1
zout
1

Ave
Data
6.9E-06
Model
6.8E-06

data/vMax
6.9E-06

ofxng
263.459

Min d/m
1.08562

ave/min d
1
ave/min m
1.06394

326_Calib_S_A

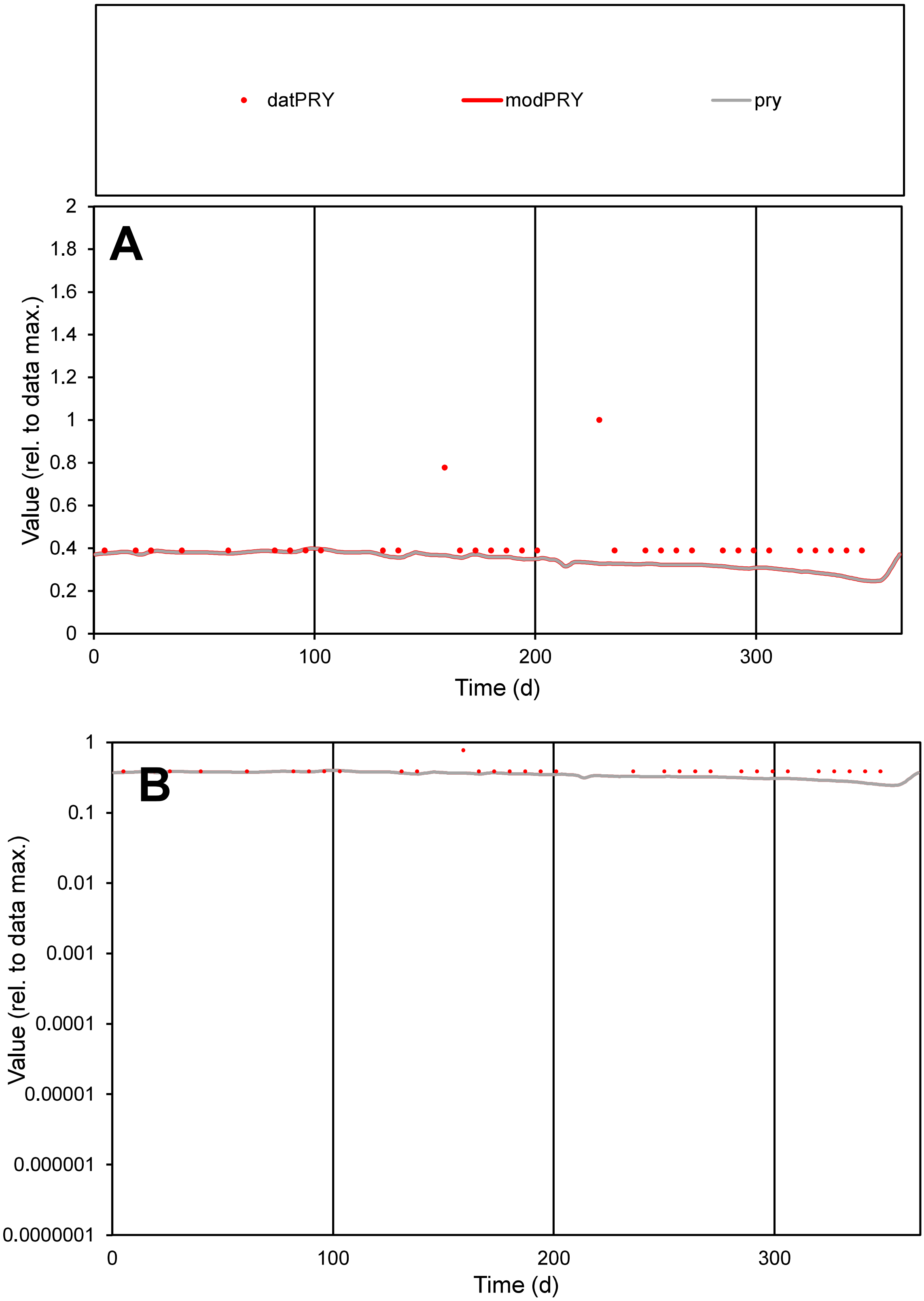

C1

Variables
PRY Provide jtd or Dst
CalibOnly?
2 1 to reproduce of
oNormalize?
2 1=no, 2=yes

ofxn calc*
0.35876
ofxn mod
0.37337

sRun
RunA1
zout
1

Ave
Data
9.5E-06
Model
7.7E-06

data/vMax
2.3E-05

ofxng
263.459

Min d/m
1.56207

ave/min d
1.07792
ave/min m
1.36693

326_Calib_S_A

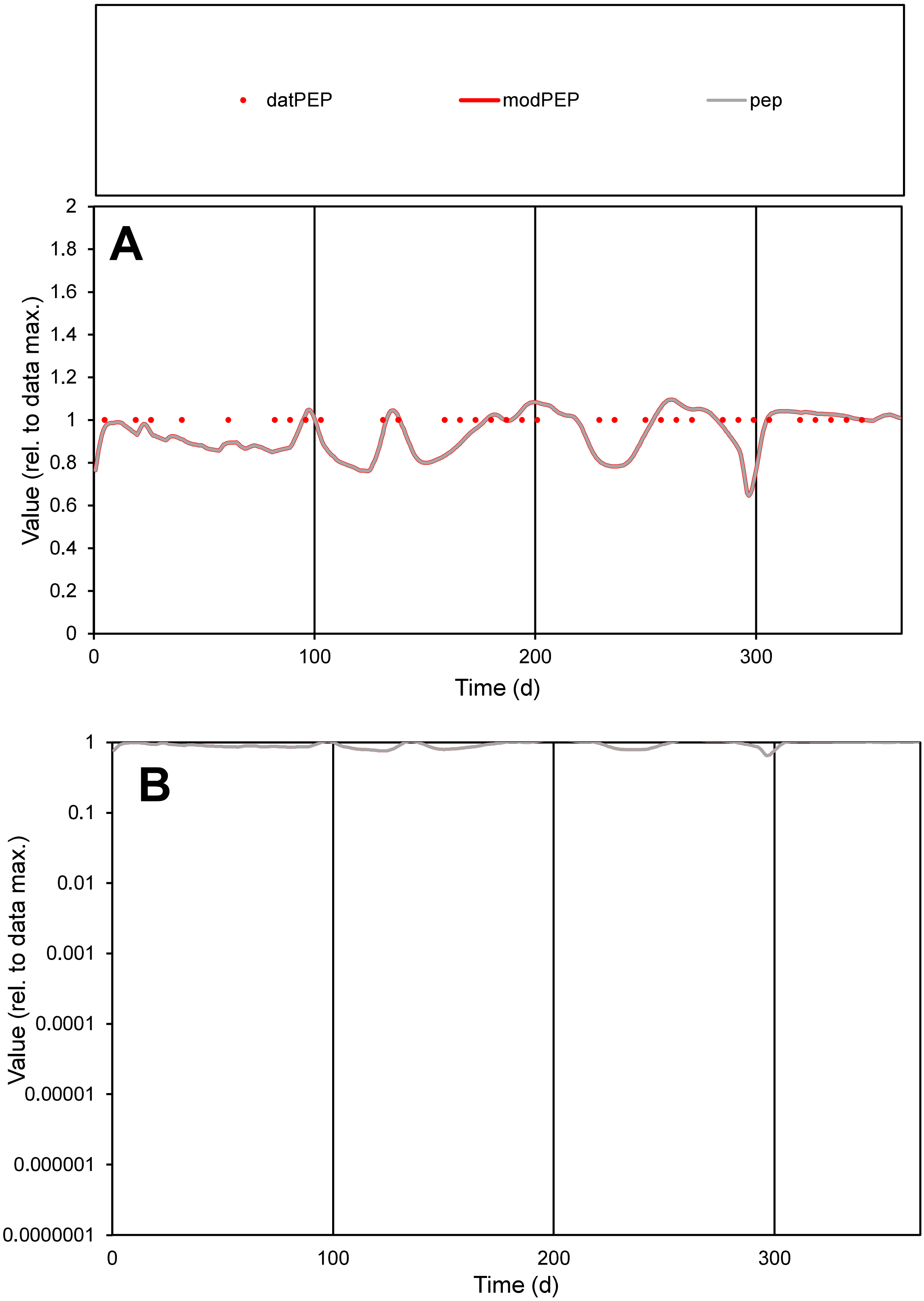

C1

Variables

PEP	Provide jtd or Dst
CalibOnly?	
2	1 to reproduce of
oNormalize?	
2	1=no, 2=yes

ofxn calc*
0.09649
ofxn mod
0.14734

sRun
RunA1
zout
1

Ave
Data
4.9E-06
Model
4.7E-06

data/vMax
4.9E-06

ofxng
263.459

Min d/m
1.27725

ave/min d
1
ave/min m
1.2257

326_Calib_S_A

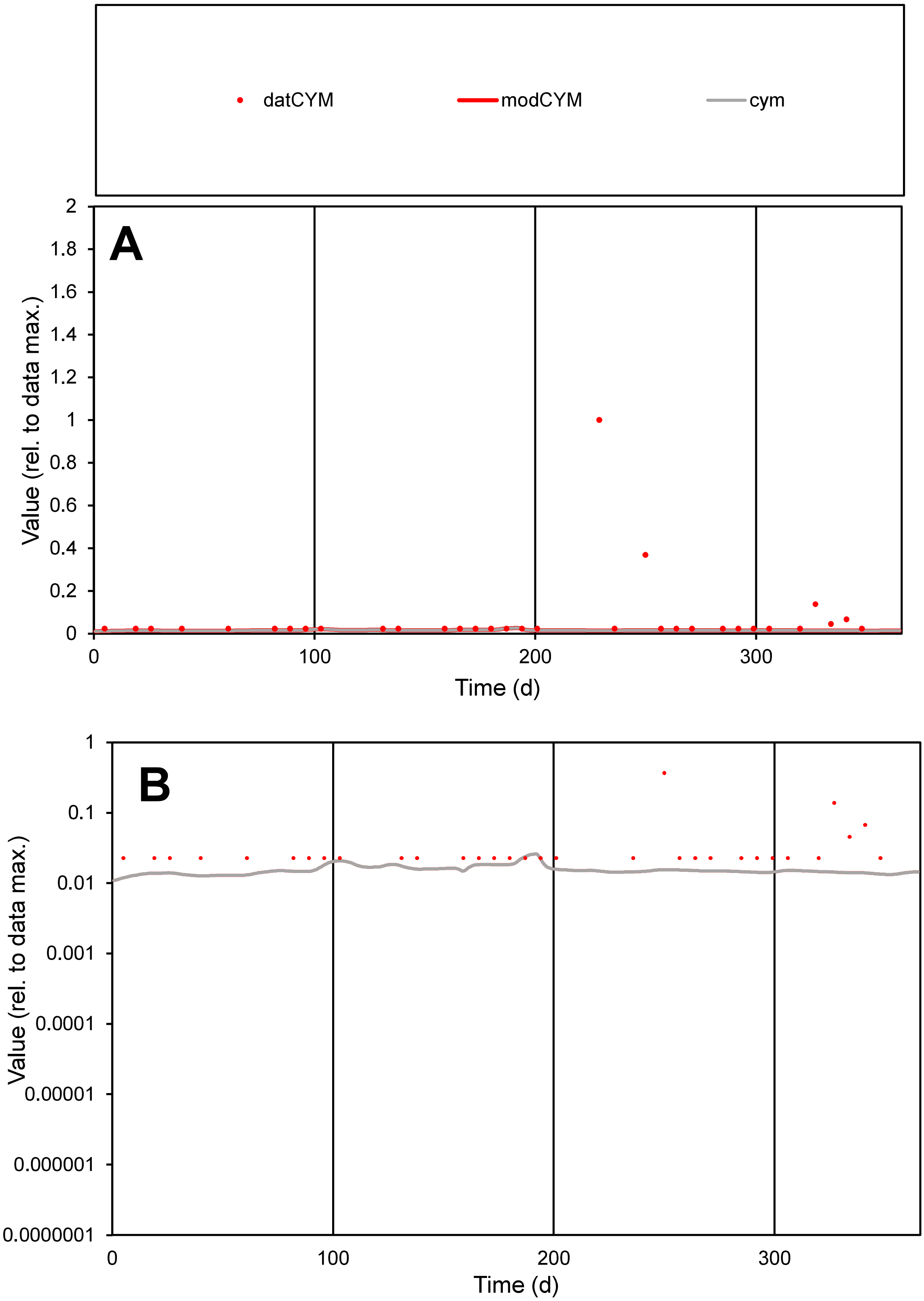

C1

Variables

CYM Provide jtd or Dst

CalibOnly?

2 1 to reproduce of

oNormalize?

2 1=no, 2=yes

ofxn calc*

2.69352

ofxn mod

2.6994

sRun

RunA1

zout

1

Ave

Data

4.1E-06

Model

9.4E-07

data/vMax

6E-05

ofxng

263.459

Min d/m

1.85195

ave/min d

3.01196

ave/min m

1.28584

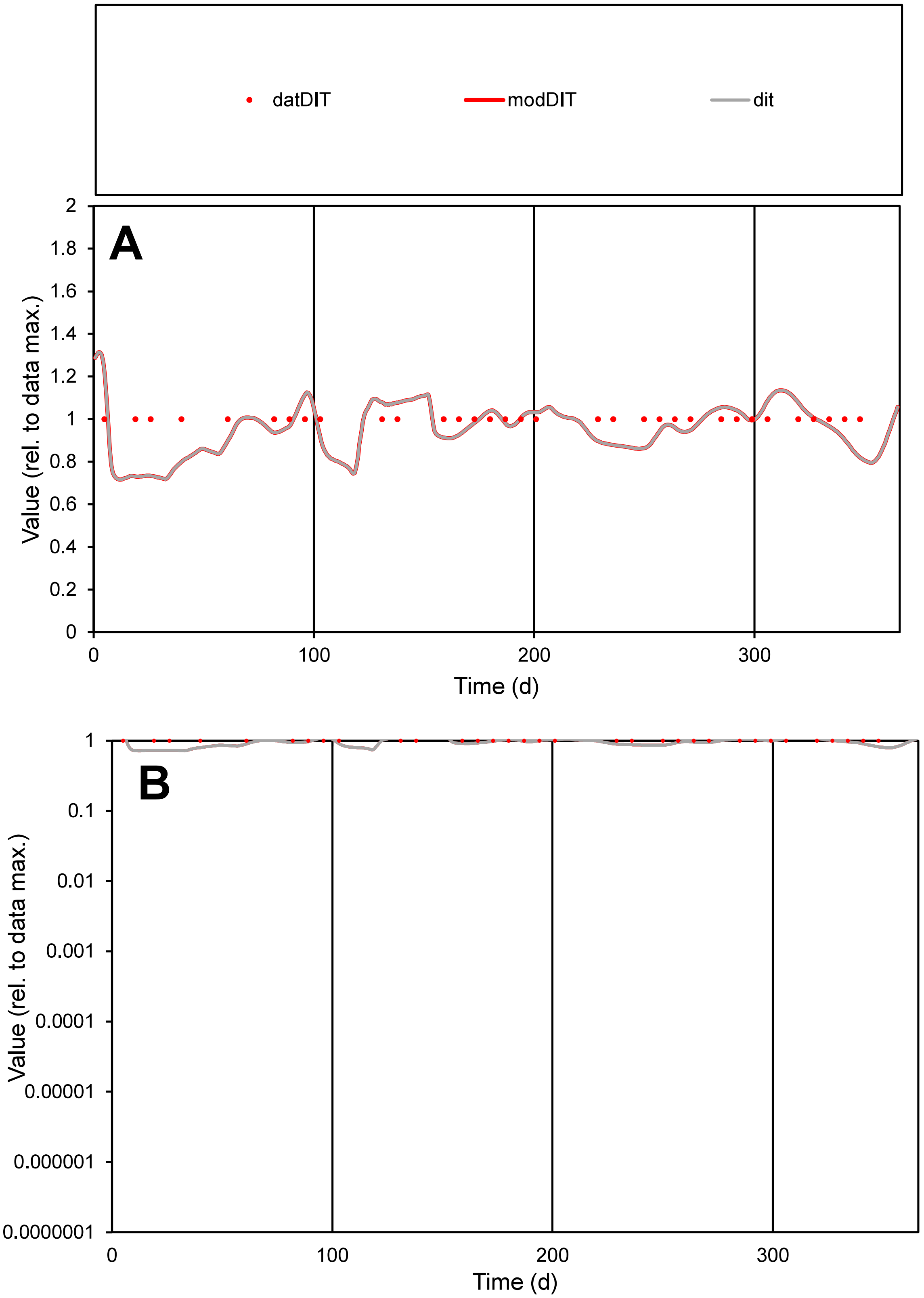

C1

Variables
DIT Provide jtd or Dst
CalibOnly?
2 1 to reproduce of
oNormalize?
2 1=no, 2=yes

ofxn calc*
0.10557
ofxn mod
0.15338

sRun
RunA1
zout
1

Ave
Data
5.1E-06
Model
4.9E-06

data/vMax
5.1E-06

ofxng
263.459

Min d/m
1.36867

ave/min d
1
ave/min m
1.31063

326_Calib_S_A

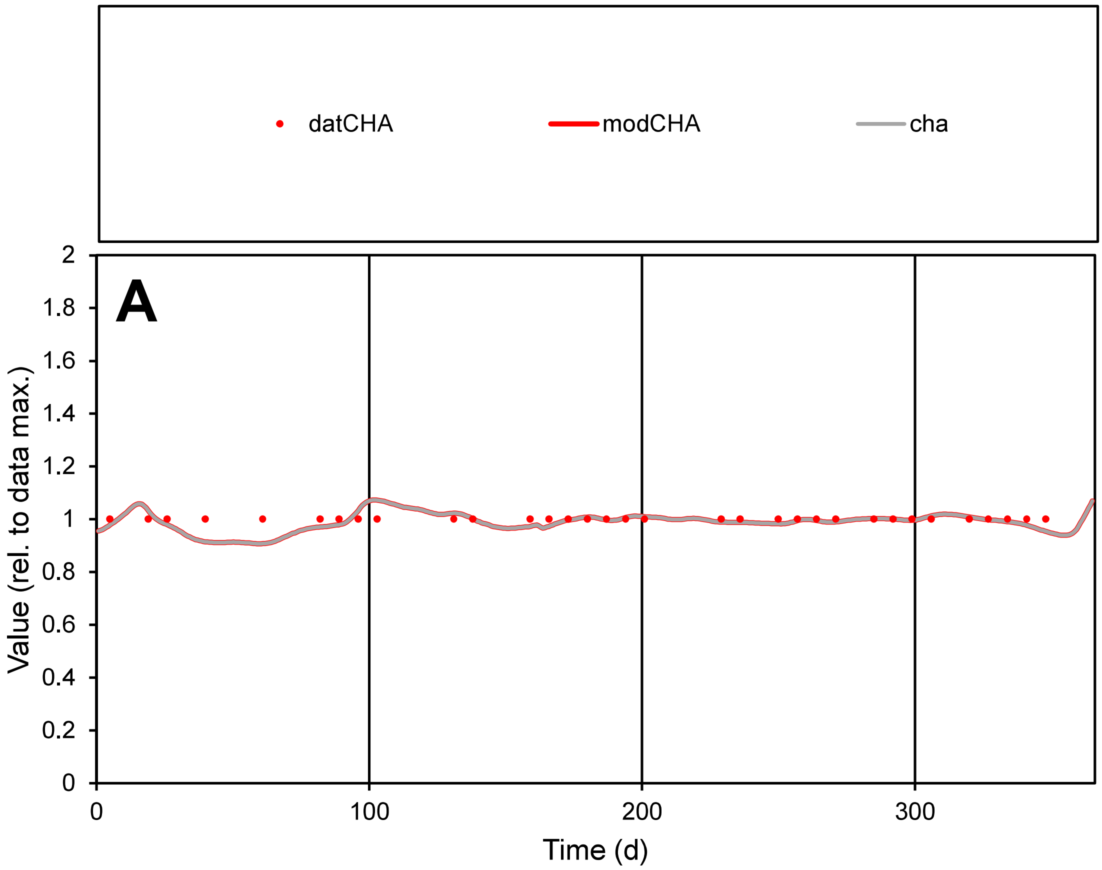

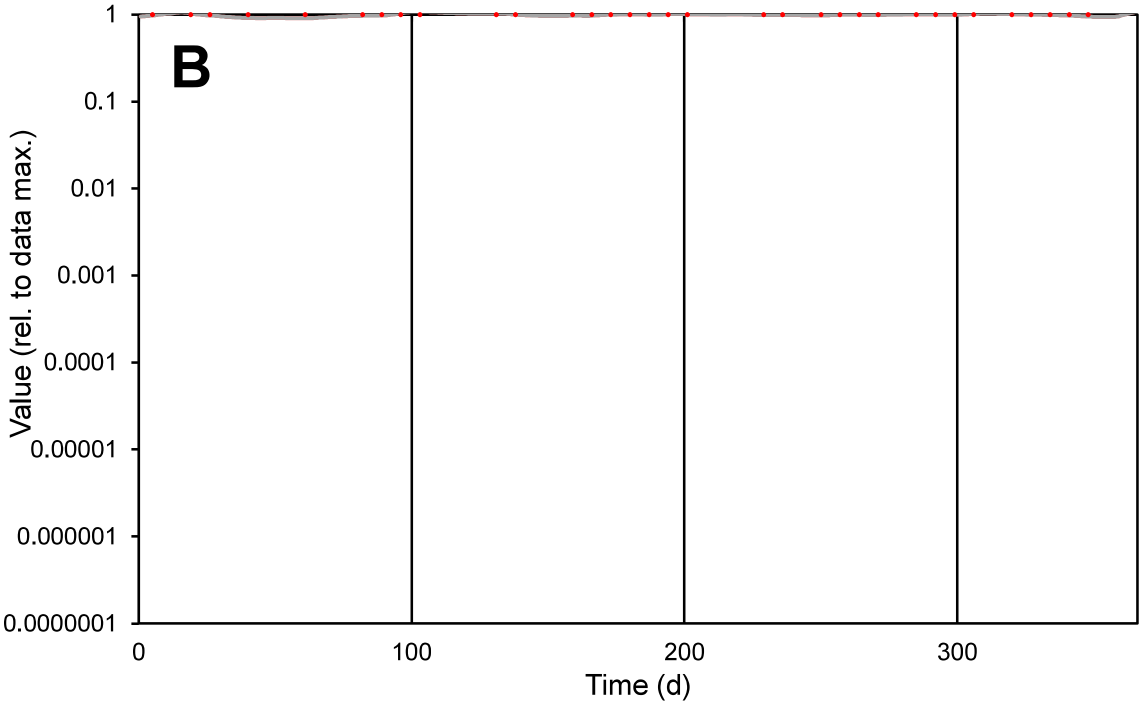

C1

Variables
CHA Provide jtd or Dst
CalibOnly?
2 1 to reproduce of
oNormalize?
2 1=no, 2=yes

ofxn calc*
0.03109
ofxn mod
0.11689

sRun
RunA1
zout
1

Ave
Data
8.7E-08
Model
8.6E-08

data/vMax
8.7E-08

ofxng
263.459

Min d/m
1.10058

ave/min d
1
ave/min m
1.09211

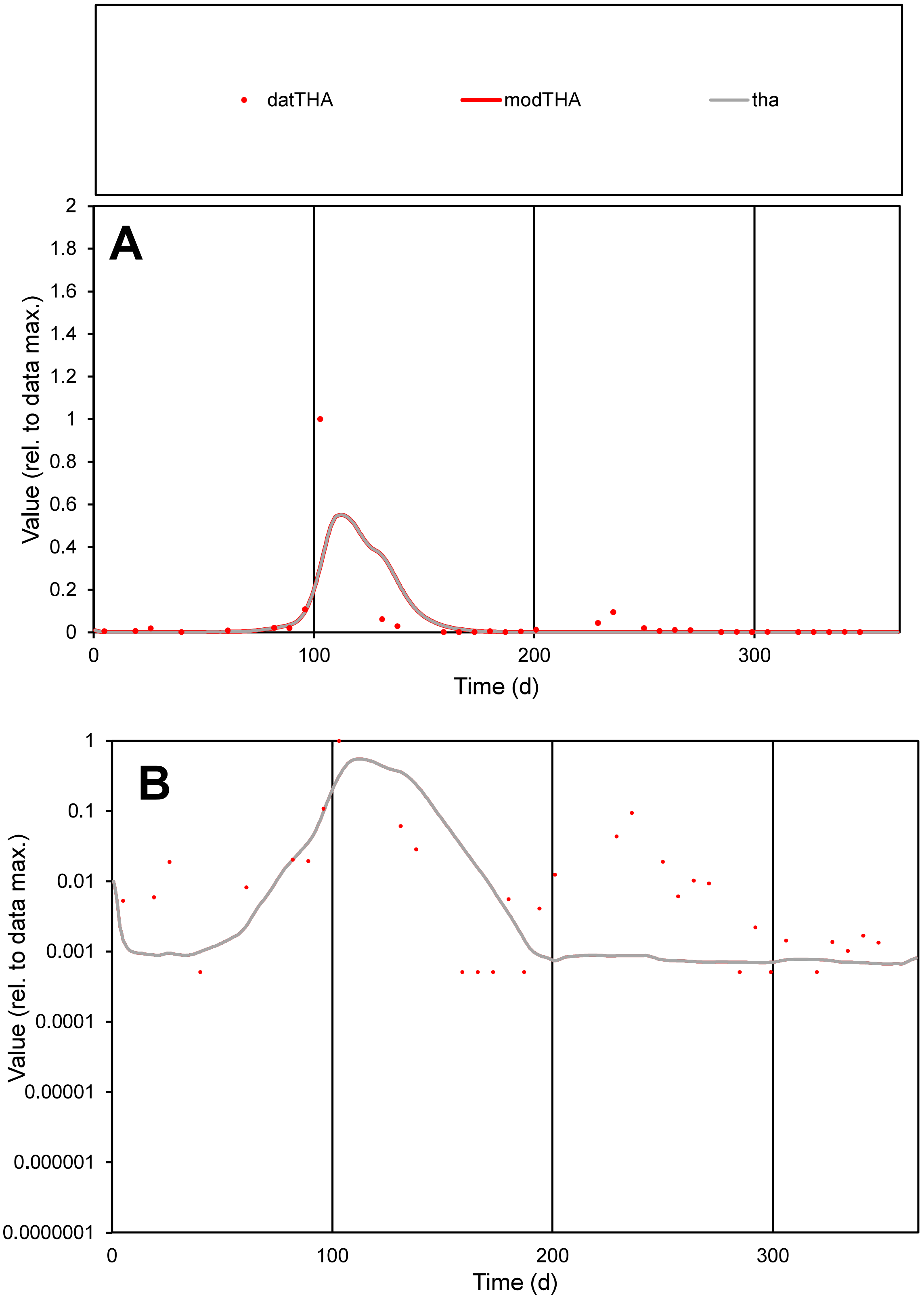

C1

Variables
THA Provide jtd or Dst
CalibOnly?
2 1 to reproduce of
oNormalize?
2 1=no, 2=yes

ofxn calc*
2.59667
ofxn mod
2.80703

sRun
RunA1
zout
1

Ave
Data
0.00024
Model
0.00019

data/vMax
0.00536

ofxng
263.459

Min d/m
0.76466

ave/min d
88.3419
ave/min m
54.1061

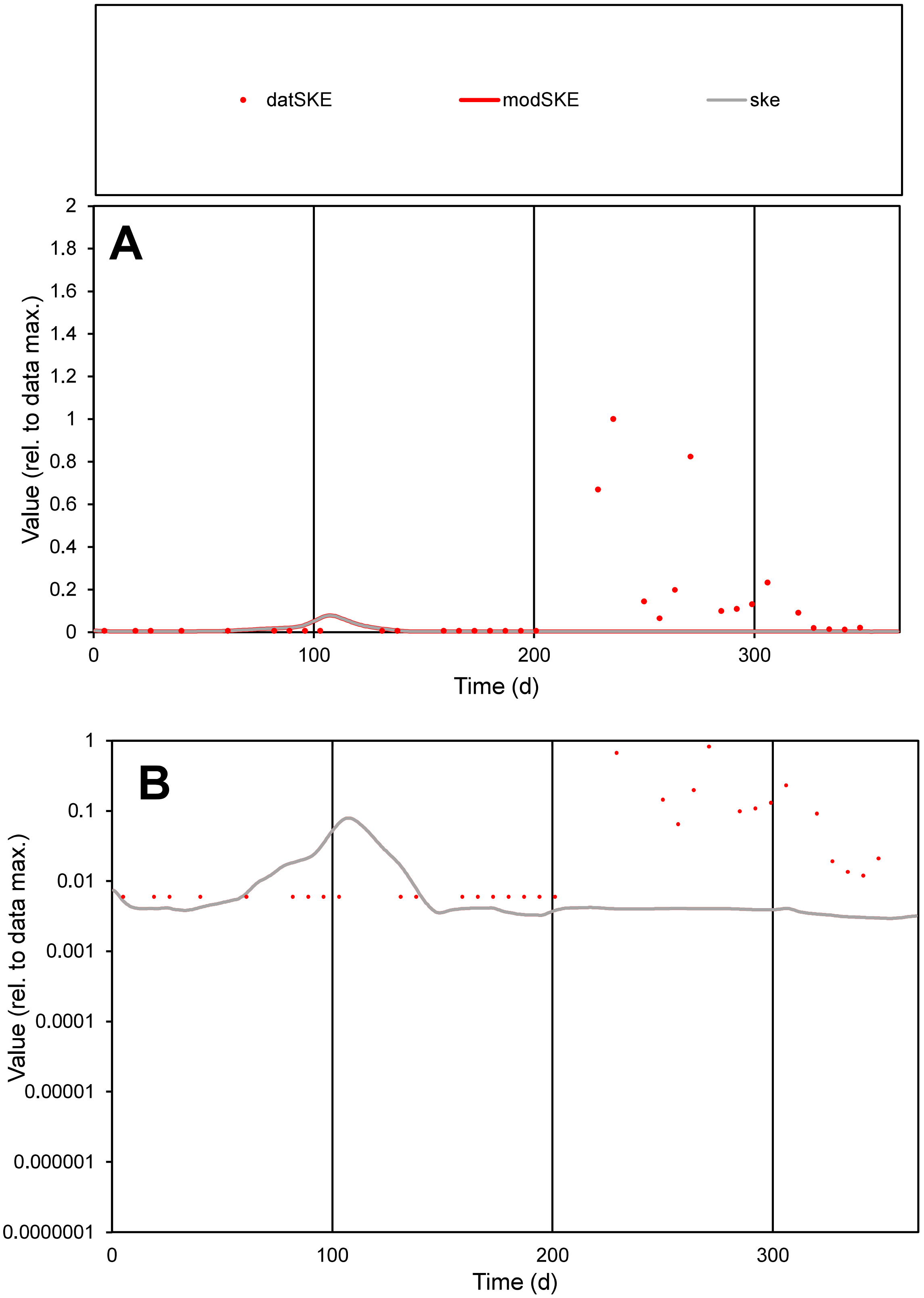

C1

Variables
SKE Provide jtd or Dst
CalibOnly?
2 1 to reproduce of
oNormalize?
2 1=no, 2=yes

ofxn calc*
2.3156
ofxn mod
2.32036

sRun
RunA1
zout
1

Ave
Data
4.4E-05
Model
3E-06

data/vMax
0.00039

ofxng
263.459

Min d/m
2.01489

ave/min d
18.9708
ave/min m
2.61508

326_Calib_S_A

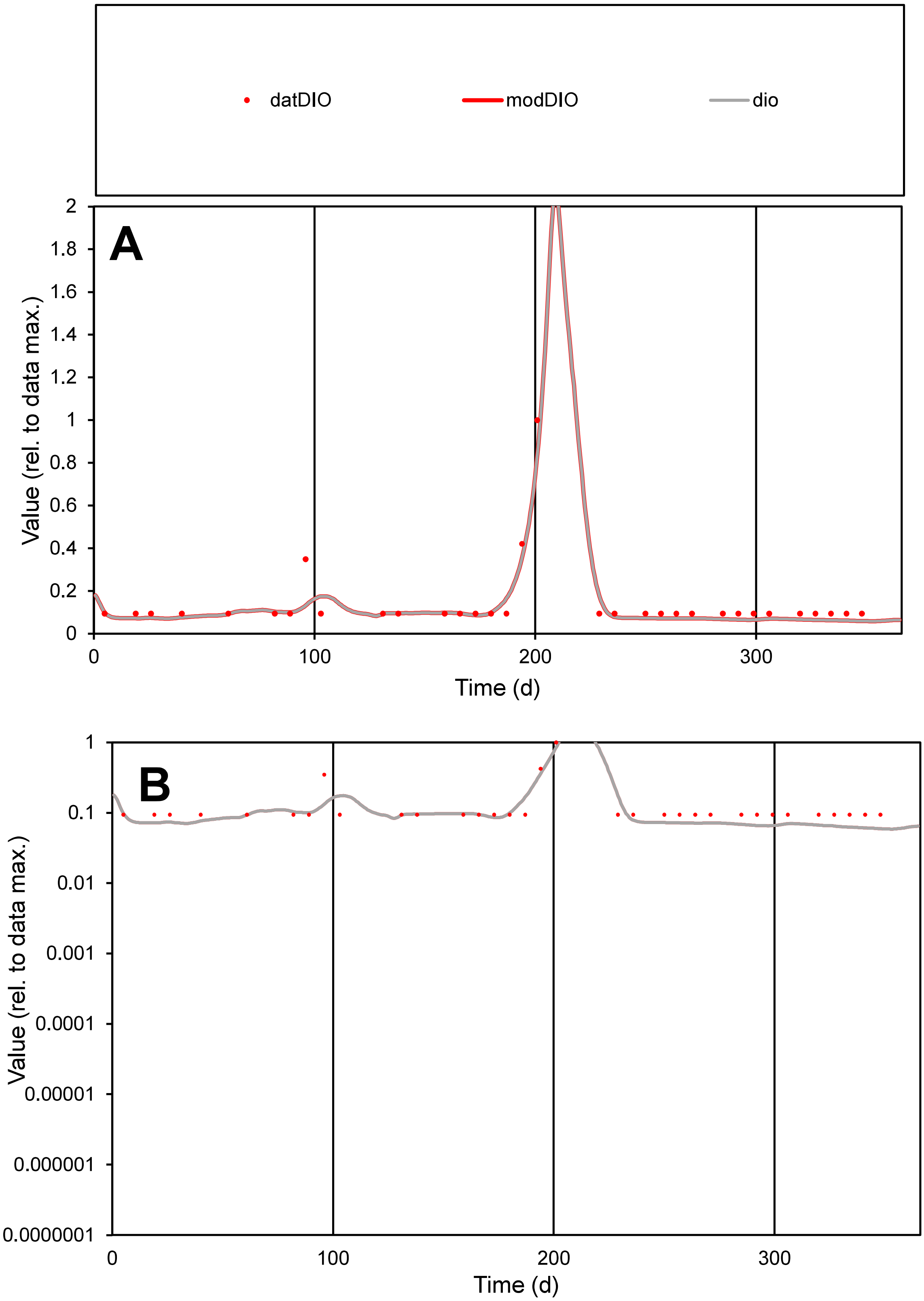

C1

Variables

DIO Provide jtd or Dst

CalibOnly?

2 1 to reproduce of

oNormalize?

2 1=no, 2=yes

ofxn calc*

0.32823

ofxn mod

0.36512

sRun

RunA1

zout

1

Ave

Data

6.9E-06

Model

6.3E-06

data/vMax

5E-05

ofxng

263.459

Min d/m

1.59406

ave/min d

1.4792

ave/min m

2.14483

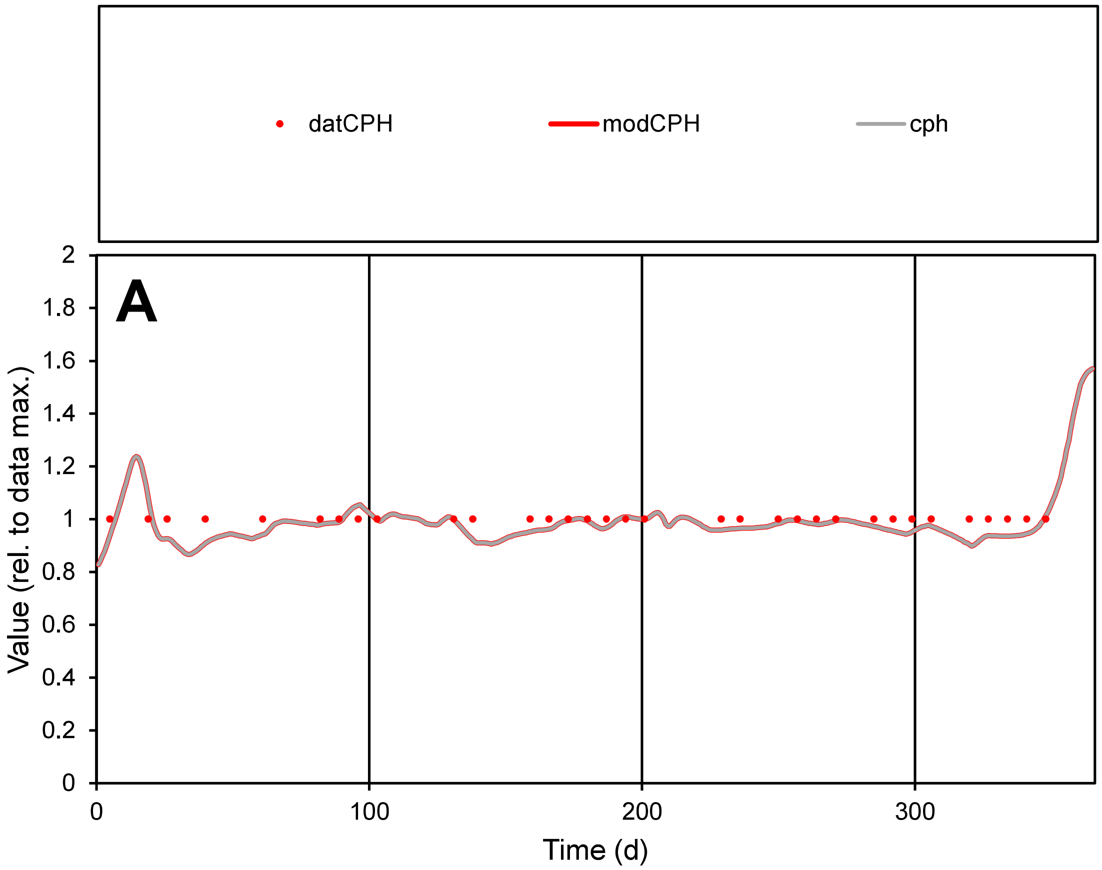

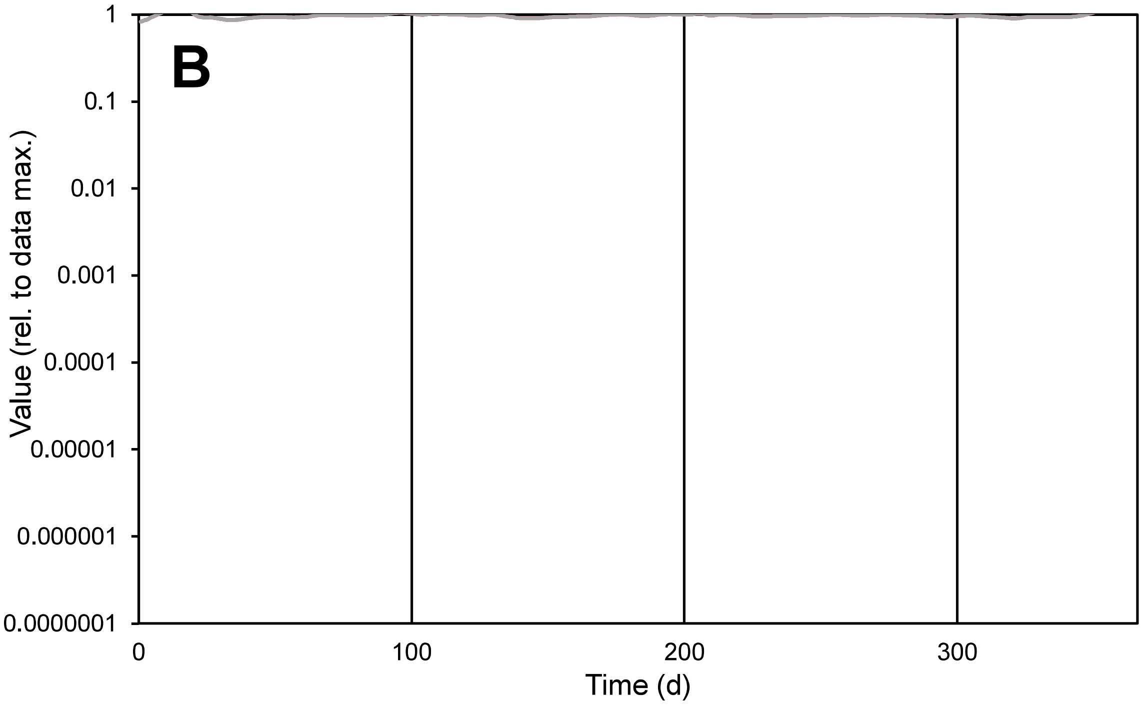

C1

Variables

CPH	Provide jtd or Dst
CalibOnly?	
2	1 to reproduce of
oNormalize?	
2	1=no, 2=yes
ofxn calc*	
0.04318	
ofxn mod	
0.11973	
sRun	
RunA1	
zout	
1	
Ave	
Data	
9.6E-06	
Model	
9.3E-06	
data/vMax	
9.6E-06	
ofxng	
263.459	
Min d/m	
1.10954	
ave/min d	
1	
ave/min m	
1.07895	

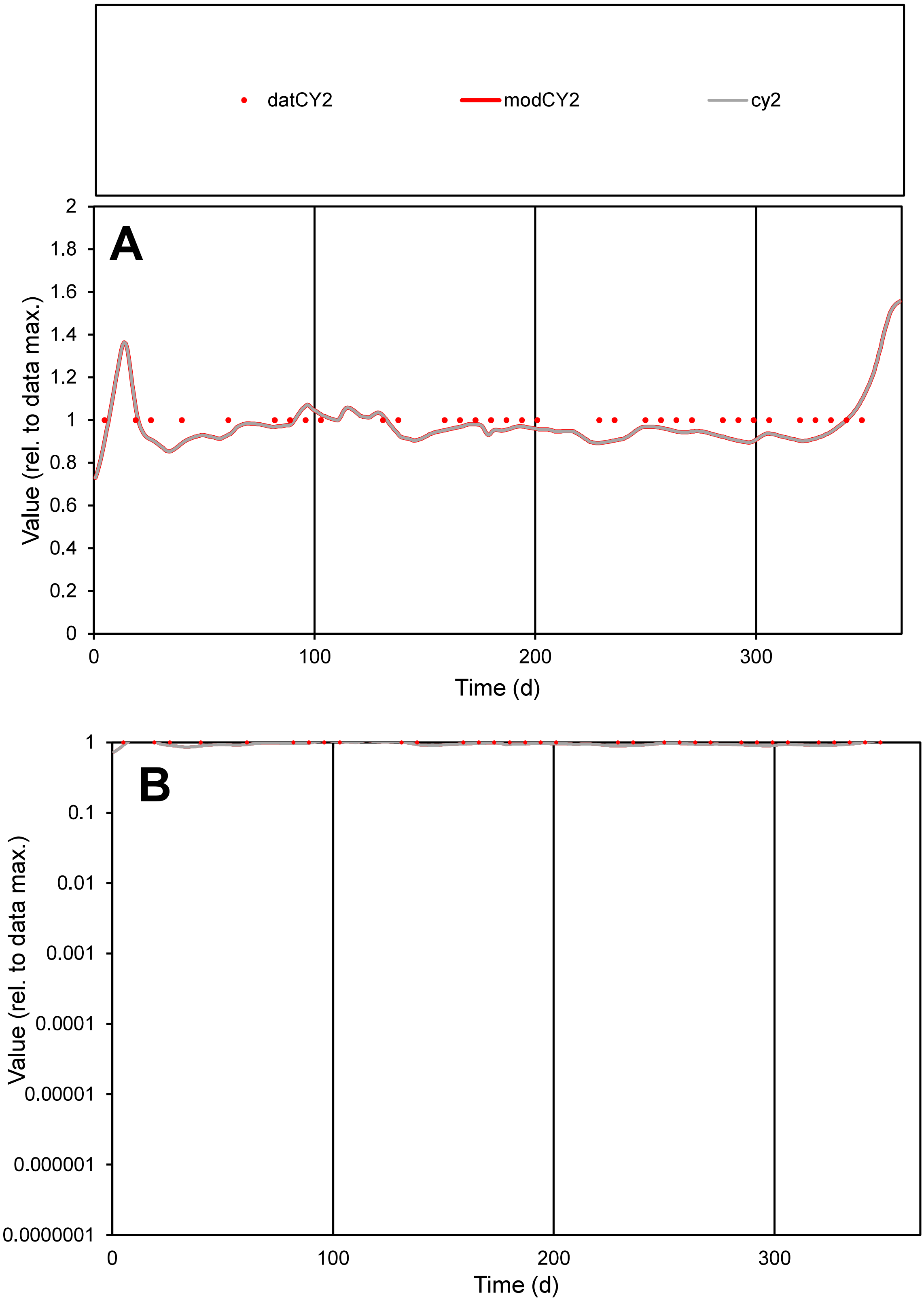

C1

Variables
CY2 Provide jtd or Dst
CalibOnly?
2 1 to reproduce of
oNormalize?
2 1=no, 2=yes

ofxn calc*
0.06437
ofxn mod
0.12788

sRun
RunA1
zout
1

Ave
Data
1.1E-05
Model
1.1E-05

data/vMax
1.1E-05

ofxng
263.459

Min d/m
1.11814

ave/min d
1
ave/min m
1.07179

326_Calib_S_A

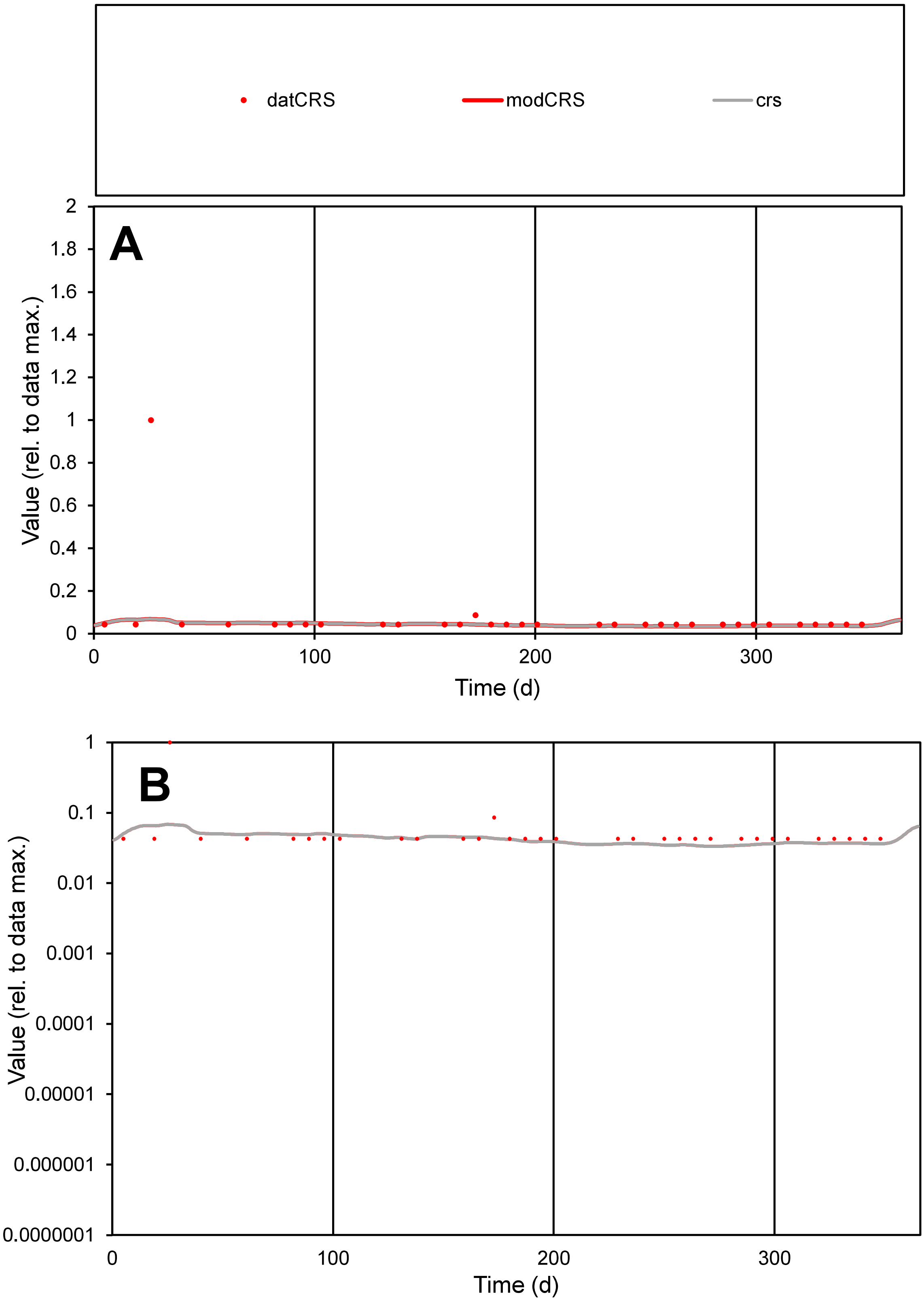

C1

Variables

CRS Provide jtd or Dst

CalibOnly?

2 1 to reproduce of

oNormalize?

2 1=no, 2=yes

ofxn calc*

2.22084

ofxn mod

2.22916

sRun

RunA1

zout

1

Ave

Data

9.2E-06

Model

5.4E-06

data/vMax

0.00013

ofxng

263.459

Min d/m

1.28443

ave/min d

1.70632

ave/min m

1.271

326_Calib_S_A

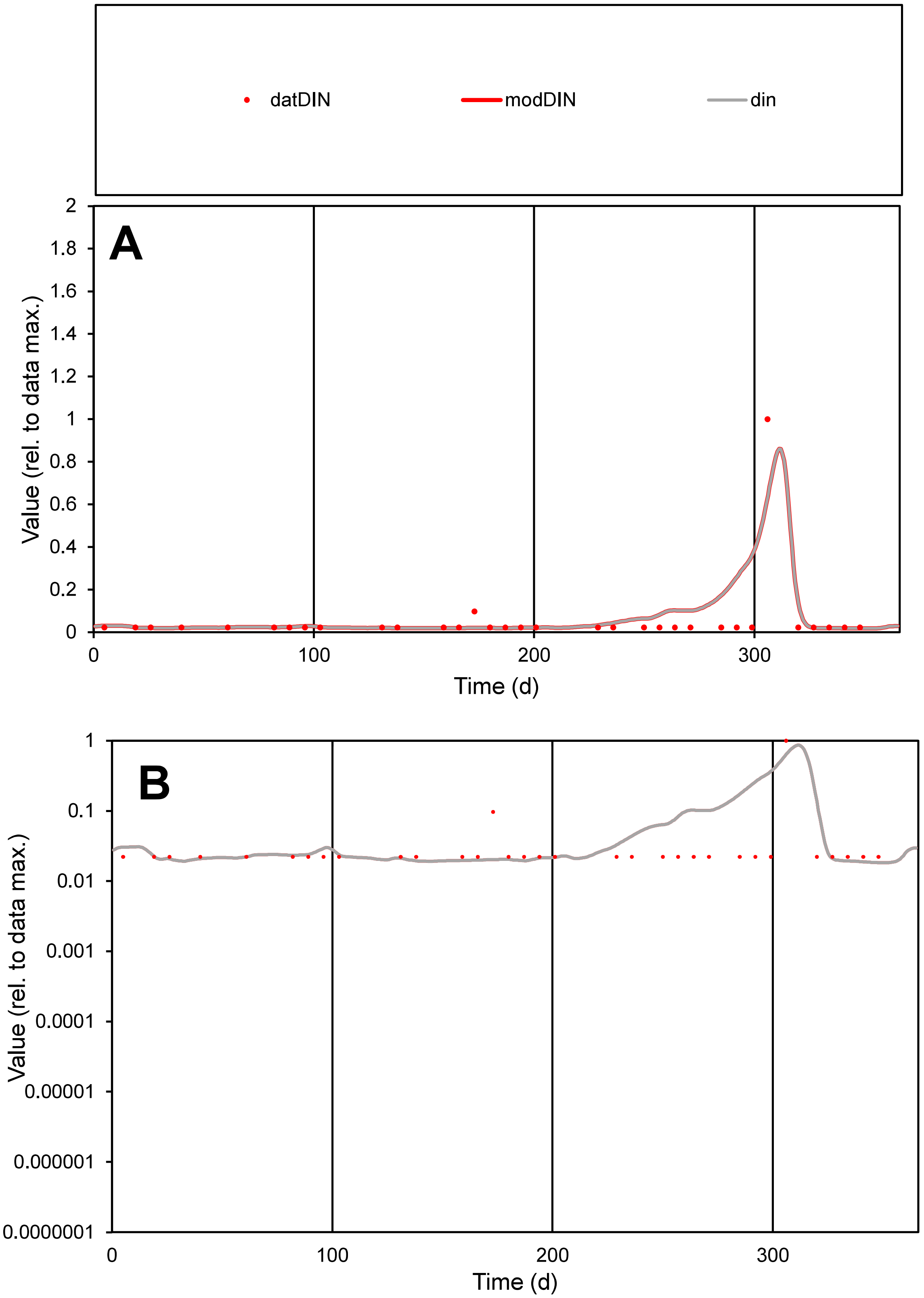

C1

Variables
DIN Provide jtd or Dst
CalibOnly?
2 1 to reproduce of
oNormalize?
2 1=no, 2=yes

ofxn calc*
1.71094
ofxn mod
1.80182

sRun
RunA1
zout
1

Ave
Data
7E-06
Model
9.6E-06

data/vMax
0.00013

ofxng
263.459

Min d/m
1.20922

ave/min d
2.43332
ave/min m
4.03847

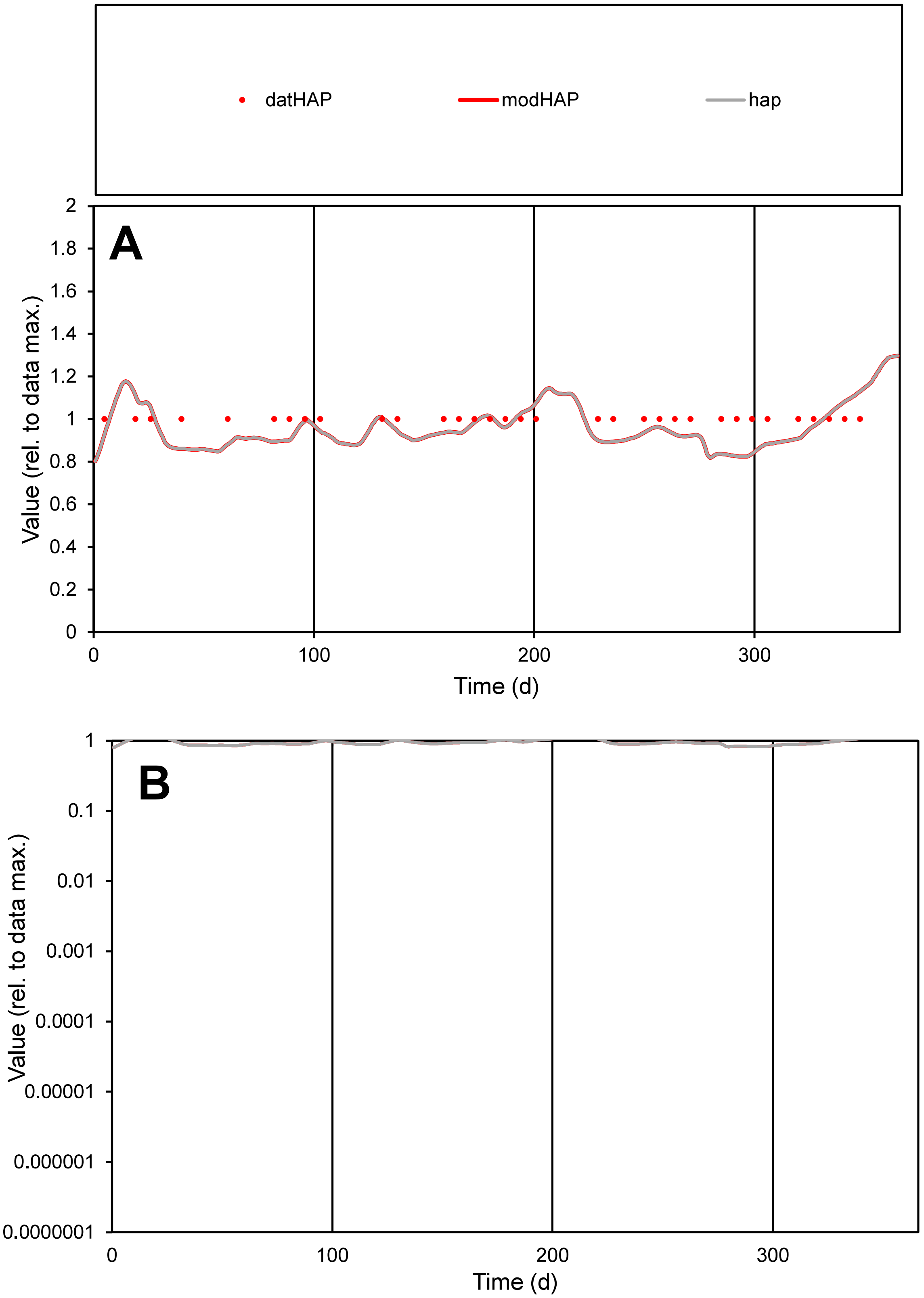

C1

Variables
HAP Provide jtd or Dst
CalibOnly?
2 1 to reproduce of
oNormalize?
2 1=no, 2=yes

ofxn calc*
0.08754
ofxn mod
0.14114

sRun
RunA1
zout
1

Ave
Data
1.1E-05
Model
1.1E-05

data/vMax
1.1E-05

ofxng
263.459

Min d/m
1.21131

ave/min d
1
ave/min m
1.15859

326_Calib_S_A

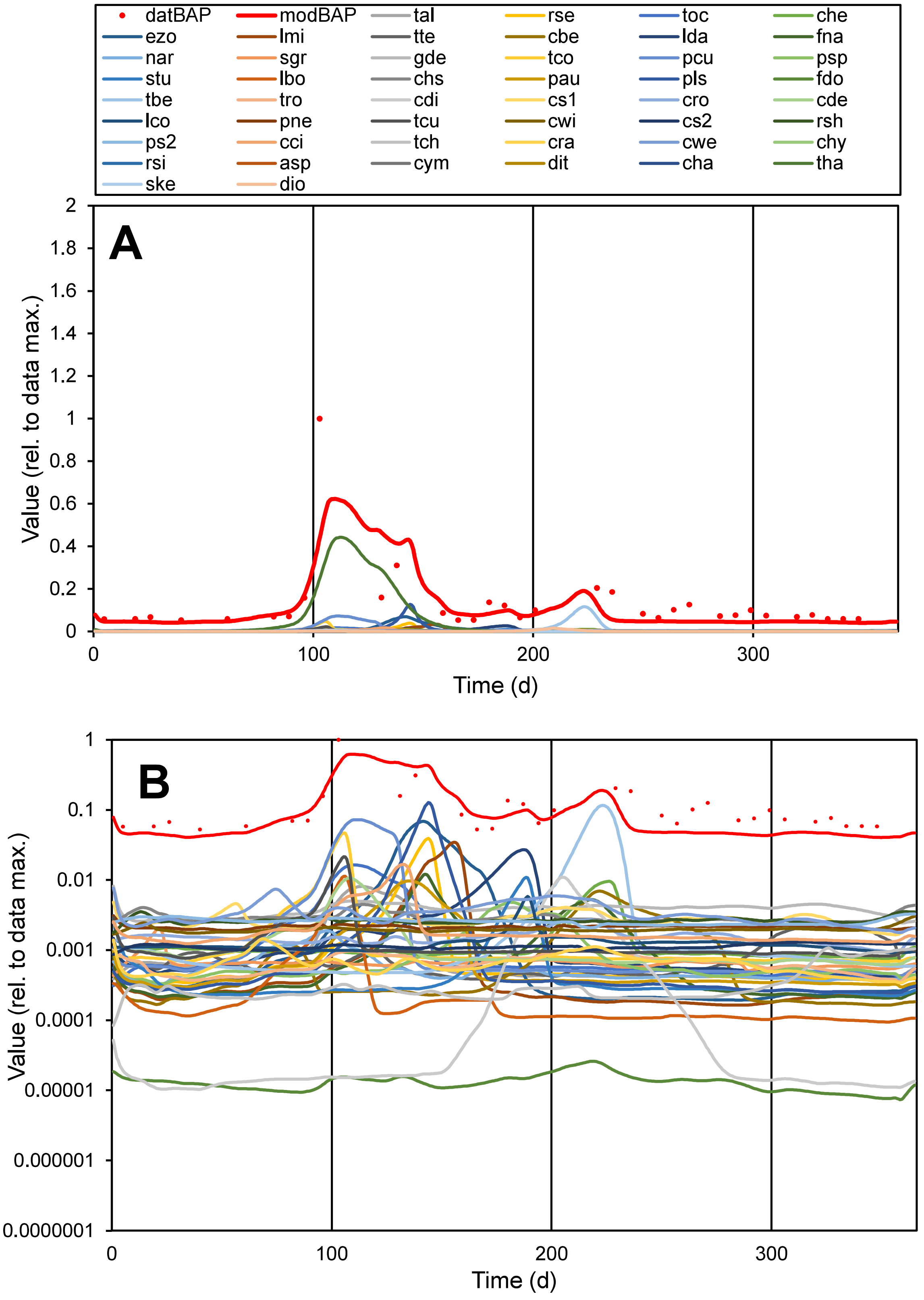

C1

Variables

BAP Provide jtd or Dst

CalibOnly?

2 1 to reproduce of

oNormalize?

2 1=no, 2=yes

ofxn calc*

0.89719

ofxn mod

0.90825

sRun

RunA1

zout

1

Ave

Data

0.00083

Model

0.00067

data/vMax

0.00669

ofxng

263.459

Min d/m

1.29764

ave/min d

2.34955

ave/min m

2.48411

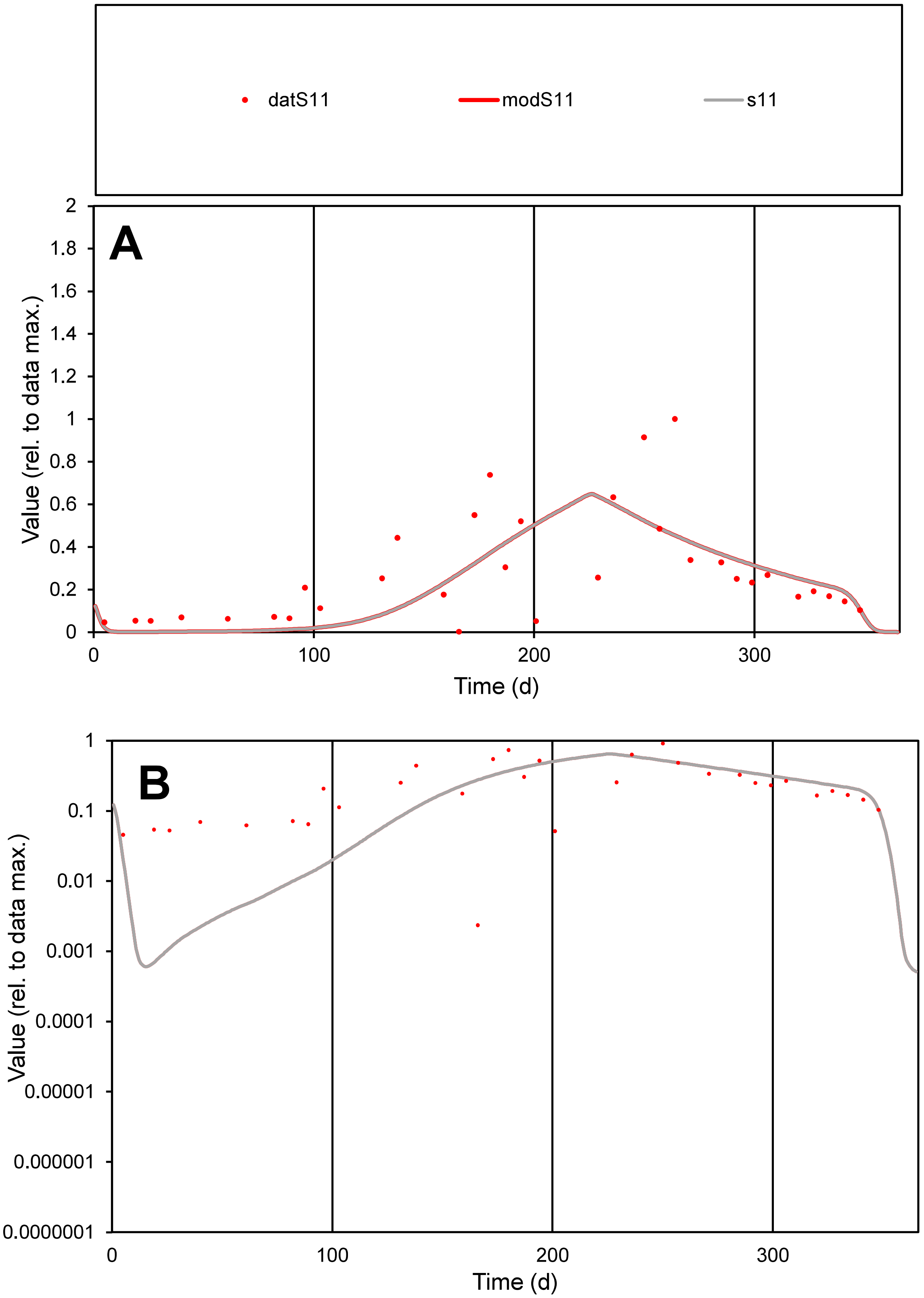

C1

Variables
S11 Provide jtd or Dst
CalibOnly?
2 1 to reproduce of
oNormalize?
2 1=no, 2=yes

ofxn calc*
0.7093
ofxn mod
0.72576

sRun
RunA1
zout
1

Ave
Data
0.0821
Model
0.07354

data/vMax
0.2927

ofxng
263.459

Min d/m
3.10921

ave/min d
119.156
ave/min m
331.876

326_Calib_S_A

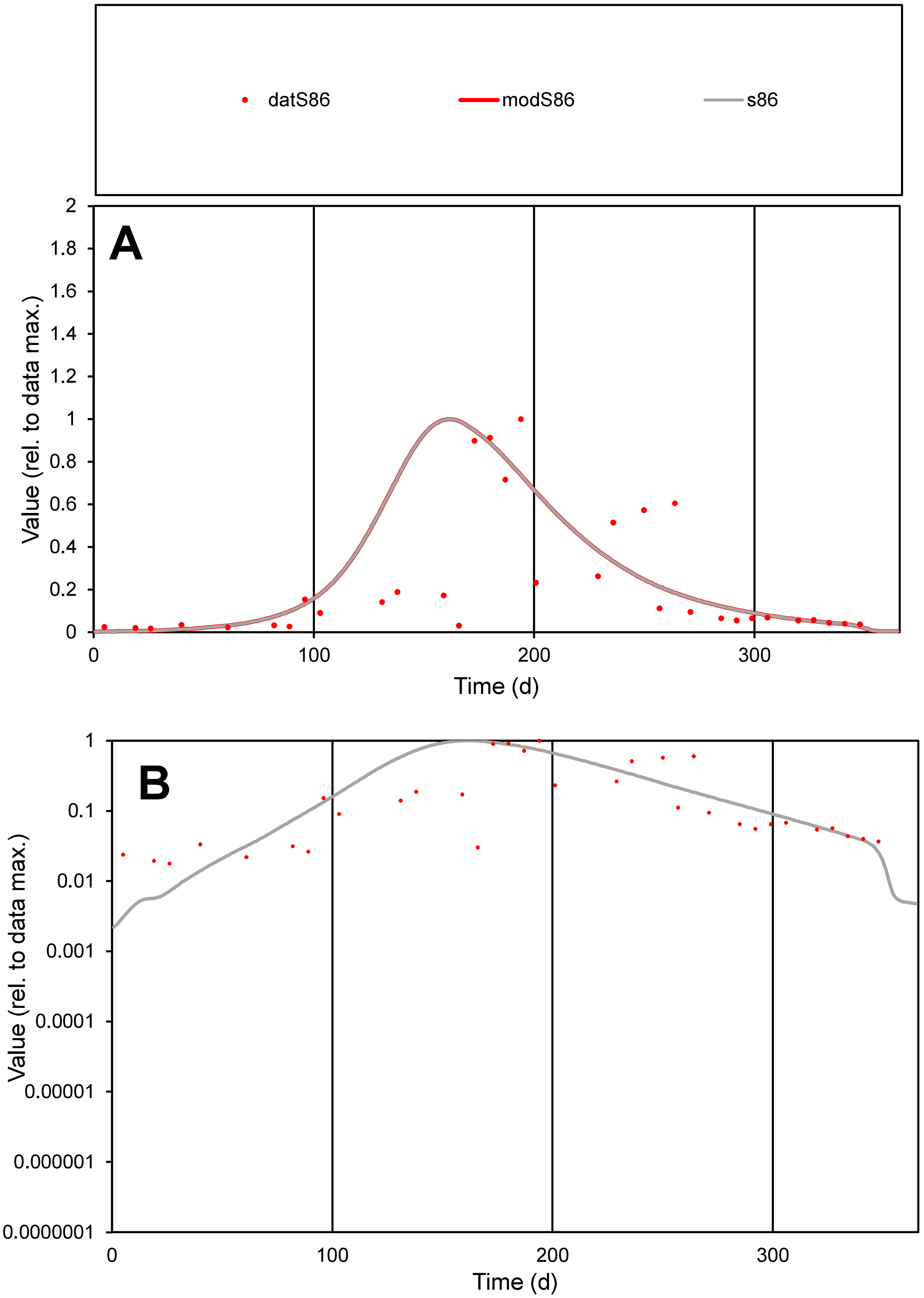

C1

Variables
S86 Provide jtd or Dst
CalibOnly?
2 1 to reproduce of
oNormalize?
2 1=no, 2=yes

ofxn calc*
1.29034
ofxn mod
1.30849

sRun
RunA1
zout
1

Ave
Data
0.04325
Model
0.05851

data/vMax
0.1942

ofxng
263.459

Min d/m
5.13289

ave/min d
12.5098
ave/min m
86.8713

326_Calib_S_A

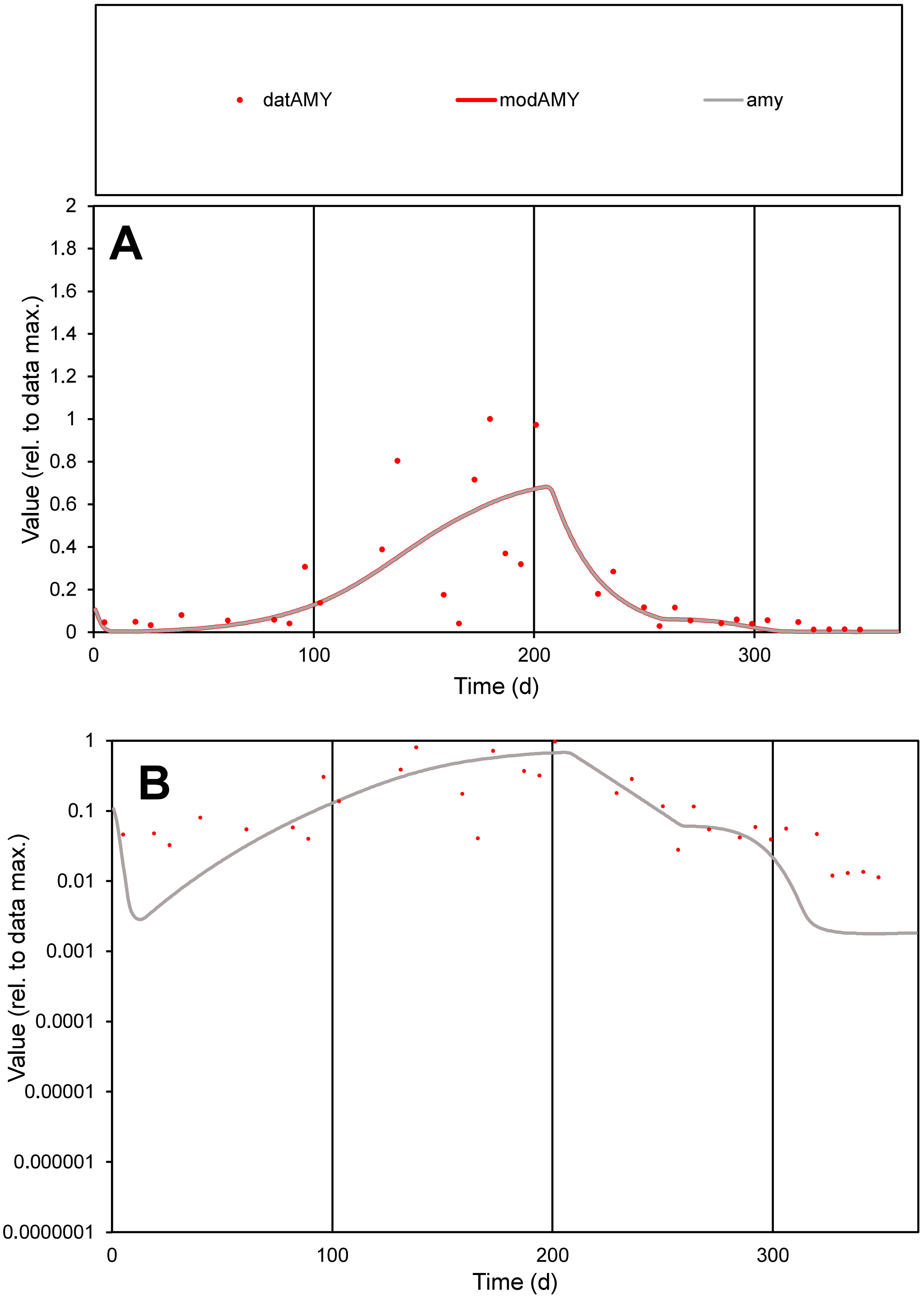

C1

Variables
AMY Provide jtd or Dst
CalibOnly?
2 1 to reproduce of
oNormalize?
2 1=no, 2=yes

ofxn calc*
0.89244
ofxn mod
0.90937

sRun
RunA1
zout
1

Ave
Data
0.0278
Model
0.02593

data/vMax
0.1377

ofxng
263.459

Min d/m
6.38628

ave/min d
17.7734
ave/min m
105.871

326_Calib_S_A

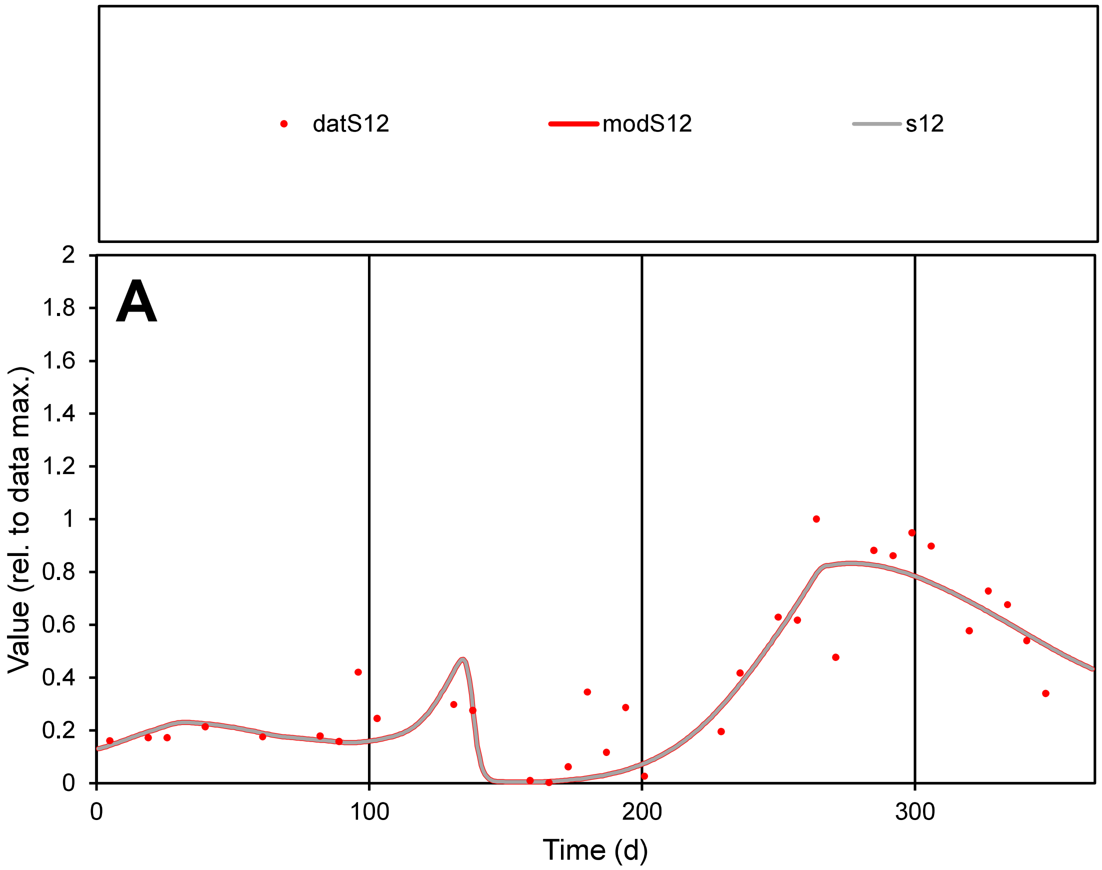

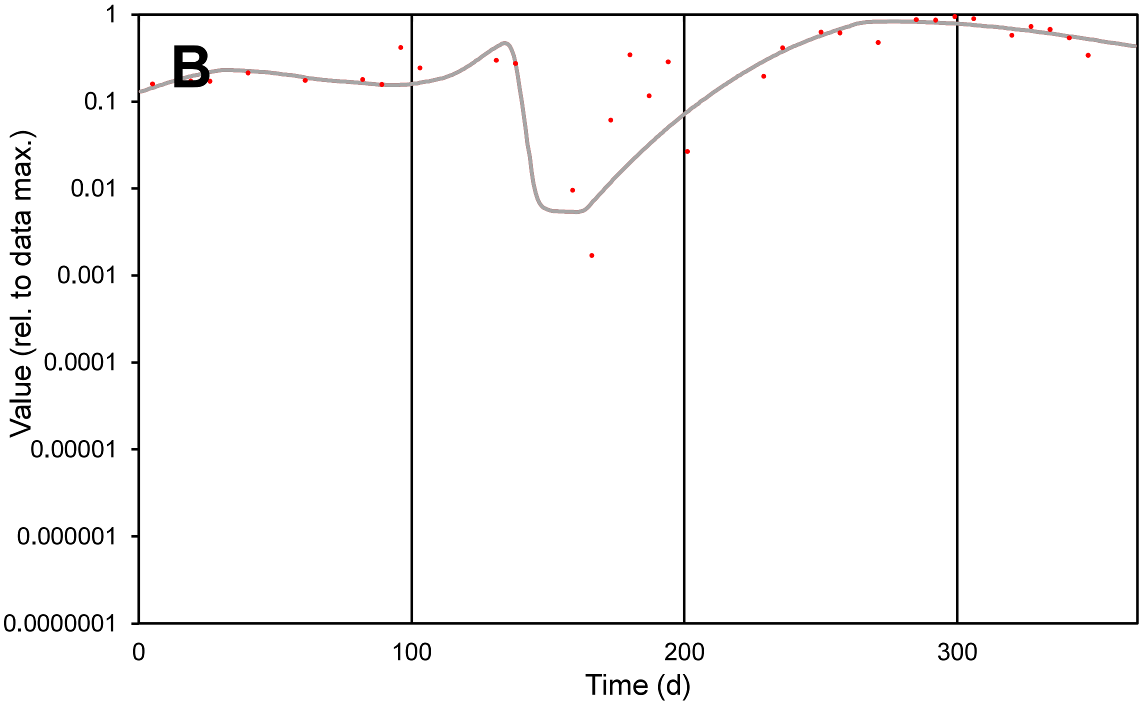

C1

Variables
S12 Provide jtd or Dst
CalibOnly?
2 1 to reproduce of
oNormalize?
2 1=no, 2=yes

ofxn calc*
0.32739
ofxn mod
0.35805

sRun
RunA1
zout
1

Ave
Data
0.0323
Model
0.03039

data/vMax
0.08138

ofxng
263.459

Min d/m
0.31732

ave/min d
232.897
ave/min m
69.5213

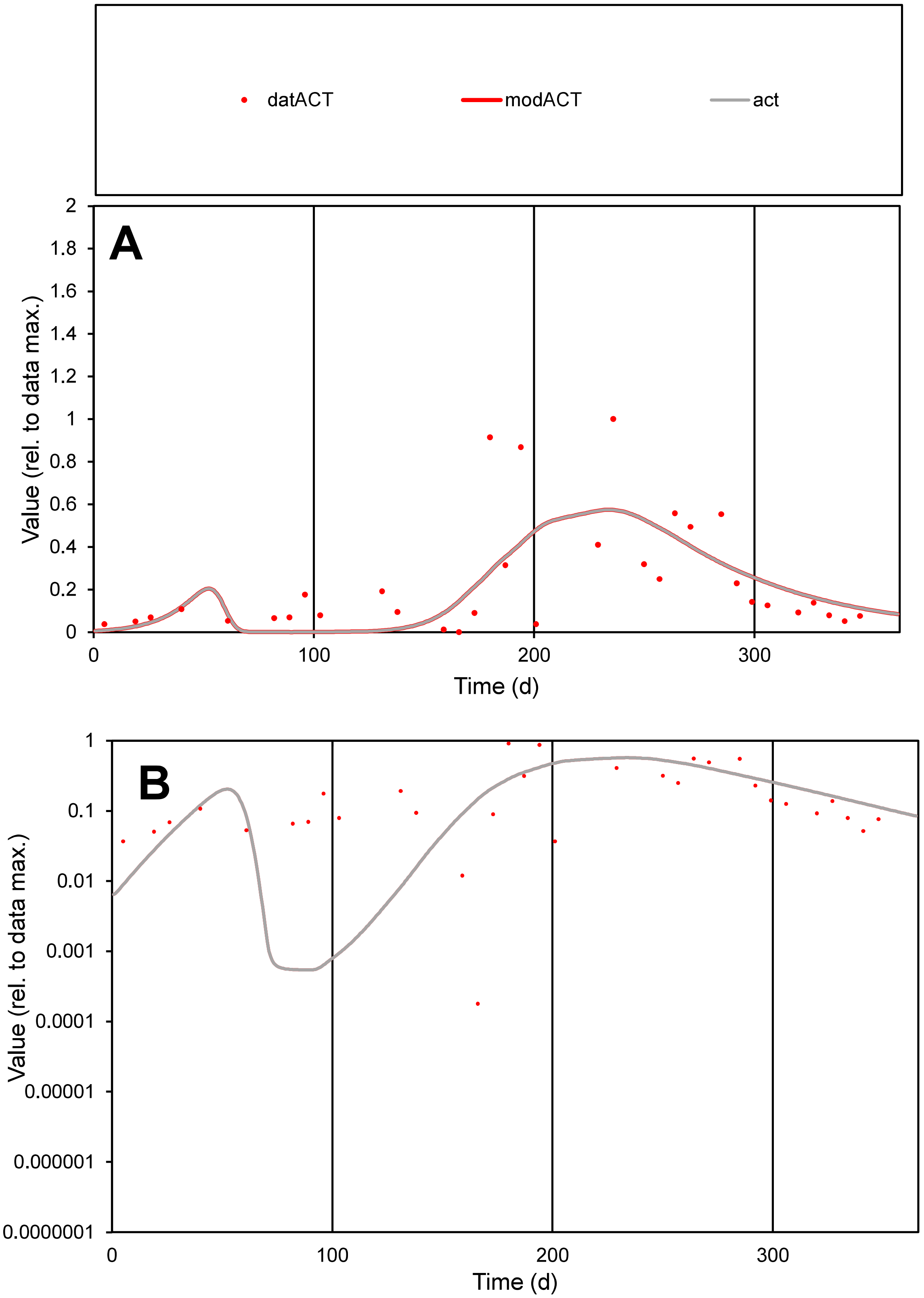

C1

Variables
ACT Provide jtd or Dst
CalibOnly?
2 1 to reproduce of
oNormalize?
2 1=no, 2=yes

ofxn calc*
0.85445
ofxn mod
0.86952

sRun
RunA1
zout
1

Ave
Data
0.03376
Model
0.03092

data/vMax
0.1438

ofxng
263.459

Min d/m
0.32697

ave/min d
1314.59
ave/min m
393.628

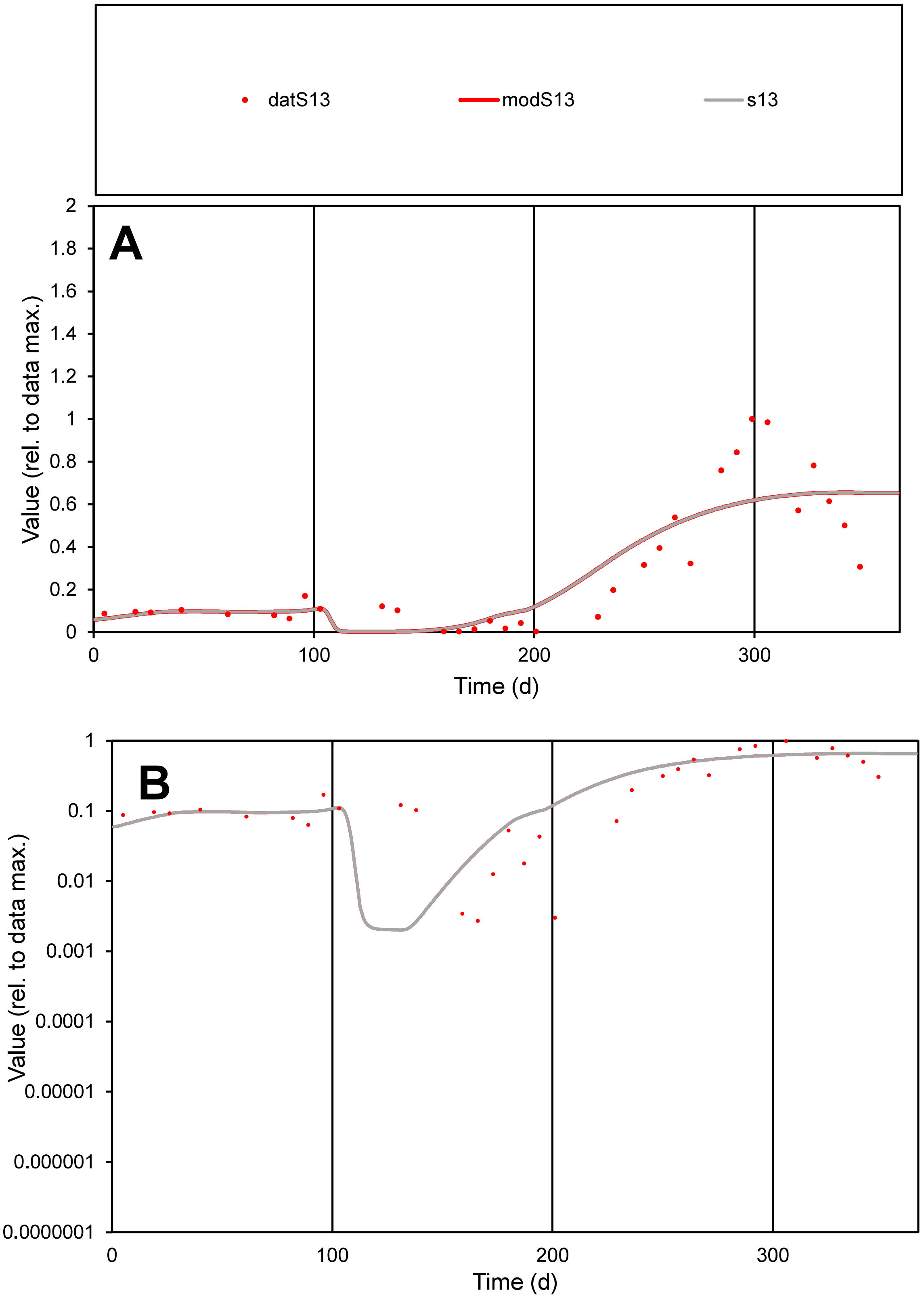

C1

Variables
S13 Provide jtd or Dst
CalibOnly?
2 1 to reproduce of
oNormalize?
2 1=no, 2=yes

ofxn calc*
0.51966
ofxn mod
0.54221

sRun
RunA1
zout
1

Ave
Data
0.02911
Model
0.02976

data/vMax
0.1017

ofxng
263.459

Min d/m
1.34208

ave/min d
105.402
ave/min m
144.621

326_Calib_S_A

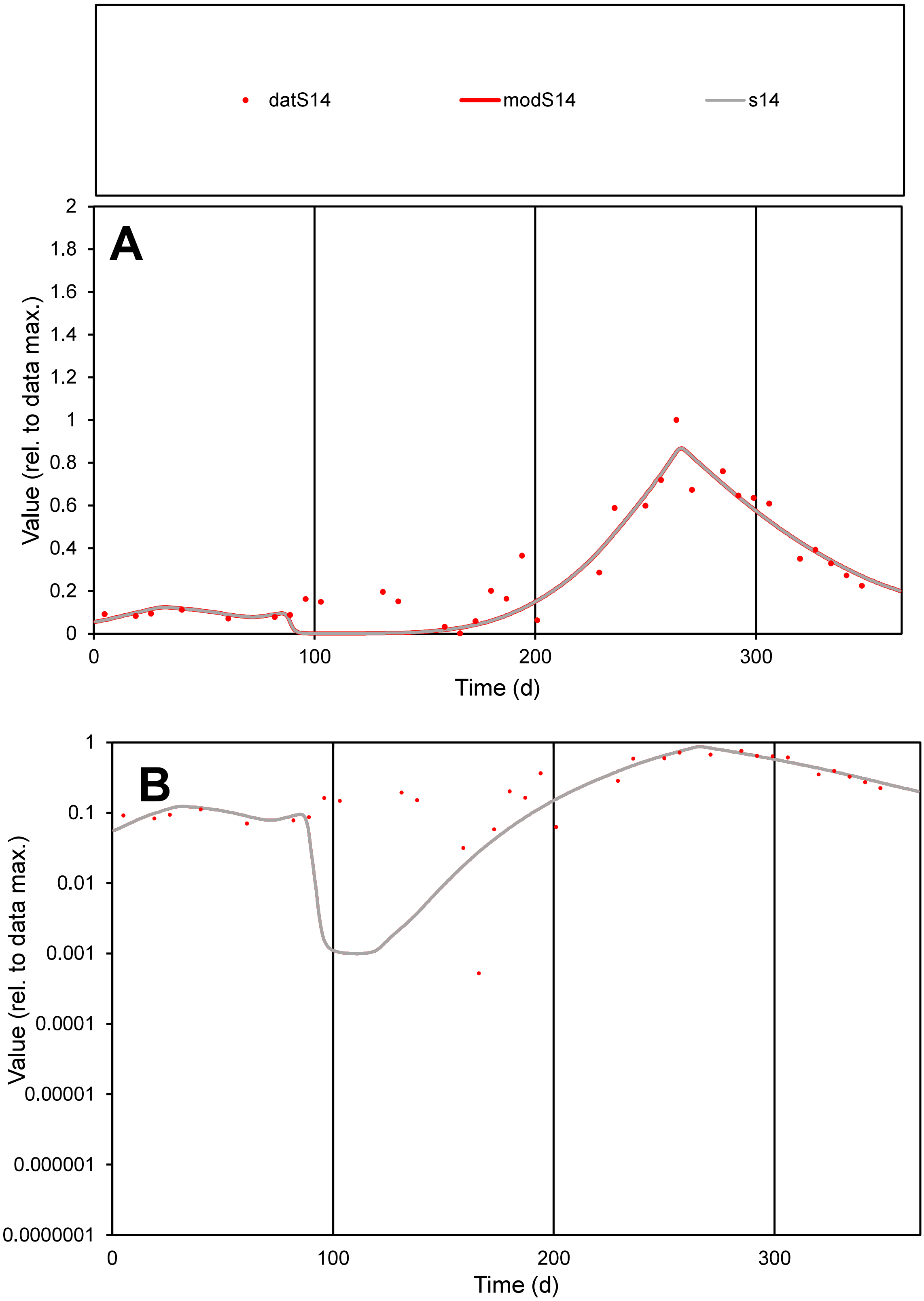

C1

Variables
S14 Provide jtd or Dst
CalibOnly?
2 1 to reproduce of
oNormalize?
2 1=no, 2=yes

ofxn calc*
0.30563
ofxn mod
0.34371

sRun
RunA1
zout
1

Ave
Data
0.02919
Model
0.02651

data/vMax
0.09409

ofxng
263.459

Min d/m
0.50998

ave/min d
591.651
ave/min m
274.069

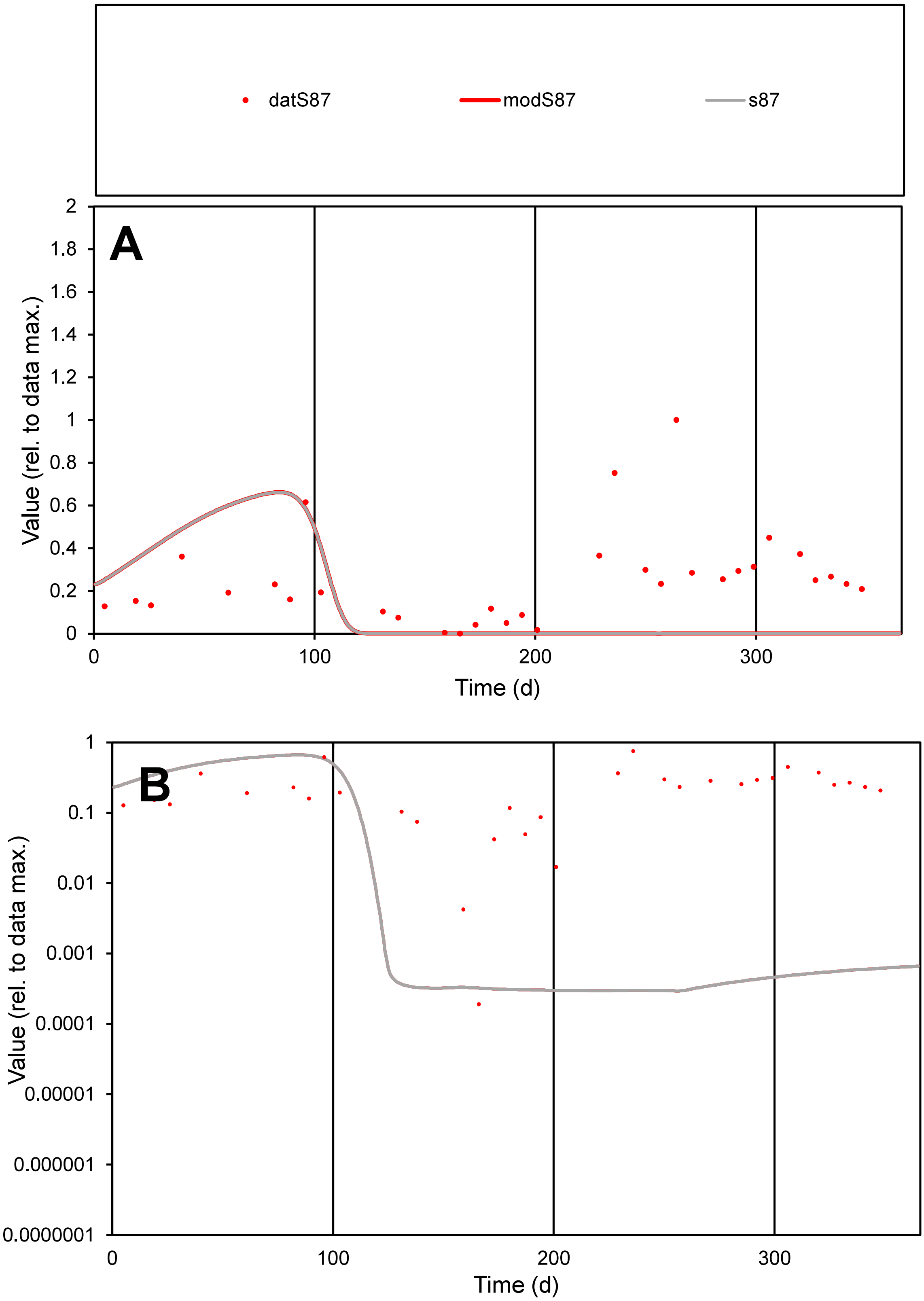

C1

Variables
S87 Provide jtd or Dst
CalibOnly?
2 1 to reproduce of
oNormalize?
2 1=no, 2=yes

ofxn calc*
1.31339
ofxn mod
1.32125

sRun
RunA1
zout
1

Ave
Data
0.023
Model
0.012

data/vMax
0.09219

ofxng
263.459

Min d/m
0.64307

ave/min d
1311.83
ave/min m
440.127

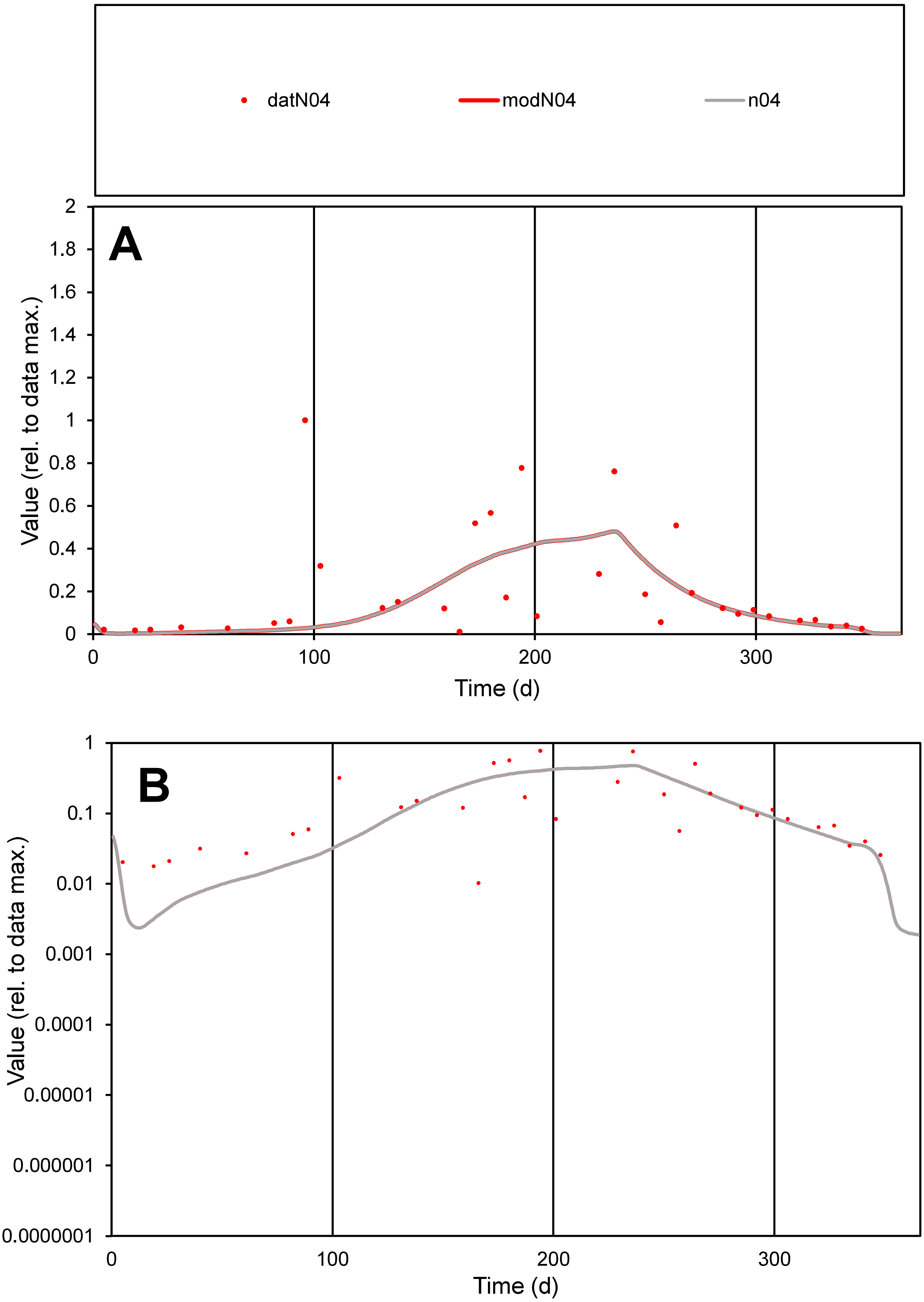

C1

Variables
N04 Provide jtd or Dst
CalibOnly?
2 1 to reproduce of
oNormalize?
2 1=no, 2=yes

ofxn calc*
1.13813
ofxn mod
1.14738

sRun
RunA1
zout
1

Ave
Data
0.01365
Model
0.01098

data/vMax
0.06724

ofxng
263.459

Min d/m
3.00218

ave/min d
19.8055
ave/min m
47.832

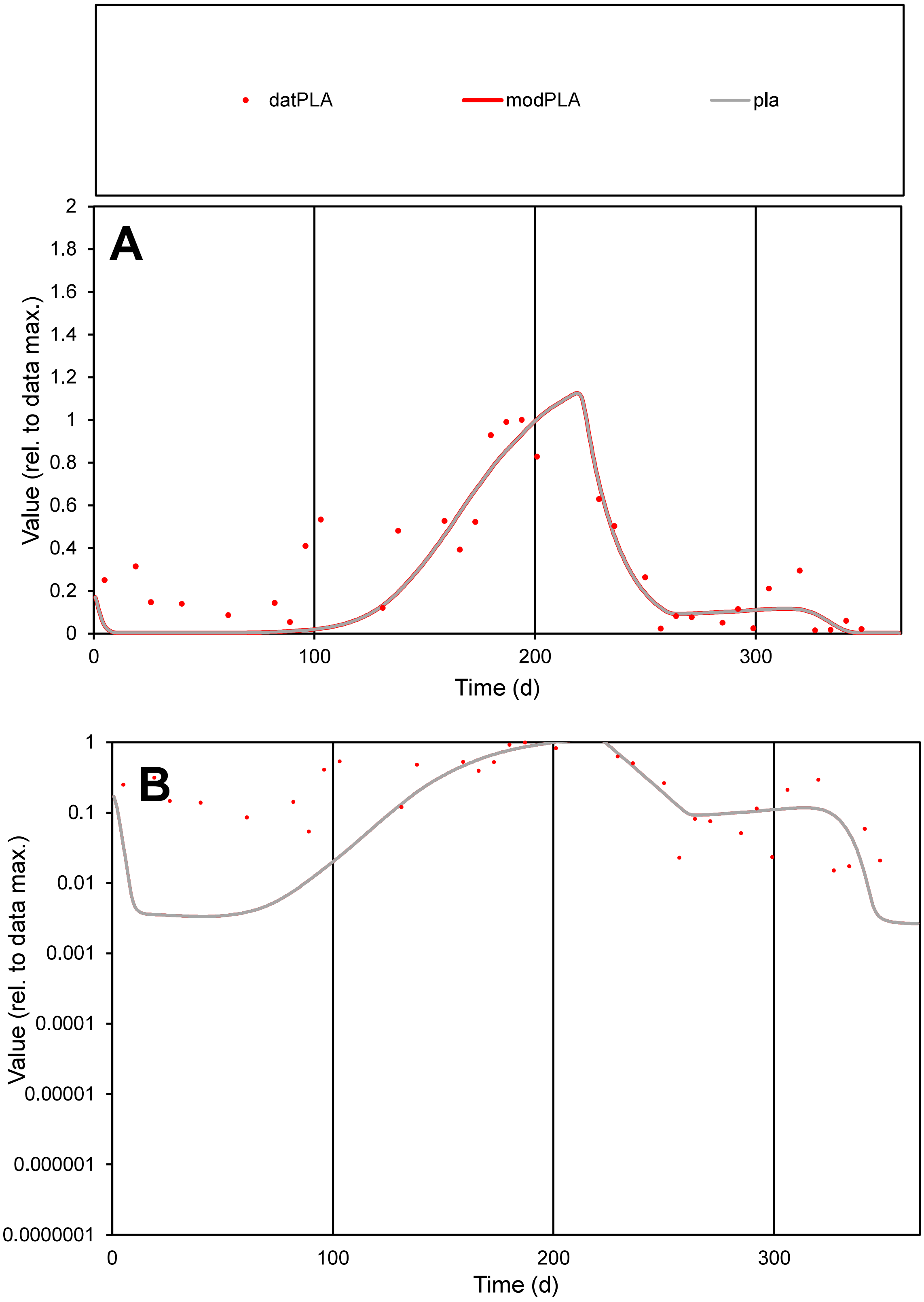

C1

Variables

PLA Provide jtd or Dst

CalibOnly?

2 1 to reproduce of

oNormalize?

2 1=no, 2=yes

ofxn calc*

0.52771

ofxn mod

0.55505

sRun

RunA1

zout

1

Ave

Data

0.01075

Model

0.00855

data/vMax

0.03462

ofxng

263.459

Min d/m

4.85634

ave/min d

20.6509

ave/min m

79.727

326_Calib_S_A

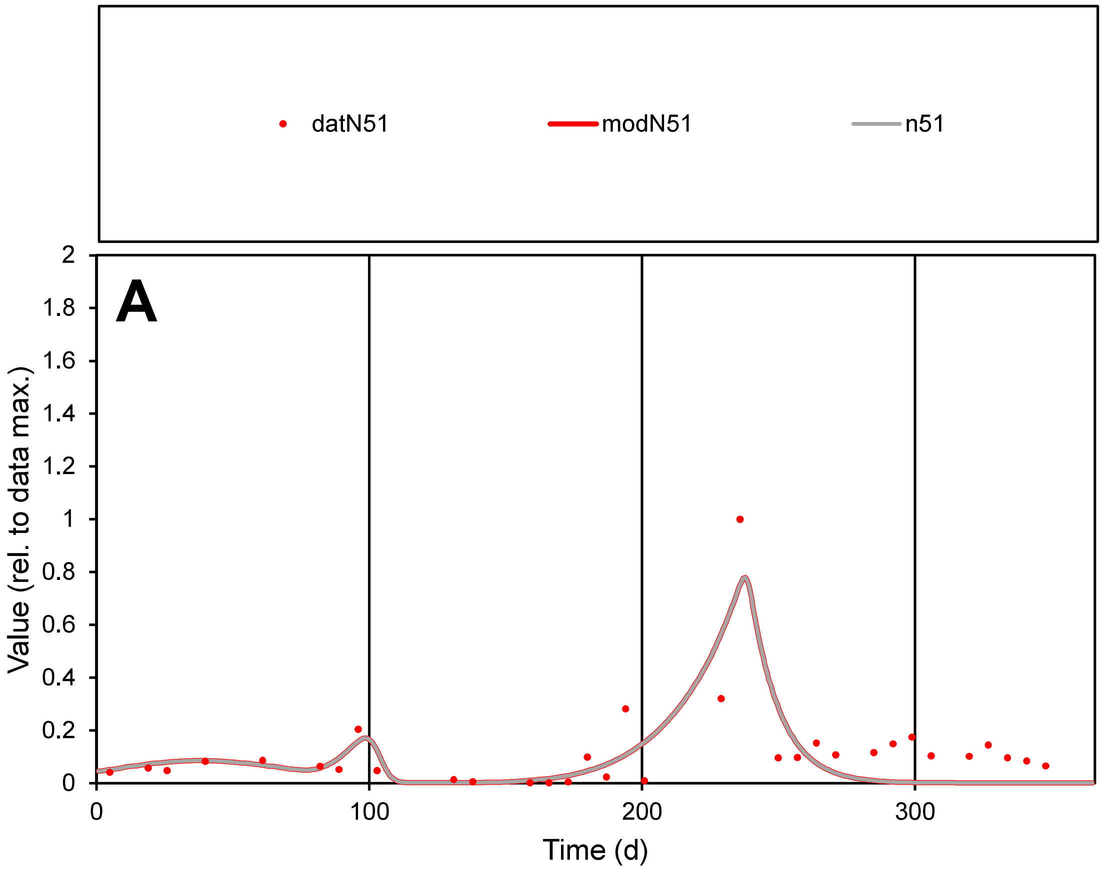

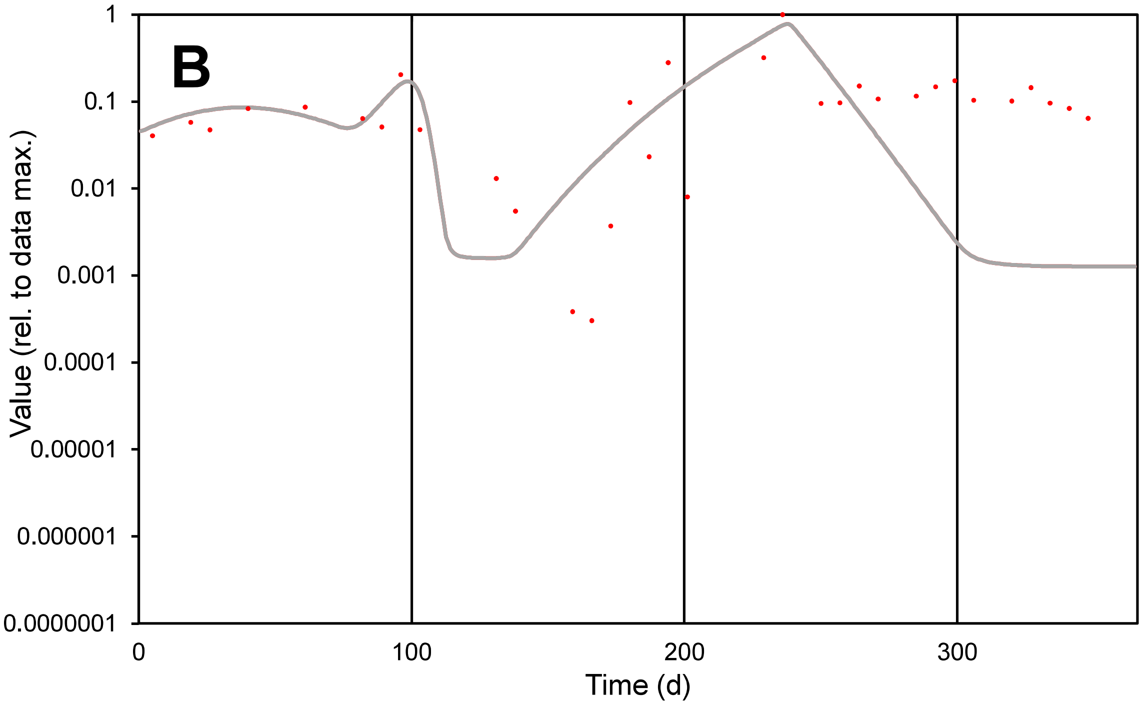

C1

Variables
N51 Provide jtd or Dst
CalibOnly?
2 1 to reproduce of
oNormalize?
2 1=no, 2=yes

ofxn calc*
0.83577
ofxn mod
0.86657

sRun
RunA1
zout
1

Ave
Data
0.01206
Model
0.00938

data/vMax
0.1016

ofxng
263.459

Min d/m
0.23897

ave/min d
393.262
ave/min m
73.1485

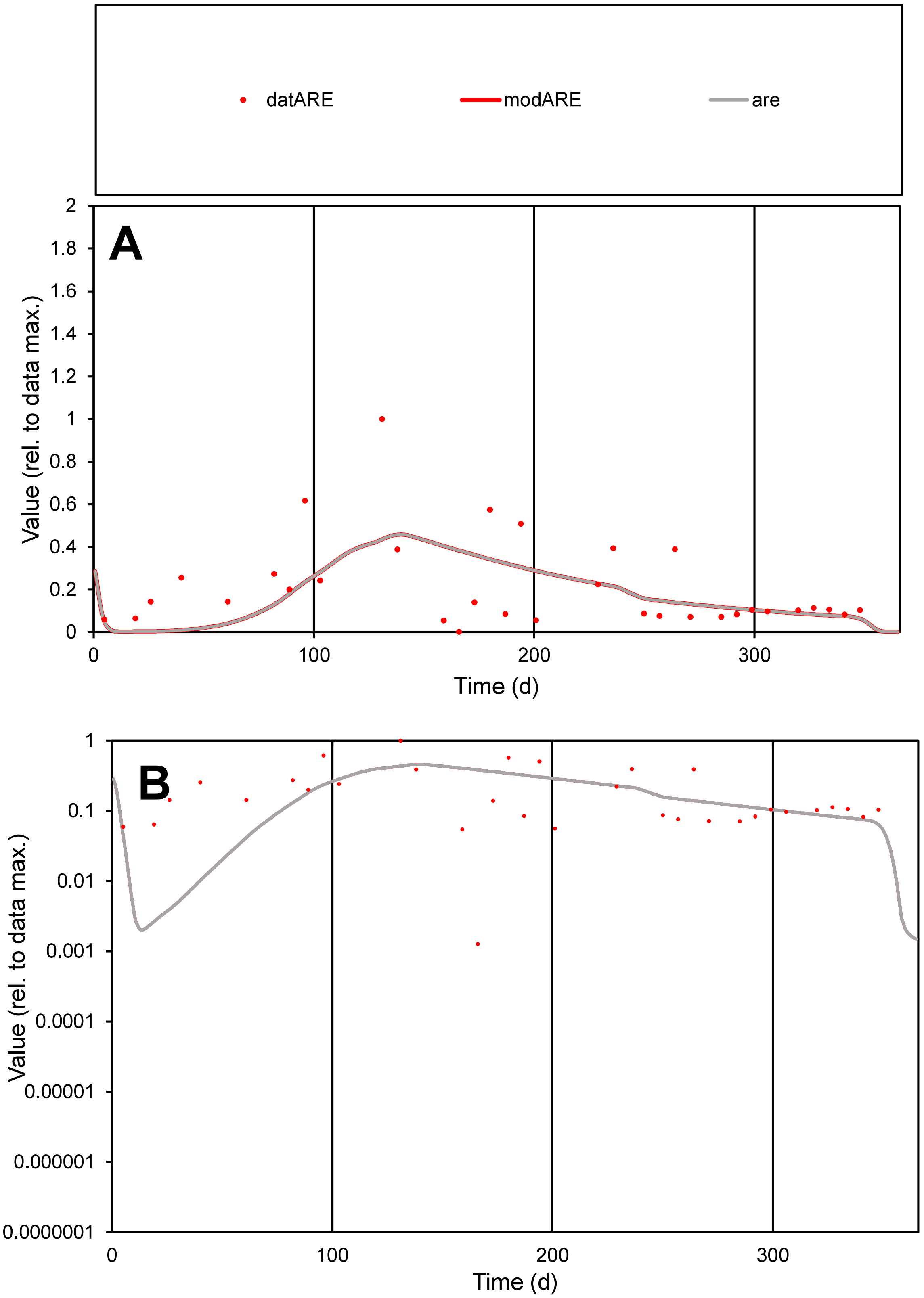

C1

Variables
ARE Provide jtd or Dst
CalibOnly?
2 1 to reproduce of
oNormalize?
2 1=no, 2=yes

ofxn calc*
0.91016
ofxn mod
0.91983

sRun
RunA1
zout
1

Ave
Data
0.00885
Model
0.00779

data/vMax
0.04227

ofxng
263.459

Min d/m
0.44322

ave/min d
165.056
ave/min m
64.4159

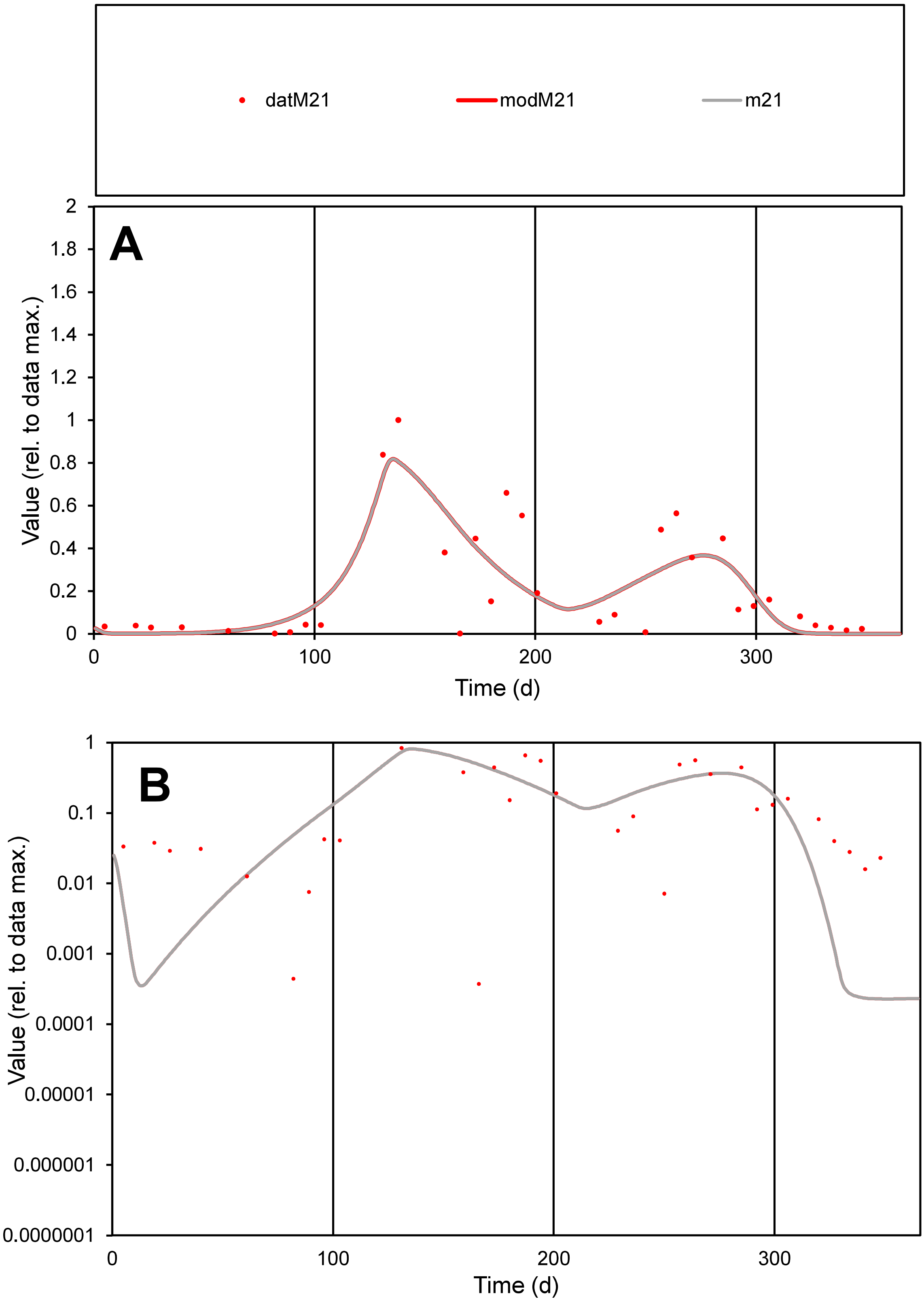

C1

Variables
M21 Provide jtd or Dst
CalibOnly?
2 1 to reproduce of
oNormalize?
2 1=no, 2=yes

ofxn calc*
0.74727
ofxn mod
0.77445

sRun
RunA1
zout
1

Ave
Data
0.01127
Model
0.01099

data/vMax
0.05272

ofxng
263.459

Min d/m
1.64387

ave/min d
571.686
ave/min m
916.876

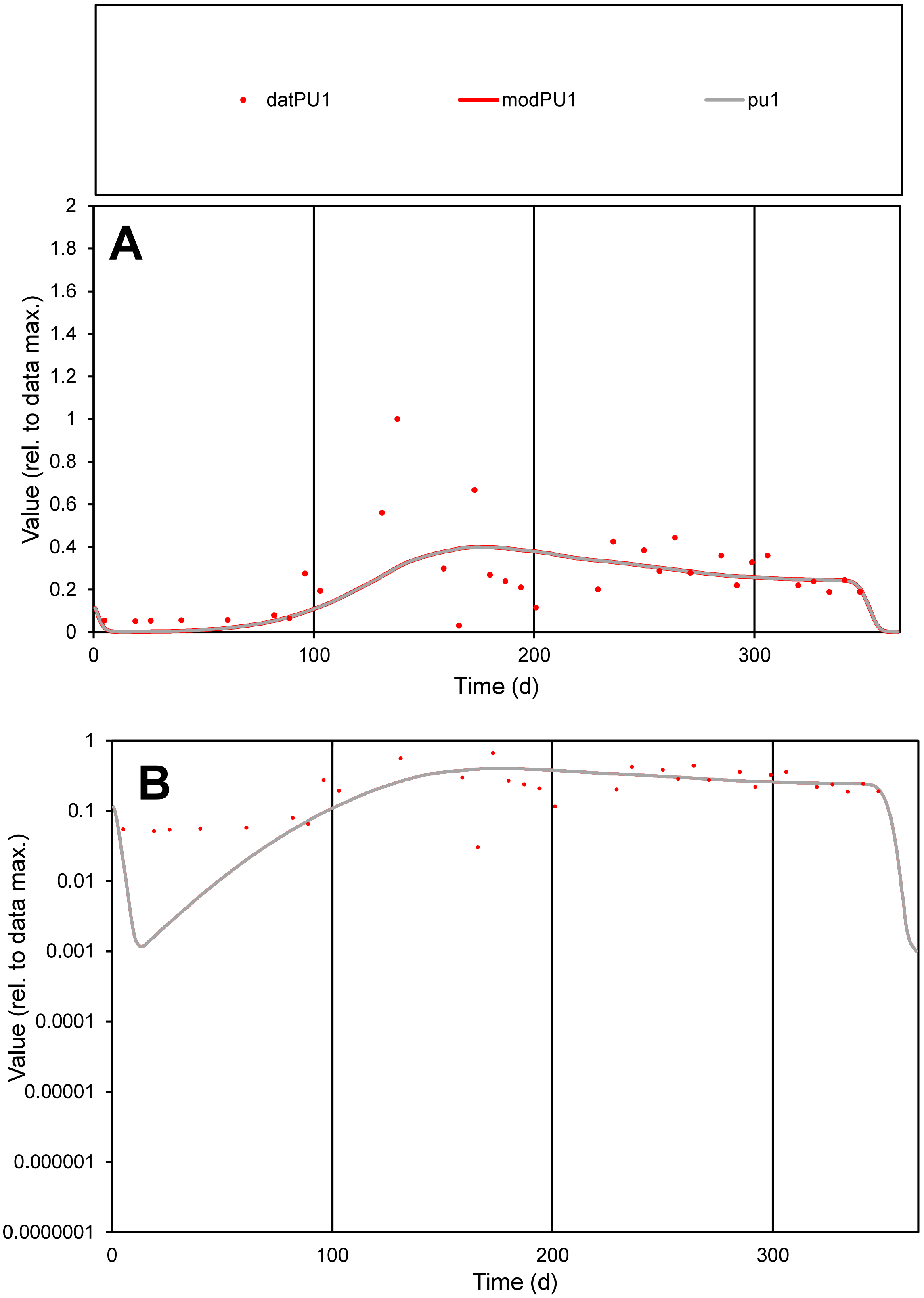

C1

Variables
PU1 Provide jtd or Dst
CalibOnly?
2 1 to reproduce of
oNormalize?
2 1=no, 2=yes

ofxn calc*
0.67707
ofxn mod
0.68925

sRun
RunA1
zout
1

Ave
Data
0.01067
Model
0.00963

data/vMax
0.04076

ofxng
263.459

Min d/m
17.2318

ave/min d
8.60662
ave/min m
133.798

326_Calib_S_A

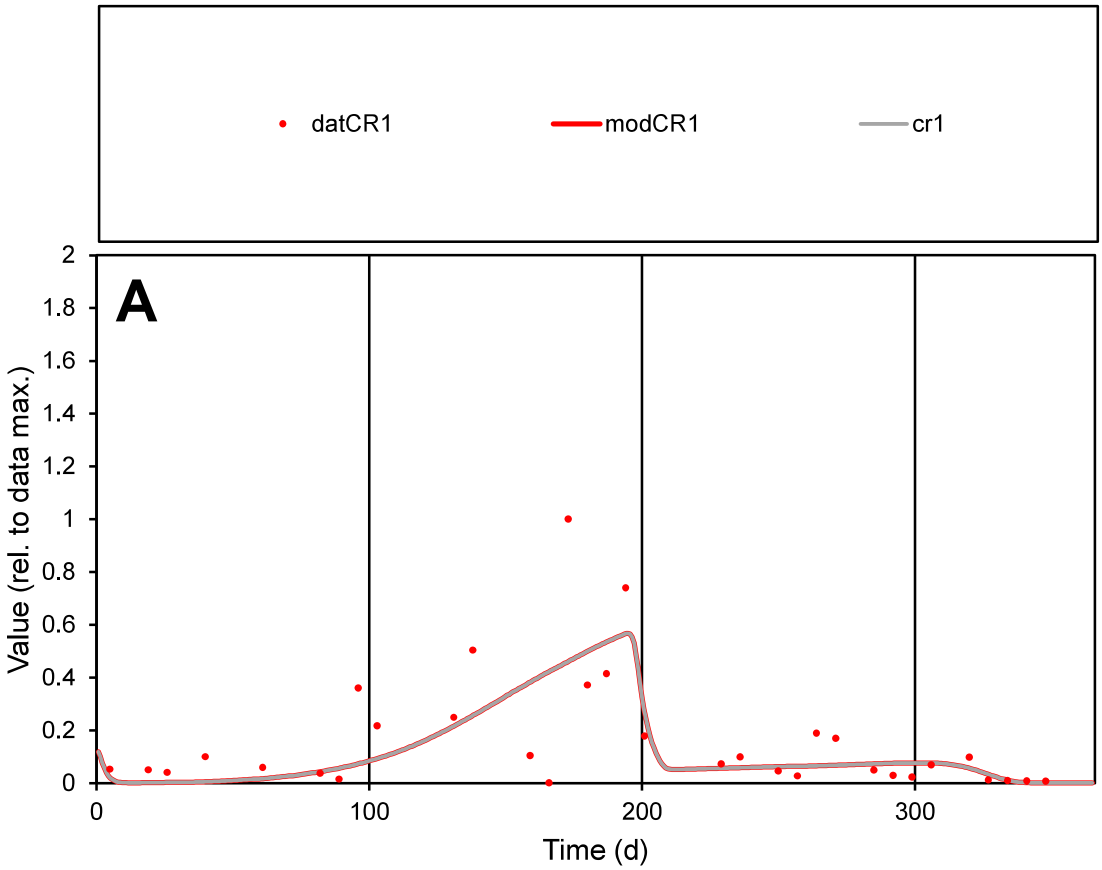

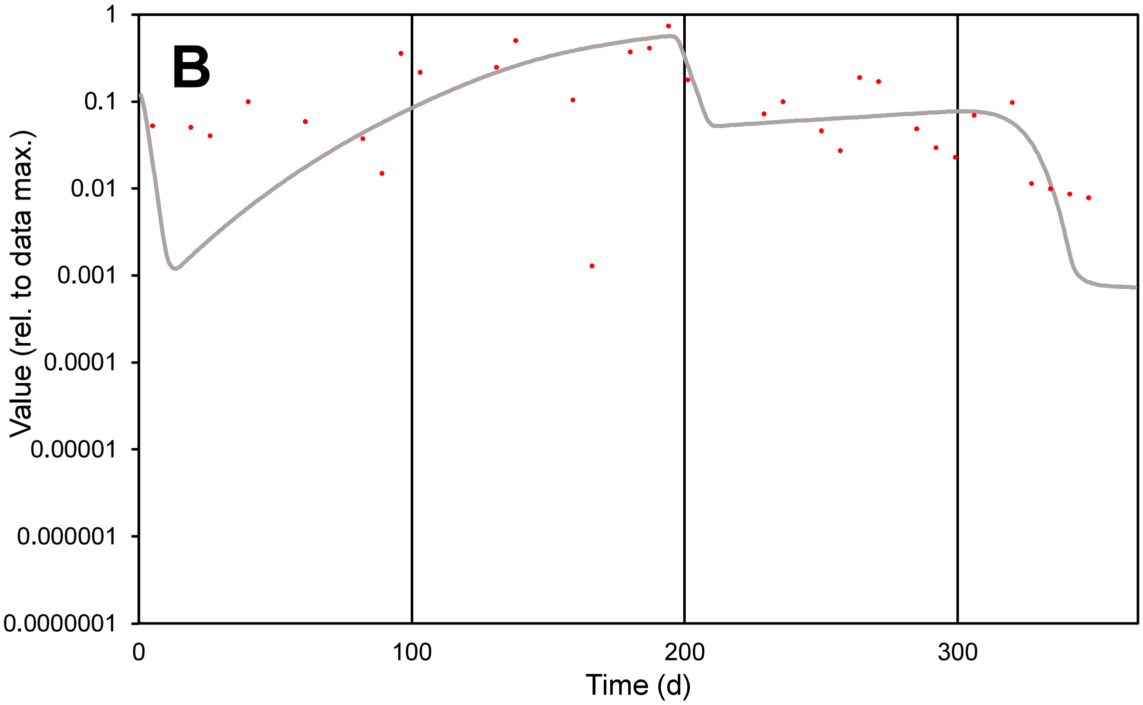

C1

Variables
CR1 Provide jtd or Dst
CalibOnly?
2 1 to reproduce of
oNormalize?
2 1=no, 2=yes

ofxn calc*
0.95651
ofxn mod
0.97538

sRun
RunA1
zout
1

Ave
Data
0.00824
Model
0.00719

data/vMax
0.05033

ofxng
263.459

Min d/m
1.57226

ave/min d
127.366
ave/min m
174.691

326_Calib_S_A

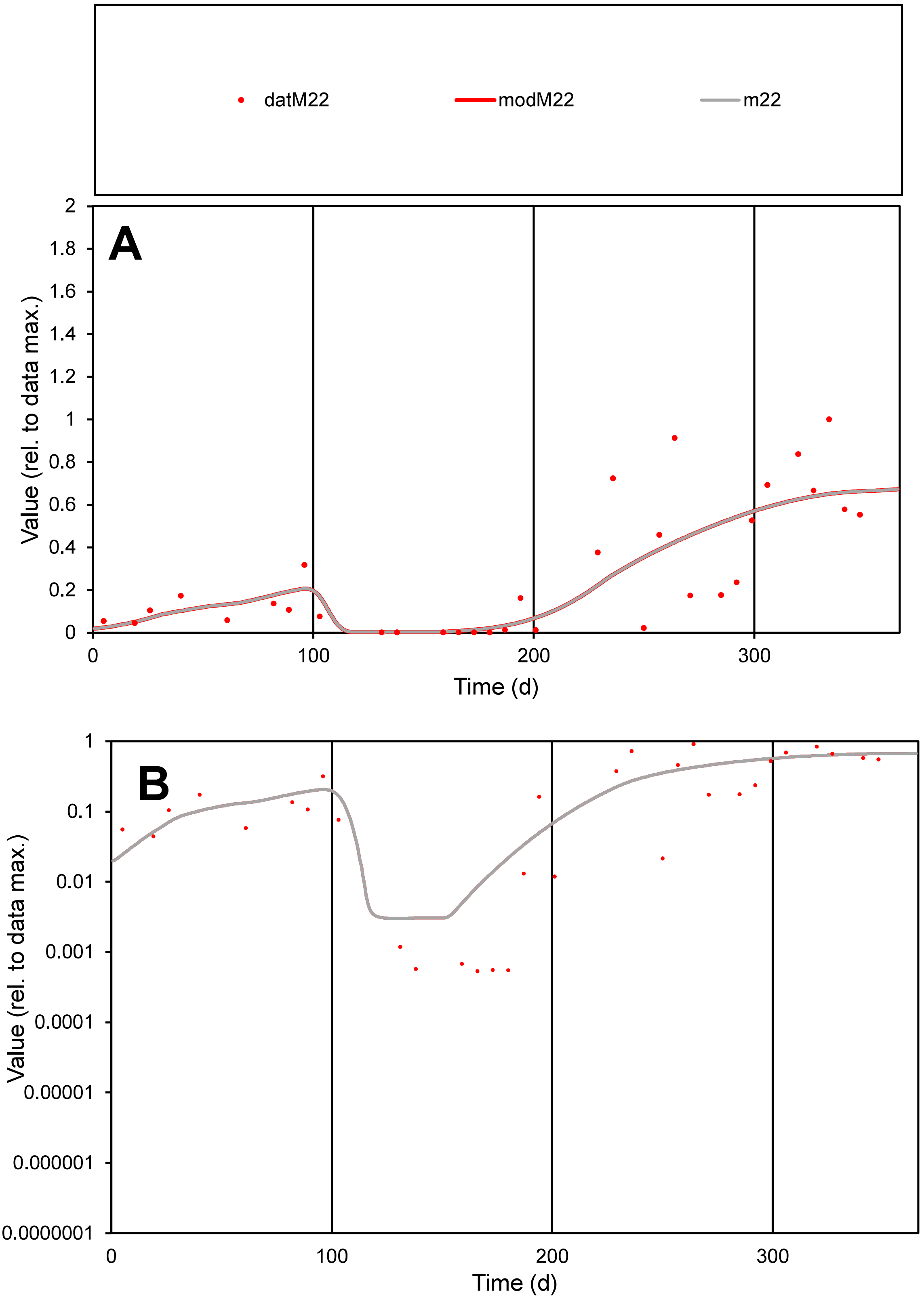

C1

Variables
M22 Provide jtd or Dst
CalibOnly?
2 1 to reproduce of
oNormalize?
2 1=no, 2=yes

ofxn calc*
0.65837
ofxn mod
0.67568

sRun
RunA1
zout
1

Ave
Data
0.01113
Model
0.01084

data/vMax
0.03993

ofxng
263.459

Min d/m
0.17745

ave/min d
521.699
ave/min m
90.1602

326_Calib_S_A

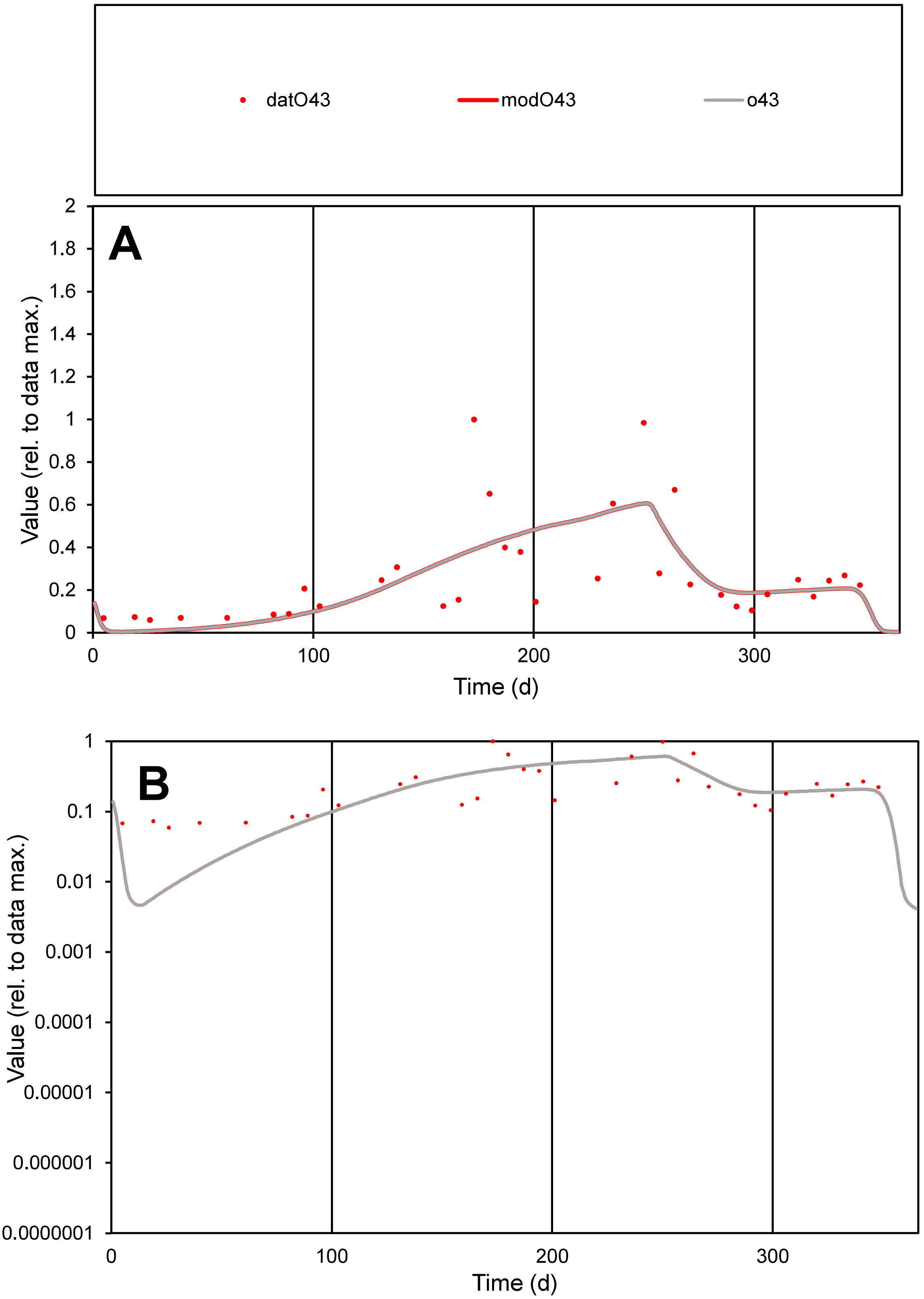

C1

Variables
O43 Provide jtd or Dst
CalibOnly?
2 1 to reproduce of
oNormalize?
2 1=no, 2=yes

ofxn calc*
0.65572
ofxn mod
0.67182

sRun
RunA1
zout
1

Ave
Data
0.01103
Model
0.01048

data/vMax
0.04049

ofxng
263.459

Min d/m
9.39442

ave/min d
4.61806
ave/min m
41.2057

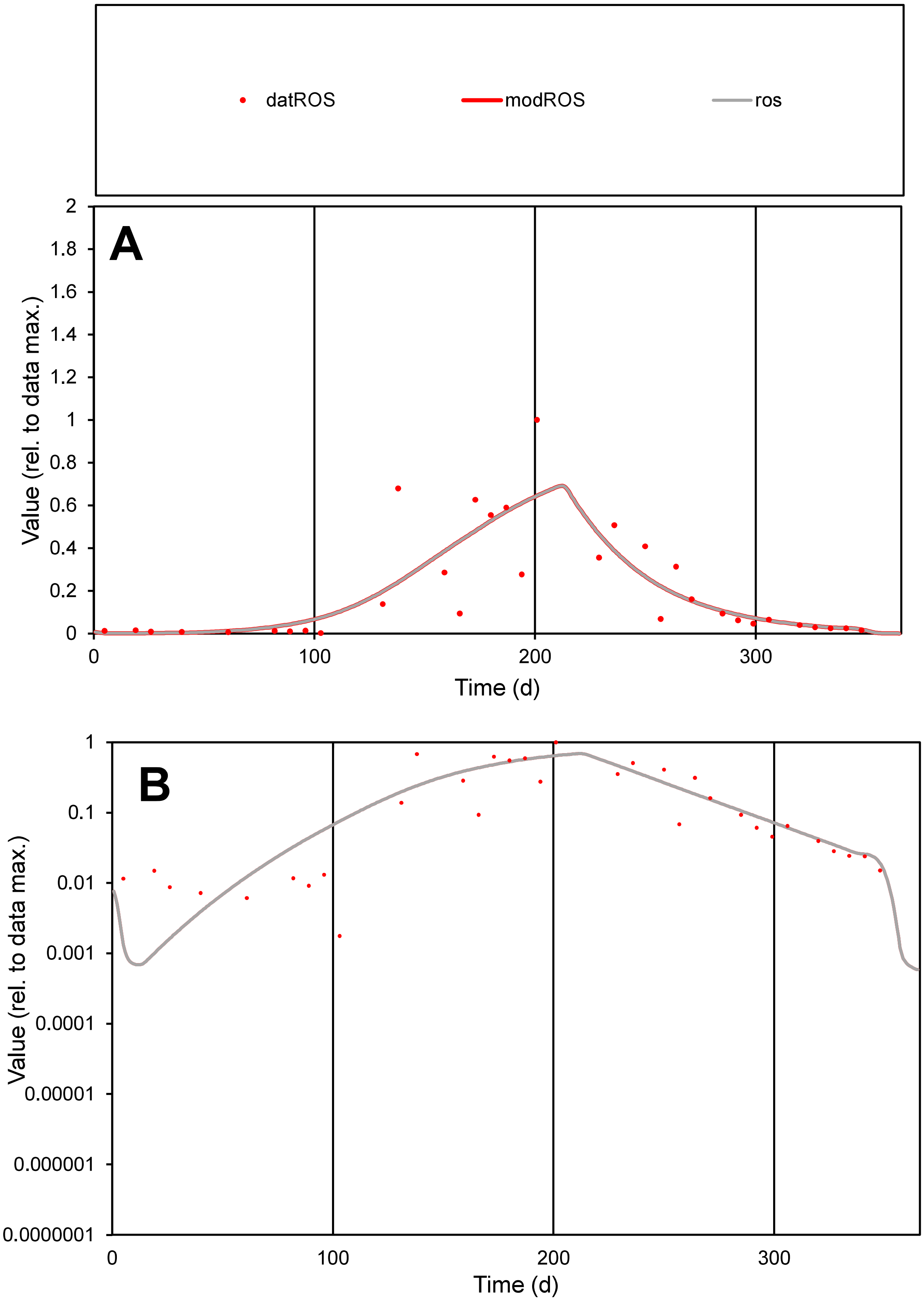

C1

Variables

ROS Provide jtd or Dst

CalibOnly?

2 1 to reproduce of

oNormalize?

2 1=no, 2=yes

ofxn calc*

0.71702

ofxn mod

0.74061

sRun

RunA1

zout

1

Ave

Data

0.0115

Model

0.0114

data/vMax

0.05804

ofxng

263.459

Min d/m

1.6015

ave/min d

112.28

ave/min m

178.334

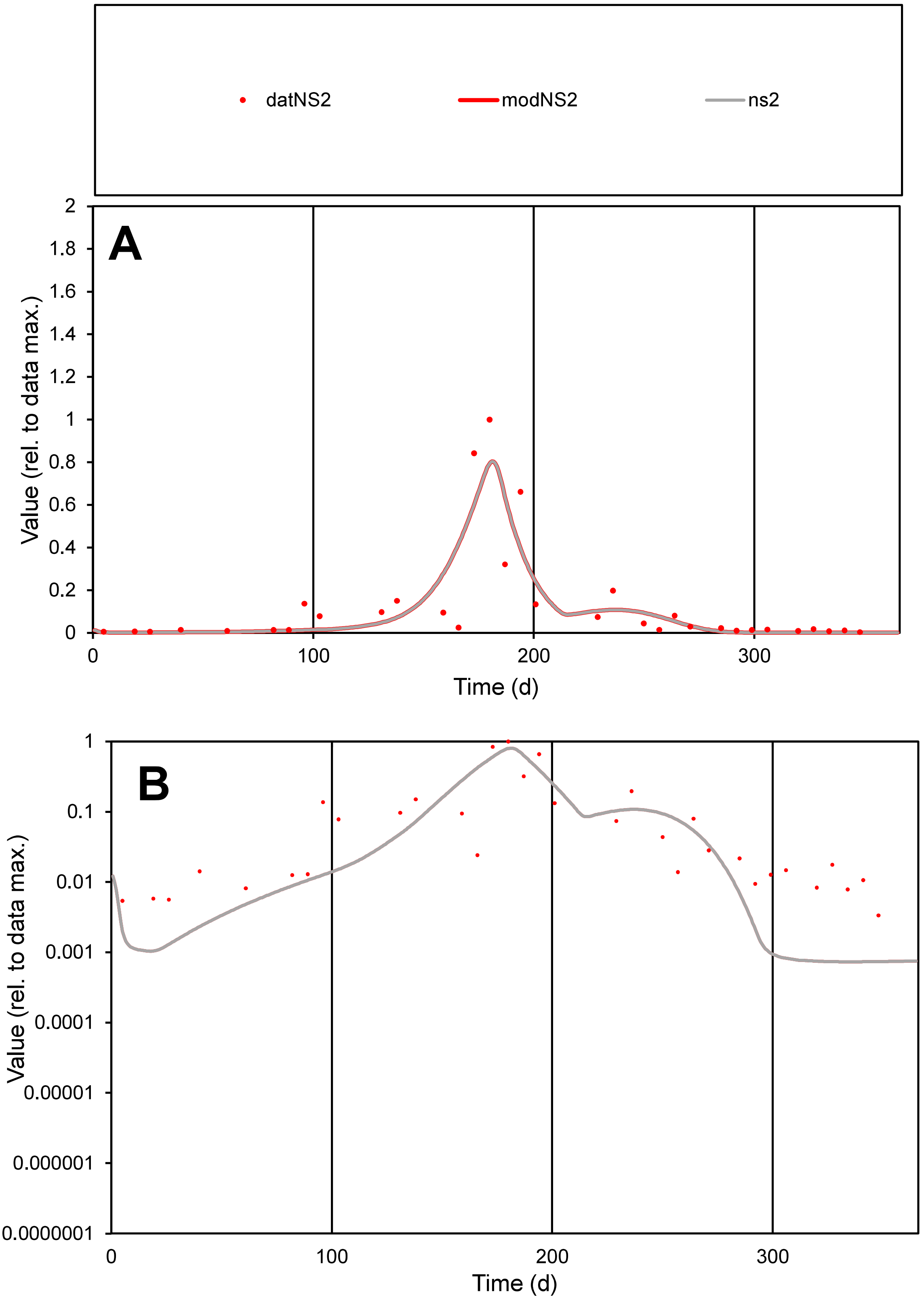

C1

Variables
NS2 Provide jtd or Dst
CalibOnly?
2 1 to reproduce of
oNormalize?
2 1=no, 2=yes

ofxn calc*
0.92146
ofxn mod
0.96594

sRun
RunA1
zout
1

Ave
Data
0.00666
Model
0.0064

data/vMax
0.05303

ofxng
263.459

Min d/m
4.57466

ave/min d
37.3924
ave/min m
164.551

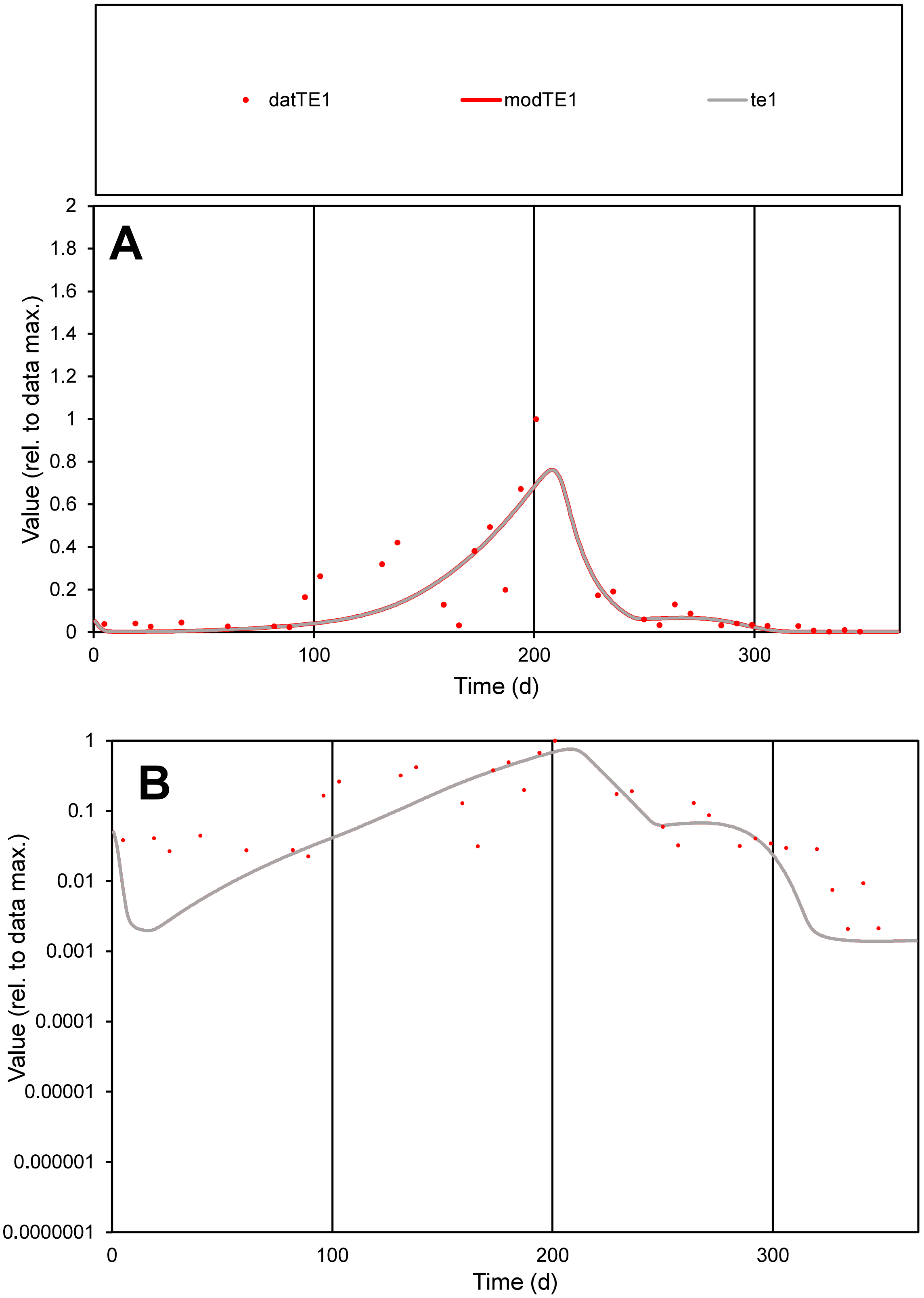

C1

Variables
TE1 Provide jtd or Dst
CalibOnly?
2 1 to reproduce of
oNormalize?
2 1=no, 2=yes

ofxn calc*
0.78204
ofxn mod
0.80528

sRun
RunA1
zout
1

Ave
Data
0.00411
Model
0.00355

data/vMax
0.0263

ofxng
263.459

Min d/m
1.48585

ave/min d
75.2468
ave/min m
96.6644

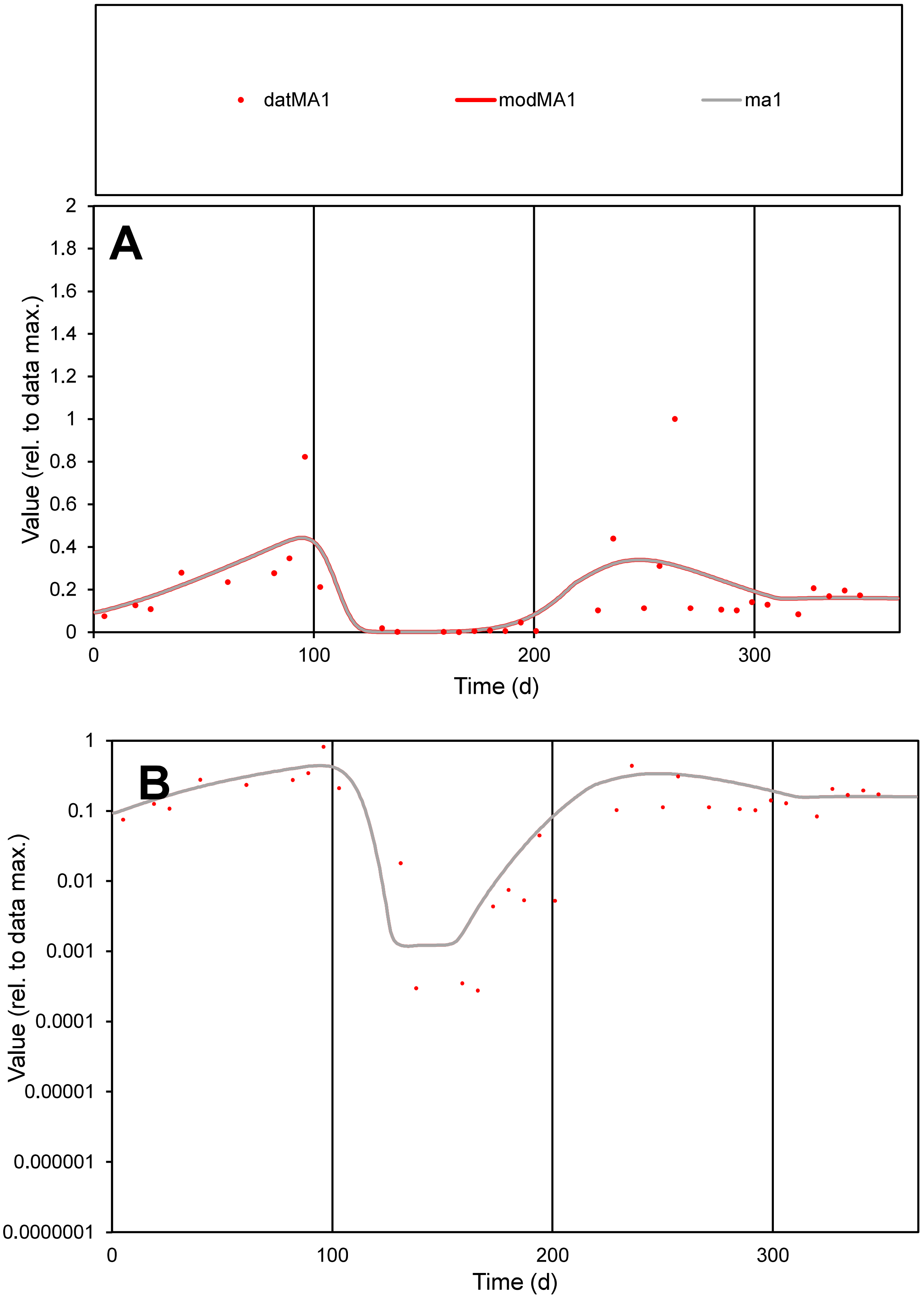

C1

Variables
MA1 Provide jtd or Dst
CalibOnly?
2 1 to reproduce of
oNormalize?
2 1=no, 2=yes

ofxn calc*
0.89915
ofxn mod
0.91395

sRun
RunA1
zout
1

Ave
Data
0.00931
Model
0.00987

data/vMax
0.05166

ofxng
263.459

Min d/m
0.23093

ave/min d
651.461
ave/min m
159.538

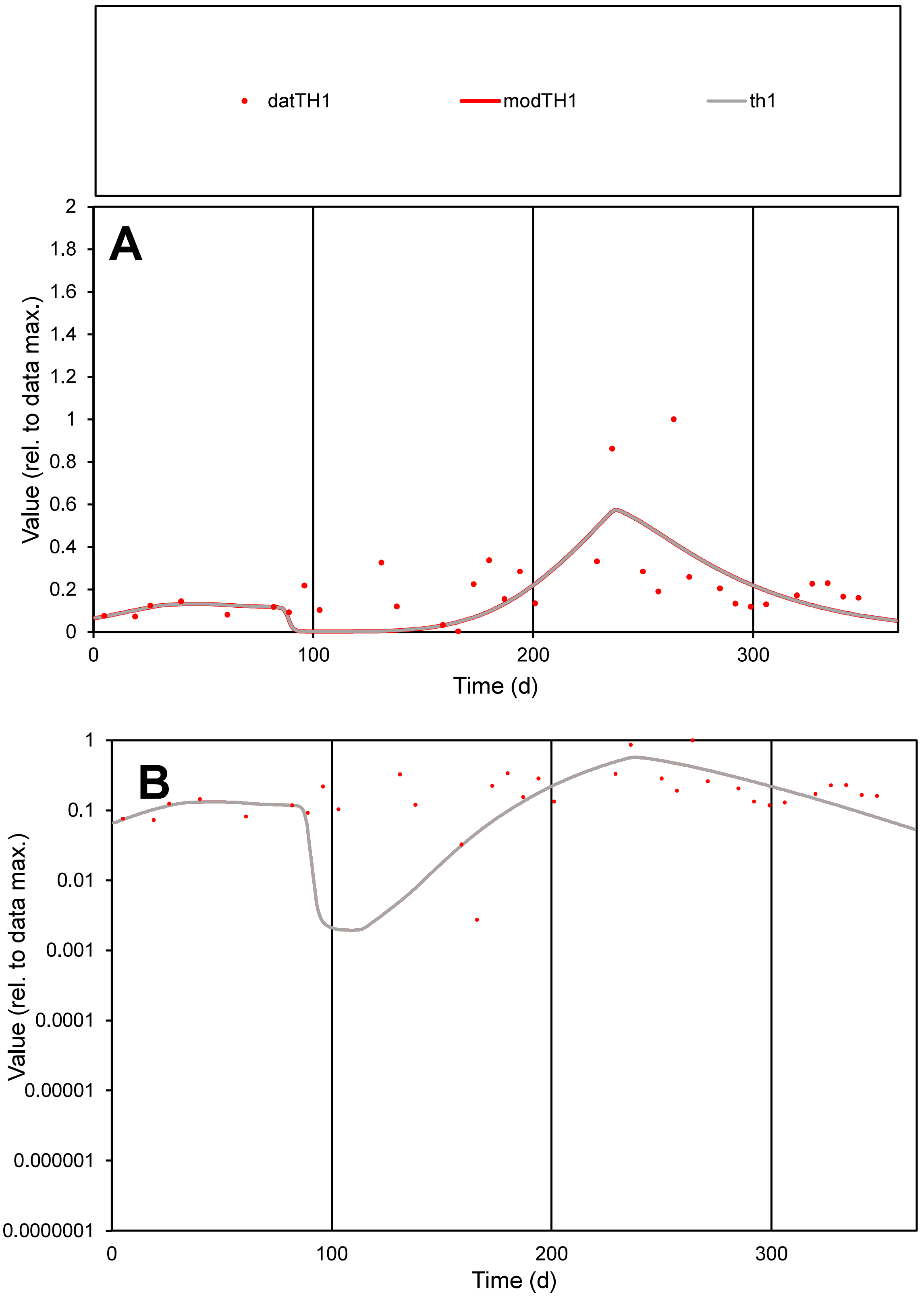

C1

Variables
TH1 Provide jtd or Dst
CalibOnly?
2 1 to reproduce of
oNormalize?
2 1=no, 2=yes

ofxn calc*
0.78033
ofxn mod
0.79343

sRun
RunA1
zout
1

Ave
Data
0.00723
Model
0.00598

data/vMax
0.03352

ofxng
263.459

Min d/m
1.38911

ave/min d
78.5384
ave/min m
90.2403

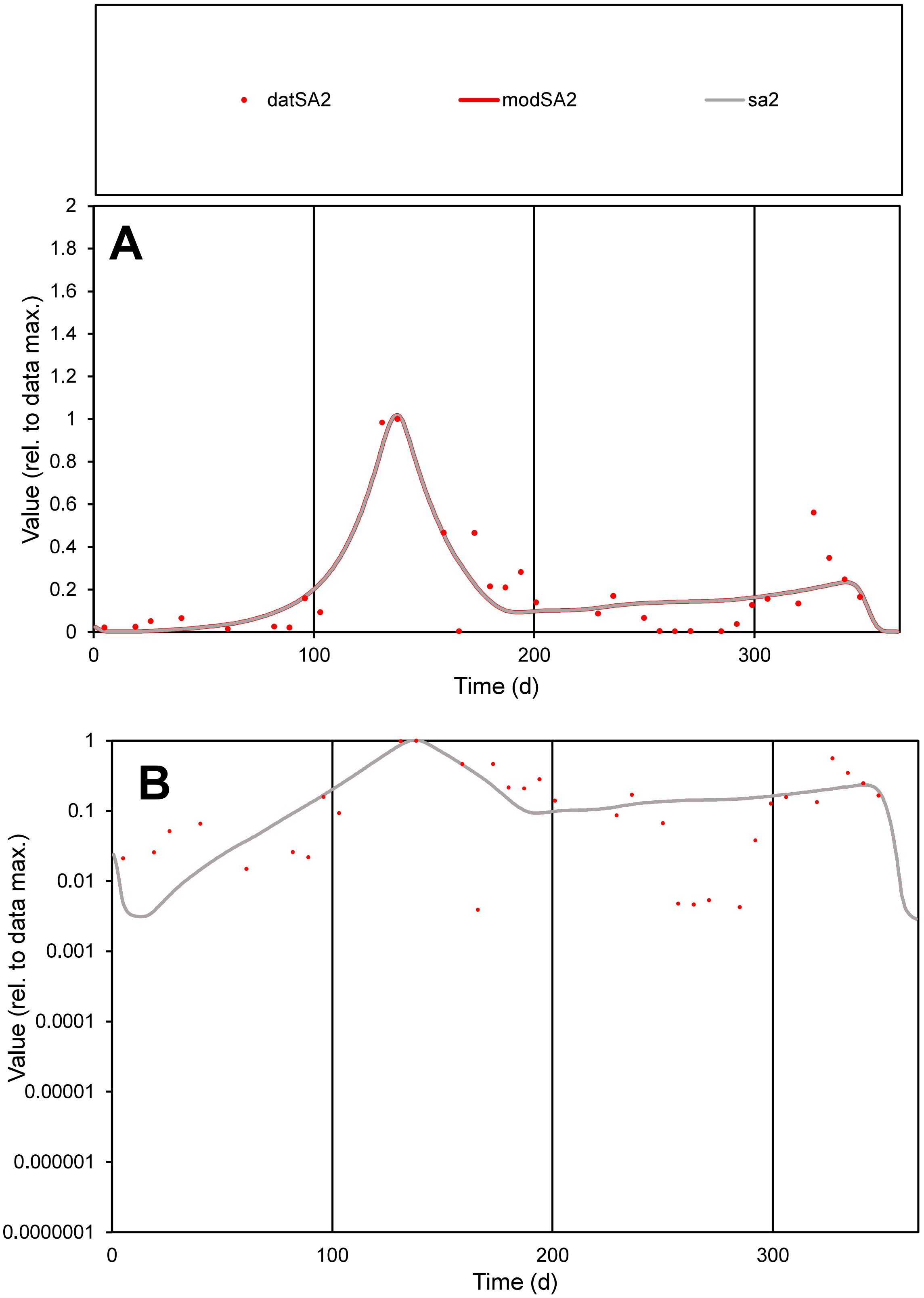

C1

Variables
SA2 Provide jtd or Dst
CalibOnly?
2 1 to reproduce of
oNormalize?
2 1=no, 2=yes

ofxn calc*
0.62197
ofxn mod
0.66026

sRun
RunA1
zout
1

Ave
Data
0.00496
Model
0.00505

data/vMax
0.0257

ofxng
263.459

Min d/m
0.94638

ave/min d
49.2954
ave/min m
47.4823

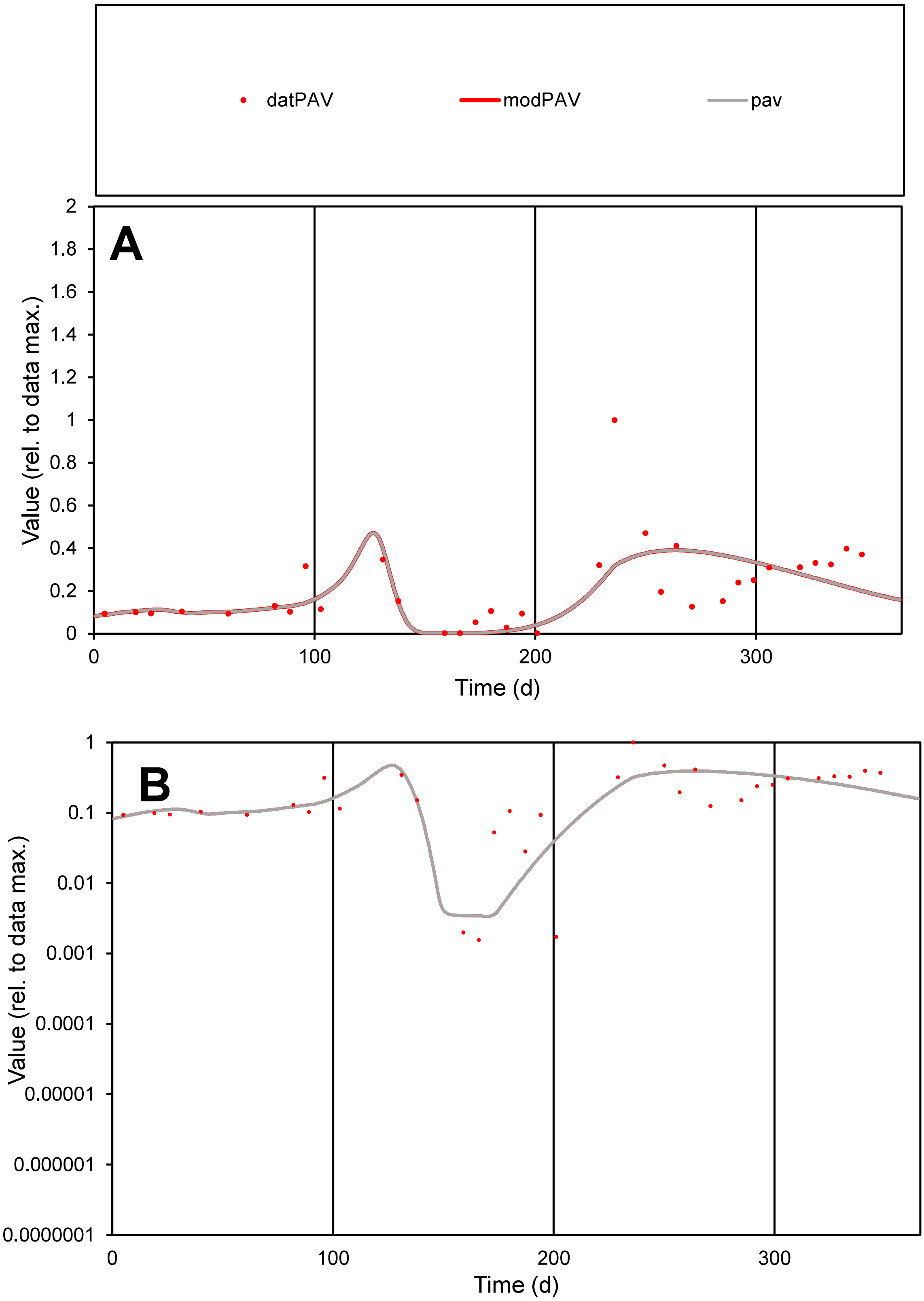

C1

Variables
PAV Provide jtd or Dst
CalibOnly?
2 1 to reproduce of
oNormalize?
2 1=no, 2=yes

ofxn calc*
0.70483
ofxn mod
0.71682

sRun
RunA1
zout
1

Ave
Data
0.00508
Model
0.00452

data/vMax
0.02349

ofxng
263.459

Min d/m
0.4598

ave/min d
137.901
ave/min m
56.4434

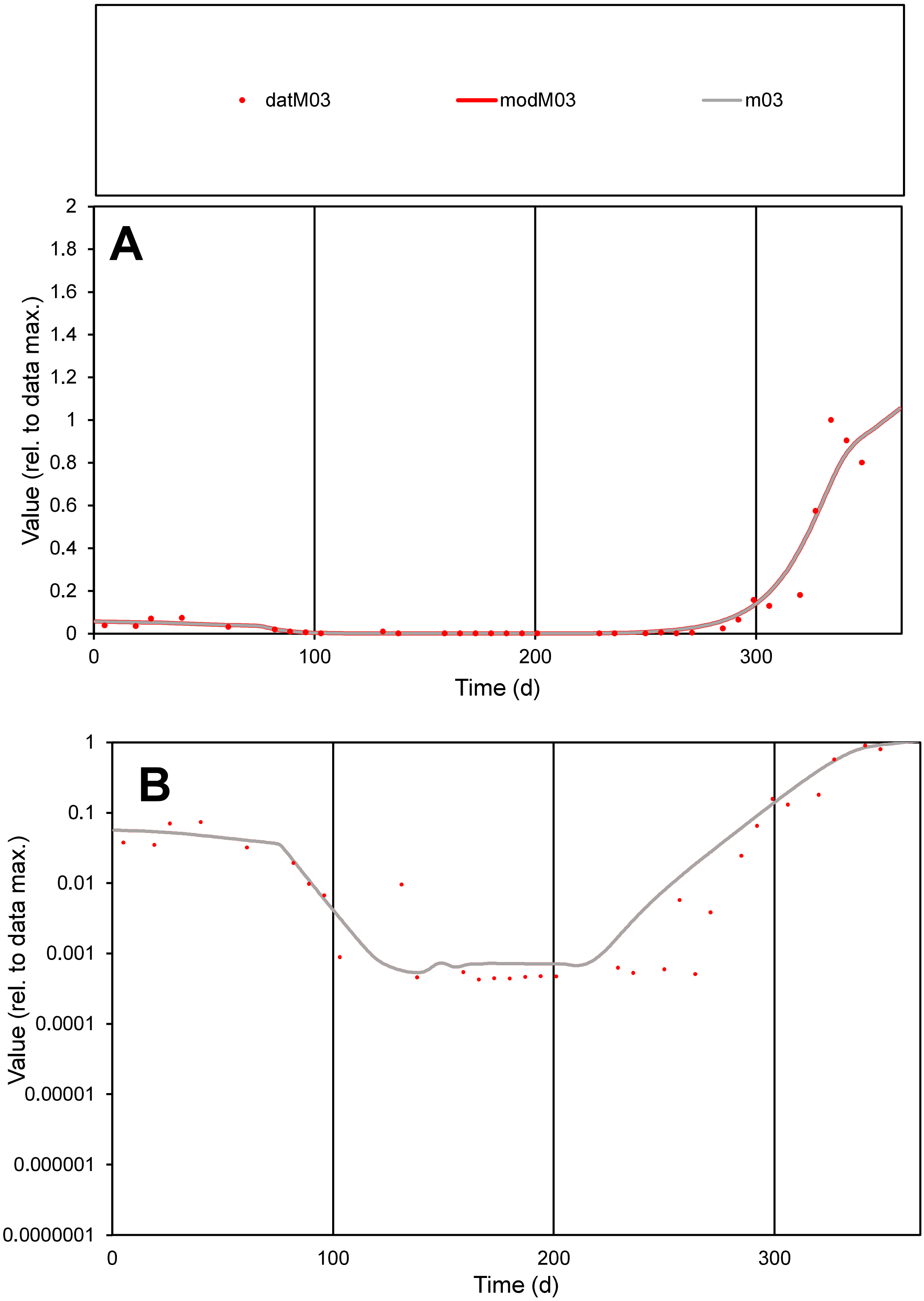

C1

Variables
M03 Provide jtd or Dst
CalibOnly?
2 1 to reproduce of
oNormalize?
2 1=no, 2=yes

ofxn calc*
0.52508
ofxn mod
0.57843

sRun
RunA1
zout
1

Ave
Data
0.0051
Model
0.00535

data/vMax
0.04056

ofxng
263.459

Min d/m
0.79652

ave/min d
293.461
ave/min m
245.007

326_Calib_S_A

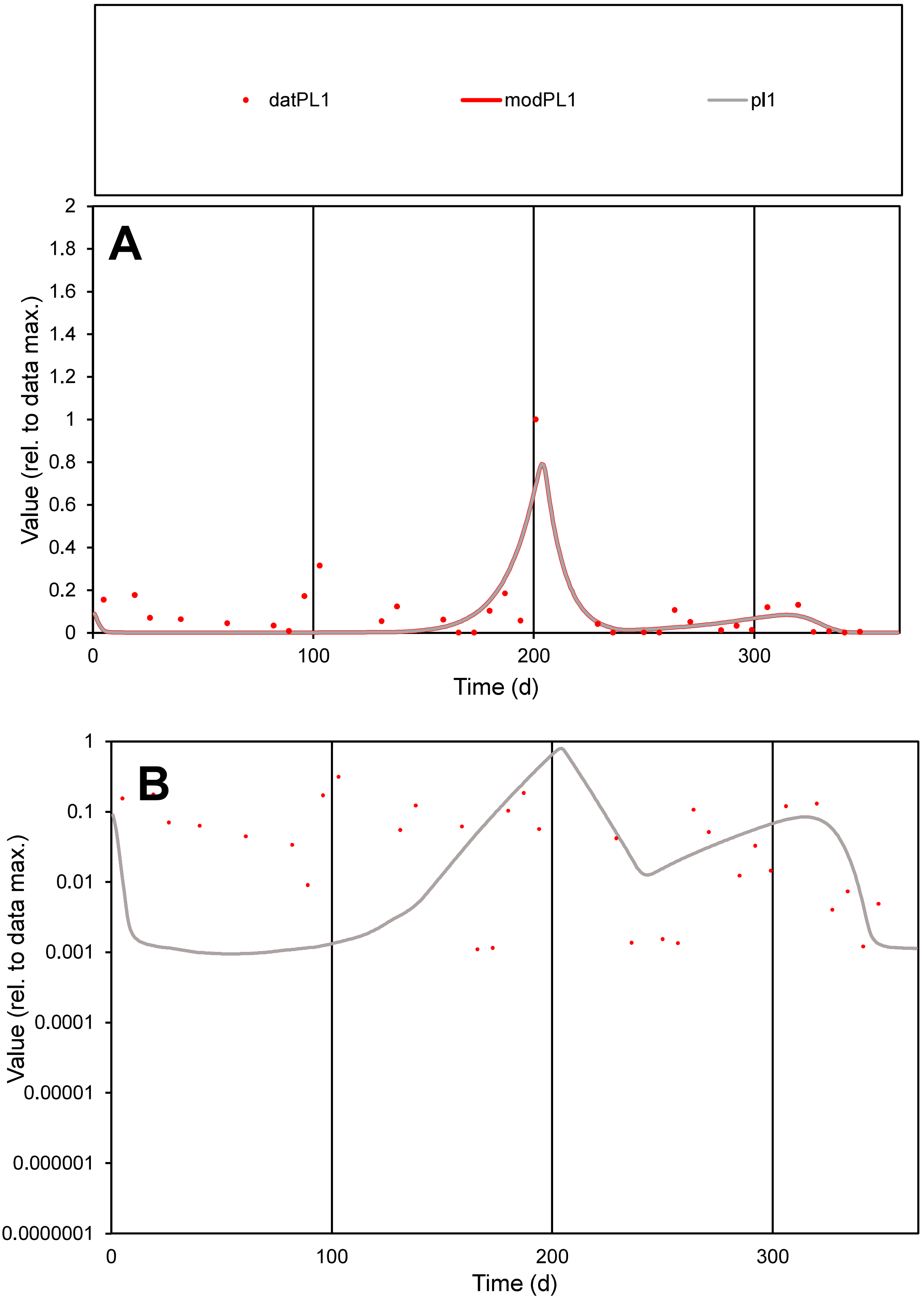

C1

Variables
PL1 Provide jtd or Dst
CalibOnly?
2 1 to reproduce of
oNormalize?
2 1=no, 2=yes

ofxn calc*
1.18376
ofxn mod
1.22826

sRun
RunA1
zout
1

Ave
Data
0.00297
Model
0.00222

data/vMax
0.03106

ofxng
263.459

Min d/m
1.15127

ave/min d
86.4604
ave/min m
74.2832

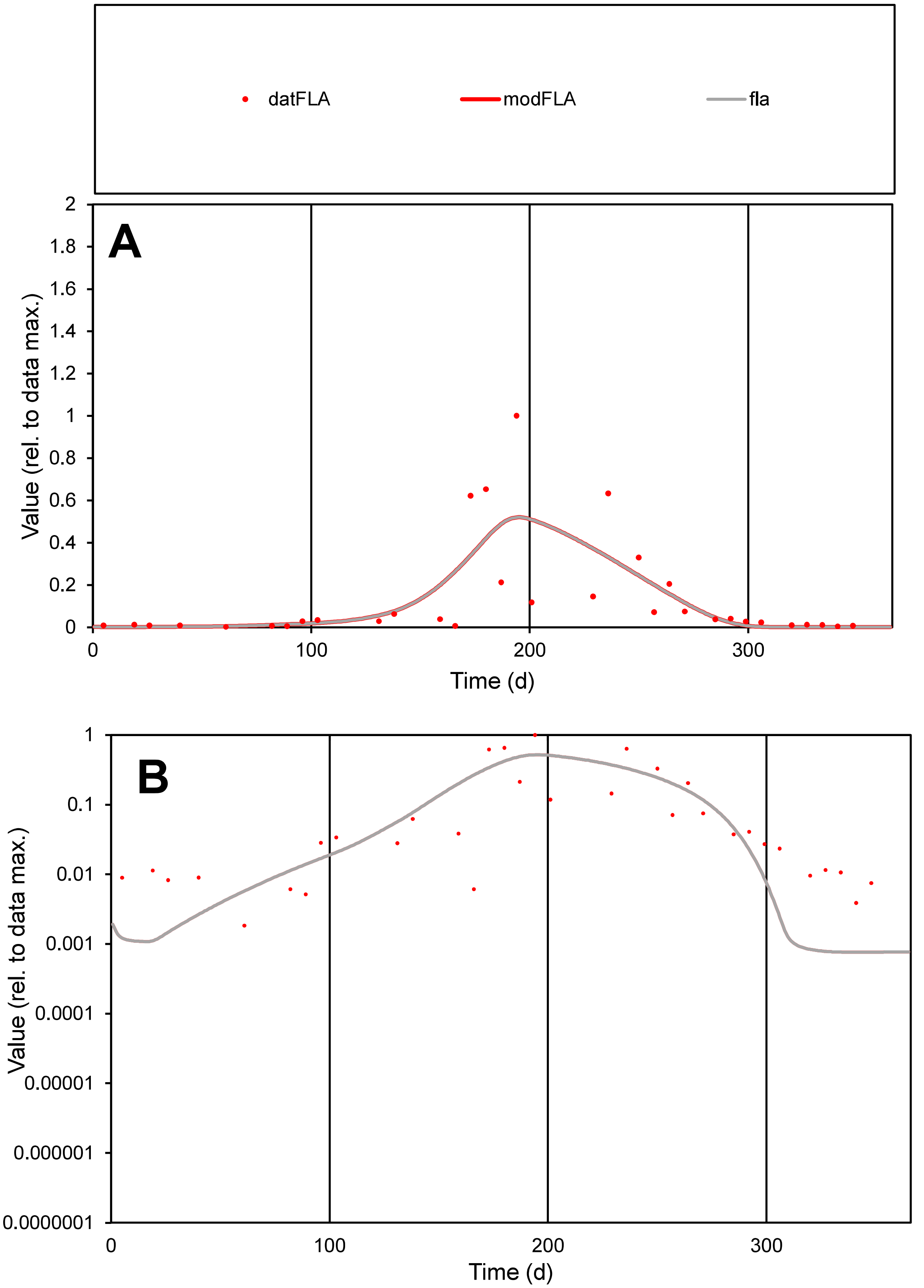

C1

Variables
FLA Provide jtd or Dst
CalibOnly?
2 1 to reproduce of
oNormalize?
2 1=no, 2=yes

ofxn calc*
1.17281
ofxn mod
1.1965

sRun
RunA1
zout
1

Ave
Data
0.00509
Model
0.00505

data/vMax
0.03746

ofxng
263.459

Min d/m
2.42254

ave/min d
73.9972
ave/min m
177.754

326_Calib_S_A

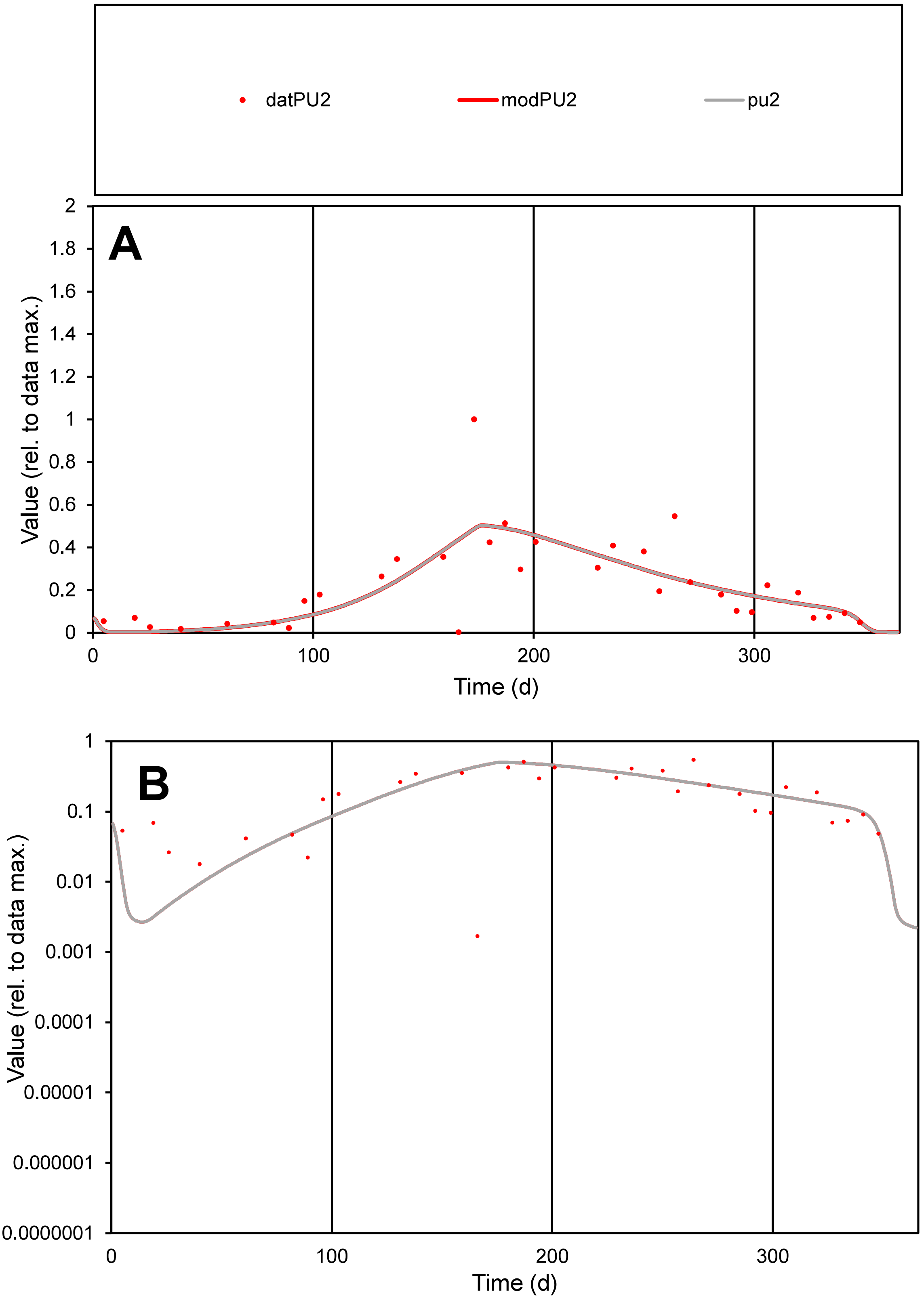

C1

Variables
PU2 Provide jtd or Dst
CalibOnly?
2 1 to reproduce of
oNormalize?
2 1=no, 2=yes

ofxn calc*
0.62689
ofxn mod
0.64417

sRun
RunA1
zout
1

Ave
Data
0.0072
Model
0.00689

data/vMax
0.03225

ofxng
263.459

Min d/m
0.49517

ave/min d
132.571
ave/min m
62.7721

326_Calib_S_A

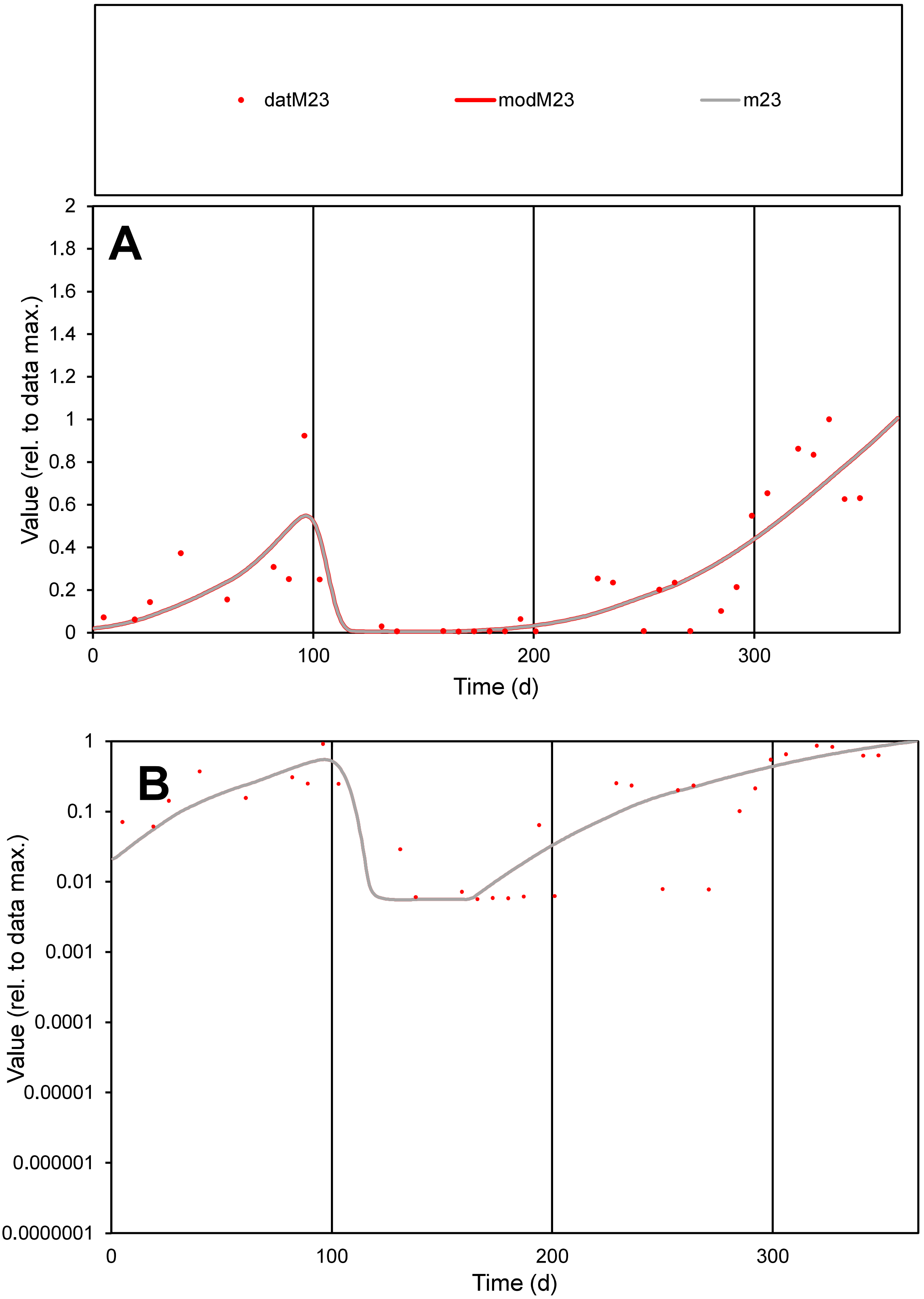

C1

Variables
M23 Provide jtd or Dst
CalibOnly?
2 1 to reproduce of
oNormalize?
2 1=no, 2=yes

ofxn calc*
0.56714
ofxn mod
0.58458

sRun
RunA1
zout
1

Ave
Data
0.00516
Model
0.0051

data/vMax
0.01876

ofxng
263.459

Min d/m
1.02111

ave/min d
48.5055
ave/min m
48.9056

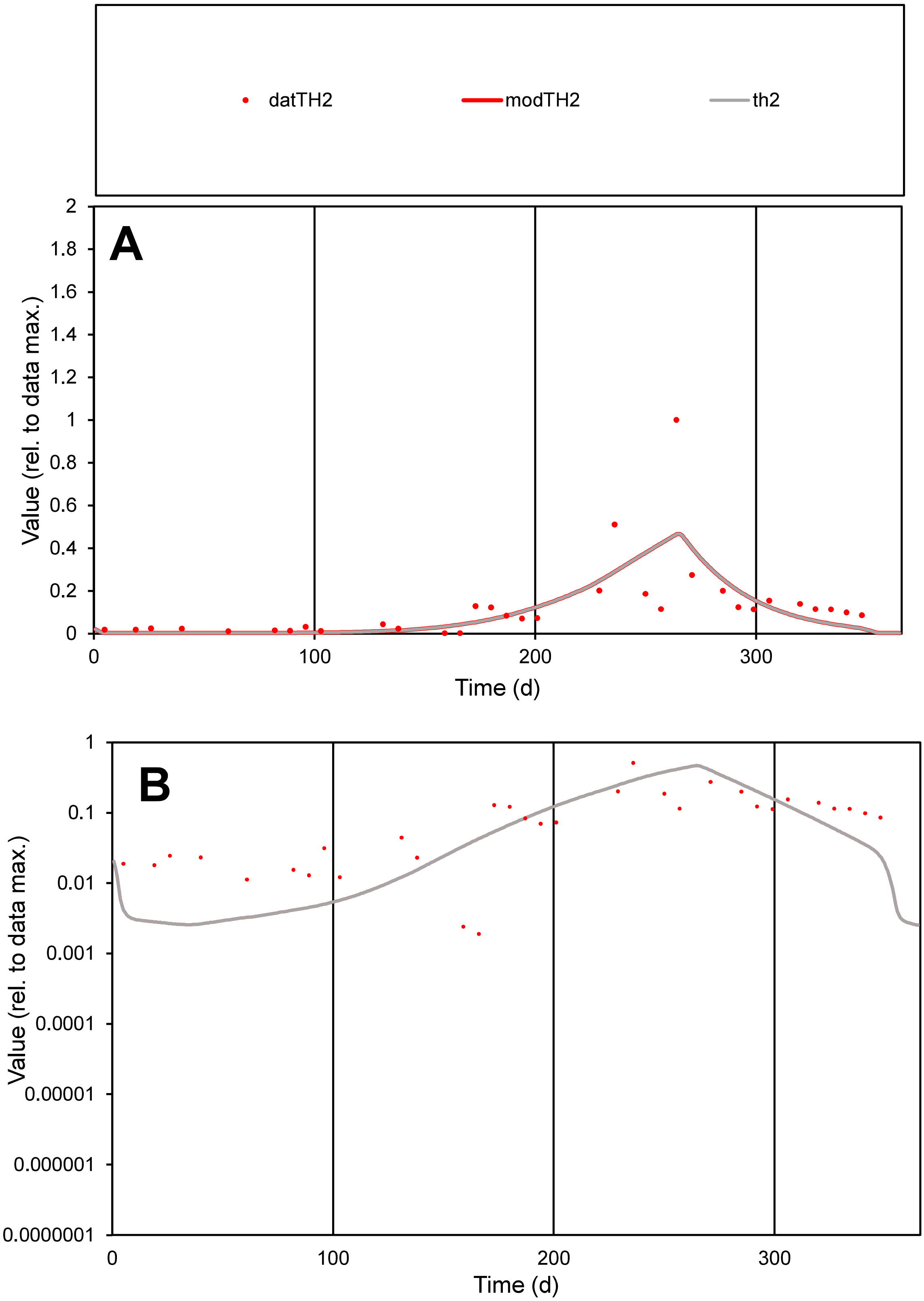

C1

Variables	
TH2	Provide jtd or Dst
CalibOnly?	
2	1 to reproduce of
oNormalize?	
2	1=no, 2=yes
ofxn calc*	
1.00409	
ofxn mod	
1.02619	
sRun	
RunA1	
zout	
1	
Ave	
Data	
0.00695	
Model	
0.00627	
data/vMax	
0.0553	
ofxng	
263.459	
Min d/m	
0.71732	
ave/min d	
66.3343	
ave/min m	
42.8885	

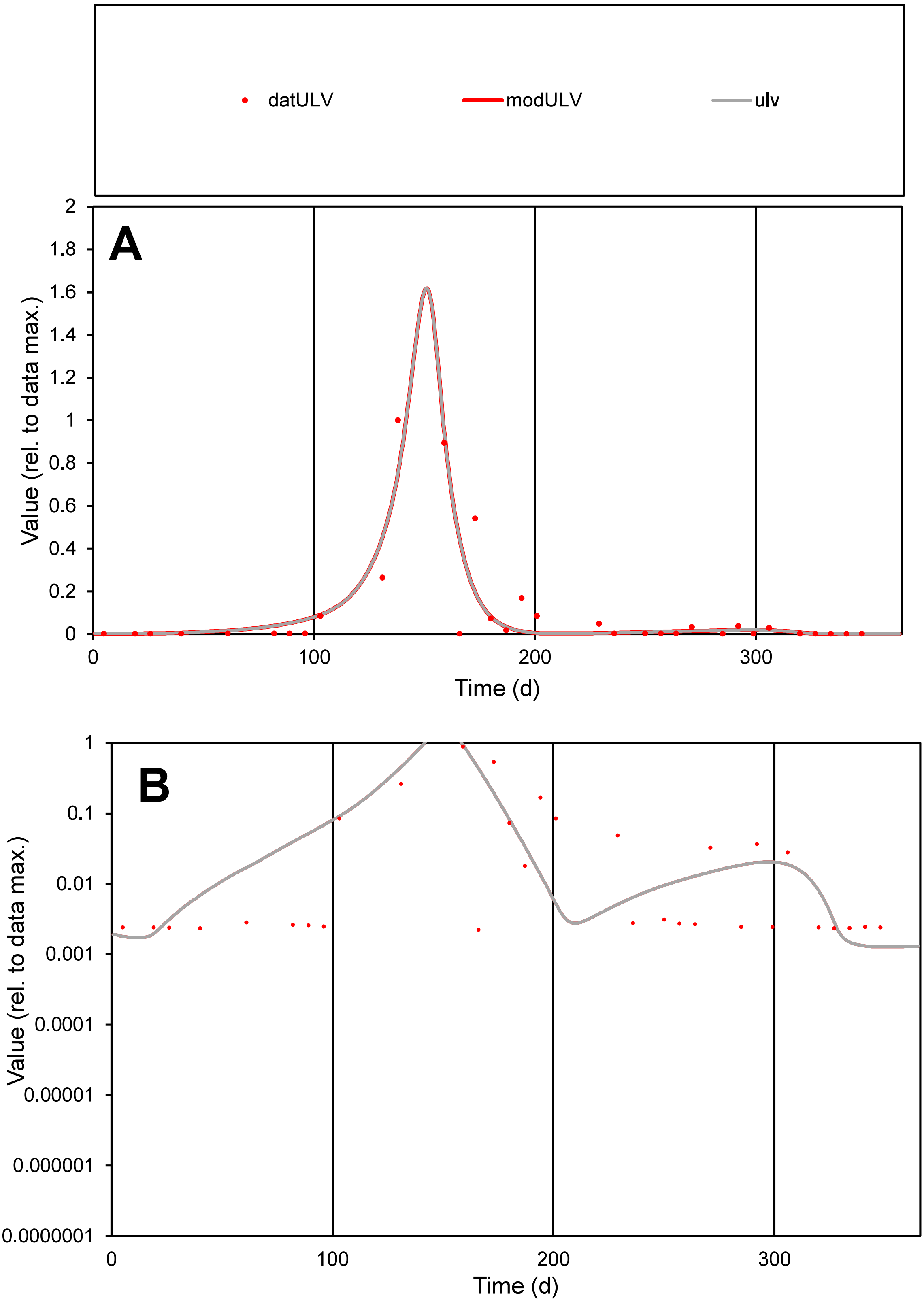

C1

Variables

ULV Provide jtd or Dst

CalibOnly?

2 1 to reproduce of

oNormalize?

2 1=no, 2=yes

ofxn calc*

0.9729

ofxn mod

1.04429

sRun

RunA1

zout

1

Ave

Data

0.00077

Model

0.00074

data/vMax

0.00763

ofxng

263.459

Min d/m

1.75128

ave/min d

45.1502

ave/min m

76.4763

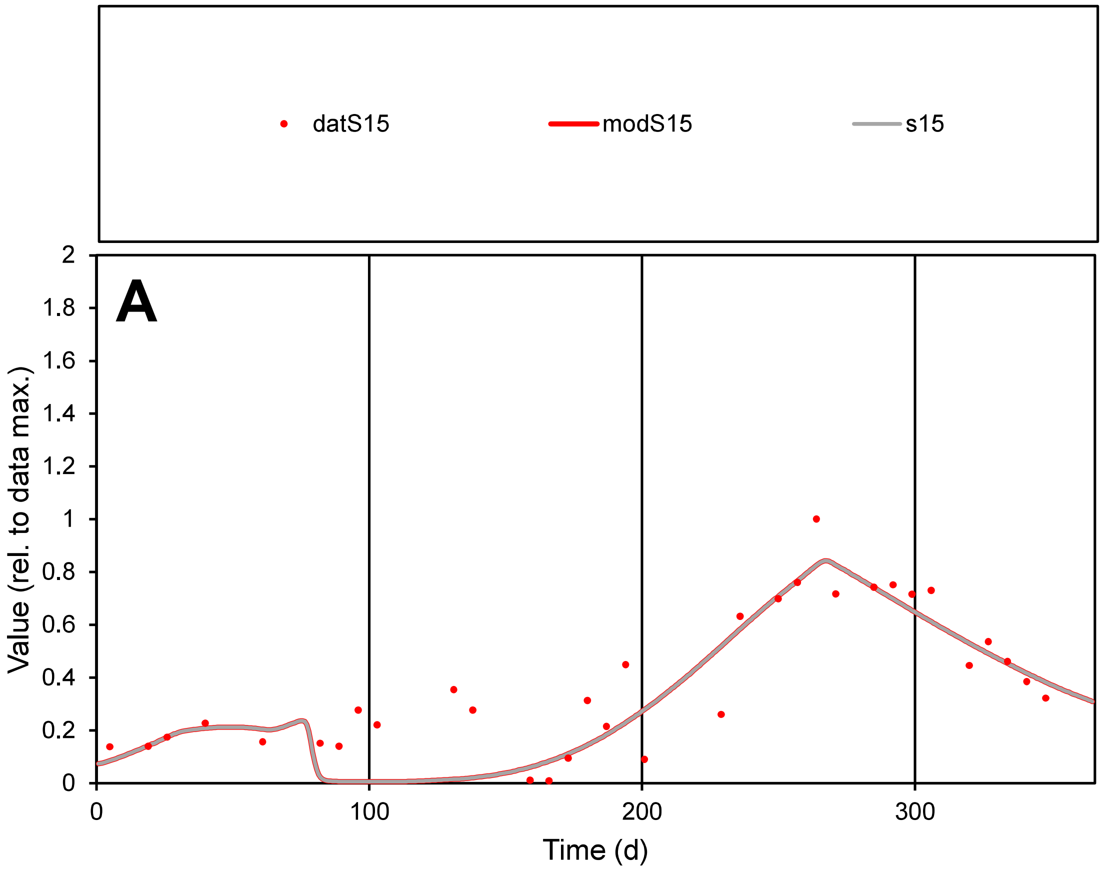

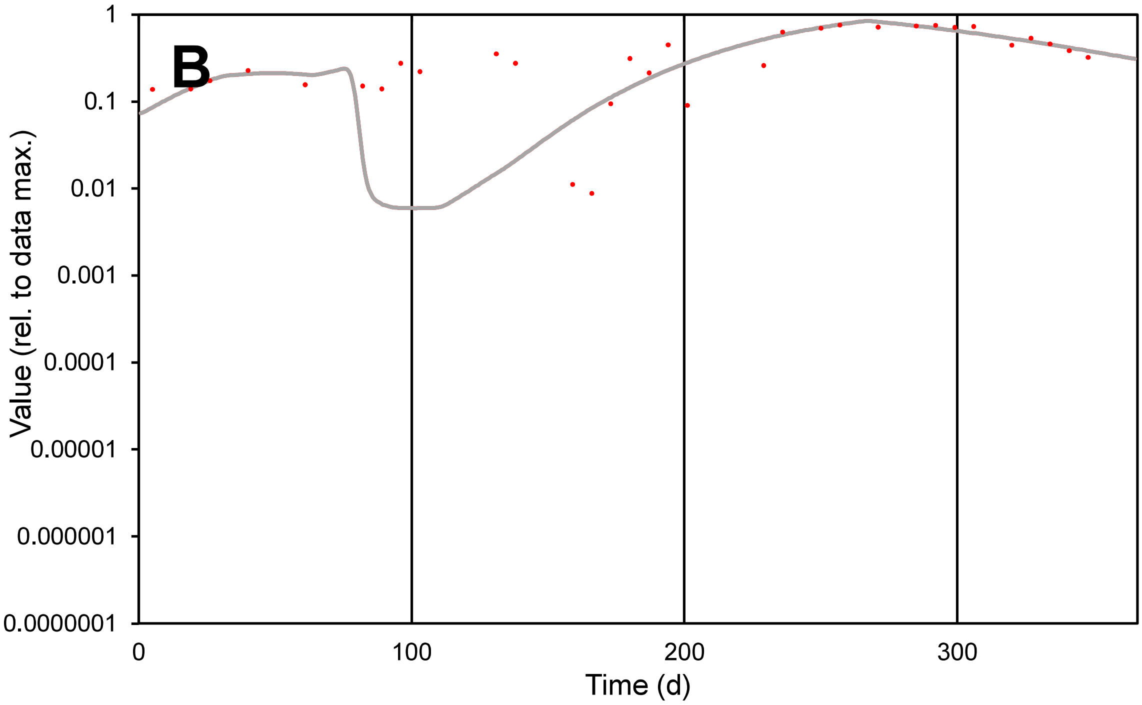

C1

Variables

S15	Provide jtd or Dst
CalibOnly?	
2	1 to reproduce of
oNormalize?	
2	1=no, 2=yes

ofxn calc*
0.34826
ofxn mod
0.37952

sRun
RunA1
zout
1

Ave
Data
0.00533
Model
0.00479

data/vMax
0.01396

ofxng
263.459

Min d/m
1.48211

ave/min d
43.3045
ave/min m
57.672

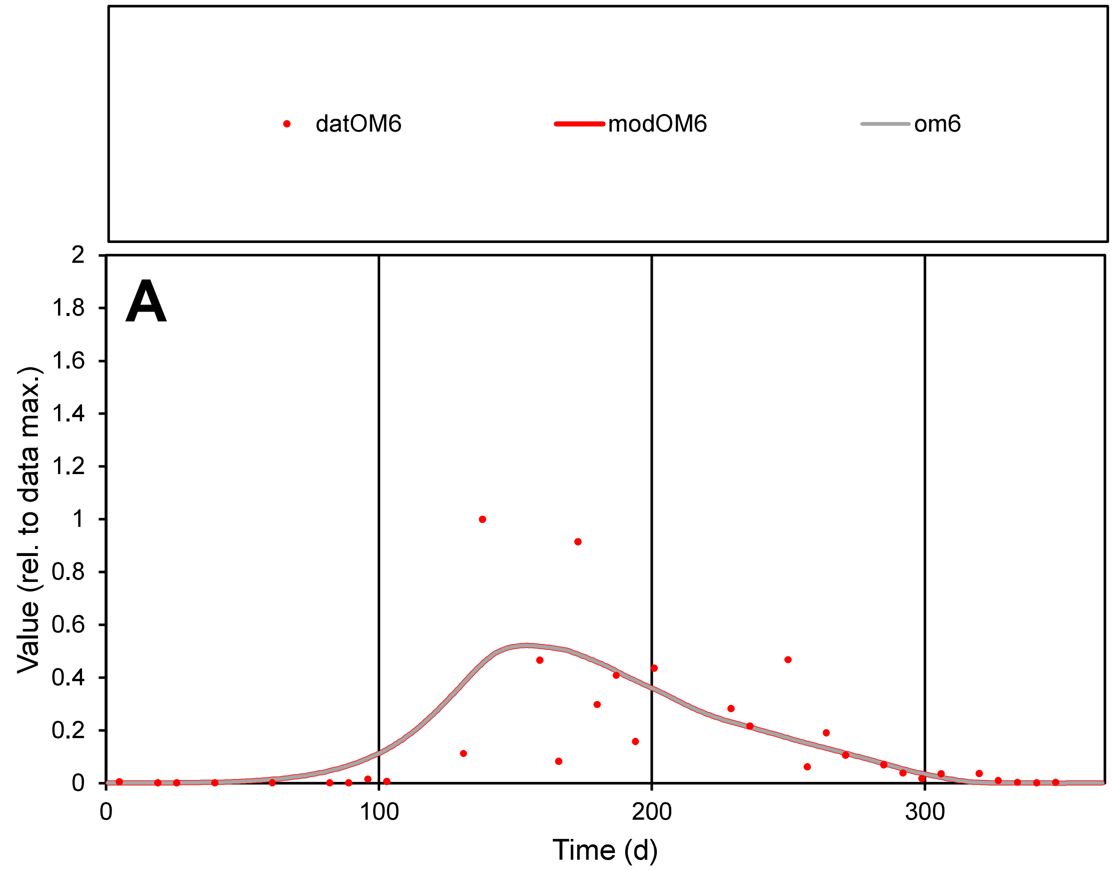

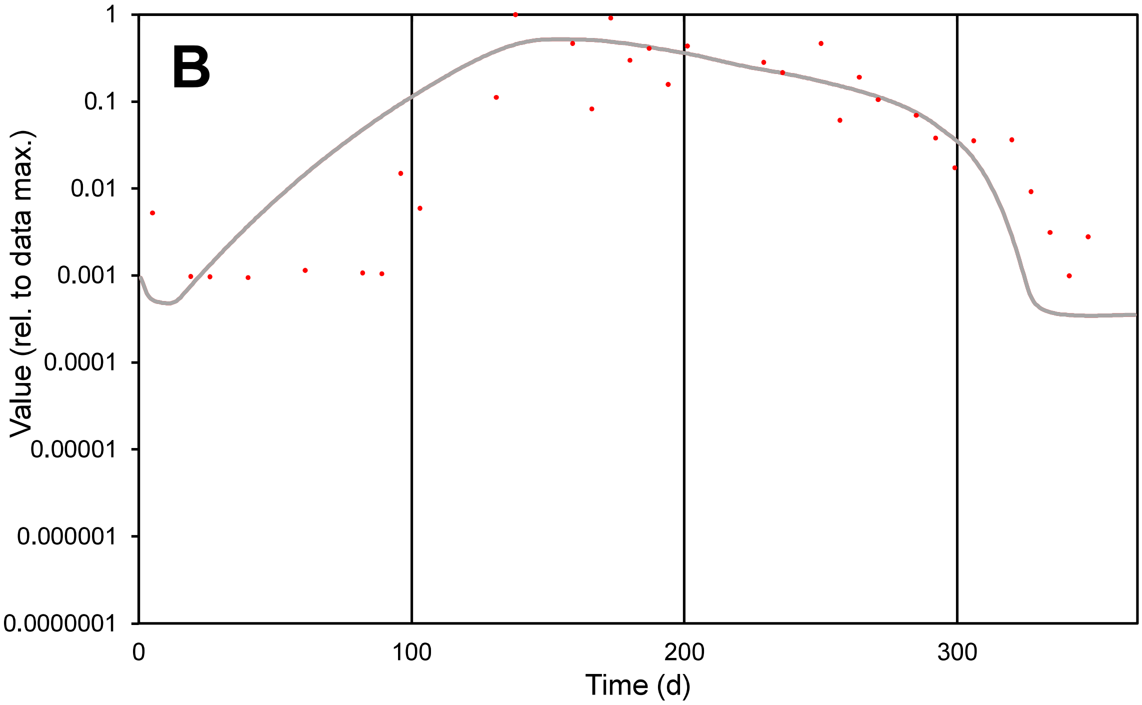

C1

Variables
OM6 Provide jtd or Dst
CalibOnly?
2 1 to reproduce of
oNormalize?
2 1=no, 2=yes

ofxn calc*
1.02356
ofxn mod
1.04235

sRun
RunA1
zout
1

Ave
Data
0.00451
Model
0.00455

data/vMax
0.02733

ofxng
263.459

Min d/m
2.75005

ave/min d
173.987
ave/min m
482.952

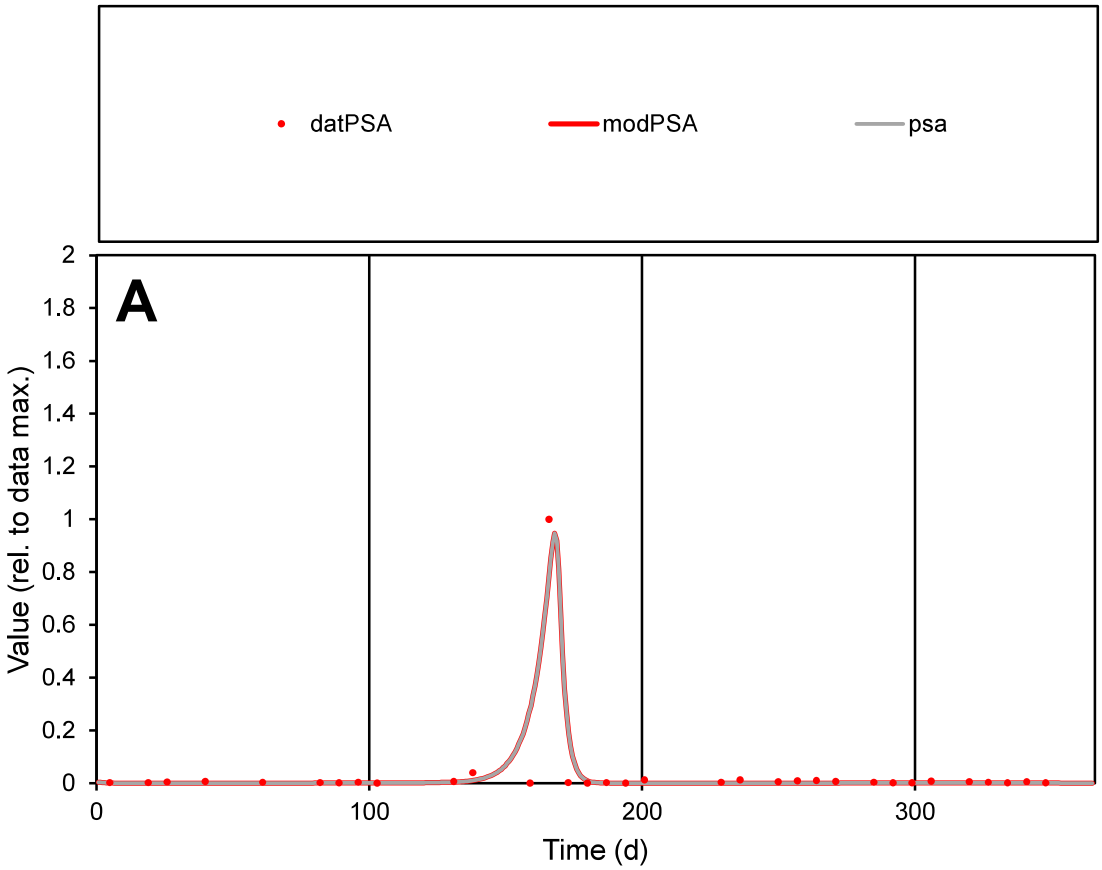

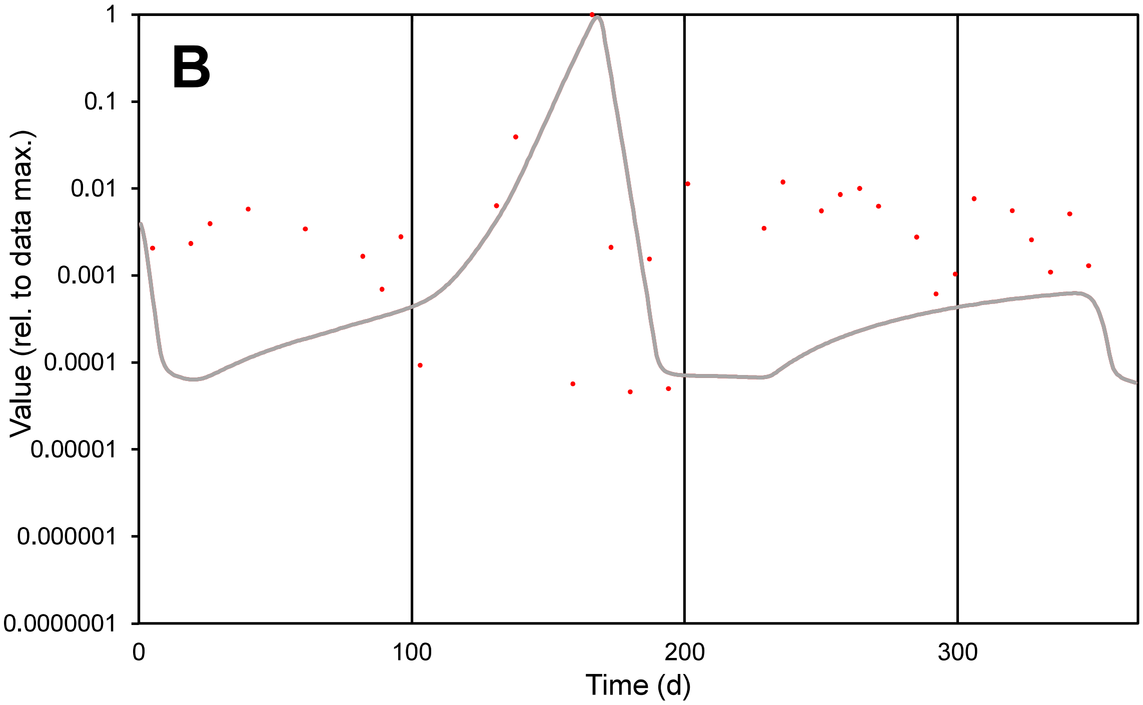

C1

Variables
PSA Provide jtd or Dst
CalibOnly?
2 1 to reproduce of
oNormalize?
2 1=no, 2=yes

ofxn calc*
1.16952
ofxn mod
1.42907

sRun
RunA1
zout
1

Ave
Data
0.00684
Model
0.00716

data/vMax
0.1951

ofxng
263.459

Min d/m
0.72623

ave/min d
757.784
ave/min m
575.811

326_Calib_S_A

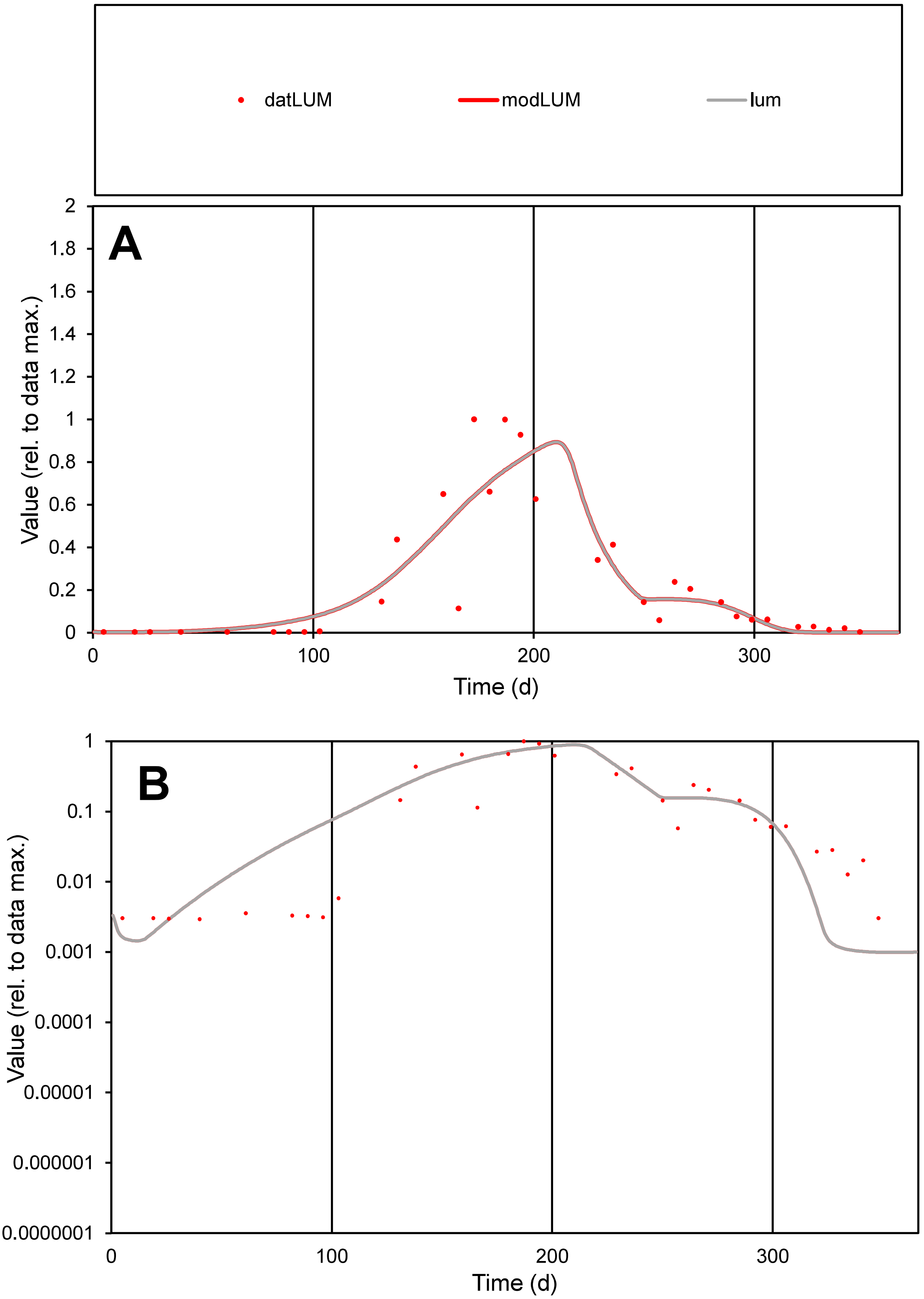

C1

Variables

LUM Provide jtd or Dst

CalibOnly?

2 1 to reproduce of

oNormalize?

2 1=no, 2=yes

ofxn calc*

0.57785

ofxn mod

0.60856

sRun

RunA1

zout

1

Ave

Data

0.00354

Model

0.00353

data/vMax

0.01575

ofxng

263.459

Min d/m

2.95156

ave/min d

76.4858

ave/min m

224.816

326_Calib_S_A

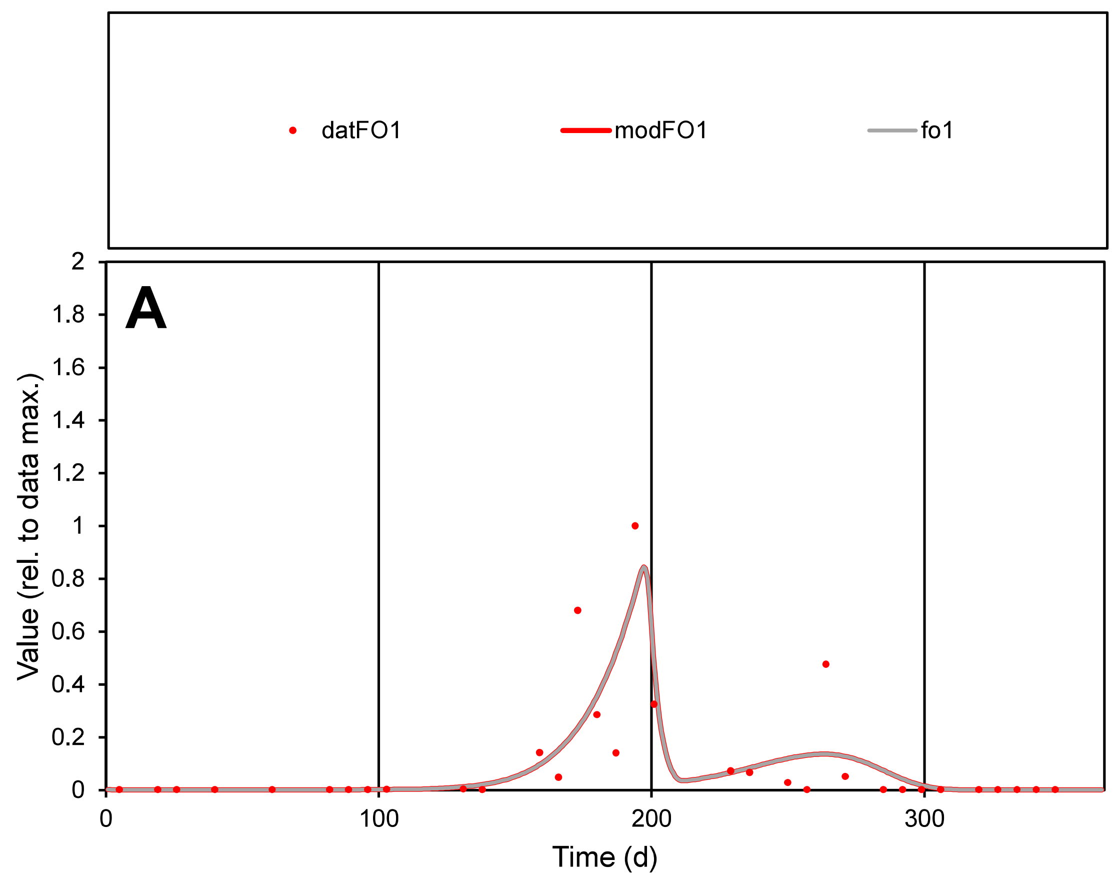

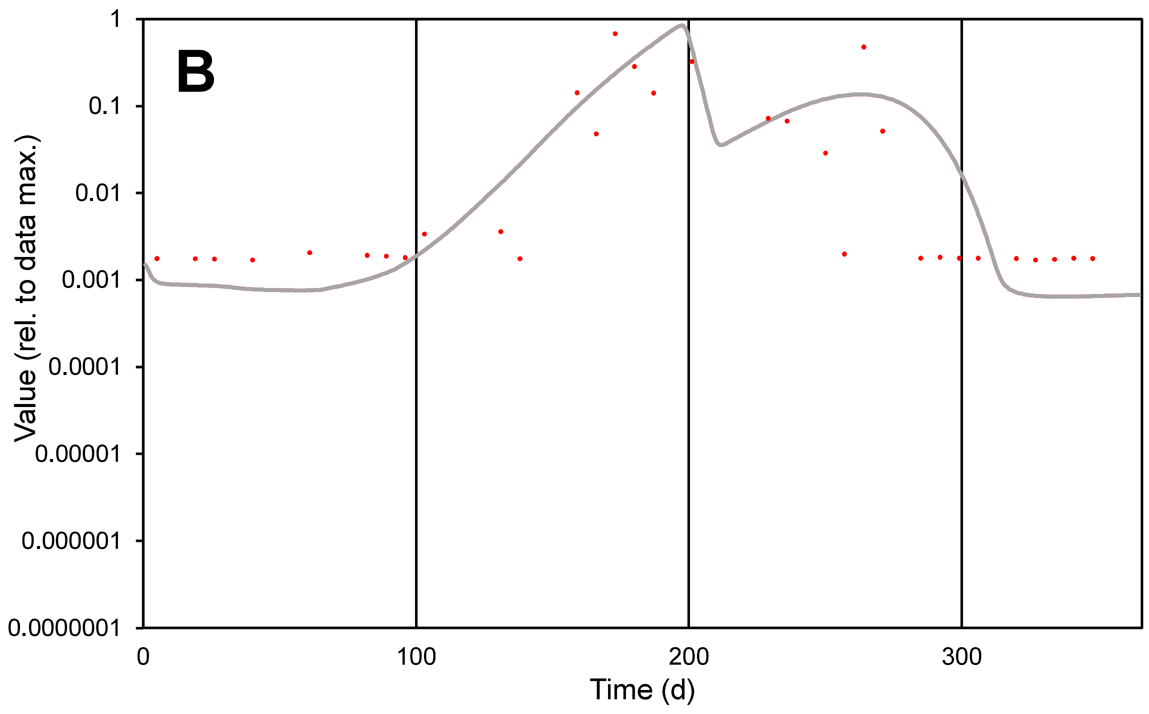

C1

Variables
FO1 Provide jtd or Dst
CalibOnly?
2 1 to reproduce of
oNormalize?
2 1=no, 2=yes

ofxn calc*
1.22656
ofxn mod
1.29225

sRun
RunA1
zout
1

Ave
Data
0.0036
Model
0.00359

data/vMax
0.03535

ofxng
263.459

Min d/m
2.63517

ave/min d
59.7386
ave/min m
157.039

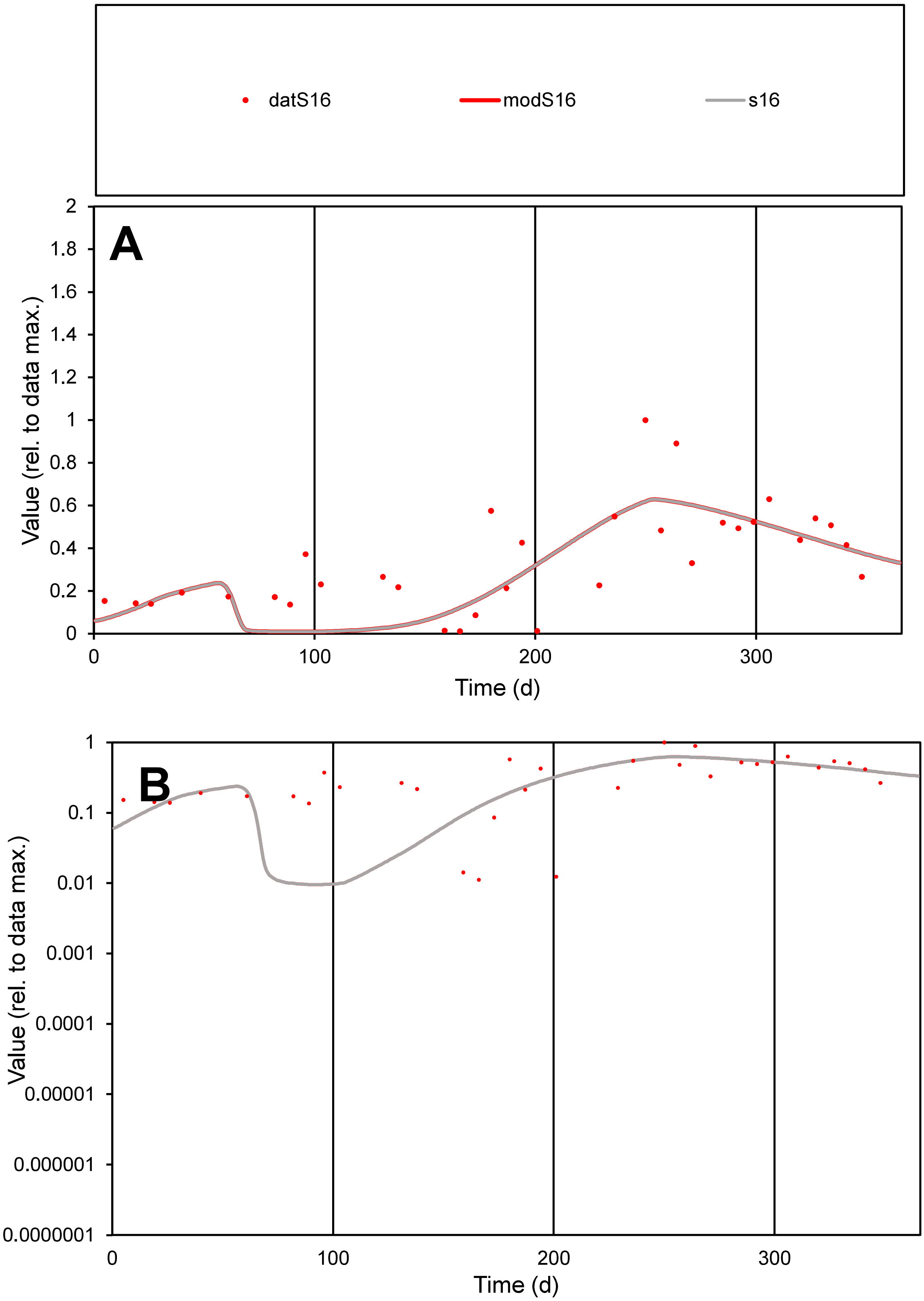

C1

Variables
S16 Provide jtd or Dst
CalibOnly?
2 1 to reproduce of
oNormalize?
2 1=no, 2=yes

ofxn calc*
0.51747
ofxn mod
0.53593

sRun
RunA1
zout
1

Ave
Data
0.00377
Model
0.00333

data/vMax
0.01099

ofxng
263.459

Min d/m
1.17479

ave/min d
30.6819
ave/min m
31.8419

326_Calib_S_A

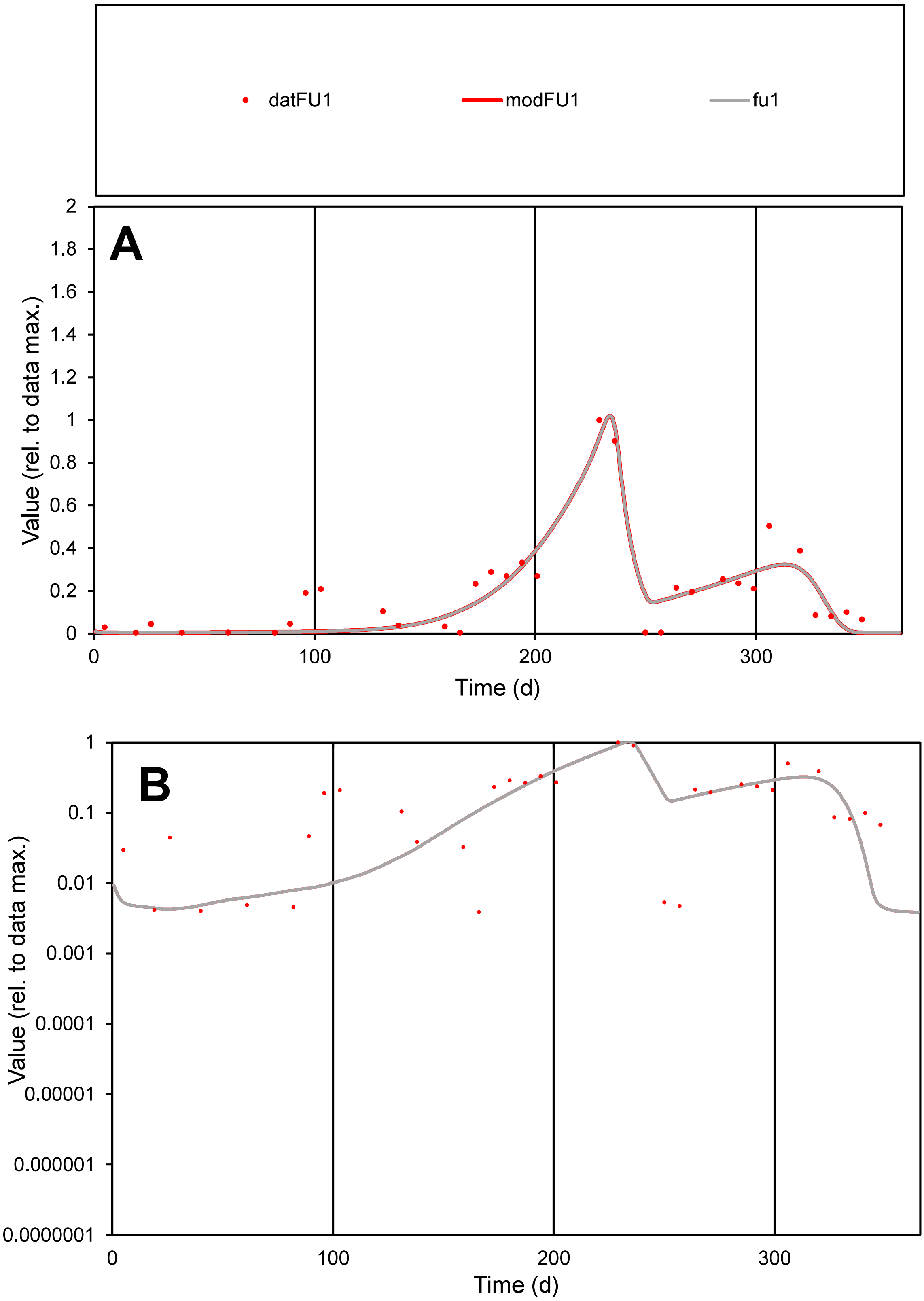

C1

Variables
FU1 Provide jtd or Dst
CalibOnly?
2 1 to reproduce of
oNormalize?
2 1=no, 2=yes

ofxn calc*
0.44814
ofxn mod
0.48391

sRun
RunA1
zout
1

Ave
Data
0.00229
Model
0.00213

data/vMax
0.01189

ofxng
263.459

Min d/m
0.90143

ave/min d
49.7227
ave/min m
41.713

326_Calib_S_A

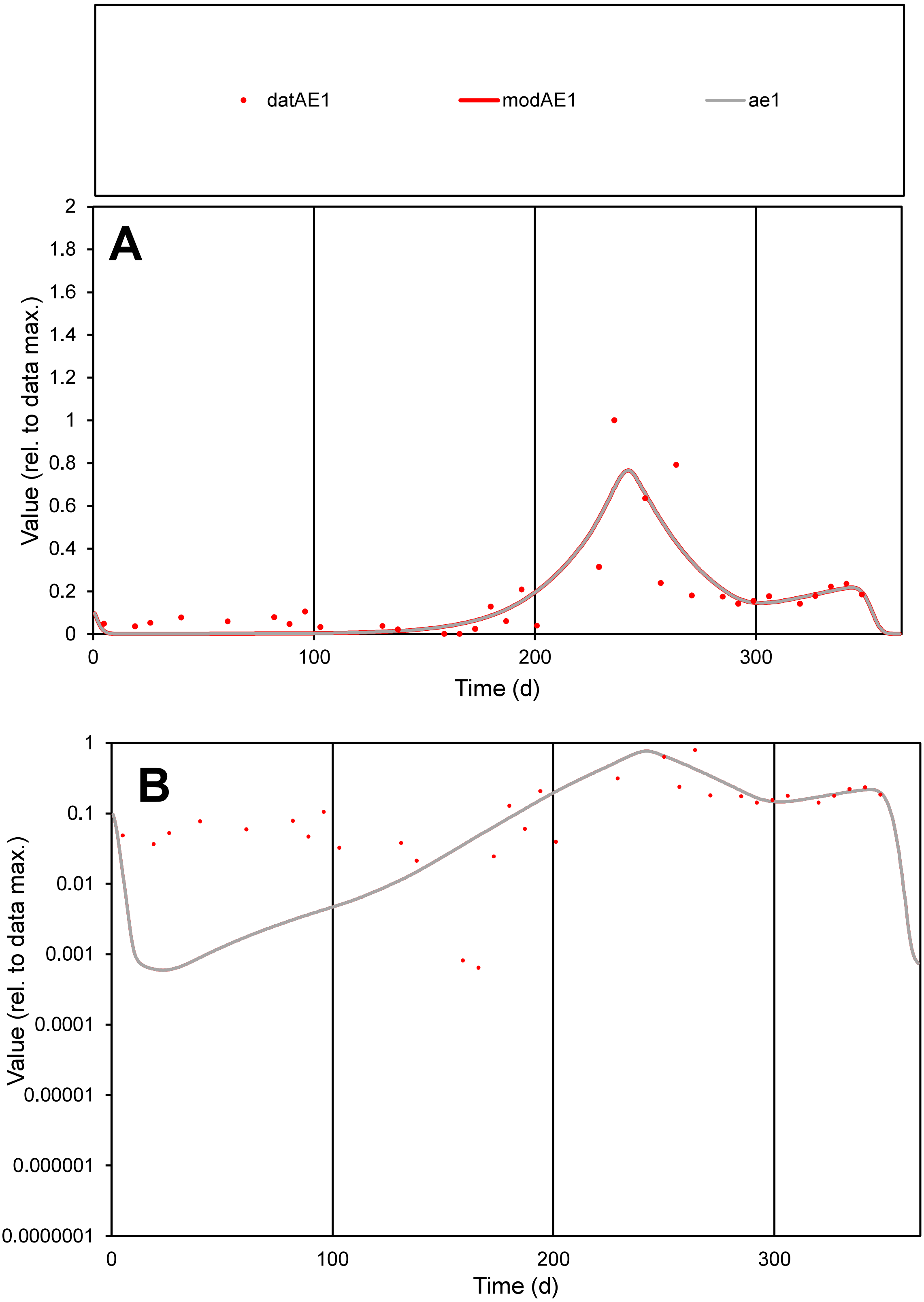

C1

Variables
AE1 Provide jtd or Dst
CalibOnly?
2 1 to reproduce of
oNormalize?
2 1=no, 2=yes

ofxn calc*
0.66092
ofxn mod
0.68858

sRun
RunA1
zout
1

Ave
Data
0.00379
Model
0.00362

data/vMax
0.02146

ofxng
263.459

Min d/m
1.06231

ave/min d
274.778
ave/min m
278.224

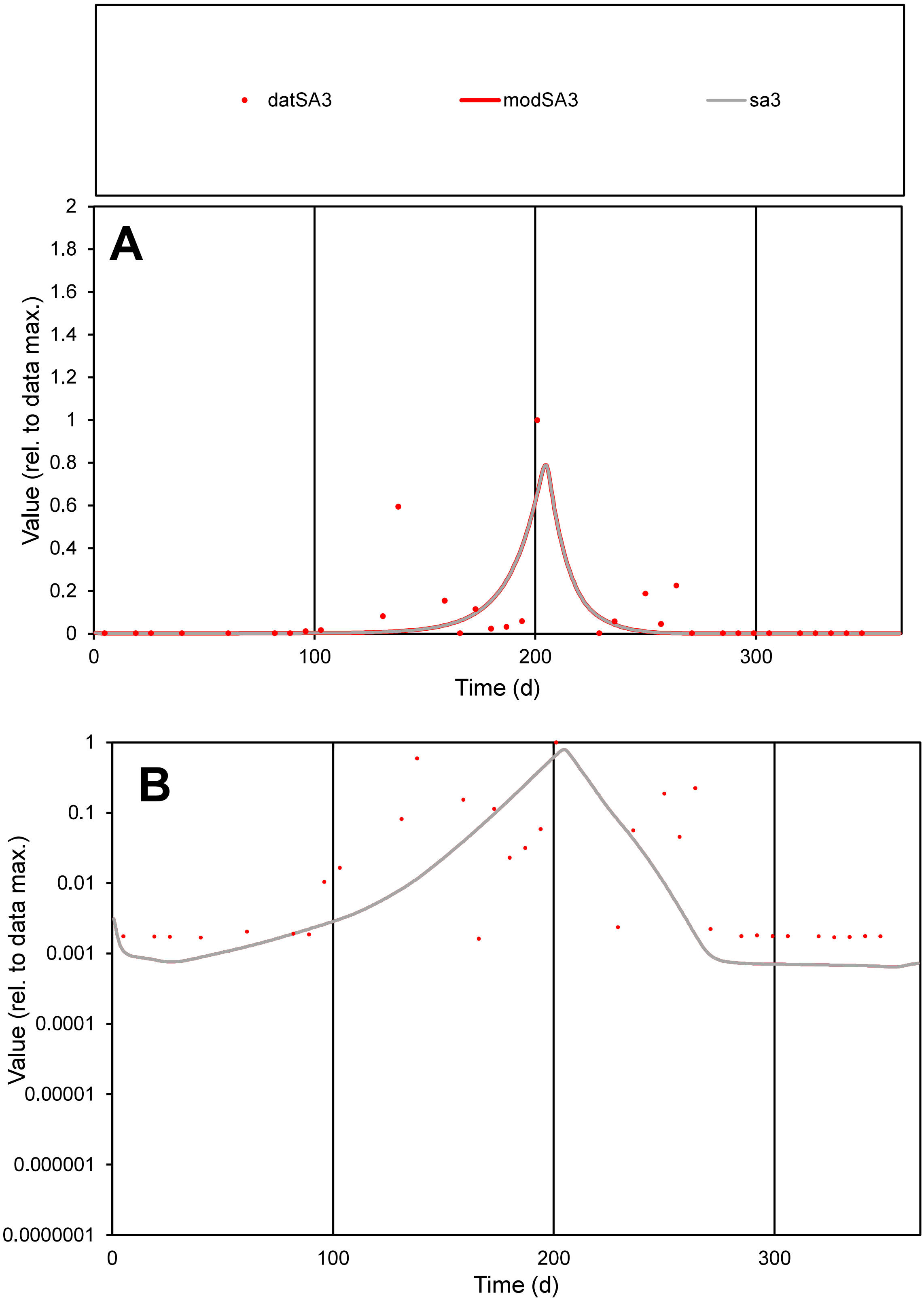

C1

Variables

SA3 Provide jtd or Dst

CalibOnly?

2 1 to reproduce of

oNormalize?

2 1=no, 2=yes

ofxn calc*

1.82321

ofxn mod

1.86147

sRun

RunA1

zout

1

Ave

Data

0.00479

Model

0.00338

data/vMax

0.06005

ofxng

263.459

Min d/m

2.46915

ave/min d

49.2467

ave/min m

85.7361

326_Calib_S_A

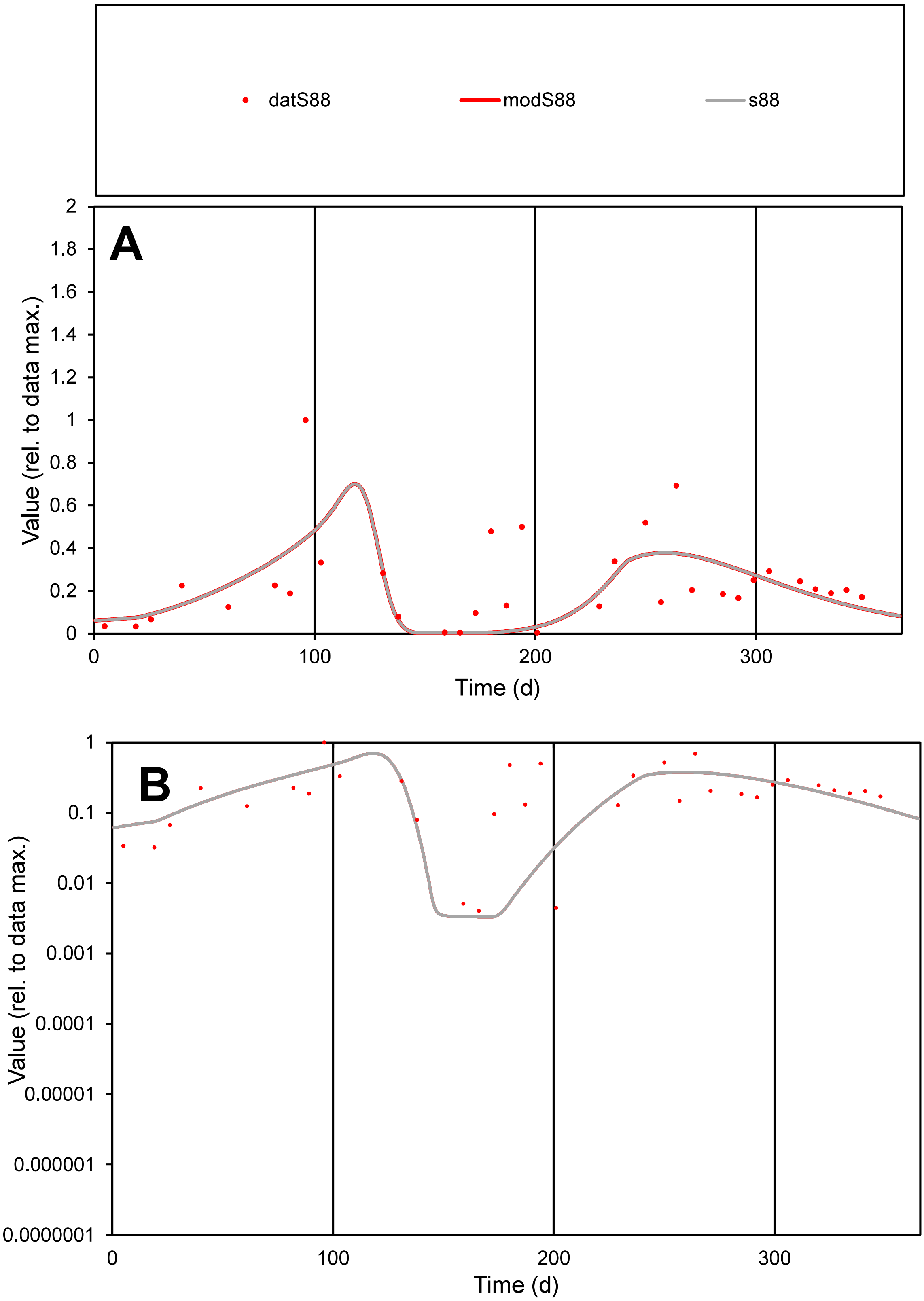

C1

Variables
S88 Provide jtd or Dst
CalibOnly?
2 1 to reproduce of
oNormalize?
2 1=no, 2=yes

ofxn calc*
0.79419
ofxn mod
0.80558

sRun
RunA1
zout
1

Ave
Data
0.00331
Model
0.00283

data/vMax
0.01411

ofxng
263.459

Min d/m
1.22011

ave/min d
57.9764
ave/min m
60.432

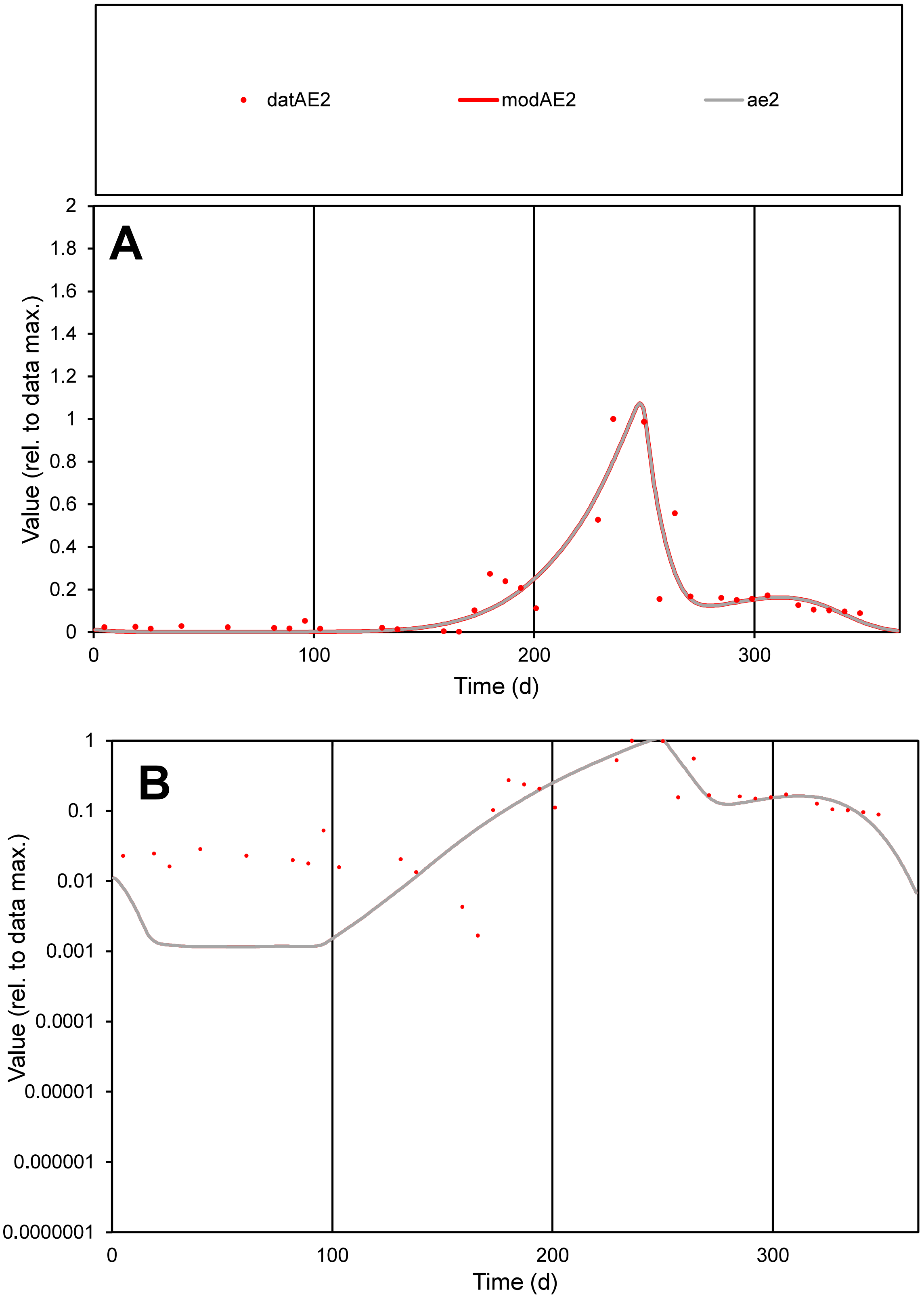

C1

Variables
AE2 Provide jtd or Dst
CalibOnly?
2 1 to reproduce of
oNormalize?
2 1=no, 2=yes

ofxn calc*
0.53876
ofxn mod
0.5947

sRun
RunA1
zout
1

Ave
Data
0.00593
Model
0.00551

data/vMax
0.03404

ofxng
263.459

Min d/m
1.45946

ave/min d
103.648
ave/min m
140.611

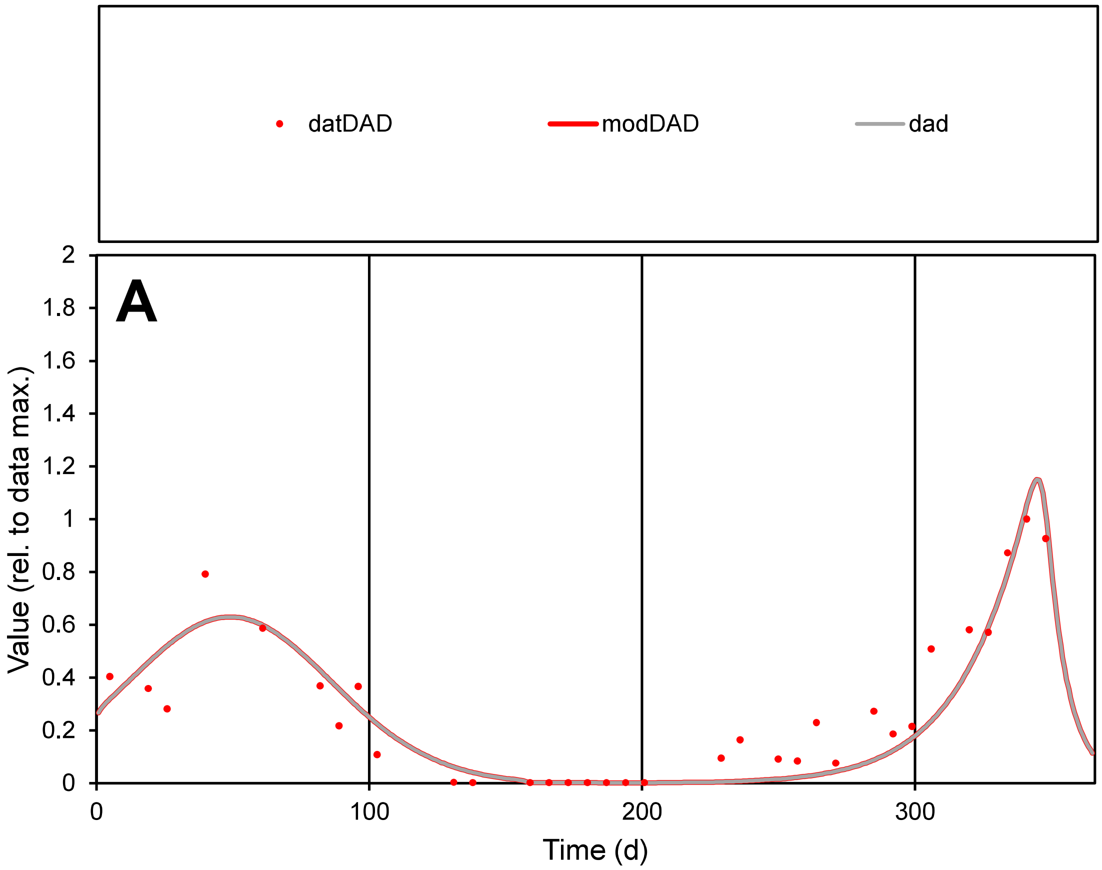

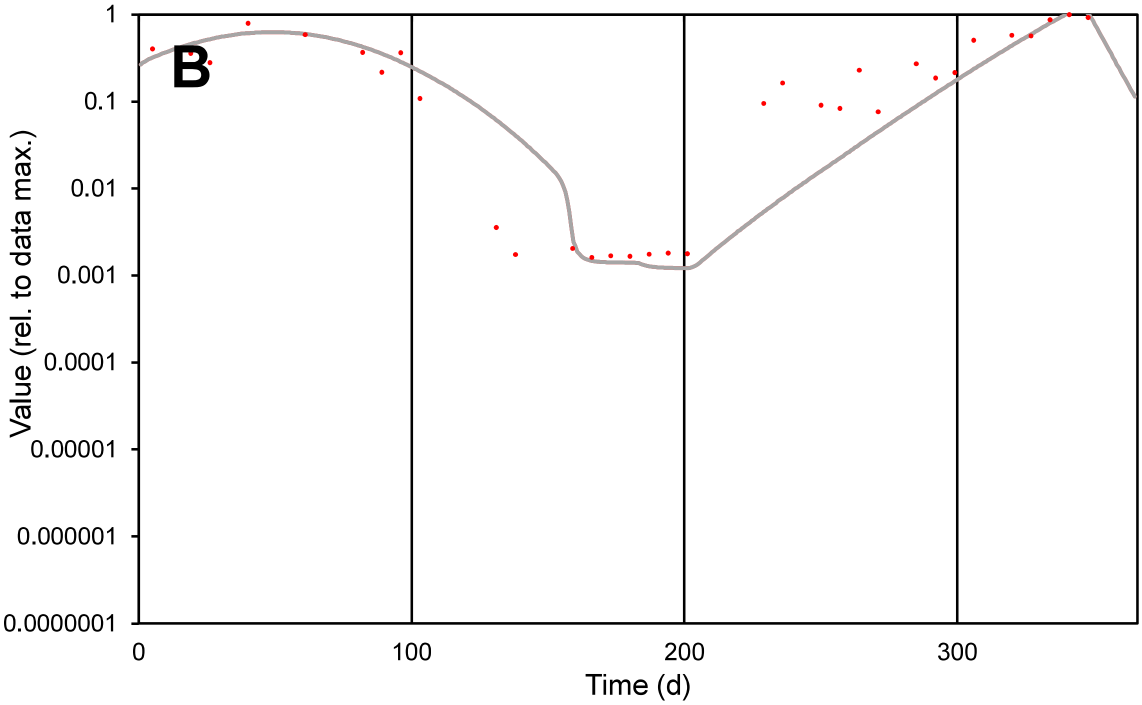

C1

Variables
DAD Provide jtd or Dst
CalibOnly?
2 1 to reproduce of
oNormalize?
2 1=no, 2=yes

ofxn calc*
0.36533
ofxn mod
0.39811

sRun
RunA1
zout
1

Ave
Data
0.00269
Model
0.00247

data/vMax
0.00946

ofxng
263.459

Min d/m
1.32239

ave/min d
175.606
ave/min m
213.537

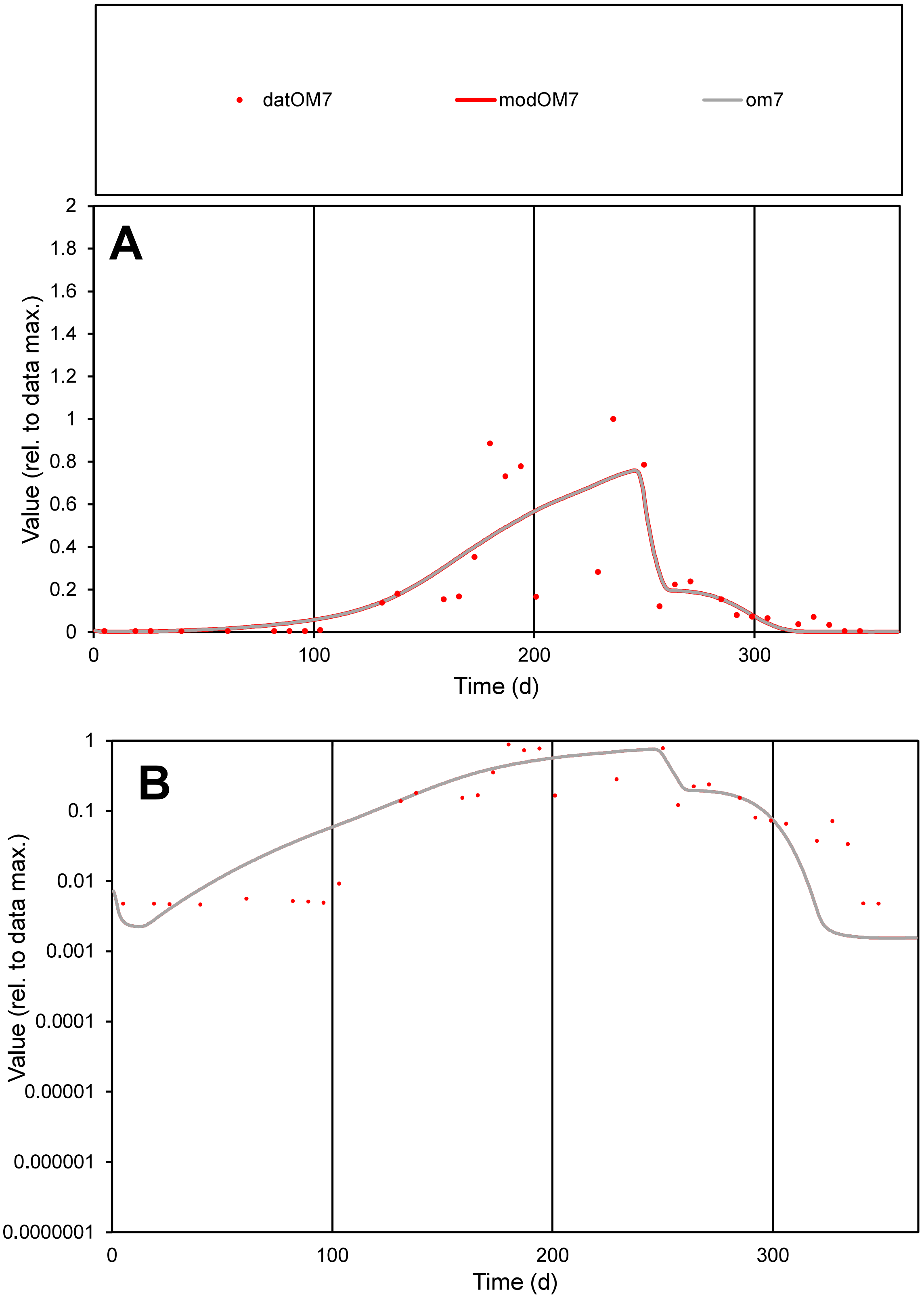

C1

Variables
OM7 Provide jtd or Dst
CalibOnly?
2 1 to reproduce of
oNormalize?
2 1=no, 2=yes

ofxn calc*
0.77604
ofxn mod
0.80176

sRun
RunA1
zout
1

Ave
Data
0.00429
Model
0.00429

data/vMax
0.02086

ofxng
263.459

Min d/m
2.99072

ave/min d
44.3389
ave/min m
132.638

326_Calib_S_A

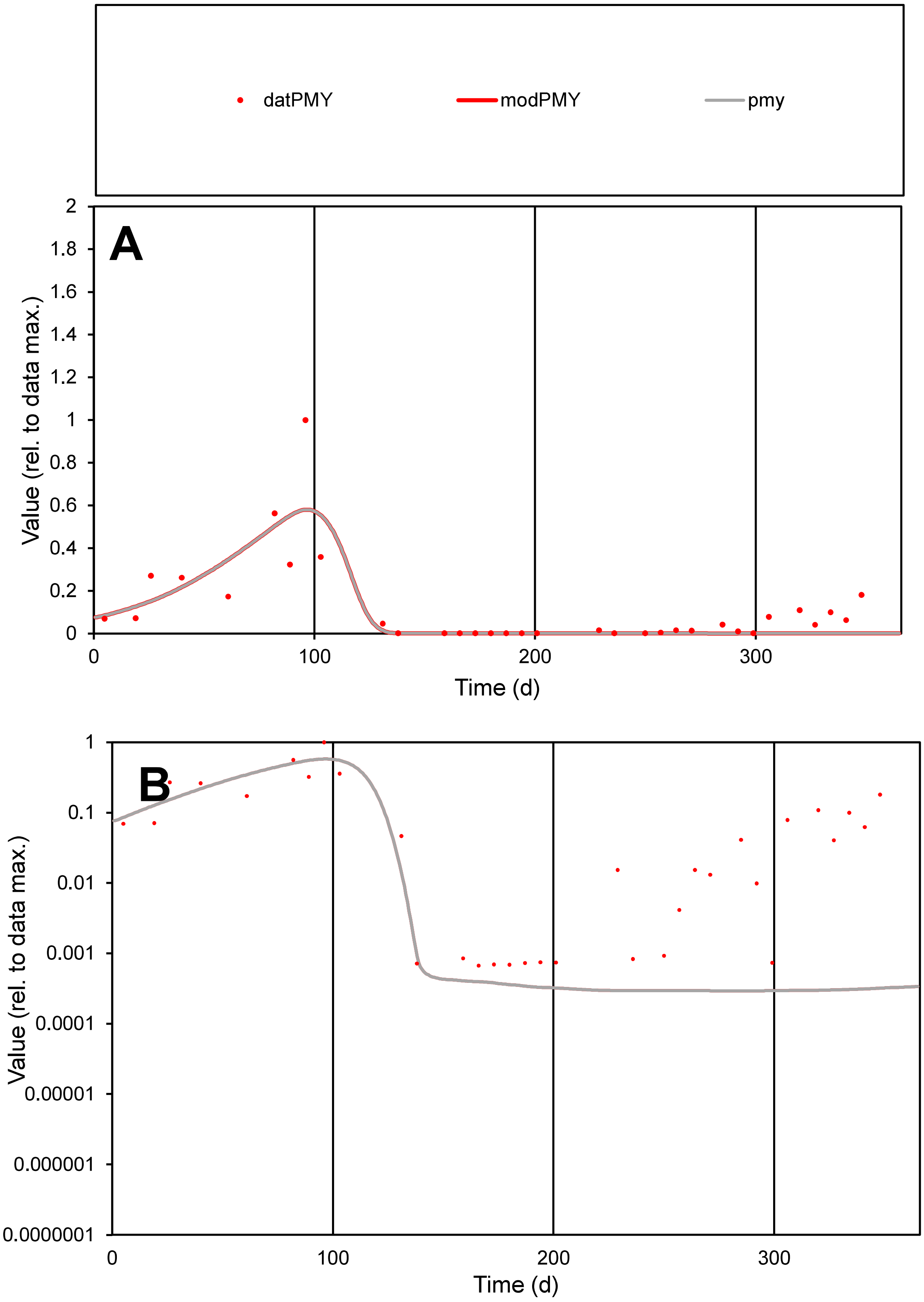

C1

Variables
PMY Provide jtd or Dst
CalibOnly?
2 1 to reproduce of
oNormalize?
2 1=no, 2=yes

ofxn calc*
0.93631
ofxn mod
0.95806

sRun
RunA1
zout
1

Ave
Data
0.00164
Model
0.00137

data/vMax
0.01422

ofxng
263.459

Min d/m
2.28997

ave/min d
172.469
ave/min m
329.001

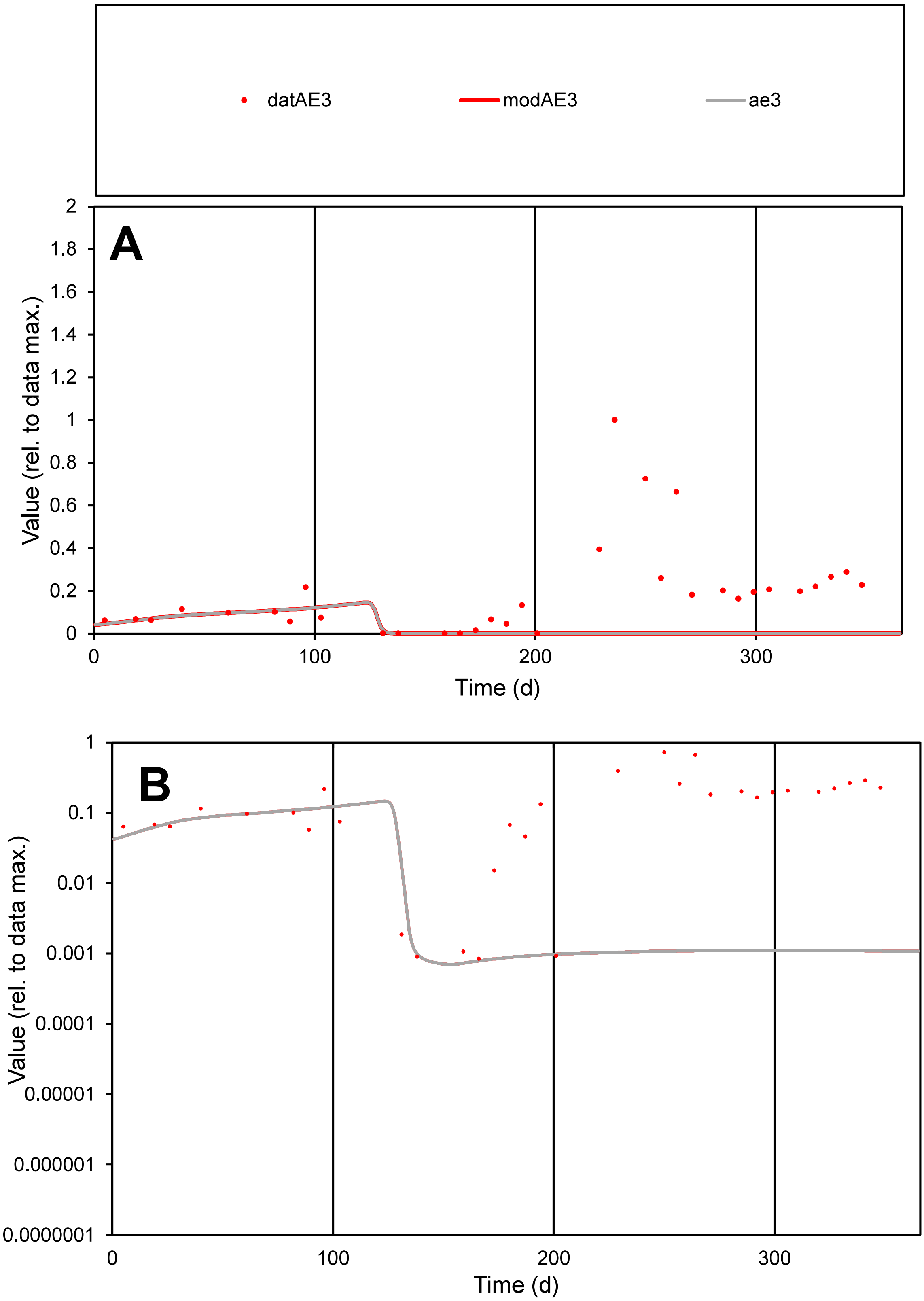

C1

Variables
AE3 Provide jtd or Dst
CalibOnly?
2 1 to reproduce of
oNormalize?
2 1=no, 2=yes

ofxn calc*
1.49246
ofxn mod
1.49521

sRun
RunA1
zout
1

Ave
Data
0.00378
Model
0.00052

data/vMax
0.01973

ofxng
263.459

Min d/m
1.14178

ave/min d
226.639
ave/min m
35.4391

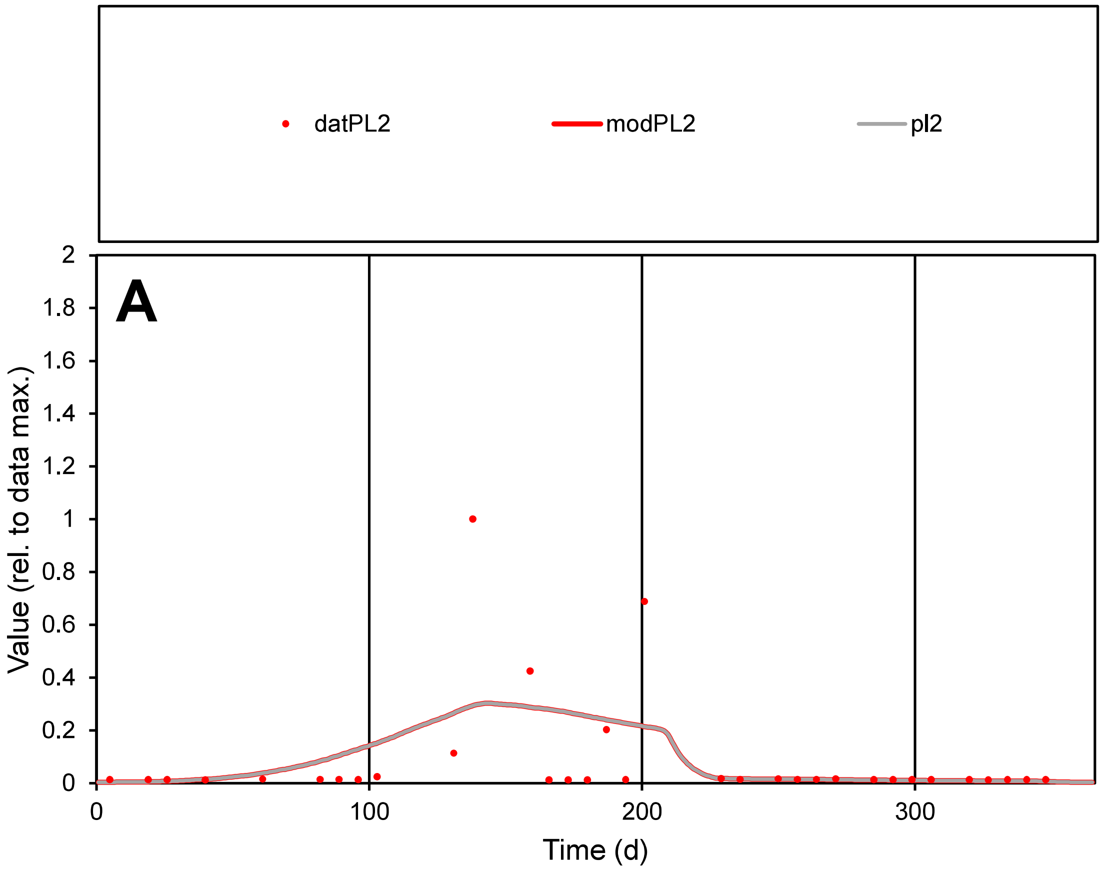

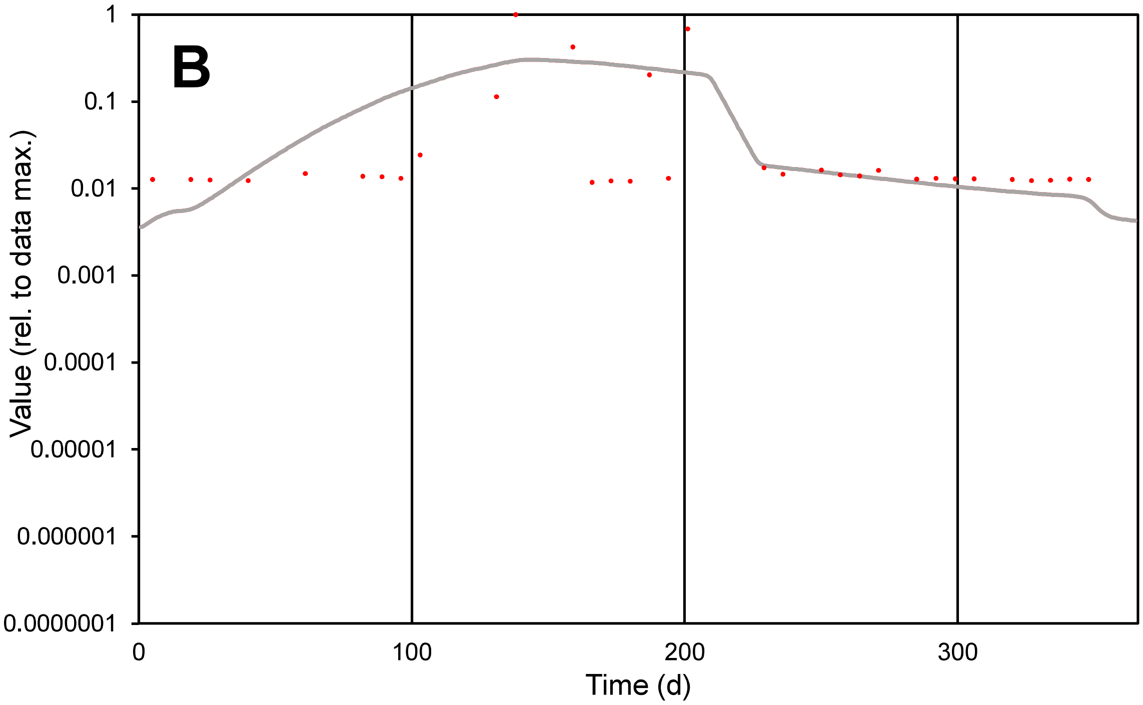

C1

Variables
PL2 Provide jtd or Dst
CalibOnly?
2 1 to reproduce of
oNormalize?
2 1=no, 2=yes

ofxn calc*
2.08329
ofxn mod
2.09664

sRun
RunA1
zout
1

Ave
Data
0.00117
Model
0.00126

data/vMax
0.01369

ofxng
263.459

Min d/m
2.5591

ave/min d
7.24428
ave/min m
20.0662

326_Calib_S_A

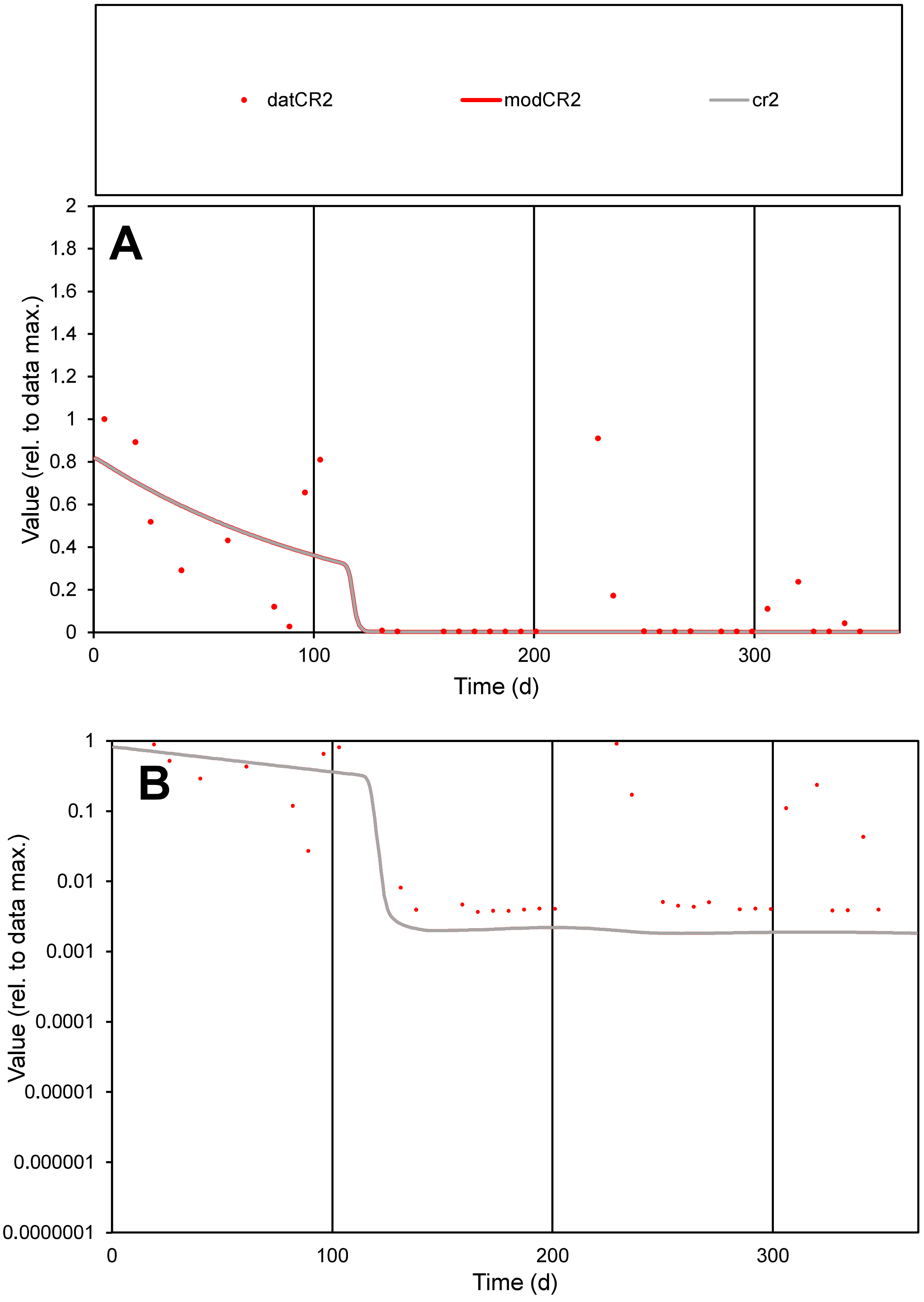

C1

Variables
CR2 Provide jtd or Dst
CalibOnly?
2 1 to reproduce of
oNormalize?
2 1=no, 2=yes

ofxn calc*
1.16429
ofxn mod
1.18034

sRun
RunA1
zout
1

Ave
Data
0.00205
Model
0.00156

data/vMax
0.01075

ofxng
263.459

Min d/m
2.01479

ave/min d
51.9507
ave/min m
79.5404

326_Calib_S_A

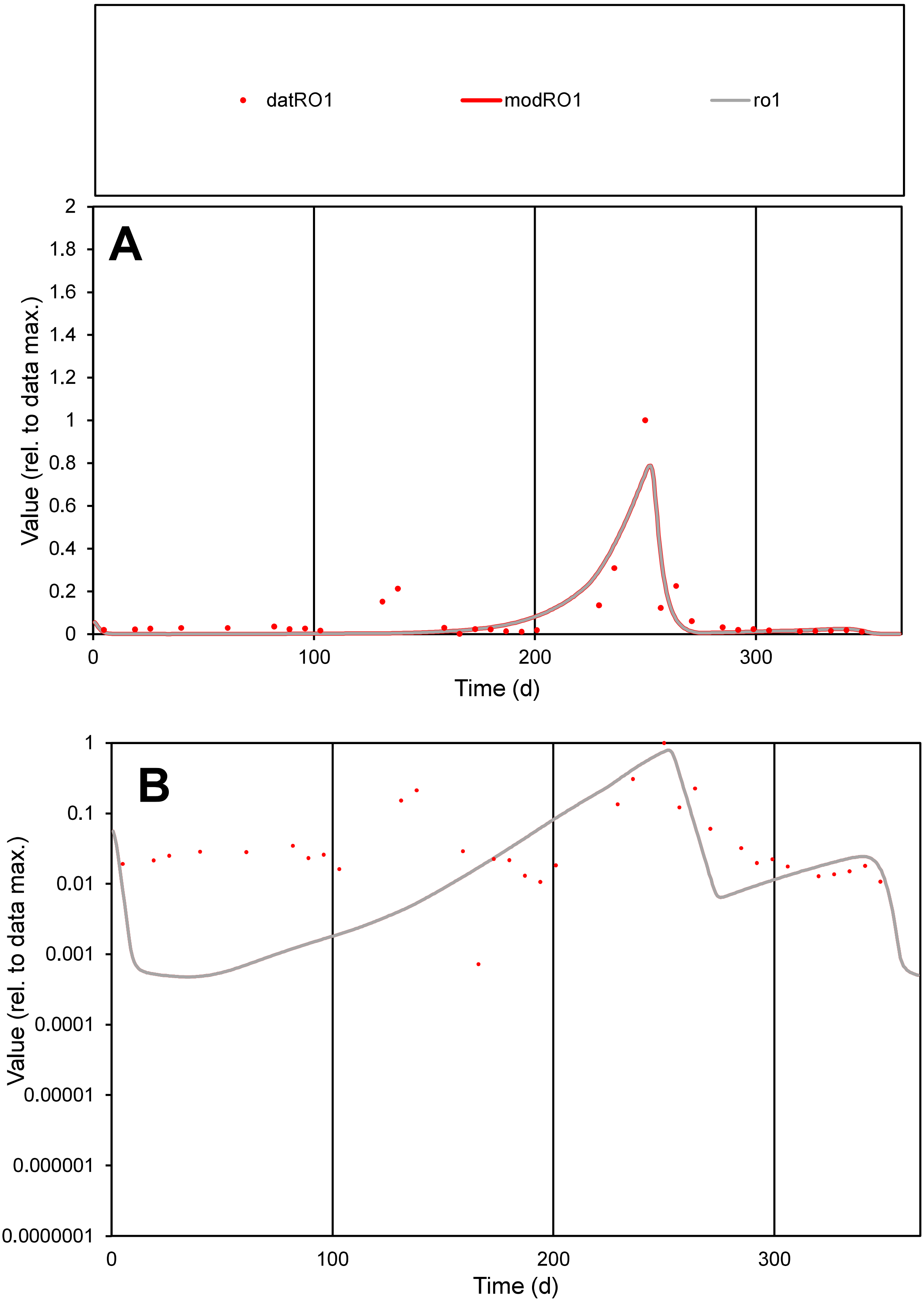

C1

Variables	
RO1	Provide jtd or Dst
CalibOnly?	
2	1 to reproduce of
oNormalize?	
2	1=no, 2=yes
ofxn calc*	
0.93296	
ofxn mod	
1.02102	
sRun	
RunA1	
zout	
1	
Ave Data	
0.00399	
Model	
0.00331	
data/vMax	
0.04848	
ofxng	
263.459	
Min d/m	
1.48475	
ave/min d	
113.904	
ave/min m	
140.358	

326_Calib_S_A

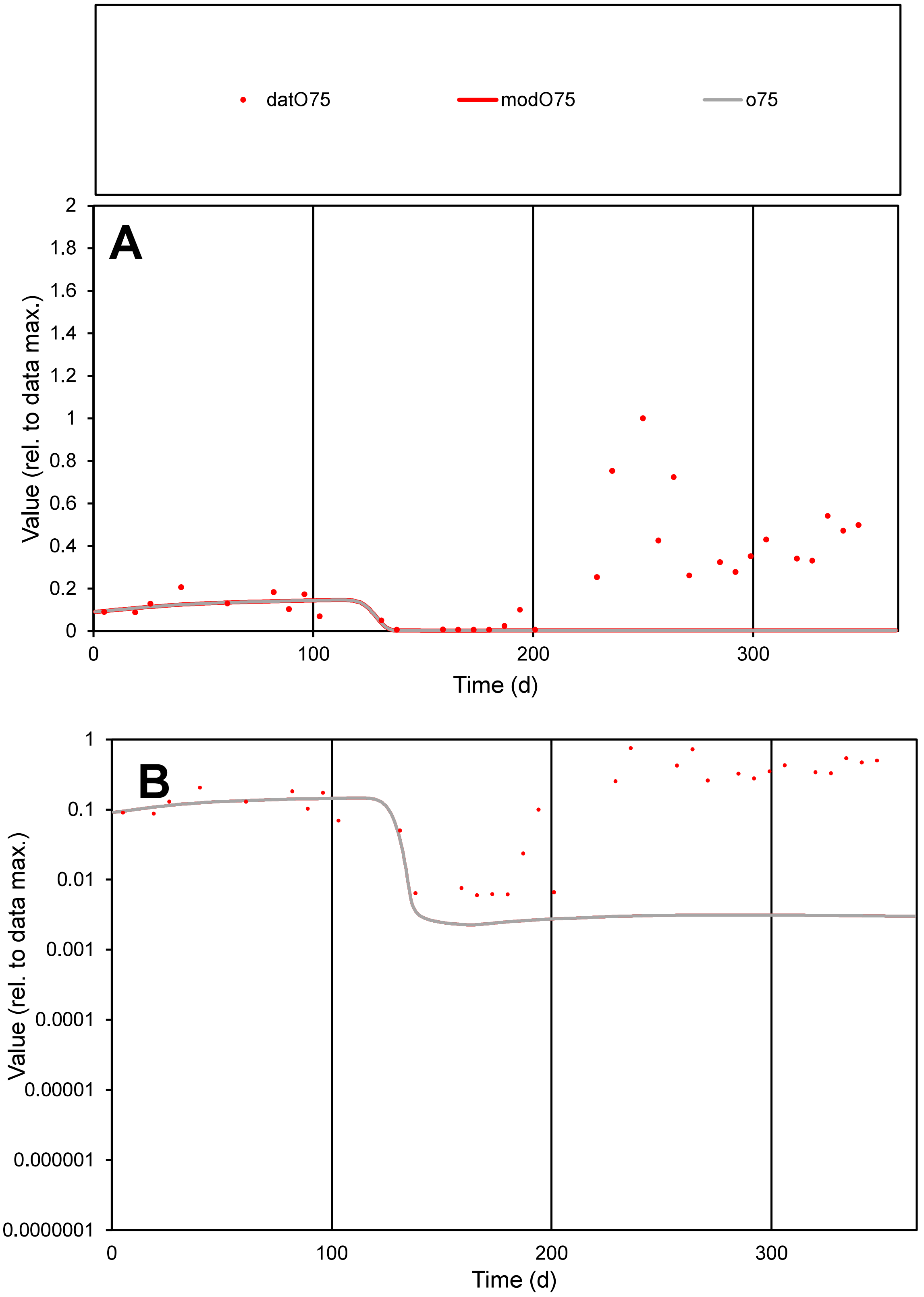

C1

Variables
O75 Provide jtd or Dst
CalibOnly?
2 1 to reproduce of
oNormalize?
2 1=no, 2=yes

ofxn calc*
1.35029
ofxn mod
1.3532

sRun
RunA1
zout
1

Ave
Data
0.00295
Model
0.00044

data/vMax
0.01163

ofxng
263.459

Min d/m
2.62798

ave/min d
42.4317
ave/min m
16.6998

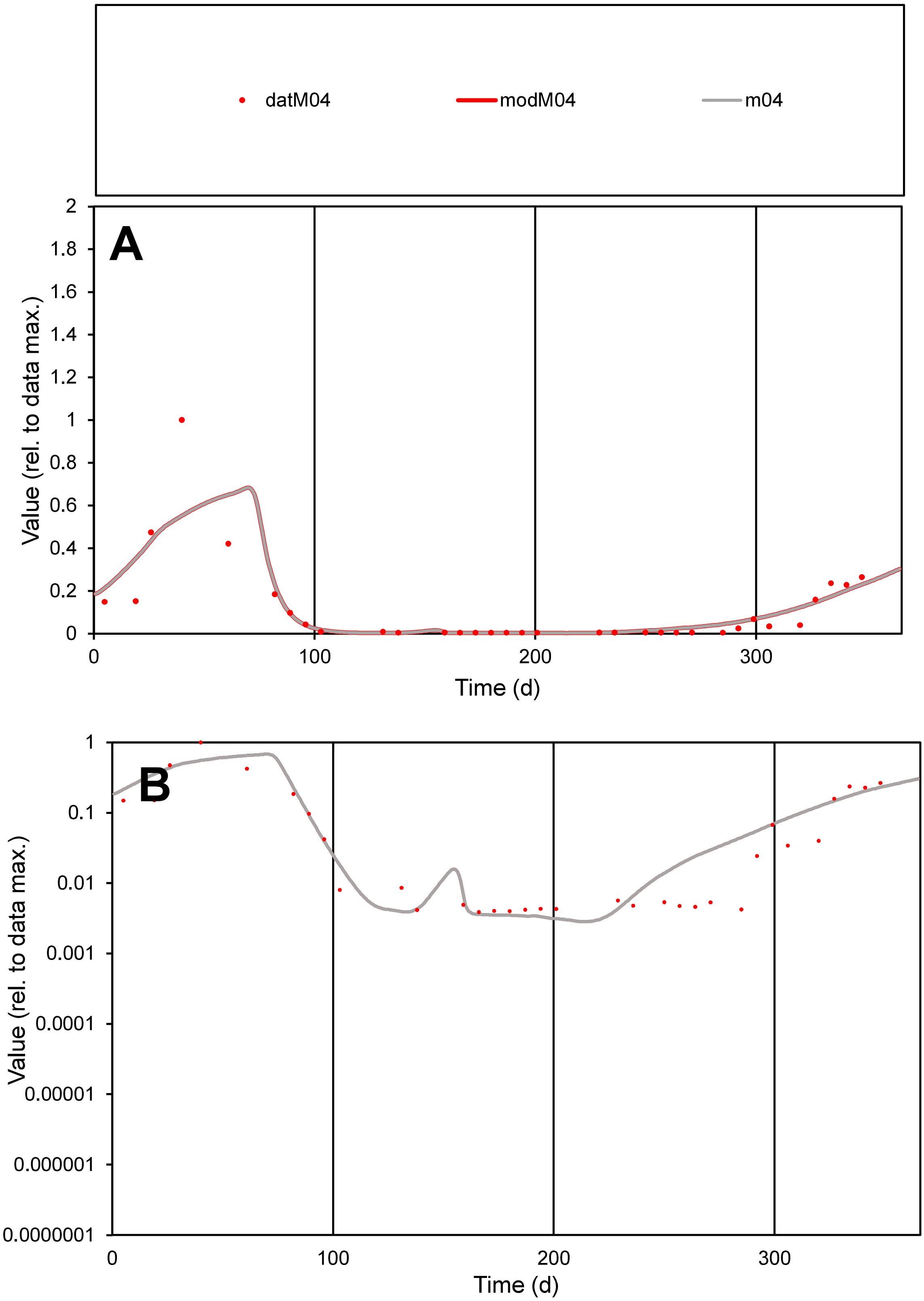

C1

Variables
M04 Provide jtd or Dst
CalibOnly?
2 1 to reproduce of
oNormalize?
2 1=no, 2=yes

ofxn calc*
0.87803
ofxn mod
0.91669

sRun
RunA1
zout
1

Ave
Data
0.001
Model
0.00105

data/vMax
0.00902

ofxng
263.459

Min d/m
1.24679

ave/min d
28.6225
ave/min m
37.5434

326_Calib_S_A

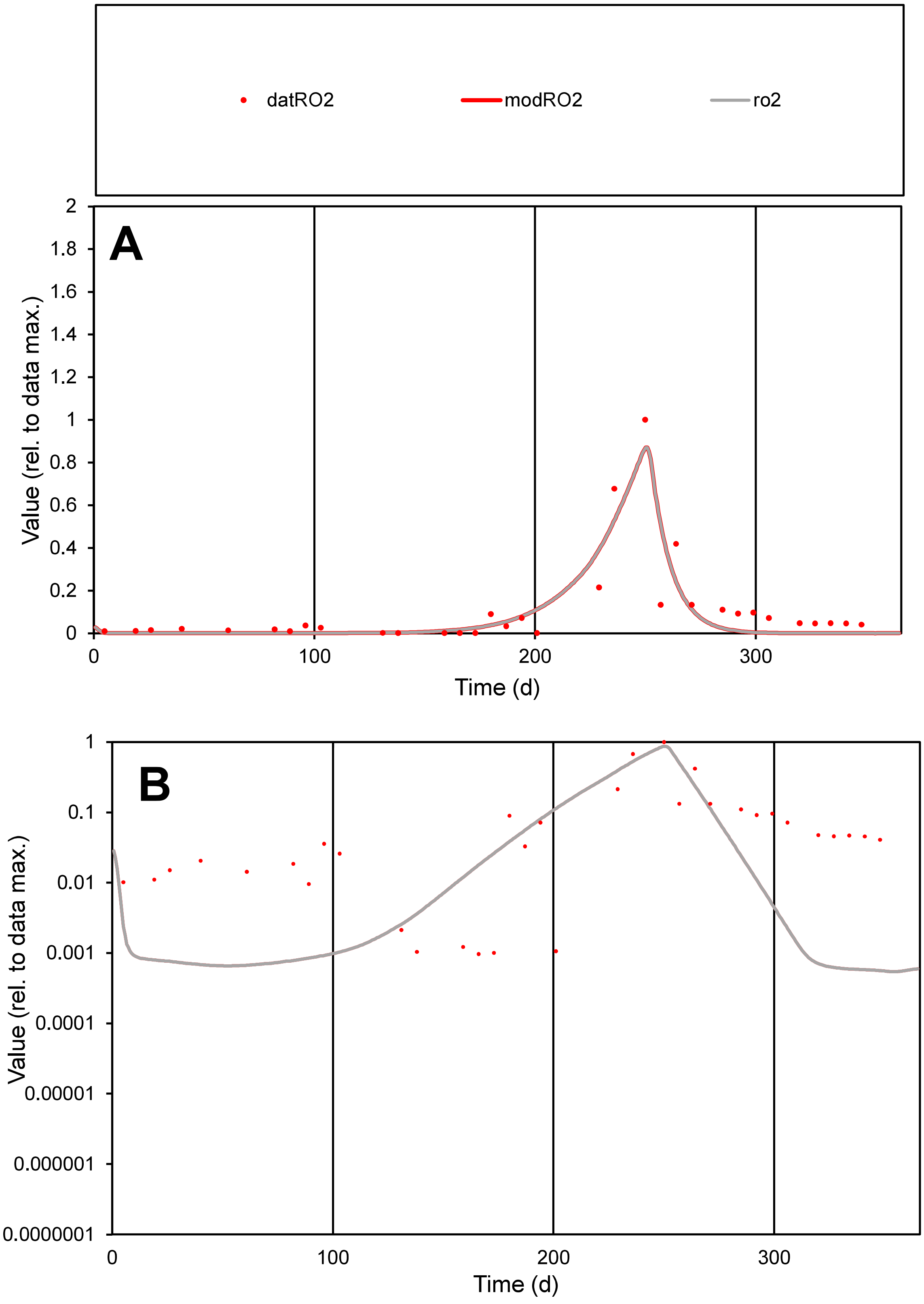

C1

Variables

RO2 Provide jtd or Dst

CalibOnly?

2 1 to reproduce of

oNormalize?

2 1=no, 2=yes

ofxn calc*

0.77187

ofxn mod

0.85318

sRun

RunA1

zout

1

Ave

Data

0.00423

Model

0.00349

data/vMax

0.03943

ofxng

263.459

Min d/m

1.72711

ave/min d

110.958

ave/min m

158.098

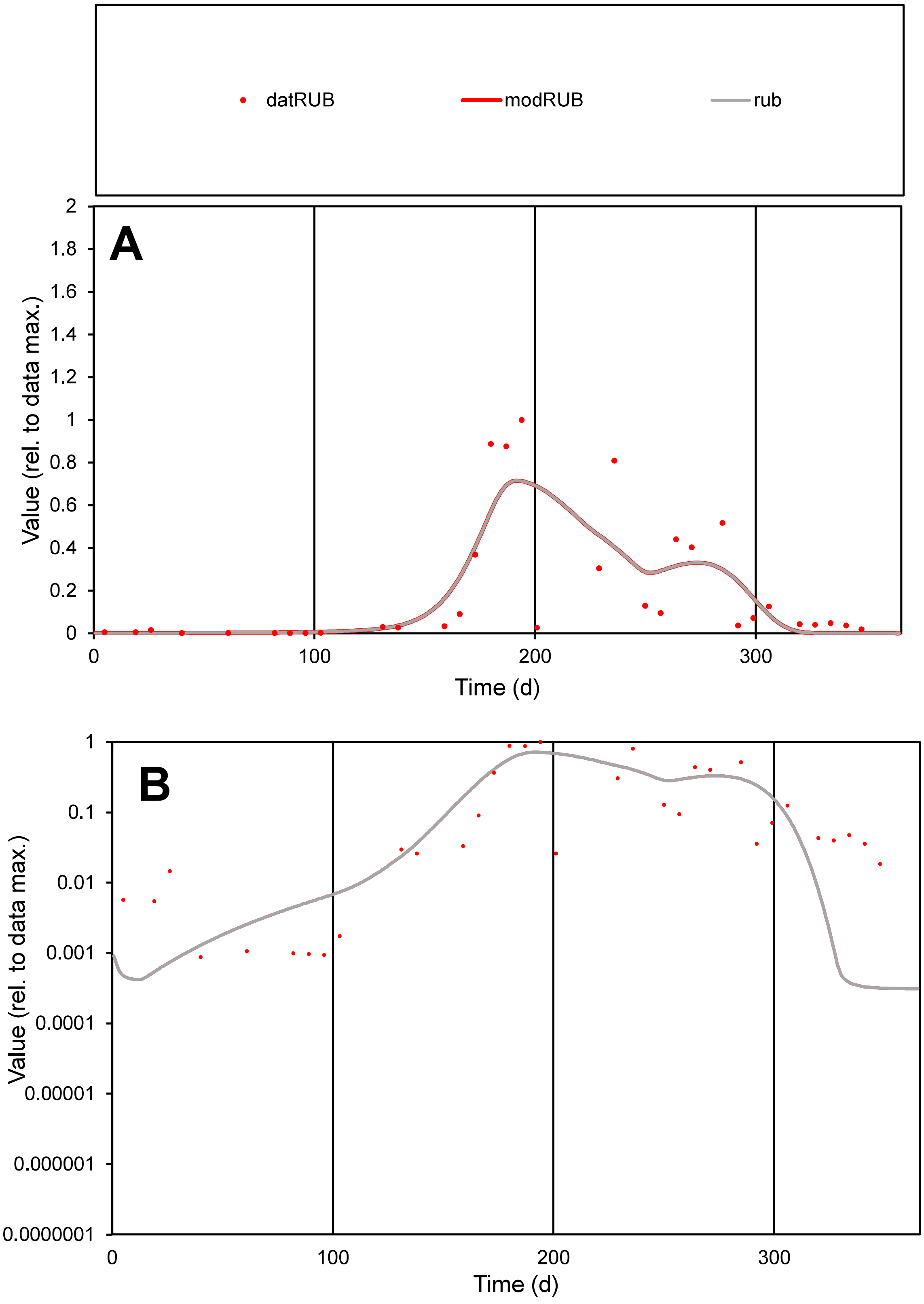

C1

Variables

RUB Provide jtd or Dst

CalibOnly?

2 1 to reproduce of

oNormalize?

2 1=no, 2=yes

ofxn calc*

0.90766

ofxn mod

0.93452

sRun

RunA1

zout

1

Ave

Data

0.0023

Model

0.0023

data/vMax

0.01173

ofxng

263.459

Min d/m

2.78092

ave/min d

223.27

ave/min m

618.893

326_Calib_S_A

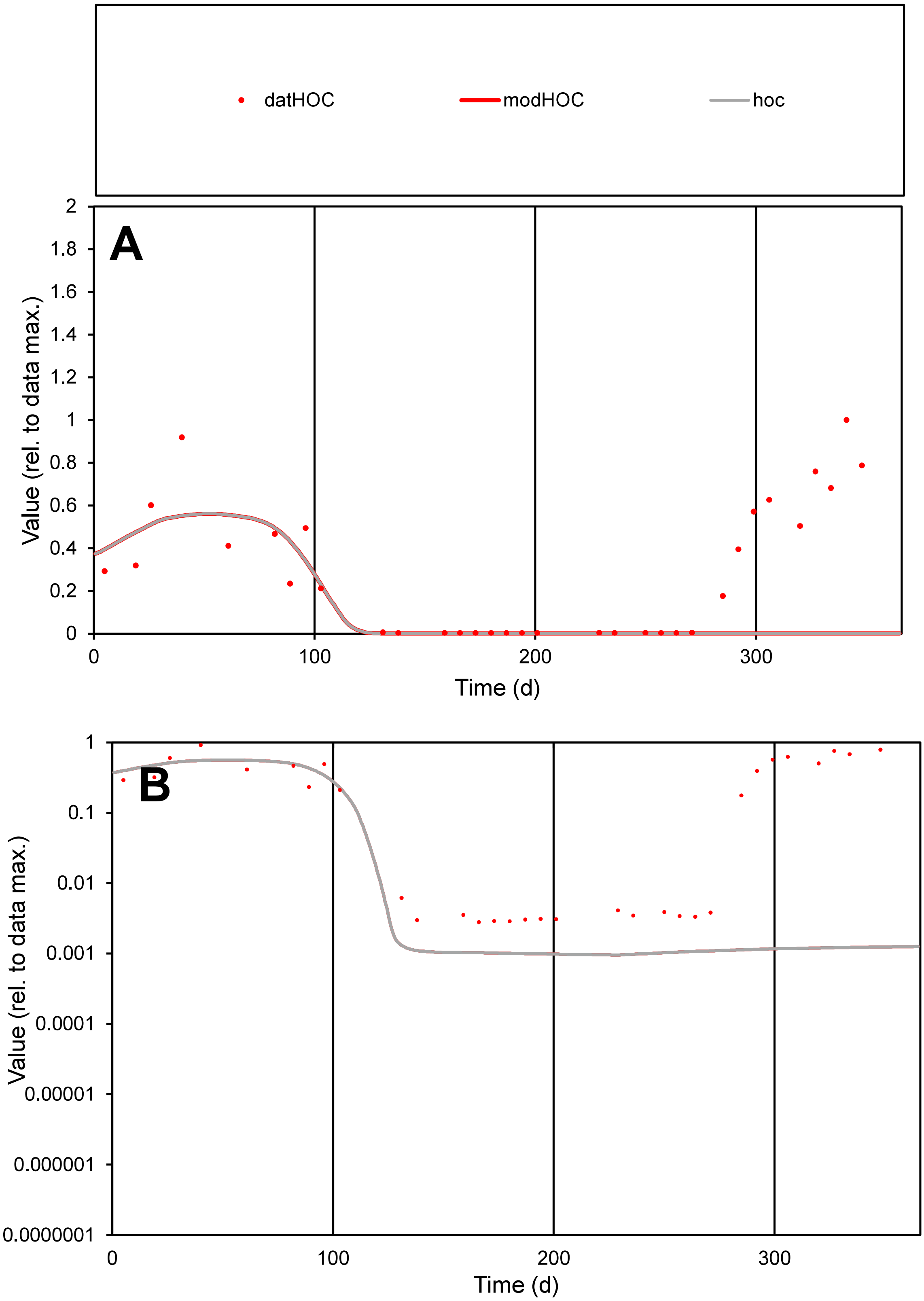

C1

Variables
HOC Provide jtd or Dst
CalibOnly?
2 1 to reproduce of
oNormalize?
2 1=no, 2=yes

ofxn calc*
1.22002
ofxn mod
1.22634

sRun
RunA1
zout
1

Ave
Data
0.00129
Model
0.00054

data/vMax
0.00447

ofxng
263.459

Min d/m
2.92174

ave/min d
103.182
ave/min m
125.94

326_Calib_S_A

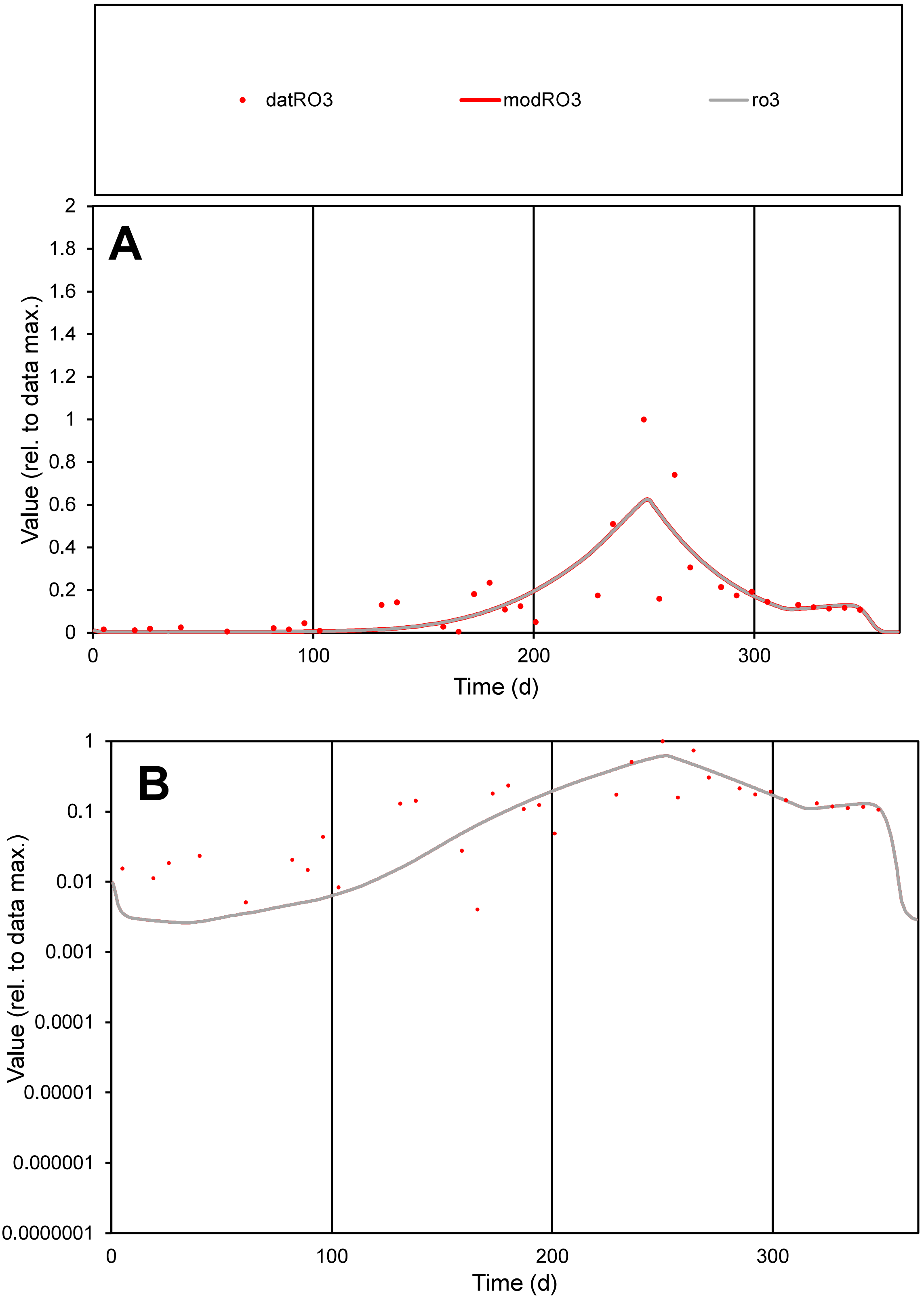

C1

Variables
RO3 Provide jtd or Dst
CalibOnly?
2 1 to reproduce of
oNormalize?
2 1=no, 2=yes

ofxn calc*
0.76831
ofxn mod
0.79496

sRun
RunA1
zout
1

Ave
Data
0.00384
Model
0.00368

data/vMax
0.02365

ofxng
263.459

Min d/m
1.51263

ave/min d
40.3002
ave/min m
58.4116

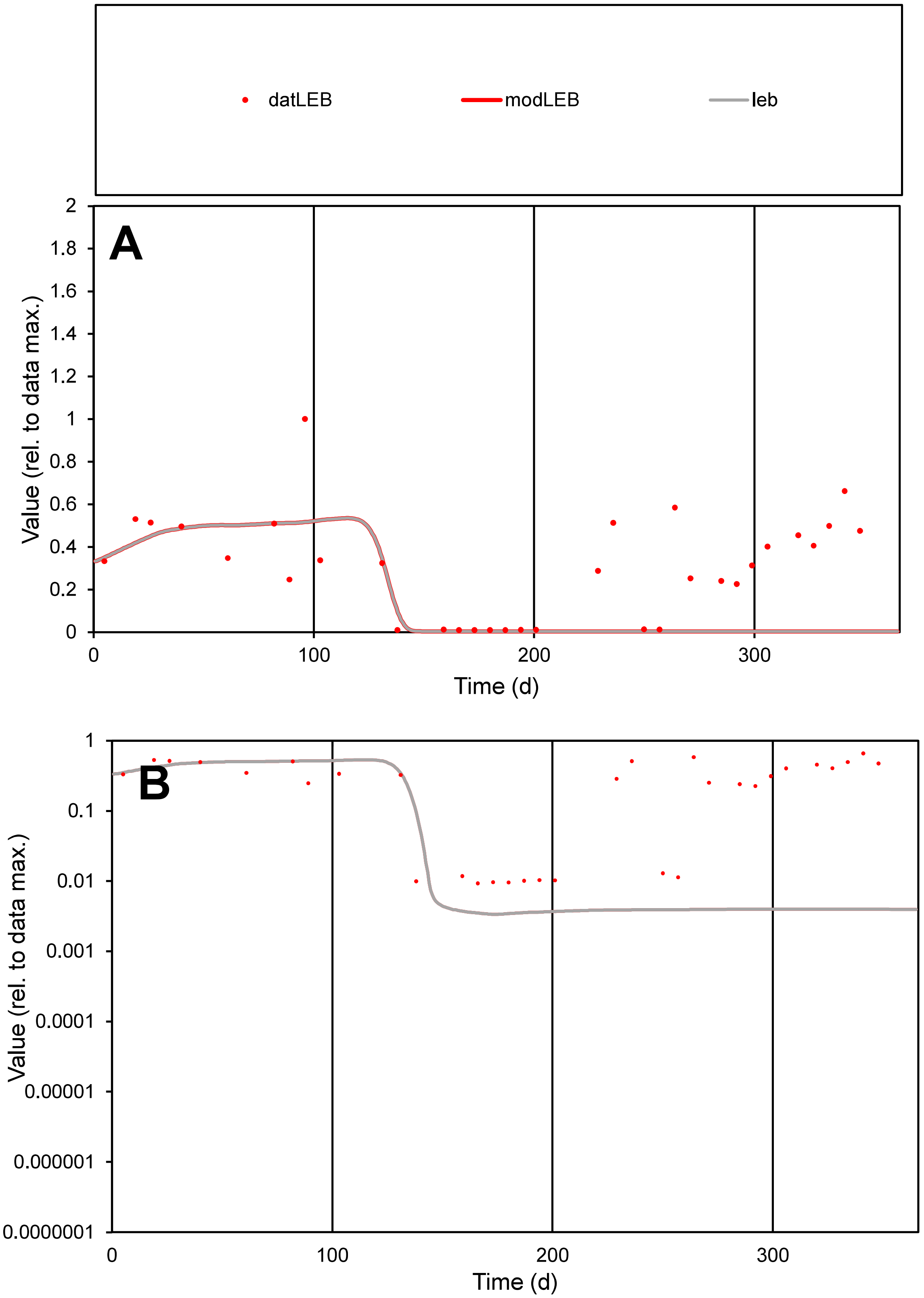

C1

Variables
LEB Provide jtd or Dst
CalibOnly?
2 1 to reproduce of
oNormalize?
2 1=no, 2=yes

ofxn calc*
0.94528
ofxn mod
0.9518

sRun
RunA1
zout
1

Ave
Data
0.00227
Model
0.00107

data/vMax
0.00744

ofxng
263.459

Min d/m
2.75968

ave/min d
32.783
ave/min m
42.5361

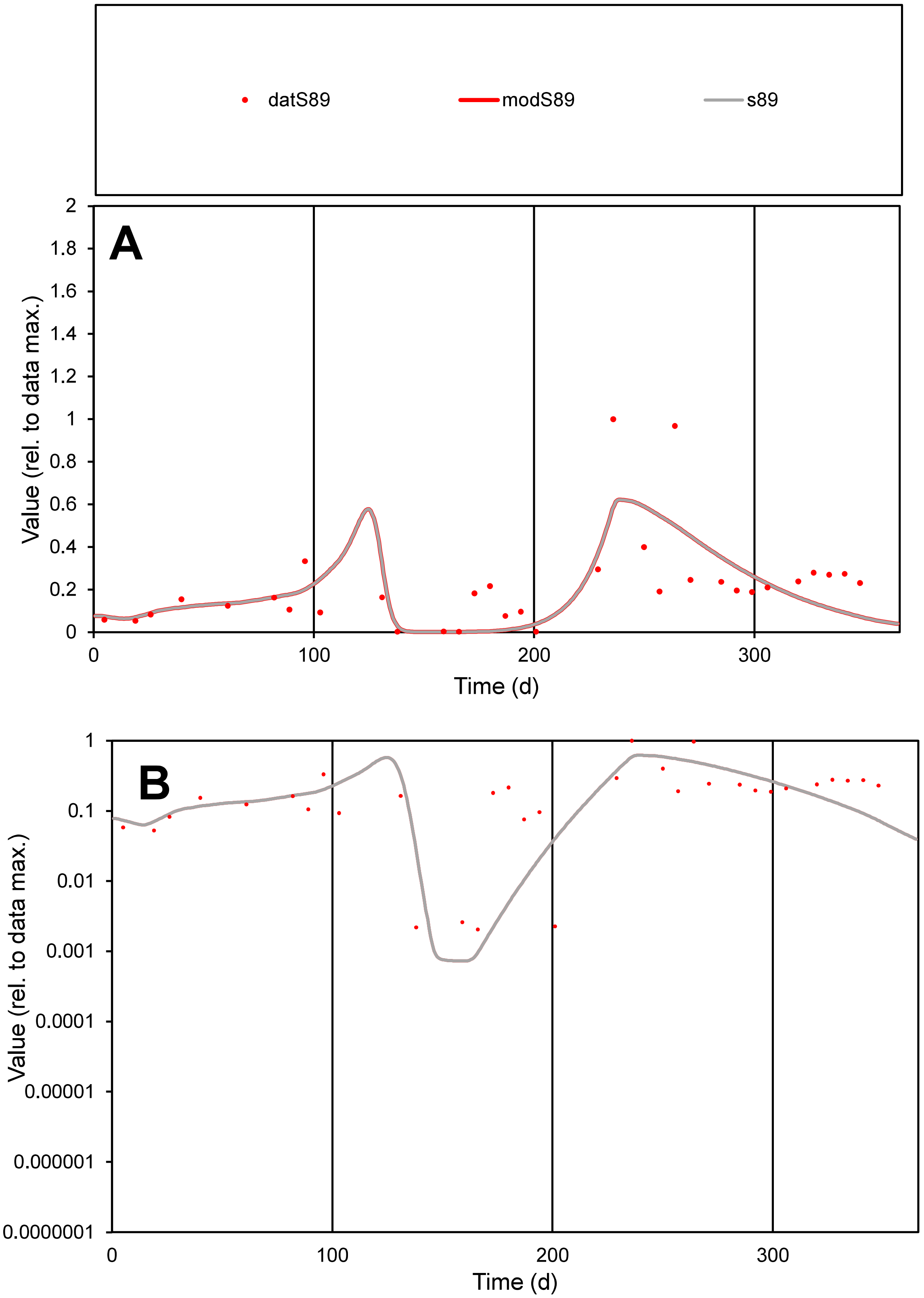

C1

Variables

S89 Provide jtd or Dst

CalibOnly?

2 1 to reproduce of

oNormalize?

2 1=no, 2=yes

ofxn calc*

0.74386

ofxn mod

0.76149

sRun

RunA1

zout

1

Ave

Data

0.00376

Model

0.00335

data/vMax

0.01741

ofxng

263.459

Min d/m

2.81039

ave/min d

105.14

ave/min m

263.397

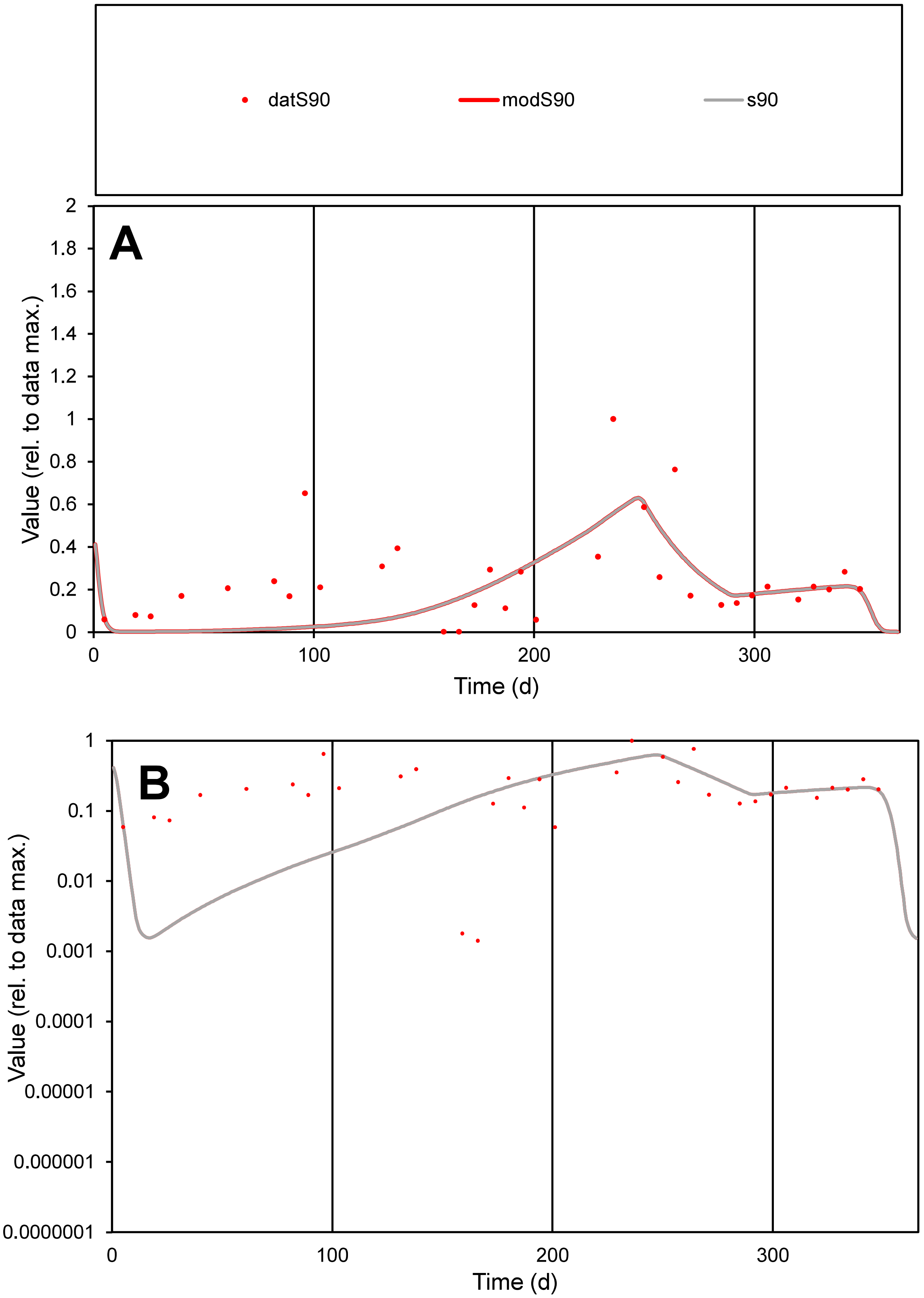

C1

Variables
S90 Provide jtd or Dst
CalibOnly?
2 1 to reproduce of
oNormalize?
2 1=no, 2=yes

ofxn calc*
0.79808
ofxn mod
0.80898

sRun
RunA1
zout
1

Ave
Data
0.0035
Model
0.00274

data/vMax
0.01395

ofxng
263.459

Min d/m
0.82914

ave/min d
177.153
ave/min m
115.023

326_Calib_S_A

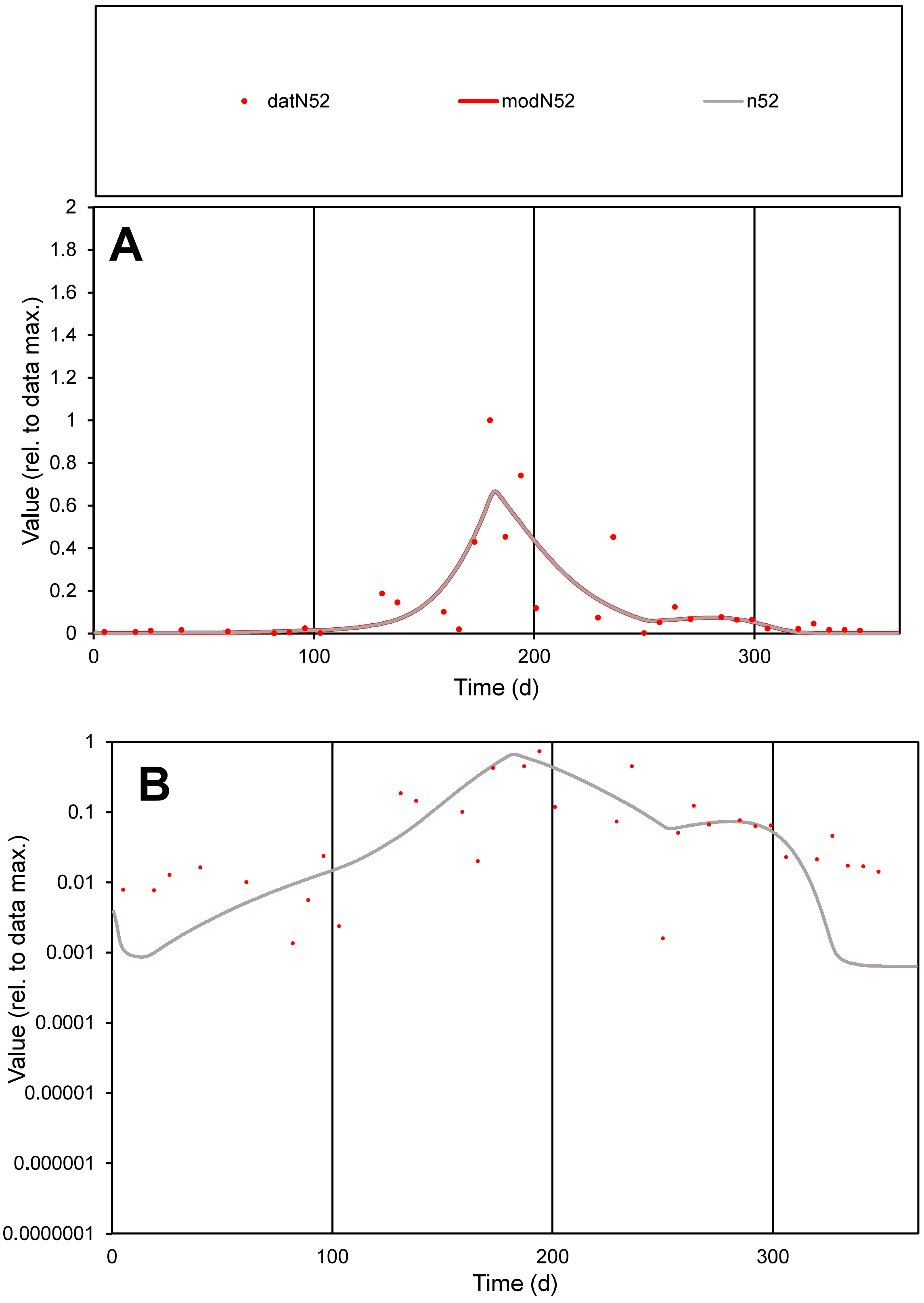

C1

Variables
N52 Provide jtd or Dst
CalibOnly?
2 1 to reproduce of
oNormalize?
2 1=no, 2=yes

ofxn calc*
0.95158
ofxn mod
0.98743

sRun
RunA1
zout
1

Ave
Data
0.00274
Model
0.00259

data/vMax
0.02059

ofxng
263.459

Min d/m
2.1306

ave/min d
97.7776
ave/min m
196.838

326_Calib_S_A

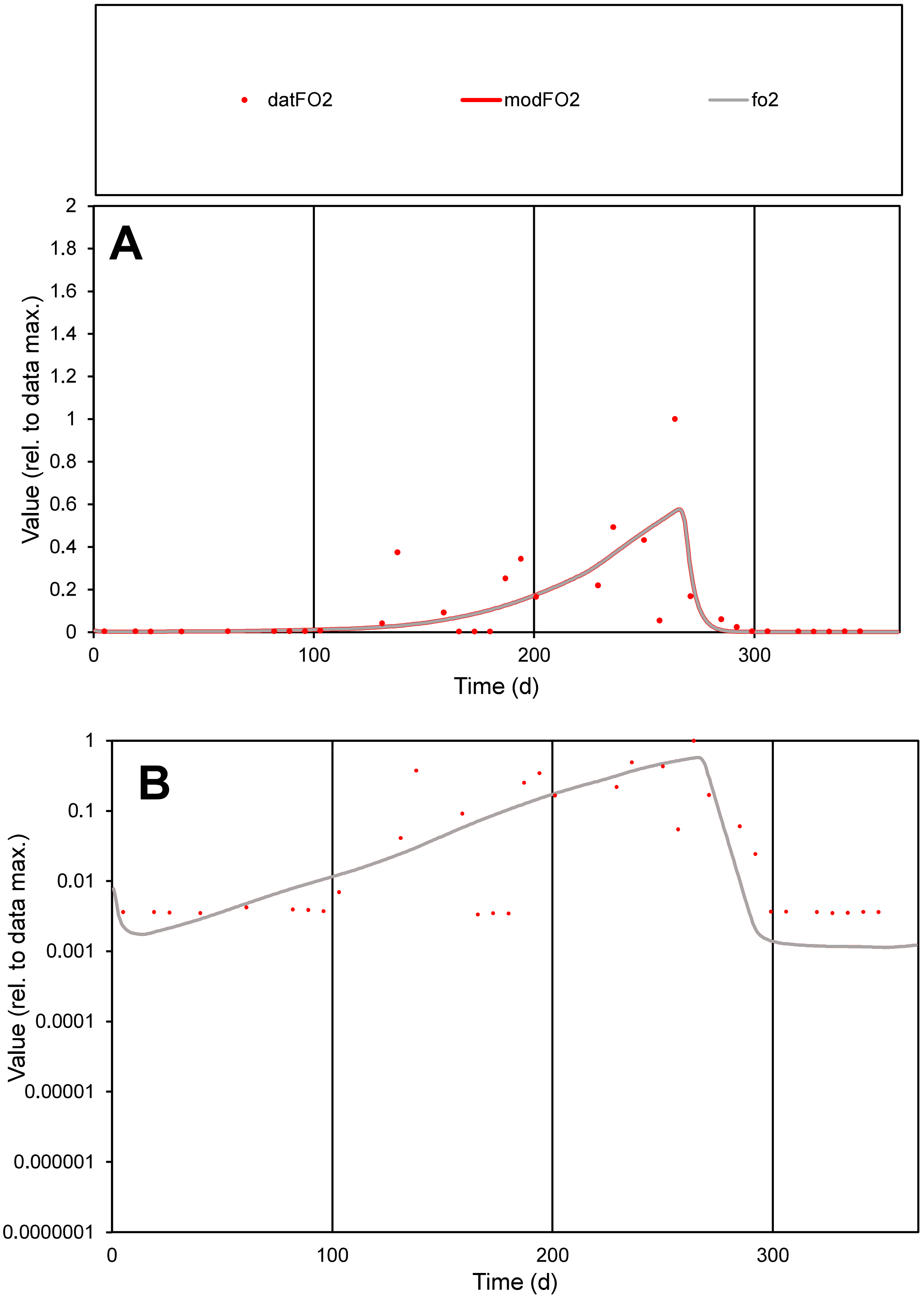

C1

Variables
FO2 Provide jtd or Dst
CalibOnly?
2 1 to reproduce of
oNormalize?
2 1=no, 2=yes

ofxn calc*
1.19417
ofxn mod
1.2197

sRun
RunA1
zout
1

Ave
Data
0.00228
Model
0.00204

data/vMax
0.01983

ofxng
263.459

Min d/m
2.94032

ave/min d
34.2963
ave/min m
90.3863

326_Calib_S_A

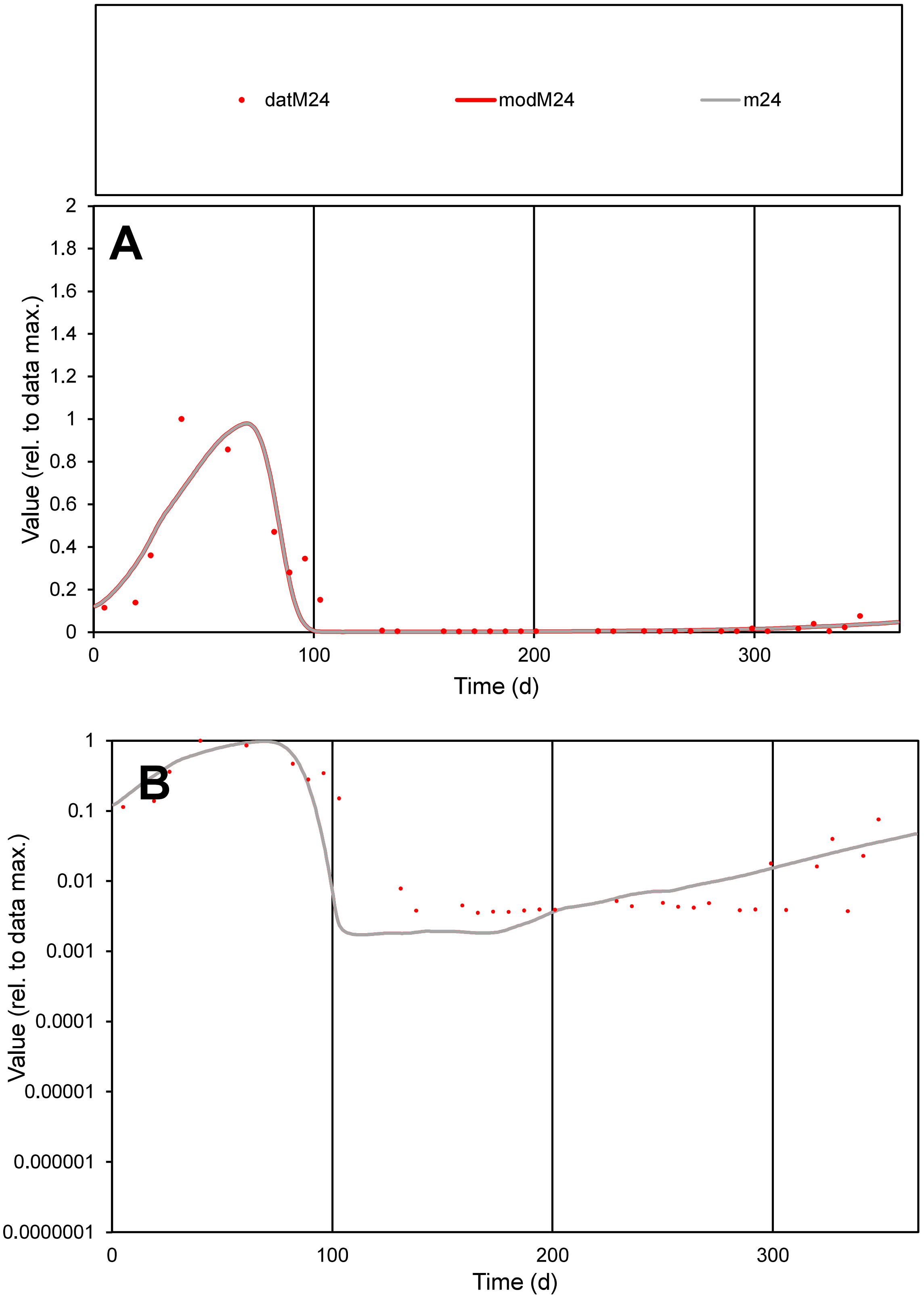

C1

Variables
M24 Provide jtd or Dst
CalibOnly?
2 1 to reproduce of
oNormalize?
2 1=no, 2=yes

ofxn calc*
0.76469
ofxn mod
0.82496

sRun
RunA1
zout
1

Ave
Data
0.00244
Model
0.00222

data/vMax
0.02023

ofxng
263.459

Min d/m
1.96782

ave/min d
34.0383
ave/min m
61.0427

326_Calib_S_A

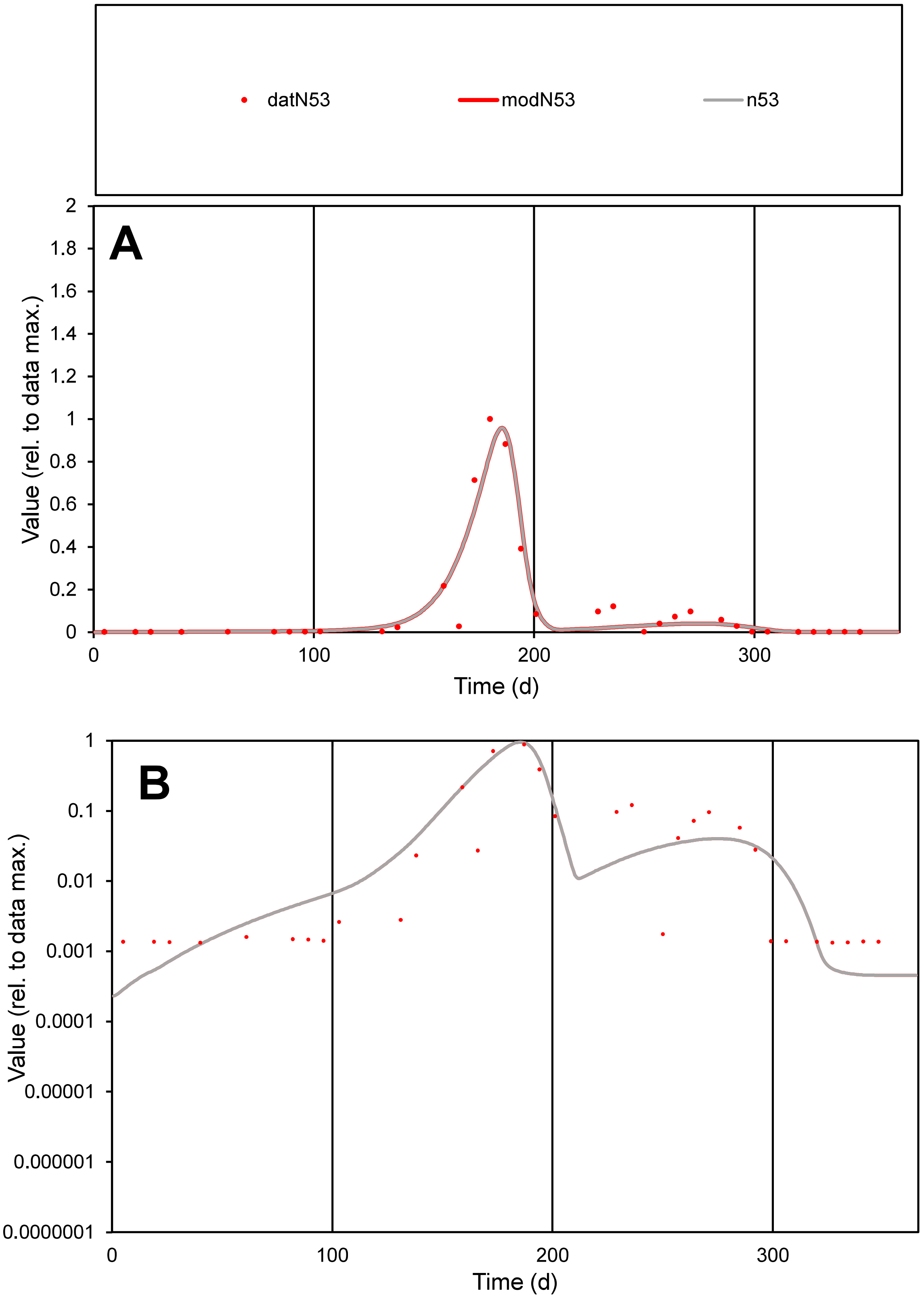

C1

Variables

N53 Provide jtd or Dst

CalibOnly?

2 1 to reproduce of

oNormalize?

2 1=no, 2=yes

ofxn calc*

0.56128

ofxn mod

0.63518

sRun

RunA1

zout

1

Ave

Data

0.00336

Model

0.00334

data/vMax

0.02856

ofxng

263.459

Min d/m

4.28377

ave/min d

88.7108

ave/min m

377.46

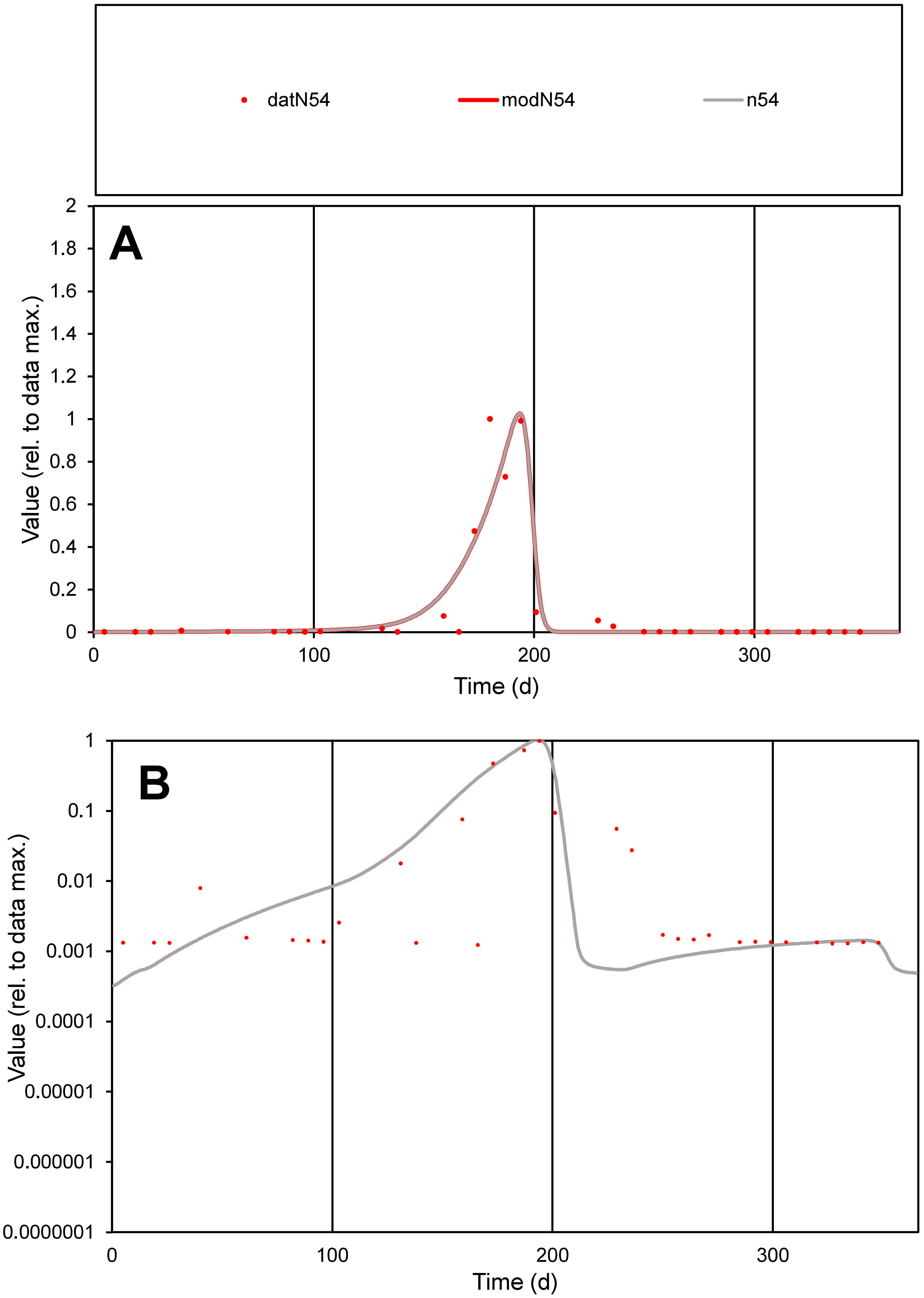

C1

Variables
N54 Provide jtd or Dst
CalibOnly?
2 1 to reproduce of
oNormalize?
2 1=no, 2=yes

ofxn calc*
0.80368
ofxn mod
0.89423

sRun
RunA1
zout
1

Ave
Data
0.00185
Model
0.00192

data/vMax
0.01738

ofxng
263.459

Min d/m
2.91446

ave/min d
86.1382
ave/min m
260.794

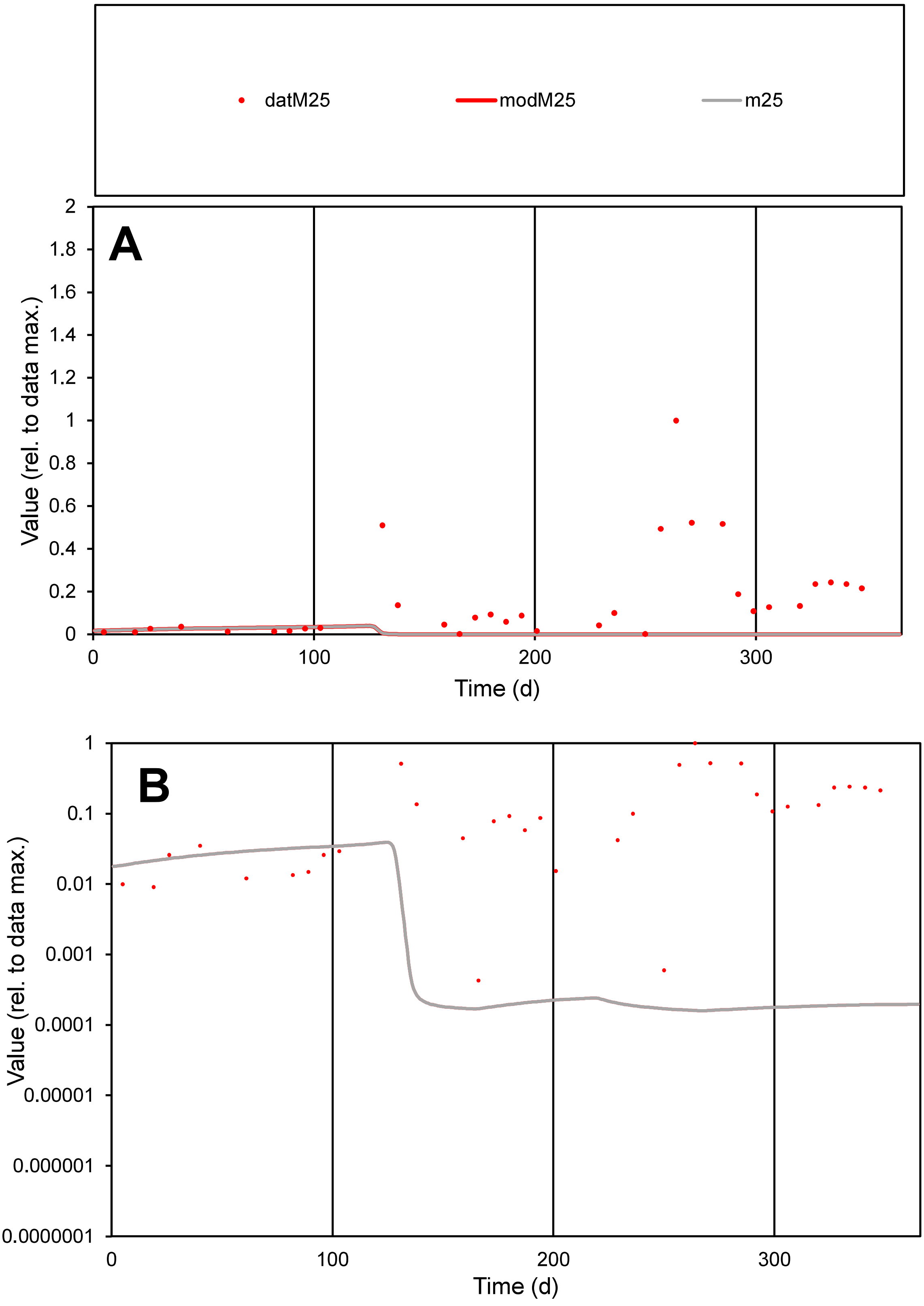

C1

Variables
M25 Provide jtd or Dst
CalibOnly?
2 1 to reproduce of
oNormalize?
2 1=no, 2=yes

ofxn calc*
1.65992
ofxn mod
1.66364

sRun
RunA1
zout
1

Ave
Data
0.00359
Model
0.00018

data/vMax
0.02217

ofxng
263.459

Min d/m
2.6866

ave/min d
377.411
ave/min m
49.7337

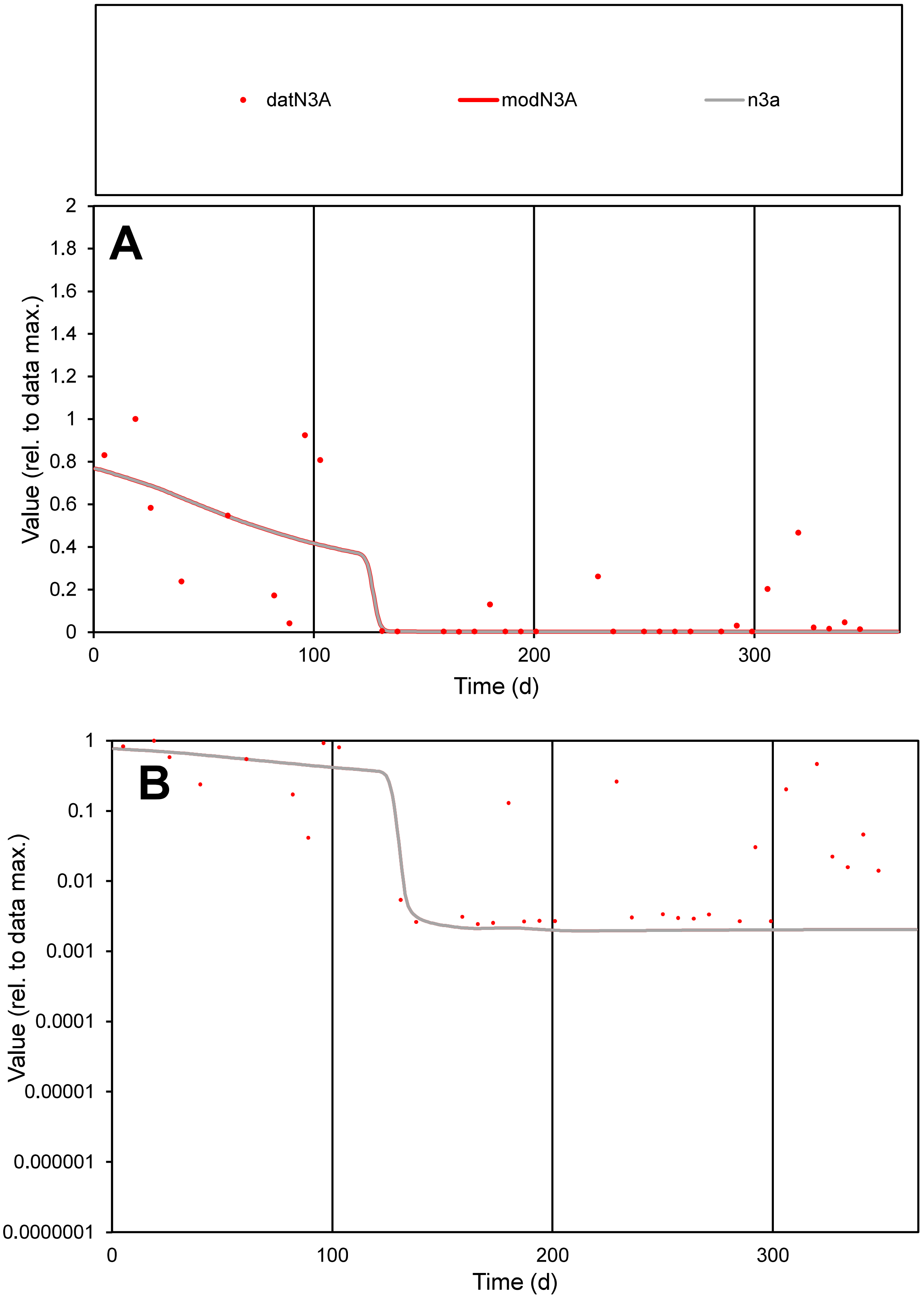

C1

Variables

N3A	Provide jtd or Dst
CalibOnly?	
2	1 to reproduce of
oNormalize?	
2	1=no, 2=yes
ofxn calc*	
1.00831	
ofxn mod	
1.02678	
sRun	
RunA1	
zout	
1	
Ave Data	
0.0014	
Model	
0.00112	
data/vMax	
0.00725	
ofxng	
263.459	
Min d/m	
1.24073	
ave/min d	
78.9915	
ave/min m	
78.3452	

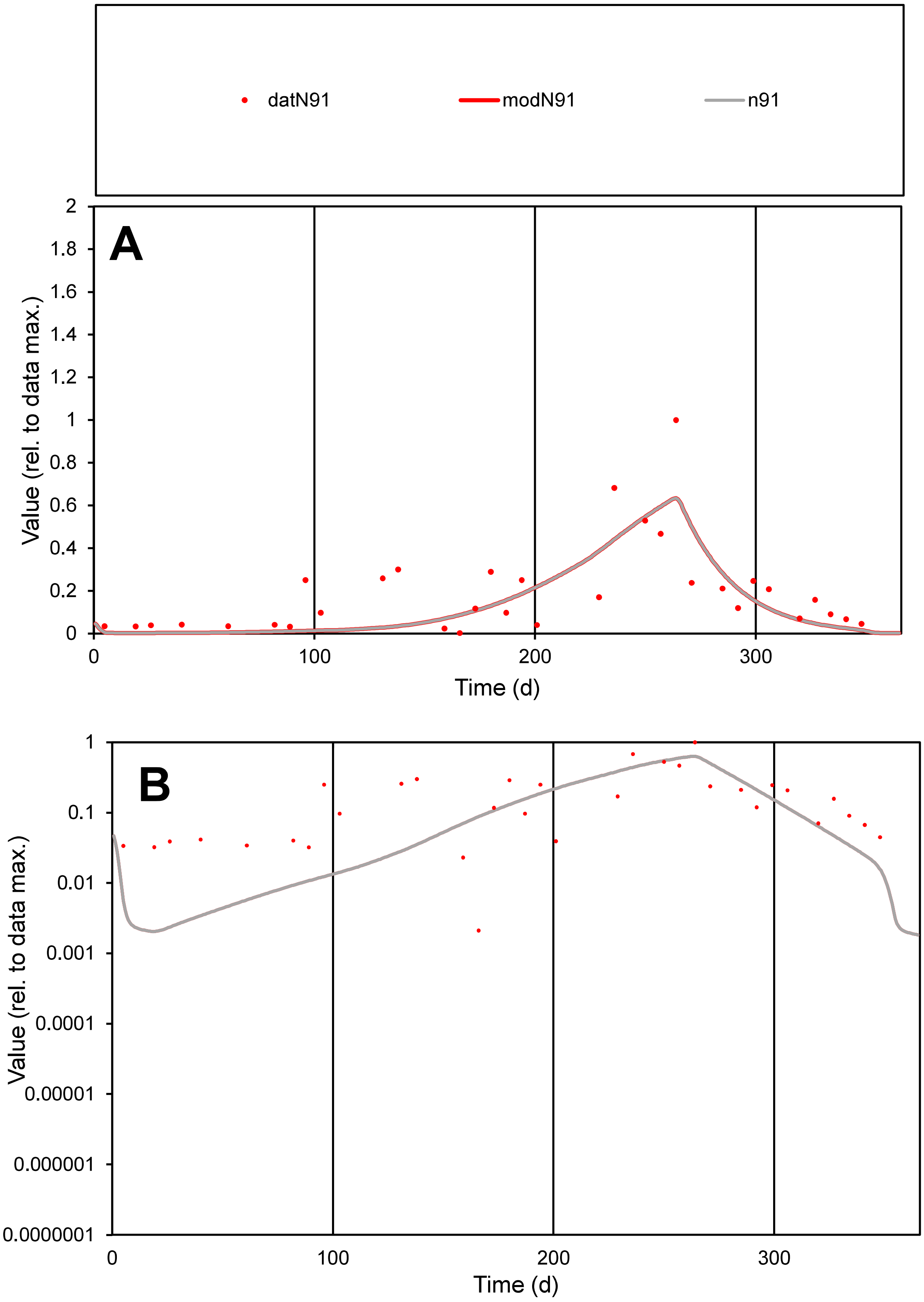

C1

Variables

N91 Provide jtd or Dst

CalibOnly?

2 1 to reproduce of

oNormalize?

2 1=no, 2=yes

ofxn calc*

0.72106

ofxn mod

0.74365

sRun

RunA1

zout

1

Ave

Data

0.00281

Model

0.00231

data/vMax

0.01478

ofxng

263.459

Min d/m

1.02457

ave/min d

89.8021

ave/min m

75.532

326_Calib_S_A

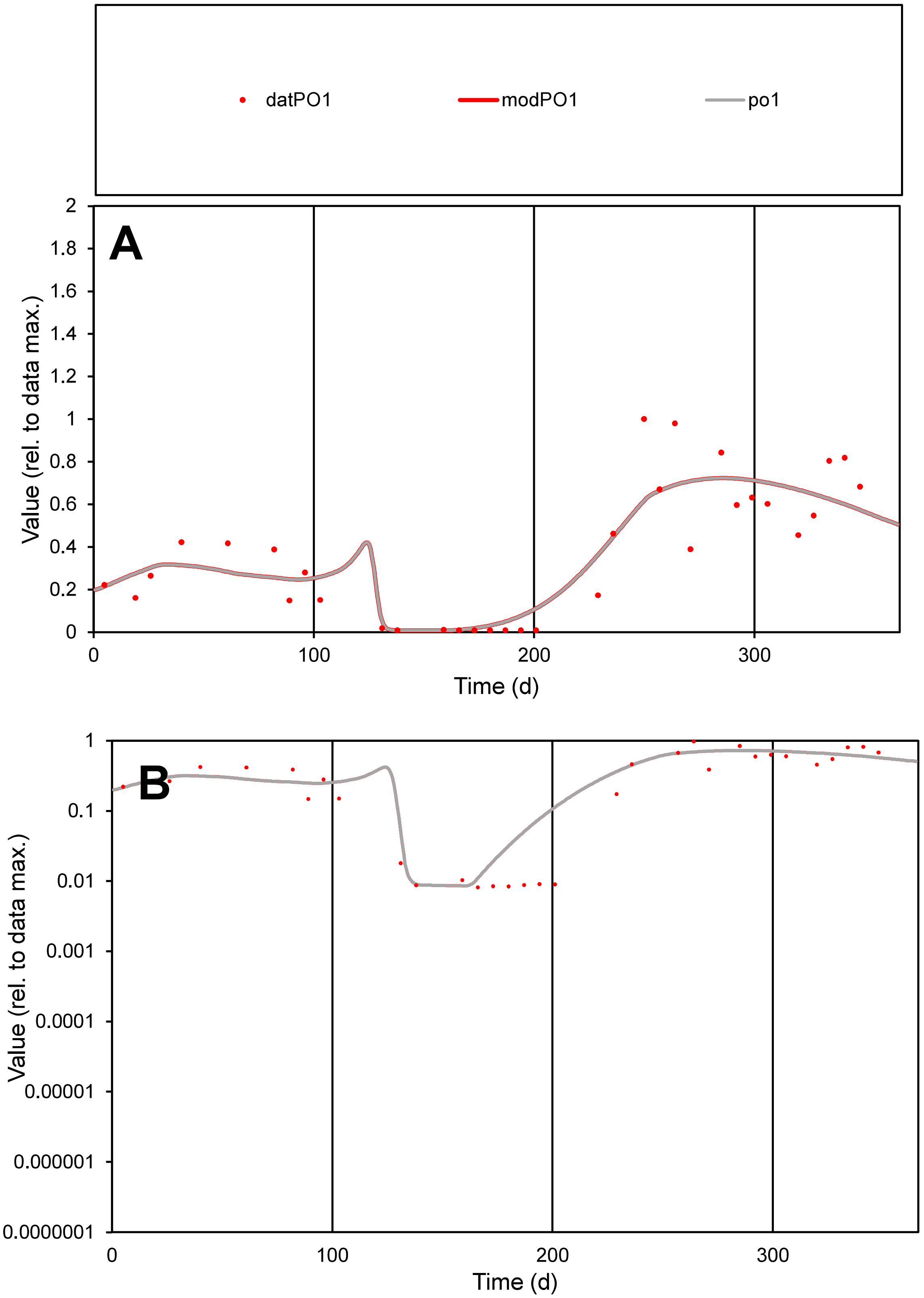

C1

Variables
PO1 Provide jtd or Dst
CalibOnly?
2 1 to reproduce of
oNormalize?
2 1=no, 2=yes

ofxn calc*
0.38135
ofxn mod
0.40954

sRun
RunA1
zout
1

Ave
Data
0.00205
Model
0.00206

data/vMax
0.00555

ofxng
263.459

Min d/m
0.94774

ave/min d
45.406
ave/min m
43.2

326_Calib_S_A

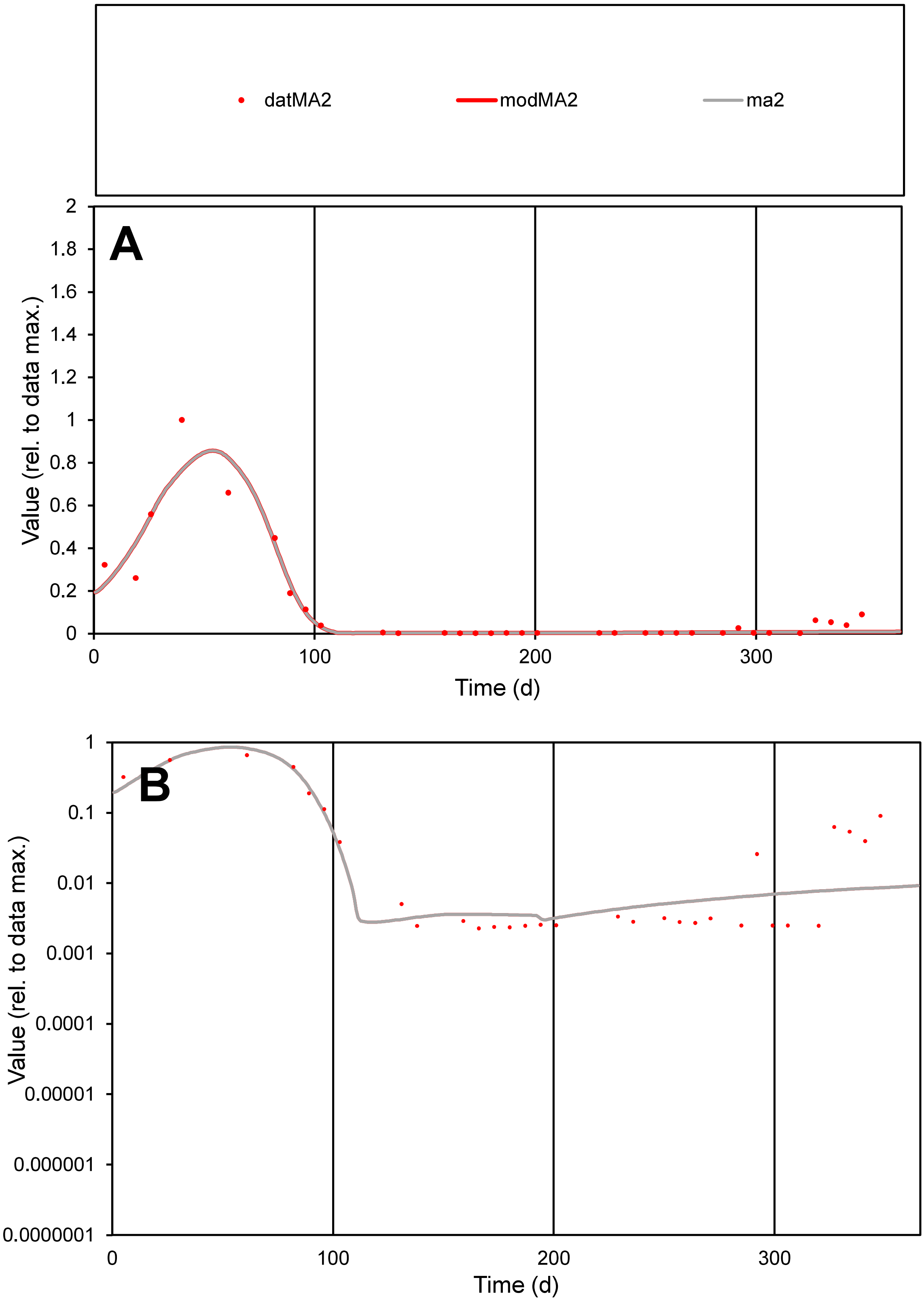

C1

Variables
MA2 Provide jtd or Dst
CalibOnly?
2 1 to reproduce of
oNormalize?
2 1=no, 2=yes

ofxn calc*
0.51906
ofxn mod
0.59663

sRun
RunA1
zout
1

Ave
Data
0.00092
Model
0.00086

data/vMax
0.00772

ofxng
263.459

Min d/m
0.75772

ave/min d
51.8078
ave/min m
37.0843

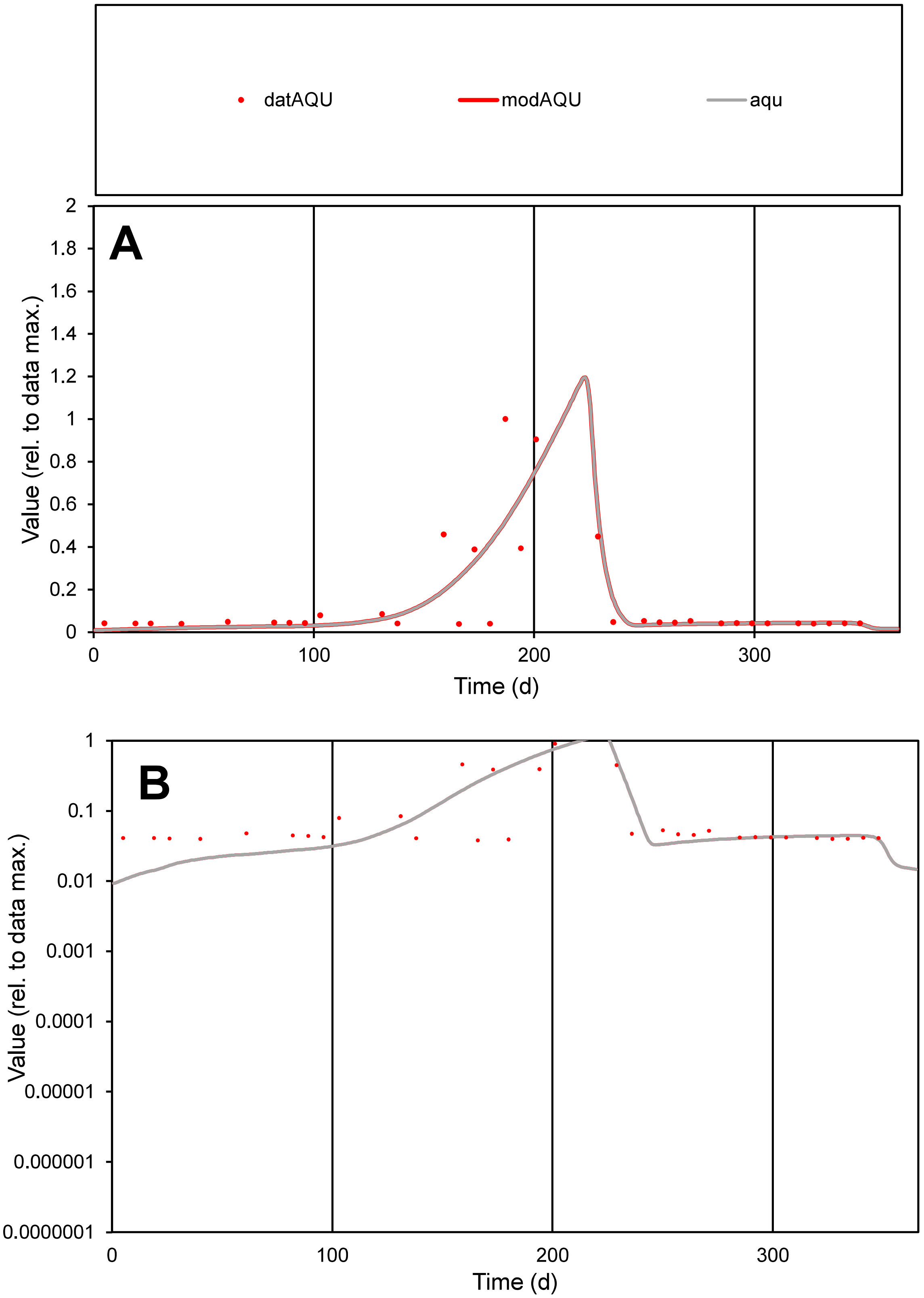

C1

Variables
AQU Provide jtd or Dst
CalibOnly?
2 1 to reproduce of
oNormalize?
2 1=no, 2=yes

ofxn calc*
0.88578
ofxn mod
0.91967

sRun
RunA1
zout
1

Ave
Data
0.0005
Model
0.00047

data/vMax
0.00339

ofxng
263.459

Min d/m
3.50748

ave/min d
3.84844
ave/min m
12.8479

326_Calib_S_A

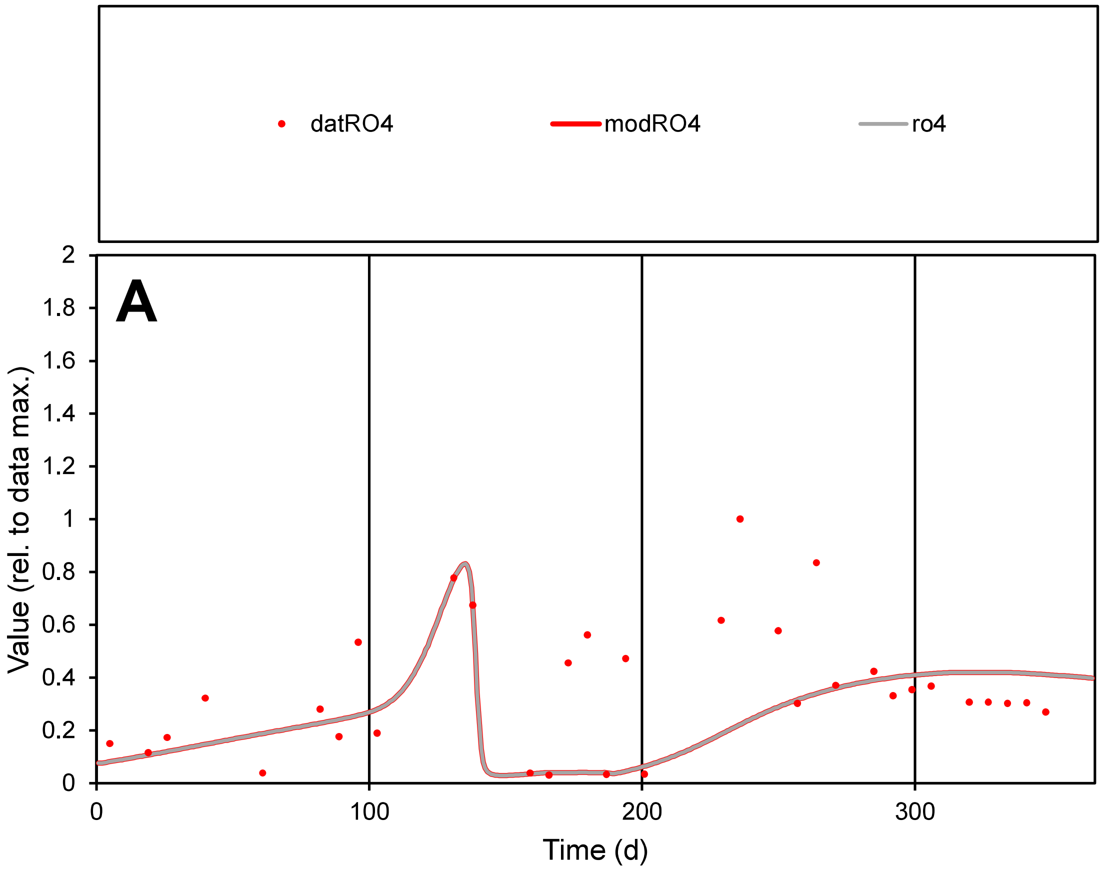

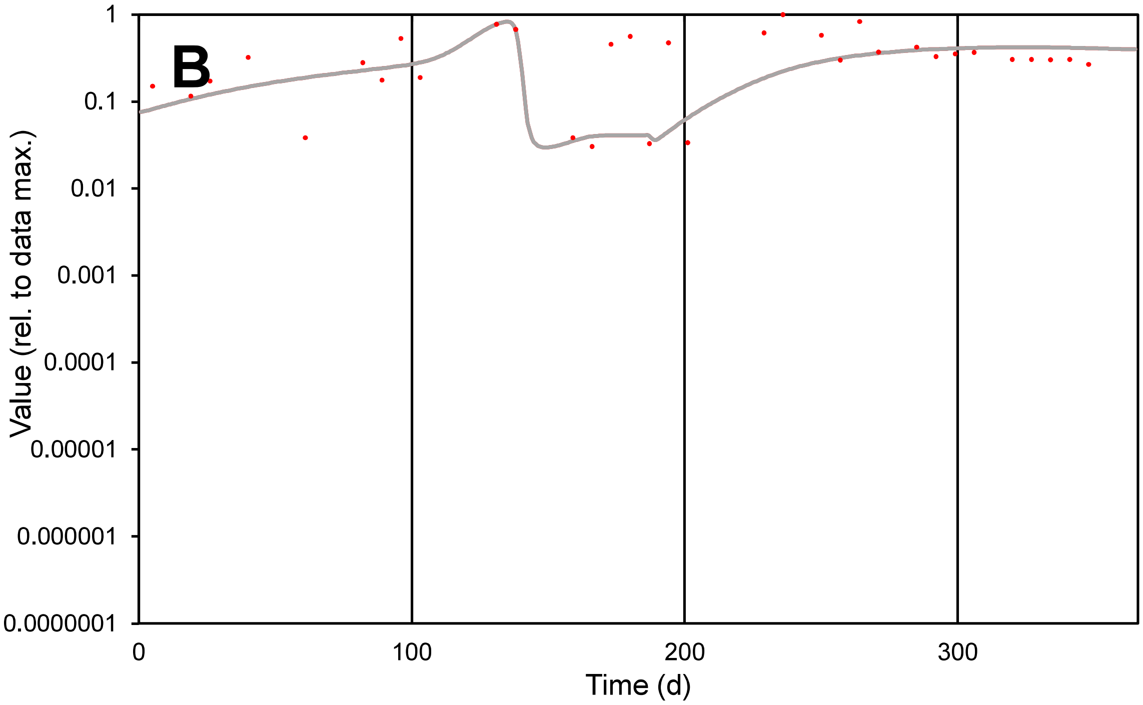

C1

Variables

RO4 Provide jtd or Dst

CalibOnly?

2 1 to reproduce of

oNormalize?

2 1=no, 2=yes

ofxn calc*

0.68484

ofxn mod

0.69753

sRun

RunA1

zout

1

Ave

Data

0.00252

Model

0.00191

data/vMax

0.00709

ofxng

263.459

Min d/m

0.84531

ave/min d

11.7018

ave/min m

7.49713

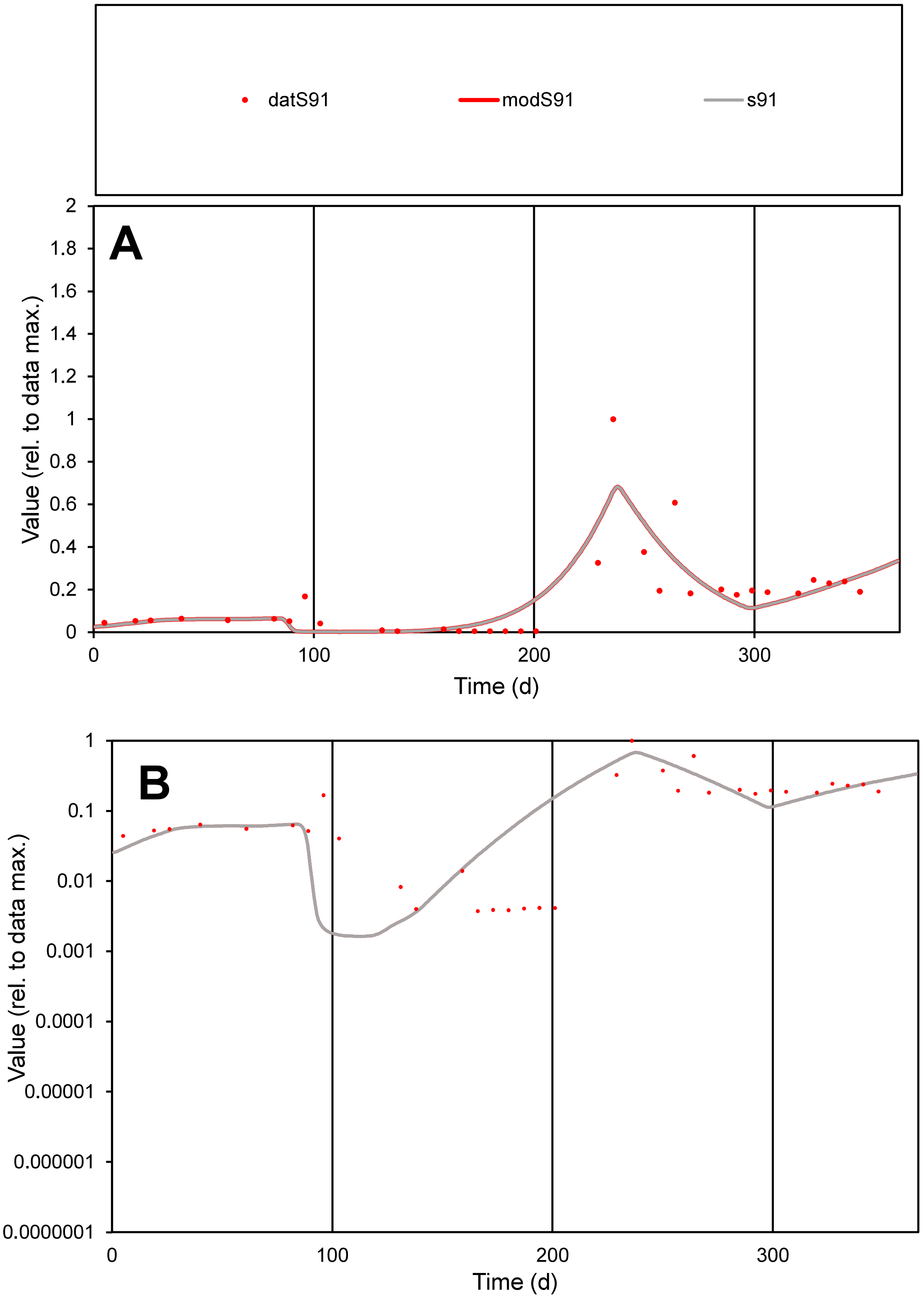

C1

Variables
S91 Provide jtd or Dst
CalibOnly?
2 1 to reproduce of
oNormalize?
2 1=no, 2=yes

ofxn calc*
0.67507
ofxn mod
0.70505

sRun
RunA1
zout
1

Ave
Data
0.00272
Model
0.00271

data/vMax
0.01735

ofxng
263.459

Min d/m
2.20197

ave/min d
41.9075
ave/min m
91.8236

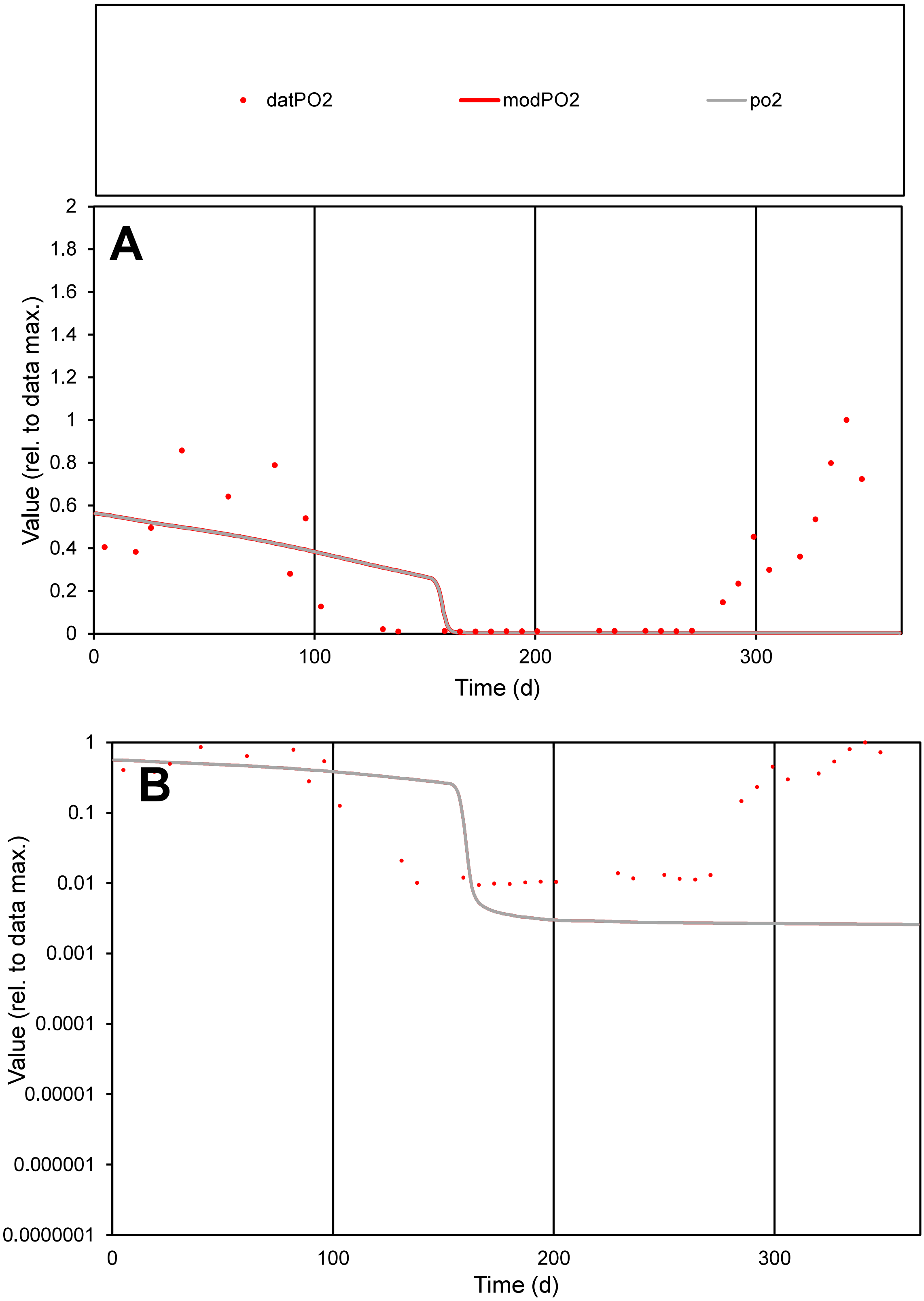

C1

Variables
PO2 Provide jtd or Dst
CalibOnly?
2 1 to reproduce of
oNormalize?
2 1=no, 2=yes

ofxn calc*
1.16718
ofxn mod
1.17441

sRun
RunA1
zout
1

Ave
Data
0.00233
Model
0.00122

data/vMax
0.00833

ofxng
263.459

Min d/m
3.61868

ave/min d
29.651
ave/min m
56.0405

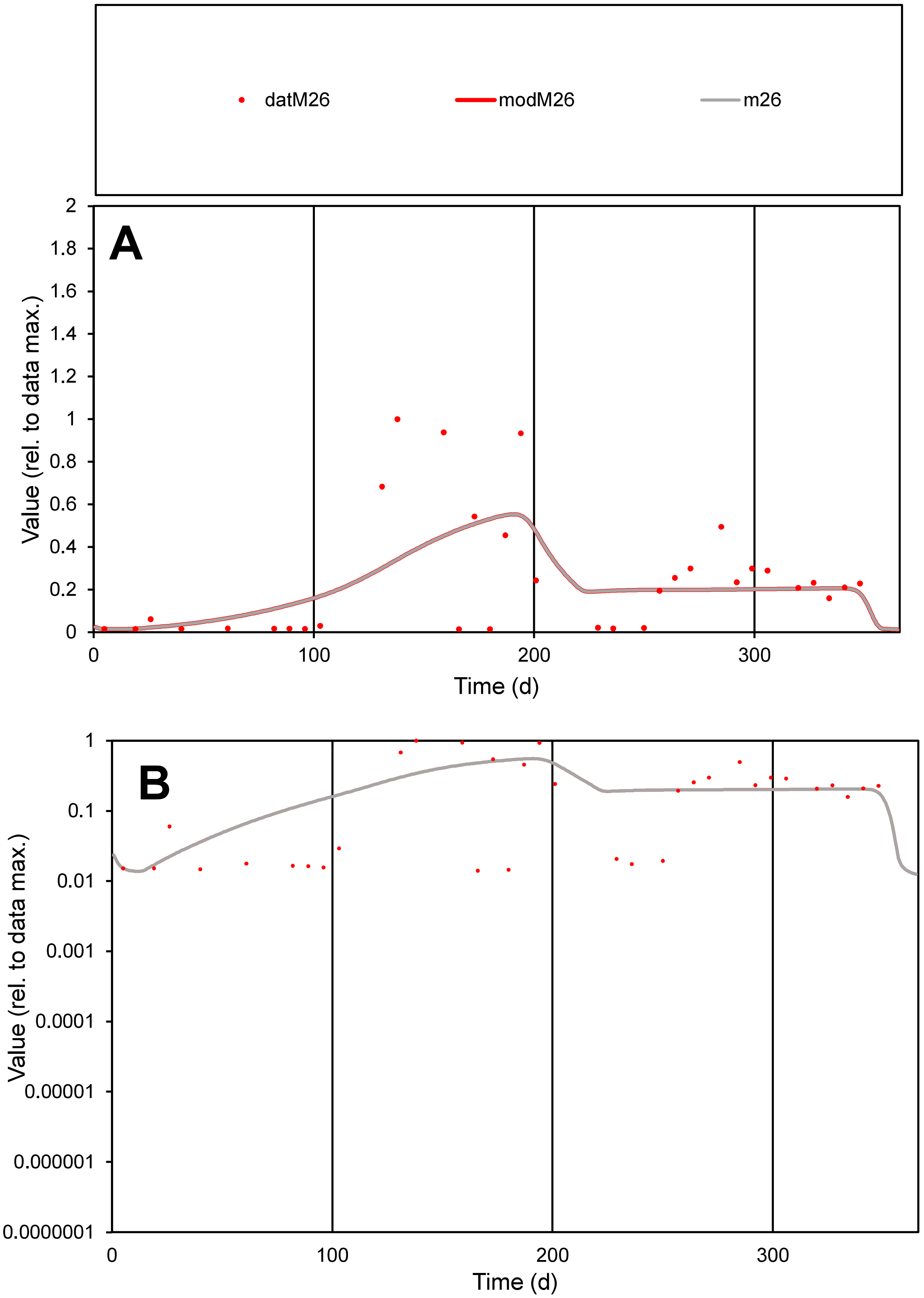

C1

Variables
M26 Provide jtd or Dst
CalibOnly?
2 1 to reproduce of
oNormalize?
2 1=no, 2=yes

ofxn calc*
0.93454
ofxn mod
0.9473

sRun
RunA1
zout
1

Ave
Data
0.00143
Model
0.00137

data/vMax
0.00575

ofxng
263.459

Min d/m
0.96496

ave/min d
17.6005
ave/min m
16.3749

326_Calib_S_A

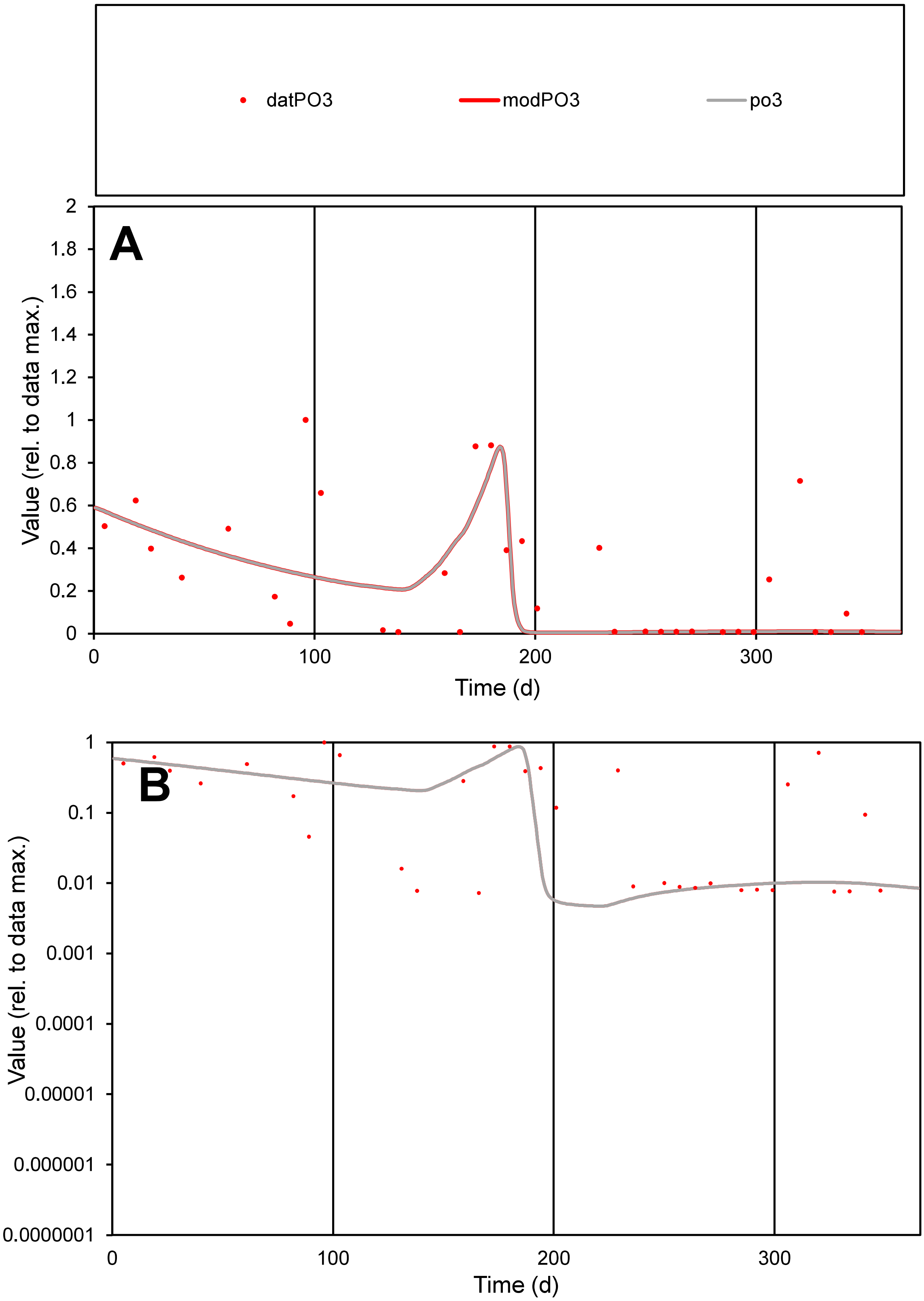

C1

Variables
PO3 Provide jtd or Dst
CalibOnly?
2 1 to reproduce of
oNormalize?
2 1=no, 2=yes

ofxn calc*
0.91456
ofxn mod
0.96268

sRun
RunA1
zout
1

Ave
Data
0.00168
Model
0.00135

data/vMax
0.00635

ofxng
263.459

Min d/m
1.34394

ave/min d
36.454
ave/min m
39.547

326_Calib_S_A

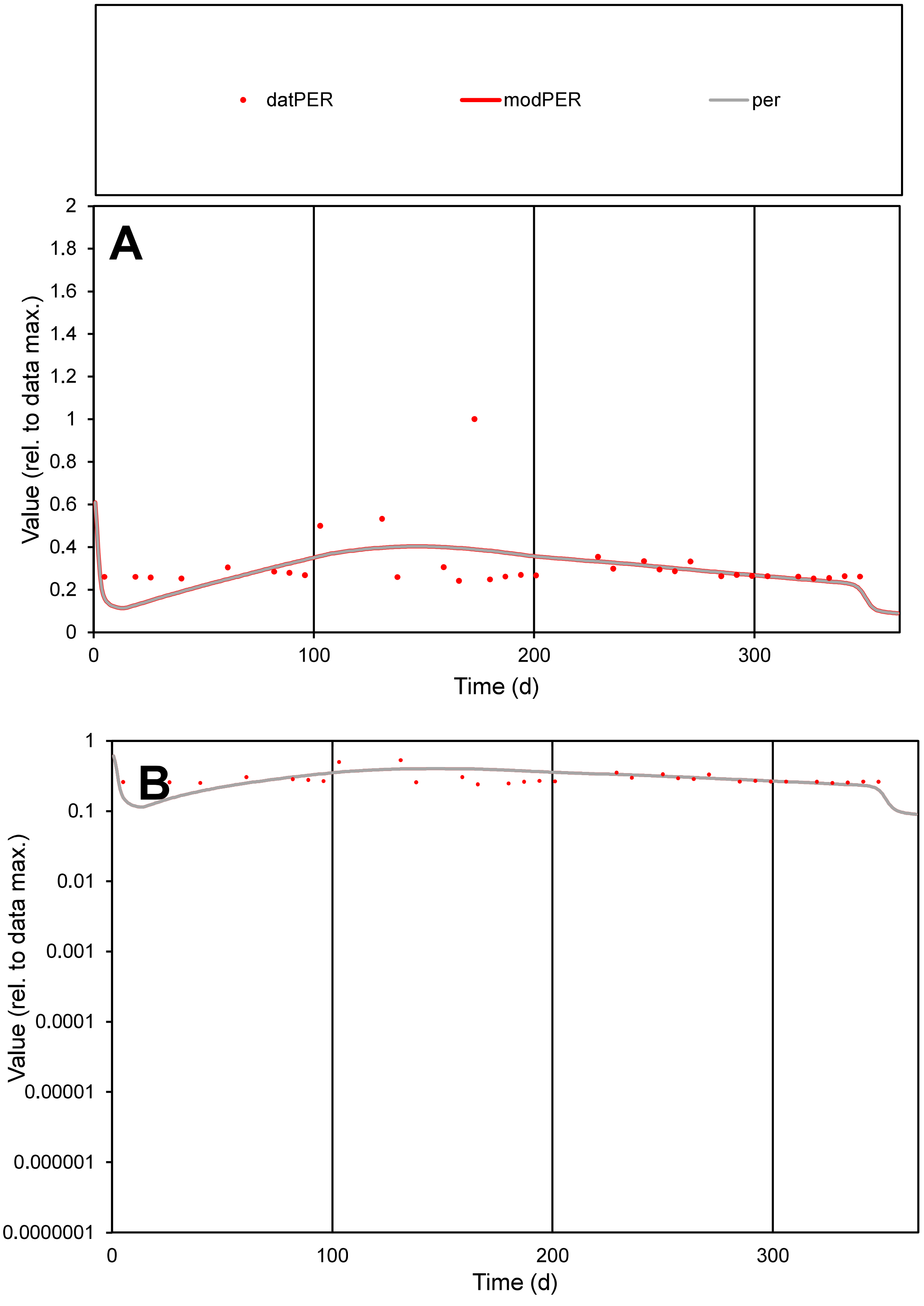

C1

Variables
PER Provide jtd or Dst
CalibOnly?
2 1 to reproduce of
oNormalize?
2 1=no, 2=yes

ofxn calc*
0.42274
ofxn mod
0.4385

sRun
RunA1
zout
1

Ave
Data
2.3E-05
Model
2.2E-05

data/vMax
7.5E-05

ofxng
263.459

Min d/m
1.8223

ave/min d
1.29545
ave/min m
2.24464

326_Calib_S_A

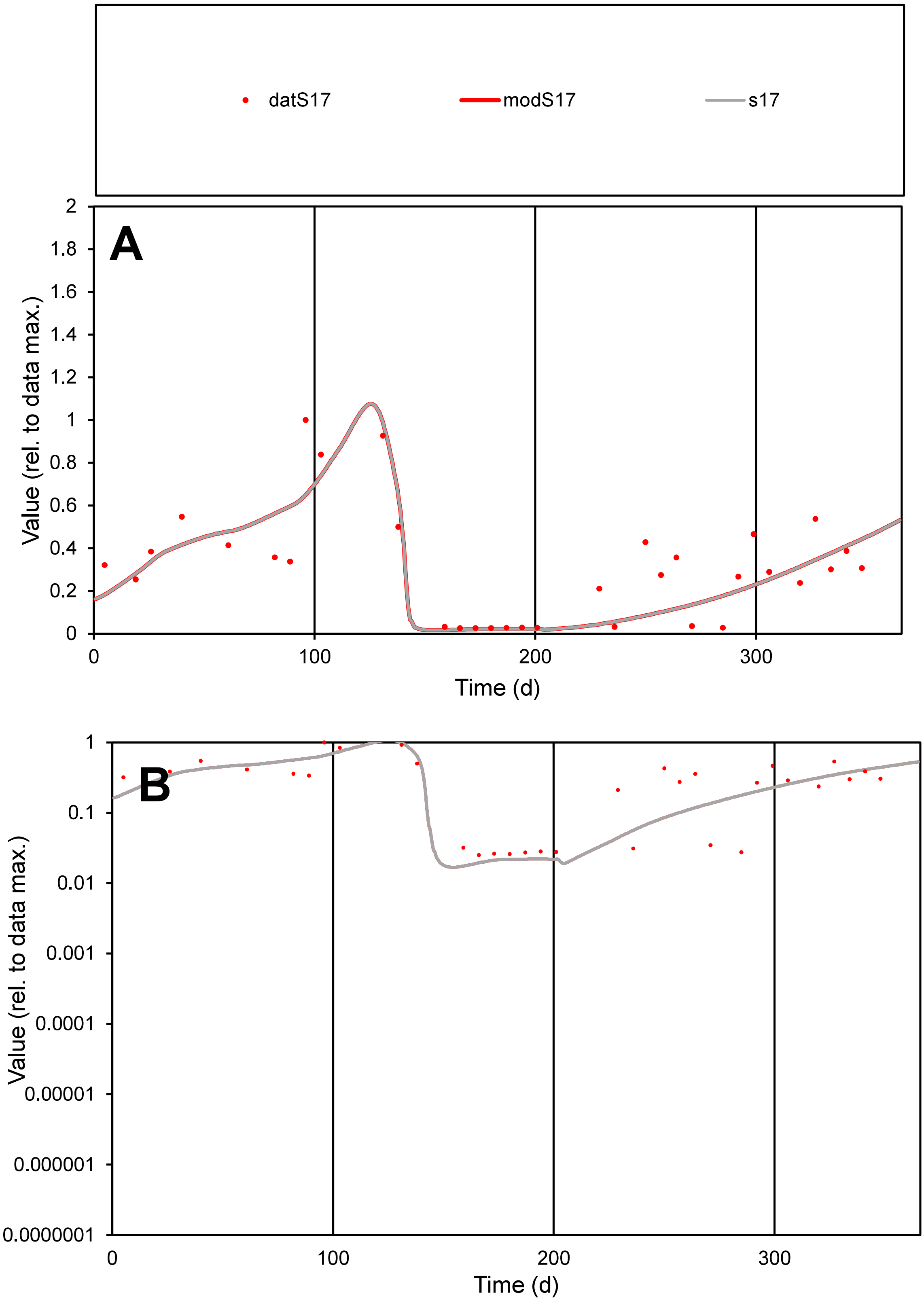

C1

Variables

S17	Provide jtd or Dst

CalibOnly?

2	1 to reproduce of

oNormalize?

2	1=no, 2=yes

ofxn calc*
0.45707
ofxn mod
0.4763

sRun
RunA1
zout
1

Ave
Data
0.00132
Model
0.0012

data/vMax
0.00428

ofxng
263.459

Min d/m
1.42234

ave/min d
12.3095
ave/min m
15.8857

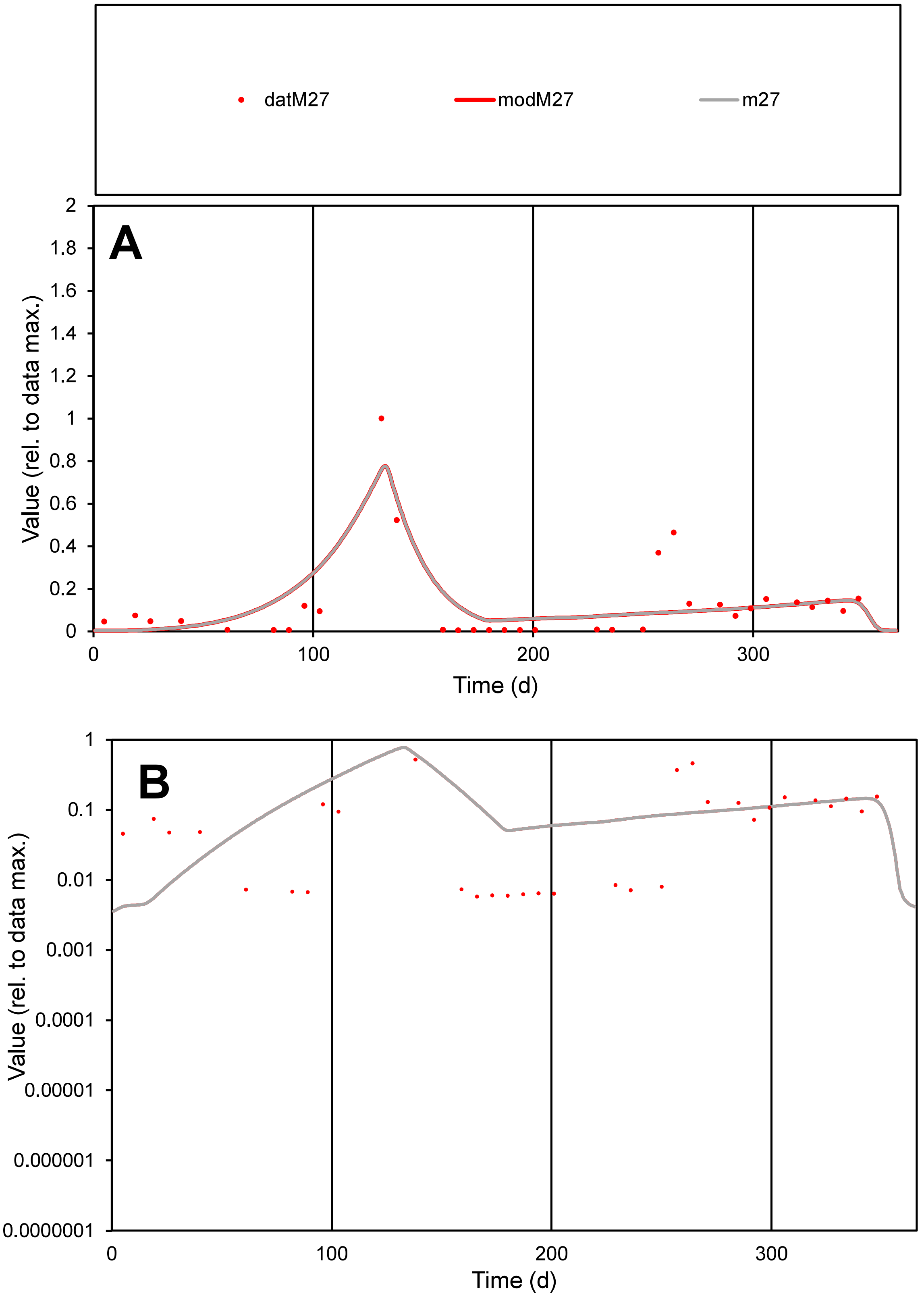

C1

Variables
M27 Provide jtd or Dst
CalibOnly?
2 1 to reproduce of
oNormalize?
2 1=no, 2=yes

ofxn calc*
0.94377
ofxn mod
0.97632

sRun
RunA1
zout
1

Ave
Data
0.00138
Model
0.00151

data/vMax
0.01111

ofxng
263.459

Min d/m
1.34999

ave/min d
21.4996
ave/min m
31.6239

326_Calib_S_A

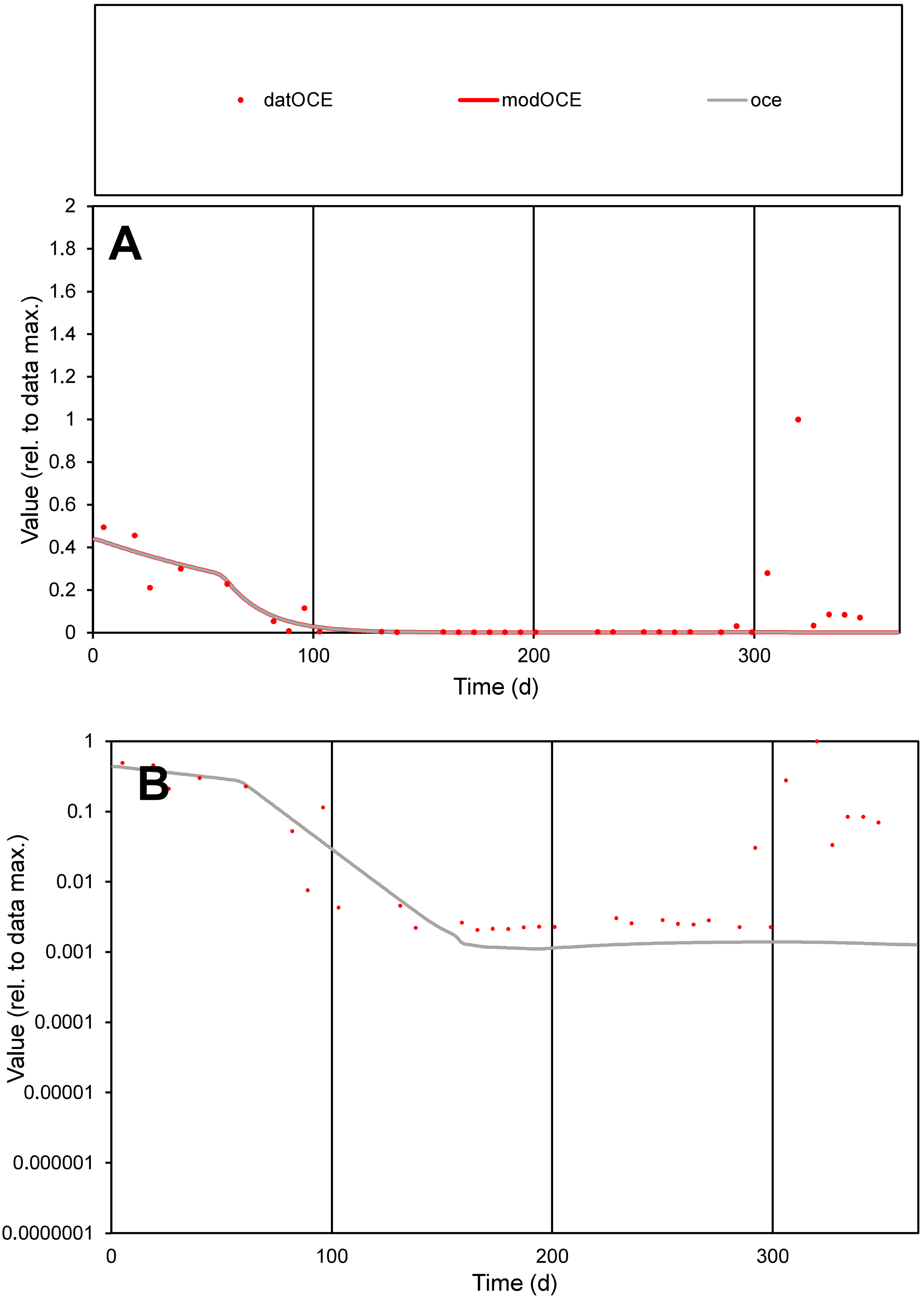

C1

Variables
OCE Provide jtd or Dst
CalibOnly?
2 1 to reproduce of
oNormalize?
2 1=no, 2=yes

ofxn calc*
1.75351
ofxn mod
1.76388

sRun
RunA1
zout
1

Ave
Data
0.00188
Model
0.00103

data/vMax
0.01779

ofxng
263.459

Min d/m
1.85693

ave/min d
51.2312
ave/min m
51.8892

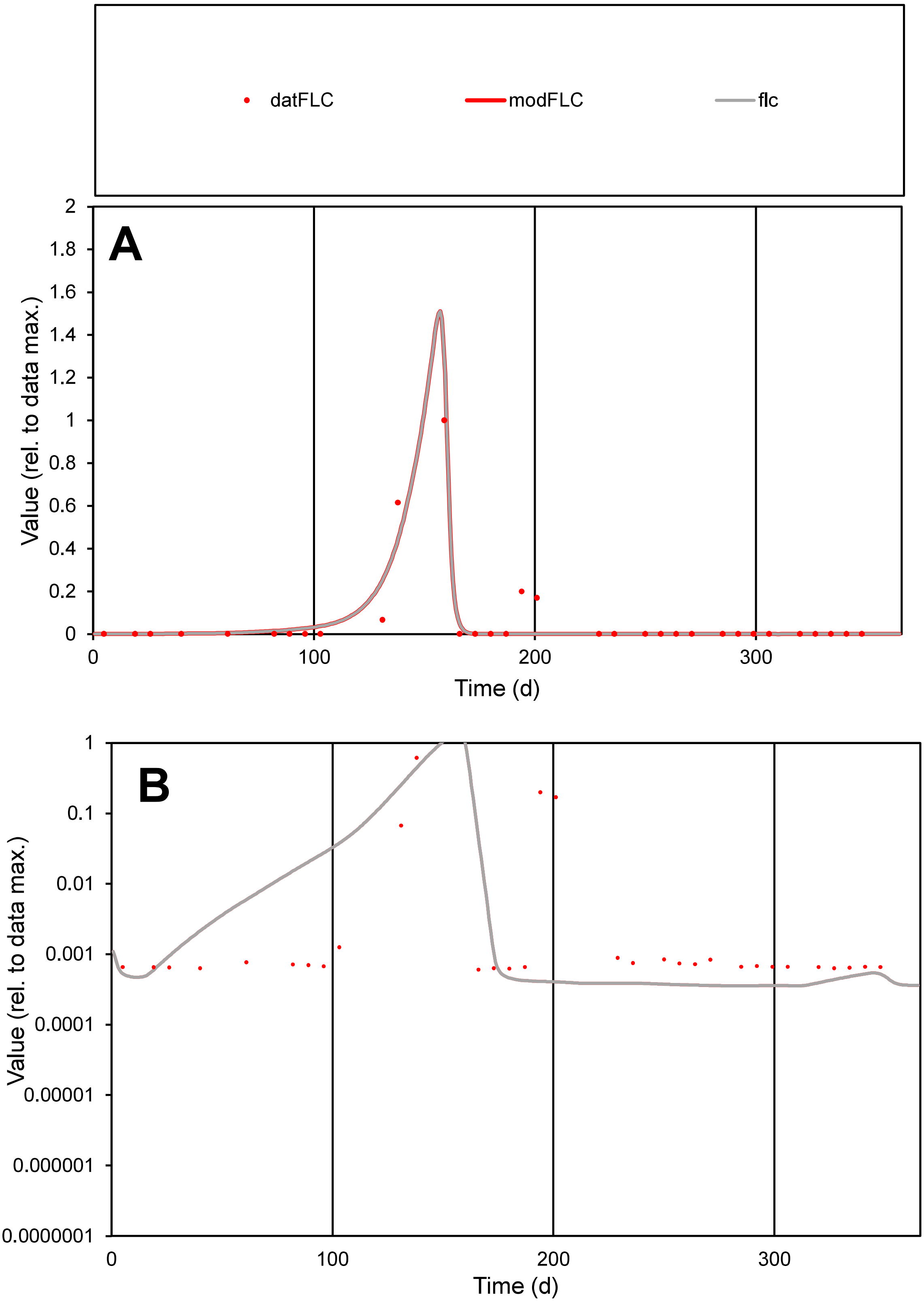

C1

Variables
FLC Provide jtd or Dst
CalibOnly?
2 1 to reproduce of
oNormalize?
2 1=no, 2=yes

ofxn calc*
0.92961
ofxn mod
0.99508

sRun
RunA1
zout
1

Ave
Data
0.00123
Model
0.00111

data/vMax
0.0196

ofxng
263.459

Min d/m
1.69973

ave/min d
103.339
ave/min m
158.389

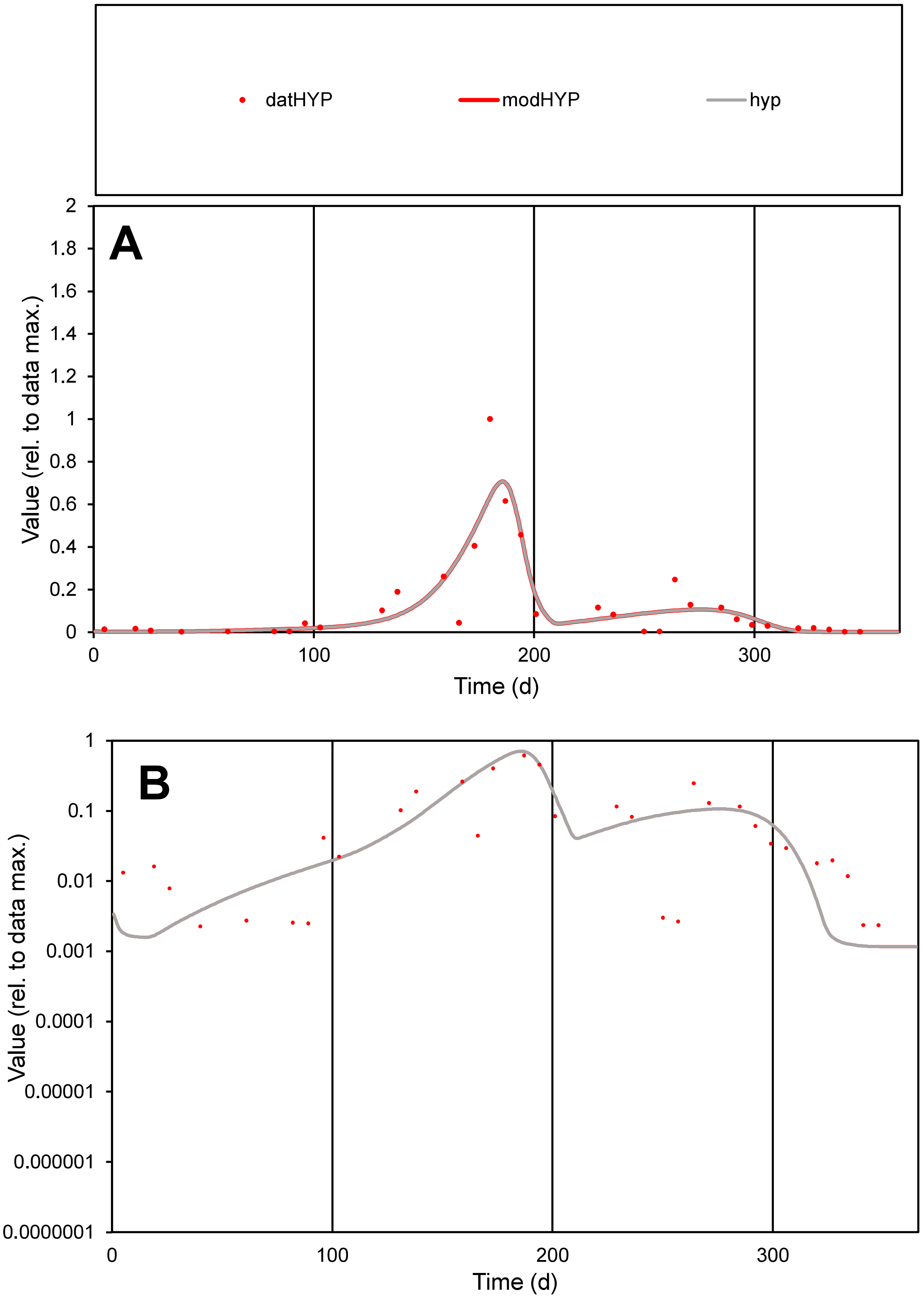

C1

Variables
HYP Provide jtd or Dst
CalibOnly?
2 1 to reproduce of
oNormalize?
2 1=no, 2=yes

ofxn calc*
0.73387
ofxn mod
0.779

sRun
RunA1
zout
1

Ave
Data
0.00091
Model
0.0009

data/vMax
0.00725

ofxng
263.459

Min d/m
1.94747

ave/min d
55.2951
ave/min m
106.749

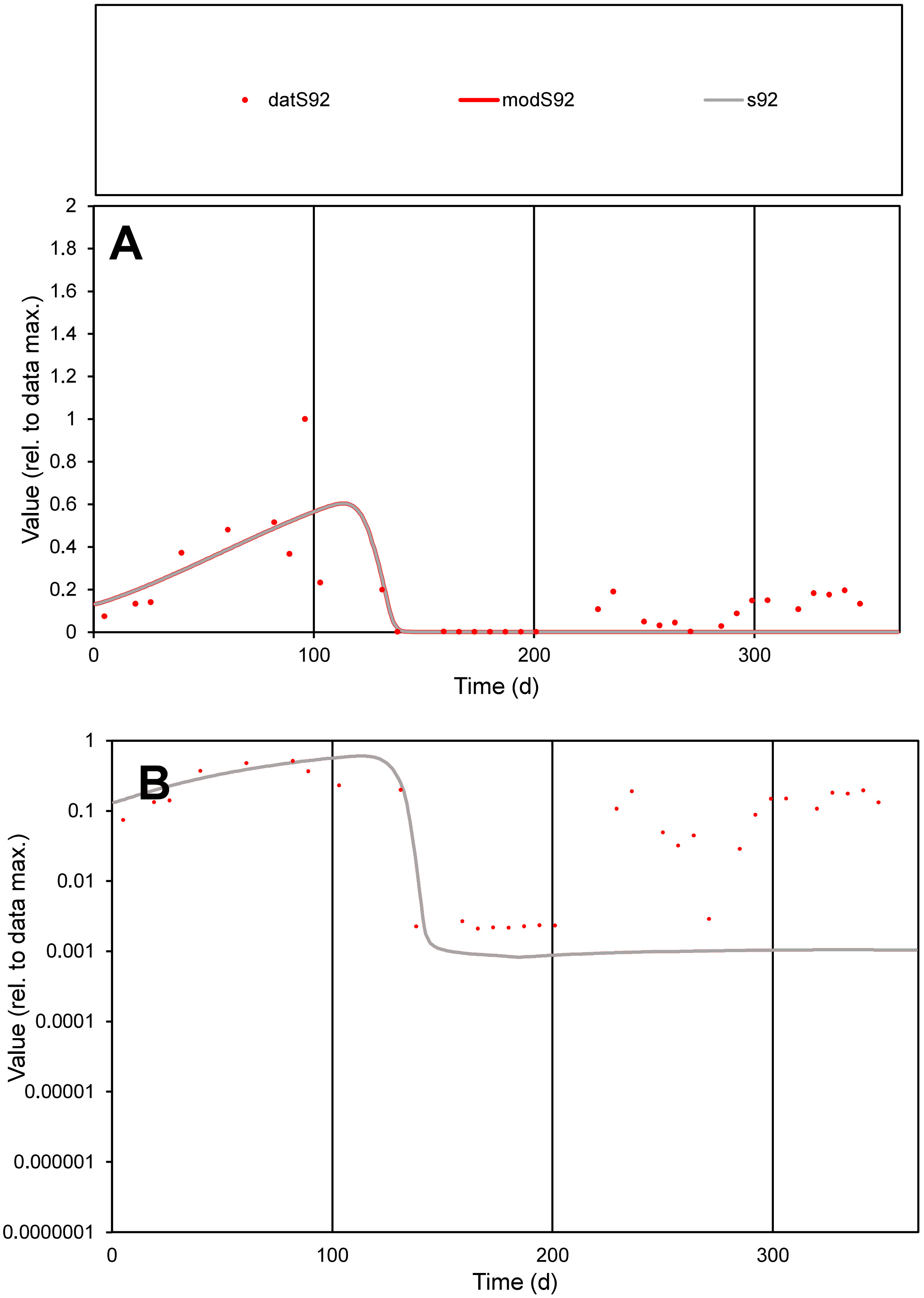

C1

Variables
S92 Provide jtd or Dst
CalibOnly?
2 1 to reproduce of
oNormalize?
2 1=no, 2=yes

ofxn calc*
0.86727
ofxn mod
0.8815

sRun
RunA1
zout
1

Ave
Data
0.00171
Model
0.0012

data/vMax
0.01092

ofxng
263.459

Min d/m
2.52109

ave/min d
74.4252
ave/min m
131.796

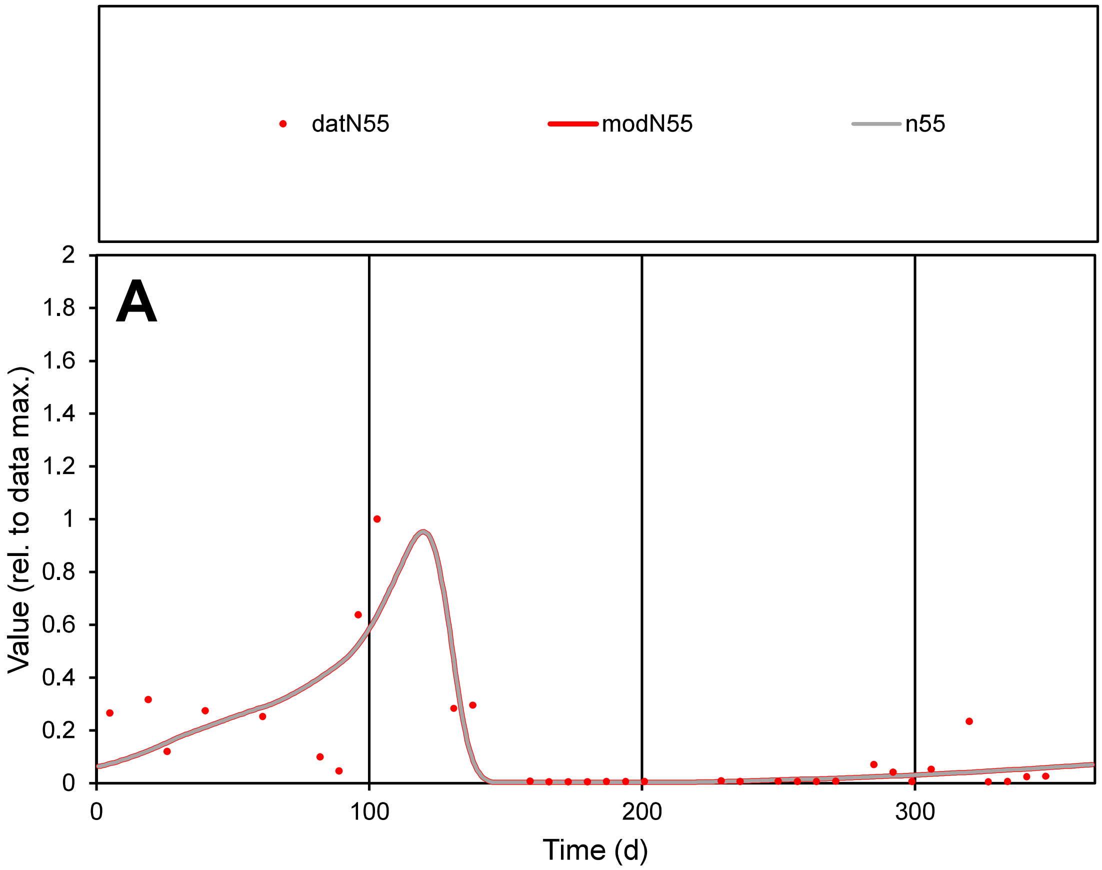

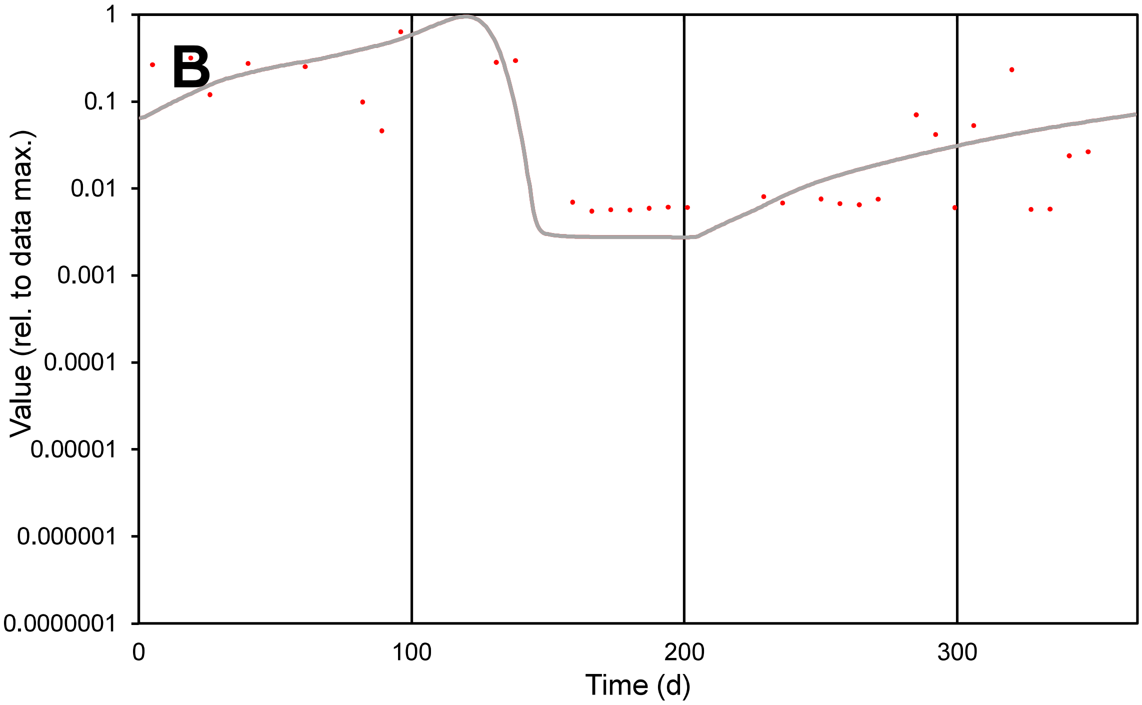

C1

Variables
N55 Provide jtd or Dst
CalibOnly?
2 1 to reproduce of
oNormalize?
2 1=no, 2=yes

ofxn calc*
1.01152
ofxn mod
1.05154

sRun
RunA1
zout
1

Ave
Data
0.00084
Model
0.0008

data/vMax
0.00671

ofxng
263.459

Min d/m
2.00054

ave/min d
22.8523
ave/min m
43.3747

326_Calib_S_A

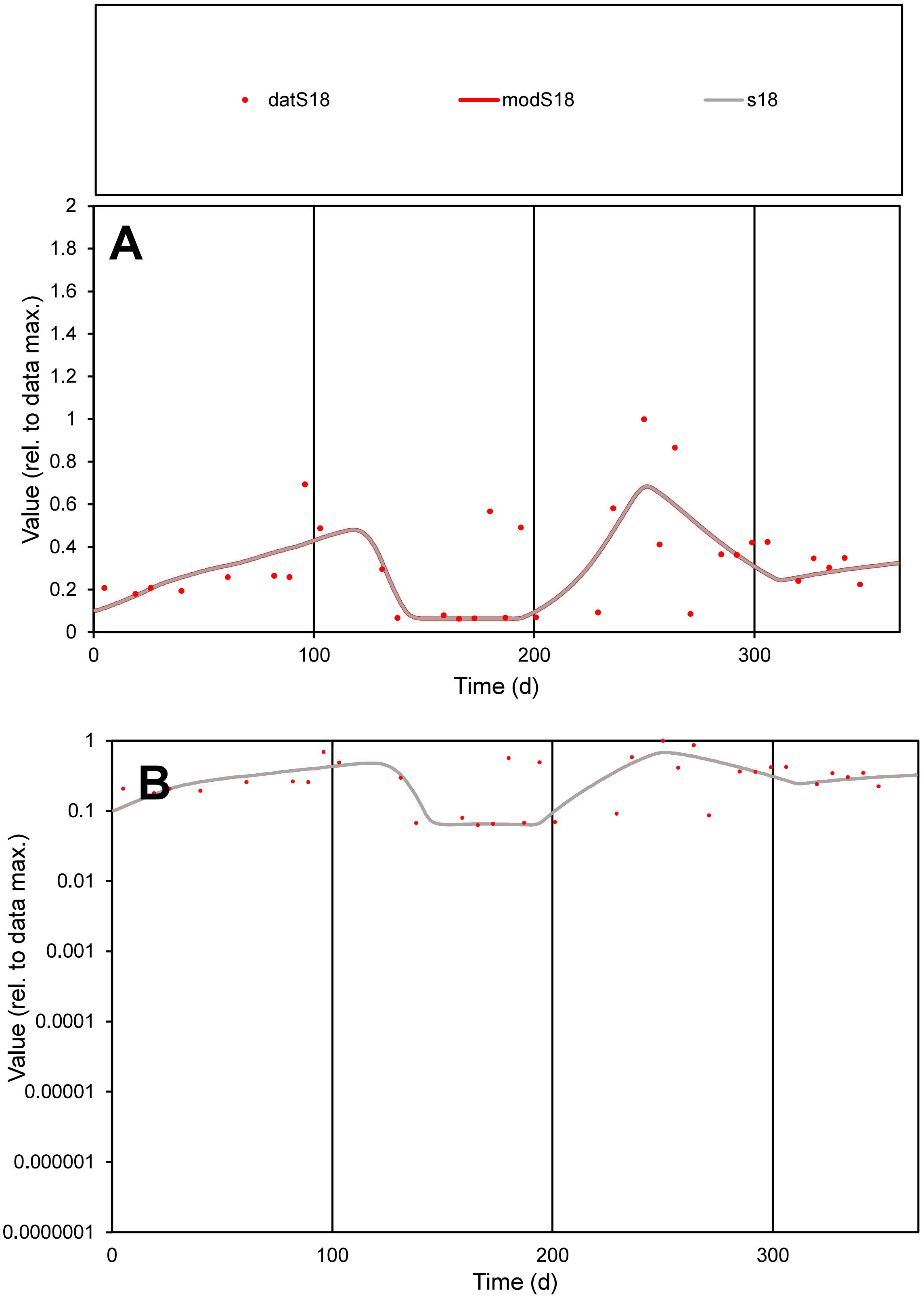

C1

Variables
S18 Provide jtd or Dst
CalibOnly?
2 1 to reproduce of
oNormalize?
2 1=no, 2=yes

ofxn calc*
0.5732
ofxn mod
0.58826

sRun
RunA1
zout
1

Ave
Data
0.00135
Model
0.00124

data/vMax
0.0042

ofxng
263.459

Min d/m
0.98874

ave/min d
5.11514
ave/min m
4.63437

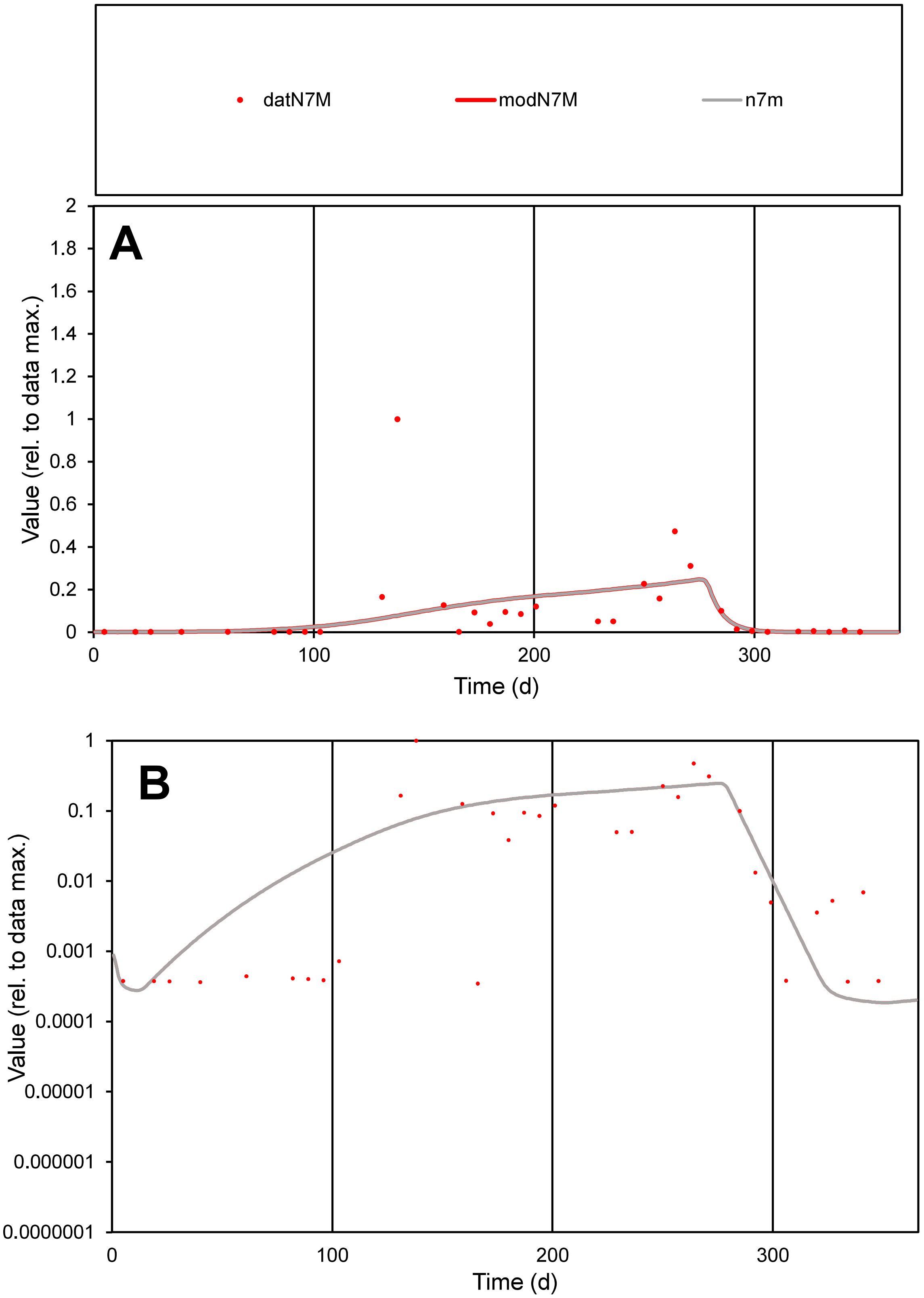

C1

Variables
N7M Provide jtd or Dst
CalibOnly?
2 1 to reproduce of
oNormalize?
2 1=no, 2=yes

ofxn calc*
1.84433
ofxn mod
1.85664

sRun
RunA1
zout
1

Ave
Data
0.00259
Model
0.00224

data/vMax
0.02733

ofxng
263.459

Min d/m
1.8737

ave/min d
272.06
ave/min m
440.241

326_Calib_S_A

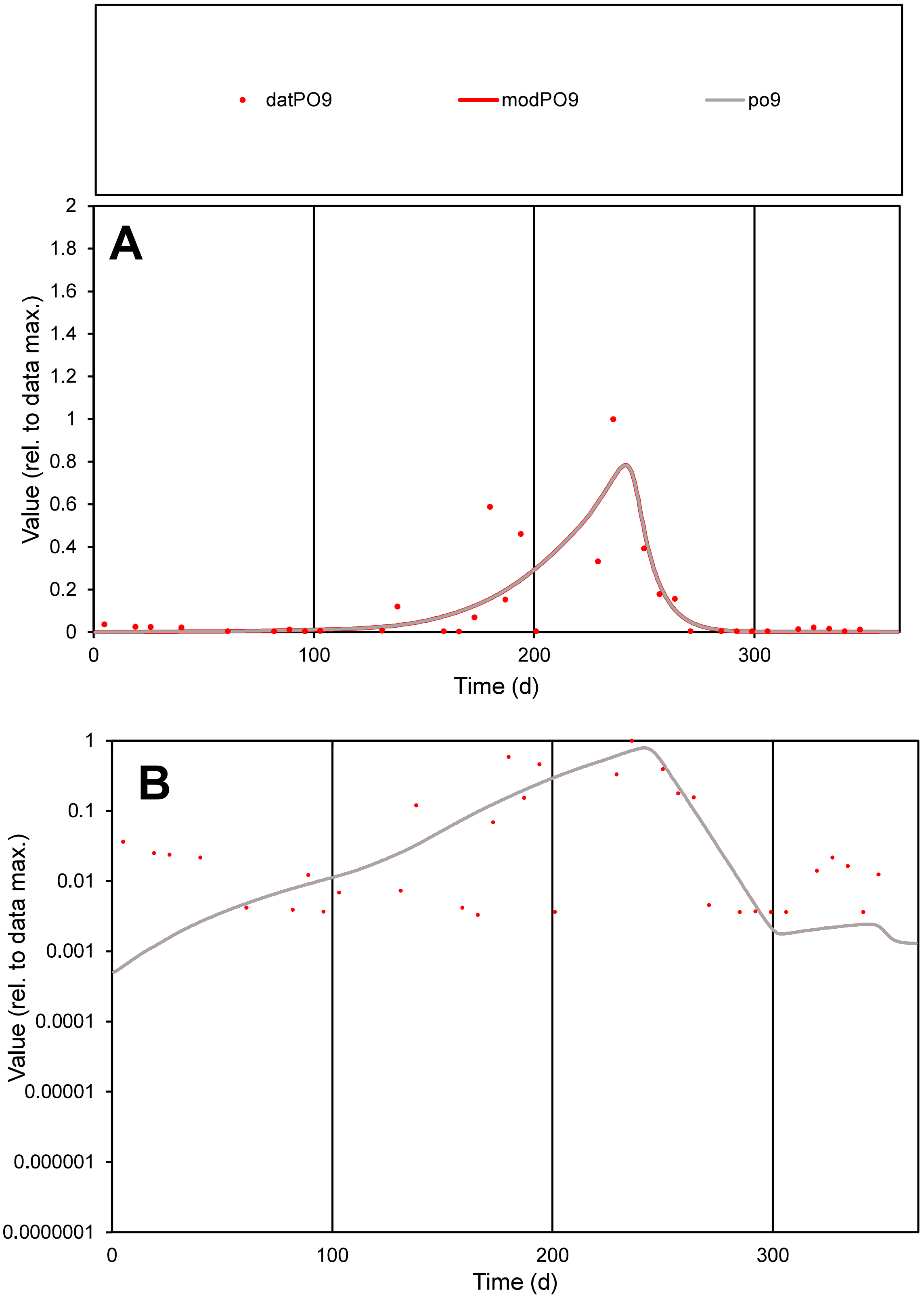

C1

Variables
PO9 Provide jtd or Dst
CalibOnly?
2 1 to reproduce of
oNormalize?
2 1=no, 2=yes

ofxn calc*
1.09233
ofxn mod
1.12117

sRun
RunA1
zout
1

Ave
Data
0.00195
Model
0.00182

data/vMax
0.01741

ofxng
263.459

Min d/m
5.01913

ave/min d
33.7671
ave/min m
158.043

326_Calib_S_A

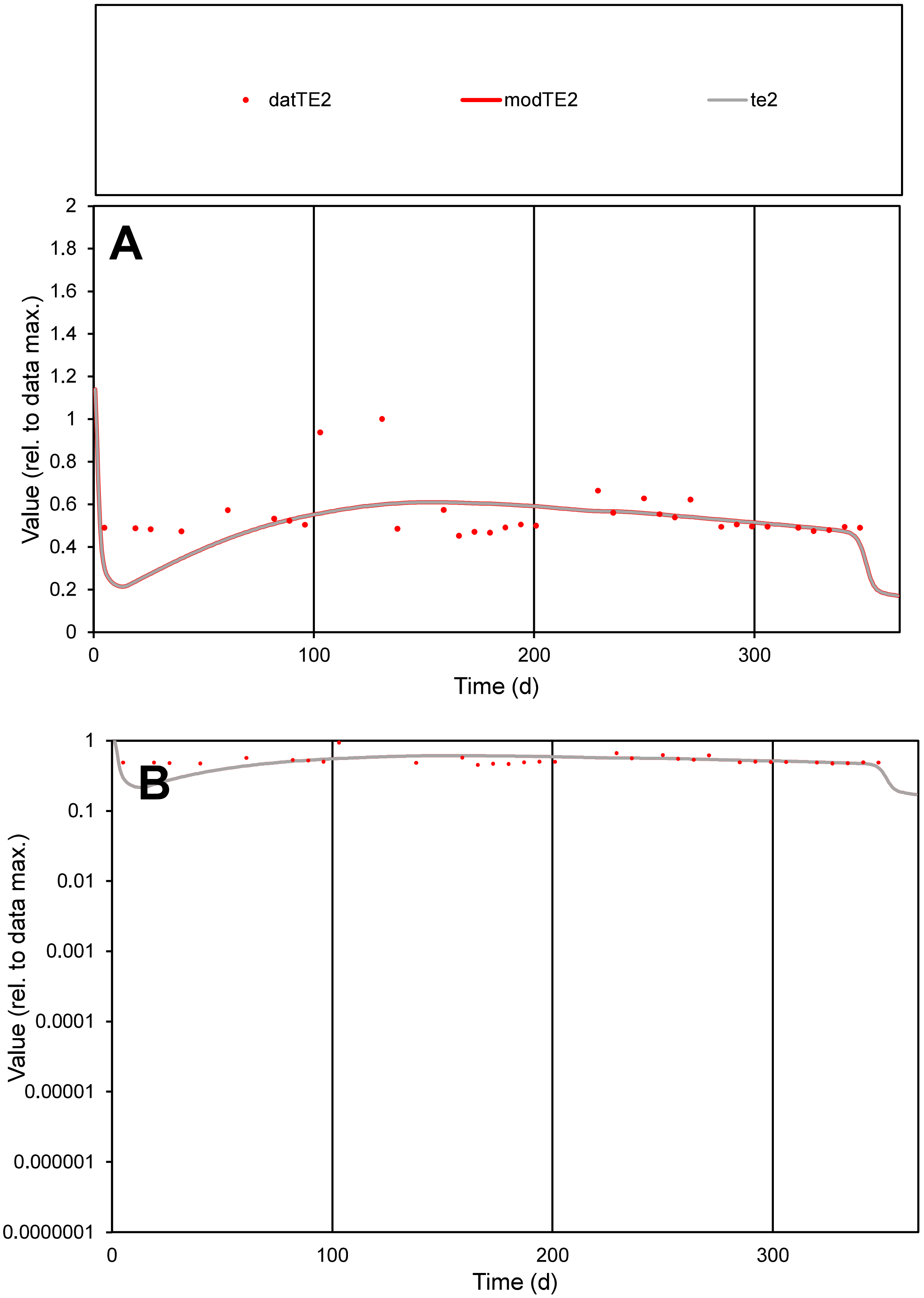

C1

Variables
TE2 Provide jtd or Dst
CalibOnly?
2 1 to reproduce of
oNormalize?
2 1=no, 2=yes

ofxn calc*
0.25047
ofxn mod
0.2746

sRun
RunA1
zout
1

Ave
Data
0.0001
Model
9.6E-05

data/vMax
0.00019

ofxng
263.459

Min d/m
1.84461

ave/min d
1.20135
ave/min m
2.08935

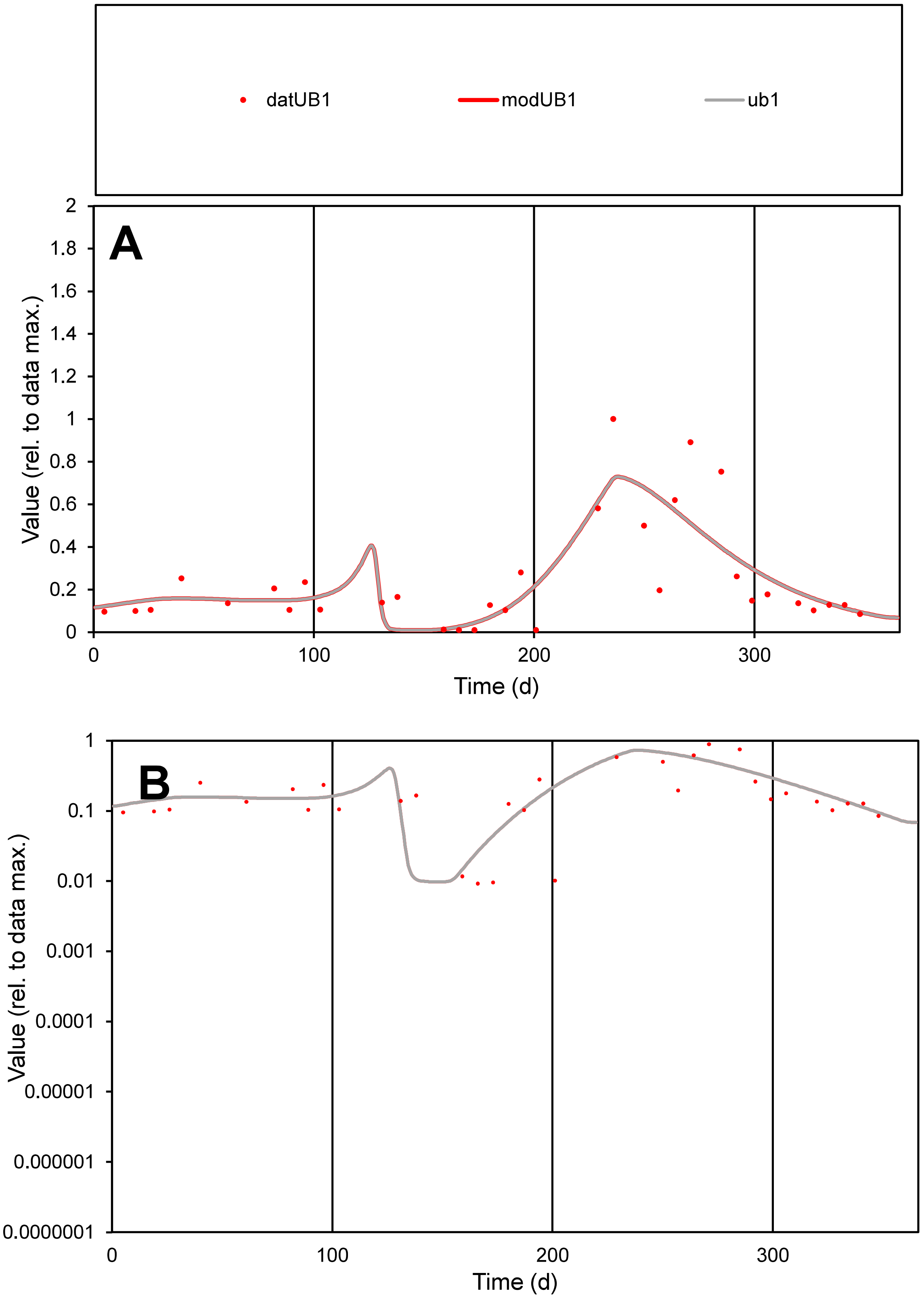

C1

Variables
UB1 Provide jtd or Dst
CalibOnly?
2 1 to reproduce of
oNormalize?
2 1=no, 2=yes

ofxn calc*
0.61706
ofxn mod
0.64352

sRun
RunA1
zout
1

Ave
Data
0.00147
Model
0.00146

data/vMax
0.00614

ofxng
263.459

Min d/m
0.90484

ave/min d
26.0102
ave/min m
23.3618

326_Calib_S_A

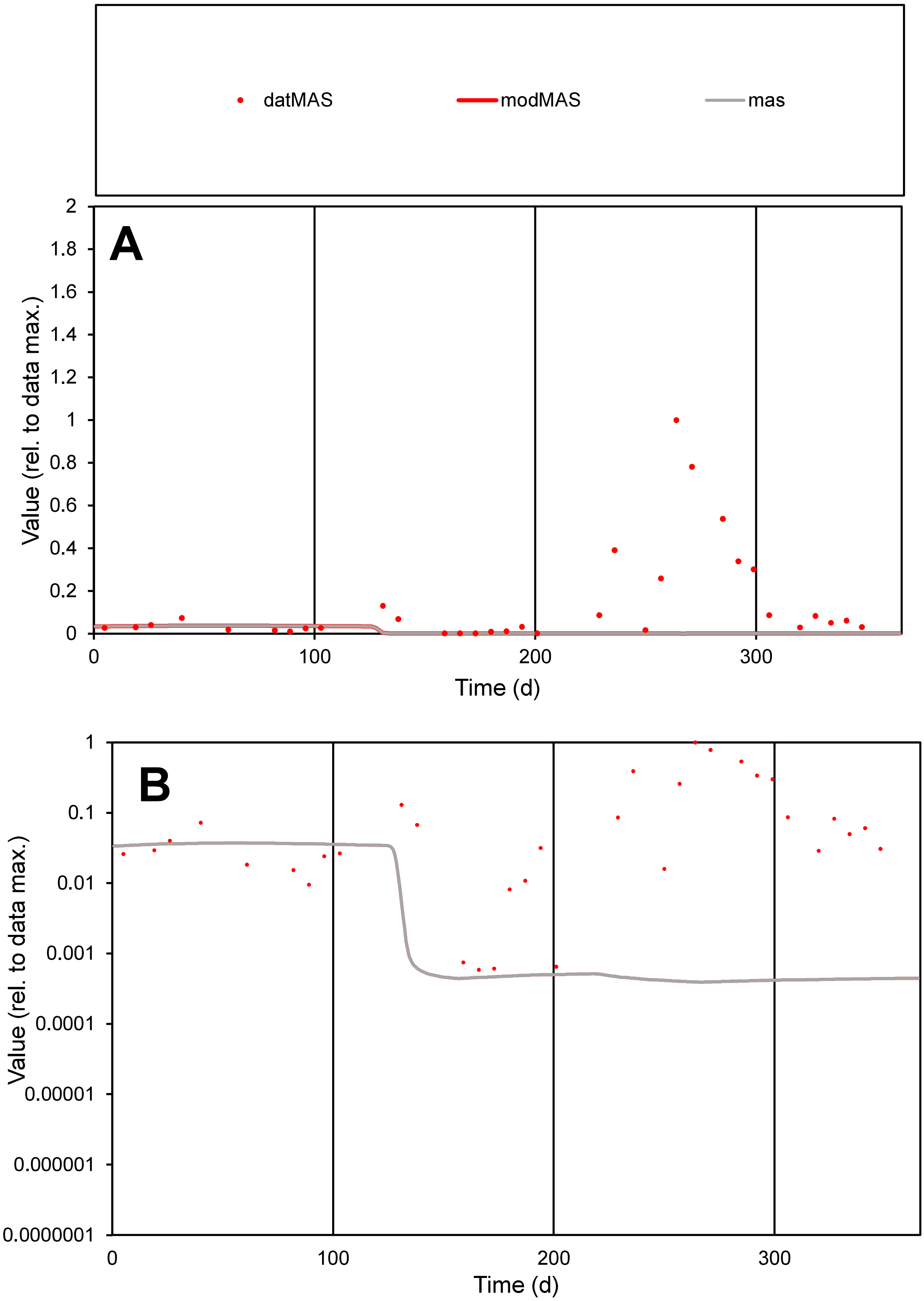

C1

Variables
MAS Provide jtd or Dst
CalibOnly?
2 1 to reproduce of
oNormalize?
2 1=no, 2=yes

ofxn calc*
1.9411
ofxn mod
1.94604

sRun
RunA1
zout
1

Ave
Data
0.00281
Model
0.00021

data/vMax
0.02032

ofxng
263.459

Min d/m
1.48708

ave/min d
235.674
ave/min m
26.4084

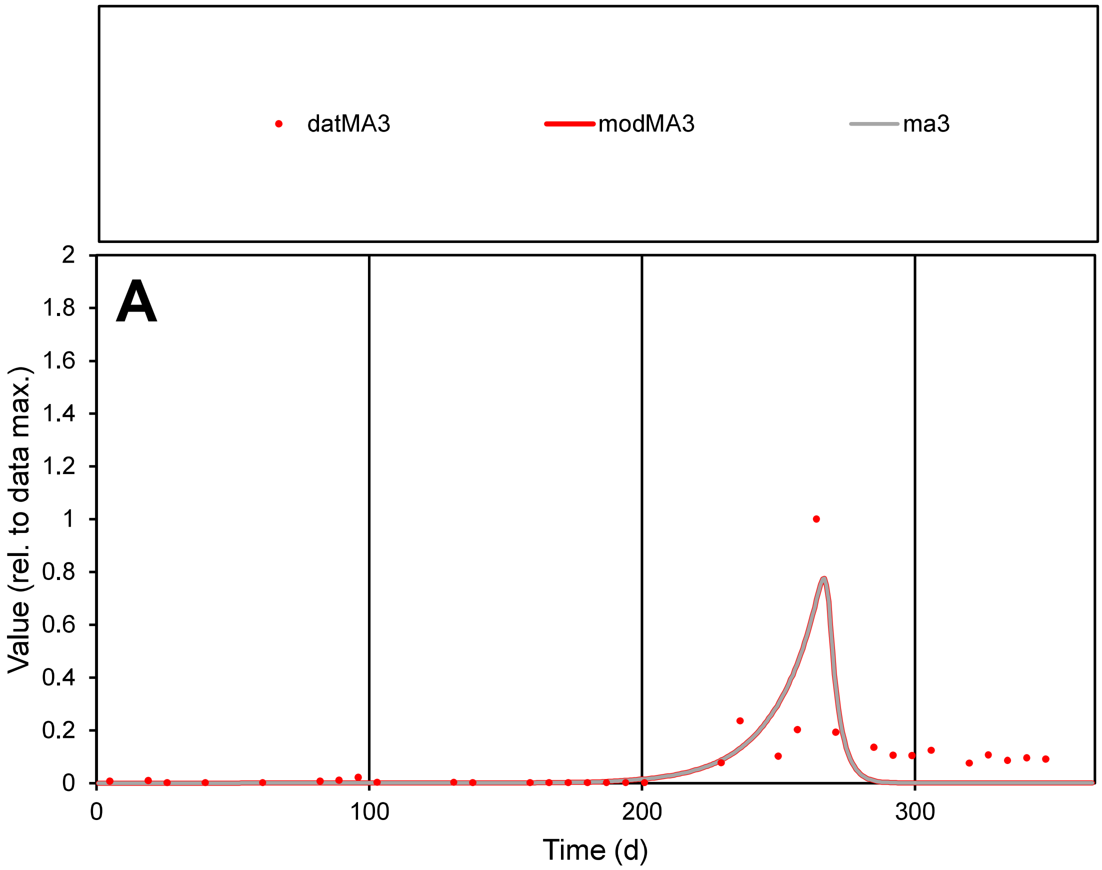

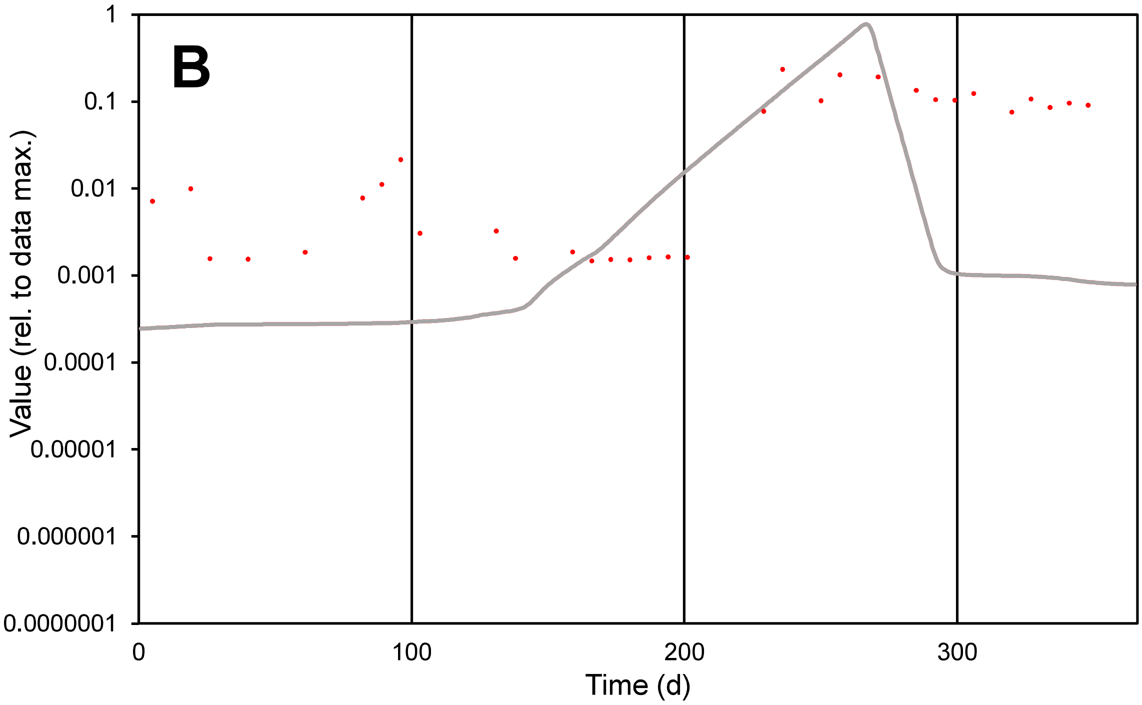

C1

Variables

MA3 Provide jtd or Dst

CalibOnly?

2 1 to reproduce of

oNormalize?

2 1=no, 2=yes

ofxn calc*

1.07667

ofxn mod

1.12053

sRun

RunA1

zout

1

Ave

Data

0.00356

Model

0.00261

data/vMax

0.04172

ofxng

263.459

Min d/m

5.87452

ave/min d

58.0396

ave/min m

249.549

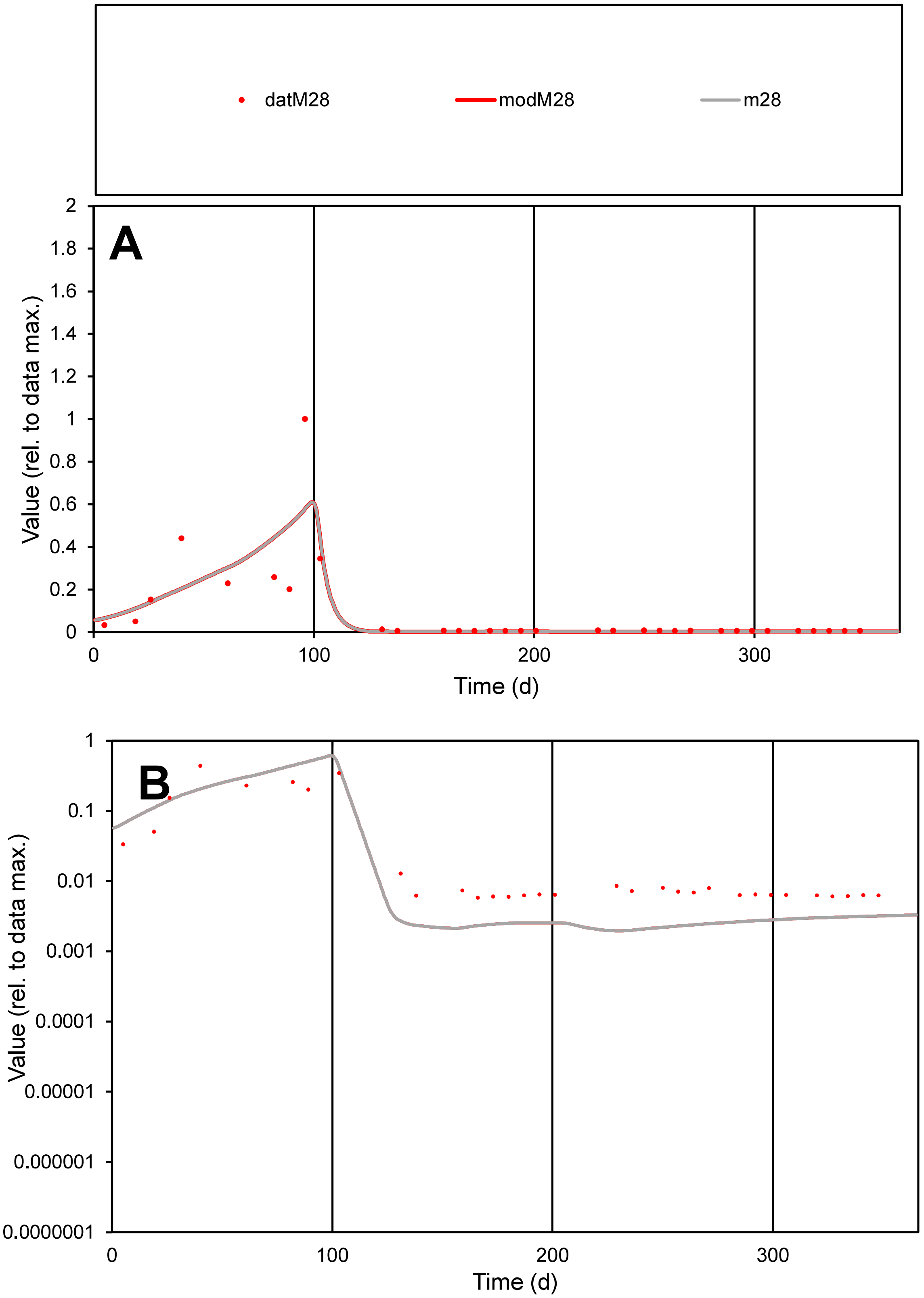

C1

Variables
M28 Provide jtd or Dst
CalibOnly?
2 1 to reproduce of
oNormalize?
2 1=no, 2=yes

ofxn calc*
1.20049
ofxn mod
1.22819

sRun
RunA1
zout
1

Ave
Data
0.0008
Model
0.00079

data/vMax
0.00921

ofxng
263.459

Min d/m
2.98099

ave/min d
15.0456
ave/min m
43.9636

326_Calib_S_A

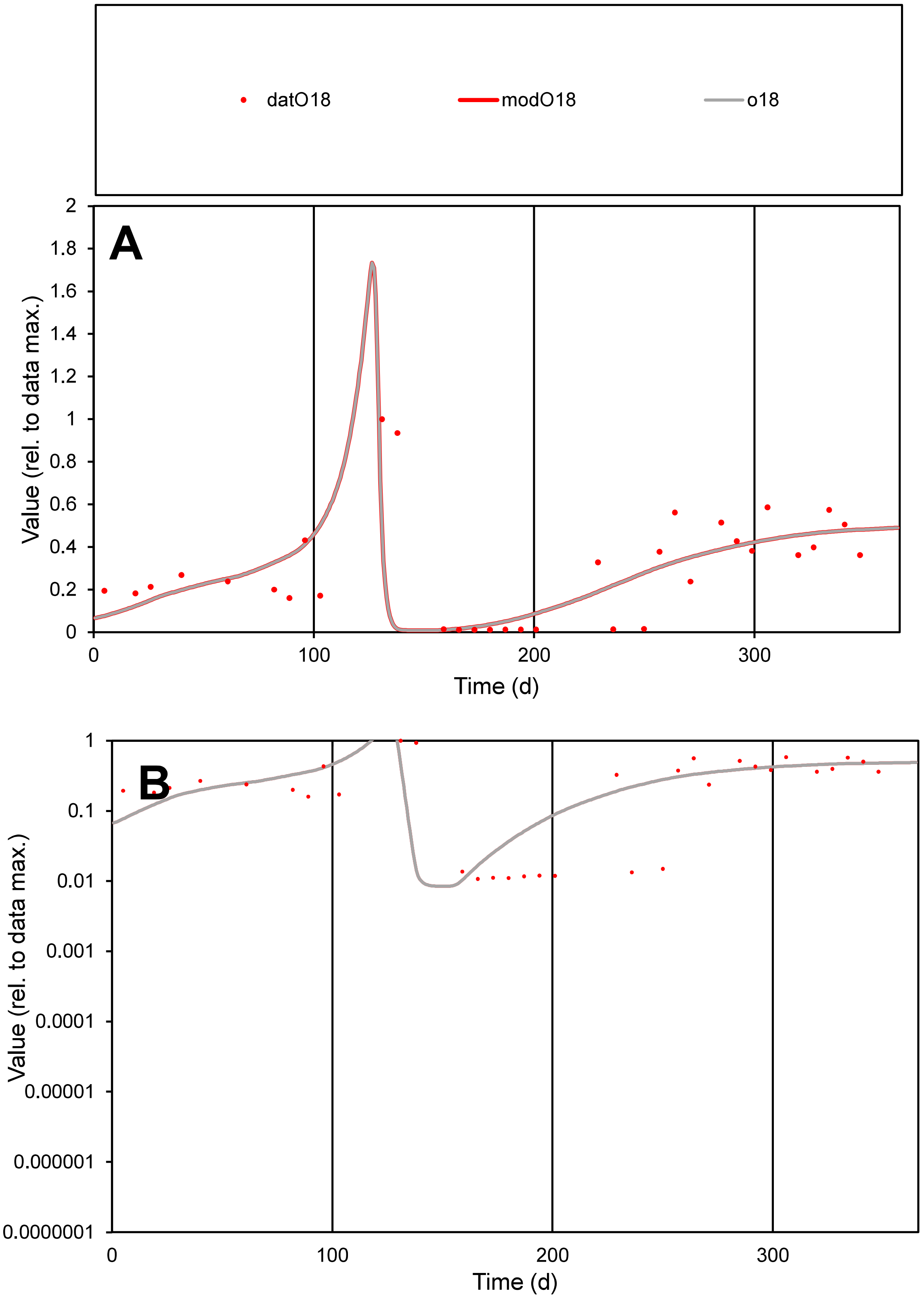

C1

Variables

O18 Provide jtd or Dst

CalibOnly?

2 1 to reproduce of

oNormalize?

2 1=no, 2=yes

ofxn calc*

0.70705

ofxn mod

0.74766

sRun

RunA1

zout

1

Ave

Data

0.00139

Model

0.00132

data/vMax

0.00474

ofxng

263.459

Min d/m

1.00631

ave/min d

27.3344

ave/min m

26.0565

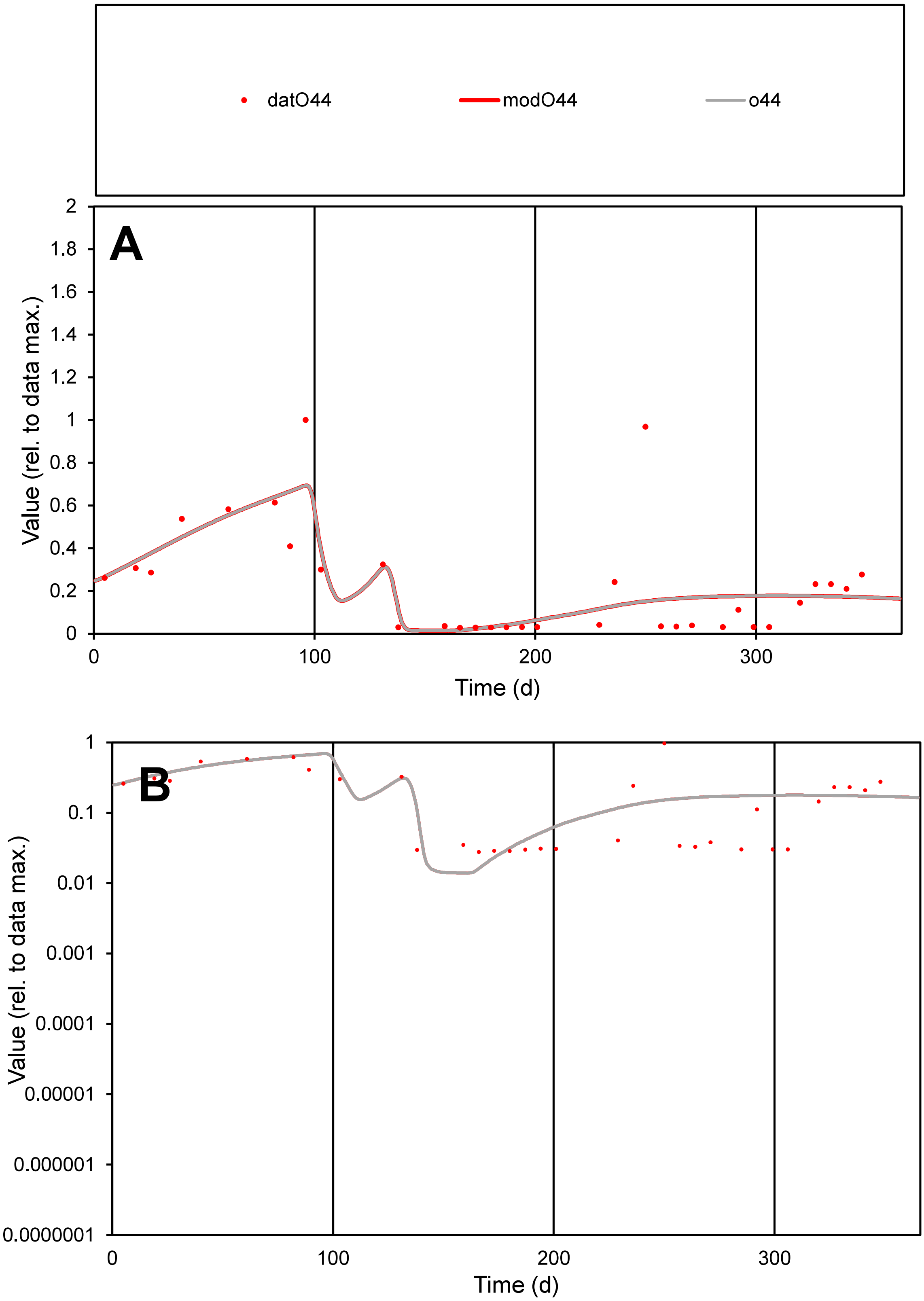

C1

Variables
O44 Provide jtd or Dst
CalibOnly?
2 1 to reproduce of
oNormalize?
2 1=no, 2=yes

ofxn calc*
0.76878
ofxn mod
0.7847

sRun
RunA1
zout
1

Ave
Data
0.0009
Model
0.00089

data/vMax
0.00395

ofxng
263.459

Min d/m
1.99562

ave/min d
8.22537
ave/min m
16.3096

326_Calib_S_A

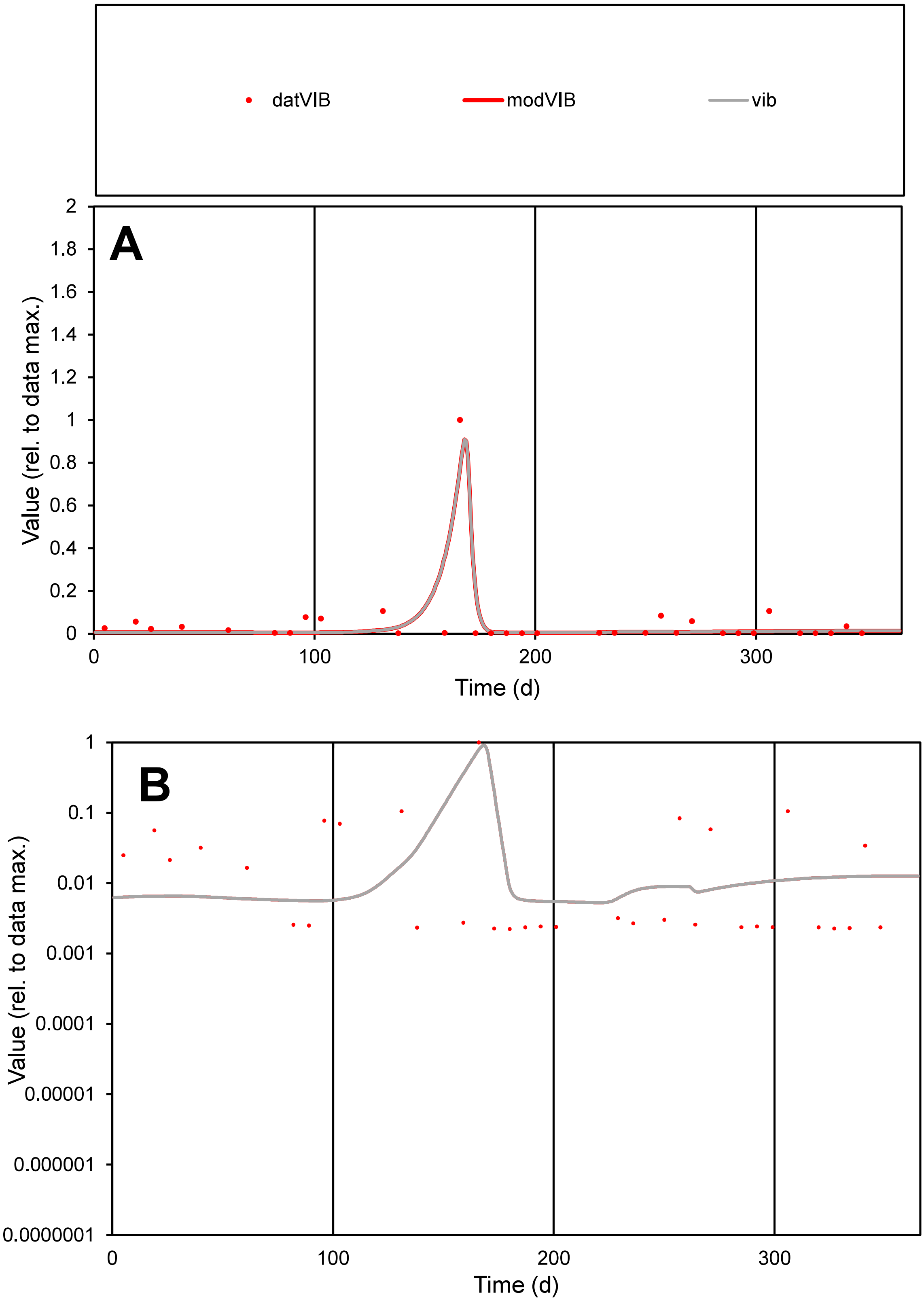

C1

Variables
VIB Provide jtd or Dst
CalibOnly?
2 1 to reproduce of
oNormalize?
2 1=no, 2=yes

ofxn calc*
1.23969
ofxn mod
1.38219

sRun
RunA1
zout
1

Ave
Data
0.00043
Model
0.00037

data/vMax
0.00808

ofxng
263.459

Min d/m
0.40943

ave/min d
23.5358
ave/min m
8.37112

326_Calib_S_A

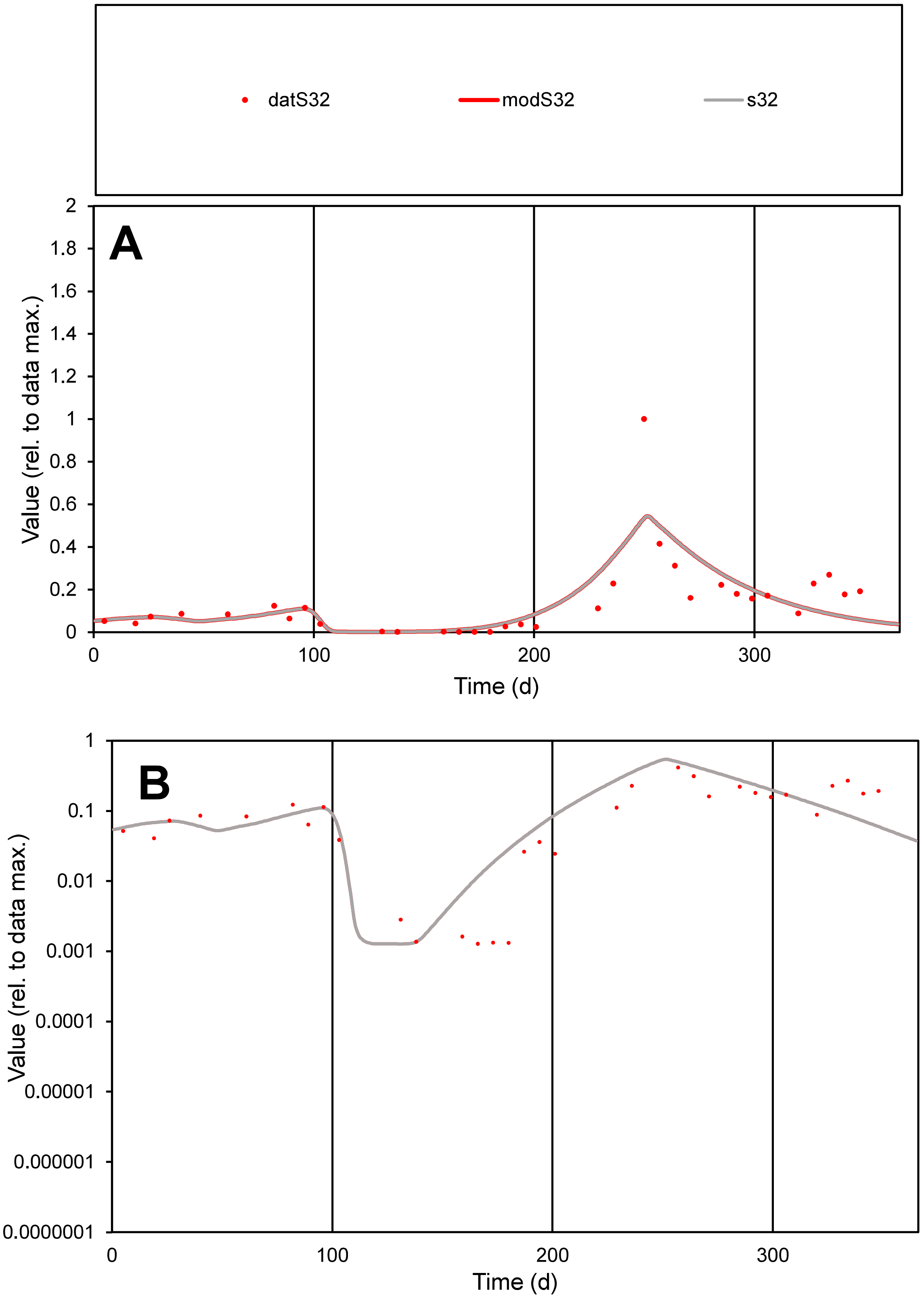

C1

Variables
S32 Provide jtd or Dst
CalibOnly?
2 1 to reproduce of
oNormalize?
2 1=no, 2=yes

ofxn calc*
0.78267
ofxn mod
0.80969

sRun
RunA1
zout
1

Ave
Data
0.00118
Model
0.00118

data/vMax
0.00835

ofxng
263.459

Min d/m
1.00471

ave/min d
111.004
ave/min m
111.064

326_Calib_S_A

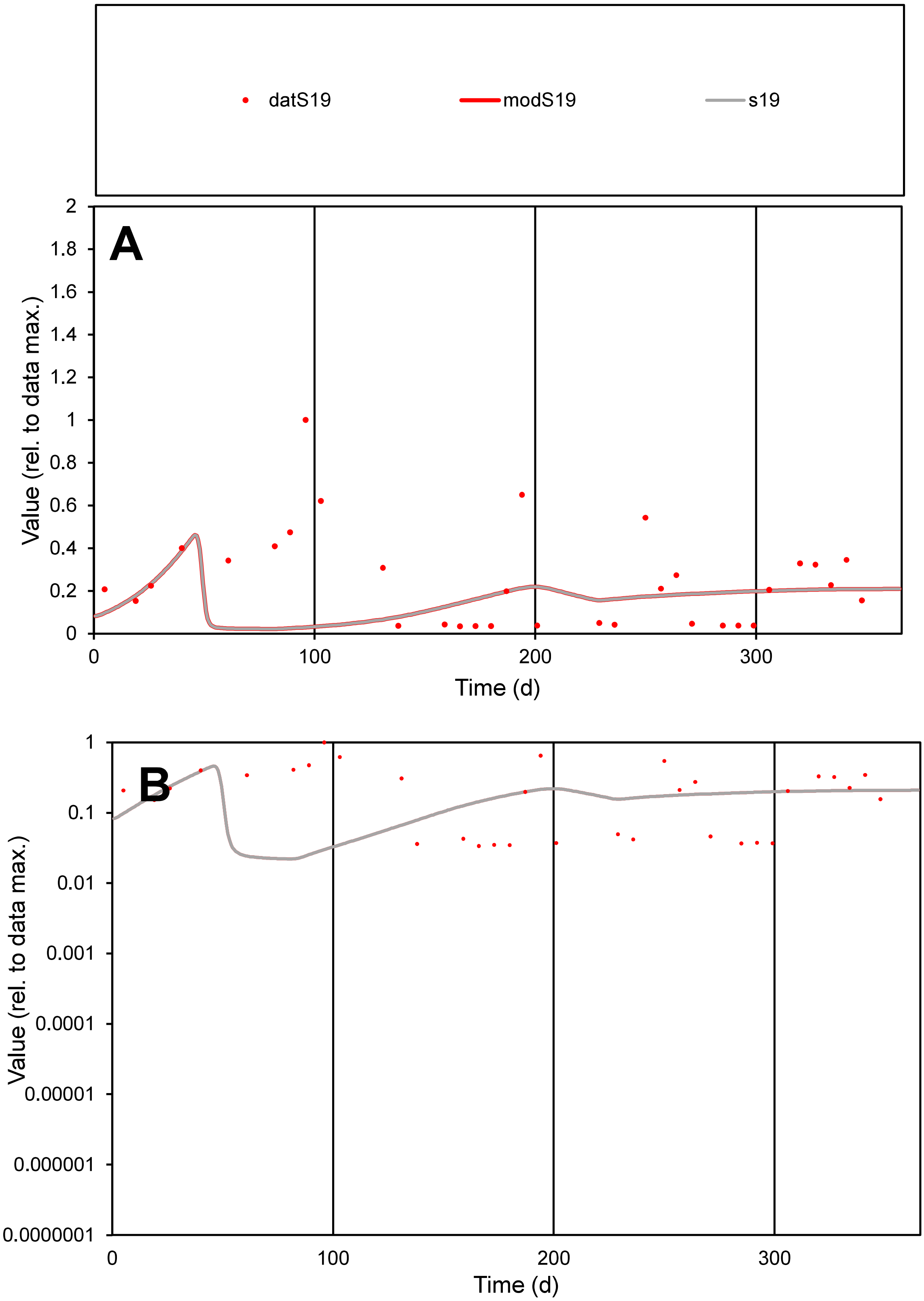

C1

Variables
S19 Provide jtd or Dst
CalibOnly?
2 1 to reproduce of
oNormalize?
2 1=no, 2=yes

ofxn calc*
1.10343
ofxn mod
1.10917

sRun
RunA1
zout
1

Ave
Data
0.00034
Model
0.00022

data/vMax
0.00139

ofxng
263.459

Min d/m
1.49856

ave/min d
7.27084
ave/min m
7.21787

326_Calib_S_A

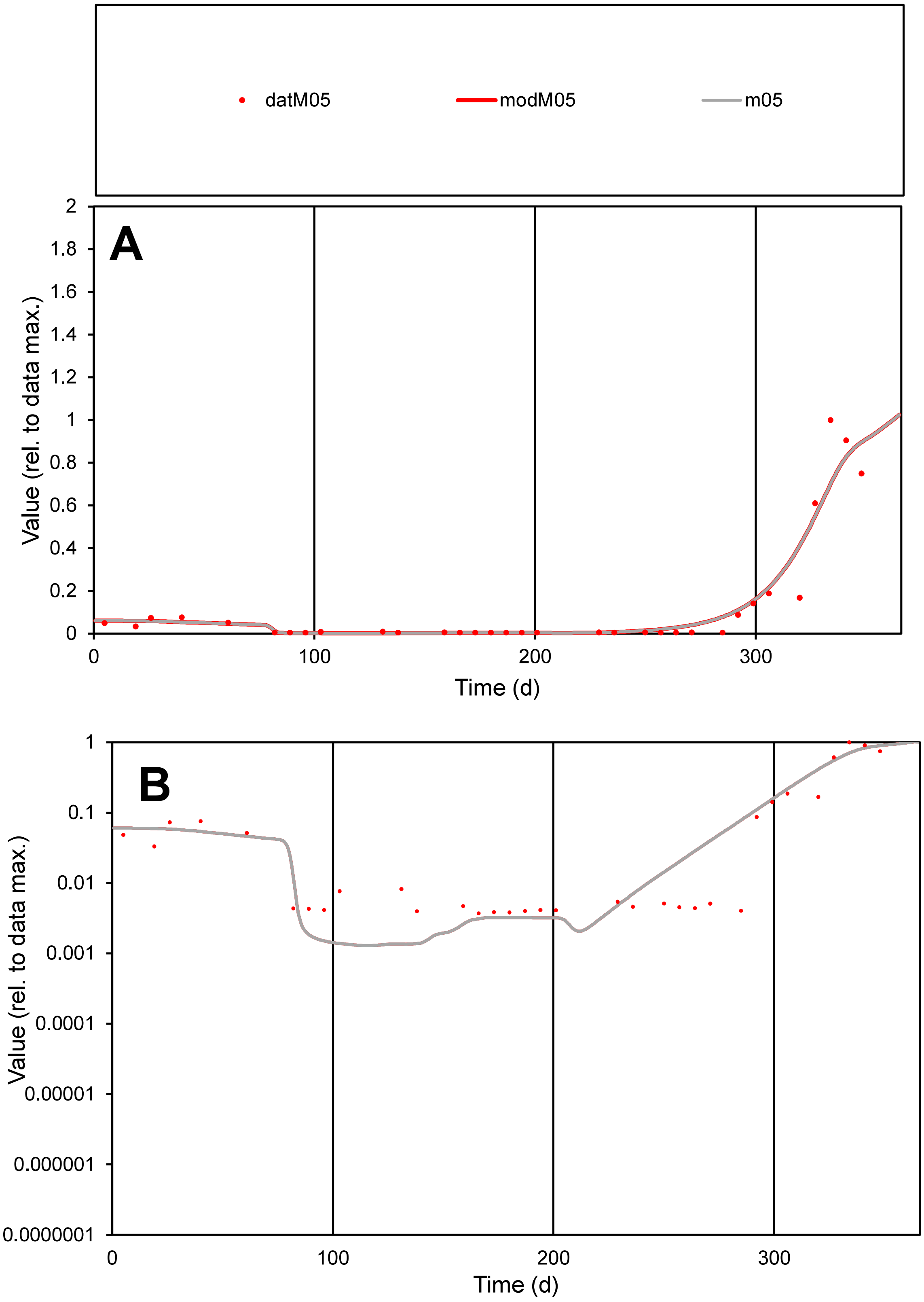

C1

Variables
M05 Provide jtd or Dst
CalibOnly?
2 1 to reproduce of
oNormalize?
2 1=no, 2=yes

ofxn calc*
0.57084
ofxn mod
0.61704

sRun
RunA1
zout
1

Ave
Data
0.00147
Model
0.00157

data/vMax
0.01153

ofxng
263.459

Min d/m
2.74005

ave/min d
34.5469
ave/min m
100.475

326_Calib_S_A

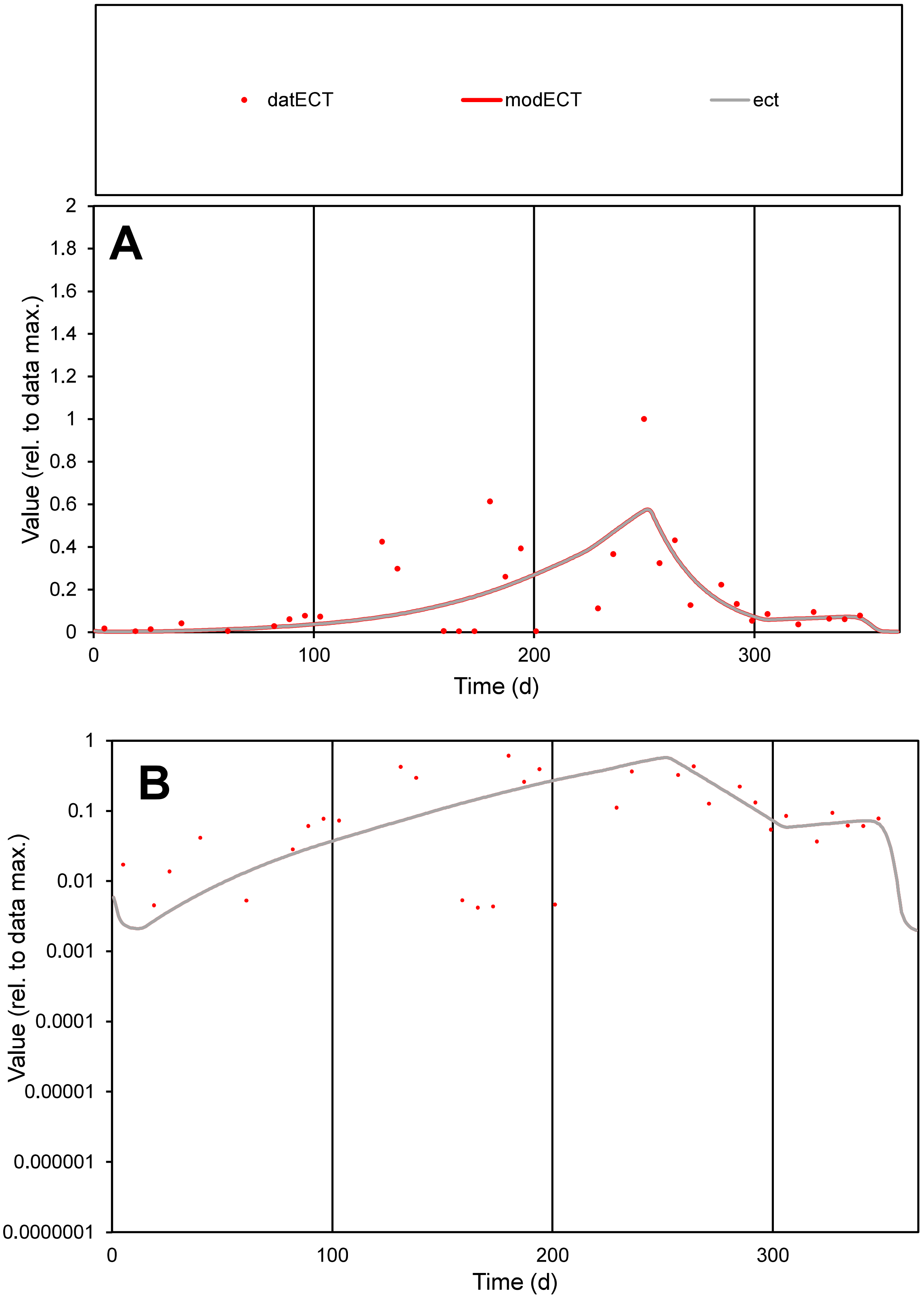

C1

Variables

ECT Provide jtd or Dst

CalibOnly?

2 1 to reproduce of

oNormalize?

2 1=no, 2=yes

ofxn calc*

0.95635

ofxn mod

0.97446

sRun

RunA1

zout

1

Ave

Data

0.00112

Model

0.001

data/vMax

0.00668

ofxng

263.459

Min d/m

1.70377

ave/min d

39.835

ave/min m

60.8629

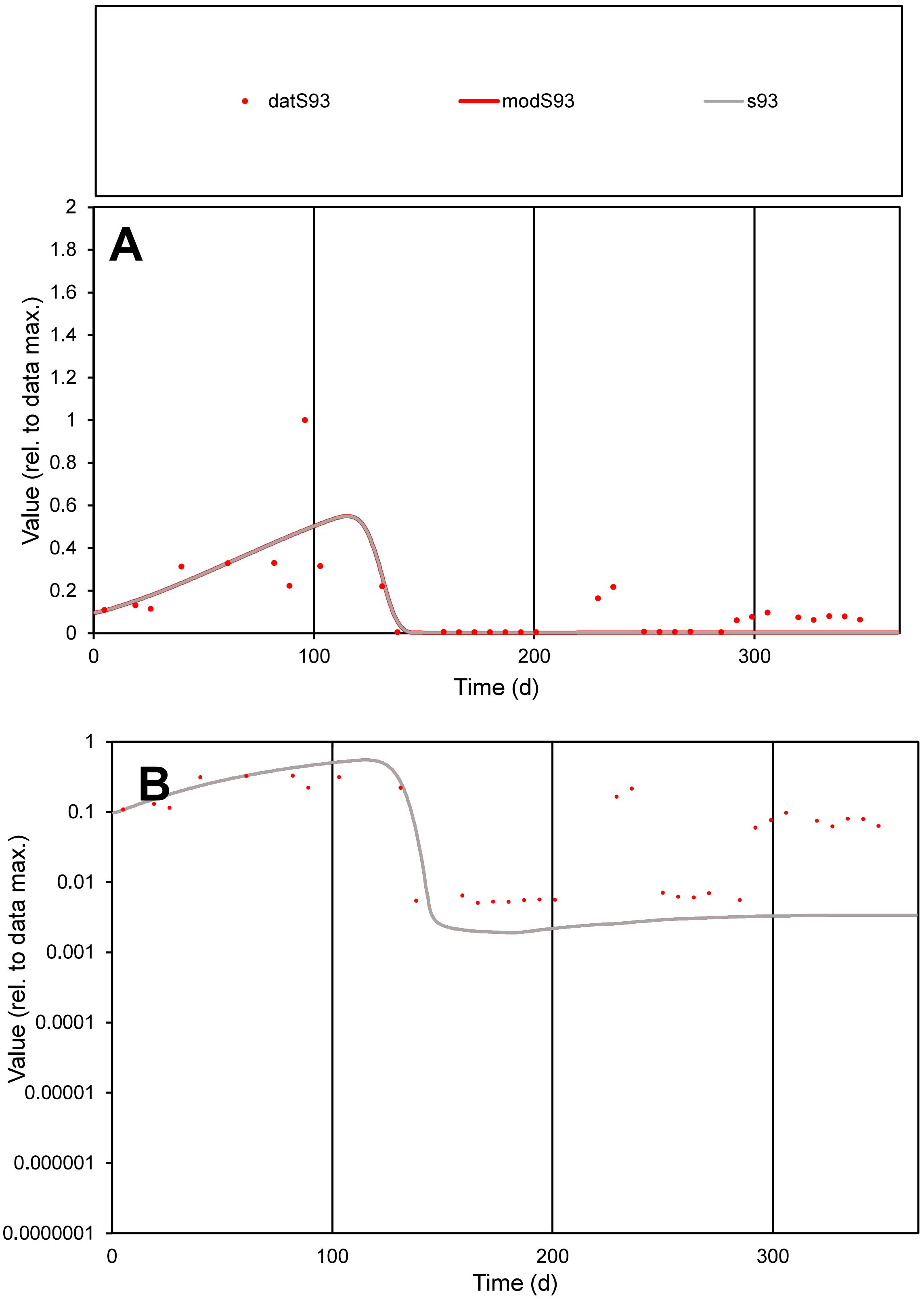

C1

Variables

S93 Provide jtd or Dst

CalibOnly?

2 1 to reproduce of

oNormalize?

2 1=no, 2=yes

ofxn calc*

0.97329

ofxn mod

0.98749

sRun

RunA1

zout

1

Ave

Data

0.00159

Model

0.00124

data/vMax

0.01266

ofxng

263.459

Min d/m

2.67621

ave/min d

24.6059

ave/min m

51.5182

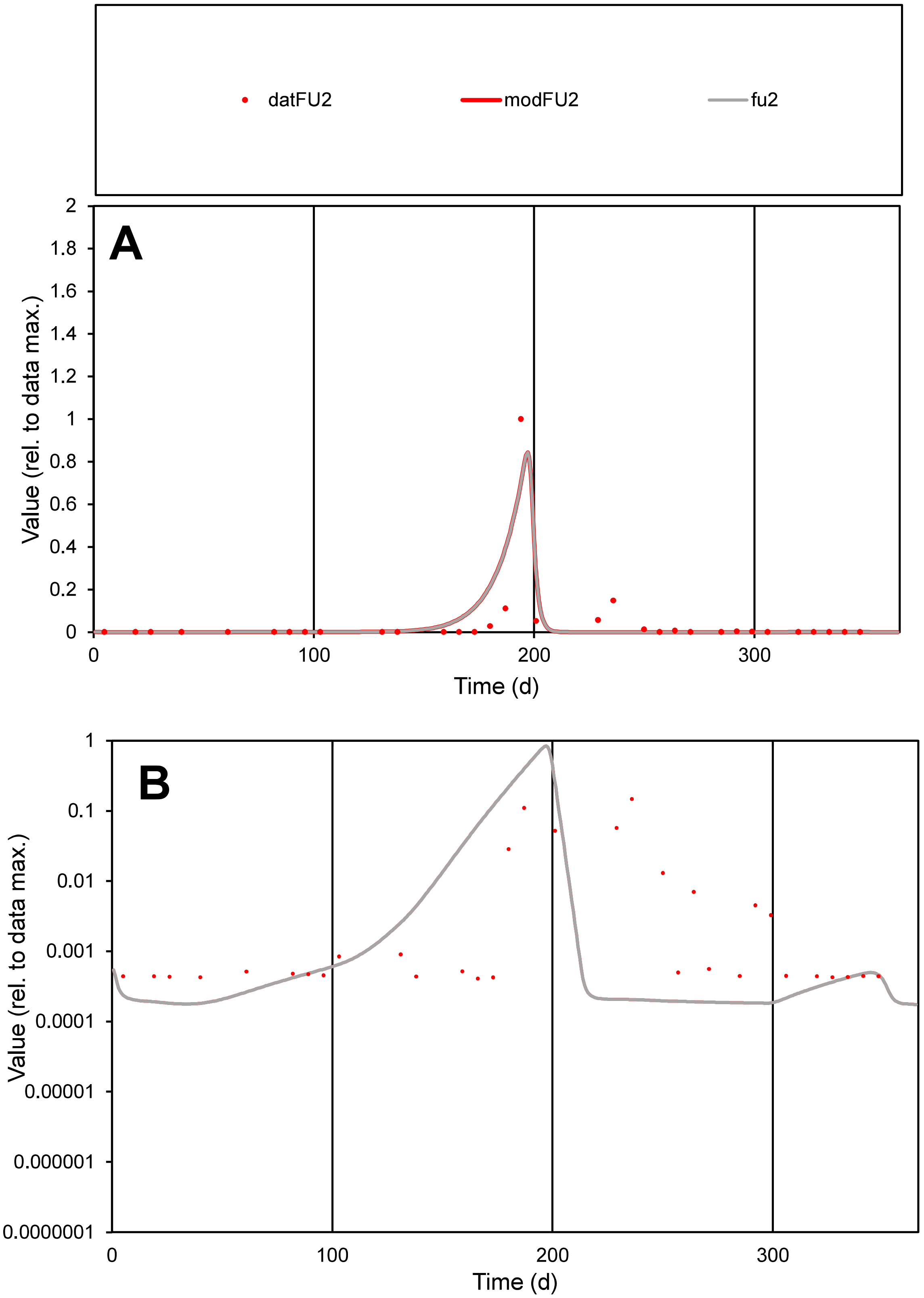

C1

Variables
FU2 Provide jtd or Dst
CalibOnly?
2 1 to reproduce of
oNormalize?
2 1=no, 2=yes

ofxn calc*
1.64977
ofxn mod
1.88799

sRun
RunA1
zout
1

Ave
Data
0.00269
Model
0.00301

data/vMax
0.0619

ofxng
263.459

Min d/m
2.25939

ave/min d
106.656
ave/min m
269.353

326_Calib_S_A

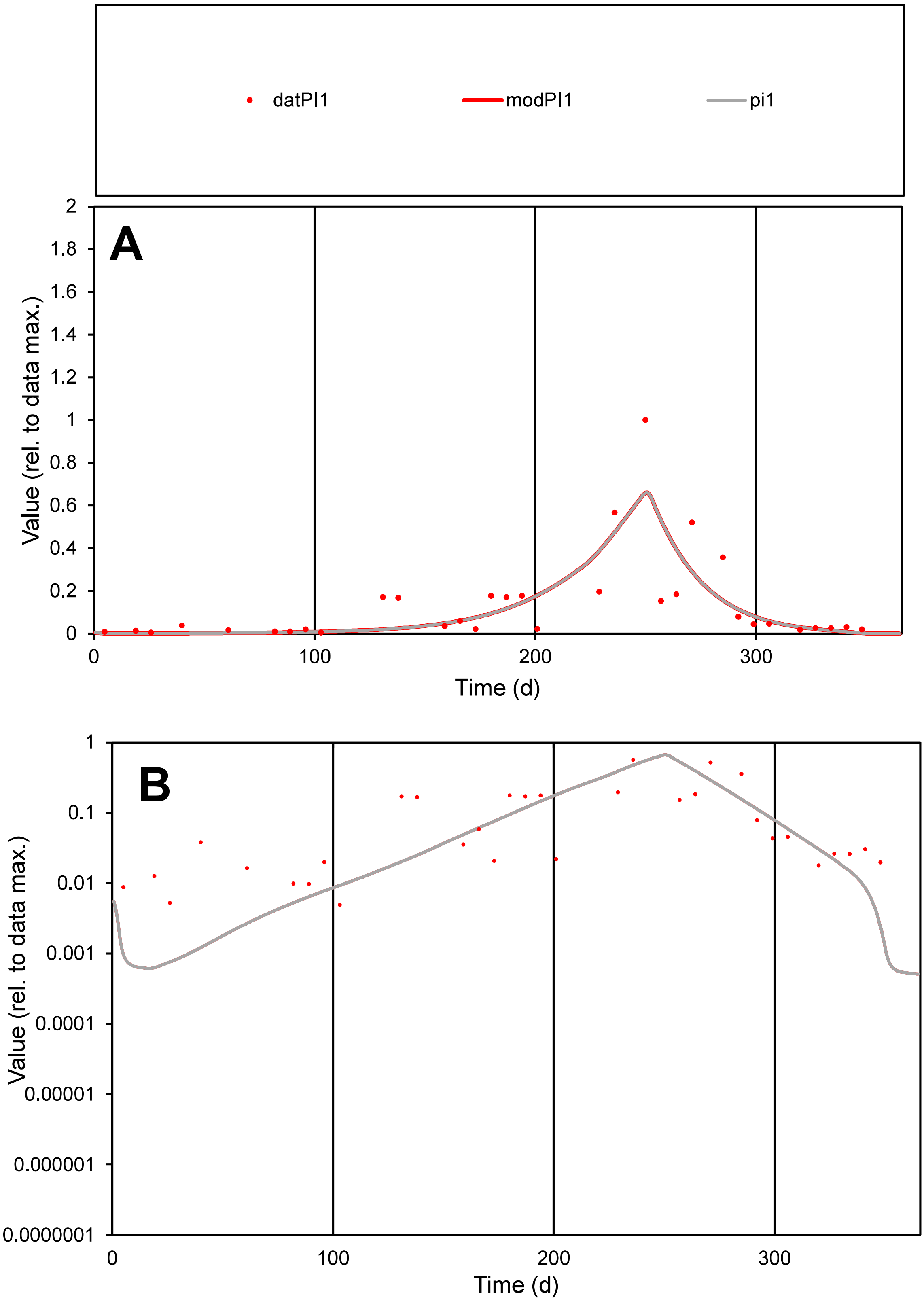

C1

Variables
PI1 Provide jtd or Dst
CalibOnly?
2 1 to reproduce of
oNormalize?
2 1=no, 2=yes

ofxn calc*
0.92711
ofxn mod
0.95914

sRun
RunA1
zout
1

Ave
Data
0.00145
Model
0.00132

data/vMax
0.01093

ofxng
263.459

Min d/m
7.60079

ave/min d
26.9795
ave/min m
186.662

326_Calib_S_A

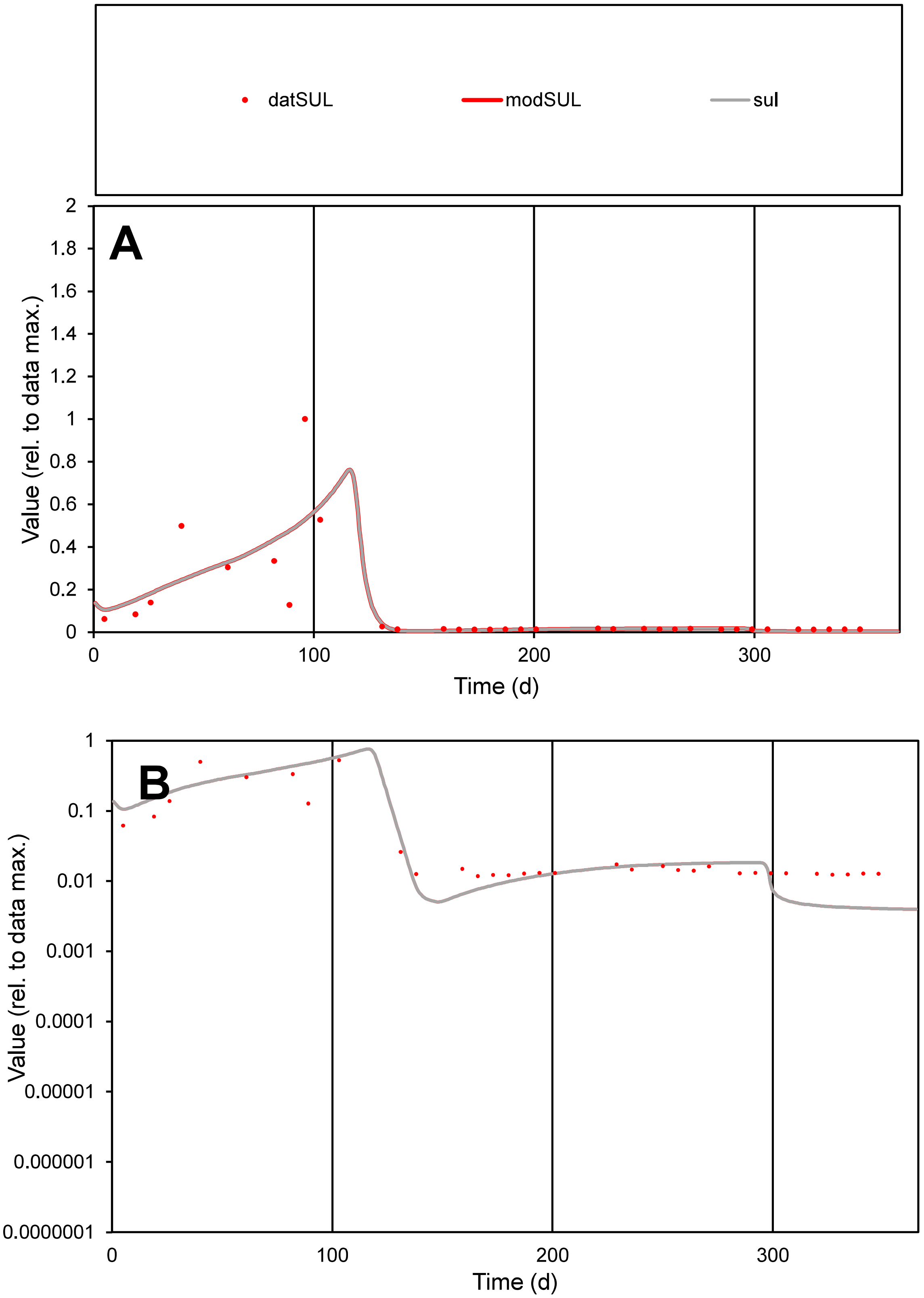

C1

Variables
SUL Provide jtd or Dst
CalibOnly?
2 1 to reproduce of
oNormalize?
2 1=no, 2=yes

ofxn calc*
1.09977
ofxn mod
1.11911

sRun
RunA1
zout
1

Ave
Data
0.00069
Model
0.00068

data/vMax
0.00665

ofxng
263.459

Min d/m
2.9066

ave/min d
8.76283
ave/min m
25.3036

326_Calib_S_A

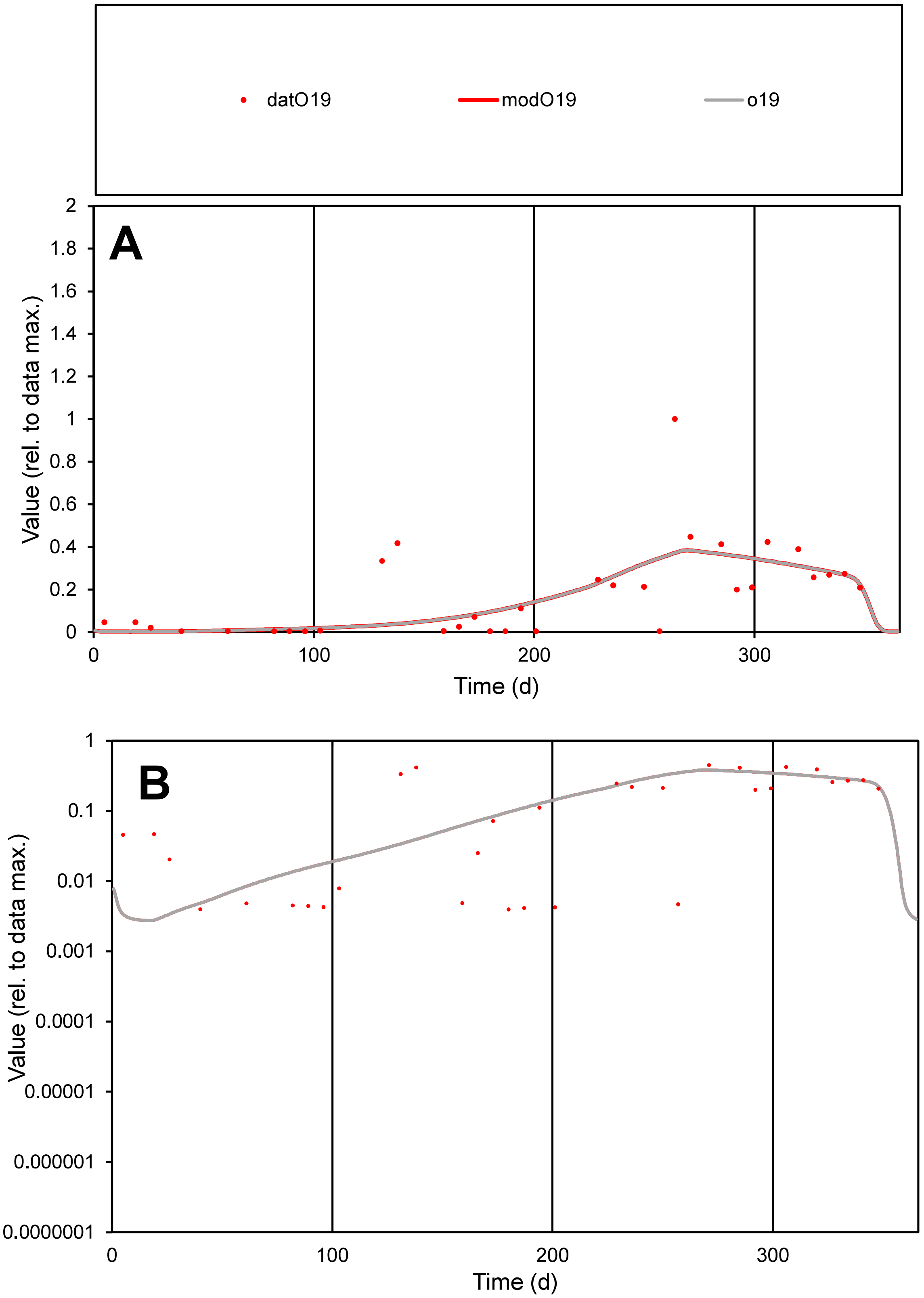

C1

Variables

O19	Provide jtd or Dst

CalibOnly?

2	1 to reproduce of

oNormalize?

2	1=no, 2=yes

ofxn calc*
0.90922
ofxn mod
0.92482

sRun
RunA1
zout
1

Ave
Data
0.00131
Model
0.00124

data/vMax
0.00733

ofxng
263.459

Min d/m
1.37464

ave/min d
45.3979
ave/min m
58.9906

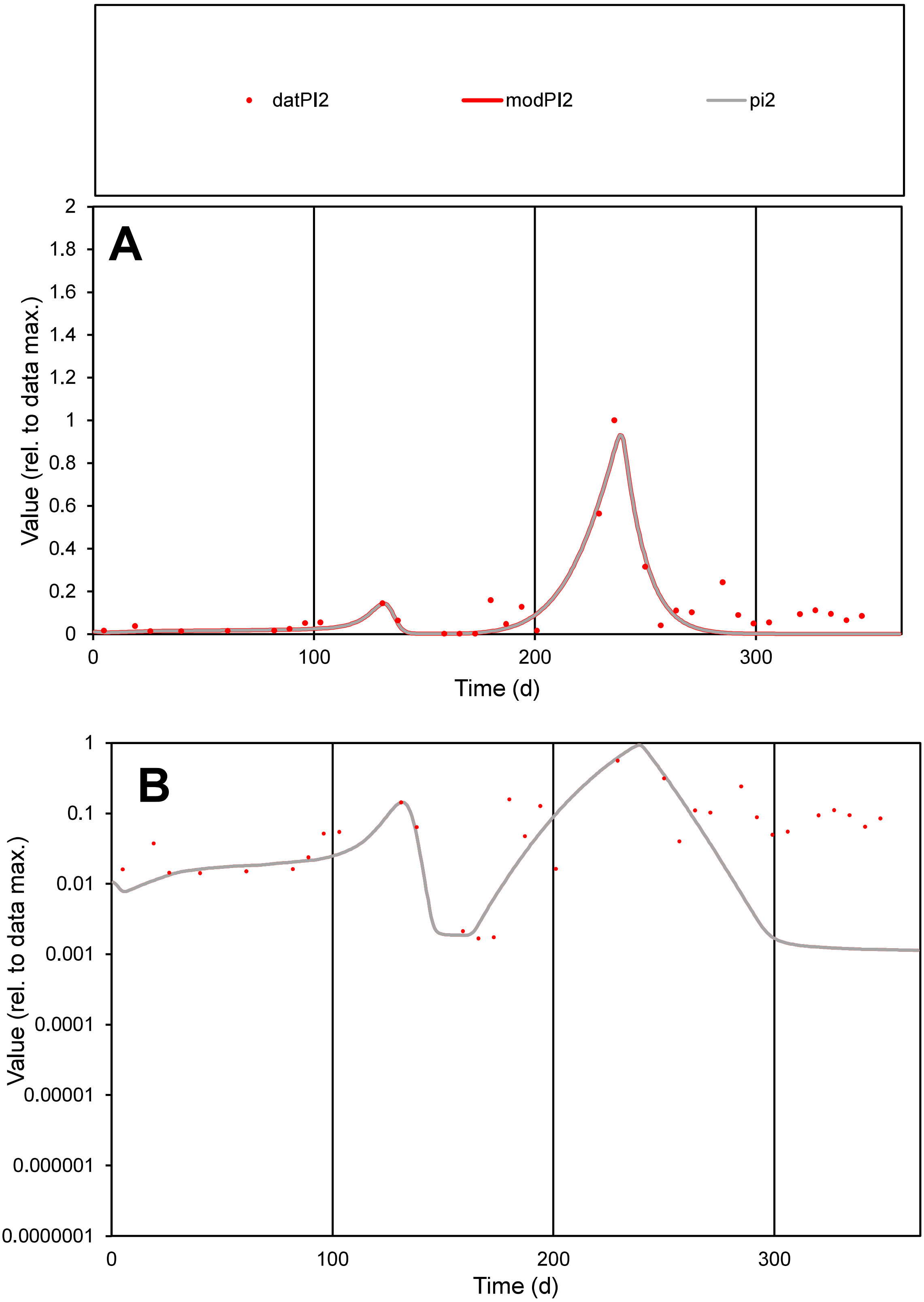

C1

Variables

PI2 Provide jtd or Dst

CalibOnly?

2 1 to reproduce of

oNormalize?

2 1=no, 2=yes

ofxn calc*

0.63171

ofxn mod

0.6637

sRun

RunA1

zout

1

Ave

Data

0.00194

Model

0.00136

data/vMax

0.01675

ofxng

263.459

Min d/m

1.43959

ave/min d

69.3452

ave/min m

69.8166

326_Calib_S_A

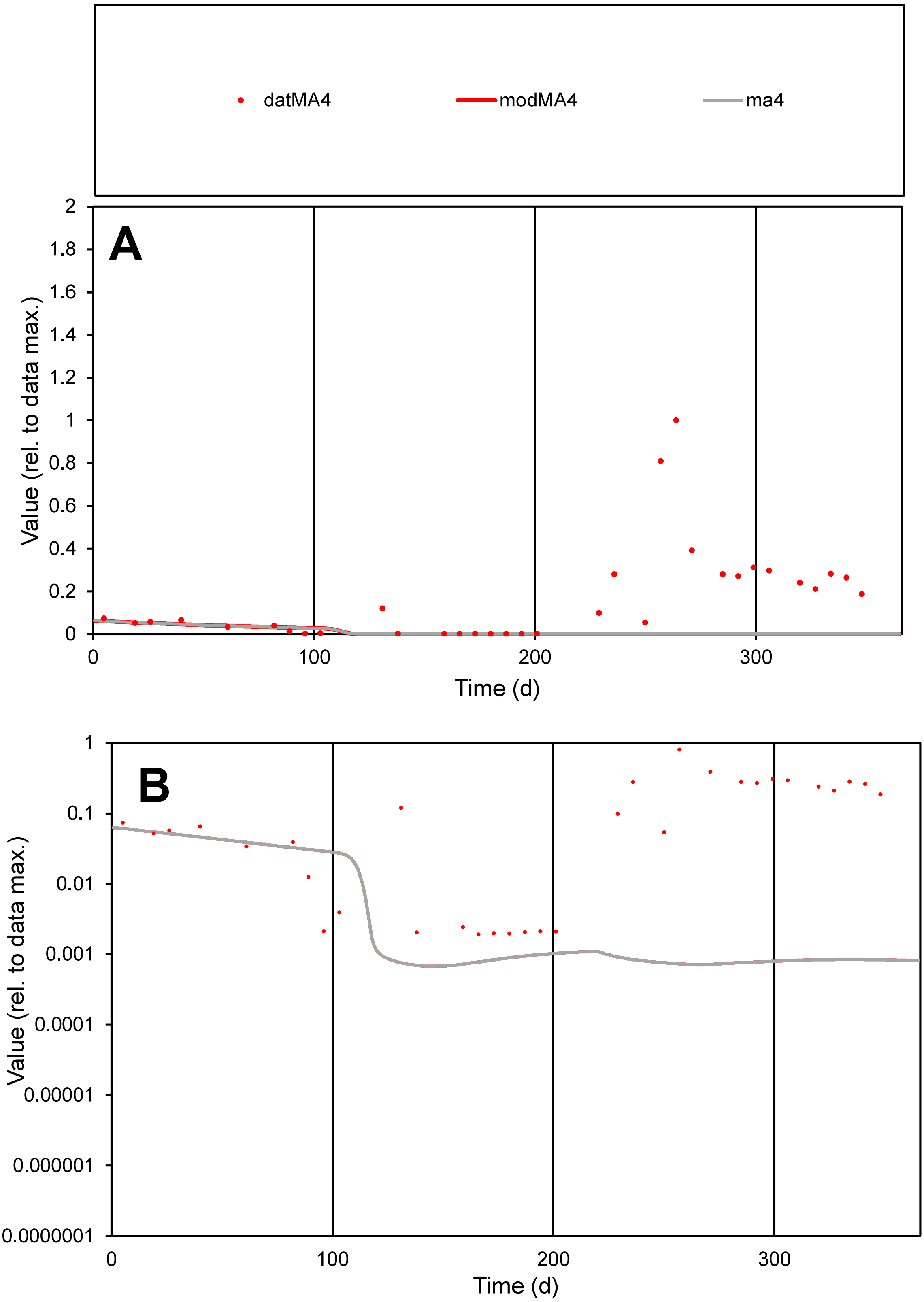

C1

Variables

MA4 Provide jtd or Dst

CalibOnly?

2 1 to reproduce of

oNormalize?

2 1=no, 2=yes

ofxn calc*

1.67553

ofxn mod

1.67881

sRun

RunA1

zout

1

Ave

Data

0.00165

Model

0.00012

data/vMax

0.01

ofxng

263.459

Min d/m

2.78053

ave/min d

86.5851

ave/min m

17.0832

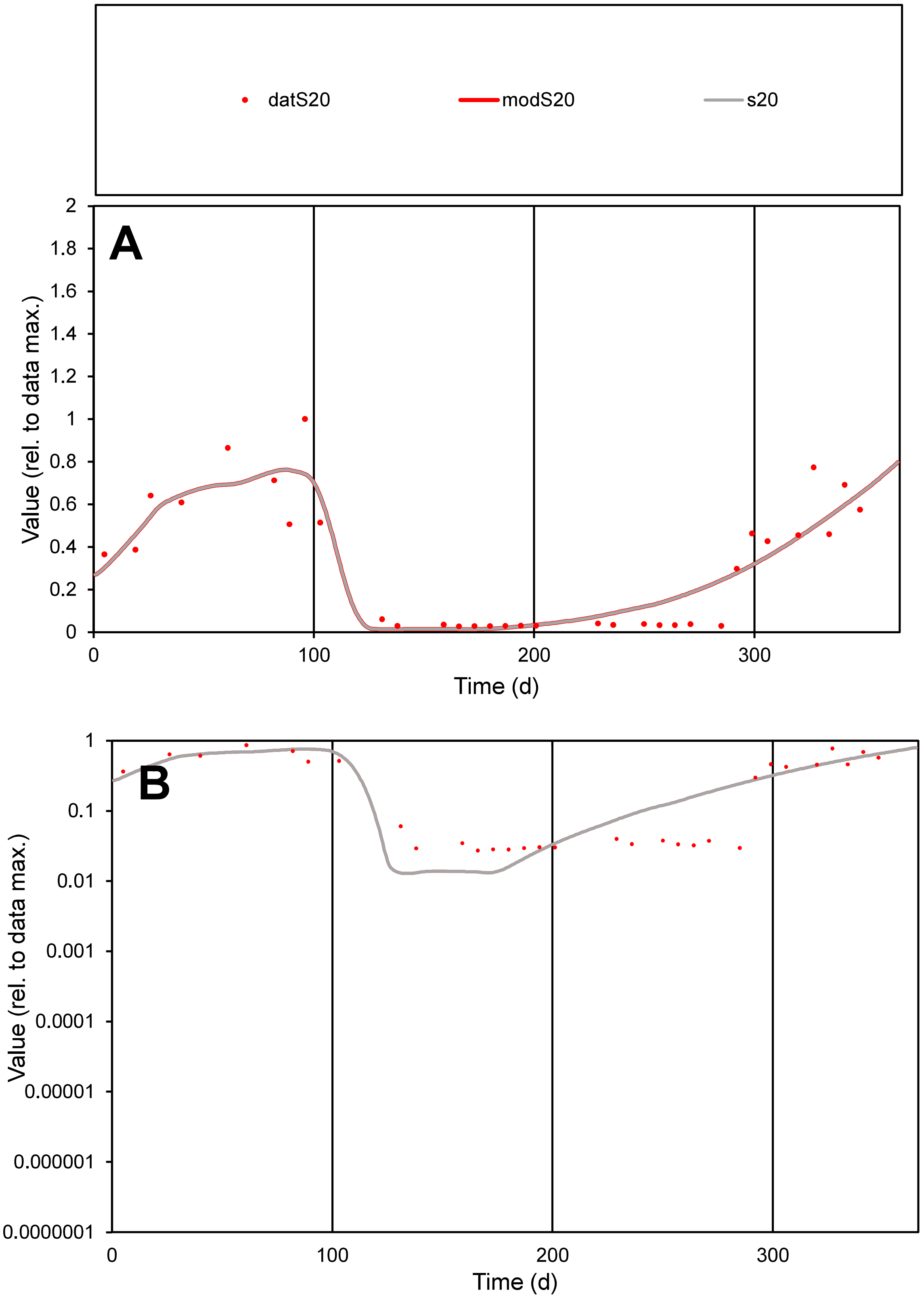

C1

Variables

S20 Provide jtd or Dst

CalibOnly?

2 1 to reproduce of

oNormalize?

2 1=no, 2=yes

ofxn calc*

0.36302

ofxn mod

0.39198

sRun

RunA1

zout

1

Ave

Data

0.00088

Model

0.0009

data/vMax

0.00283

ofxng

263.459

Min d/m

2.11647

ave/min d

11.4265

ave/min m

24.6599

326_Calib_S_A

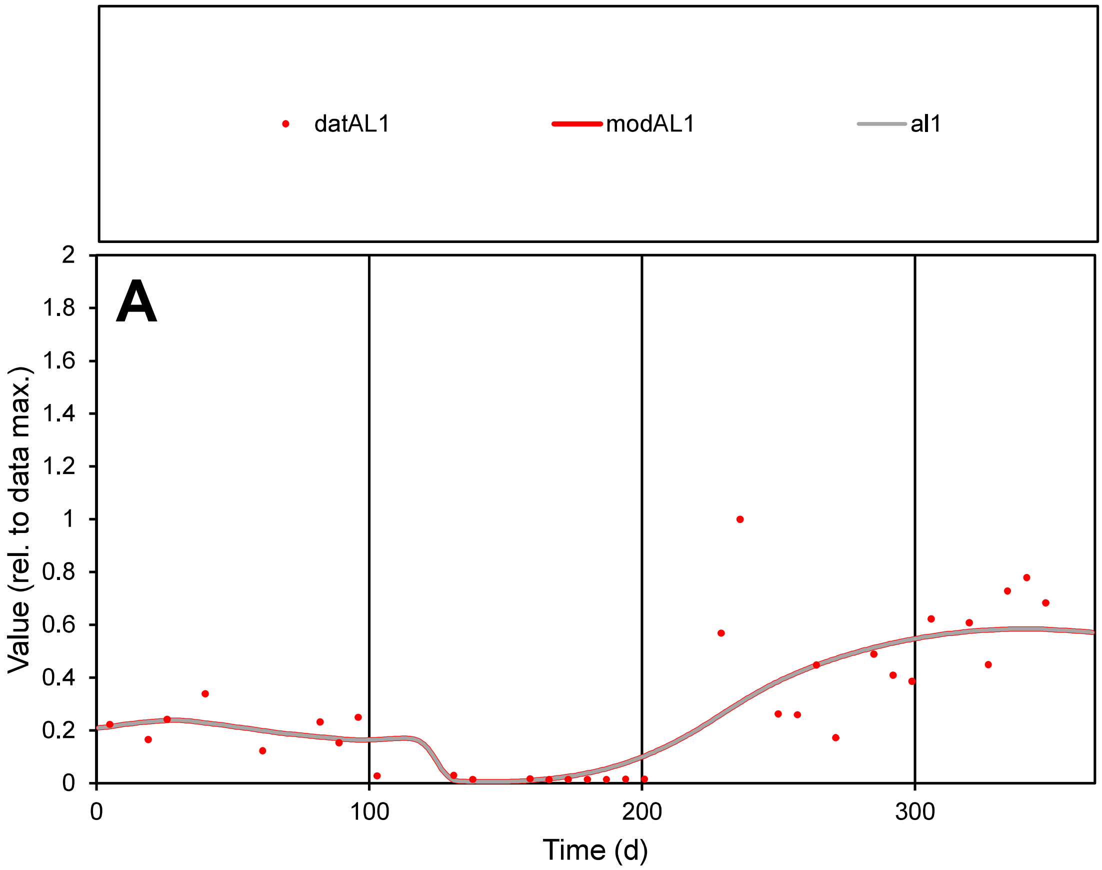

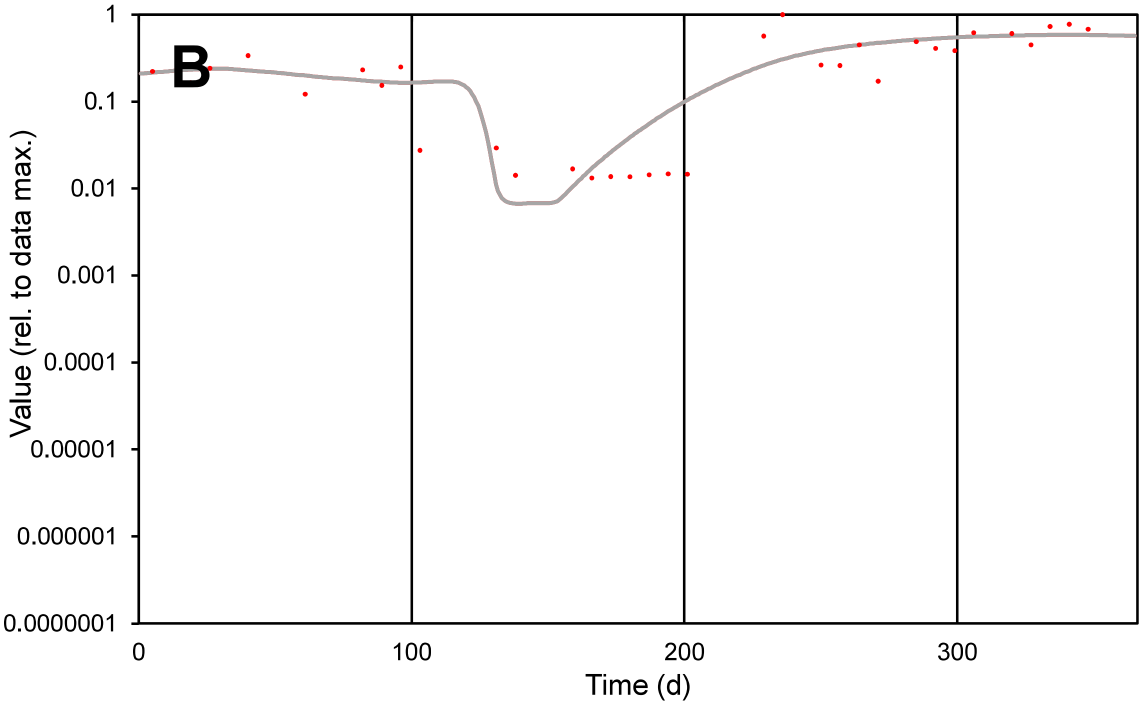

C1

Variables
AL1 Provide jtd or Dst
CalibOnly?
2 1 to reproduce of
oNormalize?
2 1=no, 2=yes

ofxn calc*
0.55767
ofxn mod
0.57414

sRun
RunA1
zout
1

Ave
Data
0.00097
Model
0.00095

data/vMax
0.00328

ofxng
263.459

Min d/m
1.98447

ave/min d
22.3182
ave/min m
43.2825

326_Calib_S_A

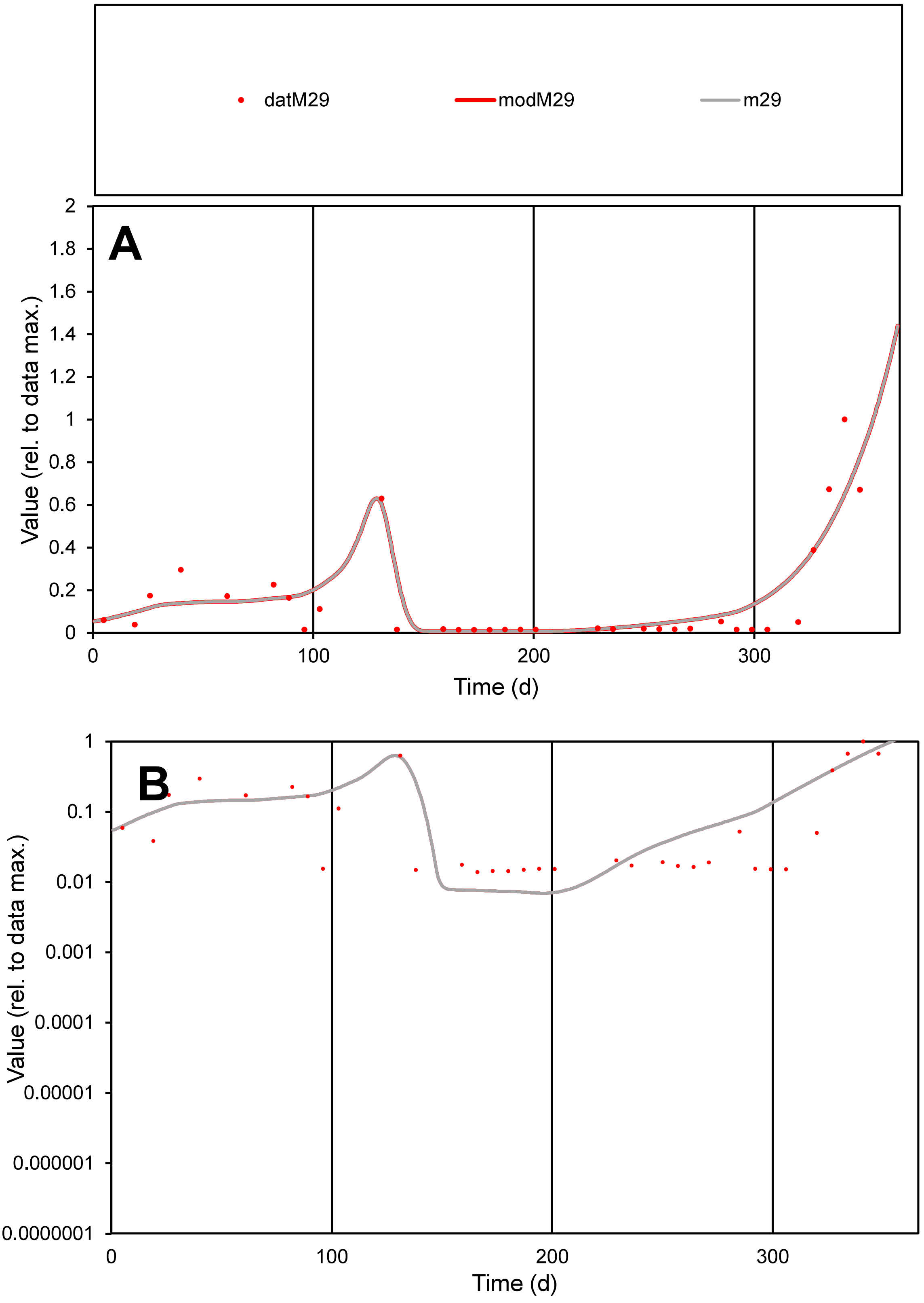

C1

Variables

M29 Provide jtd or Dst

CalibOnly?

2 1 to reproduce of

oNormalize?

2 1=no, 2=yes

ofxn calc*

0.69012

ofxn mod

0.72713

sRun

RunA1

zout

1

Ave

Data

0.00059

Model

0.00065

data/vMax

0.00389

ofxng

263.459

Min d/m

2.00223

ave/min d

10.9247

ave/min m

24.2652

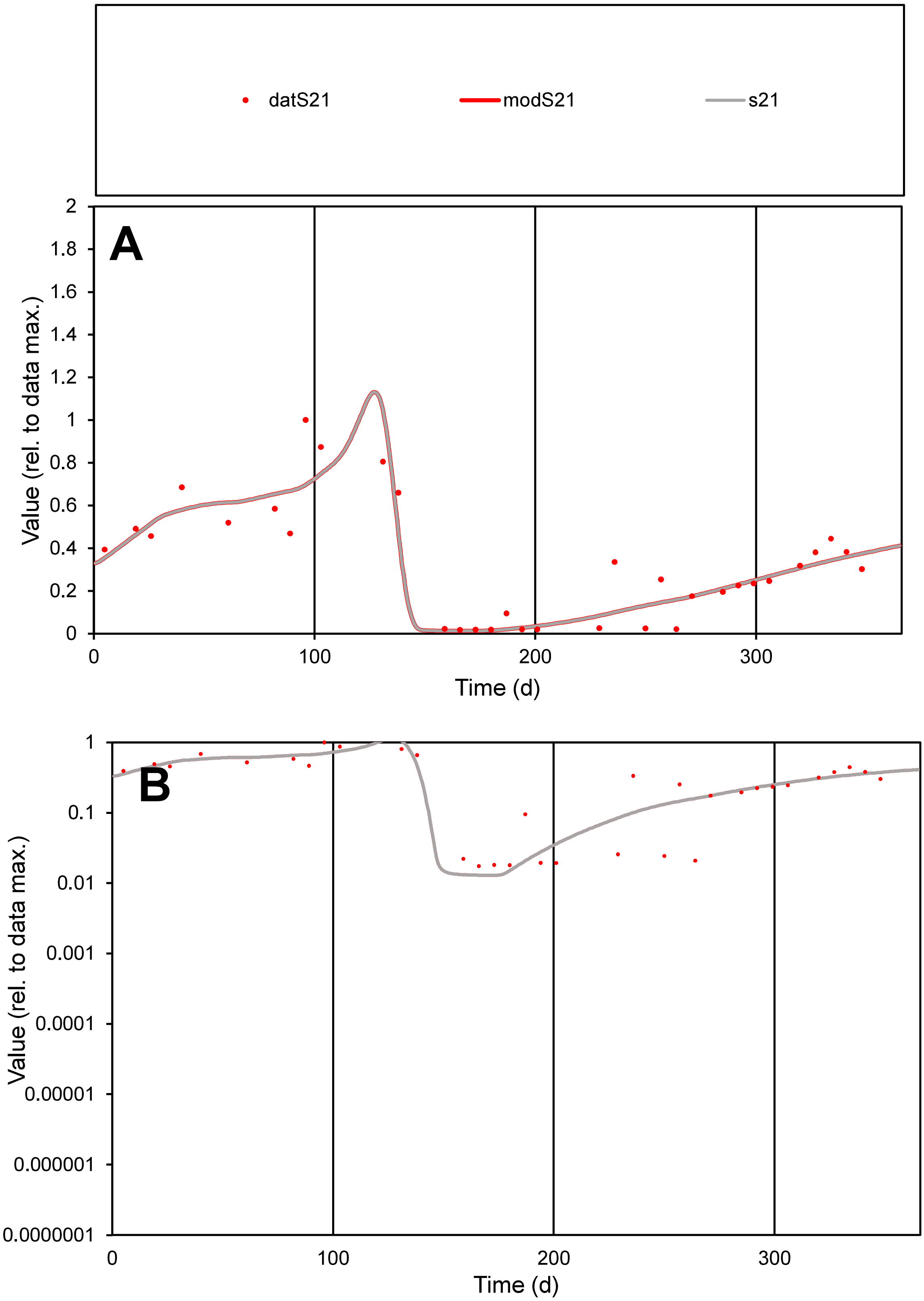

C1

Variables
S21 Provide jtd or Dst
CalibOnly?
2 1 to reproduce of
oNormalize?
2 1=no, 2=yes

ofxn calc*
0.32414
ofxn mod
0.35714

sRun
RunA1
zout
1

Ave
Data
0.00075
Model
0.00073

data/vMax
0.00232

ofxng
263.459

Min d/m
1.34485

ave/min d
18.5575
ave/min m
24.3151

326_Calib_S_A

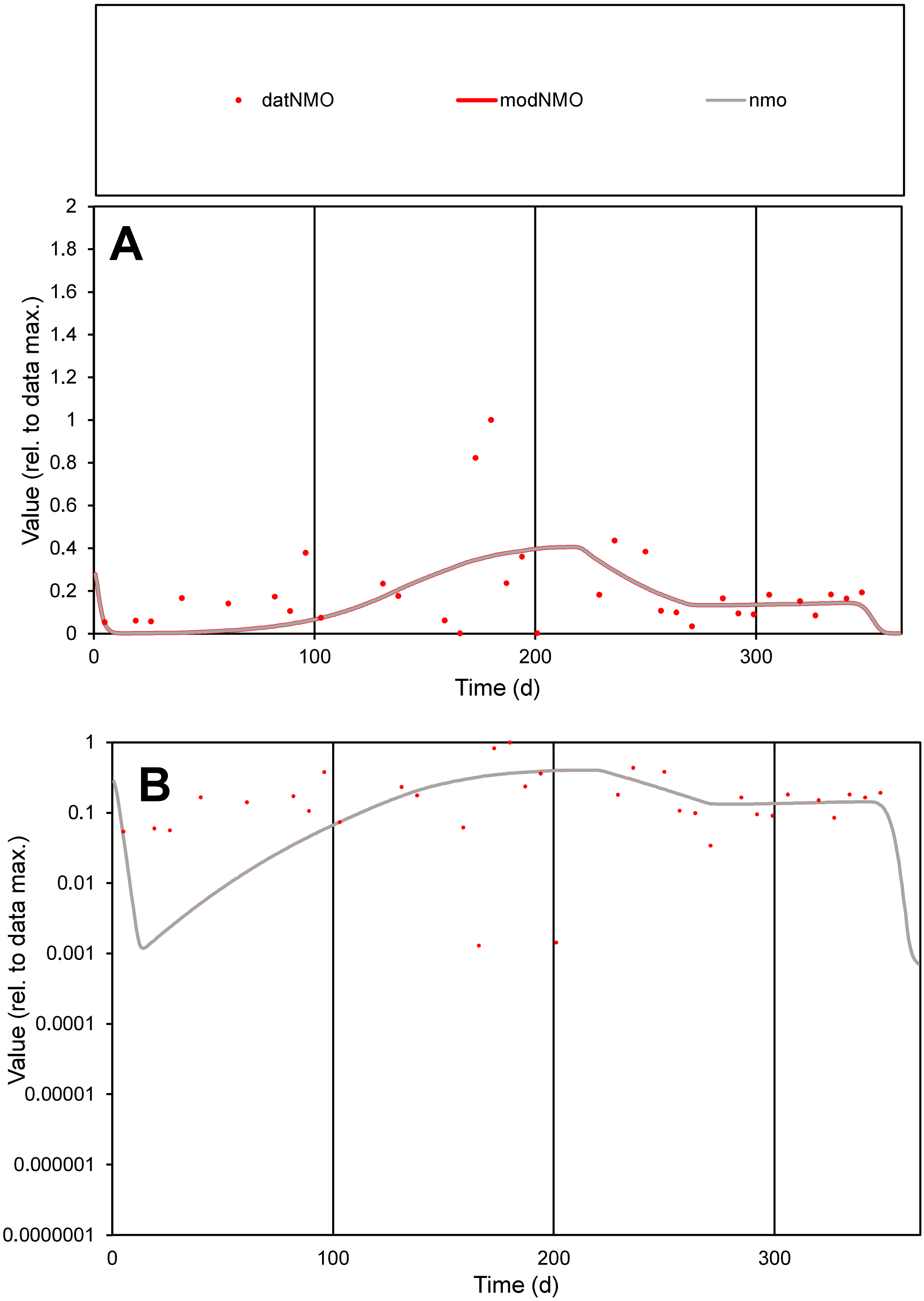

C1

Variables
NMO Provide jtd or Dst
CalibOnly?
2 1 to reproduce of
oNormalize?
2 1=no, 2=yes

ofxn calc*
0.96368
ofxn mod
0.97366

sRun
RunA1
zout
1

Ave
Data
0.0011
Model
0.00095

data/vMax
0.00548

ofxng
263.459

Min d/m
0.77481

ave/min d
154.661
ave/min m
103.007

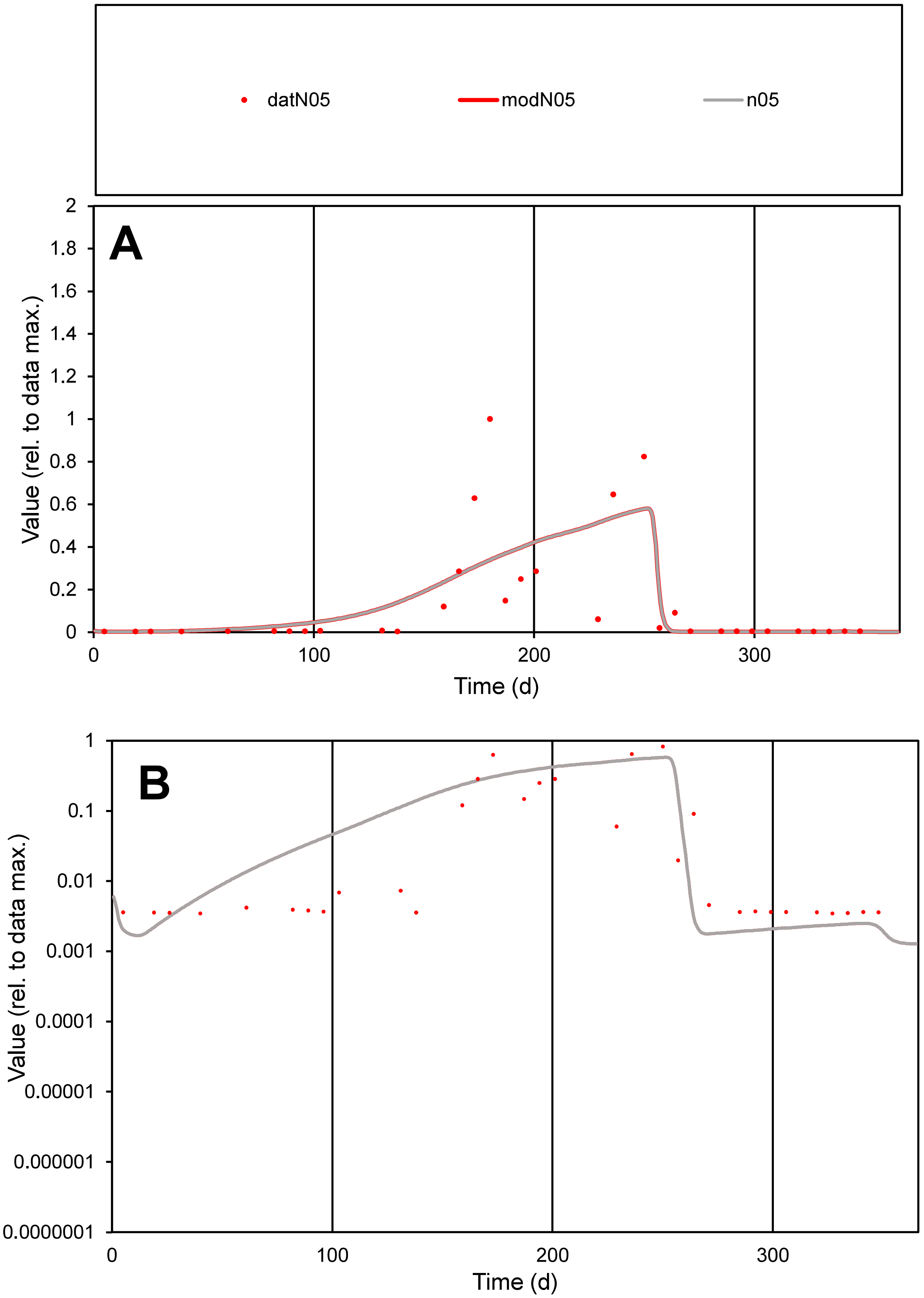

C1

Variables
N05 Provide jtd or Dst
CalibOnly?
2 1 to reproduce of
oNormalize?
2 1=no, 2=yes

ofxn calc*
1.26473
ofxn mod
1.285

sRun
RunA1
zout
1

Ave
Data
0.0016
Model
0.00163

data/vMax
0.0119

ofxng
263.459

Min d/m
1.93841

ave/min d
38.8619
ave/min m
76.5494

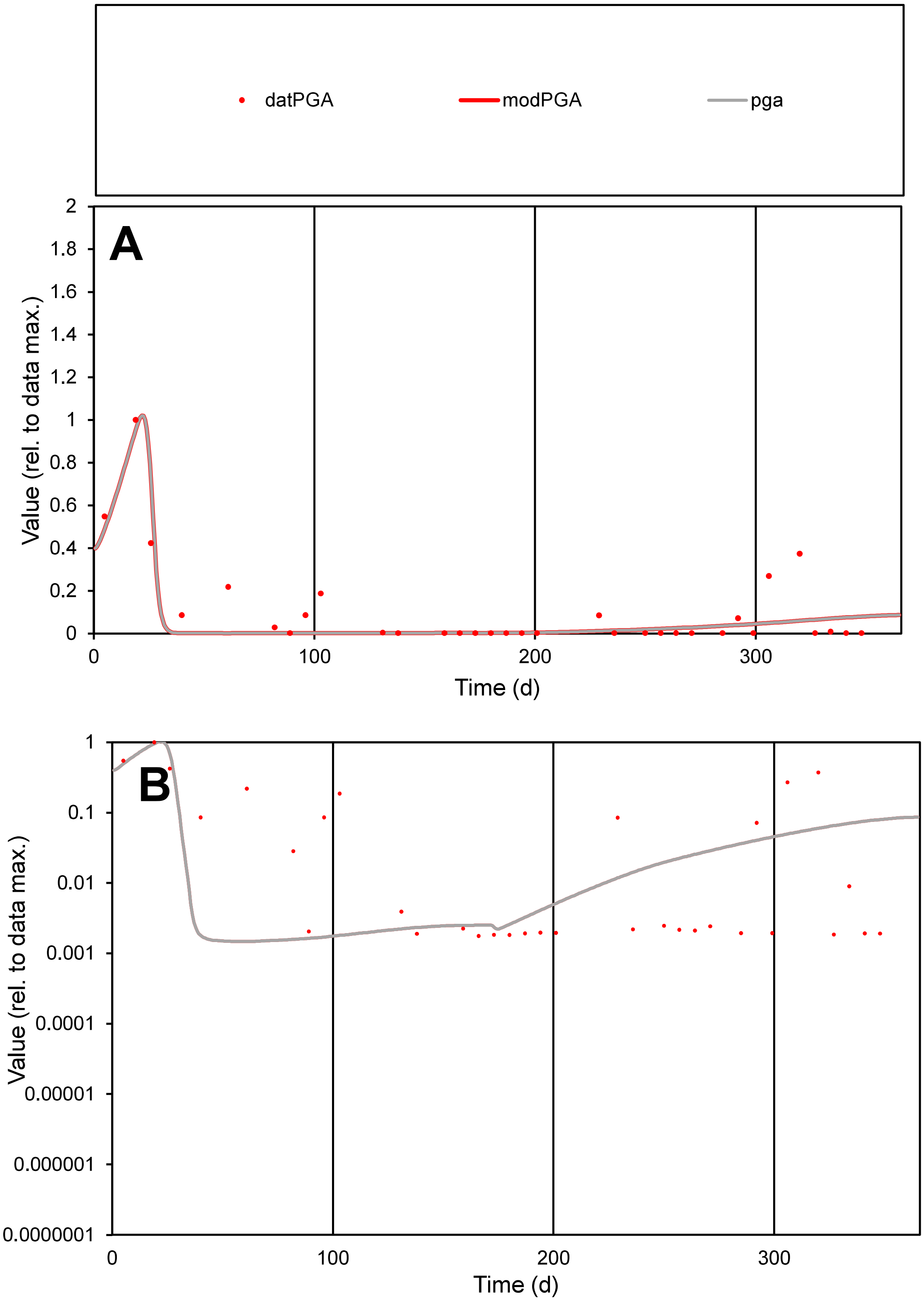

C1

Variables
PGA Provide jtd or Dst
CalibOnly?
2 1 to reproduce of
oNormalize?
2 1=no, 2=yes

ofxn calc*
0.87542
ofxn mod
0.919

sRun
RunA1
zout
1

Ave
Data
0.00099
Model
0.00076

data/vMax
0.00951

ofxng
263.459

Min d/m
1.20114

ave/min d
58.681
ave/min m
54.0694

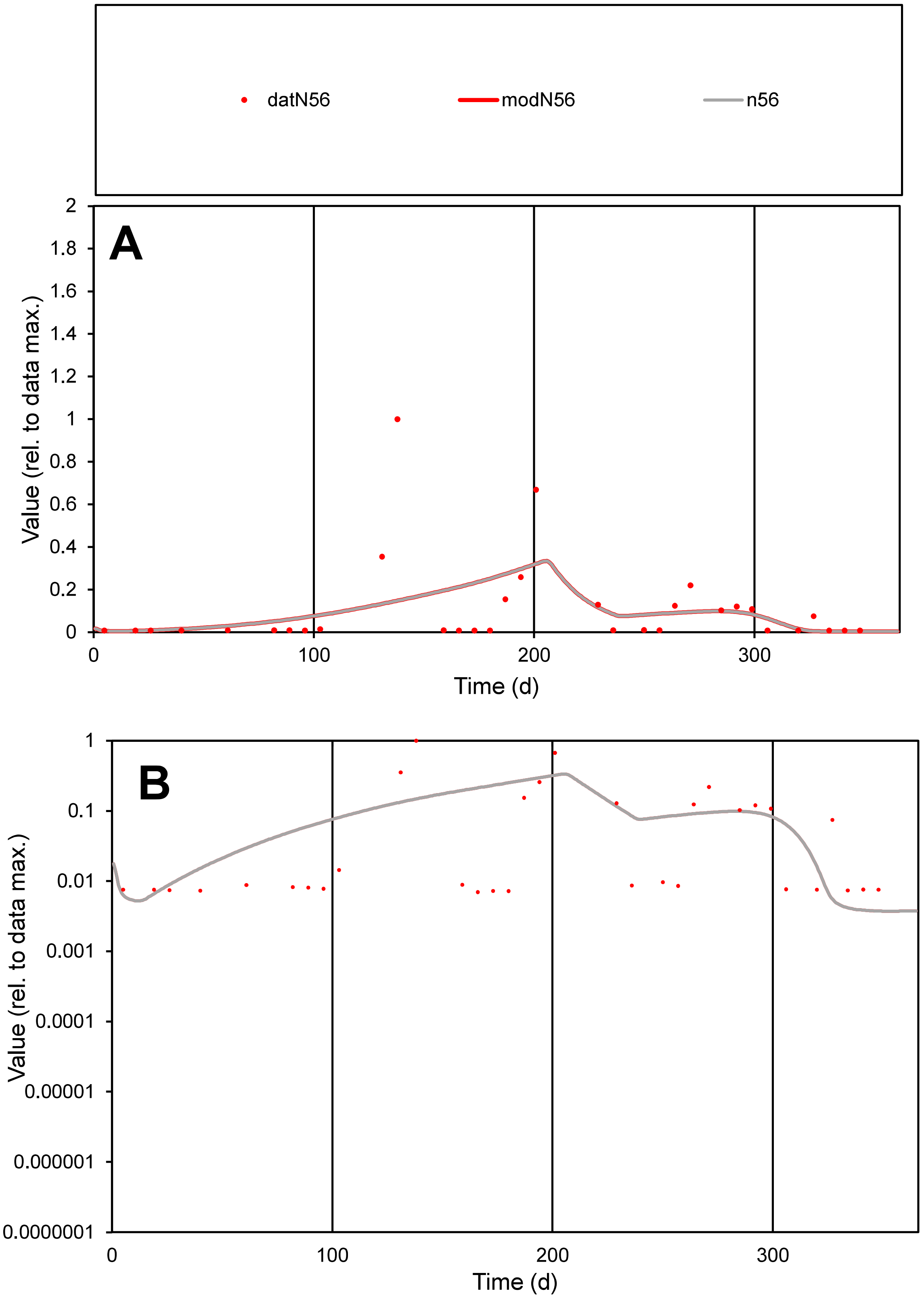

C1

Variables
N56 Provide jtd or Dst
CalibOnly?
2 1 to reproduce of
oNormalize?
2 1=no, 2=yes

ofxn calc*
1.76549
ofxn mod
1.77805

sRun
RunA1
zout
1

Ave
Data
0.00051
Model
0.00049

data/vMax
0.00484

ofxng
263.459

Min d/m
1.86504

ave/min d
15.1293
ave/min m
26.8923

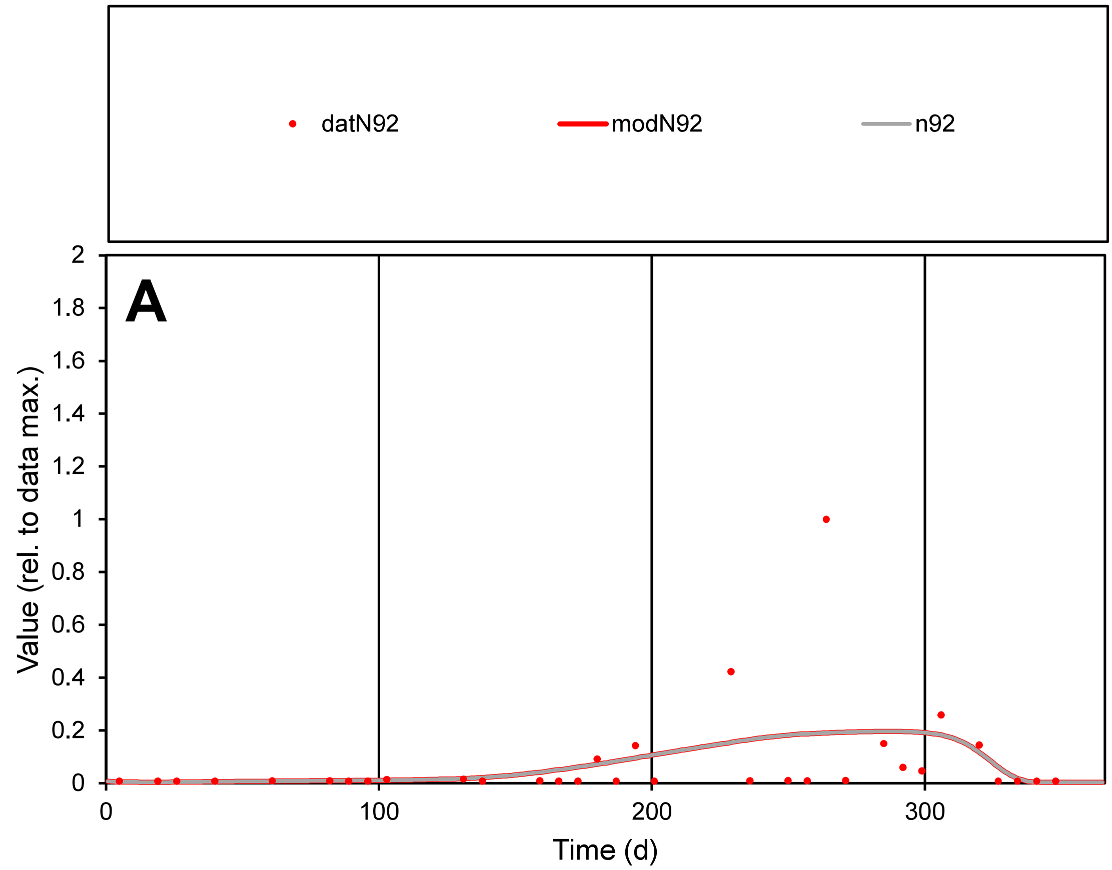

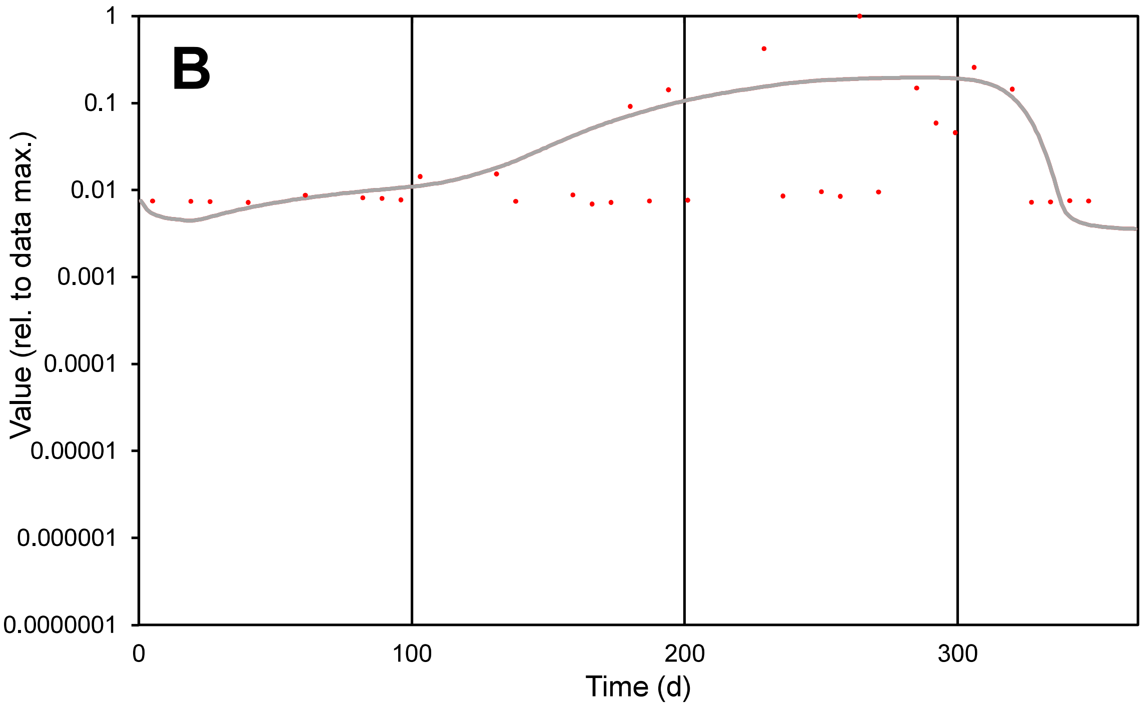

C1

Variables
N92 Provide jtd or Dst
CalibOnly?
2 1 to reproduce of
oNormalize?
2 1=no, 2=yes

ofxn calc*
2.19171
ofxn mod
2.20564

sRun
RunA1
zout
1

Ave
Data
0.00041
Model
0.00043

data/vMax
0.00543

ofxng
263.459

Min d/m
1.75585

ave/min d
11.0326
ave/min m
20.3417

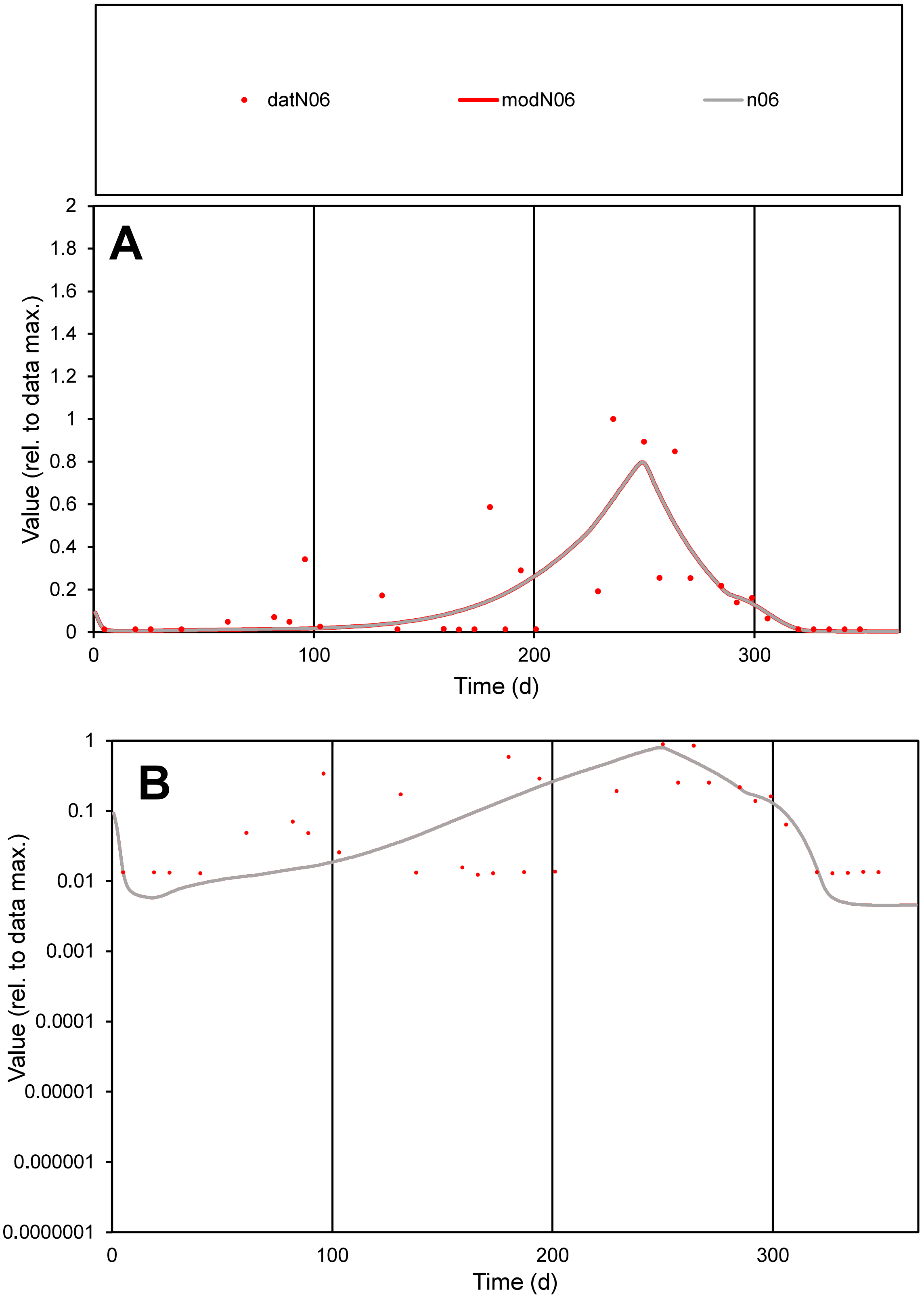

C1

Variables
N06 Provide jtd or Dst
CalibOnly?
2 1 to reproduce of
oNormalize?
2 1=no, 2=yes

ofxn calc*
0.97438
ofxn mod
0.99744

sRun
RunA1
zout
1

Ave
Data
0.00091
Model
0.00084

data/vMax
0.00519

ofxng
263.459

Min d/m
2.74199

ave/min d
14.2177
ave/min m
35.9123

326_Calib_S_A

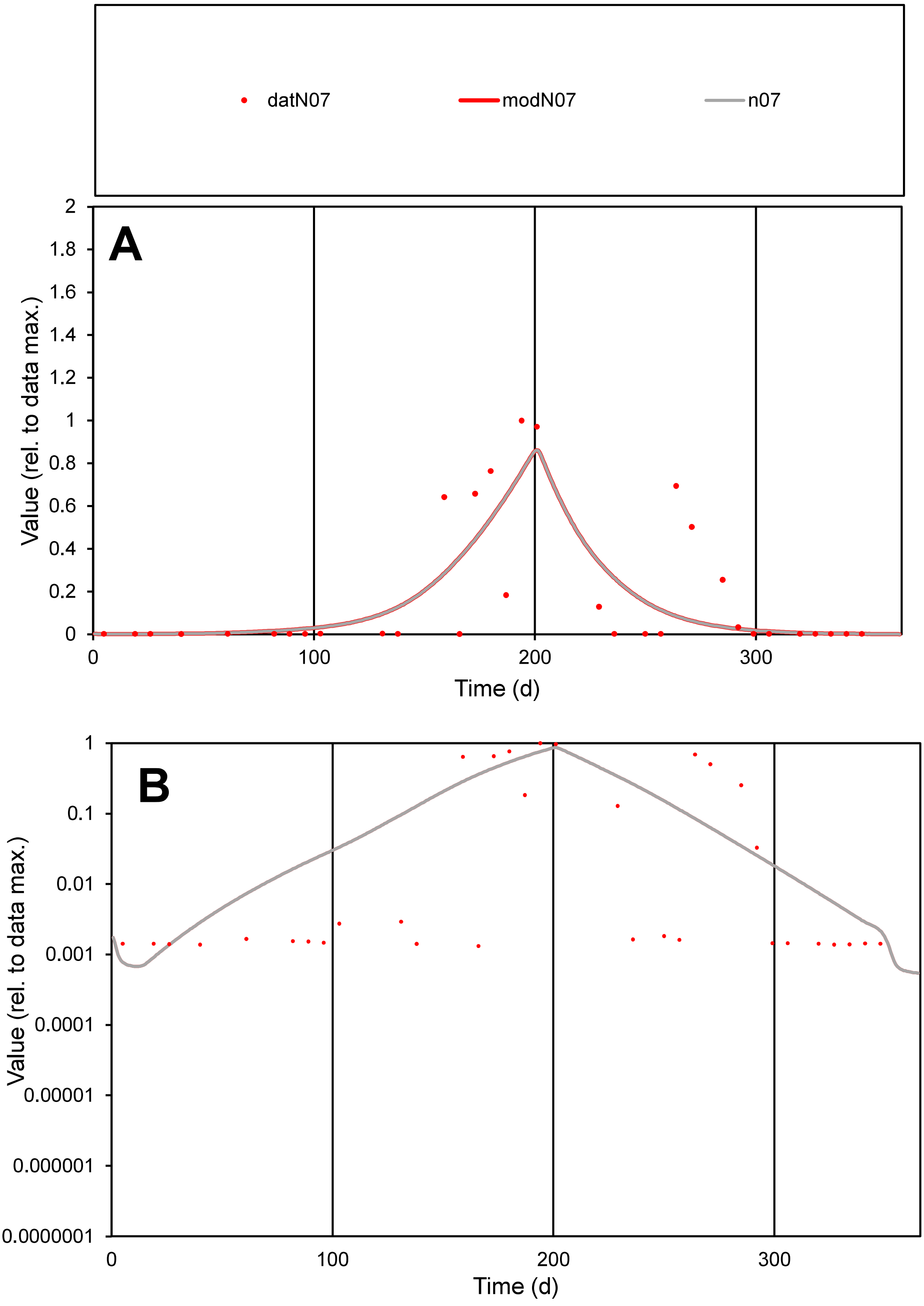

C1

Variables
N07 Provide jtd or Dst
CalibOnly?
2 1 to reproduce of
oNormalize?
2 1=no, 2=yes

ofxn calc*
1.14582
ofxn mod
1.17076

sRun
RunA1
zout
1

Ave
Data
0.00227
Model
0.00207

data/vMax
0.01276

ofxng
263.459

Min d/m
1.70084

ave/min d
134.639
ave/min m
208.715

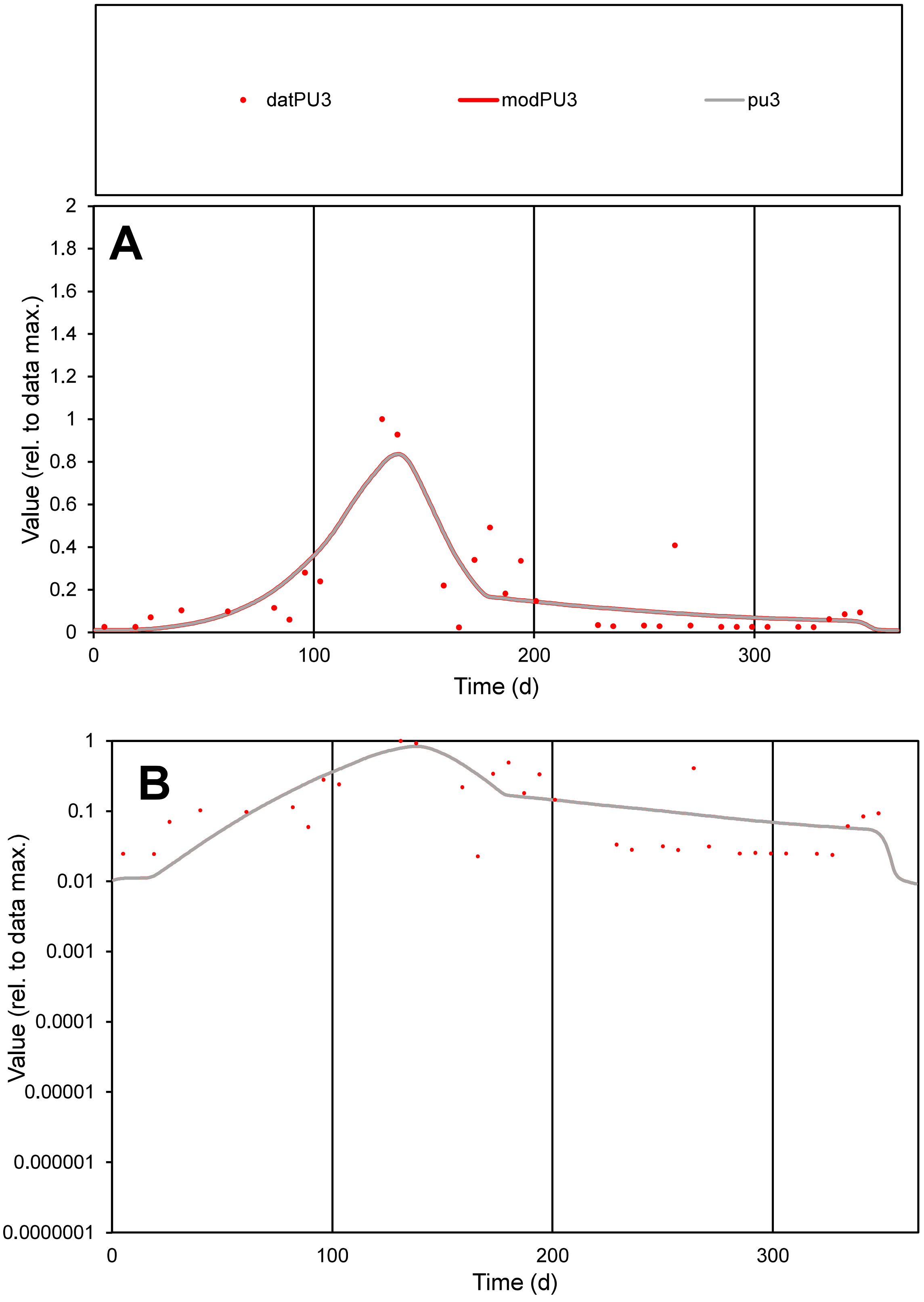

C1

Variables

PU3 Provide jtd or Dst

CalibOnly?

2 1 to reproduce of

oNormalize?

2 1=no, 2=yes

ofxn calc*

0.75227

ofxn mod

0.78775

sRun

RunA1

zout

1

Ave

Data

0.00111

Model

0.00113

data/vMax

0.00653

ofxng

263.459

Min d/m

2.04934

ave/min d

7.48422

ave/min m

15.6102

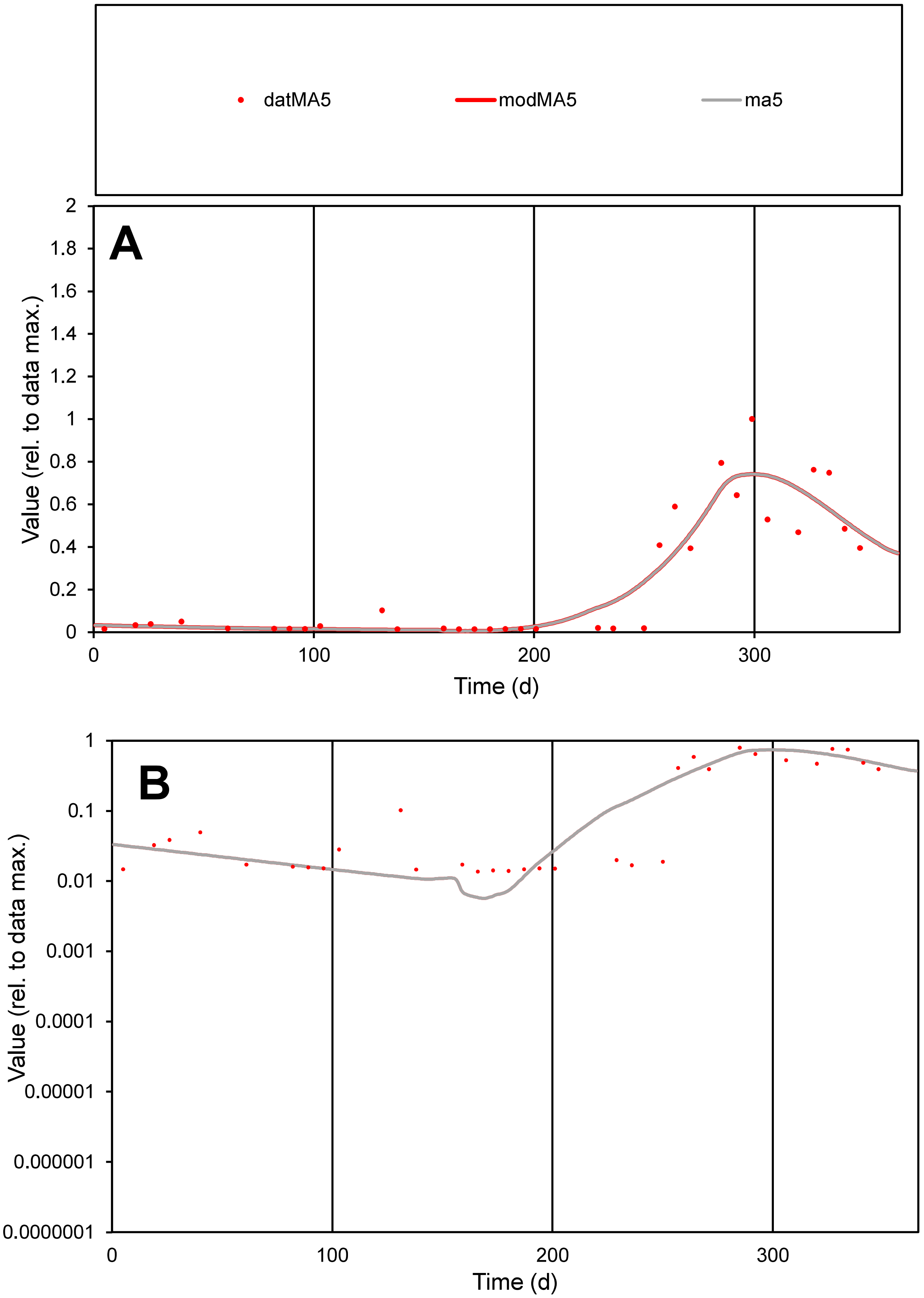

C1

Variables
MA5 Provide jtd or Dst
CalibOnly?
2 1 to reproduce of
oNormalize?
2 1=no, 2=yes

ofxn calc*
0.44703
ofxn mod
0.48077

sRun
RunA1
zout
1

Ave
Data
0.0016
Model
0.00159

data/vMax
0.00683

ofxng
263.459

Min d/m
2.37302

ave/min d
17.1901
ave/min m
40.6595

326_Calib_S_A

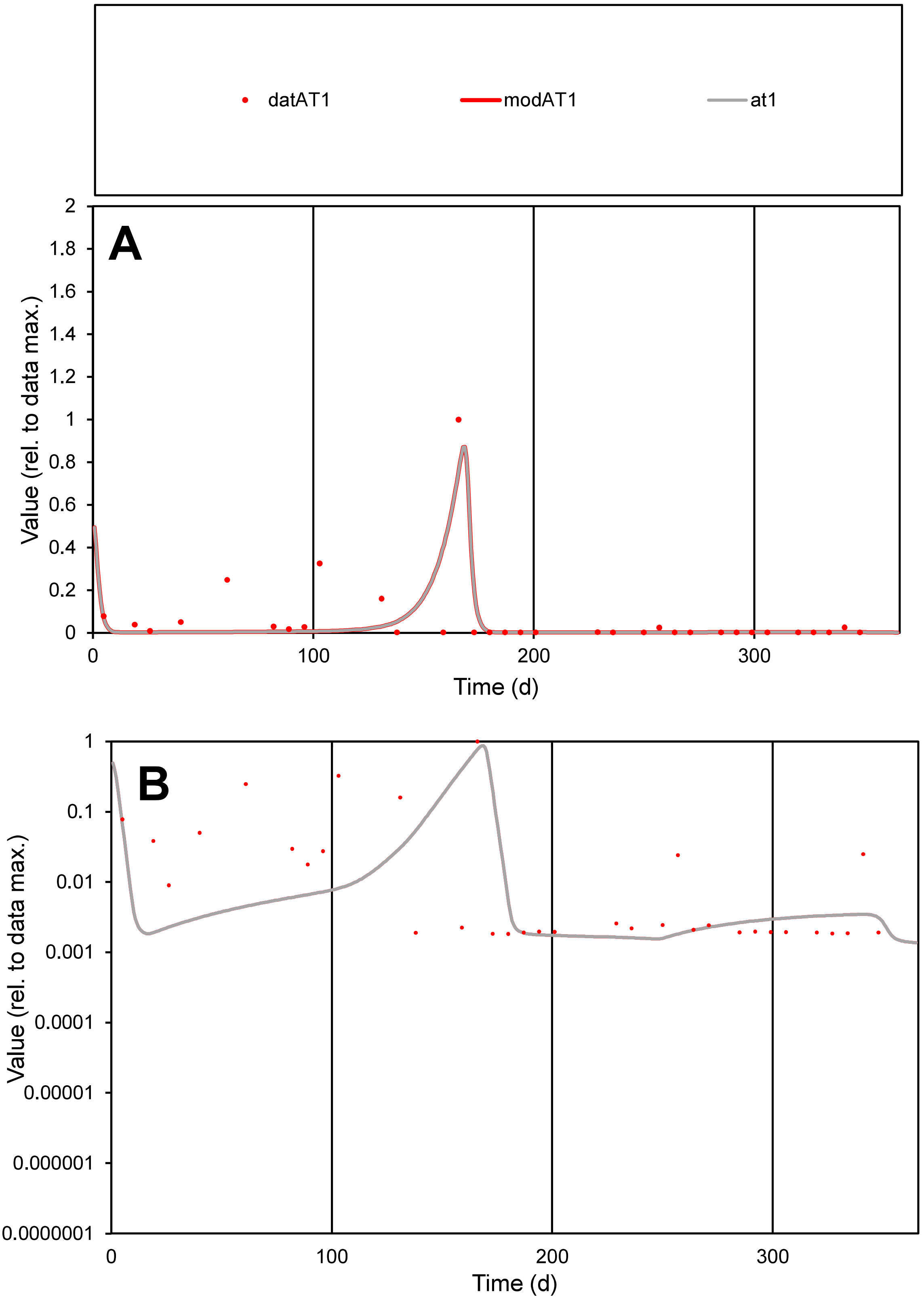

C1

Variables
AT1 Provide jtd or Dst
CalibOnly?
2 1 to reproduce of
oNormalize?
2 1=no, 2=yes

ofxn calc*
1.57907
ofxn mod
1.67477

sRun
RunA1
zout
1

Ave
Data
0.00168
Model
0.00119

data/vMax
0.02678

ofxng
263.459

Min d/m
1.13607

ave/min d
34.5026
ave/min m
27.7987

326_Calib_S_A

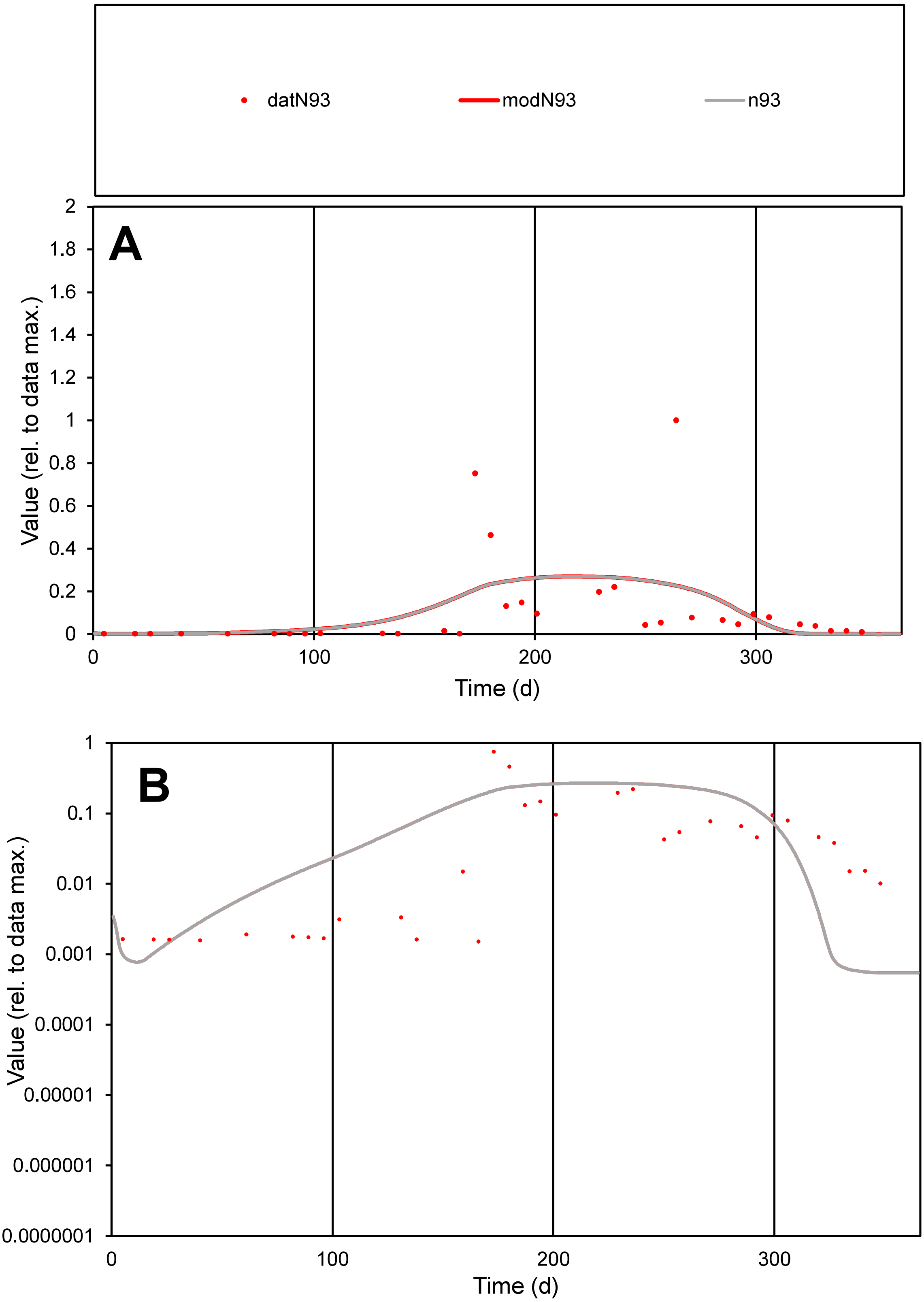

C1

Variables
N93 Provide jtd or Dst
CalibOnly?
2 1 to reproduce of
oNormalize?
2 1=no, 2=yes

ofxn calc*
1.70996
ofxn mod
1.72439

sRun
RunA1
zout
1

Ave
Data
0.00125
Model
0.00124

data/vMax
0.01135

ofxng
263.459

Min d/m
2.77221

ave/min d
72.7232
ave/min m
199.622

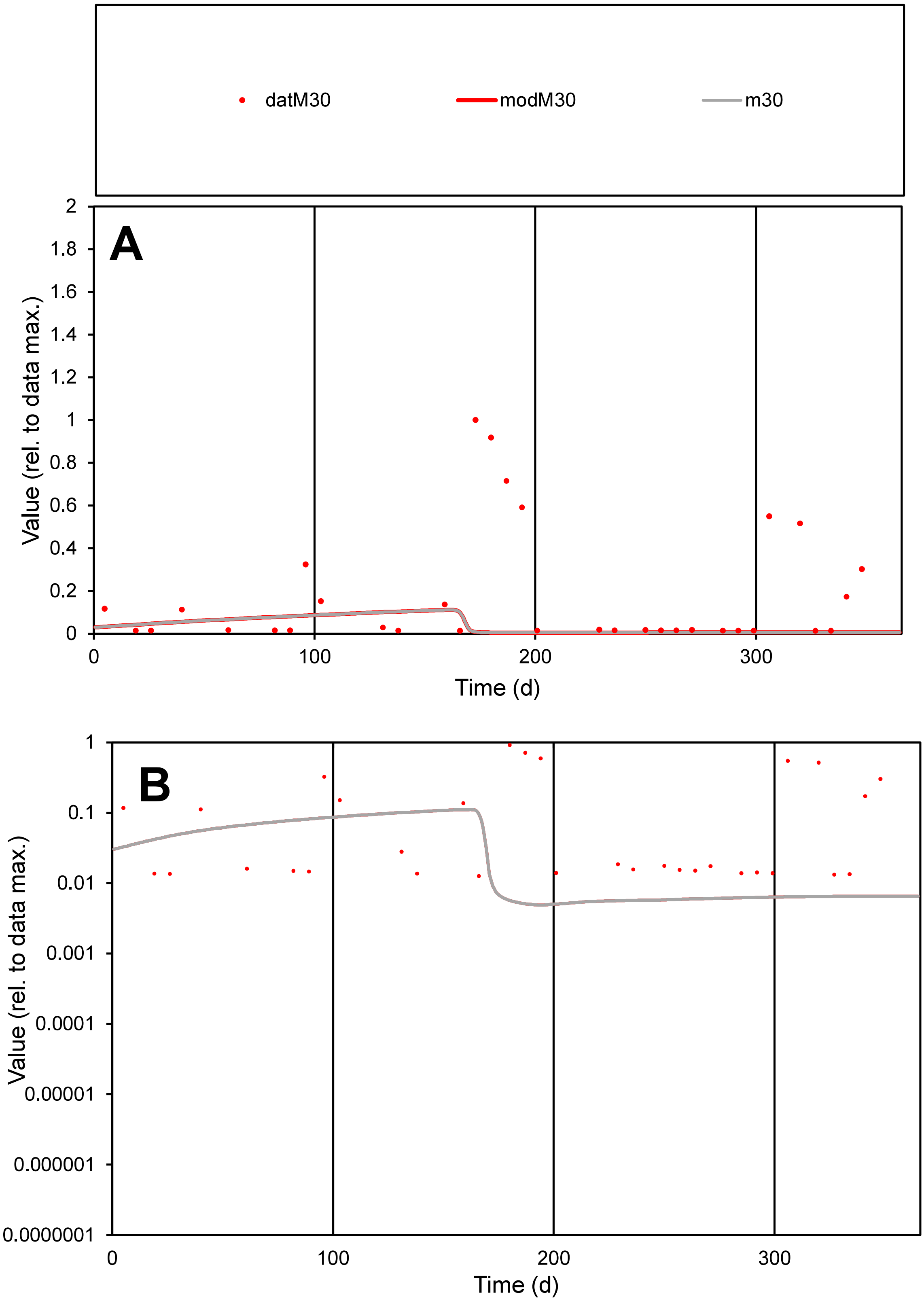

C1

Variables
M30 Provide jtd or Dst
CalibOnly?
2 1 to reproduce of
oNormalize?
2 1=no, 2=yes

ofxn calc*
1.80004
ofxn mod
1.80351

sRun
RunA1
zout
1

Ave
Data
0.00057
Model
0.0001

data/vMax
0.0032

ofxng
263.459

Min d/m
2.59155

ave/min d
14.1489
ave/min m
6.48983

326_Calib_S_A

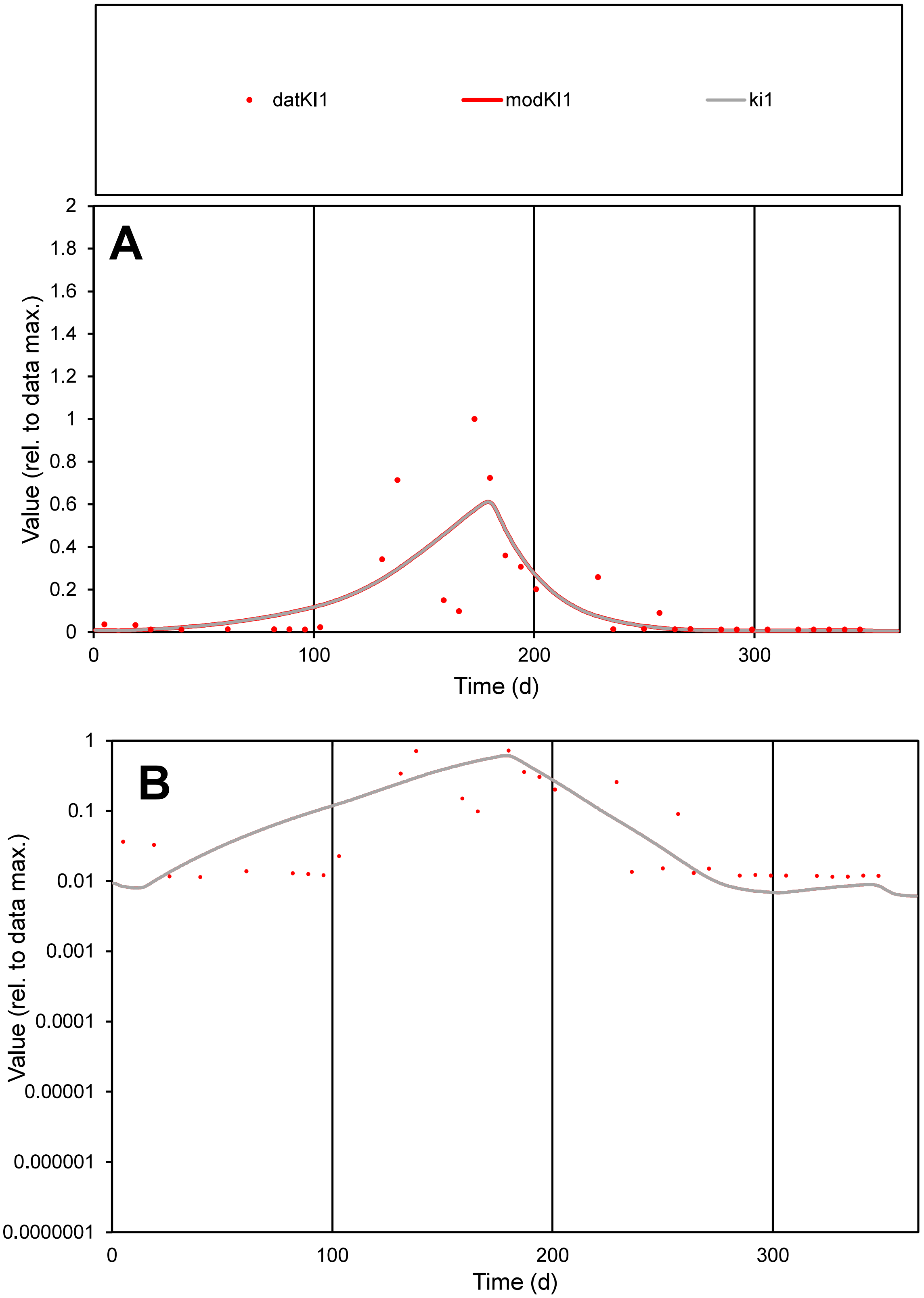

C1

Variables
KI1 Provide jtd or Dst
CalibOnly?
2 1 to reproduce of
oNormalize?
2 1=no, 2=yes

ofxn calc*
1.06493
ofxn mod
1.08842

sRun
RunA1
zout
1

Ave
Data
0.00058
Model
0.00058

data/vMax
0.00421

ofxng
263.459

Min d/m
1.67221

ave/min d
12.0879
ave/min m
20.1187

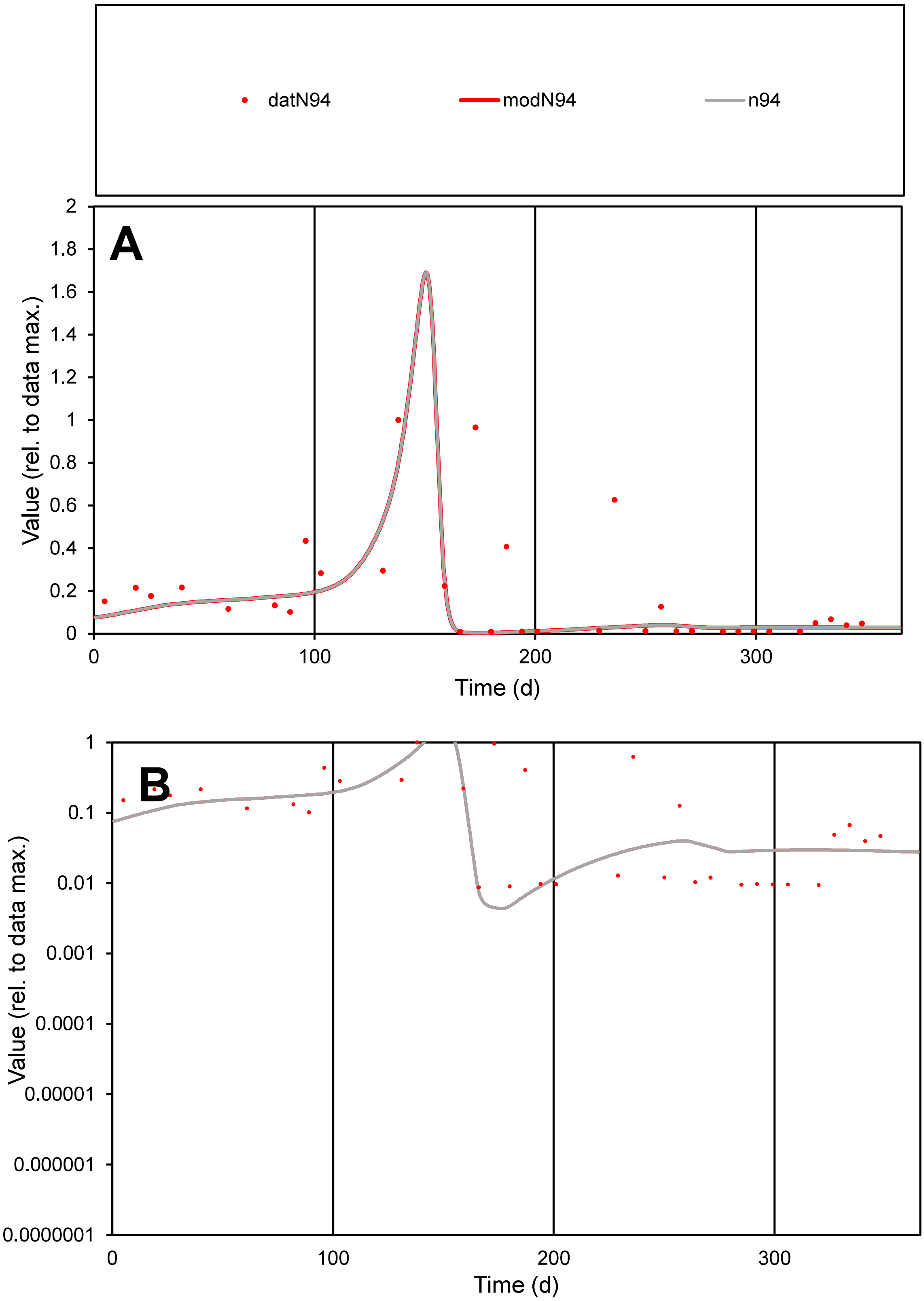

C1

Variables

N94 Provide jtd or Dst

CalibOnly?

2 1 to reproduce of

oNormalize?

2 1=no, 2=yes

ofxn calc*

1.26235

ofxn mod

1.27389

sRun

RunA1

zout

1

Ave

Data

0.0009

Model

0.00055

data/vMax

0.00514

ofxng

263.459

Min d/m

1.97837

ave/min d

20.1625

ave/min m

24.3764

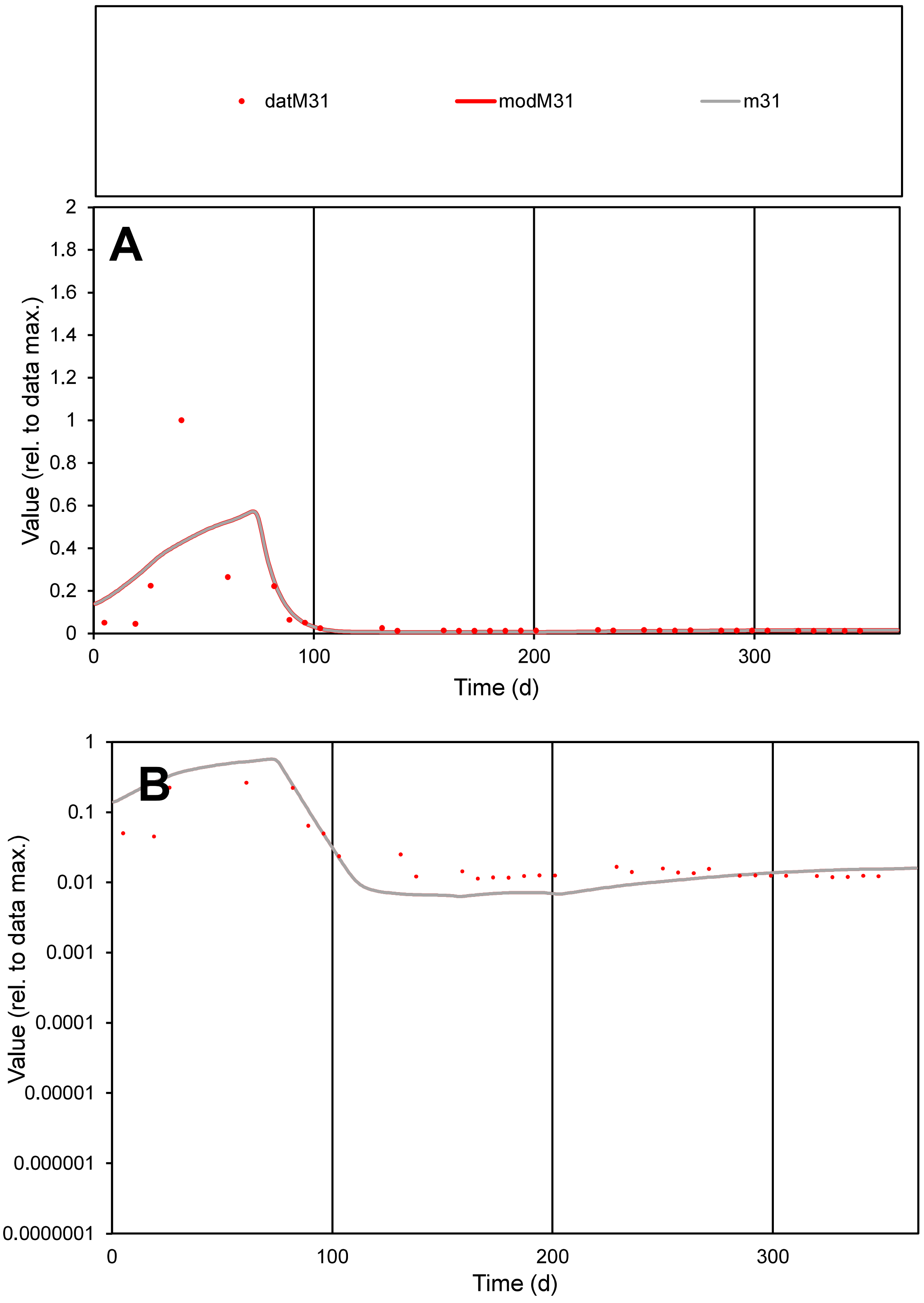

C1

Variables
M31 Provide jtd or Dst
CalibOnly?
2 1 to reproduce of
oNormalize?
2 1=no, 2=yes

ofxn calc*
1.72966
ofxn mod
1.76397

sRun
RunA1
zout
1

Ave
Data
0.00049
Model
0.0005

data/vMax
0.00708

ofxng
263.459

Min d/m
1.78473

ave/min d
6.04886
ave/min m
11.1465

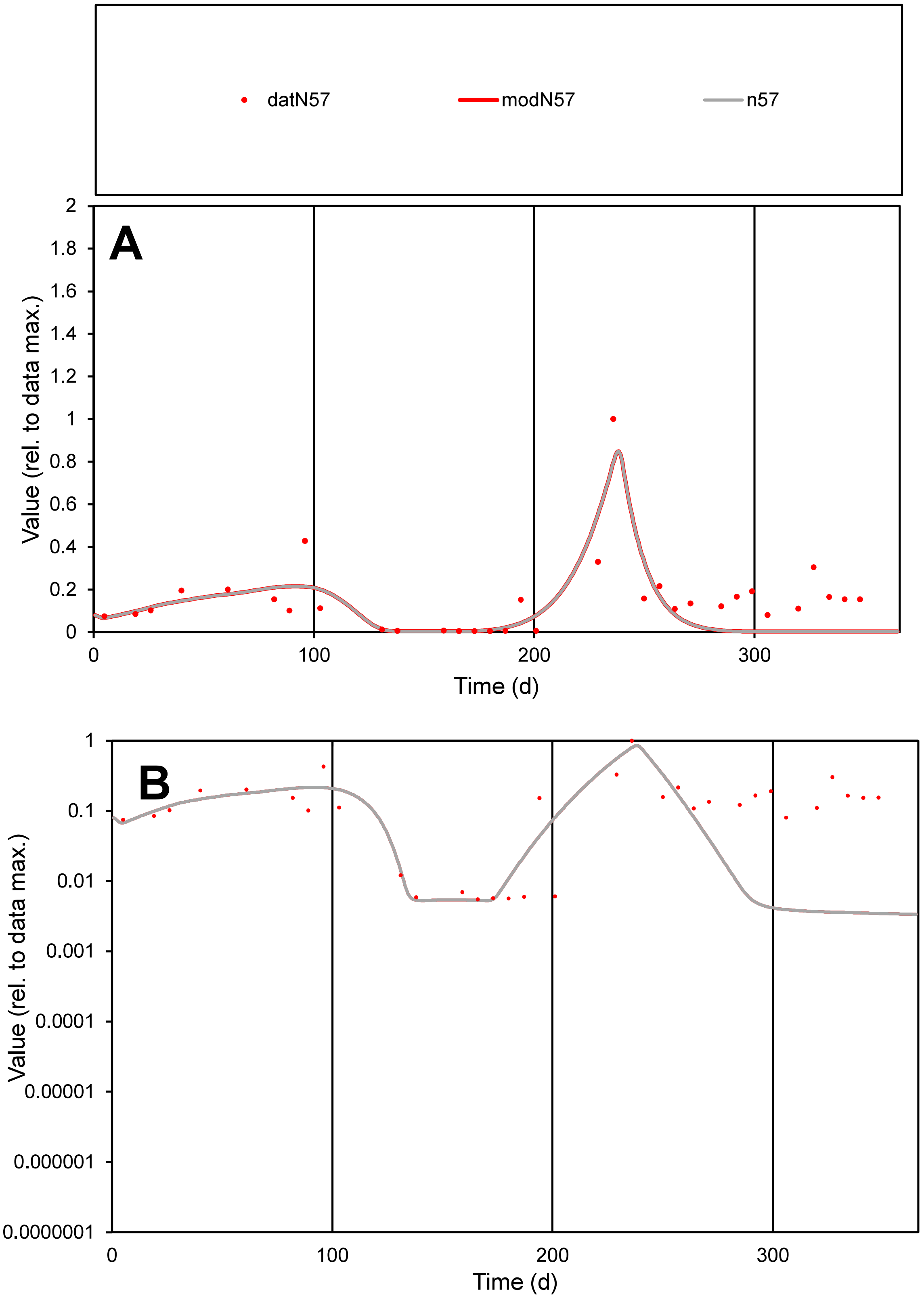

C1

Variables
N57 Provide jtd or Dst
CalibOnly?
2 1 to reproduce of
oNormalize?
2 1=no, 2=yes

ofxn calc*
0.76129
ofxn mod
0.78025

sRun
RunA1
zout
1

Ave
Data
0.001
Model
0.00072

data/vMax
0.0065

ofxng
263.459

Min d/m
1.5918

ave/min d
27.8652
ave/min m
31.8688

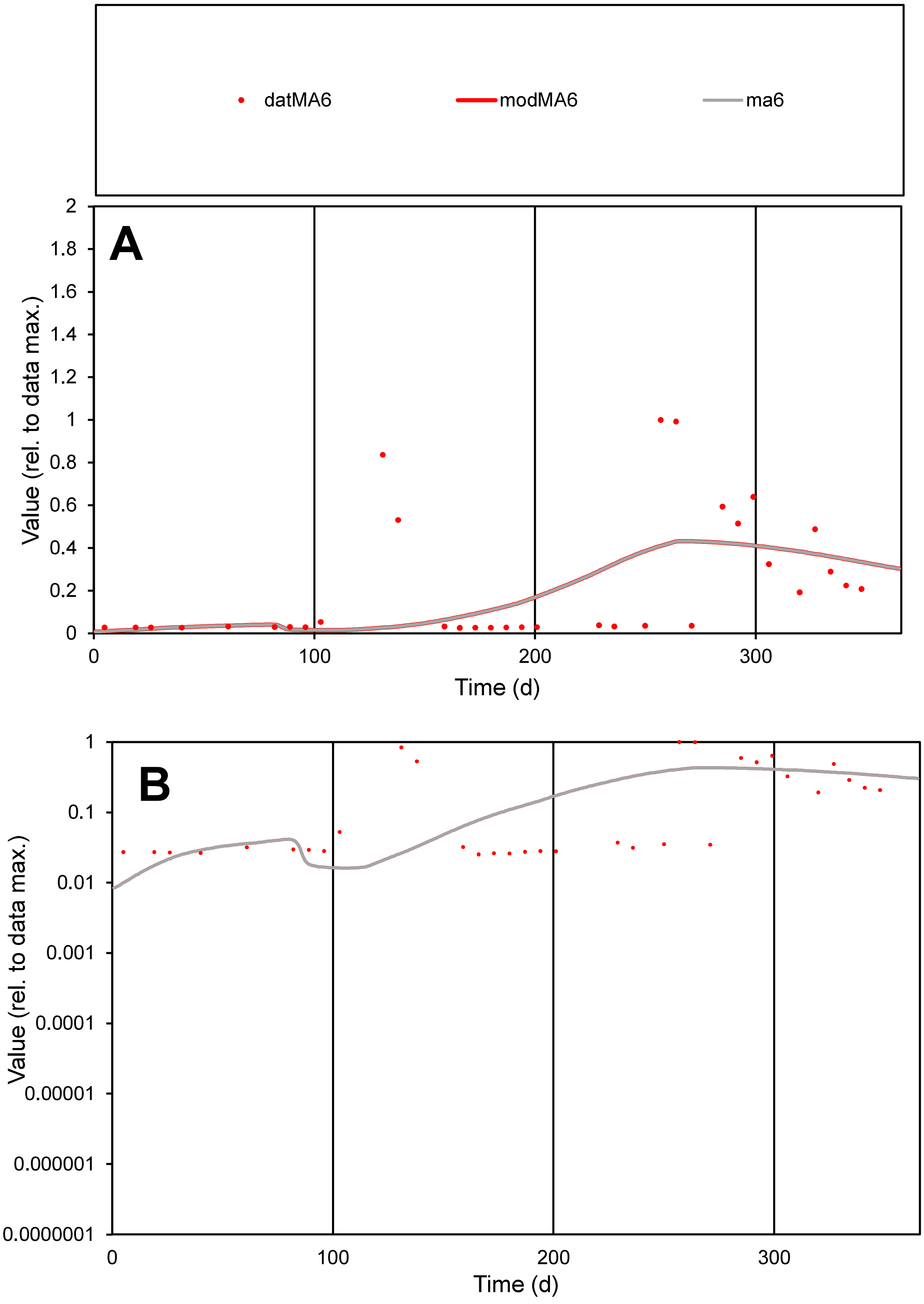

C1

Variables
MA6 Provide jtd or Dst
CalibOnly?
2 1 to reproduce of
oNormalize?
2 1=no, 2=yes

ofxn calc*
1.15457
ofxn mod
1.16506

sRun
RunA1
zout
1

Ave
Data
0.00104
Model
0.00095

data/vMax
0.0046

ofxng
263.459

Min d/m
2.37842

ave/min d
8.91232
ave/min m
19.3218

326_Calib_S_A

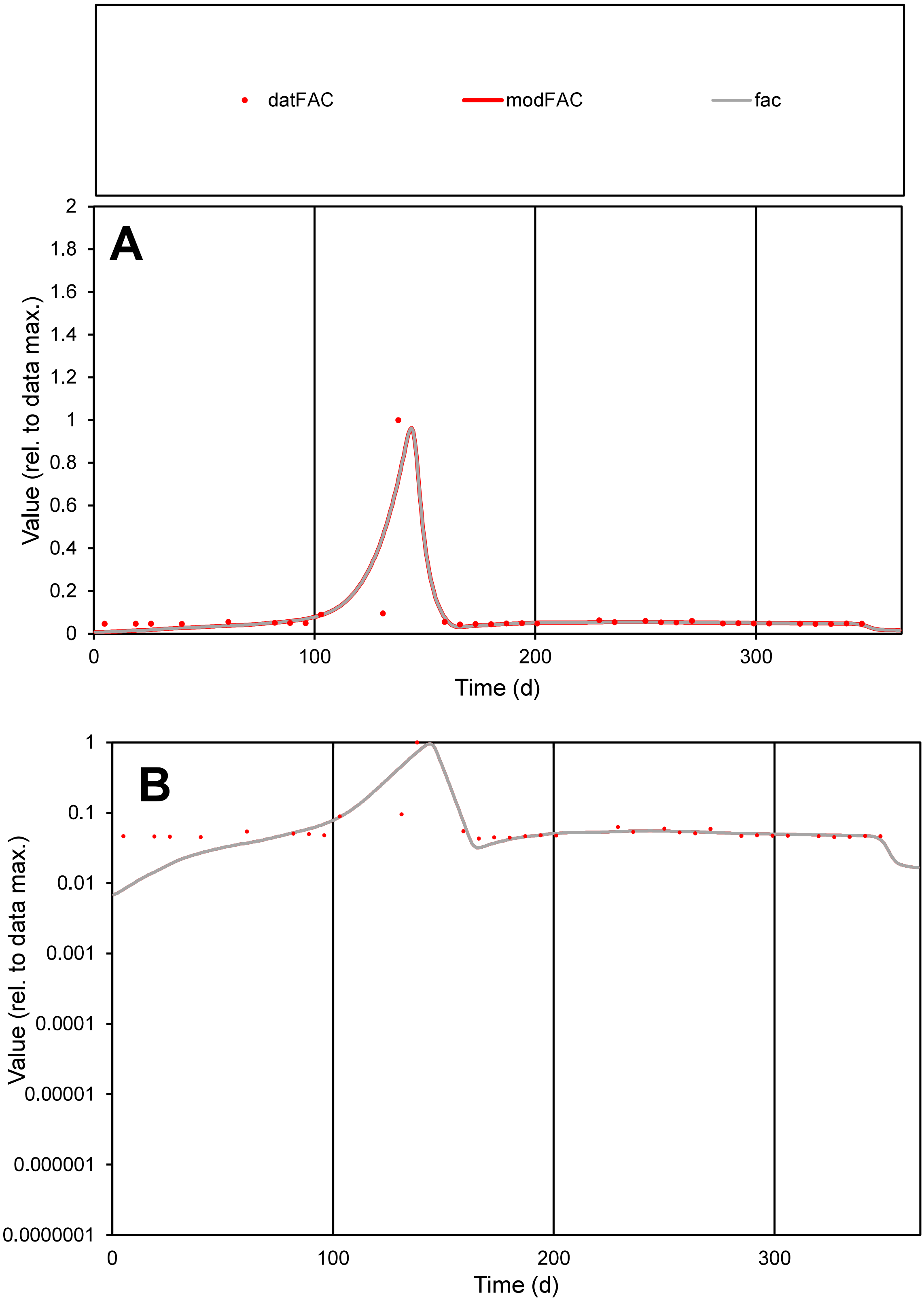

C1

Variables

FAC Provide jtd or Dst

CalibOnly?

2 1 to reproduce of

oNormalize?

2 1=no, 2=yes

ofxn calc*

0.86657

ofxn mod

0.96048

sRun

RunA1

zout

1

Ave

Data

0.00016

Model

0.00016

data/vMax

0.00199

ofxng

263.459

Min d/m

4.95183

ave/min d

1.87383

ave/min m

9.40115

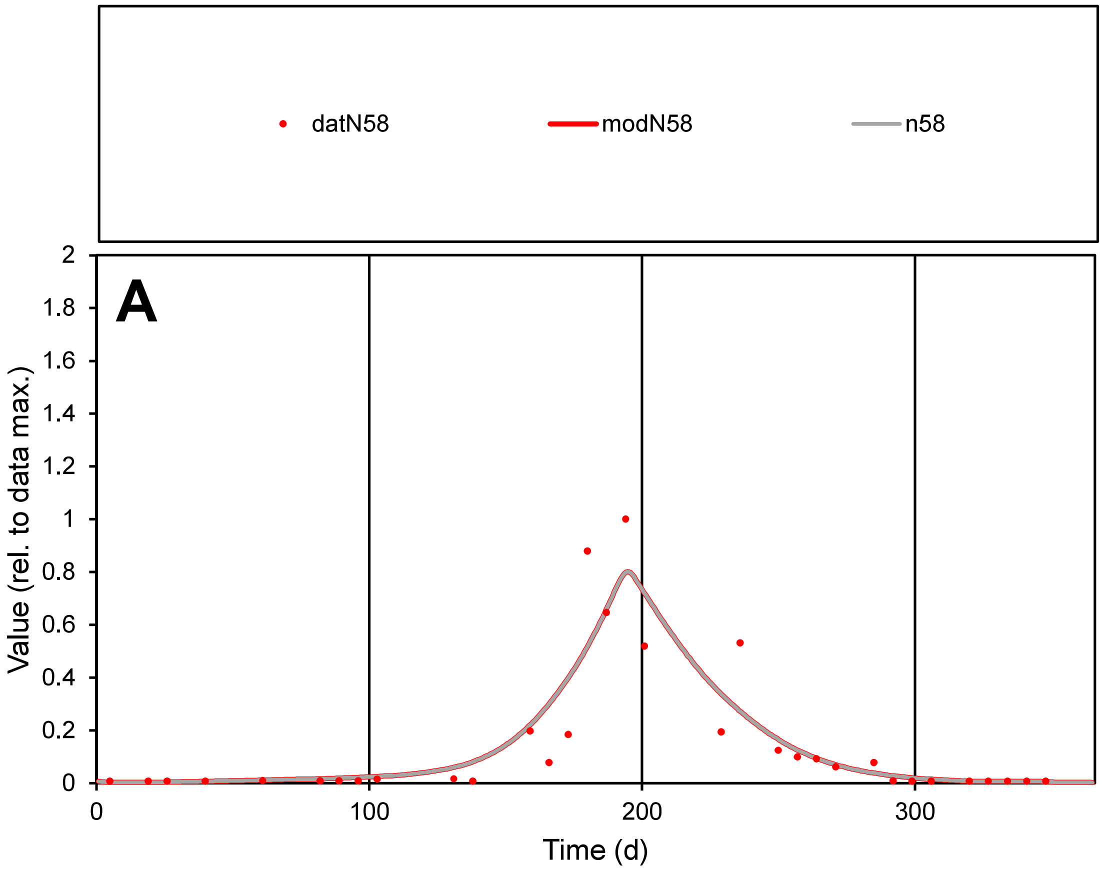

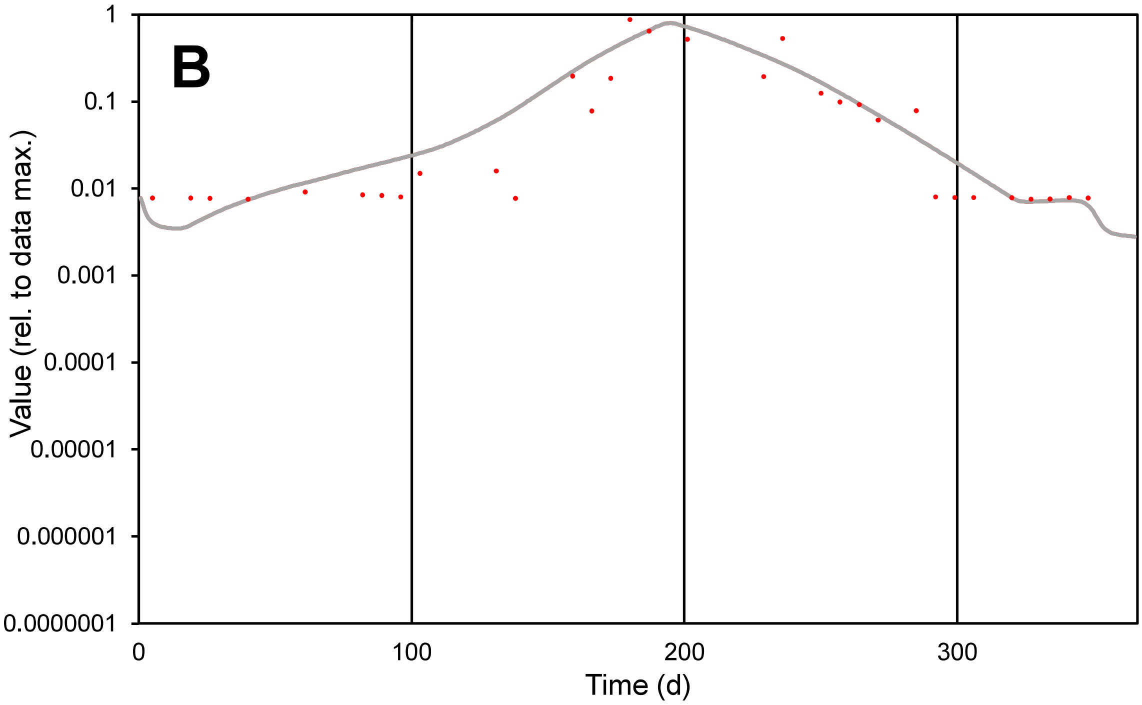

C1

Variables
N58 Provide jtd or Dst
CalibOnly?
2 1 to reproduce of
oNormalize?
2 1=no, 2=yes

ofxn calc*
0.74216
ofxn mod
0.77903

sRun
RunA1
zout
1

Ave
Data
0.00165
Model
0.00173

data/vMax
0.01121

ofxng
263.459

Min d/m
1.88765

ave/min d
19.4992
ave/min m
38.5609

326_Calib_S_A

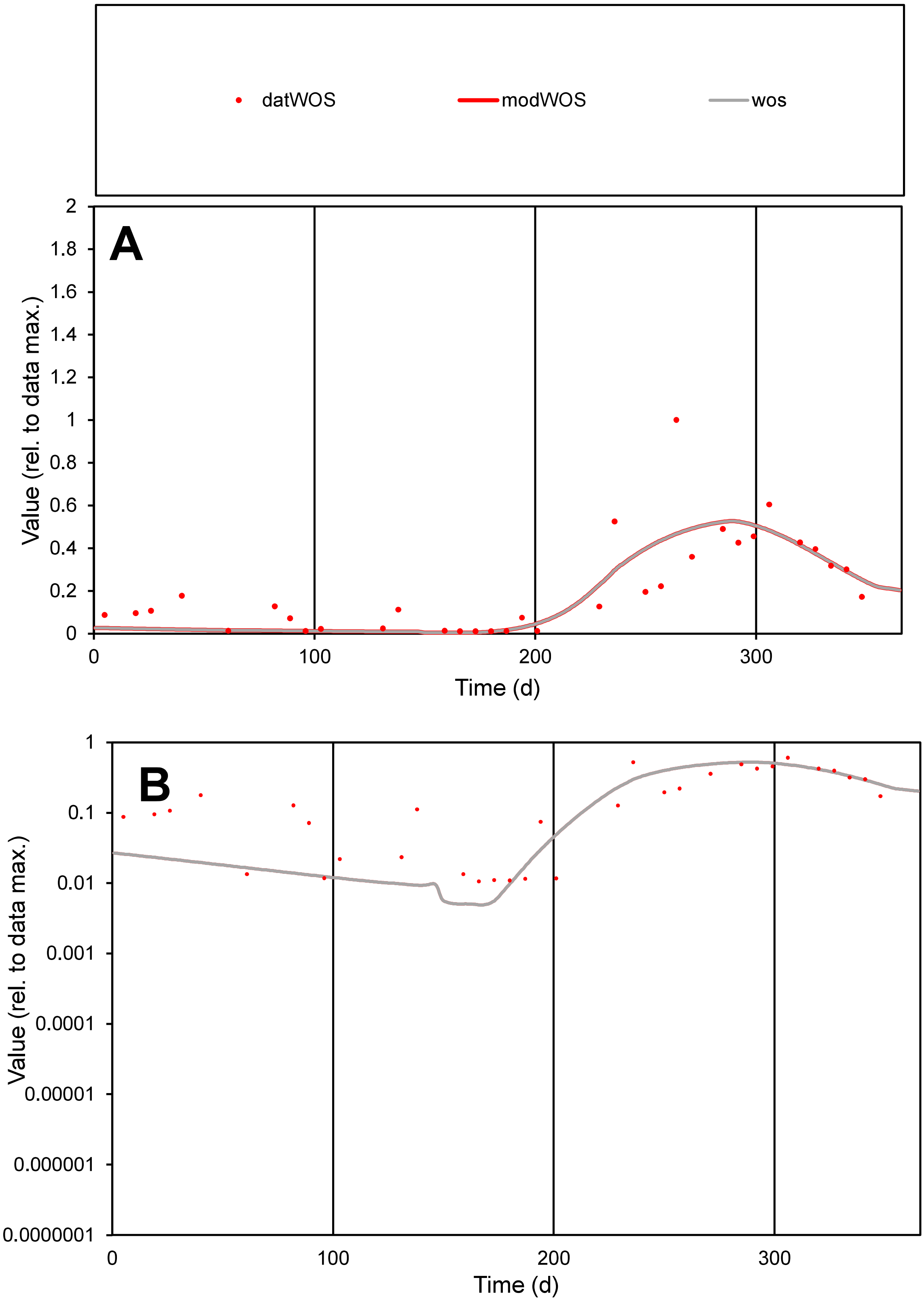

C1

Variables
WOS Provide jtd or Dst
CalibOnly?
2 1 to reproduce of
oNormalize?
2 1=no, 2=yes

ofxn calc*
0.60876
ofxn mod
0.63257

sRun
RunA1
zout
1

Ave
Data
0.00135
Model
0.00122

data/vMax
0.00636

ofxng
263.459

Min d/m
2.17634

ave/min d
19.9936
ave/min m
39.1758

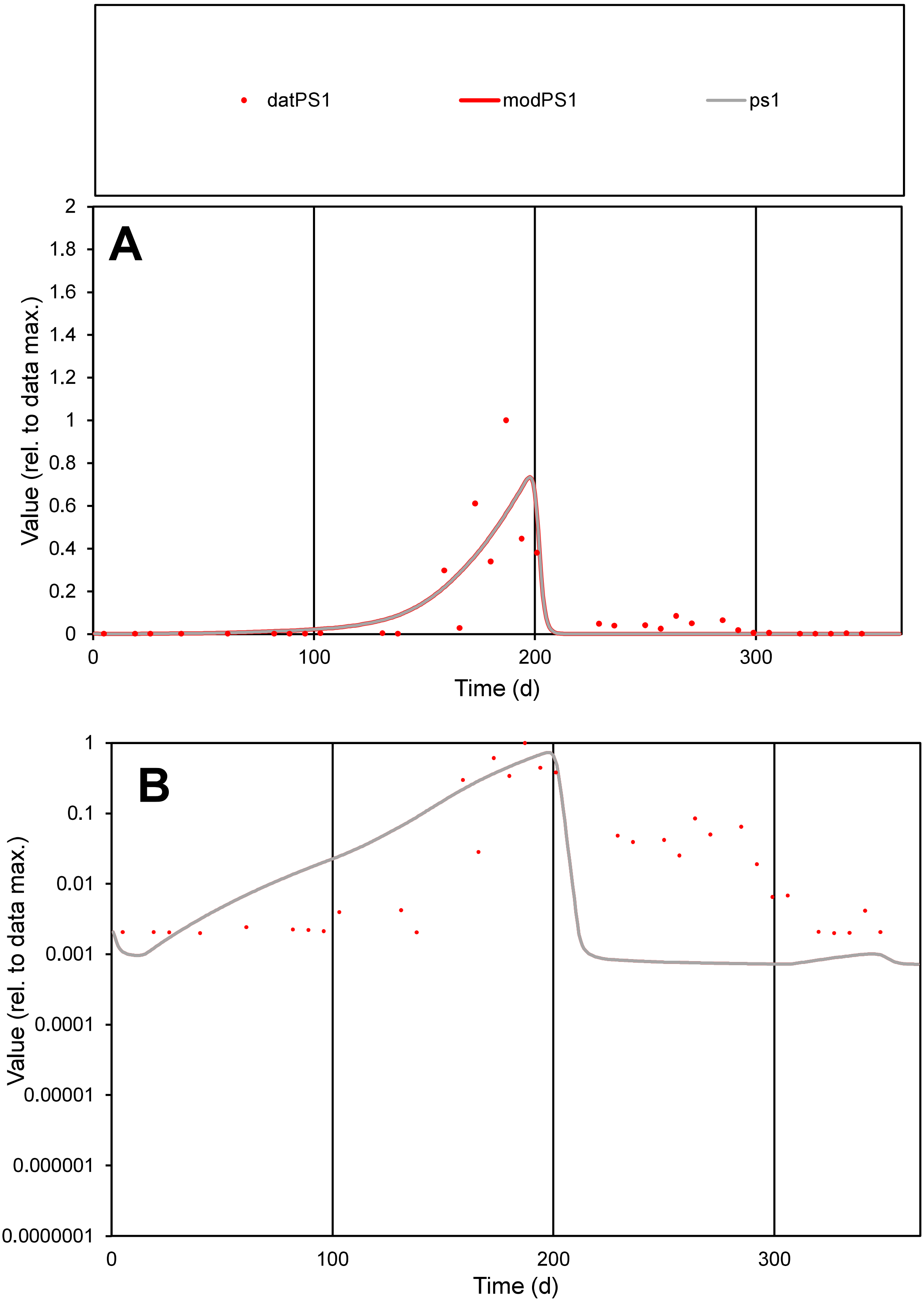

C1

Variables
PS1 Provide jtd or Dst
CalibOnly?
2 1 to reproduce of
oNormalize?
2 1=no, 2=yes

ofxn calc*
1.02276
ofxn mod
1.07025

sRun
RunA1
zout
1

Ave
Data
0.0026
Model
0.00241

data/vMax
0.02431

ofxng
263.459

Min d/m
2.75212

ave/min d
53.4941
ave/min m
136.383

326_Calib_S_A

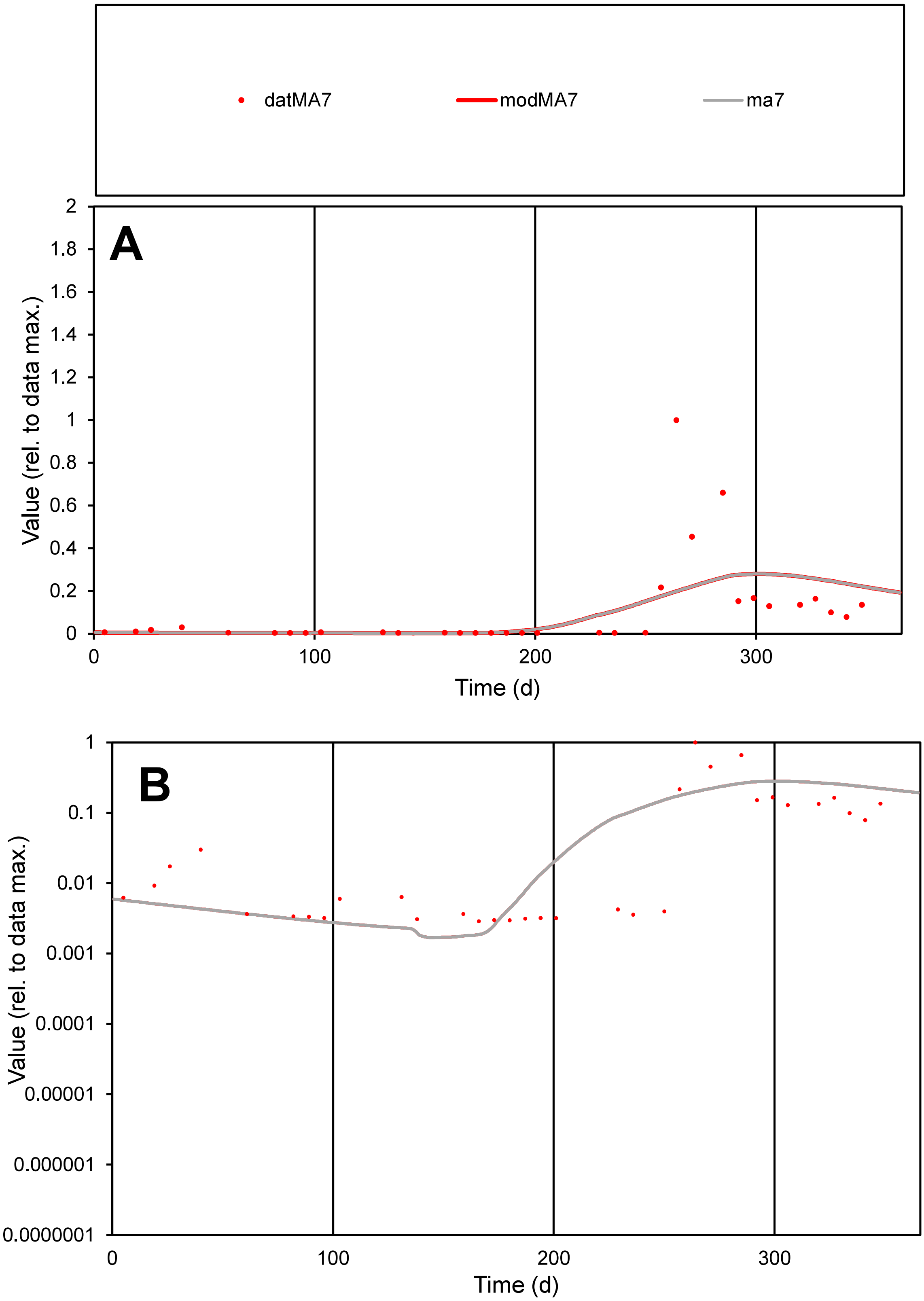

C1

Variables
MA7 Provide jtd or Dst
CalibOnly?
2 1 to reproduce of
oNormalize?
2 1=no, 2=yes

ofxn calc*
1.64522
ofxn mod
1.65961

sRun
RunA1
zout
1

Ave
Data
0.00049
Model
0.00047

data/vMax
0.00462

ofxng
263.459

Min d/m
1.61091

ave/min d
36.974
ave/min m
57.1447

326_Calib_S_A

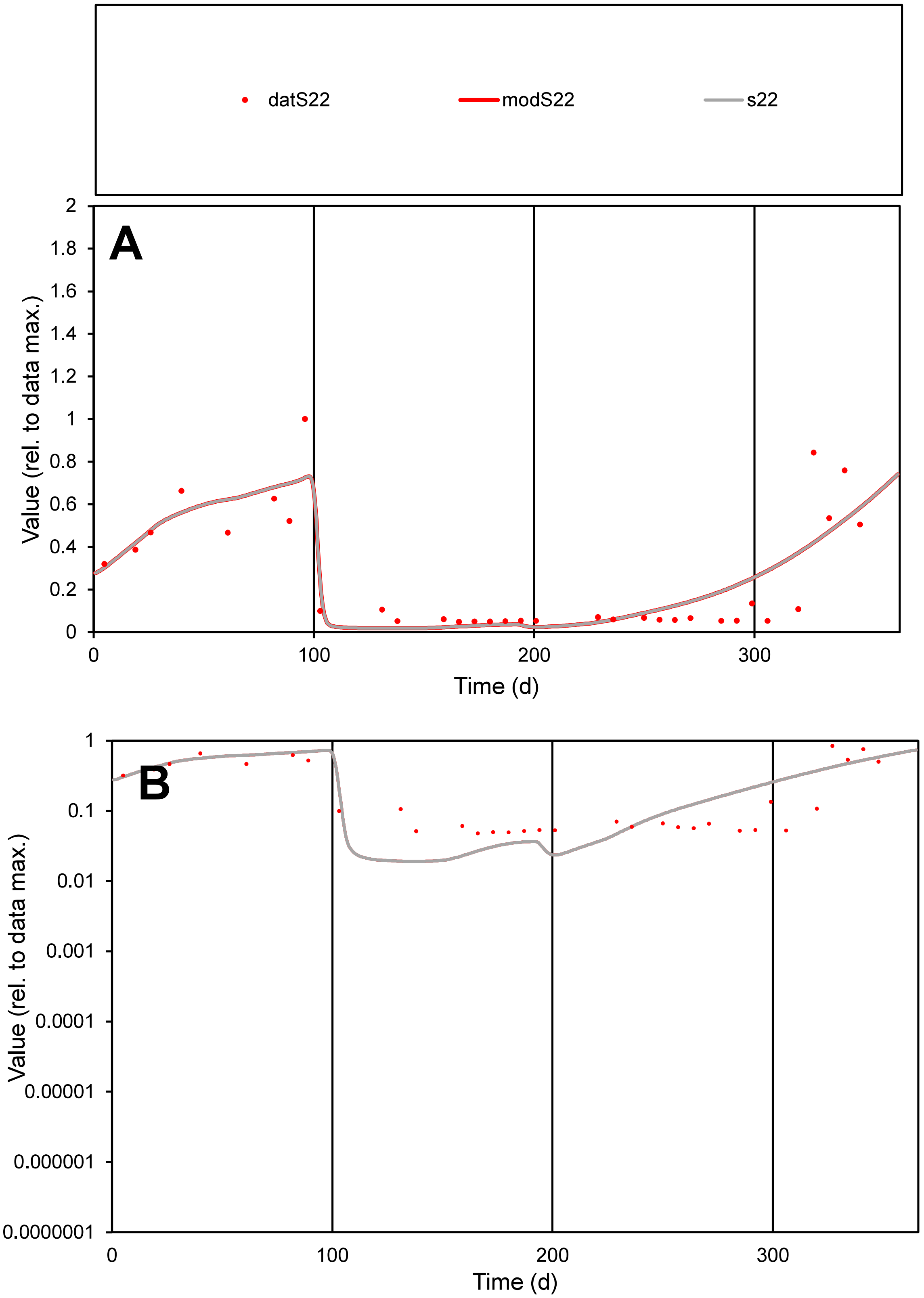

C1

Variables
S22 Provide jtd or Dst
CalibOnly?
2 1 to reproduce of
oNormalize?
2 1=no, 2=yes

ofxn calc*
0.52075
ofxn mod
0.54504

sRun
RunA1
zout
1

Ave
Data
0.00078
Model
0.00081

data/vMax
0.00302

ofxng
263.459

Min d/m
2.51039

ave/min d
5.36723
ave/min m
14.0907

326_Calib_S_A

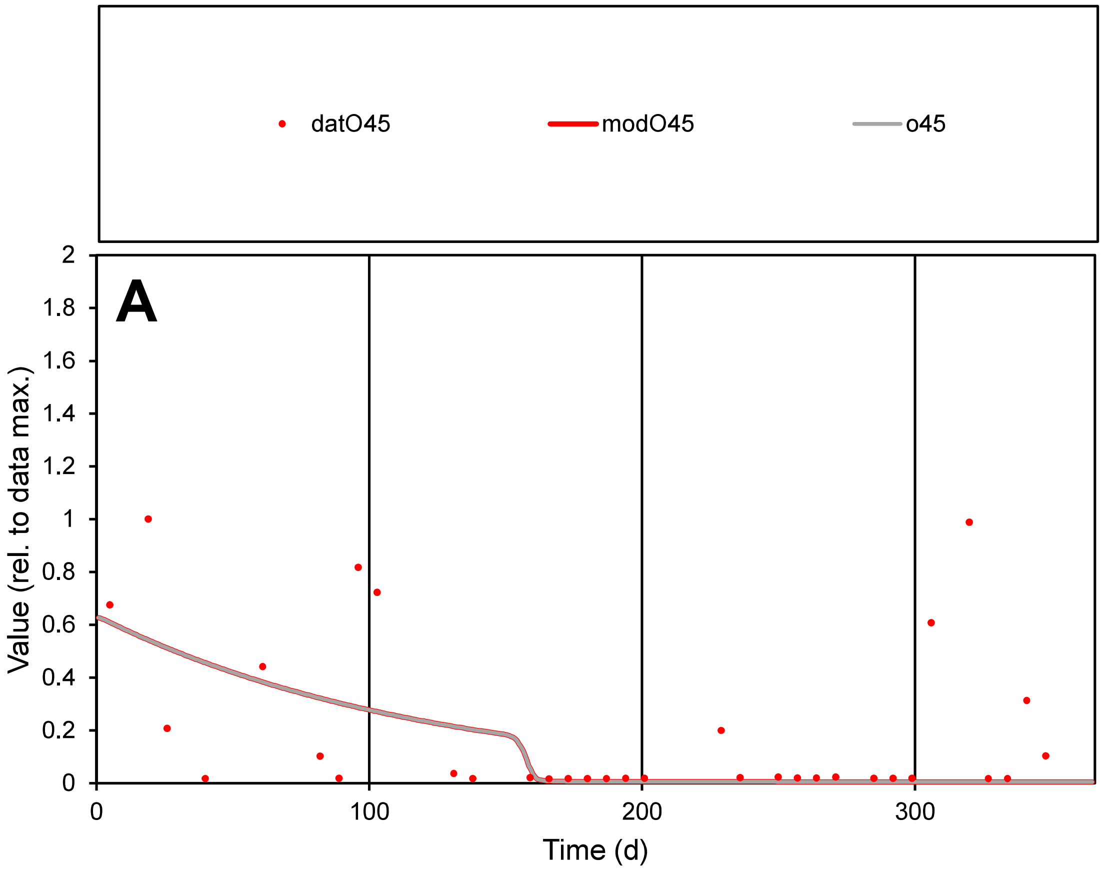

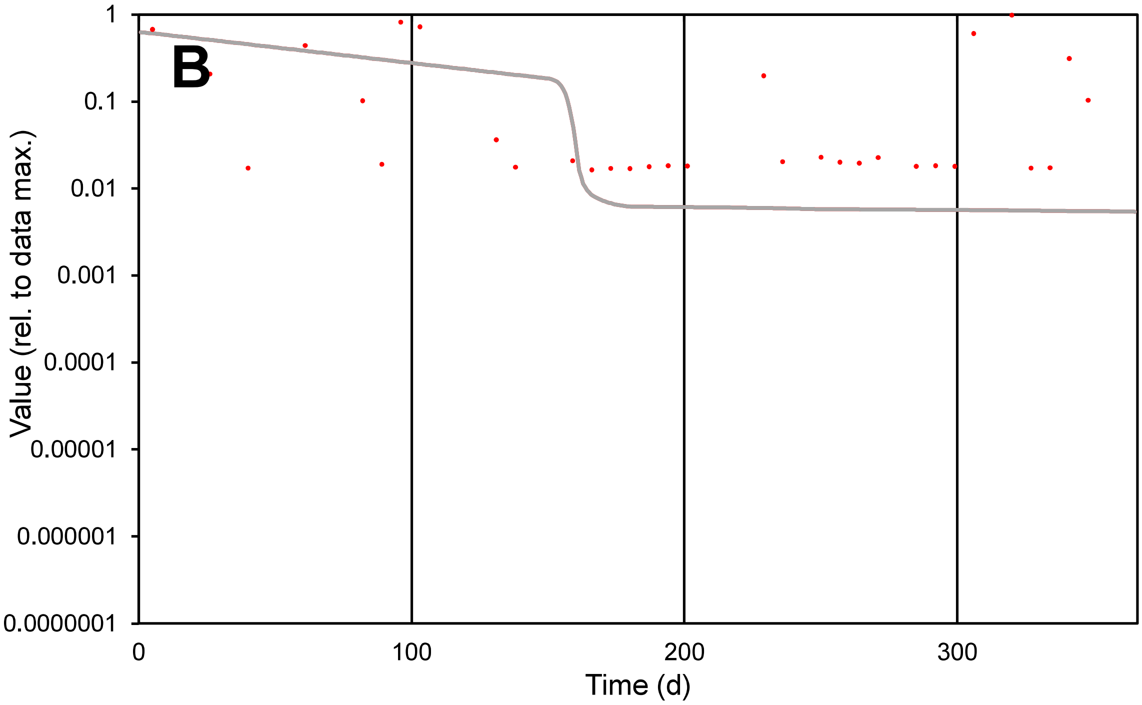

C1

Variables

O45	Provide jtd or Dst
CalibOnly?	
2	1 to reproduce of
oNormalize?	
2	1=no, 2=yes
ofxn calc*	
1.42061	
ofxn mod	
1.42904	
sRun	
RunA1	
zout	
1	
Ave	
Data	
0.00069	
Model	
0.00044	
data/vMax	
0.00343	
ofxng	
263.459	
Min d/m	
2.99629	
ave/min d	
12.135	
ave/min m	
23.2721	

326_Calib_S_A

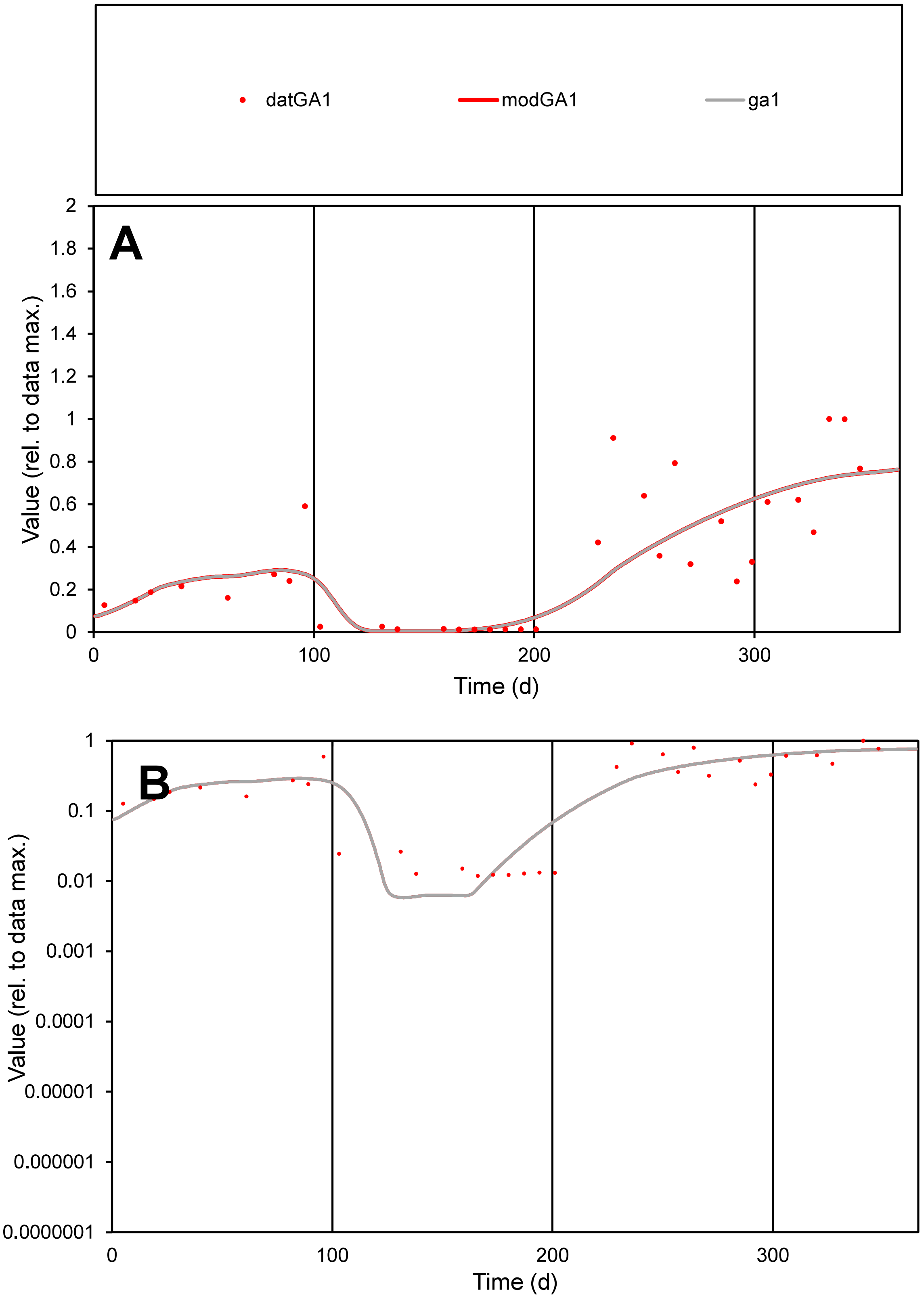

C1

Variables

GA1	Provide jtd or Dst
CalibOnly?	
2	1 to reproduce of
oNormalize?	
2	1=no, 2=yes

ofxn calc*
0.56761
ofxn mod
0.58537

sRun
RunA1
zout
1

Ave
Data
0.00099
Model
0.00095

data/vMax
0.00295

ofxng
263.459

Min d/m
2.04684

ave/min d
28.3335
ave/min m
55.469

326_Calib_S_A

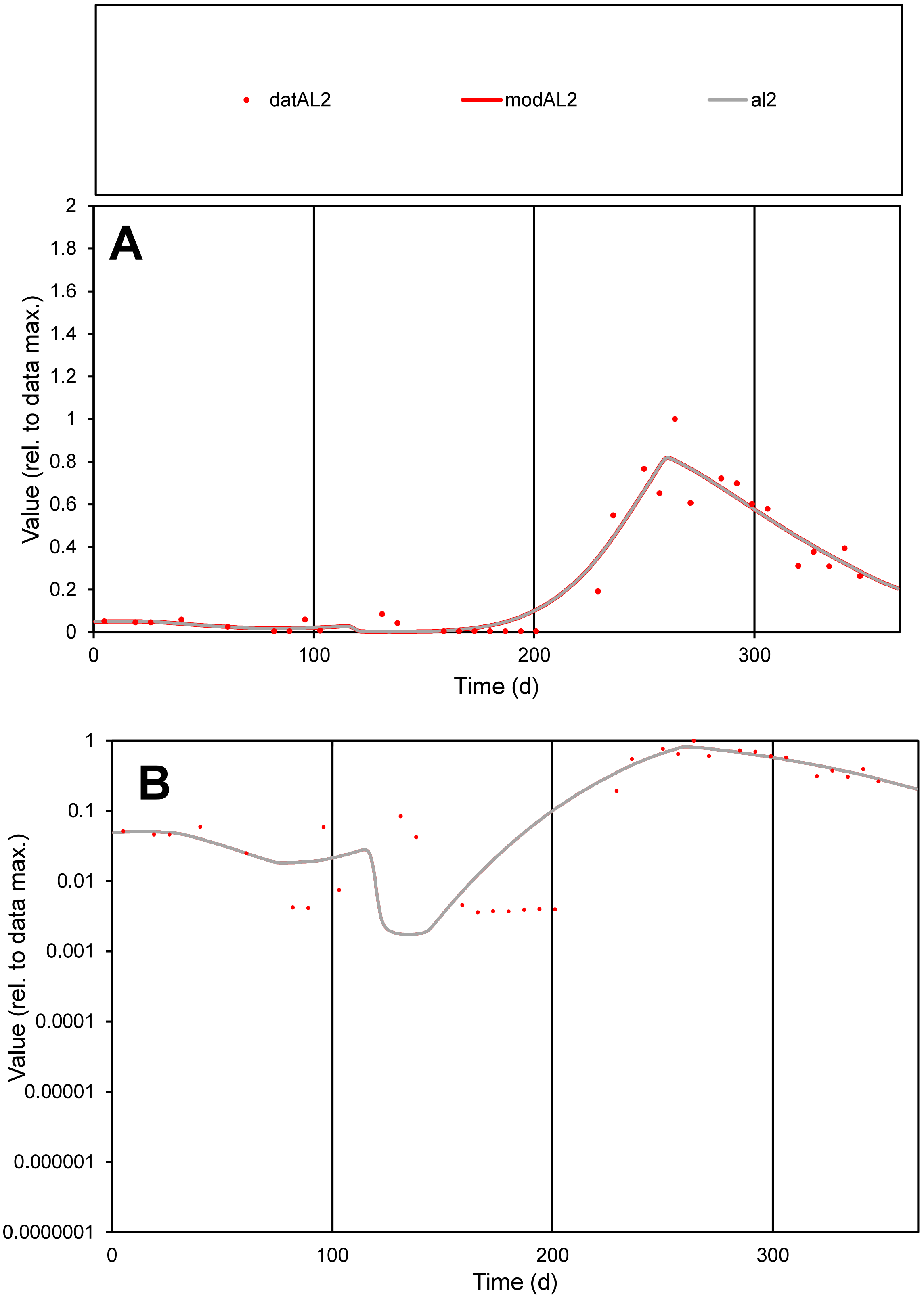

C1

Variables
AL2 Provide jtd or Dst
CalibOnly?
2 1 to reproduce of
oNormalize?
2 1=no, 2=yes

ofxn calc*
0.29136
ofxn mod
0.34702

sRun
RunA1
zout
1

Ave
Data
0.00153
Model
0.00156

data/vMax
0.00598

ofxng
263.459

Min d/m
2.06334

ave/min d
71.3936
ave/min m
149.836

326_Calib_S_A

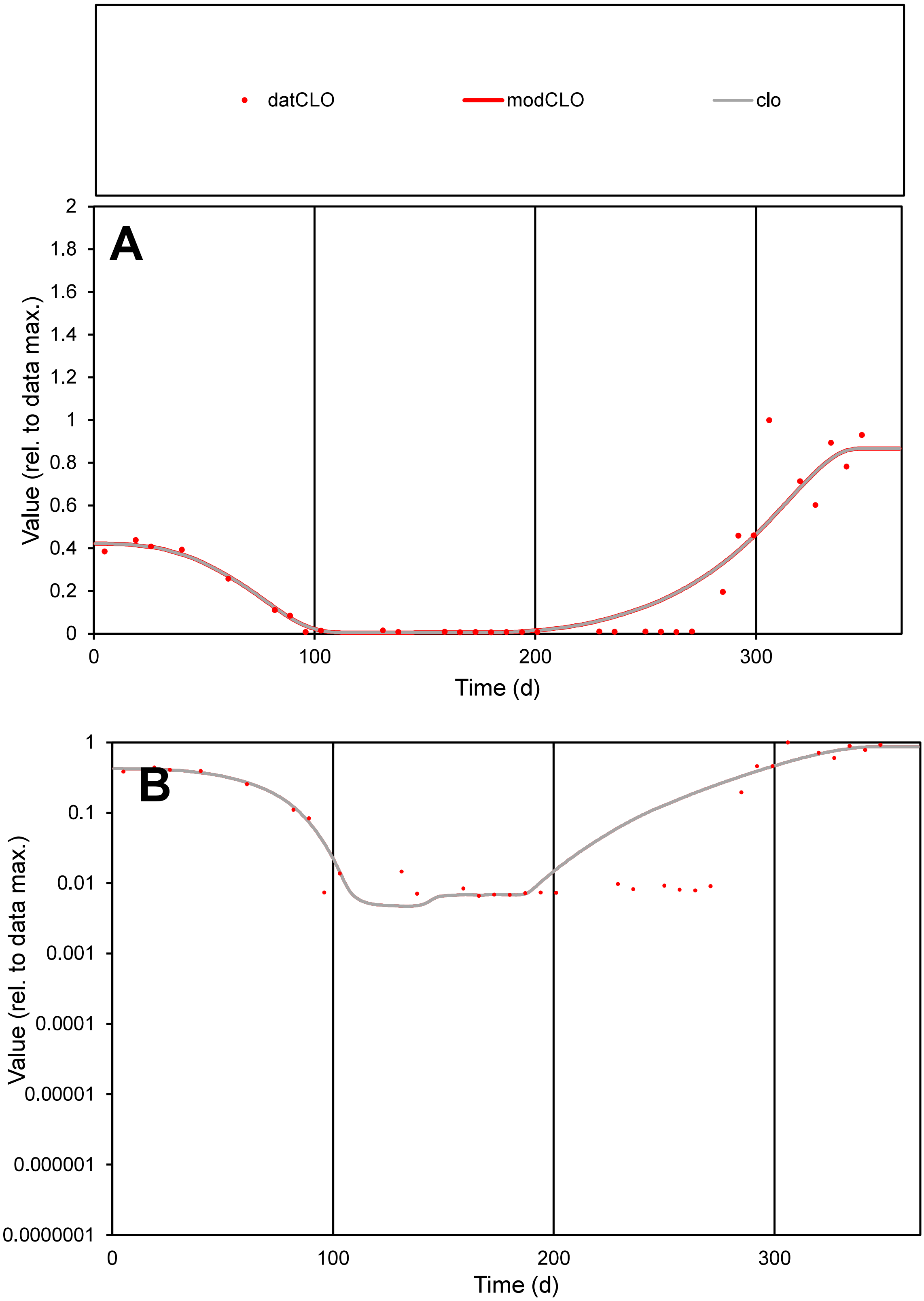

C1

Variables
CLO Provide jtd or Dst
CalibOnly?
2 1 to reproduce of
oNormalize?
2 1=no, 2=yes

ofxn calc*
0.44062
ofxn mod
0.47315

sRun
RunA1
zout
1

Ave
Data
0.00049
Model
0.00052

data/vMax
0.00197

ofxng
263.459

Min d/m
1.40606

ave/min d
37.772
ave/min m
56.4799

326_Calib_S_A

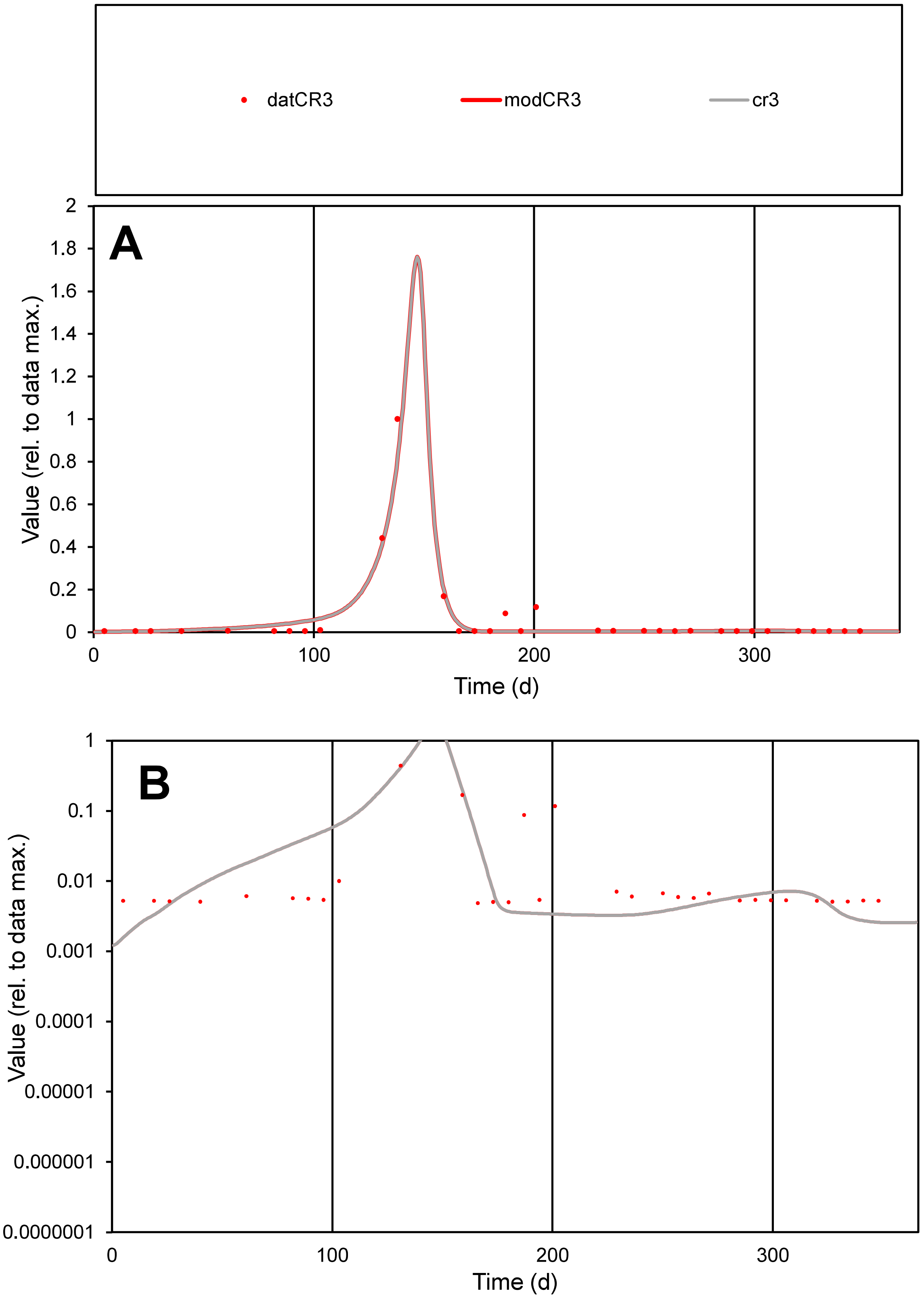

C1

Variables
CR3 Provide jtd or Dst
CalibOnly?
2 1 to reproduce of
oNormalize?
2 1=no, 2=yes

ofxn calc*
0.49767
ofxn mod
0.63264

sRun
RunA1
zout
1

Ave
Data
0.00043
Model
0.00042

data/vMax
0.00723

ofxng
263.459

Min d/m
2.81672

ave/min d
12.3353
ave/min m
34.0377

326_Calib_S_A

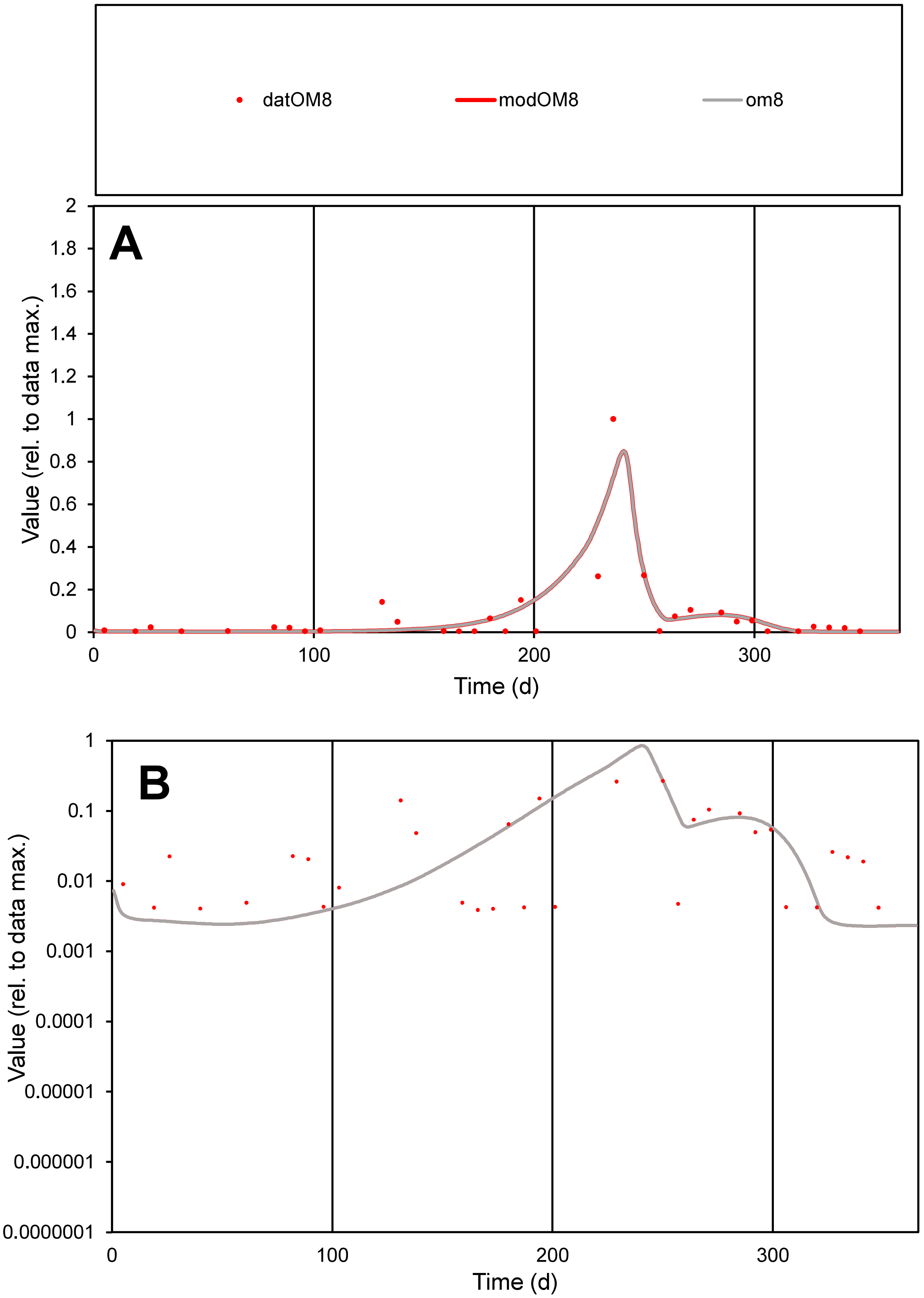

C1

Variables
OM8 Provide jtd or Dst
CalibOnly?
2 1 to reproduce of
oNormalize?
2 1=no, 2=yes

ofxn calc*
0.9738
ofxn mod
1.02205

sRun
RunA1
zout
1

Ave
Data
0.00105
Model
0.00107

data/vMax
0.01371

ofxng
263.459

Min d/m
1.70624
ave/min d
19.6175
ave/min m
34.2192

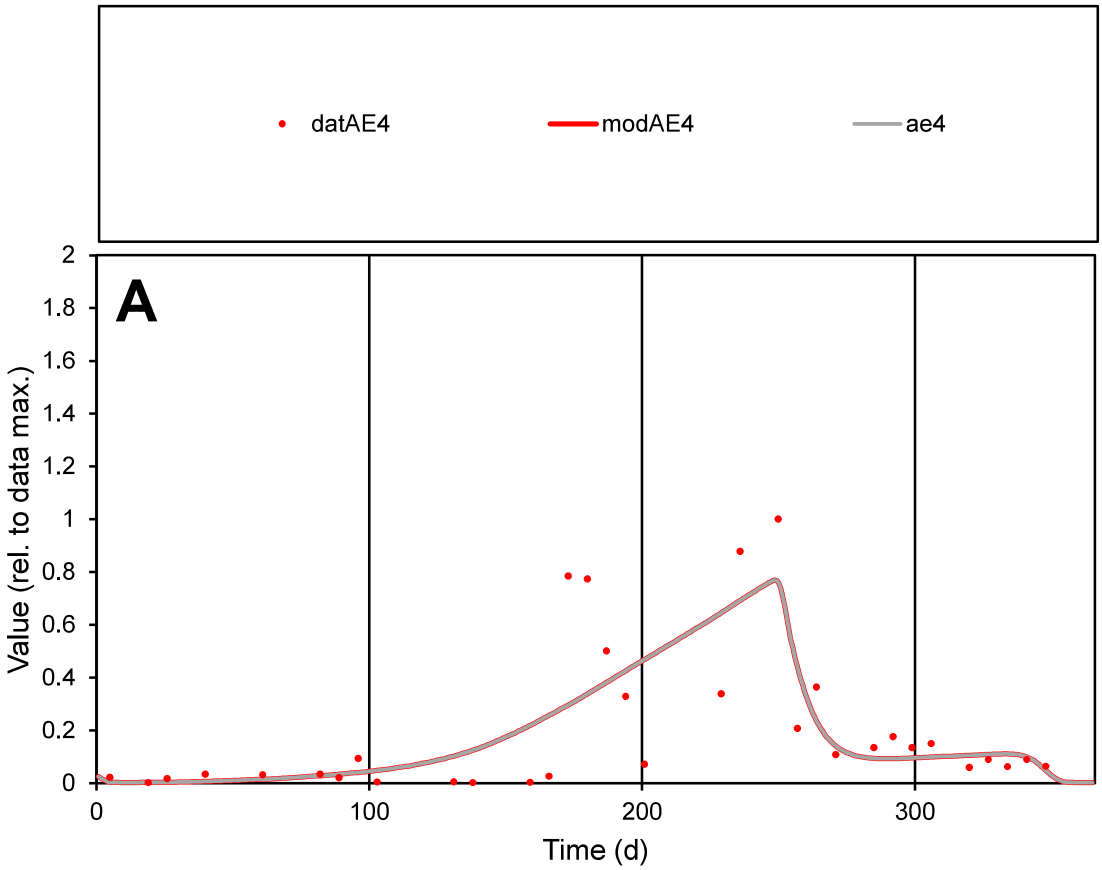

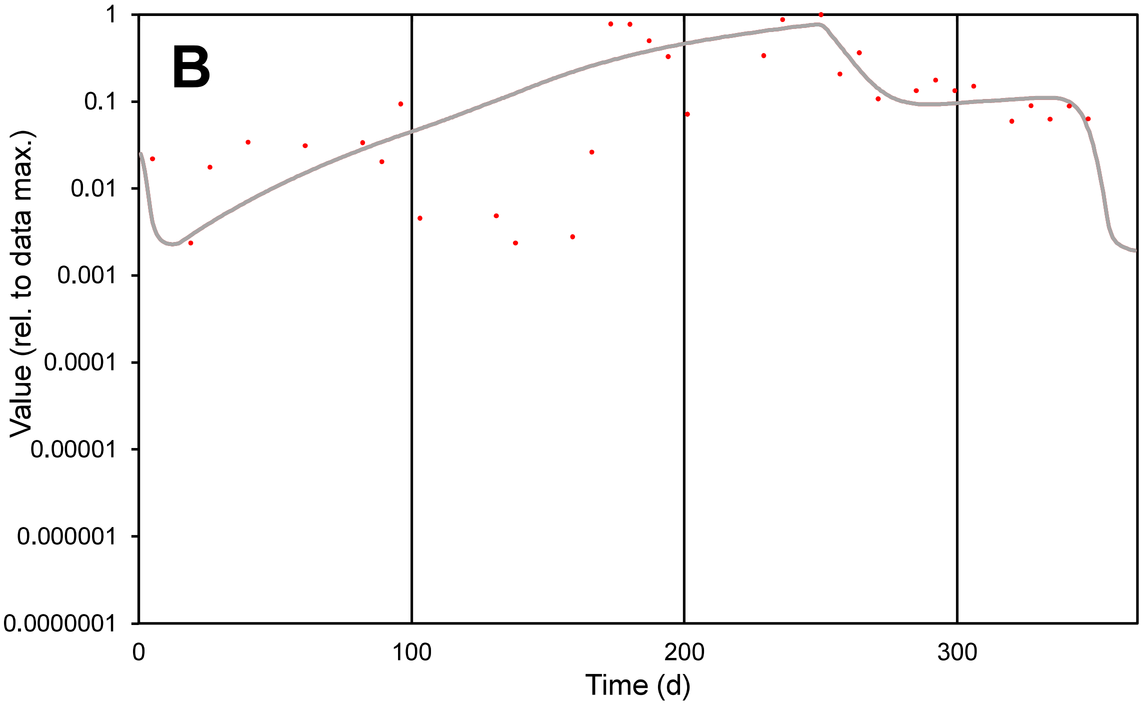

C1

Variables
AE4 Provide jtd or Dst
CalibOnly?
2 1 to reproduce of
oNormalize?
2 1=no, 2=yes

ofxn calc*
0.86625
ofxn mod
0.89122

sRun
RunA1
zout
1

Ave
Data
0.00105
Model
0.00104

data/vMax
0.00523

ofxng
263.459

Min d/m
0.79448

ave/min d
84.5892
ave/min m
66.7494

326_Calib_S_A

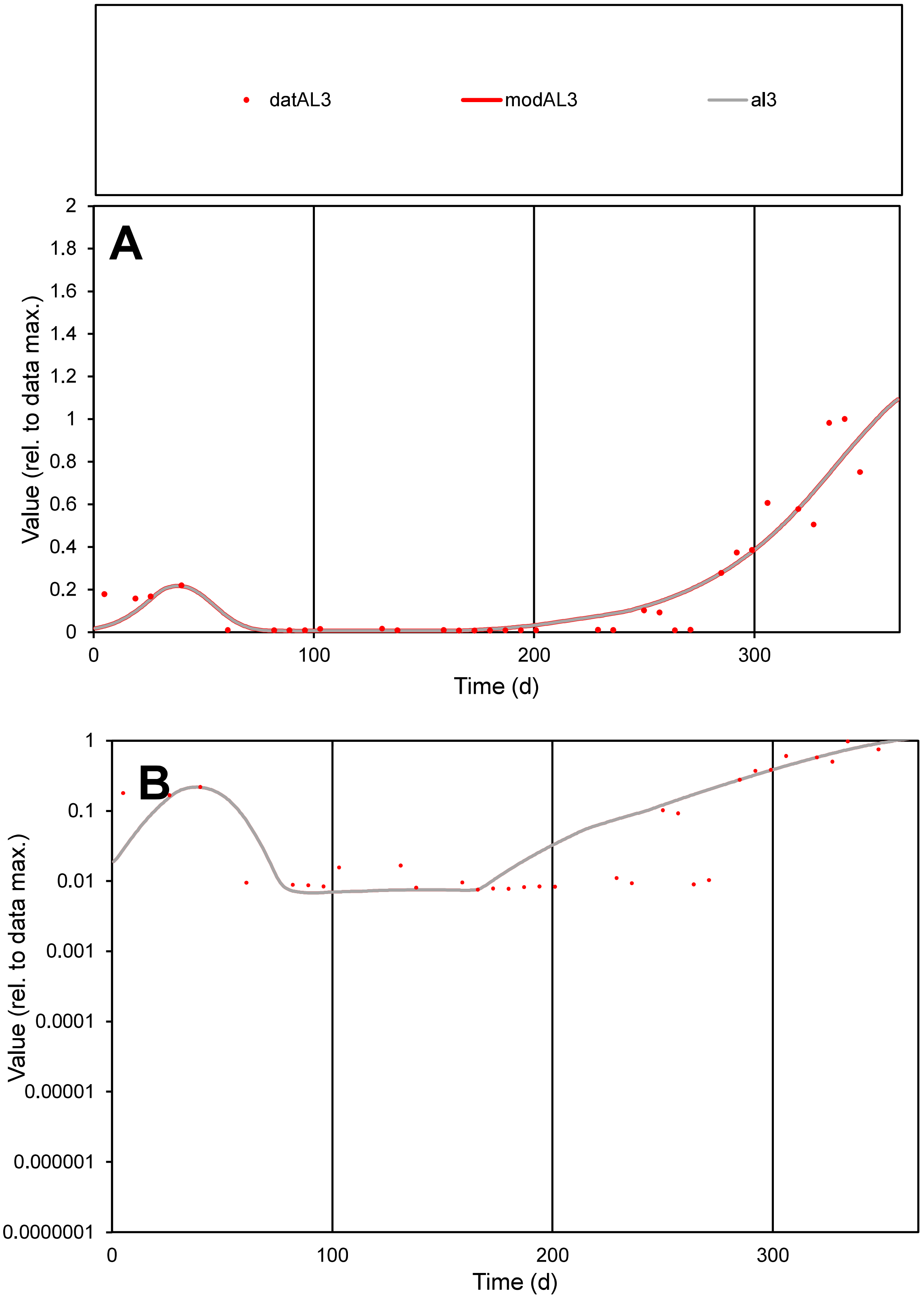

C1

Variables
AL3 Provide jtd or Dst
CalibOnly?
2 1 to reproduce of
oNormalize?
2 1=no, 2=yes

ofxn calc*
0.44599
ofxn mod
0.48086

sRun
RunA1
zout
1

Ave
Data
0.00063
Model
0.00065

data/vMax
0.00316

ofxng
263.459

Min d/m
1.11345

ave/min d
26.3344
ave/min m
30.4937

326_Calib_S_A

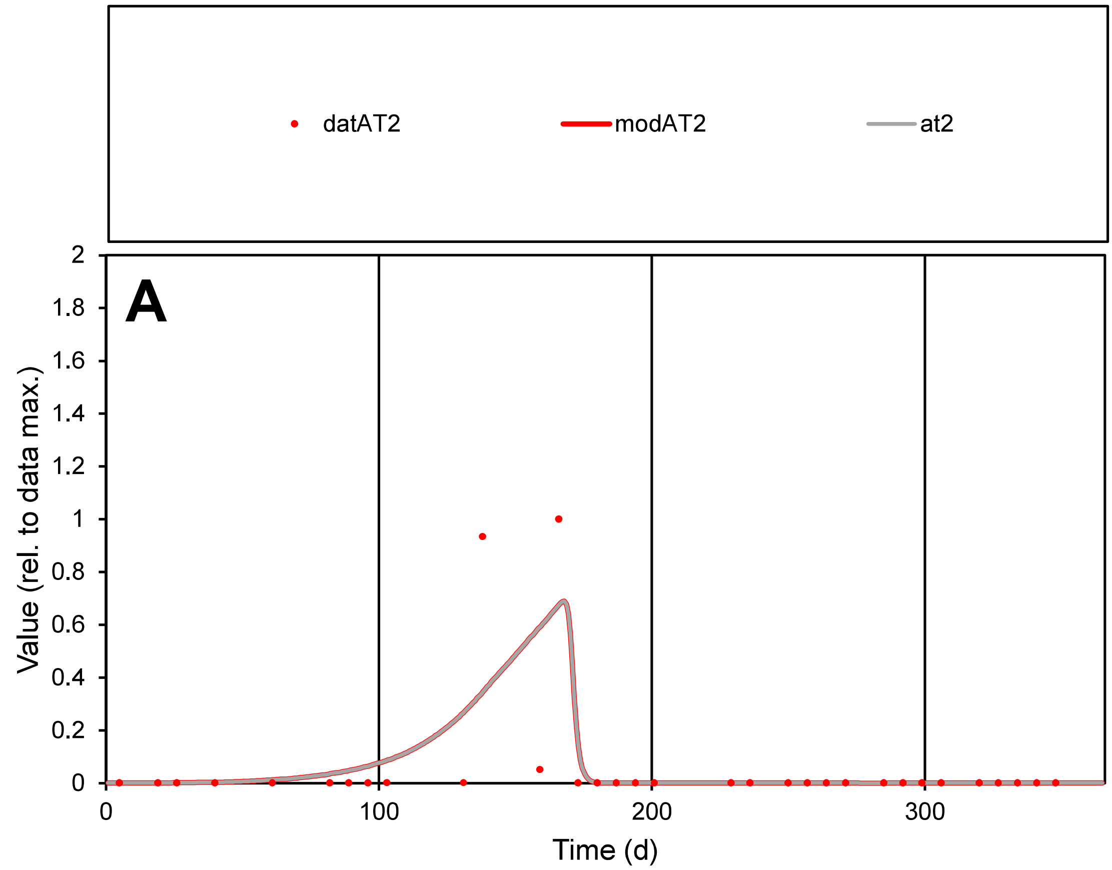

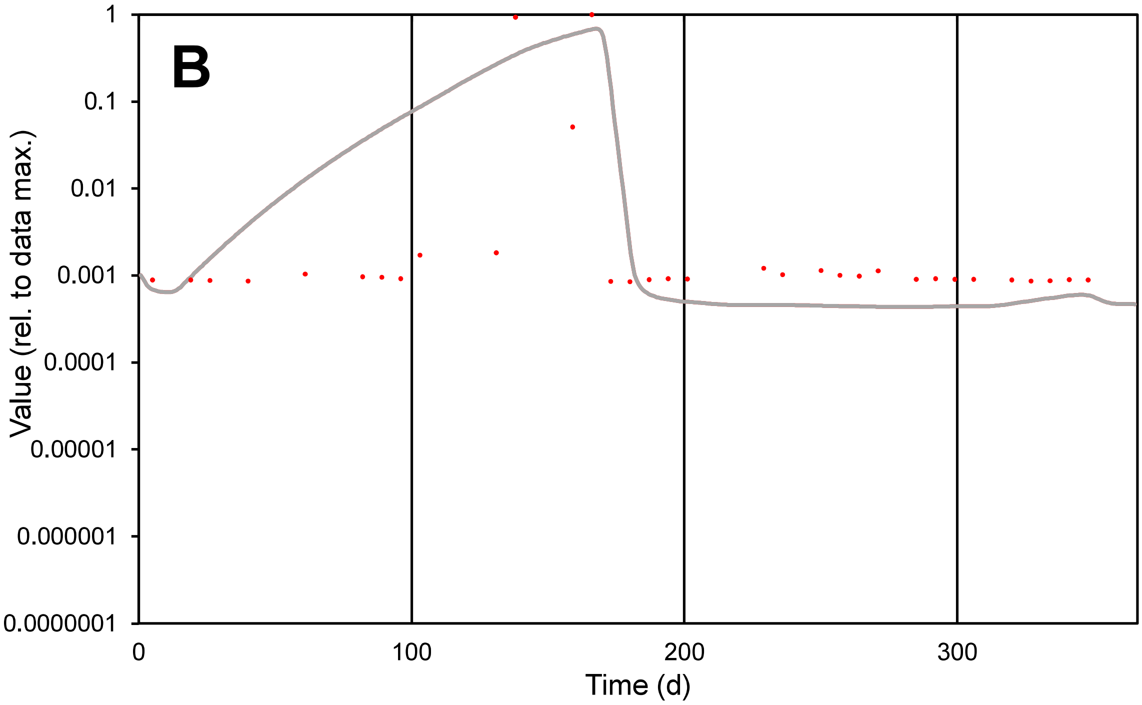

C1

Variables
AT2 Provide jtd or Dst
CalibOnly?
2 1 to reproduce of
oNormalize?
2 1=no, 2=yes

ofxn calc*
2.54857
ofxn mod
2.58908

sRun
RunA1
zout
1

Ave
Data
0.00583
Model
0.00637

data/vMax
0.0954

ofxng
263.459

Min d/m
1.9481

ave/min d
71.8511
ave/min m
153.073

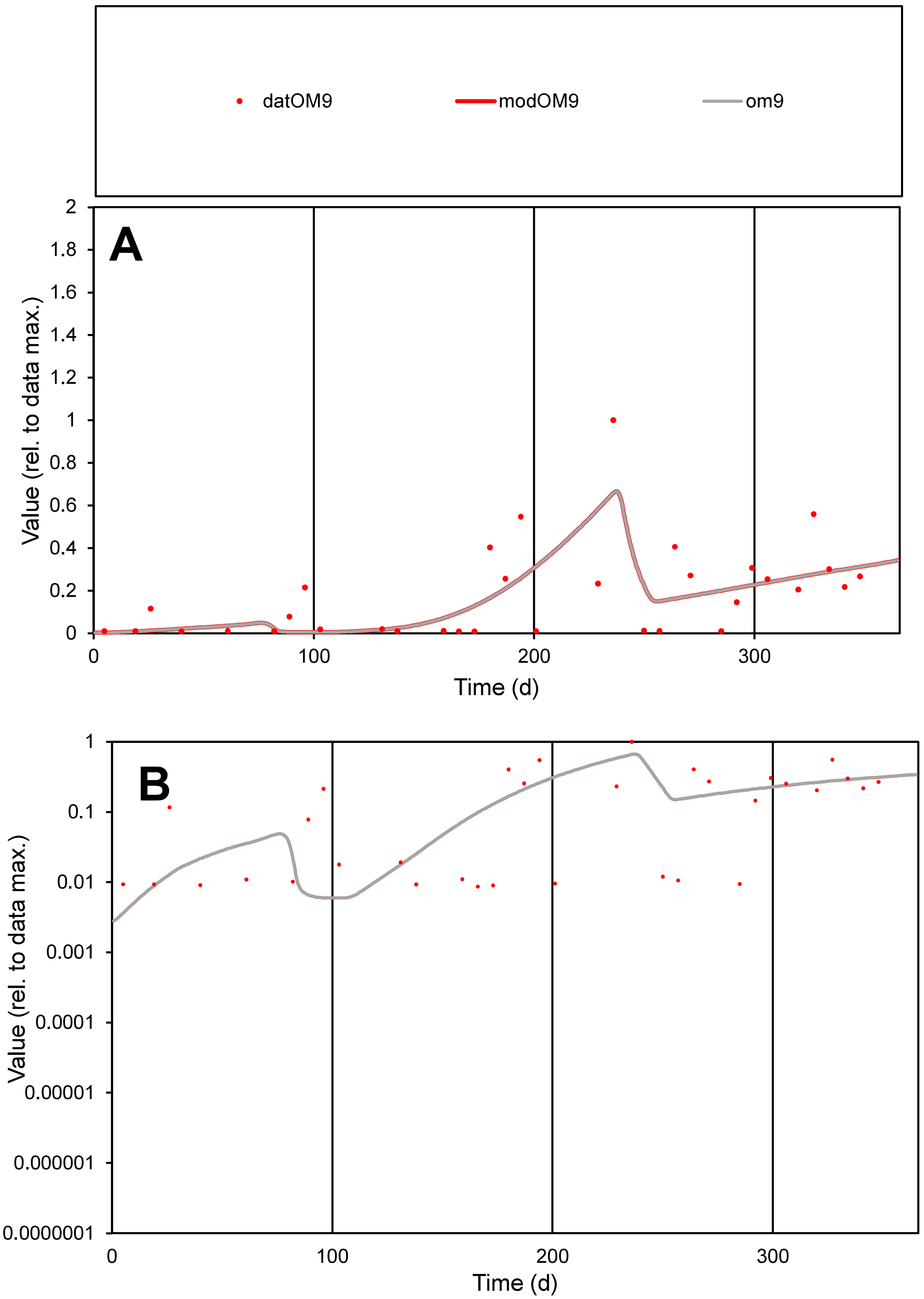

C1

Variables
OM9 Provide jtd or Dst
CalibOnly?
2 1 to reproduce of
oNormalize?
2 1=no, 2=yes

ofxn calc*
0.86354
ofxn mod
0.88017

sRun
RunA1
zout
1

Ave
Data
0.00089
Model
0.00085

data/vMax
0.00495

ofxng
263.459

Min d/m
2.14429

ave/min d
20.8219
ave/min m
42.8339

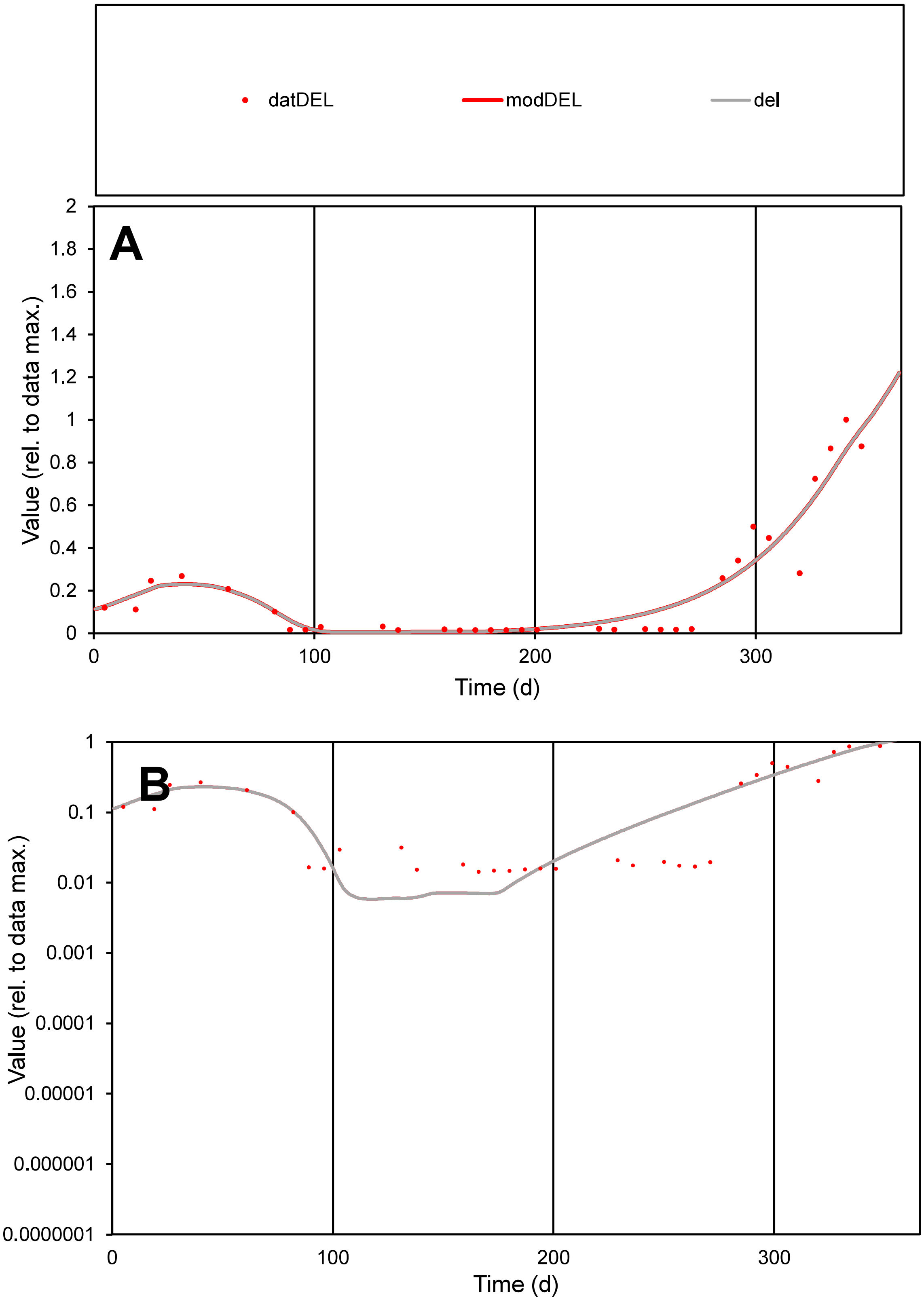

C1

Variables
DEL Provide jtd or Dst
CalibOnly?
2 1 to reproduce of
oNormalize?
2 1=no, 2=yes

ofxn calc*
0.38572
ofxn mod
0.42897

sRun
RunA1
zout
1

Ave
Data
0.00087
Model
0.00091

data/vMax
0.0043

ofxng
263.459

Min d/m
2.37007

ave/min d
14.1759
ave/min m
35.1017

326_Calib_S_A

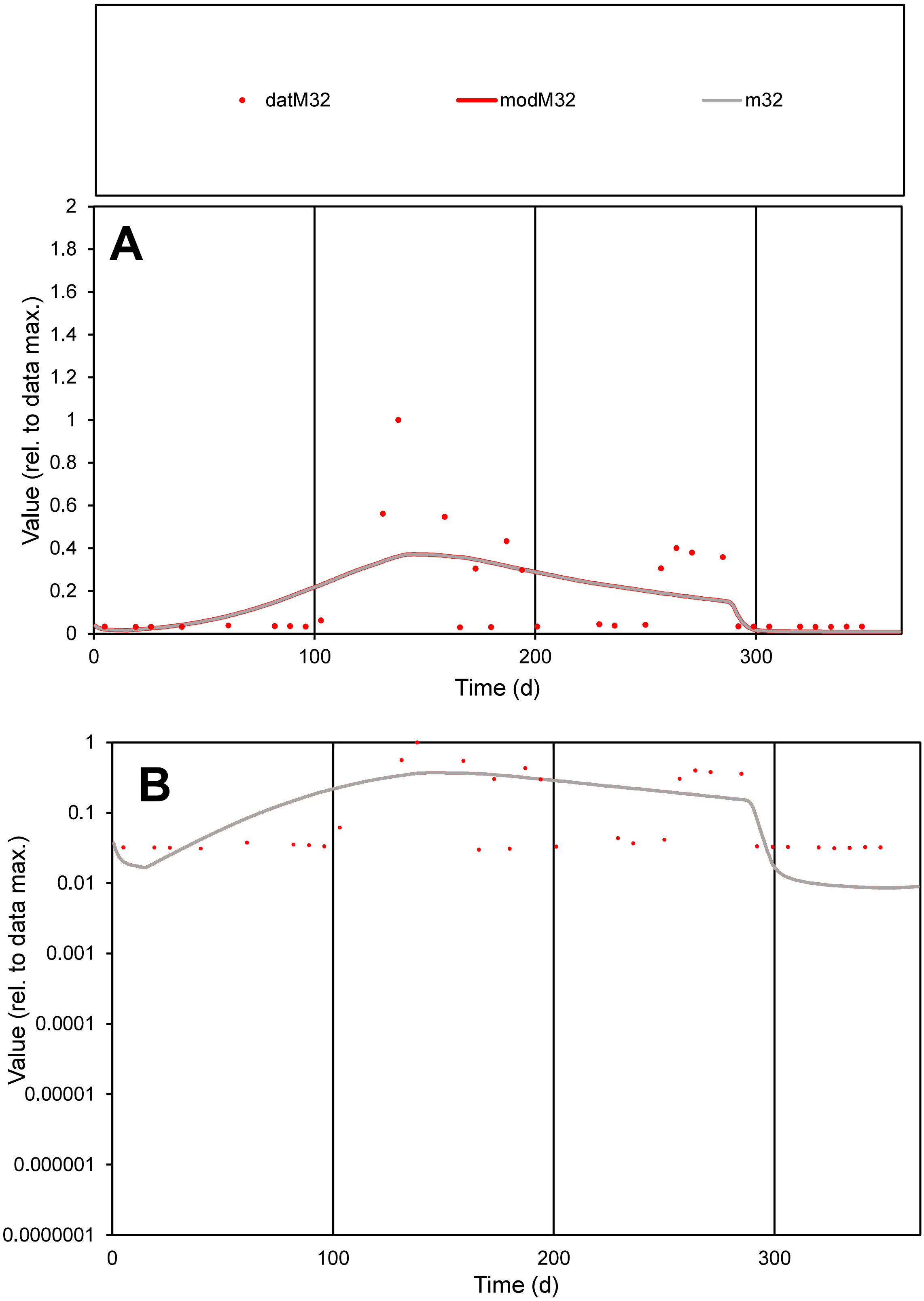

C1

Variables	
M32	Provide jtd or Dst
CalibOnly?	
2	1 to reproduce of
oNormalize?	
2	1=no, 2=yes
ofxn calc*	
1.11489	
ofxn mod	
1.1288	
sRun	
RunA1	
zout	
1	
Ave	
Data	
0.00113	
Model	
0.00114	
data/vMax	
0.00692	
ofxng	
263.459	
Min d/m	
3.48544	
ave/min d	
5.46533	
ave/min m	
19.1129	

326_Calib_S_A

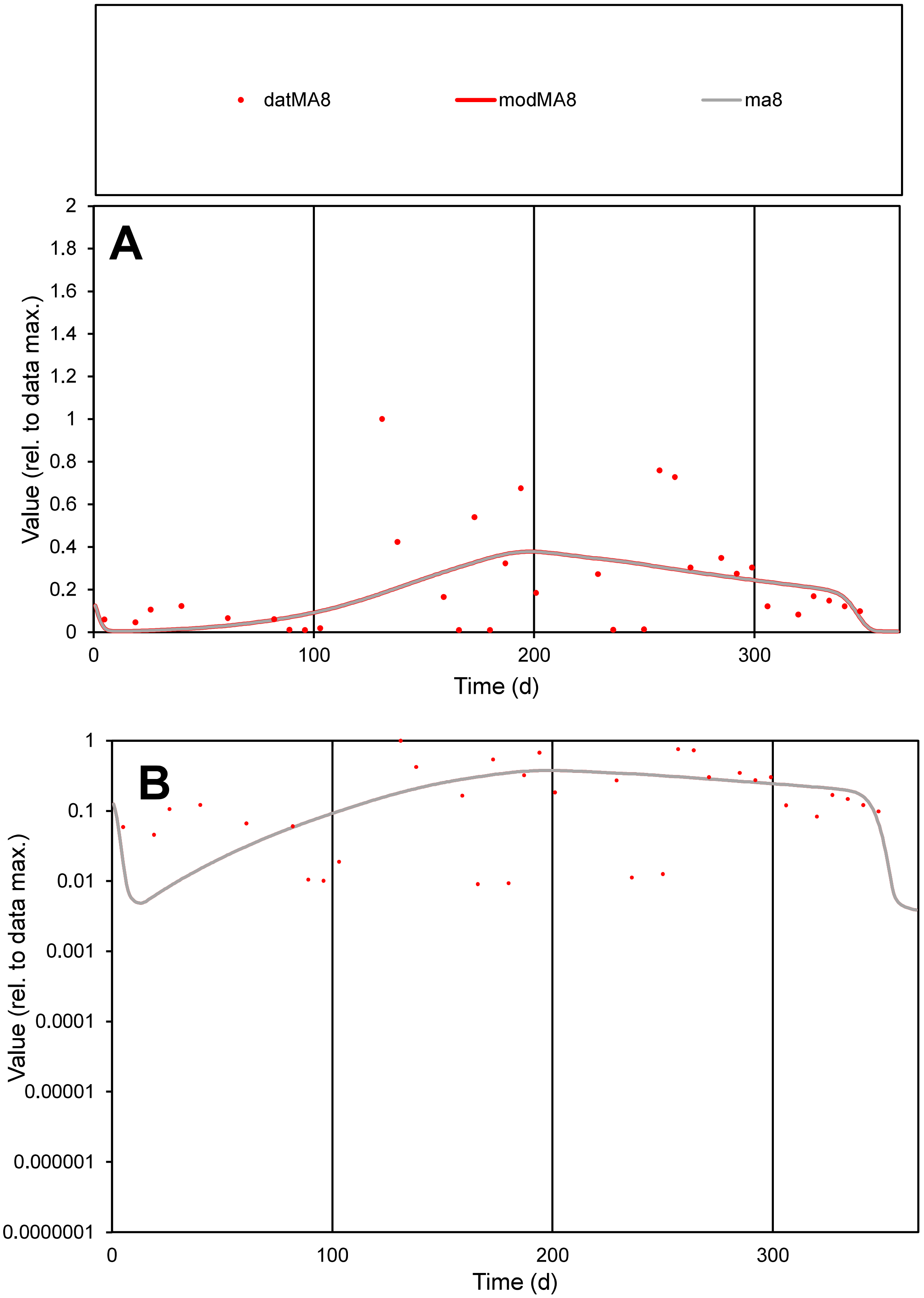

C1

Variables

MA8 Provide jtd or Dst

CalibOnly?

2 1 to reproduce of

oNormalize?

2 1=no, 2=yes

ofxn calc*

1.01064

ofxn mod

1.02151

sRun

RunA1

zout

1

Ave

Data

0.00149

Model

0.00136

data/vMax

0.00649

ofxng

263.459

Min d/m

1.39815

ave/min d

25.2122

ave/min m

32.1472

326_Calib_S_A

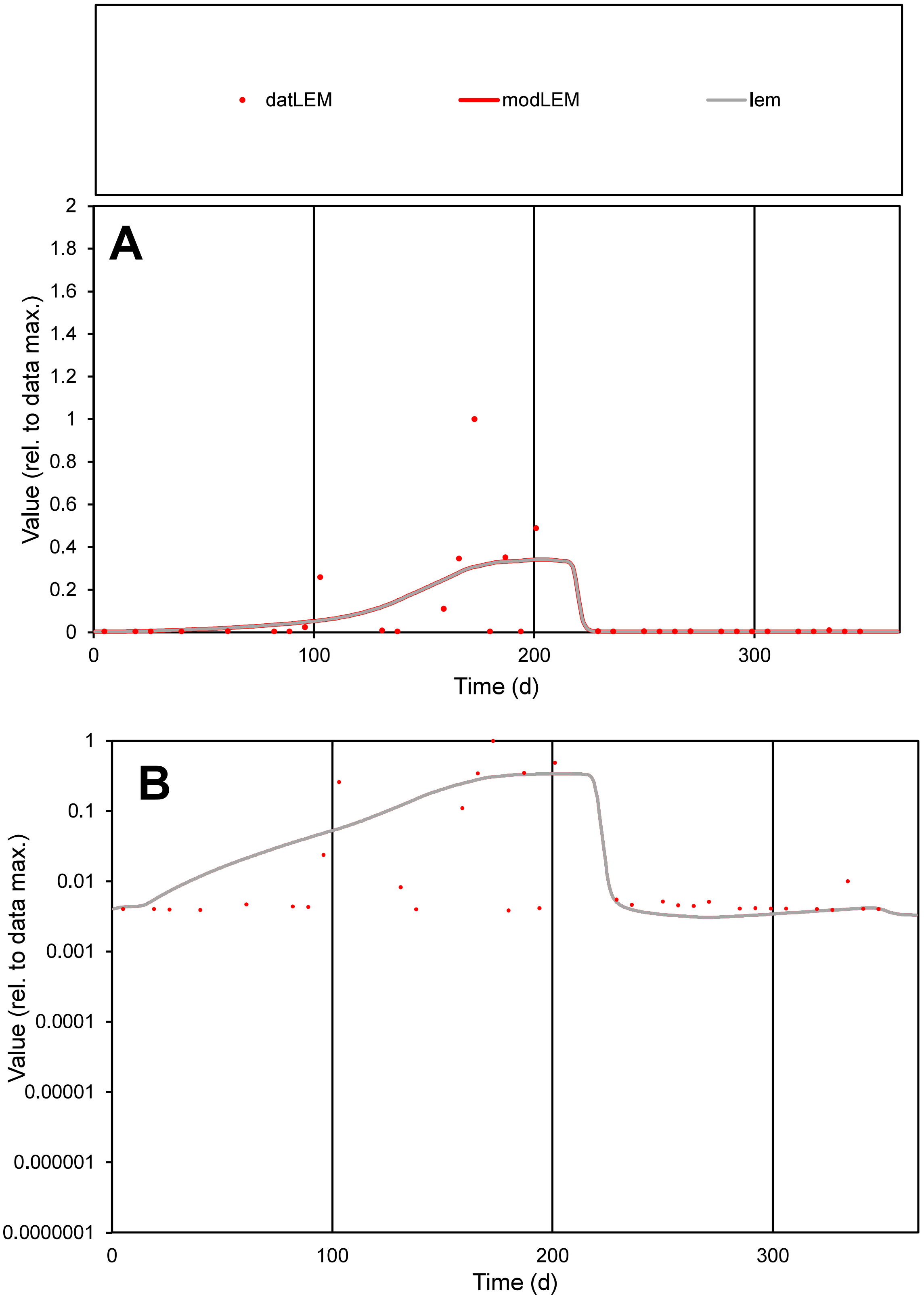

C1

Variables
LEM Provide jtd or Dst
CalibOnly?
2 1 to reproduce of
oNormalize?
2 1=no, 2=yes

ofxn calc*
1.91352
ofxn mod
1.93065

sRun
RunA1
zout
1

Ave
Data
0.00047
Model
0.00047

data/vMax
0.00571

ofxng
263.459

Min d/m
1.25128

ave/min d
21.2513
ave/min m
26.9952

326_Calib_S_A

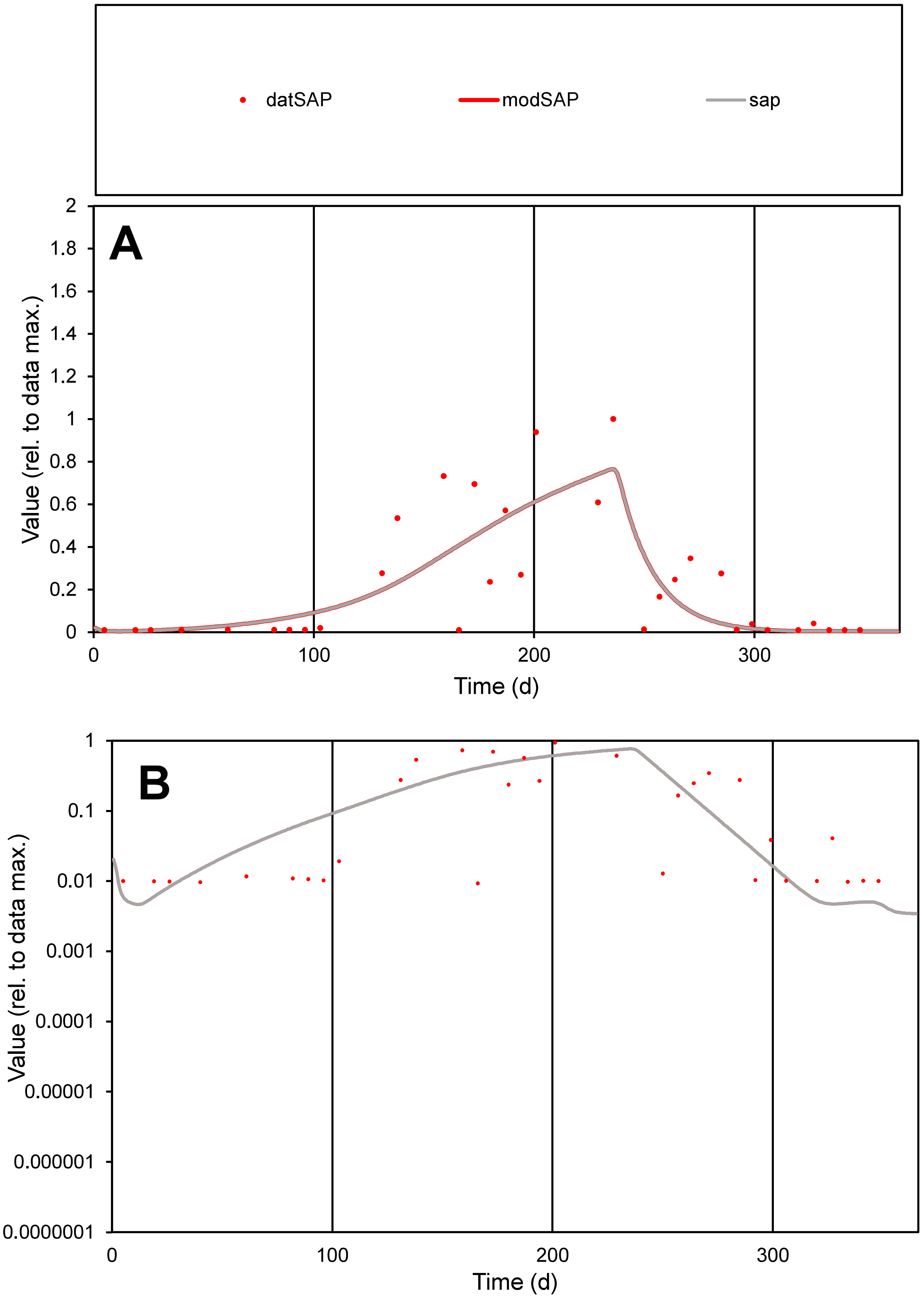

C1

Variables
SAP Provide jtd or Dst
CalibOnly?
2 1 to reproduce of
oNormalize?
2 1=no, 2=yes

ofxn calc*
0.81199
ofxn mod
0.83226

sRun
RunA1
zout
1

Ave
Data
0.00076
Model
0.00072

data/vMax
0.00352

ofxng
263.459

Min d/m
1.98536
ave/min d
23.4591
ave/min m
43.6696

326_Calib_S_A

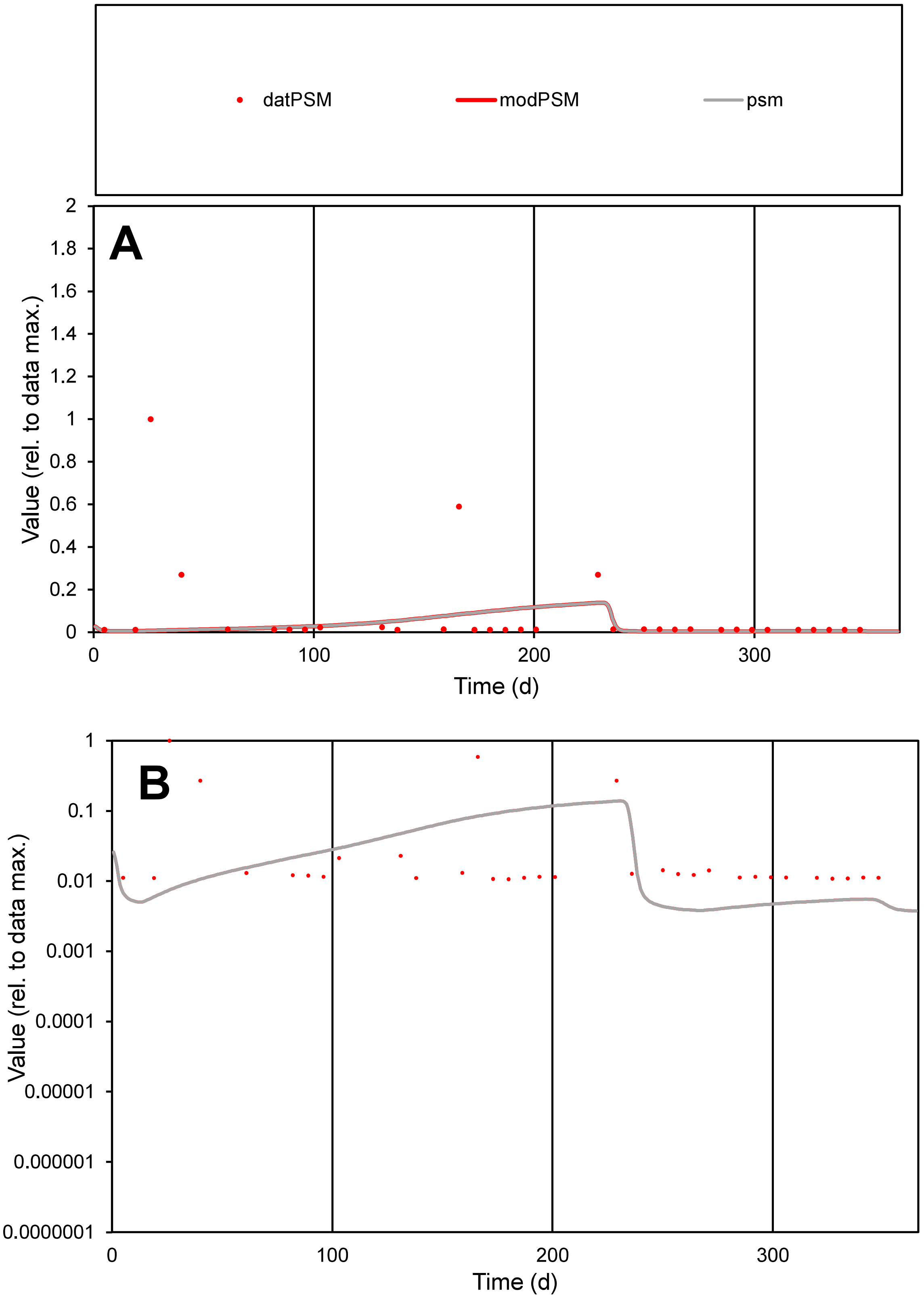

C1

Variables
PSM Provide jtd or Dst
CalibOnly?
2 1 to reproduce of
oNormalize?
2 1=no, 2=yes

ofxn calc*
2.70609
ofxn mod
2.71436

sRun
RunA1
zout
1

Ave
Data
0.0002
Model
9.3E-05

data/vMax
0.00265

ofxng
263.459

Min d/m
2.77657

ave/min d
7.09234
ave/min m
9.1474

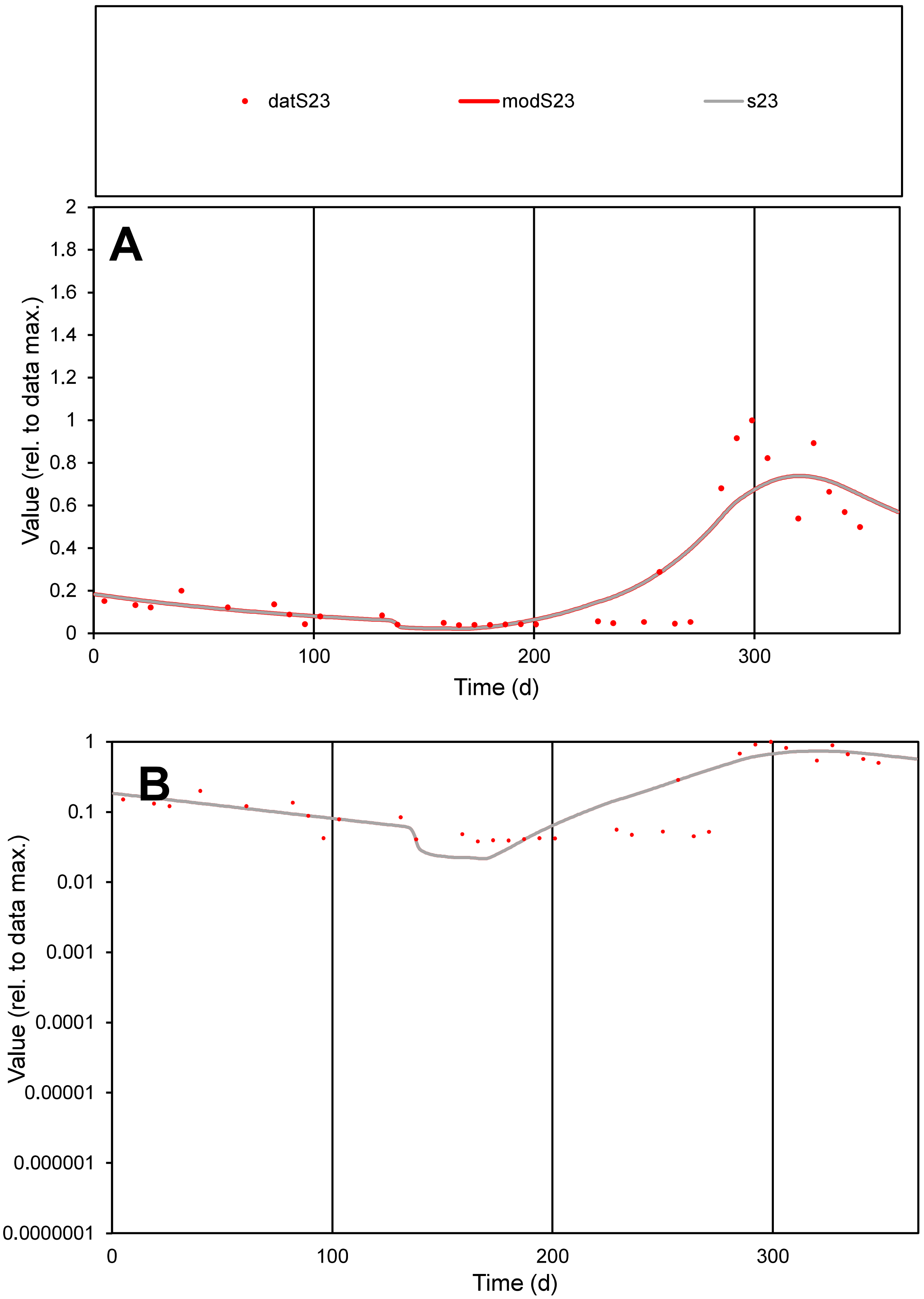

C1

Variables
S23 Provide jtd or Dst
CalibOnly?
2 1 to reproduce of
oNormalize?
2 1=no, 2=yes

ofxn calc*
0.51527
ofxn mod
0.53825

sRun
RunA1
zout
1

Ave
Data
0.00109
Model
0.00115

data/vMax
0.00418

ofxng
263.459

Min d/m
1.75497

ave/min d
6.85885
ave/min m
12.7333

326_Calib_S_A

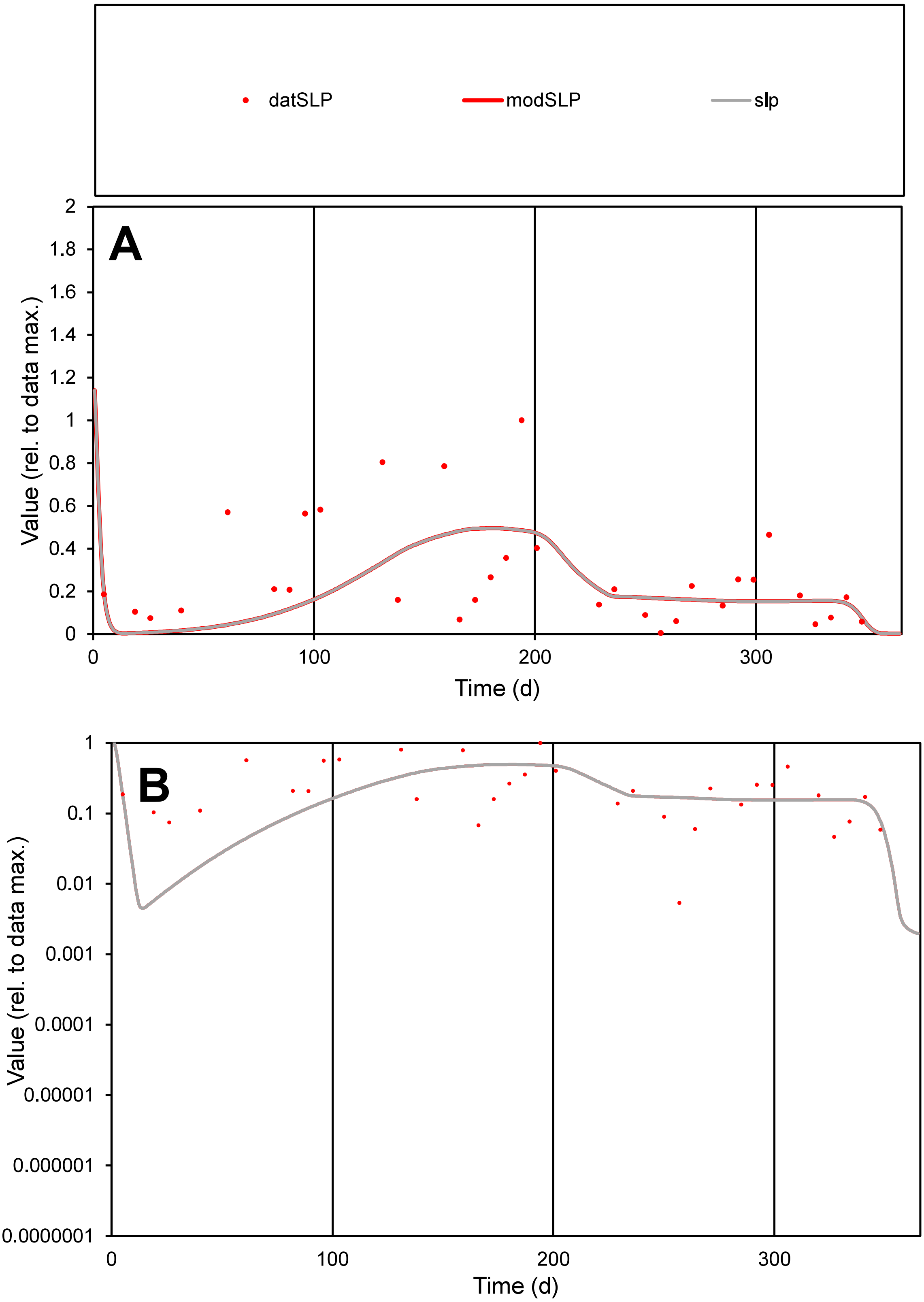

C1

Variables
SLP Provide jtd or Dst
CalibOnly?
2 1 to reproduce of
oNormalize?
2 1=no, 2=yes

ofxn calc*
0.86441
ofxn mod
0.8739

sRun
RunA1
zout
1

Ave
Data
0.00147
Model
0.00119

data/vMax
0.00541

ofxng
263.459

Min d/m
0.86408
ave/min d
50.7992
ave/min m
35.459

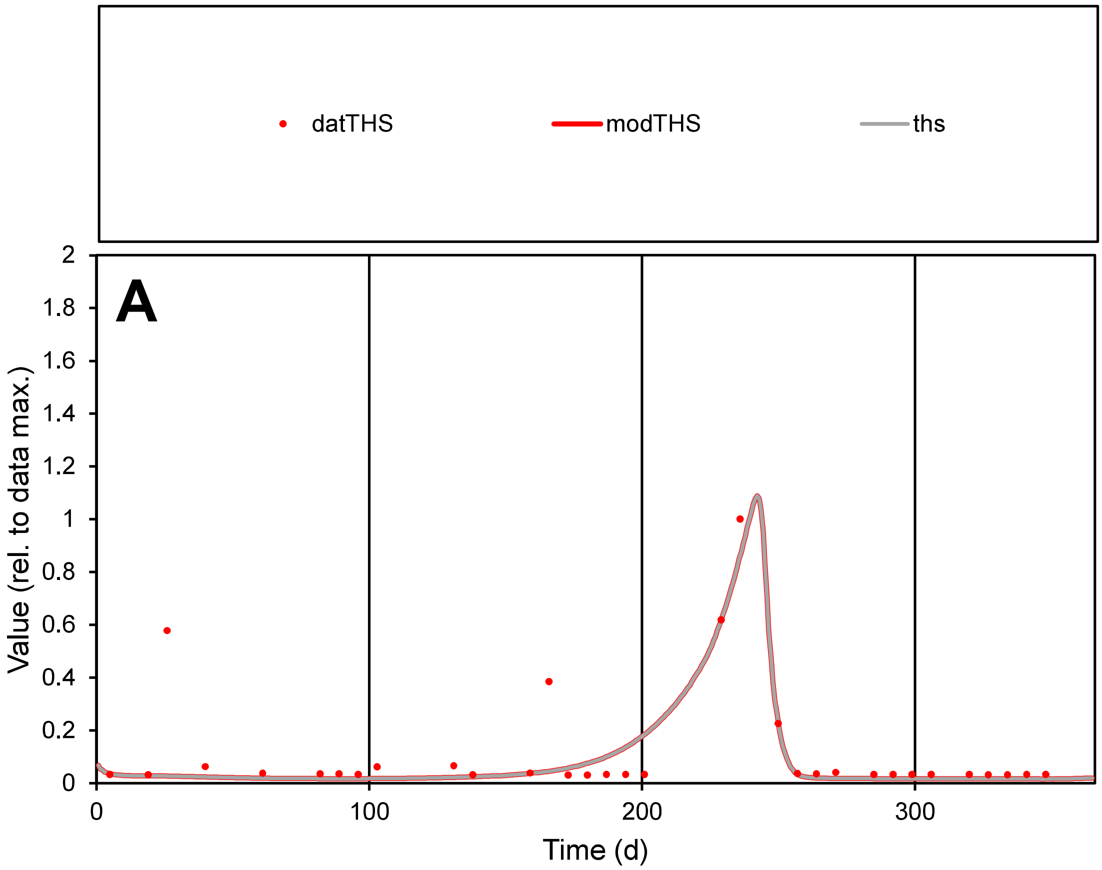

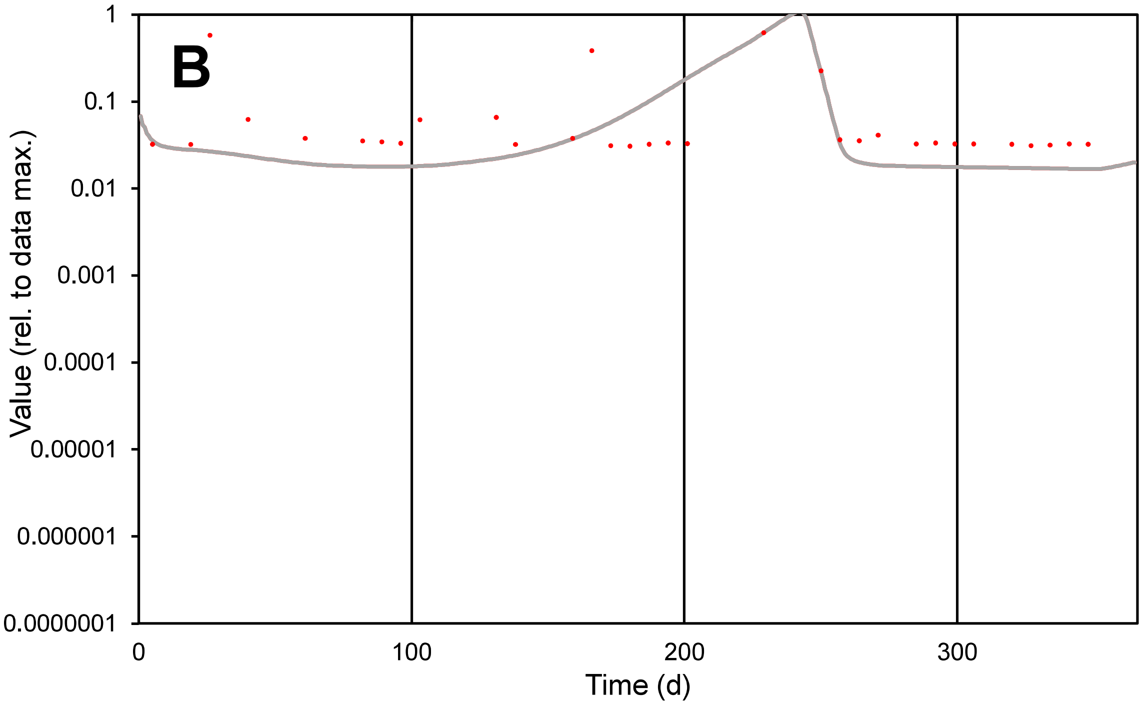

C1

Variables

THS	Provide jtd or Dst
CalibOnly?	
2	1 to reproduce of
oNormalize?	
2	1=no, 2=yes

ofxn calc*
1.03936
ofxn mod
1.05686

sRun
RunA1
zout
1

Ave
Data
0.00011
Model
8.4E-05

data/vMax
0.00095

ofxng
263.459

Min d/m
1.83021

ave/min d
3.78275
ave/min m
5.24161

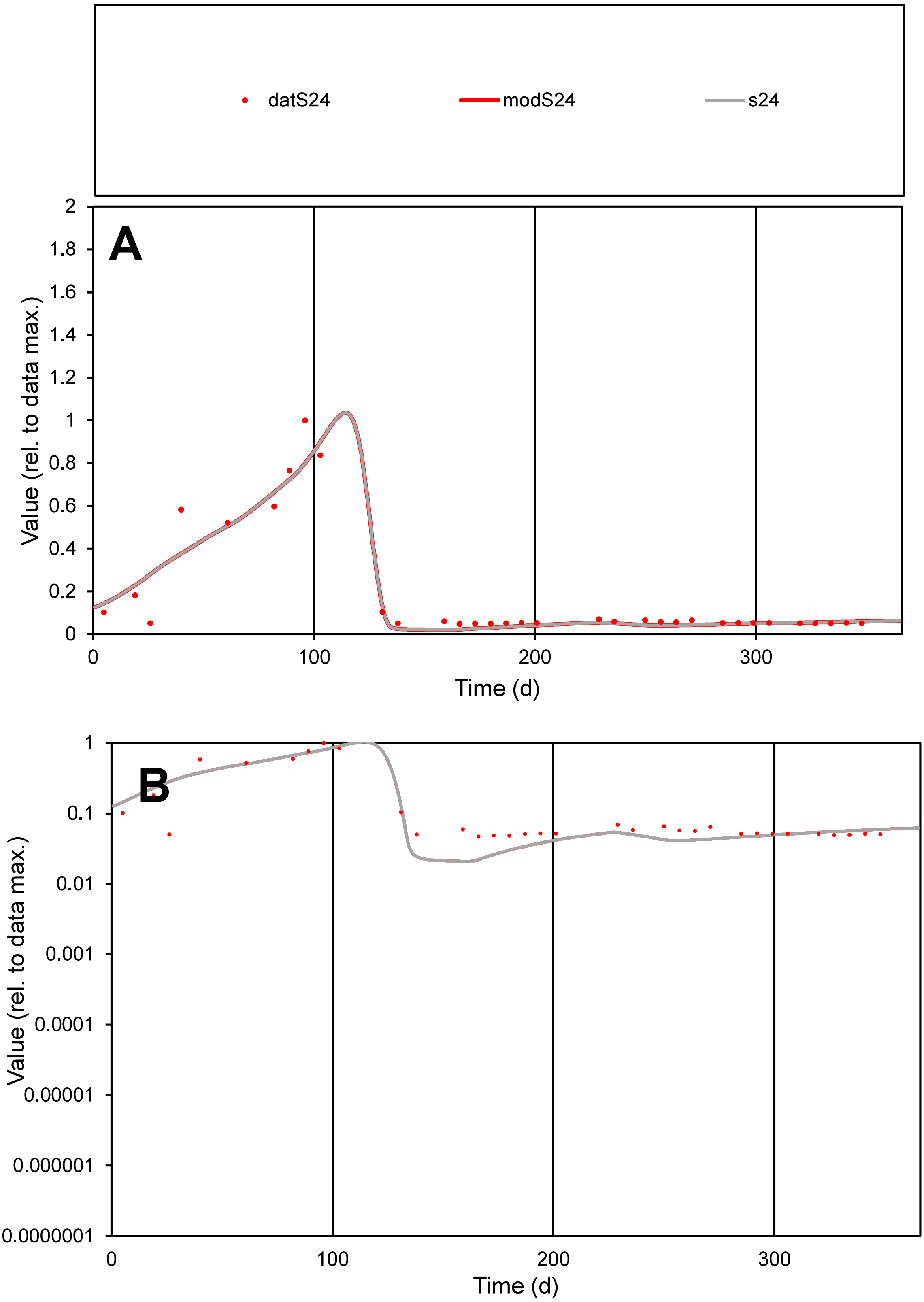

C1

Variables
S24 Provide jtd or Dst
CalibOnly?
2 1 to reproduce of
oNormalize?
2 1=no, 2=yes

ofxn calc*
0.37139
ofxn mod
0.40904

sRun
RunA1
zout
1

Ave
Data
0.00022
Model
0.00022

data/vMax
0.00123

ofxng
263.459

Min d/m
2.26169

ave/min d
3.85761
ave/min m
8.47787

326_Calib_S_A

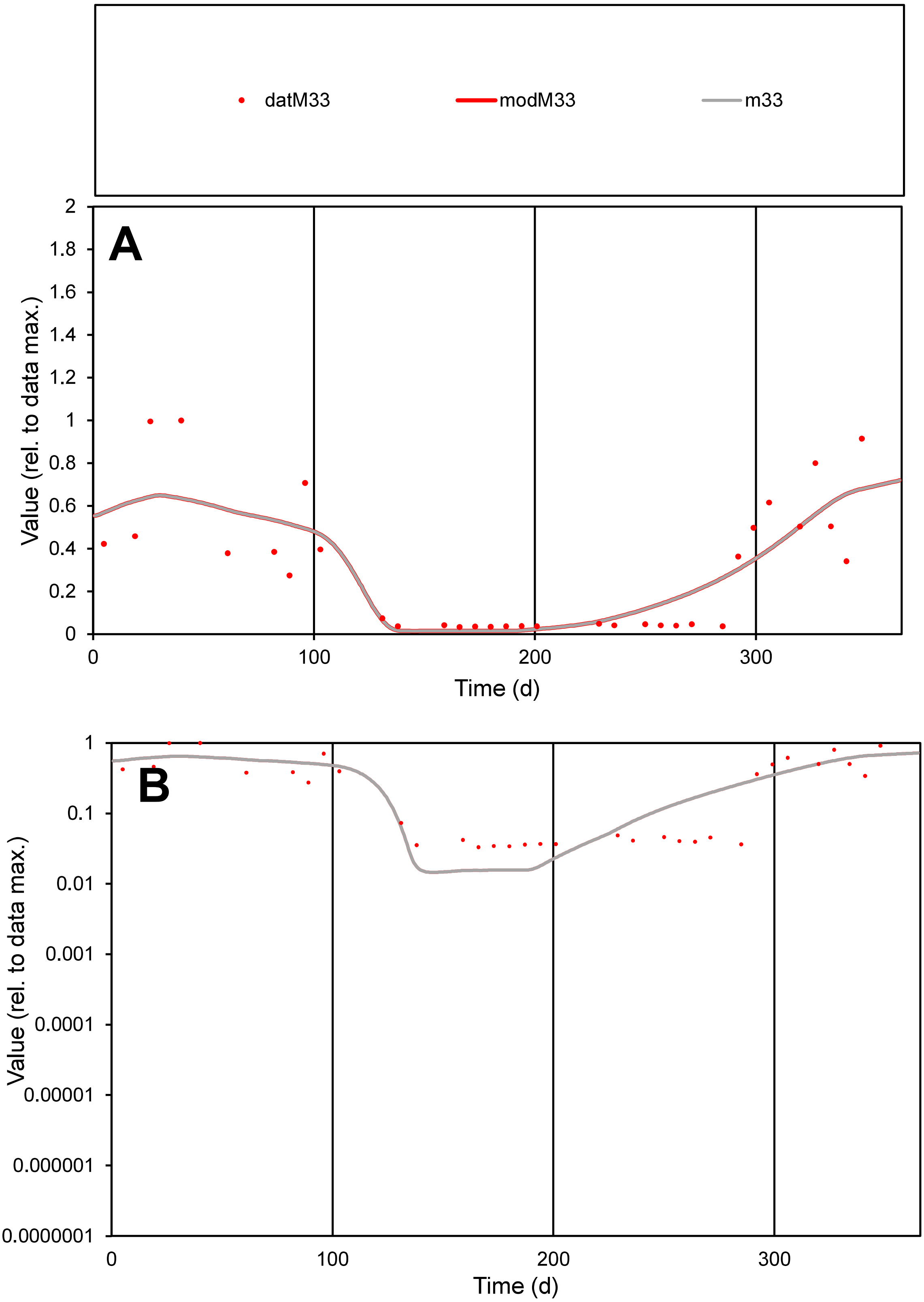

C1

Variables

M33 Provide jtd or Dst

CalibOnly?

2 1 to reproduce of

oNormalize?

2 1=no, 2=yes

ofxn calc*

0.52731

ofxn mod

0.54815

sRun

RunA1

zout

1

Ave

Data

0.00063

Model

0.00064

data/vMax

0.00204

ofxng

263.459

Min d/m

2.14707

ave/min d

9.33374

ave/min m

20.4284

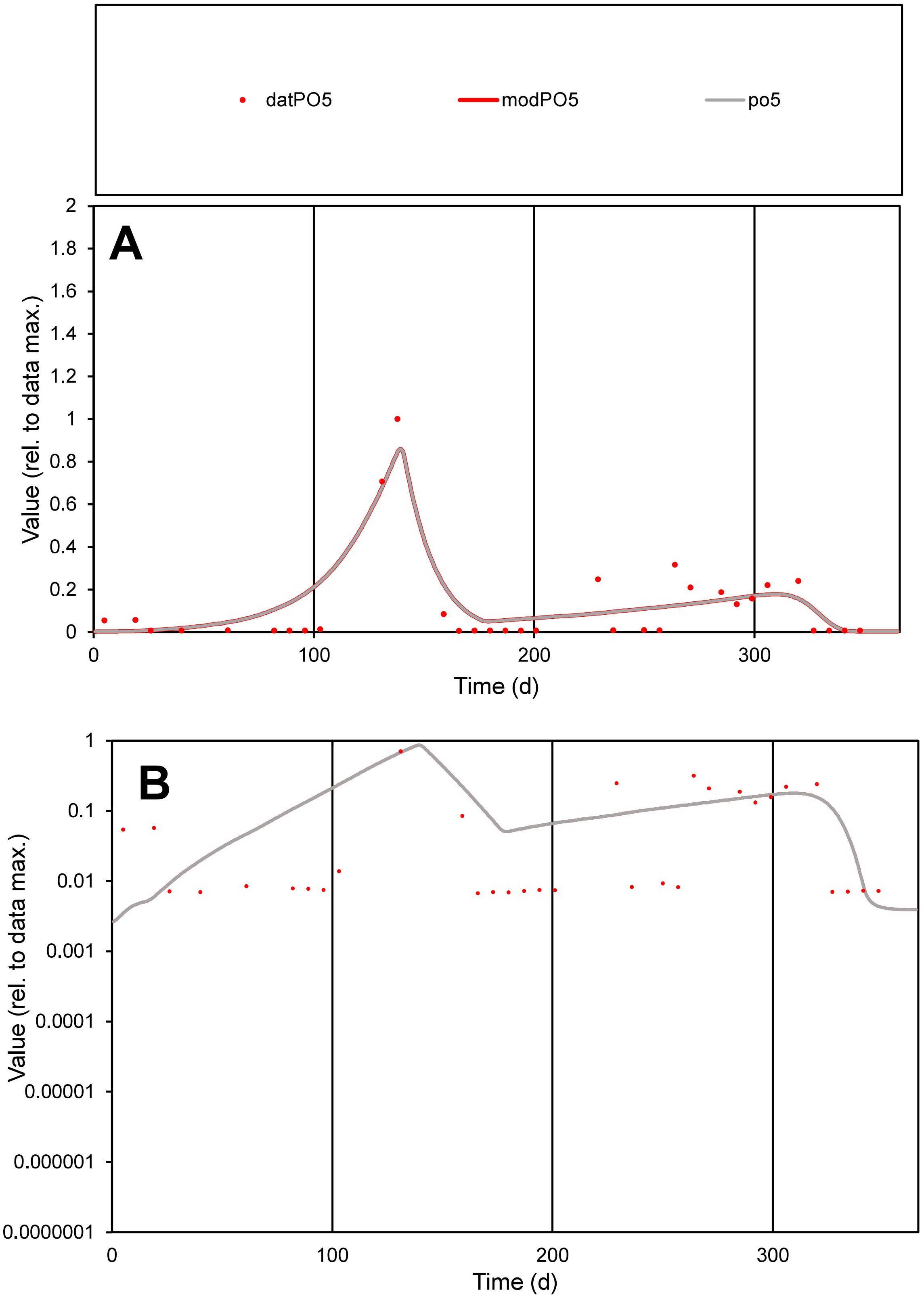

C1

Variables
PO5 Provide jtd or Dst
CalibOnly?
2 1 to reproduce of
oNormalize?
2 1=no, 2=yes

ofxn calc*
0.80294
ofxn mod
0.85751

sRun
RunA1
zout
1

Ave
Data
0.00079
Model
0.00094

data/vMax
0.00687

ofxng
263.459

Min d/m
1.75019

ave/min d
17.0577
ave/min m
35.5884

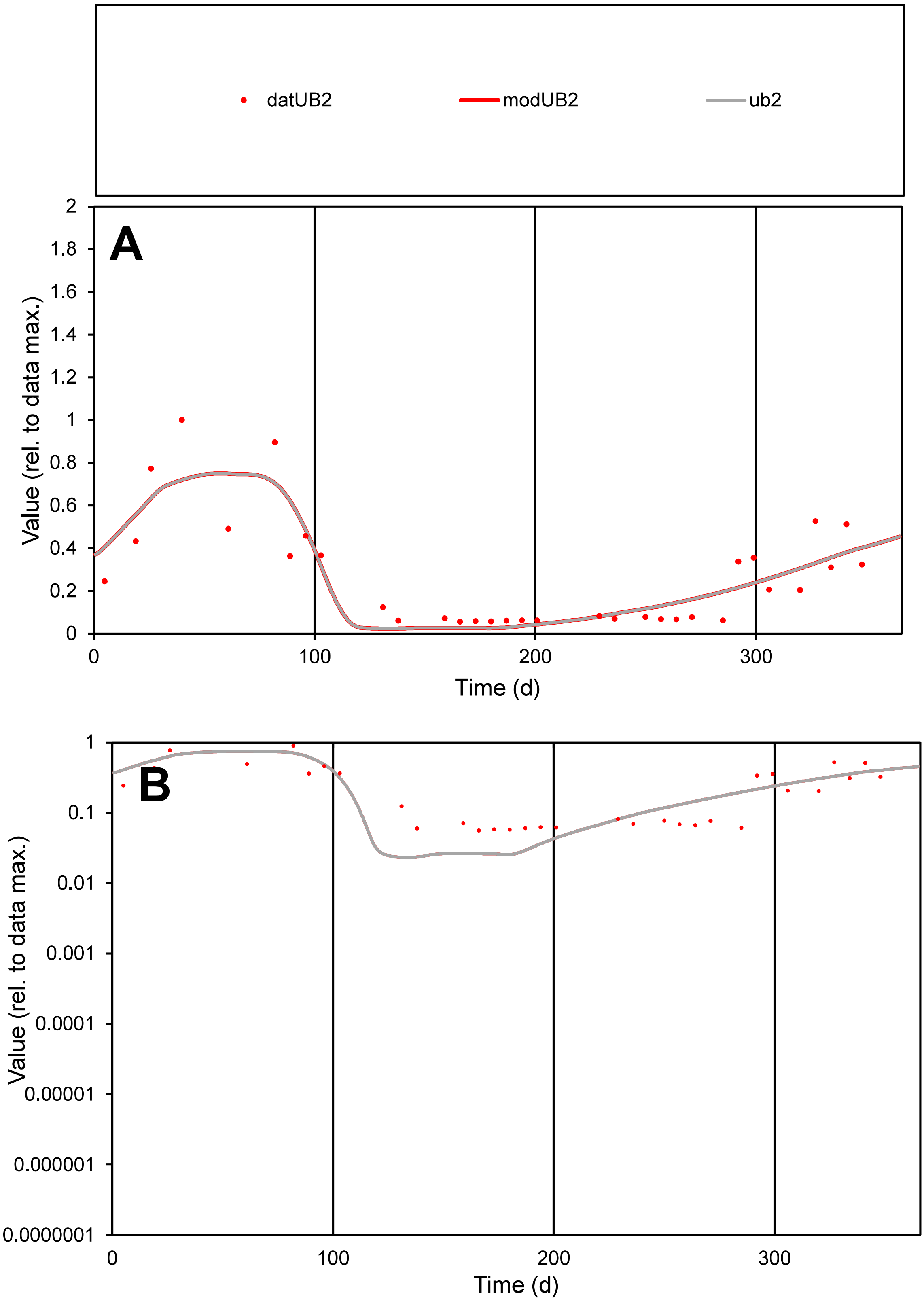

C1

Variables
UB2 Provide jtd or Dst
CalibOnly?
2 1 to reproduce of
oNormalize?
2 1=no, 2=yes

ofxn calc*
0.44004
ofxn mod
0.4709

sRun
RunA1
zout
1

Ave
Data
0.0005
Model
0.00049

data/vMax
0.00184

ofxng
263.459

Min d/m
2.42931

ave/min d
4.82753
ave/min m
11.6577

326_Calib_S_A

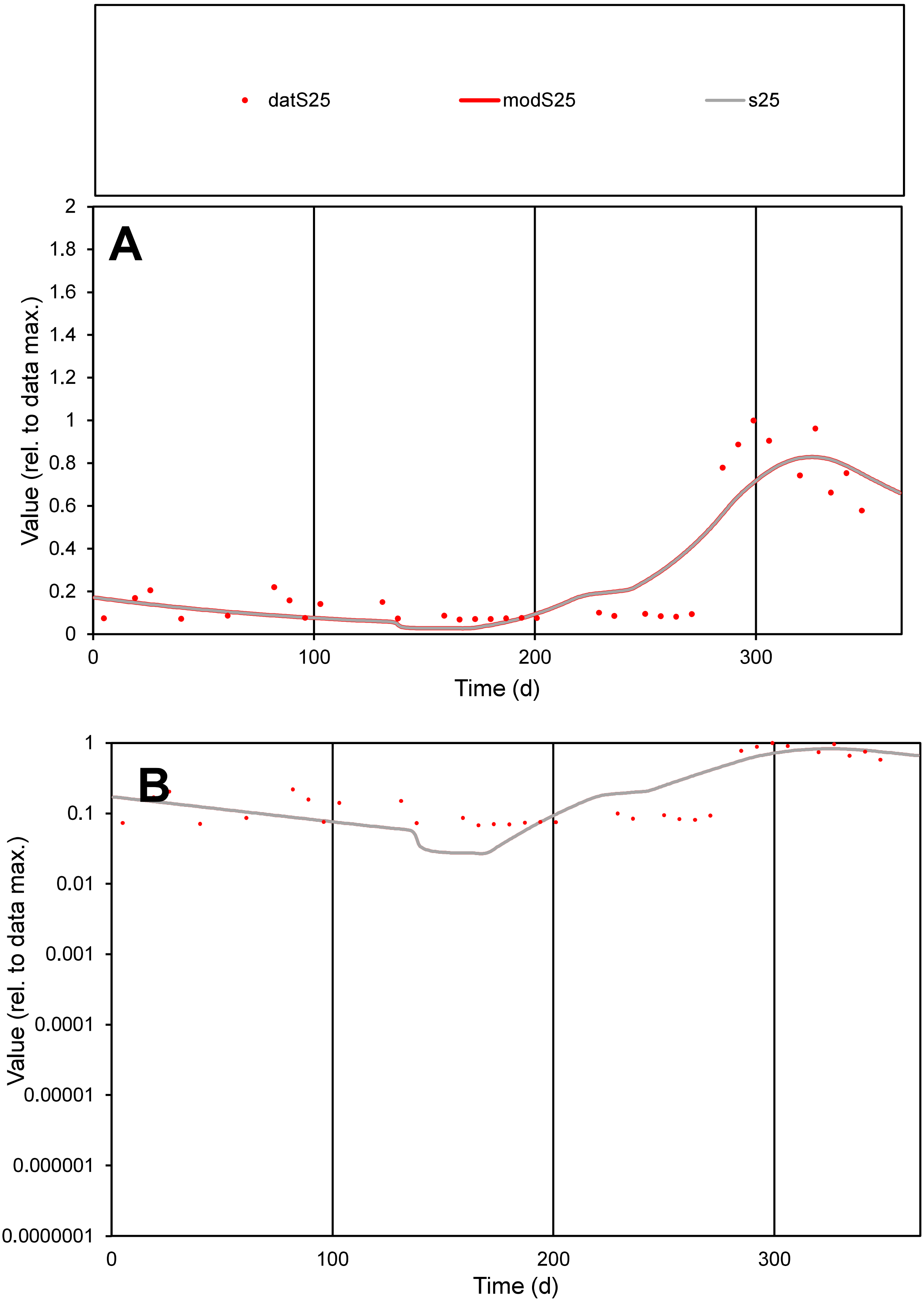

C1

Variables

S25 Provide jtd or Dst

CalibOnly?

2 1 to reproduce of

oNormalize?

2 1=no, 2=yes

ofxn calc*

0.45432

ofxn mod

0.47859

sRun

RunA1

zout

1

Ave

Data

0.00073

Model

0.00074

data/vMax

0.00246

ofxng

263.459

Min d/m

2.54291

ave/min d

4.34528

ave/min m

11.2213

326_Calib_S_A

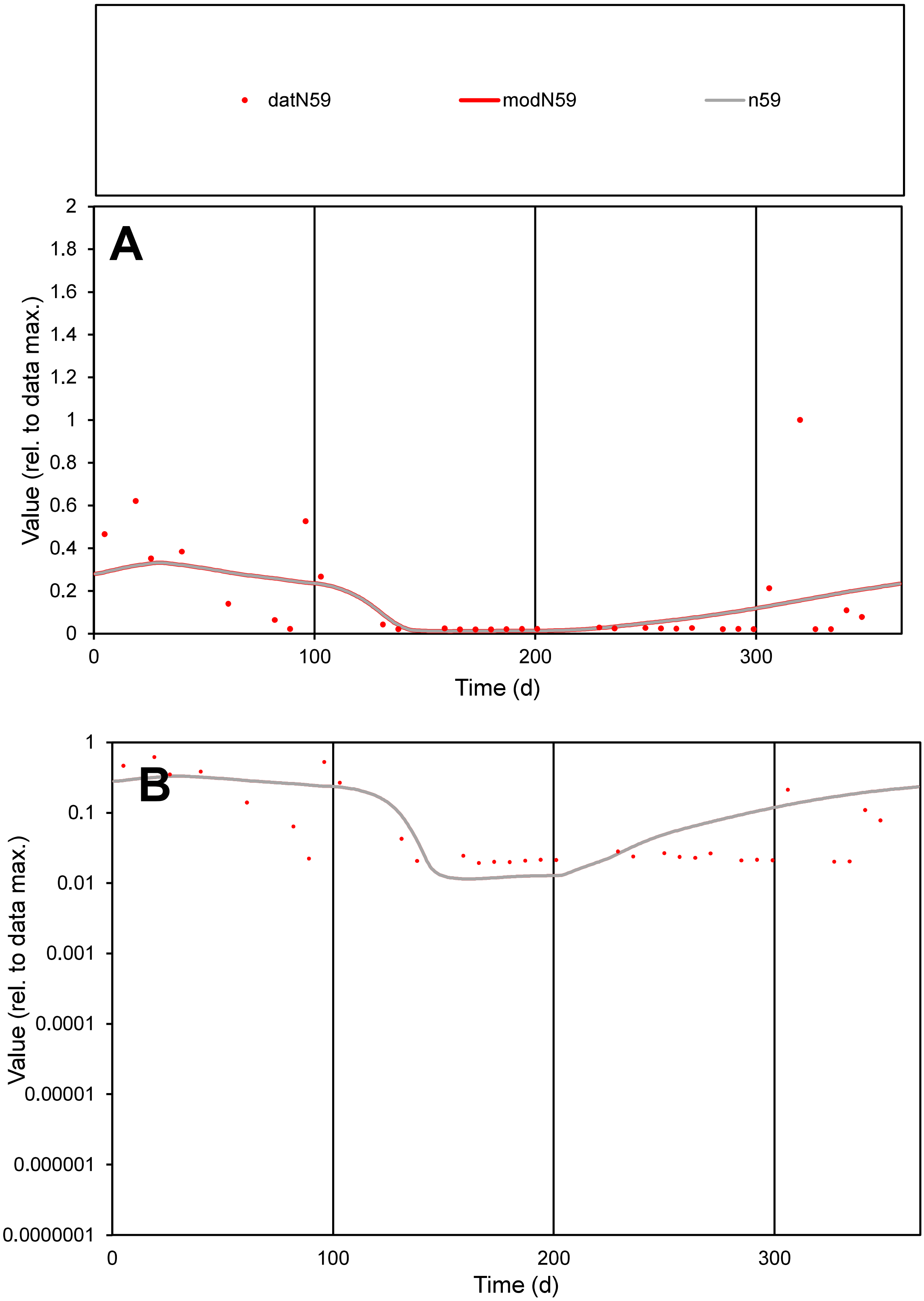

C1

Variables
N59 Provide jtd or Dst
CalibOnly?
2 1 to reproduce of
oNormalize?
2 1=no, 2=yes

ofxn calc*
1.30025
ofxn mod
1.31104

sRun
RunA1
zout
1

Ave
Data
0.0003
Model
0.00028

data/vMax
0.00212

ofxng
263.459

Min d/m
1.67857

ave/min d
7.40115
ave/min m
11.5996

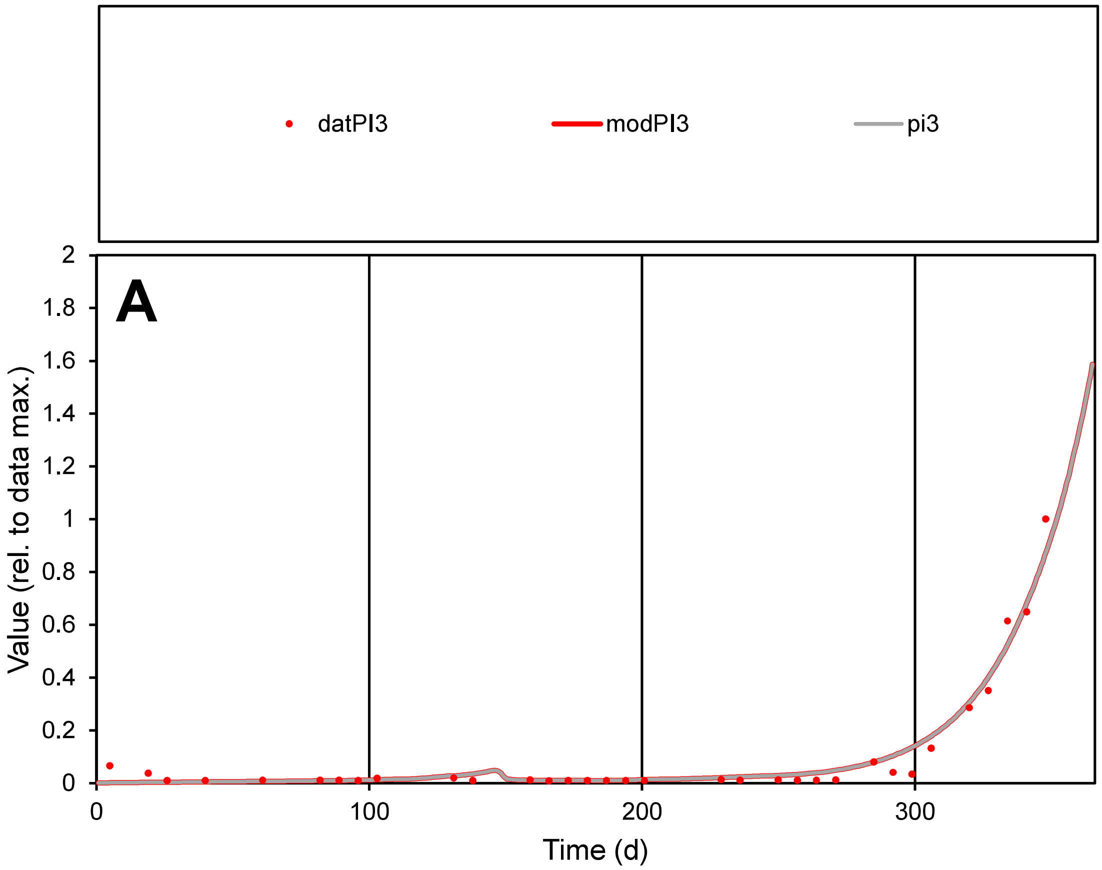

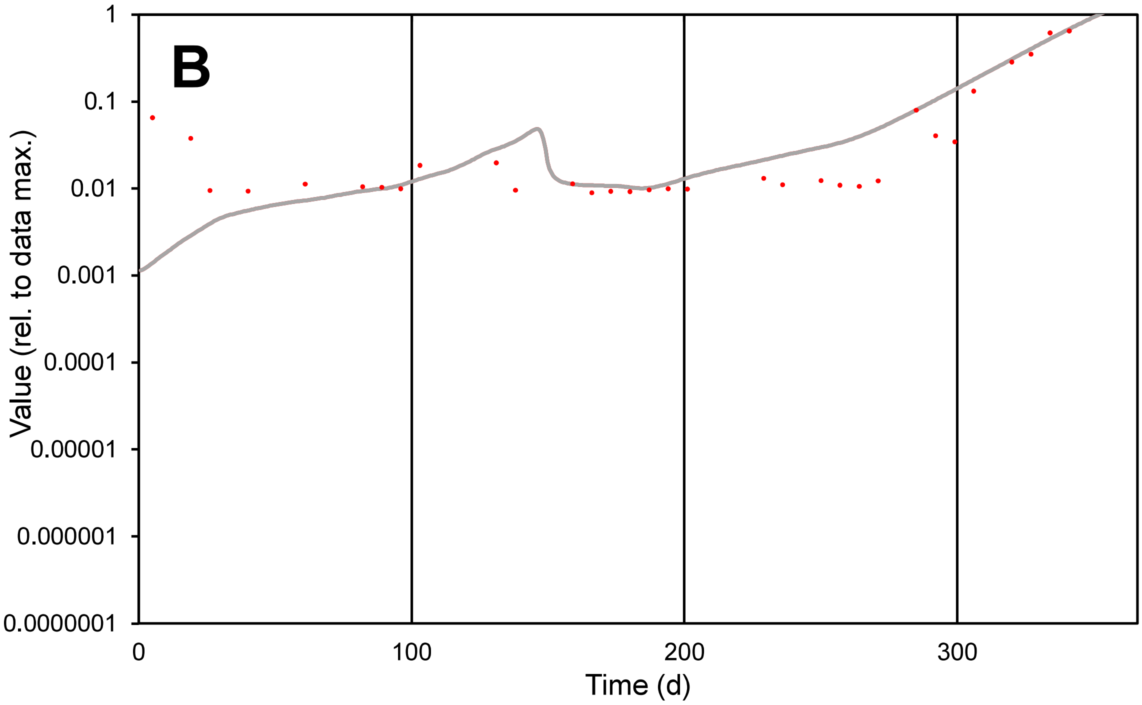

C1

Variables
PI3 Provide jtd or Dst
CalibOnly?
2 1 to reproduce of
oNormalize?
2 1=no, 2=yes

ofxn calc*
0.36774
ofxn mod
0.42575

sRun
RunA1
zout
1

Ave
Data
0.00085
Model
0.00092

data/vMax
0.00789

ofxng
263.459

Min d/m
5.8774

ave/min d
12.0039
ave/min m
76.5853

326_Calib_S_A

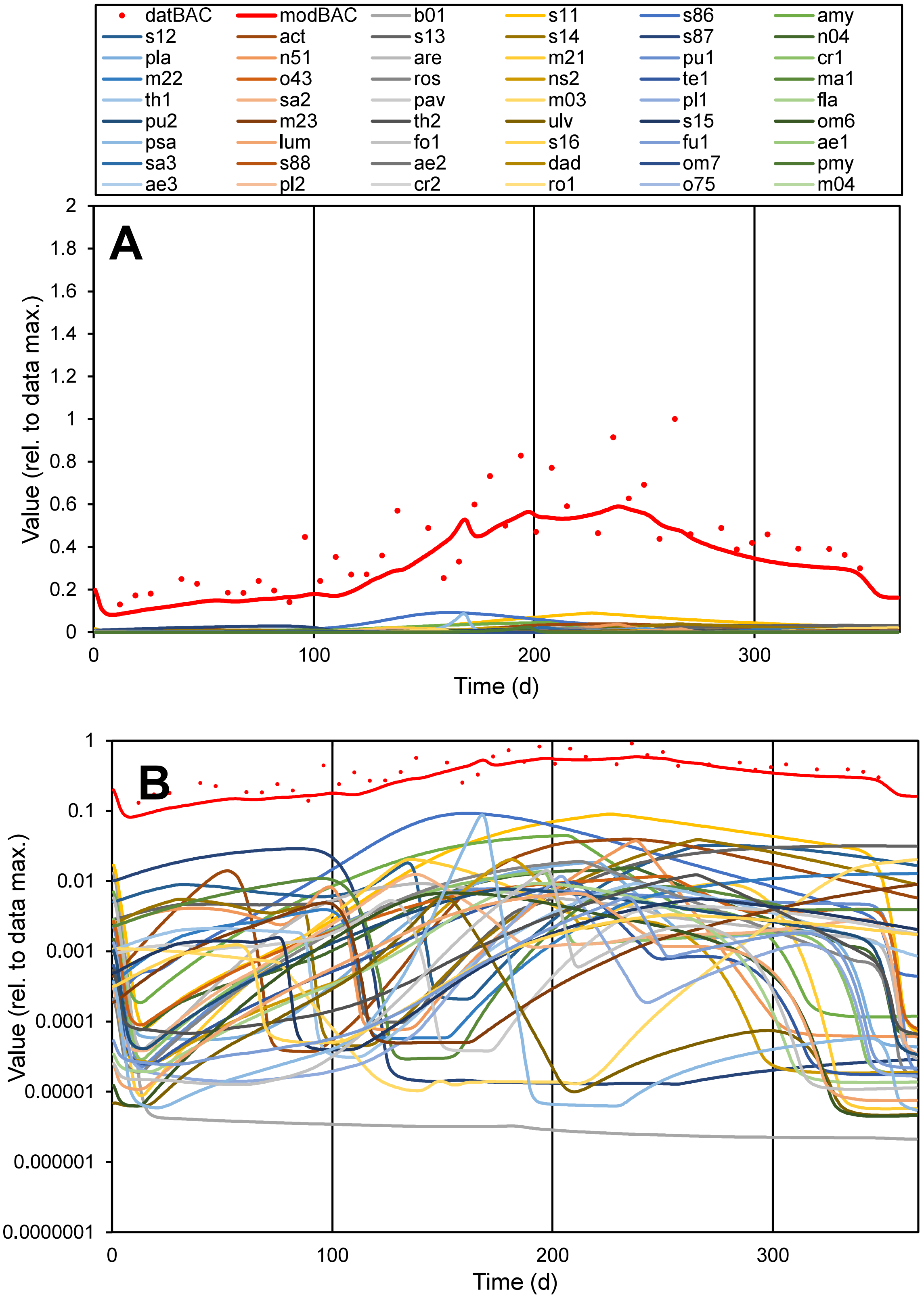

C1

Variables

BAC Provide jtd or Dst

CalibOnly?

2 1 to reproduce of

oNormalize?

2 1=no, 2=yes

ofxn calc*

0.36631

ofxn mod

0.38595

sRun

RunA1

zout

1

Ave

Data

0.88962

Model

0.70494

data/vMax

2.102

ofxng

263.459

Min d/m

1.49891

ave/min d

3.2468

ave/min m

3.85635

327_Calib_S_A

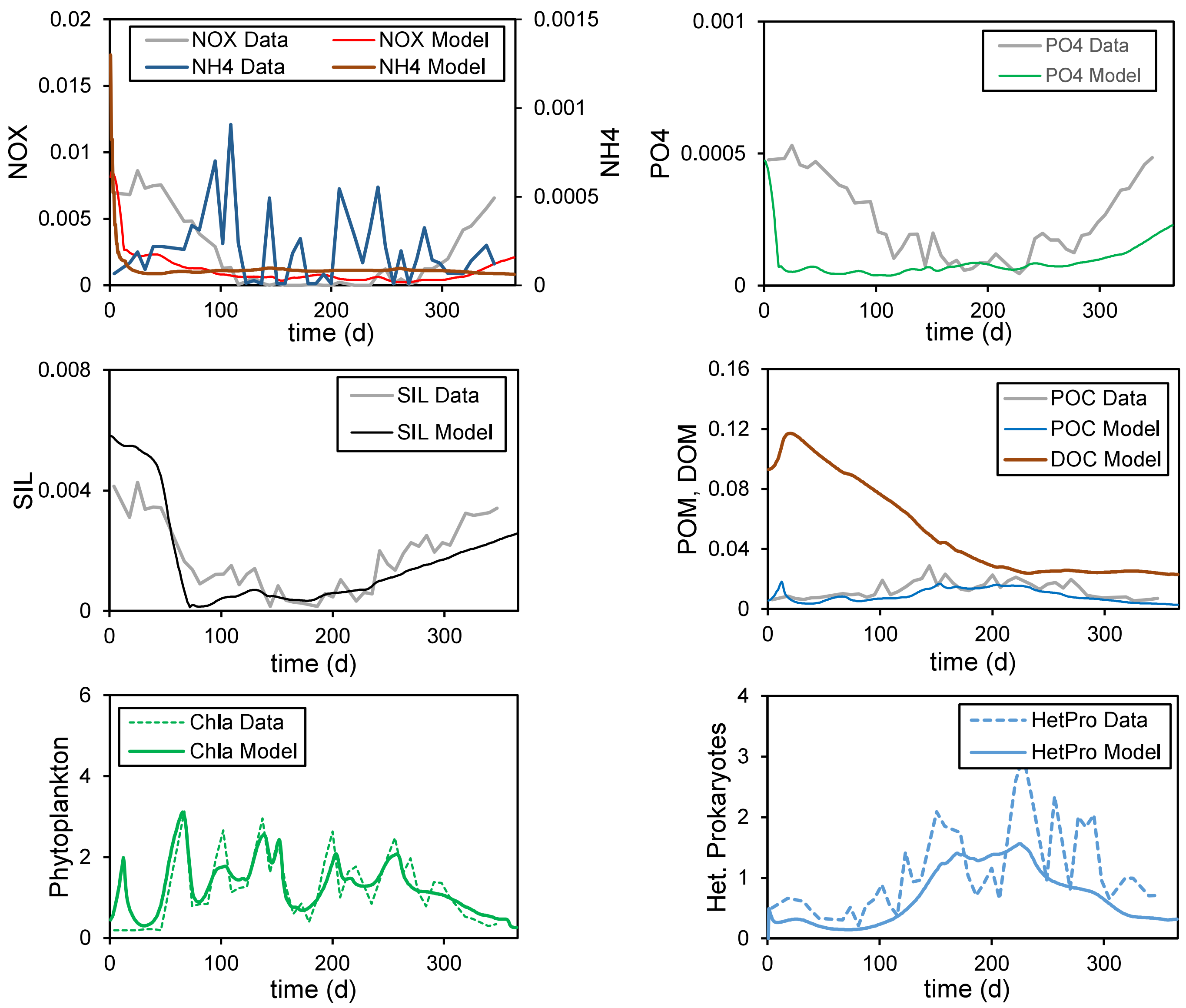

NOX Data
NOX Model
NH4 Data
NH4 Model
NOX
NH4
time (d)
PO4 Data
PO4 Model
PO4
time (d)
SIL Data
SIL Model
SIL
time (d)
POC Data
POC Model
DOC Model
POM, DOM
time (d)
Chla Data
Chla Model
Phytoplankton
time (d)
HetPro Data
HetPro Model
Het. Prokaryotes
time (d)

327_Calib_S_A

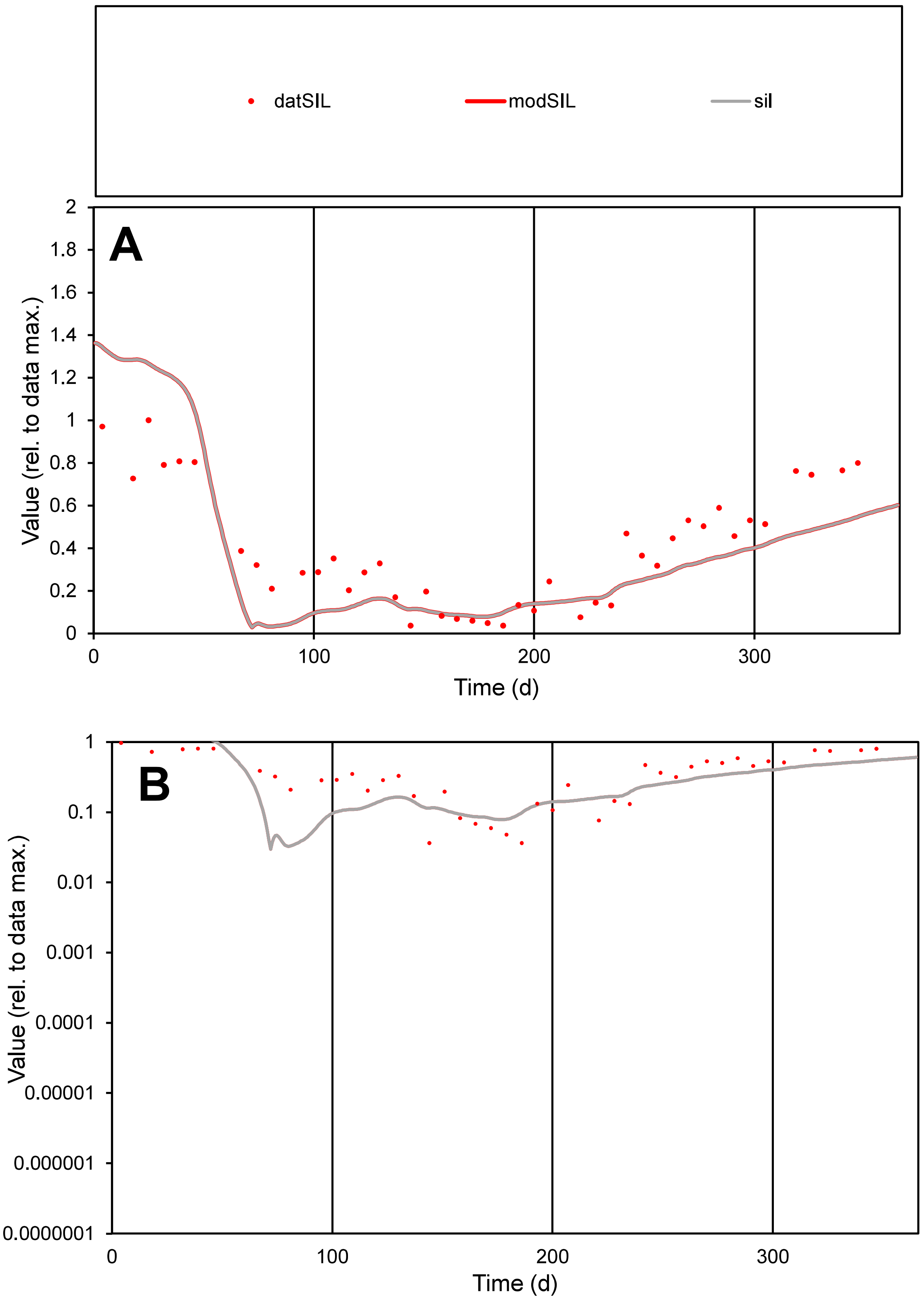

C1

Variables
SIL Provide jtd or Dst
CalibOnly?
2 1 to reproduce of
oNormalize?
2 1=no, 2=yes

ofxn calc*
1.51835
ofxn mod
1.5861

sRun
RunA1
zout
1

Ave
Data
0.0017
Model
0.00152

data/vMax
0.00427

ofxng
334.07

Min d/m
1.0697

ave/min d
10.9364
ave/min m
10.4946

327_Calib_S_A

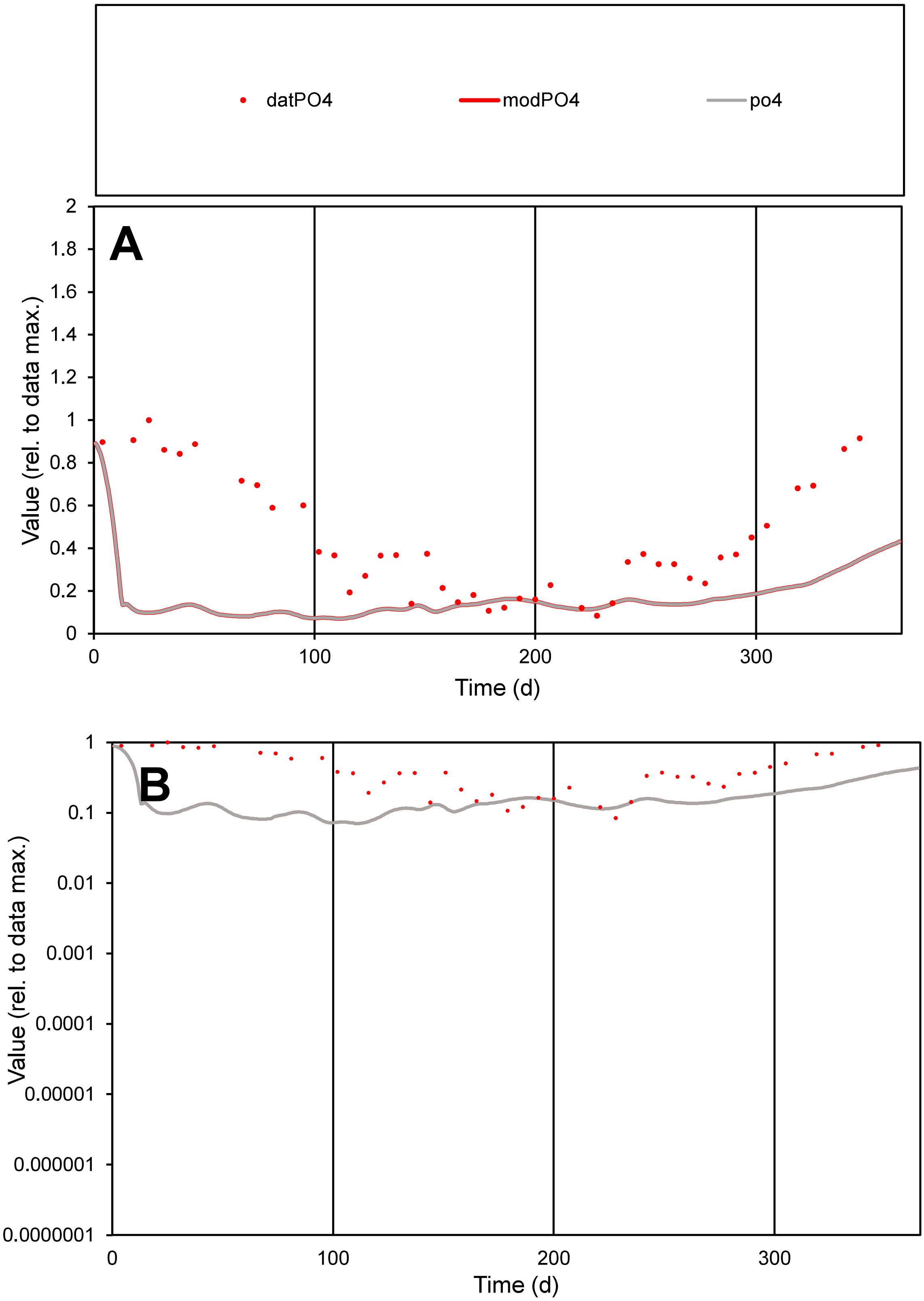

C1

Variables
PO4 Provide jtd or Dst
CalibOnly?
2 1 to reproduce of
oNormalize?
2 1=no, 2=yes

ofxn calc*
2.61757
ofxn mod
2.63116

sRun
RunA1
zout
1

Ave
Data
0.00023
Model
8.3E-05

data/vMax
0.00053

ofxng
334.07

Min d/m
1.19855

ave/min d
5.20235
ave/min m
2.23894

327_Calib_S_A

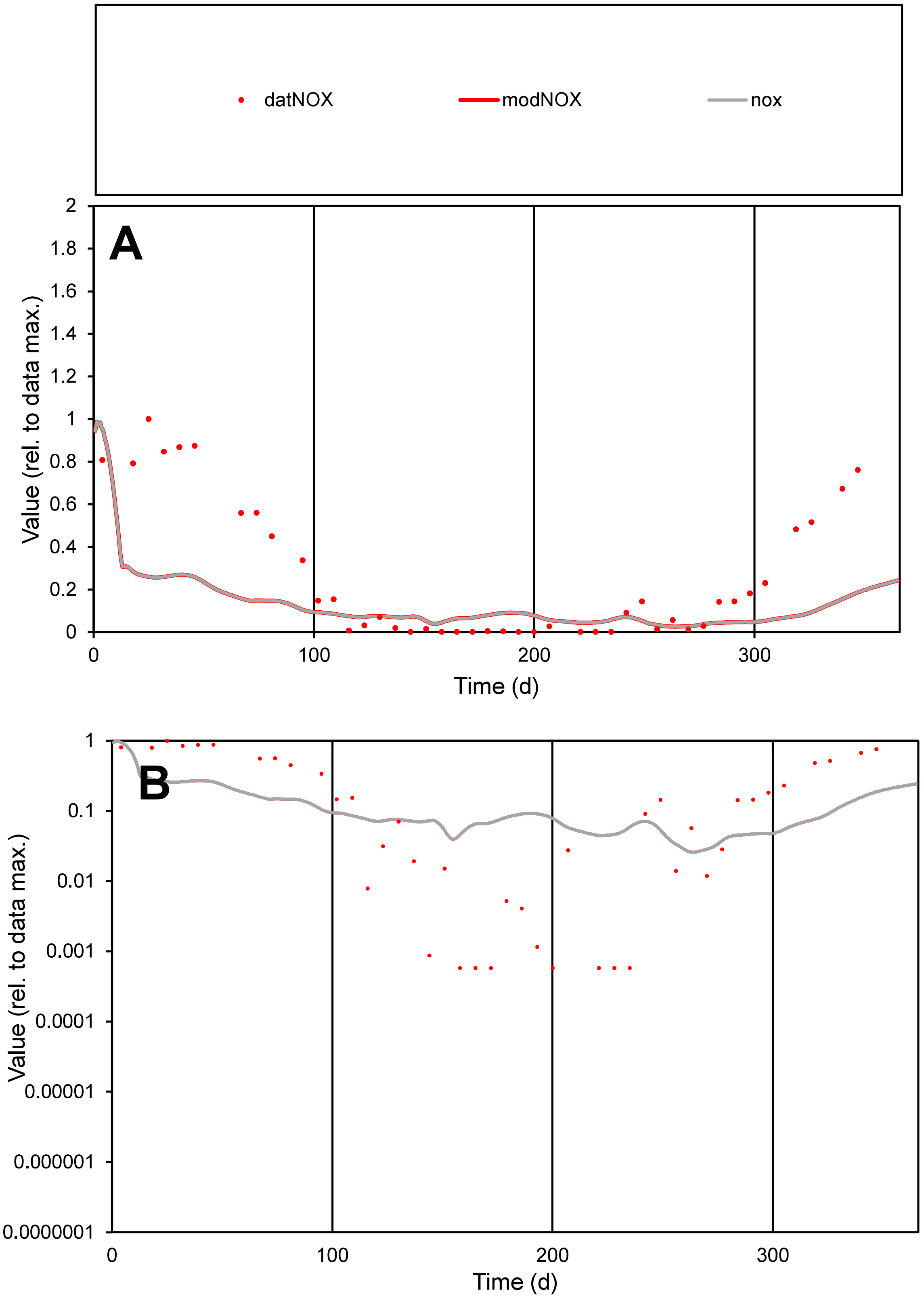

C1

Variables
NOX Provide jtd or Dst
CalibOnly?
2 1 to reproduce of
oNormalize?
2 1=no, 2=yes

ofxn calc*
3.30585
ofxn mod
3.33135

sRun
RunA1
zout
1

Ave
Data
0.00222
Model
0.00101

data/vMax
0.00862

ofxng
334.07

Min d/m
0.02229

ave/min d
443.44
ave/min m
4.51528

327_Calib_S_A

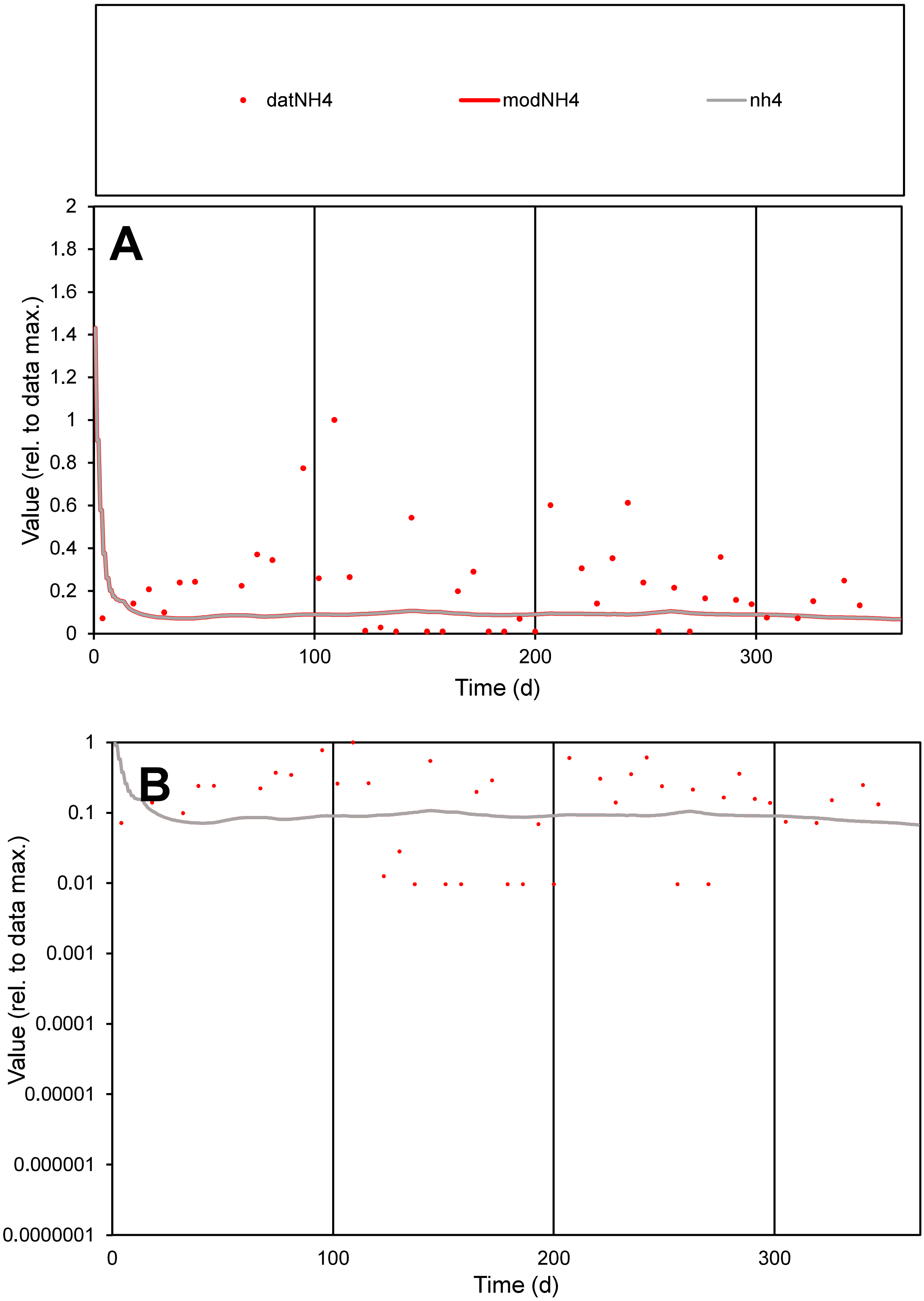

C1

Variables
NH4 Provide jtd or Dst
CalibOnly?
2 1 to reproduce of
oNormalize?
2 1=no, 2=yes

ofxn calc*
3.45237
ofxn mod
3.48088

sRun
RunA1
zout
1

Ave
Data
0.0002
Model
8.4E-05

data/vMax
0.00091

ofxng
334.07

Min d/m
0.13585

ave/min d
22.7078
ave/min m
1.30294

327_Calib_S_A

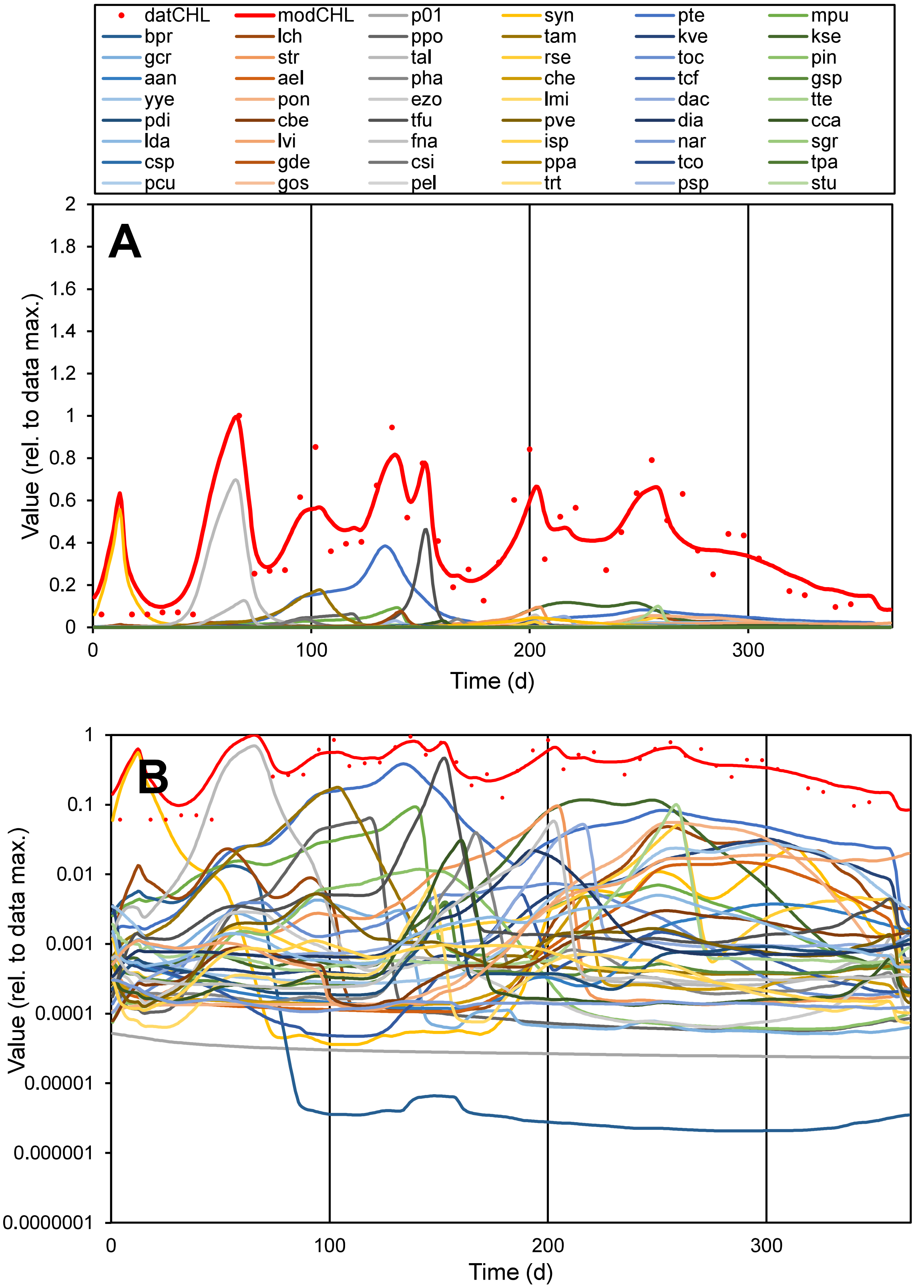

C1

Variables

CHL Provide jtd or Dst

CalibOnly?

2 1 to reproduce of

oNormalize?

2 1=no, 2=yes

ofxn calc*

2.71147

ofxn mod

3.10075

sRun

RunA1

zout

1

Ave

Data

1.24336

Model

1.25597

data/vMax

3.128

ofxng

334.07

Min d/m

0.6197

ave/min d

6.54402

ave/min m

4.09645

327_Calib_S_A

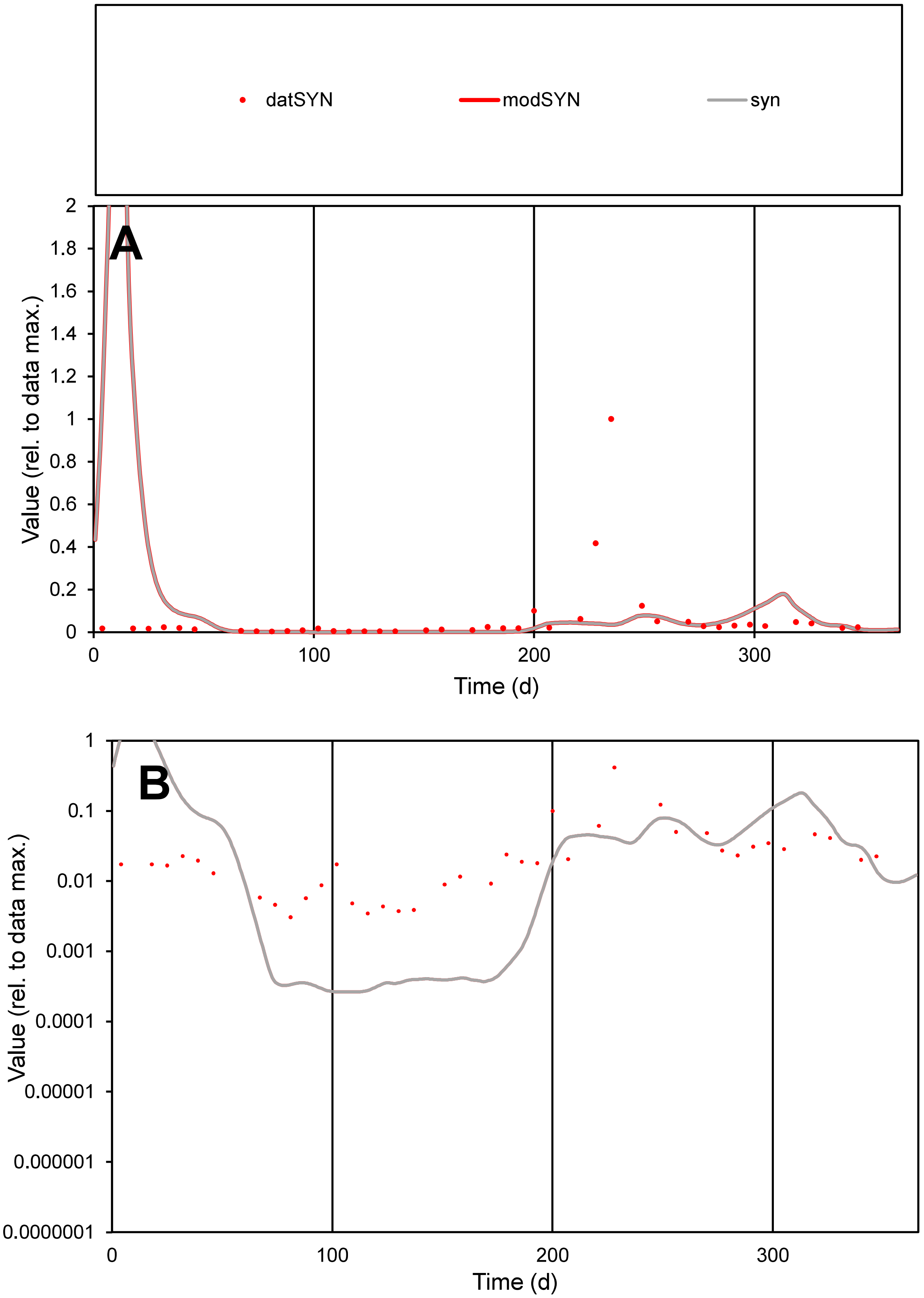

C1

Variables

SYN Provide jtd or Dst

CalibOnly?

2 1 to reproduce of

oNormalize?

2 1=no, 2=yes

ofxn calc*

3.59894

ofxn mod

4.27556

sRun

RunA1

zout

1

Ave

Data

15.004

Model

16.8841

data/vMax

254.6

ofxng

334.07

Min d/m

11.6142

ave/min d

19.2556

ave/min m

251.663

327_Calib_S_A

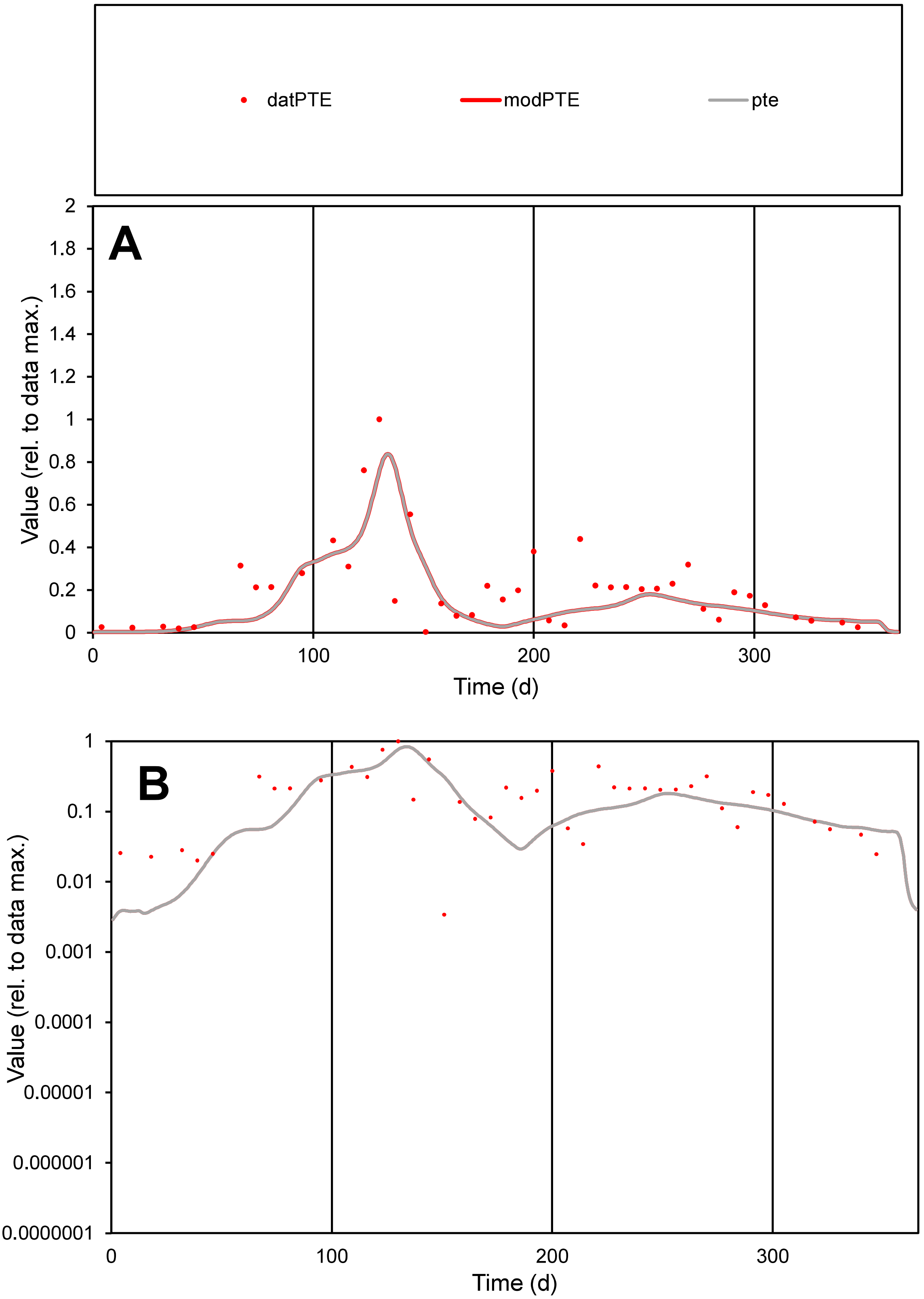

C1

Variables
PTE Provide jtd or Dst
CalibOnly?
2 1 to reproduce of
oNormalize?
2 1=no, 2=yes

ofxn calc*
0.71408
ofxn mod
0.74686

sRun
RunA1
zout
1

Ave
Data
0.0005
Model
0.00039

data/vMax
0.00245

ofxng
334.07

Min d/m
0.87539

ave/min d
60.0116
ave/min m
41.0433

327_Calib_S_A

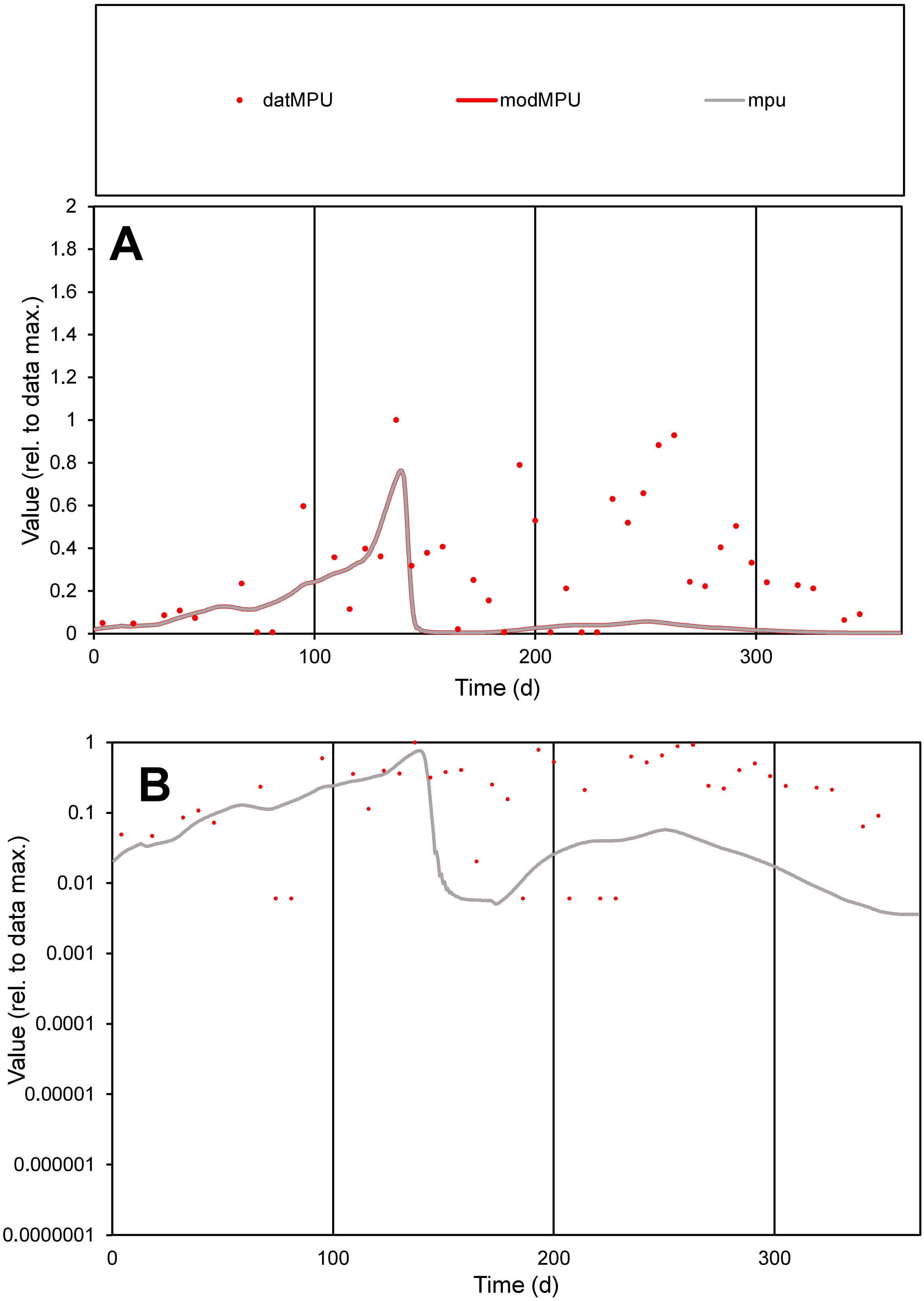

C1

Variables
MPU Provide jtd or Dst
CalibOnly?
2 1 to reproduce of
oNormalize?
2 1=no, 2=yes

ofxn calc*
1.10504
ofxn mod
1.11198

sRun
RunA1
zout
1

Ave
Data
0.0002
Model
6.5E-05

data/vMax
0.00067

ofxng
334.07

Min d/m
1.54014

ave/min d
49.6911
ave/min m
24.5795

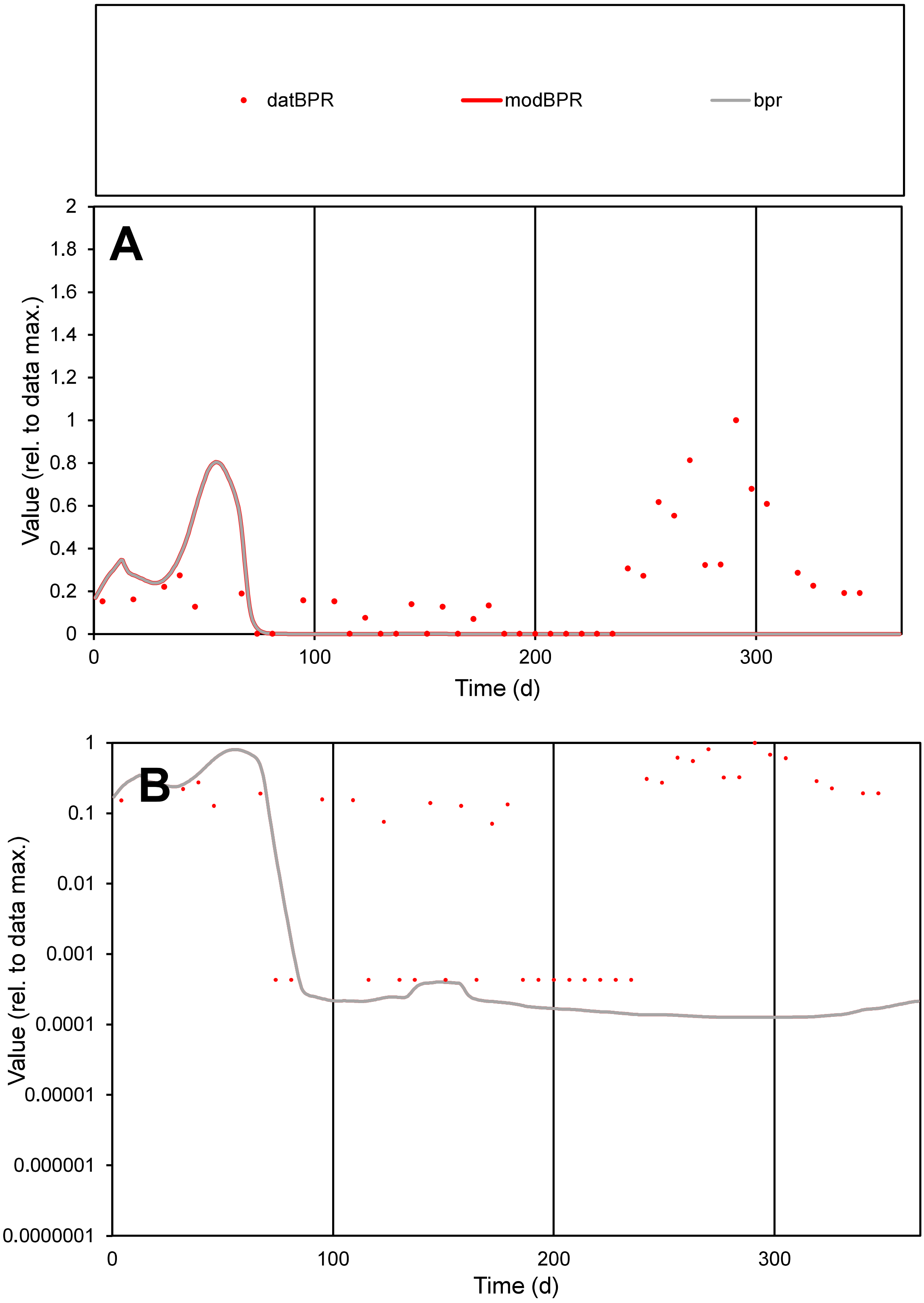

C1

Variables
BPR Provide jtd or Dst
CalibOnly?
2 1 to reproduce of
oNormalize?
2 1=no, 2=yes

ofxn calc*
1.56247
ofxn mod
1.57596

sRun
RunA1
zout
1

Ave
Data
8.6E-05
Model
2E-05

data/vMax
0.00043

ofxng
334.07

Min d/m
3.41945

ave/min d
462.23
ave/min m
370.047

327_Calib_S_A

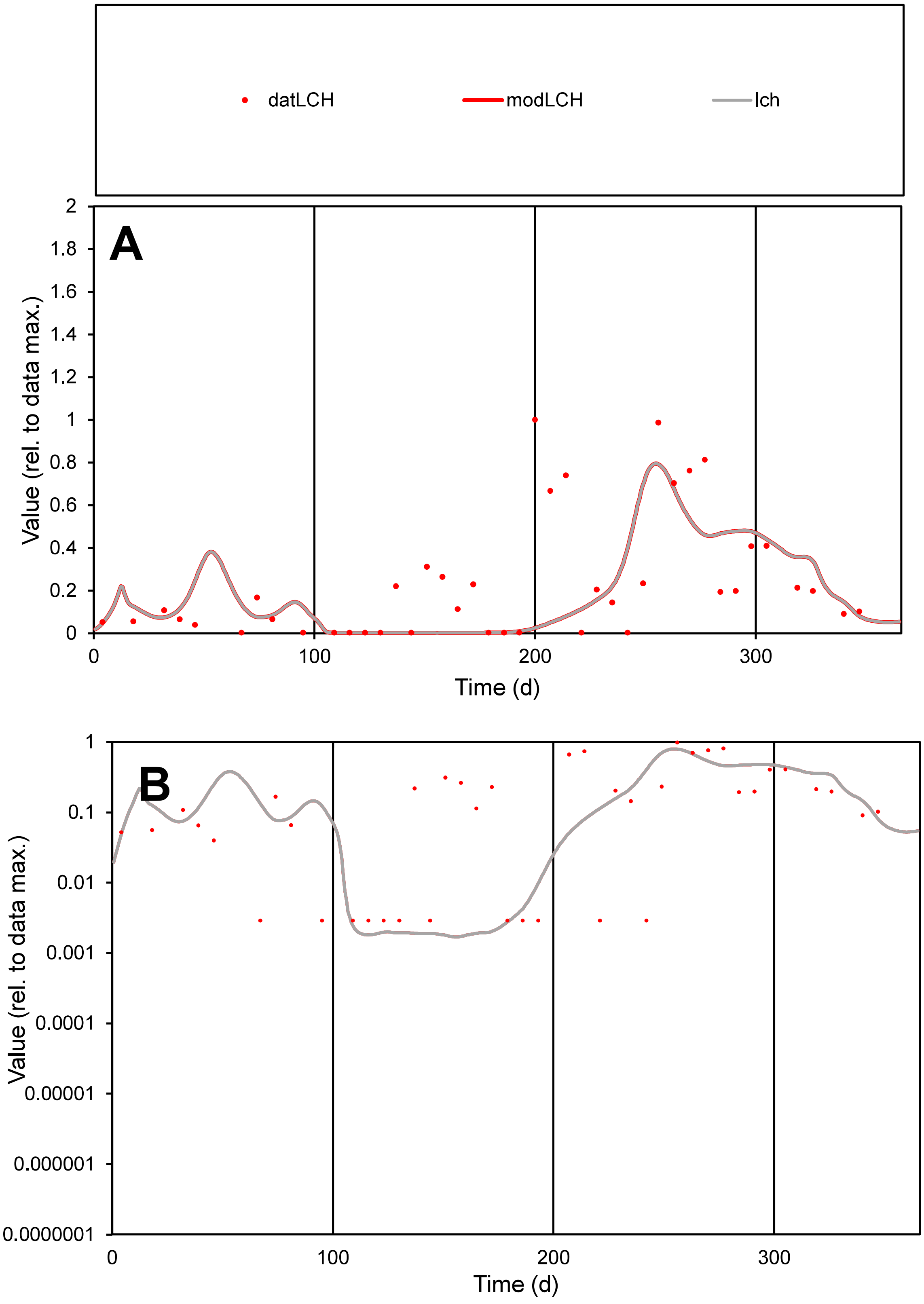

C1

Variables
LCH Provide jtd or Dst
CalibOnly?
2 1 to reproduce of
oNormalize?
2 1=no, 2=yes

ofxn calc*
1.1178
ofxn mod
1.14226

sRun
RunA1
zout
1

Ave
Data
0.0002
Model
0.00016

data/vMax
0.00085

ofxng
334.07

Min d/m
1.64936

ave/min d
80.6734
ave/min m
106.151

327_Calib_S_A

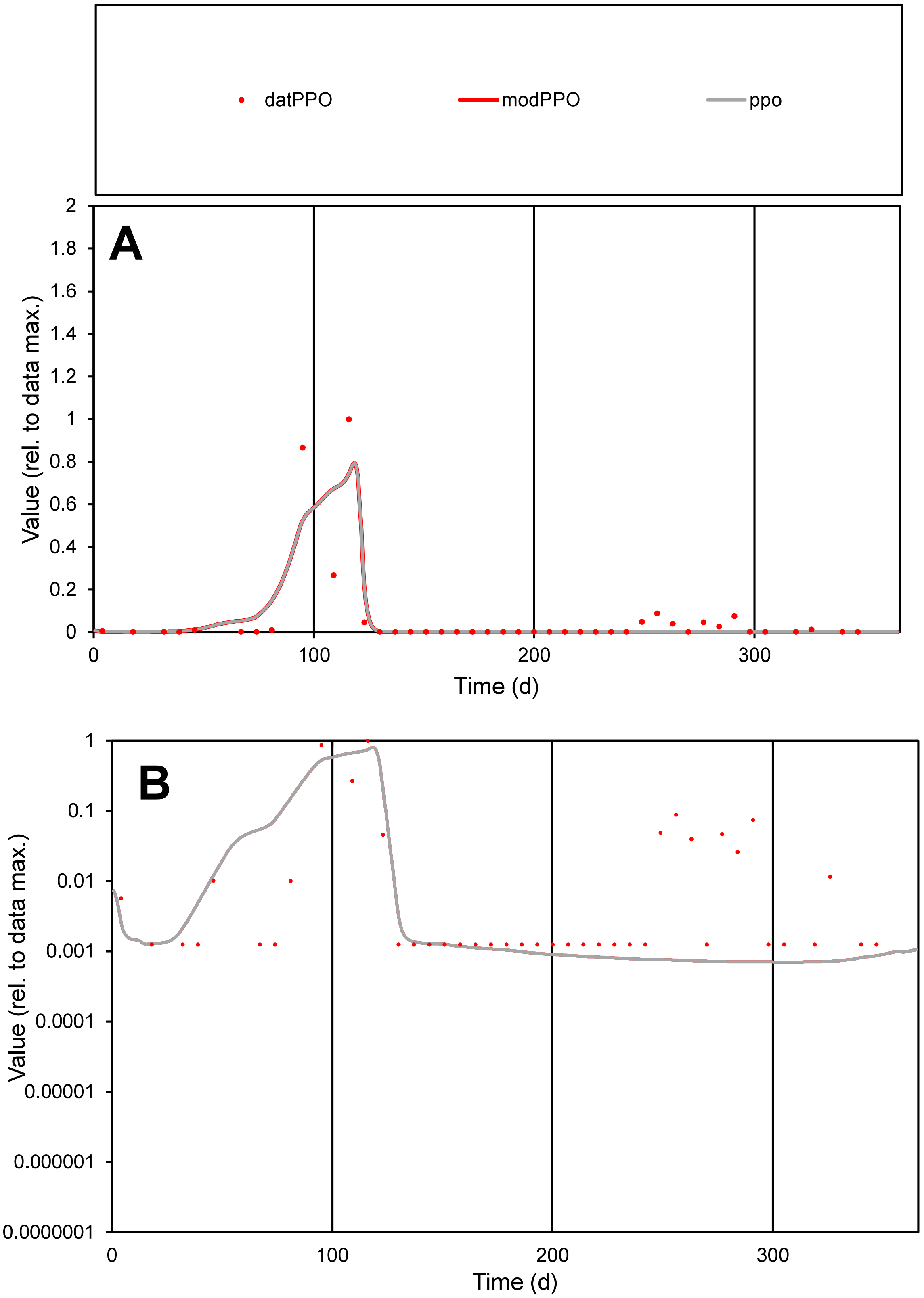

C1

Variables
PPO Provide jtd or Dst
CalibOnly?
2 1 to reproduce of
oNormalize?
2 1=no, 2=yes

ofxn calc*
1.53194
ofxn mod
1.55951

sRun
RunA1
zout
1

Ave
Data
9.4E-05
Model
8.6E-05

data/vMax
0.00153

ofxng
334.07

Min d/m
1.78119

ave/min d
49.1302
ave/min m
80.4163

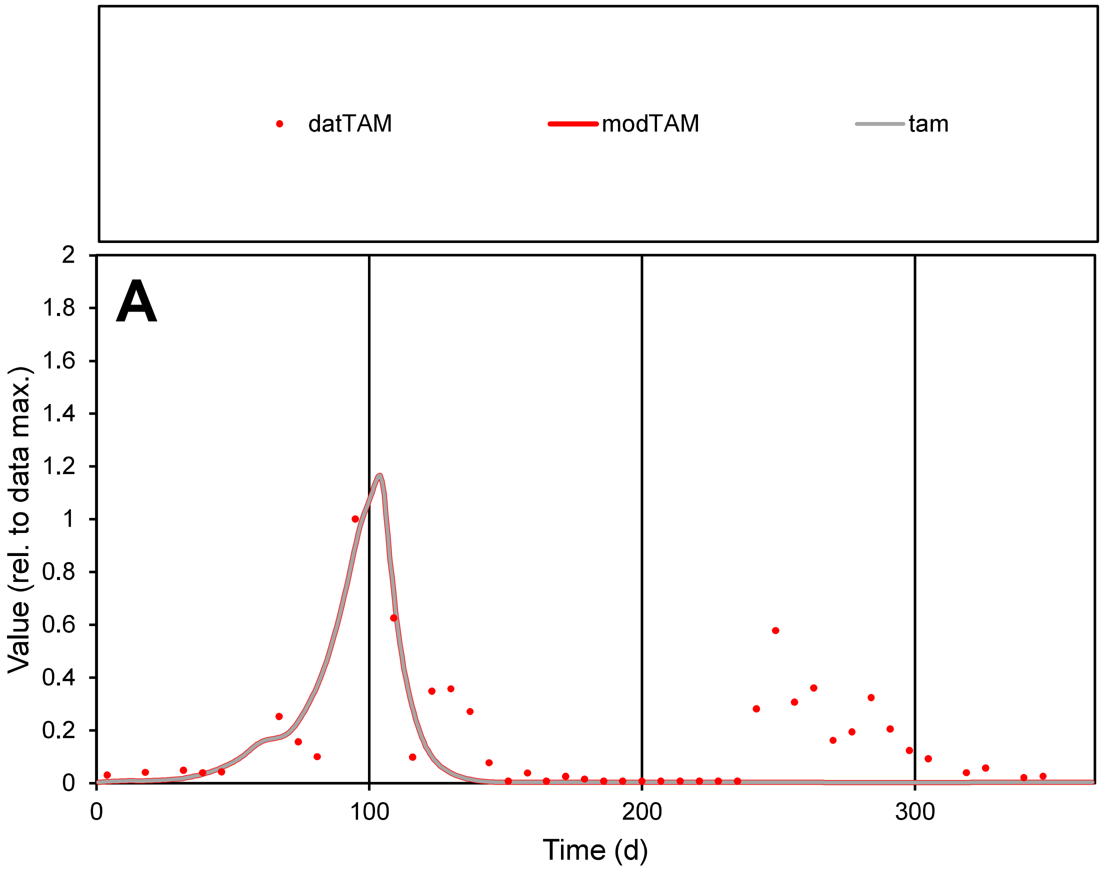

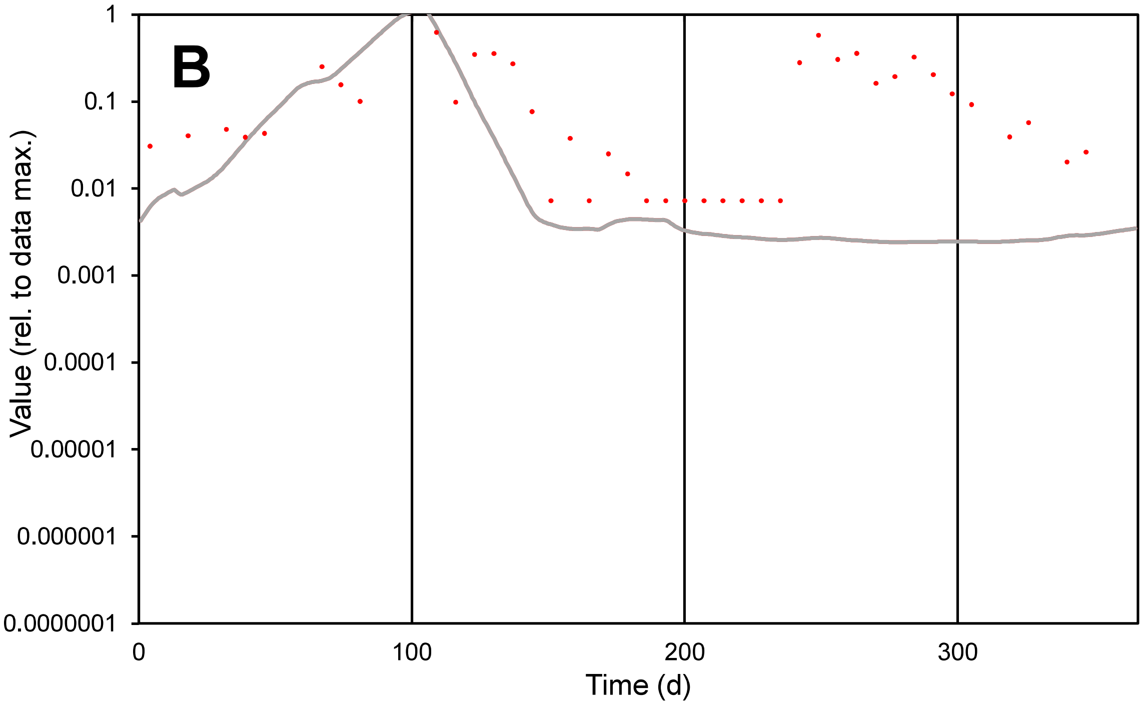

C1

Variables

TAM Provide jtd or Dst

CalibOnly?

2 1 to reproduce of

oNormalize?

2 1=no, 2=yes

ofxn calc*

1.10588

ofxn mod

1.12498

sRun

RunA1

zout

1

Ave

Data

0.00012

Model

5.4E-05

data/vMax

0.0008

ofxng

334.07

Min d/m

2.99013

ave/min d

21.0829

ave/min m

28.0988

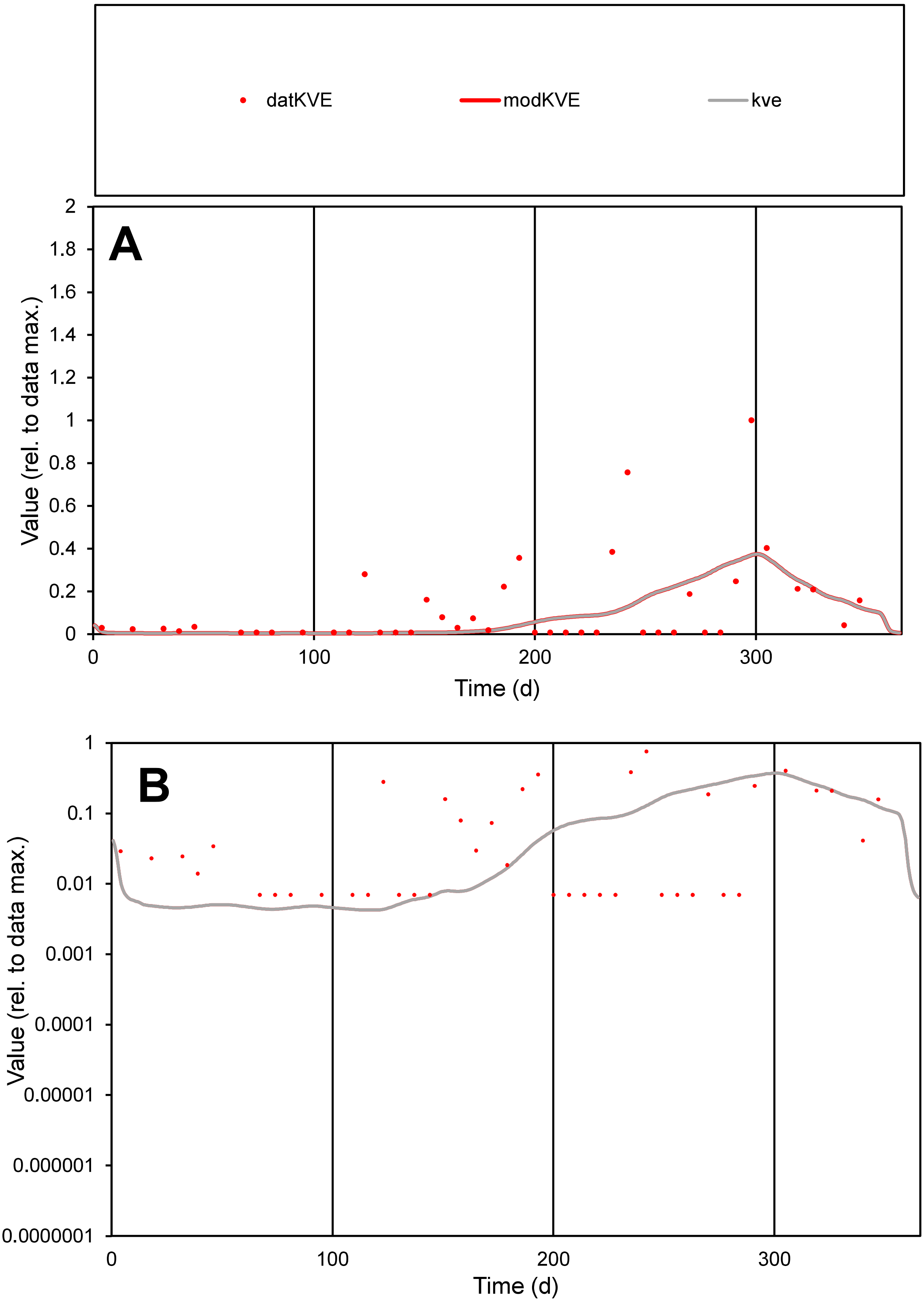

C1

Variables
KVE Provide jtd or Dst
CalibOnly?
2 1 to reproduce of
oNormalize?
2 1=no, 2=yes

ofxn calc*
1.5301
ofxn mod
1.54217

sRun
RunA1
zout
1

Ave
Data
5.4E-05
Model
4.4E-05

data/vMax
0.00045

ofxng
334.07

Min d/m
1.64443

ave/min d
17.3302
ave/min m
22.9165

327_Calib_S_A

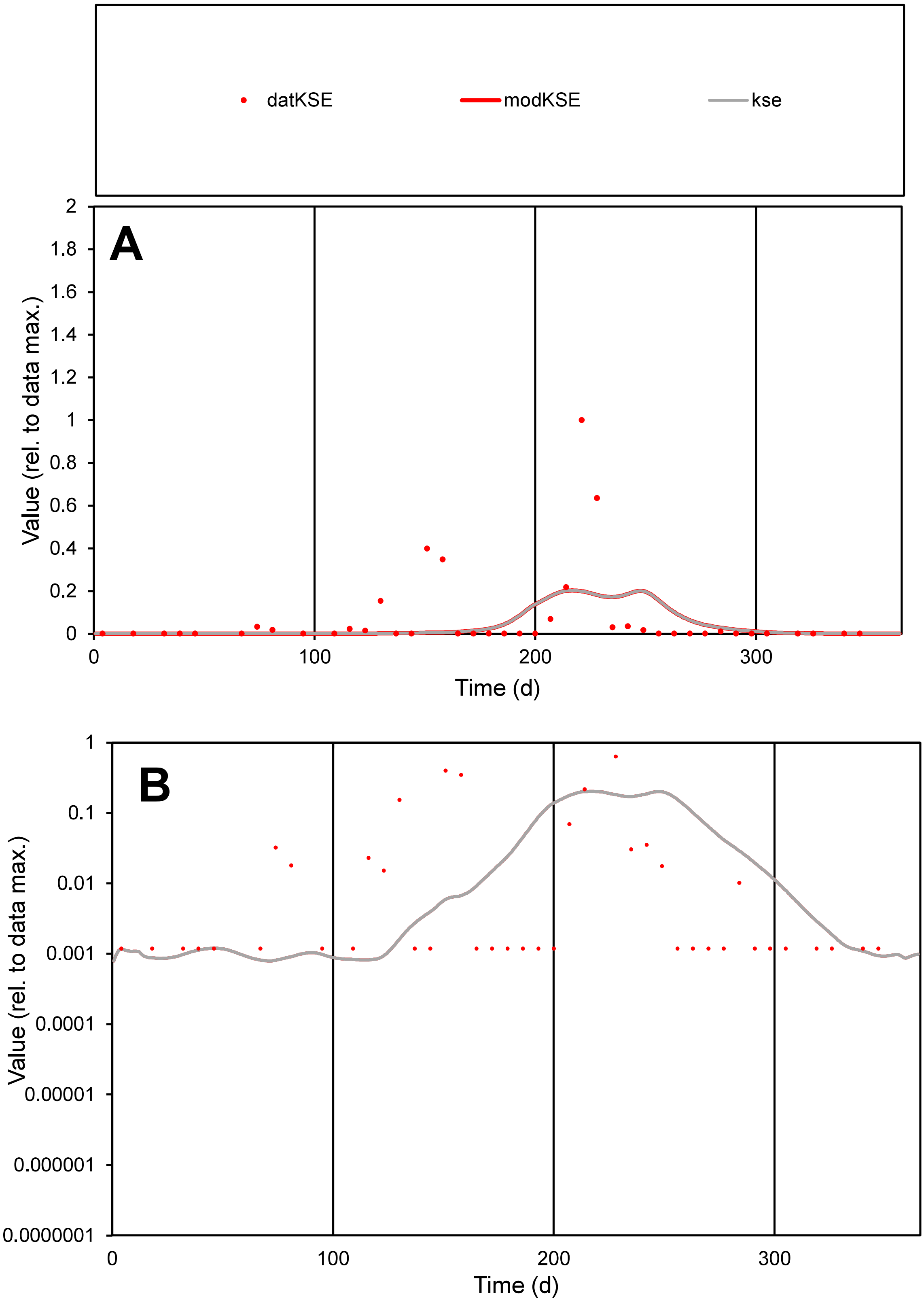

C1

Variables

KSE Provide jtd or Dst

CalibOnly?

2 1 to reproduce of

oNormalize?

2 1=no, 2=yes

ofxn calc*

2.41947

ofxn mod

2.4368

sRun

RunA1

zout

1

Ave

Data

0.00023

Model

0.00015

data/vMax

0.00316

ofxng

334.07

Min d/m

1.47501

ave/min d

61.062

ave/min m

59.2194

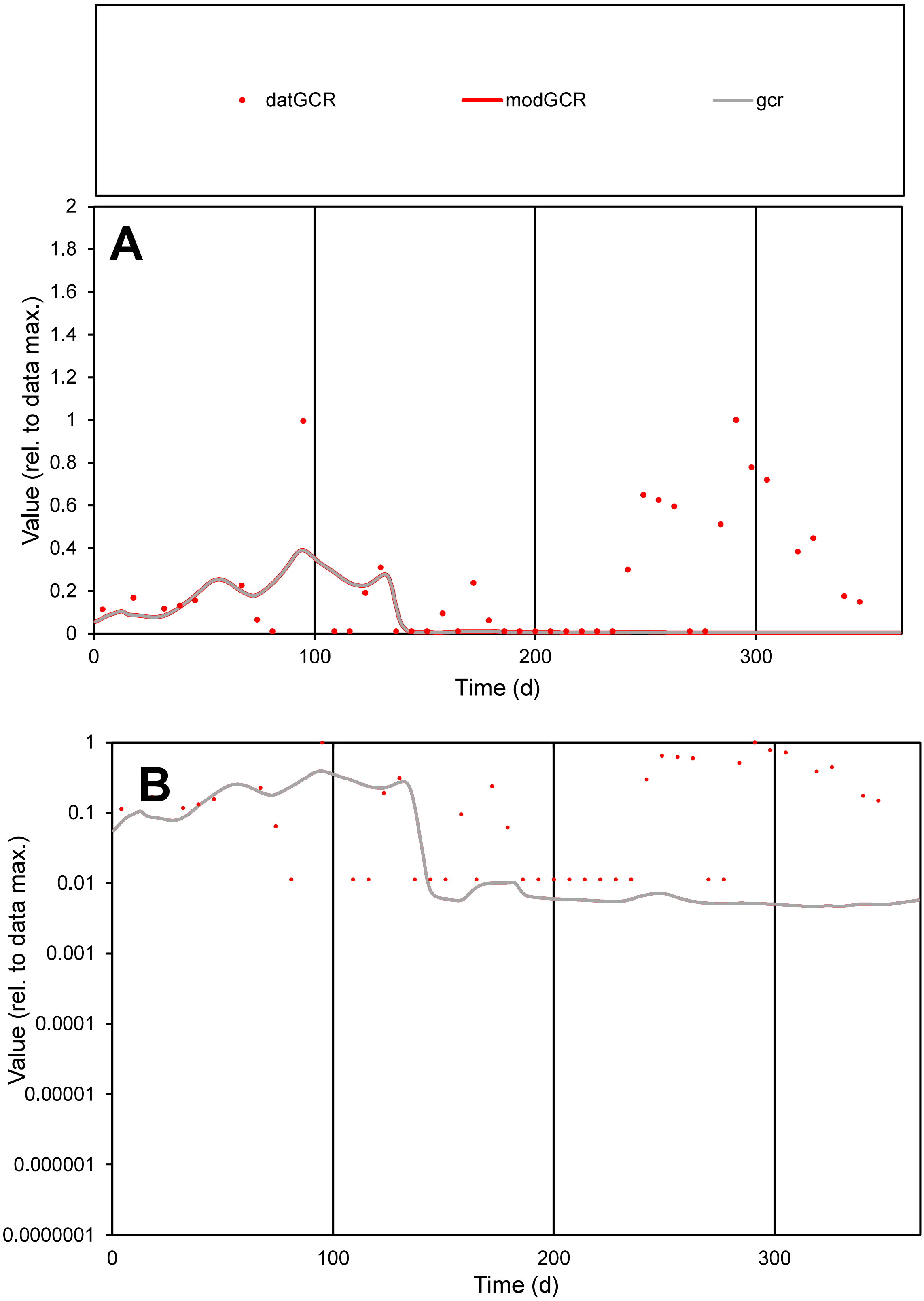

C1

Variables
GCR Provide jtd or Dst
CalibOnly?
2 1 to reproduce of
oNormalize?
2 1=no, 2=yes

ofxn calc*
1.48023
ofxn mod
1.48446

sRun
RunA1
zout
1

Ave
Data
6.2E-05
Model
1.9E-05

data/vMax
0.00028

ofxng
334.07

Min d/m
2.37965

ave/min d
19.9094
ave/min m
14.1215

327_Calib_S_A

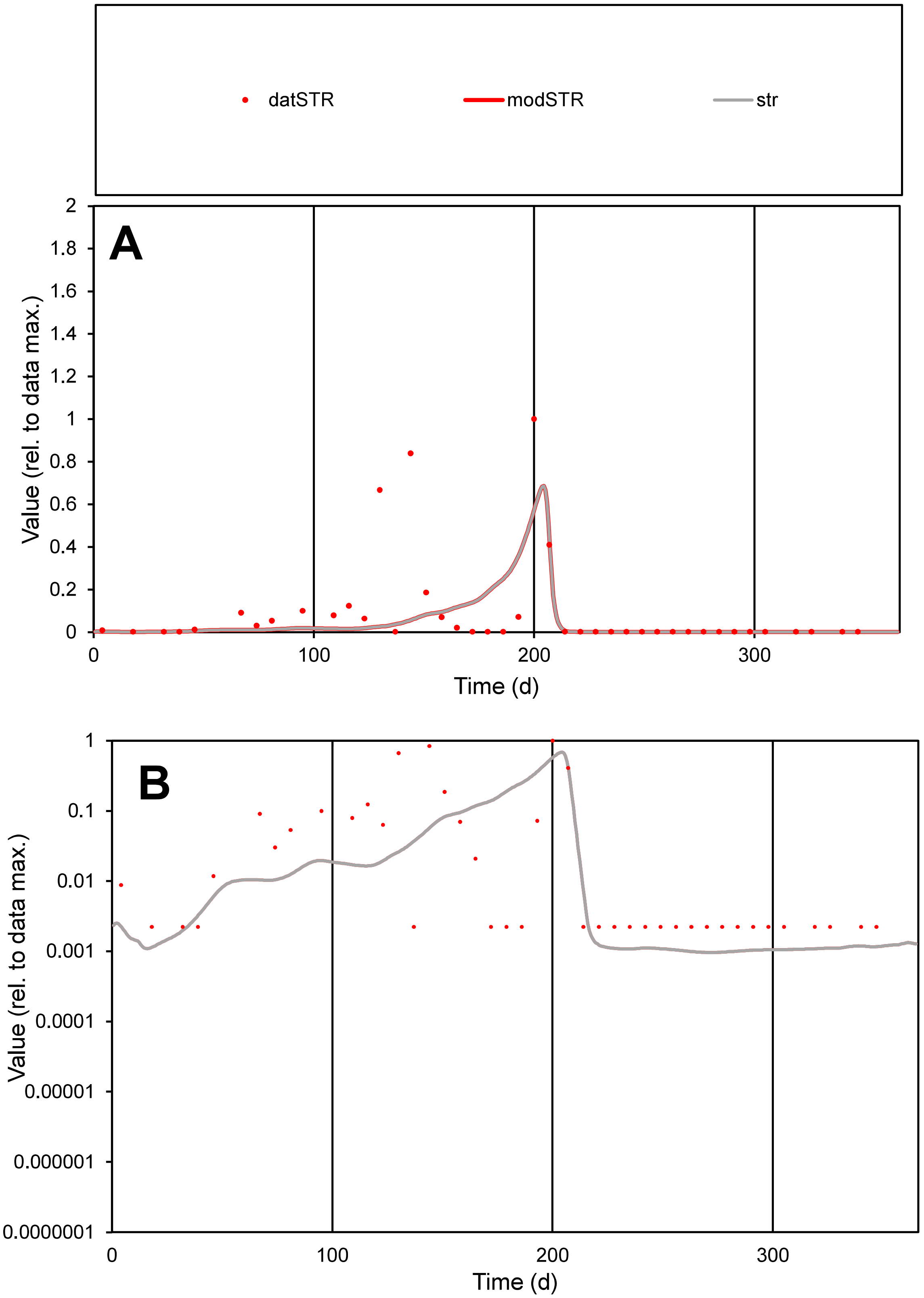

C1

Variables
STR Provide jtd or Dst
CalibOnly?
2 1 to reproduce of
oNormalize?
2 1=no, 2=yes

ofxn calc*
1.97506
ofxn mod
2.00339

sRun
RunA1
zout
1

Ave
Data
6.8E-05
Model
4.4E-05

data/vMax
0.00073

ofxng
334.07

Min d/m
2.31754

ave/min d
41.5849
ave/min m
62.1646

327_Calib_S_A

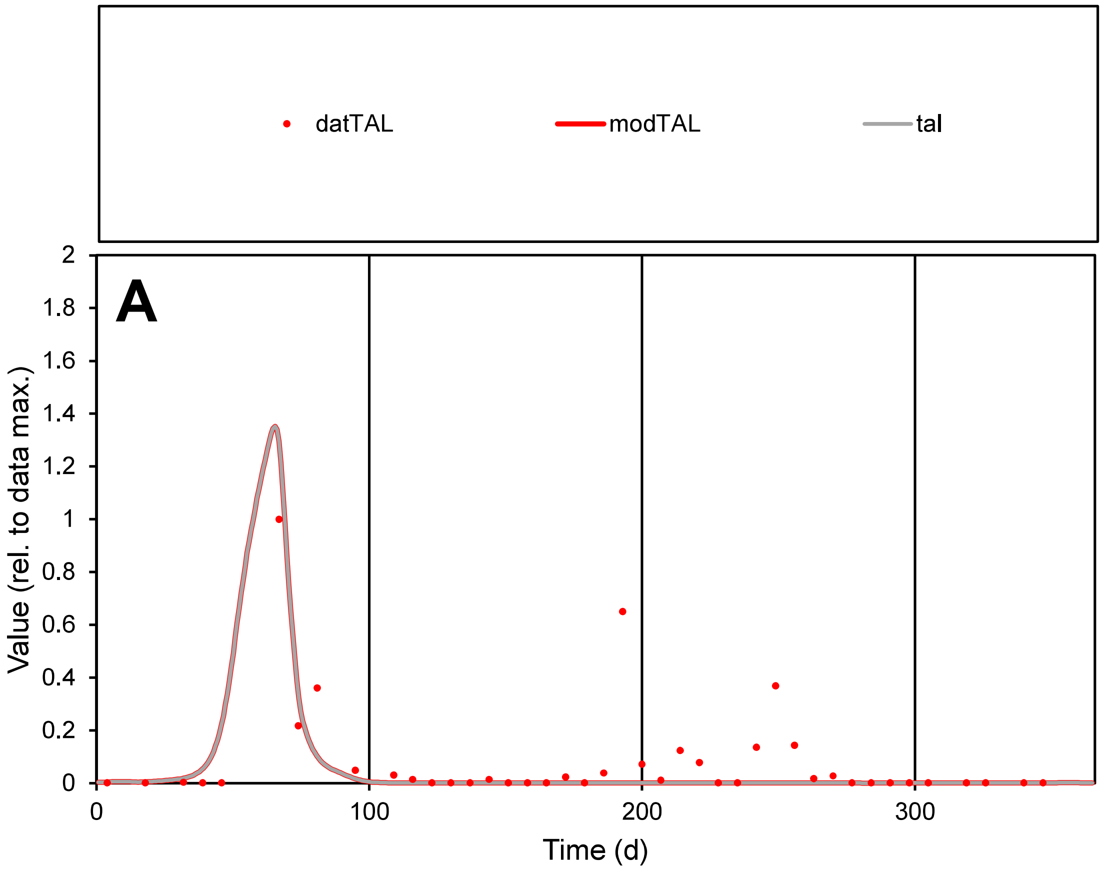

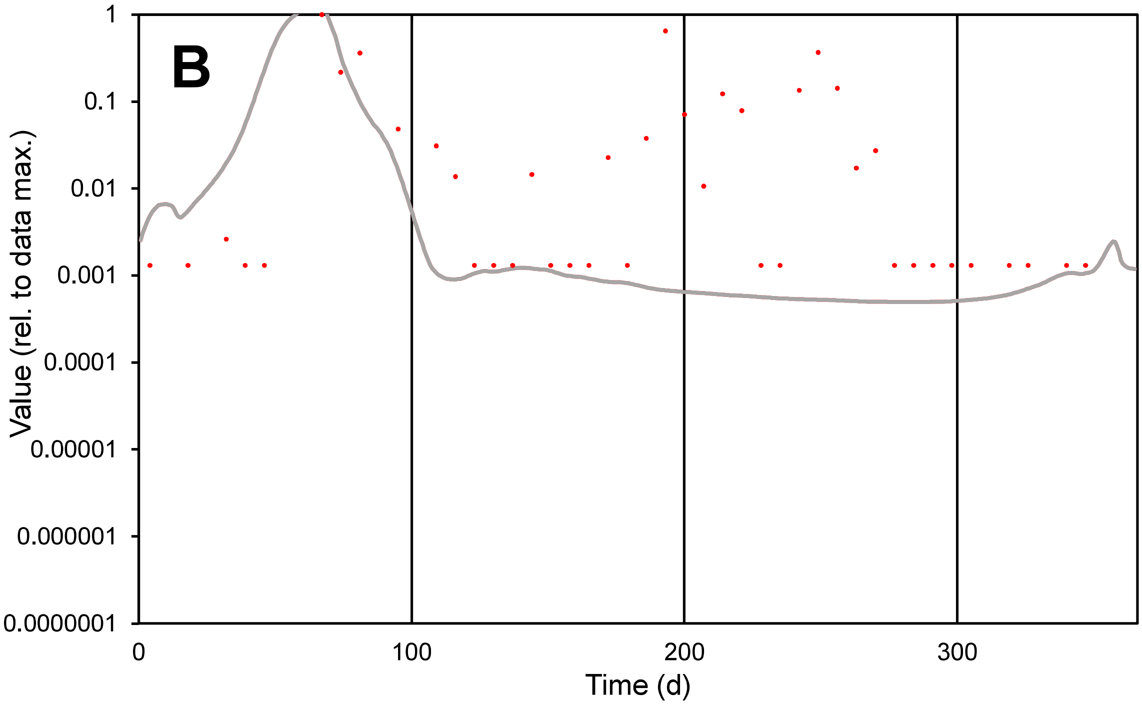

C1

Variables

TAL	Provide jtd or Dst
CalibOnly?	
2	1 to reproduce of
oNormalize?	
2	1=no, 2=yes

ofxn calc*
1.63588
ofxn mod
1.67522

sRun
RunA1
zout
1

Ave
Data
0.00026
Model
0.00013

data/vMax
0.00317

ofxng
334.07

Min d/m
2.65301

ave/min d
61.9445
ave/min m
82.5974

327_Calib_S_A

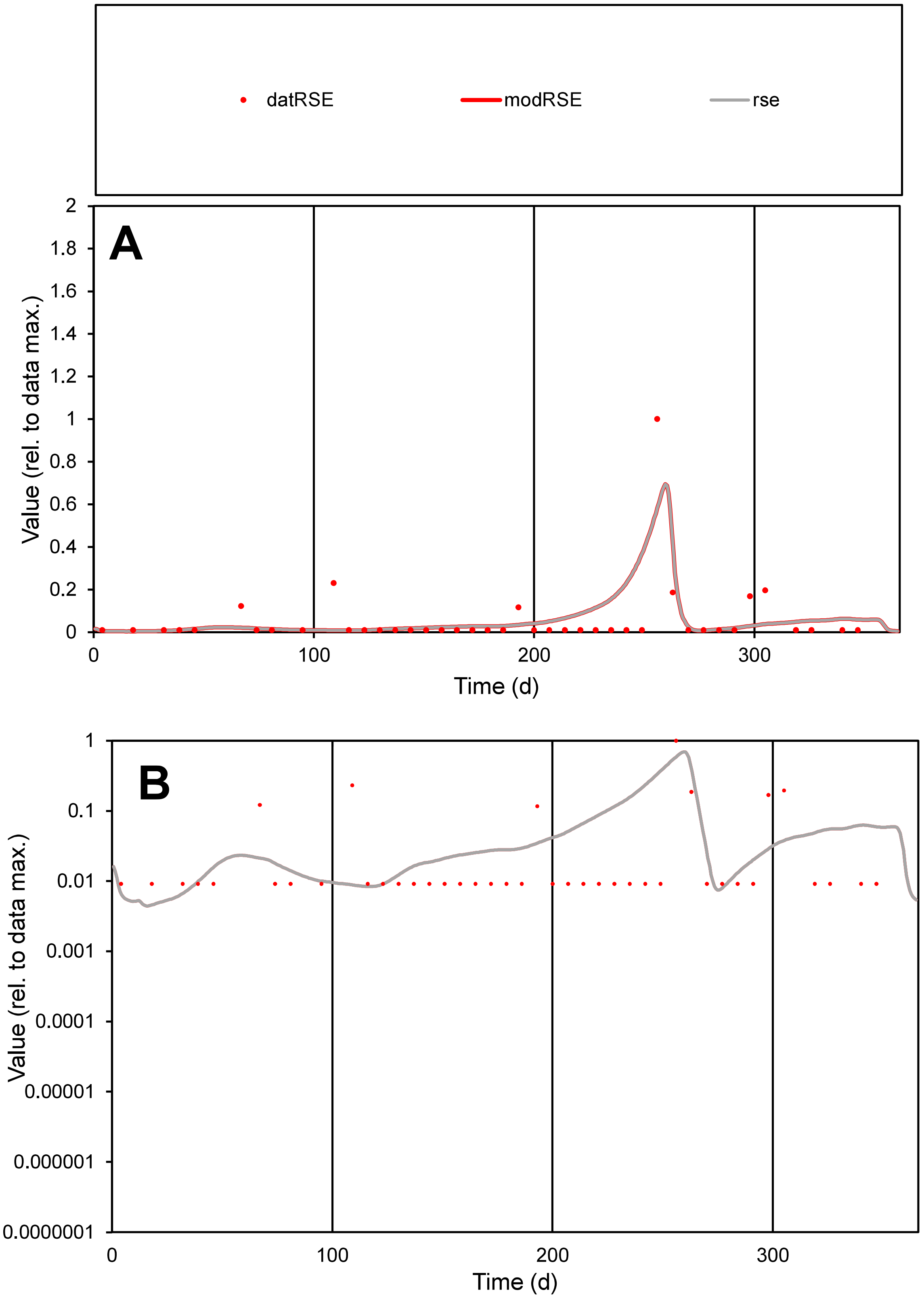

C1

Variables
RSE Provide jtd or Dst
CalibOnly?
2 1 to reproduce of
oNormalize?
2 1=no, 2=yes

ofxn calc*
1.84905
ofxn mod
1.94502

sRun
RunA1
zout
1

Ave
Data
2.8E-05
Model
3.2E-05

data/vMax
0.00051

ofxng
334.07

Min d/m
1.92051

ave/min d
6.10161
ave/min m
13.1746

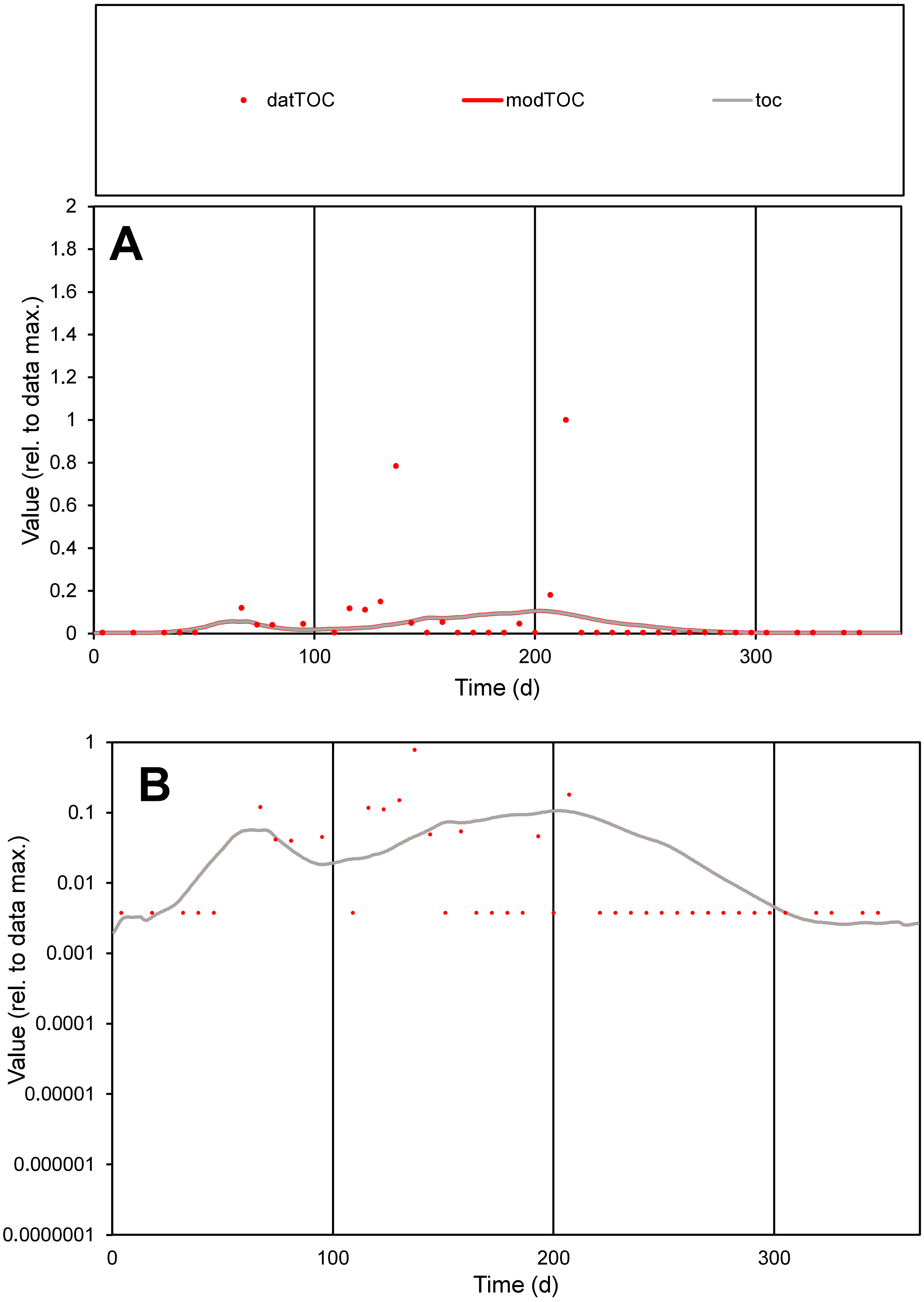

C1

Variables
TOC Provide jtd or Dst
CalibOnly?
2 1 to reproduce of
oNormalize?
2 1=no, 2=yes

ofxn calc*
2.75768
ofxn mod
2.76365

sRun
RunA1
zout
1

Ave
Data
7.8E-05
Model
4.6E-05

data/vMax
0.00115

ofxng
334.07

Min d/m
1.42405

ave/min d
17.9982
ave/min m
15.0116

327_Calib_S_A

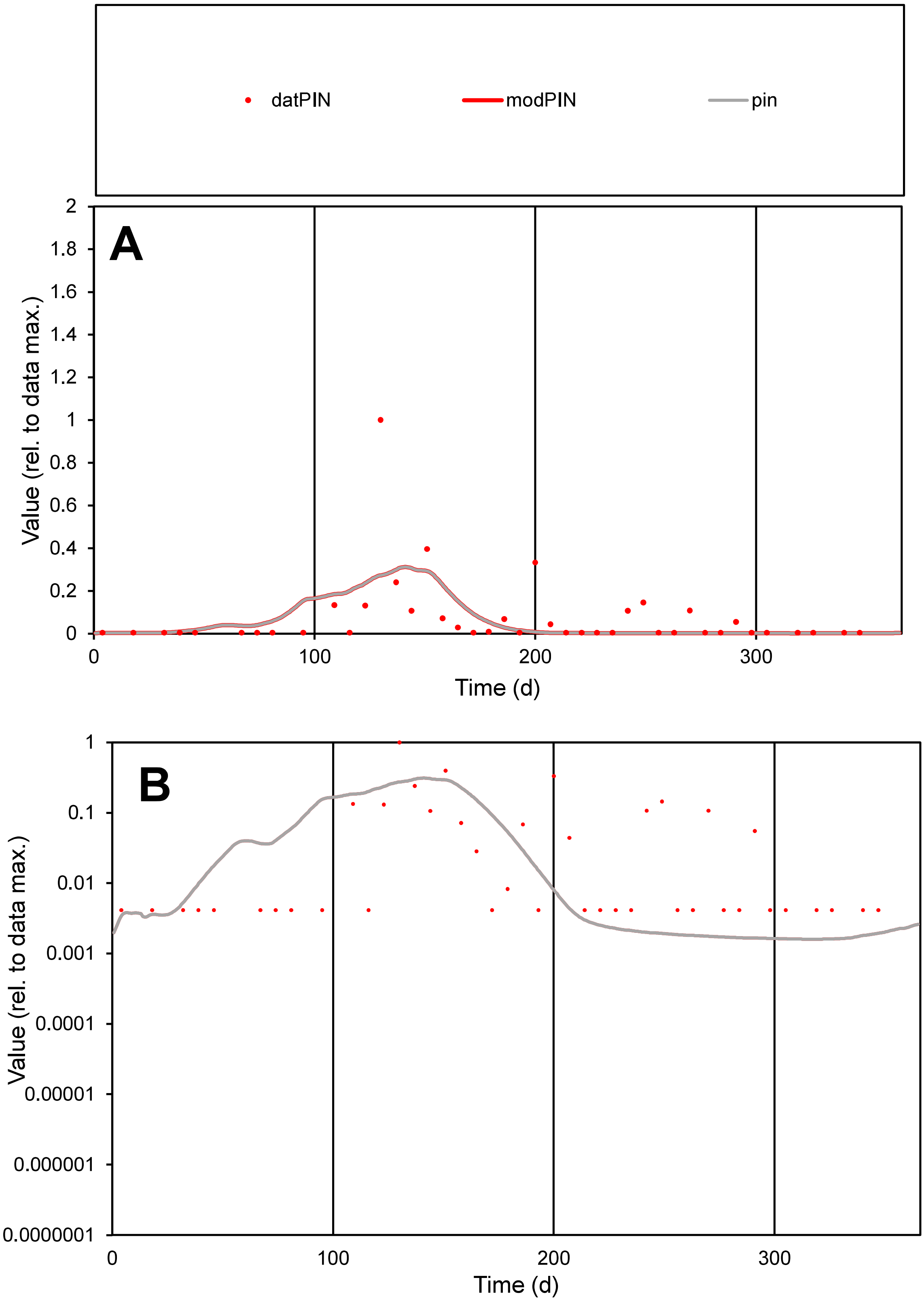

C1

Variables

PIN Provide jtd or Dst

CalibOnly?

2 1 to reproduce of

oNormalize?

2 1=no, 2=yes

ofxn calc*

1.93313

ofxn mod

1.95356

sRun

RunA1

zout

1

Ave

Data

7.1E-05

Model

6.1E-05

data/vMax

0.00097

ofxng

334.07

Min d/m

2.5871

ave/min d

17.7519

ave/min m

39.6483

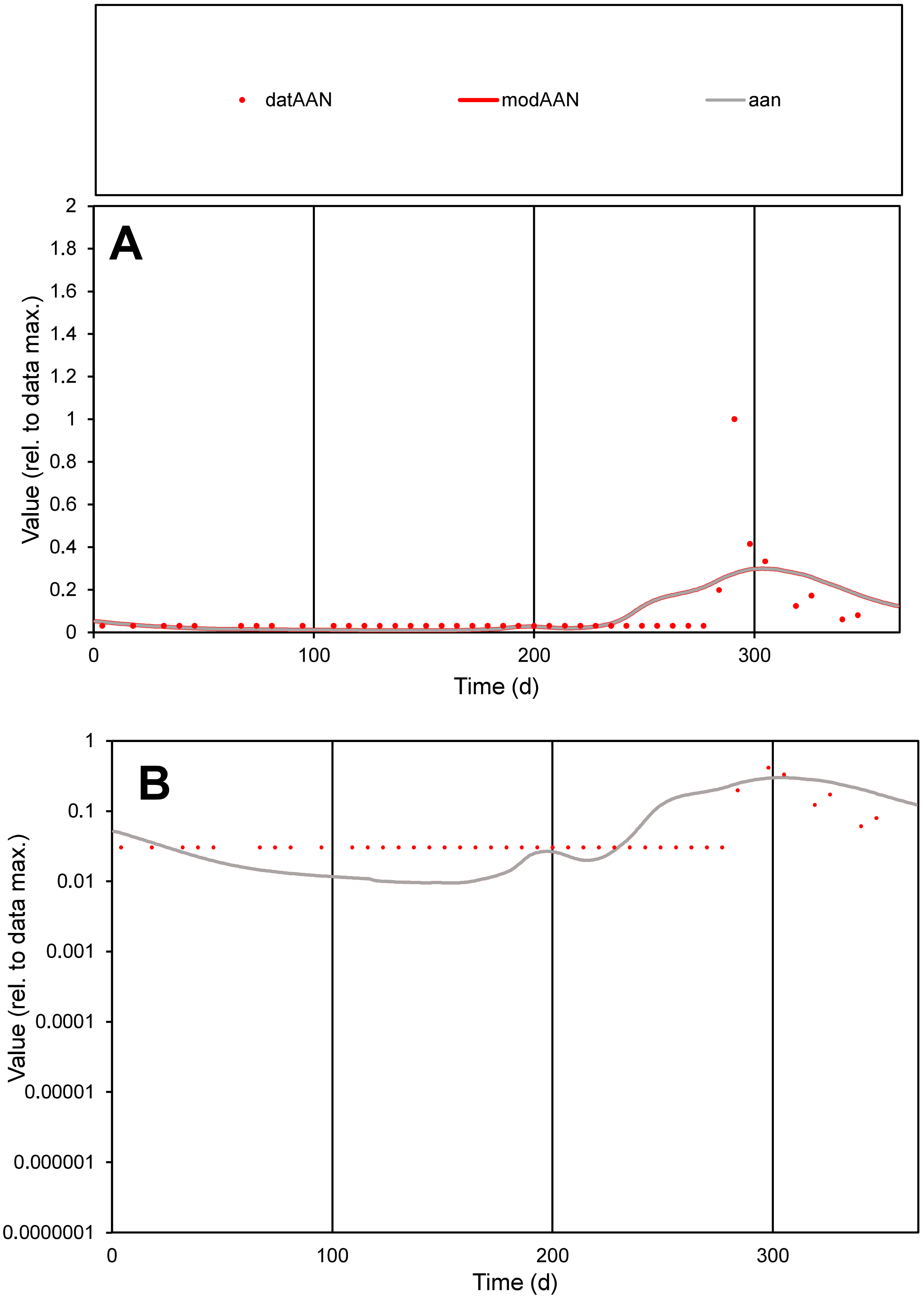

C1

Variables

AAN Provide jtd or Dst

CalibOnly?

2 1 to reproduce of

oNormalize?

2 1=no, 2=yes

ofxn calc*

1.5938

ofxn mod

1.6076

sRun

RunA1

zout

1

Ave

Data

1.5E-05

Model

1.5E-05

data/vMax

0.00018

ofxng

334.07

Min d/m

3.19473

ave/min d

2.67049

ave/min m

8.73129

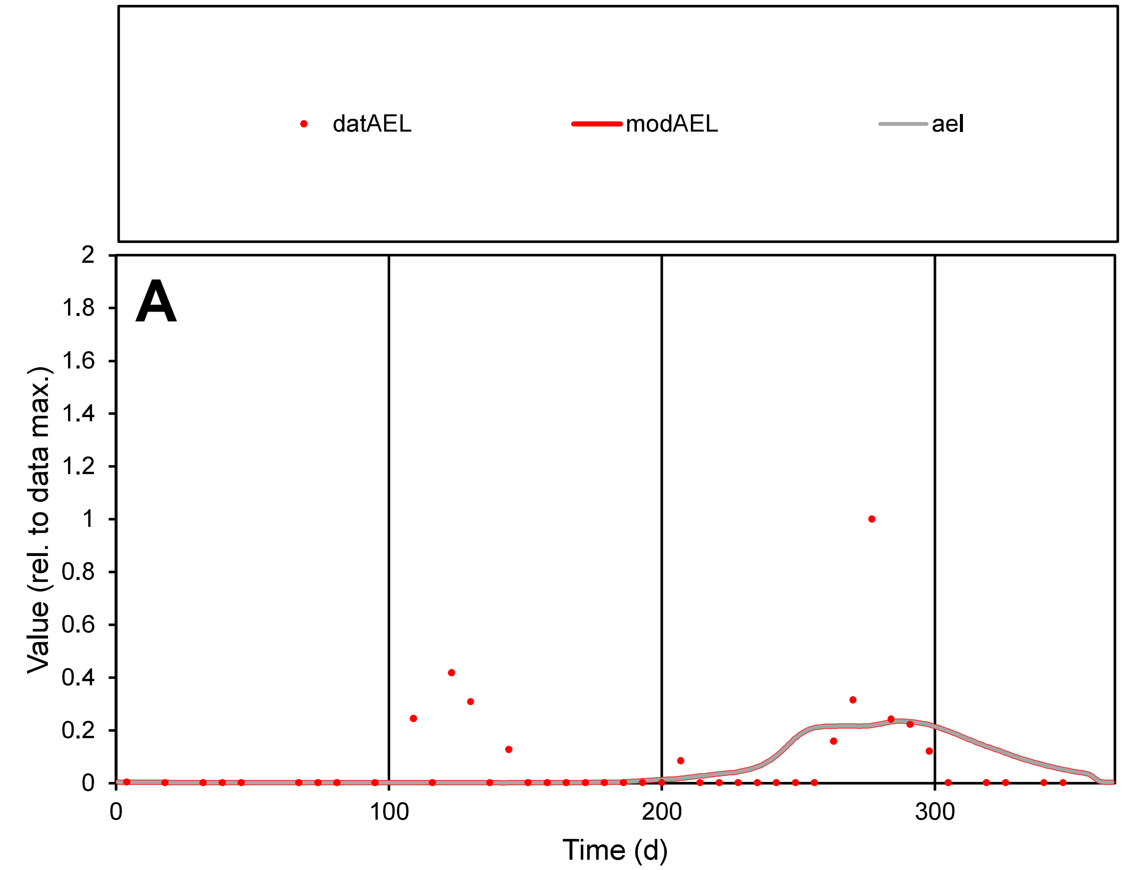

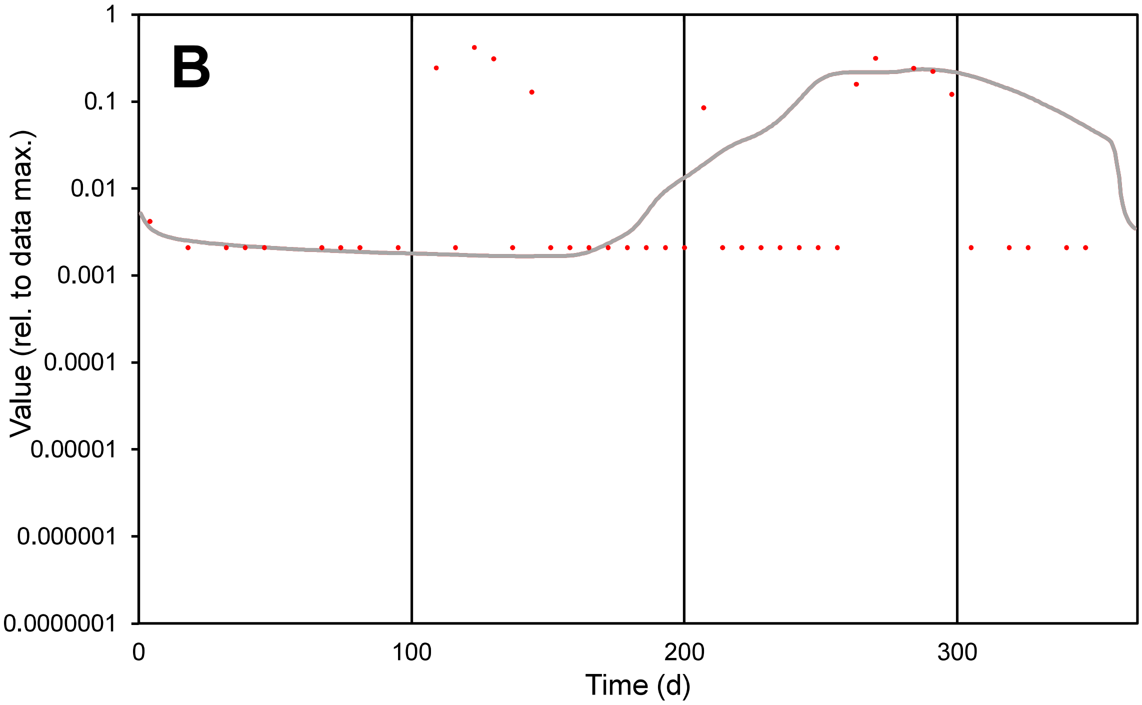

C1

Variables
AEL Provide jtd or Dst
CalibOnly?
2 1 to reproduce of
oNormalize?
2 1=no, 2=yes

ofxn calc*
2.07501
ofxn mod
2.09397

sRun
RunA1
zout
1

Ave
Data
2.6E-05
Model
2E-05

data/vMax
0.00033

ofxng
334.07

Min d/m
1.25162

ave/min d
37.6138
ave/min m
36.7362

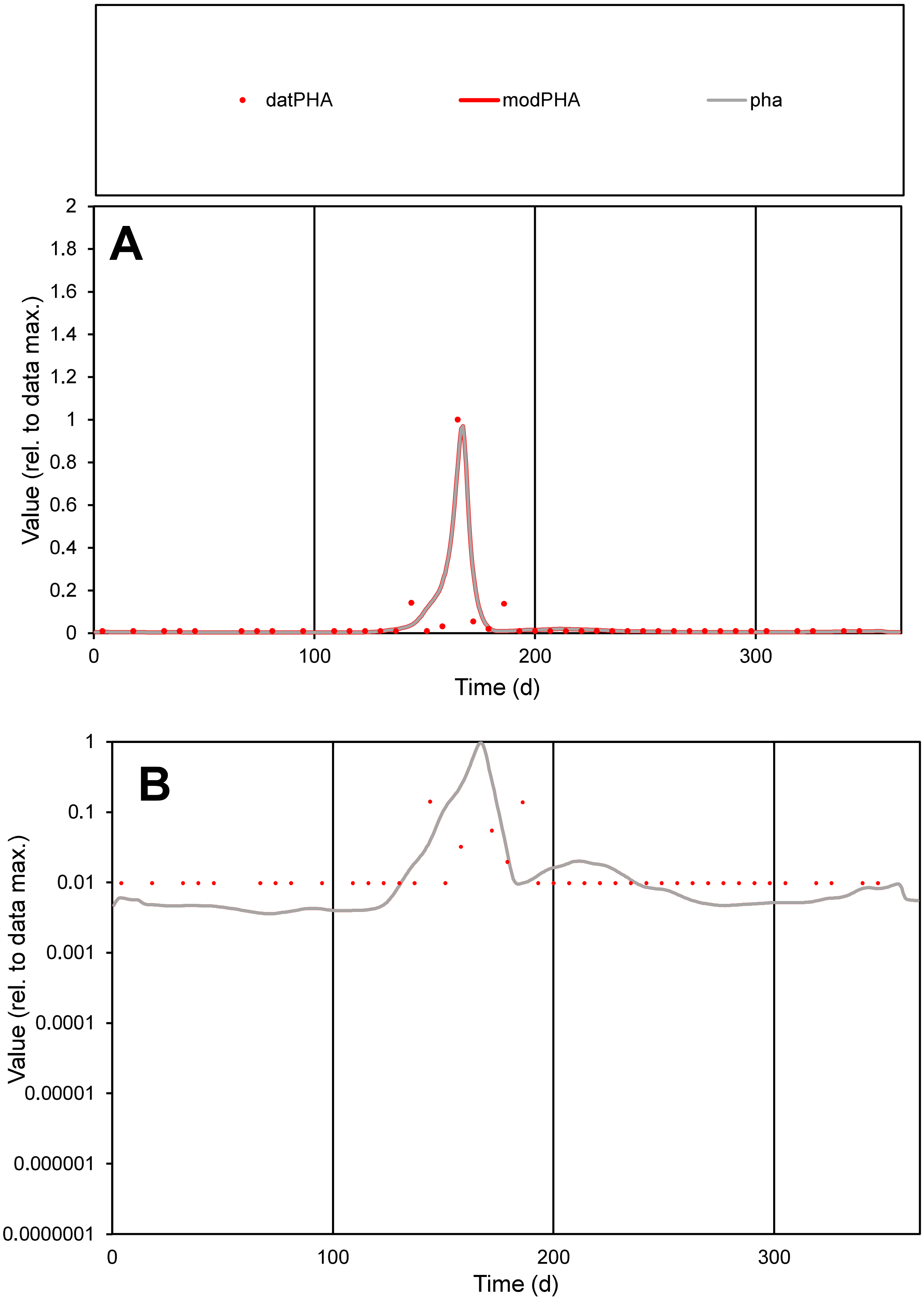

C1

Variables
PHA Provide jtd or Dst
CalibOnly?
2 1 to reproduce of
oNormalize?
2 1=no, 2=yes

ofxn calc*
0.98706
ofxn mod
1.15303

sRun
RunA1
zout
1

Ave
Data
4.4E-05
Model
4.4E-05

data/vMax
0.00106

ofxng
334.07

Min d/m
2.70109

ave/min d
4.21664
ave/min m
11.432

327_Calib_S_A

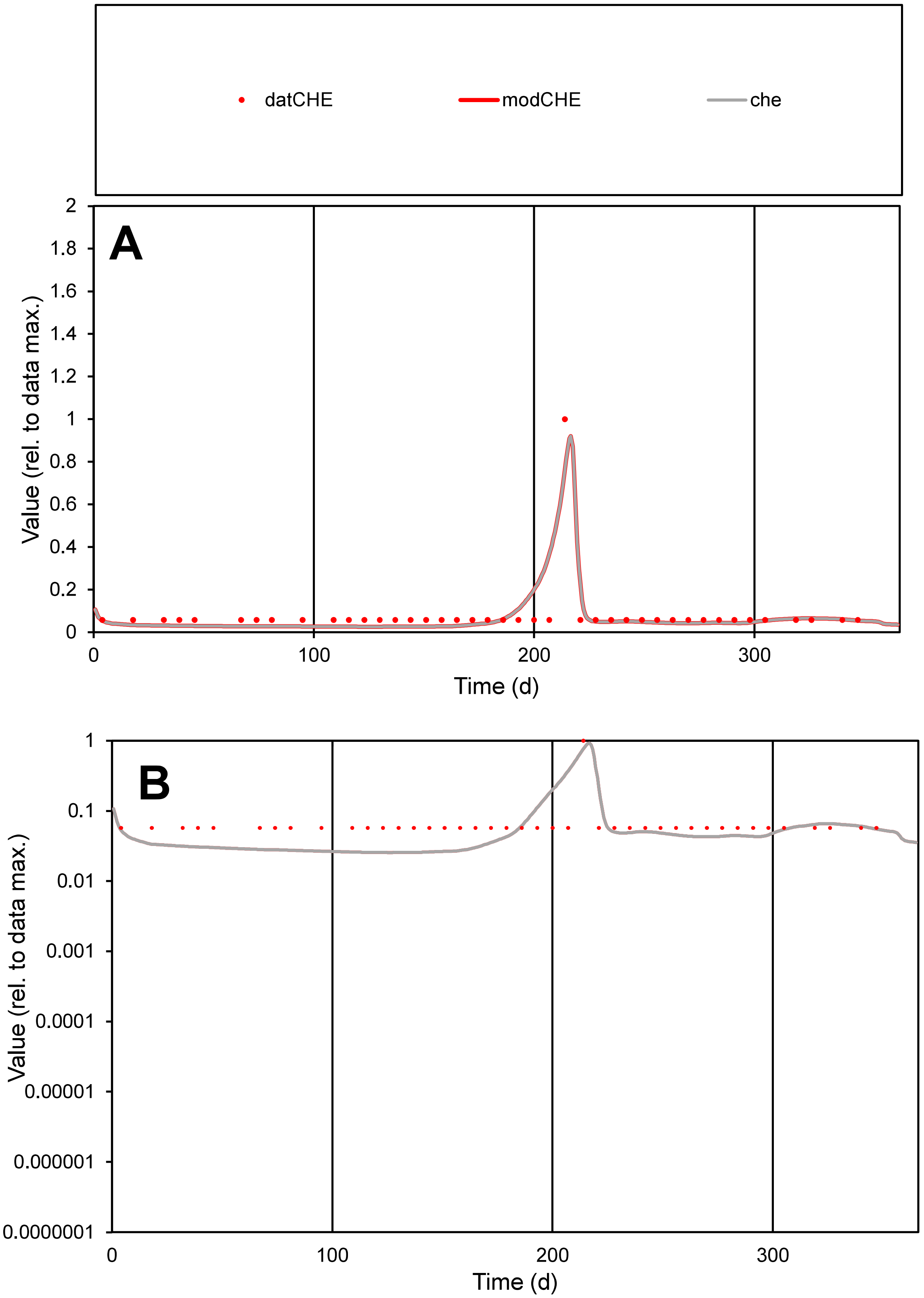

C1

Variables
CHE Provide jtd or Dst
CalibOnly?
2 1 to reproduce of
oNormalize?
2 1=no, 2=yes

ofxn calc*
0.67336
ofxn mod
0.79227

sRun
RunA1
zout
1

Ave
Data
9.8E-06
Model
8.9E-06

data/vMax
0.00012

ofxng
334.07

Min d/m
2.2464

ave/min d
1.39167
ave/min m
2.85504

327_Calib_S_A

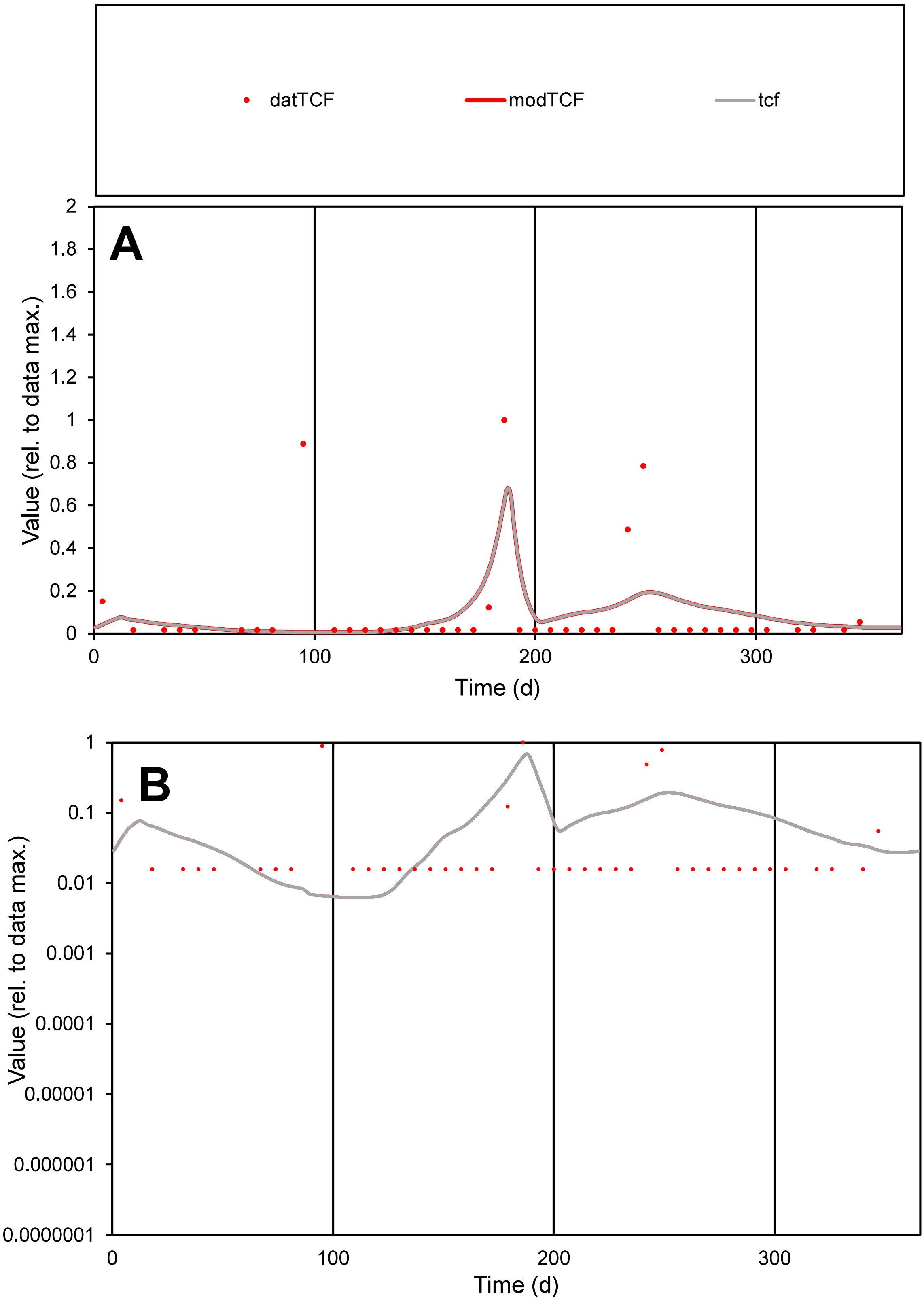

C1

Variables
TCF Provide jtd or Dst
CalibOnly?
2 1 to reproduce of
oNormalize?
2 1=no, 2=yes

ofxn calc*
1.99926
ofxn mod
2.03295

sRun
RunA1
zout
1

Ave
Data
1.9E-05
Model
1.8E-05

data/vMax
0.0002

ofxng
334.07

Min d/m
2.53577

ave/min d
6.09189
ave/min m
14.5856

327_Calib_S_A

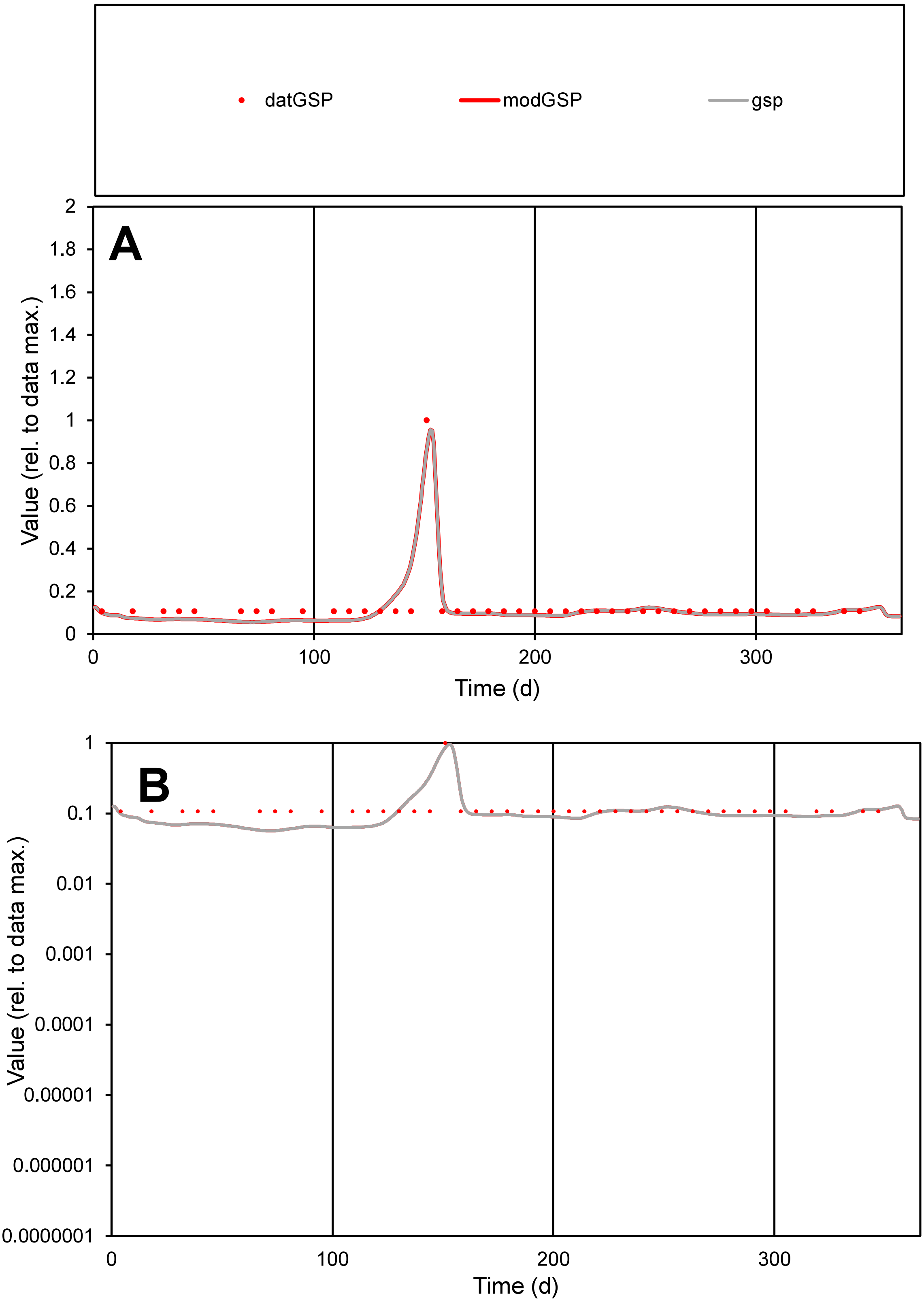

C1

Variables
GSP Provide jtd or Dst
CalibOnly?
2 1 to reproduce of
oNormalize?
2 1=no, 2=yes

ofxn calc*
0.32975
ofxn mod
0.37214

sRun
RunA1
zout
1

Ave
Data
1.1E-05
Model
1E-05

data/vMax
8.4E-05

ofxng
334.07

Min d/m
1.87398

ave/min d
1.1983
ave/min m
2.08083

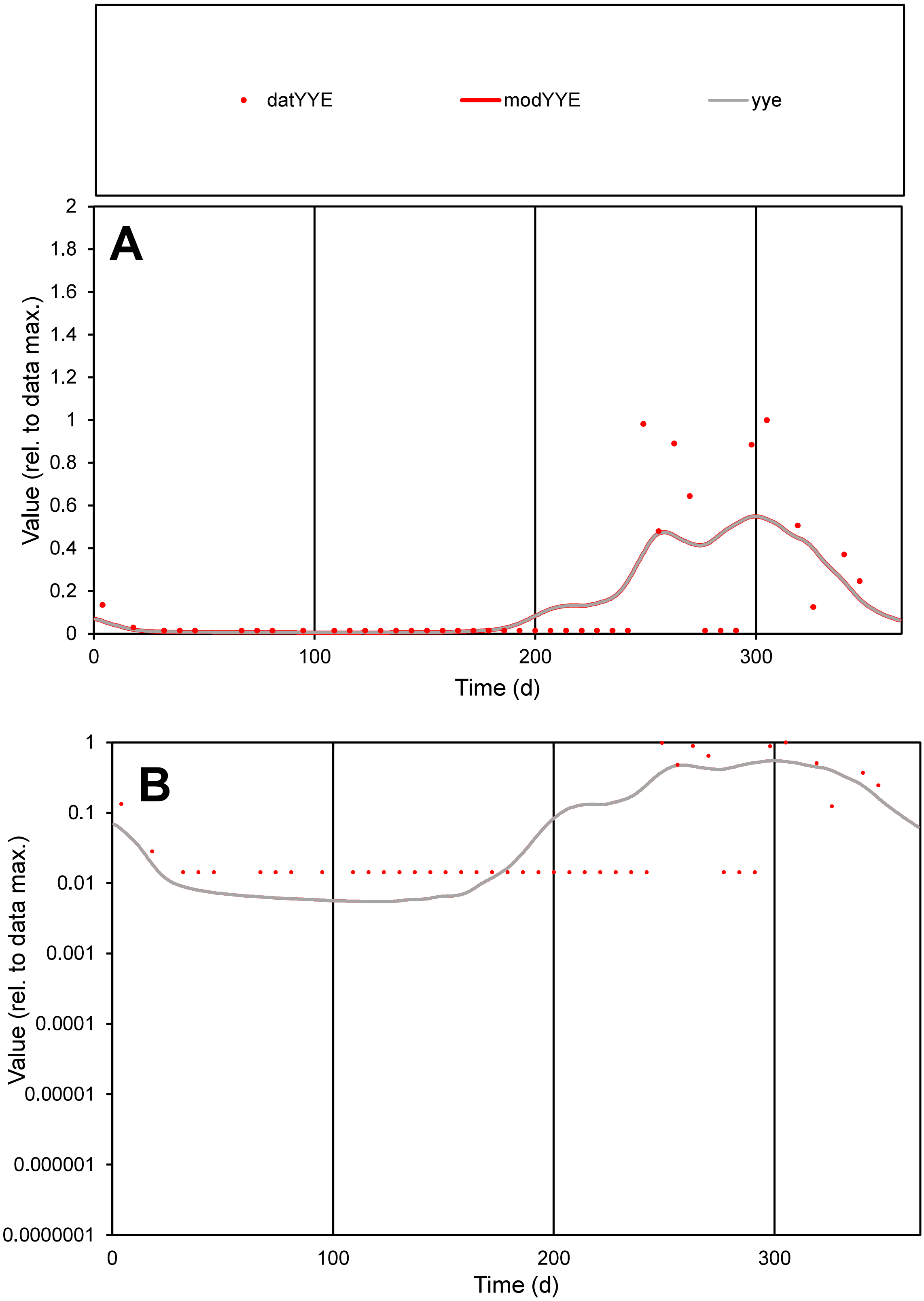

C1

Variables

YYE Provide jtd or Dst

CalibOnly?

2 1 to reproduce of

oNormalize?

2 1=no, 2=yes

ofxn calc*

1.28467

ofxn mod

1.30582

sRun

RunA1

zout

1

Ave

Data

4.2E-05

Model

4.2E-05

data/vMax

0.00026

ofxng

334.07

Min d/m

2.58981

ave/min d

11.2218

ave/min m

29.1679

327_Calib_S_A

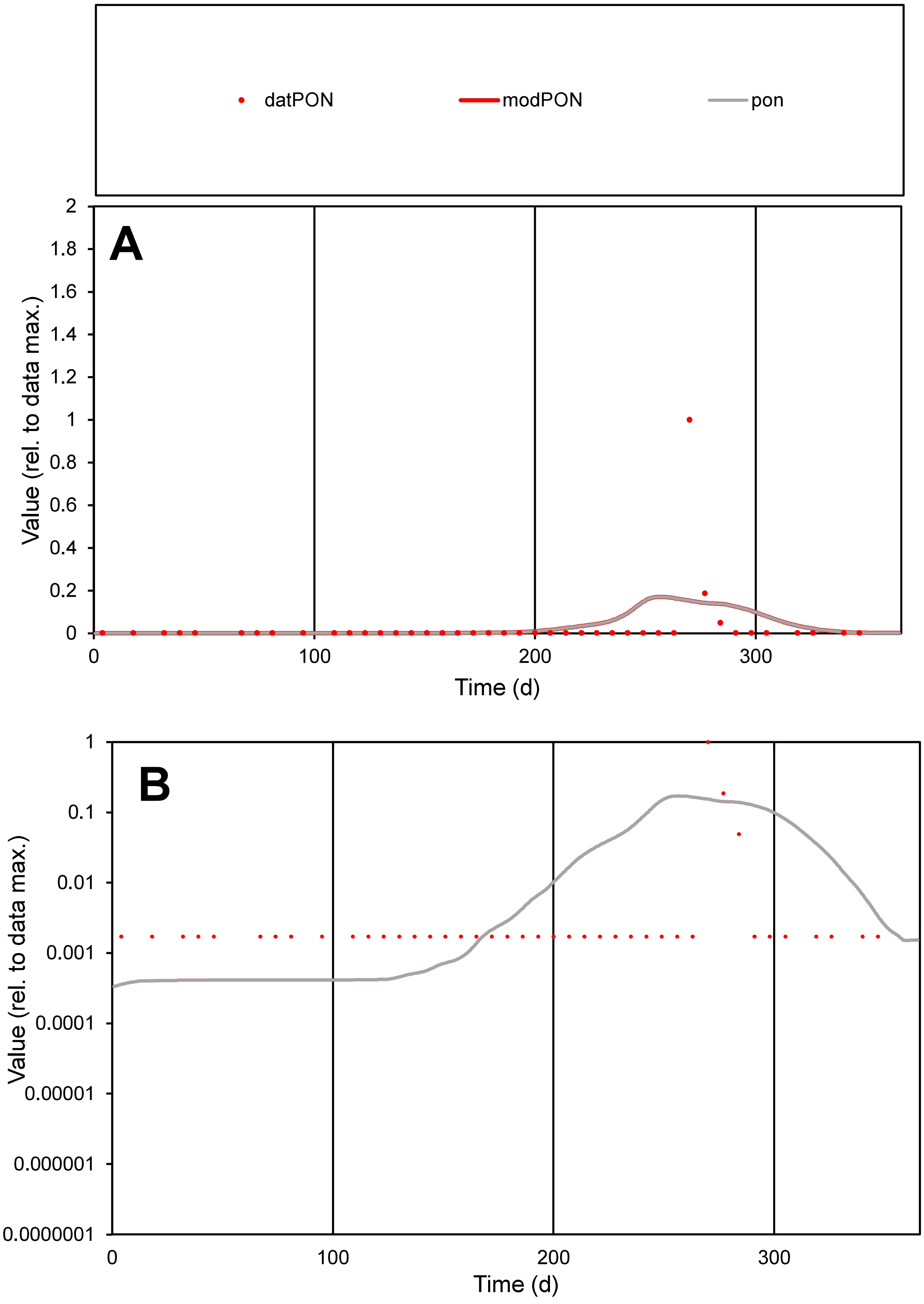

C1

Variables
PON Provide jtd or Dst
CalibOnly?
2 1 to reproduce of
oNormalize?
2 1=no, 2=yes

ofxn calc*
4.5772
ofxn mod
4.60397

sRun
RunA1
zout
1

Ave
Data
5.3E-05
Model
6.3E-05

data/vMax
0.00171

ofxng
334.07

Min d/m
4.71704

ave/min d
18.0494
ave/min m
101.203

327_Calib_S_A

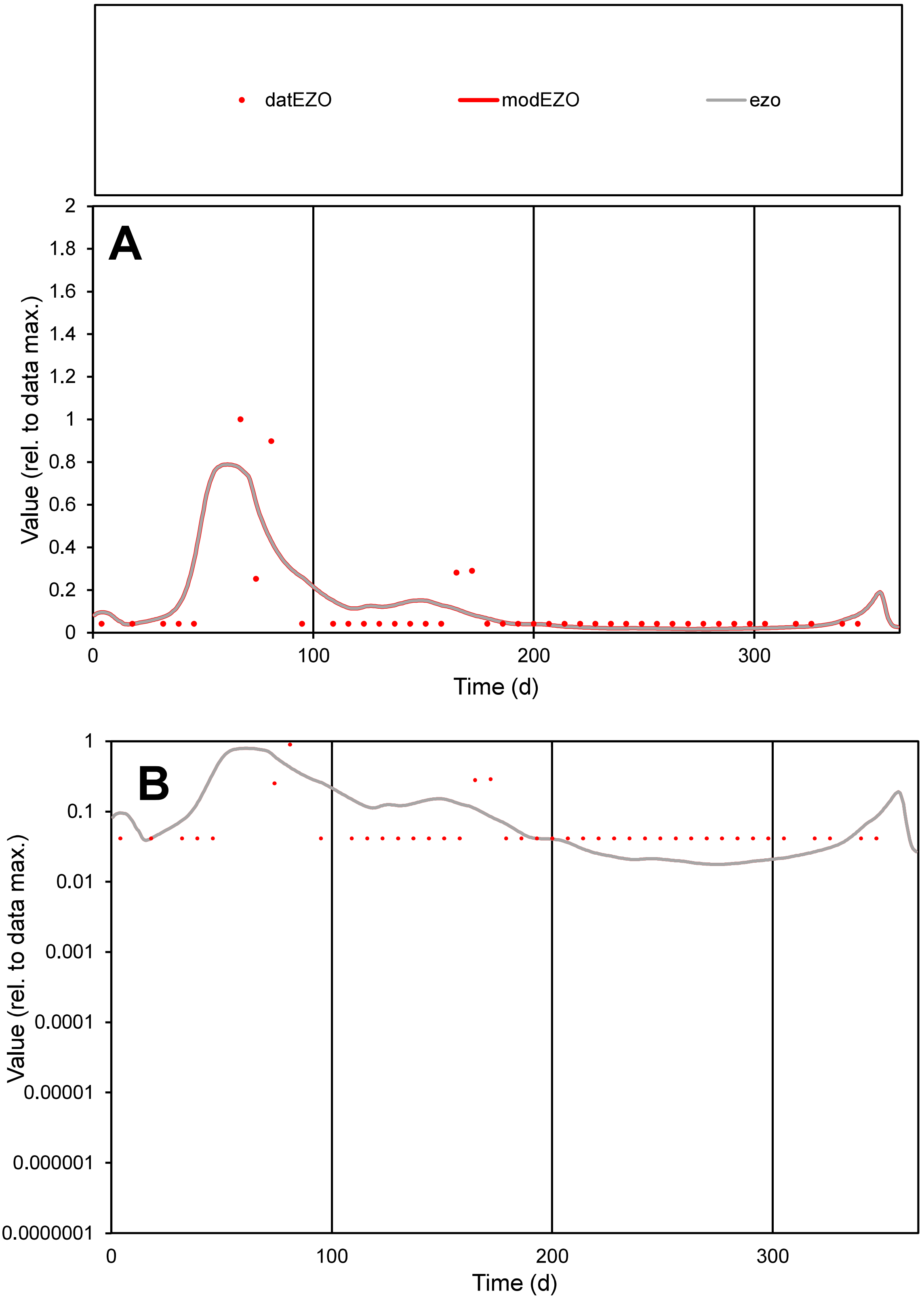

C1

Variables
EZO Provide jtd or Dst
CalibOnly?
2 1 to reproduce of
oNormalize?
2 1=no, 2=yes

ofxn calc*
1.22382
ofxn mod
1.27272

sRun
RunA1
zout
1

Ave
Data
9.6E-06
Model
1E-05

data/vMax
9.5E-05

ofxng
334.07

Min d/m
2.3359

ave/min d
2.44261
ave/min m
6.08487

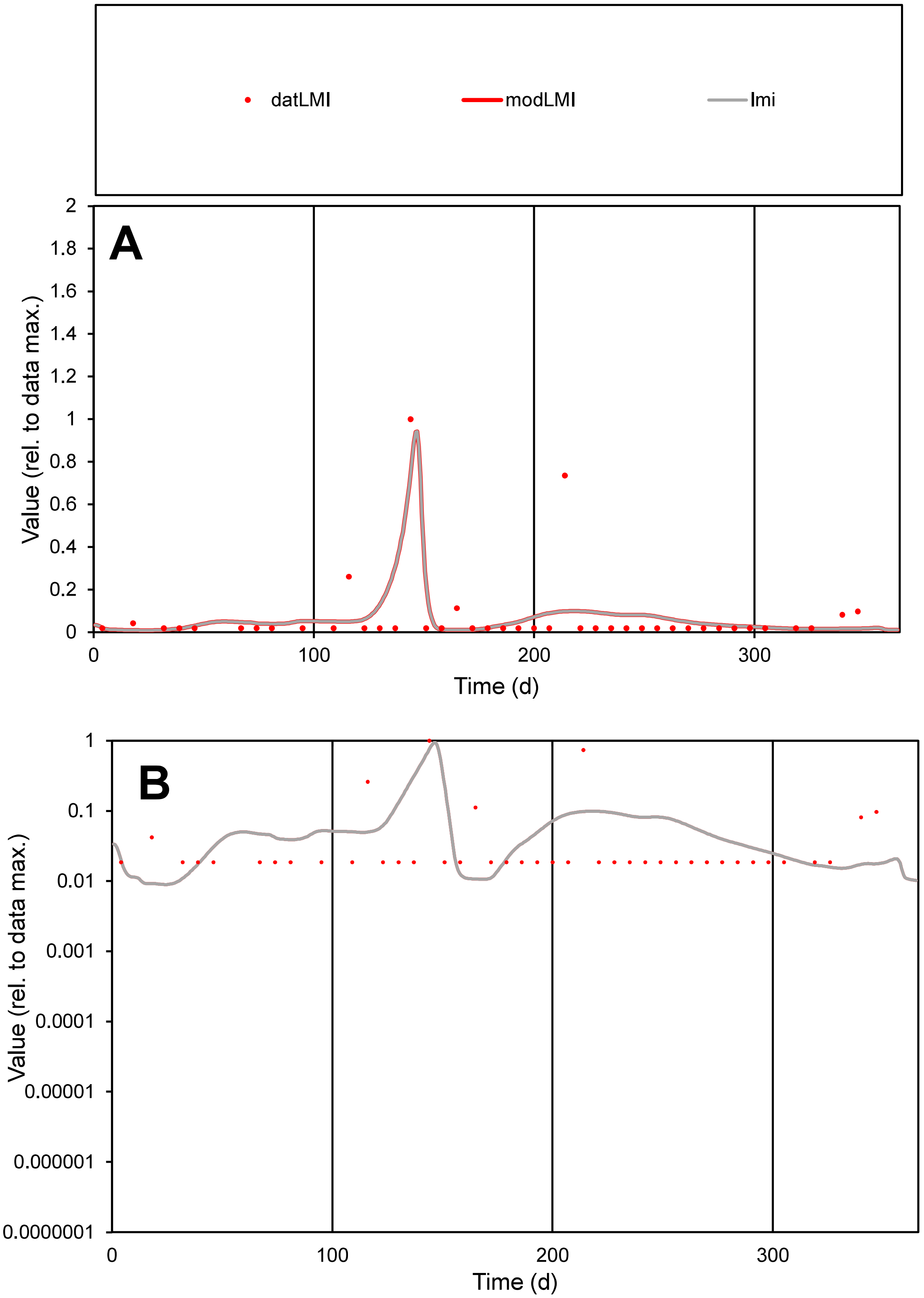

C1

Variables
LMI Provide jtd or Dst
CalibOnly?
2 1 to reproduce of
oNormalize?
2 1=no, 2=yes

ofxn calc*
1.67243
ofxn mod
1.72136

sRun
RunA1
zout
1

Ave
Data
1.1E-05
Model
1.1E-05

data/vMax
0.00016

ofxng
334.07

Min d/m
2.02759

ave/min d
3.8104
ave/min m
7.68719

327_Calib_S_A

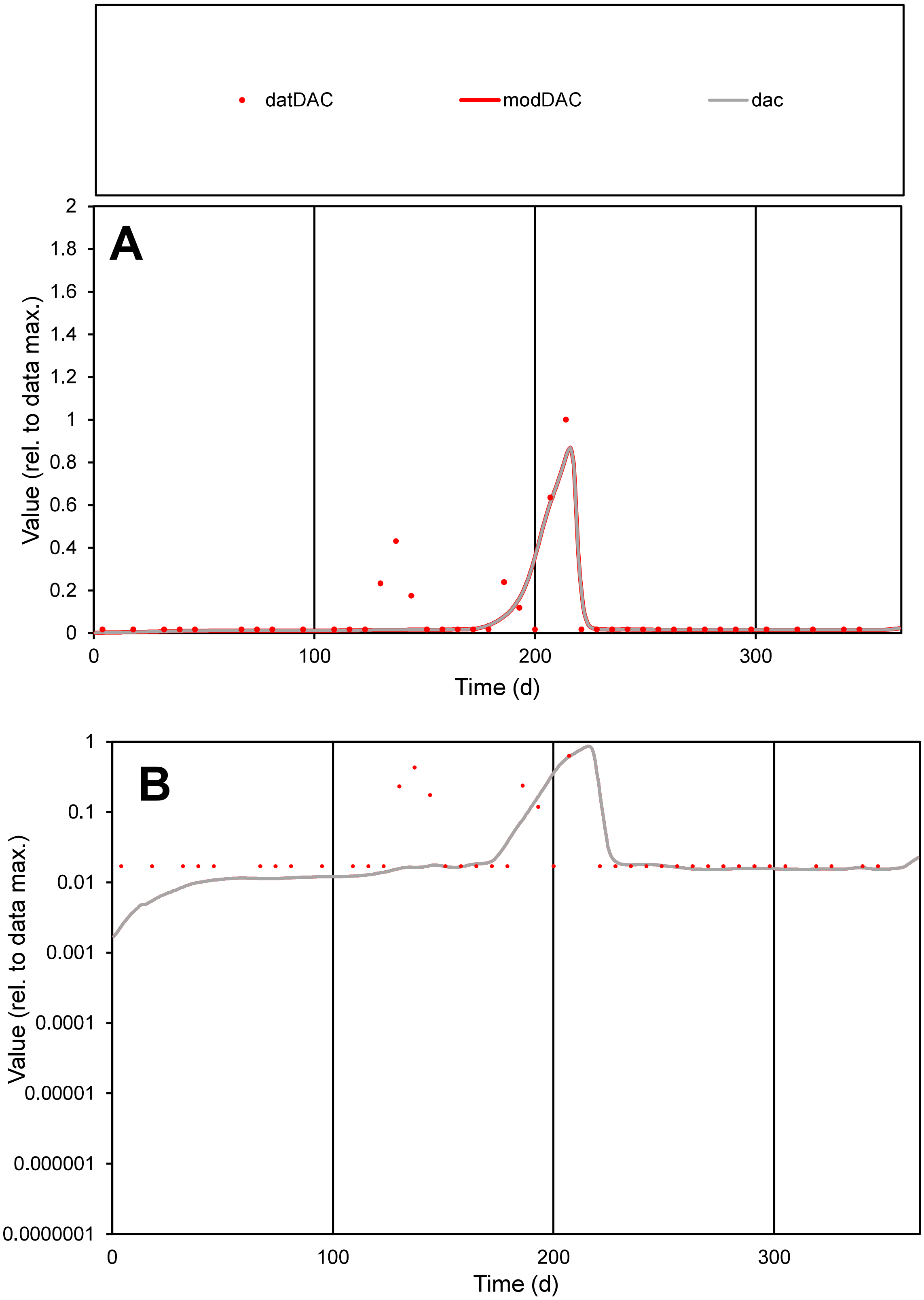

C1

Variables
DAC Provide jtd or Dst
CalibOnly?
2 1 to reproduce of
oNormalize?
2 1=no, 2=yes

ofxn calc*
1.15529
ofxn mod
1.22583

sRun
RunA1
zout
1

Ave
Data
3.7E-05
Model
2.9E-05

data/vMax
0.00045

ofxng
334.07

Min d/m
6.50295

ave/min d
4.77794
ave/min m
24.4306

327_Calib_S_A

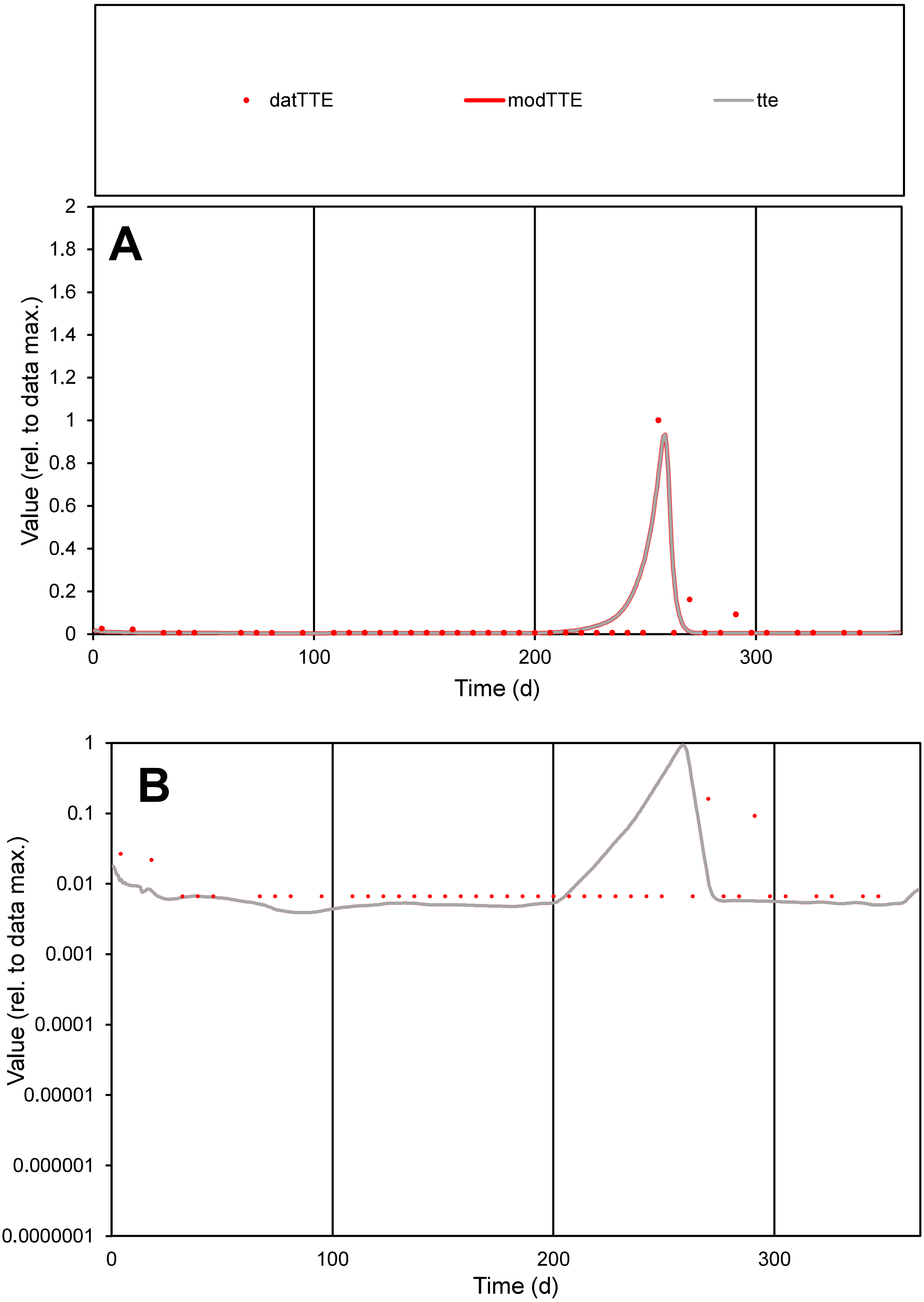

C1

Variables
TTE Provide jtd or Dst
CalibOnly?
2 1 to reproduce of
oNormalize?
2 1=no, 2=yes

ofxn calc*
1.53196
ofxn mod
1.80022

sRun
RunA1
zout
1

Ave
Data
2.1E-05
Model
2.2E-05

data/vMax
0.00056

ofxng
334.07

Min d/m
1.69034

ave/min d
5.52359
ave/min m
10.0998

327_Calib_S_A

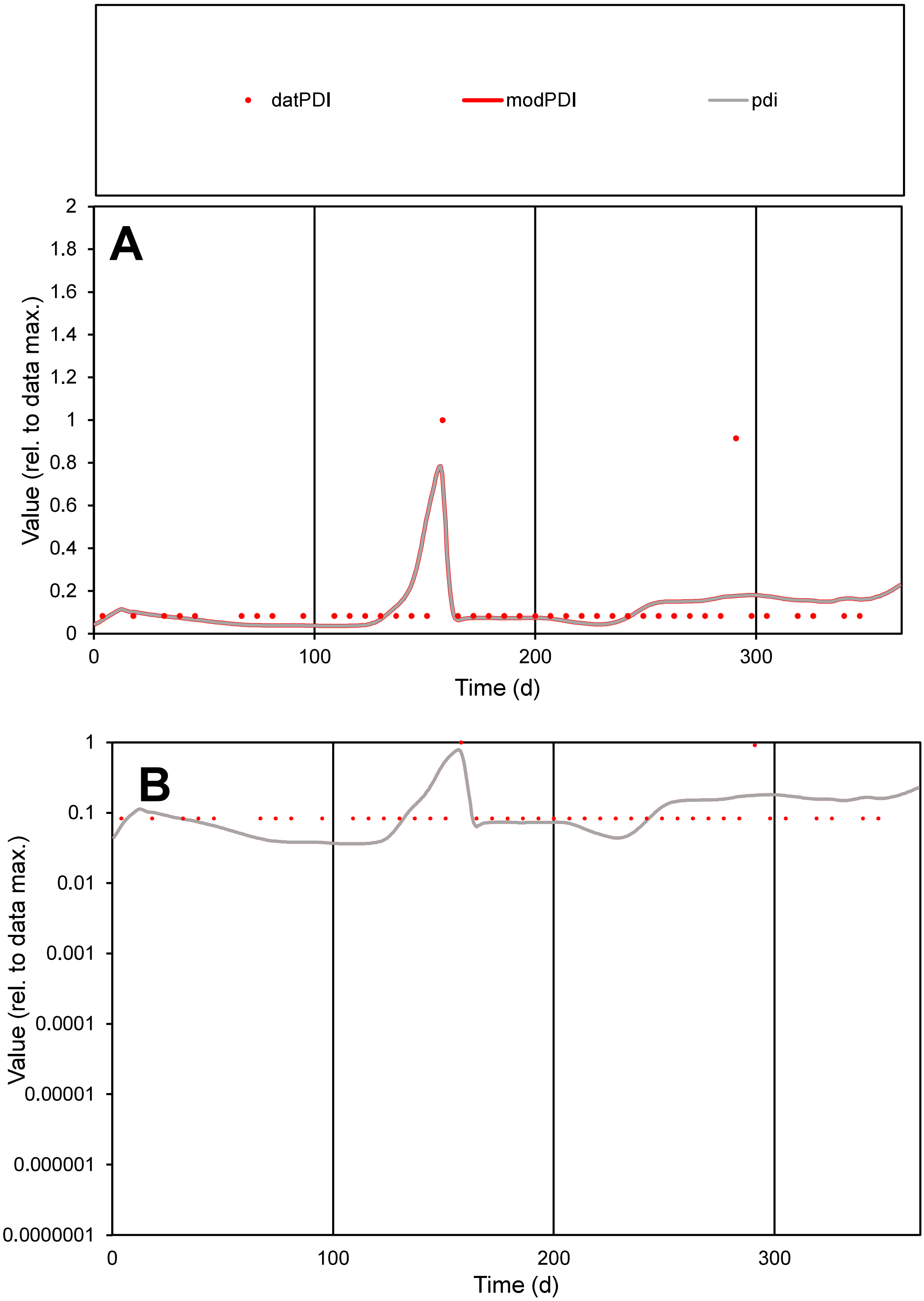

C1

Variables
PDI Provide jtd or Dst
CalibOnly?
2 1 to reproduce of
oNormalize?
2 1=no, 2=yes

ofxn calc*
1.17722
ofxn mod
1.24607

sRun
RunA1
zout
1

Ave
Data
6.6E-06
Model
6.4E-06

data/vMax
5.3E-05

ofxng
334.07

Min d/m
2.27301

ave/min d
1.50196
ave/min m
3.29787

327_Calib_S_A

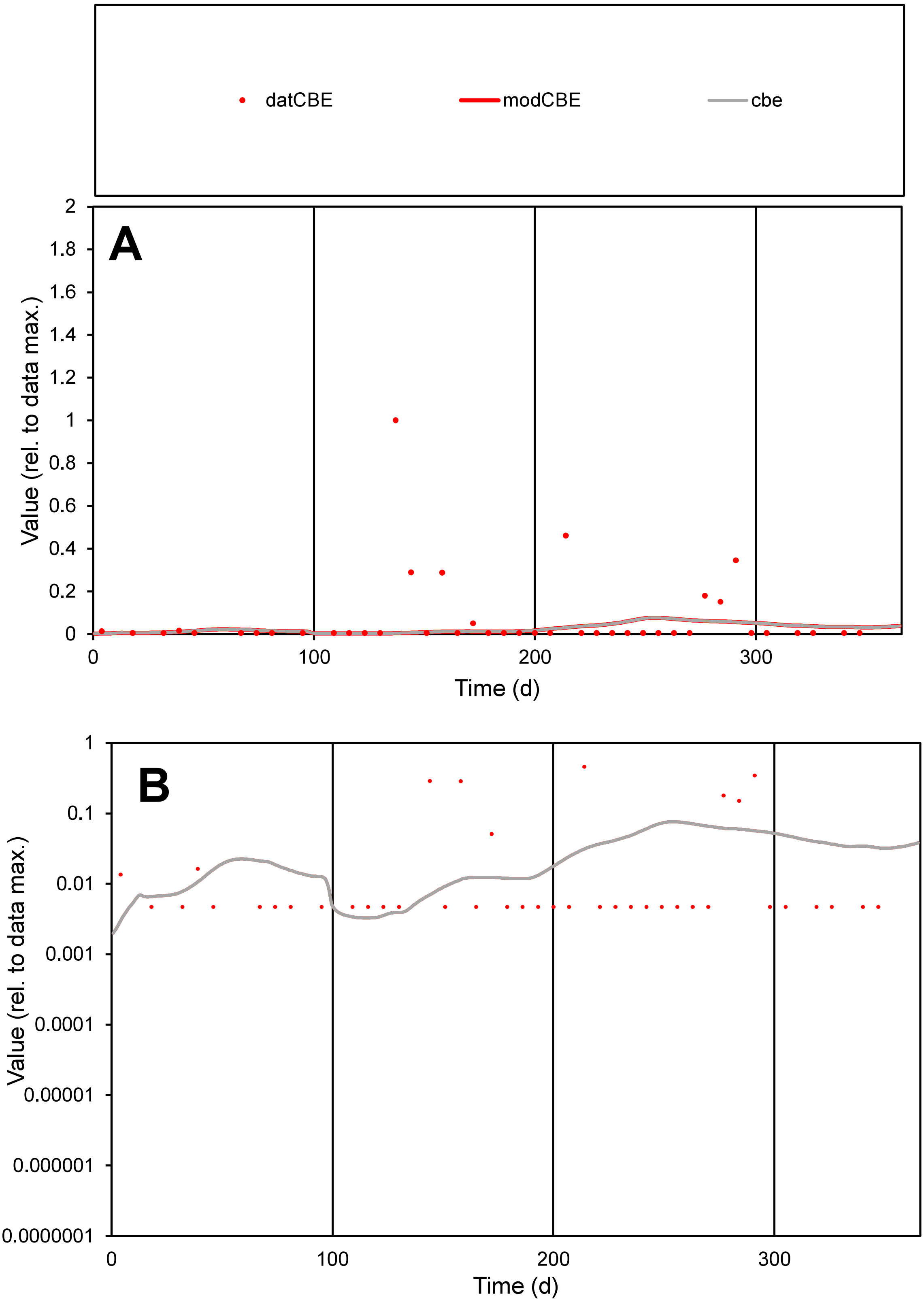

C1

Variables
CBE Provide jtd or Dst
CalibOnly?
2 1 to reproduce of
oNormalize?
2 1=no, 2=yes

ofxn calc*
2.66882
ofxn mod
2.67221

sRun
RunA1
zout
1

Ave
Data
2.5E-05
Model
1E-05

data/vMax
0.00035

ofxng
334.07

Min d/m
1.44166

ave/min d
14.8696
ave/min m
8.63629

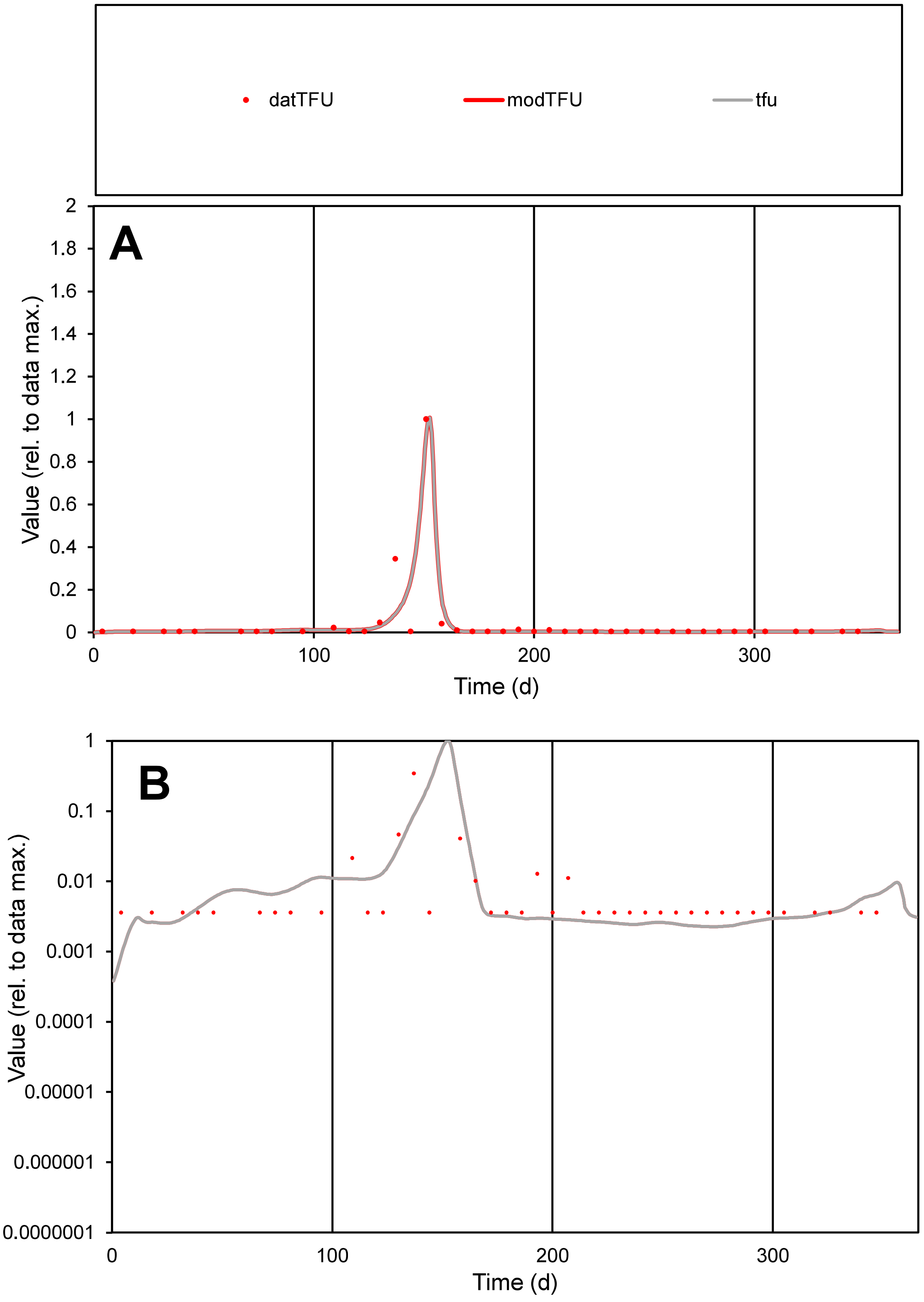

C1

Variables

TFU Provide jtd or Dst

CalibOnly?

2 1 to reproduce of

oNormalize?

2 1=no, 2=yes

ofxn calc*

1.08482

ofxn mod

1.3016

sRun

RunA1

zout

1

Ave

Data

0.00014

Model

0.00013

data/vMax

0.00355

ofxng

334.07

Min d/m

2.34074

ave/min d

10.6776

ave/min m

24.2733

327_Calib_S_A

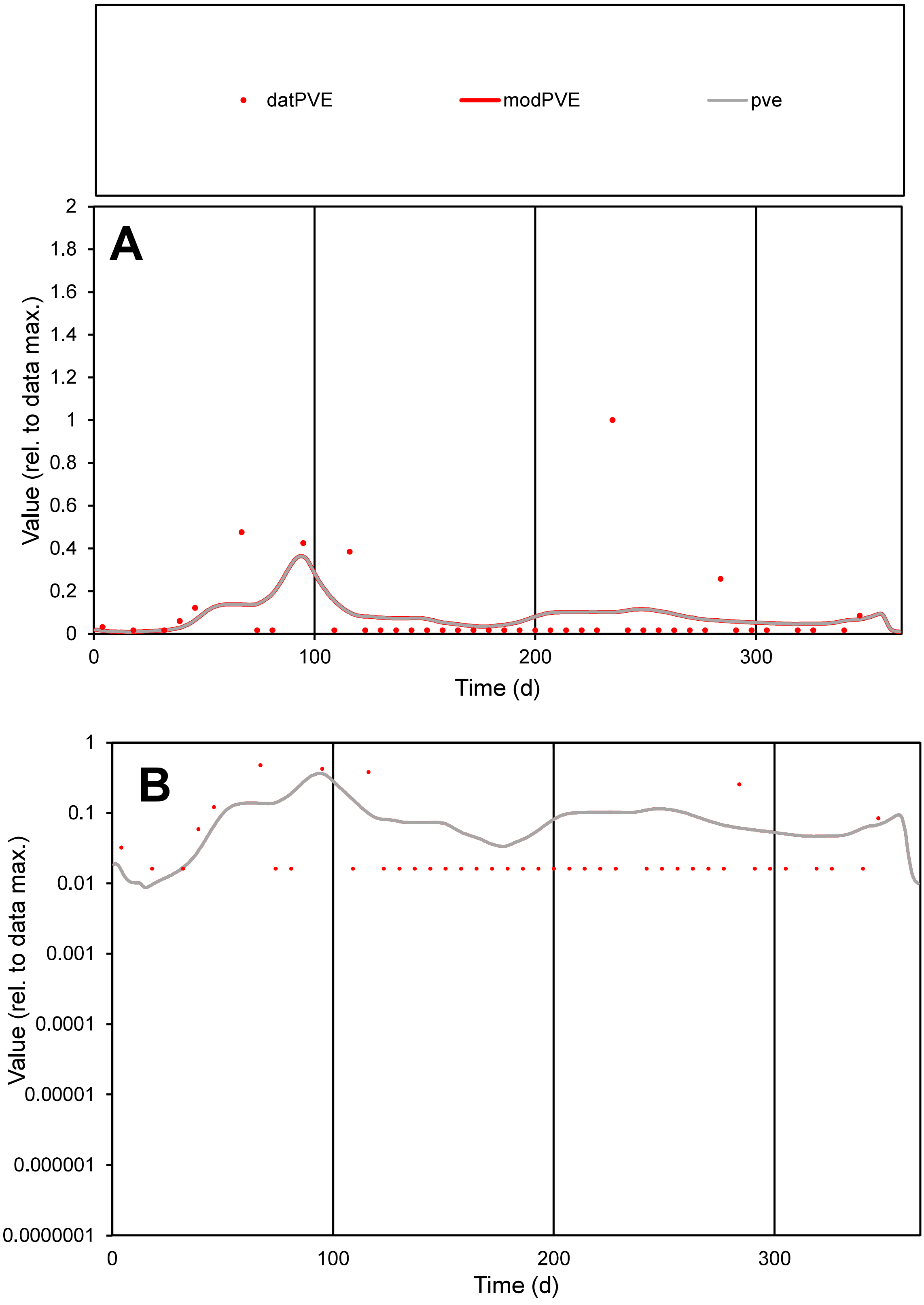

C1

Variables
PVE Provide jtd or Dst
CalibOnly?
2 1 to reproduce of
oNormalize?
2 1=no, 2=yes

ofxn calc*
2.11175
ofxn mod
2.12583

sRun
RunA1
zout
1

Ave
Data
1E-05
Model
1.1E-05

data/vMax
0.00013

ofxng
334.07

Min d/m
1.57293

ave/min d
4.97234
ave/min m
7.93636

327_Calib_S_A

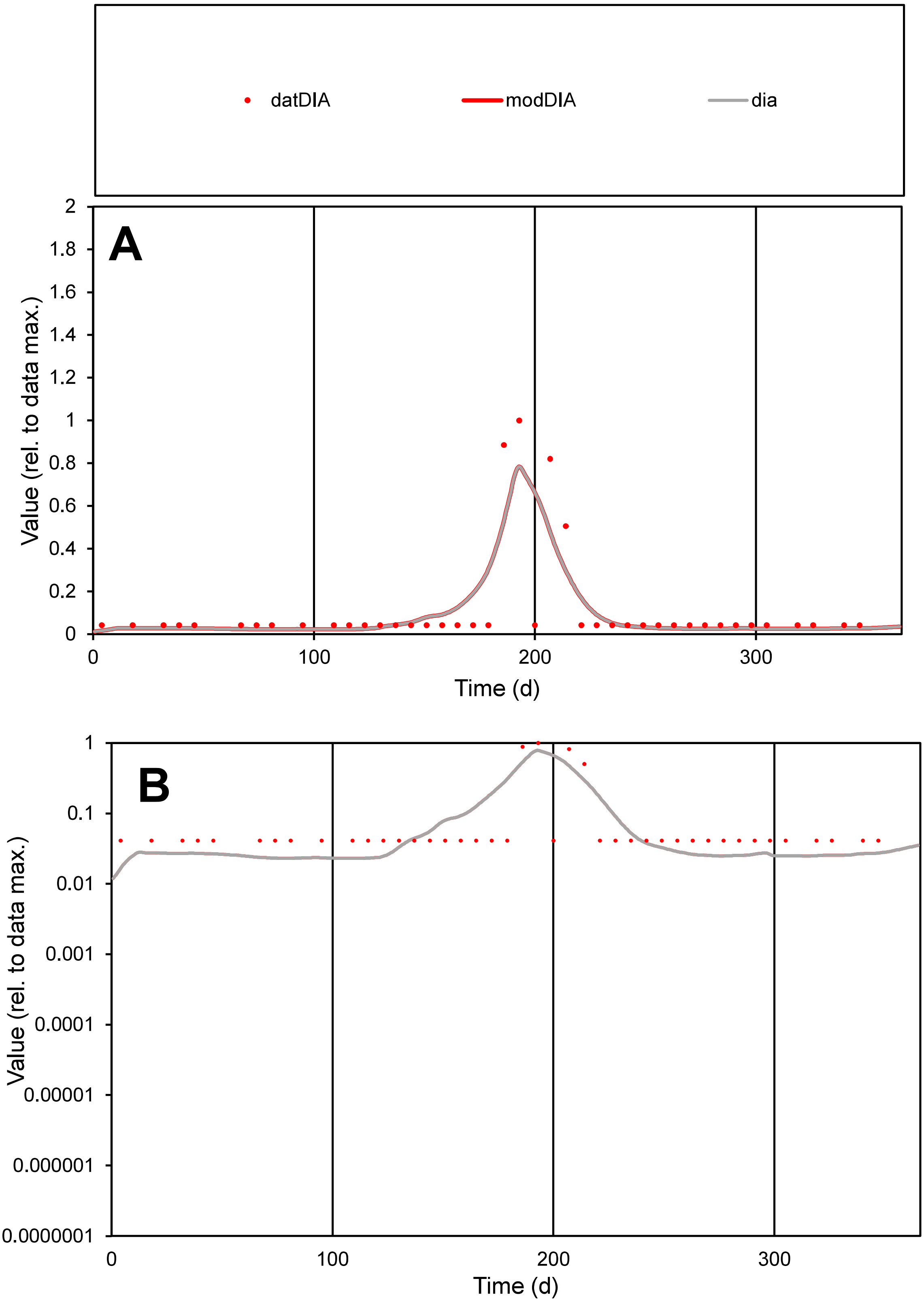

C1

Variables
DIA Provide jtd or Dst
CalibOnly?
2 1 to reproduce of
oNormalize?
2 1=no, 2=yes

ofxn calc*
1.17237
ofxn mod
1.2275

sRun
RunA1
zout
1

Ave
Data
1.7E-05
Model
1.7E-05

data/vMax
0.00015

ofxng
334.07

Min d/m
2.26849

ave/min d
2.7571
ave/min m
6.02542

327_Calib_S_A

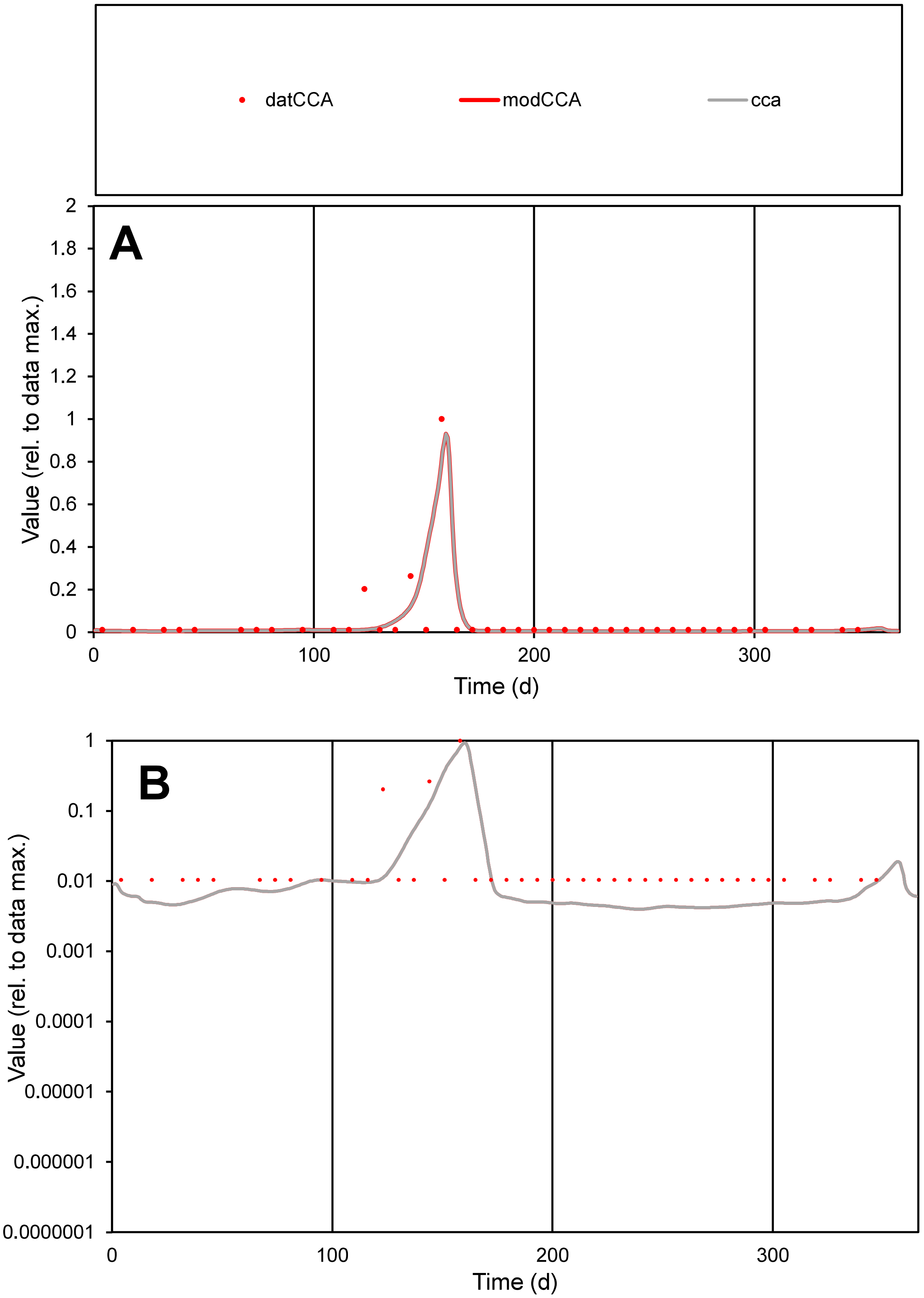

C1

Variables
CCA Provide jtd or Dst
CalibOnly?
2 1 to reproduce of
oNormalize?
2 1=no, 2=yes

ofxn calc*
1.29009
ofxn mod
1.45931

sRun
RunA1
zout
1

Ave
Data
7.8E-06
Model
7.1E-06

data/vMax
0.00018

ofxng
334.07

Min d/m
2.59034

ave/min d
4.28822
ave/min m
10.1098

327_Calib_S_A

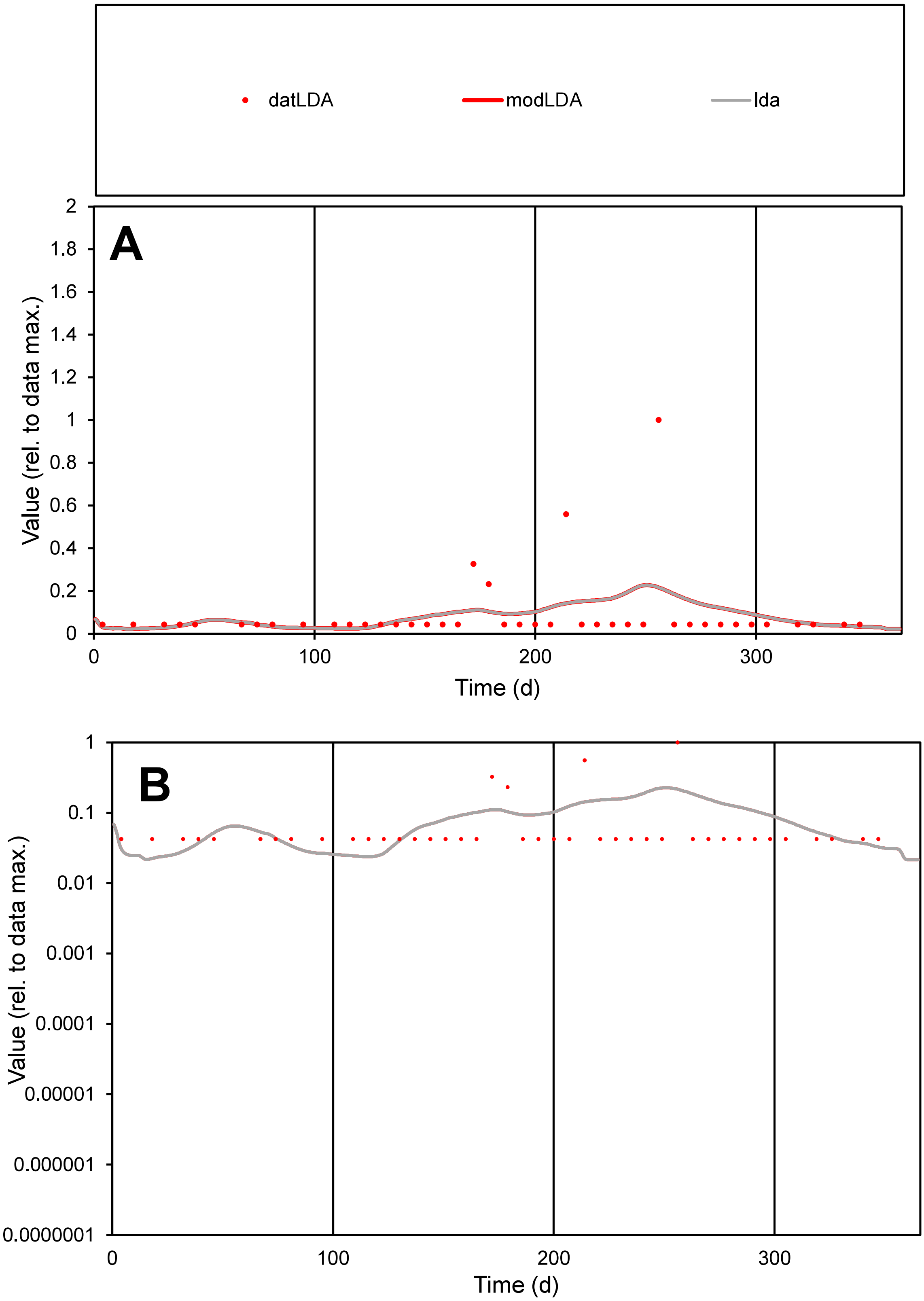

C1

Variables
LDA Provide jtd or Dst
CalibOnly?
2 1 to reproduce of
oNormalize?
2 1=no, 2=yes

ofxn calc*
1.76701
ofxn mod
1.77774

sRun
RunA1
zout
1

Ave
Data
1.4E-05
Model
1.5E-05

data/vMax
0.00016

ofxng
334.07

Min d/m
1.83901

ave/min d
2.09489
ave/min m
3.88584

327_Calib_S_A

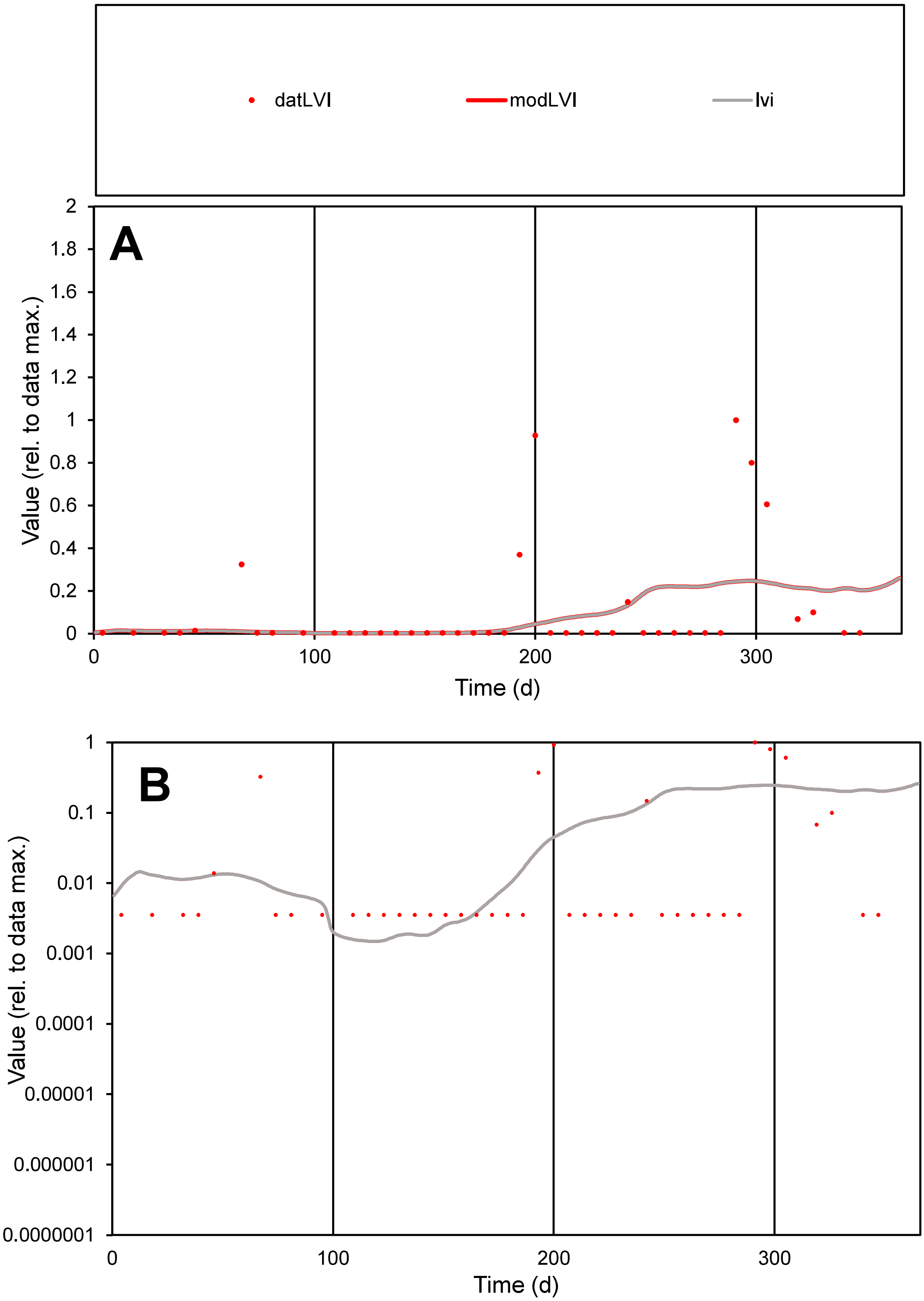

C1

Variables
LVI Provide jtd or Dst
CalibOnly?
2 1 to reproduce of
oNormalize?
2 1=no, 2=yes

ofxn calc*
2.25495
ofxn mod
2.26762

sRun
RunA1
zout
1

Ave
Data
4.3E-05
Model
3.5E-05

data/vMax
0.0004

ofxng
334.07

Min d/m
2.37753

ave/min d
30.0642
ave/min m
57.9973

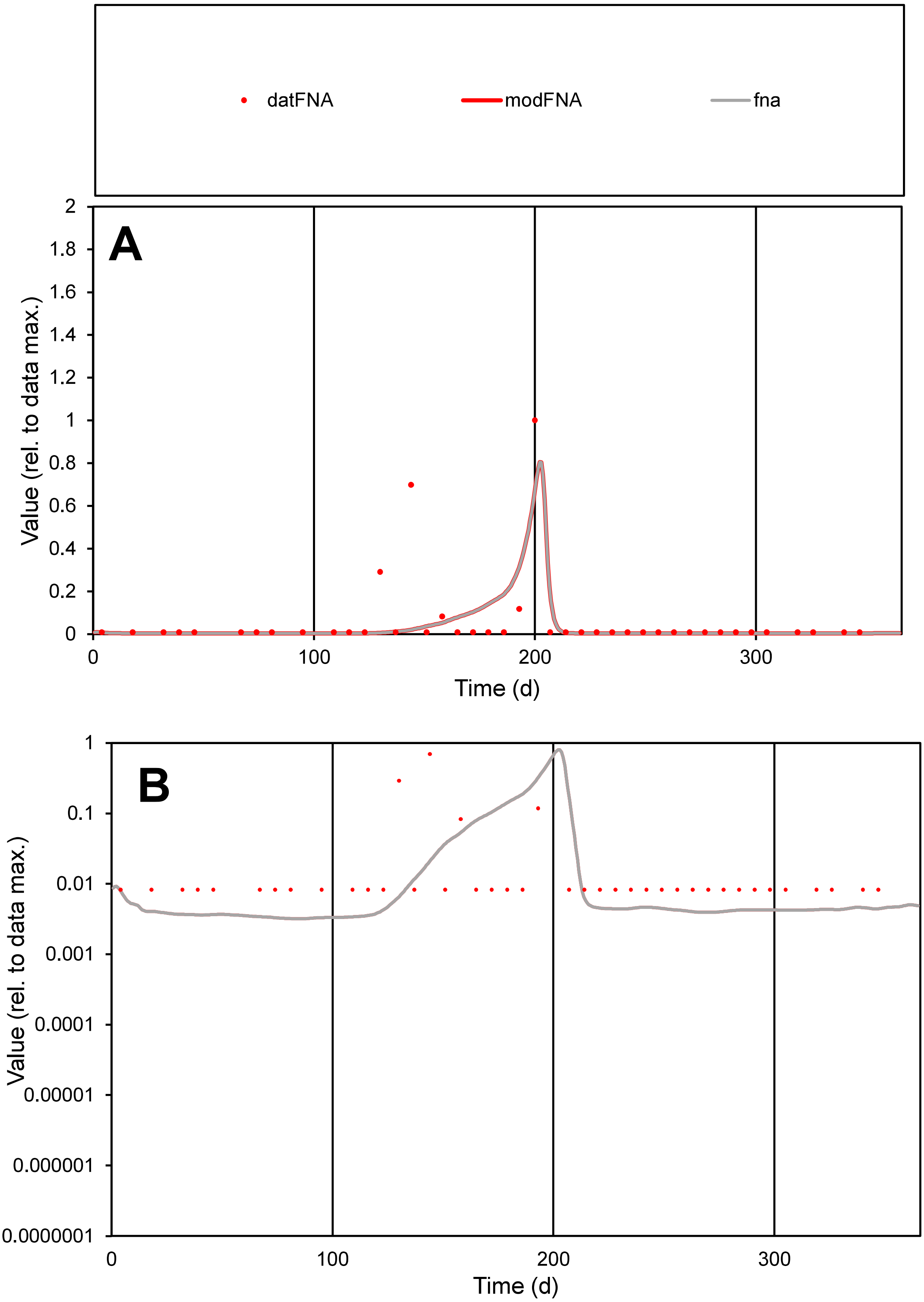

C1

Variables
FNA Provide jtd or Dst
CalibOnly?
2 1 to reproduce of
oNormalize?
2 1=no, 2=yes

ofxn calc*
2.15528
ofxn mod
2.20691

sRun
RunA1
zout
1

Ave
Data
2.2E-05
Model
1.7E-05

data/vMax
0.00038

ofxng
334.07

Min d/m
2.58541
ave/min d
7.19429
ave/min m
14.1402

327_Calib_S_A

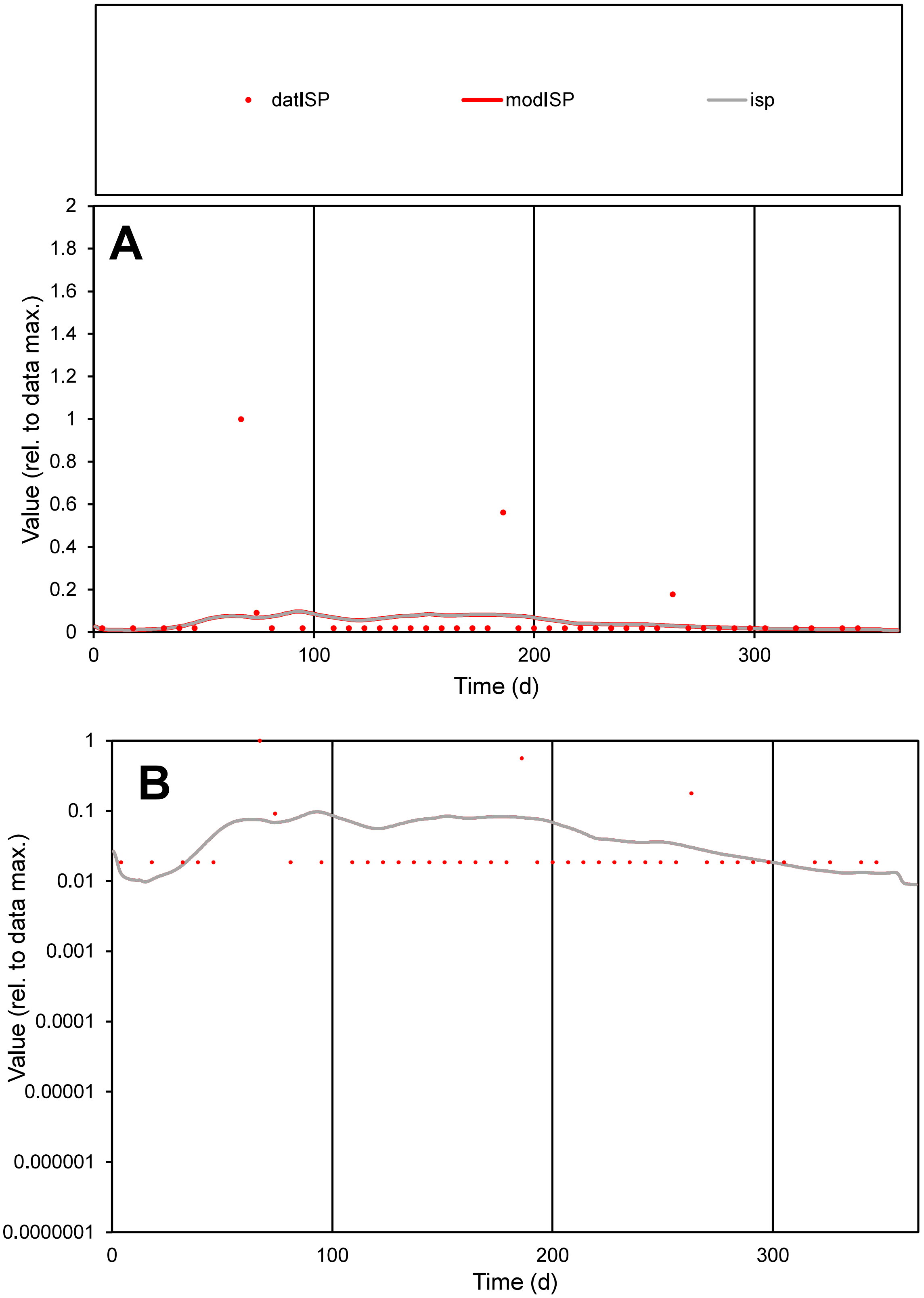

C1

Variables
ISP Provide jtd or Dst
CalibOnly?
2 1 to reproduce of
oNormalize?
2 1=no, 2=yes

ofxn calc*
2.75805
ofxn mod
2.76768

sRun
RunA1
zout
1

Ave
Data
1.6E-05
Model
1.3E-05

data/vMax
0.00027

ofxng
334.07

Min d/m
1.67397

ave/min d
3.24926
ave/min m
4.2661

327_Calib_S_A

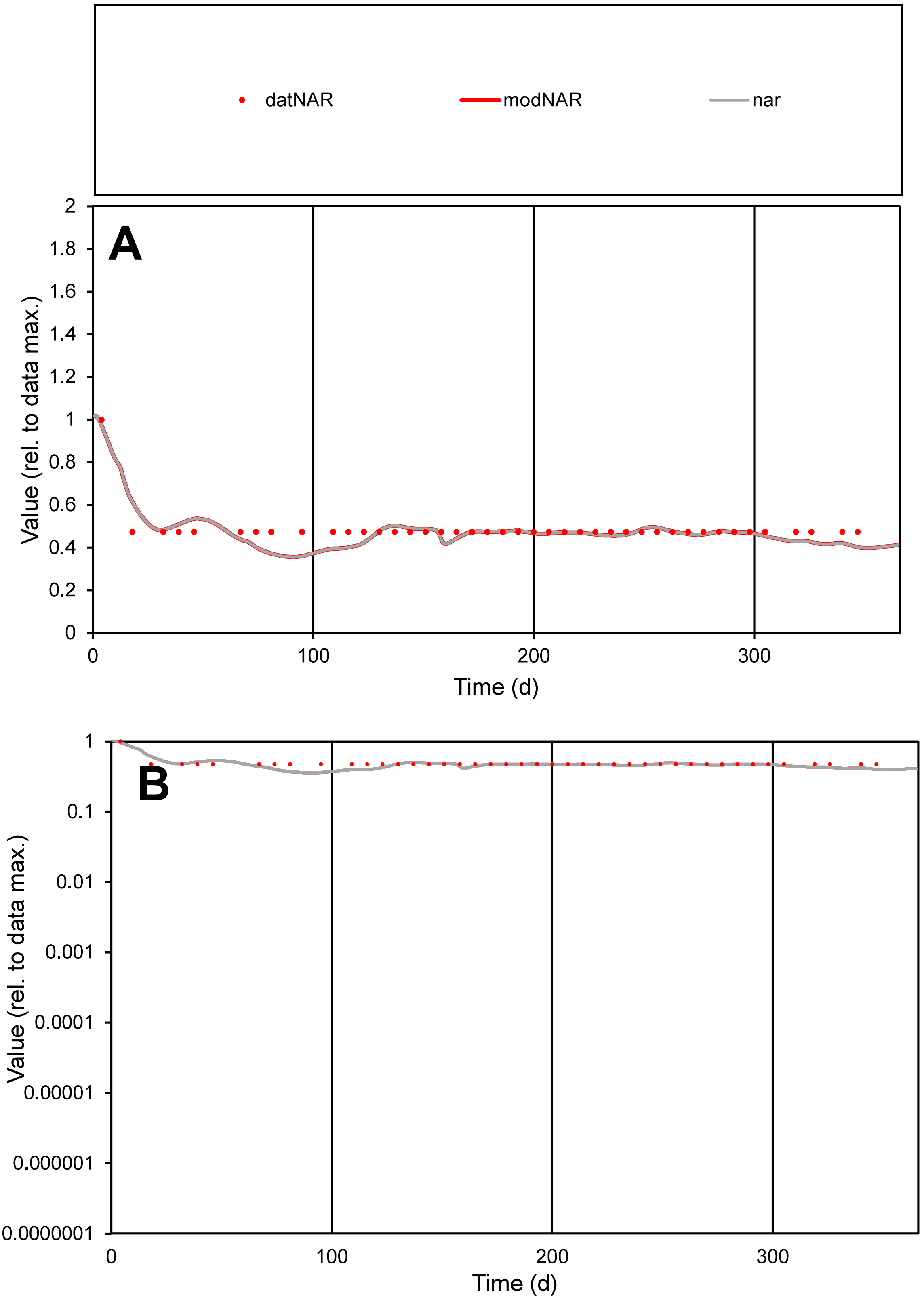

C1

Variables
NAR Provide jtd or Dst
CalibOnly?
2 1 to reproduce of
oNormalize?
2 1=no, 2=yes

ofxn calc*
0.0925
ofxn mod
0.14536

sRun
RunA1
zout
1

Ave
Data
2.9E-06
Model
2.8E-06

data/vMax
6E-06

ofxng
334.07

Min d/m
1.30304

ave/min d
1.02657
ave/min m
1.29752

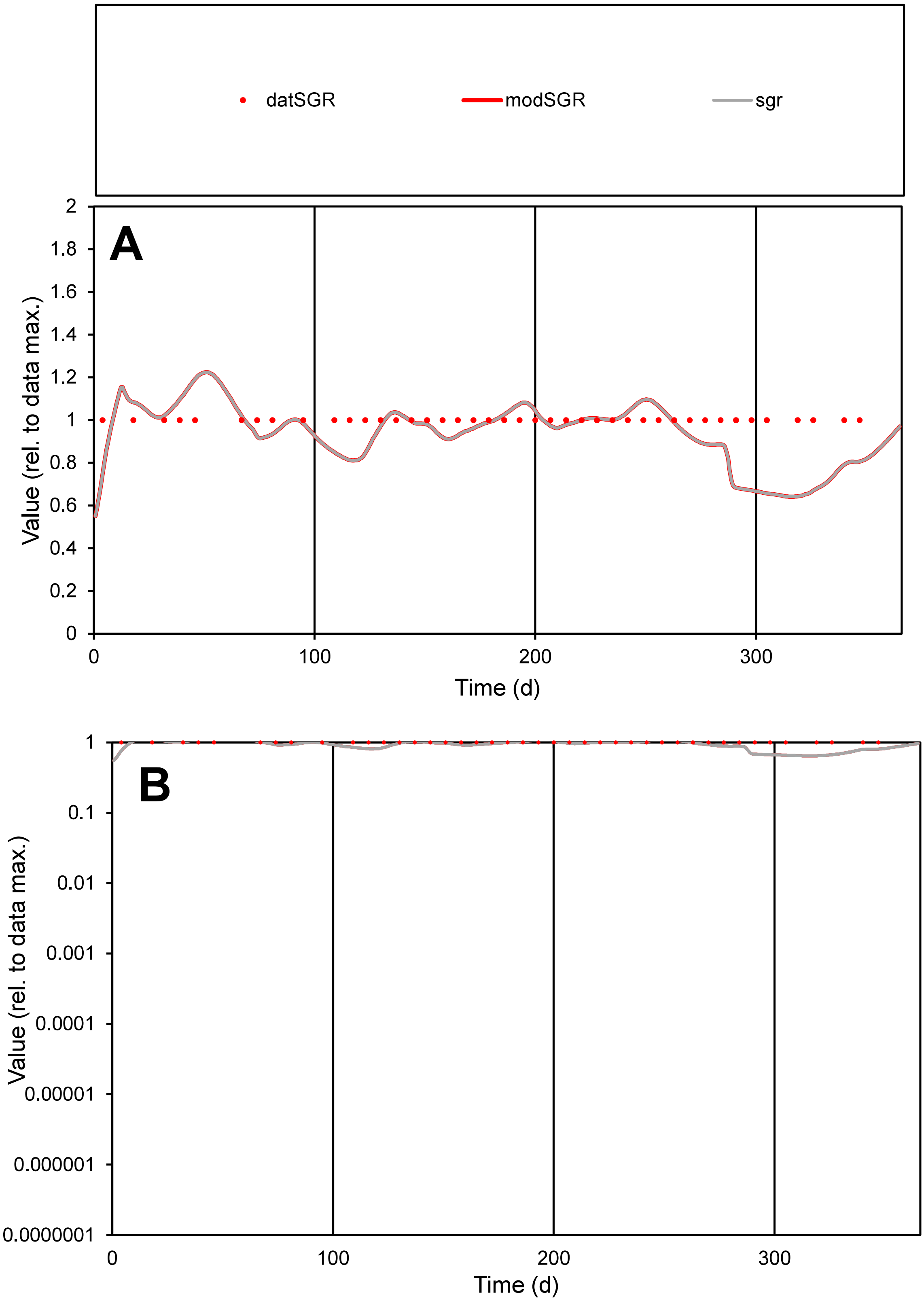

C1

Variables

SGR Provide jtd or Dst

CalibOnly?

2 1 to reproduce of

oNormalize?

2 1=no, 2=yes

ofxn calc*

0.14631

ofxn mod

0.18367

sRun

RunA1

zout

1

Ave

Data

4.5E-06

Model

4.2E-06

data/vMax

4.5E-06

ofxng

334.07

Min d/m

1.54937

ave/min d

1

ave/min m

1.45063

327_Calib_S_A

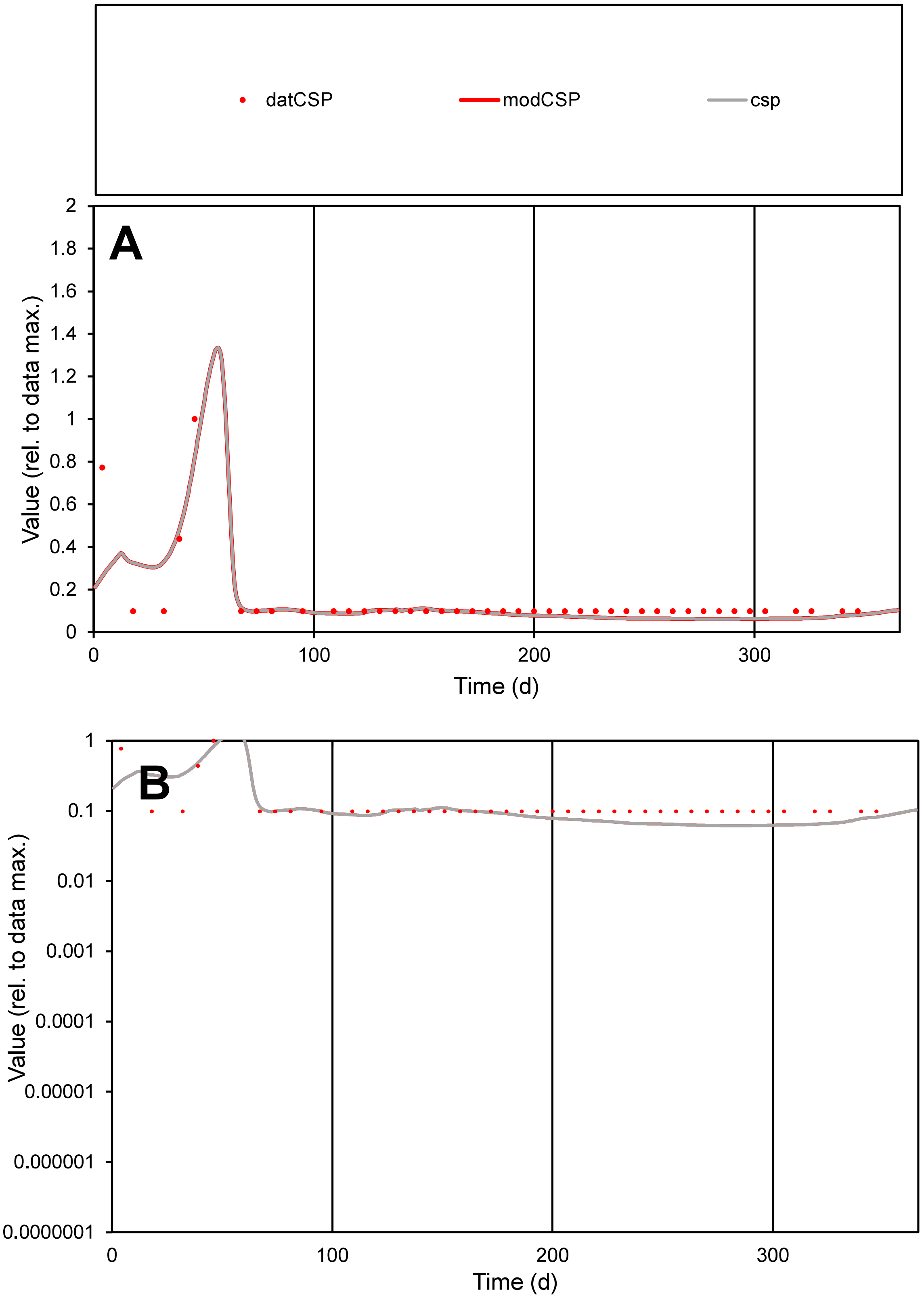

C1

Variables

CSP Provide jtd or Dst

CalibOnly?

2 1 to reproduce of

oNormalize?

2 1=no, 2=yes

ofxn calc*

0.66608

ofxn mod

0.68706

sRun

RunA1

zout

1

Ave

Data

3.5E-06

Model

3.1E-06

data/vMax

2.4E-05

ofxng

334.07

Min d/m

1.58816

ave/min d

1.46521

ave/min m

2.06248

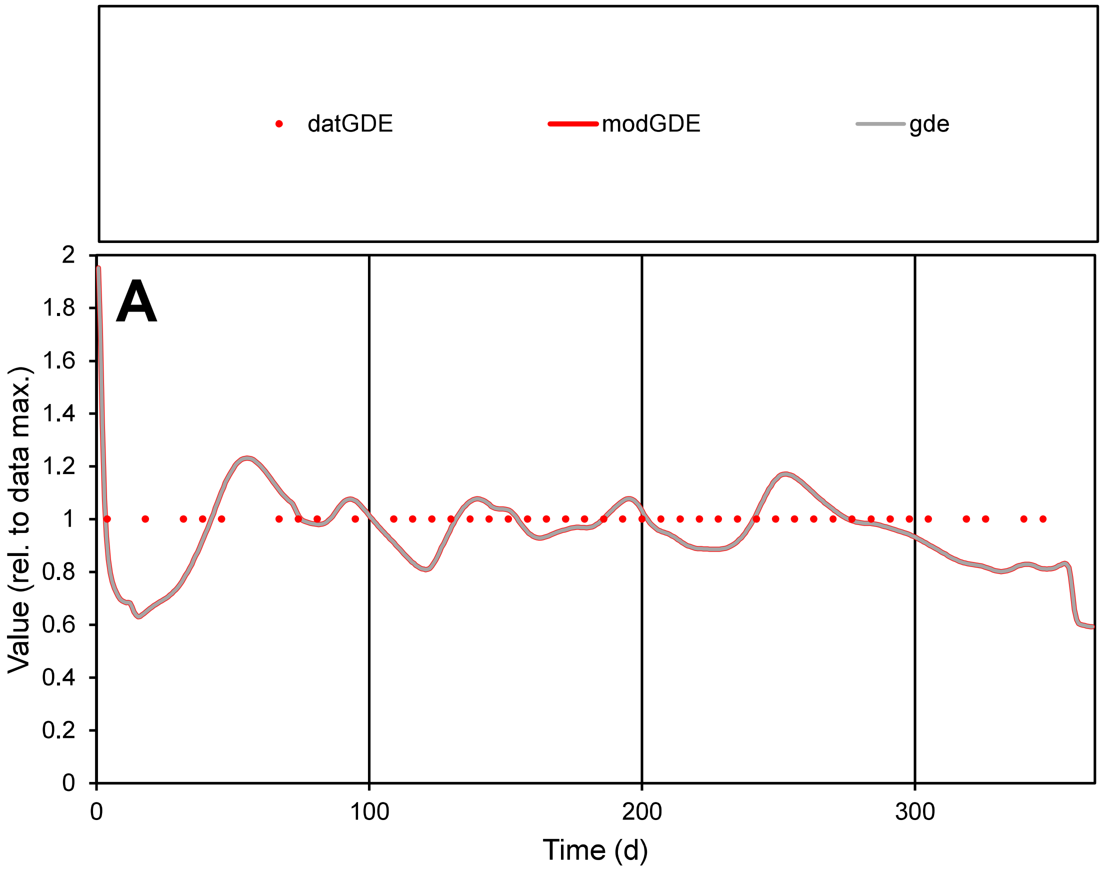

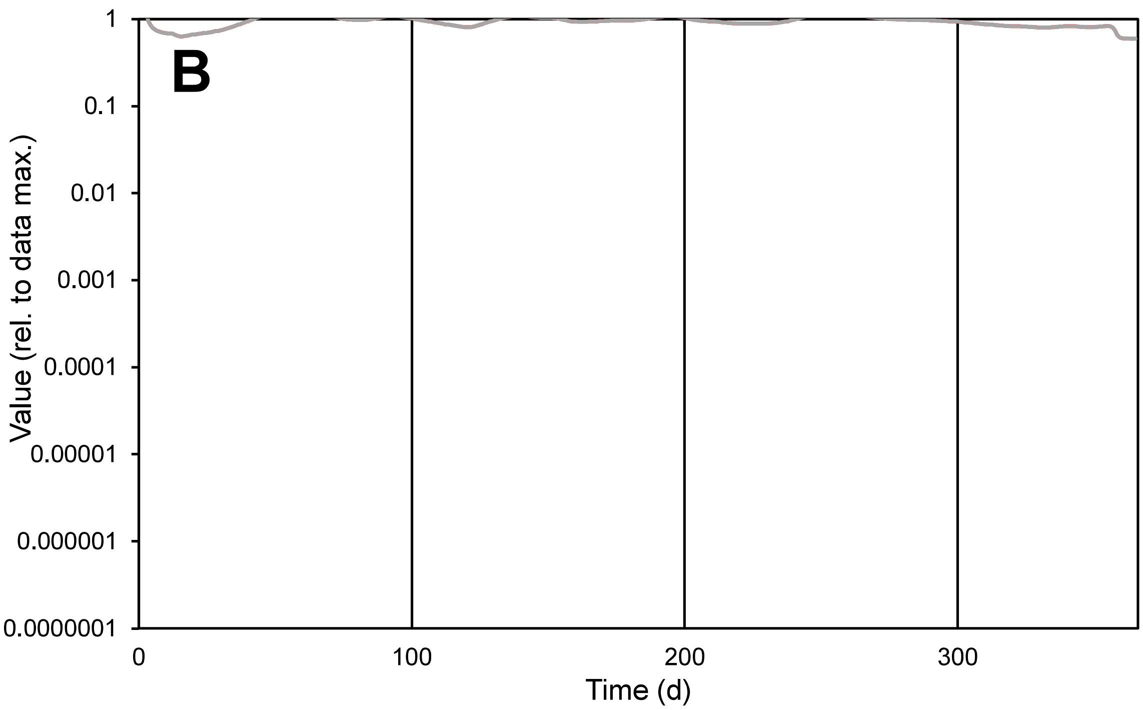

C1

Variables
GDE Provide jtd or Dst
CalibOnly?
2 1 to reproduce of
oNormalize?
2 1=no, 2=yes

ofxn calc*
0.11406
ofxn mod
0.16068

sRun
RunA1
zout
1

Ave
Data
2.7E-05
Model
2.6E-05

data/vMax
2.7E-05

ofxng
334.07

Min d/m
1.50989

ave/min d
1
ave/min m
1.44802

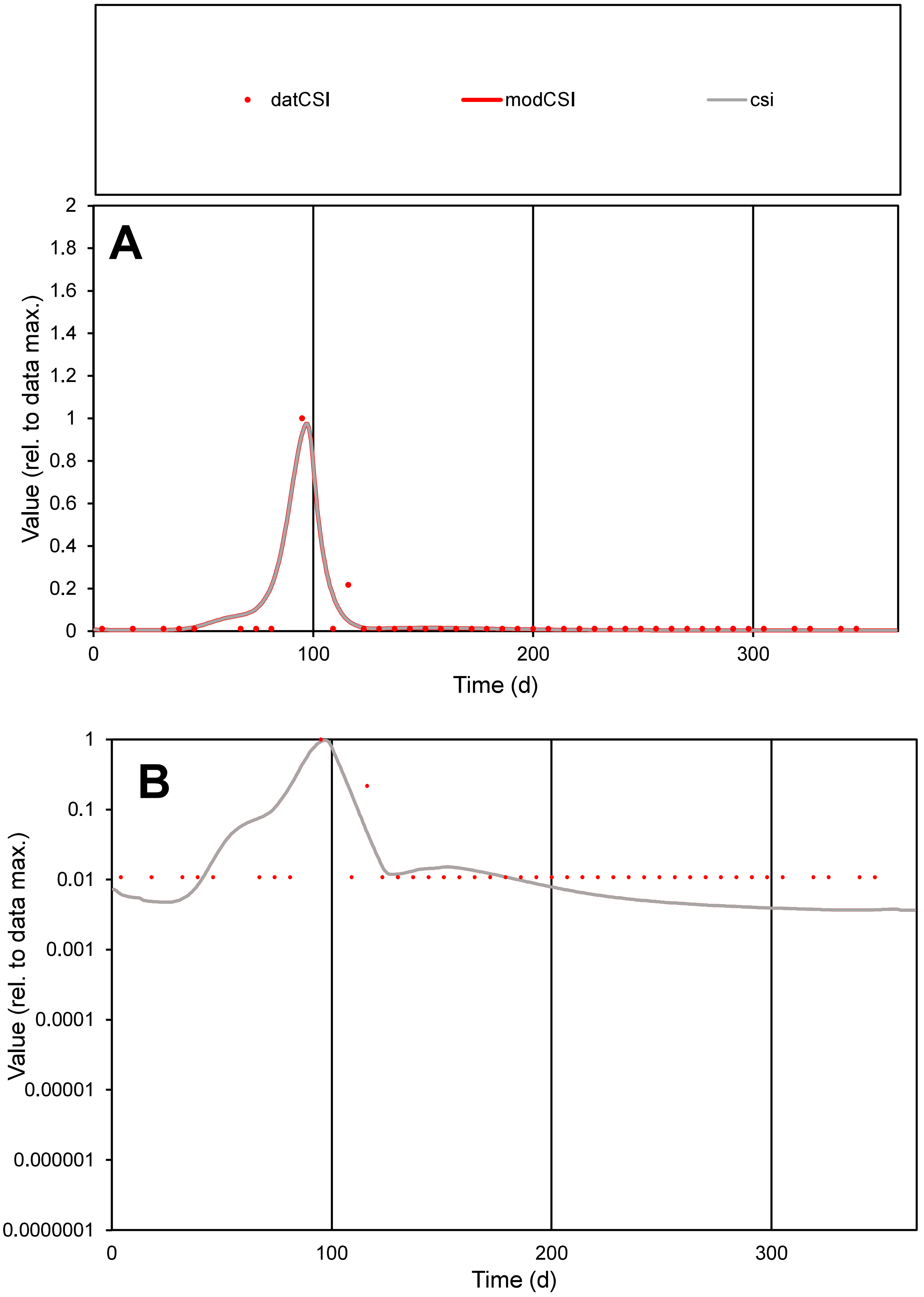

C1

Variables
CSI Provide jtd or Dst
CalibOnly?
2 1 to reproduce of
oNormalize?
2 1=no, 2=yes

ofxn calc*
1.10236
ofxn mod
1.20908

sRun
RunA1
zout
1

Ave
Data
8.4E-06
Model
8.8E-06

data/vMax
0.00021

ofxng
334.07

Min d/m
2.93557

ave/min d
3.62327
ave/min m
11.1869

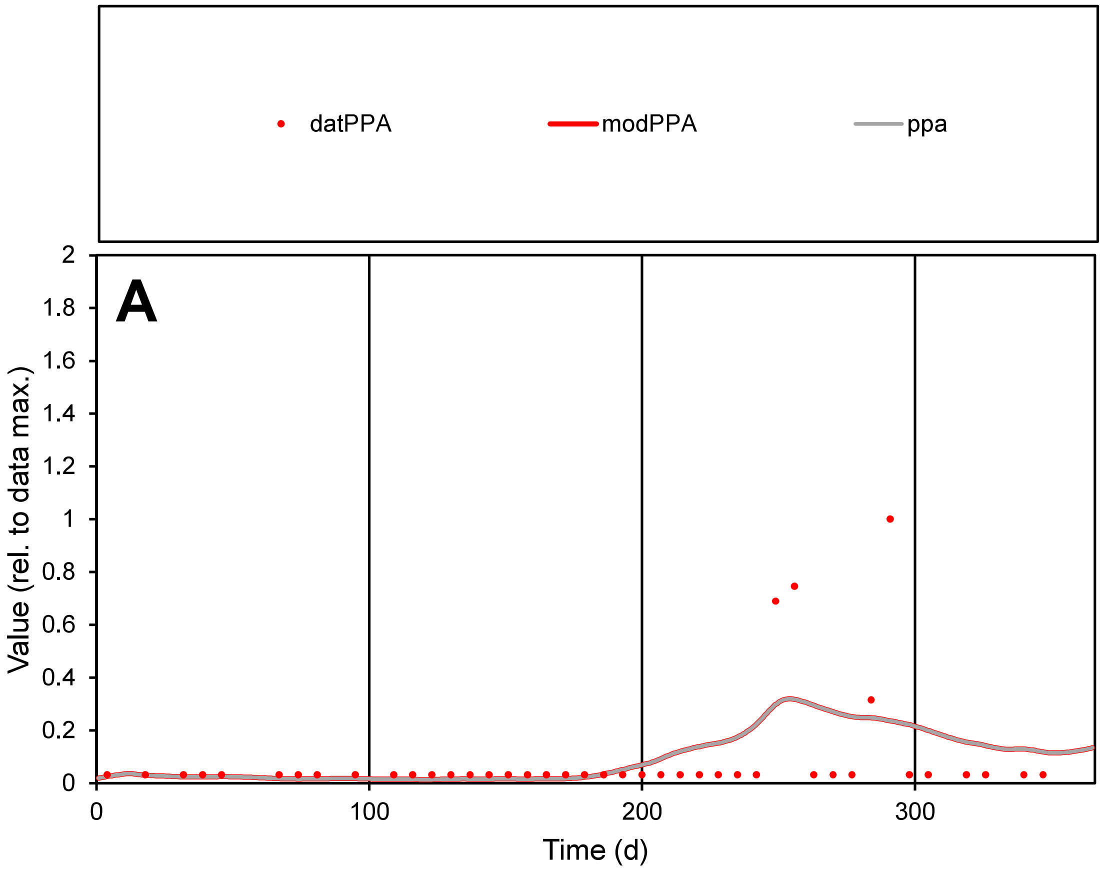

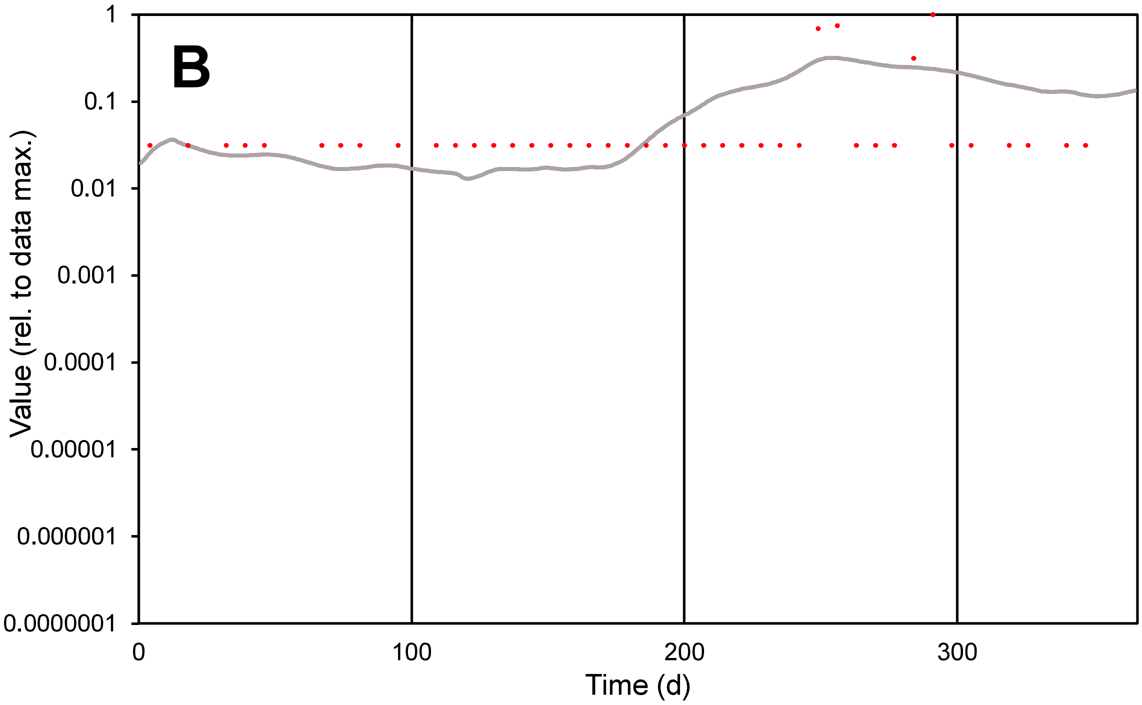

C1

Variables
PPA Provide jtd or Dst
CalibOnly?
2 1 to reproduce of
oNormalize?
2 1=no, 2=yes

ofxn calc*
1.87494
ofxn mod
1.88787

sRun
RunA1
zout
1

Ave
Data
9.6E-06
Model
1.1E-05

data/vMax
0.0001

ofxng
334.07

Min d/m
2.27247

ave/min d
2.99297
ave/min m
7.46086

327_Calib_S_A

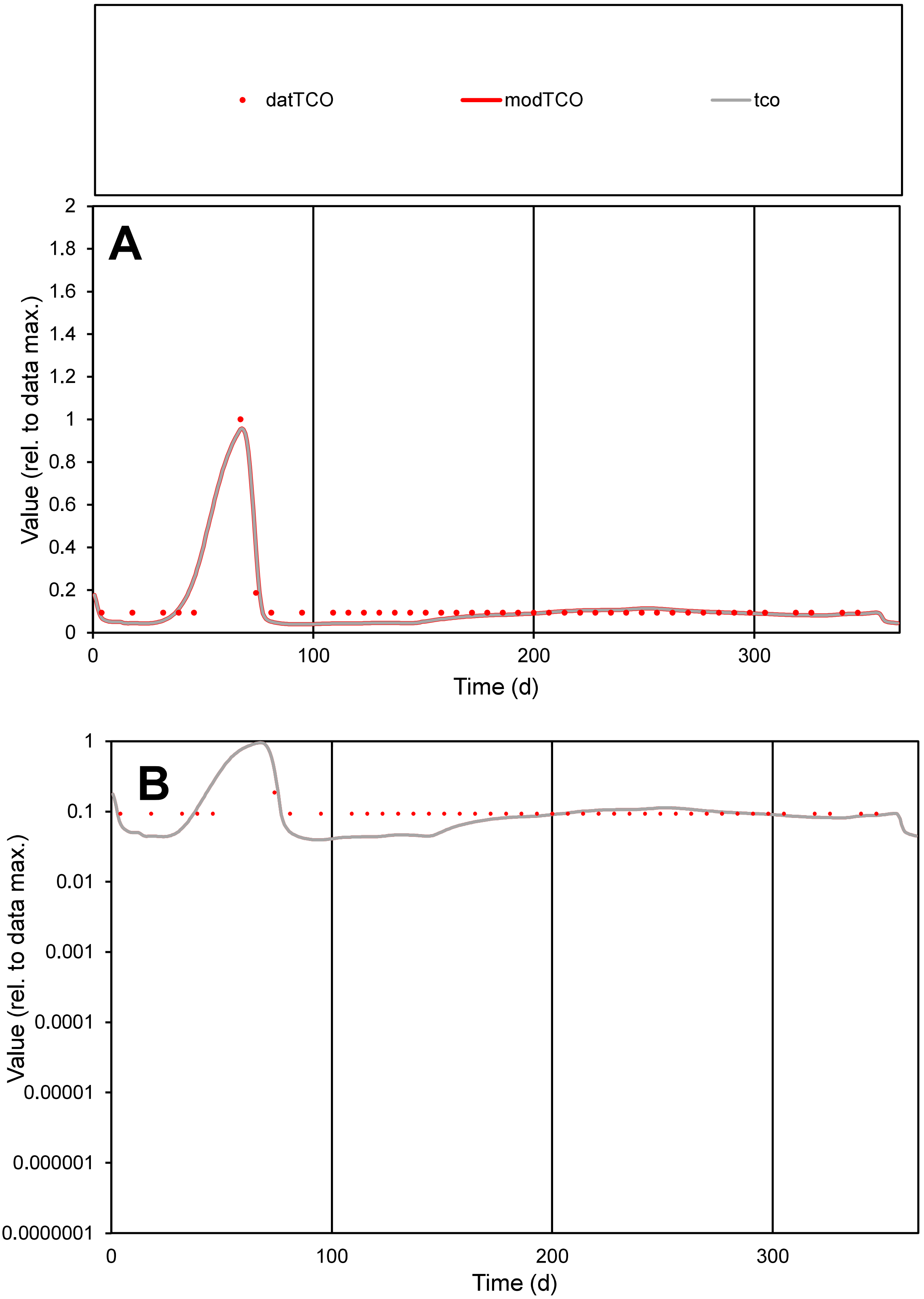

C1

Variables
TCO Provide jtd or Dst
CalibOnly?
2 1 to reproduce of
oNormalize?
2 1=no, 2=yes

ofxn calc*
0.29422
ofxn mod
0.37318

sRun
RunA1
zout
1

Ave
Data
1.1E-05
Model
9.9E-06

data/vMax
9.2E-05

ofxng
334.07

Min d/m
2.34576

ave/min d
1.25514
ave/min m
2.69227

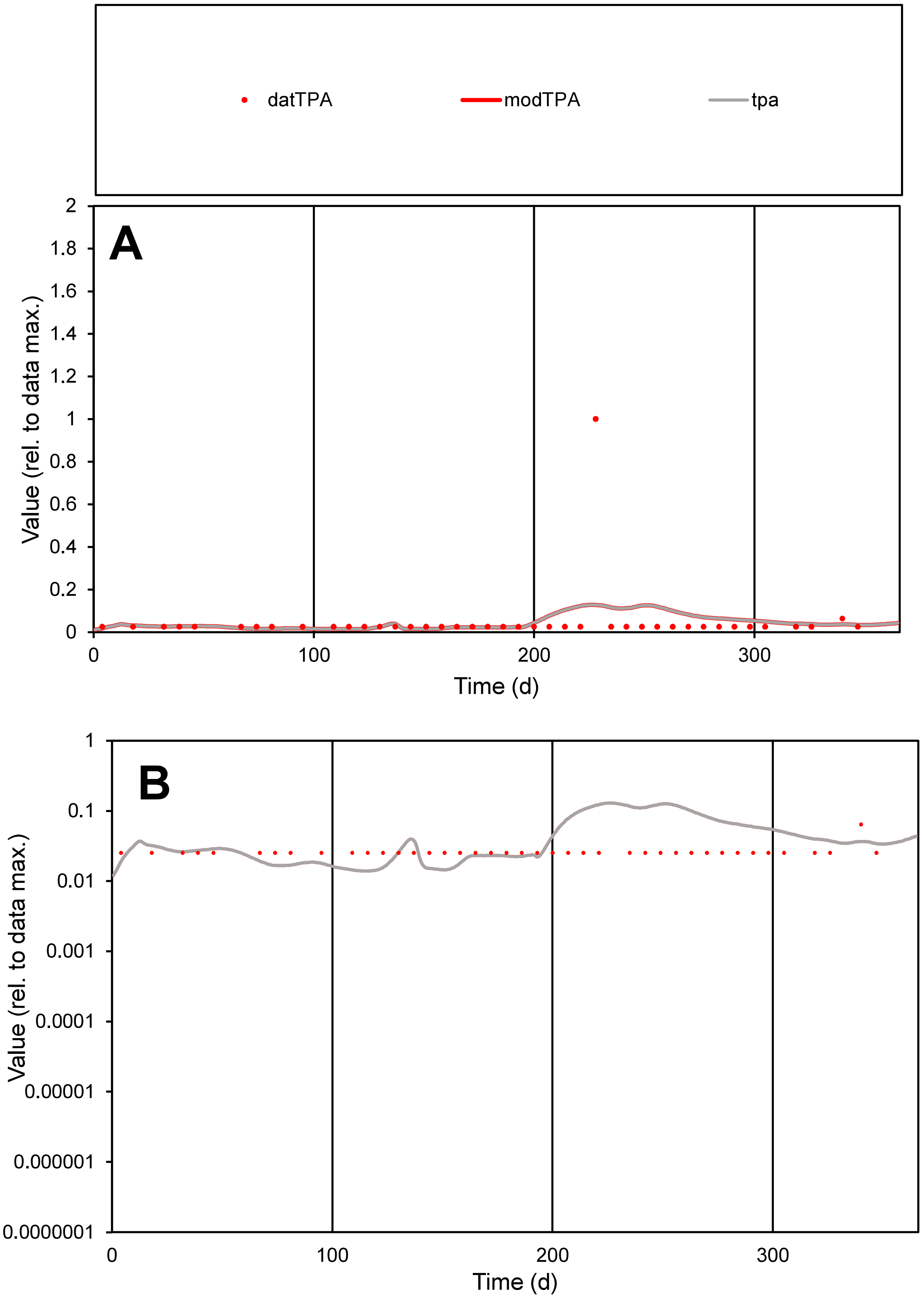

C1

Variables
TPA Provide jtd or Dst
CalibOnly?
2 1 to reproduce of
oNormalize?
2 1=no, 2=yes

ofxn calc*
2.84805
ofxn mod
2.85437

sRun
RunA1
zout
1

Ave
Data
9.2E-06
Model
9.1E-06

data/vMax
0.00019

ofxng
334.07

Min d/m
1.80719

ave/min d
1.95587
ave/min m
3.4837

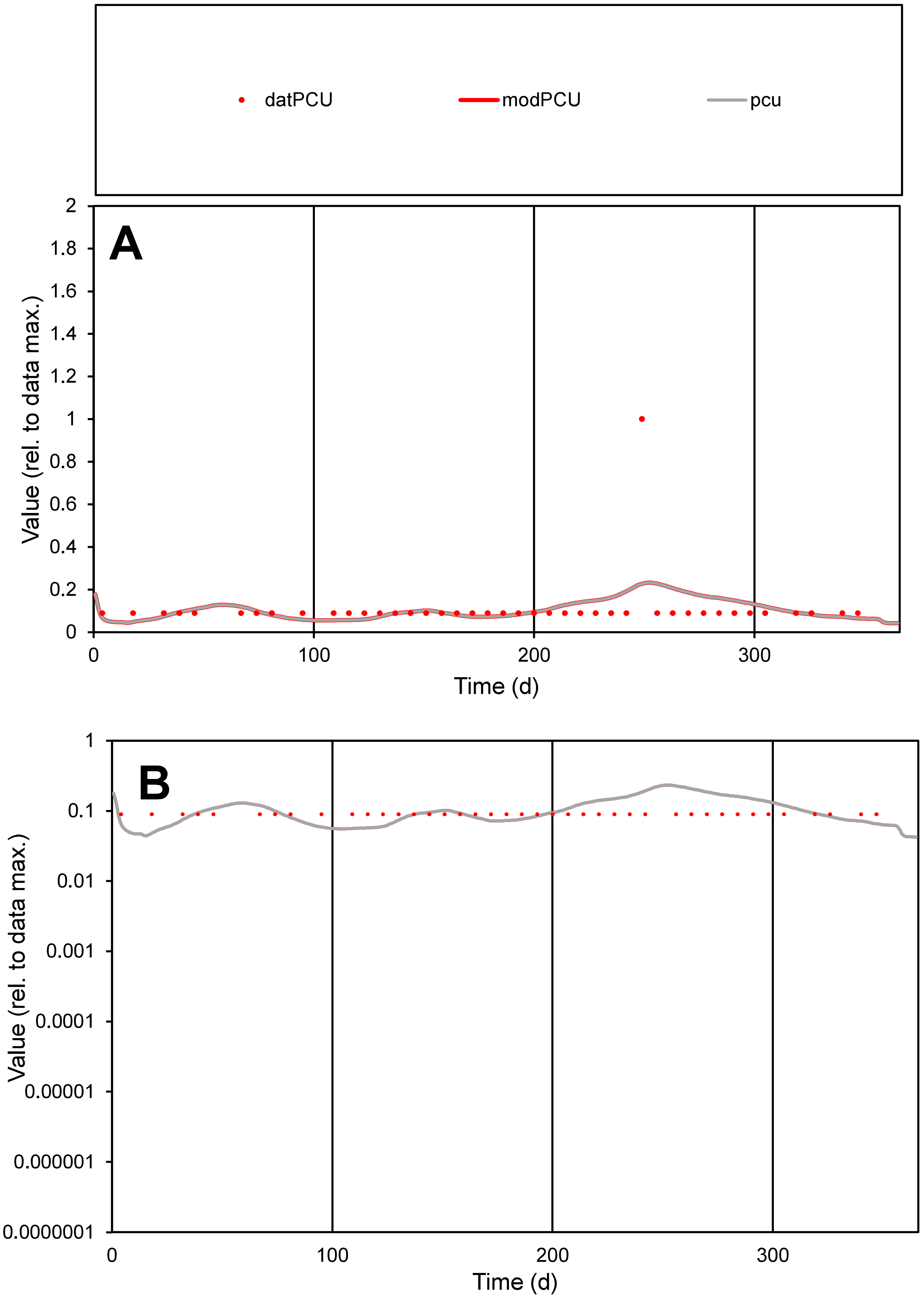

C1

Variables
PCU Provide jtd or Dst
CalibOnly?
2 1 to reproduce of
oNormalize?
2 1=no, 2=yes

ofxn calc*
1.14716
ofxn mod
1.15586

sRun
RunA1
zout
1

Ave
Data
7.7E-06
Model
7.6E-06

data/vMax
6.9E-05

ofxng
334.07

Min d/m
1.74972

ave/min d
1.24231
ave/min m
2.15426

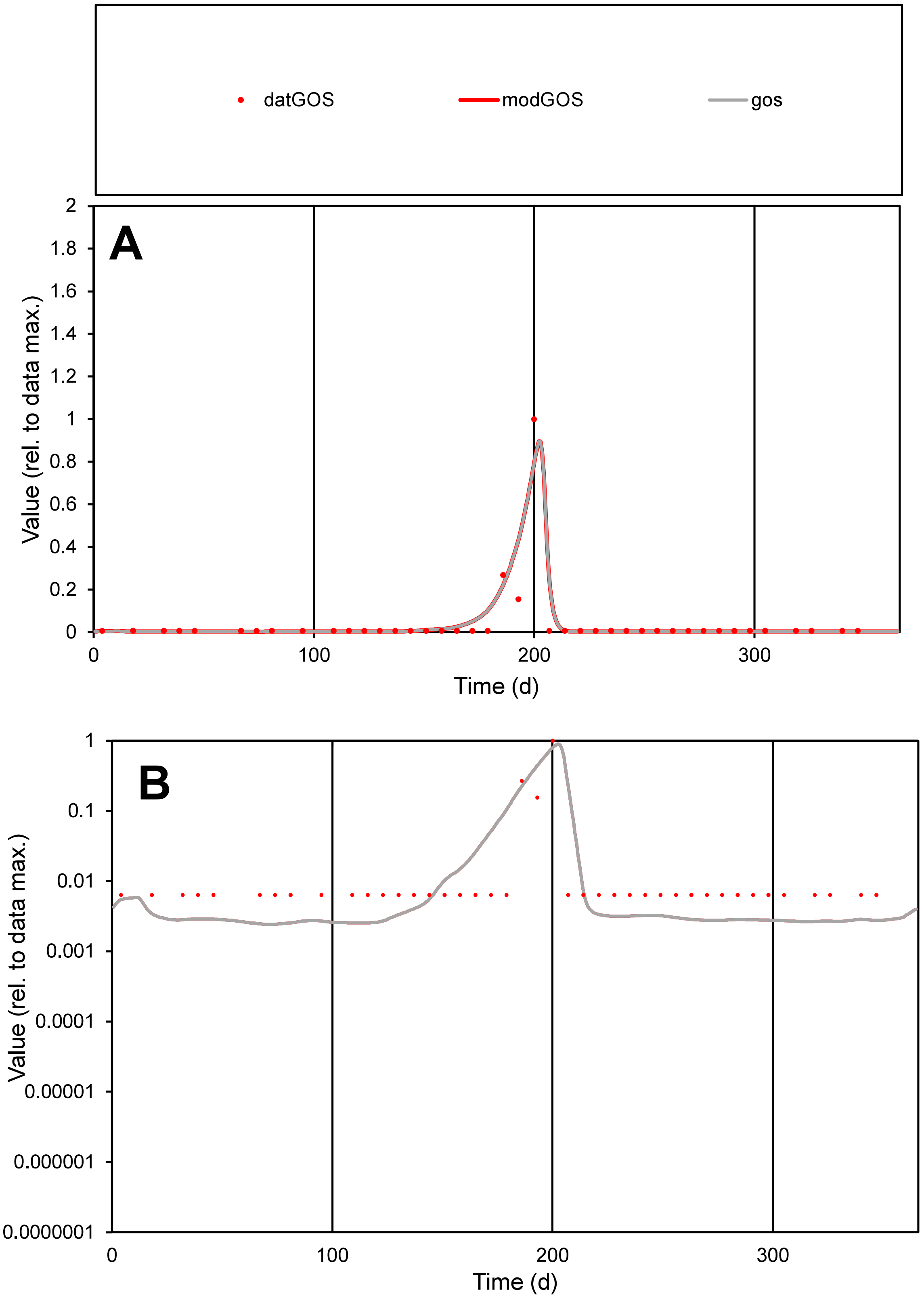

C1

Variables
GOS Provide jtd or Dst
CalibOnly?
2 1 to reproduce of
oNormalize?
2 1=no, 2=yes

ofxn calc*
1.2646
ofxn mod
1.49257

sRun
RunA1
zout
1

Ave
Data
1.1E-05
Model
1.3E-05

data/vMax
0.00028

ofxng
334.07

Min d/m
2.60945

ave/min d
6.24327
ave/min m
18.7724

327_Calib_S_A

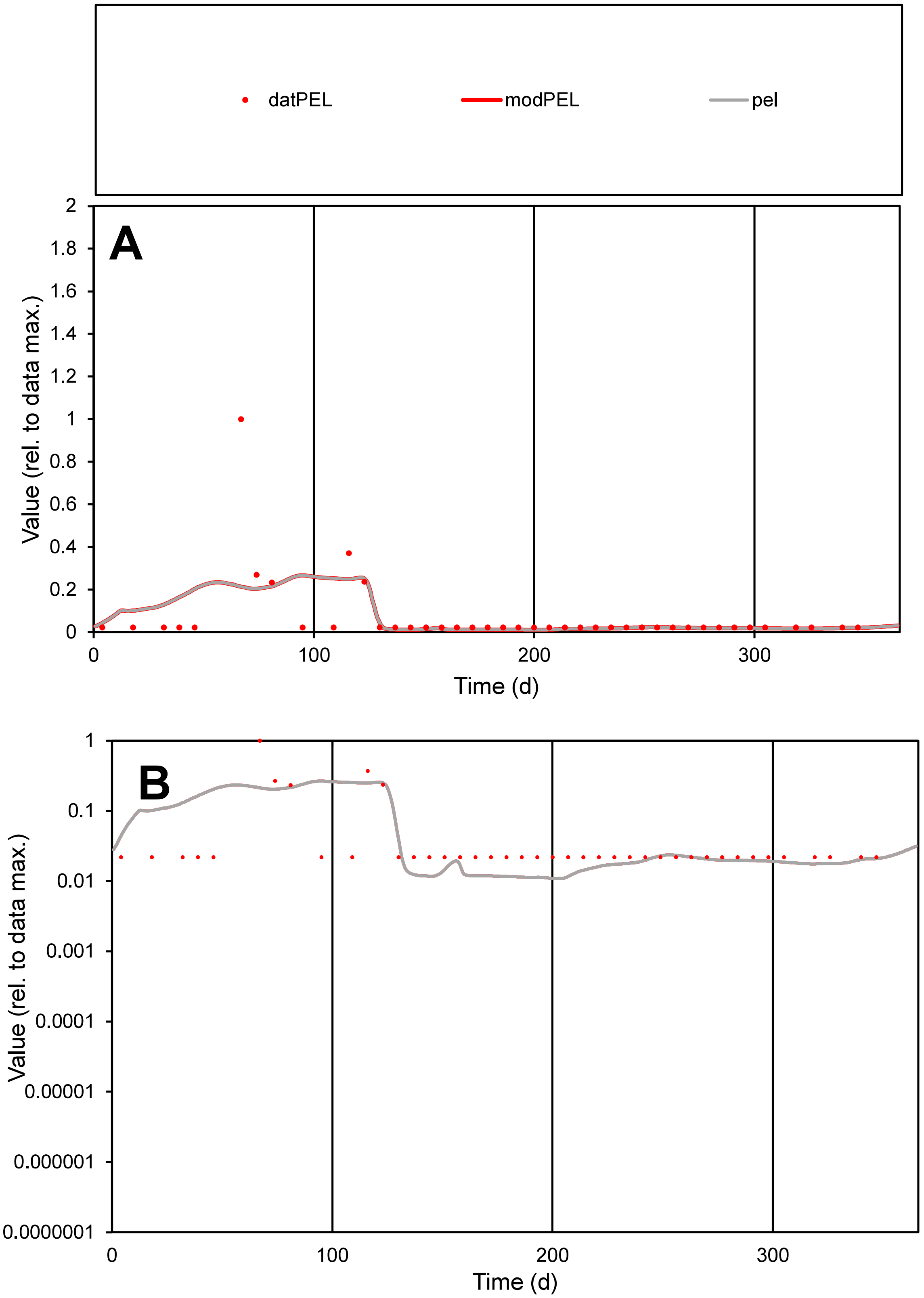

C1

Variables
PEL Provide jtd or Dst
CalibOnly?
2 1 to reproduce of
oNormalize?
2 1=no, 2=yes

ofxn calc*
2.02511
ofxn mod
2.03708

sRun
RunA1
zout
1

Ave
Data
1E-05
Model
1E-05

data/vMax
0.00015

ofxng
334.07

Min d/m
2.01597

ave/min d
3.17681
ave/min m
6.14312

327_Calib_S_A

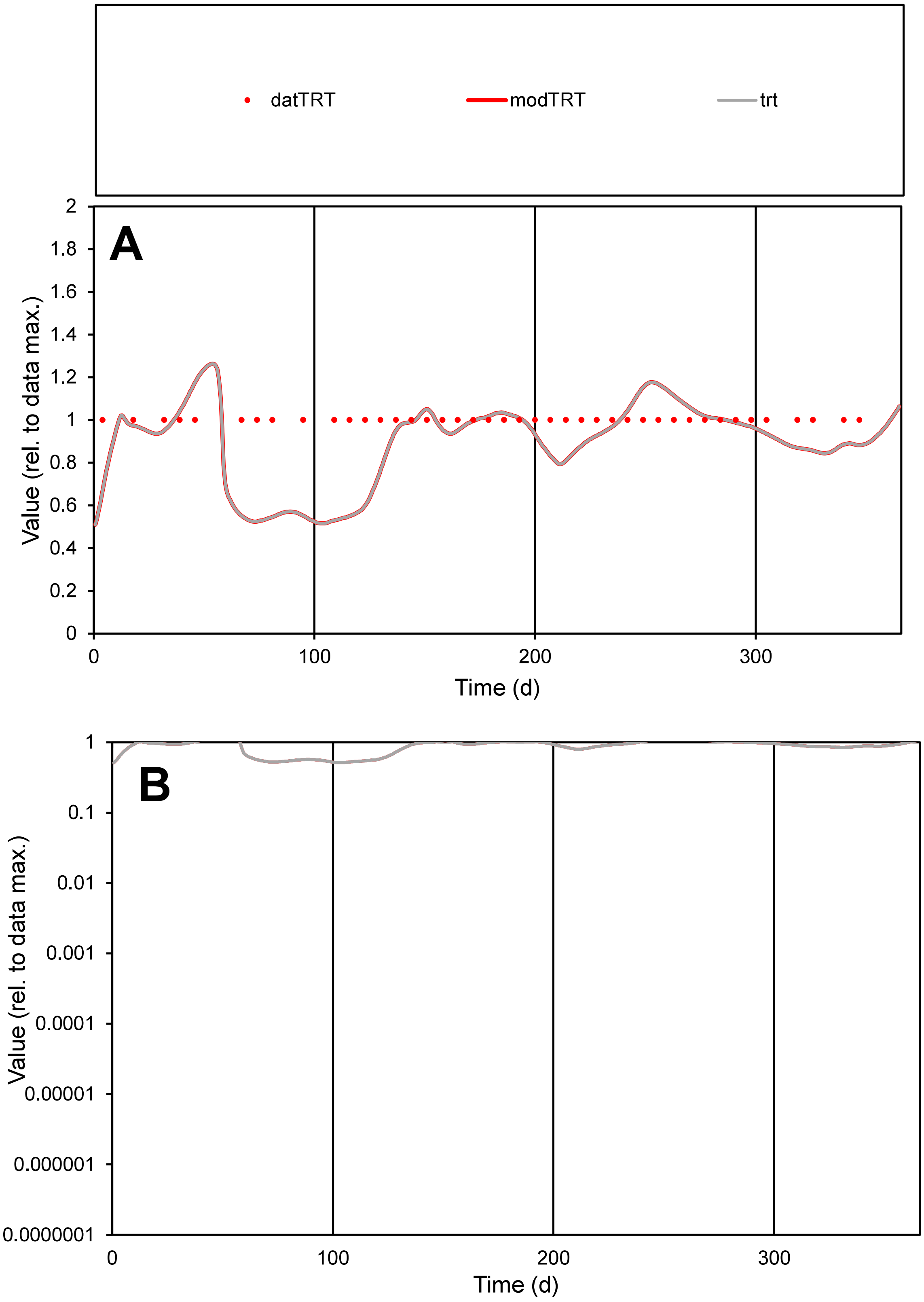

C1

Variables

TRT Provide jtd or Dst

CalibOnly?

2 1 to reproduce of

oNormalize?

2 1=no, 2=yes

ofxn calc*

0.20647

ofxn mod

0.23378

sRun

RunA1

zout

1

Ave

Data

2.3E-05

Model

2.1E-05

data/vMax

2.3E-05

ofxng

334.07

Min d/m

1.88989

ave/min d

1

ave/min m

1.70557

327_Calib_S_A

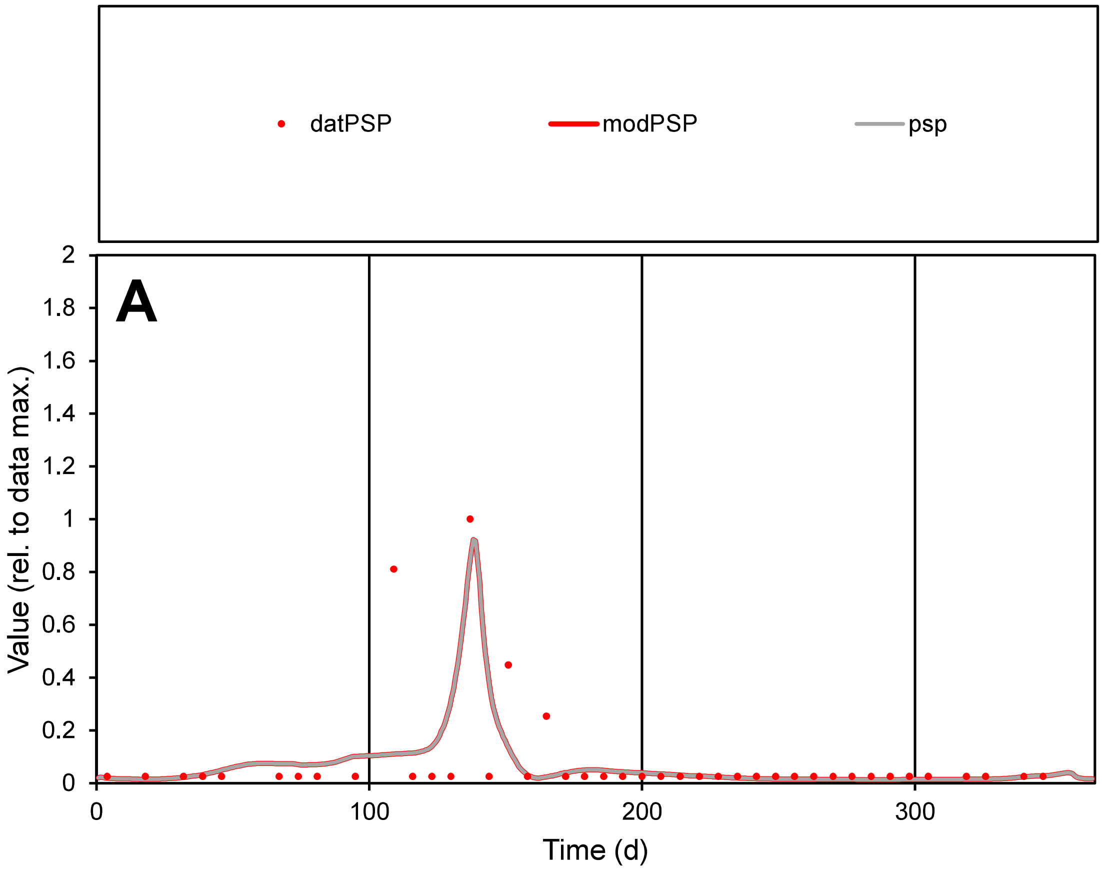

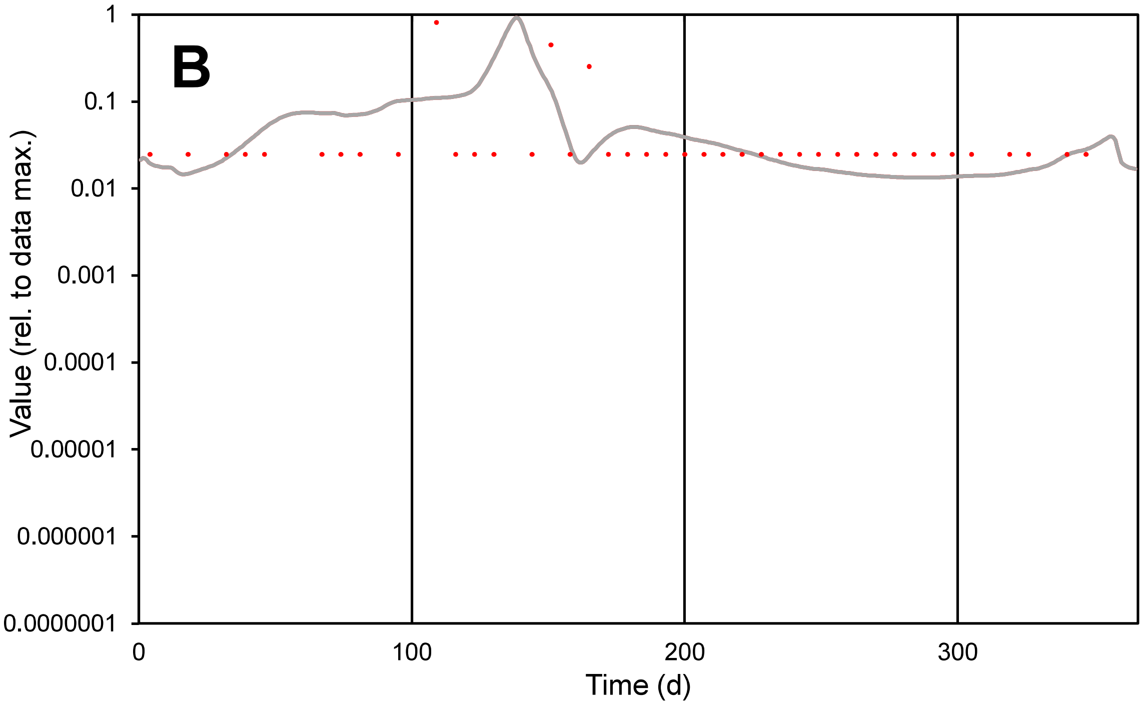

C1

Variables
PSP Provide jtd or Dst
CalibOnly?
2 1 to reproduce of
oNormalize?
2 1=no, 2=yes

ofxn calc*
1.6436
ofxn mod
1.70471

sRun
RunA1
zout
1

Ave
Data
1.4E-05
Model
1.2E-05

data/vMax
0.00018

ofxng
334.07

Min d/m
1.84336

ave/min d
3.3296
ave/min m
5.26512

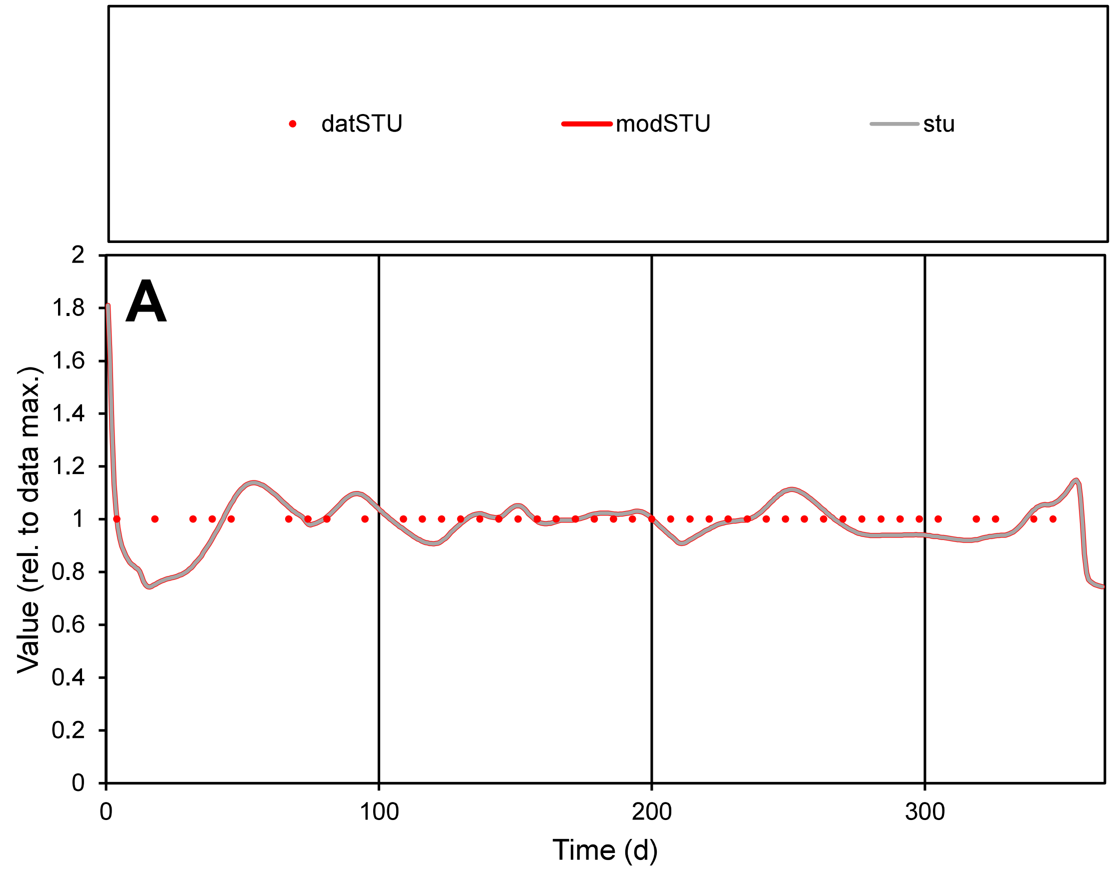

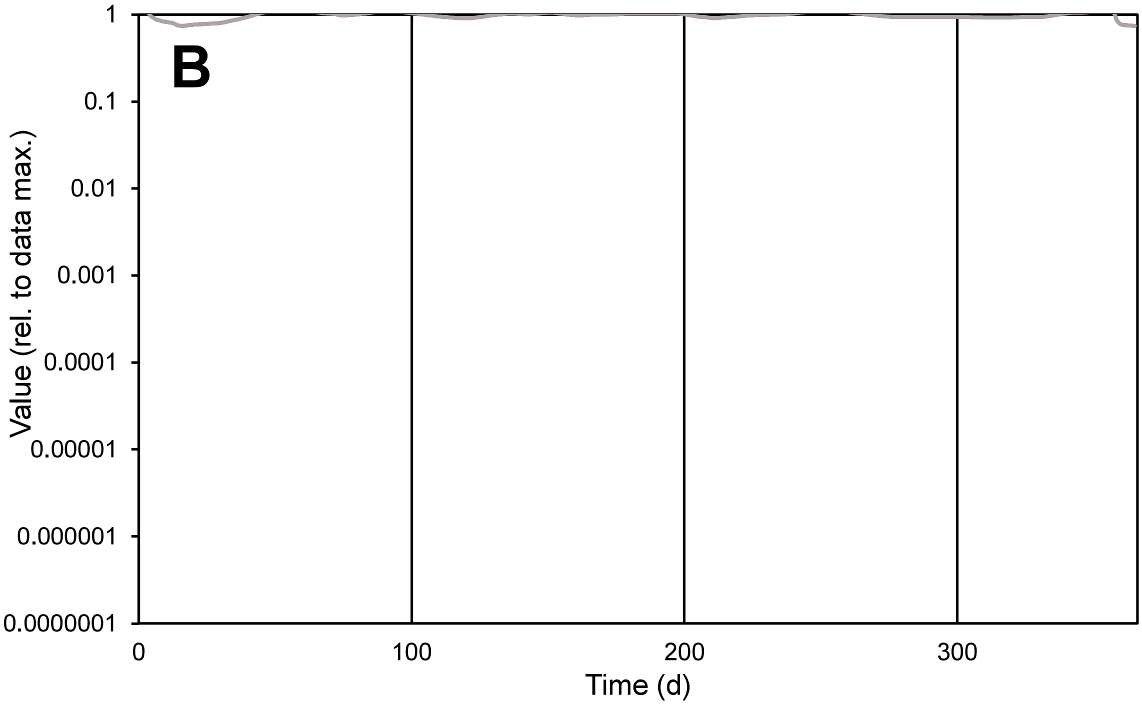

C1

Variables
STU Provide jtd or Dst
CalibOnly?
2 1 to reproduce of
oNormalize?
2 1=no, 2=yes

ofxn calc*
0.06833
ofxn mod
0.13148

sRun
RunA1
zout
1

Ave
Data
2.5E-06
Model
2.5E-06

data/vMax
2.5E-06

ofxng
334.07

Min d/m
1.31142

ave/min d
1
ave/min m
1.29102

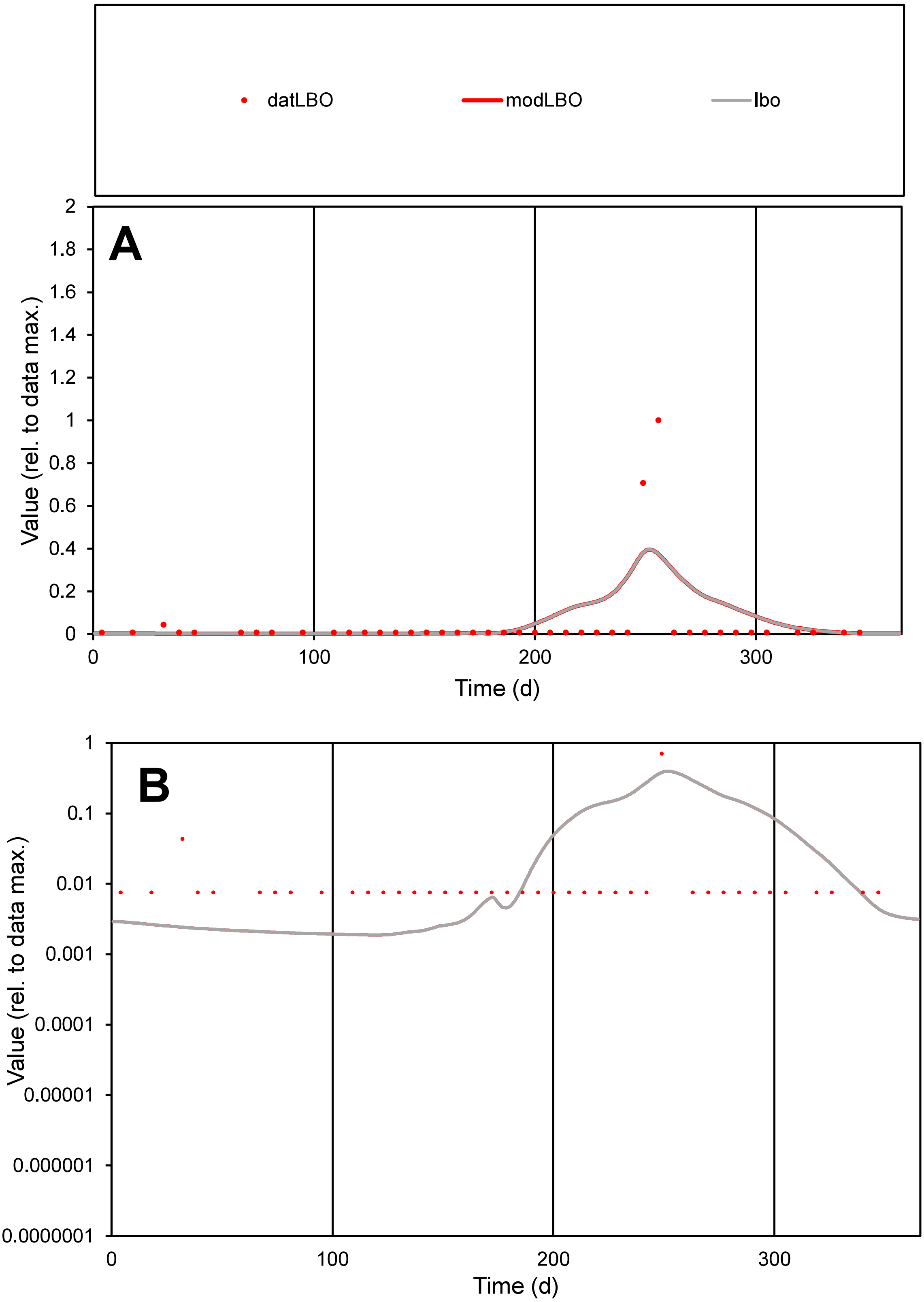

C1

Variables

LBO Provide jtd or Dst

CalibOnly?

2 1 to reproduce of

oNormalize?

2 1=no, 2=yes

ofxn calc*

2.87634

ofxn mod

2.93127

sRun

RunA1

zout

1

Ave

Data

8.9E-06

Model

1.3E-05

data/vMax

0.00018

ofxng

334.07

Min d/m

4.0308

ave/min d

6.43631

ave/min m

36.6842

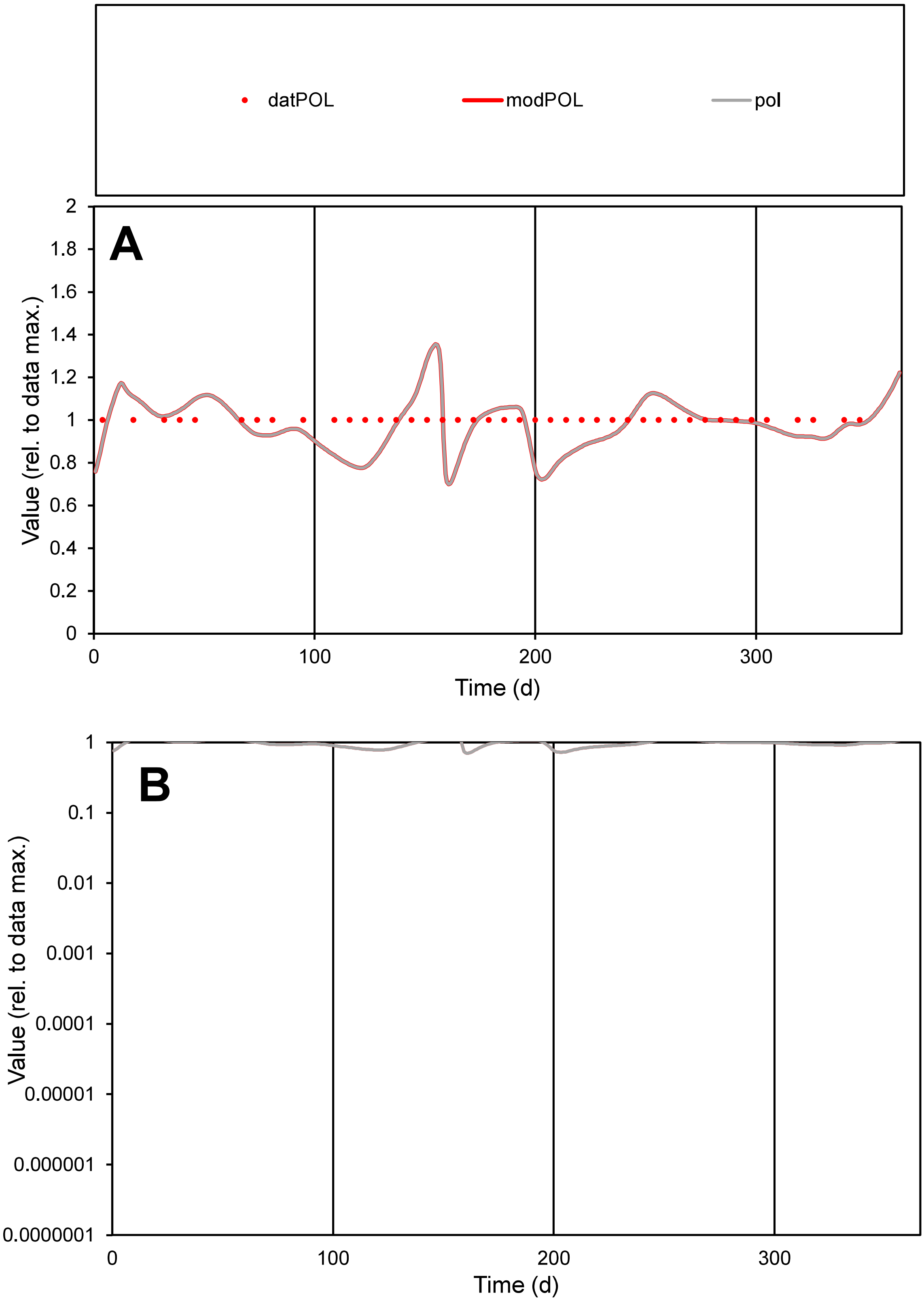

C1

Variables

POL Provide jtd or Dst

CalibOnly?

2 1 to reproduce of

oNormalize?

2 1=no, 2=yes

ofxn calc*

0.11278

ofxn mod

0.16017

sRun

RunA1

zout

1

Ave

Data

1.6E-06

Model

1.6E-06

data/vMax

1.6E-06

ofxng

334.07

Min d/m

1.3423

ave/min d

1

ave/min m

1.3041

327_Calib_S_A

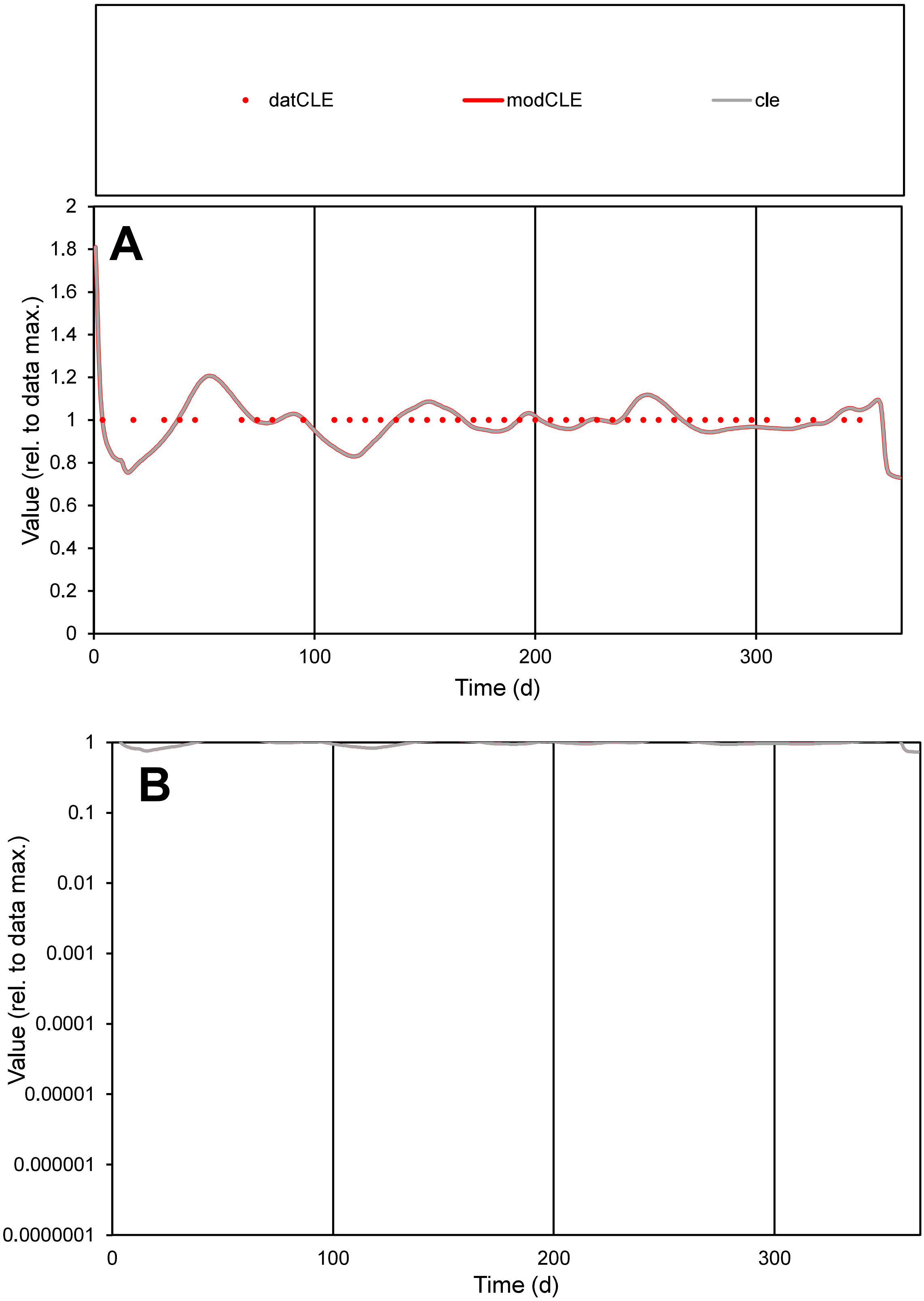

C1

Variables
CLE Provide jtd or Dst
CalibOnly?
2 1 to reproduce of
oNormalize?
2 1=no, 2=yes

ofxn calc*
0.07256
ofxn mod
0.13393

sRun
RunA1
zout
1

Ave
Data
4.7E-06
Model
4.6E-06

data/vMax
4.7E-06

ofxng
334.07

Min d/m
1.26135

ave/min d
1
ave/min m
1.24765

327_Calib_S_A

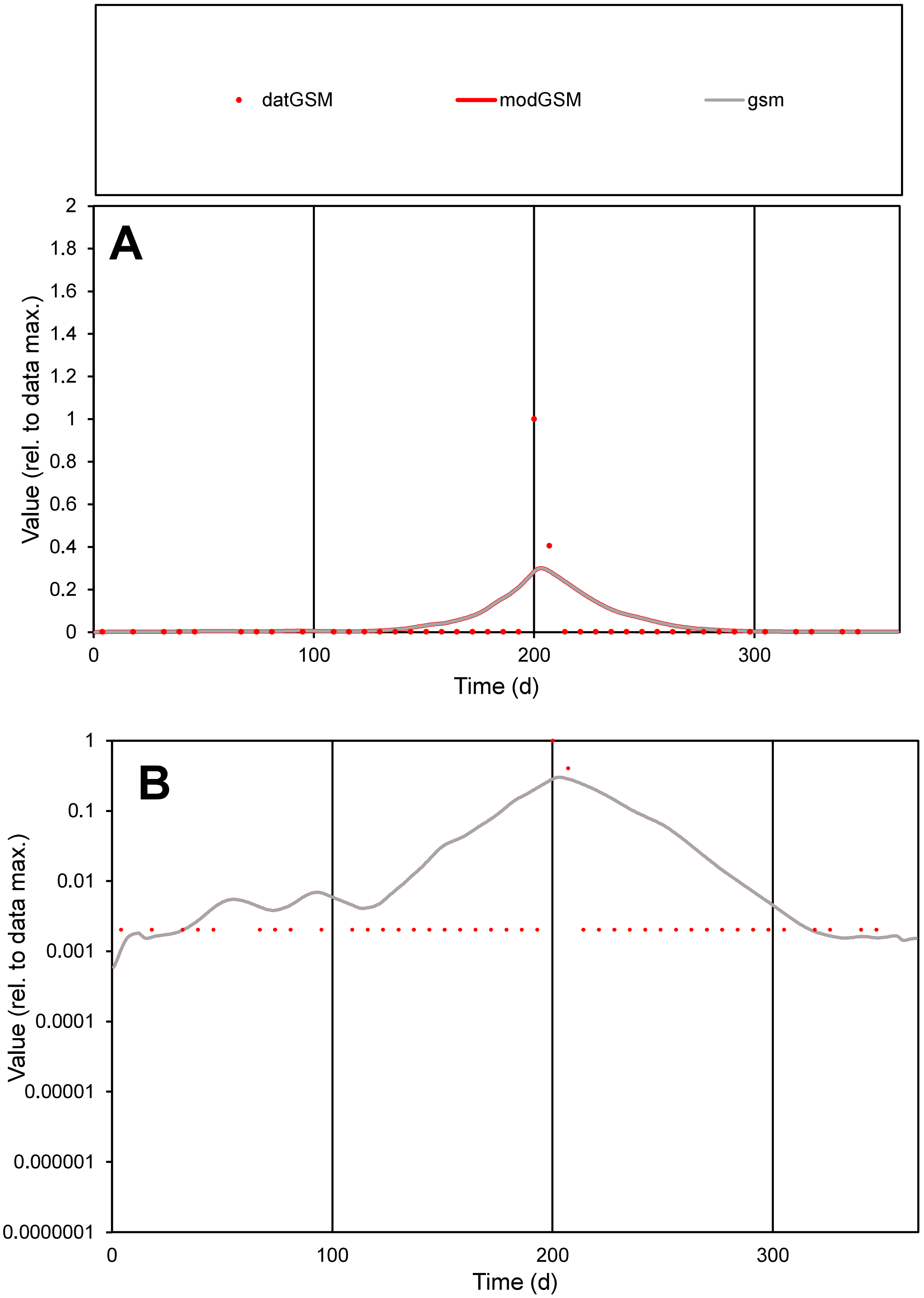

C1

Variables
GSM Provide jtd or Dst
CalibOnly?
2 1 to reproduce of
oNormalize?
2 1=no, 2=yes

ofxn calc*
3.67926
ofxn mod
3.74514

sRun
RunA1
zout
1

Ave
Data
2.8E-05
Model
4.1E-05

data/vMax
0.00078

ofxng
334.07

Min d/m
1.63458

ave/min d
17.4592
ave/min m
42.7189

327_Calib_S_A

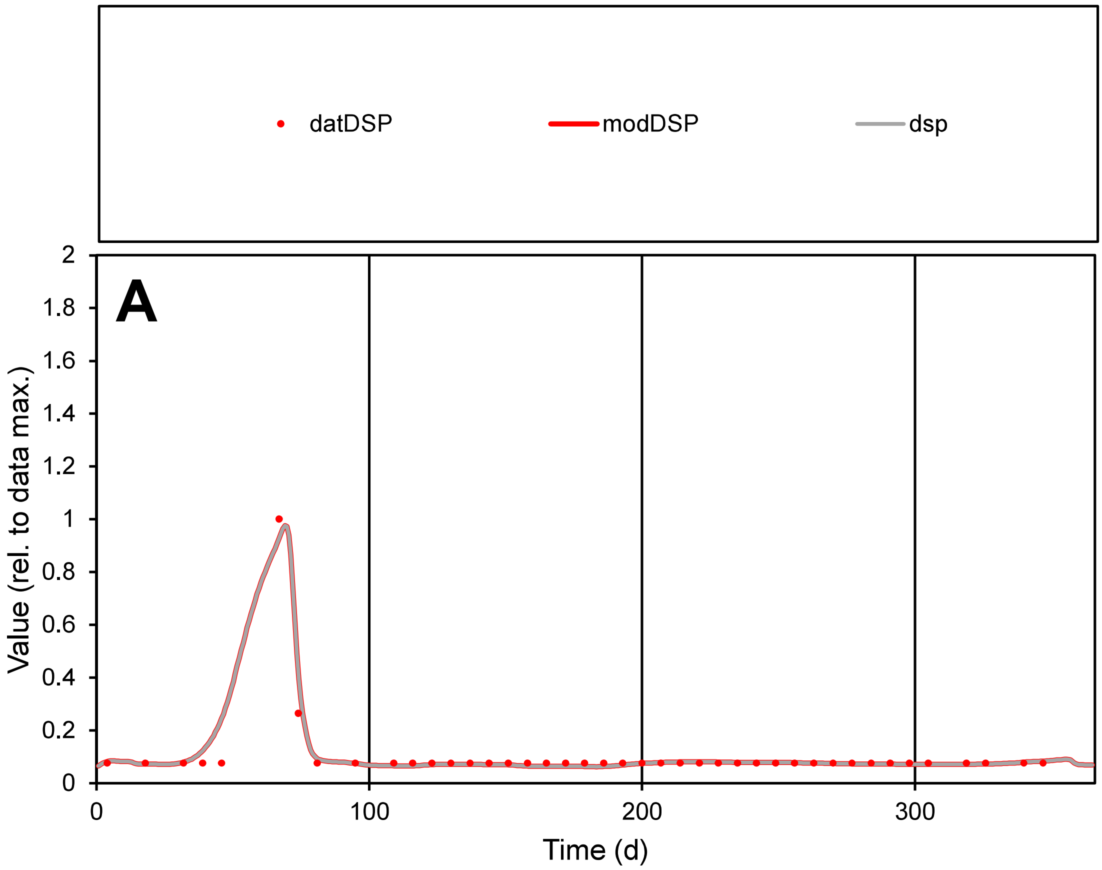

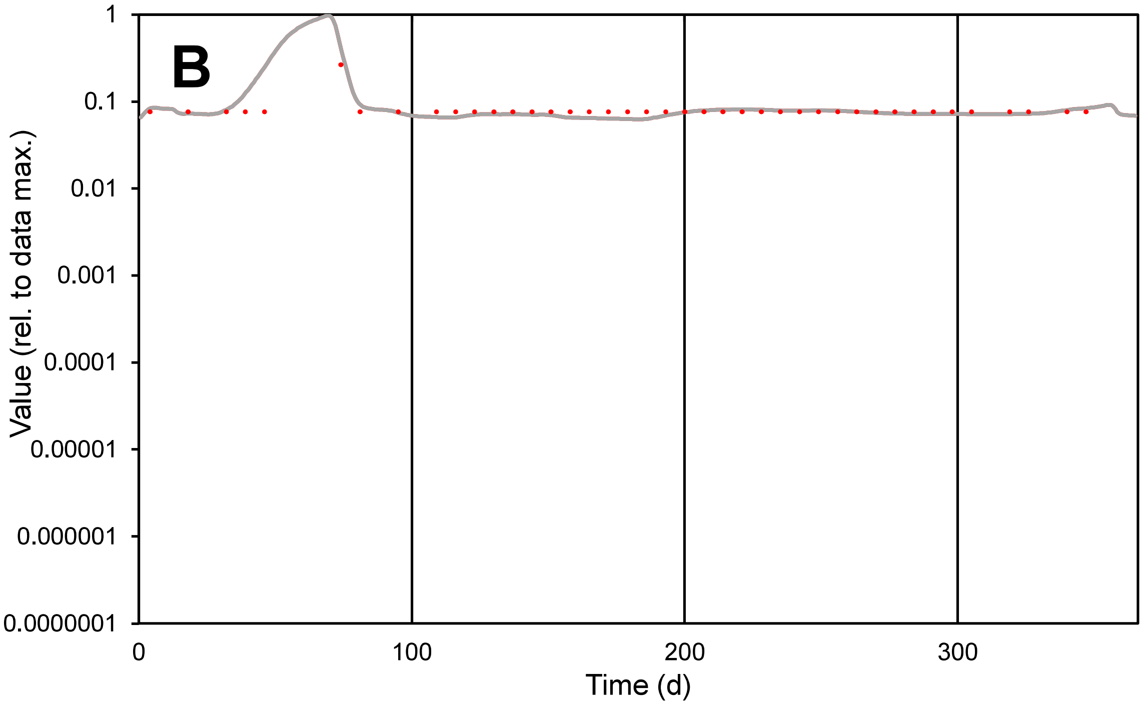

C1

Variables

DSP Provide jtd or Dst

CalibOnly?

2 1 to reproduce of

oNormalize?

2 1=no, 2=yes

ofxn calc*

0.25063

ofxn mod

0.35577

sRun

RunA1

zout

1

Ave

Data

6.7E-06

Model

6.9E-06

data/vMax

6.6E-05

ofxng

334.07

Min d/m

1.2104

ave/min d

1.34751

ave/min m

1.6603

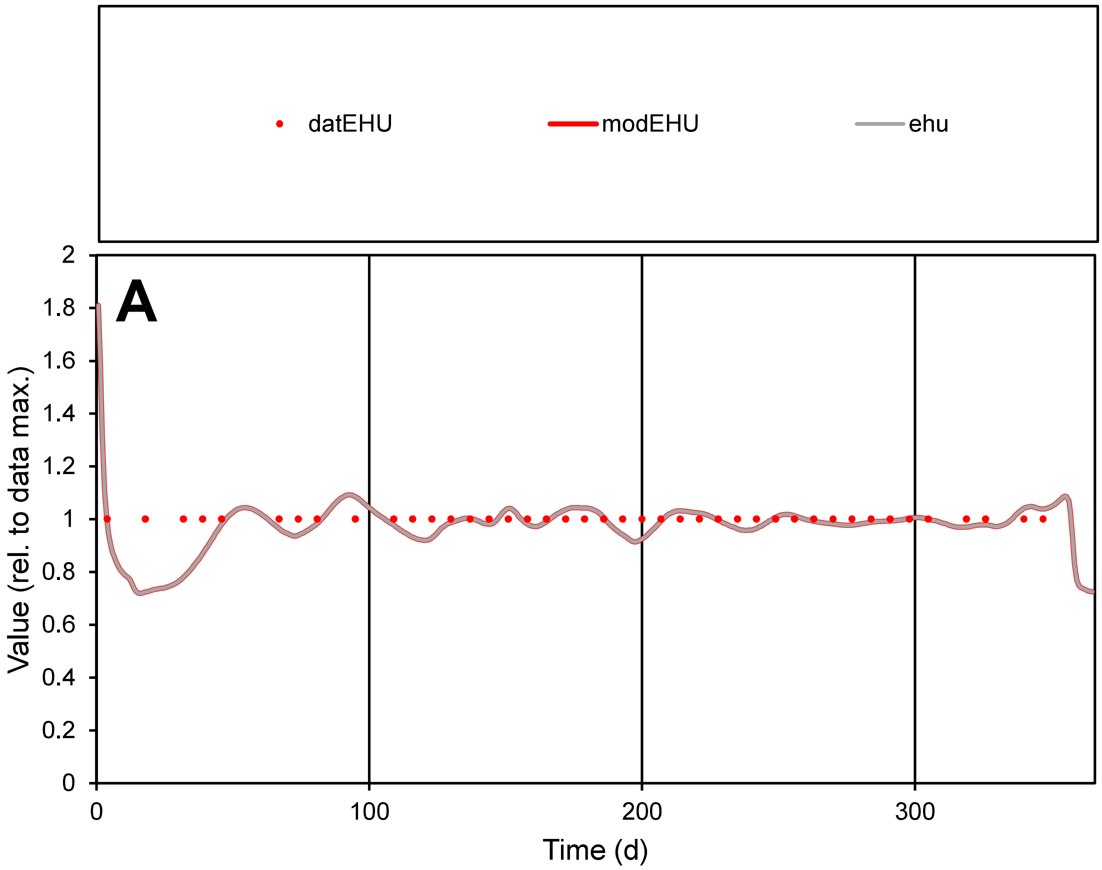

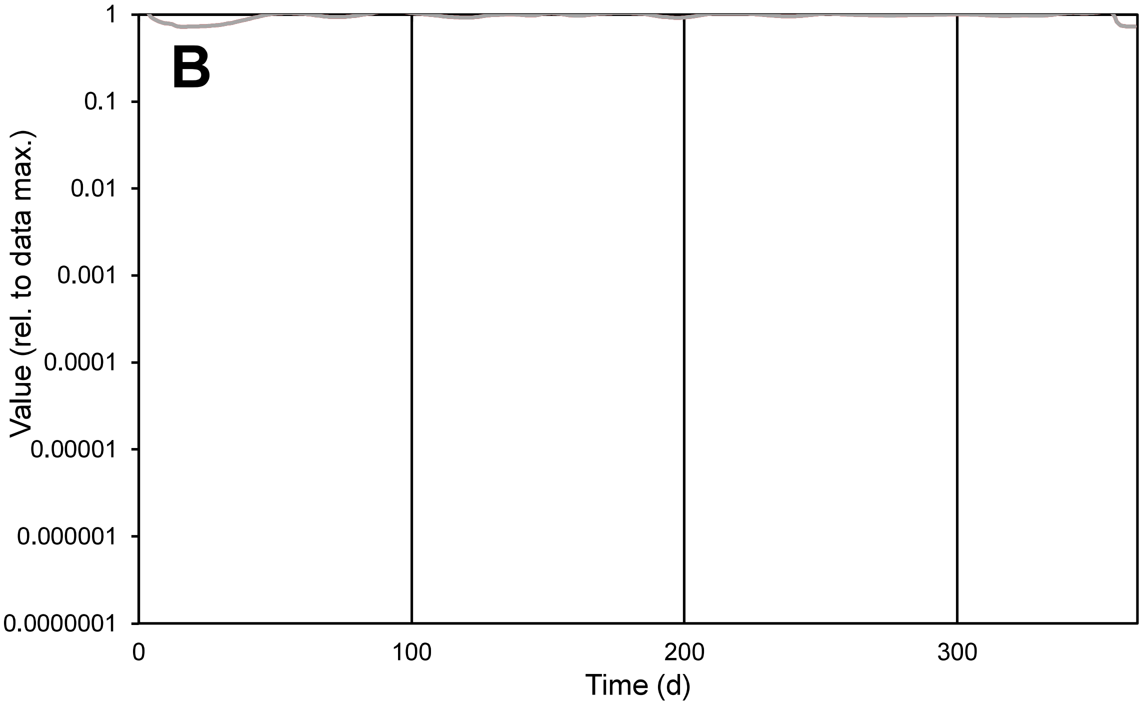

C1

Variables
EHU Provide jtd or Dst
CalibOnly?
2 1 to reproduce of
oNormalize?
2 1=no, 2=yes

ofxn calc*
0.06551
ofxn mod
0.12938

sRun
RunA1
zout
1

Ave
Data
5.8E-06
Model
5.6E-06

data/vMax
5.8E-06

ofxng
334.07

Min d/m
1.37089

ave/min d
1
ave/min m
1.33996

327_Calib_S_A

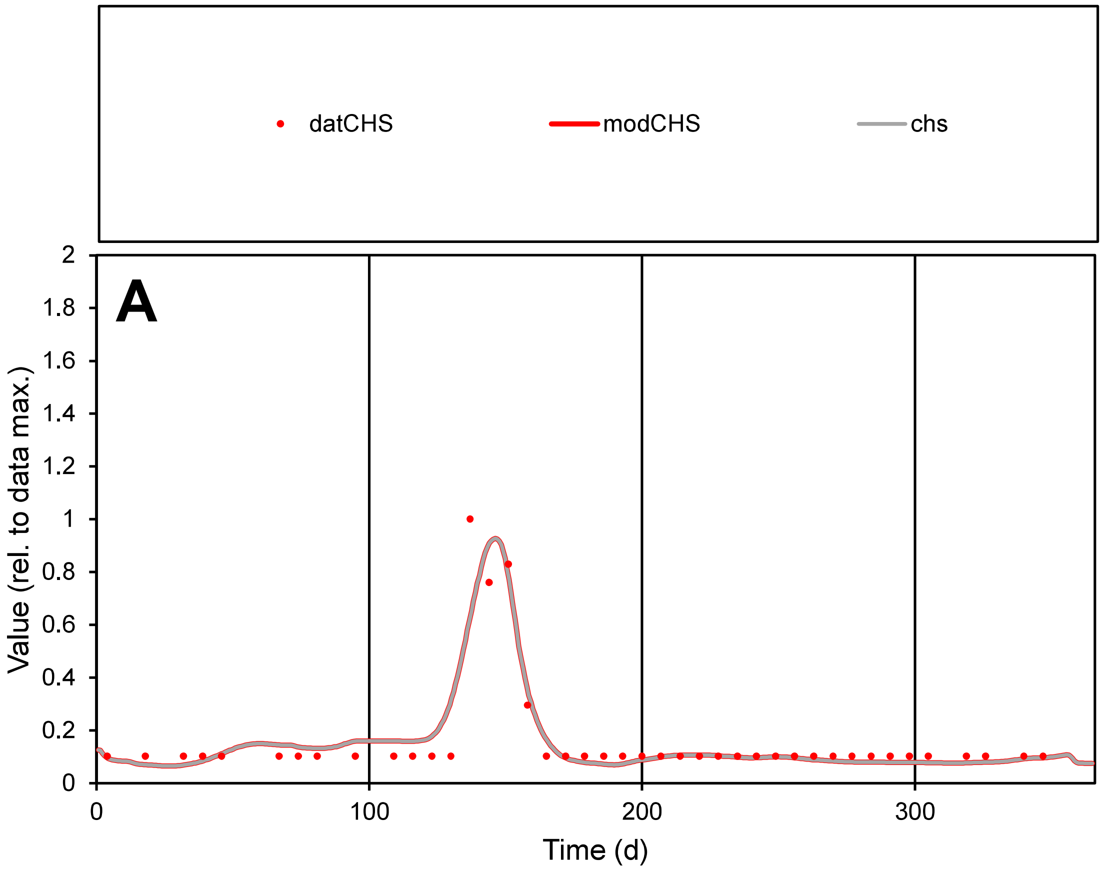

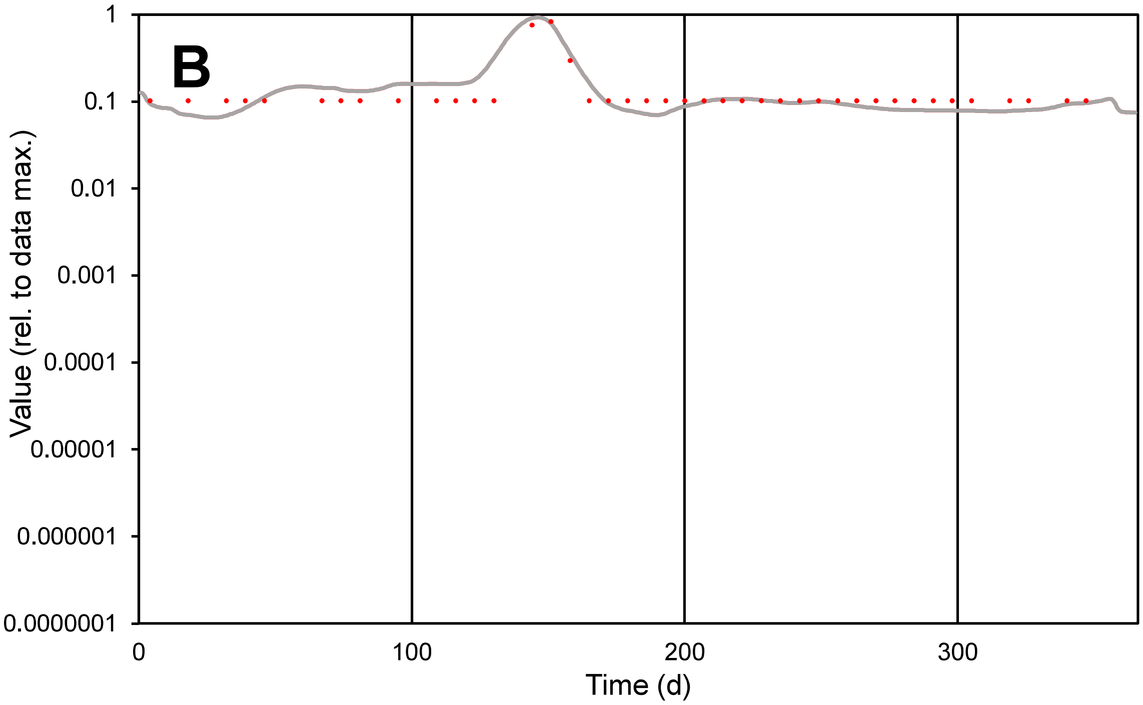

C1

Variables
CHS Provide jtd or Dst
CalibOnly?
2 1 to reproduce of
oNormalize?
2 1=no, 2=yes

ofxn calc*
0.43488
ofxn mod
0.46475

sRun
RunA1
zout
1

Ave
Data
3.4E-05
Model
3.4E-05

data/vMax
0.00021

ofxng
334.07

Min d/m
1.4701

ave/min d
1.57954
ave/min m
2.31283

327_Calib_S_A

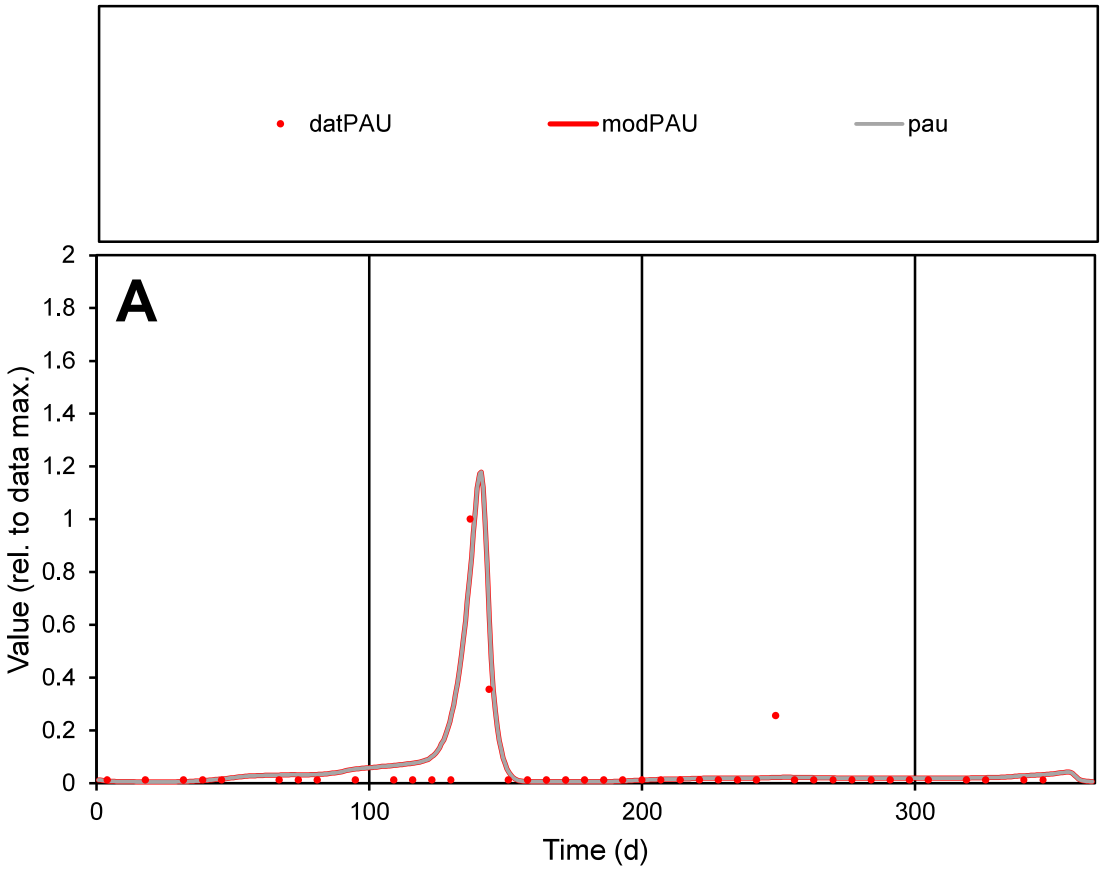

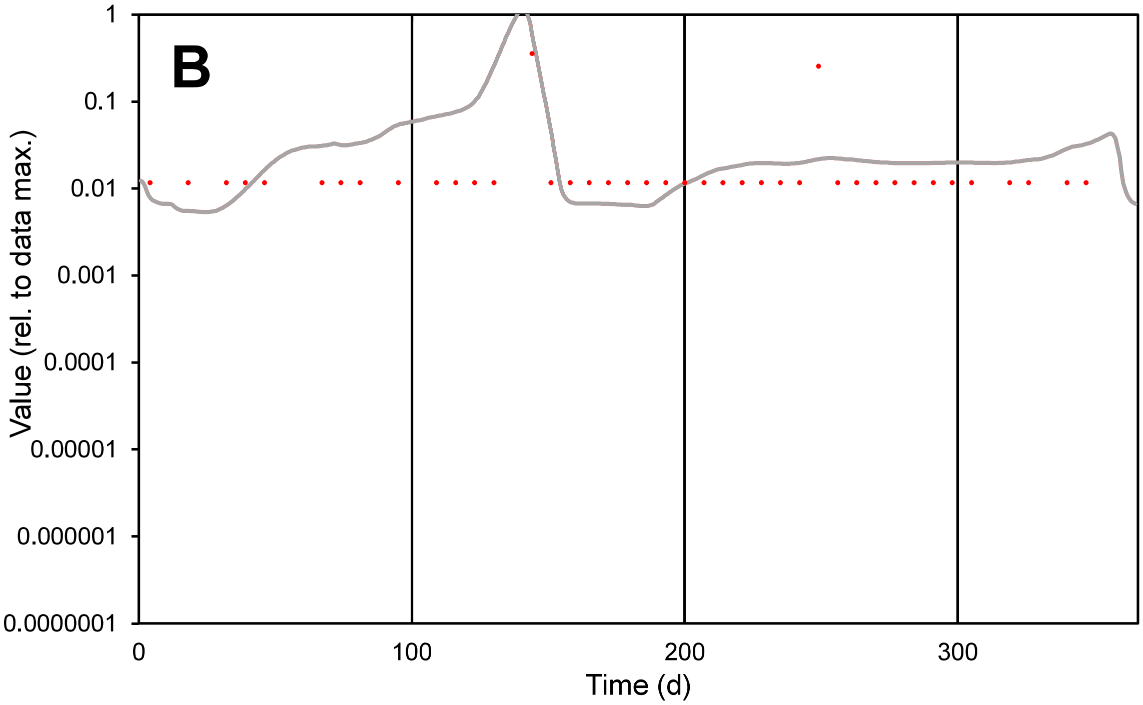

C1

Variables

PAU Provide jtd or Dst

CalibOnly?

2 1 to reproduce of

oNormalize?

2 1=no, 2=yes

ofxn calc*

1.02213

ofxn mod

1.12244

sRun

RunA1

zout

1

Ave

Data

1.7E-05

Model

1.9E-05

data/vMax

0.00034

ofxng

334.07

Min d/m

2.1279

ave/min d

4.20563

ave/min m

10.3668

327_Calib_S_A

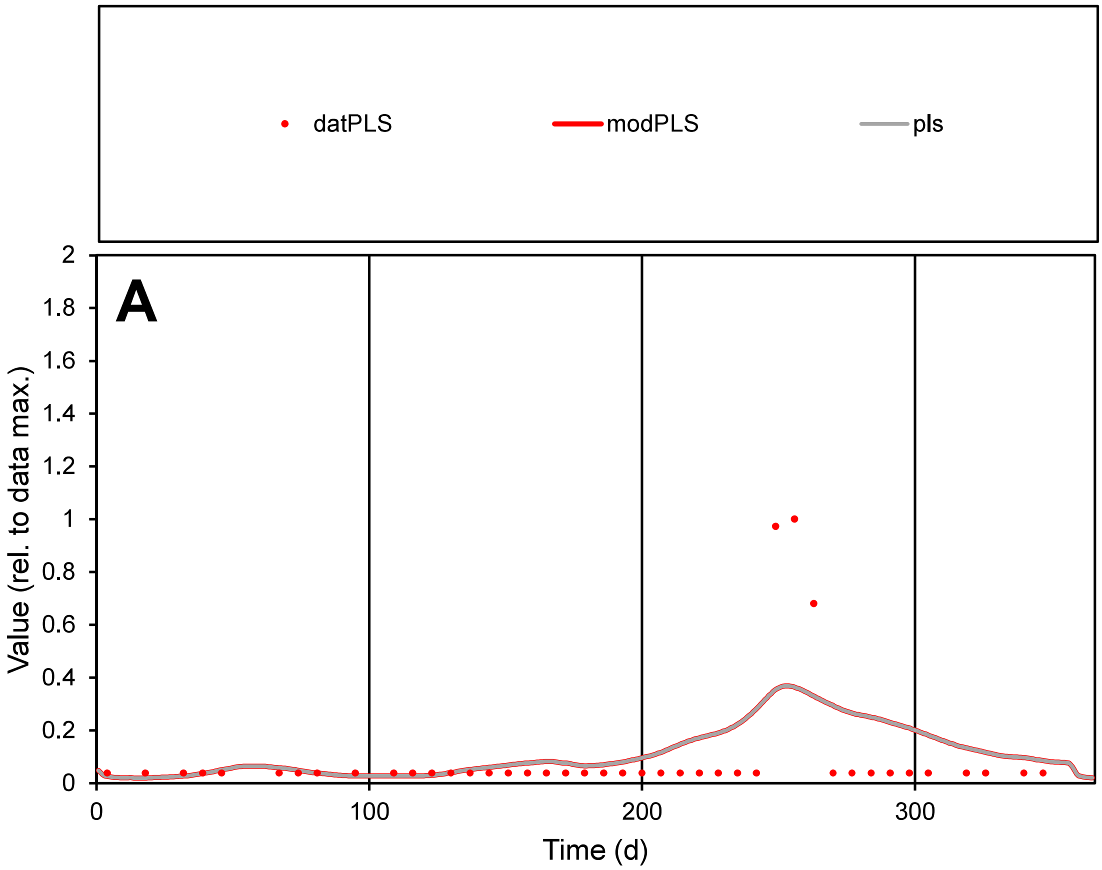

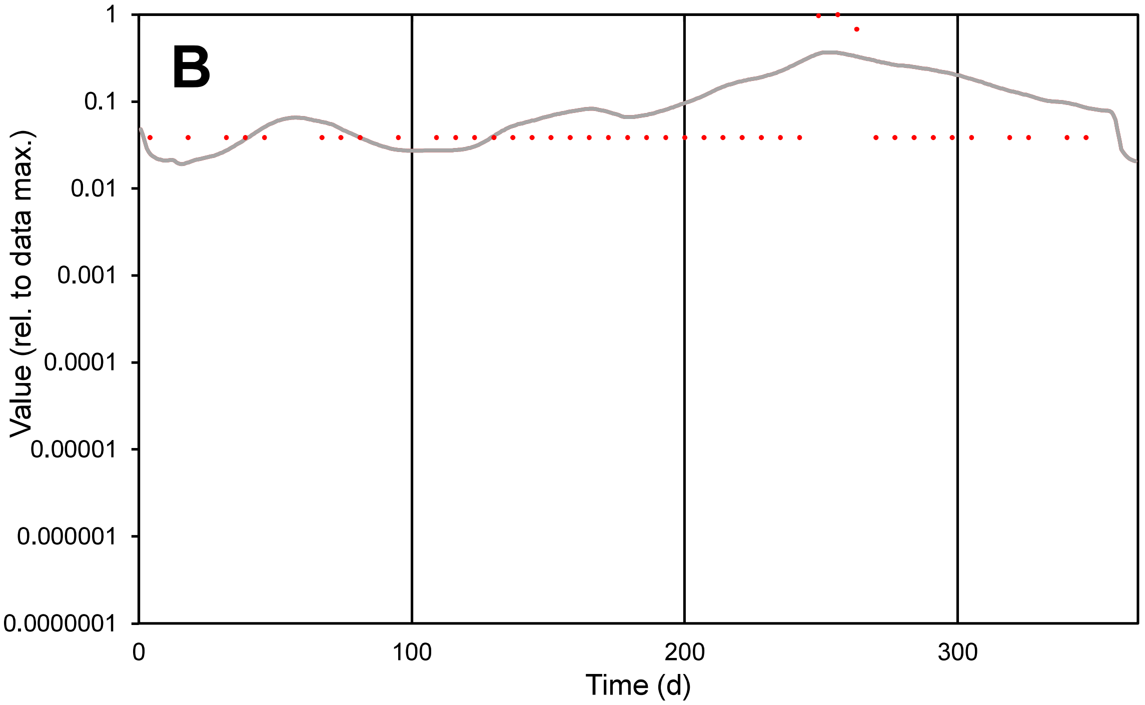

C1

Variables
PLS Provide jtd or Dst
CalibOnly?
2 1 to reproduce of
oNormalize?
2 1=no, 2=yes

ofxn calc*
1.79649
ofxn mod
1.81588

sRun
RunA1
zout
1

Ave
Data
9.8E-06
Model
1.2E-05

data/vMax
9.9E-05

ofxng
334.07

Min d/m
1.86185

ave/min d
2.55798
ave/min m
5.97681

327_Calib_S_A

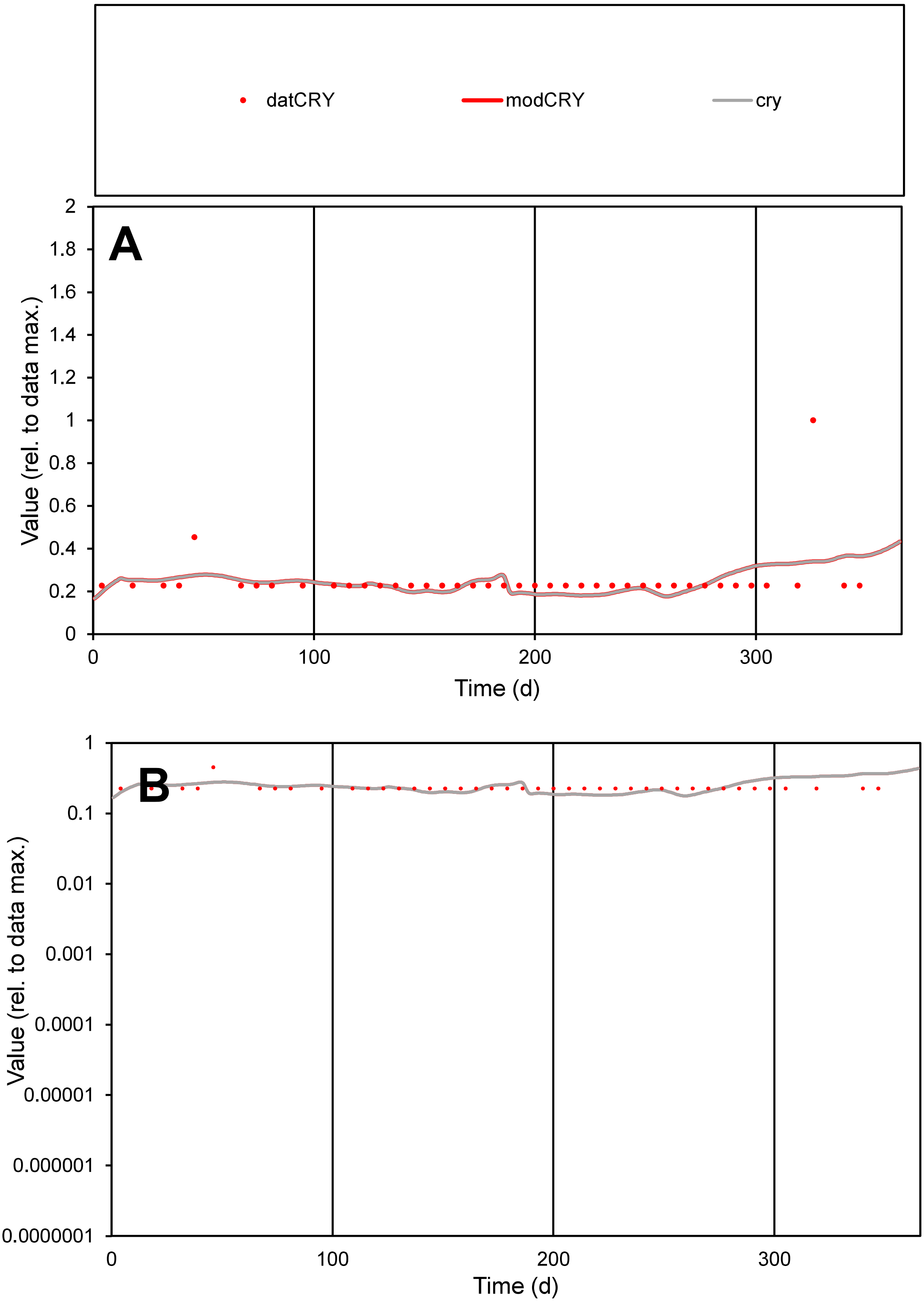

C1

Variables	
CRY	Provide jtd or Dst
CalibOnly?	
2	1 to reproduce of
oNormalize?	
2	1=no, 2=yes
ofxn calc*	
0.46299	
ofxn mod	
0.47679	
sRun	
RunA1	
zout	
1	
Ave Data	
3.3E-06	
Model	
3.2E-06	
data/vMax	
1.3E-05	
ofxng	
334.07	
Min d/m	
1.24728	
ave/min d	
1.10493	
ave/min m	
1.32265	

327_Calib_S_A

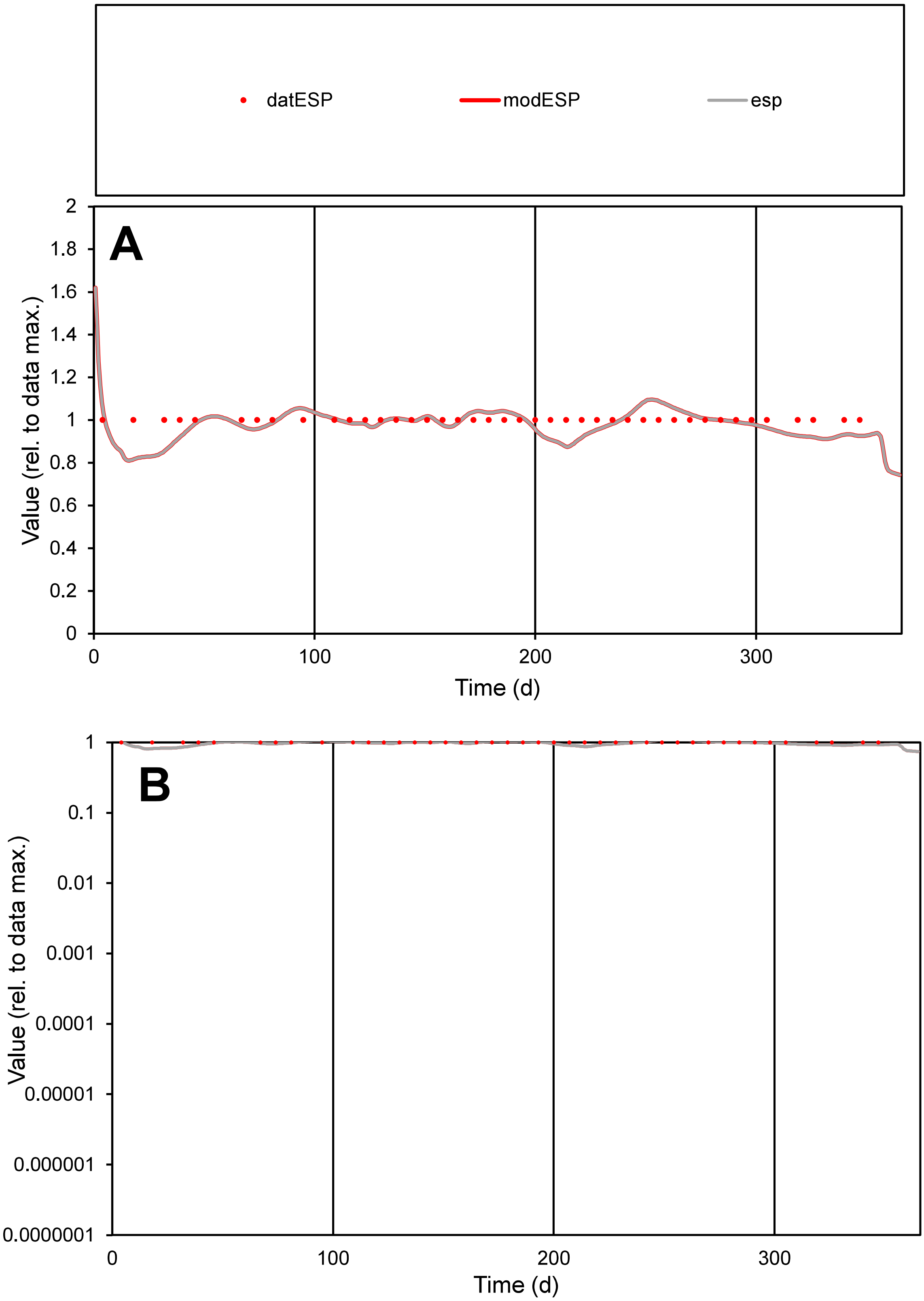

C1

Variables
ESP Provide jtd or Dst
CalibOnly?
2 1 to reproduce of
oNormalize?
2 1=no, 2=yes

ofxn calc*
0.05884
ofxn mod
0.12651

sRun
RunA1
zout
1

Ave
Data
8E-06
Model
7.9E-06

data/vMax
8E-06

ofxng
334.07

Min d/m
1.21653

ave/min d
1
ave/min m
1.19297

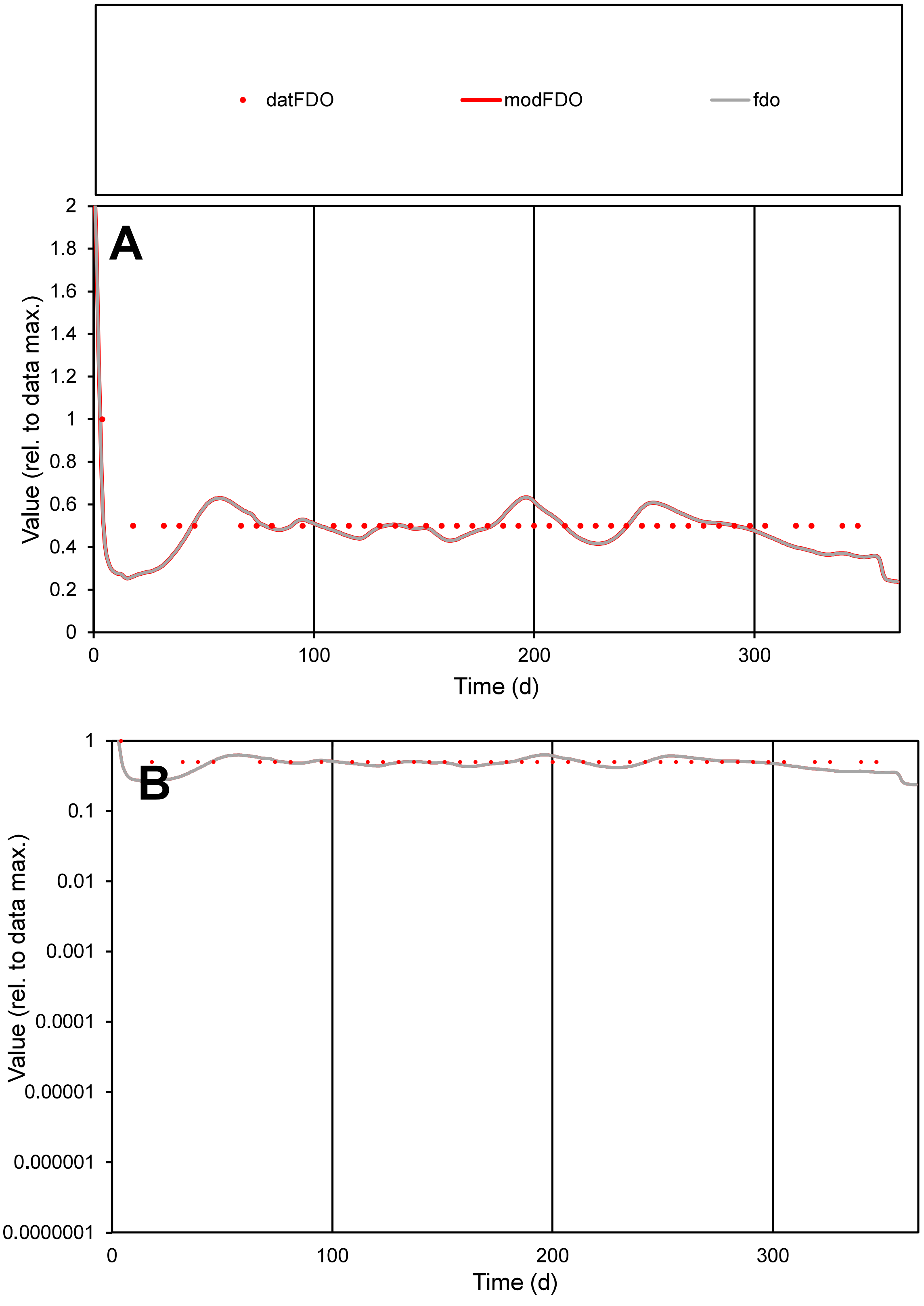

C1

Variables

FDO Provide jtd or Dst

CalibOnly?

2 1 to reproduce of

oNormalize?

2 1=no, 2=yes

ofxn calc*

0.17149

ofxn mod

0.22659

sRun

RunA1

zout

1

Ave

Data

1E-07

Model

9.7E-08

data/vMax

2E-07

ofxng

334.07

Min d/m

1.856

ave/min d

1.02382

ave/min m

1.81103

327_Calib_S_A

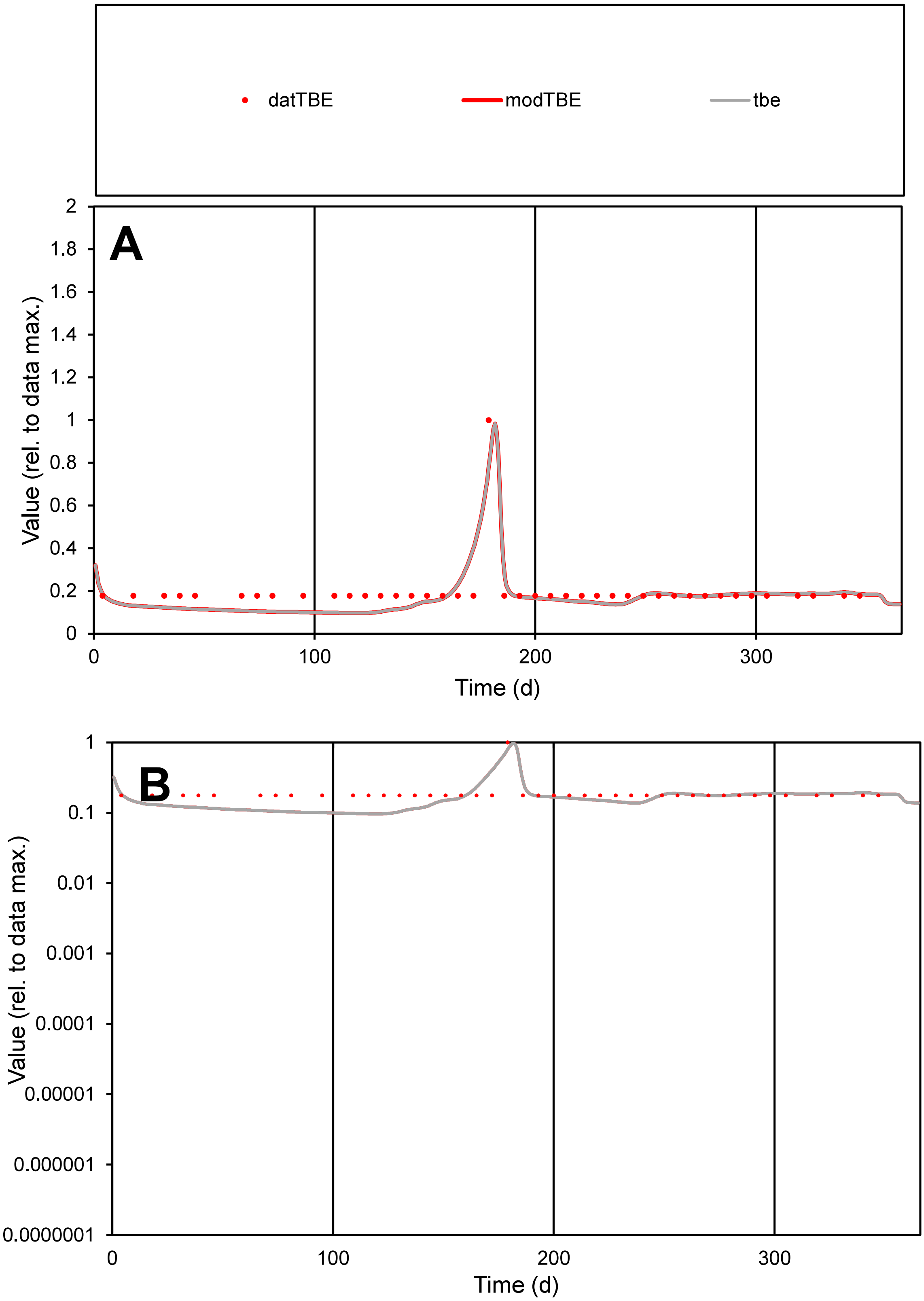

C1

Variables
TBE Provide jtd or Dst
CalibOnly?
2 1 to reproduce of
oNormalize?
2 1=no, 2=yes

ofxn calc*
0.2929
ofxn mod
0.32417

sRun
RunA1
zout
1

Ave
Data
7.4E-06
Model
6.7E-06

data/vMax
3.8E-05

ofxng
334.07

Min d/m
1.82927

ave/min d
1.11081
ave/min m
1.83147

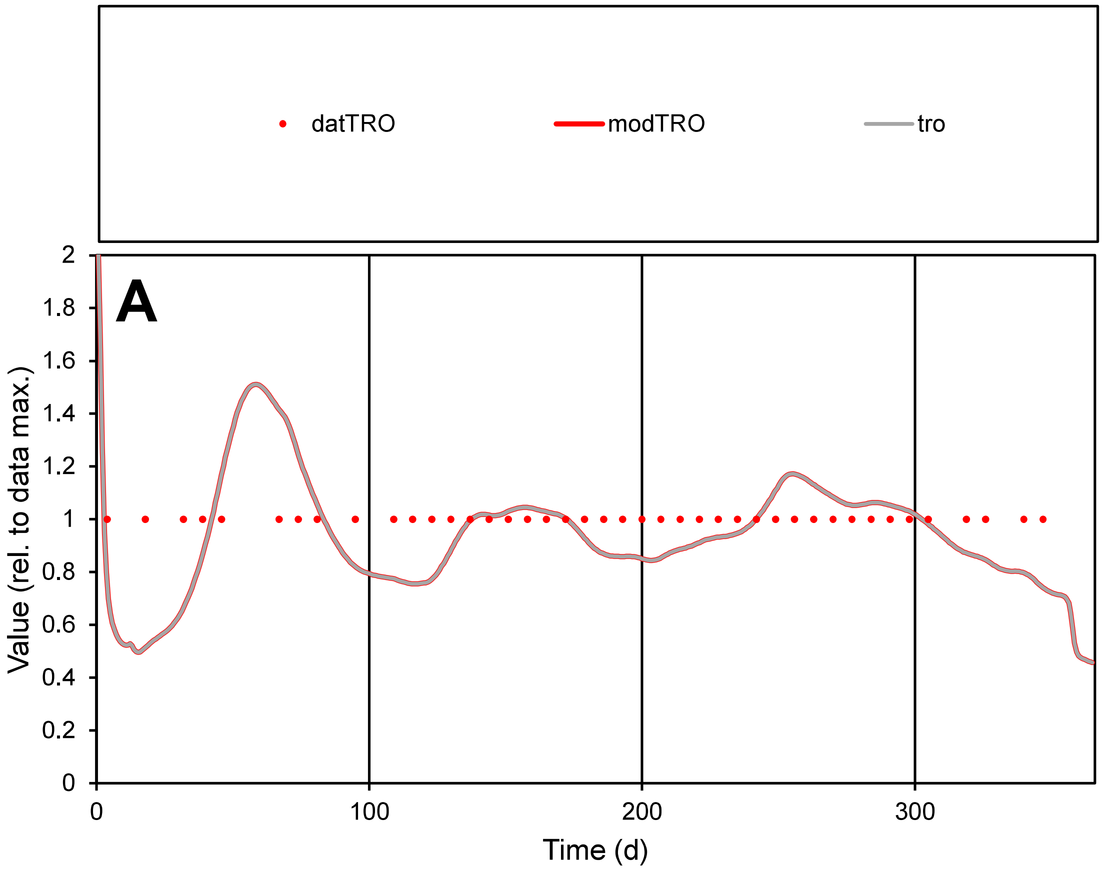

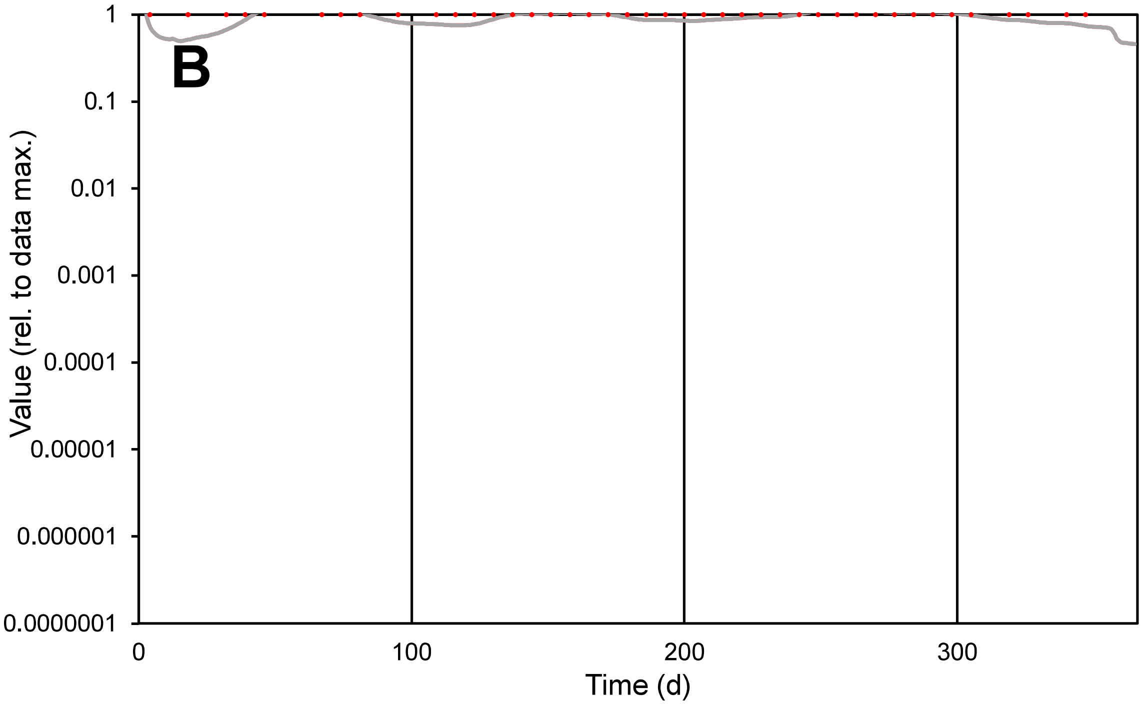

C1

Variables
TRO Provide jtd or Dst
CalibOnly?
2 1 to reproduce of
oNormalize?
2 1=no, 2=yes

ofxn calc*
0.16979
ofxn mod
0.20363

sRun
RunA1
zout
1

Ave
Data
5E-06
Model
4.8E-06

data/vMax
5E-06

ofxng
334.07

Min d/m
1.8881

ave/min d
1
ave/min m
1.78996

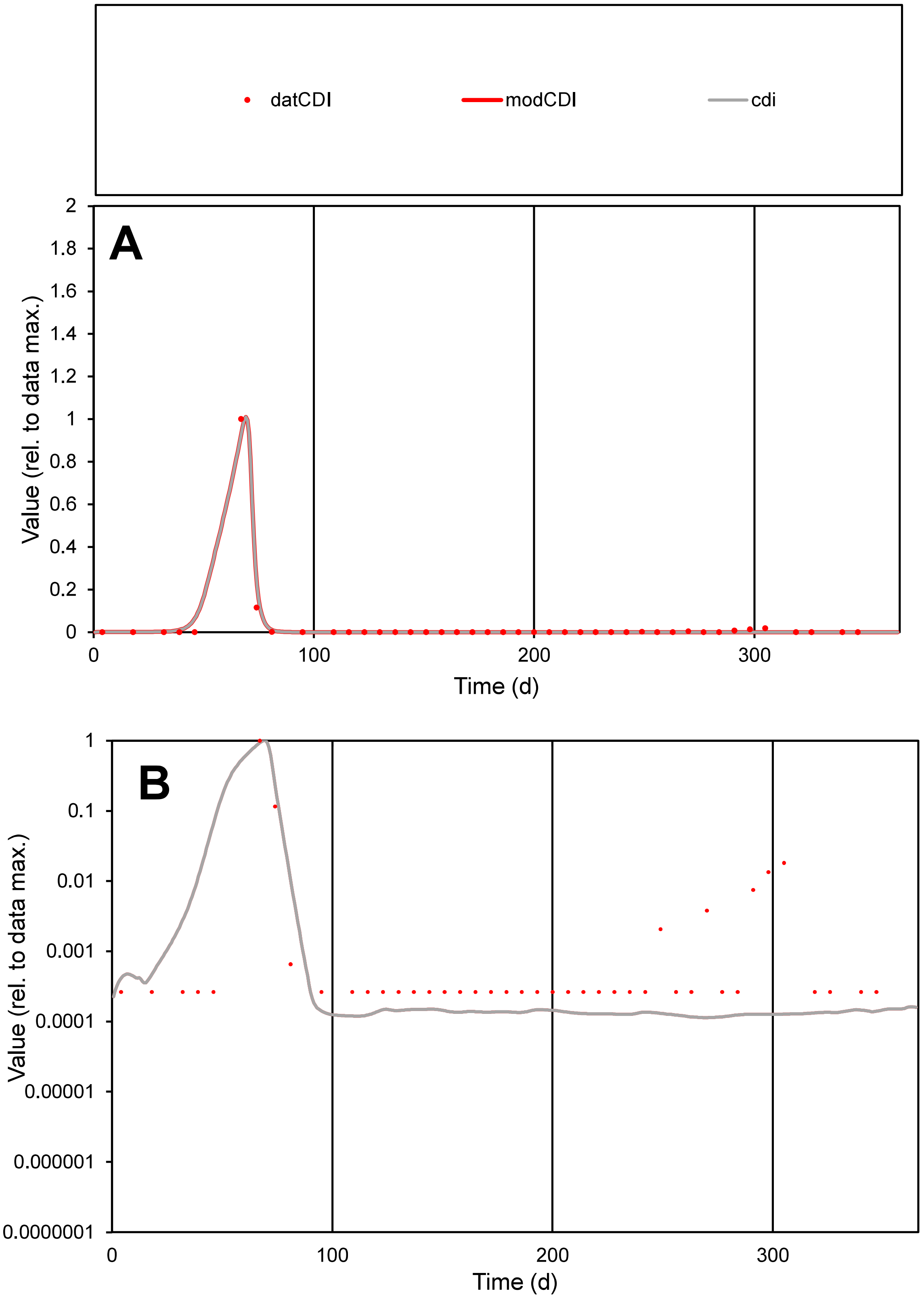

C1

Variables
CDI Provide jtd or Dst
CalibOnly?
2 1 to reproduce of
oNormalize?
2 1=no, 2=yes

ofxn calc*
0.27648
ofxn mod
0.77399

sRun
RunA1
zout
1

Ave
Data
1.8E-05
Model
1.8E-05

data/vMax
0.00066

ofxng
334.07

Min d/m
2.30749

ave/min d
106.087
ave/min m
244.182

327_Calib_S_A

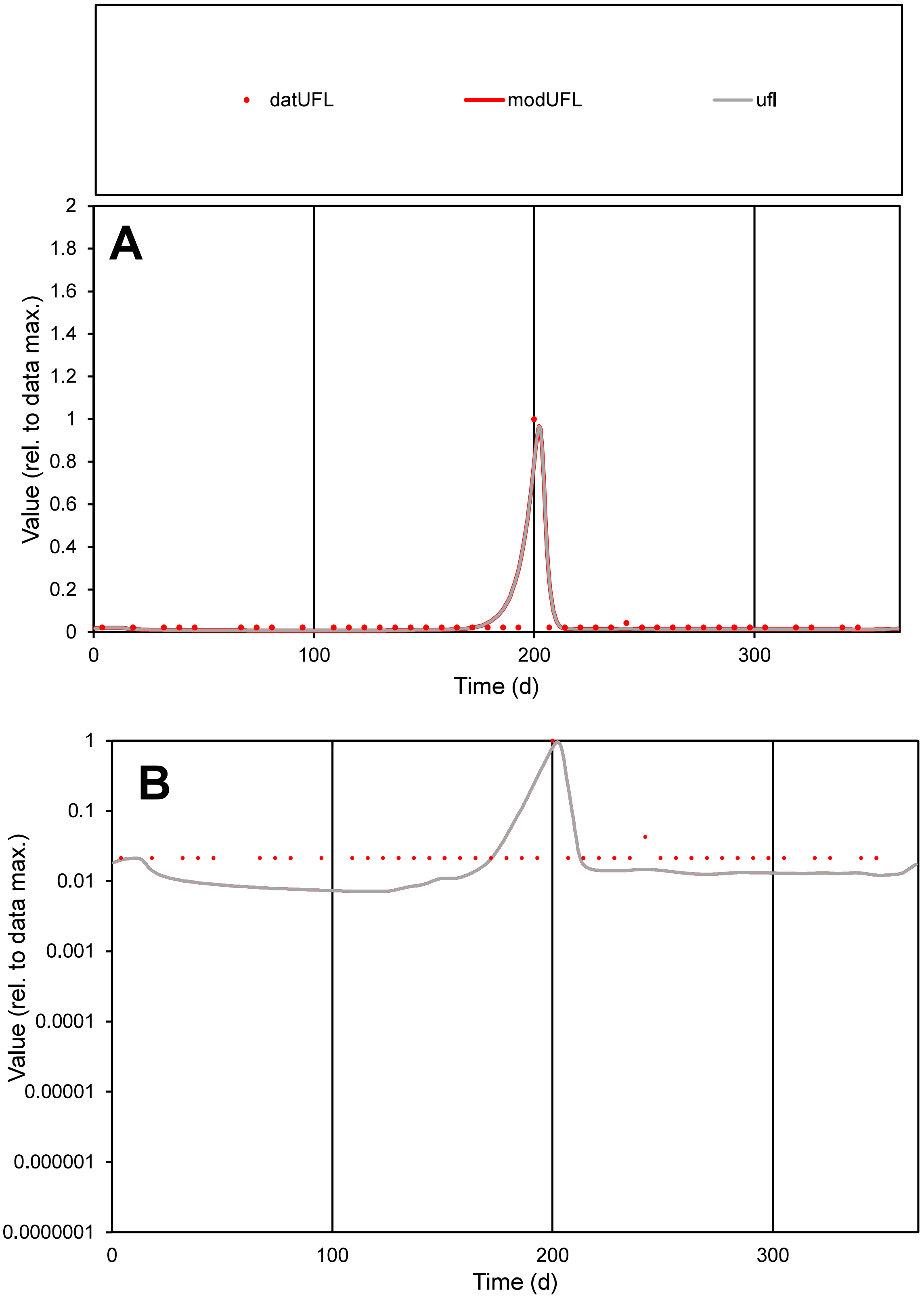

C1

Variables

UFL Provide jtd or Dst

CalibOnly?

2 1 to reproduce of

oNormalize?

2 1=no, 2=yes

ofxn calc*

0.85917

ofxn mod

1.10545

sRun

RunA1

zout

1

Ave

Data

6.5E-06

Model

6.2E-06

data/vMax

0.00014

ofxng

334.07

Min d/m

3.00786

ave/min d

2.1135

ave/min m

6.08839

327_Calib_S_A

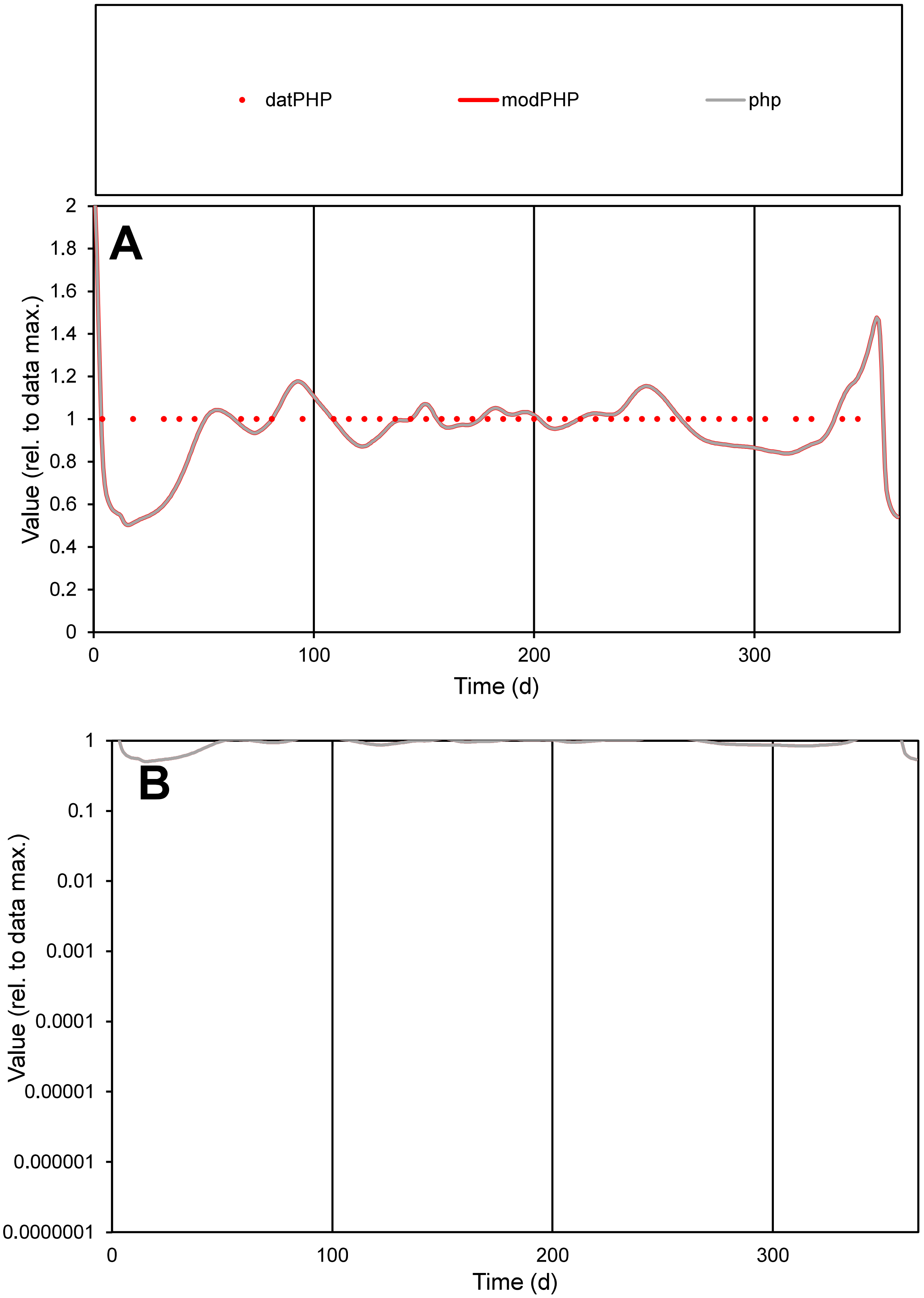

C1

Variables
PHP Provide jtd or Dst
CalibOnly?
2 1 to reproduce of
oNormalize?
2 1=no, 2=yes

ofxn calc*
0.13782
ofxn mod
0.17773

sRun
RunA1
zout
1

Ave
Data
6.9E-06
Model
6.7E-06

data/vMax
6.9E-06

ofxng
334.07

Min d/m
1.91687

ave/min d
1
ave/min m
1.8373

327_Calib_S_A

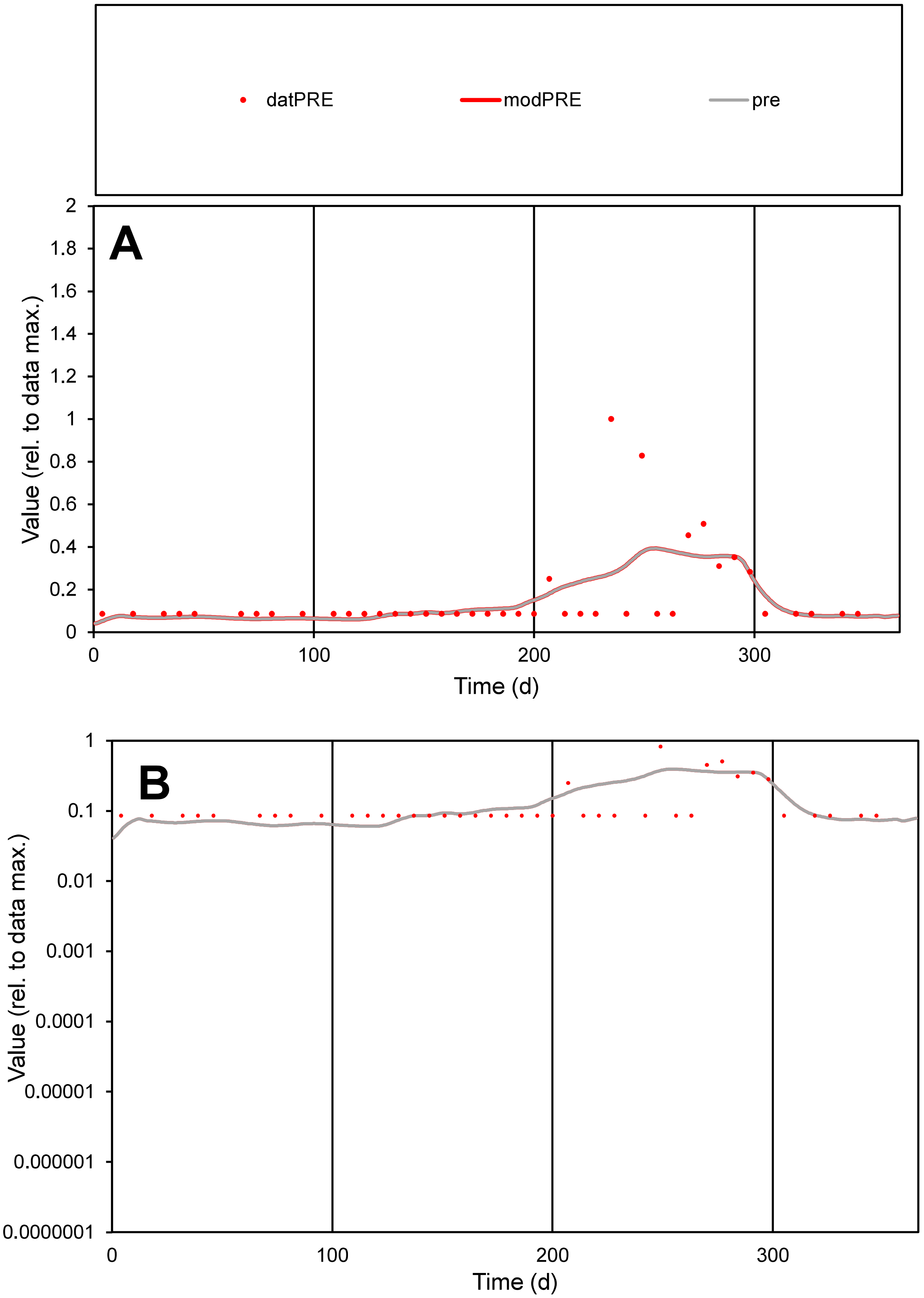

C1

Variables
PRE Provide jtd or Dst
CalibOnly?
2 1 to reproduce of
oNormalize?
2 1=no, 2=yes

ofxn calc*
0.9758
ofxn mod
0.98896

sRun
RunA1
zout
1

Ave
Data
2.6E-05
Model
2.5E-05

data/vMax
0.00016

ofxng
334.07

Min d/m
1.48891

ave/min d
1.92175
ave/min m
2.78817

327_Calib_S_A

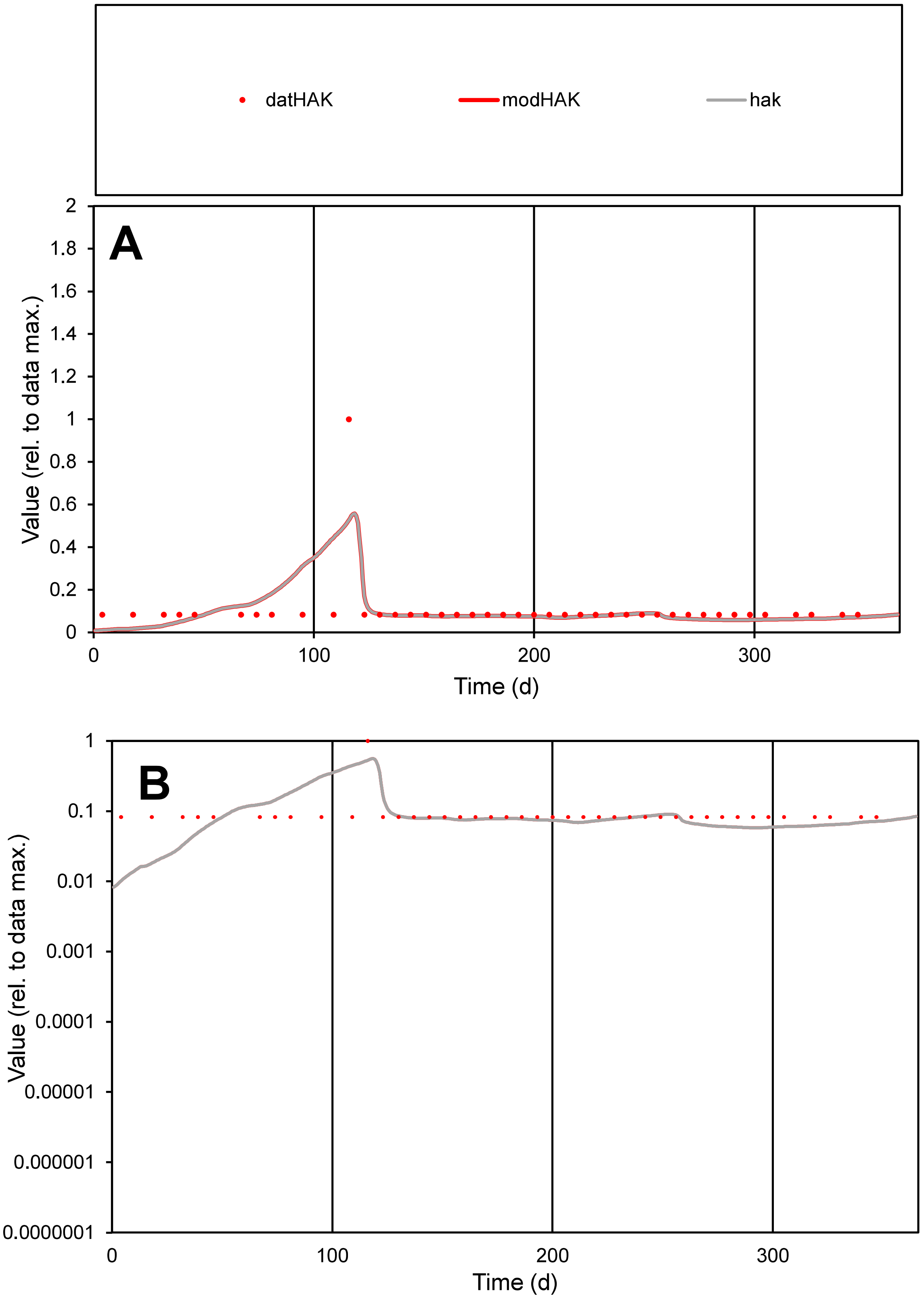

C1

Variables
HAK Provide jtd or Dst
CalibOnly?
2 1 to reproduce of
oNormalize?
2 1=no, 2=yes

ofxn calc*
0.96947
ofxn mod
0.98399

sRun
RunA1
zout
1

Ave
Data
6.5E-06
Model
6.3E-06

data/vMax
6.2E-05

ofxng
334.07

Min d/m
7.60095

ave/min d
1.26596
ave/min m
9.37001

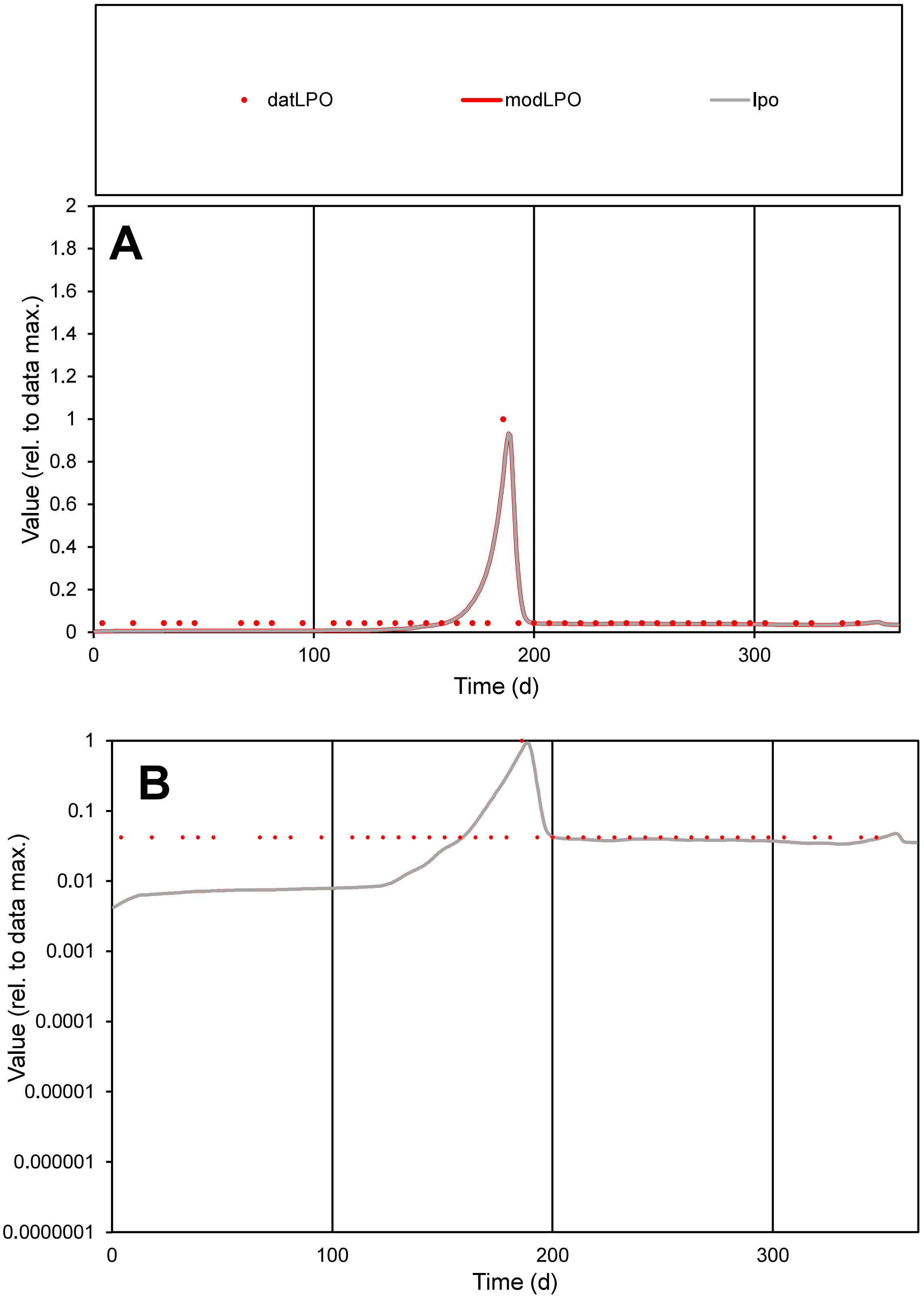

C1

Variables
LPO Provide jtd or Dst
CalibOnly?
2 1 to reproduce of
oNormalize?
2 1=no, 2=yes

ofxn calc*
0.72445
ofxn mod
0.83755

sRun
RunA1
zout
1

Ave
Data
9E-06
Model
8E-06

data/vMax
0.00014

ofxng
334.07

Min d/m
8.35021

ave/min d
1.54012
ave/min m
11.3991

327_Calib_S_A

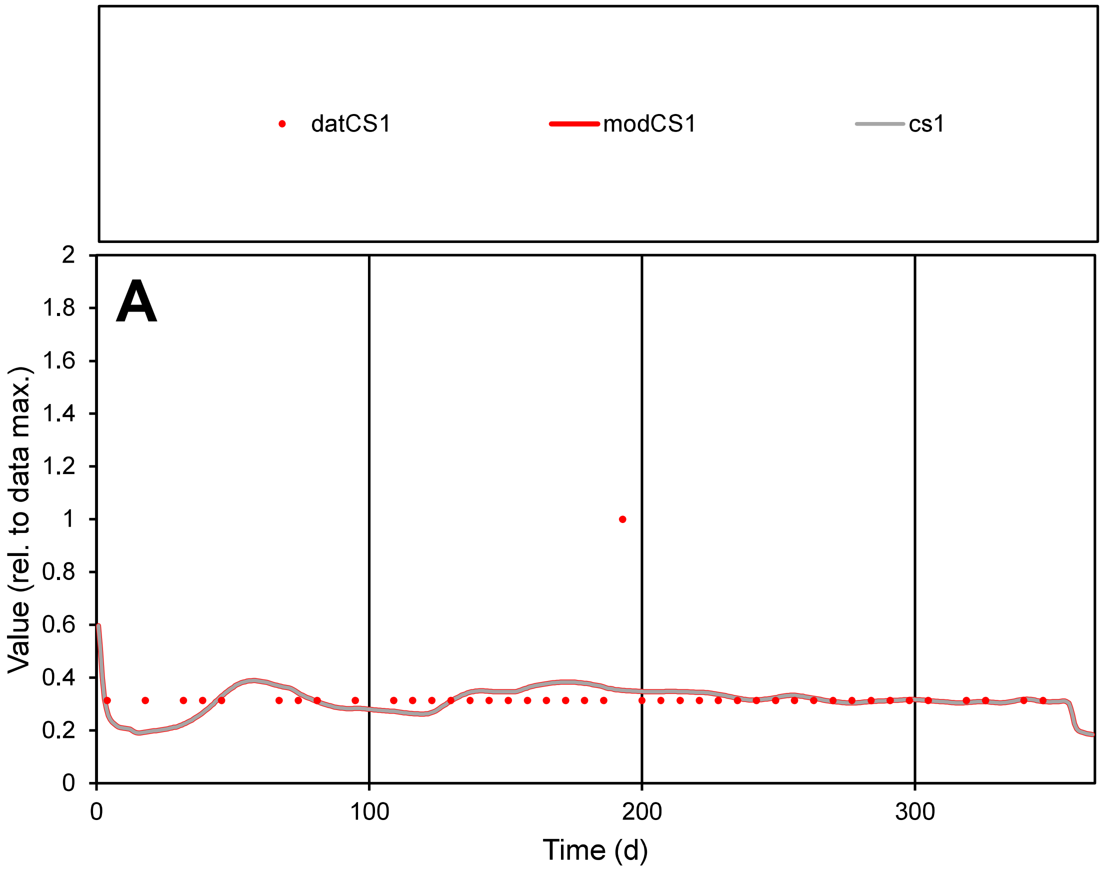

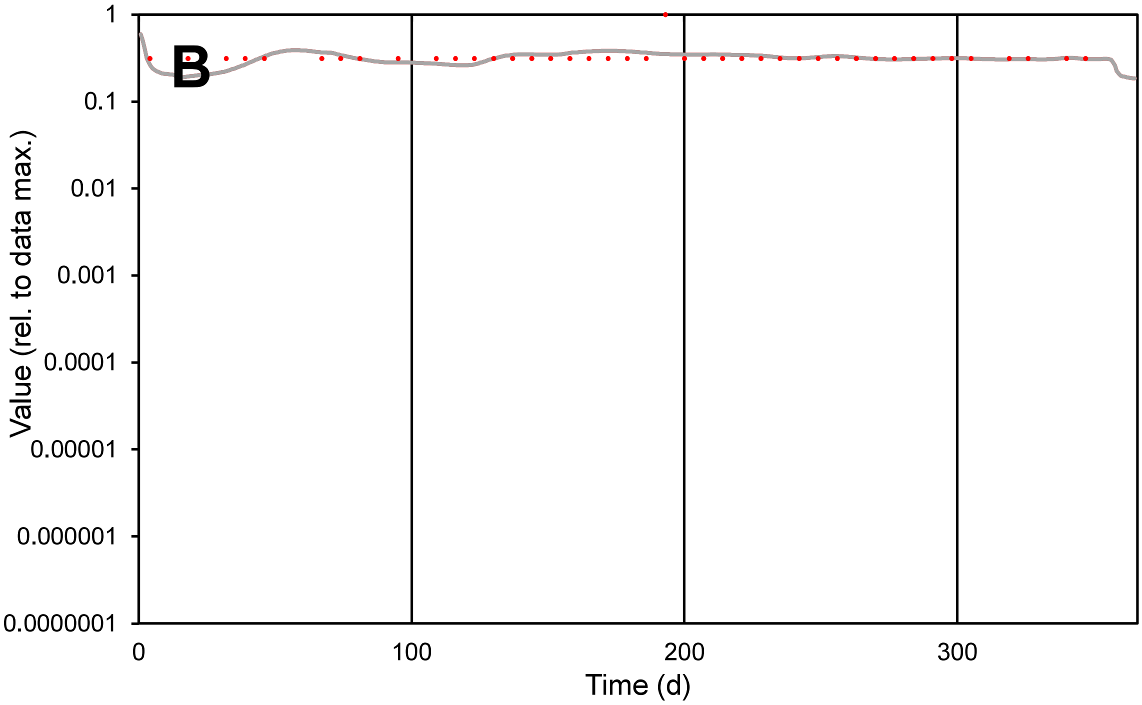

C1

Variables	
CS1	Provide jtd or Dst
CalibOnly?	
2	1 to reproduce of
oNormalize?	
2	1=no, 2=yes
ofxn calc*	
0.32553	
ofxn mod	
0.3448	
sRun	
RunA1	
zout	
1	
Ave Data	
1.8E-05	
Model	
1.8E-05	
data/vMax	
5.6E-05	
ofxng	
334.07	
Min d/m	
1.59418	
ave/min d	
1.05214	
ave/min m	
1.62488	

327_Calib_S_A

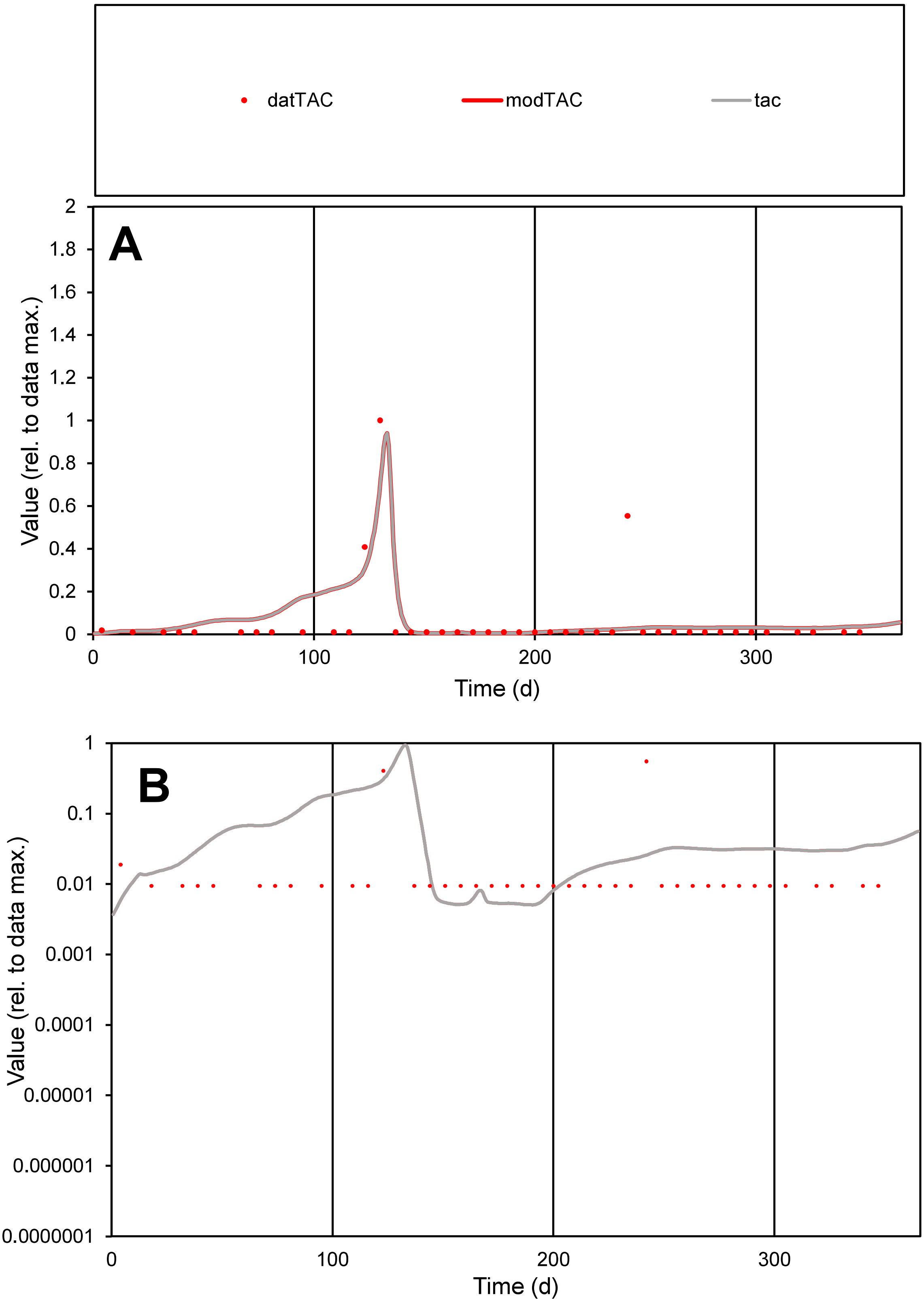

C1

Variables
TAC Provide jtd or Dst
CalibOnly?
2 1 to reproduce of
oNormalize?
2 1=no, 2=yes

ofxn calc*
1.85474
ofxn mod
1.90059

sRun
RunA1
zout
1

Ave
Data
7.7E-06
Model
9.4E-06

data/vMax
0.00014

ofxng
334.07

Min d/m
1.84394

ave/min d
5.90734
ave/min m
13.3608

327_Calib_S_A

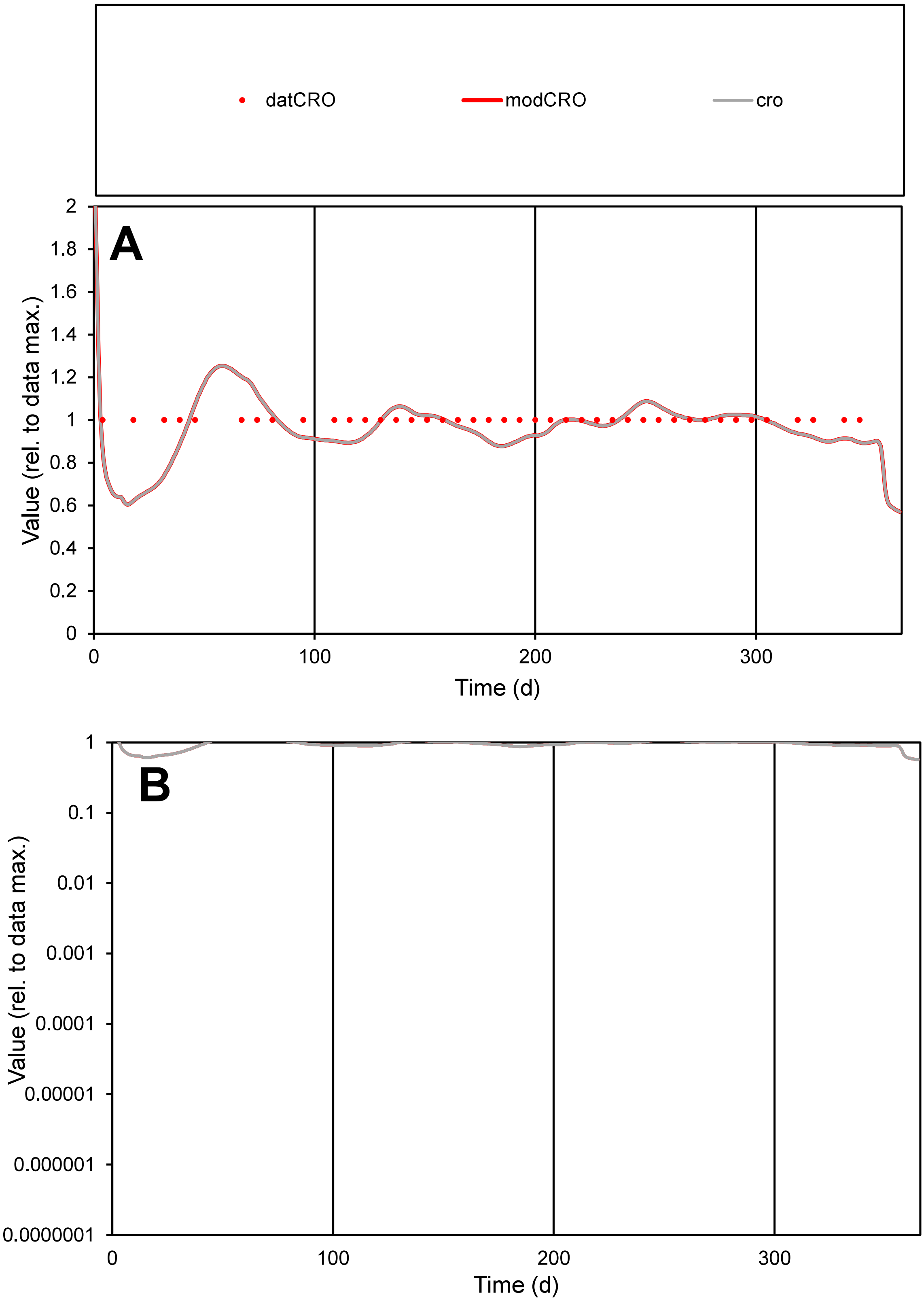

C1

Variables

CRO Provide jtd or Dst

CalibOnly?

2 1 to reproduce of

oNormalize?

2 1=no, 2=yes

ofxn calc*

0.09907

ofxn mod

0.15058

sRun

RunA1

zout

1

Ave

Data

1E-05

Model

9.9E-06

data/vMax

1E-05

ofxng

334.07

Min d/m

1.57272

ave/min d

1

ave/min m

1.52593

327_Calib_S_A

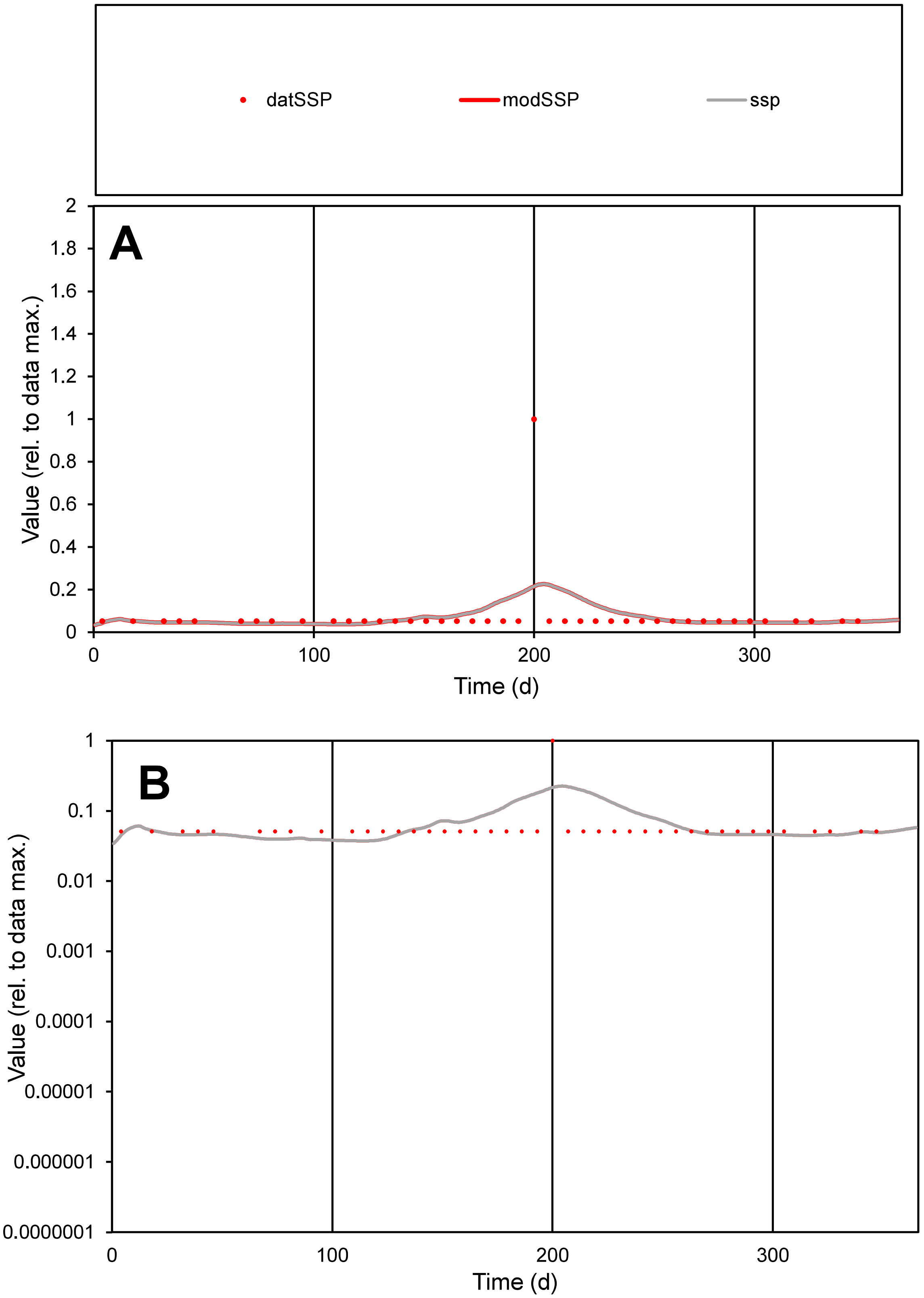

C1

Variables
SSP Provide jtd or Dst
CalibOnly?
2 1 to reproduce of
oNormalize?
2 1=no, 2=yes

ofxn calc*
1.76453
ofxn mod
1.77858

sRun
RunA1
zout
1

Ave
Data
1.9E-05
Model
2E-05

data/vMax
0.00026

ofxng
334.07

Min d/m
1.36125

ave/min d
1.44266
ave/min m
2.01944

327_Calib_S_A

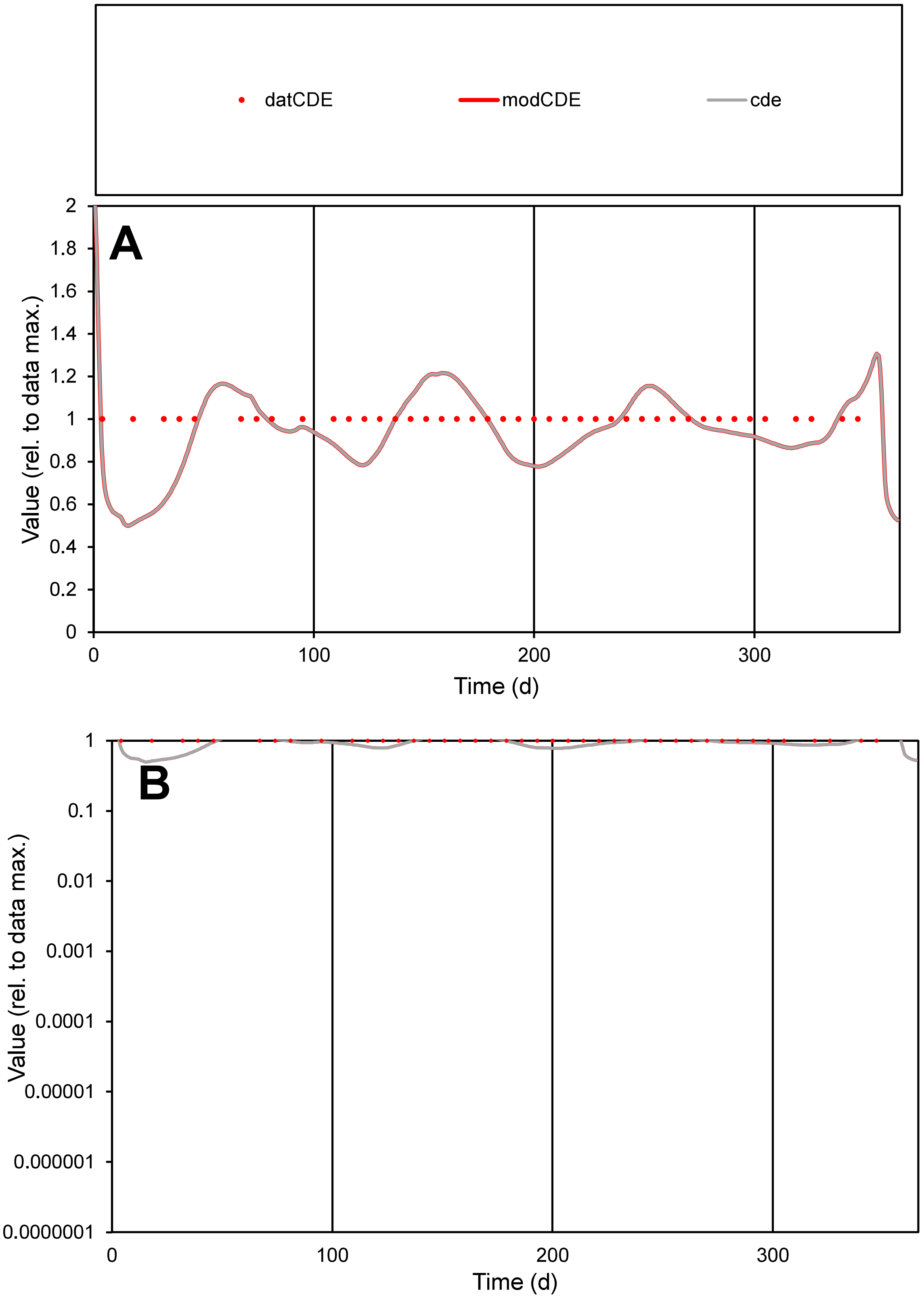

C1

Variables

CDE Provide jtd or Dst

CalibOnly?

2 1 to reproduce of

oNormalize?

2 1=no, 2=yes

ofxn calc*

0.15807

ofxn mod

0.19339

sRun

RunA1

zout

1

Ave

Data

1E-05

Model

9.5E-06

data/vMax

1E-05

ofxng

334.07

Min d/m

1.91984

ave/min d

1

ave/min m

1.82161

327_Calib_S_A

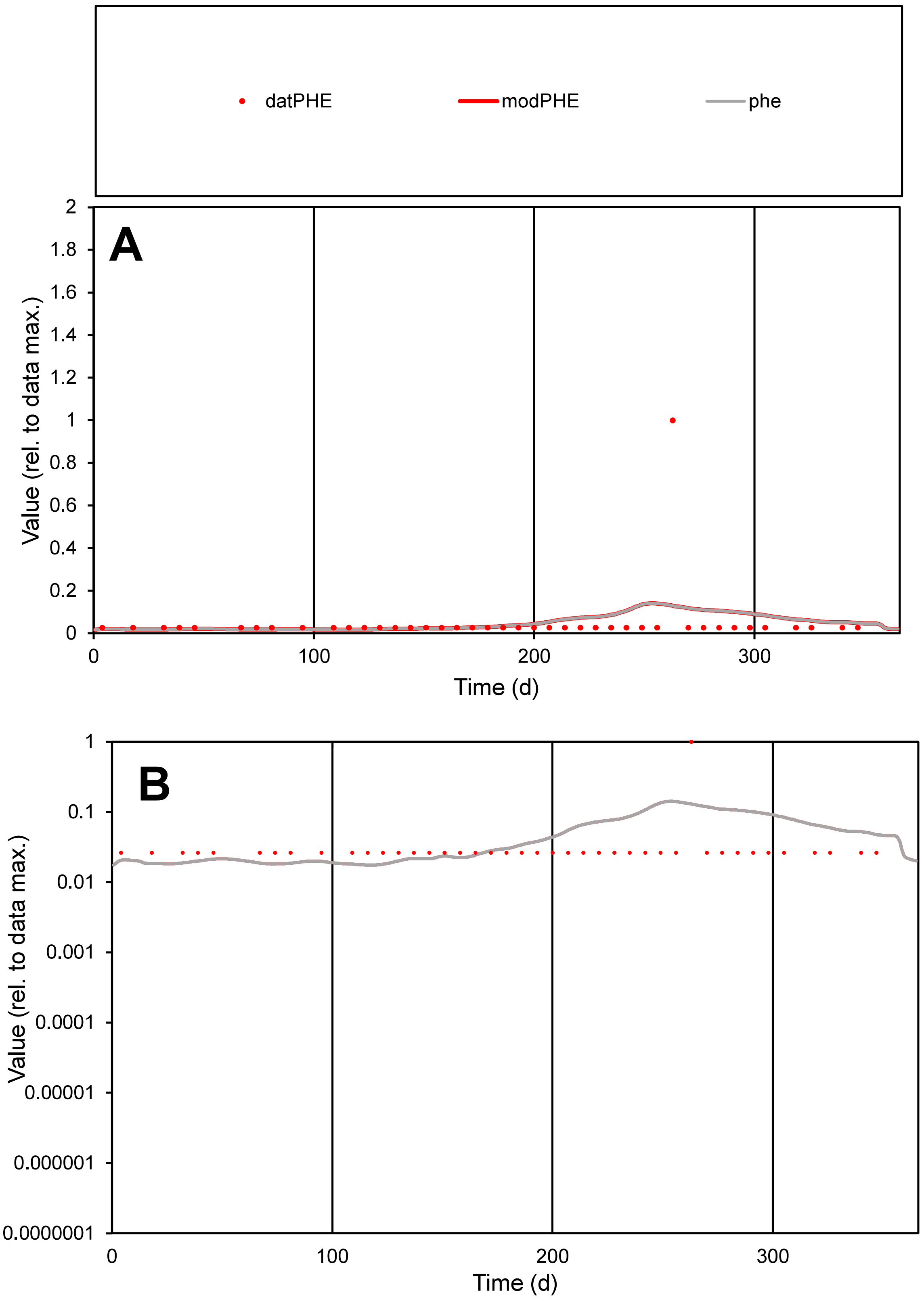

C1

Variables
PHE Provide jtd or Dst
CalibOnly?
2 1 to reproduce of
oNormalize?
2 1=no, 2=yes

ofxn calc*
2.86569
ofxn mod
2.88152

sRun
RunA1
zout
1

Ave
Data
7.5E-06
Model
8.1E-06

data/vMax
0.00015

ofxng
334.07

Min d/m
1.49924

ave/min d
1.88248
ave/min m
3.04244

327_Calib_S_A

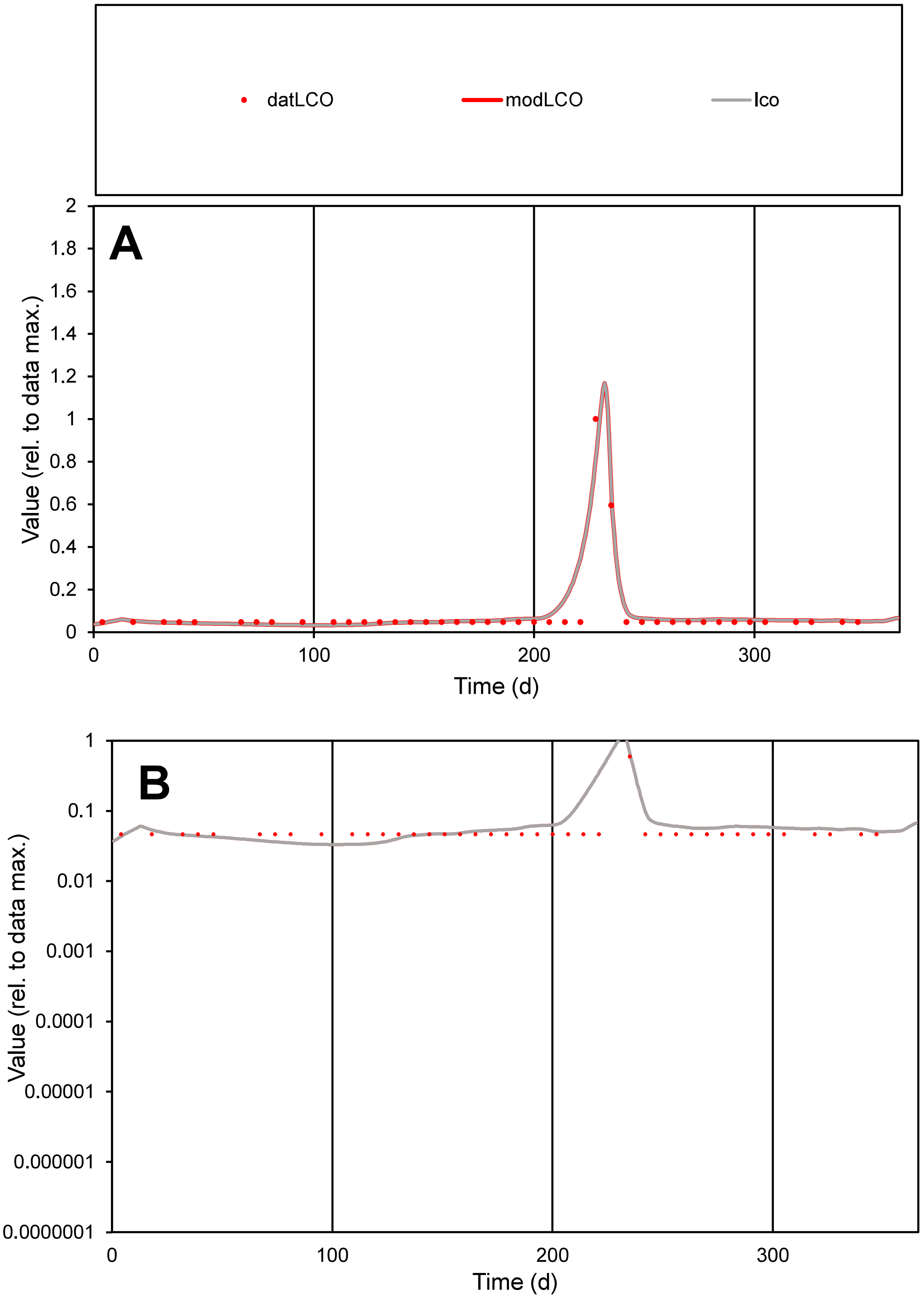

C1

Variables

LCO Provide jtd or Dst

CalibOnly?

2 1 to reproduce of

oNormalize?

2 1=no, 2=yes

ofxn calc*

0.54415

ofxn mod

0.66026

sRun

RunA1

zout

1

Ave

Data

1.3E-05

Model

1.5E-05

data/vMax

0.00016

ofxng

334.07

Min d/m

1.40604

ave/min d

1.7658

ave/min m

2.77852

327_Calib_S_A

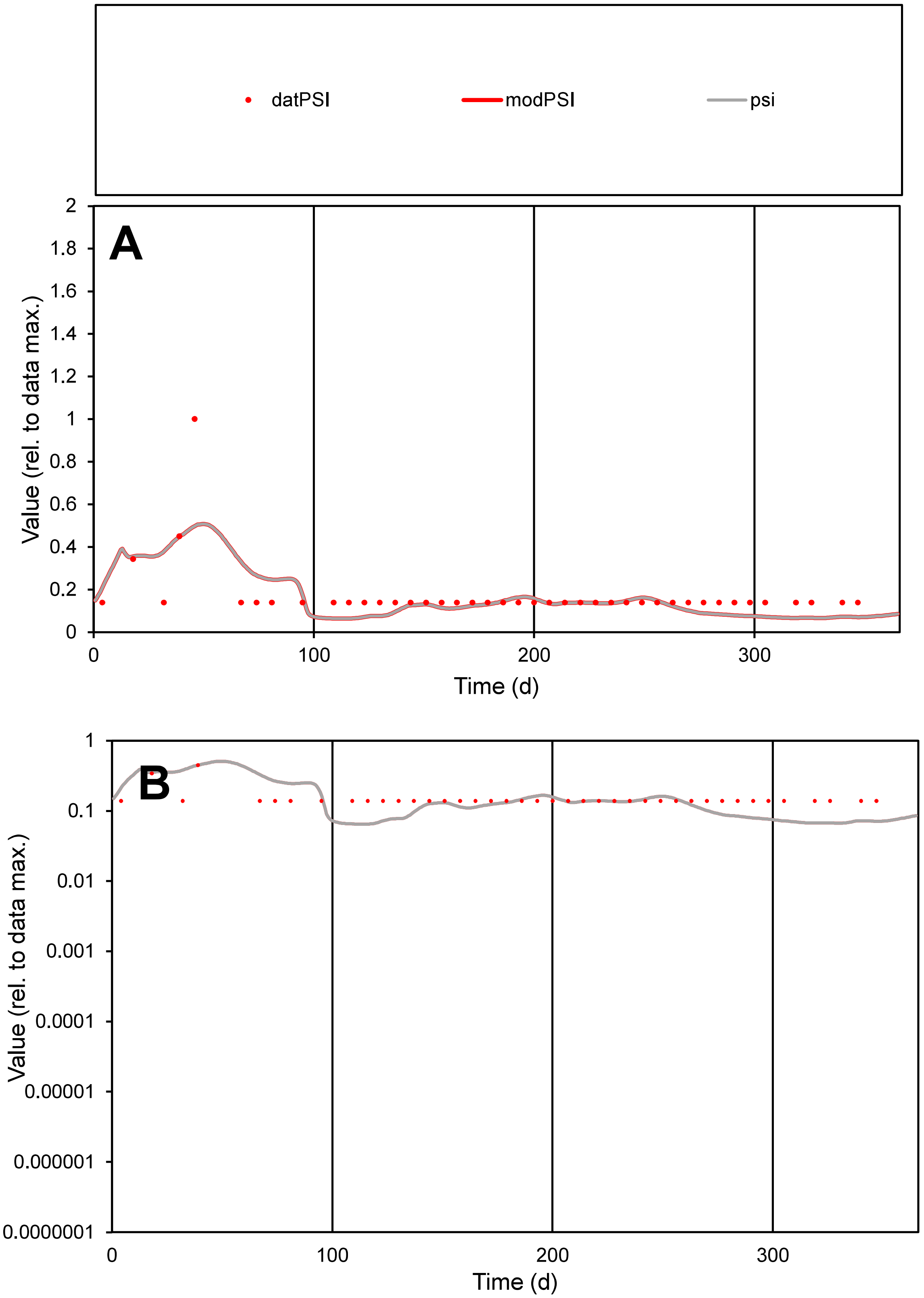

C1

Variables
PSI Provide jtd or Dst
CalibOnly?
2 1 to reproduce of
oNormalize?
2 1=no, 2=yes

ofxn calc*
0.59266
ofxn mod
0.60933

sRun
RunA1
zout
1

Ave
Data
4.4E-06
Model
4E-06

data/vMax
2.6E-05

ofxng
334.07

Min d/m
2.14019

ave/min d
1.2365
ave/min m
2.39329

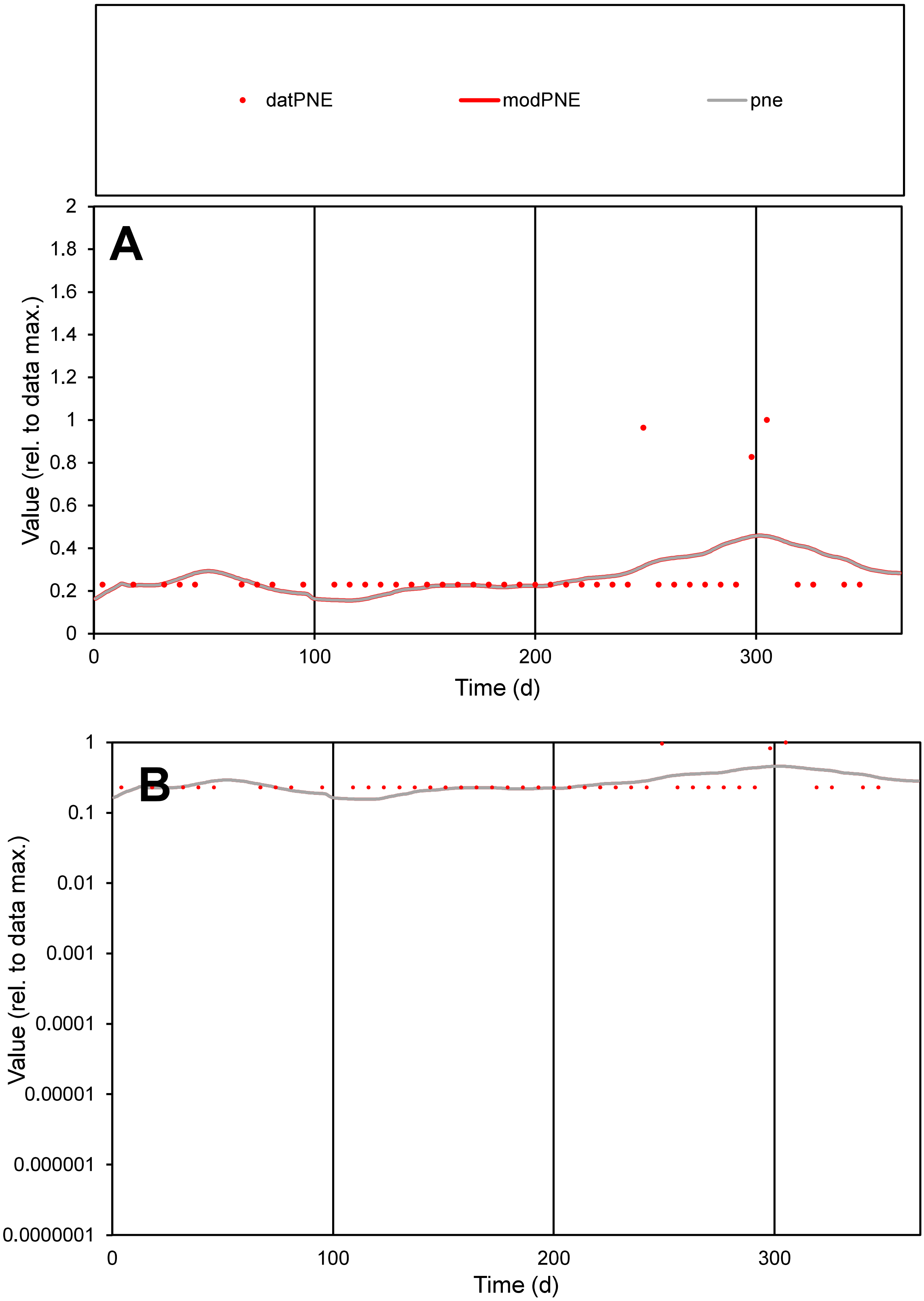

C1

Variables

PNE Provide jtd or Dst

CalibOnly?

2 1 to reproduce of

oNormalize?

2 1=no, 2=yes

ofxn calc*

0.5827

ofxn mod

0.59451

sRun

RunA1

zout

1

Ave

Data

1.8E-05

Model

1.7E-05

data/vMax

6.3E-05

ofxng

334.07

Min d/m

1.47112

ave/min d

1.21848

ave/min m

1.7555

327_Calib_S_A

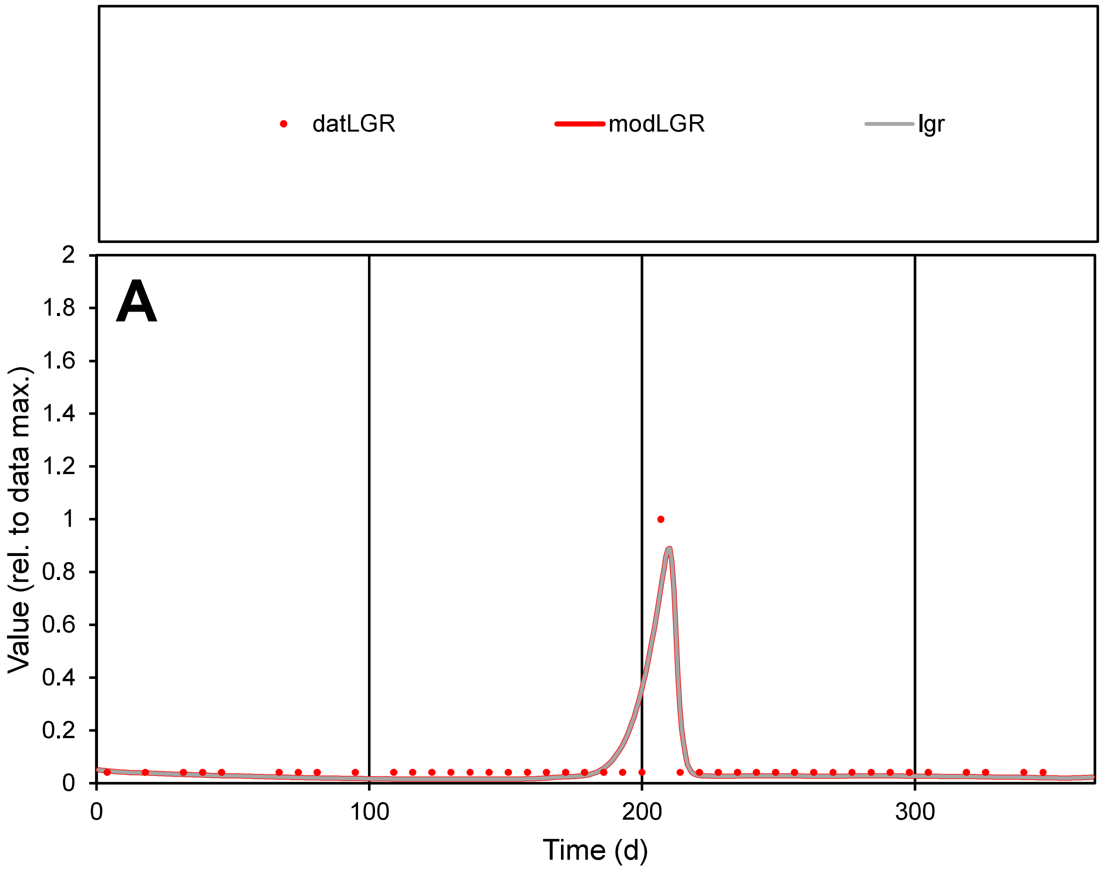

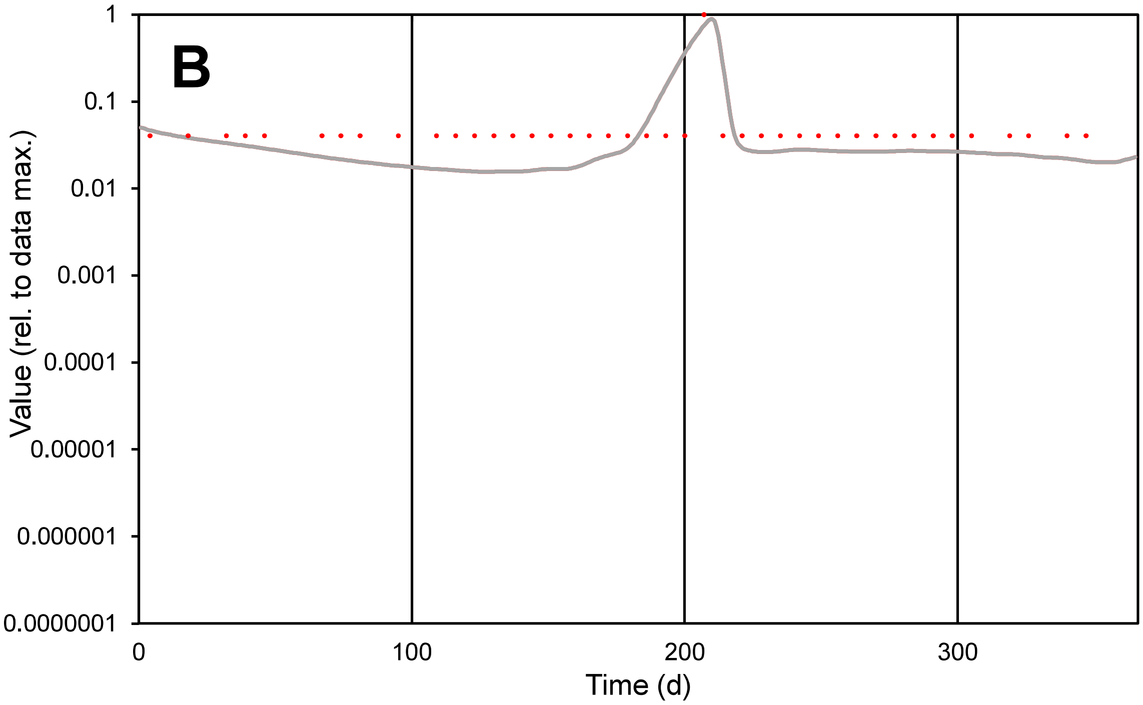

C1

Variables

LGR	Provide jtd or Dst
CalibOnly?	
2	1 to reproduce of
oNormalize?	
2	1=no, 2=yes

ofxn calc*
0.85489
ofxn mod
0.98818

sRun
RunA1
zout
1

Ave
Data
8.1E-06
Model
7.1E-06

data/vMax
0.00013

ofxng
334.07

Min d/m
2.57713

ave/min d
1.56566
ave/min m
3.55047

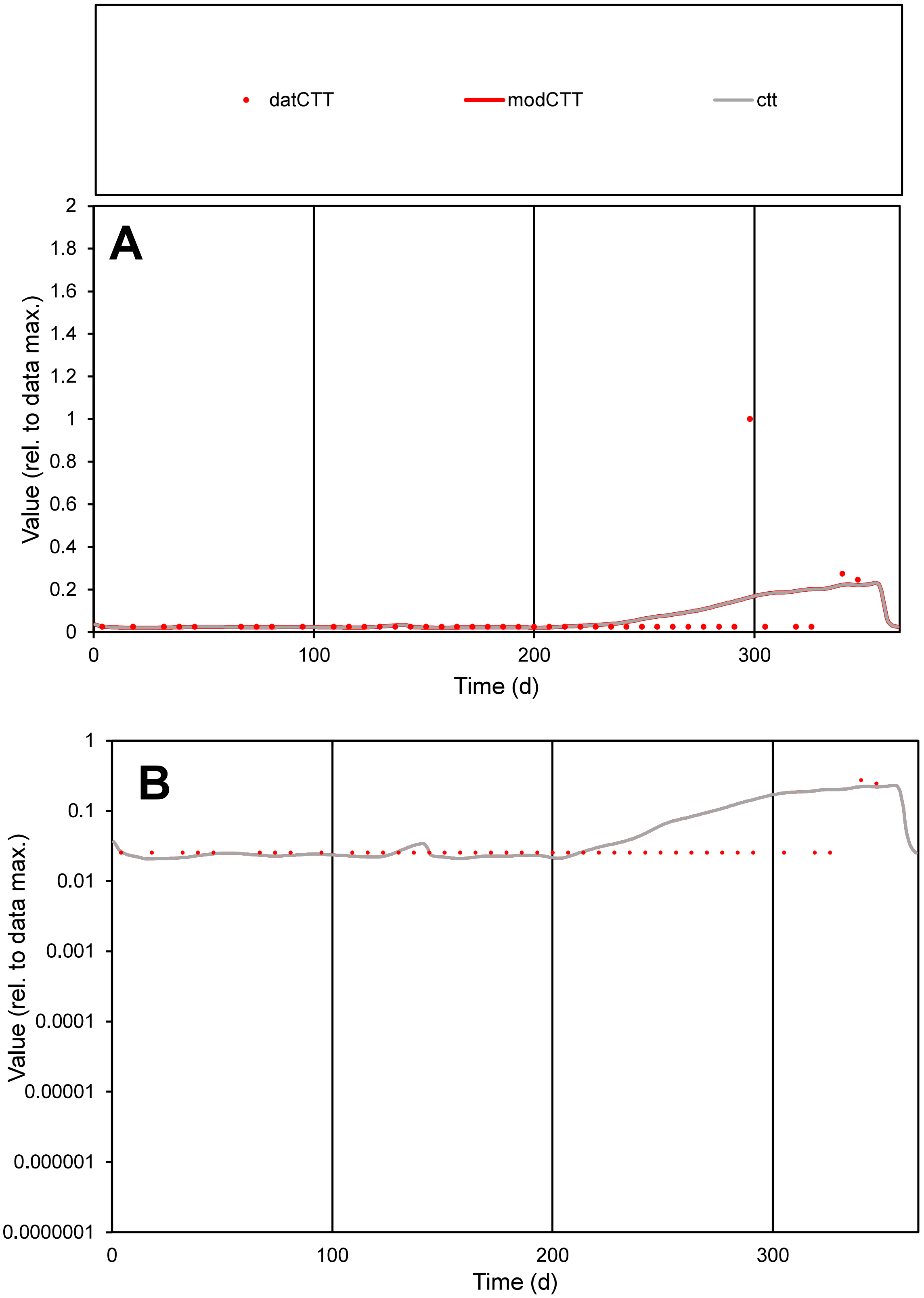

C1

Variables

CTT Provide jtd or Dst

CalibOnly?

2 1 to reproduce of

oNormalize?

2 1=no, 2=yes

ofxn calc*

2.33842

ofxn mod

2.35061

sRun

RunA1

zout

1

Ave

Data

3.5E-06

Model

3.6E-06

data/vMax

5.8E-05

ofxng

334.07

Min d/m

1.22158

ave/min d

2.34735

ave/min m

3.00167

327_Calib_S_A

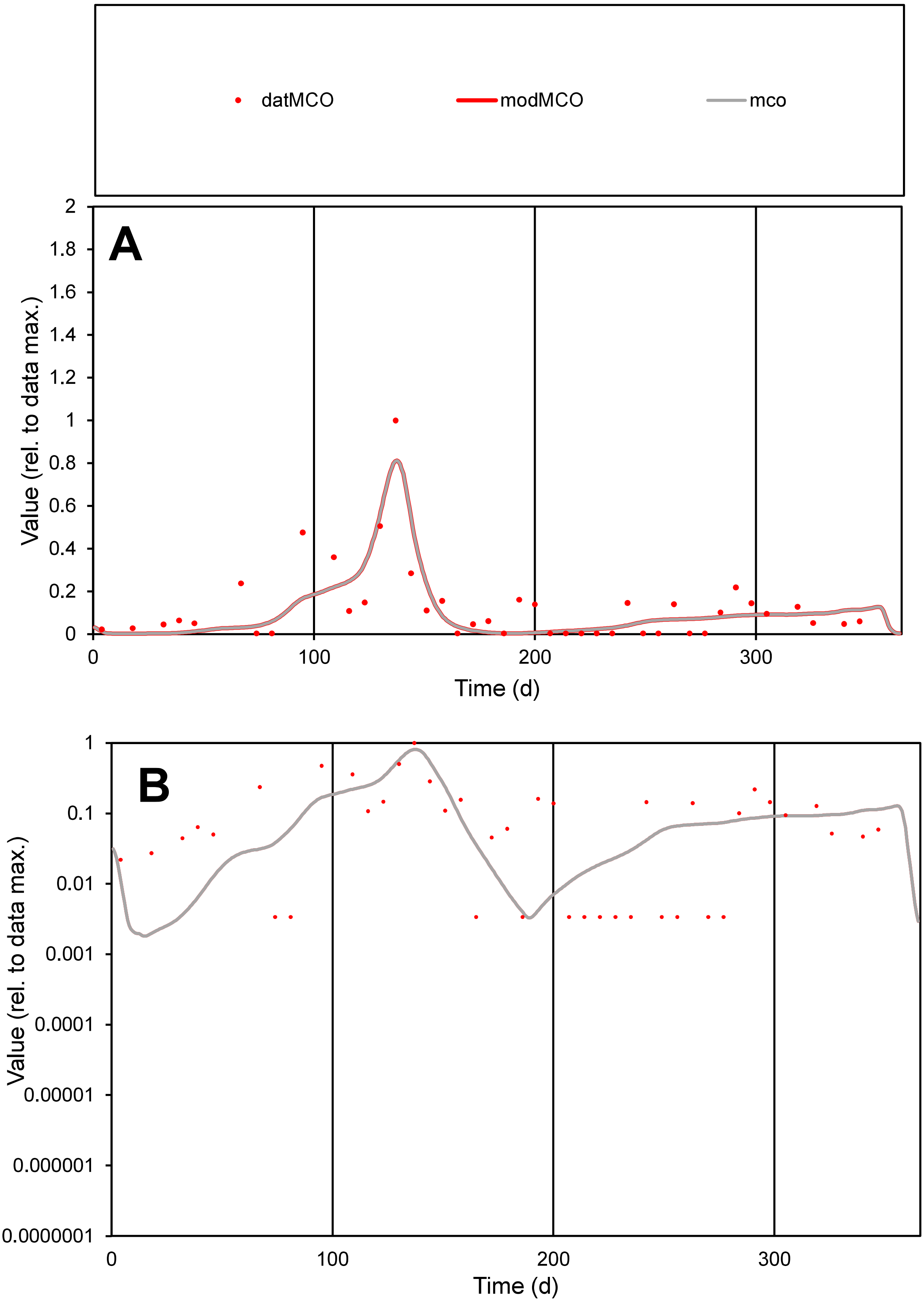

C1

Variables
MCO Provide jtd or Dst
CalibOnly?
2 1 to reproduce of
oNormalize?
2 1=no, 2=yes

ofxn calc*
0.80512
ofxn mod
0.83657

sRun
RunA1
zout
1

Ave
Data
2.2E-06
Model
2E-06

data/vMax
1.8E-05

ofxng
334.07

Min d/m
1.59786

ave/min d
36.4577
ave/min m
51.4766

327_Calib_S_A

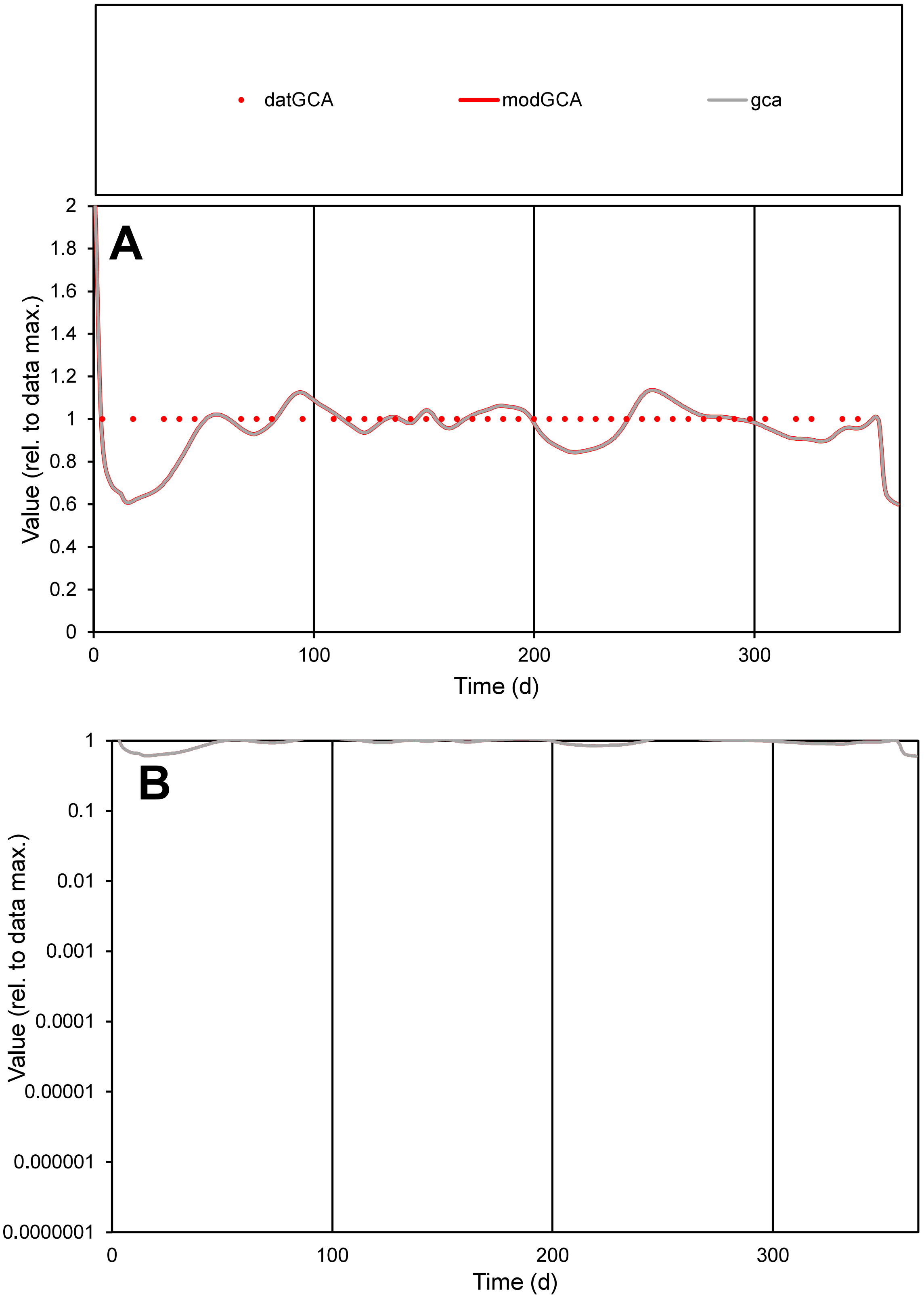

C1

Variables

GCA Provide jtd or Dst

CalibOnly?

2 1 to reproduce of

oNormalize?

2 1=no, 2=yes

ofxn calc*

0.10461

ofxn mod

0.15379

sRun

RunA1

zout

1

Ave

Data

8.4E-06

Model

8.1E-06

data/vMax

8.4E-06

ofxng

334.07

Min d/m

1.5995

ave/min d

1

ave/min m

1.54131

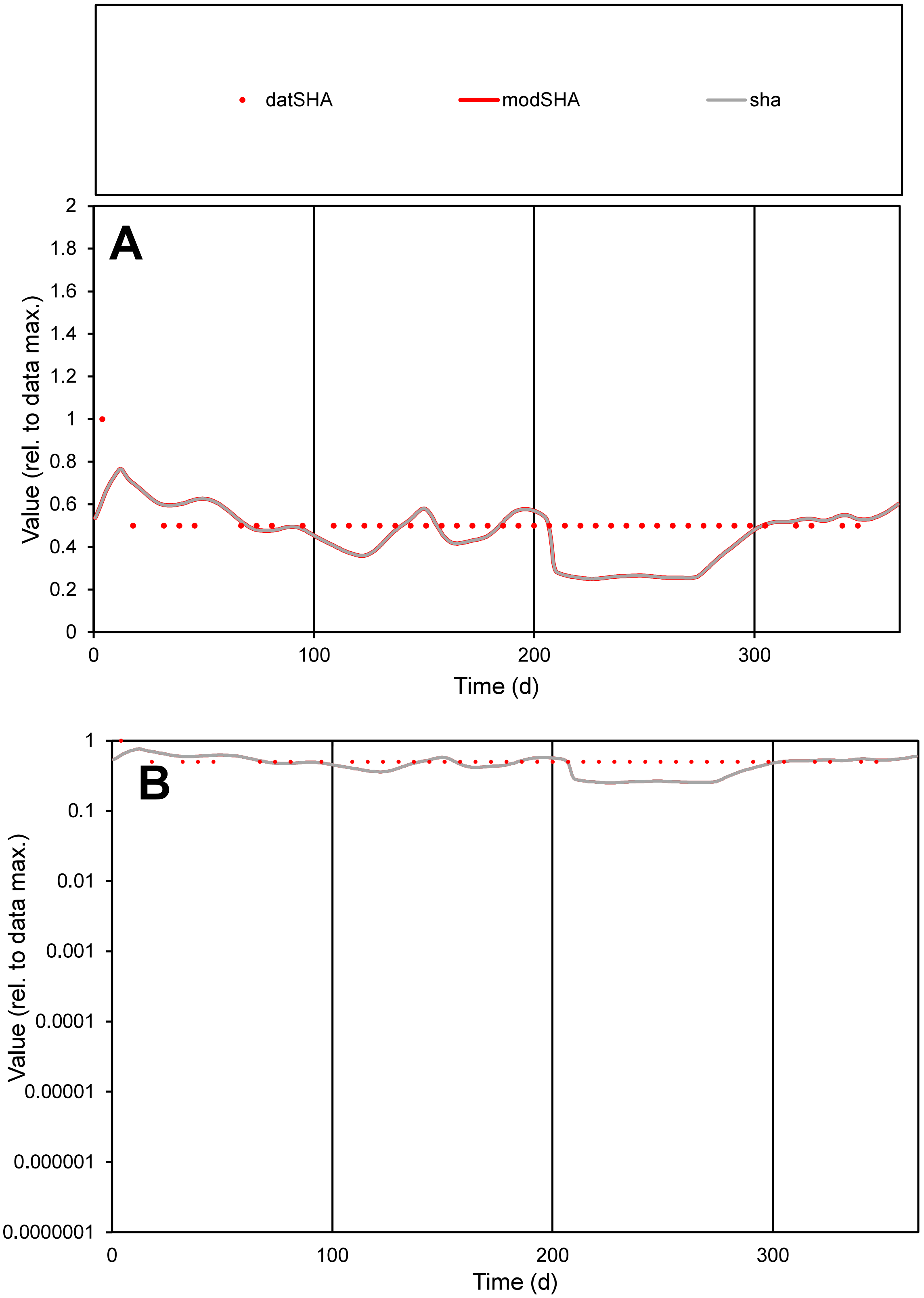

C1

Variables
SHA Provide jtd or Dst
CalibOnly?
2 1 to reproduce of
oNormalize?
2 1=no, 2=yes

ofxn calc*
0.28255
ofxn mod
0.30225

sRun
RunA1
zout
1

Ave
Data
7.1E-07
Model
6.2E-07

data/vMax
1.4E-06

ofxng
334.07

Min d/m
1.9852

ave/min d
1.02382
ave/min m
1.76747

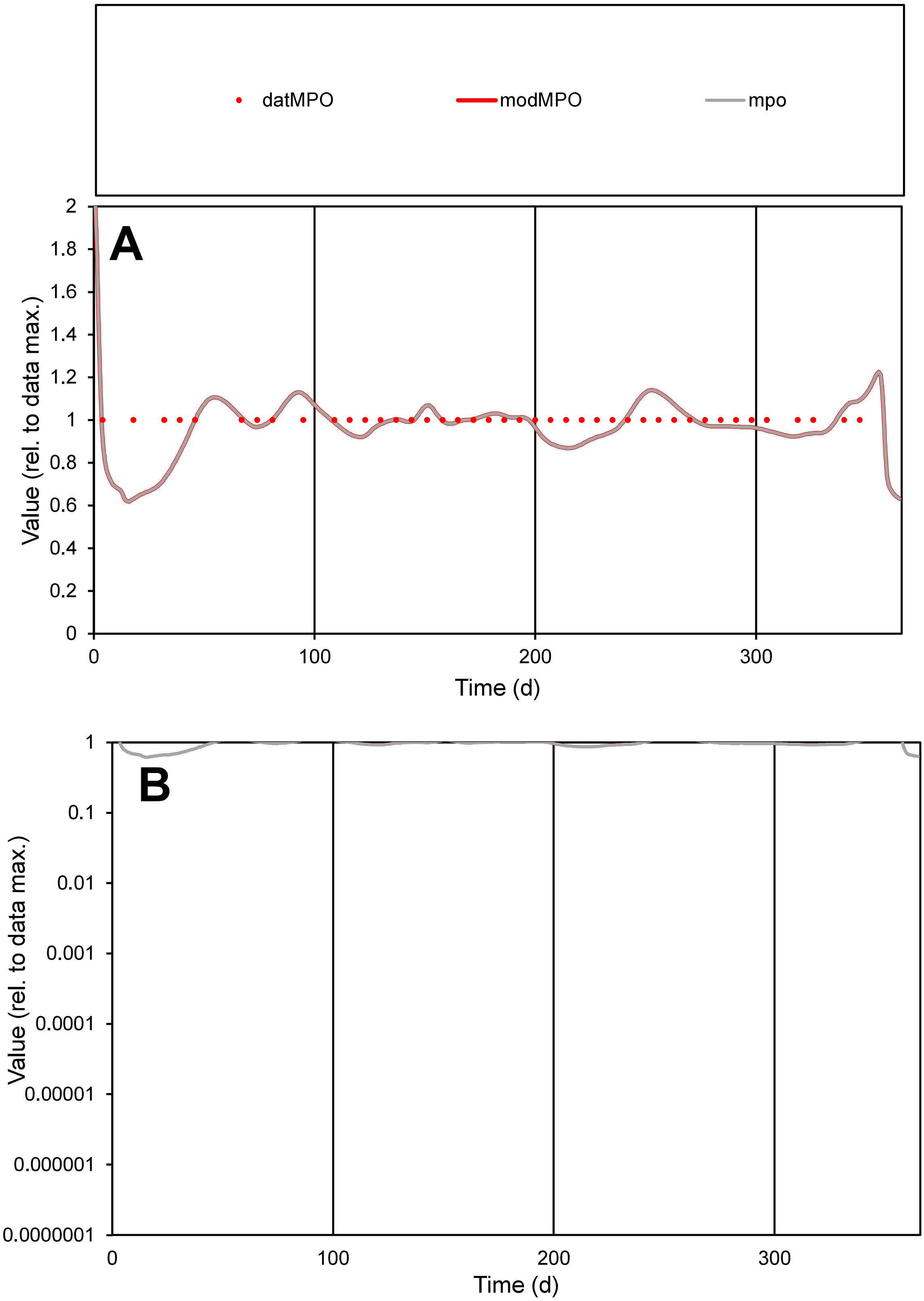

C1

Variables
MPO Provide jtd or Dst
CalibOnly?
2 1 to reproduce of
oNormalize?
2 1=no, 2=yes

ofxn calc*
0.09469
ofxn mod
0.14769

sRun
RunA1
zout
1

Ave
Data
1.2E-05
Model
1.2E-05

data/vMax
1.2E-05

ofxng
334.07

Min d/m
1.55728

ave/min d
1
ave/min m
1.51817

327_Calib_S_A

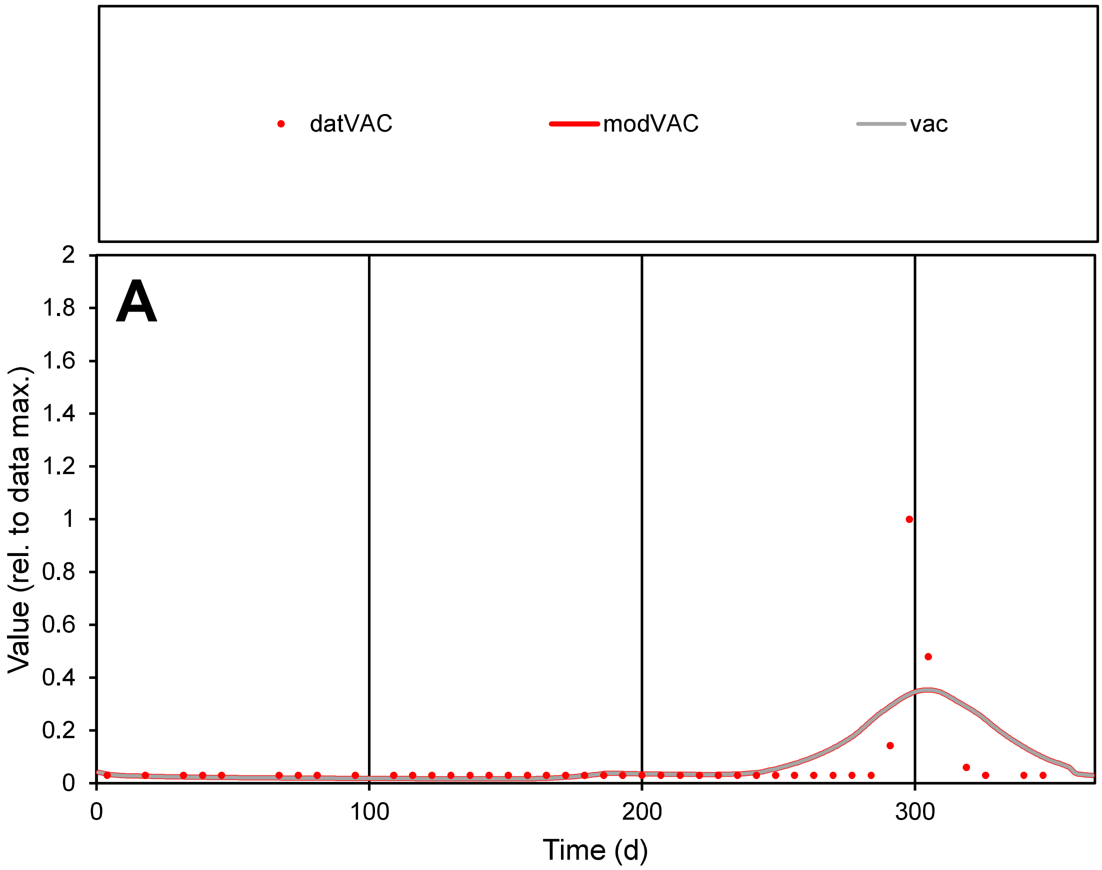

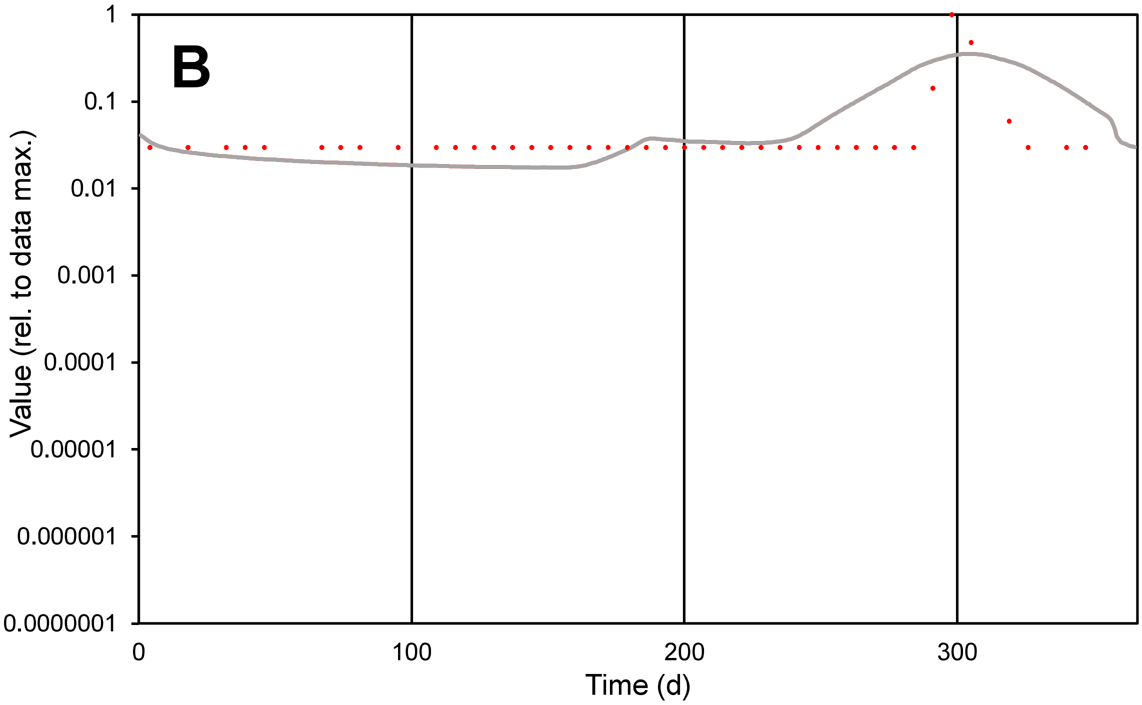

C1

Variables
VAC Provide jtd or Dst
CalibOnly?
2 1 to reproduce of
oNormalize?
2 1=no, 2=yes

ofxn calc*
1.86946
ofxn mod
1.89108

sRun
RunA1
zout
1

Ave
Data
2.1E-05
Model
2.5E-05

data/vMax
0.00032

ofxng
334.07

Min d/m
1.70526

ave/min d
2.25065
ave/min m
4.42485

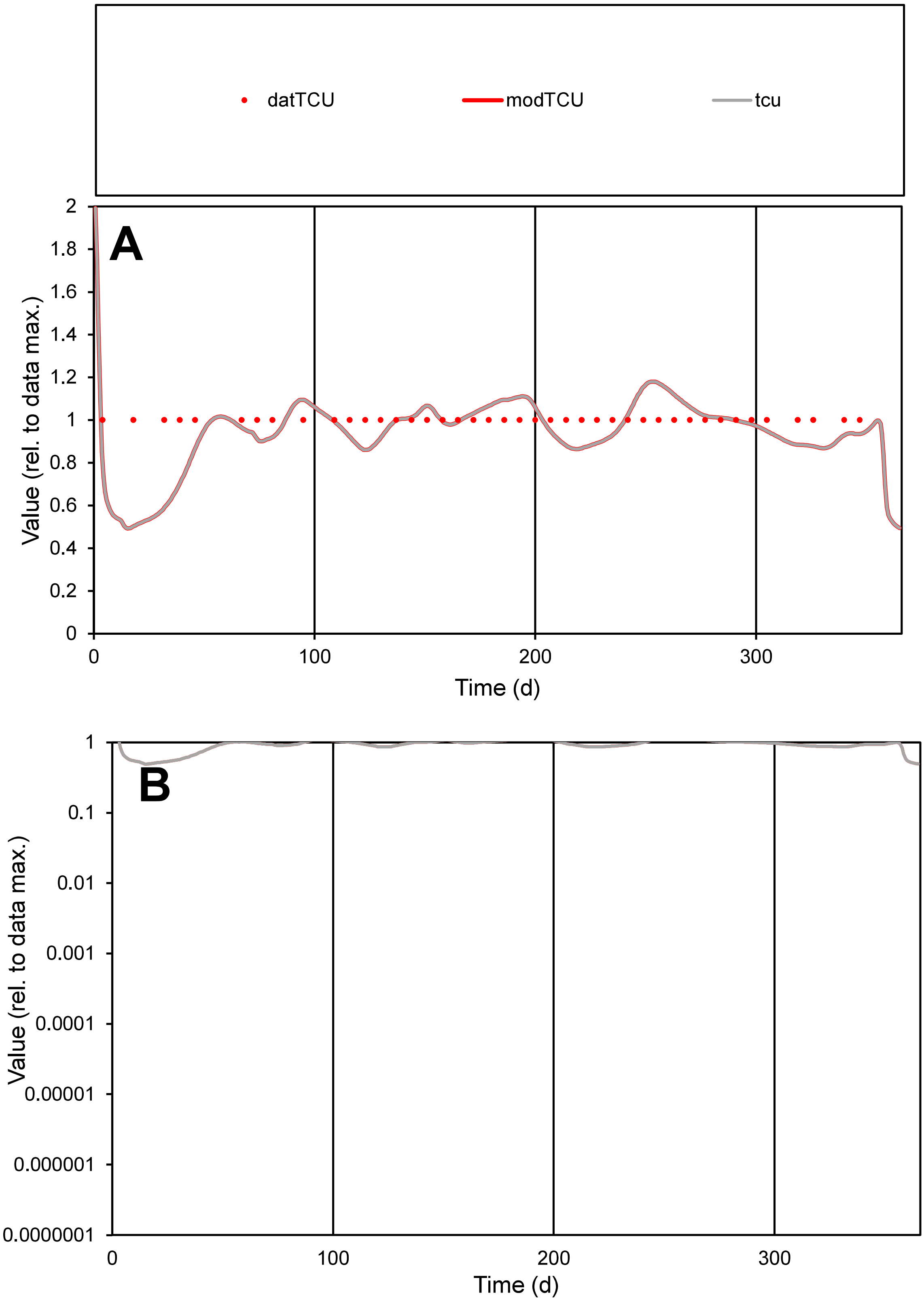

C1

Variables
TCU Provide jtd or Dst
CalibOnly?
2 1 to reproduce of
oNormalize?
2 1=no, 2=yes

ofxn calc*
0.13774
ofxn mod
0.17768

sRun
RunA1
zout
1

Ave
Data
9.8E-06
Model
9.3E-06

data/vMax
9.8E-06

ofxng
334.07

Min d/m
1.95515

ave/min d
1
ave/min m
1.86867

327_Calib_S_A

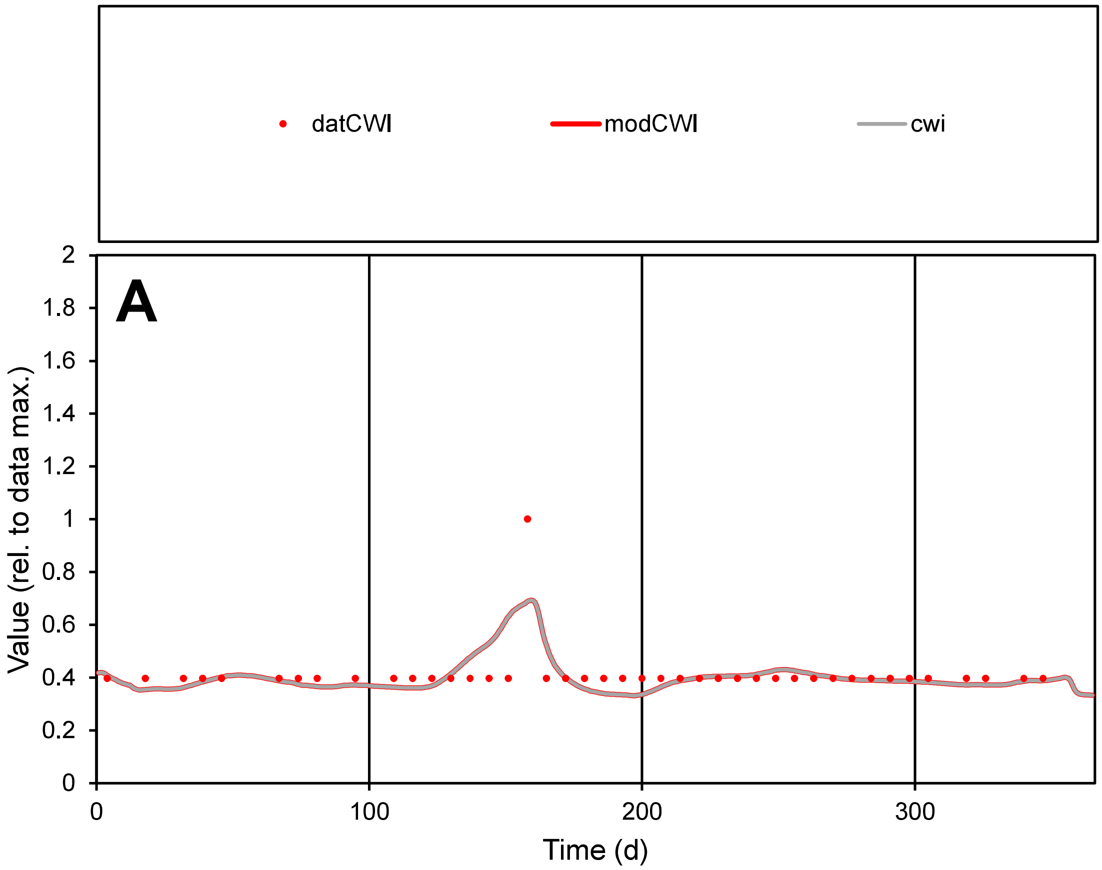

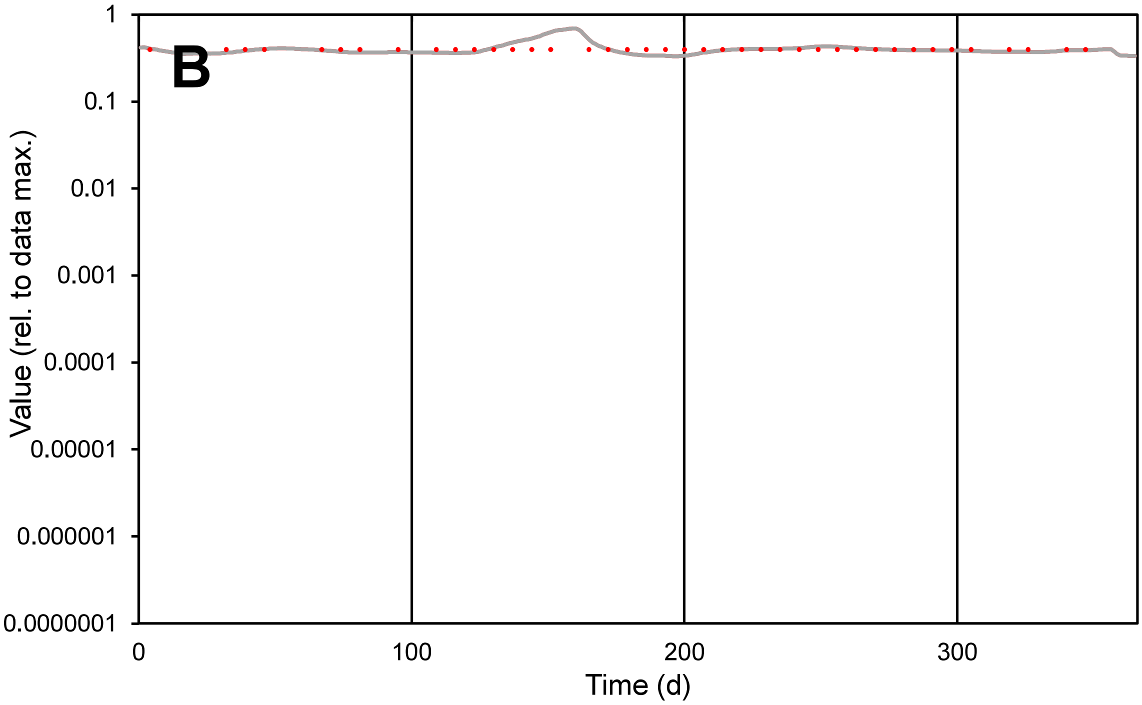

C1

Variables
CWI Provide jtd or Dst
CalibOnly?
2 1 to reproduce of
oNormalize?
2 1=no, 2=yes

ofxn calc*
0.17509
ofxn mod
0.20805

sRun
RunA1
zout
1

Ave
Data
1.4E-05
Model
1.4E-05

data/vMax
3.3E-05

ofxng
334.07

Min d/m
1.18873

ave/min d
1.0361
ave/min m
1.21467

327_Calib_S_A

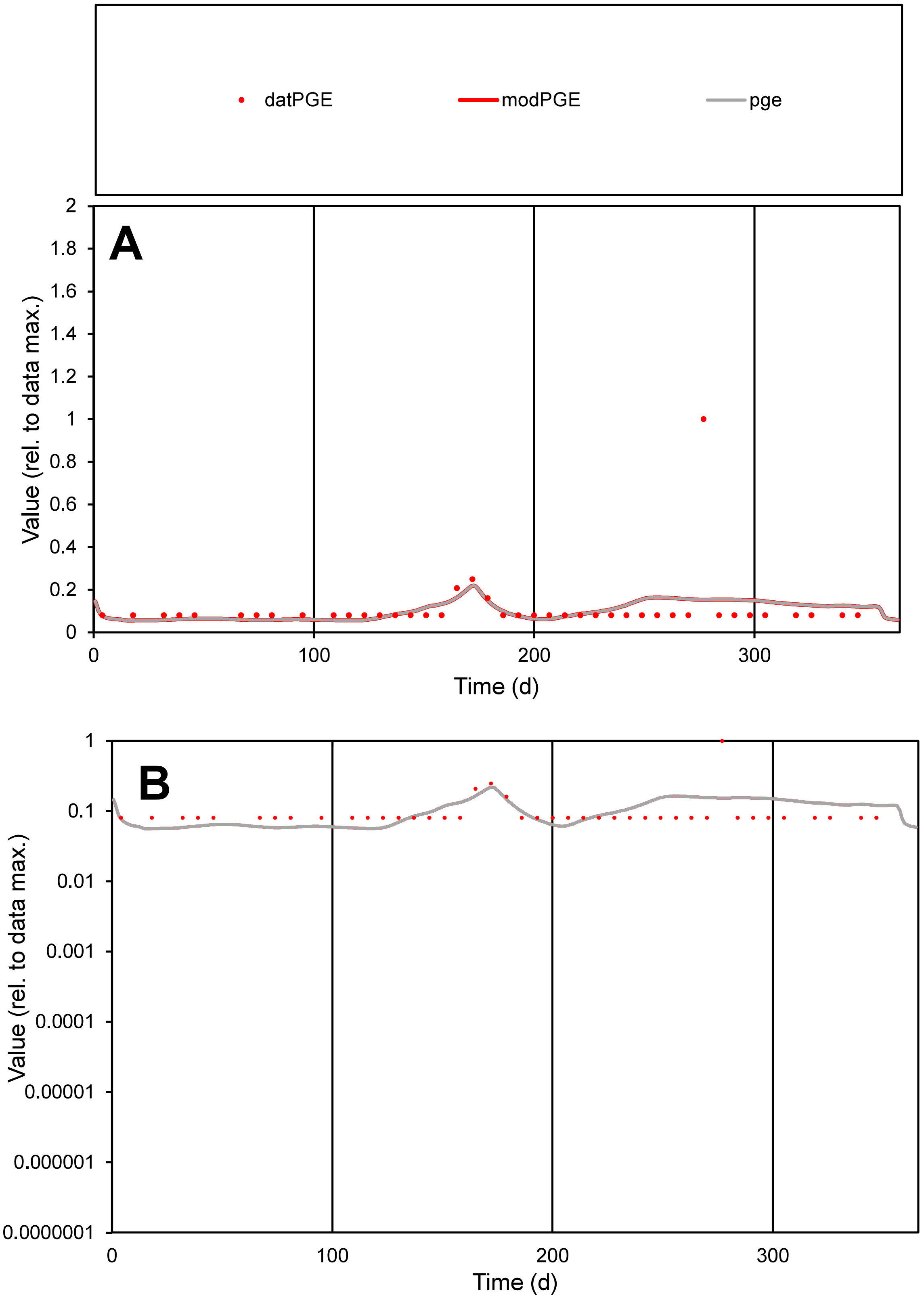

C1

Variables
PGE Provide jtd or Dst
CalibOnly?
2 1 to reproduce of
oNormalize?
2 1=no, 2=yes

ofxn calc*
1.22905
ofxn mod
1.23703

sRun
RunA1
zout
1

Ave
Data
8.1E-06
Model
7.8E-06

data/vMax
7.3E-05

ofxng
334.07

Min d/m
1.42047

ave/min d
1.38285
ave/min m
1.87291

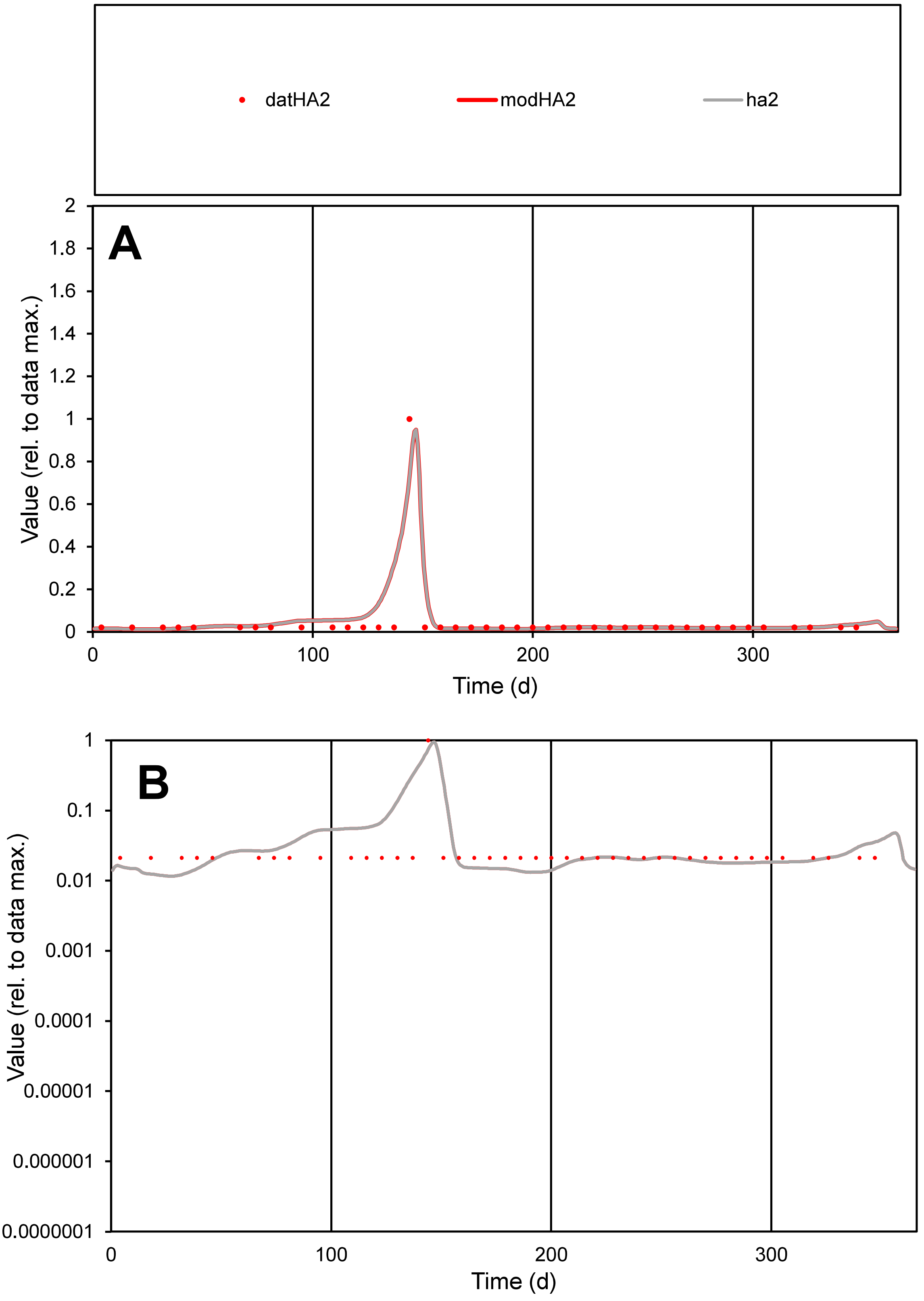

C1

Variables
HA2 Provide jtd or Dst
CalibOnly?
2 1 to reproduce of
oNormalize?
2 1=no, 2=yes

ofxn calc*
1.03274
ofxn mod
1.21851

sRun
RunA1
zout
1

Ave
Data
5.6E-06
Model
6.7E-06

data/vMax
0.00013

ofxng
334.07

Min d/m
1.7167

ave/min d
2.10268
ave/min m
4.32815

327_Calib_S_A

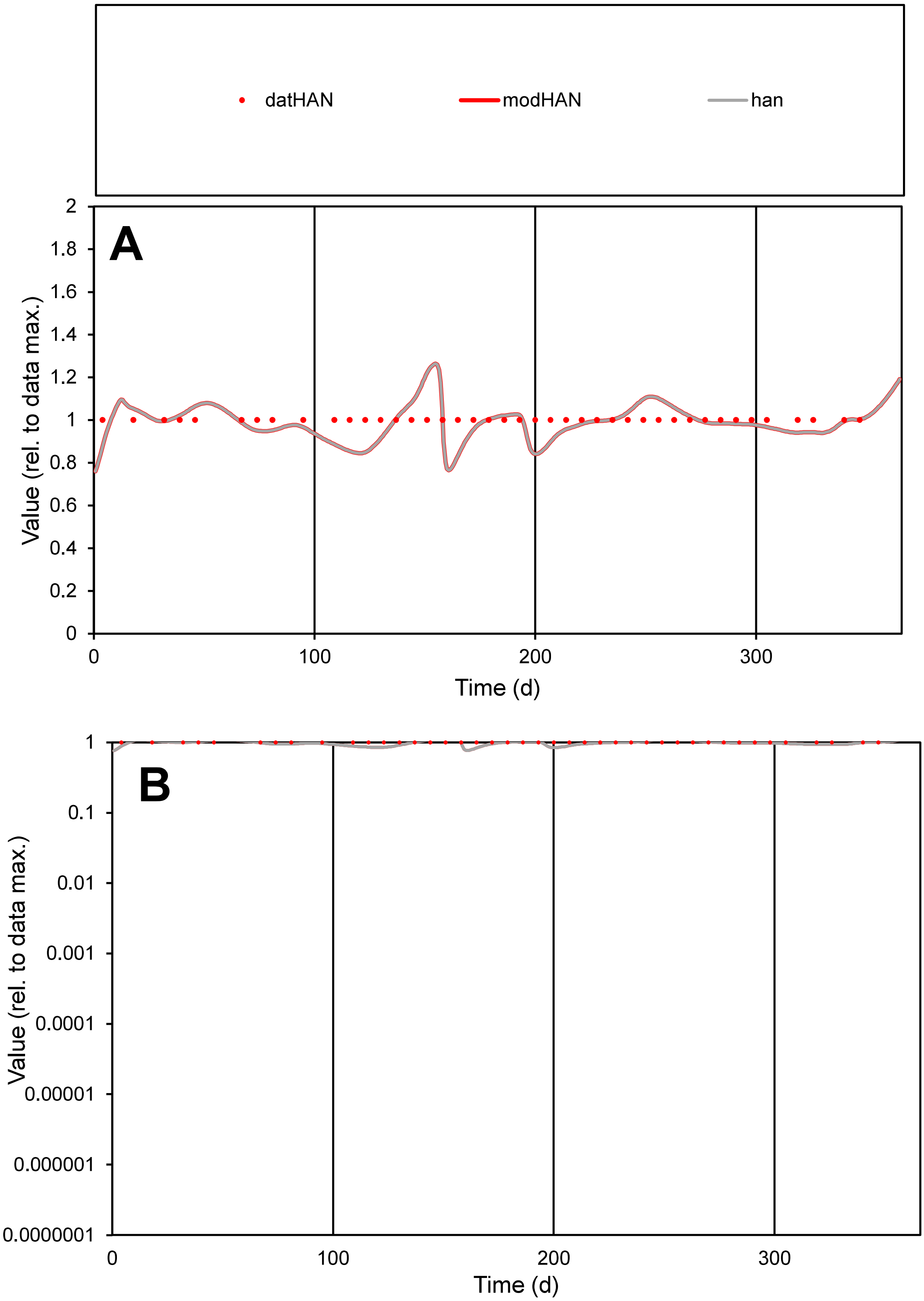

C1

Variables
HAN Provide jtd or Dst
CalibOnly?
2 1 to reproduce of
oNormalize?
2 1=no, 2=yes

ofxn calc*
0.07699
ofxn mod
0.13799

sRun
RunA1
zout
1

Ave
Data
5.1E-06
Model
5E-06

data/vMax
5.1E-06

ofxng
334.07

Min d/m
1.18886

ave/min d
1
ave/min m
1.17103

327_Calib_S_A

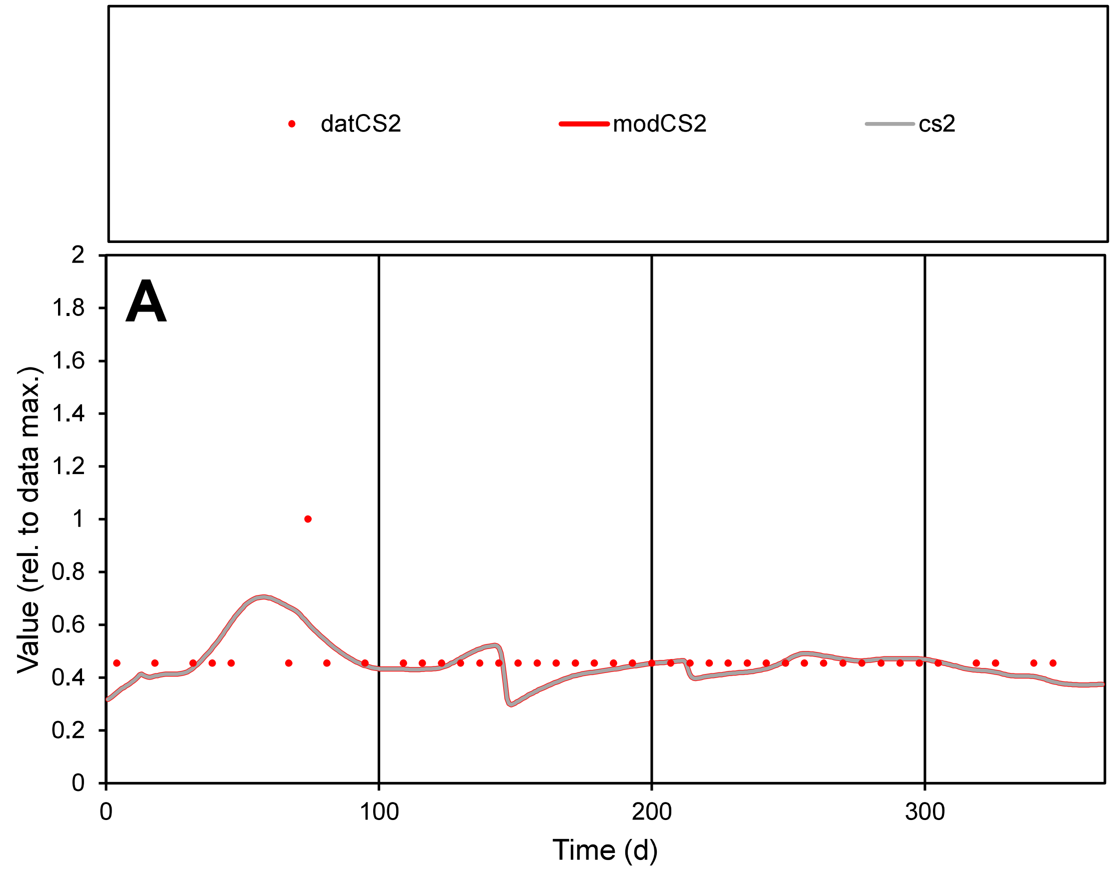

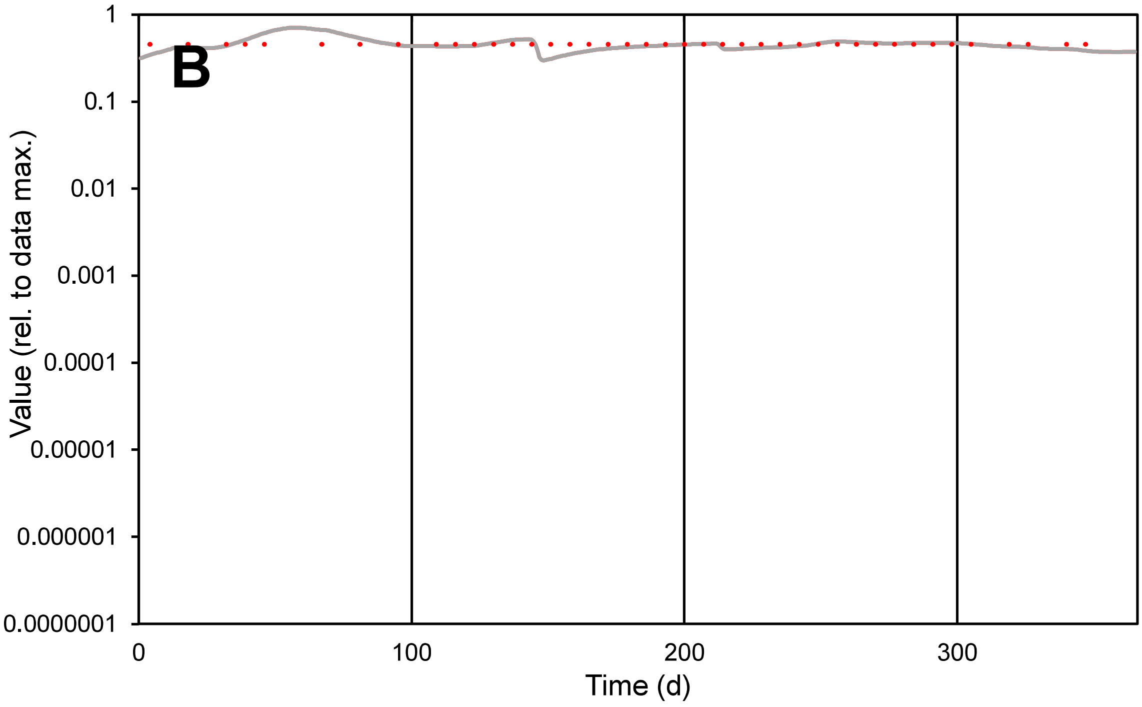

C1

Variables
CS2 Provide jtd or Dst
CalibOnly?
2 1 to reproduce of
oNormalize?
2 1=no, 2=yes

ofxn calc*
0.18982
ofxn mod
0.22152

sRun
RunA1
zout
1

Ave
Data
7.8E-06
Model
7.5E-06

data/vMax
1.7E-05

ofxng
334.07

Min d/m
1.43659

ave/min d
1.02853
ave/min m
1.42839

327_Calib_S_A

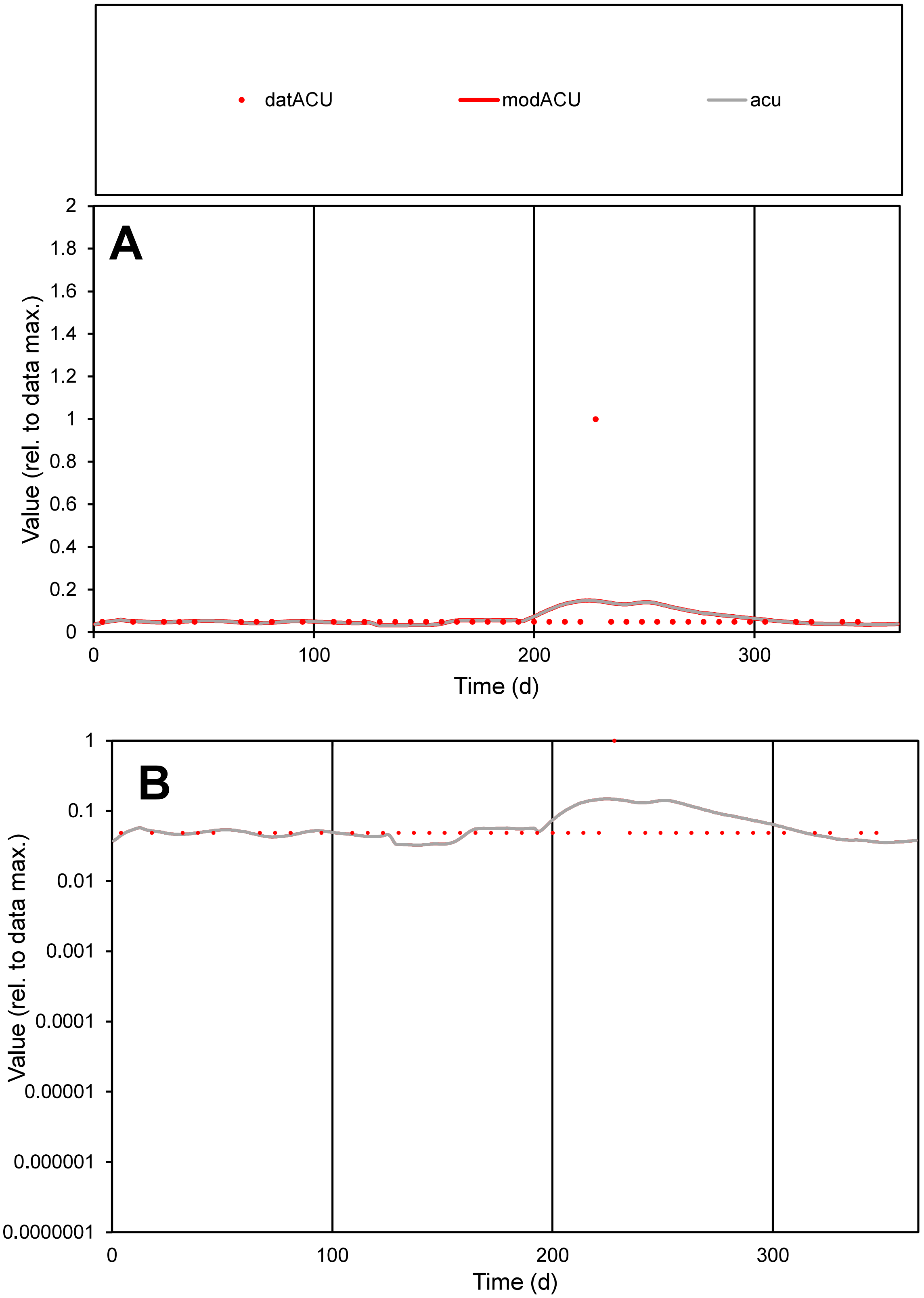

C1

Variables	
ACU	Provide jtd or Dst
CalibOnly?	
2	1 to reproduce of
oNormalize?	
2	1=no, 2=yes
ofxn calc*	
1.92425	
ofxn mod	
1.93018	
sRun	
RunA1	
zout	
1	
Ave	
Data	
1.6E-05	
Model	
1.6E-05	
data/vMax	
0.00023	
ofxng	
334.07	
Min d/m	
1.50014	
ave/min d	
1.46481	
ave/min m	
2.12846	

327_Calib_S_A

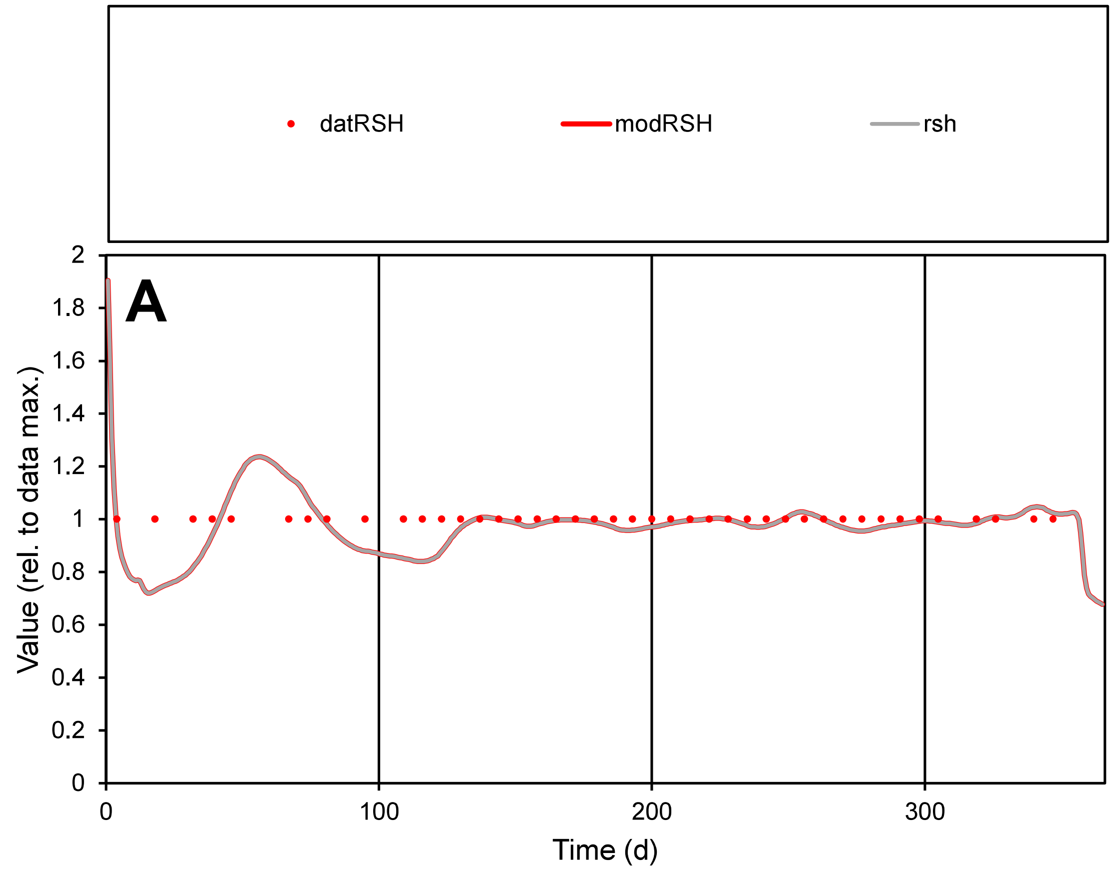

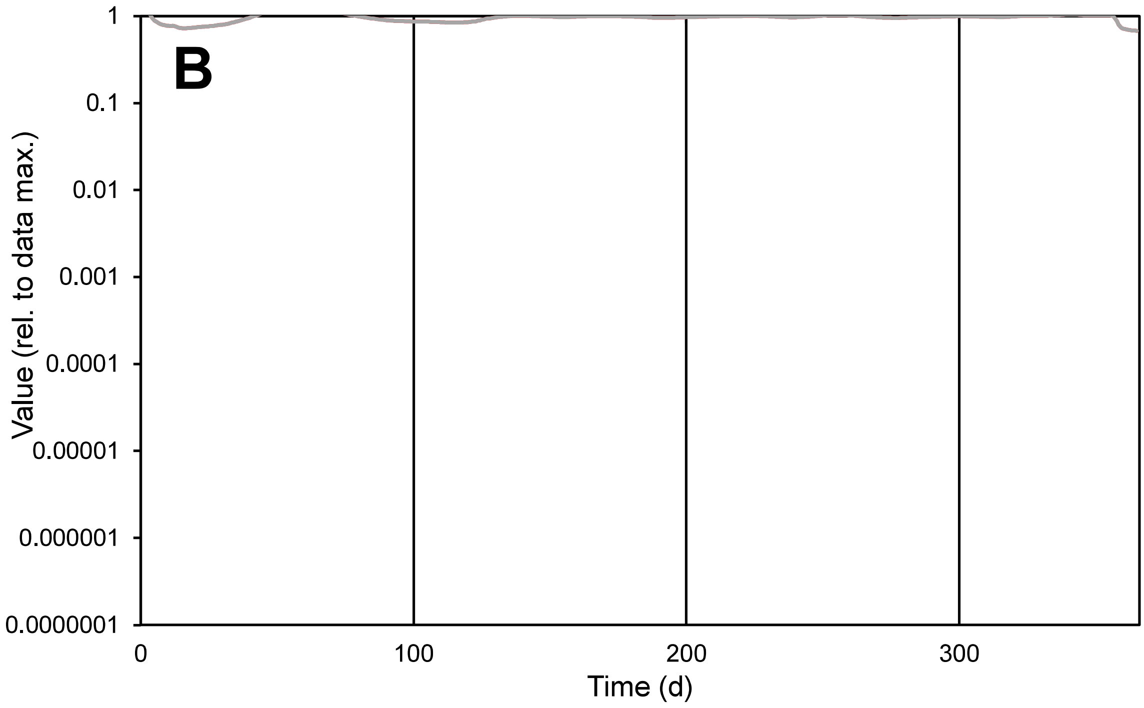

C1

Variables
RSH Provide jtd or Dst
CalibOnly?
2 1 to reproduce of
oNormalize?
2 1=no, 2=yes

ofxn calc*
0.07544
ofxn mod
0.13507

sRun
RunA1
zout
1

Ave
Data
1.8E-05
Model
1.7E-05

data/vMax
1.8E-05

ofxng
334.07

Min d/m
1.35201

ave/min d
1
ave/min m
1.31751

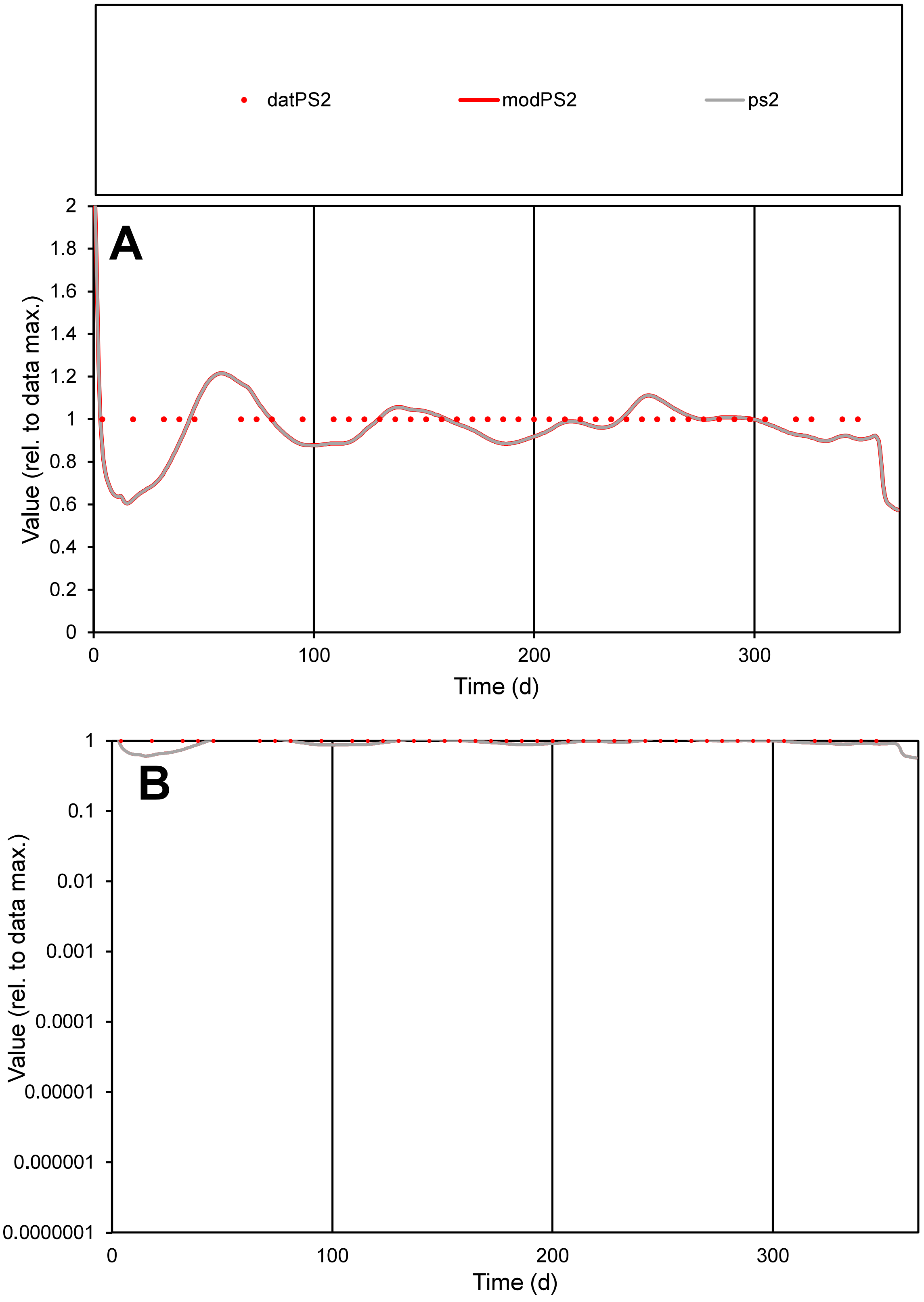

C1

Variables
PS2 Provide jtd or Dst
CalibOnly?
2 1 to reproduce of
oNormalize?
2 1=no, 2=yes

ofxn calc*
0.09808
ofxn mod
0.14979

sRun
RunA1
zout
1

Ave
Data
1.9E-05
Model
1.8E-05

data/vMax
1.9E-05

ofxng
334.07

Min d/m
1.56148

ave/min d
1
ave/min m
1.50909

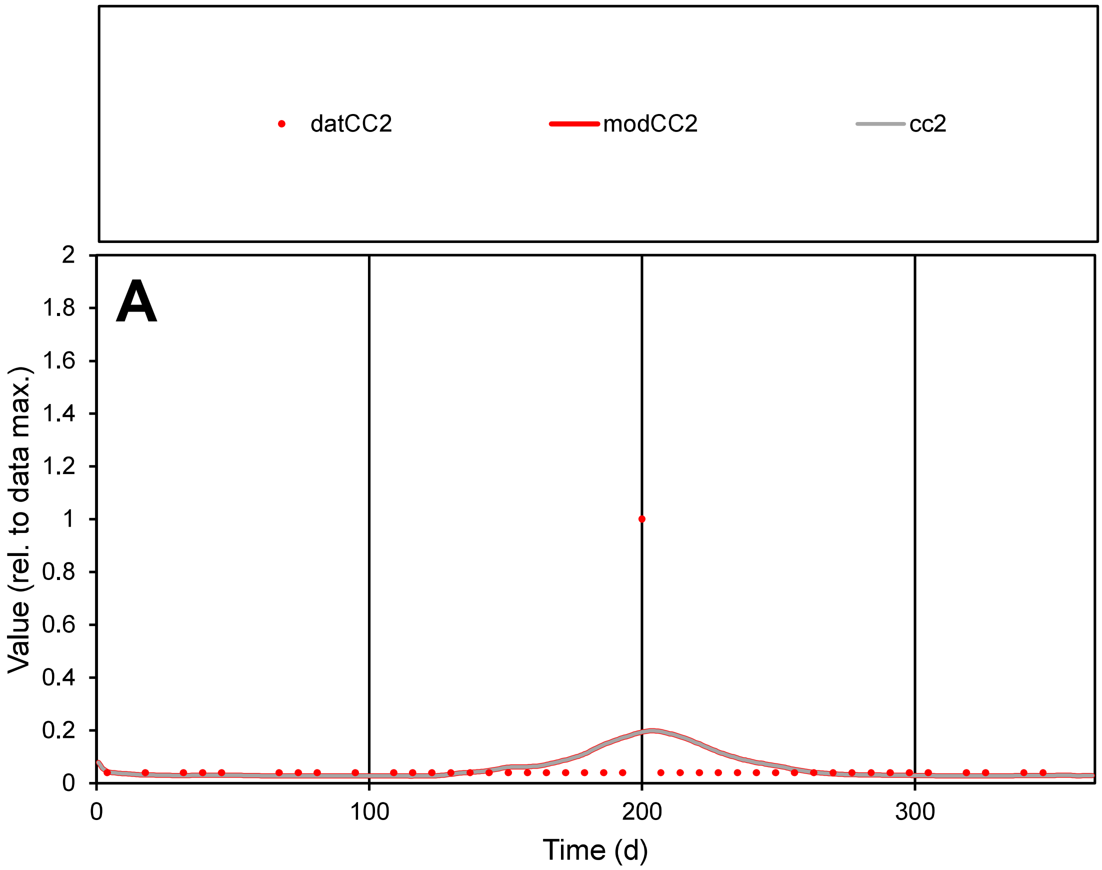

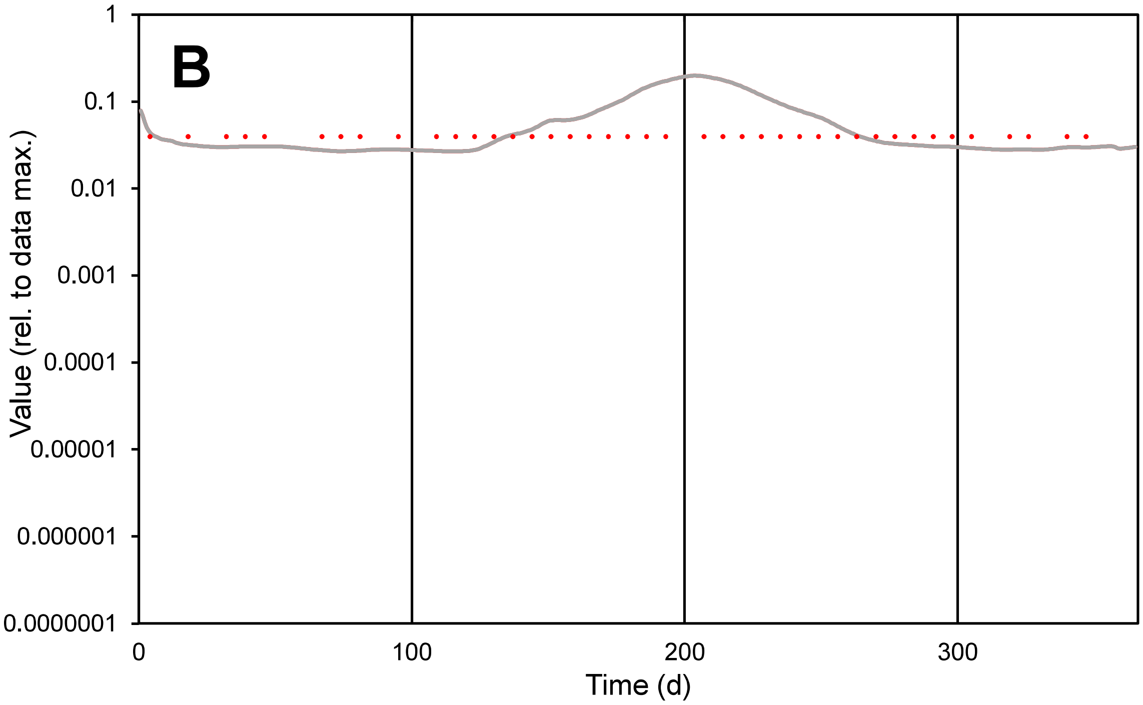

C1

Variables
CC2 Provide jtd or Dst
CalibOnly?
2 1 to reproduce of
oNormalize?
2 1=no, 2=yes

ofxn calc*
2.14234
ofxn mod
2.15714

sRun
RunA1
zout
1

Ave
Data
9.3E-06
Model
9.4E-06

data/vMax
0.00015

ofxng
334.07

Min d/m
1.47265

ave/min d
1.57934
ave/min m
2.34898

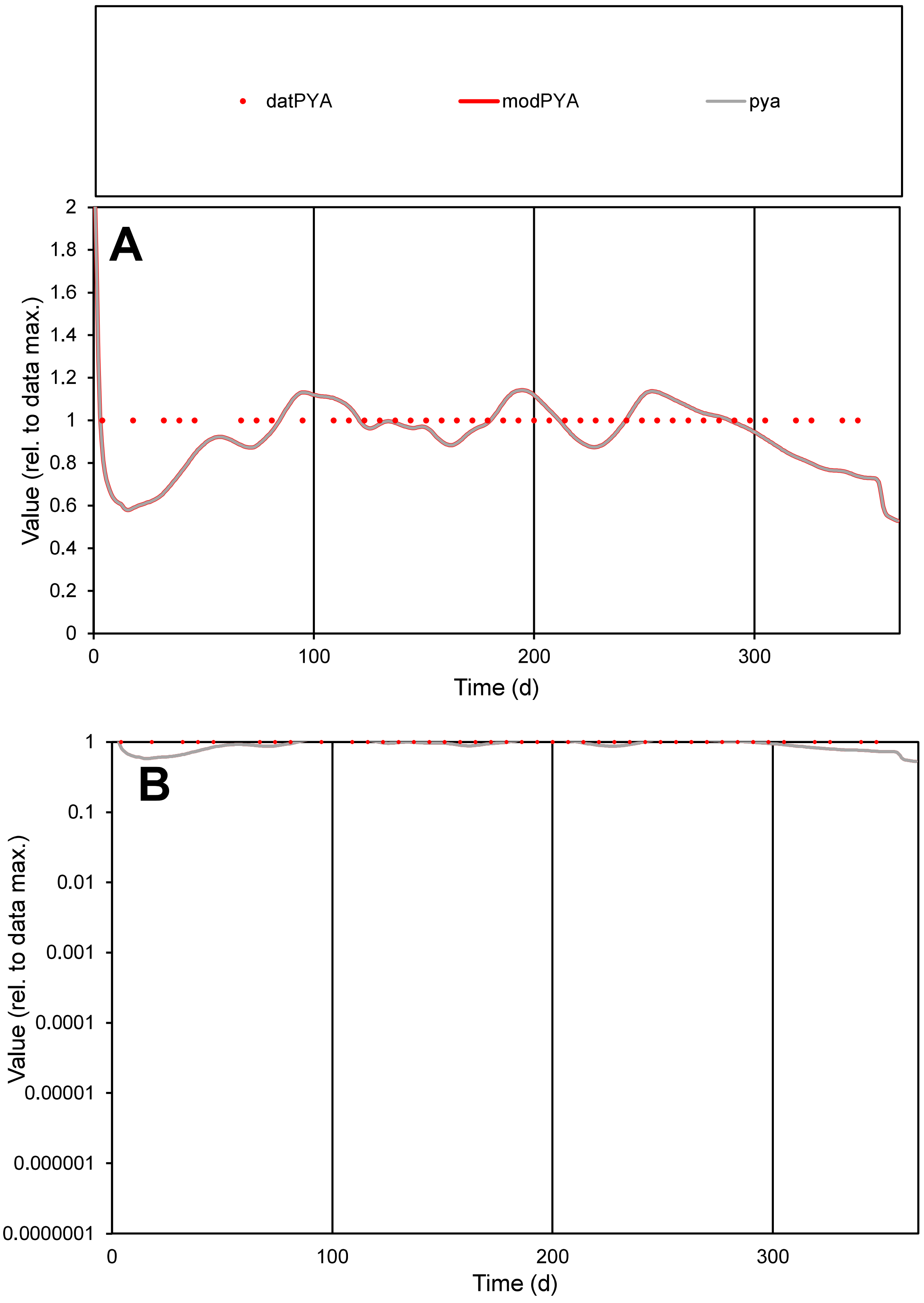

C1

Variables
PYA Provide jtd or Dst
CalibOnly?
2 1 to reproduce of
oNormalize?
2 1=no, 2=yes

ofxn calc*
0.13766
ofxn mod
0.17772

sRun
RunA1
zout
1

Ave
Data
1.8E-06
Model
1.7E-06

data/vMax
1.8E-06

ofxng
334.07

Min d/m
1.67623

ave/min d
1
ave/min m
1.58973

327_Calib_S_A

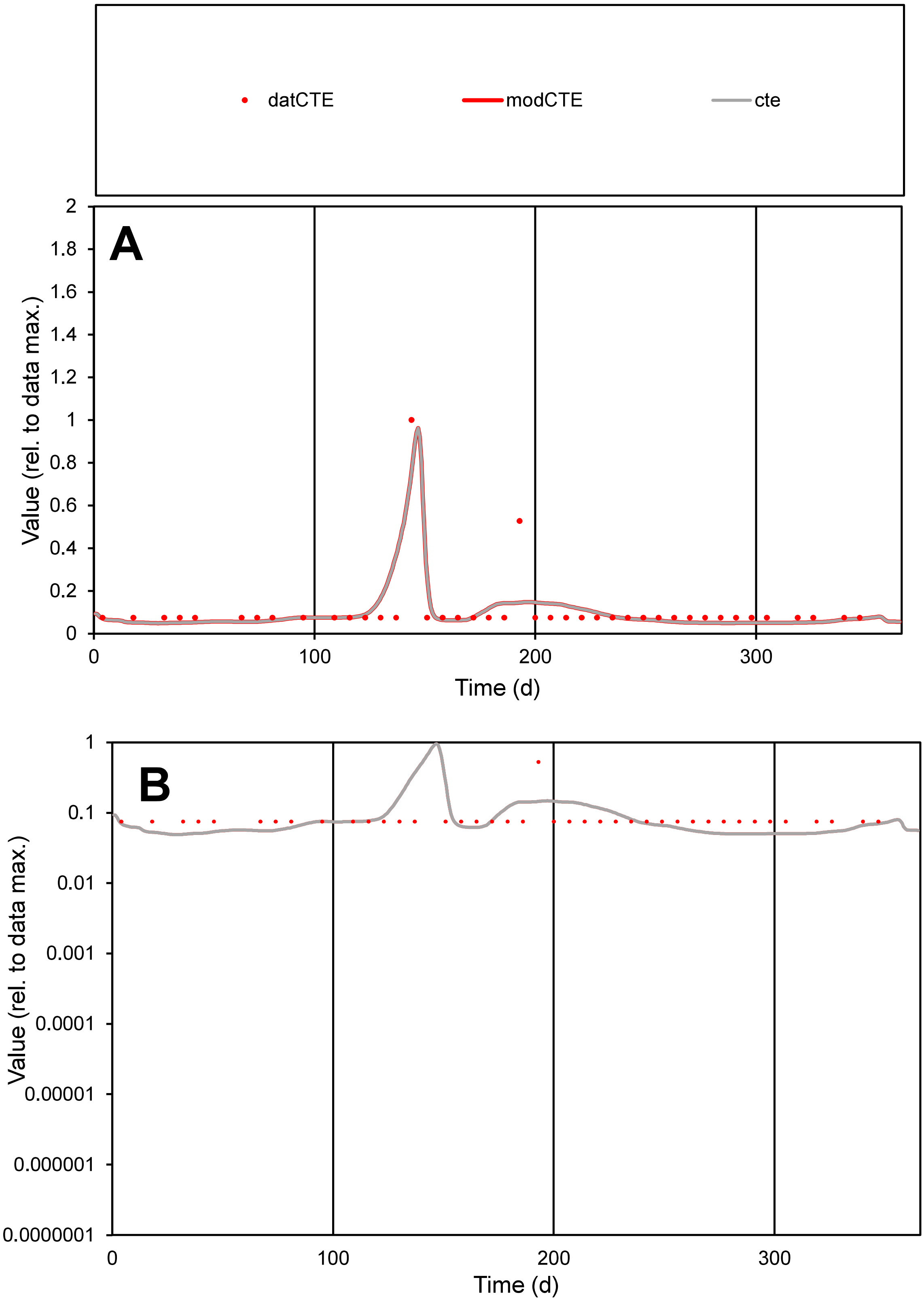

C1

Variables

CTE Provide jtd or Dst

CalibOnly?

2 1 to reproduce of

oNormalize?

2 1=no, 2=yes

ofxn calc*

0.73165

ofxn mod

0.7749

sRun

RunA1

zout

1

Ave

Data

8.8E-06

Model

8.5E-06

data/vMax

8.1E-05

ofxng

334.07

Min d/m

1.50998

ave/min d

1.43576

ave/min m

2.09389

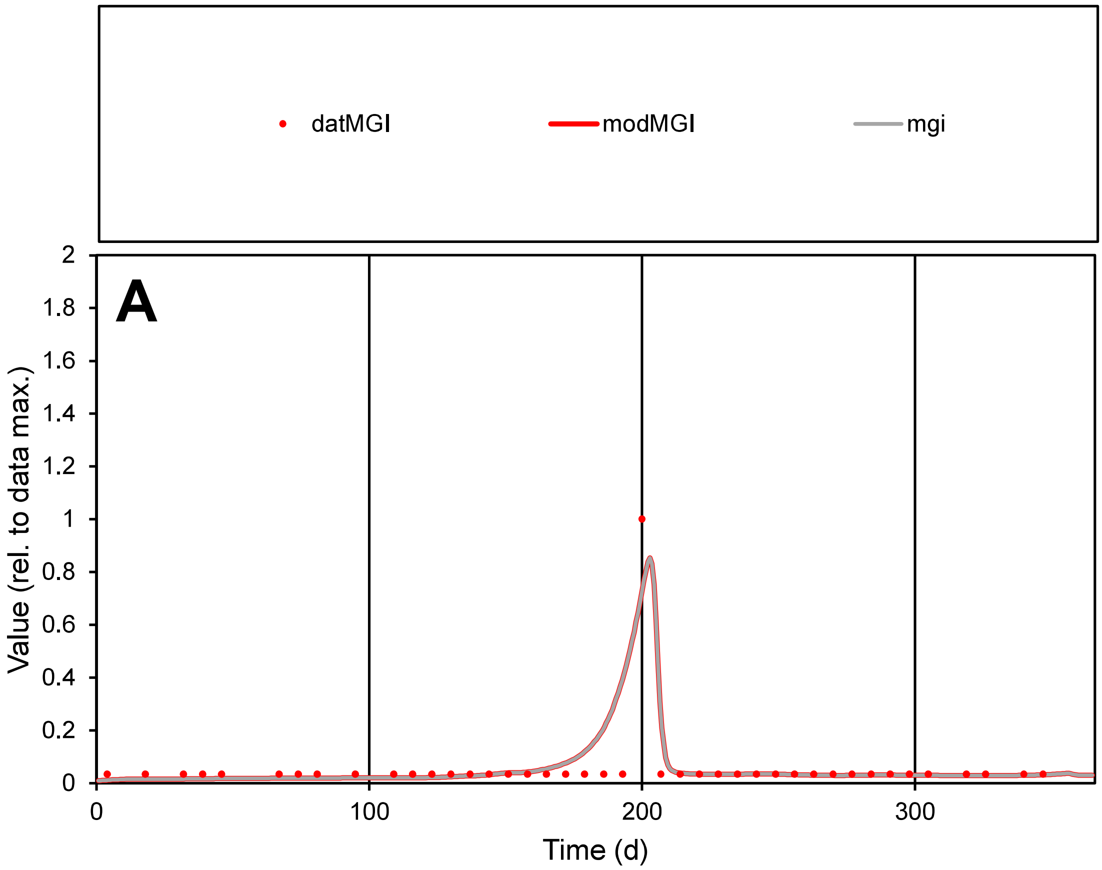

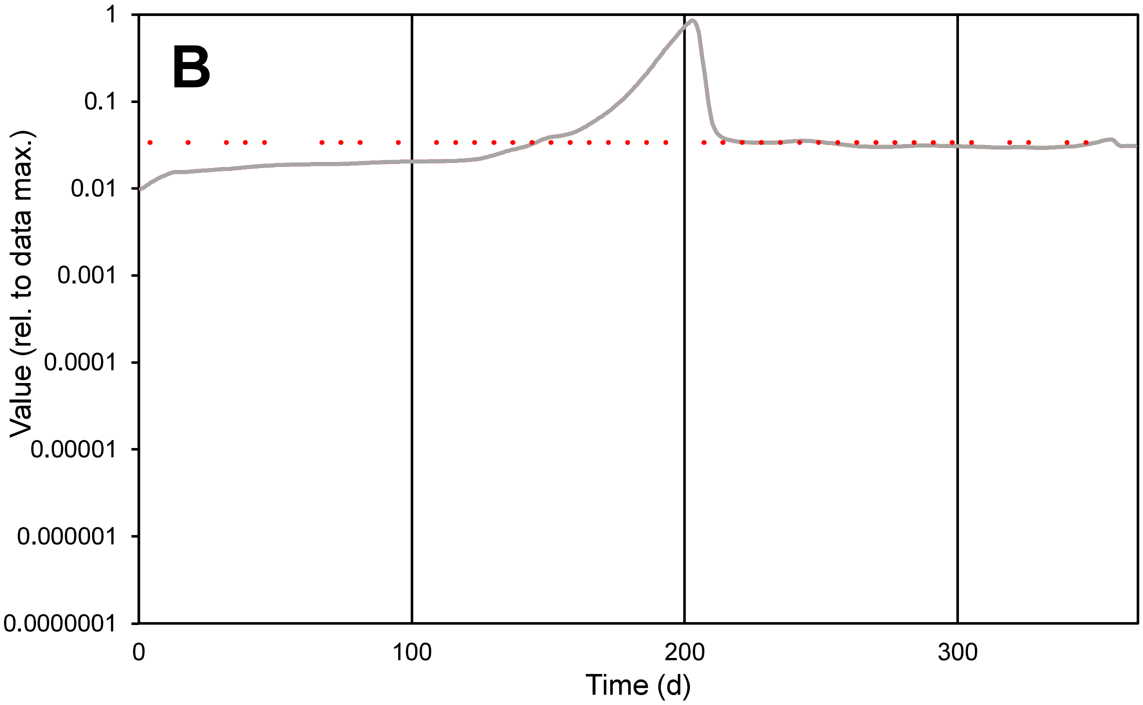

C1

Variables
MGI Provide jtd or Dst
CalibOnly?
2 1 to reproduce of
oNormalize?
2 1=no, 2=yes

ofxn calc*
1.12634
ofxn mod
1.30164

sRun
RunA1
zout
1

Ave
Data
1.7E-05
Model
1.9E-05

data/vMax
0.0003

ofxng
334.07

Min d/m
2.82662

ave/min d
1.6764
ave/min m
5.2156

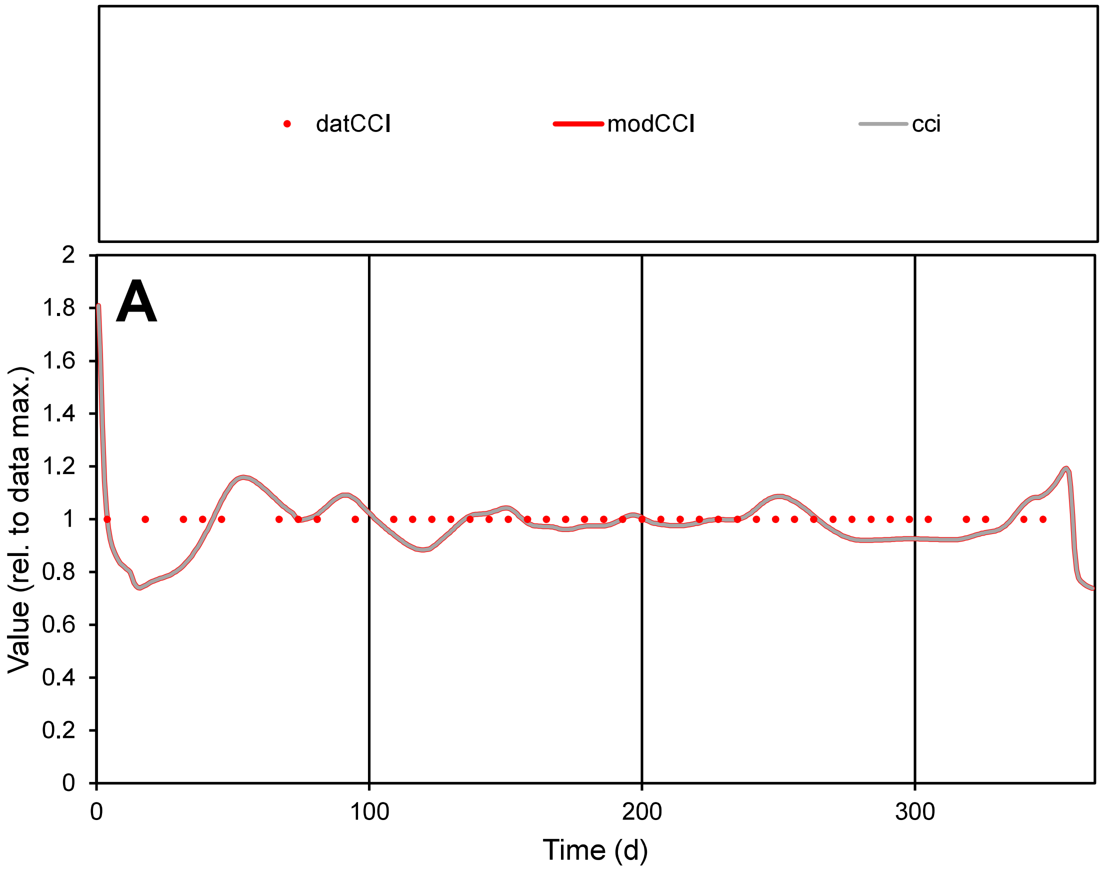

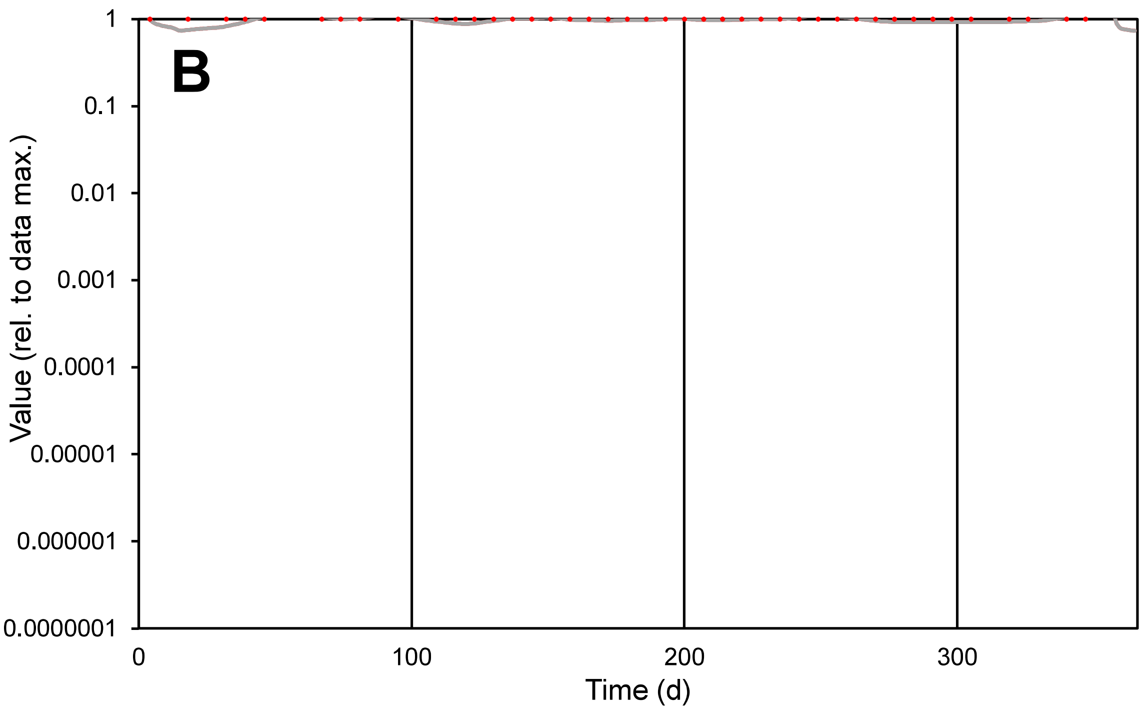

C1

Variables
CCI Provide jtd or Dst
CalibOnly?
2 1 to reproduce of
oNormalize?
2 1=no, 2=yes

ofxn calc*
0.07071
ofxn mod
0.13247

sRun
RunA1
zout
1

Ave
Data
1.1E-05
Model
1.1E-05

data/vMax
1.1E-05

ofxng
334.07

Min d/m
1.31636

ave/min d
1
ave/min m
1.28973

327_Calib_S_A

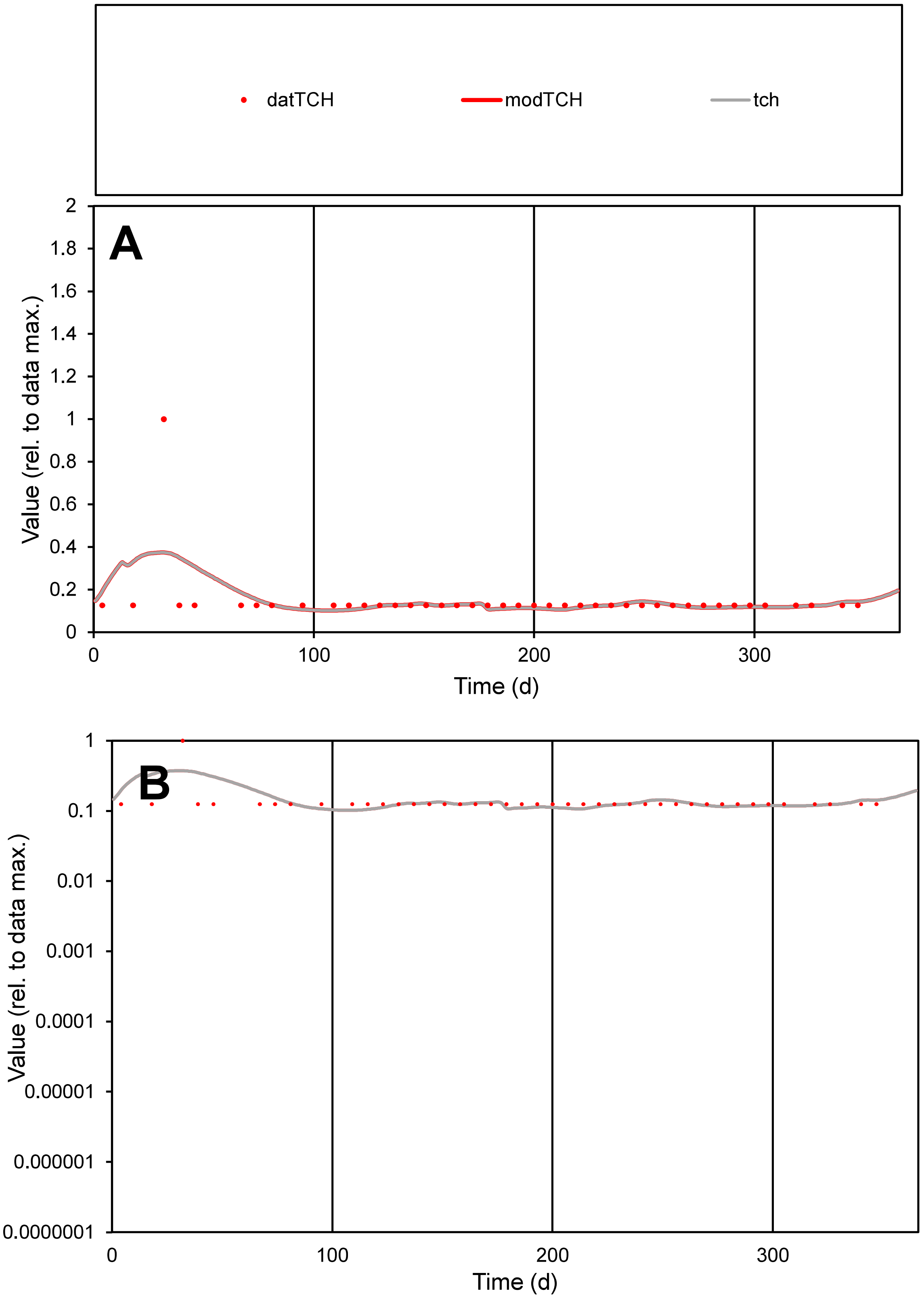

C1

Variables
TCH Provide jtd or Dst
CalibOnly?
2 1 to reproduce of
oNormalize?
2 1=no, 2=yes

ofxn calc*
0.76557
ofxn mod
0.7807

sRun
RunA1
zout
1

Ave
Data
2E-06
Model
2E-06

data/vMax
1.3E-05

ofxng
334.07

Min d/m
1.21884

ave/min d
1.1663
ave/min m
1.42345

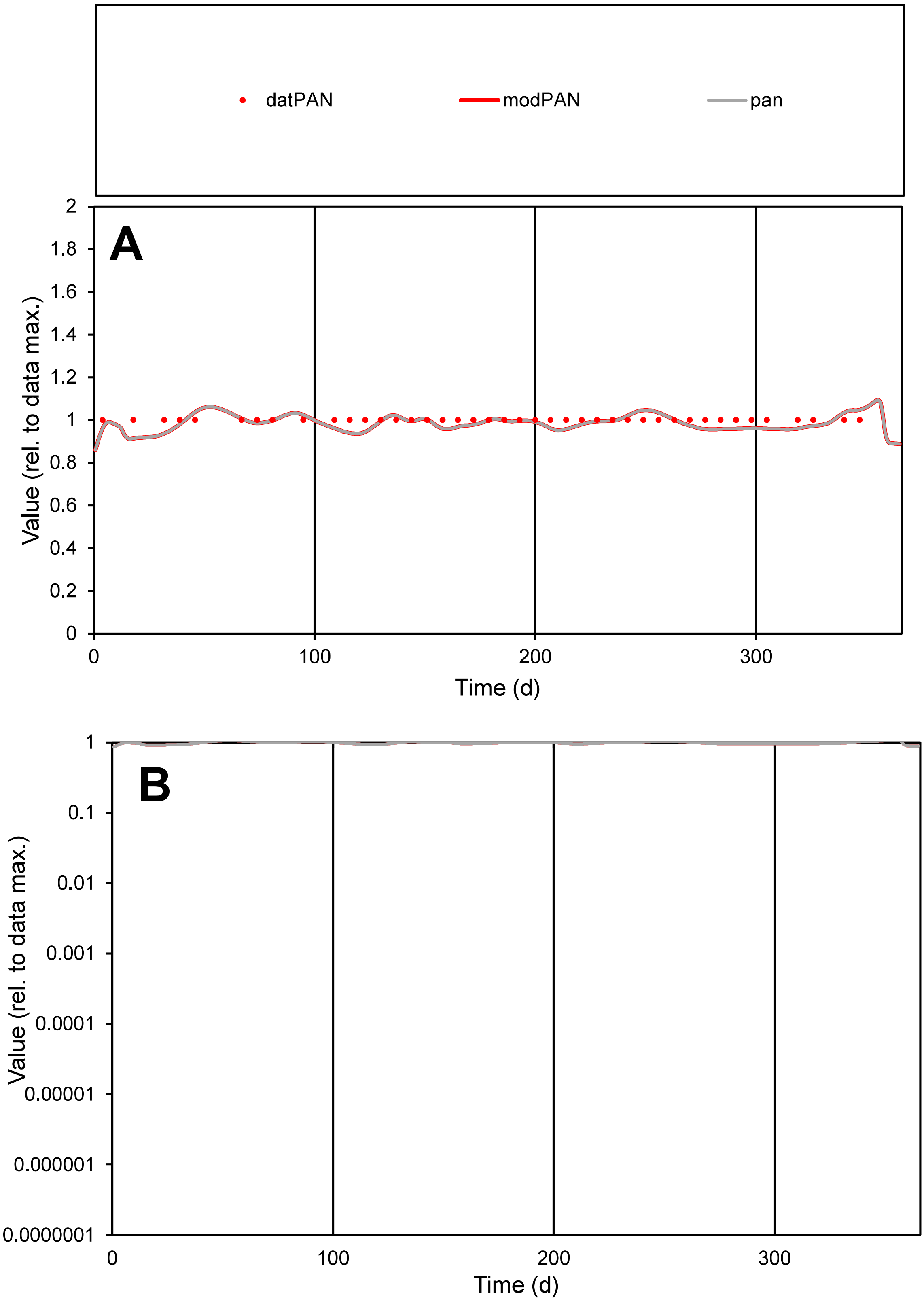

C1

Variables
PAN Provide jtd or Dst
CalibOnly?
2 1 to reproduce of
oNormalize?
2 1=no, 2=yes

ofxn calc*
0.03396
ofxn mod
0.11748

sRun
RunA1
zout
1

Ave
Data
9.1E-06
Model
9E-06

data/vMax
9.1E-06

ofxng
334.07

Min d/m
1.09117

ave/min d
1
ave/min m
1.07686

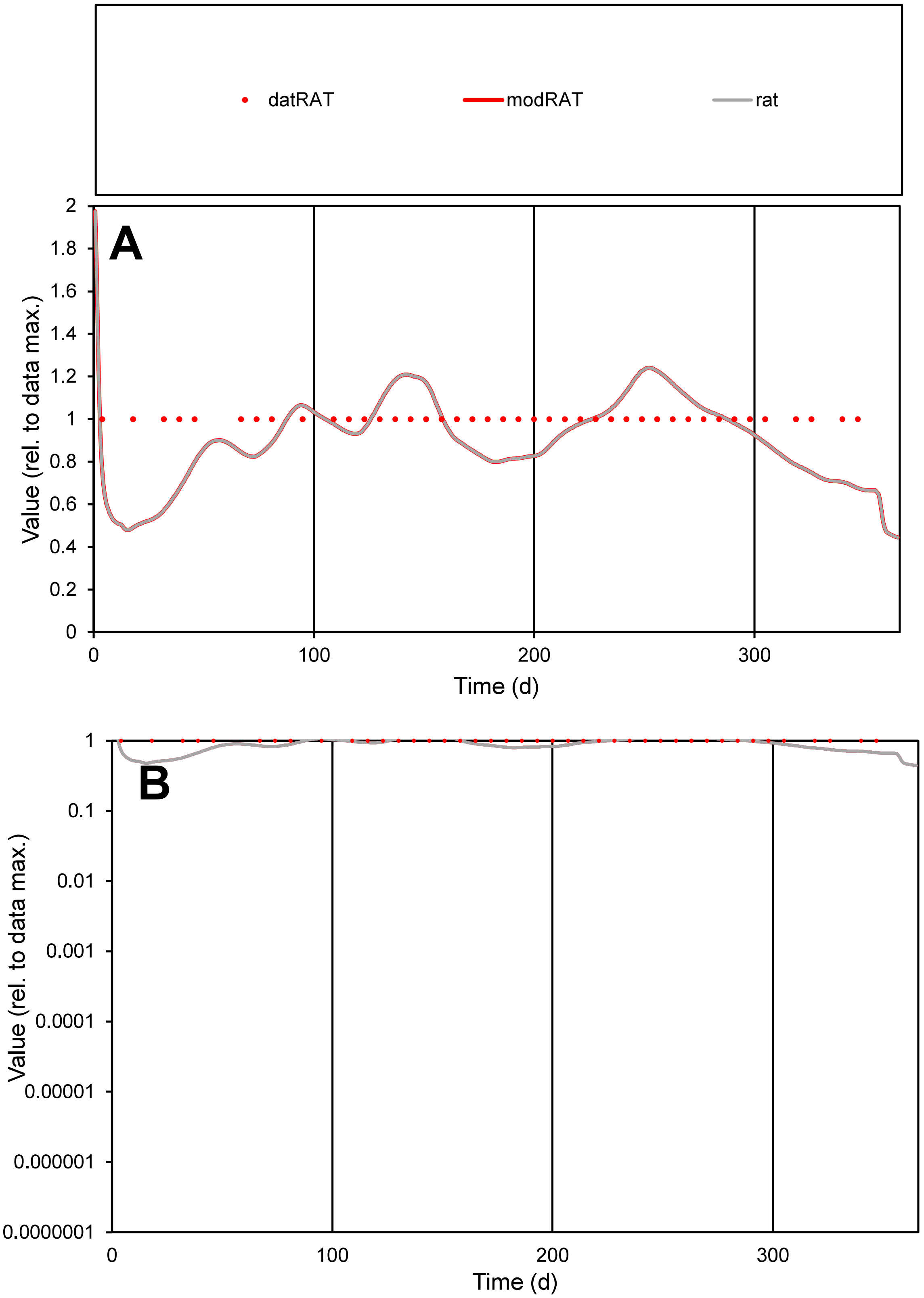

C1

Variables
RAT Provide jtd or Dst
CalibOnly?
2 1 to reproduce of
oNormalize?
2 1=no, 2=yes

ofxn calc*
0.18877
ofxn mod
0.21878

sRun
RunA1
zout
1

Ave
Data
1.1E-05
Model
1.1E-05

data/vMax
1.1E-05

ofxng
334.07

Min d/m
1.99439

ave/min d
1
ave/min m
1.85001

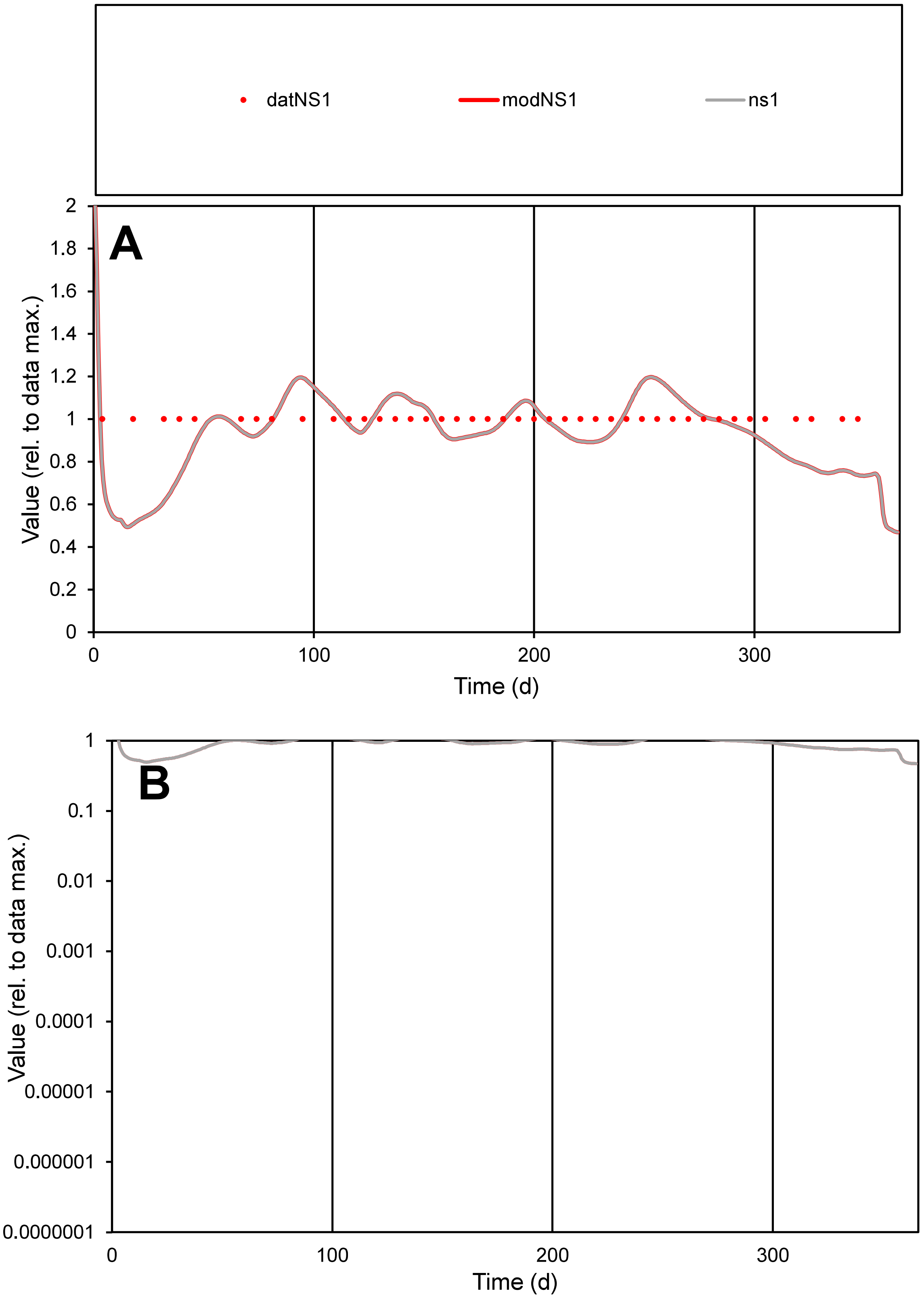

C1

Variables

NS1 Provide jtd or Dst

CalibOnly?

2 1 to reproduce of

oNormalize?

2 1=no, 2=yes

ofxn calc*

0.15286

ofxn mod

0.18953

sRun

RunA1

zout

1

Ave

Data

2.7E-05

Model

2.6E-05

data/vMax

2.7E-05

ofxng

334.07

Min d/m

1.92162

ave/min d

1

ave/min m

1.82174

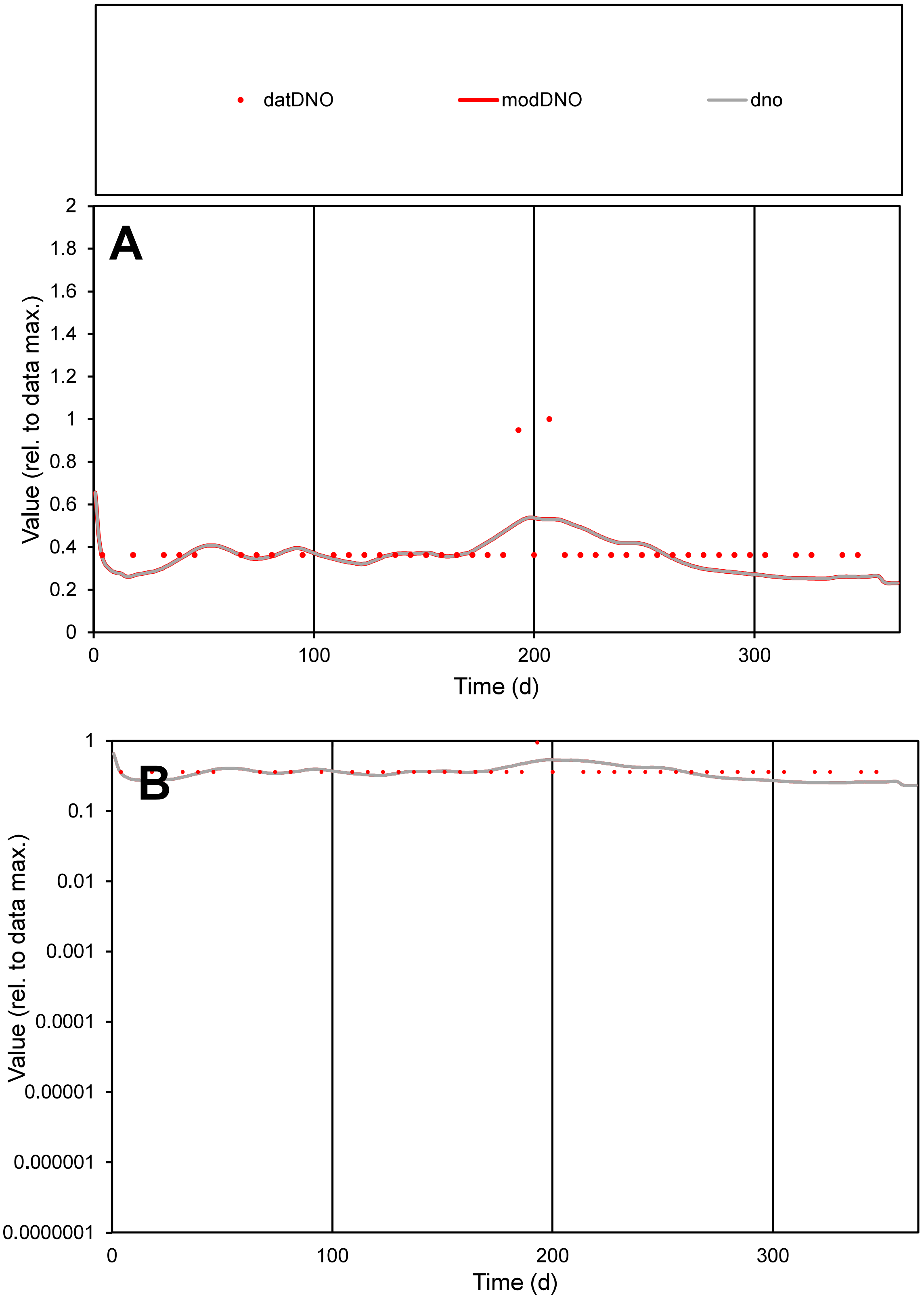

C1

Variables
DNO Provide jtd or Dst
CalibOnly?
2 1 to reproduce of
oNormalize?
2 1=no, 2=yes

ofxn calc*
0.30667
ofxn mod
0.32813

sRun
RunA1
zout
1

Ave
Data
2E-05
Model
1.8E-05

data/vMax
5E-05

ofxng
334.07

Min d/m
1.43083

ave/min d
1.08047
ave/min m
1.44425

327_Calib_S_A

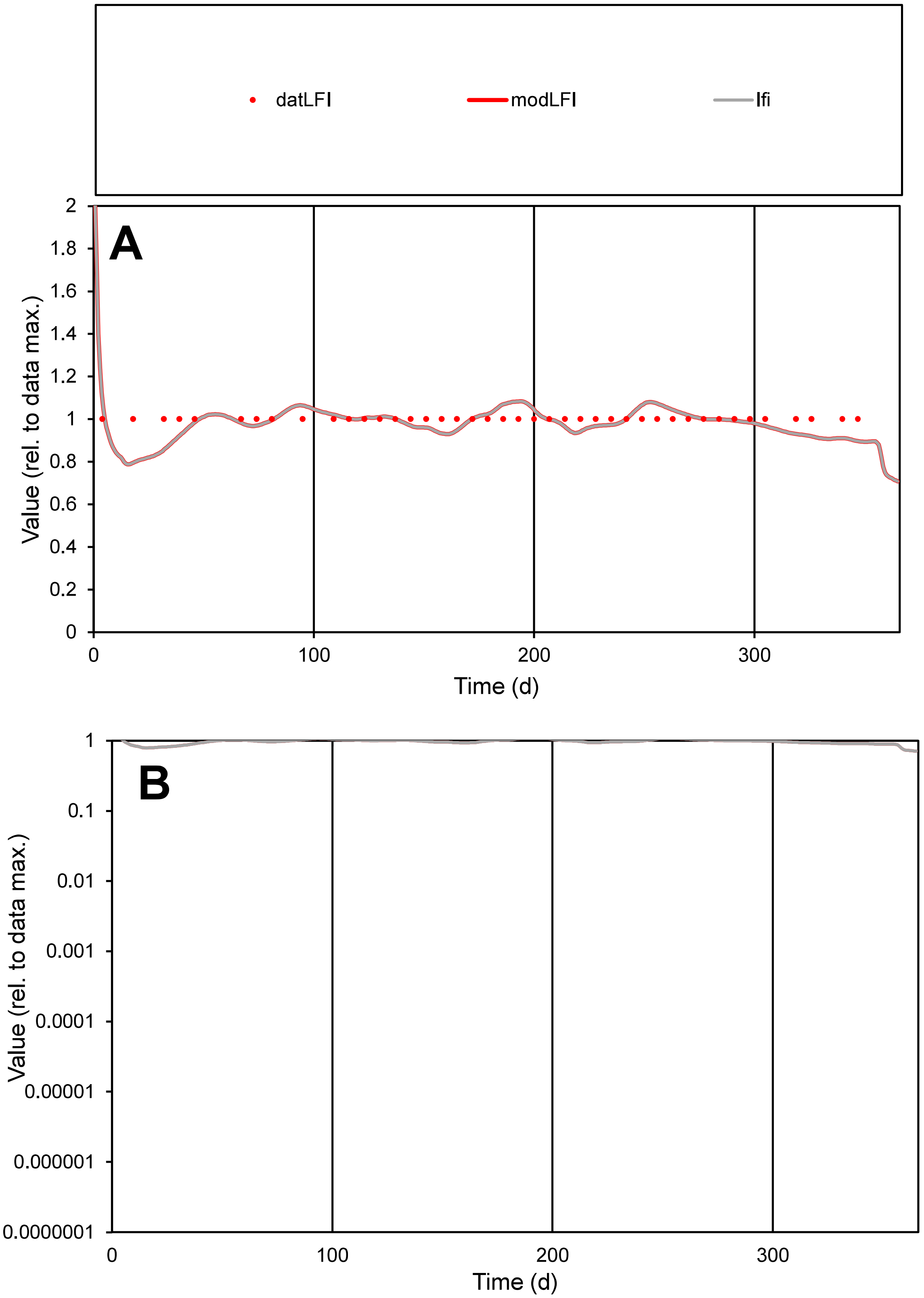

C1

Variables
LFI Provide jtd or Dst
CalibOnly?
2 1 to reproduce of
oNormalize?
2 1=no, 2=yes

ofxn calc*
0.0583
ofxn mod
0.12664

sRun
RunA1
zout
1

Ave
Data
8.3E-06
Model
8.2E-06

data/vMax
8.3E-06

ofxng
334.07

Min d/m
1.24517

ave/min d
1
ave/min m
1.22543

327_Calib_S_A

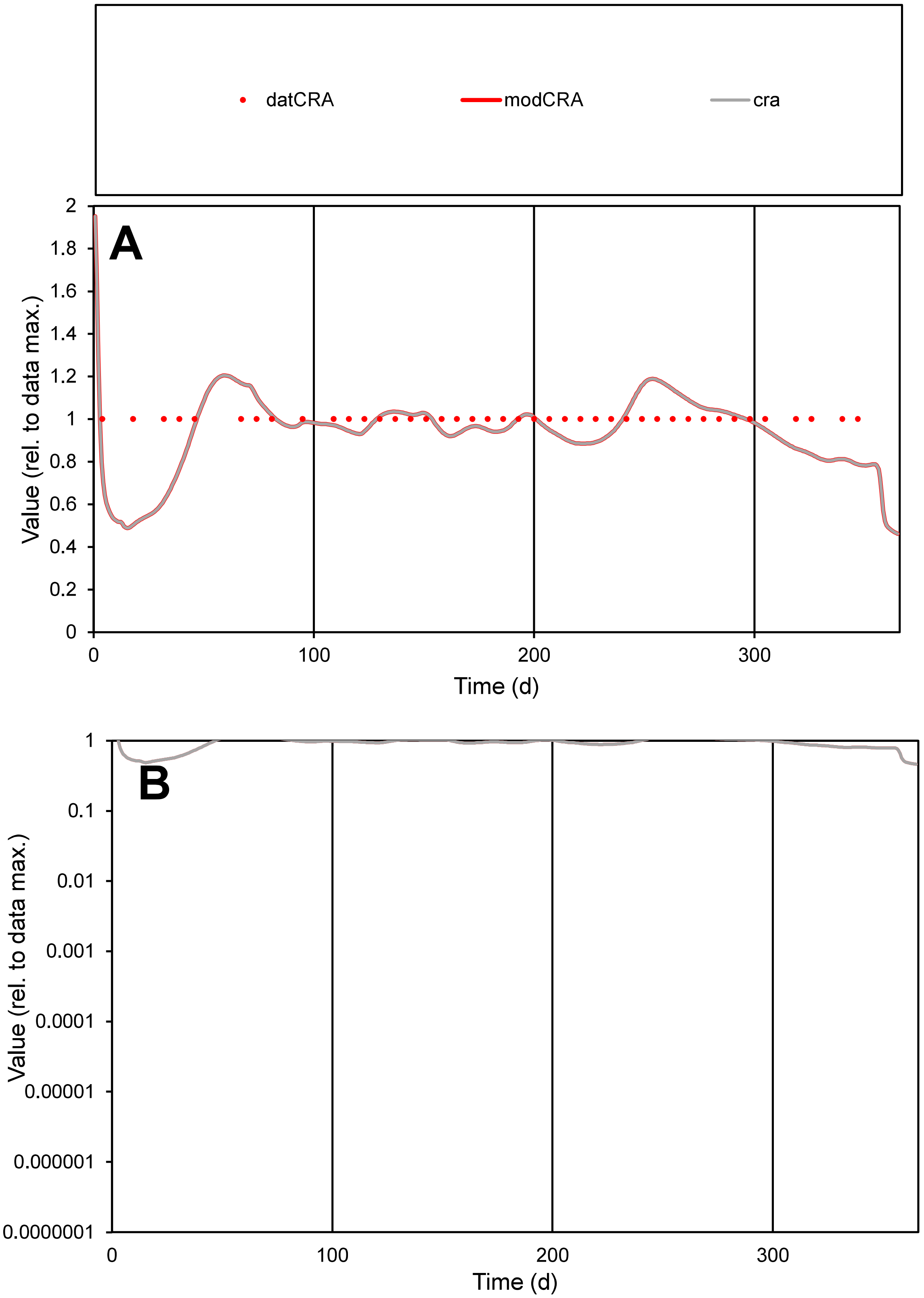

C1

Variables

CRA Provide jtd or Dst

CalibOnly?

2 1 to reproduce of

oNormalize?

2 1=no, 2=yes

ofxn calc*

0.13875

ofxn mod

0.17874

sRun

RunA1

zout

1

Ave

Data

4.9E-06

Model

4.6E-06

data/vMax

4.9E-06

ofxng

334.07

Min d/m

1.93477

ave/min d

1

ave/min m

1.84908

327_Calib_S_A

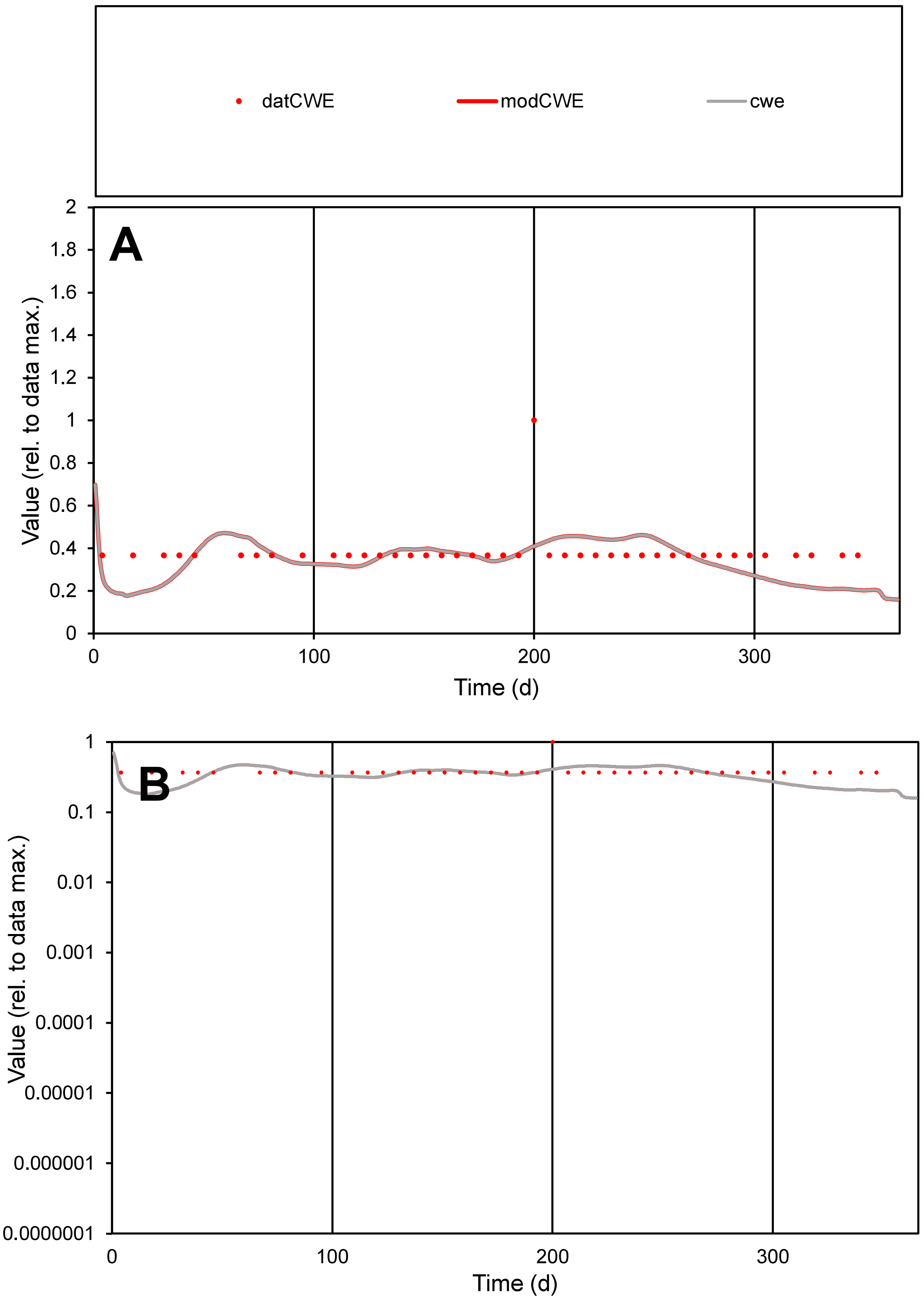

C1

Variables
CWE Provide jtd or Dst
CalibOnly?
2 1 to reproduce of
oNormalize?
2 1=no, 2=yes

ofxn calc*
0.31539
ofxn mod
0.33593

sRun
RunA1
zout
1

Ave
Data
2.7E-05
Model
2.4E-05

data/vMax
7E-05

ofxng
334.07

Min d/m
1.94064

ave/min d
1.04116
ave/min m
1.85685

327_Calib_S_A

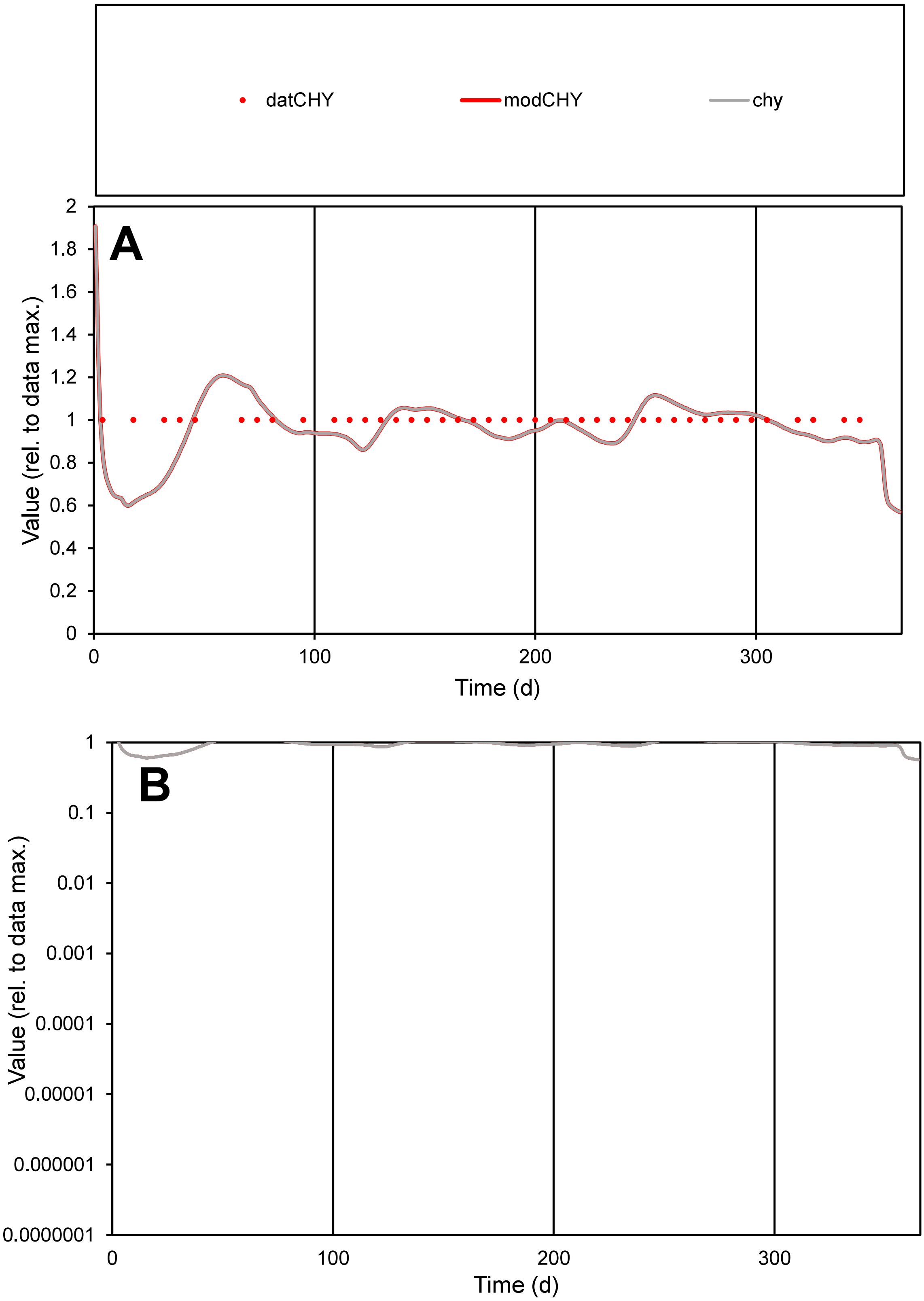

C1

Variables
CHY Provide jtd or Dst
CalibOnly?
2 1 to reproduce of
oNormalize?
2 1=no, 2=yes

ofxn calc*
0.10234
ofxn mod
0.15262

sRun
RunA1
zout
1

Ave
Data
6.7E-06
Model
6.5E-06

data/vMax
6.7E-06

ofxng
334.07

Min d/m
1.59909

ave/min d
1
ave/min m
1.54997

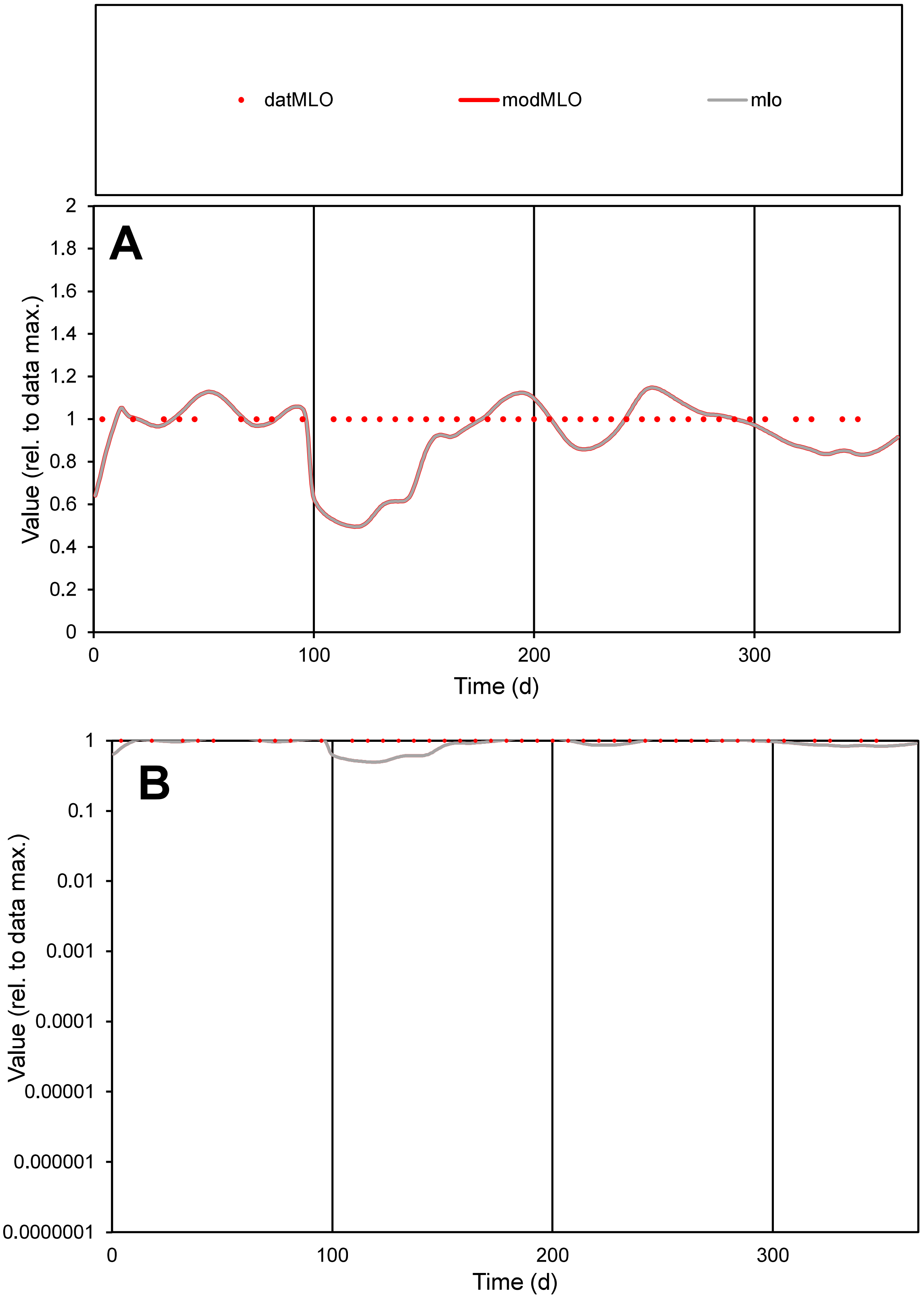

C1

Variables

MLO	Provide jtd or Dst
CalibOnly?	
2	1 to reproduce of
oNormalize?	
2	1=no, 2=yes
ofxn calc*	
0.1847	
ofxn mod	
0.2153	
sRun	
RunA1	
zout	
1	
Ave	
Data	
1E-05	
Model	
9.6E-06	
data/vMax	
1E-05	
ofxng	
334.07	
Min d/m	
2.01235	
ave/min d	
1	
ave/min m	
1.85929	

327_Calib_S_A

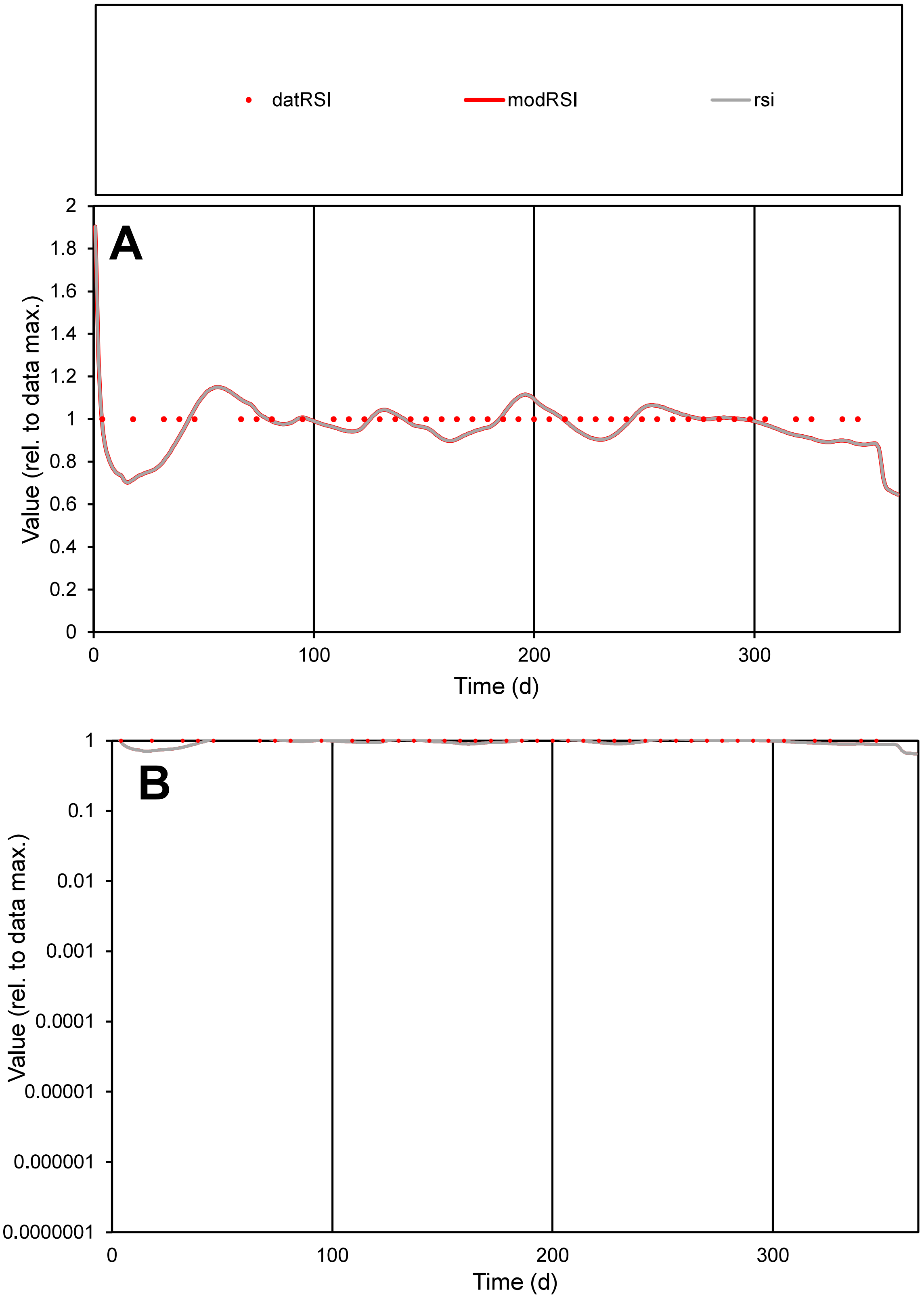

C1

Variables

RSI	Provide jtd or Dst
CalibOnly?	
2	1 to reproduce of
oNormalize?	
2	1=no, 2=yes

ofxn calc*
0.07801
ofxn mod
0.13668

sRun
RunA1
zout
1

Ave
Data
1E-05
Model
1E-05

data/vMax
1E-05

ofxng
334.07

Min d/m
1.37239

ave/min d
1
ave/min m
1.33682

327_Calib_S_A

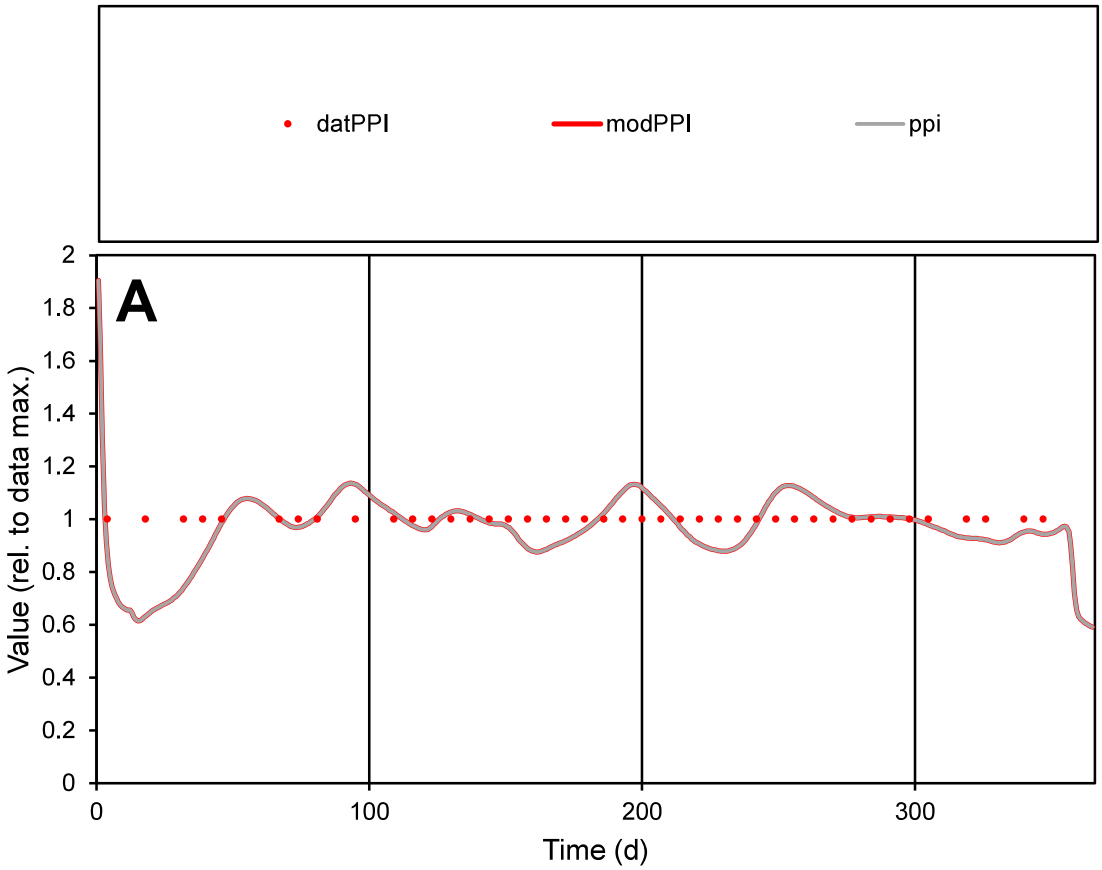

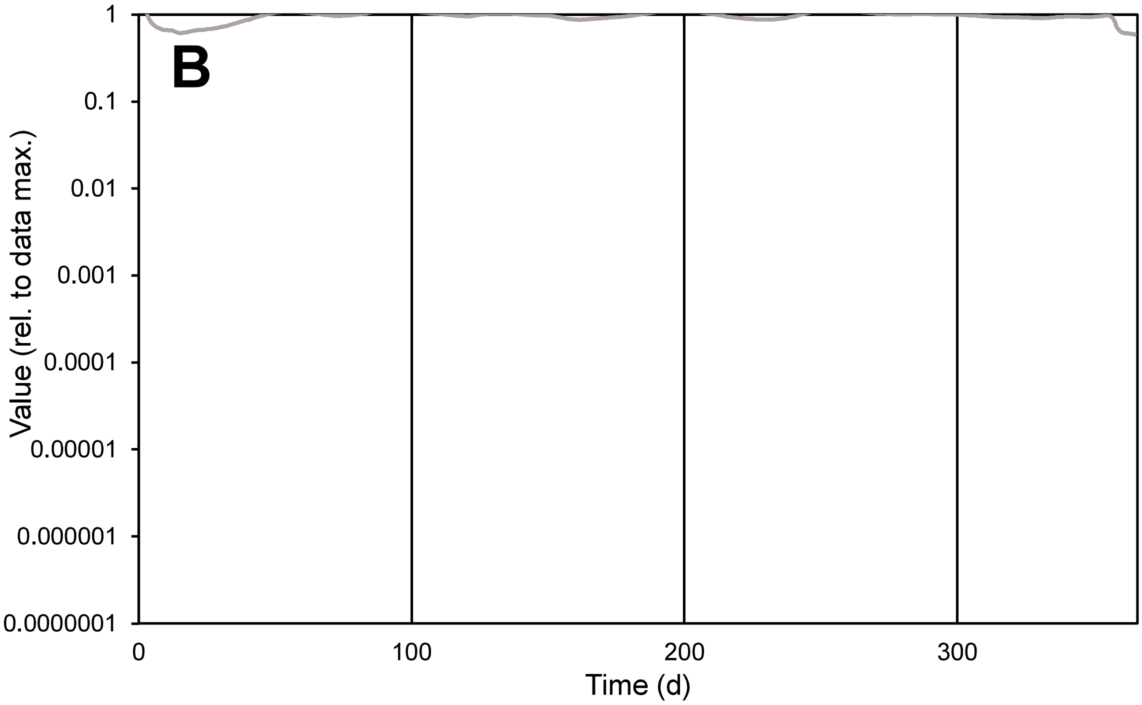

C1

Variables
PPI Provide jtd or Dst
CalibOnly?
2 1 to reproduce of
oNormalize?
2 1=no, 2=yes

ofxn calc*
0.09707
ofxn mod
0.14952

sRun
RunA1
zout
1

Ave
Data
1.4E-06
Model
1.4E-06

data/vMax
1.4E-06

ofxng
334.07

Min d/m
1.54735

ave/min d
1
ave/min m
1.50703

327_Calib_S_A

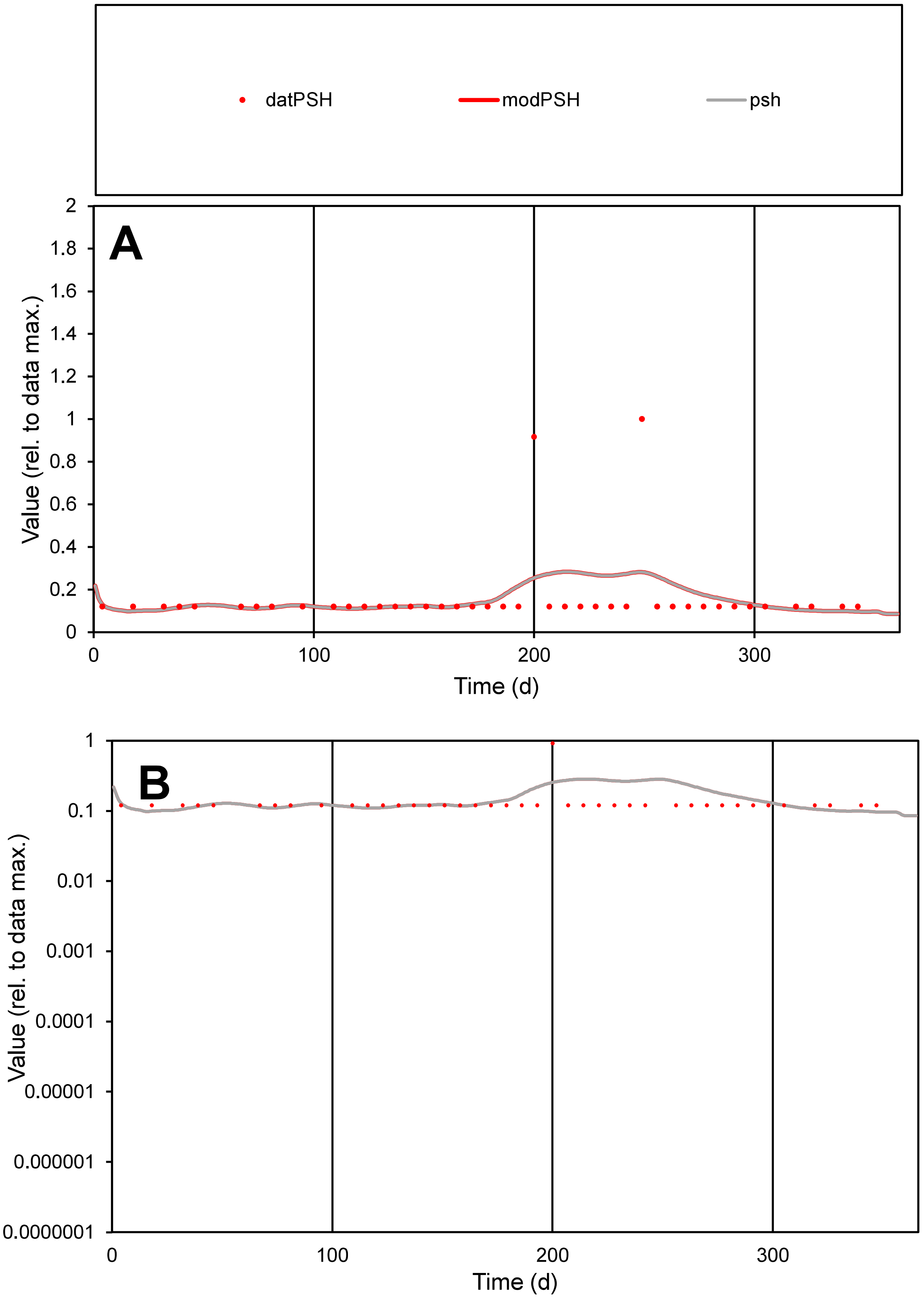

C1

Variables
PSH Provide jtd or Dst
CalibOnly?
2 1 to reproduce of
oNormalize?
2 1=no, 2=yes

ofxn calc*
1.03083
ofxn mod
1.04182

sRun
RunA1
zout
1

Ave
Data
1.7E-05
Model
1.7E-05

data/vMax
0.00011

ofxng
334.07

Min d/m
1.25

ave/min d
1.33171
ave/min m
1.66796

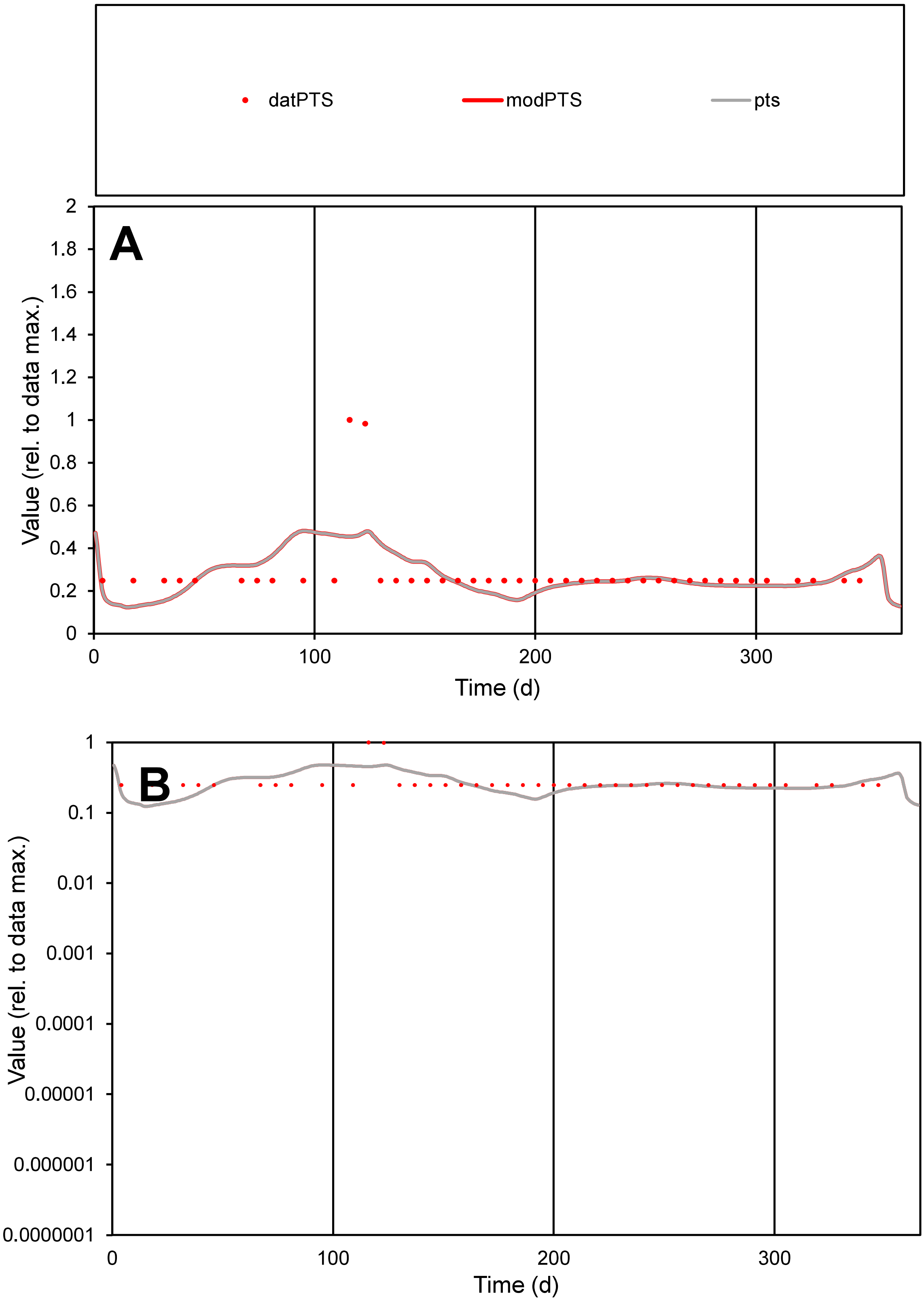

C1

Variables
PTS Provide jtd or Dst
CalibOnly?
2 1 to reproduce of
oNormalize?
2 1=no, 2=yes

ofxn calc*
0.48542
ofxn mod
0.4988

sRun
RunA1
zout
1

Ave
Data
1.1E-05
Model
1.1E-05

data/vMax
3.9E-05

ofxng
334.07

Min d/m
1.91667

ave/min d
1.14285
ave/min m
2.08327

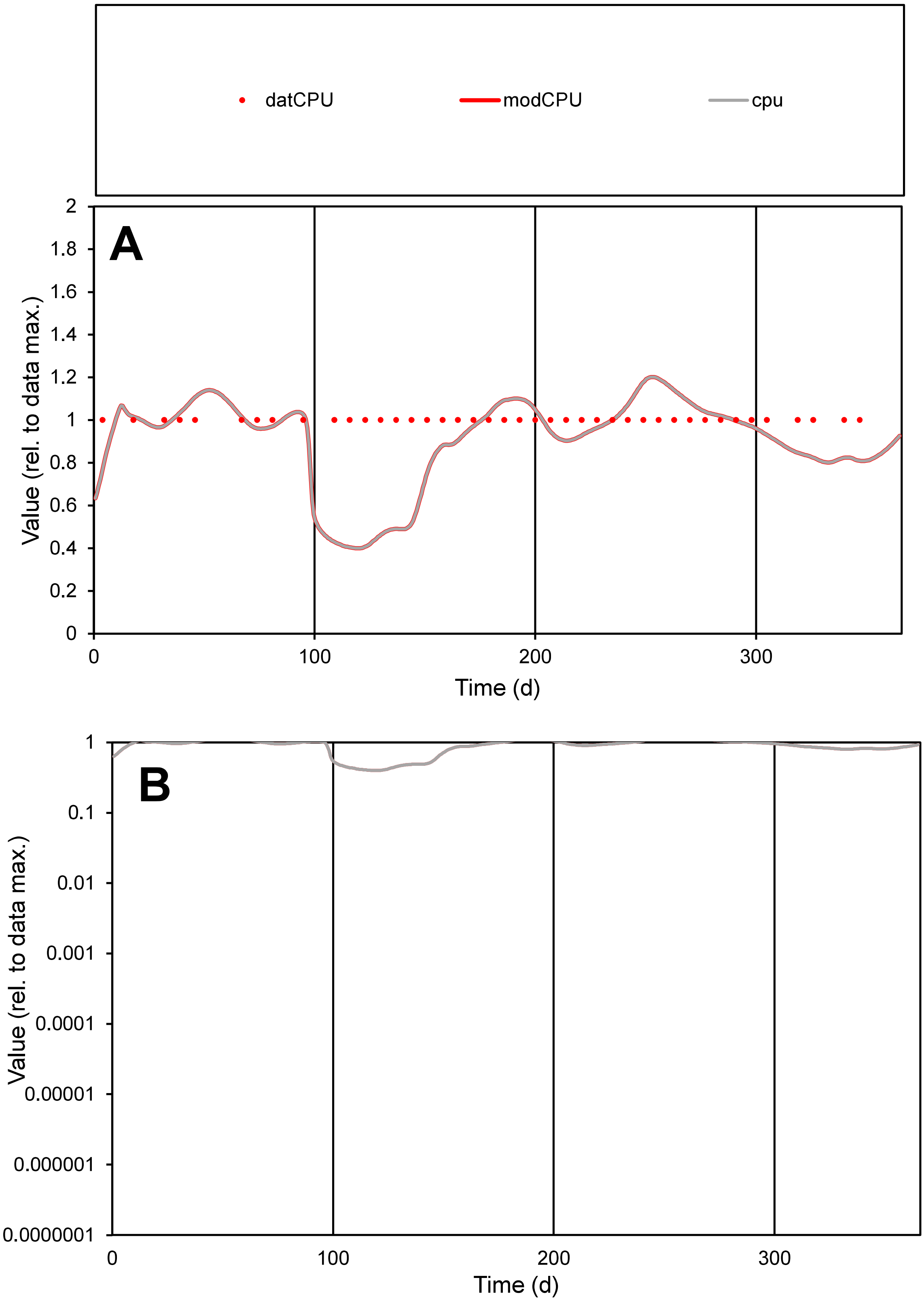

C1

Variables
CPU Provide jtd or Dst
CalibOnly?
2 1 to reproduce of
oNormalize?
2 1=no, 2=yes

ofxn calc*
0.22728
ofxn mod
0.25269

sRun
RunA1
zout
1

Ave
Data
4.4E-07
Model
4E-07

data/vMax
4.4E-07

ofxng
334.07

Min d/m
2.48567

ave/min d
1
ave/min m
2.25913

327_Calib_S_A

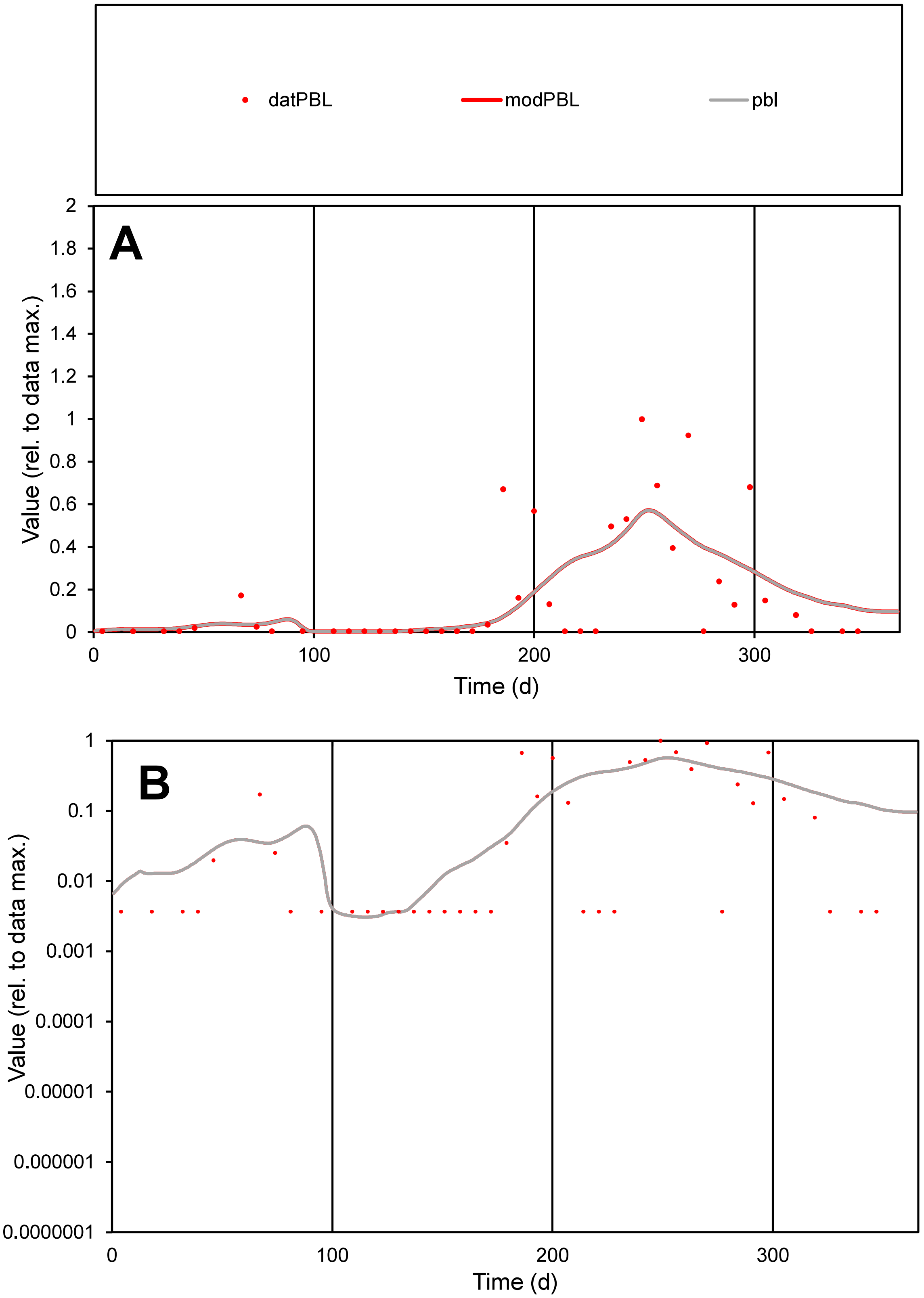

C1

Variables
PBL Provide jtd or Dst
CalibOnly?
2 1 to reproduce of
oNormalize?
2 1=no, 2=yes

ofxn calc*
1.19597
ofxn mod
1.21581

sRun
RunA1
zout
1

Ave
Data
2E-06
Model
2E-06

data/vMax
1.2E-05

ofxng
334.07

Min d/m
1.19383

ave/min d
46.3317
ave/min m
55.5847

327_Calib_S_A

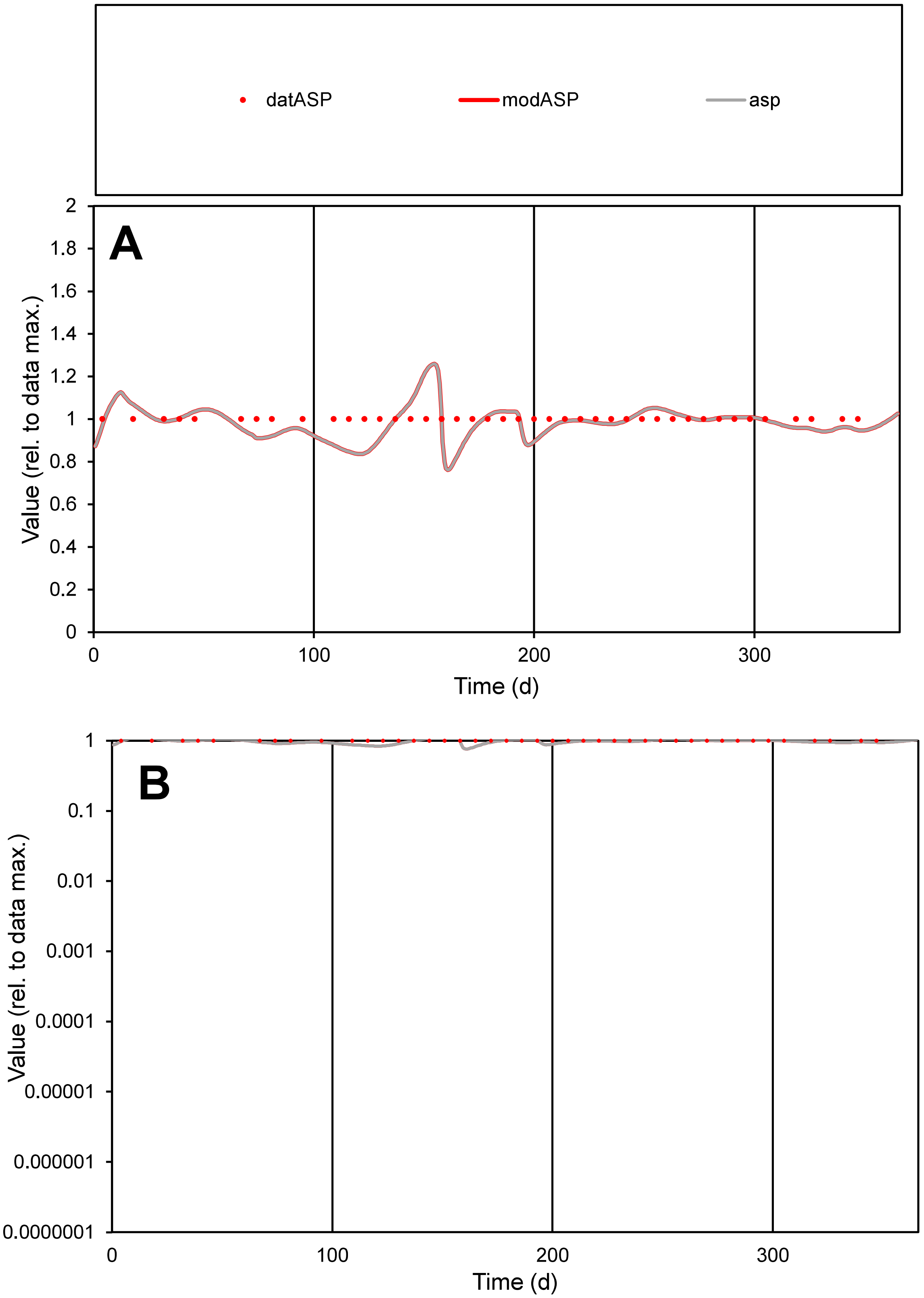

C1

Variables
ASP Provide jtd or Dst
CalibOnly?
2 1 to reproduce of
oNormalize?
2 1=no, 2=yes

ofxn calc*
0.07353
ofxn mod
0.13588

sRun
RunA1
zout
1

Ave
Data
5.8E-06
Model
5.7E-06

data/vMax
5.8E-06

ofxng
334.07

Min d/m
1.18569

ave/min d
1
ave/min m
1.1626

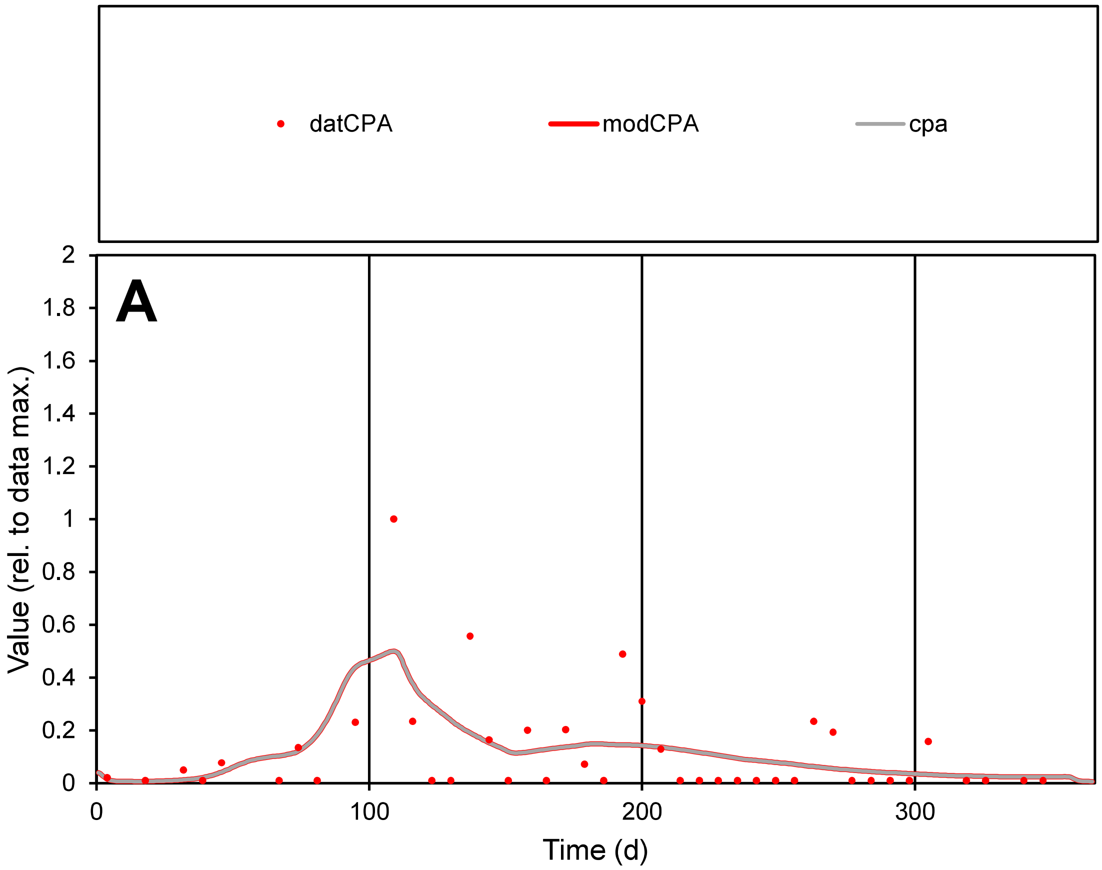

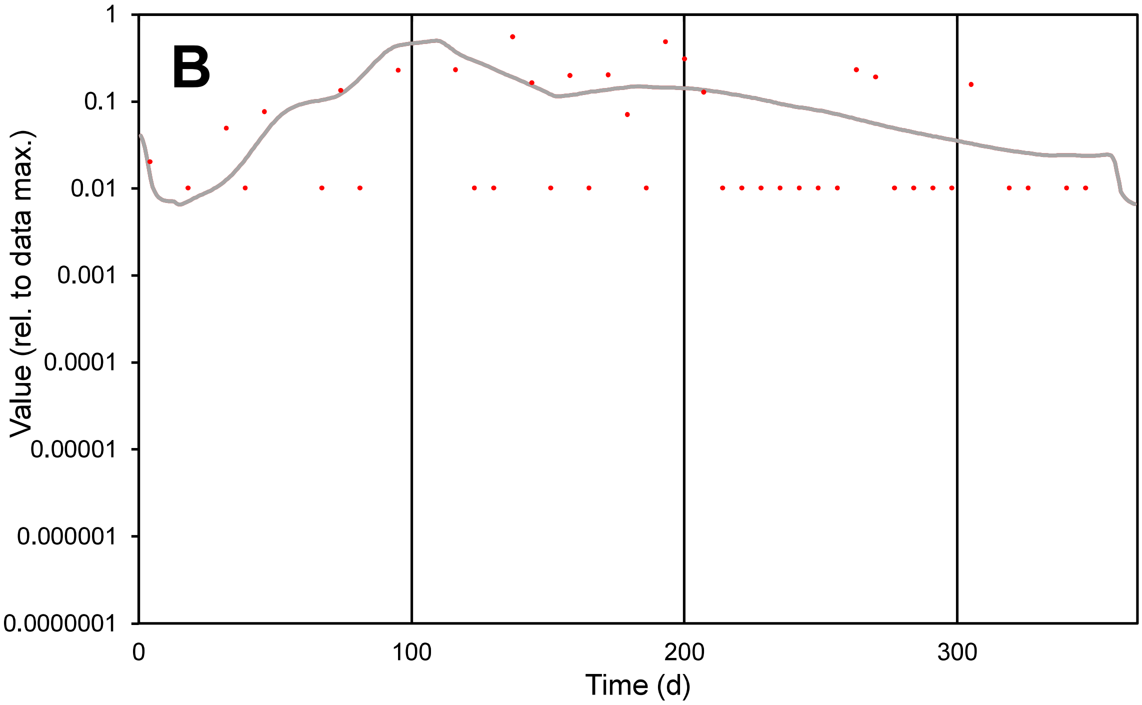

C1

Variables
CPA Provide jtd or Dst
CalibOnly?
2 1 to reproduce of
oNormalize?
2 1=no, 2=yes

ofxn calc*
1.34071
ofxn mod
1.356

sRun
RunA1
zout
1

Ave
Data
7.3E-07
Model
7.7E-07

data/vMax
6.5E-06

ofxng
334.07

Min d/m
1.3489

ave/min d
10.9585
ave/min m
15.5925

327_Calib_S_A

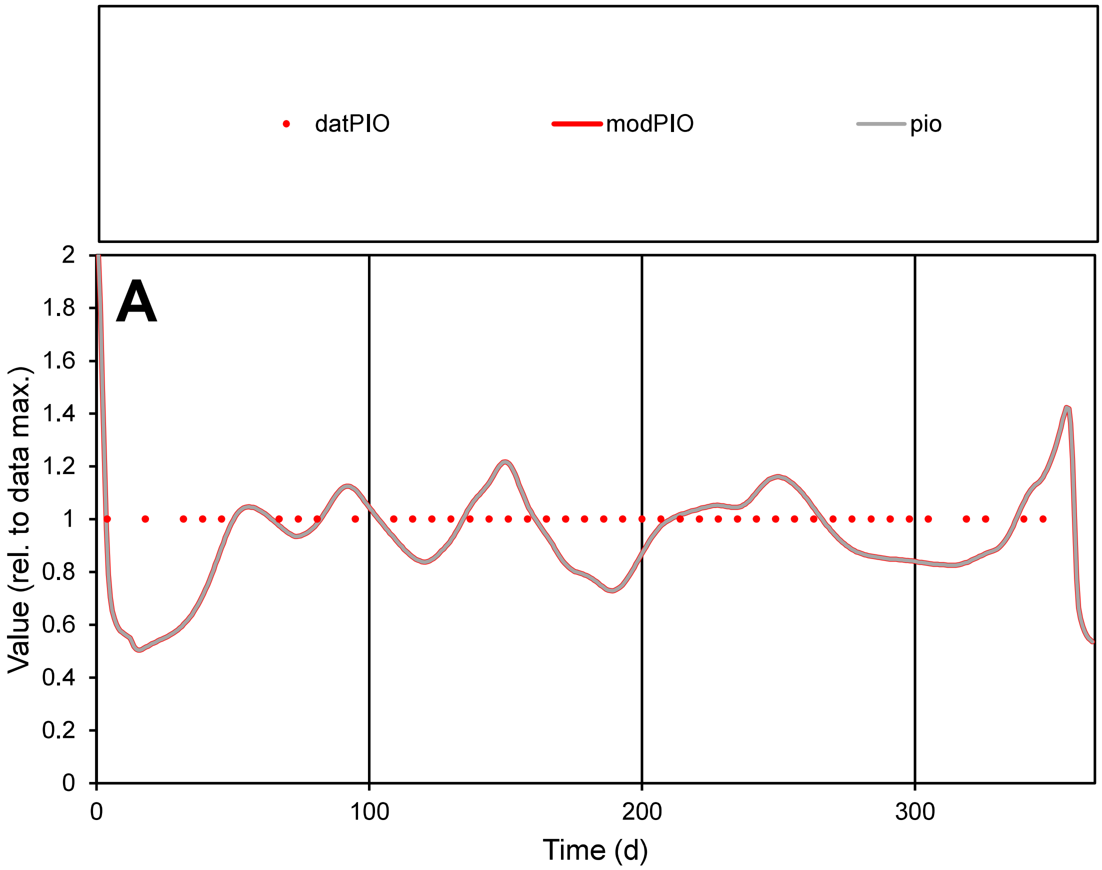

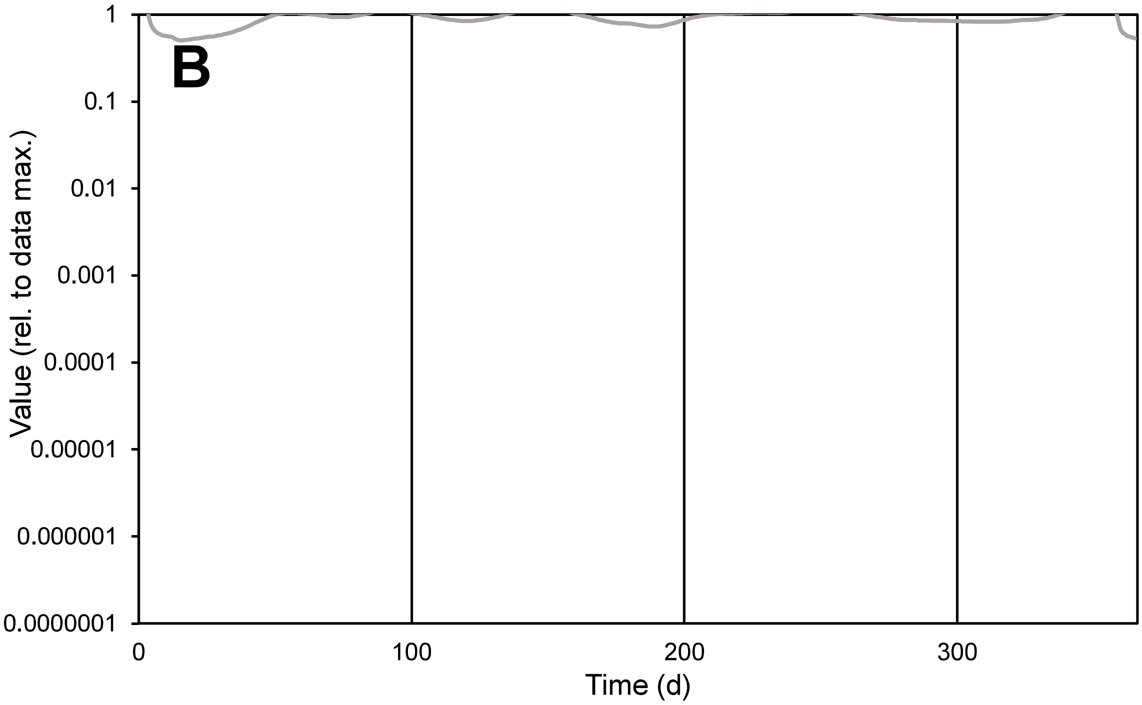

C1

Variables
PIO Provide jtd or Dst
CalibOnly?
2 1 to reproduce of
oNormalize?
2 1=no, 2=yes

ofxn calc*
0.16246
ofxn mod
0.19678

sRun
RunA1
zout
1

Ave
Data
1.5E-07
Model
1.4E-07

data/vMax
1.5E-07

ofxng
334.07

Min d/m
1.90802

ave/min d
1
ave/min m
1.78631

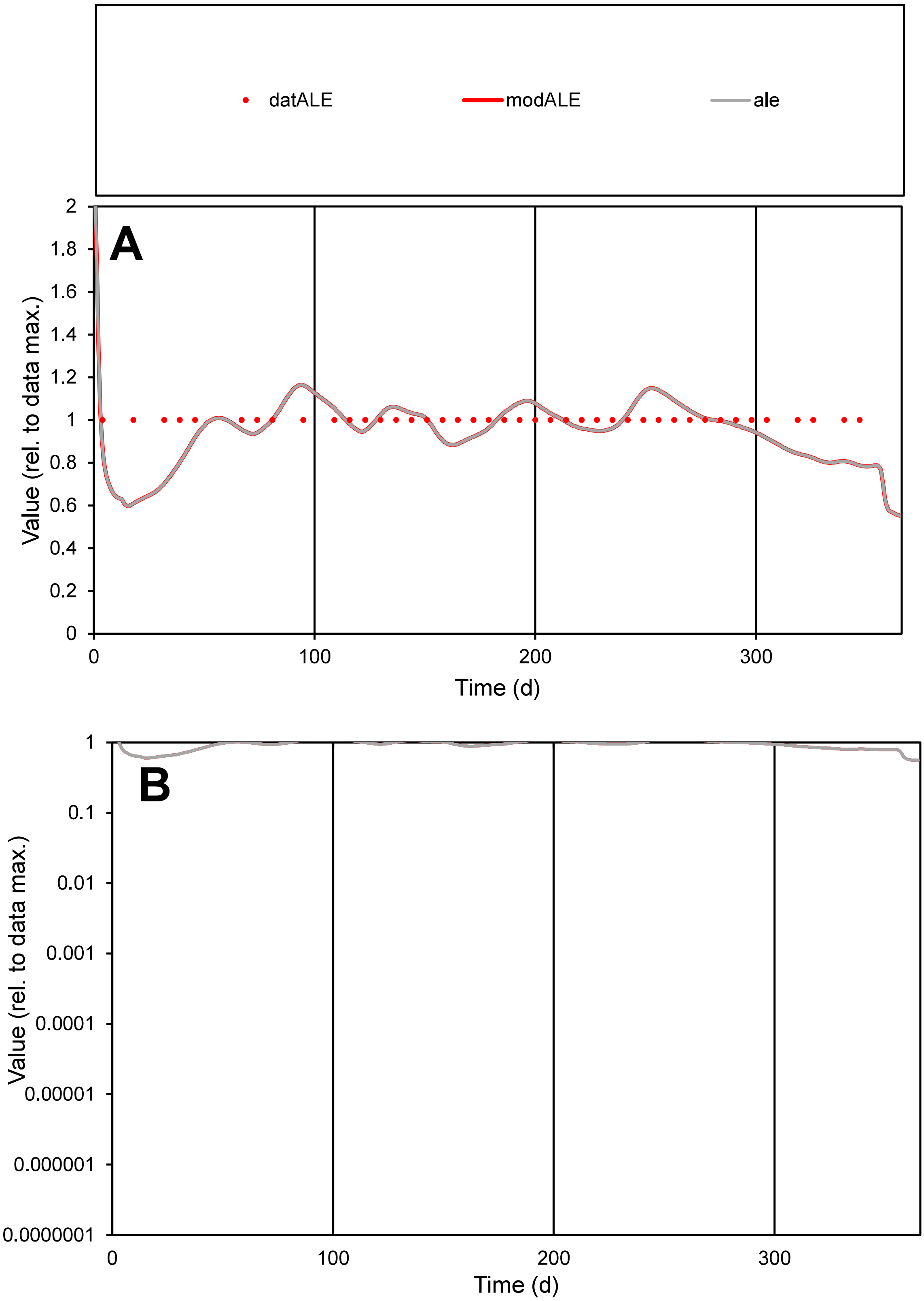

C1

Variables
ALE Provide jtd or Dst
CalibOnly?
2 1 to reproduce of
oNormalize?
2 1=no, 2=yes

ofxn calc*
0.11741
ofxn mod
0.1631

sRun
RunA1
zout
1

Ave
Data
1.1E-05
Model
1.1E-05

data/vMax
1.1E-05

ofxng
334.07

Min d/m
1.61248

ave/min d
1
ave/min m
1.5516

327_Calib_S_A

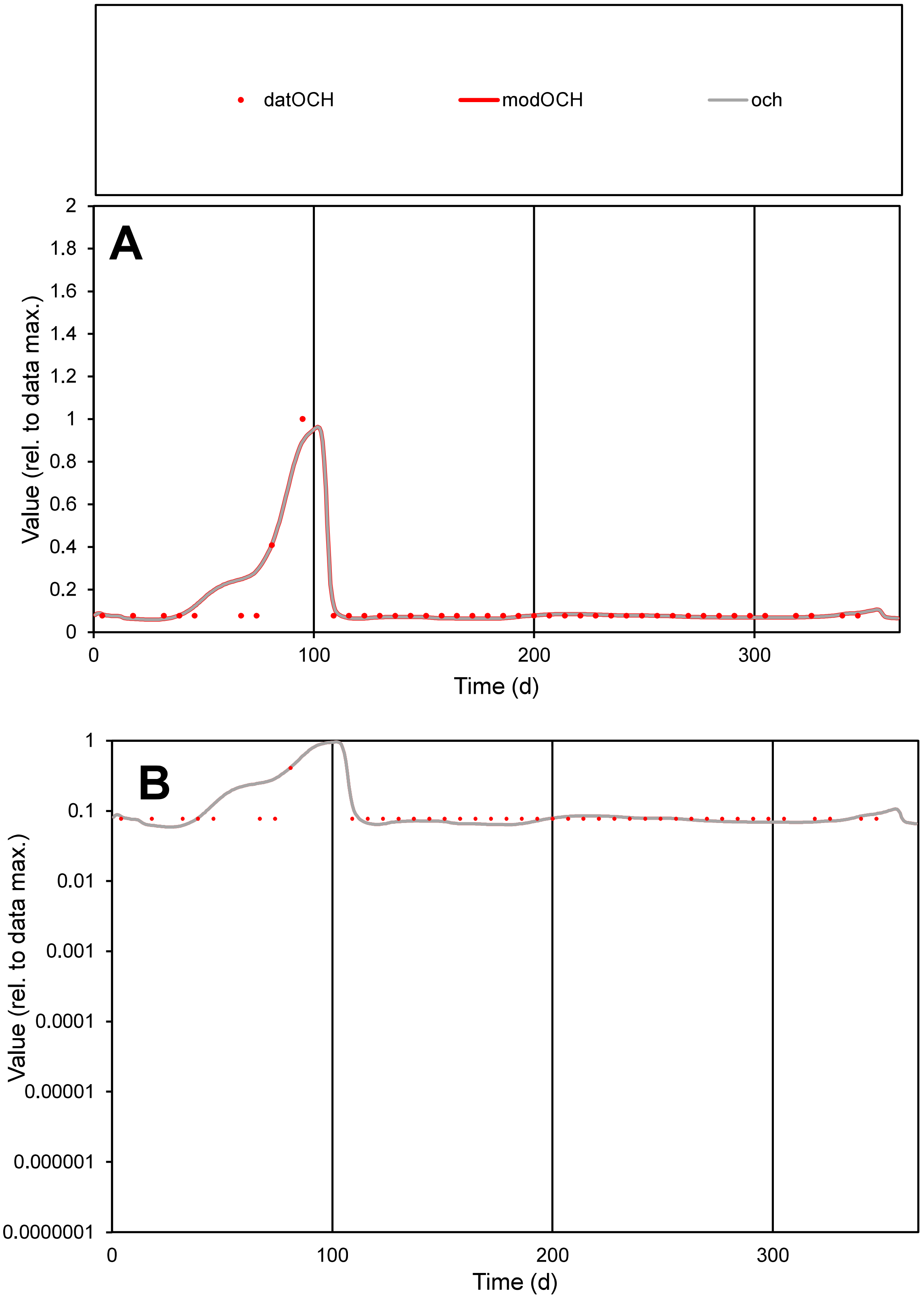

C1

Variables
OCH Provide jtd or Dst
CalibOnly?
2 1 to reproduce of
oNormalize?
2 1=no, 2=yes

ofxn calc*
0.43204
ofxn mod
0.45258

sRun
RunA1
zout
1

Ave
Data
6.1E-06
Model
6.5E-06

data/vMax
5.7E-05

ofxng
334.07

Min d/m
1.25629

ave/min d
1.38606
ave/min m
1.84568

327_Calib_S_A

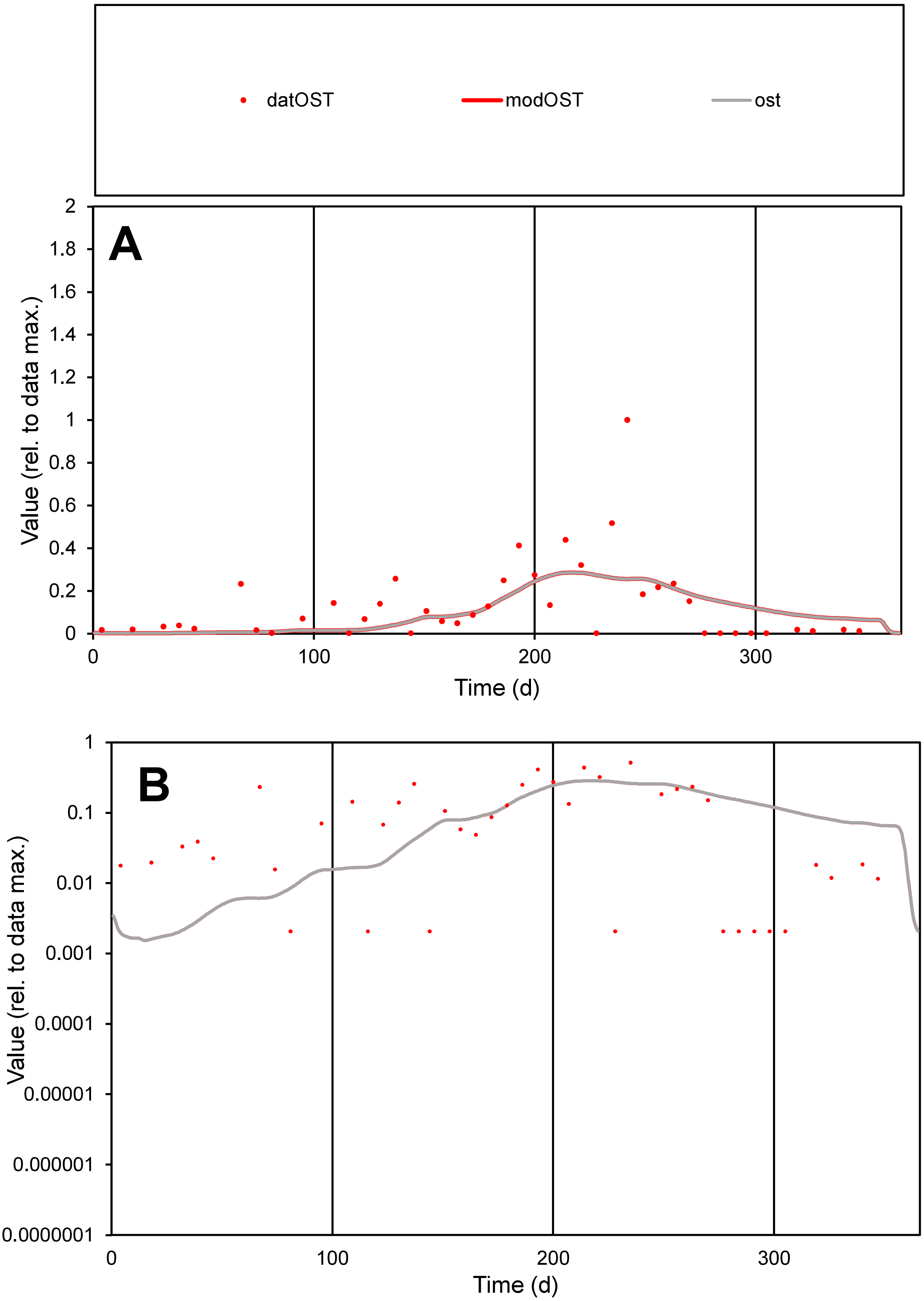

C1

Variables
OST Provide jtd or Dst
CalibOnly?
2 1 to reproduce of
oNormalize?
2 1=no, 2=yes

ofxn calc*
1.15613
ofxn mod
1.1695

sRun
RunA1
zout
1

Ave
Data
0.00014
Model
0.00012

data/vMax
0.00105

ofxng
334.07

Min d/m
1.25188

ave/min d
65.6878
ave/min m
69.9127

327_Calib_S_A

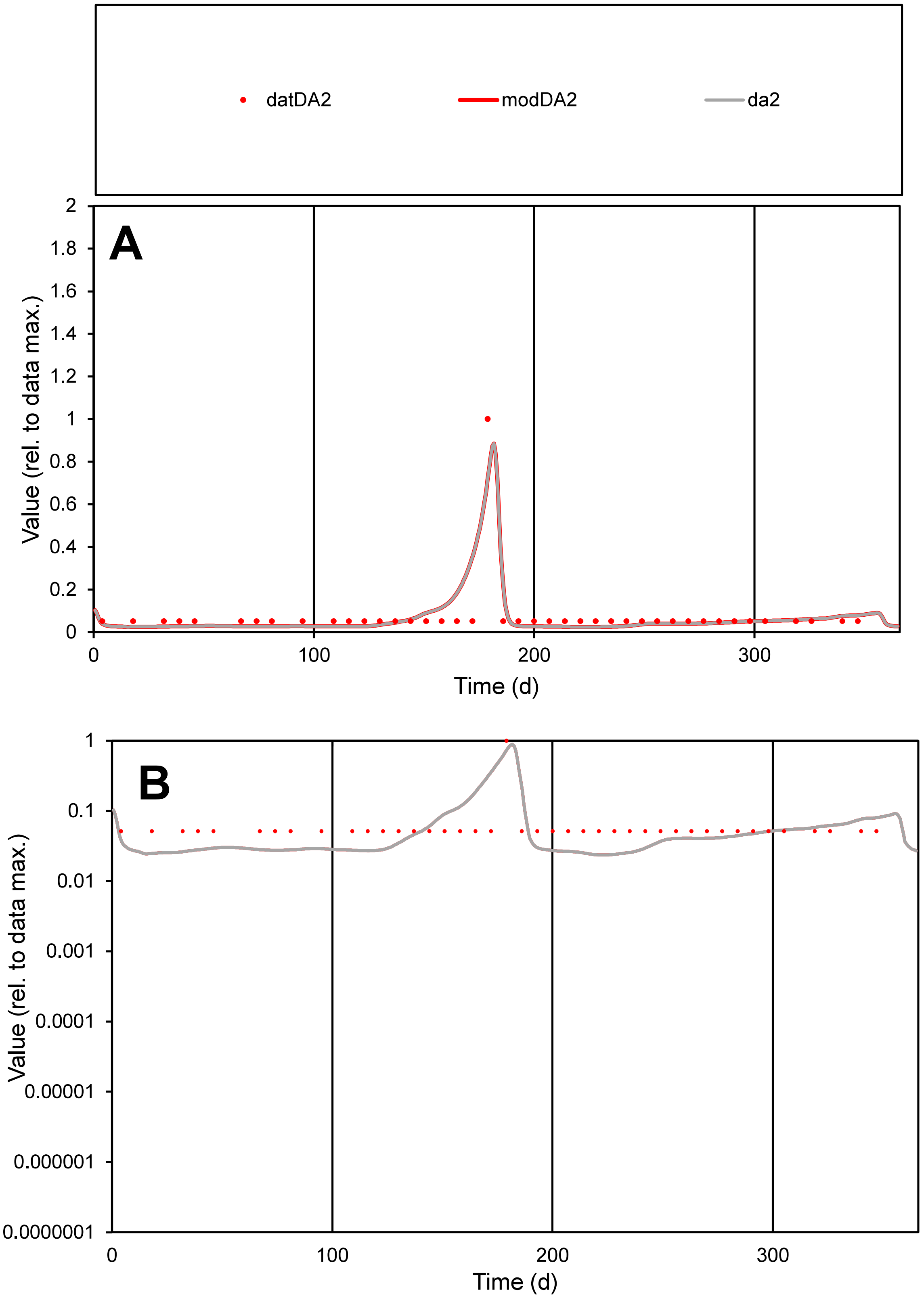

C1

Variables
DA2 Provide jtd or Dst
CalibOnly?
2 1 to reproduce of
oNormalize?
2 1=no, 2=yes

ofxn calc*
0.74034
ofxn mod
0.86452

sRun
RunA1
zout
1

Ave
Data
4.5E-06
Model
4.3E-06

data/vMax
6E-05

ofxng
334.07

Min d/m
2.17877

ave/min d
1.43765
ave/min m
3.00515

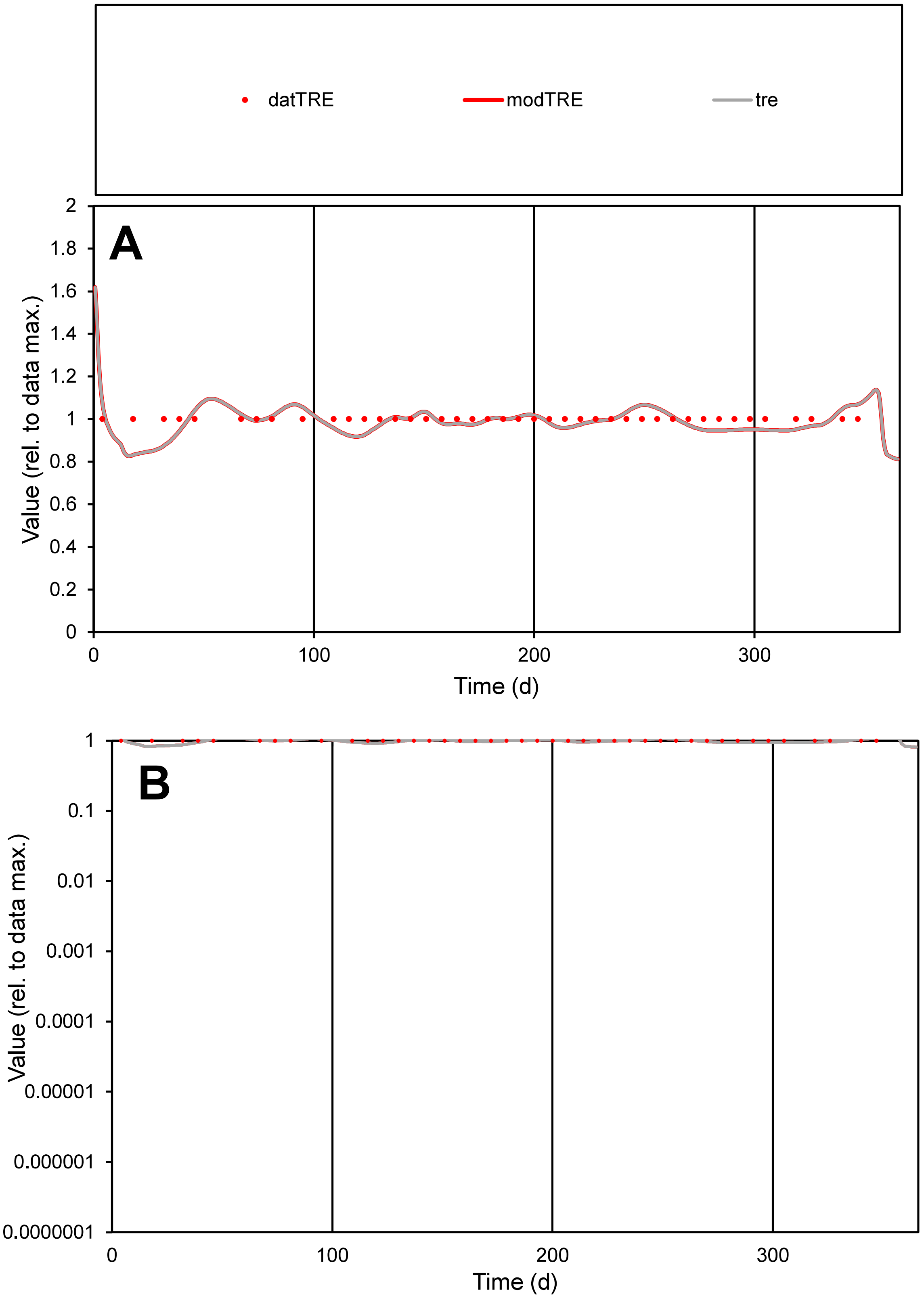

C1

Variables
TRE Provide jtd or Dst
CalibOnly?
2 1 to reproduce of
oNormalize?
2 1=no, 2=yes

ofxn calc*
0.04946
ofxn mod
0.12278

sRun
RunA1
zout
1

Ave
Data
6.9E-06
Model
6.8E-06

data/vMax
6.9E-06

ofxng
334.07

Min d/m
1.19506

ave/min d
1
ave/min m
1.18013

327_Calib_S_A

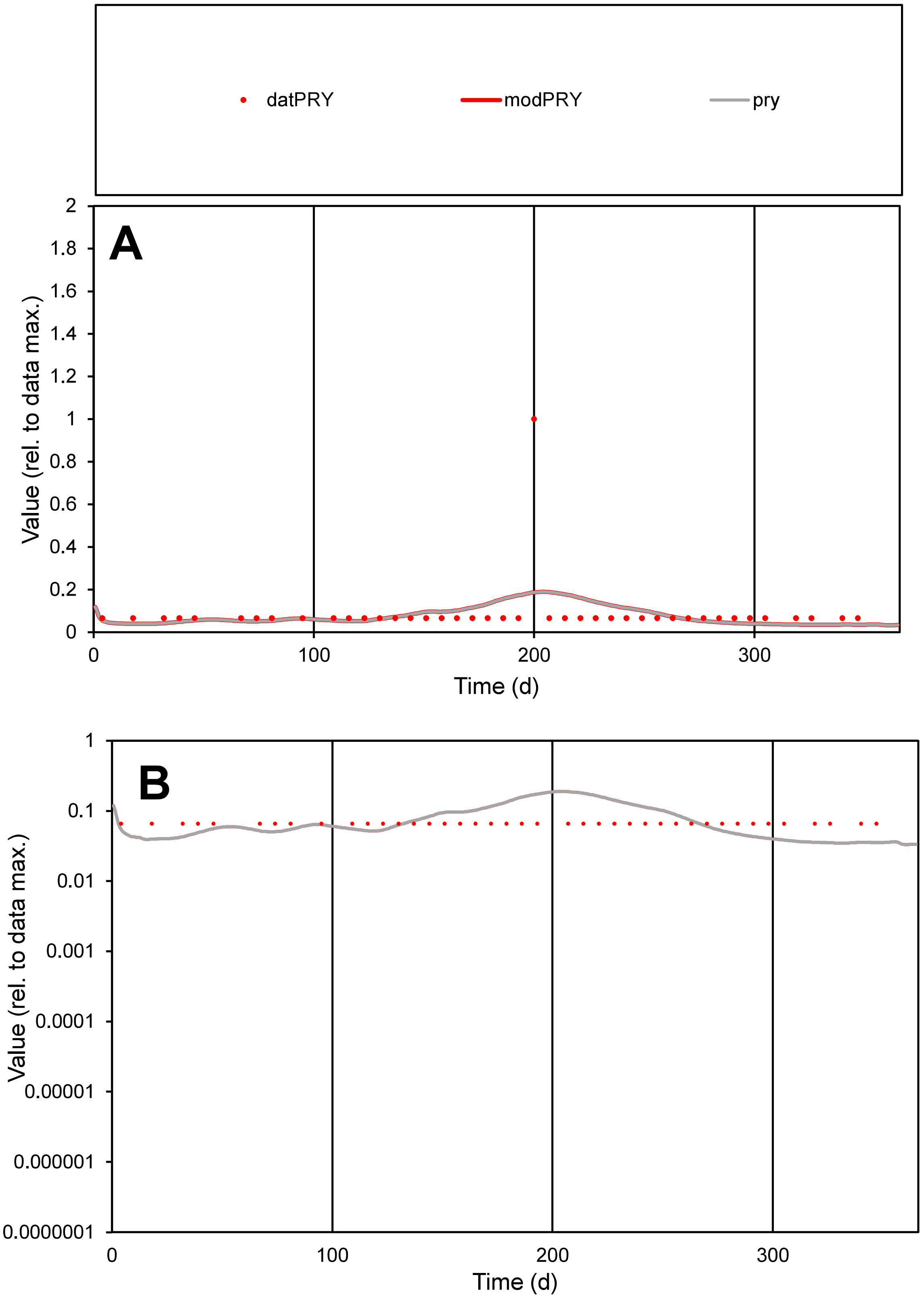

C1

Variables
PRY Provide jtd or Dst
CalibOnly?
2 1 to reproduce of
oNormalize?
2 1=no, 2=yes

ofxn calc*
1.51305
ofxn mod
1.52484

sRun
RunA1
zout
1

Ave
Data
1.2E-05
Model
1.1E-05

data/vMax
0.00013

ofxng
334.07

Min d/m
1.87989

ave/min d
1.33783
ave/min m
2.37303

327_Calib_S_A

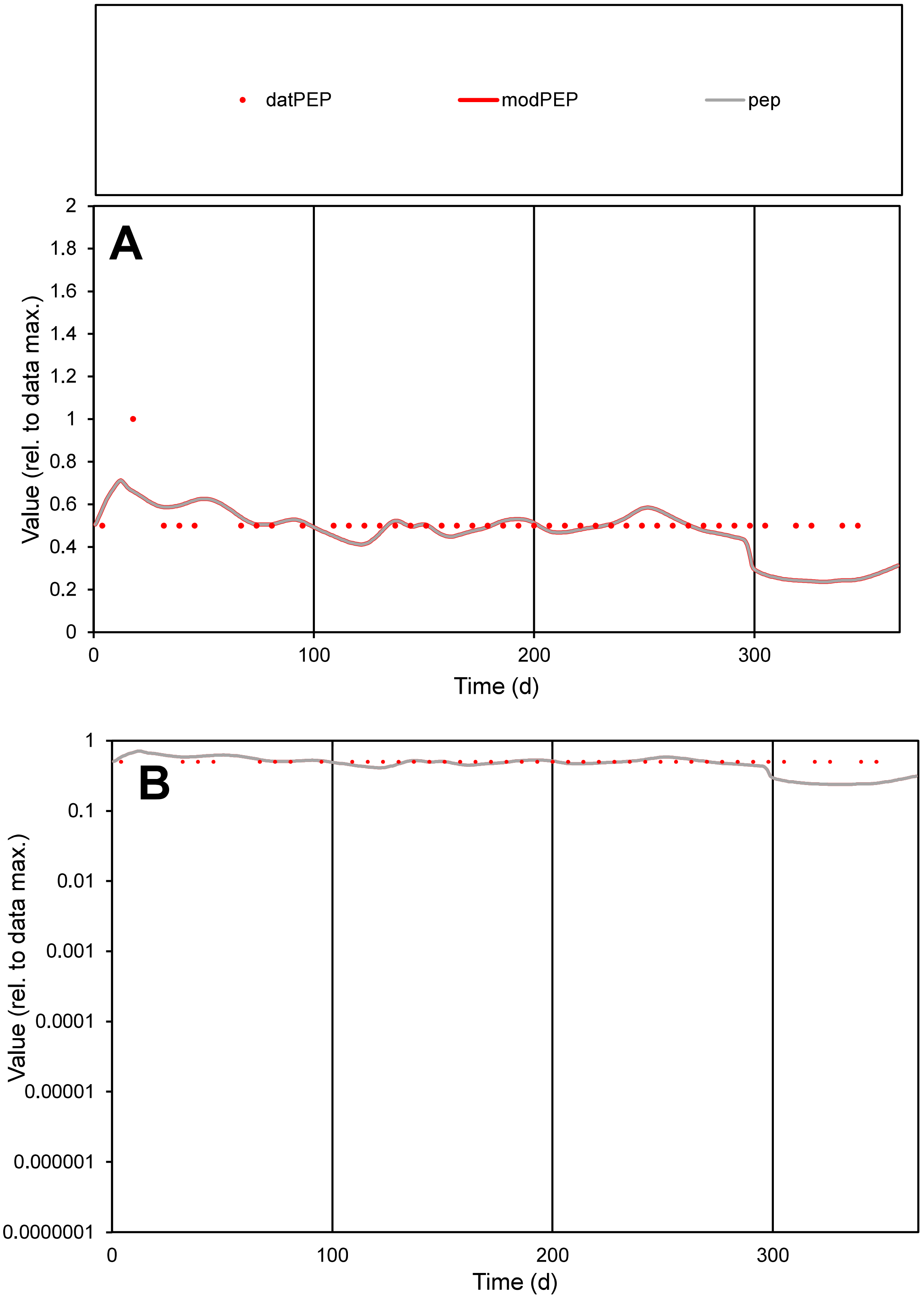

C1

Variables

PEP Provide jtd or Dst

CalibOnly?

2 1 to reproduce of

oNormalize?

2 1=no, 2=yes

ofxn calc*

0.22445

ofxn mod

0.25259

sRun

RunA1

zout

1

Ave

Data

5.1E-06

Model

4.7E-06

data/vMax

9.9E-06

ofxng

334.07

Min d/m

2.09334

ave/min d

1.02381

ave/min m

1.99303

327_Calib_S_A

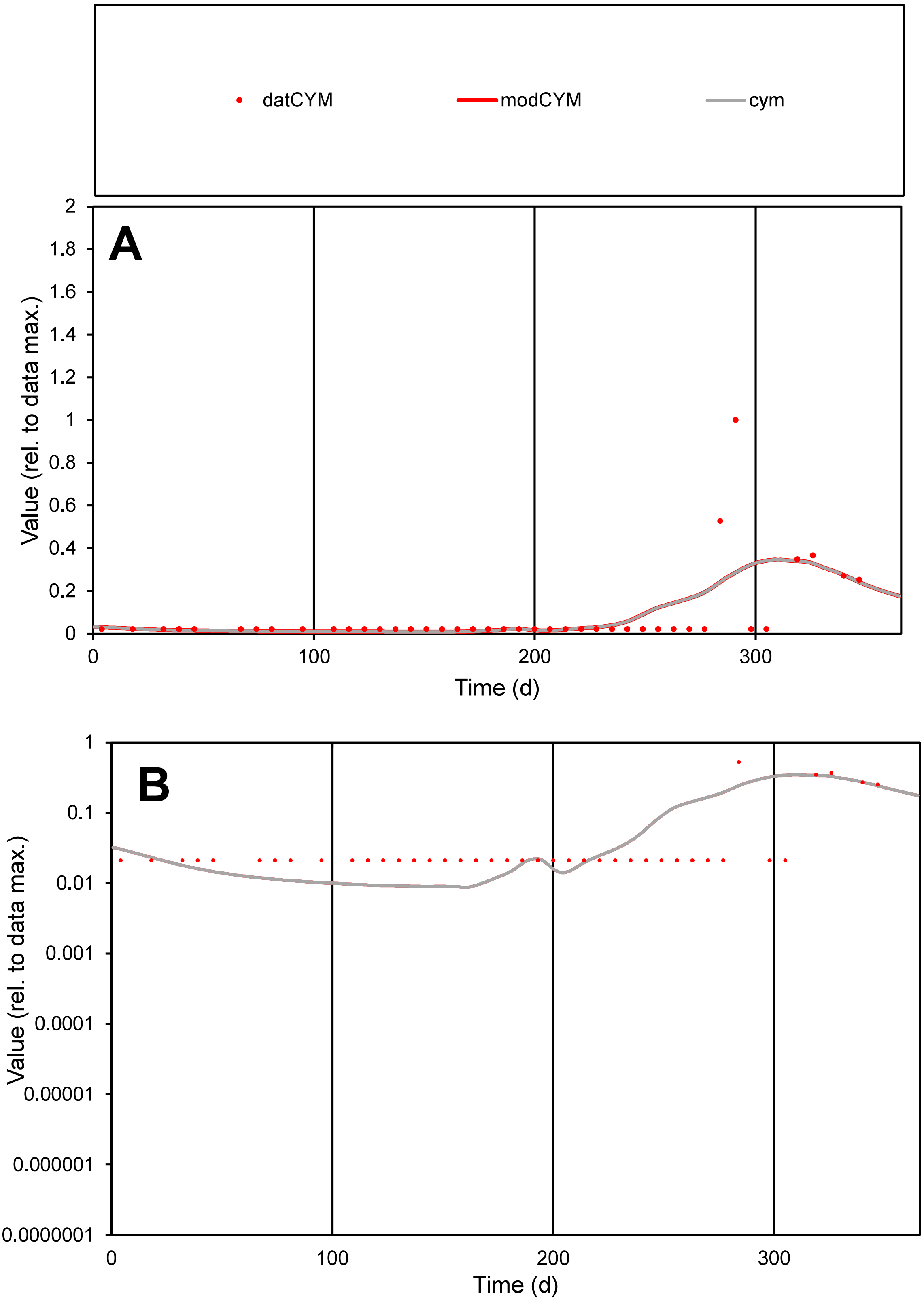

C1

Variables
CYM Provide jtd or Dst
CalibOnly?
2 1 to reproduce of
oNormalize?
2 1=no, 2=yes

ofxn calc*
1.70712
ofxn mod
1.72001

sRun
RunA1
zout
1

Ave
Data
5.4E-06
Model
5.6E-06

data/vMax
6.5E-05

ofxng
334.07

Min d/m
2.40842

ave/min d
3.98999
ave/min m
9.85587

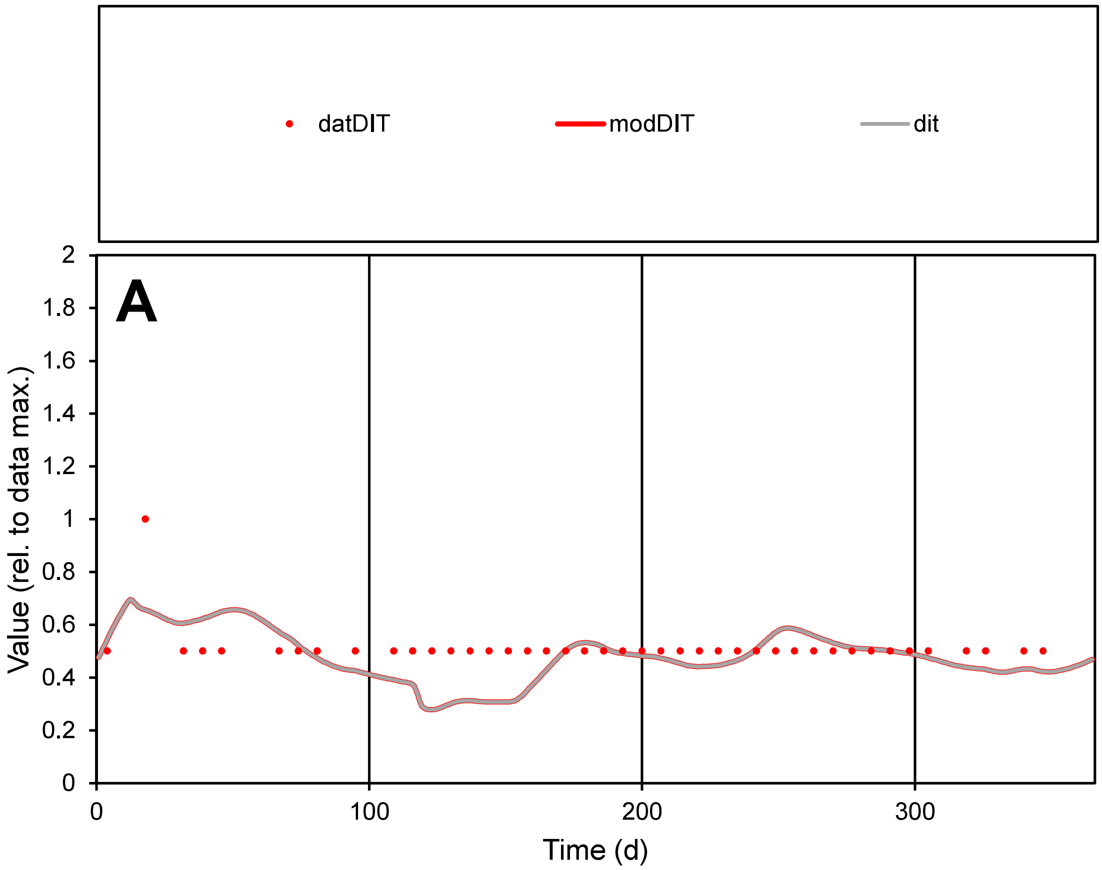

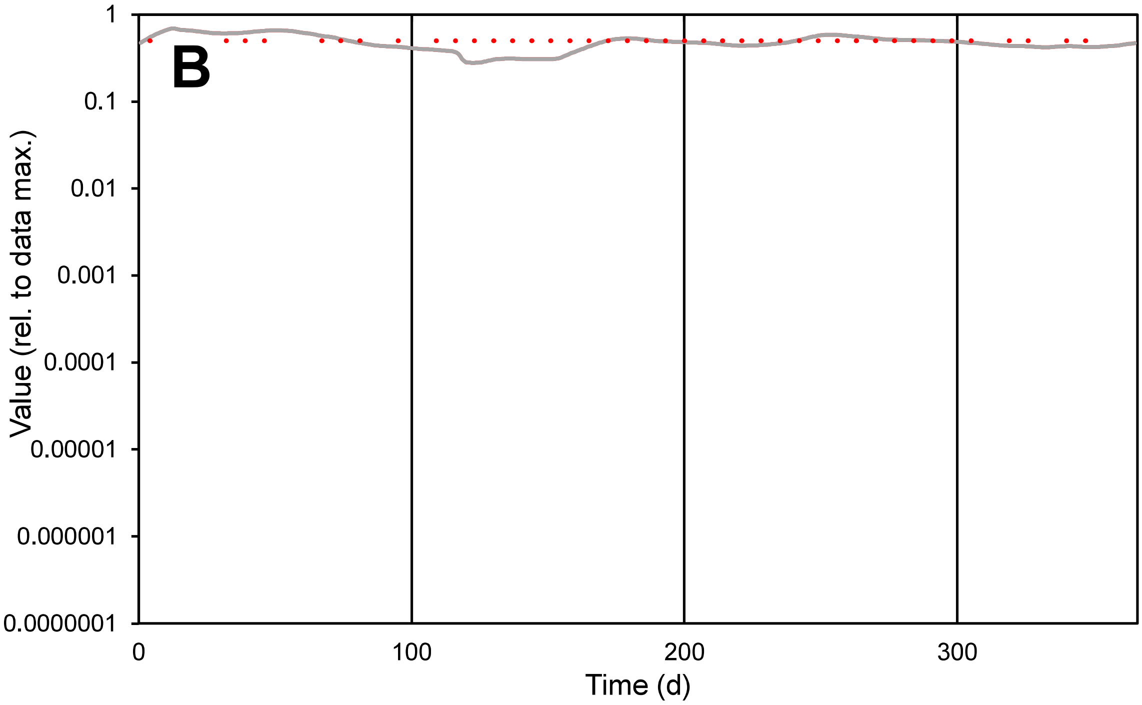

C1

Variables
DIT Provide jtd or Dst
CalibOnly?
2 1 to reproduce of
oNormalize?
2 1=no, 2=yes

ofxn calc*
0.21055
ofxn mod
0.23923

sRun
RunA1
zout
1

Ave
Data
5.2E-06
Model
4.8E-06

data/vMax
1E-05

ofxng
334.07

Min d/m
1.78492

ave/min d
1.02379
ave/min m
1.6921

327_Calib_S_A

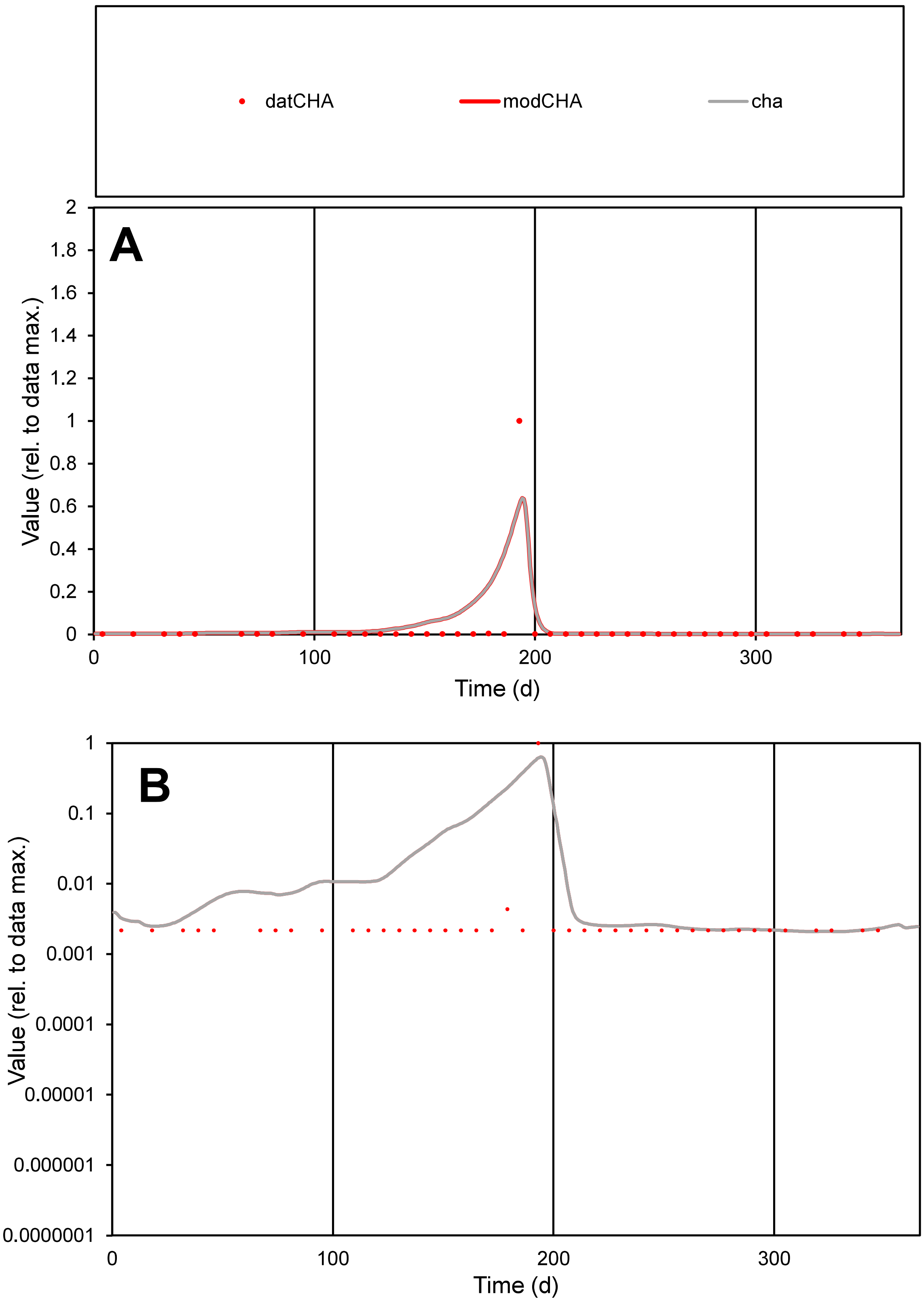

C1

Variables

CHA Provide jtd or Dst

CalibOnly?

2 1 to reproduce of

oNormalize?

2 1=no, 2=yes

ofxn calc*

3.41742

ofxn mod

3.613

sRun

RunA1

zout

1

Ave

Data

1E-06

Model

1.7E-06

data/vMax

4E-05

ofxng

334.07

Min d/m

1.04064

ave/min d

11.9295

ave/min m

20.4439

327_Calib_S_A

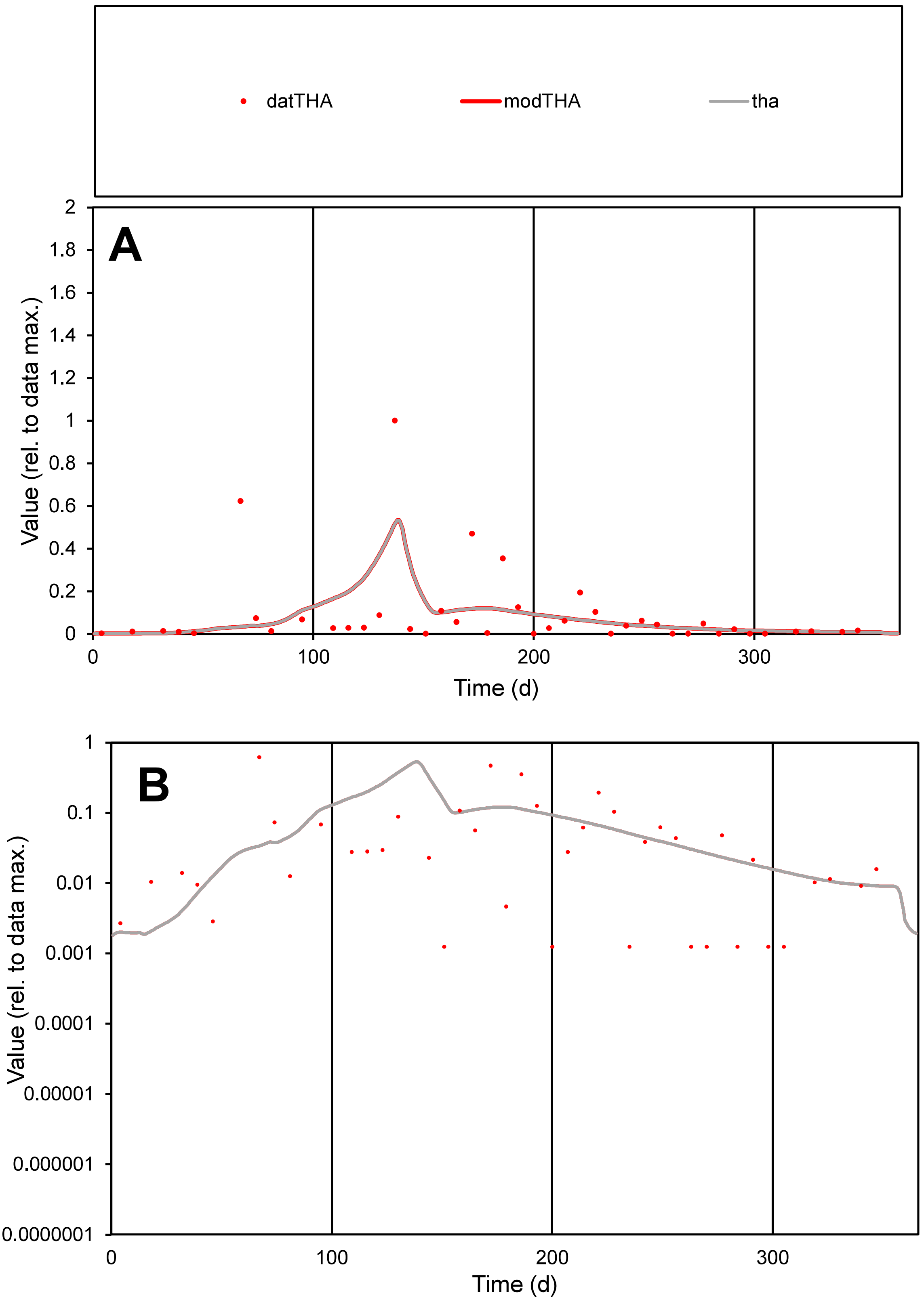

C1

Variables
THA Provide jtd or Dst
CalibOnly?
2 1 to reproduce of
oNormalize?
2 1=no, 2=yes

ofxn calc*
1.73824
ofxn mod
1.76795

sRun
RunA1
zout
1

Ave
Data
0.0002
Model
0.00019

data/vMax
0.00222

ofxng
334.07

Min d/m
0.61676

ave/min d
72.8737
ave/min m
41.6441

327_Calib_S_A

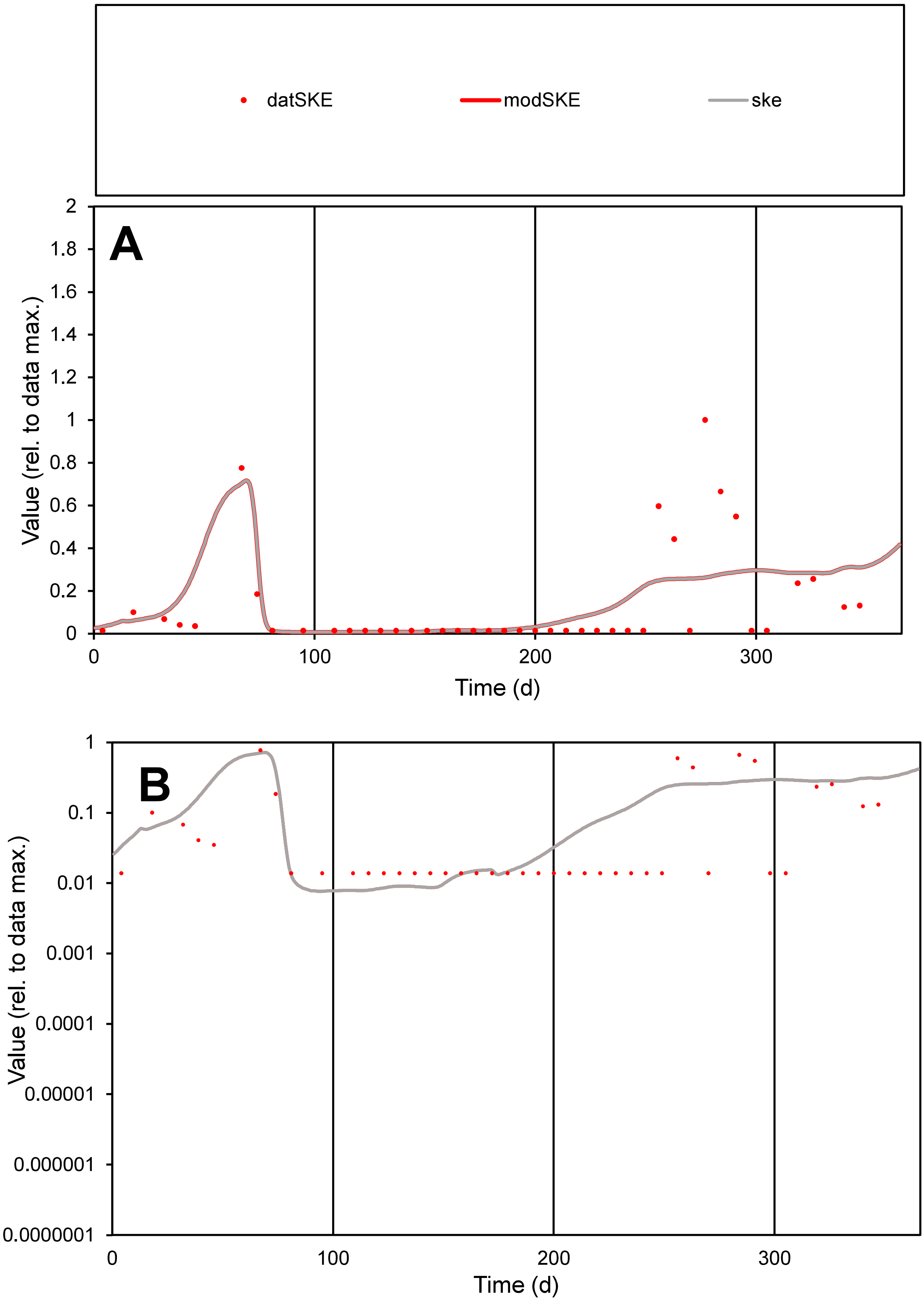

C1

Variables
SKE Provide jtd or Dst
CalibOnly?
2 1 to reproduce of
oNormalize?
2 1=no, 2=yes

ofxn calc*
1.3617
ofxn mod
1.38382

sRun
RunA1
zout
1

Ave
Data
2.2E-05
Model
2.4E-05

data/vMax
0.00017

ofxng
334.07

Min d/m
1.80754

ave/min d
9.57992
ave/min m
18.491

327_Calib_S_A

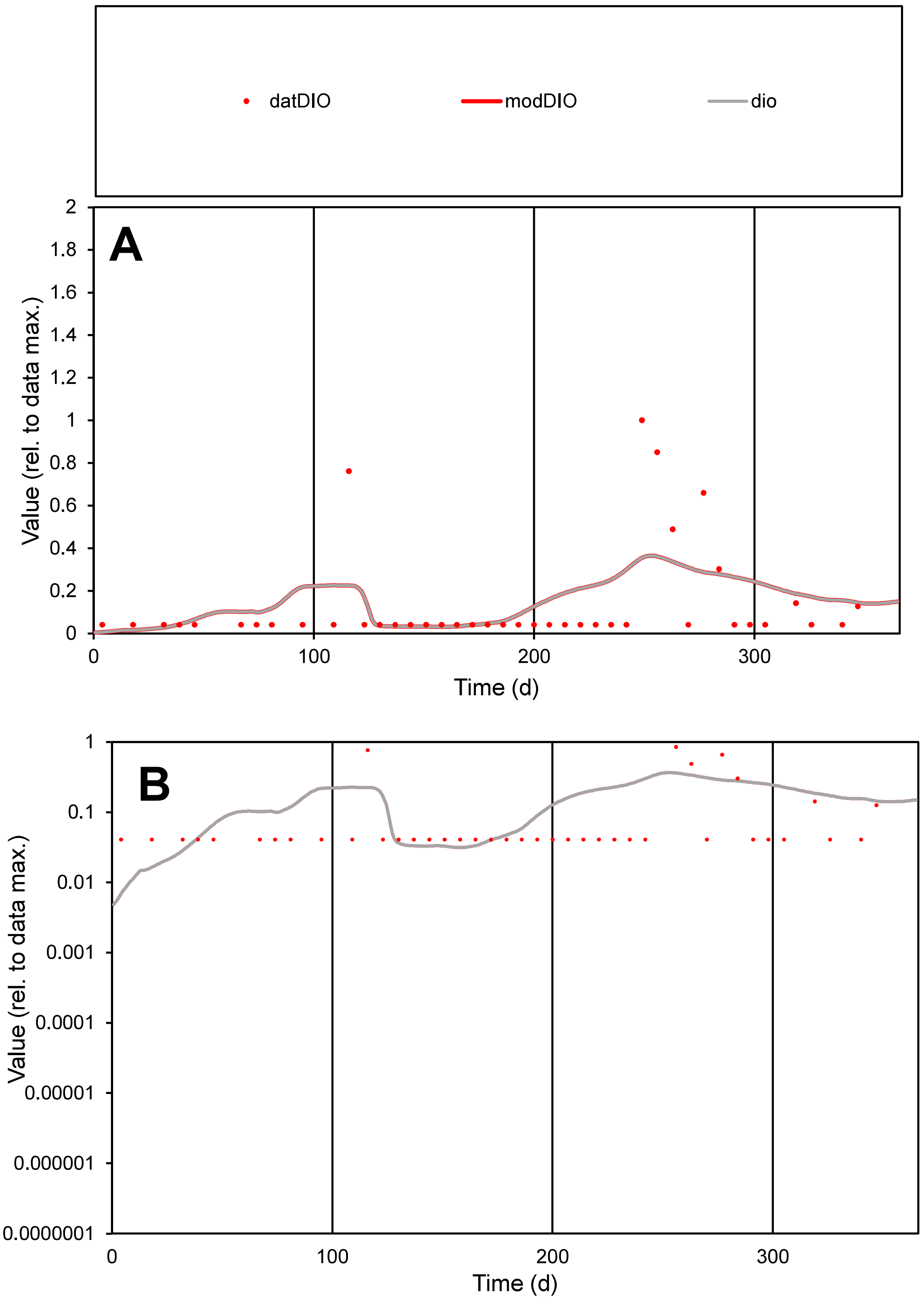

C1

Variables

DIO Provide jtd or Dst

CalibOnly?

2 1 to reproduce of

oNormalize?

2 1=no, 2=yes

ofxn calc*

1.45019

ofxn mod

1.46478

sRun

RunA1

zout

1

Ave

Data

1.6E-05

Model

1.8E-05

data/vMax

0.00011

ofxng

334.07

Min d/m

5.37841

ave/min d

3.32775

ave/min m

20.1813

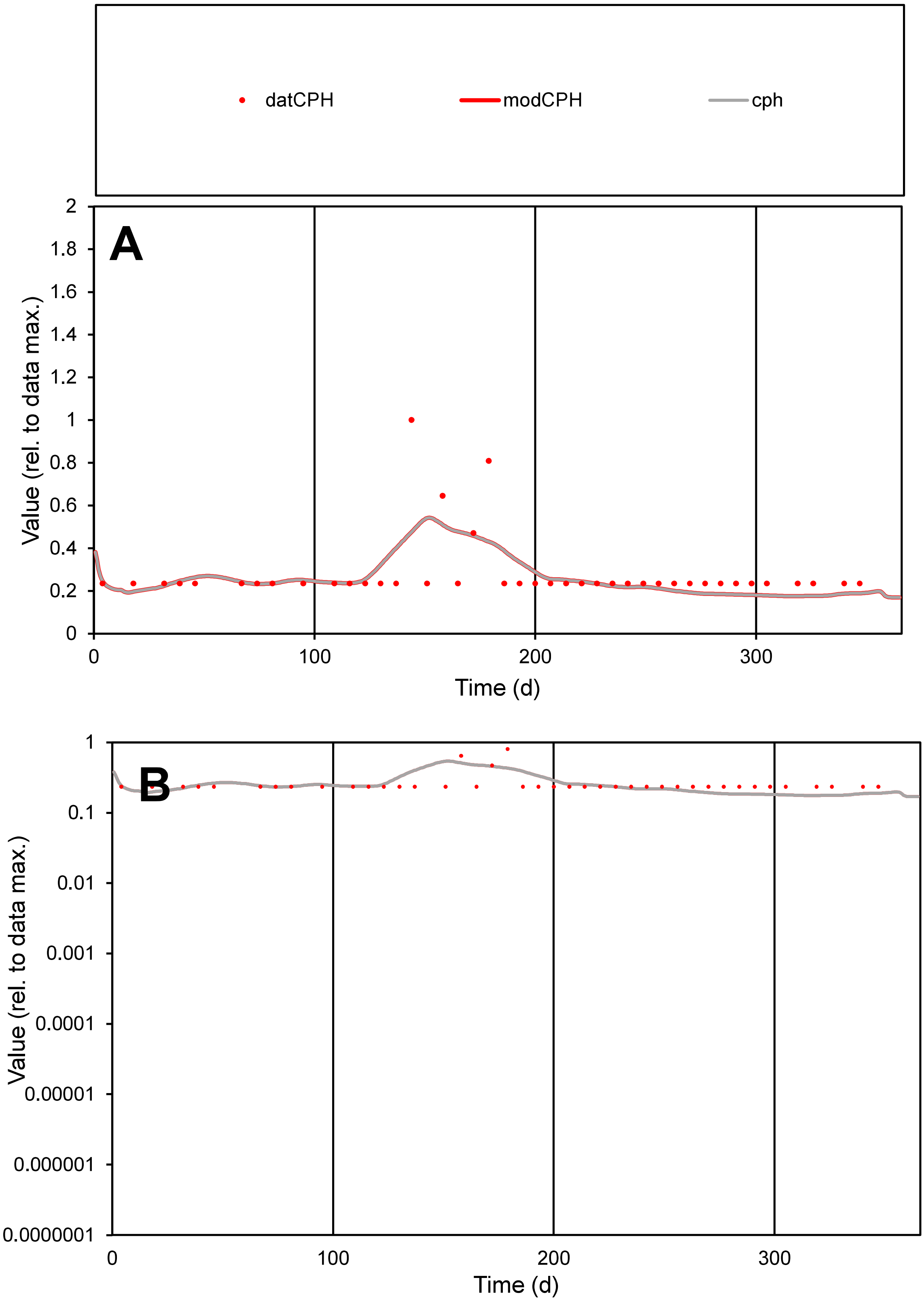

C1

Variables
CPH Provide jtd or Dst
CalibOnly?
2 1 to reproduce of
oNormalize?
2 1=no, 2=yes

ofxn calc*
0.45081
ofxn mod
0.46617

sRun
RunA1
zout
1

Ave
Data
1.2E-05
Model
1.1E-05

data/vMax
4.1E-05

ofxng
334.07

Min d/m
1.33435

ave/min d
1.20075
ave/min m
1.53604

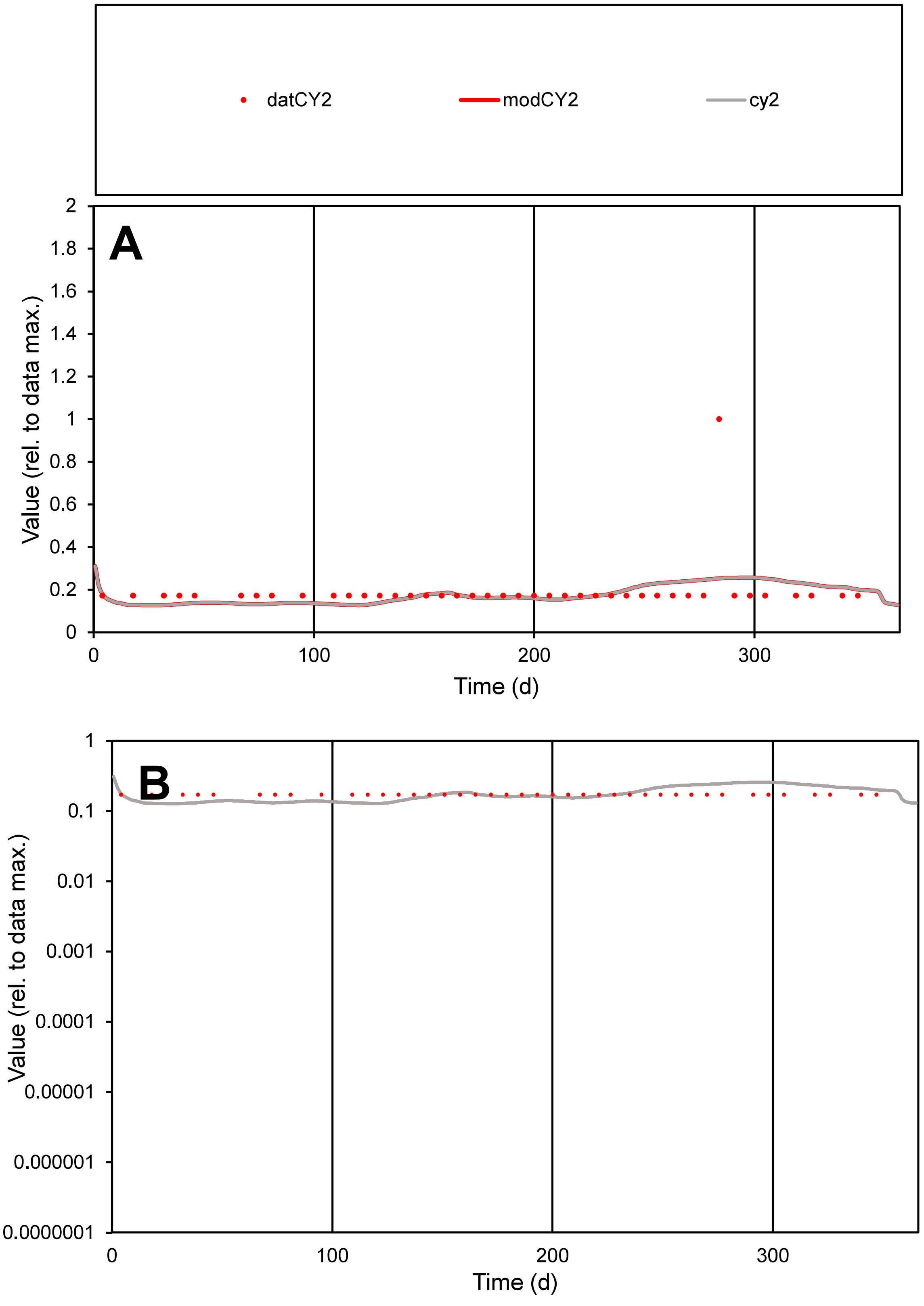

C1

Variables	
CY2	Provide jtd or Dst
CalibOnly?	
2	1 to reproduce of
oNormalize?	
2	1=no, 2=yes
ofxn calc*	
0.63903	
ofxn mod	
0.64868	
sRun	
RunA1	
zout	
1	
Ave	
Data	
1.2E-05	
Model	
1.2E-05	
data/vMax	
6.5E-05	
ofxng	
334.07	
Min d/m	
1.33333	
ave/min d	
1.11503	
ave/min m	
1.39496	

327_Calib_S_A

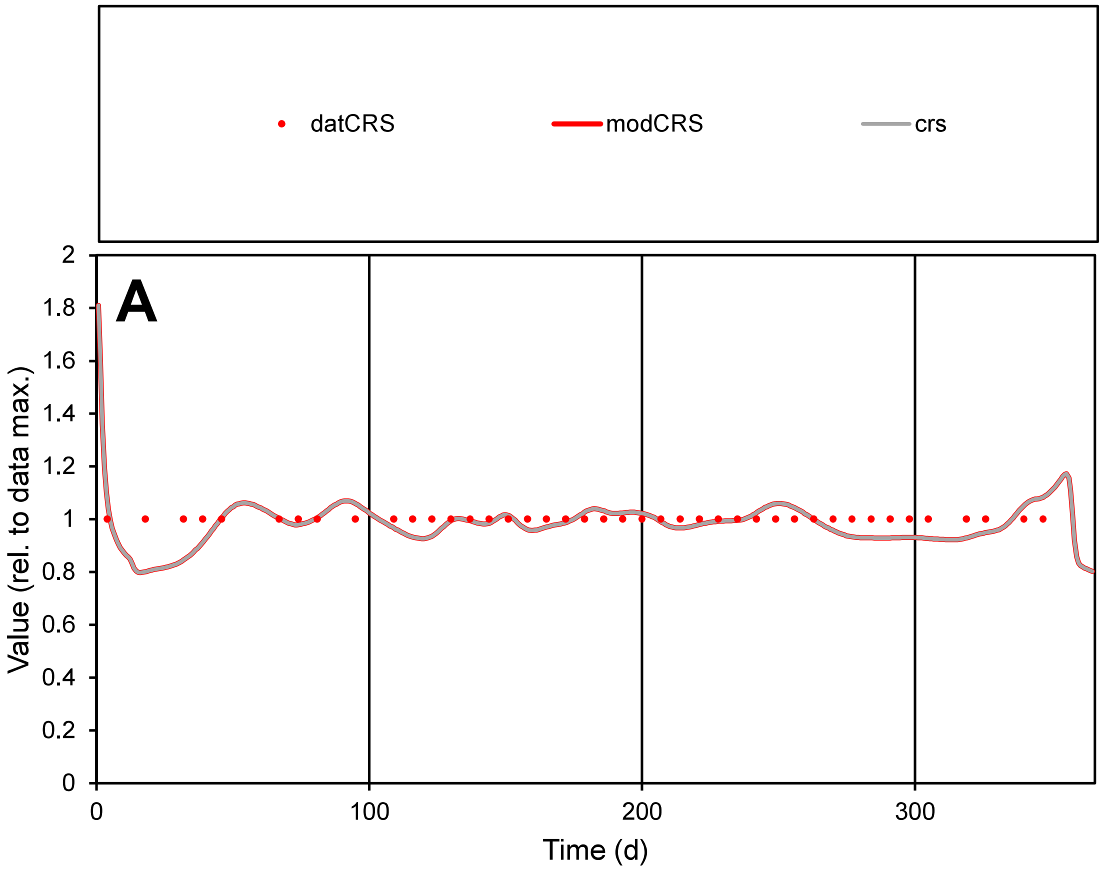

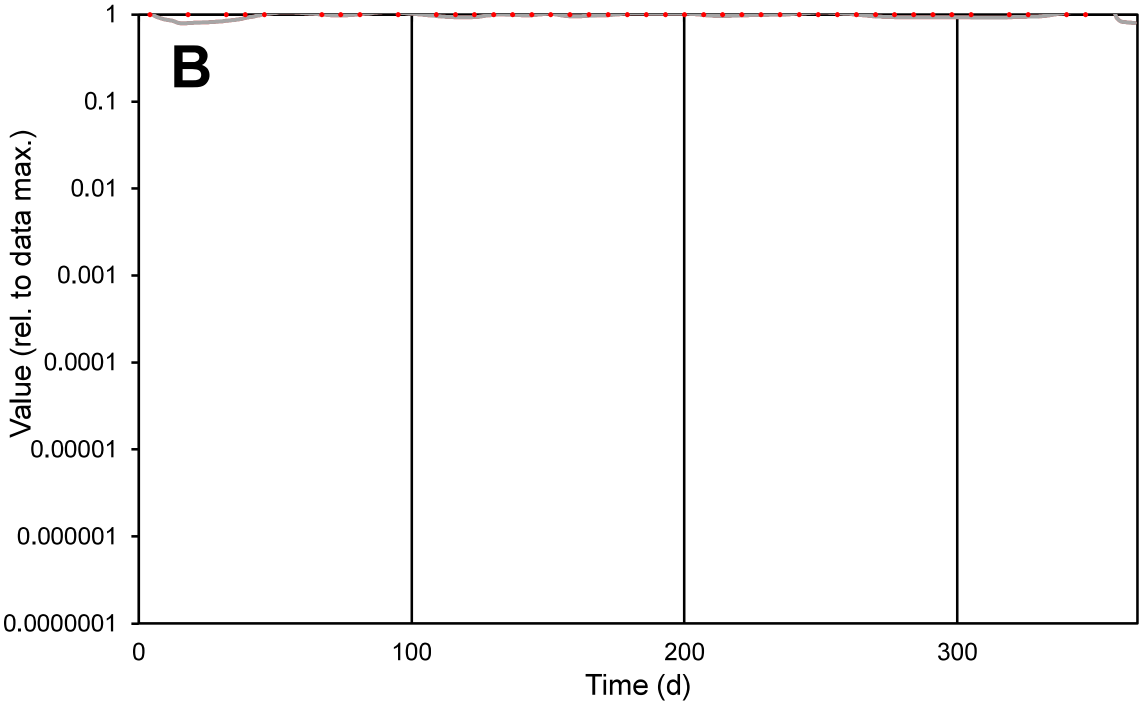

C1

Variables
CRS Provide jtd or Dst
CalibOnly?
2 1 to reproduce of
oNormalize?
2 1=no, 2=yes

ofxn calc*
0.05735
ofxn mod
0.12586

sRun
RunA1
zout
1

Ave
Data
5.4E-06
Model
5.3E-06

data/vMax
5.4E-06

ofxng
334.07

Min d/m
1.24054

ave/min d
1
ave/min m
1.21683

327_Calib_S_A

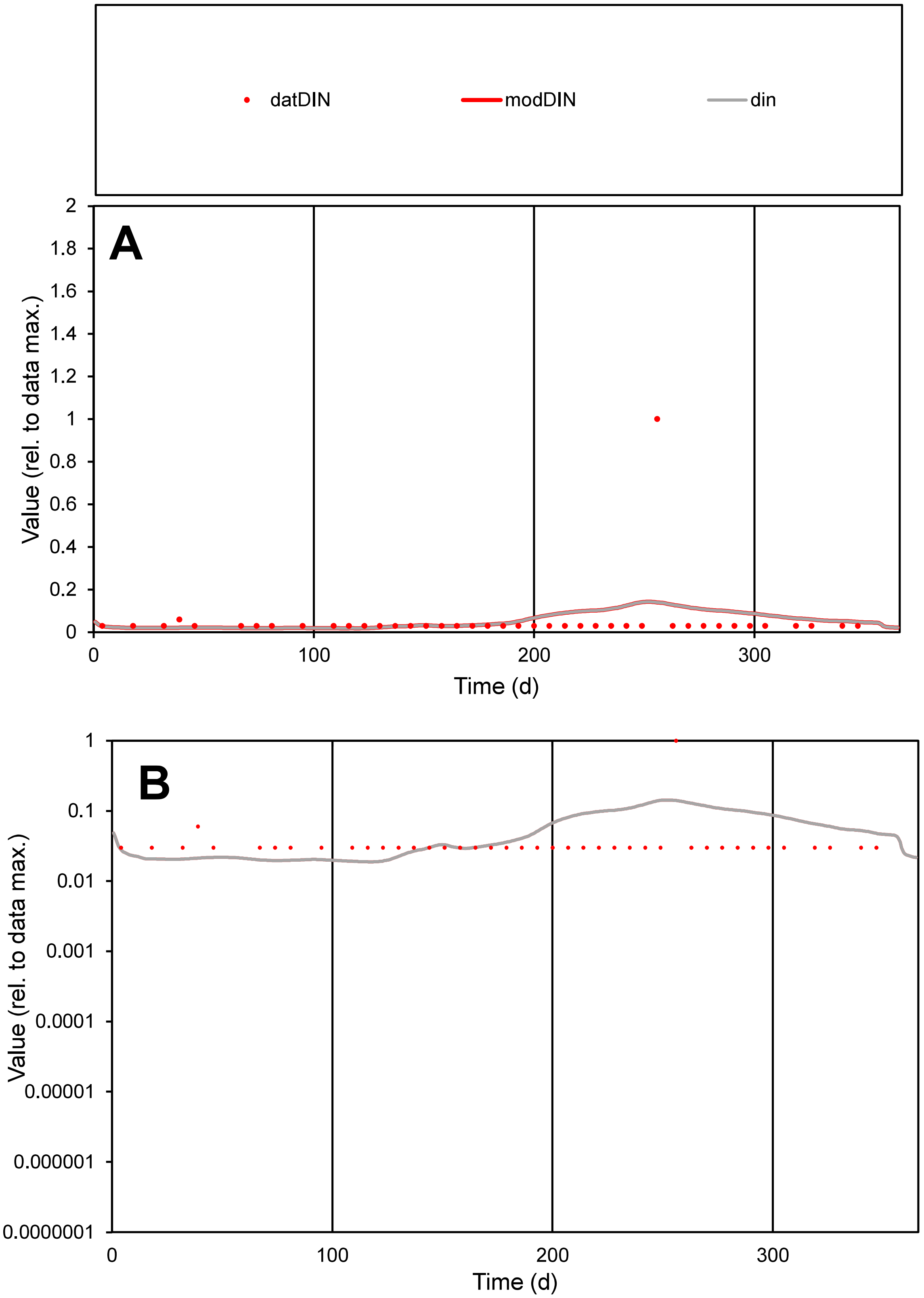

C1

Variables
DIN Provide jtd or Dst
CalibOnly?
2 1 to reproduce of
oNormalize?
2 1=no, 2=yes

ofxn calc*
2.61771
ofxn mod
2.63049

sRun
RunA1
zout
1

Ave
Data
5.2E-06
Model
5.7E-06

data/vMax
9.6E-05

ofxng
334.07

Min d/m
1.58549

ave/min d
1.79618
ave/min m
3.12914

327_Calib_S_A

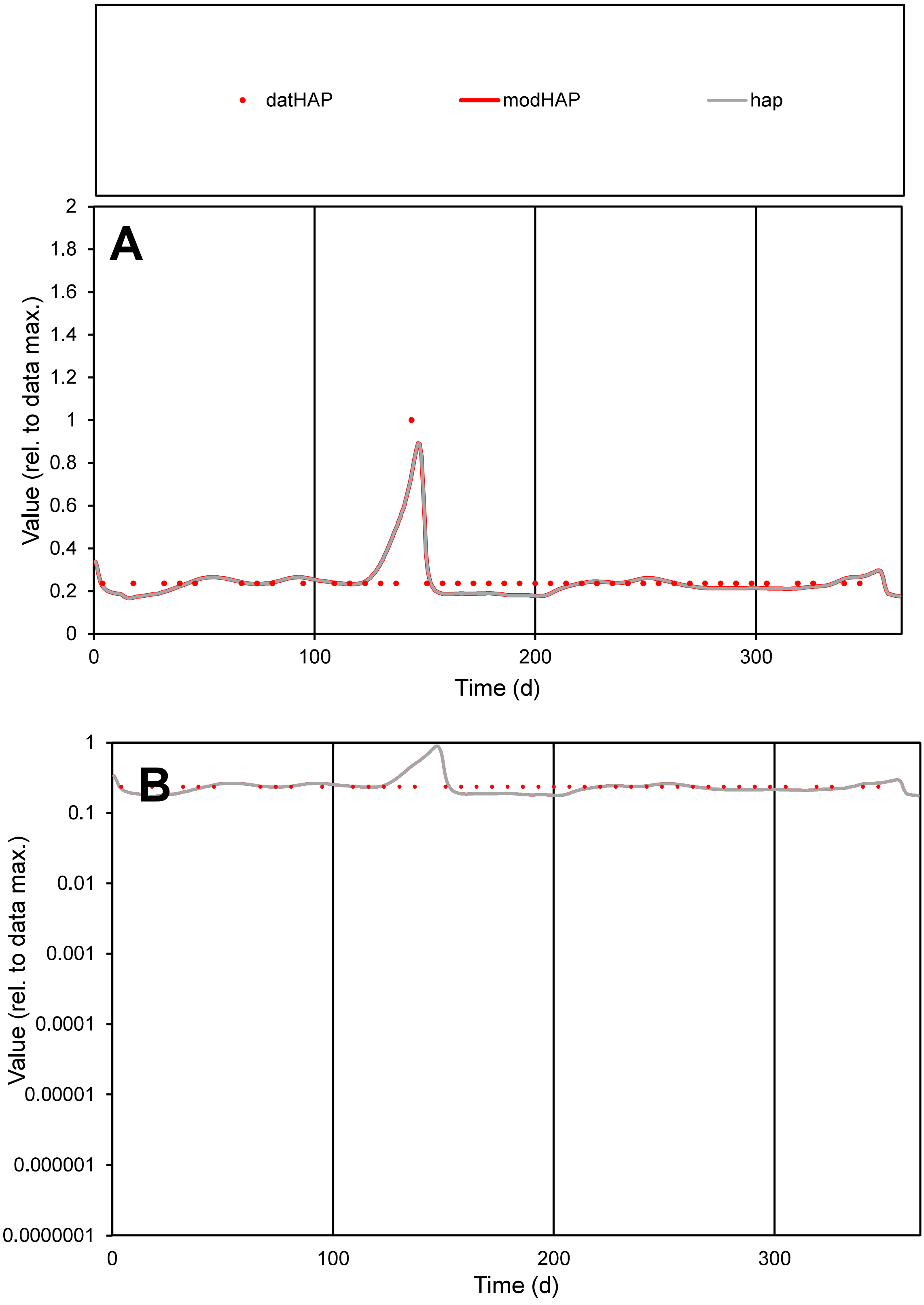

C1

Variables
HAP Provide jtd or Dst
CalibOnly?
2 1 to reproduce of
oNormalize?
2 1=no, 2=yes

ofxn calc*
0.23314
ofxn mod
0.26504

sRun
RunA1
zout
1

Ave
Data
1.2E-05
Model
1.2E-05

data/vMax
4.8E-05

ofxng
334.07

Min d/m
1.35944

ave/min d
1.07692
ave/min m
1.42344

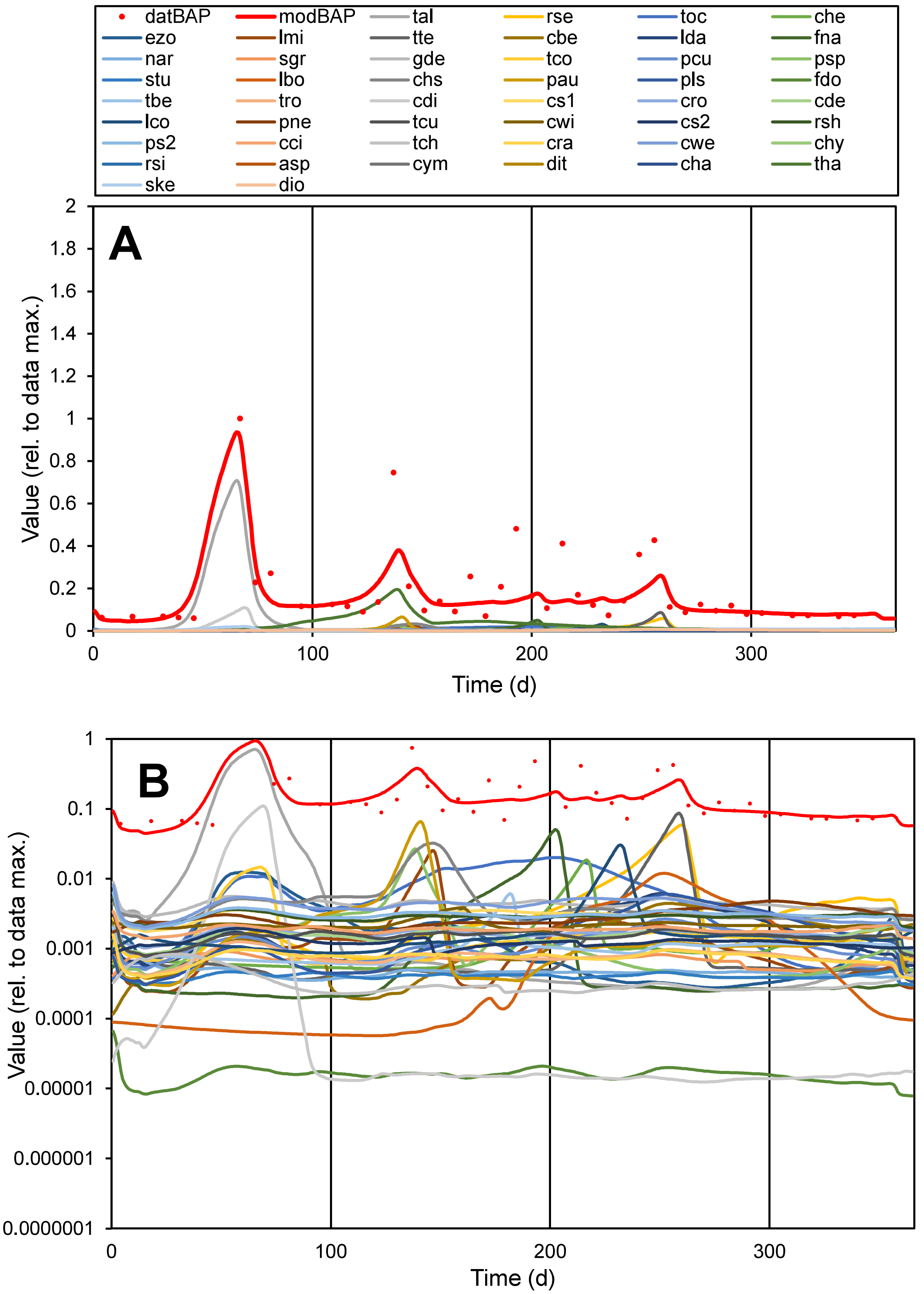

C1

Variables

BAP Provide jtd or Dst

CalibOnly?

2 1 to reproduce of

oNormalize?

2 1=no, 2=yes

ofxn calc*

0.59405

ofxn mod

0.61949

sRun

RunA1

zout

1

Ave

Data

0.0011

Model

0.00094

data/vMax

0.00605

ofxng

334.07

Min d/m

1.2758

ave/min d

3.07346

ave/min m

3.34659

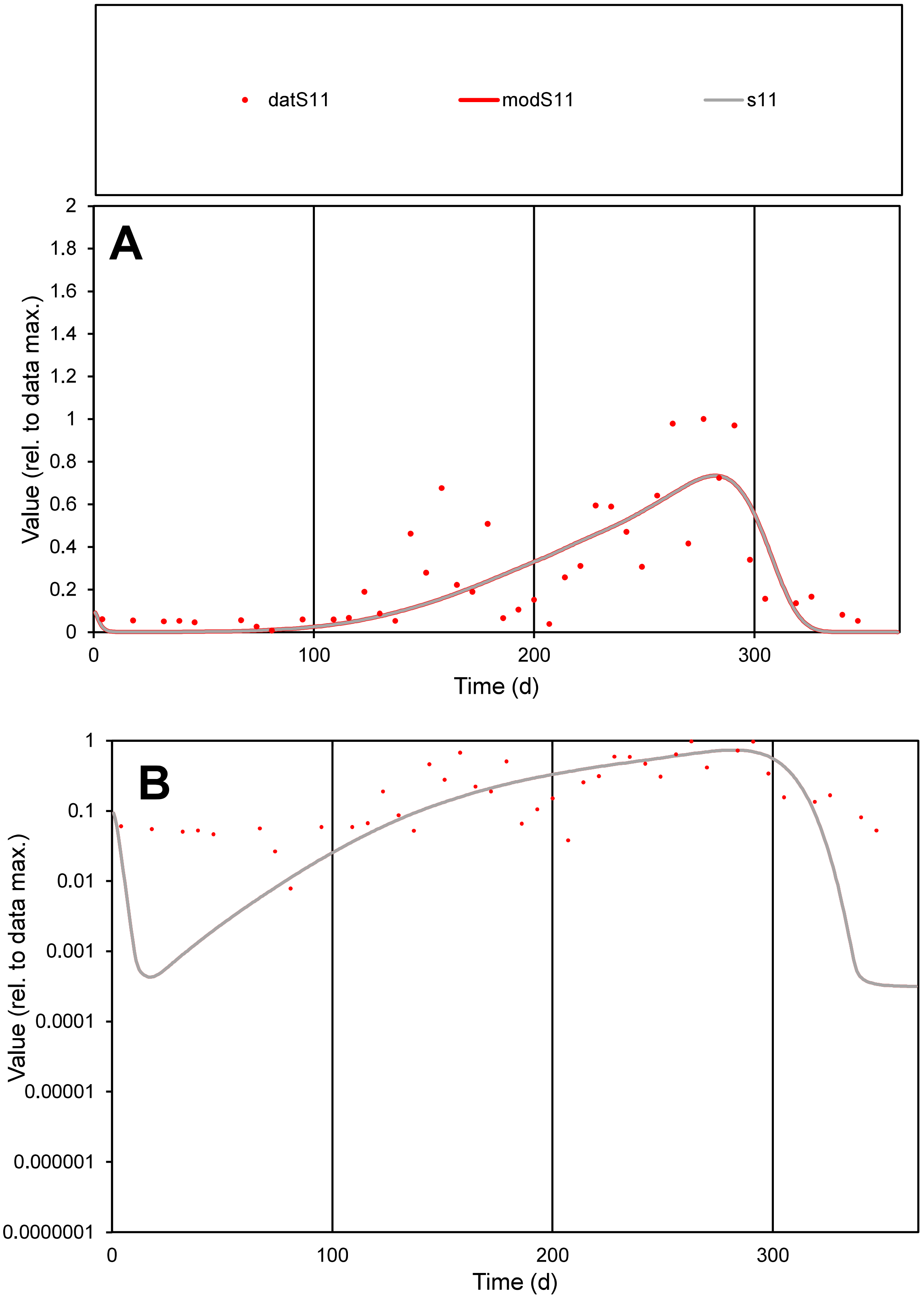

C1

Variables
S11 Provide jtd or Dst
CalibOnly?
2 1 to reproduce of
oNormalize?
2 1=no, 2=yes

ofxn calc*
0.63398
ofxn mod
0.65809

sRun
RunA1
zout
1

Ave
Data
0.10213
Model
0.09419

data/vMax
0.3649

ofxng
334.07

Min d/m
23.4069

ave/min d
35.6486
ave/min m
769.521

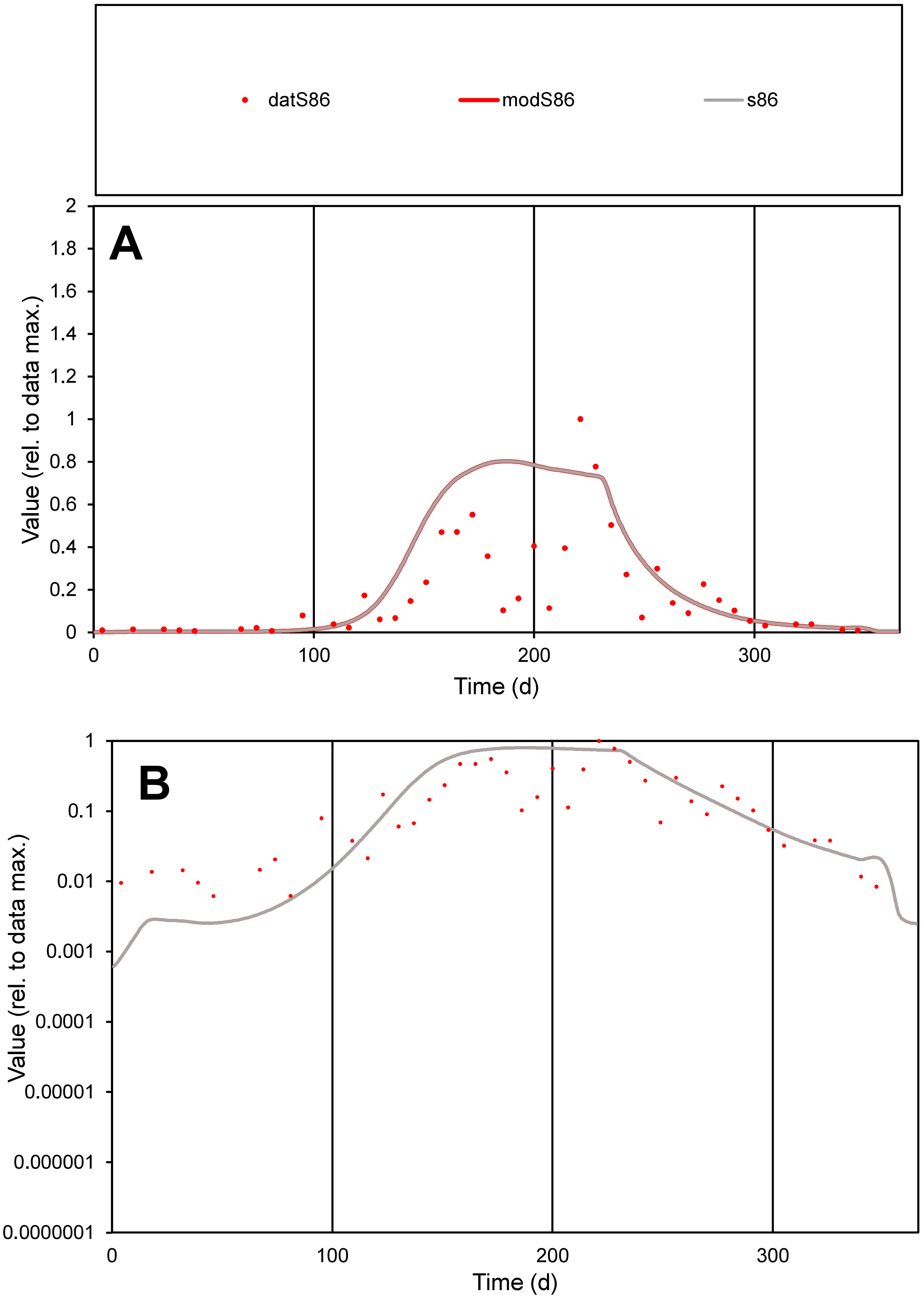

C1

Variables
S86 Provide jtd or Dst
CalibOnly?
2 1 to reproduce of
oNormalize?
2 1=no, 2=yes

ofxn calc*
1.27719
ofxn mod
1.30232

sRun
RunA1
zout
1

Ave
Data
0.07546
Model
0.11945

data/vMax
0.4092

ofxng
334.07

Min d/m
6.71558

ave/min d
29.9801
ave/min m
318.71

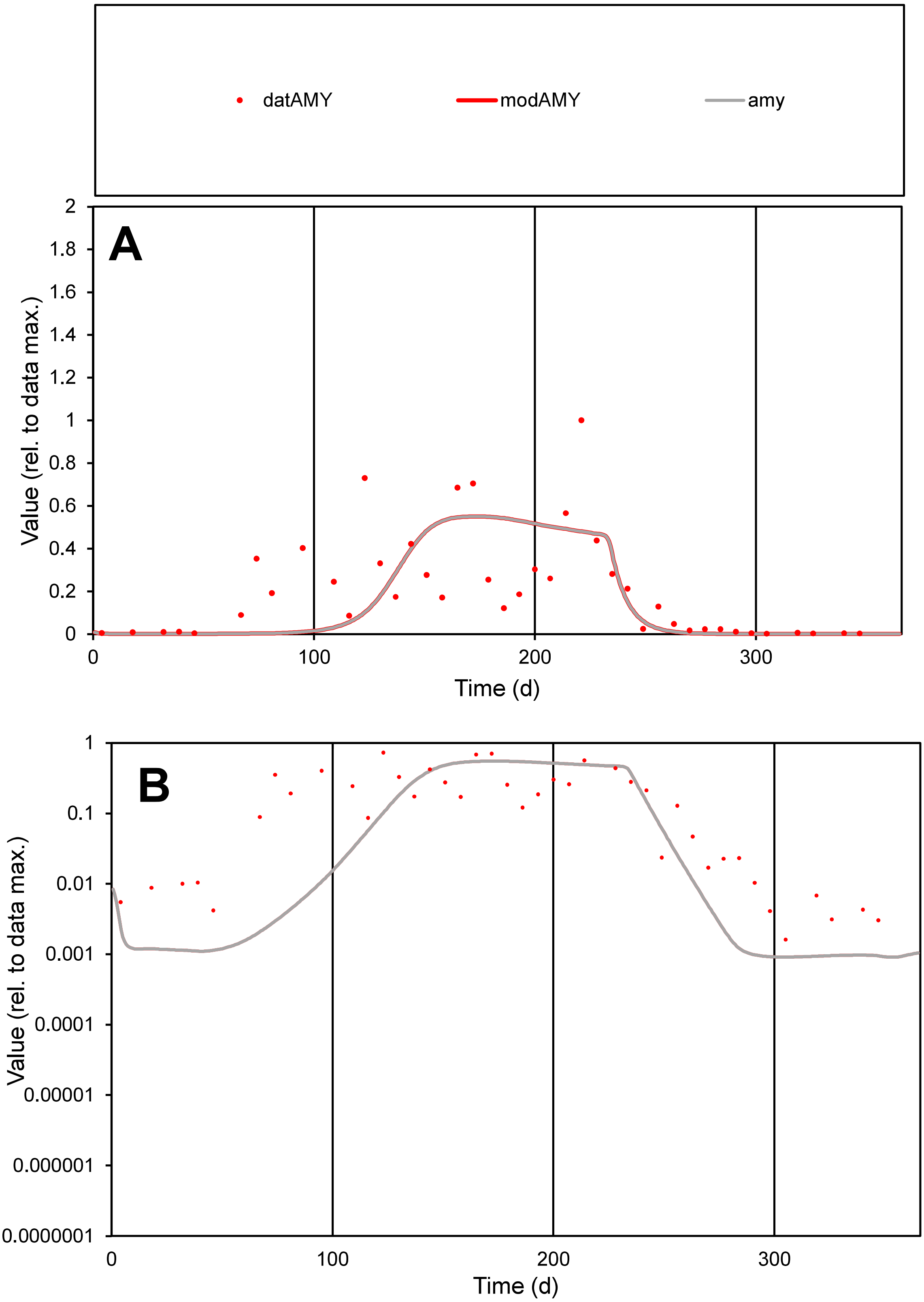

C1

Variables
AMY Provide jtd or Dst
CalibOnly?
2 1 to reproduce of
oNormalize?
2 1=no, 2=yes

ofxn calc*
0.97853
ofxn mod
0.99134

sRun
RunA1
zout
1

Ave
Data
0.06911
Model
0.06197

data/vMax
0.3291

ofxng
334.07

Min d/m
1.75895

ave/min d
130.266
ave/min m
205.483

327_Calib_S_A

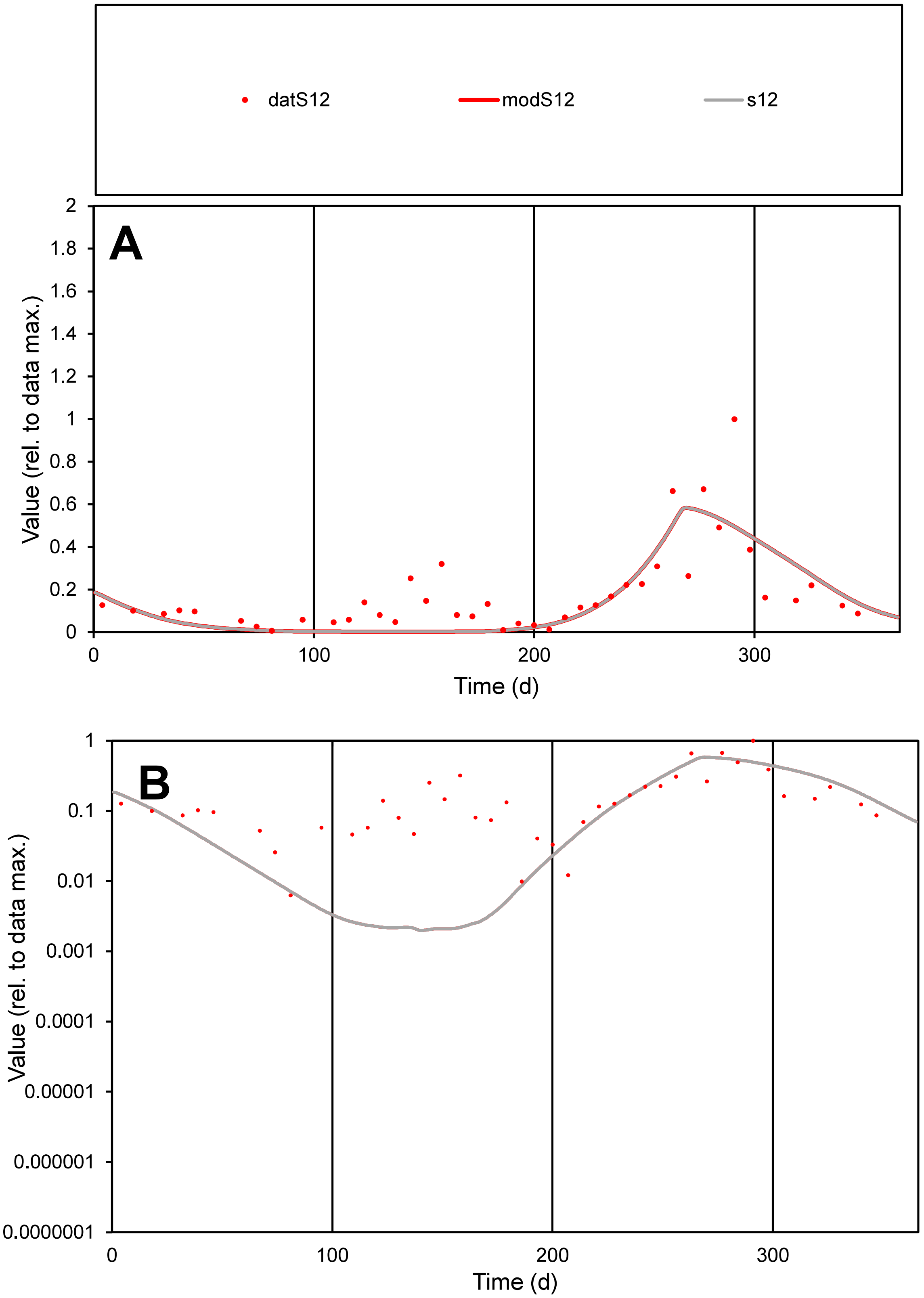

C1

Variables
S12 Provide jtd or Dst
CalibOnly?
2 1 to reproduce of
oNormalize?
2 1=no, 2=yes

ofxn calc*
0.74519
ofxn mod
0.76345

sRun
RunA1
zout
1

Ave
Data
0.0284
Model
0.0238

data/vMax
0.1575

ofxng
334.07

Min d/m
3.03606

ave/min d
28.8299
ave/min m
73.3417

327_Calib_S_A

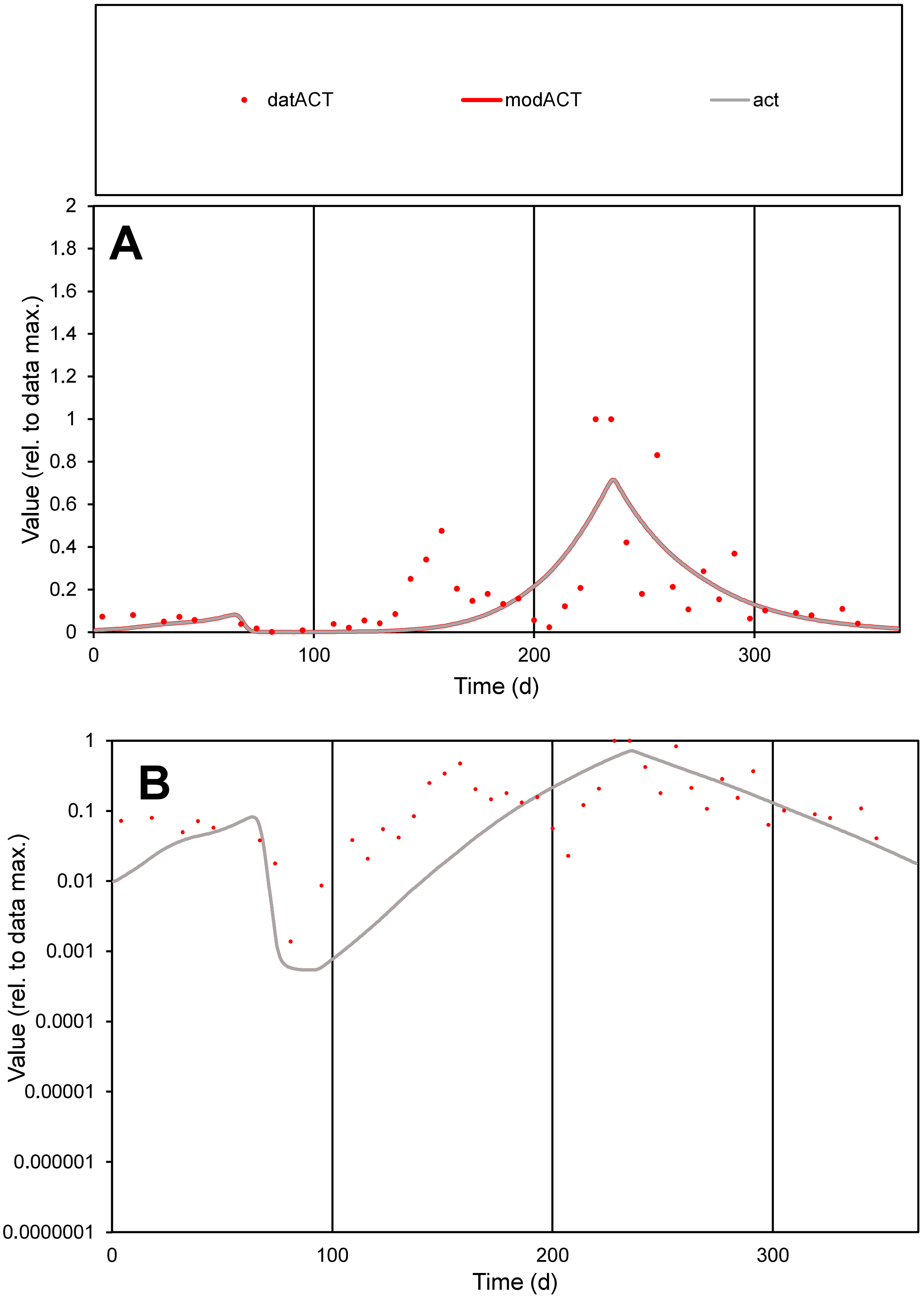

C1

Variables
ACT Provide jtd or Dst
CalibOnly?
2 1 to reproduce of
oNormalize?
2 1=no, 2=yes

ofxn calc*
0.92213
ofxn mod
0.93914

sRun
RunA1
zout
1

Ave
Data
0.02612
Model
0.02182

data/vMax
0.1376

ofxng
334.07

Min d/m
2.42123

ave/min d
137.599
ave/min m
278.373

327_Calib_S_A

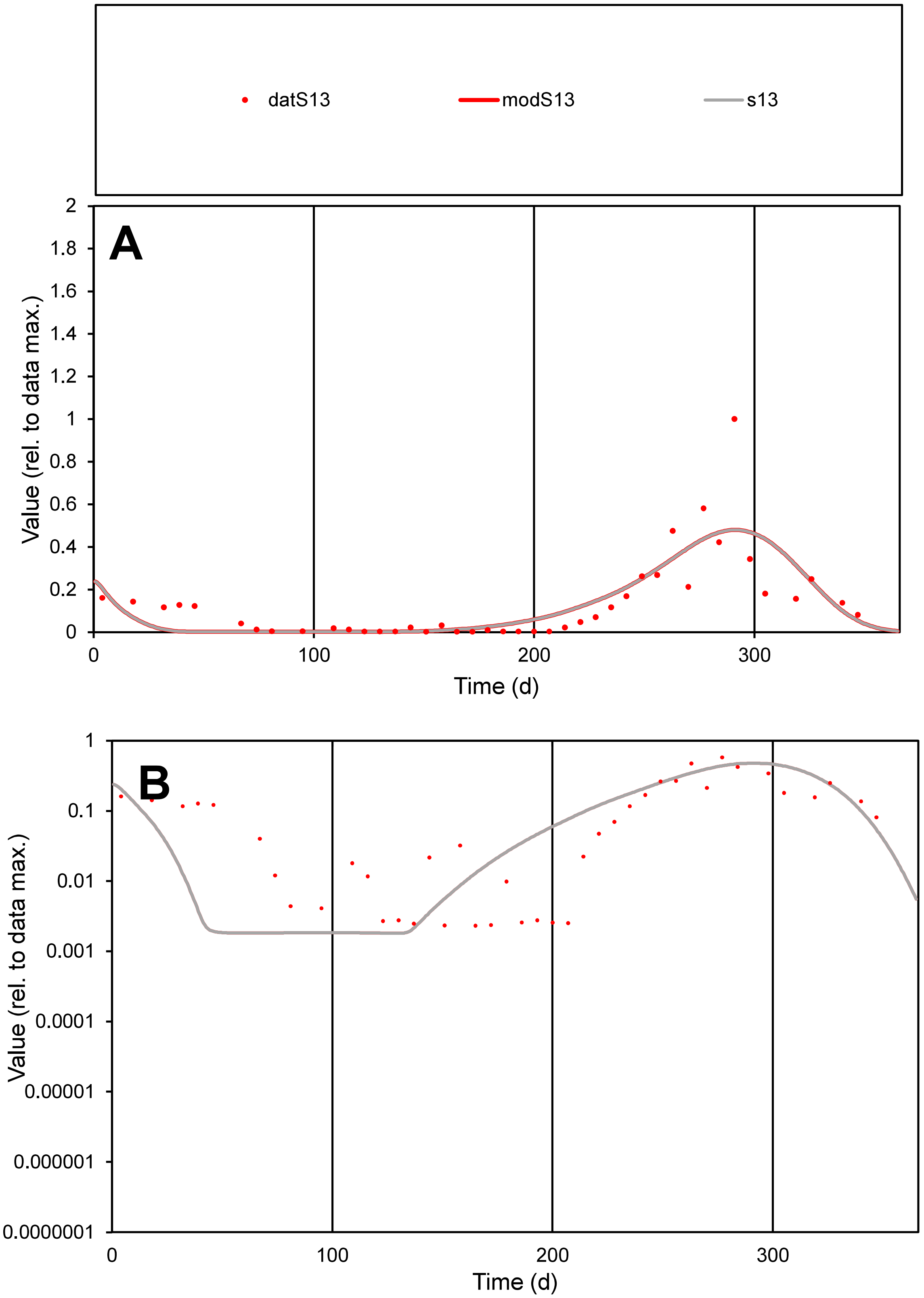

C1

Variables
S13 Provide jtd or Dst
CalibOnly?
2 1 to reproduce of
oNormalize?
2 1=no, 2=yes

ofxn calc*
0.83512
ofxn mod
0.85819

sRun
RunA1
zout
1

Ave
Data
0.01604
Model
0.01602

data/vMax
0.1194

ofxng
334.07

Min d/m
1.27852

ave/min d
57.9536
ave/min m
73.9938

327_Calib_S_A

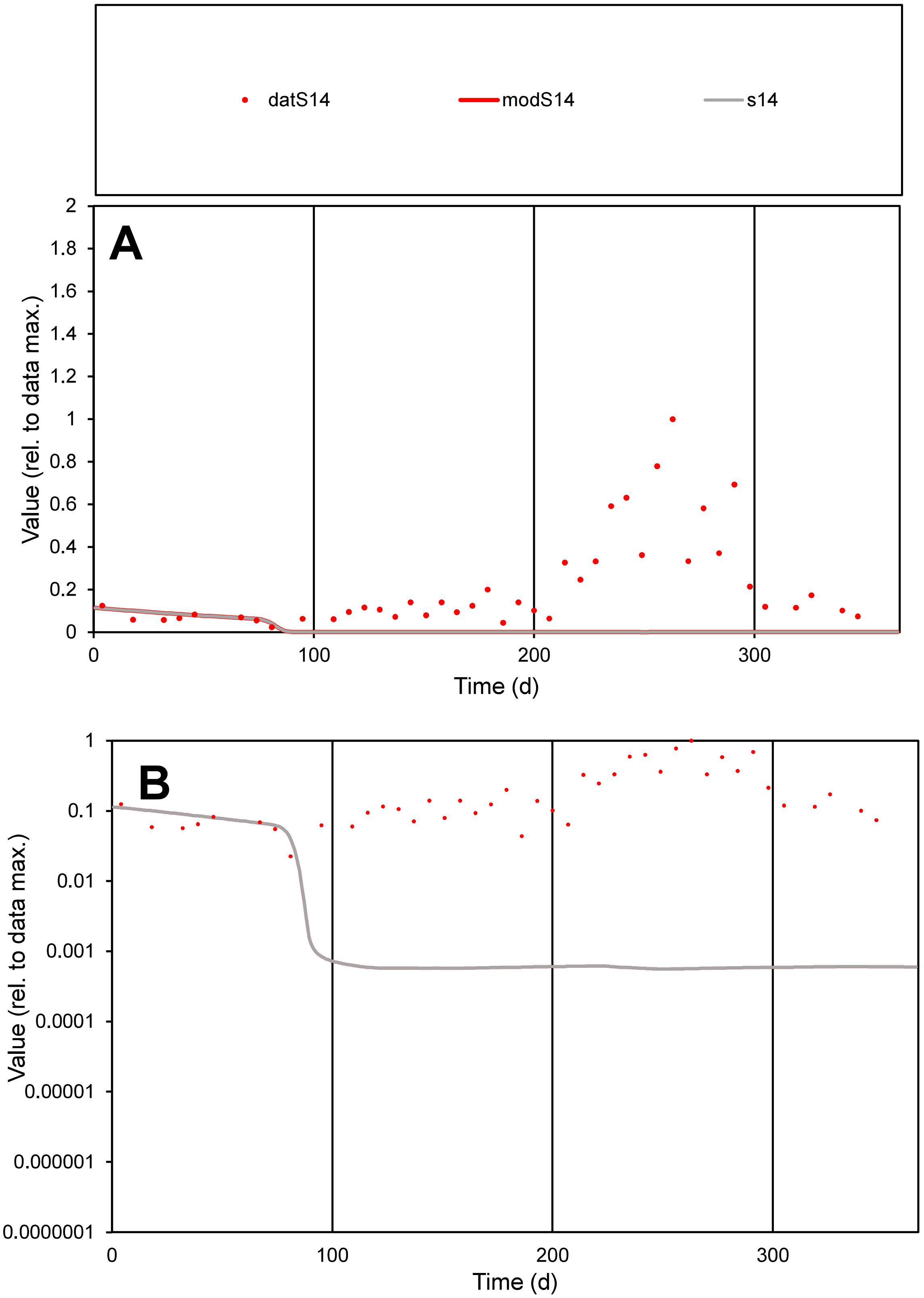

C1

Variables

S14 Provide jtd or Dst

CalibOnly?

2 1 to reproduce of

oNormalize?

2 1=no, 2=yes

ofxn calc*

1.43072

ofxn mod

1.43394

sRun

RunA1

zout

1

Ave

Data

0.02486

Model

0.00173

data/vMax

0.1134

ofxng

334.07

Min d/m

40.3279

ave/min d

9.71678

ave/min m

27.284

327_Calib_S_A

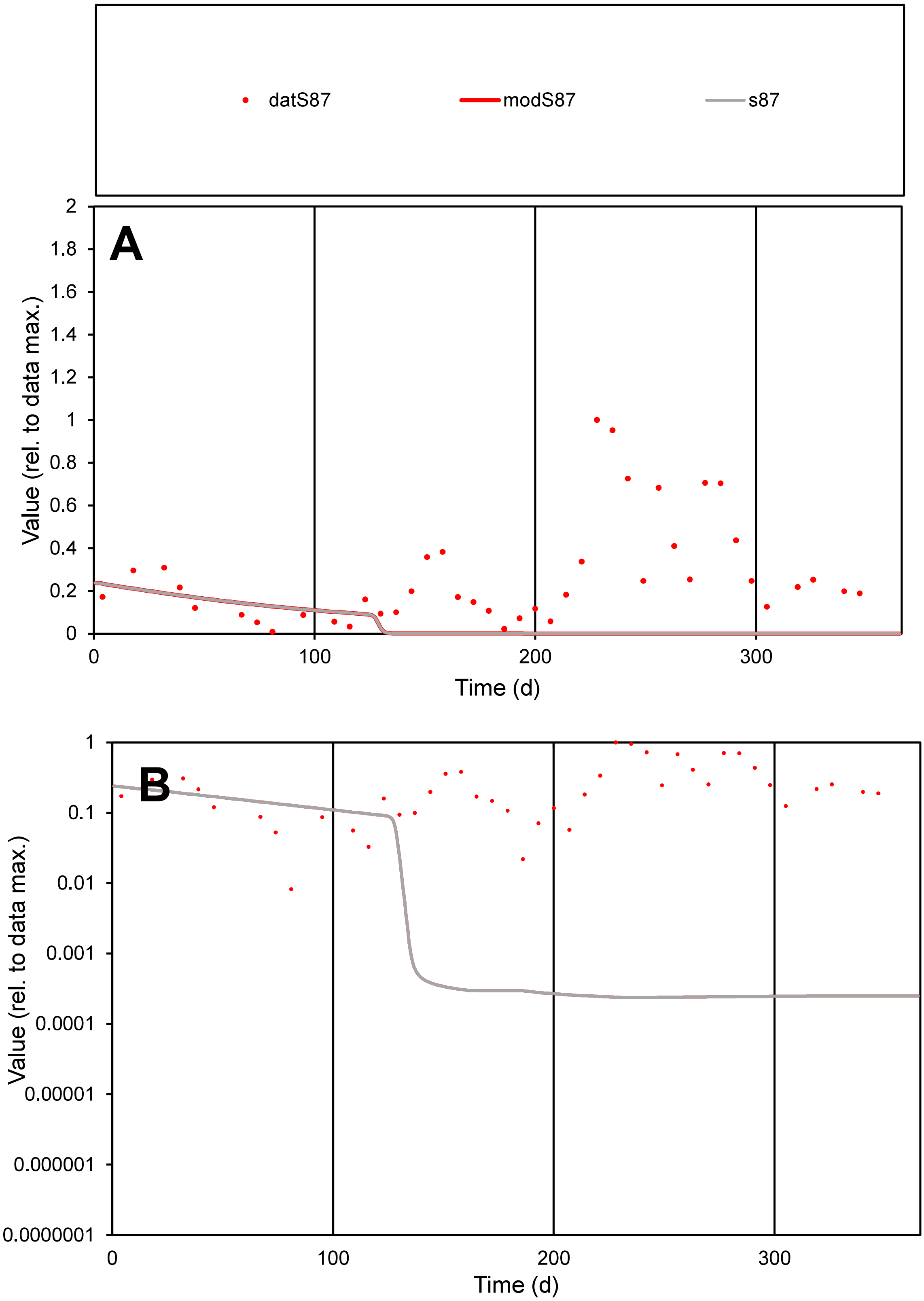

C1

Variables

S87 Provide jtd or Dst

CalibOnly?

2 1 to reproduce of

oNormalize?

2 1=no, 2=yes

ofxn calc*

1.31619

ofxn mod

1.3192

sRun

RunA1

zout

1

Ave

Data

0.02168

Model

0.00355

data/vMax

0.08068

ofxng

334.07

Min d/m

34.4903

ave/min d

32.8657

ave/min m

185.605

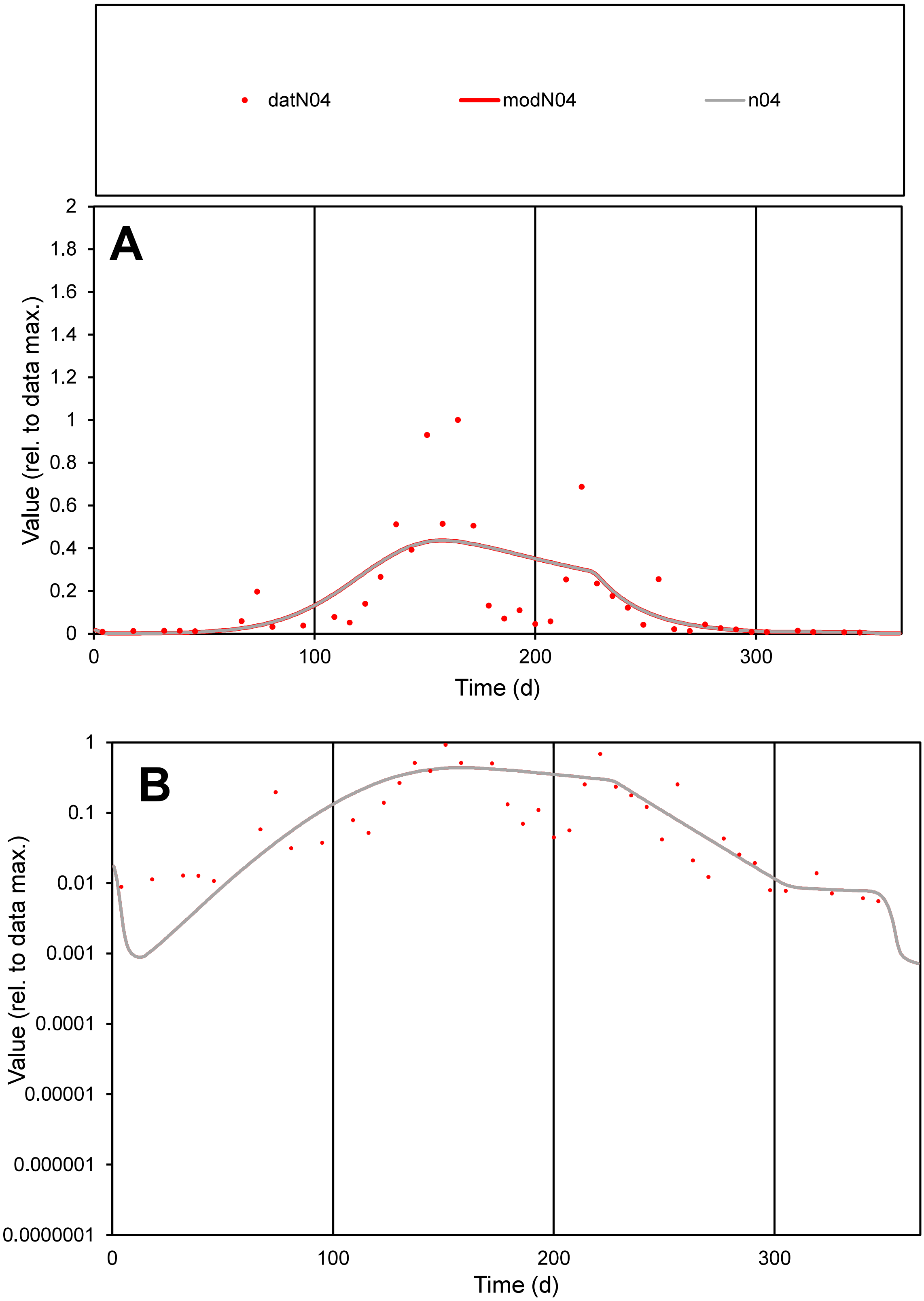

C1

Variables
N04 Provide jtd or Dst
CalibOnly?
2 1 to reproduce of
oNormalize?
2 1=no, 2=yes

ofxn calc*
1.041
ofxn mod
1.053

sRun
RunA1
zout
1

Ave
Data
0.02944
Model
0.03

data/vMax
0.1737

ofxng
334.07

Min d/m
4.54541

ave/min d
30.4825
ave/min m
141.185

327_Calib_S_A

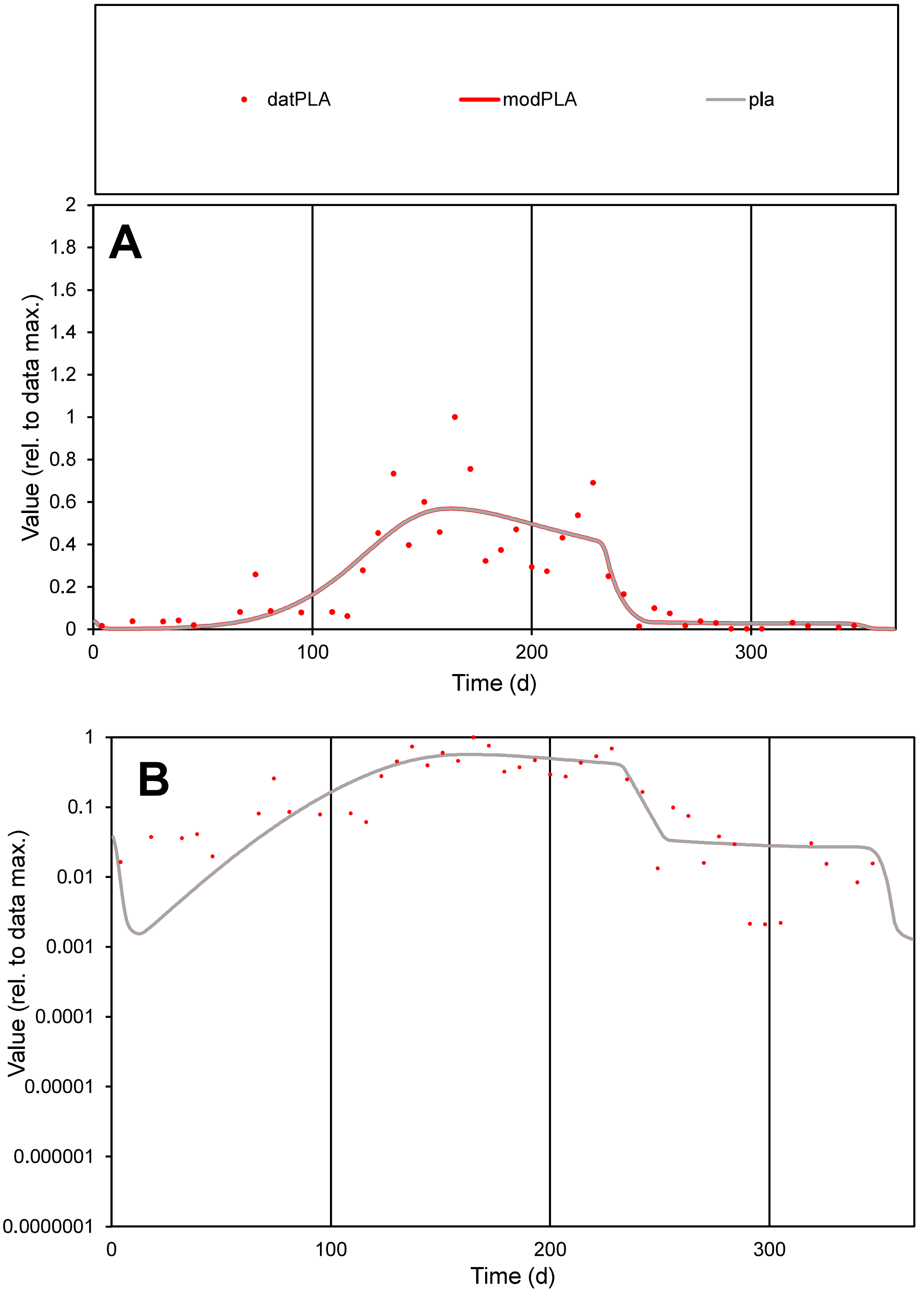

C1

Variables
PLA Provide jtd or Dst
CalibOnly?
2 1 to reproduce of
oNormalize?
2 1=no, 2=yes

ofxn calc*
0.56434
ofxn mod
0.58506

sRun
RunA1
zout
1

Ave
Data
0.01813
Model
0.01794

data/vMax
0.07909

ofxng
334.07

Min d/m
0.98128

ave/min d
108.095
ave/min m
104.965

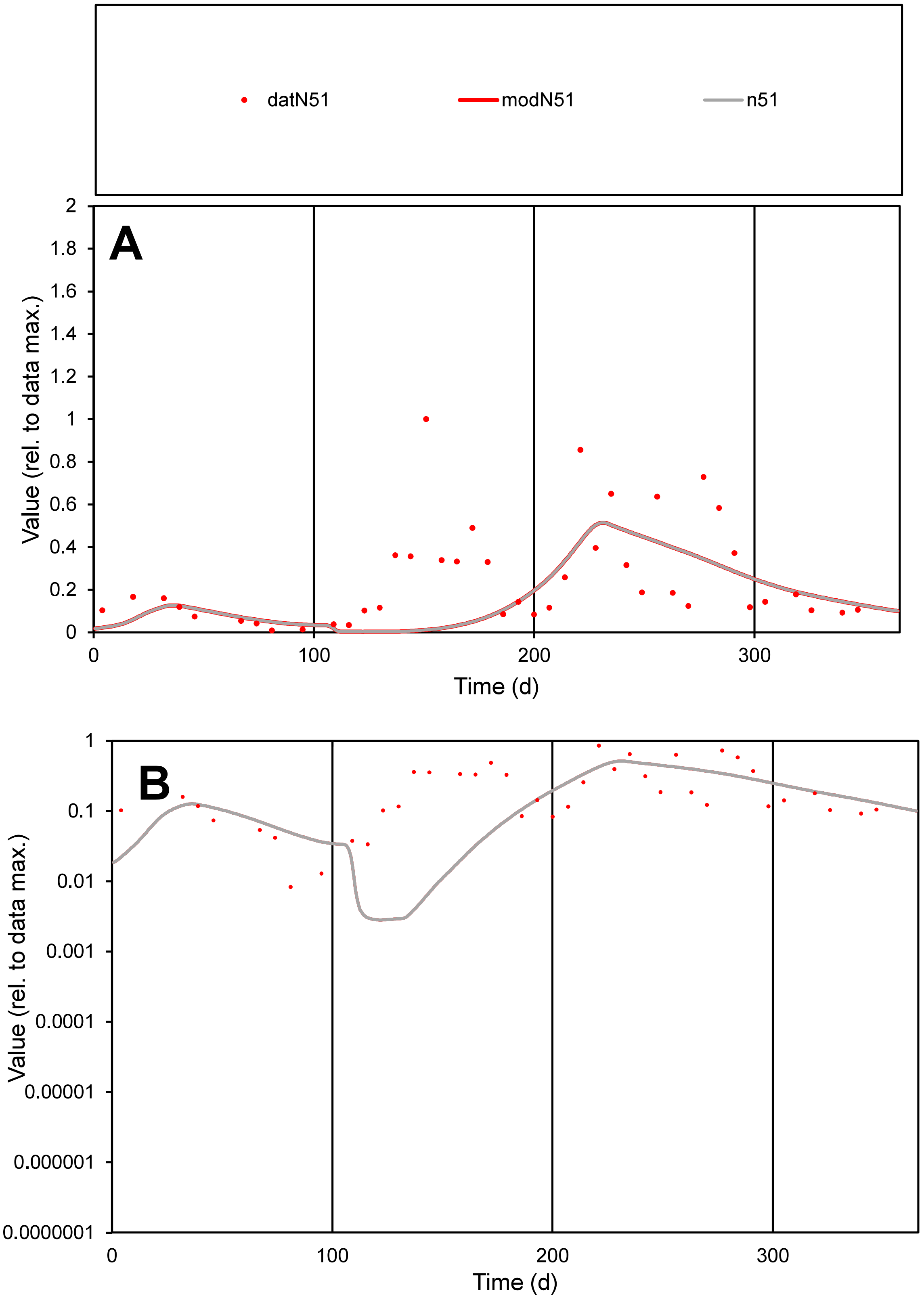

C1

Variables
N51 Provide jtd or Dst
CalibOnly?
2 1 to reproduce of
oNormalize?
2 1=no, 2=yes

ofxn calc*
0.95172
ofxn mod
0.95974

sRun
RunA1
zout
1

Ave
Data
0.01438
Model
0.01018

data/vMax
0.05647

ofxng
334.07

Min d/m
2.9247

ave/min d
30.5891
ave/min m
63.332

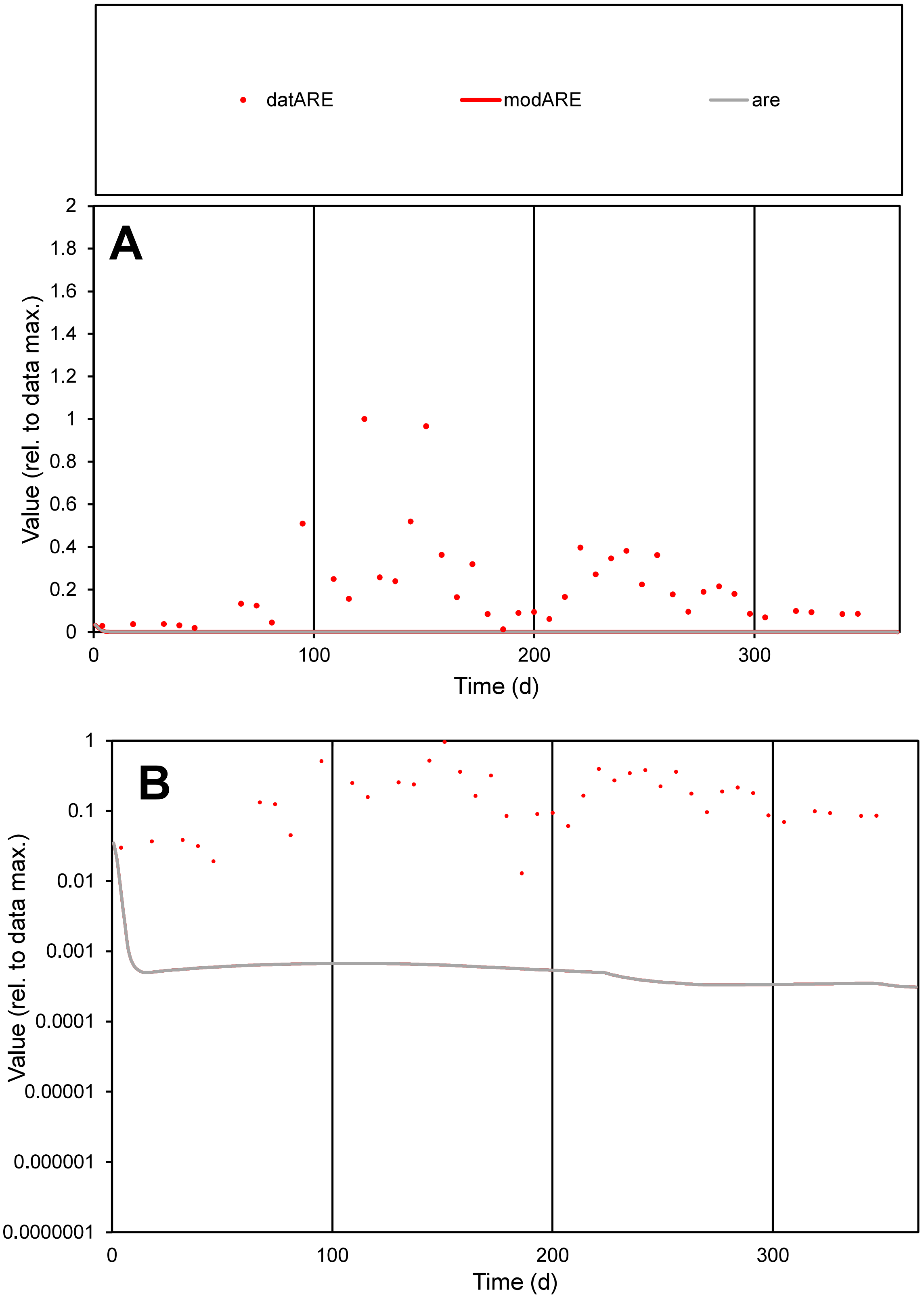

C1

Variables
ARE Provide jtd or Dst
CalibOnly?
2 1 to reproduce of
oNormalize?
2 1=no, 2=yes

ofxn calc*
1.41092
ofxn mod
1.41277

sRun
RunA1
zout
1

Ave
Data
0.02832
Model
0.00013

data/vMax
0.1312

ofxng
334.07

Min d/m
38.8953

ave/min d
16.6905
ave/min m
2.96176

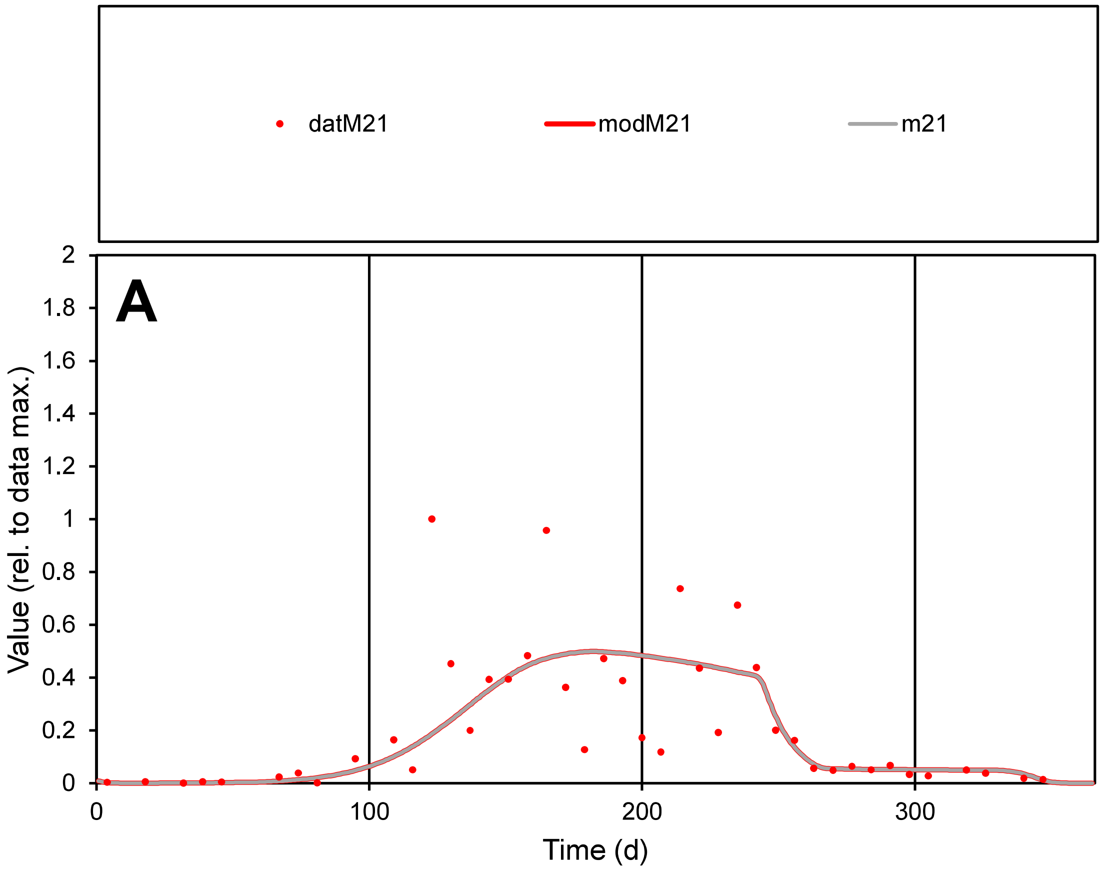

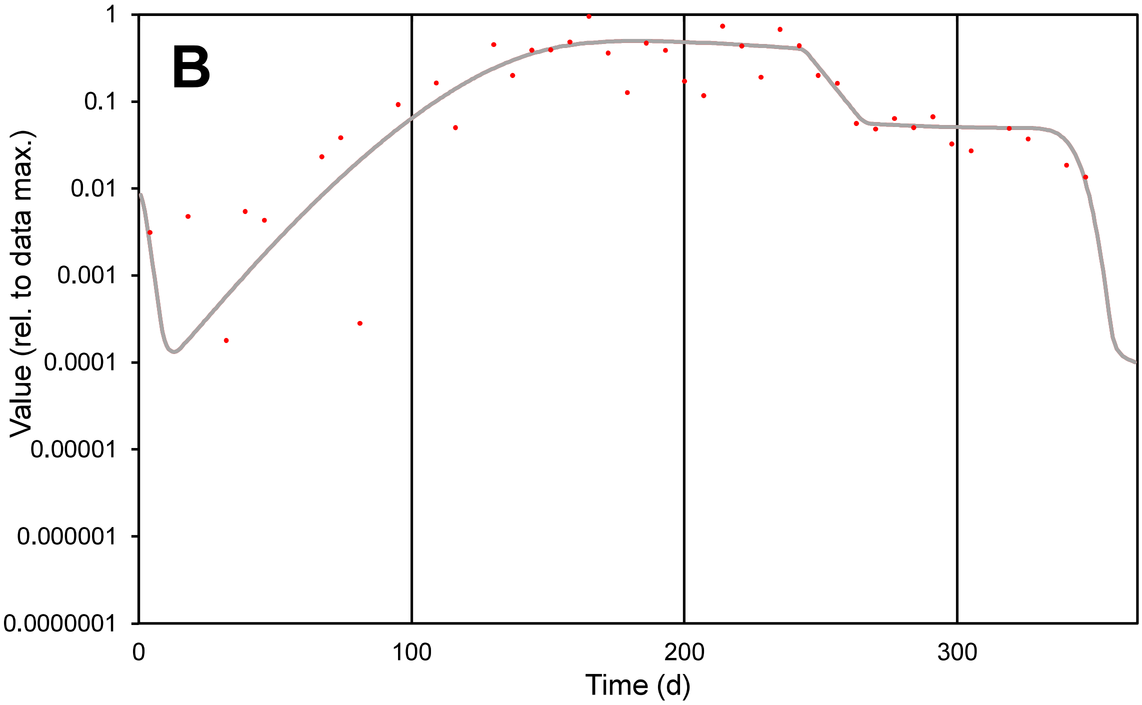

C1

Variables
M21 Provide jtd or Dst
CalibOnly?
2 1 to reproduce of
oNormalize?
2 1=no, 2=yes

ofxn calc*
0.87285
ofxn mod
0.887

sRun
RunA1
zout
1

Ave
Data
0.02906
Model
0.02769

data/vMax
0.1326

ofxng
334.07

Min d/m
0.86812

ave/min d
1219.5
ave/min m
1008.61

327_Calib_S_A

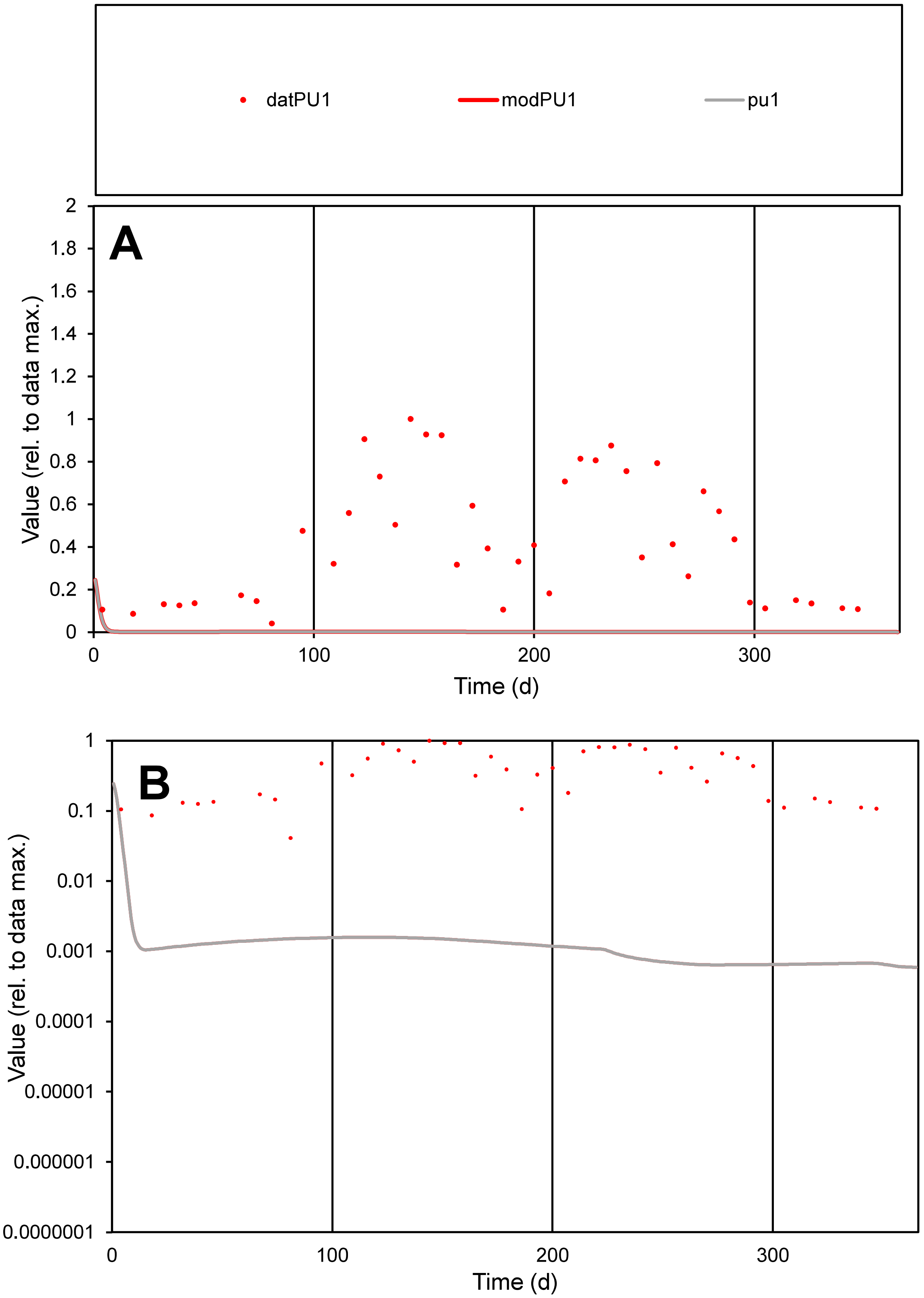

C1

Variables
PU1 Provide jtd or Dst
CalibOnly?
2 1 to reproduce of
oNormalize?
2 1=no, 2=yes

ofxn calc*
1.21479
ofxn mod
1.21665

sRun
RunA1
zout
1

Ave
Data
0.01692
Model
0.00011

data/vMax
0.0399

ofxng
334.07

Min d/m
64.1066

ave/min d
10.344
ave/min m
4.12559

327_Calib_S_A

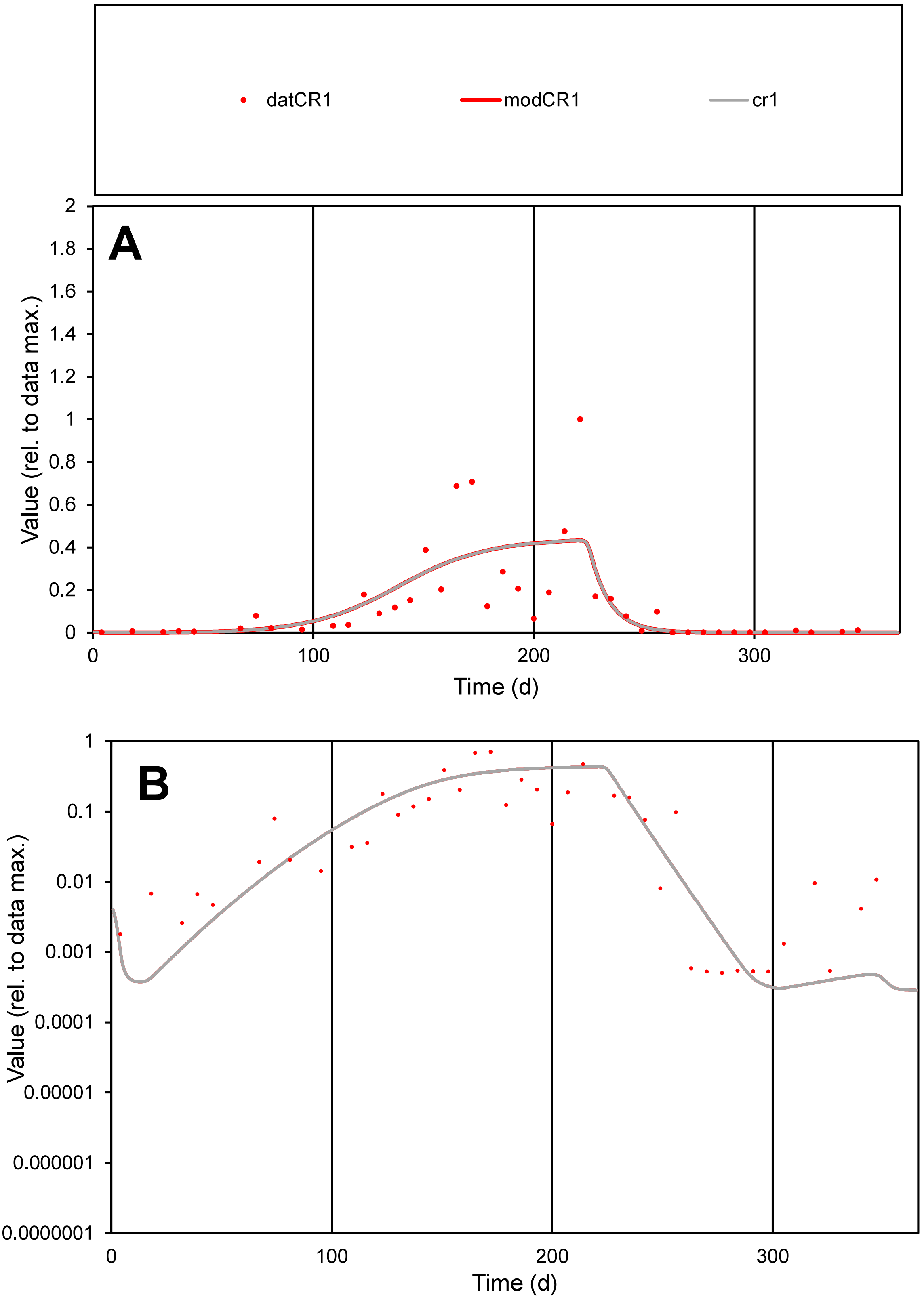

C1

Variables
CR1 Provide jtd or Dst
CalibOnly?
2 1 to reproduce of
oNormalize?
2 1=no, 2=yes

ofxn calc*
1.11687
ofxn mod
1.13355

sRun
RunA1
zout
1

Ave
Data
0.01799
Model
0.01858

data/vMax
0.1342

ofxng
334.07

Min d/m
1.61932

ave/min d
265.707
ave/min m
444.386

327_Calib_S_A

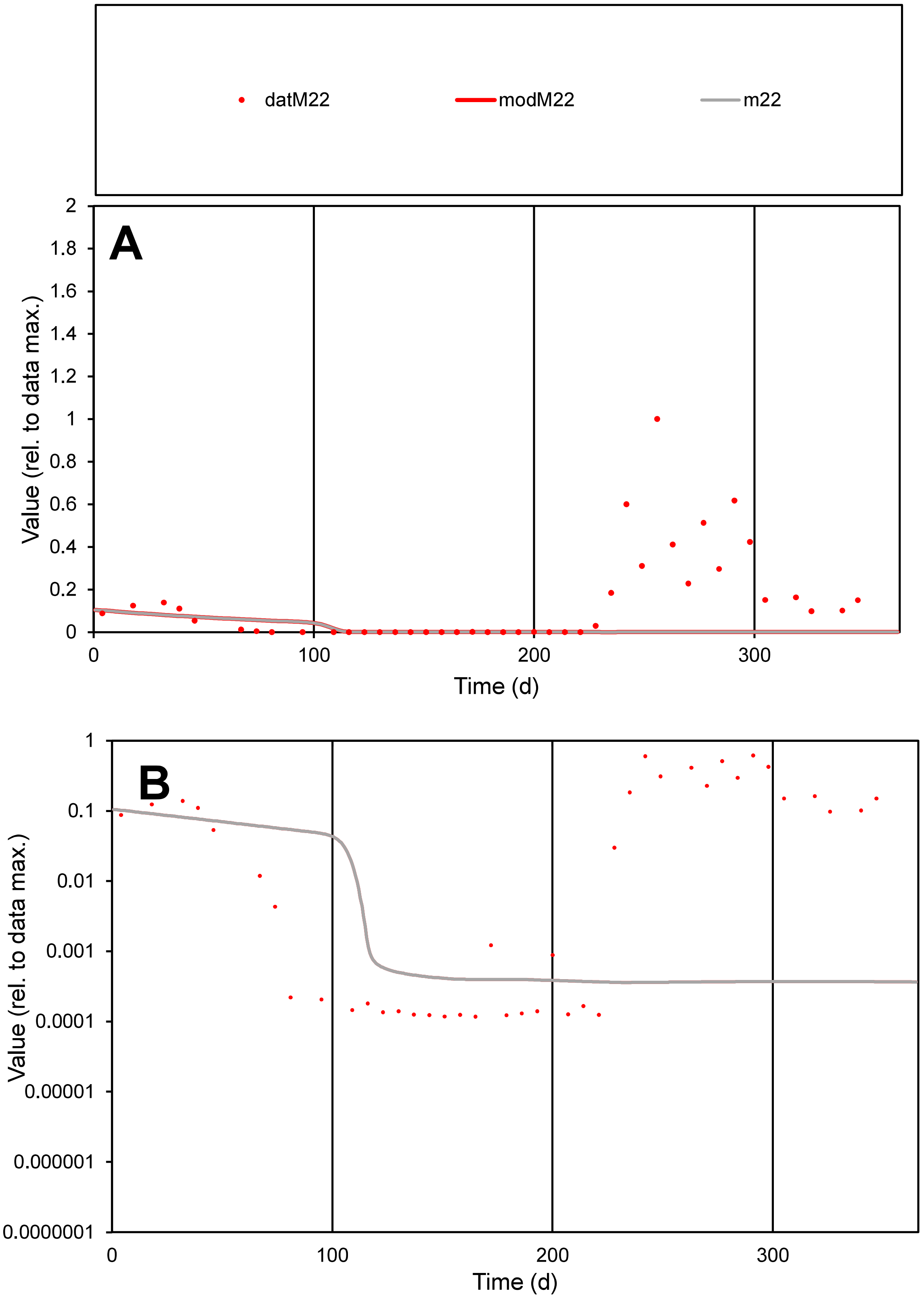

C1

Variables
M22 Provide jtd or Dst
CalibOnly?
2 1 to reproduce of
oNormalize?
2 1=no, 2=yes

ofxn calc*
1.84192
ofxn mod
1.84627

sRun
RunA1
zout
1

Ave
Data
0.02531
Model
0.00286

data/vMax
0.1829

ofxng
334.07

Min d/m
0.3232

ave/min d
1184.29
ave/min m
43.2599

327_Calib_S_A

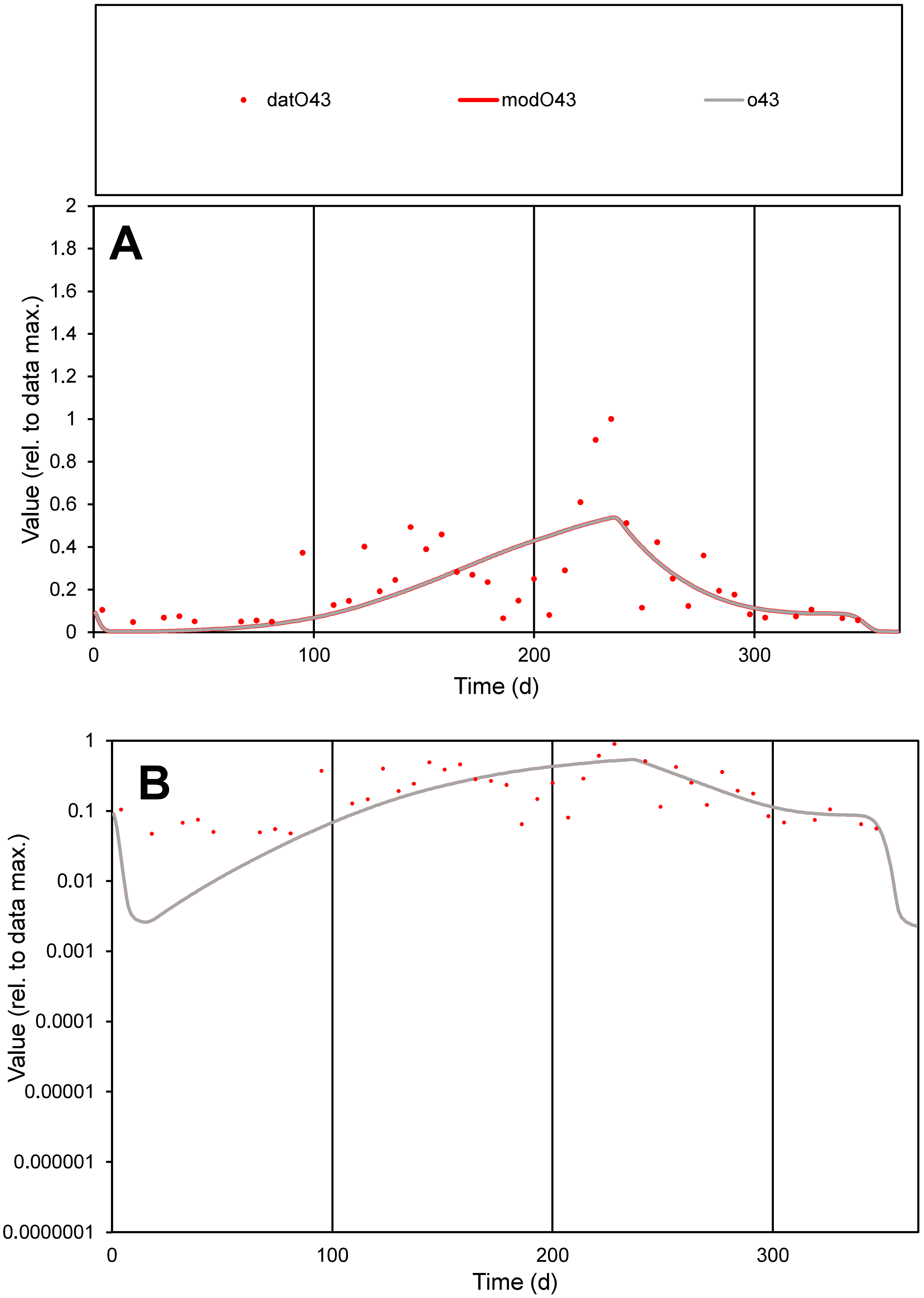

C1

Variables
O43 Provide jtd or Dst
CalibOnly?
2 1 to reproduce of
oNormalize?
2 1=no, 2=yes

ofxn calc*
0.71437
ofxn mod
0.72883

sRun
RunA1
zout
1

Ave
Data
0.01631
Model
0.01454

data/vMax
0.0681

ofxng
334.07

Min d/m
16.046

ave/min d
5.08741
ave/min m
72.7964

327_Calib_S_A

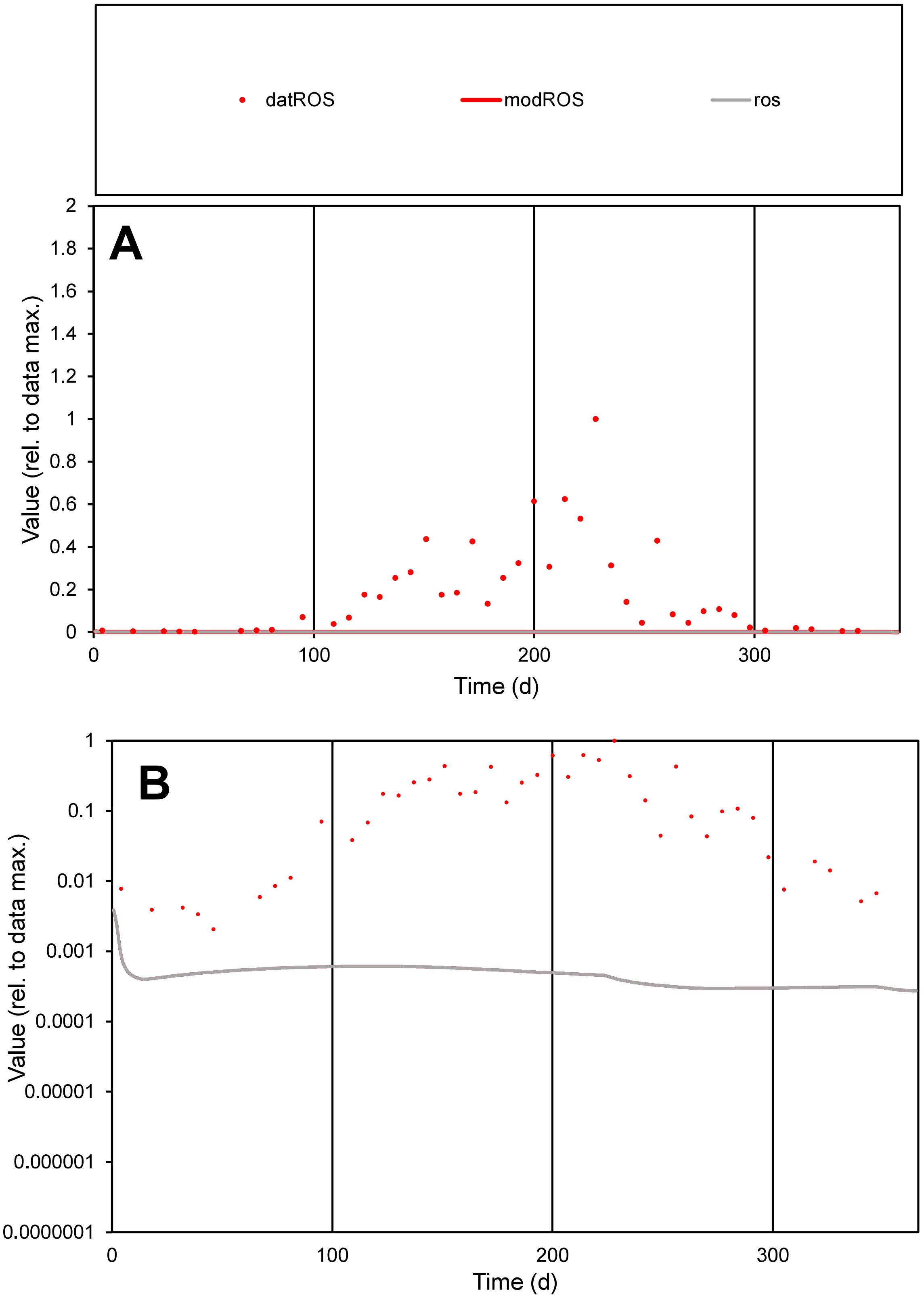

C1

Variables
ROS Provide jtd or Dst
CalibOnly?
2 1 to reproduce of
oNormalize?
2 1=no, 2=yes

ofxn calc*
1.56782
ofxn mod
1.57001

sRun
RunA1
zout
1

Ave
Data
0.01489
Model
4.3E-05

data/vMax
0.08307

ofxng
334.07

Min d/m
7.02084

ave/min d
86.669
ave/min m
1.76282

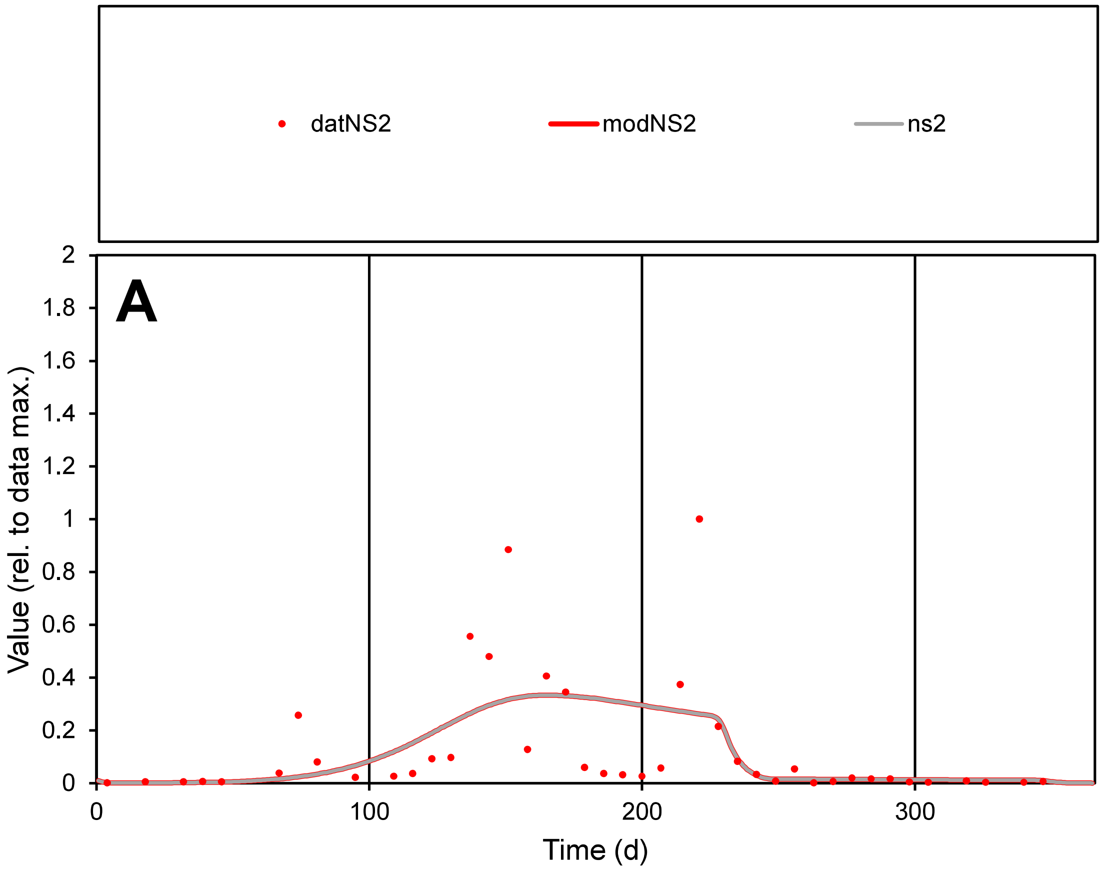

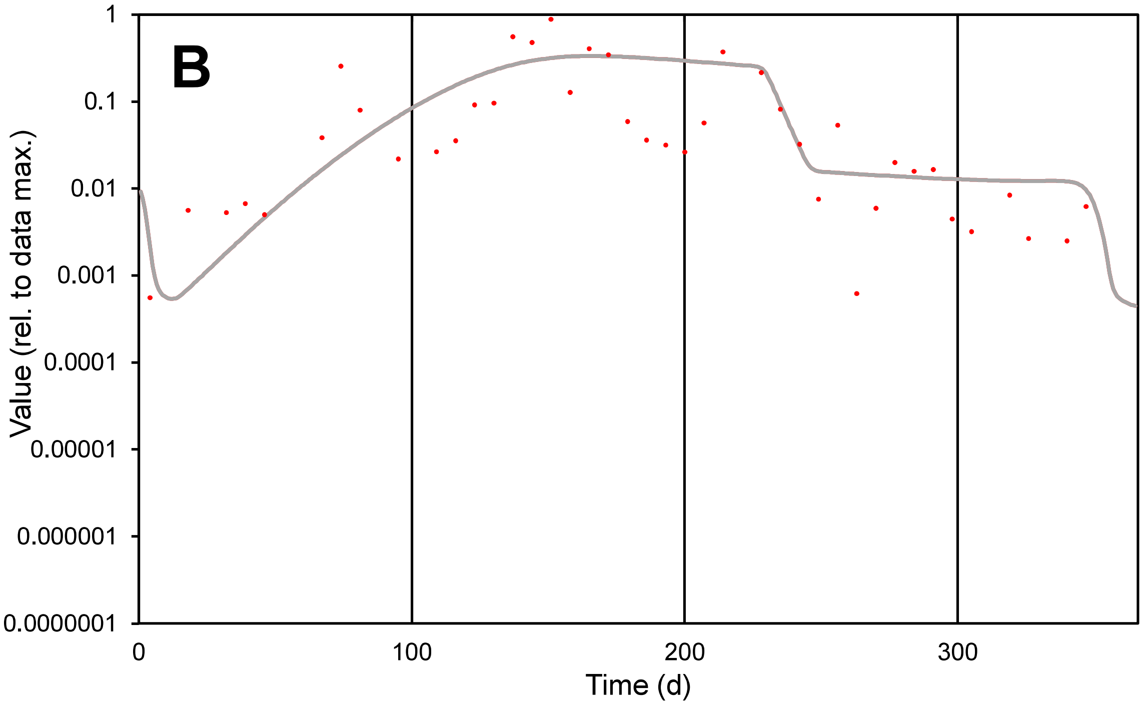

C1

Variables
NS2 Provide jtd or Dst
CalibOnly?
2 1 to reproduce of
oNormalize?
2 1=no, 2=yes

ofxn calc*
1.43169
ofxn mod
1.44094

sRun
RunA1
zout
1

Ave
Data
0.01457
Model
0.01395

data/vMax
0.1107

ofxng
334.07

Min d/m
0.71394

ave/min d
235.933
ave/min m
161.243

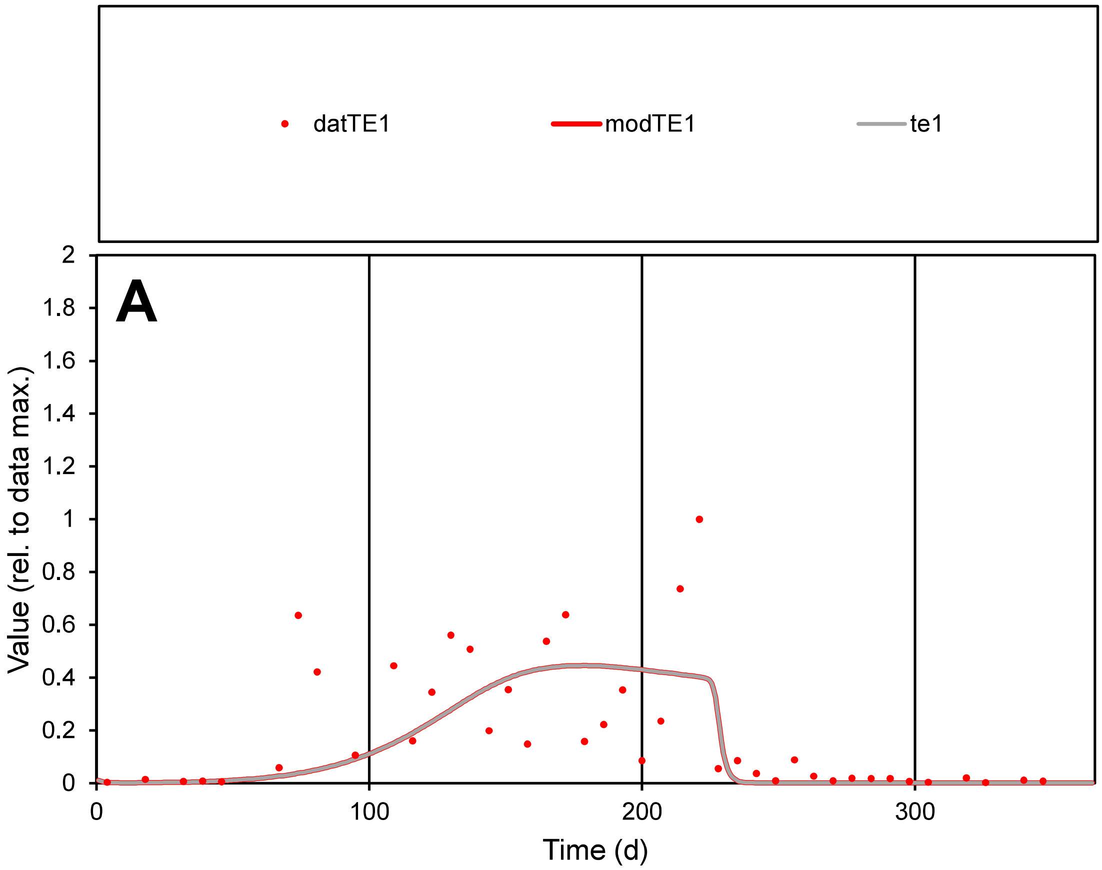

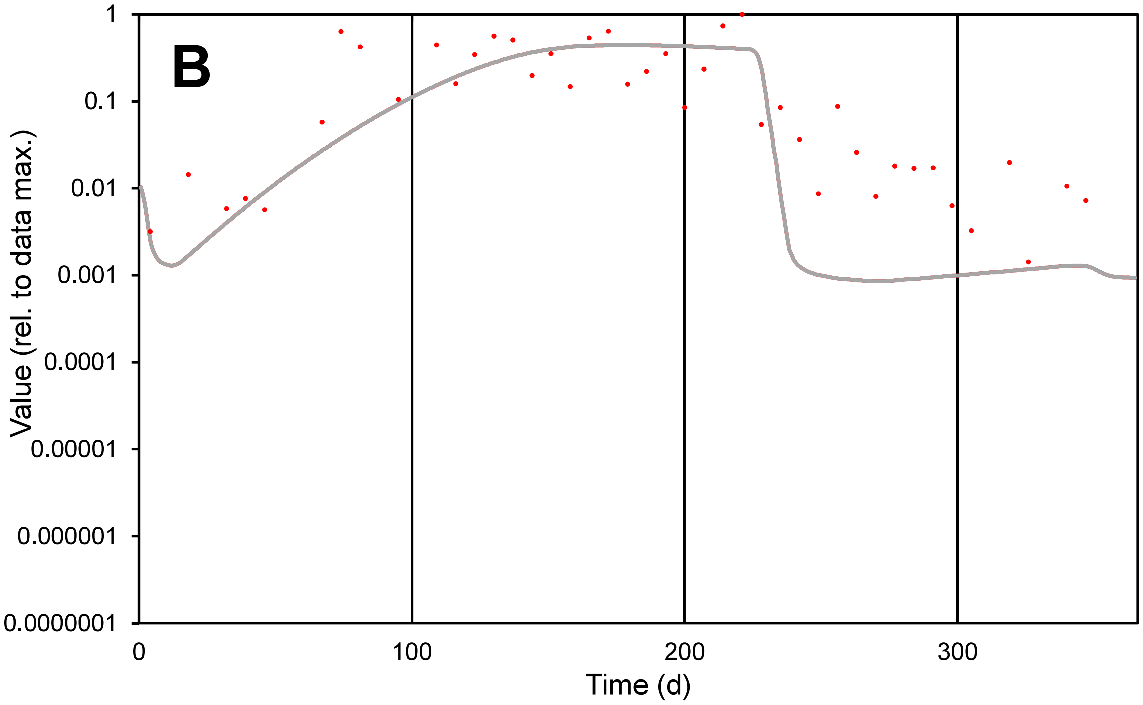

C1

Variables
TE1 Provide jtd or Dst
CalibOnly?
2 1 to reproduce of
oNormalize?
2 1=no, 2=yes

ofxn calc*
0.98657
ofxn mod
0.99972

sRun
RunA1
zout
1

Ave
Data
0.00808
Model
0.00647

data/vMax
0.04069

ofxng
334.07

Min d/m
1.66839

ave/min d
139.397
ave/min m
186.339

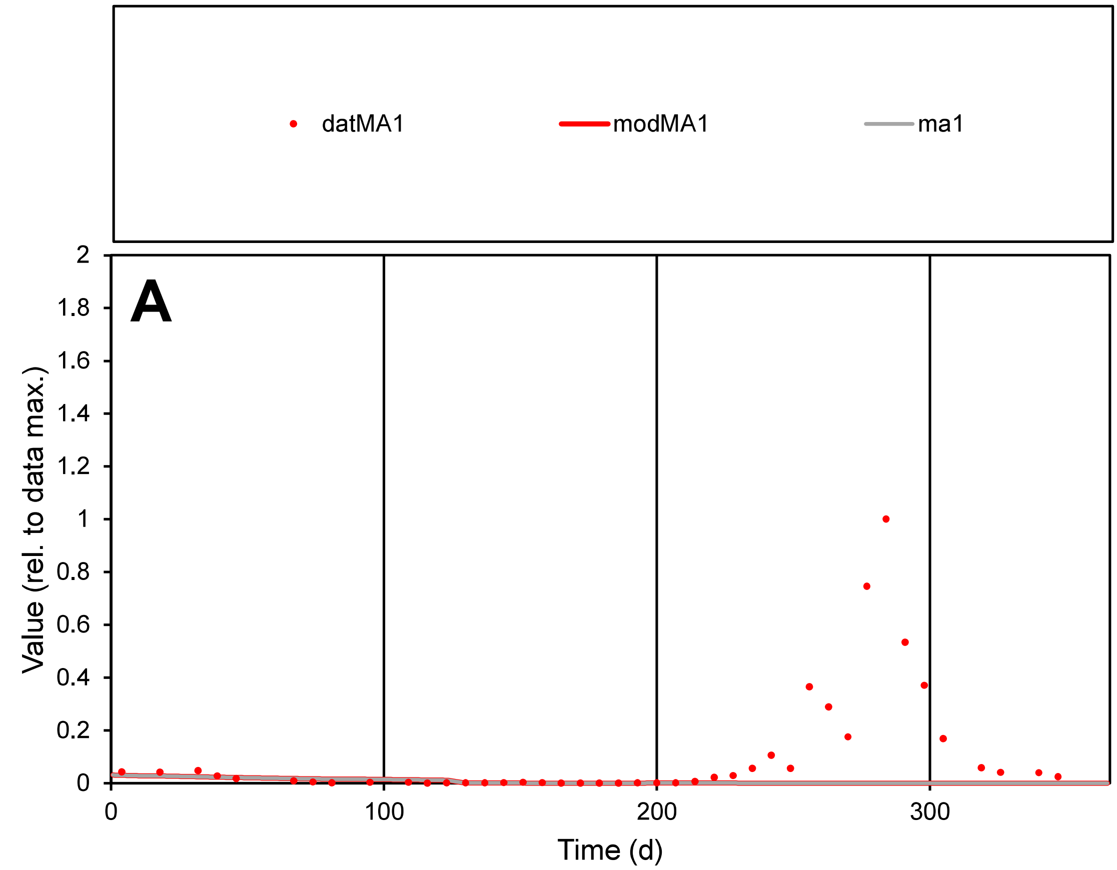

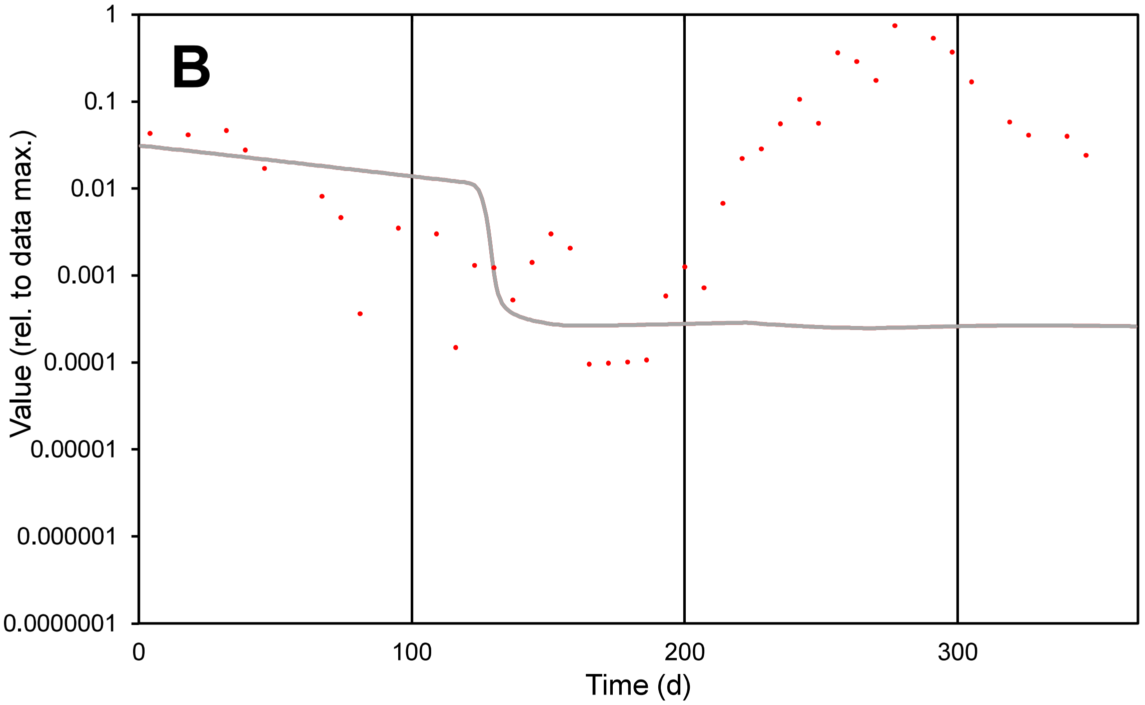

C1

Variables
MA1 Provide jtd or Dst
CalibOnly?
2 1 to reproduce of
oNormalize?
2 1=no, 2=yes

ofxn calc*
2.27891
ofxn mod
2.28228

sRun
RunA1
zout
1

Ave
Data
0.01525
Model
0.00082

data/vMax
0.1492

ofxng
334.07

Min d/m
0.38724

ave/min d
1065.19
ave/min m
22.1004

327_Calib_S_A

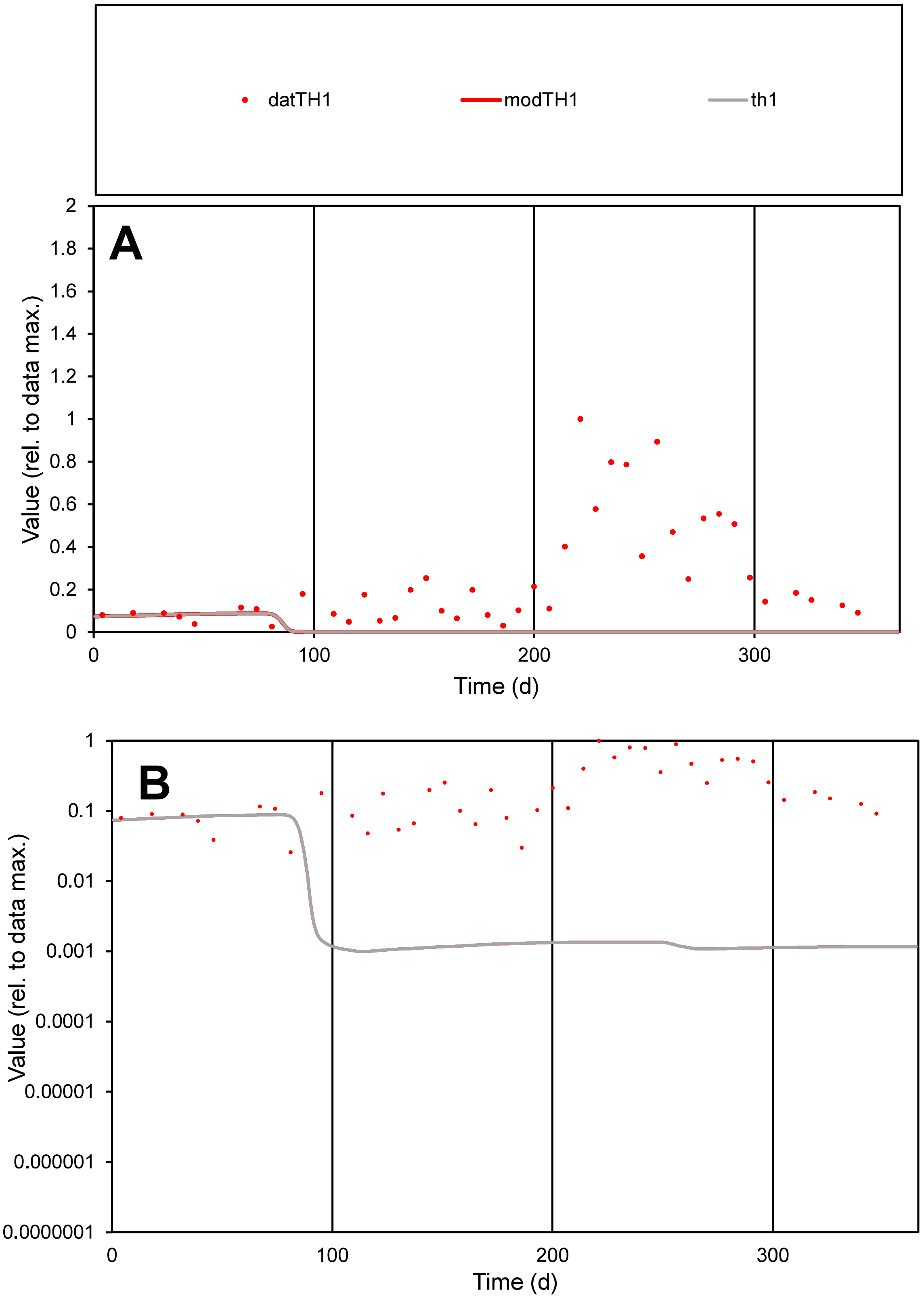

C1

Variables

TH1 Provide jtd or Dst

CalibOnly?

2 1 to reproduce of

oNormalize?

2 1=no, 2=yes

ofxn calc*

1.39542

ofxn mod

1.39825

sRun

RunA1

zout

1

Ave

Data

0.01344

Model

0.00087

data/vMax

0.05295

ofxng

334.07

Min d/m

25.6242

ave/min d

9.84516

ave/min m

16.3162

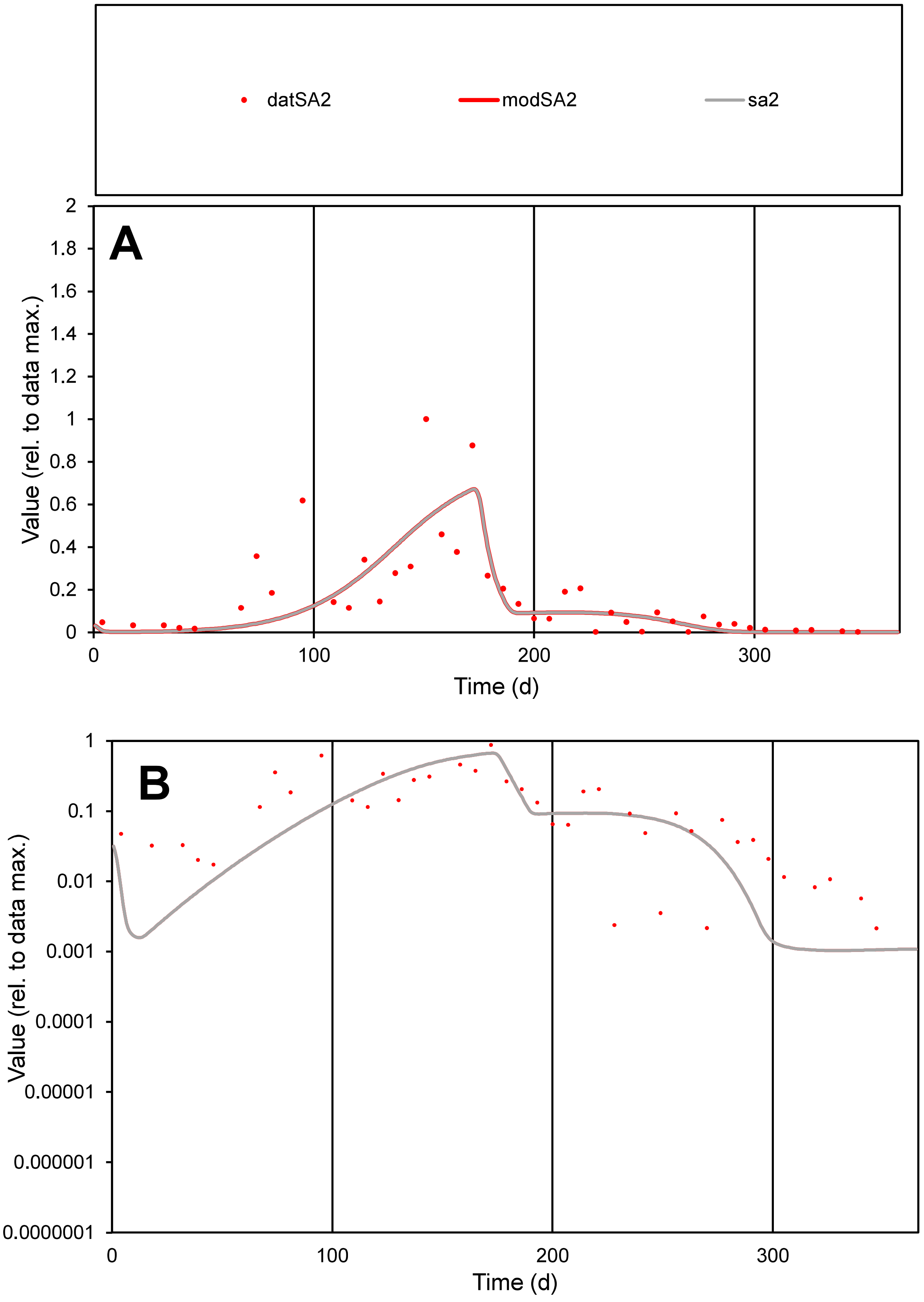

C1

Variables
SA2 Provide jtd or Dst
CalibOnly?
2 1 to reproduce of
oNormalize?
2 1=no, 2=yes

ofxn calc*
0.8581
ofxn mod
0.87038

sRun
RunA1
zout
1

Ave
Data
0.00863
Model
0.00747

data/vMax
0.05106

ofxng
334.07

Min d/m
2.07601

ave/min d
78.6261
ave/min m
141.194

327_Calib_S_A

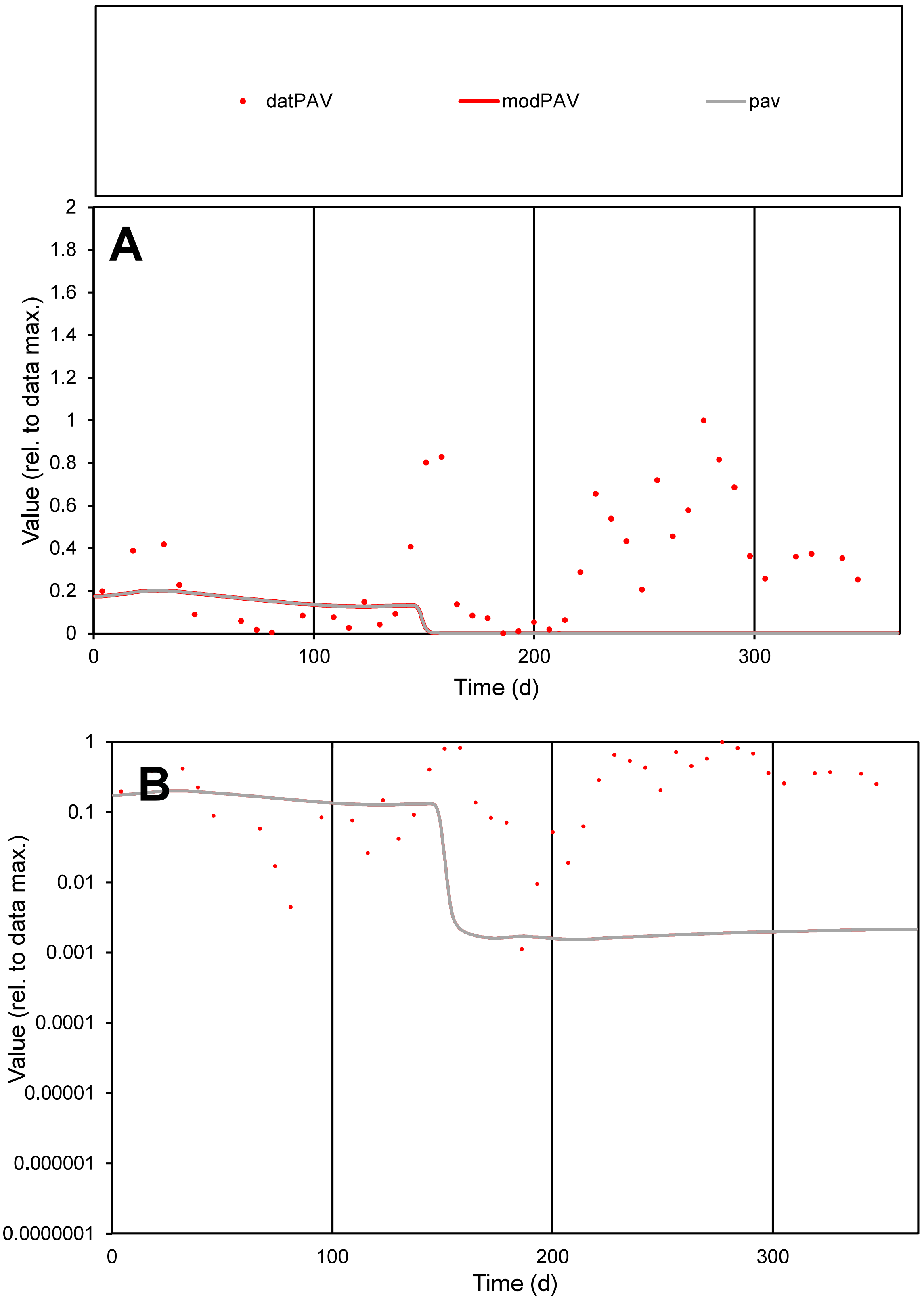

C1

Variables
PAV Provide jtd or Dst
CalibOnly?
2 1 to reproduce of
oNormalize?
2 1=no, 2=yes

ofxn calc*
1.2968
ofxn mod
1.29994

sRun
RunA1
zout
1

Ave
Data
0.01103
Model
0.00209

data/vMax
0.03656

ofxng
334.07

Min d/m
0.73248

ave/min d
267.859
ave/min m
37.238

327_Calib_S_A

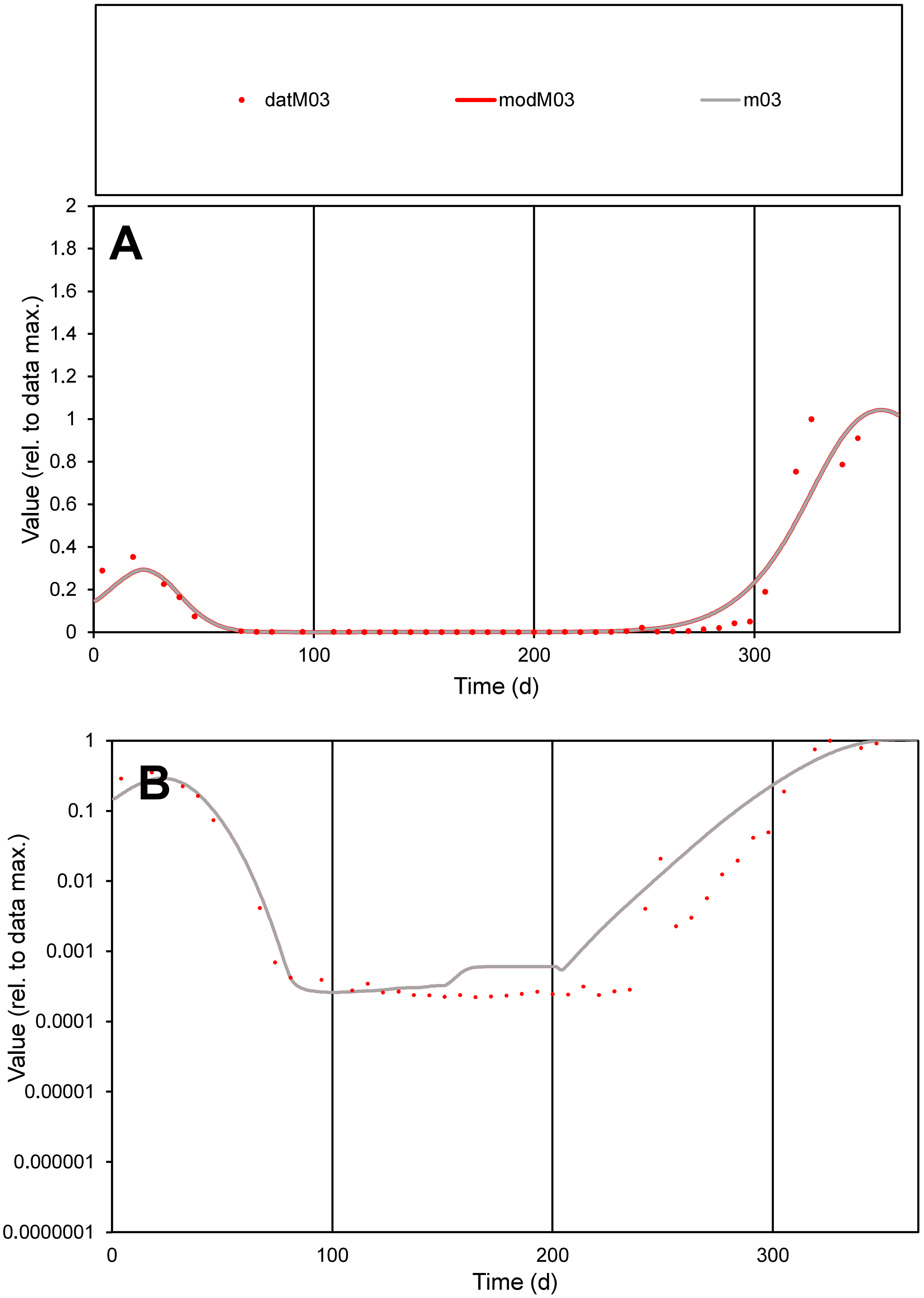

C1

Variables
M03 Provide jtd or Dst
CalibOnly?
2 1 to reproduce of
oNormalize?
2 1=no, 2=yes

ofxn calc*
0.66485
ofxn mod
0.71136

sRun
RunA1
zout
1

Ave
Data
0.00915
Model
0.00946

data/vMax
0.07822

ofxng
334.07

Min d/m
0.84769

ave/min d
525.319
ave/min m
460.226

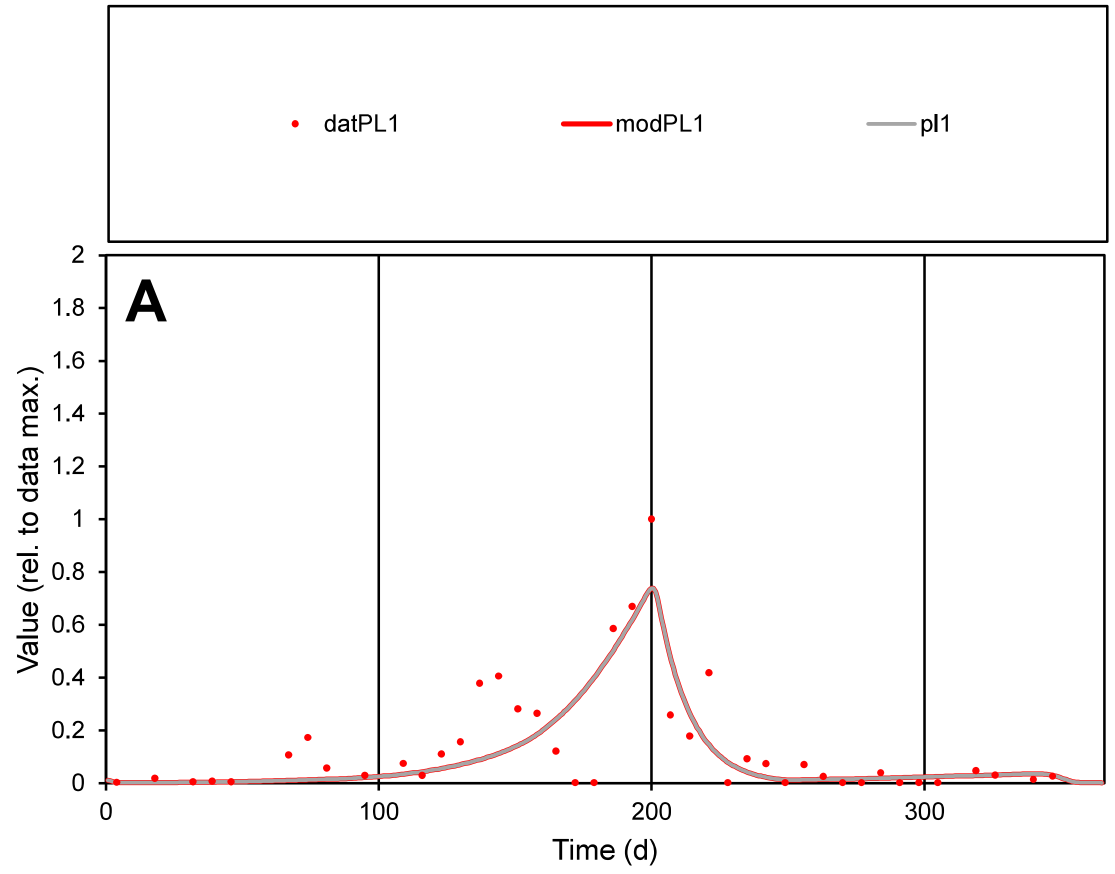

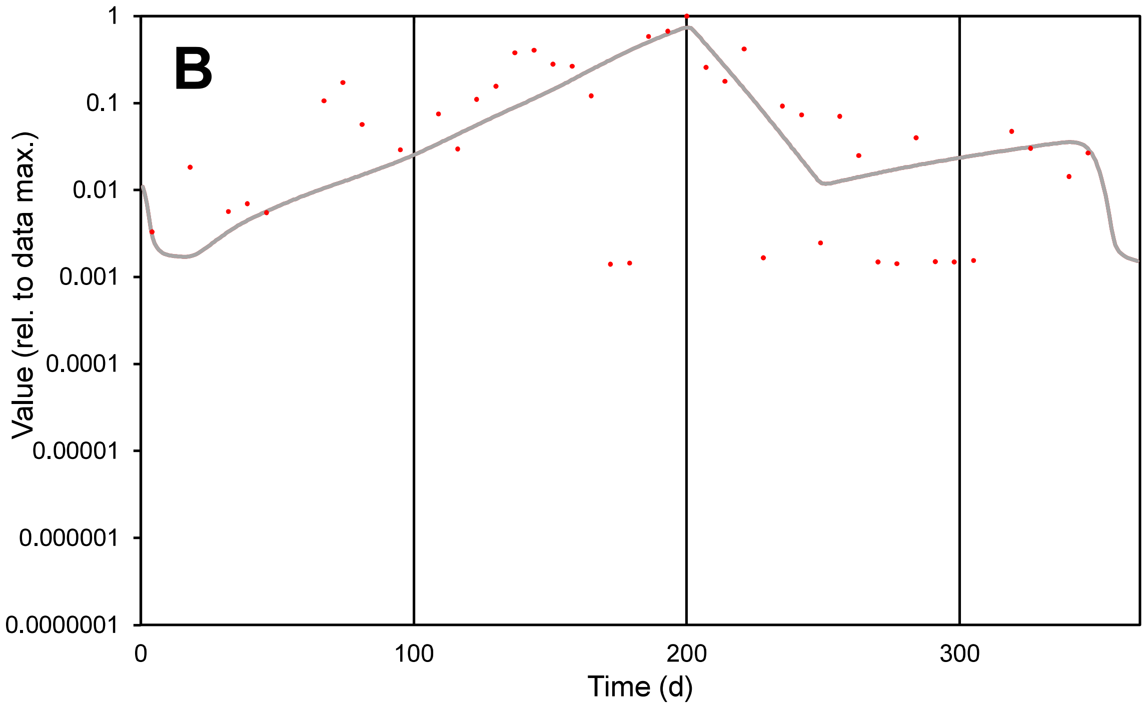

C1

Variables

PL1 Provide jtd or Dst

CalibOnly?

2 1 to reproduce of

oNormalize?

2 1=no, 2=yes

ofxn calc*

0.92964

ofxn mod

0.96622

sRun

RunA1

zout

1

Ave

Data

0.00345

Model

0.00294

data/vMax

0.02515

ofxng

334.07

Min d/m

0.78004

ave/min d

98.2041

ave/min m

65.1212

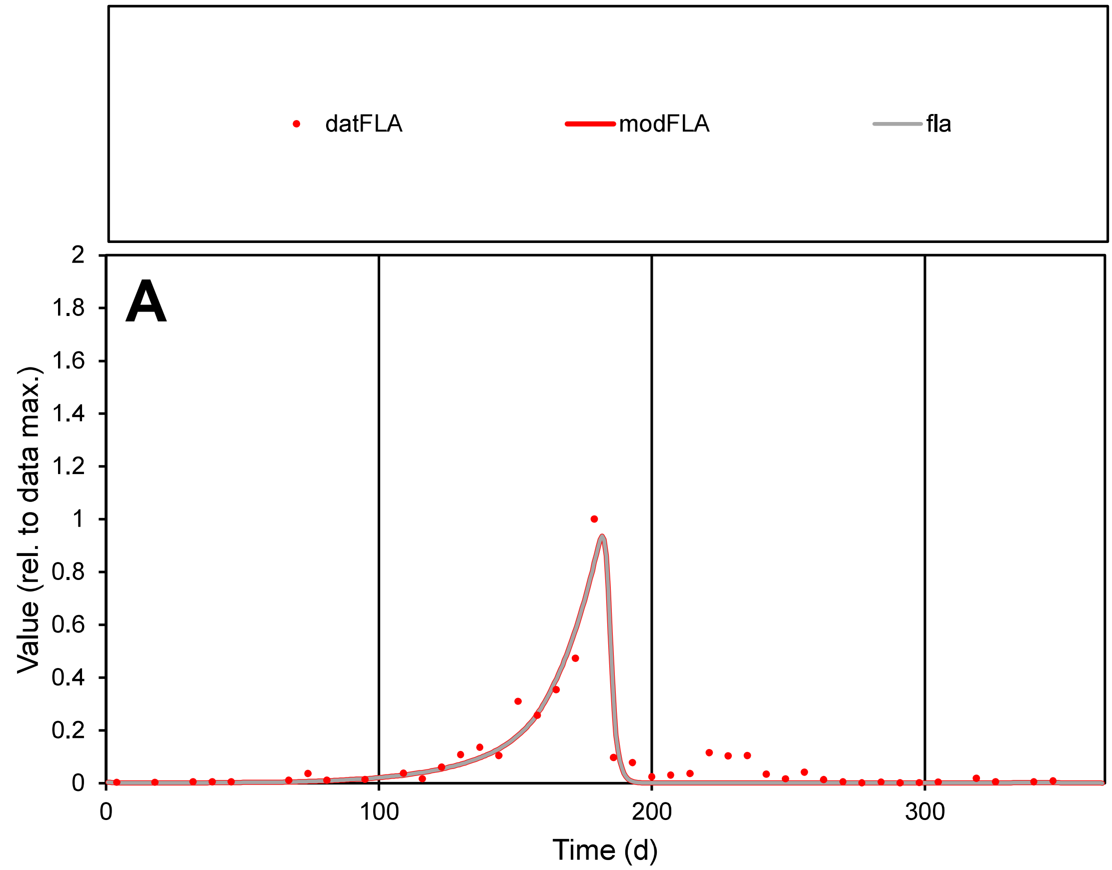

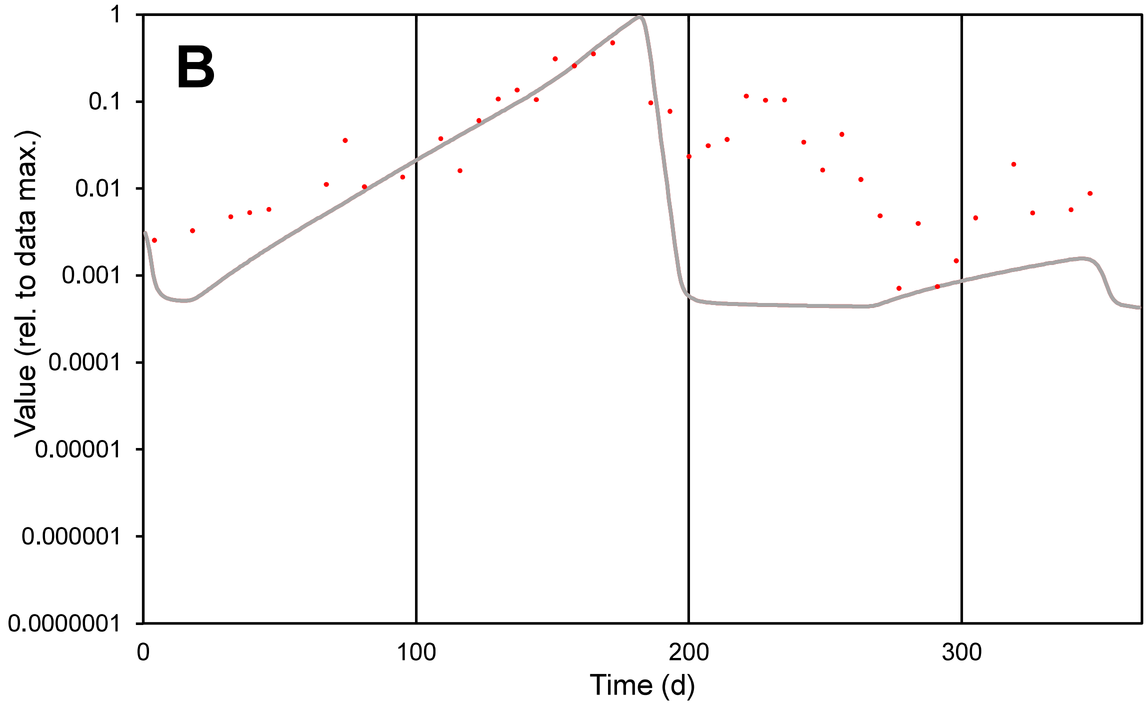

C1

Variables
FLA Provide jtd or Dst
CalibOnly?
2 1 to reproduce of
oNormalize?
2 1=no, 2=yes

ofxn calc*
0.55237
ofxn mod
0.61002

sRun
RunA1
zout
1

Ave
Data
0.00706
Model
0.00592

data/vMax
0.08017

ofxng
334.07

Min d/m
1.61172

ave/min d
124.045
ave/min m
167.78

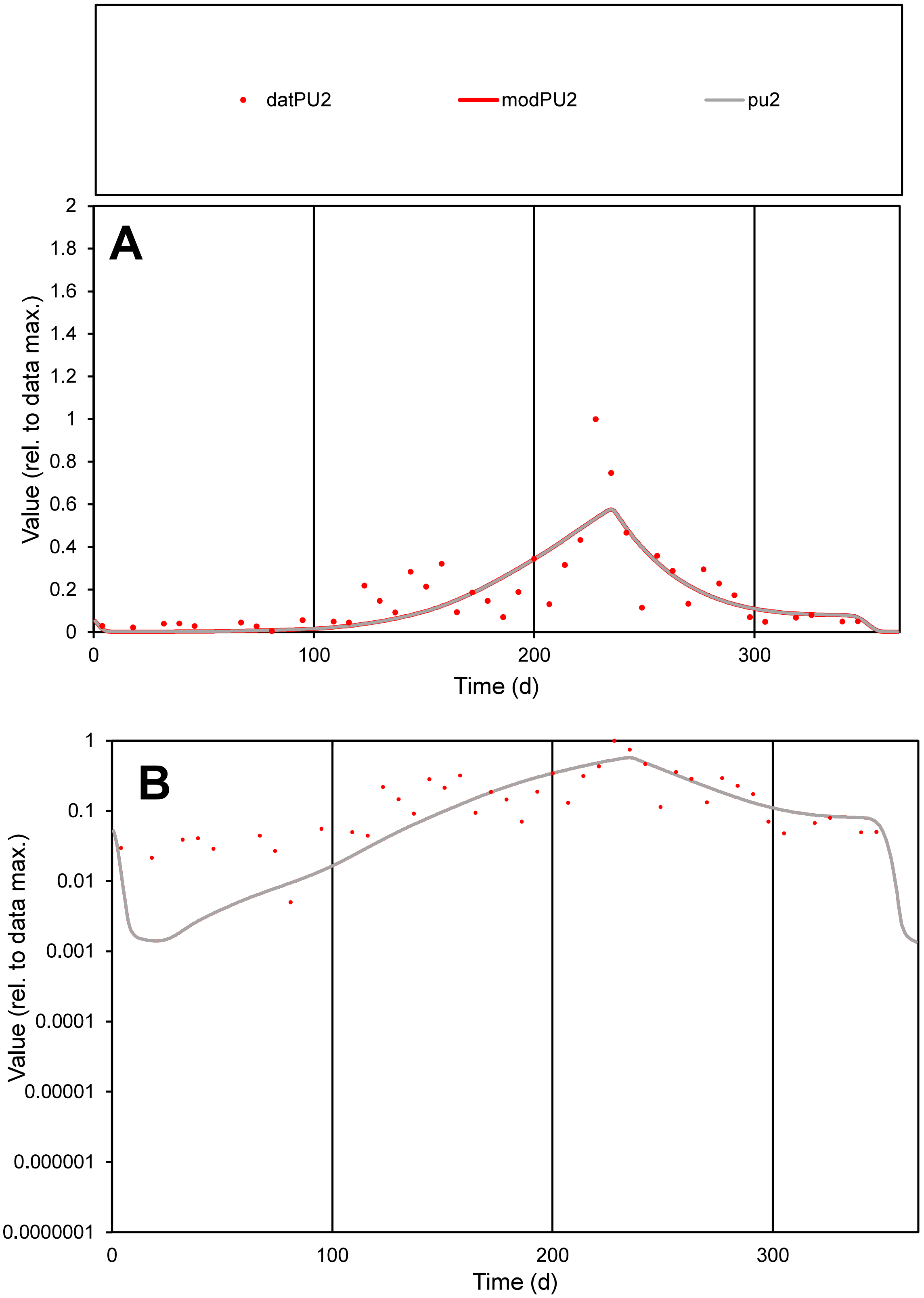

C1

Variables
PU2 Provide jtd or Dst
CalibOnly?
2 1 to reproduce of
oNormalize?
2 1=no, 2=yes

ofxn calc*
0.6688
ofxn mod
0.68832

sRun
RunA1
zout
1

Ave
Data
0.0106
Model
0.00977

data/vMax
0.05754

ofxng
334.07

Min d/m
3.56553

ave/min d
36.6453
ave/min m
120.478

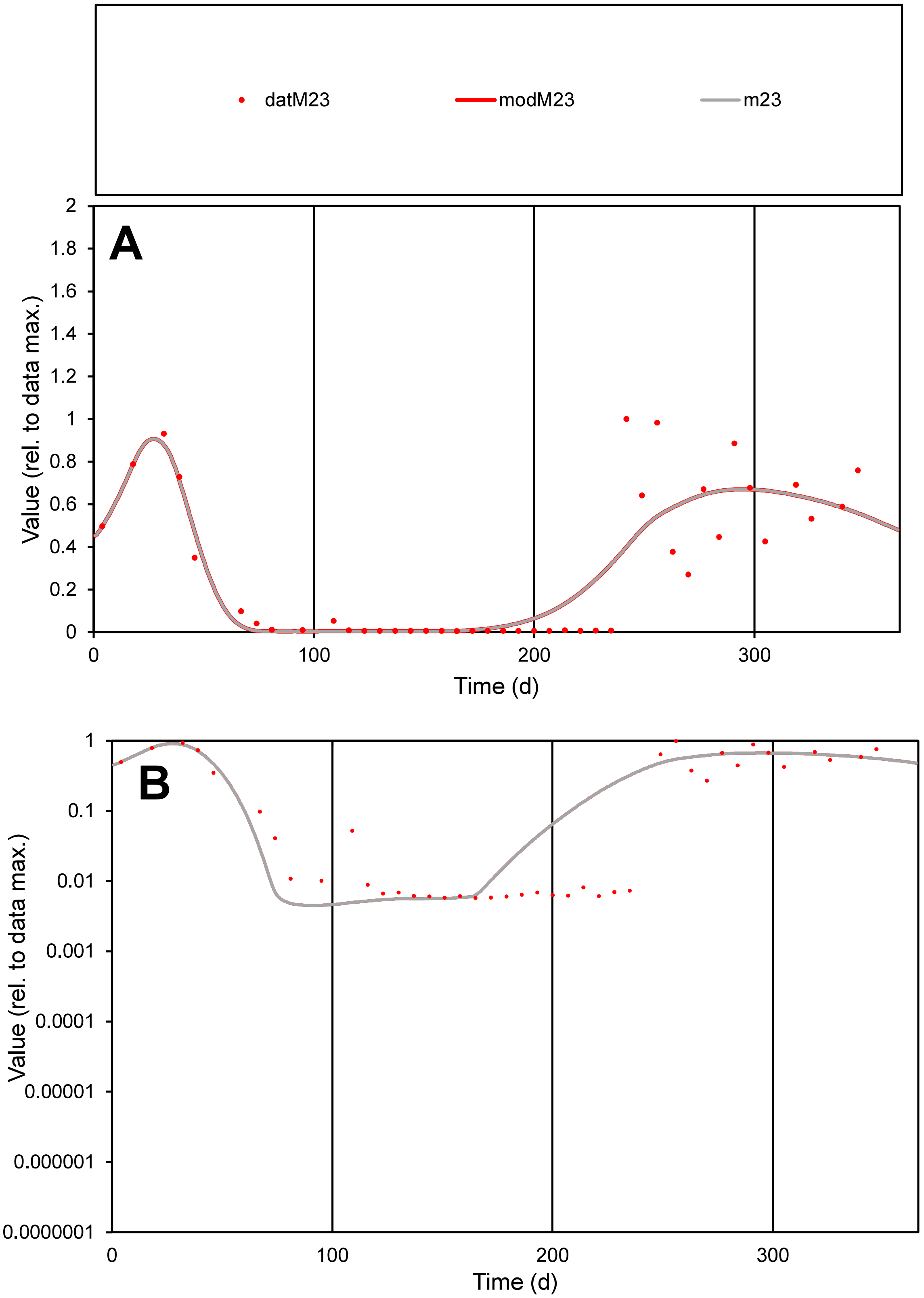

C1

Variables
M23 Provide jtd or Dst
CalibOnly?
2 1 to reproduce of
oNormalize?
2 1=no, 2=yes

ofxn calc*
0.55235
ofxn mod
0.58398

sRun
RunA1
zout
1

Ave
Data
0.00557
Model
0.00571

data/vMax
0.01859

ofxng
334.07

Min d/m
1.26467

ave/min d
52.1647
ave/min m
67.7161

327_Calib_S_A

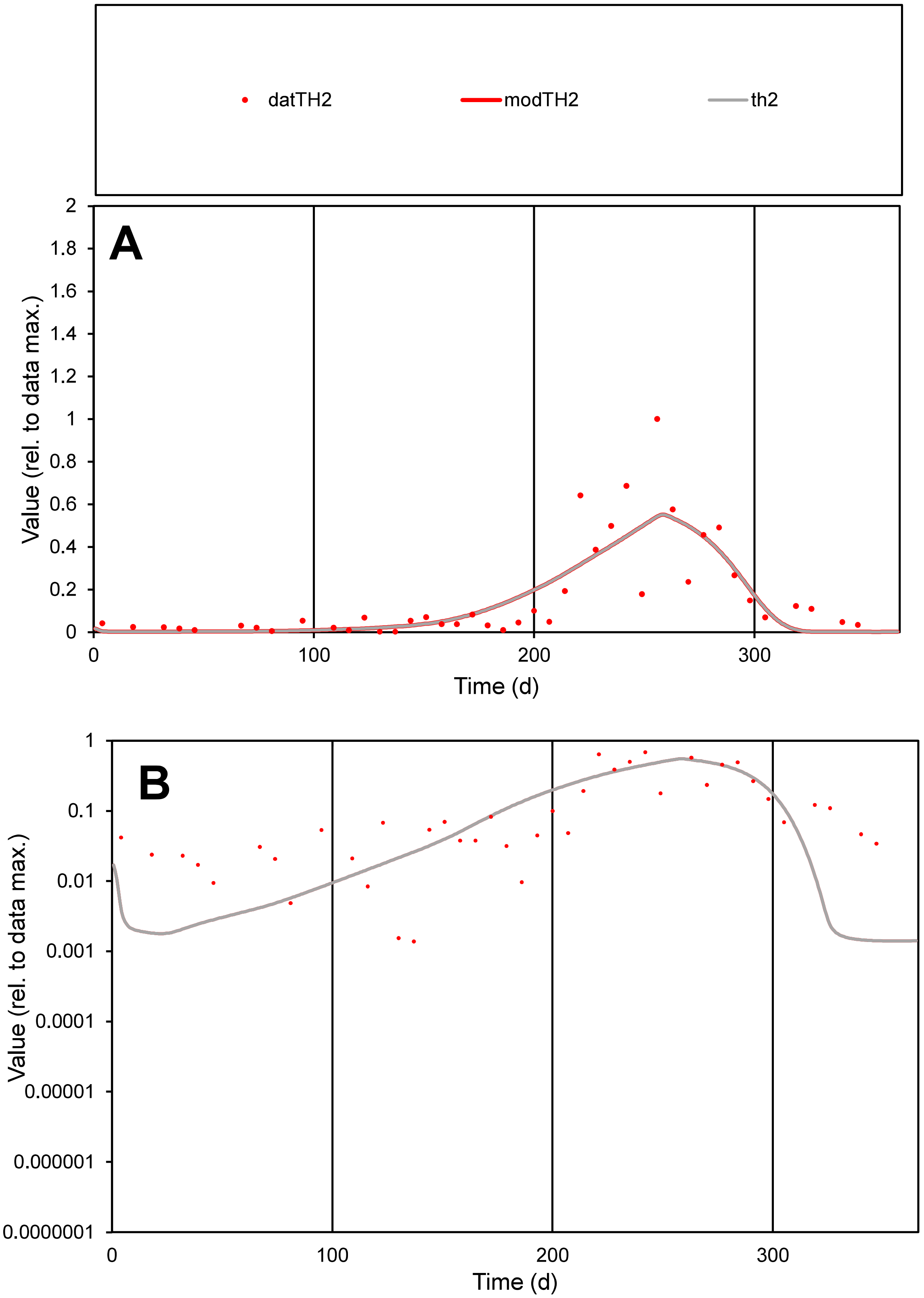

C1

Variables
TH2 Provide jtd or Dst
CalibOnly?
2 1 to reproduce of
oNormalize?
2 1=no, 2=yes

ofxn calc*
0.76252
ofxn mod
0.78963

sRun
RunA1
zout
1

Ave
Data
0.01355
Model
0.01277

data/vMax
0.08154

ofxng
334.07

Min d/m
0.97489

ave/min d
120.377
ave/min m
110.568

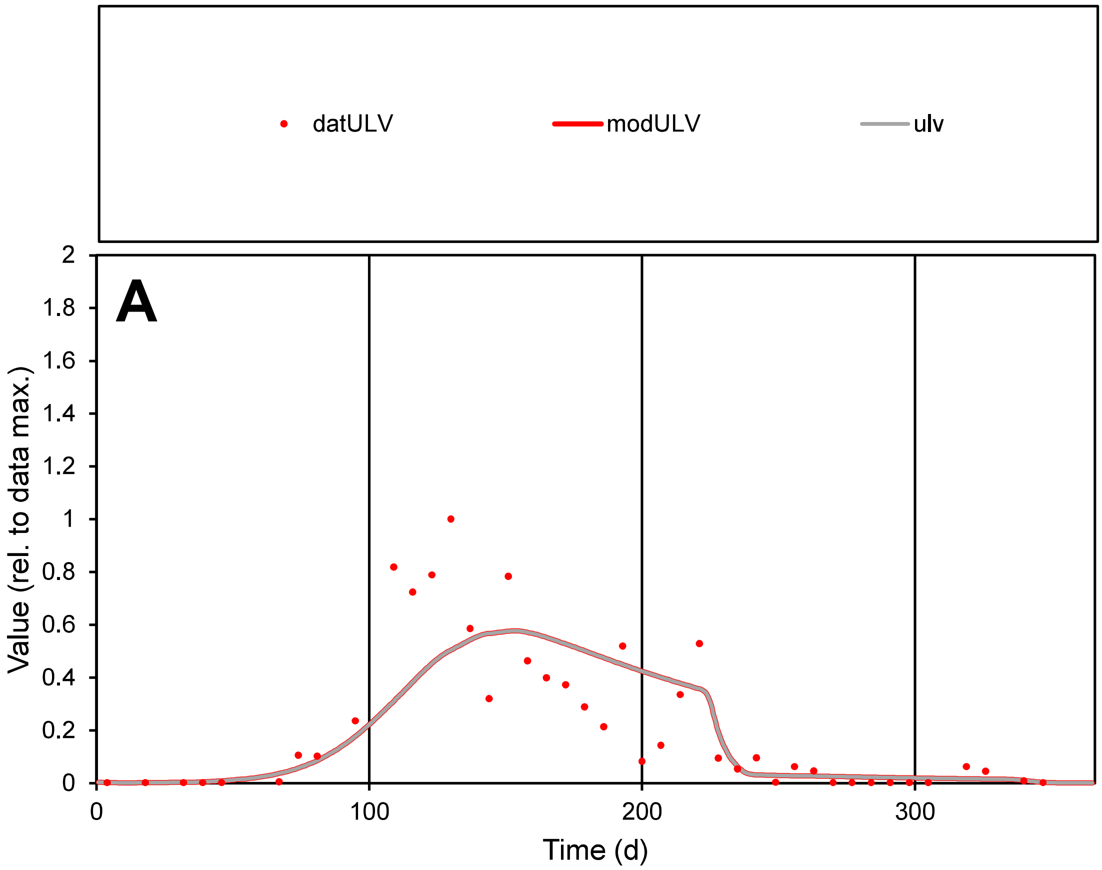

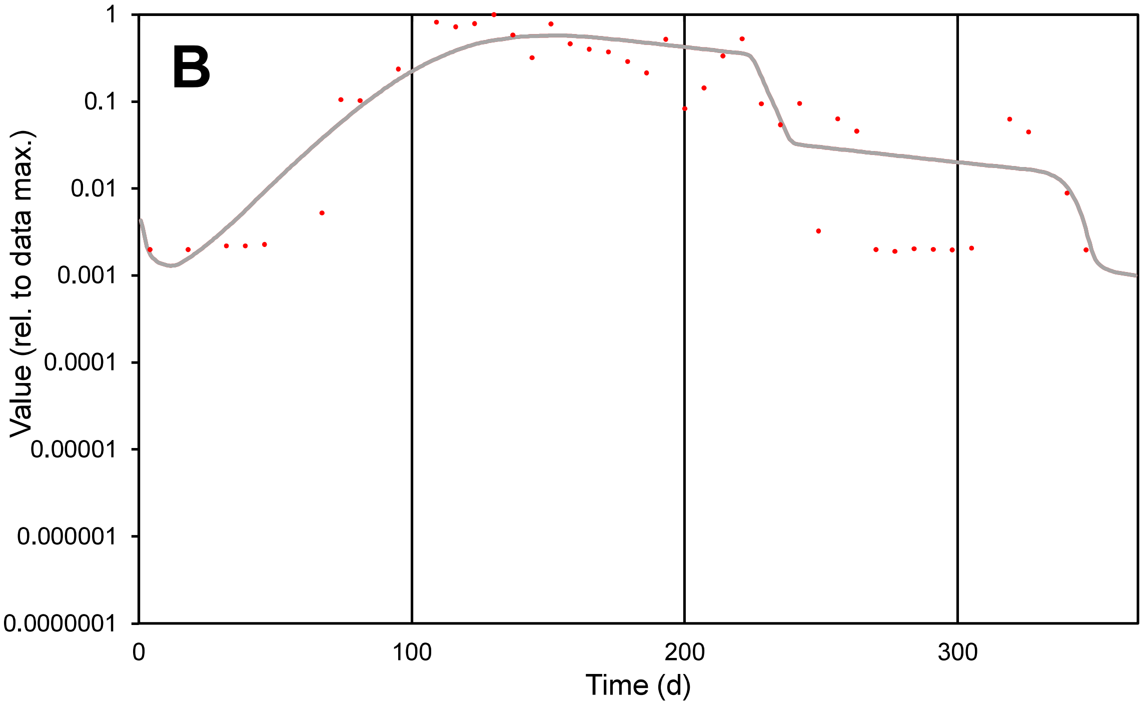

C1

Variables

ULV Provide jtd or Dst

CalibOnly?

2 1 to reproduce of

oNormalize?

2 1=no, 2=yes

ofxn calc*

0.76066

ofxn mod

0.77707

sRun

RunA1

zout

1

Ave

Data

0.00208

Model

0.00198

data/vMax

0.0094

ofxng

334.07

Min d/m

1.11584

ave/min d

116.989

ave/min m

124.234

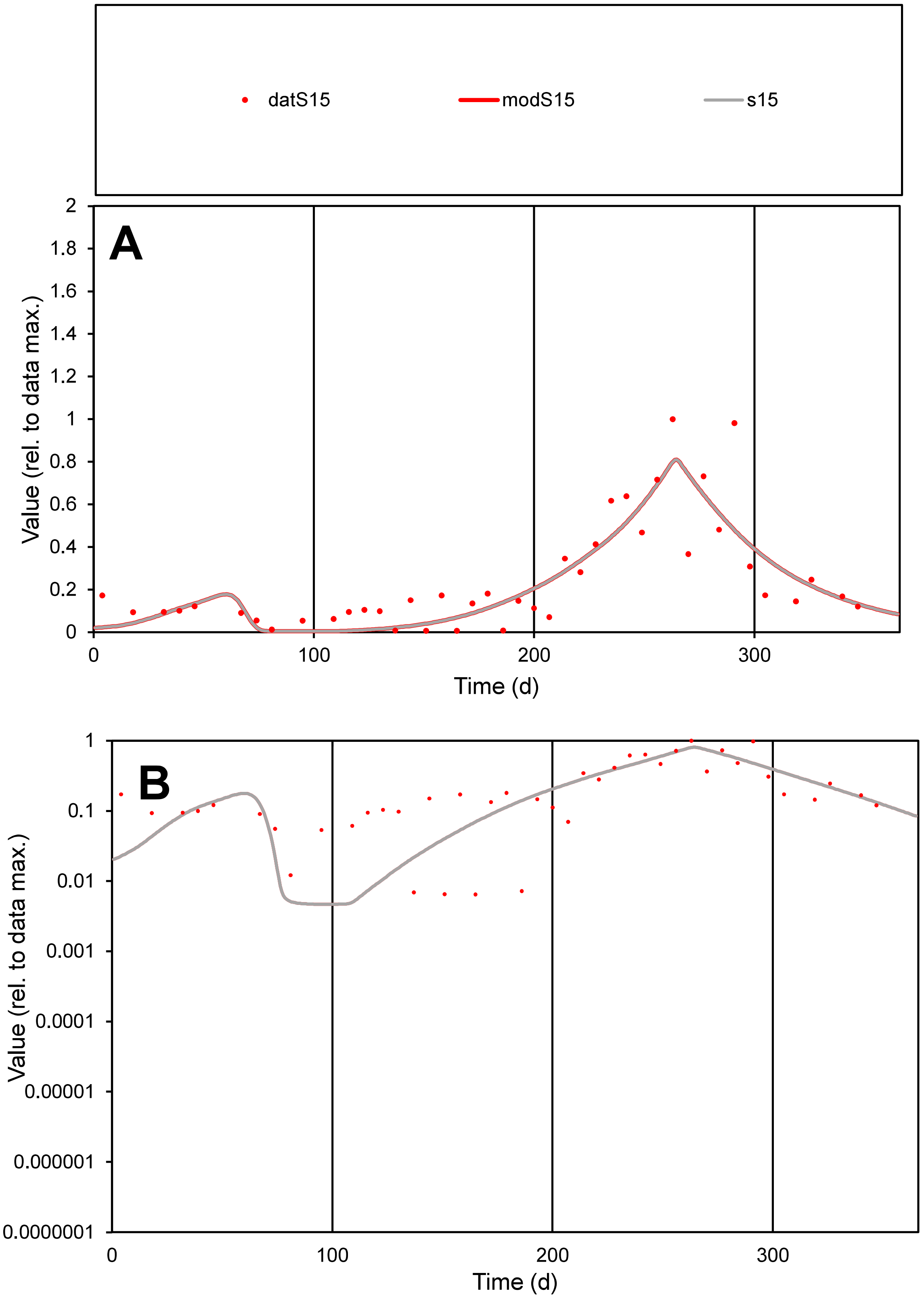

C1

Variables
S15 Provide jtd or Dst
CalibOnly?
2 1 to reproduce of
oNormalize?
2 1=no, 2=yes

ofxn calc*
0.52476
ofxn mod
0.55496

sRun
RunA1
zout
1

Ave
Data
0.00471
Model
0.00447

data/vMax
0.01914

ofxng
334.07

Min d/m
1.37046

ave/min d
38.2036
ave/min m
49.6864

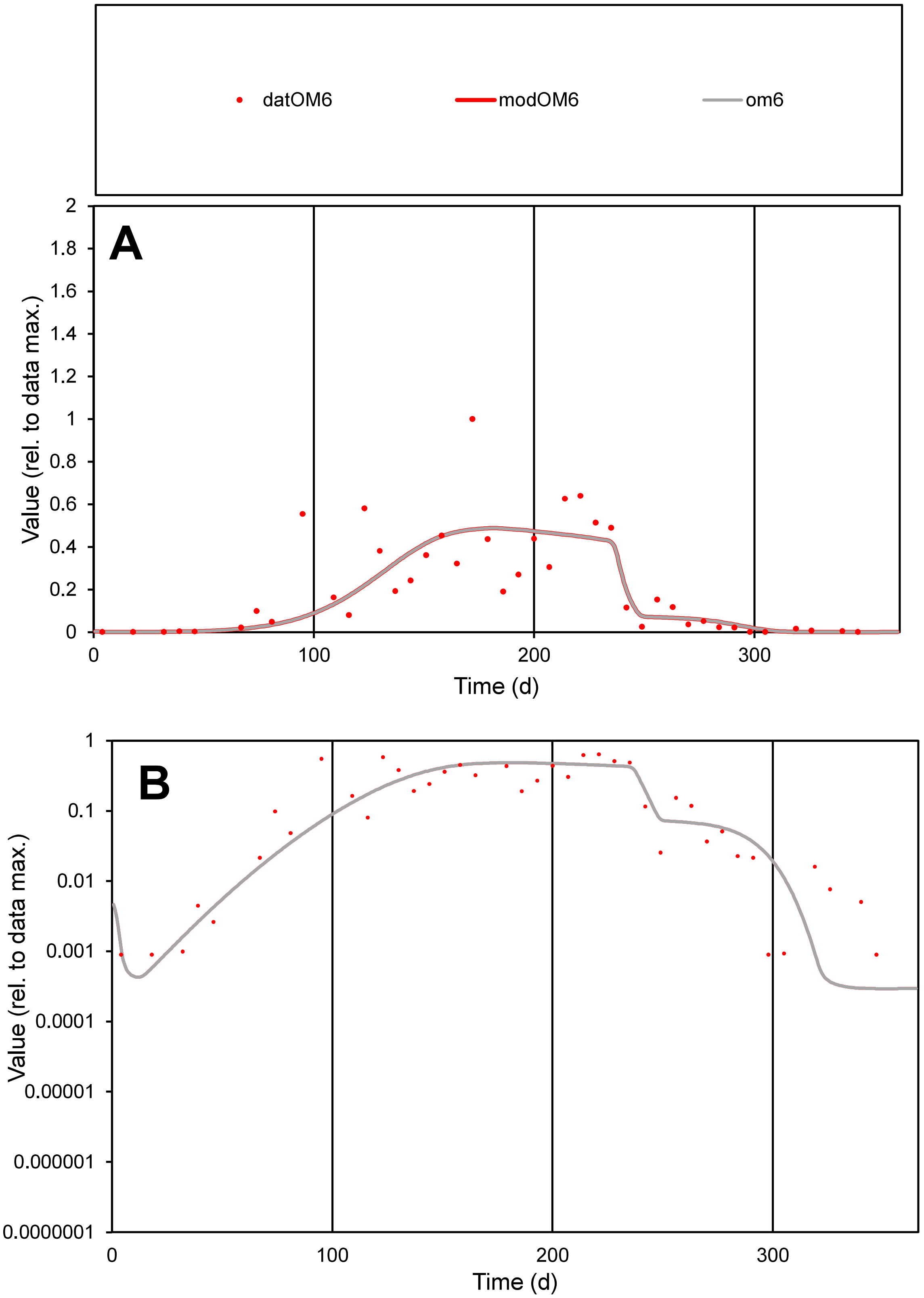

C1

Variables
OM6 Provide jtd or Dst
CalibOnly?
2 1 to reproduce of
oNormalize?
2 1=no, 2=yes

ofxn calc*
0.70736
ofxn mod
0.72327

sRun
RunA1
zout
1

Ave
Data
0.00647
Model
0.00596

data/vMax
0.0302

ofxng
334.07

Min d/m
3.05104

ave/min d
239.46
ave/min m
673.003

327_Calib_S_A

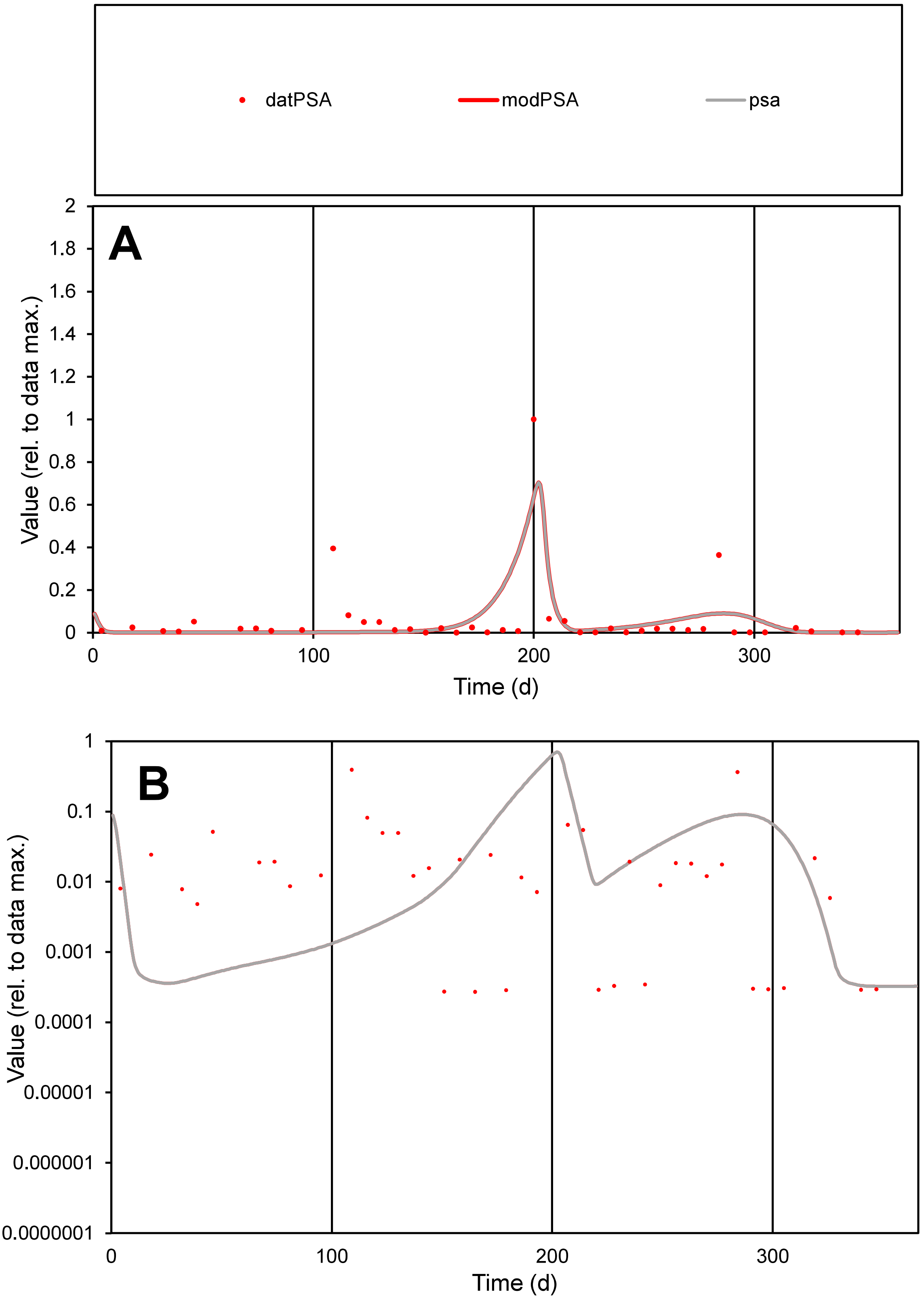

C1

Variables
PSA Provide jtd or Dst
CalibOnly?
2 1 to reproduce of
oNormalize?
2 1=no, 2=yes

ofxn calc*
1.92368
ofxn mod
2.02883

sRun
RunA1
zout
1

Ave
Data
0.00187
Model
0.00177

data/vMax
0.03227

ofxng
334.07

Min d/m
0.83981

ave/min d
212.795
ave/min m
169.418

327_Calib_S_A

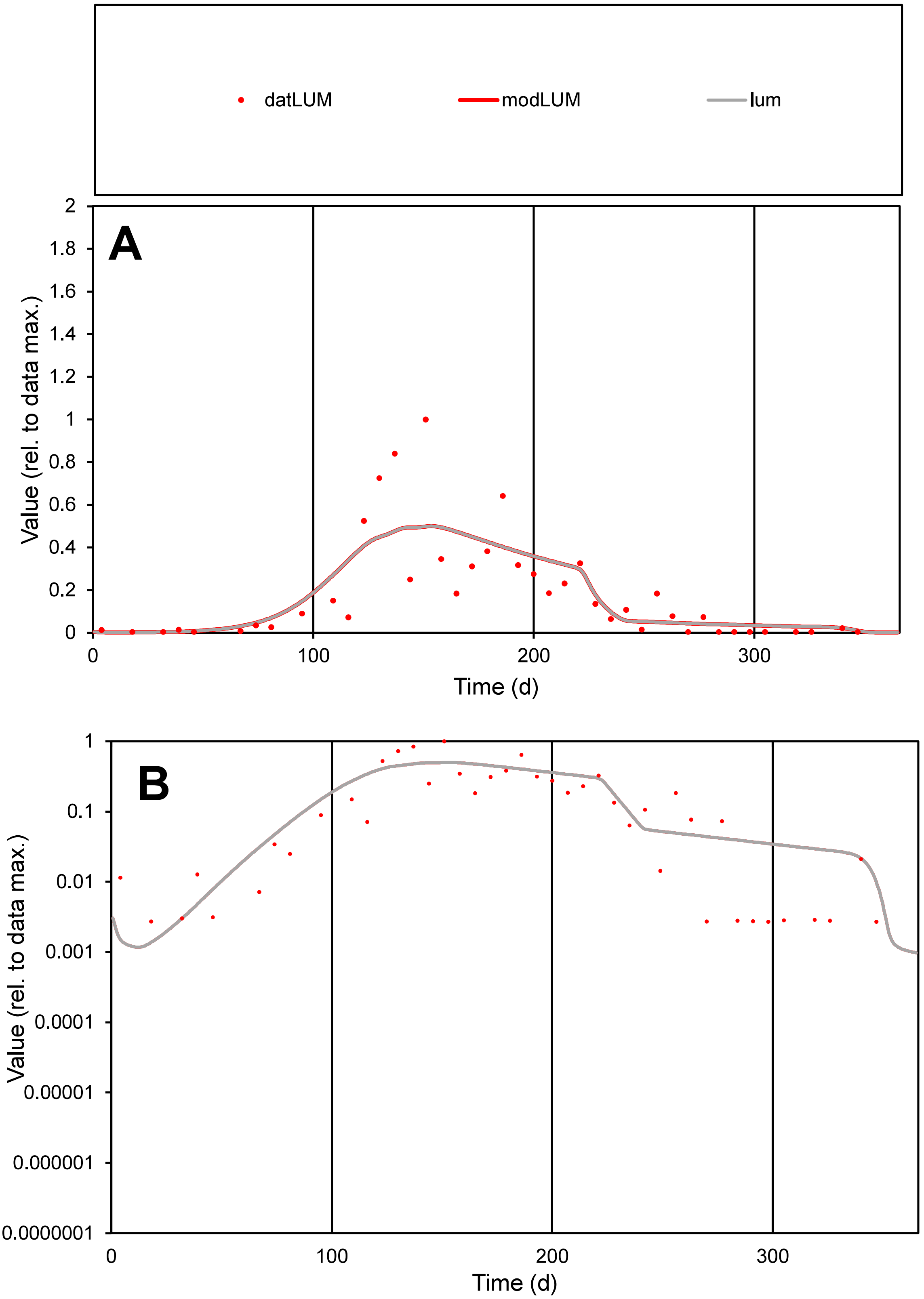

C1

Variables
LUM Provide jtd or Dst
CalibOnly?
2 1 to reproduce of
oNormalize?
2 1=no, 2=yes

ofxn calc*
0.79456
ofxn mod
0.81213

sRun
RunA1
zout
1

Ave
Data
0.00324
Model
0.00337

data/vMax
0.01784

ofxng
334.07

Min d/m
1.83378

ave/min d
67.0998
ave/min m
127.796

327_Calib_S_A

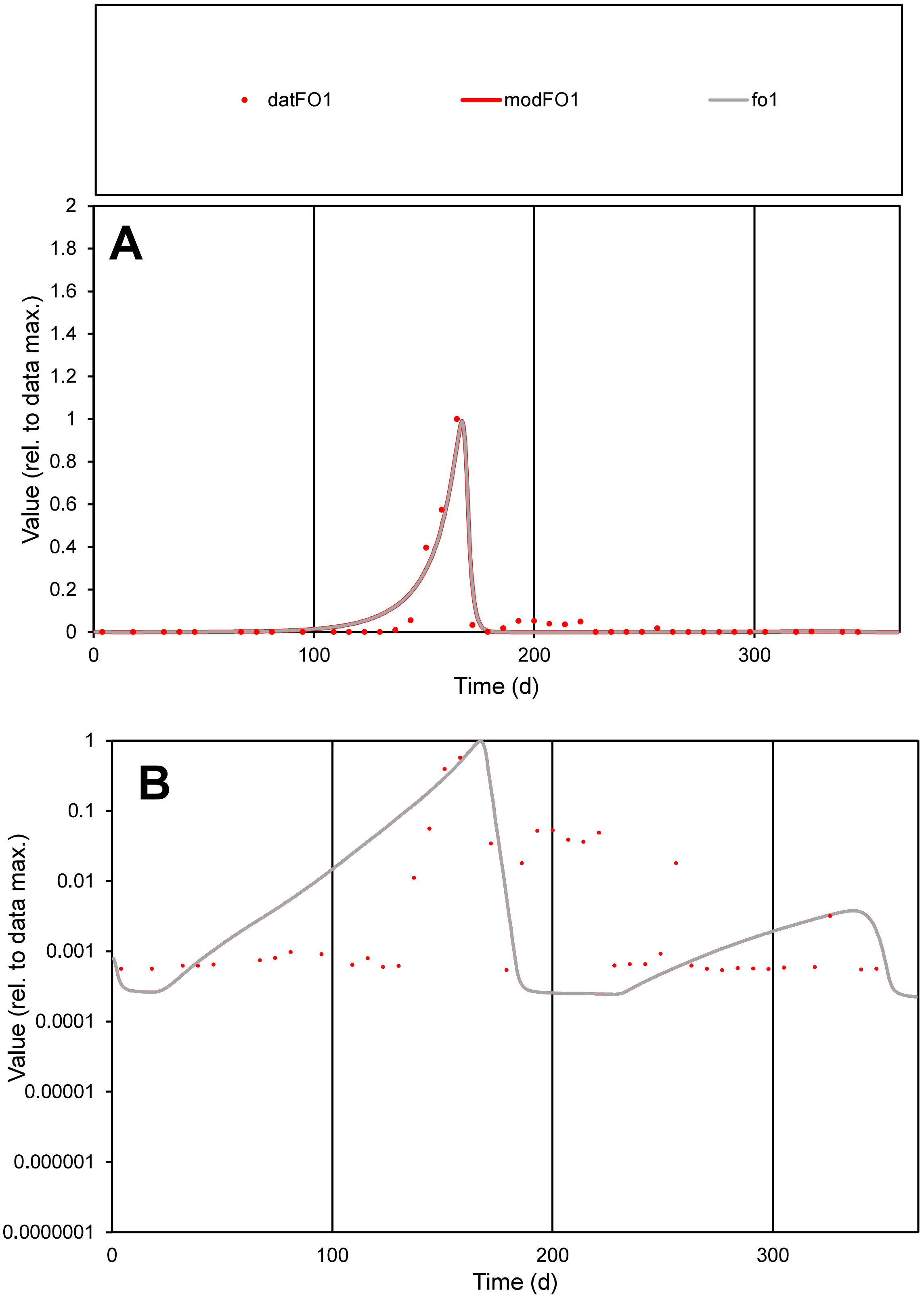

C1

Variables
FO1 Provide jtd or Dst
CalibOnly?
2 1 to reproduce of
oNormalize?
2 1=no, 2=yes

ofxn calc*
0.65479
ofxn mod
0.77075

sRun
RunA1
zout
1

Ave
Data
0.00628
Model
0.00646

data/vMax
0.1117

ofxng
334.07

Min d/m
2.20608

ave/min d
104.167
ave/min m
236.598

327_Calib_S_A

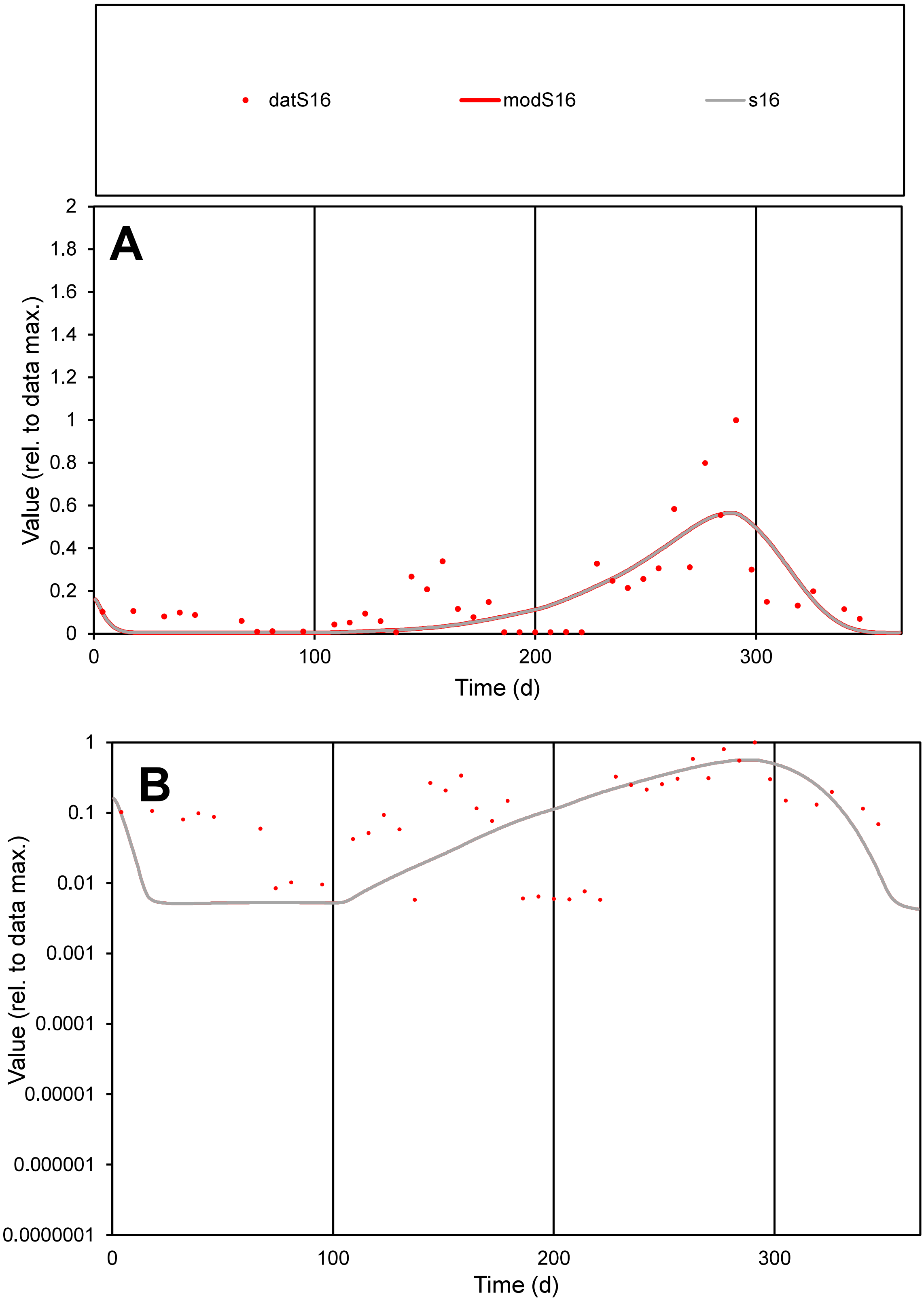

C1

Variables
S16 Provide jtd or Dst
CalibOnly?
2 1 to reproduce of
oNormalize?
2 1=no, 2=yes

ofxn calc*
0.7796
ofxn mod
0.79911

sRun
RunA1
zout
1

Ave
Data
0.00409
Model
0.00364

data/vMax
0.02272

ofxng
334.07

Min d/m
1.12543

ave/min d
31.0254
ave/min m
31.0335

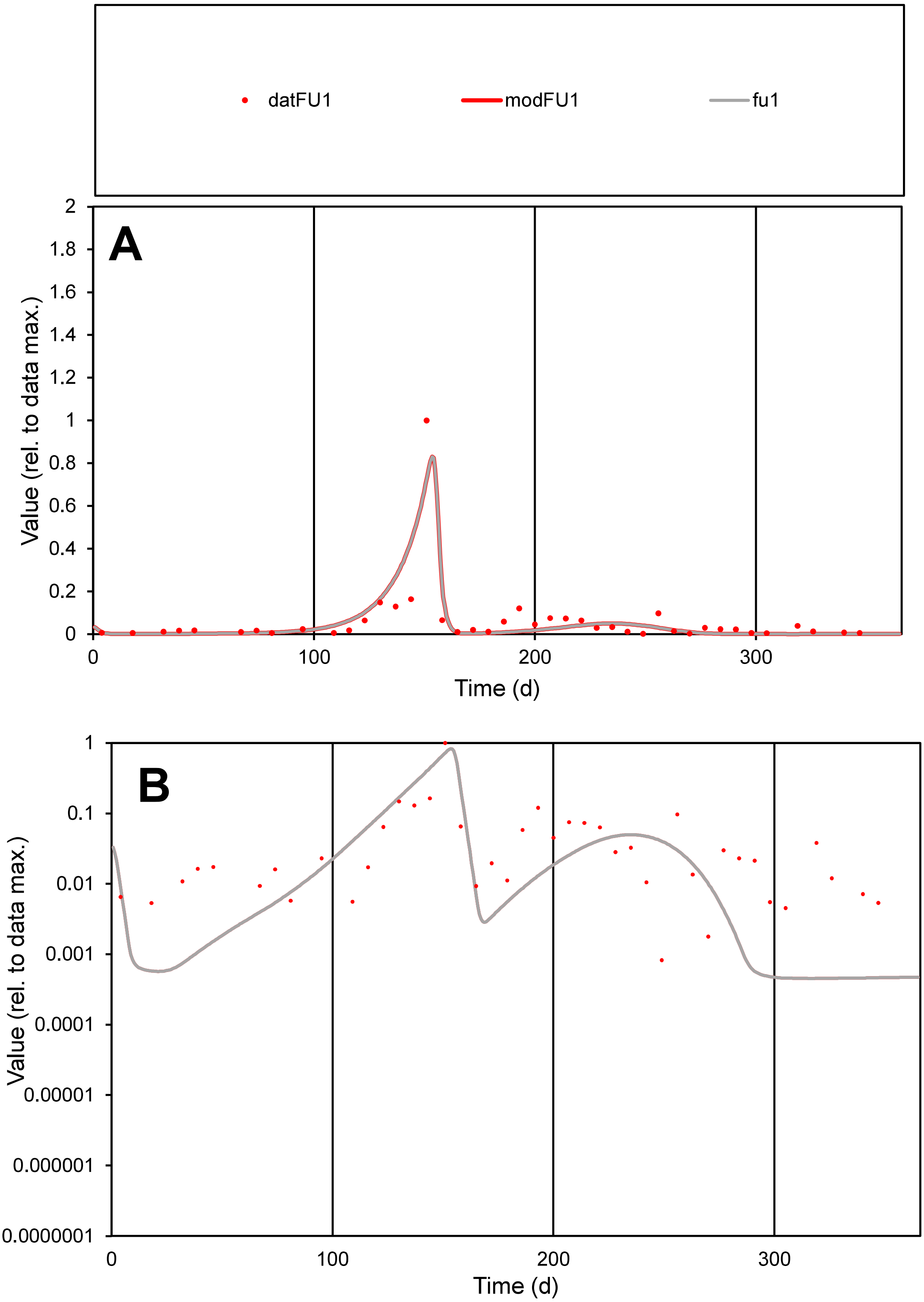

C1

Variables
FU1 Provide jtd or Dst
CalibOnly?
2 1 to reproduce of
oNormalize?
2 1=no, 2=yes

ofxn calc*
1.10753
ofxn mod
1.14631

sRun
RunA1
zout
1

Ave
Data
0.00602
Model
0.00578

data/vMax
0.1007

ofxng
334.07

Min d/m
1.82064

ave/min d
72.5596
ave/min m
126.791

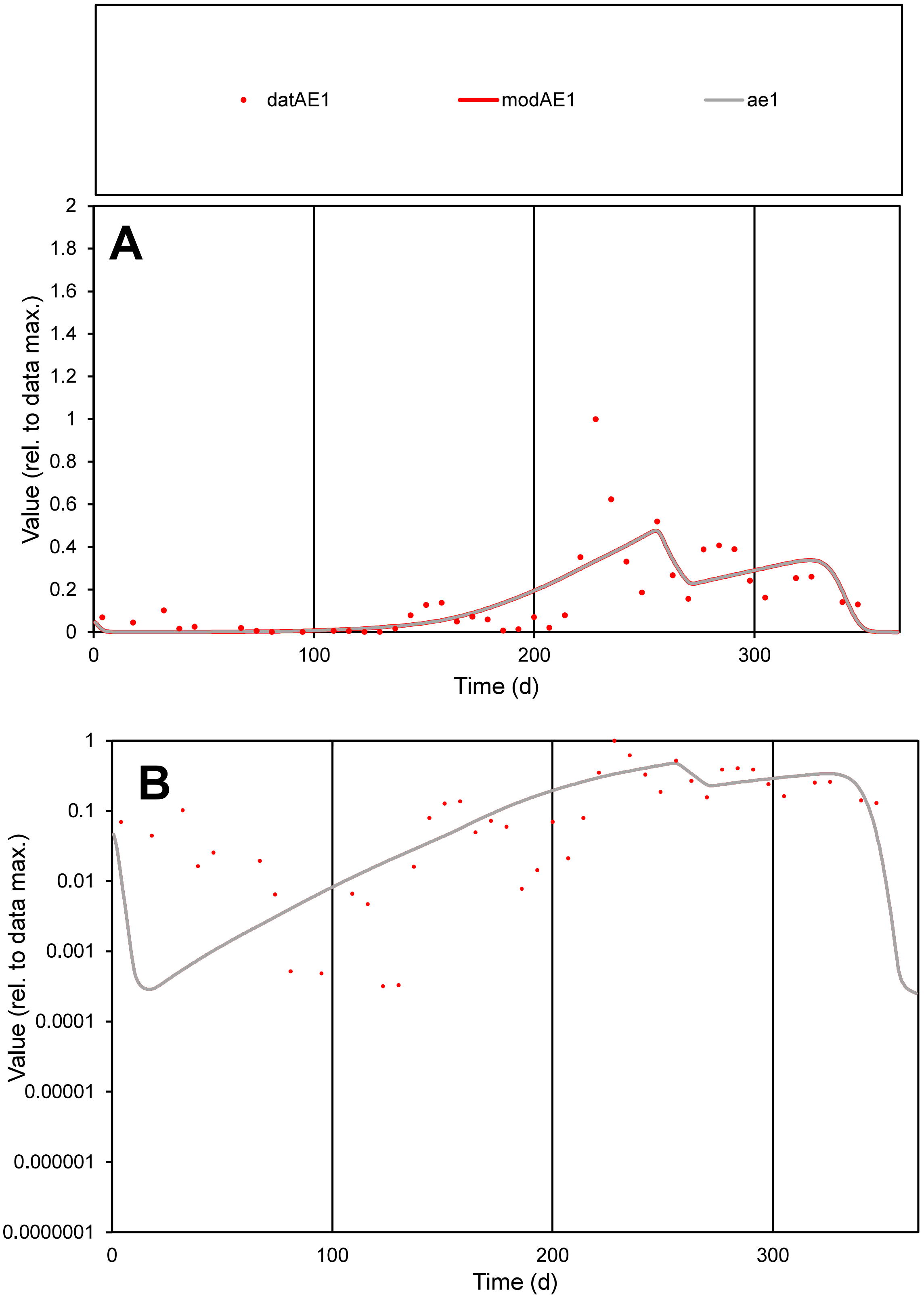

C1

Variables
AE1 Provide jtd or Dst
CalibOnly?
2 1 to reproduce of
oNormalize?
2 1=no, 2=yes

ofxn calc*
0.8727
ofxn mod
0.89006

sRun
RunA1
zout
1

Ave
Data
0.0082
Model
0.00807

data/vMax
0.05036

ofxng
334.07

Min d/m
1.05851

ave/min d
509.277
ave/min m
530.854

327_Calib_S_A

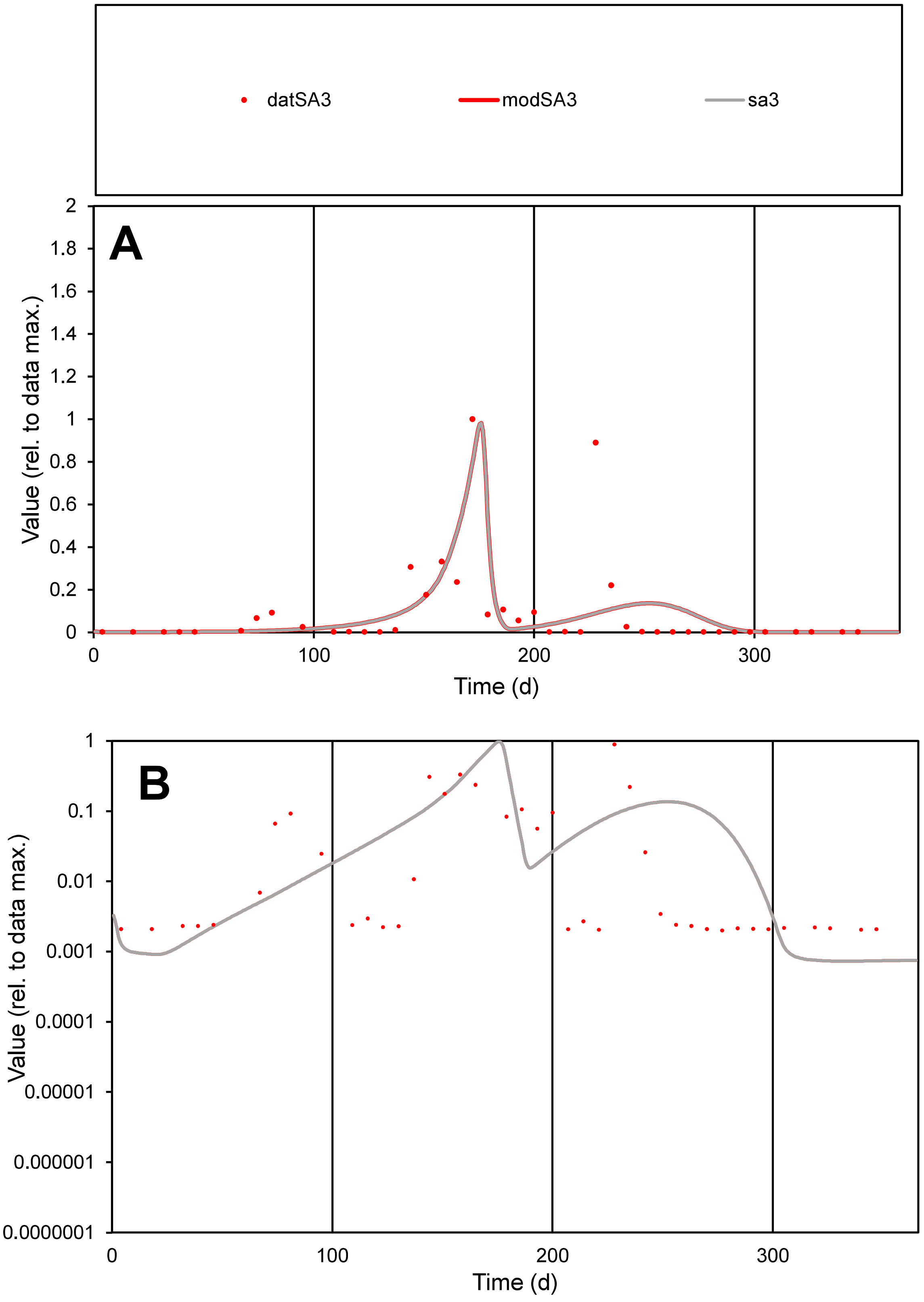

C1

Variables
SA3 Provide jtd or Dst
CalibOnly?
2 1 to reproduce of
oNormalize?
2 1=no, 2=yes

ofxn calc*
1.61209
ofxn mod
1.63708

sRun
RunA1
zout
1

Ave
Data
0.00459
Model
0.00438

data/vMax
0.05091

ofxng
334.07

Min d/m
2.7263

ave/min d
45.09
ave/min m
117.194

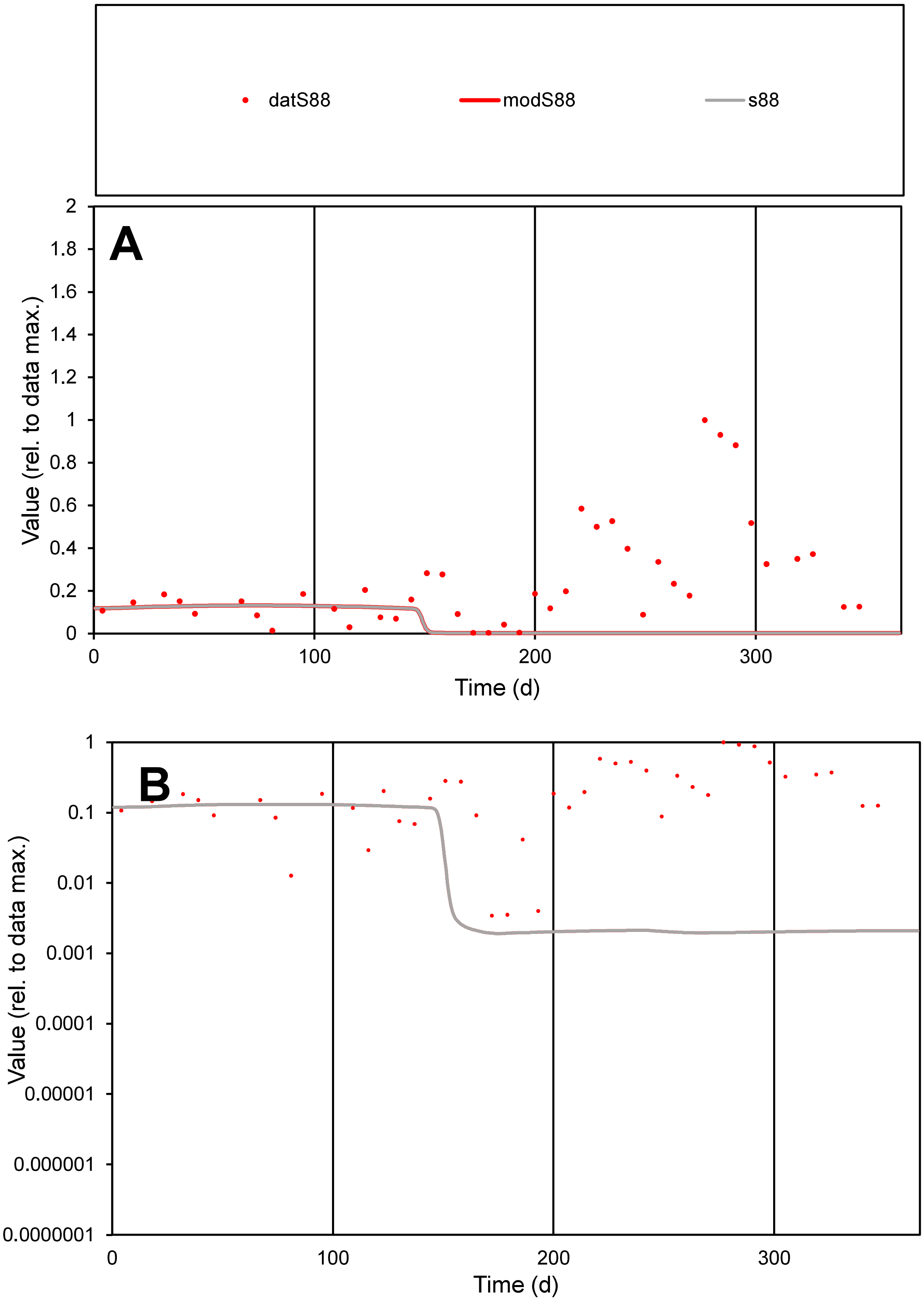

C1

Variables

S88 Provide jtd or Dst

CalibOnly?

2 1 to reproduce of

oNormalize?

2 1=no, 2=yes

ofxn calc*

1.35718

ofxn mod

1.36183

sRun

RunA1

zout

1

Ave

Data

0.00423

Model

0.00081

data/vMax

0.01702

ofxng

334.07

Min d/m

1.7902

ave/min d

72.3612

ave/min m

24.7843

327_Calib_S_A

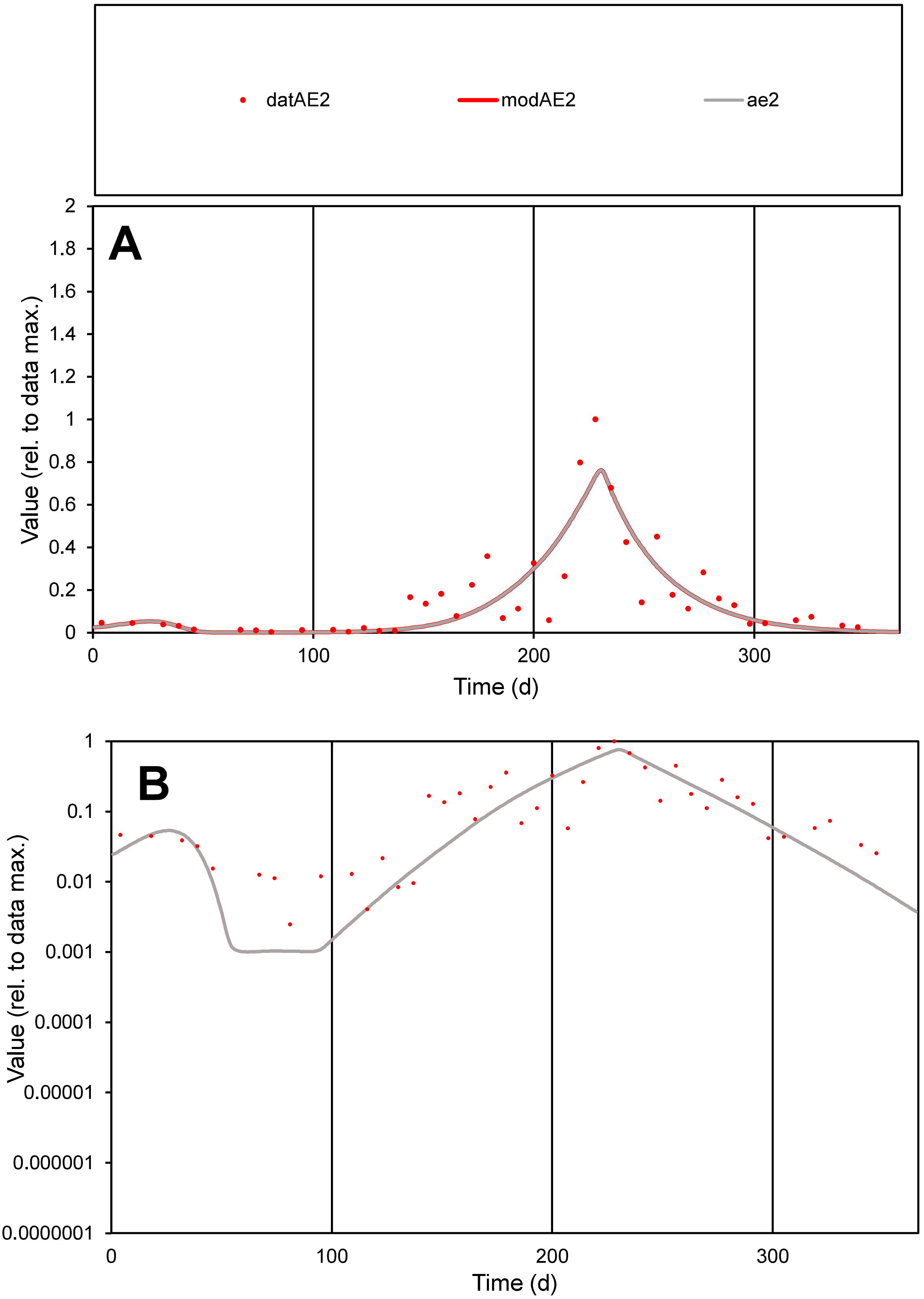

C1

Variables

AE2 Provide jtd or Dst

CalibOnly?

2 1 to reproduce of

oNormalize?

2 1=no, 2=yes

ofxn calc*

0.67356

ofxn mod

0.70117

sRun

RunA1

zout

1

Ave

Data

0.00714

Model

0.00656

data/vMax

0.04362

ofxng

334.07

Min d/m

2.43171

ave/min d

65.7685

ave/min m

146.976

327_Calib_S_A

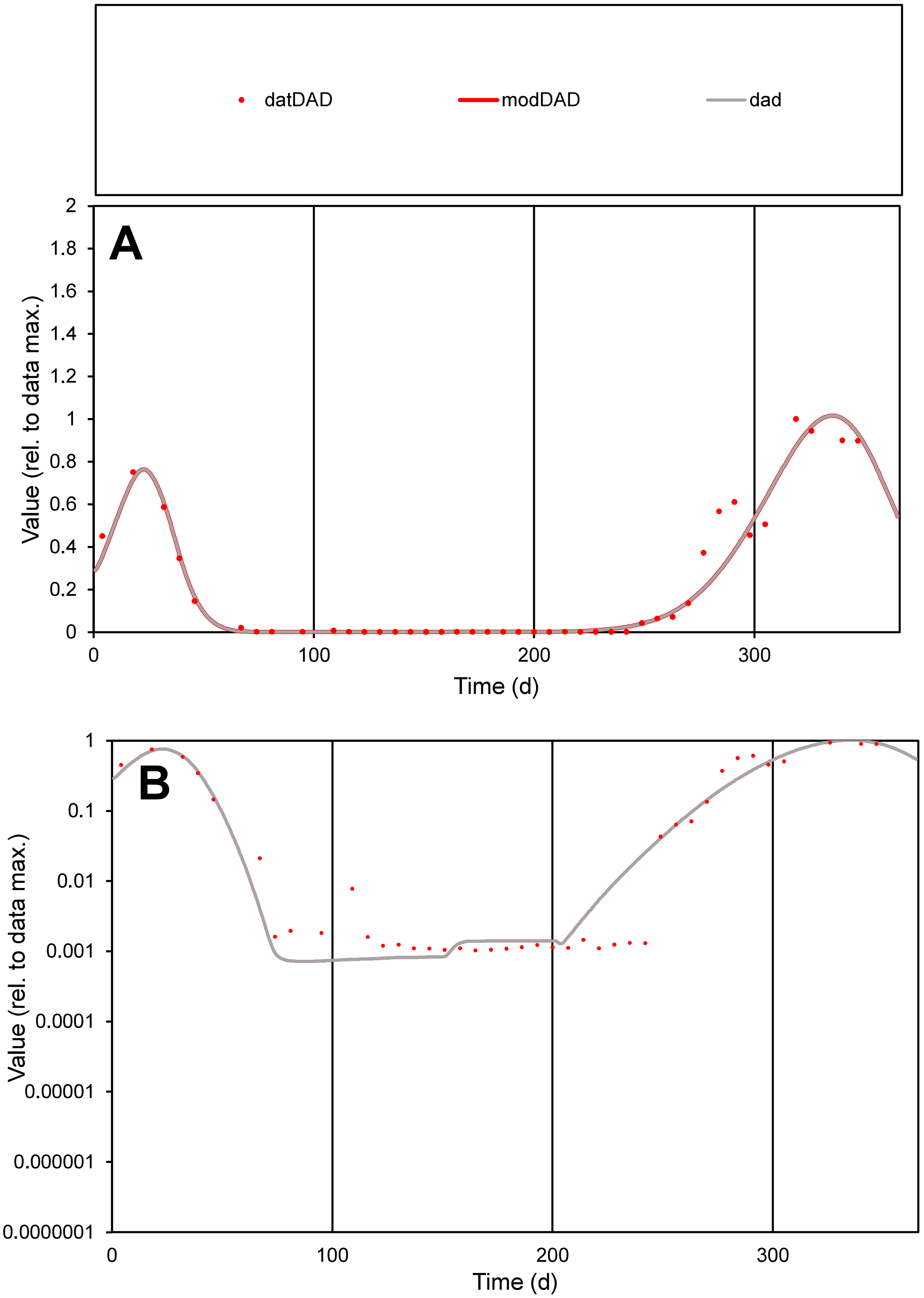

C1

Variables

DAD Provide jtd or Dst

CalibOnly?

2 1 to reproduce of

oNormalize?

2 1=no, 2=yes

ofxn calc*

0.31929

ofxn mod

0.36836

sRun

RunA1

zout

1

Ave

Data

0.00315

Model

0.00299

data/vMax

0.01484

ofxng

334.07

Min d/m

1.4234

ave/min d

205.29

ave/min m

277.796

327_Calib_S_A

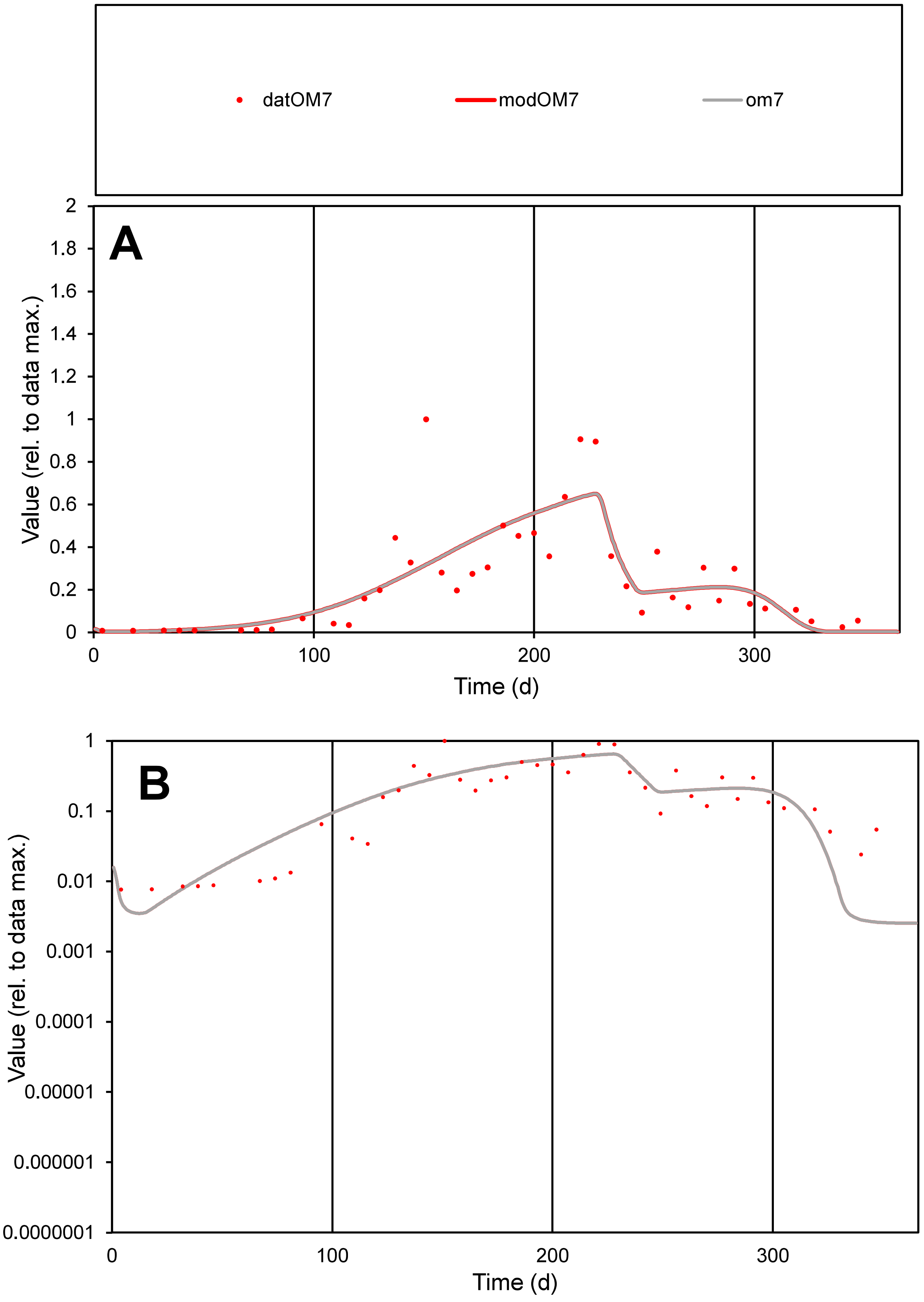

C1

Variables
OM7 Provide jtd or Dst
CalibOnly?
2 1 to reproduce of
oNormalize?
2 1=no, 2=yes

ofxn calc*
0.61187
ofxn mod
0.62974

sRun
RunA1
zout
1

Ave
Data
0.00319
Model
0.00316

data/vMax
0.01318

ofxng
334.07

Min d/m
2.93639

ave/min d
31.5425
ave/min m
91.6445

327_Calib_S_A

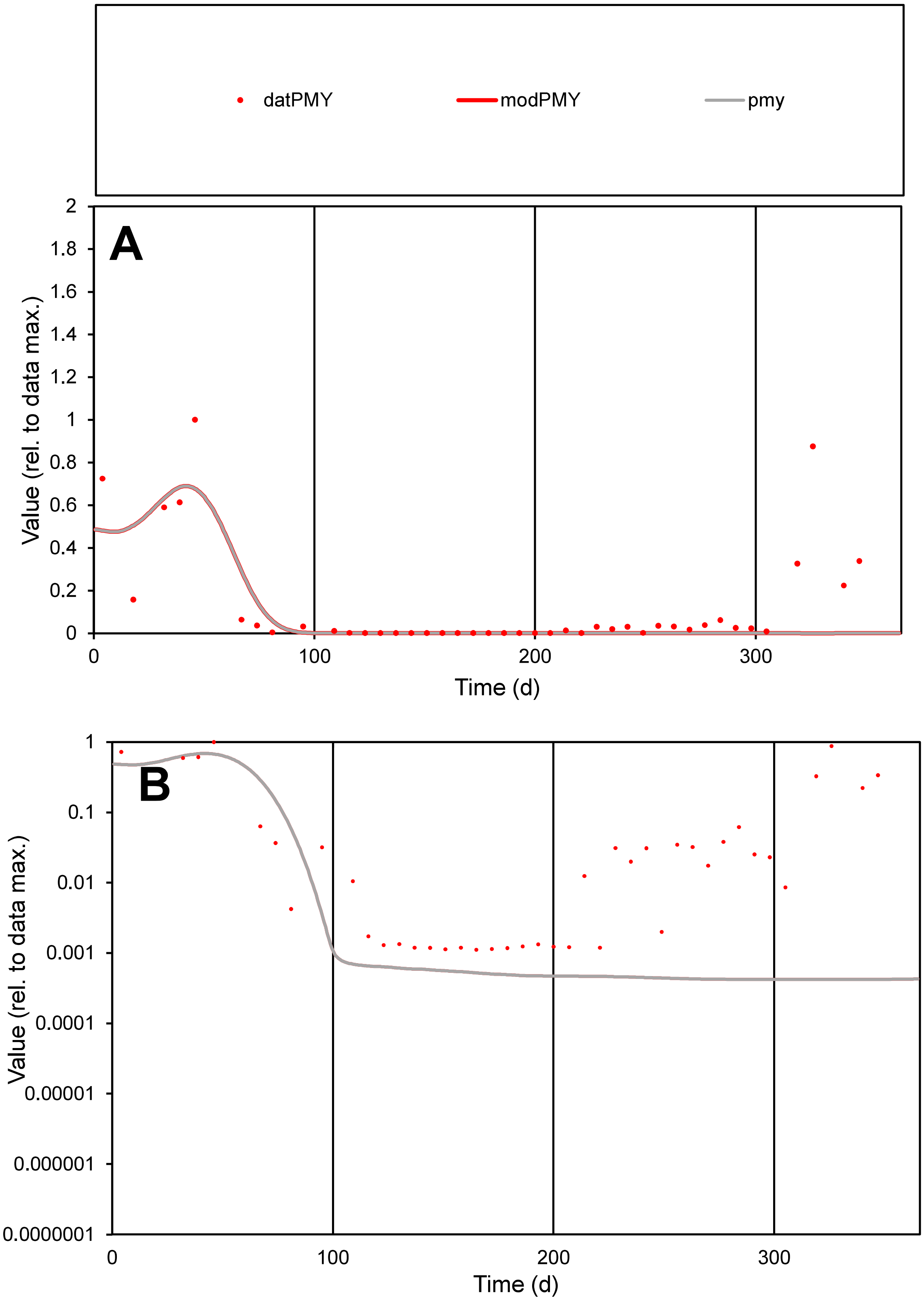

C1

Variables
PMY Provide jtd or Dst
CalibOnly?
2 1 to reproduce of
oNormalize?
2 1=no, 2=yes

ofxn calc*
1.42688
ofxn mod
1.44752

sRun
RunA1
zout
1

Ave
Data
0.00109
Model
0.00069

data/vMax
0.00856

ofxng
334.07

Min d/m
2.65628

ave/min d
114.228
ave/min m
192.79

327_Calib_S_A

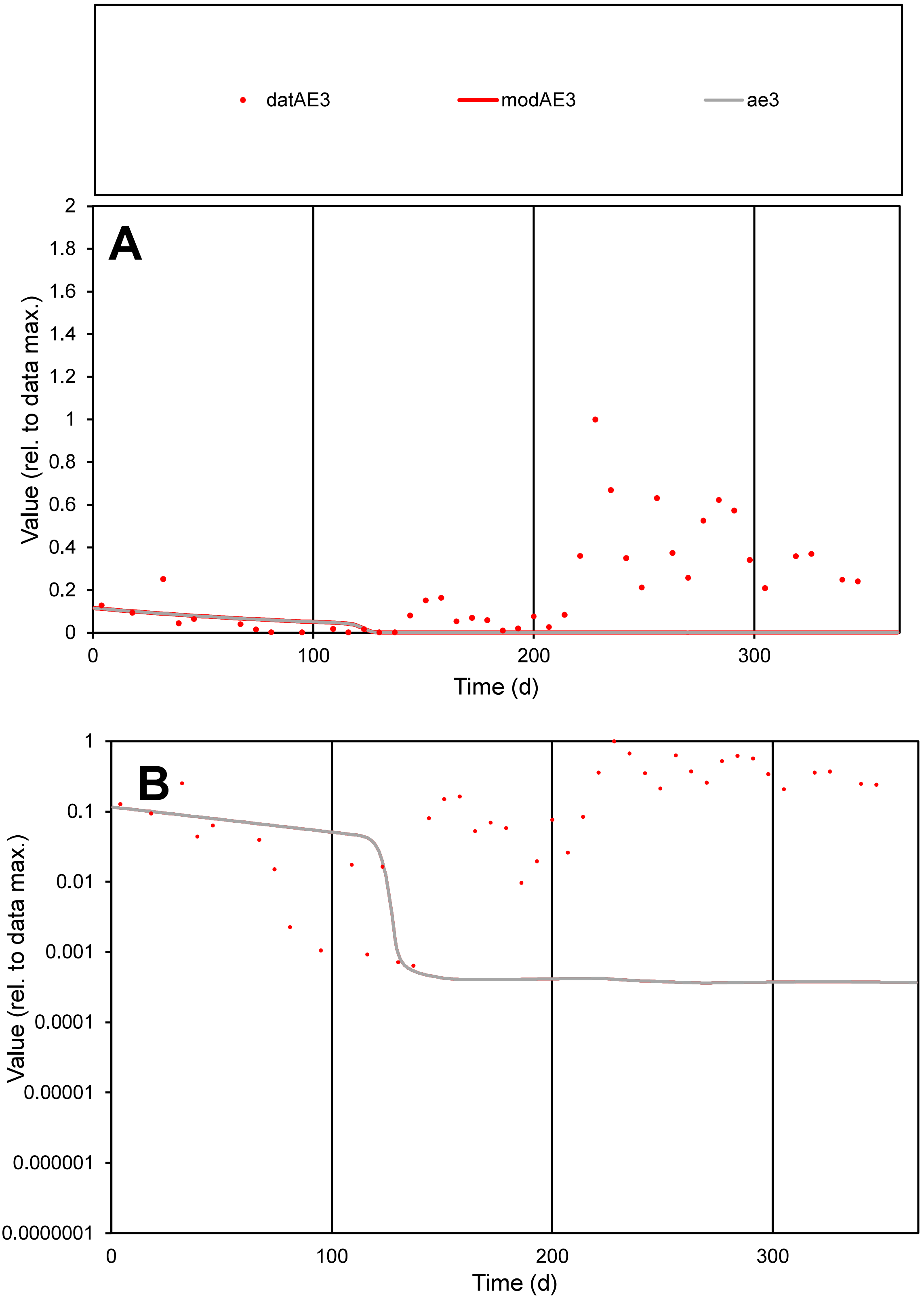

C1

Variables
AE3 Provide jtd or Dst
CalibOnly?
2 1 to reproduce of
oNormalize?
2 1=no, 2=yes

ofxn calc*
1.47184
ofxn mod
1.47423

sRun
RunA1
zout
1

Ave
Data
0.00585
Model
0.00054

data/vMax
0.02793

ofxng
334.07

Min d/m
1.76106

ave/min d
326.704
ave/min m
53

327_Calib_S_A

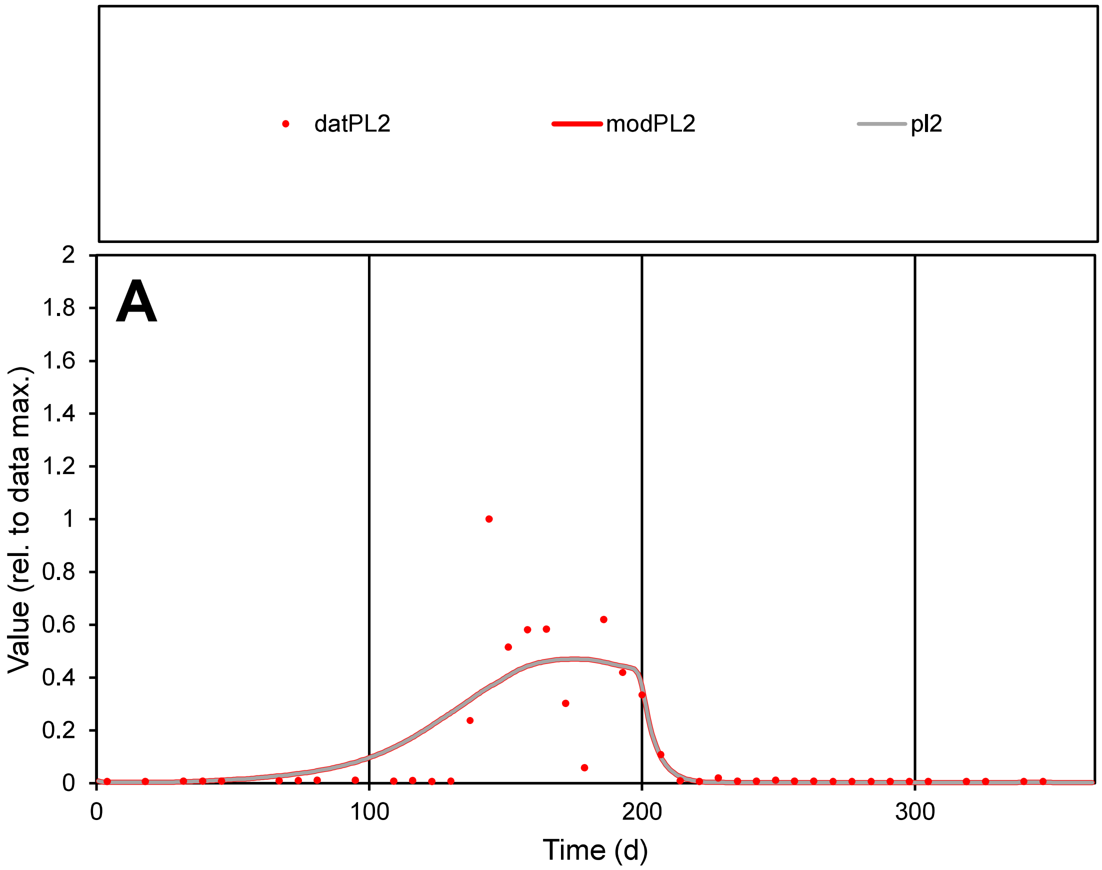

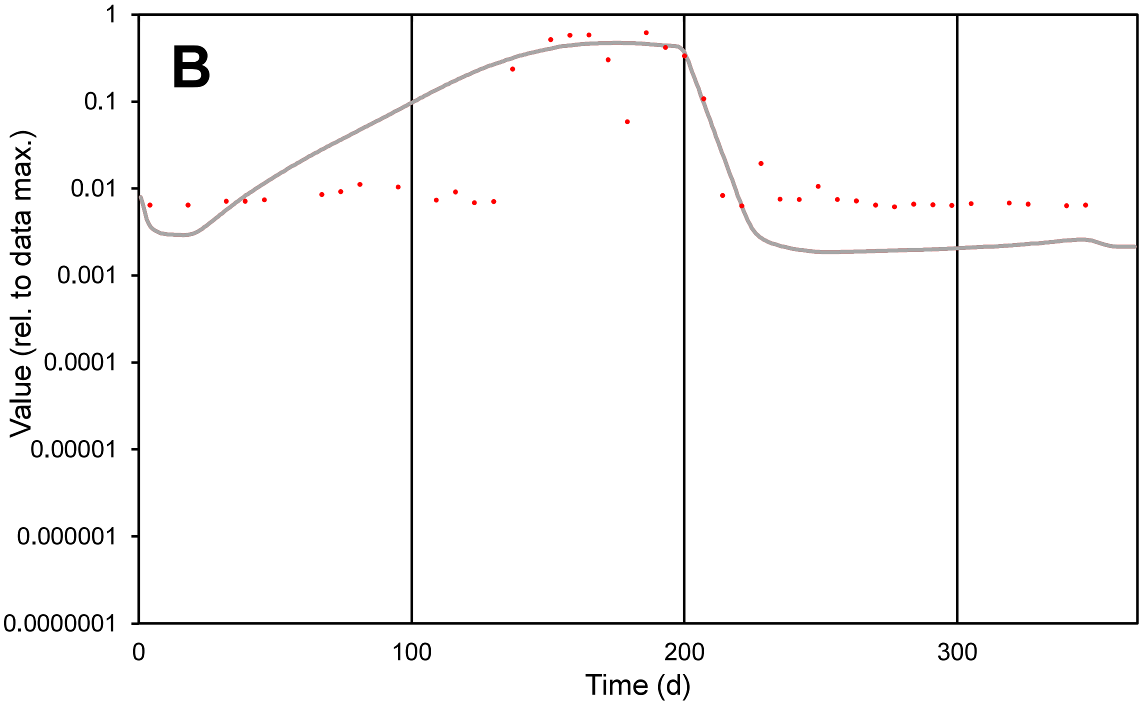

C1

Variables
PL2 Provide jtd or Dst
CalibOnly?
2 1 to reproduce of
oNormalize?
2 1=no, 2=yes

ofxn calc*
1.18079
ofxn mod
1.2018

sRun
RunA1
zout
1

Ave
Data
0.00326
Model
0.00349

data/vMax
0.02733

ofxng
334.07

Min d/m
3.31174

ave/min d
19.2973
ave/min m
68.4506

327_Calib_S_A

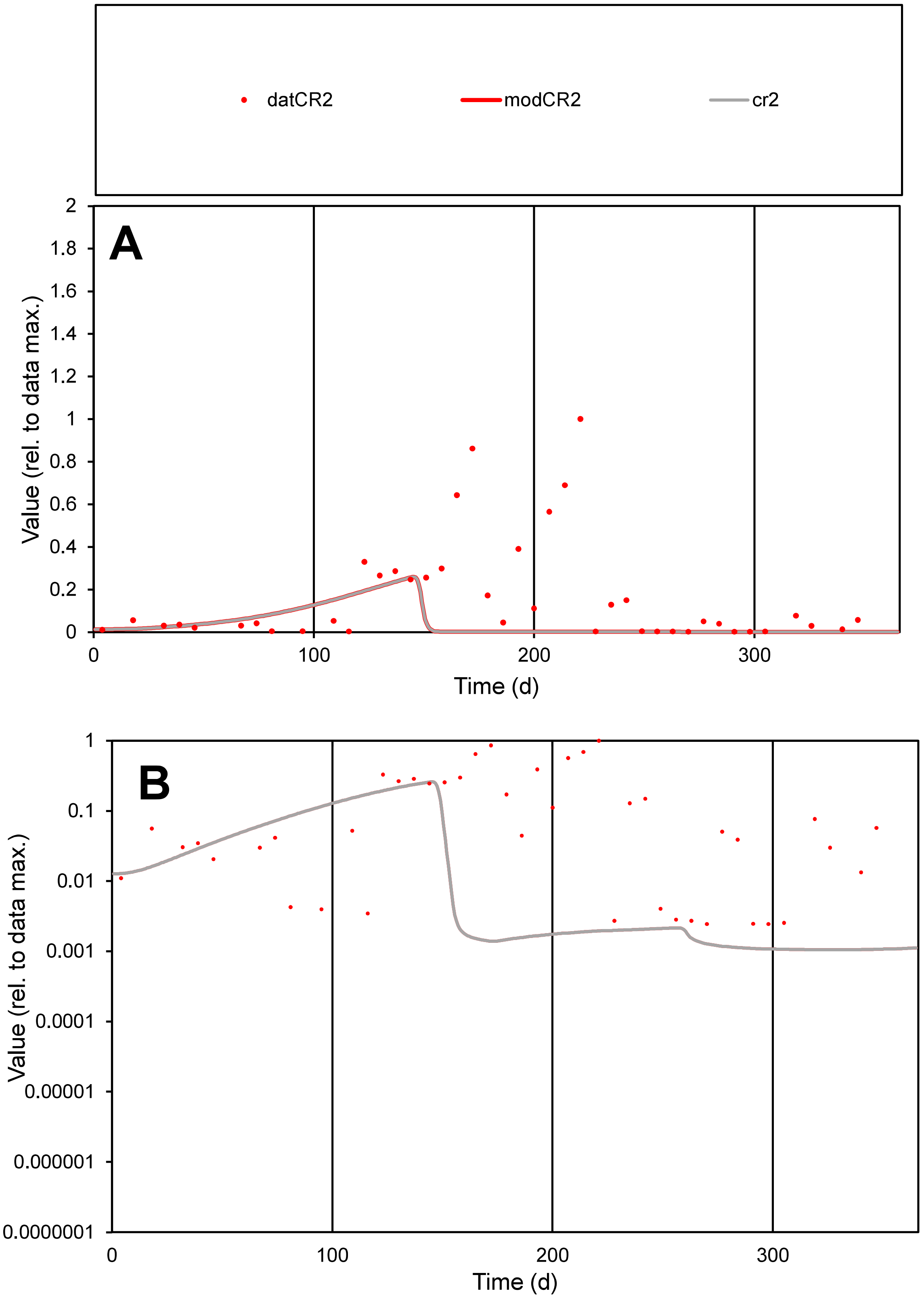

C1

Variables
CR2 Provide jtd or Dst
CalibOnly?
2 1 to reproduce of
oNormalize?
2 1=no, 2=yes

ofxn calc*
1.69508
ofxn mod
1.70247

sRun
RunA1
zout
1

Ave
Data
0.00295
Model
0.00079

data/vMax
0.01765

ofxng
334.07

Min d/m
2.3247

ave/min d
68.3841
ave/min m
42.7066

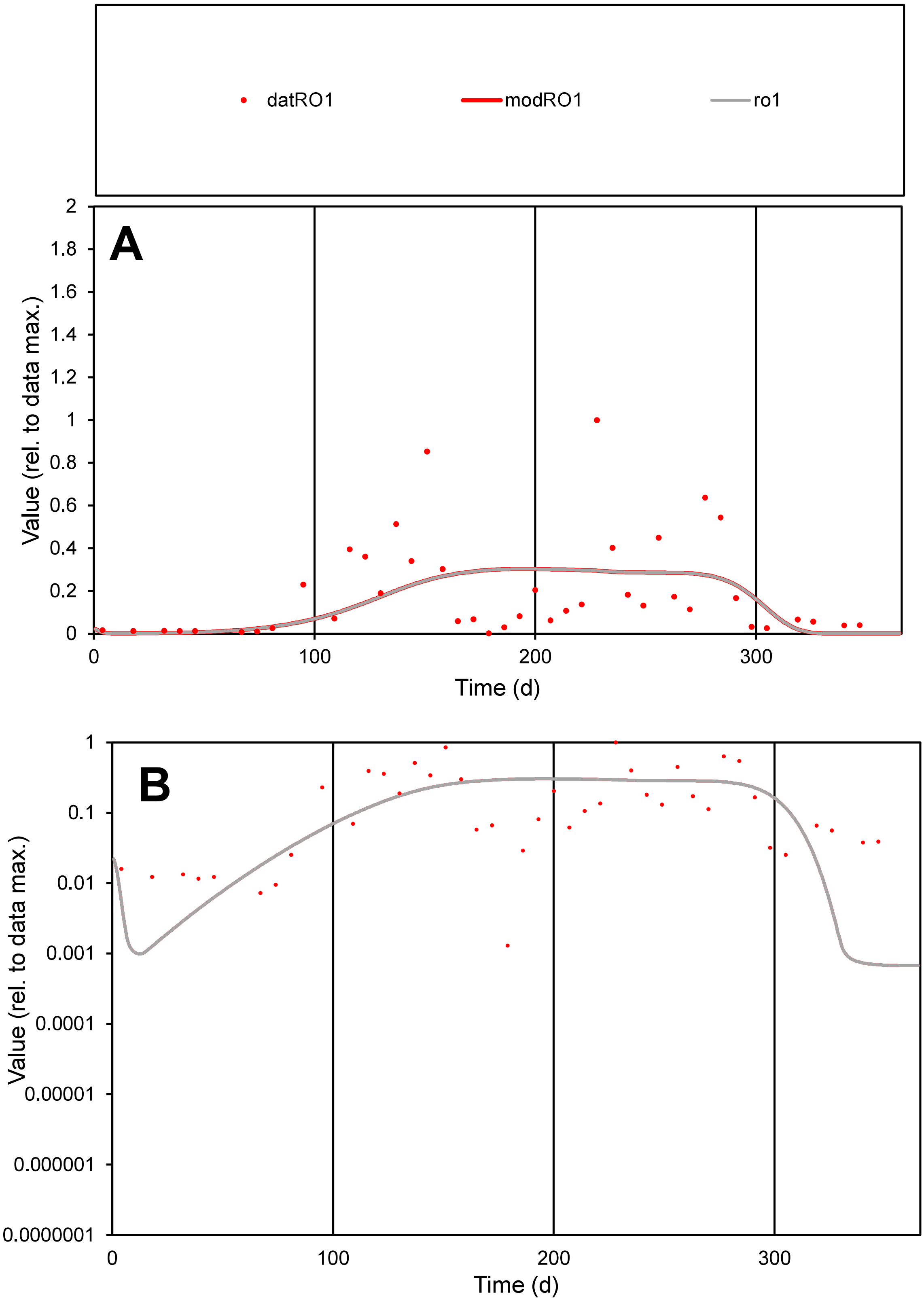

C1

Variables

RO1 Provide jtd or Dst

CalibOnly?

2 1 to reproduce of

oNormalize?

2 1=no, 2=yes

ofxn calc*

1.10173

ofxn mod

1.10943

sRun

RunA1

zout

1

Ave

Data

0.00554

Model

0.00503

data/vMax

0.02855

ofxng

334.07

Min d/m

1.88878

ave/min d

149.694

ave/min m

256.857

327_Calib_S_A

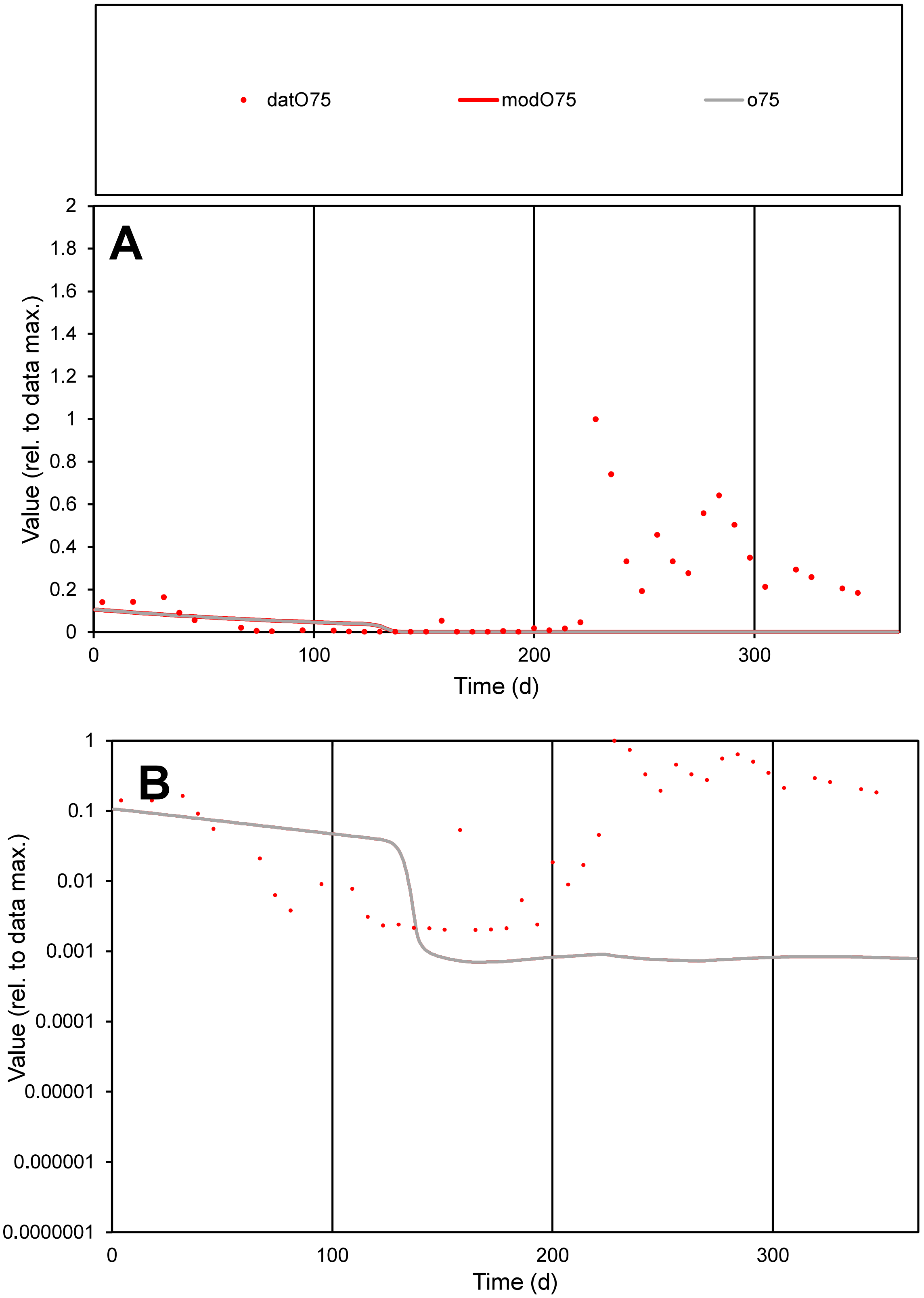

C1

Variables
O75 Provide jtd or Dst
CalibOnly?
2 1 to reproduce of
oNormalize?
2 1=no, 2=yes

ofxn calc*
1.64564
ofxn mod
1.64825

sRun
RunA1
zout
1

Ave
Data
0.00605
Model
0.00067

data/vMax
0.03458

ofxng
334.07

Min d/m
2.88884

ave/min d
86.8731
ave/min m
27.6932

327_Calib_S_A

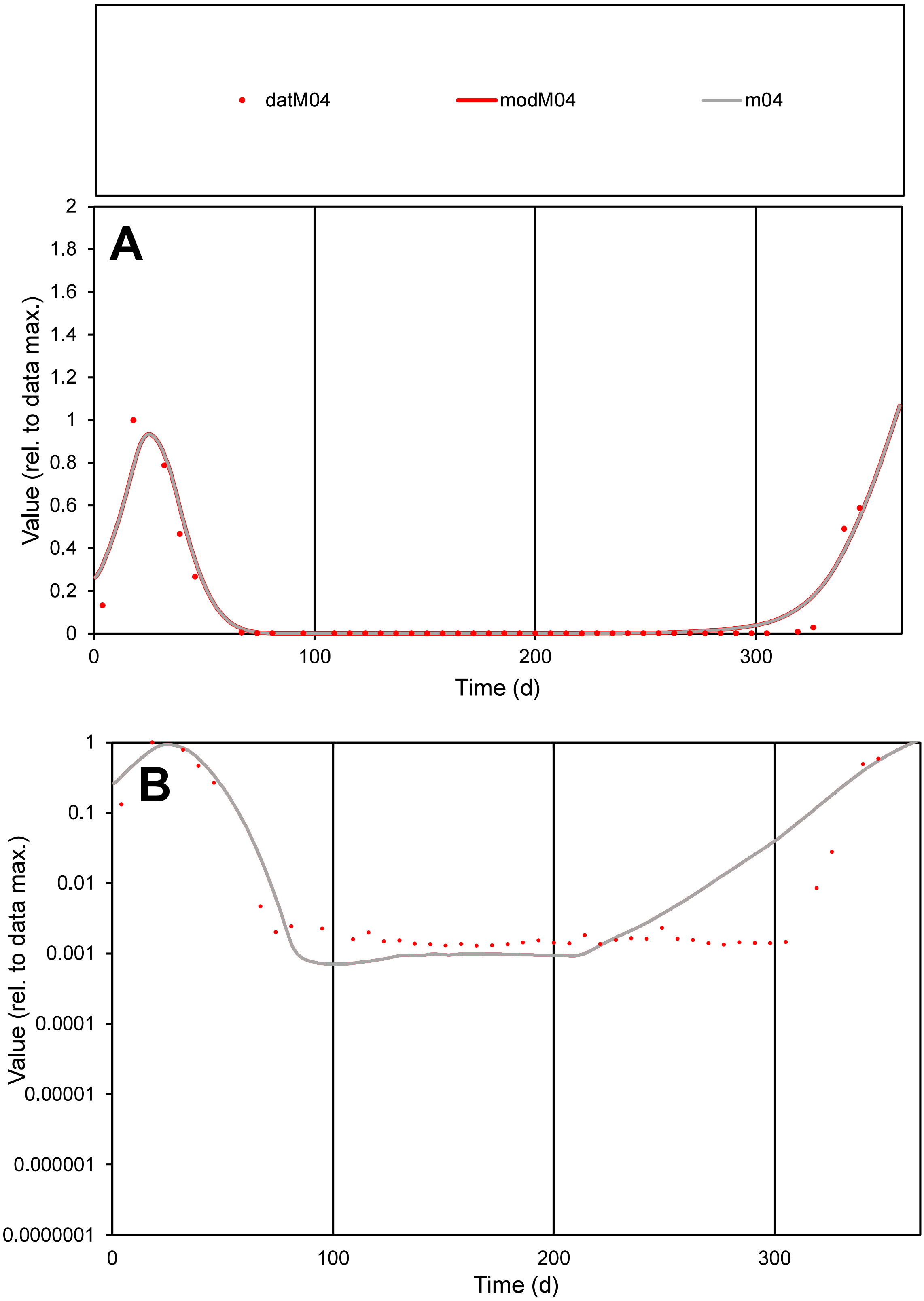

C1

Variables
M04 Provide jtd or Dst
CalibOnly?
2 1 to reproduce of
oNormalize?
2 1=no, 2=yes

ofxn calc*
0.56139
ofxn mod
0.68022

sRun
RunA1
zout
1

Ave
Data
0.00248
Model
0.00278

data/vMax
0.02722

ofxng
334.07

Min d/m
1.80103

ave/min d
70.7506
ave/min m
143.054

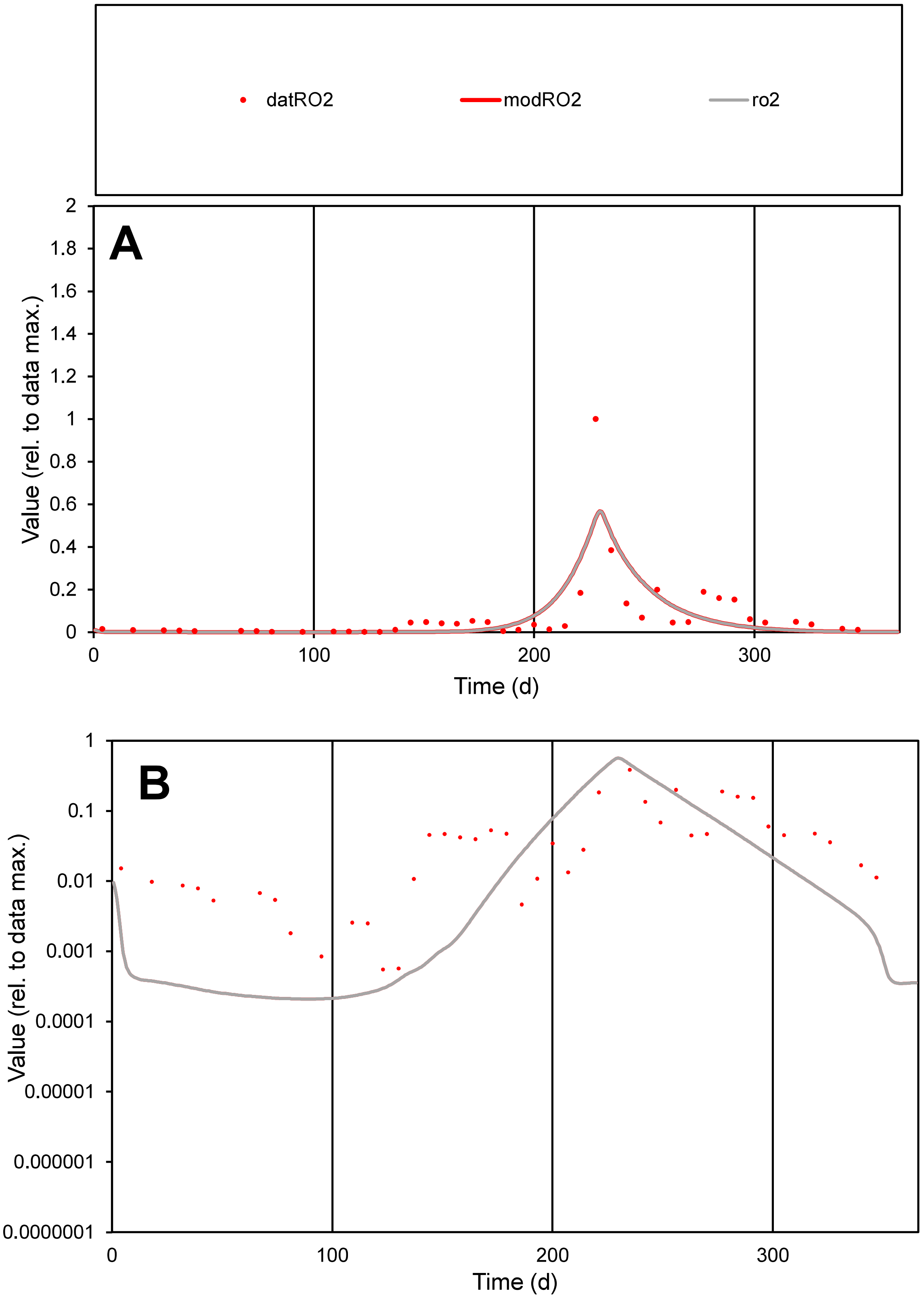

C1

Variables

RO2 Provide jtd or Dst

CalibOnly?

2 1 to reproduce of

oNormalize?

2 1=no, 2=yes

ofxn calc*

1.24528

ofxn mod

1.28722

sRun

RunA1

zout

1

Ave

Data

0.00616

Model

0.0056

data/vMax

0.08014

ofxng

334.07

Min d/m

2.63777

ave/min d

138.568

ave/min m

332.414

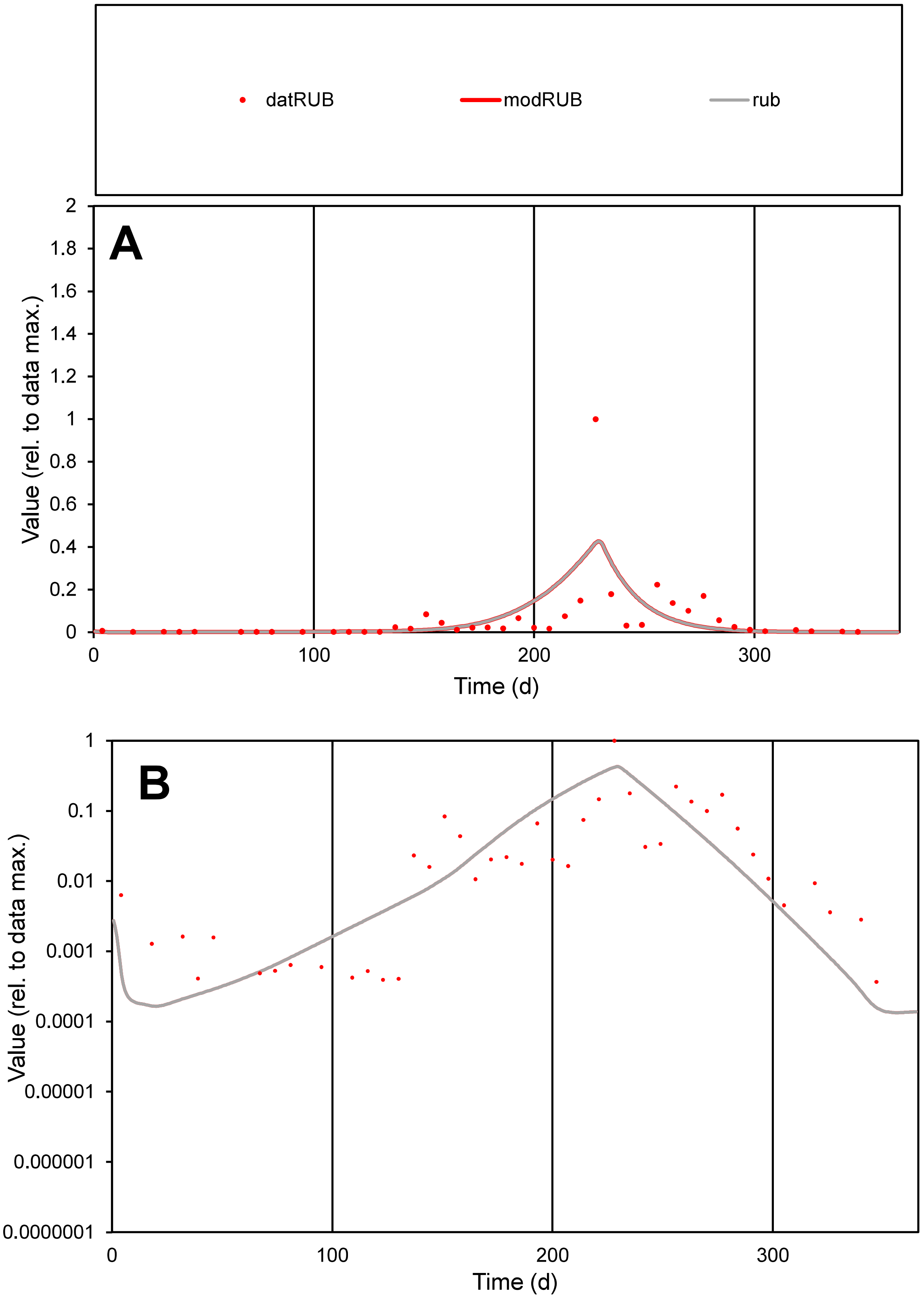

C1

Variables
RUB Provide jtd or Dst
CalibOnly?
2 1 to reproduce of
oNormalize?
2 1=no, 2=yes

ofxn calc*
1.8966
ofxn mod
1.92383

sRun
RunA1
zout
1

Ave
Data
0.00178
Model
0.00181

data/vMax
0.02926

ofxng
334.07

Min d/m
2.39024

ave/min d
165.534
ave/min m
400.598

327_Calib_S_A

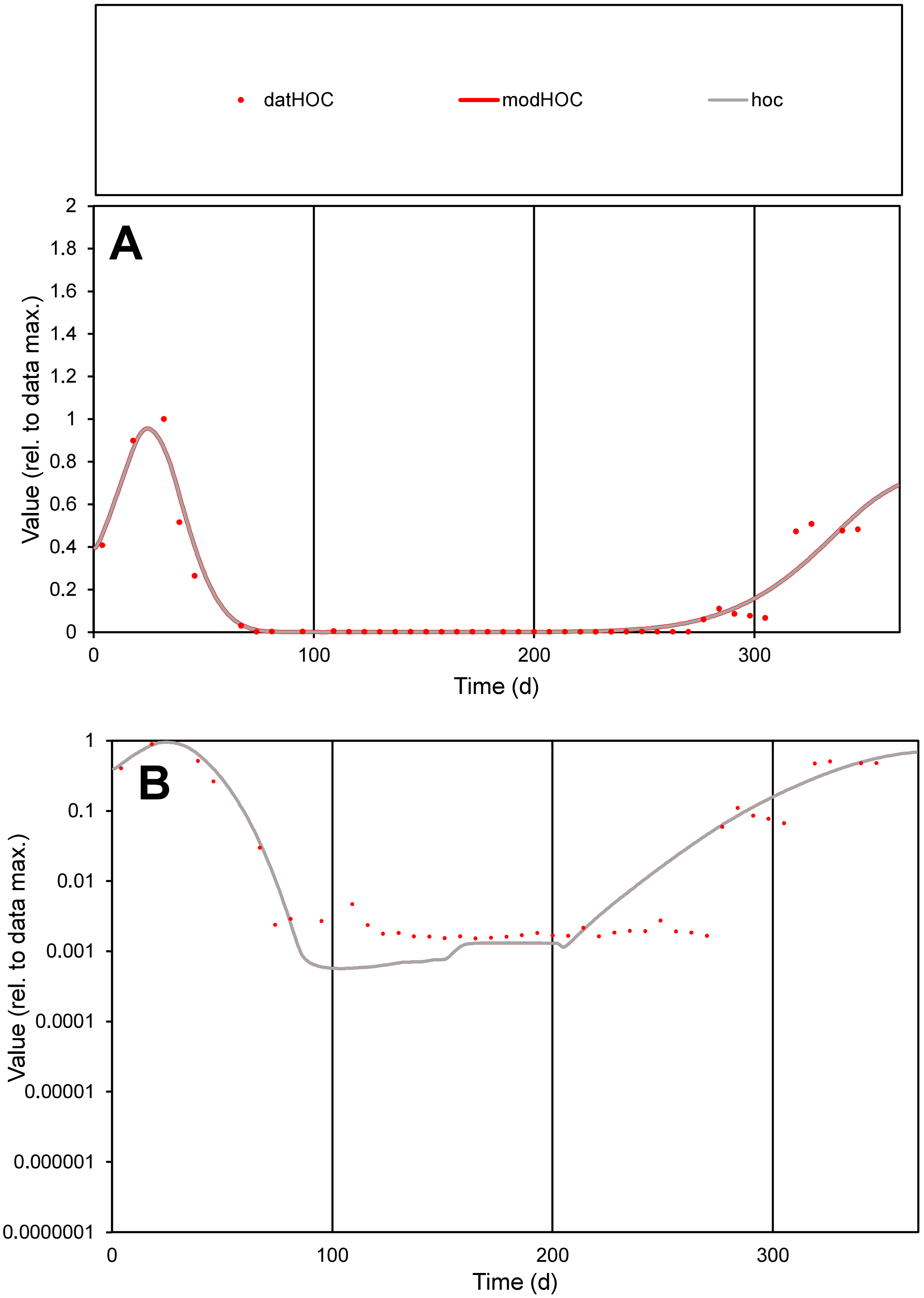

C1

Variables
HOC Provide jtd or Dst
CalibOnly?
2 1 to reproduce of
oNormalize?
2 1=no, 2=yes

ofxn calc*
0.41861
ofxn mod
0.49961

sRun
RunA1
zout
1

Ave
Data
0.00107
Model
0.00111

data/vMax
0.00817

ofxng
334.07

Min d/m
2.63948

ave/min d
85.8817
ave/min m
235.43

327_Calib_S_A

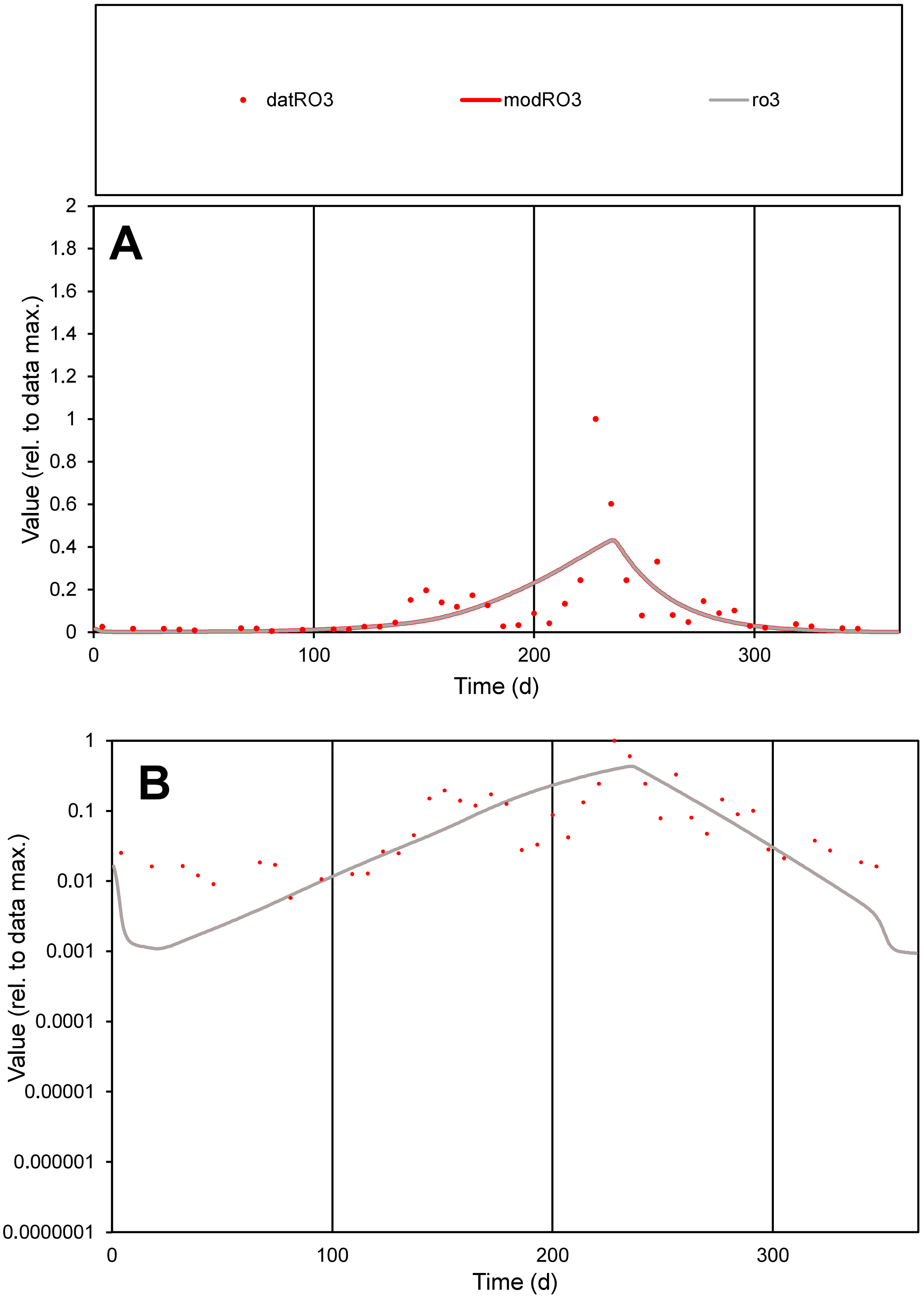

C1

Variables

RO3 Provide jtd or Dst

CalibOnly?

2 1 to reproduce of

oNormalize?

2 1=no, 2=yes

ofxn calc*

1.15649

ofxn mod

1.17189

sRun

RunA1

zout

1

Ave

Data

0.00685

Model

0.00651

data/vMax

0.06268

ofxng

334.07

Min d/m

5.26346

ave/min d

18.9486

ave/min m

94.7119

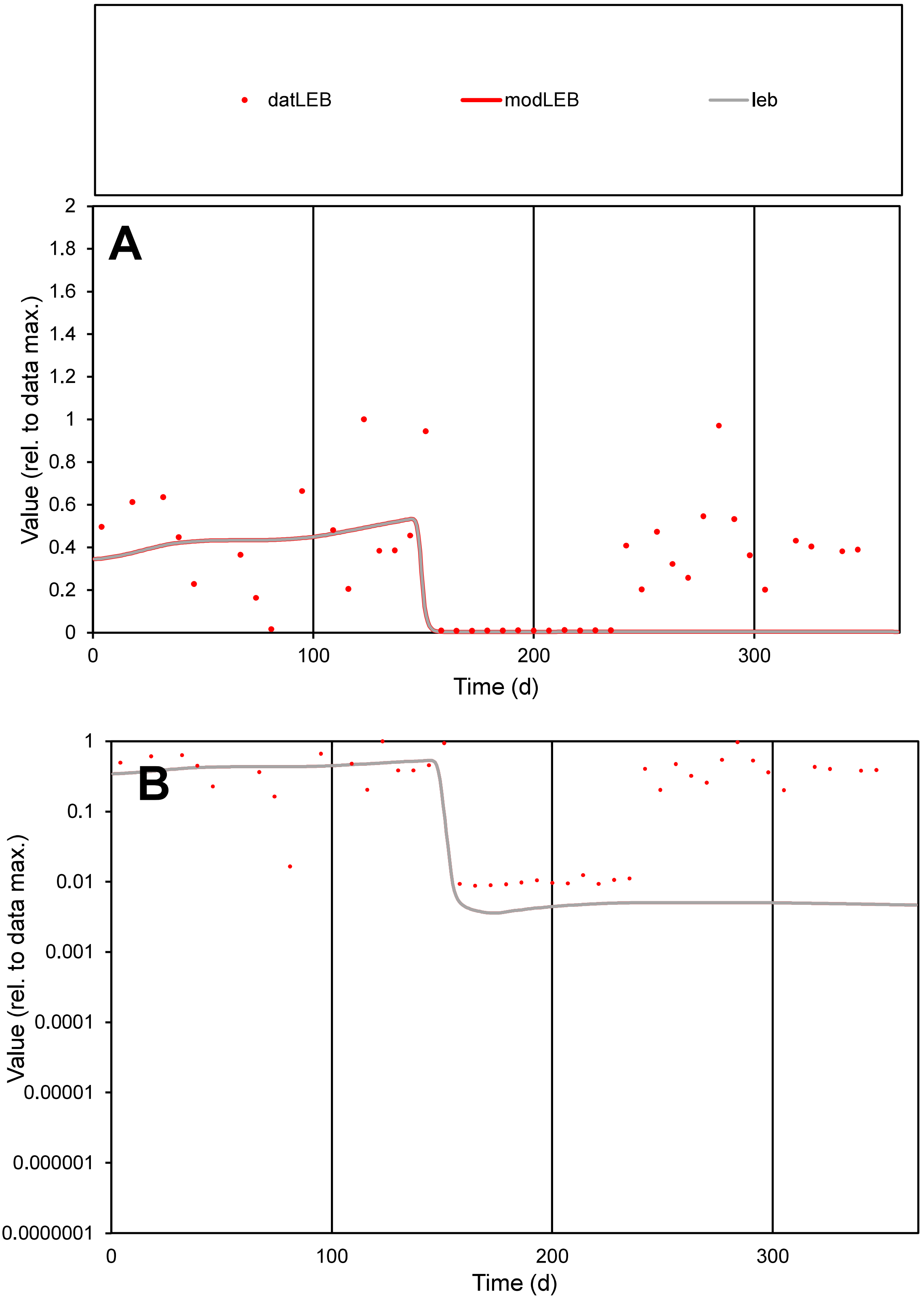

C1

Variables		
LEB	Provide jtd or Dst	
CalibOnly?		
2	1 to reproduce of	
oNormalize?		
2	1=no, 2=yes	
ofxn calc*		
0.98043		
ofxn mod		
1.00706		
sRun		
RunA1		
zout		
1		
Ave		
Data		
0.00254		
Model		
0.00135		
data/vMax		
0.0079		
ofxng		
334.07		
Min d/m		
2.44339		
ave/min d		
36.6306		
ave/min m		
47.4924		

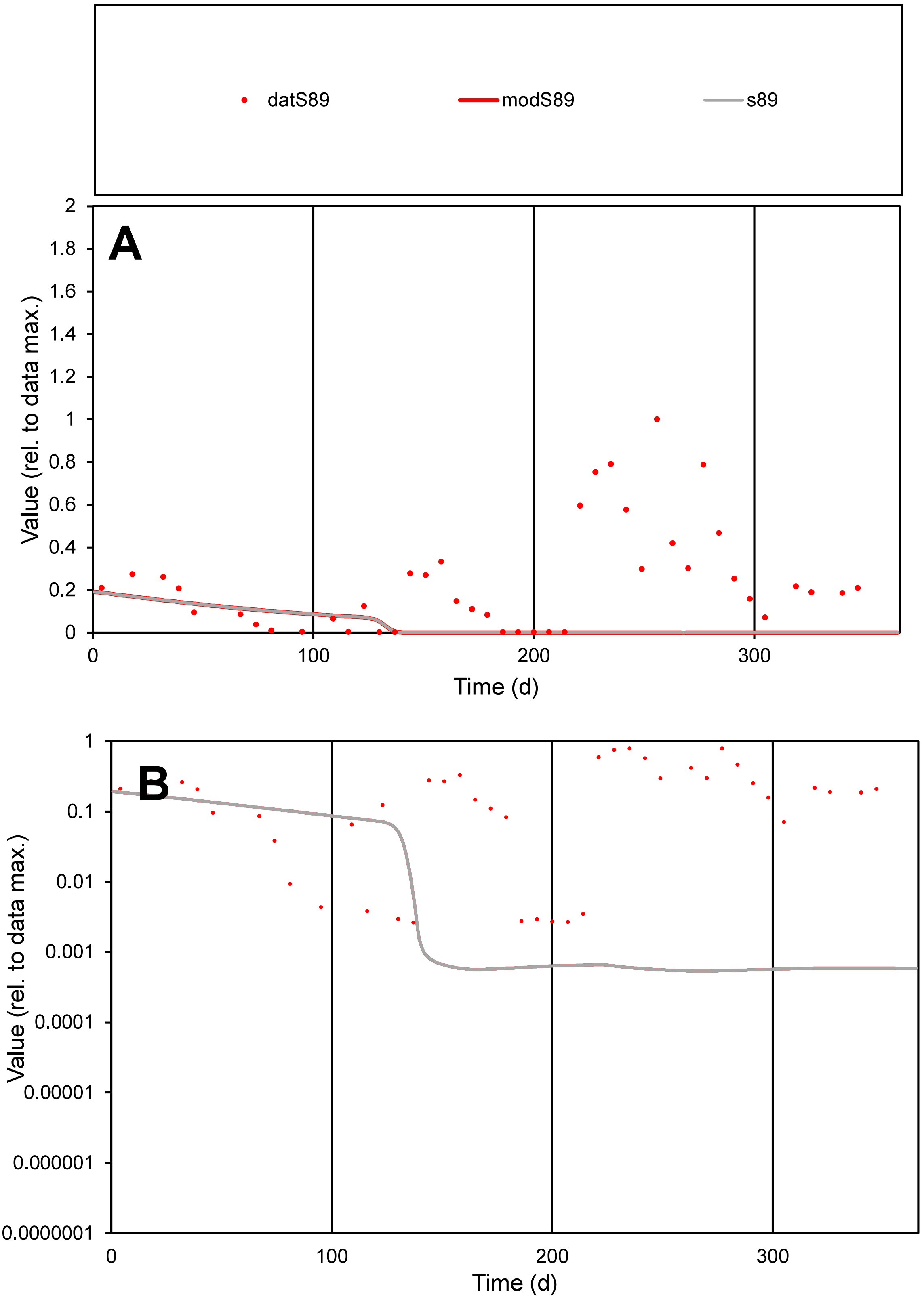

C1

Variables
S89 Provide jtd or Dst
CalibOnly?
2 1 to reproduce of
oNormalize?
2 1=no, 2=yes

ofxn calc*
1.41624
ofxn mod
1.41947

sRun
RunA1
zout
1

Ave
Data
0.00341
Model
0.0005

data/vMax
0.01448

ofxng
334.07

Min d/m
4.9593

ave/min d
88.922
ave/min m
64.8197

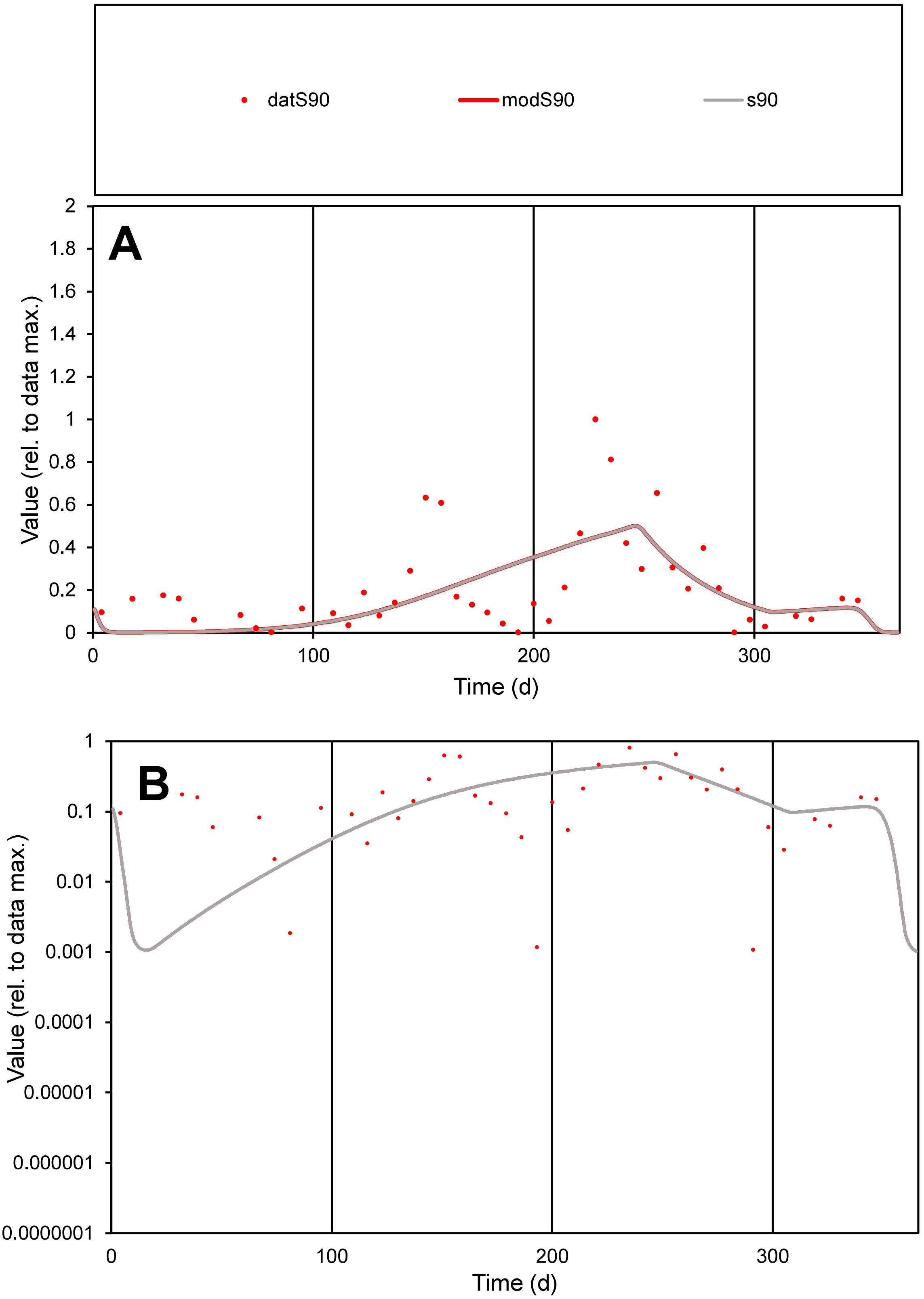

C1

Variables
S90 Provide jtd or Dst
CalibOnly?
2 1 to reproduce of
oNormalize?
2 1=no, 2=yes

ofxn calc*
0.87822
ofxn mod
0.89092

sRun
RunA1
zout
1

Ave
Data
0.00435
Model
0.00393

data/vMax
0.02012

ofxng
334.07

Min d/m
0.92015

ave/min d
199.854
ave/min m
165.95

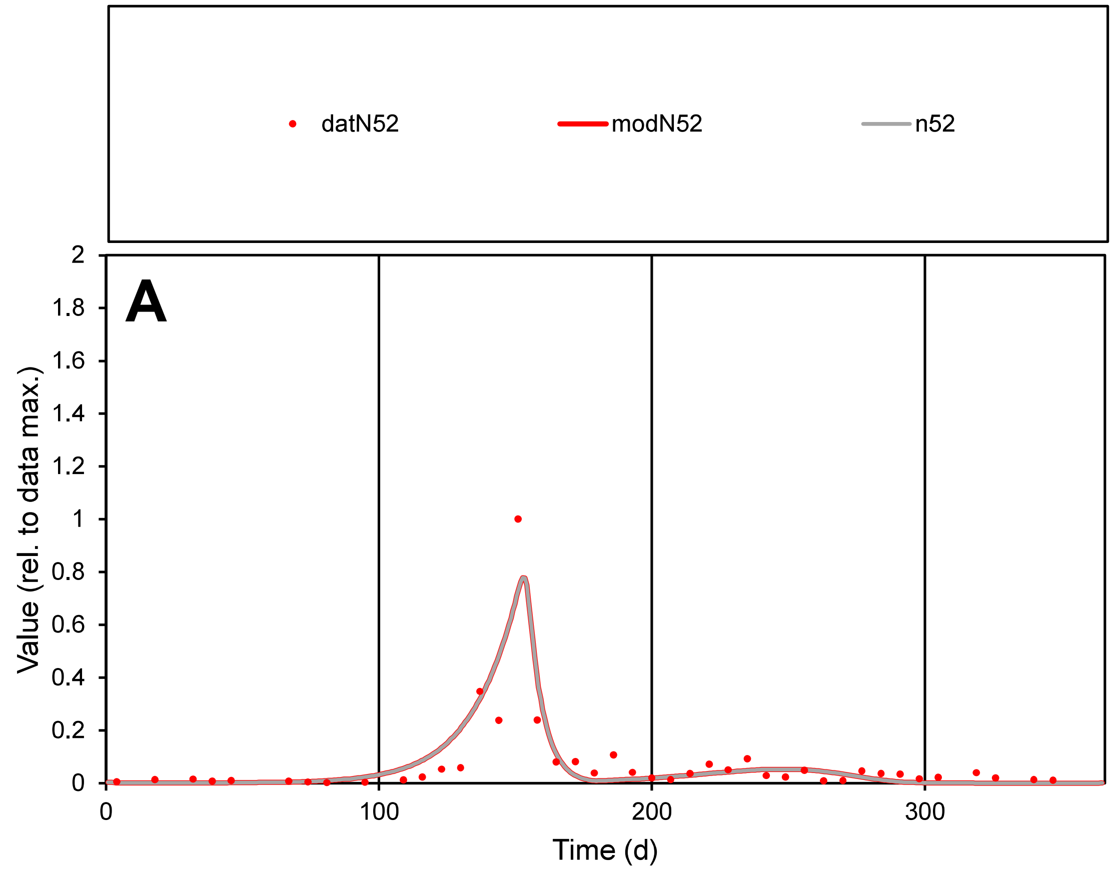

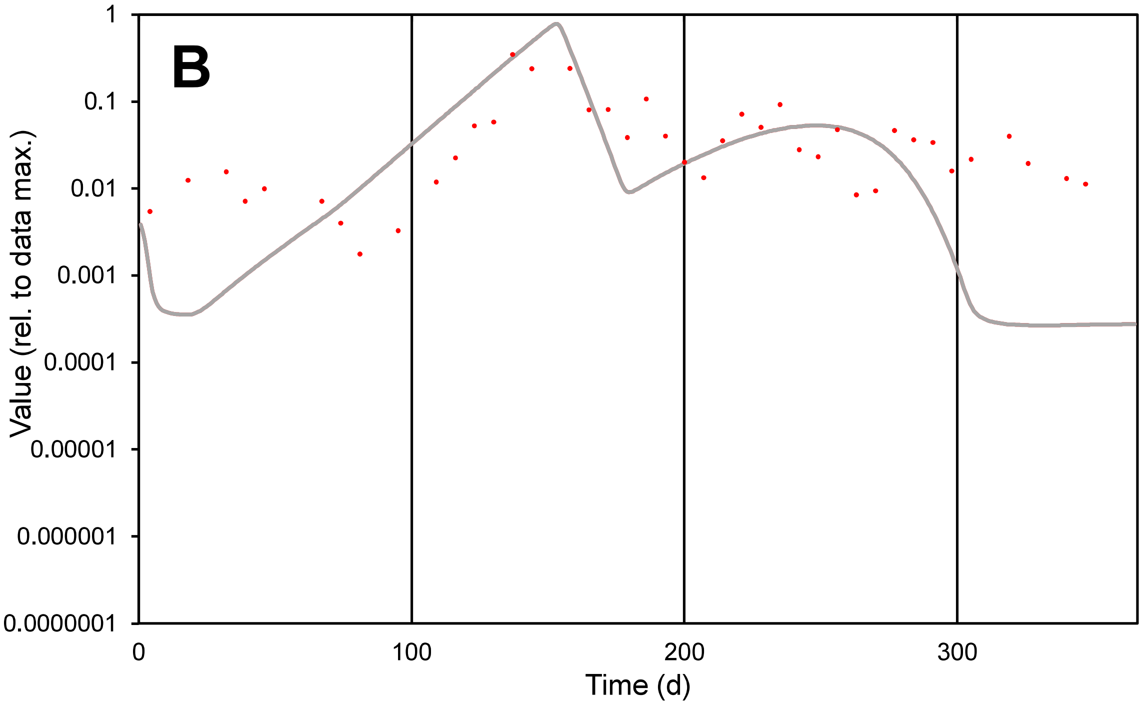

C1

Variables

N52 Provide jtd or Dst

CalibOnly?

2 1 to reproduce of

oNormalize?

2 1=no, 2=yes

ofxn calc*

0.9247

ofxn mod

0.95859

sRun

RunA1

zout

1

Ave

Data

0.00366

Model

0.00373

data/vMax

0.05091

ofxng

334.07

Min d/m

6.64632

ave/min d

40.5458

ave/min m

274.53

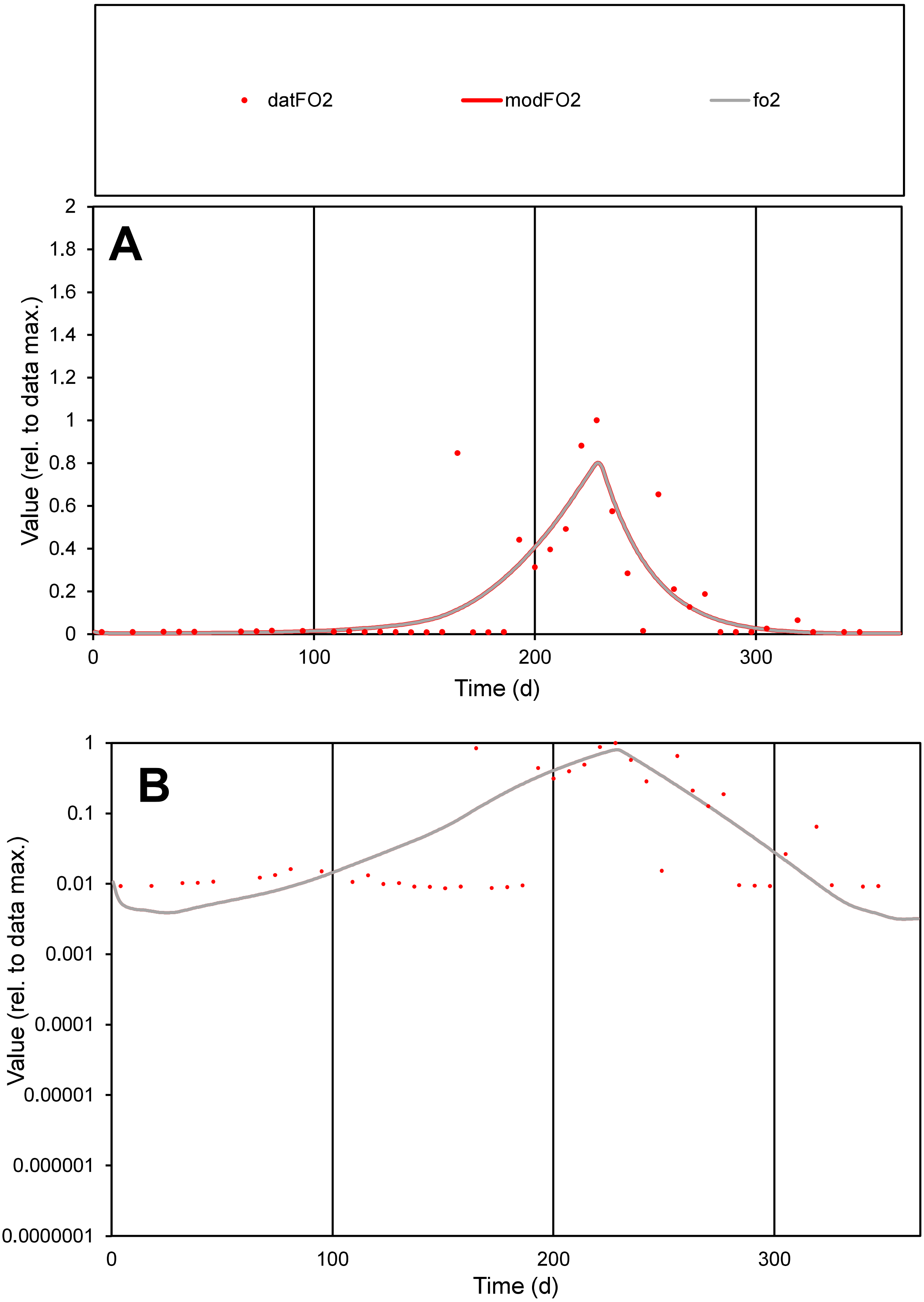

C1

Variables
FO2 Provide jtd or Dst
CalibOnly?
2 1 to reproduce of
oNormalize?
2 1=no, 2=yes

ofxn calc*
0.98307
ofxn mod
1.01214

sRun
RunA1
zout
1

Ave
Data
0.00126
Model
0.00124

data/vMax
0.0078

ofxng
334.07

Min d/m
2.34179

ave/min d
18.7182
ave/min m
43.0272

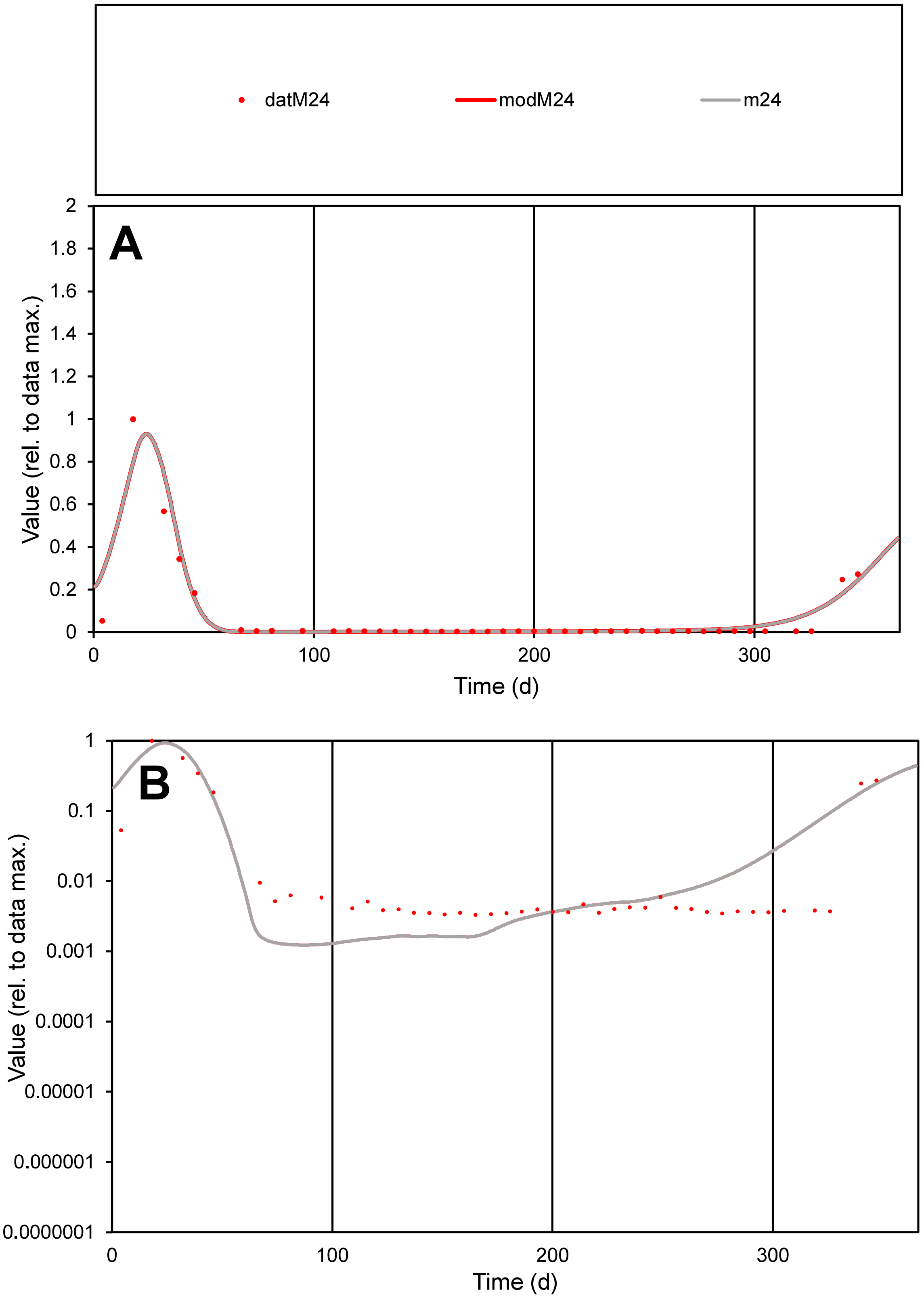

C1

Variables
M24 Provide jtd or Dst
CalibOnly?
2 1 to reproduce of
oNormalize?
2 1=no, 2=yes

ofxn calc*
0.67372
ofxn mod
0.84884

sRun
RunA1
zout
1

Ave
Data
0.00145
Model
0.00157

data/vMax
0.02167

ofxng
334.07

Min d/m
2.66803

ave/min d
20.2427
ave/min m
58.4772

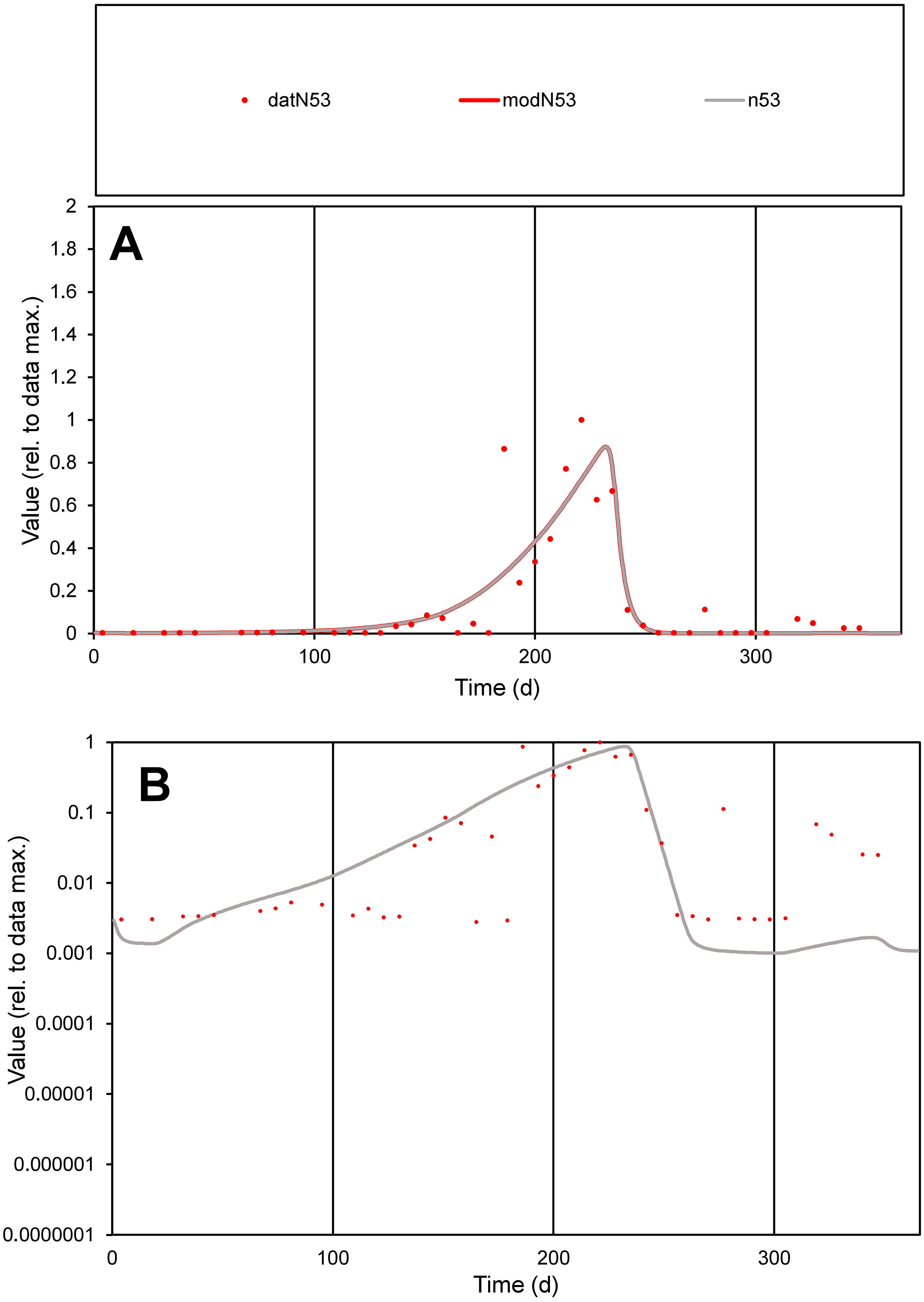

C1

Variables

N53	Provide jtd or Dst

CalibOnly?

2	1 to reproduce of

oNormalize?

2	1=no, 2=yes

ofxn calc*
0.86432
ofxn mod
0.90375

sRun
RunA1
zout
1

Ave
Data
0.00177
Model
0.00174

data/vMax
0.01301

ofxng
334.07

Min d/m
2.76104

ave/min d
48.9054
ave/min m
132.168

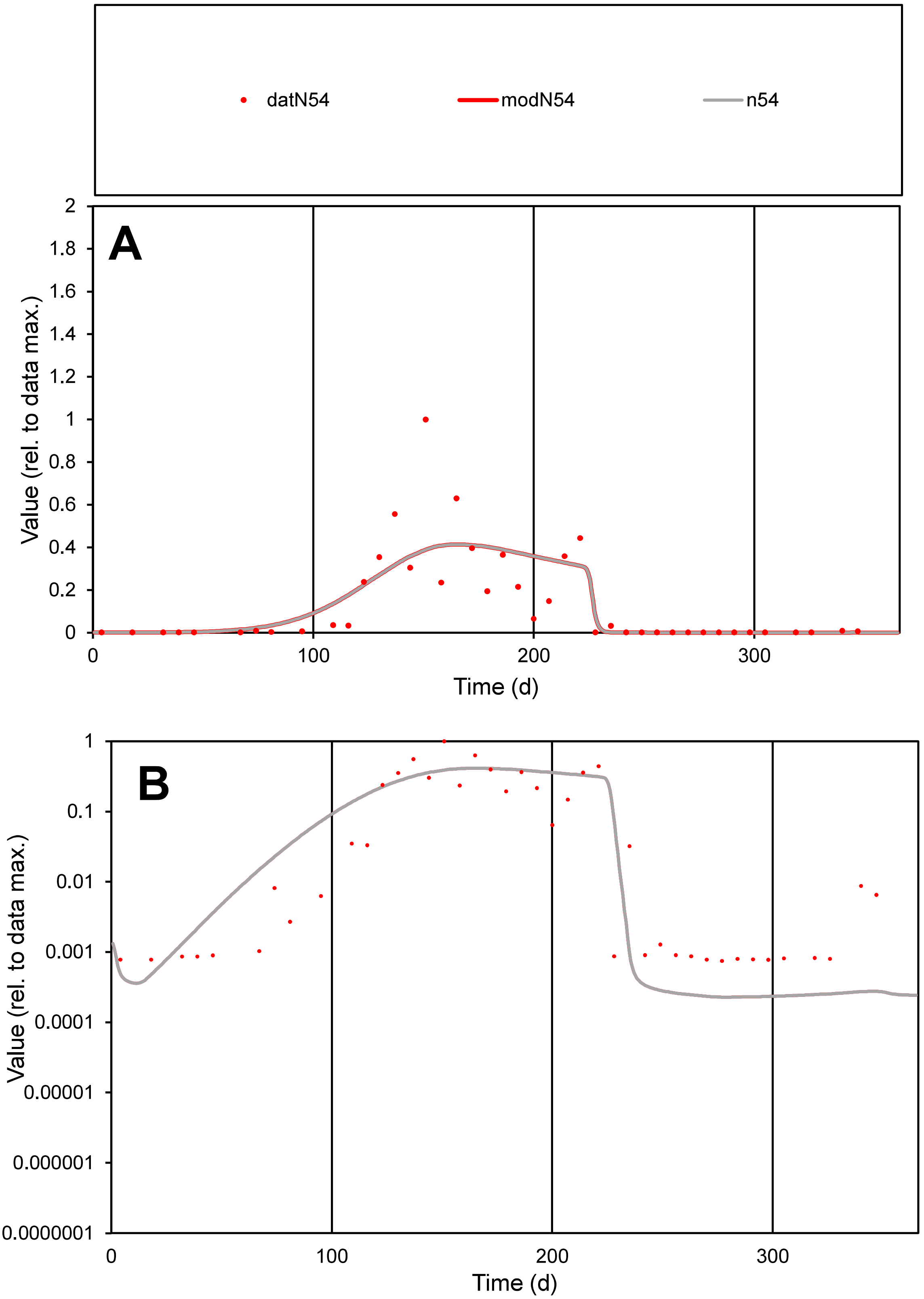

C1

Variables
N54 Provide jtd or Dst
CalibOnly?
2 1 to reproduce of
oNormalize?
2 1=no, 2=yes

ofxn calc*
0.99611
ofxn mod
1.01185

sRun
RunA1
zout
1

Ave
Data
0.00405
Model
0.00416

data/vMax
0.03007

ofxng
334.07

Min d/m
3.28114

ave/min d
180.445
ave/min m
608.384

327_Calib_S_A

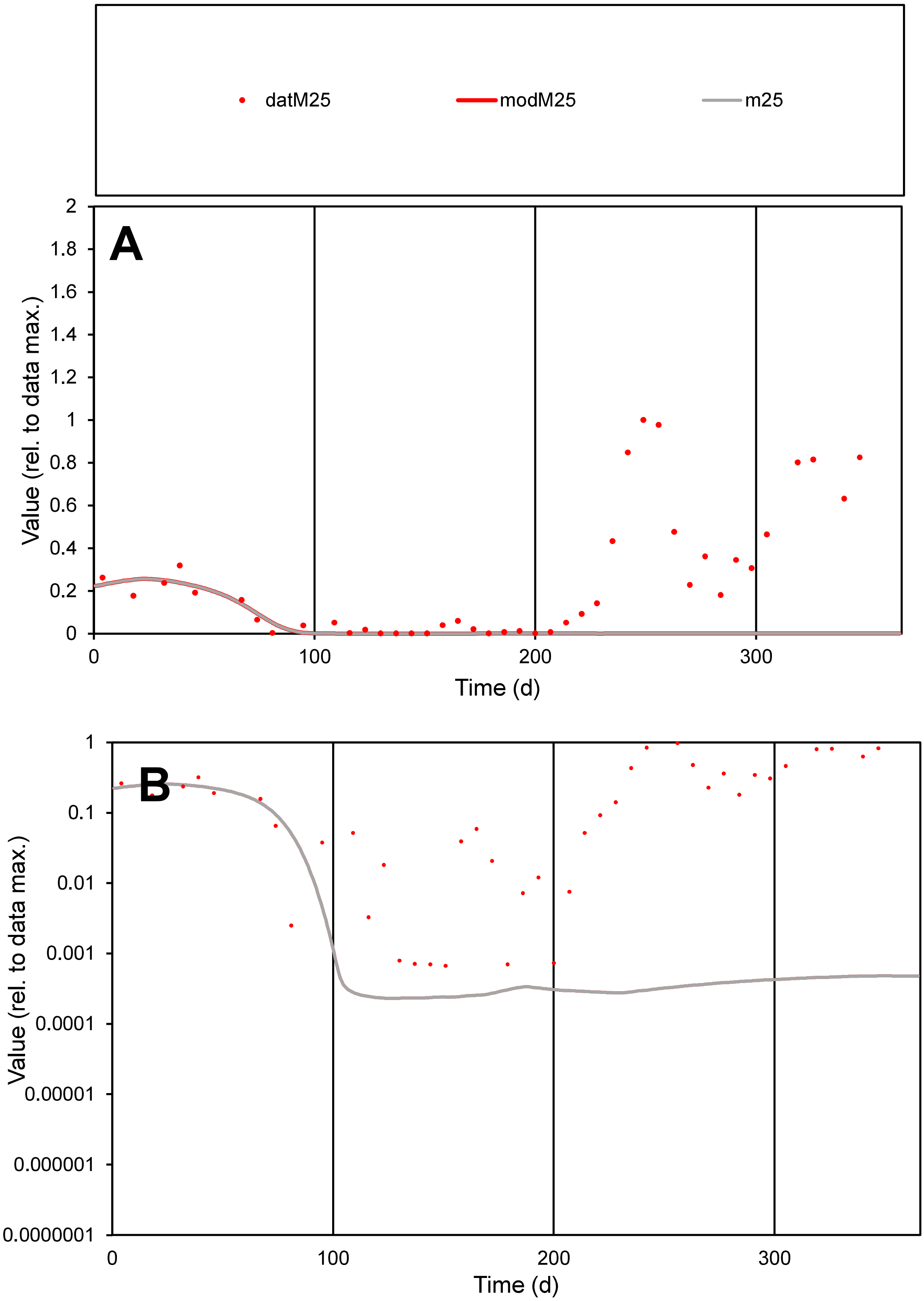

C1

Variables

M25	Provide jtd or Dst
CalibOnly?	
2	1 to reproduce of
oNormalize?	
2	1=no, 2=yes

ofxn calc*
1.51126
ofxn mod
1.5145

sRun
RunA1
zout
1

Ave
Data
0.00365
Model
0.0005

data/vMax
0.01439

ofxng
334.07

Min d/m
2.88686

ave/min d
378.446
ave/min m
149.659

327_Calib_S_A

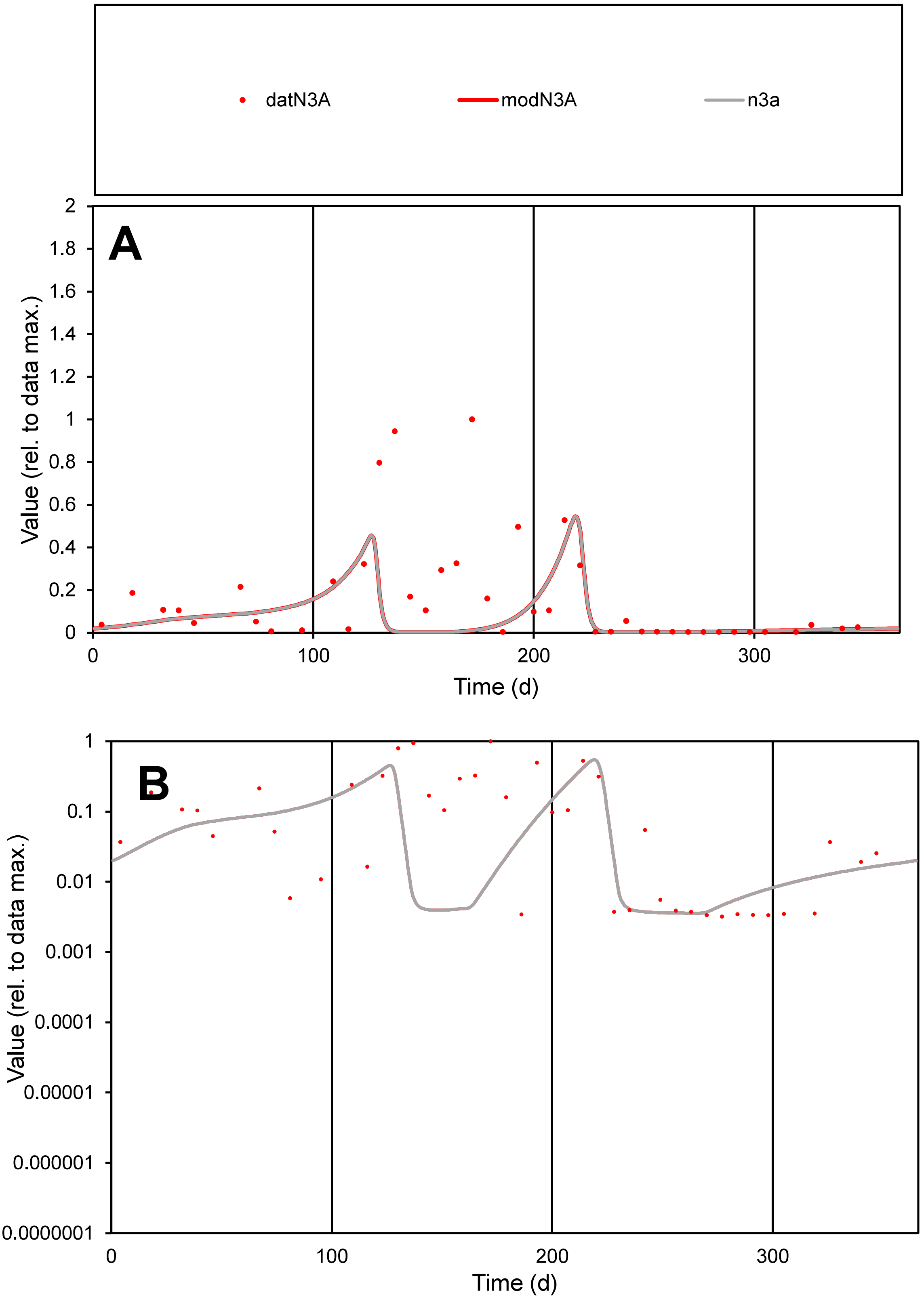

C1

Variables
N3A Provide jtd or Dst
CalibOnly?
2 1 to reproduce of
oNormalize?
2 1=no, 2=yes

ofxn calc*
1.52414
ofxn mod
1.57916

sRun
RunA1
zout
1

Ave
Data
0.00094
Model
0.00048

data/vMax
0.00577

ofxng
334.07

Min d/m
0.90709

ave/min d
50.8023
ave/min m
23.6441

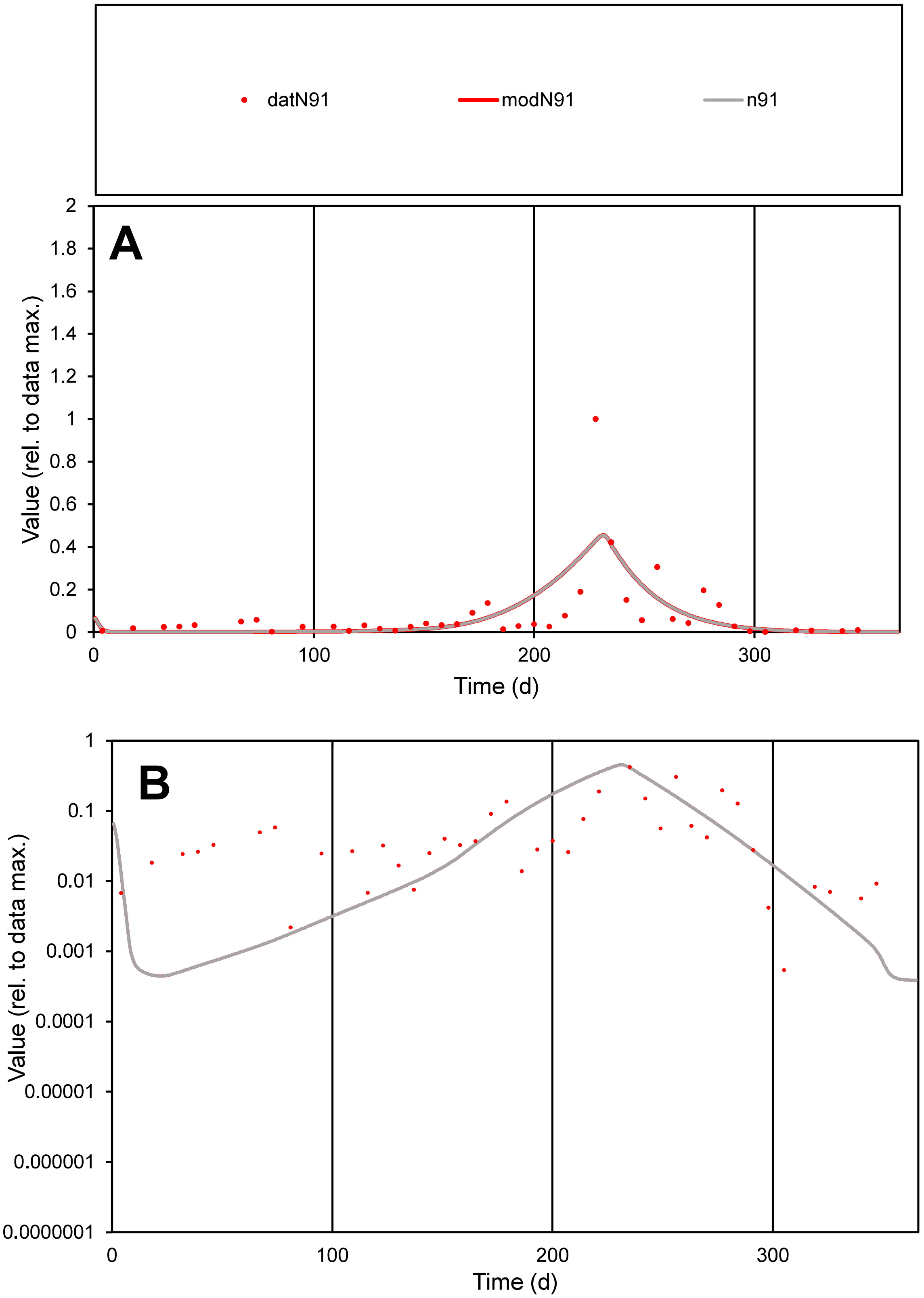

C1

Variables
N91 Provide jtd or Dst
CalibOnly?
2 1 to reproduce of
oNormalize?
2 1=no, 2=yes

ofxn calc*
1.39972
ofxn mod
1.4236

sRun
RunA1
zout
1

Ave
Data
0.00548
Model
0.00526

data/vMax
0.06587

ofxng
334.07

Min d/m
1.19858

ave/min d
154.099
ave/min m
177.183

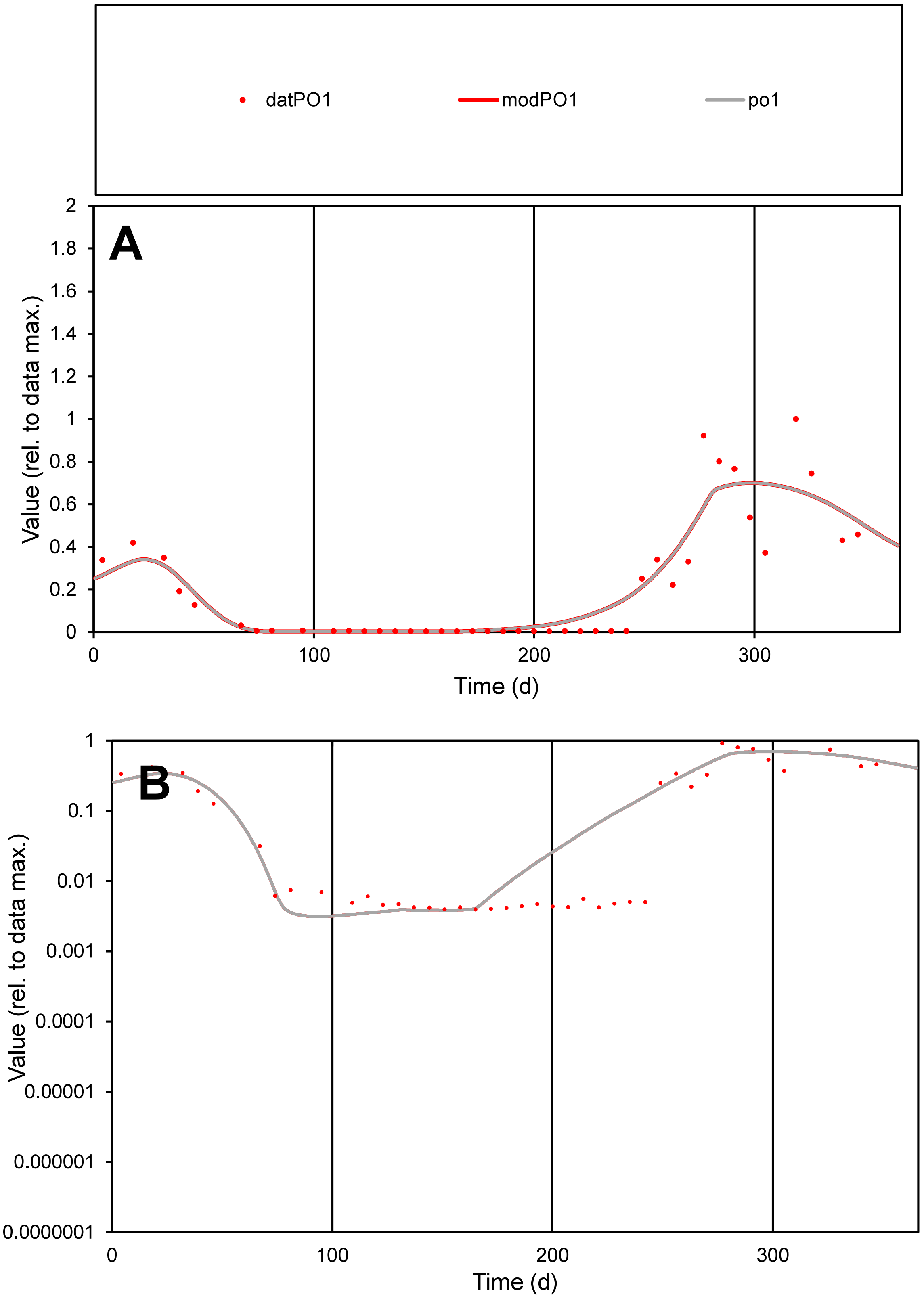

C1

Variables

PO1	Provide jtd or Dst
CalibOnly?	
2	1 to reproduce of
oNormalize?	
2	1=no, 2=yes

ofxn calc*
0.52481
ofxn mod
0.55605

sRun
RunA1
zout
1

Ave
Data
0.00239
Model
0.00249

data/vMax
0.01148

ofxng
334.07

Min d/m
1.2559

ave/min d
52.8421
ave/min m
69.091

327_Calib_S_A

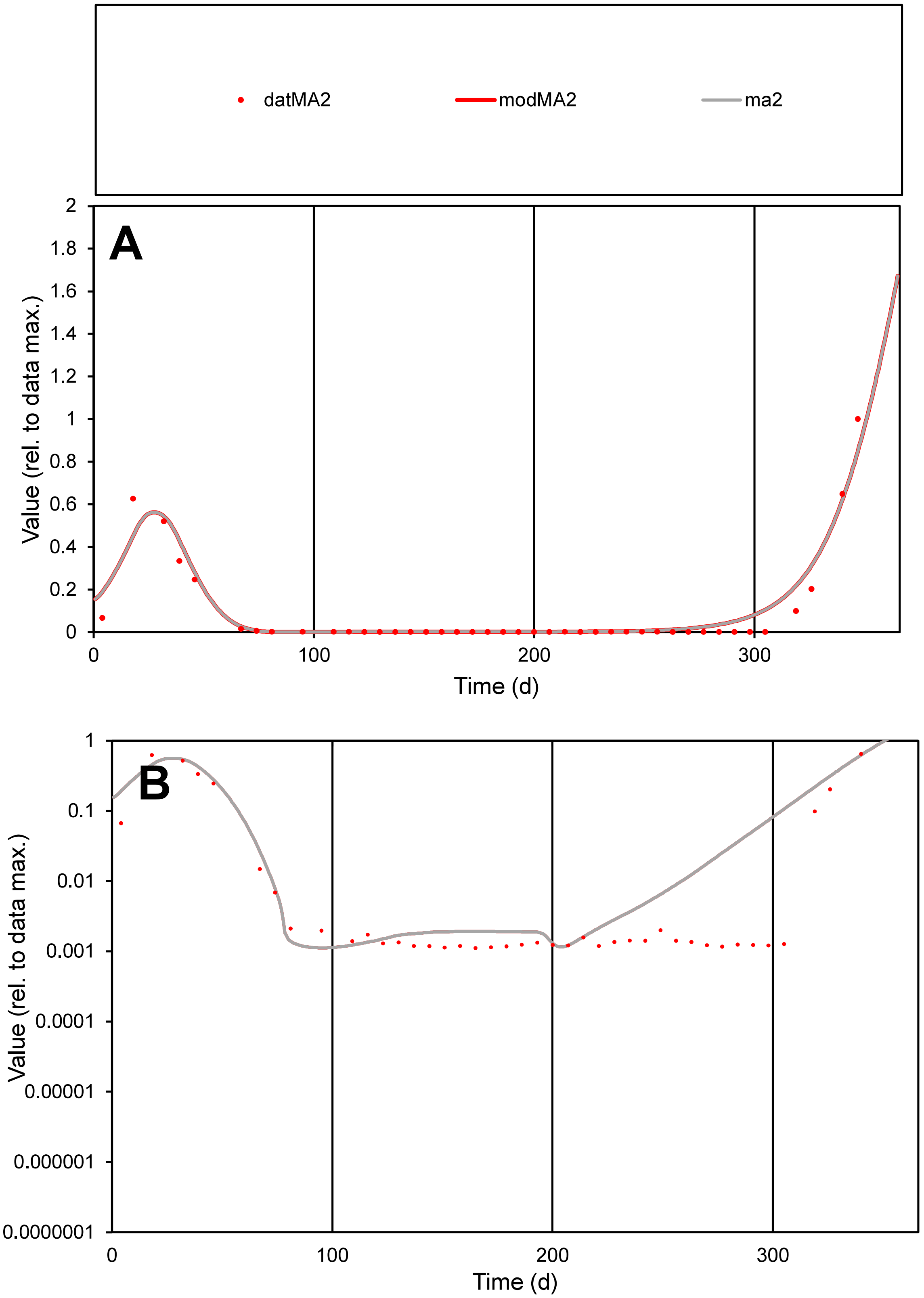

C1

Variables		
MA2	Provide jtd or Dst	
CalibOnly?		
2	1 to reproduce of	
oNormalize?		
2	1=no, 2=yes	
ofxn calc*		
0.53154		
ofxn mod		
0.62505		
sRun		
RunA1		
zout		
1		
Ave		
Data		
0.00144		
Model		
0.00164		
data/vMax		
0.01586		
ofxng		
334.07		
Min d/m		
0.99775		
ave/min d		
81.1841		
ave/min m		
92.5872		

327_Calib_S_A

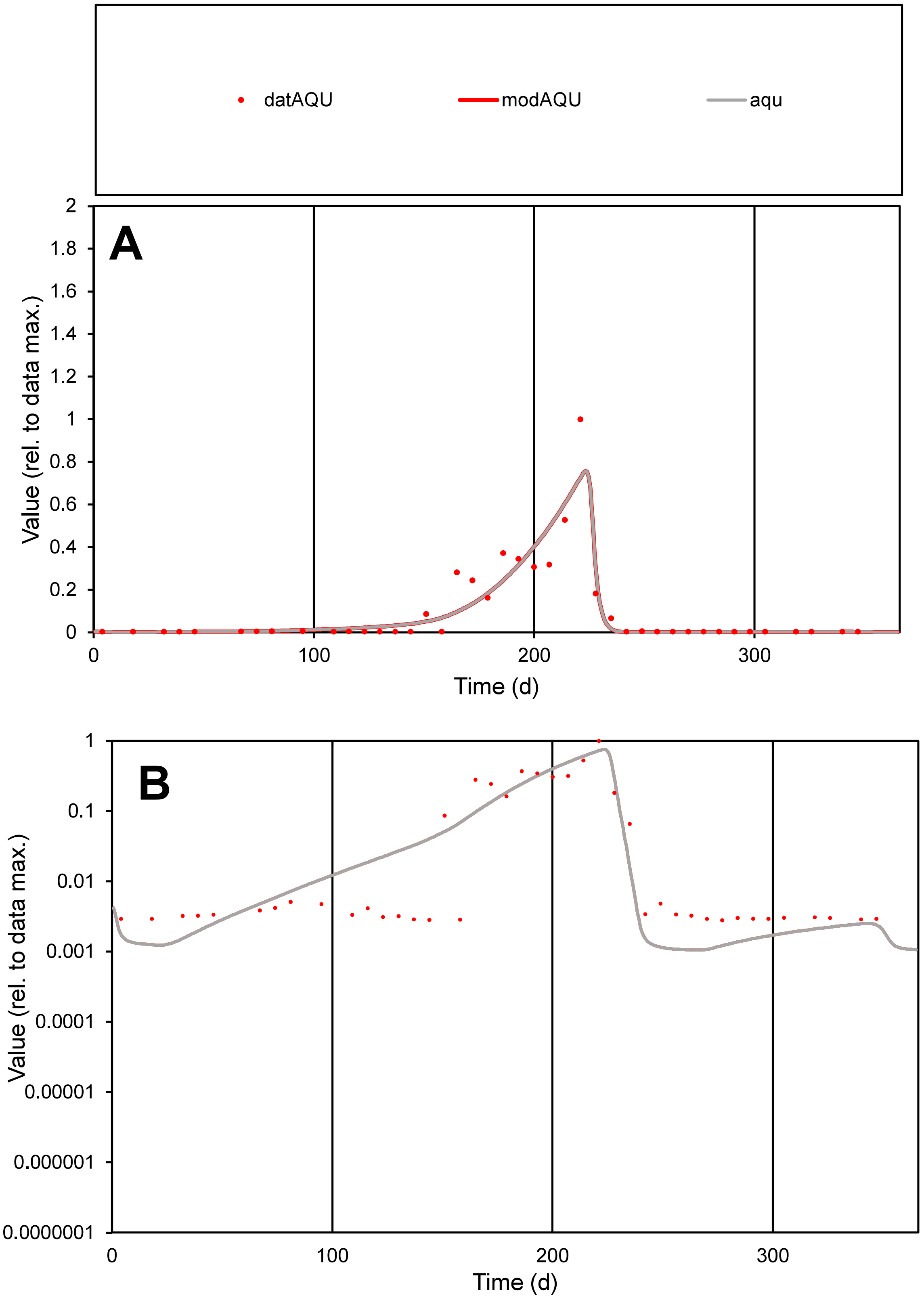

C1

Variables
AQU Provide jtd or Dst
CalibOnly?
2 1 to reproduce of
oNormalize?
2 1=no, 2=yes

ofxn calc*
0.69313
ofxn mod
0.74669

sRun
RunA1
zout
1

Ave
Data
0.00458
Model
0.00446

data/vMax
0.04823

ofxng
334.07

Min d/m
2.66653

ave/min d
33.9598
ave/min m
88.0776

327_Calib_S_A

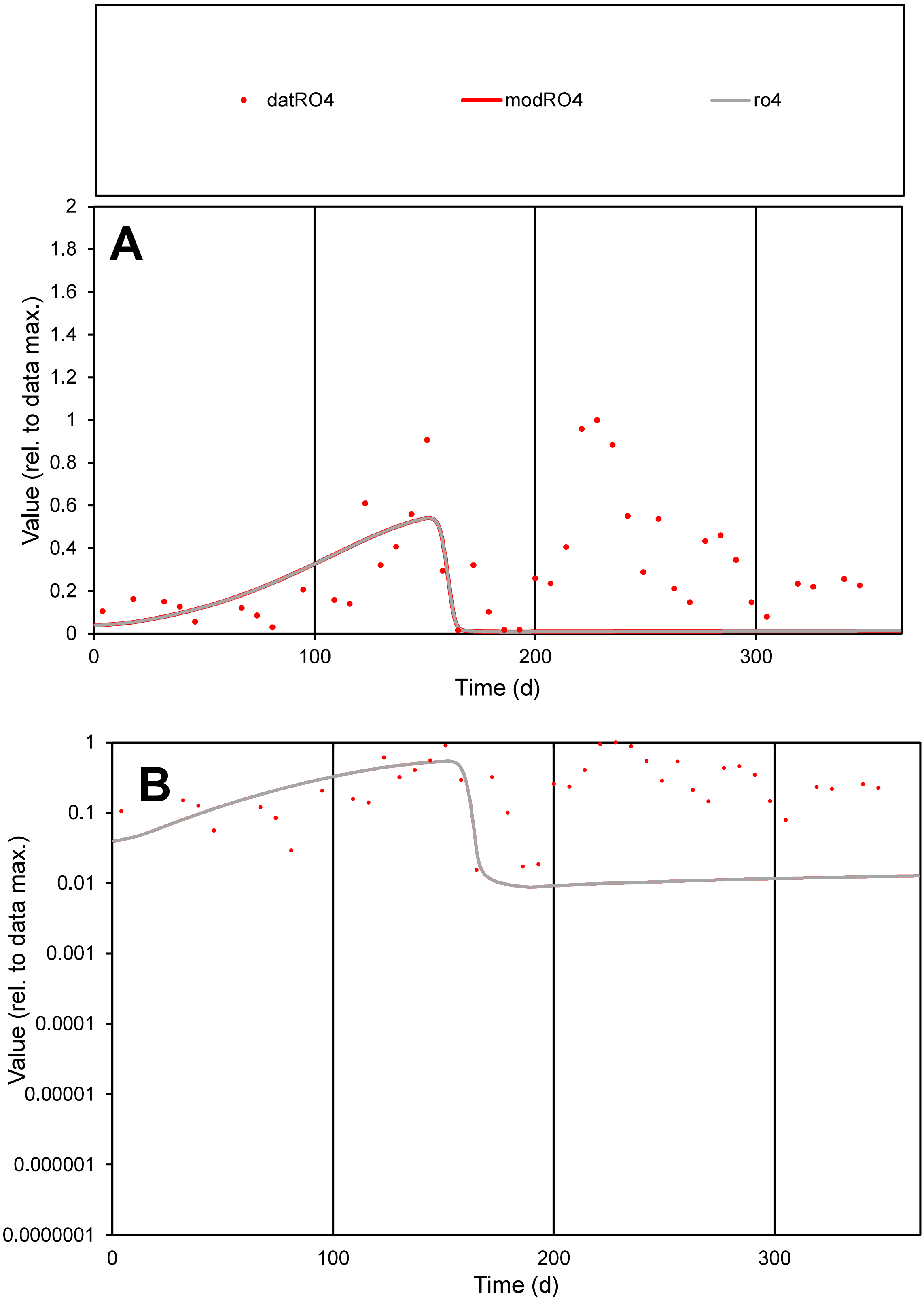

C1

Variables

RO4 Provide jtd or Dst

CalibOnly?

2 1 to reproduce of

oNormalize?

2 1=no, 2=yes

ofxn calc*

1.11635

ofxn mod

1.12055

sRun

RunA1

zout

1

Ave

Data

0.00424

Model

0.00174

data/vMax

0.01392

ofxng

334.07

Min d/m

1.75223

ave/min d

19.6386

ave/min m

14.127

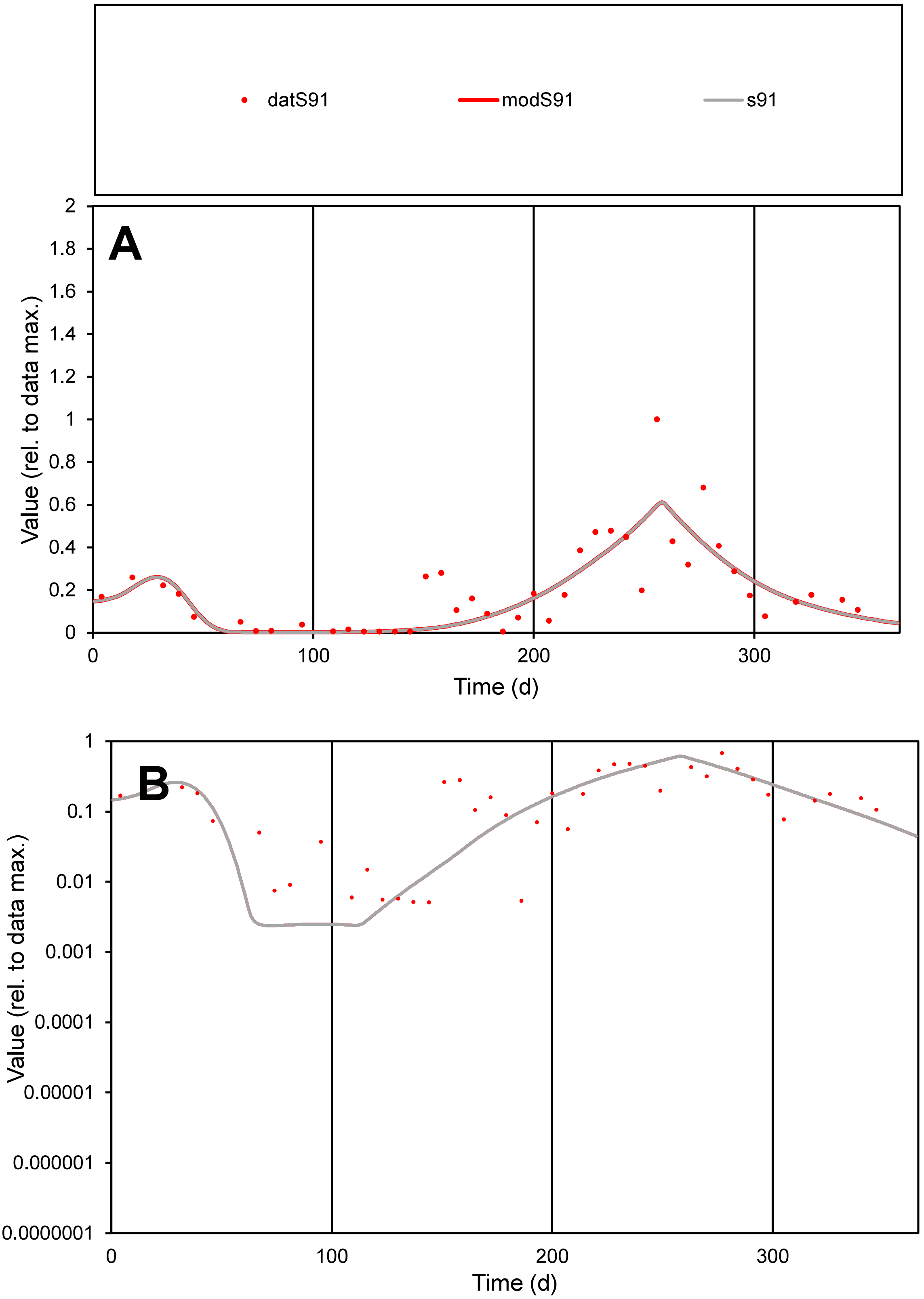

C1

Variables
S91 Provide jtd or Dst
CalibOnly?
2 1 to reproduce of
oNormalize?
2 1=no, 2=yes

ofxn calc*
0.60885
ofxn mod
0.63291

sRun
RunA1
zout
1

Ave
Data
0.0027
Model
0.00246

data/vMax
0.01353

ofxng
334.07

Min d/m
2.13199

ave/min d
39.1232
ave/min m
76.1248

327_Calib_S_A

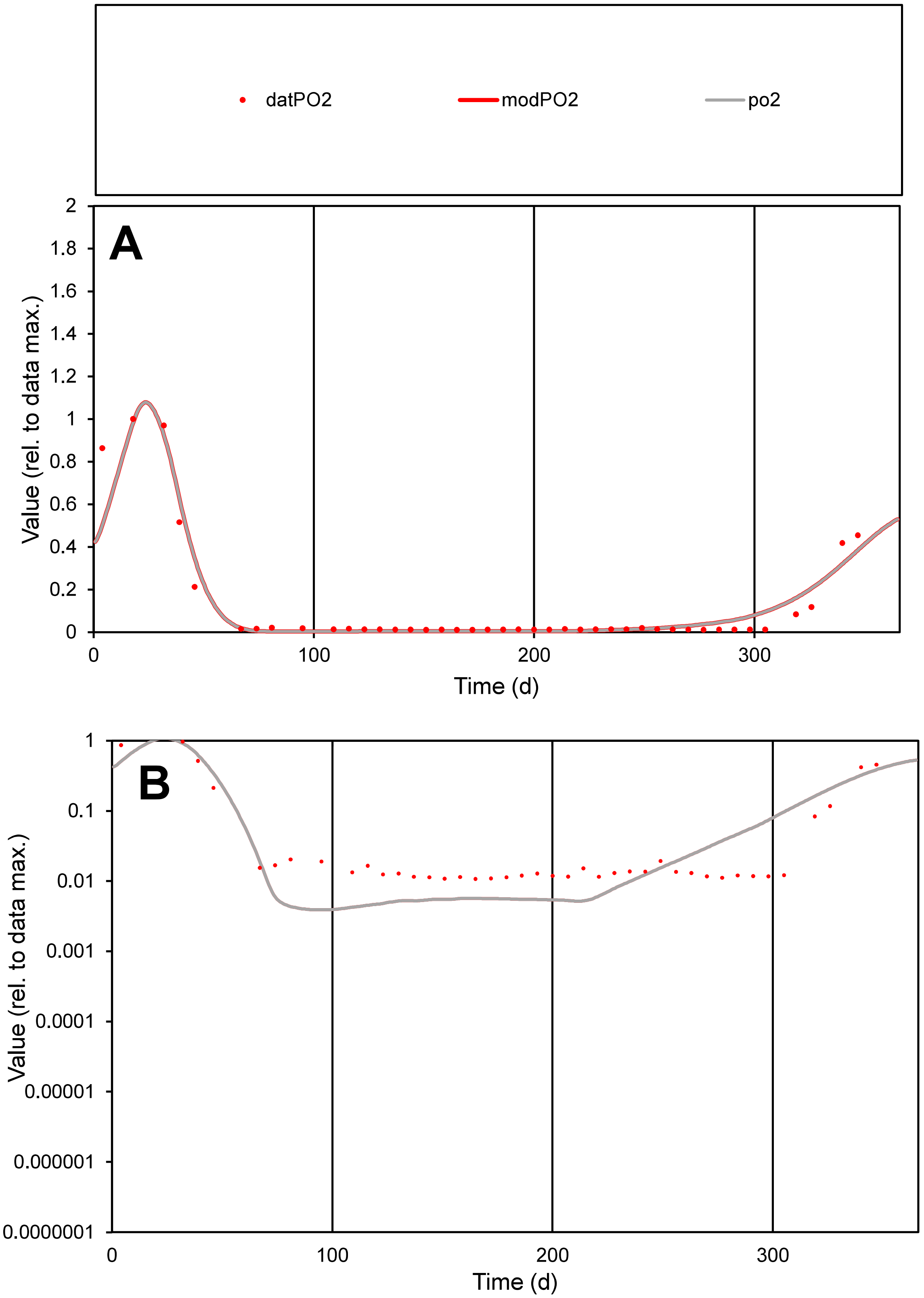

C1

Variables
PO2 Provide jtd or Dst
CalibOnly?
2 1 to reproduce of
oNormalize?
2 1=no, 2=yes

ofxn calc*
0.51743
ofxn mod
0.59973

sRun
RunA1
zout
1

Ave
Data
0.00089
Model
0.00087

data/vMax
0.00735

ofxng
334.07

Min d/m
2.74956

ave/min d
11.259
ave/min m
30.3477

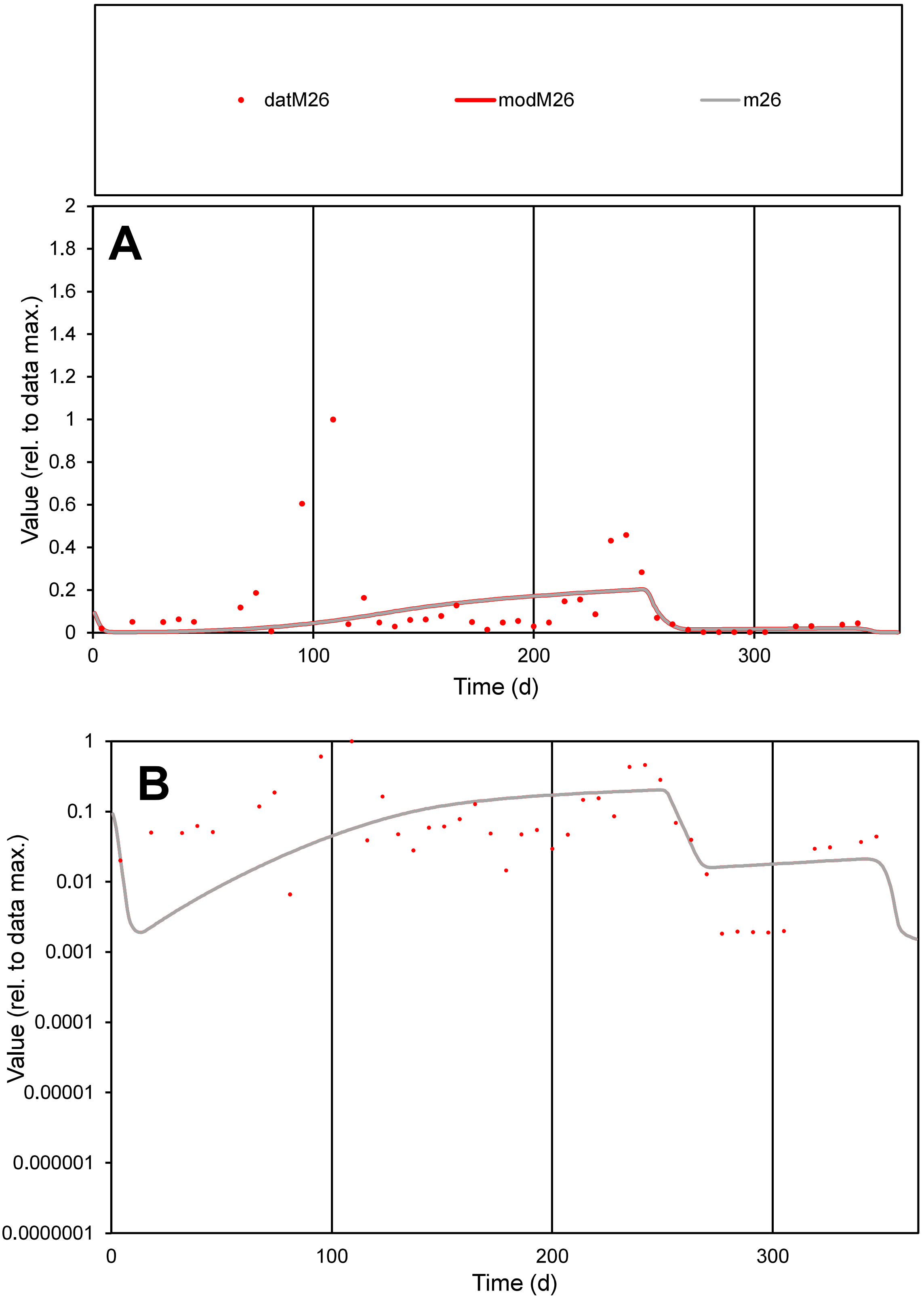

C1

Variables
M26 Provide jtd or Dst
CalibOnly?
2 1 to reproduce of
oNormalize?
2 1=no, 2=yes

ofxn calc*
1.65127
ofxn mod
1.65813

sRun
RunA1
zout
1

Ave
Data
0.00534
Model
0.00386

data/vMax
0.04647

ofxng
334.07

Min d/m
0.74634

ave/min d
63.0322
ave/min m
33.9797

327_Calib_S_A

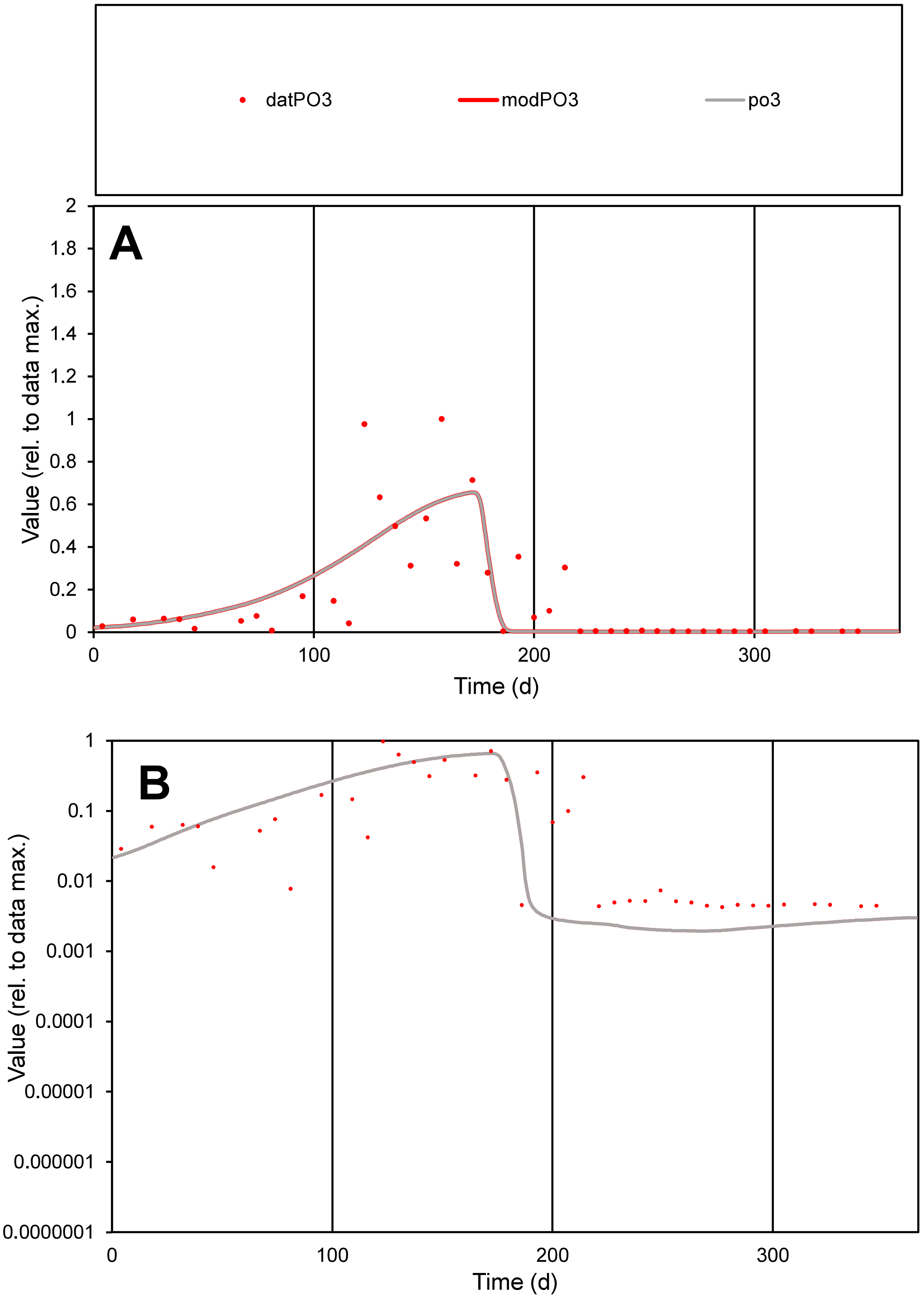

C1

Variables
PO3 Provide jtd or Dst
CalibOnly?
2 1 to reproduce of
oNormalize?
2 1=no, 2=yes

ofxn calc*
0.96876
ofxn mod
0.98504

sRun
RunA1
zout
1

Ave
Data
0.00185
Model
0.00173

data/vMax
0.01127

ofxng
334.07

Min d/m
2.2011

ave/min d
38.4542
ave/min m
79.1829

327_Calib_S_A

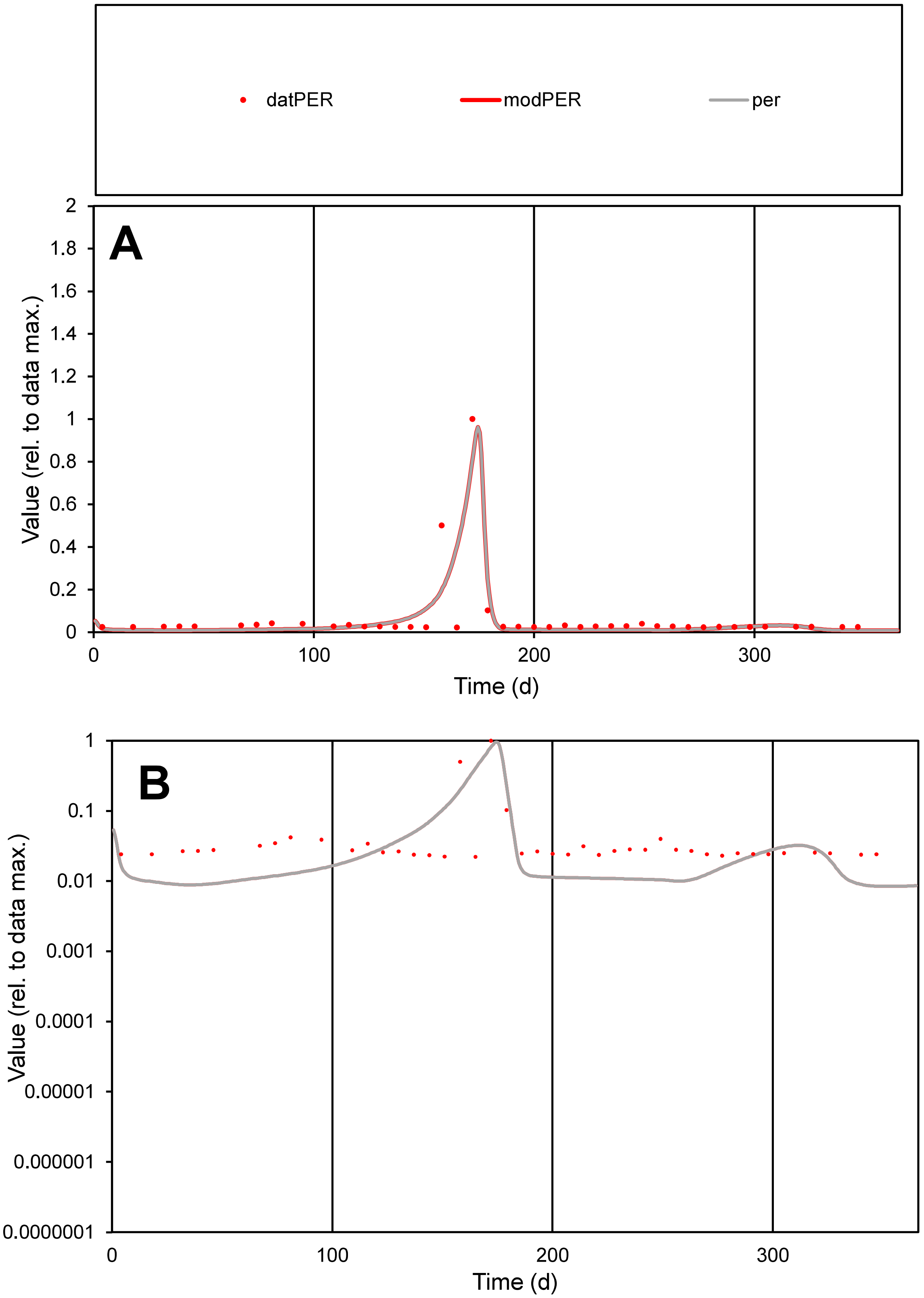

C1

Variables
PER Provide jtd or Dst
CalibOnly?
2 1 to reproduce of
oNormalize?
2 1=no, 2=yes

ofxn calc*
1.12855
ofxn mod
1.21628

sRun
RunA1
zout
1

Ave
Data
5.2E-05
Model
4.6E-05

data/vMax
0.00082

ofxng
334.07

Min d/m
2.62245

ave/min d
2.86232
ave/min m
6.67494

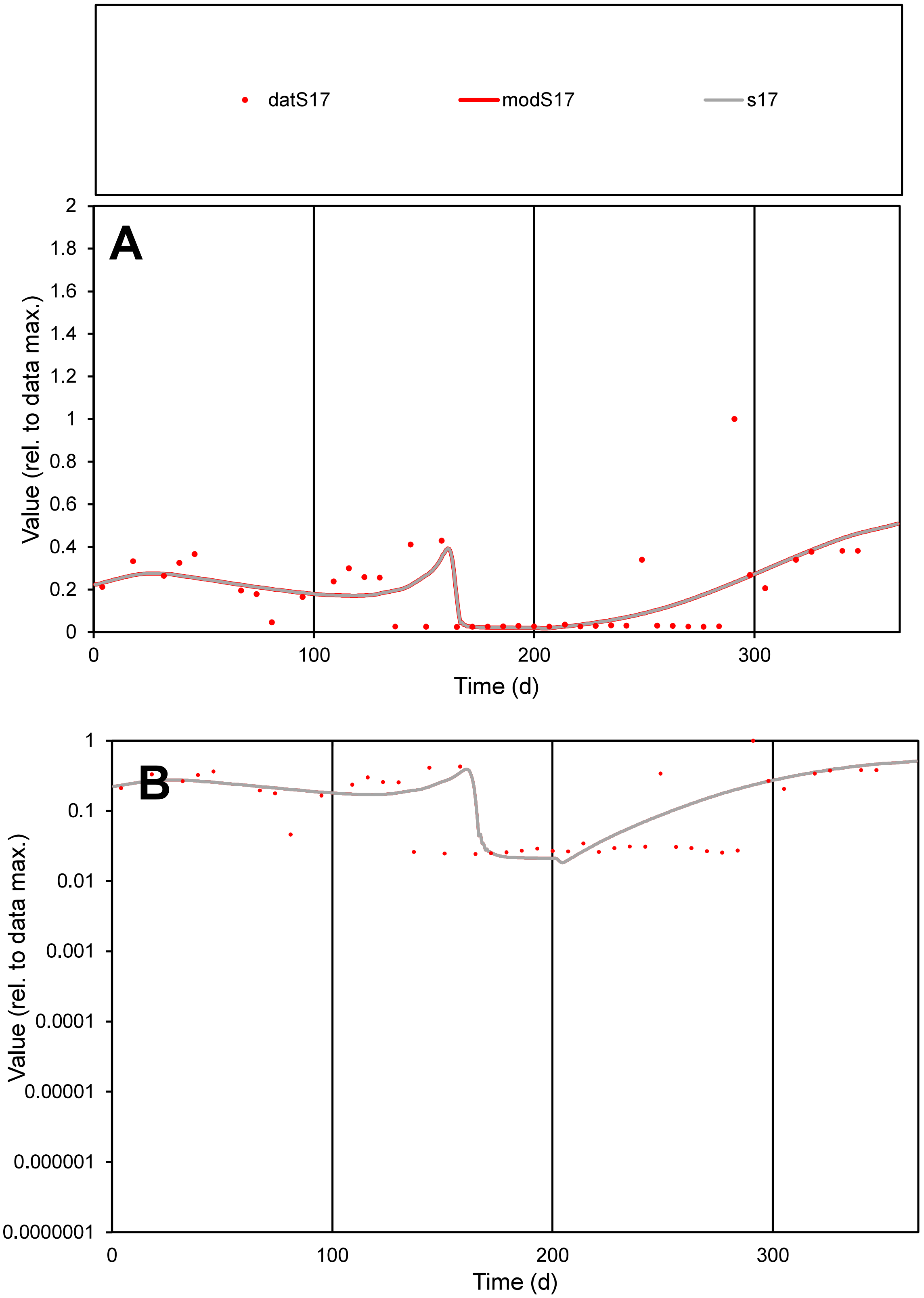

C1

Variables
S17 Provide jtd or Dst
CalibOnly?
2 1 to reproduce of
oNormalize?
2 1=no, 2=yes

ofxn calc*
0.81221
ofxn mod
0.8247

sRun
RunA1
zout
1

Ave
Data
0.00082
Model
0.0008

data/vMax
0.00443

ofxng
334.07

Min d/m
1.15872

ave/min d
7.61331
ave/min m
8.63274

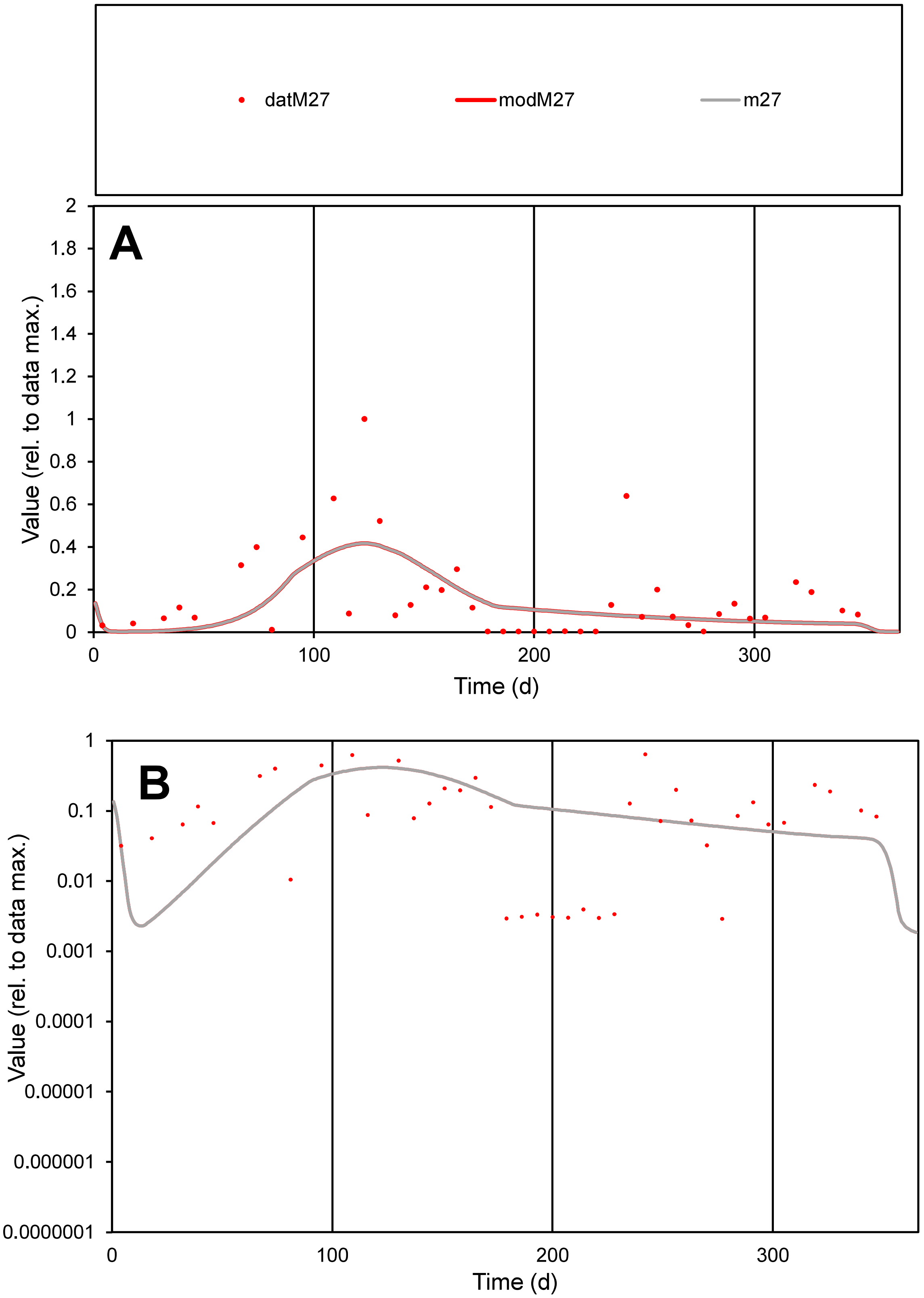

C1

Variables
M27 Provide jtd or Dst
CalibOnly?
2 1 to reproduce of
oNormalize?
2 1=no, 2=yes

ofxn calc*
1.09416
ofxn mod
1.10517

sRun
RunA1
zout
1

Ave
Data
0.00379
Model
0.00311

data/vMax
0.02315

ofxng
334.07

Min d/m
0.94163

ave/min d
56.3442
ave/min m
43.4848

327_Calib_S_A

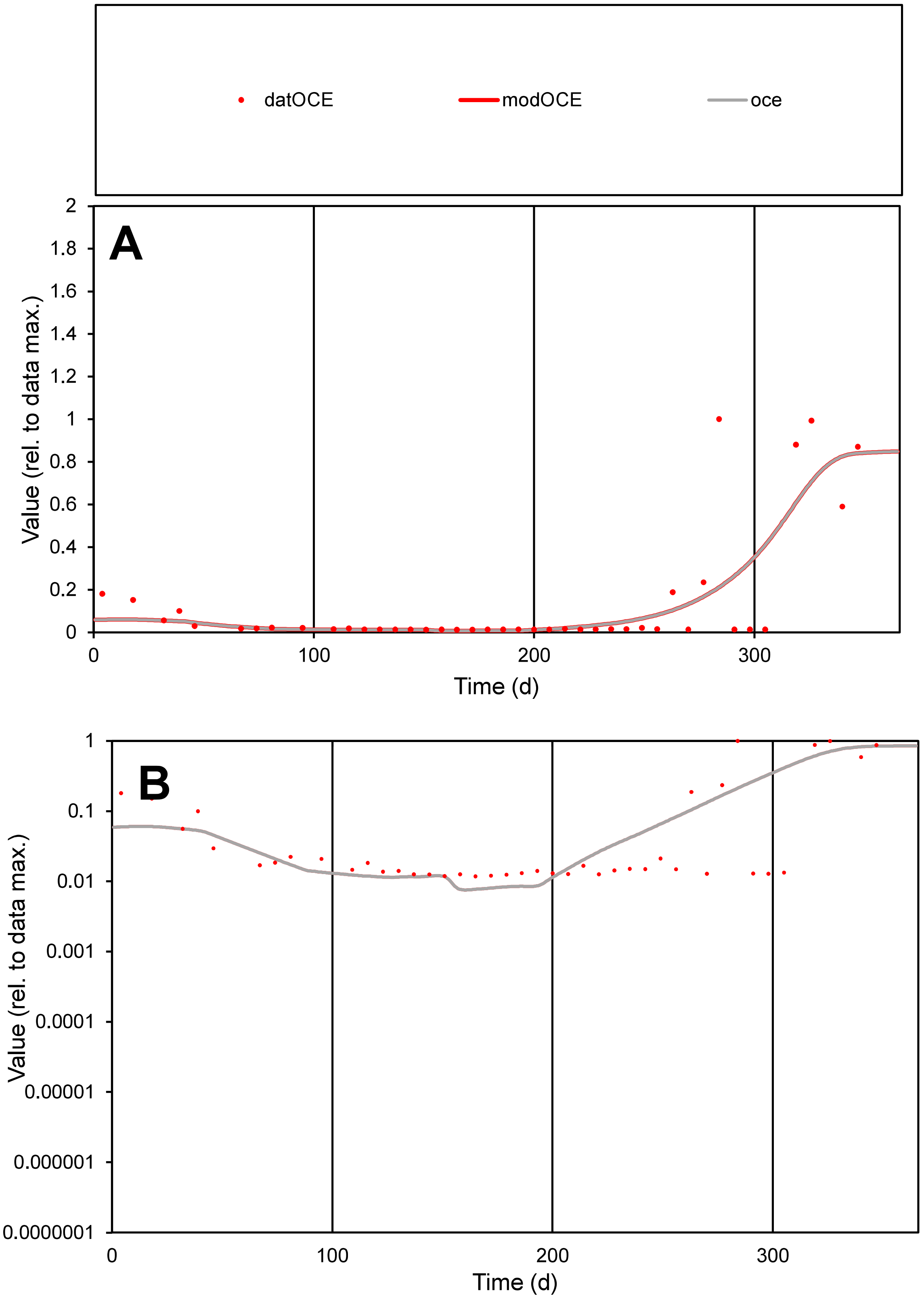

C1

Variables

OCE Provide jtd or Dst

CalibOnly?

2 1 to reproduce of

oNormalize?

2 1=no, 2=yes

ofxn calc*

1.22928

ofxn mod

1.25459

sRun

RunA1

zout

1

Ave

Data

0.00042

Model

0.00041

data/vMax

0.00312

ofxng

334.07

Min d/m

1.56064

ave/min d

11.5448

ave/min m

17.2737

327_Calib_S_A

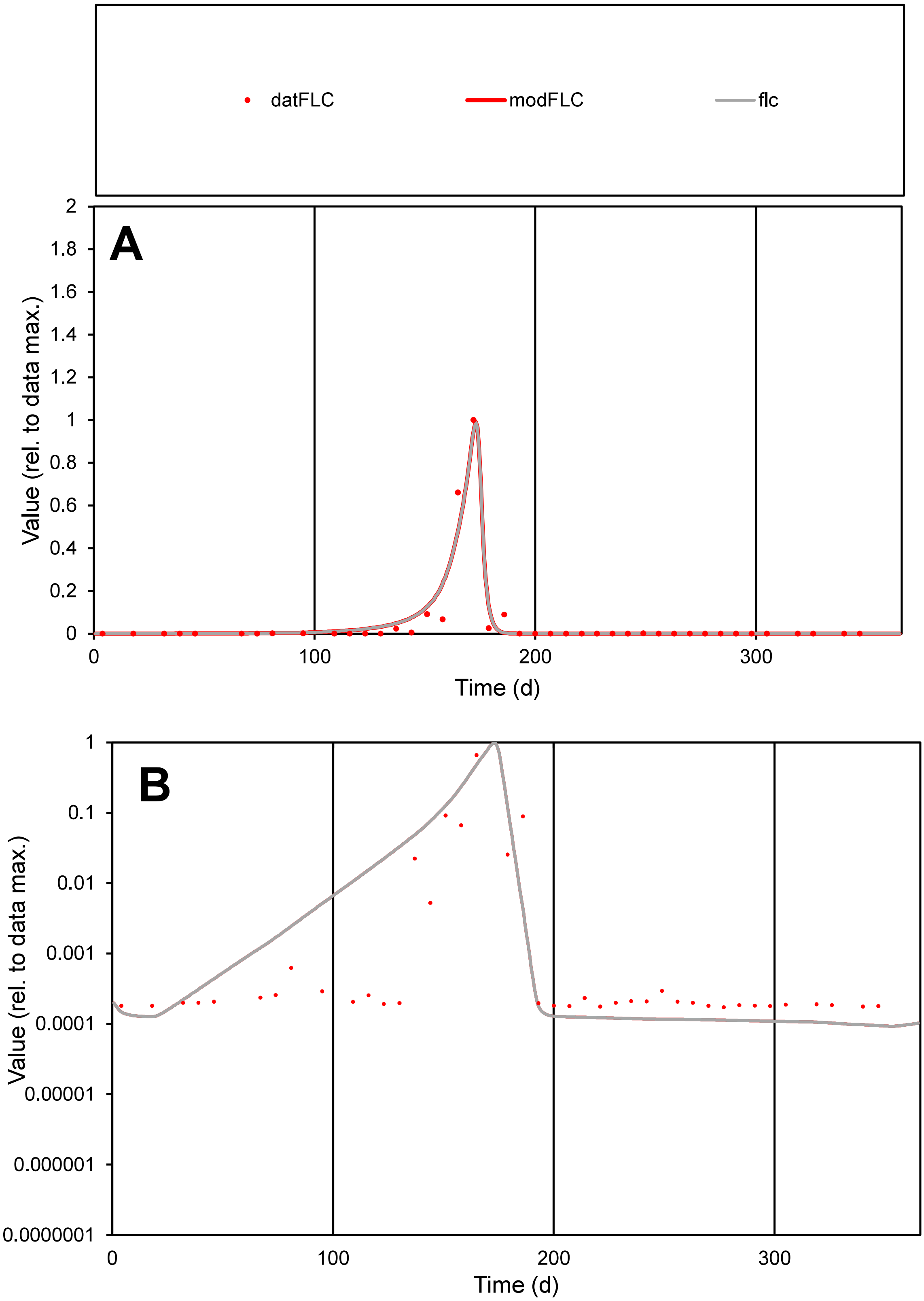

C1

Variables
FLC Provide jtd or Dst
CalibOnly?
2 1 to reproduce of
oNormalize?
2 1=no, 2=yes

ofxn calc*
0.71606
ofxn mod
0.8448

sRun
RunA1
zout
1

Ave
Data
0.00339
Model
0.00371

data/vMax
0.07236

ofxng
334.07

Min d/m
1.83047

ave/min d
272.041
ave/min m
545.369

327_Calib_S_A

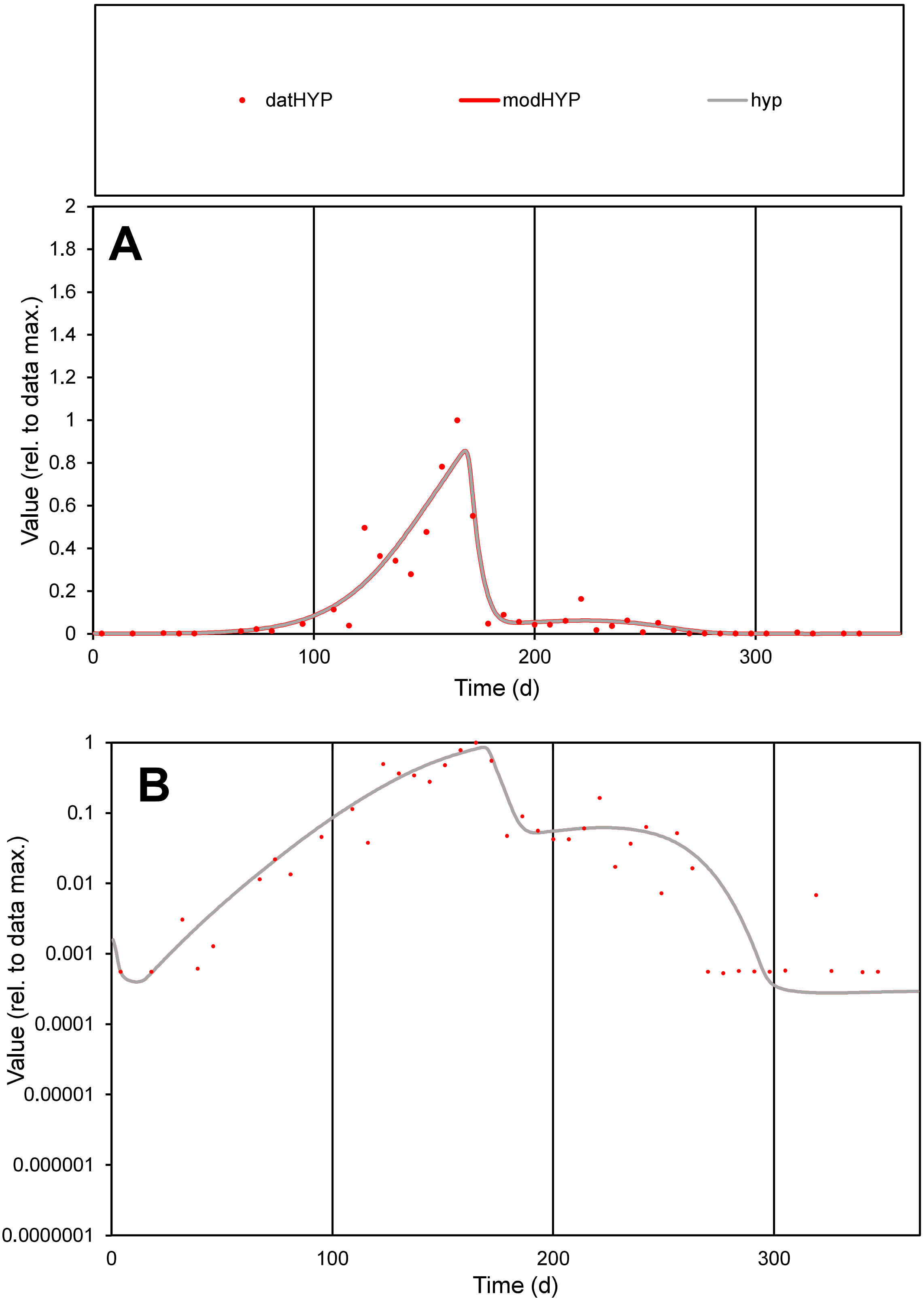

C1

Variables
HYP Provide jtd or Dst
CalibOnly?
2 1 to reproduce of
oNormalize?
2 1=no, 2=yes

ofxn calc*
0.56268
ofxn mod
0.61092

sRun
RunA1
zout
1

Ave
Data
0.00387
Model
0.00402

data/vMax
0.03095

ofxng
334.07

Min d/m
1.90868

ave/min d
234.709
ave/min m
465.884

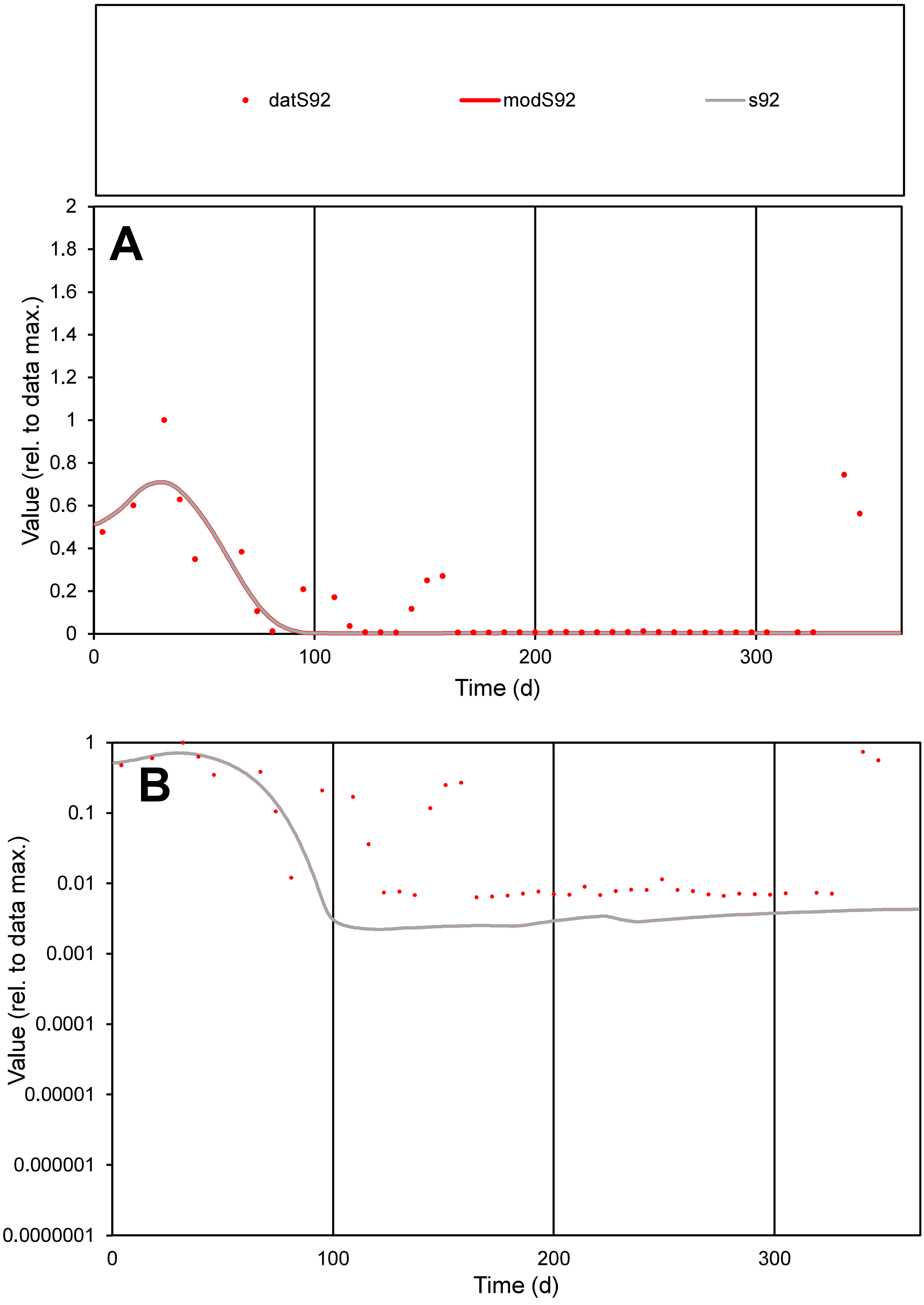

C1

Variables
S92 Provide jtd or Dst
CalibOnly?
2 1 to reproduce of
oNormalize?
2 1=no, 2=yes

ofxn calc*
1.18275
ofxn mod
1.20267

sRun
RunA1
zout
1

Ave
Data
0.00053
Model
0.00031

data/vMax
0.00361

ofxng
334.07

Min d/m
2.8575

ave/min d
22.7769
ave/min m
38.813

327_Calib_S_A

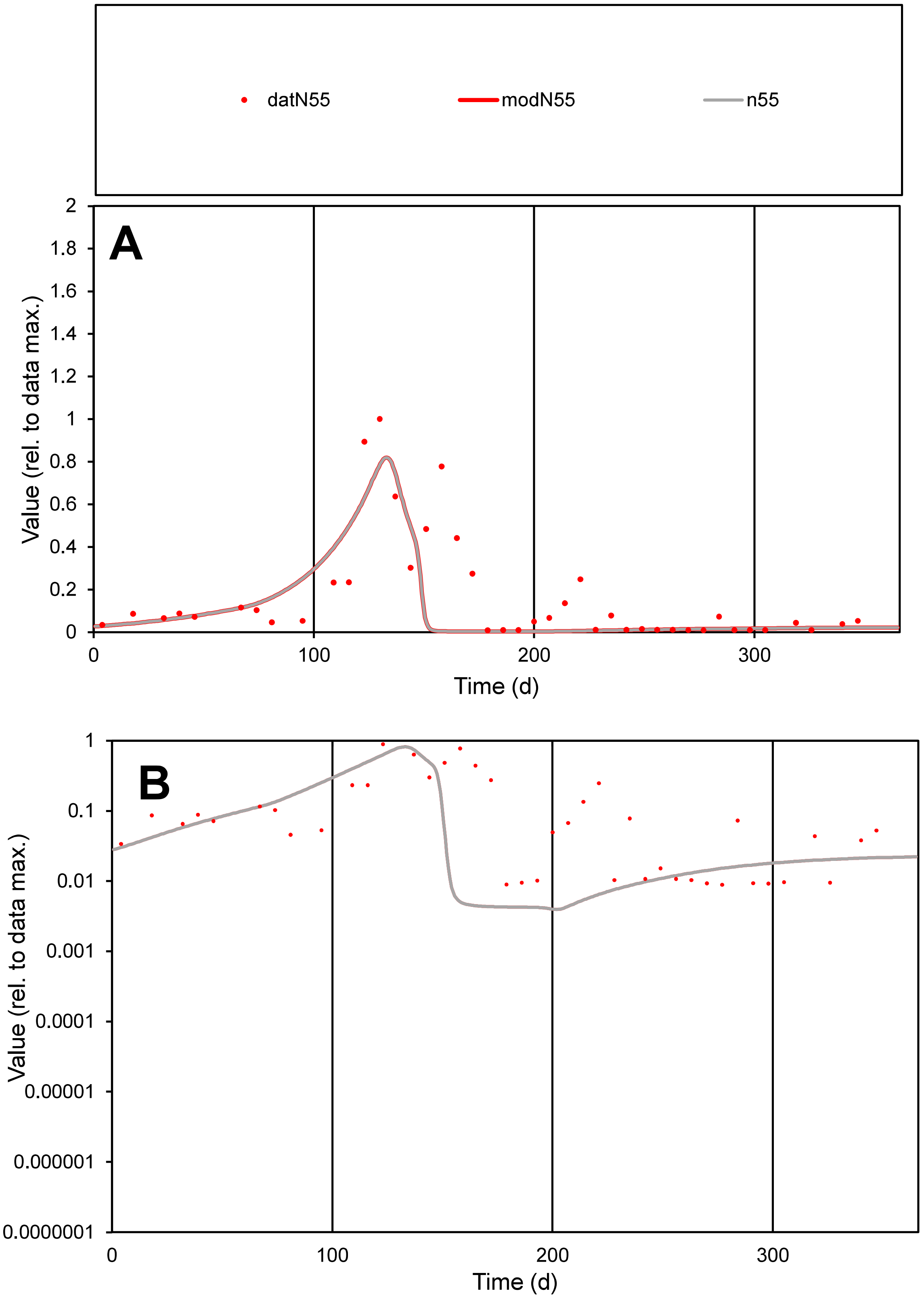

C1

Variables

N55 Provide jtd or Dst

CalibOnly?

2 1 to reproduce of

oNormalize?

2 1=no, 2=yes

ofxn calc*

1.06663

ofxn mod

1.11632

sRun

RunA1

zout

1

Ave

Data

0.00071

Model

0.00052

data/vMax

0.00435

ofxng

334.07

Min d/m

2.23006

ave/min d

18.426

ave/min m

30.1969

327_Calib_S_A

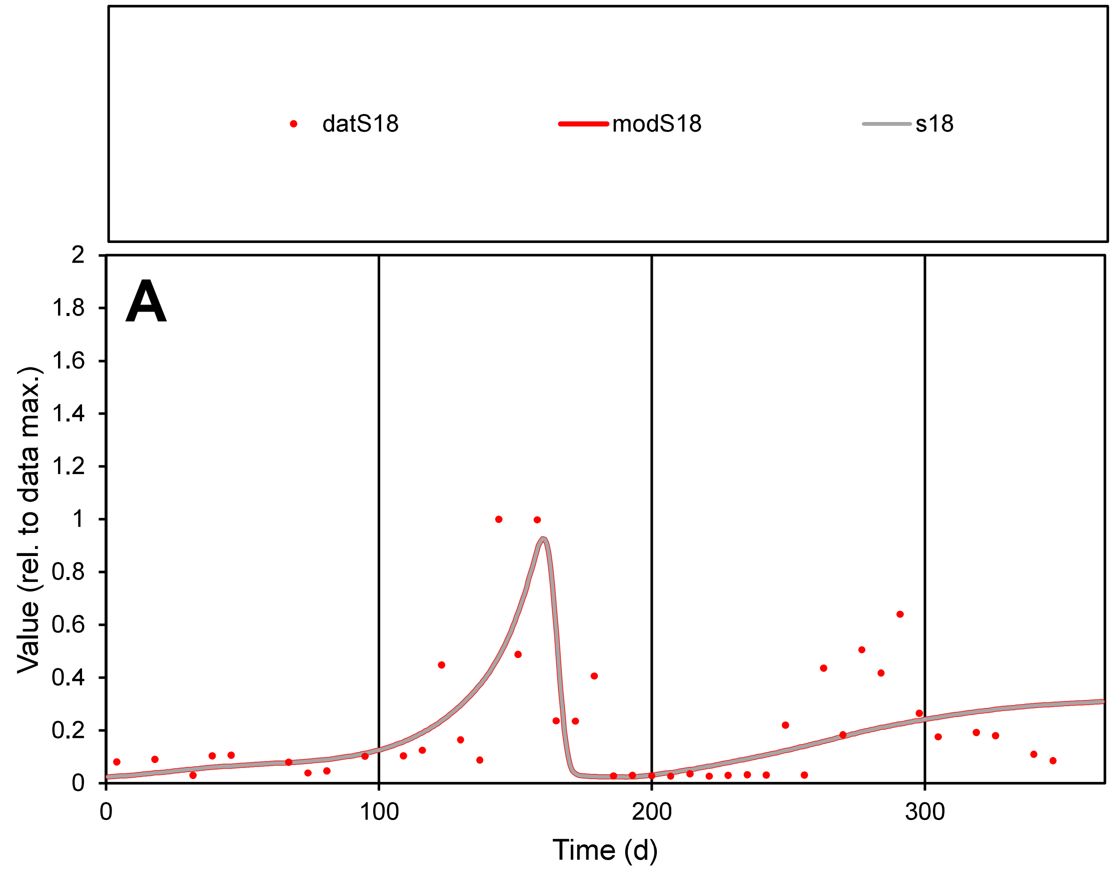

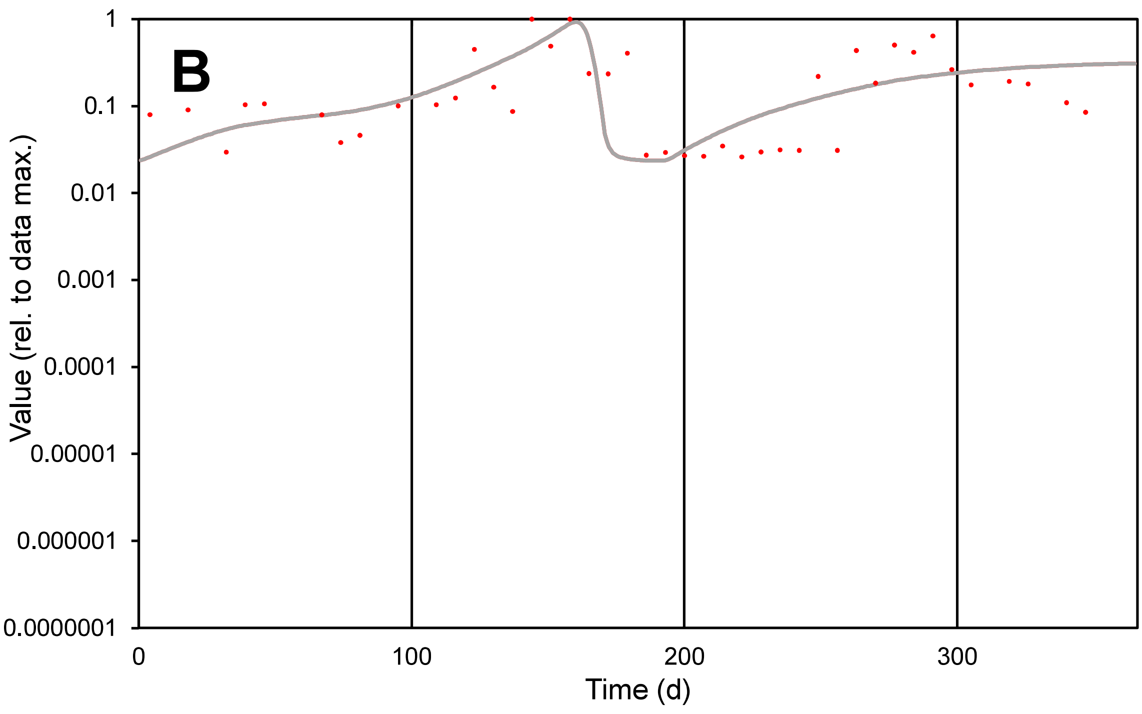

C1

Variables
S18 Provide jtd or Dst
CalibOnly?
2 1 to reproduce of
oNormalize?
2 1=no, 2=yes

ofxn calc*
0.79491
ofxn mod
0.82099

sRun
RunA1
zout
1

Ave
Data
0.00222
Model
0.00206

data/vMax
0.01079

ofxng
334.07

Min d/m
1.10958

ave/min d
7.87563
ave/min m
8.07162

327_Calib_S_A

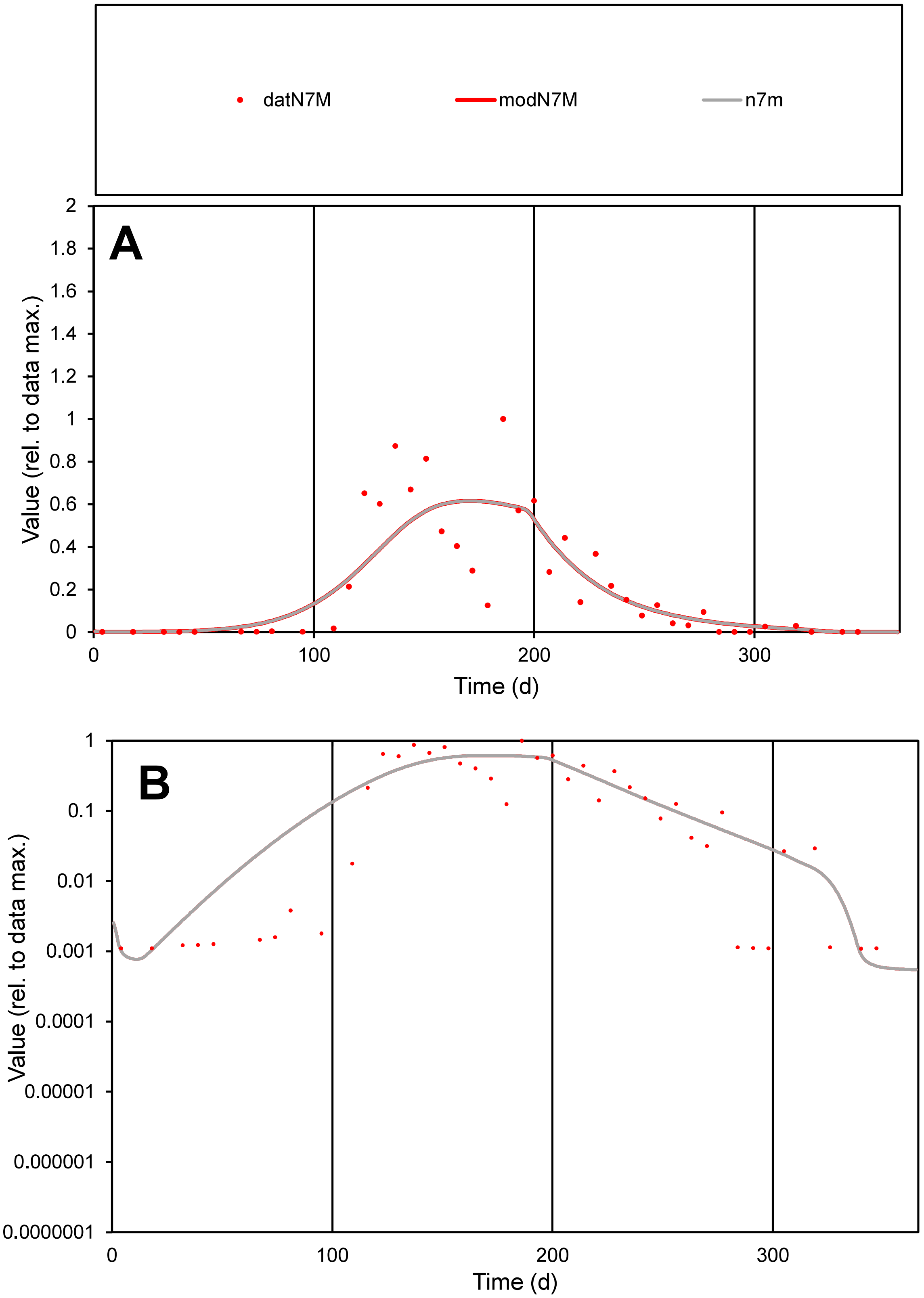

C1

Variables
N7M Provide jtd or Dst
CalibOnly?
2 1 to reproduce of
oNormalize?
2 1=no, 2=yes

ofxn calc*
0.71706
ofxn mod
0.73604

sRun
RunA1
zout
1

Ave
Data
0.0021
Model
0.00207

data/vMax
0.00941

ofxng
334.07

Min d/m
1.80409

ave/min d
205.132
ave/min m
365.369

327_Calib_S_A

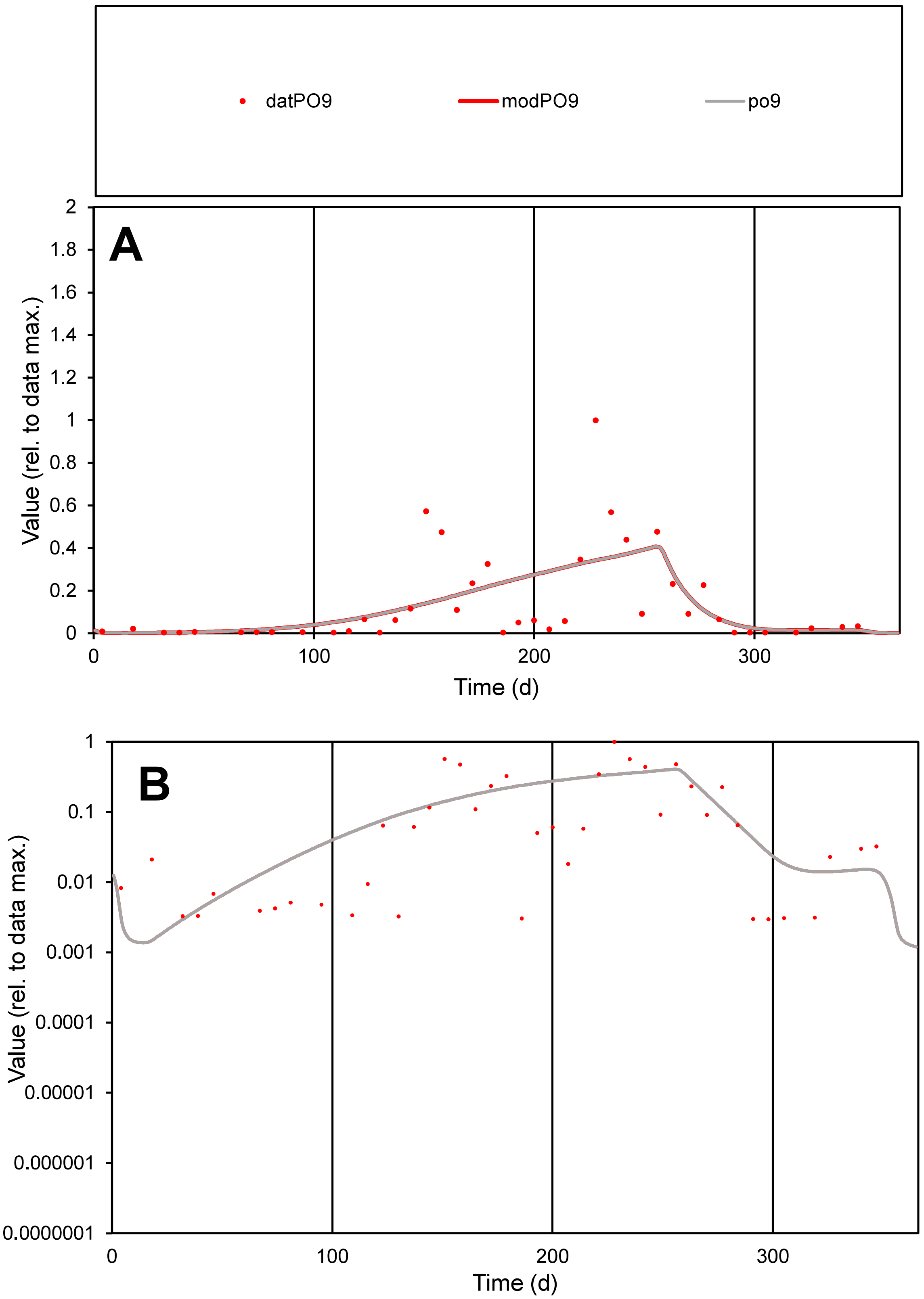

C1

Variables
PO9 Provide jtd or Dst
CalibOnly?
2 1 to reproduce of
oNormalize?
2 1=no, 2=yes

ofxn calc*
1.20948
ofxn mod
1.22316

sRun
RunA1
zout
1

Ave
Data
0.00297
Model
0.00297

data/vMax
0.02128

ofxng
334.07

Min d/m
1.85643

ave/min d
47.1241
ave/min m
87.4716

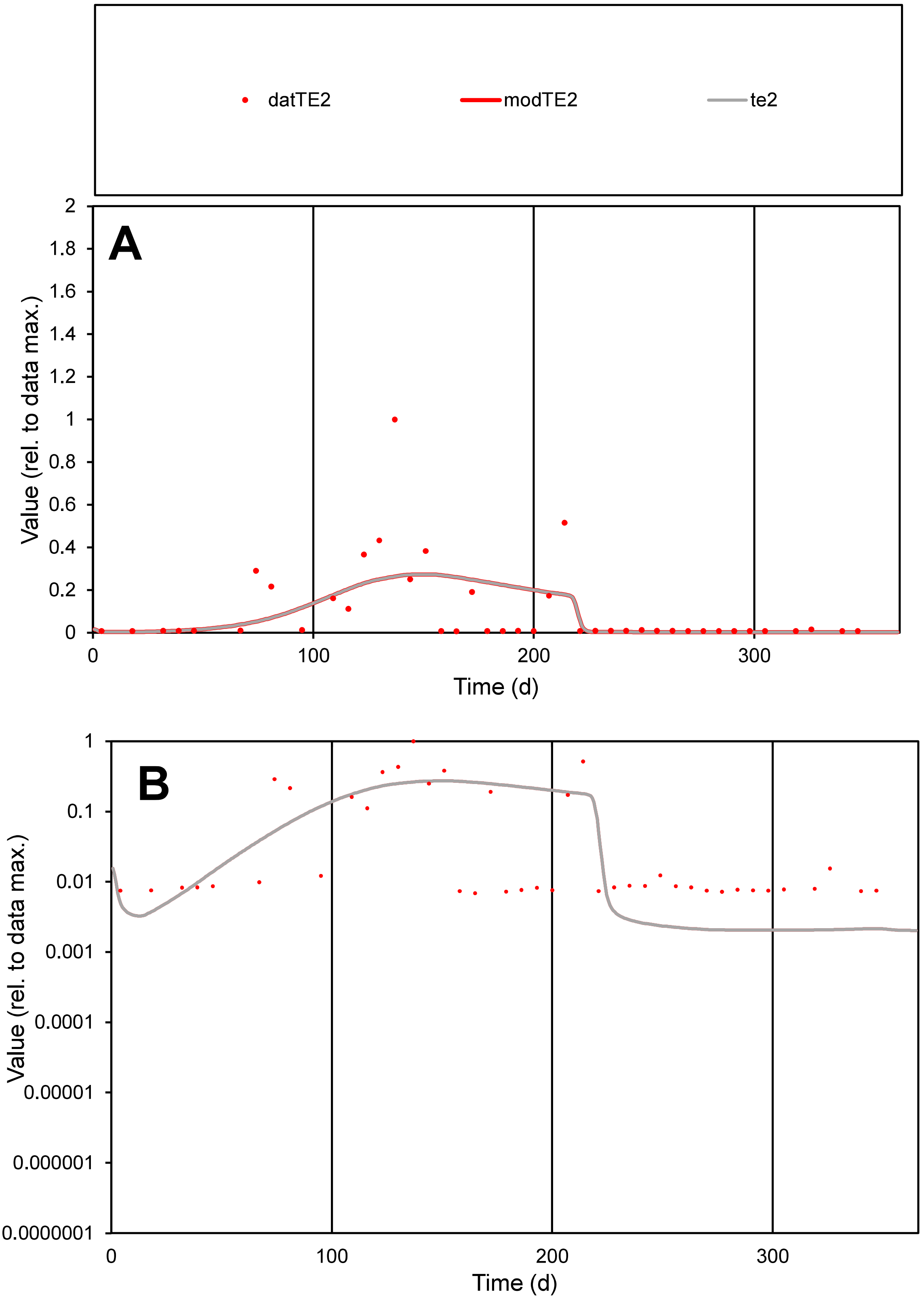

C1

Variables
TE2 Provide jtd or Dst
CalibOnly?
2 1 to reproduce of
oNormalize?
2 1=no, 2=yes

ofxn calc*
1.58025
ofxn mod
1.5882

sRun
RunA1
zout
1

Ave
Data
0.00127
Model
0.00119

data/vMax
0.01232

ofxng
334.07

Min d/m
3.34386

ave/min d
15.0383
ave/min m
47.1029

327_Calib_S_A

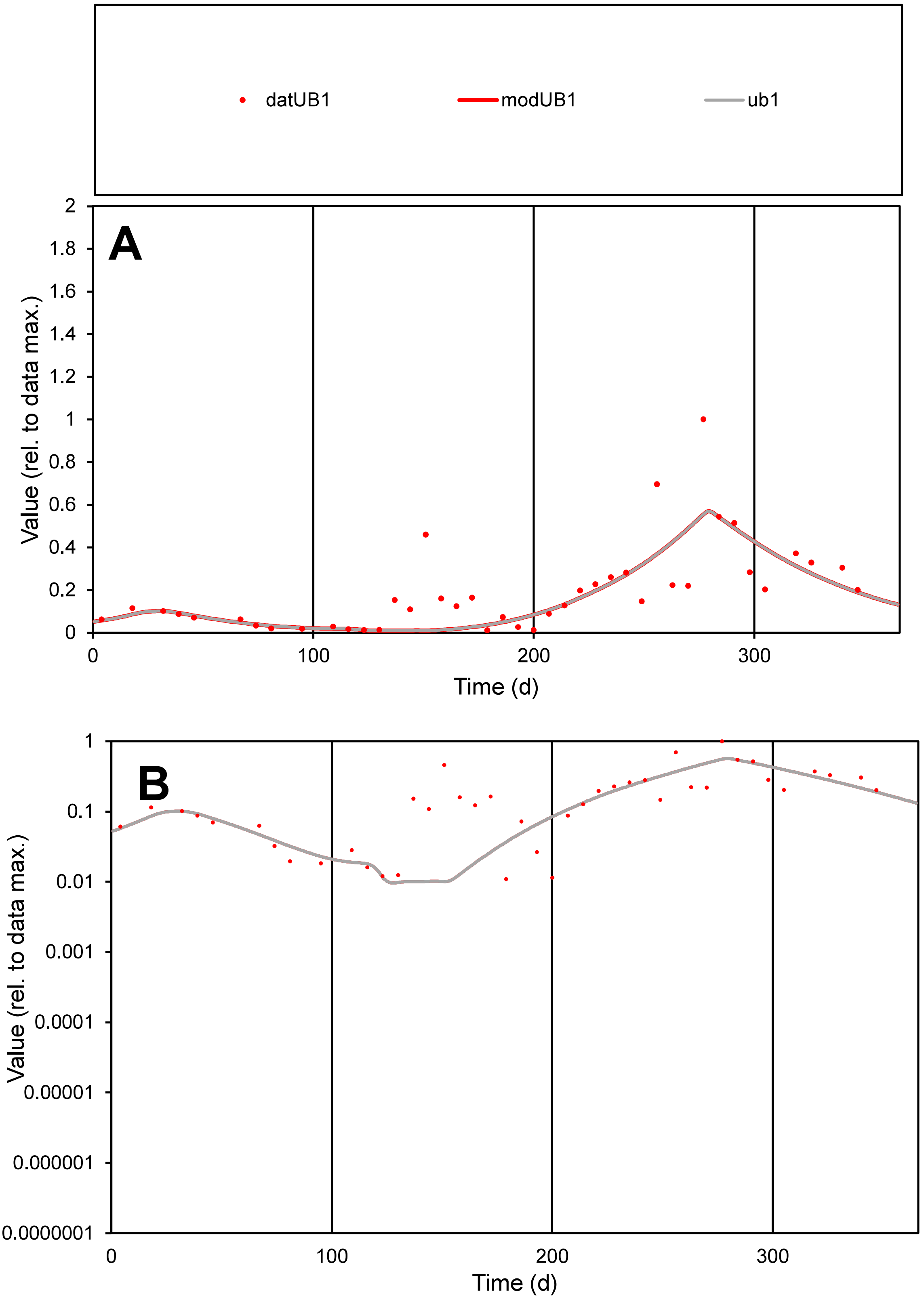

C1

Variables

UB1 Provide jtd or Dst

CalibOnly?

2 1 to reproduce of

oNormalize?

2 1=no, 2=yes

ofxn calc*

0.71321

ofxn mod

0.73116

sRun

RunA1

zout

1

Ave

Data

0.00106

Model

0.00092

data/vMax

0.00546

ofxng

334.07

Min d/m

1.10744

ave/min d

17.7151

ave/min m

17.066

327_Calib_S_A

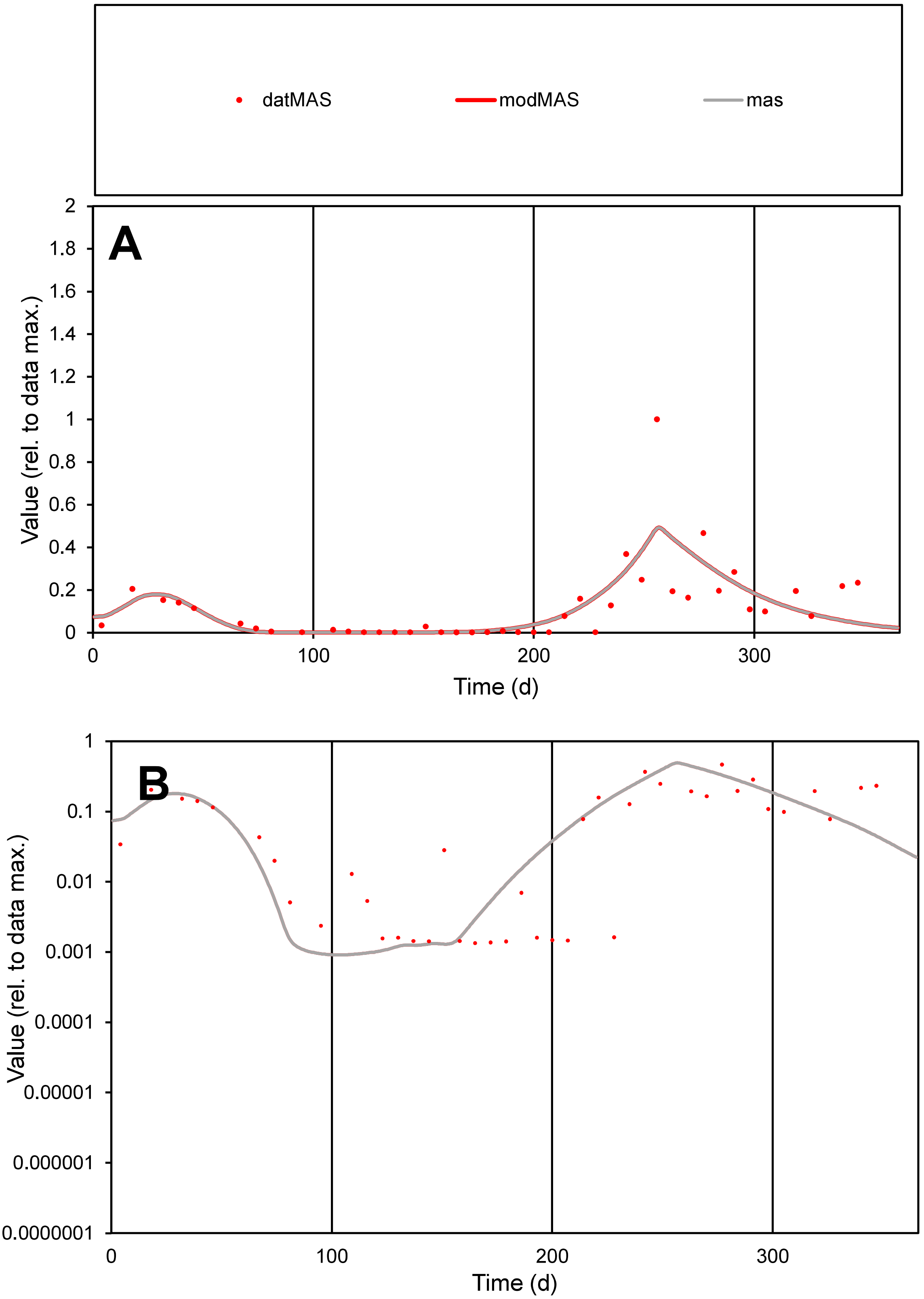

C1

Variables
MAS Provide jtd or Dst
CalibOnly?
2 1 to reproduce of
oNormalize?
2 1=no, 2=yes

ofxn calc*
0.94757
ofxn mod
0.97595

sRun
RunA1
zout
1

Ave
Data
0.00106
Model
0.00104

data/vMax
0.0089

ofxng
334.07

Min d/m
1.43562
ave/min d
88.95
ave/min m
124.867

327_Calib_S_A

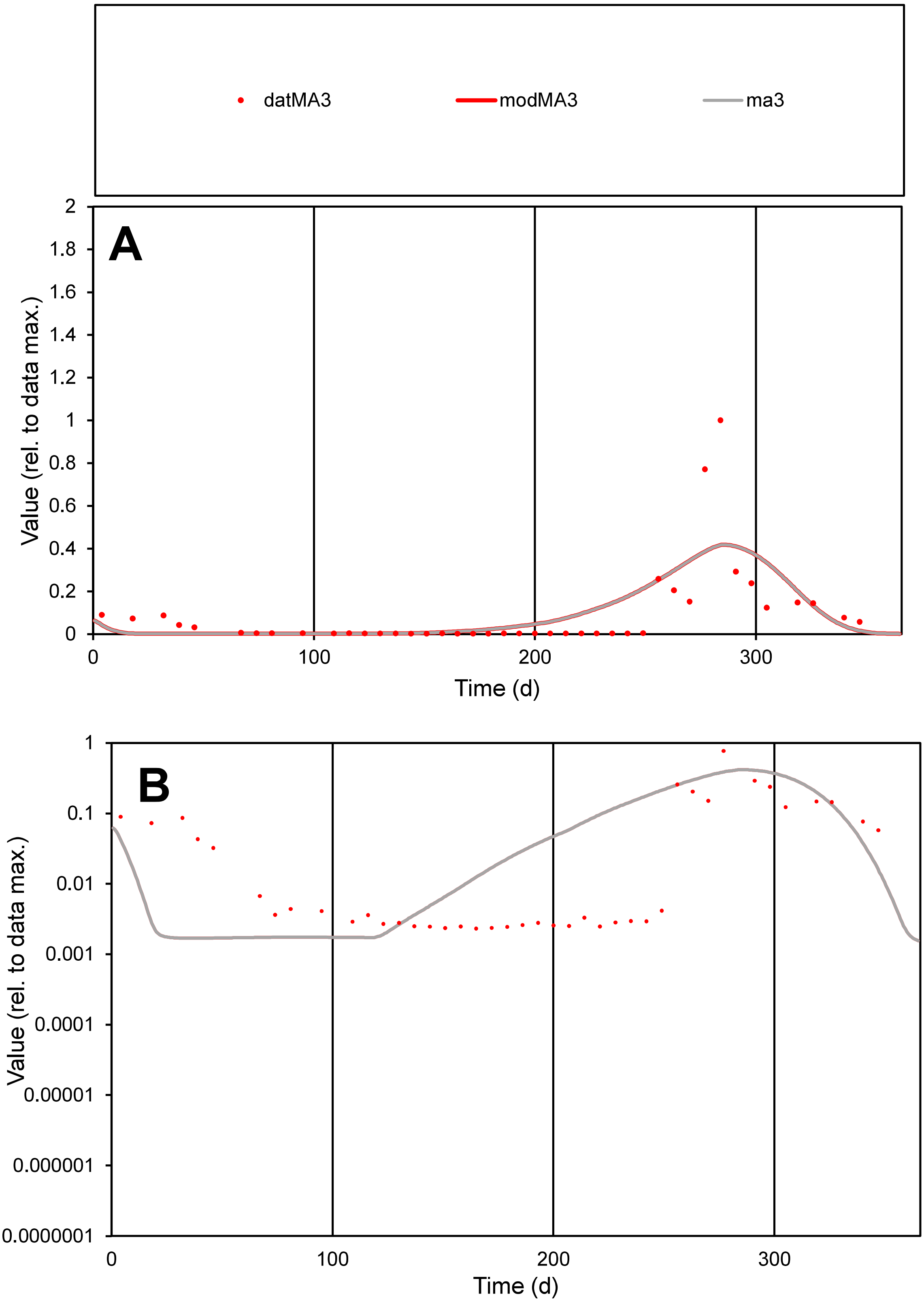

C1

Variables

MA3	Provide jtd or Dst
CalibOnly?	
2	1 to reproduce of
oNormalize?	
2	1=no, 2=yes

ofxn calc*
1.45255
ofxn mod
1.4734

sRun
RunA1
zout
1

Ave
Data
0.00243
Model
0.00272

data/vMax
0.02641

ofxng
334.07

Min d/m
1.37073

ave/min d
39.5704
ave/min m
60.648

327_Calib_S_A

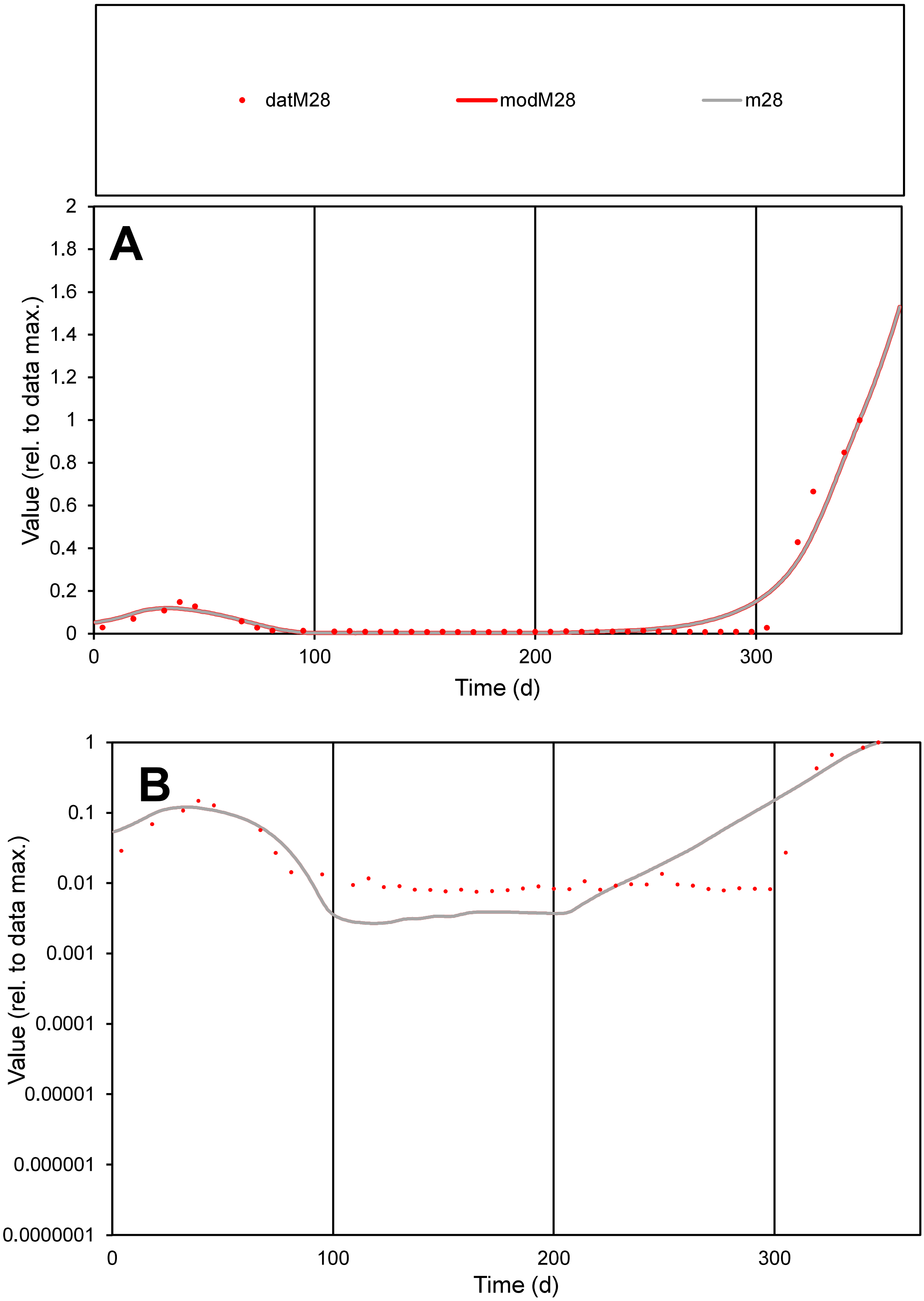

C1

Variables
M28 Provide jtd or Dst
CalibOnly?
2 1 to reproduce of
oNormalize?
2 1=no, 2=yes

ofxn calc*
0.52359
ofxn mod
0.59776

sRun
RunA1
zout
1

Ave
Data
0.00064
Model
0.0007

data/vMax
0.00707

ofxng
334.07

Min d/m
2.8156

ave/min d
12.0023
ave/min m
36.7304

327_Calib_S_A

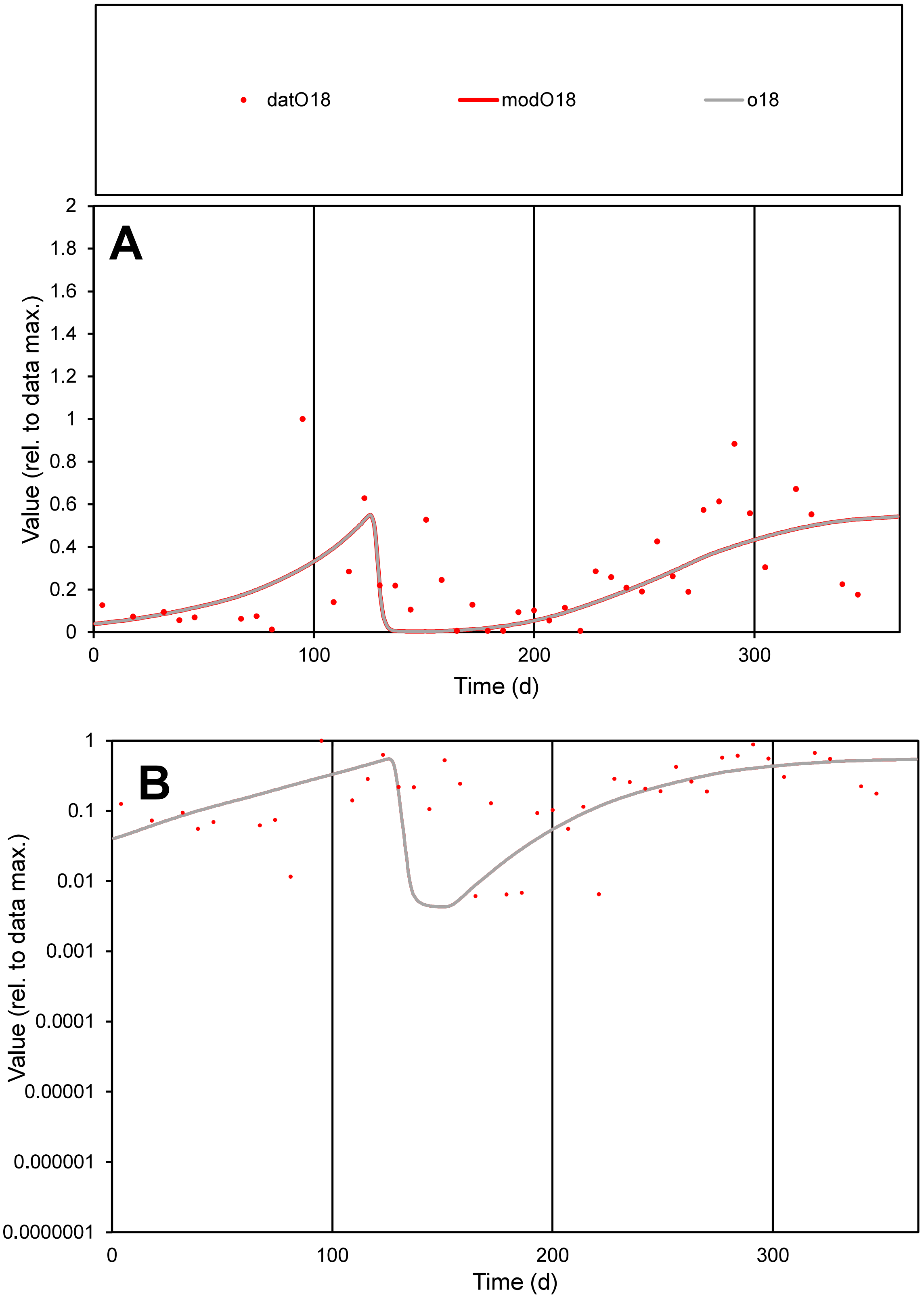

C1

Variables
O18 Provide jtd or Dst
CalibOnly?
2 1 to reproduce of
oNormalize?
2 1=no, 2=yes

ofxn calc*
0.79792
ofxn mod
0.80806

sRun
RunA1
zout
1

Ave
Data
0.00216
Model
0.00181

data/vMax
0.00837

ofxng
334.07

Min d/m
1.40467

ave/min d
42.2304
ave/min m
49.7871

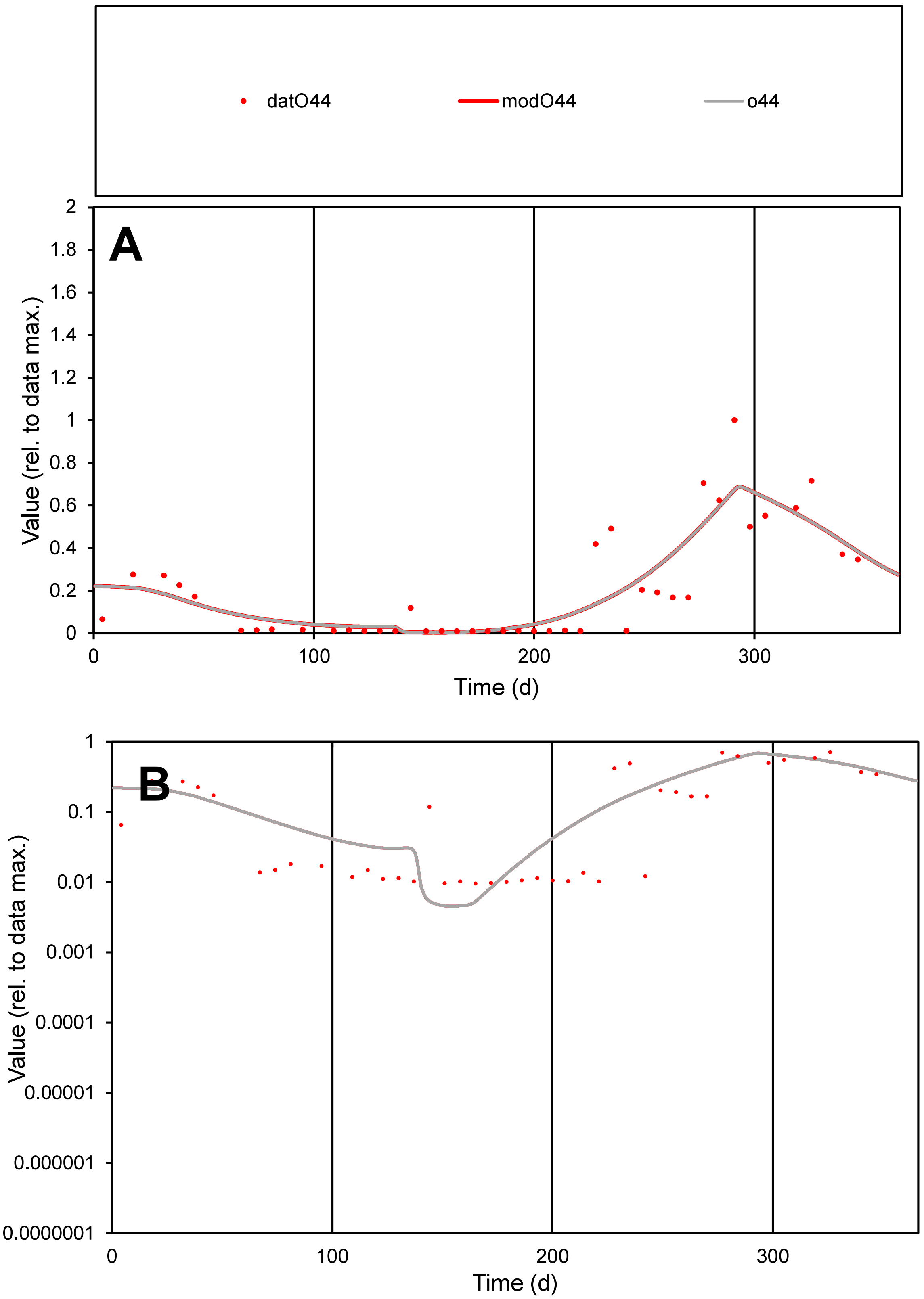

C1

Variables
O44 Provide jtd or Dst
CalibOnly?
2 1 to reproduce of
oNormalize?
2 1=no, 2=yes

ofxn calc*
0.61885
ofxn mod
0.64204

sRun
RunA1
zout
1

Ave
Data
0.00229
Model
0.00235

data/vMax
0.01144

ofxng
334.07

Min d/m
2.09249

ave/min d
20.947
ave/min m
44.8991

327_Calib_S_A

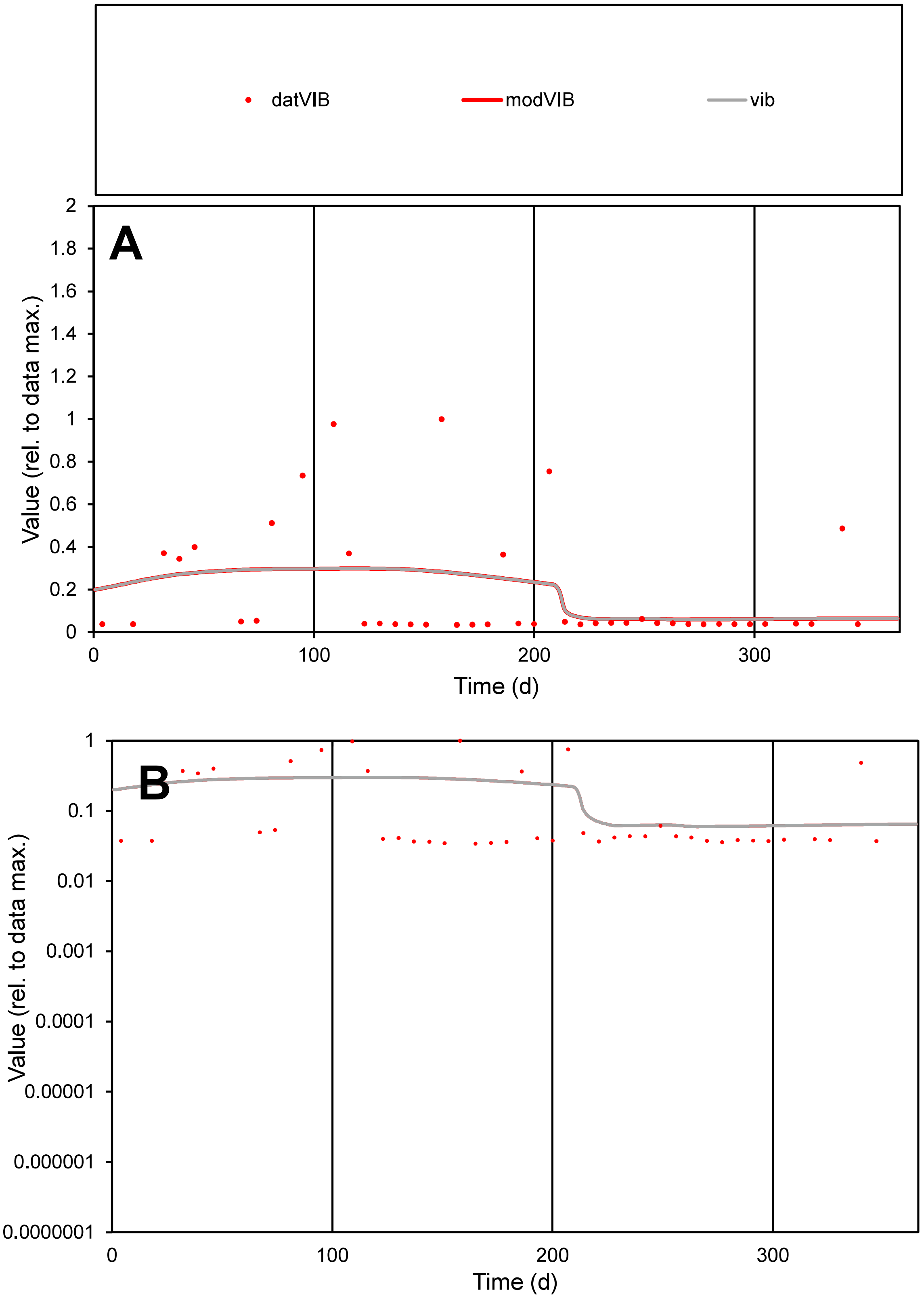

C1

Variables

VIB Provide jtd or Dst

CalibOnly?

2 1 to reproduce of

oNormalize?

2 1=no, 2=yes

ofxn calc*

1.35149

ofxn mod

1.36013

sRun

RunA1

zout

1

Ave

Data

9.2E-05

Model

9.4E-05

data/vMax

0.00051

ofxng

334.07

Min d/m

0.57536

ave/min d

5.25466

ave/min m

3.07835

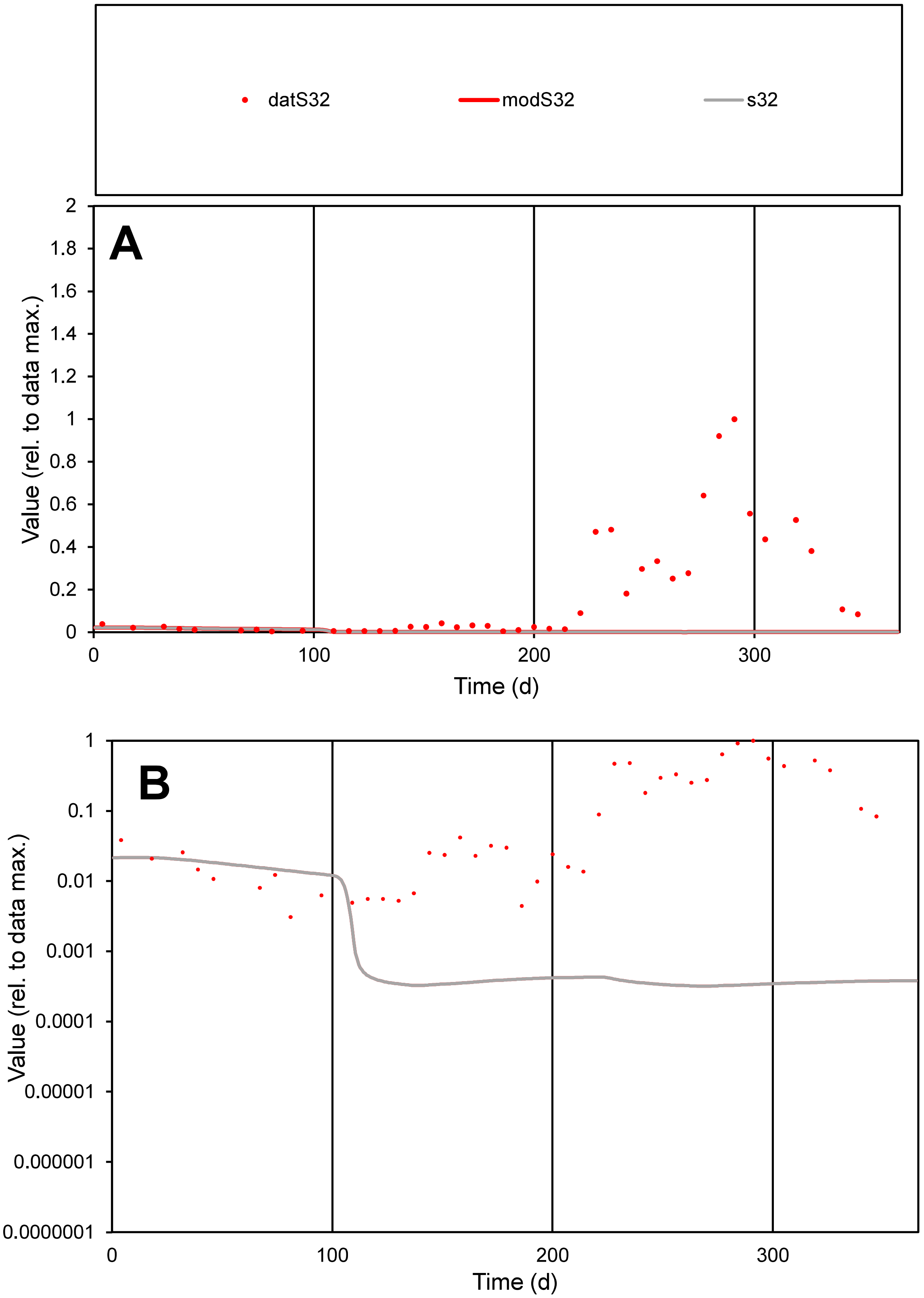

C1

Variables
S32 Provide jtd or Dst
CalibOnly?
2 1 to reproduce of
oNormalize?
2 1=no, 2=yes

ofxn calc*
1.74877
ofxn mod
1.75123

sRun
RunA1
zout
1

Ave
Data
0.00414
Model
9.5E-05

data/vMax
0.02337

ofxng
334.07

Min d/m
9.70083

ave/min d
57.225
ave/min m
12.8108

327_Calib_S_A

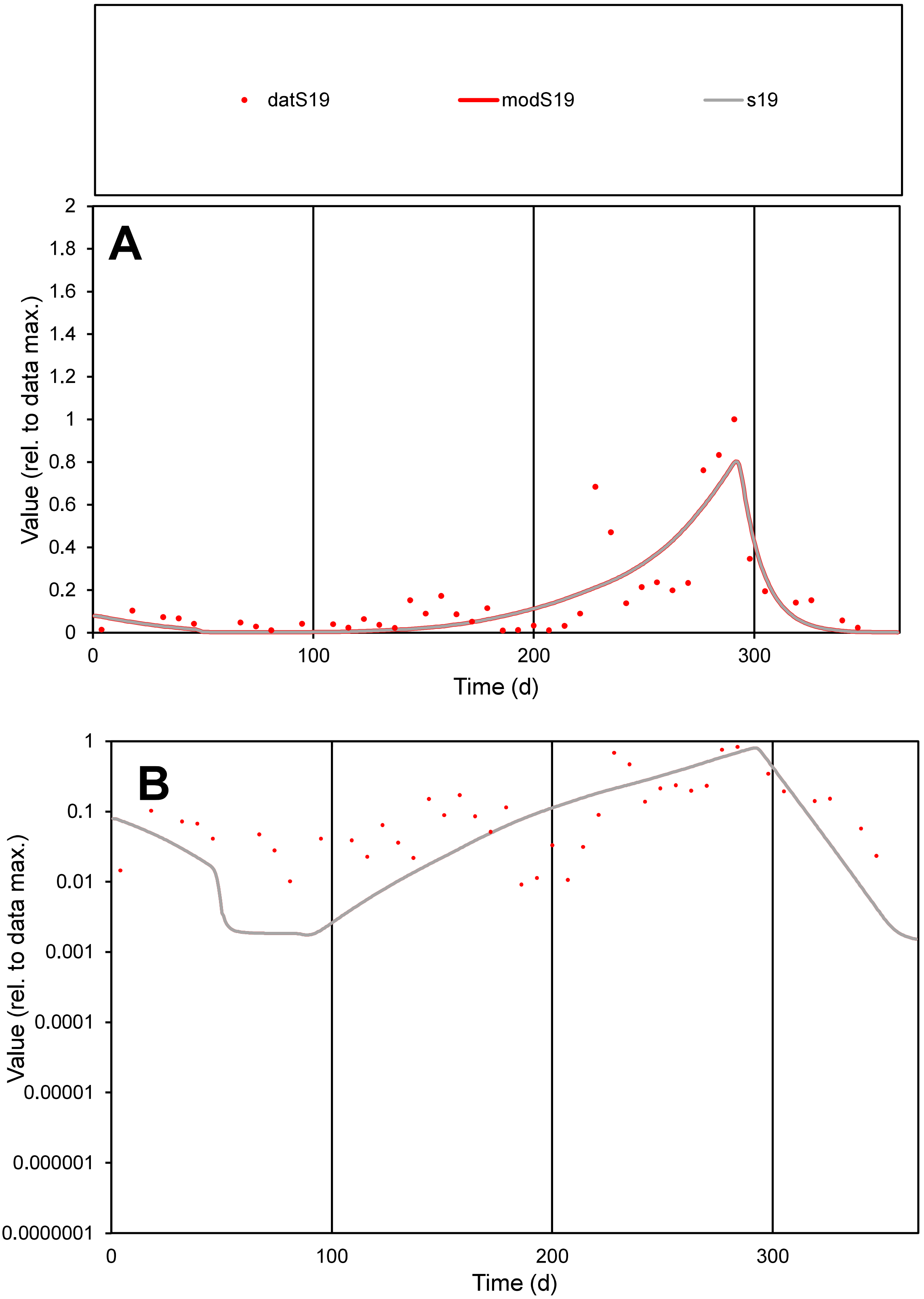

C1

Variables

S19 Provide jtd or Dst

CalibOnly?

2 1 to reproduce of

oNormalize?

2 1=no, 2=yes

ofxn calc*

0.75475

ofxn mod

0.77932

sRun

RunA1

zout

1

Ave

Data

0.00346

Model

0.00317

data/vMax

0.02034

ofxng

334.07

Min d/m

4.96401

ave/min d

18.5697

ave/min m

84.4442

327_Calib_S_A

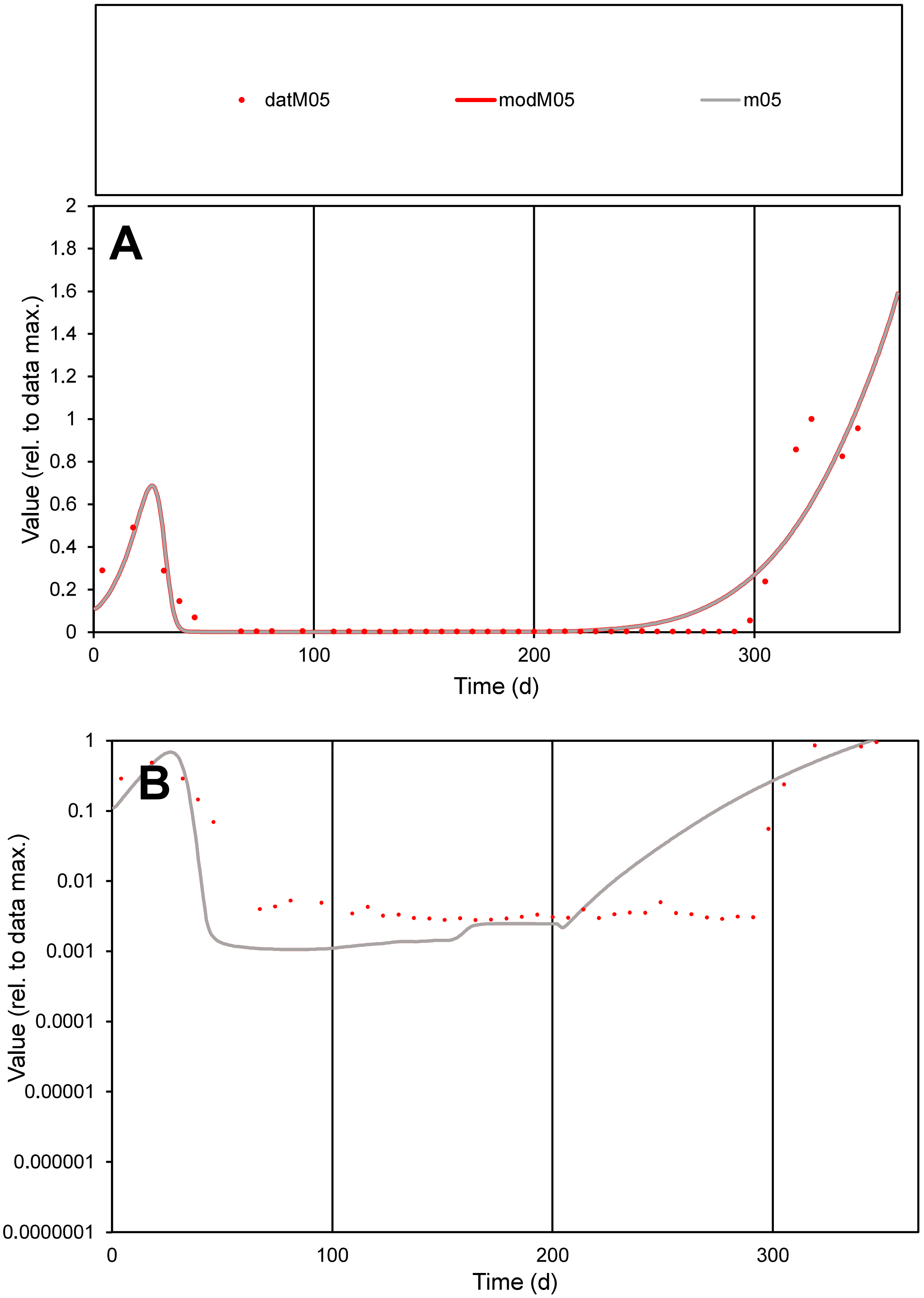

C1

Variables
M05 Provide jtd or Dst
CalibOnly?
2 1 to reproduce of
oNormalize?
2 1=no, 2=yes

ofxn calc*
0.80022
ofxn mod
0.84524

sRun
RunA1
zout
1

Ave
Data
0.00195
Model
0.00203

data/vMax
0.01536

ofxng
334.07

Min d/m
2.63362

ave/min d
45.5252
ave/min m
125.048

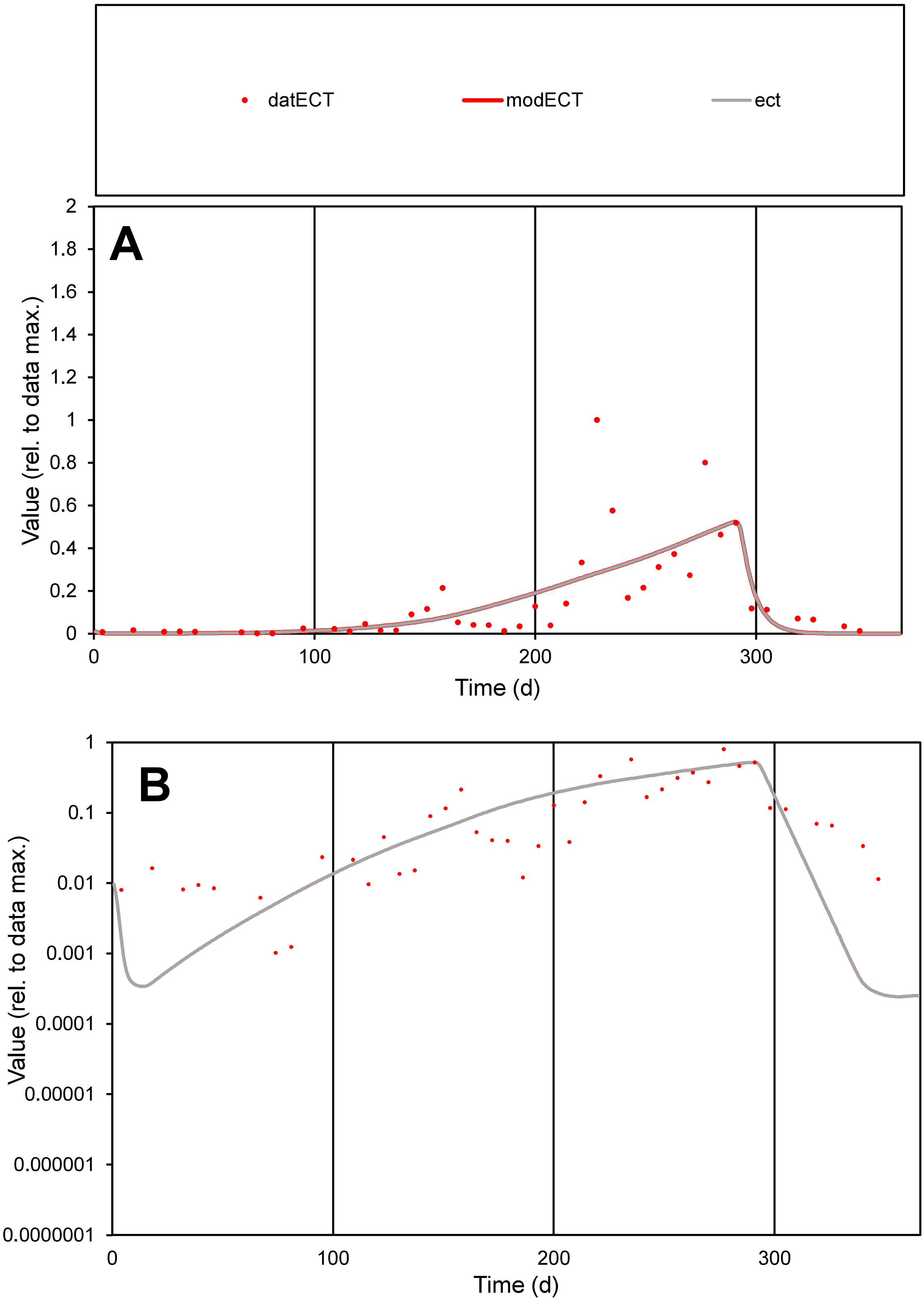

C1

Variables
ECT Provide jtd or Dst
CalibOnly?
2 1 to reproduce of
oNormalize?
2 1=no, 2=yes

ofxn calc*
0.94566
ofxn mod
0.96268

sRun
RunA1
zout
1

Ave
Data
0.00666
Model
0.00637

data/vMax
0.04282

ofxng
334.07

Min d/m
3.86596

ave/min d
152.017
ave/min m
561.74

327_Calib_S_A

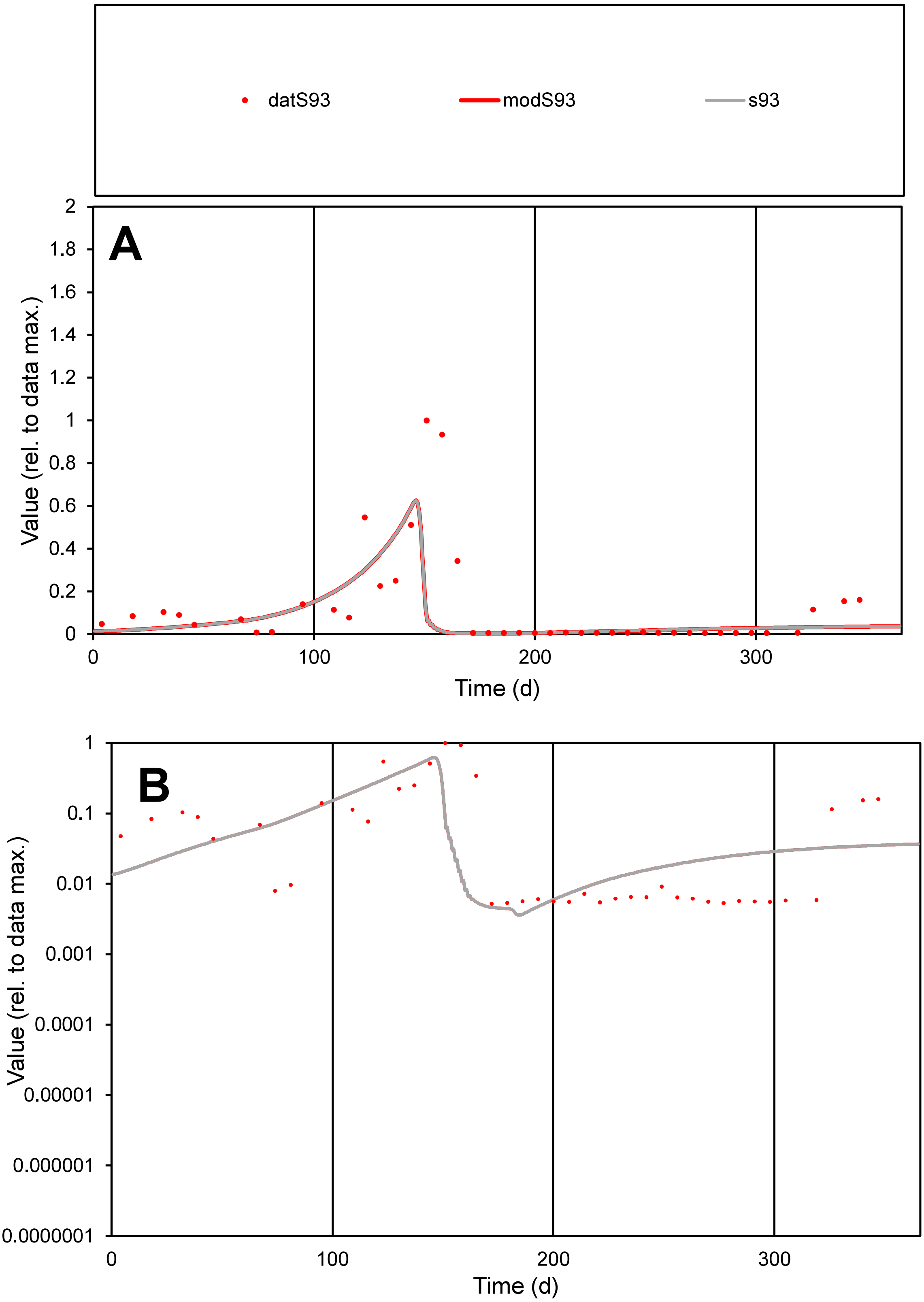

C1

Variables

S93 Provide jtd or Dst

CalibOnly?

2 1 to reproduce of

oNormalize?

2 1=no, 2=yes

ofxn calc*

1.52811

ofxn mod

1.69964

sRun

RunA1

zout

1

Ave

Data

0.00155

Model

0.00111

data/vMax

0.01268

ofxng

334.07

Min d/m

1.3281

ave/min d

23.557

ave/min m

22.3984

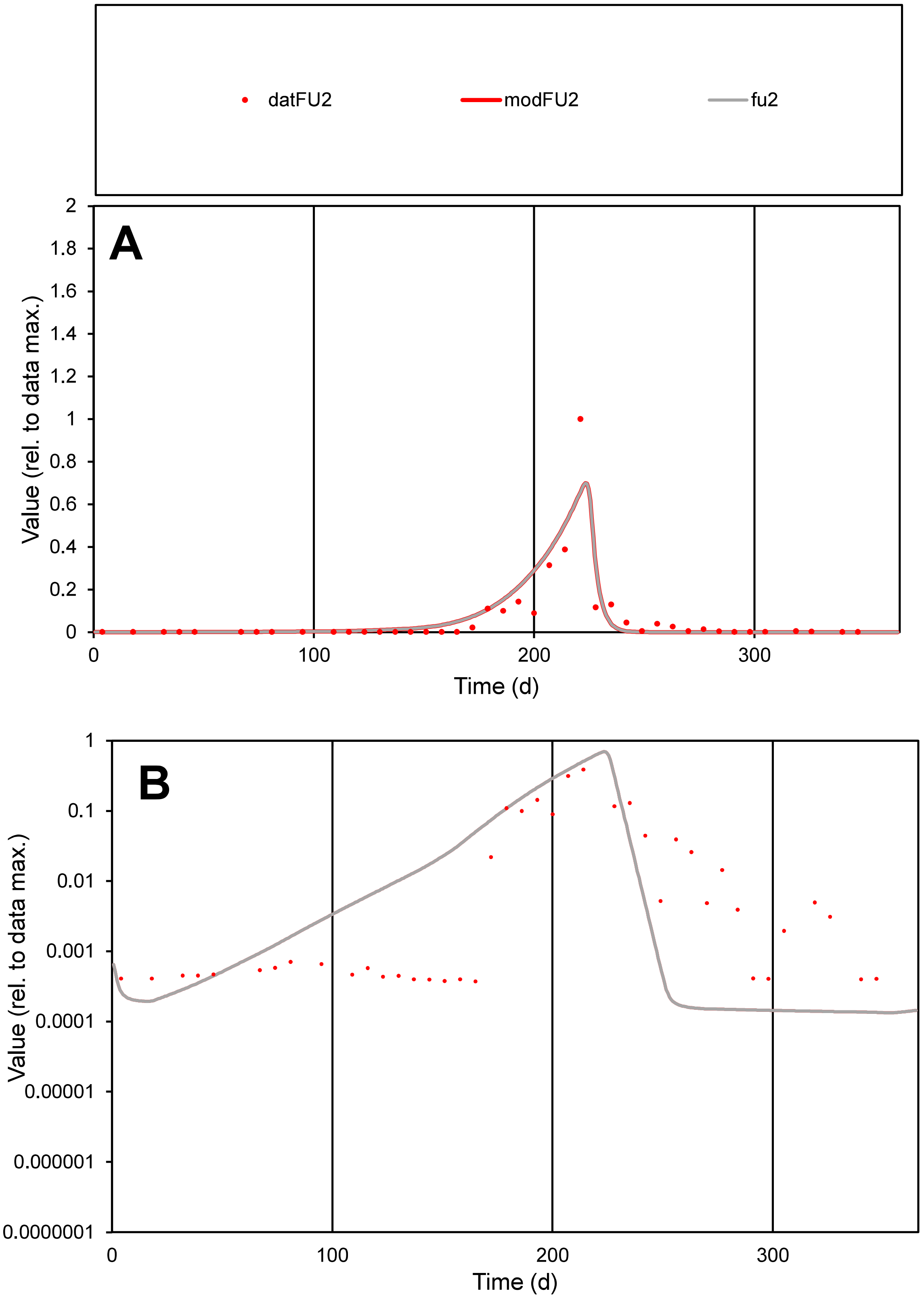

C1

Variables
FU2 Provide jtd or Dst
CalibOnly?
2 1 to reproduce of
oNormalize?
2 1=no, 2=yes

ofxn calc*
1.1113
ofxn mod
1.19013

sRun
RunA1
zout
1

Ave
Data
0.00415
Model
0.00461

data/vMax
0.06778

ofxng
334.07

Min d/m
2.78101

ave/min d
163.935
ave/min m
506.217

327_Calib_S_A

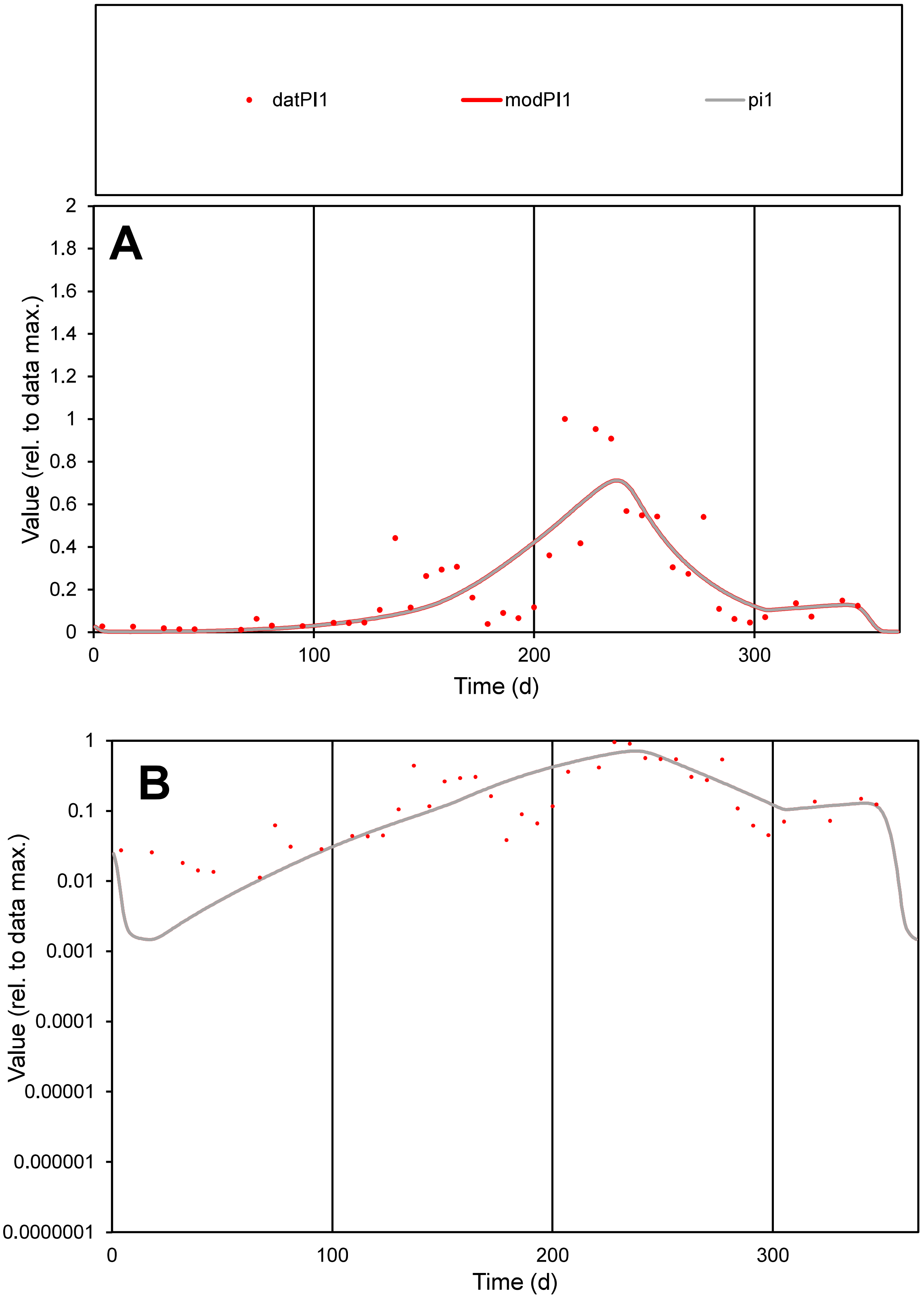

C1

Variables
PI1 Provide jtd or Dst
CalibOnly?
2 1 to reproduce of
oNormalize?
2 1=no, 2=yes

ofxn calc*
0.67517
ofxn mod
0.698

sRun
RunA1
zout
1

Ave
Data
0.00099
Model
0.00097

data/vMax
0.00437

ofxng
334.07

Min d/m
7.42413

ave/min d
20.2107
ave/min m
146.054

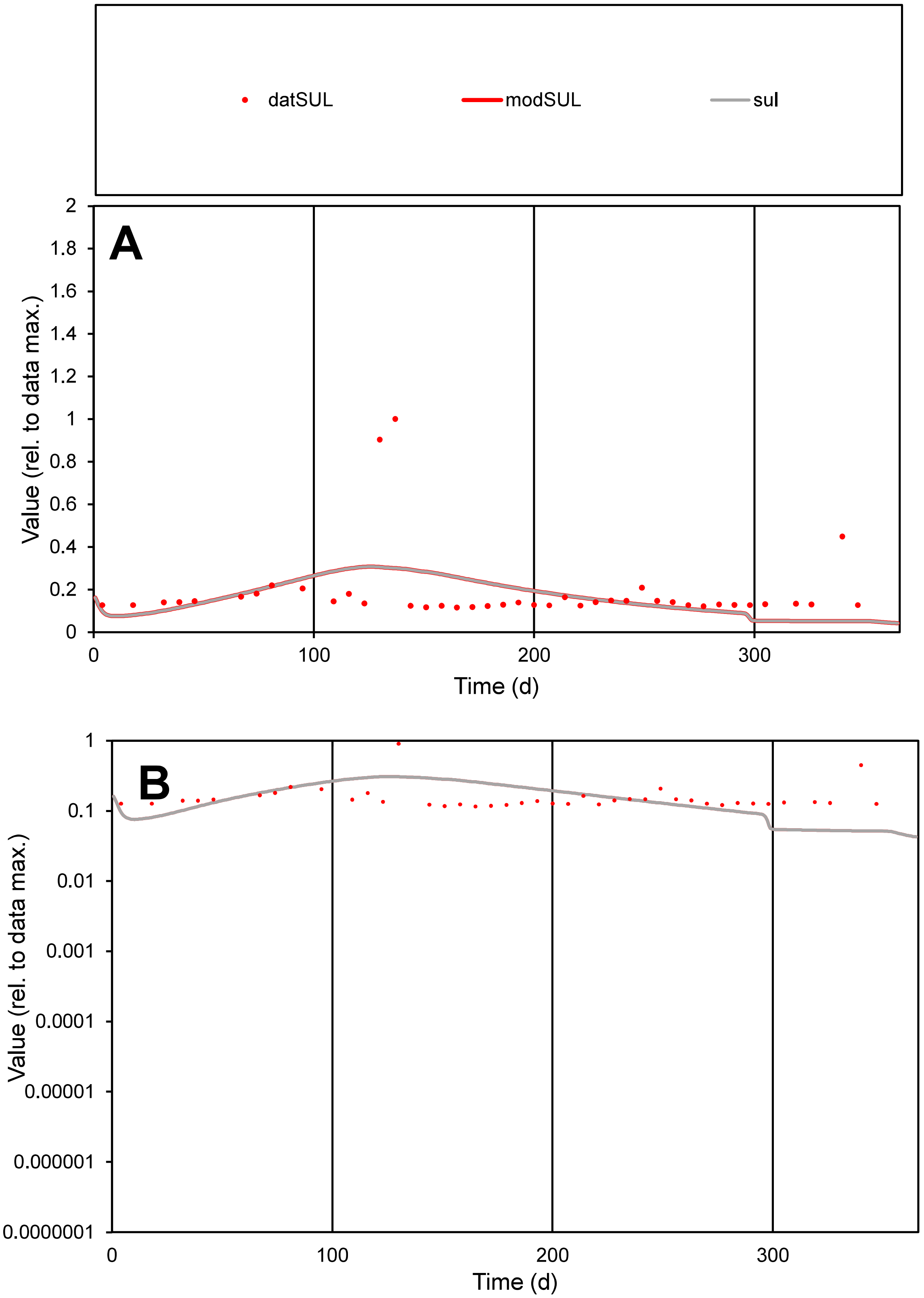

C1

Variables
SUL Provide jtd or Dst
CalibOnly?
2 1 to reproduce of
oNormalize?
2 1=no, 2=yes

ofxn calc*
0.92453
ofxn mod
0.93241

sRun
RunA1
zout
1

Ave
Data
0.00013
Model
0.00011

data/vMax
0.00068

ofxng
334.07

Min d/m
2.22985

ave/min d
1.6183
ave/min m
3.25538

327_Calib_S_A

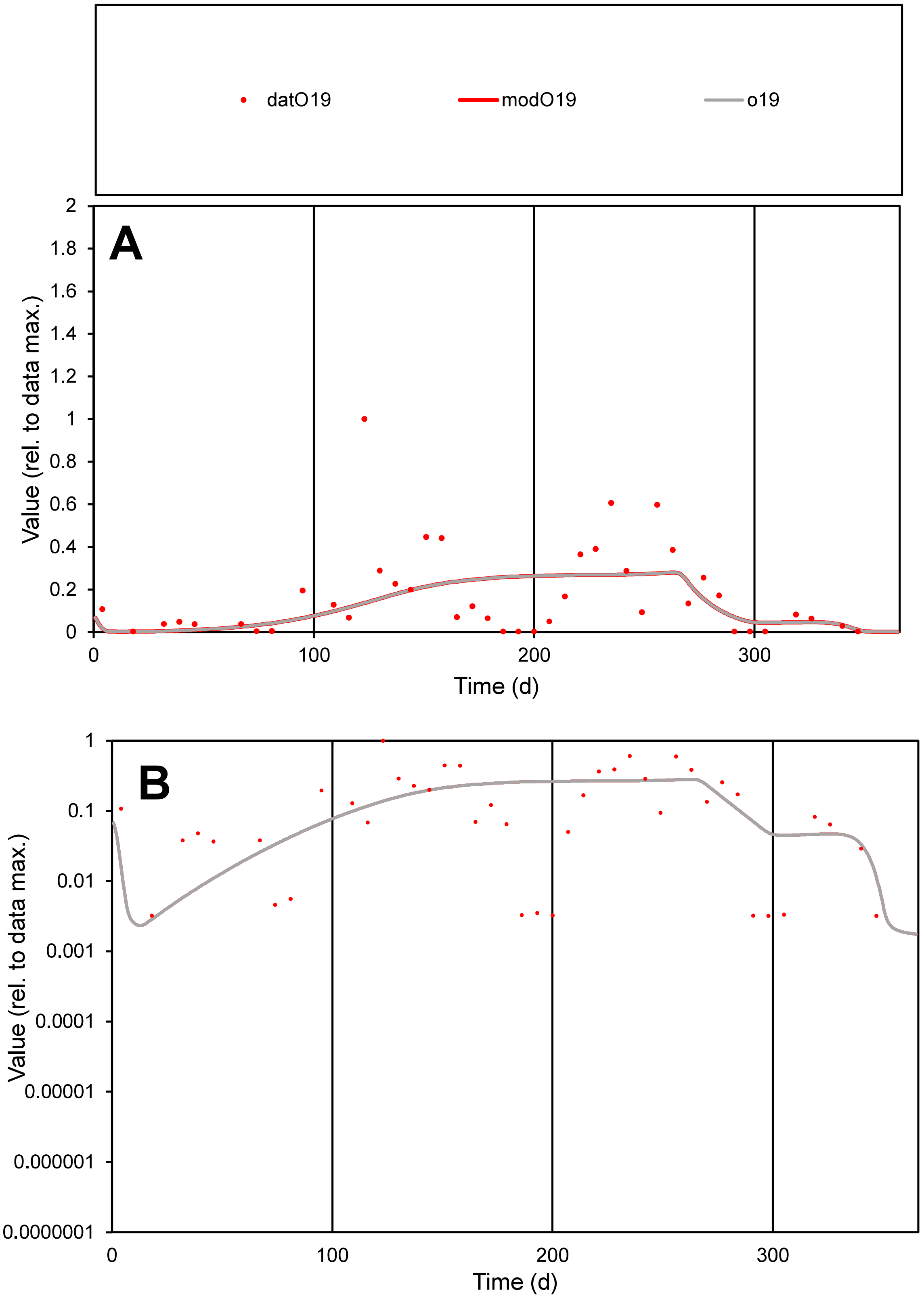

C1

Variables

O19 Provide jtd or Dst

CalibOnly?

2 1 to reproduce of

oNormalize?

2 1=no, 2=yes

ofxn calc*

1.1077

ofxn mod

1.11934

sRun

RunA1

zout

1

Ave

Data

0.00164

Model

0.00144

data/vMax

0.00954

ofxng

334.07

Min d/m

1.03142

ave/min d

53.844

ave/min m

48.6362

327_Calib_S_A

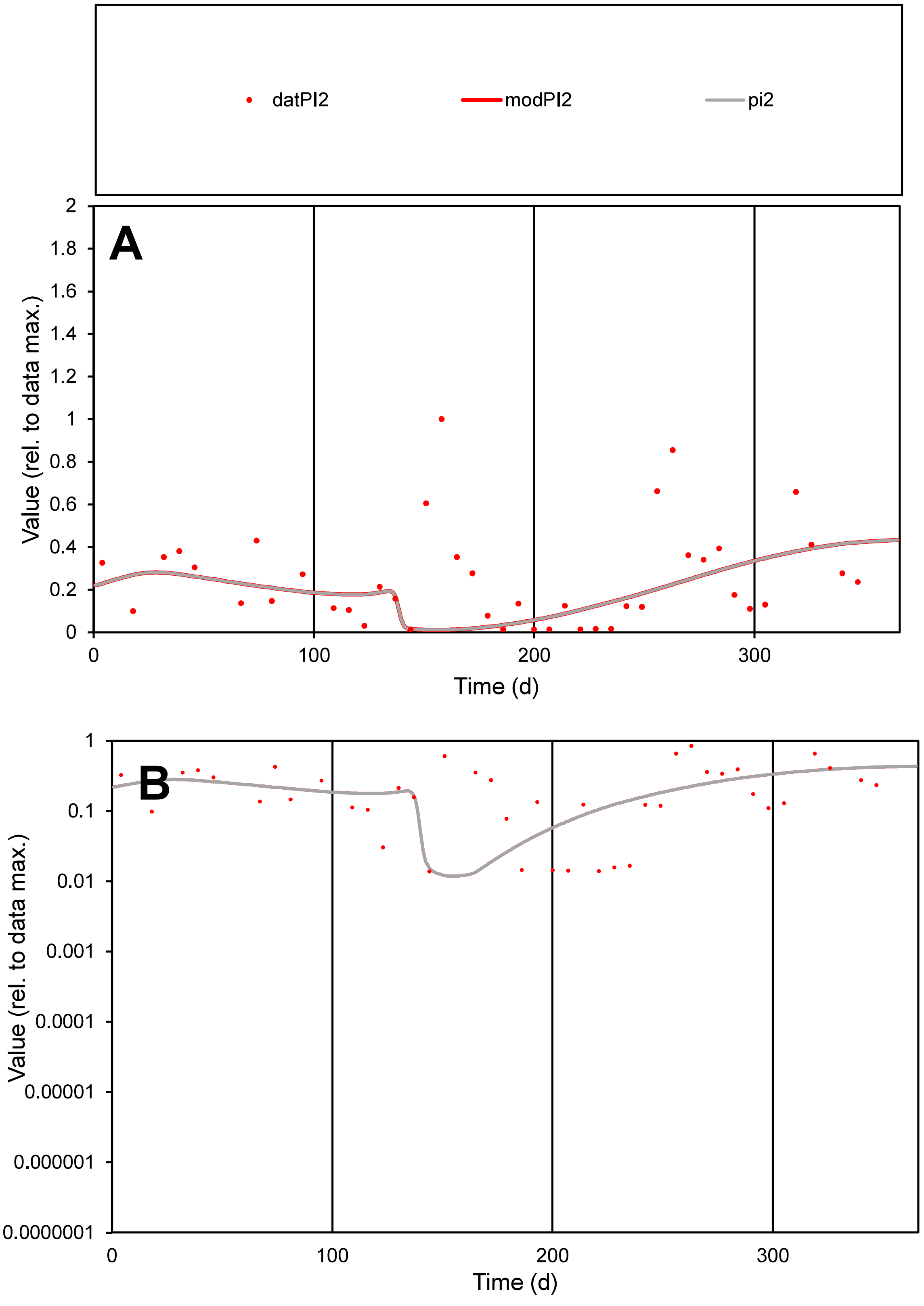

C1

Variables
PI2 Provide jtd or Dst
CalibOnly?
2 1 to reproduce of
oNormalize?
2 1=no, 2=yes

ofxn calc*
0.98483
ofxn mod
0.99253

sRun
RunA1
zout
1

Ave
Data
0.00054
Model
0.00041

data/vMax
0.00215

ofxng
334.07

Min d/m
1.15432

ave/min d
18.236
ave/min m
15.9863

327_Calib_S_A

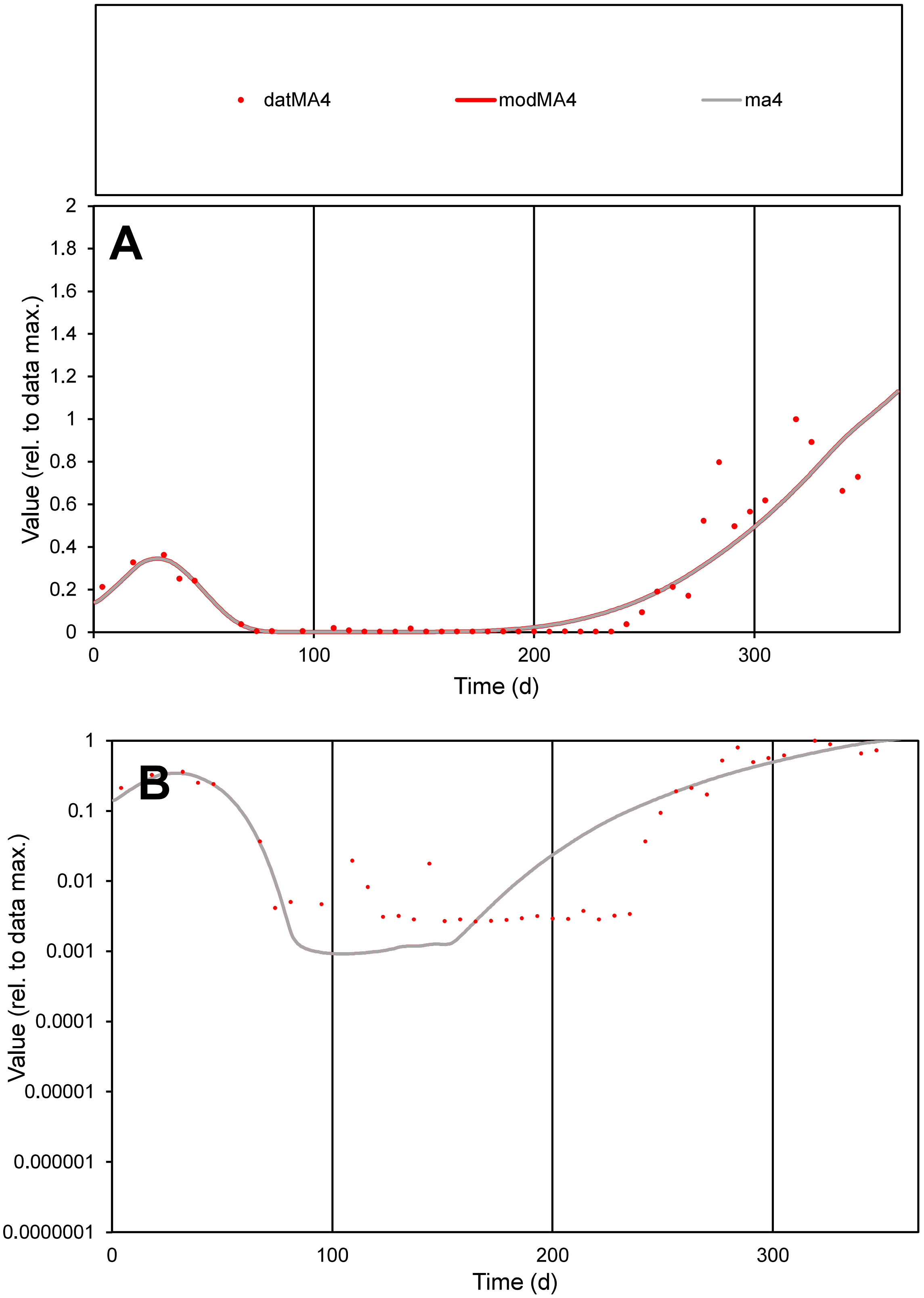

C1

Variables

MA4 Provide jtd or Dst

CalibOnly?

2 1 to reproduce of

oNormalize?

2 1=no, 2=yes

ofxn calc*

0.55048

ofxn mod

0.57555

sRun

RunA1

zout

1

Ave

Data

0.00146

Model

0.00141

data/vMax

0.00717

ofxng

334.07

Min d/m

2.85629

ave/min d

76.1403

ave/min m

210.74

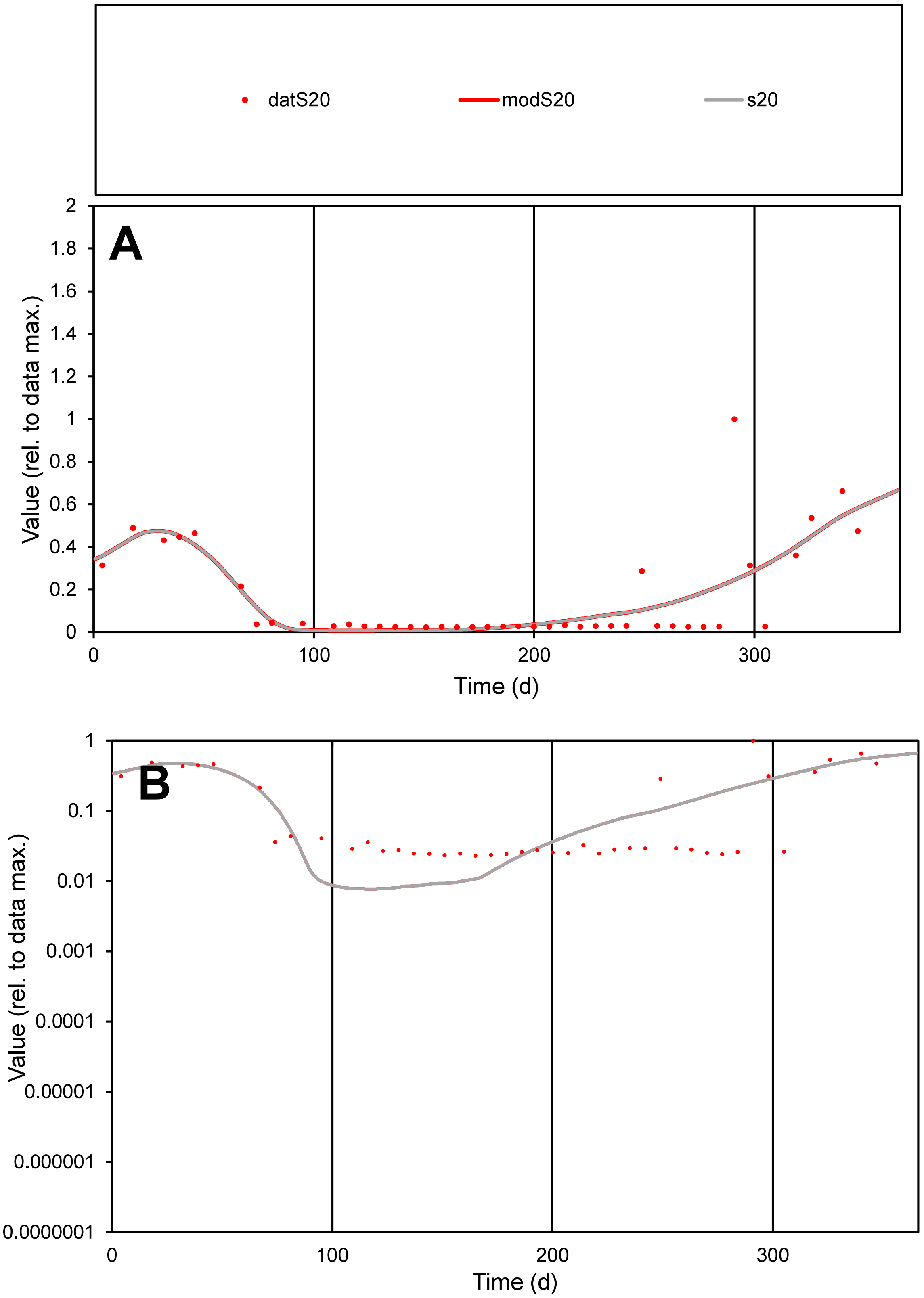

C1

Variables
S20 Provide jtd or Dst
CalibOnly?
2 1 to reproduce of
oNormalize?
2 1=no, 2=yes

ofxn calc*
0.87558
ofxn mod
0.89477

sRun
RunA1
zout
1

Ave
Data
0.00054
Model
0.00055

data/vMax
0.00335

ofxng
334.07

Min d/m
3.00622

ave/min d
7.00642
ave/min m
21.3749

327_Calib_S_A

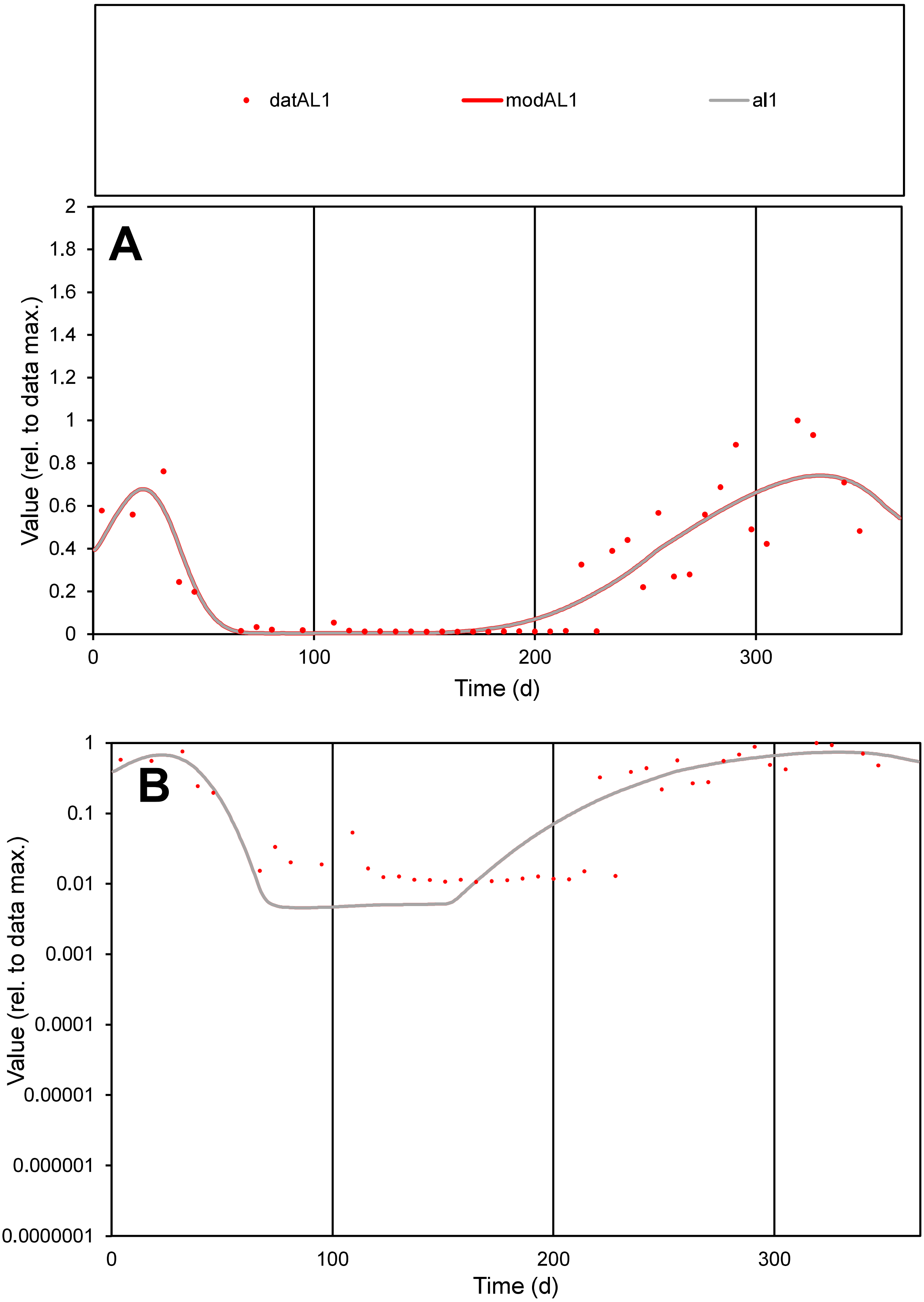

C1

Variables
AL1 Provide jtd or Dst
CalibOnly?
2 1 to reproduce of
oNormalize?
2 1=no, 2=yes

ofxn calc*
0.462
ofxn mod
0.4901

sRun
RunA1
zout
1

Ave
Data
0.0011
Model
0.0011

data/vMax
0.00408

ofxng
334.07

Min d/m
2.33405

ave/min d
25.2894
ave/min m
59.062

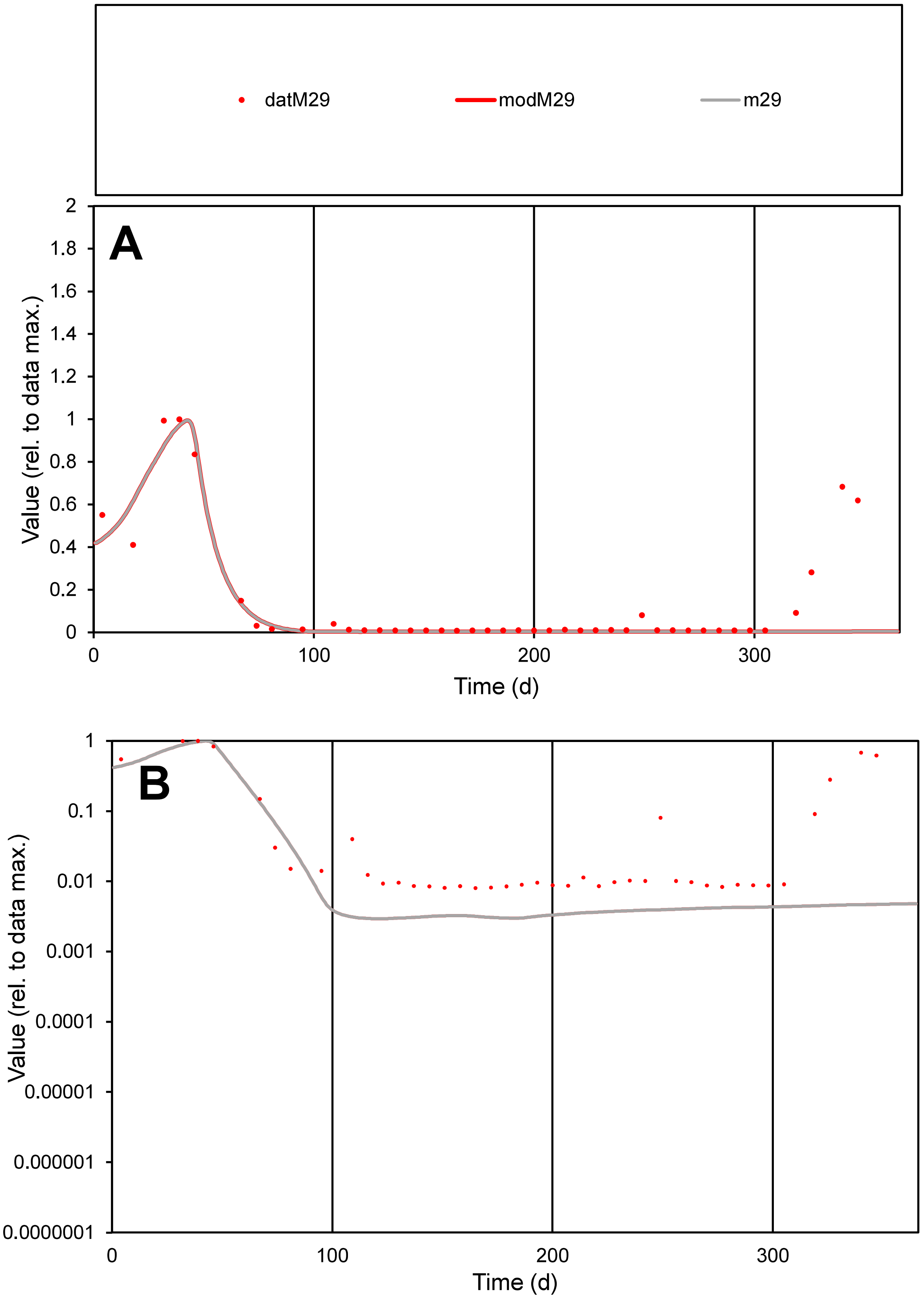

C1

Variables
M29 Provide jtd or Dst
CalibOnly?
2 1 to reproduce of
oNormalize?
2 1=no, 2=yes

ofxn calc*
1.07036
ofxn mod
1.10576

sRun
RunA1
zout
1

Ave
Data
0.00097
Model
0.00066

data/vMax
0.00675

ofxng
334.07

Min d/m
2.72264

ave/min d
17.9719
ave/min m
33.4075

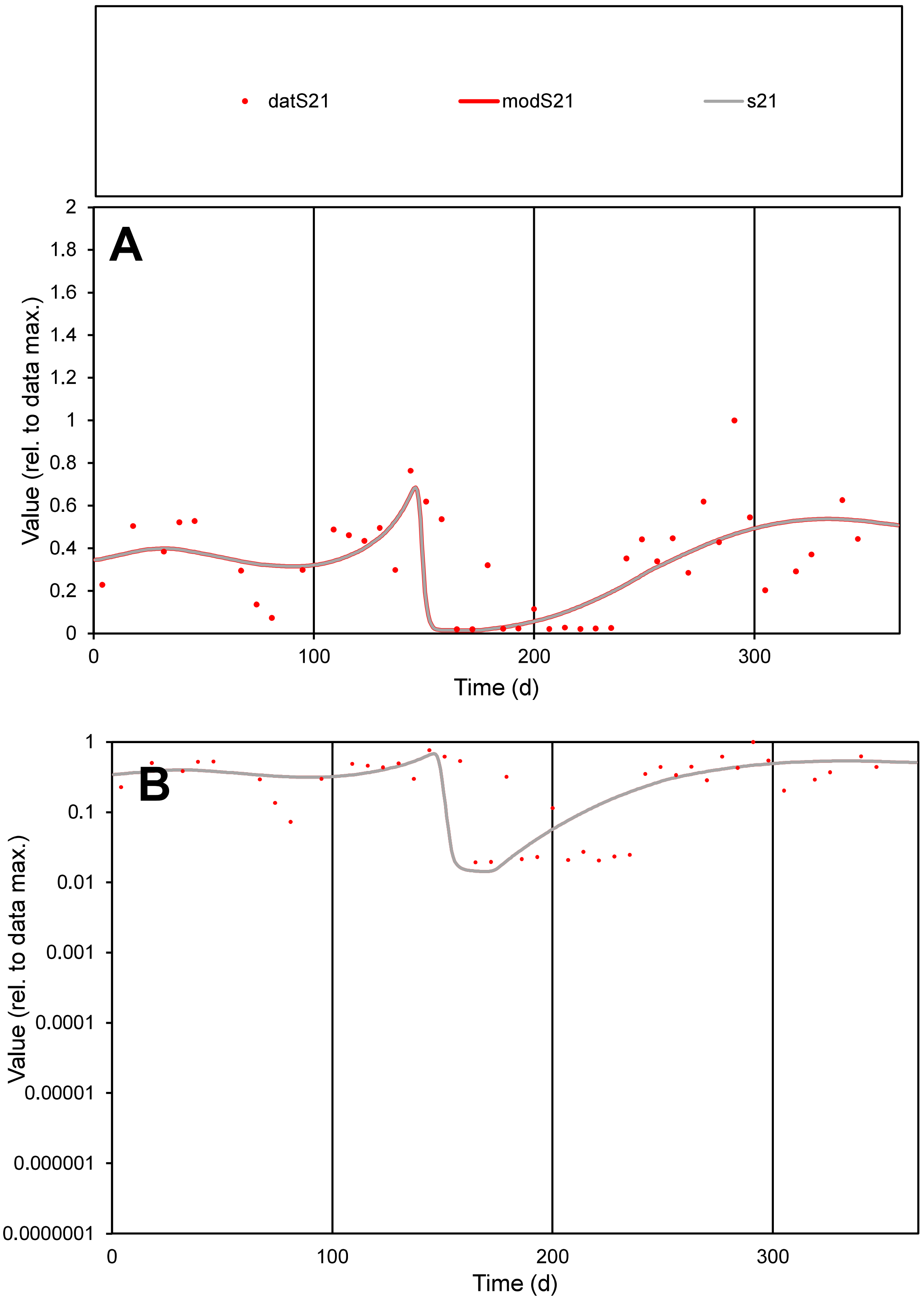

C1

Variables
S21 Provide jtd or Dst
CalibOnly?
2 1 to reproduce of
oNormalize?
2 1=no, 2=yes

ofxn calc*
0.52712
ofxn mod
0.55974

sRun
RunA1
zout
1

Ave
Data
0.00071
Model
0.00067

data/vMax
0.00211

ofxng
334.07

Min d/m
1.33421

ave/min d
17.3982
ave/min m
22.0052

327_Calib_S_A

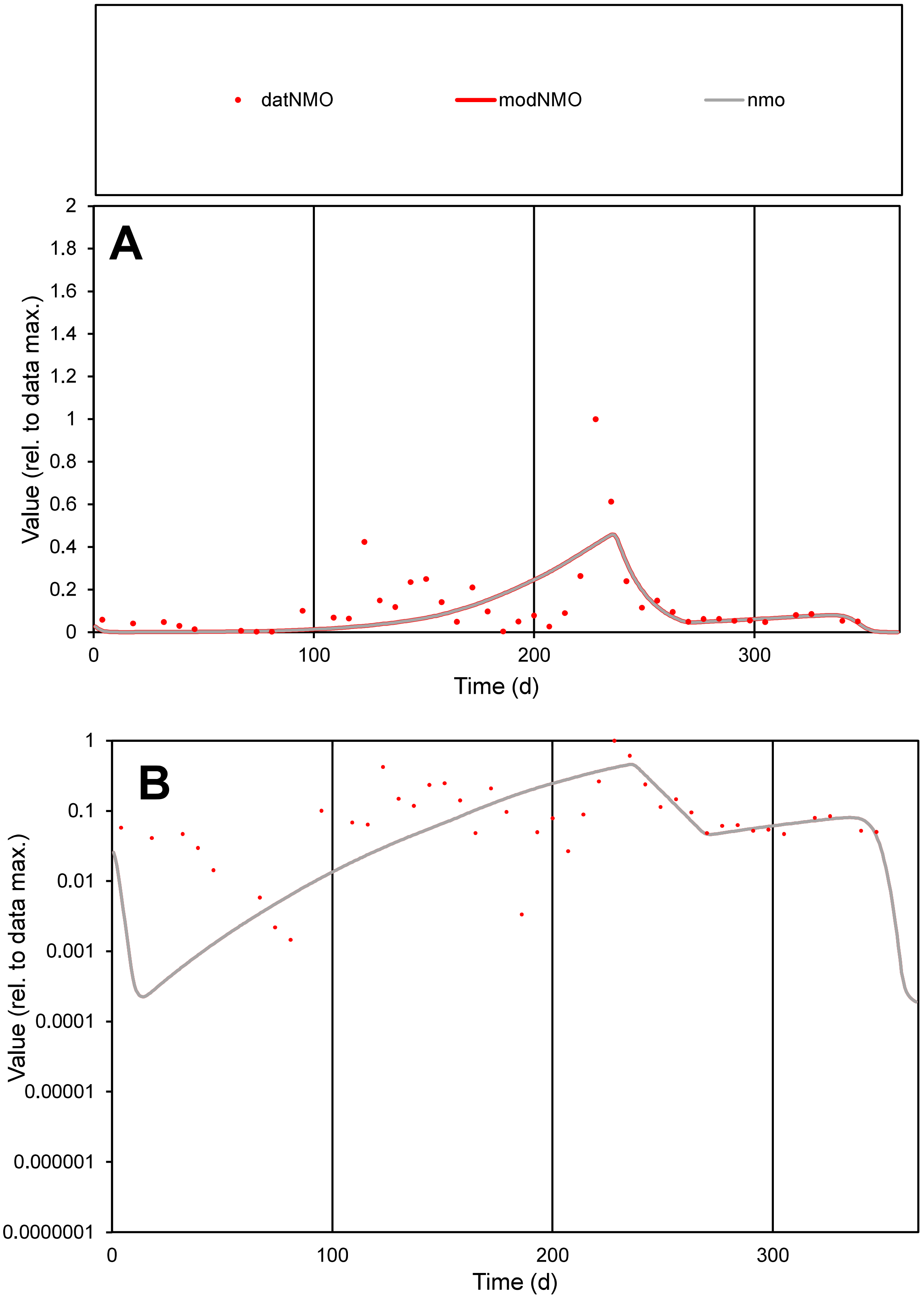

C1

Variables
NMO Provide jtd or Dst
CalibOnly?
2 1 to reproduce of
oNormalize?
2 1=no, 2=yes

ofxn calc*
1.11165
ofxn mod
1.12371

sRun
RunA1
zout
1

Ave
Data
0.00239
Model
0.00202

data/vMax
0.01853

ofxng
334.07

Min d/m
4.98896

ave/min d
88.0708
ave/min m
371.348

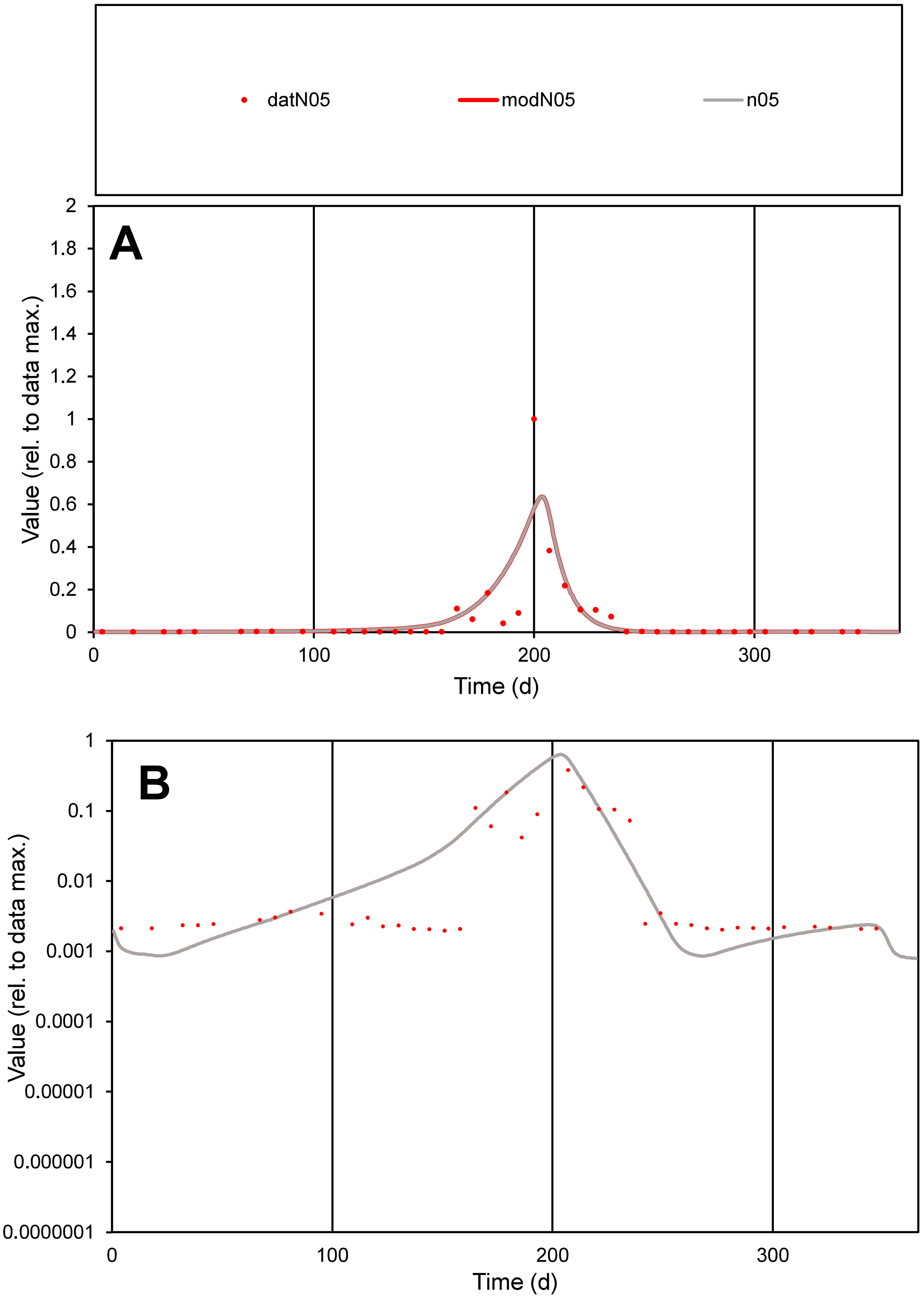

C1

Variables
N05 Provide jtd or Dst
CalibOnly?
2 1 to reproduce of
oNormalize?
2 1=no, 2=yes

ofxn calc*
1.52102
ofxn mod
1.58484

sRun
RunA1
zout
1

Ave
Data
0.00118
Model
0.00132

data/vMax
0.02031

ofxng
334.07

Min d/m
2.2592

ave/min d
29.6118
ave/min m
74.6163

327_Calib_S_A

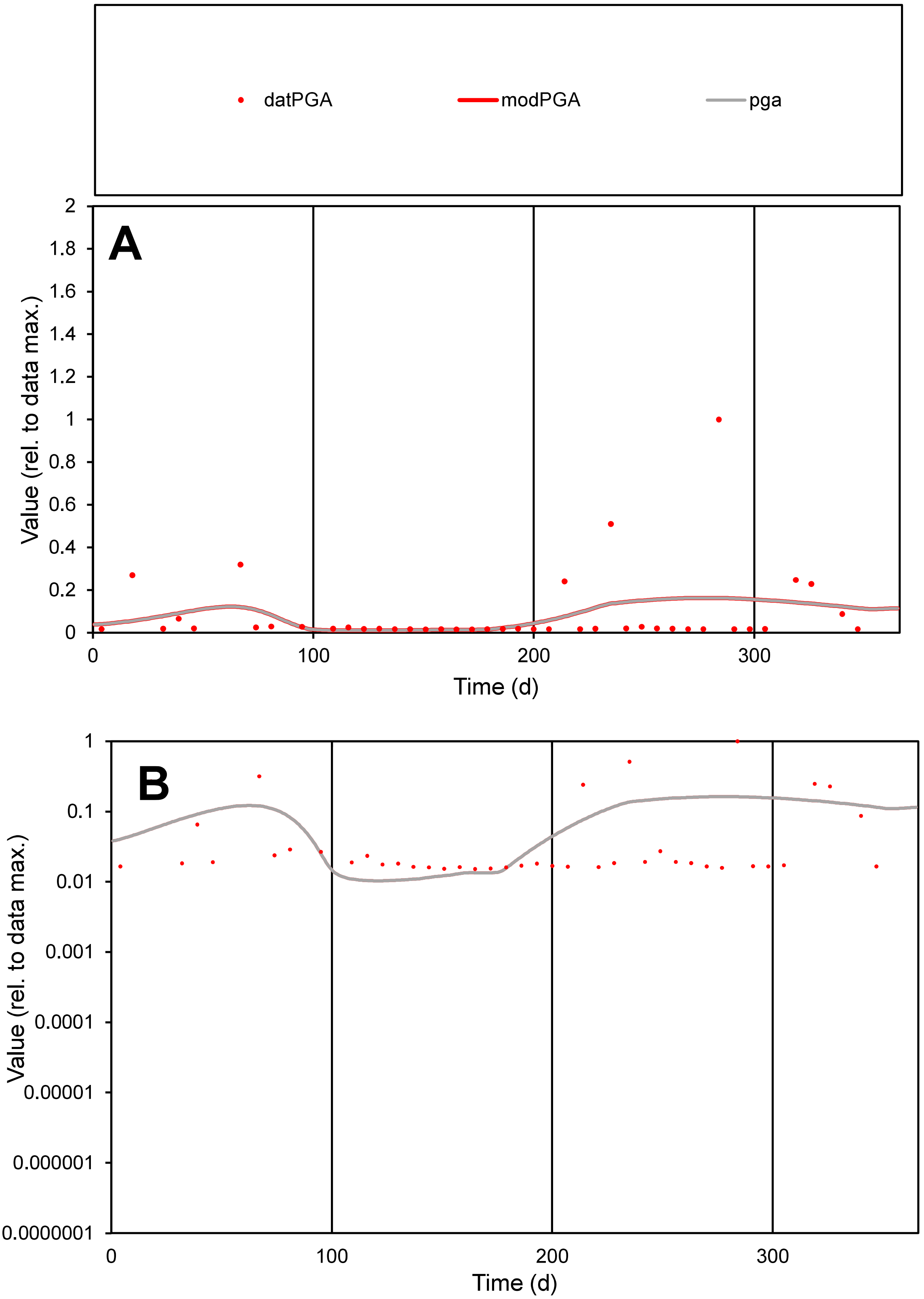

C1

Variables
PGA Provide jtd or Dst
CalibOnly?
2 1 to reproduce of
oNormalize?
2 1=no, 2=yes

ofxn calc*
1.98008
ofxn mod
1.98701

sRun
RunA1
zout
1

Ave
Data
9.5E-05
Model
9.3E-05

data/vMax
0.00111

ofxng
334.07

Min d/m
1.46401

ave/min d
5.60445
ave/min m
8.03316

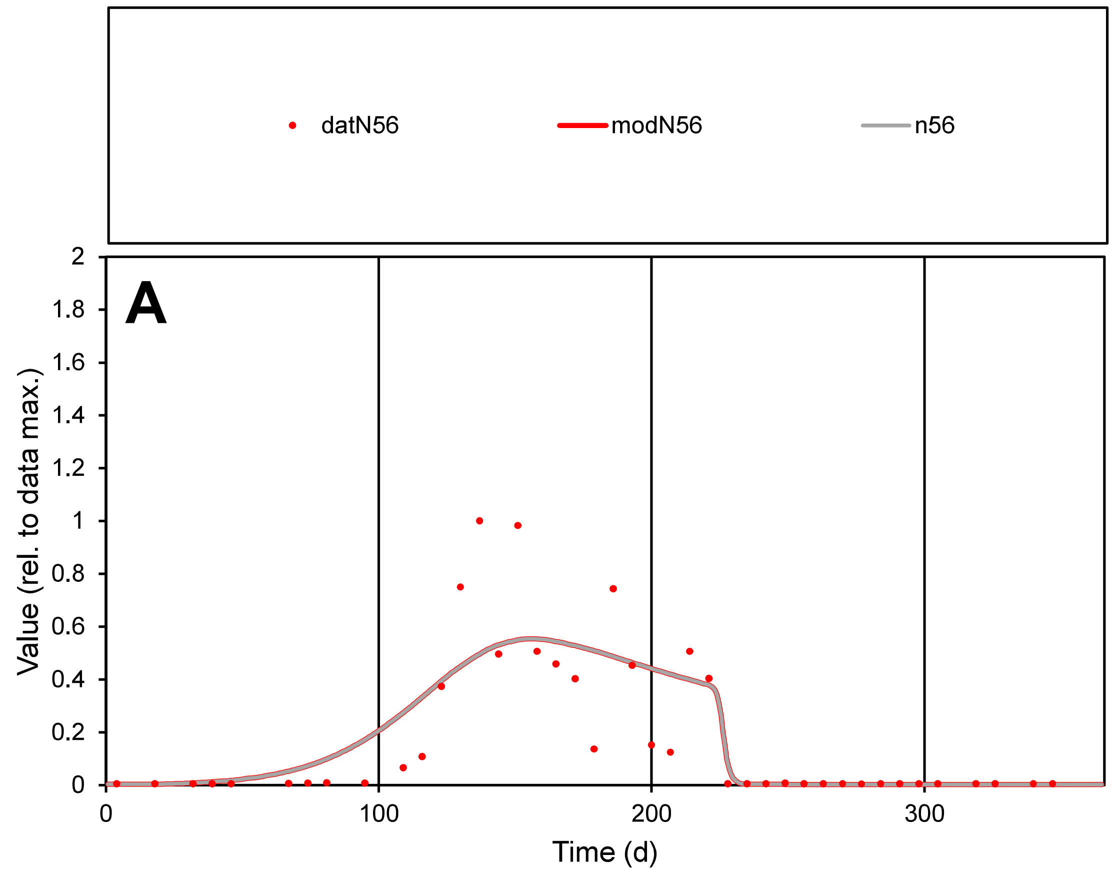

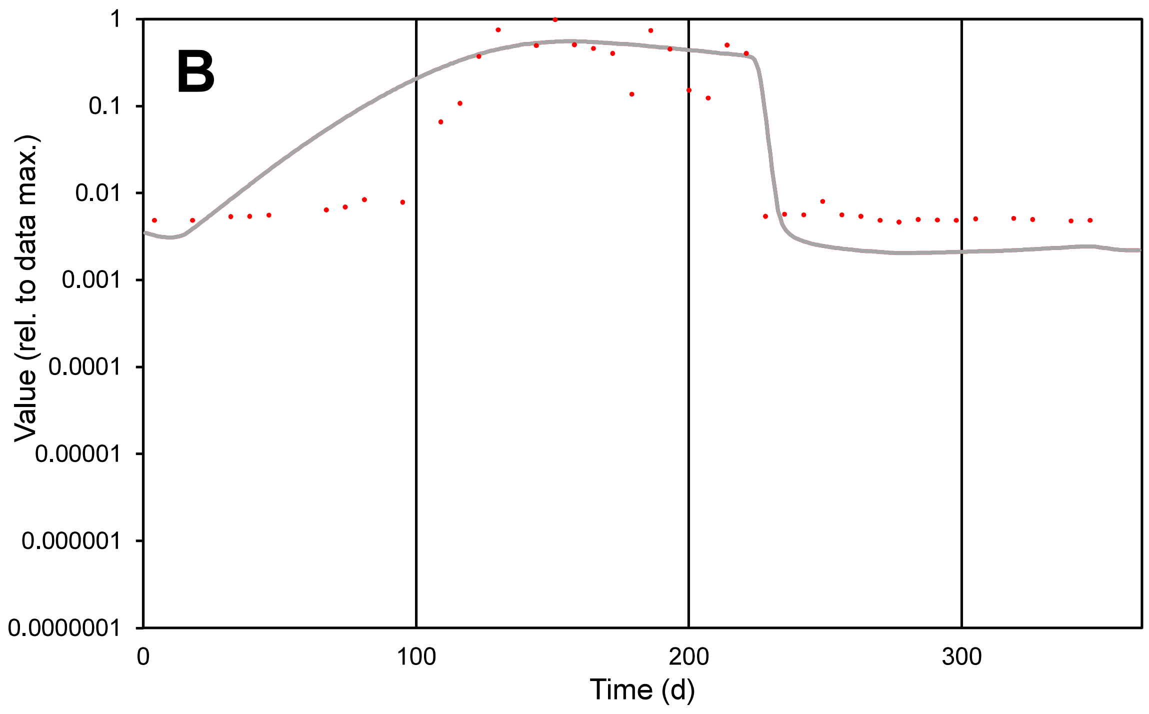

C1

Variables
N56 Provide jtd or Dst
CalibOnly?
2 1 to reproduce of
oNormalize?
2 1=no, 2=yes

ofxn calc*
0.85692
ofxn mod
0.87585

sRun
RunA1
zout
1

Ave
Data
0.00141
Model
0.00149

data/vMax
0.00759

ofxng
334.07

Min d/m
2.27613

ave/min d
39.991
ave/min m
96.2321

327_Calib_S_A

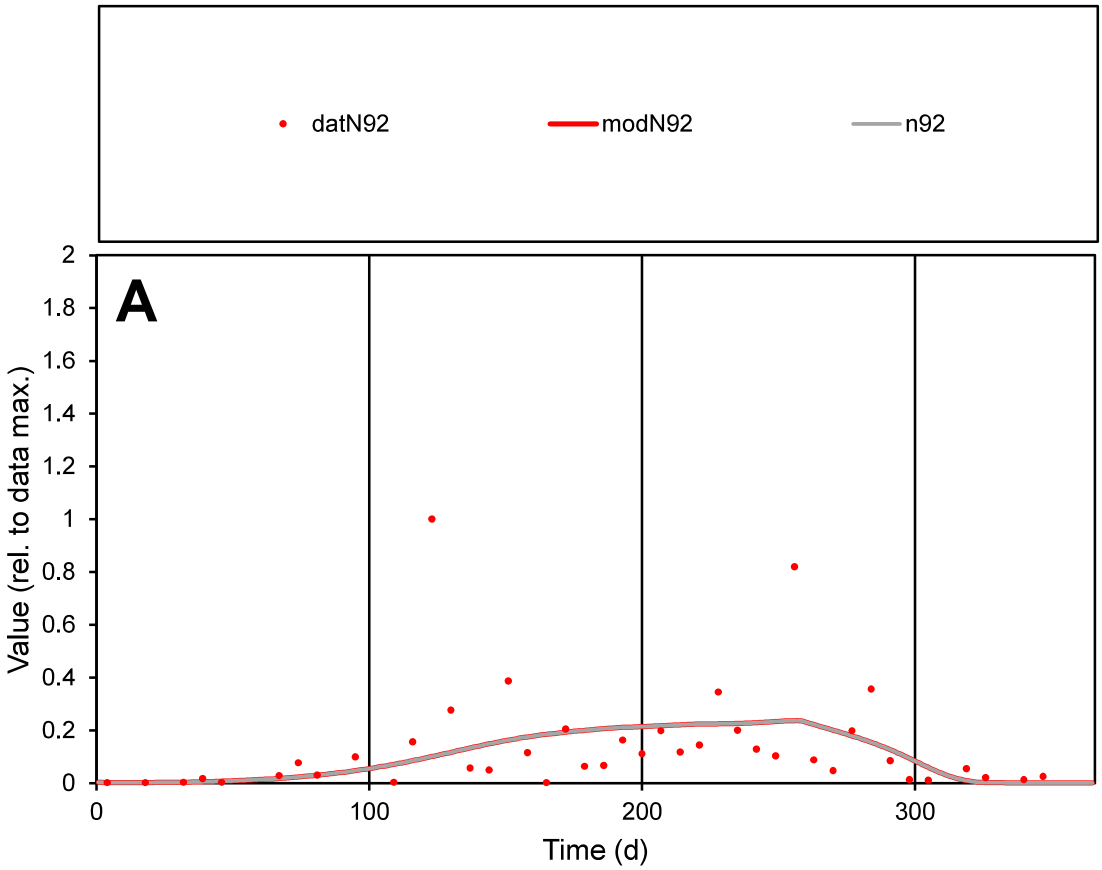

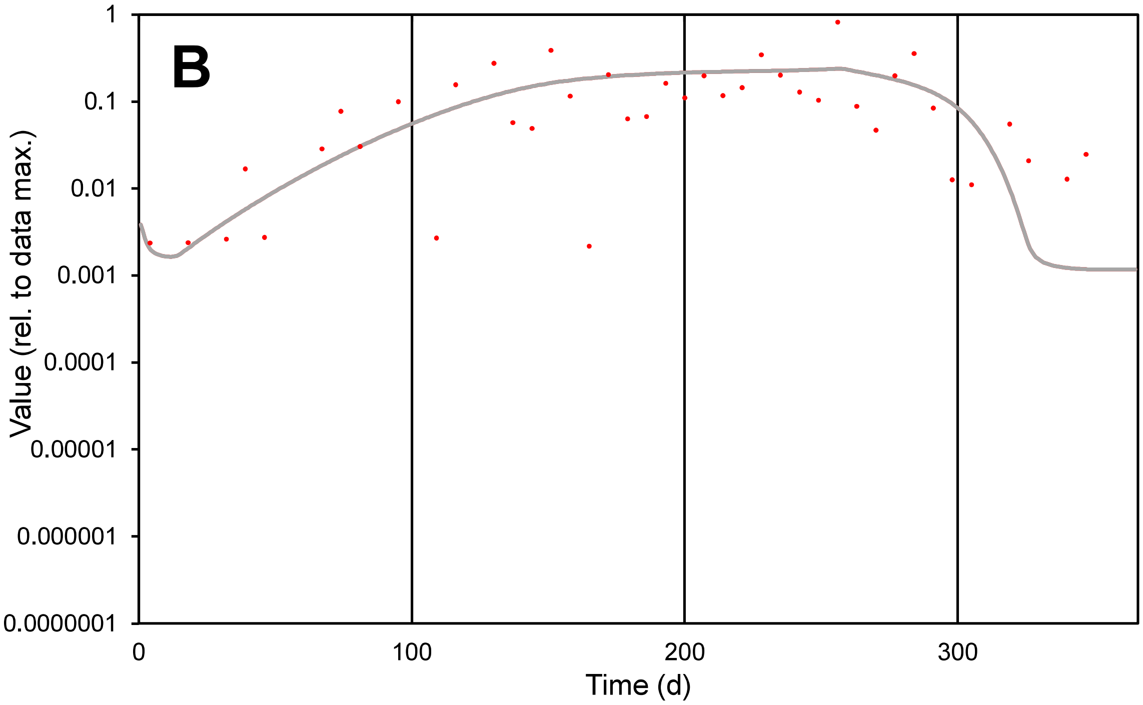

C1

Variables

N92 Provide jtd or Dst

CalibOnly?

2 1 to reproduce of

oNormalize?

2 1=no, 2=yes

ofxn calc*

1.3423

ofxn mod

1.35184

sRun

RunA1

zout

1

Ave

Data

0.00242

Model

0.00213

data/vMax

0.01724

ofxng

334.07

Min d/m

1.84363

ave/min d

64.2618

ave/min m

104.635

327_Calib_S_A

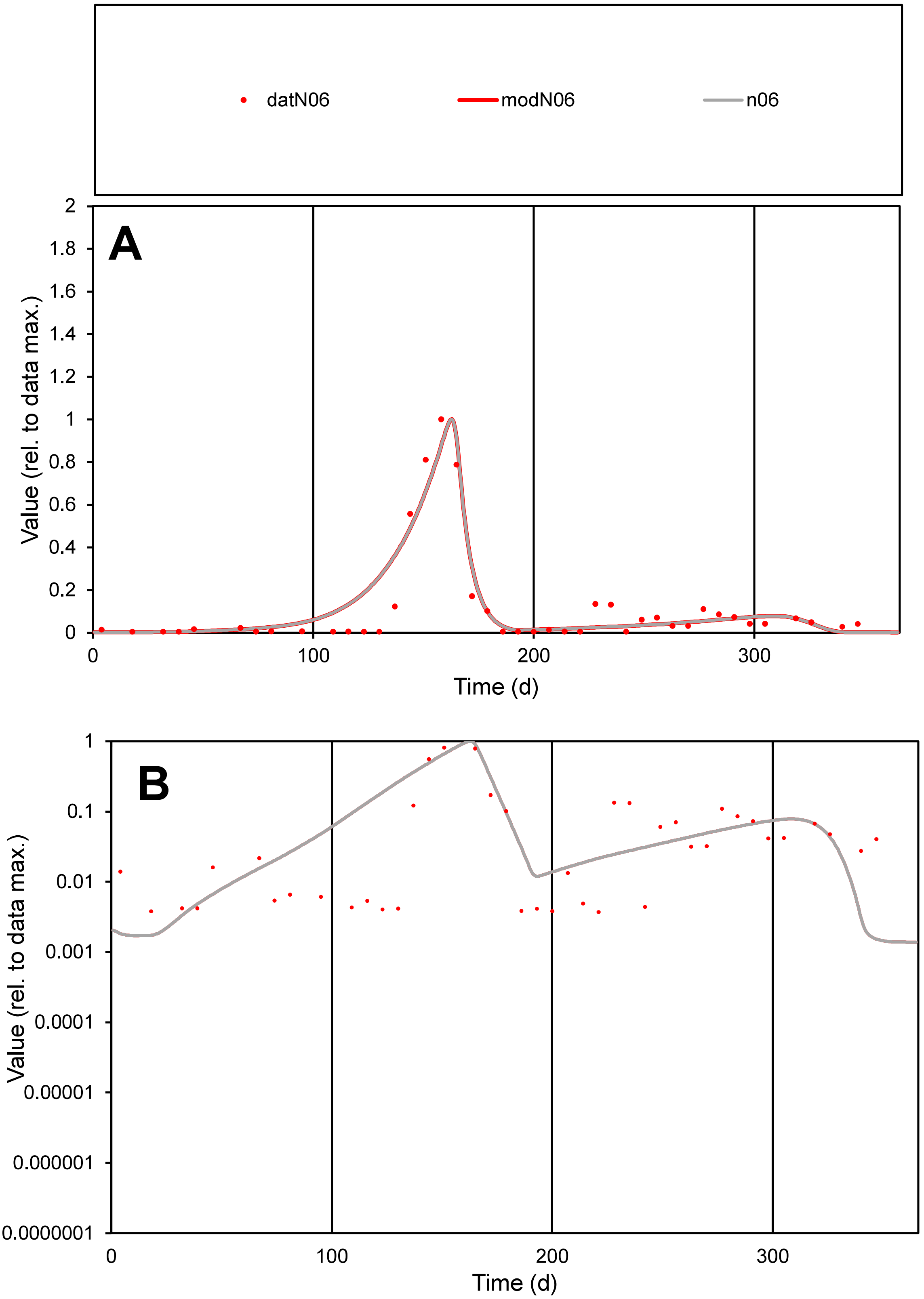

C1

Variables
N06 Provide jtd or Dst
CalibOnly?
2 1 to reproduce of
oNormalize?
2 1=no, 2=yes

ofxn calc*
0.68779
ofxn mod
0.73602

sRun
RunA1
zout
1

Ave
Data
0.00207
Model
0.0023

data/vMax
0.01855

ofxng
334.07

Min d/m
2.49982

ave/min d
30.0402
ave/min m
83.3829

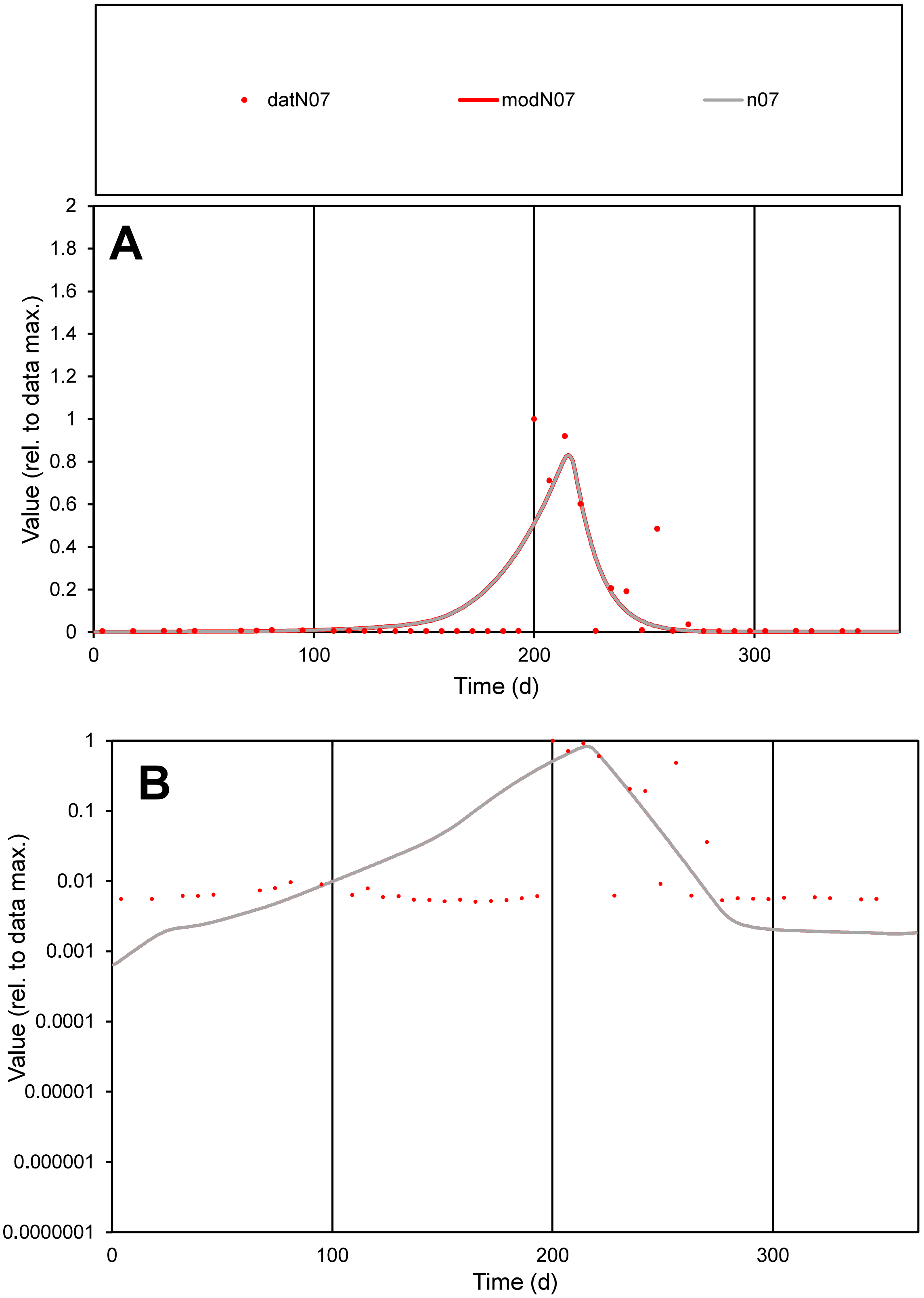

C1

Variables
N07 Provide jtd or Dst
CalibOnly?
2 1 to reproduce of
oNormalize?
2 1=no, 2=yes

ofxn calc*
1.33726
ofxn mod
1.39161

sRun
RunA1
zout
1

Ave
Data
0.00034
Model
0.00037

data/vMax
0.00331

ofxng
334.07

Min d/m
6.47239

ave/min d
20.3383
ave/min m
140.595

327_Calib_S_A

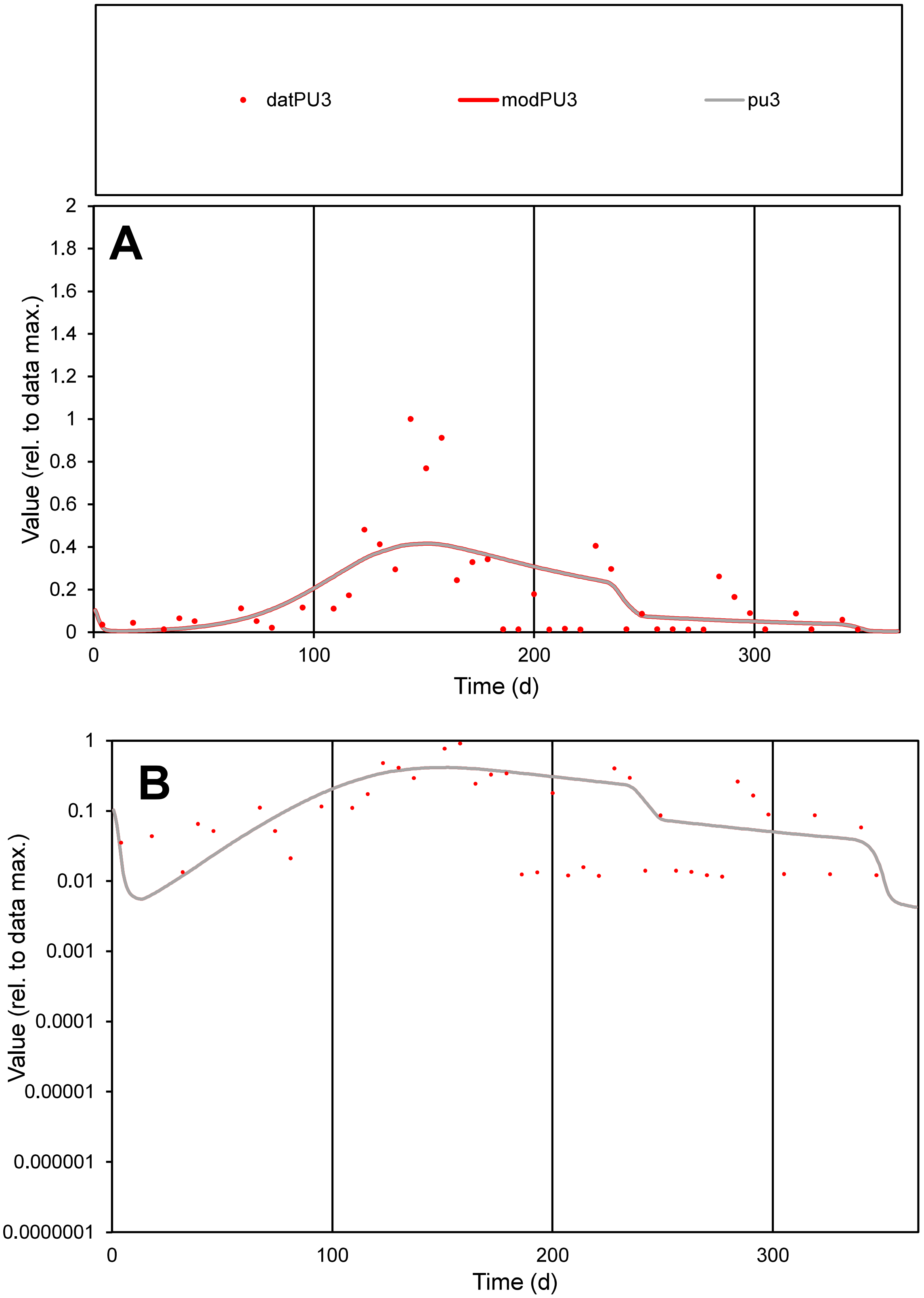

C1

Variables

PU3 Provide jtd or Dst

CalibOnly?

2 1 to reproduce of

oNormalize?

2 1=no, 2=yes

ofxn calc*

1.02924

ofxn mod

1.04024

sRun

RunA1

zout

1

Ave

Data

0.00235

Model

0.00243

data/vMax

0.01337

ofxng

334.07

Min d/m

1.70689

ave/min d

15.0812

ave/min m

26.6981

327_Calib_S_A

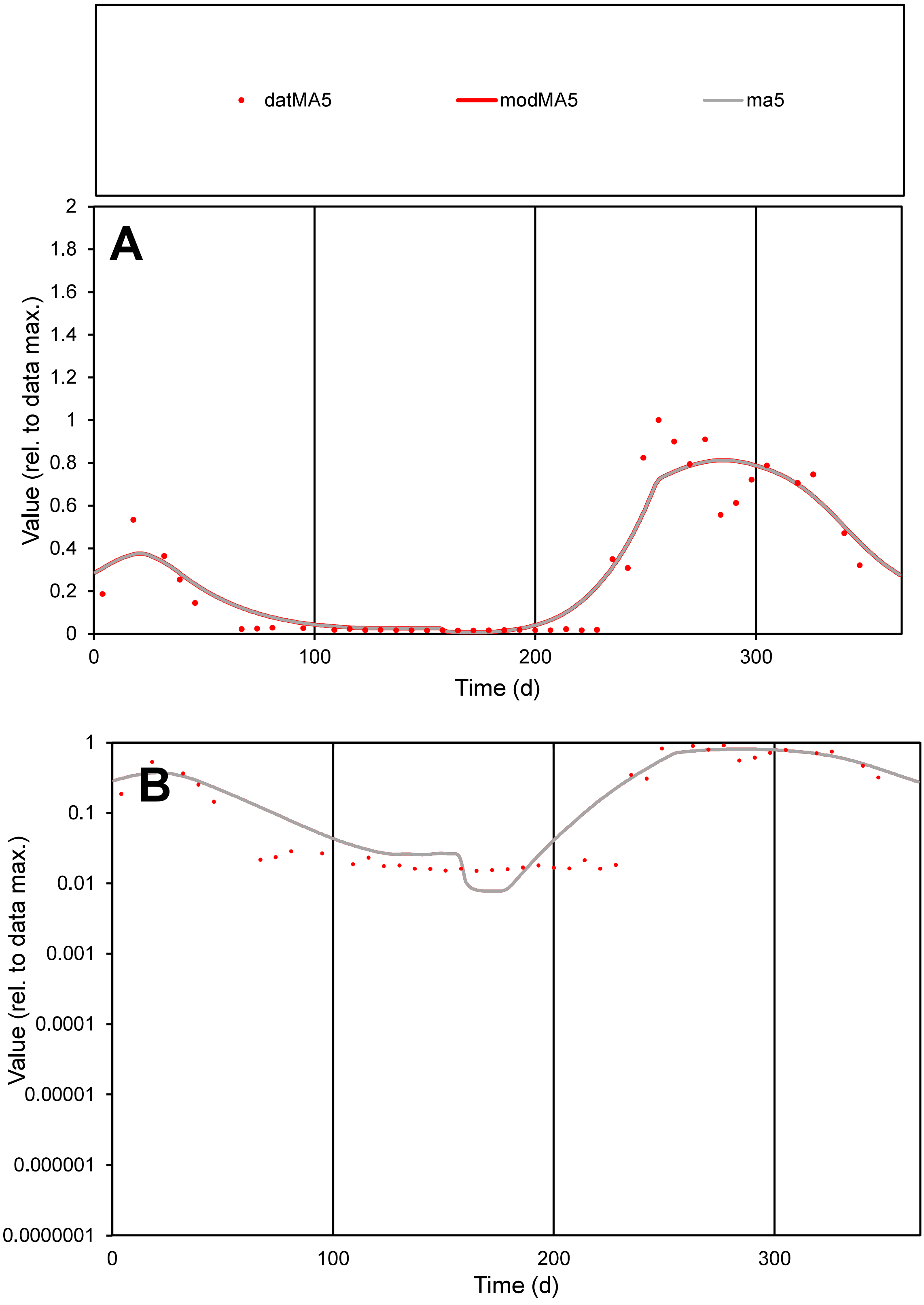

C1

Variables
MA5 Provide jtd or Dst
CalibOnly?
2 1 to reproduce of
oNormalize?
2 1=no, 2=yes

ofxn calc*
0.35669
ofxn mod
0.40043

sRun
RunA1
zout
1

Ave
Data
0.00175
Model
0.00184

data/vMax
0.00617

ofxng
334.07

Min d/m
1.94301

ave/min d
18.792
ave/min m
38.495

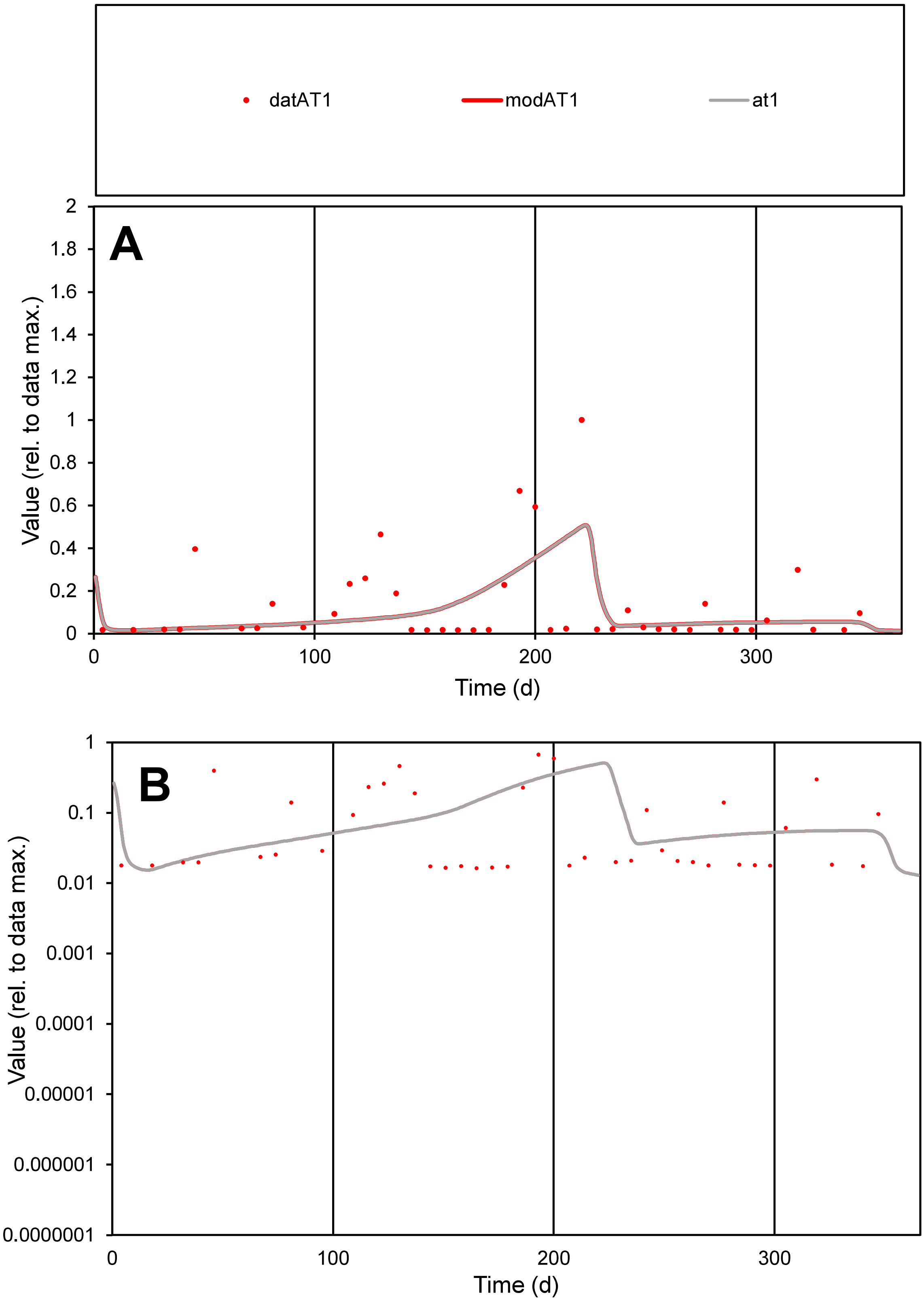

C1

Variables
AT1 Provide jtd or Dst
CalibOnly?
2 1 to reproduce of
oNormalize?
2 1=no, 2=yes

ofxn calc*
1.3694
ofxn mod
1.38376

sRun
RunA1
zout
1

Ave
Data
0.00038
Model
0.00032

data/vMax
0.0029

ofxng
334.07

Min d/m
1.01608

ave/min d
7.98814
ave/min m
6.9174

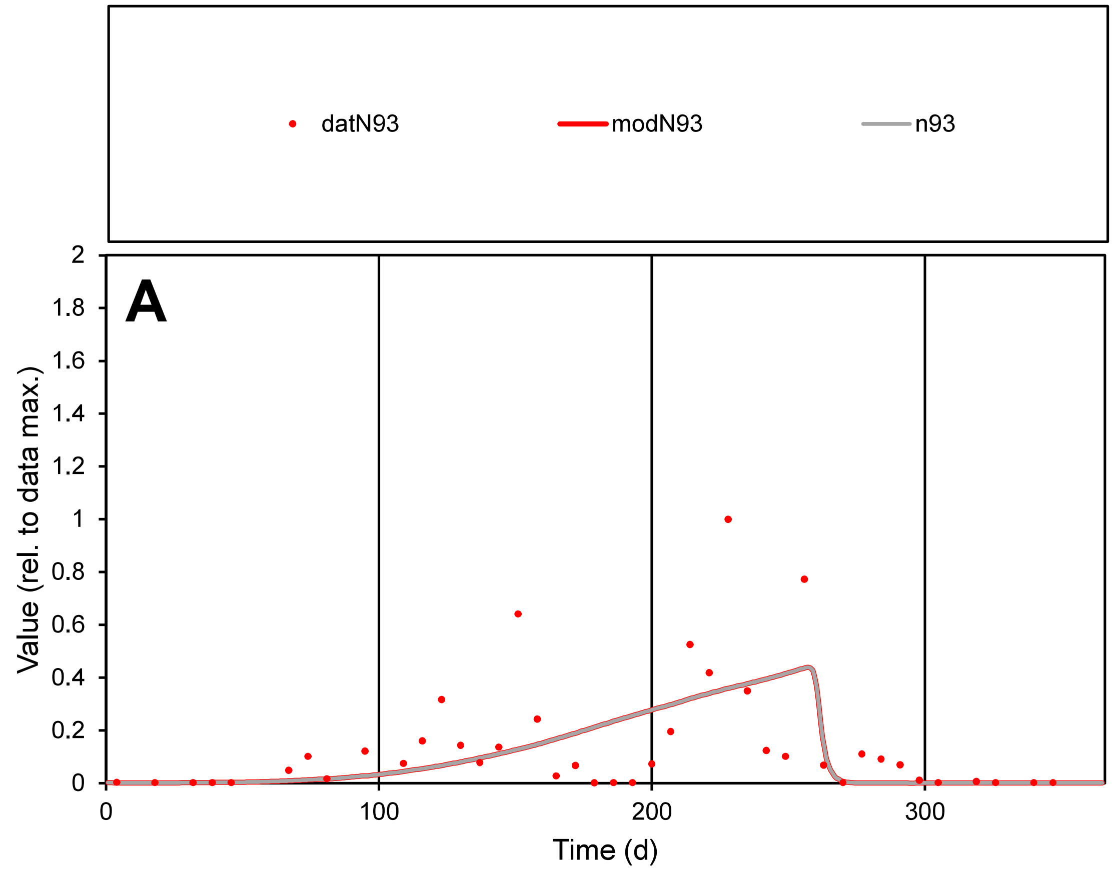

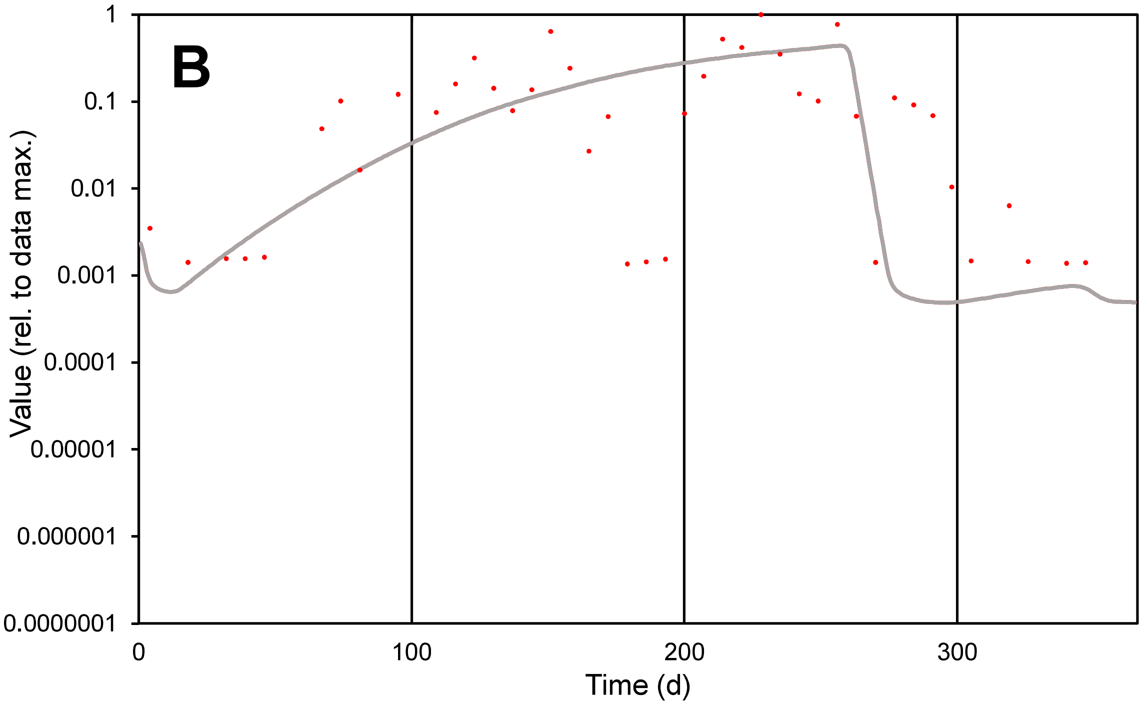

C1

Variables
N93 Provide jtd or Dst
CalibOnly?
2 1 to reproduce of
oNormalize?
2 1=no, 2=yes

ofxn calc*
1.24699
ofxn mod
1.25938

sRun
RunA1
zout
1

Ave
Data
0.00193
Model
0.00165

data/vMax
0.0133

ofxng
334.07

Min d/m
2.79009

ave/min d
106.684
ave/min m
254.402

327_Calib_S_A

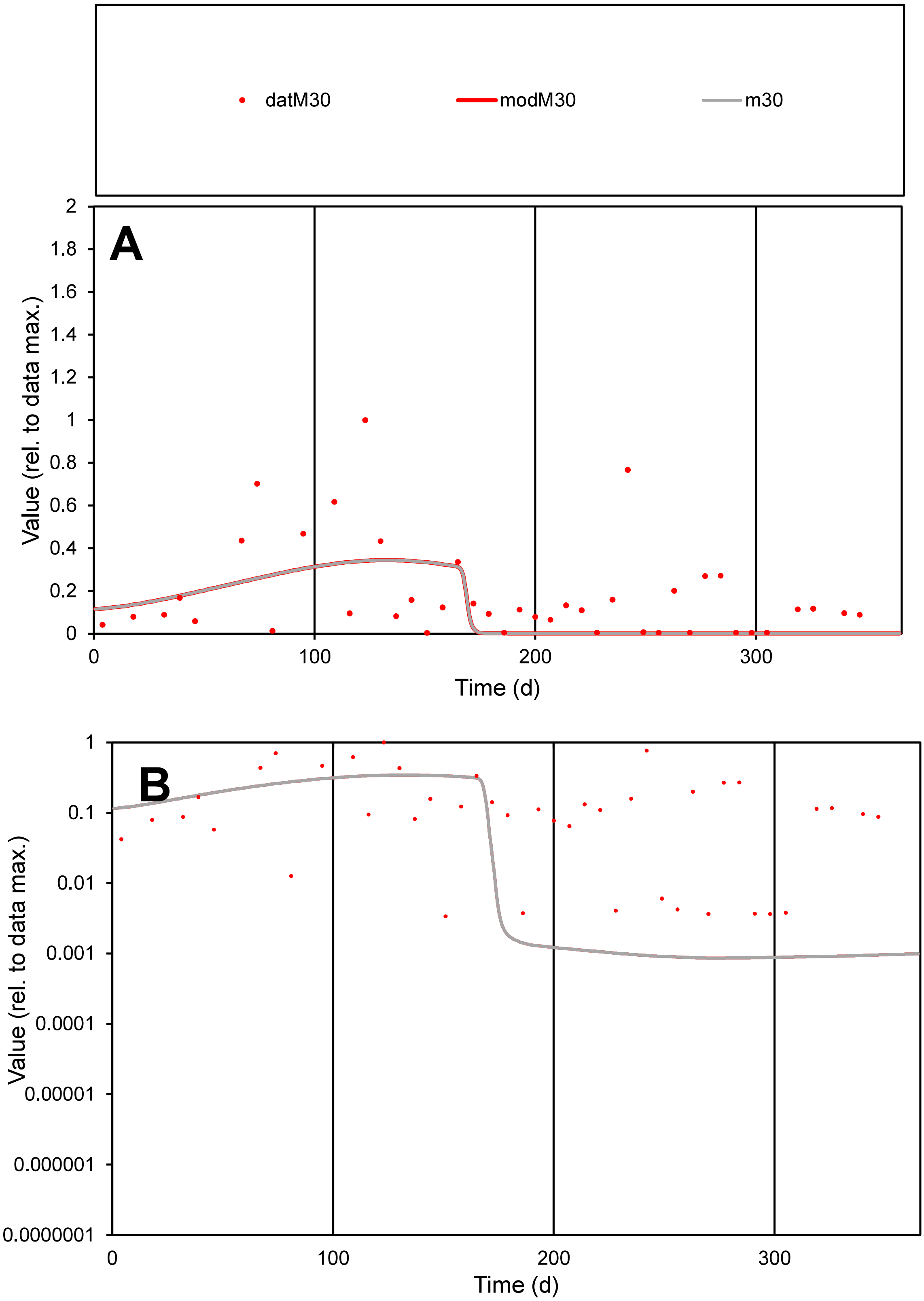

C1

Variables

M30 Provide jtd or Dst

CalibOnly?

2 1 to reproduce of

oNormalize?

2 1=no, 2=yes

ofxn calc*

1.21153

ofxn mod

1.22107

sRun

RunA1

zout

1

Ave

Data

0.00223

Model

0.00144

data/vMax

0.01212

ofxng

334.07

Min d/m

3.91874

ave/min d

54.4747

ave/min m

137.437

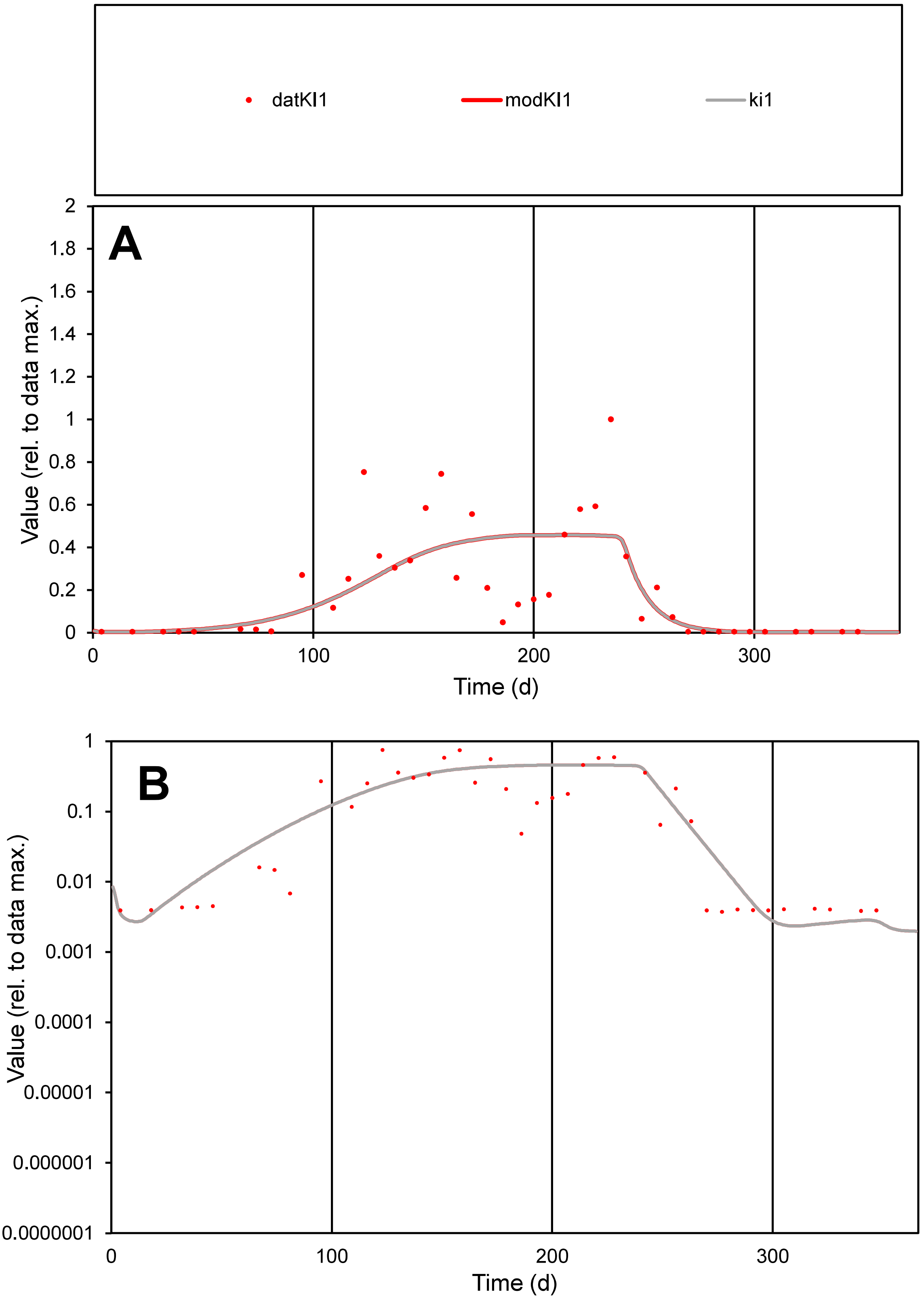

C1

Variables
Kl1 Provide jtd or Dst
CalibOnly?
2 1 to reproduce of
oNormalize?
2 1=no, 2=yes

ofxn calc*
0.86701
ofxn mod
0.88182

sRun
RunA1
zout
1

Ave
Data
0.00266
Model
0.00255

data/vMax
0.01287

ofxng
334.07

Min d/m
1.56481

ave/min d
55.1833
ave/min m
82.6656

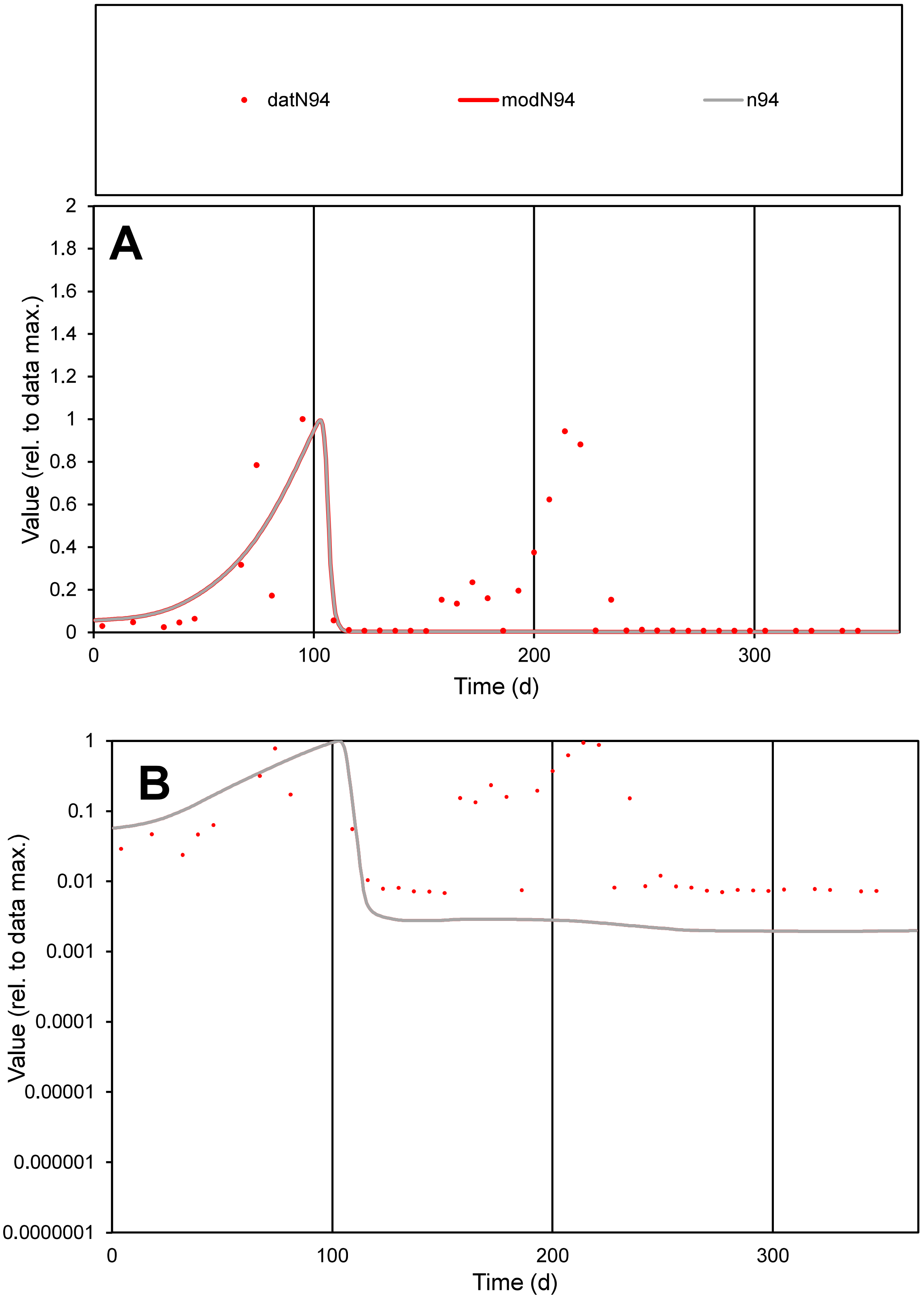

C1

Variables
N94 Provide jtd or Dst
CalibOnly?
2 1 to reproduce of
oNormalize?
2 1=no, 2=yes

ofxn calc*
1.61053
ofxn mod
1.6185

sRun
RunA1
zout
1

Ave
Data
0.00104
Model
0.00047

data/vMax
0.00667

ofxng
334.07

Min d/m
3.51783

ave/min d
22.9854
ave/min m
36.1394

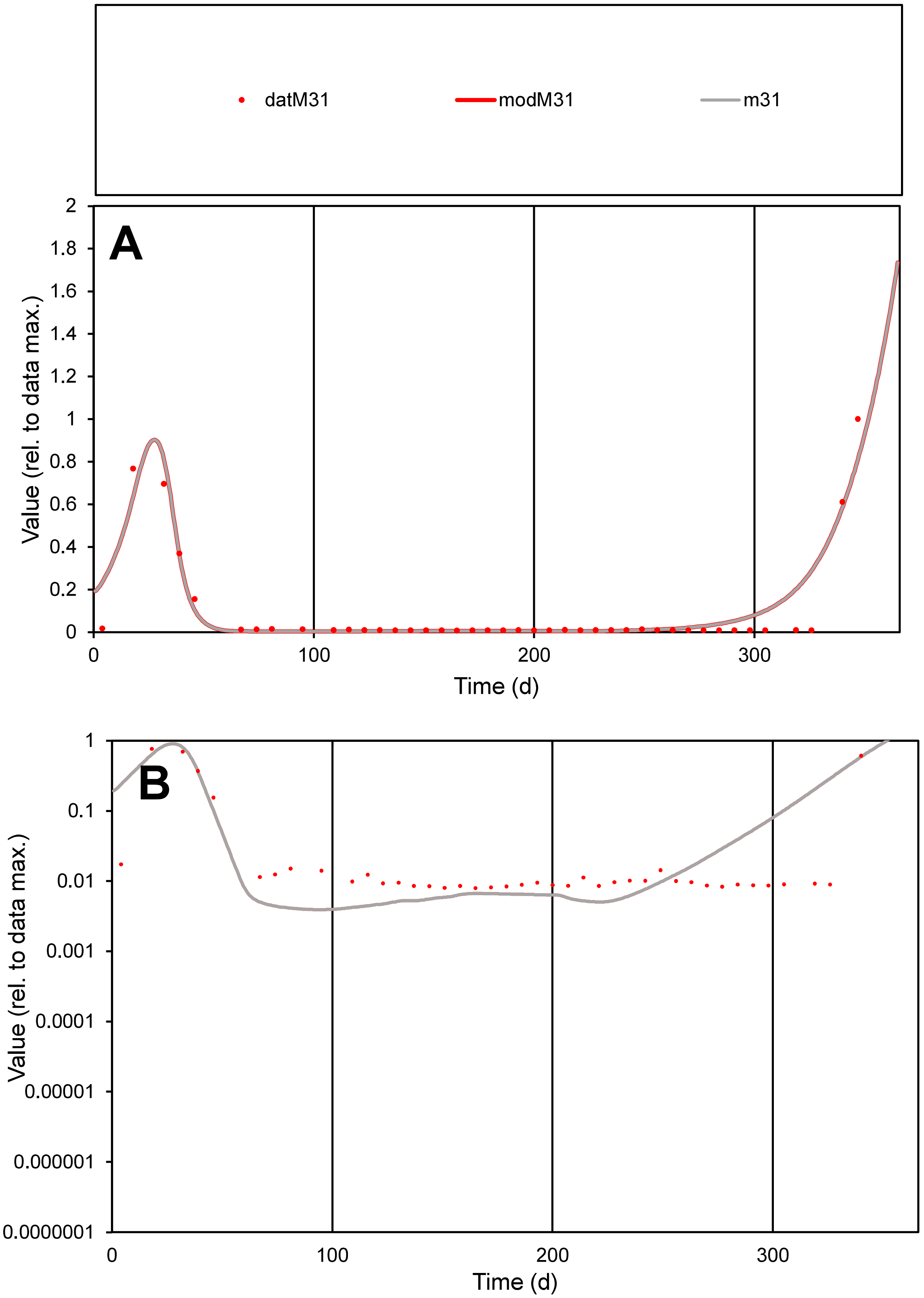

C1

Variables
M31 Provide jtd or Dst
CalibOnly?
2 1 to reproduce of
oNormalize?
2 1=no, 2=yes

ofxn calc*
0.71135
ofxn mod
0.82117

sRun
RunA1
zout
1

Ave
Data
0.00095
Model
0.00109

data/vMax
0.01013

ofxng
334.07

Min d/m
2.01955

ave/min d
11.8463
ave/min m
27.3571

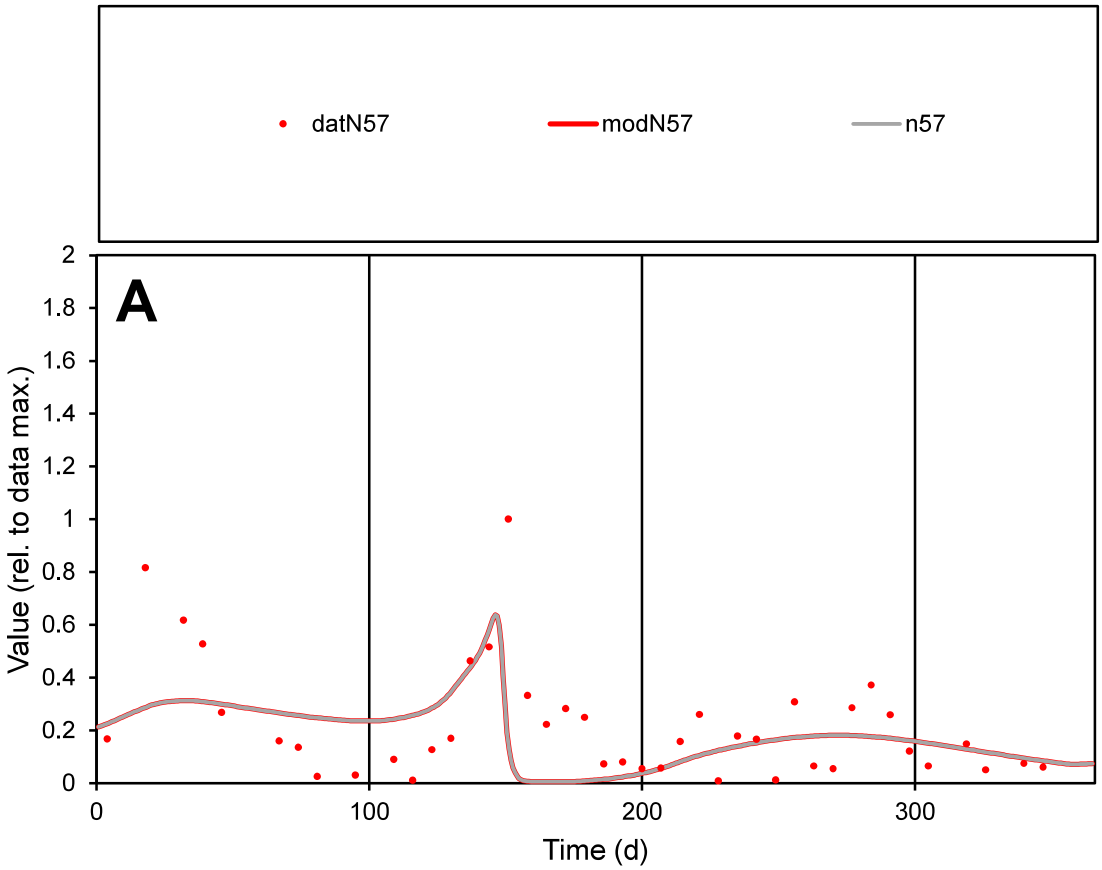

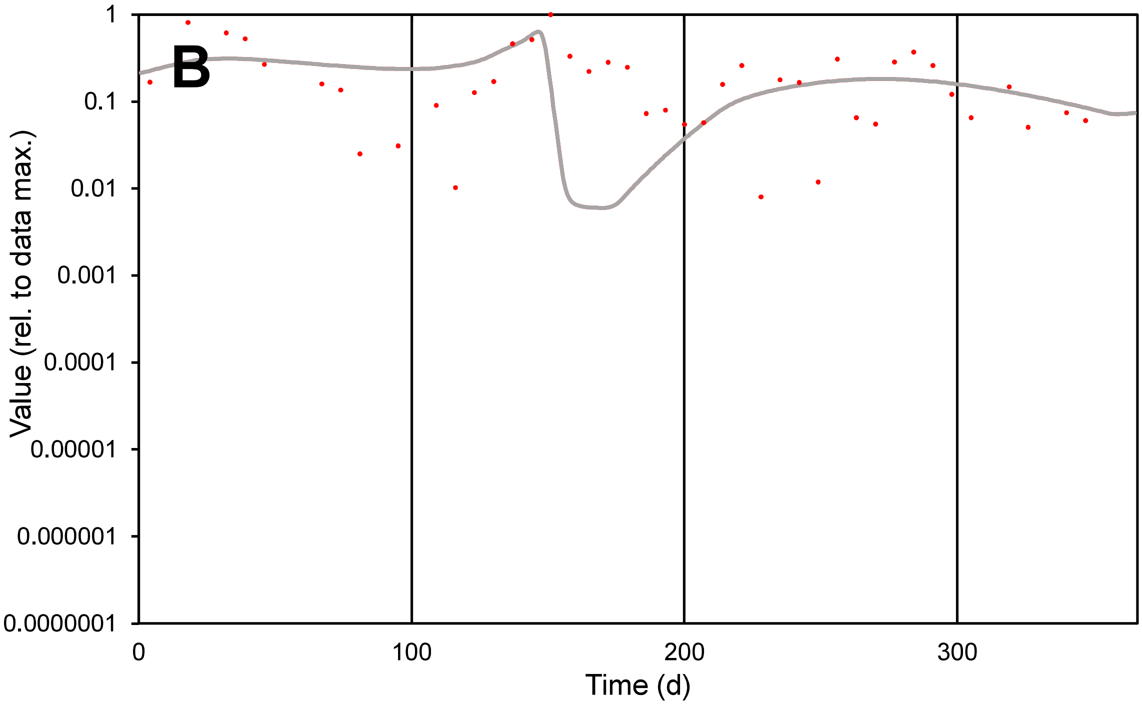

C1

Variables
N57 Provide jtd or Dst
CalibOnly?
2 1 to reproduce of
oNormalize?
2 1=no, 2=yes

ofxn calc*
0.87621
ofxn mod
0.94606

sRun
RunA1
zout
1

Ave
Data
0.00117
Model
0.00101

data/vMax
0.00541

ofxng
334.07

Min d/m
1.32601

ave/min d
27.0217
ave/min m
30.7248

327_Calib_S_A

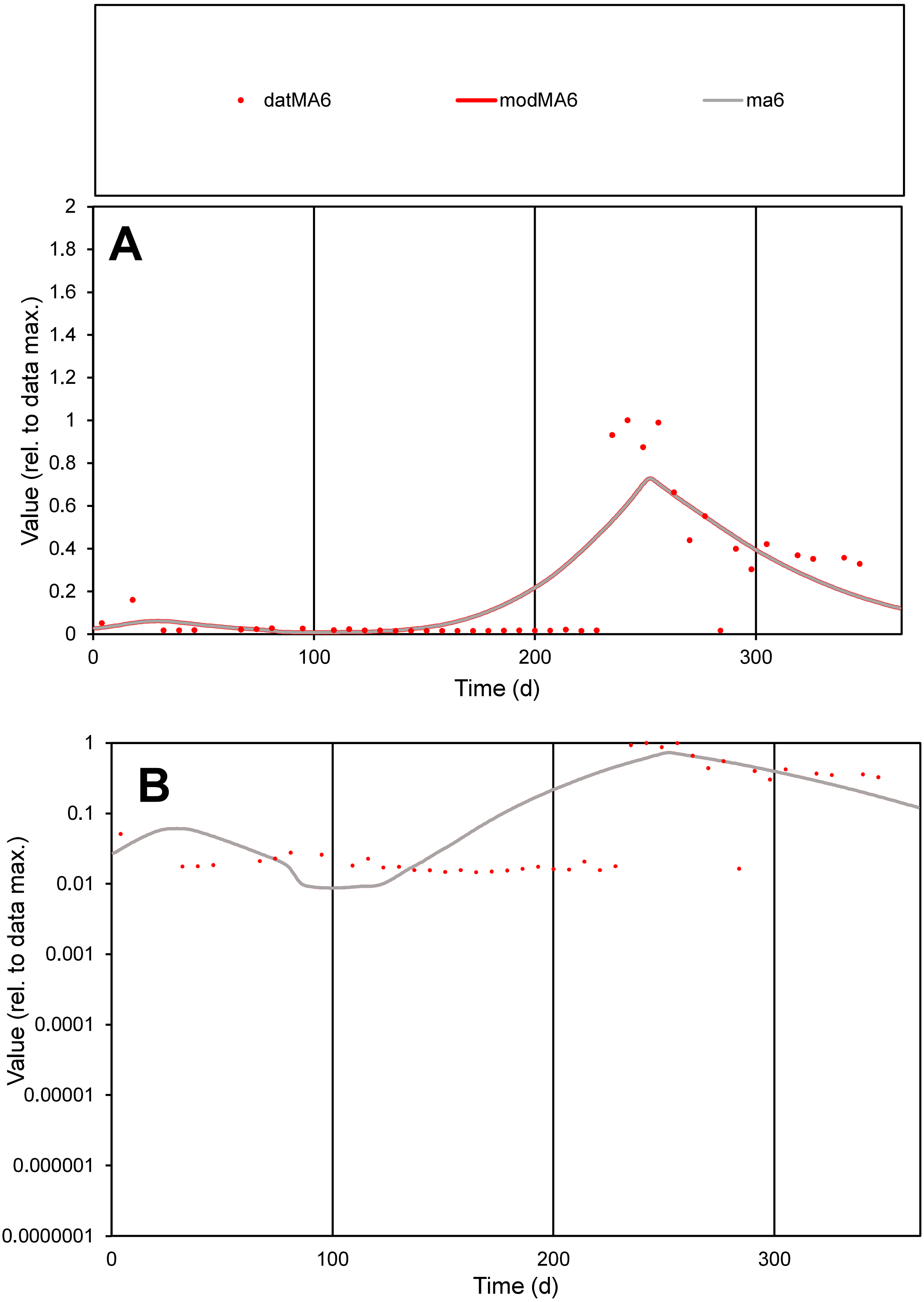

C1

Variables	
MA6	Provide jtd or Dst
CalibOnly?	
2	1 to reproduce of
oNormalize?	
2	1=no, 2=yes
ofxn calc*	
0.86173	
ofxn mod	
0.88999	
sRun	
RunA1	
zout	
1	
Ave	
Data	
0.00164	
Model	
0.00183	
data/vMax	
0.00798	
ofxng	
334.07	
Min d/m	
1.66786	
ave/min d	
14.0873	
ave/min m	
26.1853	

327_Calib_S_A

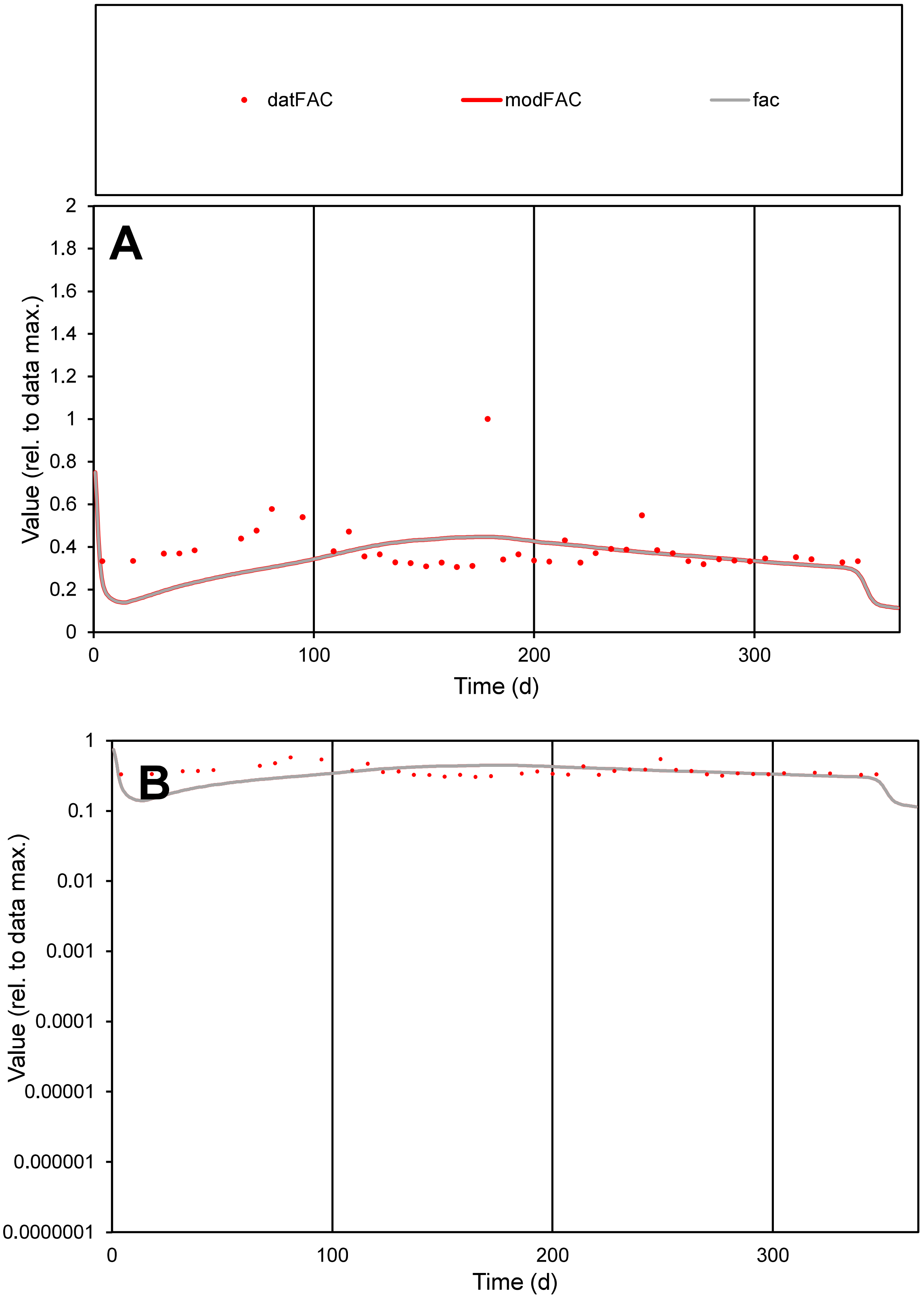

C1

Variables

FAC Provide jtd or Dst

CalibOnly?

2 1 to reproduce of

oNormalize?

2 1=no, 2=yes

ofxn calc*

0.35054

ofxn mod

0.36802

sRun

RunA1

zout

1

Ave

Data

0.00011

Model

1E-04

data/vMax

0.00028

ofxng

334.07

Min d/m

1.97939

ave/min d

1.26736

ave/min m

2.31097

327_Calib_S_A

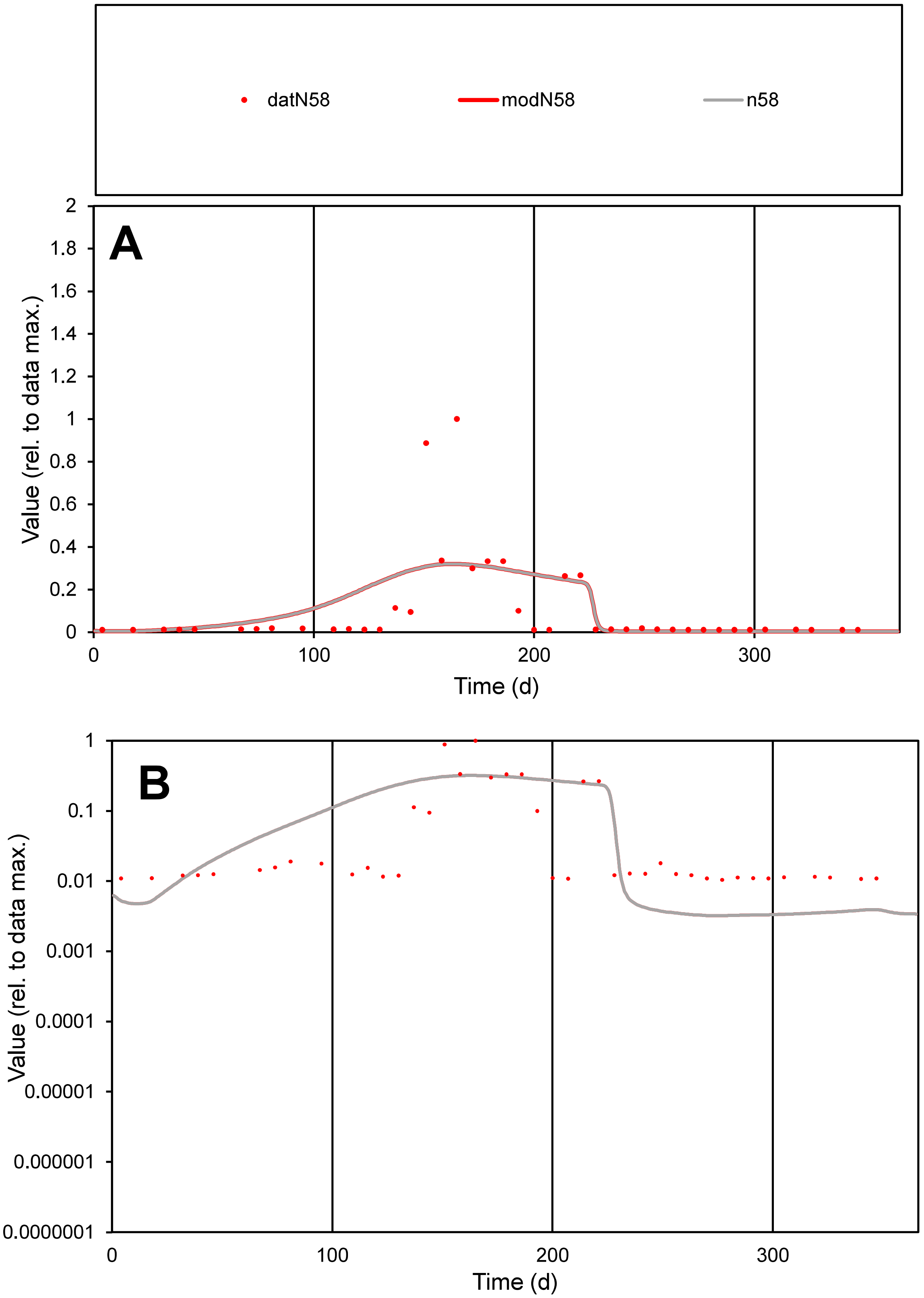

C1

Variables
N58 Provide jtd or Dst
CalibOnly?
2 1 to reproduce of
oNormalize?
2 1=no, 2=yes

ofxn calc*
1.58715
ofxn mod
1.59924

sRun
RunA1
zout
1

Ave
Data
0.00085
Model
0.00094

data/vMax
0.00808

ofxng
334.07

Min d/m
3.25568

ave/min d
10.0504
ave/min m
36.0655

327_Calib_S_A

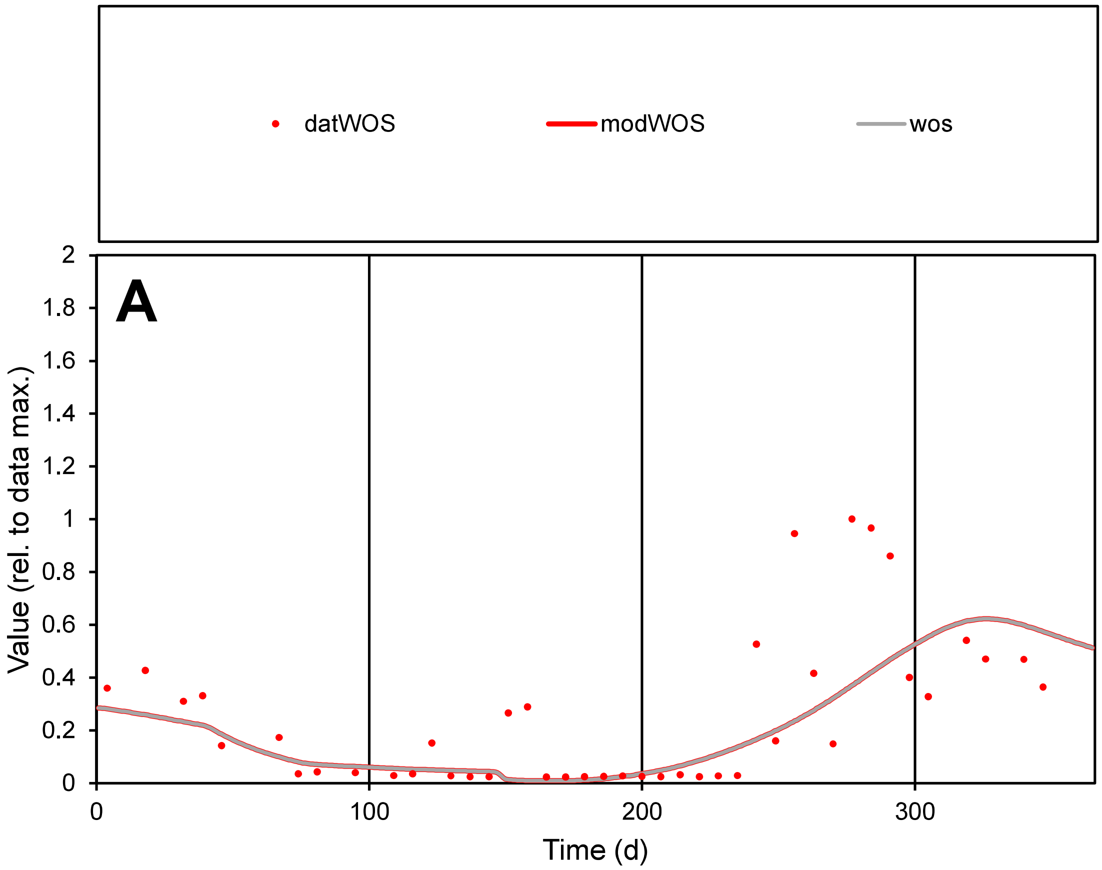

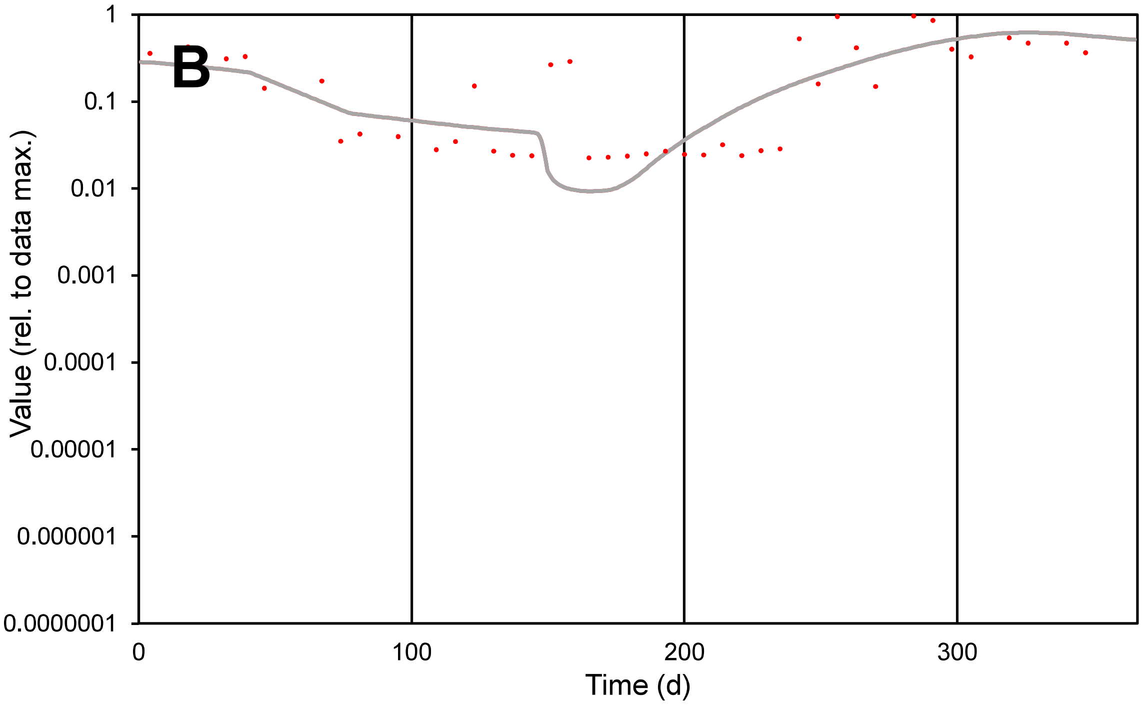

C1

Variables

WOS Provide jtd or Dst

CalibOnly?

2 1 to reproduce of

oNormalize?

2 1=no, 2=yes

ofxn calc*

0.8418

ofxn mod

0.85374

sRun

RunA1

zout

1

Ave

Data

0.00076

Model

0.0006

data/vMax

0.00301

ofxng

334.07

Min d/m

2.40064

ave/min d

11.2155

ave/min m

21.2933

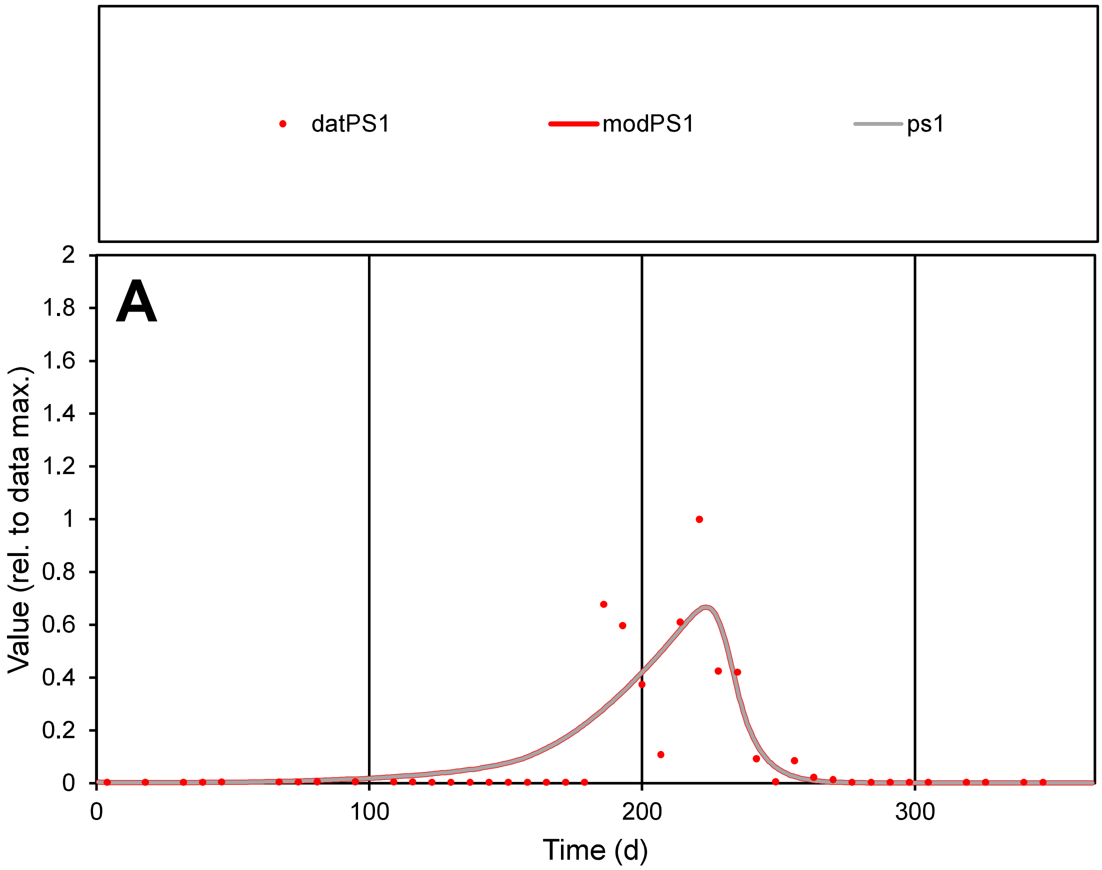

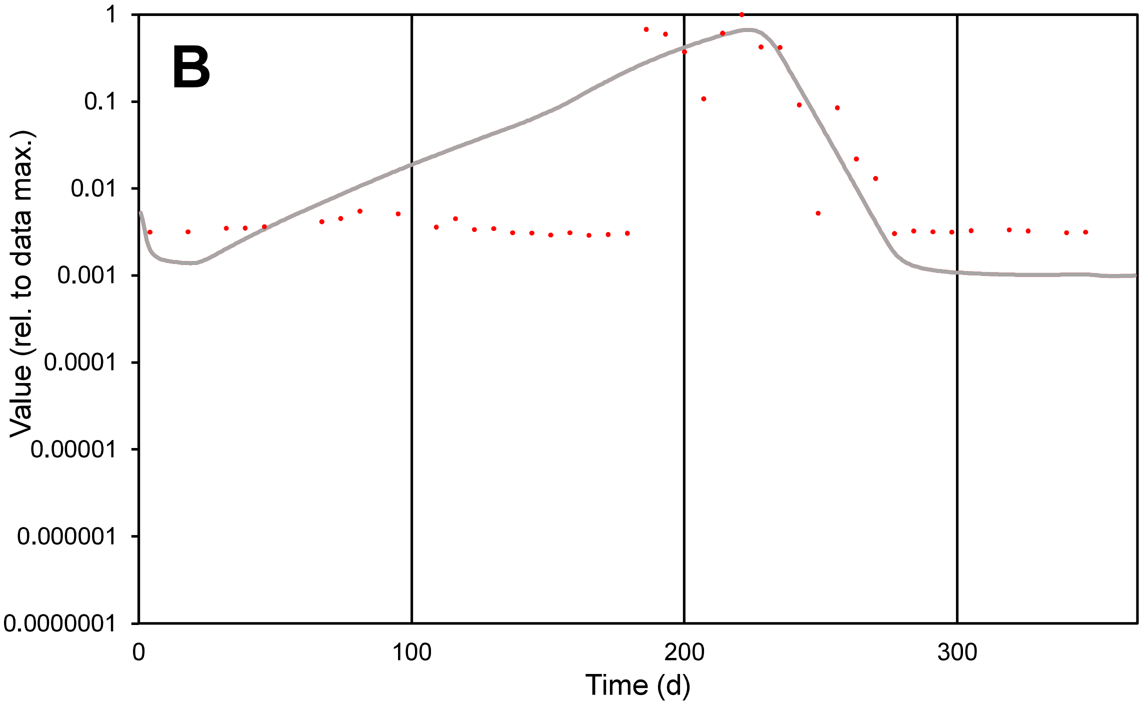

C1

Variables

PS1	Provide jtd or Dst
CalibOnly?	
2	1 to reproduce of
oNormalize?	
2	1=no, 2=yes

ofxn calc*
1.13336
ofxn mod
1.1697

sRun
RunA1
zout
1

Ave
Data
0.00173
Model
0.0019

data/vMax
0.01608

ofxng
334.07

Min d/m
2.8432

ave/min d
37.2022
ave/min m
116.029

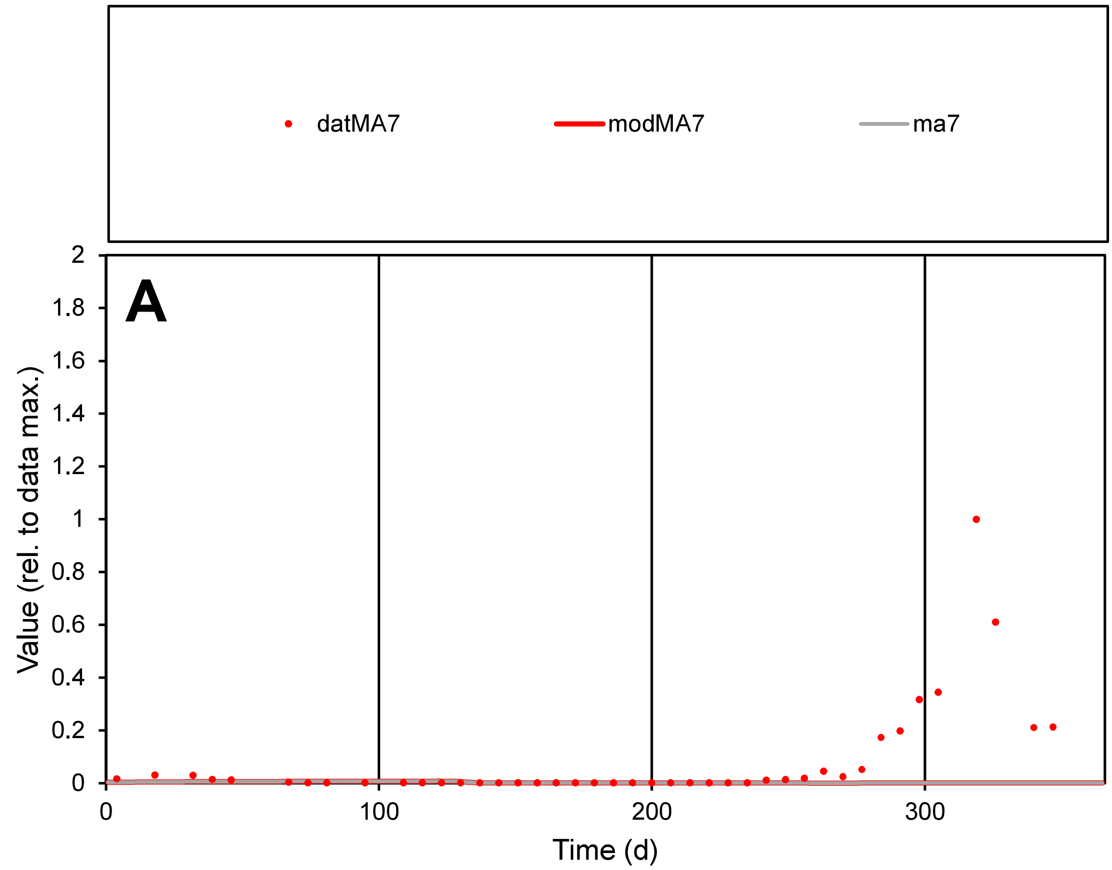

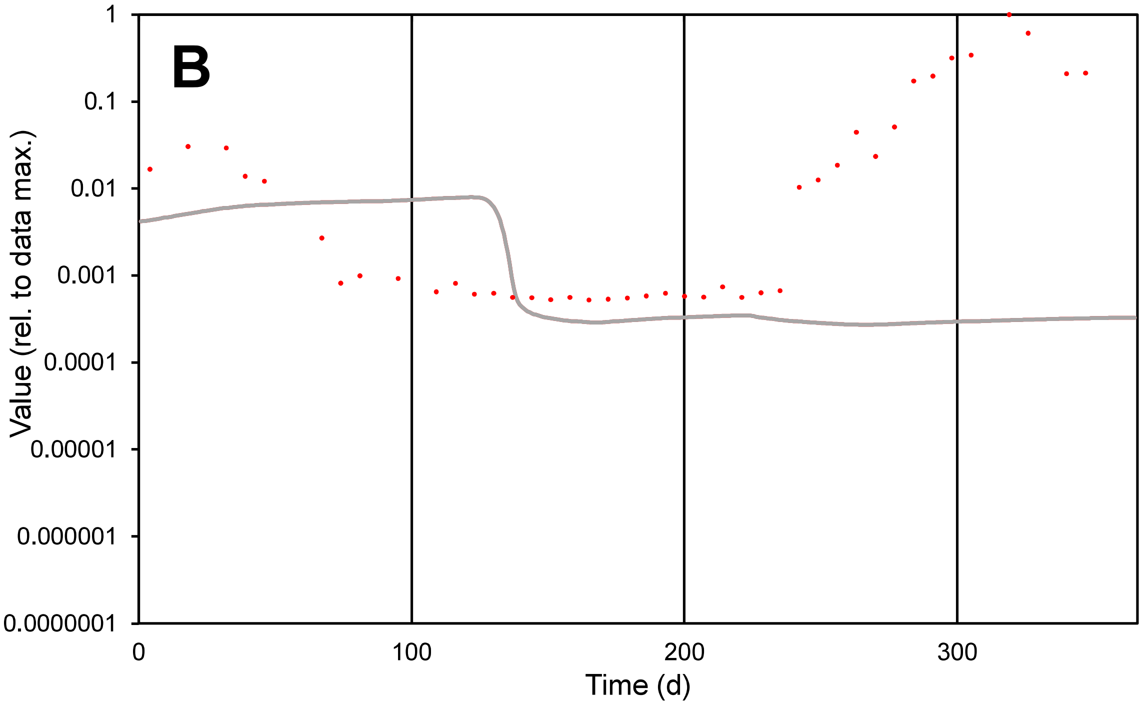

C1

Variables
MA7 Provide jtd or Dst
CalibOnly?
2 1 to reproduce of
oNormalize?
2 1=no, 2=yes

ofxn calc*
2.56763
ofxn mod
2.56948

sRun
RunA1
zout
1

Ave
Data
0.00203
Model
5.7E-05

data/vMax
0.02546

ofxng
334.07

Min d/m
1.92759

ave/min d
152.235
ave/min m
8.18941

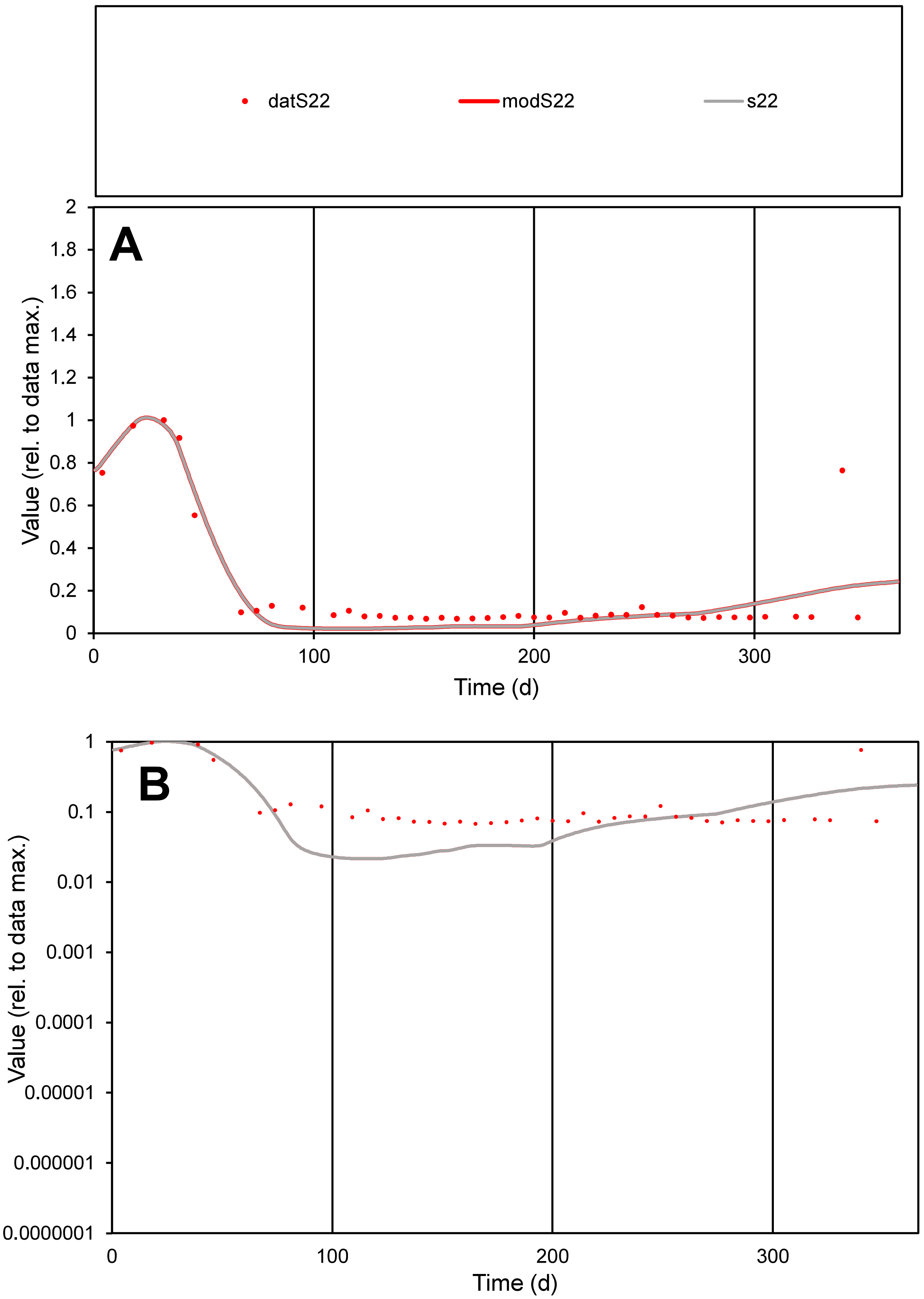

C1

Variables
S22 Provide jtd or Dst
CalibOnly?
2 1 to reproduce of
oNormalize?
2 1=no, 2=yes

ofxn calc*
0.54162
ofxn mod
0.5842

sRun
RunA1
zout
1

Ave
Data
0.0004
Model
0.00036

data/vMax
0.00214

ofxng
334.07

Min d/m
3.15309

ave/min d
2.78875
ave/min m
7.86885

327_Calib_S_A

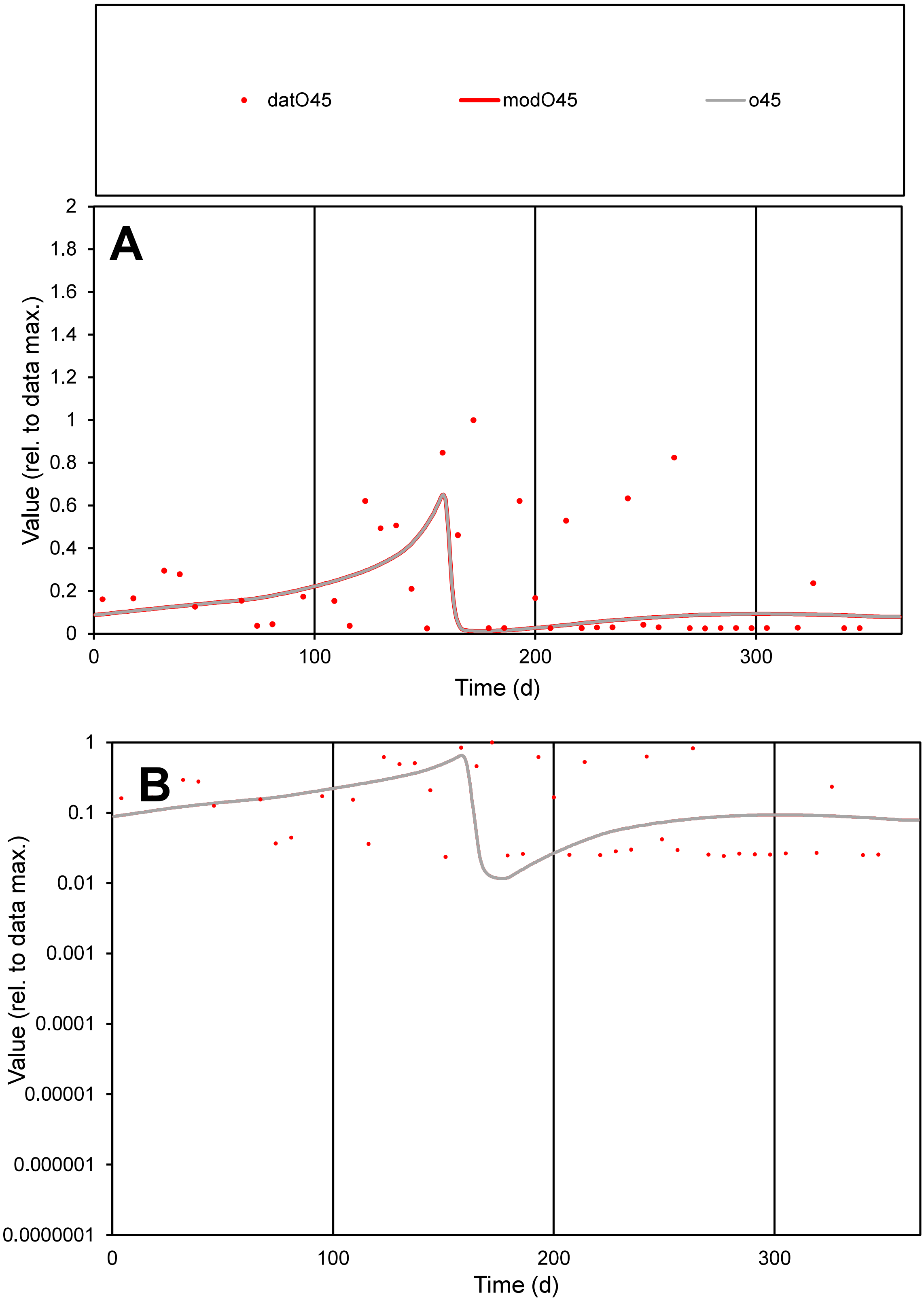

C1

Variables

O45 Provide jtd or Dst

CalibOnly?

2 1 to reproduce of

oNormalize?

2 1=no, 2=yes

ofxn calc*

1.2558

ofxn mod

1.27923

sRun

RunA1

zout

1

Ave

Data

0.00053

Model

0.00036

data/vMax

0.00242

ofxng

334.07

Min d/m

1.96631

ave/min d

9.32538

ave/min m

12.2414

327_Calib_S_A

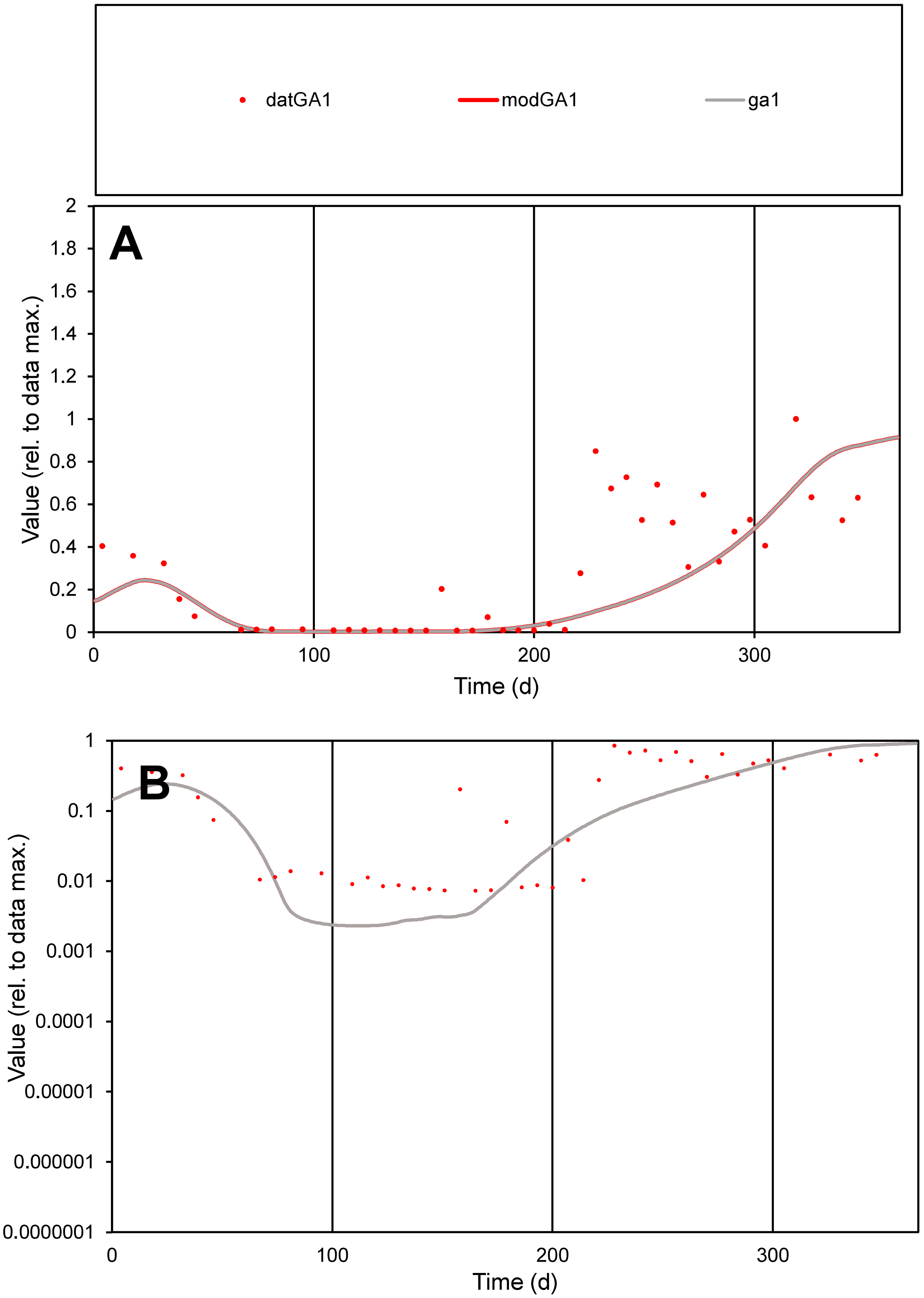

C1

Variables

GA1 Provide jtd or Dst

CalibOnly?

2 1 to reproduce of

oNormalize?

2 1=no, 2=yes

ofxn calc*

0.83805

ofxn mod

0.84817

sRun

RunA1

zout

1

Ave

Data

0.00132

Model

0.0009

data/vMax

0.0048

ofxng

334.07

Min d/m

3.17301

ave/min d

37.5751

ave/min m

81.5595

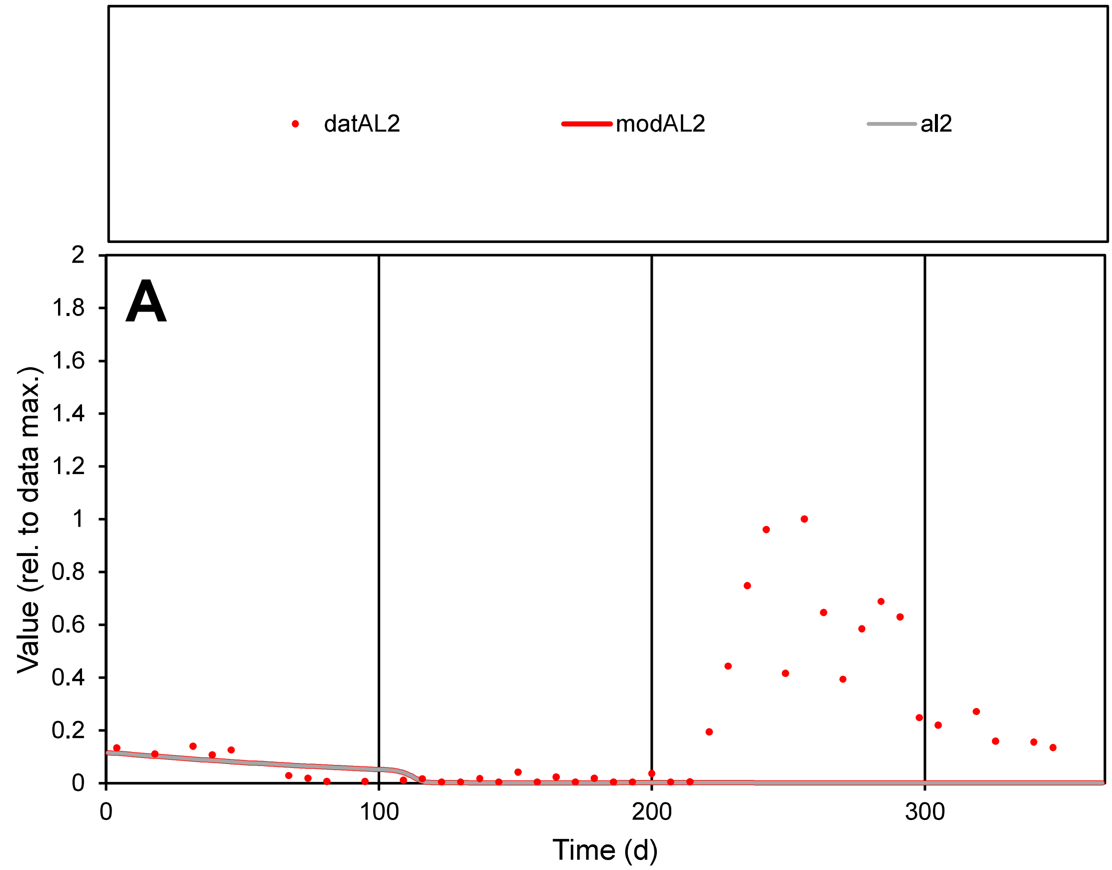

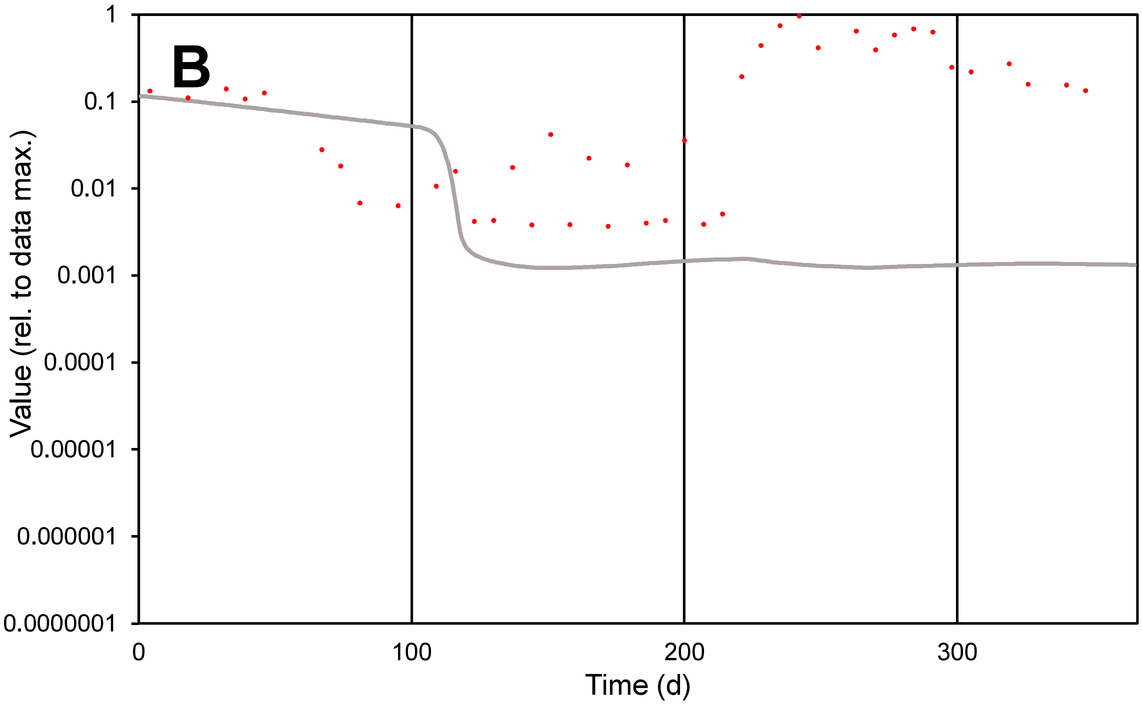

C1

Variables
AL2 Provide jtd or Dst
CalibOnly?
2 1 to reproduce of
oNormalize?
2 1=no, 2=yes

ofxn calc*
1.64047
ofxn mod
1.64399

sRun
RunA1
zout
1

Ave
Data
0.00125
Model
0.00011

data/vMax
0.00599

ofxng
334.07

Min d/m
2.99224

ave/min d
56.8828
ave/min m
15.5017

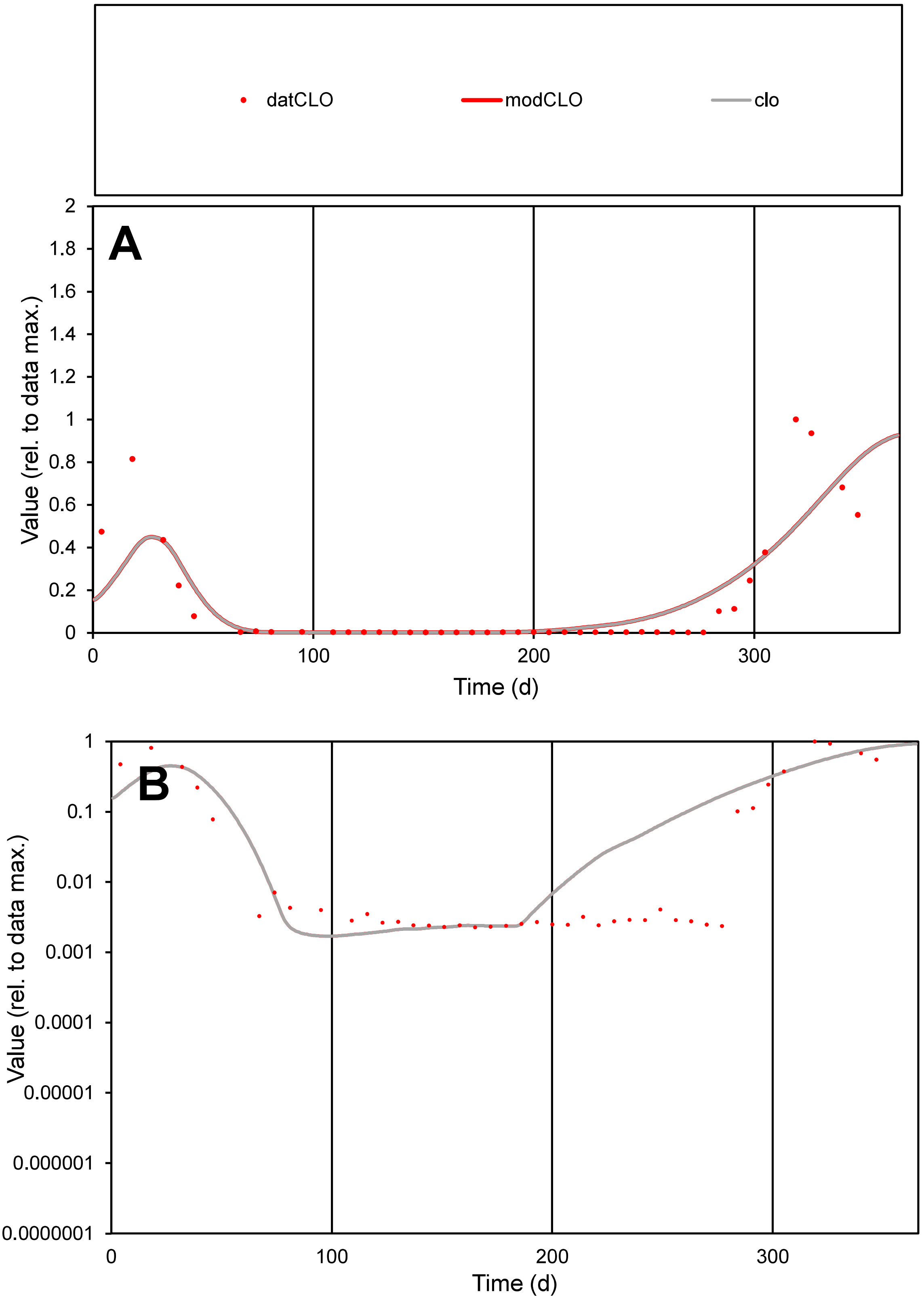

C1

Variables
CLO Provide jtd or Dst
CalibOnly?
2 1 to reproduce of
oNormalize?
2 1=no, 2=yes

ofxn calc*
0.95071
ofxn mod
0.9751

sRun
RunA1
zout
1

Ave
Data
0.00084
Model
0.00084

data/vMax
0.00575

ofxng
334.07

Min d/m
1.33306

ave/min d
64.1122
ave/min m
85.5609

327_Calib_S_A

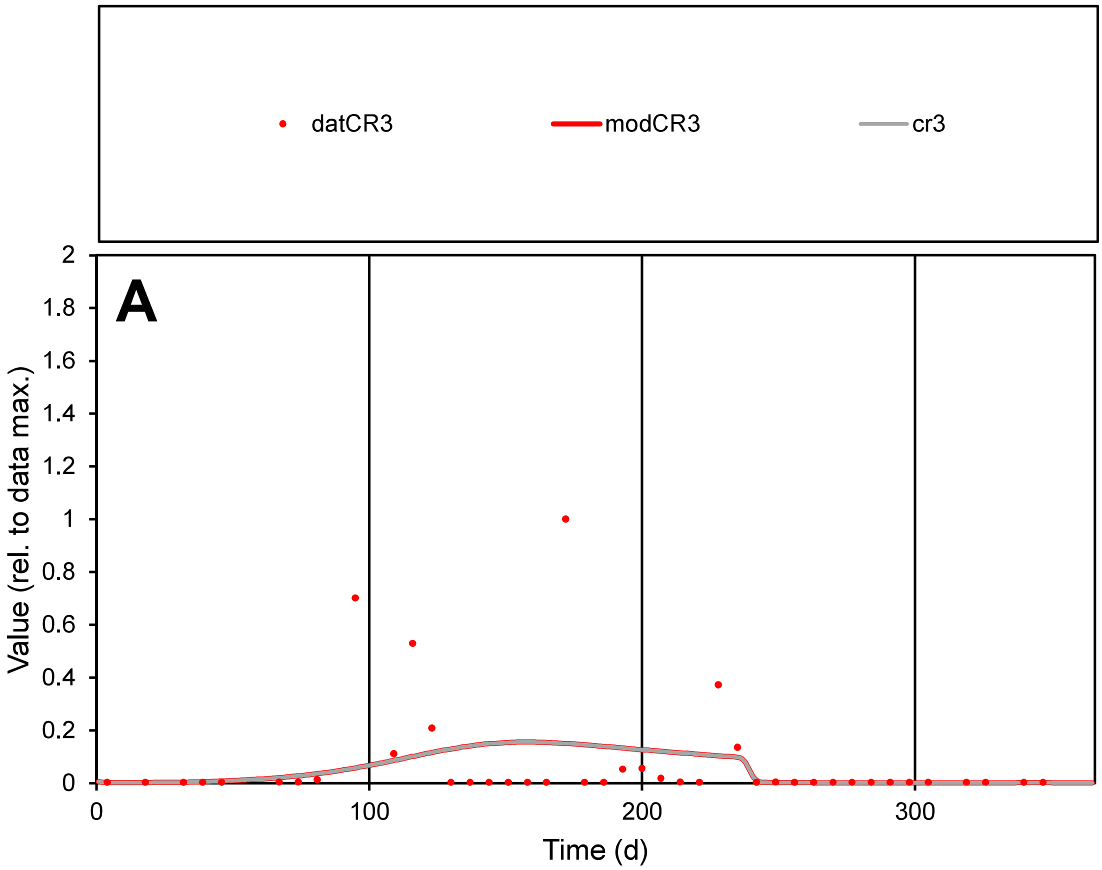

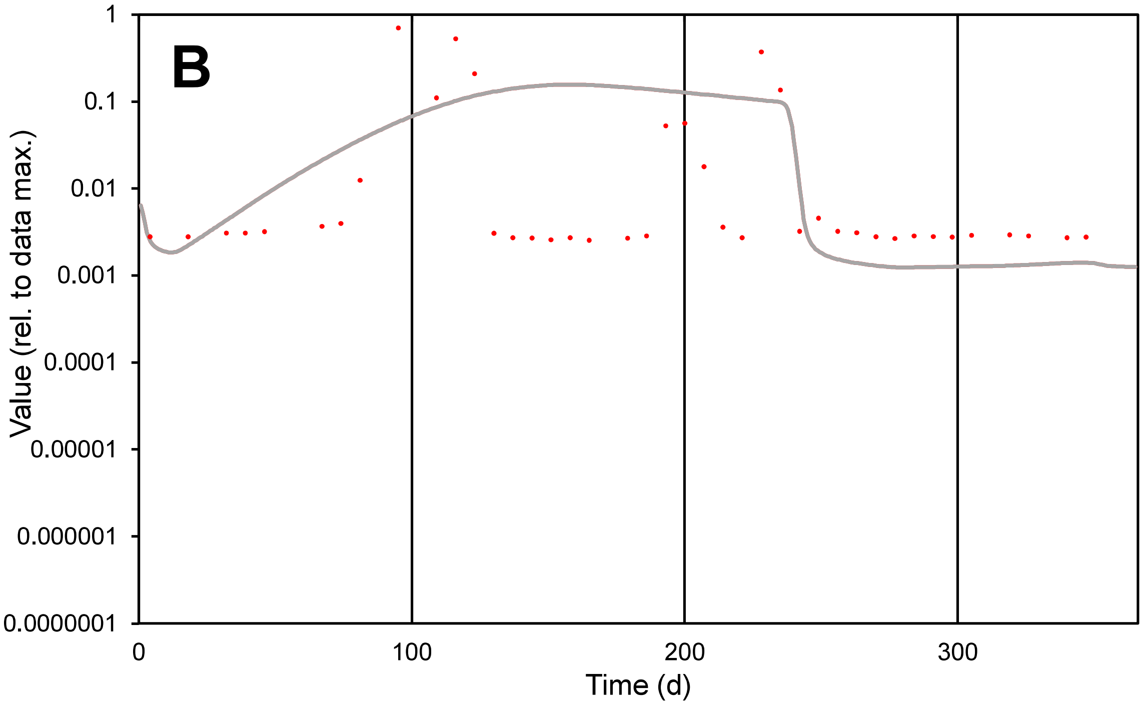

C1

Variables

CR3 Provide jtd or Dst

CalibOnly?

2 1 to reproduce of

oNormalize?

2 1=no, 2=yes

ofxn calc*

2.49793

ofxn mod

2.50224

sRun

RunA1

zout

1

Ave

Data

0.00108

Model

0.00086

data/vMax

0.01378

ofxng

334.07

Min d/m

2.05442

ave/min d

30.7546

ave/min m

50.3592

327_Calib_S_A

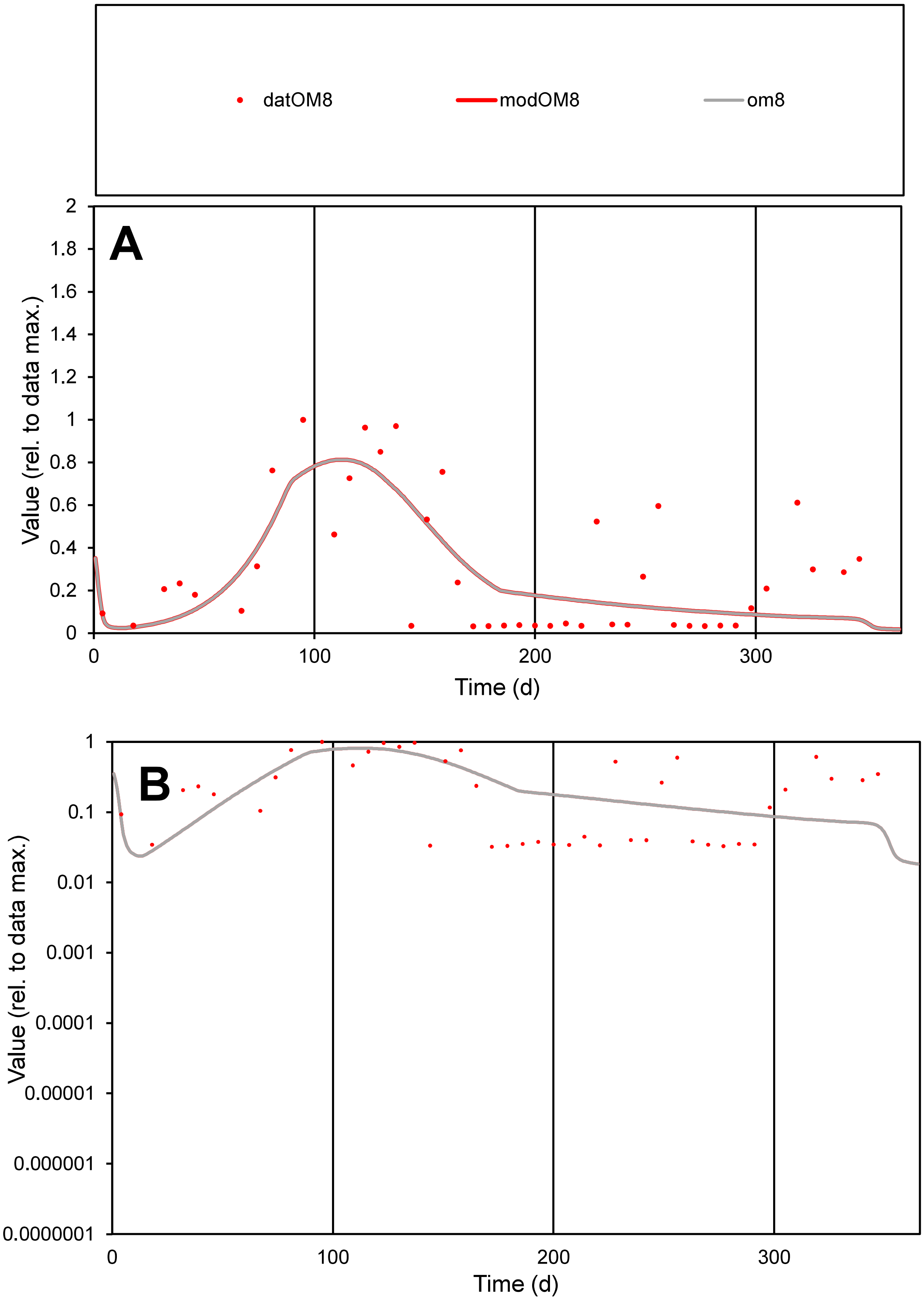

C1

Variables

OM8 Provide jtd or Dst

CalibOnly?

2 1 to reproduce of

oNormalize?

2 1=no, 2=yes

ofxn calc*

0.75027

ofxn mod

0.76258

sRun

RunA1

zout

1

Ave

Data

0.00049

Model

0.00045

data/vMax

0.0017

ofxng

334.07

Min d/m

1.0613

ave/min d

9.0621

ave/min m

8.73771

327_Calib_S_A

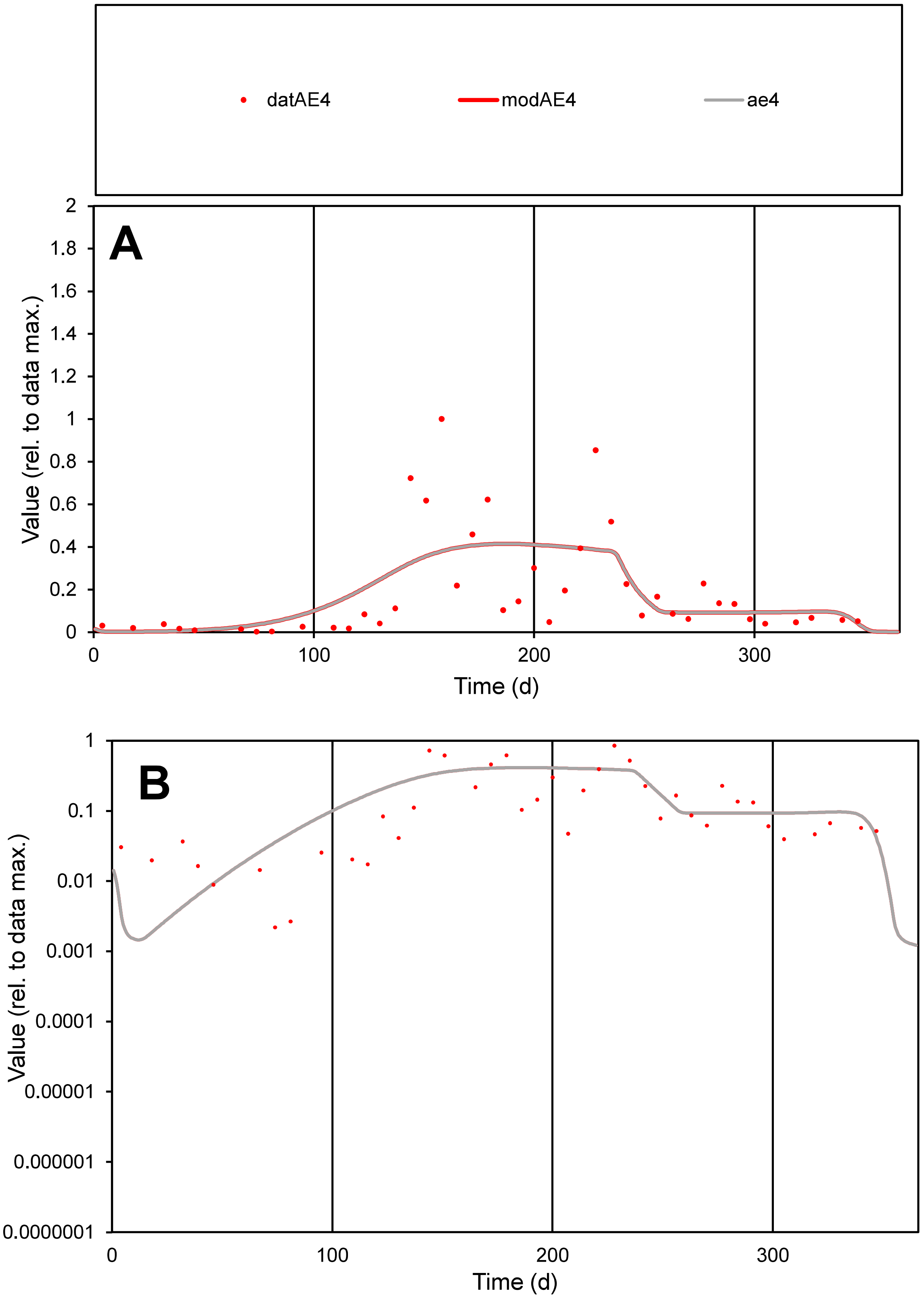

C1

Variables
AE4 Provide jtd or Dst
CalibOnly?
2 1 to reproduce of
oNormalize?
2 1=no, 2=yes

ofxn calc*
0.96691
ofxn mod
0.97857

sRun
RunA1
zout
1

Ave
Data
0.00157
Model
0.00161

data/vMax
0.0082

ofxng
334.07

Min d/m
1.07198

ave/min d
87.3634
ave/min m
95.8359

327_Calib_S_A

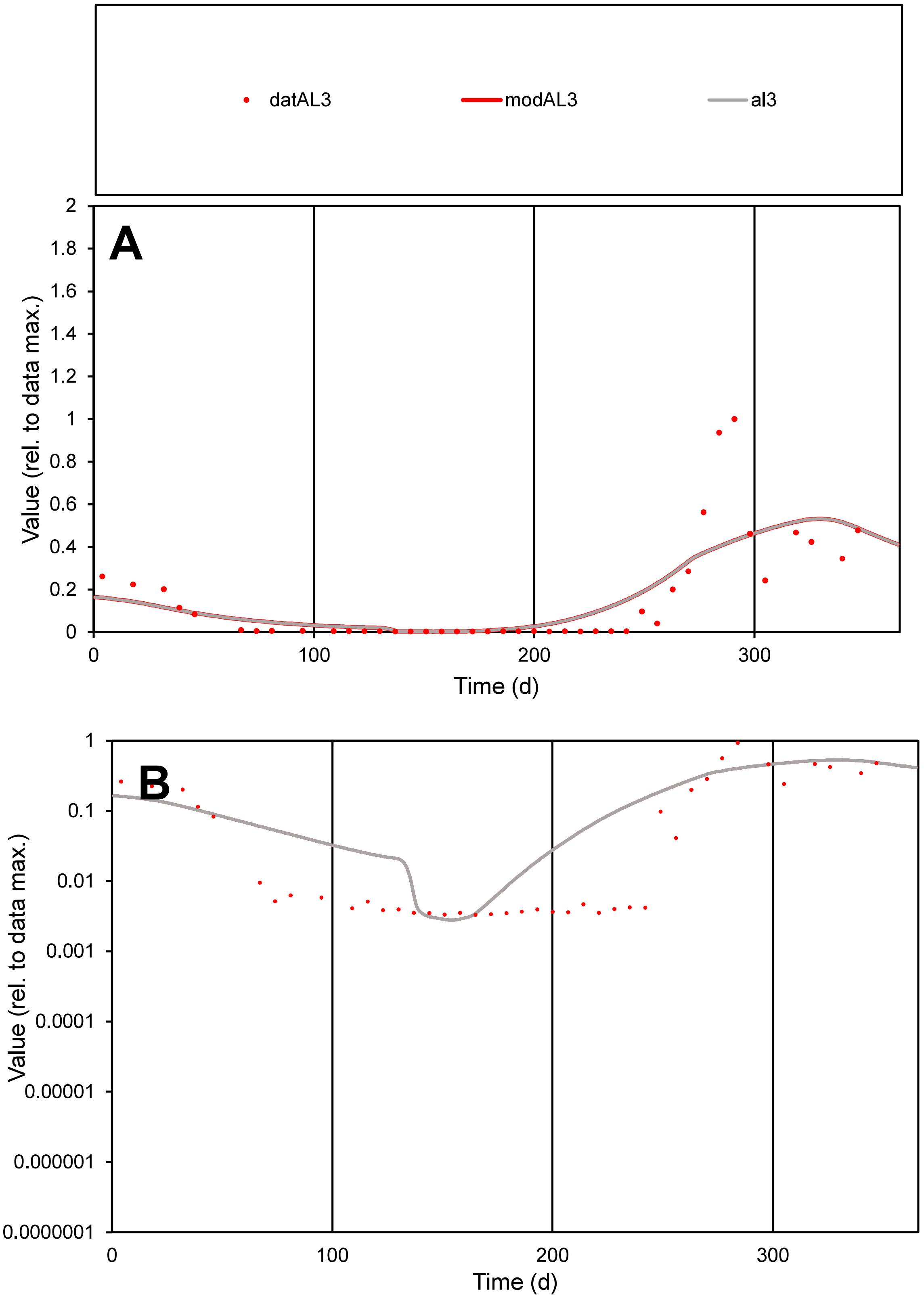

C1

Variables
AL3 Provide jtd or Dst
CalibOnly?
2 1 to reproduce of
oNormalize?
2 1=no, 2=yes

ofxn calc*
0.92788
ofxn mod
0.94556

sRun
RunA1
zout
1

Ave
Data
0.00112
Model
0.00117

data/vMax
0.00723

ofxng
334.07

Min d/m
1.17329

ave/min d
47.0399
ave/min m
57.1936

327_Calib_S_A

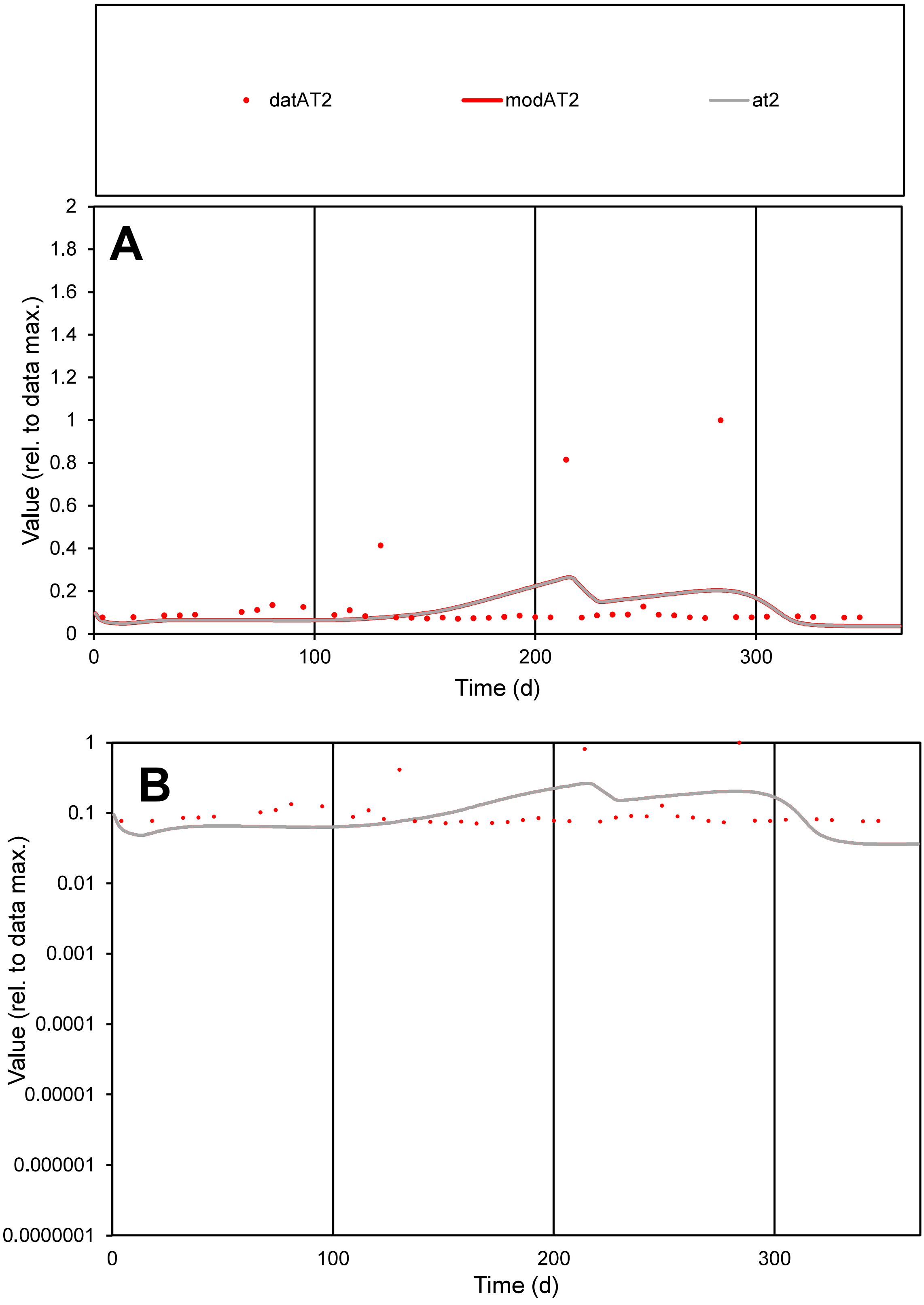

C1

Variables
AT2 Provide jtd or Dst
CalibOnly?
2 1 to reproduce of
oNormalize?
2 1=no, 2=yes

ofxn calc*
1.31127
ofxn mod
1.31848

sRun
RunA1
zout
1

Ave
Data
0.00015
Model
0.00014

data/vMax
0.00111

ofxng
334.07

Min d/m
1.96829

ave/min d
1.87387
ave/min m
3.48183

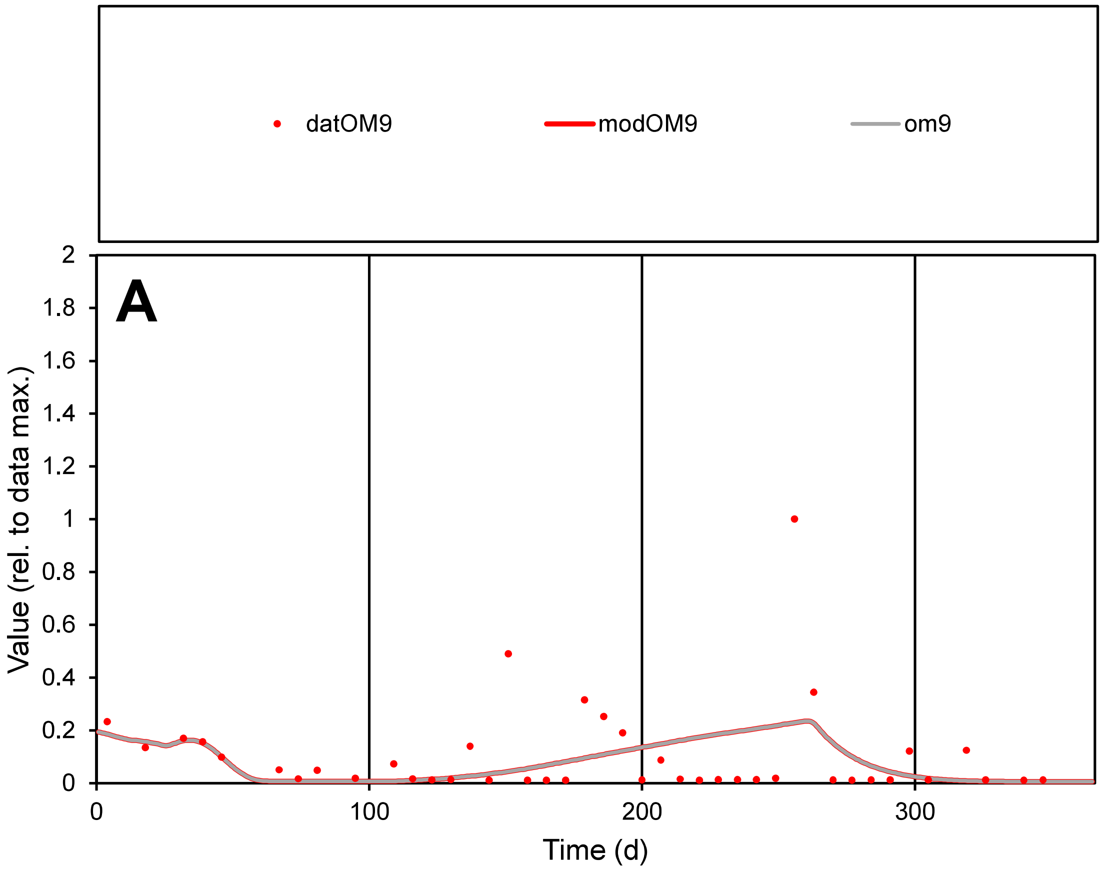

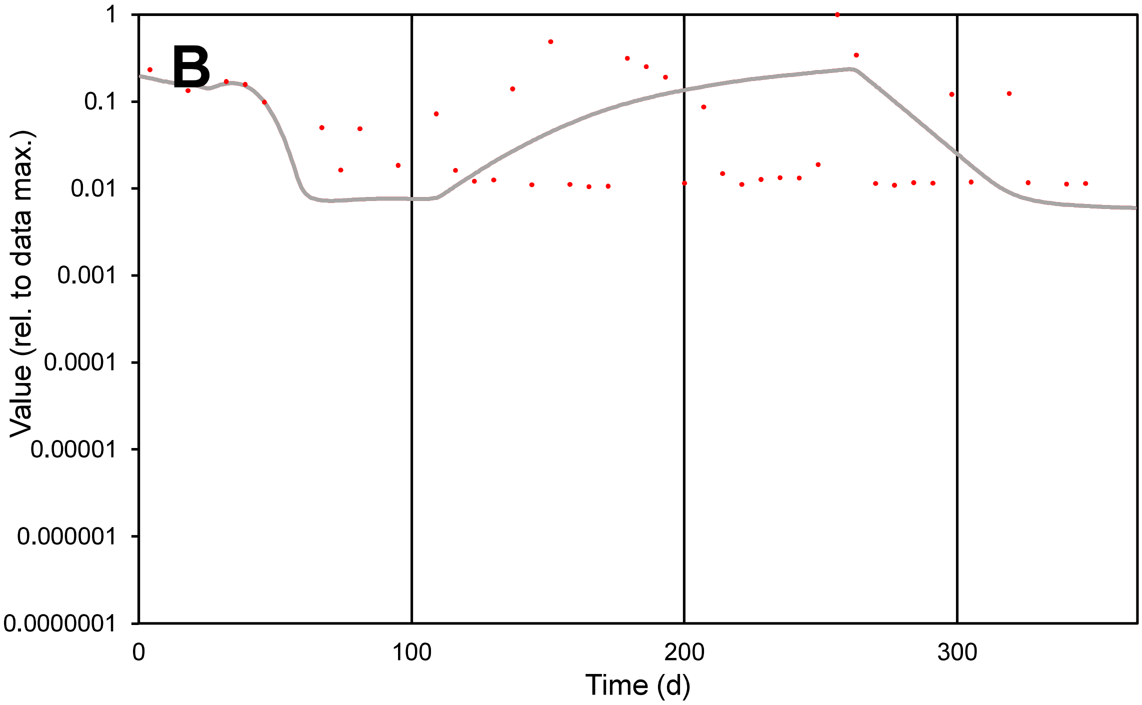

C1

Variables
OM9 Provide jtd or Dst
CalibOnly?
2 1 to reproduce of
oNormalize?
2 1=no, 2=yes

ofxn calc*
1.61251
ofxn mod
1.62682

sRun
RunA1
zout
1

Ave
Data
0.00042
Model
0.00036

data/vMax
0.0041

ofxng
334.07

Min d/m
1.67082

ave/min d
9.85089
ave/min m
14.2128

327_Calib_S_A

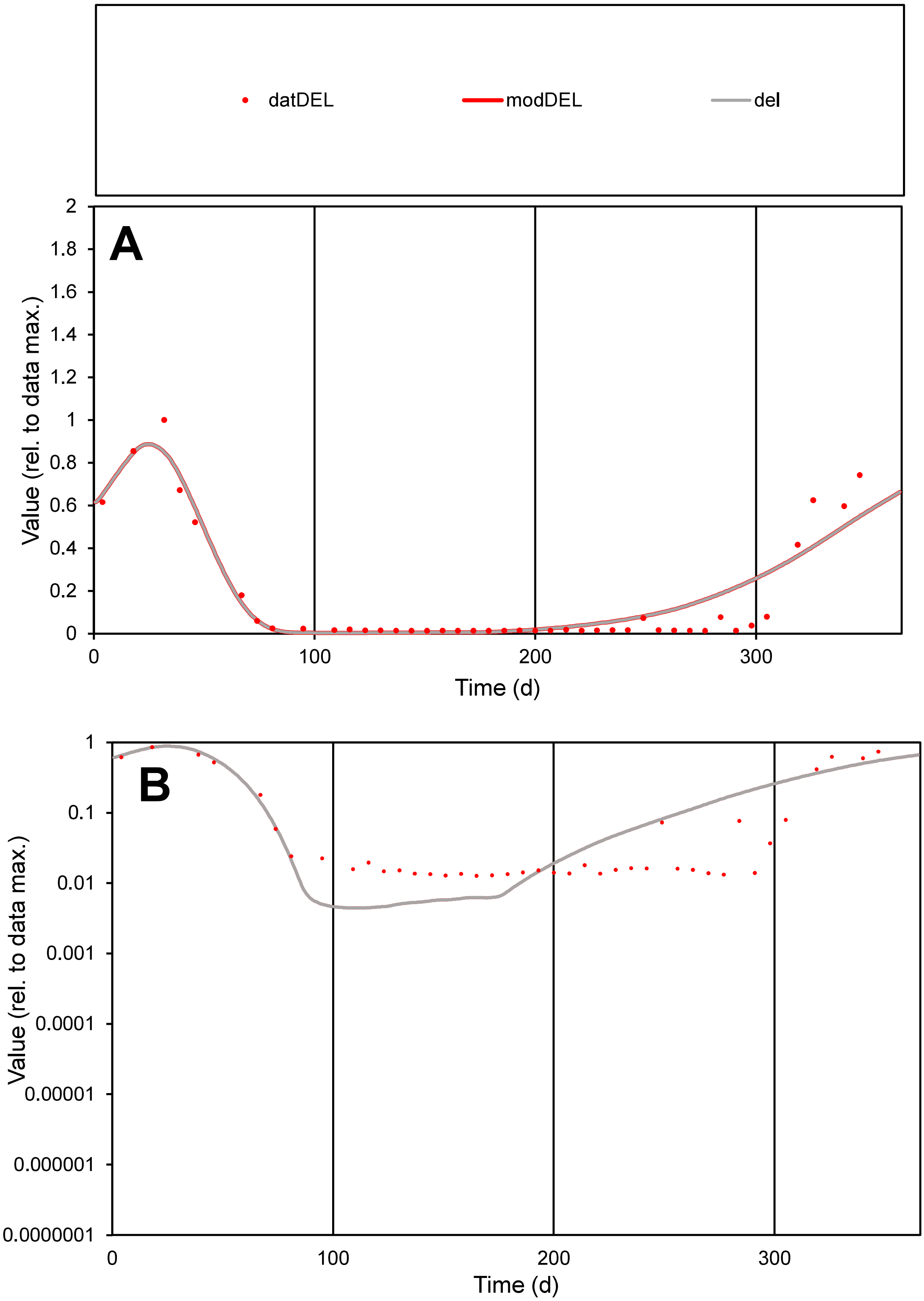

C1

Variables

DEL	Provide jtd or Dst
CalibOnly?	
2	1 to reproduce of
oNormalize?	
2	1=no, 2=yes

ofxn calc*
0.53014
ofxn mod
0.58215

sRun
RunA1
zout
1

Ave
Data
0.0008
Model
0.00088

data/vMax
0.00483

ofxng
334.07

Min d/m
2.86829

ave/min d
13.0338
ave/min m
40.9471

327_Calib_S_A

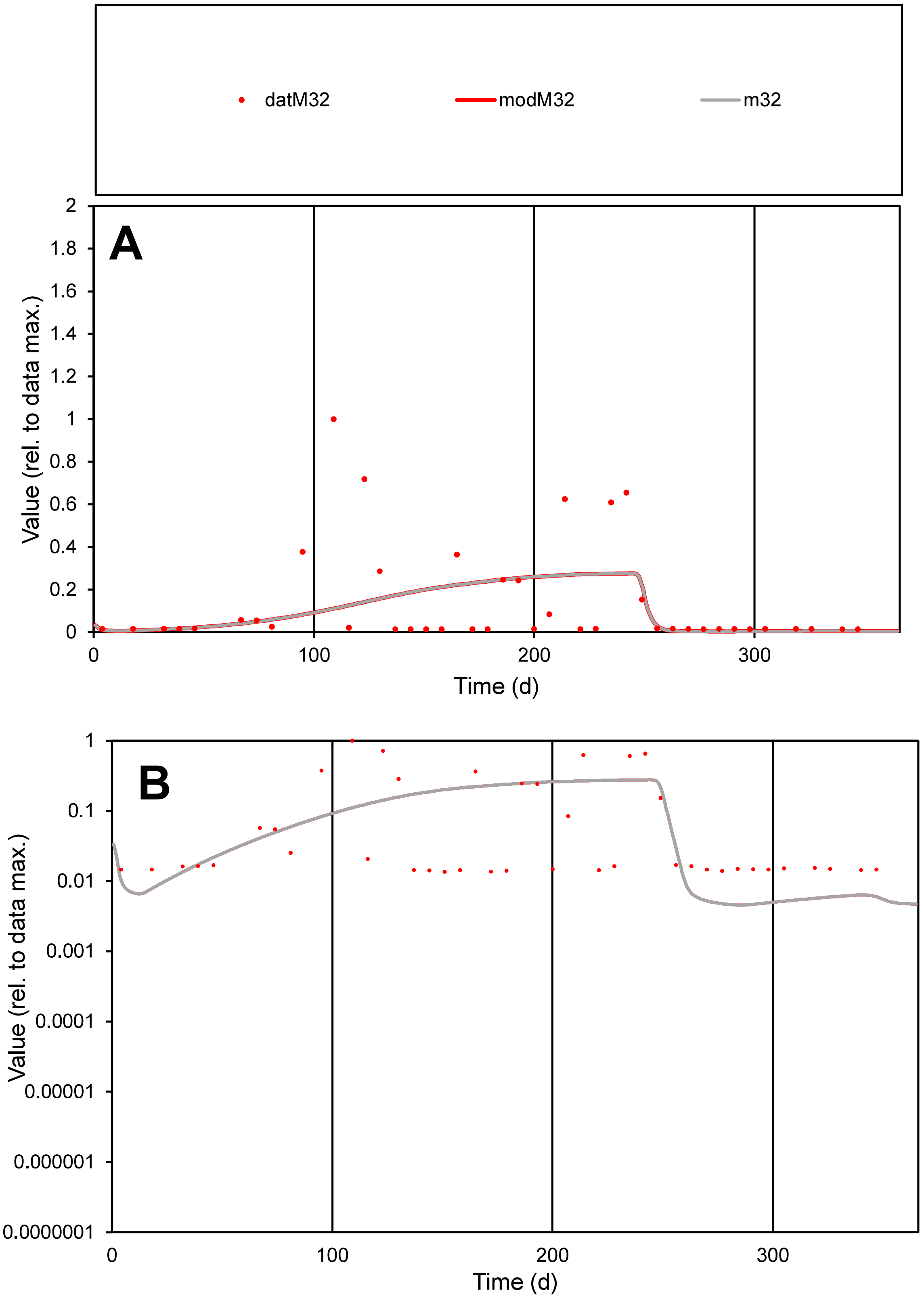

C1

Variables
M32 Provide jtd or Dst
CalibOnly?
2 1 to reproduce of
oNormalize?
2 1=no, 2=yes

ofxn calc*
1.58404
ofxn mod
1.59391

sRun
RunA1
zout
1

Ave
Data
0.00217
Model
0.00183

data/vMax
0.01547

ofxng
334.07

Min d/m
2.98151

ave/min d
10.3765
ave/min m
26.0413

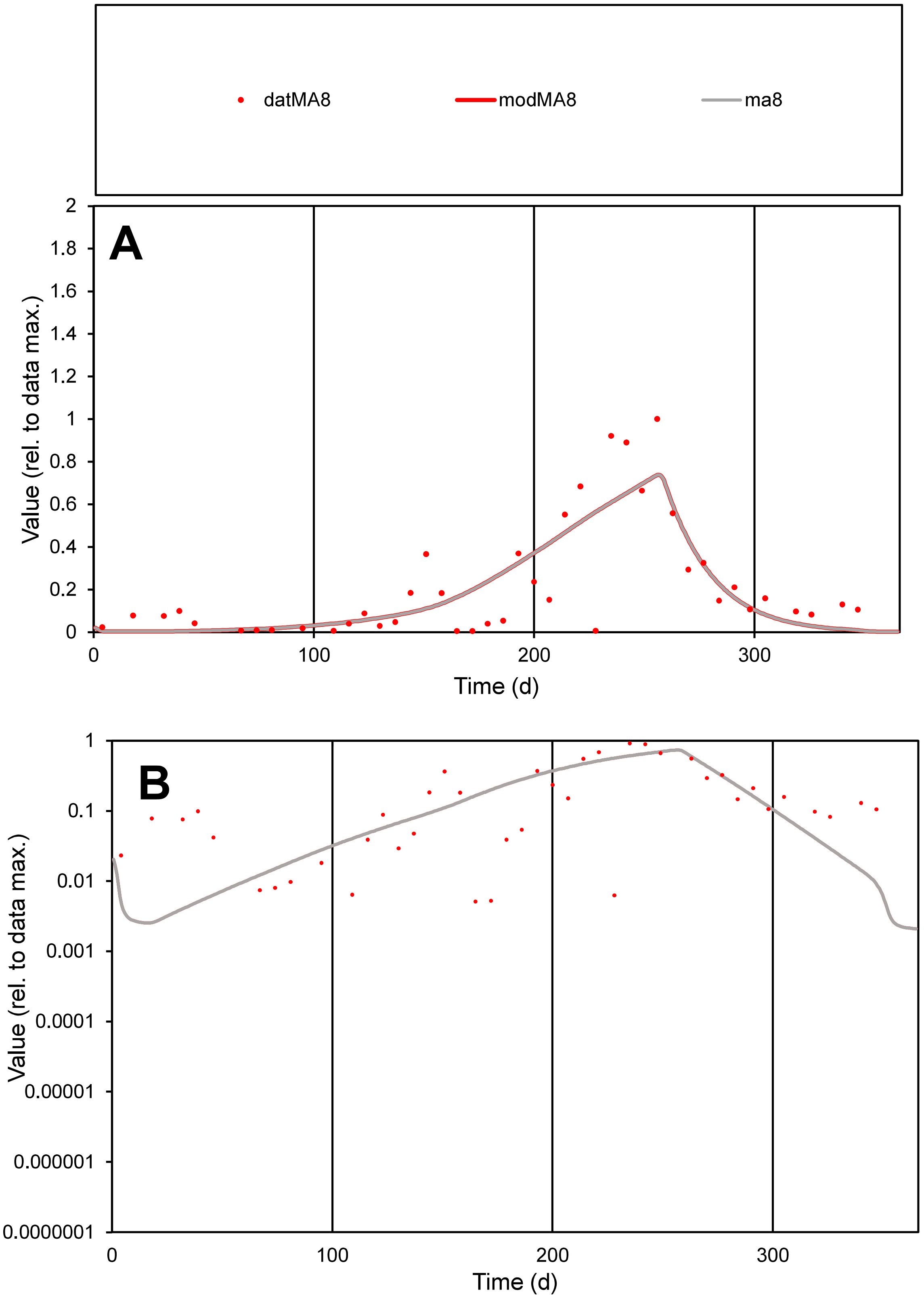

C1

Variables
MA8 Provide jtd or Dst
CalibOnly?
2 1 to reproduce of
oNormalize?
2 1=no, 2=yes

ofxn calc*
0.70009
ofxn mod
0.72686

sRun
RunA1
zout
1

Ave
Data
0.00249
Model
0.00246

data/vMax
0.01151

ofxng
334.07

Min d/m
1.94291

ave/min d
42.0931
ave/min m
80.7091

327_Calib_S_A

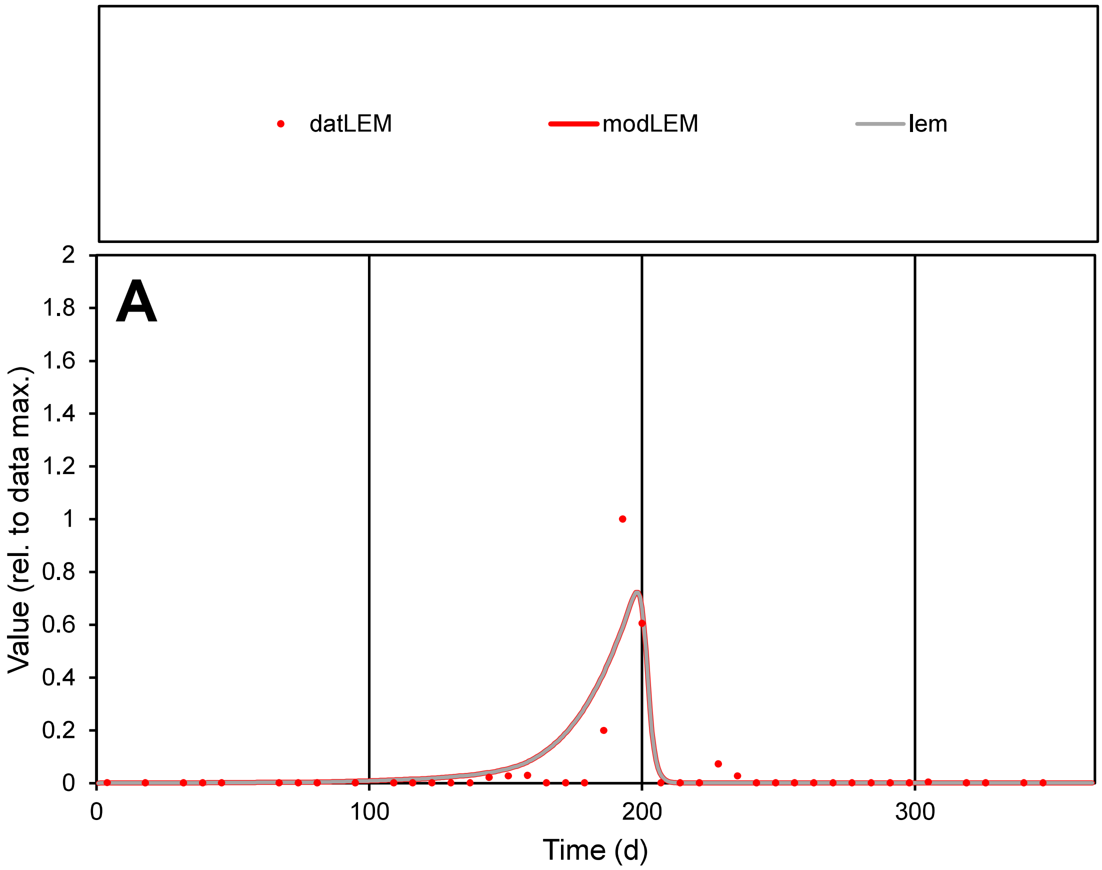

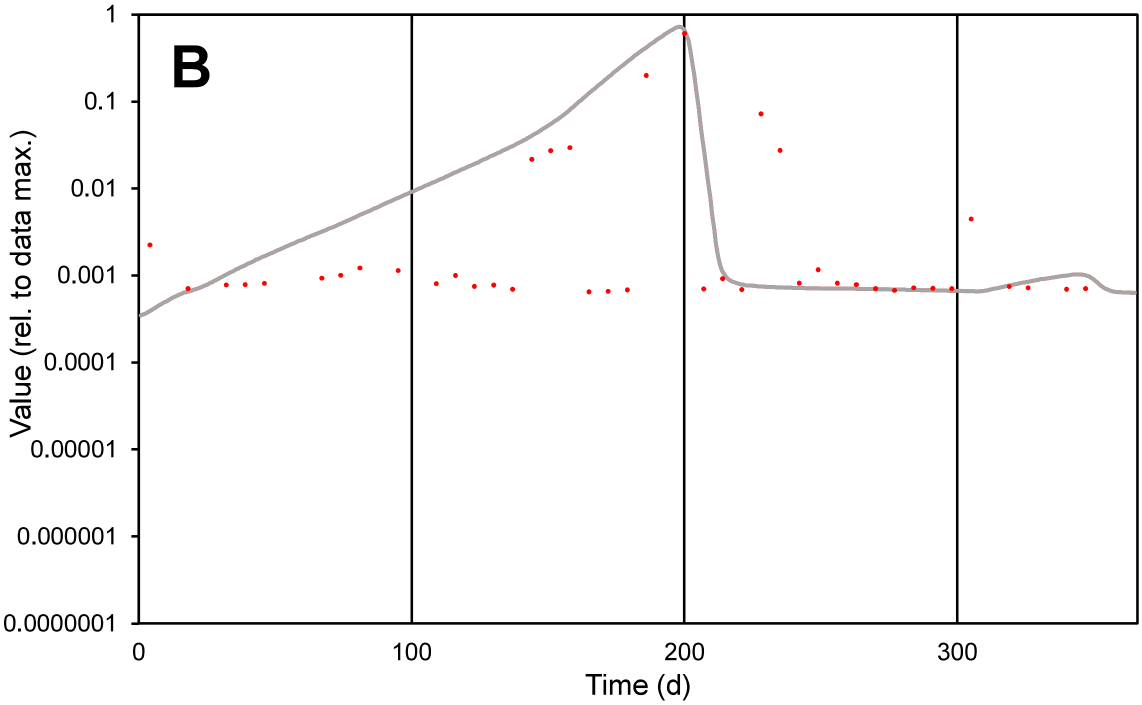

C1

Variables
LEM Provide jtd or Dst
CalibOnly?
2 1 to reproduce of
oNormalize?
2 1=no, 2=yes

ofxn calc*
1.80915
ofxn mod
1.897

sRun
RunA1
zout
1

Ave
Data
0.00159
Model
0.002

data/vMax
0.03304

ofxng
334.07

Min d/m
1.58401

ave/min d
74.0732
ave/min m
147.873

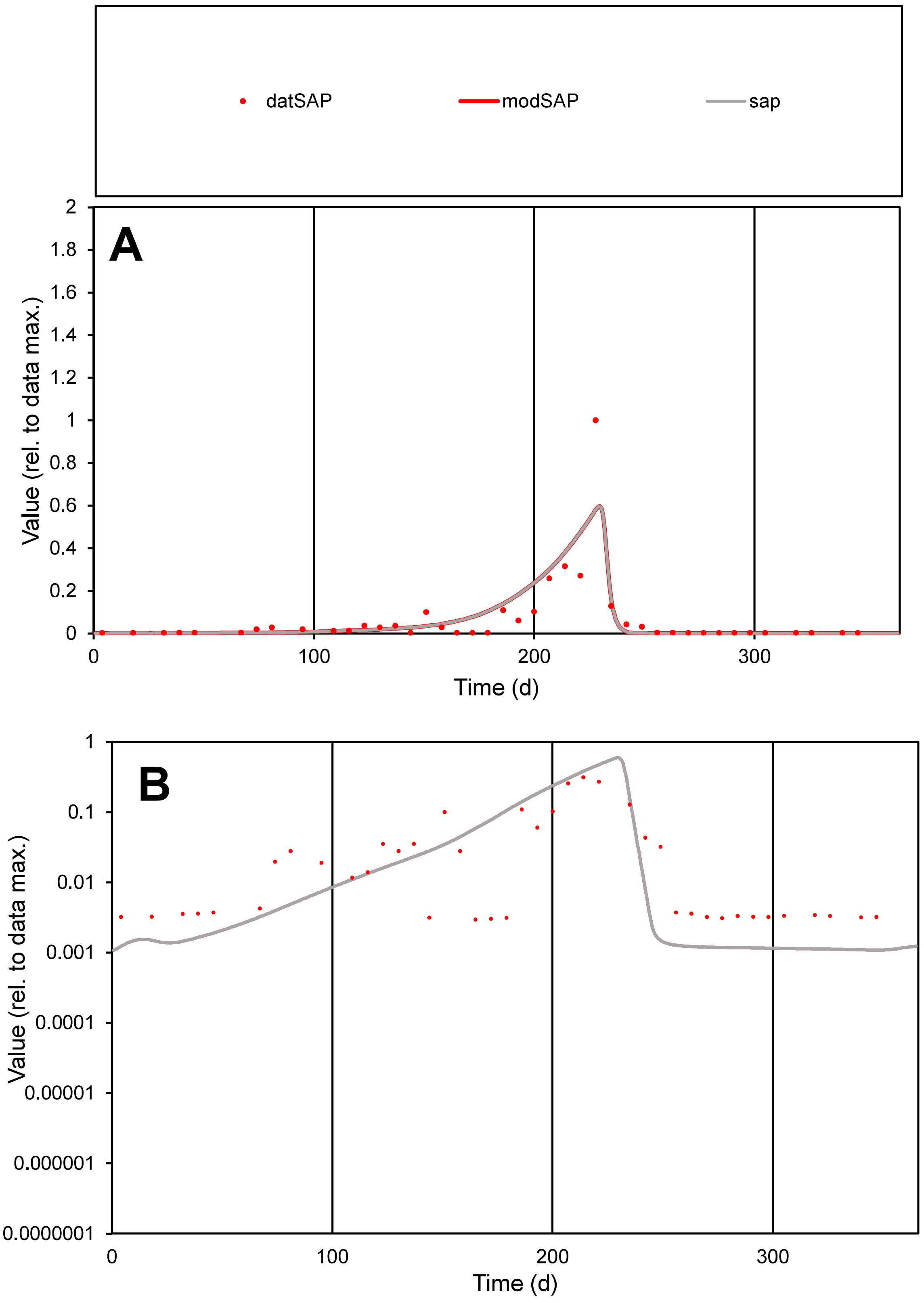

C1

Variables
SAP Provide jtd or Dst
CalibOnly?
2 1 to reproduce of
oNormalize?
2 1=no, 2=yes

ofxn calc*
1.25966
ofxn mod
1.30331

sRun
RunA1
zout
1

Ave
Data
0.00071
Model
0.00078

data/vMax
0.01099

ofxng
334.07

Min d/m
2.71893

ave/min d
21.7918
ave/min m
64.8259

327_Calib_S_A

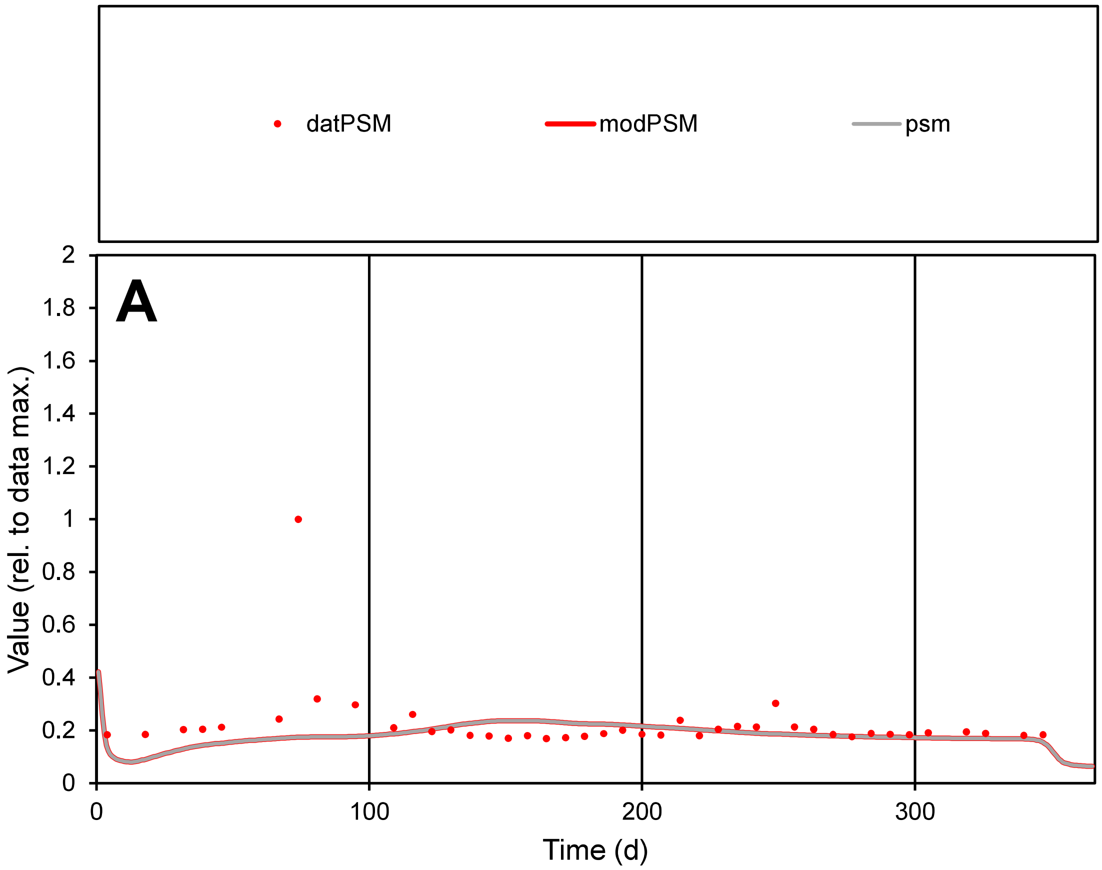

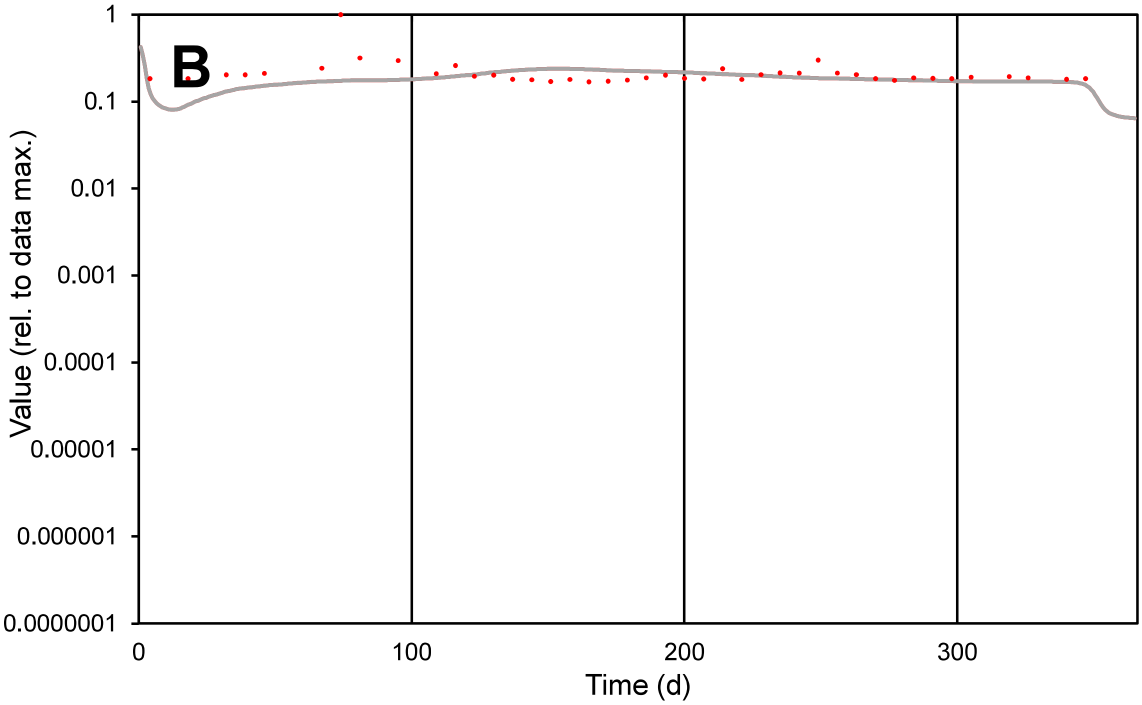

C1

Variables

PSM Provide jtd or Dst

CalibOnly?

2 1 to reproduce of

oNormalize?

2 1=no, 2=yes

ofxn calc*

0.62089

ofxn mod

0.63108

sRun

RunA1

zout

1

Ave

Data

3.6E-05

Model

3.1E-05

data/vMax

0.00016

ofxng

334.07

Min d/m

1.74539

ave/min d

1.319

ave/min m

1.95414

327_Calib_S_A

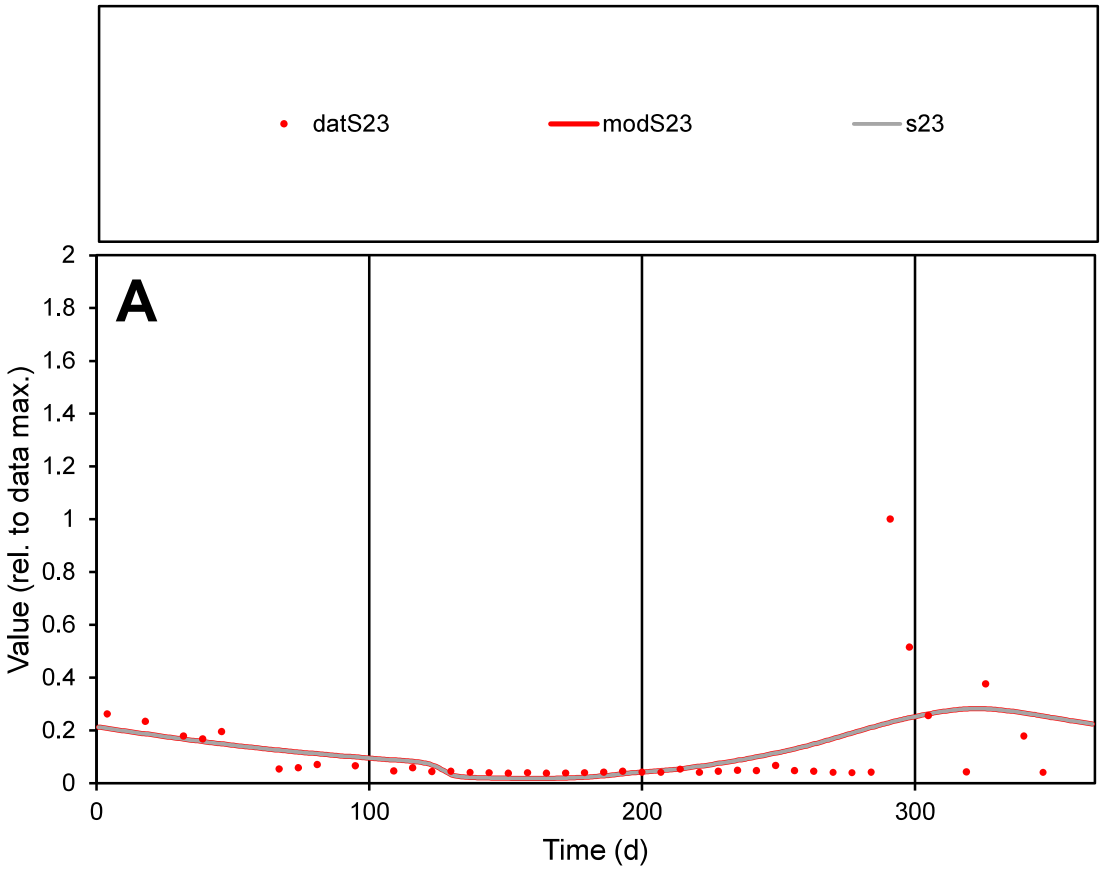

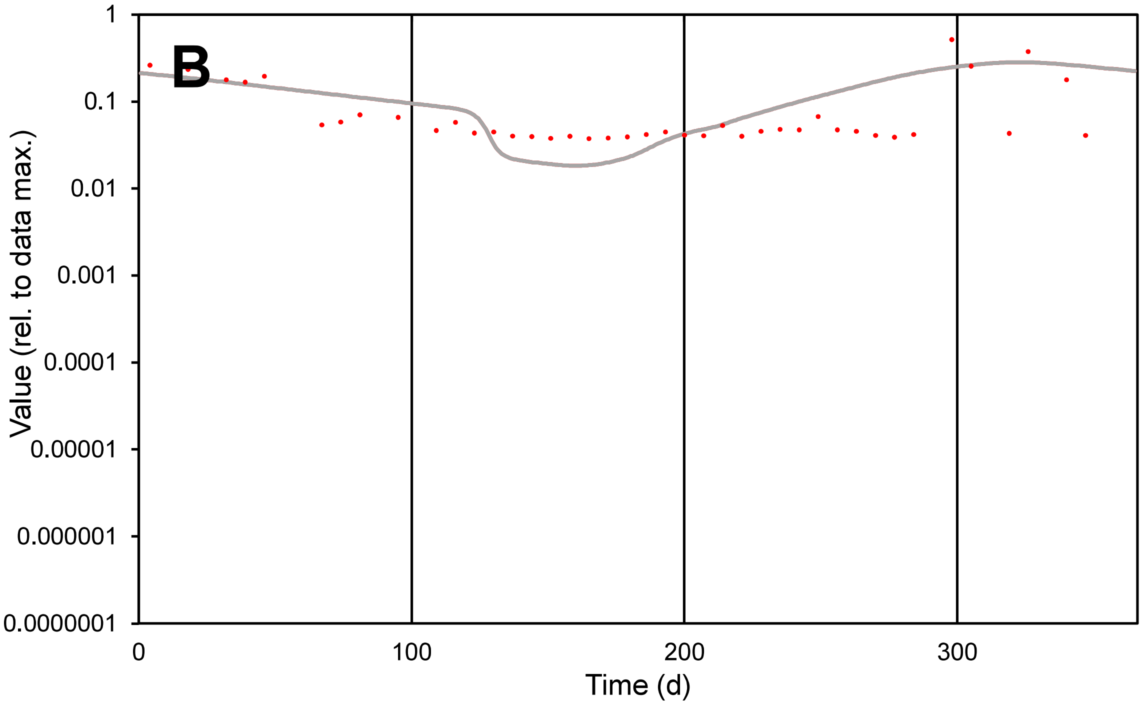

C1

Variables
S23 Provide jtd or Dst
CalibOnly?
2 1 to reproduce of
oNormalize?
2 1=no, 2=yes

ofxn calc*
1.26452
ofxn mod
1.27619

sRun
RunA1
zout
1

Ave
Data
0.00049
Model
0.00051

data/vMax
0.00426

ofxng
334.07

Min d/m
2.03213

ave/min d
3.0852
ave/min m
6.54729

327_Calib_S_A

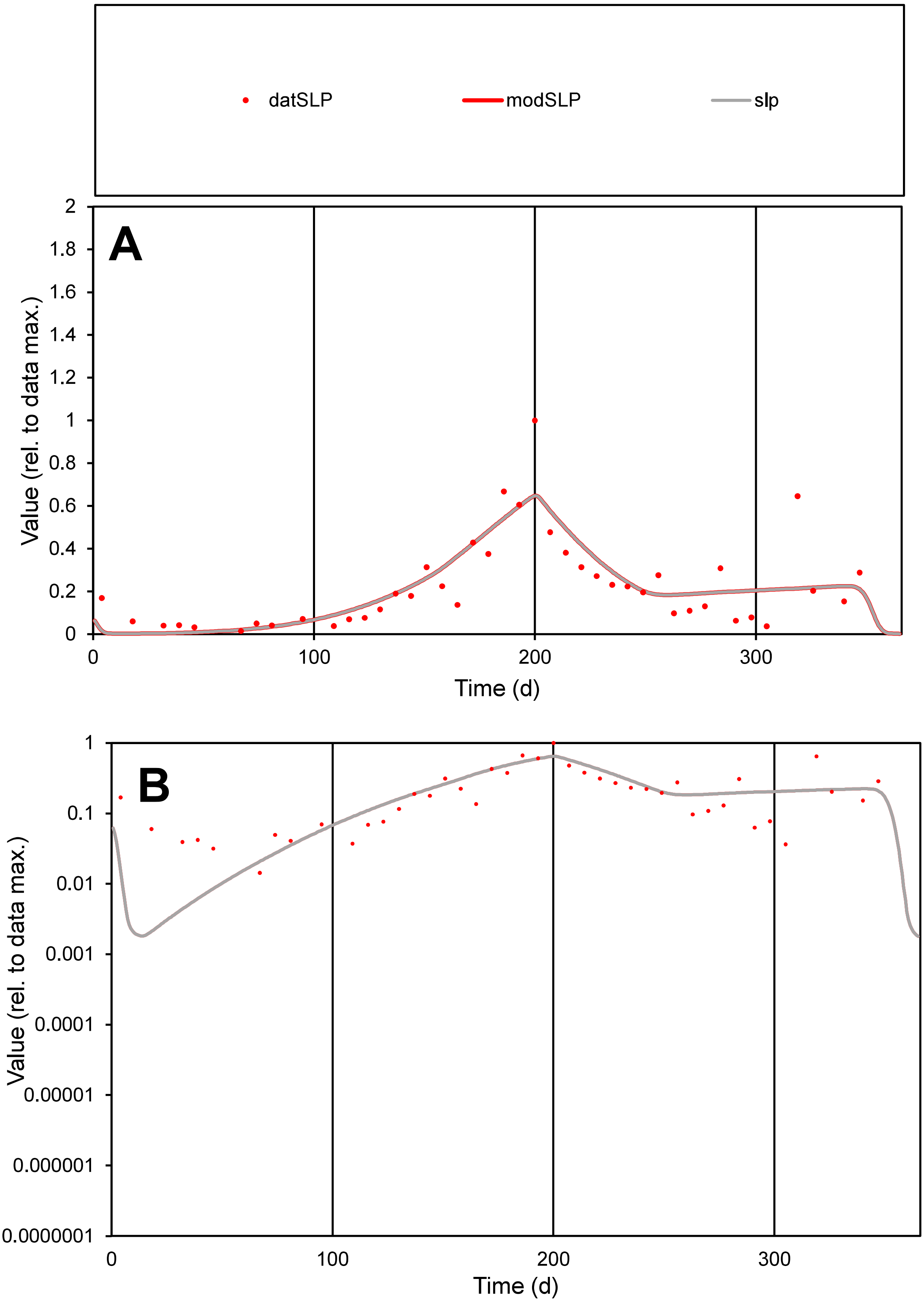

C1

Variables

SLP Provide jtd or Dst

CalibOnly?

2 1 to reproduce of

oNormalize?

2 1=no, 2=yes

ofxn calc*

0.52753

ofxn mod

0.55355

sRun

RunA1

zout

1

Ave

Data

0.00154

Model

0.0016

data/vMax

0.00688

ofxng

334.07

Min d/m

6.23921

ave/min d

15.6637

ave/min m

101.322

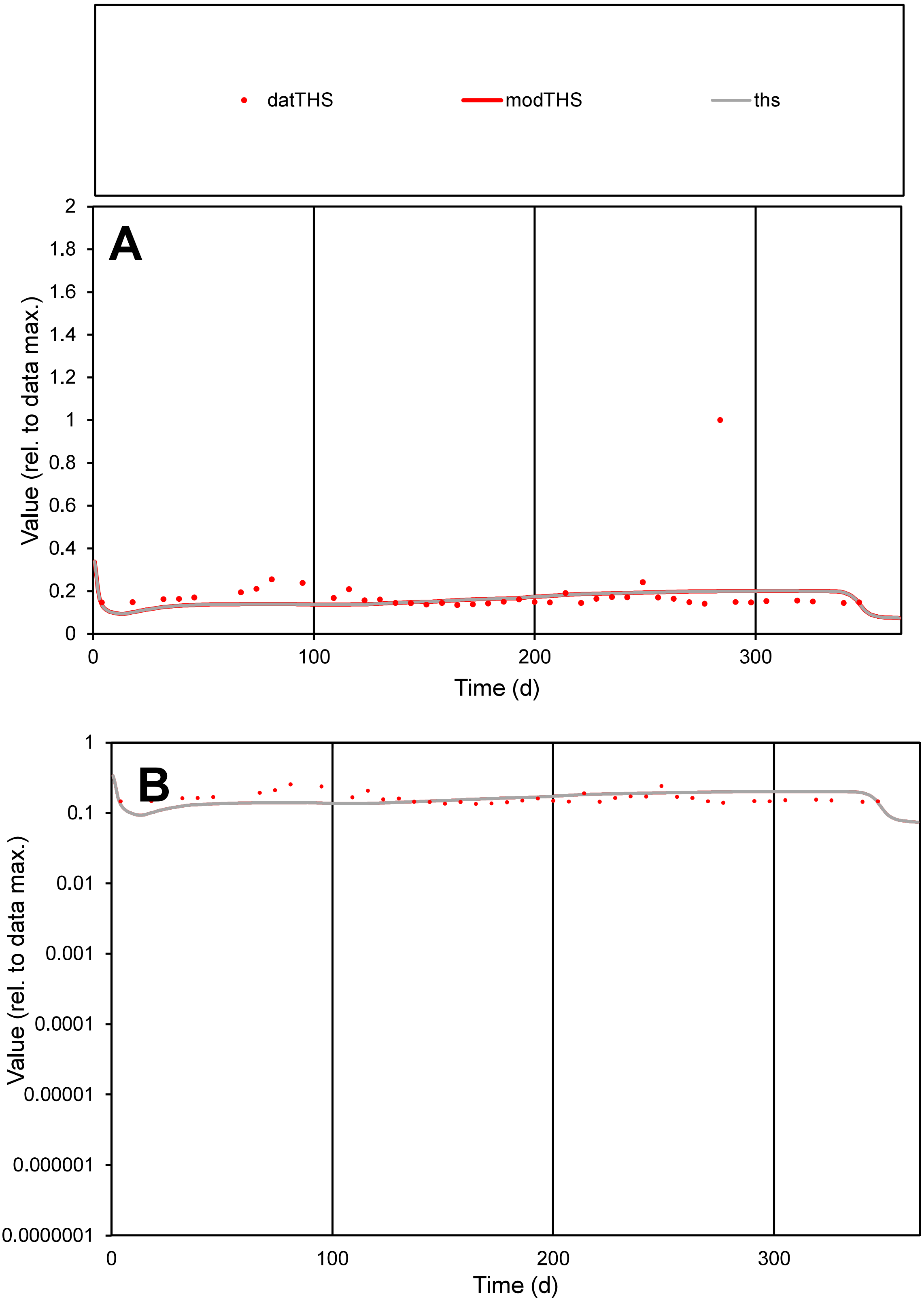

C1

Variables

THS Provide jtd or Dst

CalibOnly?

2 1 to reproduce of

oNormalize?

2 1=no, 2=yes

ofxn calc*

0.71023

ofxn mod

0.7188

sRun

RunA1

zout

1

Ave

Data

3.9E-05

Model

3.5E-05

data/vMax

0.00021

ofxng

334.07

Min d/m

1.29063

ave/min d

1.36443

ave/min m

1.58876

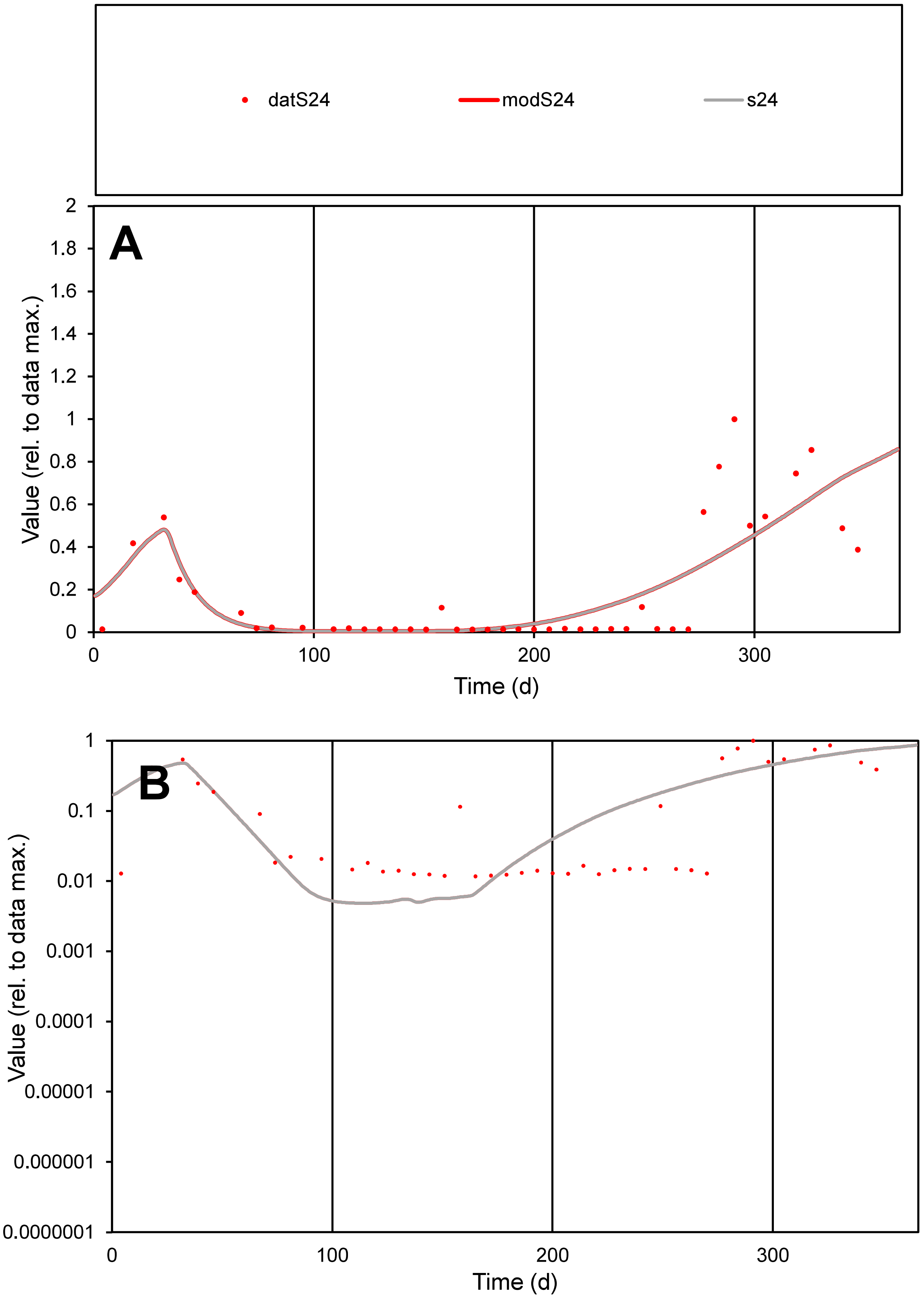

C1

Variables
S24 Provide jtd or Dst
CalibOnly?
2 1 to reproduce of
oNormalize?
2 1=no, 2=yes

ofxn calc*
0.86859
ofxn mod
0.88636

sRun
RunA1
zout
1

Ave
Data
0.00093
Model
0.00094

data/vMax
0.00491

ofxng
334.07

Min d/m
2.42107

ave/min d
16.1064
ave/min m
39.5247

327_Calib_S_A

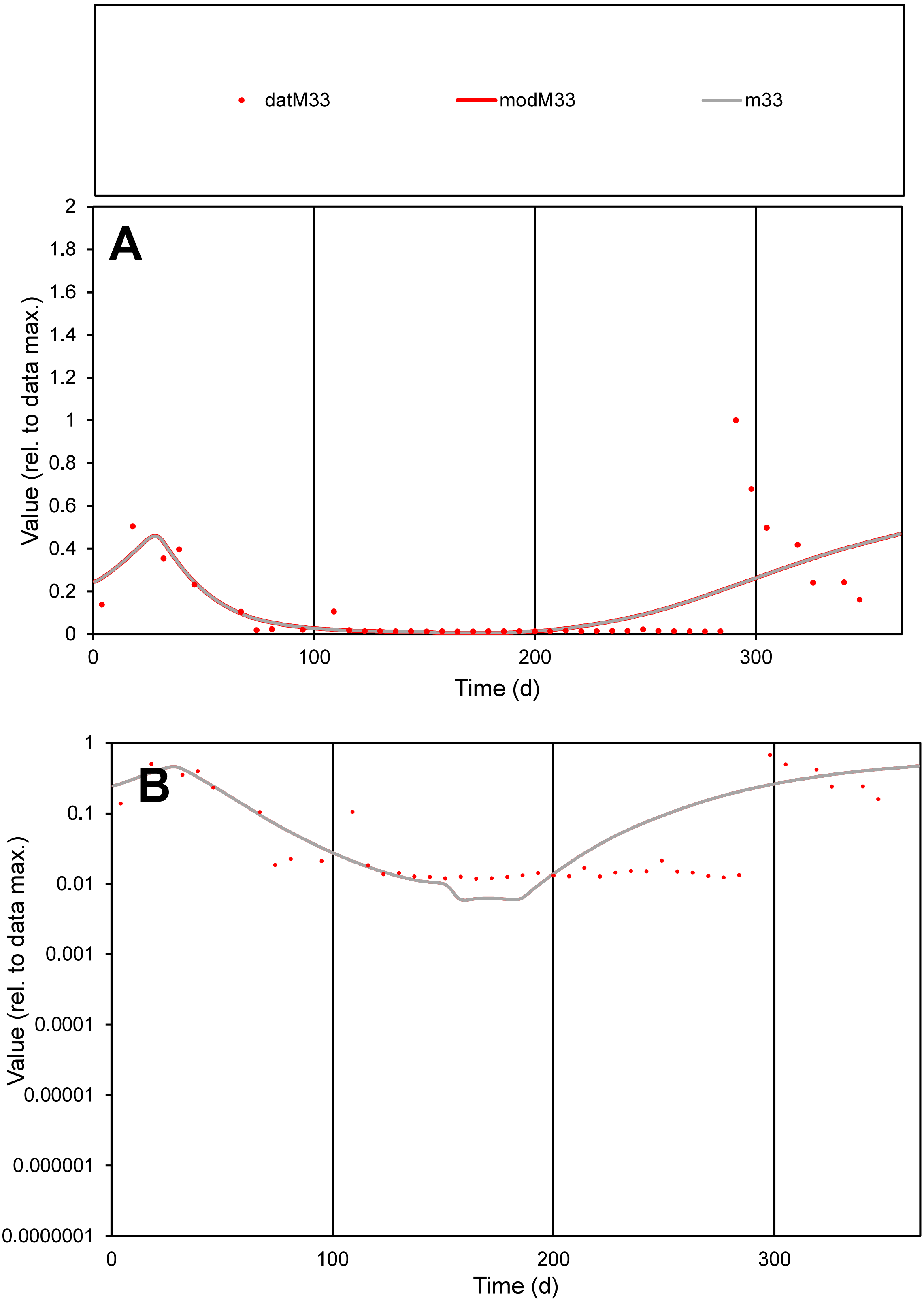

C1

Variables
M33 Provide jtd or Dst
CalibOnly?
2 1 to reproduce of
oNormalize?
2 1=no, 2=yes

ofxn calc*
1.23479
ofxn mod
1.25127

sRun
RunA1
zout
1

Ave
Data
0.00075
Model
0.00075

data/vMax
0.00572

ofxng
334.07

Min d/m
2.02869

ave/min d
10.9953
ave/min m
22.271

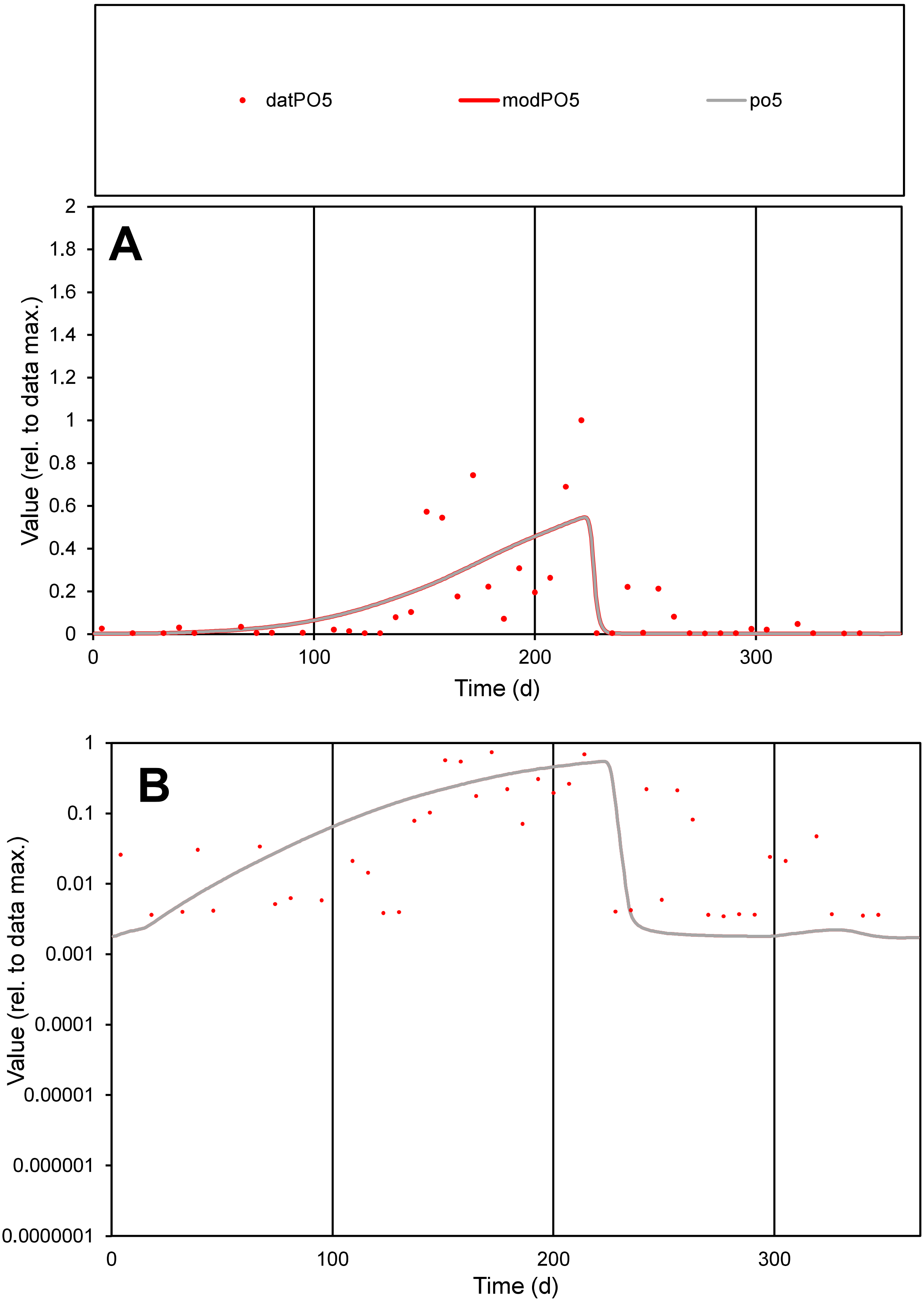

C1

Variables
PO5 Provide jtd or Dst
CalibOnly?
2 1 to reproduce of
oNormalize?
2 1=no, 2=yes

ofxn calc*
1.15054
ofxn mod
1.16827

sRun
RunA1
zout
1

Ave
Data
0.00191
Model
0.00179

data/vMax
0.01392

ofxng
334.07

Min d/m
1.96411

ave/min d
39.7572
ave/min m
72.8672

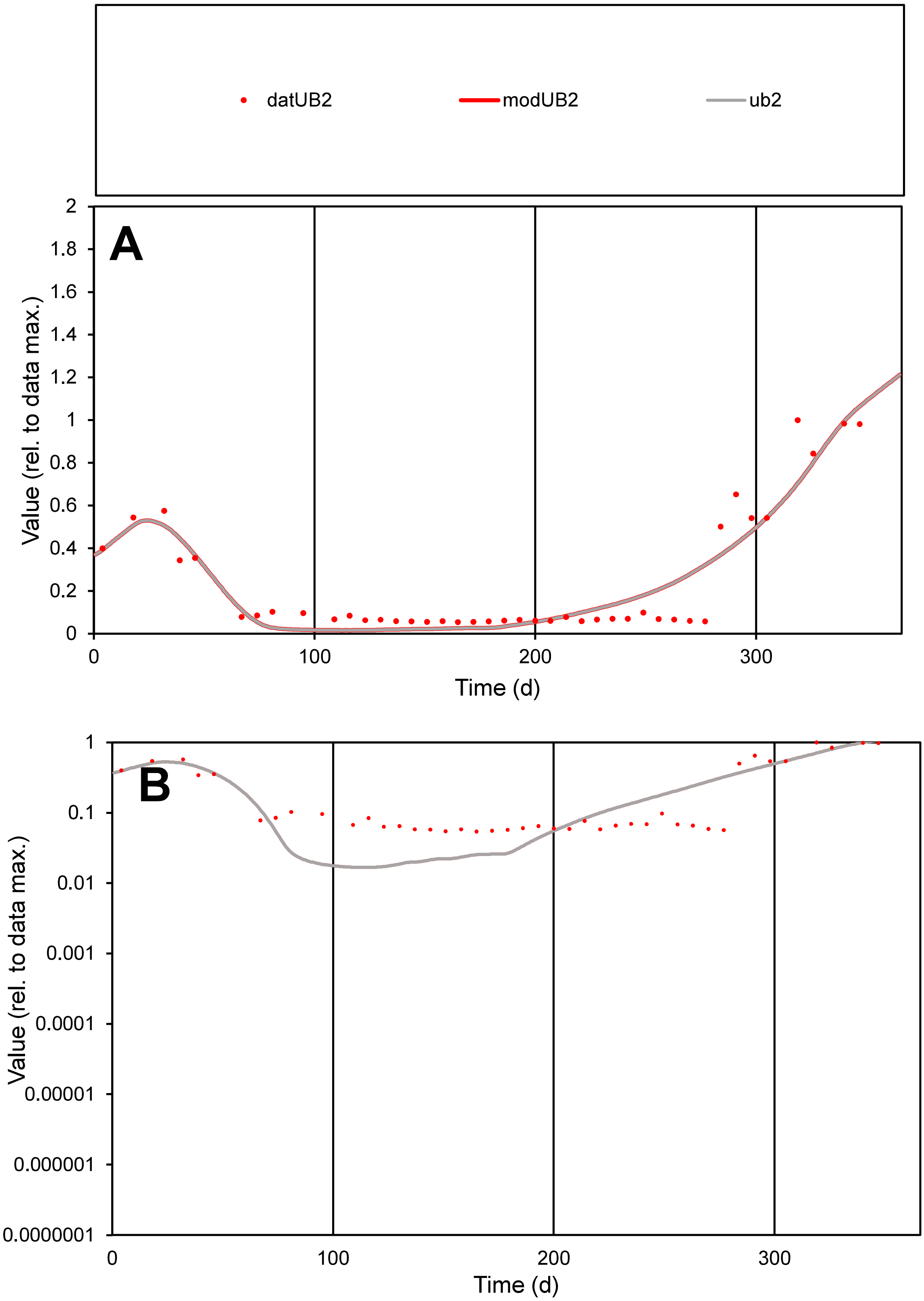

C1

Variables

UB2 Provide jtd or Dst

CalibOnly?

2 1 to reproduce of

oNormalize?

2 1=no, 2=yes

ofxn calc*

0.39943

ofxn mod

0.43107

sRun

RunA1

zout

1

Ave

Data

0.00046

Model

0.00046

data/vMax

0.0019

ofxng

334.07

Min d/m

3.22309

ave/min d

4.48403

ave/min m

14.3671

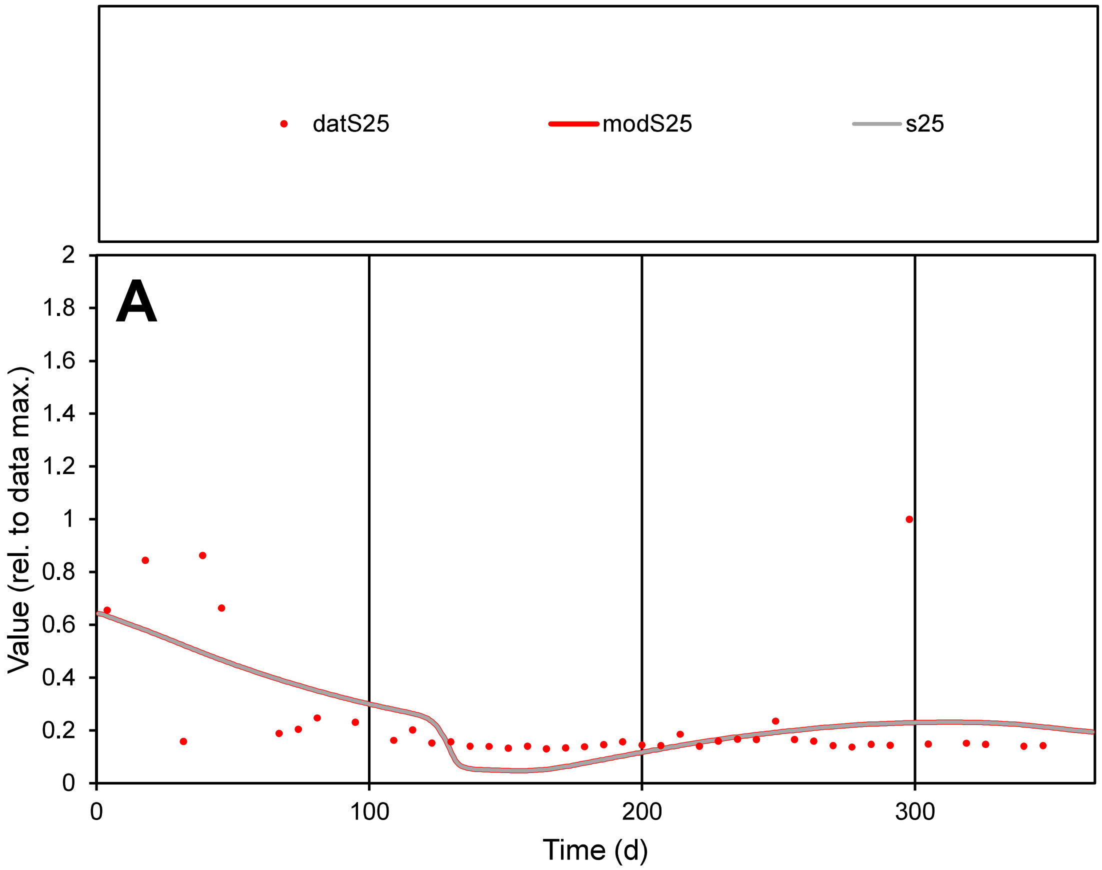

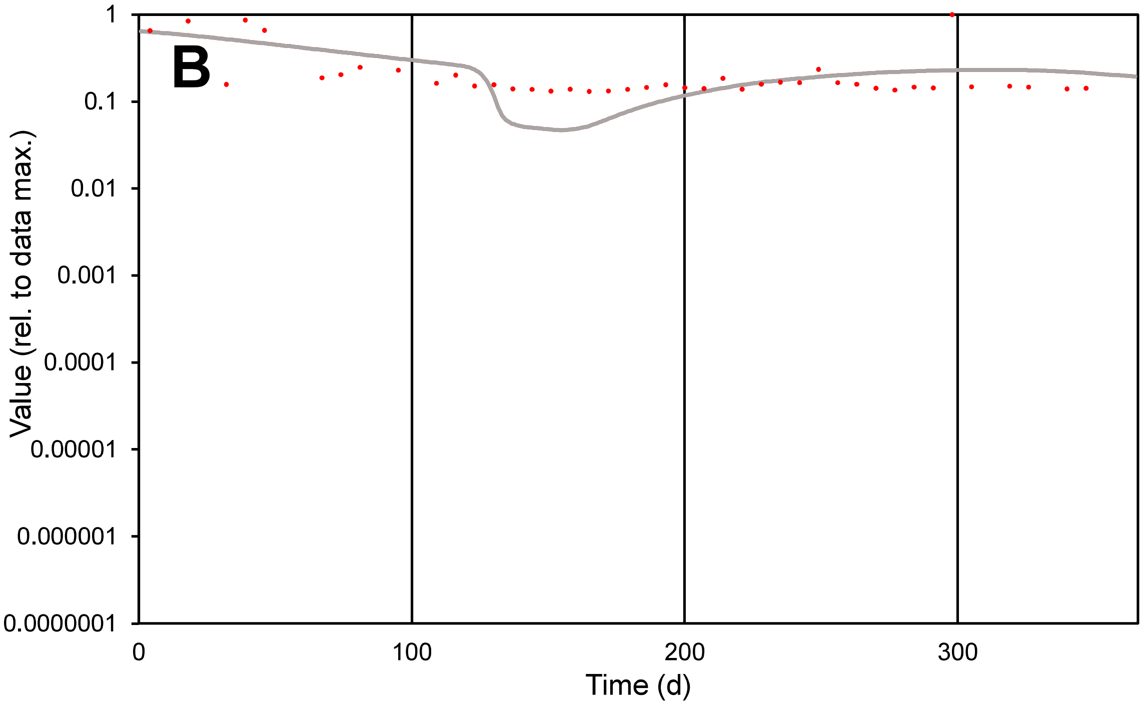

C1

Variables
S25 Provide jtd or Dst
CalibOnly?
2 1 to reproduce of
oNormalize?
2 1=no, 2=yes

ofxn calc*
0.71461
ofxn mod
0.72999

sRun
RunA1
zout
1

Ave
Data
0.0003
Model
0.00029

data/vMax
0.00129

ofxng
334.07

Min d/m
2.74273

ave/min d
1.81154
ave/min m
4.74421

327_Calib_S_A

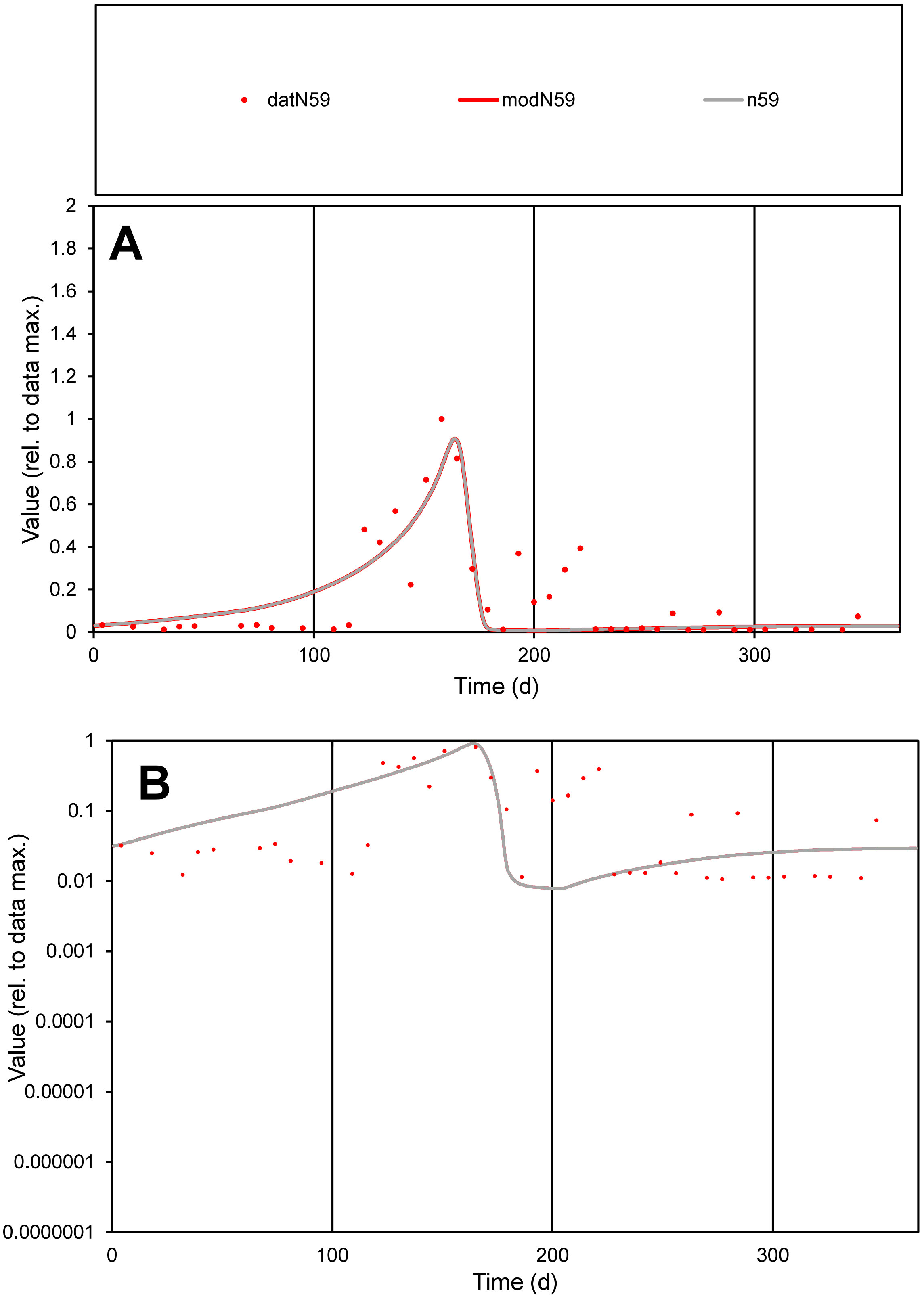

C1

Variables
N59 Provide jtd or Dst
CalibOnly?
2 1 to reproduce of
oNormalize?
2 1=no, 2=yes

ofxn calc*
0.83287
ofxn mod
0.85735

sRun
RunA1
zout
1

Ave
Data
0.00064
Model
0.00057

data/vMax
0.004

ofxng
334.07

Min d/m
1.3568

ave/min d
14.8766
ave/min m
18.2225

327_Calib_S_A

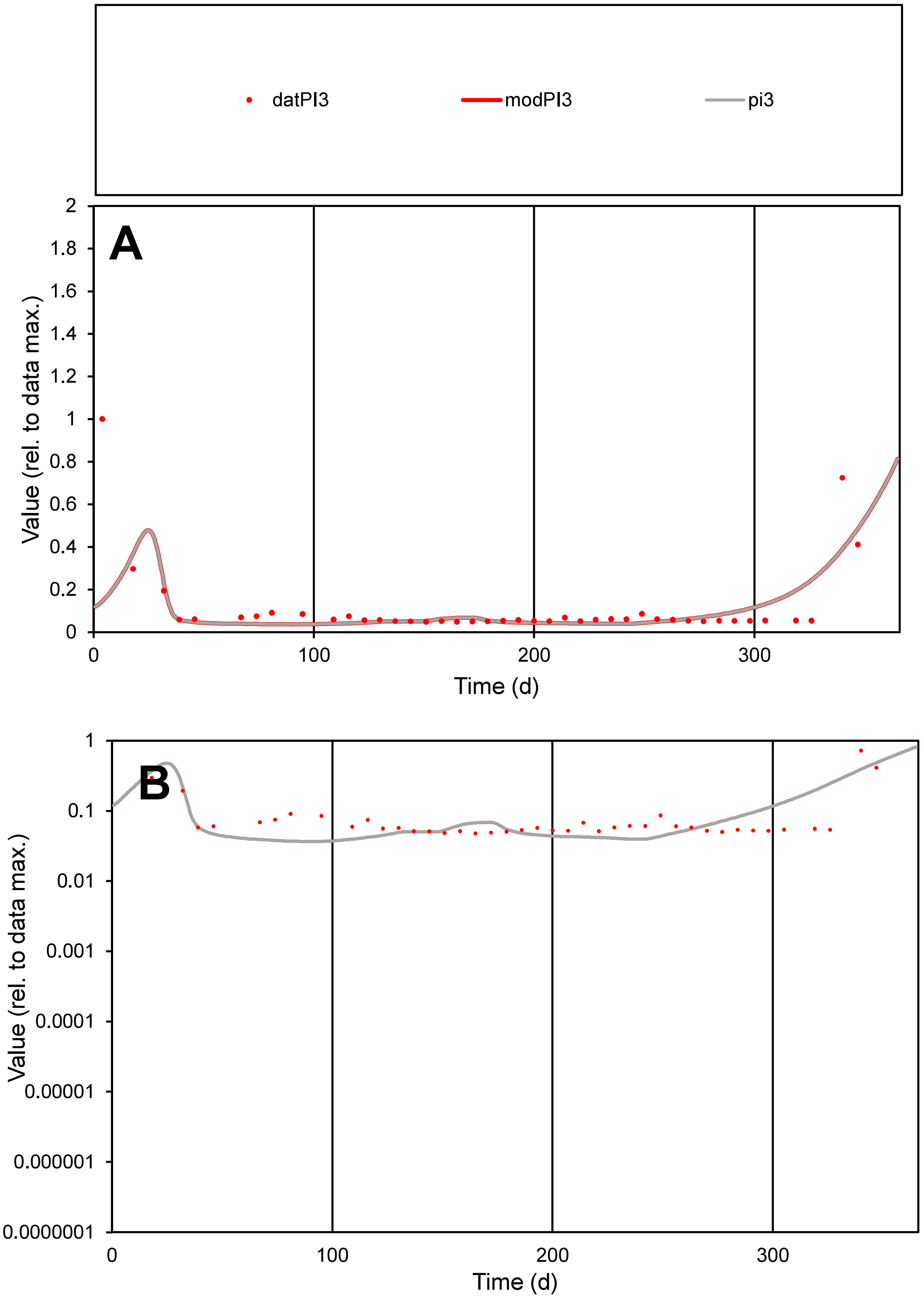

C1

Variables
PI3 Provide jtd or Dst
CalibOnly?
2 1 to reproduce of
oNormalize?
2 1=no, 2=yes

ofxn calc*
1.28785
ofxn mod
1.29913

sRun
RunA1
zout
1

Ave
Data
0.00017
Model
0.00014

data/vMax
0.00147

ofxng
334.07

Min d/m
1.30007

ave/min d
2.38492
ave/min m
2.59131

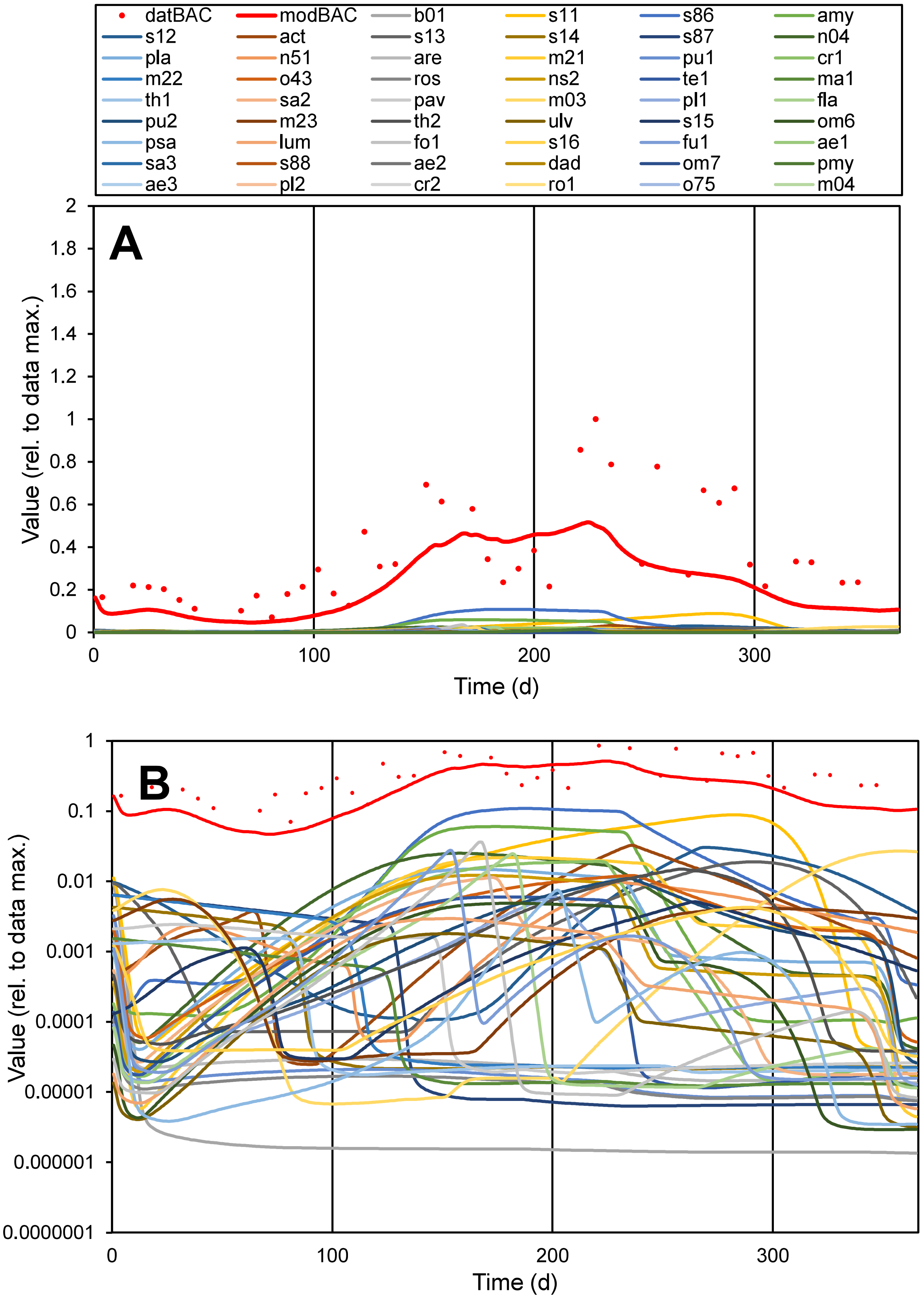

C1

Variables

BAC Provide jtd or Dst

CalibOnly?

2 1 to reproduce of

oNormalize?

2 1=no, 2=yes

ofxn calc*

0.59319

ofxn mod

0.6028

sRun

RunA1

zout

1

Ave

Data

1.09306

Model

0.69903

data/vMax

3.019

ofxng

334.07

Min d/m

1.46552

ave/min d

5.14379

ave/min m

4.82086

328_Calib_S_A

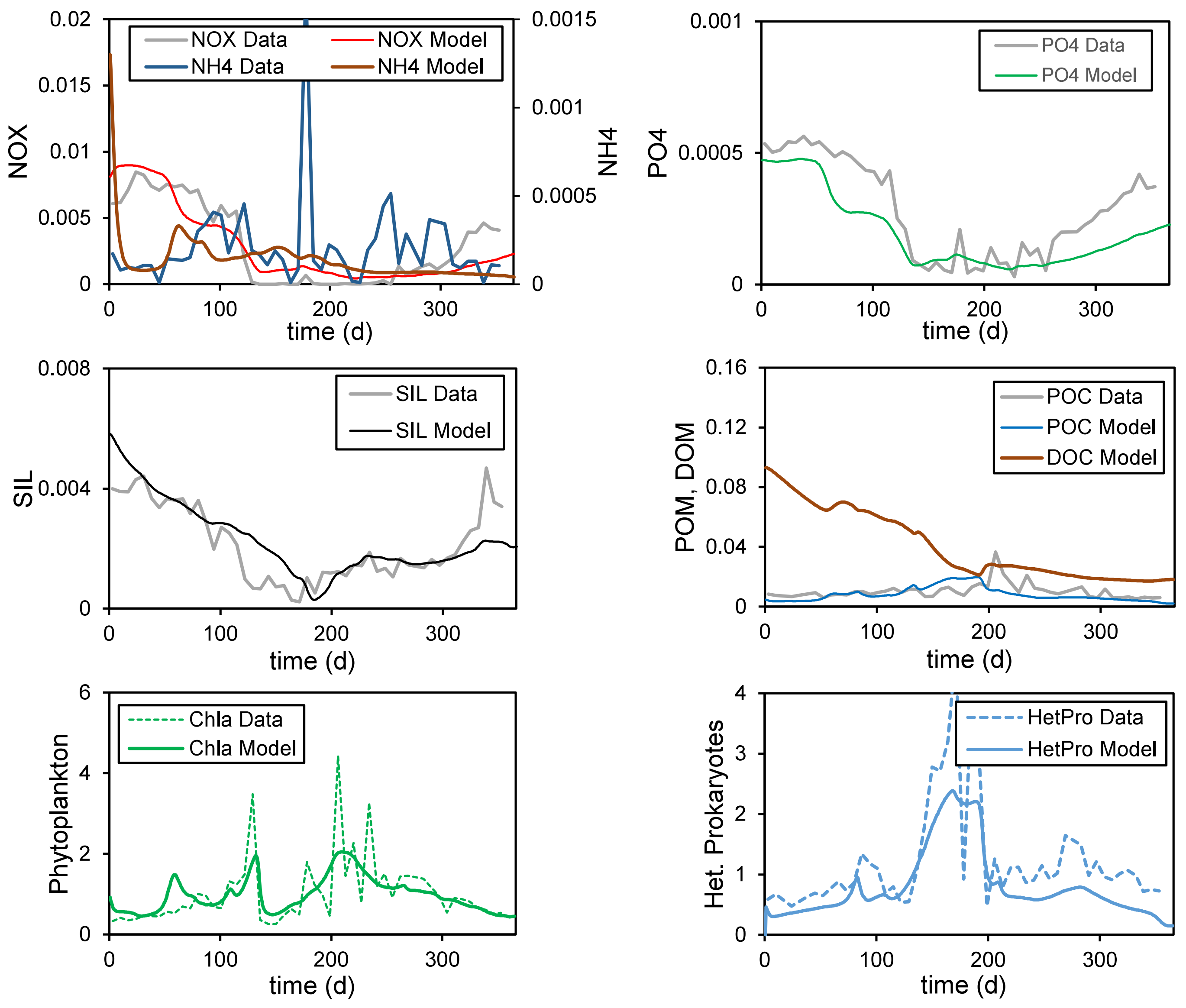

NOX Data
NOX Model
NH4 Data
NH4 Model
NOX
NH4
time (d)
PO4 Data
PO4 Model
PO4
time (d)
SIL Data
SIL Model
SIL
time (d)
POC Data
POC Model
DOC Model
POM, DOM
time (d)
Chla Data
Chla Model
Phytoplankton
time (d)
HetPro Data
HetPro Model
Het. Prokaryotes
time (d)

328_Calib_S_A

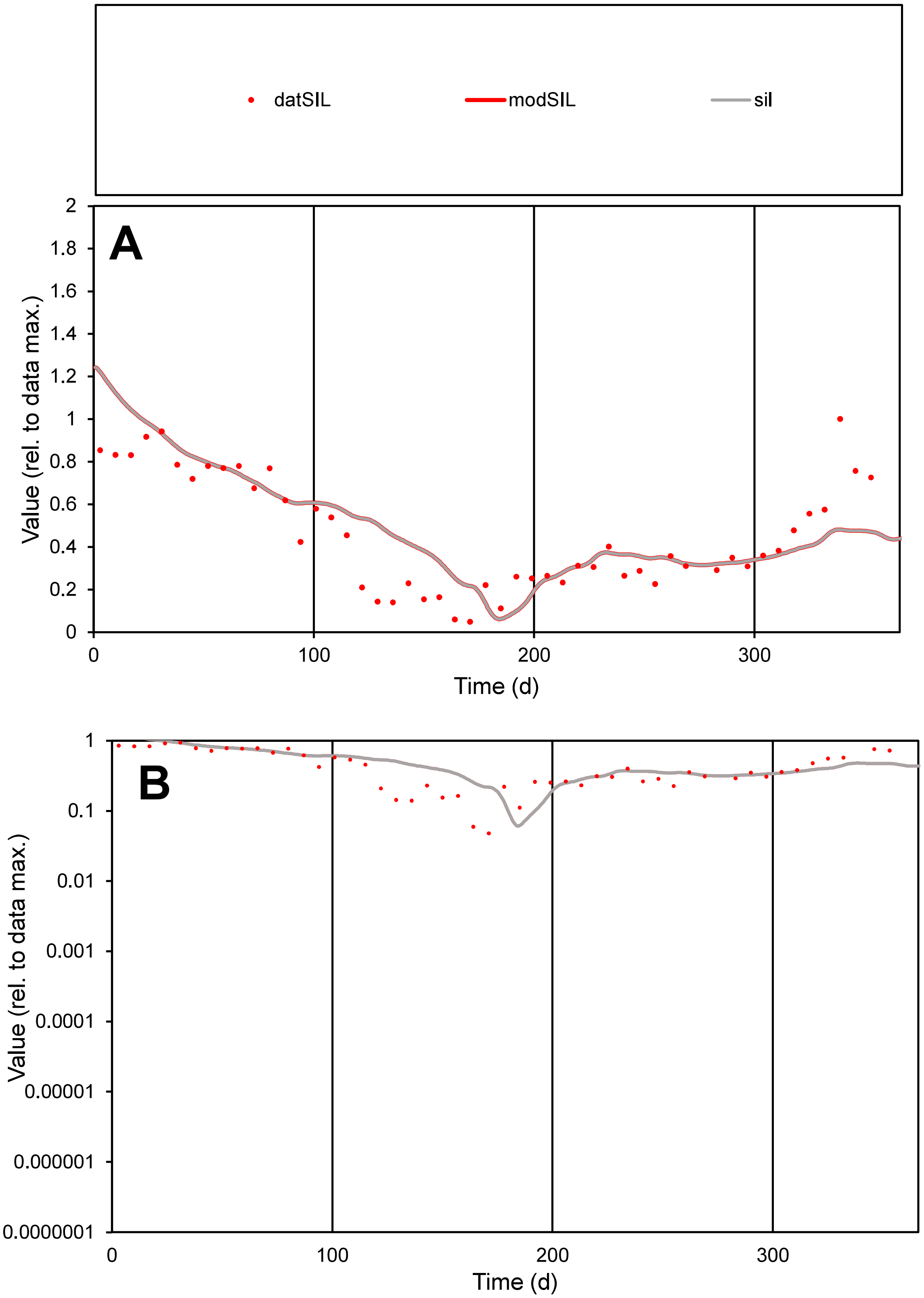

C1

Variables

SIL Provide jtd or Dst

CalibOnly?

2 1 to reproduce of

oNormalize?

2 1=no, 2=yes

ofxn calc*

1.06863

ofxn mod

1.14607

sRun

RunA1

zout

1

Ave

Data

0.00216

Model

0.00232

data/vMax

0.00469

ofxng

369.894

Min d/m

0.72464

ave/min d

9.58178

ave/min m

7.47688

328_Calib_S_A

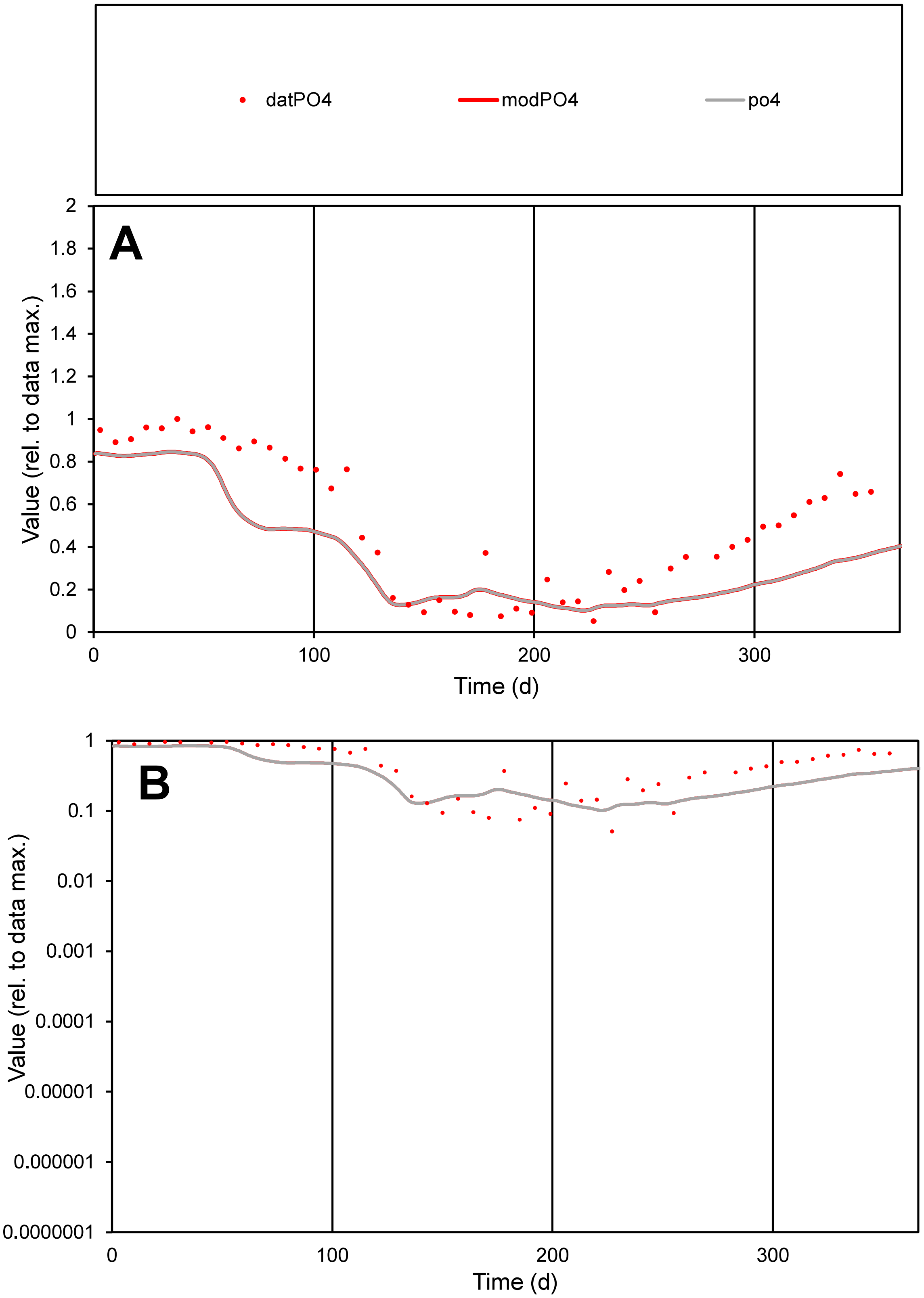

C1

Variables

PO4 Provide jtd or Dst

CalibOnly?

2 1 to reproduce of

oNormalize?

2 1=no, 2=yes

ofxn calc*

1.23068

ofxn mod

1.28719

sRun

RunA1

zout

1

Ave

Data

0.00028

Model

0.0002

data/vMax

0.00056

ofxng

369.894

Min d/m

0.49853

ave/min d

9.84803

ave/min m

3.4299

328_Calib_S_A

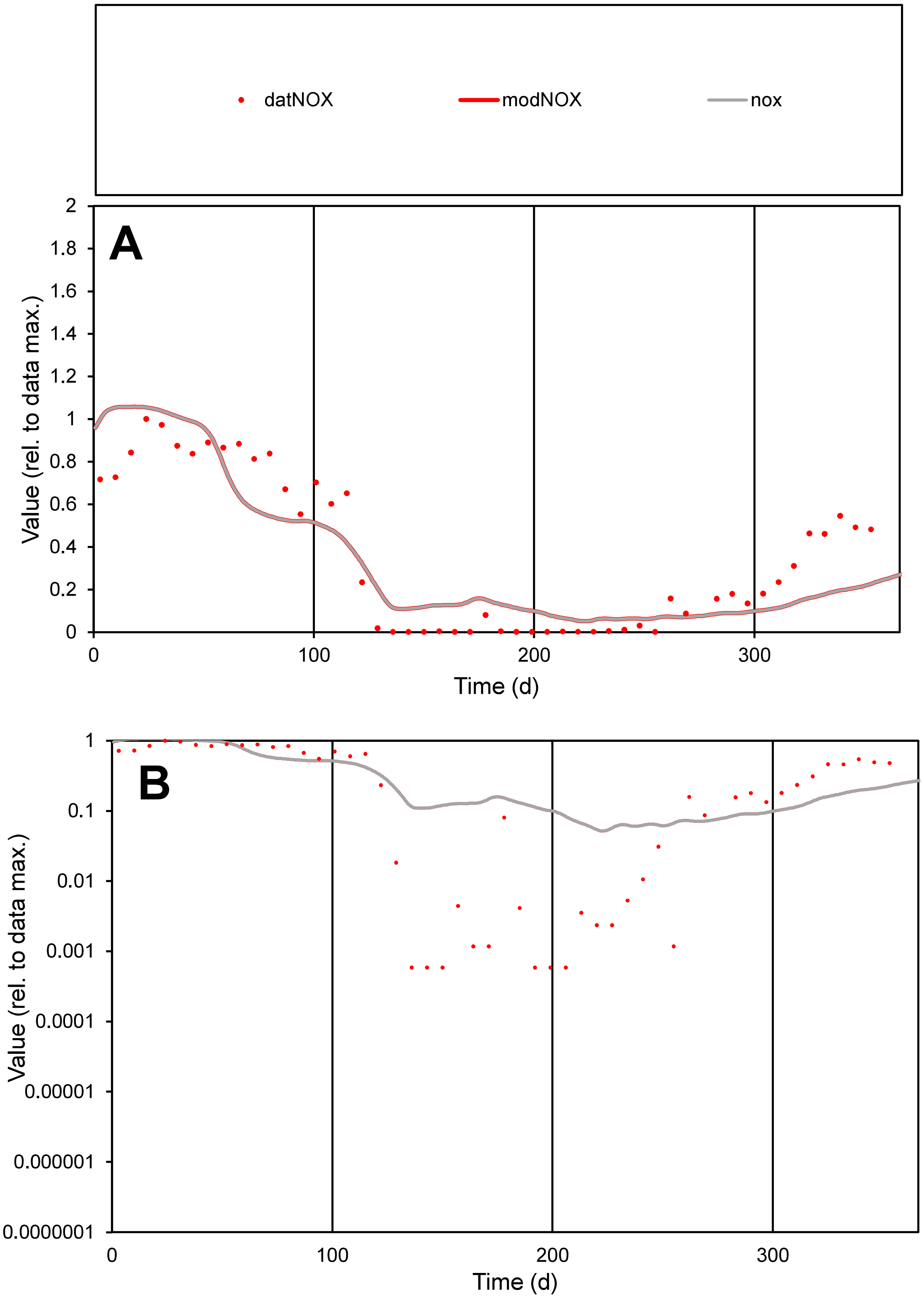

C1

Variables
NOX Provide jtd or Dst
CalibOnly?
2 1 to reproduce of
oNormalize?
2 1=no, 2=yes

ofxn calc*
1.38353
ofxn mod
1.4705

sRun
RunA1
zout
1

Ave
Data
0.00301
Model
0.00288

data/vMax
0.00848

ofxng
369.894

Min d/m
0.01119

ave/min d
601.322
ave/min m
6.44786

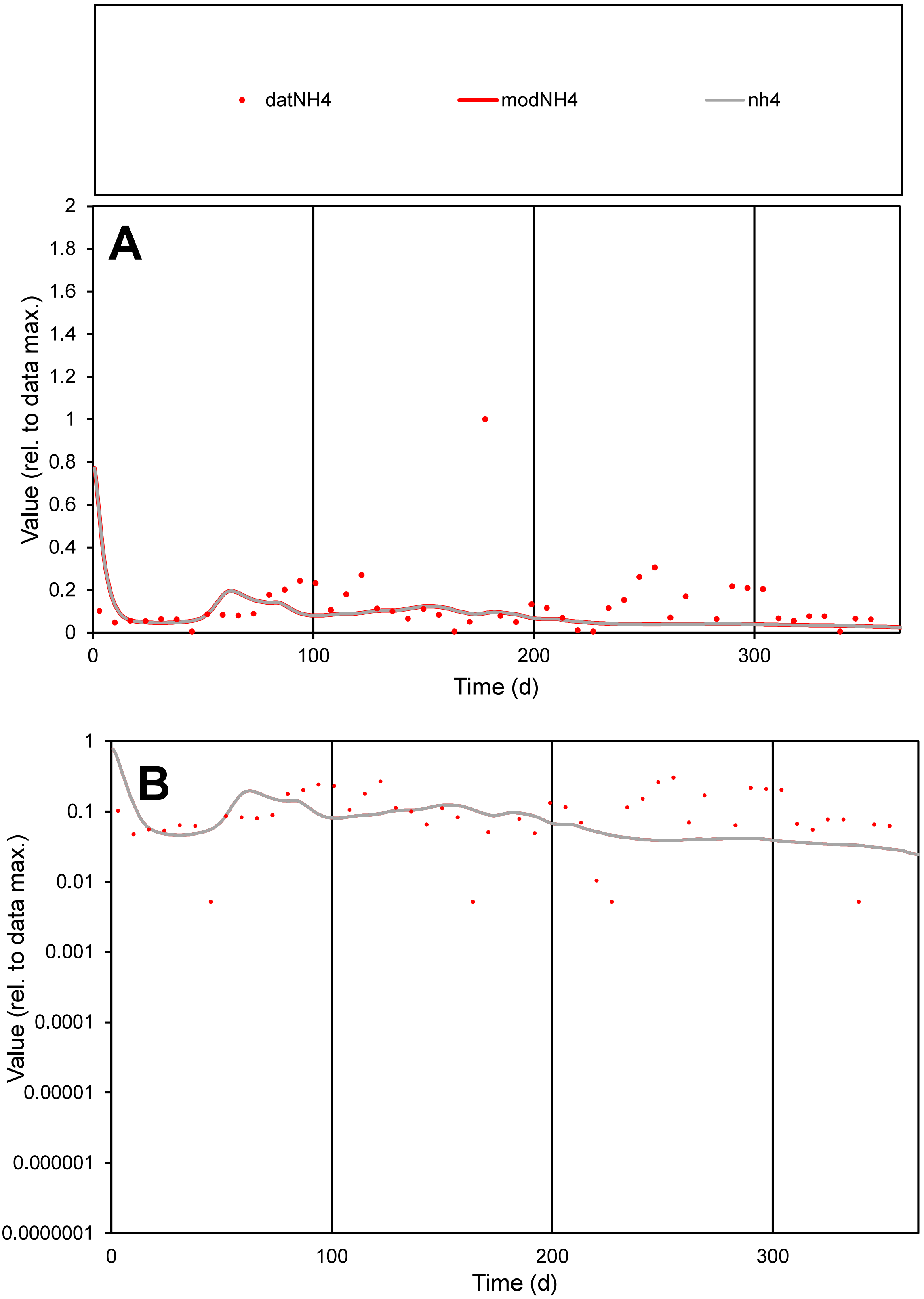

C1

Variables
NH4 Provide jtd or Dst
CalibOnly?
2 1 to reproduce of
oNormalize?
2 1=no, 2=yes

ofxn calc*
3.74893
ofxn mod
3.83291

sRun
RunA1
zout
1

Ave
Data
0.00021
Model
0.00013

data/vMax
0.00168

ofxng
369.894

Min d/m
0.17989

ave/min d
24.2421
ave/min m
2.67309

328_Calib_S_A

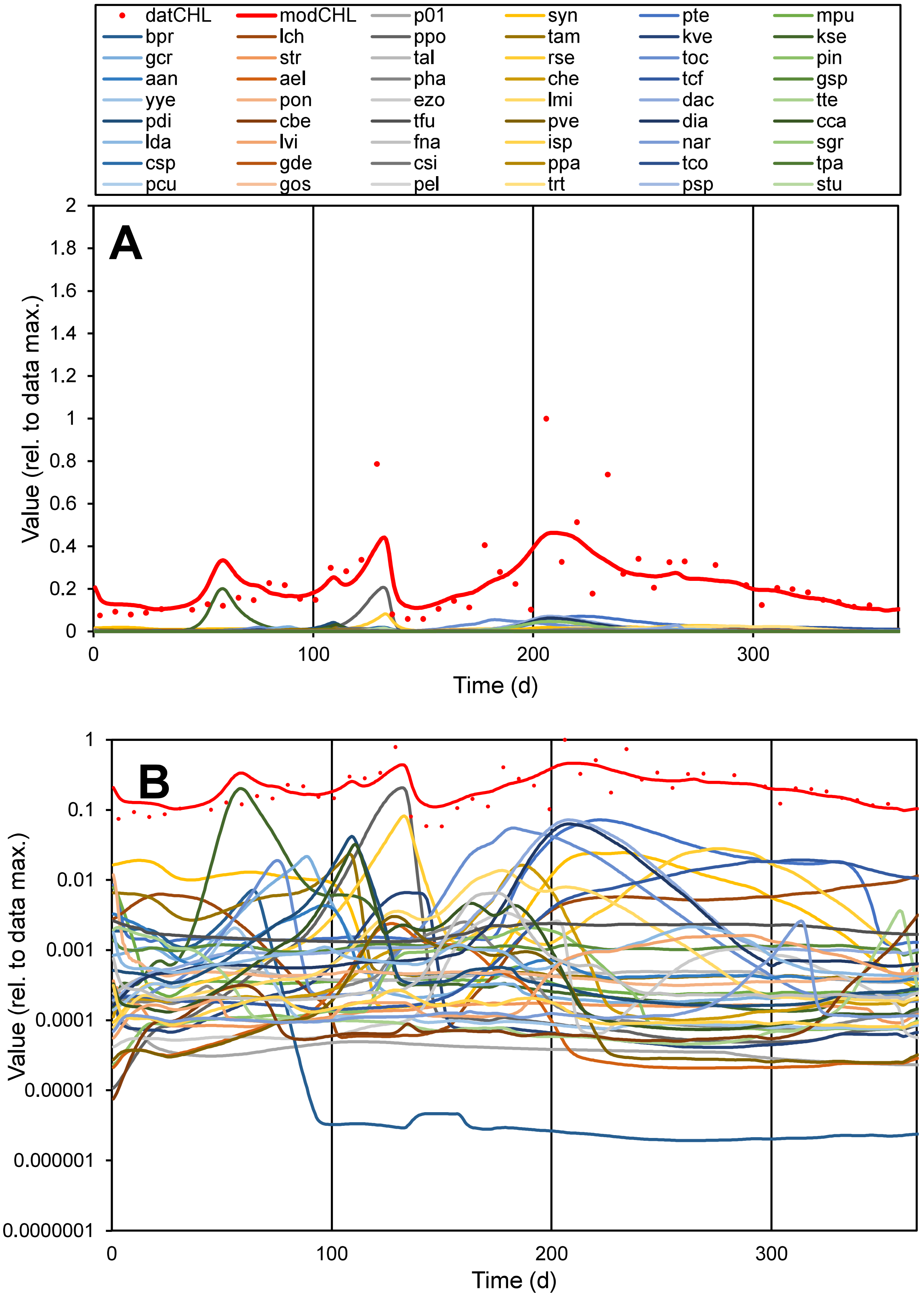

C1

Variables

CHL Provide jtd or Dst

CalibOnly?

2 1 to reproduce of

oNormalize?

2 1=no, 2=yes

ofxn calc*

5.94195

ofxn mod

6.11282

sRun

RunA1

zout

1

Ave

Data

1.02276

Model

0.9878

data/vMax

4.427

ofxng

369.894

Min d/m

0.55135

ave/min d

3.96879

ave/min m

2.1134

328_Calib_S_A

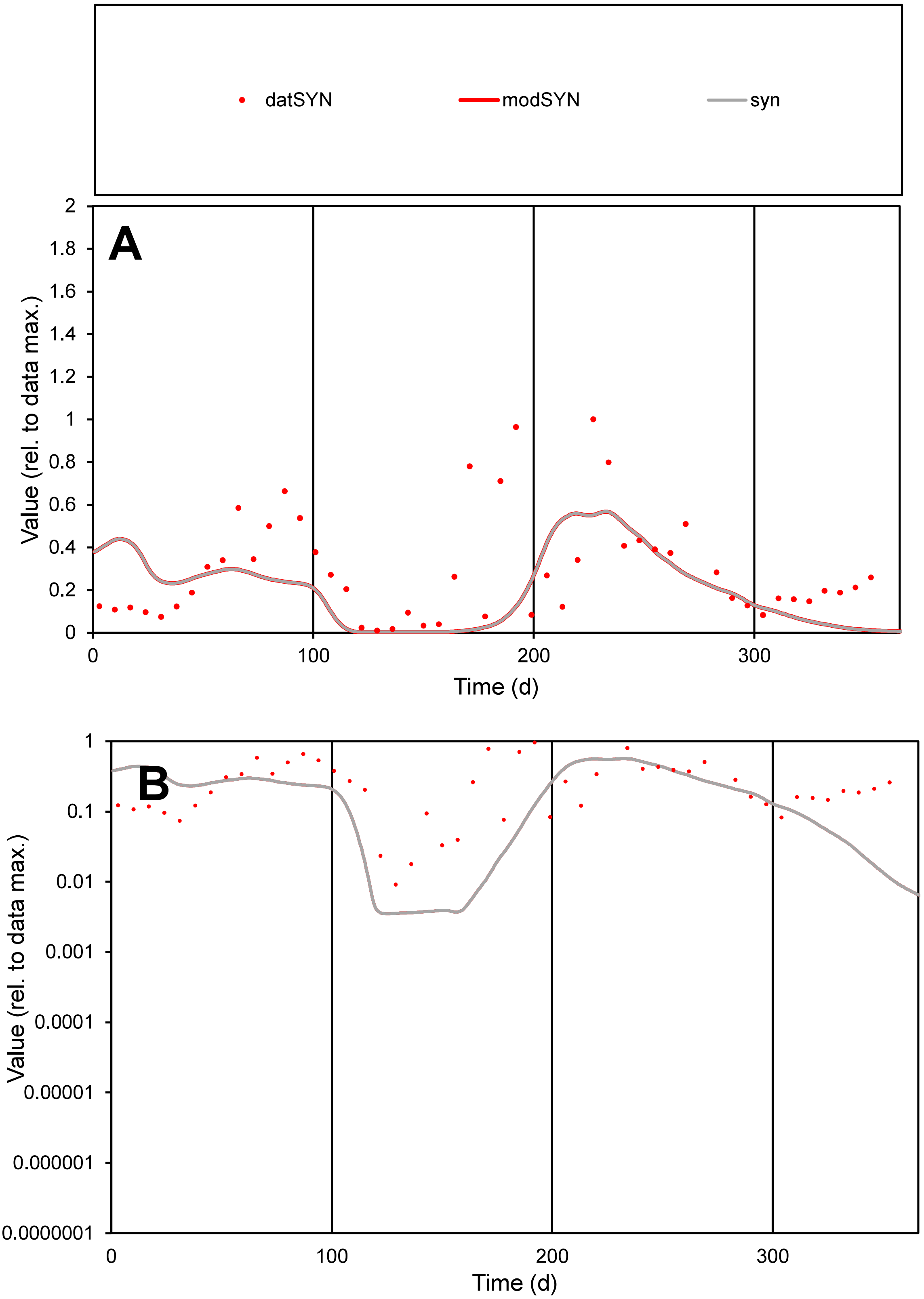

C1

Variables
SYN Provide jtd or Dst
CalibOnly?
2 1 to reproduce of
oNormalize?
2 1=no, 2=yes

ofxn calc*
0.89167
ofxn mod
0.90257

sRun
RunA1
zout
1

Ave
Data
6.69811
Model
4.77344

data/vMax
22.84

ofxng
369.894

Min d/m
2.60085

ave/min d
32.0637
ave/min m
59.4303

328_Calib_S_A

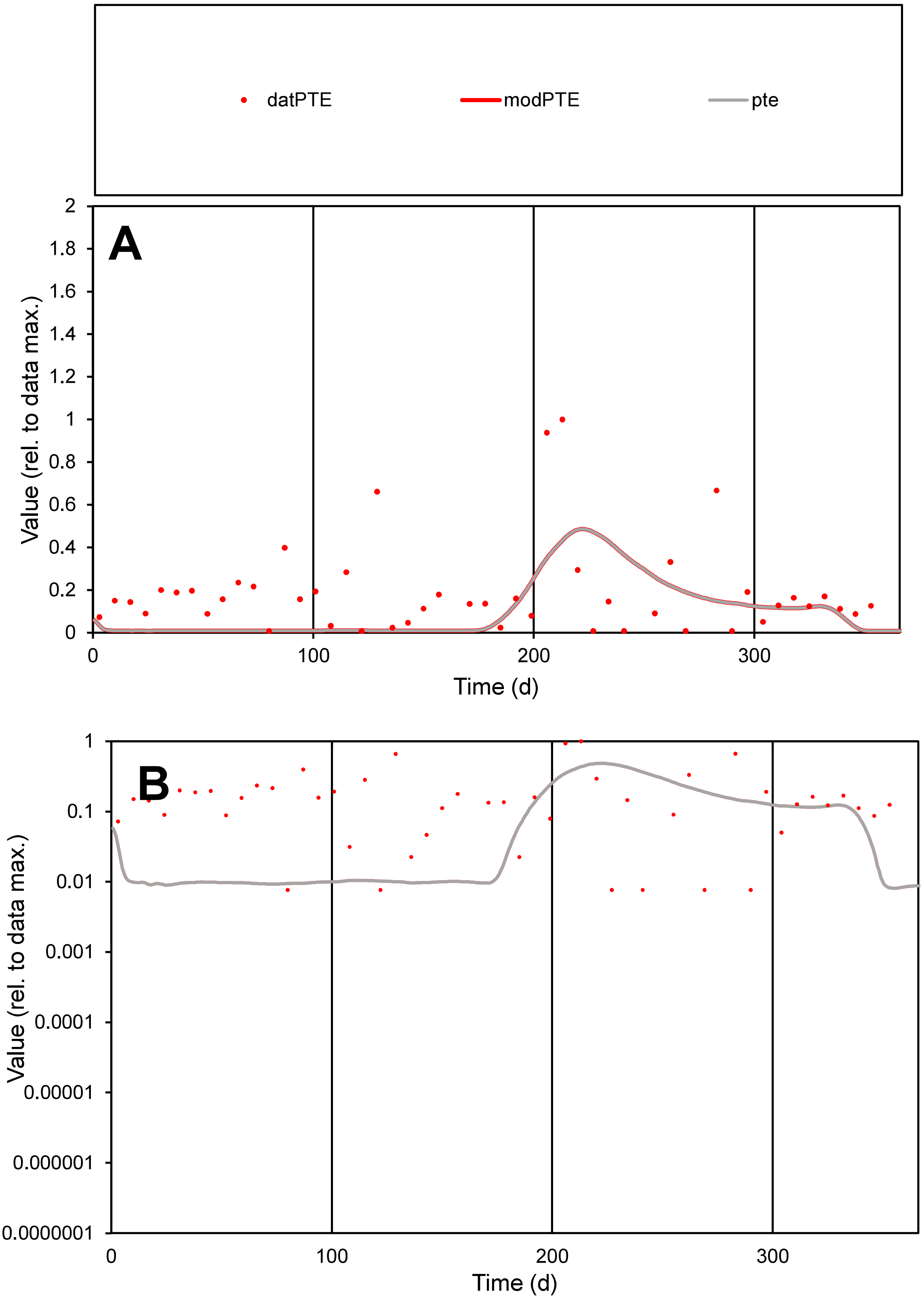

C1

Variables

PTE Provide jtd or Dst

CalibOnly?

2 1 to reproduce of

oNormalize?

2 1=no, 2=yes

ofxn calc*

1.22065

ofxn mod

1.22895

sRun

RunA1

zout

1

Ave

Data

0.0002

Model

0.00011

data/vMax

0.00109

ofxng

369.894

Min d/m

0.94795

ave/min d

24.4552

ave/min m

12.9709

328_Calib_S_A

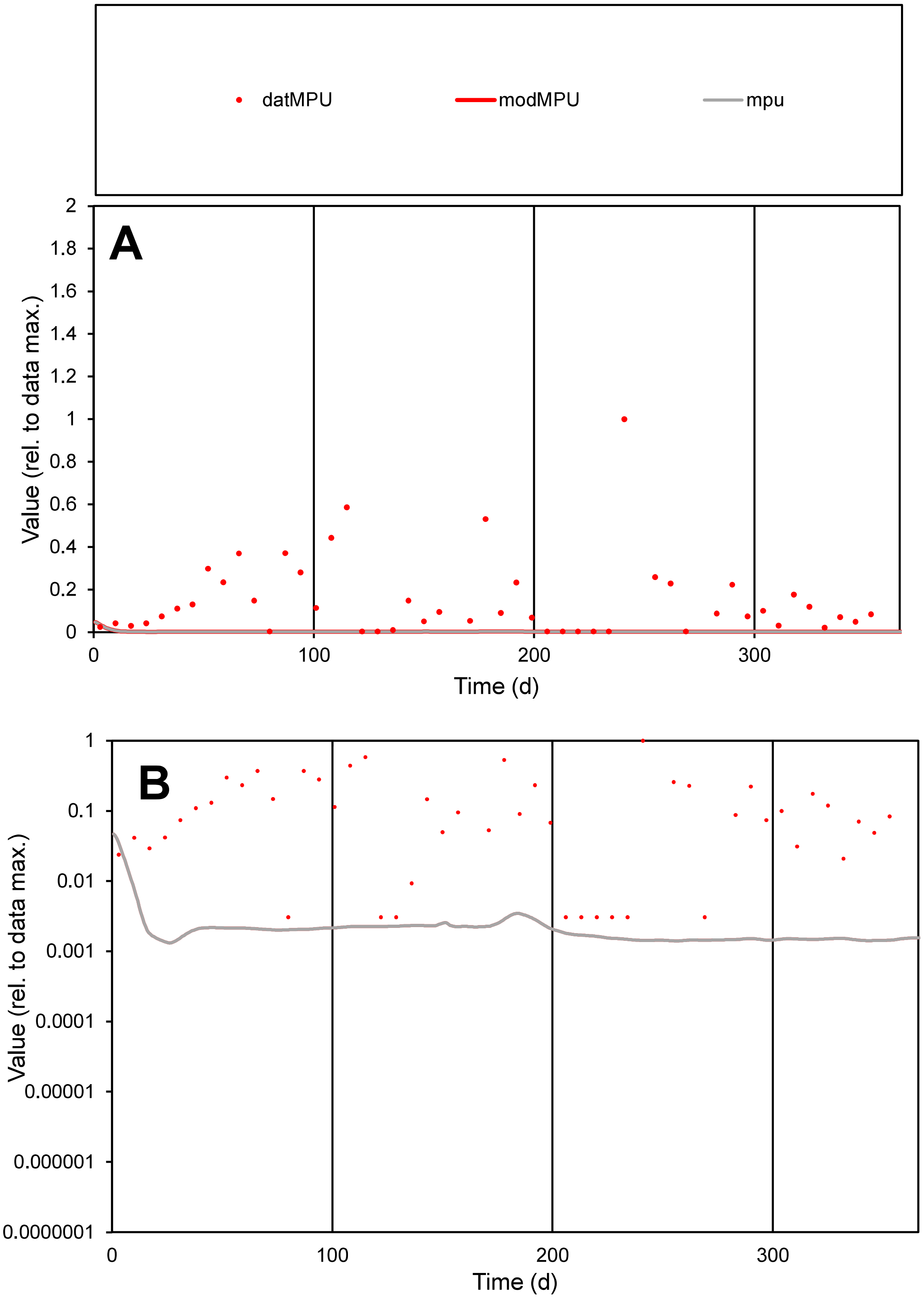

C1

Variables

MPU	Provide jtd or Dst
CalibOnly?	
2	1 to reproduce of
oNormalize?	
2	1=no, 2=yes

ofxn calc*
1.60477
ofxn mod
1.60717

sRun
RunA1
zout
1

Ave
Data
0.0002
Model
3.4E-06

data/vMax
0.00133

ofxng
369.894

Min d/m
2.28396

ave/min d
48.311
ave/min m
1.89546

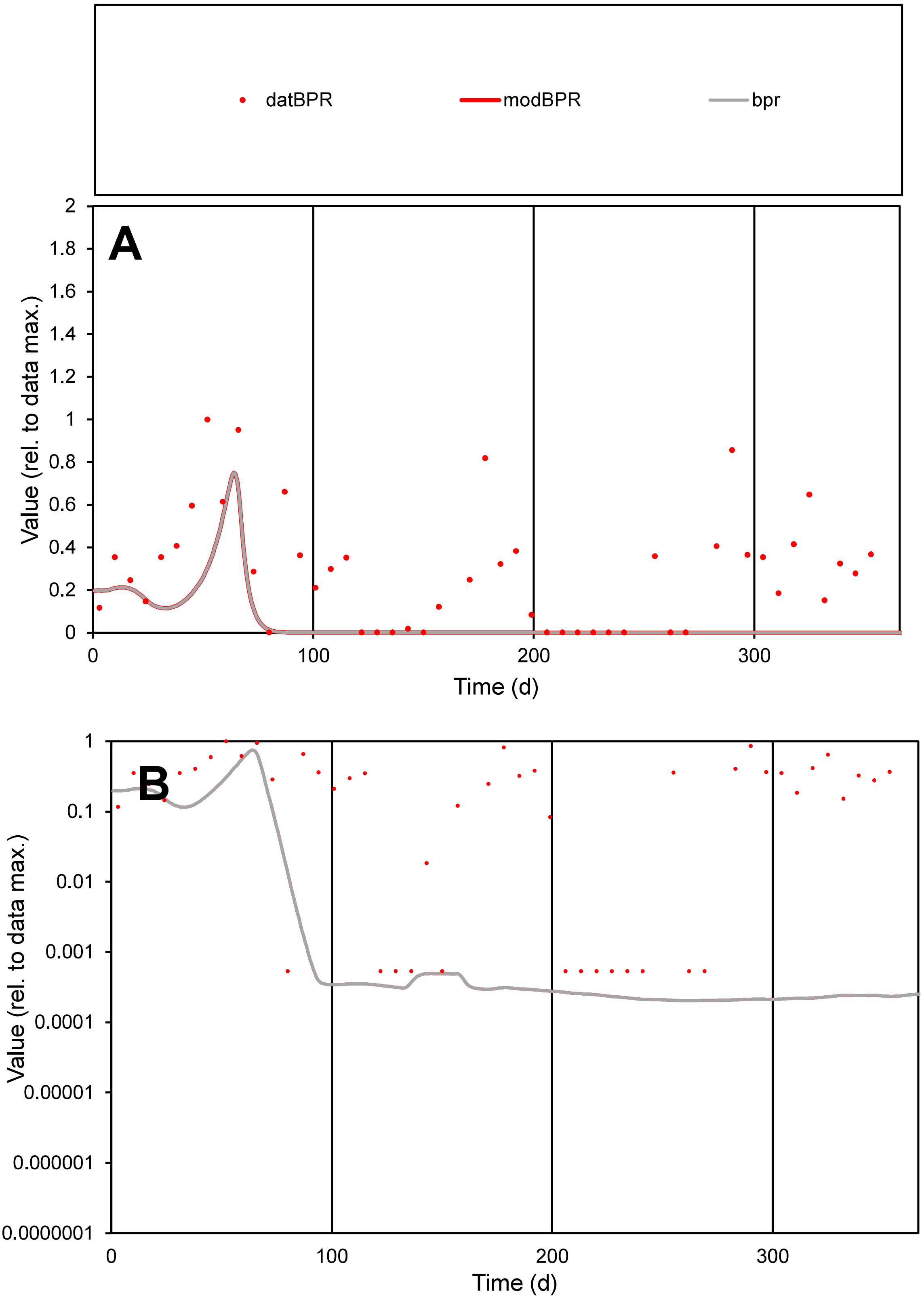

C1

Variables
BPR Provide jtd or Dst
CalibOnly?
2 1 to reproduce of
oNormalize?
2 1=no, 2=yes

ofxn calc*
1.12696
ofxn mod
1.13442

sRun
RunA1
zout
1

Ave
Data
9.8E-05
Model
2.1E-05

data/vMax
0.00035

ofxng
369.894

Min d/m
2.62465

ave/min d
531.292
ave/min m
302.64

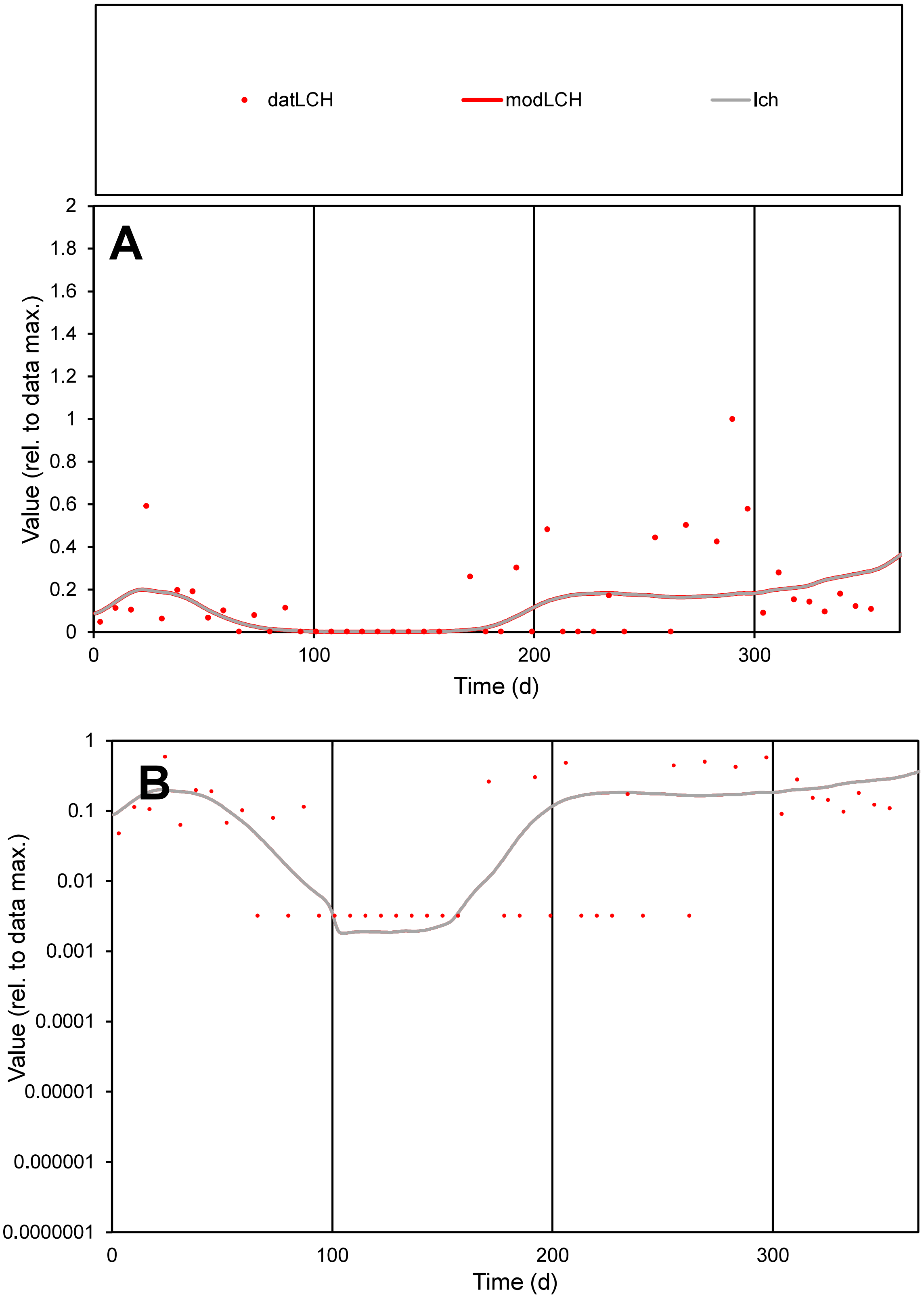

C1

Variables
LCH Provide jtd or Dst
CalibOnly?
2 1 to reproduce of
oNormalize?
2 1=no, 2=yes

ofxn calc*
1.30187
ofxn mod
1.31206

sRun
RunA1
zout
1

Ave
Data
0.00011
Model
8.9E-05

data/vMax
0.00076

ofxng
369.894

Min d/m
1.72496

ave/min d
45.7322
ave/min m
62.6031

328_Calib_S_A

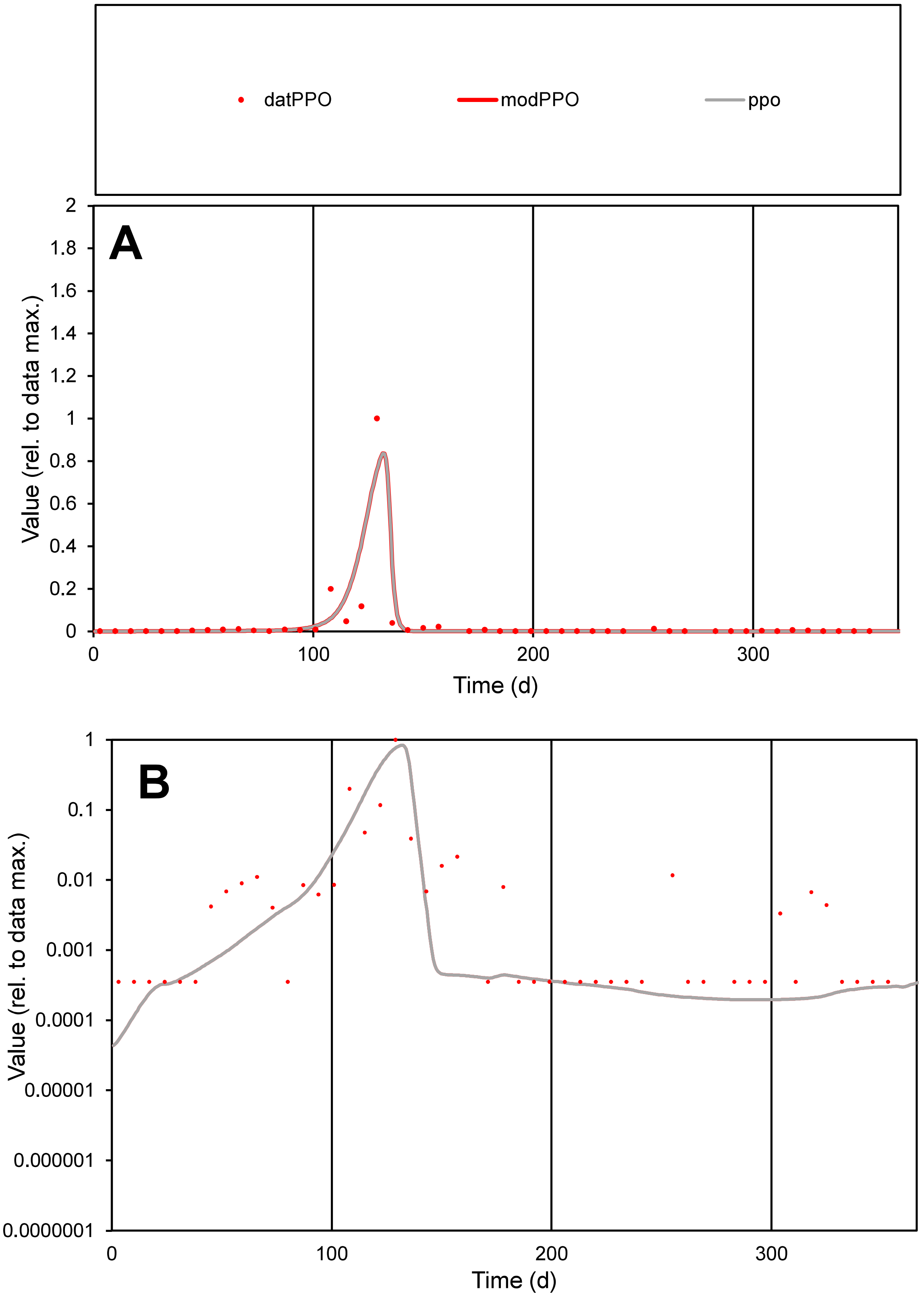

C1

Variables
PPO Provide jtd or Dst
CalibOnly?
2 1 to reproduce of
oNormalize?
2 1=no, 2=yes

ofxn calc*
1.6882
ofxn mod
1.883

sRun
RunA1
zout
1

Ave
Data
0.00018
Model
0.00018

data/vMax
0.00542

ofxng
369.894

Min d/m
5.86091

ave/min d
91.5531
ave/min m
549.703

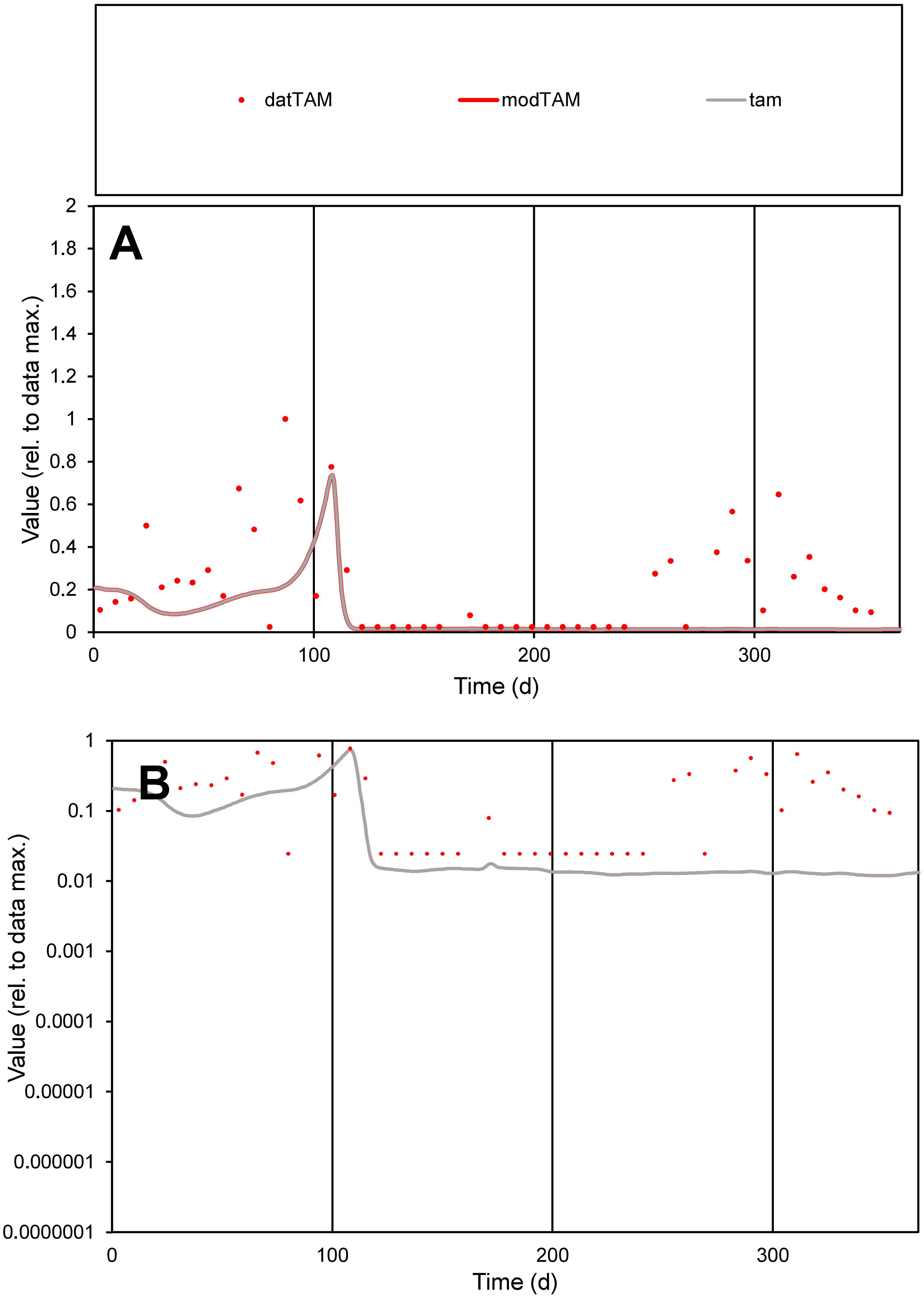

C1

Variables
TAM Provide jtd or Dst
CalibOnly?
2 1 to reproduce of
oNormalize?
2 1=no, 2=yes

ofxn calc*
1.10908
ofxn mod
1.12173

sRun
RunA1
zout
1

Ave
Data
5.1E-05
Model
2E-05

data/vMax
0.00023

ofxng
369.894

Min d/m
2.04292

ave/min d
8.81852
ave/min m
7.25314

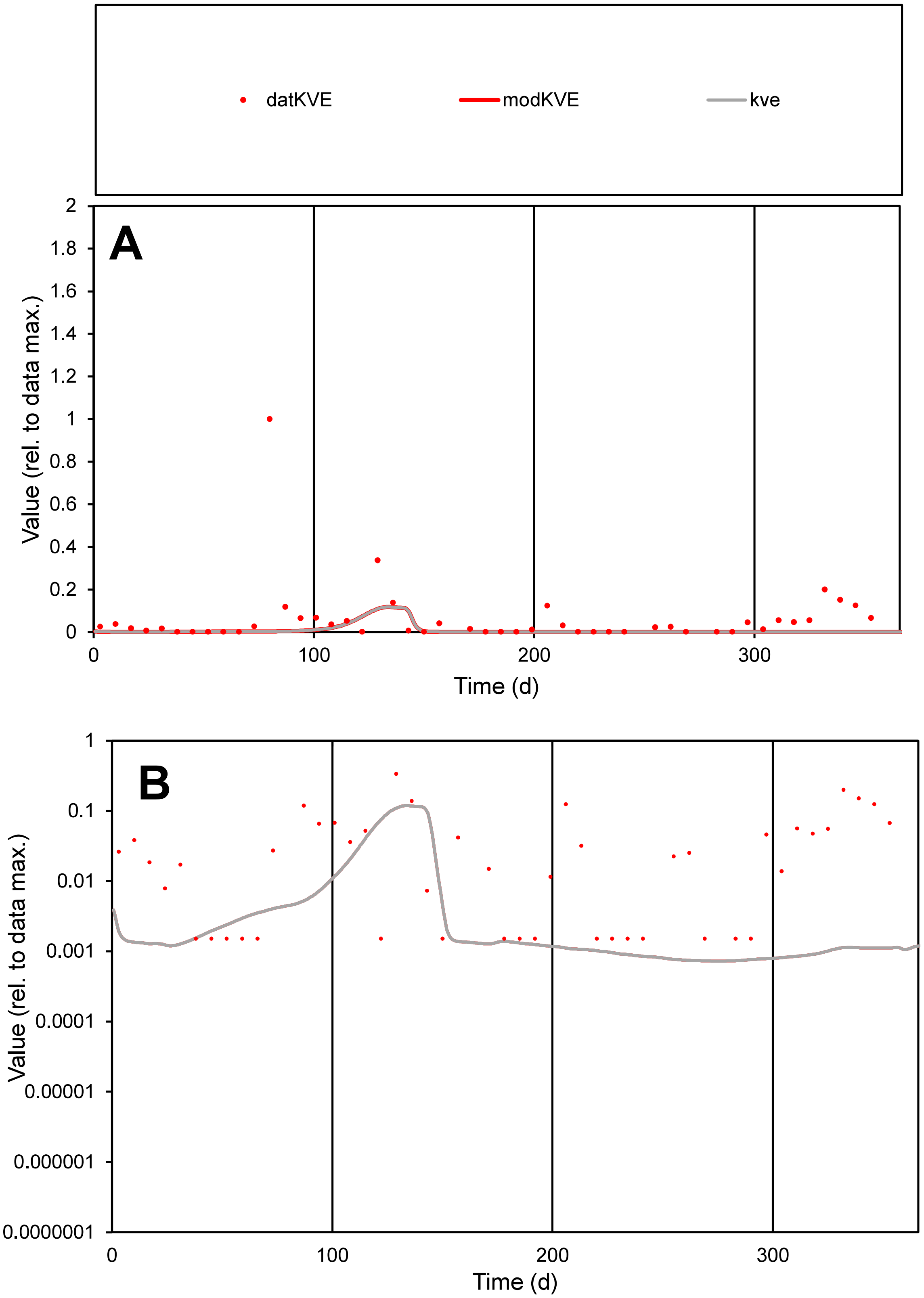

C1

Variables
KVE Provide jtd or Dst
CalibOnly?
2 1 to reproduce of
oNormalize?
2 1=no, 2=yes

ofxn calc*
2.49062
ofxn mod
2.49616

sRun
RunA1
zout
1

Ave
Data
0.00013
Model
2E-05

data/vMax
0.00206

ofxng
369.894

Min d/m
2.07902

ave/min d
41.4851
ave/min m
13.5695

328_Calib_S_A

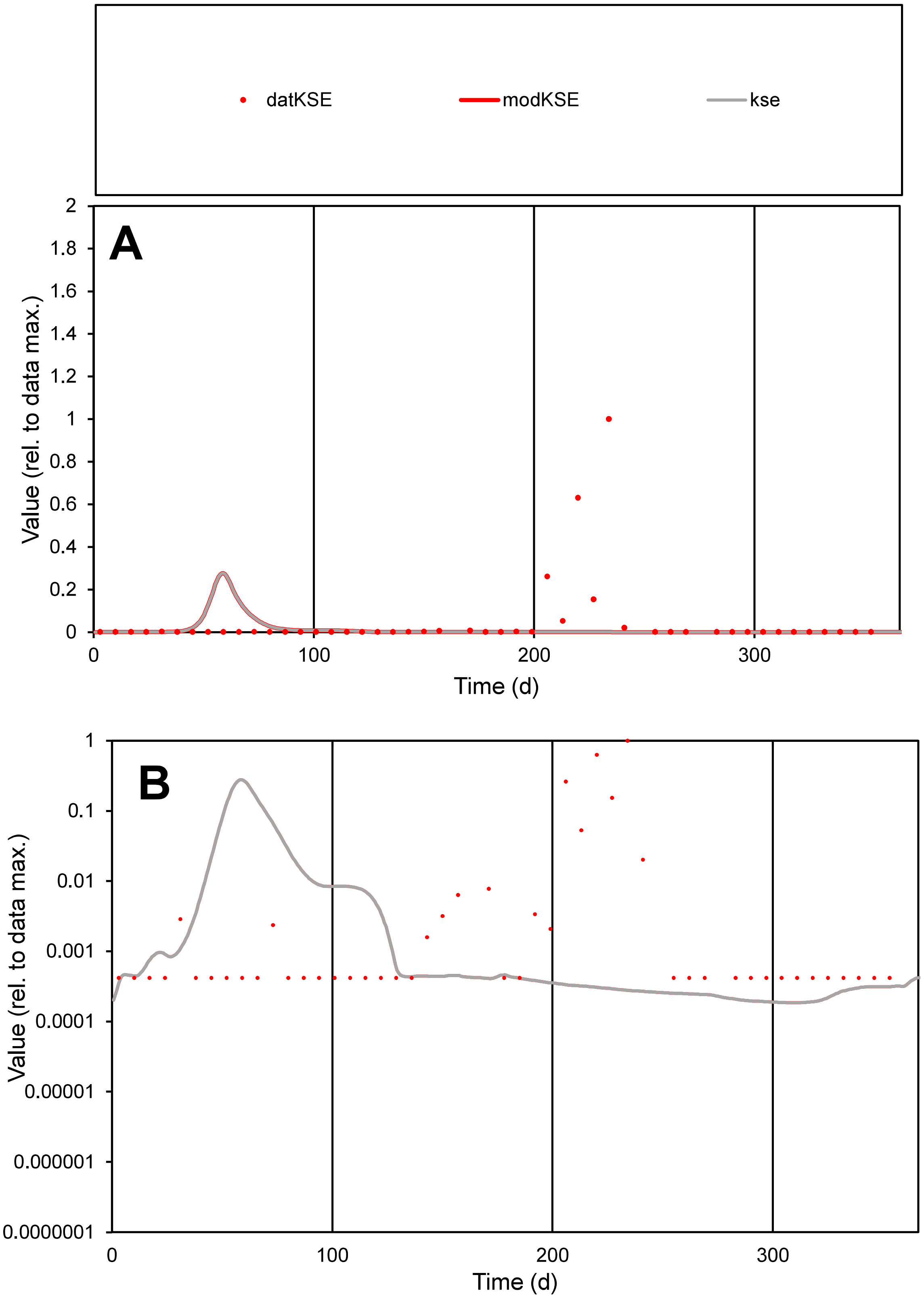

C1

Variables
KSE Provide jtd or Dst
CalibOnly?
2 1 to reproduce of
oNormalize?
2 1=no, 2=yes

ofxn calc*
3.99874
ofxn mod
4.0269

sRun
RunA1
zout
1

Ave
Data
0.0004
Model
1E-04

data/vMax
0.00893

ofxng
369.894

Min d/m
2.25783

ave/min d
107.325
ave/min m
60.0561

328_Calib_S_A

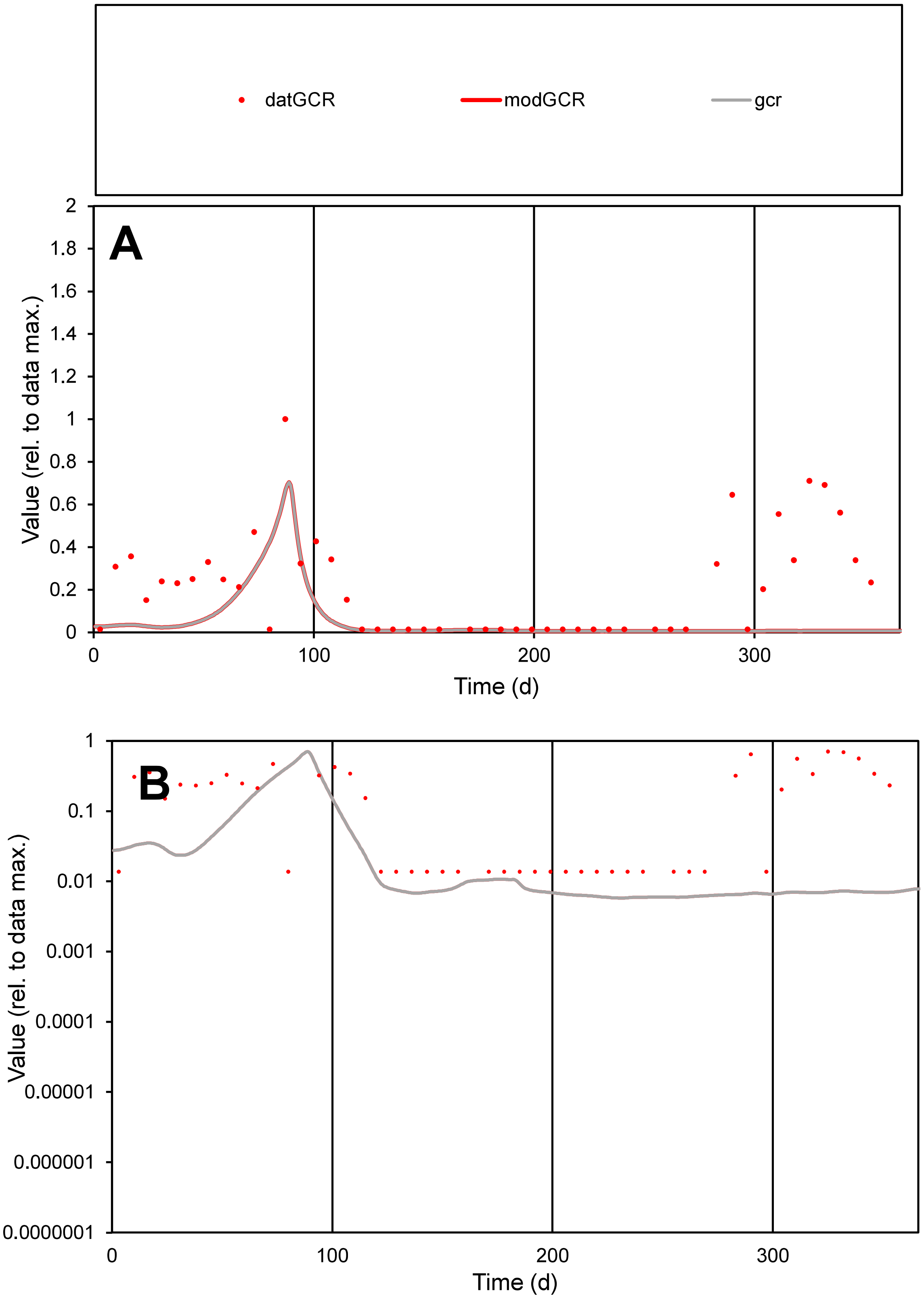

C1

Variables

GCR	Provide jtd or Dst

CalibOnly?

2	1 to reproduce of

oNormalize?

2	1=no, 2=yes

ofxn calc*
1.25542
ofxn mod
1.26149

sRun
RunA1
zout
1

Ave
Data
4.7E-05
Model
1.3E-05

data/vMax
0.00023

ofxng
369.894

Min d/m
2.3635

ave/min d
15.0859
ave/min m
9.94452

328_Calib_S_A

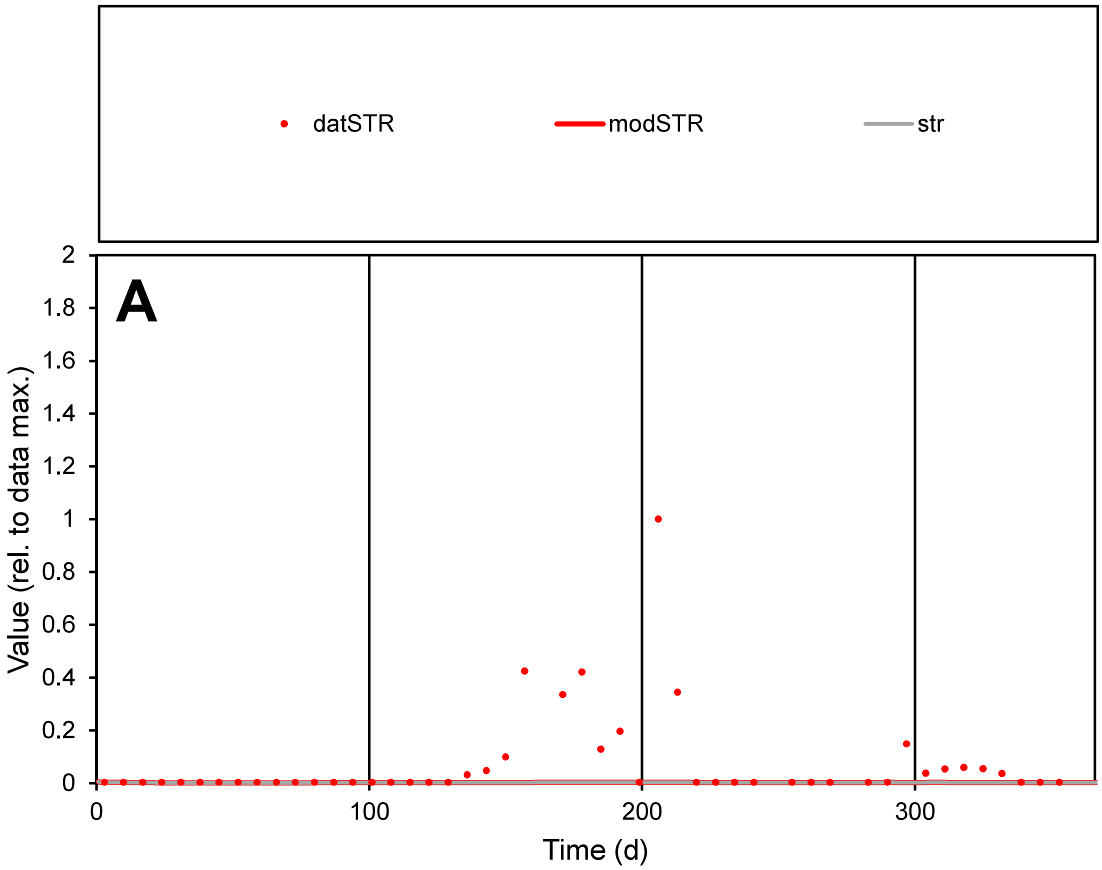

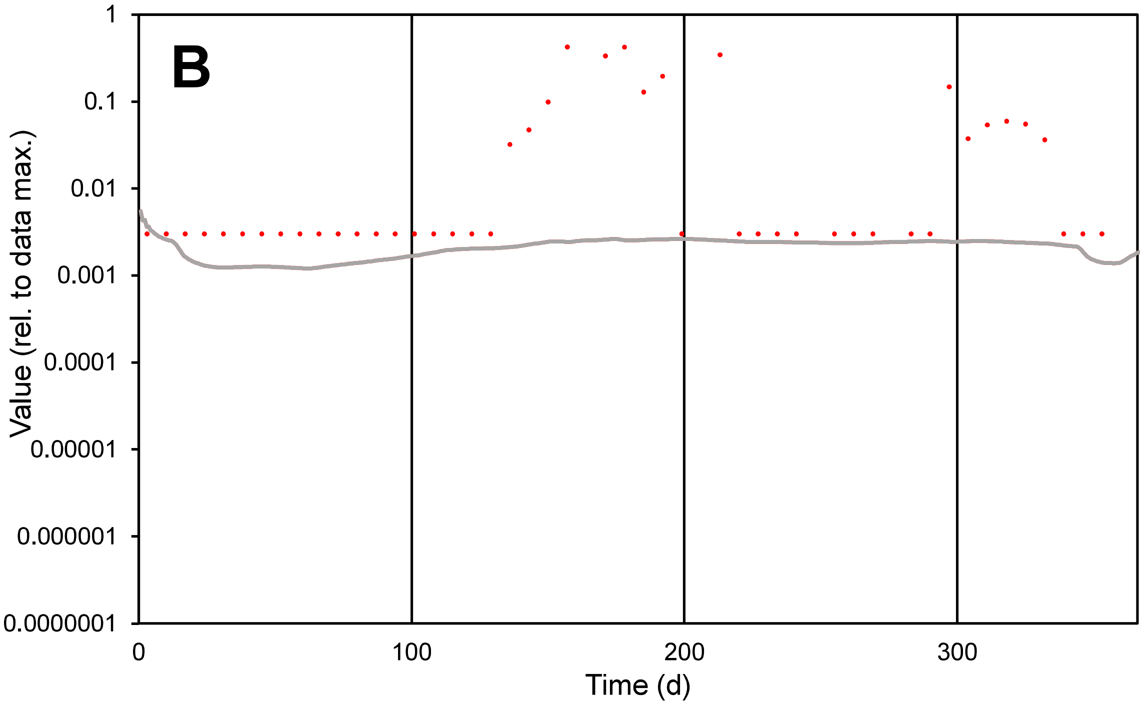

C1

Variables
STR Provide jtd or Dst
CalibOnly?
2 1 to reproduce of
oNormalize?
2 1=no, 2=yes

ofxn calc*
2.54904
ofxn mod
2.55301

sRun
RunA1
zout
1

Ave
Data
3.9E-05
Model
1.1E-06

data/vMax
0.00054

ofxng
369.894

Min d/m
2.49502

ave/min d
24.2247
ave/min m
1.73449

328_Calib_S_A

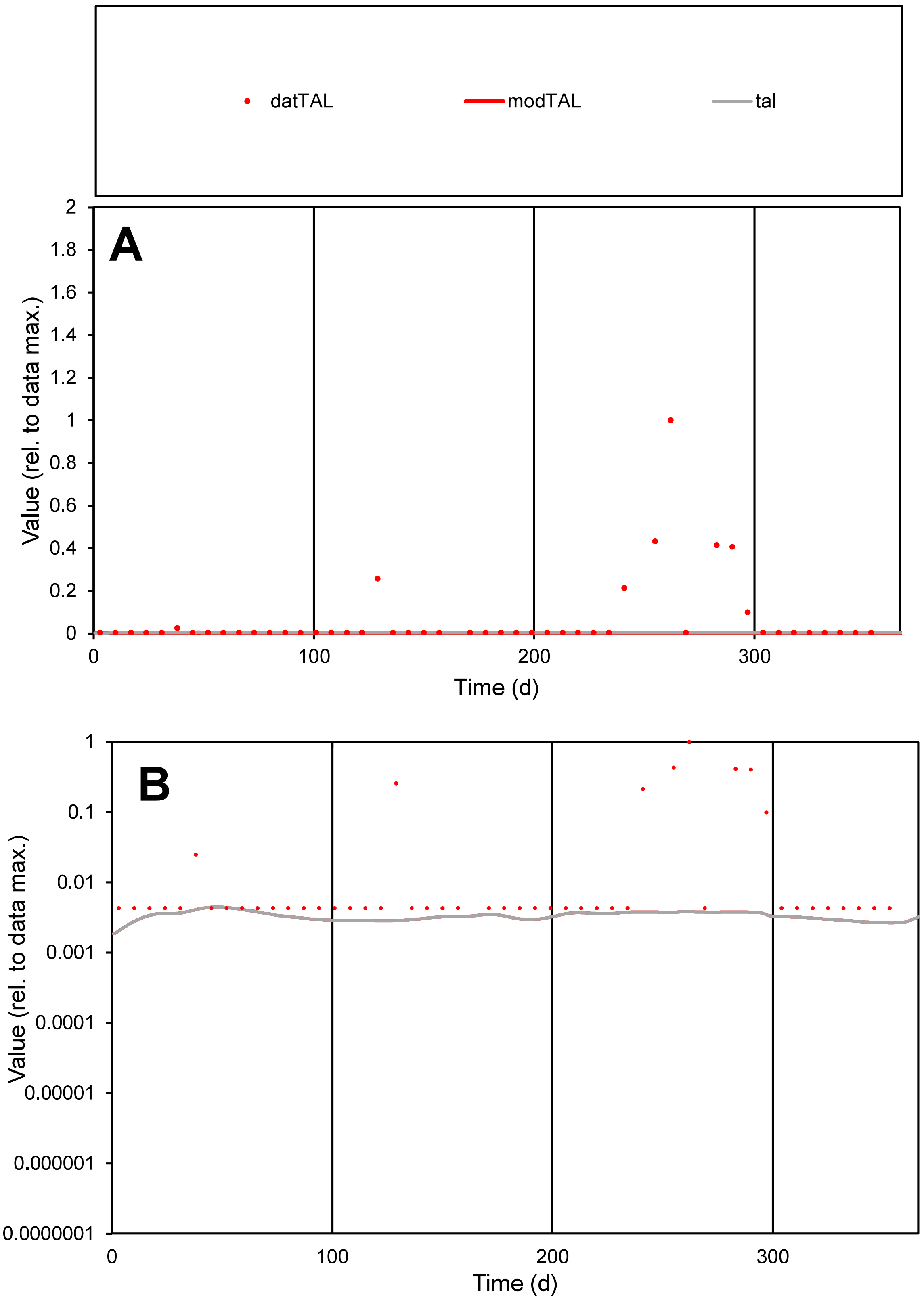

C1

Variables
TAL Provide jtd or Dst
CalibOnly?
2 1 to reproduce of
oNormalize?
2 1=no, 2=yes

ofxn calc*
2.92455
ofxn mod
2.92948

sRun
RunA1
zout
1

Ave
Data
6.1E-05
Model
3.2E-06

data/vMax
0.00097

ofxng
369.894

Min d/m
1.97804

ave/min d
14.6651
ave/min m
1.5387

328_Calib_S_A

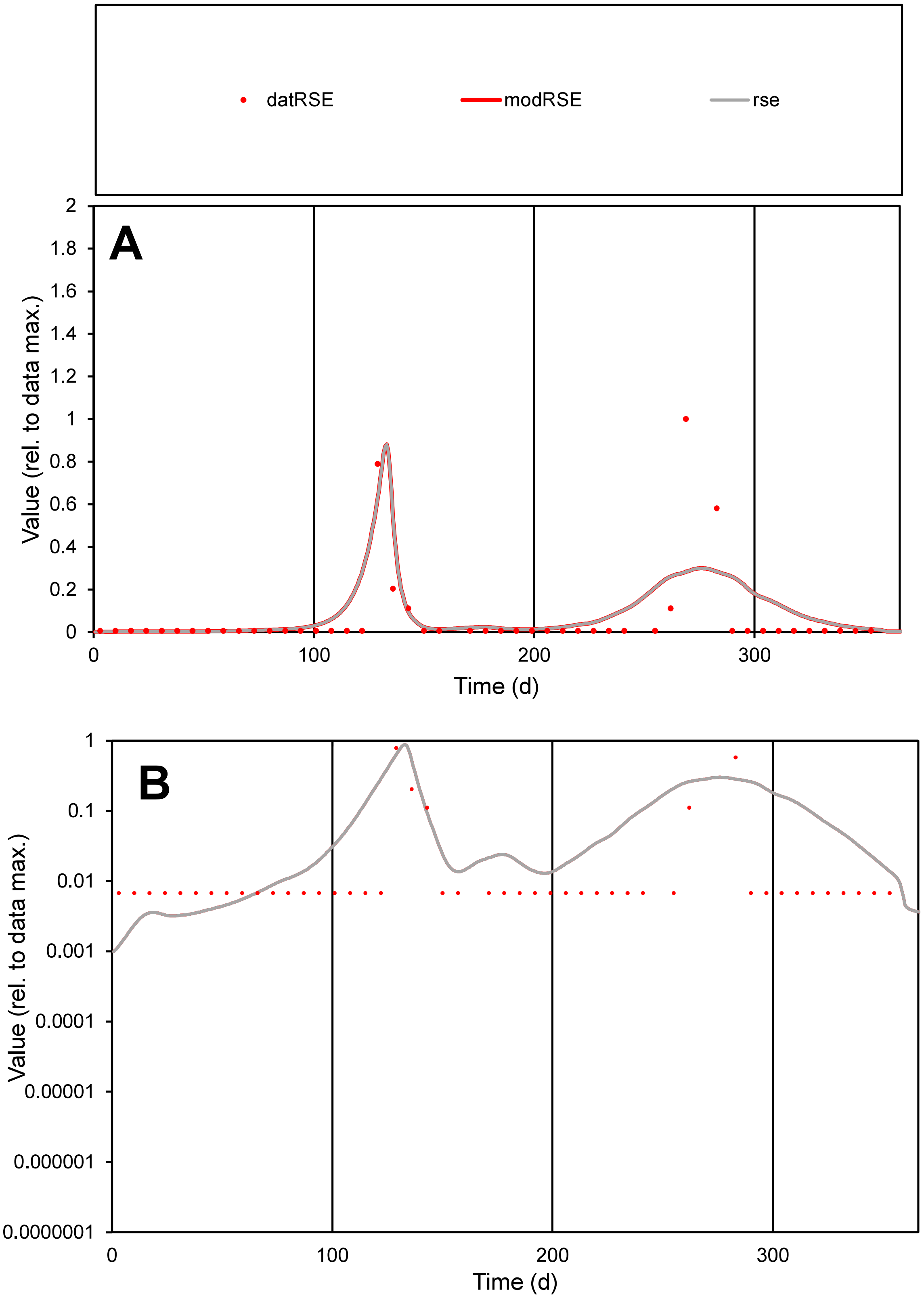

C1

Variables
RSE Provide jtd or Dst
CalibOnly?
2 1 to reproduce of
oNormalize?
2 1=no, 2=yes

ofxn calc*
2.13715
ofxn mod
2.18751

sRun
RunA1
zout
1

Ave
Data
4.4E-05
Model
5.9E-05

data/vMax
0.00069

ofxng
369.894

Min d/m
5.02858

ave/min d
9.49722
ave/min m
63.5259

328_Calib_S_A

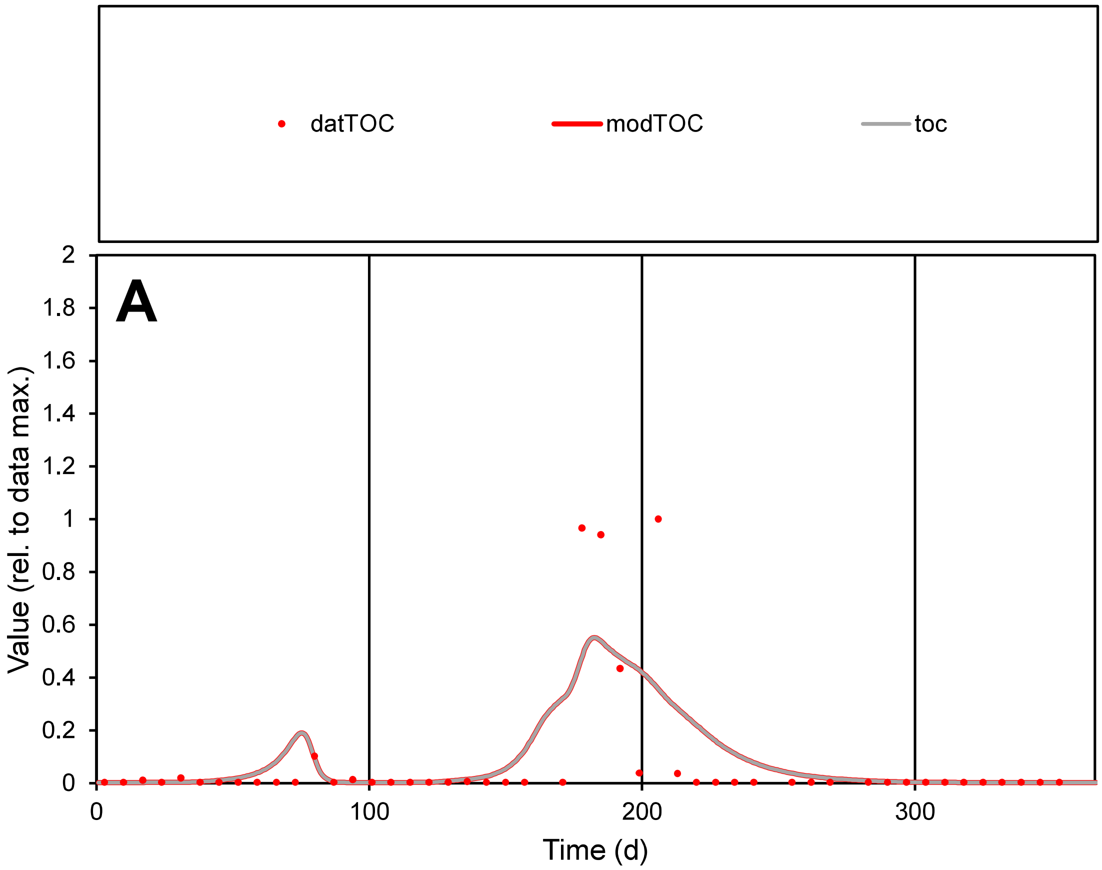

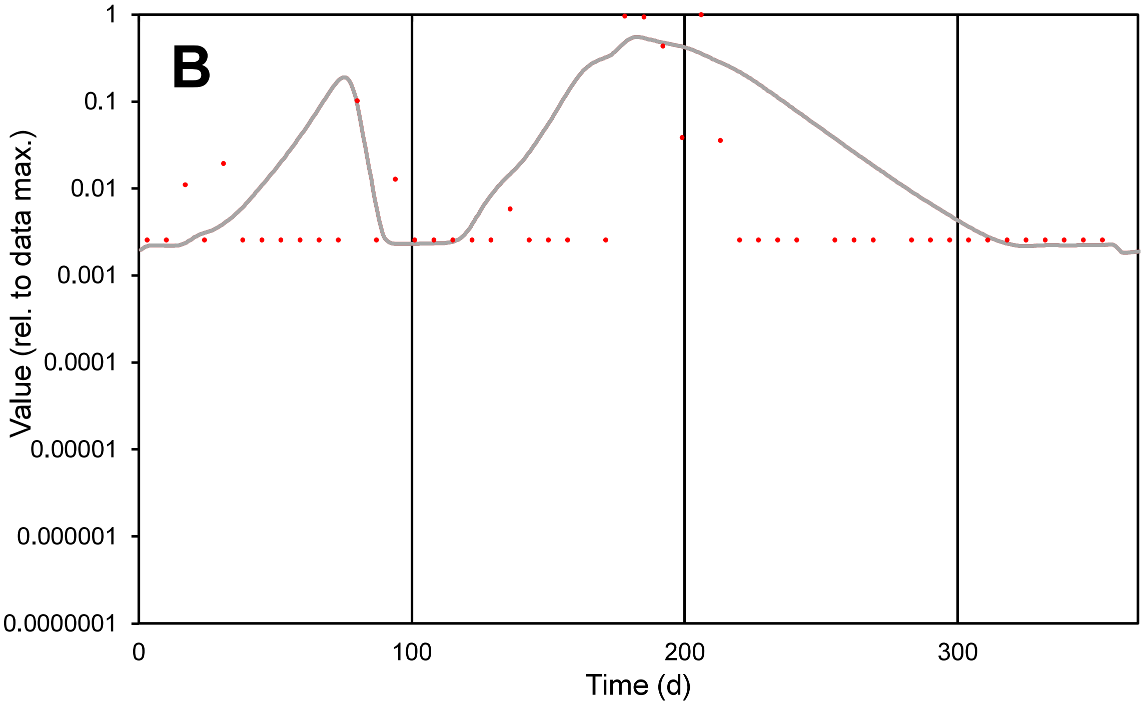

C1

Variables
TOC Provide jtd or Dst
CalibOnly?
2 1 to reproduce of
oNormalize?
2 1=no, 2=yes

ofxn calc*
2.0642
ofxn mod
2.11862

sRun
RunA1
zout
1

Ave
Data
0.00013
Model
0.00014

data/vMax
0.00169

ofxng
369.894

Min d/m
1.17159

ave/min d
29.7564
ave/min m
38.6301

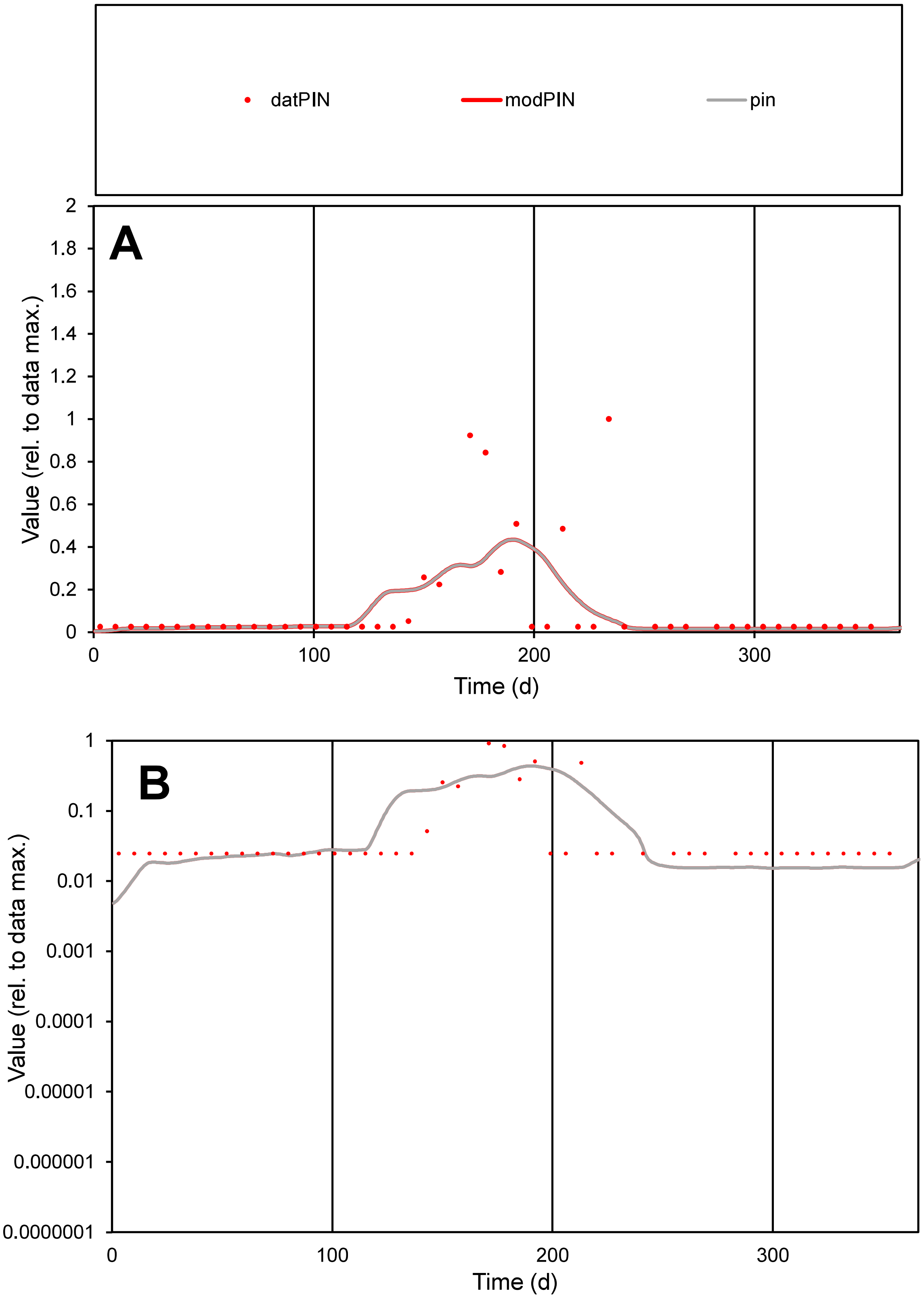

C1

Variables
PIN Provide jtd or Dst
CalibOnly?
2 1 to reproduce of
oNormalize?
2 1=no, 2=yes

ofxn calc*
1.70451
ofxn mod
1.72704

sRun
RunA1
zout
1

Ave
Data
1.9E-05
Model
1.5E-05

data/vMax
0.00016

ofxng
369.894

Min d/m
3.92752

ave/min d
4.65188
ave/min m
14.5758

328_Calib_S_A

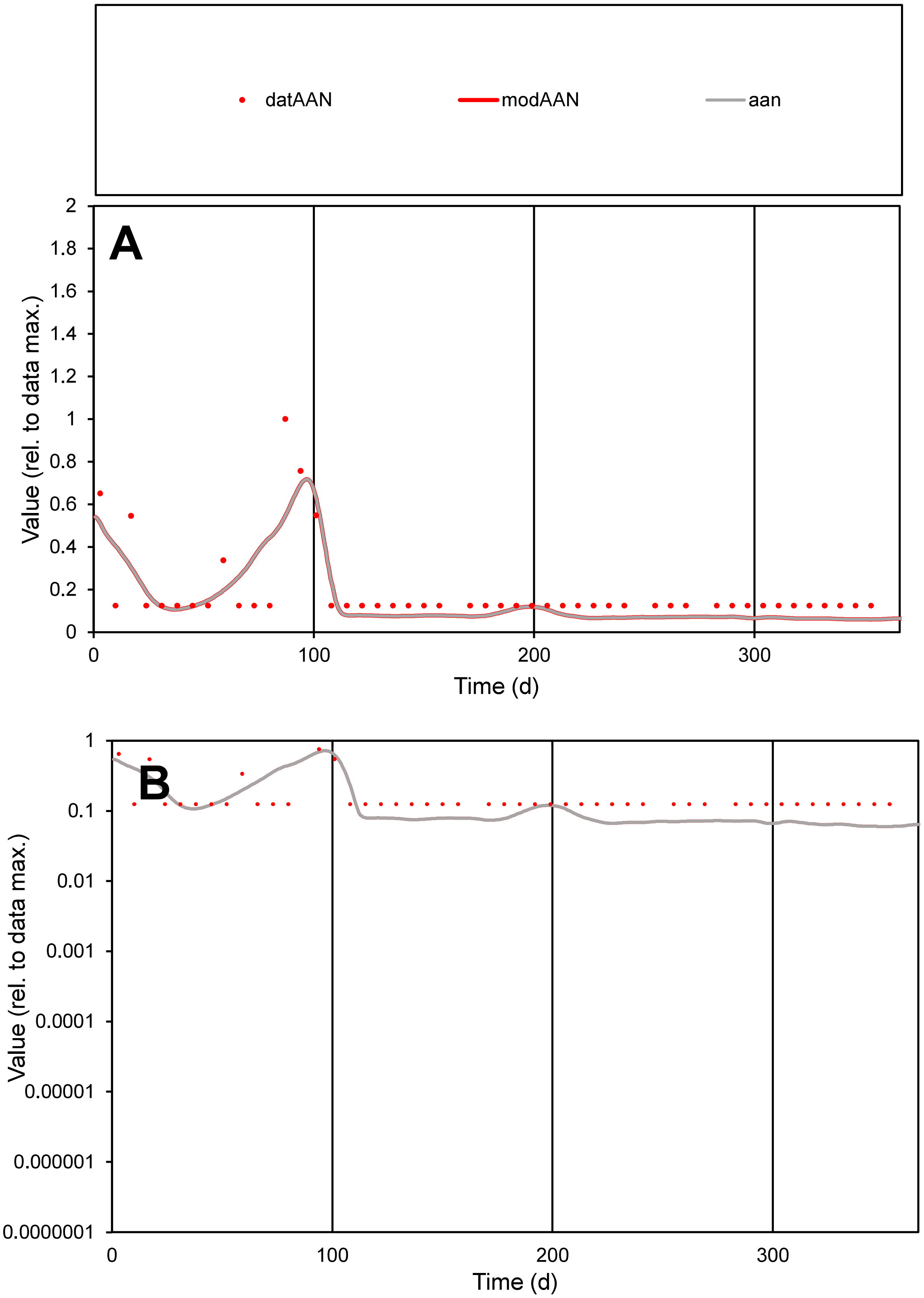

C1

Variables
AAN Provide jtd or Dst
CalibOnly?
2 1 to reproduce of
oNormalize?
2 1=no, 2=yes

ofxn calc*
0.60454
ofxn mod
0.62323

sRun
RunA1
zout
1

Ave
Data
8.5E-06
Model
7.1E-06

data/vMax
4.5E-05

ofxng
369.894

Min d/m
2.08057

ave/min d
1.51613
ave/min m
2.62975

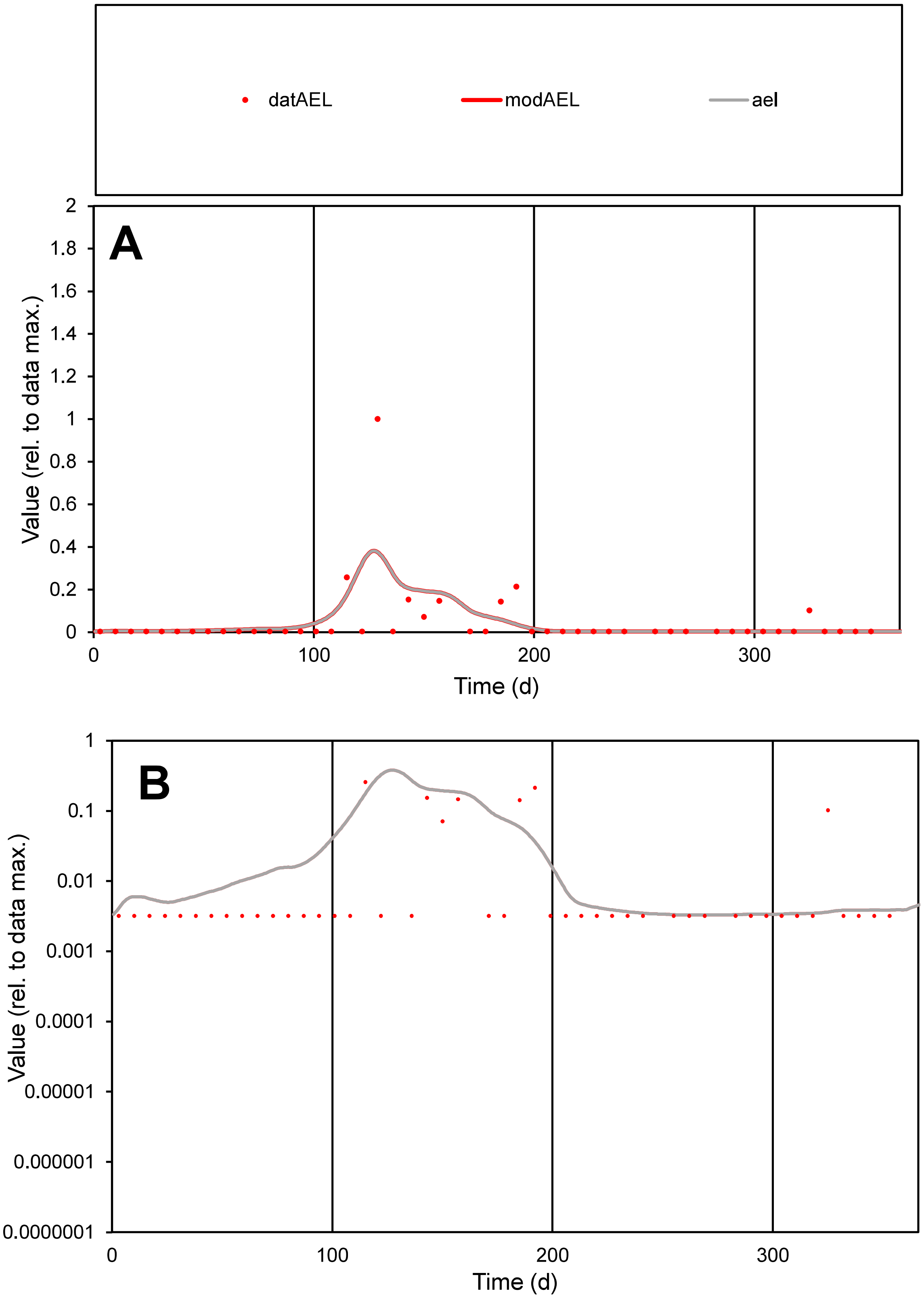

C1

Variables

AEL Provide jtd or Dst

CalibOnly?

2 1 to reproduce of

oNormalize?

2 1=no, 2=yes

ofxn calc*

2.48175

ofxn mod

2.53843

sRun

RunA1

zout

1

Ave

Data

1E-05

Model

1E-05

data/vMax

0.00022

ofxng

369.894

Min d/m

0.97348

ave/min d

14.3884

ave/min m

14.3456

328_Calib_S_A

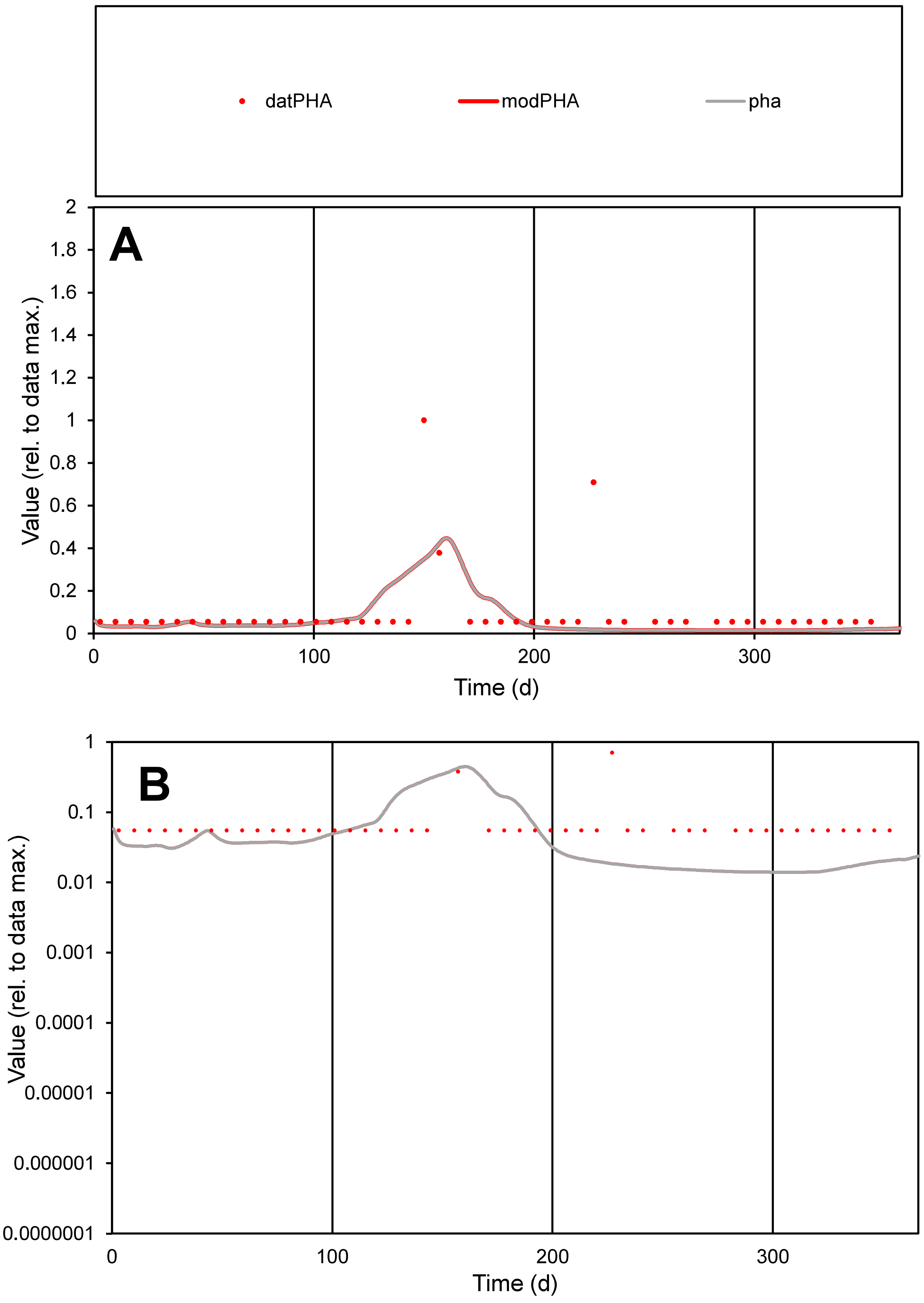

C1

Variables

PHA Provide jtd or Dst

CalibOnly?

2 1 to reproduce of

oNormalize?

2 1=no, 2=yes

ofxn calc*

1.56596

ofxn mod

1.58025

sRun

RunA1

zout

1

Ave

Data

1.8E-05

Model

1.3E-05

data/vMax

0.00019

ofxng

369.894

Min d/m

3.95817

ave/min d

1.72709

ave/min m

4.77327

328_Calib_S_A

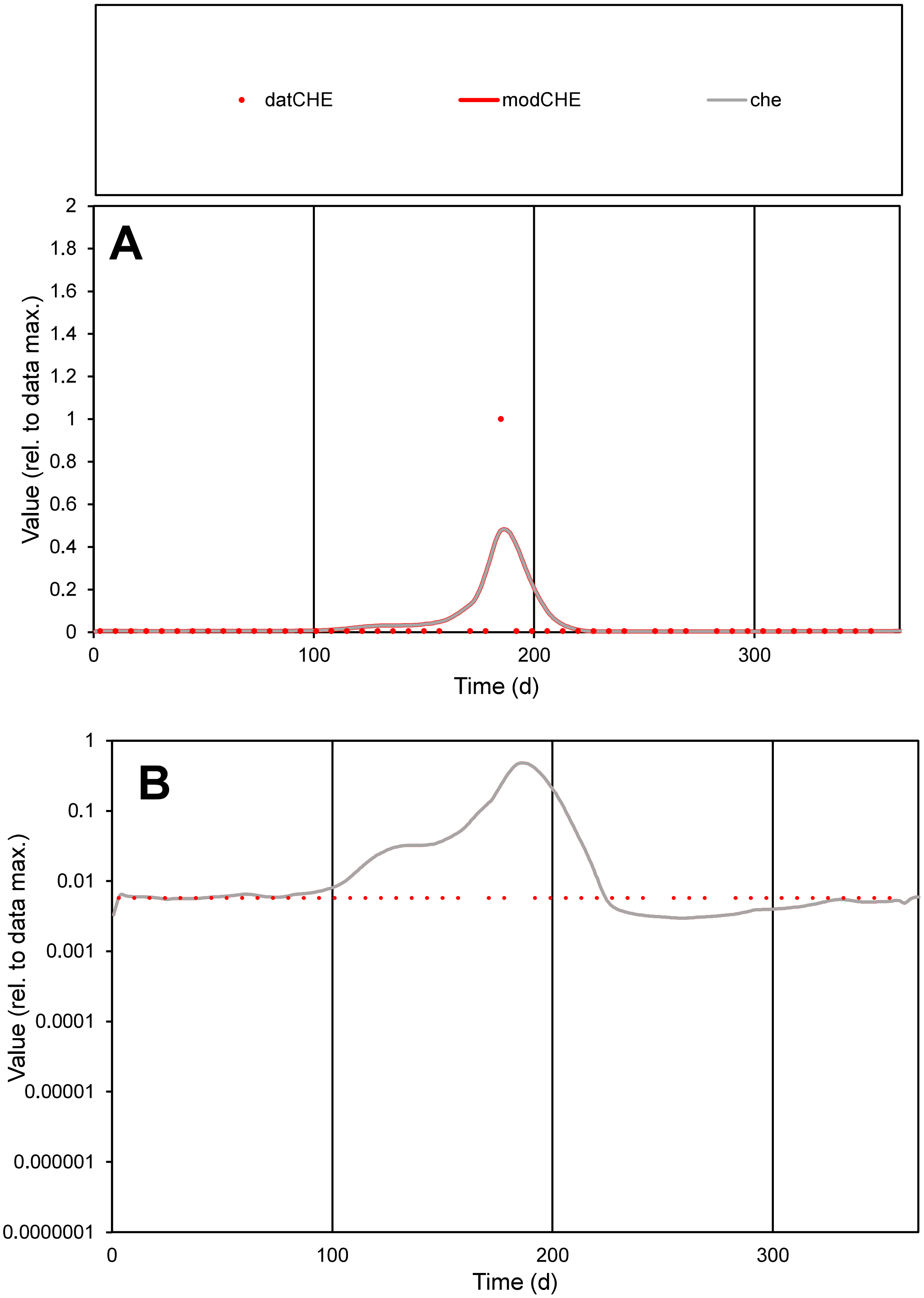

C1

Variables

CHE Provide jtd or Dst

CalibOnly?

2 1 to reproduce of

oNormalize?

2 1=no, 2=yes

ofxn calc*

3.66936

ofxn mod

3.8707

sRun

RunA1

zout

1

Ave

Data

3.2E-05

Model

4.6E-05

data/vMax

0.00122

ofxng

369.894

Min d/m

1.94137

ave/min d

4.59087

ave/min m

12.6069

328_Calib_S_A

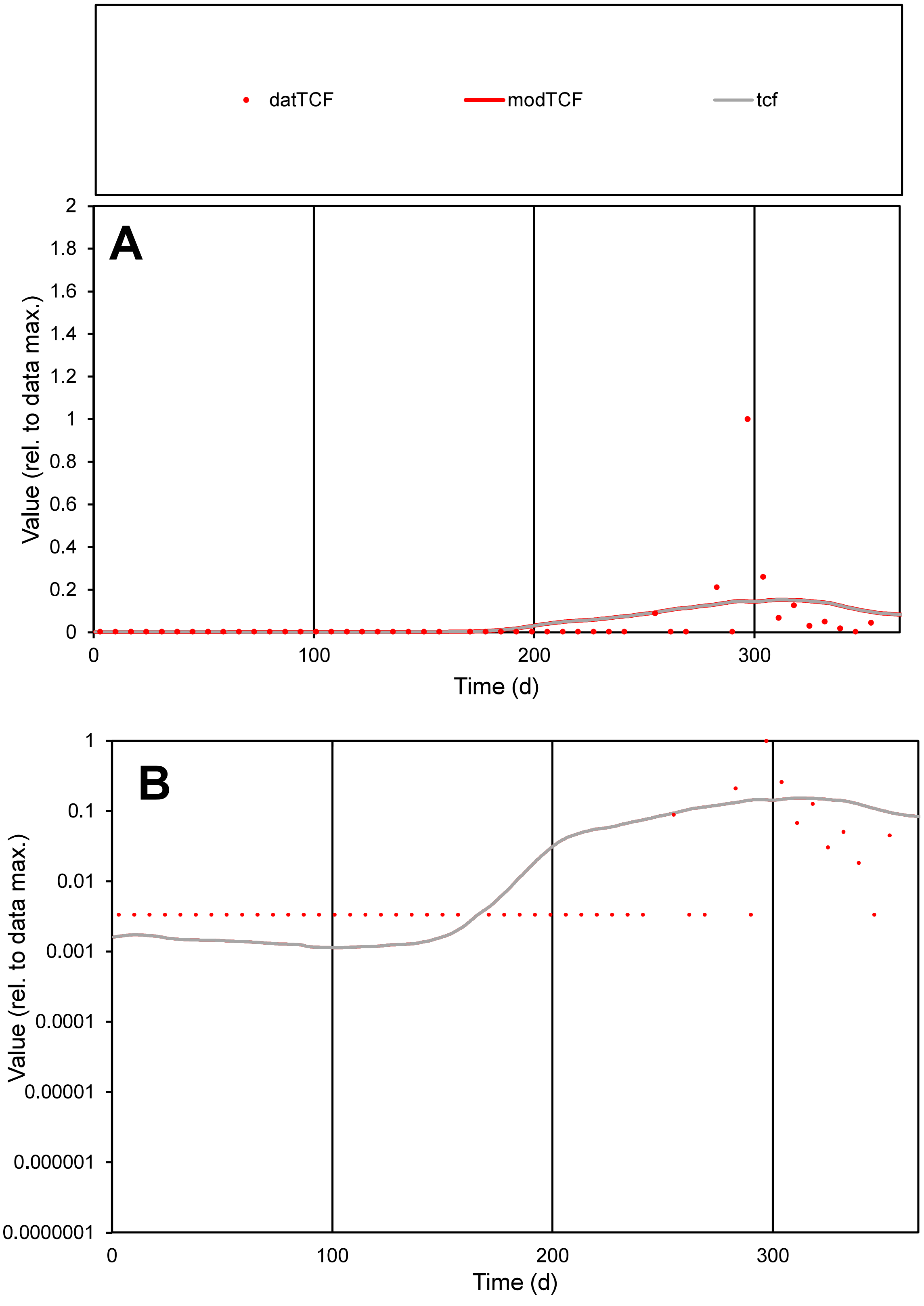

C1

Variables
TCF Provide jtd or Dst
CalibOnly?
2 1 to reproduce of
oNormalize?
2 1=no, 2=yes

ofxn calc*
3.18151
ofxn mod
3.18971

sRun
RunA1
zout
1

Ave
Data
3.9E-05
Model
4.4E-05

data/vMax
0.00093

ofxng
369.894

Min d/m
2.93691

ave/min d
12.6247
ave/min m
41.1734

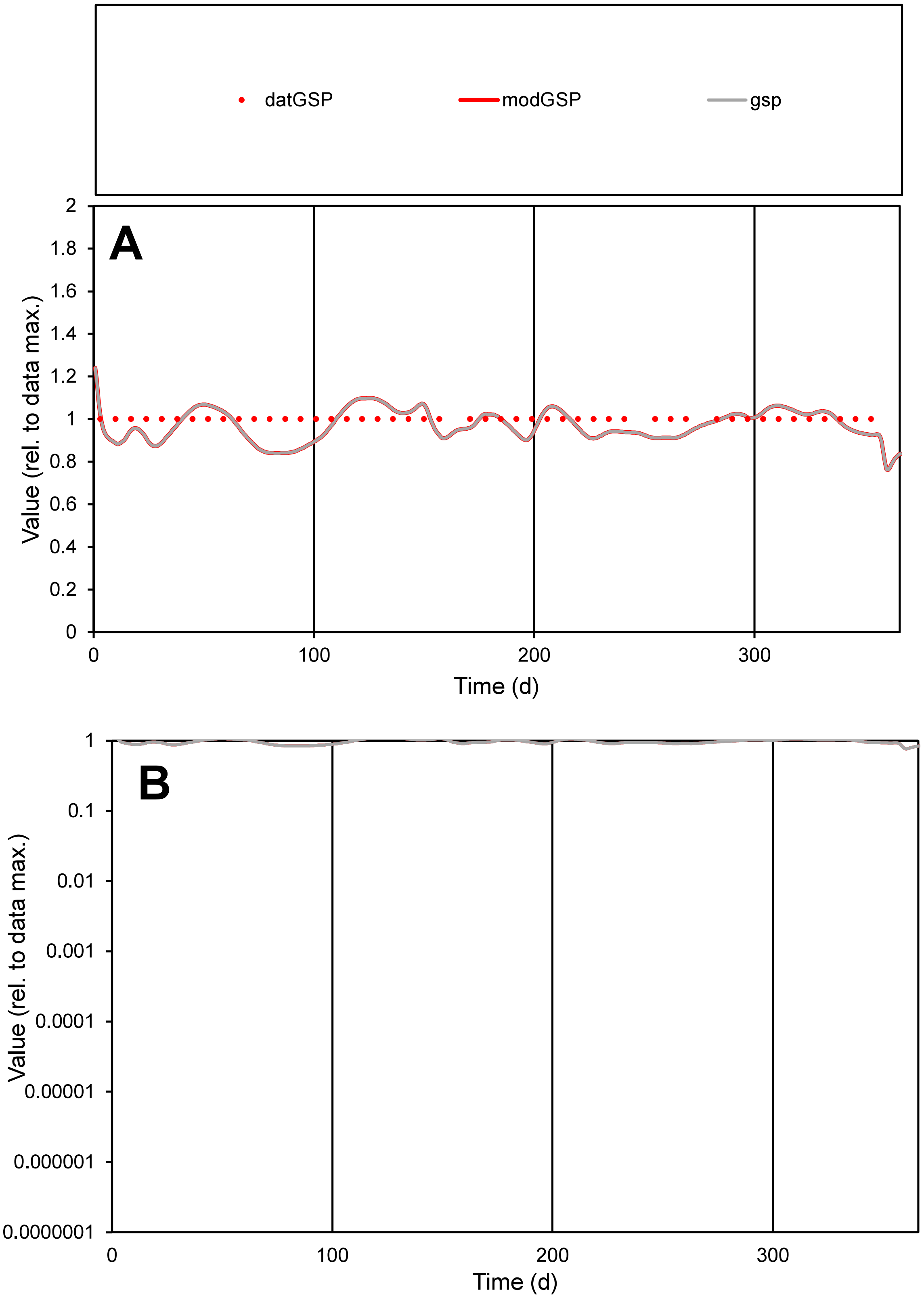

C1

Variables

GSP	Provide jtd or Dst
CalibOnly?	
2	1 to reproduce of
oNormalize?	
2	1=no, 2=yes

ofxn calc*
0.07129
ofxn mod
0.13221

sRun
RunA1
zout
1

Ave
Data
9E-06
Model
8.8E-06

data/vMax
9E-06

ofxng
369.894

Min d/m
1.18882

ave/min d
1
ave/min m
1.15974

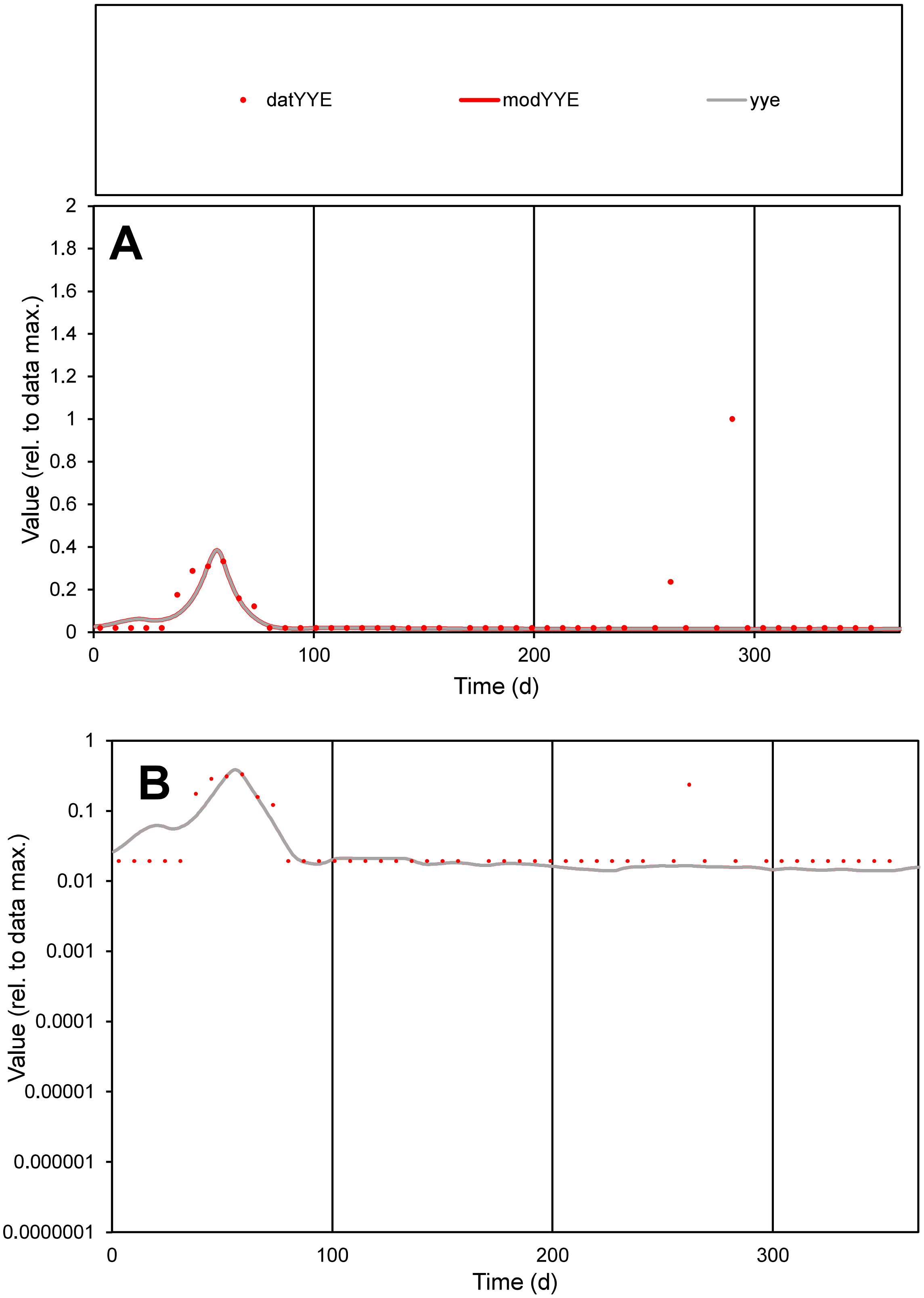

C1

Variables
YYE Provide jtd or Dst
CalibOnly?
2 1 to reproduce of
oNormalize?
2 1=no, 2=yes

ofxn calc*
2.08939
ofxn mod
2.09987

sRun
RunA1
zout
1

Ave
Data
1.4E-05
Model
8.2E-06

data/vMax
0.0002

ofxng
369.894

Min d/m
1.37286

ave/min d
3.66823
ave/min m
3.00579

328_Calib_S_A

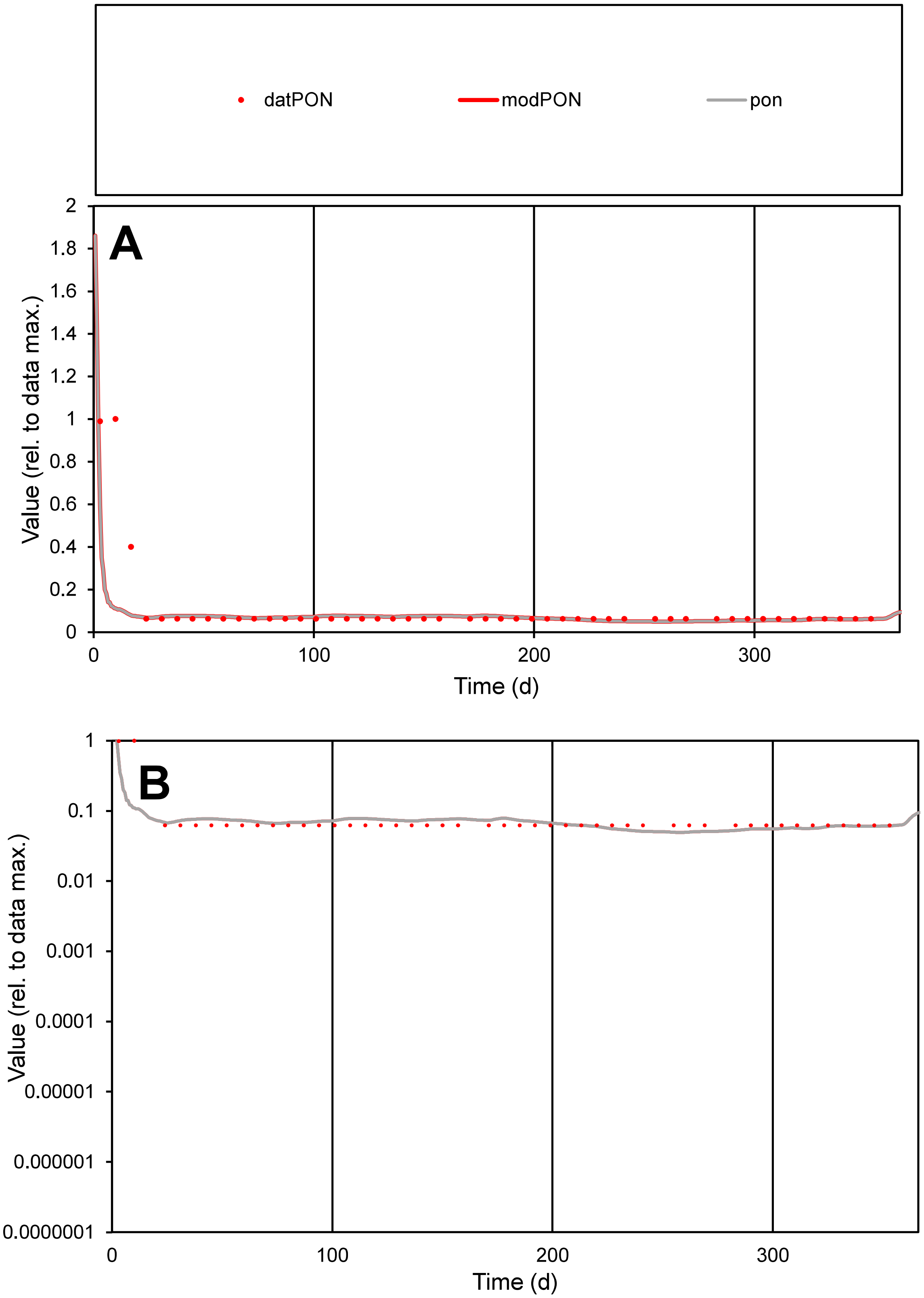

C1

Variables
PON Provide jtd or Dst
CalibOnly?
2 1 to reproduce of
oNormalize?
2 1=no, 2=yes

ofxn calc*
1.26858
ofxn mod
1.34049

sRun
RunA1
zout
1

Ave
Data
5.1E-06
Model
4E-06

data/vMax
4.7E-05

ofxng
369.894

Min d/m
1.26302

ave/min d
1.7364
ave/min m
1.7432

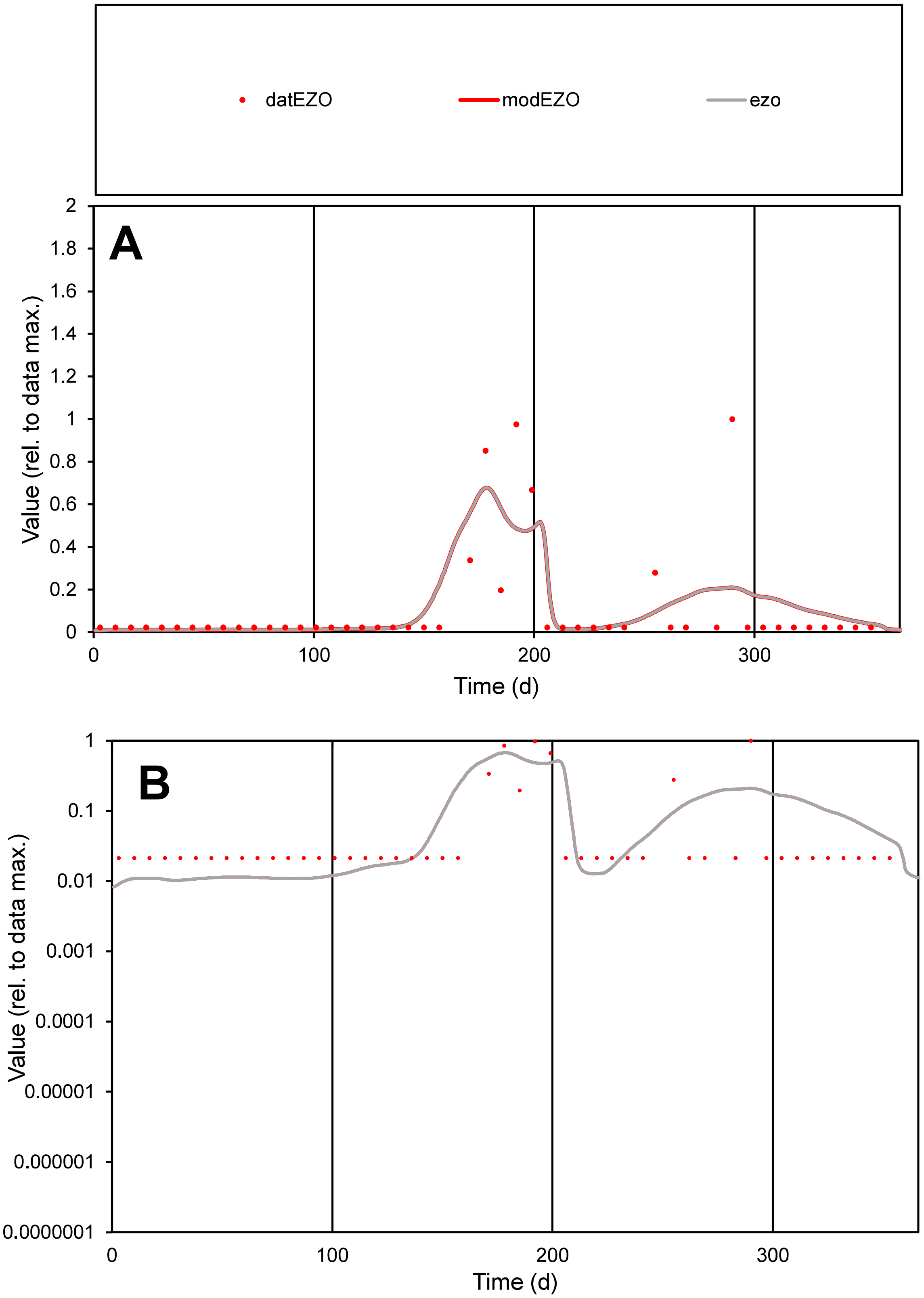

C1

Variables
EZO Provide jtd or Dst
CalibOnly?
2 1 to reproduce of
oNormalize?
2 1=no, 2=yes

ofxn calc*
1.5448
ofxn mod
1.58006

sRun
RunA1
zout
1

Ave
Data
2E-05
Model
2E-05

data/vMax
0.00018

ofxng
369.894

Min d/m
2.22768

ave/min d
5.05808
ave/min m
11.495

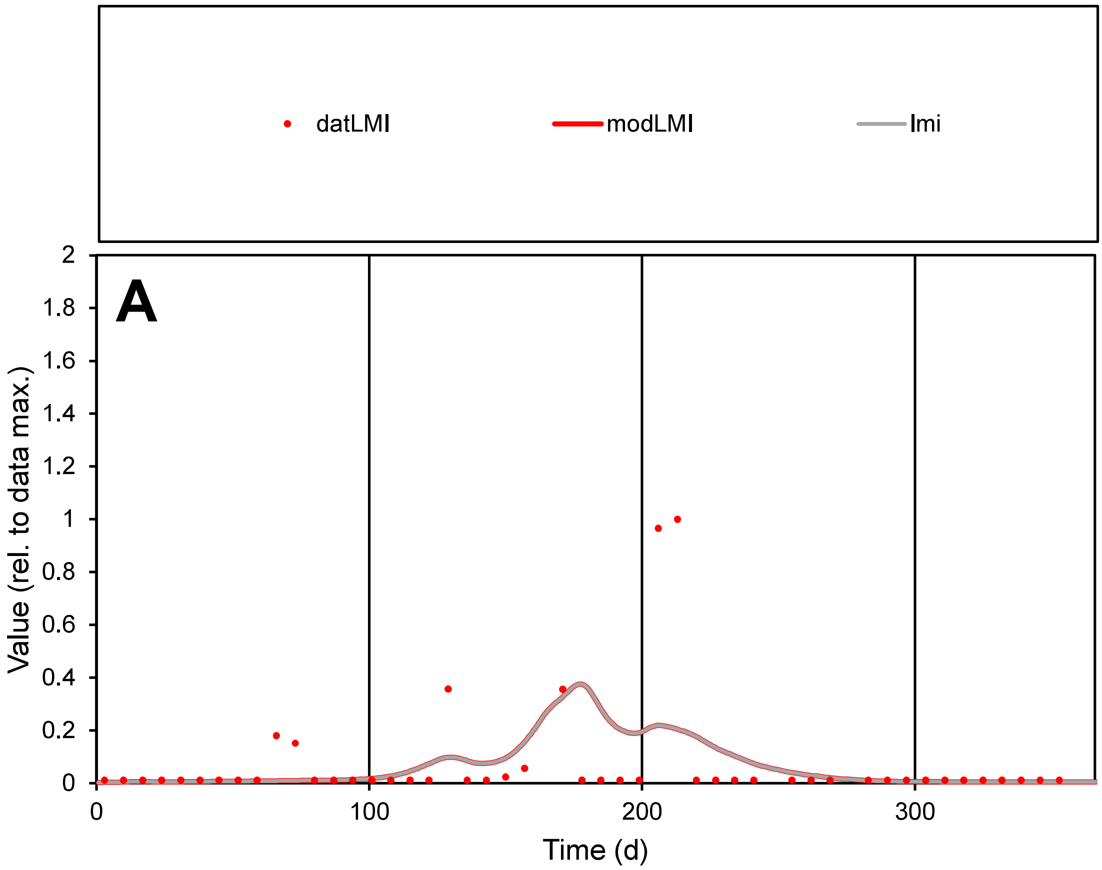

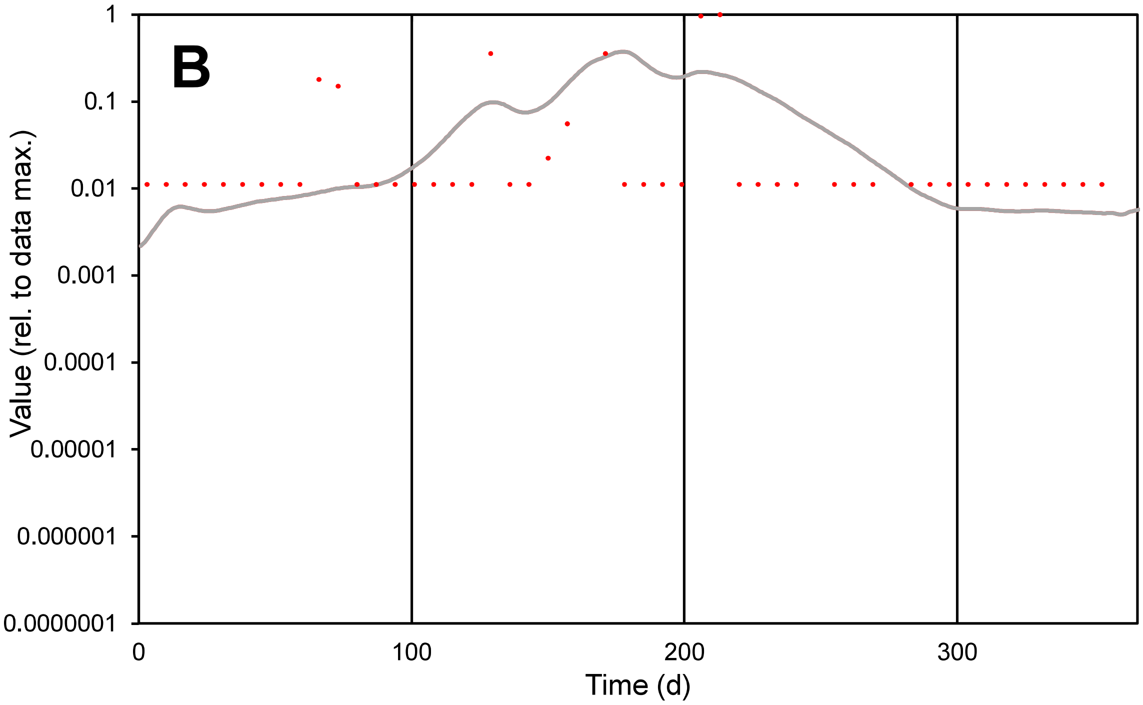

C1

Variables
LMI Provide jtd or Dst
CalibOnly?
2 1 to reproduce of
oNormalize?
2 1=no, 2=yes

ofxn calc*
2.50792
ofxn mod
2.52432

sRun
RunA1
zout
1

Ave
Data
2E-05
Model
1.8E-05

data/vMax
0.00027

ofxng
369.894

Min d/m
3.75826

ave/min d
6.56534
ave/min m
21.8705

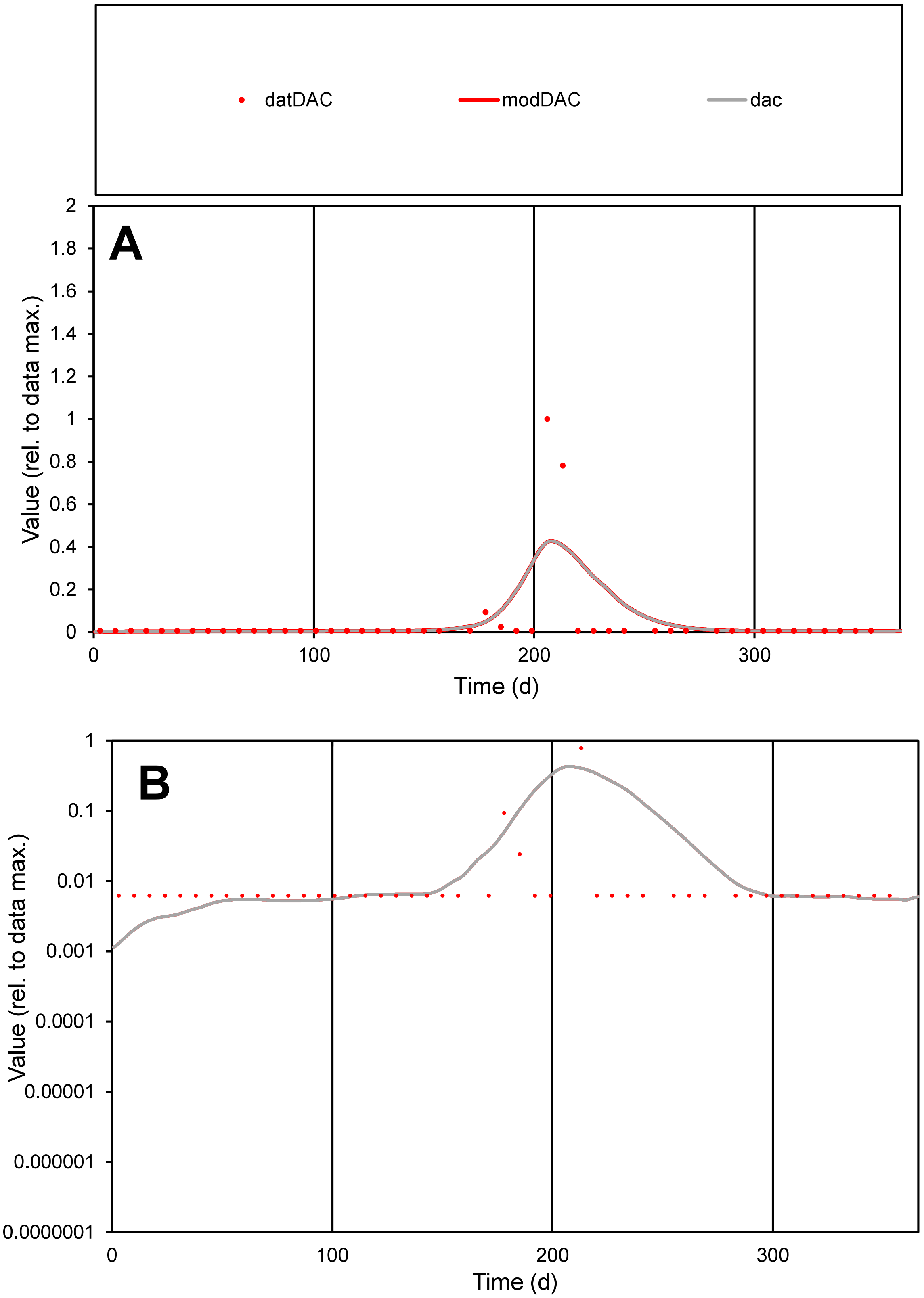

C1

Variables
DAC Provide jtd or Dst
CalibOnly?
2 1 to reproduce of
oNormalize?
2 1=no, 2=yes

ofxn calc*
2.81929
ofxn mod
2.90015

sRun
RunA1
zout
1

Ave
Data
5.6E-05
Model
6.7E-05

data/vMax
0.00124

ofxng
369.894

Min d/m
4.38339

ave/min d
7.2787
ave/min m
37.968

328_Calib_S_A

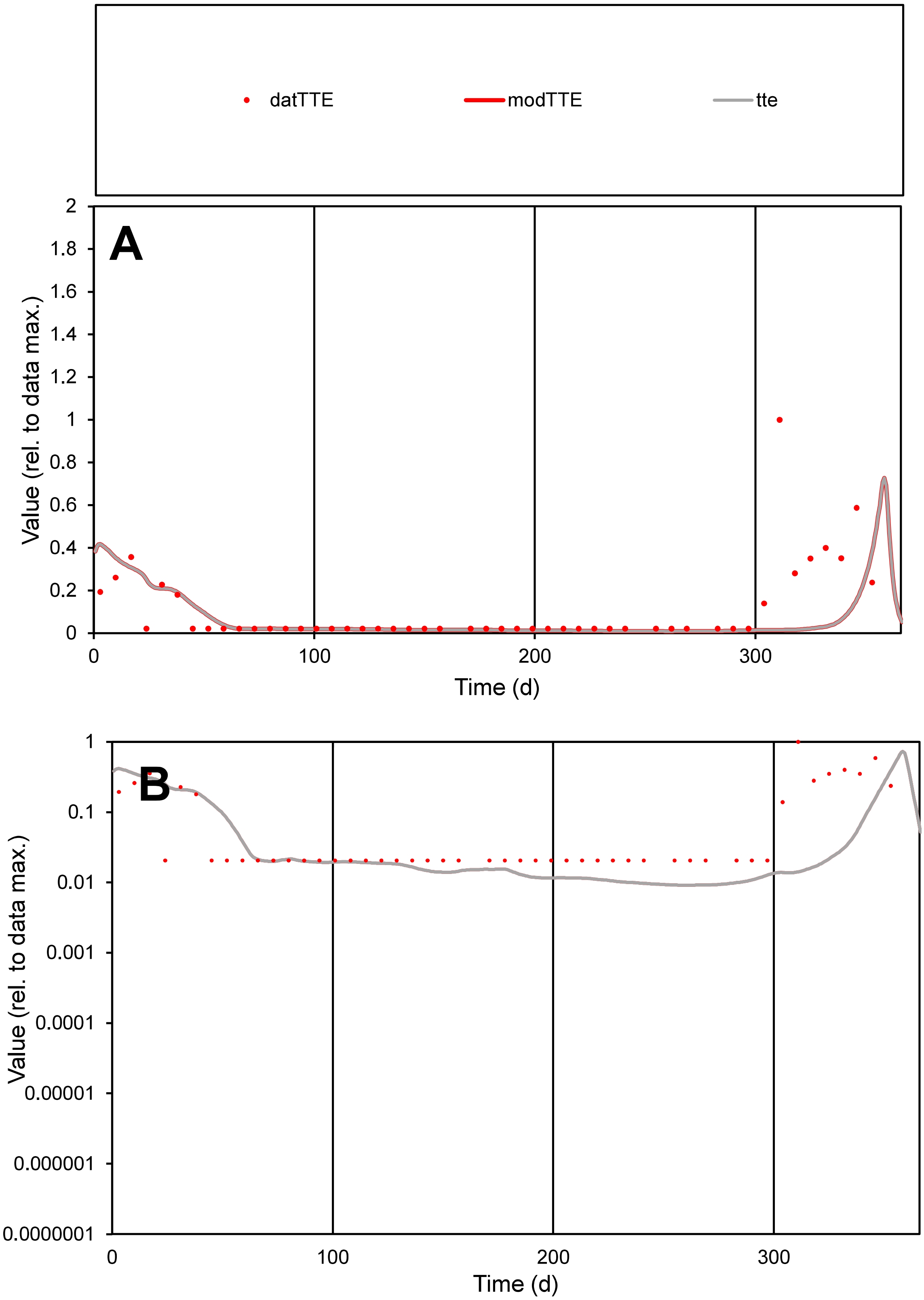

C1

Variables

TTE	Provide jtd or Dst
CalibOnly?	
2	1 to reproduce of
oNormalize?	
2	1=no, 2=yes
ofxn calc*	
1.69393	
ofxn mod	
1.70376	
sRun	
RunA1	
zout	
1	
Ave	
Data	
2E-05	
Model	
1.2E-05	
data/vMax	
0.00018	
ofxng	
369.894	
Min d/m	
2.25886	
ave/min d	
5.34103	
ave/min m	
7.10294	

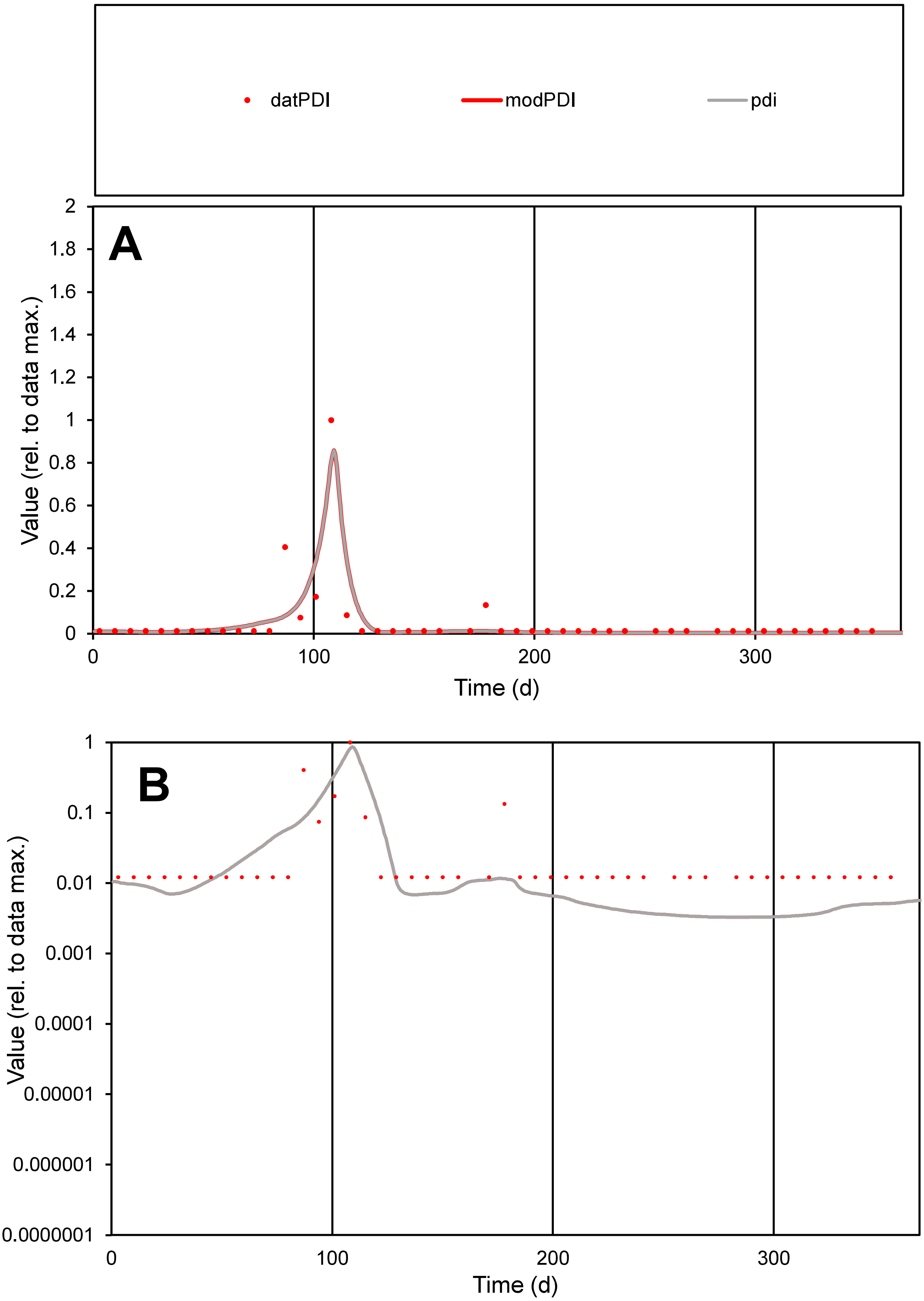

C1

Variables

PDI Provide jtd or Dst

CalibOnly?

2 1 to reproduce of

oNormalize?

2 1=no, 2=yes

ofxn calc*

1.28603

ofxn mod

1.34439

sRun

RunA1

zout

1

Ave

Data

1.8E-05

Model

1.6E-05

data/vMax

0.00036

ofxng

369.894

Min d/m

3.73492

ave/min d

4.07512

ave/min m

13.4449

328_Calib_S_A

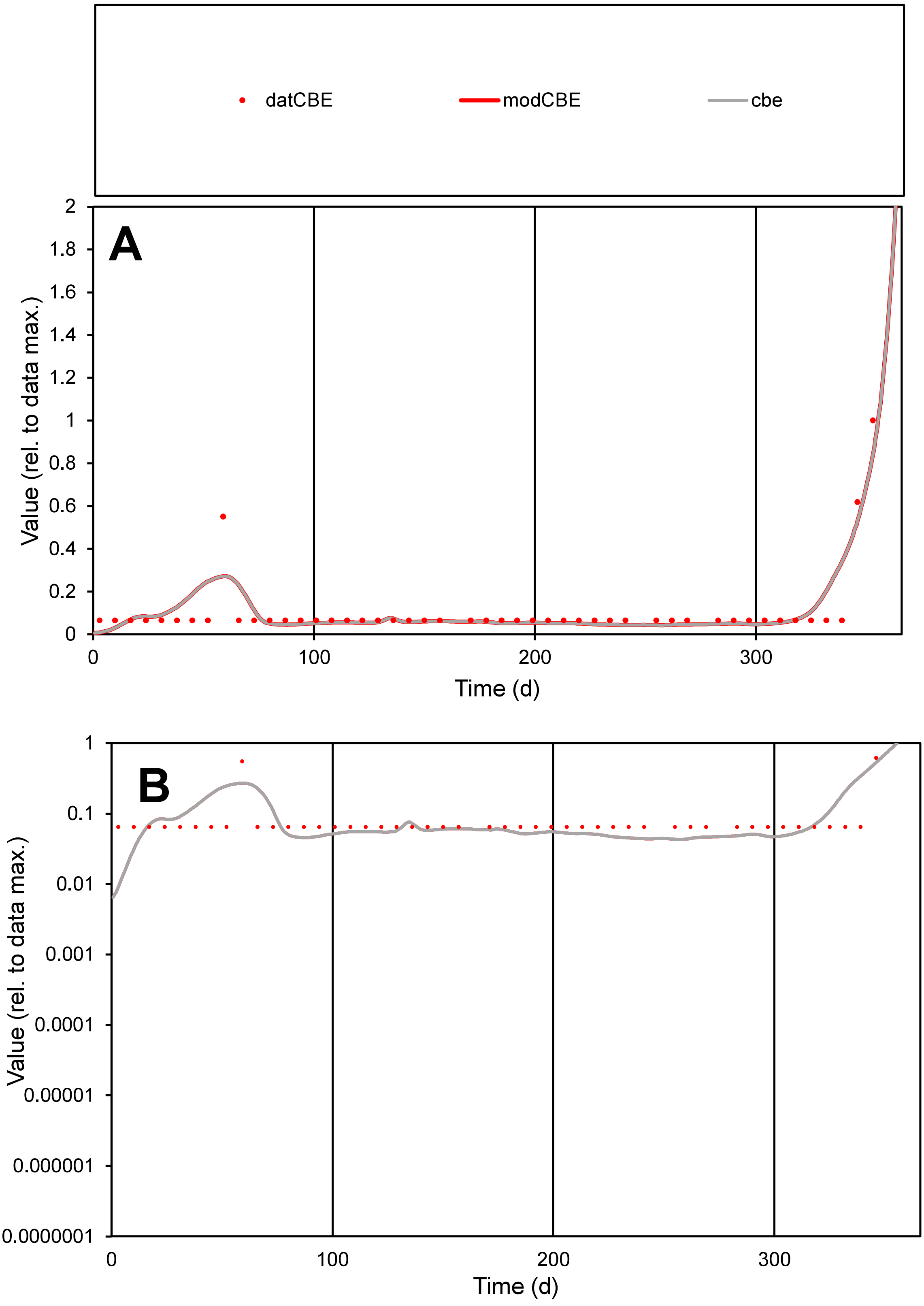

C1

Variables

CBE Provide jtd or Dst

CalibOnly?

2 1 to reproduce of

oNormalize?

2 1=no, 2=yes

ofxn calc*

0.69205

ofxn mod

0.73667

sRun

RunA1

zout

1

Ave

Data

2.7E-06

Model

2.8E-06

data/vMax

2.6E-05

ofxng

369.894

Min d/m

5.63514

ave/min d

1.63532

ave/min m

9.5599

328_Calib_S_A

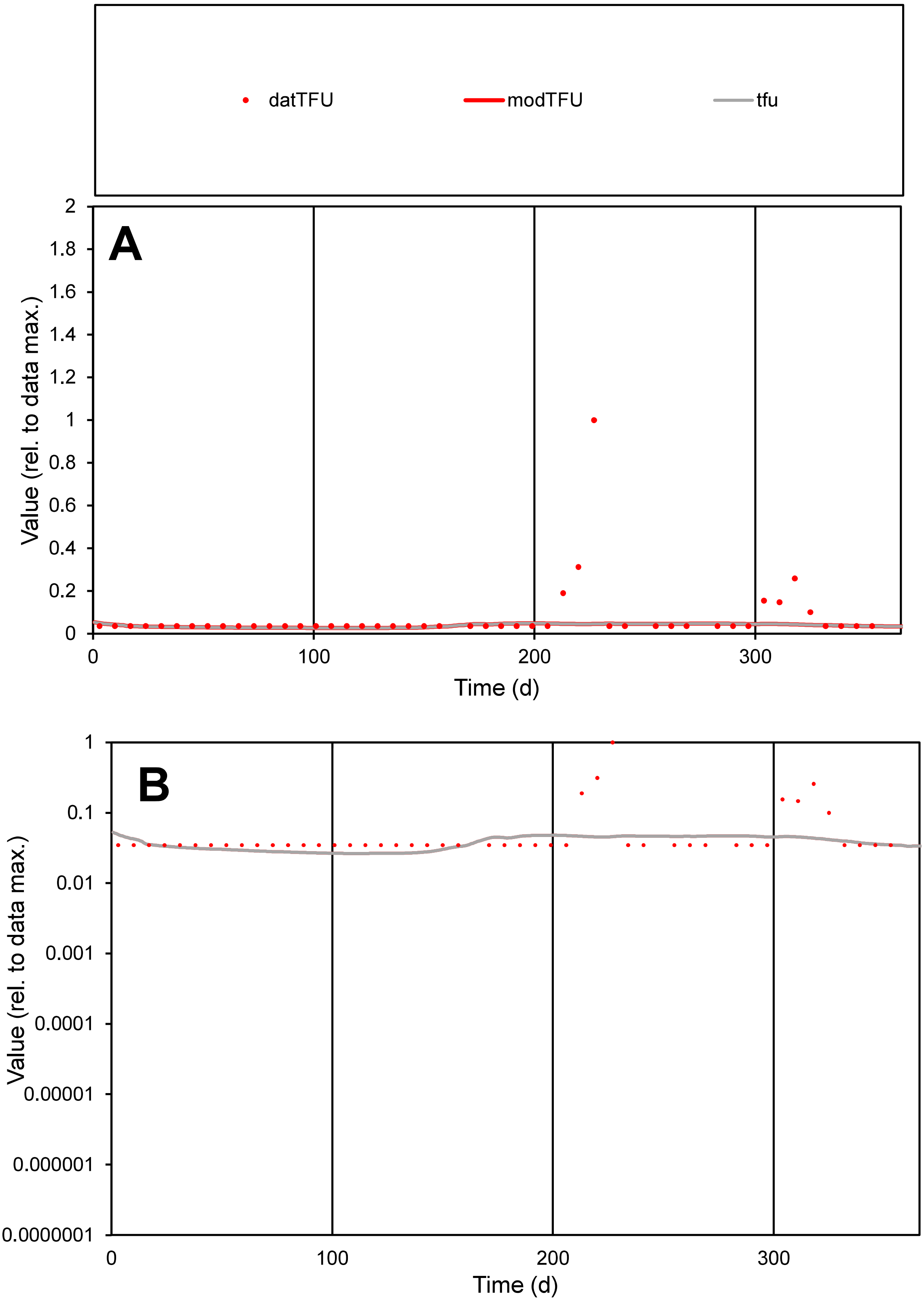

C1

Variables

TFU	Provide jtd or Dst
CalibOnly?	
2	1 to reproduce of
oNormalize?	
2	1=no, 2=yes

ofxn calc*
2.00743
ofxn mod
2.01318

sRun
RunA1
zout
1

Ave
Data
2.7E-05
Model
1.4E-05

data/vMax
0.00037

ofxng
369.894

Min d/m
1.3085

ave/min d
2.15423
ave/min m
1.42379

328_Calib_S_A

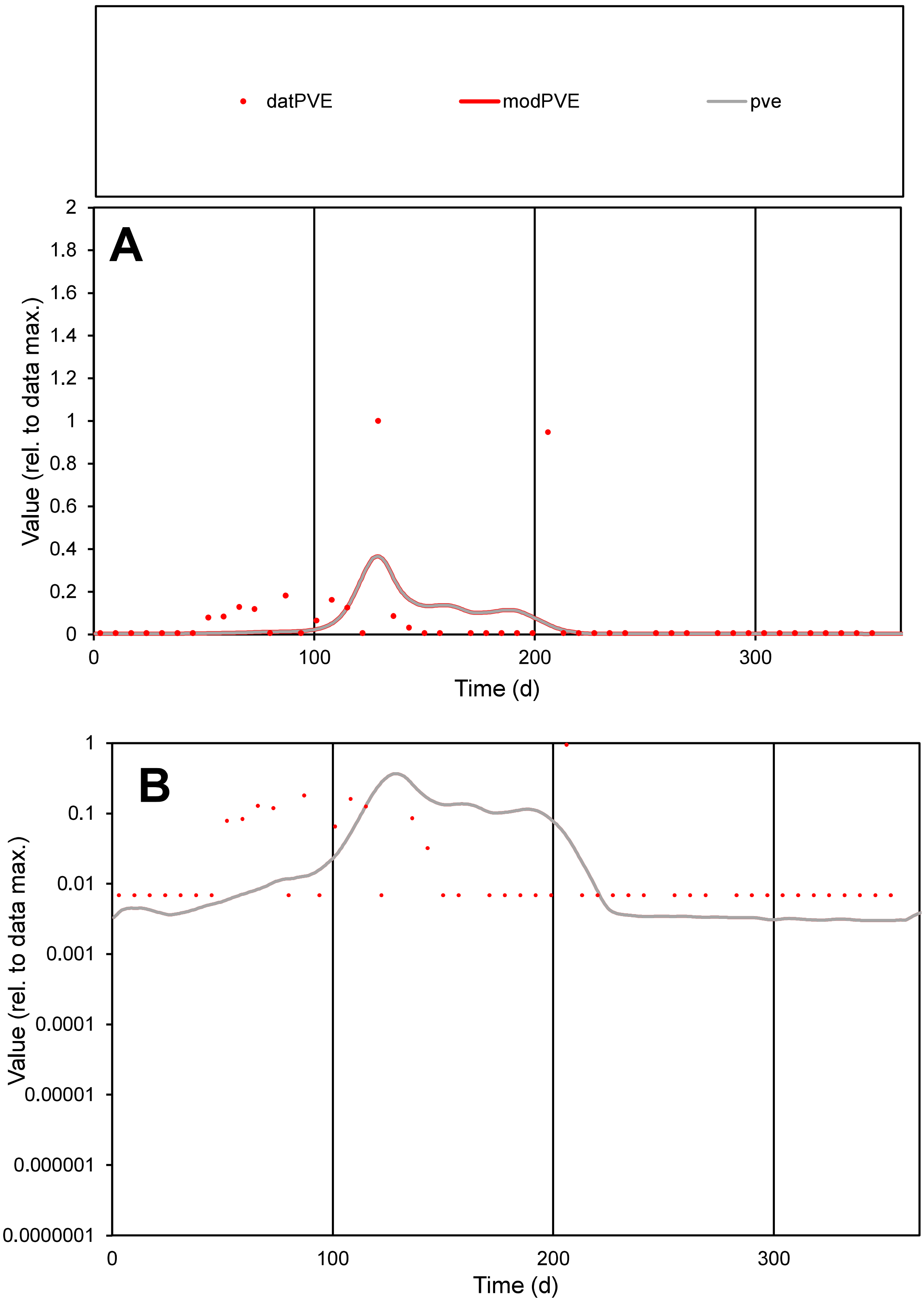

C1

Variables

PVE Provide jtd or Dst

CalibOnly?

2 1 to reproduce of

oNormalize?

2 1=no, 2=yes

ofxn calc*

2.57077

ofxn mod

2.59788

sRun

RunA1

zout

1

Ave

Data

2.1E-05

Model

1.4E-05

data/vMax

0.0003

ofxng

369.894

Min d/m

2.28559

ave/min d

9.86218

ave/min m

15.2401

328_Calib_S_A

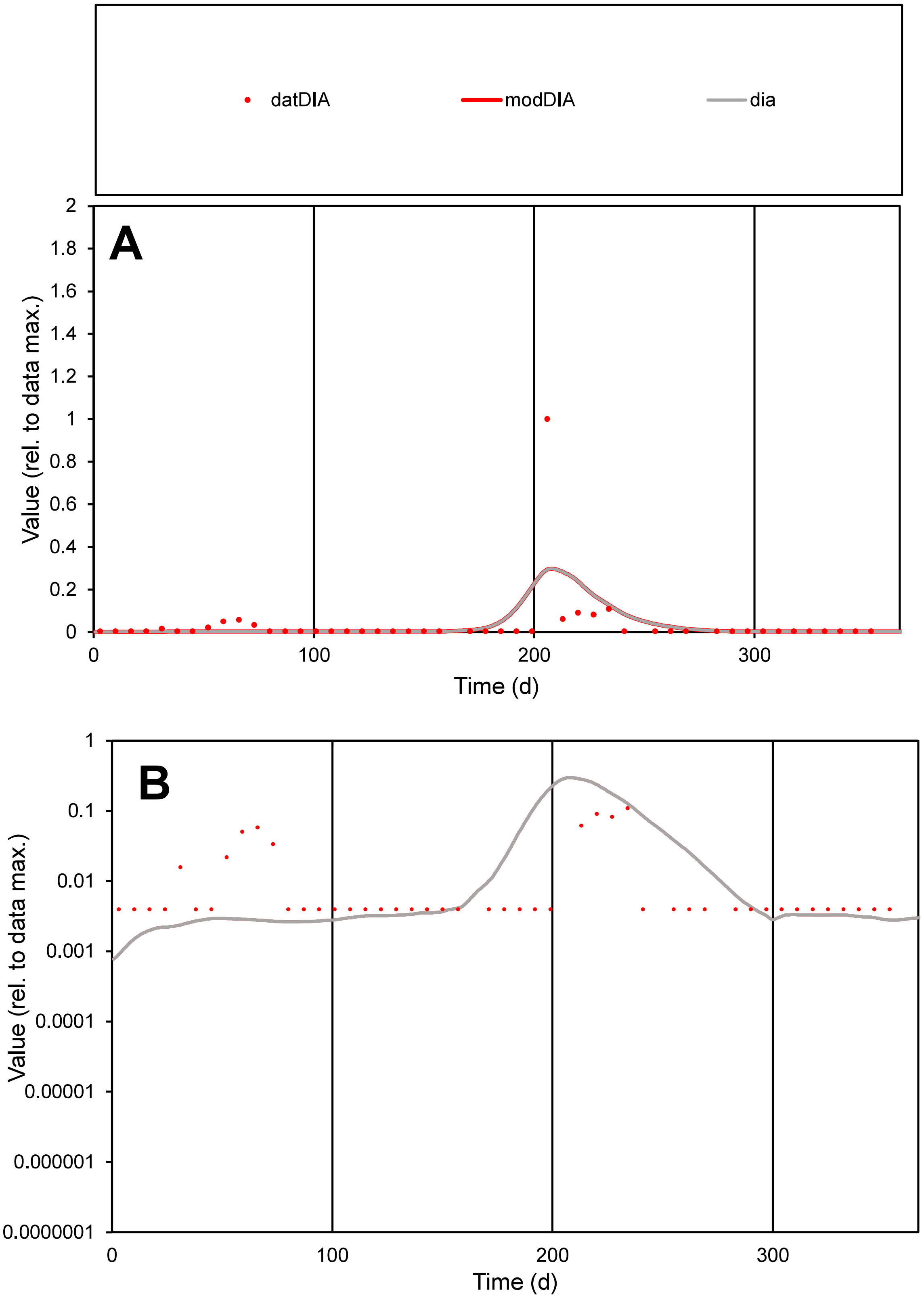

C1

Variables

DIA	Provide jtd or Dst
CalibOnly?	
2	1 to reproduce of
oNormalize?	
2	1=no, 2=yes
ofxn calc*	
3.25456	
ofxn mod	
3.2998	
sRun	
RunA1	
zout	
1	
Ave	
Data	
5.5E-05	
Model	
5.5E-05	
data/vMax	
0.00157	
ofxng	
369.894	
Min d/m	
3.98146	
ave/min d	
8.77474	
ave/min m	
34.9811	

328_Calib_S_A

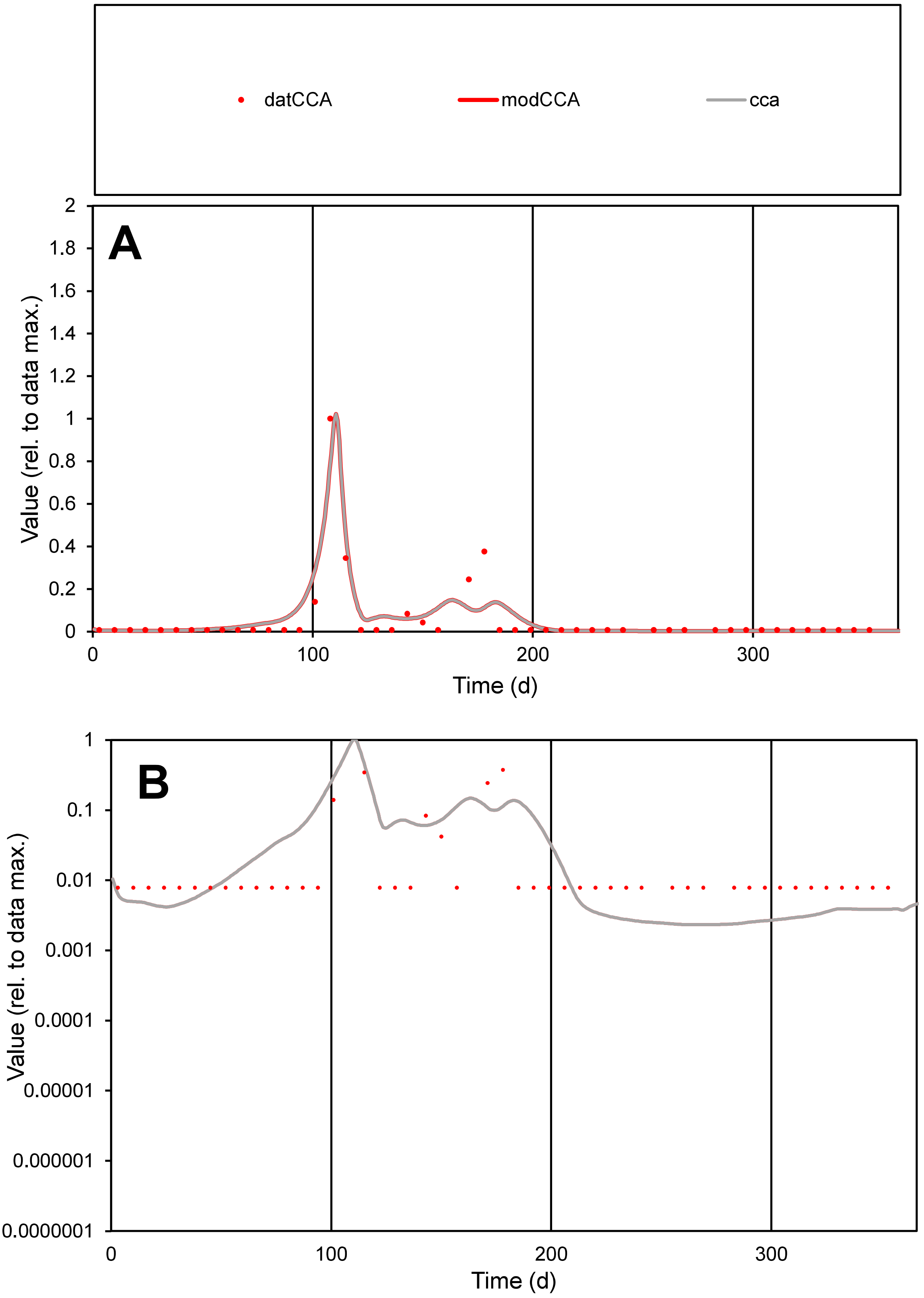

C1

Variables
CCA Provide jtd or Dst
CalibOnly?
2 1 to reproduce of
oNormalize?
2 1=no, 2=yes

ofxn calc*
1.08762
ofxn mod
1.14574

sRun
RunA1
zout
1

Ave
Data
1.2E-05
Model
1.4E-05

data/vMax
0.00023

ofxng
369.894

Min d/m
3.34615

ave/min d
6.79641
ave/min m
24.8313

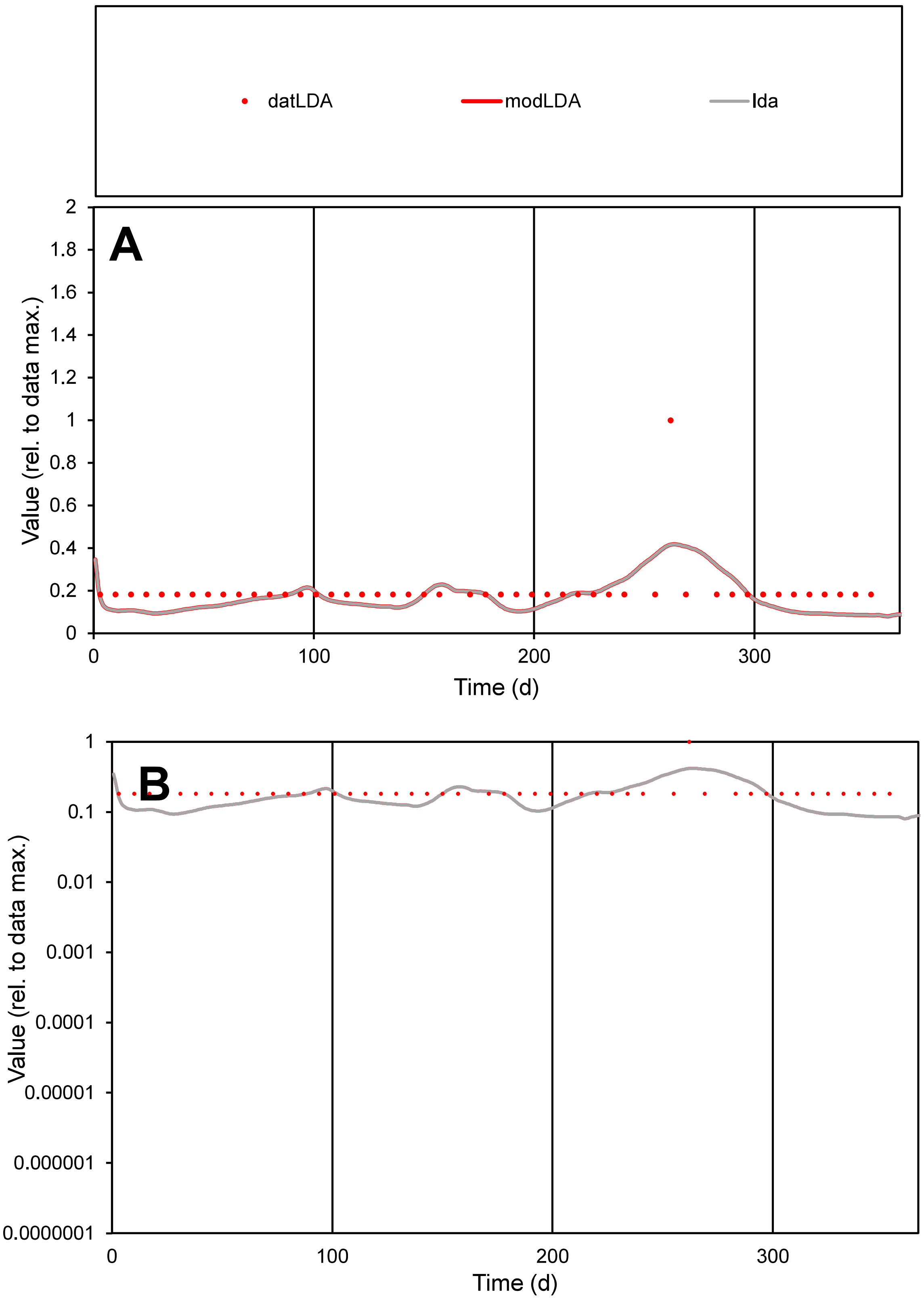
datLDA
modLDA
lda
A
Value (rel. to data max.)
Time (d)
B

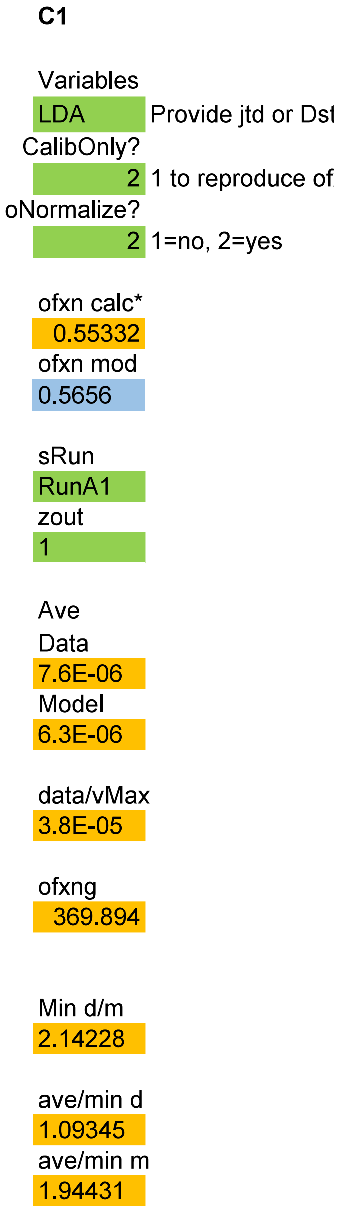
C1
Variables
LDA
Provide jtd or Dst
CalibOnly?
2
1 to reproduce of
oNormalize?
2
1=no, 2=yes
ofxn calc*
0.55332
ofxn mod
0.5656
sRun
RunA1
zout
1
Ave
Data
7.6E-06
Model
6.3E-06
data/vMax
3.8E-05
ofxng
369.894
Min d/m
2.14228
ave/min d
1.09345
ave/min m
1.94431

328_Calib_S_A

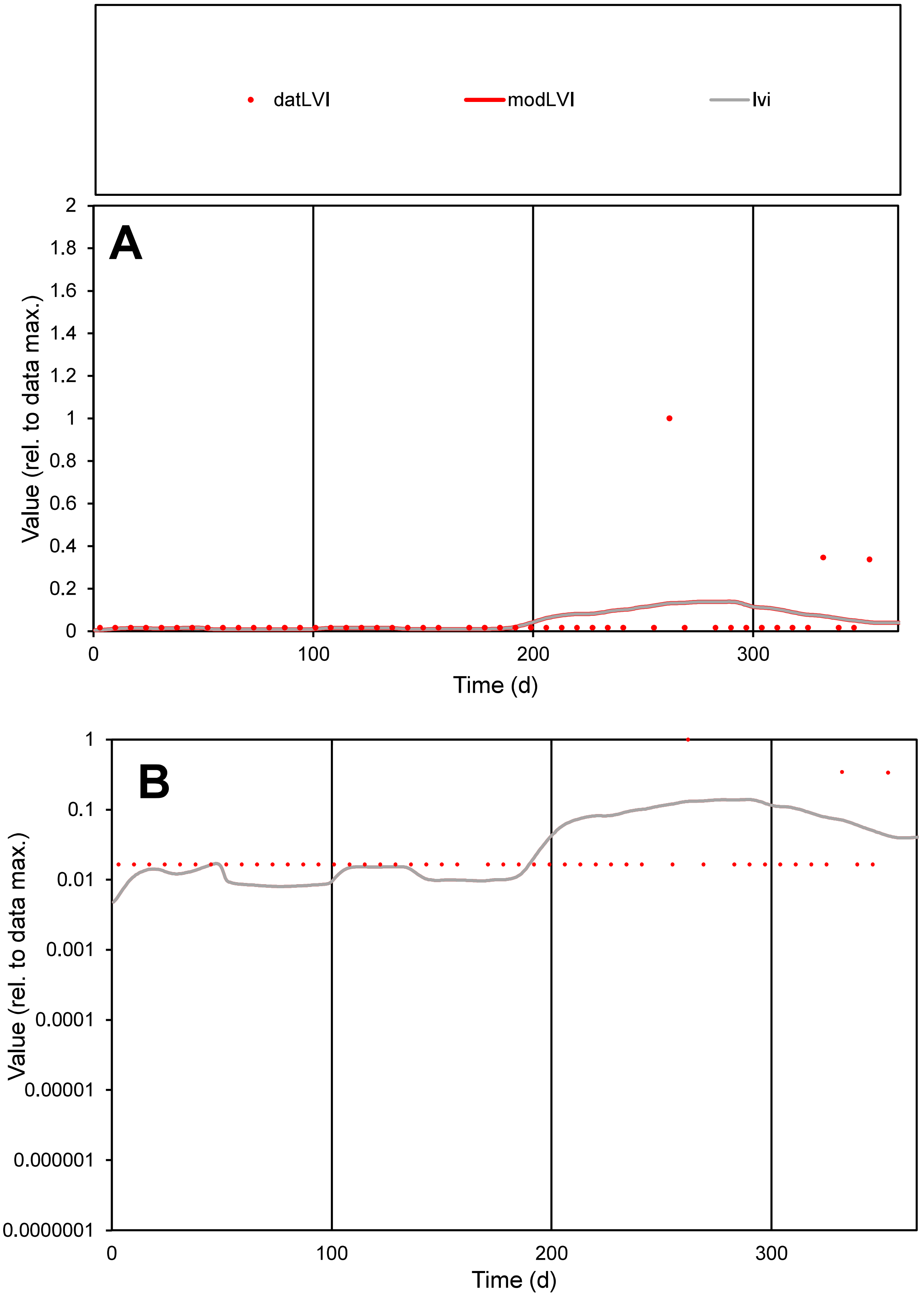

C1

Variables
LVI Provide jtd or Dst
CalibOnly?
2 1 to reproduce of
oNormalize?
2 1=no, 2=yes

ofxn calc*
2.90698
ofxn mod
2.91378

sRun
RunA1
zout
1

Ave
Data
4.3E-06
Model
4E-06

data/vMax
8.6E-05

ofxng
369.894

Min d/m
2.42538

ave/min d
3.05805
ave/min m
6.81646

328_Calib_S_A

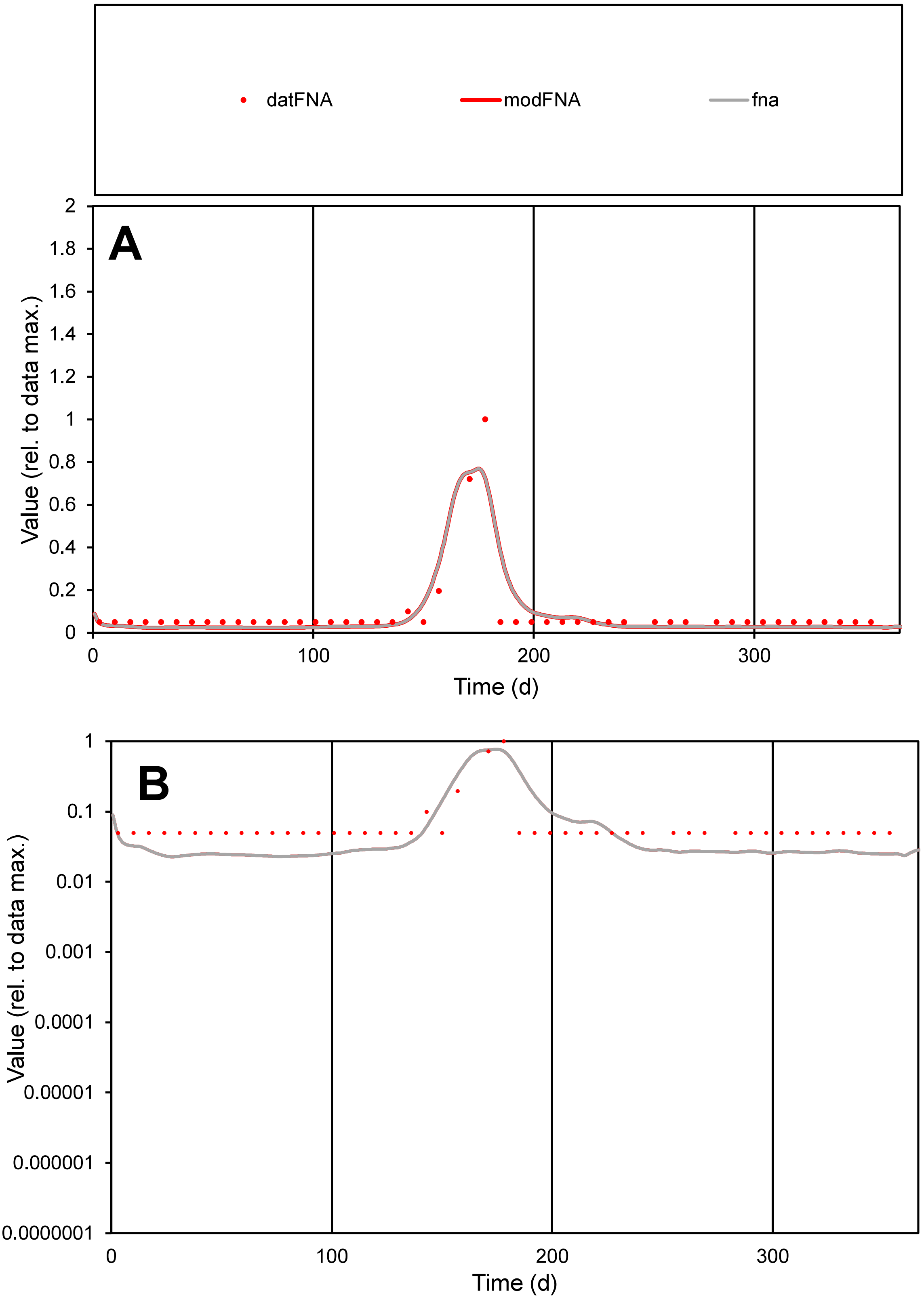

C1

Variables

FNA Provide jtd or Dst

CalibOnly?

2 1 to reproduce of

oNormalize?

2 1=no, 2=yes

ofxn calc*

0.71669

ofxn mod

0.82819

sRun

RunA1

zout

1

Ave

Data

5.5E-06

Model

4.9E-06

data/vMax

6.3E-05

ofxng

369.894

Min d/m

2.15331

ave/min d

1.76654

ave/min m

3.40637

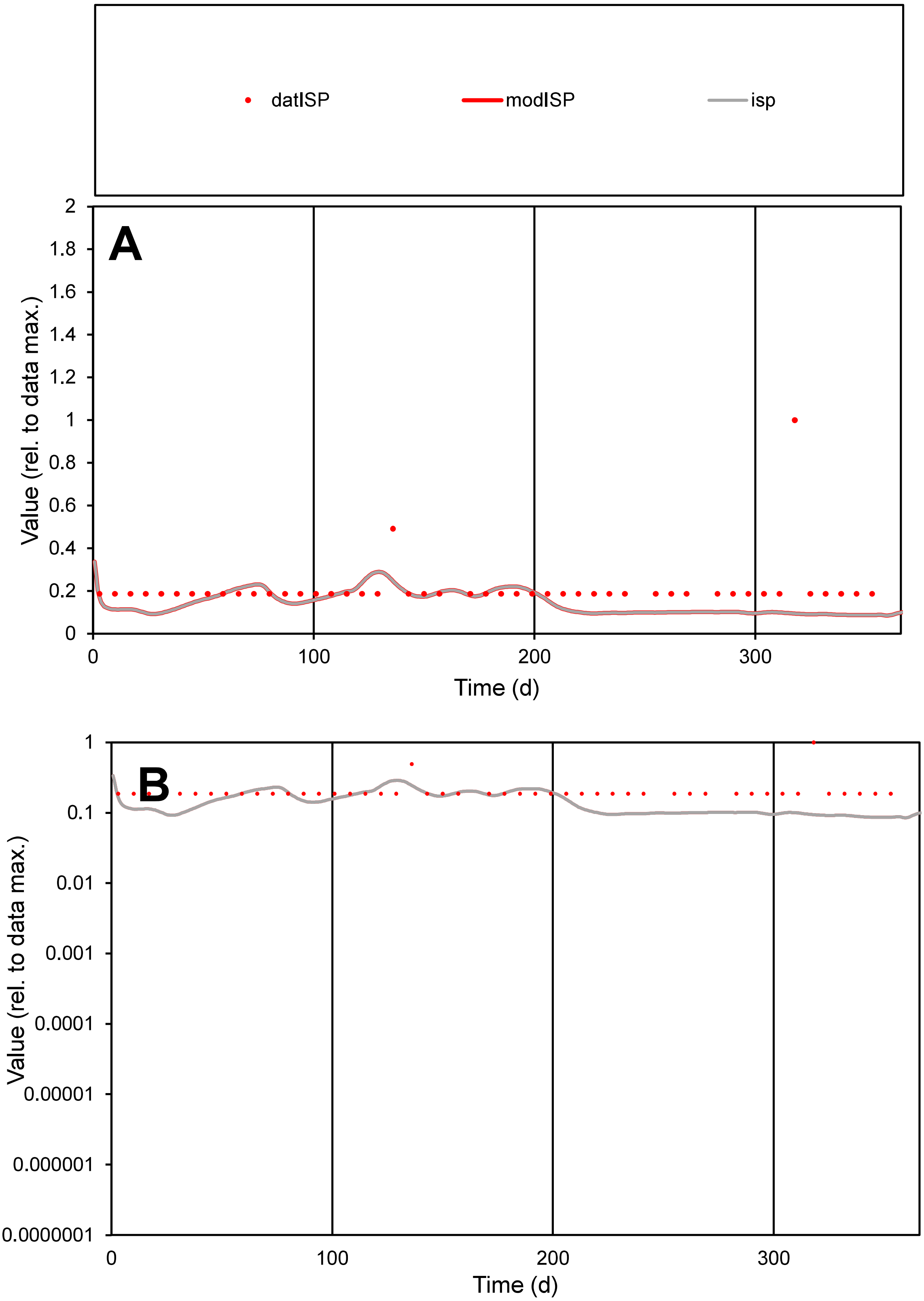

C1

Variables
ISP Provide jtd or Dst
CalibOnly?
2 1 to reproduce of
oNormalize?
2 1=no, 2=yes

ofxn calc*
0.71798
ofxn mod
0.72765

sRun
RunA1
zout
1

Ave
Data
5.7E-06
Model
3.9E-06

data/vMax
2.7E-05

ofxng
369.894

Min d/m
2.14827

ave/min d
1.12518
ave/min m
1.67842

328_Calib_S_A

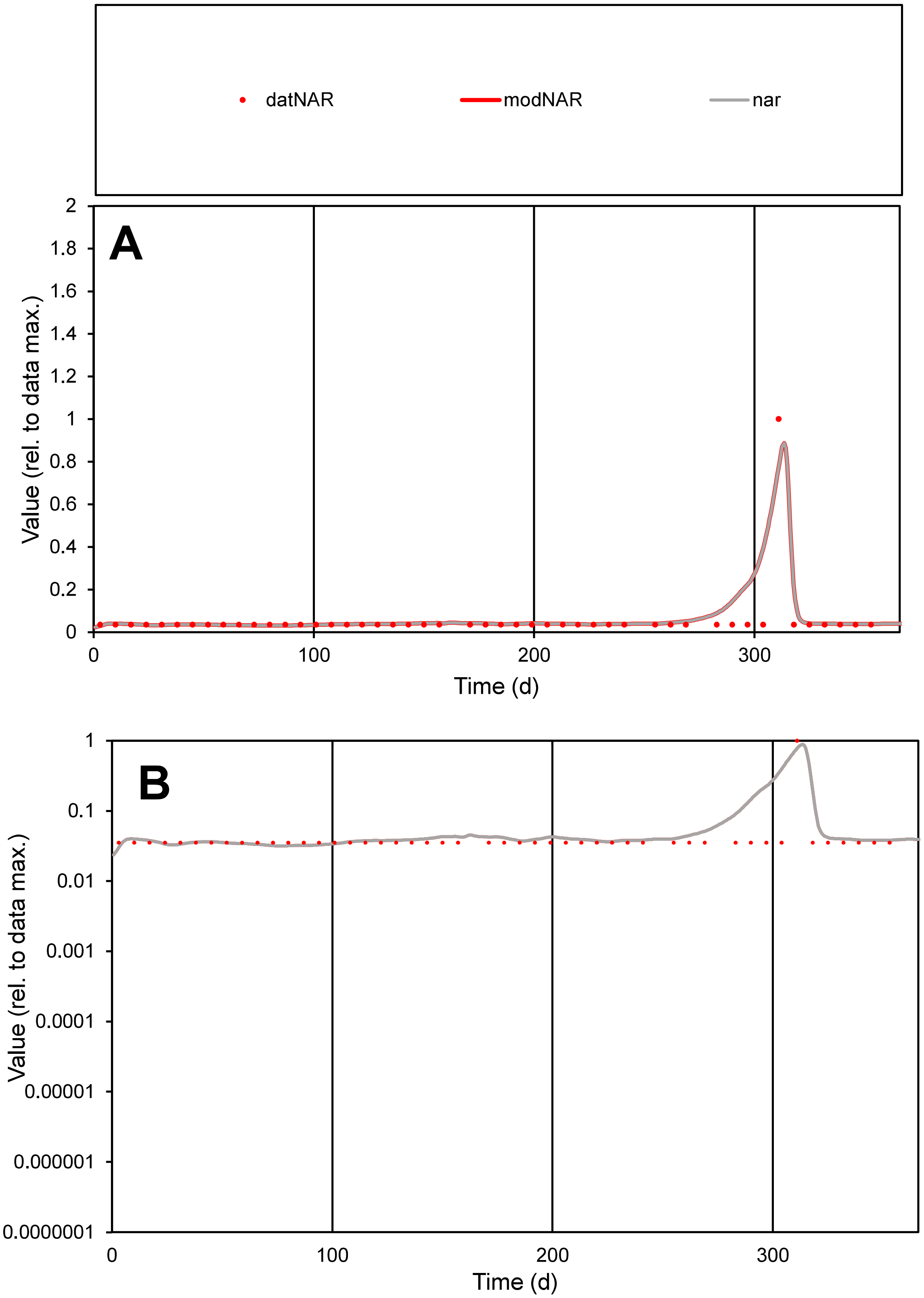

C1

Variables
NAR Provide jtd or Dst
CalibOnly?
2 1 to reproduce of
oNormalize?
2 1=no, 2=yes

ofxn calc*
1.12778
ofxn mod
1.19005

sRun
RunA1
zout
1

Ave
Data
4.4E-06
Model
5.6E-06

data/vMax
8E-05

ofxng
369.894

Min d/m
1.10697

ave/min d
1.57002
ave/min m
2.17693

328_Calib_S_A

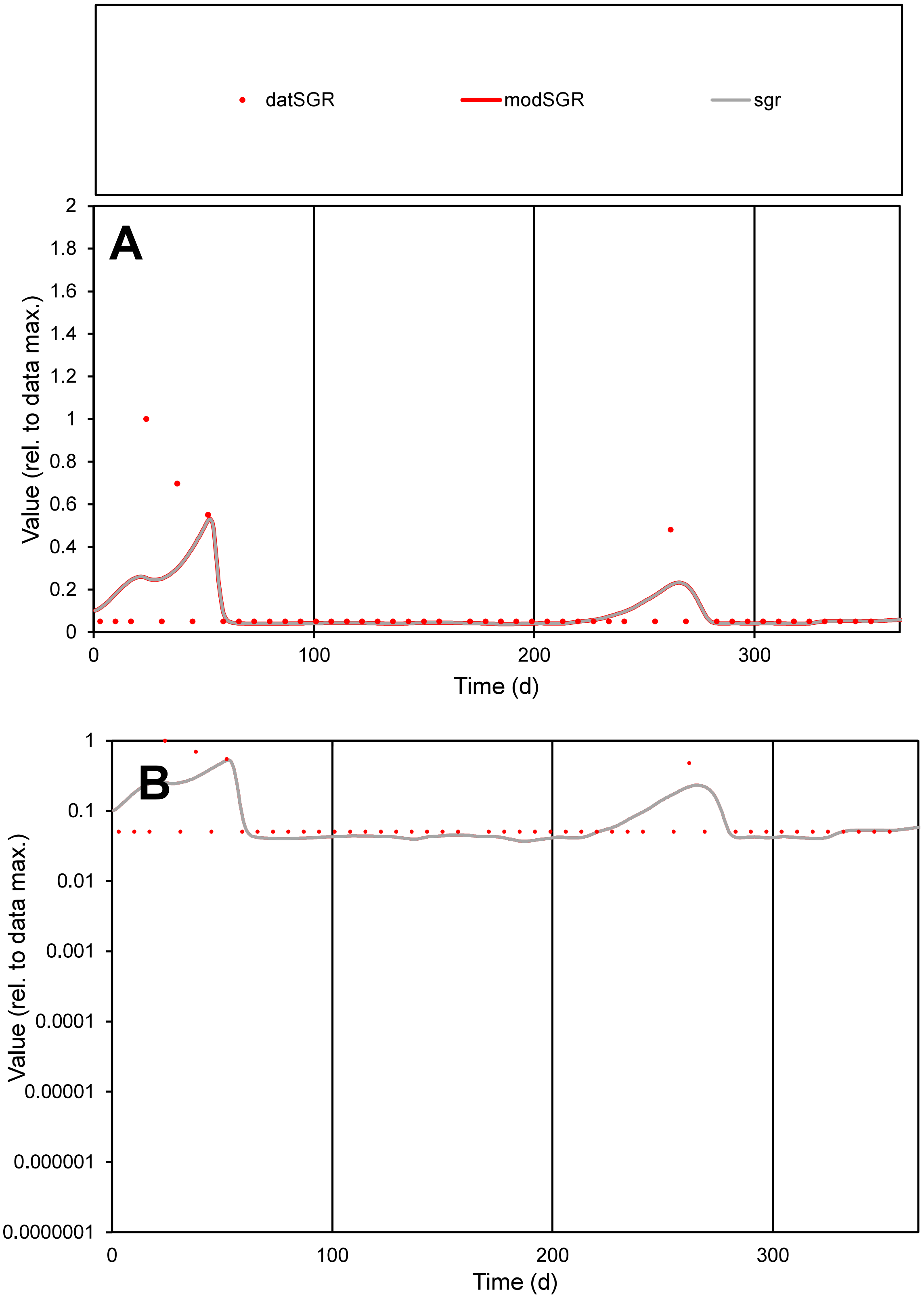

C1

Variables
SGR Provide jtd or Dst
CalibOnly?
2 1 to reproduce of
oNormalize?
2 1=no, 2=yes

ofxn calc*
1.42728
ofxn mod
1.44097

sRun
RunA1
zout
1

Ave
Data
9.3E-06
Model
8.7E-06

data/vMax
9E-05

ofxng
369.894

Min d/m
1.36145

ave/min d
2.0418
ave/min m
2.60109

328_Calib_S_A

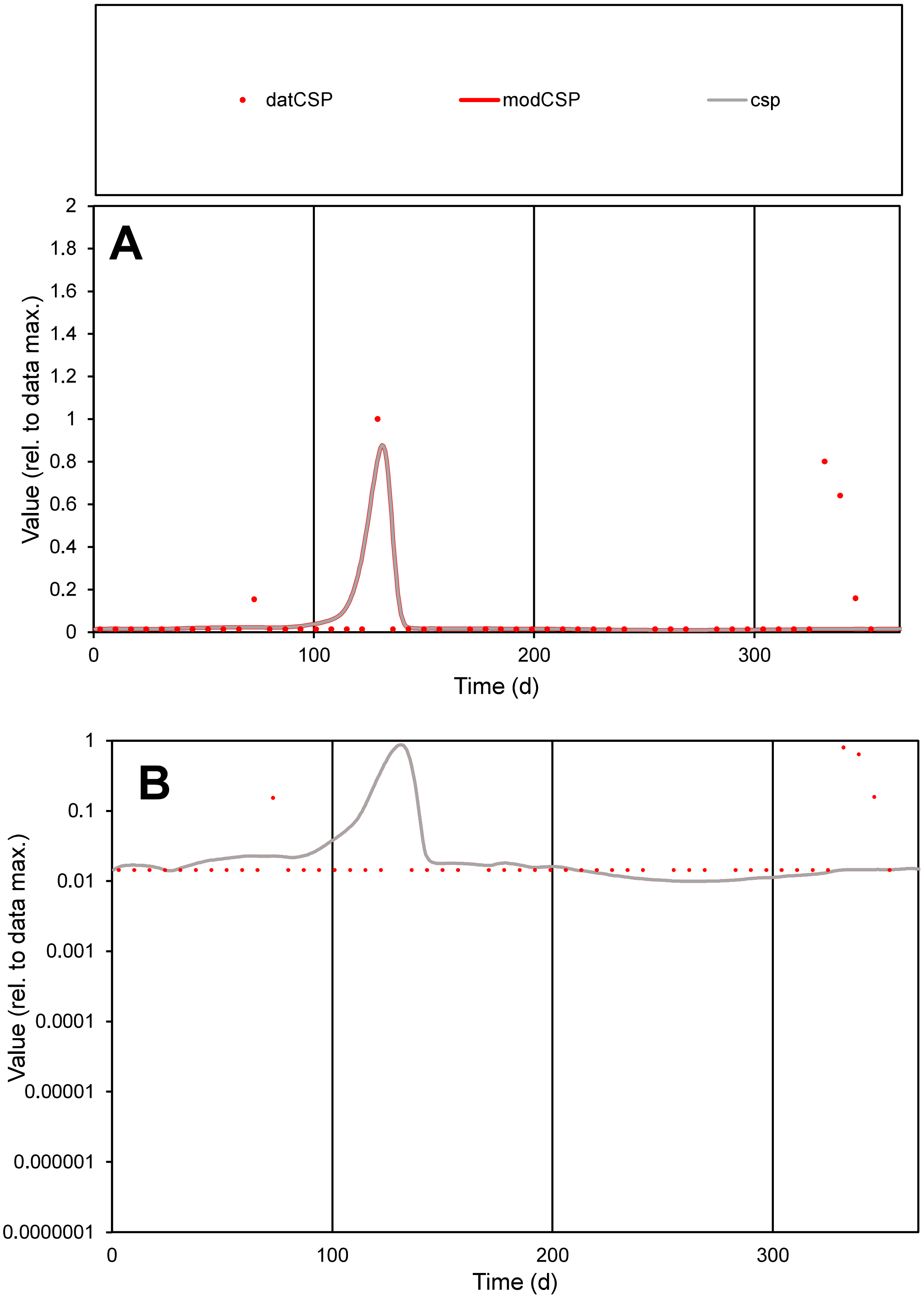

C1

Variables

CSP Provide jtd or Dst

CalibOnly?

2 1 to reproduce of

oNormalize?

2 1=no, 2=yes

ofxn calc*

2.19929

ofxn mod

2.25987

sRun

RunA1

zout

1

Ave

Data

1.2E-05

Model

7.2E-06

data/vMax

0.00017

ofxng

369.894

Min d/m

1.45994

ave/min d

4.8711

ave/min m

4.40449

328_Calib_S_A

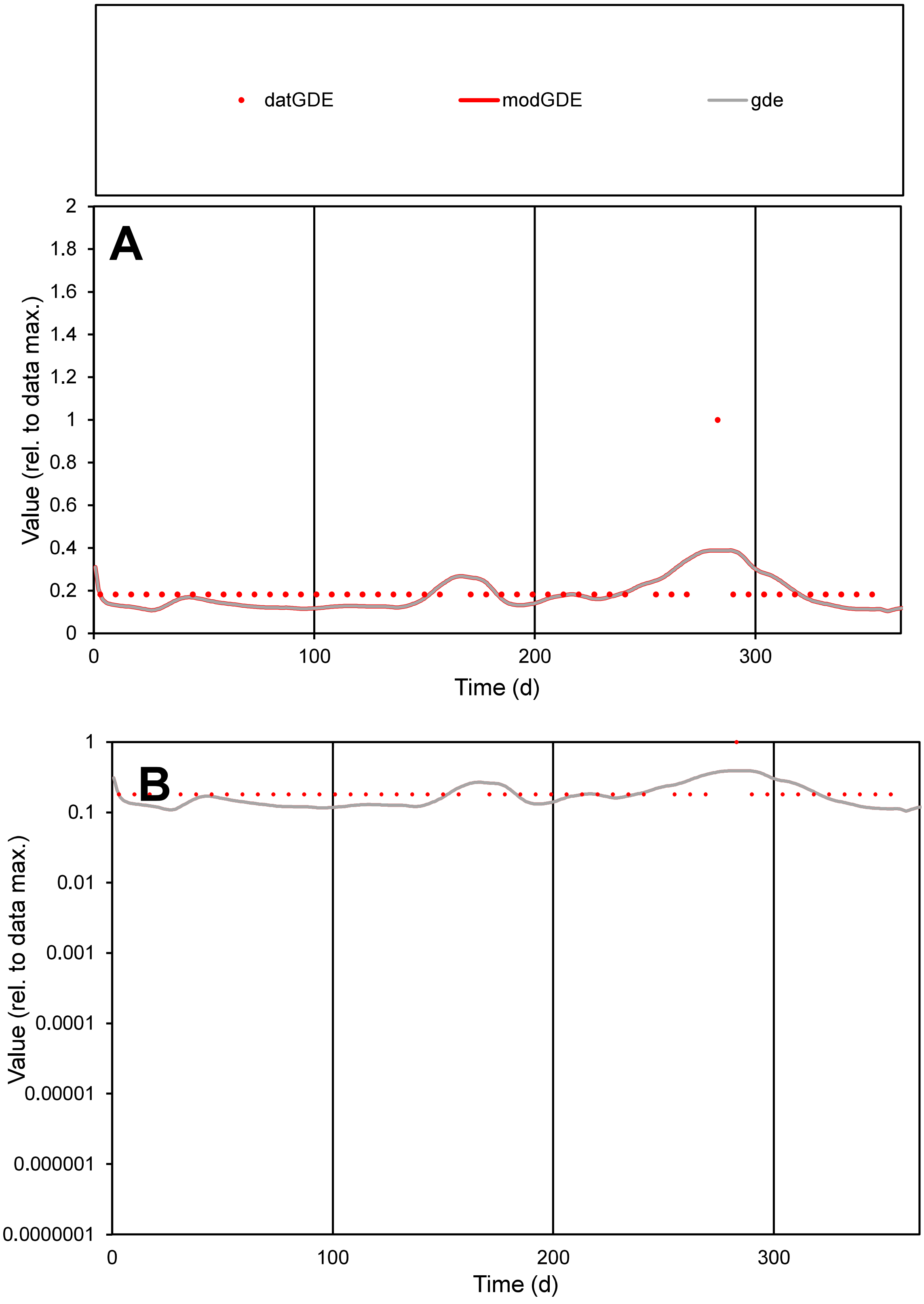

C1

Variables
GDE Provide jtd or Dst
CalibOnly?
2 1 to reproduce of
oNormalize?
2 1=no, 2=yes

ofxn calc*
0.55337
ofxn mod
0.5681

sRun
RunA1
zout
1

Ave
Data
3E-05
Model
2.7E-05

data/vMax
0.00015

ofxng
369.894

Min d/m
1.64946

ave/min d
1.0941
ave/min m
1.60314

328_Calib_S_A

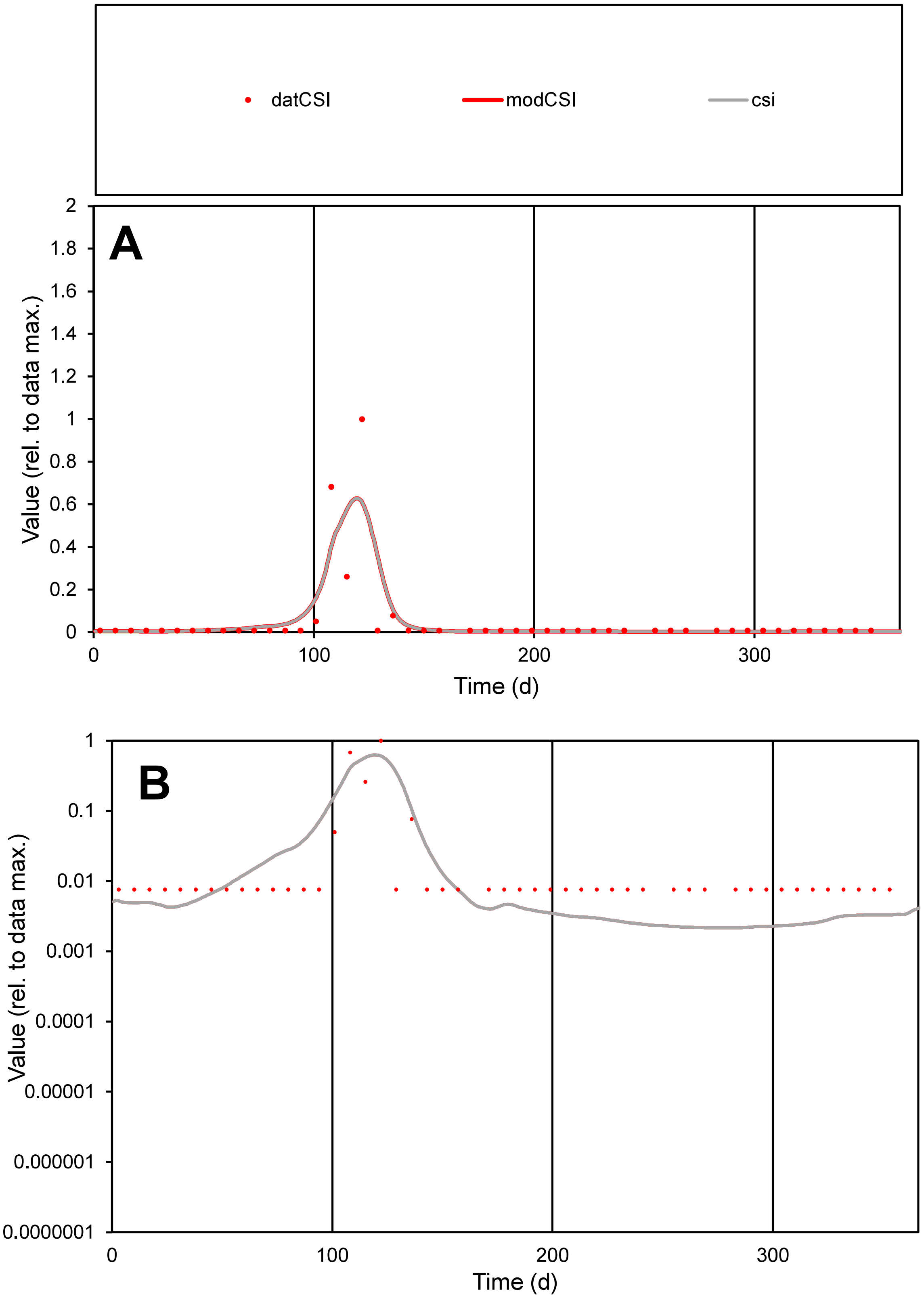

C1

Variables
CSI Provide jtd or Dst
CalibOnly?
2 1 to reproduce of
oNormalize?
2 1=no, 2=yes

ofxn calc*
1.77867
ofxn mod
1.91006

sRun
RunA1
zout
1

Ave
Data
1.5E-05
Model
1.5E-05

data/vMax
0.0003

ofxng
369.894

Min d/m
3.50797

ave/min d
6.55285
ave/min m
23.2168

328_Calib_S_A

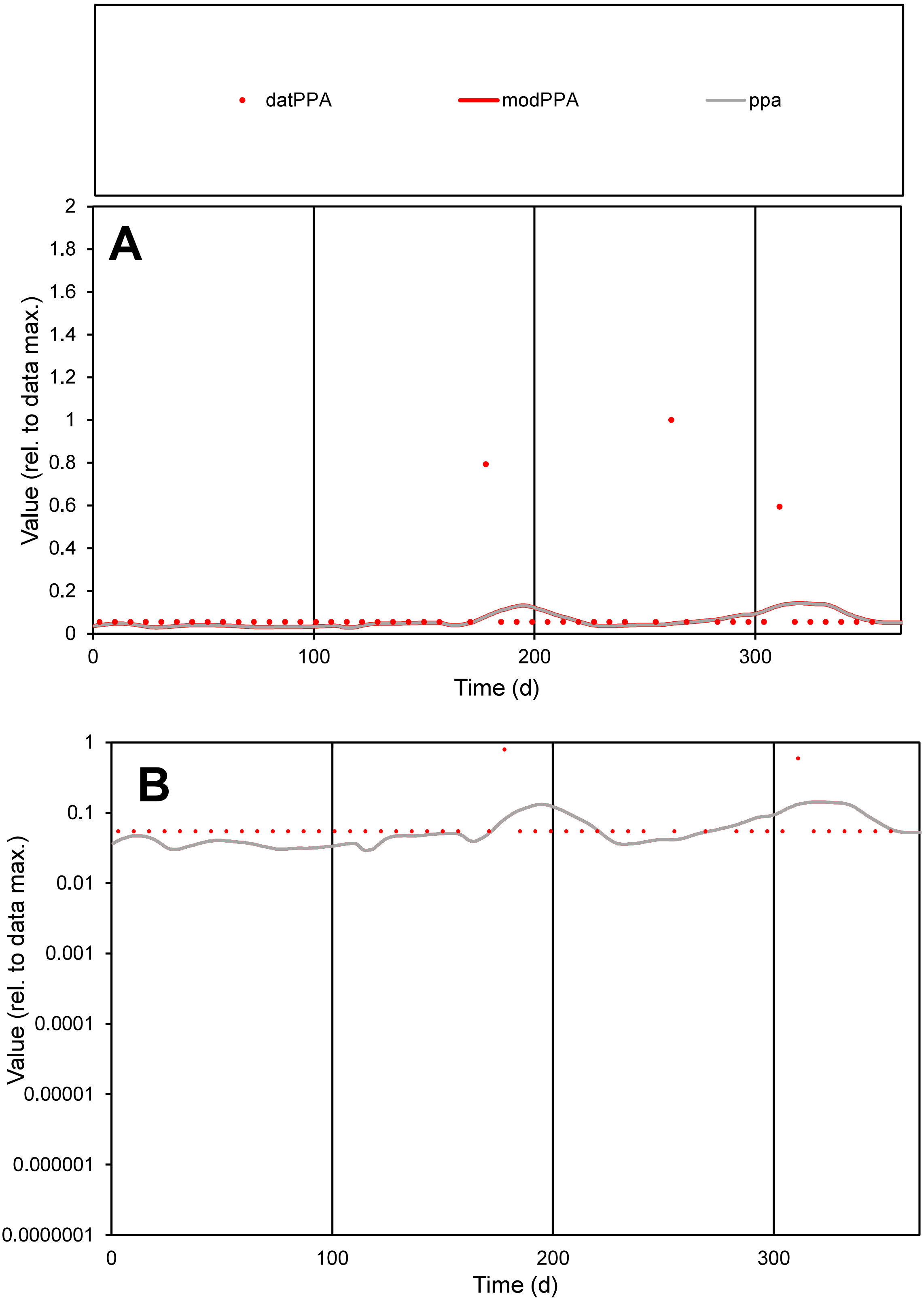

C1

Variables
PPA Provide jtd or Dst
CalibOnly?
2 1 to reproduce of
oNormalize?
2 1=no, 2=yes

ofxn calc*
1.84893
ofxn mod
1.85395

sRun
RunA1
zout
1

Ave
Data
5.9E-06
Model
3.7E-06

data/vMax
5.9E-05

ofxng
369.894

Min d/m
1.85393

ave/min d
1.85025
ave/min m
2.12931

328_Calib_S_A

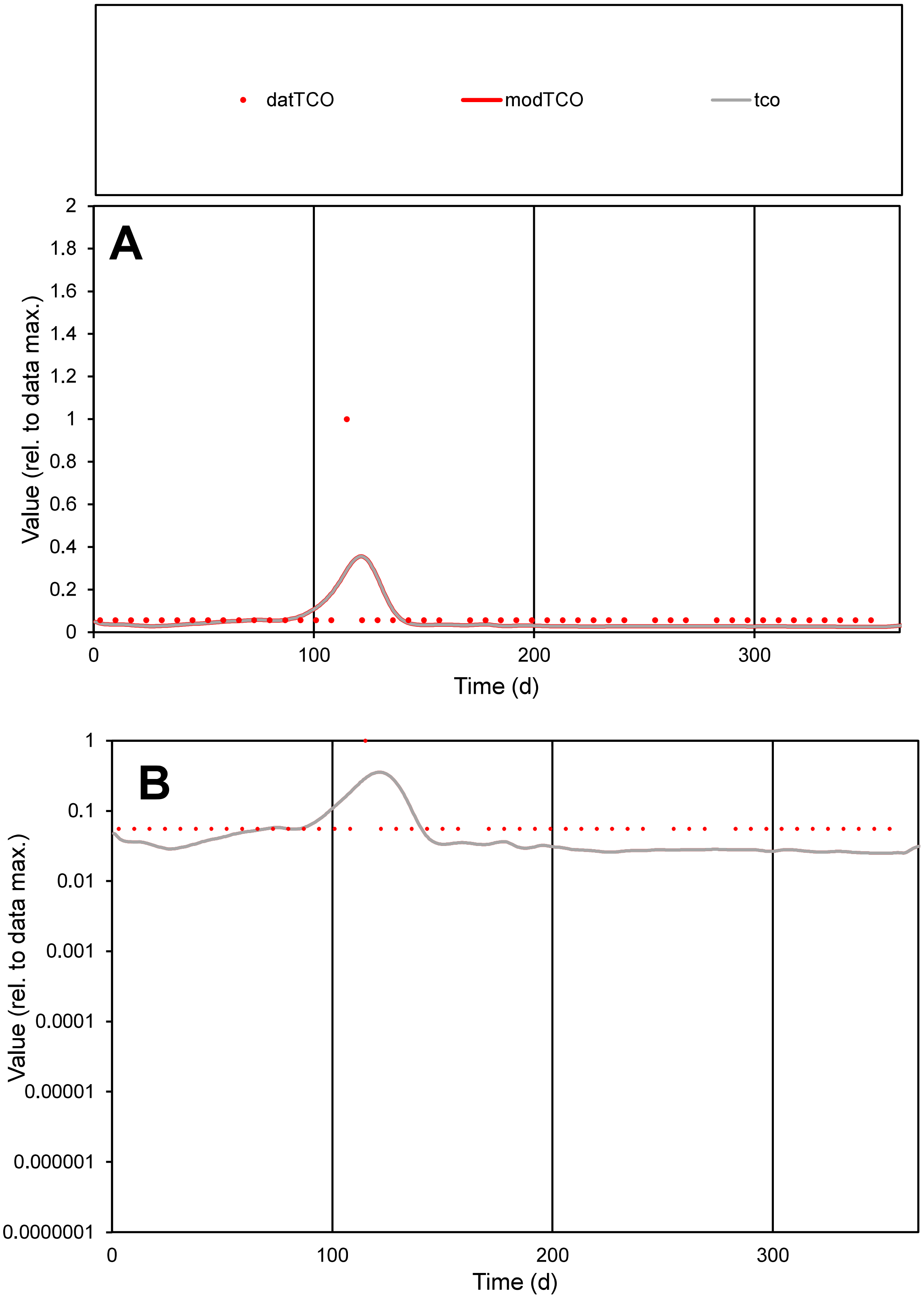

C1

Variables

TCO Provide jtd or Dst

CalibOnly?

2 1 to reproduce of

oNormalize?

2 1=no, 2=yes

ofxn calc*

1.50735

ofxn mod

1.5229

sRun

RunA1

zout

1

Ave

Data

1.2E-05

Model

8.7E-06

data/vMax

0.00015

ofxng

369.894

Min d/m

2.22759

ave/min d

1.35317

ave/min m

2.2421

328_Calib_S_A

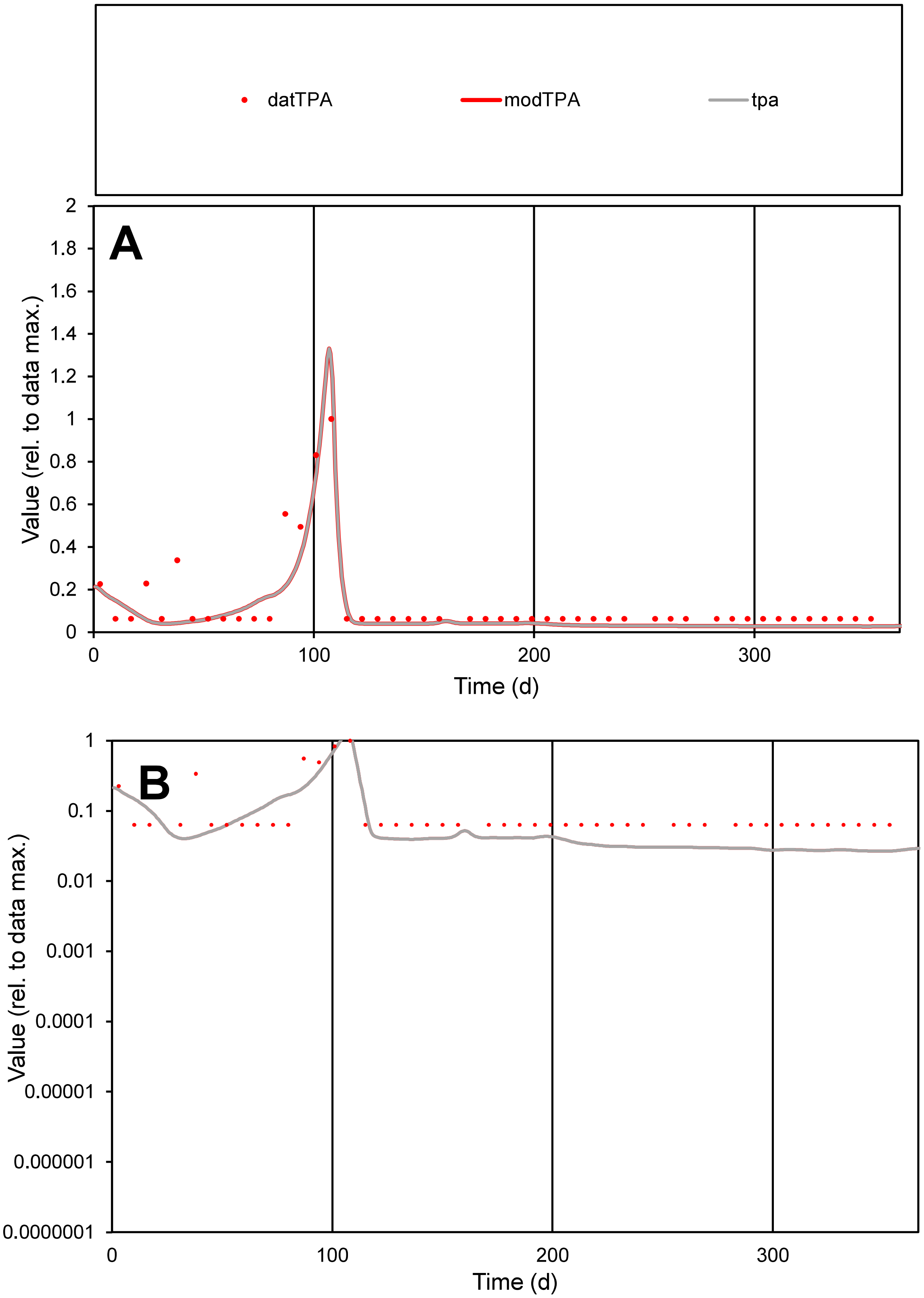

C1

Variables
TPA Provide jtd or Dst
CalibOnly?
2 1 to reproduce of
oNormalize?
2 1=no, 2=yes

ofxn calc*
0.59361
ofxn mod
0.6163

sRun
RunA1
zout
1

Ave
Data
9.8E-06
Model
7.5E-06

data/vMax
7.5E-05

ofxng
369.894

Min d/m
2.35494

ave/min d
2.06545
ave/min m
3.73382

328_Calib_S_A

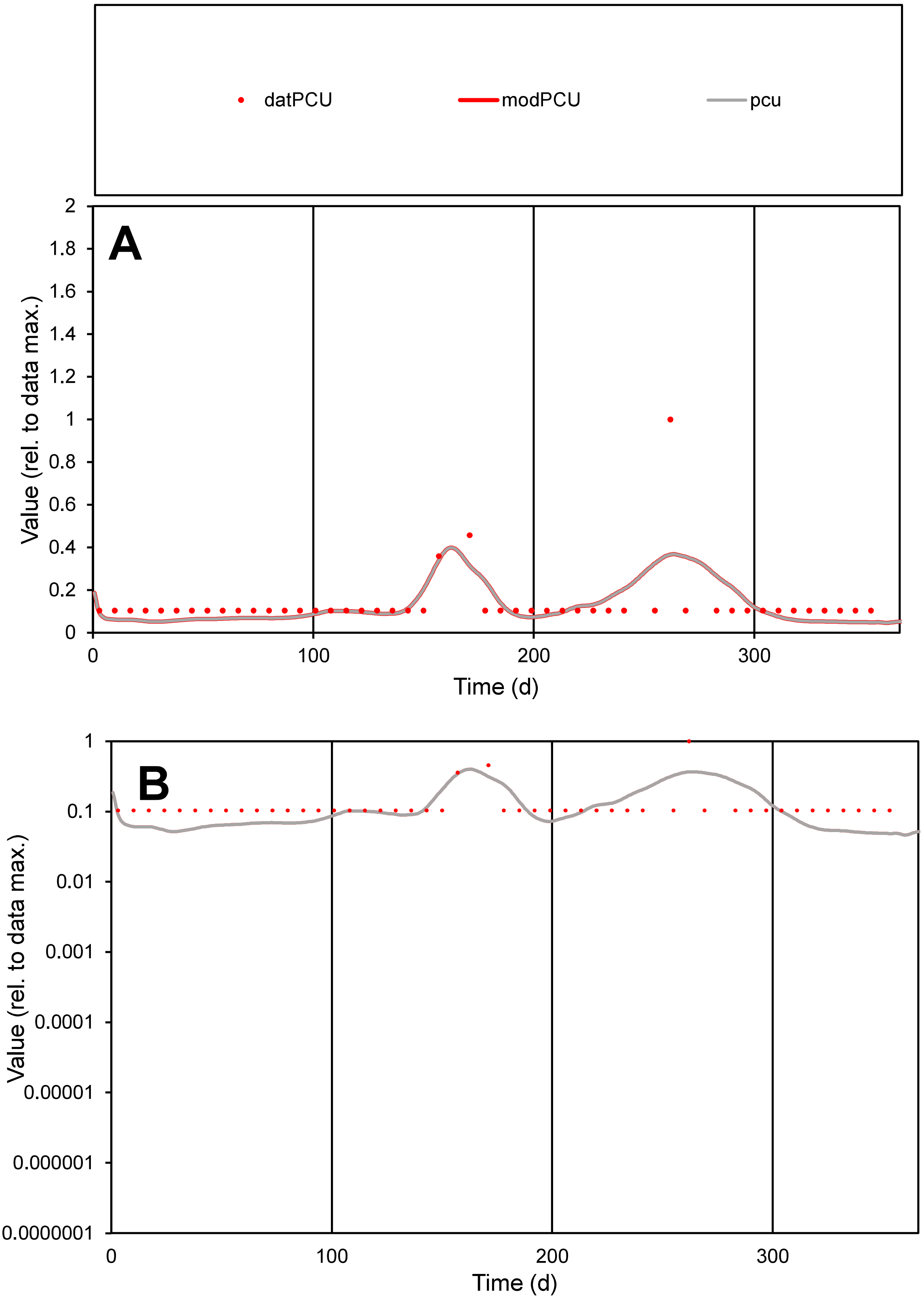

C1

Variables

PCU Provide jtd or Dst

CalibOnly?

2 1 to reproduce of

oNormalize?

2 1=no, 2=yes

ofxn calc*

0.85997

ofxn mod

0.8734

sRun

RunA1

zout

1

Ave

Data

8.1E-06

Model

7.4E-06

data/vMax

6E-05

ofxng

369.894

Min d/m

2.12999

ave/min d

1.3017

ave/min m

2.53551

328_Calib_S_A

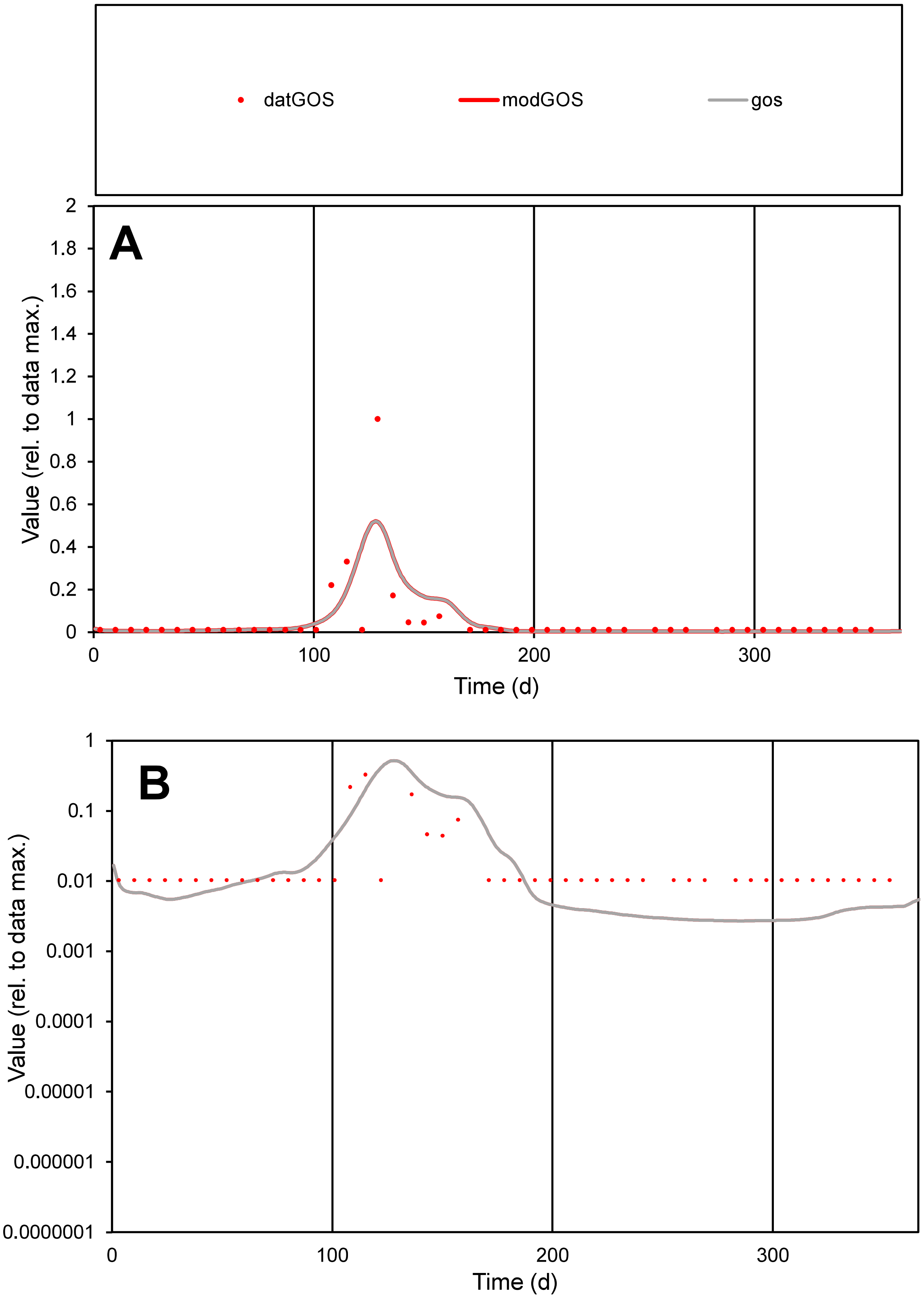

C1

Variables
GOS Provide jtd or Dst
CalibOnly?
2 1 to reproduce of
oNormalize?
2 1=no, 2=yes

ofxn calc*
1.99044
ofxn mod
2.08928

sRun
RunA1
zout
1

Ave
Data
8.4E-06
Model
8.6E-06

data/vMax
0.00017

ofxng
369.894

Min d/m
3.83898

ave/min d
4.64688
ave/min m
18.1451

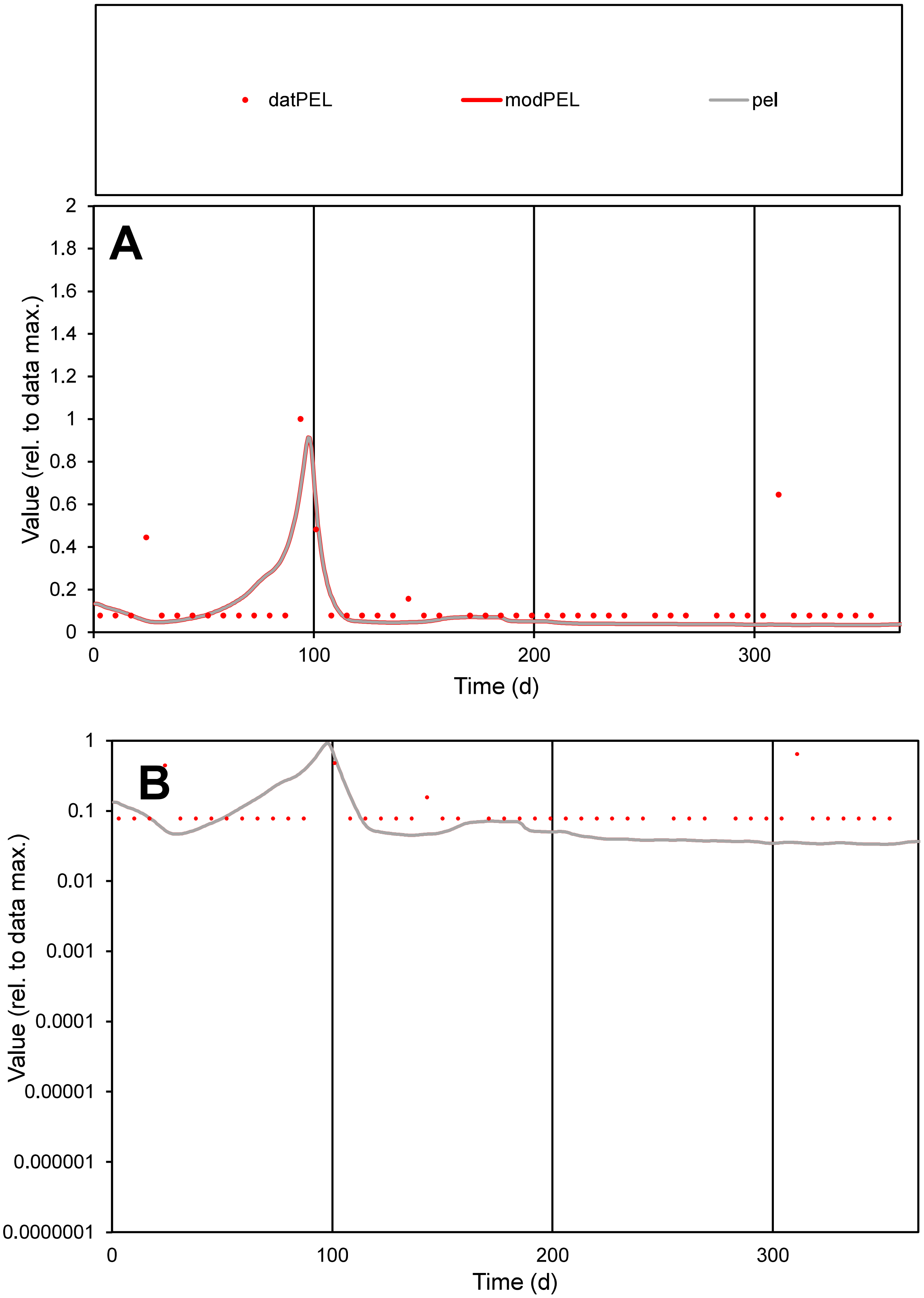

C1

Variables
PEL Provide jtd or Dst
CalibOnly?
2 1 to reproduce of
oNormalize?
2 1=no, 2=yes

ofxn calc*
1.01044
ofxn mod
1.03177

sRun
RunA1
zout
1

Ave
Data
5.3E-06
Model
3.9E-06

data/vMax
4.2E-05

ofxng
369.894

Min d/m
2.3178

ave/min d
1.62445
ave/min m
2.77629

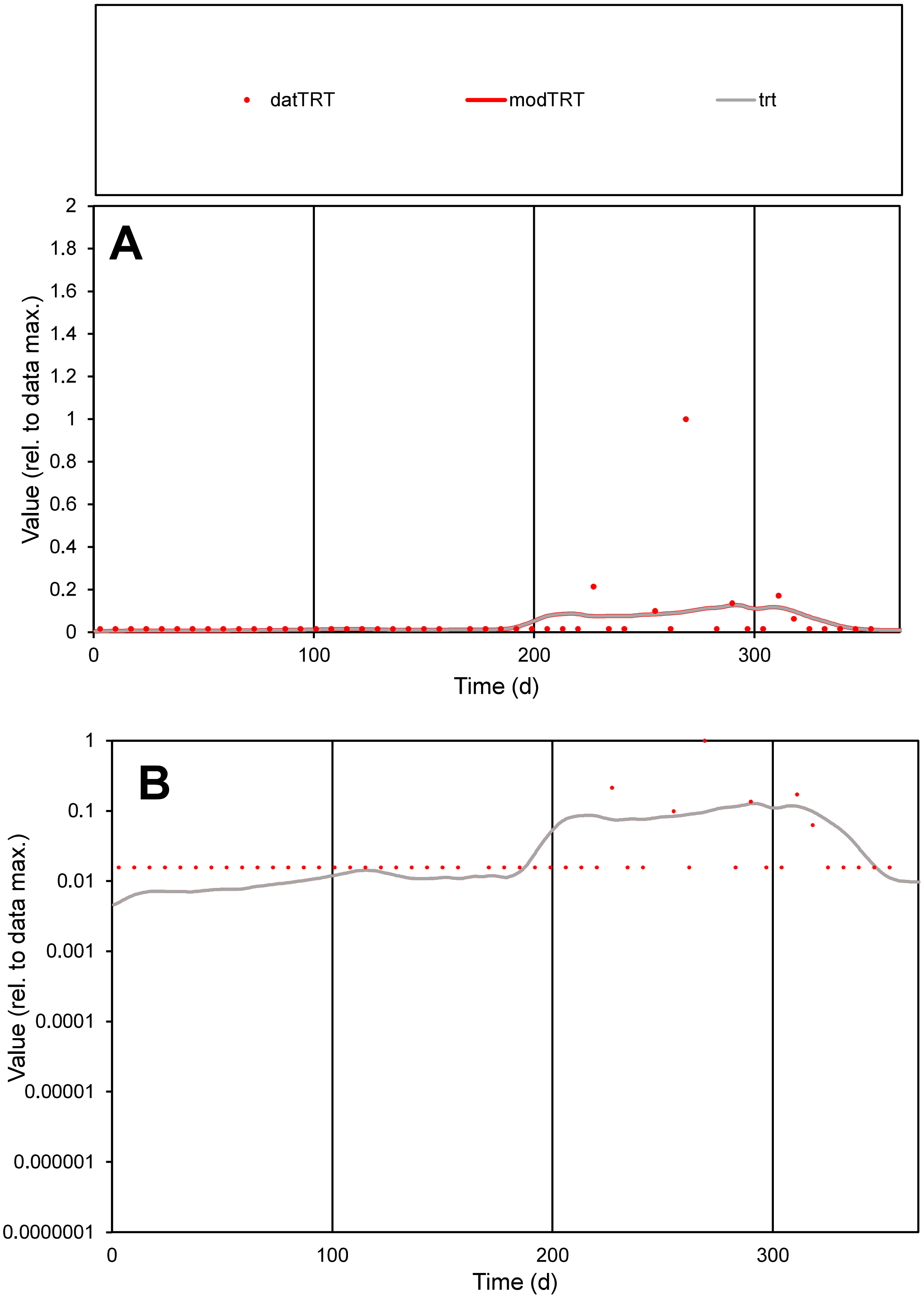

C1

Variables

TRT Provide jtd or Dst

CalibOnly?

2 1 to reproduce of

oNormalize?

2 1=no, 2=yes

ofxn calc*

2.80449

ofxn mod

2.81421

sRun

RunA1

zout

1

Ave

Data

7.2E-05

Model

5.9E-05

data/vMax

0.00148

ofxng

369.894

Min d/m

2.99354

ave/min d

3.11152

ave/min m

7.65396

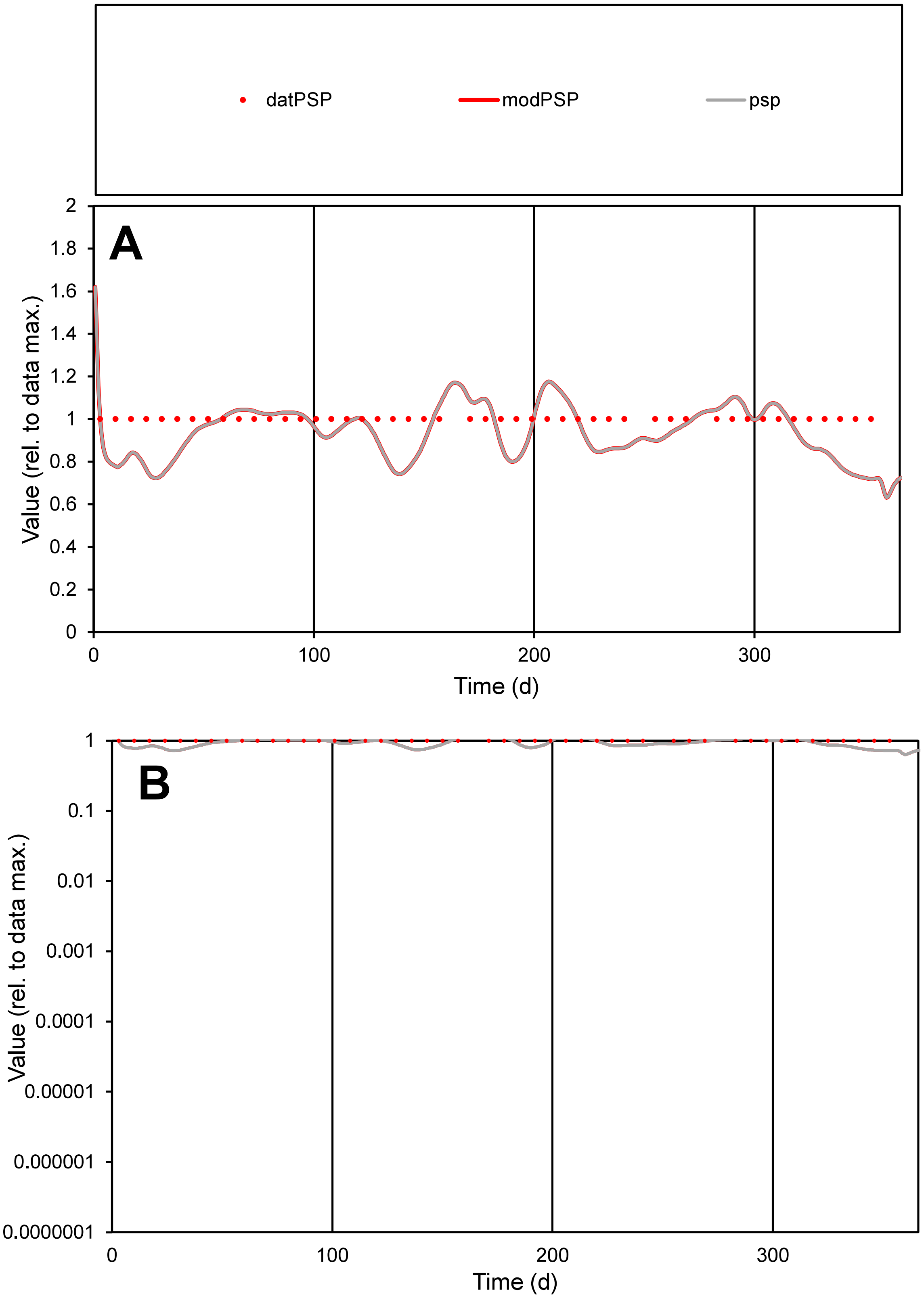

C1

Variables
PSP Provide jtd or Dst
CalibOnly?
2 1 to reproduce of
oNormalize?
2 1=no, 2=yes

ofxn calc*
0.1324
ofxn mod
0.17176

sRun
RunA1
zout
1

Ave
Data
4.3E-06
Model
4E-06

data/vMax
4.3E-06

ofxng
369.894

Min d/m
1.38964

ave/min d
1
ave/min m
1.29976

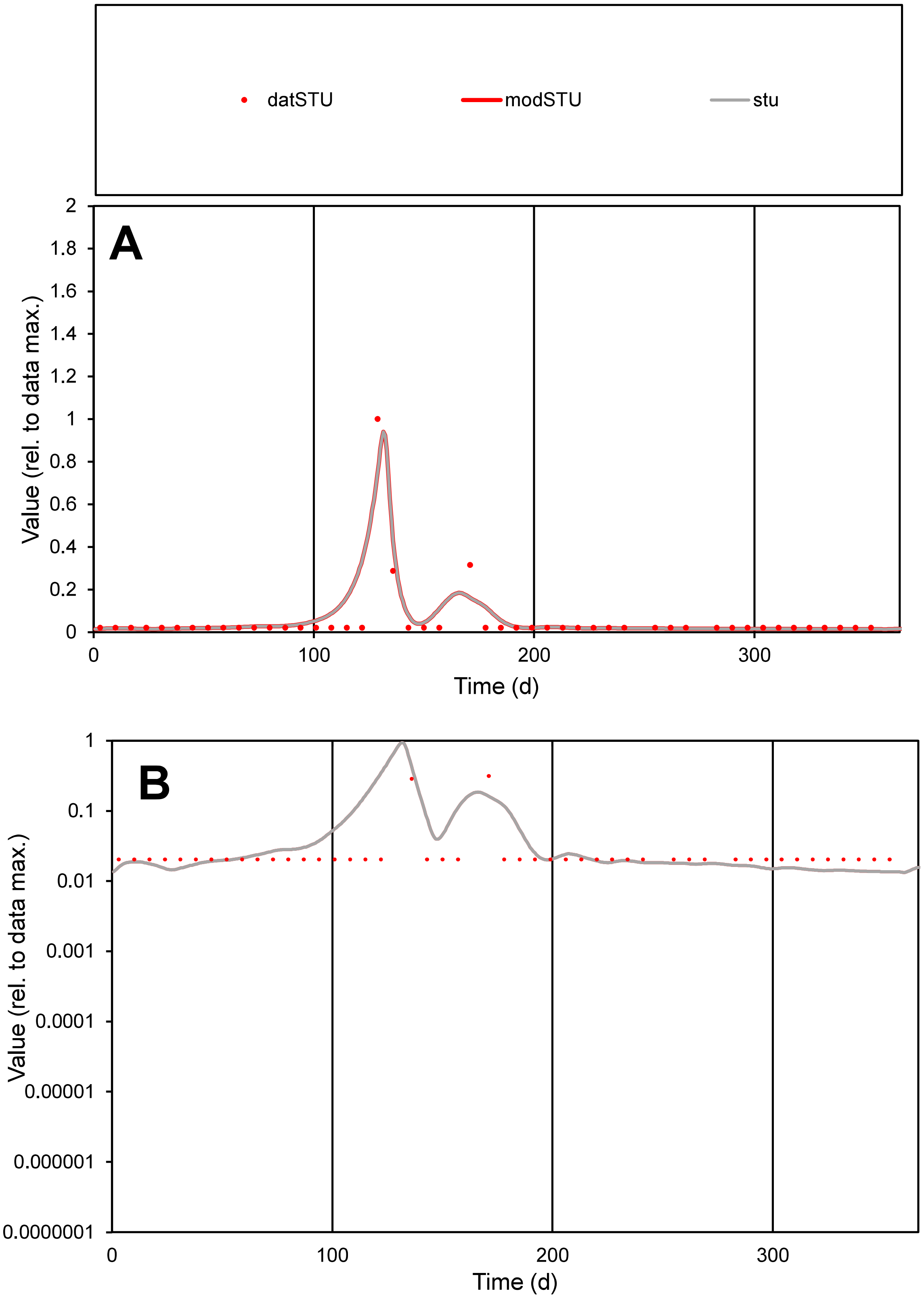

C1

Variables
STU Provide jtd or Dst
CalibOnly?
2 1 to reproduce of
oNormalize?
2 1=no, 2=yes

ofxn calc*
1.05787
ofxn mod
1.18627

sRun
RunA1
zout
1

Ave
Data
6.4E-06
Model
7.4E-06

data/vMax
0.00012

ofxng
369.894

Min d/m
1.5009

ave/min d
2.57751
ave/min m
4.46508

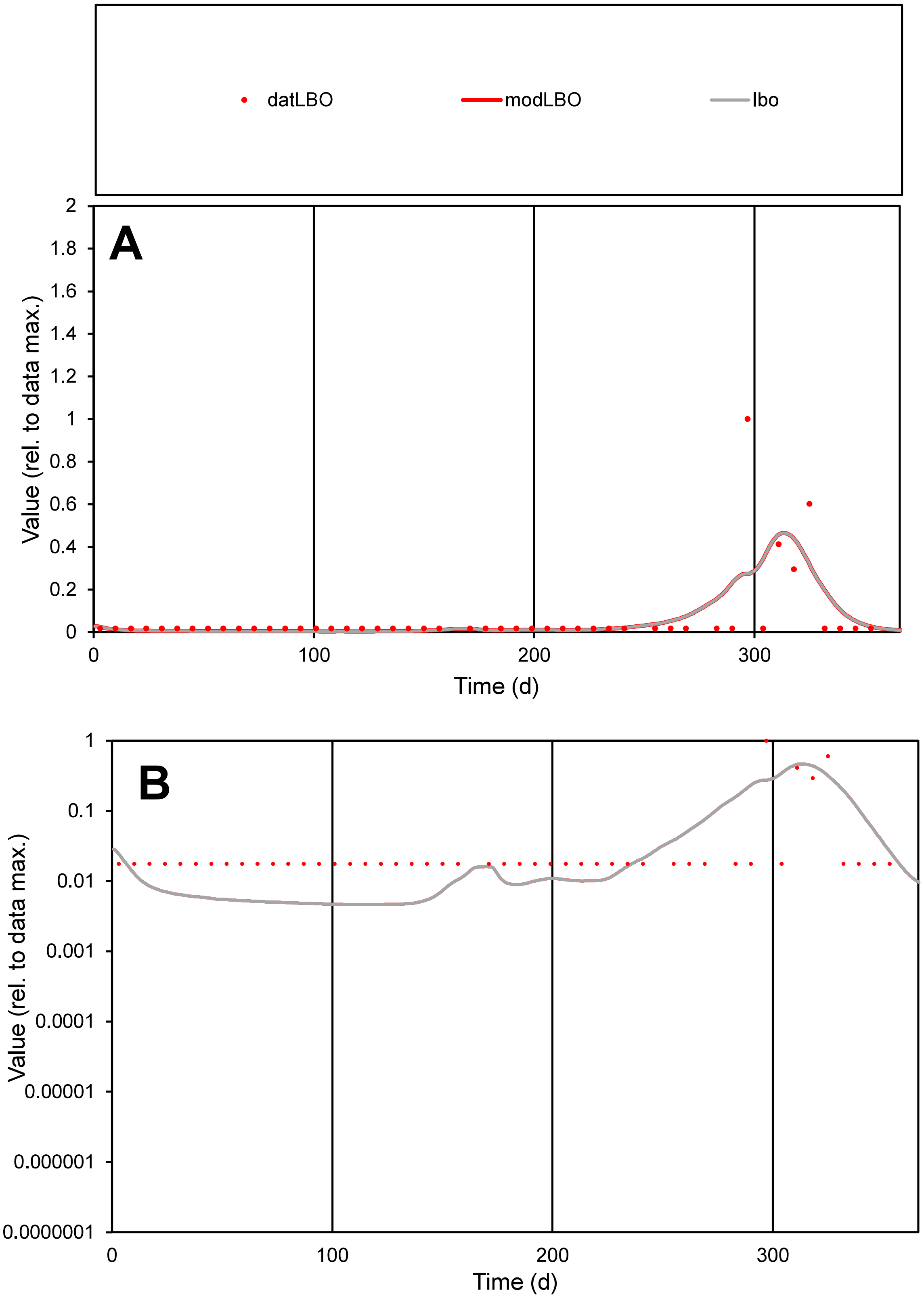

C1

Variables
LBO Provide jtd or Dst
CalibOnly?
2 1 to reproduce of
oNormalize?
2 1=no, 2=yes

ofxn calc*
2.02783
ofxn mod
2.06845

sRun
RunA1
zout
1

Ave
Data
5.1E-06
Model
4.8E-06

data/vMax
7.9E-05

ofxng
369.894

Min d/m
3.7721

ave/min d
3.65174
ave/min m
13.1872

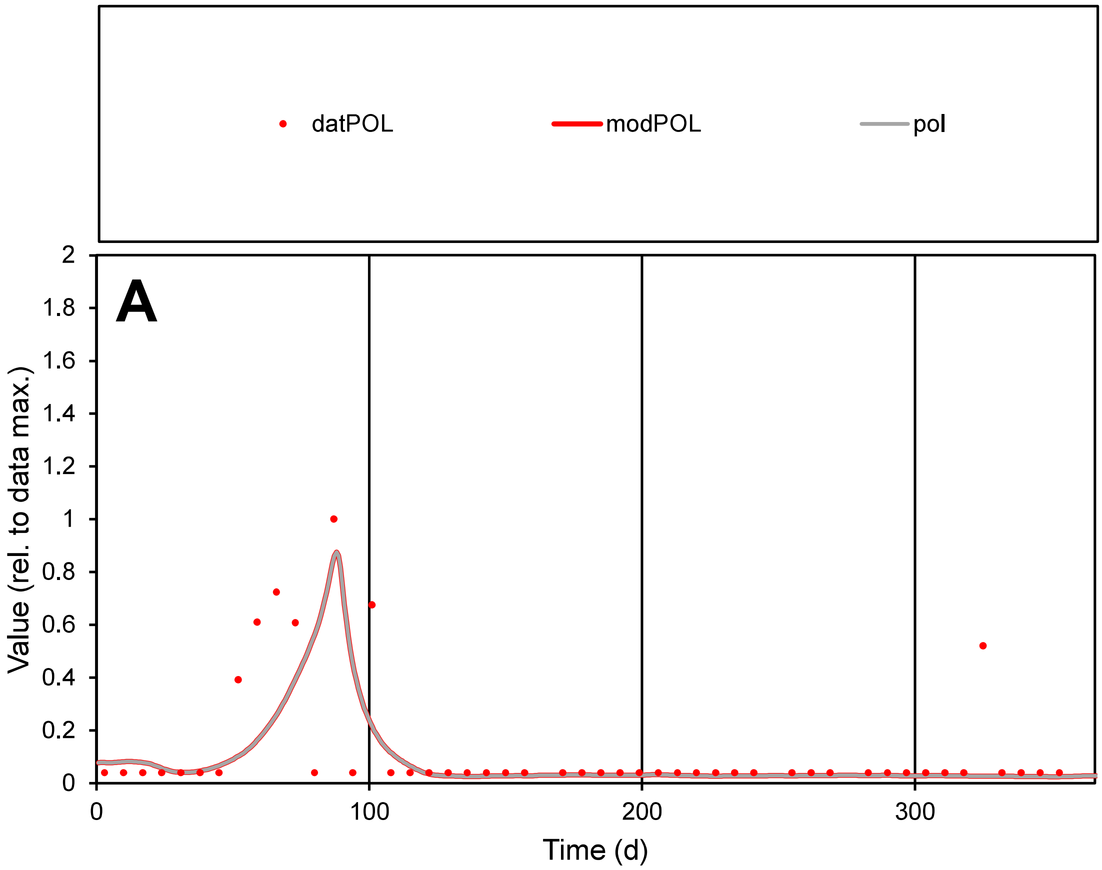

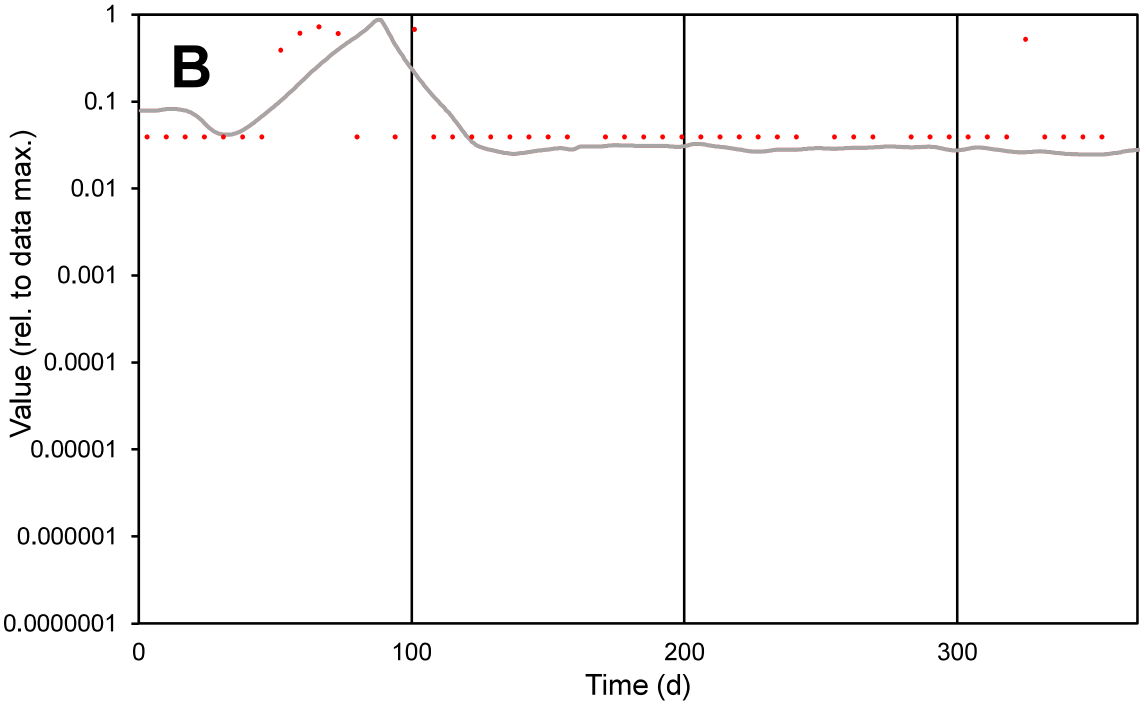

C1

Variables
POL Provide jtd or Dst
CalibOnly?
2 1 to reproduce of
oNormalize?
2 1=no, 2=yes

ofxn calc*
1.28876
ofxn mod
1.32446

sRun
RunA1
zout
1

Ave
Data
5.2E-06
Model
3.8E-06

data/vMax
4.1E-05

ofxng
369.894

Min d/m
1.58964

ave/min d
3.26136
ave/min m
3.73616

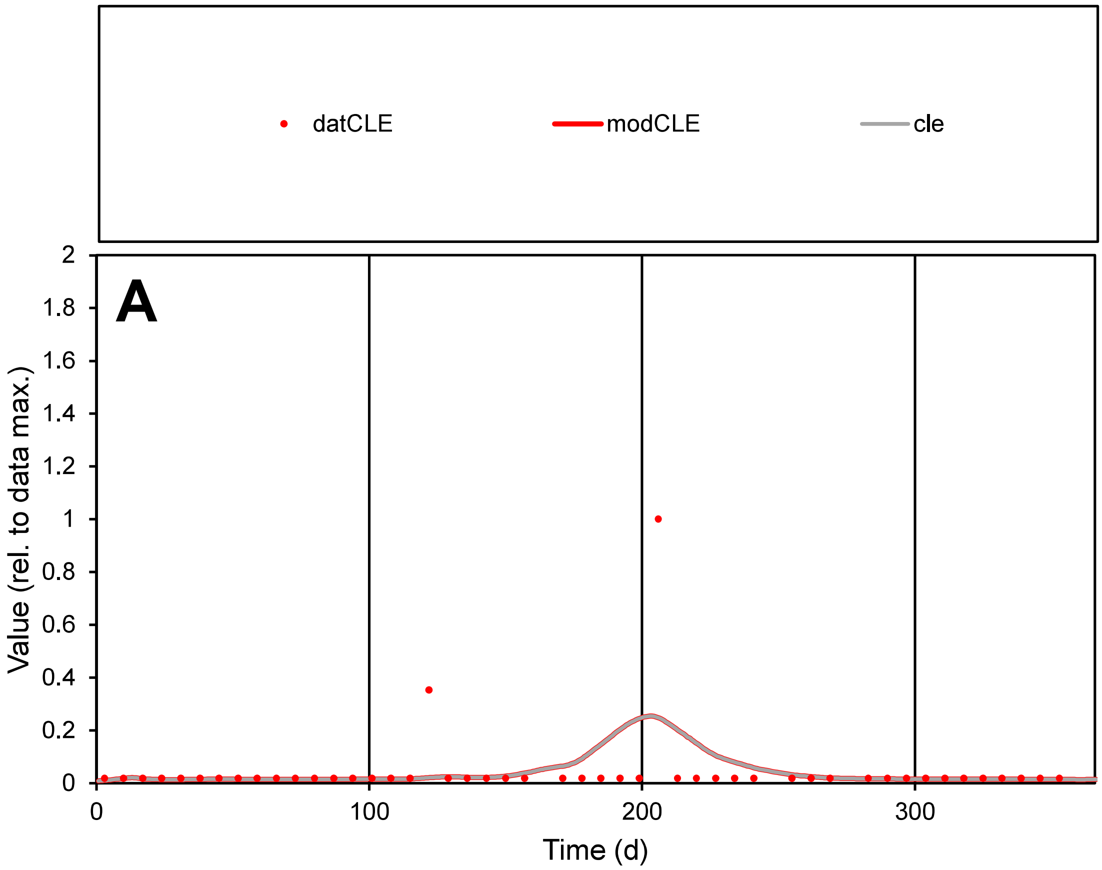

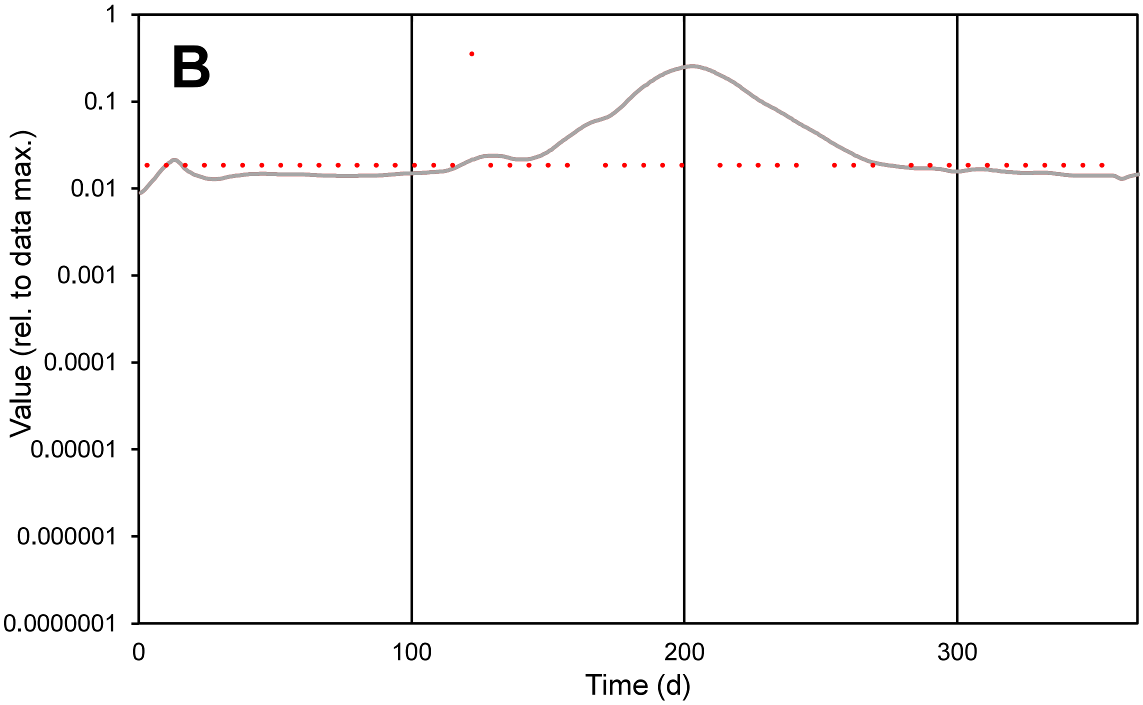

C1

Variables
CLE Provide jtd or Dst
CalibOnly?
2 1 to reproduce of
oNormalize?
2 1=no, 2=yes

ofxn calc*
2.88355
ofxn mod
2.90881

sRun
RunA1
zout
1

Ave
Data
1.2E-05
Model
1.2E-05

data/vMax
0.00025

ofxng
369.894

Min d/m
1.60767

ave/min d
2.4754
ave/min m
3.9392

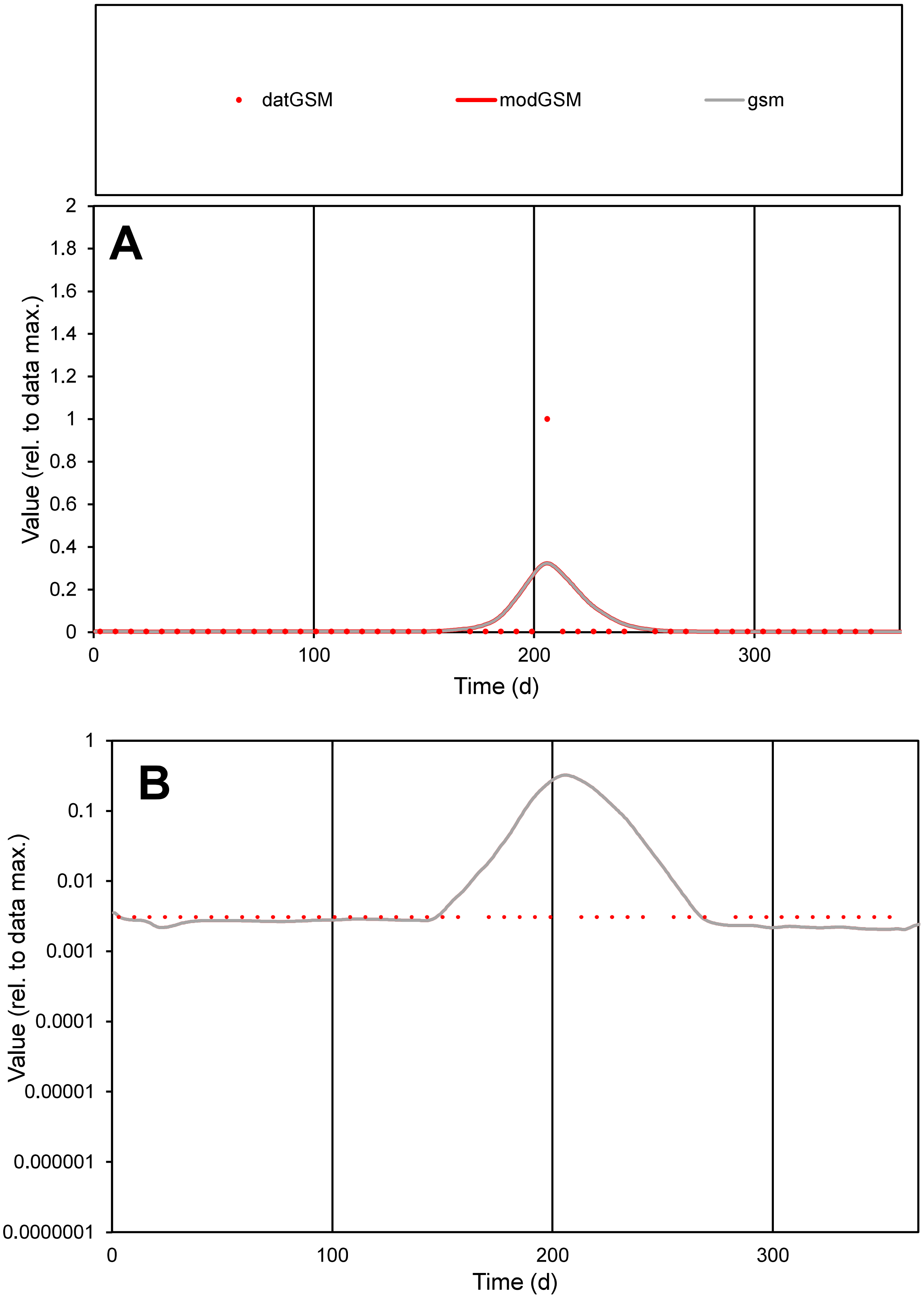

C1

Variables

GSM Provide jtd or Dst

CalibOnly?

2 1 to reproduce of

oNormalize?

2 1=no, 2=yes

ofxn calc*

4.82436

ofxn mod

4.93742

sRun

RunA1

zout

1

Ave

Data

1.2E-05

Model

1.6E-05

data/vMax

0.00051

ofxng

369.894

Min d/m

1.49385

ave/min d

7.73582

ave/min m

15.1842

328_Calib_S_A

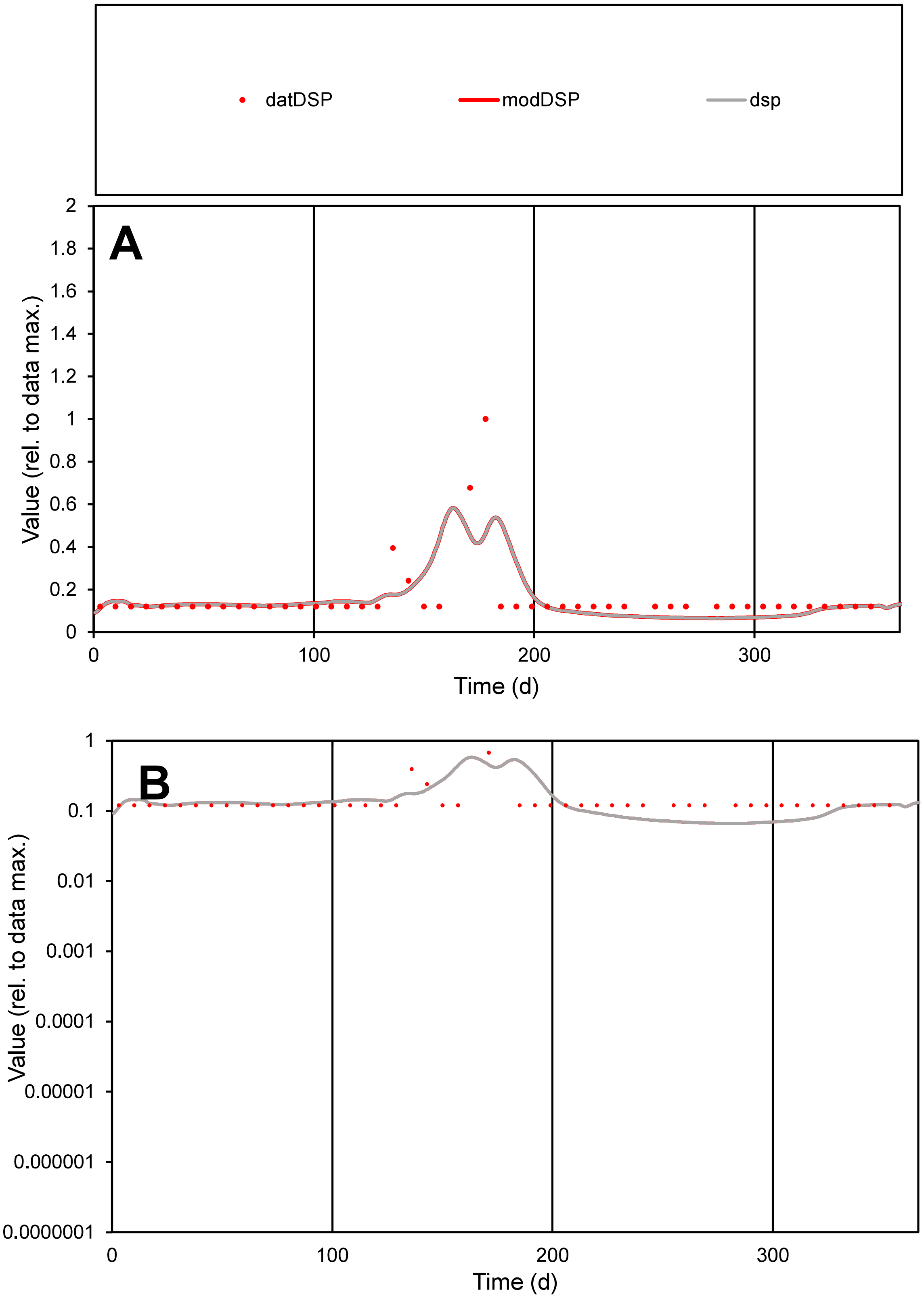

C1

Variables

DSP Provide jtd or Dst

CalibOnly?

2 1 to reproduce of

oNormalize?

2 1=no, 2=yes

ofxn calc*

0.72666

ofxn mod

0.76354

sRun

RunA1

zout

1

Ave

Data

6.6E-06

Model

6.2E-06

data/vMax

4.2E-05

ofxng

369.894

Min d/m

1.81472

ave/min d

1.3166

ave/min m

2.2603

328_Calib_S_A

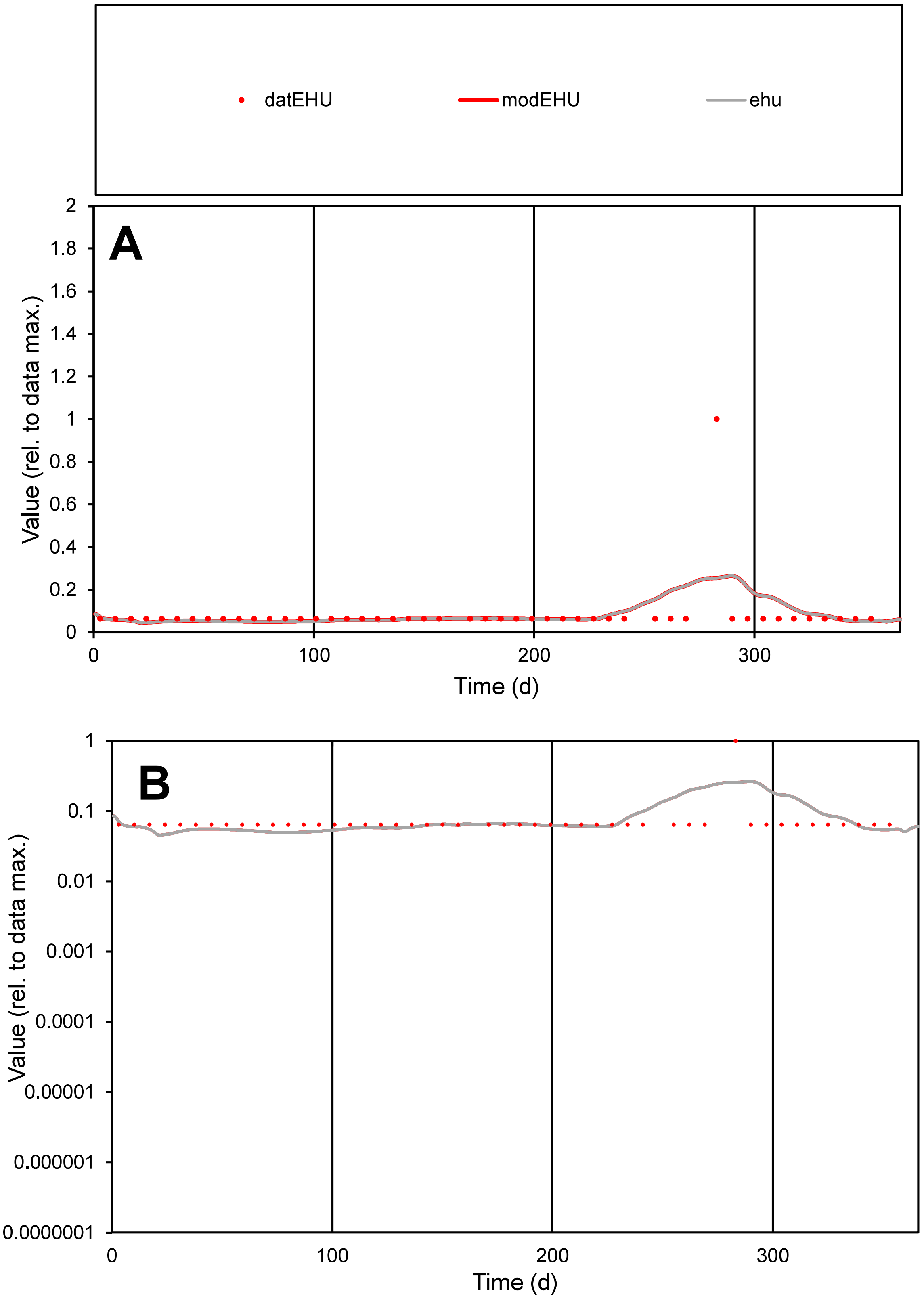

C1

Variables
EHU Provide jtd or Dst
CalibOnly?
2 1 to reproduce of
oNormalize?
2 1=no, 2=yes

ofxn calc*
1.42577
ofxn mod
1.43767

sRun
RunA1
zout
1

Ave
Data
7.5E-06
Model
7.6E-06

data/vMax
9E-05

ofxng
369.894

Min d/m
1.36504

ave/min d
1.30349
ave/min m
1.80464

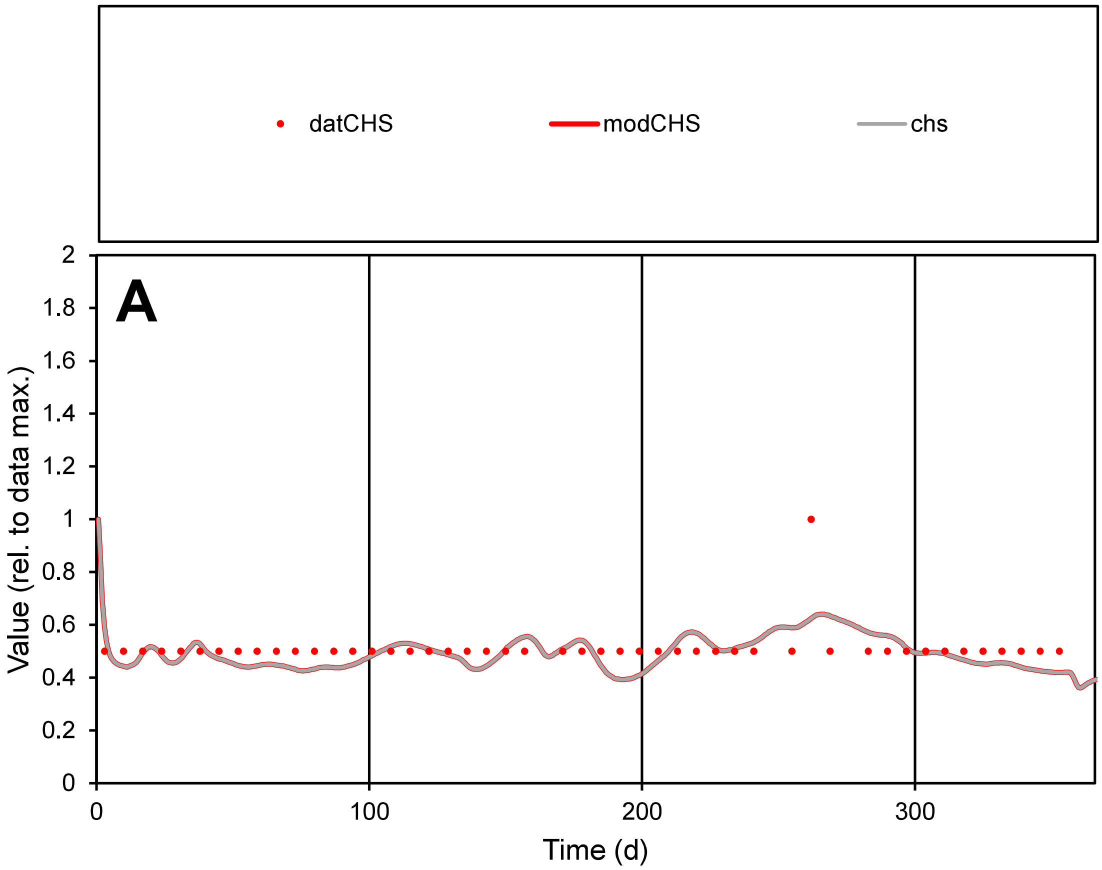

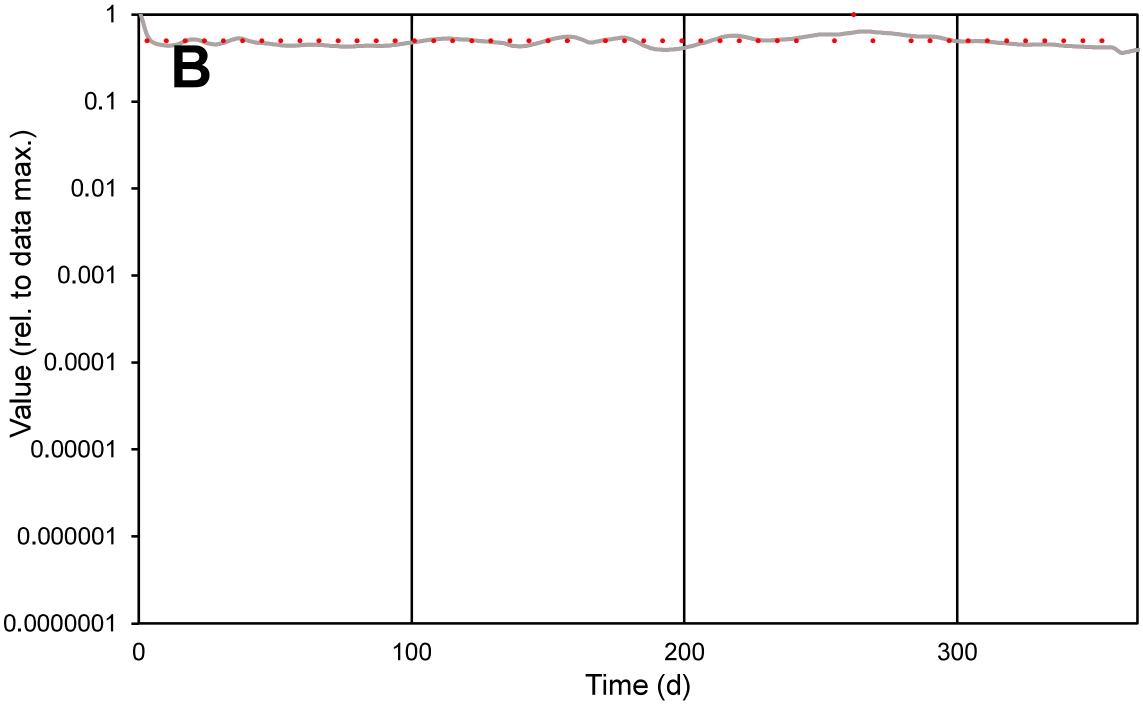

C1

Variables
CHS Provide jtd or Dst
CalibOnly?
2 1 to reproduce of
oNormalize?
2 1=no, 2=yes

ofxn calc*
0.14504
ofxn mod
0.18294

sRun
RunA1
zout
1

Ave
Data
2.2E-05
Model
2.1E-05

data/vMax
4.3E-05

ofxng
369.894

Min d/m
1.27246

ave/min d
1.02084
ave/min m
1.24772

328_Calib_S_A

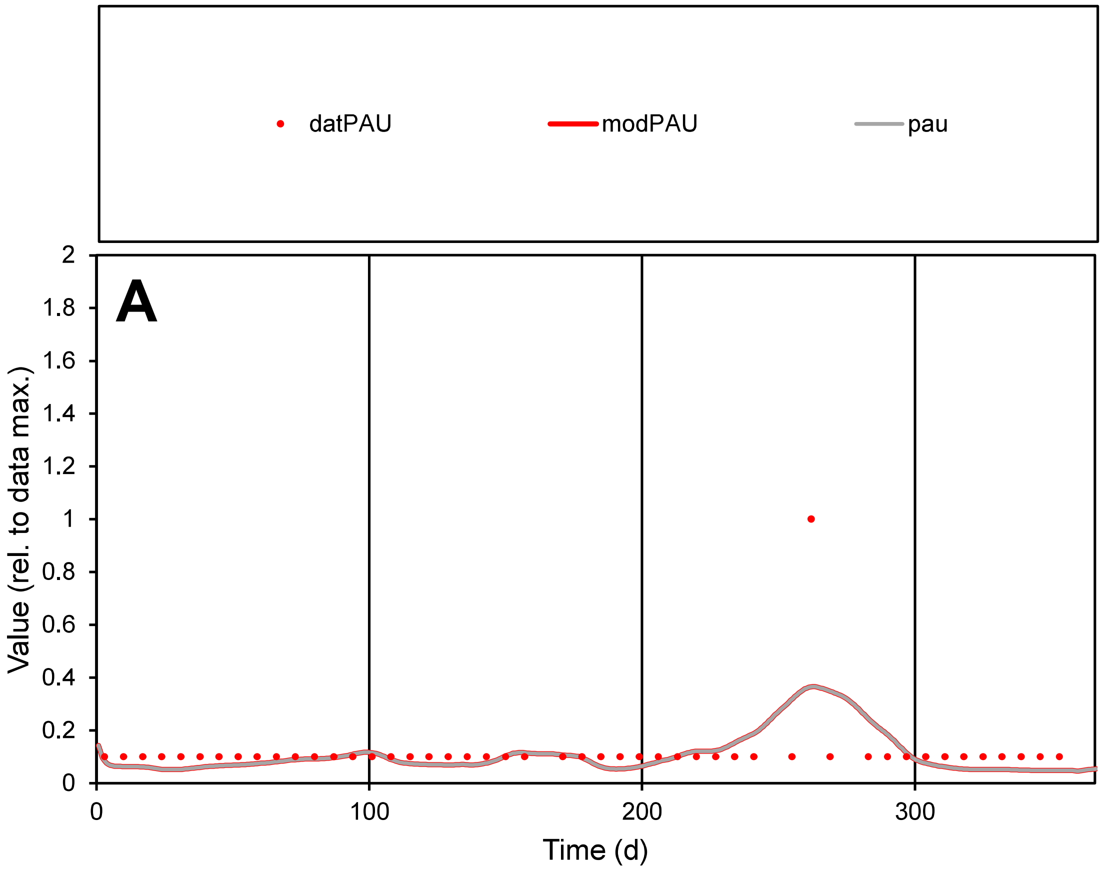

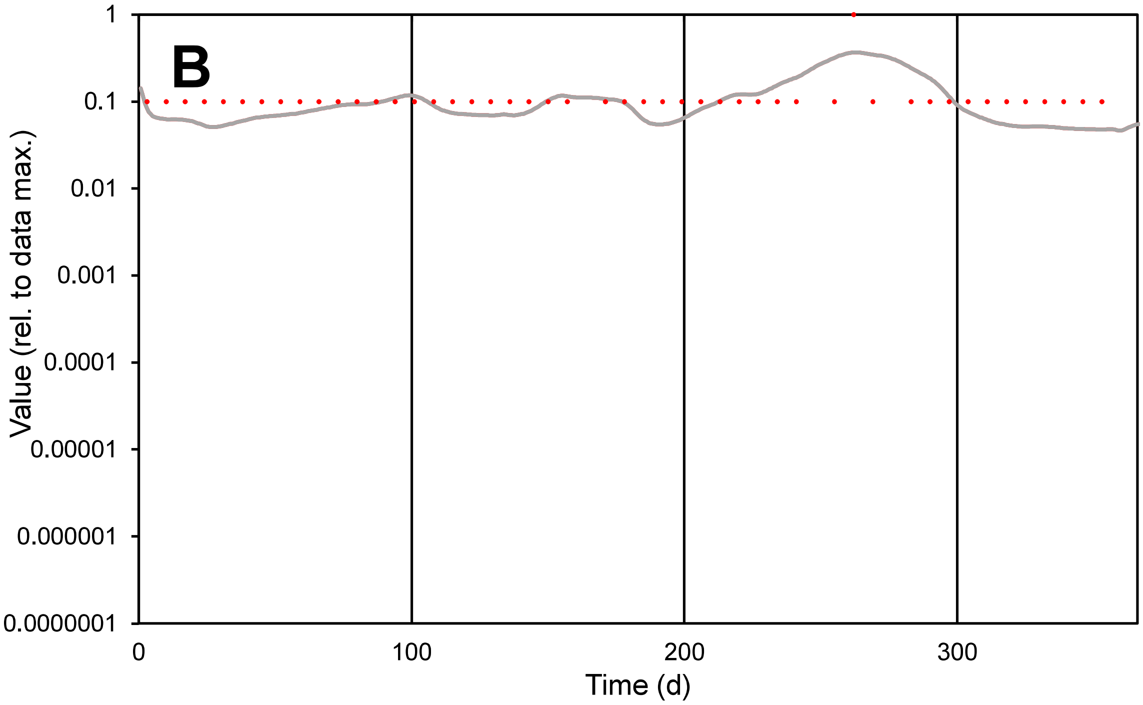

C1

Variables
PAU Provide jtd or Dst
CalibOnly?
2 1 to reproduce of
oNormalize?
2 1=no, 2=yes

ofxn calc*
0.92806
ofxn mod
0.93844

sRun
RunA1
zout
1

Ave
Data
4.7E-06
Model
4.1E-06

data/vMax
3.9E-05

ofxng
369.894

Min d/m
2.08514

ave/min d
1.18739
ave/min m
2.15778

328_Calib_S_A

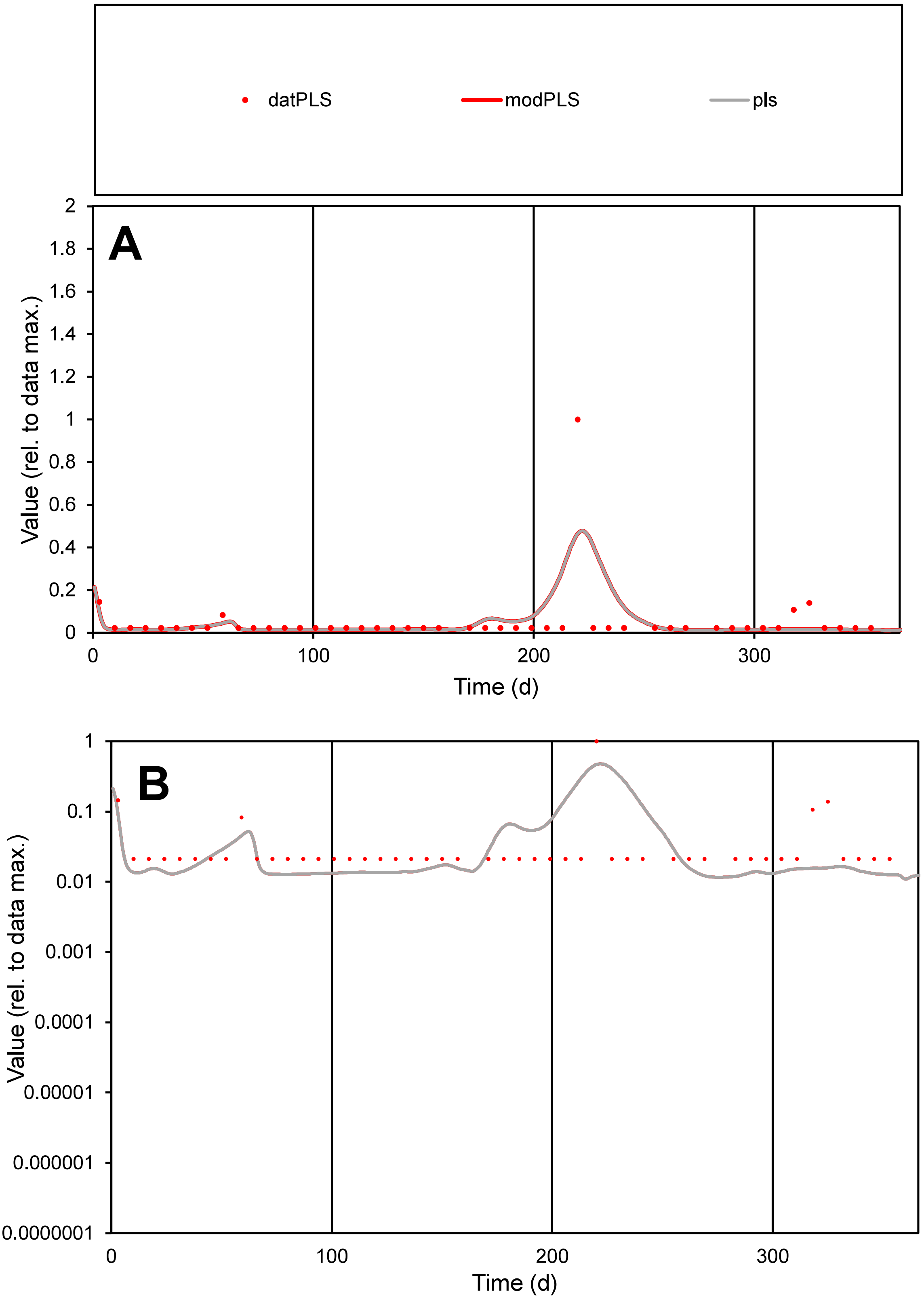

C1

Variables
PLS Provide jtd or Dst
CalibOnly?
2 1 to reproduce of
oNormalize?
2 1=no, 2=yes

ofxn calc*
2.05544
ofxn mod
2.15317

sRun
RunA1
zout
1

Ave
Data
9E-06
Model
9.3E-06

data/vMax
0.00018

ofxng
369.894

Min d/m
1.78984

ave/min d
2.33817
ave/min m
4.3507

328_Calib_S_A

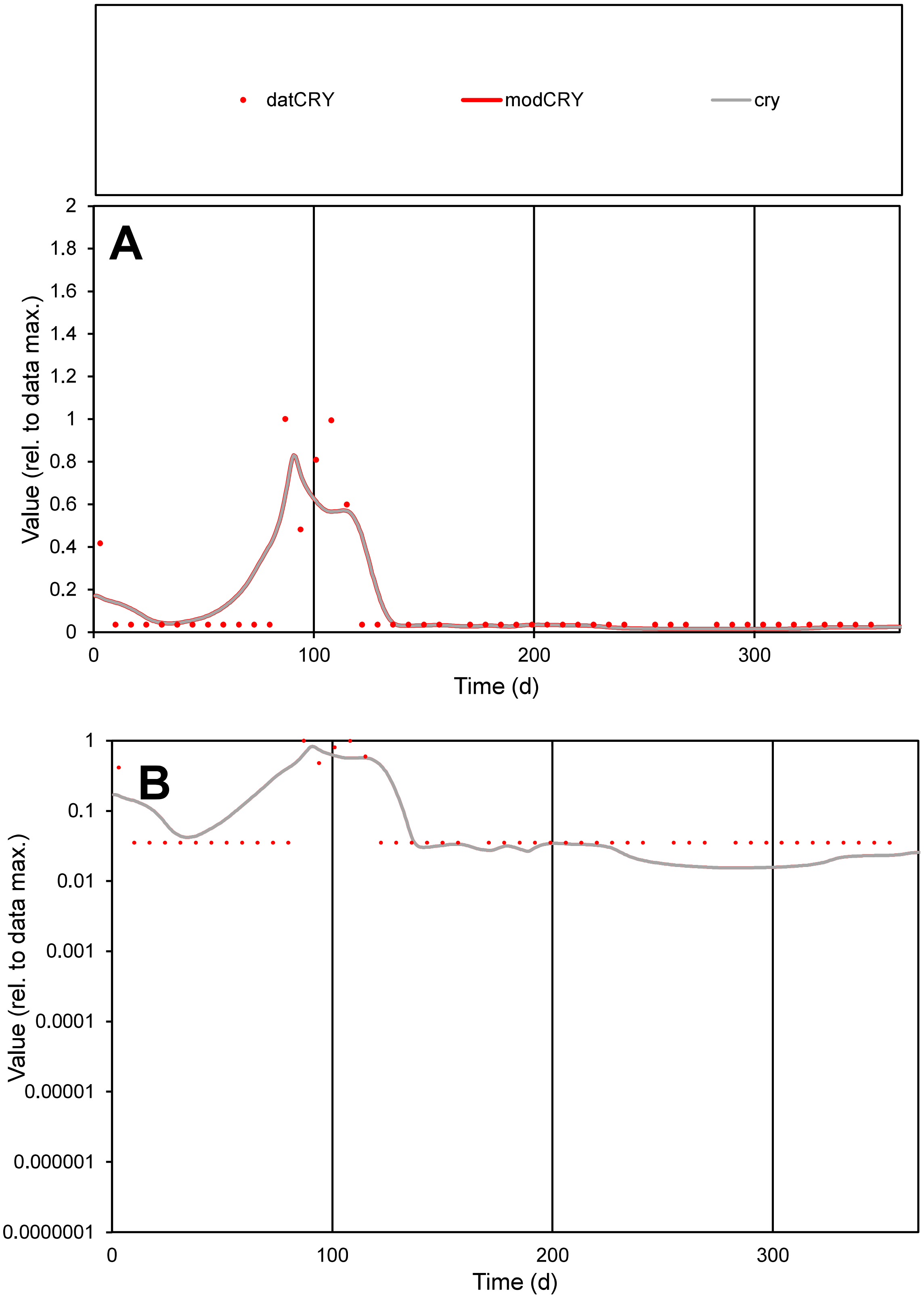

C1

Variables
CRY Provide jtd or Dst
CalibOnly?
2 1 to reproduce of
oNormalize?
2 1=no, 2=yes

ofxn calc*
1.04692
ofxn mod
1.09796

sRun
RunA1
zout
1

Ave
Data
1E-05
Model
1E-05

data/vMax
8.5E-05

ofxng
369.894

Min d/m
2.29516

ave/min d
3.41498
ave/min m
8.00796

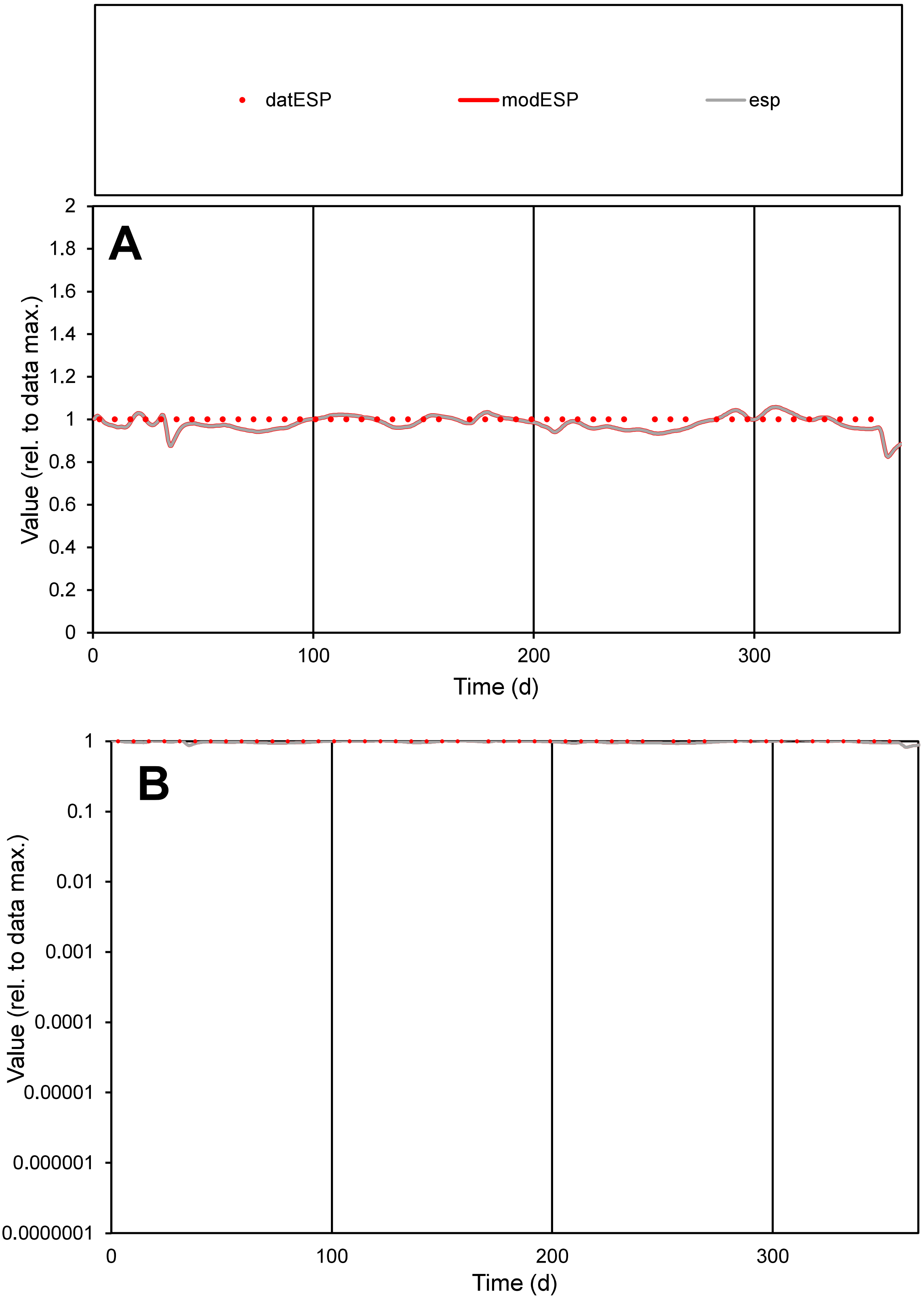

C1

Variables
ESP Provide jtd or Dst
CalibOnly?
2 1 to reproduce of
oNormalize?
2 1=no, 2=yes

ofxn calc*
0.0319
ofxn mod
0.11671

sRun
RunA1
zout
1

Ave
Data
8E-06
Model
7.9E-06

data/vMax
8E-06

ofxng
369.894

Min d/m
1.07118

ave/min d
1
ave/min m
1.05637

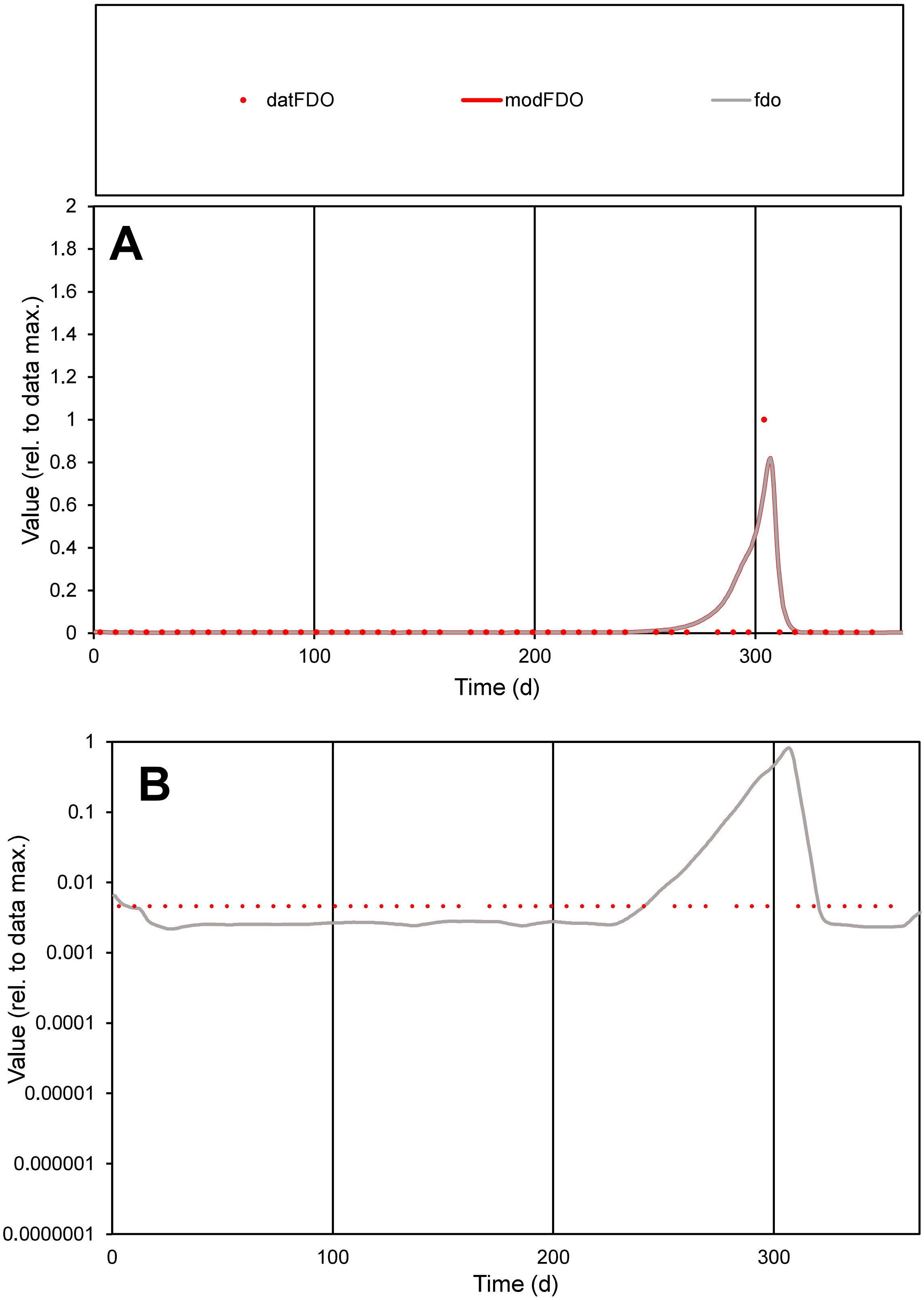

C1

Variables
FDO Provide jtd or Dst
CalibOnly?
2 1 to reproduce of
oNormalize?
2 1=no, 2=yes

ofxn calc*
2.72681
ofxn mod
2.91462

sRun
RunA1
zout
1

Ave
Data
5.5E-07
Model
7.6E-07

data/vMax
2.2E-05

ofxng
369.894

Min d/m
2.07519

ave/min d
5.51888
ave/min m
15.9153

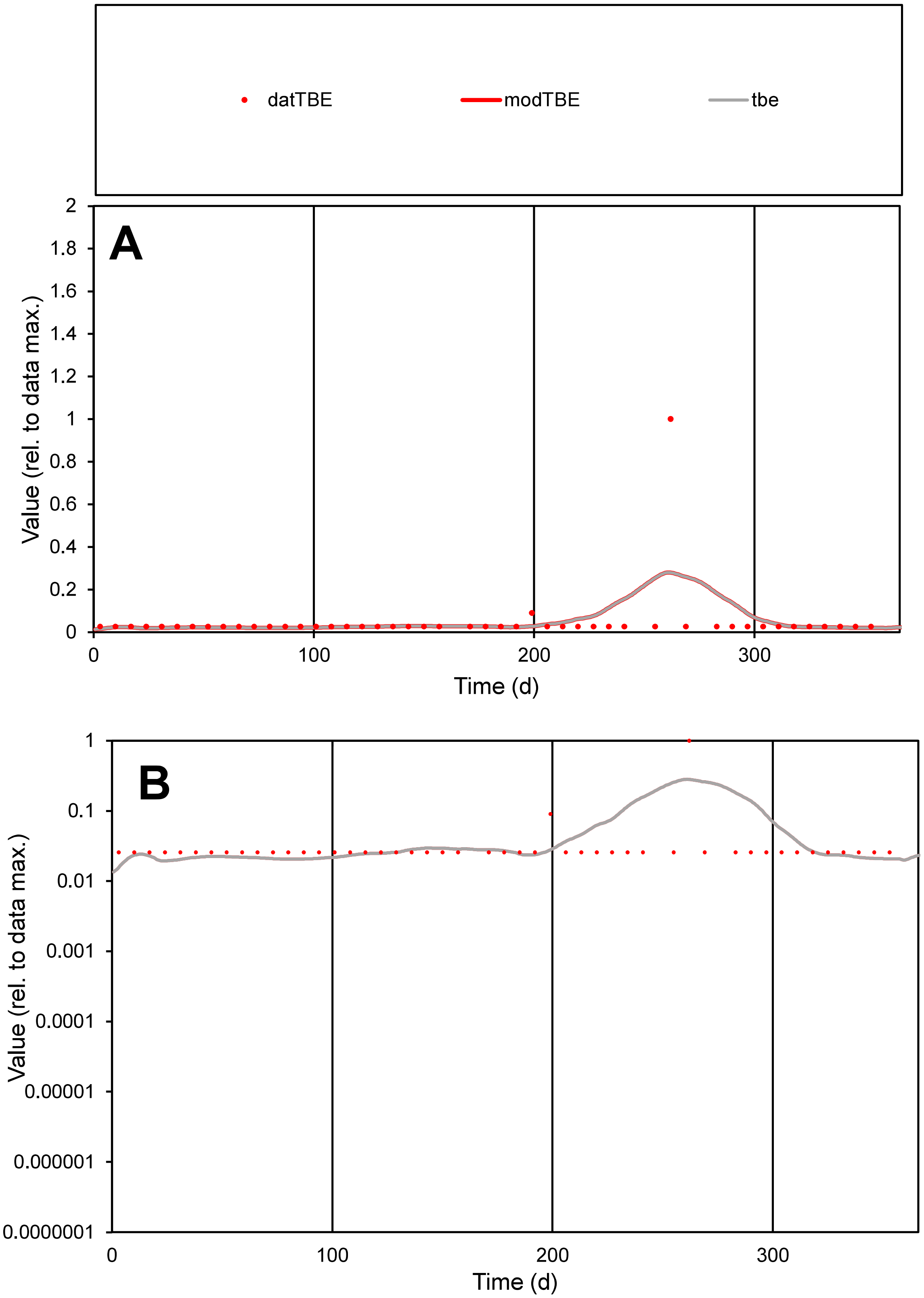

C1

Variables
TBE Provide jtd or Dst
CalibOnly?
2 1 to reproduce of
oNormalize?
2 1=no, 2=yes

ofxn calc*
2.54393
ofxn mod
2.56066

sRun
RunA1
zout
1

Ave
Data
1.2E-05
Model
1.4E-05

data/vMax
0.00026

ofxng
369.894

Min d/m
1.47318

ave/min d
1.84341
ave/min m
3.03934

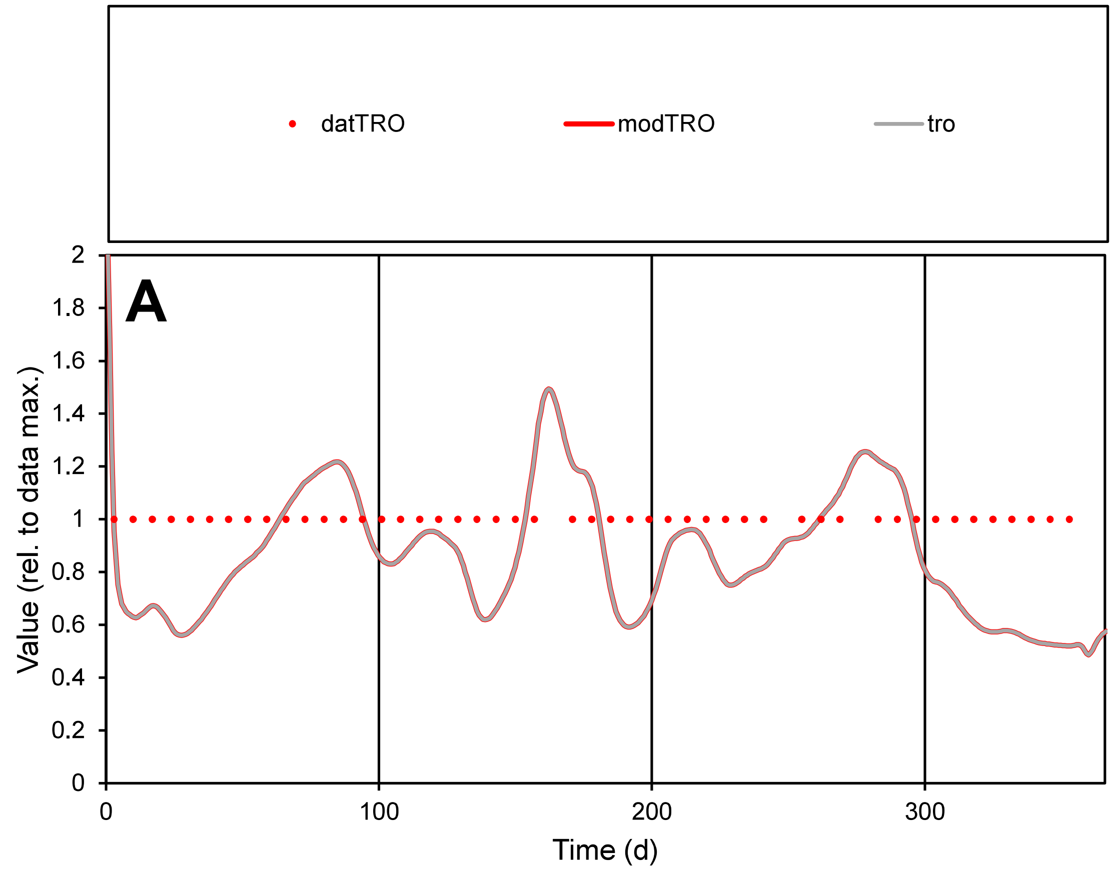

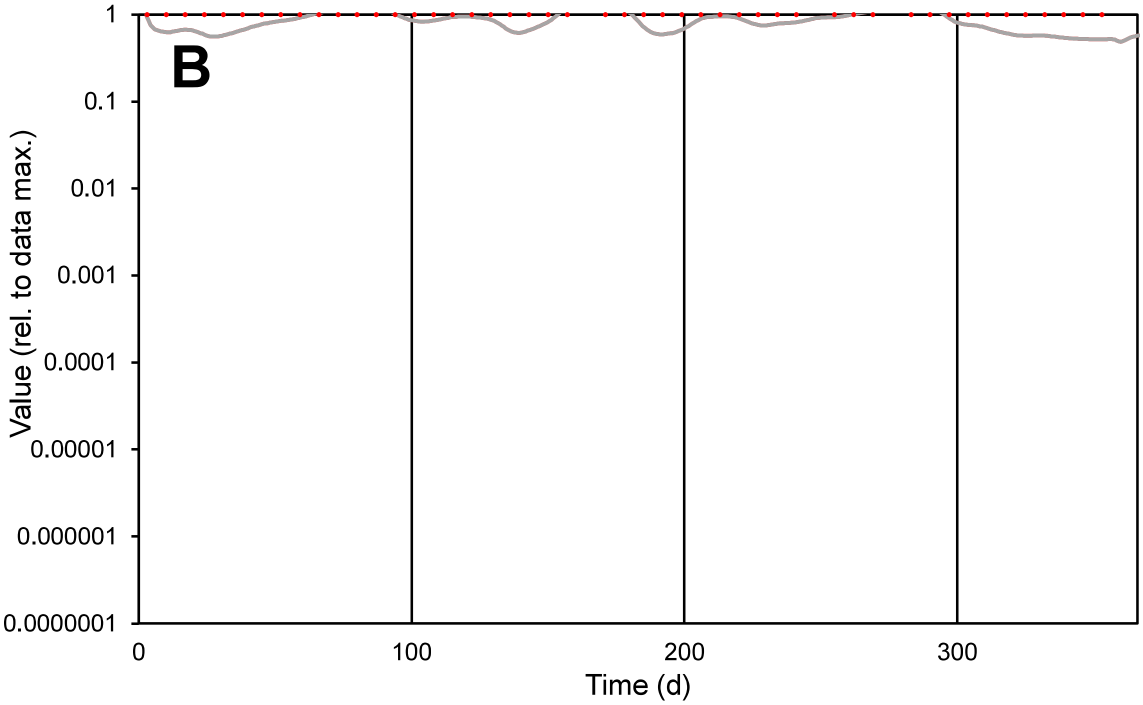

C1

Variables
TRO Provide jtd or Dst
CalibOnly?
2 1 to reproduce of
oNormalize?
2 1=no, 2=yes

ofxn calc*
0.26115
ofxn mod
0.28348

sRun
RunA1
zout
1

Ave
Data
5E-06
Model
4.2E-06

data/vMax
5E-06

ofxng
369.894

Min d/m
1.91762

ave/min d
1
ave/min m
1.61891

328_Calib_S_A

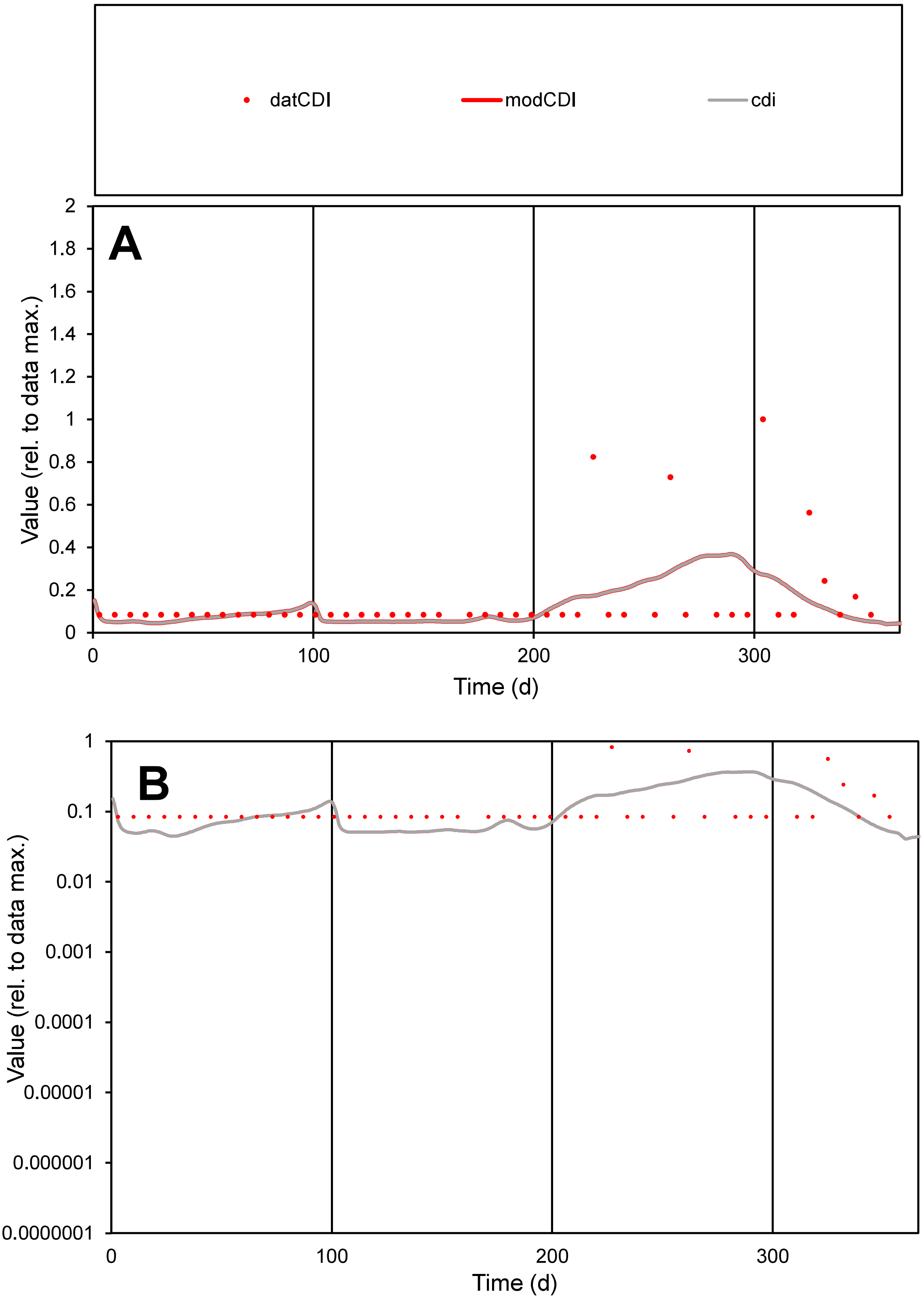

C1

Variables
CDI Provide jtd or Dst
CalibOnly?
2 1 to reproduce of
oNormalize?
2 1=no, 2=yes

ofxn calc*
1.29001
ofxn mod
1.30023

sRun
RunA1
zout
1

Ave
Data
3E-07
Model
2.5E-07

data/vMax
2.1E-06

ofxng
369.894

Min d/m
1.8036

ave/min d
1.74844
ave/min m
2.63561

328_Calib_S_A

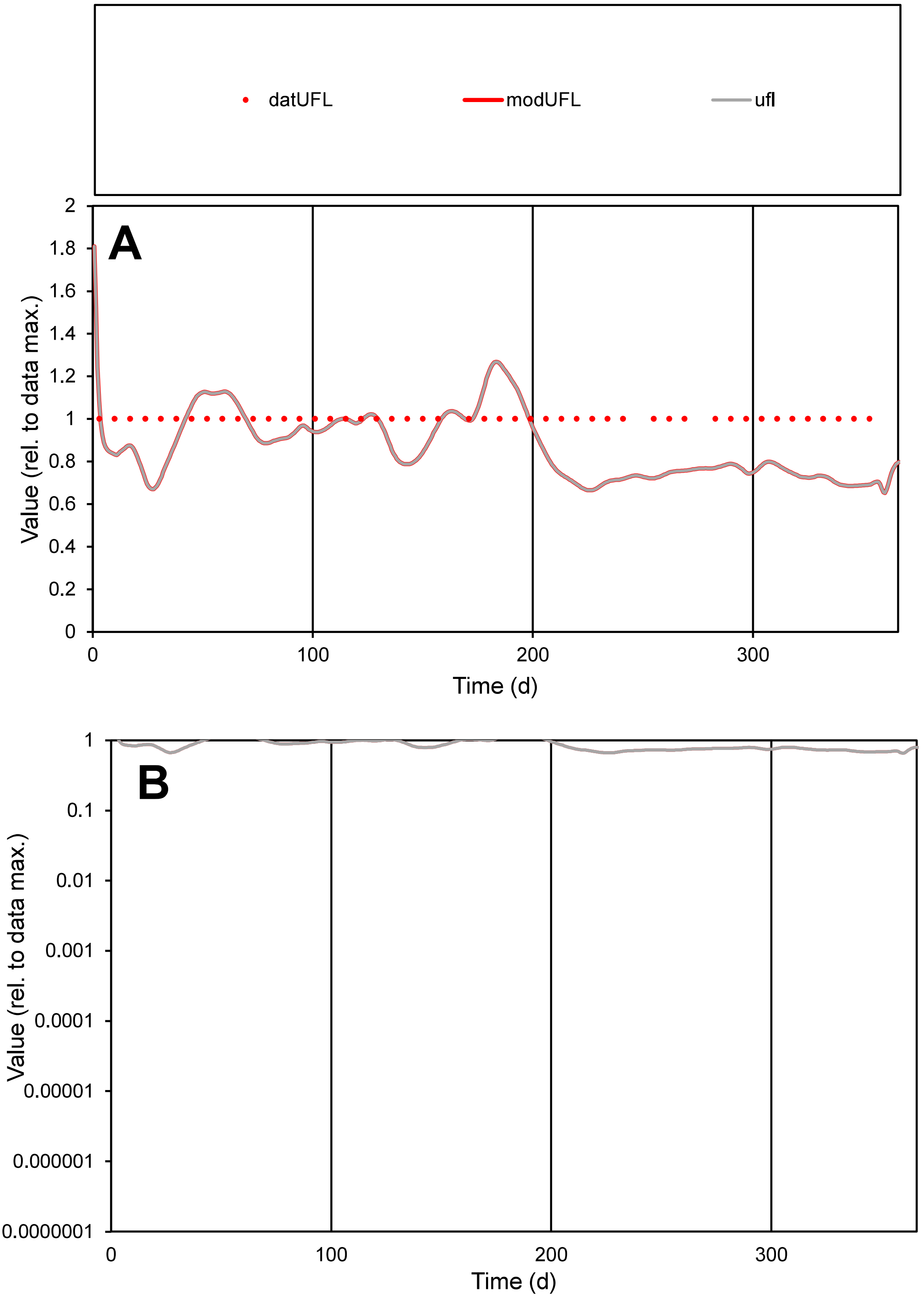

C1

Variables
UFL Provide jtd or Dst
CalibOnly?
2 1 to reproduce of
oNormalize?
2 1=no, 2=yes

ofxn calc*
0.19993
ofxn mod
0.22651

sRun
RunA1
zout
1

Ave
Data
3.1E-06
Model
2.7E-06

data/vMax
3.1E-06

ofxng
369.894

Min d/m
1.4922

ave/min d
1
ave/min m
1.29825

328_Calib_S_A

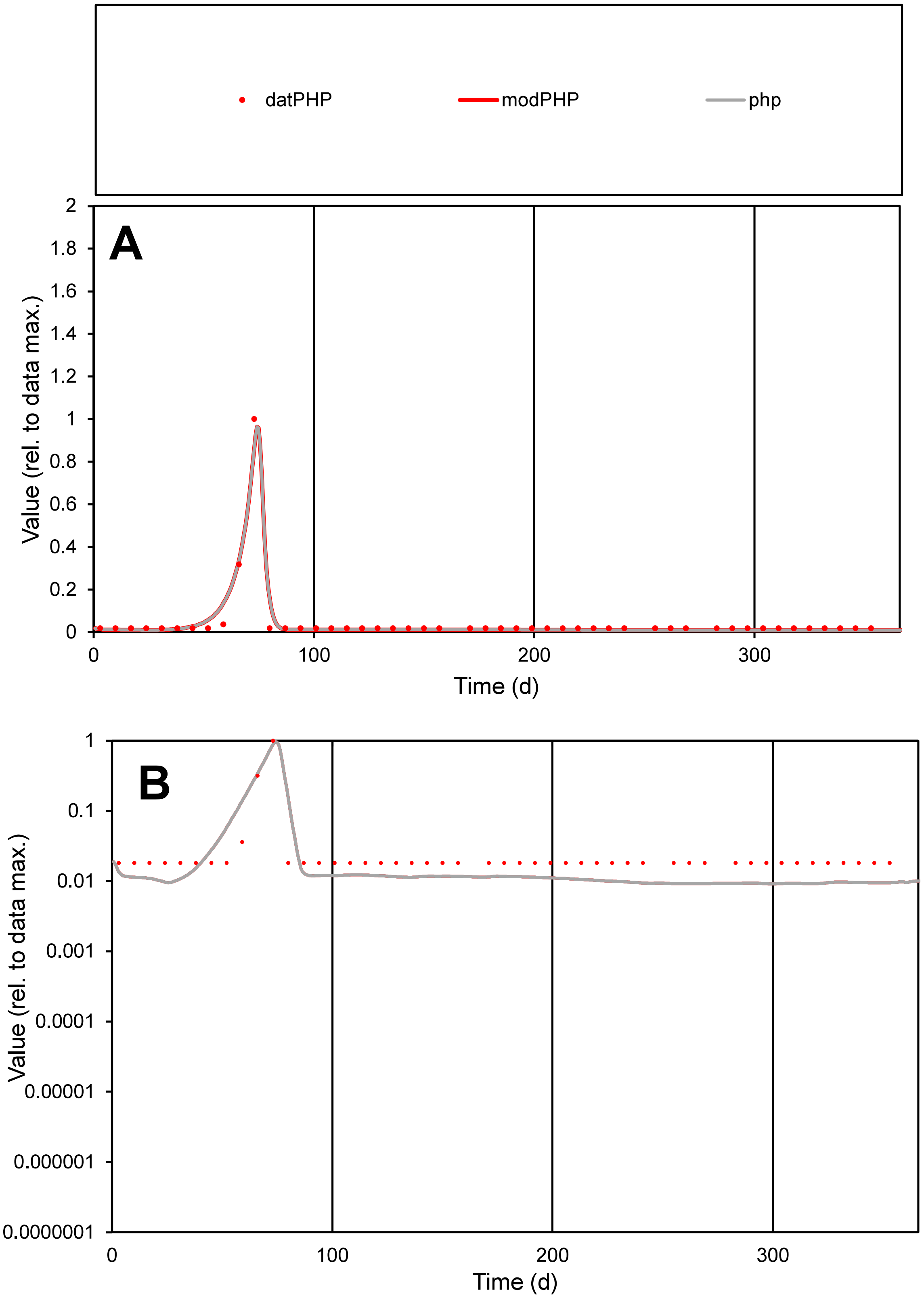

C1

Variables

PHP Provide jtd or Dst

CalibOnly?

2 1 to reproduce of

oNormalize?

2 1=no, 2=yes

ofxn calc*

0.39131

ofxn mod

0.53422

sRun

RunA1

zout

1

Ave

Data

1.7E-05

Model

1.6E-05

data/vMax

0.00038

ofxng

369.894

Min d/m

1.98088

ave/min d

2.49641

ave/min m

4.6186

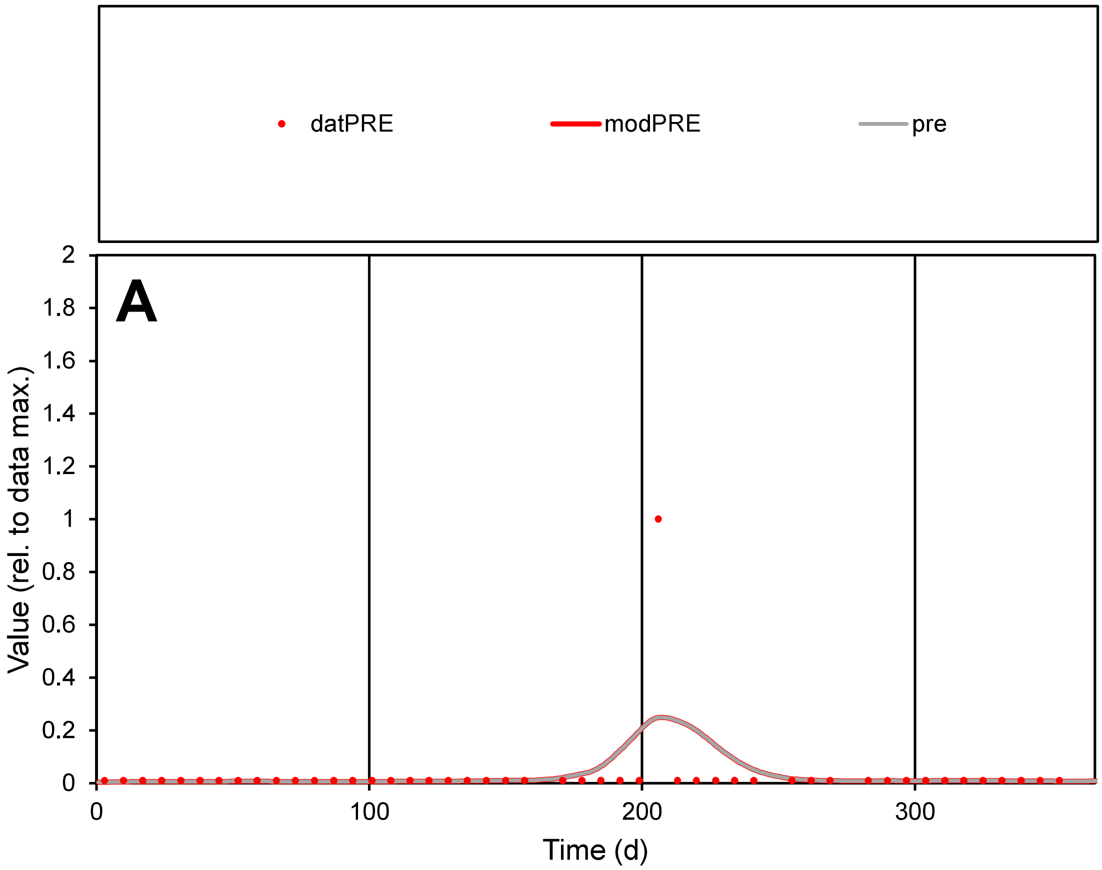

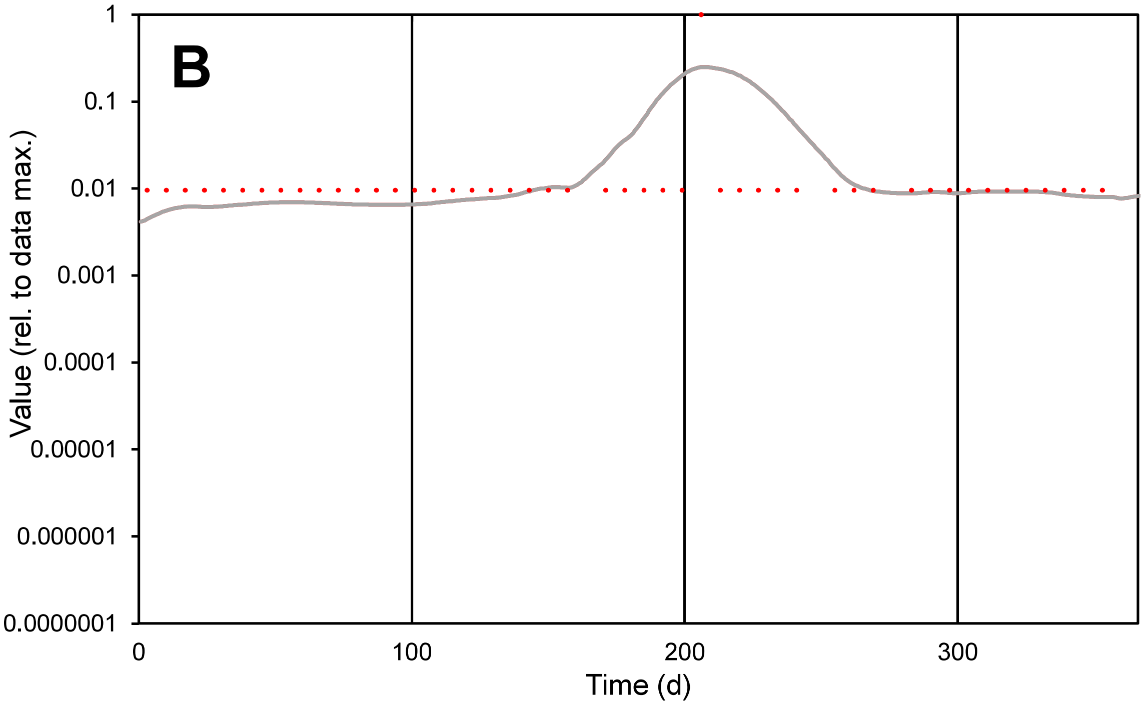

C1

Variables
PRE Provide jtd or Dst
CalibOnly?
2 1 to reproduce of
oNormalize?
2 1=no, 2=yes

ofxn calc*
3.99445
ofxn mod
4.04941

sRun
RunA1
zout
1

Ave
Data
4.2E-05
Model
4.7E-05

data/vMax
0.0014

ofxng
369.894

Min d/m
2.05258

ave/min d
3.14937
ave/min m
7.10754

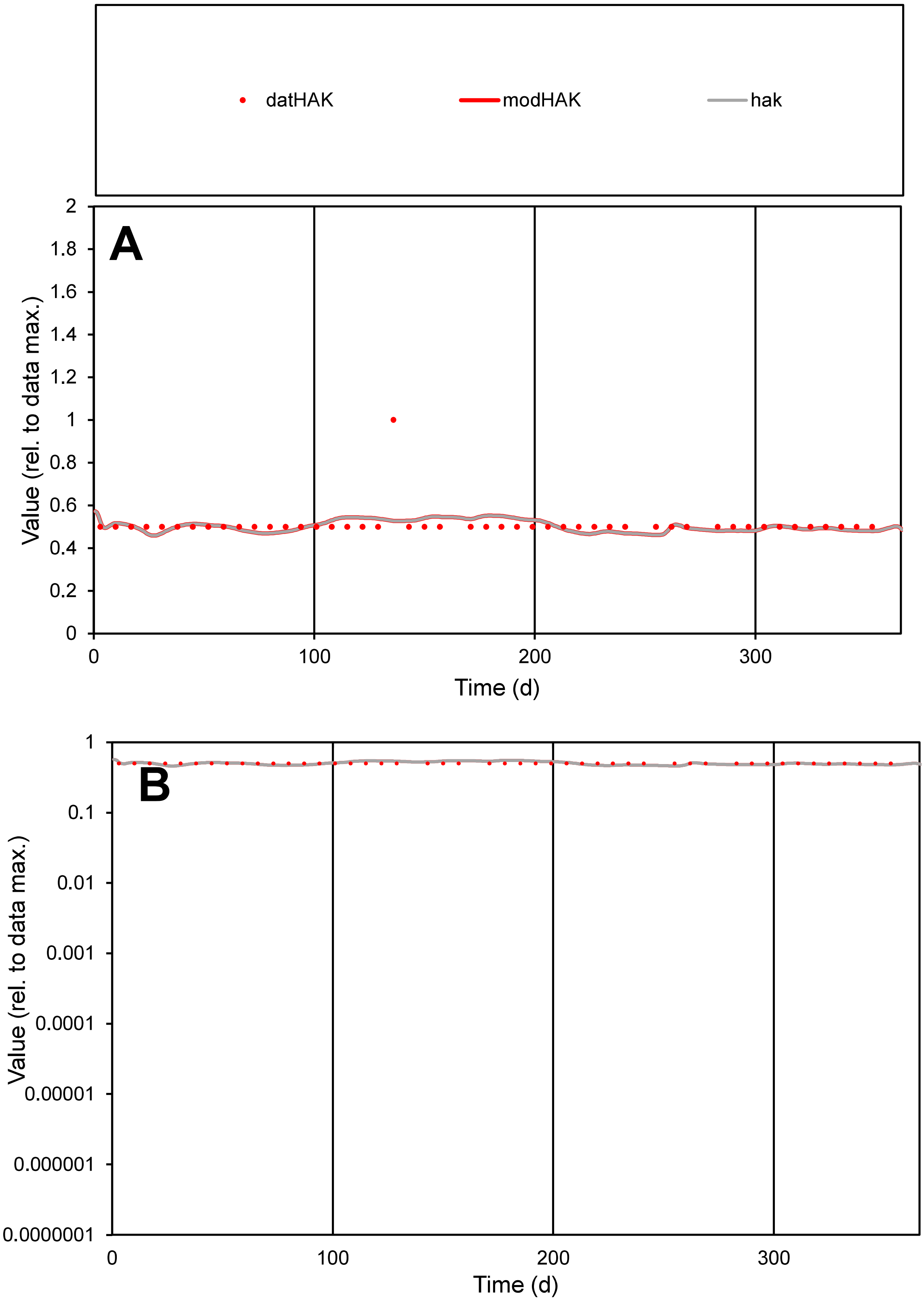

C1

Variables
HAK Provide jtd or Dst
CalibOnly?
2 1 to reproduce of
oNormalize?
2 1=no, 2=yes

ofxn calc*
0.14304
ofxn mod
0.18173

sRun
RunA1
zout
1

Ave
Data
5.2E-06
Model
5.1E-06

data/vMax
1E-05

ofxng
369.894

Min d/m
1.08141

ave/min d
1.02083
ave/min m
1.08711

328_Calib_S_A

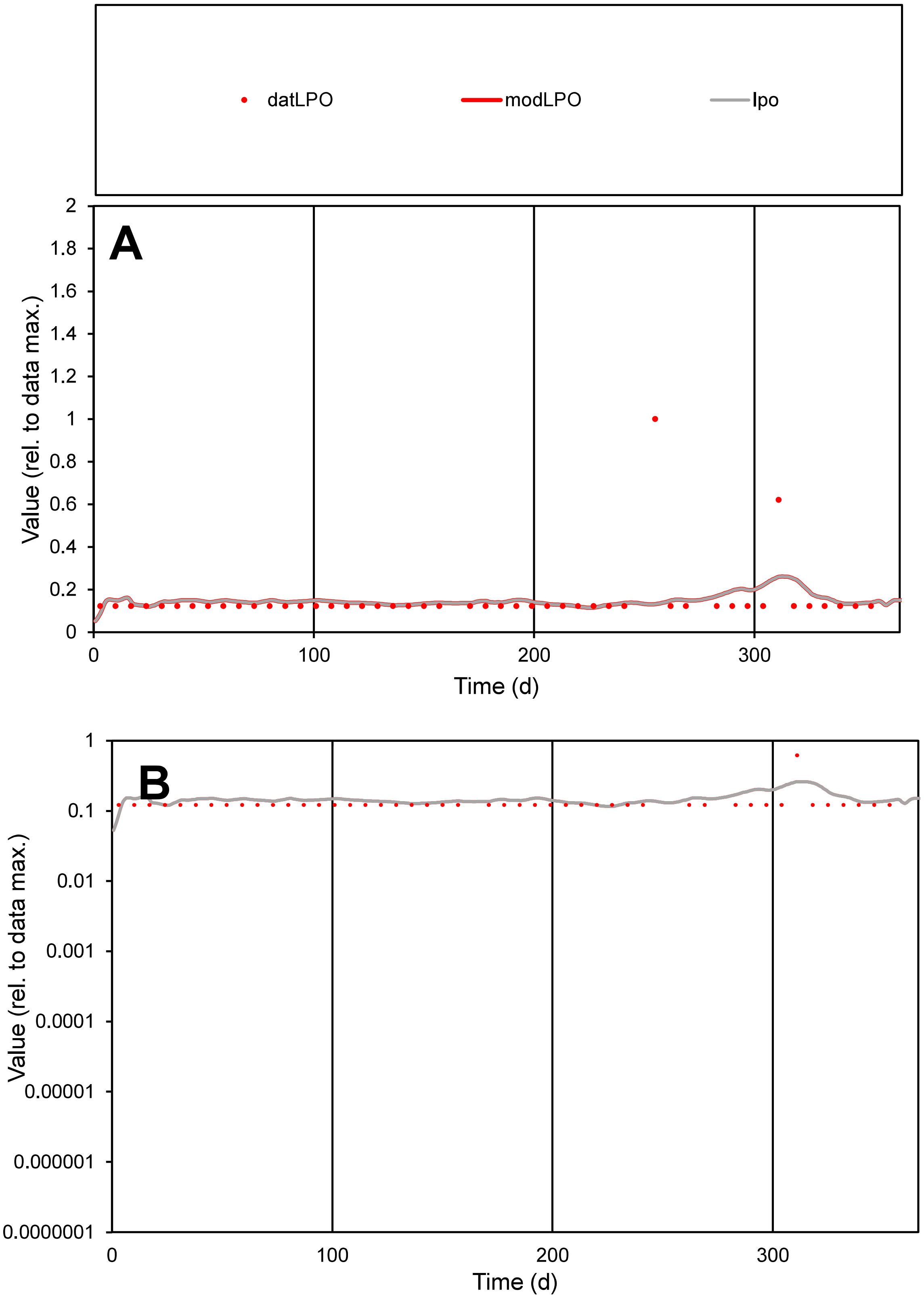

C1

Variables
LPO Provide jtd or Dst
CalibOnly?
2 1 to reproduce of
oNormalize?
2 1=no, 2=yes

ofxn calc*
0.92832
ofxn mod
0.93859

sRun
RunA1
zout
1

Ave
Data
7.2E-06
Model
7.2E-06

data/vMax
4.8E-05

ofxng
369.894

Min d/m
1.0383

ave/min d
1.23561
ave/min m
1.27113

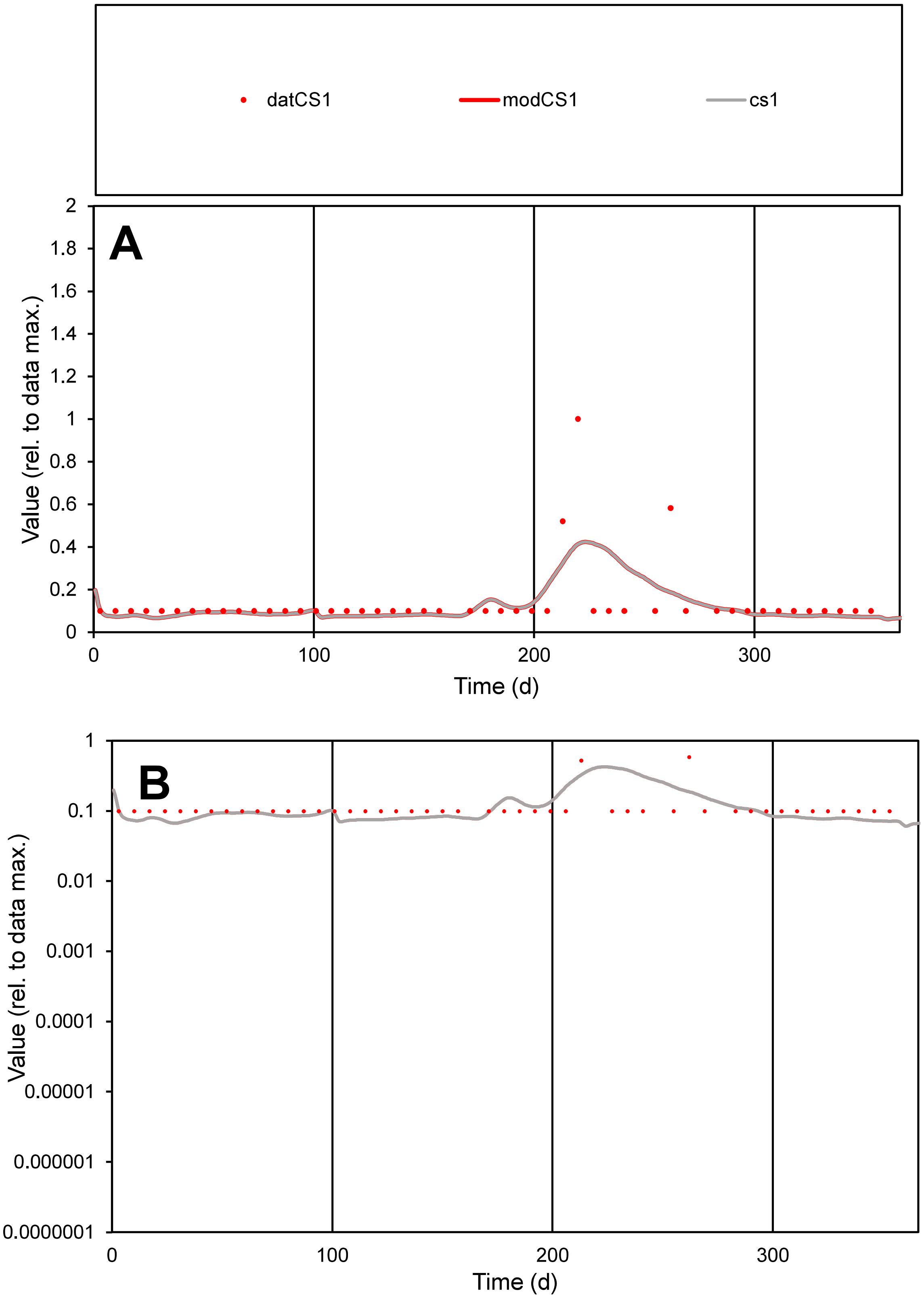

C1

Variables
CS1 Provide jtd or Dst
CalibOnly?
2 1 to reproduce of
oNormalize?
2 1=no, 2=yes

ofxn calc*
0.93745
ofxn mod
0.94755

sRun
RunA1
zout
1

Ave
Data
2.4E-05
Model
2.2E-05

data/vMax
0.00018

ofxng
369.894

Min d/m
1.4016

ave/min d
1.38108
ave/min m
1.79738

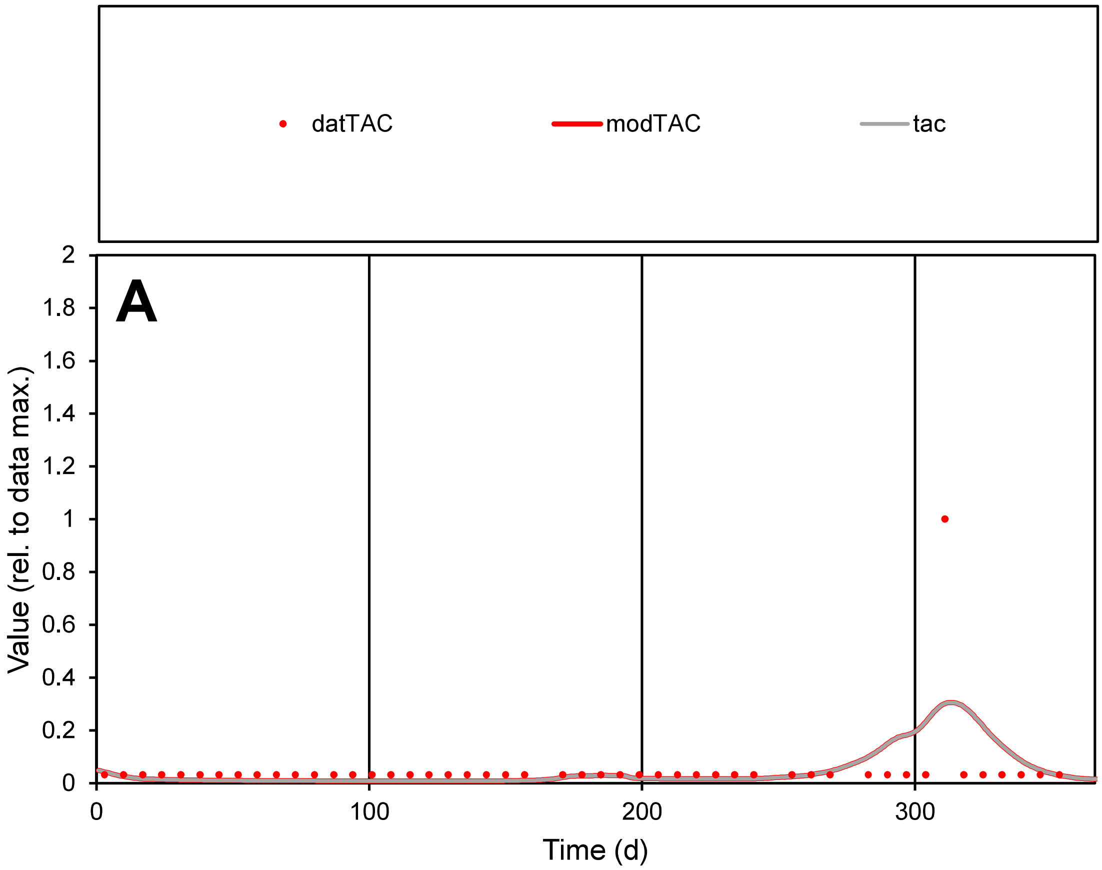

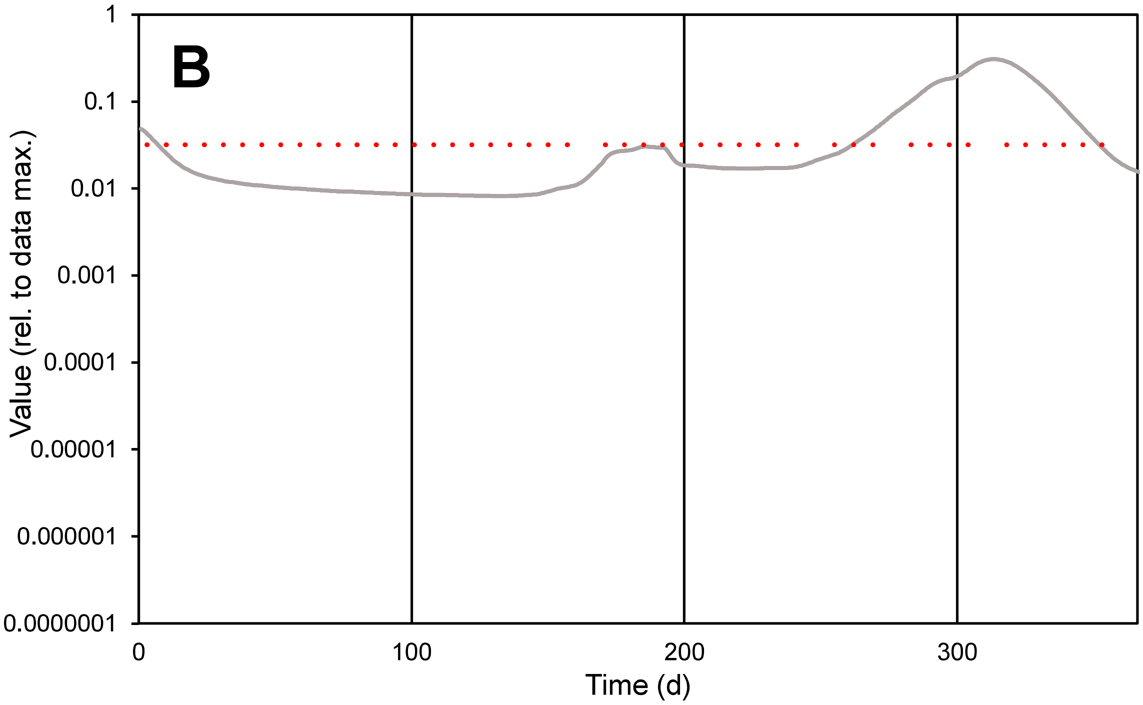

C1

Variables
TAC Provide jtd or Dst
CalibOnly?
2 1 to reproduce of
oNormalize?
2 1=no, 2=yes

ofxn calc*
2.26369
ofxn mod
2.28866

sRun
RunA1
zout
1

Ave
Data
2.1E-06
Model
2E-06

data/vMax
4.1E-05

ofxng
369.894

Min d/m
3.88655

ave/min d
1.63264
ave/min m
5.85609

328_Calib_S_A

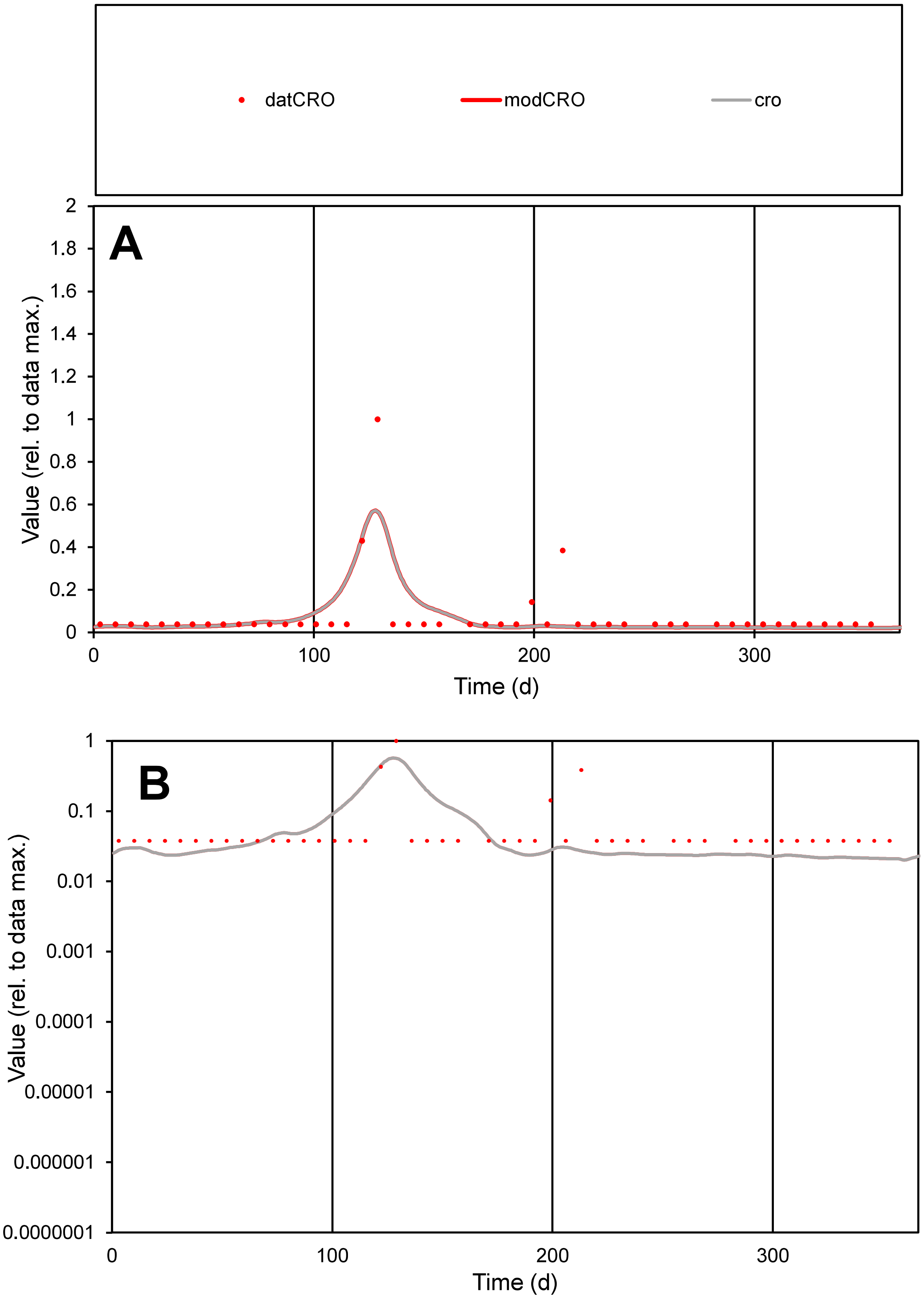

C1

Variables
CRO Provide jtd or Dst
CalibOnly?
2 1 to reproduce of
oNormalize?
2 1=no, 2=yes

ofxn calc*
1.33457
ofxn mod
1.3867

sRun
RunA1
zout
1

Ave
Data
2E-05
Model
1.9E-05

data/vMax
0.00027

ofxng
369.894

Min d/m
1.79744

ave/min d
1.99799
ave/min m
3.26882

328_Calib_S_A

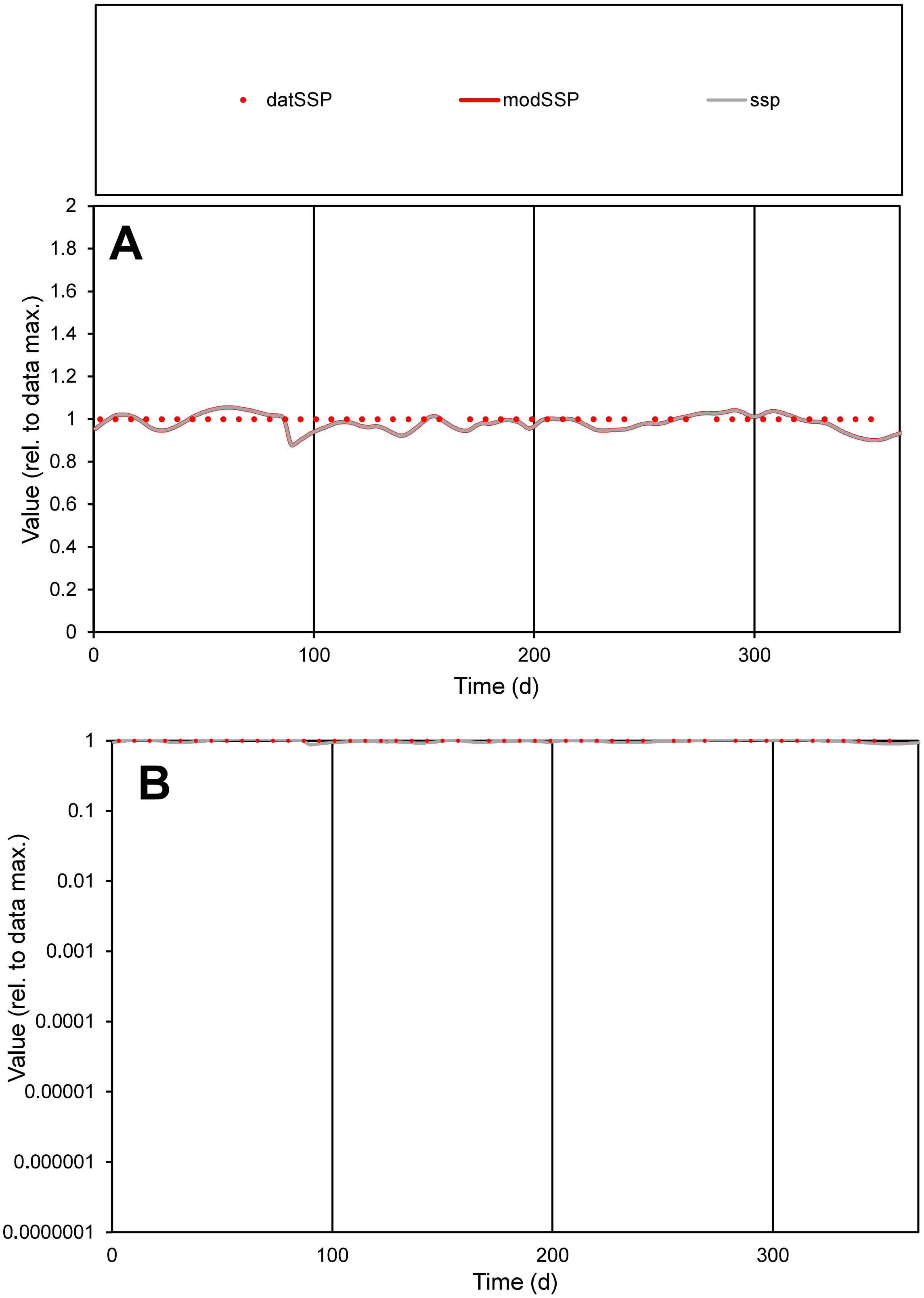

C1

Variables
SSP Provide jtd or Dst
CalibOnly?
2 1 to reproduce of
oNormalize?
2 1=no, 2=yes

ofxn calc*
0.04017
ofxn mod
0.11915

sRun
RunA1
zout
1

Ave
Data
1.3E-05
Model
1.3E-05

data/vMax
1.3E-05

ofxng
369.894

Min d/m
1.1099

ave/min d
1
ave/min m
1.09324

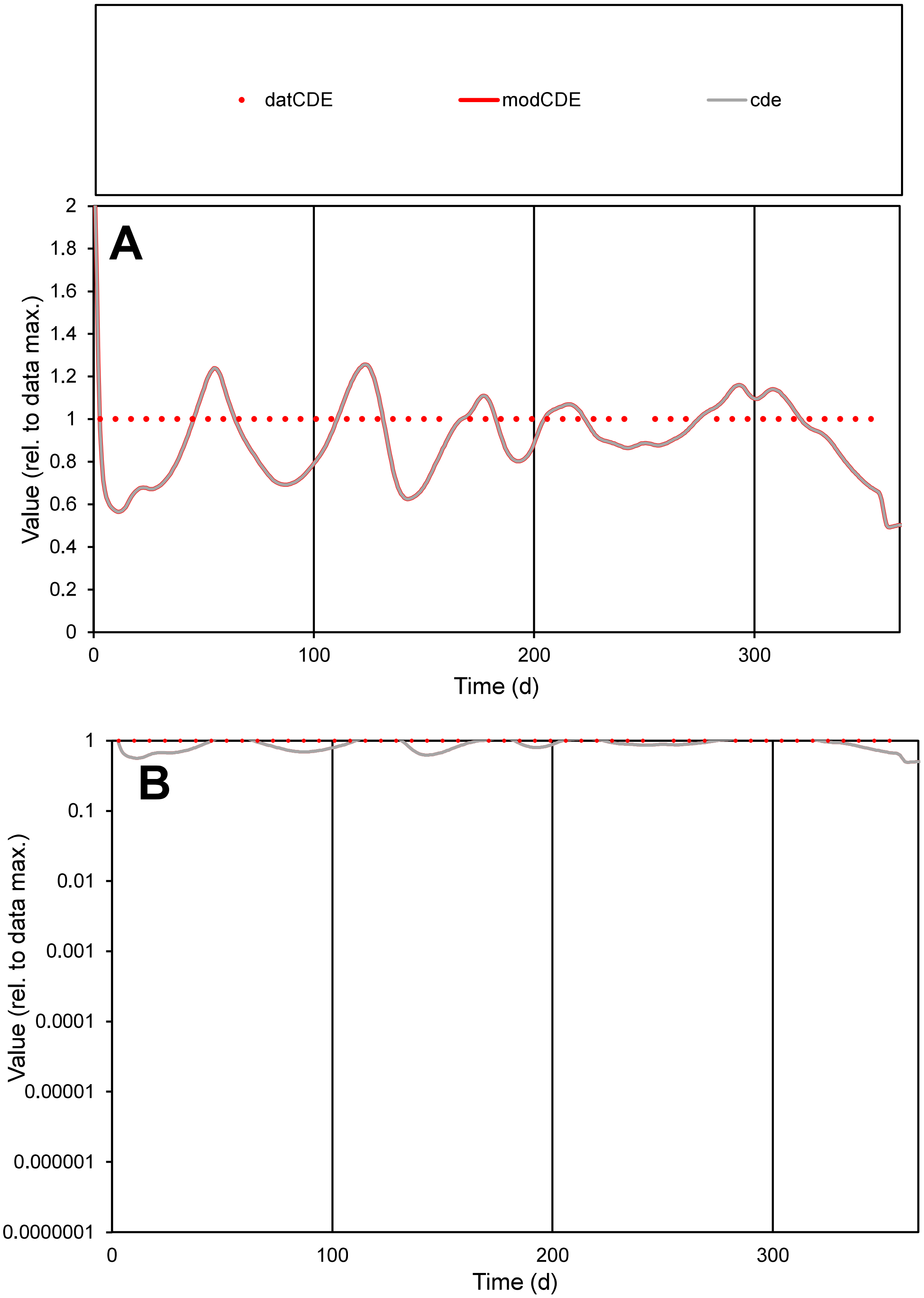

C1

Variables
CDE Provide jtd or Dst
CalibOnly?
2 1 to reproduce of
oNormalize?
2 1=no, 2=yes

ofxn calc*
0.19447
ofxn mod
0.22252

sRun
RunA1
zout
1

Ave
Data
1E-05
Model
9.1E-06

data/vMax
1E-05

ofxng
369.894

Min d/m
1.77043

ave/min d
1
ave/min m
1.6088

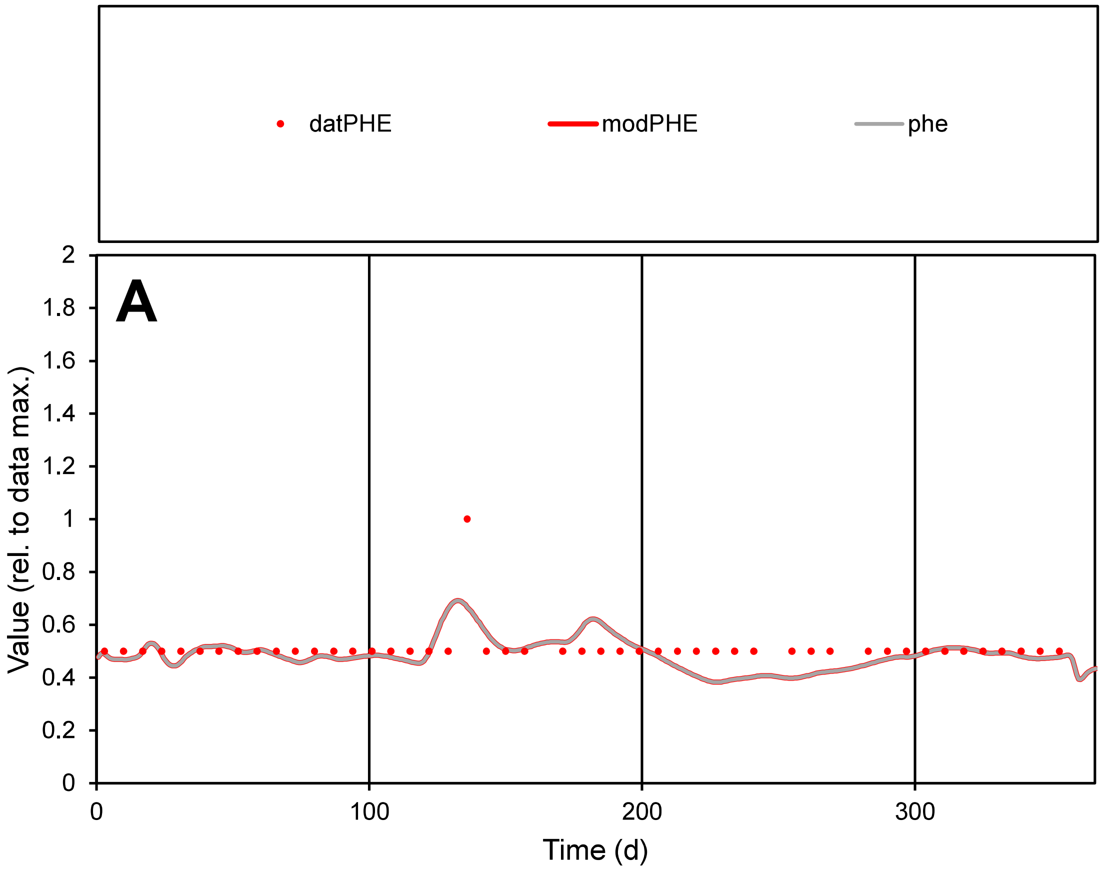

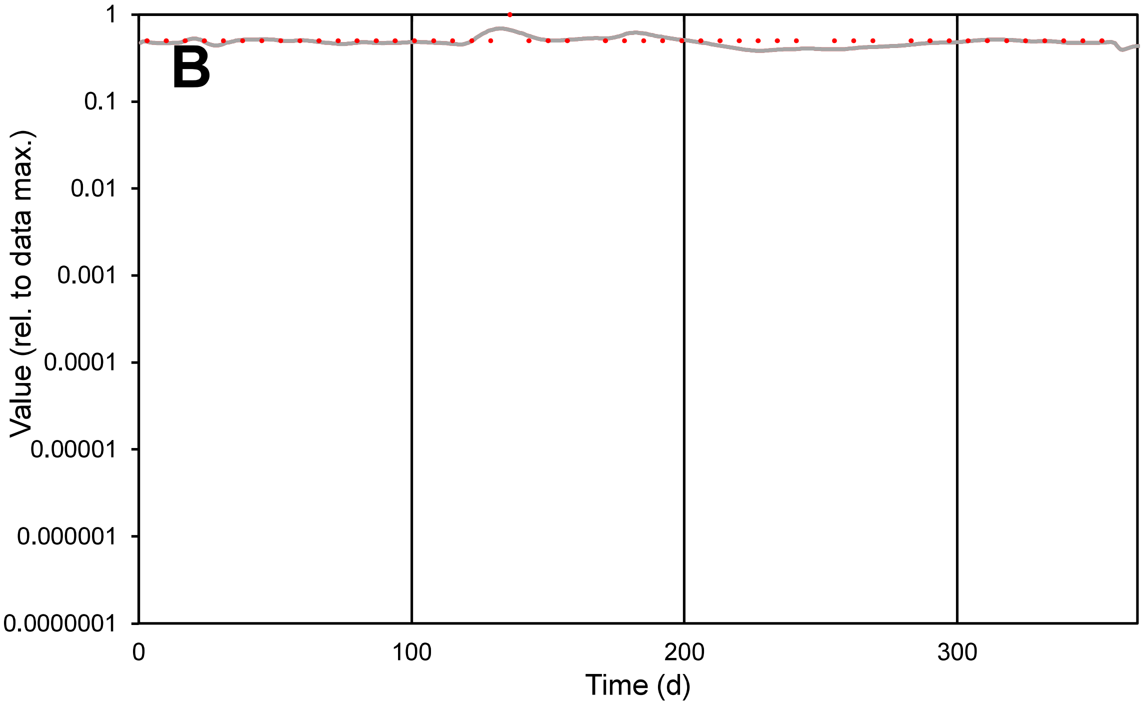

C1

Variables
PHE Provide jtd or Dst
CalibOnly?
2 1 to reproduce of
oNormalize?
2 1=no, 2=yes

ofxn calc*
0.14755
ofxn mod
0.18416

sRun
RunA1
zout
1

Ave
Data
4E-06
Model
3.9E-06

data/vMax
7.9E-06

ofxng
369.894

Min d/m
1.30365

ave/min d
1.02084
ave/min m
1.28004

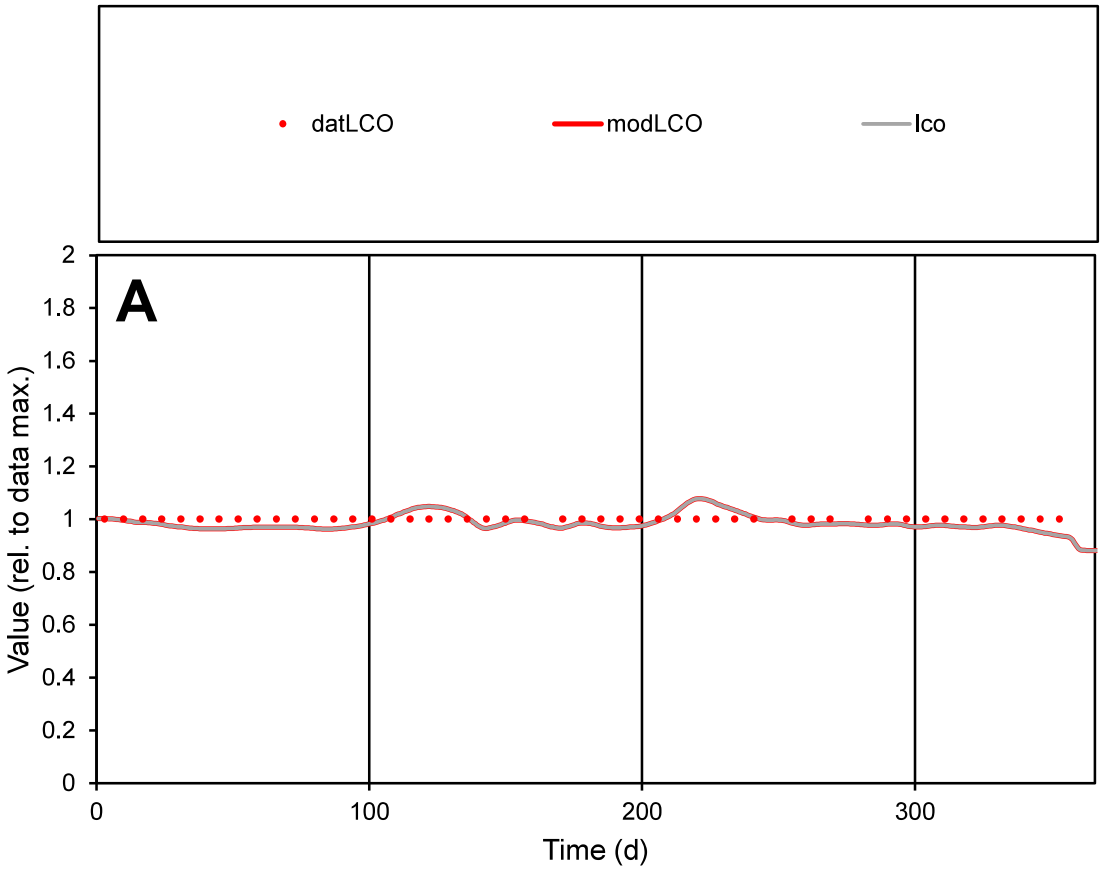

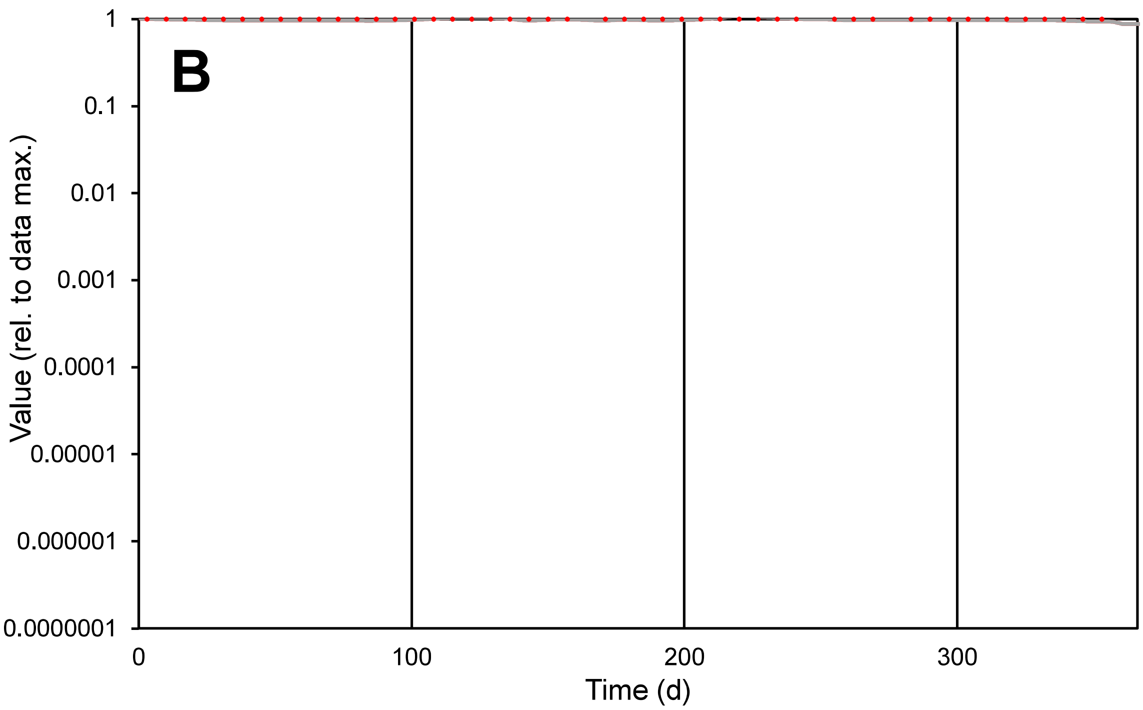

C1

Variables

LCO Provide jtd or Dst

CalibOnly?

2 1 to reproduce of

oNormalize?

2 1=no, 2=yes

ofxn calc*

0.03157

ofxn mod

0.11673

sRun

RunA1

zout

1

Ave

Data

7.4E-06

Model

7.3E-06

data/vMax

7.4E-06

ofxng

369.894

Min d/m

1.06791

ave/min d

1

ave/min m

1.05402

328_Calib_S_A

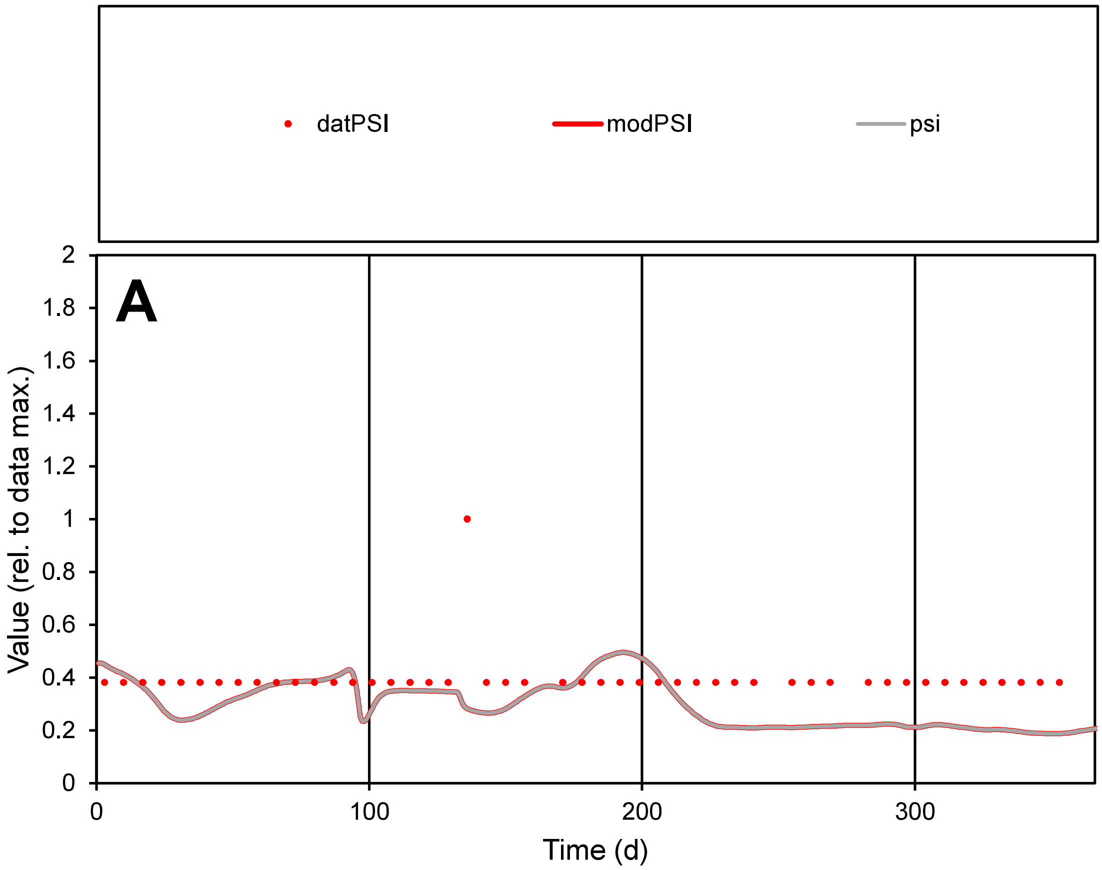

**C1**	
Variables	
PSI	Provide jtd or Dst
CalibOnly?	
2	1 to reproduce of
oNormalize?	
2	1=no, 2=yes
ofxn calc*	
0.3975	
ofxn mod	
0.41021	
sRun	
RunA1	
zout	
1	
Ave	
Data	
3.7E-06	
Model	
2.8E-06	
data/vMax	
9.3E-06	
ofxng	
369.894	
Min d/m	
2.01873	
ave/min d	
1.03379	
ave/min m	
1.59907	

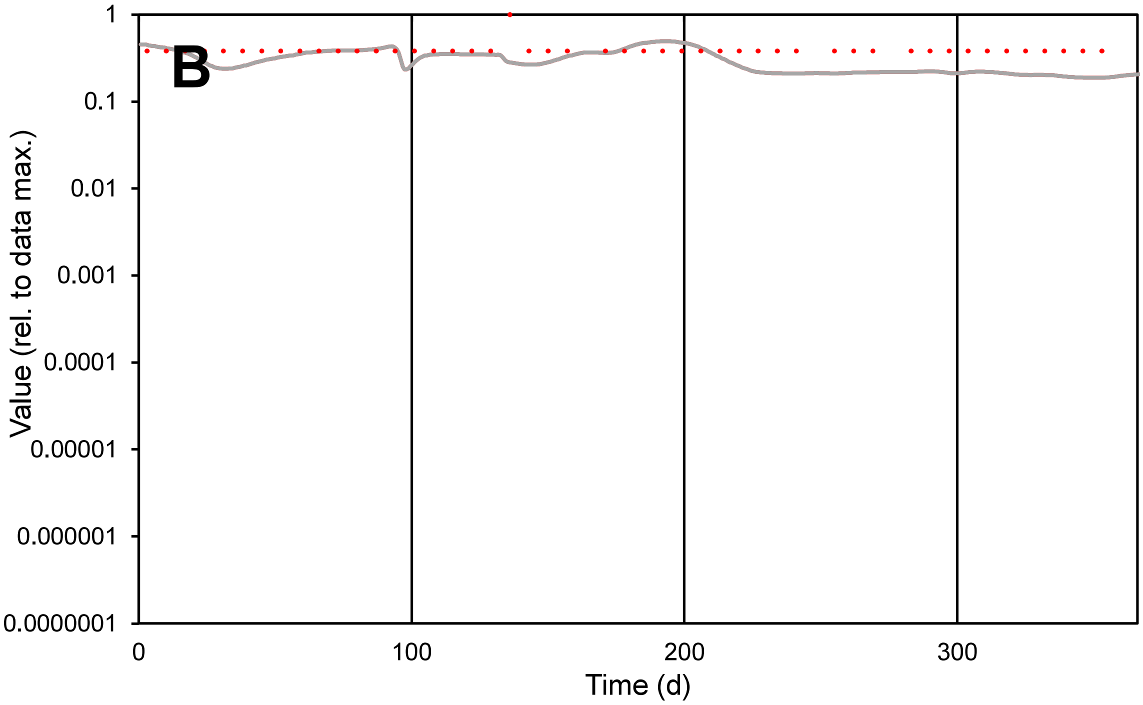

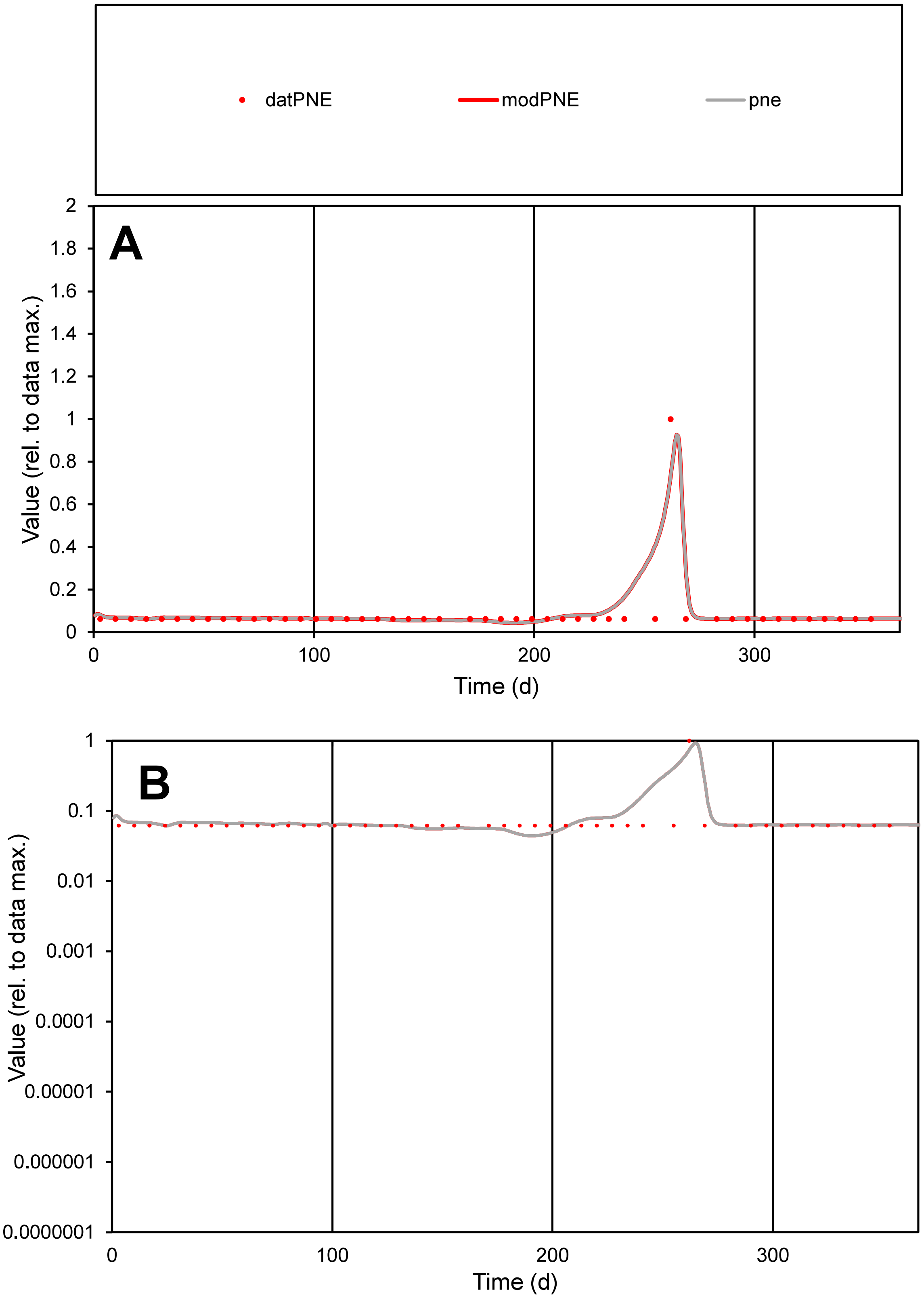

C1

Variables
PNE Provide jtd or Dst
CalibOnly?
2 1 to reproduce of
oNormalize?
2 1=no, 2=yes

ofxn calc*
0.70743
ofxn mod
0.75895

sRun
RunA1
zout
1

Ave
Data
1.9E-05
Model
2.1E-05

data/vMax
0.00023

ofxng
369.894

Min d/m
1.38418

ave/min d
1.31577
ave/min m
2.03023

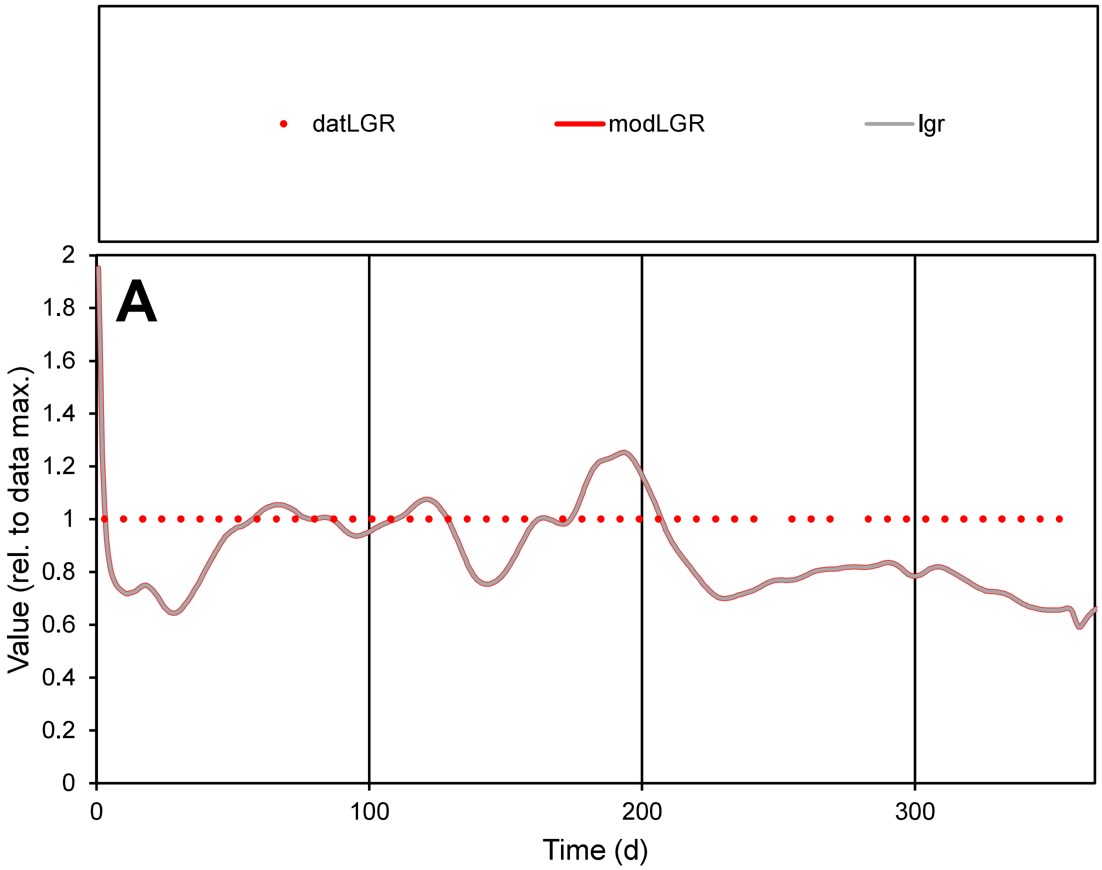

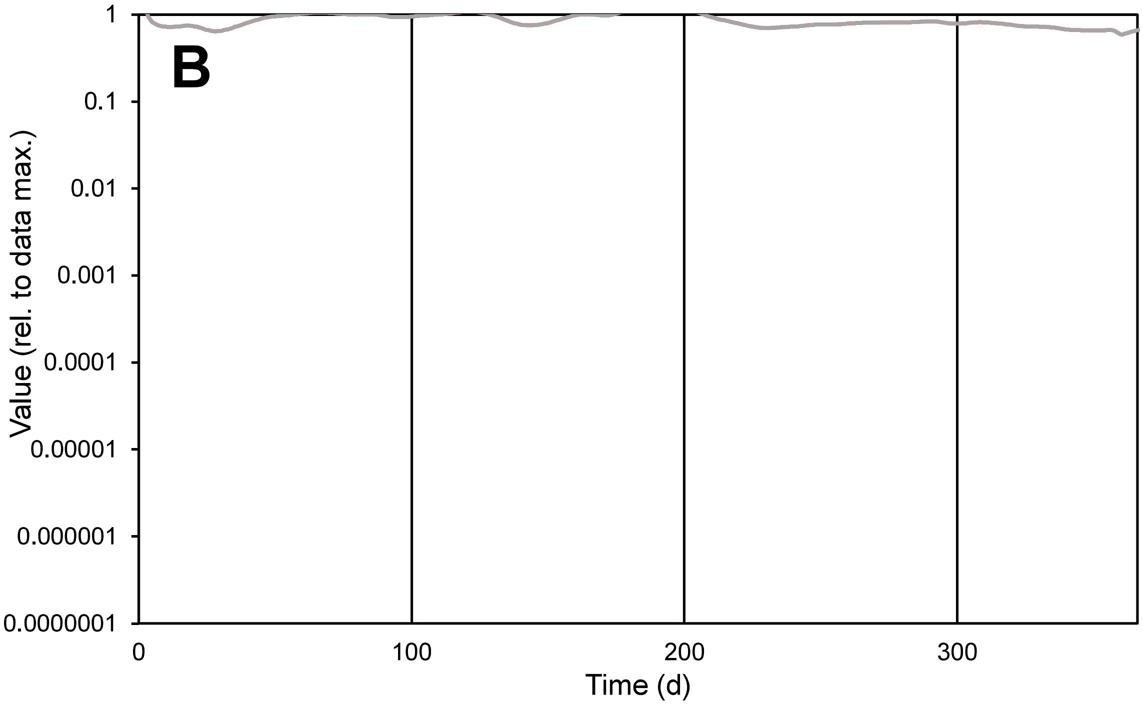

C1

Variables
LGR Provide jtd or Dst
CalibOnly?
2 1 to reproduce of
oNormalize?
2 1=no, 2=yes

ofxn calc*
0.19767
ofxn mod
0.22466

sRun
RunA1
zout
1

Ave
Data
5.2E-06
Model
4.5E-06

data/vMax
5.2E-06

ofxng
369.894

Min d/m
1.52137

ave/min d
1
ave/min m
1.3326

328_Calib_S_A

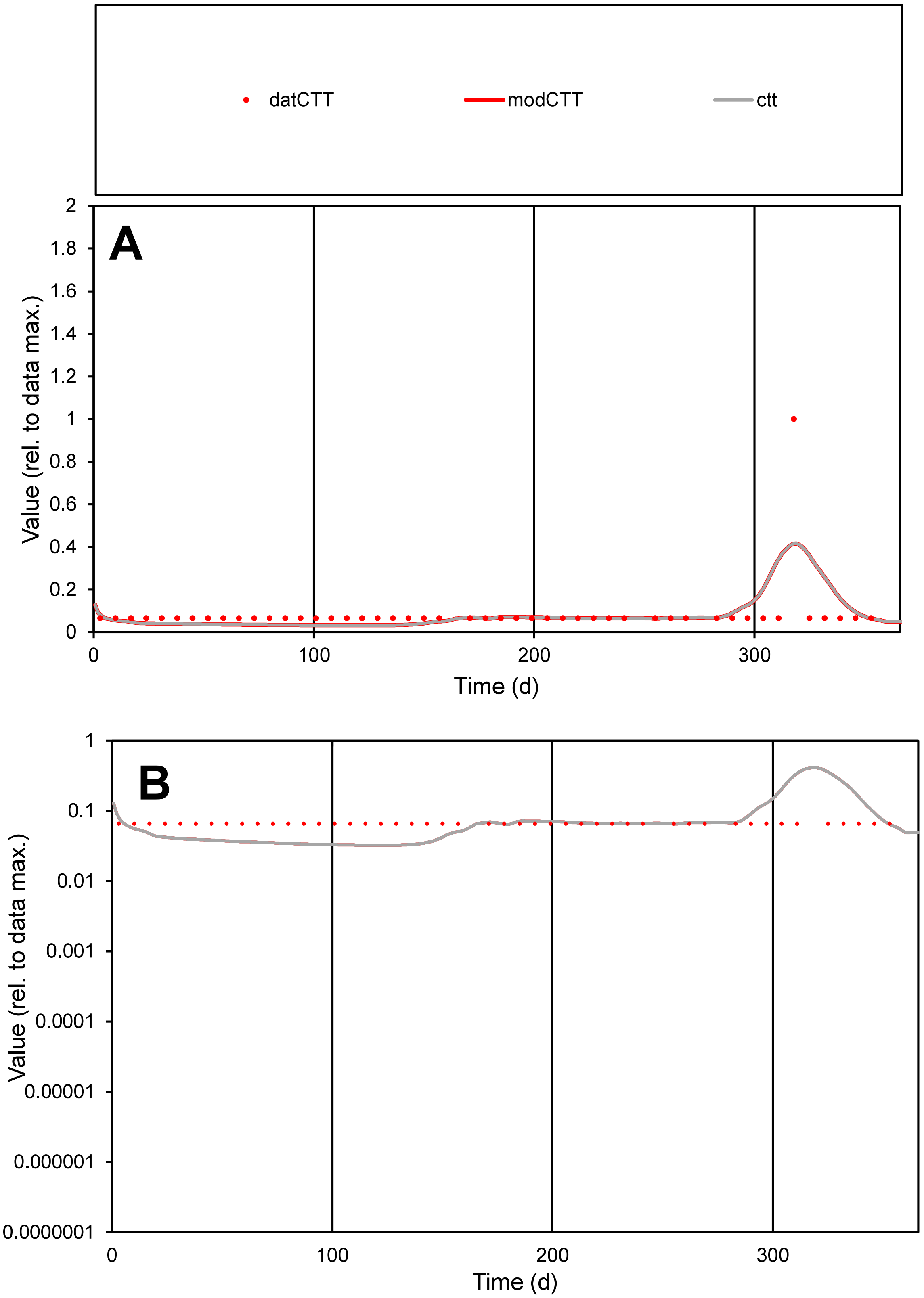

C1

Variables
CTT Provide jtd or Dst
CalibOnly?
2 1 to reproduce of
oNormalize?
2 1=no, 2=yes

ofxn calc*
1.27402
ofxn mod
1.29194

sRun
RunA1
zout
1

Ave
Data
1.9E-06
Model
1.9E-06

data/vMax
2.3E-05

ofxng
369.894

Min d/m
2.02568

ave/min d
1.29581
ave/min m
2.54042

328_Calib_S_A

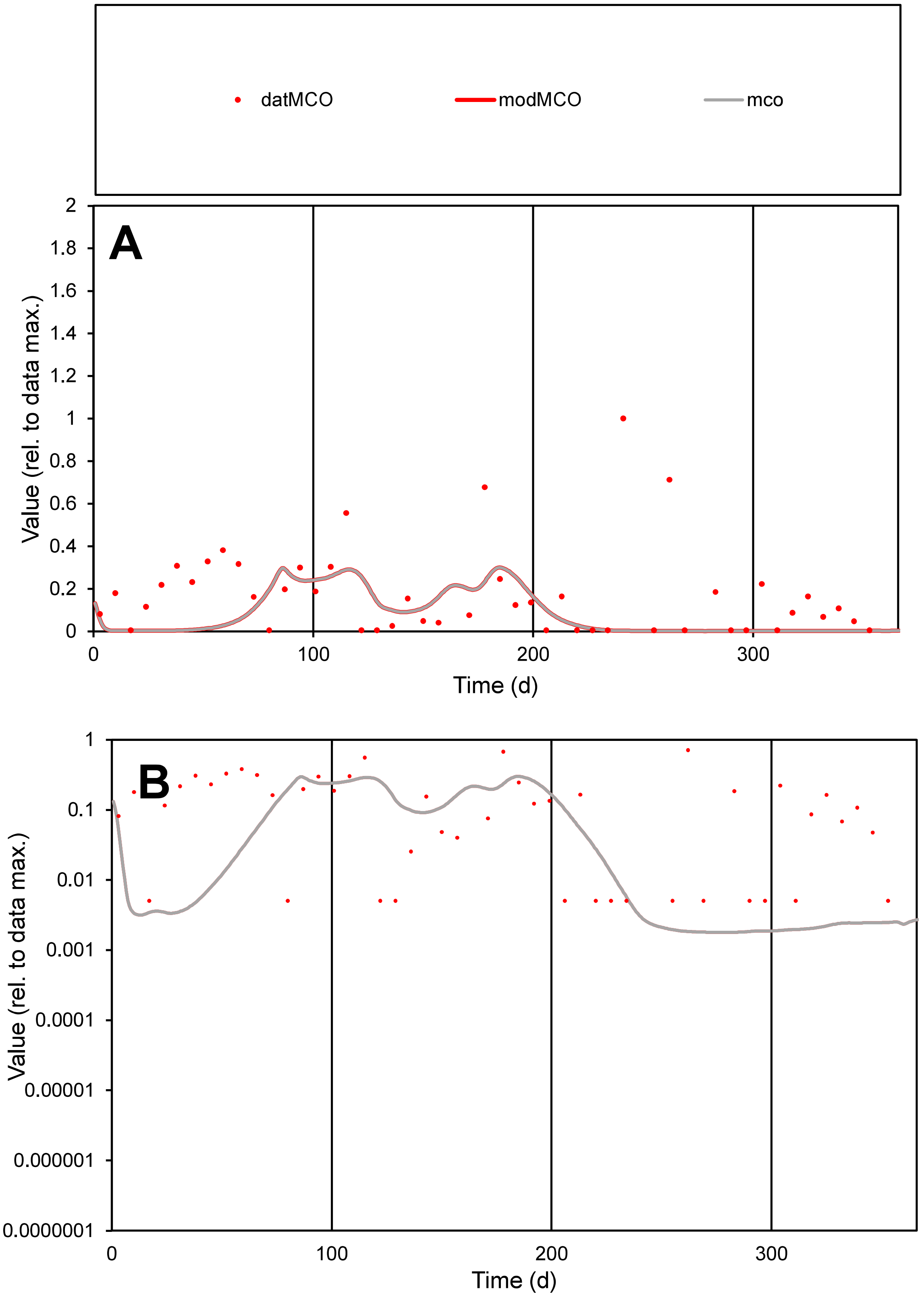

C1

Variables

MCO Provide jtd or Dst

CalibOnly?

2 1 to reproduce of

oNormalize?

2 1=no, 2=yes

ofxn calc*

1.34837

ofxn mod

1.36019

sRun

RunA1

zout

1

Ave

Data

2.1E-06

Model

1E-06

data/vMax

1.2E-05

ofxng

369.894

Min d/m

2.8102

ave/min d

33.9431

ave/min m

45.7761

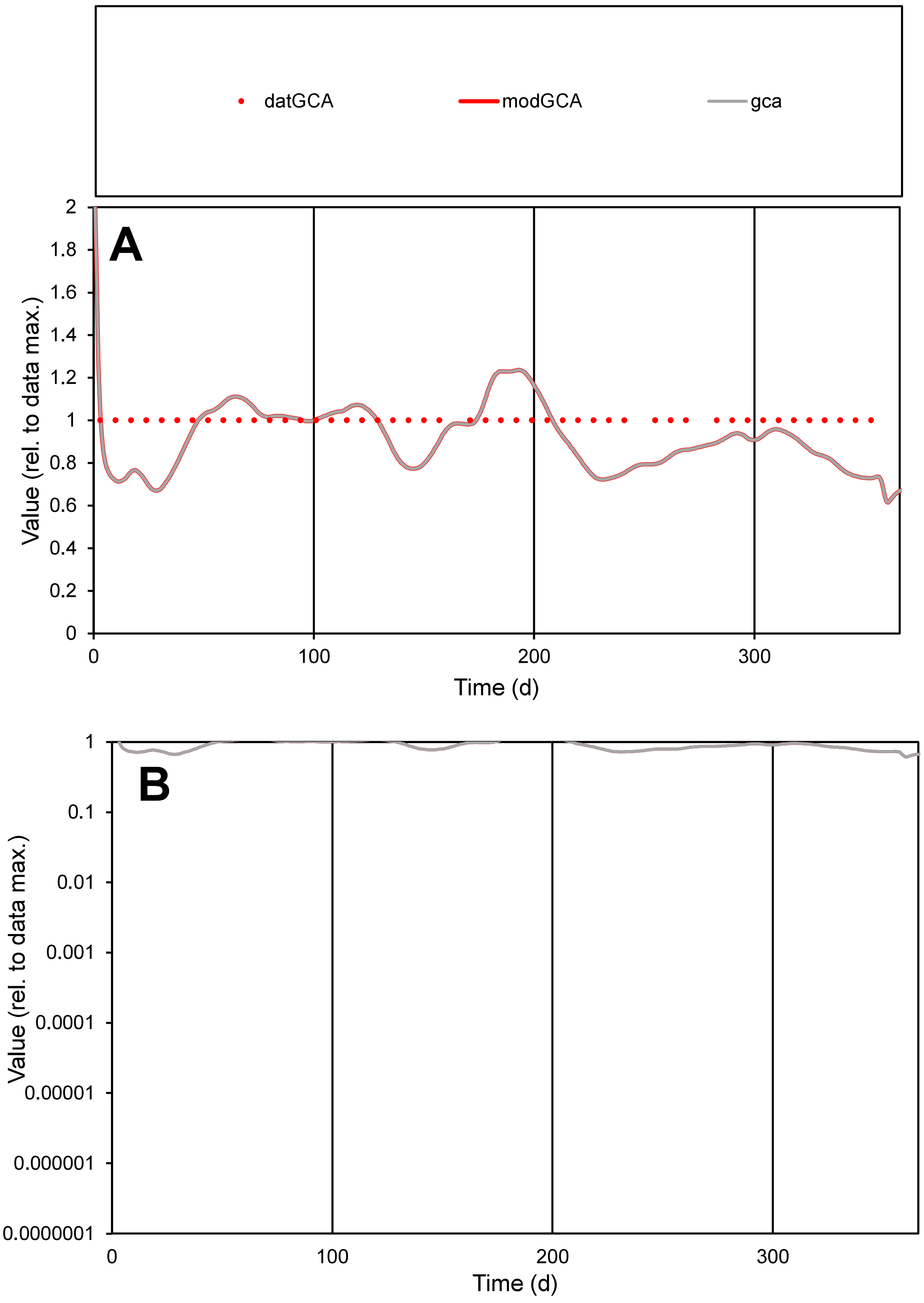

C1

Variables
GCA Provide jtd or Dst
CalibOnly?
2 1 to reproduce of
oNormalize?
2 1=no, 2=yes

ofxn calc*
0.16267
ofxn mod
0.1957

sRun
RunA1
zout
1

Ave
Data
8.4E-06
Model
7.7E-06

data/vMax
8.4E-06

ofxng
369.894

Min d/m
1.4326

ave/min d
1
ave/min m
1.31791

328_Calib_S_A

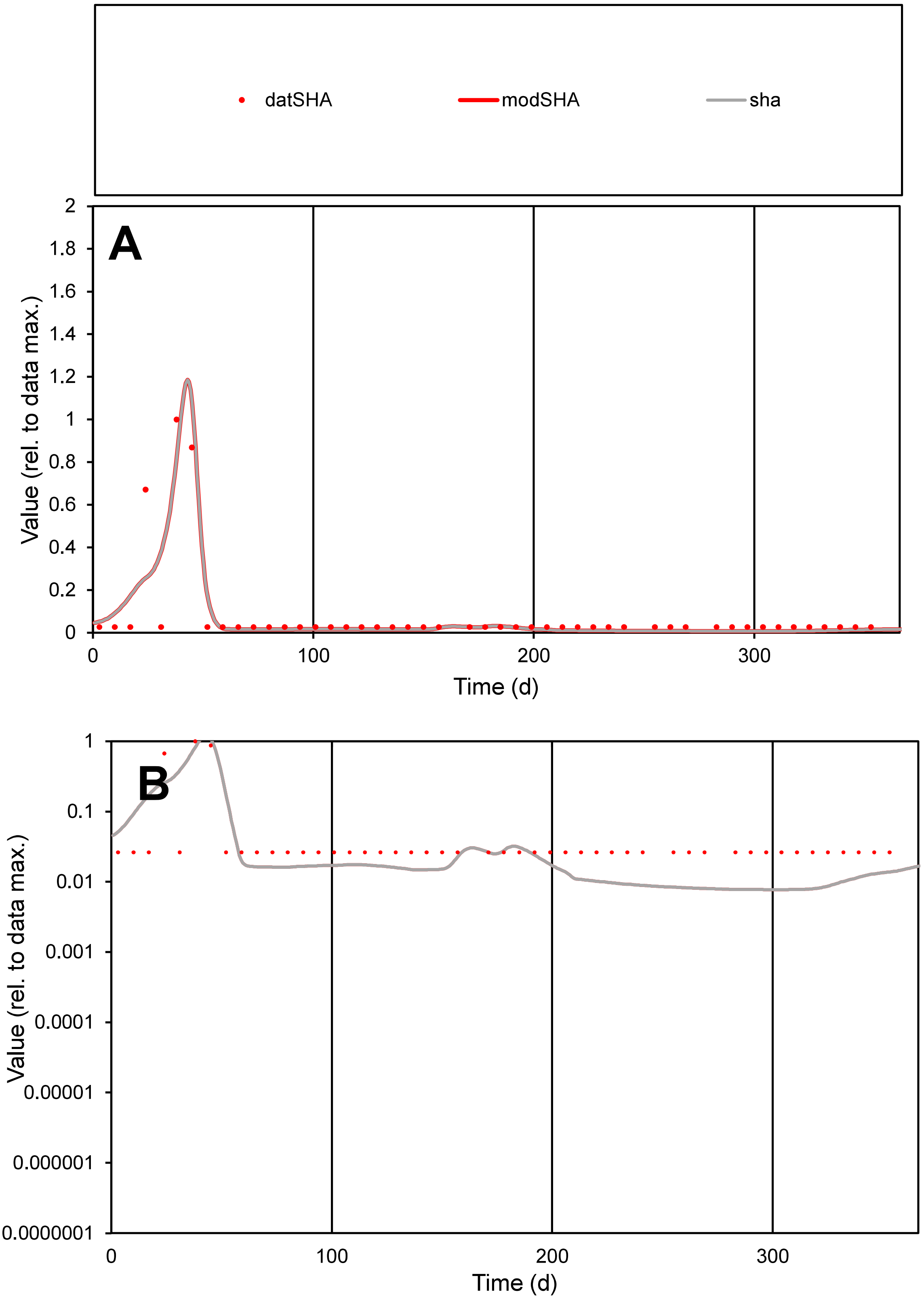

C1

Variables
SHA Provide jtd or Dst
CalibOnly?
2 1 to reproduce of
oNormalize?
2 1=no, 2=yes

ofxn calc*
0.99234
ofxn mod
1.10167

sRun
RunA1
zout
1

Ave
Data
2.1E-06
Model
1.8E-06

data/vMax
2.6E-05

ofxng
369.894

Min d/m
3.42703

ave/min d
2.94227
ave/min m
9.05464

328_Calib_S_A

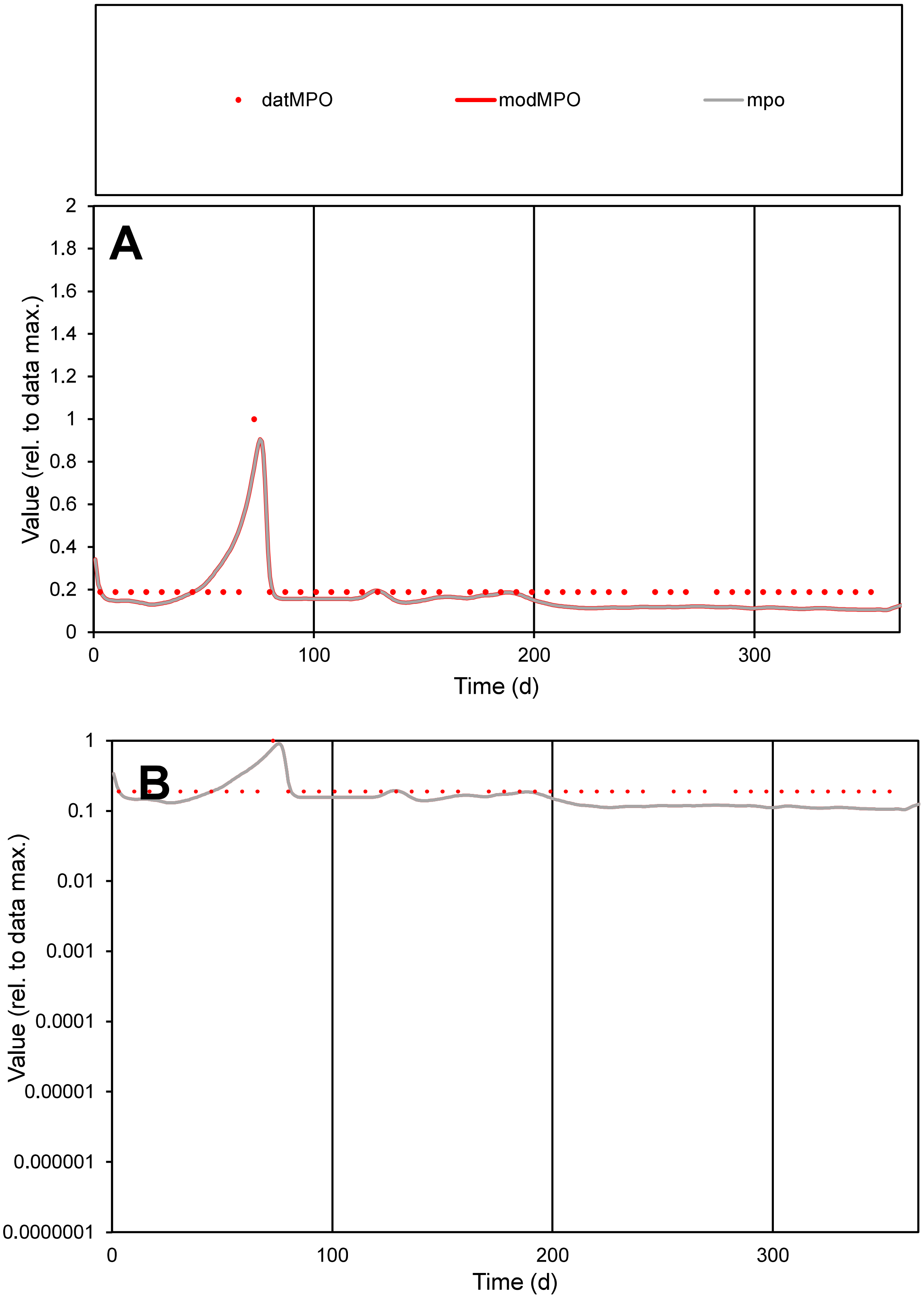

C1

Variables

MPO Provide jtd or Dst

CalibOnly?

2 1 to reproduce of

oNormalize?

2 1=no, 2=yes

ofxn calc*

0.36716

ofxn mod

0.38451

sRun

RunA1

zout

1

Ave

Data

1.4E-05

Model

1.1E-05

data/vMax

6.6E-05

ofxng

369.894

Min d/m

1.78253

ave/min d

1.08965

ave/min m

1.59823

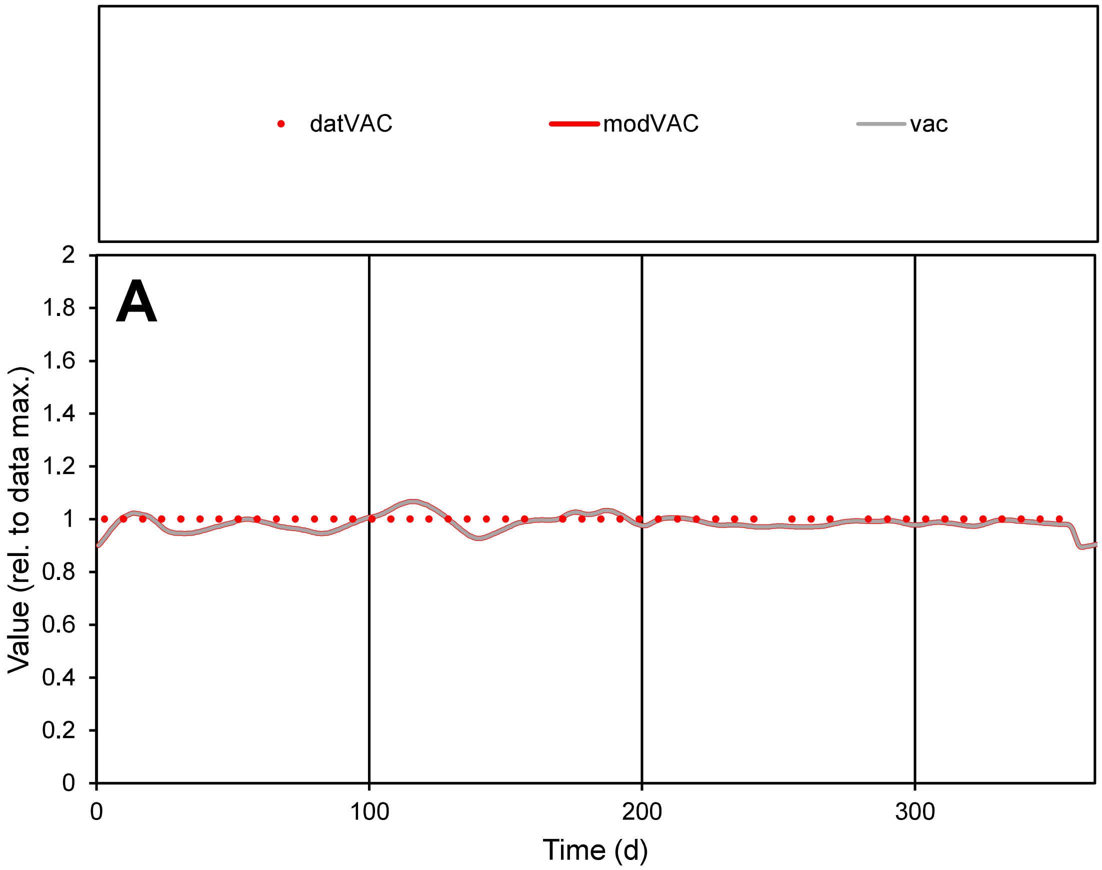

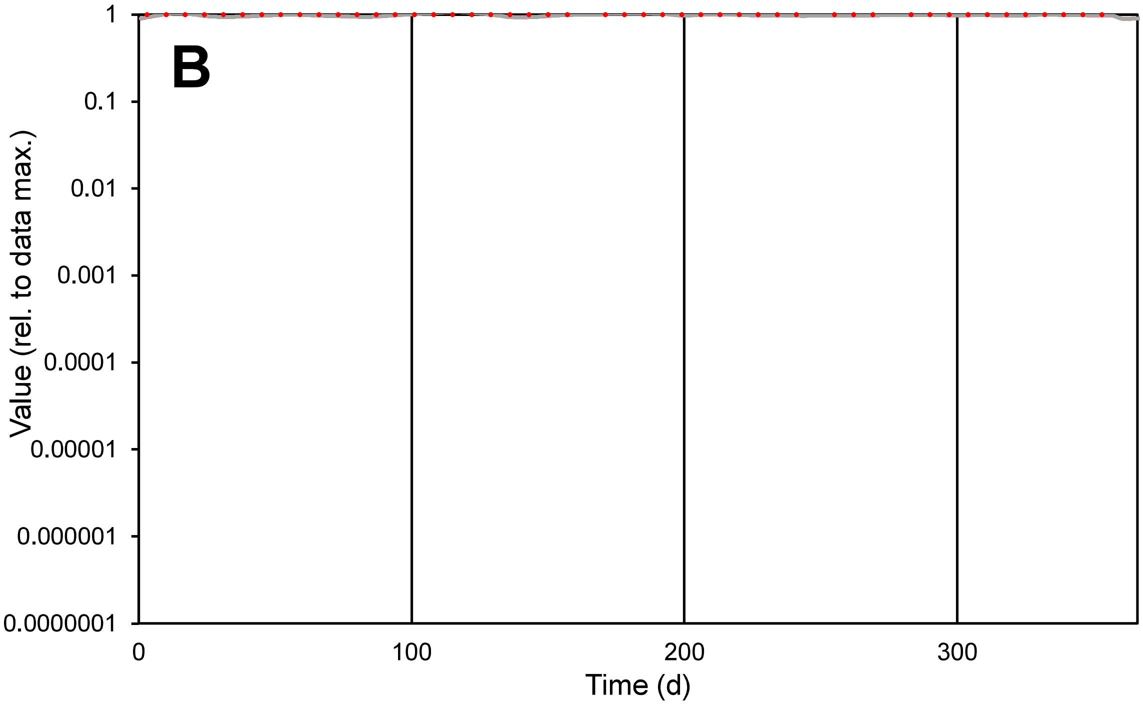

C1

Variables
VAC Provide jtd or Dst
CalibOnly?
2 1 to reproduce of
oNormalize?
2 1=no, 2=yes

ofxn calc*
0.02992
ofxn mod
0.11628

sRun
RunA1
zout
1

Ave
Data
9.5E-06
Model
9.4E-06

data/vMax
9.5E-06

ofxng
369.894

Min d/m
1.07066

ave/min d
1
ave/min m
1.05712

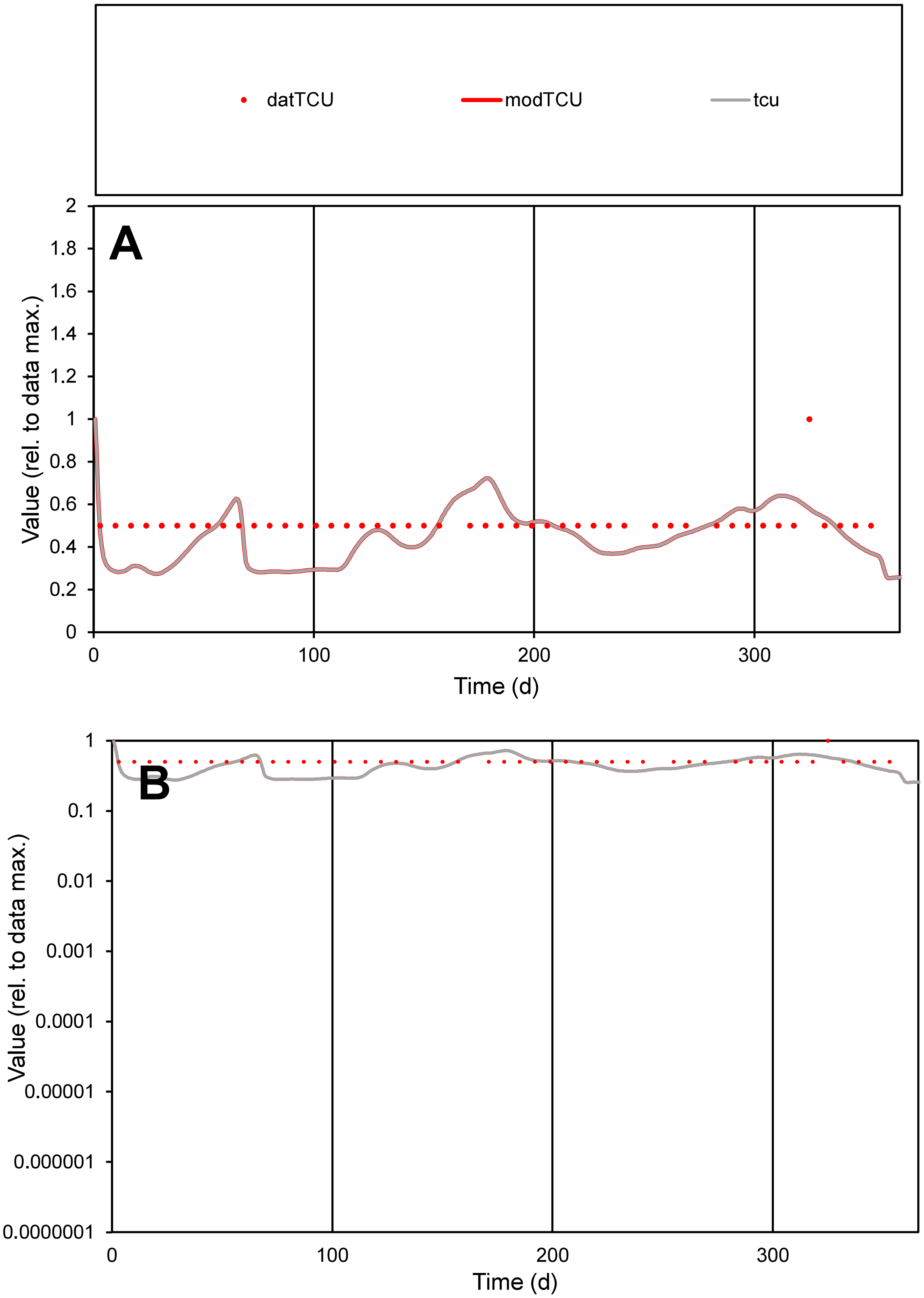

C1

Variables

TCU Provide jtd or Dst

CalibOnly?

2 1 to reproduce of

oNormalize?

2 1=no, 2=yes

ofxn calc*

0.27717

ofxn mod

0.29741

sRun

RunA1

zout

1

Ave

Data

1E-05

Model

8.7E-06

data/vMax

2E-05

ofxng

369.894

Min d/m

1.77463

ave/min d

1.02084

ave/min m

1.58783

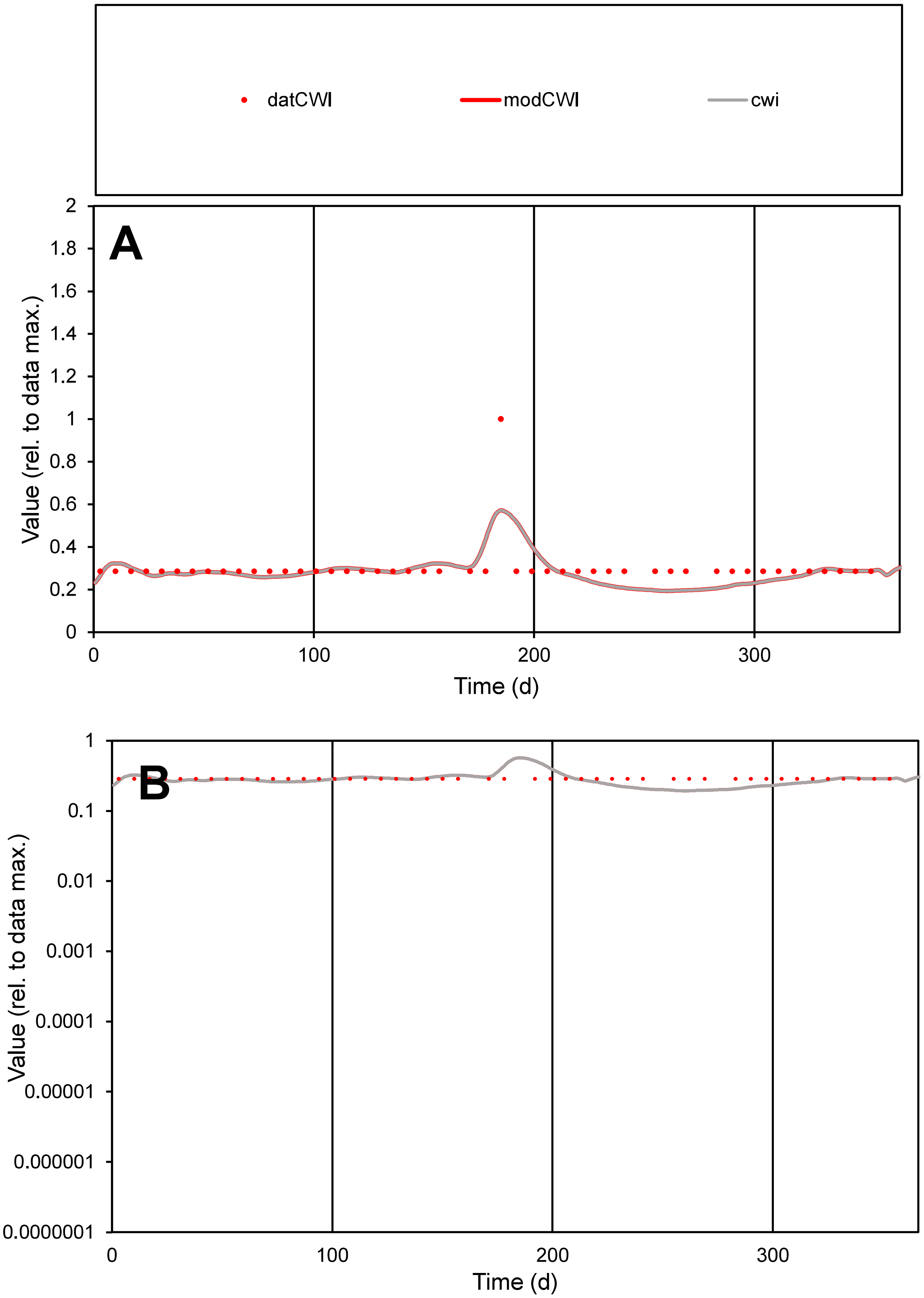

C1

Variables
CWI Provide jtd or Dst
CalibOnly?
2 1 to reproduce of
oNormalize?
2 1=no, 2=yes

ofxn calc*
0.27509
ofxn mod
0.29944

sRun
RunA1
zout
1

Ave
Data
1.4E-05
Model
1.3E-05

data/vMax
4.6E-05

ofxng
369.894

Min d/m
1.47143

ave/min d
1.052
ave/min m
1.46815

328_Calib_S_A

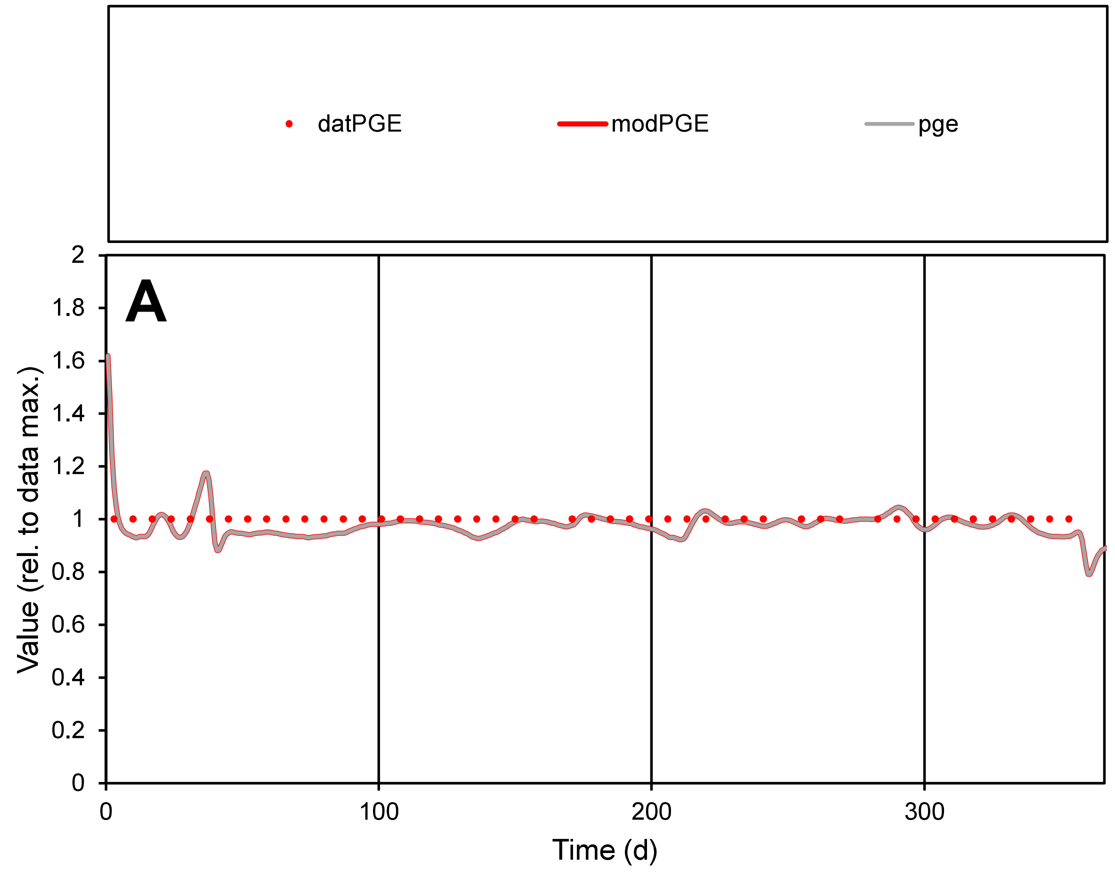

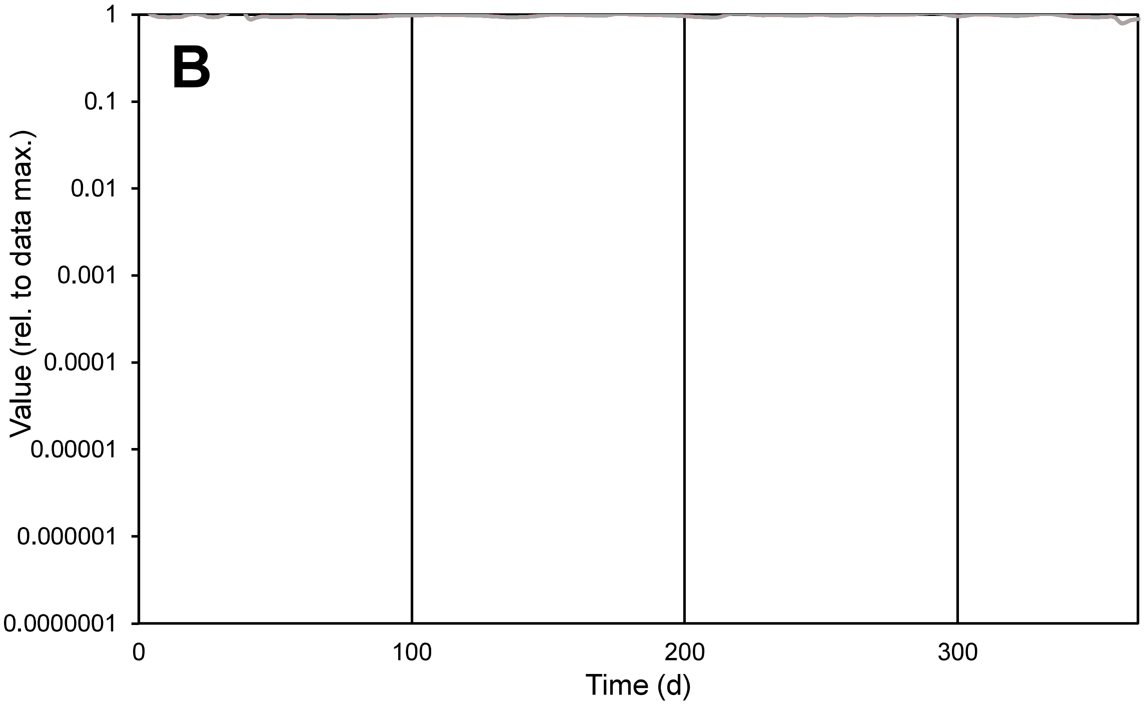

C1

Variables
PGE Provide jtd or Dst
CalibOnly?
2 1 to reproduce of
oNormalize?
2 1=no, 2=yes

ofxn calc*
0.03725
ofxn mod
0.11793

sRun
RunA1
zout
1

Ave
Data
5.9E-06
Model
5.7E-06

data/vMax
5.9E-06

ofxng
369.894

Min d/m
1.07685

ave/min d
1
ave/min m
1.05178

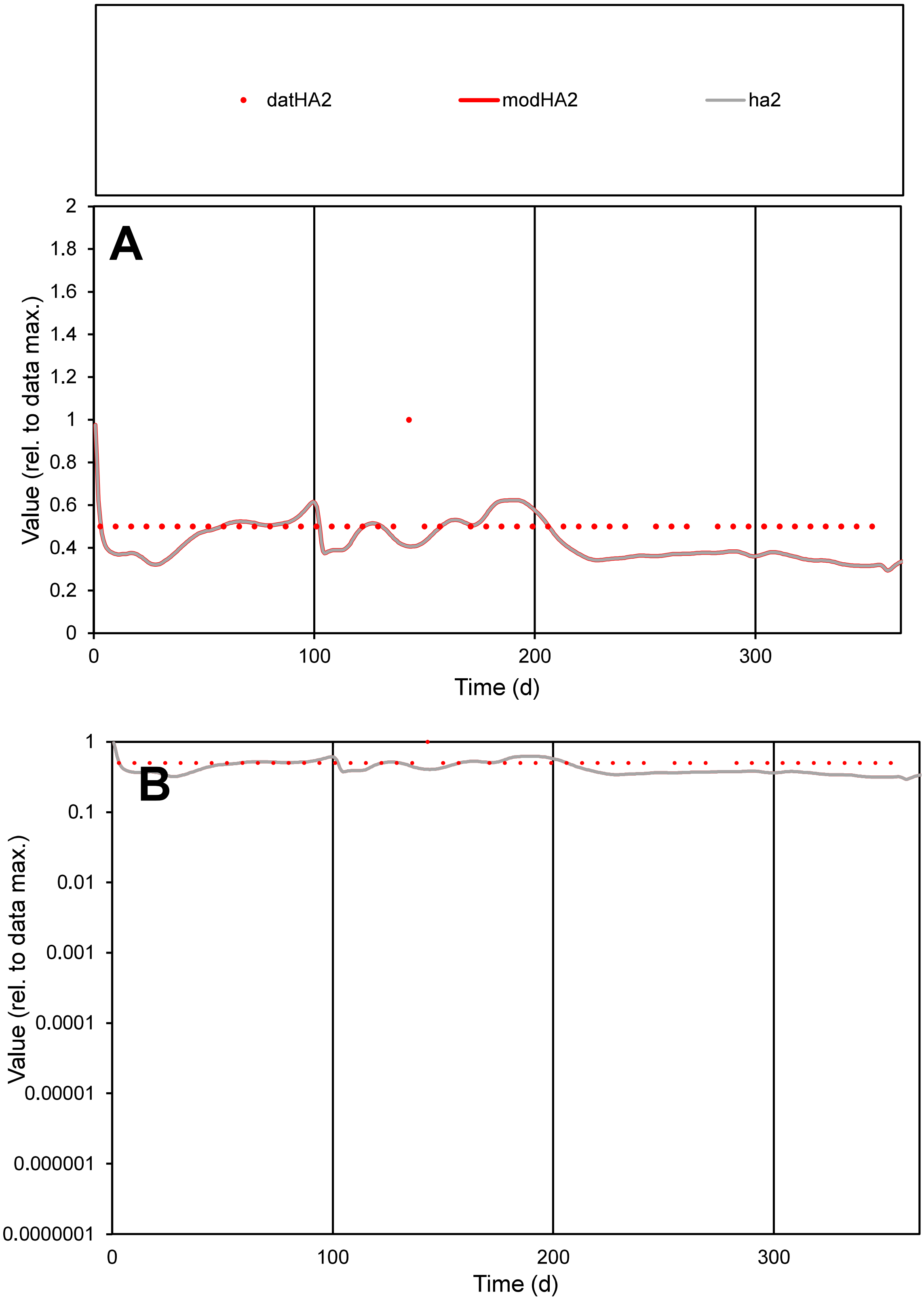

C1

Variables
HA2 Provide jtd or Dst
CalibOnly?
2 1 to reproduce of
oNormalize?
2 1=no, 2=yes

ofxn calc*
0.2718
ofxn mod
0.29101

sRun
RunA1
zout
1

Ave
Data
2.7E-06
Model
2.3E-06

data/vMax
5.3E-06

ofxng
369.894

Min d/m
1.58393

ave/min d
1.02083
ave/min m
1.36258

328_Calib_S_A

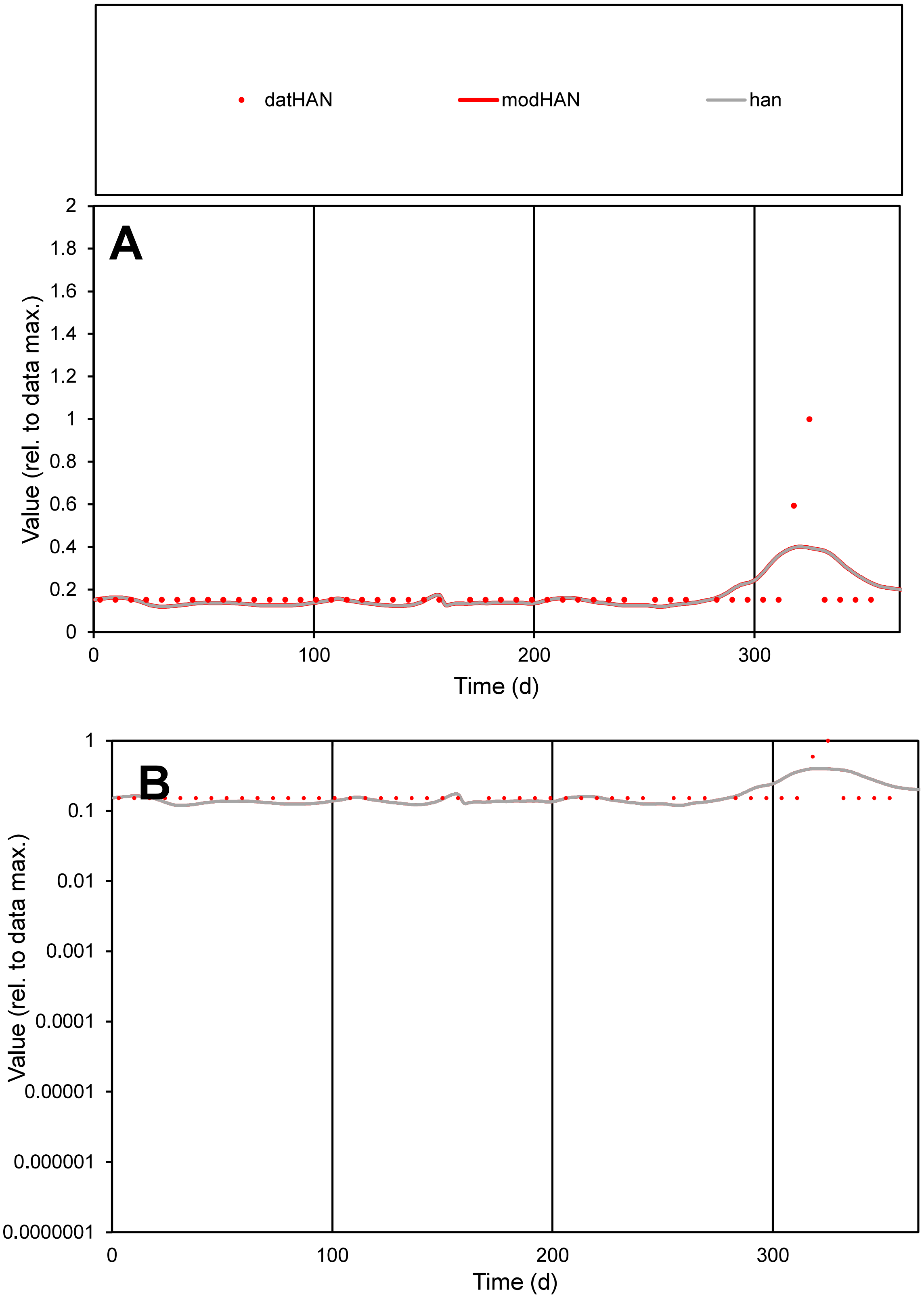

C1

Variables
HAN Provide jtd or Dst
CalibOnly?
2 1 to reproduce of
oNormalize?
2 1=no, 2=yes

ofxn calc*
0.61636
ofxn mod
0.62996

sRun
RunA1
zout
1

Ave
Data
6E-06
Model
5.8E-06

data/vMax
3.4E-05

ofxng
369.894

Min d/m
1.26957

ave/min d
1.17594
ave/min m
1.44566

328_Calib_S_A

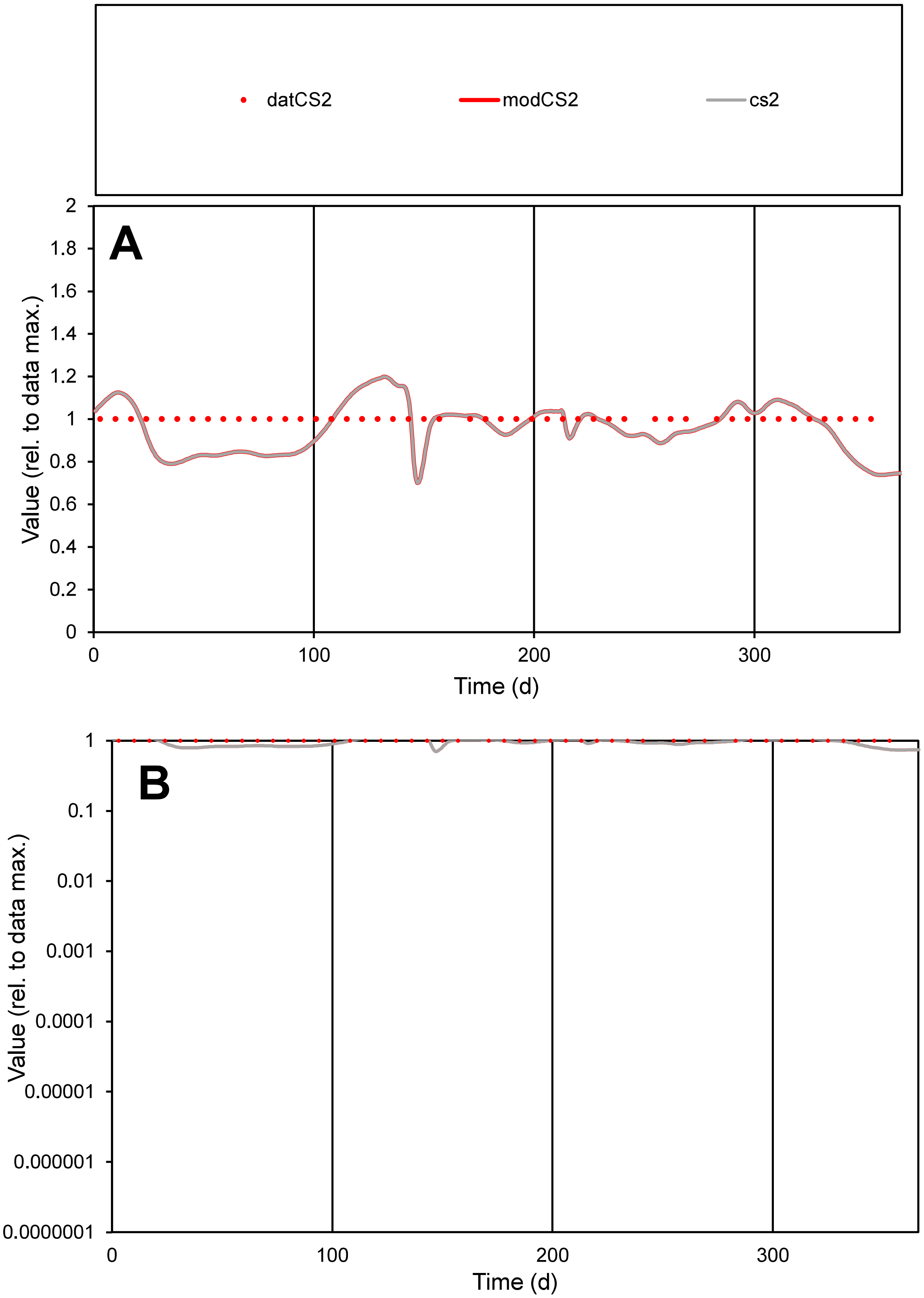

C1

Variables
CS2 Provide jtd or Dst
CalibOnly?
2 1 to reproduce of
oNormalize?
2 1=no, 2=yes

ofxn calc*
0.11601
ofxn mod
0.16054

sRun
RunA1
zout
1

Ave
Data
7.6E-06
Model
7.3E-06

data/vMax
7.6E-06

ofxng
369.894

Min d/m
1.34433

ave/min d
1
ave/min m
1.2928

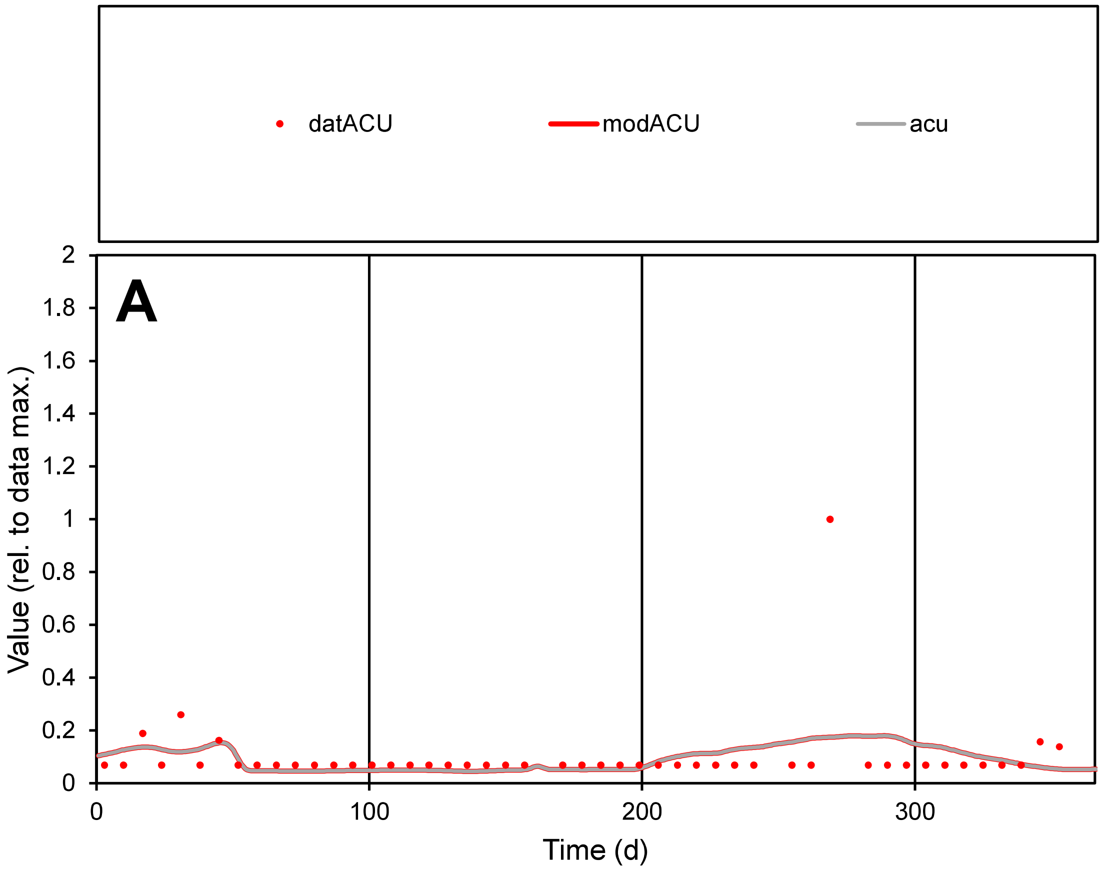

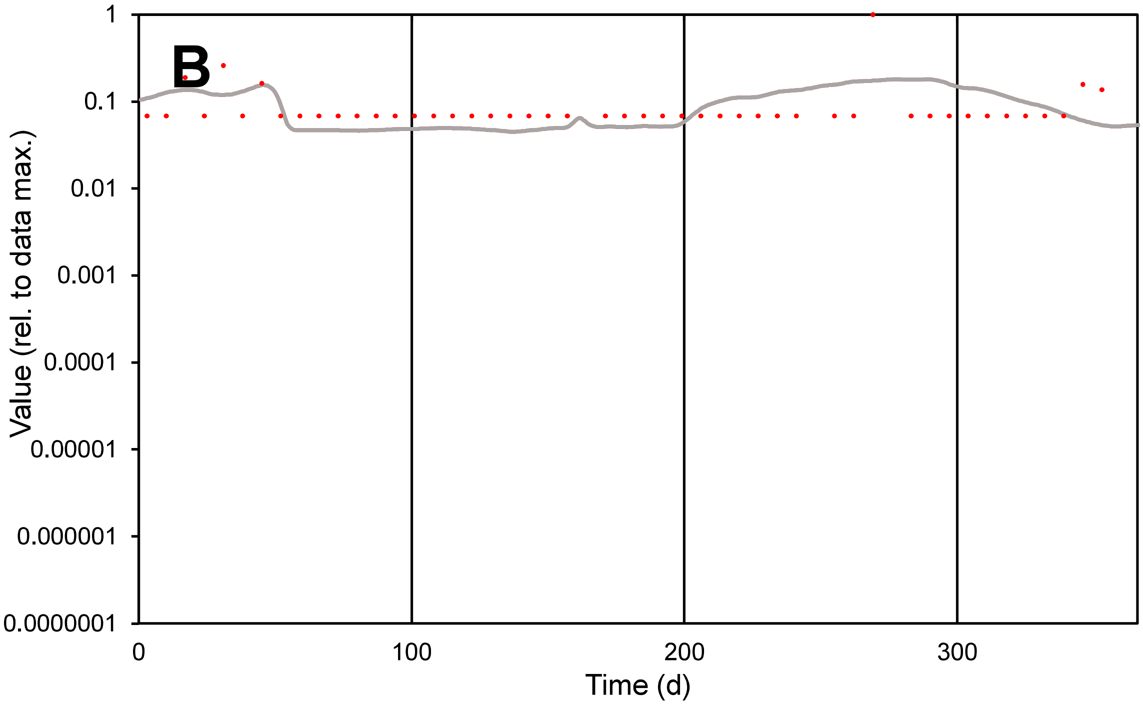

C1

Variables
ACU Provide jtd or Dst
CalibOnly?
2 1 to reproduce of
oNormalize?
2 1=no, 2=yes

ofxn calc*
1.30663
ofxn mod
1.31589

sRun
RunA1
zout
1

Ave
Data
1.6E-05
Model
1.5E-05

data/vMax
0.00016

ofxng
369.894

Min d/m
1.52505

ave/min d
1.45314
ave/min m
2.04036

328_Calib_S_A

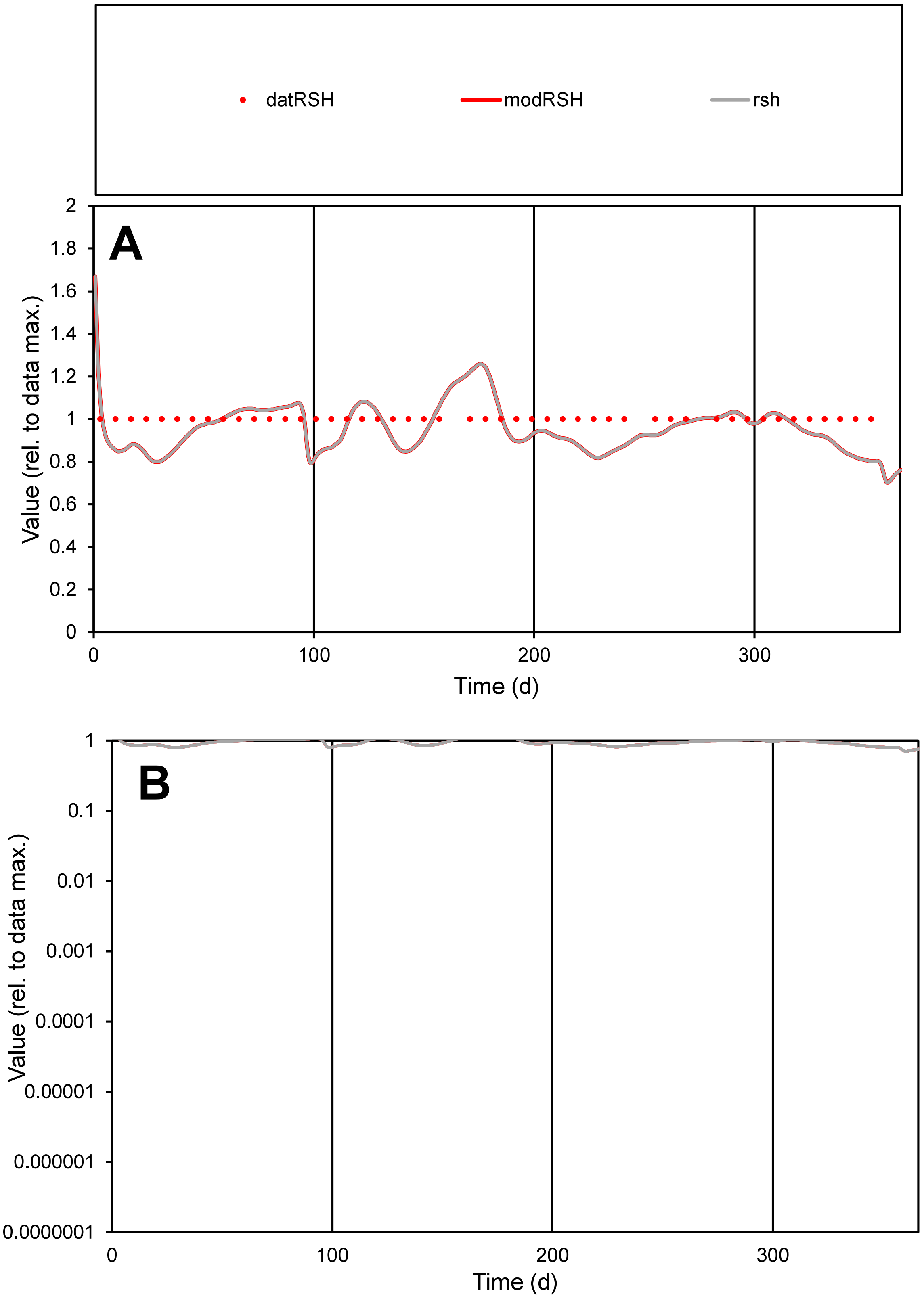

C1

Variables
RSH Provide jtd or Dst
CalibOnly?
2 1 to reproduce of
oNormalize?
2 1=no, 2=yes

ofxn calc*
0.10733
ofxn mod
0.15395

sRun
RunA1
zout
1

Ave
Data
1.8E-05
Model
1.7E-05

data/vMax
1.8E-05

ofxng
369.894

Min d/m
1.24547

ave/min d
1
ave/min m
1.18778

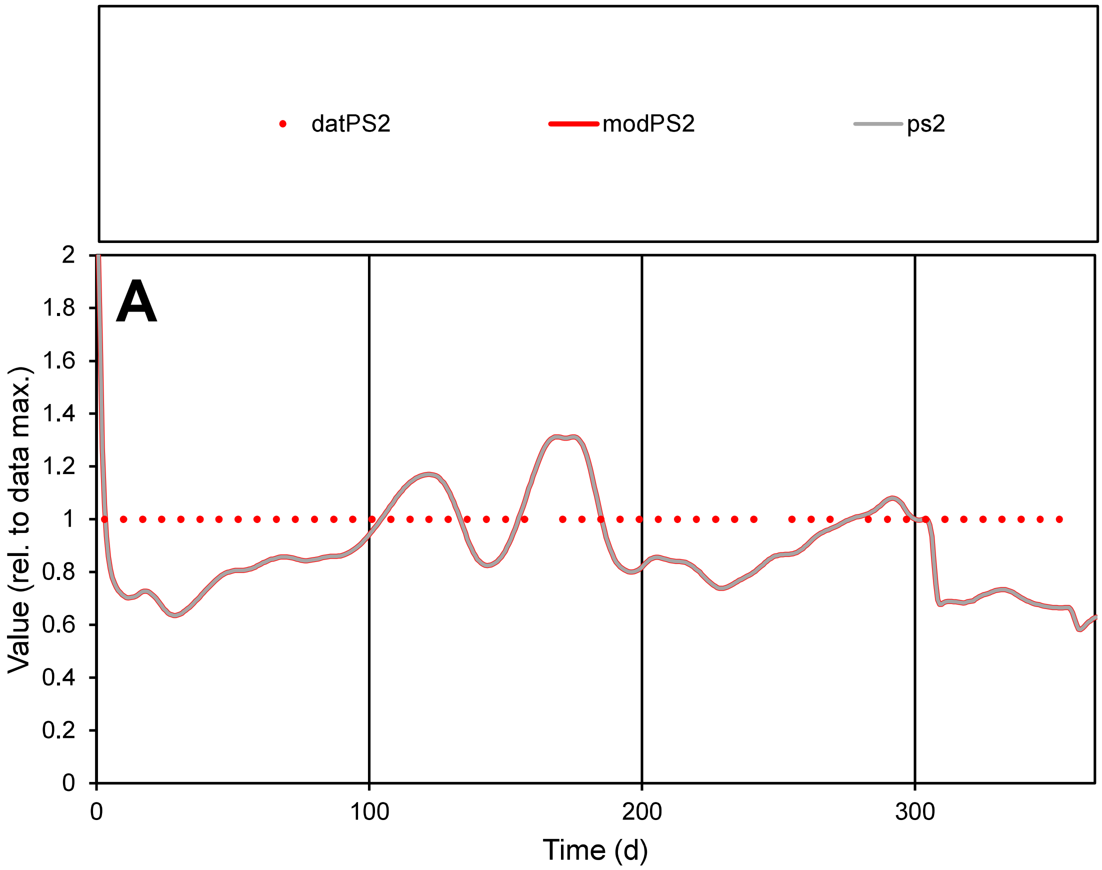

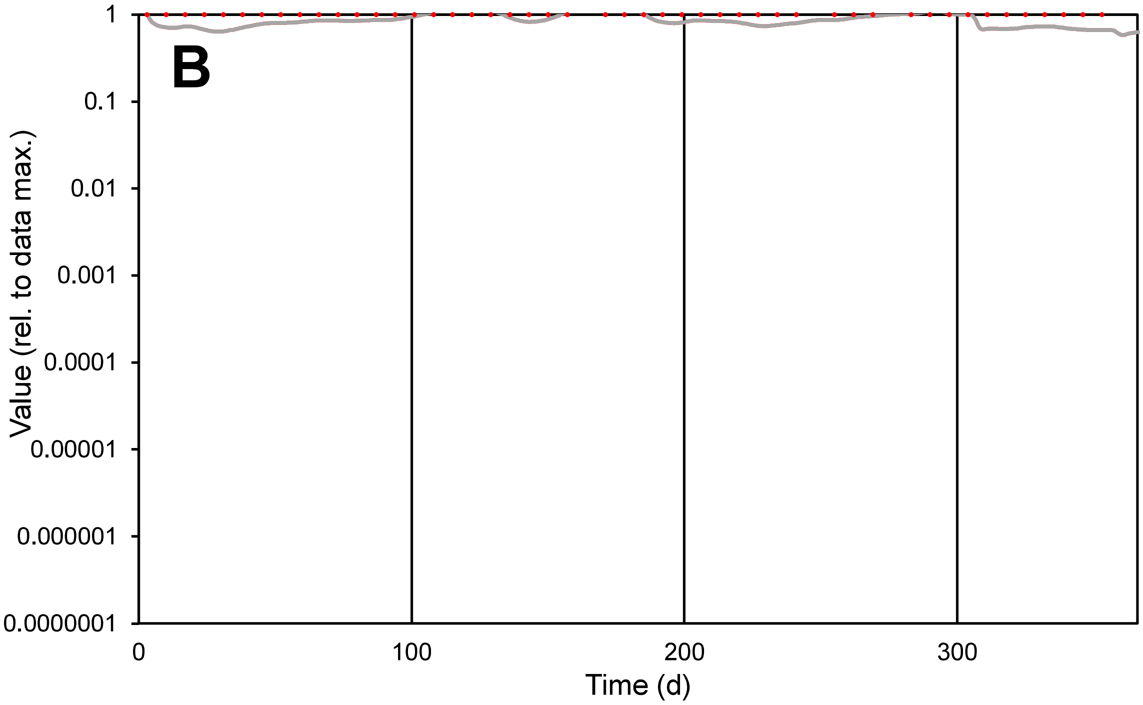

C1

Variables
PS2 Provide jtd or Dst
CalibOnly?
2 1 to reproduce of
oNormalize?
2 1=no, 2=yes

ofxn calc*
0.20258
ofxn mod
0.22875

sRun
RunA1
zout
1

Ave
Data
1.9E-05
Model
1.7E-05

data/vMax
1.9E-05

ofxng
369.894

Min d/m
1.52644

ave/min d
1
ave/min m
1.33599

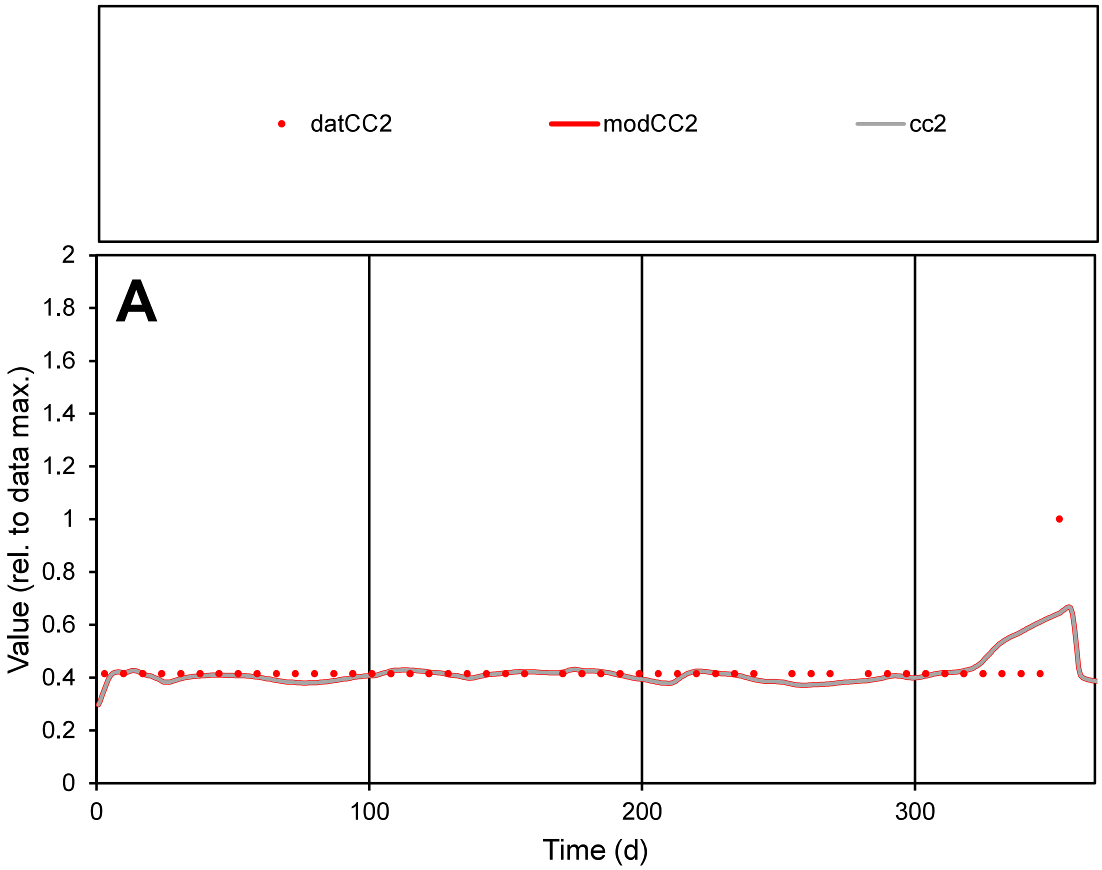

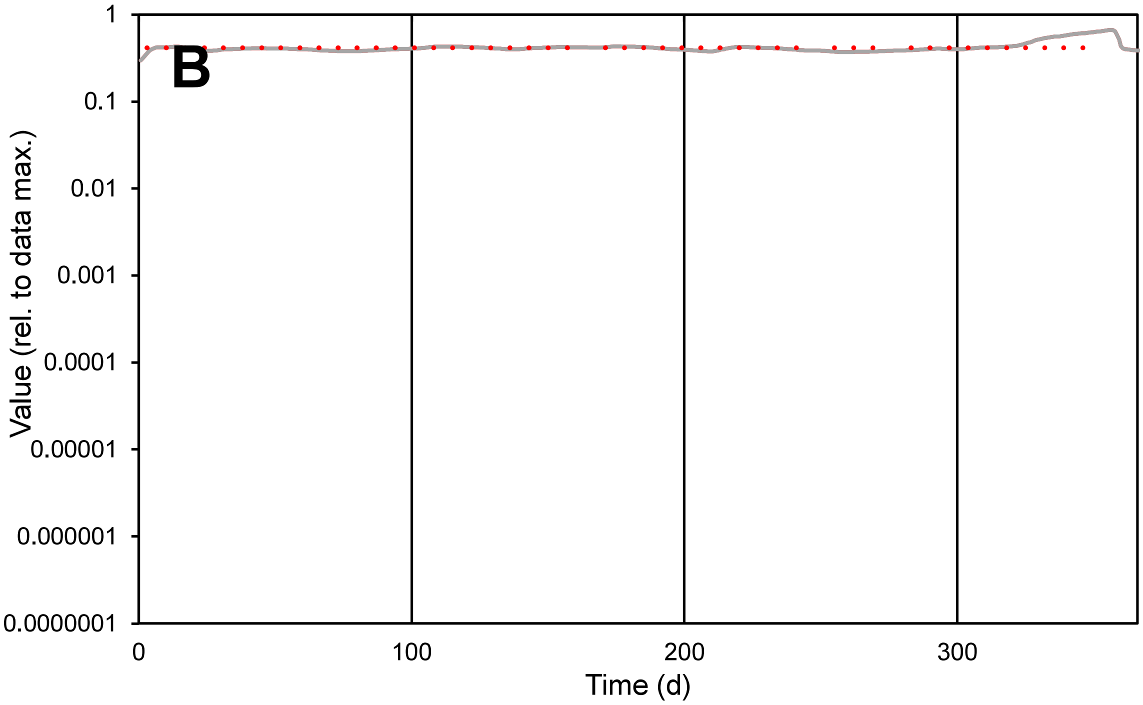

C1

Variables
CC2 Provide jtd or Dst
CalibOnly?
2 1 to reproduce of
oNormalize?
2 1=no, 2=yes

ofxn calc*
0.16113
ofxn mod
0.19598

sRun
RunA1
zout
1

Ave
Data
6E-06
Model
6E-06

data/vMax
1.4E-05

ofxng
369.894

Min d/m
1.1109

ave/min d
1.02946
ave/min m
1.13227

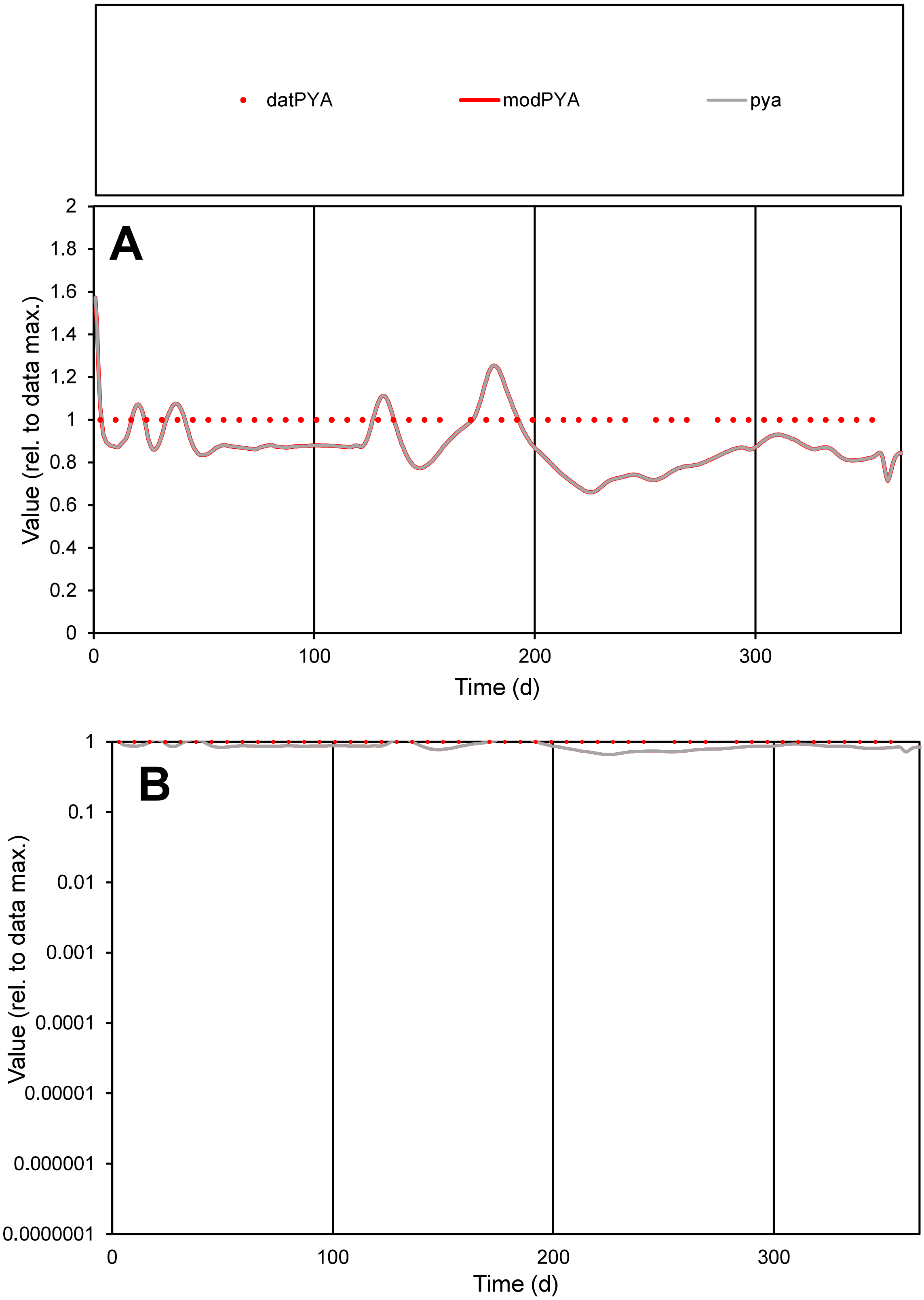

C1

Variables
PYA Provide jtd or Dst
CalibOnly?
2 1 to reproduce of
oNormalize?
2 1=no, 2=yes

ofxn calc*
0.16554
ofxn mod
0.19665

sRun
RunA1
zout
1

Ave
Data
1.8E-06
Model
1.6E-06

data/vMax
1.8E-06

ofxng
369.894

Min d/m
1.49136

ave/min d
1
ave/min m
1.31187

328_Calib_S_A

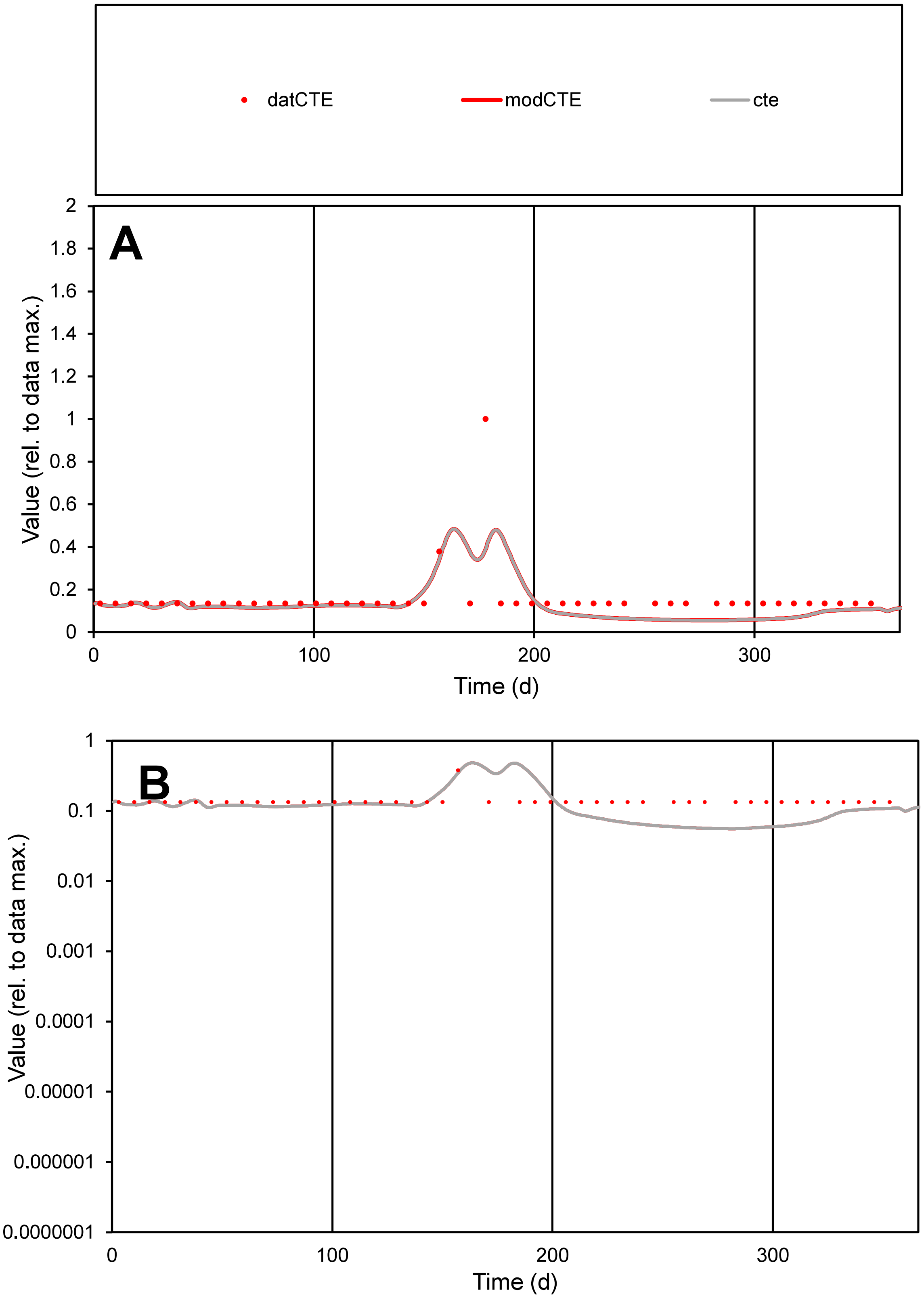

C1

Variables
CTE Provide jtd or Dst
CalibOnly?
2 1 to reproduce of
oNormalize?
2 1=no, 2=yes

ofxn calc*
0.66974
ofxn mod
0.69294

sRun
RunA1
zout
1

Ave
Data
7.2E-06
Model
6E-06

data/vMax
4.6E-05

ofxng
369.894

Min d/m
2.39578

ave/min d
1.17231
ave/min m
2.34521

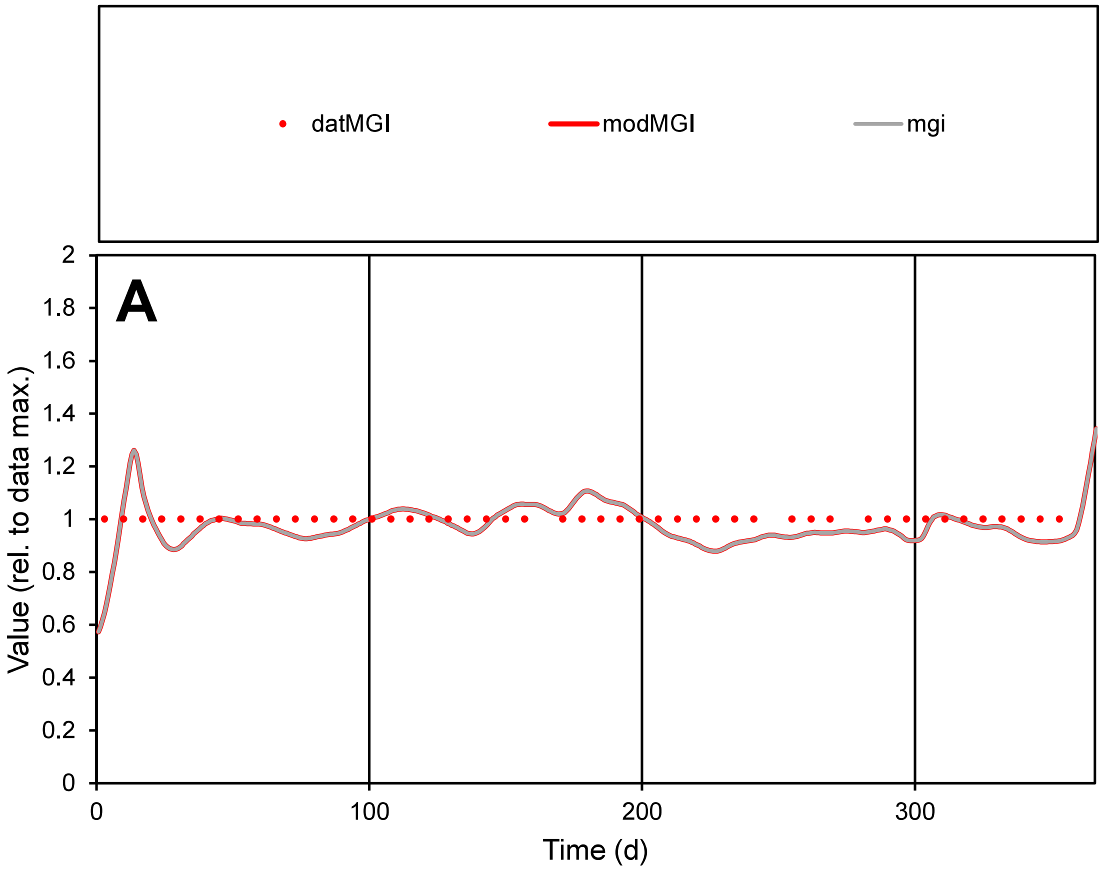

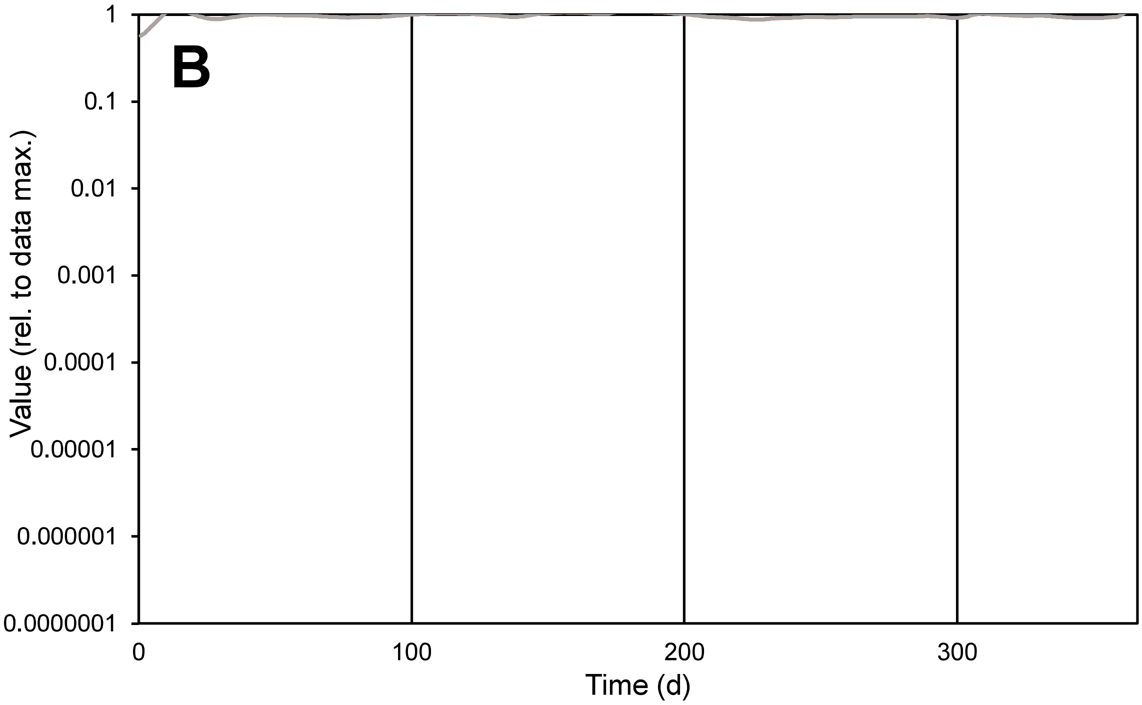

C1

Variables
MGI Provide jtd or Dst
CalibOnly?
2 1 to reproduce of
oNormalize?
2 1=no, 2=yes

ofxn calc*
0.07412
ofxn mod
0.13368

sRun
RunA1
zout
1

Ave
Data
1E-05
Model
1E-05

data/vMax
1E-05

ofxng
369.894

Min d/m
1.40046

ave/min d
1
ave/min m
1.36147

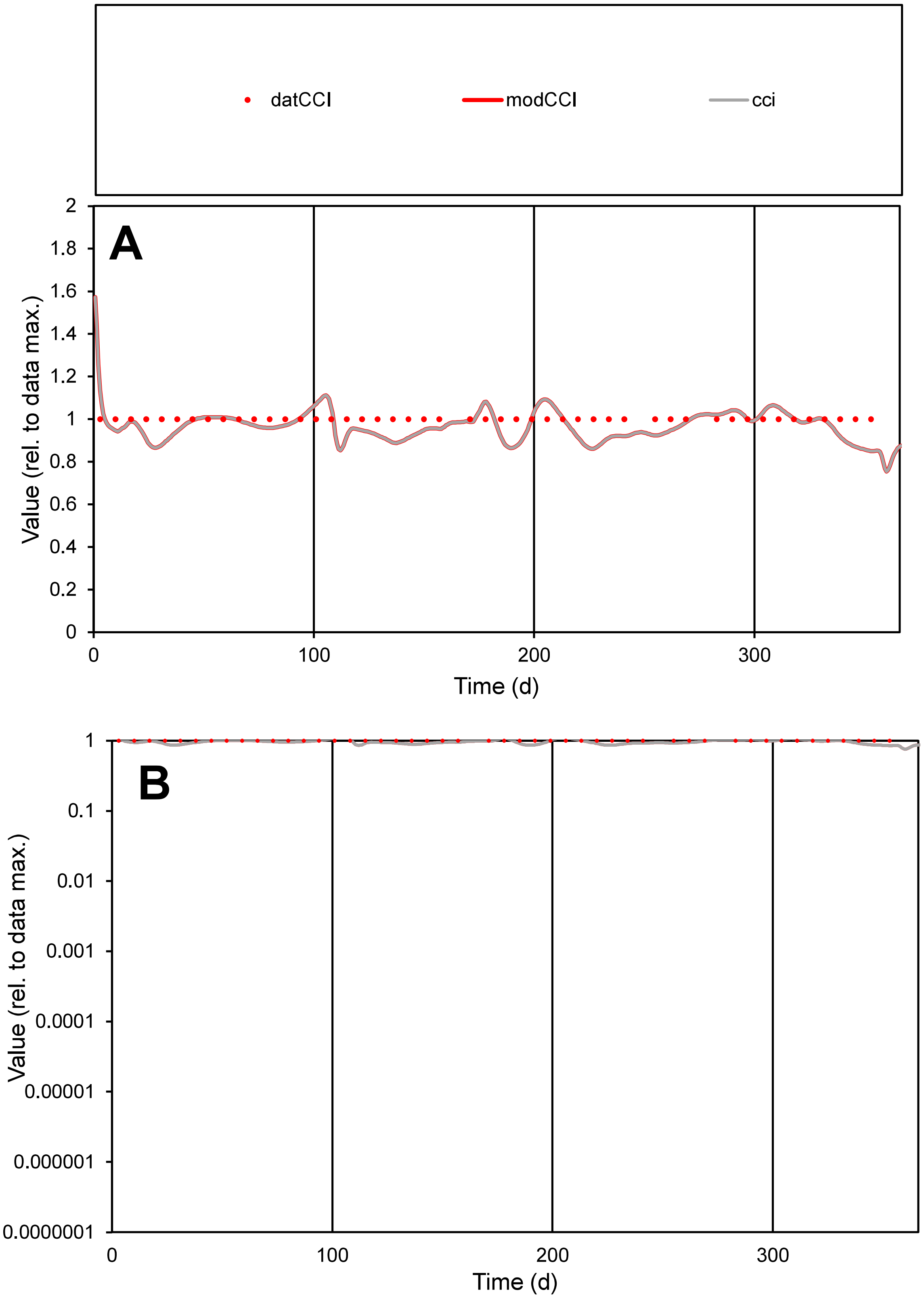

C1

Variables
CCI Provide jtd or Dst
CalibOnly?
2 1 to reproduce of
oNormalize?
2 1=no, 2=yes

ofxn calc*
0.06794
ofxn mod
0.13024

sRun
RunA1
zout
1

Ave
Data
1.1E-05
Model
1.1E-05

data/vMax
1.1E-05

ofxng
369.894

Min d/m
1.17563

ave/min d
1
ave/min m
1.13607

328_Calib_S_A

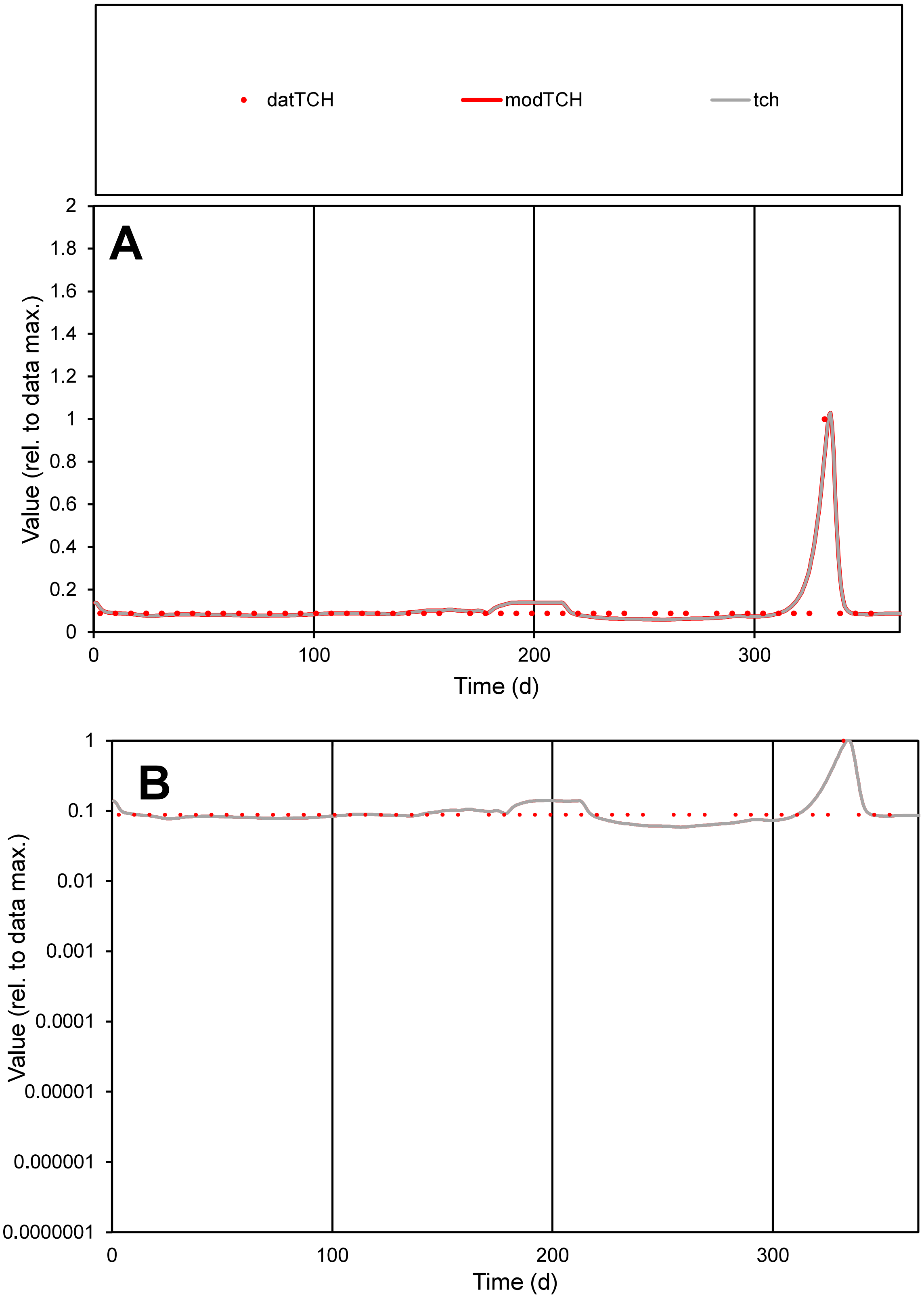

C1

Variables
TCH Provide jtd or Dst
CalibOnly?
2 1 to reproduce of
oNormalize?
2 1=no, 2=yes

ofxn calc*
0.31845
ofxn mod
0.37481

sRun
RunA1
zout
1

Ave
Data
2E-06
Model
2.1E-06

data/vMax
1.9E-05

ofxng
369.894

Min d/m
1.49511

ave/min d
1.21611
ave/min m
1.89122

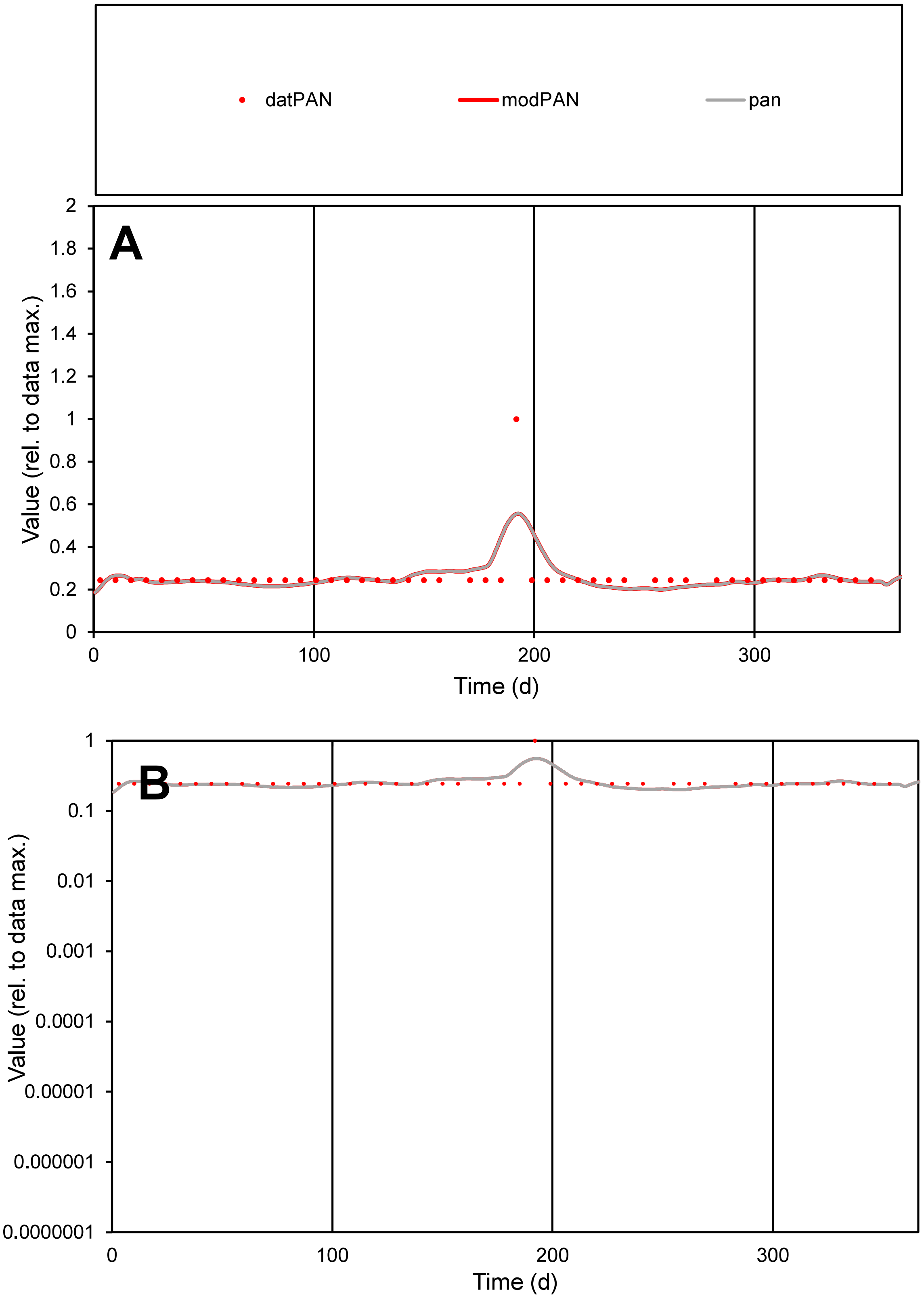

C1

Variables
PAN Provide jtd or Dst
CalibOnly?
2 1 to reproduce of
oNormalize?
2 1=no, 2=yes

ofxn calc*
0.31191
ofxn mod
0.33621

sRun
RunA1
zout
1

Ave
Data
9.7E-06
Model
9.7E-06

data/vMax
3.7E-05

ofxng
369.894

Min d/m
1.21132

ave/min d
1.06472
ave/min m
1.28409

328_Calib_S_A

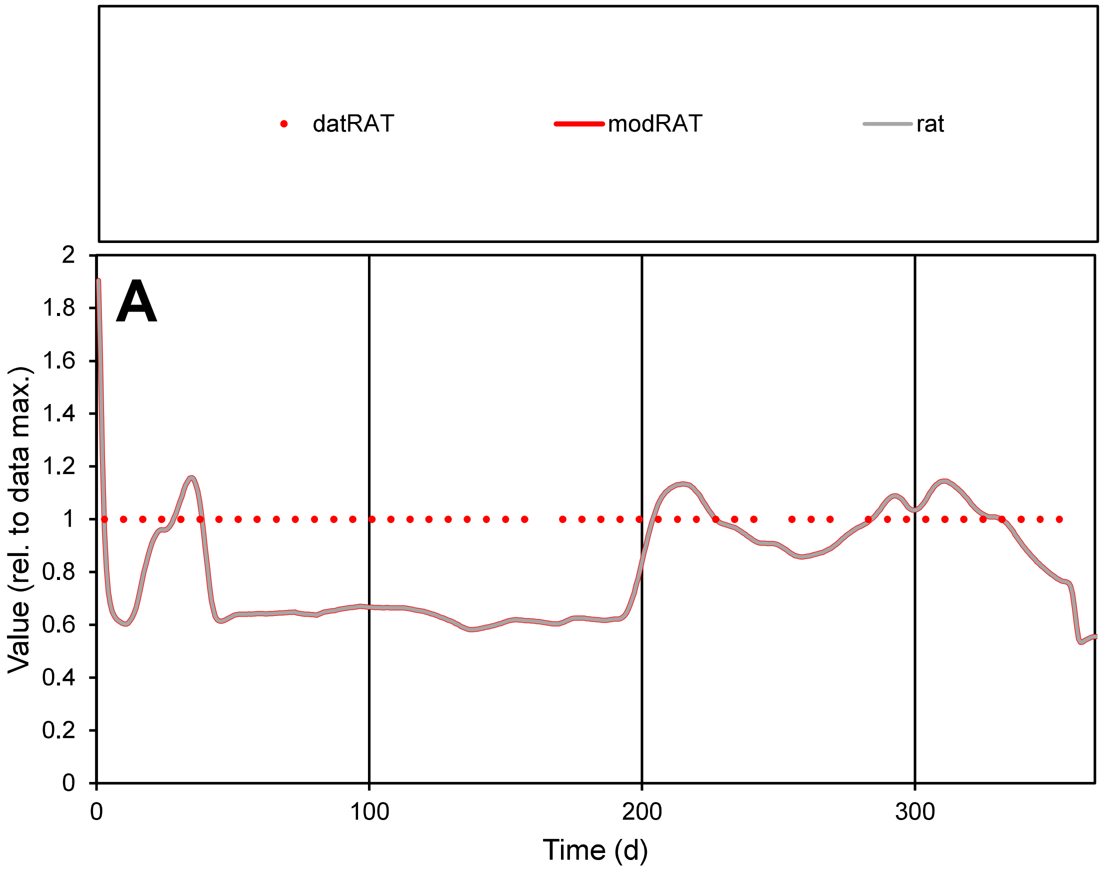

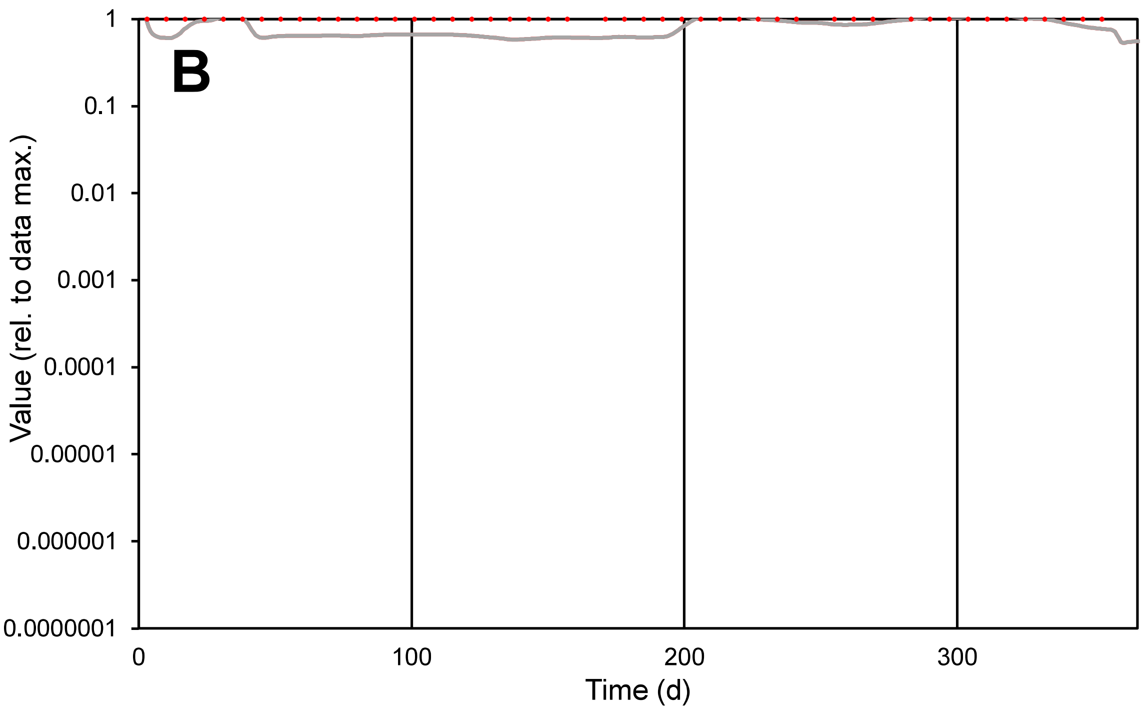

C1

Variables
RAT Provide jtd or Dst
CalibOnly?
2 1 to reproduce of
oNormalize?
2 1=no, 2=yes

ofxn calc*
0.26367
ofxn mod
0.28314

sRun
RunA1
zout
1

Ave
Data
1.1E-05
Model
9.3E-06

data/vMax
1.1E-05

ofxng
369.894

Min d/m
1.71726

ave/min d
1
ave/min m
1.40106

328_Calib_S_A

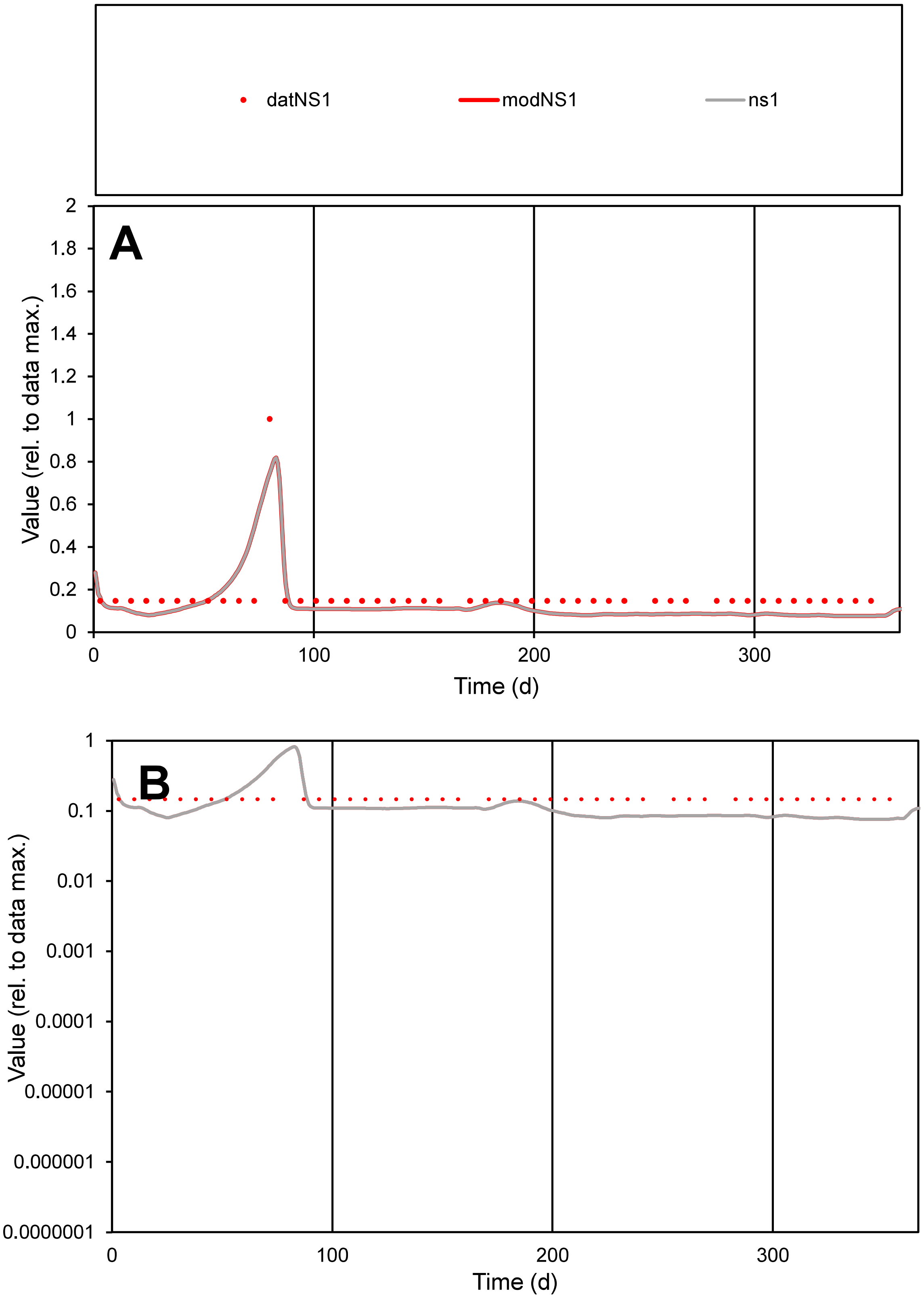

C1

Variables

NS1 Provide jtd or Dst

CalibOnly?

2 1 to reproduce of

oNormalize?

2 1=no, 2=yes

ofxn calc*

0.46553

ofxn mod

0.50599

sRun

RunA1

zout

1

Ave

Data

3.1E-05

Model

2.4E-05

data/vMax

0.00019

ofxng

369.894

Min d/m

1.93244

ave/min d

1.12096

ave/min m

1.6988

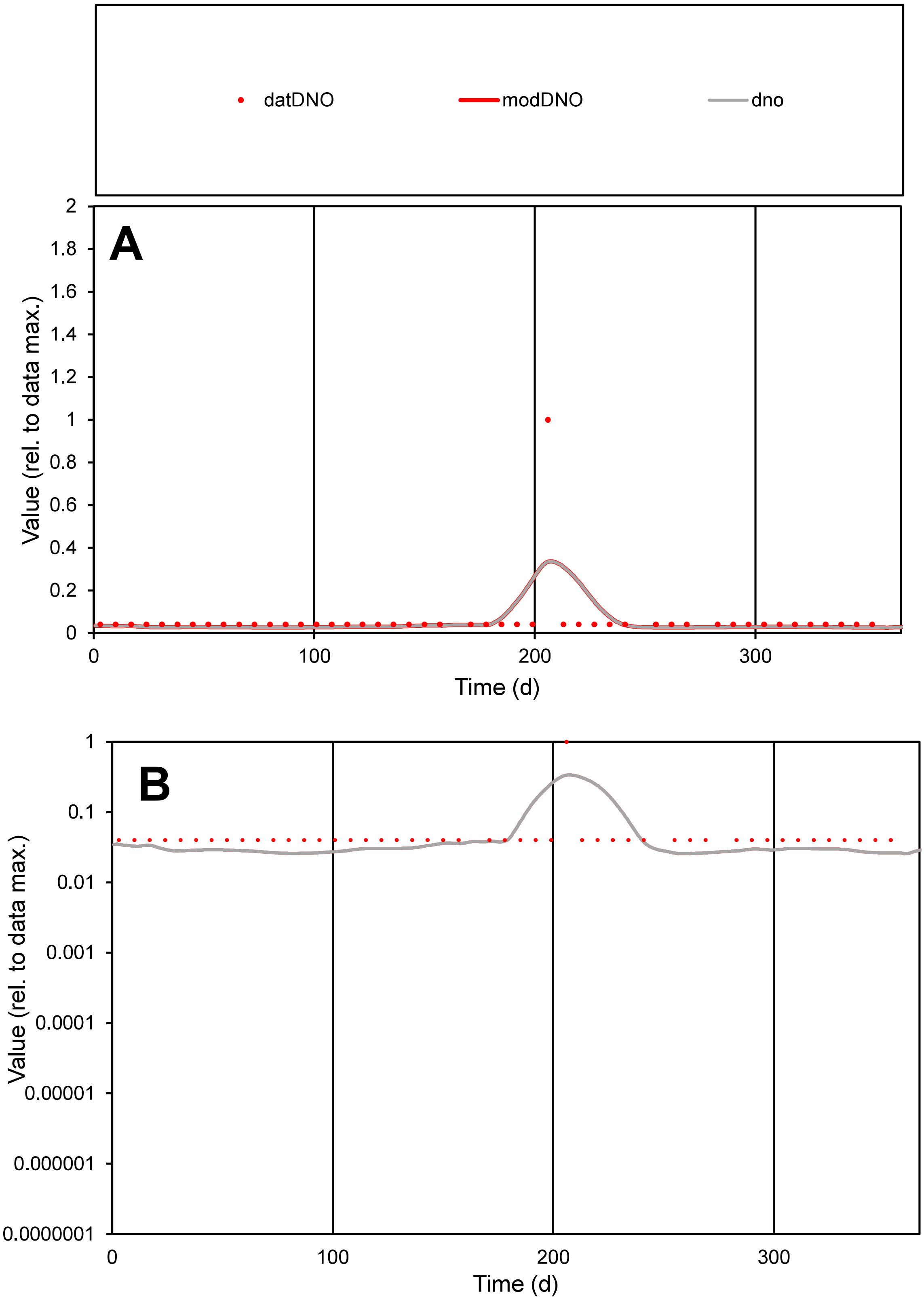

C1

Variables
DNO Provide jtd or Dst
CalibOnly?
2 1 to reproduce of
oNormalize?
2 1=no, 2=yes

ofxn calc*
1.85598
ofxn mod
1.88287

sRun
RunA1
zout
1

Ave
Data
2.7E-05
Model
2.5E-05

data/vMax
0.00045

ofxng
369.894

Min d/m
1.55498

ave/min d
1.49724
ave/min m
2.16849

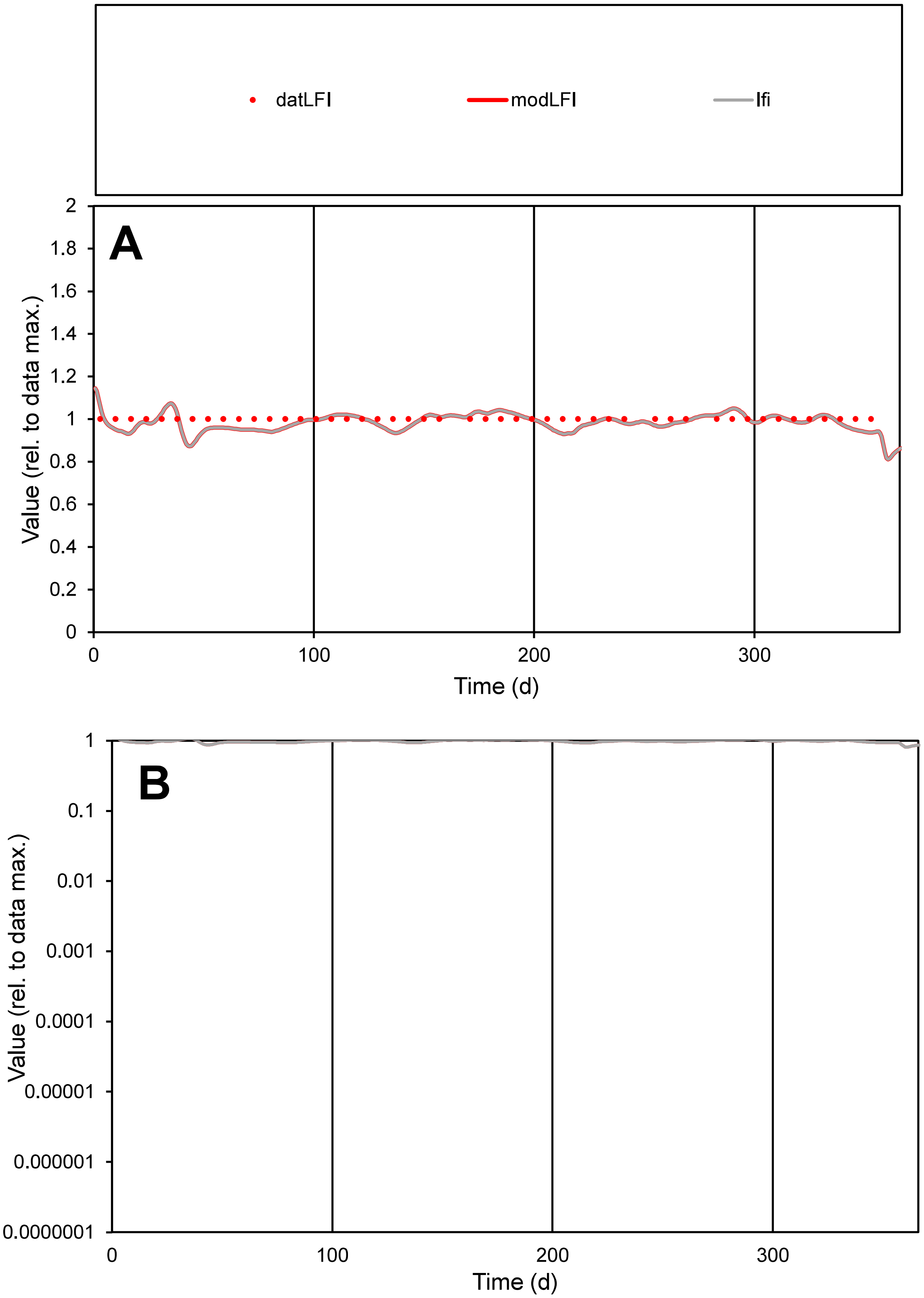

C1

Variables
LFI Provide jtd or Dst
CalibOnly?
2 1 to reproduce of
oNormalize?
2 1=no, 2=yes

ofxn calc*
0.03673
ofxn mod
0.11826

sRun
RunA1
zout
1

Ave
Data
8.3E-06
Model
8.2E-06

data/vMax
8.3E-06

ofxng
369.894

Min d/m
1.1092

ave/min d
1
ave/min m
1.09233

328_Calib_S_A

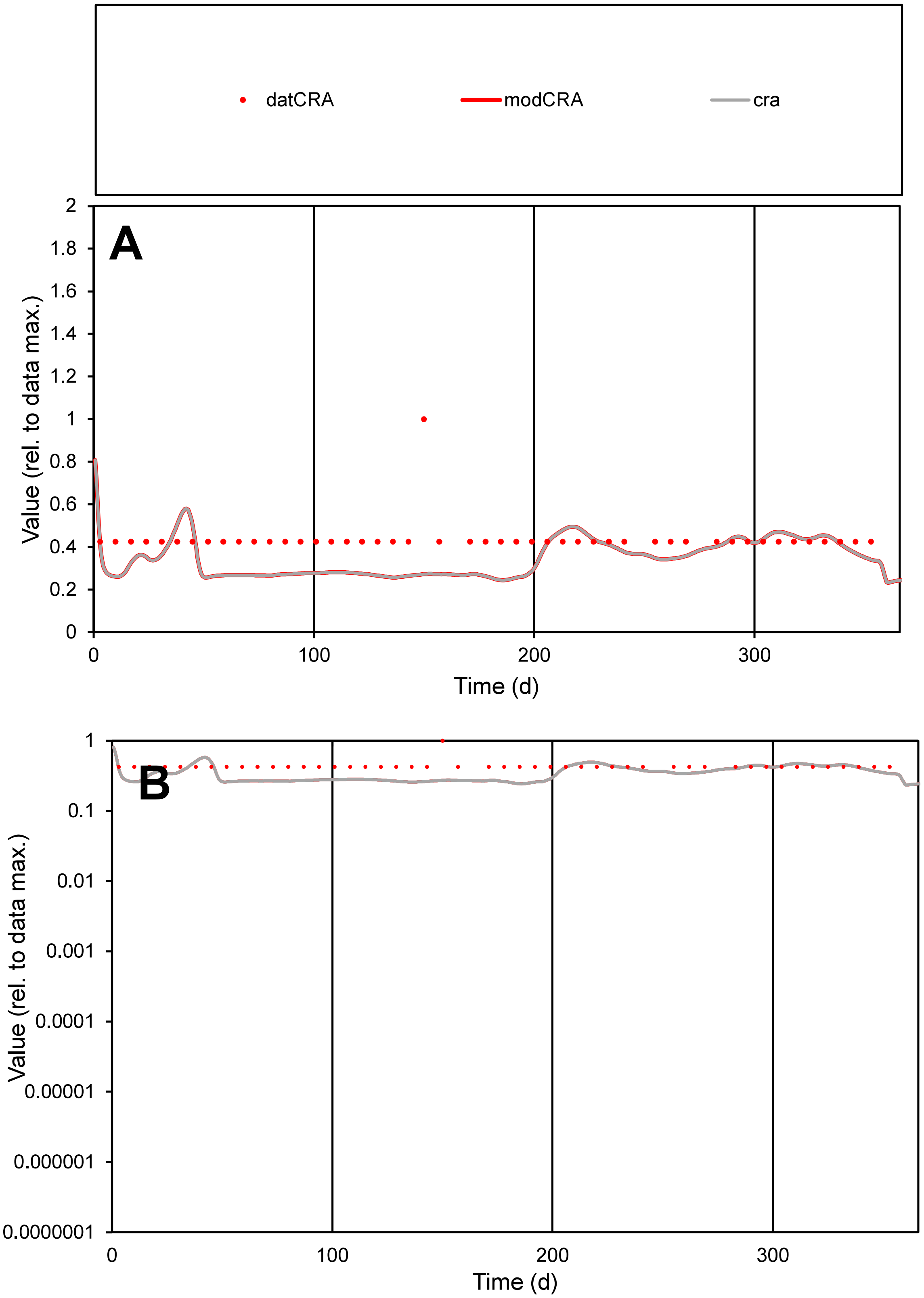

C1

Variables
CRA Provide jtd or Dst
CalibOnly?
2 1 to reproduce of
oNormalize?
2 1=no, 2=yes

ofxn calc*
0.349
ofxn mod
0.36349

sRun
RunA1
zout
1

Ave
Data
5E-06
Model
4E-06

data/vMax
1.1E-05

ofxng
369.894

Min d/m
1.74025

ave/min d
1.02829
ave/min m
1.41893

328_Calib_S_A

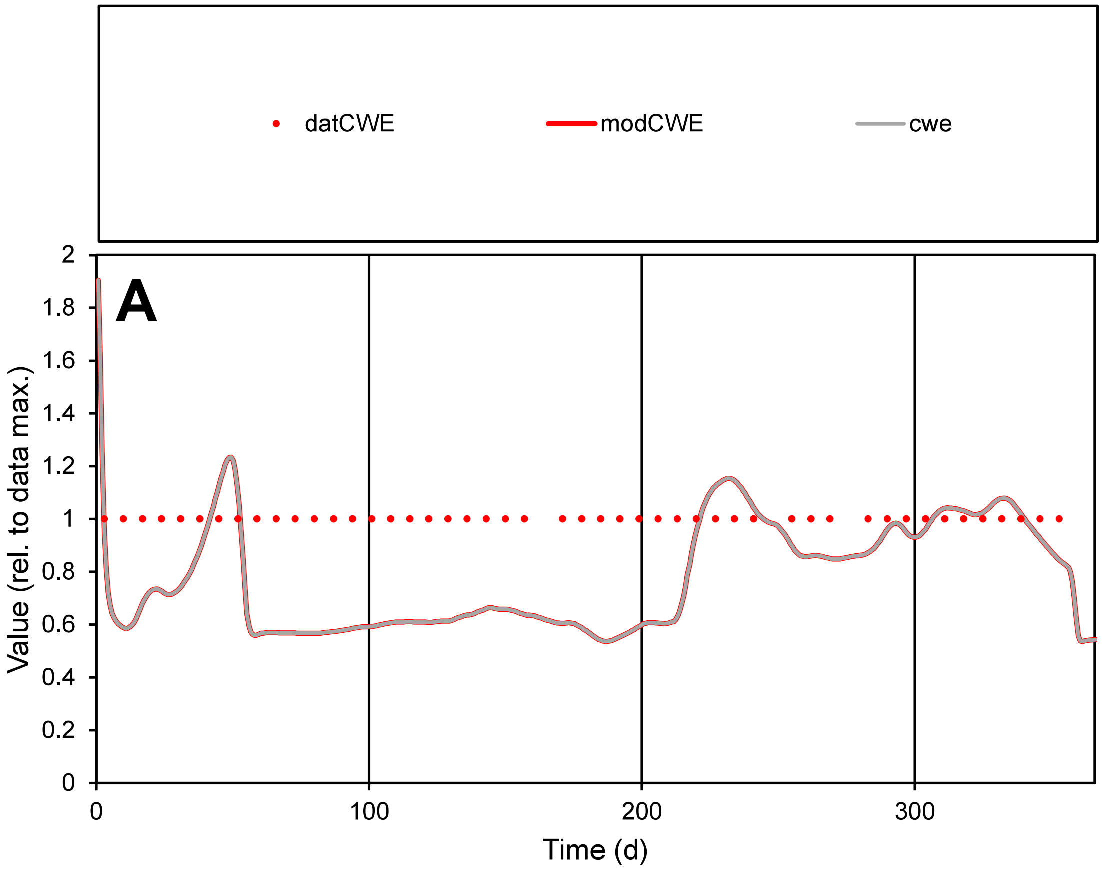

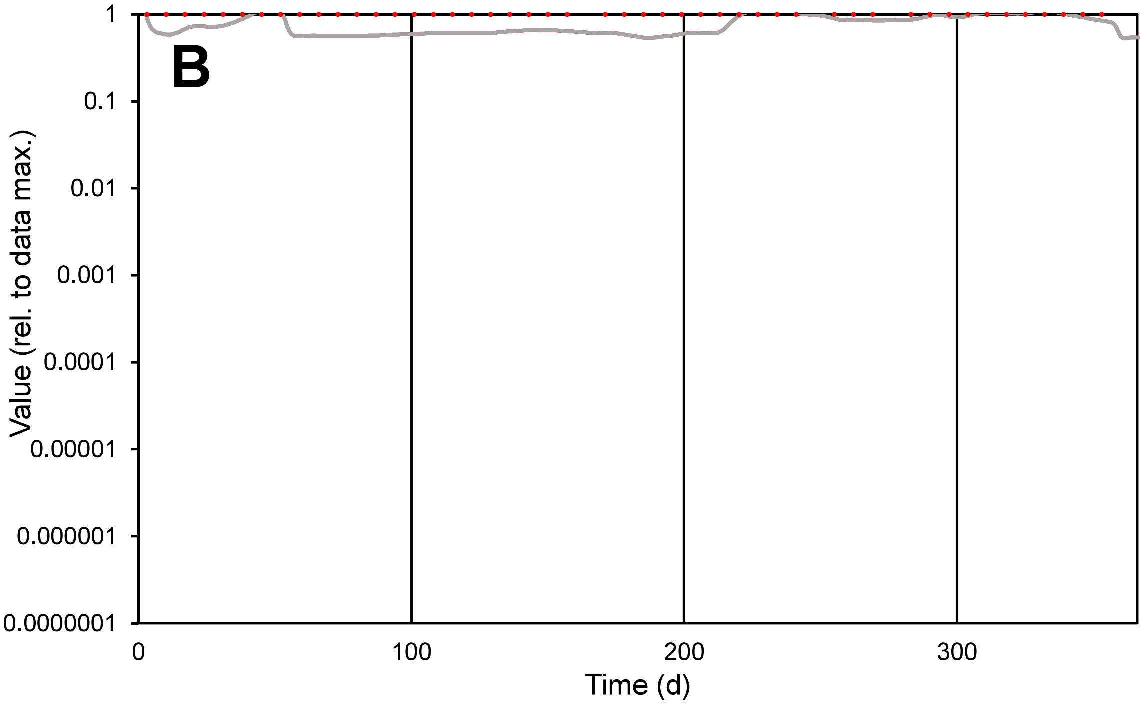

C1

Variables
CWE Provide jtd or Dst
CalibOnly?
2 1 to reproduce of
oNormalize?
2 1=no, 2=yes

ofxn calc*
0.29394
ofxn mod
0.31084

sRun
RunA1
zout
1

Ave
Data
2.6E-05
Model
2E-05

data/vMax
2.6E-05

ofxng
369.894

Min d/m
1.86267

ave/min d
1
ave/min m
1.45384

328_Calib_S_A

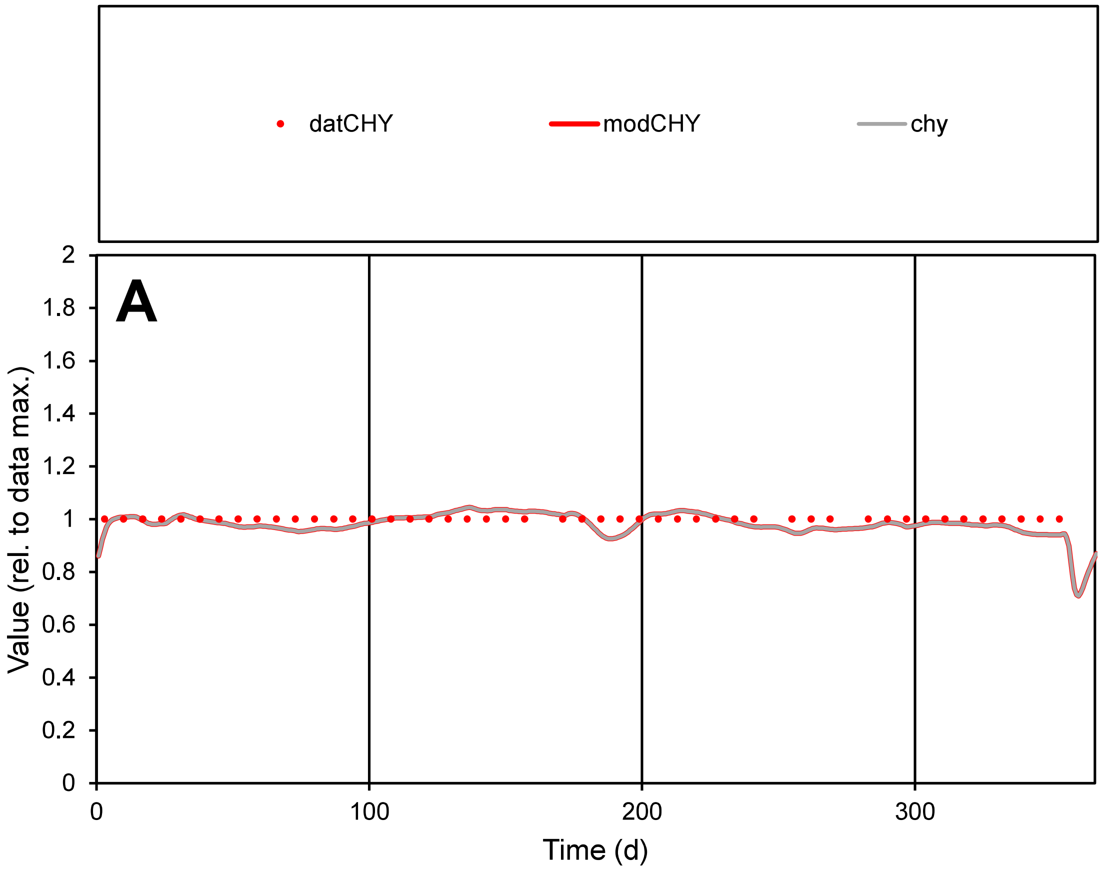

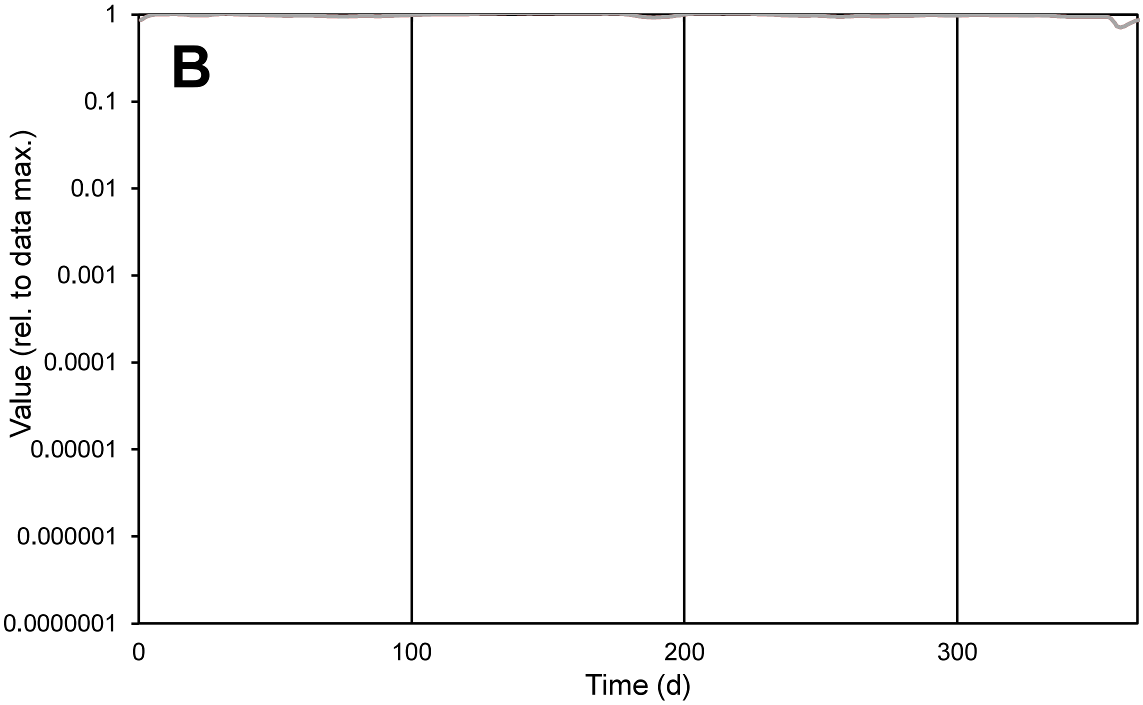

C1

Variables
CHY Provide jtd or Dst
CalibOnly?
2 1 to reproduce of
oNormalize?
2 1=no, 2=yes

ofxn calc*
0.03087
ofxn mod
0.11643

sRun
RunA1
zout
1

Ave
Data
6.7E-06
Model
6.6E-06

data/vMax
6.7E-06

ofxng
369.894

Min d/m
1.07255

ave/min d
1
ave/min m
1.05863

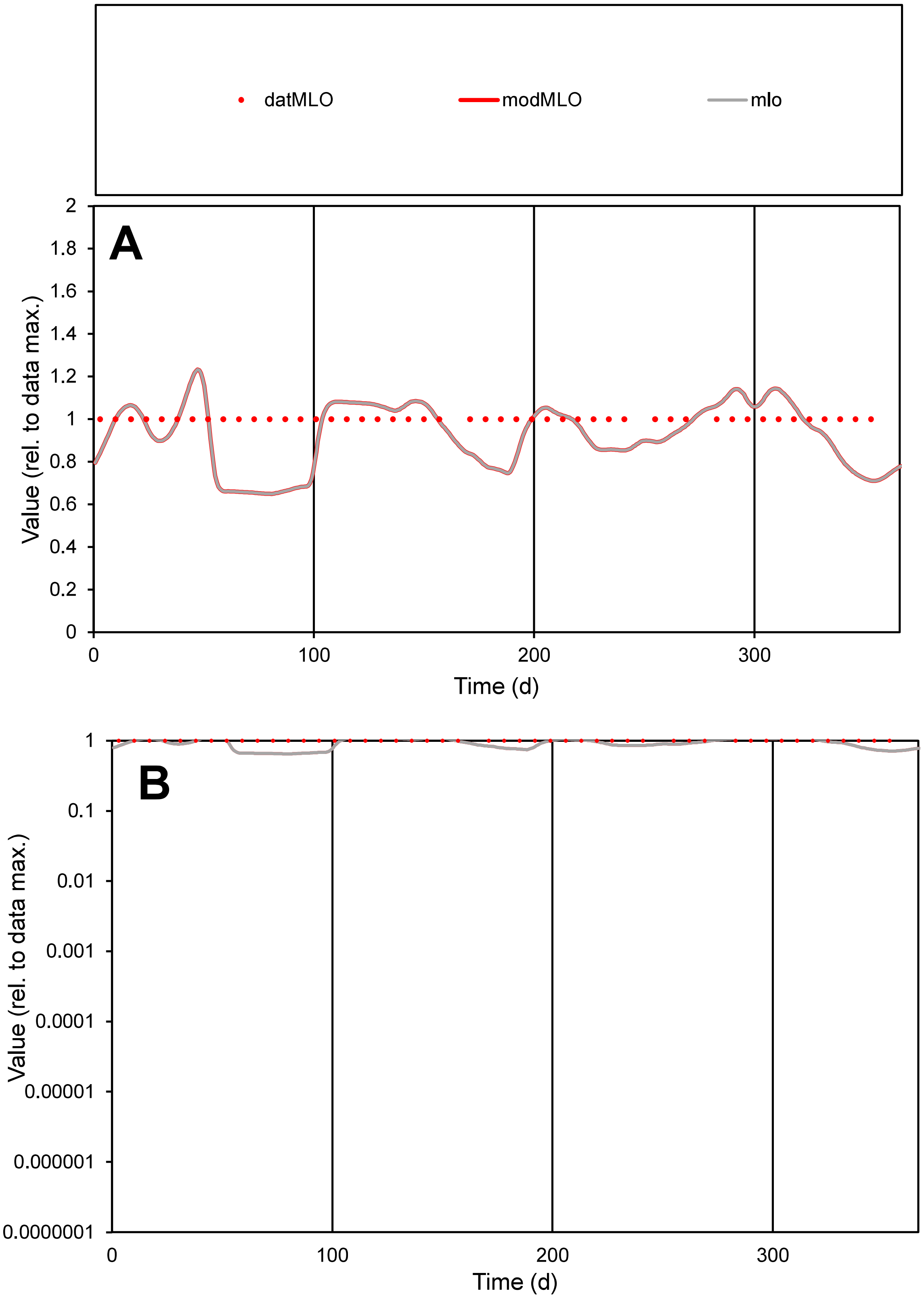

C1

Variables
MLO Provide jtd or Dst
CalibOnly?
2 1 to reproduce of
oNormalize?
2 1=no, 2=yes

ofxn calc*
0.16599
ofxn mod
0.19862

sRun
RunA1
zout
1

Ave
Data
1E-05
Model
9.7E-06

data/vMax
1E-05

ofxng
369.894

Min d/m
1.53948

ave/min d
1
ave/min m
1.43326

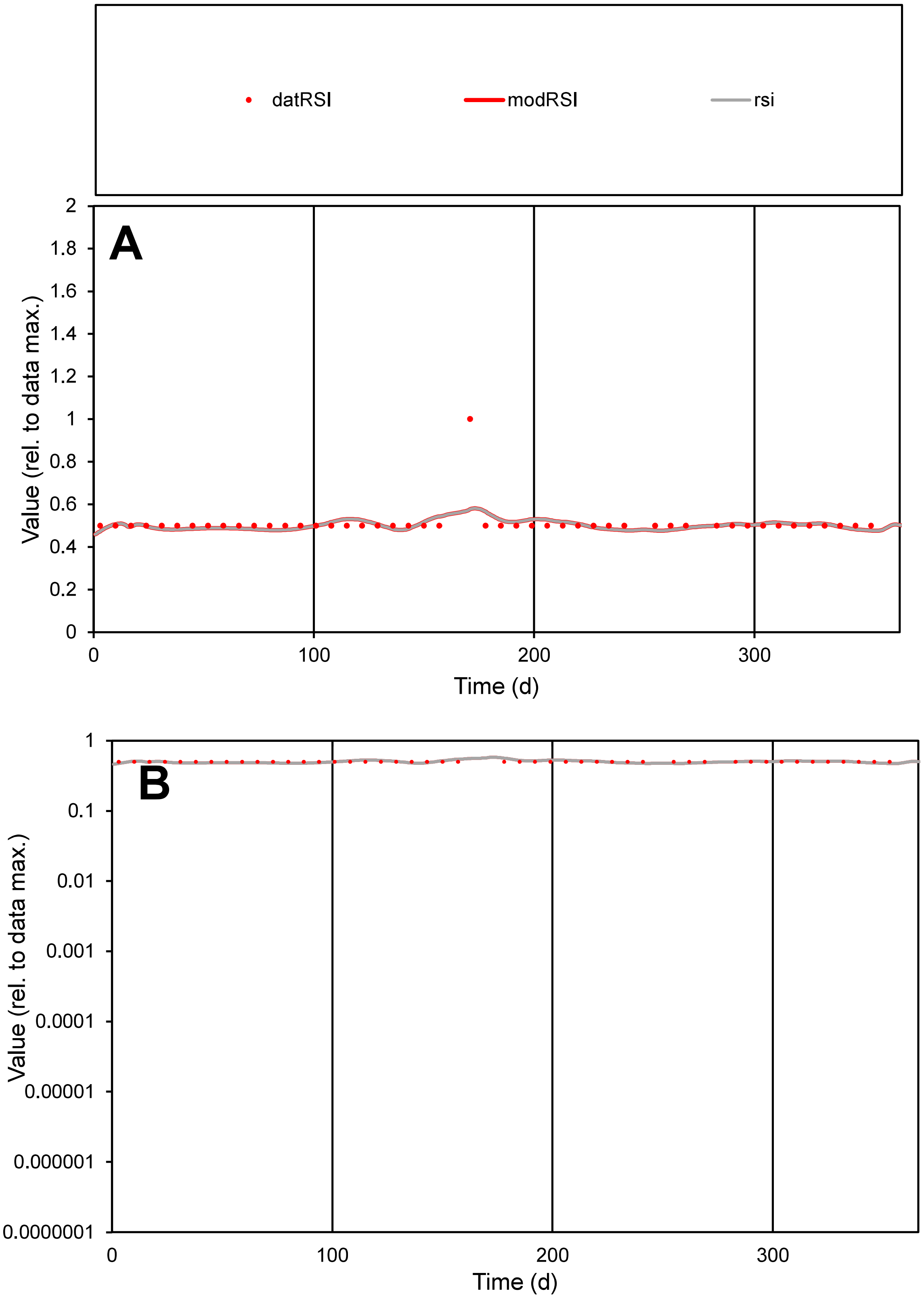

C1

Variables
RSI Provide jtd or Dst
CalibOnly?
2 1 to reproduce of
oNormalize?
2 1=no, 2=yes

ofxn calc*
0.12409
ofxn mod
0.16785

sRun
RunA1
zout
1

Ave
Data
1.1E-05
Model
1E-05

data/vMax
2.1E-05

ofxng
369.894

Min d/m
1.05044

ave/min d
1.02081
ave/min m
1.05522

328_Calib_S_A

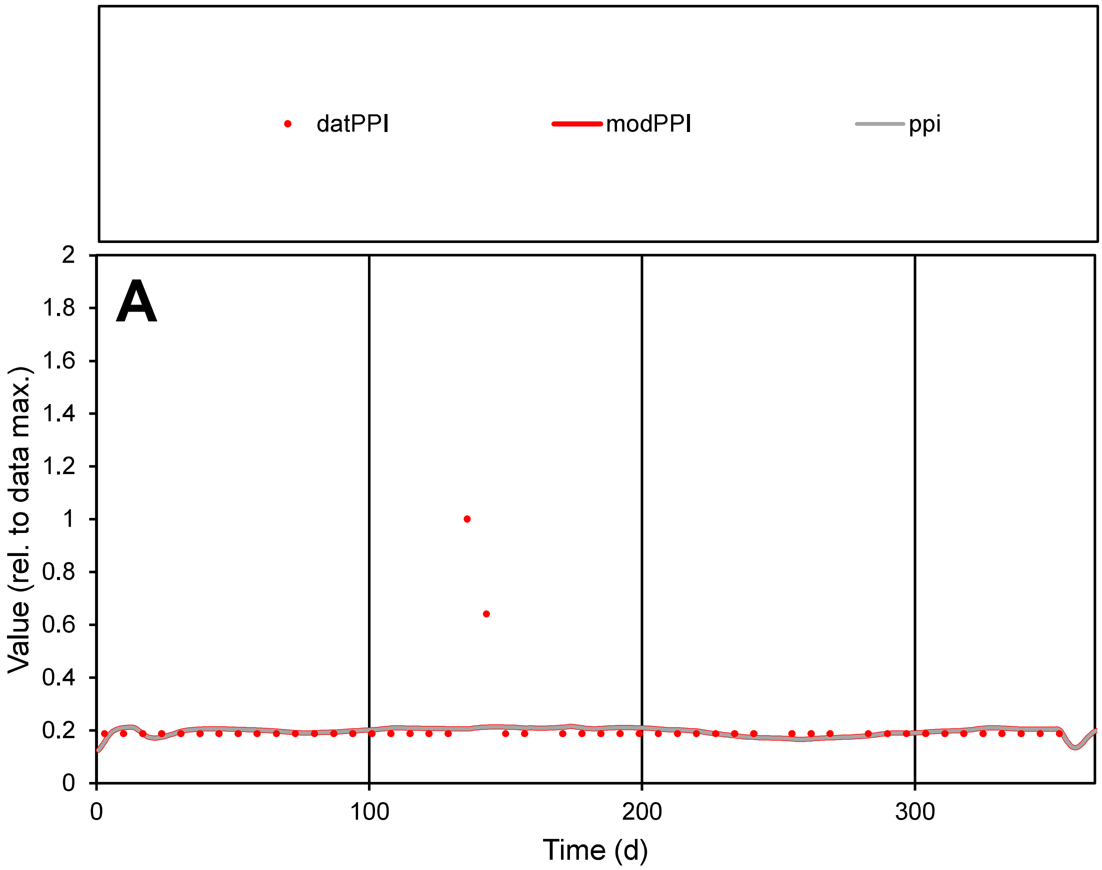

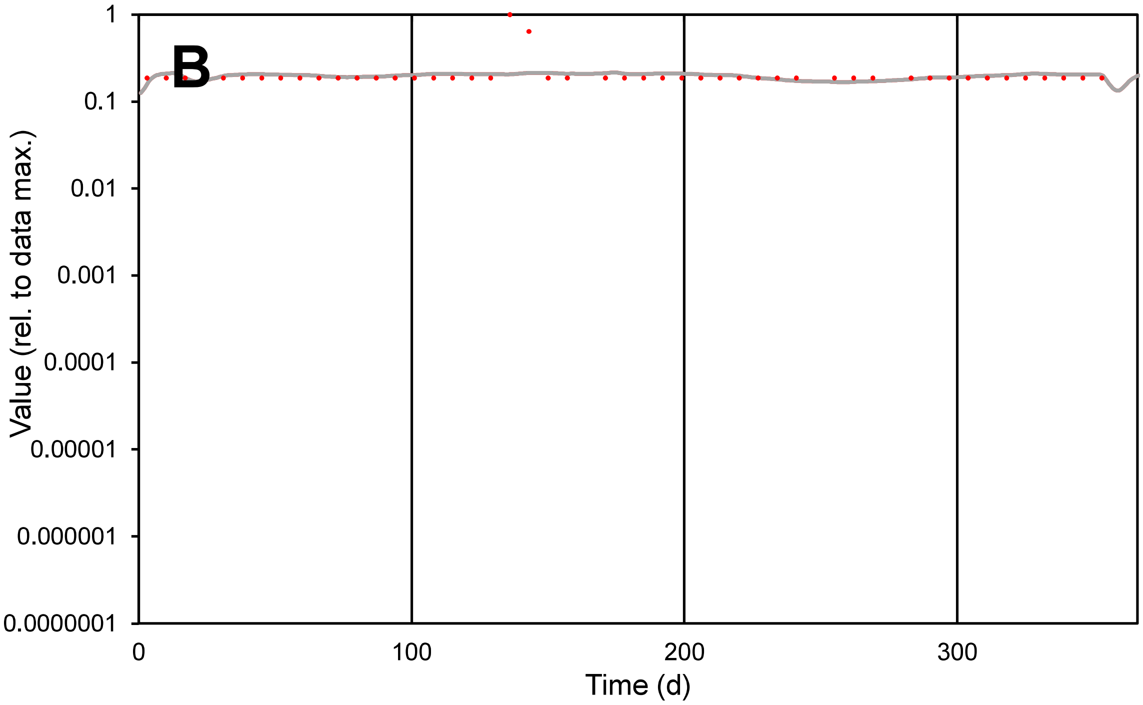

C1

Variables
PPI Provide jtd or Dst
CalibOnly?
2 1 to reproduce of
oNormalize?
2 1=no, 2=yes

ofxn calc*
0.6136
ofxn mod
0.62303

sRun
RunA1
zout
1

Ave
Data
1.7E-06
Model
1.5E-06

data/vMax
7.7E-06

ofxng
369.894

Min d/m
1.12267

ave/min d
1.14108
ave/min m
1.18465

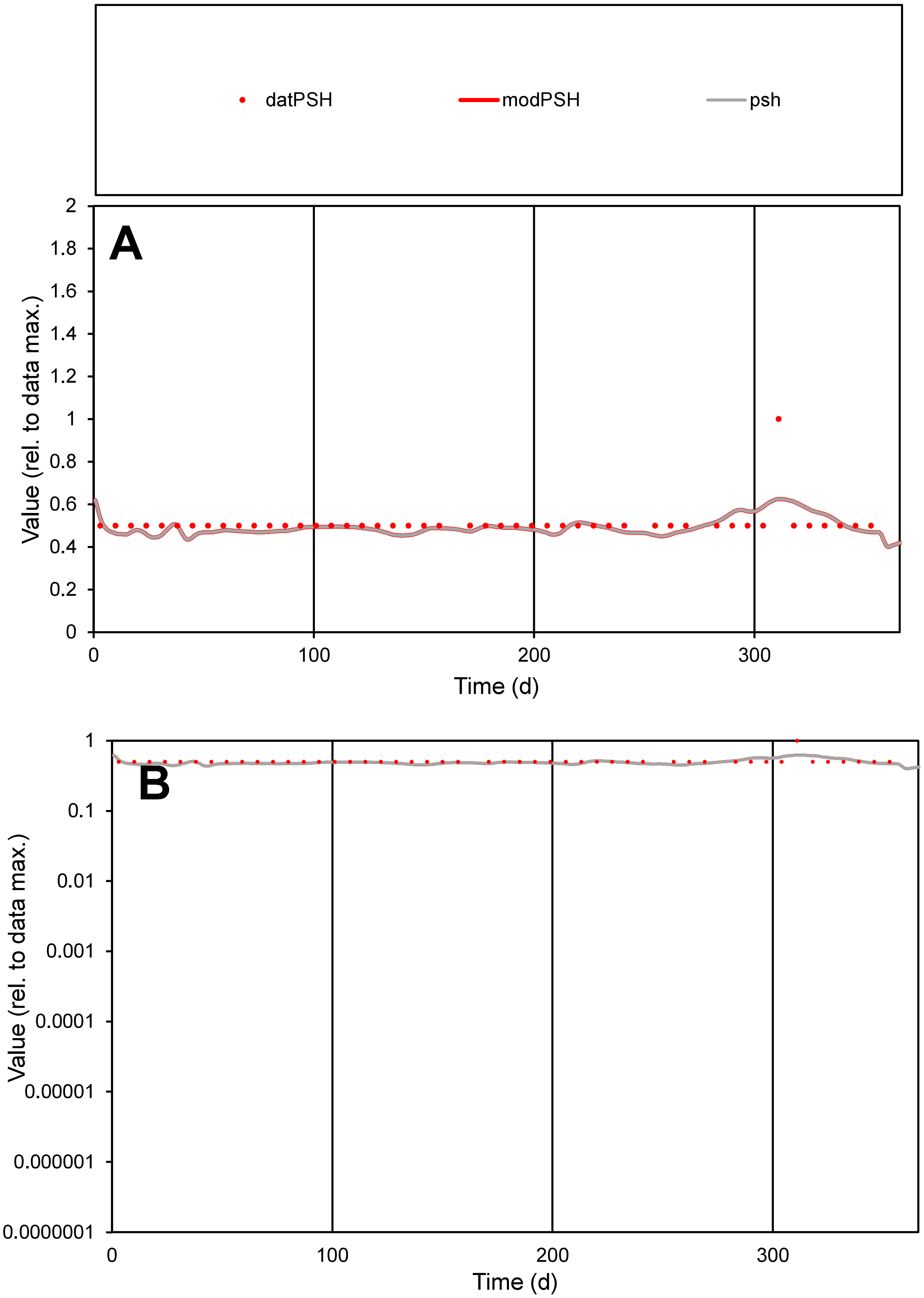

C1

Variables
PSH Provide jtd or Dst
CalibOnly?
2 1 to reproduce of
oNormalize?
2 1=no, 2=yes

ofxn calc*
0.12828
ofxn mod
0.16901

sRun
RunA1
zout
1

Ave
Data
1.3E-05
Model
1.3E-05

data/vMax
2.6E-05

ofxng
369.894

Min d/m
1.1092

ave/min d
1.02085
ave/min m
1.09913

328_Calib_S_A

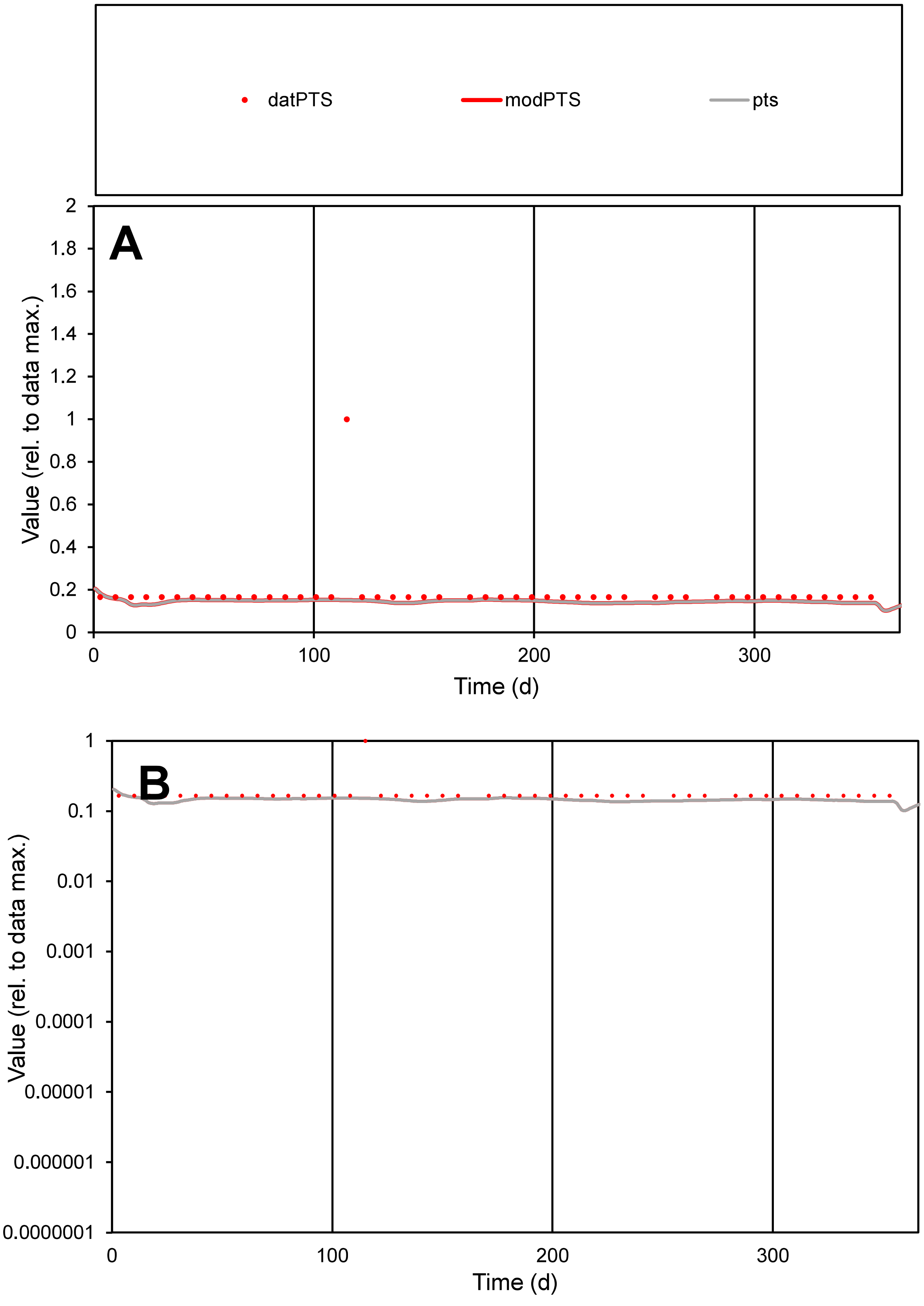

C1

Variables

PTS Provide jtd or Dst

CalibOnly?

2 1 to reproduce of

oNormalize?

2 1=no, 2=yes

ofxn calc*

0.67841

ofxn mod

0.68722

sRun

RunA1

zout

1

Ave

Data

1.1E-05

Model

8.5E-06

data/vMax

5.8E-05

ofxng

369.894

Min d/m

1.2966

ave/min d

1.1049

ave/min m

1.144

328_Calib_S_A

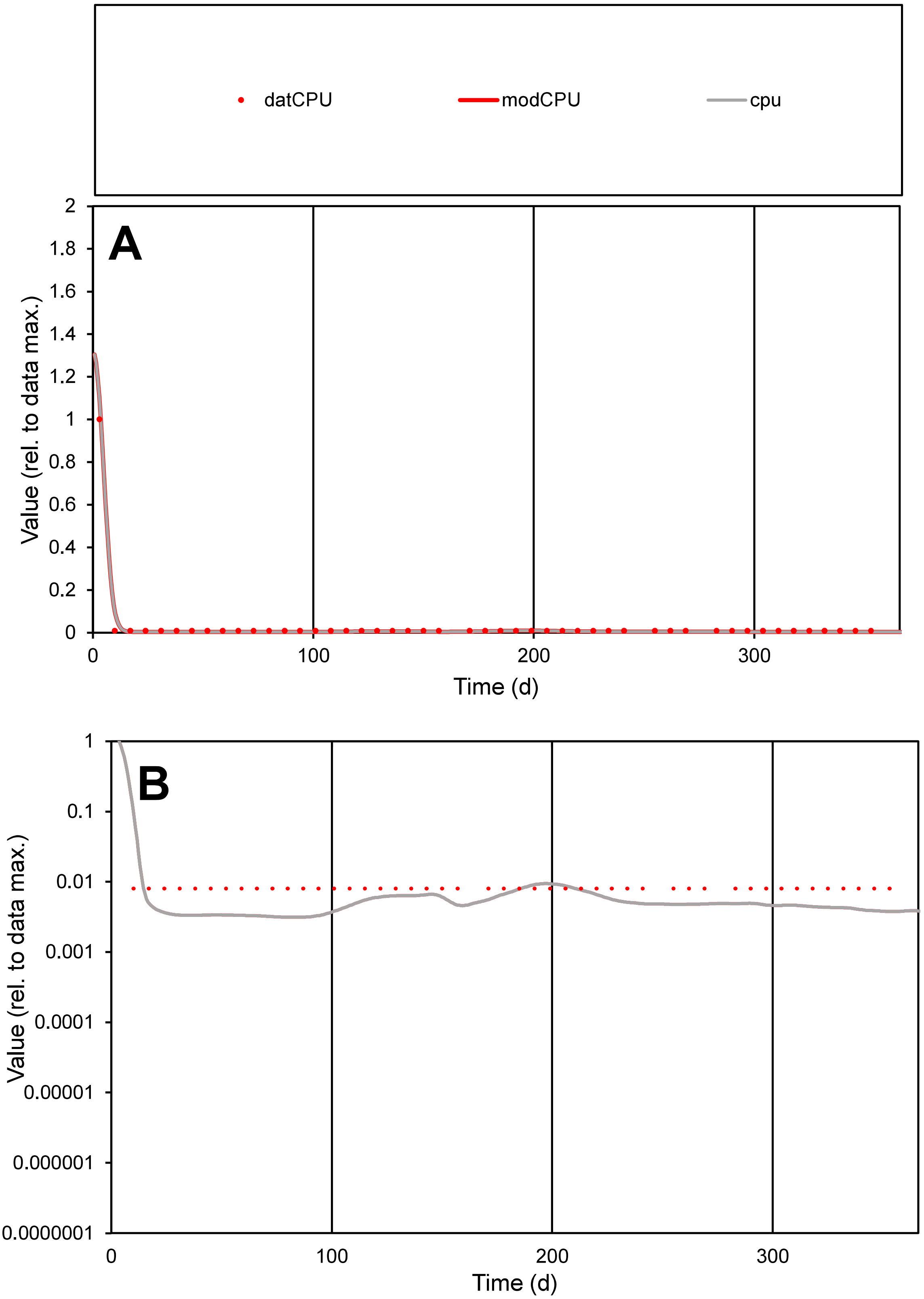

C1

Variables
CPU Provide jtd or Dst
CalibOnly?
2 1 to reproduce of
oNormalize?
2 1=no, 2=yes

ofxn calc*
0.1559
ofxn mod
0.70754

sRun
RunA1
zout
1

Ave
Data
1.6E-06
Model
1.4E-06

data/vMax
5.5E-05

ofxng
369.894

Min d/m
2.56645

ave/min d
3.56661
ave/min m
8.28019

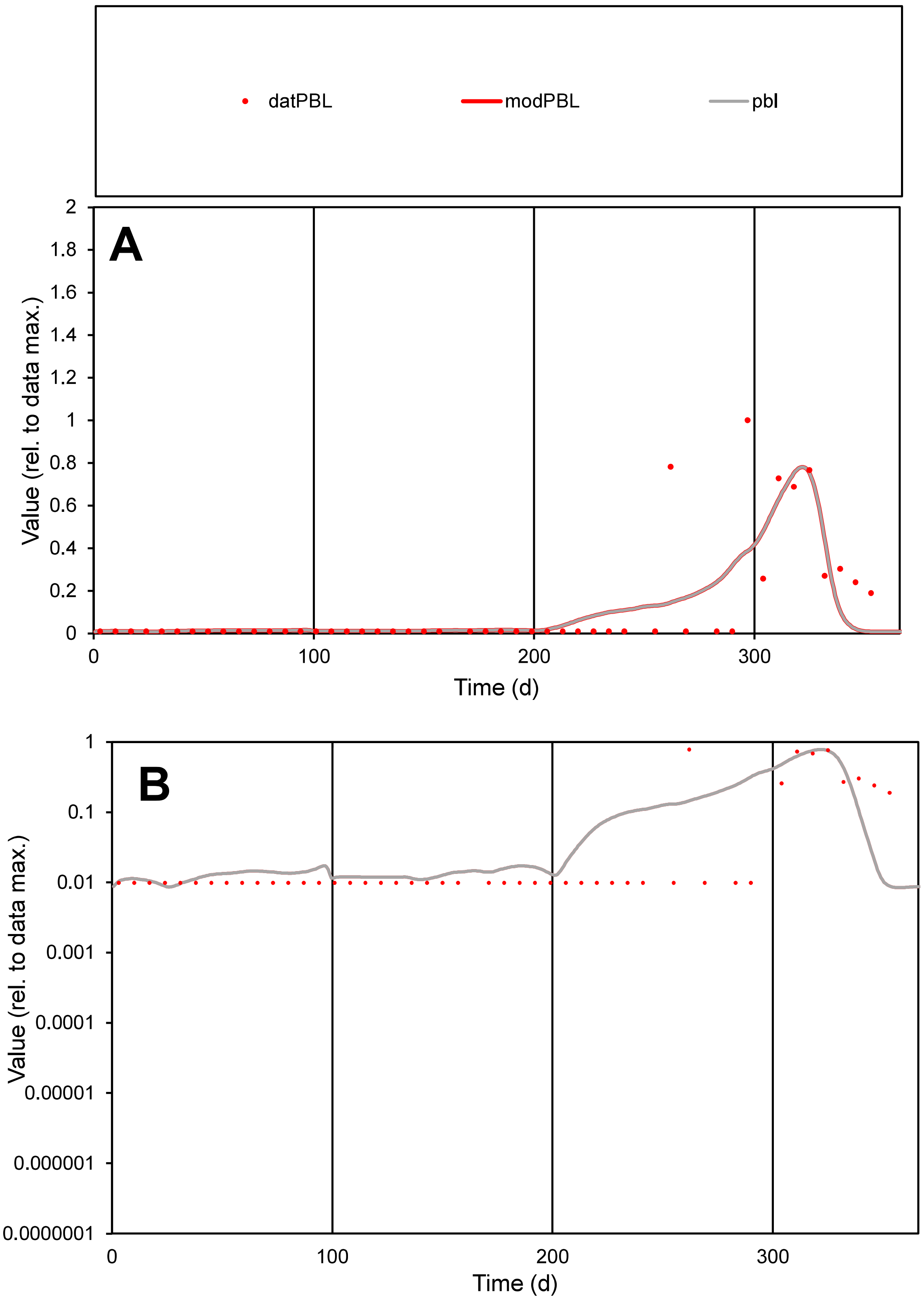

C1

Variables
PBL Provide jtd or Dst
CalibOnly?
2 1 to reproduce of
oNormalize?
2 1=no, 2=yes

ofxn calc*
1.33293
ofxn mod
1.36315

sRun
RunA1
zout
1

Ave
Data
5E-07
Model
4.8E-07

data/vMax
4.3E-06

ofxng
369.894

Min d/m
1.13827

ave/min d
11.8327
ave/min m
12.7528

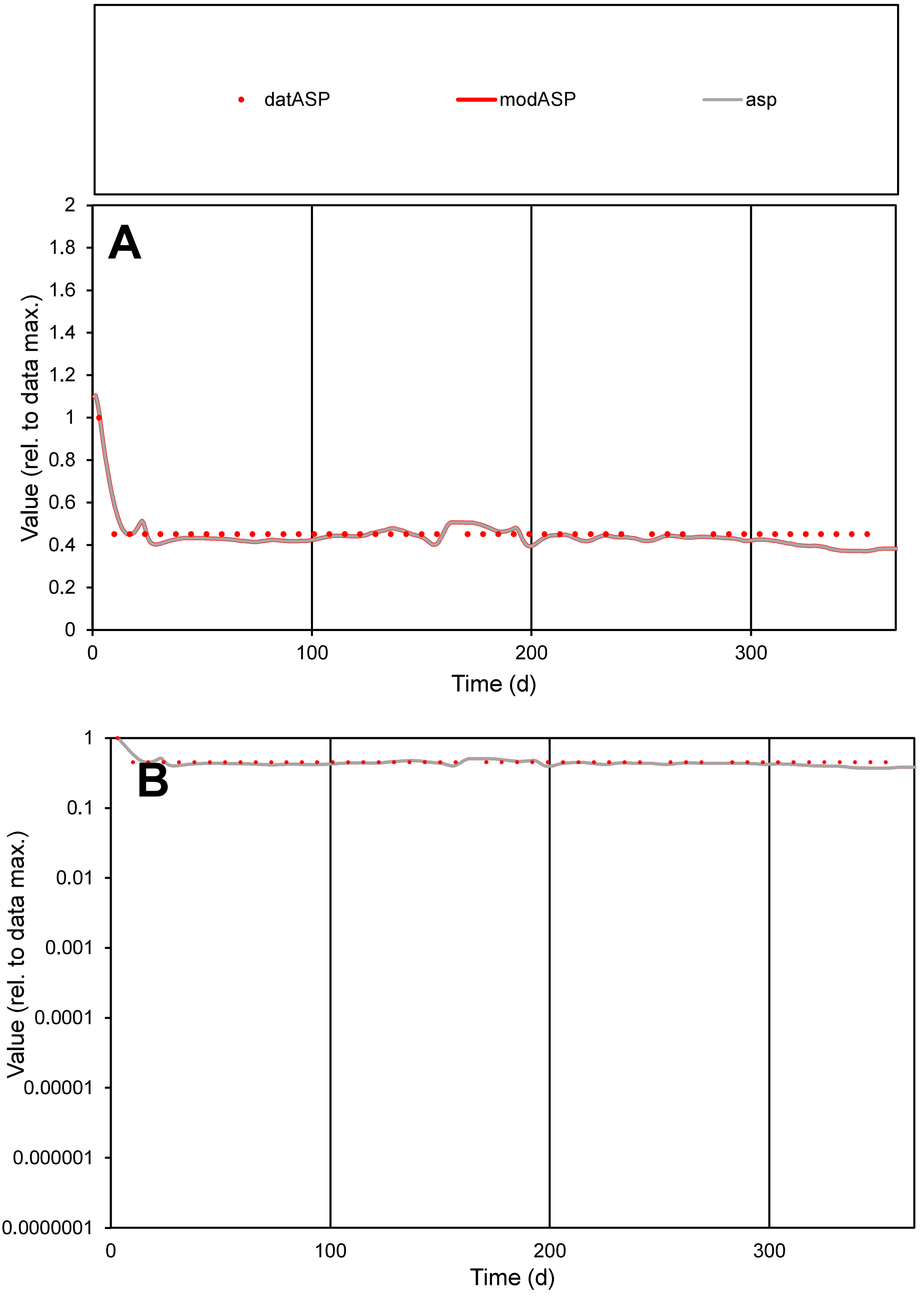

C1

Variables
ASP Provide jtd or Dst
CalibOnly?
2 1 to reproduce of
oNormalize?
2 1=no, 2=yes

ofxn calc*
0.07527
ofxn mod
0.13725

sRun
RunA1
zout
1

Ave
Data
5.9E-06
Model
5.7E-06

data/vMax
1.3E-05

ofxng
369.894

Min d/m
1.21094

ave/min d
1.02534
ave/min m
1.19432

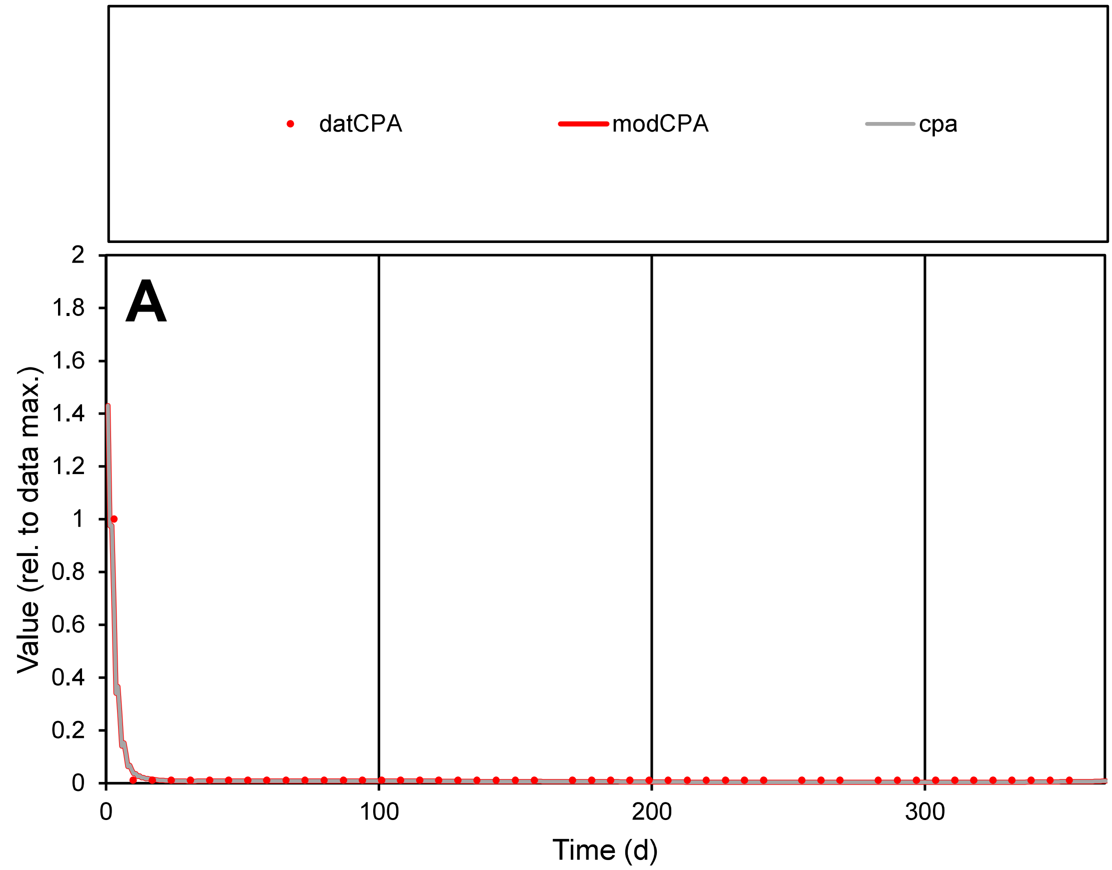

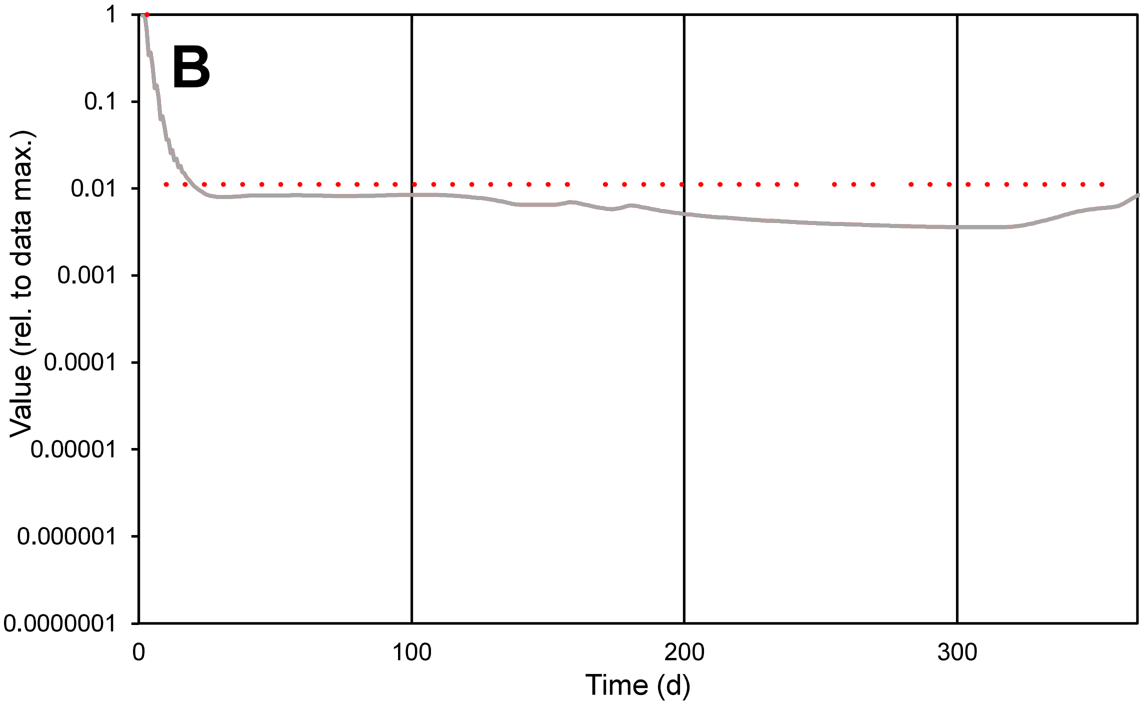

C1

Variables

CPA Provide jtd or Dst

CalibOnly?

2 1 to reproduce of

oNormalize?

2 1=no, 2=yes

ofxn calc*

0.20942

ofxn mod

1.42658

sRun

RunA1

zout

1

Ave

Data

1.9E-07

Model

1.6E-07

data/vMax

5.9E-06

ofxng

369.894

Min d/m

3.11831

ave/min d

2.83917

ave/min m

7.45752

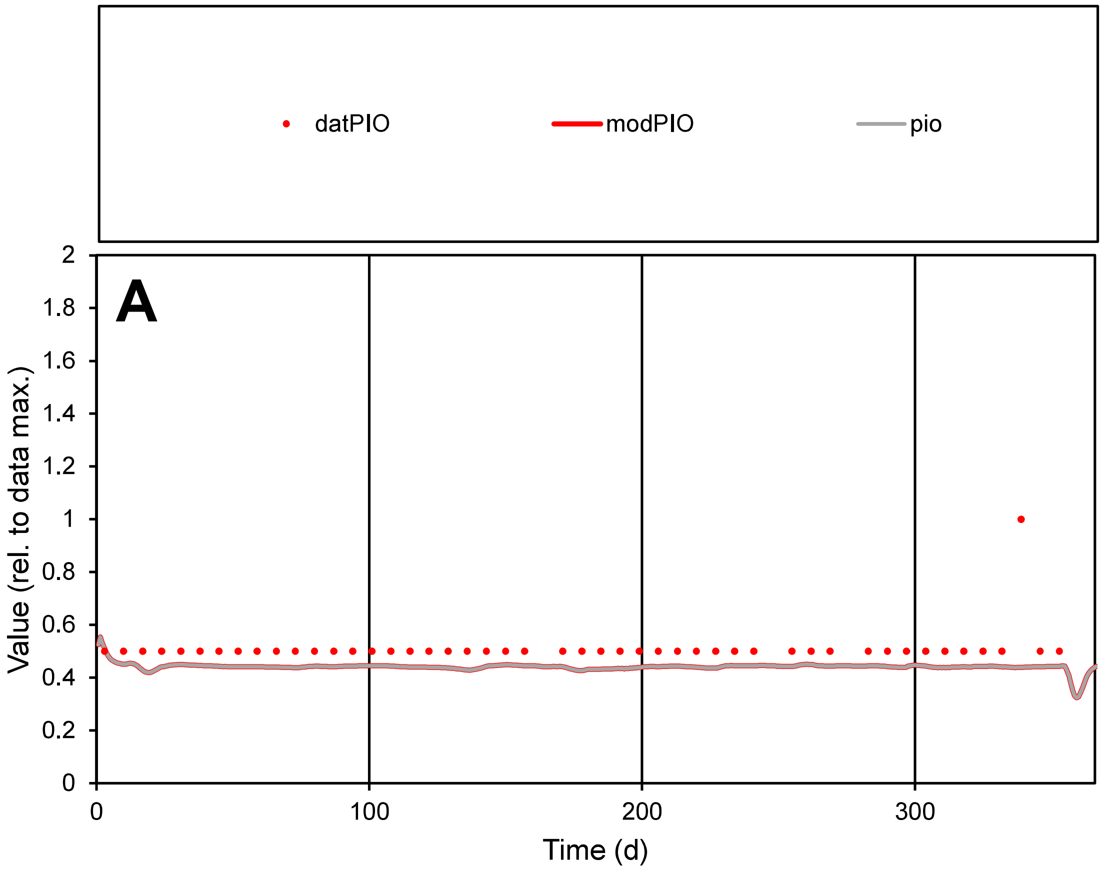

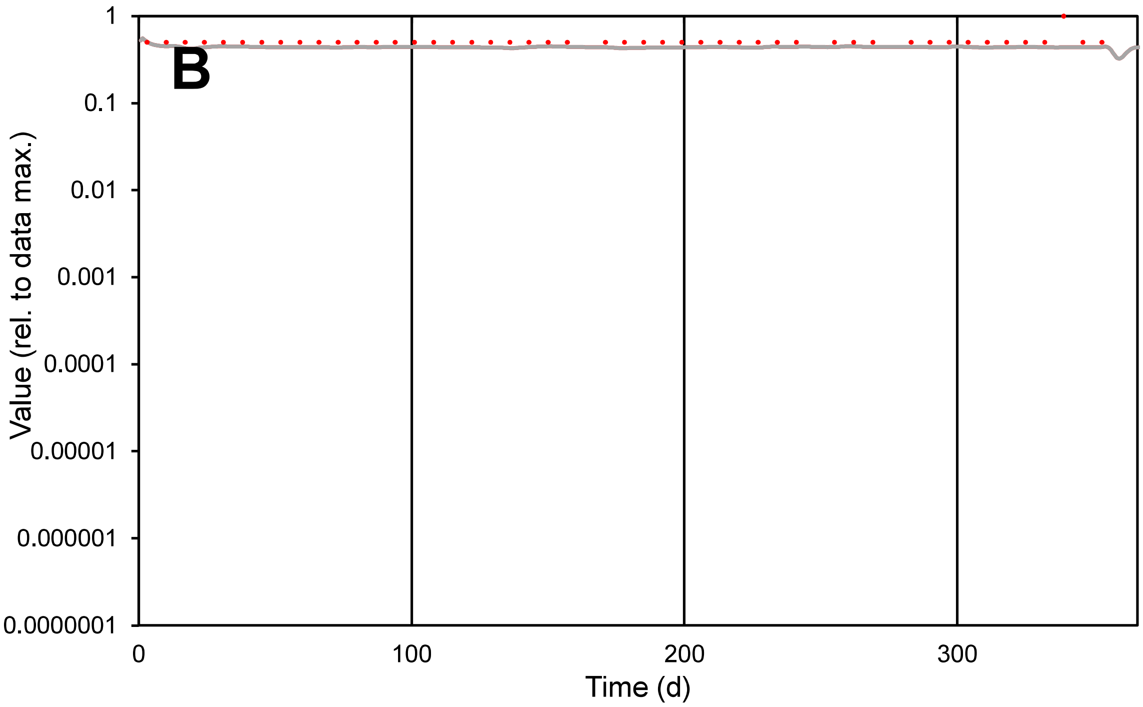

C1

Variables
PIO Provide jtd or Dst
CalibOnly?
2 1 to reproduce of
oNormalize?
2 1=no, 2=yes

ofxn calc*
0.19471
ofxn mod
0.22178

sRun
RunA1
zout
1

Ave
Data
1.5E-07
Model
1.3E-07

data/vMax
2.9E-07

ofxng
369.894

Min d/m
1.18627

ave/min d
1.02085
ave/min m
1.04958

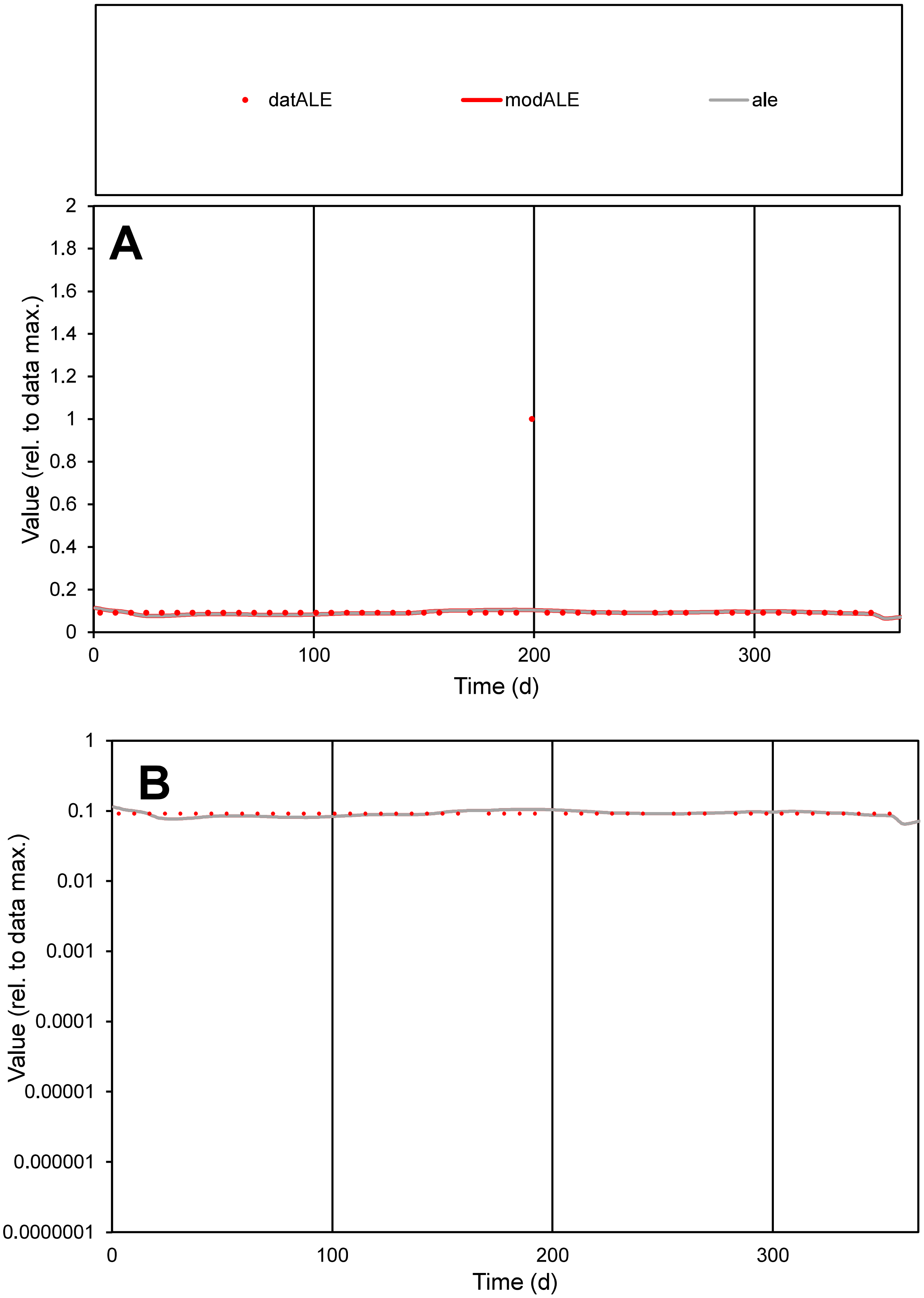

C1

Variables
ALE Provide jtd or Dst
CalibOnly?
2 1 to reproduce of
oNormalize?
2 1=no, 2=yes

ofxn calc*
1.17032
ofxn mod
1.17916

sRun
RunA1
zout
1

Ave
Data
1.3E-05
Model
1.1E-05

data/vMax
0.00012

ofxng
369.894

Min d/m
1.19271

ave/min d
1.20615
ave/min m
1.1904

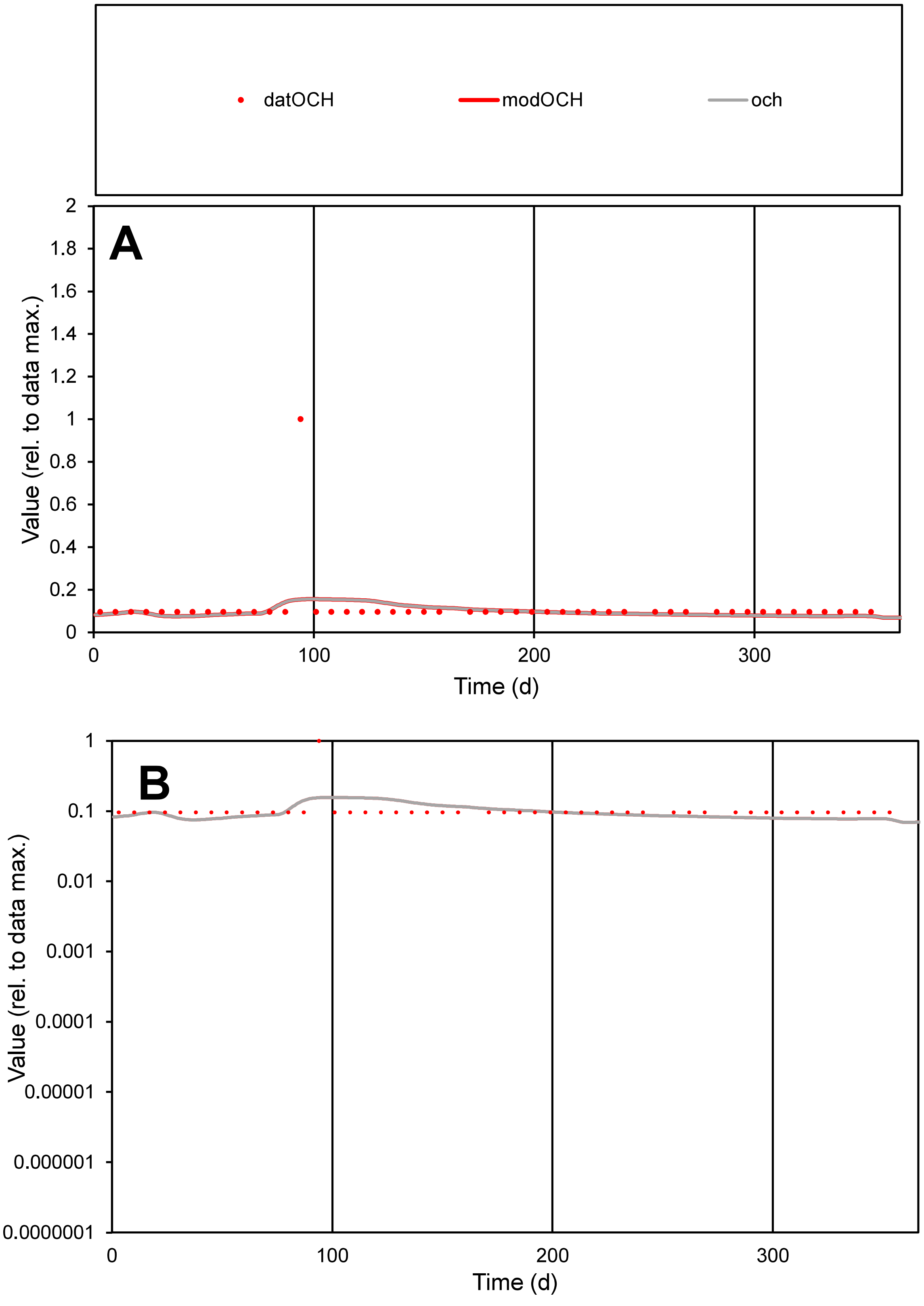

C1

Variables
OCH Provide jtd or Dst
CalibOnly?
2 1 to reproduce of
oNormalize?
2 1=no, 2=yes

ofxn calc*
1.08298
ofxn mod
1.08945

sRun
RunA1
zout
1

Ave
Data
5.3E-06
Model
4.5E-06

data/vMax
4.6E-05

ofxng
369.894

Min d/m
1.27486

ave/min d
1.1965
ave/min m
1.31843

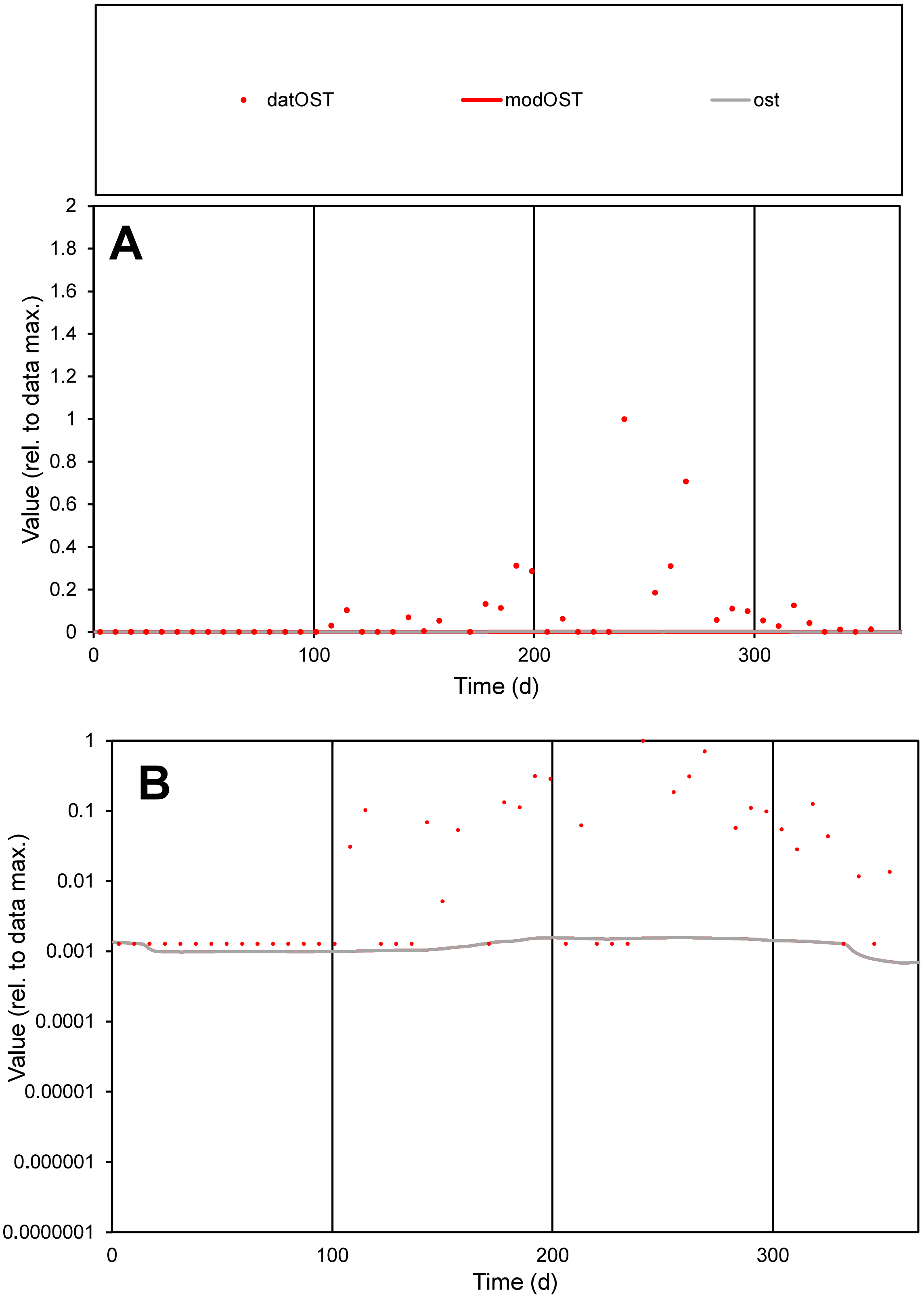

C1

Variables
OST Provide jtd or Dst
CalibOnly?
2 1 to reproduce of
oNormalize?
2 1=no, 2=yes

ofxn calc*
2.41962
ofxn mod
2.42328

sRun
RunA1
zout
1

Ave
Data
0.00014
Model
2.1E-06

data/vMax
0.00169

ofxng
369.894

Min d/m
1.81795

ave/min d
64.255
ave/min m
1.73264

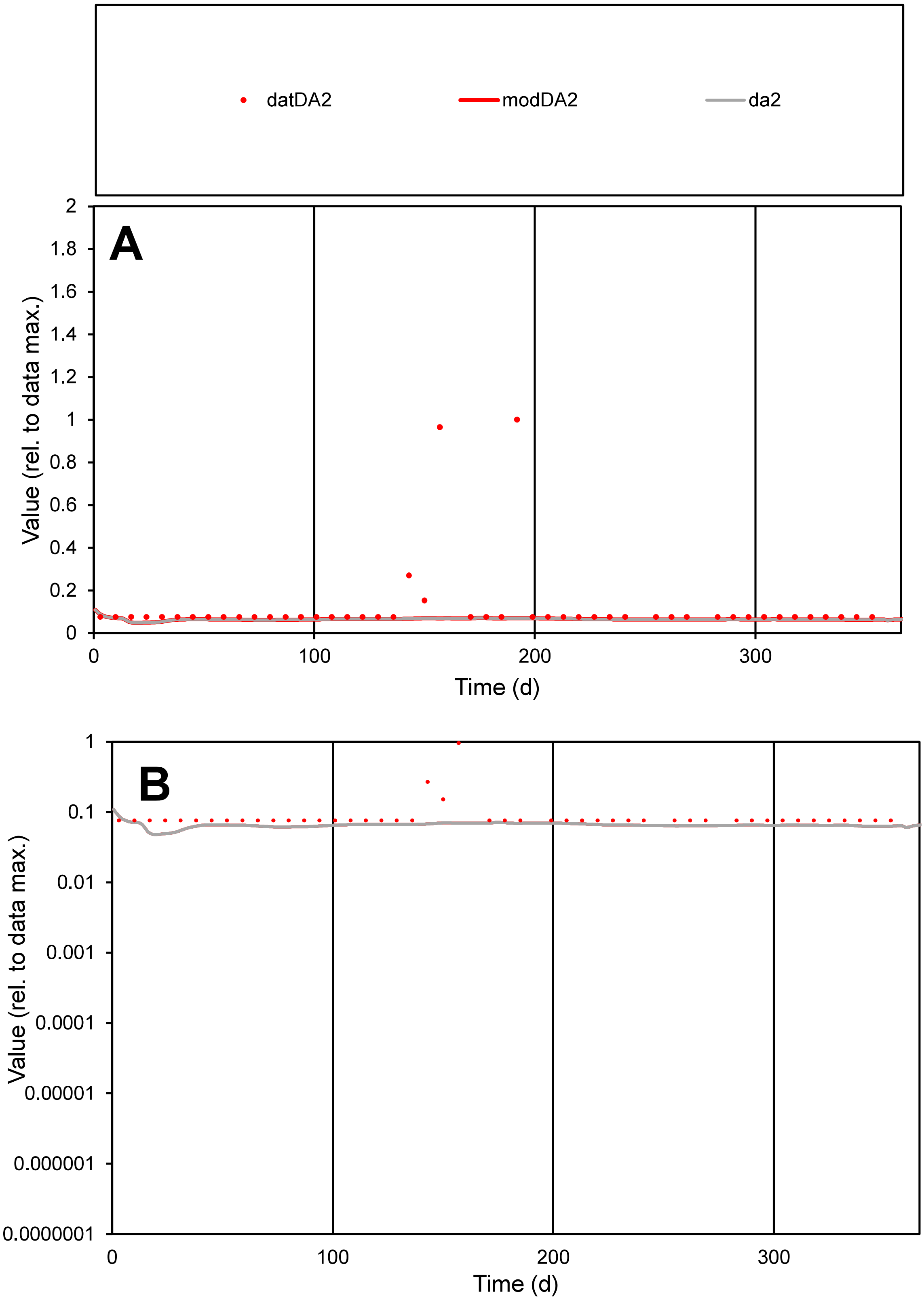

C1

Variables
DA2 Provide jtd or Dst
CalibOnly?
2 1 to reproduce of
oNormalize?
2 1=no, 2=yes

ofxn calc*
1.58112
ofxn mod
1.58691

sRun
RunA1
zout
1

Ave
Data
4.9E-06
Model
2.7E-06

data/vMax
4.1E-05

ofxng
369.894

Min d/m
1.56863

ave/min d
1.56804
ave/min m
1.34372

328_Calib_S_A

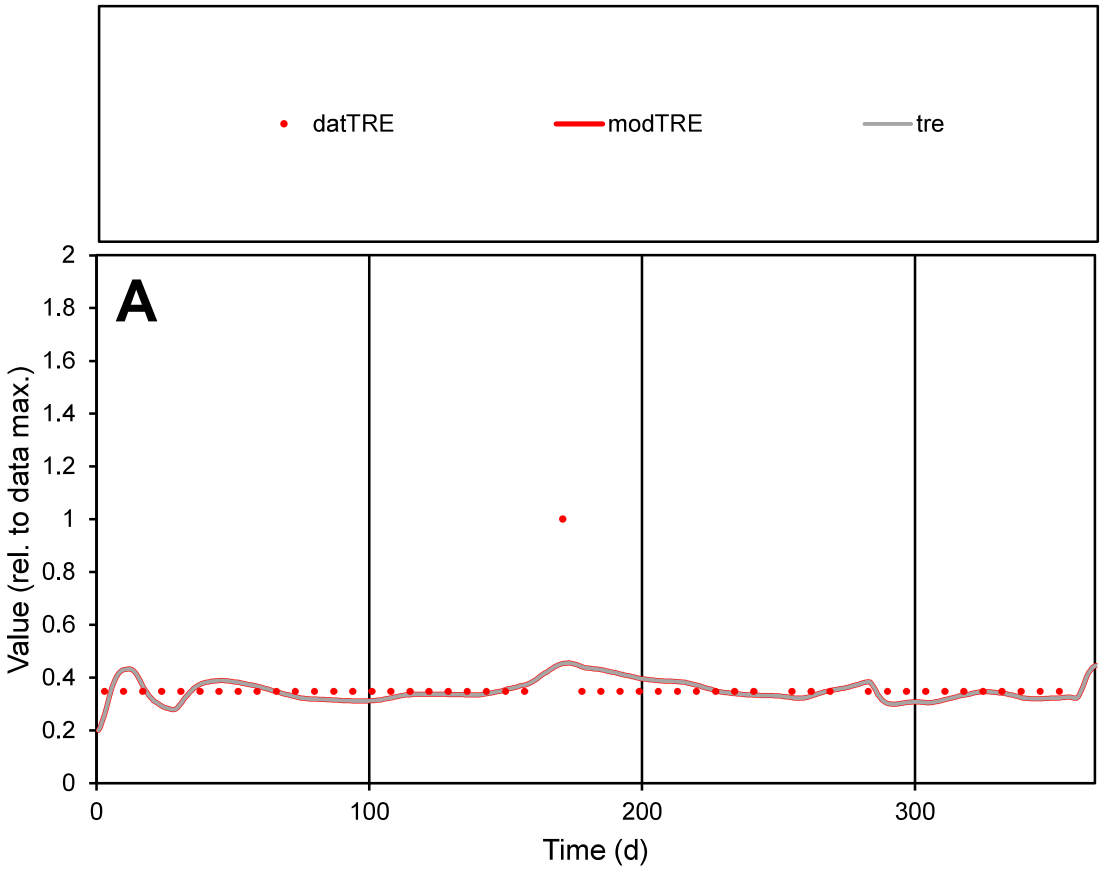

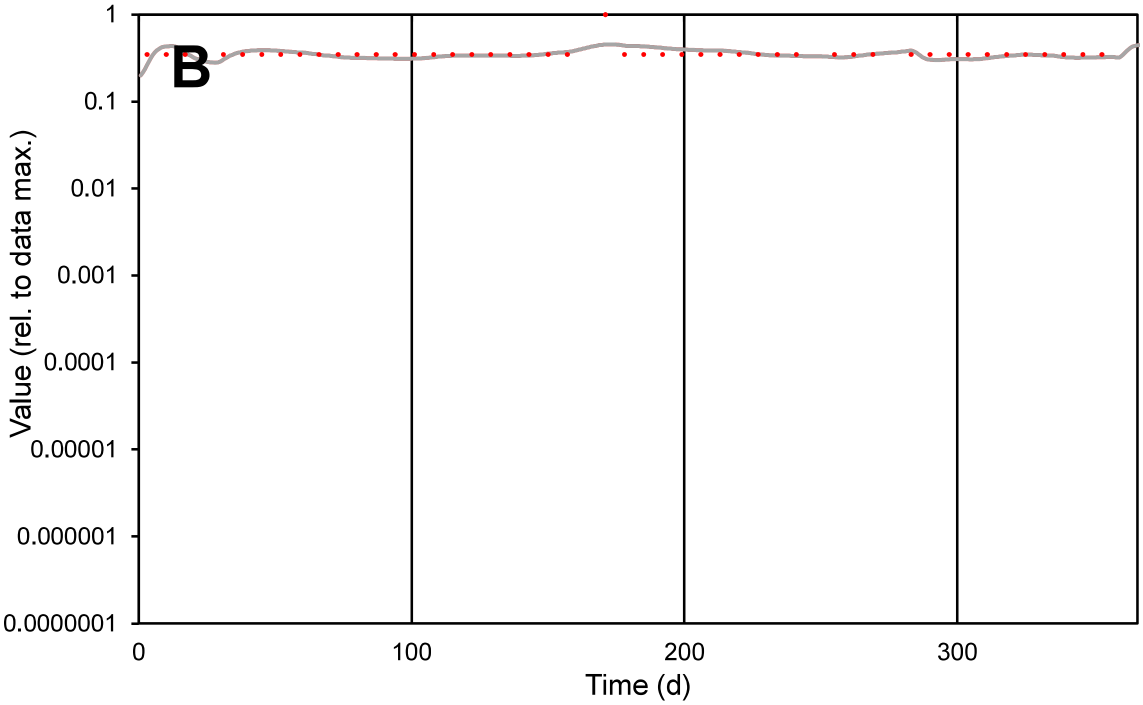

C1

Variables
TRE Provide jtd or Dst
CalibOnly?
2 1 to reproduce of
oNormalize?
2 1=no, 2=yes

ofxn calc*
0.23799
ofxn mod
0.26369

sRun
RunA1
zout
1

Ave
Data
7.2E-06
Model
7E-06

data/vMax
2E-05

ofxng
369.894

Min d/m
1.20484

ave/min d
1.03911
ave/min m
1.21205

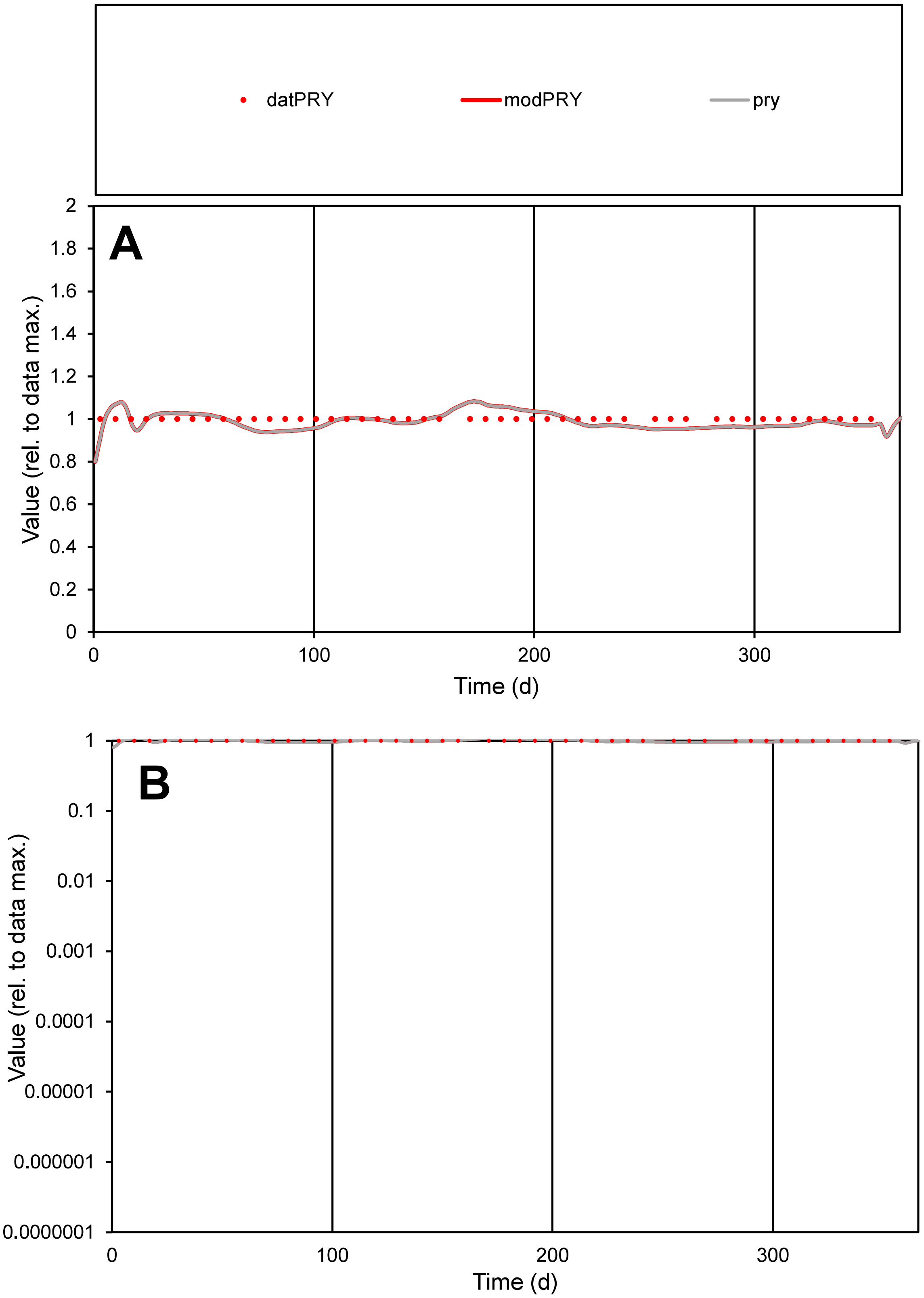

C1

Variables
PRY Provide jtd or Dst
CalibOnly?
2 1 to reproduce of
oNormalize?
2 1=no, 2=yes

ofxn calc*
0.03659
ofxn mod
0.11859

sRun
RunA1
zout
1

Ave
Data
8.8E-06
Model
8.7E-06

data/vMax
8.8E-06

ofxng
369.894

Min d/m
1.06296

ave/min d
1
ave/min m
1.05331

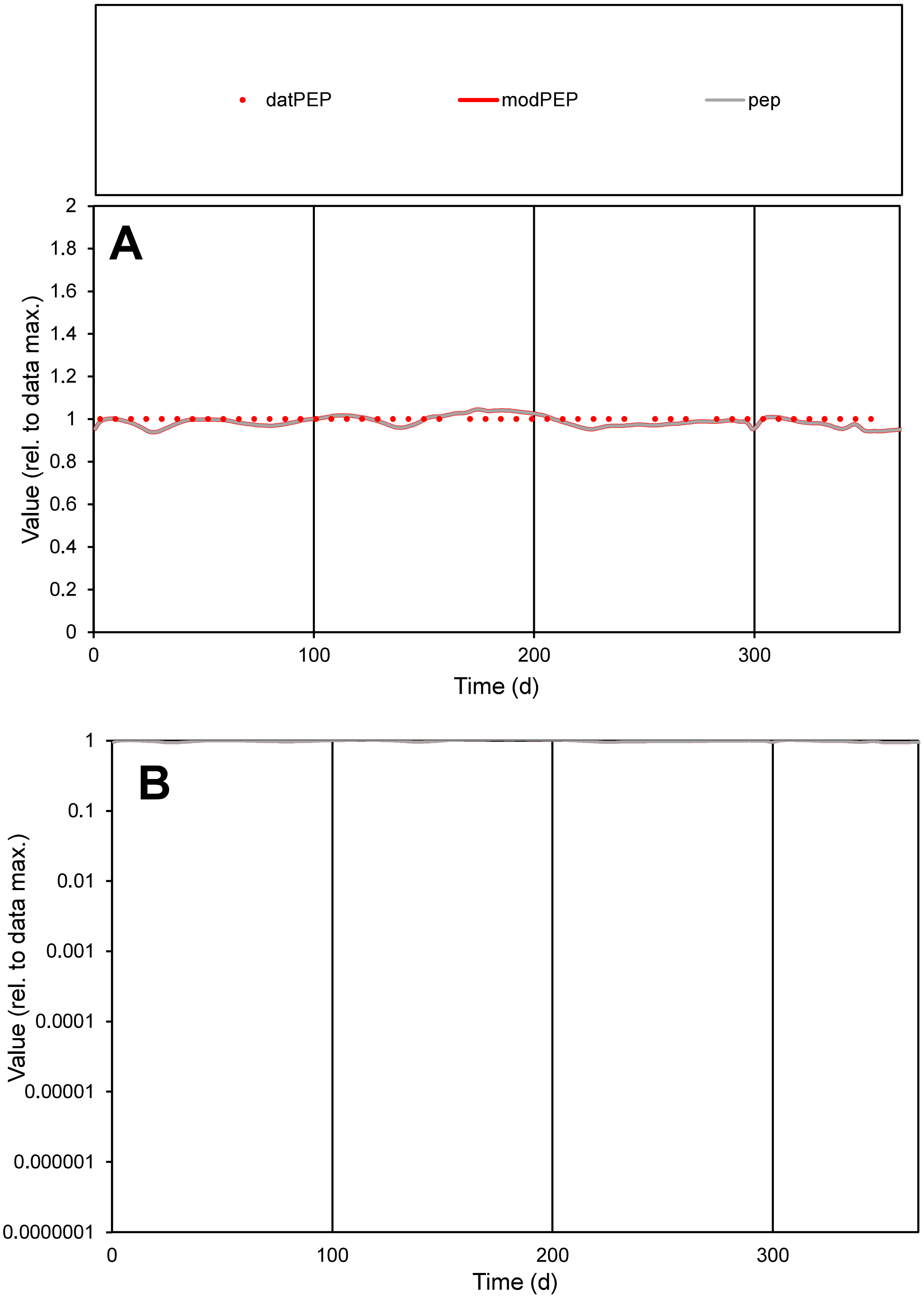

C1

Variables
PEP Provide jtd or Dst
CalibOnly?
2 1 to reproduce of
oNormalize?
2 1=no, 2=yes

ofxn calc*
0.02699
ofxn mod
0.11568

sRun
RunA1
zout
1

Ave
Data
4.9E-06
Model
4.9E-06

data/vMax
4.9E-06

ofxng
369.894

Min d/m
1.06062

ave/min d
1
ave/min m
1.04846

328_Calib_S_A

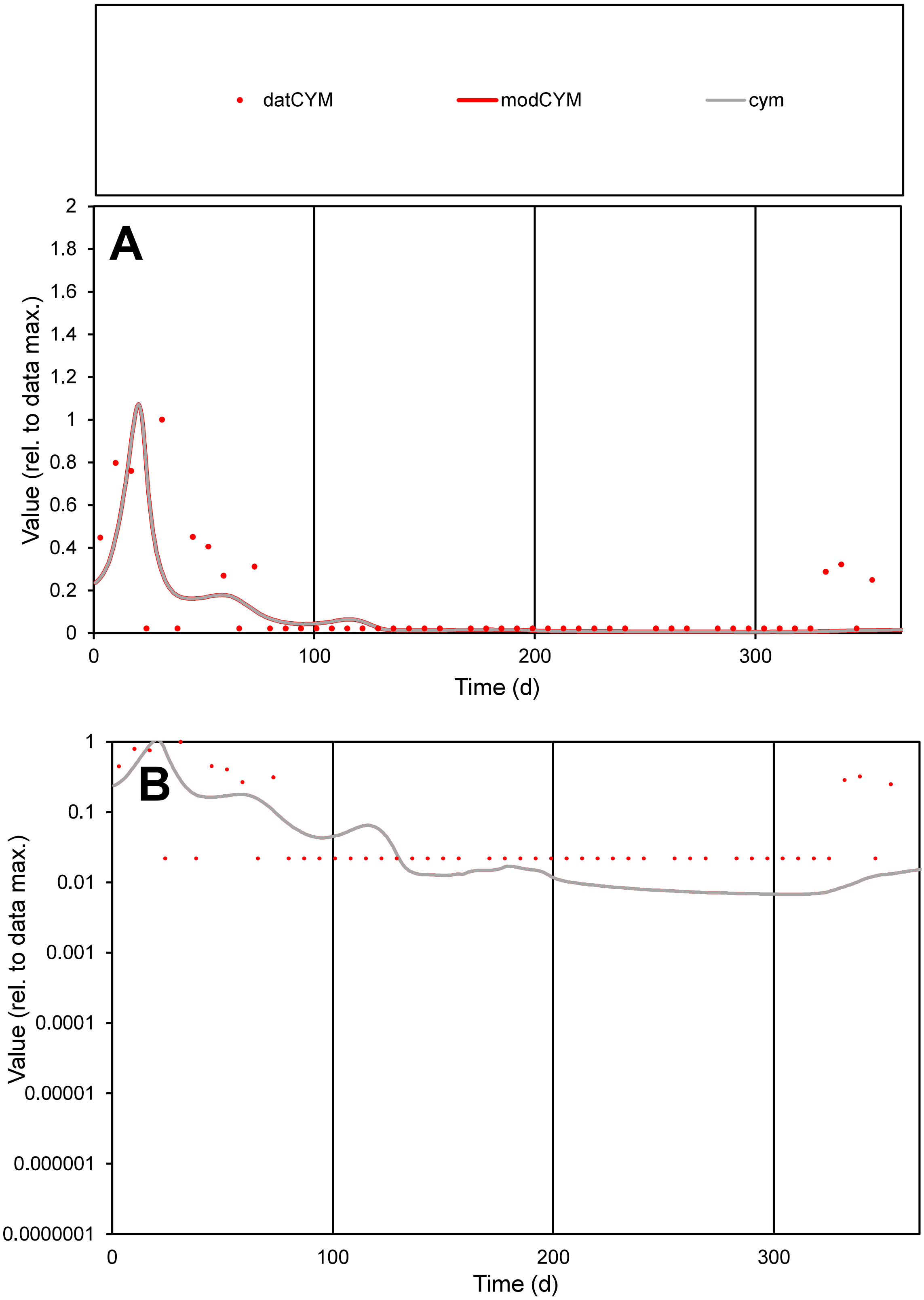

C1

Variables
CYM Provide jtd or Dst
CalibOnly?
2 1 to reproduce of
oNormalize?
2 1=no, 2=yes

ofxn calc*
1.28261
ofxn mod
1.33844

sRun
RunA1
zout
1

Ave
Data
7.9E-06
Model
5.2E-06

data/vMax
6.2E-05

ofxng
369.894

Min d/m
3.23508

ave/min d
5.78568
ave/min m
12.4349

328_Calib_S_A

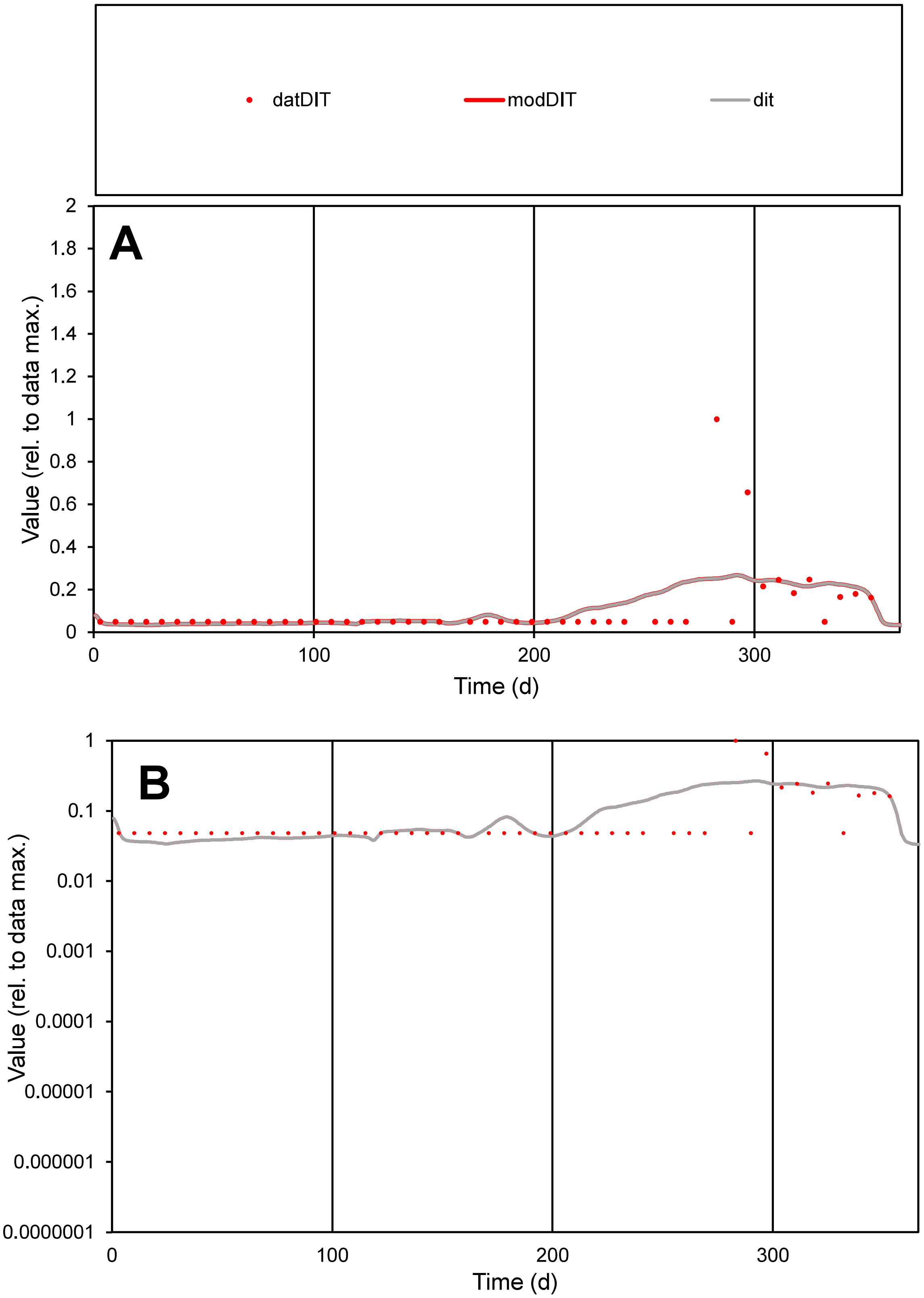

C1

Variables
DIT Provide jtd or Dst
CalibOnly?
2 1 to reproduce of
oNormalize?
2 1=no, 2=yes

ofxn calc*
1.34014
ofxn mod
1.35152

sRun
RunA1
zout
1

Ave
Data
1.1E-05
Model
1.1E-05

data/vMax
0.00011

ofxng
369.894

Min d/m
1.43354

ave/min d
2.12257
ave/min m
3.07489

328_Calib_S_A

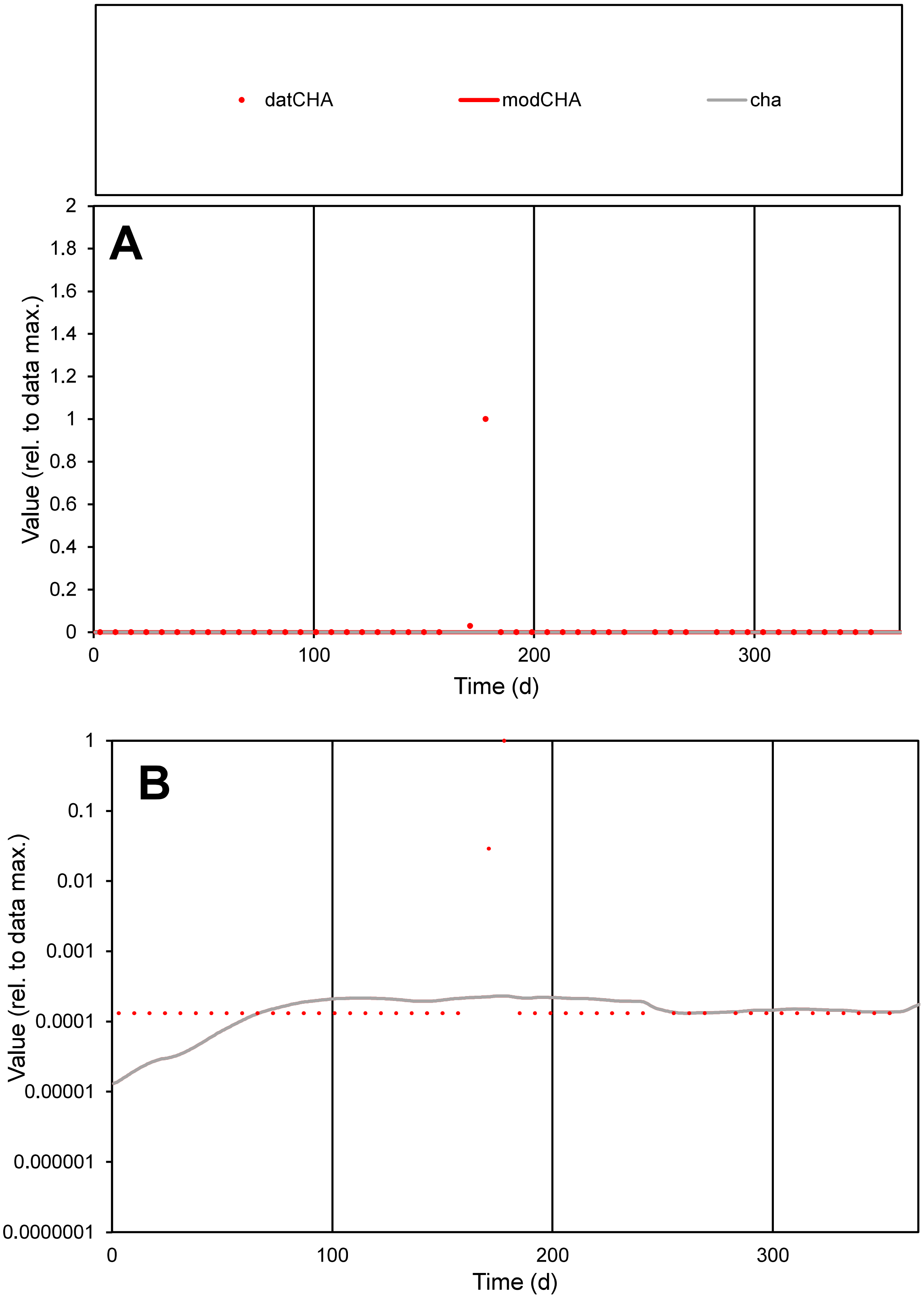

C1

Variables
CHA Provide jtd or Dst
CalibOnly?
2 1 to reproduce of
oNormalize?
2 1=no, 2=yes

ofxn calc*
6.69427
ofxn mod
6.70893

sRun
RunA1
zout
1

Ave
Data
1.4E-05
Model
1E-07

data/vMax
0.00066

ofxng
369.894

Min d/m
8.71513

ave/min d
164.301
ave/min m
10.315

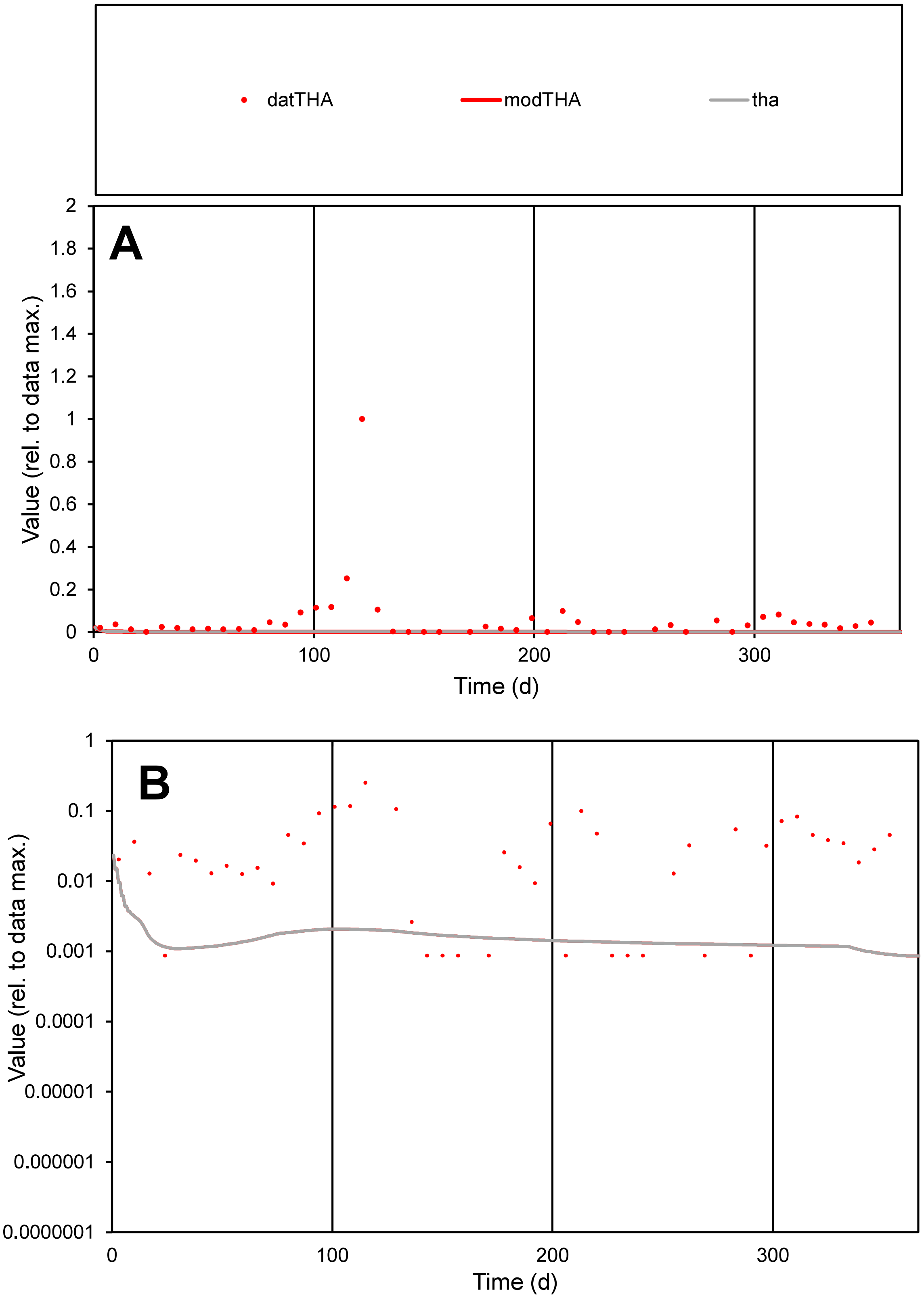

C1

Variables
THA Provide jtd or Dst
CalibOnly?
2 1 to reproduce of
oNormalize?
2 1=no, 2=yes

ofxn calc*
2.73521
ofxn mod
2.7413

sRun
RunA1
zout
1

Ave
Data
0.00018
Model
5.6E-06

data/vMax
0.00315

ofxng
369.894

Min d/m
0.97589

ave/min d
64.8519
ave/min m
1.97551

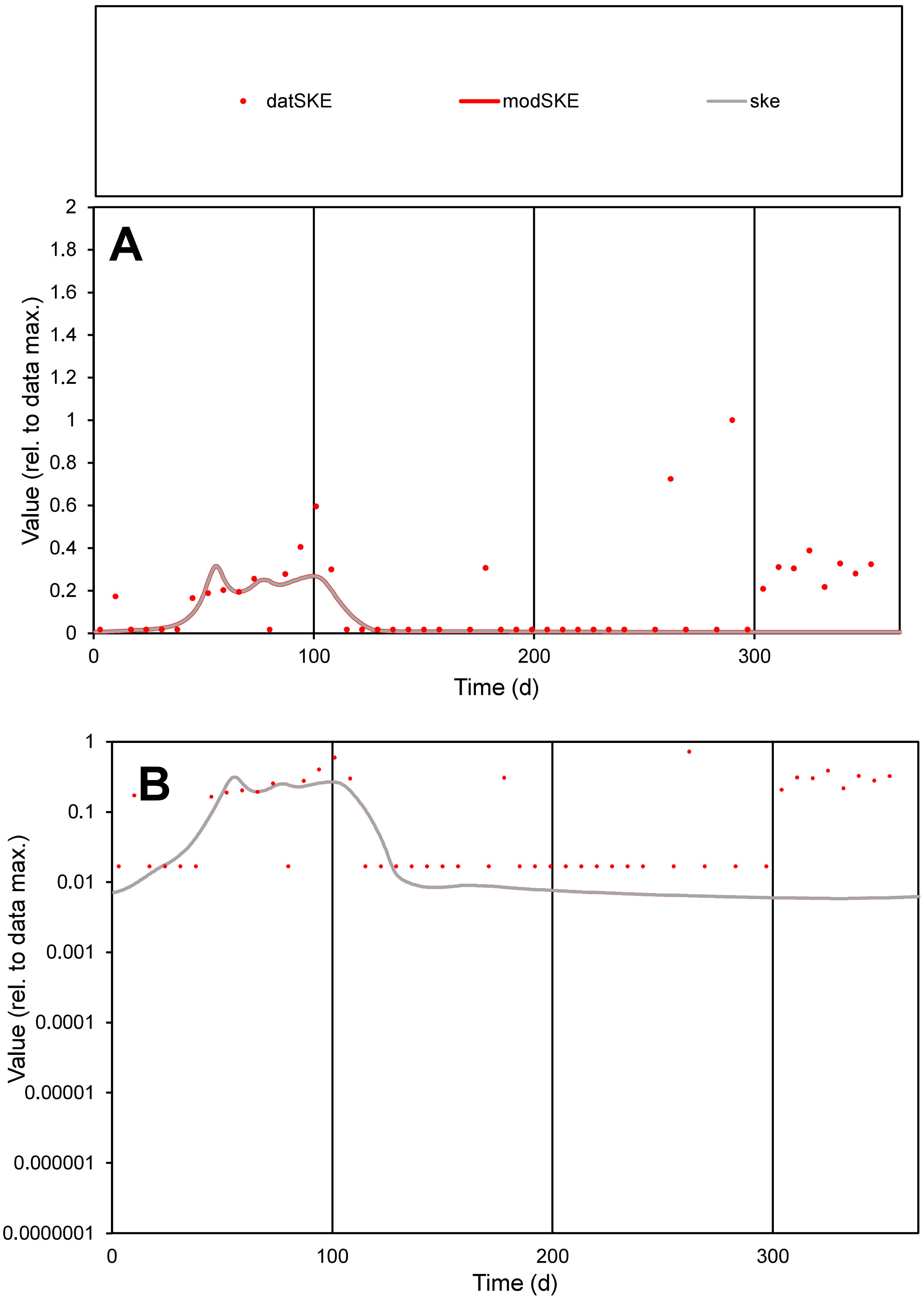
datSKE
modSKE
ske
A
Value (rel. to data max.)
2
1.8
1.6
1.4
1.2
1
0.8
0.6
0.4
0.2
0
0
100
200
300
Time (d)
B
1
0.1
0.01
0.001
0.0001
0.00001
0.000001
0.0000001
Time (d)

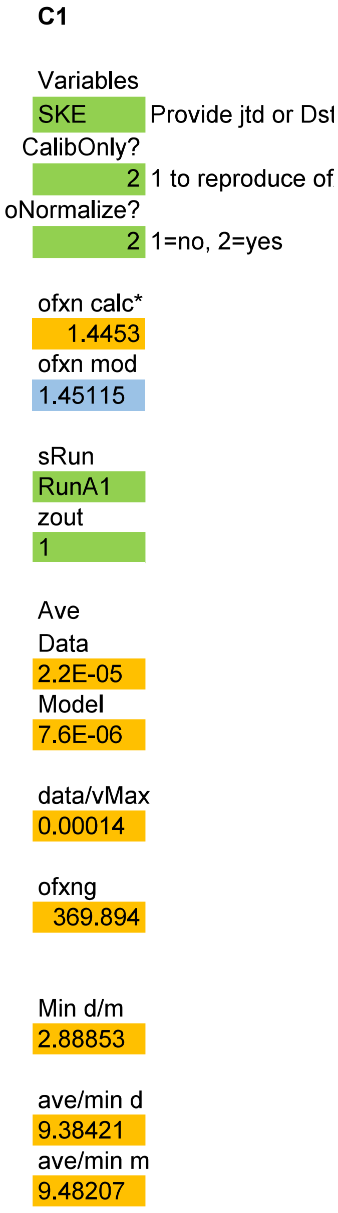
C1
Variables
SKE
Provide jtd or Dst
CalibOnly?
2
1 to reproduce of
oNormalize?
2
1=no, 2=yes
ofxn calc*
1.4453
ofxn mod
1.45115
sRun
RunA1
zout
1
Ave
Data
2.2E-05
Model
7.6E-06
data/vMax
0.00014
ofxng
369.894
Min d/m
2.88853
ave/min d
9.38421
ave/min m
9.48207

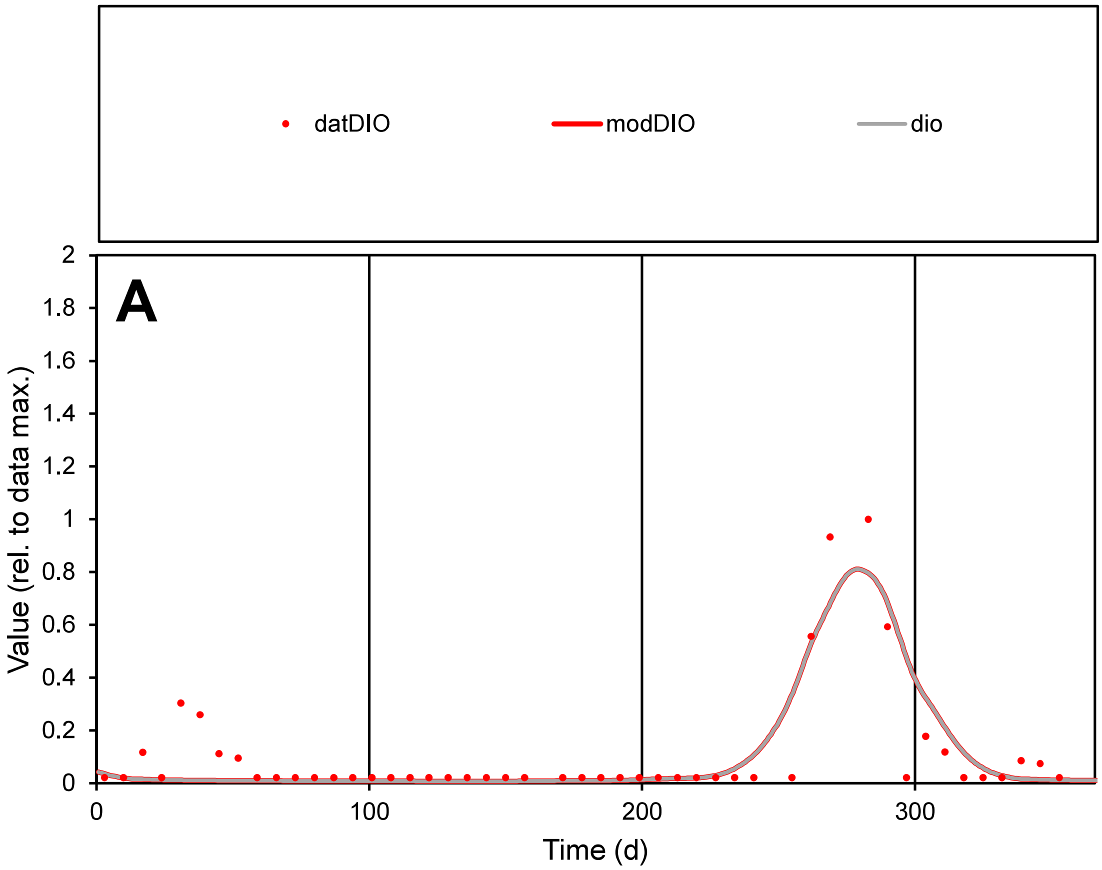

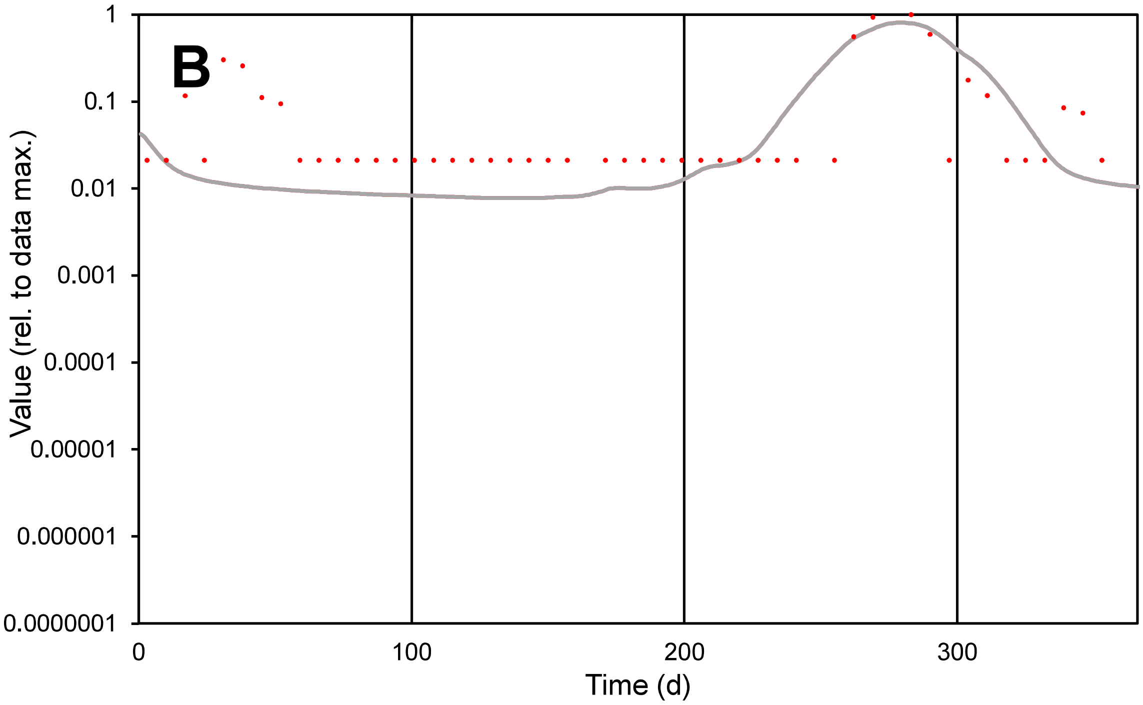

C1

Variables
DIO Provide jtd or Dst
CalibOnly?
2 1 to reproduce of
oNormalize?
2 1=no, 2=yes

ofxn calc*
0.98263
ofxn mod
1.04753

sRun
RunA1
zout
1

Ave
Data
2.4E-05
Model
2.1E-05

data/vMax
0.00022

ofxng
369.894

Min d/m
2.73361

ave/min d
5.08494
ave/min m
12.4445

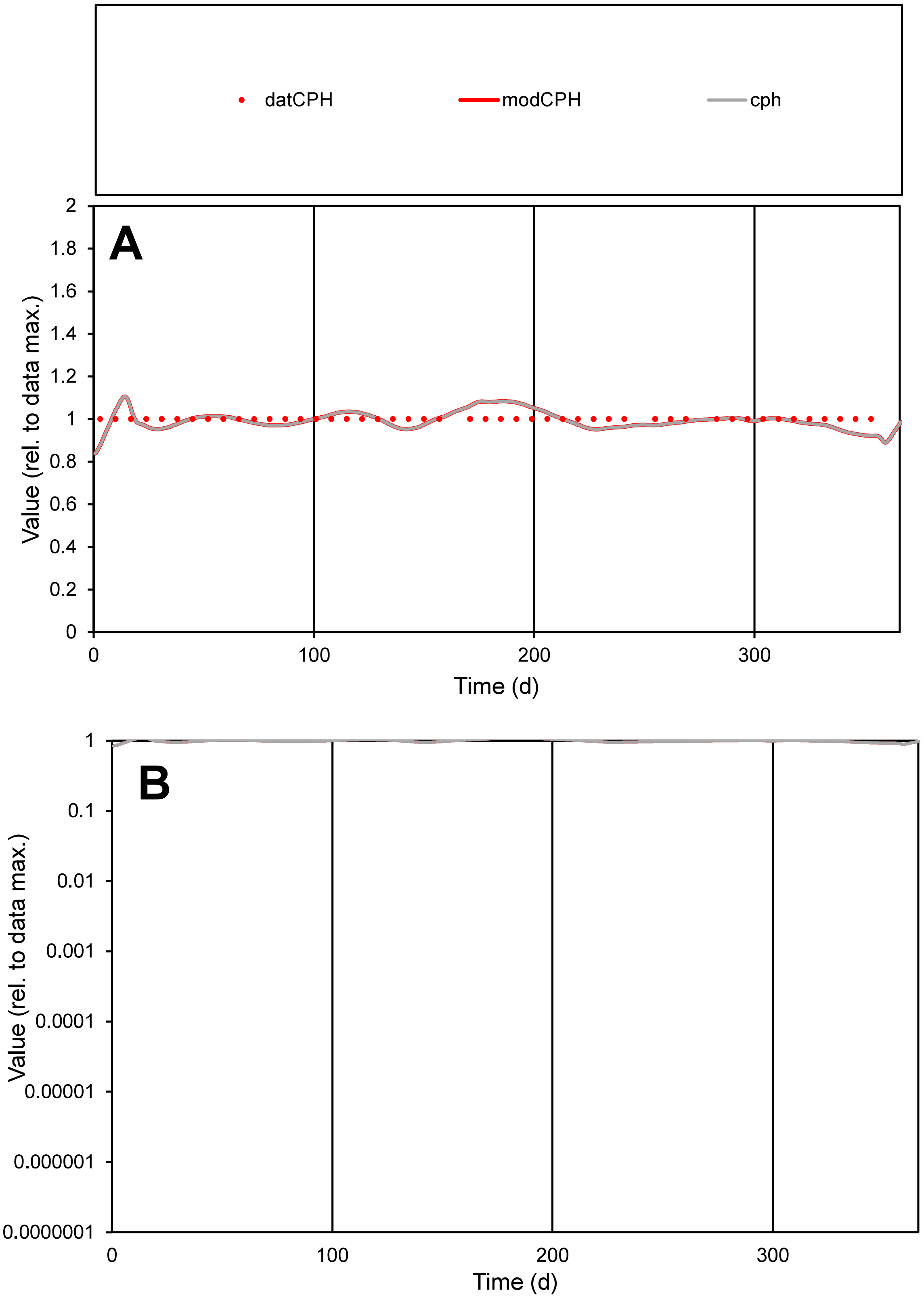

C1

Variables
CPH Provide jtd or Dst
CalibOnly?
2 1 to reproduce of
oNormalize?
2 1=no, 2=yes

ofxn calc*
0.04006
ofxn mod
0.11977

sRun
RunA1
zout
1

Ave
Data
9.6E-06
Model
9.5E-06

data/vMax
9.6E-06

ofxng
369.894

Min d/m
1.09936

ave/min d
1
ave/min m
1.0934

328_Calib_S_A

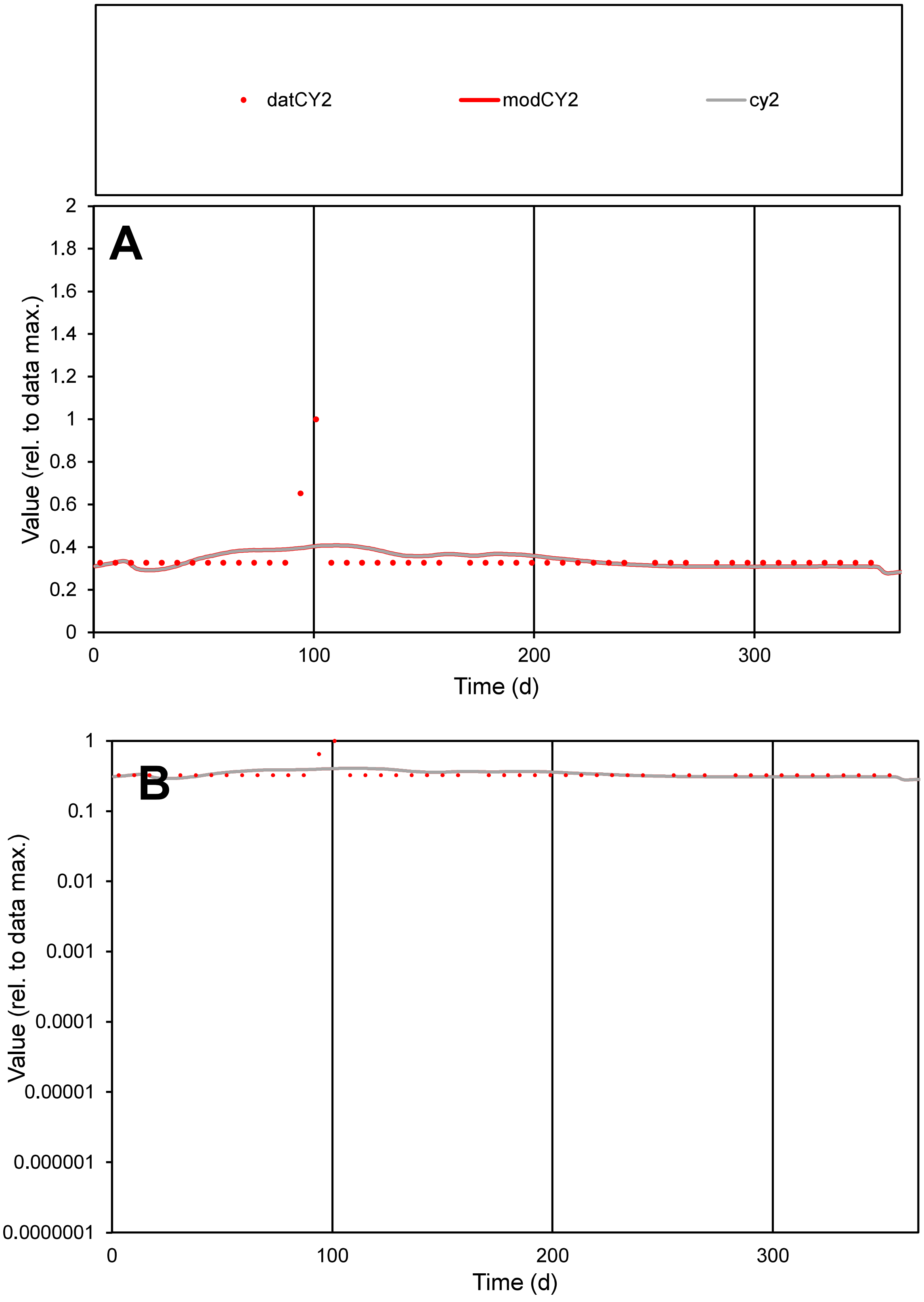

C1

Variables
CY2 Provide jtd or Dst
CalibOnly?
2 1 to reproduce of
oNormalize?
2 1=no, 2=yes

ofxn calc*
0.28754
ofxn mod
0.30933

sRun
RunA1
zout
1

Ave
Data
1.2E-05
Model
1.2E-05

data/vMax
3.4E-05

ofxng
369.894

Min d/m
1.11702

ave/min d
1.0639
ave/min m
1.17706

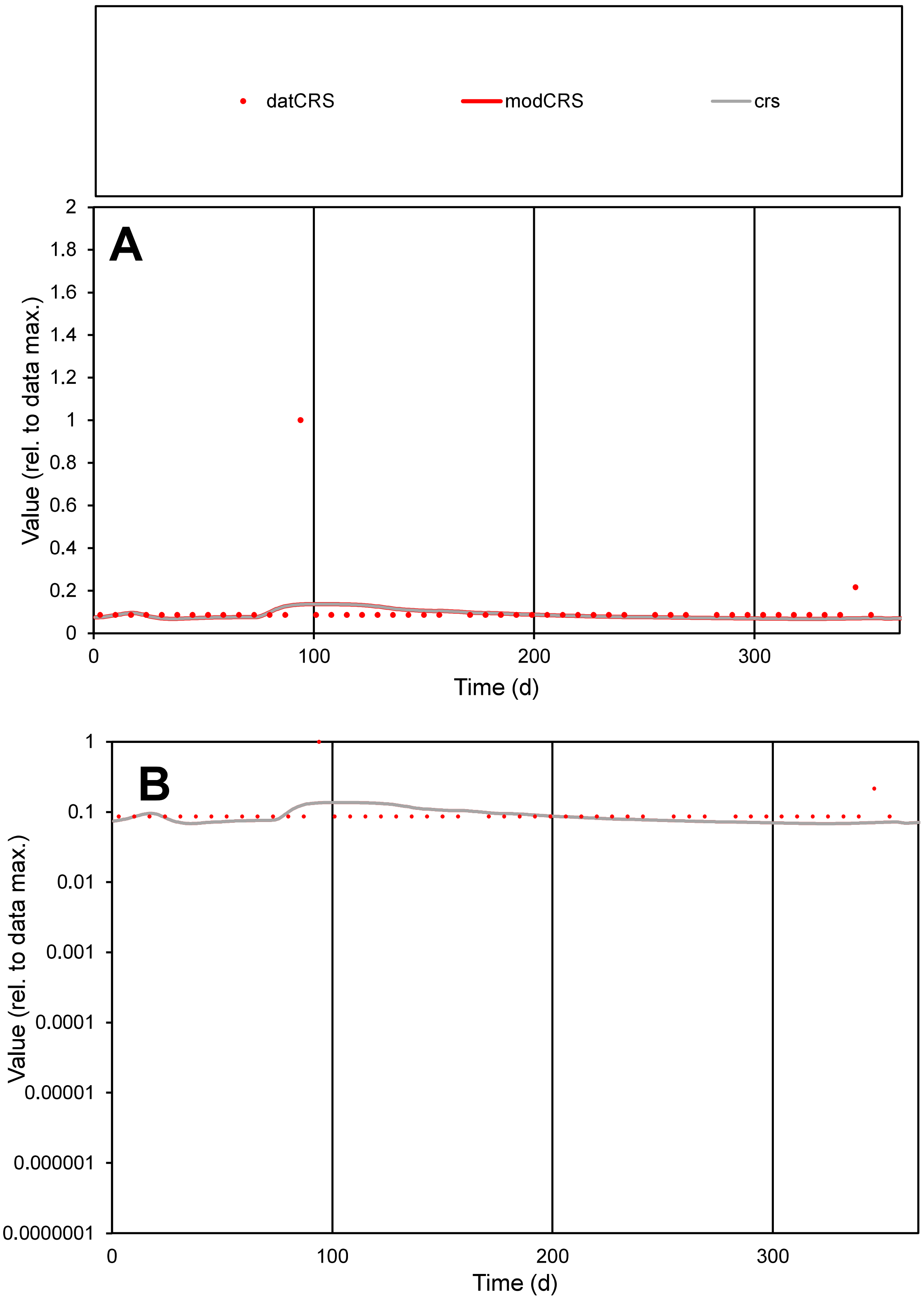

C1

Variables
CRS Provide jtd or Dst
CalibOnly?
2 1 to reproduce of
oNormalize?
2 1=no, 2=yes

ofxn calc*
1.18638
ofxn mod
1.1924

sRun
RunA1
zout
1

Ave
Data
6.8E-06
Model
5.6E-06

data/vMax
6.3E-05

ofxng
369.894

Min d/m
1.26284

ave/min d
1.25181
ave/min m
1.29898

328_Calib_S_A

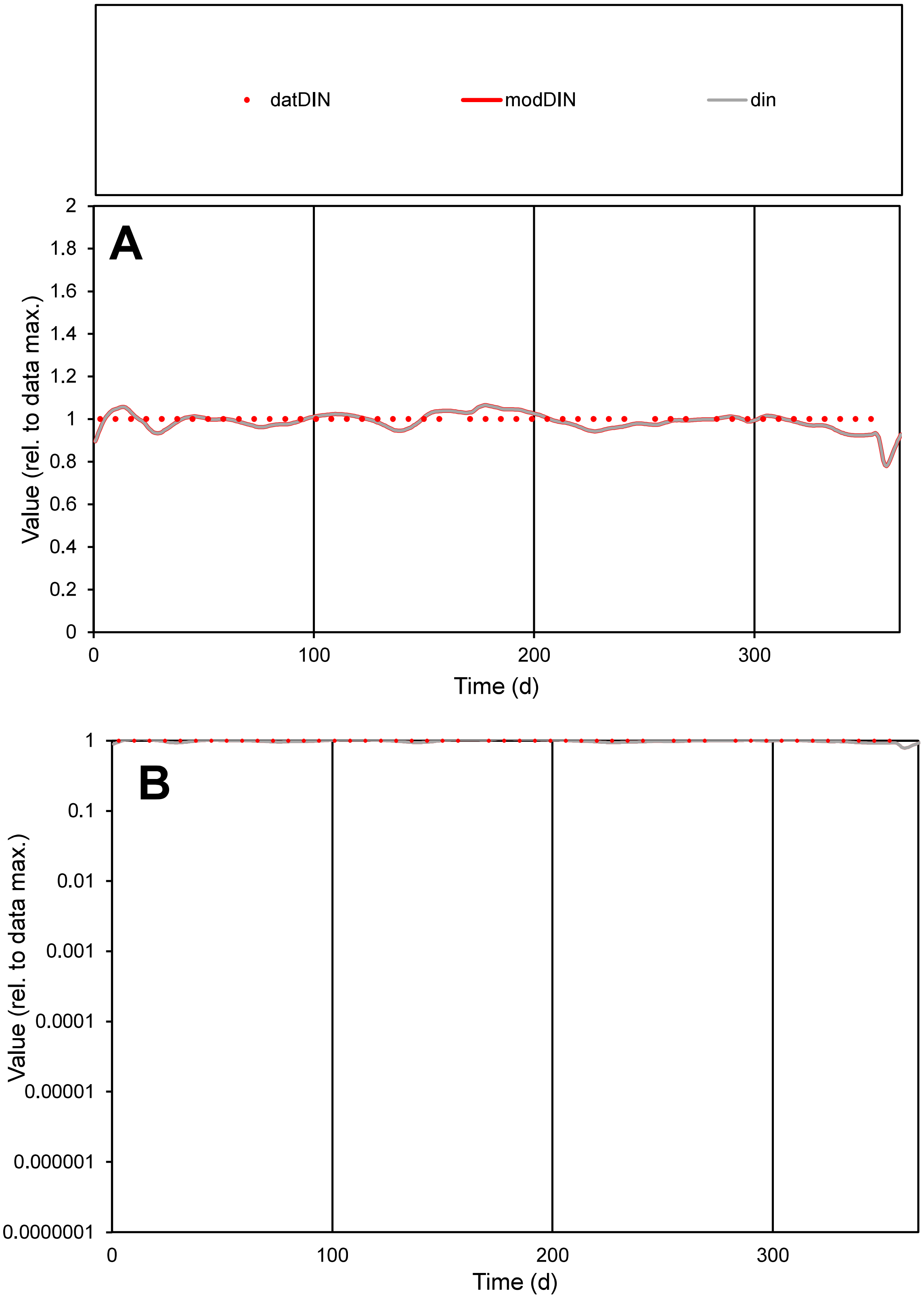

C1

Variables
DIN Provide jtd or Dst
CalibOnly?
2 1 to reproduce of
oNormalize?
2 1=no, 2=yes

ofxn calc*
0.03365
ofxn mod
0.11758

sRun
RunA1
zout
1

Ave
Data
2.9E-06
Model
2.9E-06

data/vMax
2.9E-06

ofxng
369.894

Min d/m
1.08218

ave/min d
1
ave/min m
1.07333

328_Calib_S_A

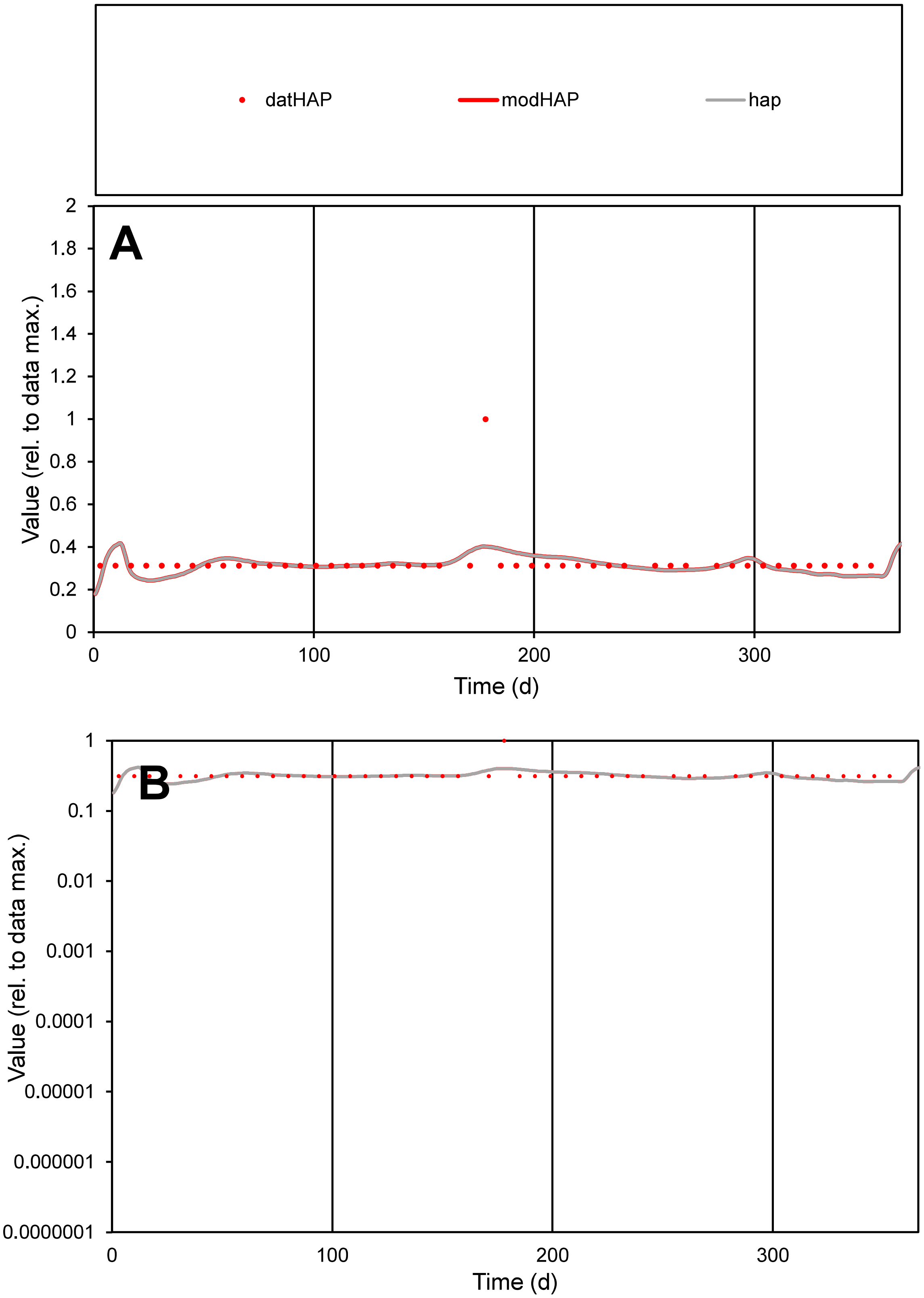

C1

Variables

HAP	Provide jtd or Dst
CalibOnly?	
2	1 to reproduce of
oNormalize?	
2	1=no, 2=yes

ofxn calc*
0.28642
ofxn mod
0.3085

sRun
RunA1
zout
1

Ave
Data
1.2E-05
Model
1.1E-05

data/vMax
3.6E-05

ofxng
369.894

Min d/m
1.28641

ave/min d
1.04591
ave/min m
1.30236

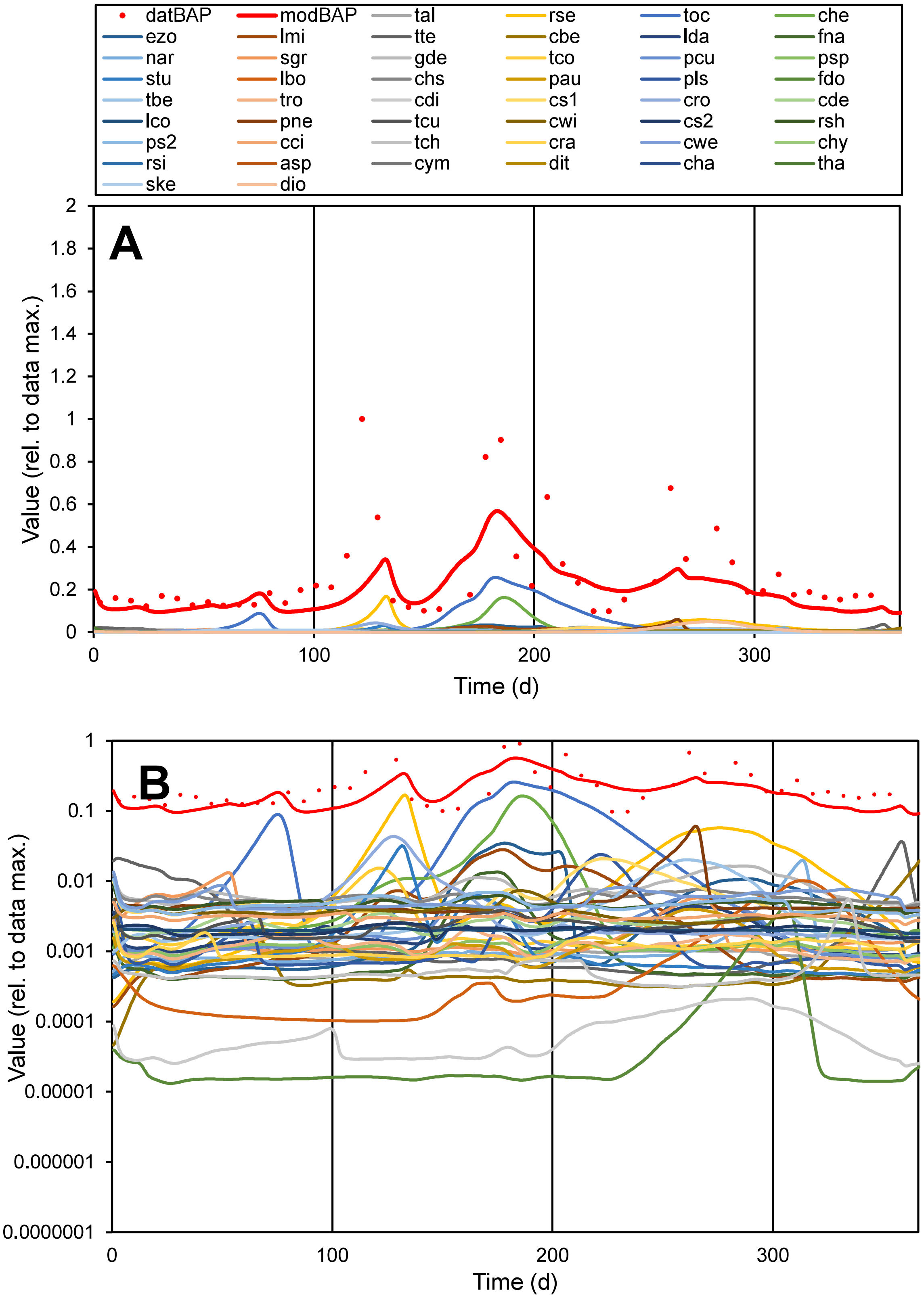

C1

Variables

BAP Provide jtd or Dst

CalibOnly?

2 1 to reproduce of

oNormalize?

2 1=no, 2=yes

ofxn calc*

0.65408

ofxn mod

0.66731

sRun

RunA1

zout

1

Ave

Data

0.00095

Model

0.00072

data/vMax

0.00361

ofxng

369.894

Min d/m

1.0121

ave/min d

2.69779

ave/min m

2.06267

328_Calib_S_A

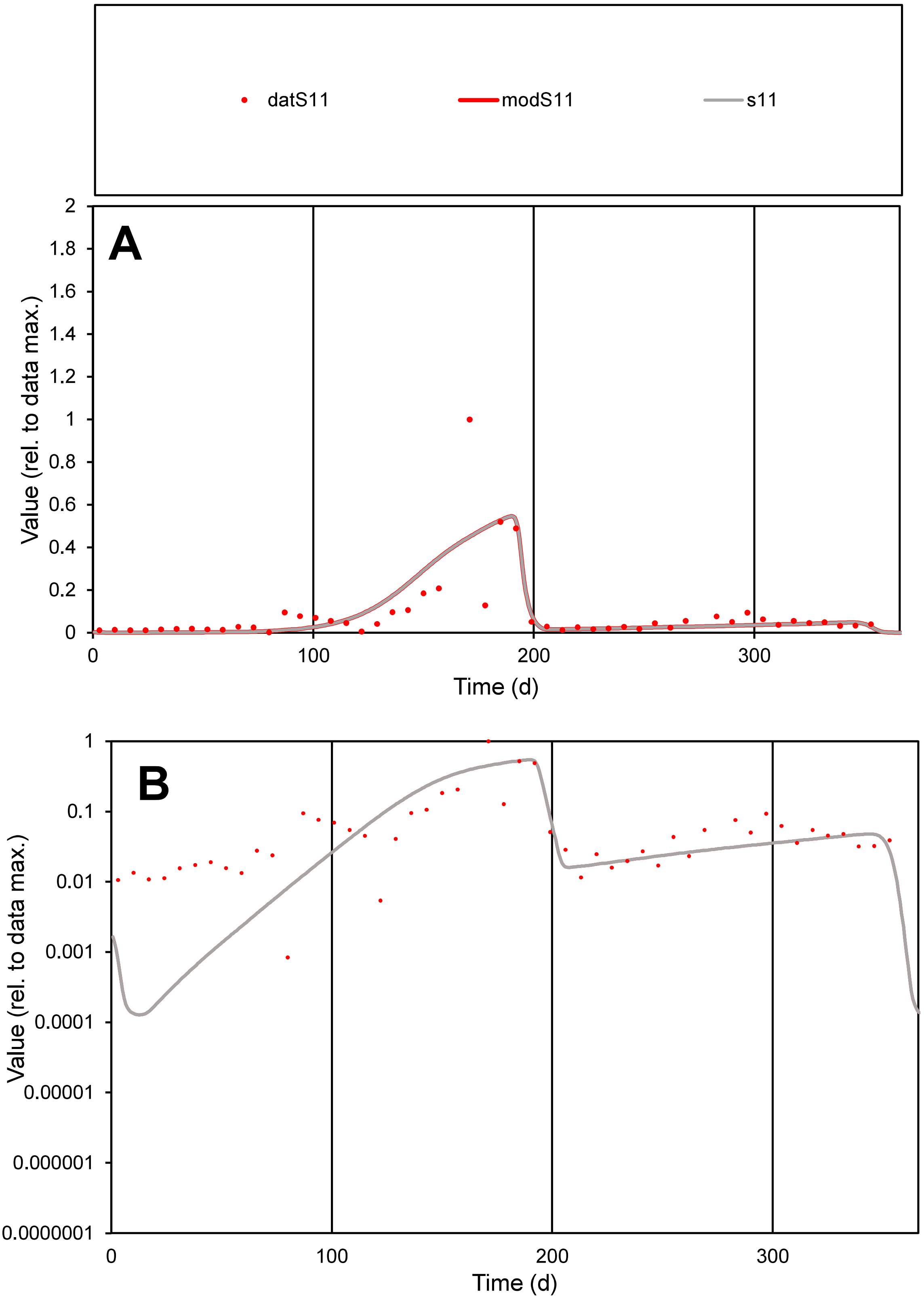

C1

Variables

S11 Provide jtd or Dst

CalibOnly?

2 1 to reproduce of

oNormalize?

2 1=no, 2=yes

ofxn calc*

1.20764

ofxn mod

1.24163

sRun

RunA1

zout

1

Ave

Data

0.10645

Model

0.10527

data/vMax

1.248

ofxng

369.894

Min d/m

6.46621

ave/min d

102.066

ave/min m

652.645

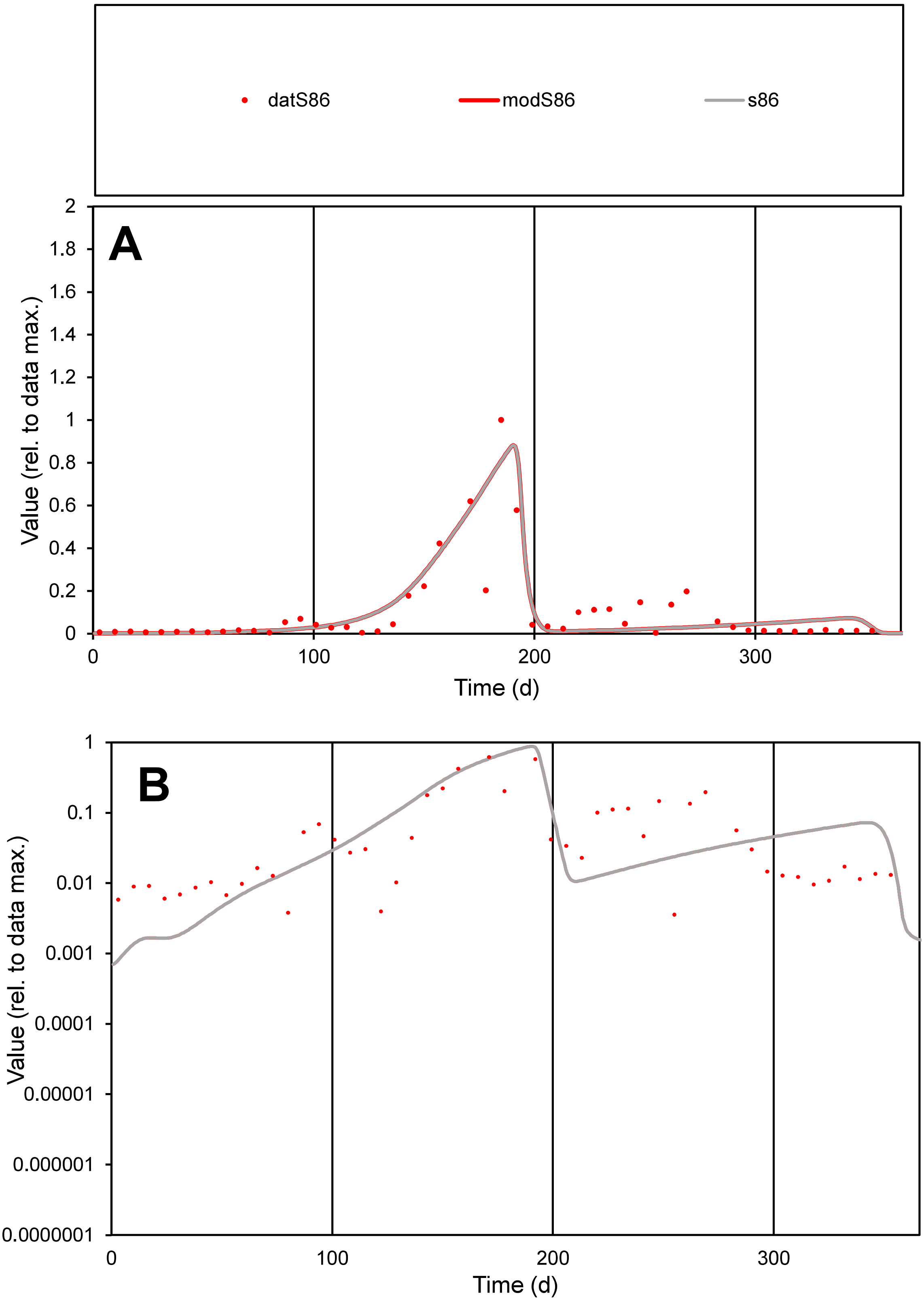

C1

Variables

S86	Provide jtd or Dst

CalibOnly?

2	1 to reproduce of

oNormalize?

2	1=no, 2=yes

ofxn calc*
0.96485
ofxn mod
1.01008

sRun
RunA1
zout
1

Ave
Data
0.06287
Model
0.06799

data/vMax
0.6468

ofxng
369.894

Min d/m
3.97829

ave/min d
27.23
ave/min m
117.15

328_Calib_S_A

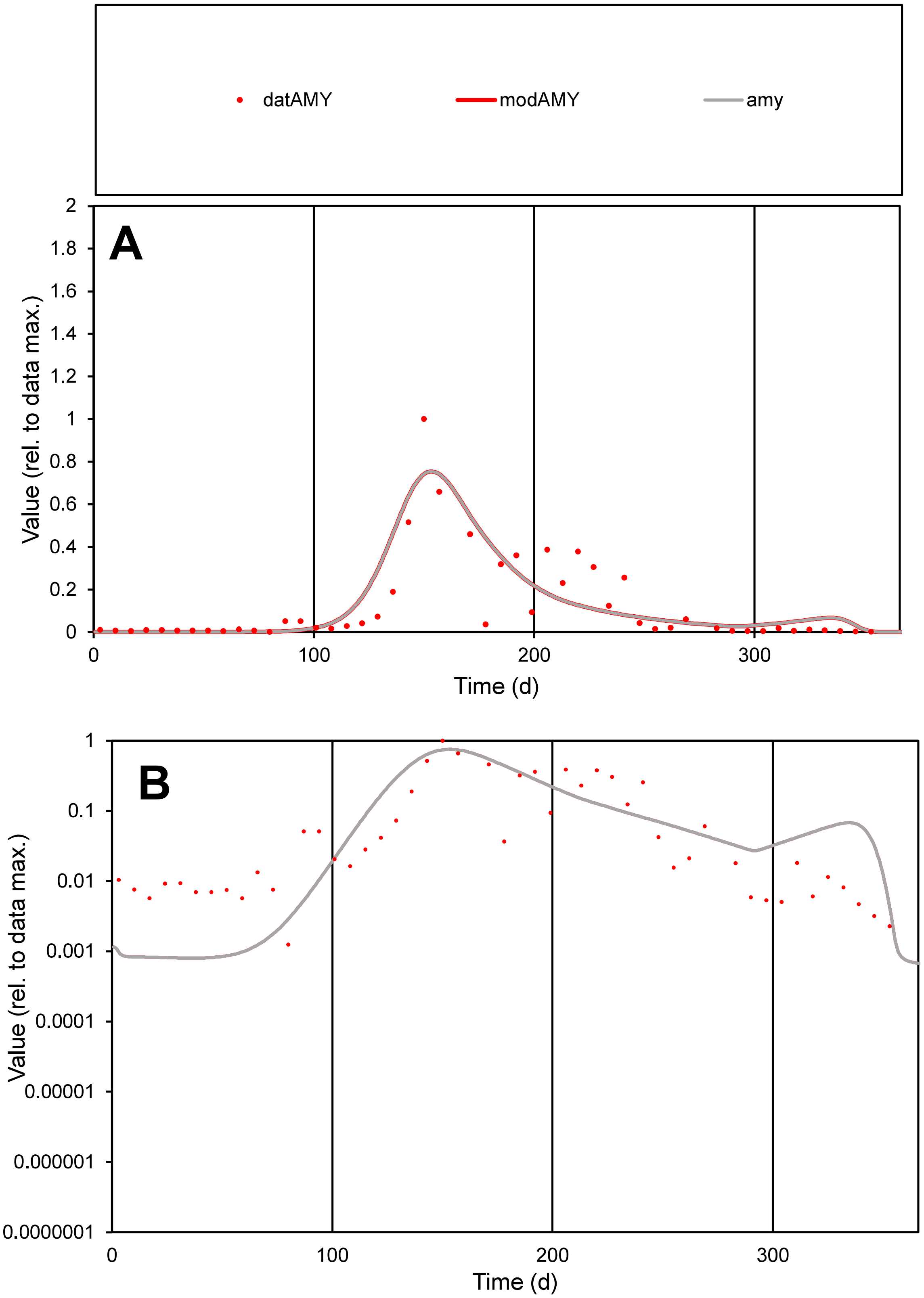

C1

Variables
AMY Provide jtd or Dst
CalibOnly?
2 1 to reproduce of
oNormalize?
2 1=no, 2=yes

ofxn calc*
0.91977
ofxn mod
0.95697

sRun
RunA1
zout
1

Ave
Data
0.05814
Model
0.06236

data/vMax
0.4817

ofxng
369.894

Min d/m
1.56491

ave/min d
96.2793
ave/min m
161.602

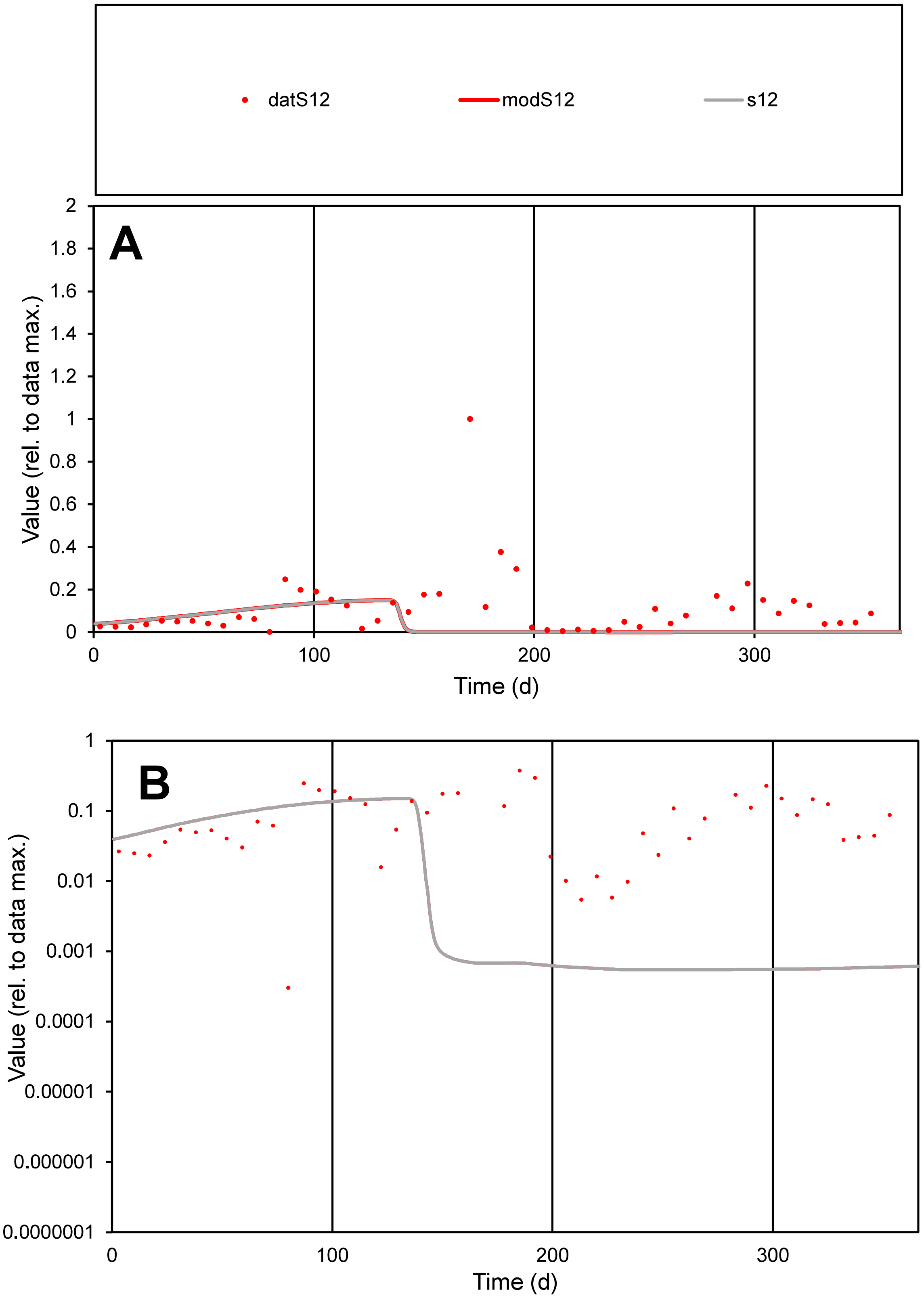

C1

Variables
S12 Provide jtd or Dst
CalibOnly?
2 1 to reproduce of
oNormalize?
2 1=no, 2=yes

ofxn calc*
1.61895
ofxn mod
1.62536

sRun
RunA1
zout
1

Ave
Data
0.05142
Model
0.02027

data/vMax
0.4641

ofxng
369.894

Min d/m
0.5519

ave/min d
364.908
ave/min m
79.3919

328_Calib_S_A

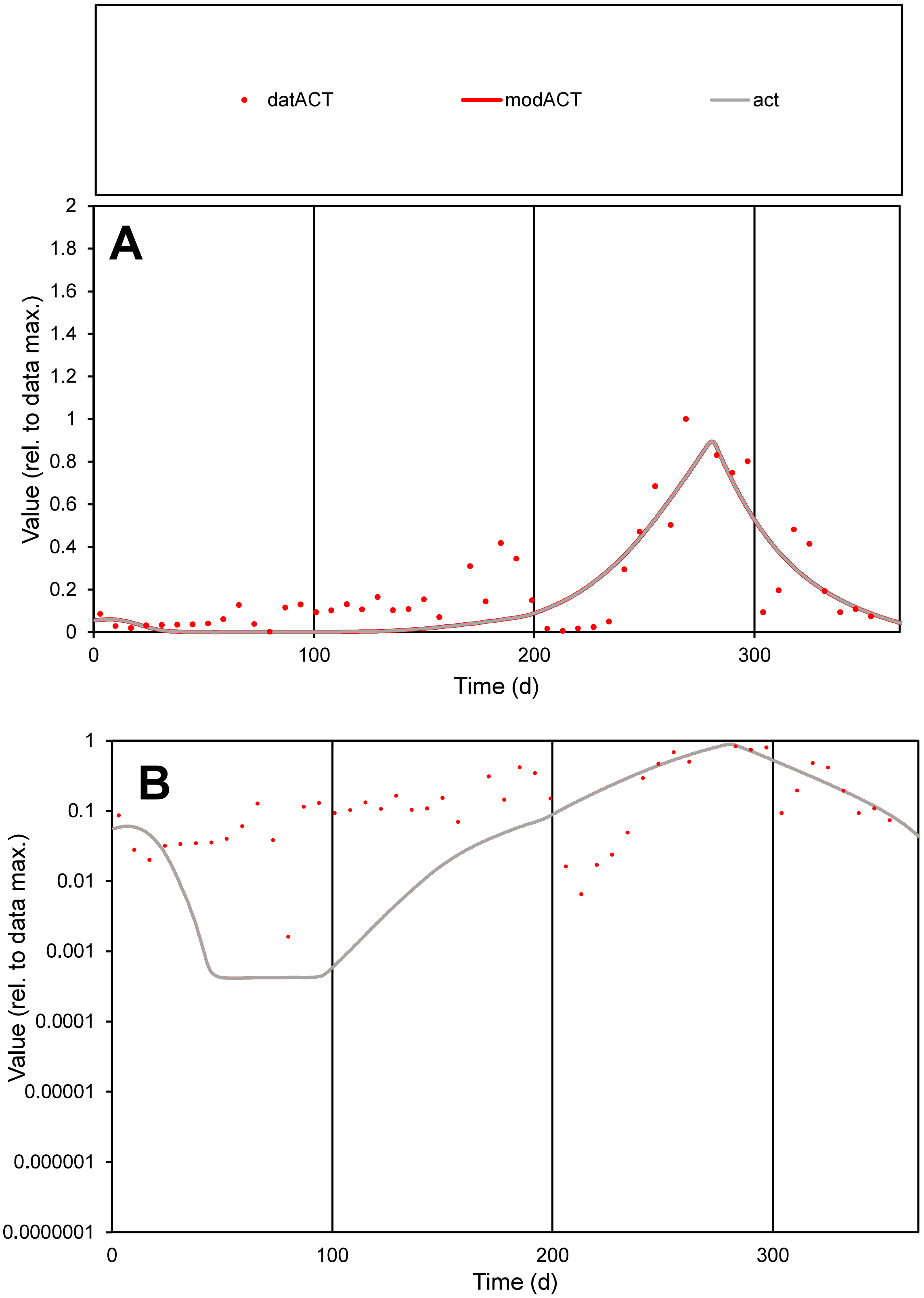

C1

Variables

ACT Provide jtd or Dst

CalibOnly?

2 1 to reproduce of

oNormalize?

2 1=no, 2=yes

ofxn calc*

0.68441

ofxn mod

0.70721

sRun

RunA1

zout

1

Ave

Data

0.03821

Model

0.03069

data/vMax

0.182

ofxng

369.894

Min d/m

3.89413

ave/min d

130.484

ave/min m

408.104

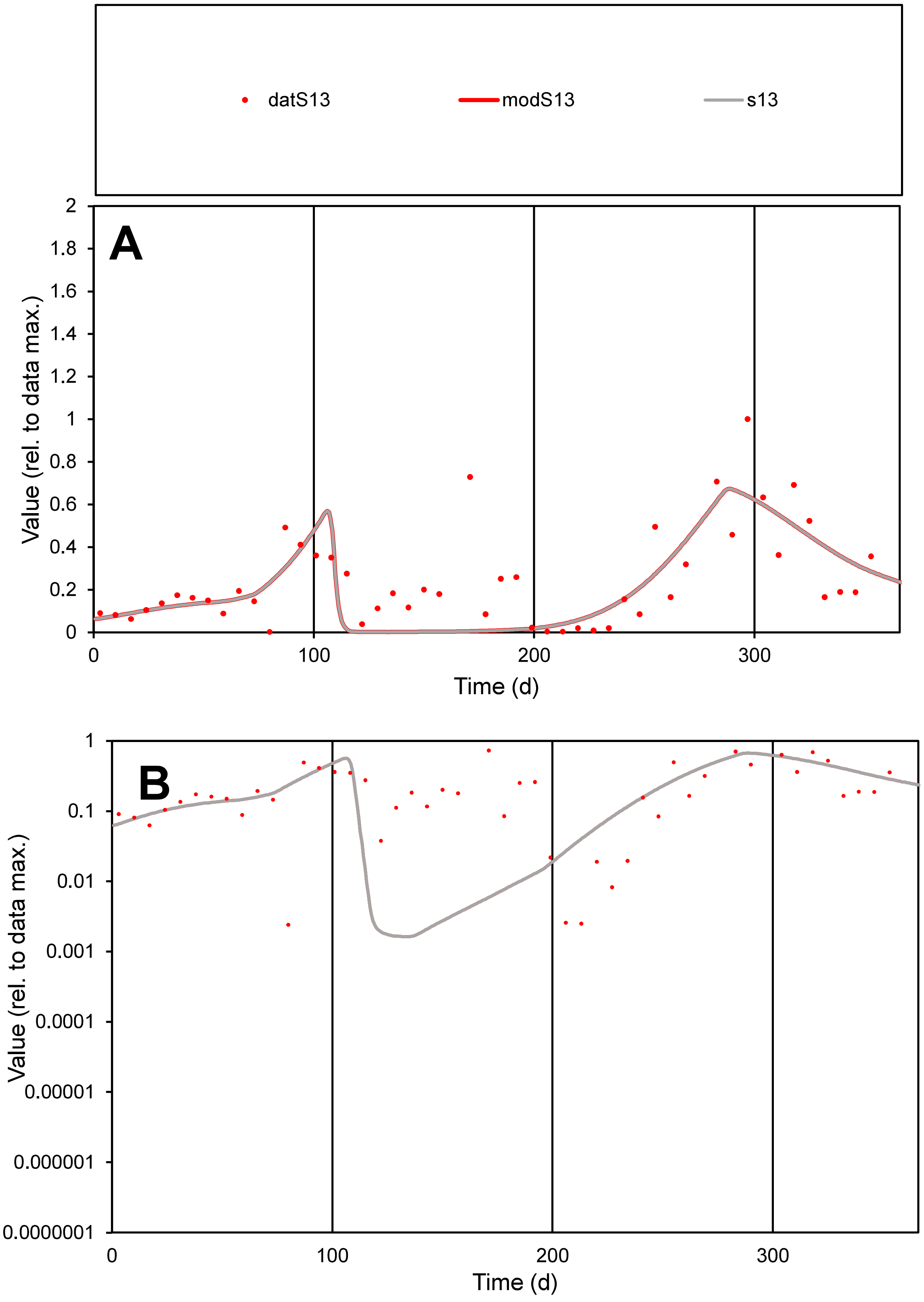

C1

Variables

S13 Provide jtd or Dst

CalibOnly?

2 1 to reproduce of

oNormalize?

2 1=no, 2=yes

ofxn calc*

0.69162

ofxn mod

0.71612

sRun

RunA1

zout

1

Ave

Data

0.02859

Model

0.02506

data/vMax

0.1167

ofxng

369.894

Min d/m

1.46122

ave/min d

101.837

ave/min m

130.476

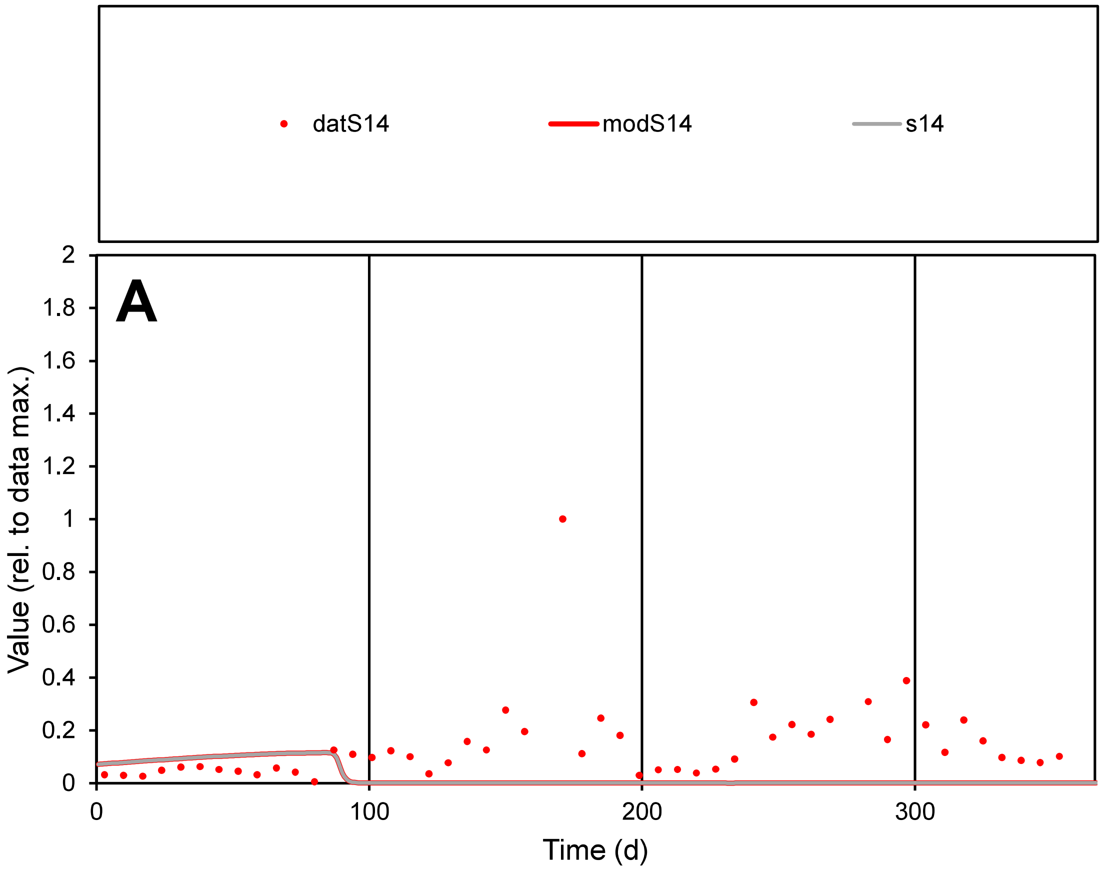

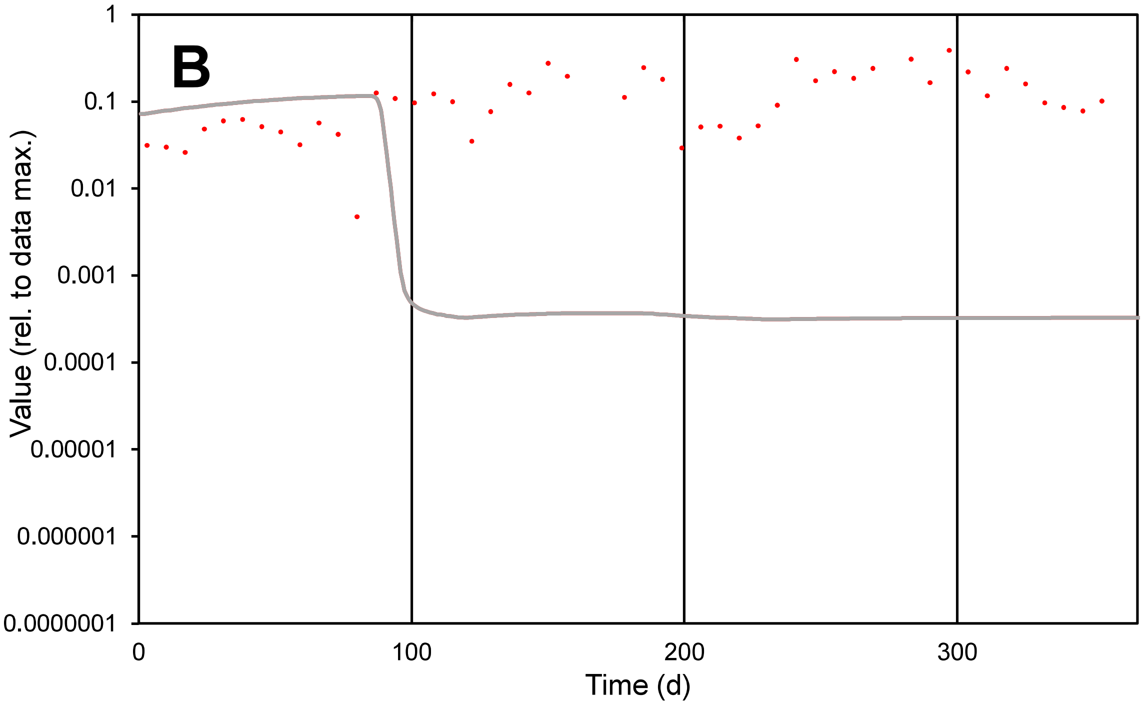

C1

Variables
S14 Provide jtd or Dst
CalibOnly?
2 1 to reproduce of
oNormalize?
2 1=no, 2=yes

ofxn calc*
1.47498
ofxn mod
1.4802

sRun
RunA1
zout
1

Ave
Data
0.03187
Model
0.00623

data/vMax
0.228

ofxng
369.894

Min d/m
15.1028

ave/min d
29.5059
ave/min m
87.1312

328_Calib_S_A

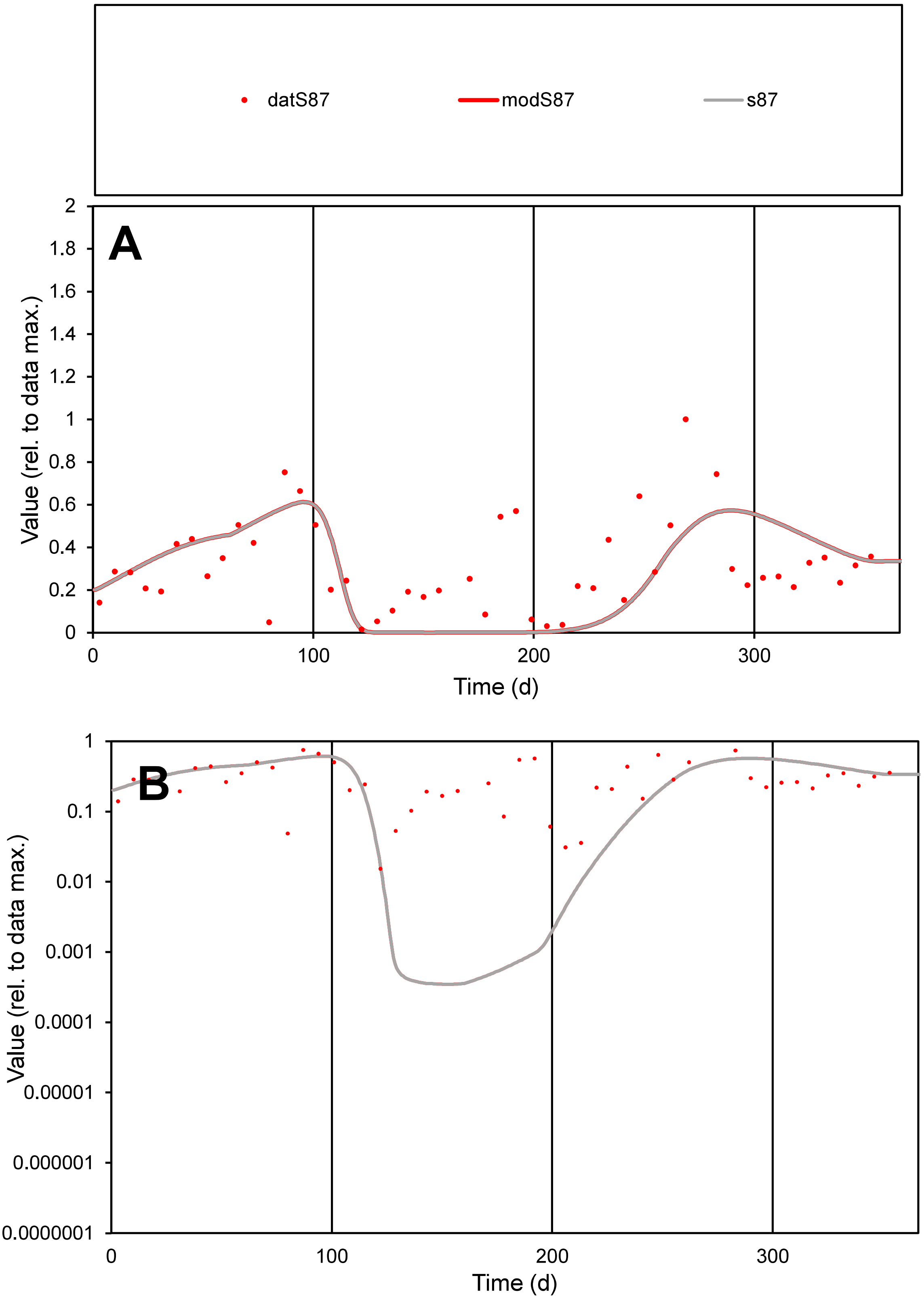

C1

Variables
S87 Provide jtd or Dst
CalibOnly?
2 1 to reproduce of
oNormalize?
2 1=no, 2=yes

ofxn calc*
0.70352
ofxn mod
0.71792

sRun
RunA1
zout
1

Ave
Data
0.02533
Model
0.02306

data/vMax
0.08137

ofxng
369.894

Min d/m
44.2872

ave/min d
20.2307
ave/min m
815.864

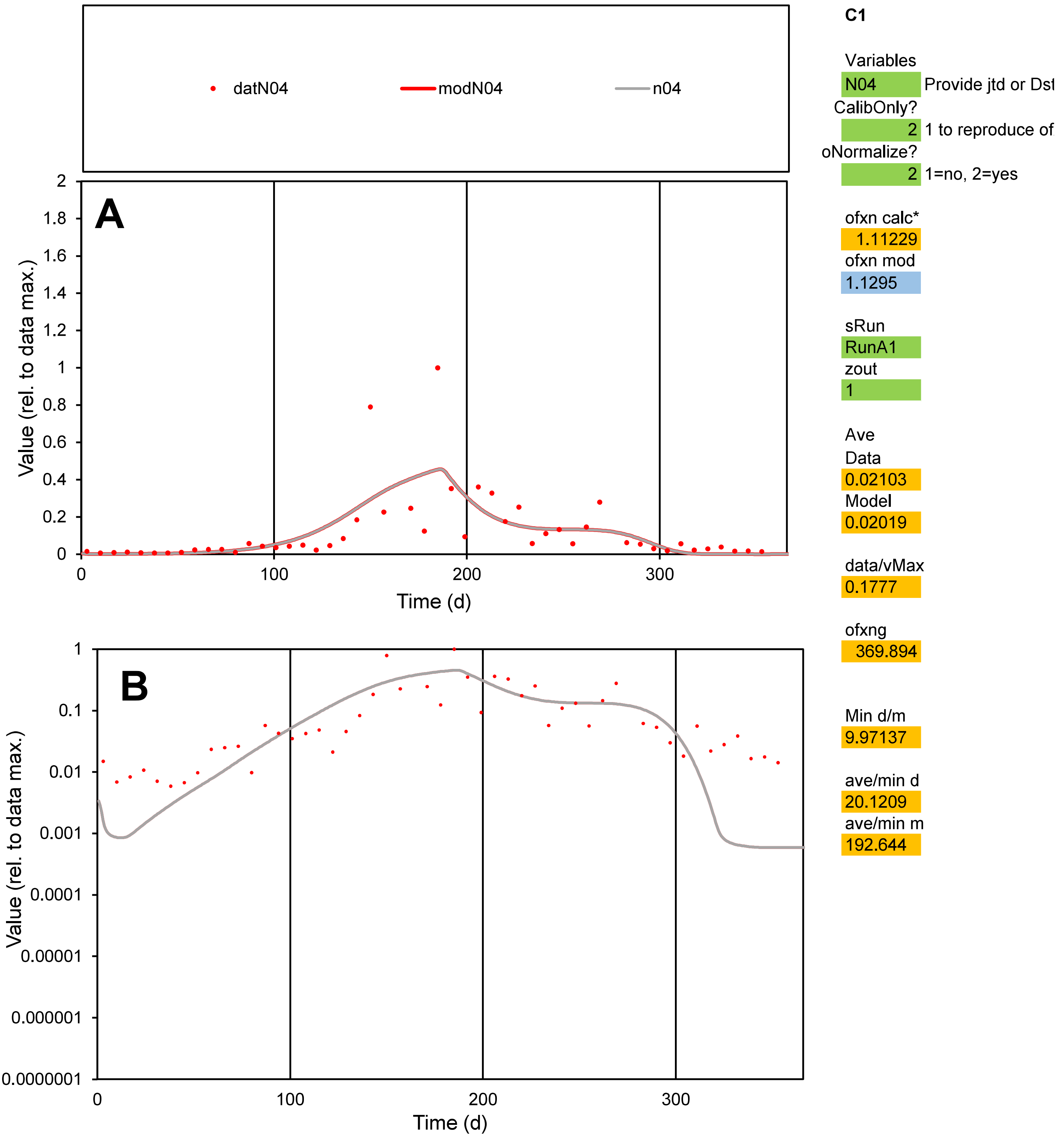
datN04
modN04
n04
A
Value (rel. to data max.)
Time (d)
B
Value (rel. to data max.)
Time (d)
C1
Variables
N04
Provide jtd or Dst
CalibOnly?
2
1 to reproduce of
oNormalize?
2
1=no, 2=yes
ofxn calc*
1.11229
ofxn mod
1.1295
sRun
RunA1
zout
1
Ave
Data
0.02103
Model
0.02019
data/vMax
0.1777
ofxng
369.894
Min d/m
9.97137
ave/min d
20.1209
ave/min m
192.644

328_Calib_S_A

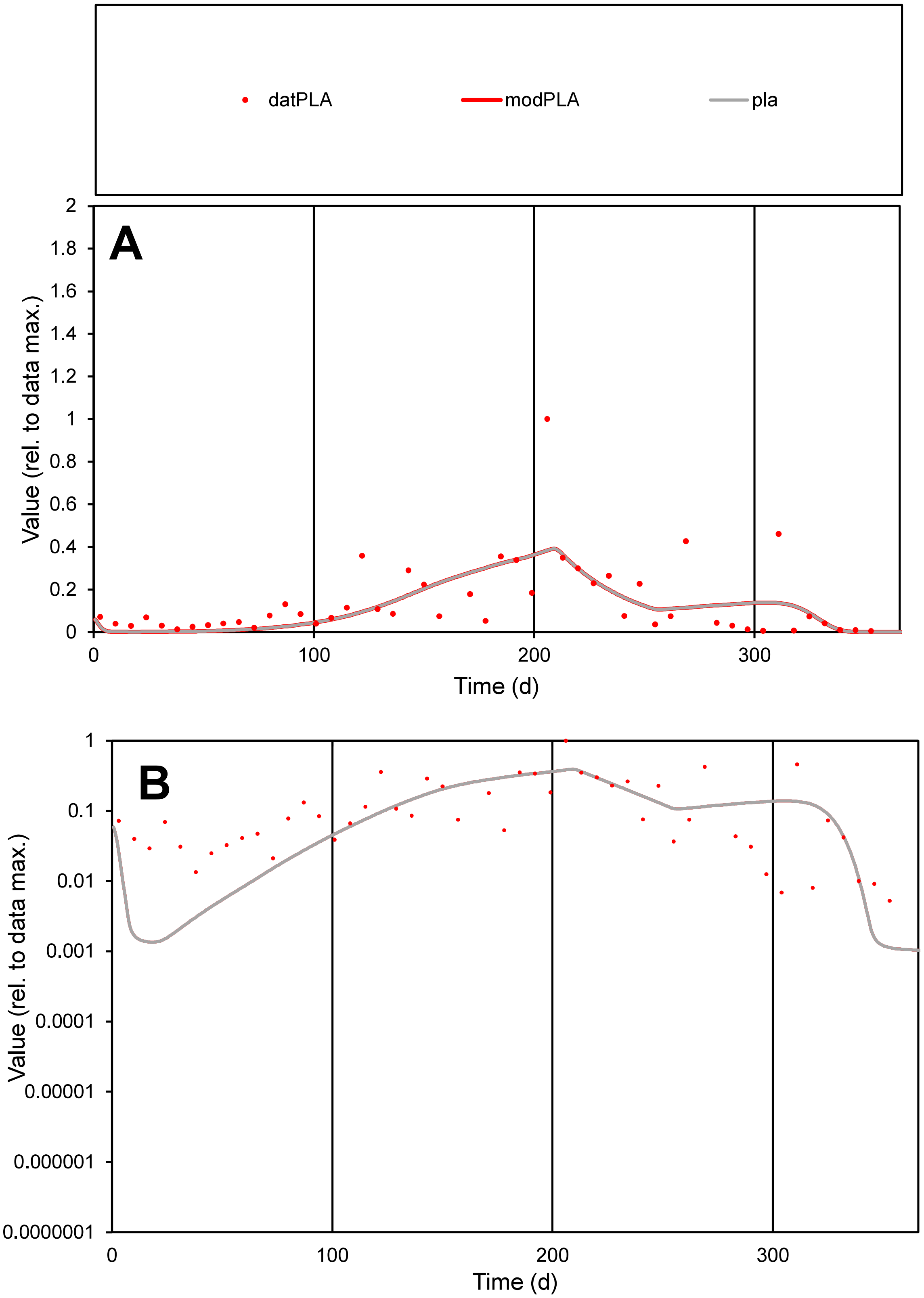

C1

Variables

PLA Provide jtd or Dst

CalibOnly?

2 1 to reproduce of

oNormalize?

2 1=no, 2=yes

ofxn calc*

0.96982

ofxn mod

0.9843

sRun

RunA1

zout

1

Ave

Data

0.01201

Model

0.01046

data/vMax

0.0856

ofxng

369.894

Min d/m

4.73375

ave/min d

26.6458

ave/min m

109.819

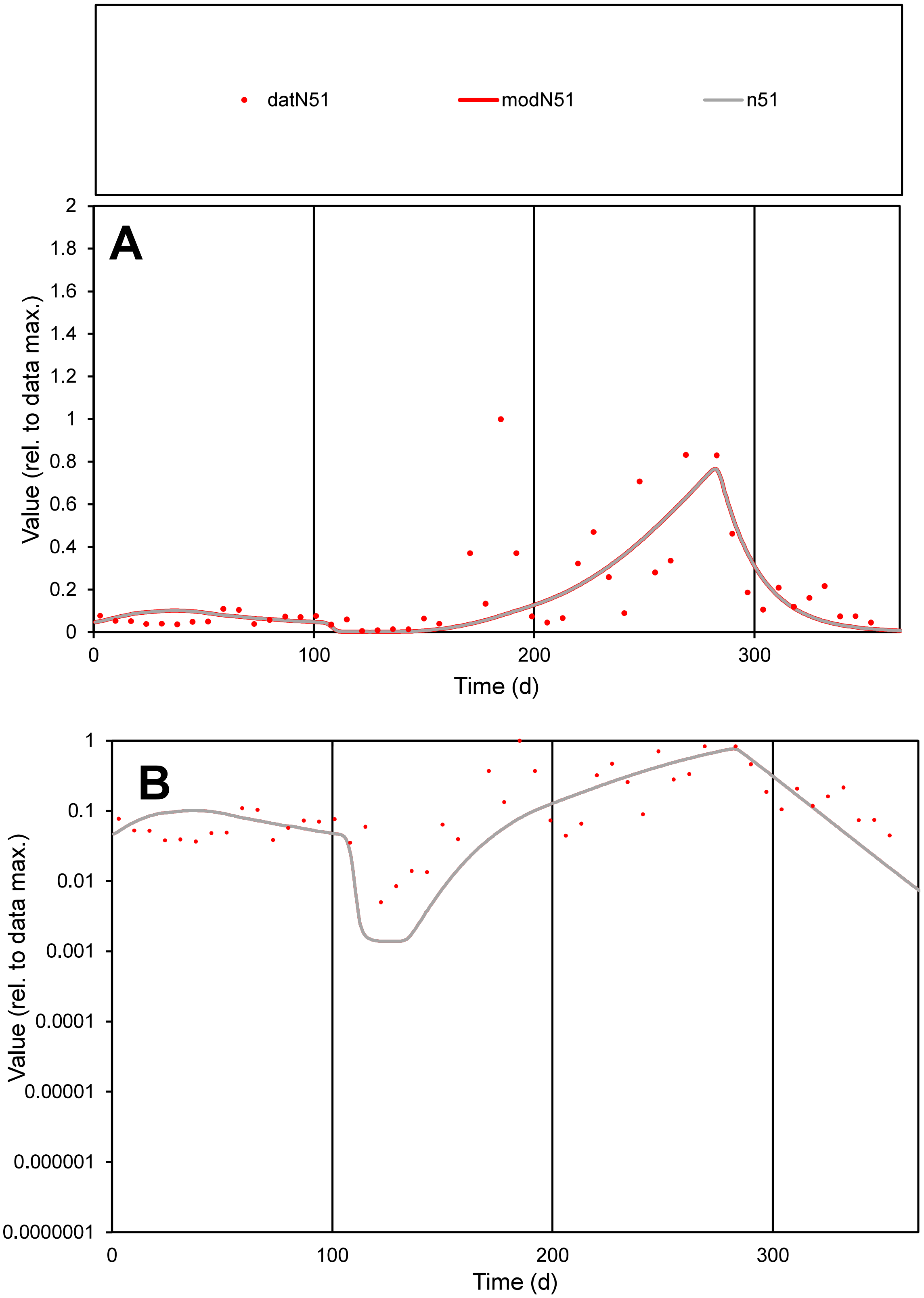

C1

Variables
N51 Provide jtd or Dst
CalibOnly?
2 1 to reproduce of
oNormalize?
2 1=no, 2=yes

ofxn calc*
0.95735
ofxn mod
0.97401

sRun
RunA1
zout
1

Ave
Data
0.02179
Model
0.01825

data/vMax
0.1187

ofxng
369.894

Min d/m
3.59612

ave/min d
36.7408
ave/min m
110.654

328_Calib_S_A

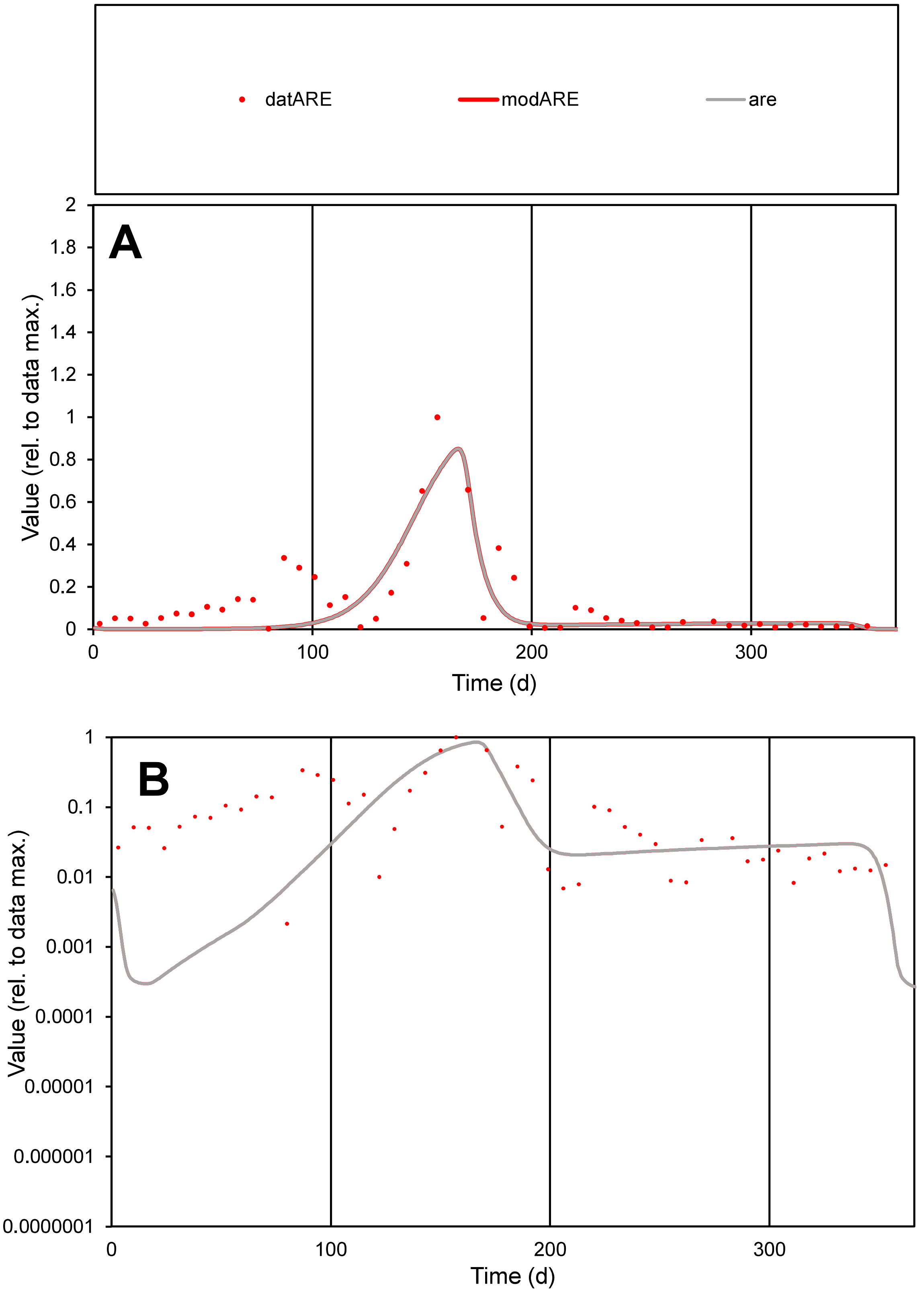

C1

Variables

ARE Provide jtd or Dst

CalibOnly?

2 1 to reproduce of

oNormalize?

2 1=no, 2=yes

ofxn calc*

0.90047

ofxn mod

0.93157

sRun

RunA1

zout

1

Ave

Data

0.02855

Model

0.01999

data/vMax

0.2299

ofxng

369.894

Min d/m

6.99759

ave/min d

57.7884

ave/min m

283.066

328_Calib_S_A

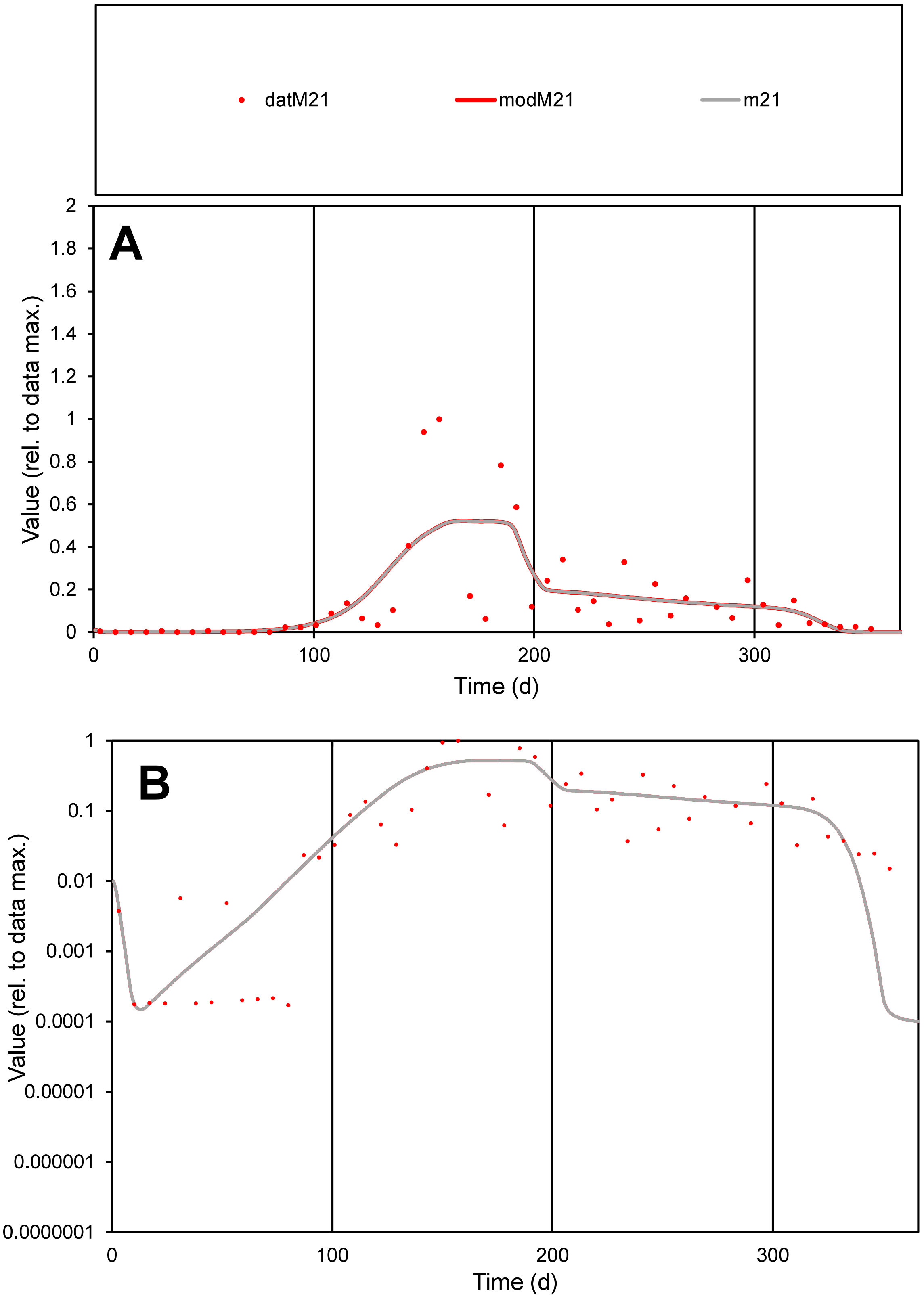

C1

Variables

M21	Provide jtd or Dst
CalibOnly?	
2	1 to reproduce of
oNormalize?	
2	1=no, 2=yes

ofxn calc*
1.03952
ofxn mod
1.05861

sRun
RunA1
zout
1

Ave
Data
0.01722
Model
0.0171

data/vMax
0.1175

ofxng
369.894

Min d/m
1.35246

ave/min d
859.701
ave/min m
1154.84

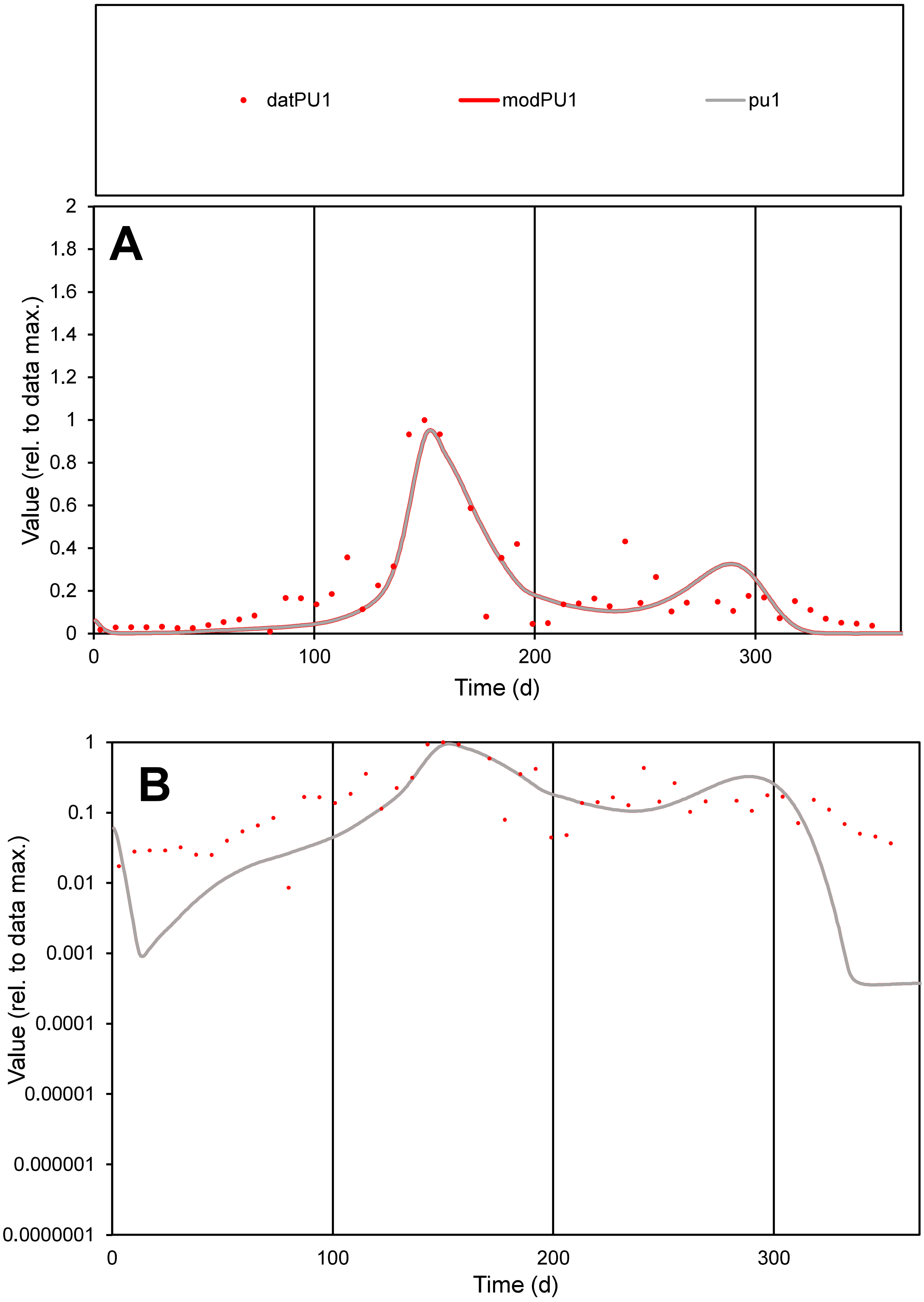

C1

Variables
PU1 Provide jtd or Dst
CalibOnly?
2 1 to reproduce of
oNormalize?
2 1=no, 2=yes

ofxn calc*
0.61933
ofxn mod
0.63996

sRun
RunA1
zout
1

Ave
Data
0.01932
Model
0.01635

data/vMax
0.1019

ofxng
369.894

Min d/m
24.0066

ave/min d
21.9948
ave/min m
446.951

328_Calib_S_A

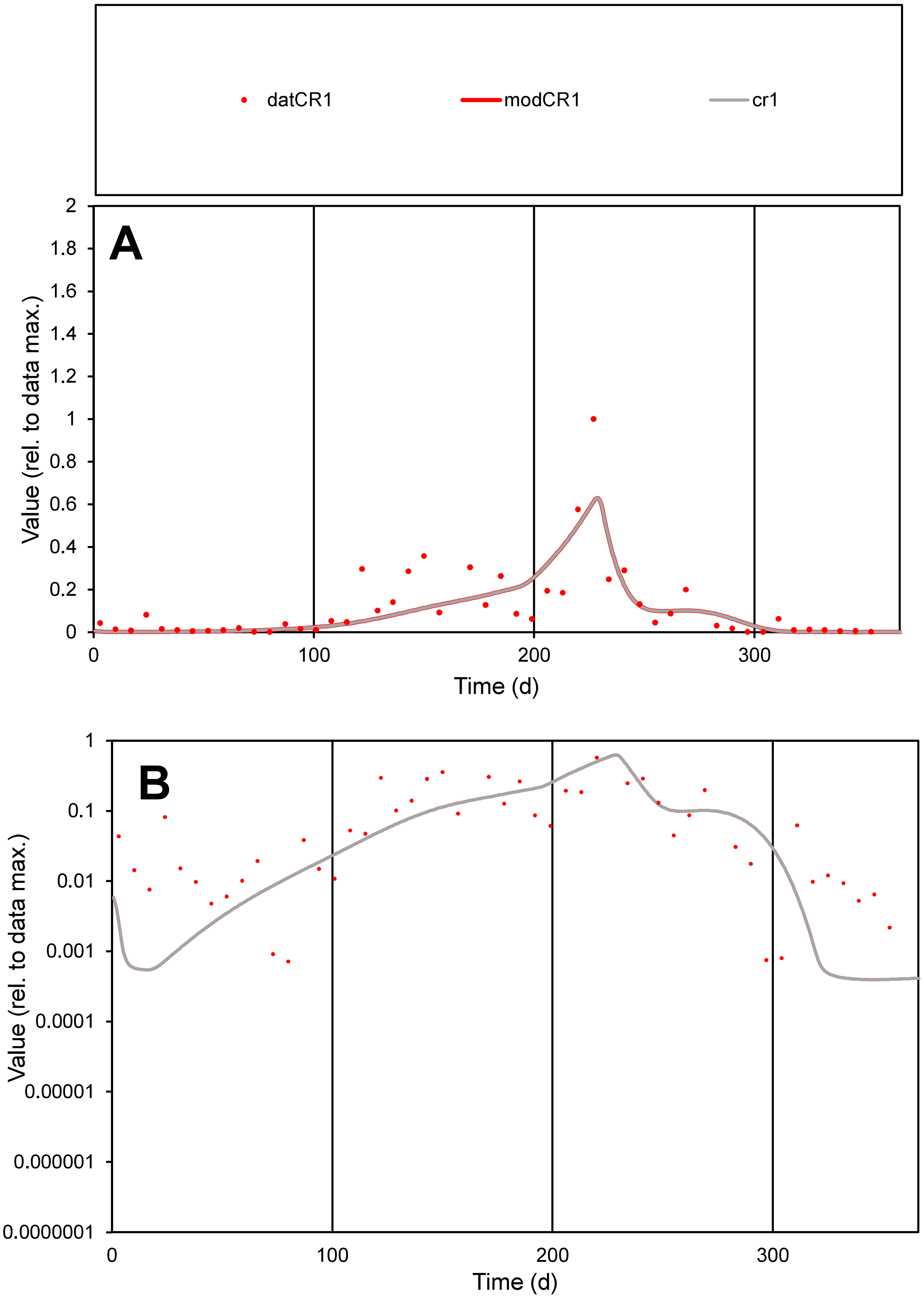

C1

Variables
CR1 Provide jtd or Dst
CalibOnly?
2 1 to reproduce of
oNormalize?
2 1=no, 2=yes

ofxn calc*
0.88029
ofxn mod
0.90562

sRun
RunA1
zout
1

Ave
Data
0.01047
Model
0.00896

data/vMax
0.09154

ofxng
369.894

Min d/m
1.81412

ave/min d
159.201
ave/min m
246.976

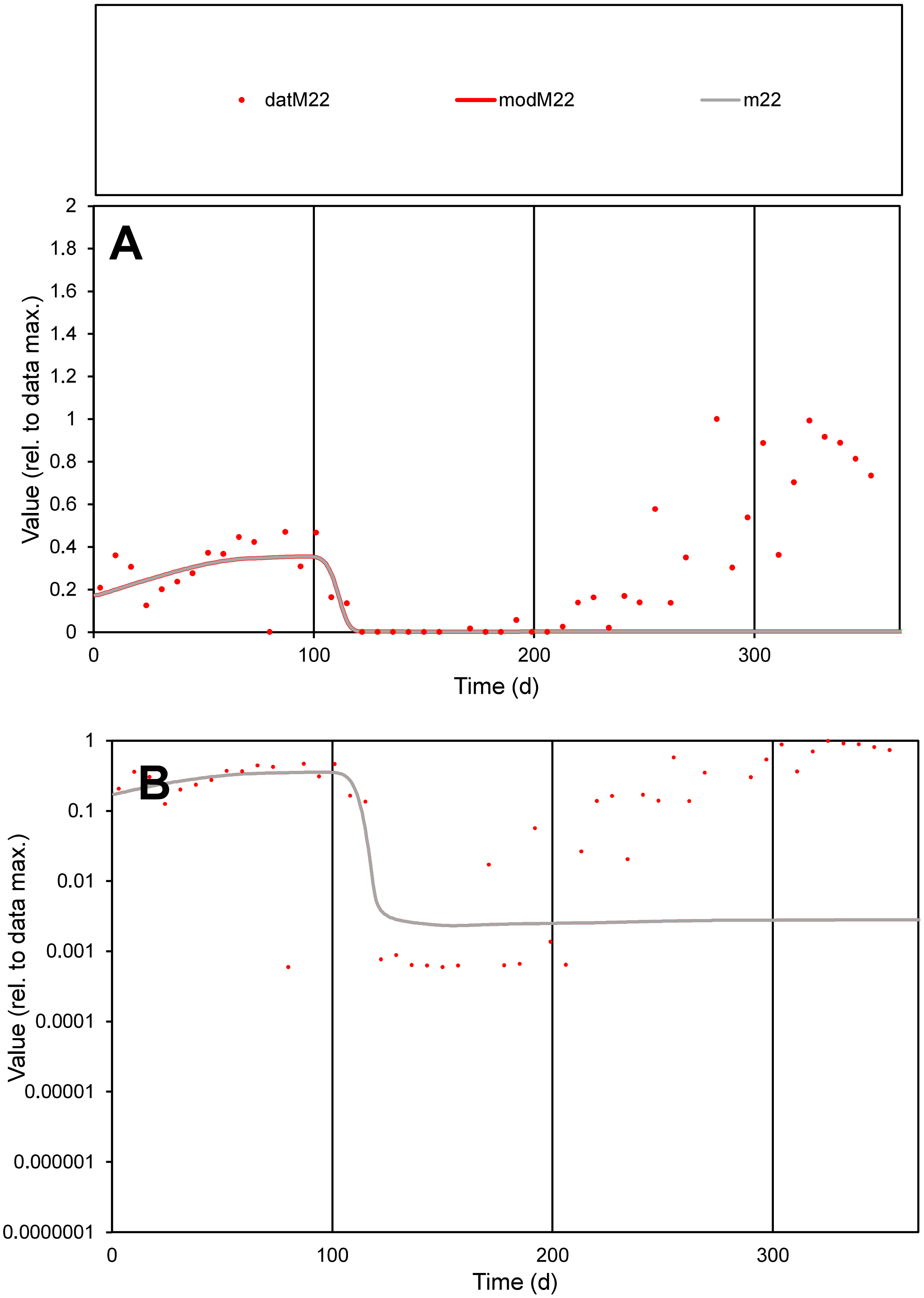

C1

Variables
M22 Provide jtd or Dst
CalibOnly?
2 1 to reproduce of
oNormalize?
2 1=no, 2=yes

ofxn calc*
1.28215
ofxn mod
1.2859

sRun
RunA1
zout
1

Ave
Data
0.01095
Model
0.00362

data/vMax
0.0362

ofxng
369.894

Min d/m
0.2556

ave/min d
507.991
ave/min m
42.8677

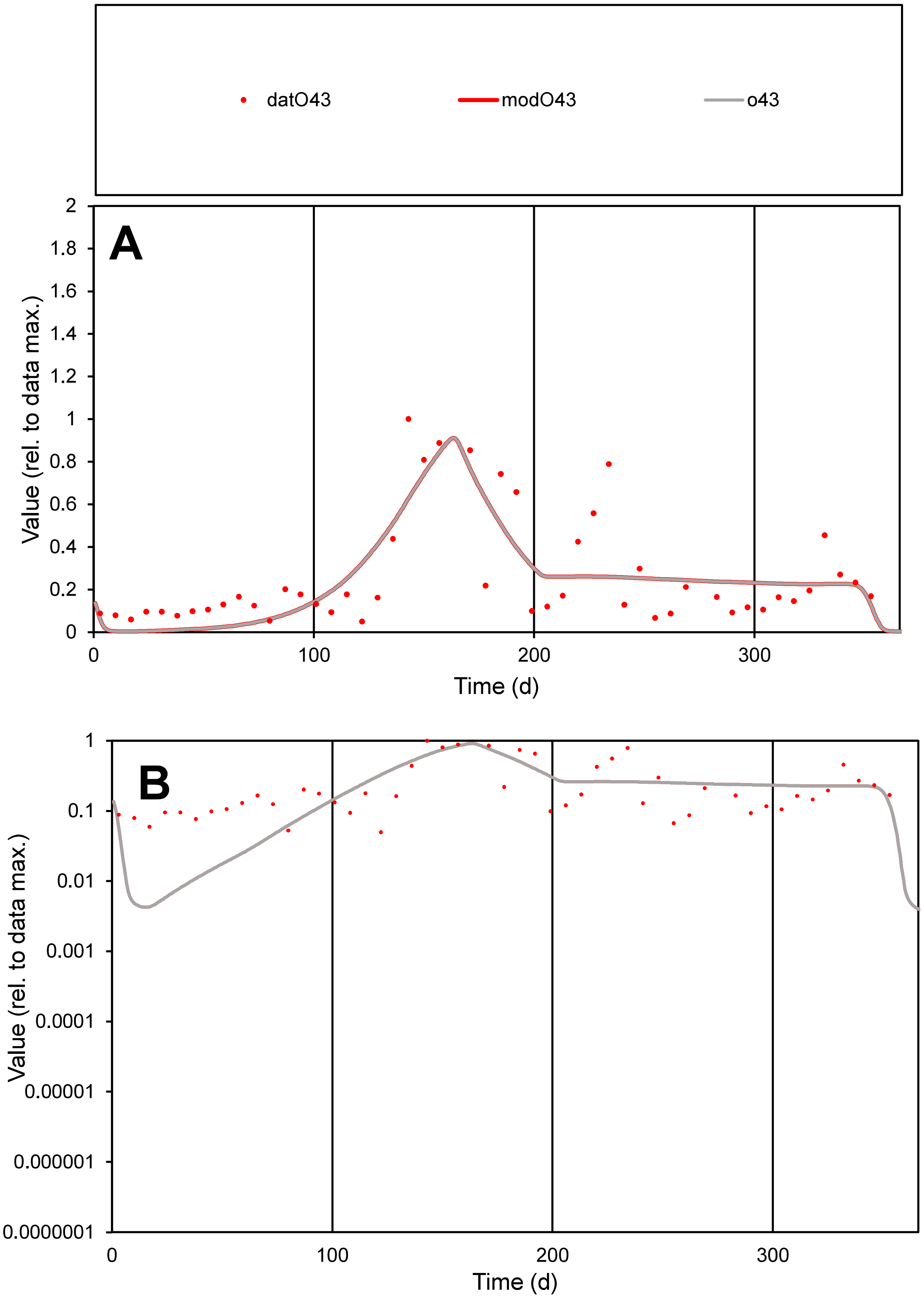

C1

Variables
O43 Provide jtd or Dst
CalibOnly?
2 1 to reproduce of
oNormalize?
2 1=no, 2=yes

ofxn calc*
0.63192
ofxn mod
0.64998

sRun
RunA1
zout
1

Ave
Data
0.01099
Model
0.01042

data/vMax
0.04196

ofxng
369.894

Min d/m
11.2265

ave/min d
5.26733
ave/min m
56.0428

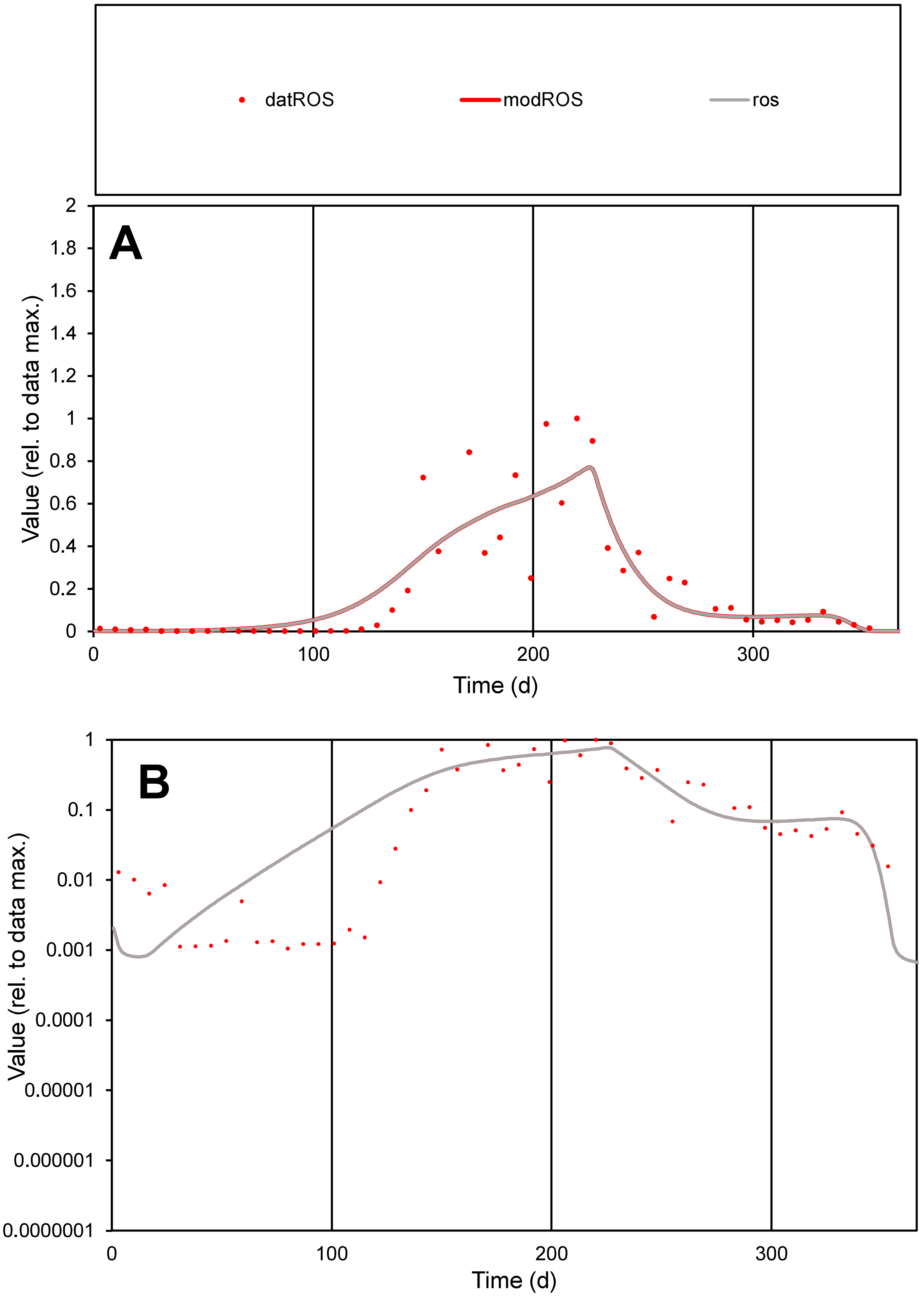

C1

Variables

ROS Provide jtd or Dst

CalibOnly?

2 1 to reproduce of

oNormalize?

2 1=no, 2=yes

ofxn calc*

0.64043

ofxn mod

0.67404

sRun

RunA1

zout

1

Ave

Data

0.00952

Model

0.00968

data/vMax

0.04744

ofxng

369.894

Min d/m

1.31767

ave/min d

189.685

ave/min m

254.207

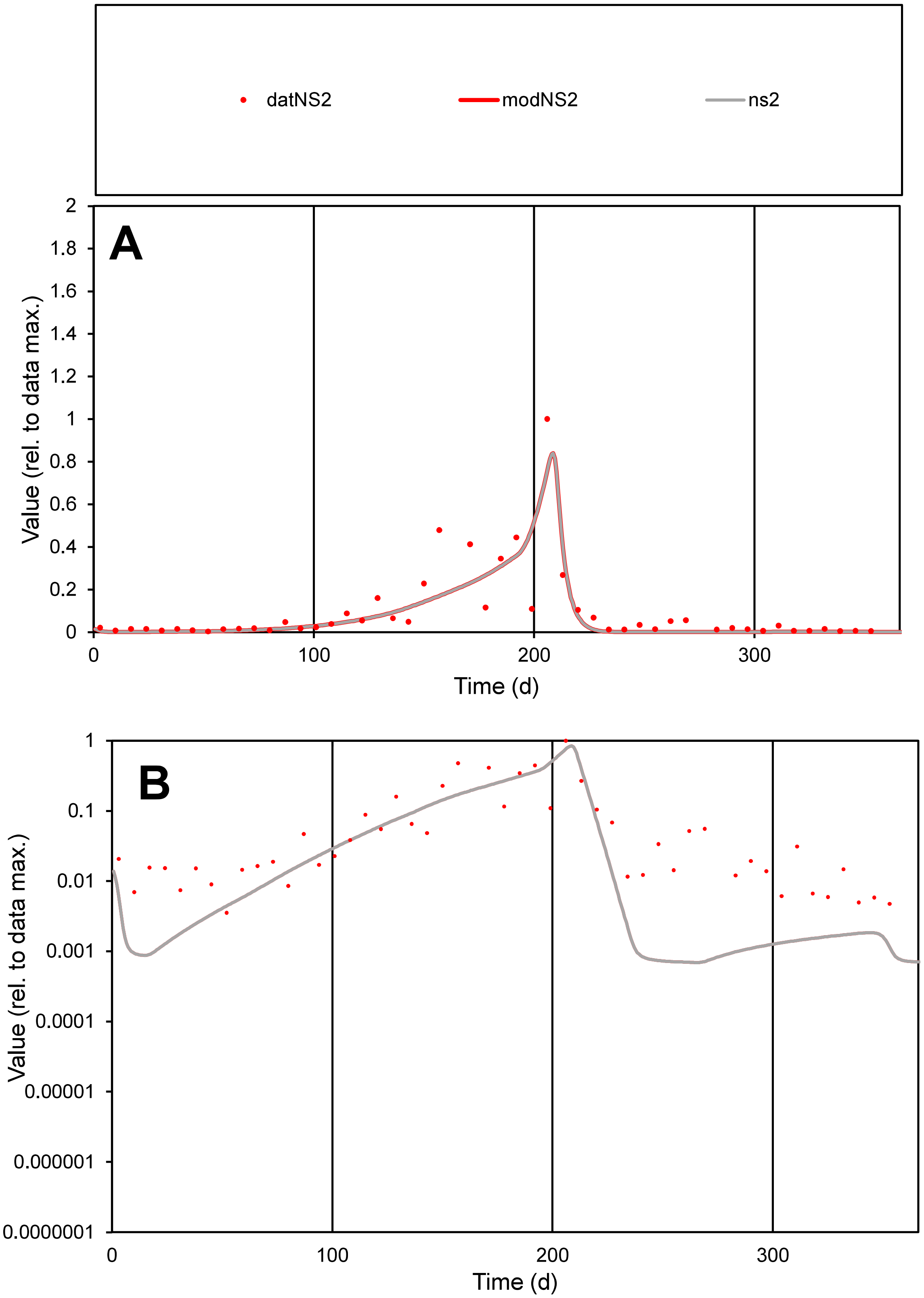

C1

Variables
NS2 Provide jtd or Dst
CalibOnly?
2 1 to reproduce of
oNormalize?
2 1=no, 2=yes

ofxn calc*
0.9306
ofxn mod
0.96117

sRun
RunA1
zout
1

Ave
Data
0.00619
Model
0.00515

data/vMax
0.06632

ofxng
369.894

Min d/m
5.09898

ave/min d
26.3952
ave/min m
111.957

328_Calib_S_A

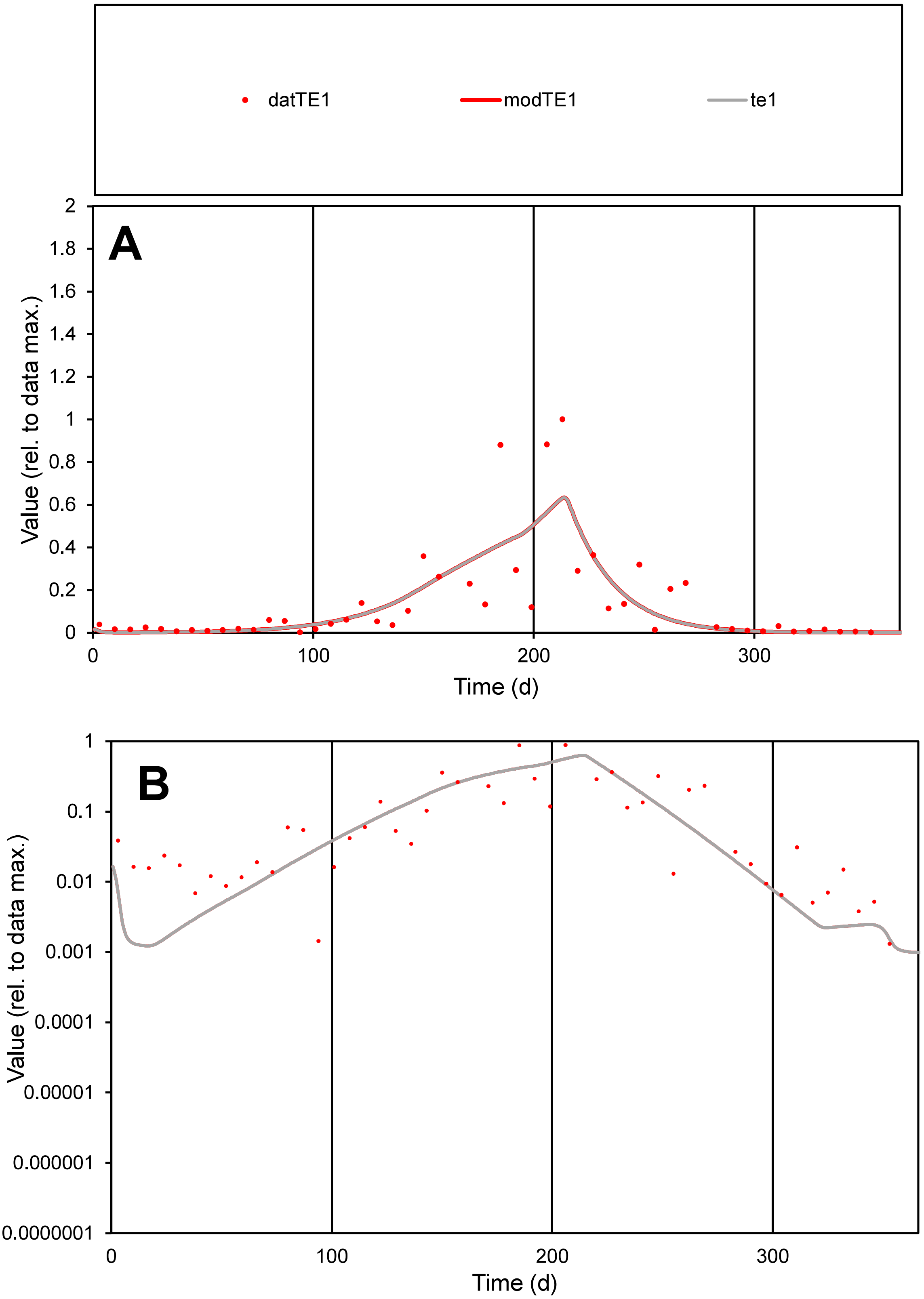

C1

Variables
TE1 Provide jtd or Dst
CalibOnly?
2 1 to reproduce of
oNormalize?
2 1=no, 2=yes

ofxn calc*
0.98242
ofxn mod
1.00457

sRun
RunA1
zout
1

Ave
Data
0.00578
Model
0.00534

data/vMax
0.04225

ofxng
369.894

Min d/m
1.0569

ave/min d
104.728
ave/min m
102.348

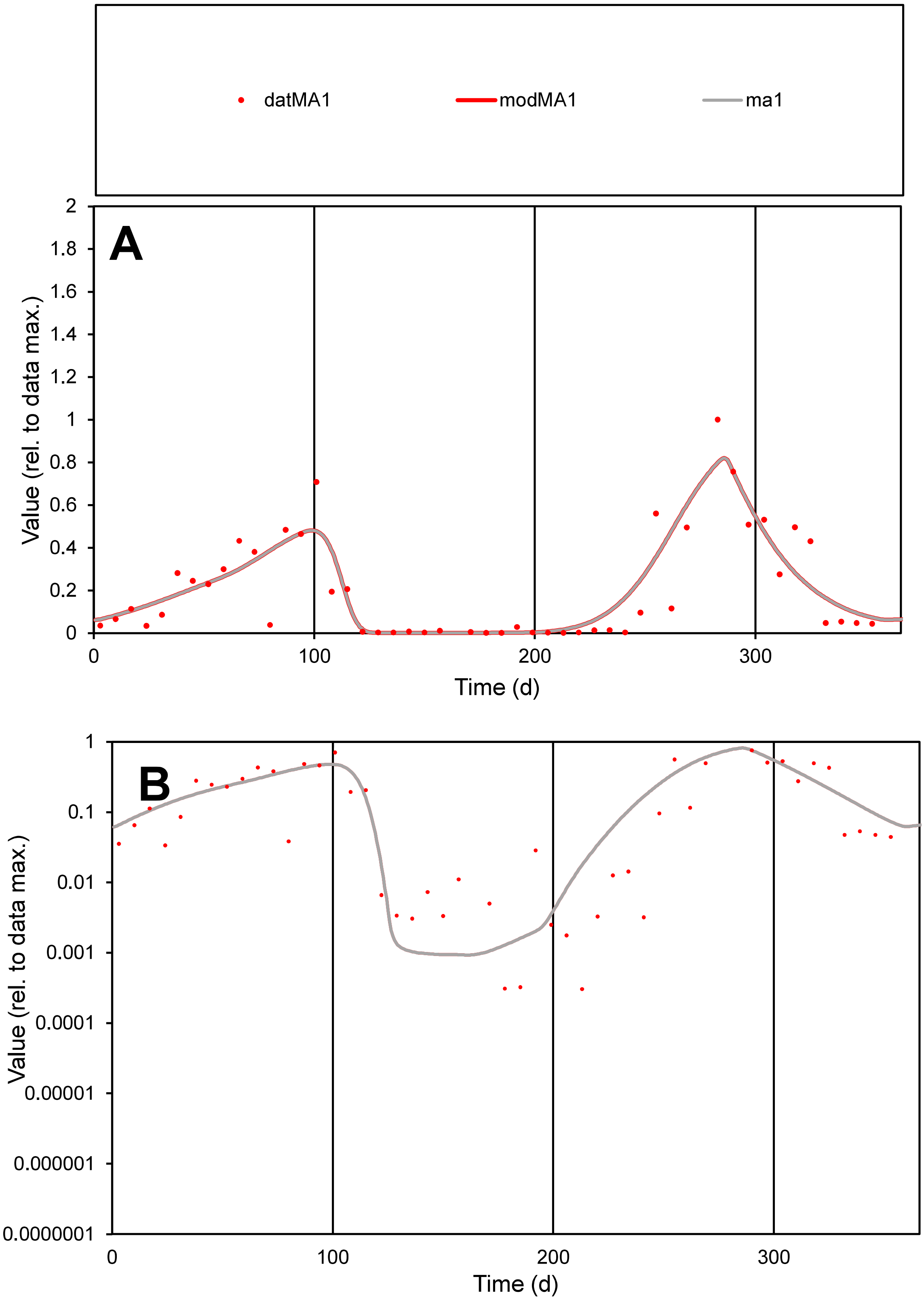

C1

Variables
MA1 Provide jtd or Dst
CalibOnly?
2 1 to reproduce of
oNormalize?
2 1=no, 2=yes

ofxn calc*
0.58193
ofxn mod
0.61296

sRun
RunA1
zout
1

Ave
Data
0.00999
Model
0.01051

data/vMax
0.04964

ofxng
369.894

Min d/m
0.32544

ave/min d
661.042
ave/min m
226.408

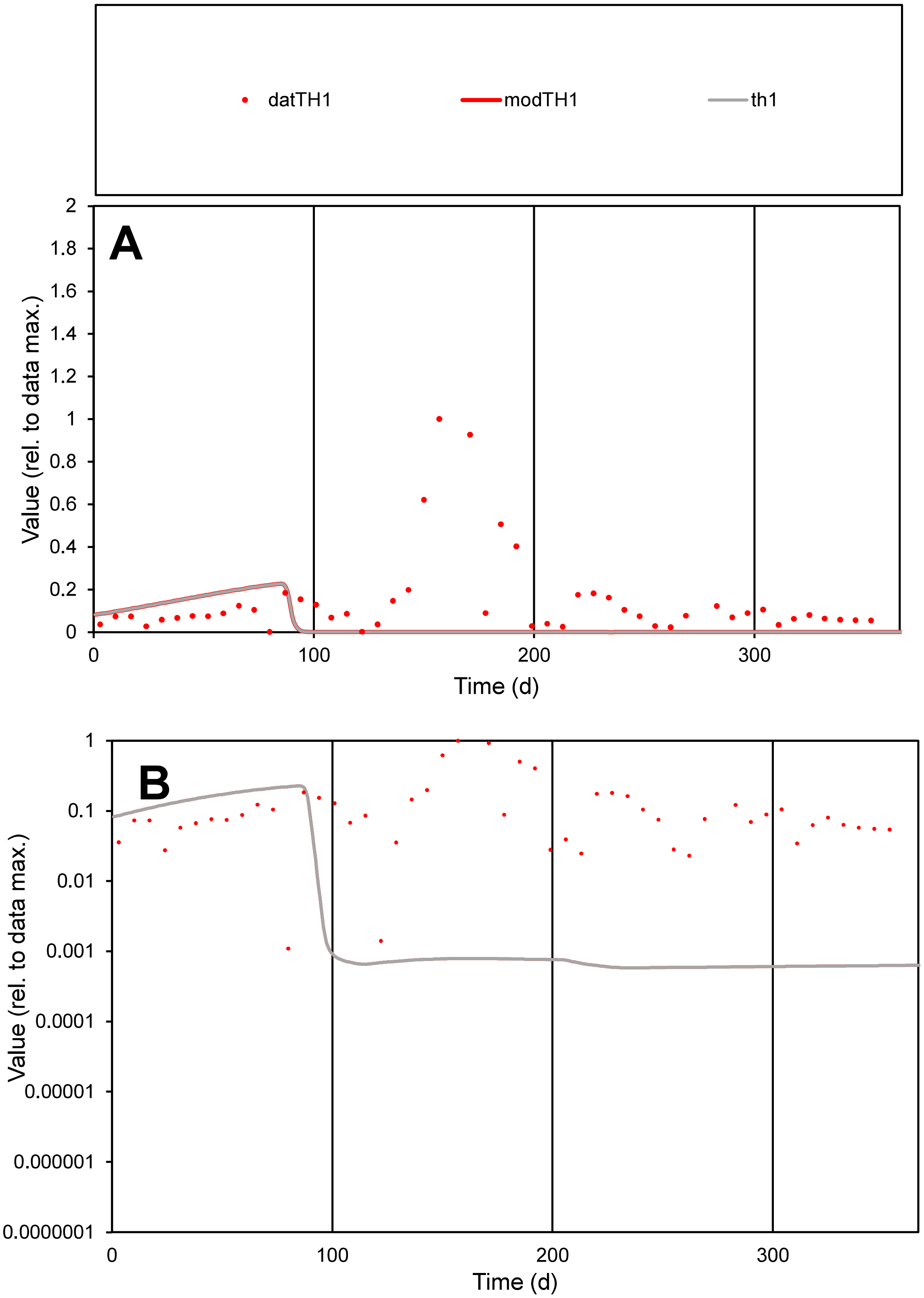

C1

Variables
TH1 Provide jtd or Dst
CalibOnly?
2 1 to reproduce of
oNormalize?
2 1=no, 2=yes

ofxn calc*
1.73433
ofxn mod
1.74169

sRun
RunA1
zout
1

Ave
Data
0.01231
Model
0.00379

data/vMax
0.08543

ofxng
369.894

Min d/m
1.89221

ave/min d
131.56
ave/min m
76.7052

328_Calib_S_A

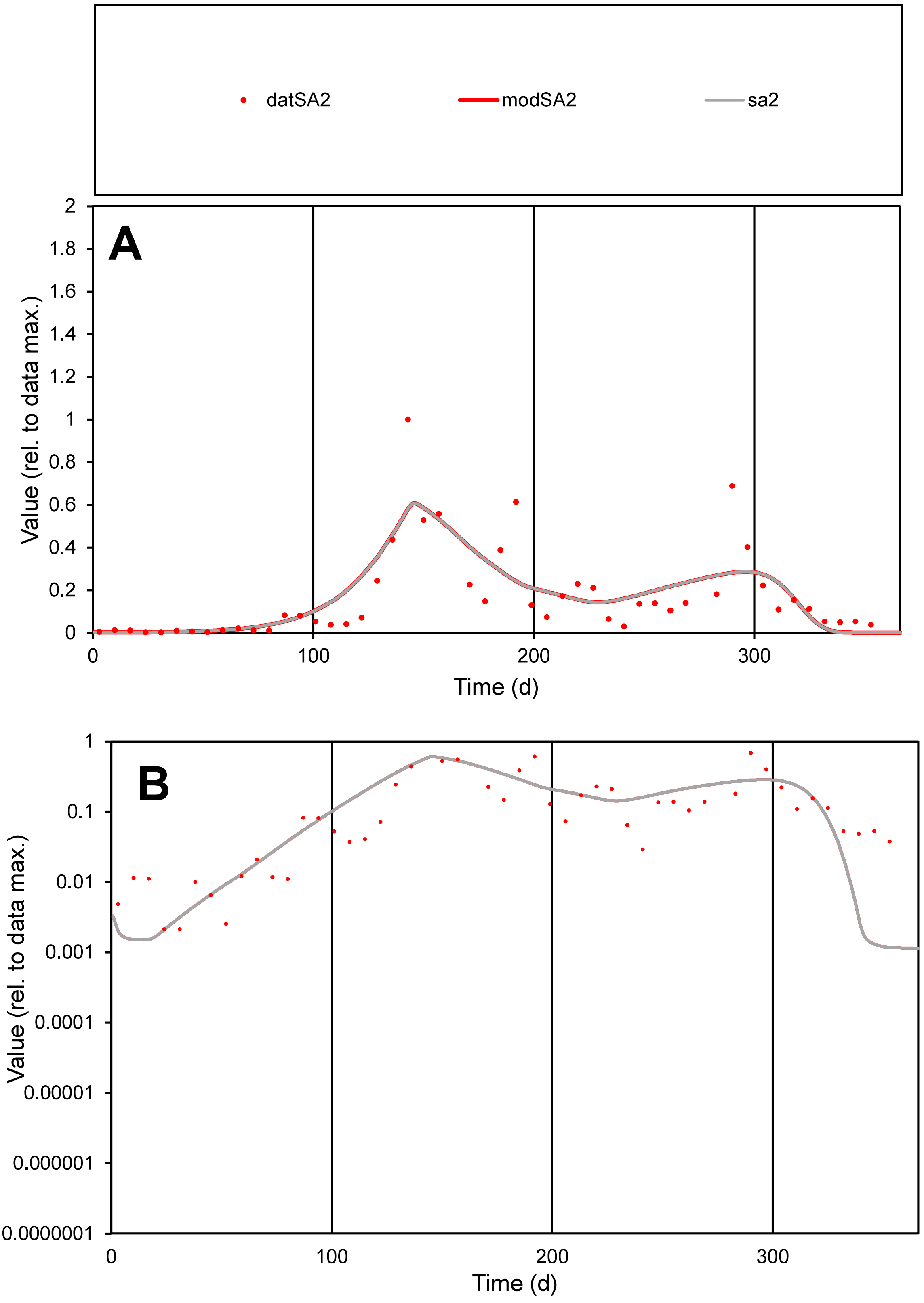

C1

Variables

SA2 Provide jtd or Dst

CalibOnly?

2 1 to reproduce of

oNormalize?

2 1=no, 2=yes

ofxn calc*

0.75905

ofxn mod

0.77702

sRun

RunA1

zout

1

Ave

Data

0.00845

Model

0.00874

data/vMax

0.05111

ofxng

369.894

Min d/m

1.82489

ave/min d

77.6683

ave/min m

146.599

328_Calib_S_A

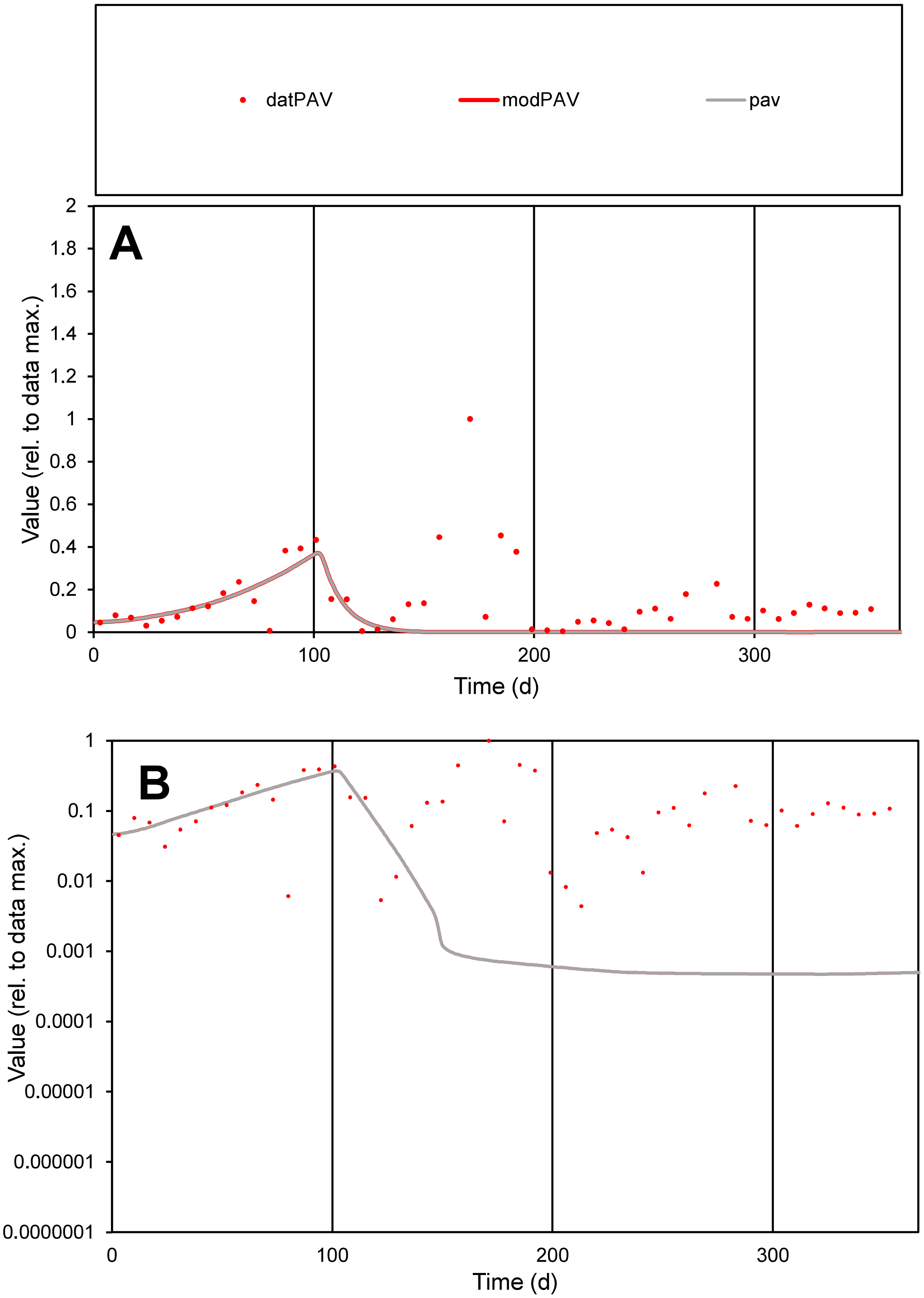

C1

Variables
PAV Provide jtd or Dst
CalibOnly?
2 1 to reproduce of
oNormalize?
2 1=no, 2=yes

ofxn calc*
1.34511
ofxn mod
1.35027

sRun
RunA1
zout
1

Ave
Data
0.01128
Model
0.00457

data/vMax
0.0775

ofxng
369.894

Min d/m
9.31316

ave/min d
33.0215
ave/min m
124.543

328_Calib_S_A

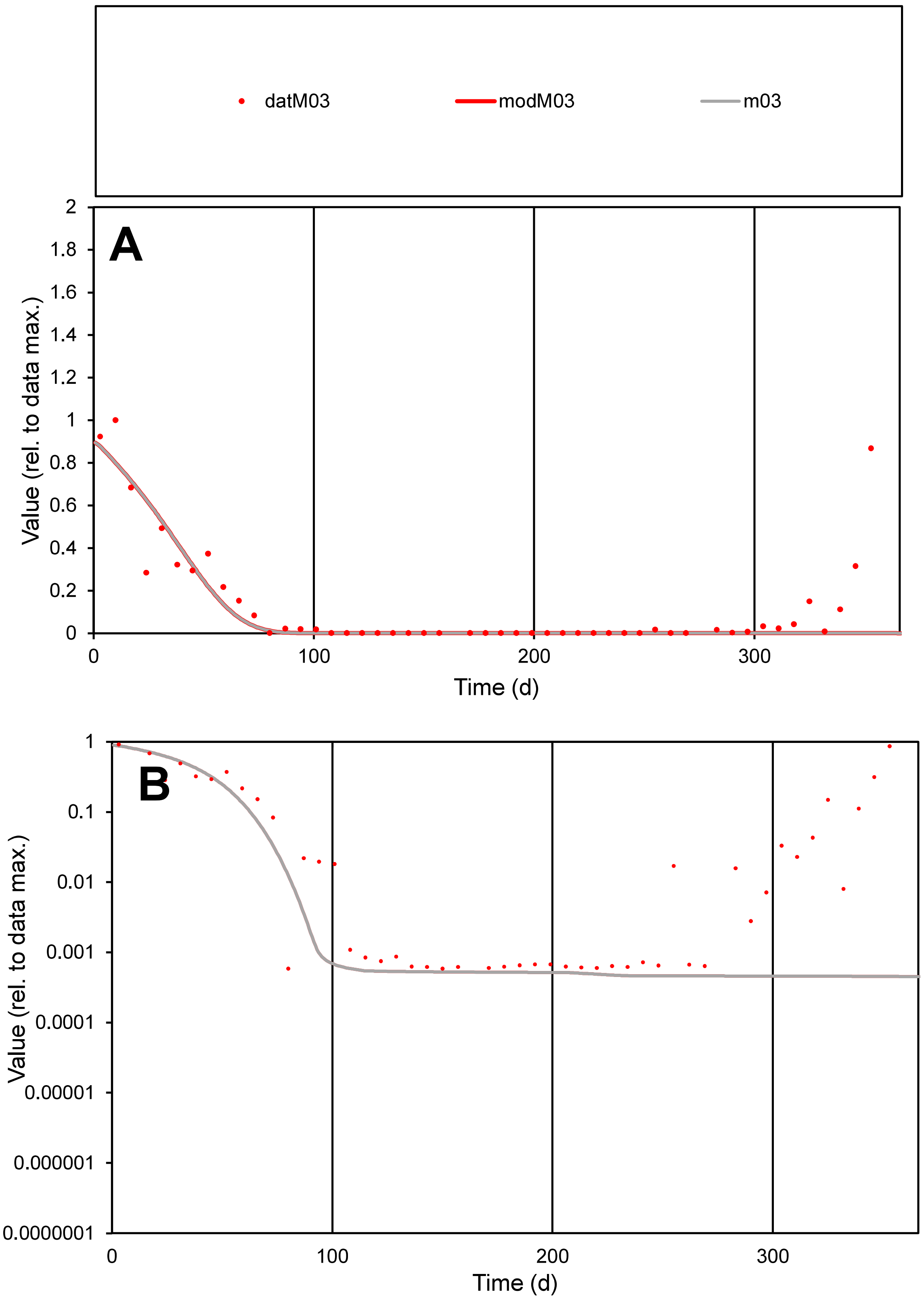

C1

Variables
M03 Provide jtd or Dst
CalibOnly?
2 1 to reproduce of
oNormalize?
2 1=no, 2=yes

ofxn calc*
1.12688
ofxn mod
1.154

sRun
RunA1
zout
1

Ave
Data
0.00398
Model
0.00284

data/vMax
0.02999

ofxng
369.894

Min d/m
1.29477

ave/min d
226.357
ave/min m
209.264

328_Calib_S_A

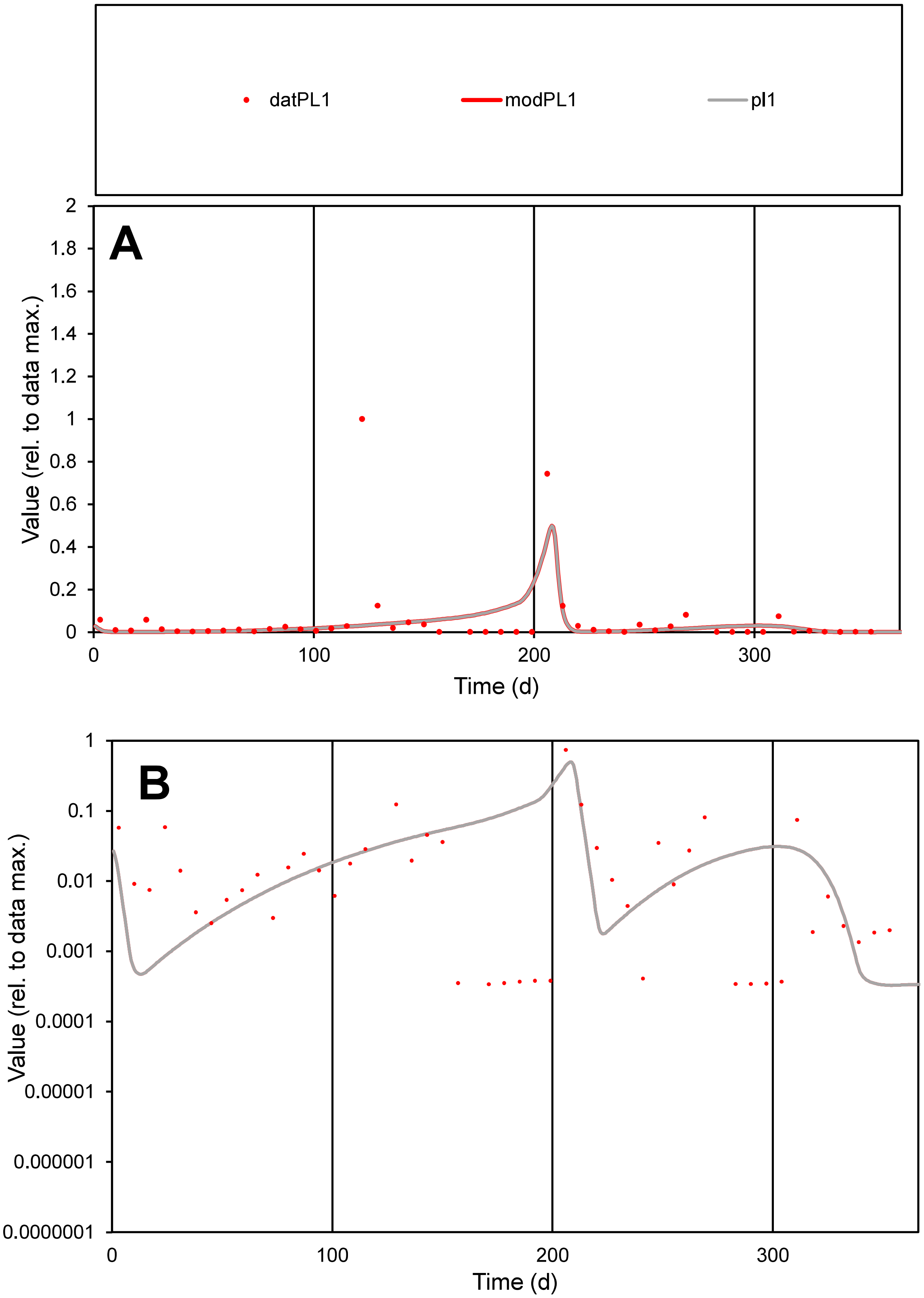

C1

Variables
PL1 Provide jtd or Dst
CalibOnly?
2 1 to reproduce of
oNormalize?
2 1=no, 2=yes

ofxn calc*
2.75819
ofxn mod
2.78286

sRun
RunA1
zout
1

Ave
Data
0.00571
Model
0.00397

data/vMax
0.1048

ofxng
369.894

Min d/m
1.03158

ave/min d
160.457
ave/min m
114.925

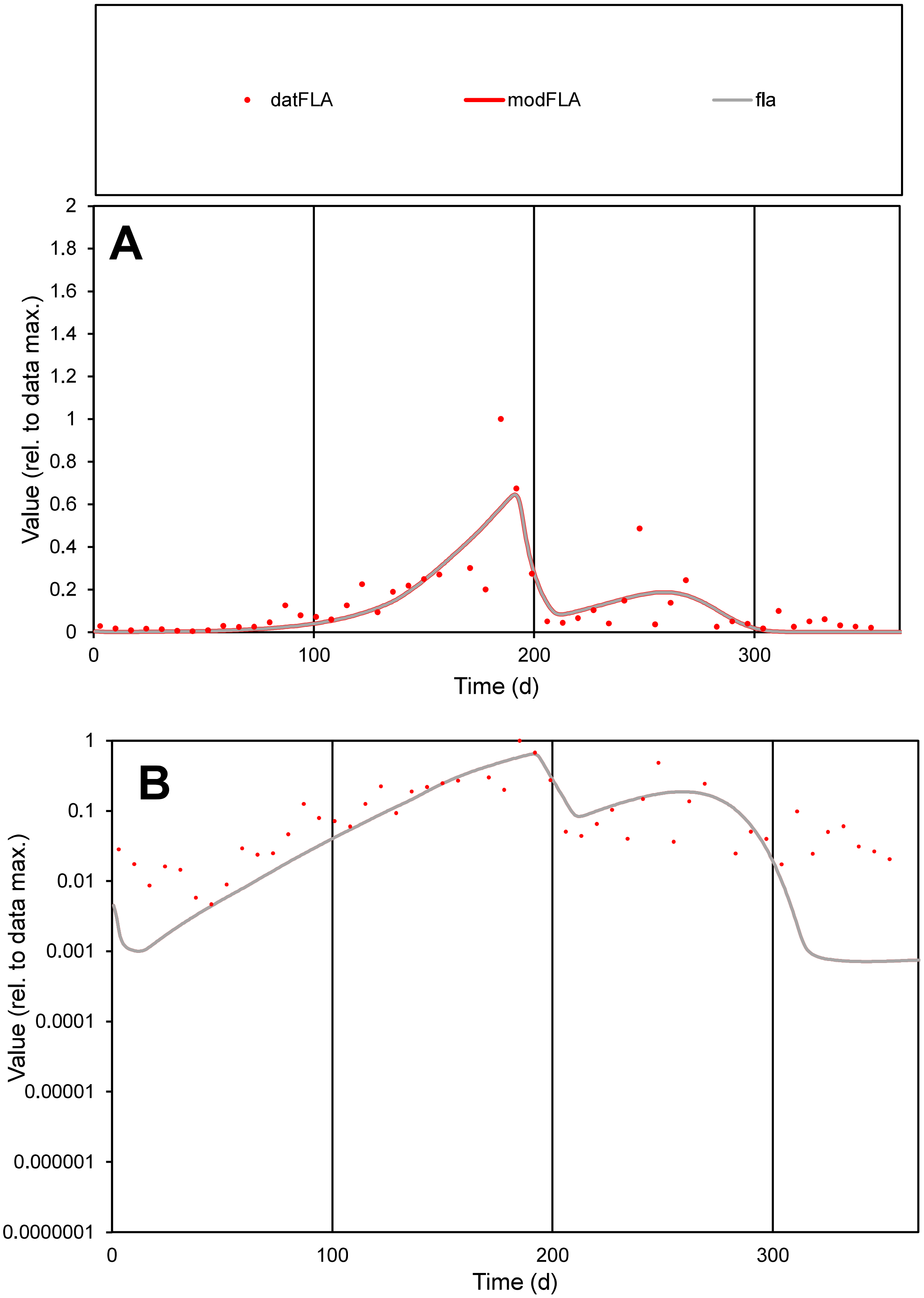

C1

Variables
FLA Provide jtd or Dst
CalibOnly?
2 1 to reproduce of
oNormalize?
2 1=no, 2=yes

ofxn calc*
0.78582
ofxn mod
0.81575

sRun
RunA1
zout
1

Ave
Data
0.00522
Model
0.00464

data/vMax
0.04138

ofxng
369.894

Min d/m
6.51178
ave/min d
27.0051
ave/min m
156.139

328_Calib_S_A

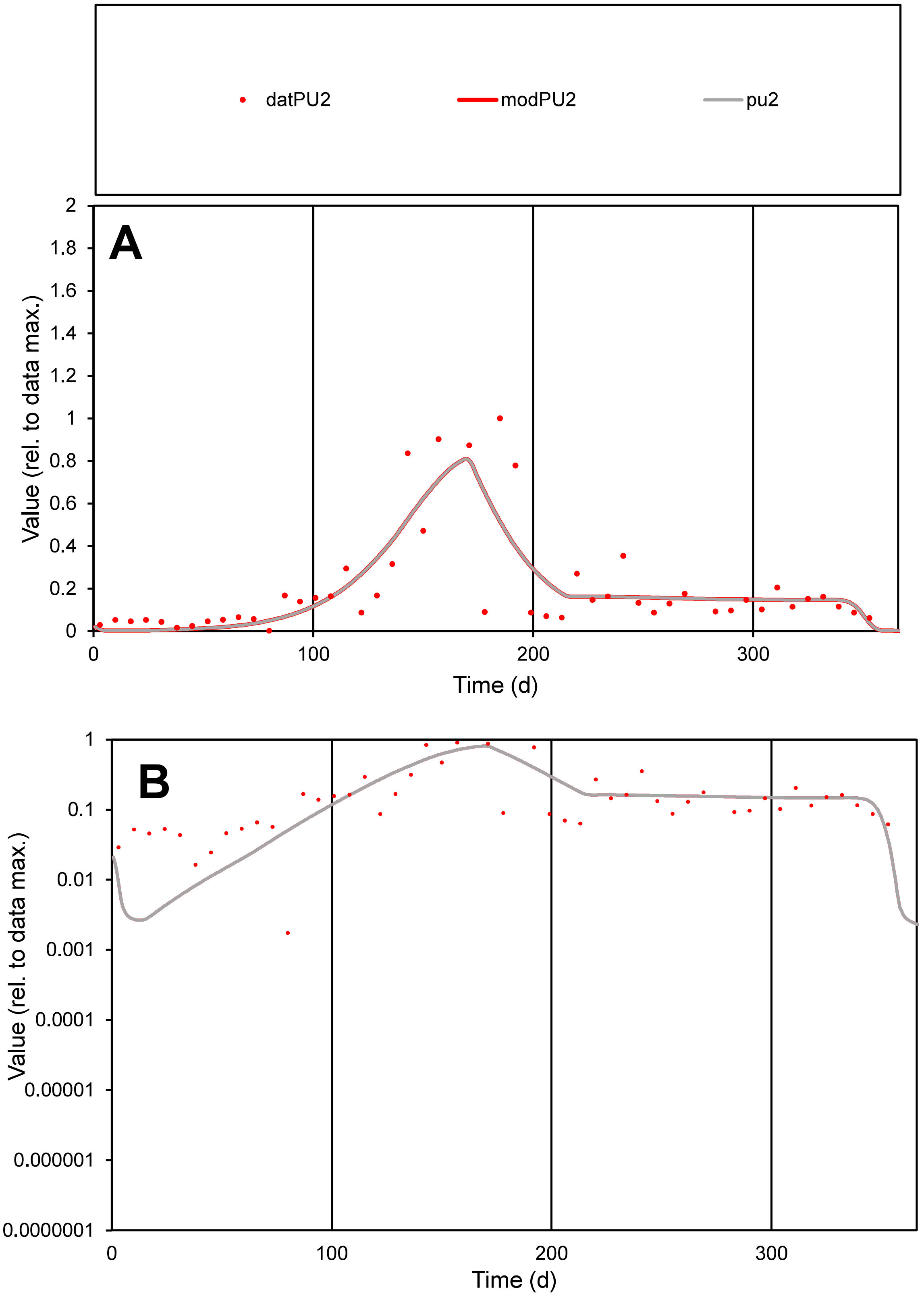

C1

Variables
PU2 Provide jtd or Dst
CalibOnly?
2 1 to reproduce of
oNormalize?
2 1=no, 2=yes

ofxn calc*
0.73513
ofxn mod
0.75538

sRun
RunA1
zout
1

Ave
Data
0.00641
Model
0.00619

data/vMax
0.0316

ofxng
369.894

Min d/m
0.658

ave/min d
116.208
ave/min m
73.8344

328_Calib_S_A

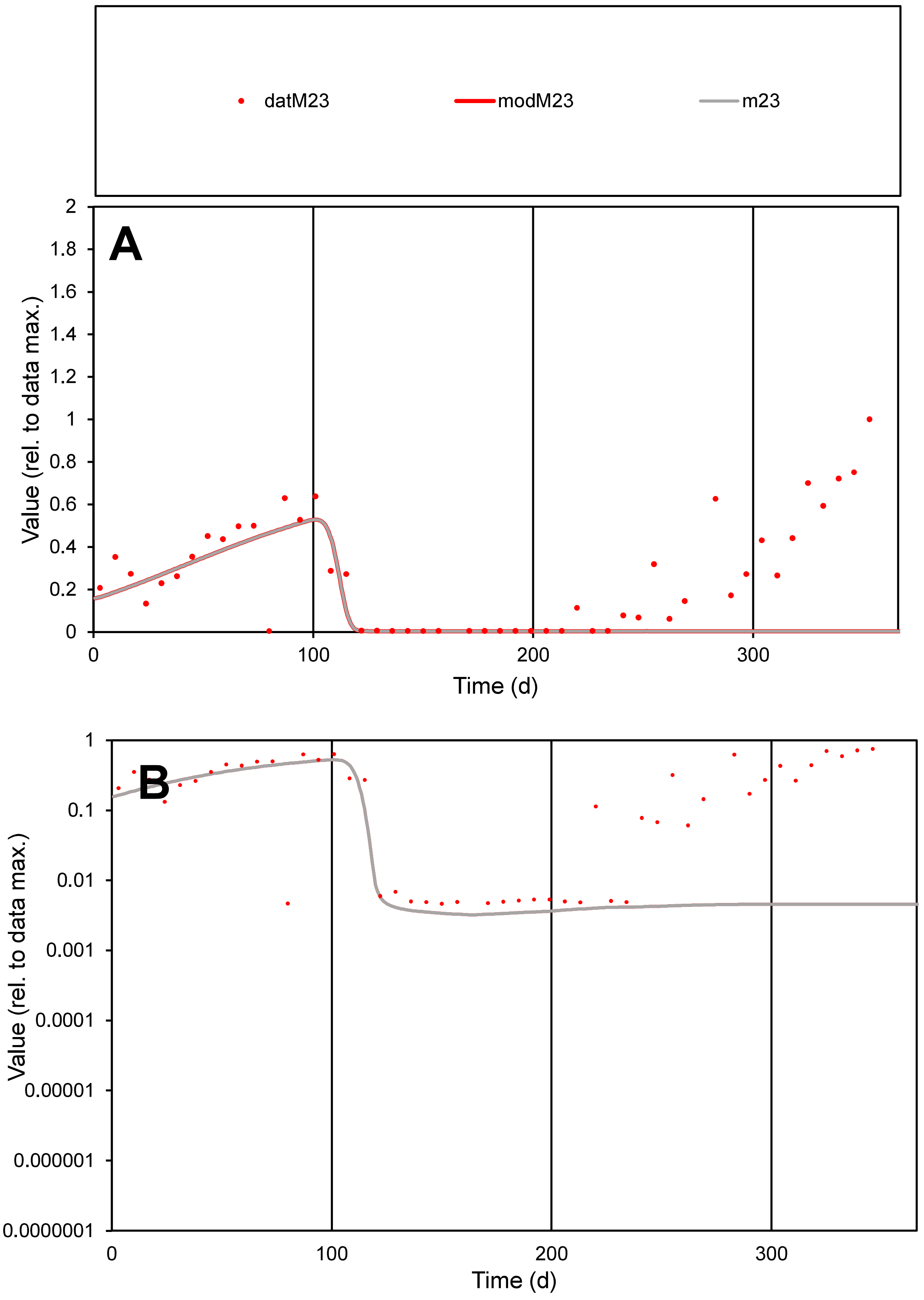

C1

Variables

M23 Provide jtd or Dst

CalibOnly?

2 1 to reproduce of

oNormalize?

2 1=no, 2=yes

ofxn calc*

1.12648

ofxn mod

1.13293

sRun

RunA1

zout

1

Ave

Data

0.00609

Model

0.00285

data/vMax

0.02315

ofxng

369.894

Min d/m

1.42084

ave/min d

56.5997

ave/min m

37.67

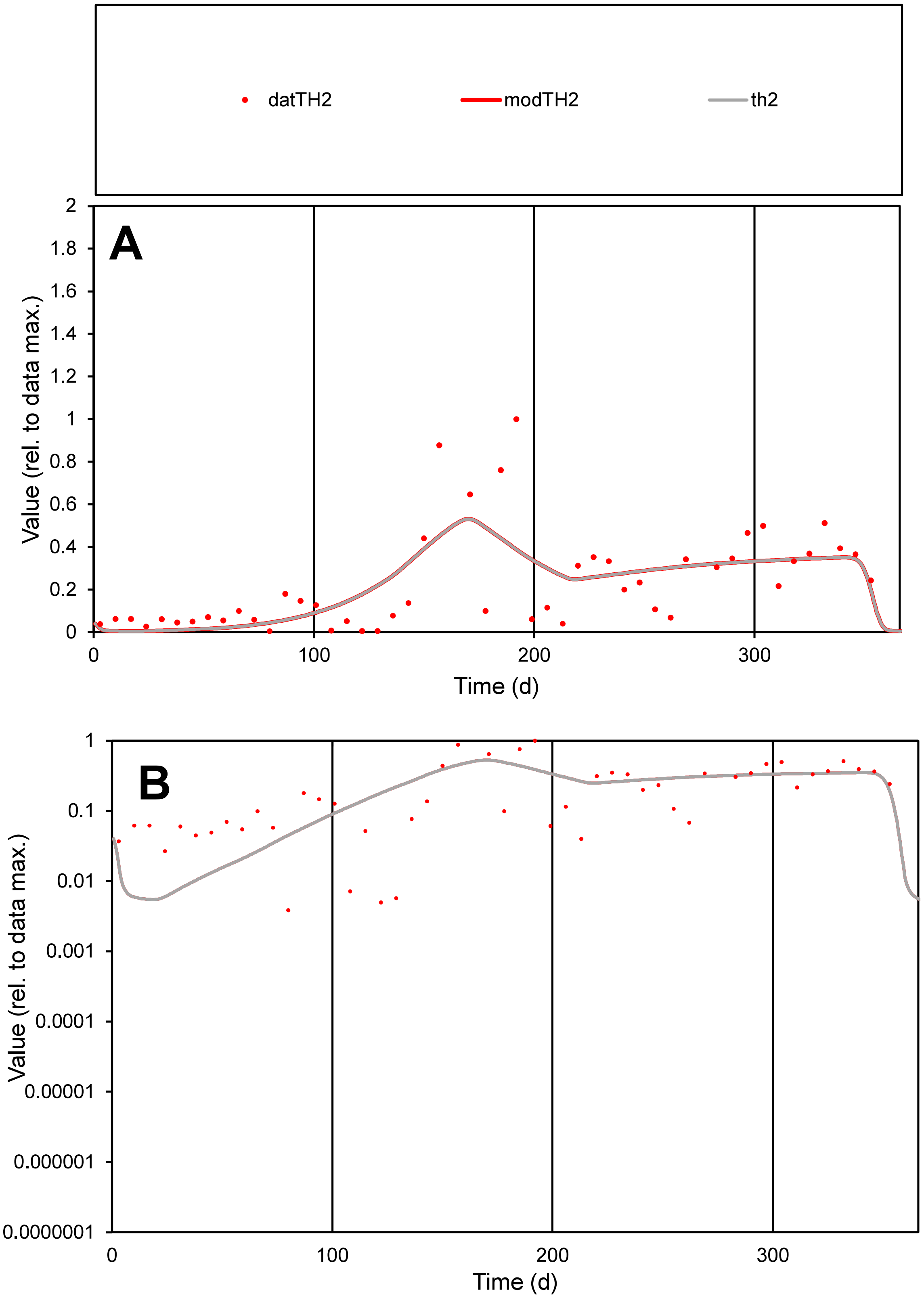

C1

Variables
TH2 Provide jtd or Dst
CalibOnly?
2 1 to reproduce of
oNormalize?
2 1=no, 2=yes

ofxn calc*
0.72558
ofxn mod
0.74215

sRun
RunA1
zout
1

Ave
Data
0.0064
Model
0.00623

data/vMax
0.02752

ofxng
369.894

Min d/m
0.71095

ave/min d
60.0677
ave/min m
41.5839

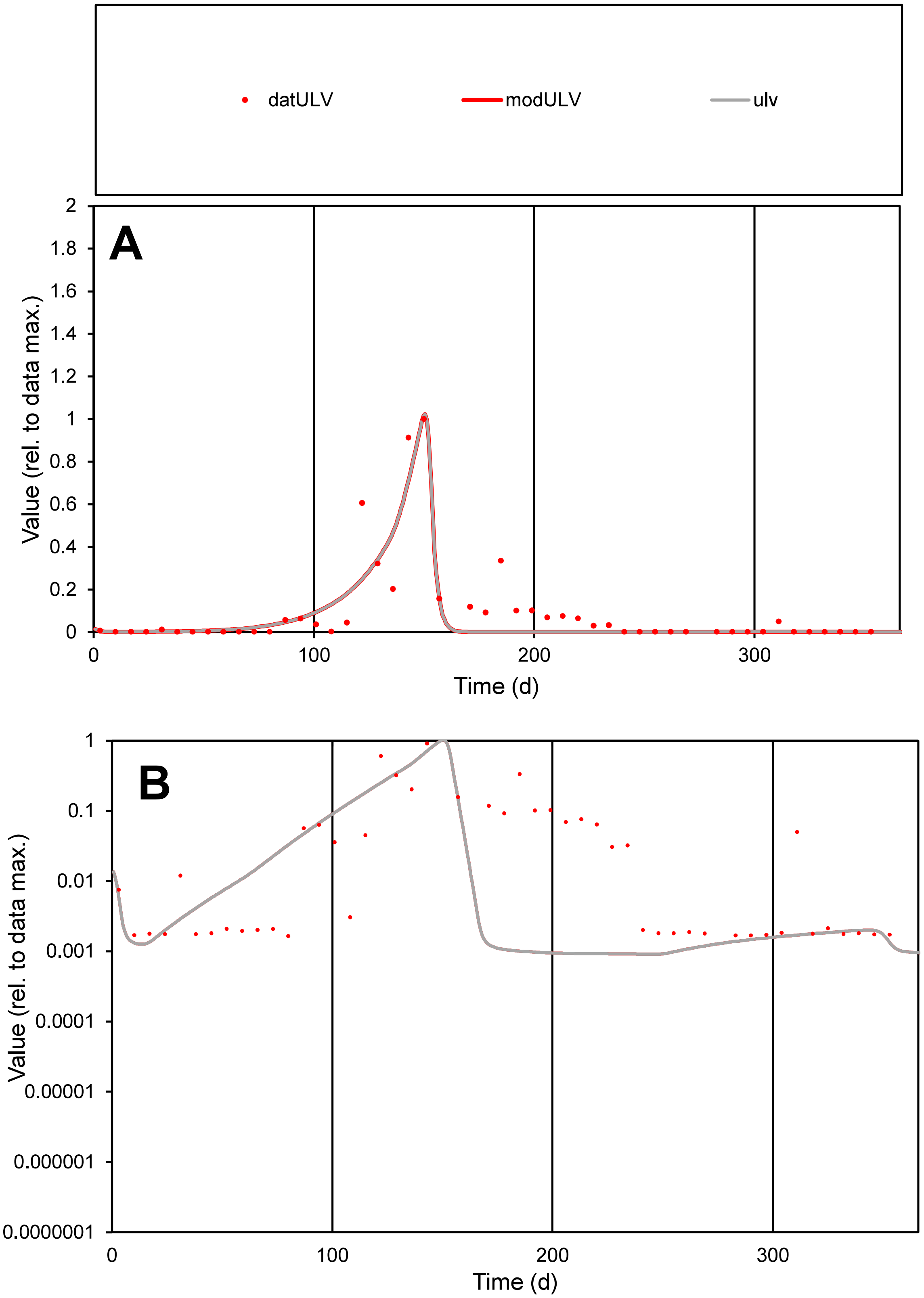

C1

Variables
ULV Provide jtd or Dst
CalibOnly?
2 1 to reproduce of
oNormalize?
2 1=no, 2=yes

ofxn calc*
1.00481
ofxn mod
1.0355

sRun
RunA1
zout
1

Ave
Data
0.00097
Model
0.00079

data/vMax
0.0105

ofxng
369.894

Min d/m
1.79882

ave/min d
56.2643
ave/min m
81.9563

328_Calib_S_A

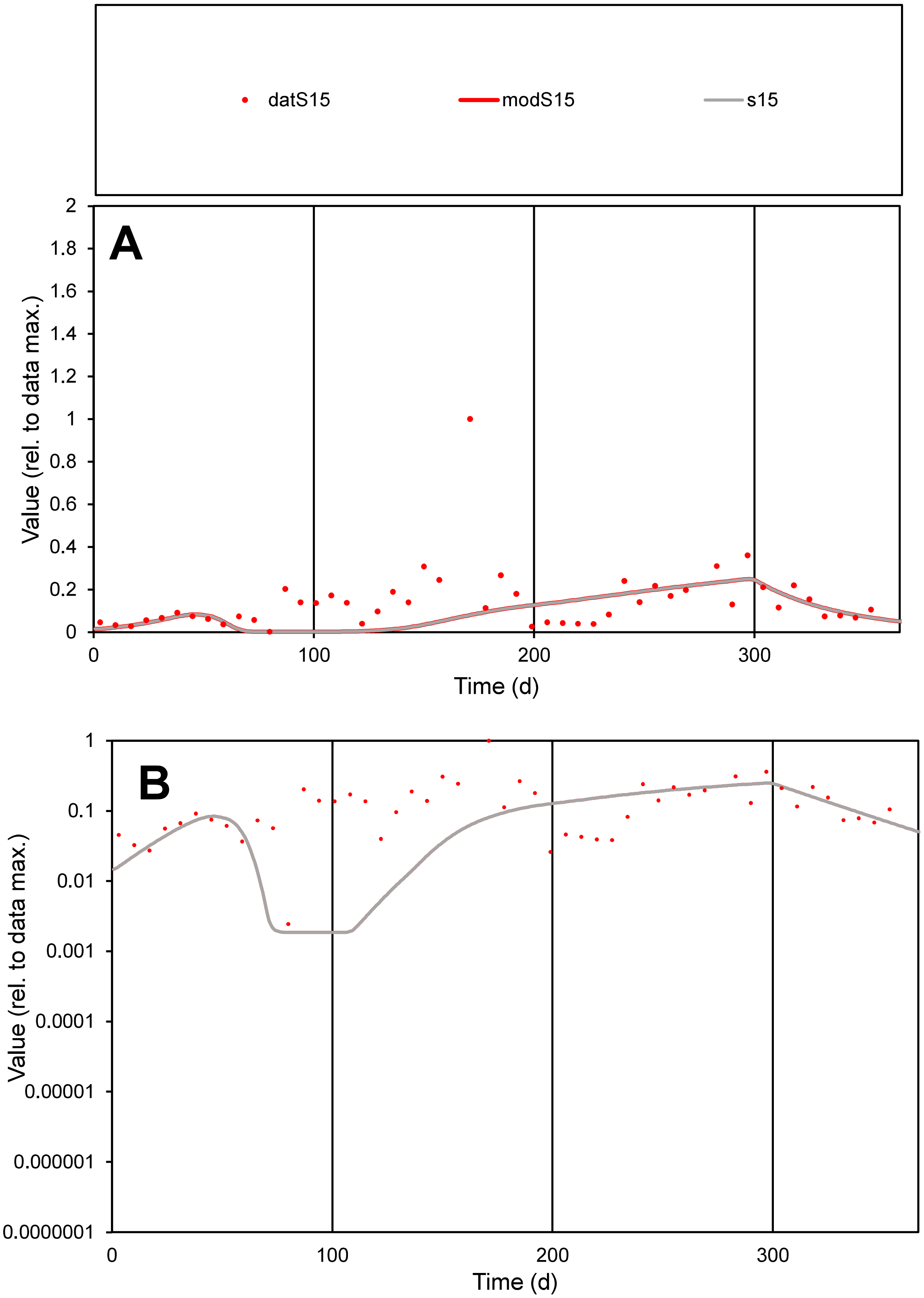

C1

Variables

S15 Provide jtd or Dst

CalibOnly?

2 1 to reproduce of

oNormalize?

2 1=no, 2=yes

ofxn calc*

1.12187

ofxn mod

1.12877

sRun

RunA1

zout

1

Ave

Data

0.00736

Model

0.00491

data/vMax

0.05113

ofxng

369.894

Min d/m

1.31372

ave/min d

58.9125

ave/min m

51.5915

328_Calib_S_A

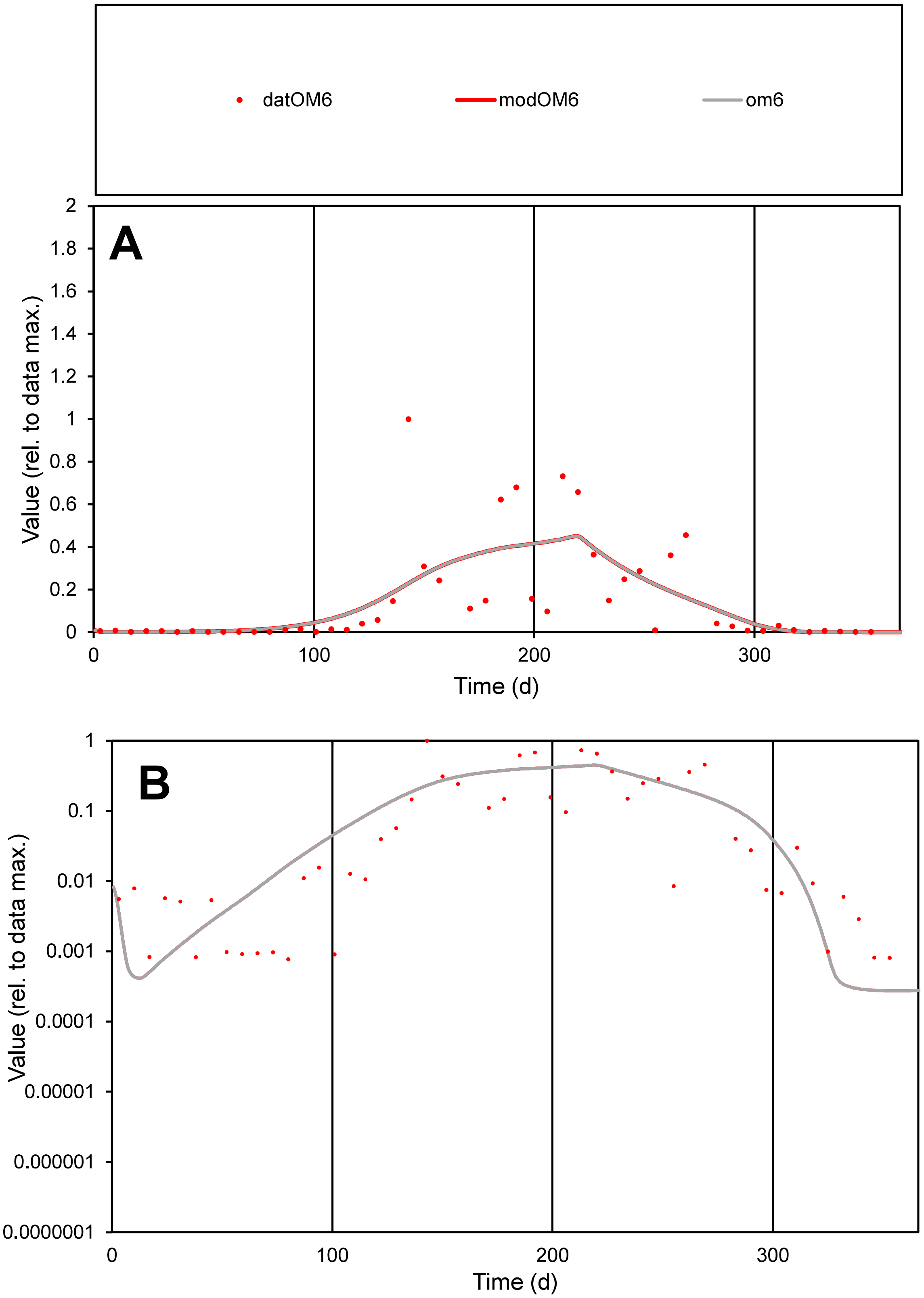

C1

Variables
OM6 Provide jtd or Dst
CalibOnly?
2 1 to reproduce of
oNormalize?
2 1=no, 2=yes

ofxn calc*
1.15697
ofxn mod
1.17257

sRun
RunA1
zout
1

Ave
Data
0.00473
Model
0.00465

data/vMax
0.03274

ofxng
369.894

Min d/m
2.79436

ave/min d
187.987
ave/min m
515.912

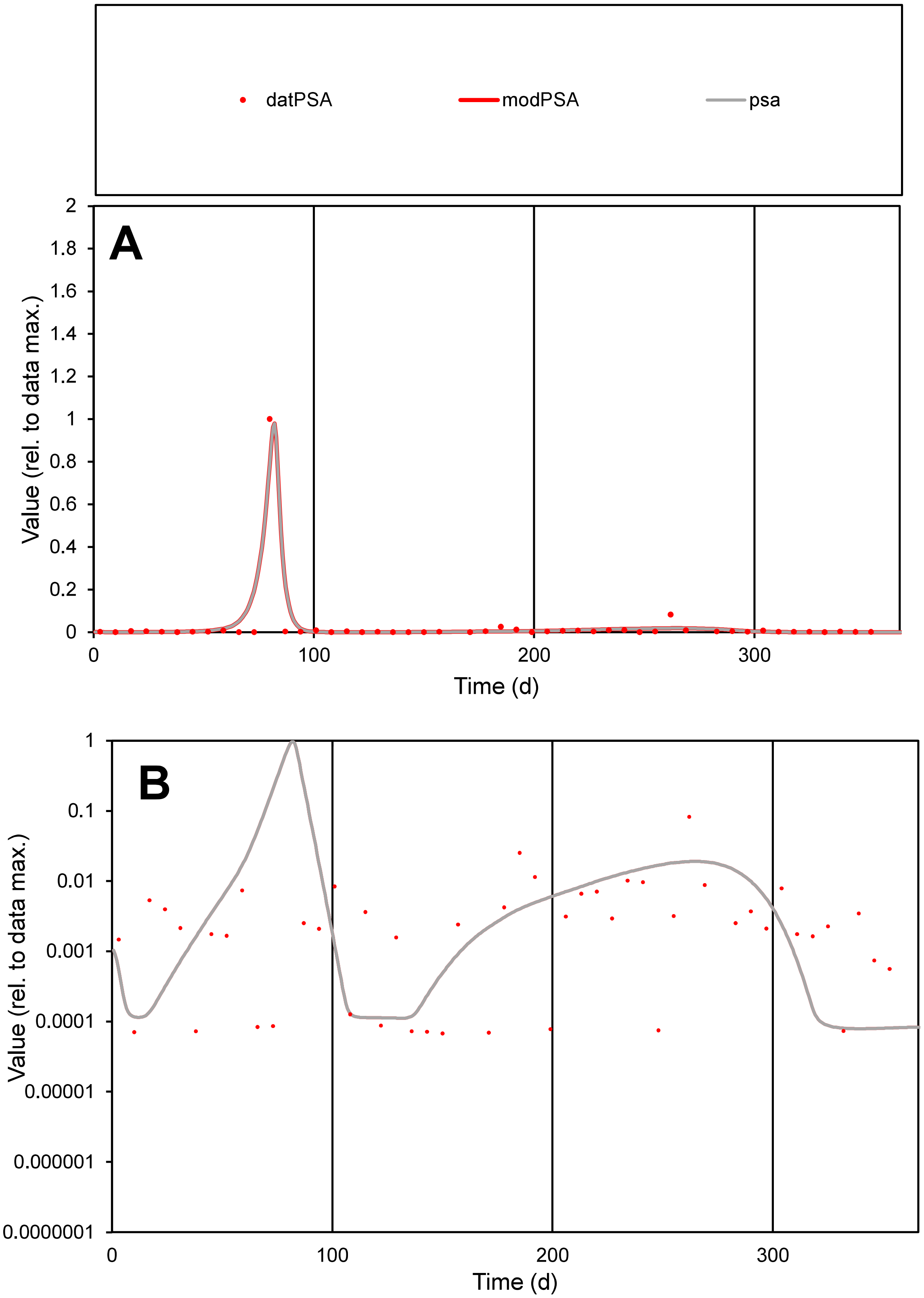

C1

Variables

PSA Provide jtd or Dst

CalibOnly?

2 1 to reproduce of

oNormalize?

2 1=no, 2=yes

ofxn calc*

1.02876

ofxn mod

1.31263

sRun

RunA1

zout

1

Ave

Data

0.00332

Model

0.00382

data/vMax

0.1304

ofxng

369.894

Min d/m

0.85971

ave/min d

374.781

ave/min m

371.29

328_Calib_S_A

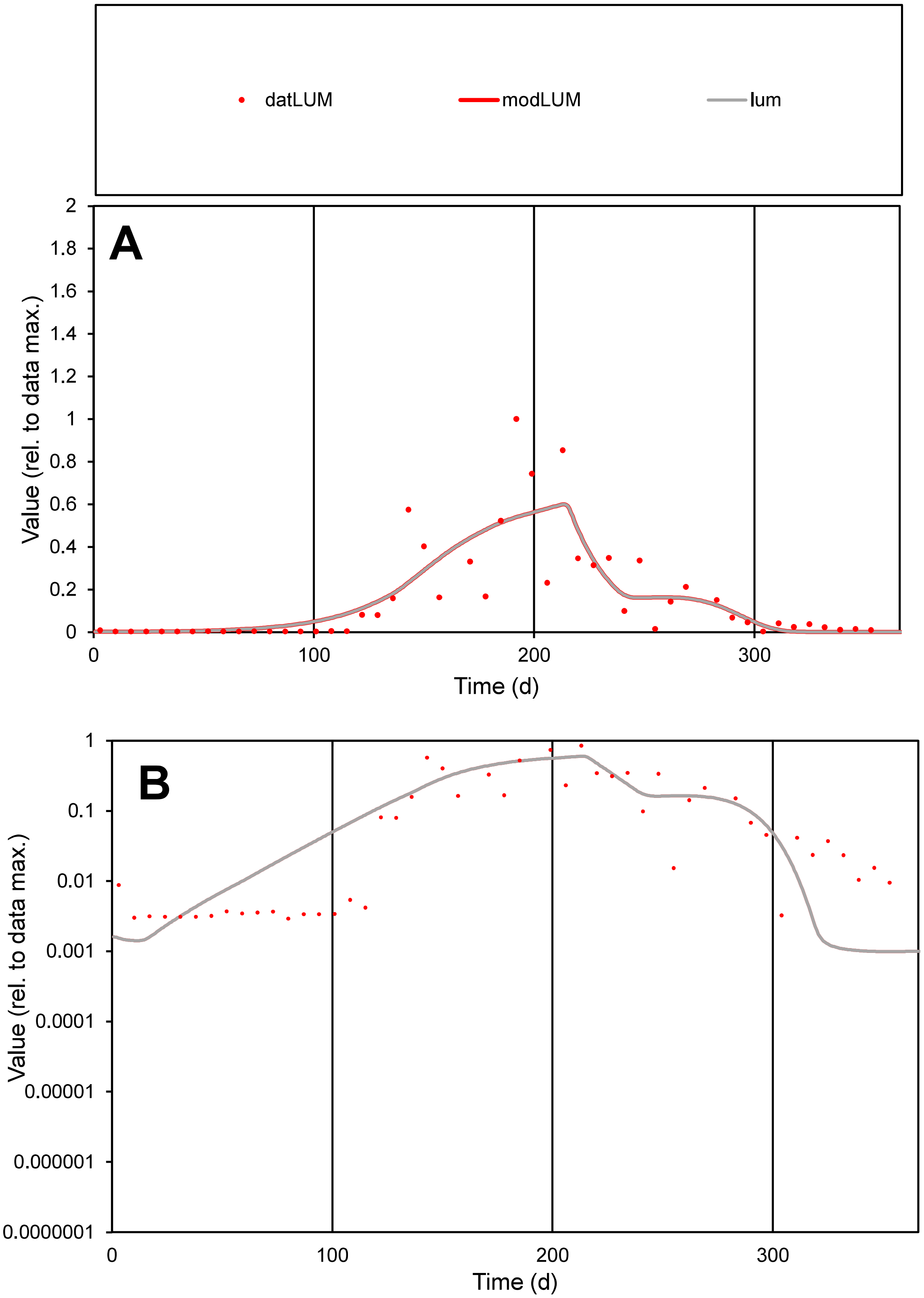

C1

Variables
LUM Provide jtd or Dst
CalibOnly?
2 1 to reproduce of
oNormalize?
2 1=no, 2=yes

ofxn calc*
0.8274
ofxn mod
0.85743

sRun
RunA1
zout
1

Ave
Data
0.0024
Model
0.00239

data/vMax
0.01543

ofxng
369.894

Min d/m
2.92653

ave/min d
53.2915
ave/min m
155.398

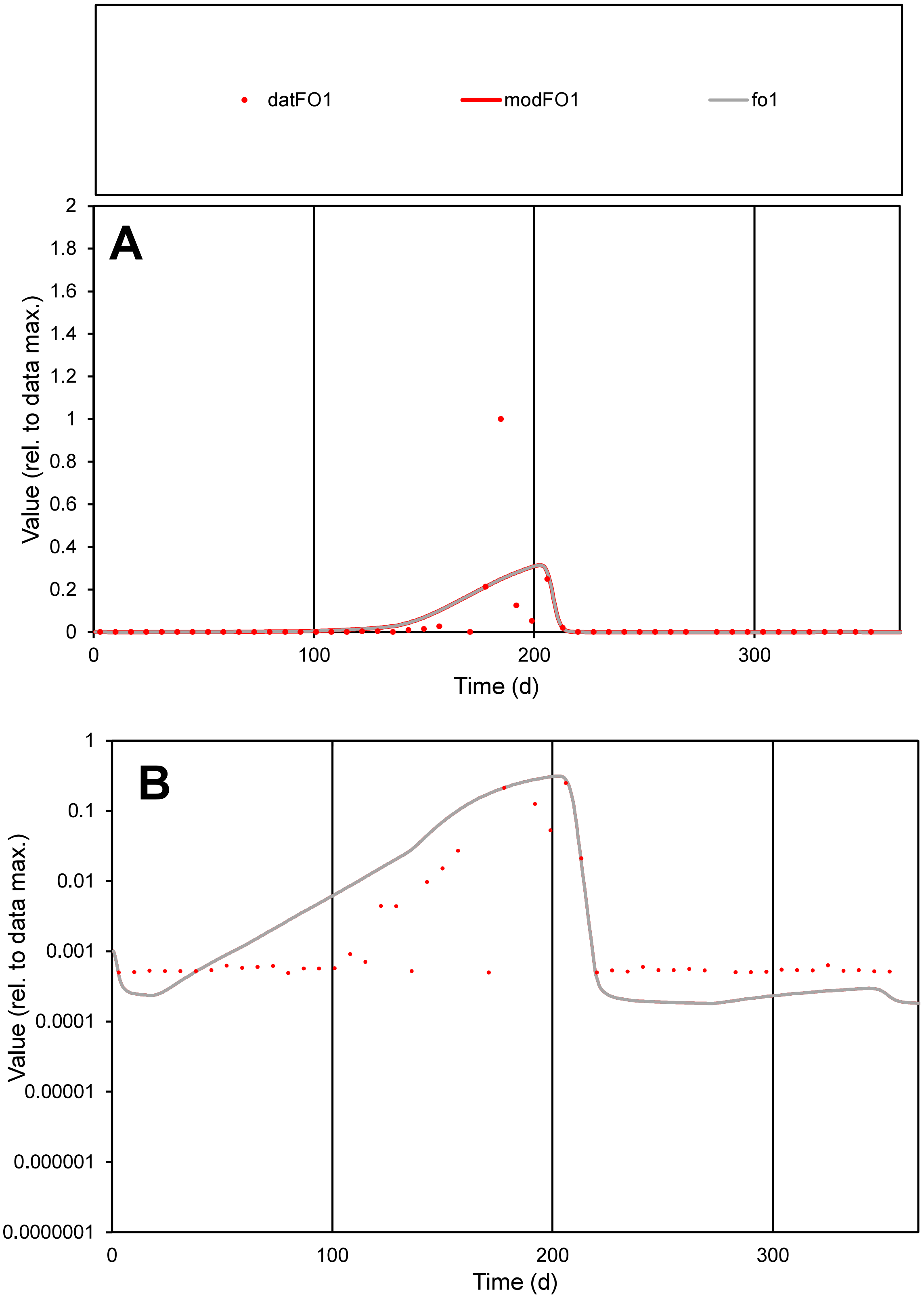

C1

Variables
FO1 Provide jtd or Dst
CalibOnly?
2 1 to reproduce of
oNormalize?
2 1=no, 2=yes

ofxn calc*
3.31892
ofxn mod
3.34544

sRun
RunA1
zout
1

Ave
Data
0.00424
Model
0.00442

data/vMax
0.1191

ofxng
369.894

Min d/m
2.70564

ave/min d
72.4533
ave/min m
204.086

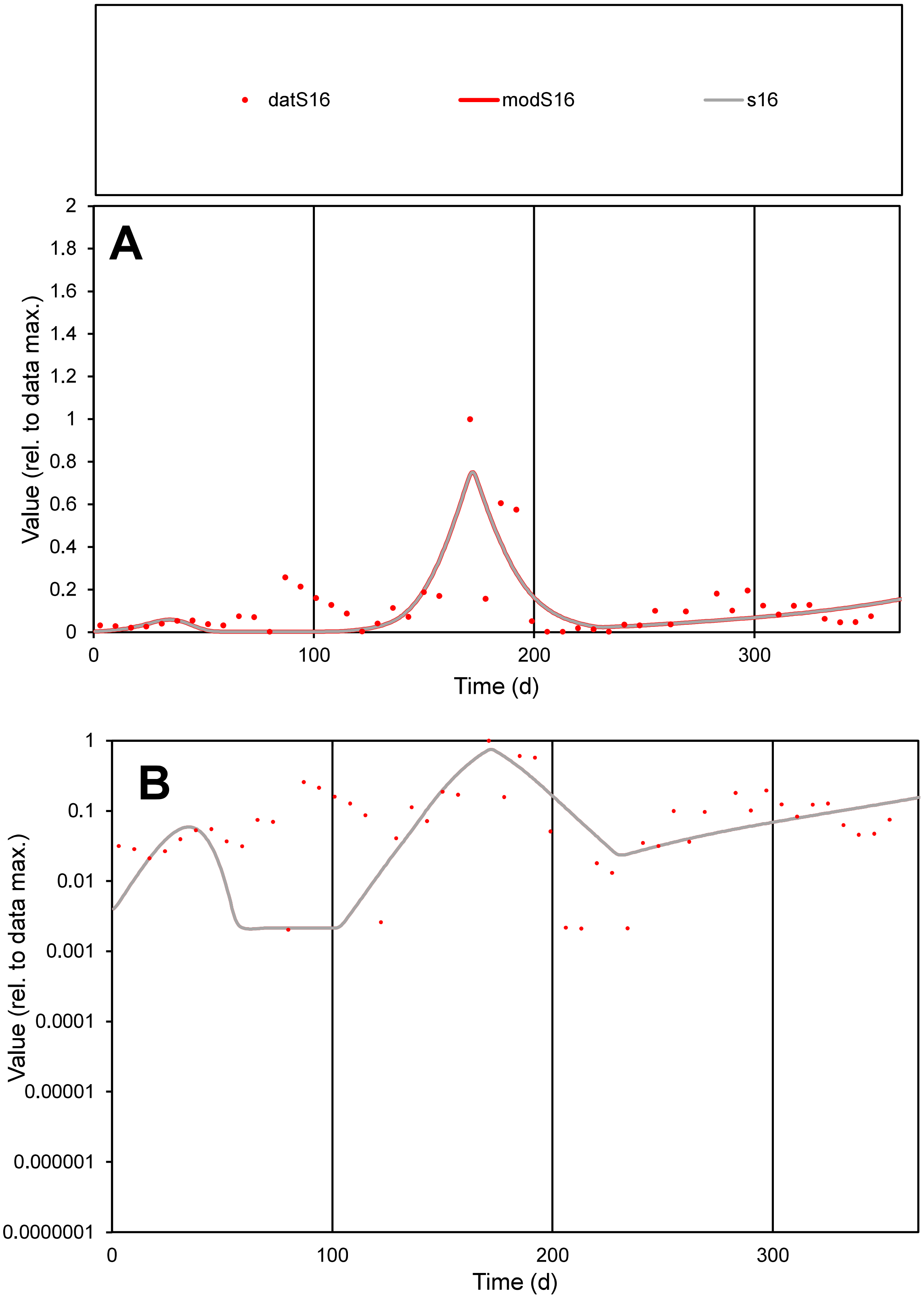

C1

Variables
S16 Provide jtd or Dst
CalibOnly?
2 1 to reproduce of
oNormalize?
2 1=no, 2=yes

ofxn calc*
0.9684
ofxn mod
0.99165

sRun
RunA1
zout
1

Ave
Data
0.00728
Model
0.00574

data/vMax
0.06158

ofxng
369.894

Min d/m
0.96228

ave/min d
58.2565
ave/min m
44.1507

328_Calib_S_A

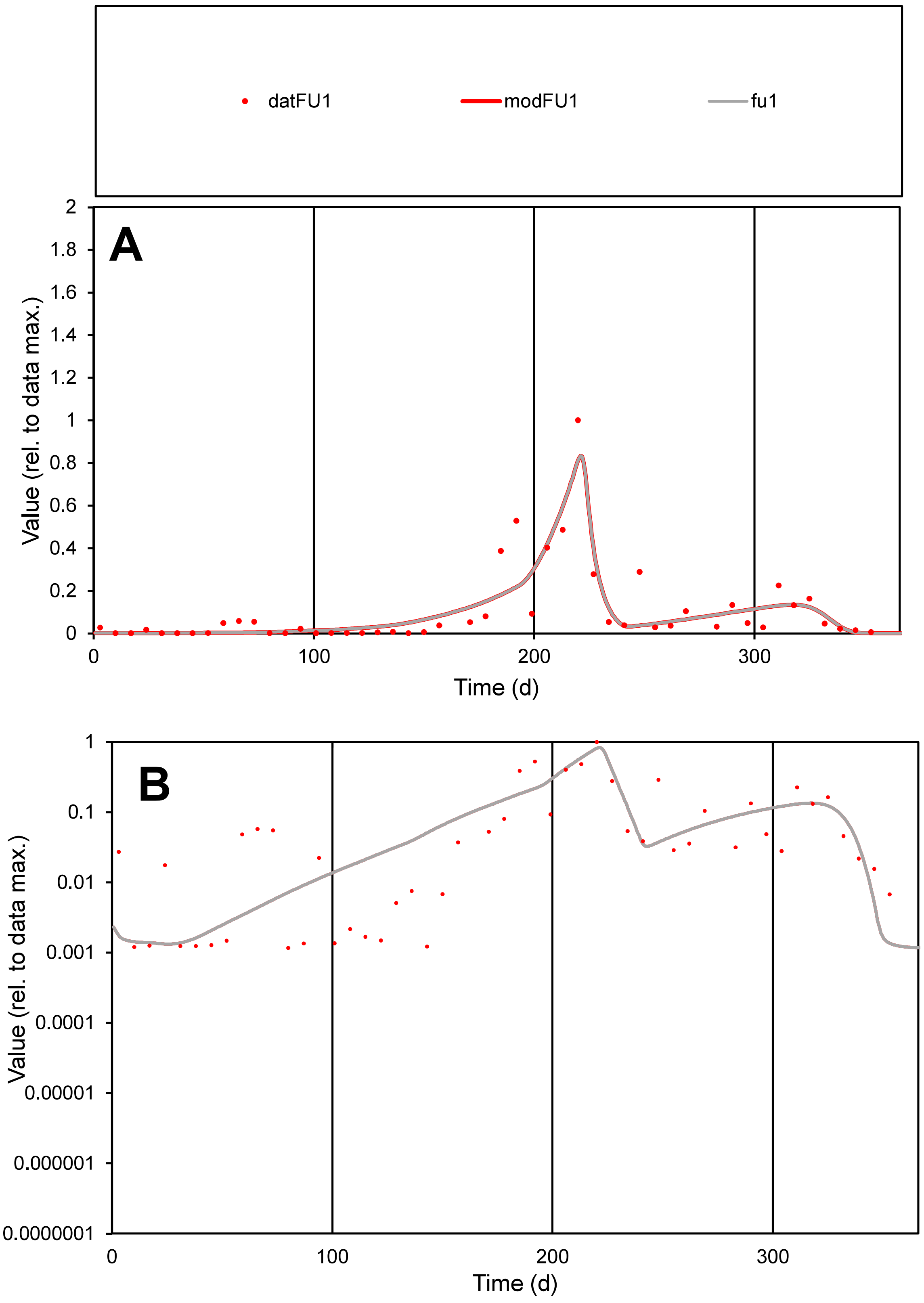

C1

Variables
FU1 Provide jtd or Dst
CalibOnly?
2 1 to reproduce of
oNormalize?
2 1=no, 2=yes

ofxn calc*
0.81215
ofxn mod
0.8502

sRun
RunA1
zout
1

Ave
Data
0.00412
Model
0.00398

data/vMax
0.04026

ofxng
369.894

Min d/m
0.88628

ave/min d
87.9408
ave/min m
75.3144

328_Calib_S_A

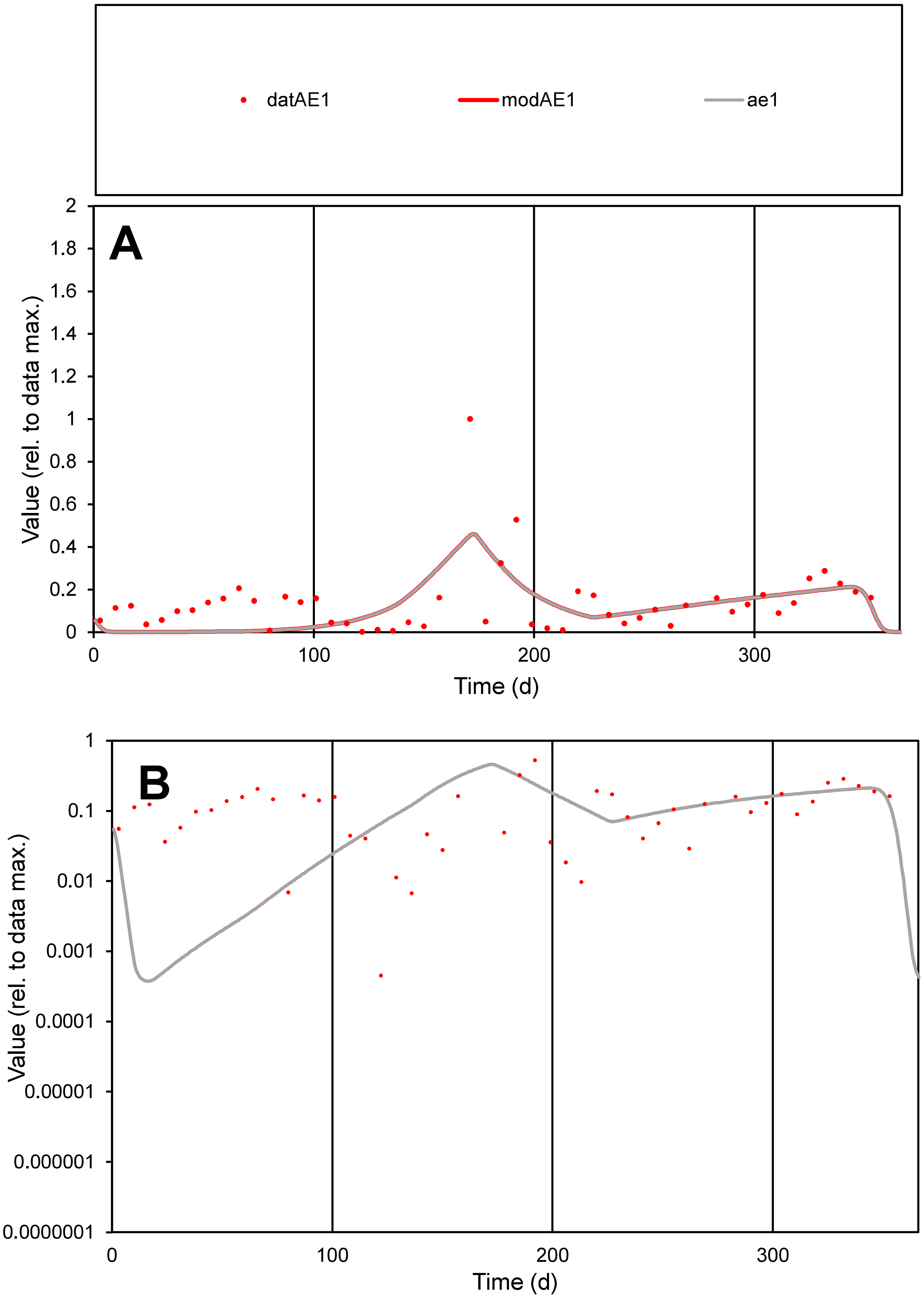

C1

Variables

AE1 Provide jtd or Dst

CalibOnly?

2 1 to reproduce of

oNormalize?

2 1=no, 2=yes

ofxn calc*

0.98353

ofxn mod

1.00113

sRun

RunA1

zout

1

Ave

Data

0.00545

Model

0.00456

data/vMax

0.03968

ofxng

369.894

Min d/m

1.16688

ave/min d

303.253

ave/min m

296.418

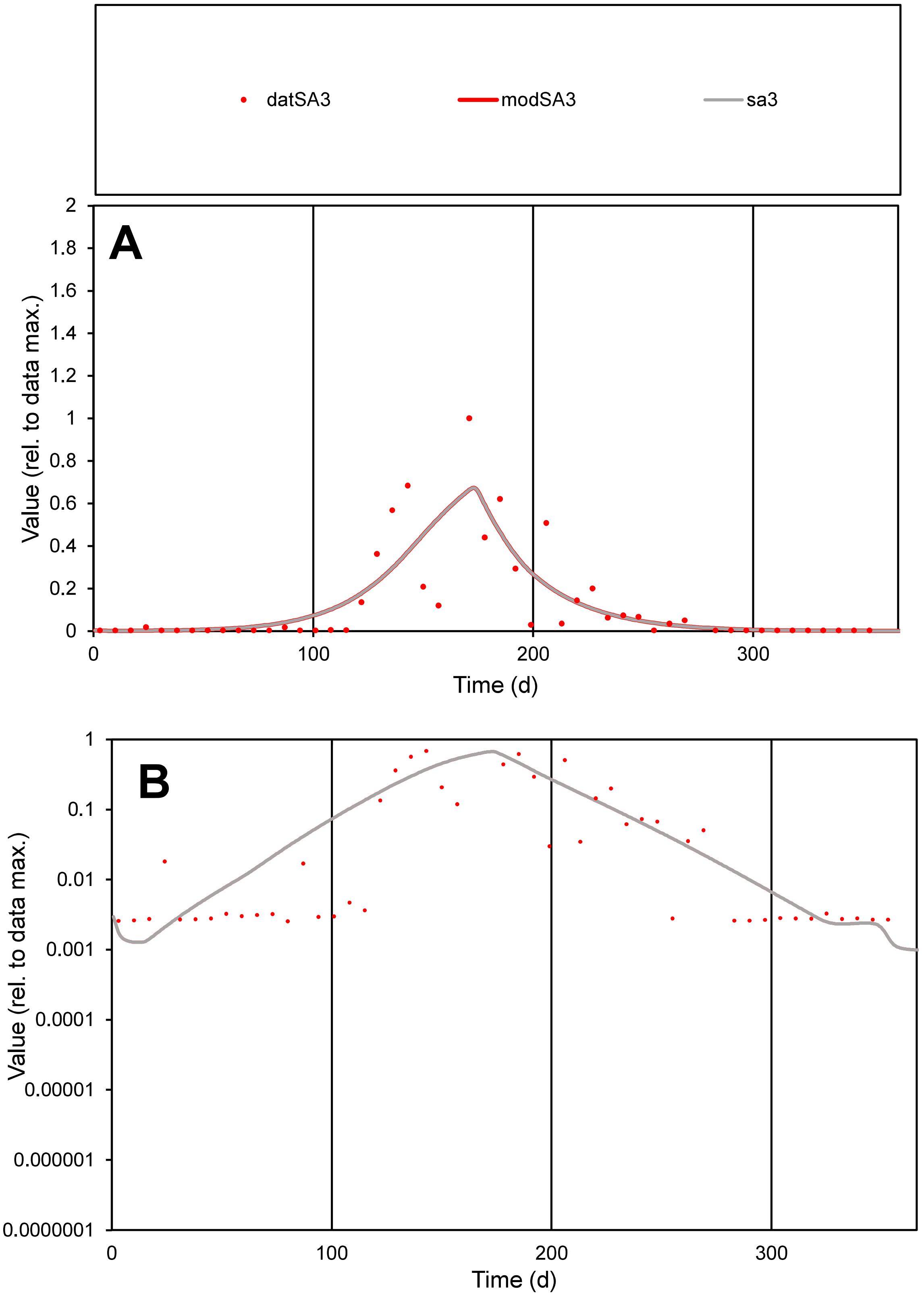

C1

Variables

SA3 Provide jtd or Dst

CalibOnly?

2 1 to reproduce of

oNormalize?

2 1=no, 2=yes

ofxn calc*

1.06124

ofxn mod

1.09266

sRun

RunA1

zout

1

Ave

Data

0.00456

Model

0.00463

data/vMax

0.03881

ofxng

369.894

Min d/m

2.00487

ave/min d

46.1029

ave/min m

93.8766

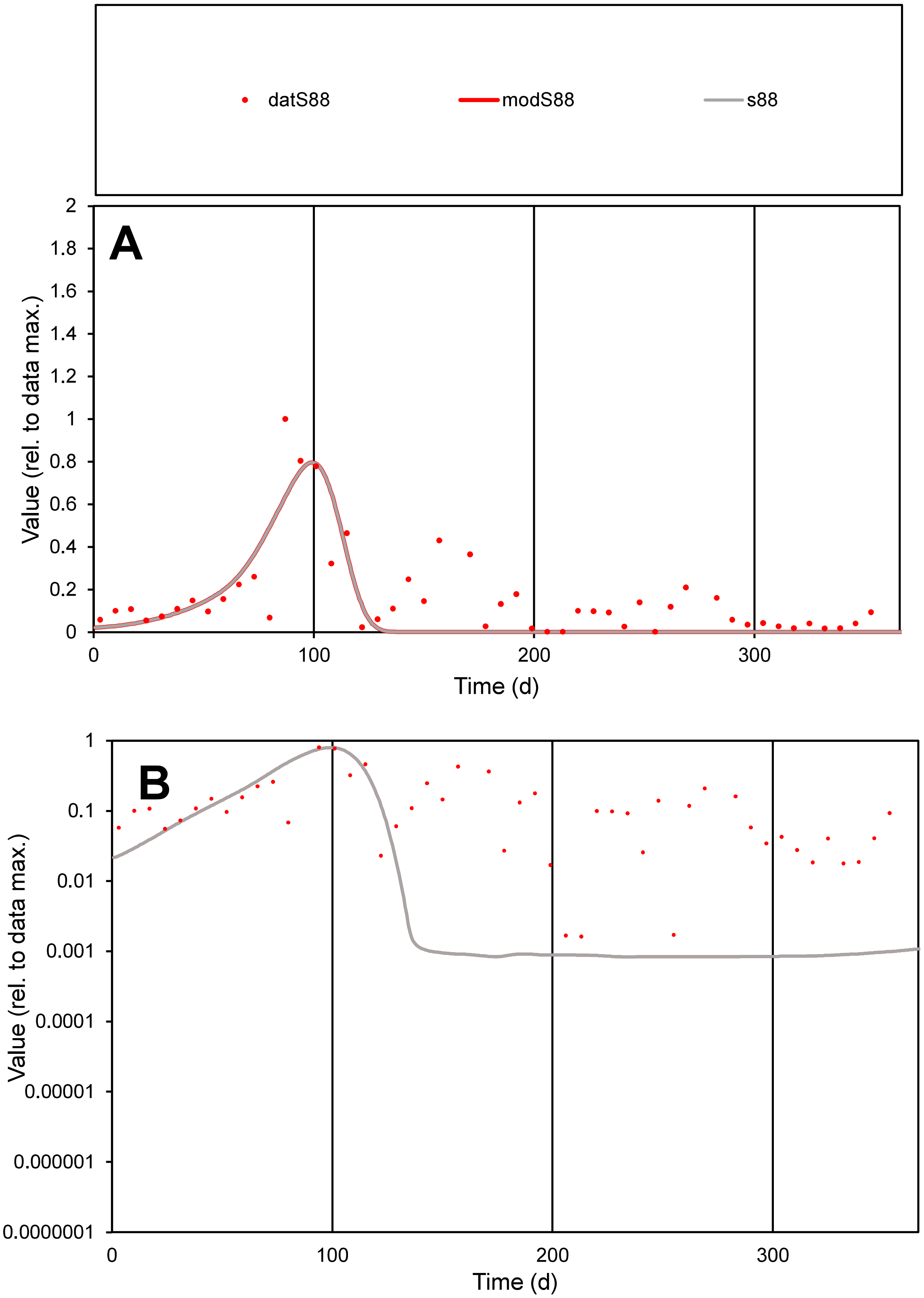

C1

Variables
S88 Provide jtd or Dst
CalibOnly?
2 1 to reproduce of
oNormalize?
2 1=no, 2=yes

ofxn calc*
0.90749
ofxn mod
0.92274

sRun
RunA1
zout
1

Ave
Data
0.00599
Model
0.00397

data/vMax
0.03713

ofxng
369.894

Min d/m
1.96042

ave/min d
99.1568
ave/min m
128.857

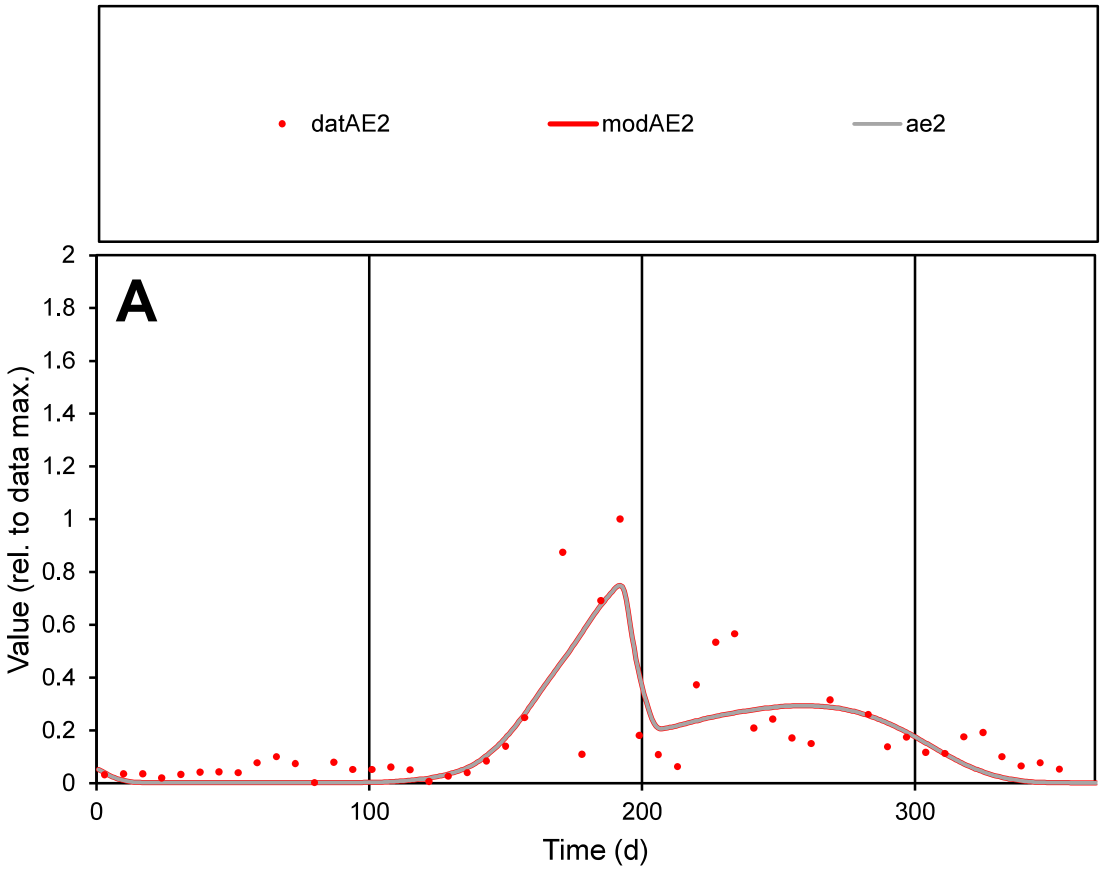

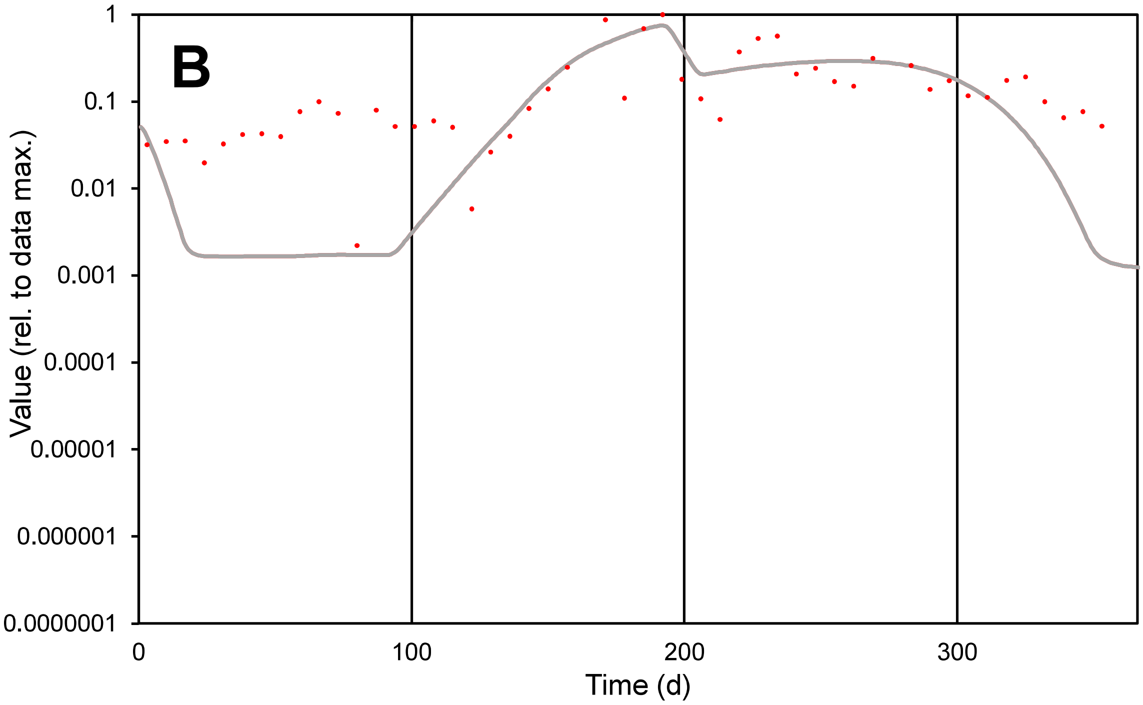

C1

Variables
AE2 Provide jtd or Dst
CalibOnly?
2 1 to reproduce of
oNormalize?
2 1=no, 2=yes

ofxn calc*
0.75475
ofxn mod
0.77888

sRun
RunA1
zout
1

Ave
Data
0.00452
Model
0.00392

data/vMax
0.02631

ofxng
369.894

Min d/m
1.47415

ave/min d
77.6879
ave/min m
99.2802

328_Calib_S_A

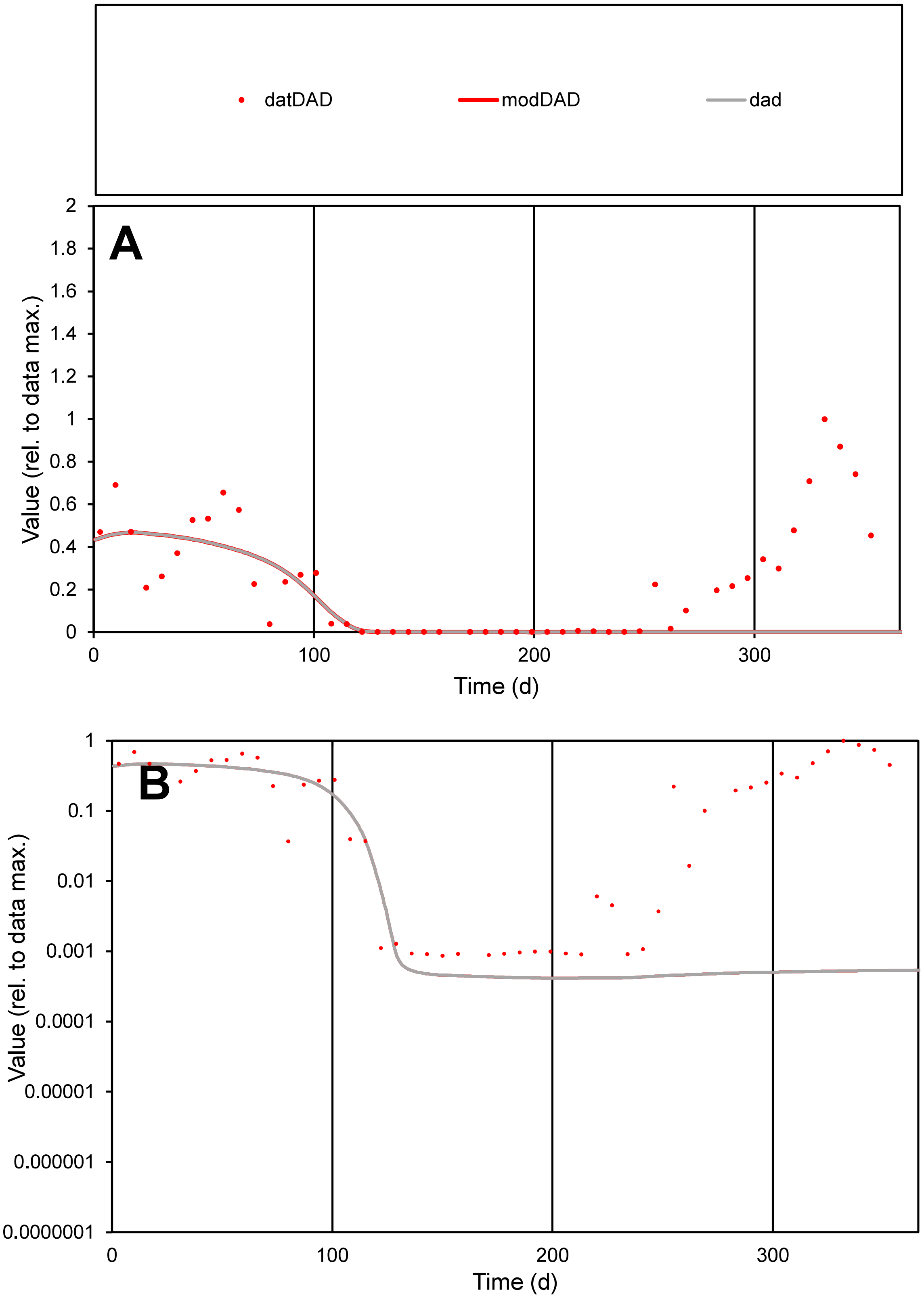

C1

Variables
DAD Provide jtd or Dst
CalibOnly?
2 1 to reproduce of
oNormalize?
2 1=no, 2=yes

ofxn calc*
1.19317
ofxn mod
1.20029

sRun
RunA1
zout
1

Ave
Data
0.0043
Model
0.00212

data/vMax
0.01785

ofxng
369.894

Min d/m
2.09252

ave/min d
278.057
ave/min m
287.034

328_Calib_S_A

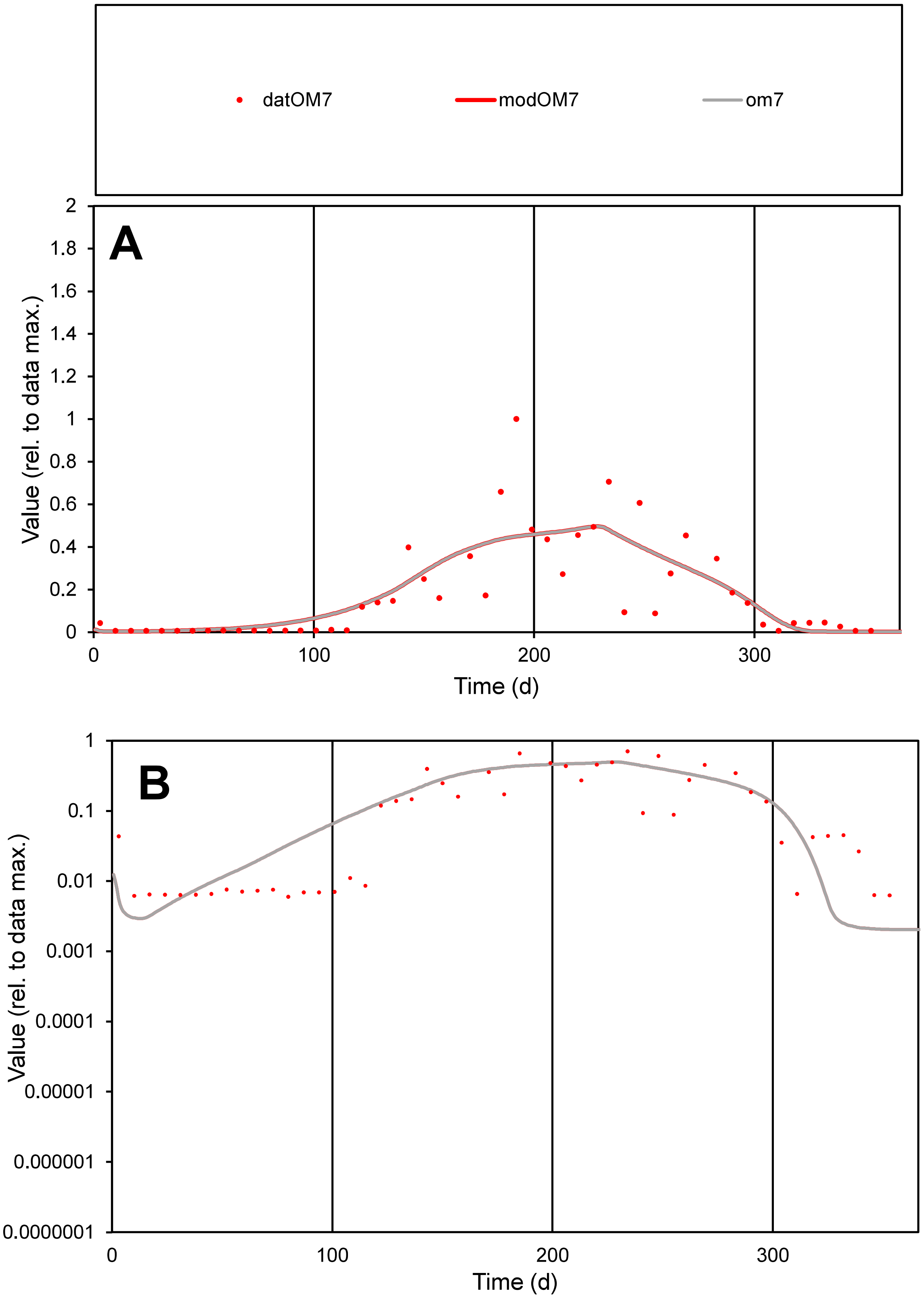

C1

Variables
OM7 Provide jtd or Dst
CalibOnly?
2 1 to reproduce of
oNormalize?
2 1=no, 2=yes

ofxn calc*
0.74536
ofxn mod
0.77007

sRun
RunA1
zout
1

Ave
Data
0.00282
Model
0.00288

data/vMax
0.01571

ofxng
369.894

Min d/m
2.92284
ave/min d
30.0292
ave/min m
89.6317

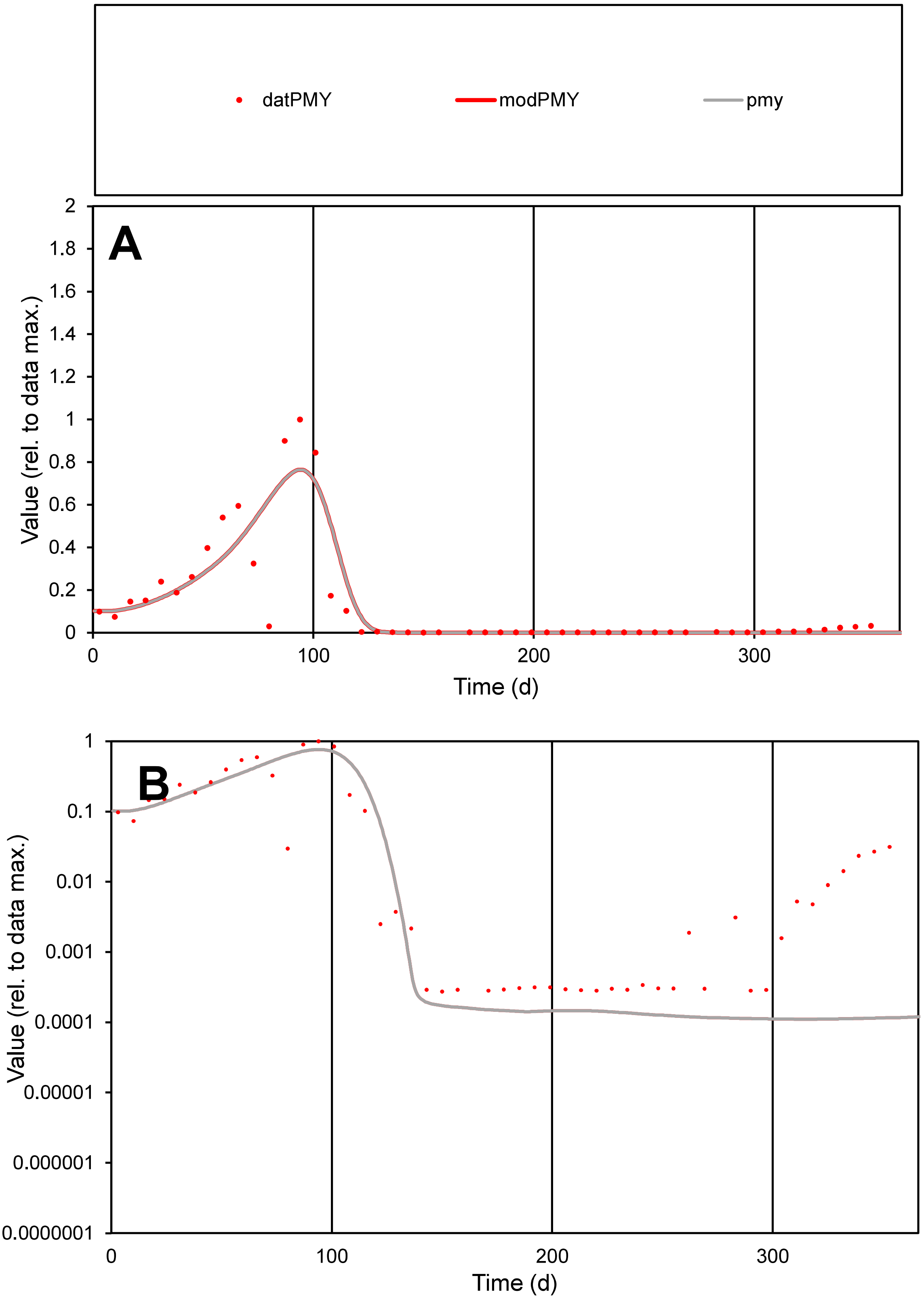

C1

Variables

PMY Provide jtd or Dst

CalibOnly?

2 1 to reproduce of

oNormalize?

2 1=no, 2=yes

ofxn calc*

0.91171

ofxn mod

0.95434

sRun

RunA1

zout

1

Ave

Data

0.00443

Model

0.00442

data/vMax

0.03504

ofxng

369.894

Min d/m

2.47621

ave/min d

459.831

ave/min m

1136.1

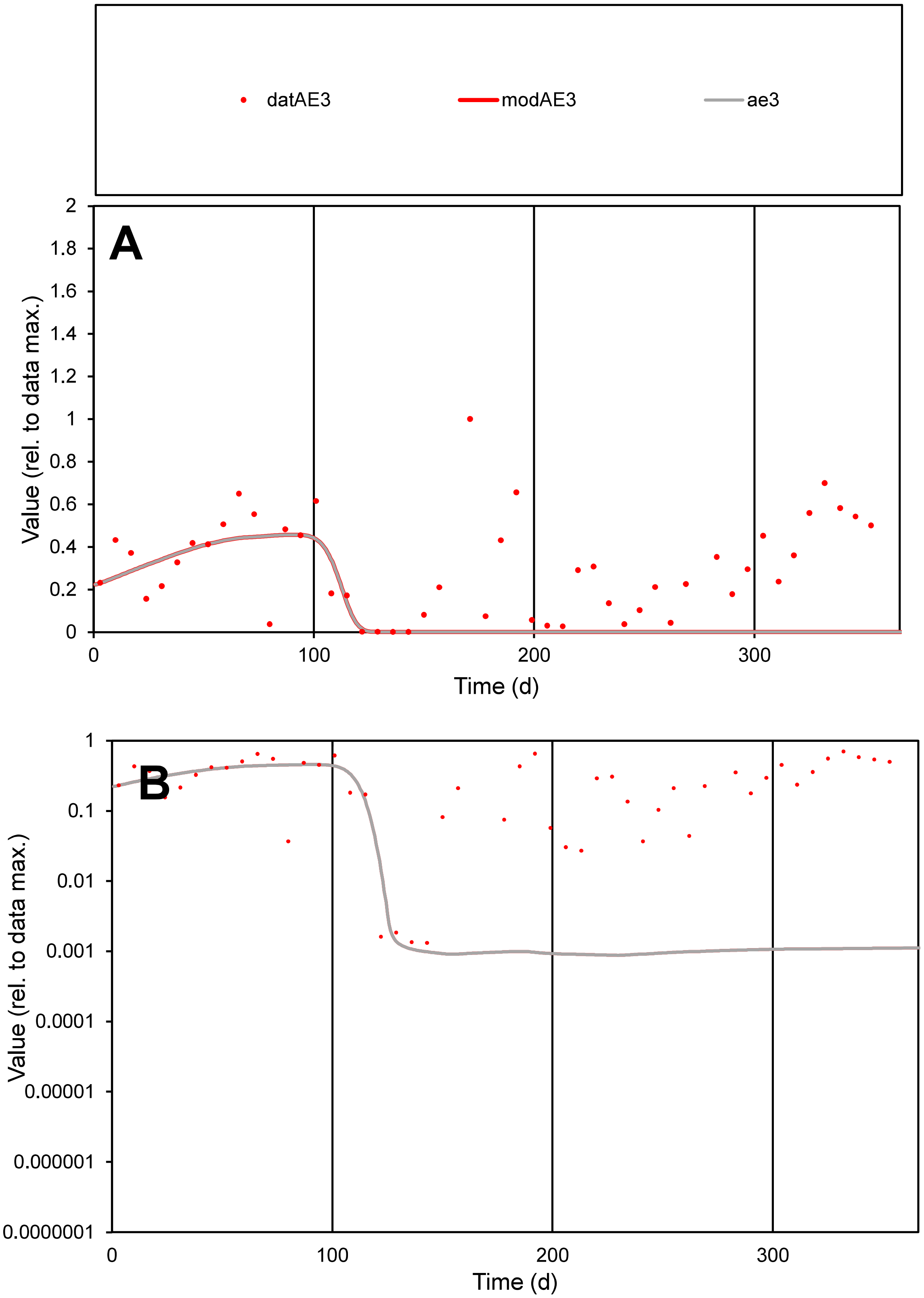

C1

Variables
AE3 Provide jtd or Dst
CalibOnly?
2 1 to reproduce of
oNormalize?
2 1=no, 2=yes

ofxn calc*
1.01304
ofxn mod
1.01812

sRun
RunA1
zout
1

Ave
Data
0.00409
Model
0.00171

data/vMax
0.01343

ofxng
369.894

Min d/m
1.50763

ave/min d
229.707
ave/min m
144.547

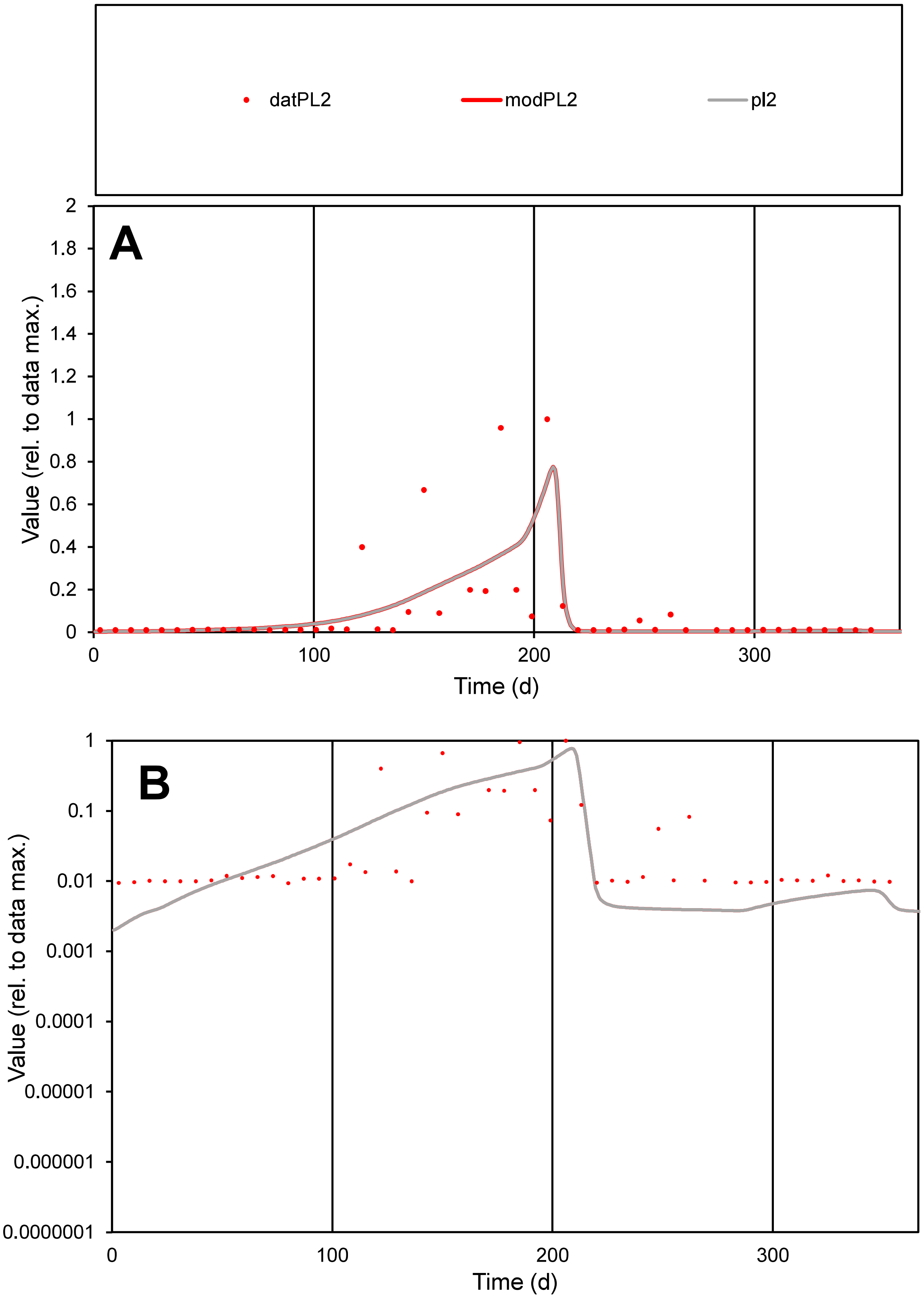

C1

Variables
PL2 Provide jtd or Dst
CalibOnly?
2 1 to reproduce of
oNormalize?
2 1=no, 2=yes

ofxn calc*
1.59648
ofxn mod
1.61532

sRun
RunA1
zout
1

Ave
Data
0.00162
Model
0.00147

data/vMax
0.01756

ofxng
369.894

Min d/m
4.03993

ave/min d
9.87404
ave/min m
36.2412

328_Calib_S_A

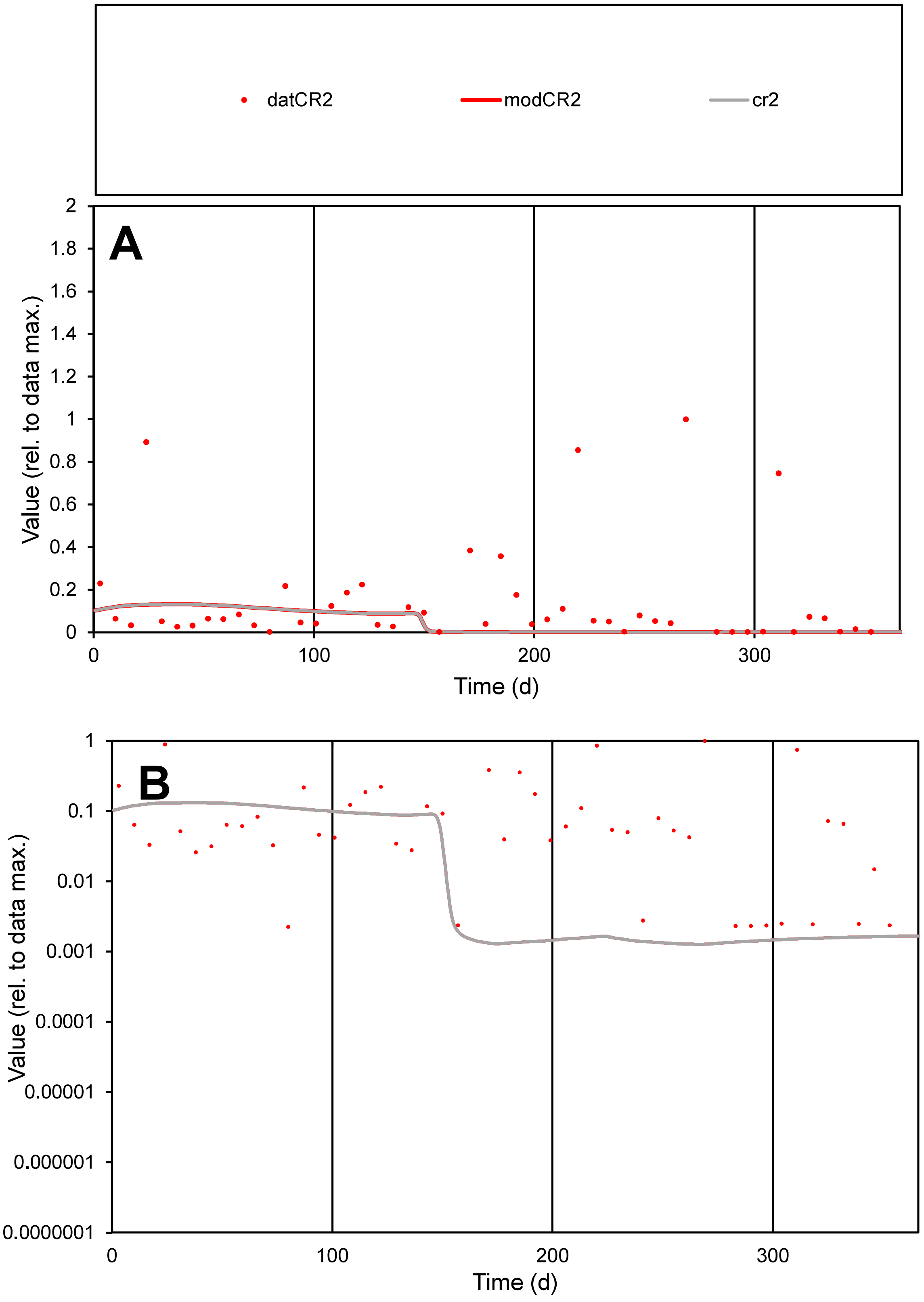

C1

Variables
CR2 Provide jtd or Dst
CalibOnly?
2 1 to reproduce of
oNormalize?
2 1=no, 2=yes

ofxn calc*
1.85438
ofxn mod
1.85849

sRun
RunA1
zout
1

Ave
Data
0.00251
Model
0.00088

data/vMax
0.01781

ofxng
369.894

Min d/m
1.77028

ave/min d
62.495
ave/min m
38.936

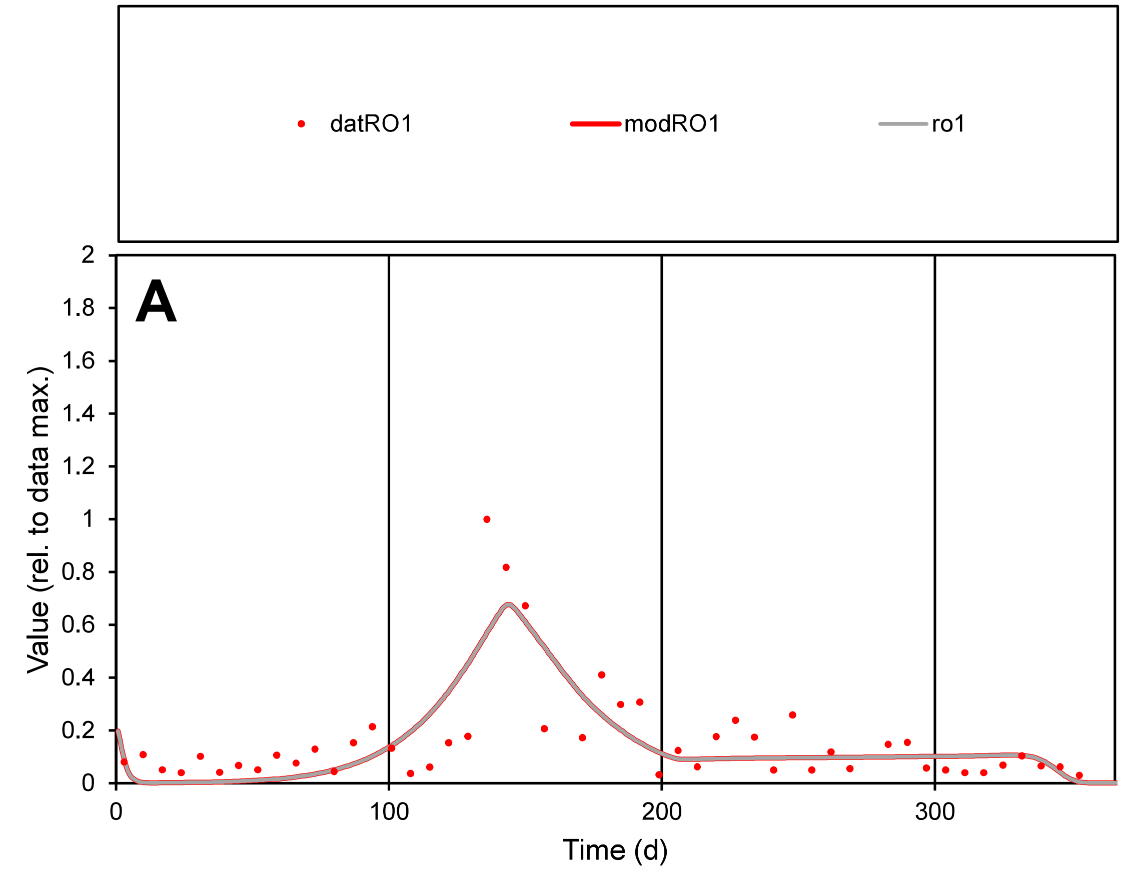

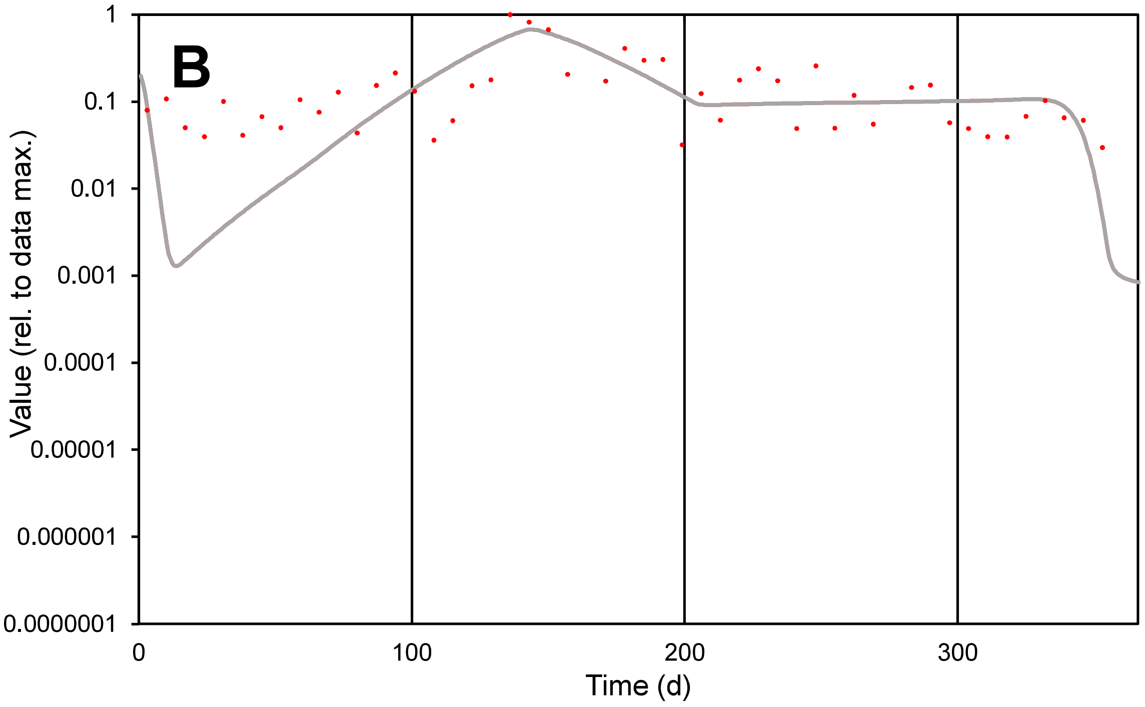

C1

Variables
RO1 Provide jtd or Dst
CalibOnly?
2 1 to reproduce of
oNormalize?
2 1=no, 2=yes

ofxn calc*
0.74808
ofxn mod
0.76656

sRun
RunA1
zout
1

Ave
Data
0.00427
Model
0.0039

data/vMax
0.0267

ofxng
369.894

Min d/m
18.2832

ave/min d
5.36581
ave/min m
89.4485

328_Calib_S_A

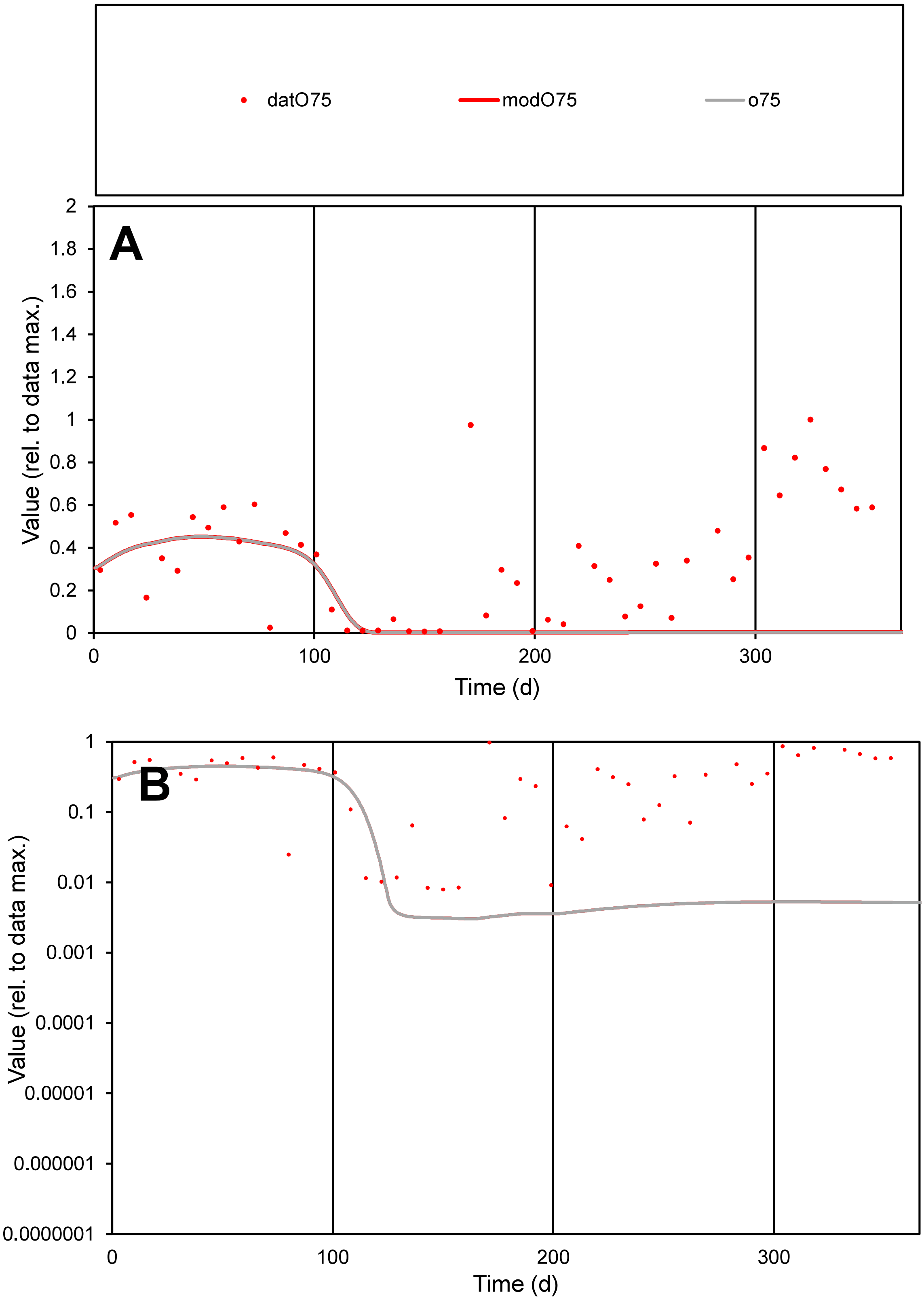

C1

Variables

O75 Provide jtd or Dst

CalibOnly?

2 1 to reproduce of

oNormalize?

2 1=no, 2=yes

ofxn calc*

1.08604

ofxn mod

1.09024

sRun

RunA1

zout

1

Ave

Data

0.00305

Model

0.00116

data/vMax

0.00881

ofxng

369.894

Min d/m

2.60296

ave/min d

43.4431

ave/min m

42.9243

328_Calib_S_A

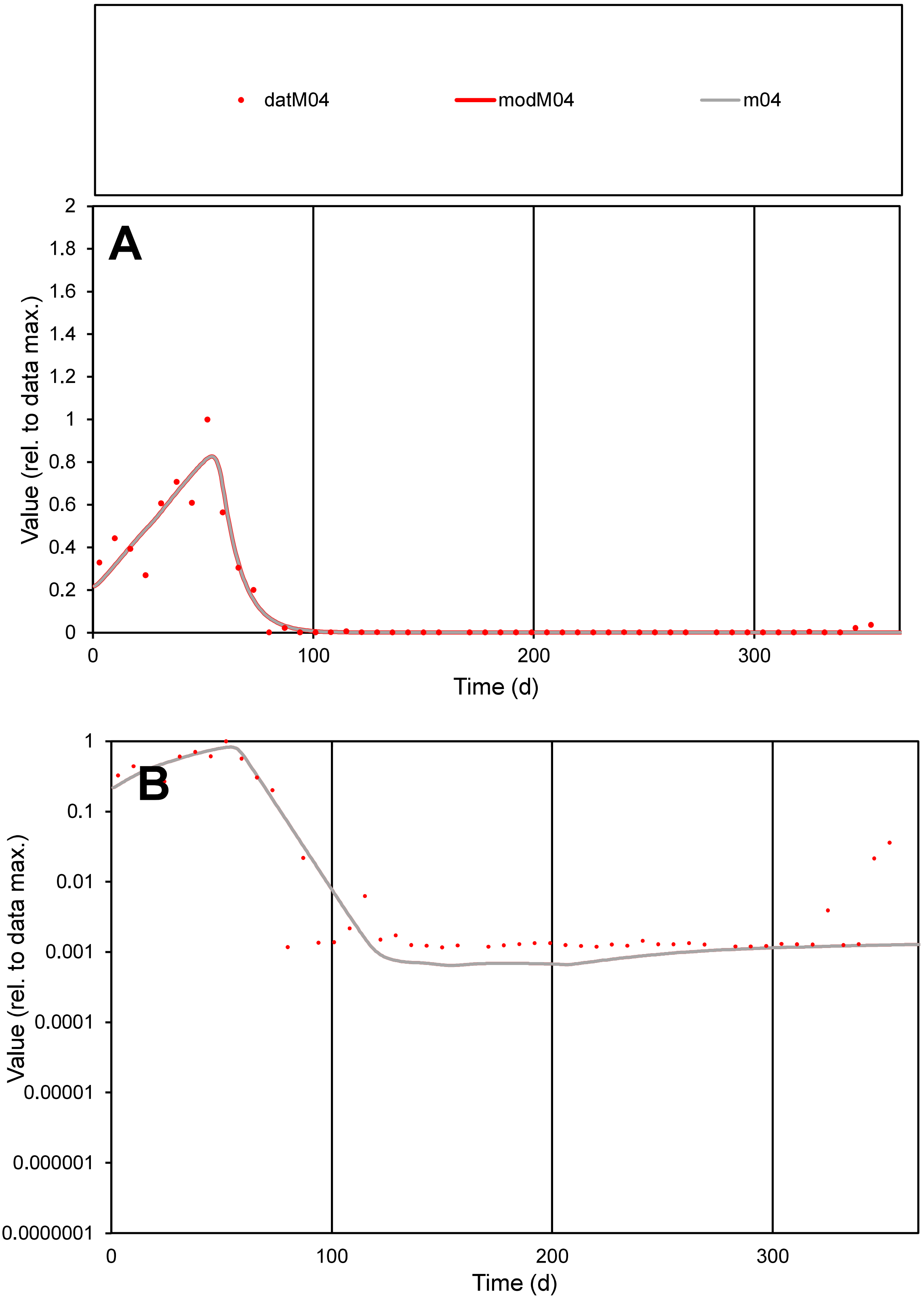

C1

Variables	
M04	Provide jtd or Dst
CalibOnly?	
2	1 to reproduce of
oNormalize?	
2	1=no, 2=yes
ofxn calc*	
0.45867	
ofxn mod	
0.52901	
sRun	
RunA1	
zout	
1	
Ave	
Data	
0.00343	
Model	
0.00339	
data/vMax	
0.03023	
ofxng	
369.894	
Min d/m	
1.7926	
ave/min d	
96.9991	
ave/min m	
171.914	

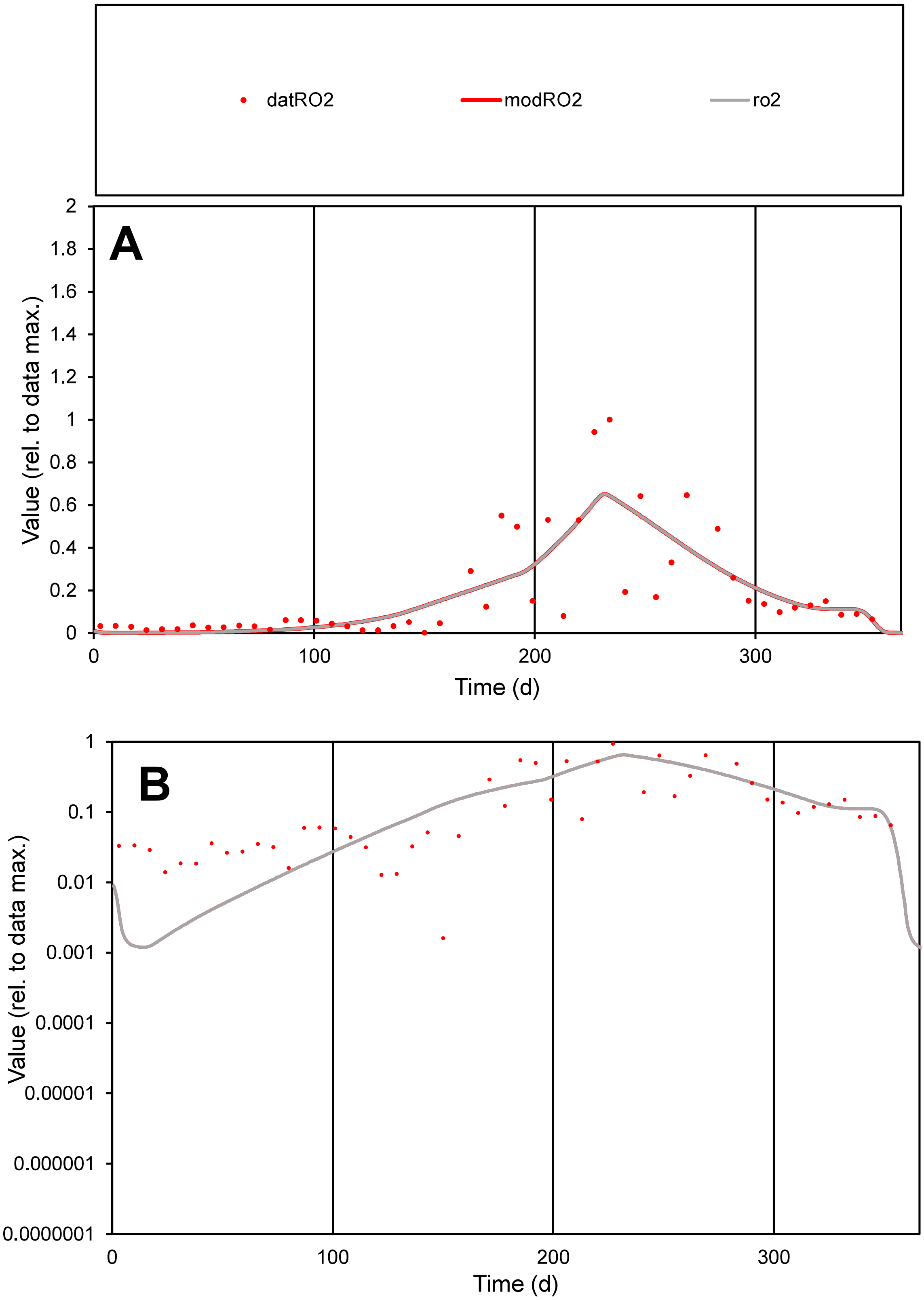

C1

Variables
RO2 Provide jtd or Dst
CalibOnly?
2 1 to reproduce of
oNormalize?
2 1=no, 2=yes

ofxn calc*
0.76745
ofxn mod
0.79251

sRun
RunA1
zout
1

Ave
Data
0.00449
Model
0.00438

data/vMax
0.02395

ofxng
369.894

Min d/m
1.32008

ave/min d
116.495
ave/min m
150.066

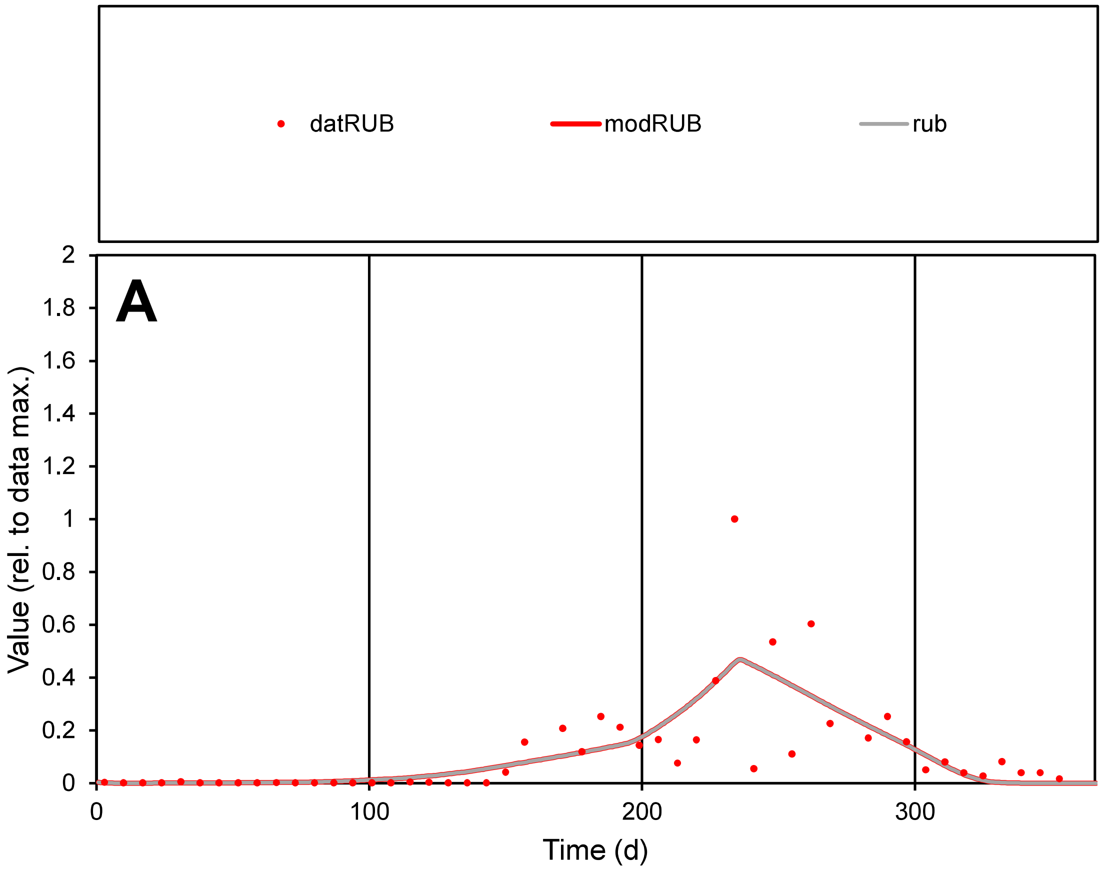

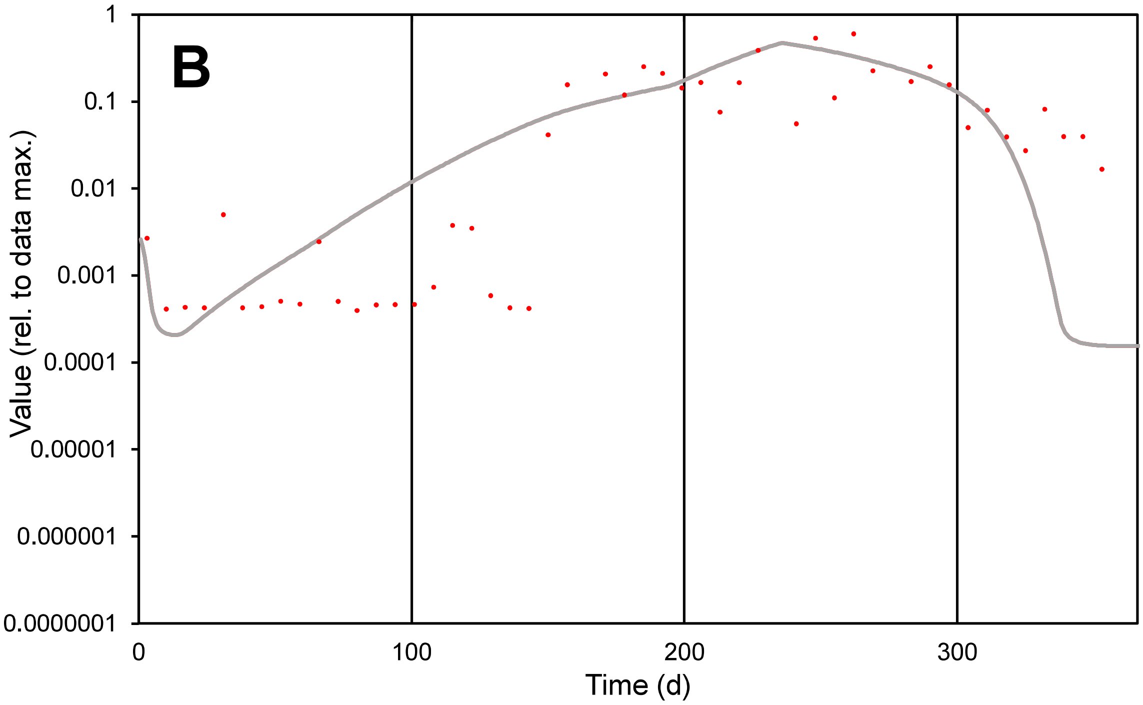

C1

Variables
RUB Provide jtd or Dst
CalibOnly?
2 1 to reproduce of
oNormalize?
2 1=no, 2=yes

ofxn calc*
1.09371
ofxn mod
1.11779

sRun
RunA1
zout
1

Ave
Data
0.0028
Model
0.00272

data/vMax
0.02522

ofxng
369.894

Min d/m
2.54892

ave/min d
278.723
ave/min m
691.604

328_Calib_S_A

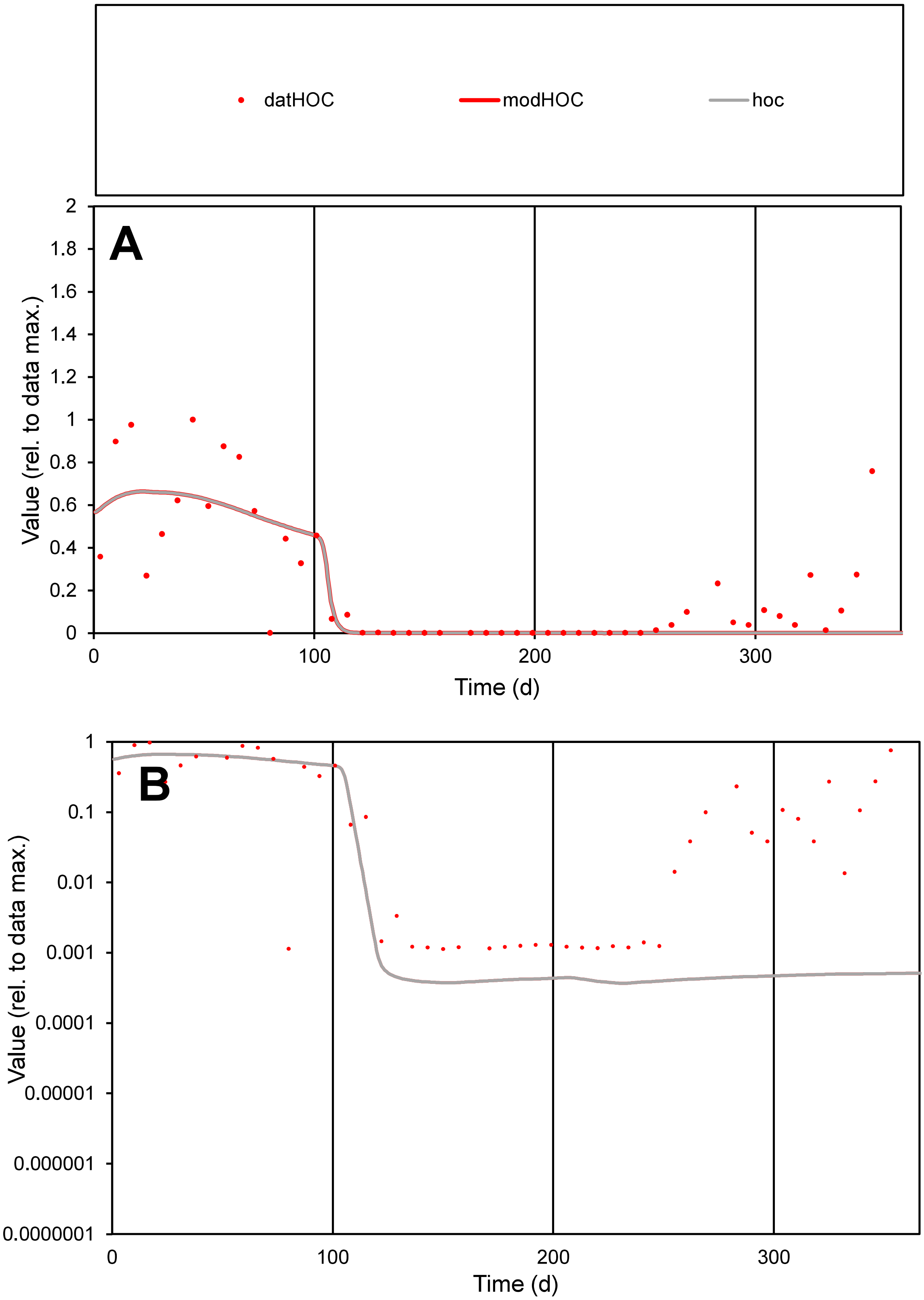

C1

Variables
HOC Provide jtd or Dst
CalibOnly?
2 1 to reproduce of
oNormalize?
2 1=no, 2=yes

ofxn calc*
0.85673
ofxn mod
0.87529

sRun
RunA1
zout
1

Ave
Data
0.00249
Model
0.00201

data/vMax
0.01111

ofxng
369.894

Min d/m
3.05031

ave/min d
197.569
ave/min m
487.286

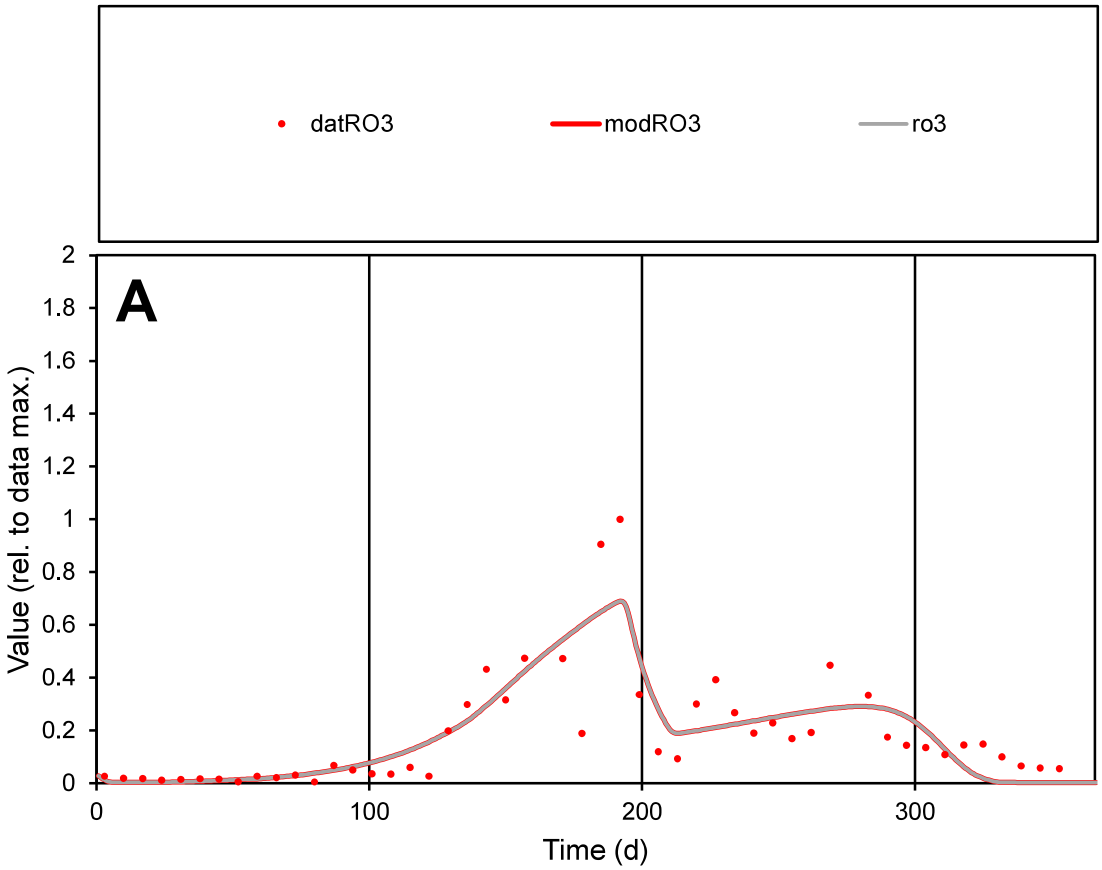

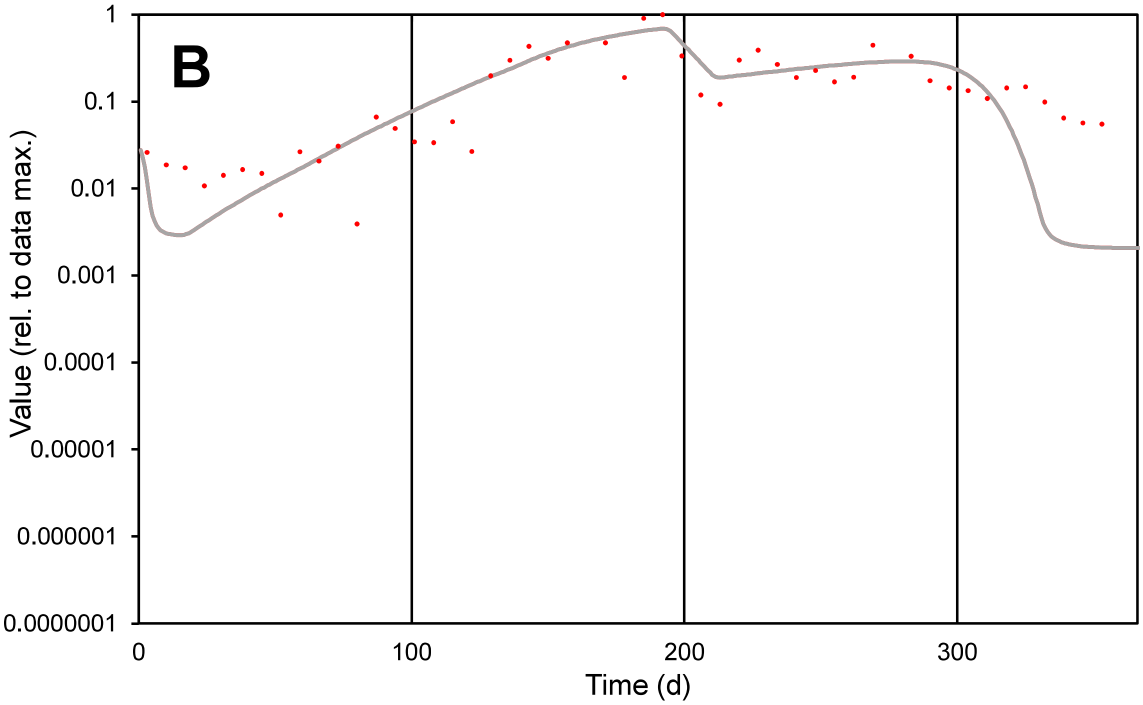

C1

Variables
RO3 Provide jtd or Dst
CalibOnly?
2 1 to reproduce of
oNormalize?
2 1=no, 2=yes

ofxn calc*
0.59603
ofxn mod
0.62371

sRun
RunA1
zout
1

Ave
Data
0.00452
Model
0.00446

data/vMax
0.02475

ofxng
369.894

Min d/m
1.87362

ave/min d
46.6717
ave/min m
86.3866

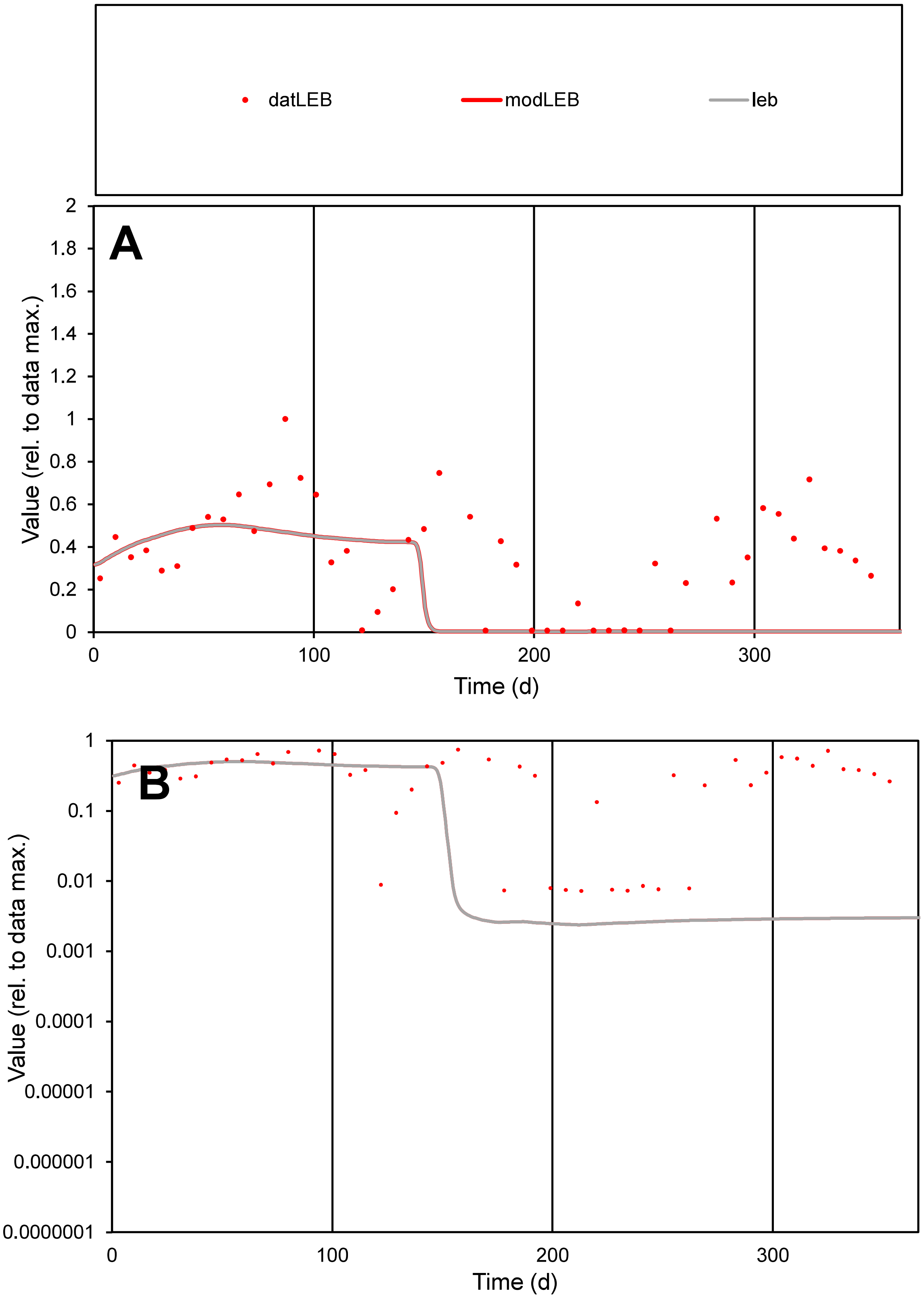

C1

Variables
LEB Provide jtd or Dst
CalibOnly?
2 1 to reproduce of
oNormalize?
2 1=no, 2=yes

ofxn calc*
0.8601
ofxn mod
0.86834

sRun
RunA1
zout
1

Ave
Data
0.00356
Model
0.00201

data/vMax
0.01011

ofxng
369.894

Min d/m
3.01195

ave/min d
48.762
ave/min m
82.9018

328_Calib_S_A

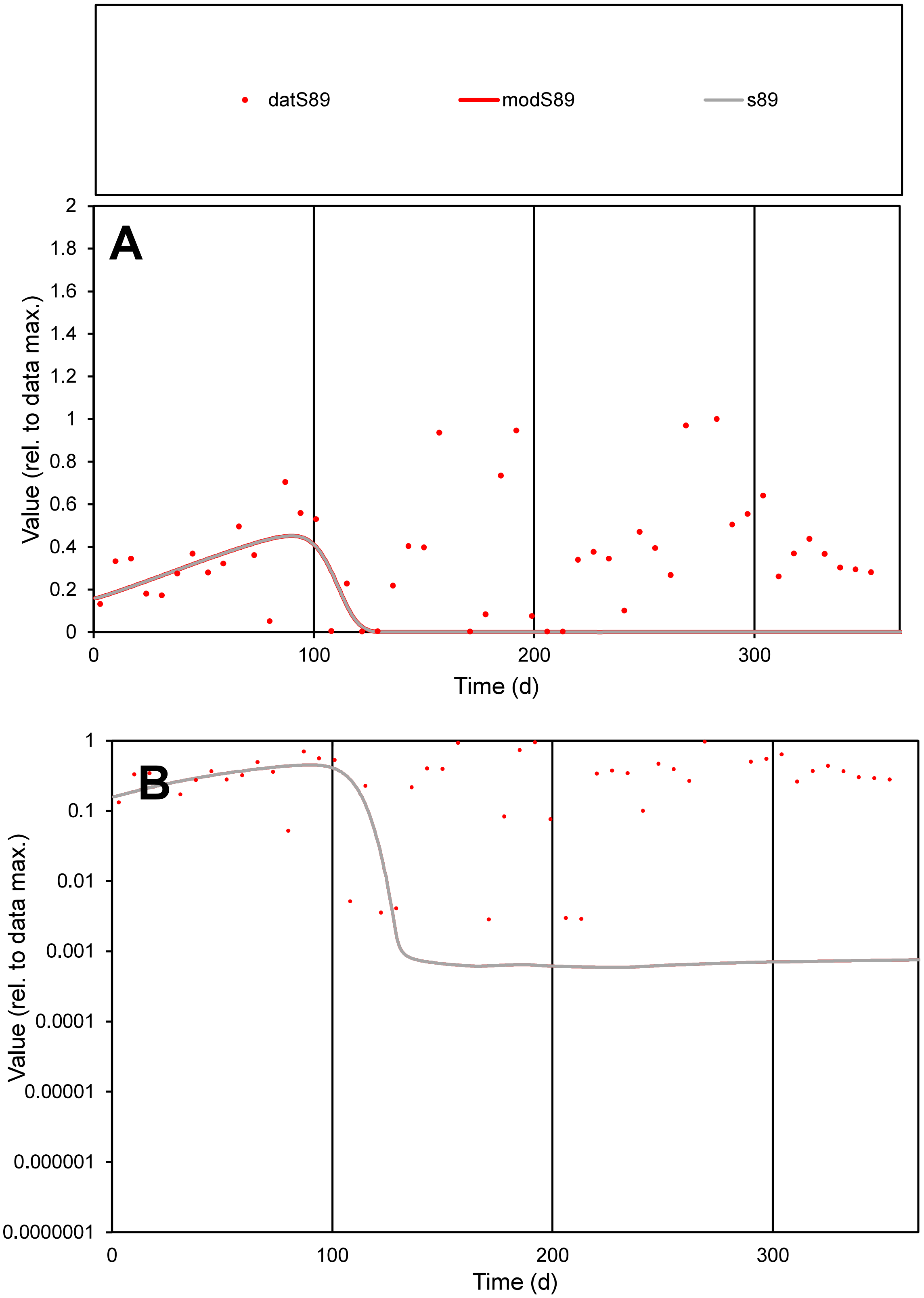

C1

Variables
S89 Provide jtd or Dst
CalibOnly?
2 1 to reproduce of
oNormalize?
2 1=no, 2=yes

ofxn calc*
1.10613
ofxn mod
1.11037

sRun
RunA1
zout
1

Ave
Data
0.00463
Model
0.00141

data/vMax
0.013

ofxng
369.894

Min d/m
4.8138

ave/min d
125.146
ave/min m
184.014

328_Calib_S_A

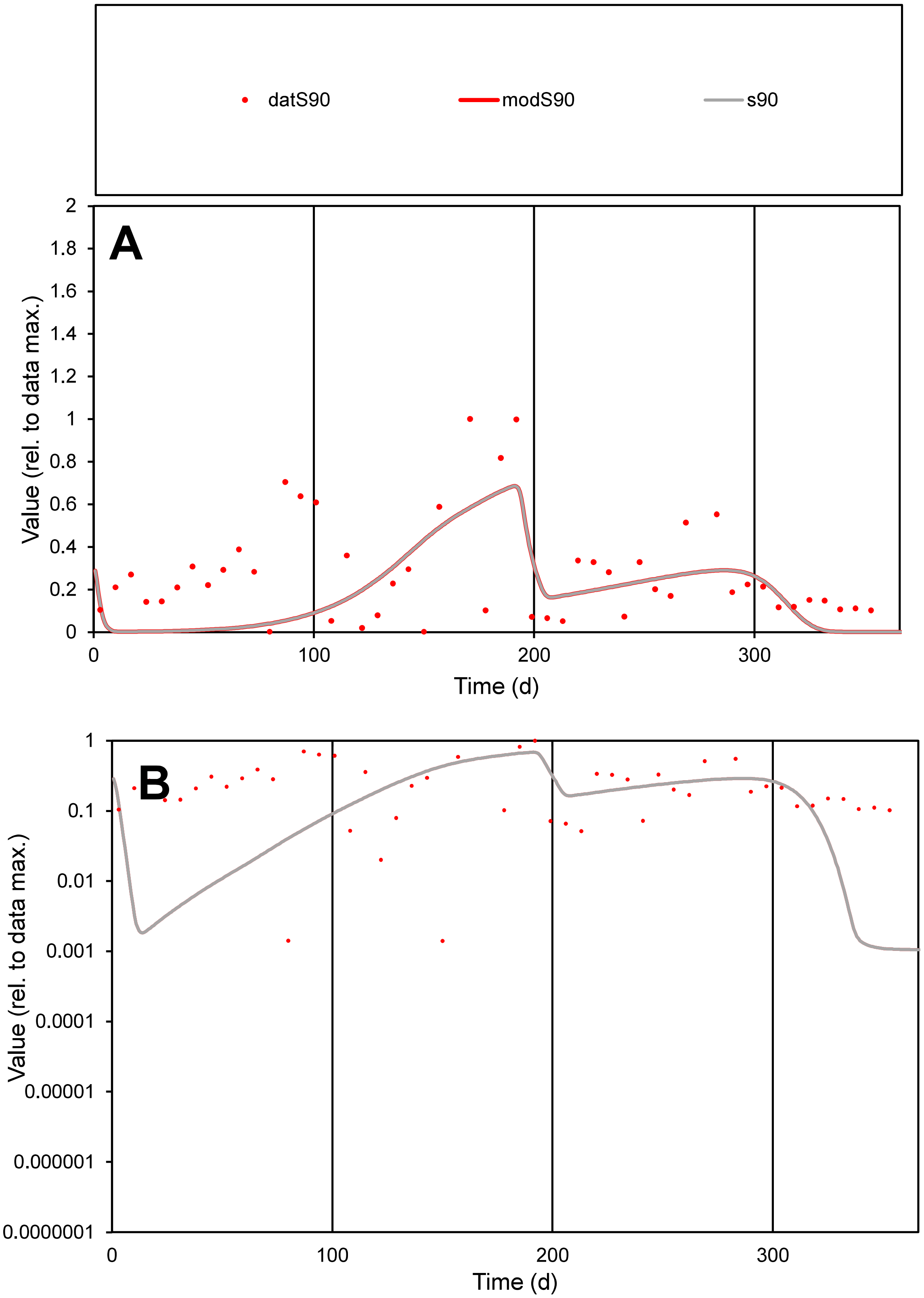

C1

Variables
S90 Provide jtd or Dst
CalibOnly?
2 1 to reproduce of
oNormalize?
2 1=no, 2=yes

ofxn calc*
0.87166
ofxn mod
0.88329

sRun
RunA1
zout
1

Ave
Data
0.00391
Model
0.00268

data/vMax
0.01418

ofxng
369.894

Min d/m
1.31209

ave/min d
195.889
ave/min m
176.153

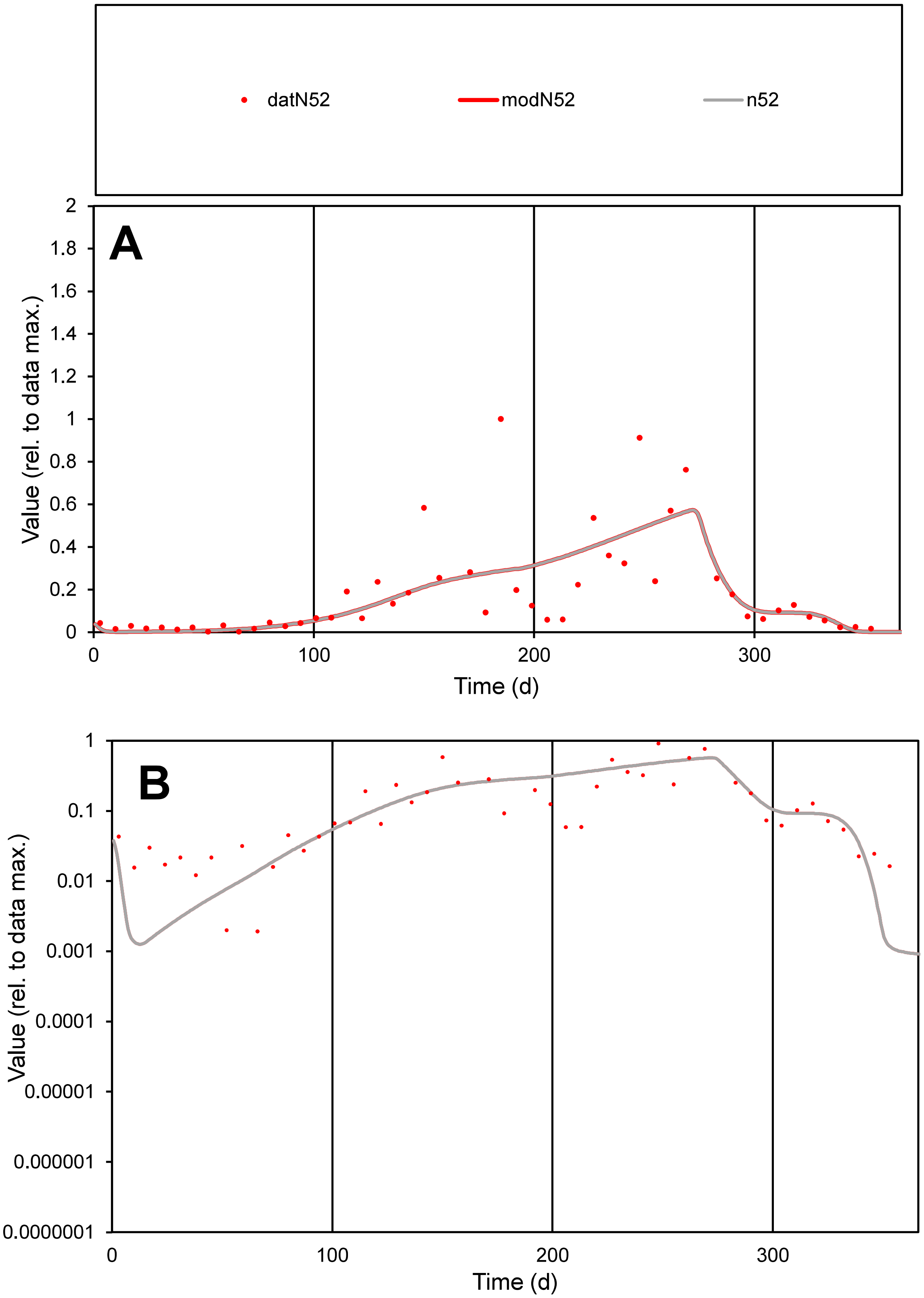

C1

Variables
N52 Provide jtd or Dst
CalibOnly?
2 1 to reproduce of
oNormalize?
2 1=no, 2=yes

ofxn calc*
0.89295
ofxn mod
0.91169

sRun
RunA1
zout
1

Ave
Data
0.00279
Model
0.00267

data/vMax
0.01546

ofxng
369.894

Min d/m
1.726

ave/min d
93.9187
ave/min m
155.35

328_Calib_S_A

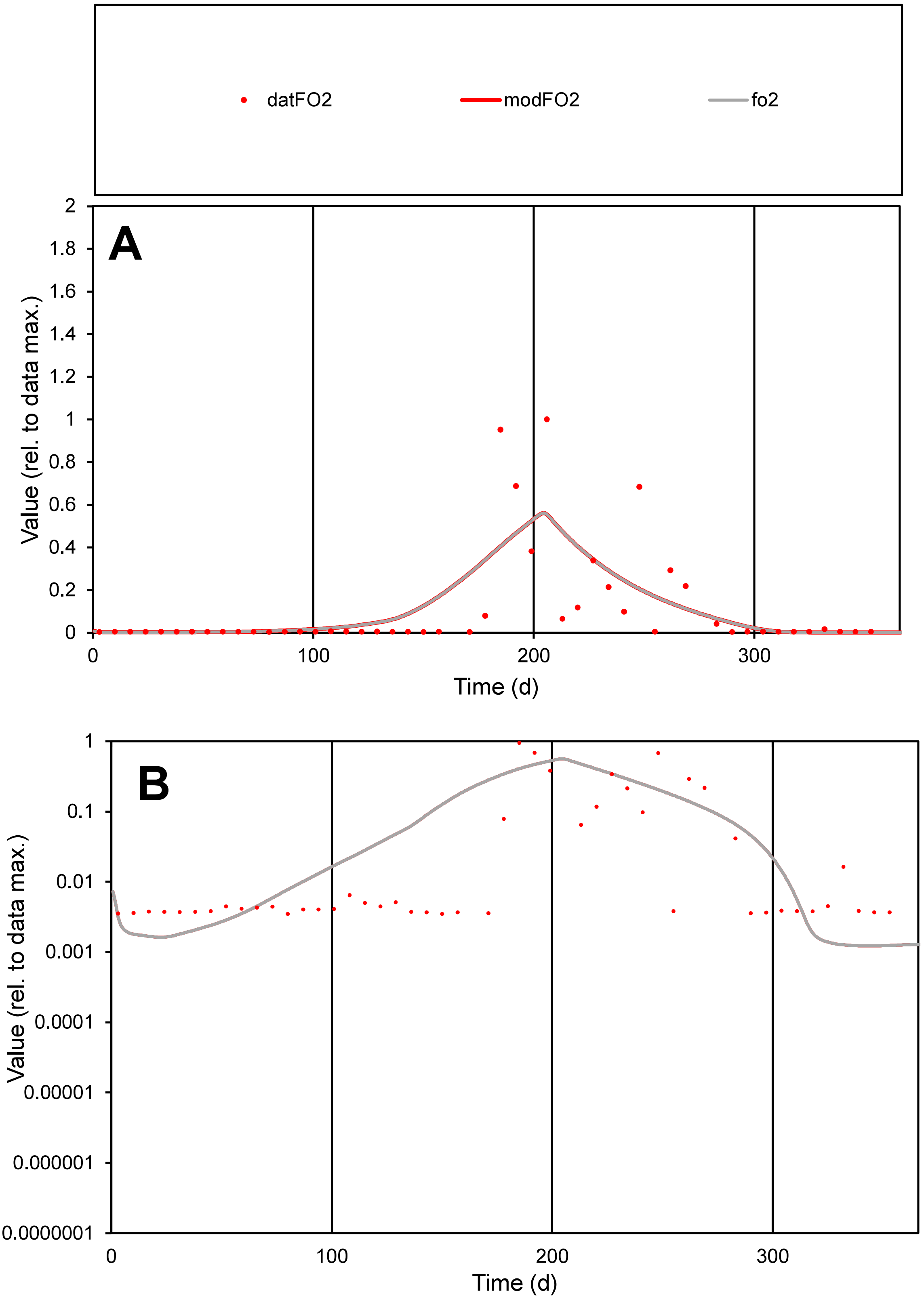

C1

Variables
FO2 Provide jtd or Dst
CalibOnly?
2 1 to reproduce of
oNormalize?
2 1=no, 2=yes

ofxn calc*
1.5053
ofxn mod
1.53399

sRun
RunA1
zout
1

Ave
Data
0.0021
Model
0.00224

data/vMax
0.01934

ofxng
369.894

Min d/m
2.83354

ave/min d
31.2399
ave/min m
94.5573

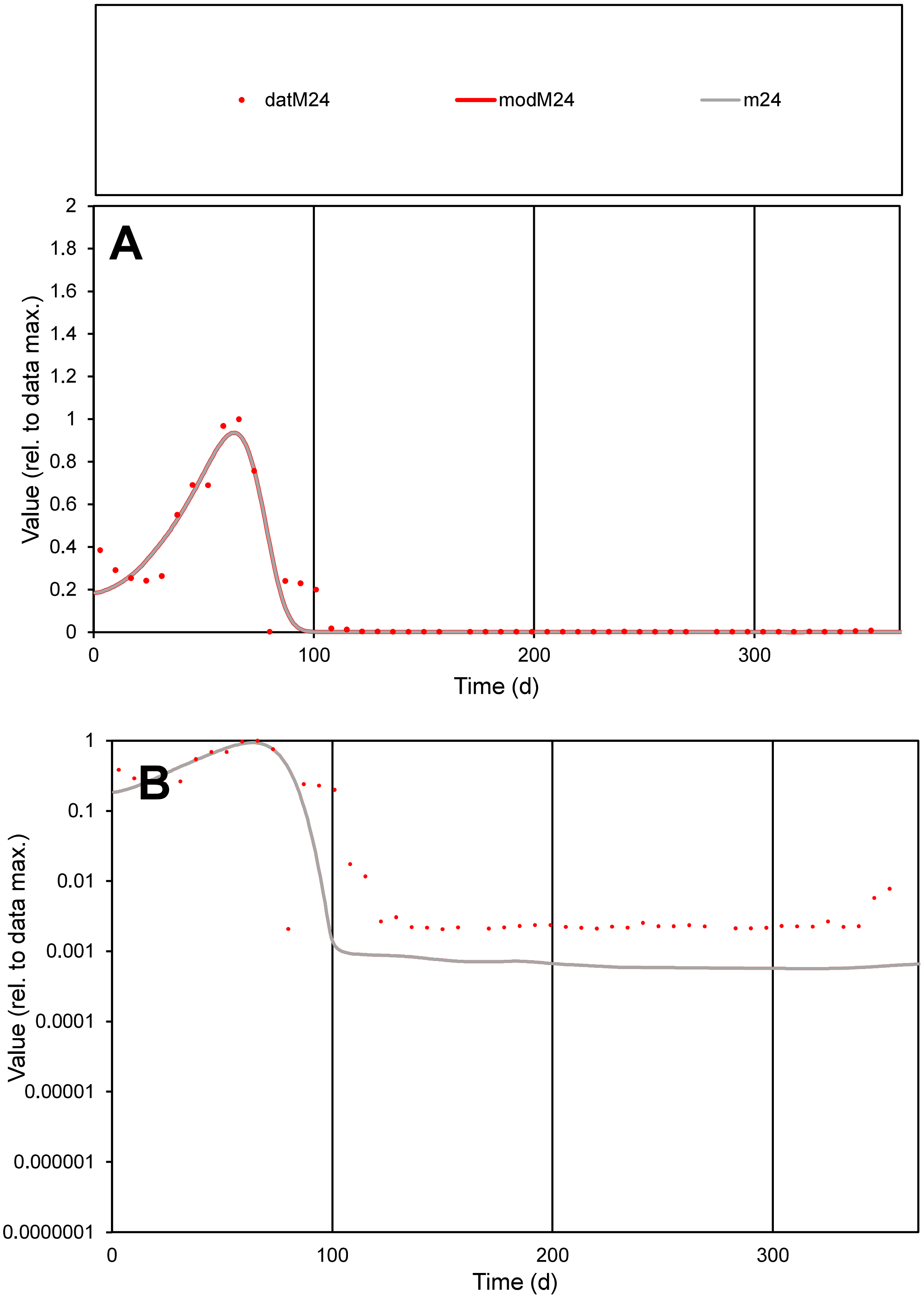

C1

Variables
M24 Provide jtd or Dst
CalibOnly?
2 1 to reproduce of
oNormalize?
2 1=no, 2=yes

ofxn calc*
0.52575
ofxn mod
0.60347

sRun
RunA1
zout
1

Ave
Data
0.00492
Model
0.0046

data/vMax
0.03507

ofxng
369.894

Min d/m
3.63518

ave/min d
67.995
ave/min m
231.047

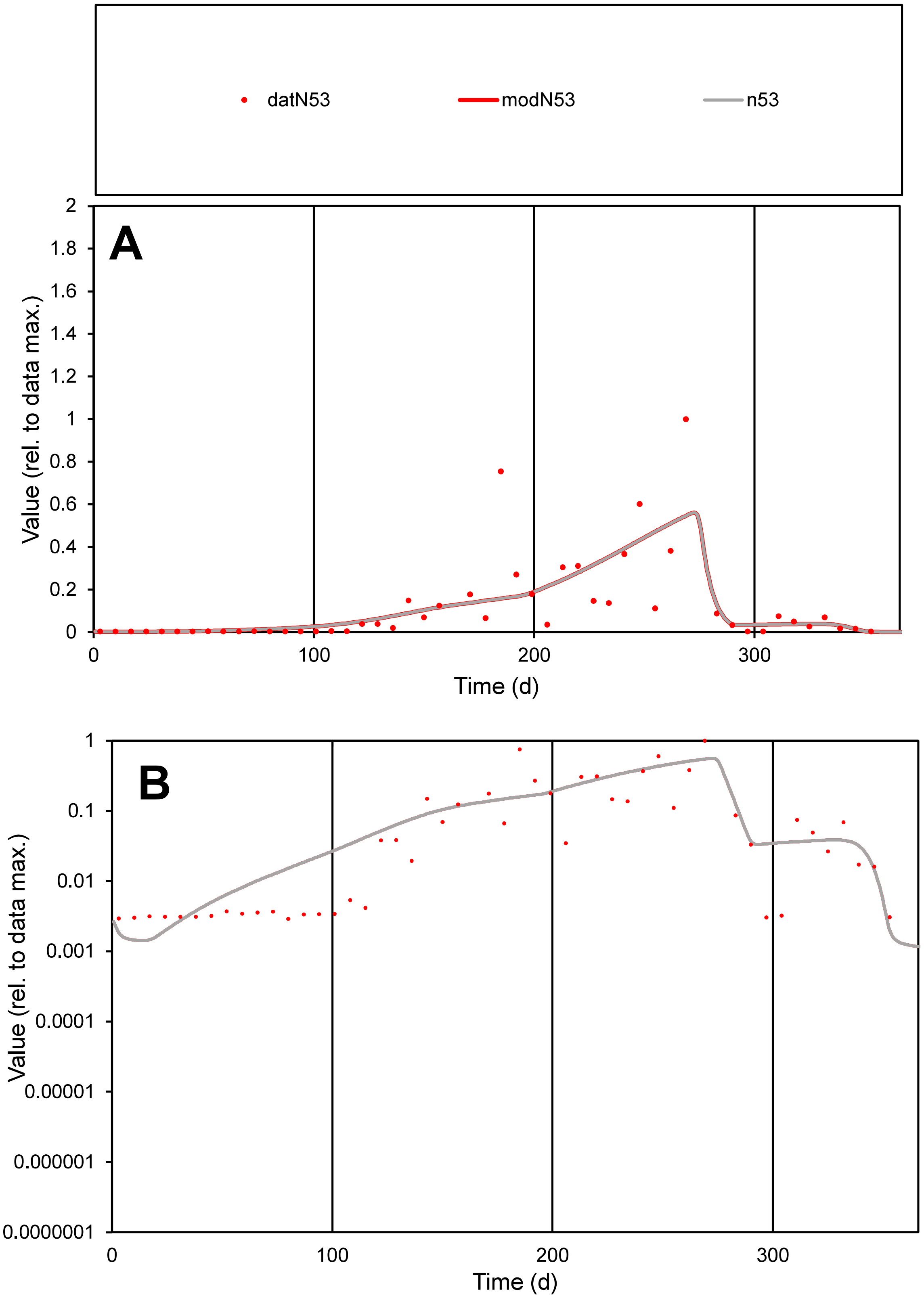

C1

Variables
N53 Provide jtd or Dst
CalibOnly?
2 1 to reproduce of
oNormalize?
2 1=no, 2=yes

ofxn calc*
1.14853
ofxn mod
1.1698

sRun
RunA1
zout
1

Ave
Data
0.00148
Model
0.00149

data/vMax
0.01265

ofxng
369.894

Min d/m
2.02978

ave/min d
40.1104
ave/min m
82.0572

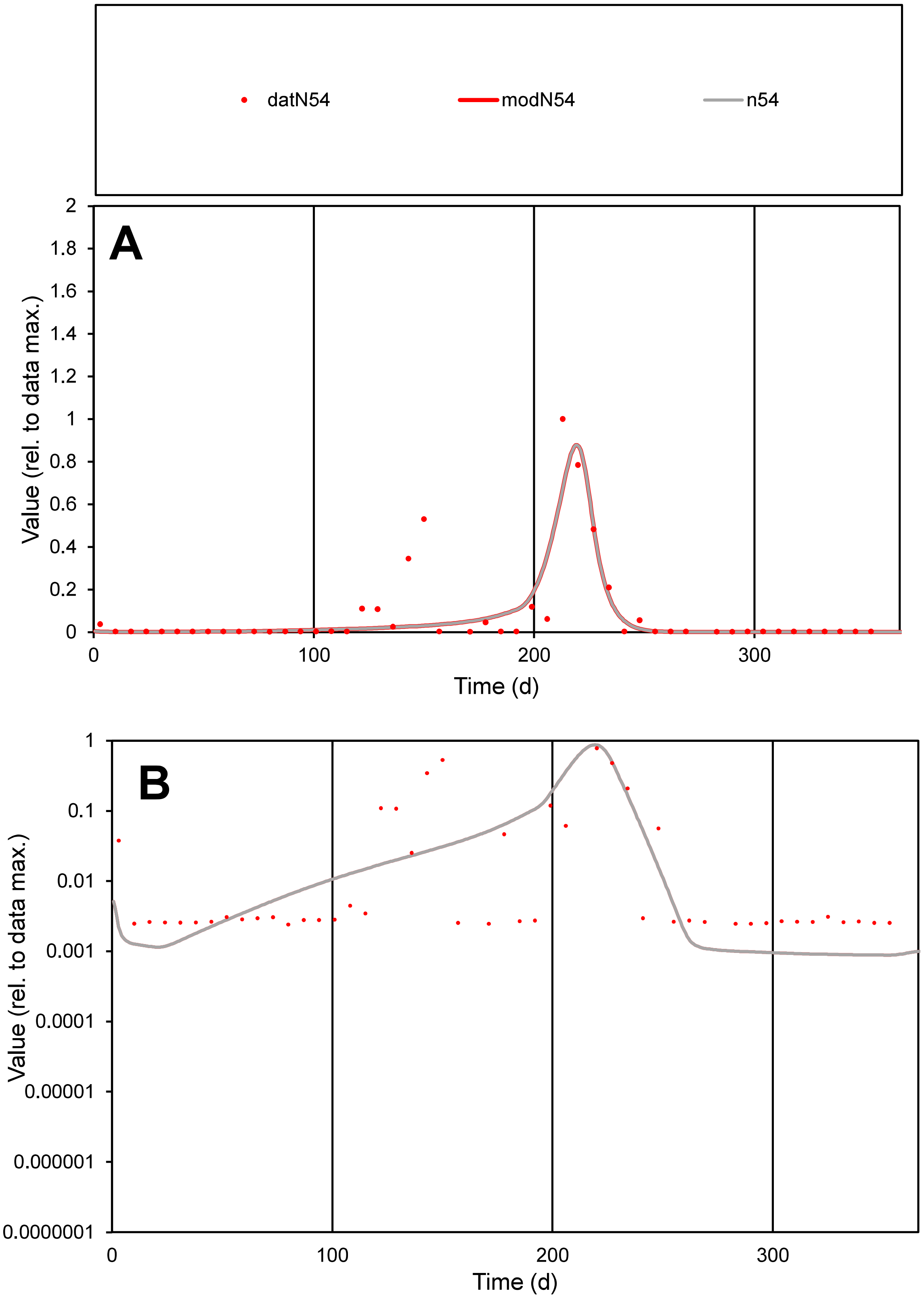

C1

Variables
N54 Provide jtd or Dst
CalibOnly?
2 1 to reproduce of
oNormalize?
2 1=no, 2=yes

ofxn calc*
1.27876
ofxn mod
1.32385

sRun
RunA1
zout
1

Ave
Data
0.00074
Model
0.00062

data/vMax
0.00902

ofxng
369.894

Min d/m
2.73069

ave/min d
33.9059
ave/min m
77.158

328_Calib_S_A

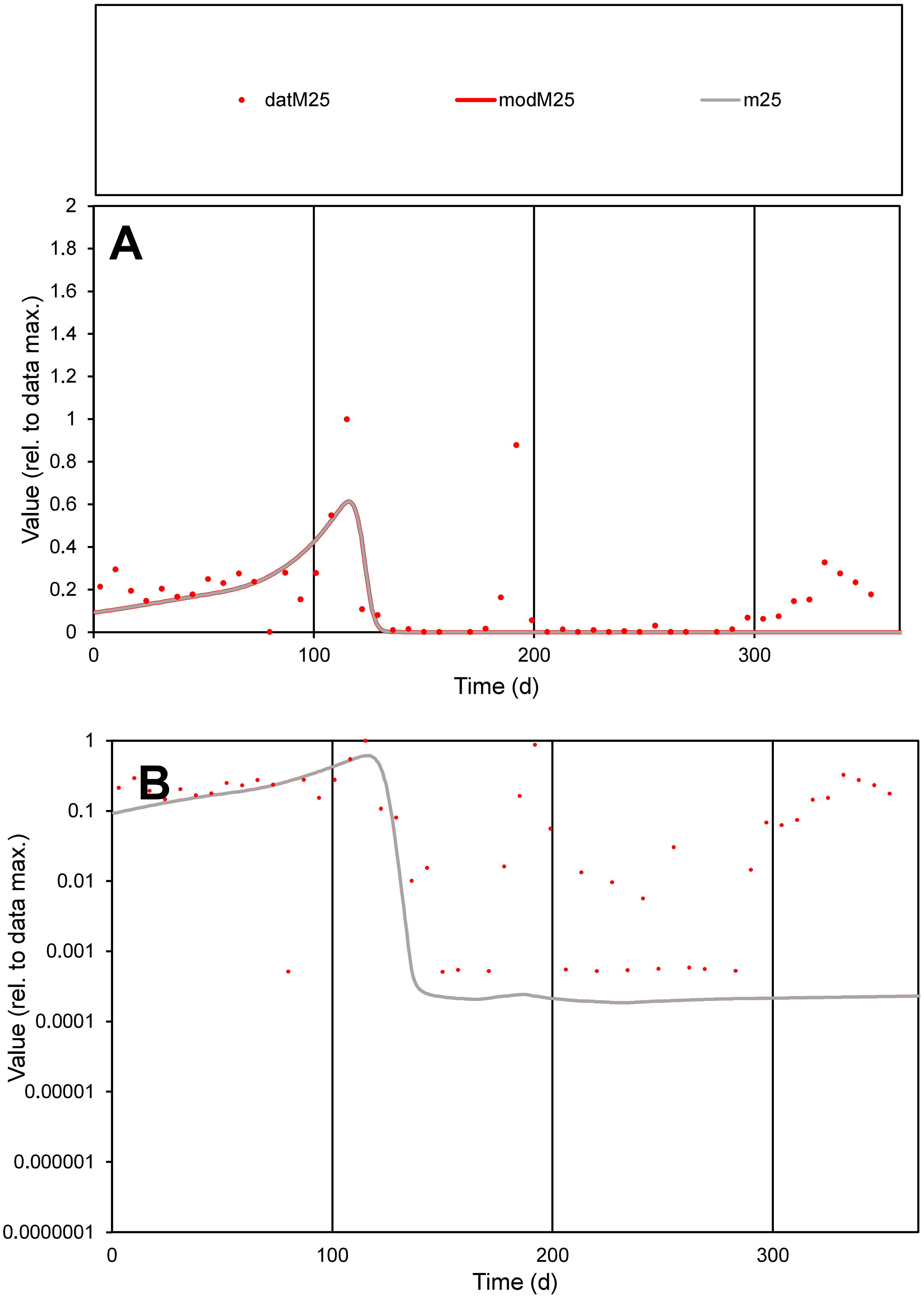

C1

Variables
M25 Provide jtd or Dst
CalibOnly?
2 1 to reproduce of
oNormalize?
2 1=no, 2=yes

ofxn calc*
1.13455
ofxn mod
1.15079

sRun
RunA1
zout
1

Ave
Data
0.0029
Model
0.00176

data/vMax
0.01879

ofxng
369.894

Min d/m
2.75221

ave/min d
301.554
ave/min m
502.927

328_Calib_S_A

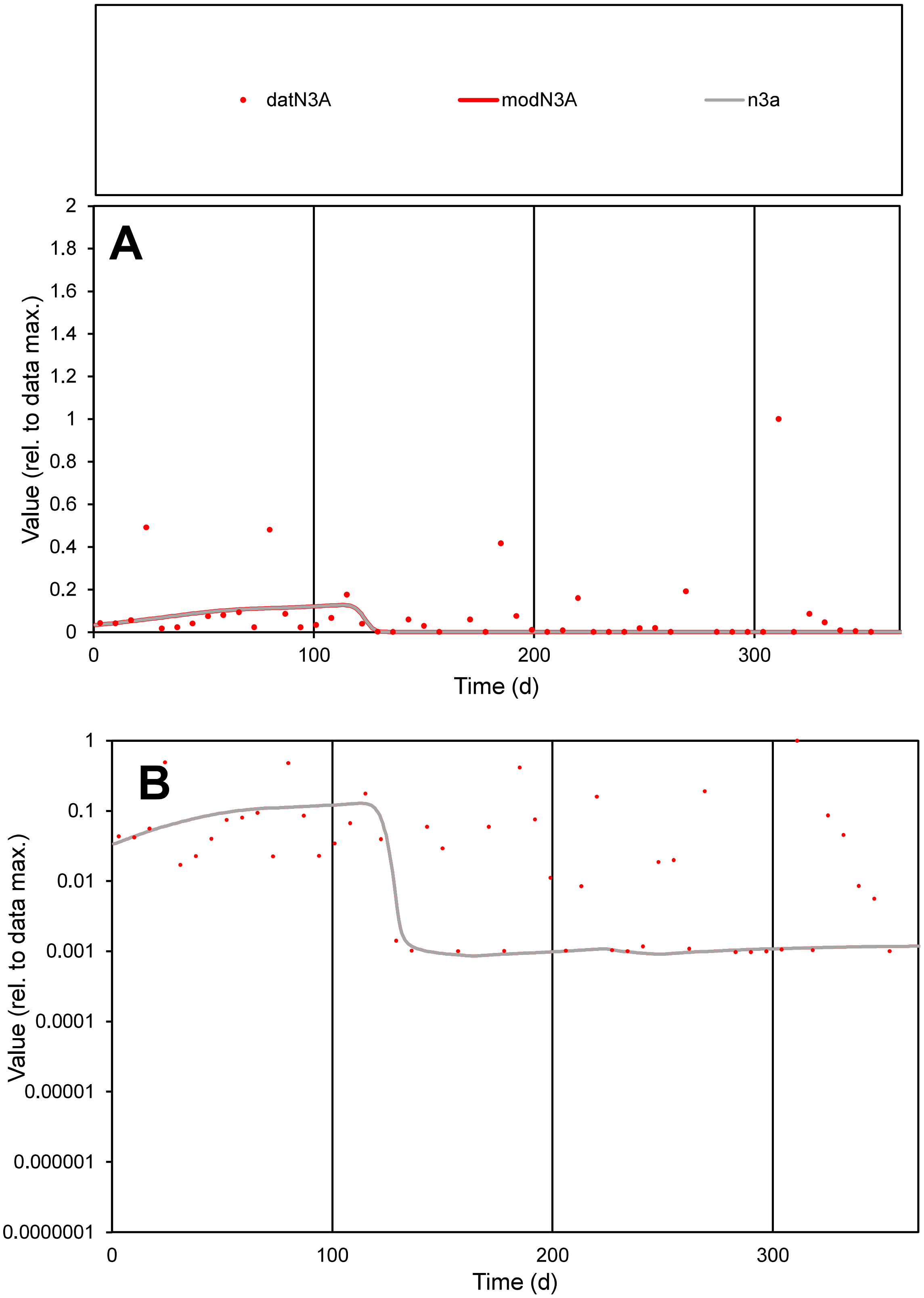

C1

Variables
N3A Provide jtd or Dst
CalibOnly?
2 1 to reproduce of
oNormalize?
2 1=no, 2=yes

ofxn calc*
2.16927
ofxn mod
2.17278

sRun
RunA1
zout
1

Ave
Data
0.00158
Model
0.00064

data/vMax
0.01883

ofxng
369.894

Min d/m
1.11339

ave/min d
85.4093
ave/min m
38.5375

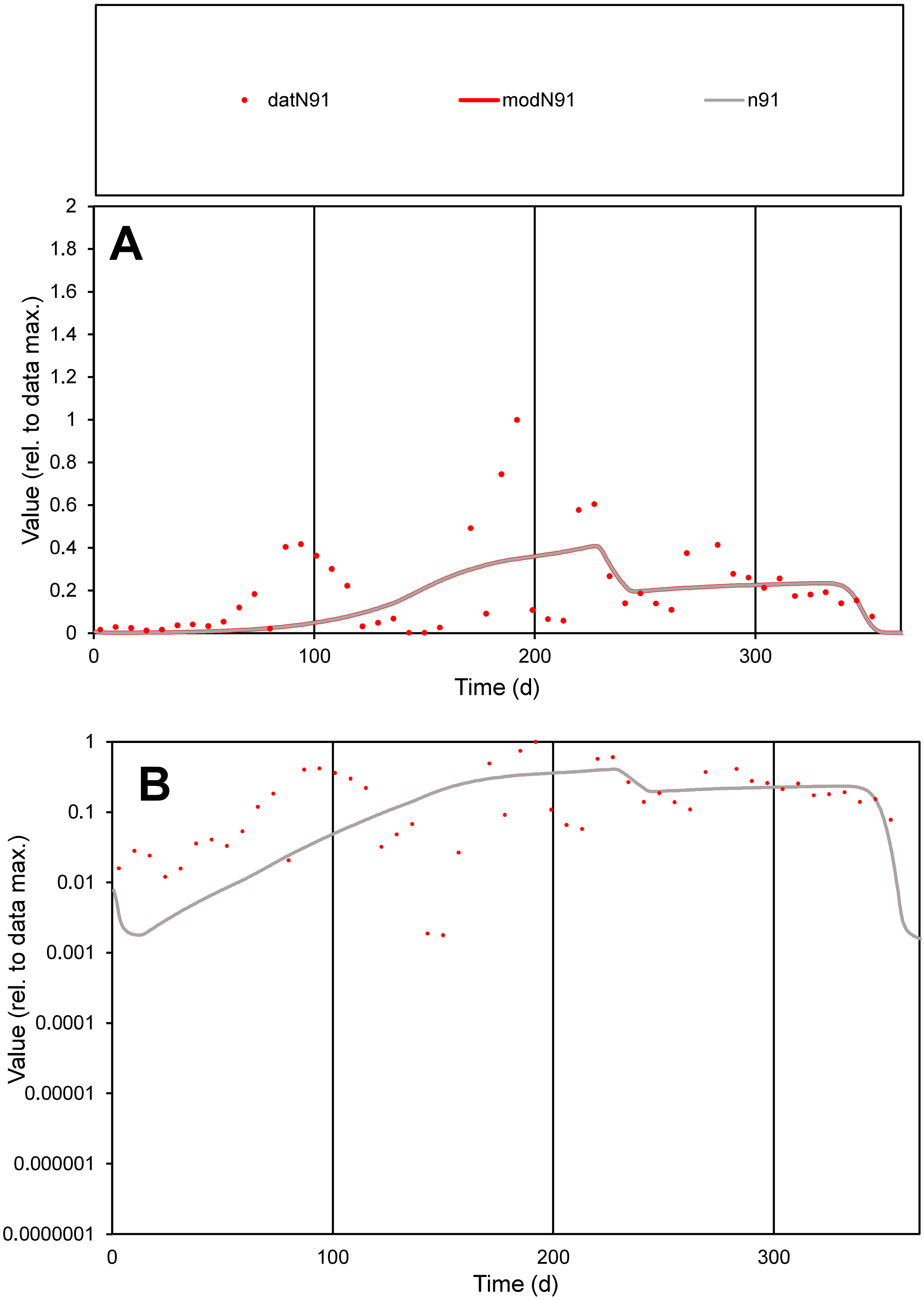

C1

Variables

N91 Provide jtd or Dst

CalibOnly?

2 1 to reproduce of

oNormalize?

2 1=no, 2=yes

ofxn calc*

0.94517

ofxn mod

0.9581

sRun

RunA1

zout

1

Ave

Data

0.00353

Model

0.00295

data/vMax

0.01771

ofxng

369.894

Min d/m

1.00381

ave/min d

111.617

ave/min m

93.6006

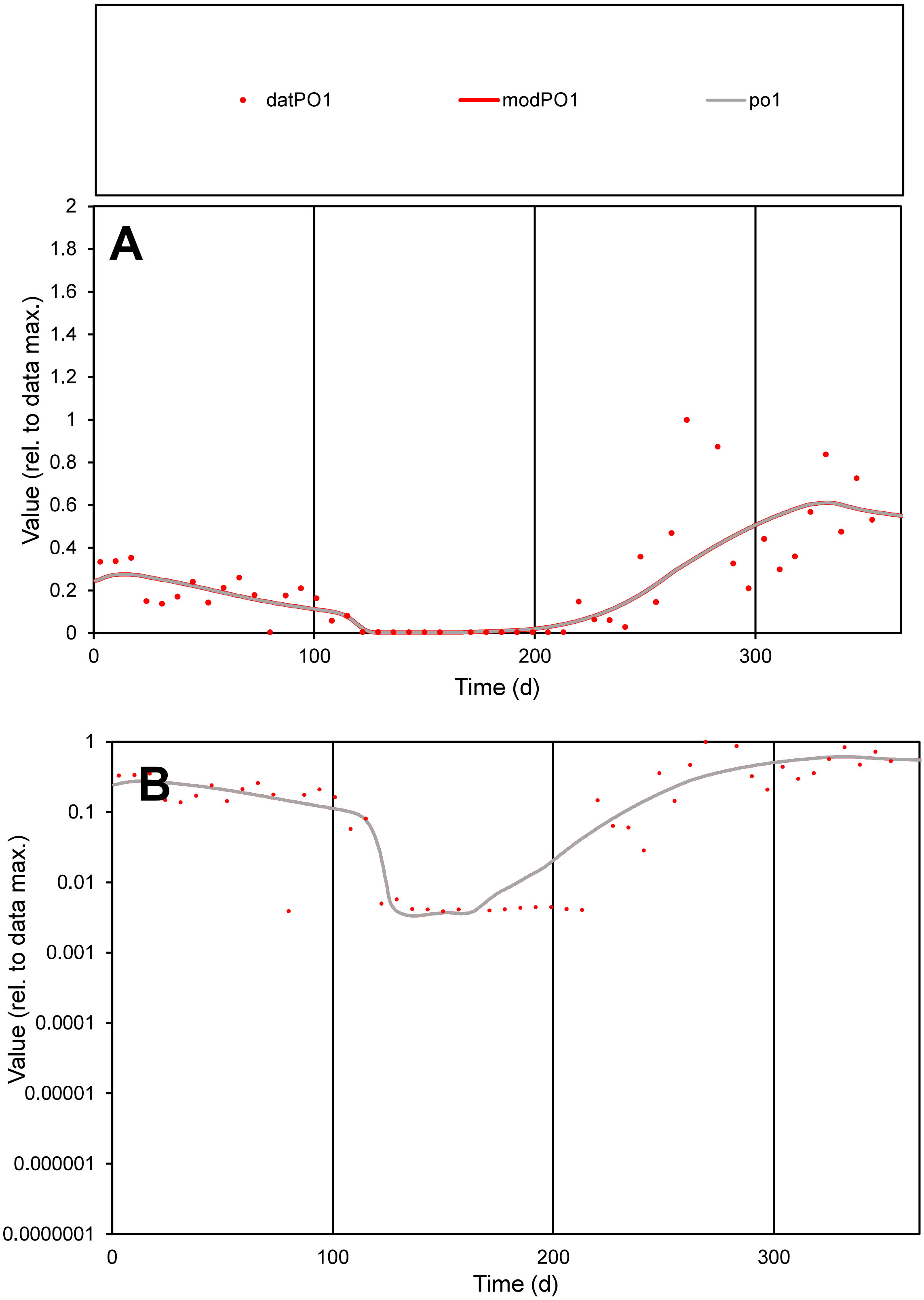

C1

Variables
PO1 Provide jtd or Dst
CalibOnly?
2 1 to reproduce of
oNormalize?
2 1=no, 2=yes

ofxn calc*
0.67018
ofxn mod
0.68689

sRun
RunA1
zout
1

Ave
Data
0.00267
Model
0.00258

data/vMax
0.01171

ofxng
369.894

Min d/m
1.16272

ave/min d
58.5451
ave/min m
65.664

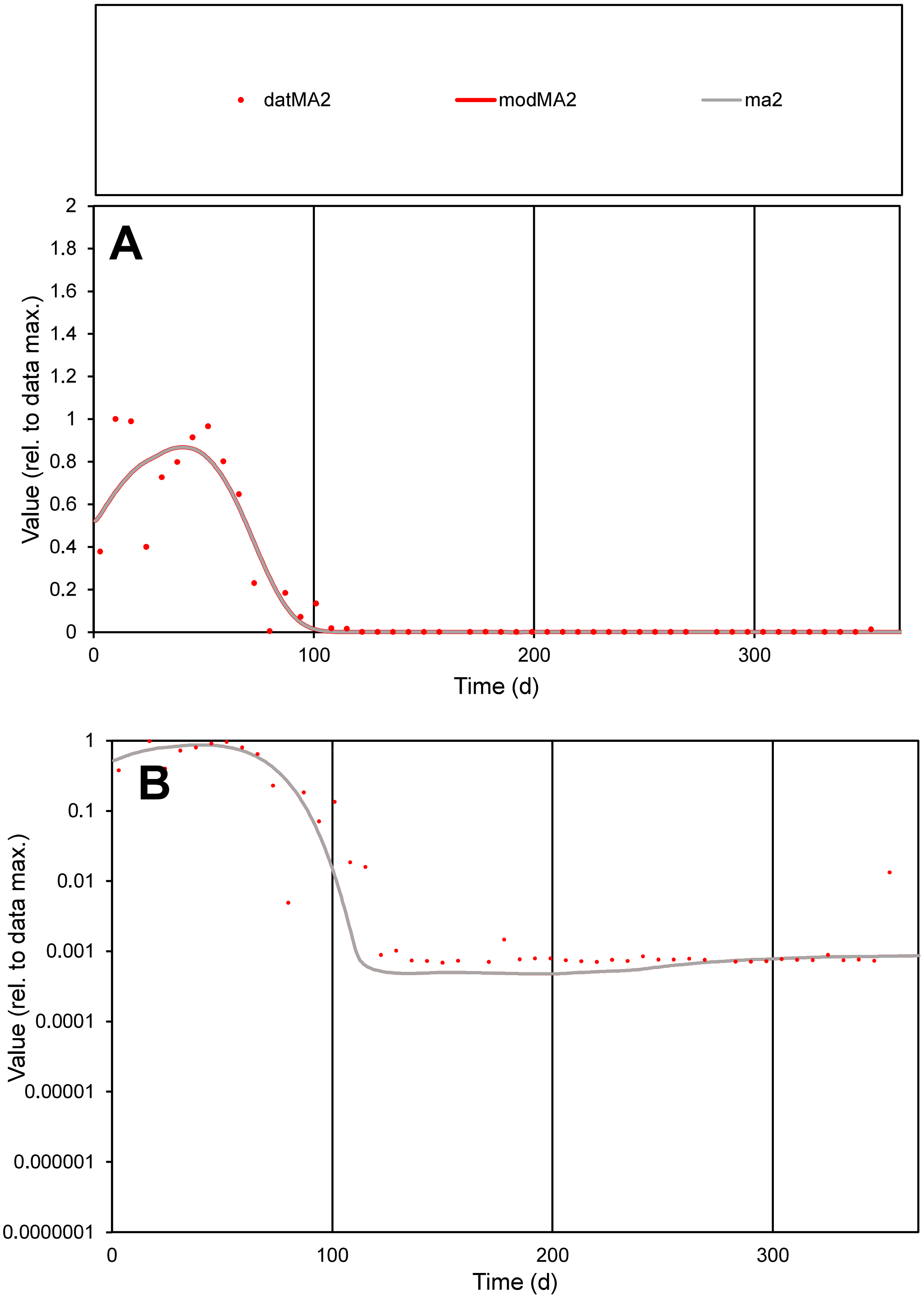

C1

Variables
MA2 Provide jtd or Dst
CalibOnly?
2 1 to reproduce of
oNormalize?
2 1=no, 2=yes

ofxn calc*
0.59511
ofxn mod
0.65115

sRun
RunA1
zout
1

Ave
Data
0.00439
Model
0.00433

data/vMax
0.02587

ofxng
369.894

Min d/m
1.44498

ave/min d
245.929
ave/min m
350.41

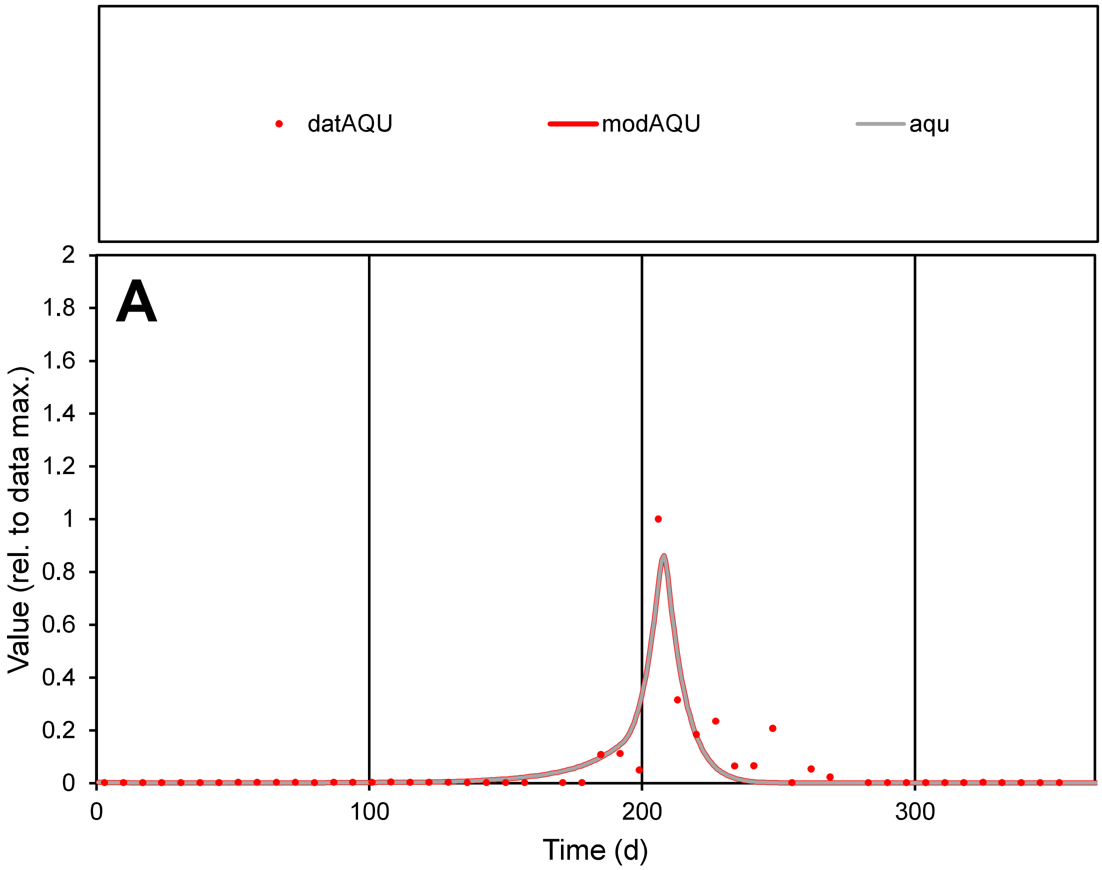

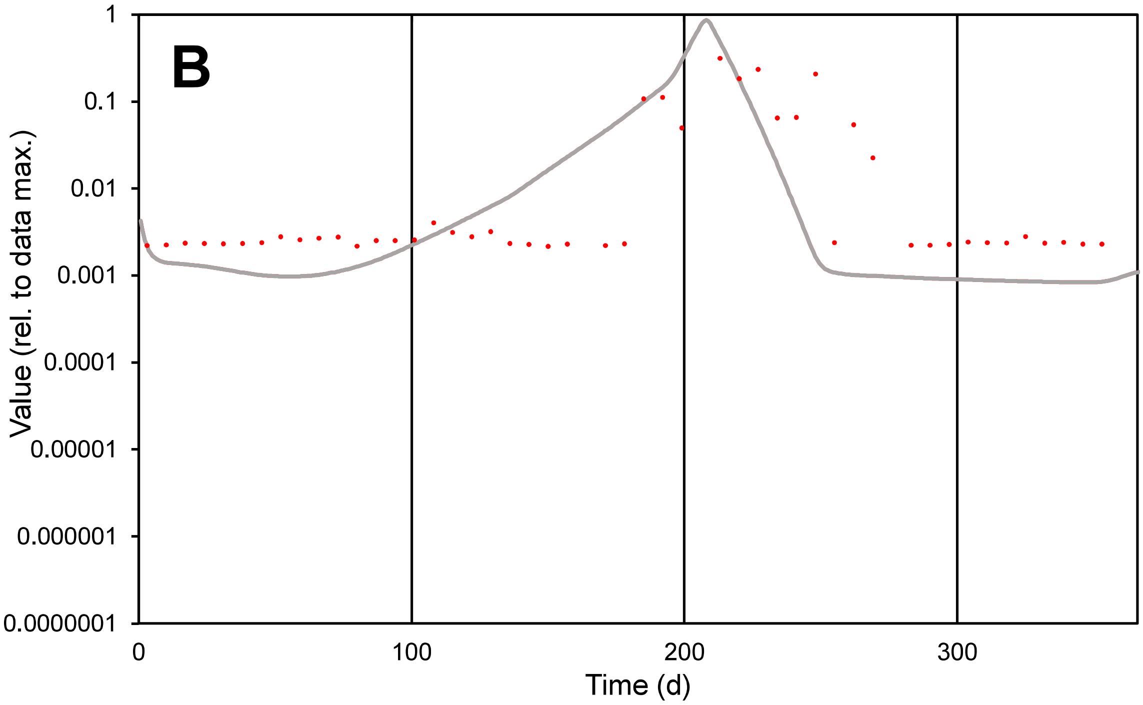

C1

Variables
AQU Provide jtd or Dst
CalibOnly?
2 1 to reproduce of
oNormalize?
2 1=no, 2=yes

ofxn calc*
1.07695
ofxn mod
1.21206

sRun
RunA1
zout
1

Ave
Data
0.00308
Model
0.0027

data/vMax
0.06009

ofxng
369.894

Min d/m
2.58679

ave/min d
23.6065
ave/min m
53.493

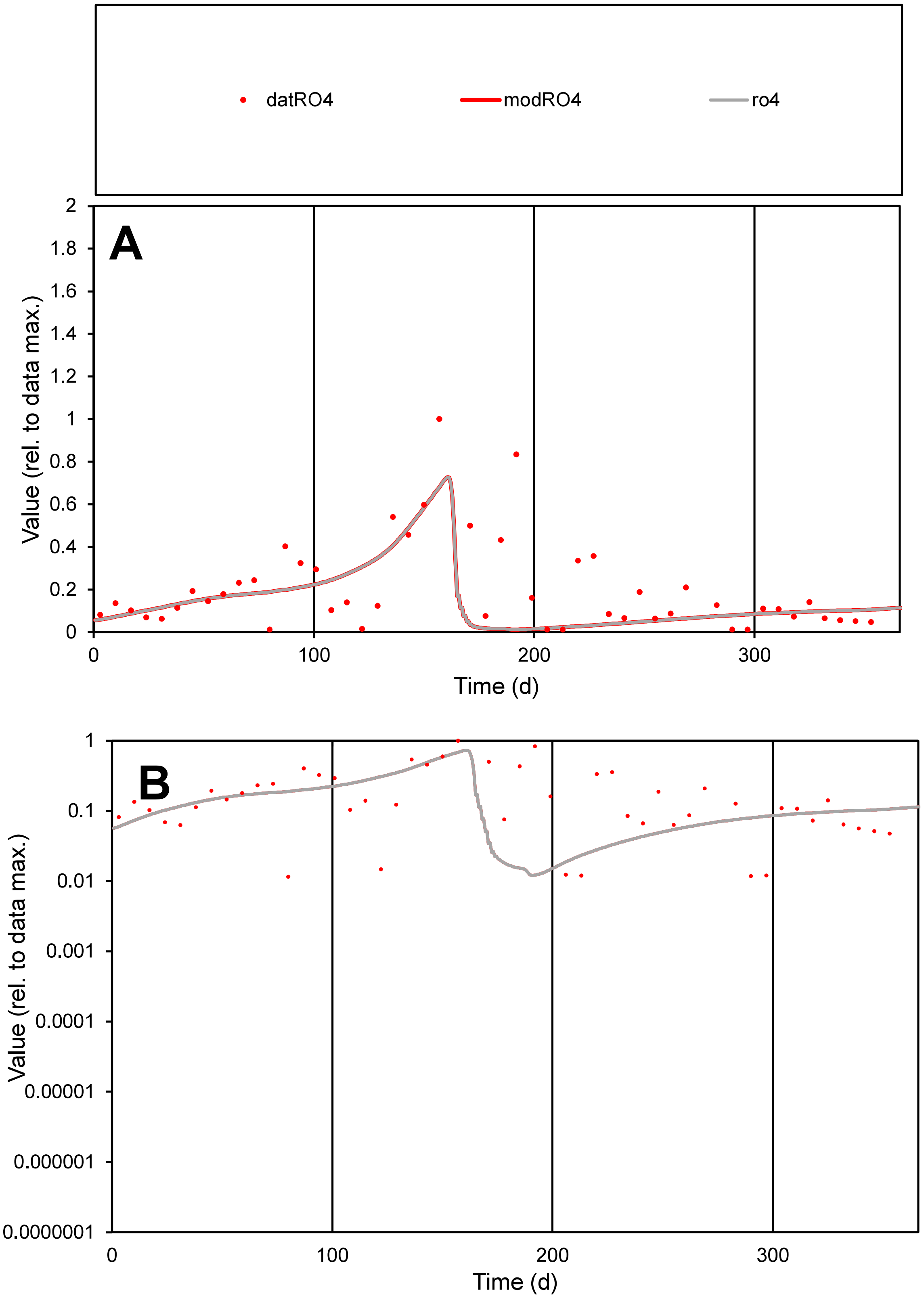

C1

Variables
RO4 Provide jtd or Dst
CalibOnly?
2 1 to reproduce of
oNormalize?
2 1=no, 2=yes

ofxn calc*
0.94443
ofxn mod
0.95404

sRun
RunA1
zout
1

Ave
Data
0.00378
Model
0.00279

data/vMax
0.01894

ofxng
369.894

Min d/m
0.93704

ave/min d
17.285
ave/min m
11.9428

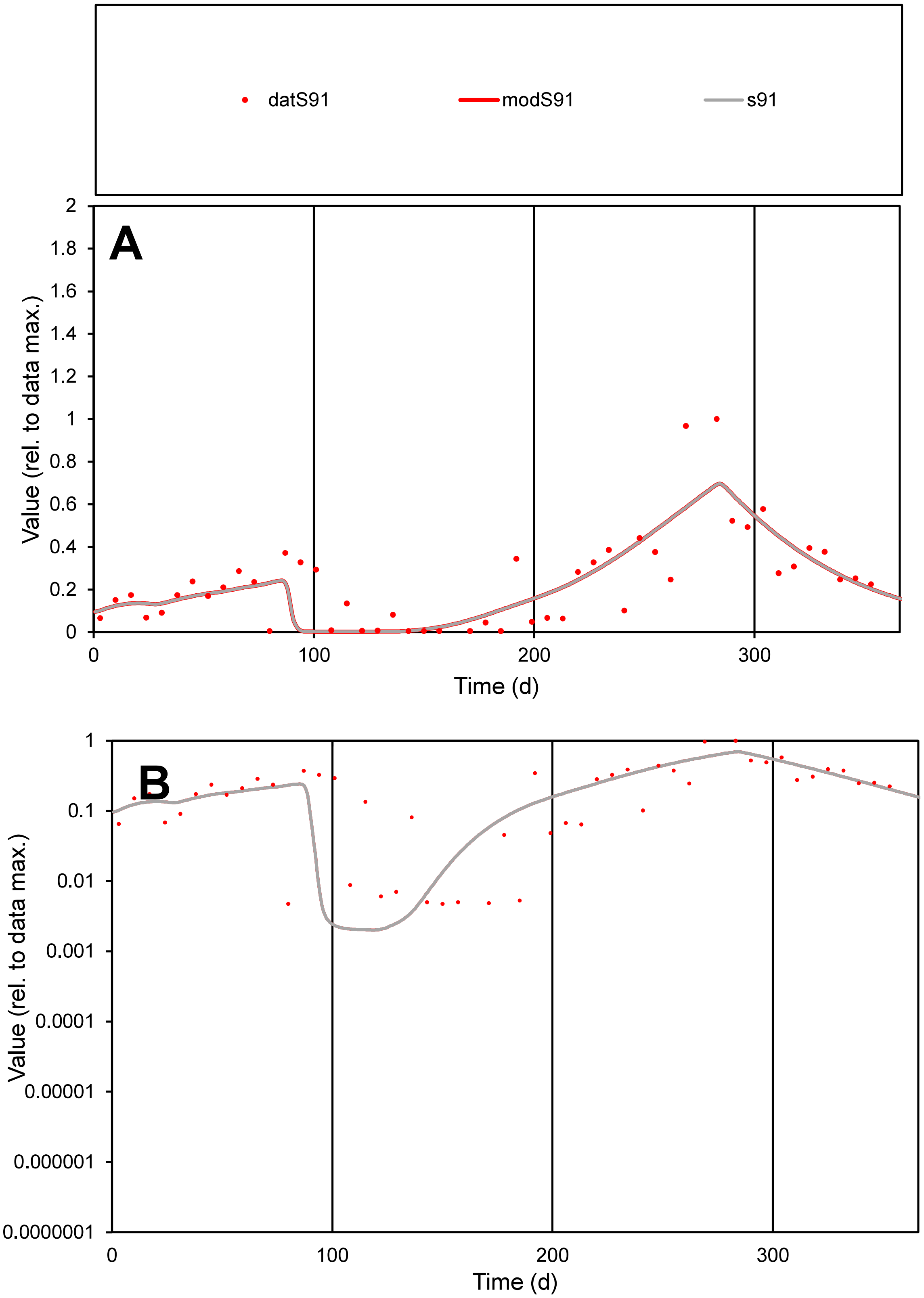

C1

Variables
S91 Provide jtd or Dst
CalibOnly?
2 1 to reproduce of
oNormalize?
2 1=no, 2=yes

ofxn calc*
0.5738
ofxn mod
0.59504

sRun
RunA1
zout
1

Ave
Data
0.00325
Model
0.00313

data/vMax
0.01384

ofxng
369.894

Min d/m
2.33999

ave/min d
49.5202
ave/min m
111.841

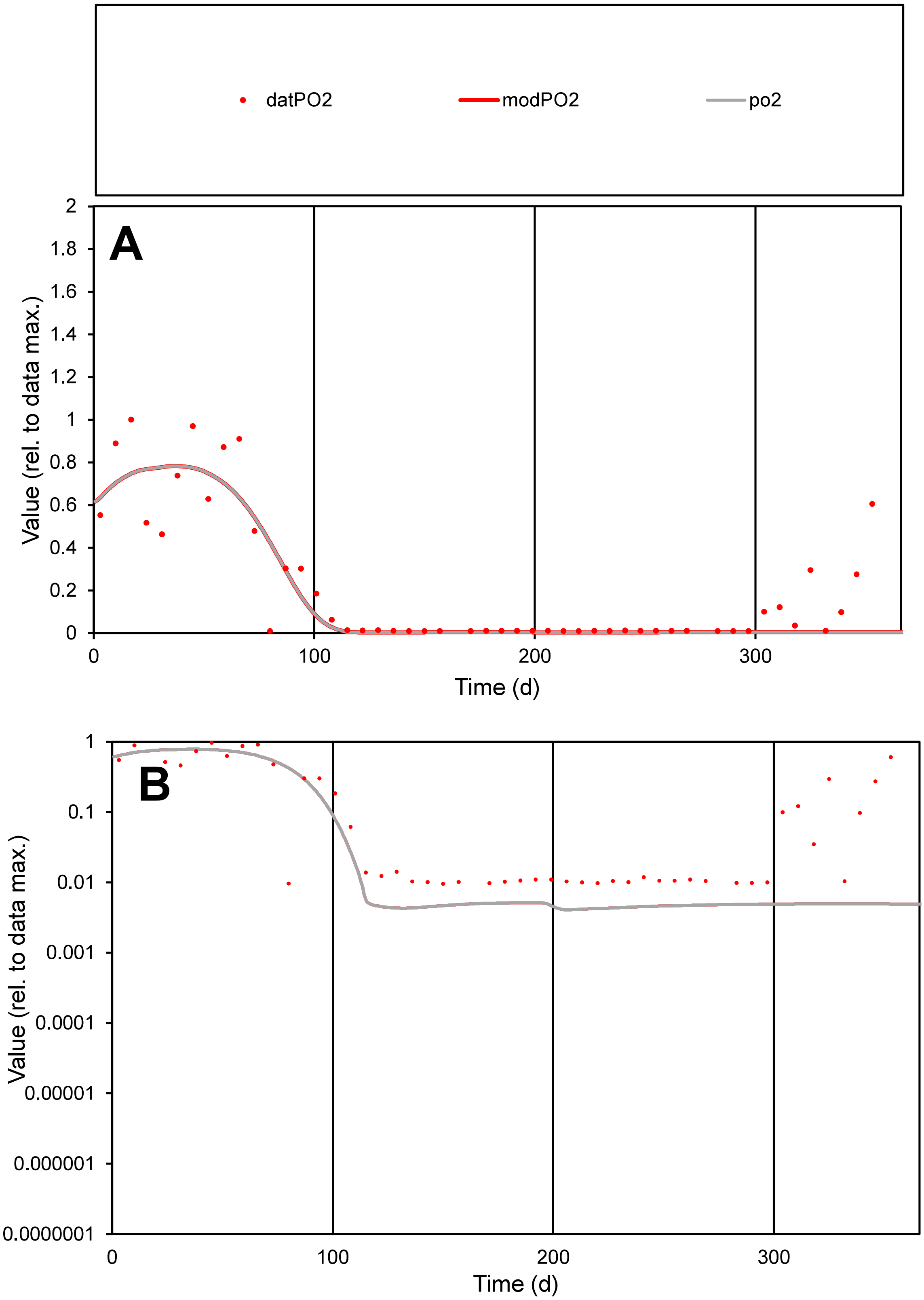

C1

Variables

PO2 Provide jtd or Dst

CalibOnly?

2 1 to reproduce of

oNormalize?

2 1=no, 2=yes

ofxn calc*

0.70035

ofxn mod

0.73534

sRun

RunA1

zout

1

Ave

Data

0.00181

Model

0.0015

data/vMax

0.00829

ofxng

369.894

Min d/m

2.3477

ave/min d

22.7295

ave/min m

44.2006

328_Calib_S_A

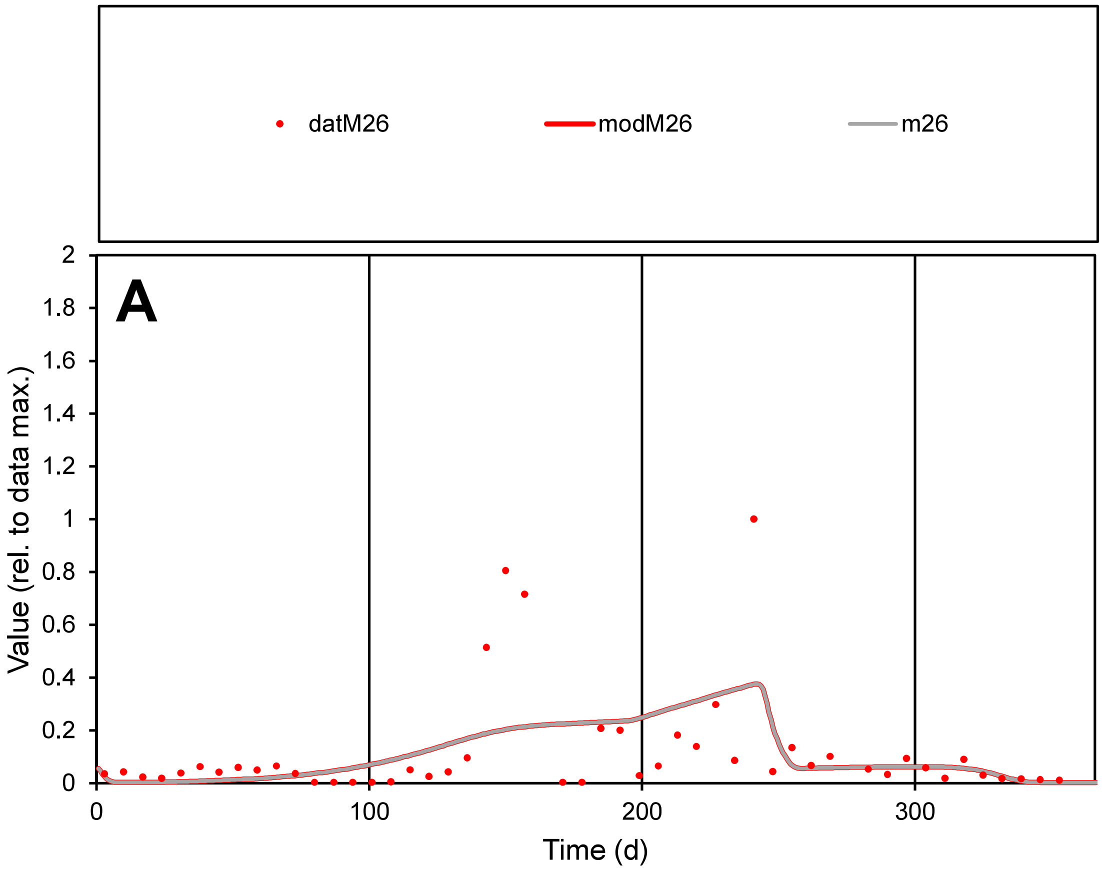

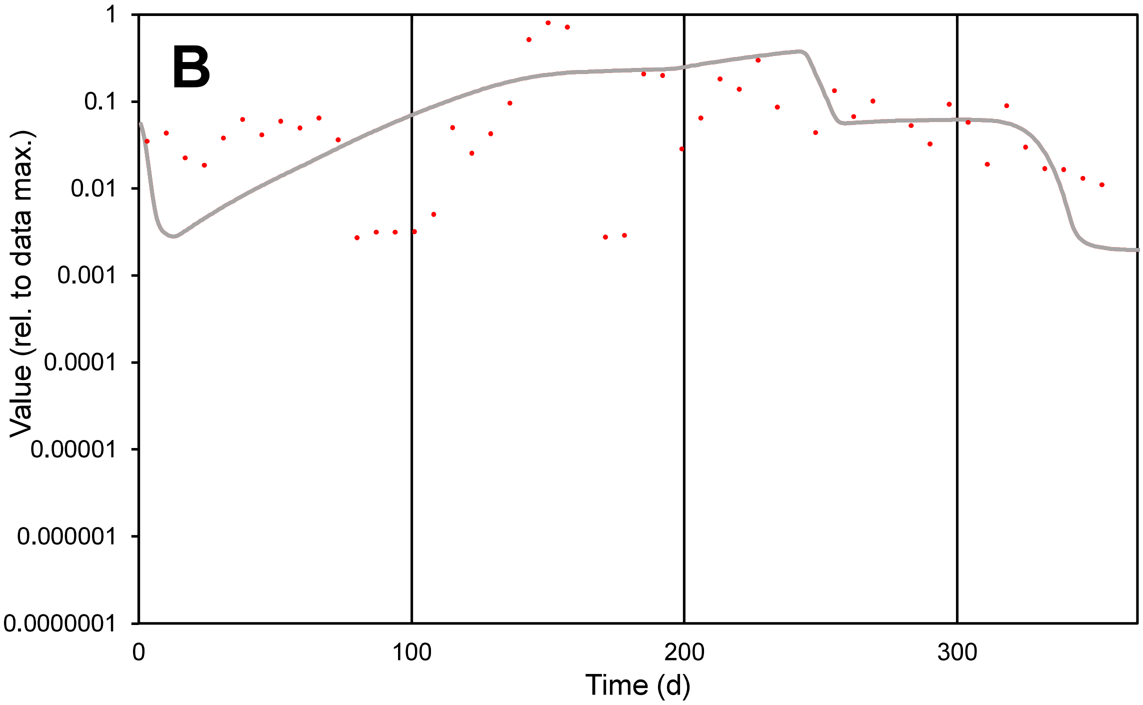

C1

Variables

M26	Provide jtd or Dst

CalibOnly?

2	1 to reproduce of

oNormalize?

2	1=no, 2=yes

ofxn calc*
1.49148
ofxn mod
1.50594

sRun
RunA1
zout
1

Ave
Data
0.00353
Model
0.00338

data/vMax
0.03018

ofxng
369.894

Min d/m
1.31938

ave/min d
42.8937
ave/min m
54.2439

328_Calib_S_A

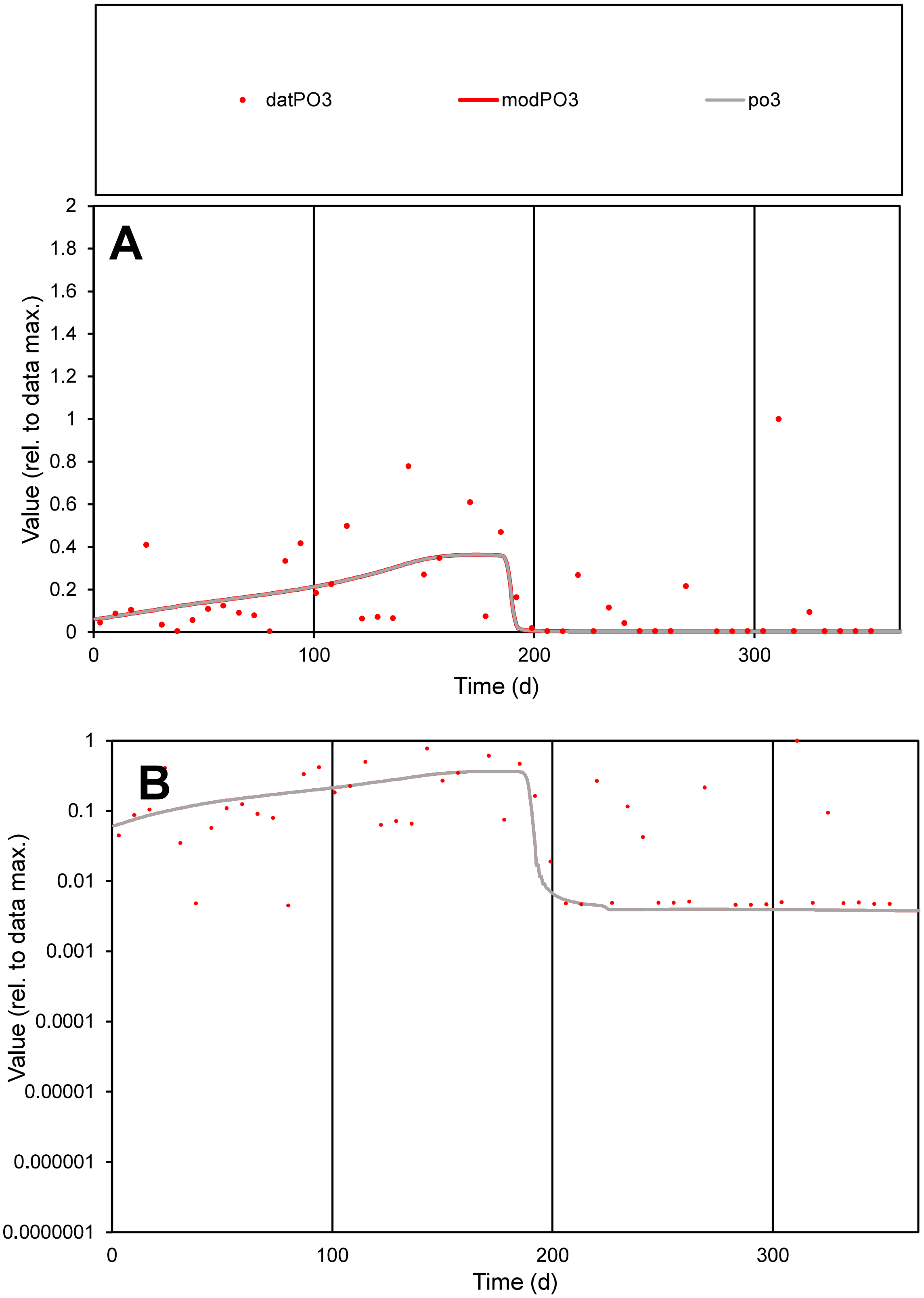

C1

Variables

PO3 Provide jtd or Dst

CalibOnly?

2 1 to reproduce of

oNormalize?

2 1=no, 2=yes

ofxn calc*

1.28921

ofxn mod

1.29814

sRun

RunA1

zout

1

Ave

Data

0.0016

Model

0.00122

data/vMax

0.0104

ofxng

369.894

Min d/m

1.184

ave/min d

34.2992

ave/min m

30.9101

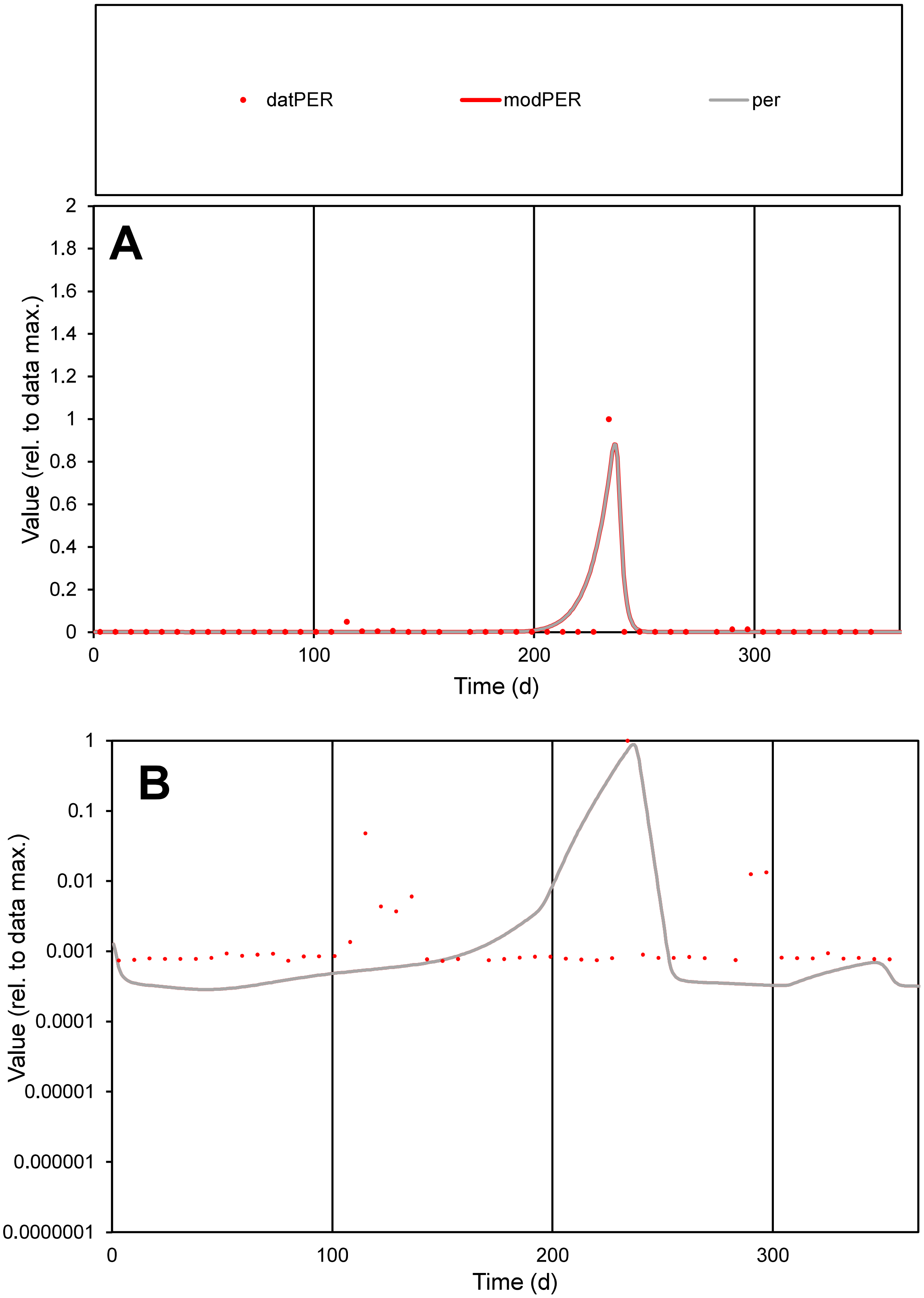

C1

Variables
PER Provide jtd or Dst
CalibOnly?
2 1 to reproduce of
oNormalize?
2 1=no, 2=yes

ofxn calc*
2.22784
ofxn mod
2.57097

sRun
RunA1
zout
1

Ave
Data
0.00058
Model
0.00072

data/vMax
0.02513

ofxng
369.894

Min d/m
2.54376

ave/min d
31.4359
ave/min m
100.583

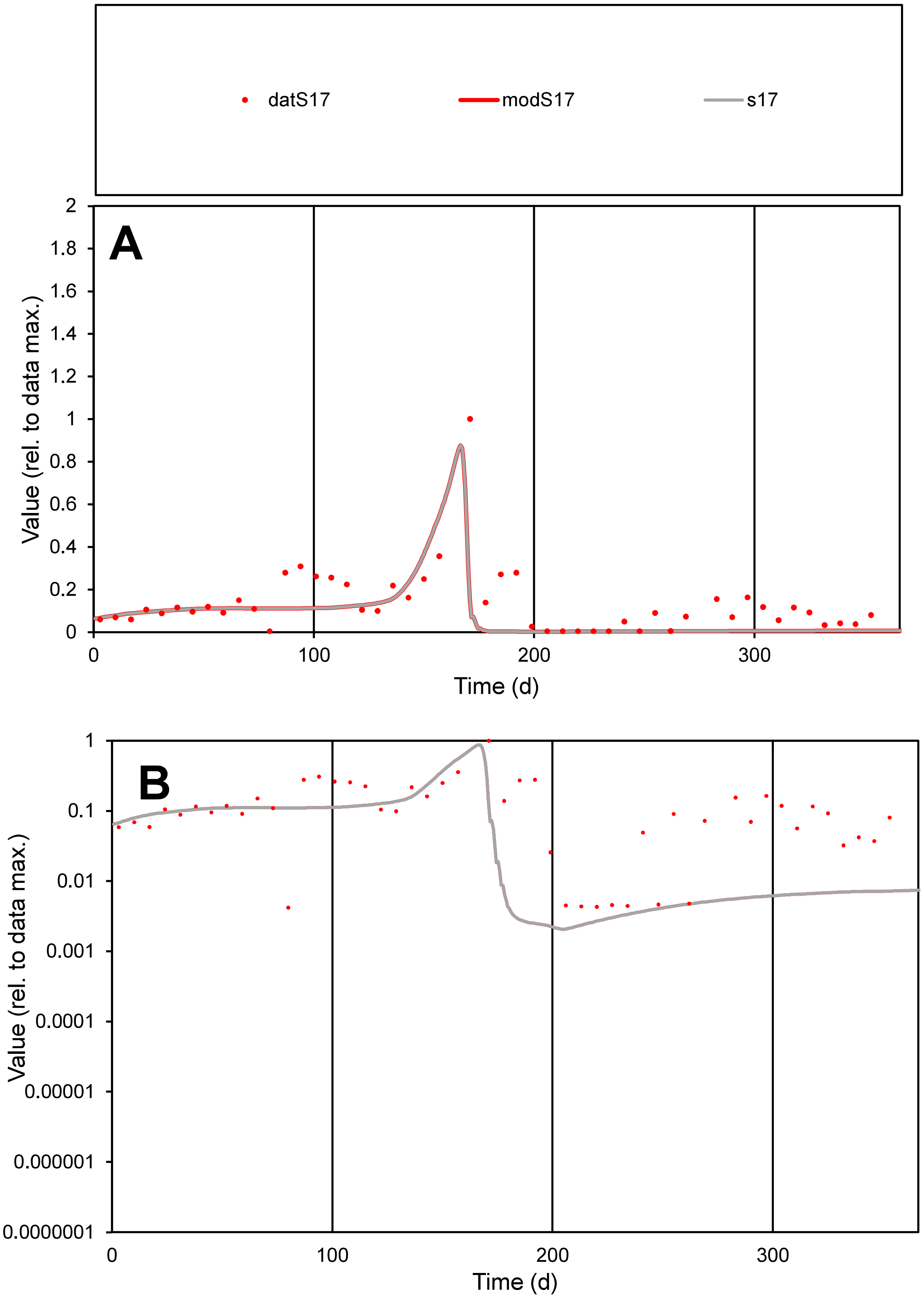

C1

Variables
S17 Provide jtd or Dst
CalibOnly?
2 1 to reproduce of
oNormalize?
2 1=no, 2=yes

ofxn calc*
0.77061
ofxn mod
1.02786

sRun
RunA1
zout
1

Ave
Data
0.00345
Model
0.00229

data/vMax
0.026

ofxng
369.894

Min d/m
1.98945

ave/min d
31.544
ave/min m
41.6595

328_Calib_S_A

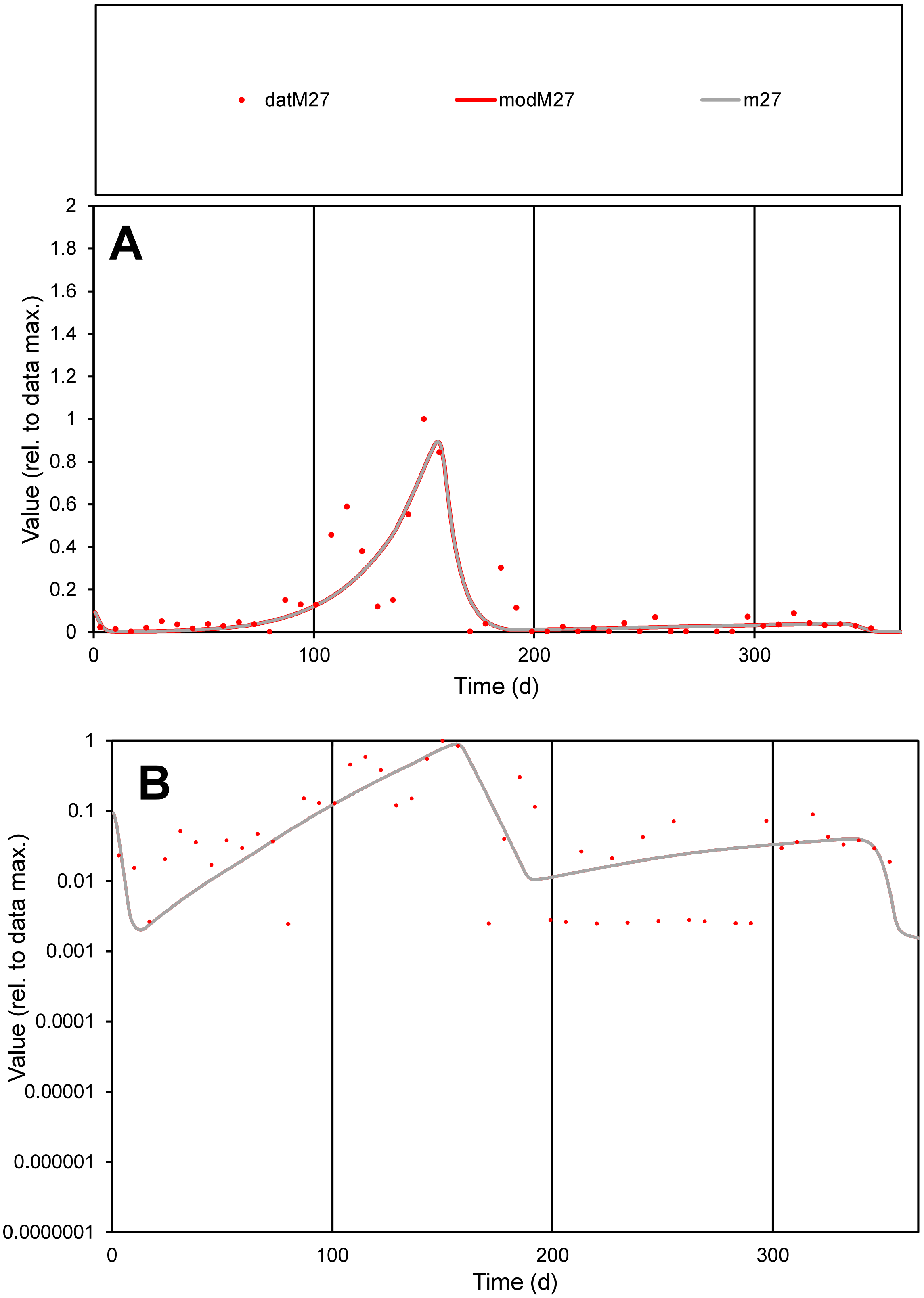

C1

Variables
M27 Provide jtd or Dst
CalibOnly?
2 1 to reproduce of
oNormalize?
2 1=no, 2=yes

ofxn calc*
0.88174
ofxn mod
0.91123

sRun
RunA1
zout
1

Ave
Data
0.00319
Model
0.00274

data/vMax
0.02673

ofxng
369.894

Min d/m
1.07289

ave/min d
48.8736
ave/min m
45.0523

328_Calib_S_A

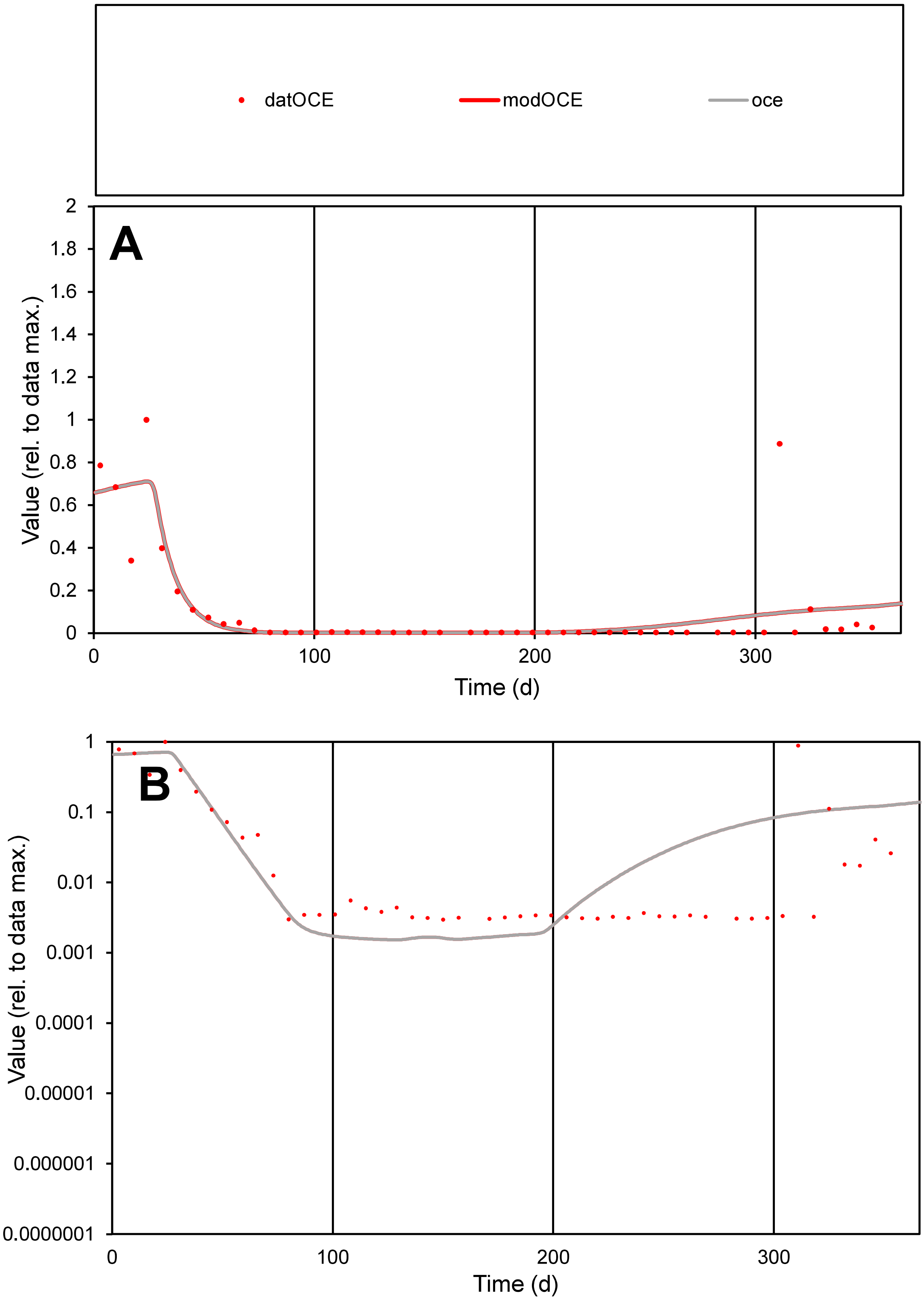

C1

Variables
OCE Provide jtd or Dst
CalibOnly?
2 1 to reproduce of
oNormalize?
2 1=no, 2=yes

ofxn calc*
1.37685
ofxn mod
1.40826

sRun
RunA1
zout
1

Ave
Data
0.00125
Model
0.00125

data/vMax
0.01248

ofxng
369.894

Min d/m
1.93992

ave/min d
33.578
ave/min m
65.2985

328_Calib_S_A

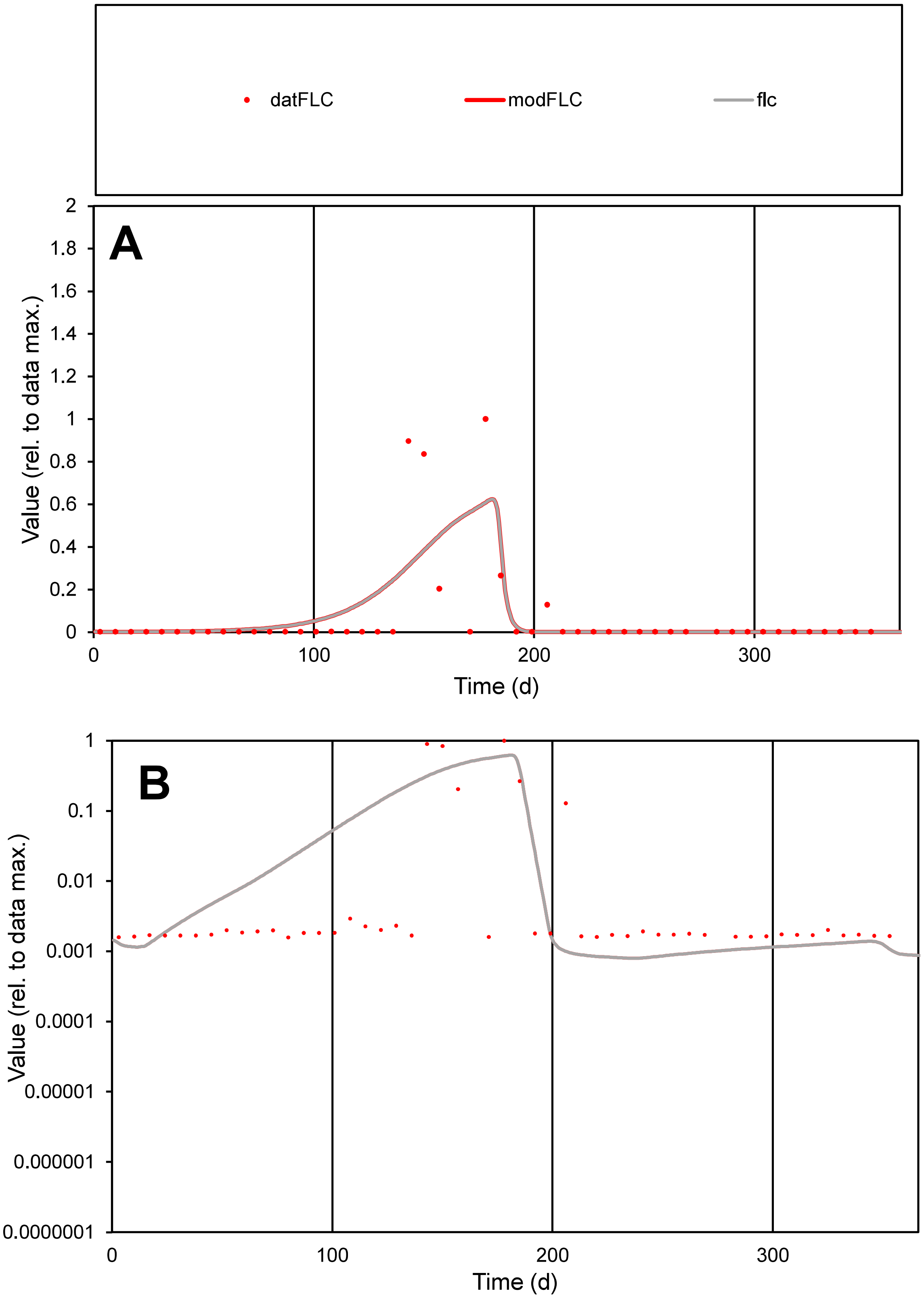

C1

Variables

FLC Provide jtd or Dst

CalibOnly?

2 1 to reproduce of

oNormalize?

2 1=no, 2=yes

ofxn calc*

2.24338

ofxn mod

2.27568

sRun

RunA1

zout

1

Ave

Data

0.00054

Model

0.00056

data/vMax

0.0077

ofxng

369.894

Min d/m

1.97133

ave/min d

44.2727

ave/min m

91.5973

328_Calib_S_A

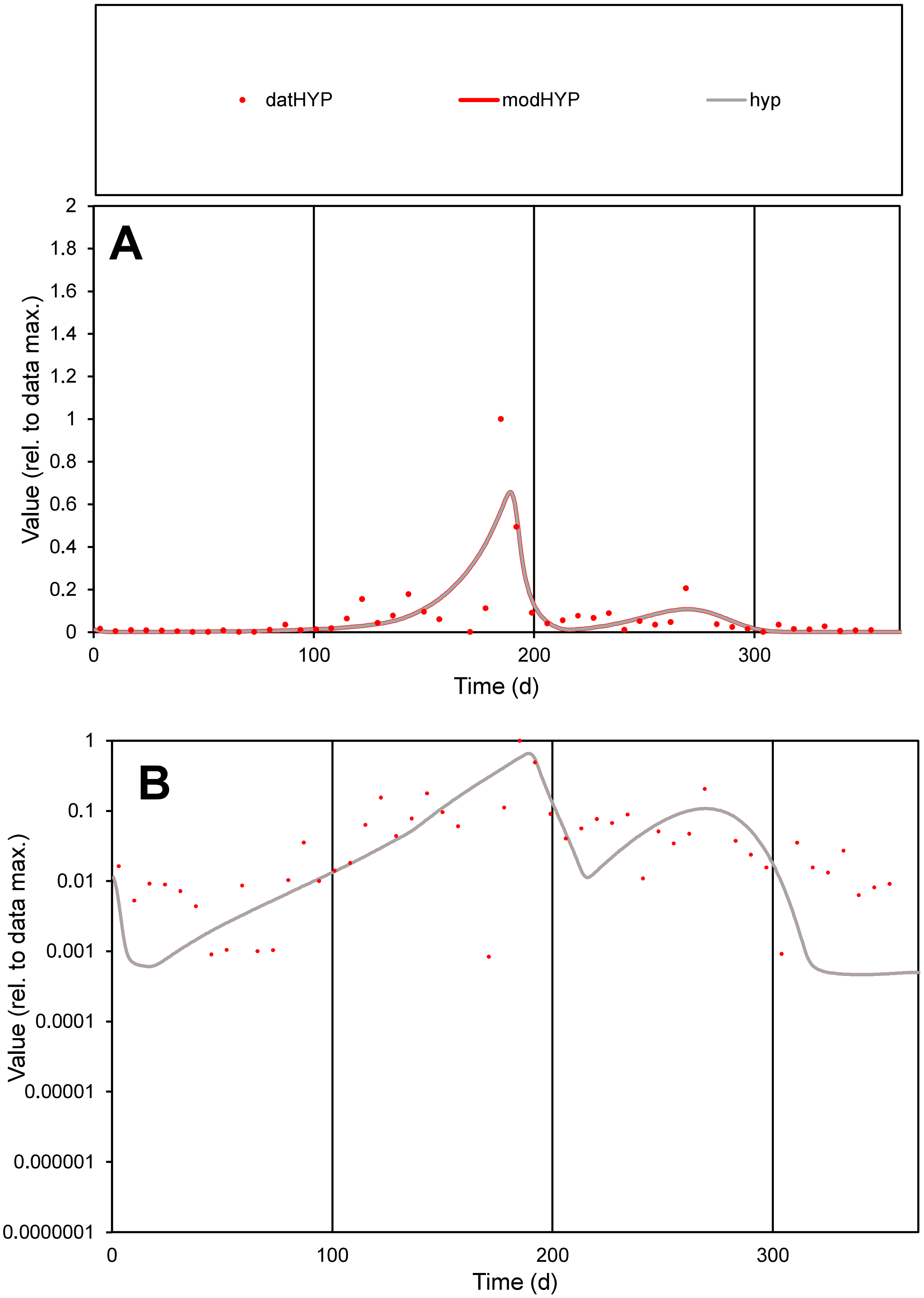

C1

Variables
HYP Provide jtd or Dst
CalibOnly?
2 1 to reproduce of
oNormalize?
2 1=no, 2=yes

ofxn calc*
1.31604
ofxn mod
1.35435

sRun
RunA1
zout
1

Ave
Data
0.00135
Model
0.00127

data/vMax
0.01944

ofxng
369.894

Min d/m
1.79326

ave/min d
82.7697
ave/min m
140.211

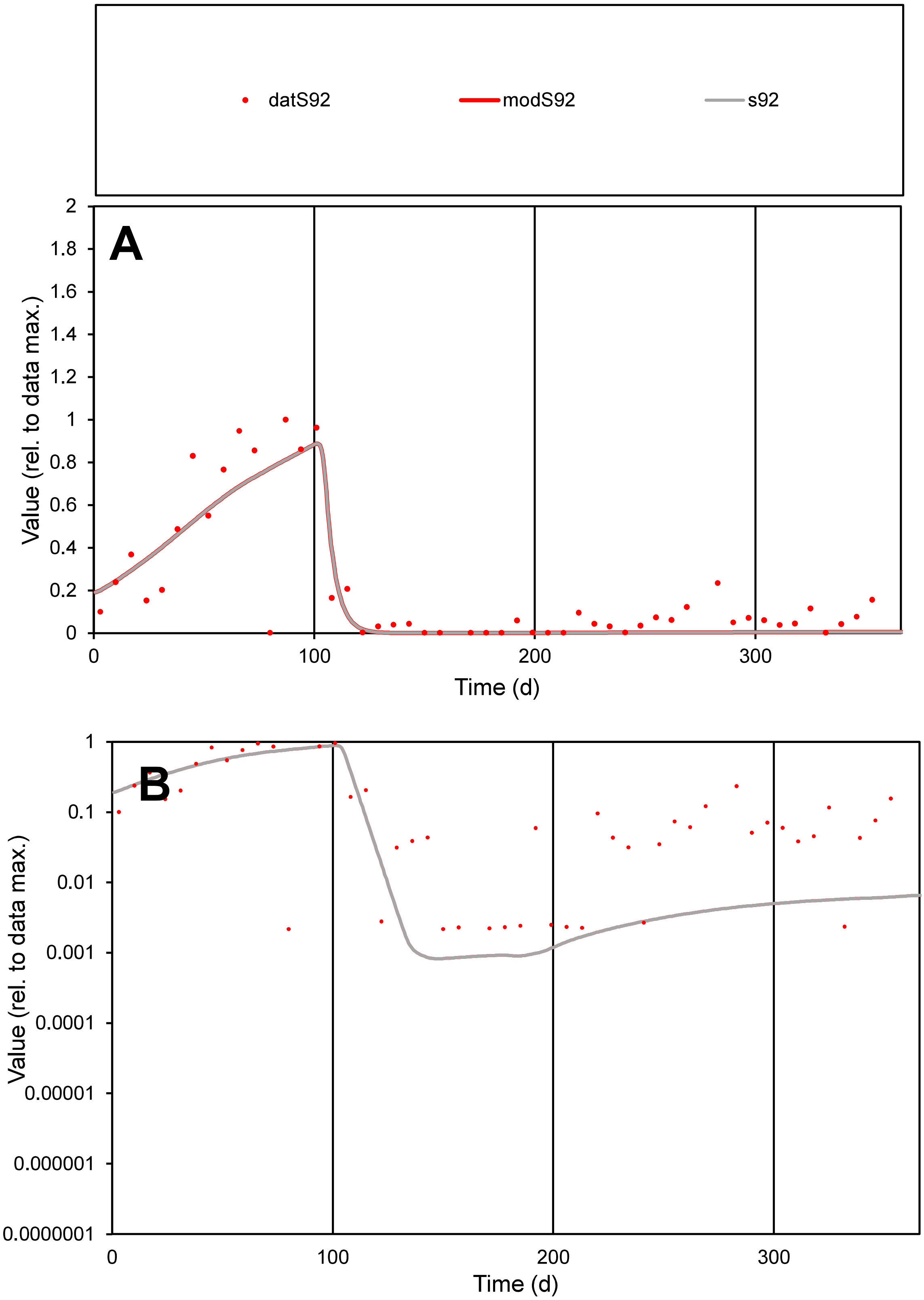

C1

Variables
S92 Provide jtd or Dst
CalibOnly?
2 1 to reproduce of
oNormalize?
2 1=no, 2=yes

ofxn calc*
0.70615
ofxn mod
0.73628

sRun
RunA1
zout
1

Ave
Data
0.00224
Model
0.00196

data/vMax
0.01071

ofxng
369.894

Min d/m
2.61343

ave/min d
96.3626
ave/min m
220.342

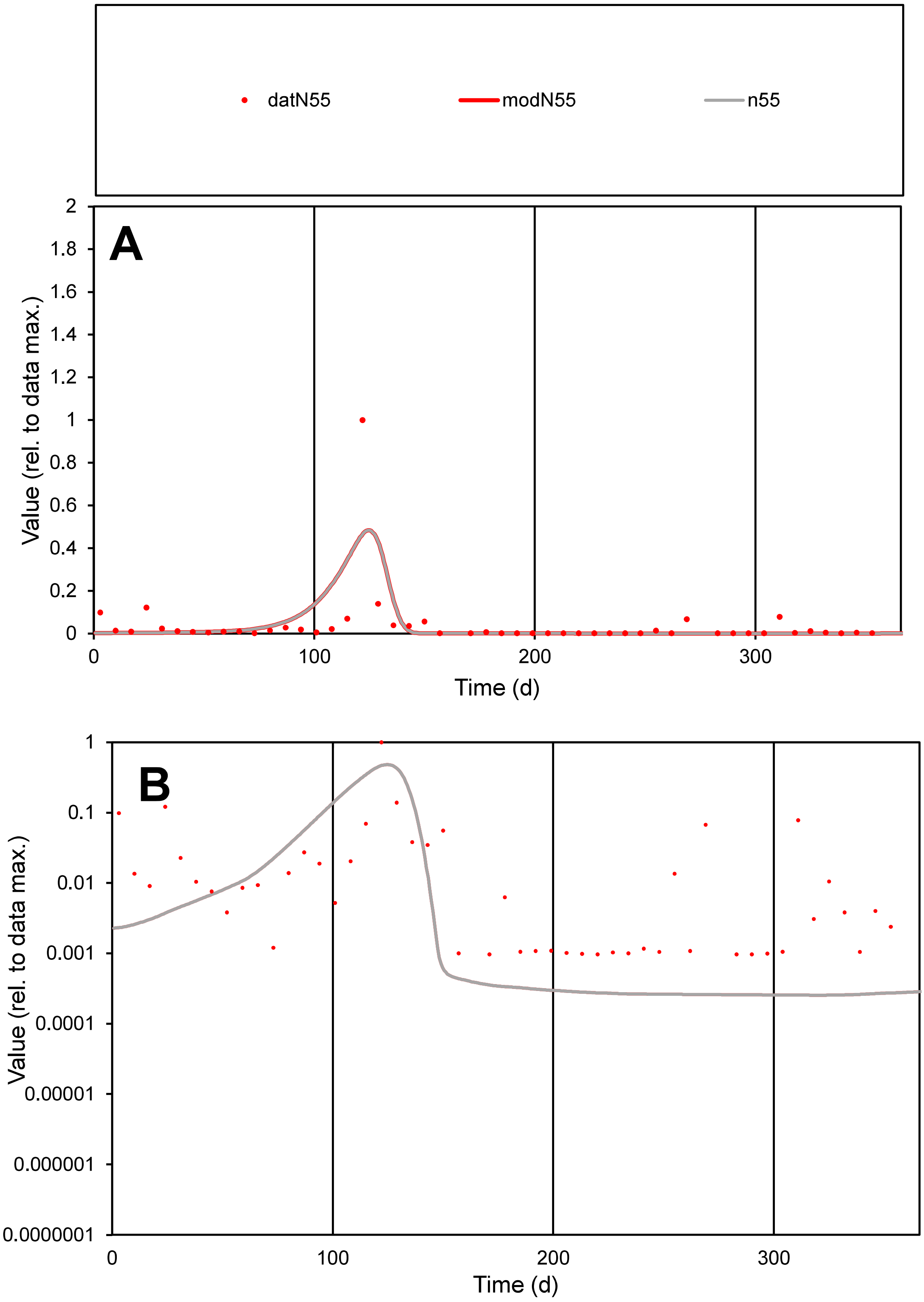

C1

Variables
N55 Provide jtd or Dst
CalibOnly?
2 1 to reproduce of
oNormalize?
2 1=no, 2=yes

ofxn calc*
2.49323
ofxn mod
2.62553

sRun
RunA1
zout
1

Ave
Data
0.00156
Model
0.00152

data/vMax
0.03945

ofxng
369.894

Min d/m
3.79047

ave/min d
40.8422
ave/min m
150.51

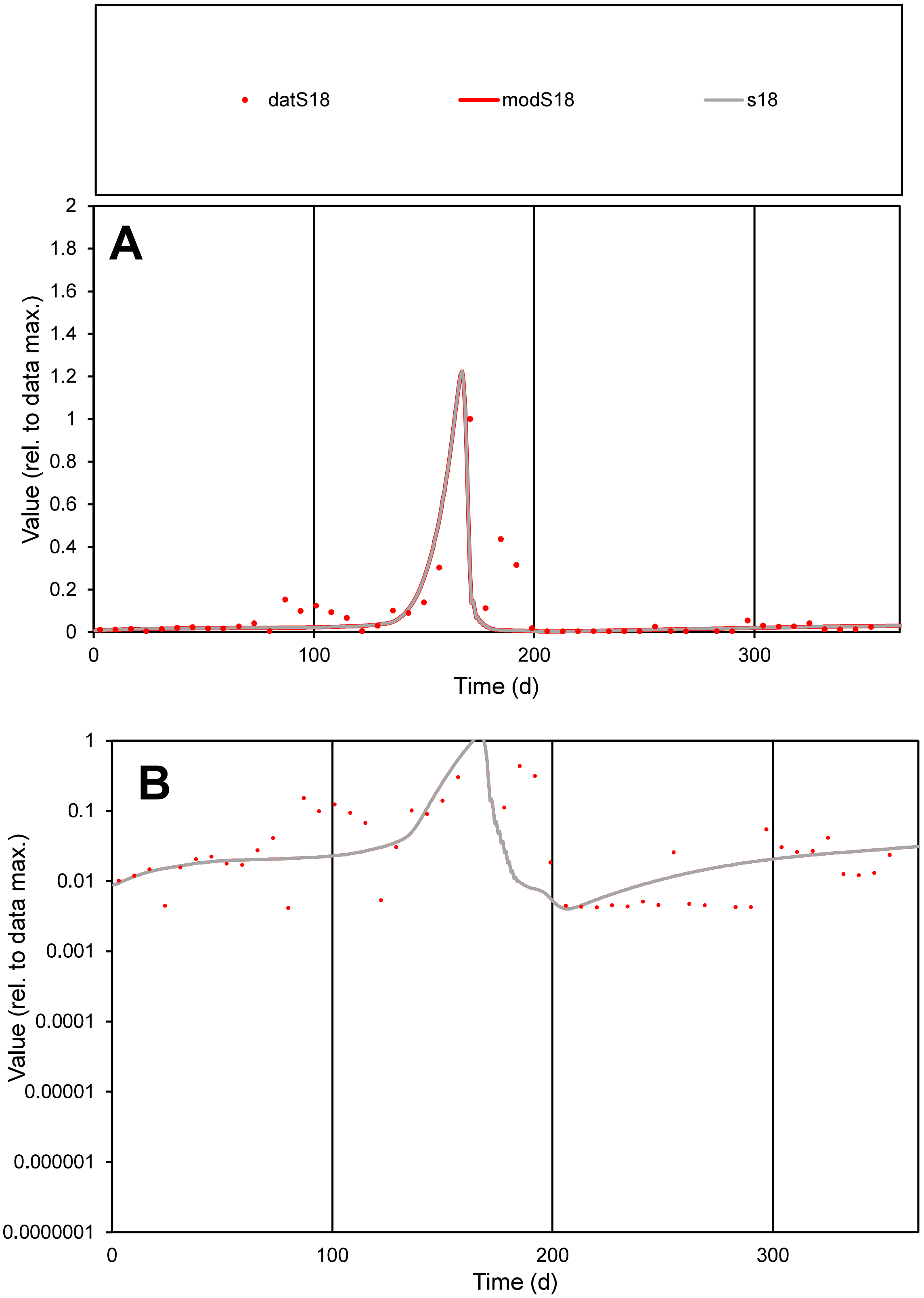

C1

Variables
S18 Provide jtd or Dst
CalibOnly?
2 1 to reproduce of
oNormalize?
2 1=no, 2=yes

ofxn calc*
1.28308
ofxn mod
1.57709

sRun
RunA1
zout
1

Ave
Data
0.00474
Model
0.00339

data/vMax
0.06431

ofxng
369.894

Min d/m
1.03398

ave/min d
17.7121
ave/min m
13.0849

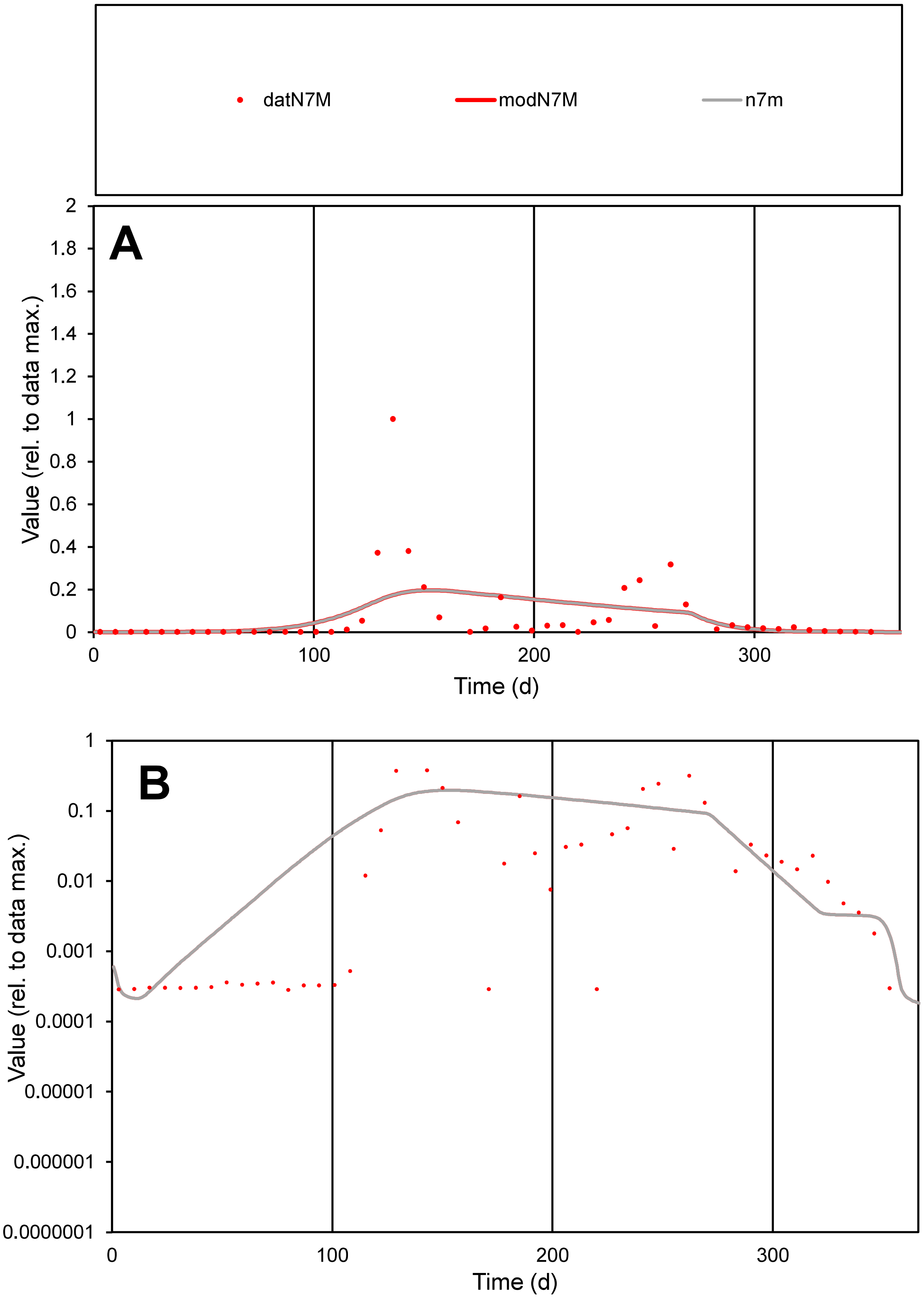

C1

Variables
N7M Provide jtd or Dst
CalibOnly?
2 1 to reproduce of
oNormalize?
2 1=no, 2=yes

ofxn calc*
1.99091
ofxn mod
2.00022

sRun
RunA1
zout
1

Ave
Data
0.00249
Model
0.00241

data/vMax
0.03424

ofxng
369.894

Min d/m
1.3224

ave/min d
256.983
ave/min m
328.843

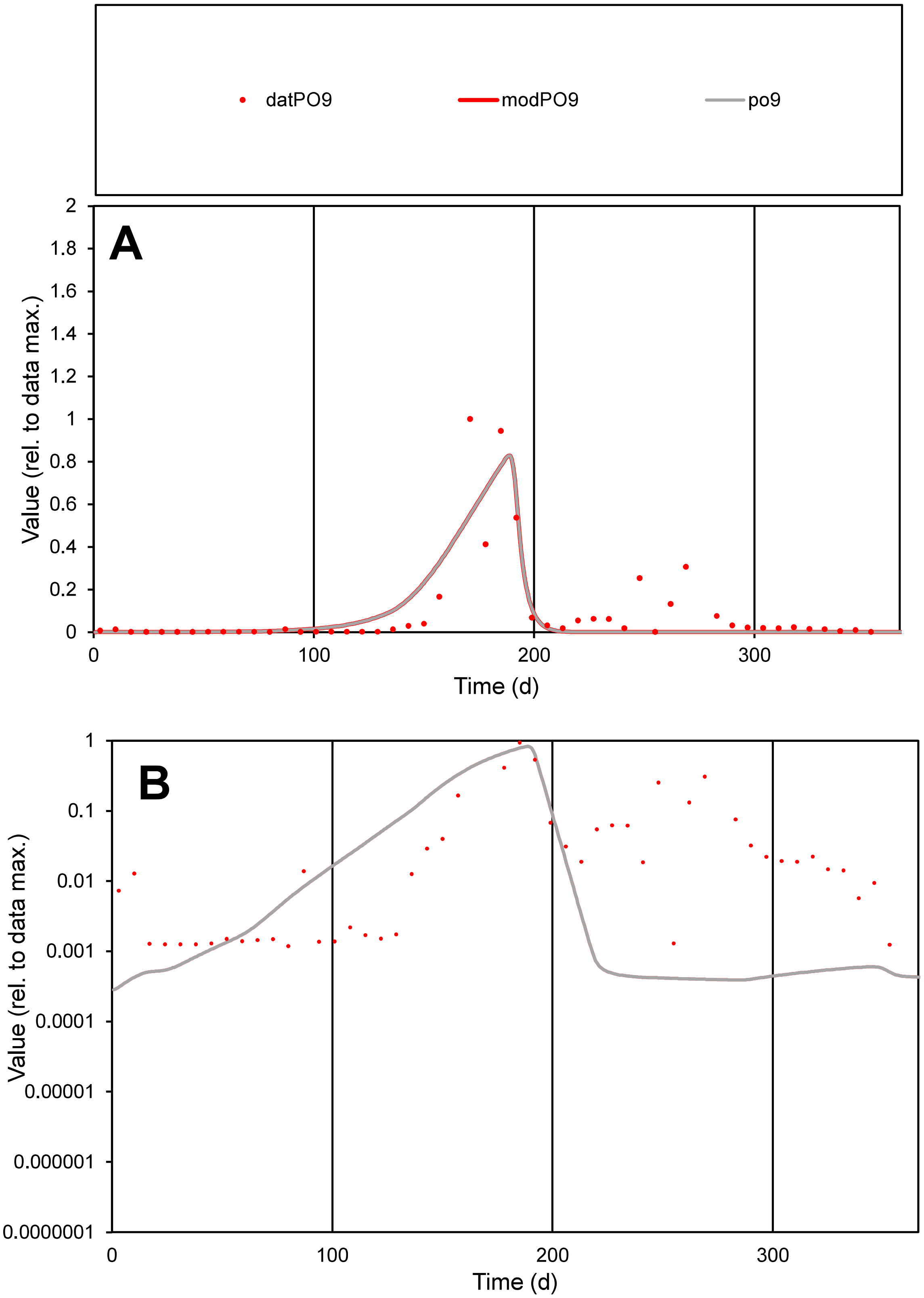

C1

Variables

PO9 Provide jtd or Dst

CalibOnly?

2 1 to reproduce of

oNormalize?

2 1=no, 2=yes

ofxn calc*

1.17211

ofxn mod

1.22484

sRun

RunA1

zout

1

Ave

Data

0.0045

Model

0.00377

data/vMax

0.04963

ofxng

369.894

Min d/m

3.58777

ave/min d

76.7193

ave/min m

230.297

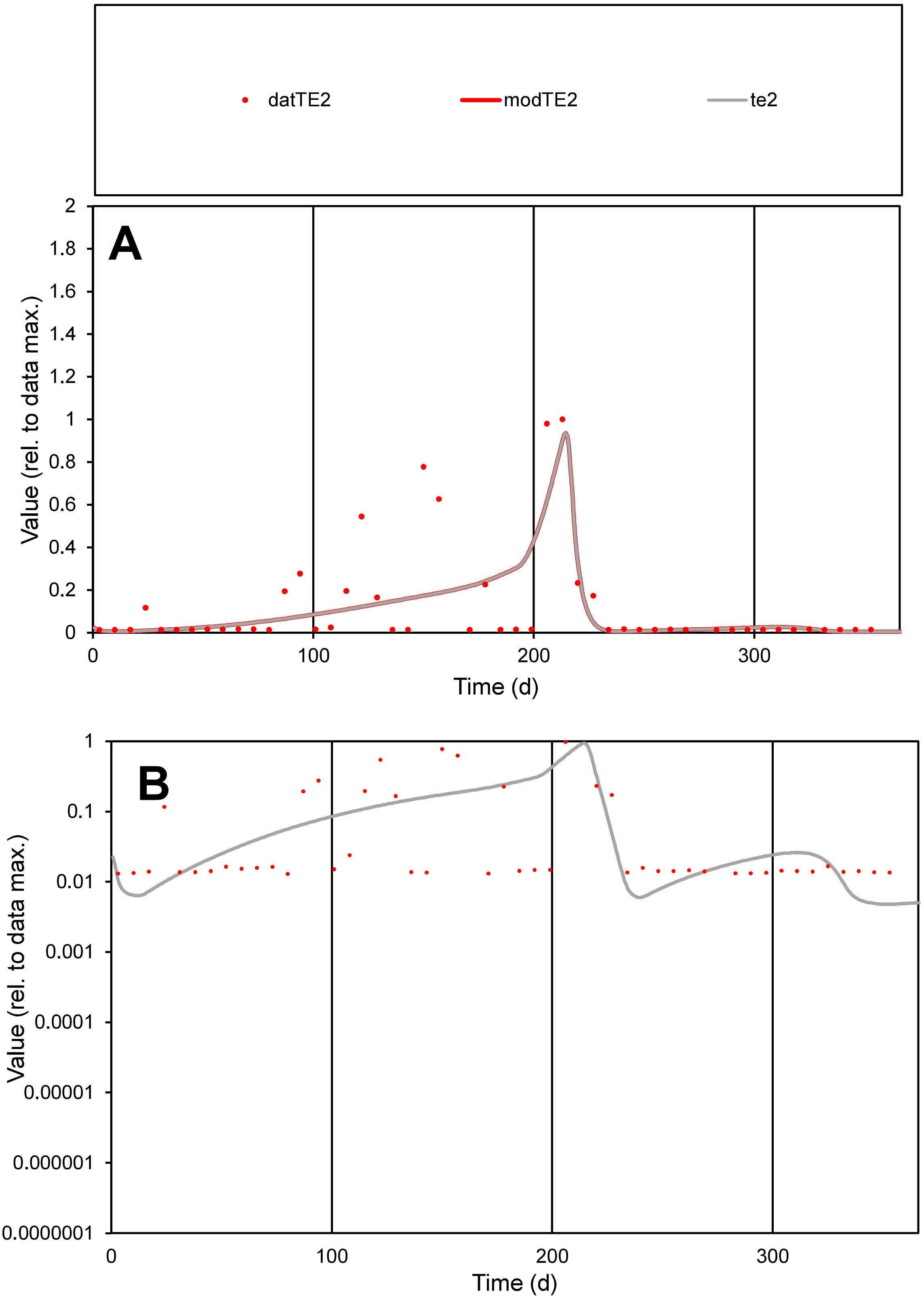

C1

Variables
TE2 Provide jtd or Dst
CalibOnly?
2 1 to reproduce of
oNormalize?
2 1=no, 2=yes

ofxn calc*
1.31835
ofxn mod
1.35116

sRun
RunA1
zout
1

Ave
Data
0.00082
Model
0.00071

data/vMax
0.00665

ofxng
369.894

Min d/m
2.68858

ave/min d
9.52788
ave/min m
22.2308

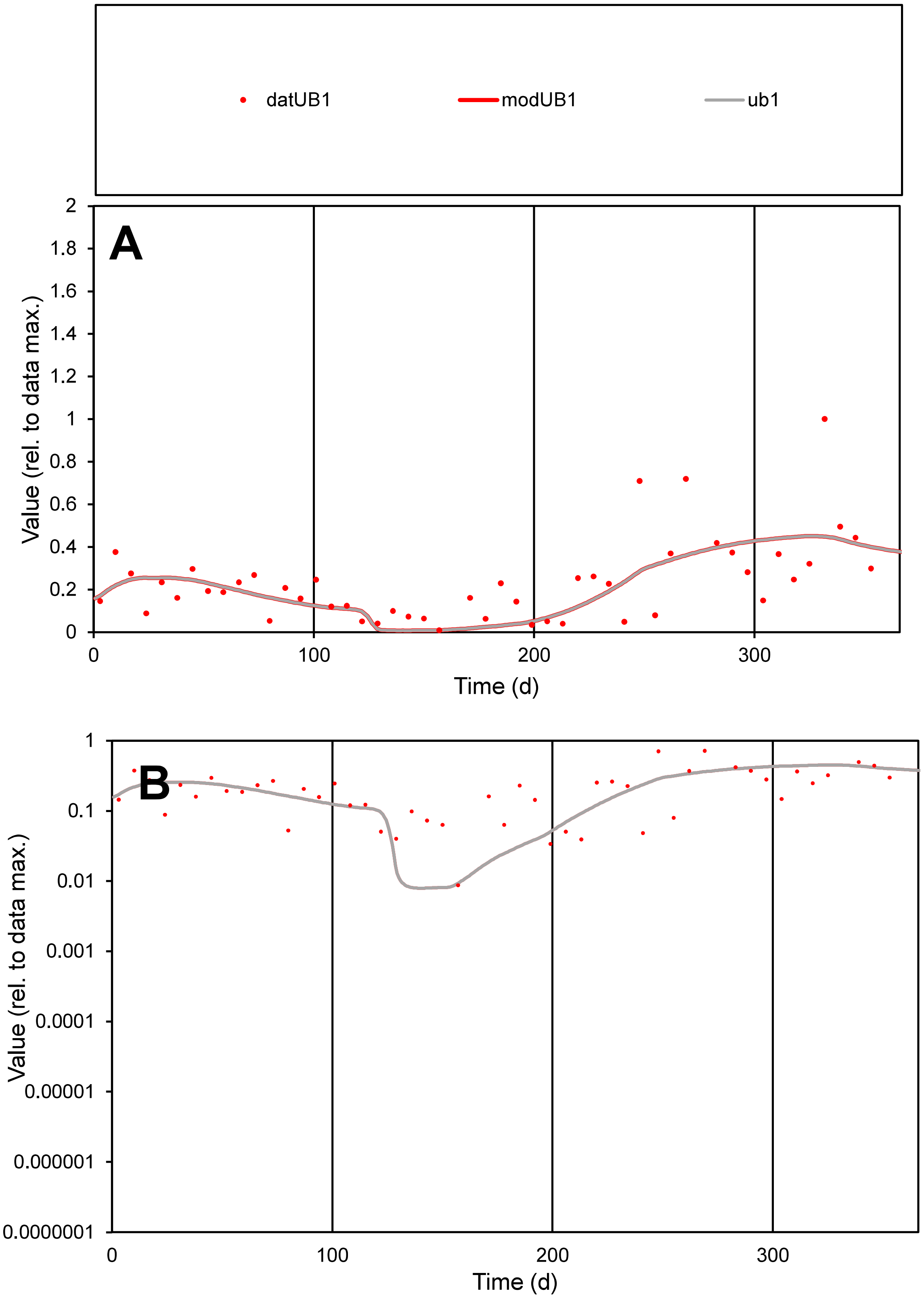

C1

Variables
UB1 Provide jtd or Dst
CalibOnly?
2 1 to reproduce of
oNormalize?
2 1=no, 2=yes

ofxn calc*
0.64002
ofxn mod
0.6534

sRun
RunA1
zout
1

Ave
Data
0.00163
Model
0.00149

data/vMax
0.00695

ofxng
369.894

Min d/m
1.09132

ave/min d
26.8619
ave/min m
26.7543

328_Calib_S_A

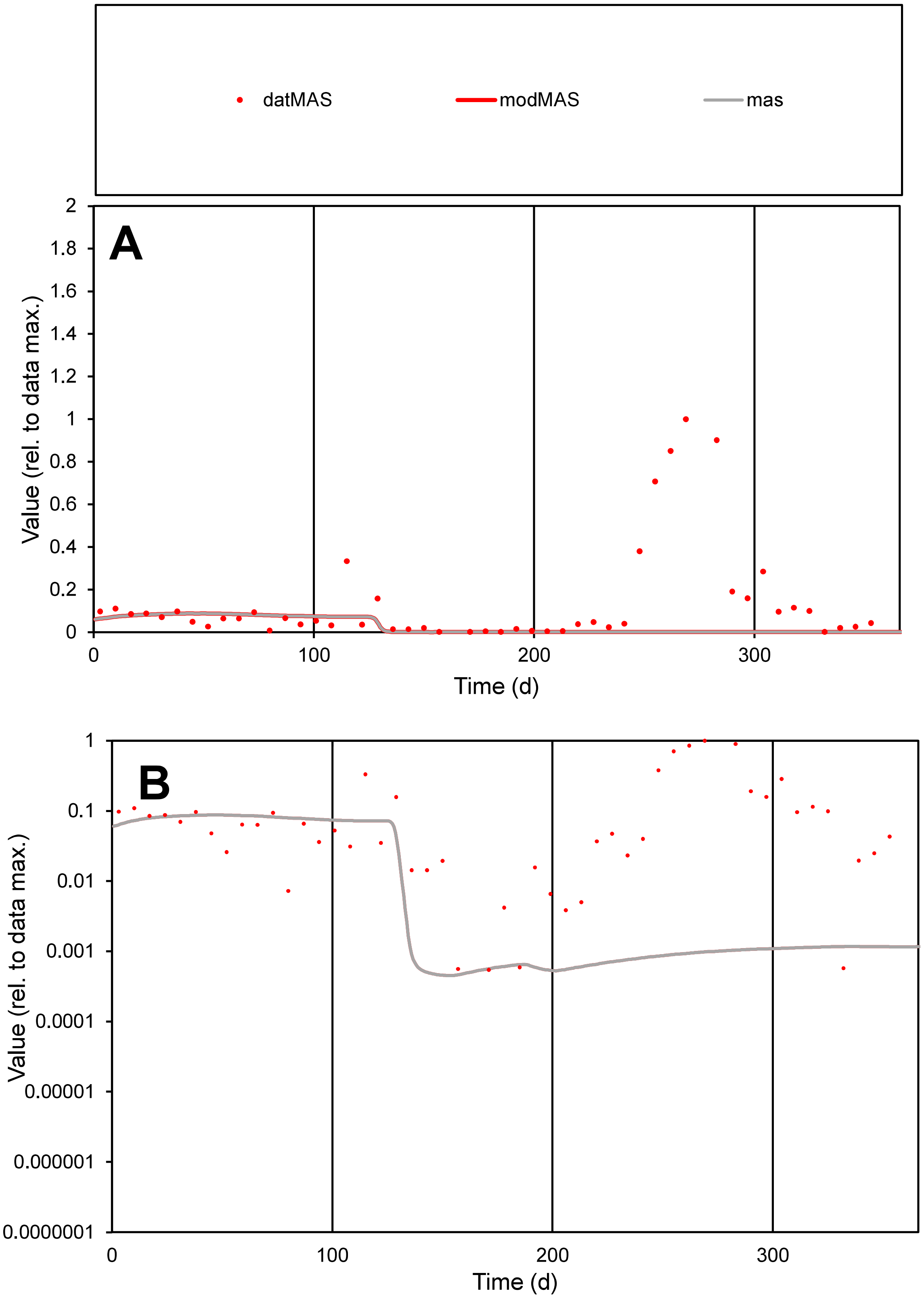

C1

Variables
MAS Provide jtd or Dst
CalibOnly?
2 1 to reproduce of
oNormalize?
2 1=no, 2=yes

ofxn calc*
1.95175
ofxn mod
1.95679

sRun
RunA1
zout
1

Ave
Data
0.00308
Model
0.0007

data/vMax
0.02268

ofxng
369.894

Min d/m
1.19961

ave/min d
250.286
ave/min m
68.27

328_Calib_S_A

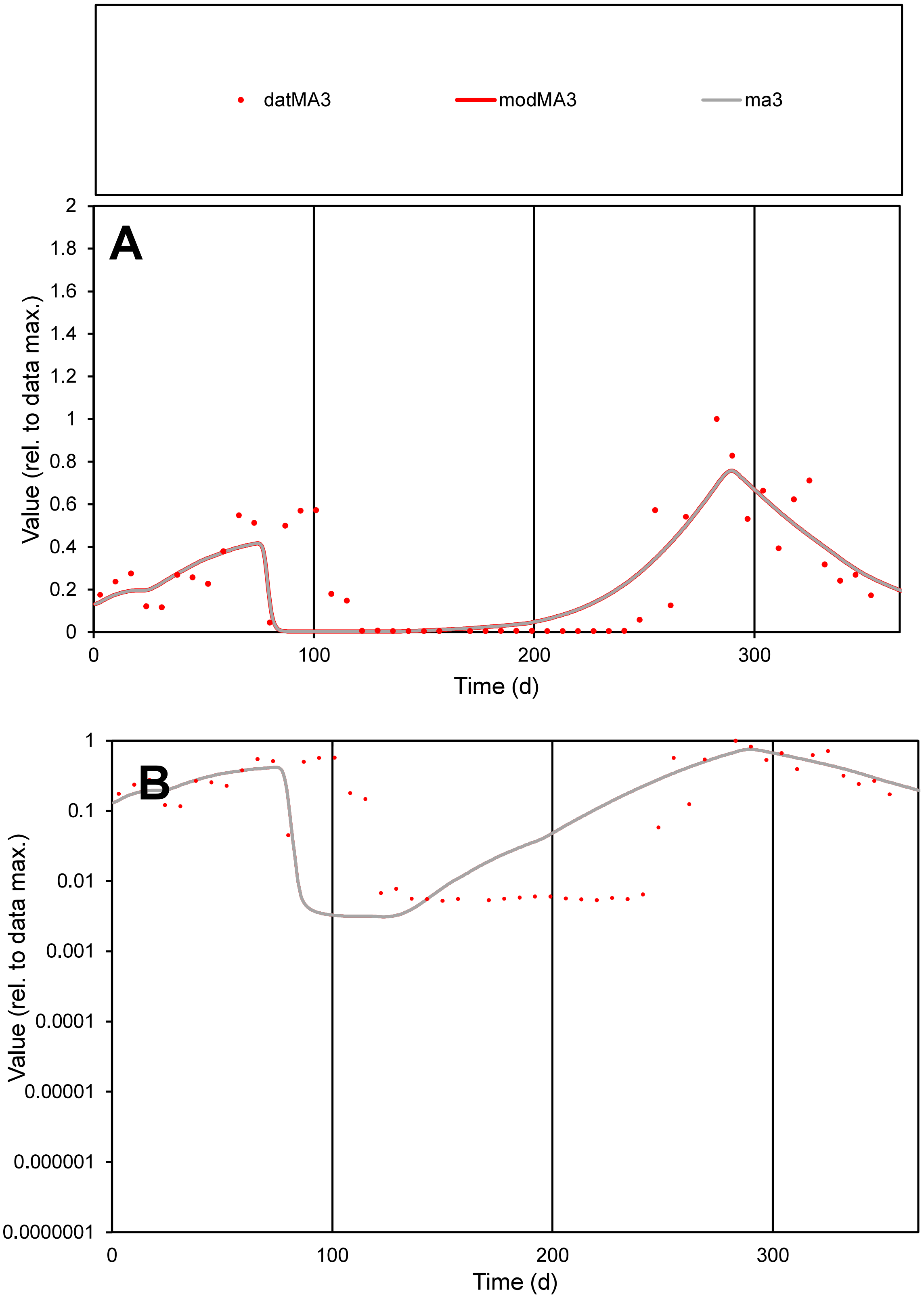

C1

Variables
MA3 Provide jtd or Dst
CalibOnly?
2 1 to reproduce of
oNormalize?
2 1=no, 2=yes

ofxn calc*
0.71614
ofxn mod
0.73286

sRun
RunA1
zout
1

Ave
Data
0.00296
Model
0.00272

data/vMax
0.0118

ofxng
369.894

Min d/m
1.69306

ave/min d
47.701
ave/min m
74.2619

328_Calib_S_A

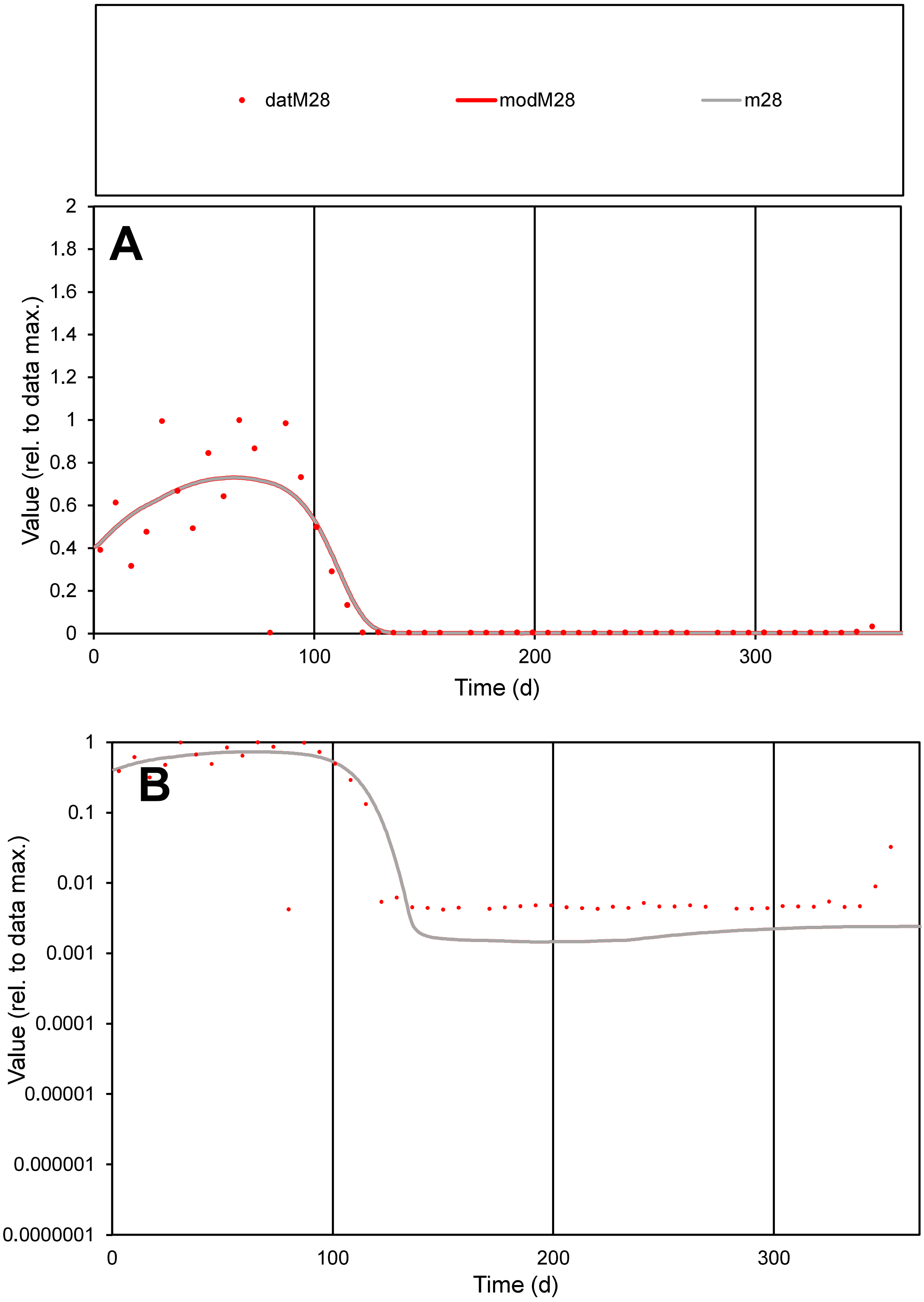

C1

Variables
M28 Provide jtd or Dst
CalibOnly?
2 1 to reproduce of
oNormalize?
2 1=no, 2=yes

ofxn calc*
0.68502
ofxn mod
0.71535

sRun
RunA1
zout
1

Ave
Data
0.00265
Model
0.00265

data/vMax
0.01282

ofxng
369.894

Min d/m
2.88806

ave/min d
49.1741
ave/min m
142.003

328_Calib_S_A

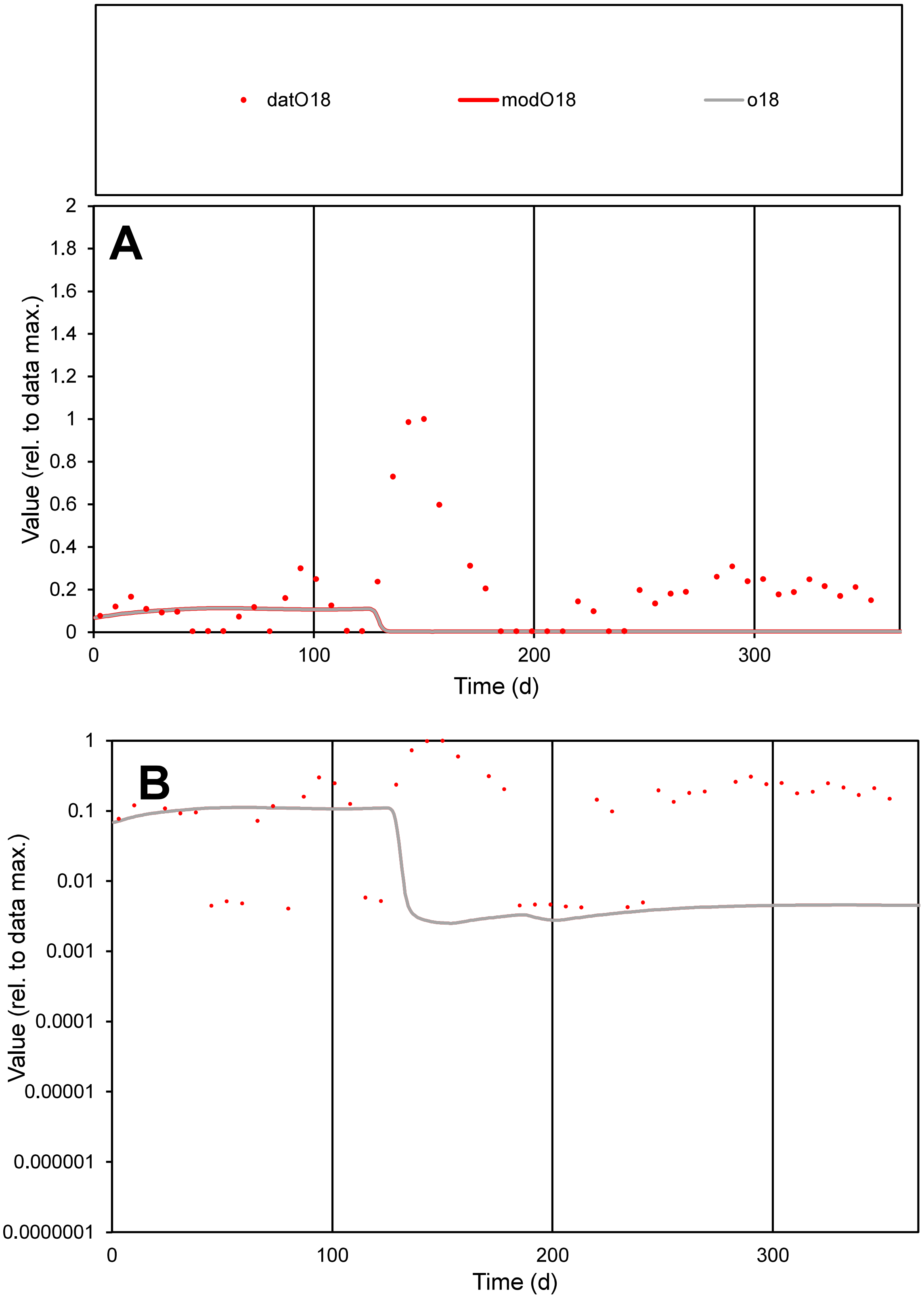

C1

Variables
O18 Provide jtd or Dst
CalibOnly?
2 1 to reproduce of
oNormalize?
2 1=no, 2=yes

ofxn calc*
1.49104
ofxn mod
1.4944

sRun
RunA1
zout
1

Ave
Data
0.00239
Model
0.00054

data/vMax
0.01276

ofxng
369.894

Min d/m
1.61608

ave/min d
46.0685
ave/min m
16.8367

328_Calib_S_A

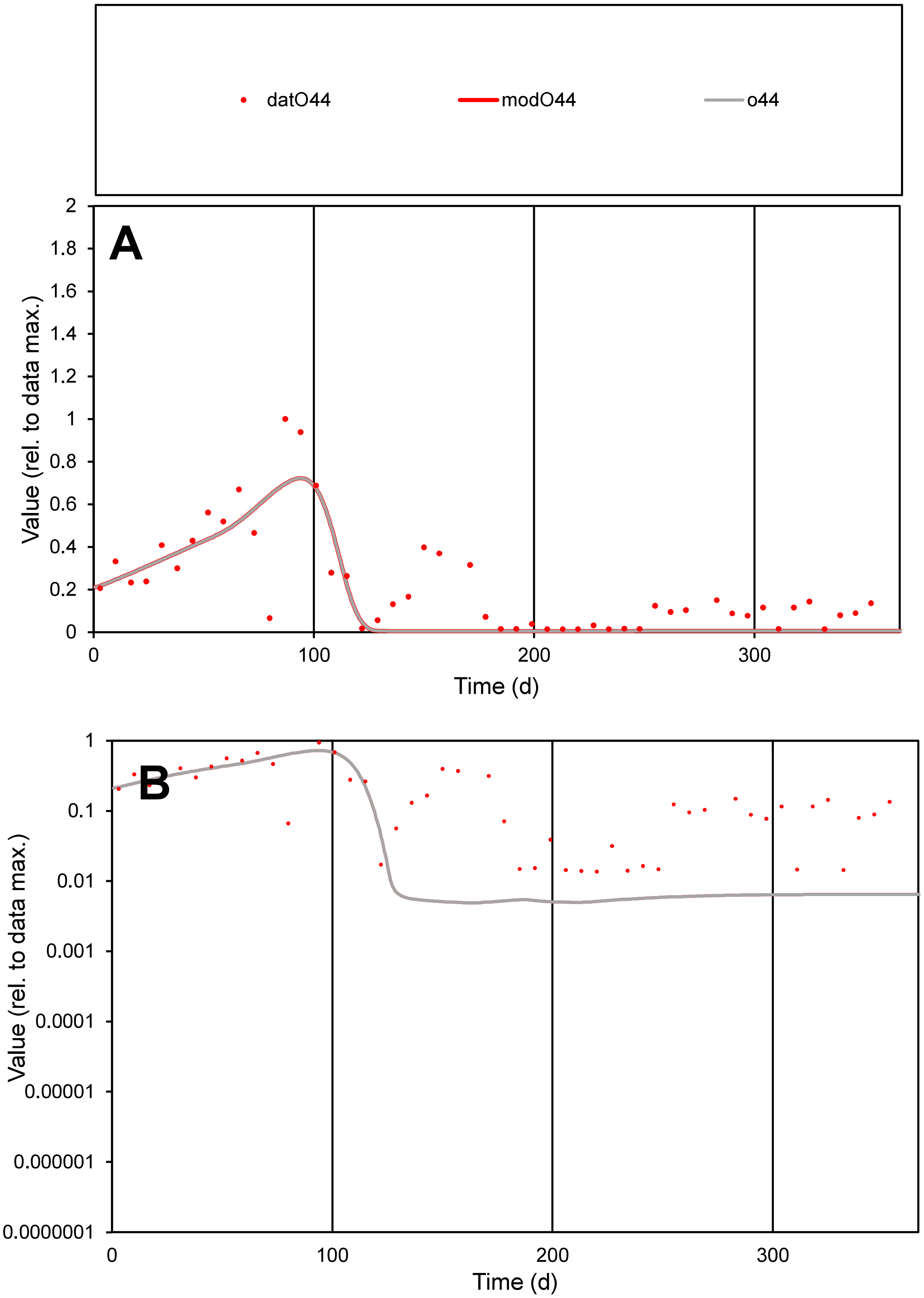

C1

Variables

O44	Provide jtd or Dst
CalibOnly?	
2	1 to reproduce of
oNormalize?	
2	1=no, 2=yes

ofxn calc*
0.7038
ofxn mod
0.72014

sRun
RunA1
zout
1

Ave
Data
0.0018
Model
0.00132

data/vMax
0.00829

ofxng
369.894

Min d/m
2.76502

ave/min d
15.9183
ave/min m
32.3343

328_Calib_S_A

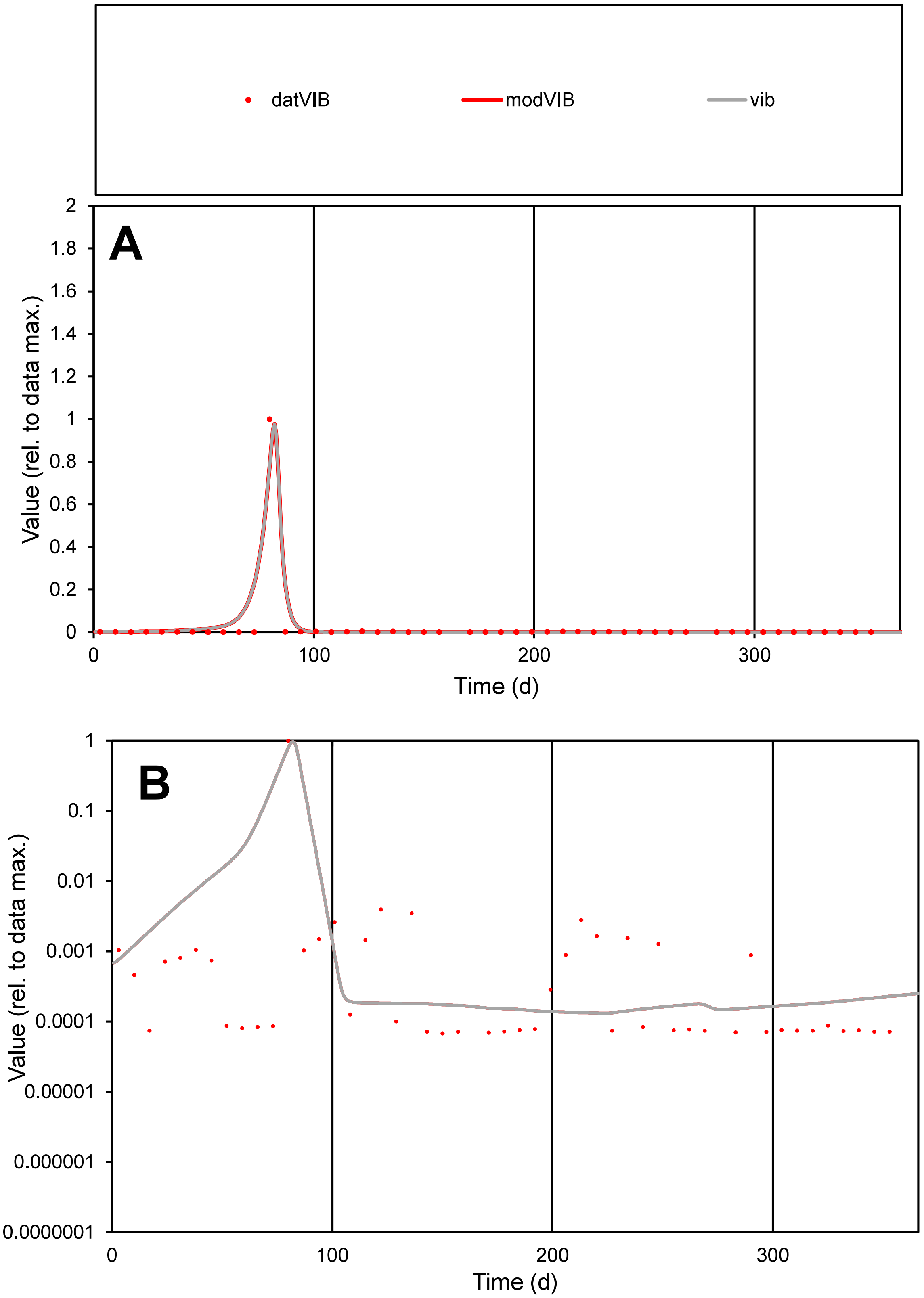

C1

Variables
VIB Provide jtd or Dst
CalibOnly?
2 1 to reproduce of
oNormalize?
2 1=no, 2=yes

ofxn calc*
1.35665
ofxn mod
1.71779

sRun
RunA1
zout
1

Ave
Data
0.0055
Model
0.00704

data/vMax
0.2617

ofxng
369.894

Min d/m
0.51512

ave/min d
310.559
ave/min m
204.524

328_Calib_S_A

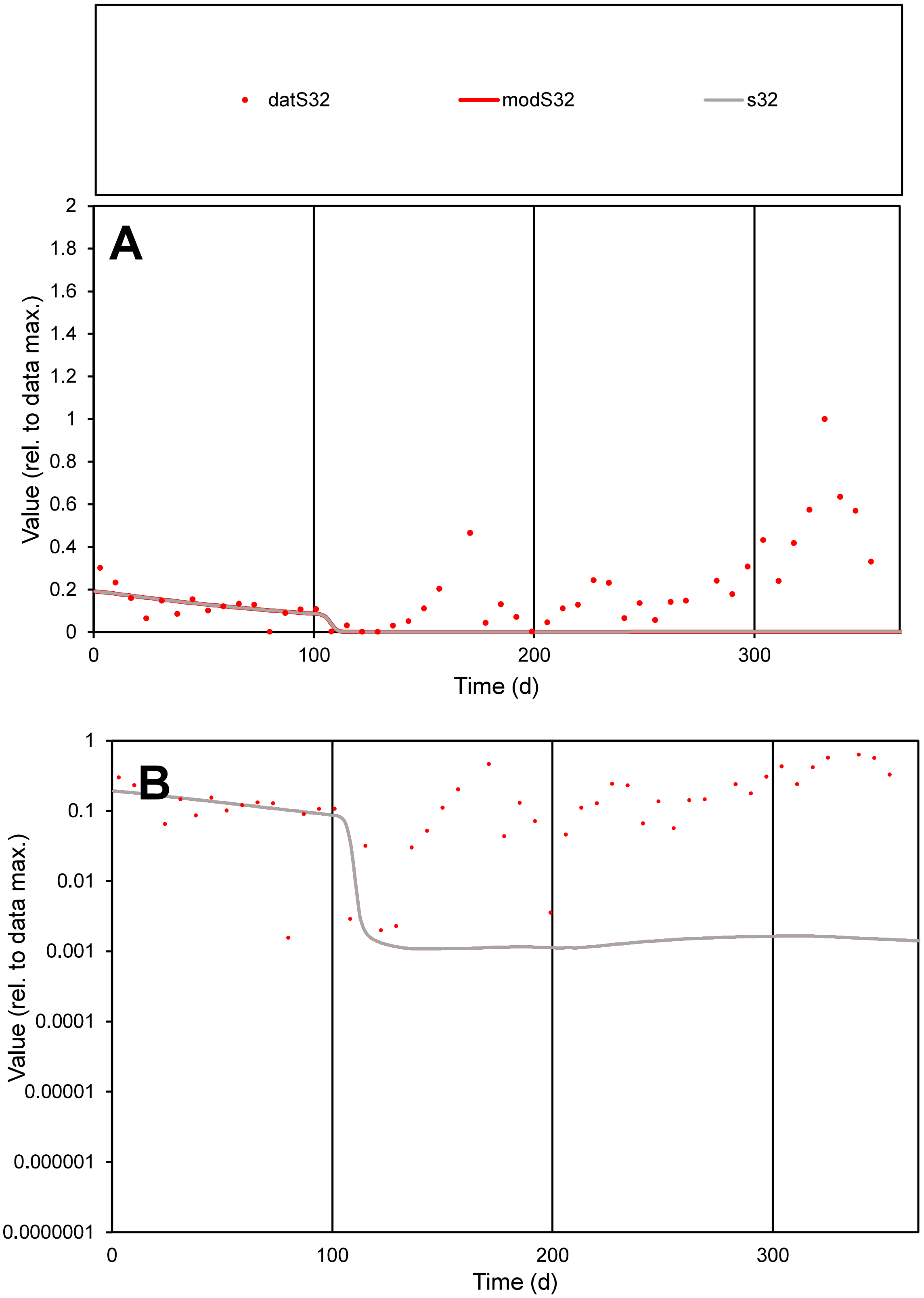

C1

Variables
S32 Provide jtd or Dst
CalibOnly?
2 1 to reproduce of
oNormalize?
2 1=no, 2=yes

ofxn calc*
1.36362
ofxn mod
1.3664

sRun
RunA1
zout
1

Ave
Data
0.00132
Model
0.00029

data/vMax
0.00695

ofxng
369.894

Min d/m
1.42669

ave/min d
122.004
ave/min m
37.7948

328_Calib_S_A

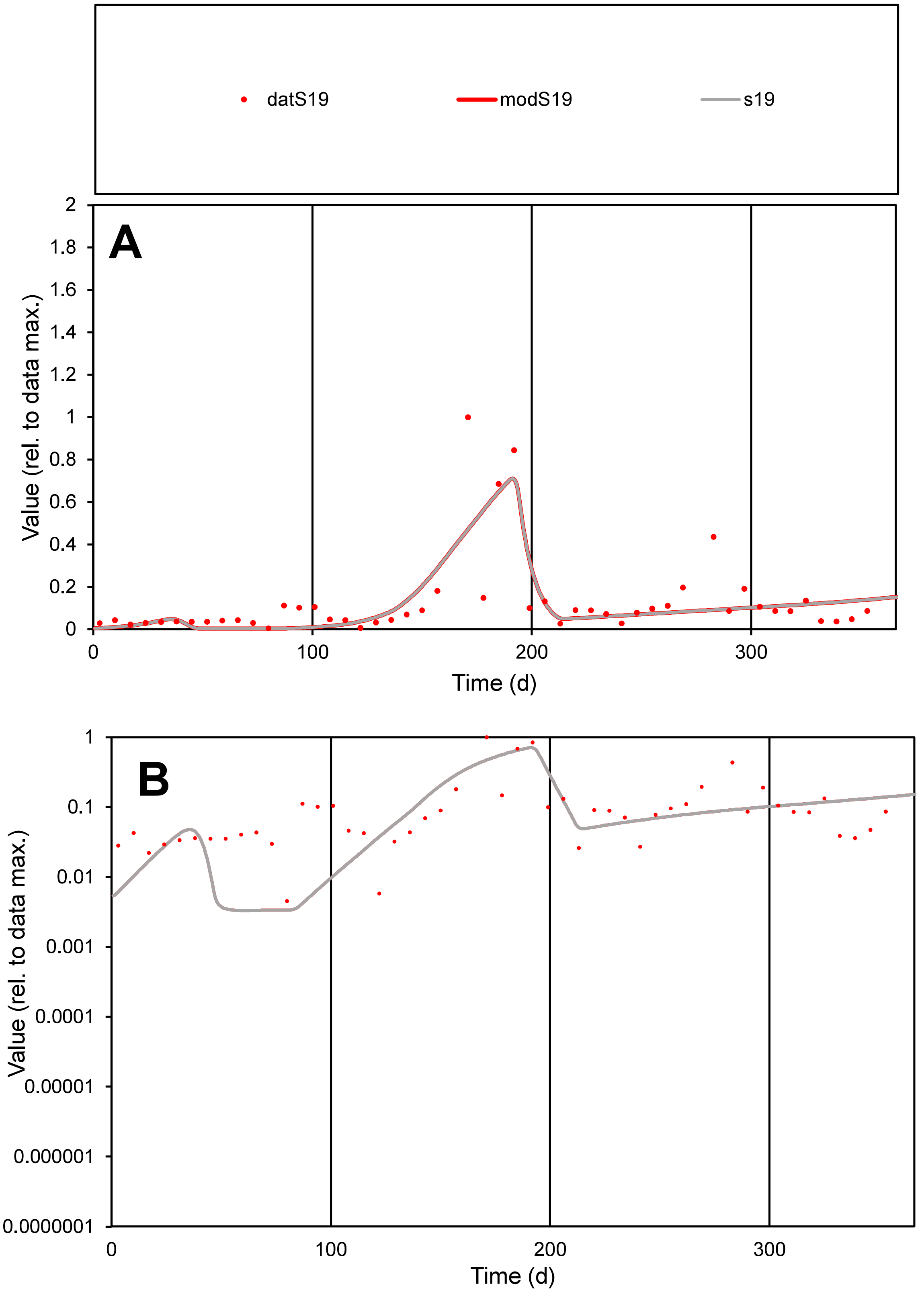

C1

Variables
S19 Provide jtd or Dst
CalibOnly?
2 1 to reproduce of
oNormalize?
2 1=no, 2=yes

ofxn calc*
0.95774
ofxn mod
0.98392

sRun
RunA1
zout
1

Ave
Data
0.00133
Model
0.00122

data/vMax
0.01045

ofxng
369.894

Min d/m
1.36557

ave/min d
28.0048
ave/min m
35.0254

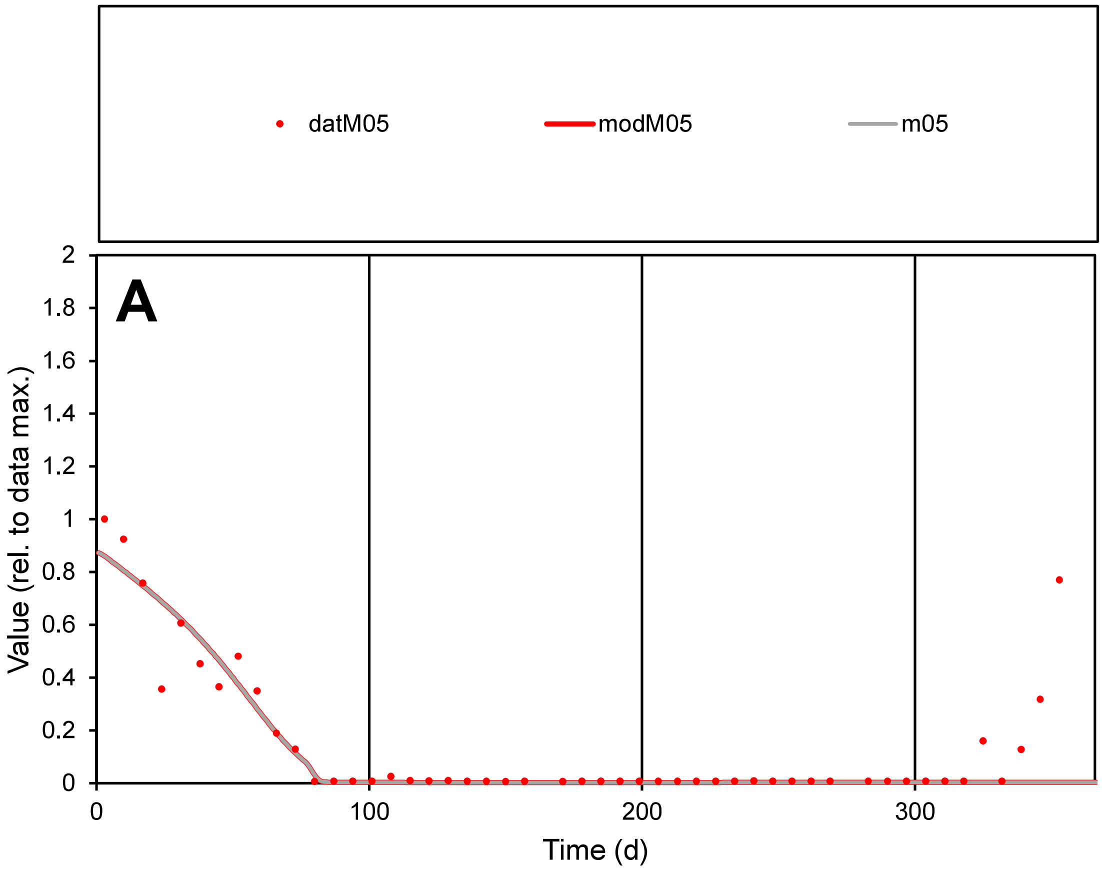

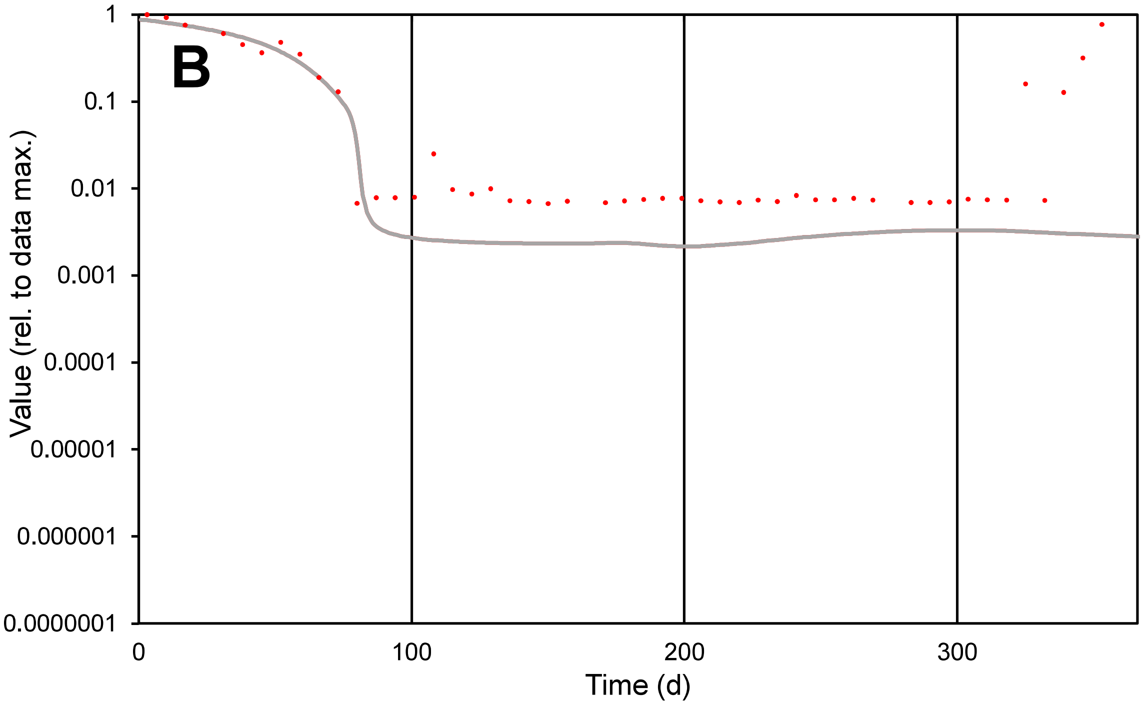

C1

Variables
M05 Provide jtd or Dst
CalibOnly?
2 1 to reproduce of
oNormalize?
2 1=no, 2=yes

ofxn calc*
0.91657
ofxn mod
0.94703

sRun
RunA1
zout
1

Ave
Data
0.00095
Model
0.00074

data/vMax
0.00641

ofxng
369.894

Min d/m
3.11175

ave/min d
22.002
ave/min m
53.3927

328_Calib_S_A

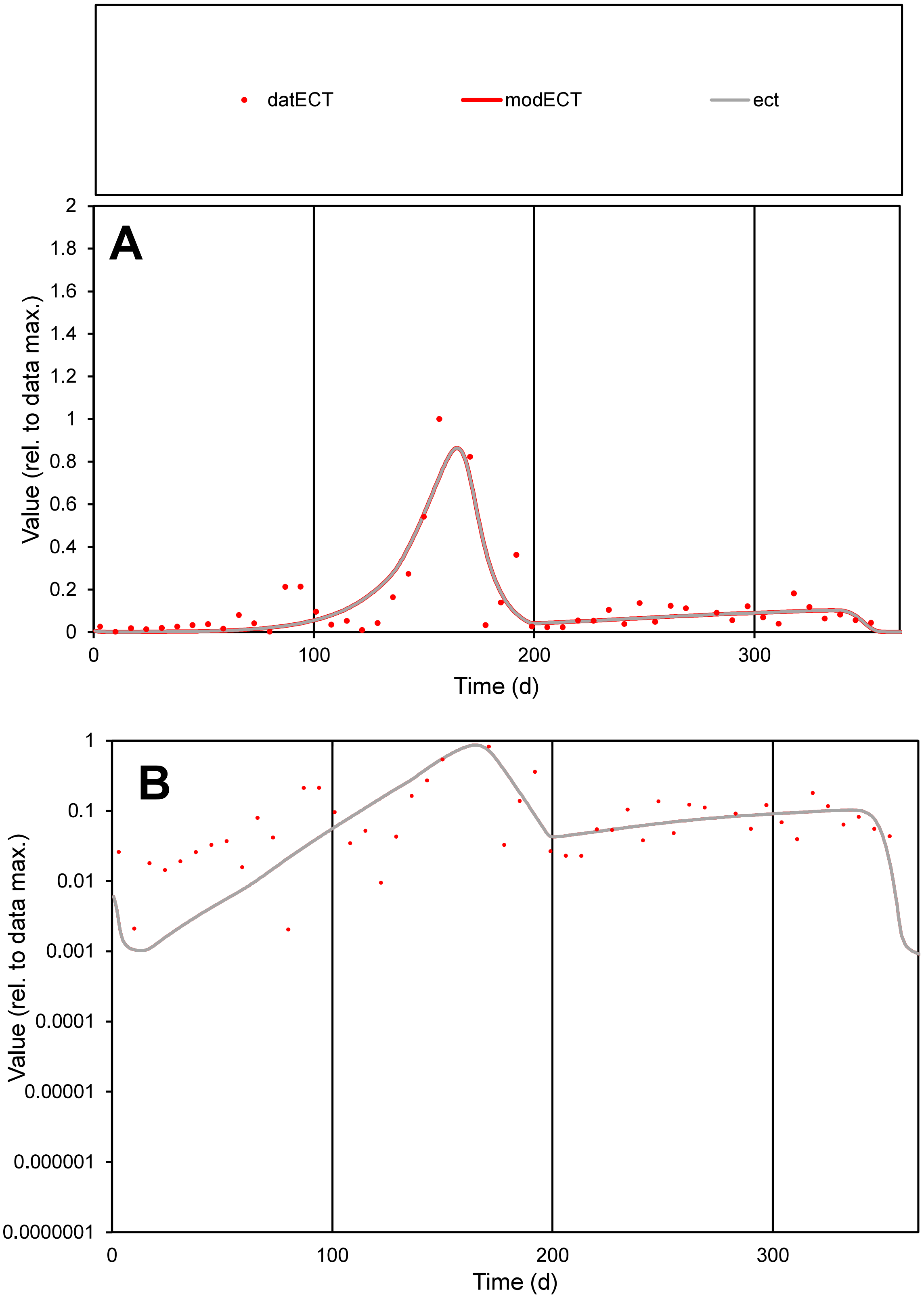

C1

Variables
ECT Provide jtd or Dst
CalibOnly?
2 1 to reproduce of
oNormalize?
2 1=no, 2=yes

ofxn calc*
0.75303
ofxn mod
0.79461

sRun
RunA1
zout
1

Ave
Data
0.0017
Model
0.00161

data/vMax
0.01391

ofxng
369.894

Min d/m
1.98536

ave/min d
59.5983
ave/min m
111.936

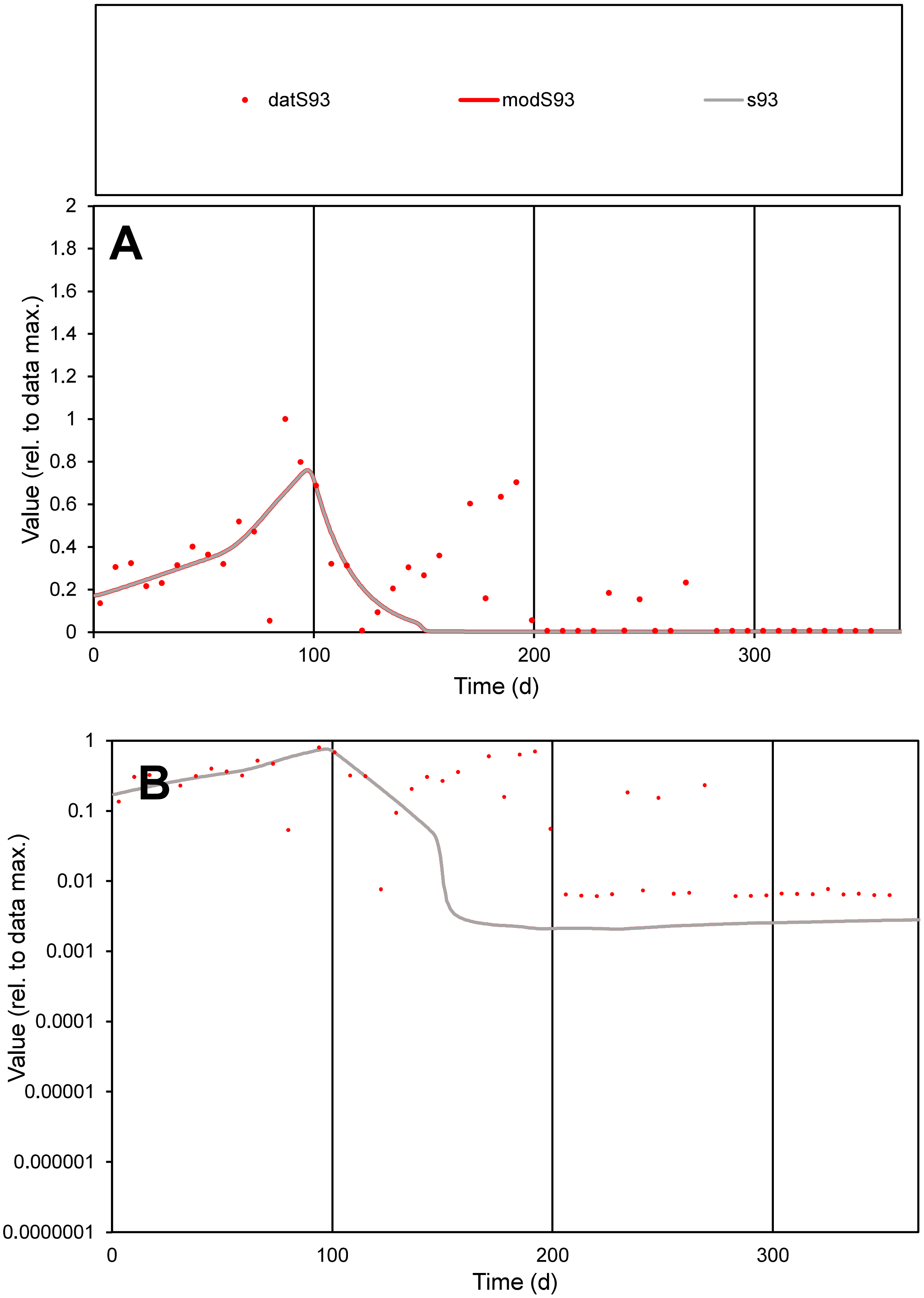

C1

Variables
S93 Provide jtd or Dst
CalibOnly?
2 1 to reproduce of
oNormalize?
2 1=no, 2=yes

ofxn calc*
0.9408
ofxn mod
0.95297

sRun
RunA1
zout
1

Ave
Data
0.00242
Model
0.00164

data/vMax
0.01092

ofxng
369.894

Min d/m
2.95619

ave/min d
36.2158
ave/min m
72.3499

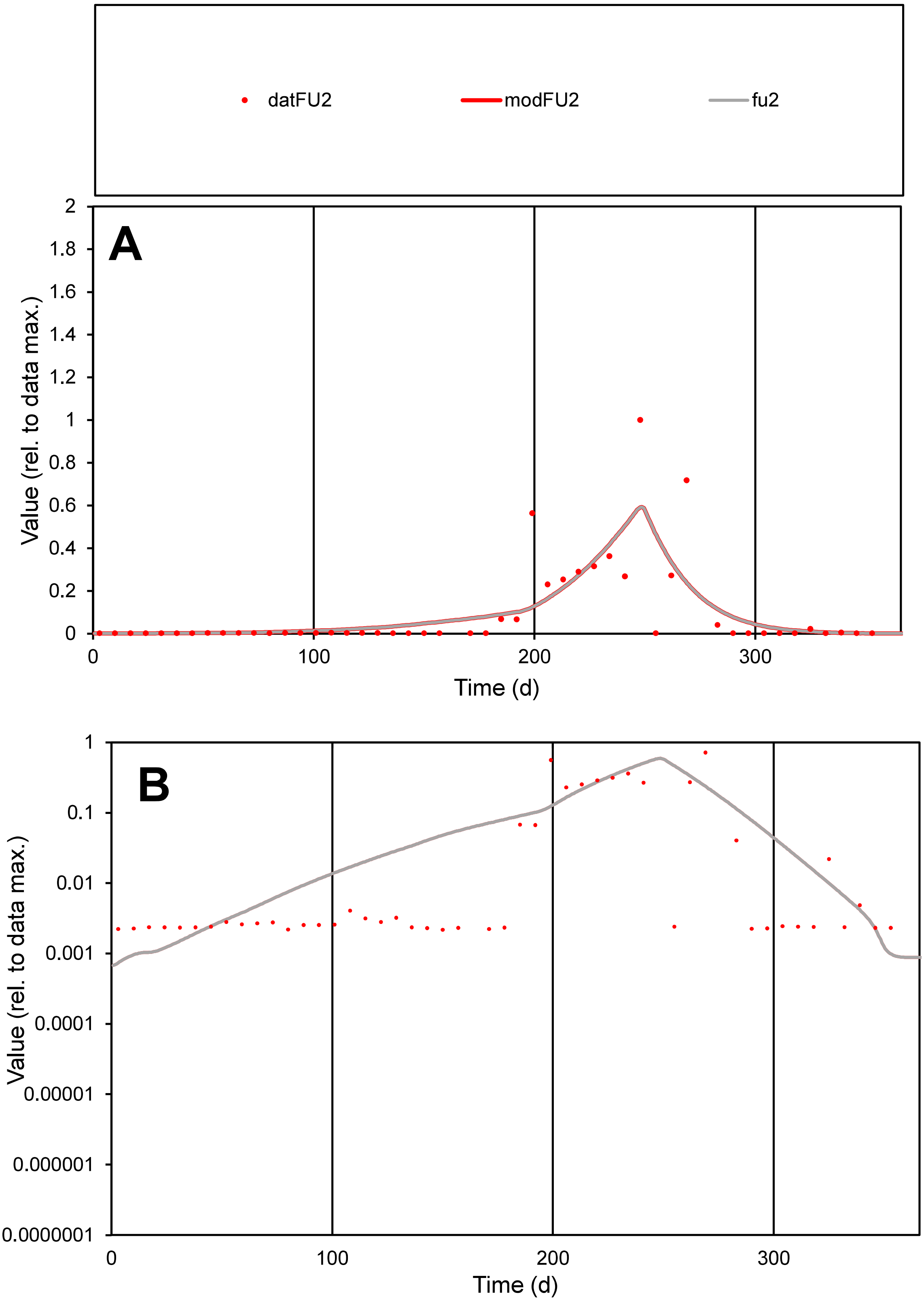

C1

Variables
FU2 Provide jtd or Dst
CalibOnly?
2 1 to reproduce of
oNormalize?
2 1=no, 2=yes

ofxn calc*
1.42312
ofxn mod
1.46391

sRun
RunA1
zout
1

Ave
Data
0.00109
Model
0.00112

data/vMax
0.01169

ofxng
369.894

Min d/m
2.78973

ave/min d
42.5975
ave/min m
121.958

328_Calib_S_A

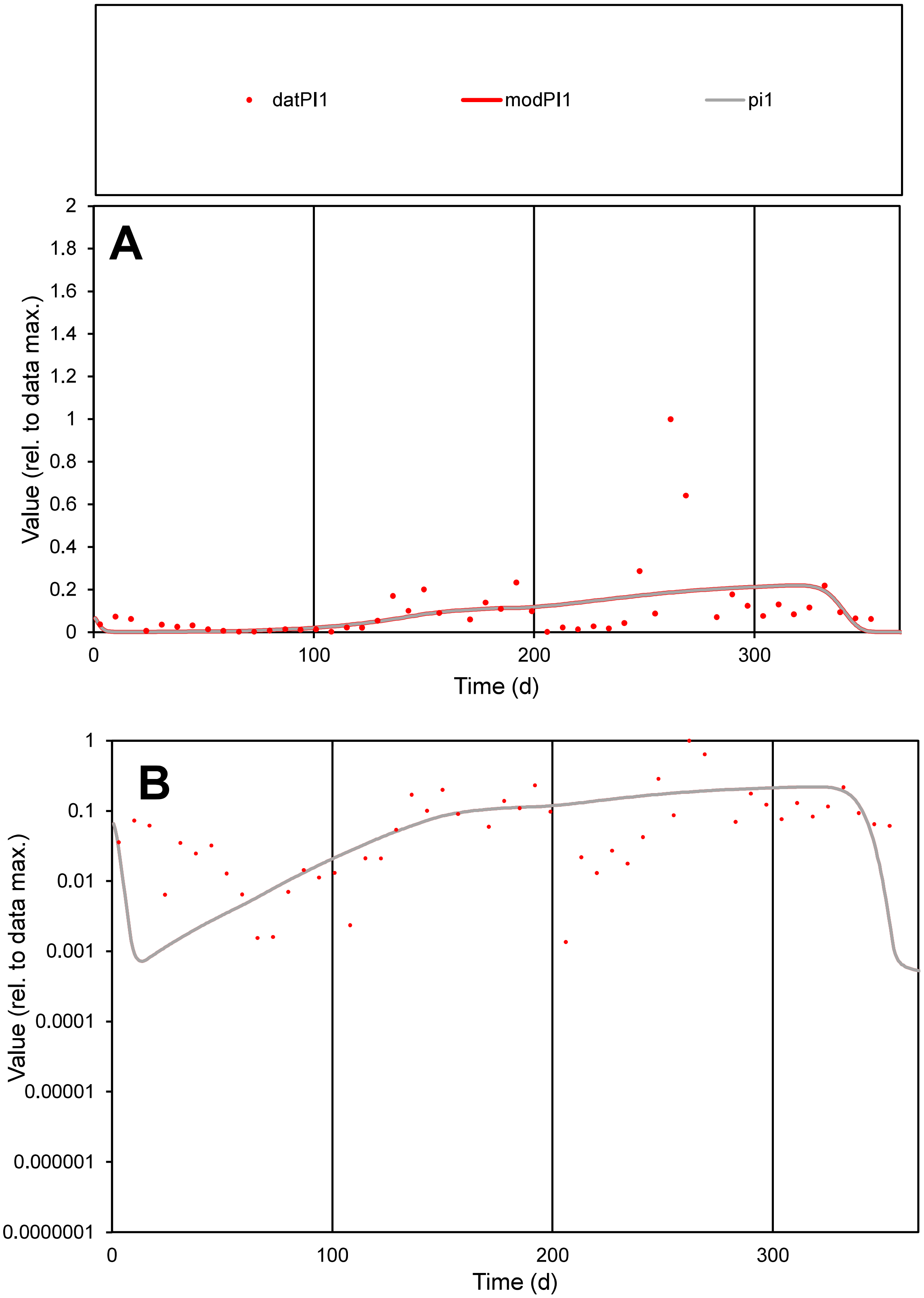

C1

Variables
PI1 Provide jtd or Dst
CalibOnly?
2 1 to reproduce of
oNormalize?
2 1=no, 2=yes

ofxn calc*
1.48343
ofxn mod
1.49243

sRun
RunA1
zout
1

Ave
Data
0.00106
Model
0.00098

data/vMax
0.01042

ofxng
369.894

Min d/m
1.76127

ave/min d
74.7279
ave/min m
121.289

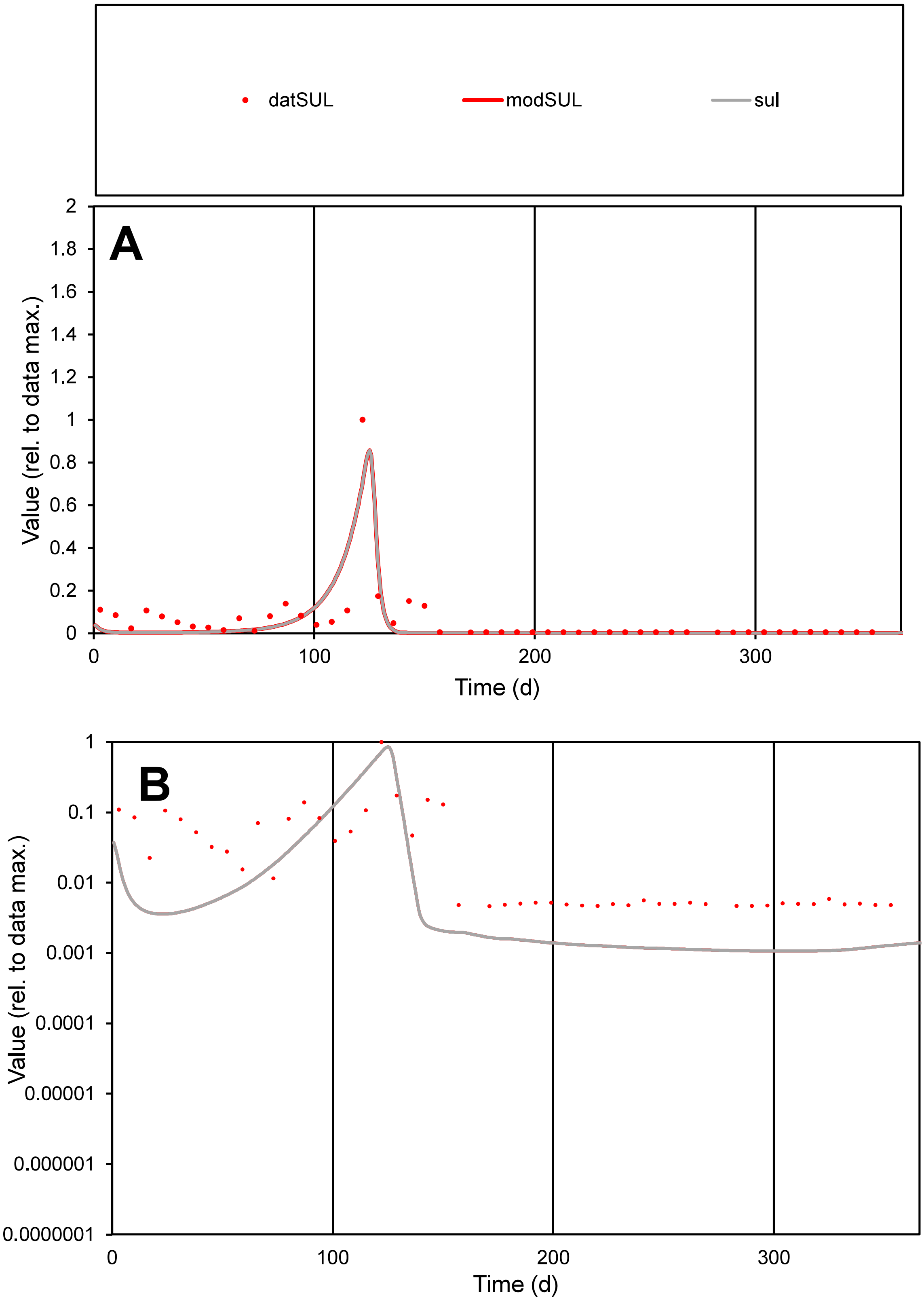

C1

Variables

SUL Provide jtd or Dst

CalibOnly?

2 1 to reproduce of

oNormalize?

2 1=no, 2=yes

ofxn calc*

1.21692

ofxn mod

1.31944

sRun

RunA1

zout

1

Ave

Data

0.00098

Model

0.00073

data/vMax

0.01744

ofxng

369.894

Min d/m

4.36686

ave/min d

12.064

ave/min m

39.3474

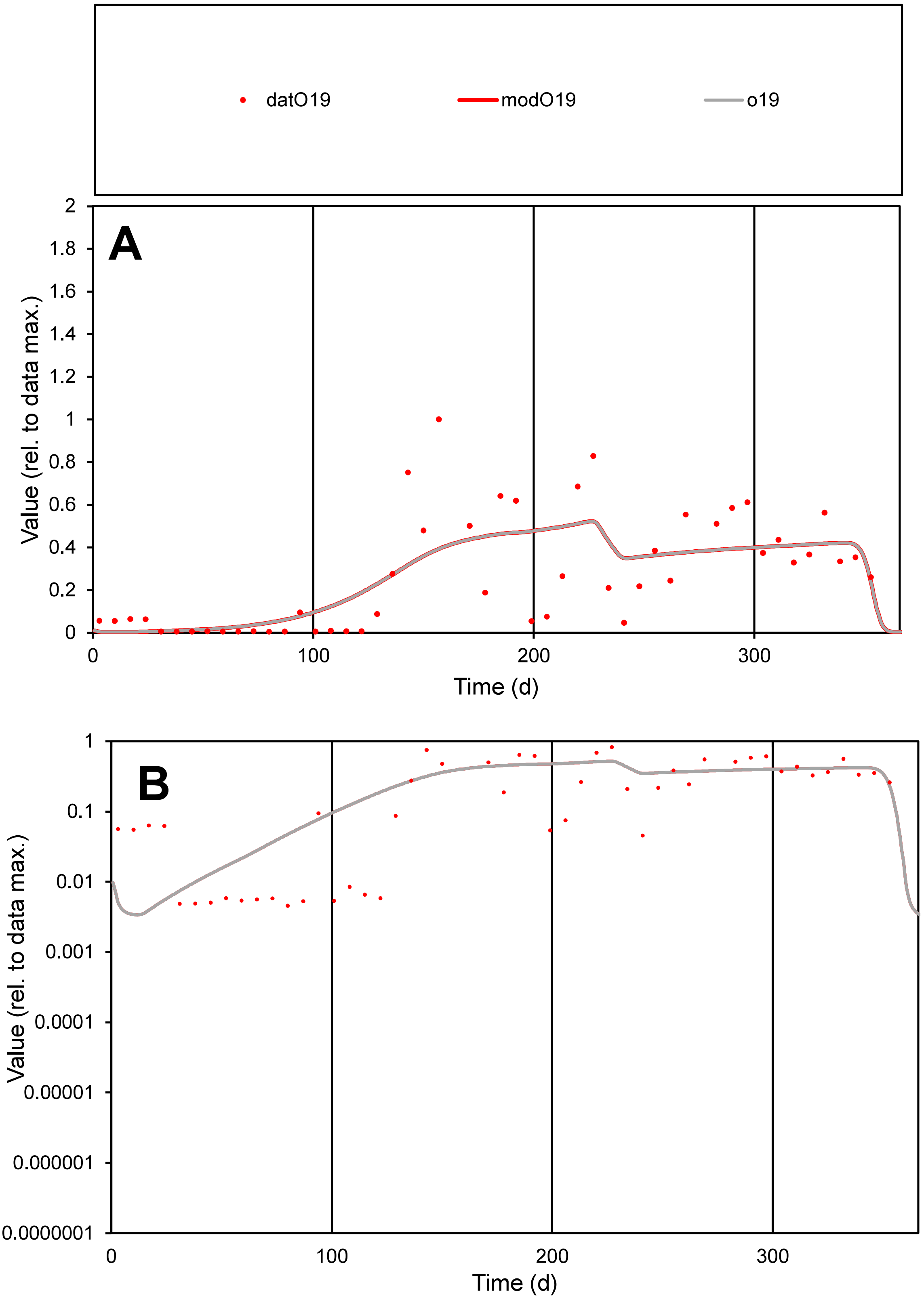

C1

Variables
O19 Provide jtd or Dst
CalibOnly?
2 1 to reproduce of
oNormalize?
2 1=no, 2=yes

ofxn calc*
0.68398
ofxn mod
0.69976

sRun
RunA1
zout
1

Ave
Data
0.00168
Model
0.00169

data/vMax
0.00621

ofxng
369.894

Min d/m
1.35493

ave/min d
58.9111
ave/min m
80.4191

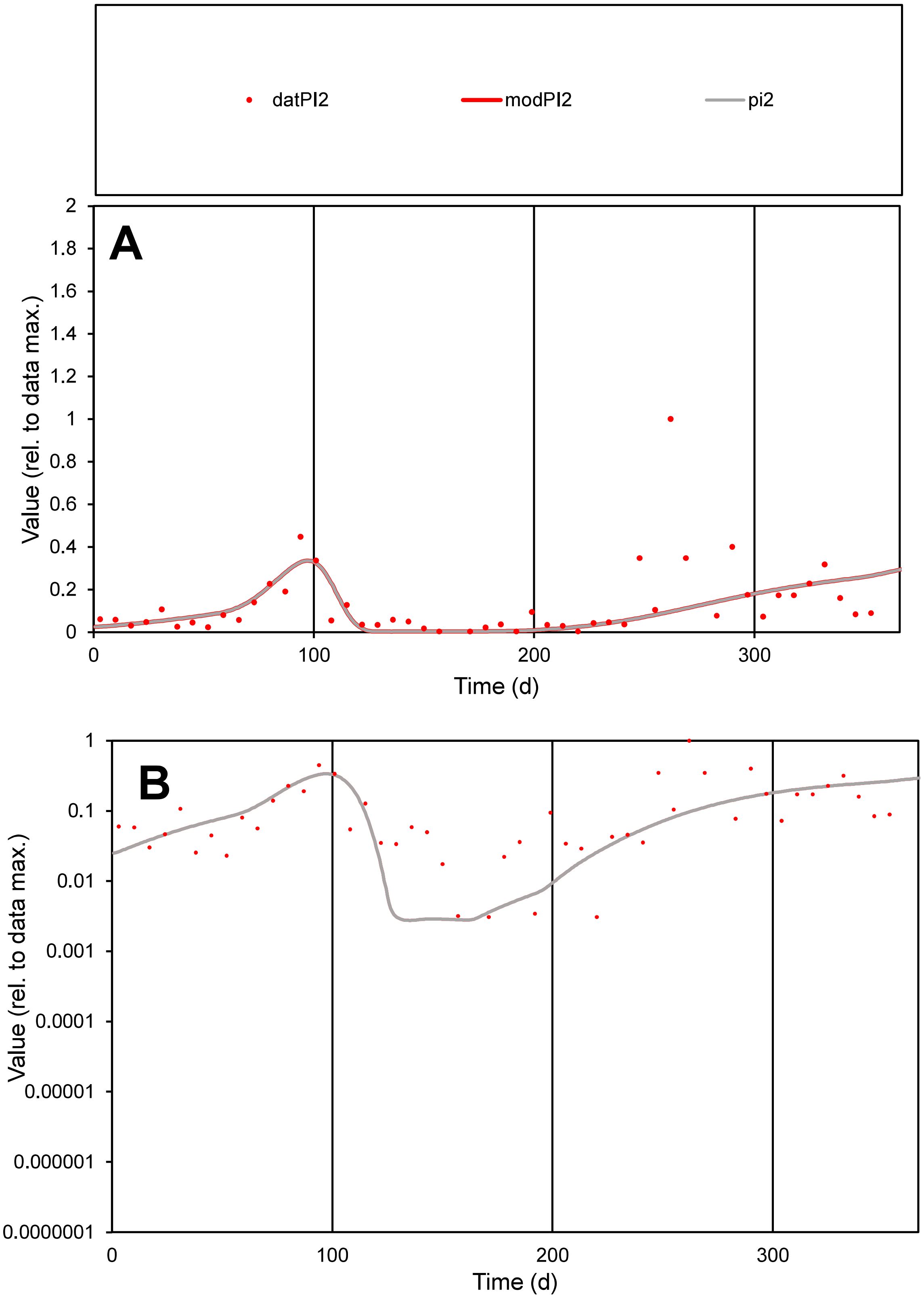

C1

Variables
PI2 Provide jtd or Dst
CalibOnly?
2 1 to reproduce of
oNormalize?
2 1=no, 2=yes

ofxn calc*
1.19063
ofxn mod
1.20007

sRun
RunA1
zout
1

Ave
Data
0.00122
Model
0.001

data/vMax
0.00943

ofxng
369.894

Min d/m
1.10526

ave/min d
42.1791
ave/min m
38.0994

328_Calib_S_A

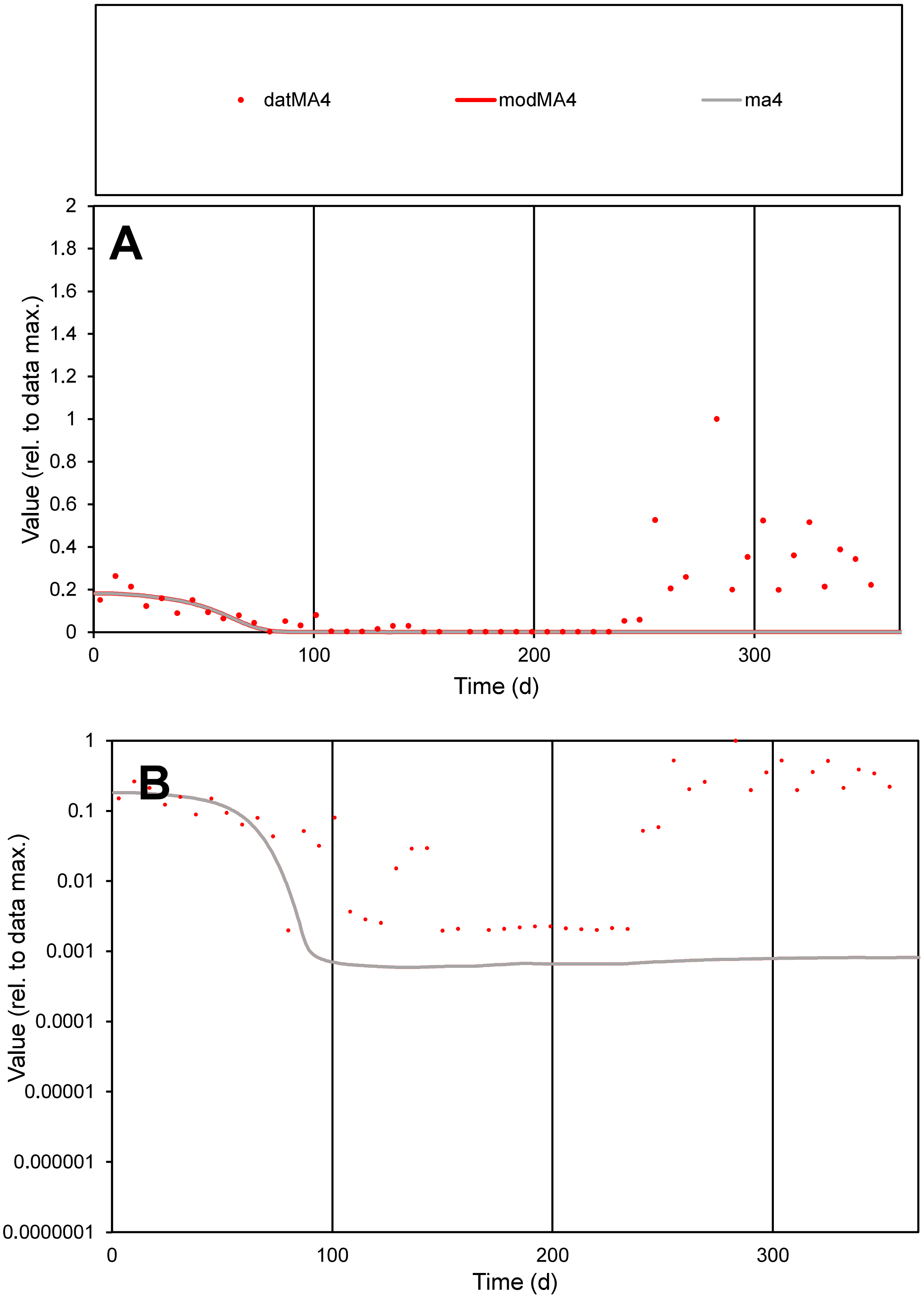

C1

Variables

MA4 Provide jtd or Dst

CalibOnly?

2 1 to reproduce of

oNormalize?

2 1=no, 2=yes

ofxn calc*

1.59972

ofxn mod

1.60374

sRun

RunA1

zout

1

Ave

Data

0.00142

Model

0.00029

data/vMax

0.0098

ofxng

369.894

Min d/m

3.31273

ave/min d

73.8067

ave/min m

49.2845

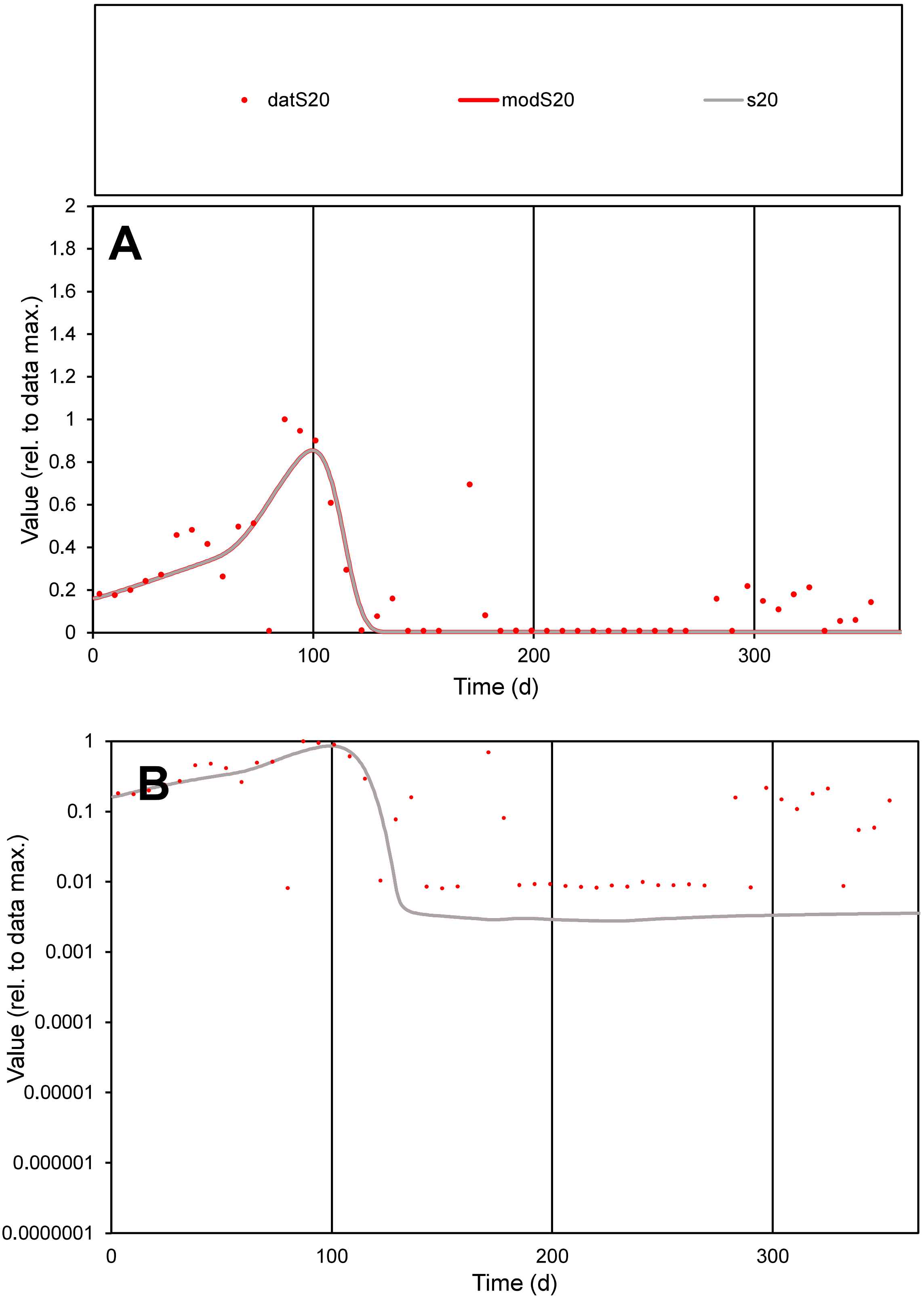

C1

Variables
S20 Provide jtd or Dst
CalibOnly?
2 1 to reproduce of
oNormalize?
2 1=no, 2=yes

ofxn calc*
0.79022
ofxn mod
0.80629

sRun
RunA1
zout
1

Ave
Data
0.00196
Model
0.00149

data/vMax
0.00967

ofxng
369.894

Min d/m
2.92106

ave/min d
25.0716
ave/min m
55.7543

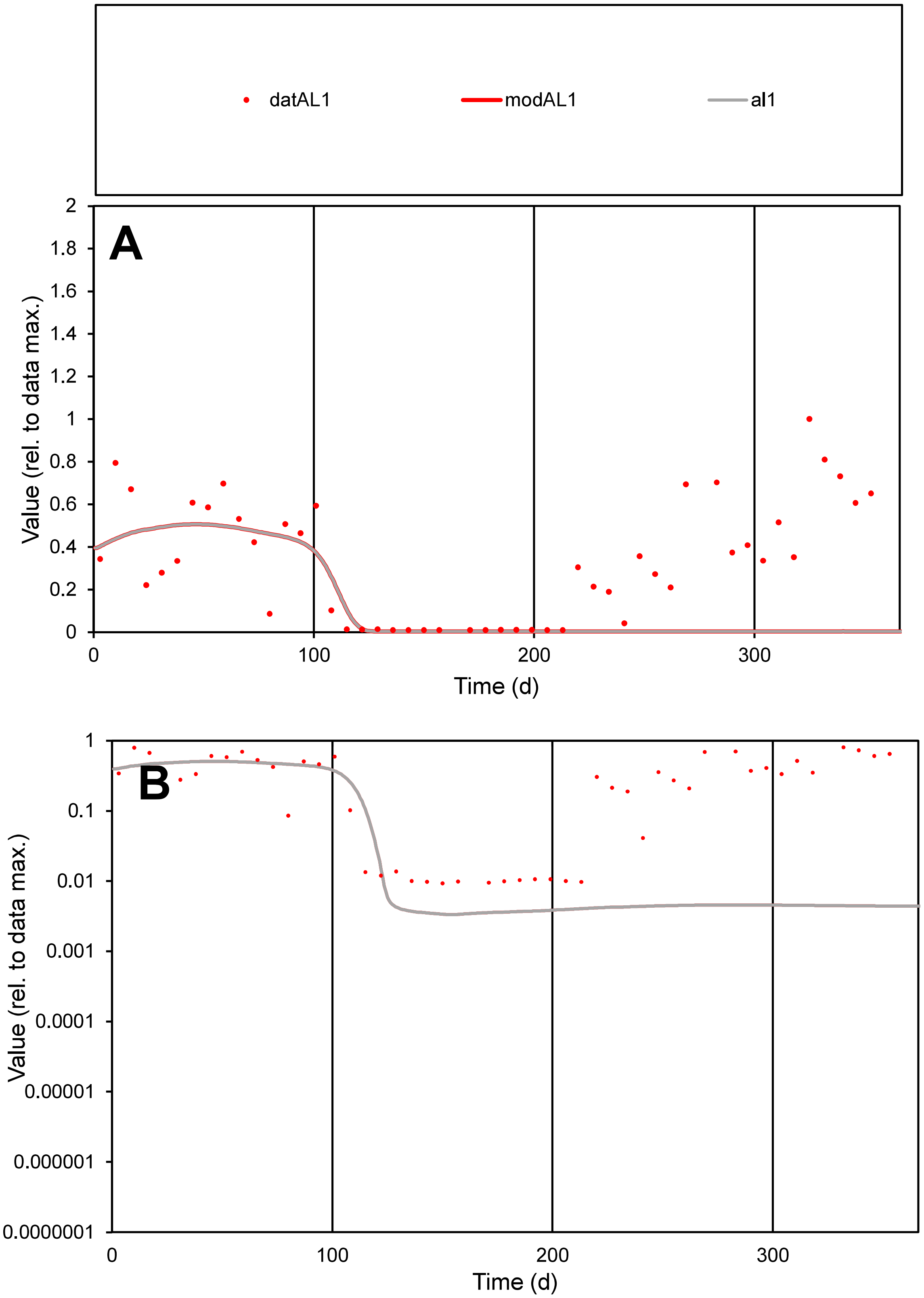

C1

Variables
AL1 Provide jtd or Dst
CalibOnly?
2 1 to reproduce of
oNormalize?
2 1=no, 2=yes

ofxn calc*
1.0342
ofxn mod
1.04041

sRun
RunA1
zout
1

Ave
Data
0.00156
Model
0.00071

data/vMax
0.00472

ofxng
369.894

Min d/m
2.76749

ave/min d
35.4075
ave/min m
44.4926

328_Calib_S_A

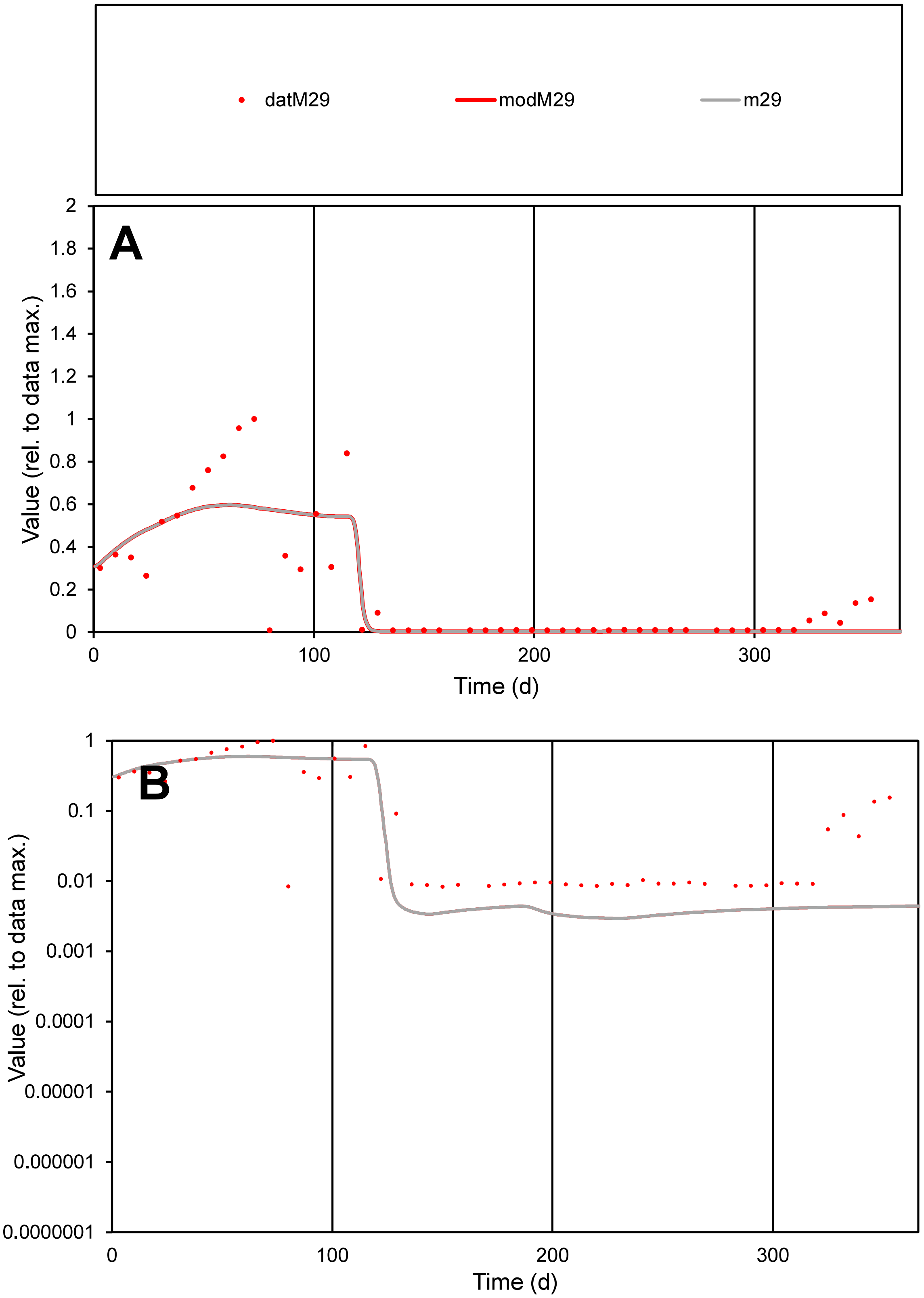

C1

Variables
M29 Provide jtd or Dst
CalibOnly?
2 1 to reproduce of
oNormalize?
2 1=no, 2=yes

ofxn calc*
0.75231
ofxn mod
0.77539

sRun
RunA1
zout
1

Ave
Data
0.00129
Model
0.00122

data/vMax
0.00652

ofxng
369.894

Min d/m
2.84439

ave/min d
23.7665
ave/min m
63.6787

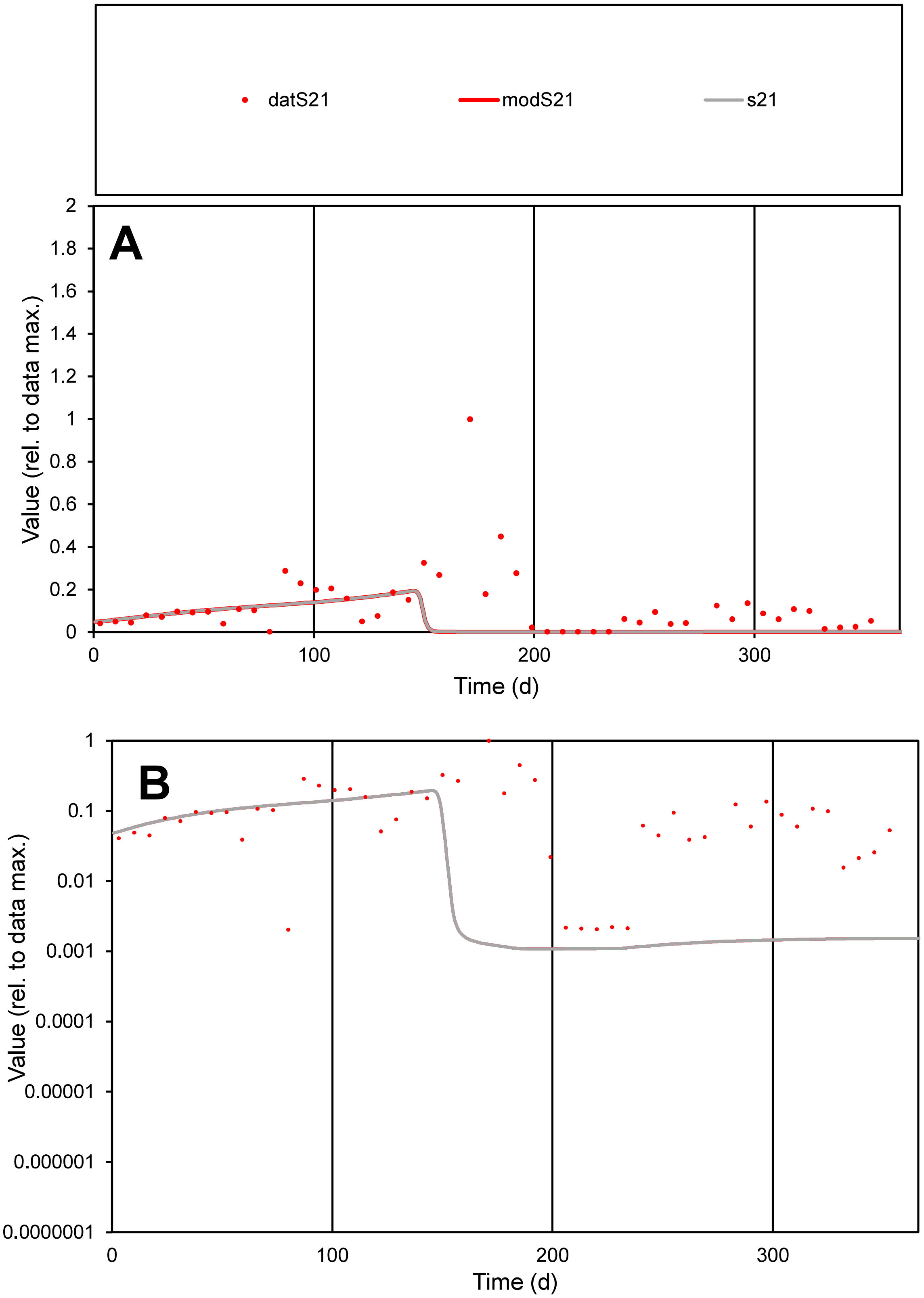

C1

Variables
S21 Provide jtd or Dst
CalibOnly?
2 1 to reproduce of
oNormalize?
2 1=no, 2=yes

ofxn calc*
1.47963
ofxn mod
1.48822

sRun
RunA1
zout
1

Ave
Data
0.00247
Model
0.00112

data/vMax
0.02028

ofxng
369.894

Min d/m
1.87768

ave/min d
60.0418
ave/min m
50.9282

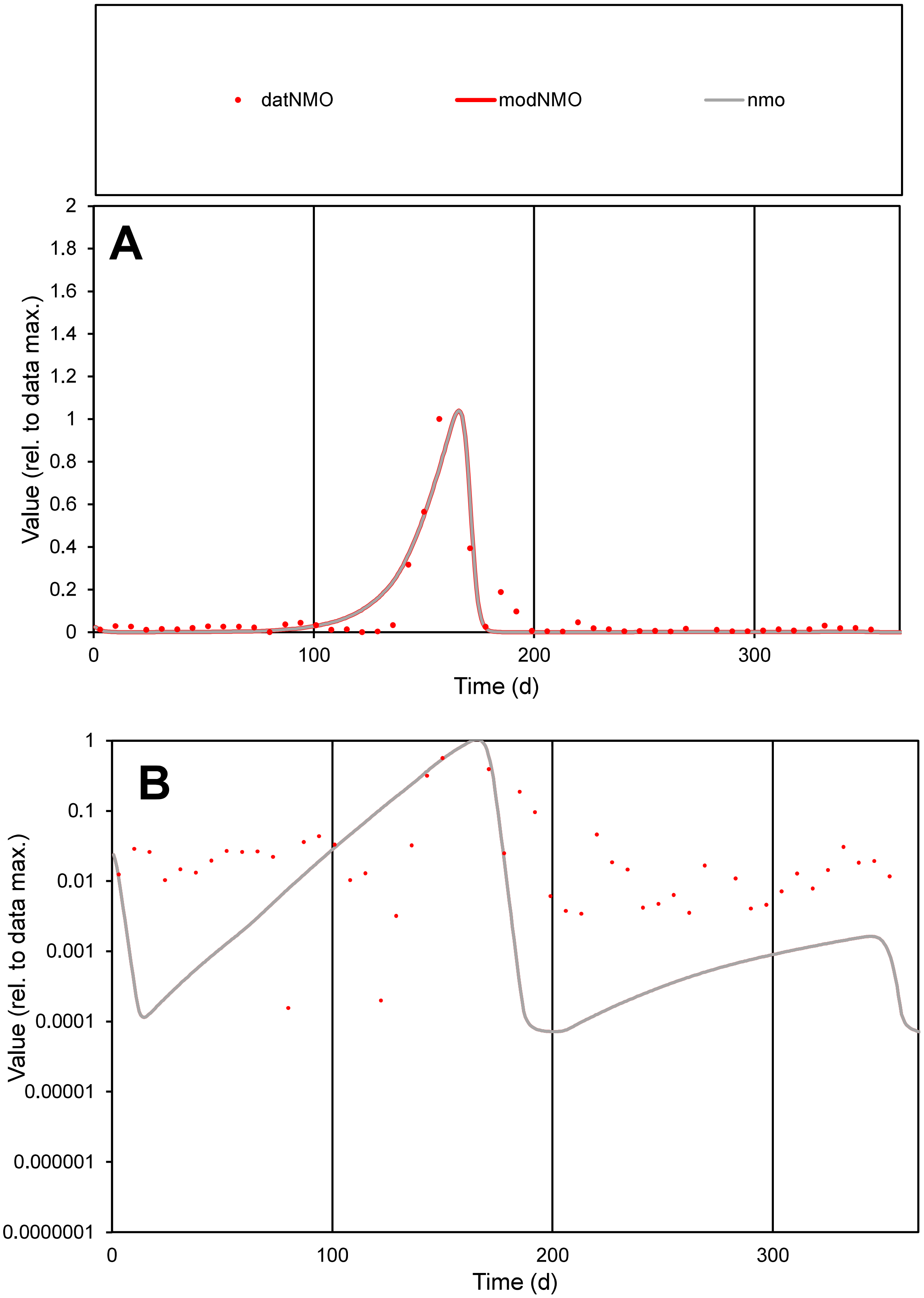

C1

Variables
NMO Provide jtd or Dst
CalibOnly?
2 1 to reproduce of
oNormalize?
2 1=no, 2=yes

ofxn calc*
0.87317
ofxn mod
0.94234

sRun
RunA1
zout
1

Ave
Data
0.00309
Model
0.00276

data/vMax
0.04652

ofxng
369.894

Min d/m
2.173

ave/min d
425.404
ave/min m
827.283

328_Calib_S_A

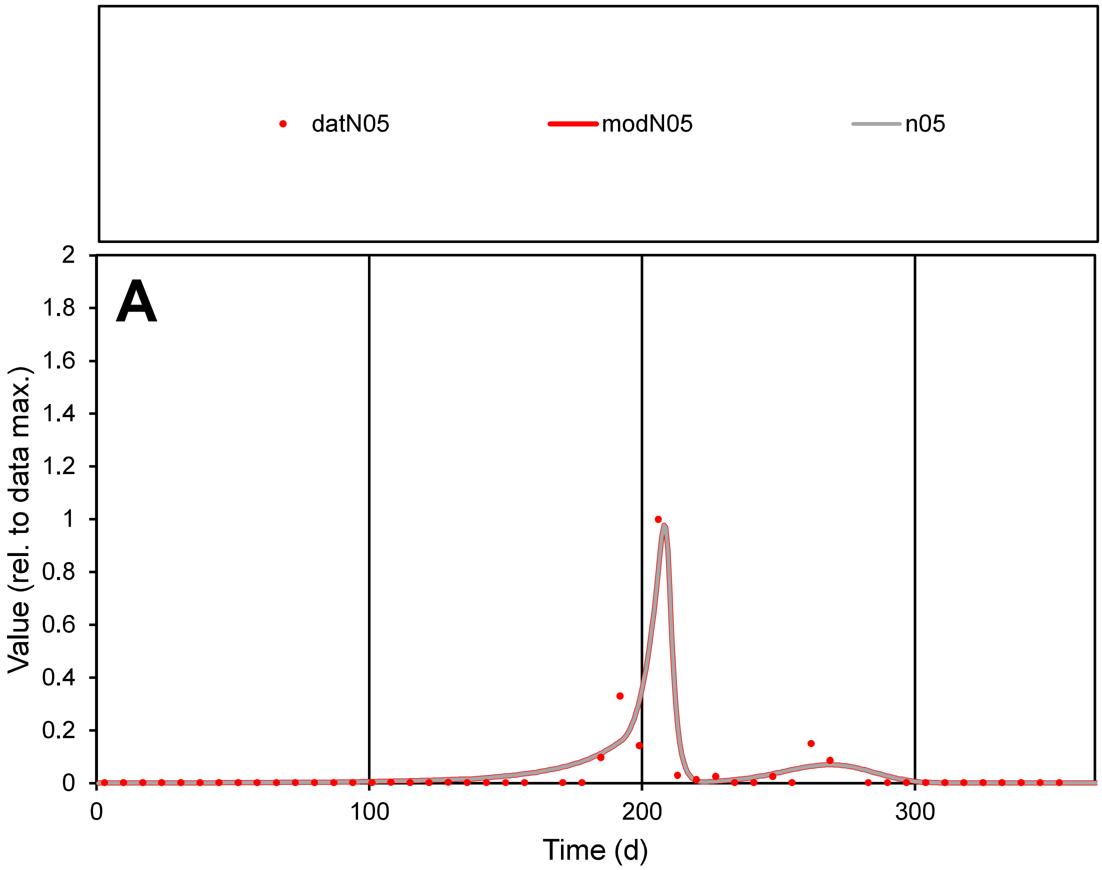

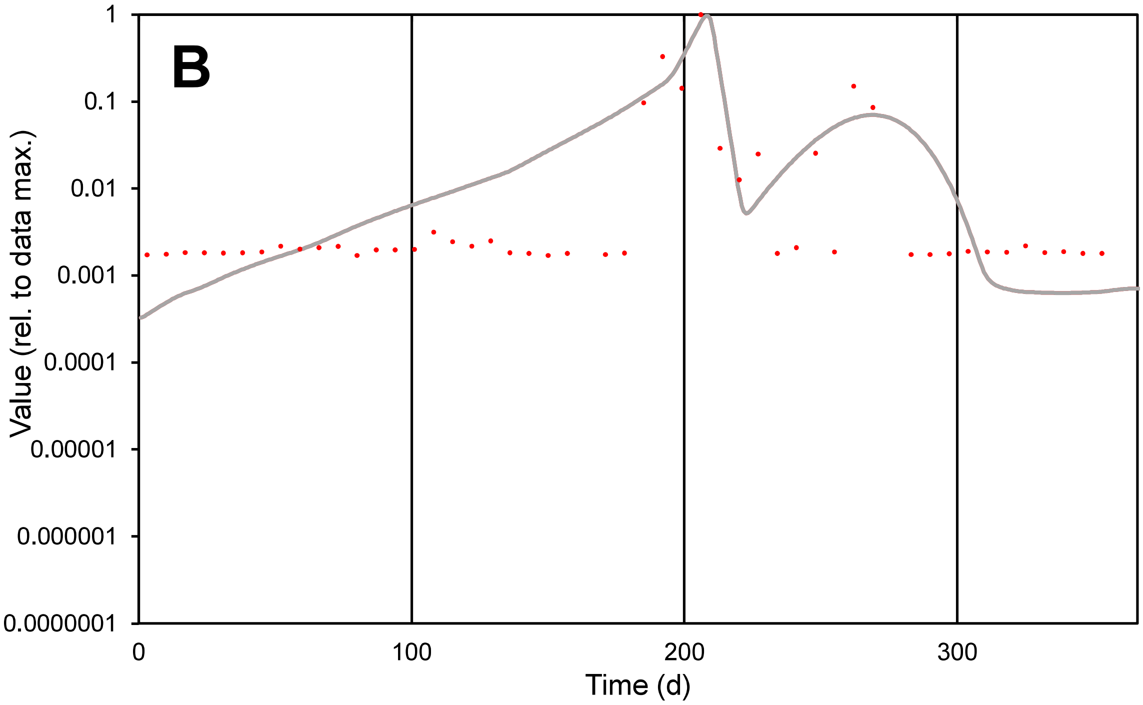

C1

Variables

N05 Provide jtd or Dst

CalibOnly?

2 1 to reproduce of

oNormalize?

2 1=no, 2=yes

ofxn calc*

1.05682

ofxn mod

1.1527

sRun

RunA1

zout

1

Ave

Data

0.00095

Model

0.0011

data/vMax

0.02349

ofxng

369.894

Min d/m

4.49431

ave/min d

23.7188

ave/min m

123.49

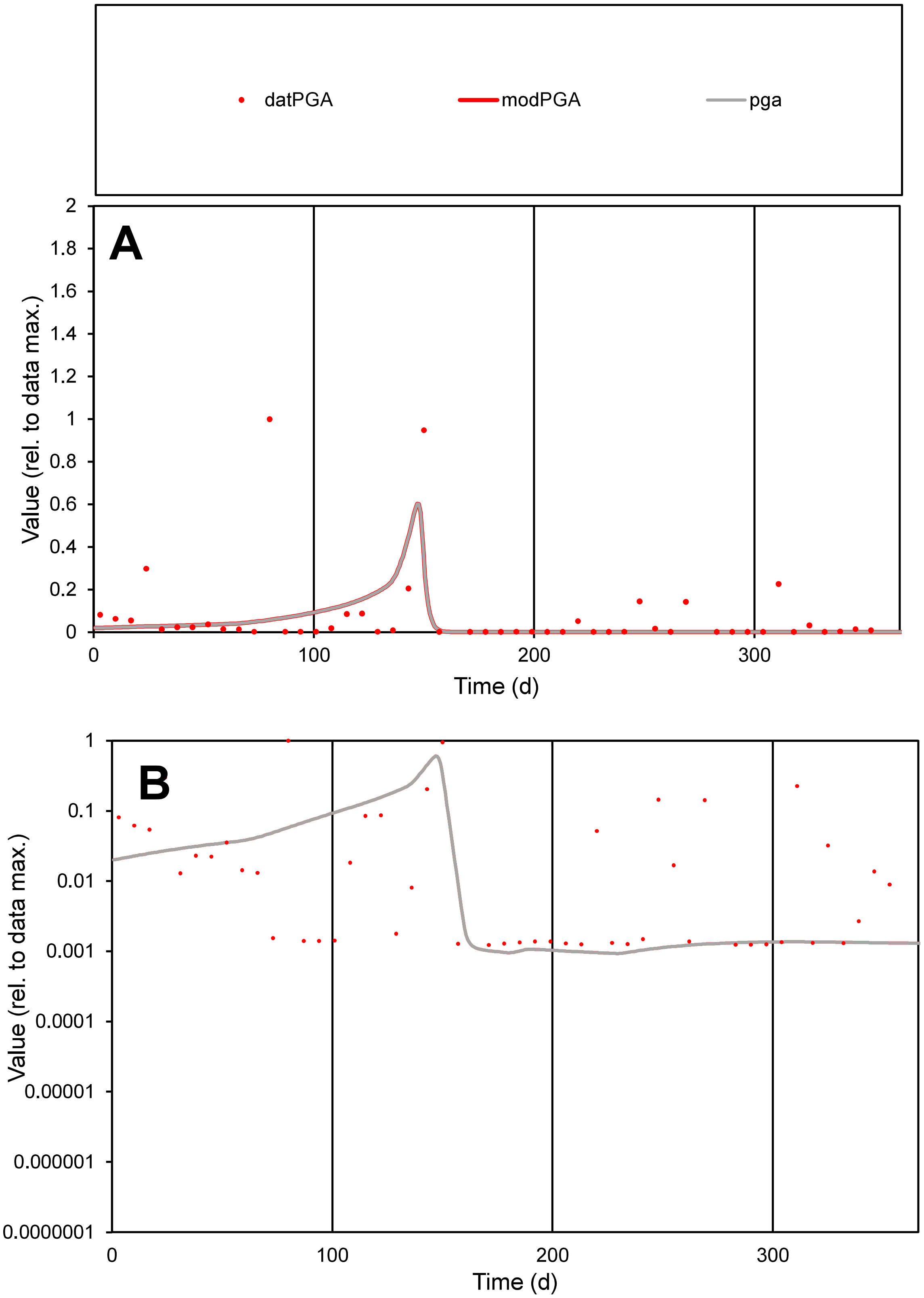

C1

Variables
PGA Provide jtd or Dst
CalibOnly?
2 1 to reproduce of
oNormalize?
2 1=no, 2=yes

ofxn calc*
2.32971
ofxn mod
2.4215

sRun
RunA1
zout
1

Ave
Data
0.00105
Model
0.00071

data/vMax
0.01411

ofxng
369.894

Min d/m
1.32346

ave/min d
60.086
ave/min m
53.706

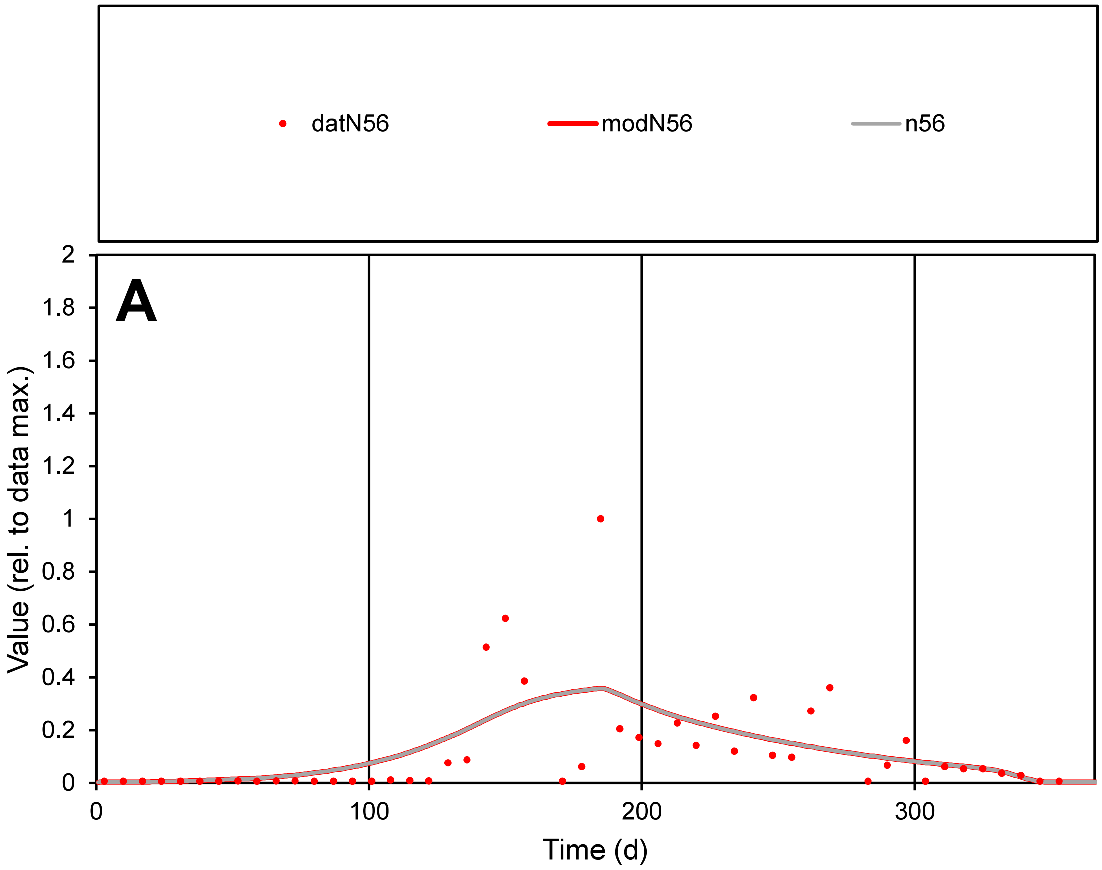

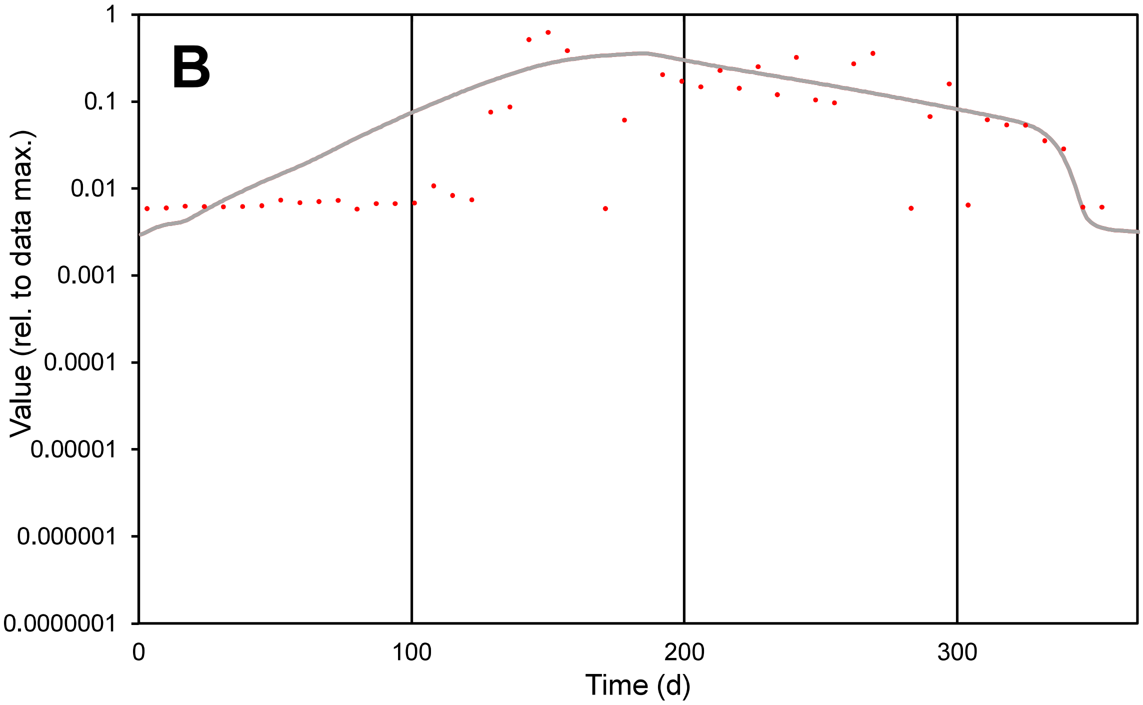

C1

Variables
N56 Provide jtd or Dst
CalibOnly?
2 1 to reproduce of
oNormalize?
2 1=no, 2=yes

ofxn calc*
1.24674
ofxn mod
1.26039

sRun
RunA1
zout
1

Ave
Data
0.0007
Model
0.00073

data/vMax
0.00591

ofxng
369.894

Min d/m
1.73695

ave/min d
20.3711
ave/min m
37.205

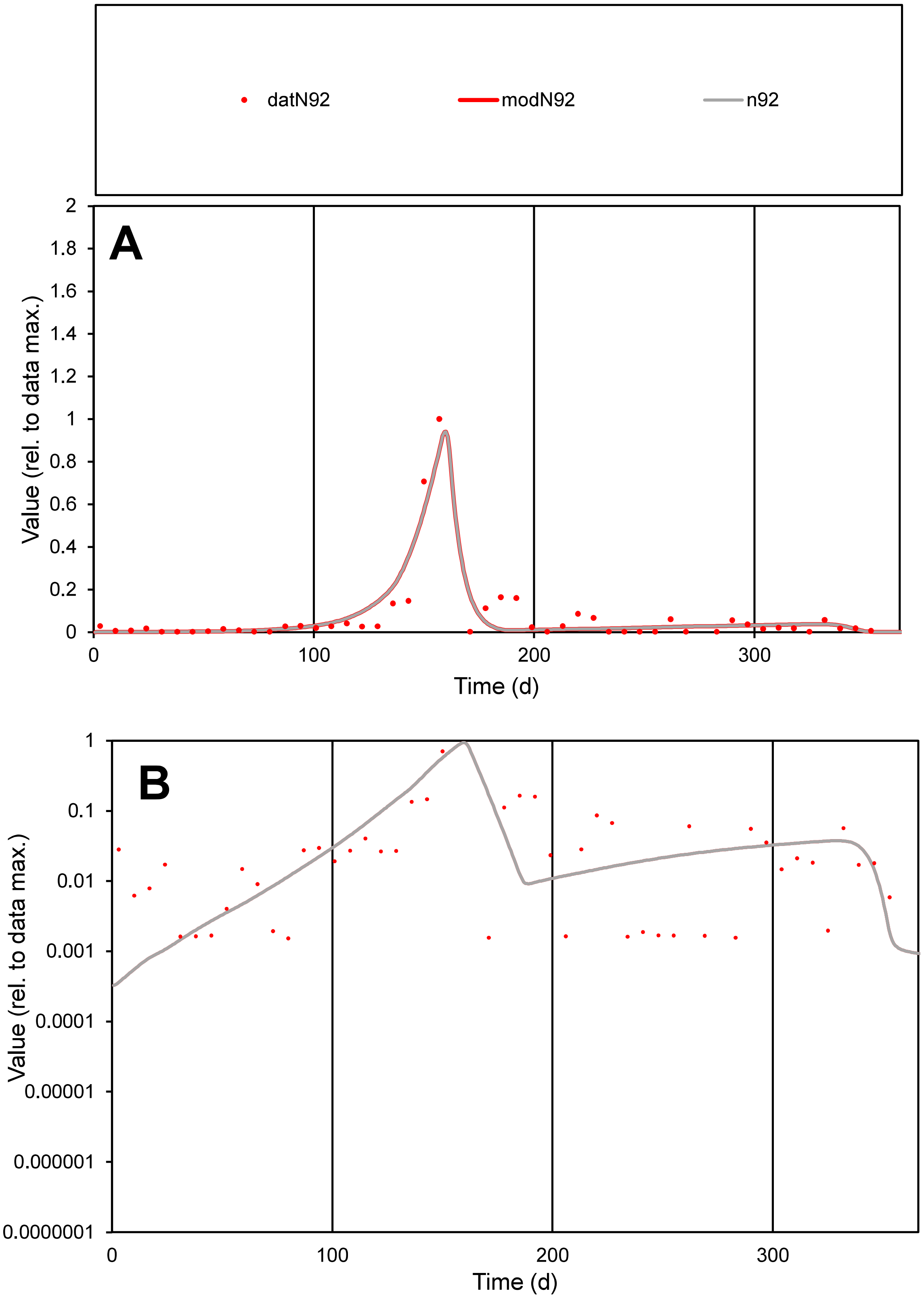

C1

Variables
N92 Provide jtd or Dst
CalibOnly?
2 1 to reproduce of
oNormalize?
2 1=no, 2=yes

ofxn calc*
0.8661
ofxn mod
0.93732

sRun
RunA1
zout
1

Ave
Data
0.00165
Model
0.00165

data/vMax
0.02491

ofxng
369.894

Min d/m
3.91402

ave/min d
43.1959
ave/min m
169.782

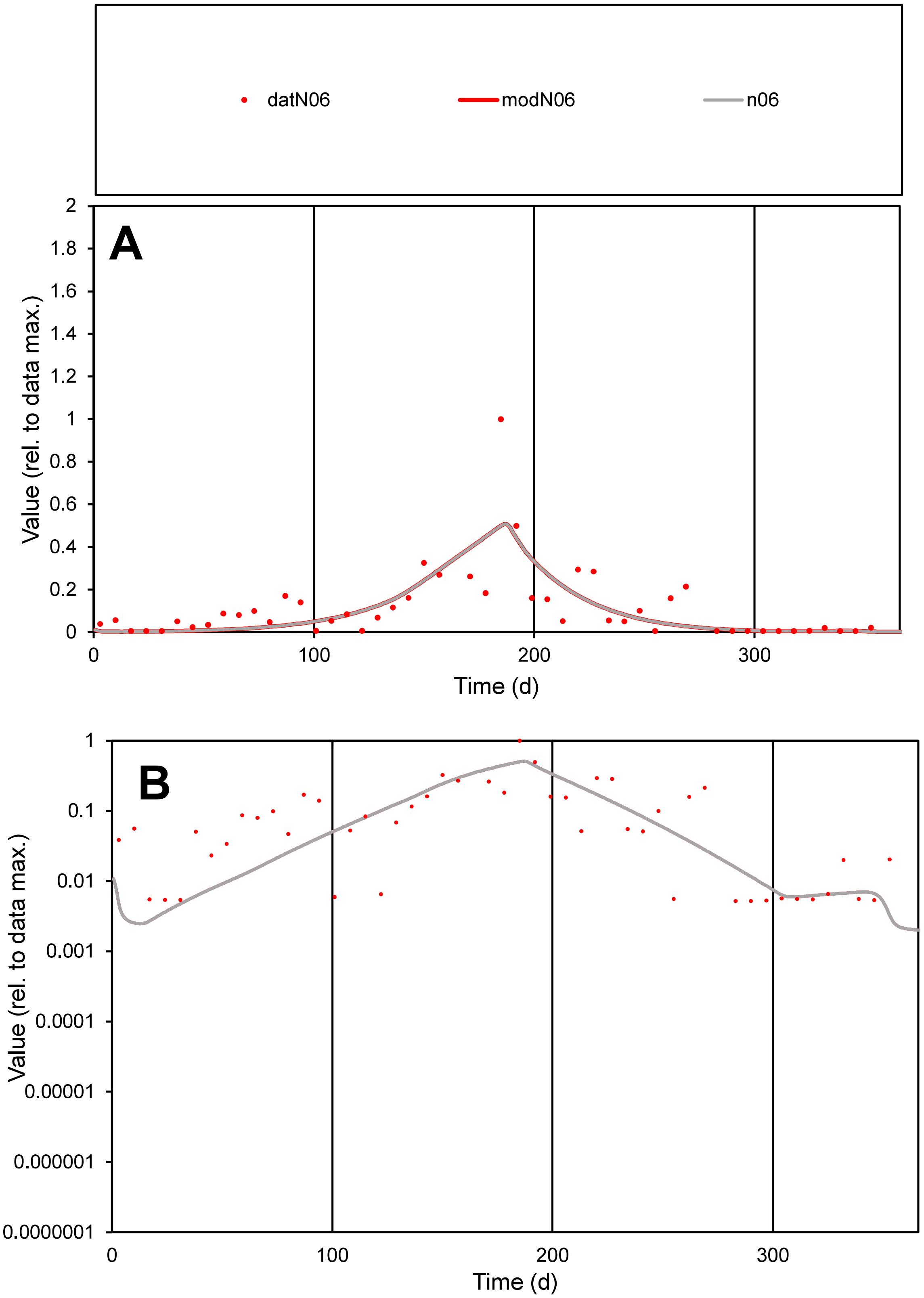

C1

Variables

N06 Provide jtd or Dst

CalibOnly?

2 1 to reproduce of

oNormalize?

2 1=no, 2=yes

ofxn calc*

0.96567

ofxn mod

0.98735

sRun

RunA1

zout

1

Ave

Data

0.00144

Model

0.00128

data/vMax

0.01285

ofxng

369.894

Min d/m

2.09302

ave/min d

21.5347

ave/min m

39.9781

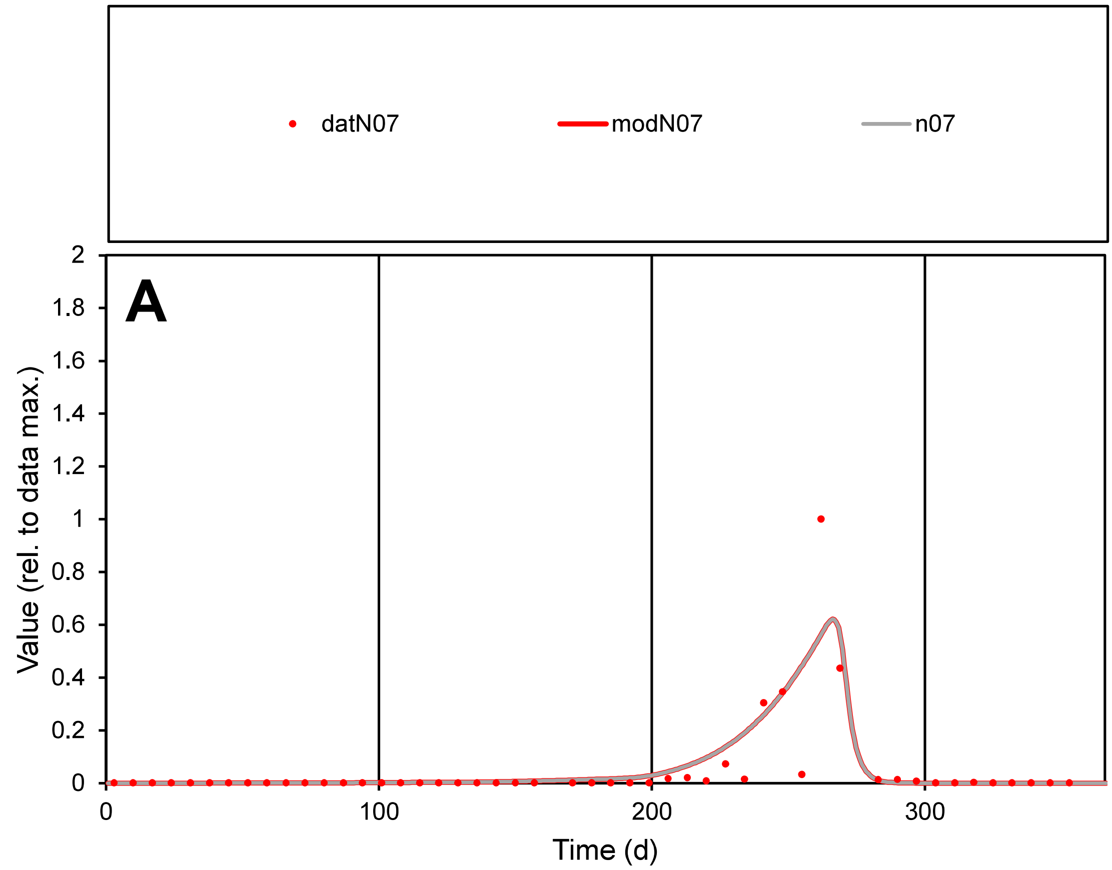

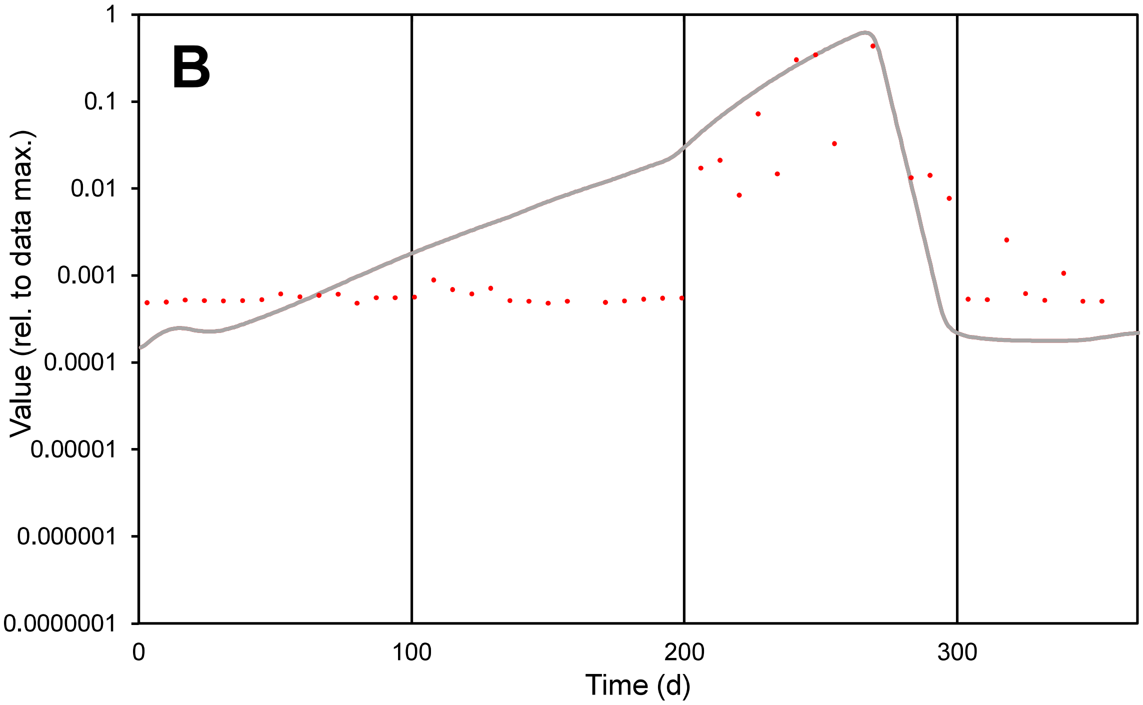

C1

Variables
N07 Provide jtd or Dst
CalibOnly?
2 1 to reproduce of
oNormalize?
2 1=no, 2=yes

ofxn calc*
1.90111
ofxn mod
1.94575

sRun
RunA1
zout
1

Ave
Data
0.00167
Model
0.00202

data/vMax
0.03552

ofxng
369.894

Min d/m
2.71351

ave/min d
98.3089
ave/min m
322.408

328_Calib_S_A

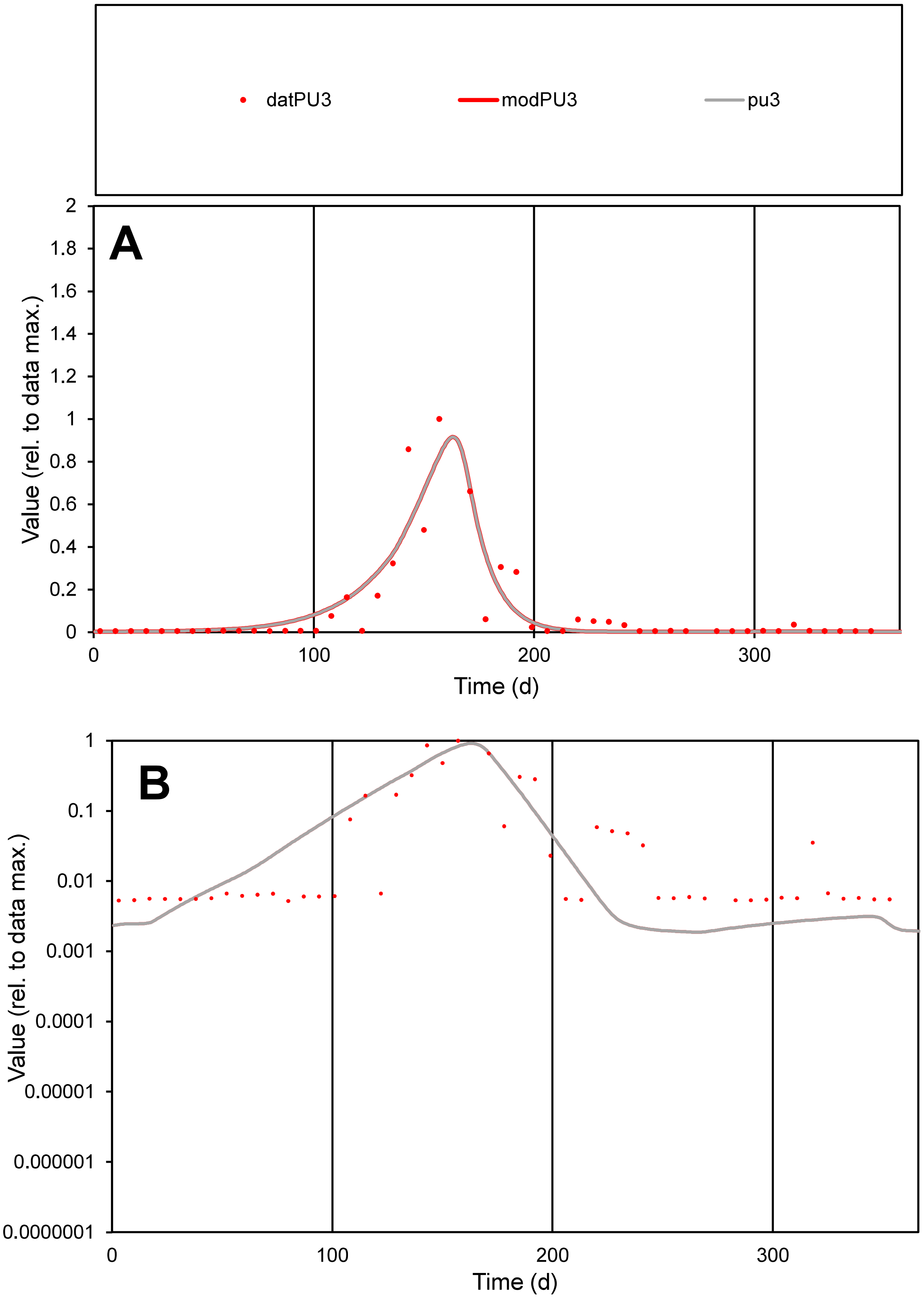

C1

Variables

PU3 Provide jtd or Dst

CalibOnly?

2 1 to reproduce of

oNormalize?

2 1=no, 2=yes

ofxn calc*

0.88106

ofxn mod

0.92822

sRun

RunA1

zout

1

Ave

Data

0.00285

Model

0.00288

data/vMax

0.029

ofxng

369.894

Min d/m

2.77228

ave/min d

18.8463

ave/min m

52.7622

328_Calib_S_A

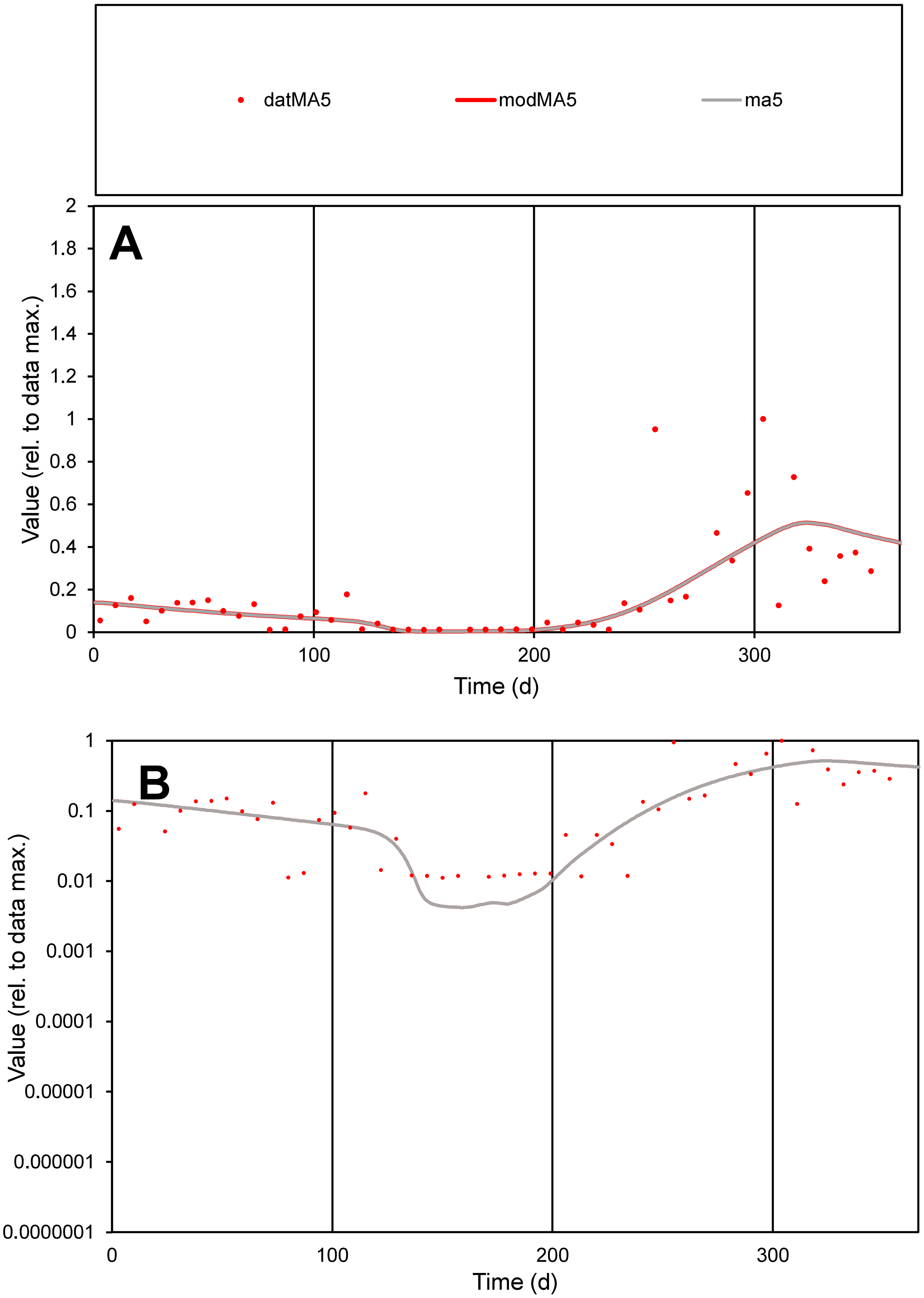

C1

Variables
MA5 Provide jtd or Dst
CalibOnly?
2 1 to reproduce of
oNormalize?
2 1=no, 2=yes

ofxn calc*
0.97376
ofxn mod
0.98663

sRun
RunA1
zout
1

Ave
Data
0.00144
Model
0.00131

data/vMax
0.00837

ofxng
369.894

Min d/m
2.67397

ave/min d
15.3331
ave/min m
37.3856

328_Calib_S_A

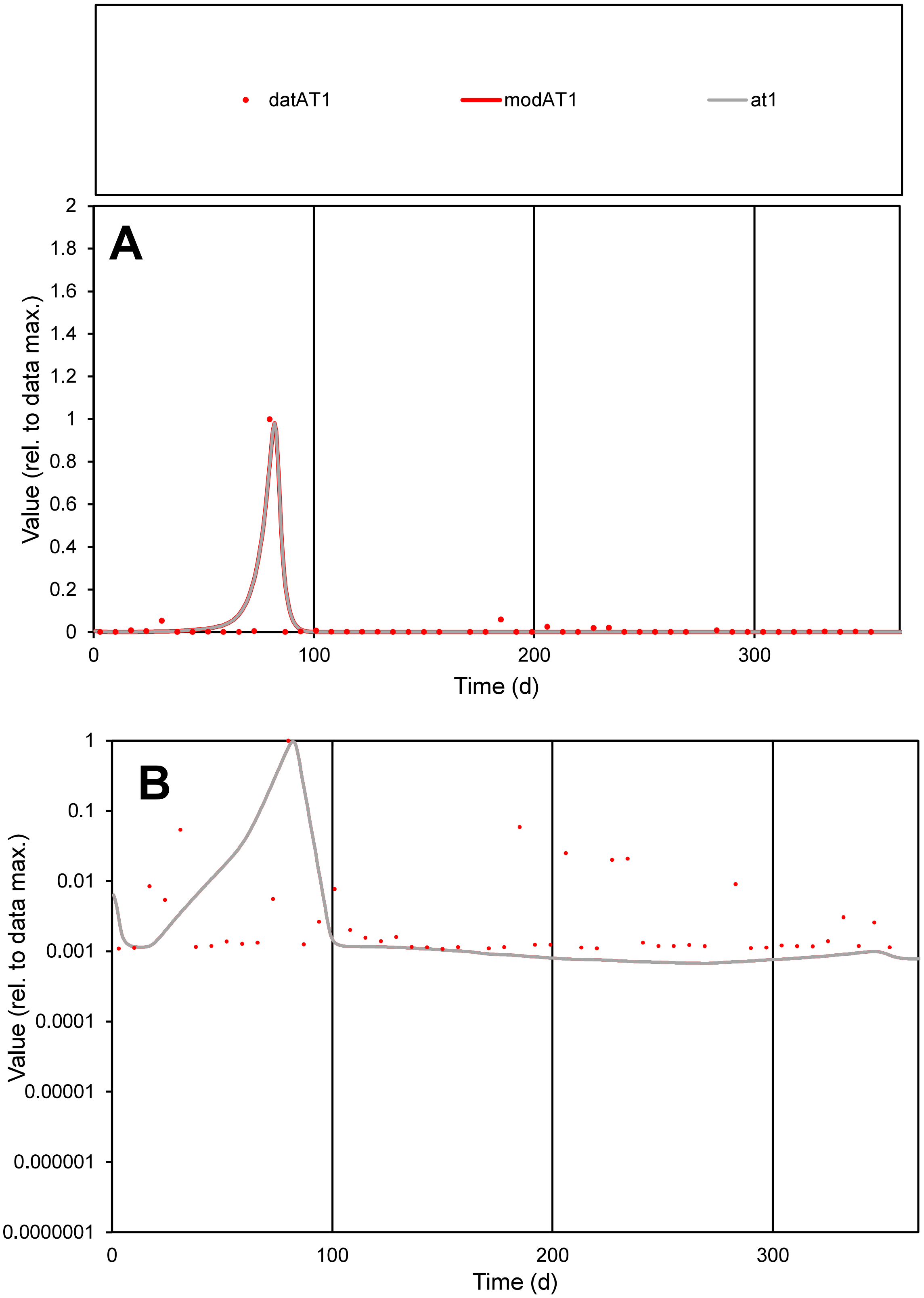

C1

Variables

AT1 Provide jtd or Dst

CalibOnly?

2 1 to reproduce of

oNormalize?

2 1=no, 2=yes

ofxn calc*

1.30412

ofxn mod

1.55767

sRun

RunA1

zout

1

Ave

Data

0.00115

Model

0.00126

data/vMax

0.04429

ofxng

369.894

Min d/m

1.60074

ave/min d

23.9431

ave/min m

42.0622

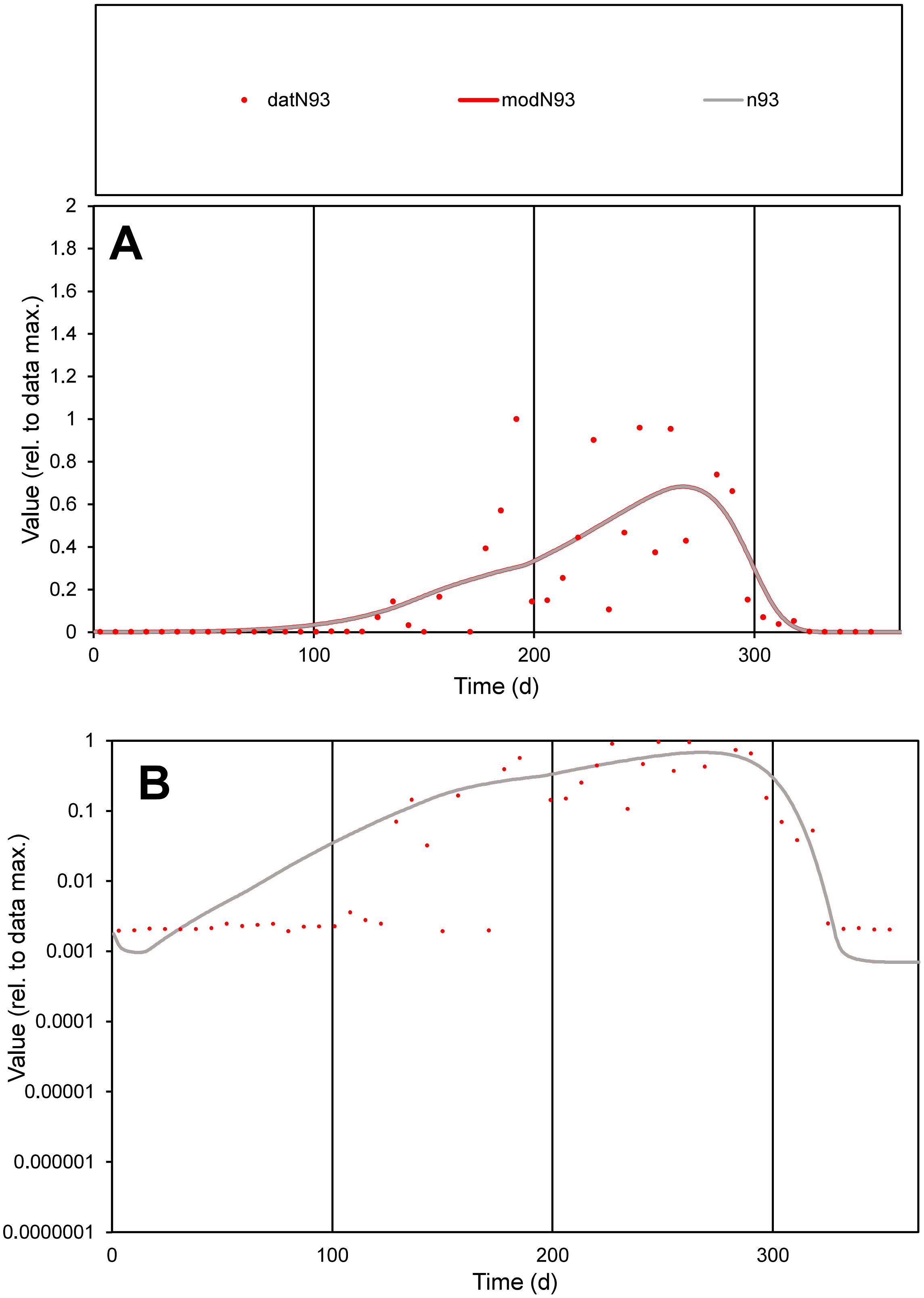

C1

Variables
N93 Provide jtd or Dst
CalibOnly?
2 1 to reproduce of
oNormalize?
2 1=no, 2=yes

ofxn calc*
0.94285
ofxn mod
0.96976

sRun
RunA1
zout
1

Ave
Data
0.00171
Model
0.00176

data/vMax
0.00896

ofxng
369.894

Min d/m
2.77422

ave/min d
98.4163
ave/min m
281.677

328_Calib_S_A

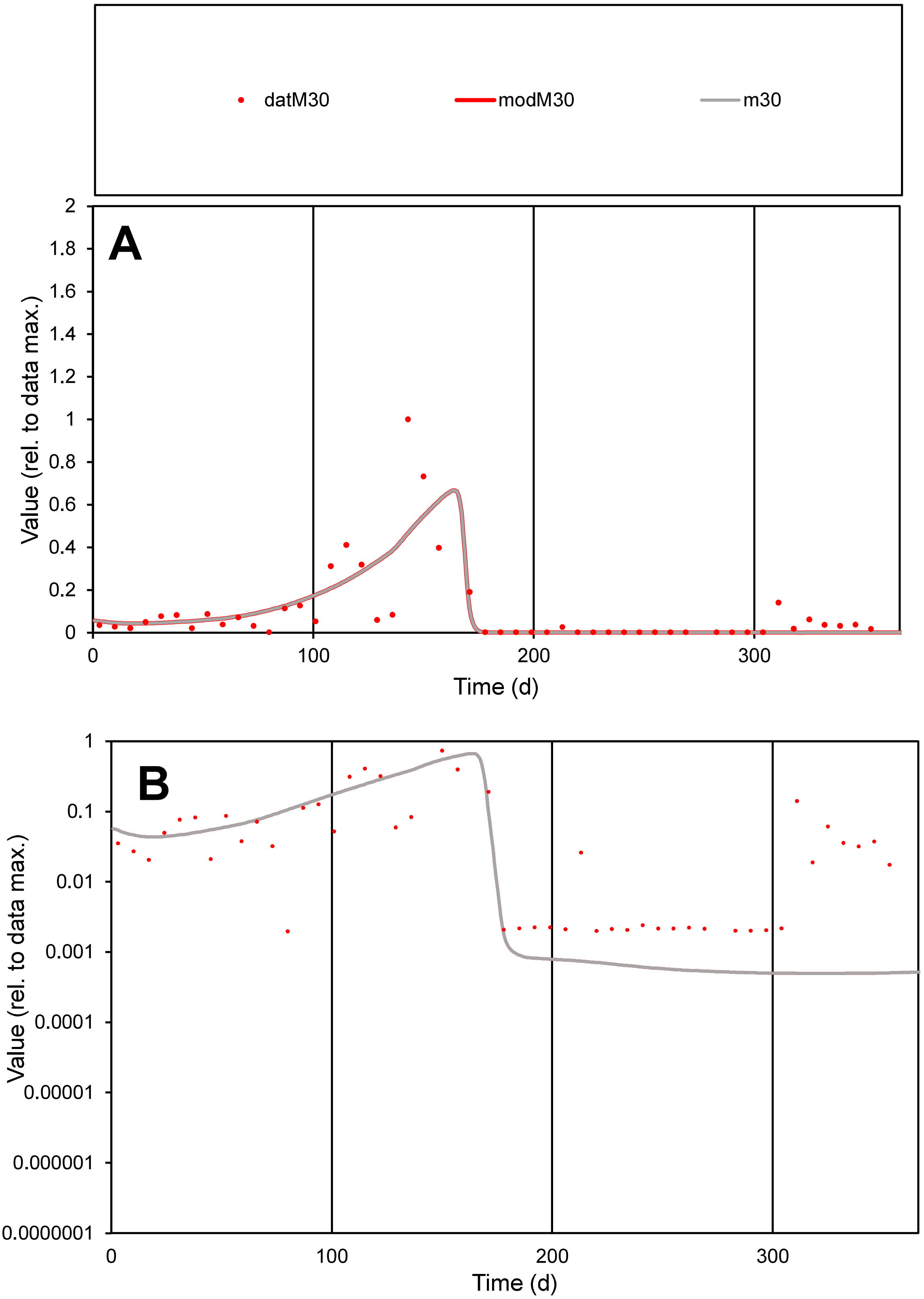

C1

Variables
M30 Provide jtd or Dst
CalibOnly?
2 1 to reproduce of
oNormalize?
2 1=no, 2=yes

ofxn calc*
1.16589
ofxn mod
1.18888

sRun
RunA1
zout
1

Ave
Data
0.00203
Model
0.00195

data/vMax
0.02094

ofxng
369.894

Min d/m
3.97872

ave/min d
49.3168
ave/min m
188.382

328_Calib_S_A

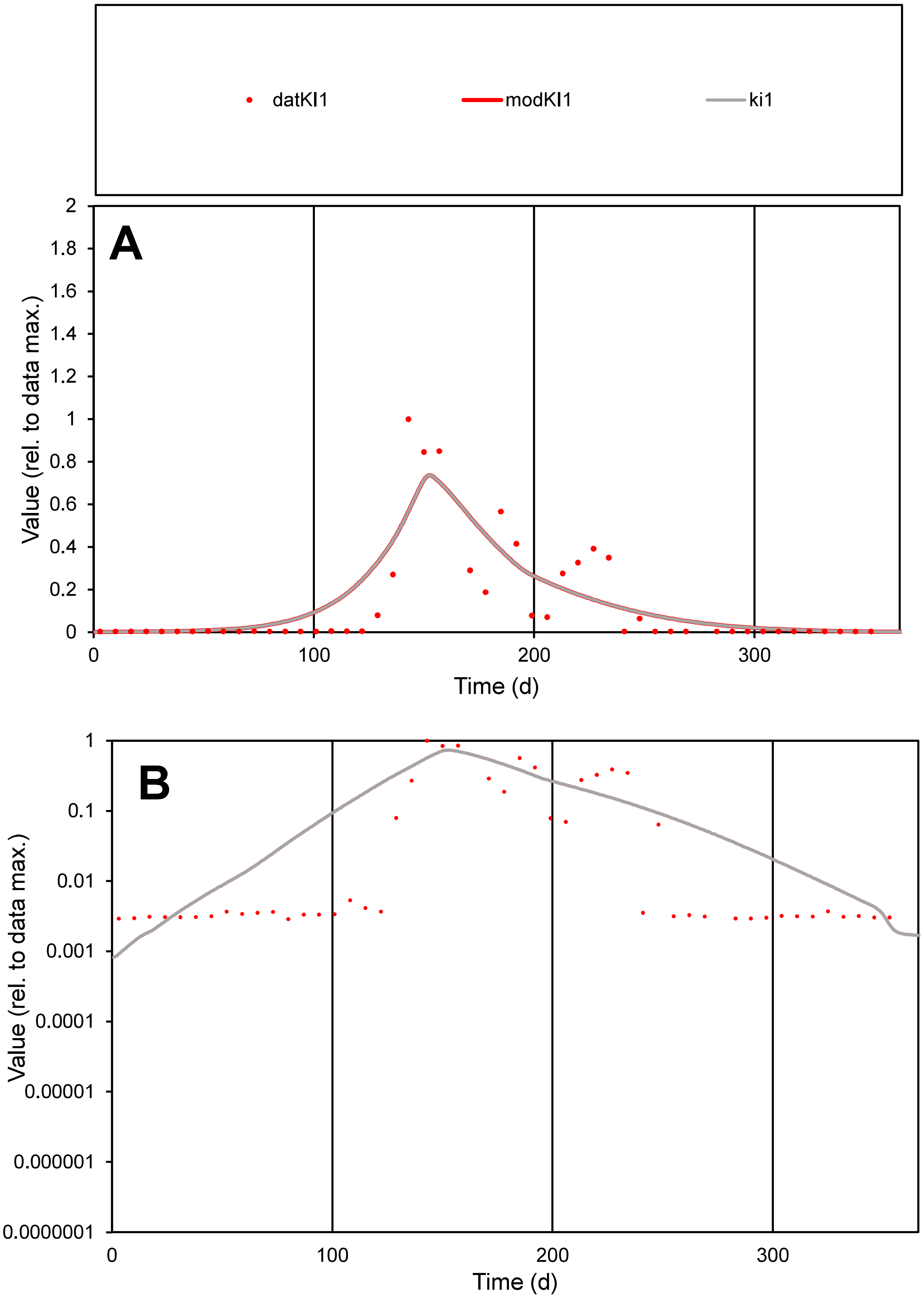

C1

Variables
KI1 Provide jtd or Dst
CalibOnly?
2 1 to reproduce of
oNormalize?
2 1=no, 2=yes

ofxn calc*
1.01608
ofxn mod
1.04806

sRun
RunA1
zout
1

Ave
Data
0.00205
Model
0.00232

data/vMax
0.01628

ofxng
369.894

Min d/m
2.90763

ave/min d
43.6743
ave/min m
143.65

328_Calib_S_A

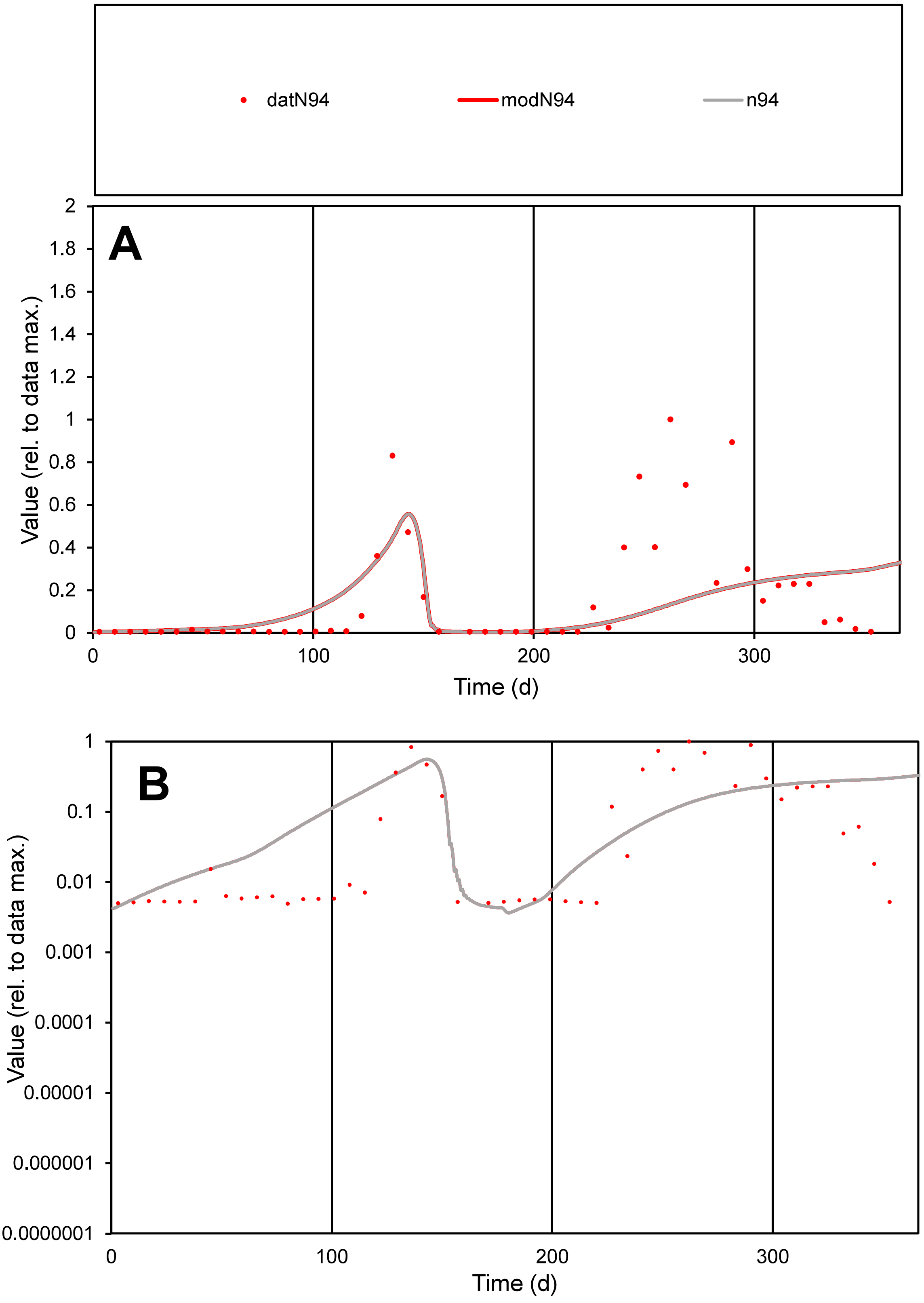

C1

Variables

N94 Provide jtd or Dst

CalibOnly?

2 1 to reproduce of

oNormalize?

2 1=no, 2=yes

ofxn calc*

1.44827

ofxn mod

1.45774

sRun

RunA1

zout

1

Ave

Data

0.00147

Model

0.00118

data/vMax

0.0092

ofxng

369.894

Min d/m

1.21928

ave/min d

32.2744

ave/min m

31.5578

328_Calib_S_A

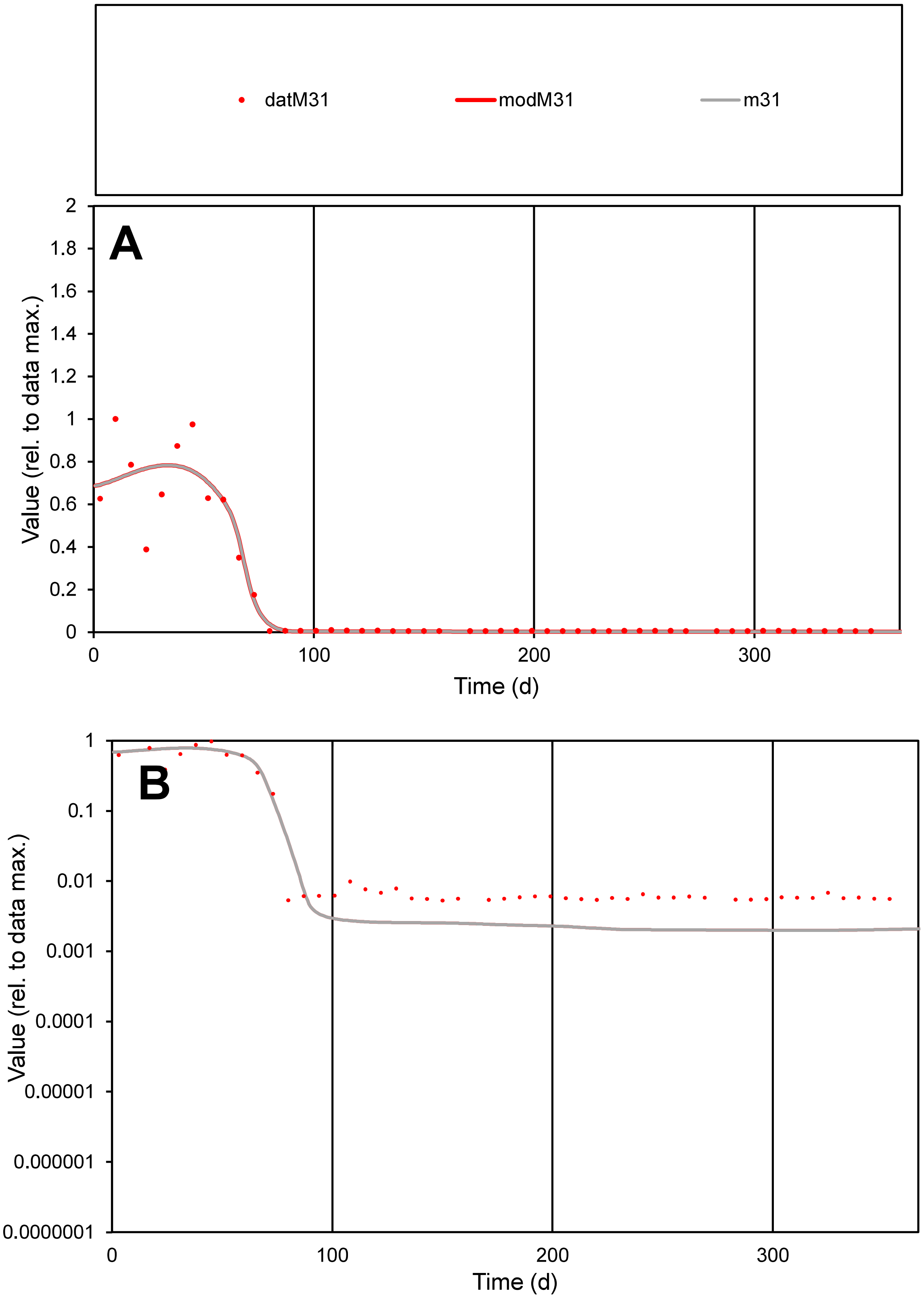

C1

Variables	
M31	Provide jtd or Dst
CalibOnly?	
2	1 to reproduce of
oNormalize?	
2	1=no, 2=yes
ofxn calc*	
0.53977	
ofxn mod	
0.60232	
sRun	
RunA1	
zout	
1	
Ave	
Data	
0.00229	
Model	
0.00224	
data/vMax	
0.01539	
ofxng	
369.894	
Min d/m	
2.64821	
ave/min d	
28.1982	
ave/min m	
72.9902	

328_Calib_S_A

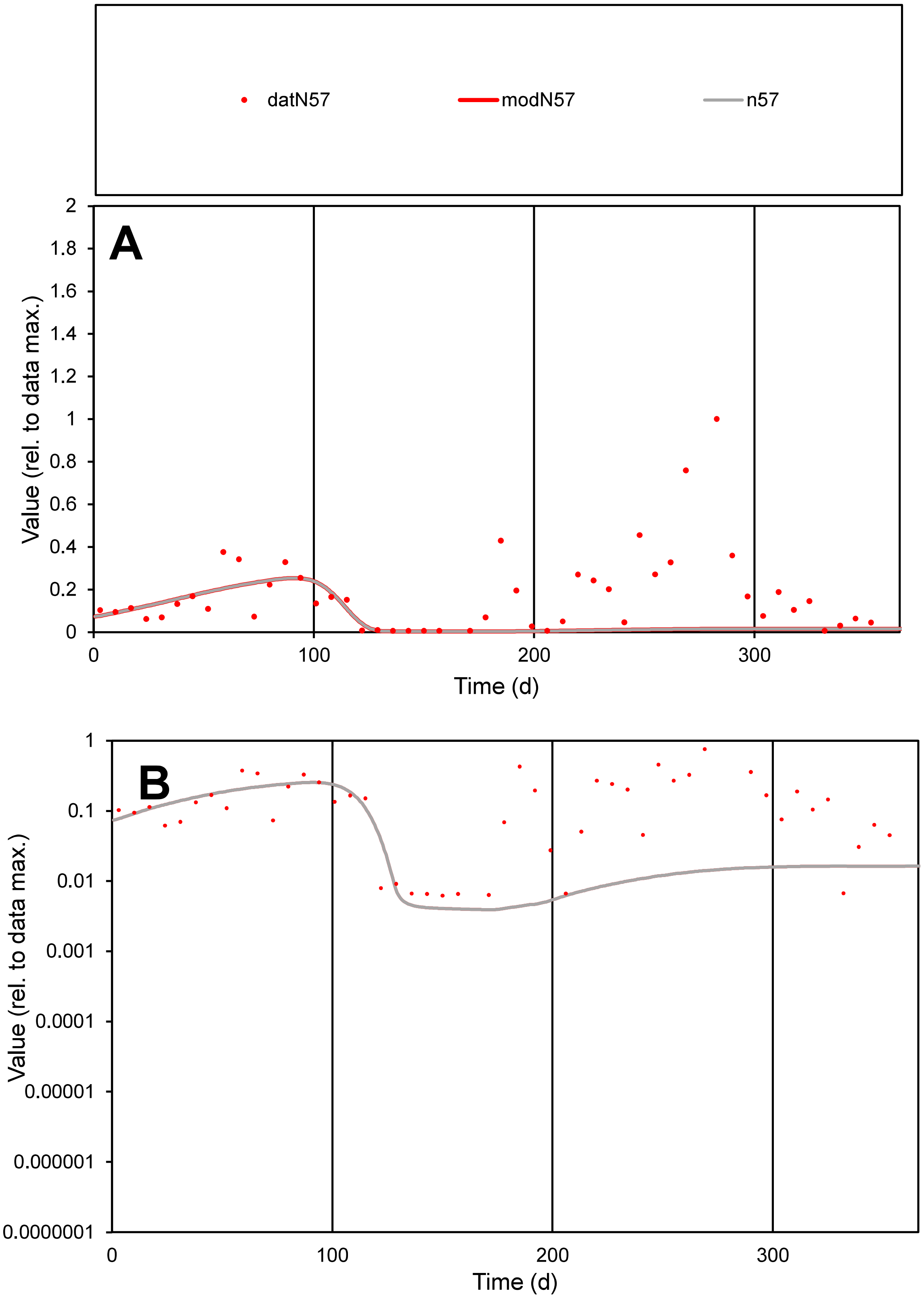

C1

Variables

N57 Provide jtd or Dst

CalibOnly?

2 1 to reproduce of

oNormalize?

2 1=no, 2=yes

ofxn calc*

1.32315

ofxn mod

1.32778

sRun

RunA1

zout

1

Ave

Data

0.00101

Model

0.0004

data/vMax

0.00583

ofxng

369.894

Min d/m

1.57479

ave/min d

27.9602

ave/min m

17.3825

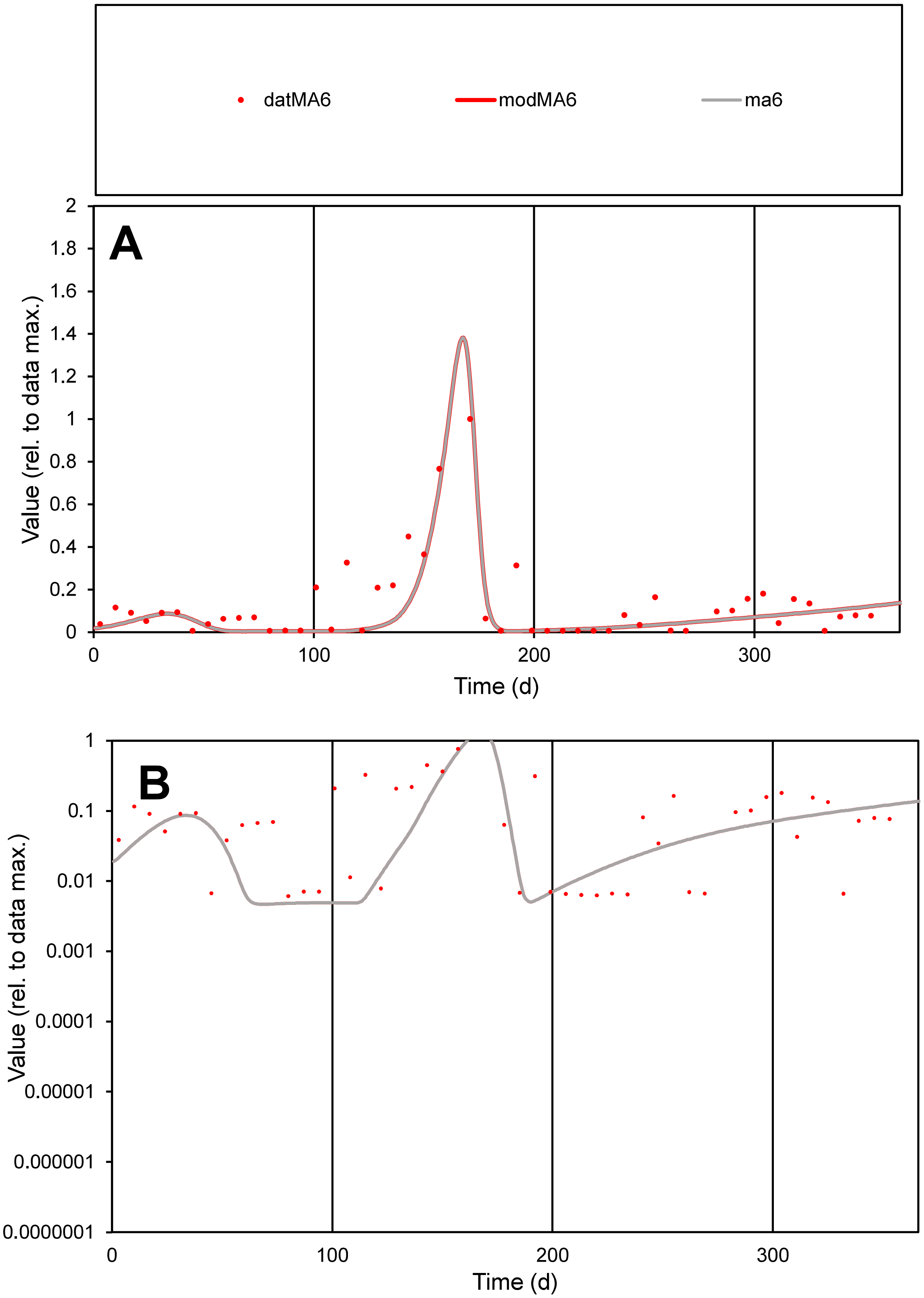

C1

Variables
MA6 Provide jtd or Dst
CalibOnly?
2 1 to reproduce of
oNormalize?
2 1=no, 2=yes

ofxn calc*
0.76715
ofxn mod
0.80255

sRun
RunA1
zout
1

Ave
Data
0.00241
Model
0.00168

data/vMax
0.01928

ofxng
369.894

Min d/m
1.30848

ave/min d
20.3713
ave/min m
18.5314

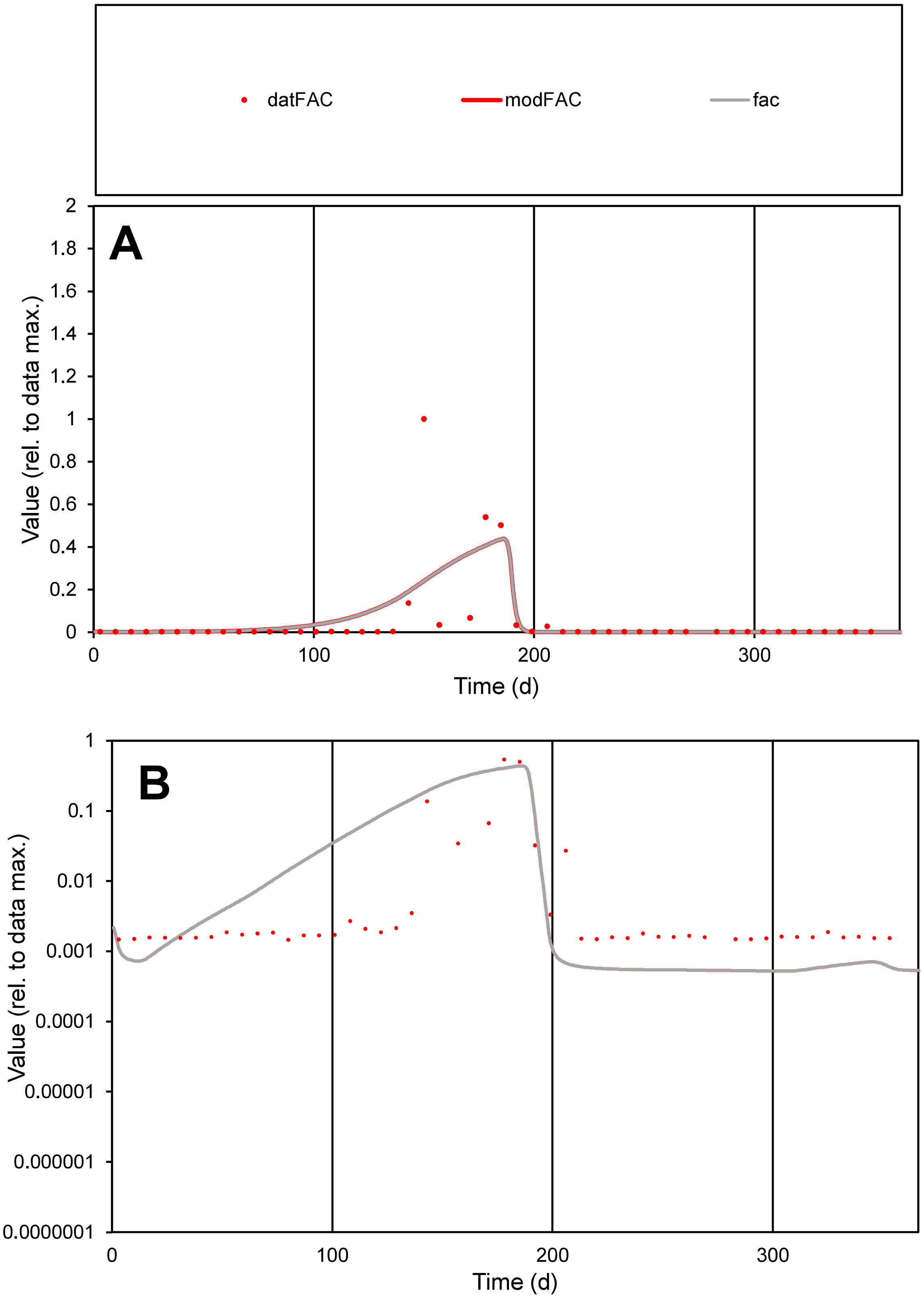

C1

Variables
FAC Provide jtd or Dst
CalibOnly?
2 1 to reproduce of
oNormalize?
2 1=no, 2=yes

ofxn calc*
2.58681
ofxn mod
2.61377

sRun
RunA1
zout
1

Ave
Data
0.00292
Model
0.00315

data/vMax
0.05934

ofxng
369.894

Min d/m
2.78631

ave/min d
33.6656
ave/min m
101.08

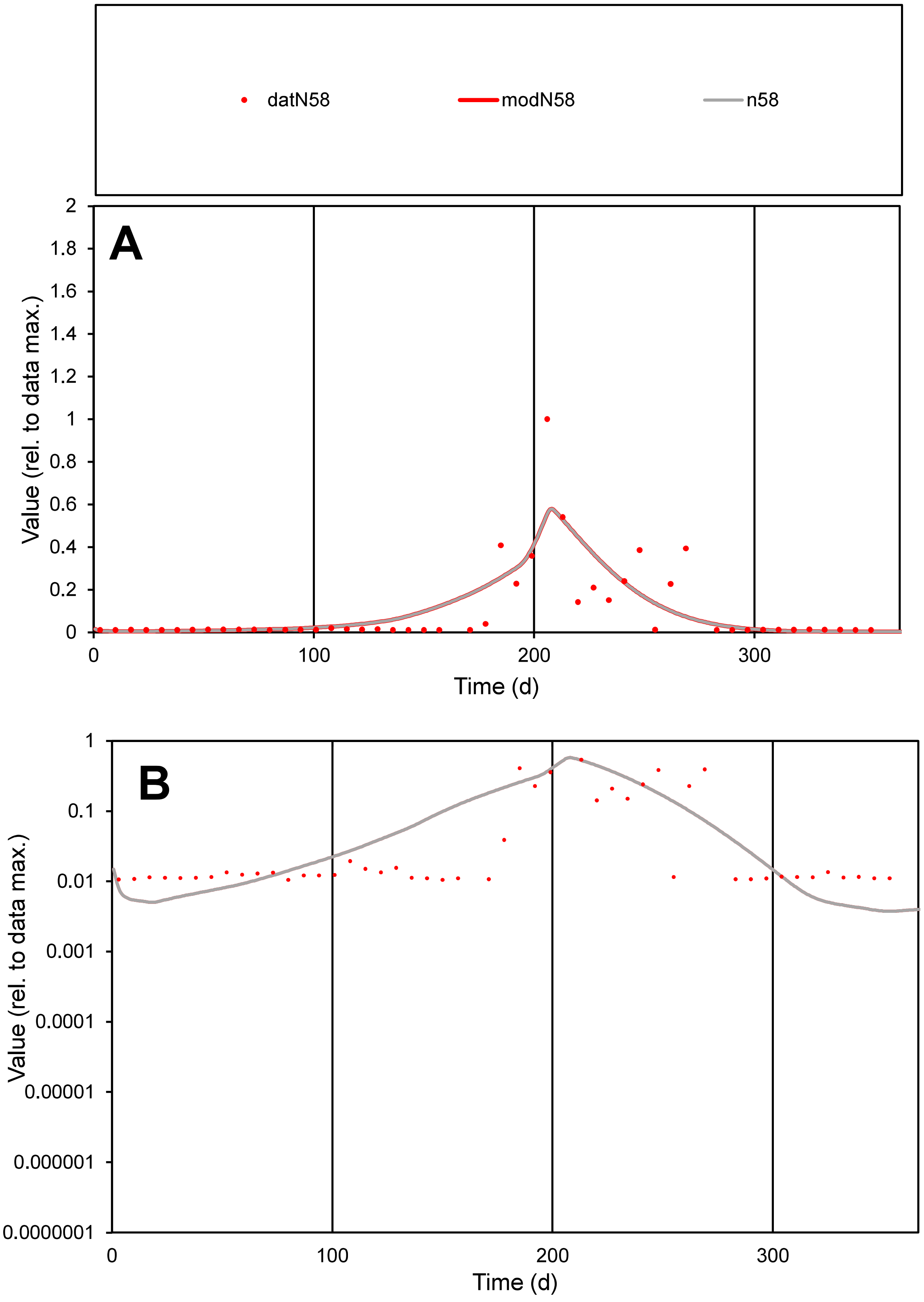

C1

Variables
N58 Provide jtd or Dst
CalibOnly?
2 1 to reproduce of
oNormalize?
2 1=no, 2=yes

ofxn calc*
1.14089
ofxn mod
1.169

sRun
RunA1
zout
1

Ave
Data
0.00076
Model
0.0008

data/vMax
0.0078

ofxng
369.894

Min d/m
2.79733

ave/min d
9.26706
ave/min m
27.3425

328_Calib_S_A

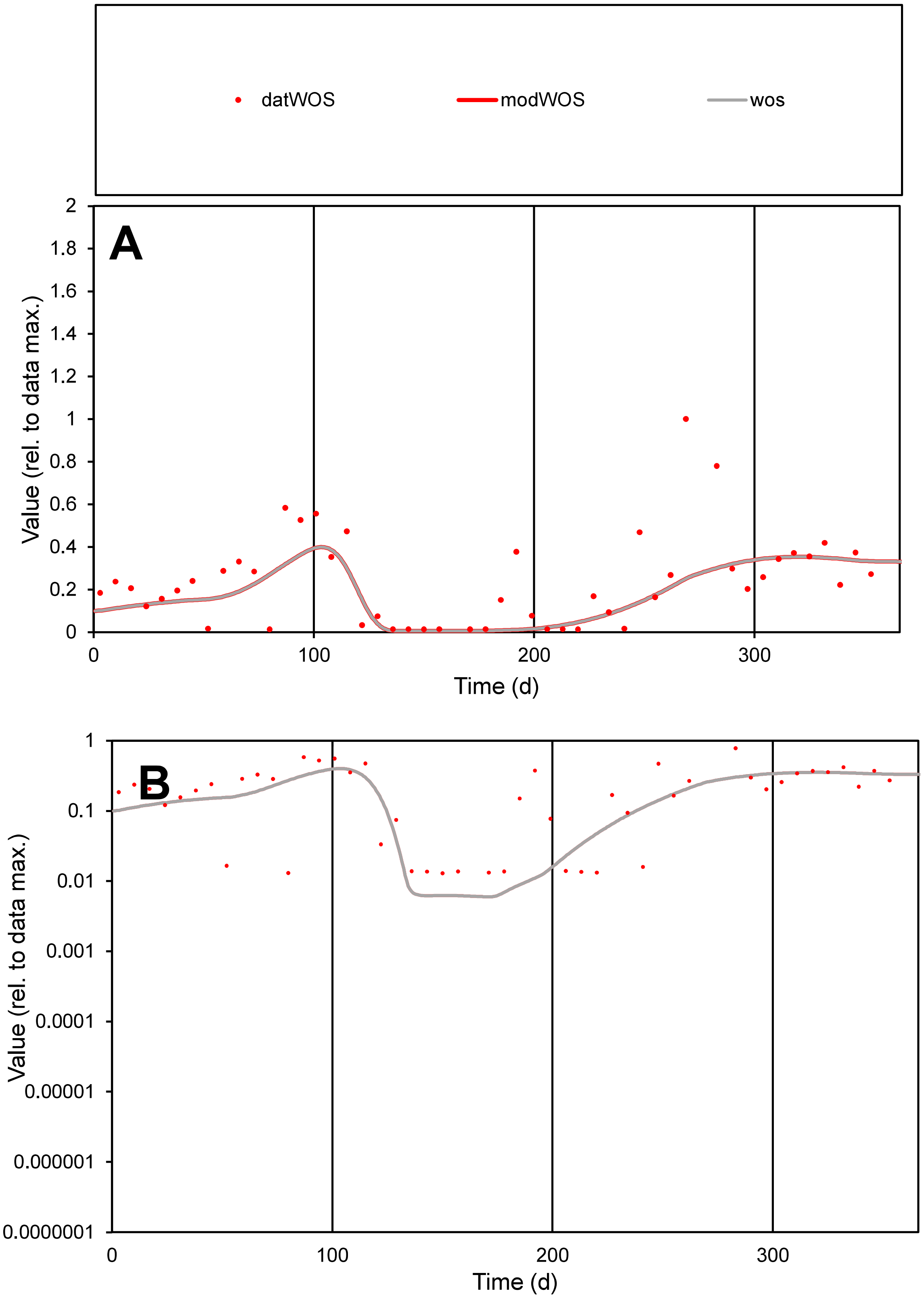

C1

Variables
WOS Provide jtd or Dst
CalibOnly?
2 1 to reproduce of
oNormalize?
2 1=no, 2=yes

ofxn calc*
0.71574
ofxn mod
0.72725

sRun
RunA1
zout
1

Ave
Data
0.00126
Model
0.00095

data/vMax
0.00528

ofxng
369.894

Min d/m
2.153

ave/min d
18.4256
ave/min m
30.1046

328_Calib_S_A

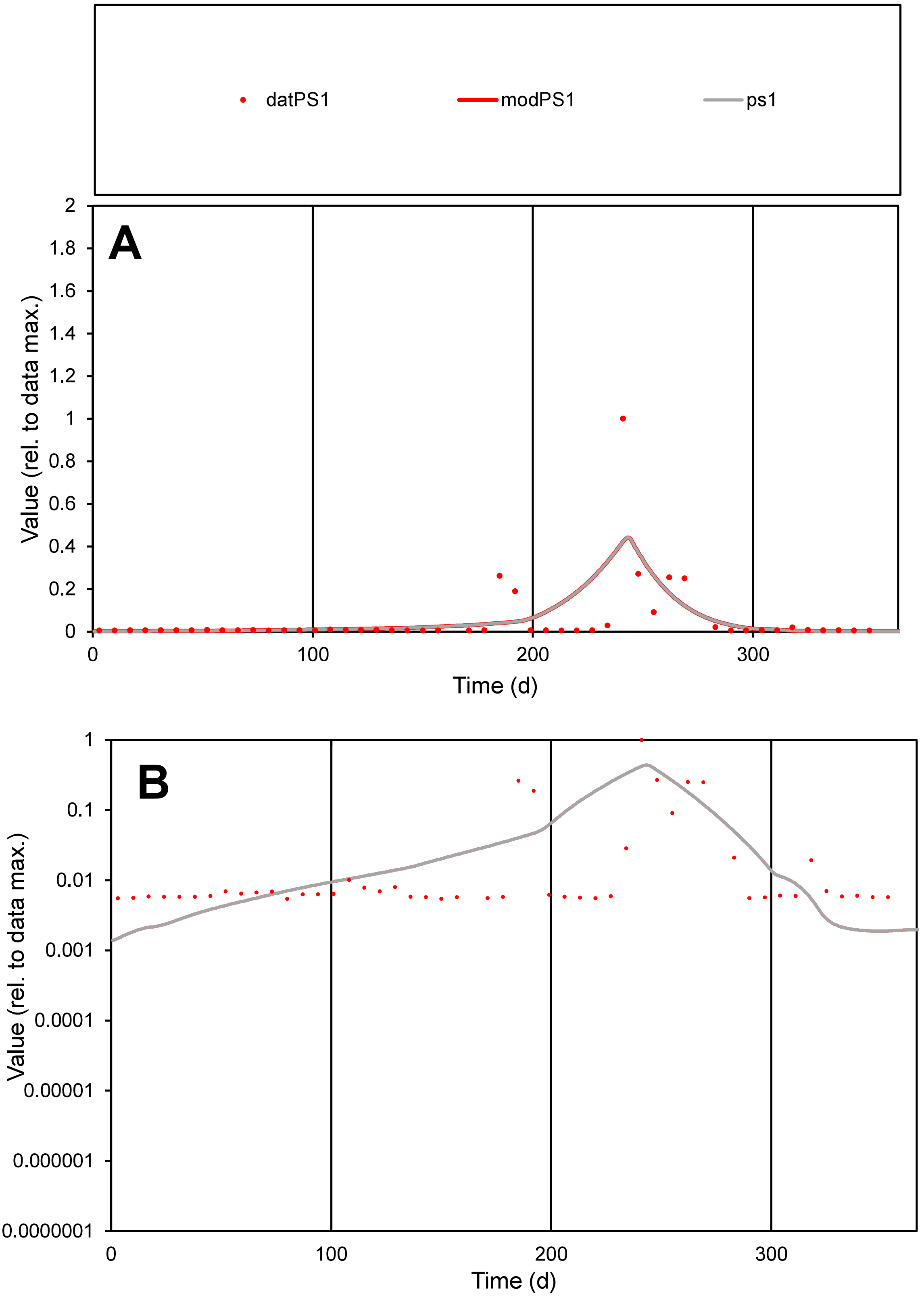

C1

Variables
PS1 Provide jtd or Dst
CalibOnly?
2 1 to reproduce of
oNormalize?
2 1=no, 2=yes

ofxn calc*
2.14203
ofxn mod
2.17784

sRun
RunA1
zout
1

Ave
Data
0.00046
Model
0.0005

data/vMax
0.00865

ofxng
369.894

Min d/m
3.50373

ave/min d
9.87783
ave/min m
37.3082

328_Calib_S_A

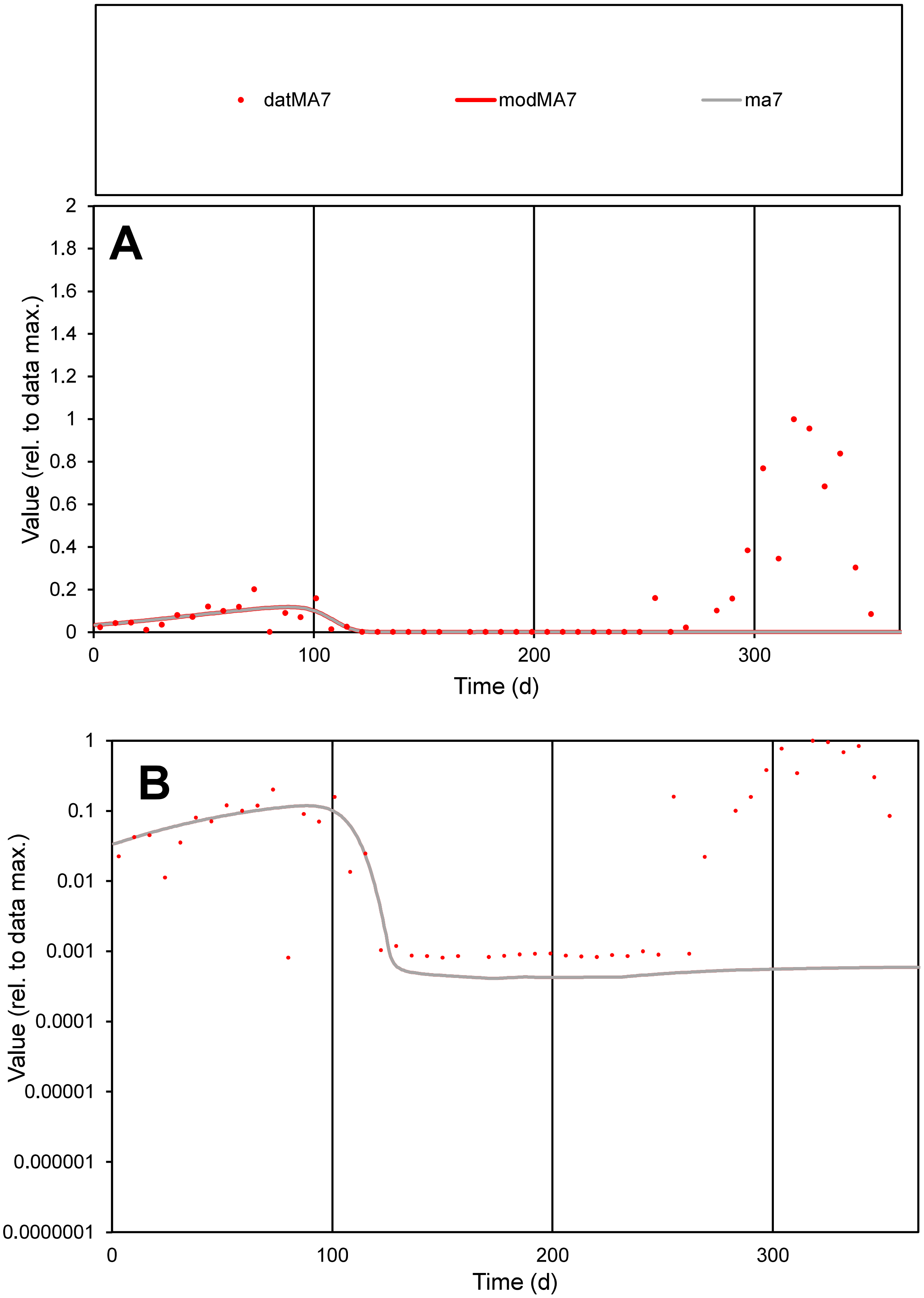

C1

Variables
MA7 Provide jtd or Dst
CalibOnly?
2 1 to reproduce of
oNormalize?
2 1=no, 2=yes

ofxn calc*
2.02331
ofxn mod
2.02666

sRun
RunA1
zout
1

Ave
Data
0.00238
Model
0.00045

data/vMax
0.01659

ofxng
369.894

Min d/m
1.97036

ave/min d
177.168
ave/min m
65.8696

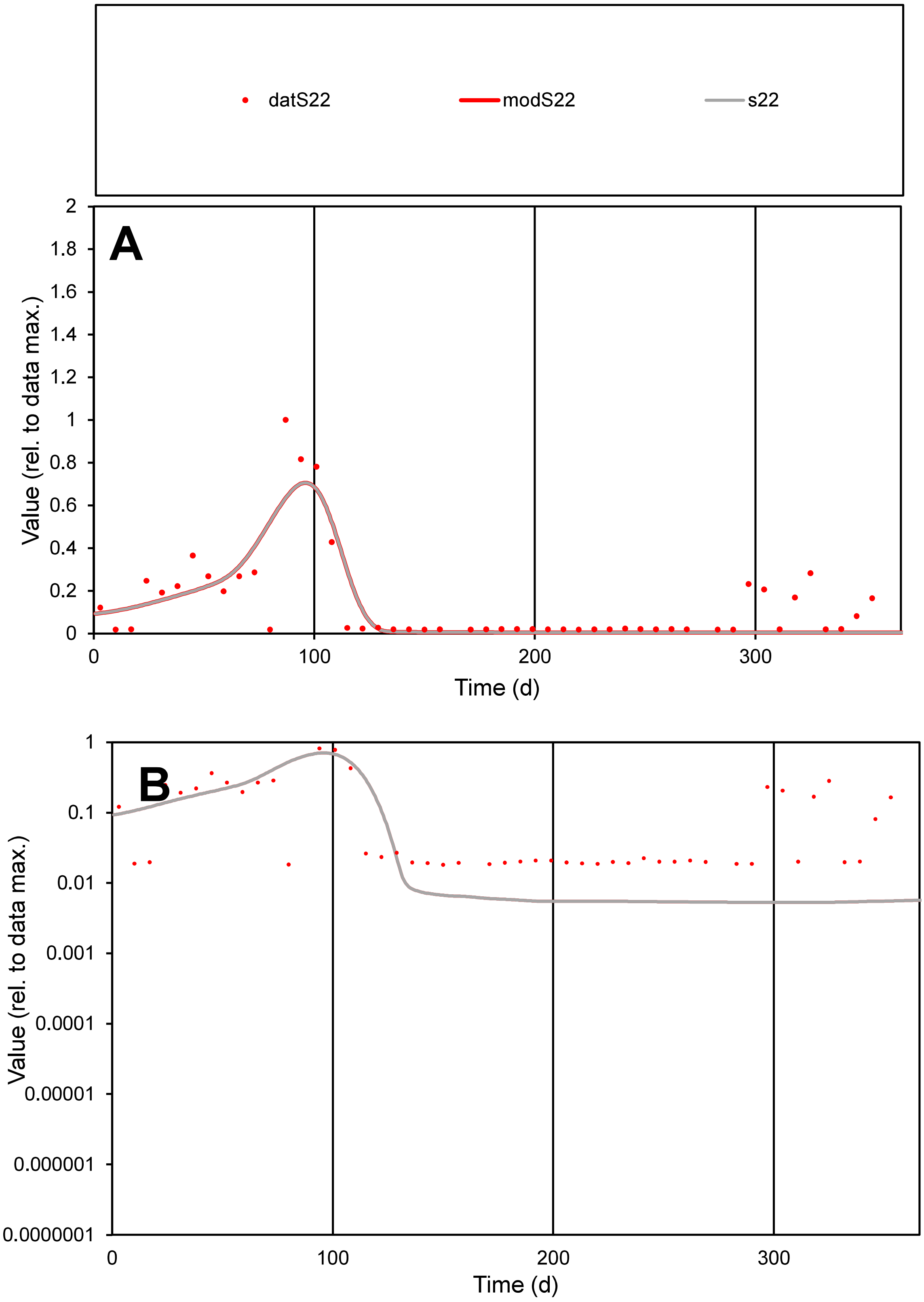

C1

Variables
S22 Provide jtd or Dst
CalibOnly?
2 1 to reproduce of
oNormalize?
2 1=no, 2=yes

ofxn calc*
0.87891
ofxn mod
0.9036

sRun
RunA1
zout
1

Ave
Data
0.00114
Model
0.00094

data/vMax
0.00805

ofxng
369.894

Min d/m
3.44293

ave/min d
7.76655
ave/min m
22.0345

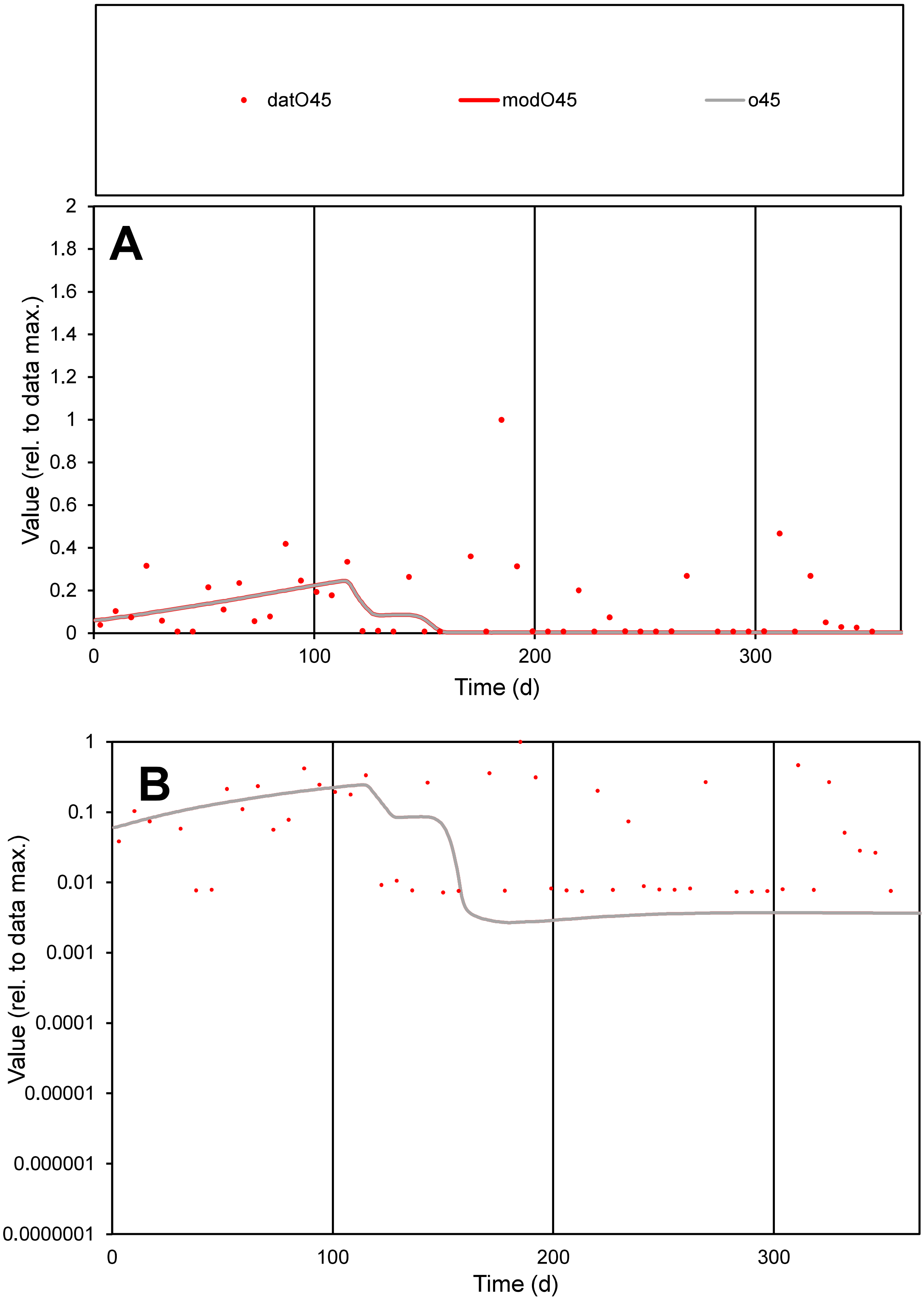

C1

Variables
O45 Provide jtd or Dst
CalibOnly?
2 1 to reproduce of
oNormalize?
2 1=no, 2=yes

ofxn calc*
1.55049
ofxn mod
1.5559

sRun
RunA1
zout
1

Ave
Data
0.001
Model
0.00051

data/vMax
0.00796

ofxng
369.894

Min d/m
2.67494
ave/min d
17.4756
ave/min m
23.6625

328_Calib_S_A

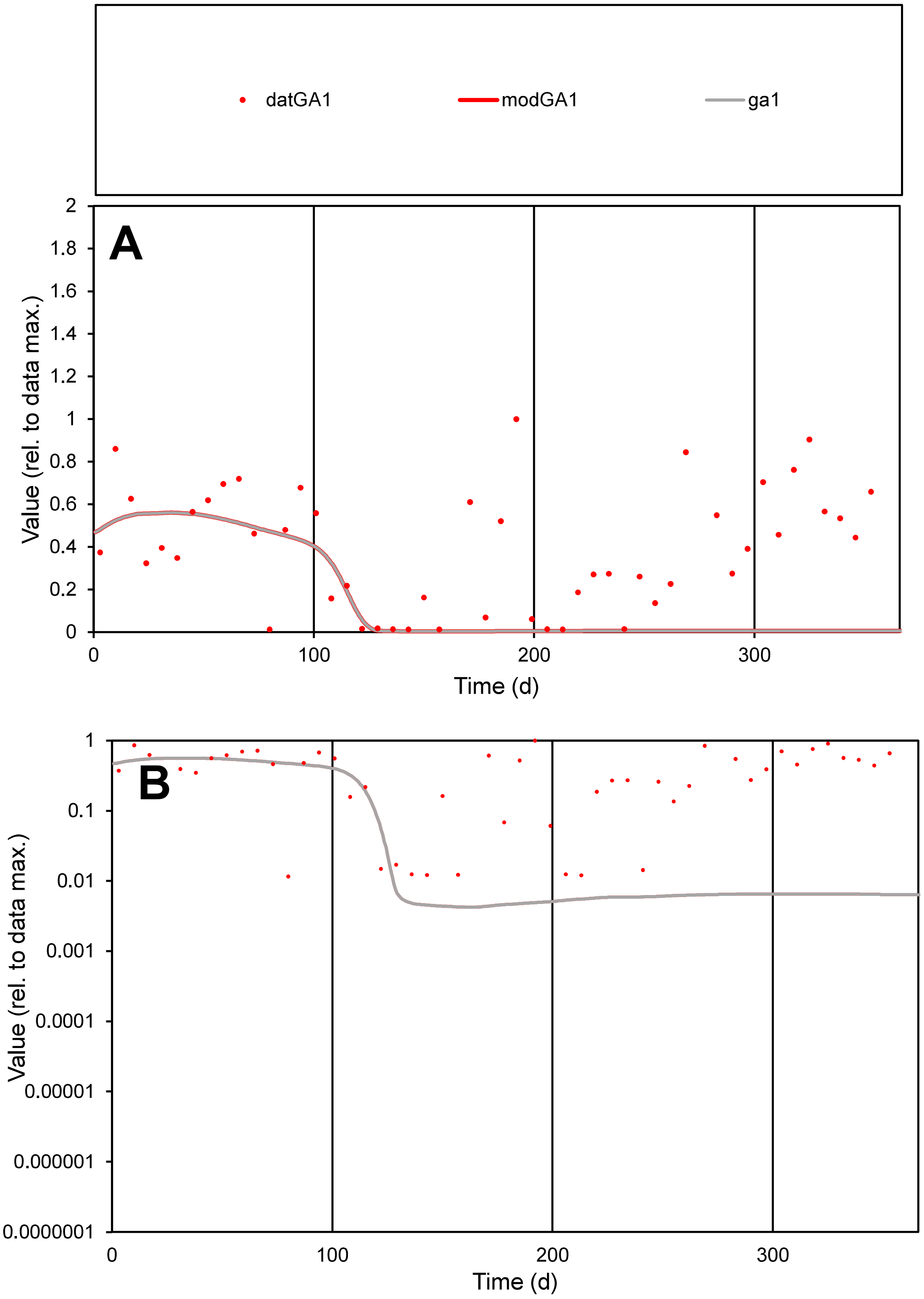

C1

Variables

GA1 Provide jtd or Dst

CalibOnly?

2 1 to reproduce of

oNormalize?

2 1=no, 2=yes

ofxn calc*

0.97944

ofxn mod

0.98486

sRun

RunA1

zout

1

Ave

Data

0.00119

Model

0.00052

data/vMax

0.00306

ofxng

369.894

Min d/m

2.73098

ave/min d

33.4499

ave/min m

39.7822

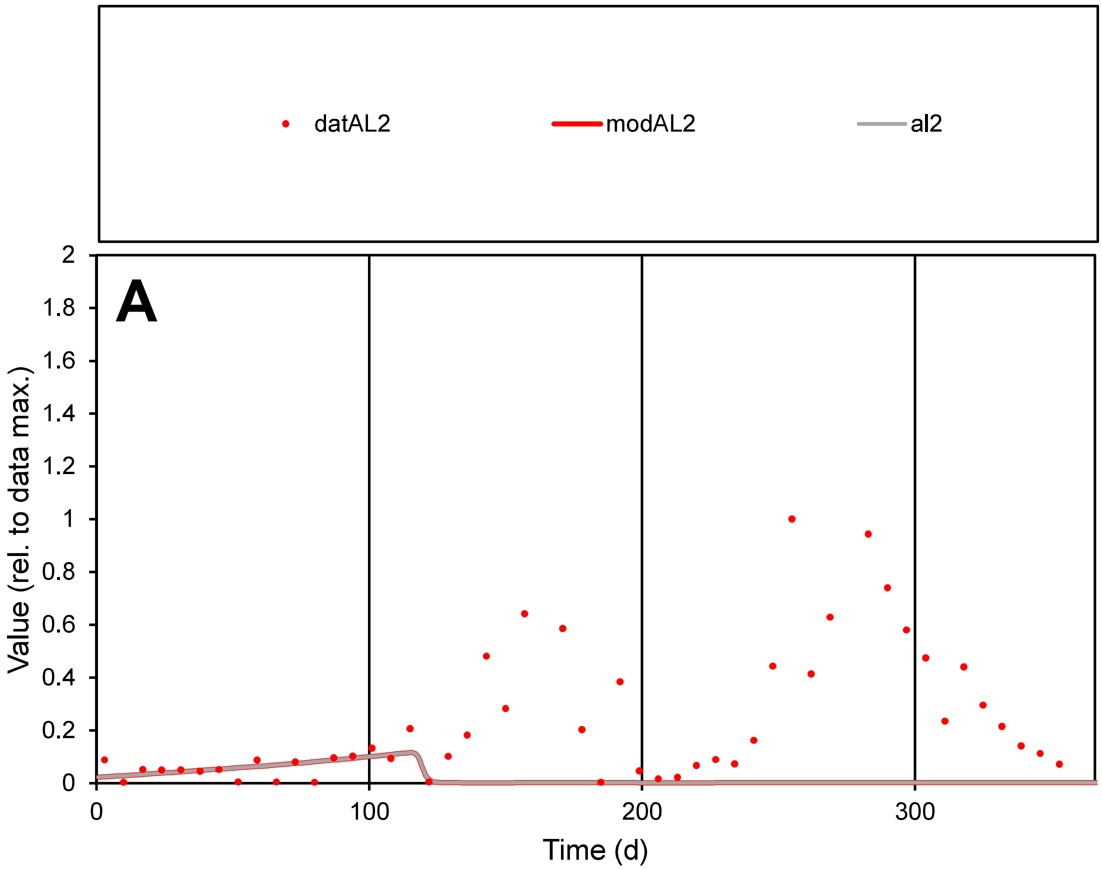

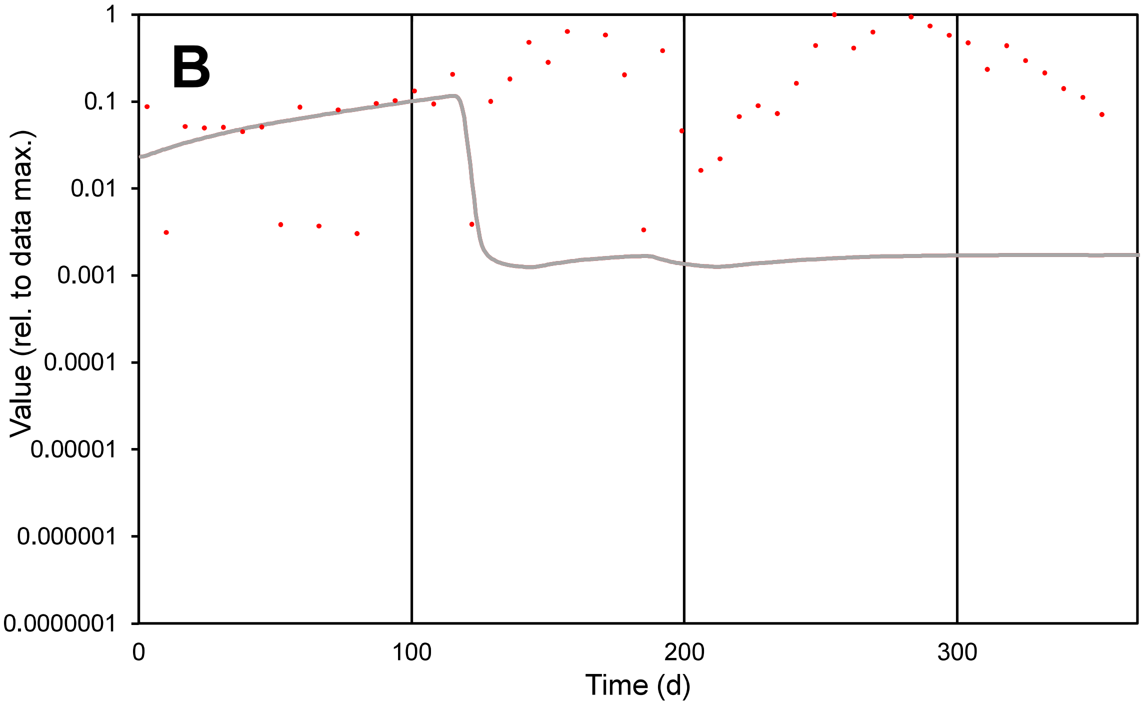

C1

Variables
AL2 Provide jtd or Dst
CalibOnly?
2 1 to reproduce of
oNormalize?
2 1=no, 2=yes

ofxn calc*
1.46582
ofxn mod
1.46884

sRun
RunA1
zout
1

Ave
Data
0.00165
Model
0.00018

data/vMax
0.00722

ofxng
369.894

Min d/m
2.42589

ave/min d
75.7032
ave/min m
19.5063

328_Calib_S_A

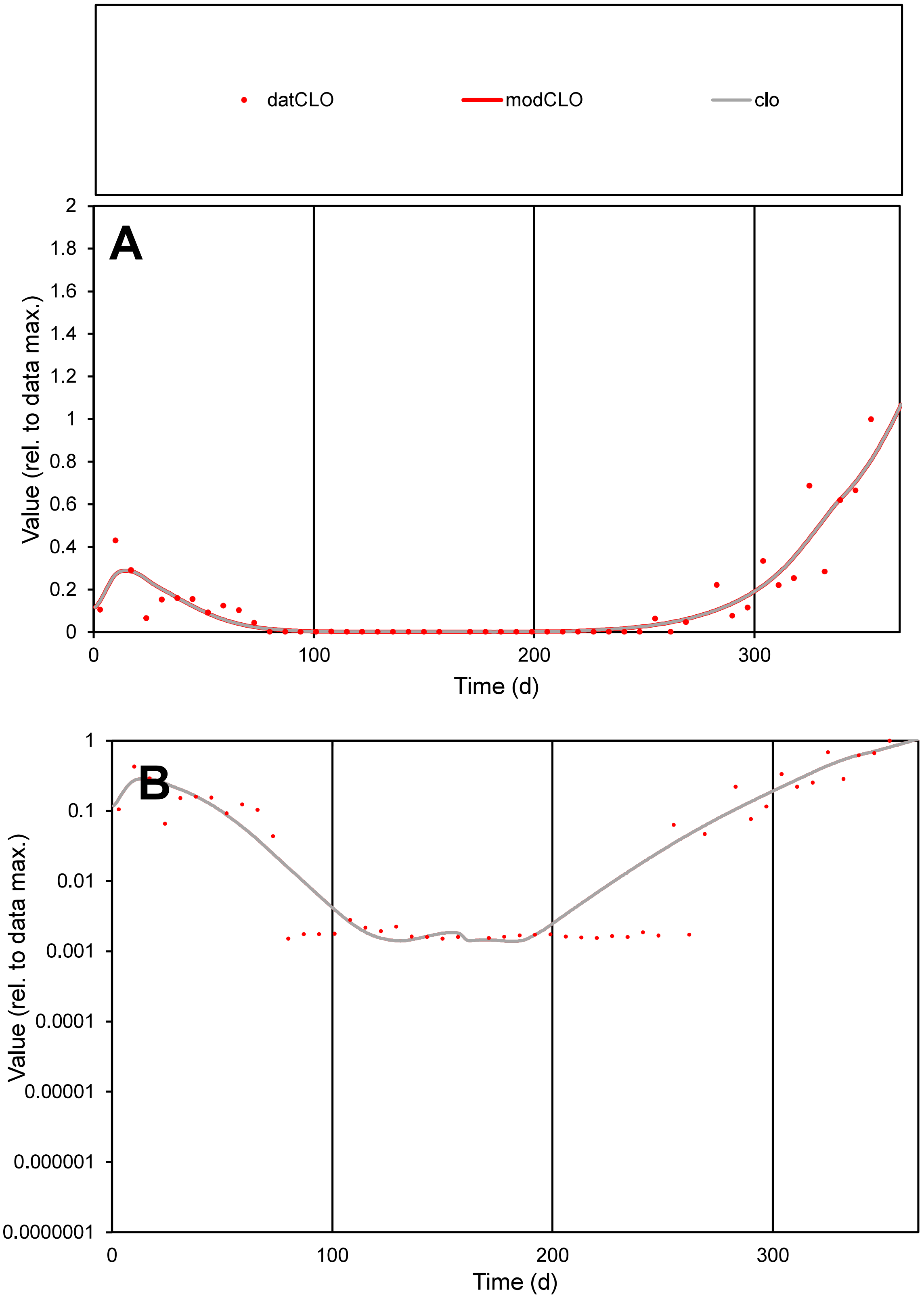

C1

Variables

CLO Provide jtd or Dst

CalibOnly?

2 1 to reproduce of

oNormalize?

2 1=no, 2=yes

ofxn calc*

0.57081

ofxn mod

0.60933

sRun

RunA1

zout

1

Ave

Data

0.00113

Model

0.00114

data/vMax

0.00871

ofxng

369.894

Min d/m

1.08217

ave/min d

85.792

ave/min m

93.7189

328_Calib_S_A

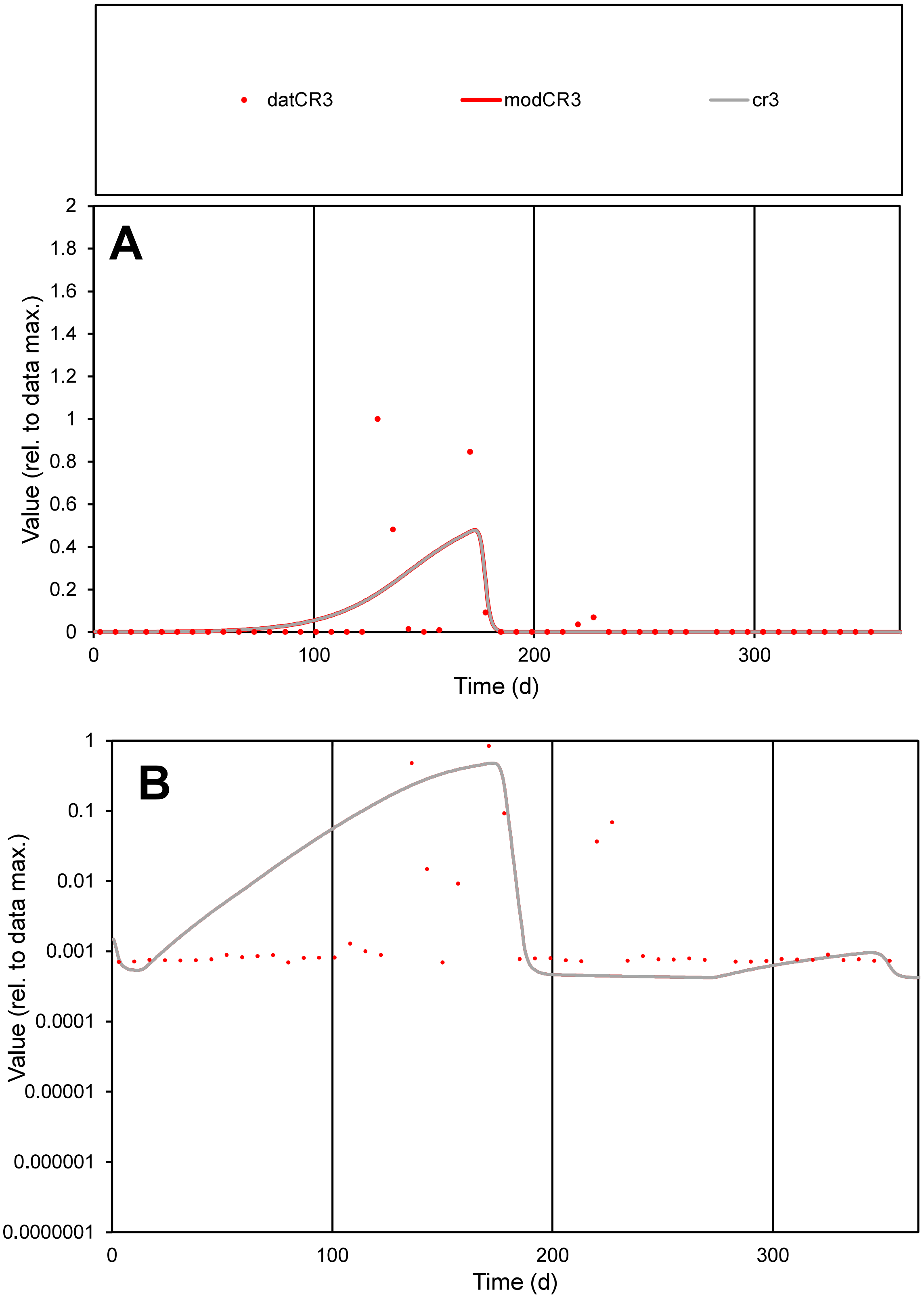

C1

Variables
CR3 Provide jtd or Dst
CalibOnly?
2 1 to reproduce of
oNormalize?
2 1=no, 2=yes

ofxn calc*
2.99867
ofxn mod
3.02782

sRun
RunA1
zout
1

Ave
Data
0.00269
Model
0.00265

data/vMax
0.05103

ofxng
369.894

Min d/m
1.64332

ave/min d
75.9162
ave/min m
122.877

328_Calib_S_A

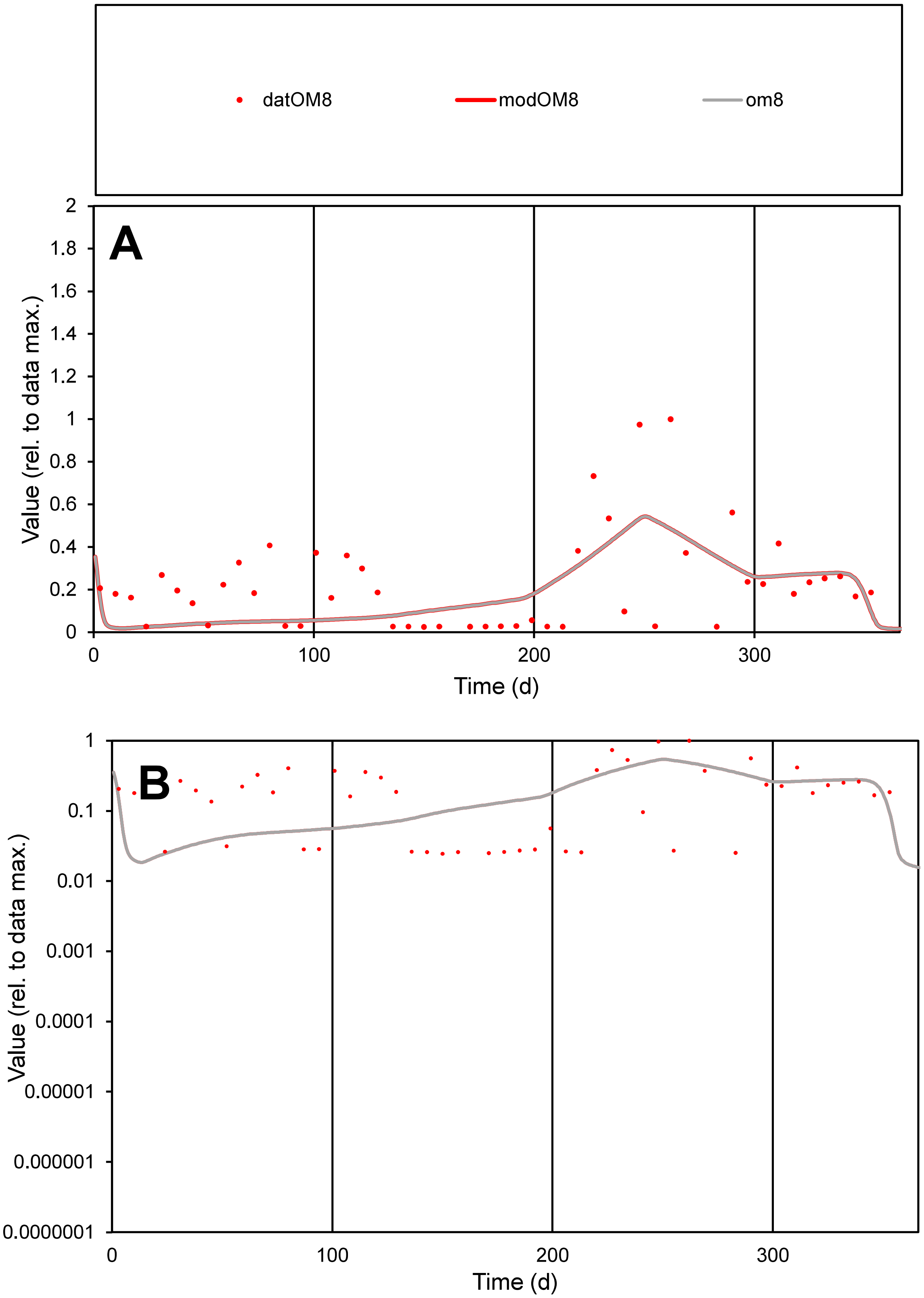

C1

Variables
OM8 Provide jtd or Dst
CalibOnly?
2 1 to reproduce of
oNormalize?
2 1=no, 2=yes

ofxn calc*
0.91219
ofxn mod
0.9261

sRun
RunA1
zout
1

Ave
Data
0.00049
Model
0.00041

data/vMax
0.0022

ofxng
369.894

Min d/m
1.29872

ave/min d
9.13191
ave/min m
9.86267

328_Calib_S_A

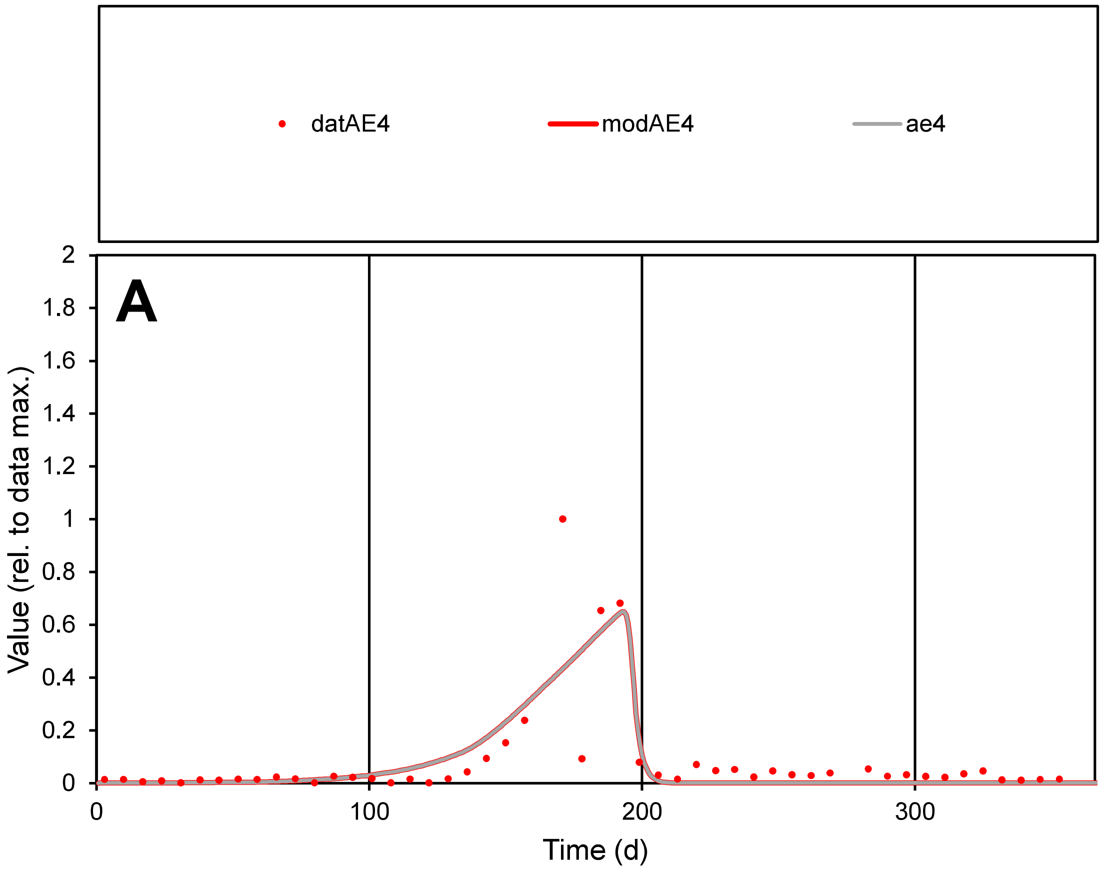

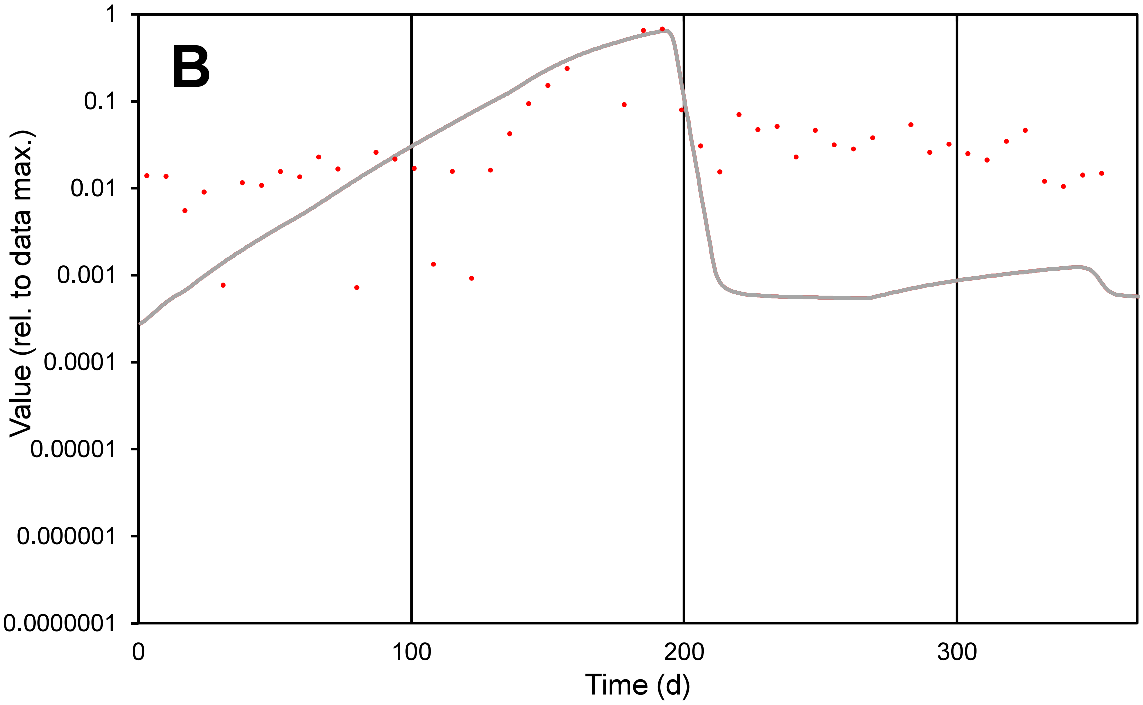

C1

Variables
AE4 Provide jtd or Dst
CalibOnly?
2 1 to reproduce of
oNormalize?
2 1=no, 2=yes

ofxn calc*
1.31746
ofxn mod
1.35244

sRun
RunA1
zout
1

Ave
Data
0.0013
Model
0.00117

data/vMax
0.01617

ofxng
369.894

Min d/m
2.12292

ave/min d
111.101
ave/min m
212.349

328_Calib_S_A

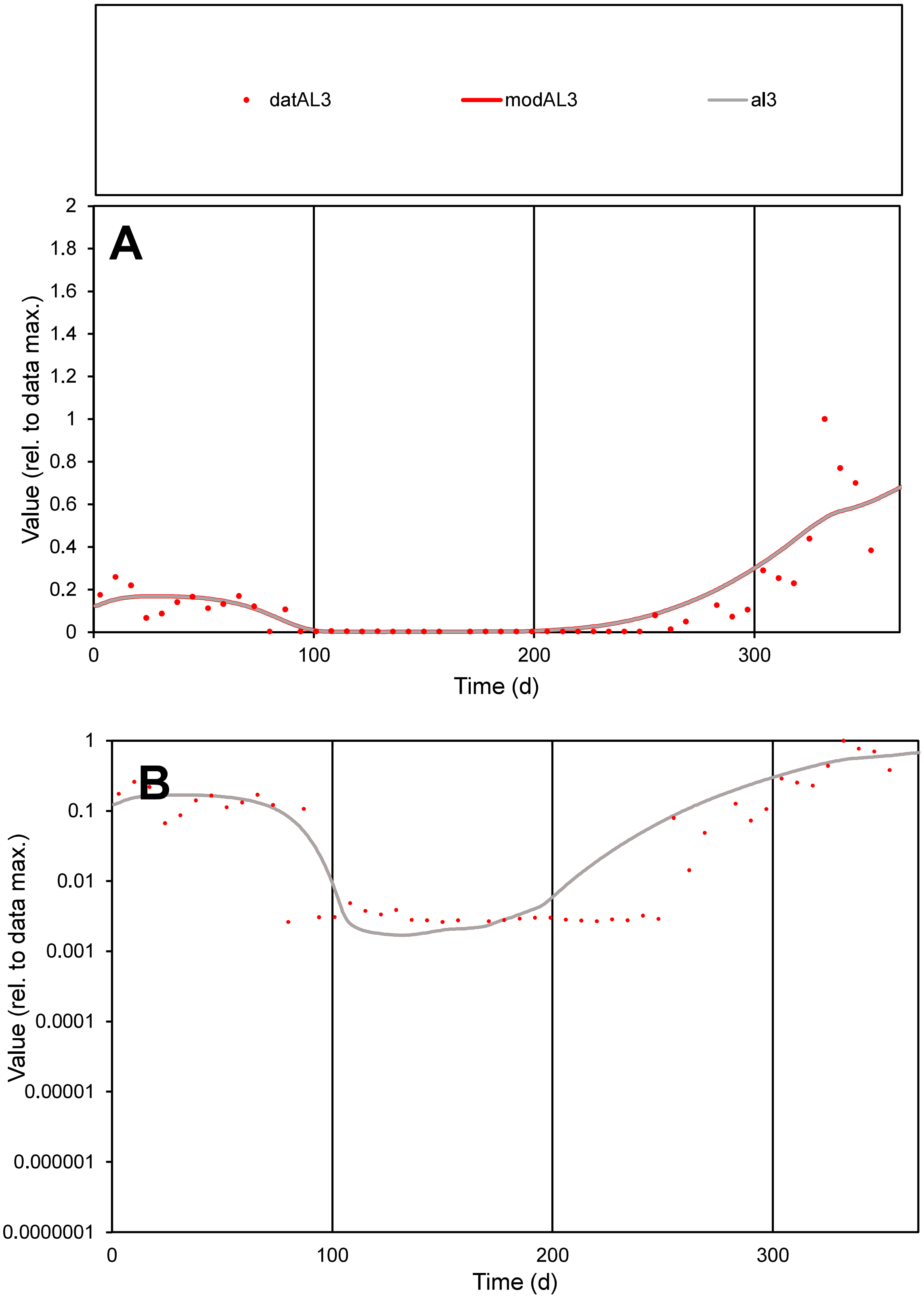

C1

Variables

AL3	Provide jtd or Dst

CalibOnly?

2	1 to reproduce of

oNormalize?

2	1=no, 2=yes

ofxn calc*
0.79453
ofxn mod
0.82055

sRun
RunA1
zout
1

Ave
Data
0.00119
Model
0.00134

data/vMax
0.00922

ofxng
369.894

Min d/m
1.53827

ave/min d
49.4302
ave/min m
85.1429

328_Calib_S_A

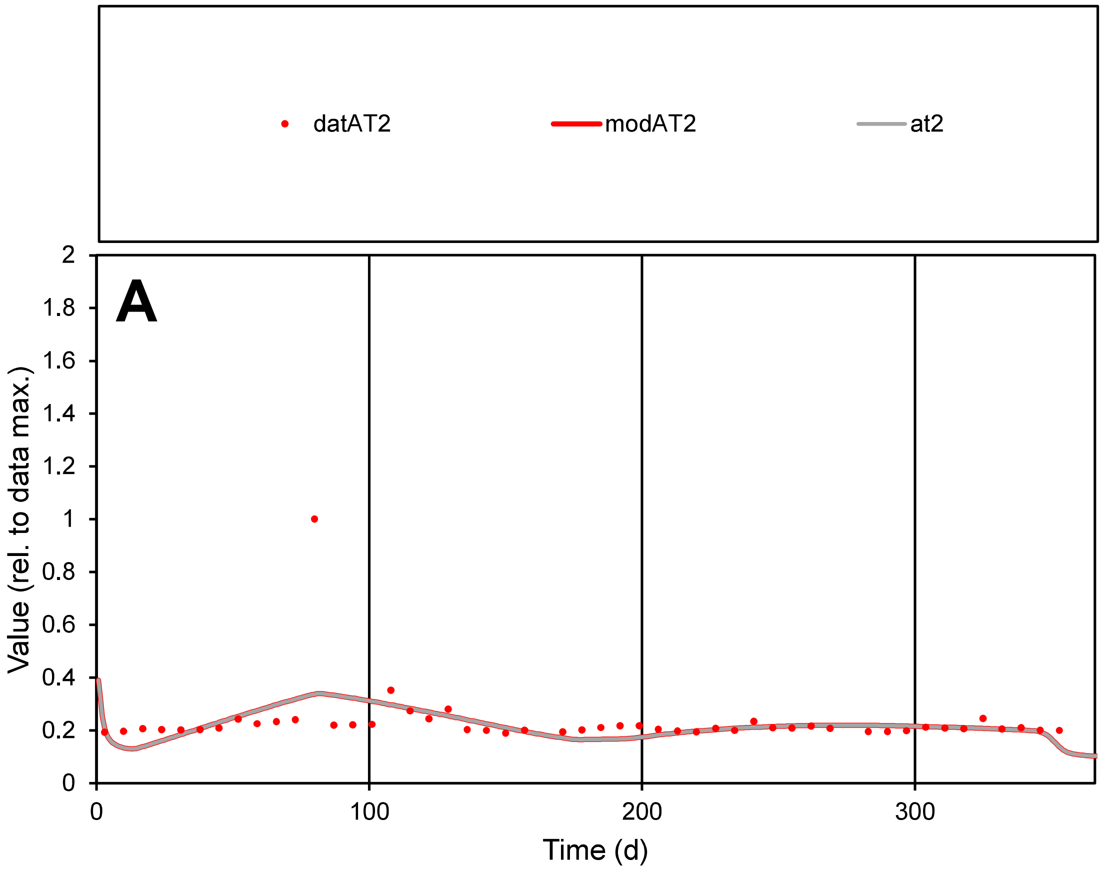

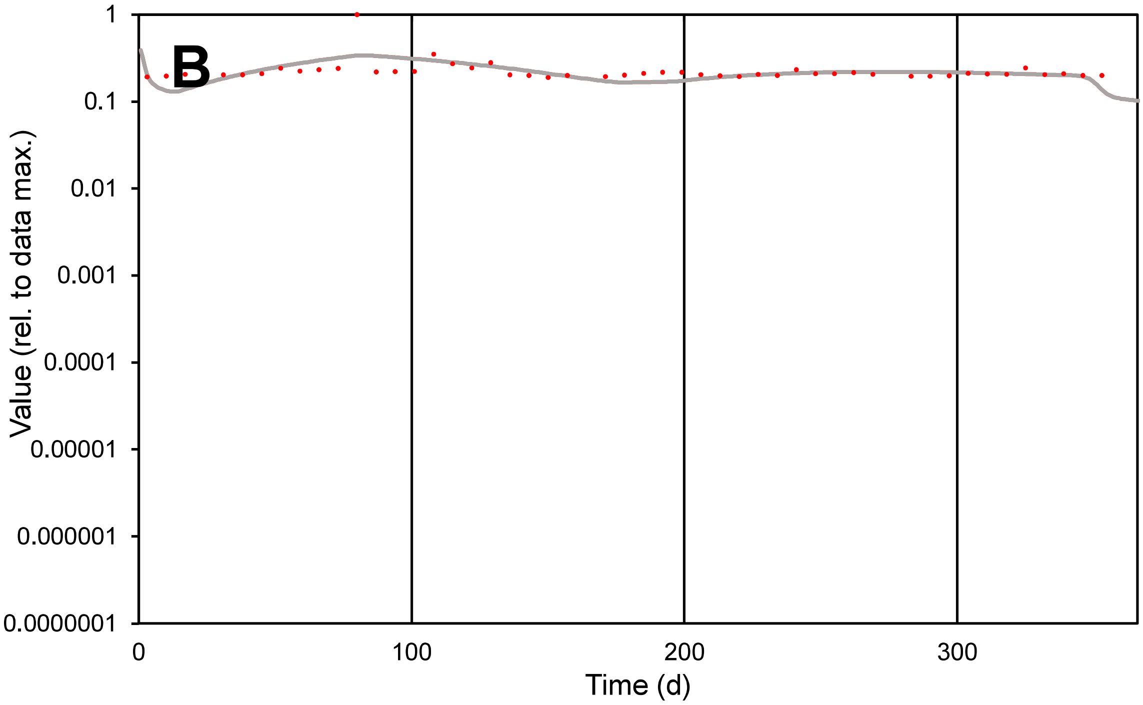

C1

Variables
AT2 Provide jtd or Dst
CalibOnly?
2 1 to reproduce of
oNormalize?
2 1=no, 2=yes

ofxn calc*
0.443
ofxn mod
0.45692

sRun
RunA1
zout
1

Ave
Data
9.7E-05
Model
9.3E-05

data/vMax
0.00042

ofxng
369.894

Min d/m
1.48451

ave/min d
1.22475
ave/min m
1.73399

328_Calib_S_A

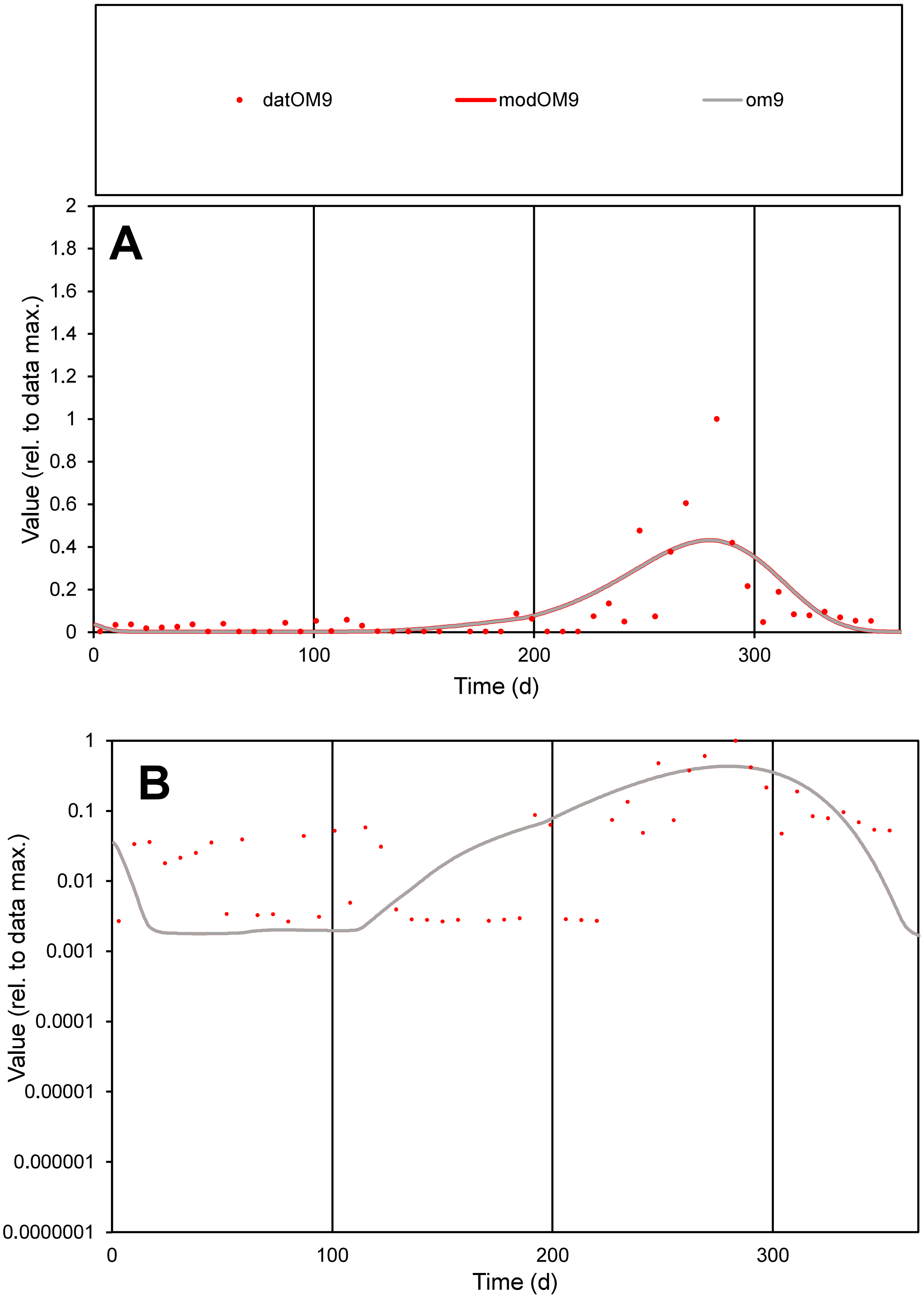

C1

Variables
OM9 Provide jtd or Dst
CalibOnly?
2 1 to reproduce of
oNormalize?
2 1=no, 2=yes

ofxn calc*
1.25313
ofxn mod
1.27709

sRun
RunA1
zout
1

Ave
Data
0.00156
Model
0.00169

data/vMax
0.01628

ofxng
369.894

Min d/m
1.49155
ave/min d
36.0989
ave/min m
58.3337

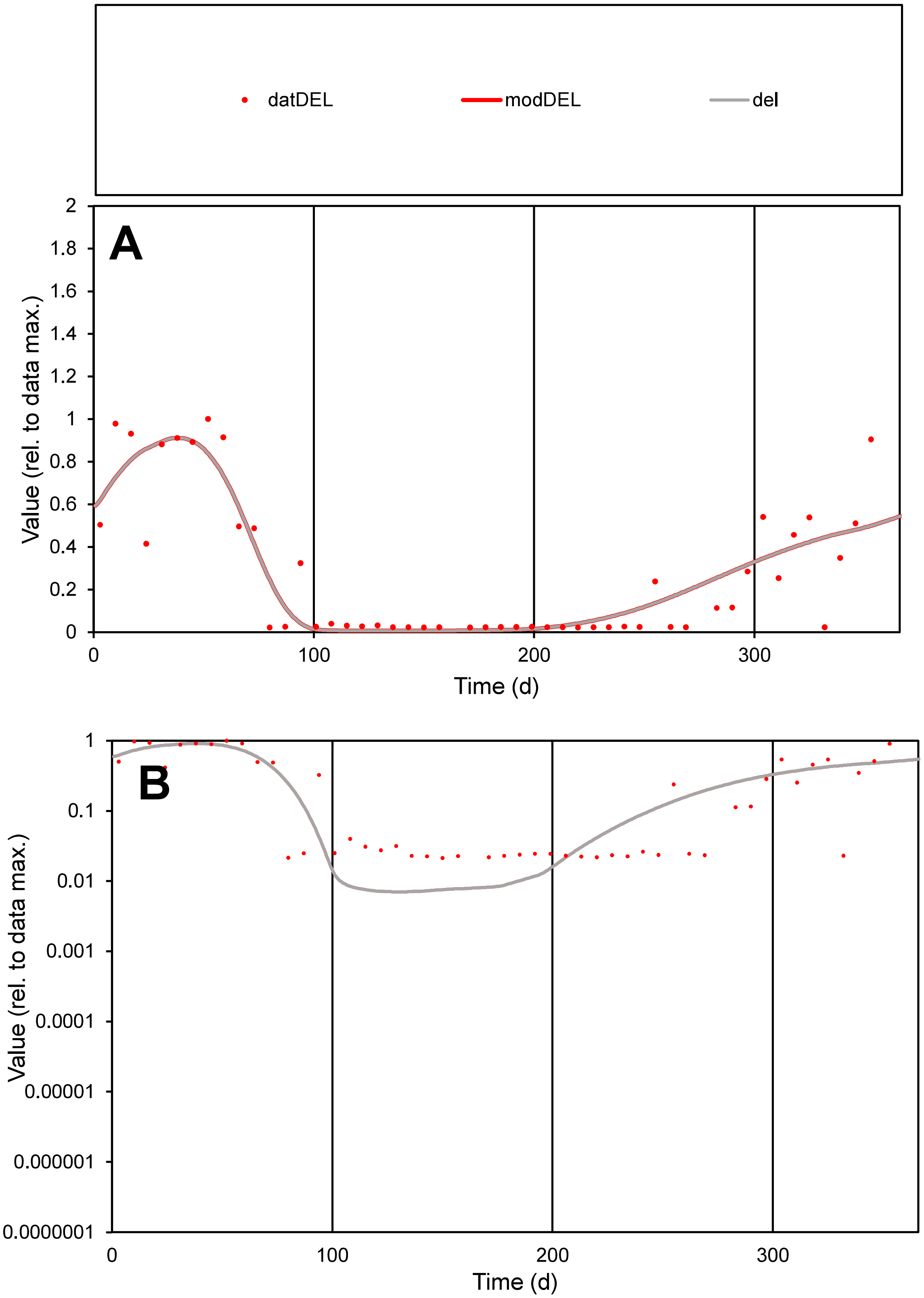

C1

Variables
DEL Provide jtd or Dst
CalibOnly?
2 1 to reproduce of
oNormalize?
2 1=no, 2=yes

ofxn calc*
0.51811
ofxn mod
0.55202

sRun
RunA1
zout
1

Ave
Data
0.00081
Model
0.00082

data/vMax
0.0029

ofxng
369.894

Min d/m
3.05372

ave/min d
13.0539
ave/min m
40.6067

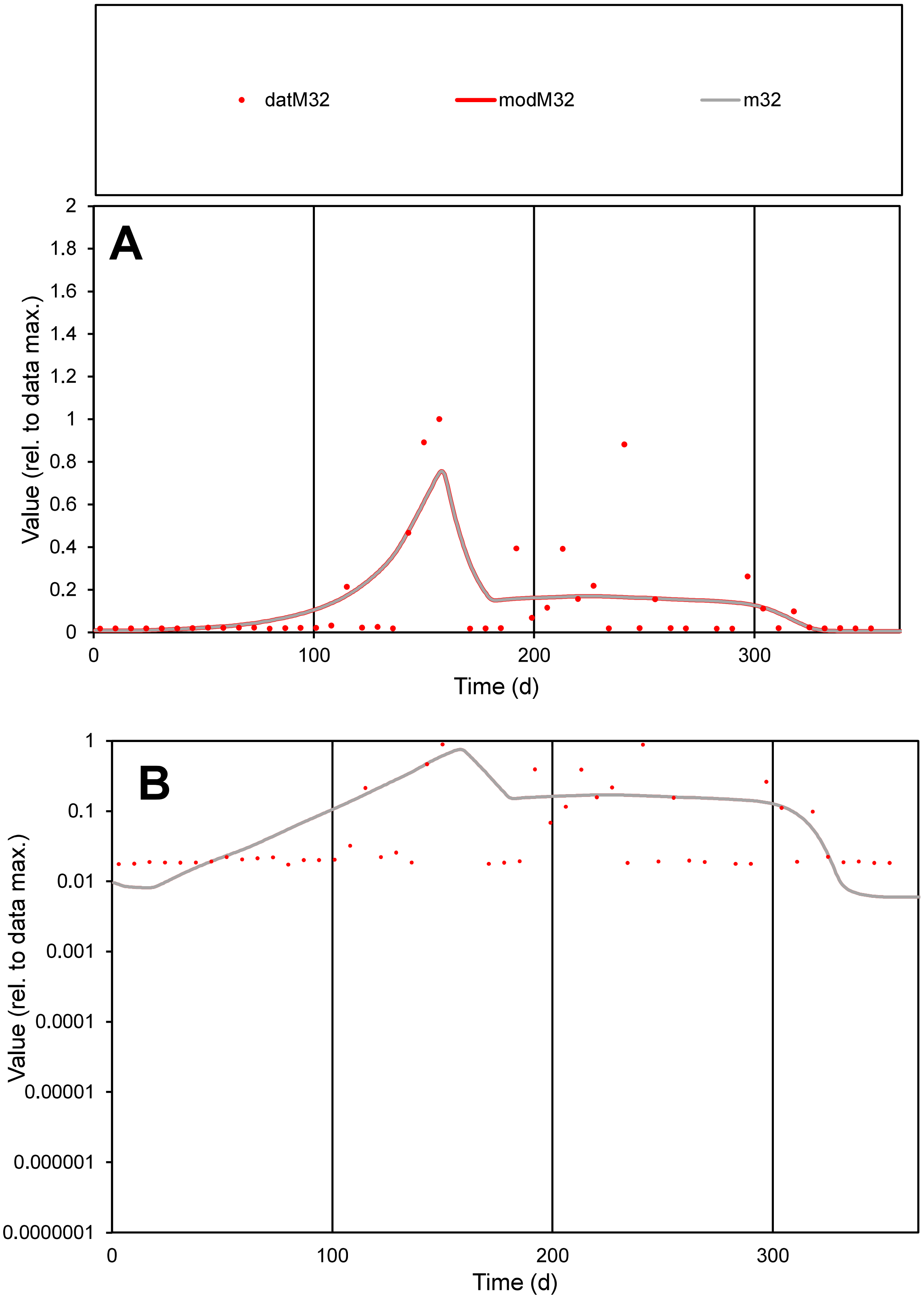

C1

Variables
M32 Provide jtd or Dst
CalibOnly?
2 1 to reproduce of
oNormalize?
2 1=no, 2=yes

ofxn calc*
1.27048
ofxn mod
1.29791

sRun
RunA1
zout
1

Ave
Data
0.00151
Model
0.00168

data/vMax
0.0121

ofxng
369.894

Min d/m
2.91194

ave/min d
7.16253
ave/min m
23.2994

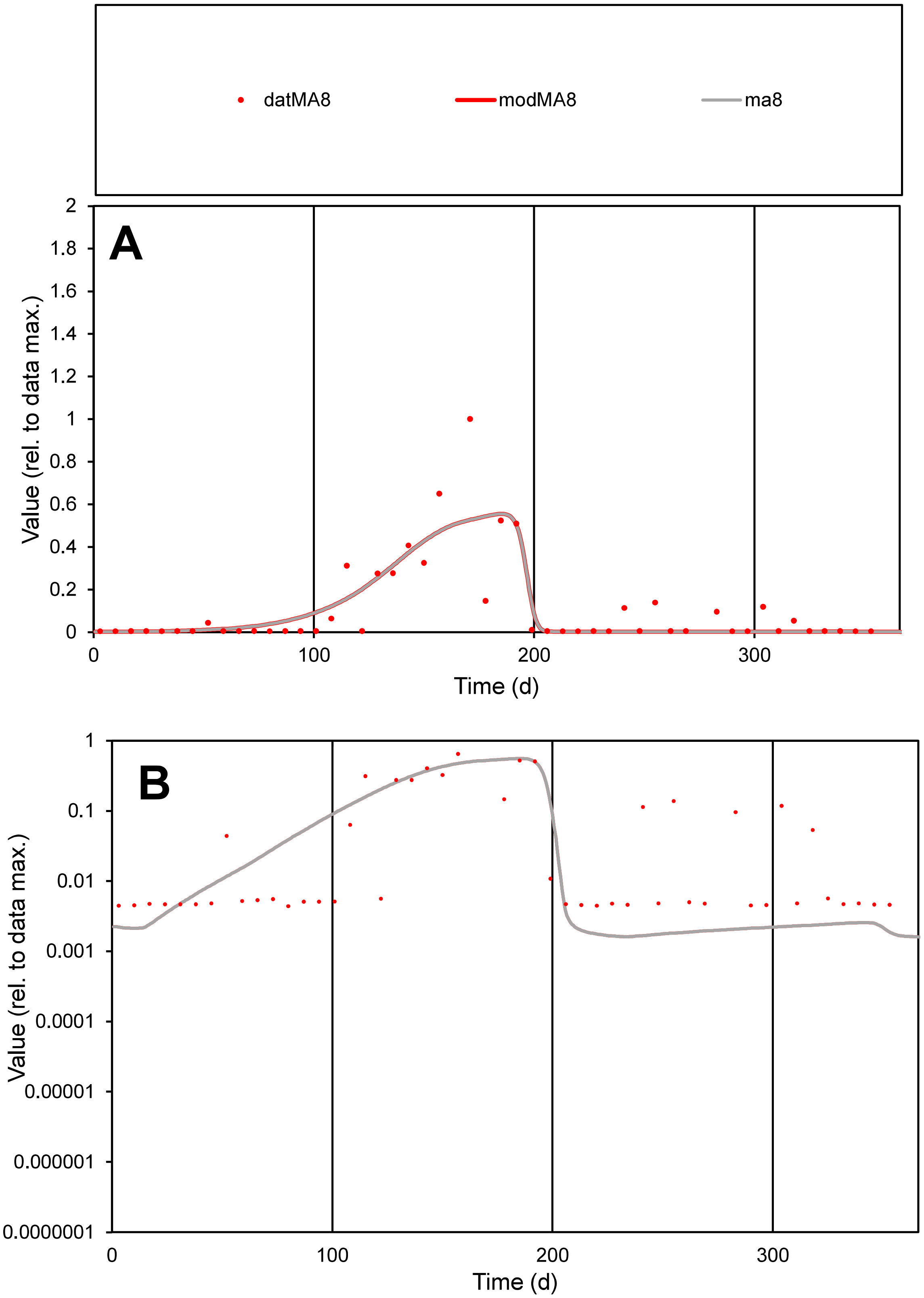

C1

Variables
MA8 Provide jtd or Dst
CalibOnly?
2 1 to reproduce of
oNormalize?
2 1=no, 2=yes

ofxn calc*
1.00901
ofxn mod
1.03544

sRun
RunA1
zout
1

Ave
Data
0.00146
Model
0.00138

data/vMax
0.01368

ofxng
369.894

Min d/m
2.71151

ave/min d
24.2353
ave/min m
62.2941

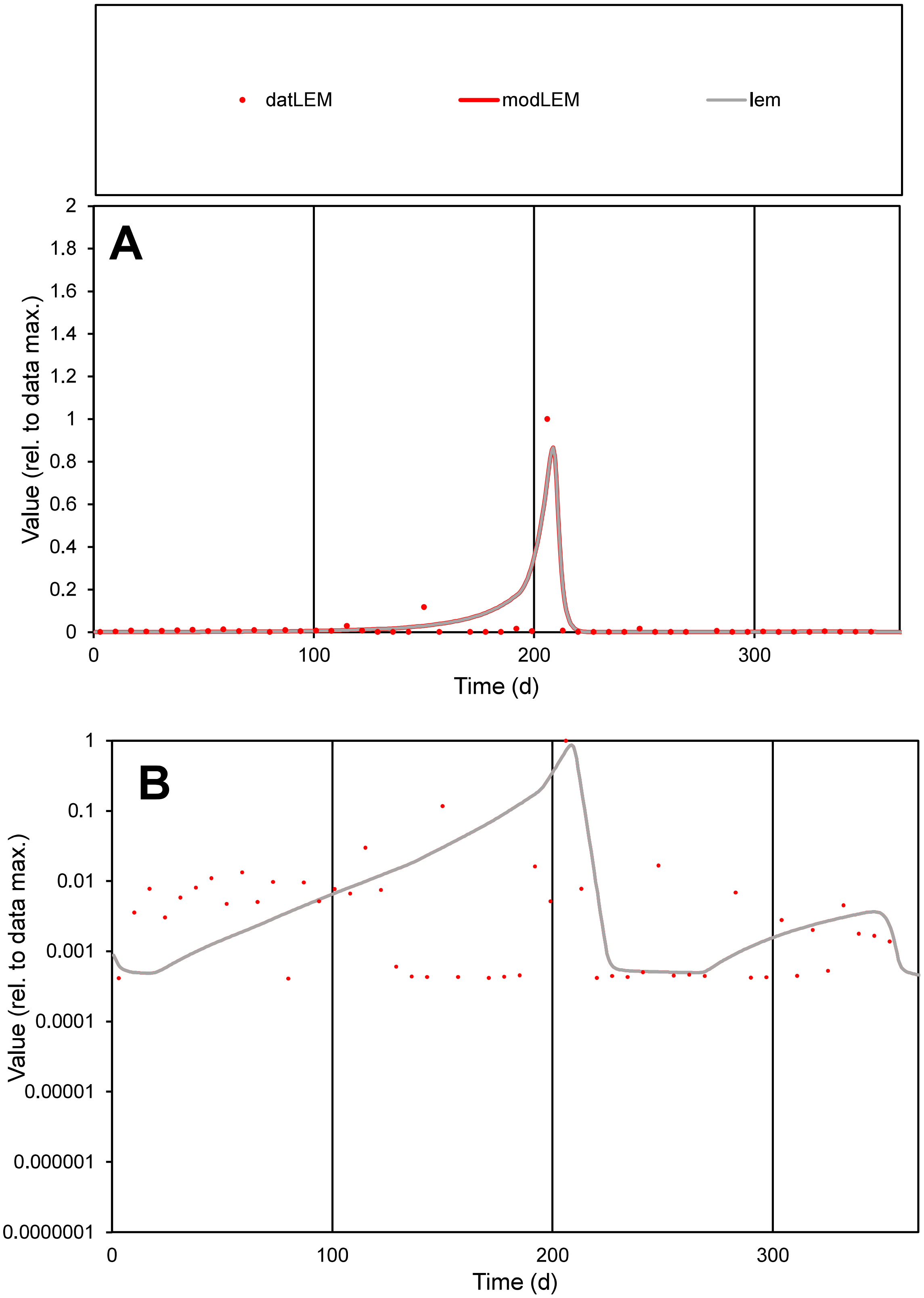

C1

Variables
LEM Provide jtd or Dst
CalibOnly?
2 1 to reproduce of
oNormalize?
2 1=no, 2=yes

ofxn calc*
2.10417
ofxn mod
2.31689

sRun
RunA1
zout
1

Ave
Data
0.00144
Model
0.00194

data/vMax
0.05291

ofxng
369.894

Min d/m
0.84017

ave/min d
66.252
ave/min m
75.1992

328_Calib_S_A

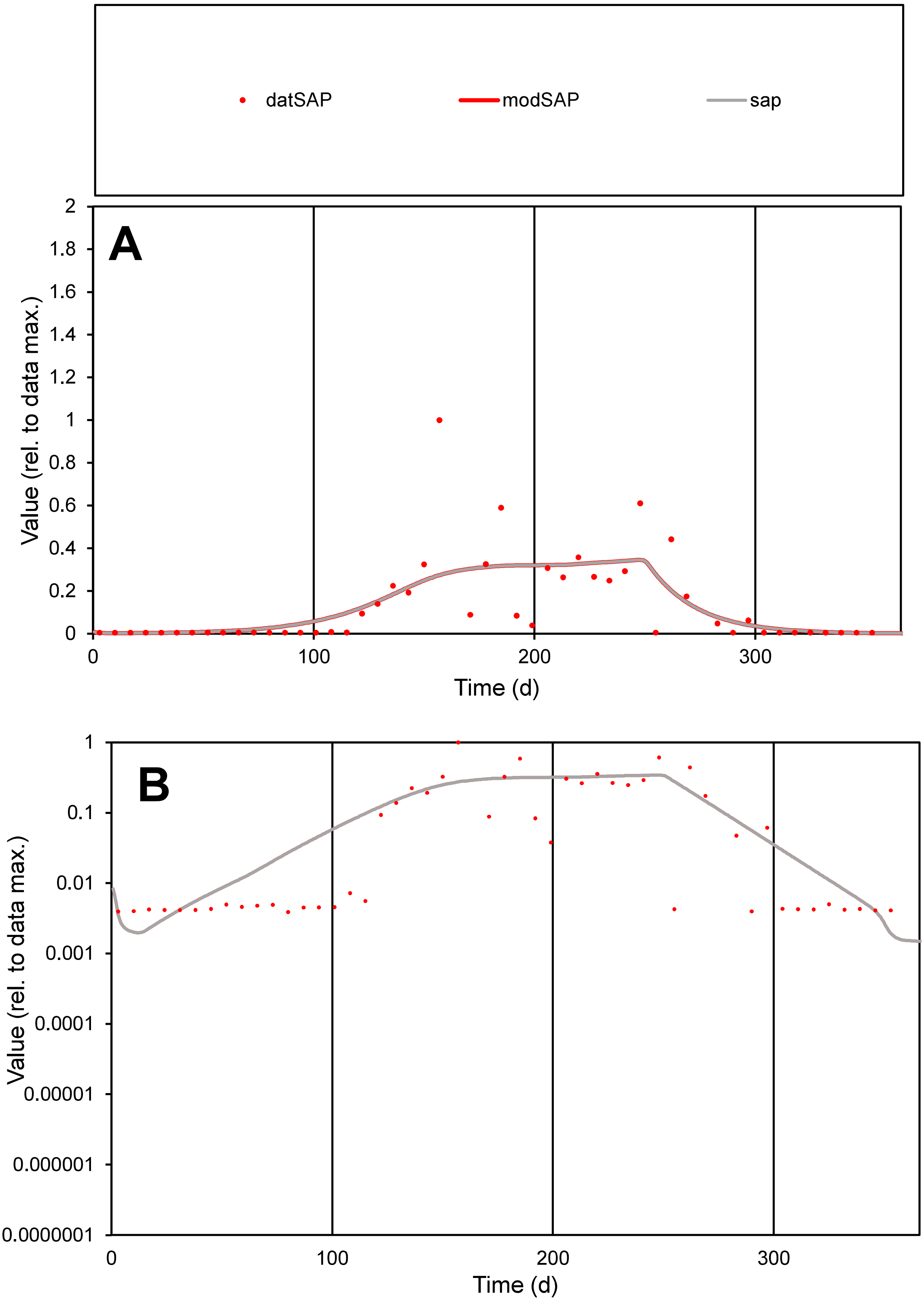

C1

Variables
SAP Provide jtd or Dst
CalibOnly?
2 1 to reproduce of
oNormalize?
2 1=no, 2=yes

ofxn calc*
1.125
ofxn mod
1.14373

sRun
RunA1
zout
1

Ave
Data
0.00109
Model
0.00111

data/vMax
0.00849

ofxng
369.894

Min d/m
2.07076

ave/min d
32.9075
ave/min m
69.4075

328_Calib_S_A

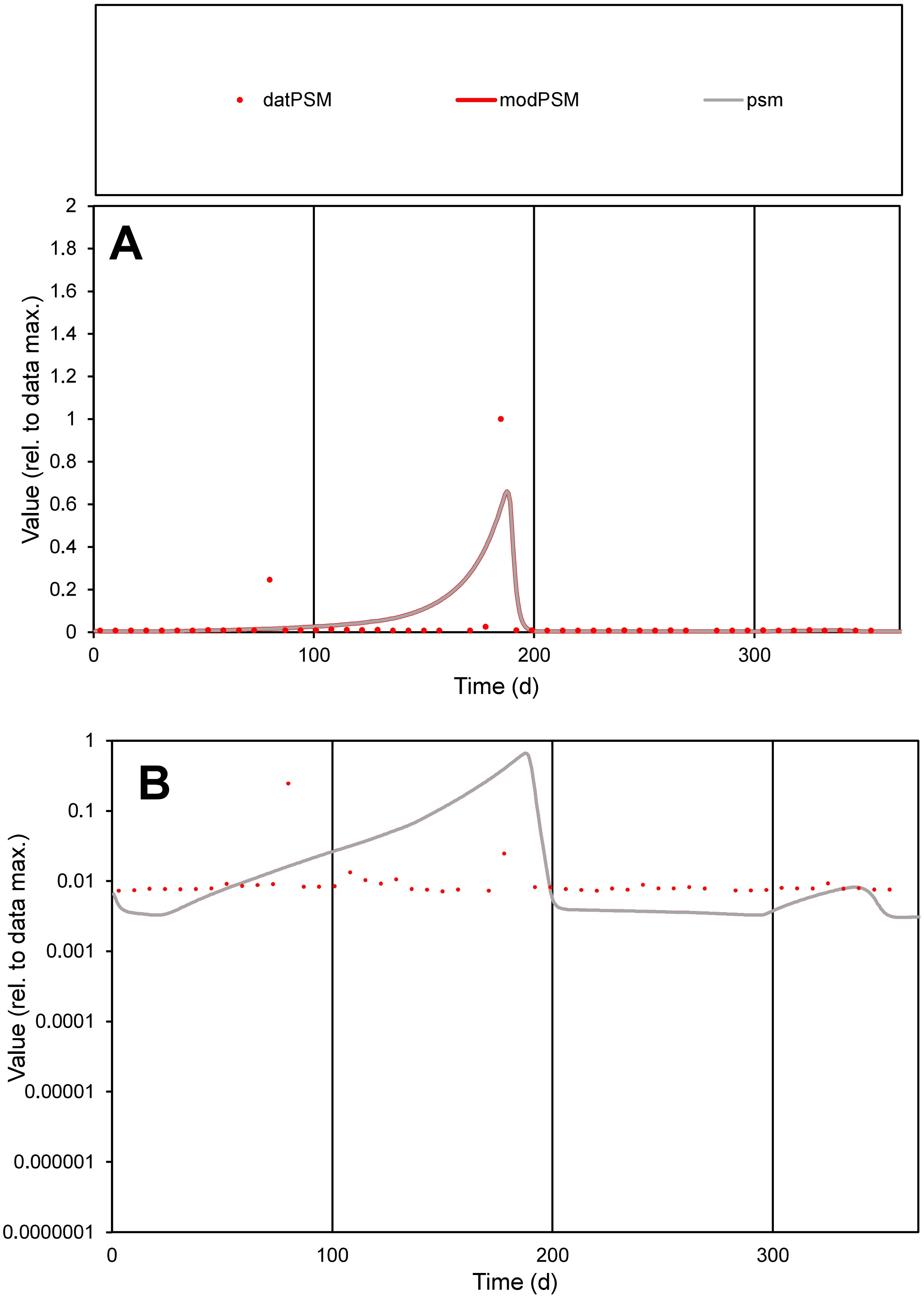

C1

Variables
PSM Provide jtd or Dst
CalibOnly?
2 1 to reproduce of
oNormalize?
2 1=no, 2=yes

ofxn calc*
2.79866
ofxn mod
2.89353

sRun
RunA1
zout
1

Ave
Data
0.00013
Model
0.00017

data/vMax
0.00387

ofxng
369.894

Min d/m
2.29246

ave/min d
4.69504
ave/min m
13.9888

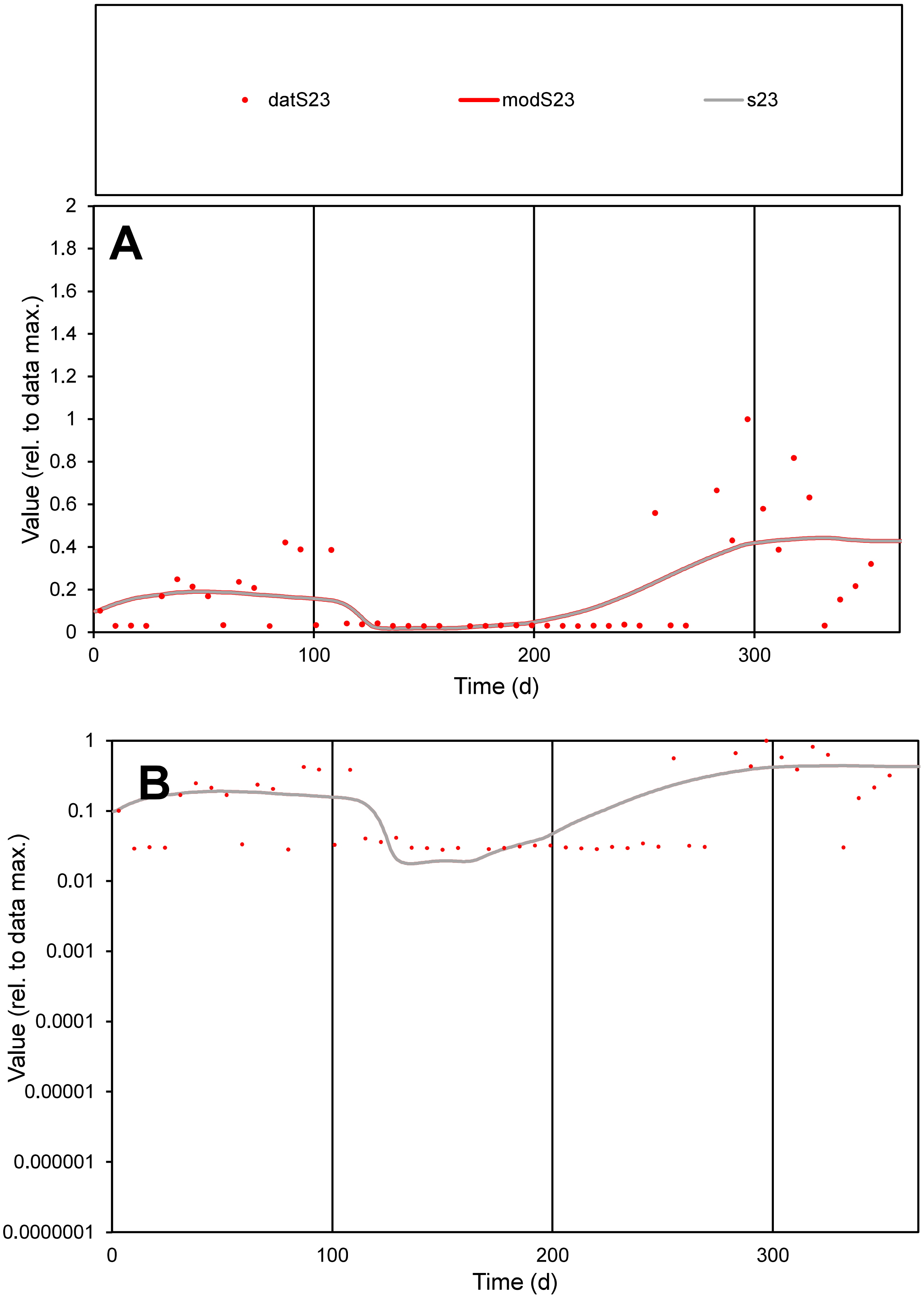

C1

Variables
S23 Provide jtd or Dst
CalibOnly?
2 1 to reproduce of
oNormalize?
2 1=no, 2=yes

ofxn calc*
0.94881
ofxn mod
0.96046

sRun
RunA1
zout
1

Ave
Data
0.00108
Model
0.00111

data/vMax
0.00575

ofxng
369.894

Min d/m
1.57493

ave/min d
6.69307
ave/min m
10.832

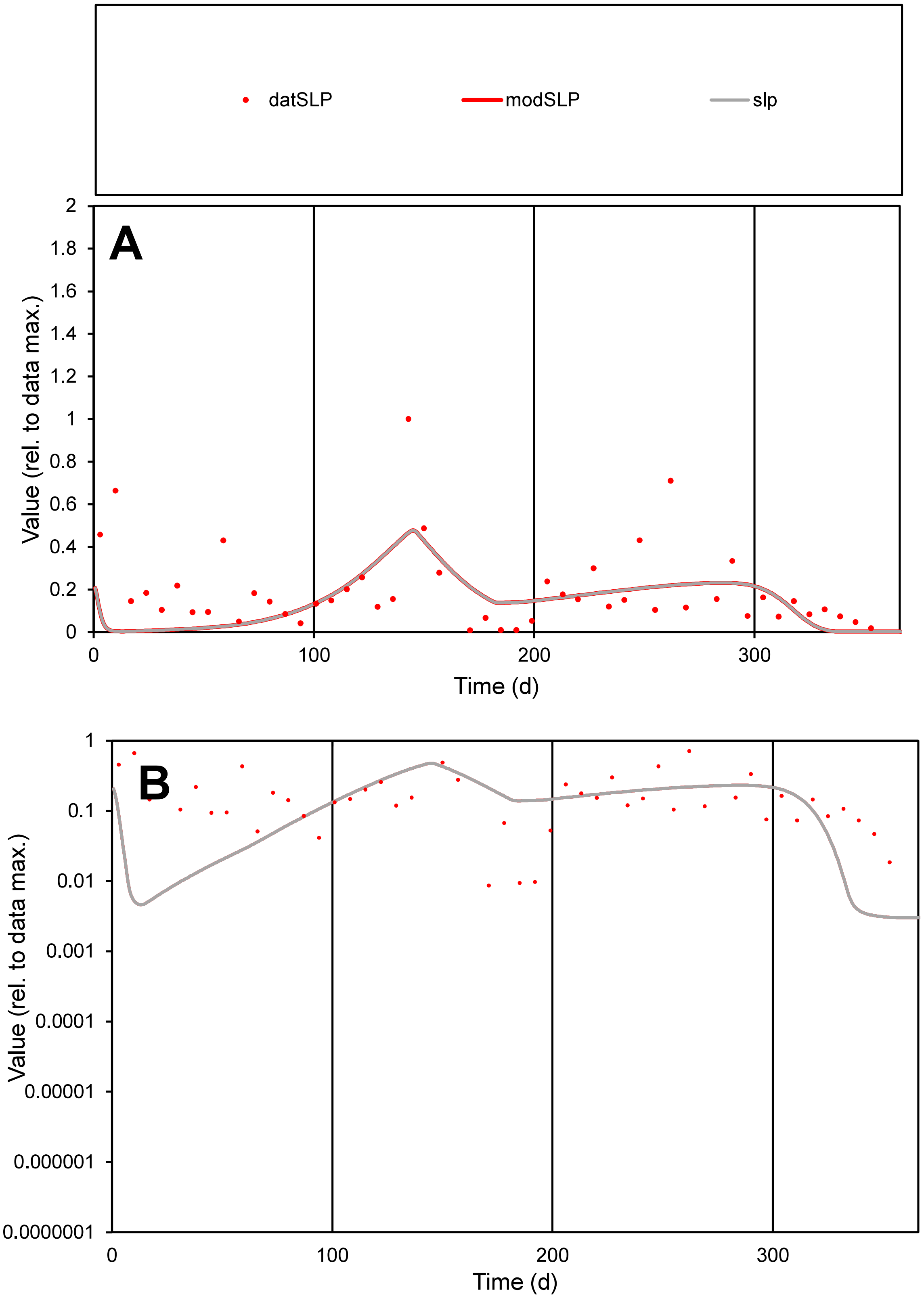

C1

Variables
SLP Provide jtd or Dst
CalibOnly?
2 1 to reproduce of
oNormalize?
2 1=no, 2=yes

ofxn calc*
0.95209
ofxn mod
0.96987

sRun
RunA1
zout
1

Ave
Data
0.00055
Model
0.00042

data/vMax
0.00282

ofxng
369.894

Min d/m
2.85034

ave/min d
22.5403
ave/min m
49.1576

328_Calib_S_A

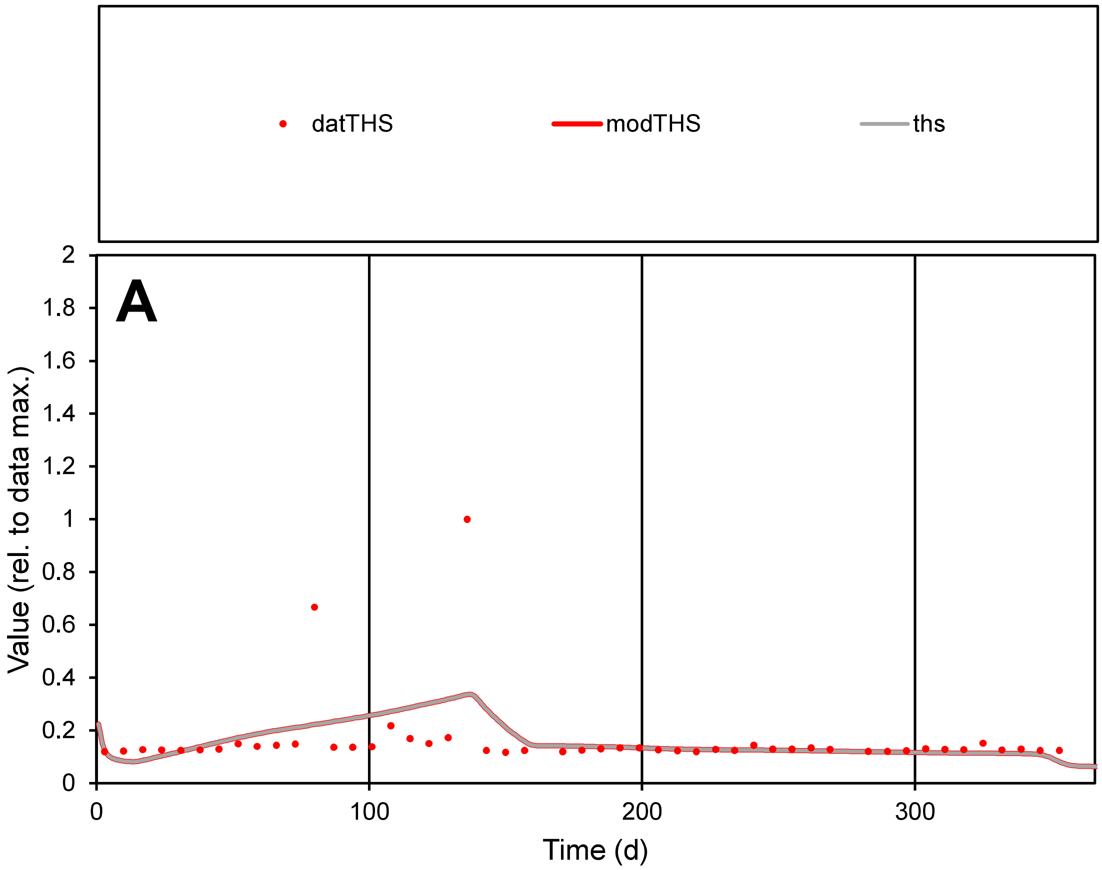

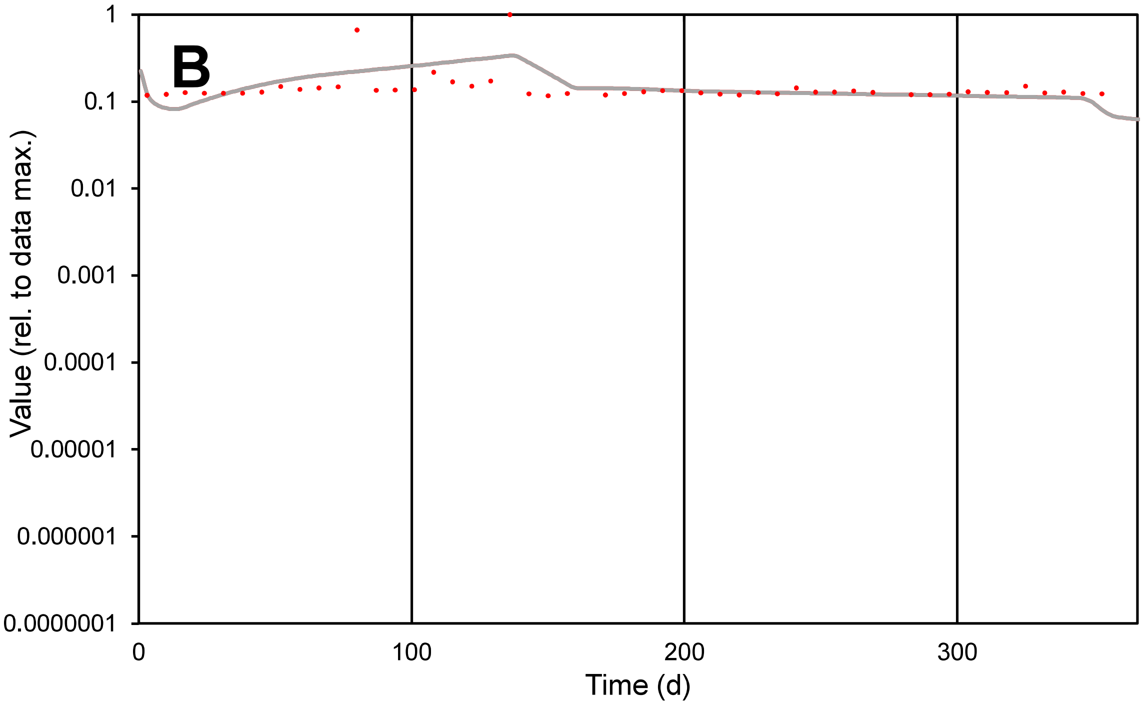

C1

Variables
THS Provide jtd or Dst
CalibOnly?
2 1 to reproduce of
oNormalize?
2 1=no, 2=yes

ofxn calc*
0.78695
ofxn mod
0.79596

sRun
RunA1
zout
1

Ave
Data
4E-05
Model
4E-05

data/vMax
0.00025

ofxng
369.894

Min d/m
1.5355

ave/min d
1.3873
ave/min m
2.12281

328_Calib_S_A

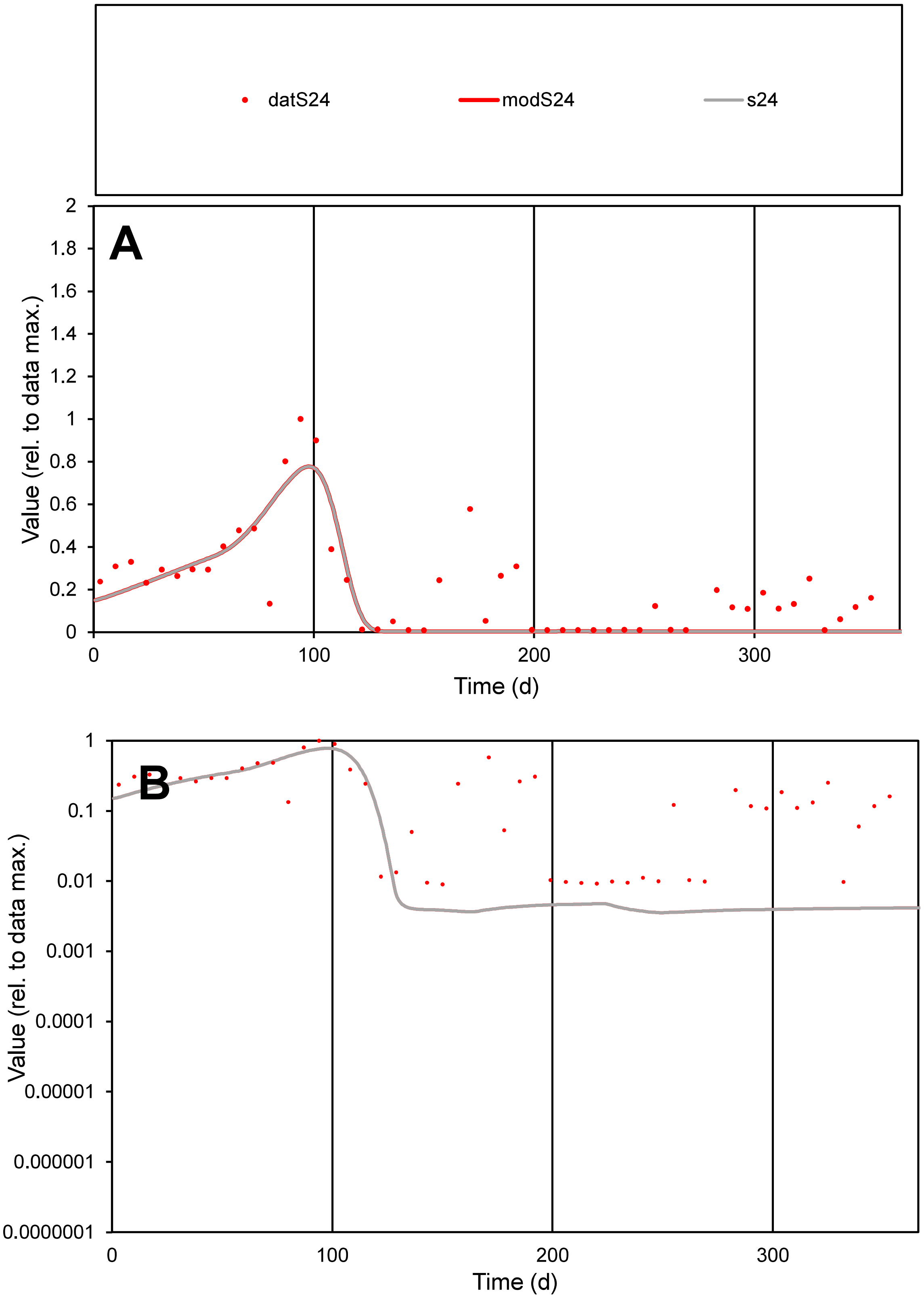

C1

Variables
S24 Provide jtd or Dst
CalibOnly?
2 1 to reproduce of
oNormalize?
2 1=no, 2=yes

ofxn calc*
0.73402
ofxn mod
0.74947

sRun
RunA1
zout
1

Ave
Data
0.00136
Model
0.00095

data/vMax
0.00645

ofxng
369.894

Min d/m
2.54661

ave/min d
23.3061
ave/min m
41.3825

328_Calib_S_A

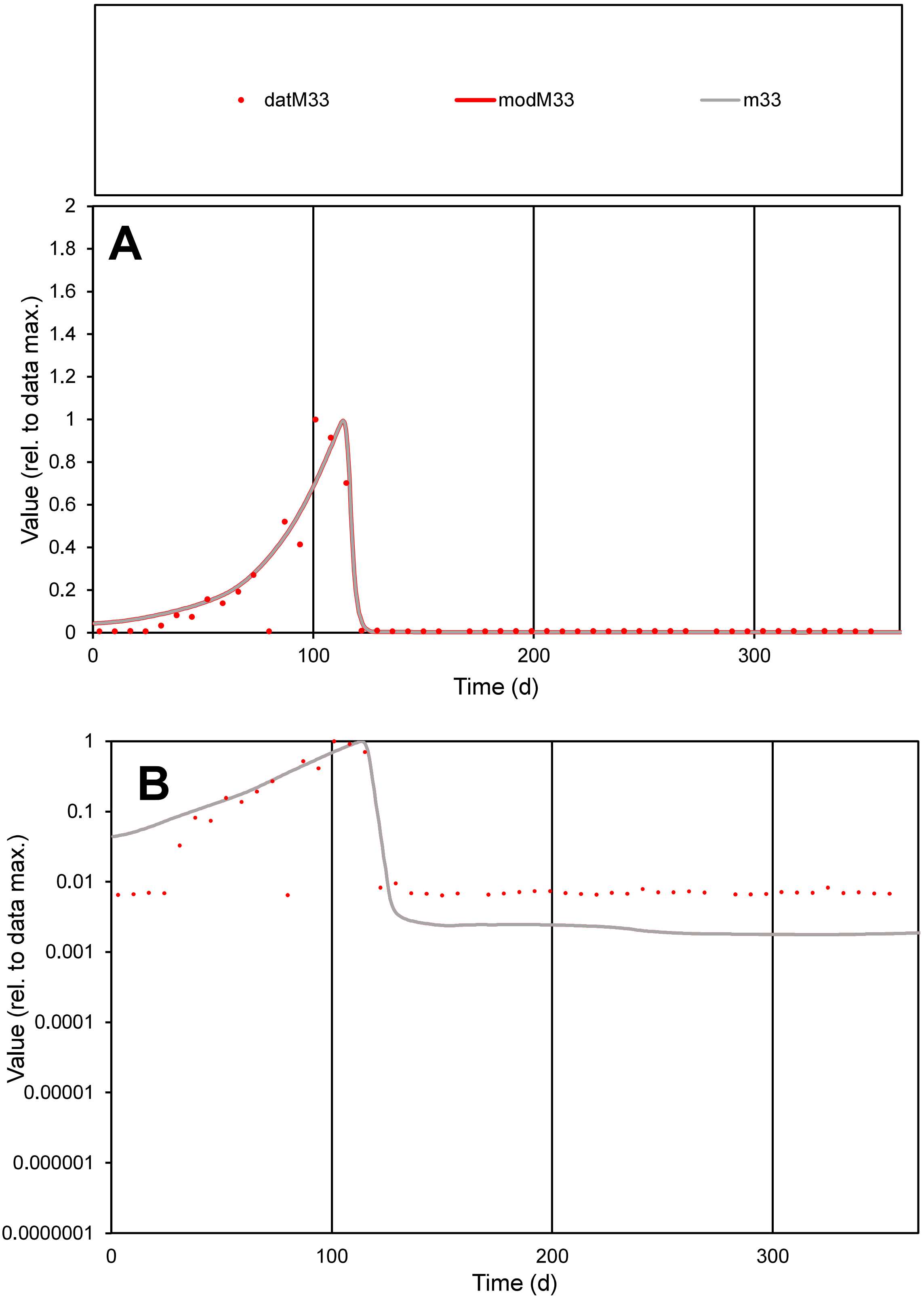

C1

Variables
M33 Provide jtd or Dst
CalibOnly?
2 1 to reproduce of
oNormalize?
2 1=no, 2=yes

ofxn calc*
0.72925
ofxn mod
0.77544

sRun
RunA1
zout
1

Ave
Data
0.00104
Model
0.00115

data/vMax
0.01069

ofxng
369.894

Min d/m
3.6315

ave/min d
15.1476
ave/min m
60.8316

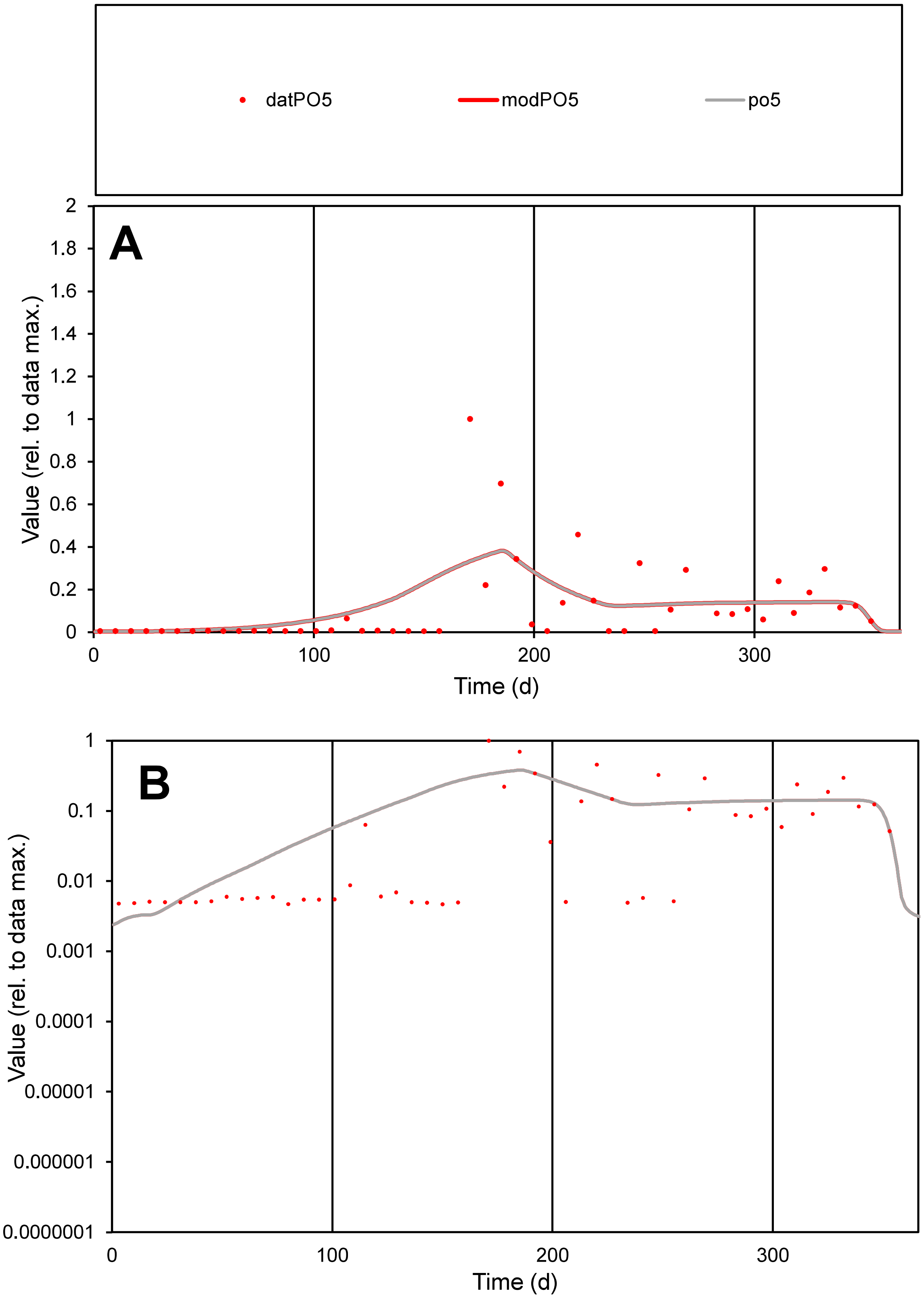

C1

Variables
PO5 Provide jtd or Dst
CalibOnly?
2 1 to reproduce of
oNormalize?
2 1=no, 2=yes

ofxn calc*
1.37378
ofxn mod
1.39164

sRun
RunA1
zout
1

Ave
Data
0.0011
Model
0.00125

data/vMax
0.00995

ofxng
369.894

Min d/m
1.70664

ave/min d
23.5985
ave/min m
45.8603

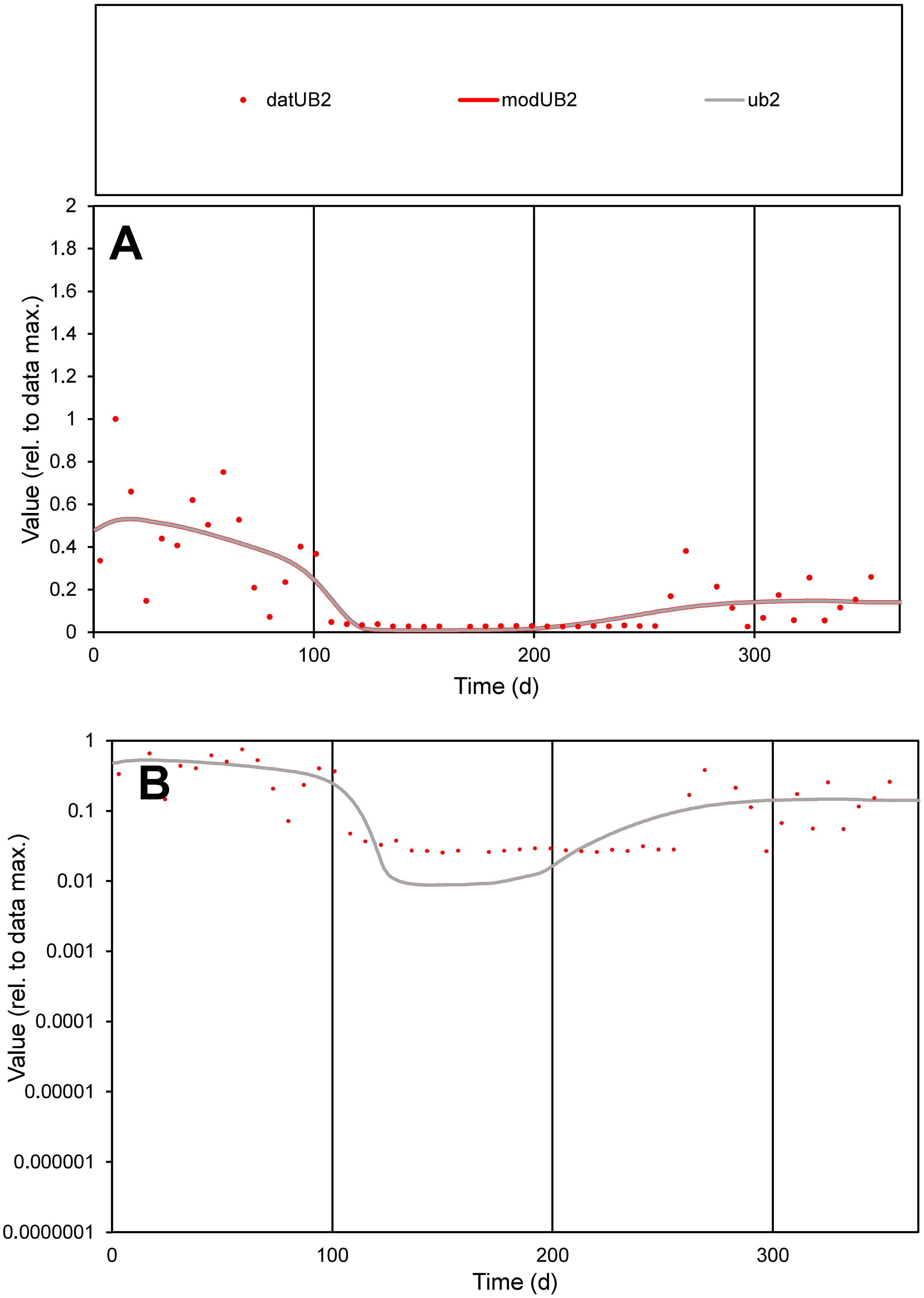

C1

Variables
UB2 Provide jtd or Dst
CalibOnly?
2 1 to reproduce of
oNormalize?
2 1=no, 2=yes

ofxn calc*
0.70178
ofxn mod
0.7241

sRun
RunA1
zout
1

Ave
Data
0.00078
Model
0.00075

data/vMax
0.00409

ofxng
369.894

Min d/m
2.89928

ave/min d
7.47432
ave/min m
20.9792

328_Calib_S_A

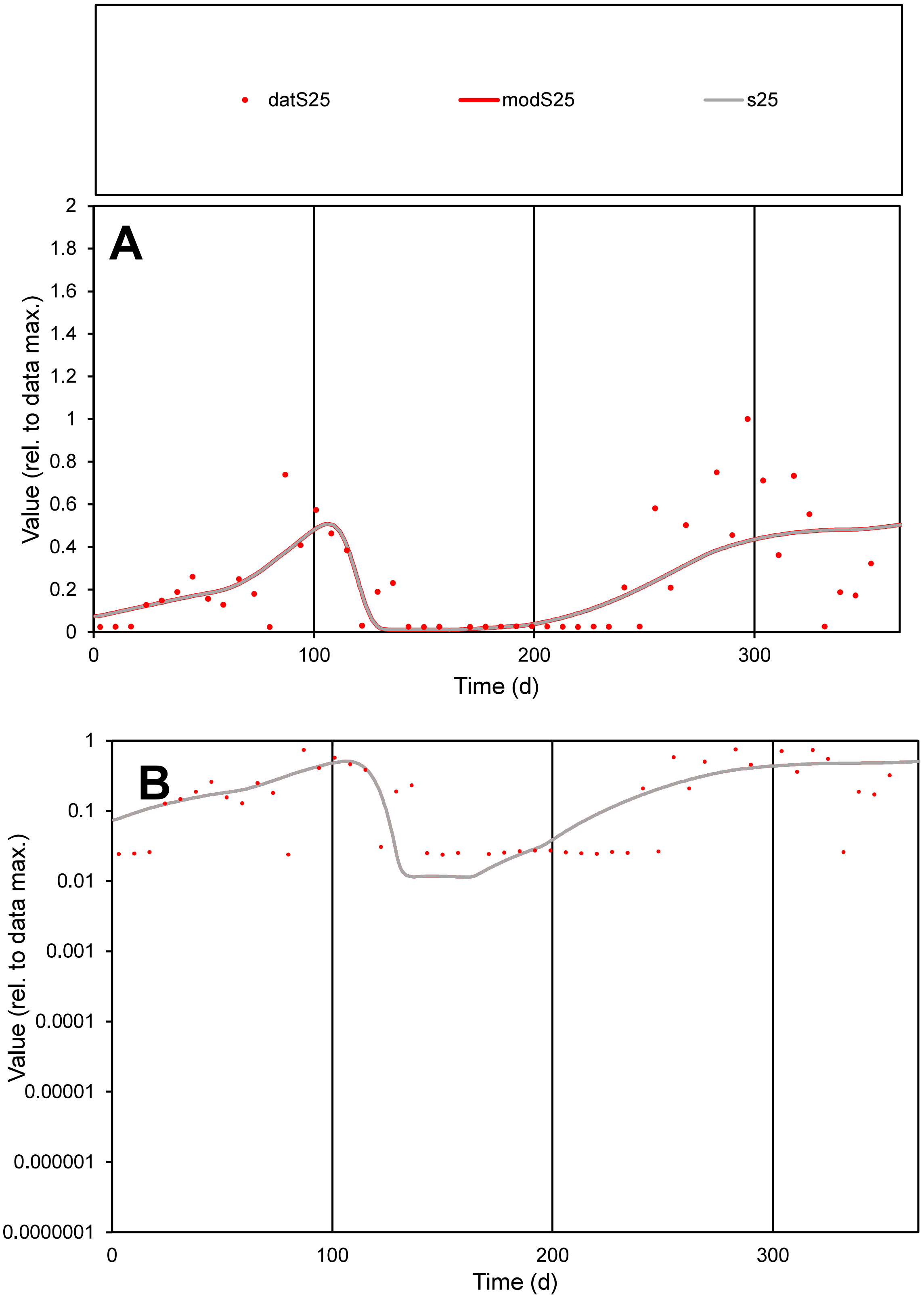

C1

Variables
S25 Provide jtd or Dst
CalibOnly?
2 1 to reproduce of
oNormalize?
2 1=no, 2=yes

ofxn calc*
0.75194
ofxn mod
0.76548

sRun
RunA1
zout
1

Ave
Data
0.00169
Model
0.00164

data/vMax
0.00709

ofxng
369.894

Min d/m
2.08652

ave/min d
9.98985
ave/min m
20.2142

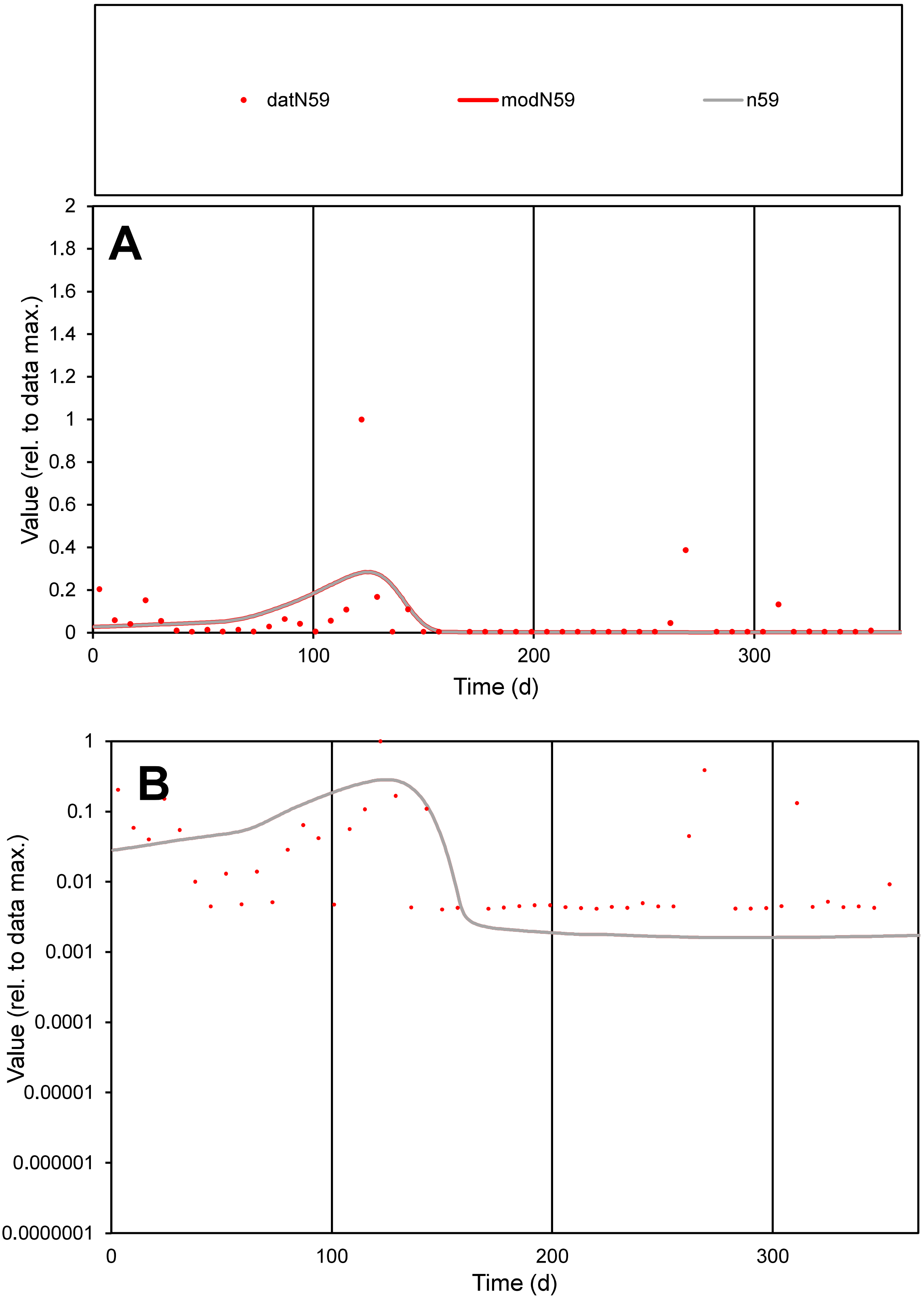

C1

Variables
N59 Provide jtd or Dst
CalibOnly?
2 1 to reproduce of
oNormalize?
2 1=no, 2=yes

ofxn calc*
2.33495
ofxn mod
2.35996

sRun
RunA1
zout
1

Ave
Data
0.00059
Model
0.00052

data/vMax
0.01026

ofxng
369.894

Min d/m
2.50697

ave/min d
14.3026
ave/min m
31.3594

328_Calib_S_A

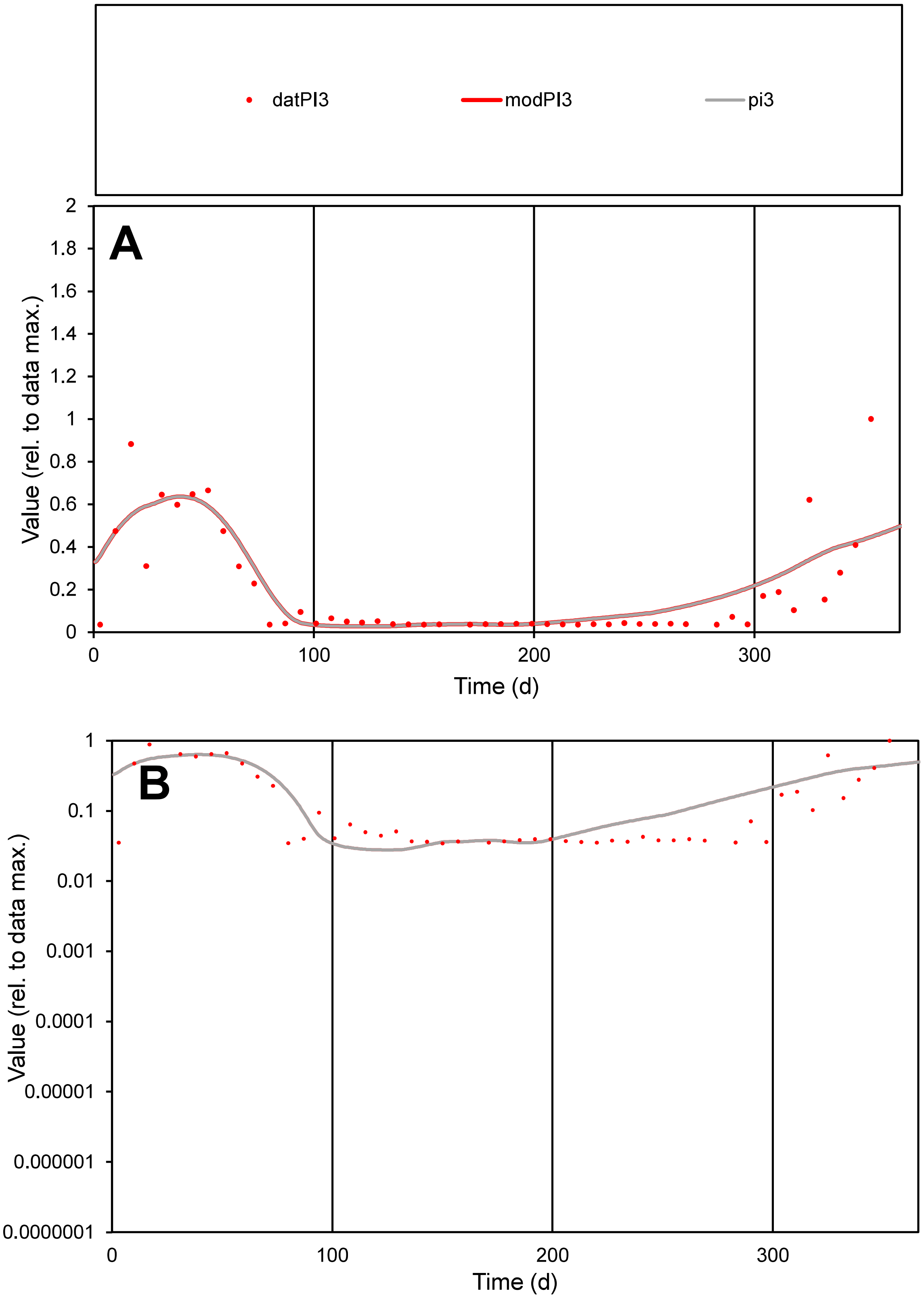

C1

Variables

PI3 Provide jtd or Dst

CalibOnly?

2 1 to reproduce of

oNormalize?

2 1=no, 2=yes

ofxn calc*

0.70179

ofxn mod

0.72865

sRun

RunA1

zout

1

Ave

Data

0.0004

Model

0.00044

data/vMax

0.00207

ofxng

369.894

Min d/m

1.24519

ave/min d

5.59873

ave/min m

7.76468

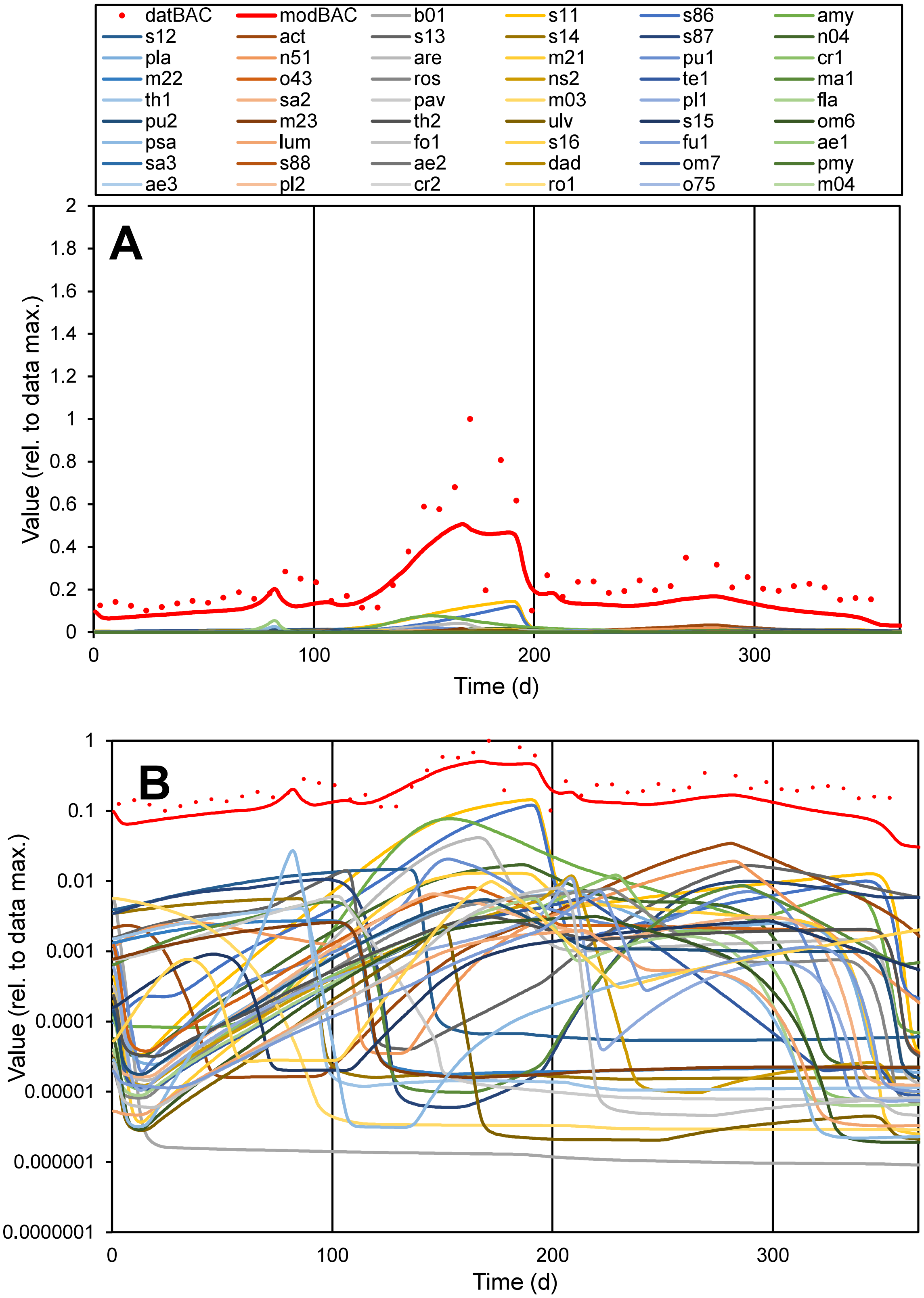

C1

Variables

BAC Provide jtd or Dst

CalibOnly?

2 1 to reproduce of

oNormalize?

2 1=no, 2=yes

ofxn calc*

0.52353

ofxn mod

0.53573

sRun

RunA1

zout

1

Ave

Data

1.20369

Model

0.82423

data/vMax

4.709

ofxng

369.894

Min d/m

2.11249

ave/min d

2.53355

ave/min m

3.66488

330_Calib_S_A

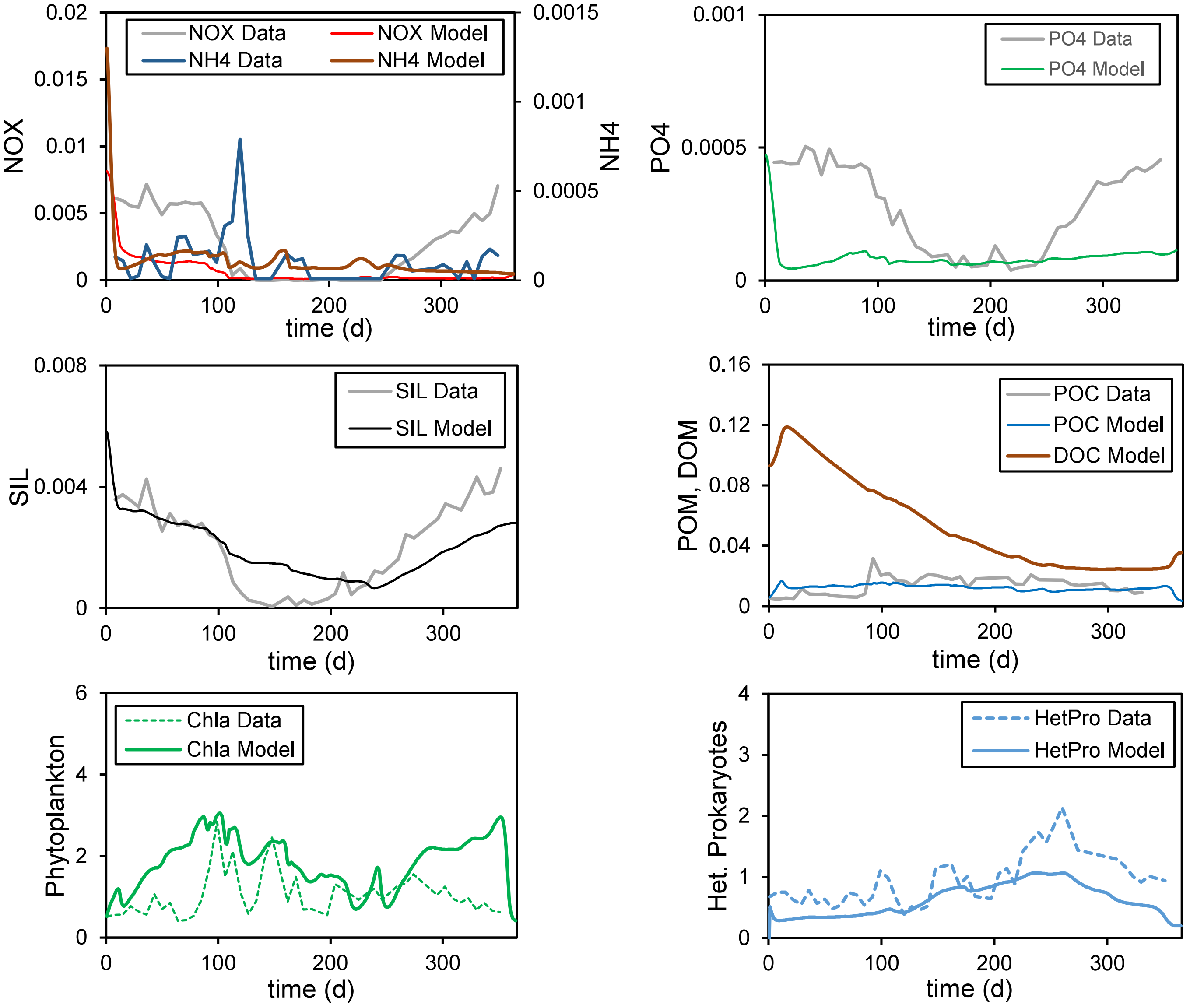
NOX Data
NOX Model
NH4 Data
NH4 Model
NOX
NH4
0.02
0.015
0.01
0.005
0
0.0015
0.001
0.0005
0
0
100
200
300
time (d)
PO4 Data
PO4 Model
PO4
0.001
0.0005
0
0
100
200
300
time (d)
SIL Data
SIL Model
SIL
0.008
0.004
0
0
100
200
300
time (d)
POC Data
POC Model
DOC Model
POM, DOM
0.16
0.12
0.08
0.04
0
0
100
200
300
time (d)
Chla Data
Chla Model
Phytoplankton
6
4
2
0
0
100
200
300
time (d)
HetPro Data
HetPro Model
Het. Prokaryotes
4
3
2
1
0
0
100
200
300
time (d)

330_Calib_S_A

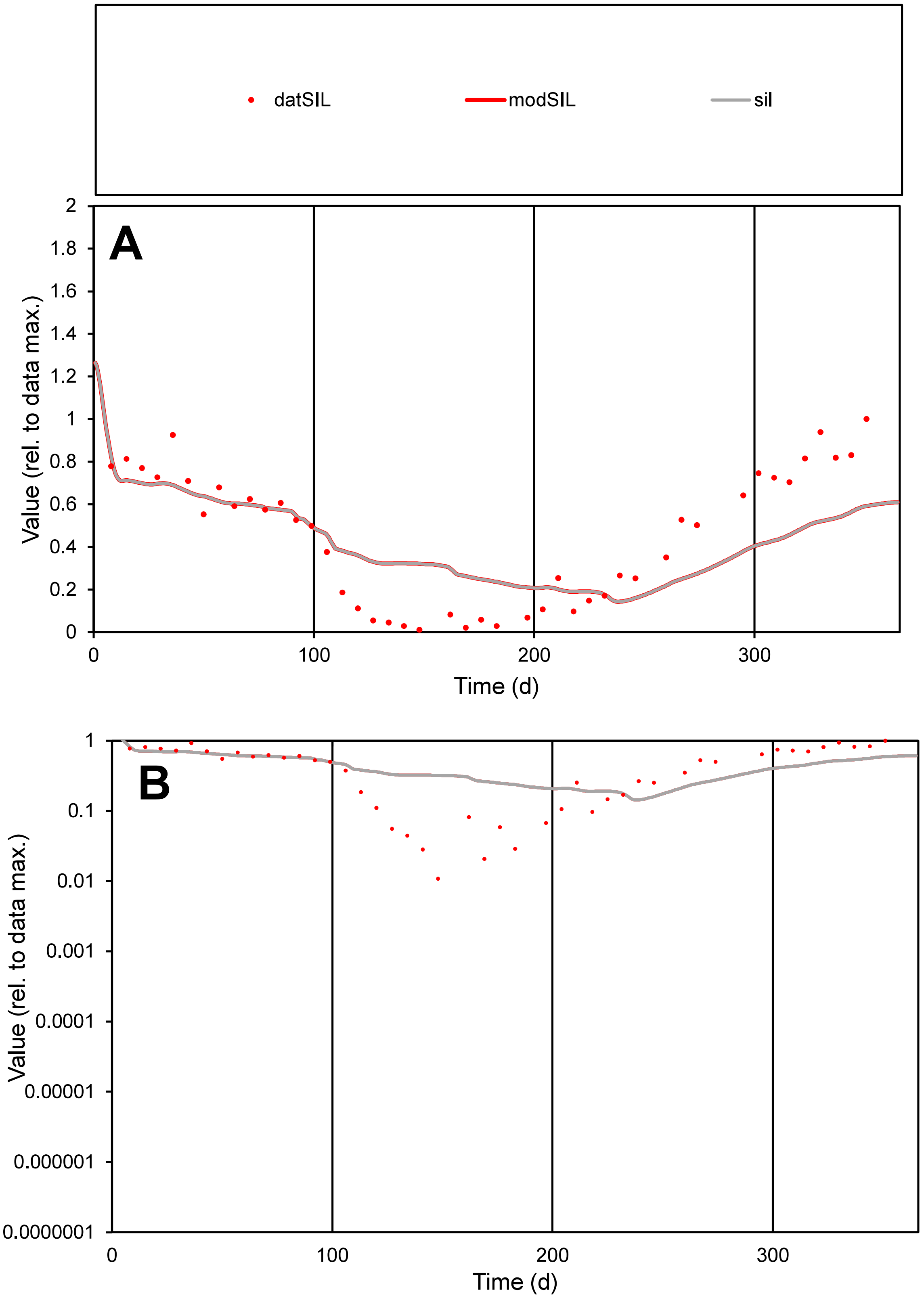

C1

Variables
SIL Provide jtd or Dst
CalibOnly?
2 1 to reproduce of
oNormalize?
2 1=no, 2=yes

ofxn calc*
1.3292
ofxn mod
1.37596

sRun
RunA1
zout
1

Ave
Data
0.00208
Model
0.00194

data/vMax
0.00461

ofxng
319.391

Min d/m
0.07452

ave/min d
41.6195
ave/min m
2.89385

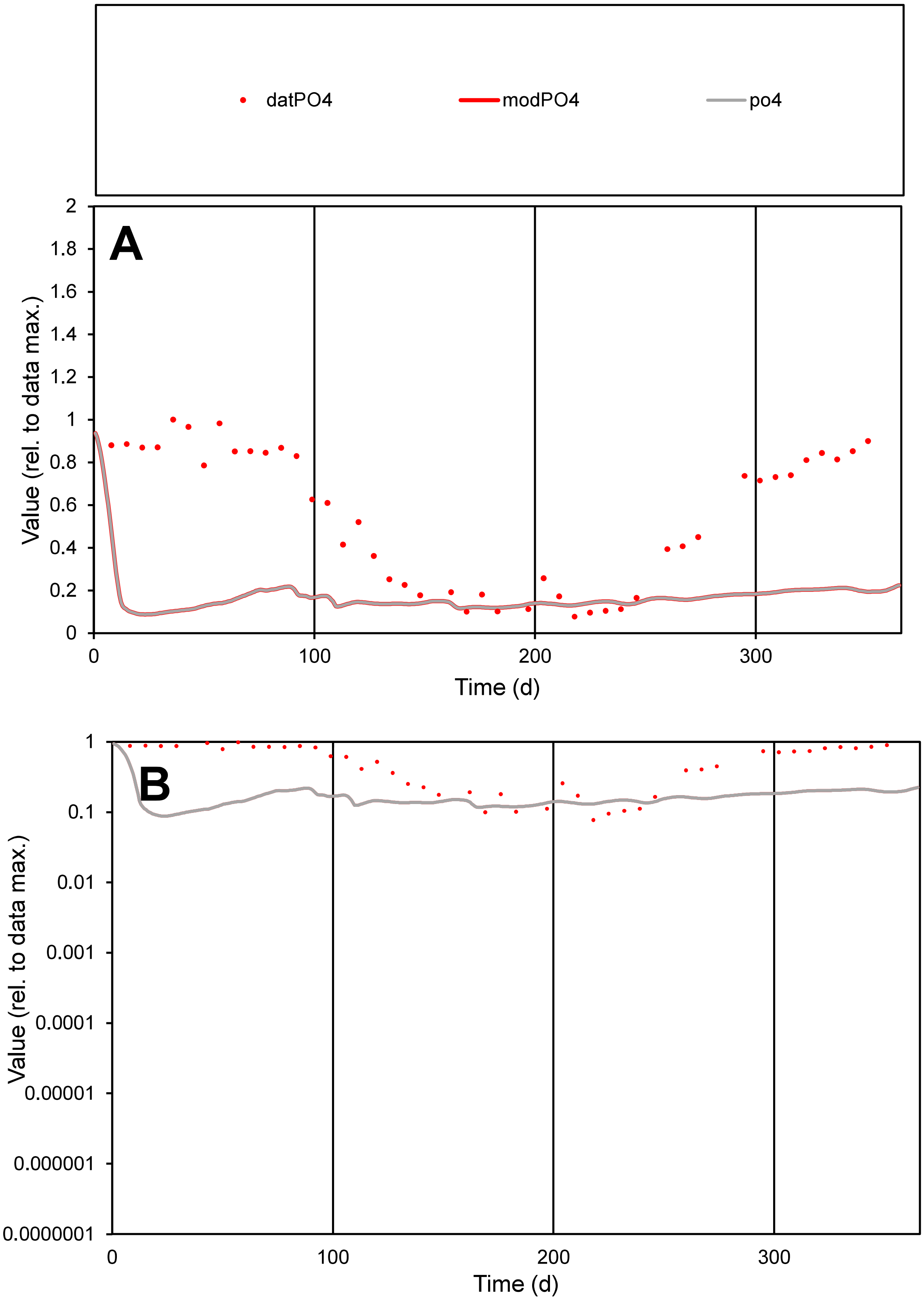

C1

Variables

PO4 Provide jtd or Dst

CalibOnly?

2 1 to reproduce of

oNormalize?

2 1=no, 2=yes

ofxn calc*

2.69248

ofxn mod

2.70722

sRun

RunA1

zout

1

Ave

Data

0.00028

Model

8.2E-05

data/vMax

0.0005

ofxng

319.391

Min d/m

0.87903

ave/min d

7.12216

ave/min m

1.84068

330_Calib_S_A

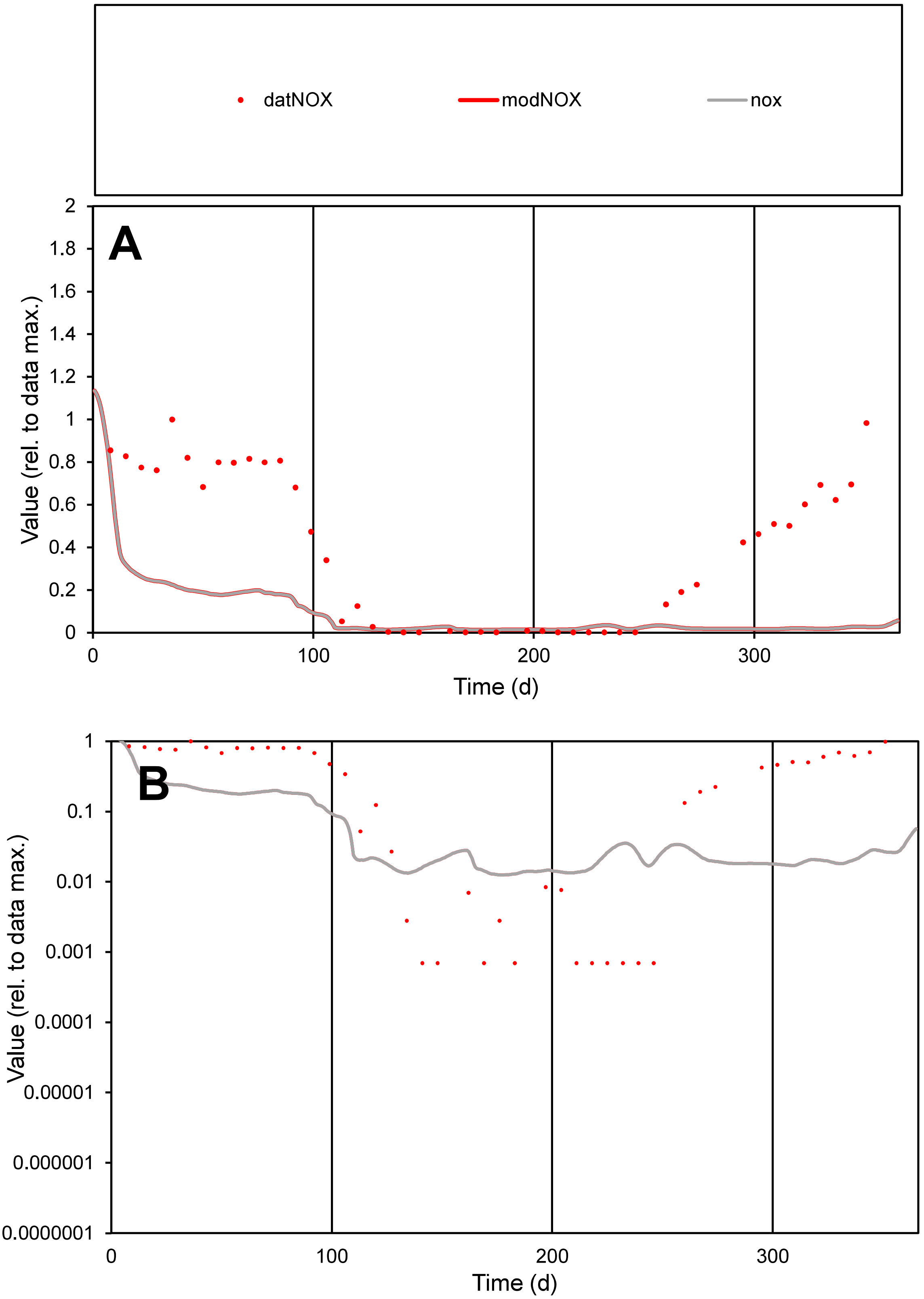

C1

Variables
NOX Provide jtd or Dst
CalibOnly?
2 1 to reproduce of
oNormalize?
2 1=no, 2=yes

ofxn calc*
3.235
ofxn mod
3.25667

sRun
RunA1
zout
1

Ave
Data
0.00279
Model
0.00063

data/vMax
0.00717

ofxng
319.391

Min d/m
0.05592

ave/min d
557.673
ave/min m
7.07371

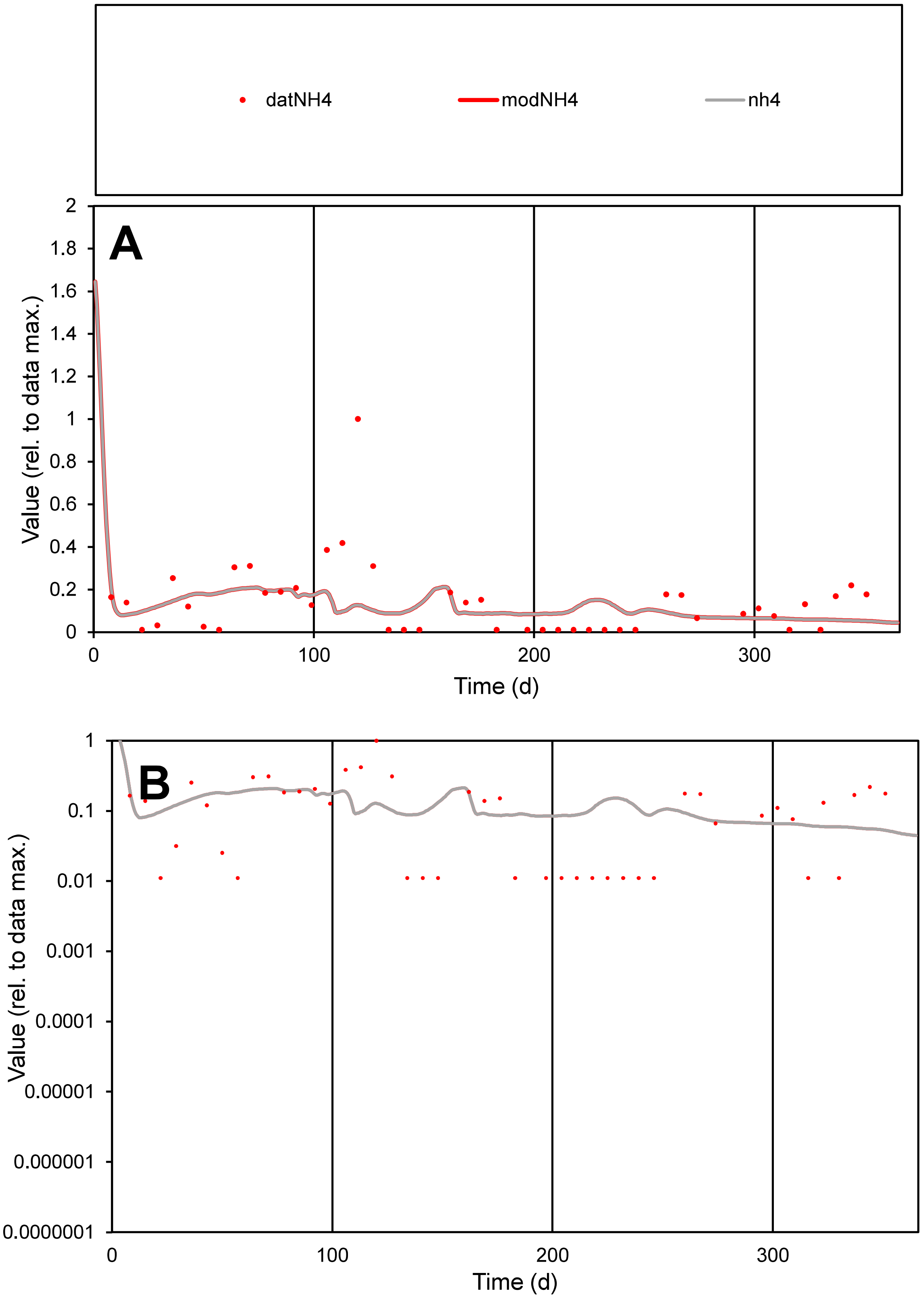

C1

Variables
NH4 Provide jtd or Dst
CalibOnly?
2 1 to reproduce of
oNormalize?
2 1=no, 2=yes

ofxn calc*
3.63603
ofxn mod
3.6656

sRun
RunA1
zout
1

Ave
Data
0.00011
Model
9E-05

data/vMax
0.00079

ofxng
319.391

Min d/m
0.21125

ave/min d
12.4612
ave/min m
2.17571

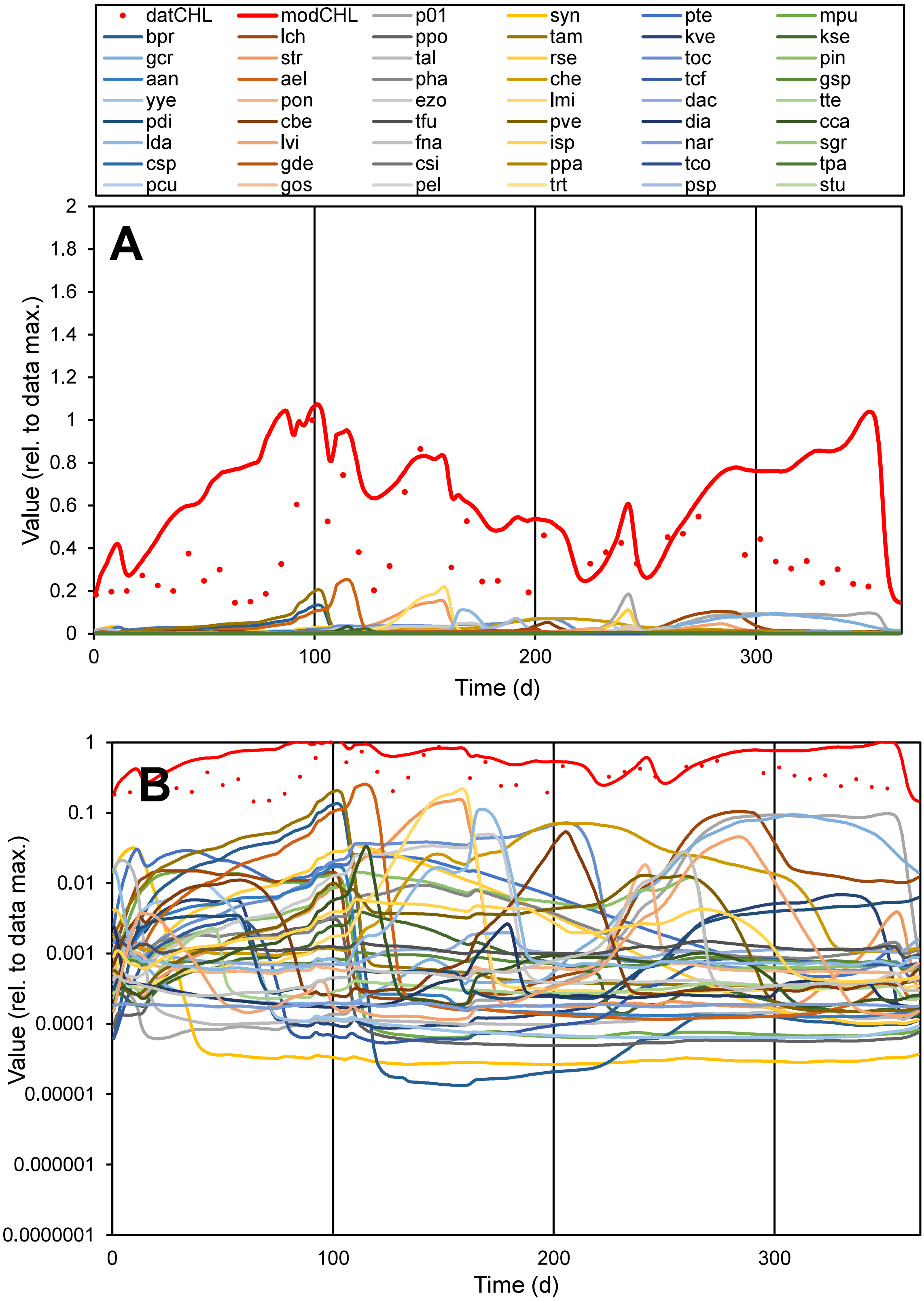

C1

Variables
CHL Provide jtd or Dst
CalibOnly?
2 1 to reproduce of
oNormalize?
2 1=no, 2=yes

ofxn calc*
2.7198
ofxn mod
2.98239

sRun
RunA1
zout
1

Ave
Data
1.03269
Model
1.03124

data/vMax
2.841

ofxng
319.391

Min d/m
0.70277

ave/min d
2.51445
ave/min m
1.76461

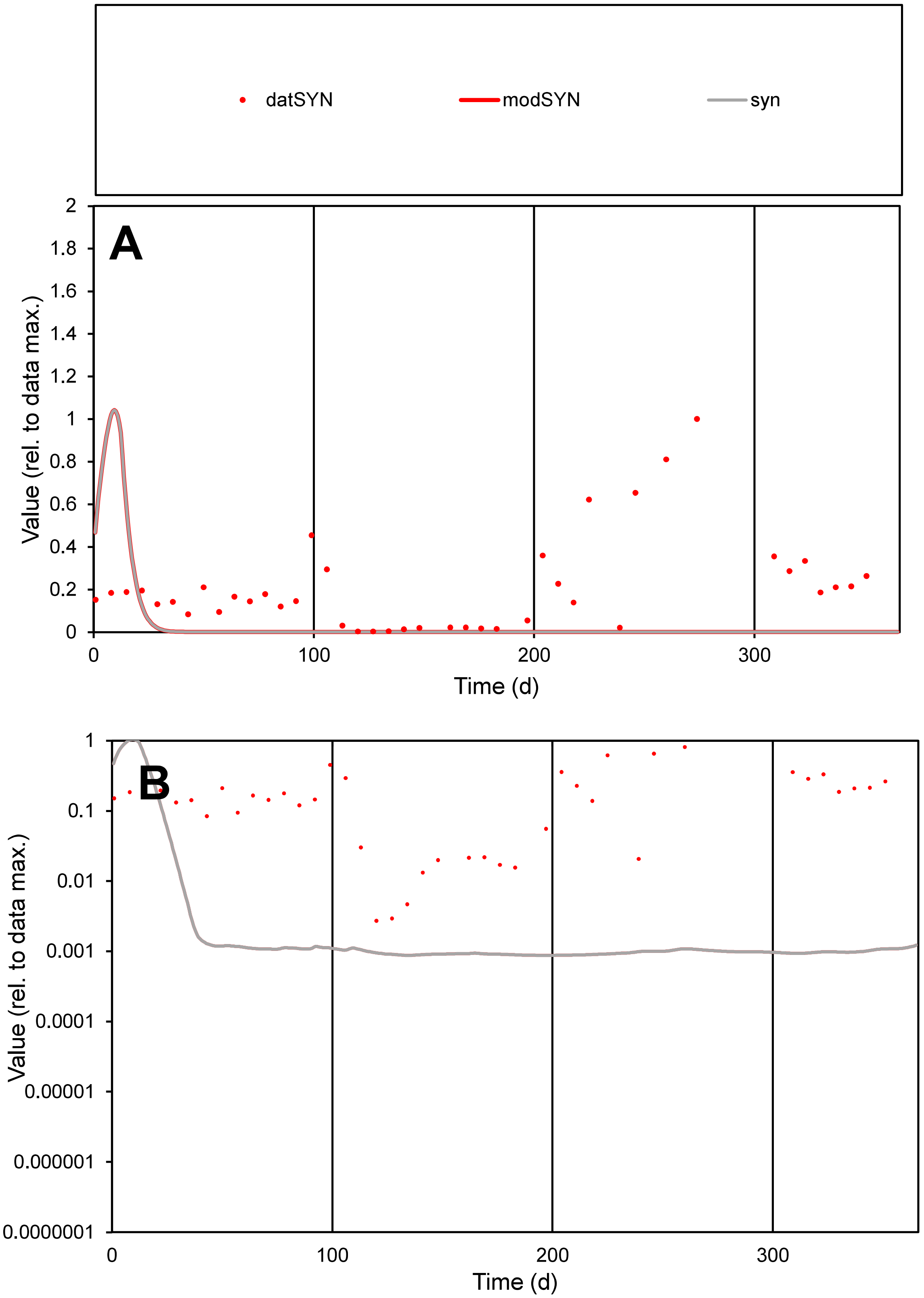

C1

Variables
SYN Provide jtd or Dst
CalibOnly?
2 1 to reproduce of
oNormalize?
2 1=no, 2=yes

ofxn calc*
1.58195
ofxn mod
1.58985

sRun
RunA1
zout
1

Ave
Data
10.7184
Model
2.67643

data/vMax
51.32

ofxng
319.391

Min d/m
3.10913

ave/min d
76.7796
ave/min m
59.6087

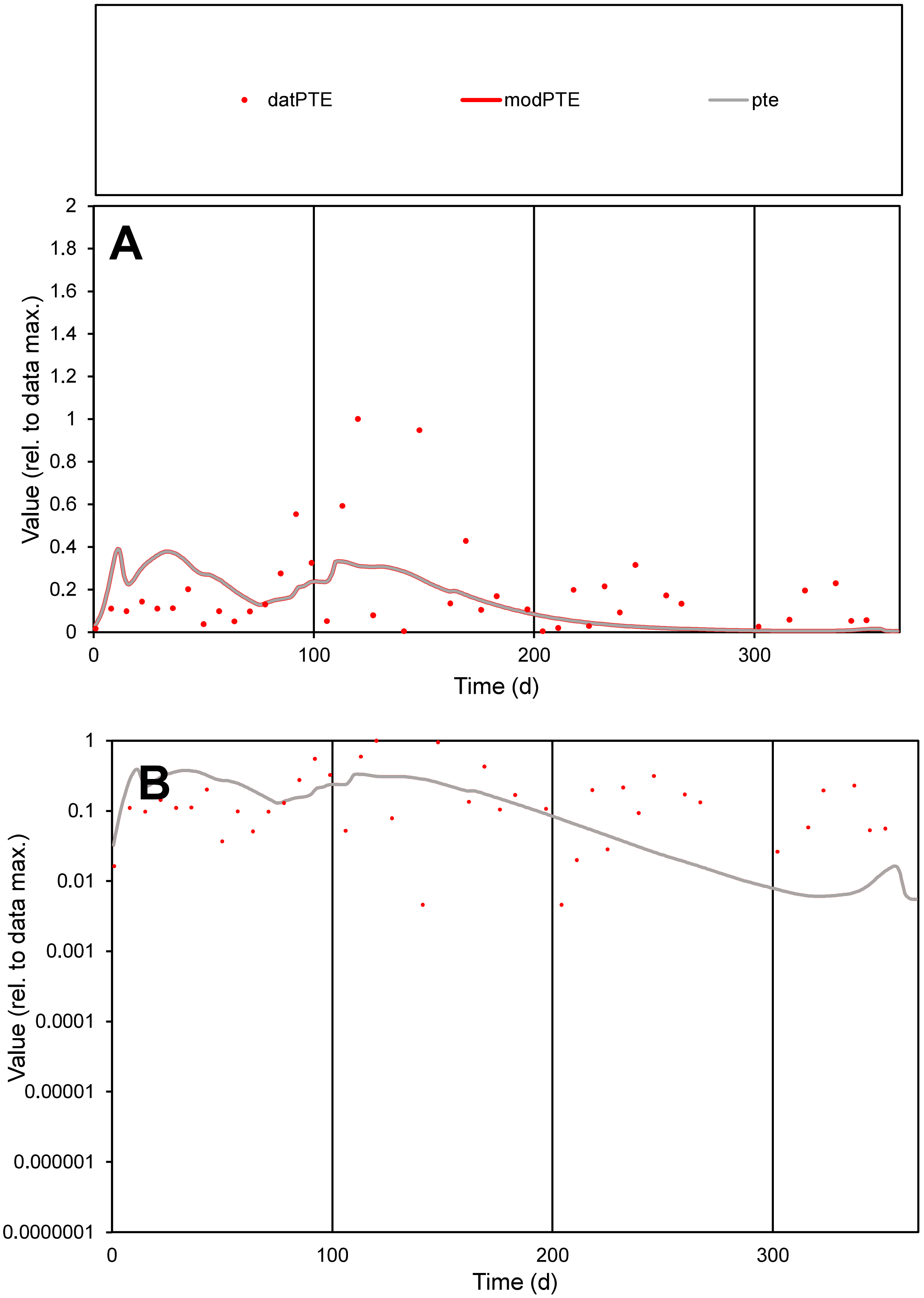

C1

Variables
PTE Provide jtd or Dst
CalibOnly?
2 1 to reproduce of
oNormalize?
2 1=no, 2=yes

ofxn calc*
1.16004
ofxn mod
1.16942

sRun
RunA1
zout
1

Ave
Data
0.00035
Model
0.00028

data/vMax
0.00182

ofxng
319.391

Min d/m
0.74843

ave/min d
41.3317
ave/min m
25.0112

330_Calib_S_A

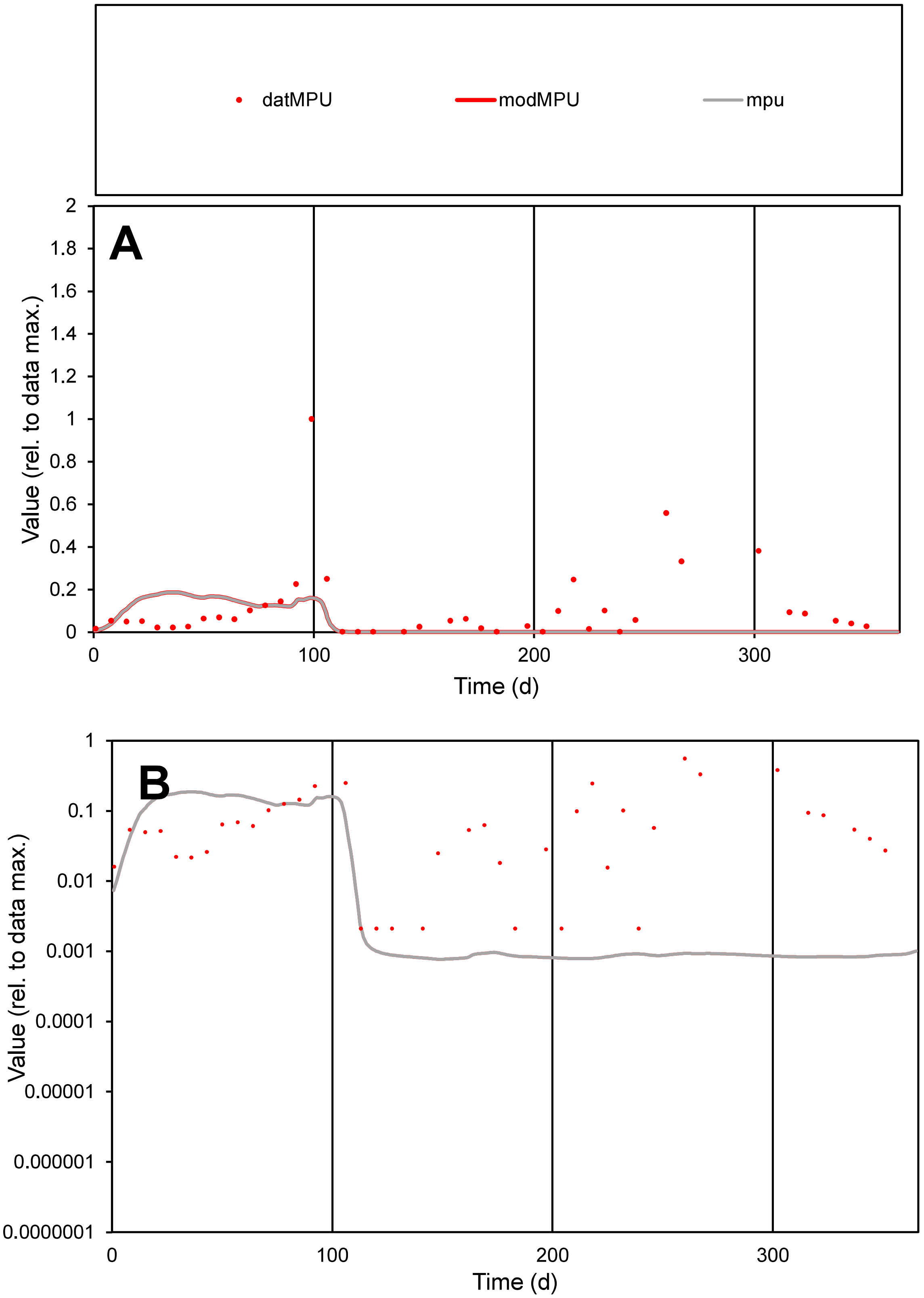

C1

Variables

MPU Provide jtd or Dst

CalibOnly?

2 1 to reproduce of

oNormalize?

2 1=no, 2=yes

ofxn calc*

1.73049

ofxn mod

1.73968

sRun

RunA1

zout

1

Ave

Data

0.00022

Model

0.0001

data/vMax

0.00193

ofxng

319.391

Min d/m

2.74412

ave/min d

52.7038

ave/min m

68.248

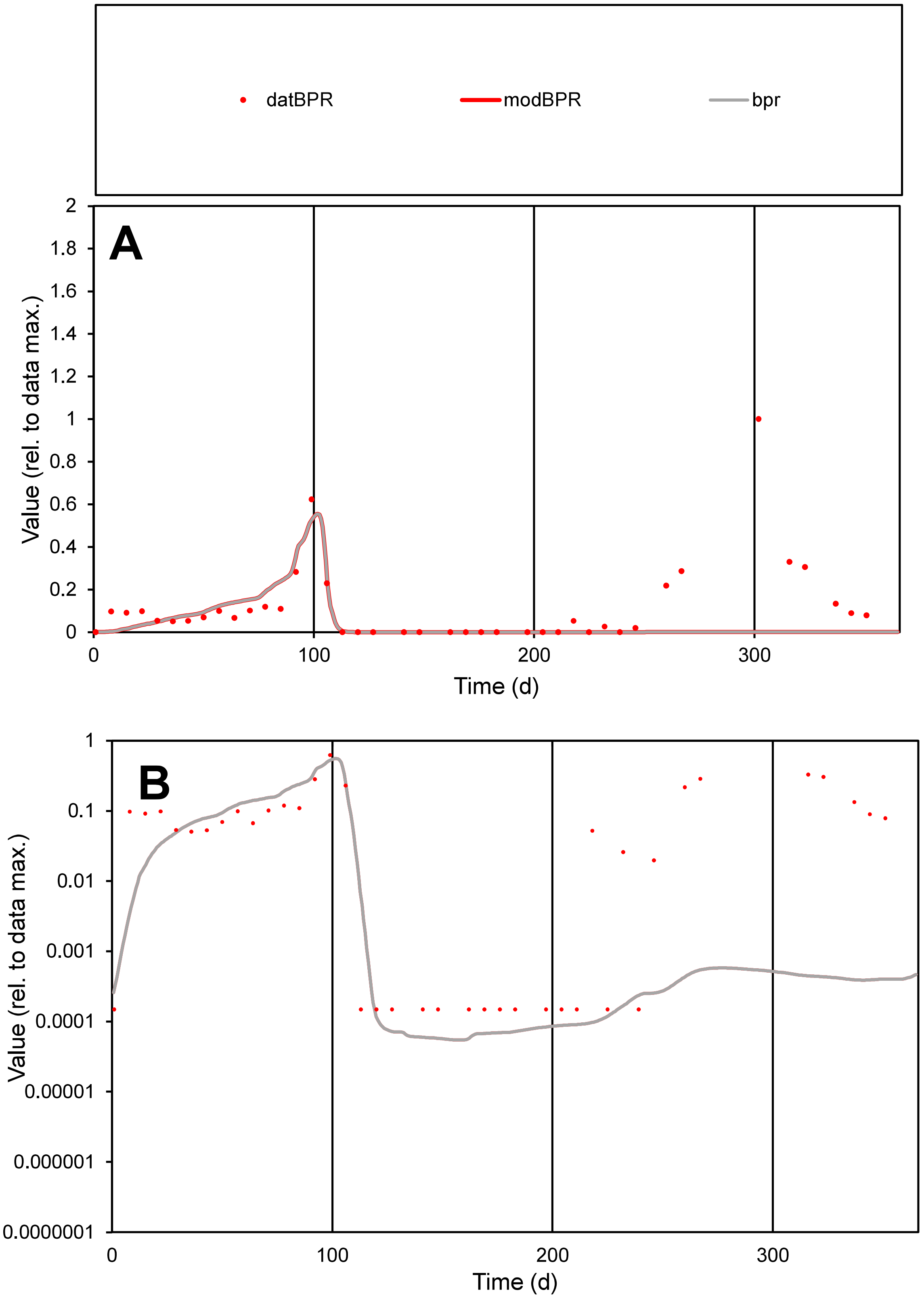

C1

Variables
BPR Provide jtd or Dst
CalibOnly?
2 1 to reproduce of
oNormalize?
2 1=no, 2=yes

ofxn calc*
1.6356
ofxn mod
1.64607

sRun
RunA1
zout
1

Ave
Data
0.00014
Model
7.3E-05

data/vMax
0.00125

ofxng
319.391

Min d/m
2.59706

ave/min d
770.152
ave/min m
1021.19

330_Calib_S_A

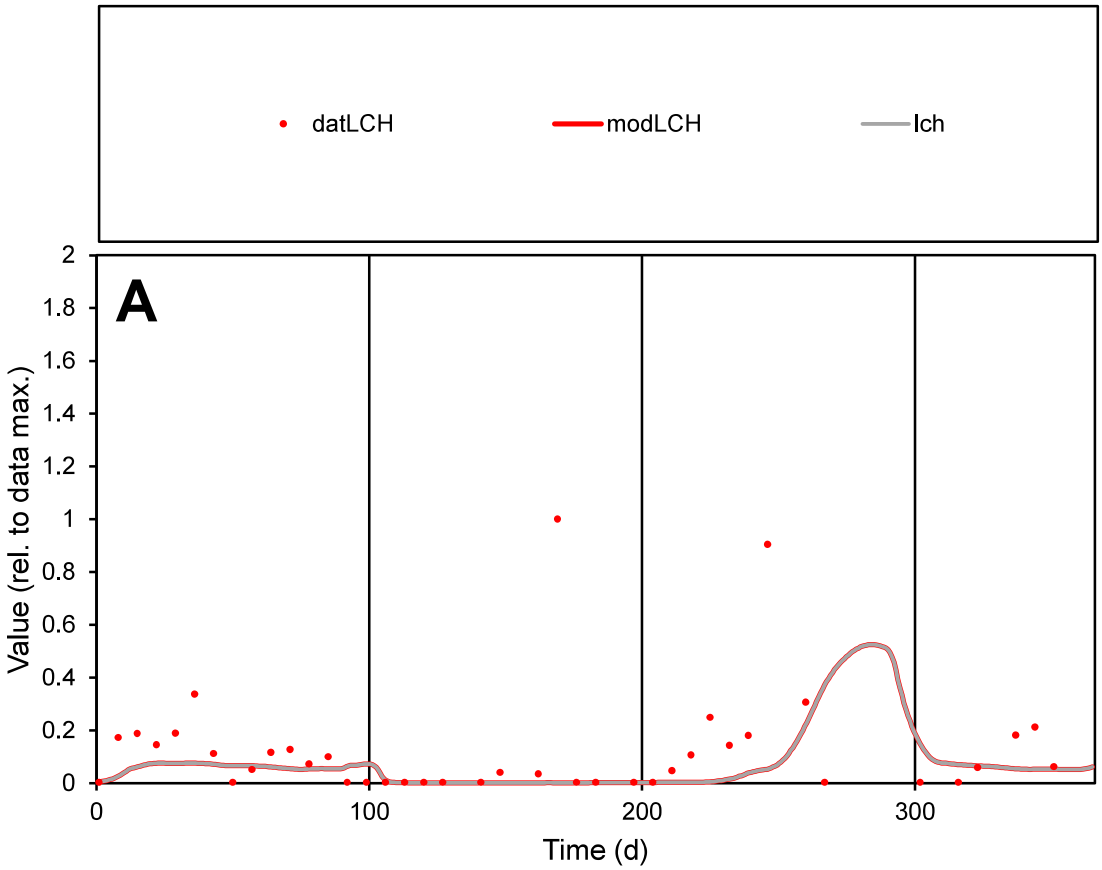

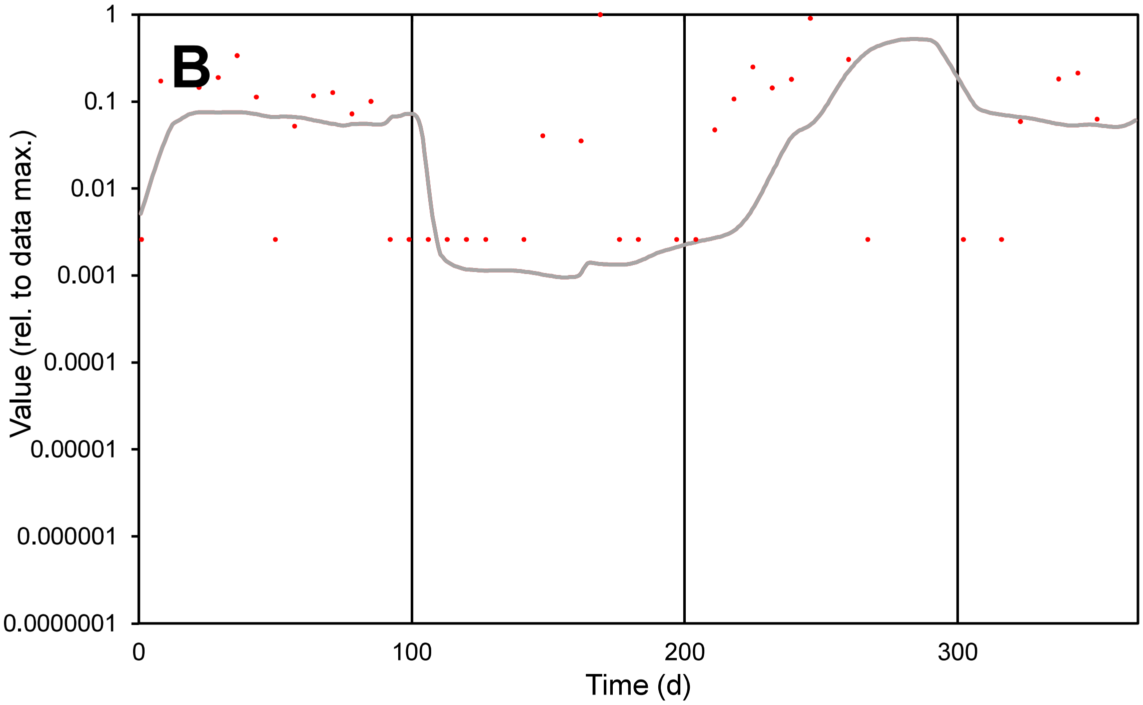

C1

Variables
LCH Provide jtd or Dst
CalibOnly?
2 1 to reproduce of
oNormalize?
2 1=no, 2=yes

ofxn calc*
1.80851
ofxn mod
1.81819

sRun
RunA1
zout
1

Ave
Data
0.00012
Model
4.6E-05

data/vMax
0.00094

ofxng
319.391

Min d/m
2.56906

ave/min d
48.7042
ave/min m
48.5516

330_Calib_S_A

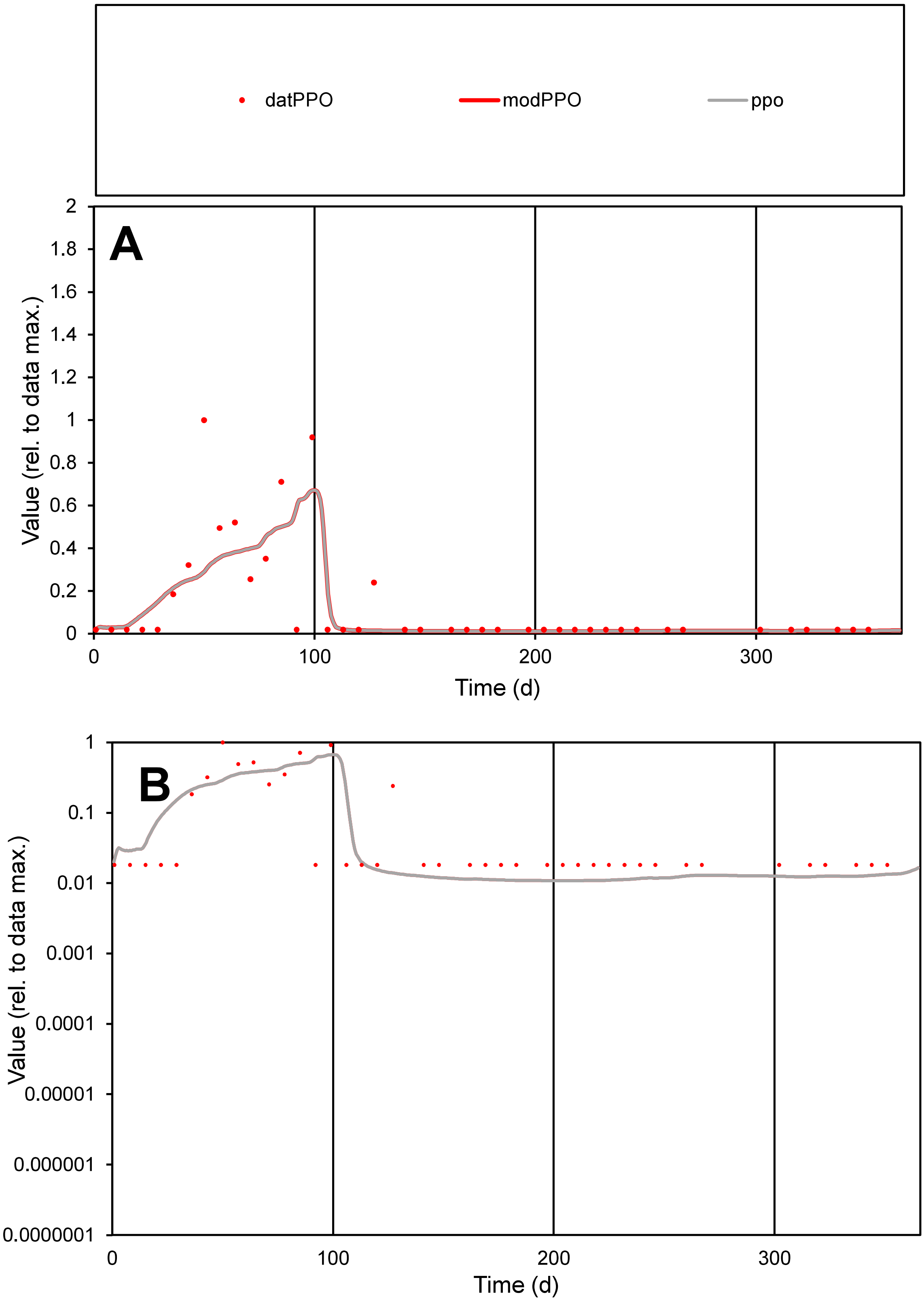

C1

Variables
PPO Provide jtd or Dst
CalibOnly?
2 1 to reproduce of
oNormalize?
2 1=no, 2=yes

ofxn calc*
1.14928
ofxn mod
1.20793

sRun
RunA1
zout
1

Ave
Data
1.4E-05
Model
1.2E-05

data/vMax
0.00011

ofxng
319.391

Min d/m
1.67513

ave/min d
7.48554
ave/min m
10.8179

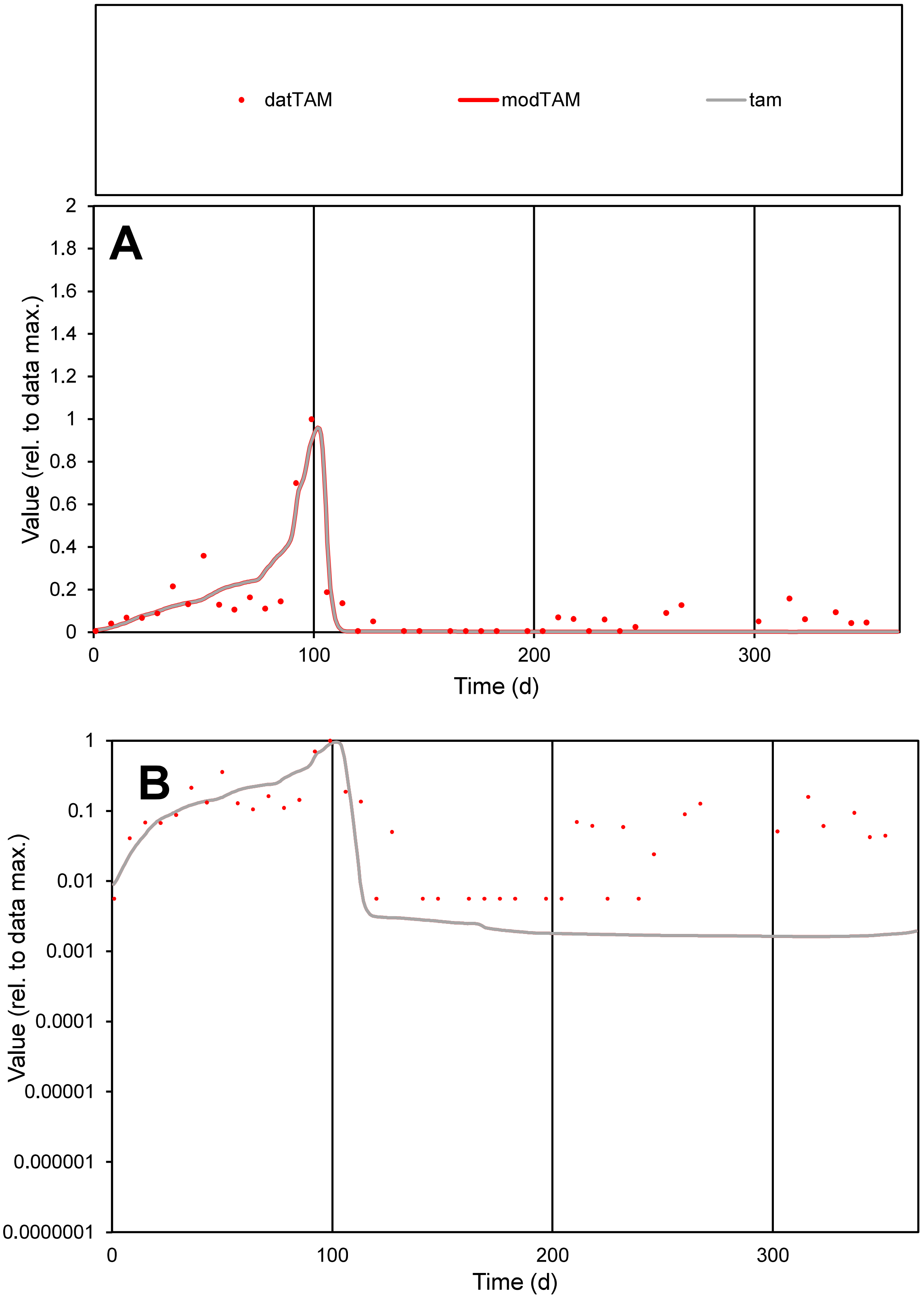

C1

Variables
TAM Provide jtd or Dst
CalibOnly?
2 1 to reproduce of
oNormalize?
2 1=no, 2=yes

ofxn calc*
0.6822
ofxn mod
0.7308

sRun
RunA1
zout
1

Ave
Data
0.00012
Model
9.9E-05

data/vMax
0.00102

ofxng
319.391

Min d/m
3.47346

ave/min d
20.115
ave/min m
59.923

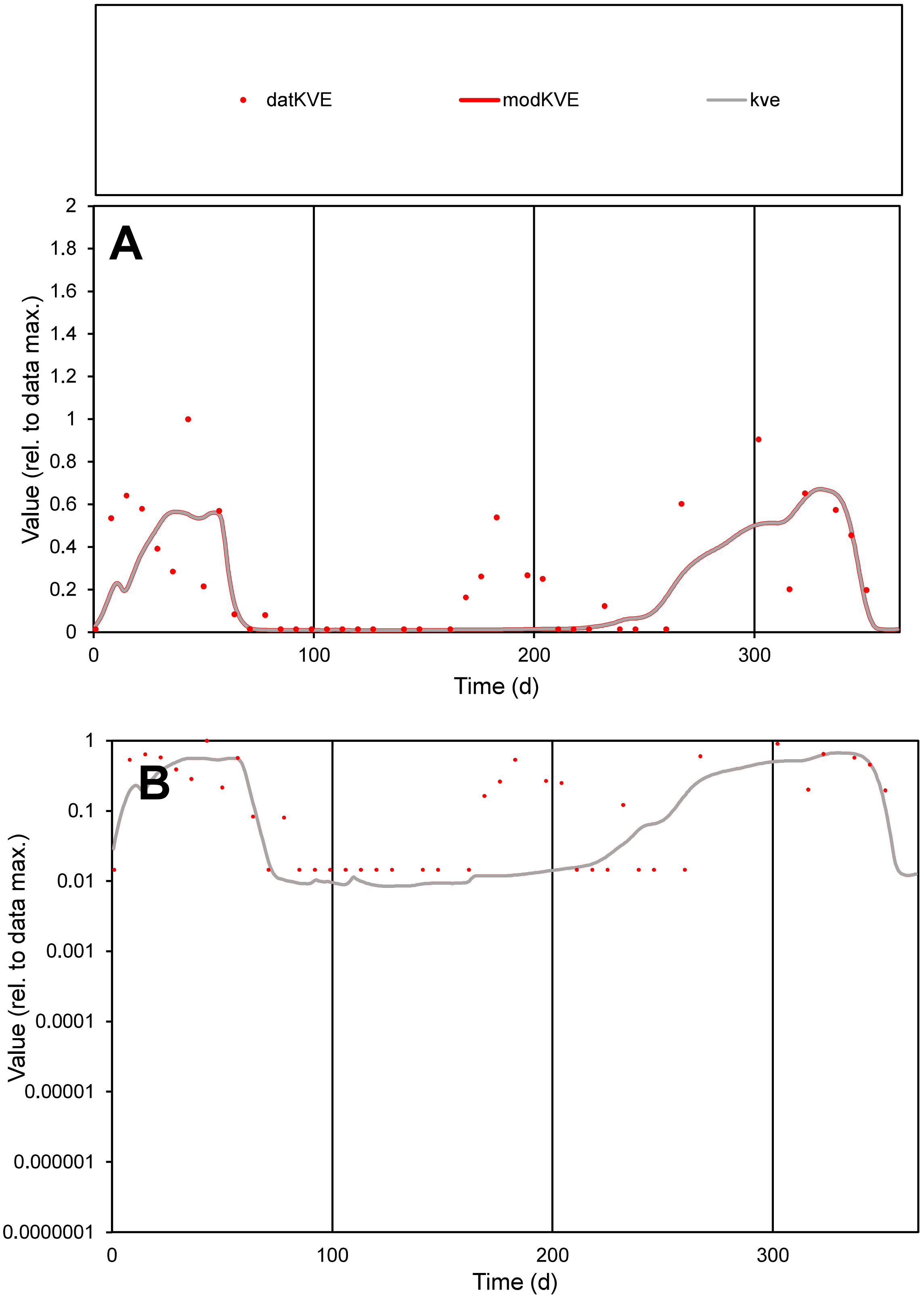

C1

Variables
KVE Provide jtd or Dst
CalibOnly?
2 1 to reproduce of
oNormalize?
2 1=no, 2=yes

ofxn calc*
0.82374
ofxn mod
0.84093

sRun
RunA1
zout
1

Ave
Data
5.2E-05
Model
3.9E-05

data/vMax
0.00022

ofxng
319.391

Min d/m
1.70348

ave/min d
16.5004
ave/min m
21.1056

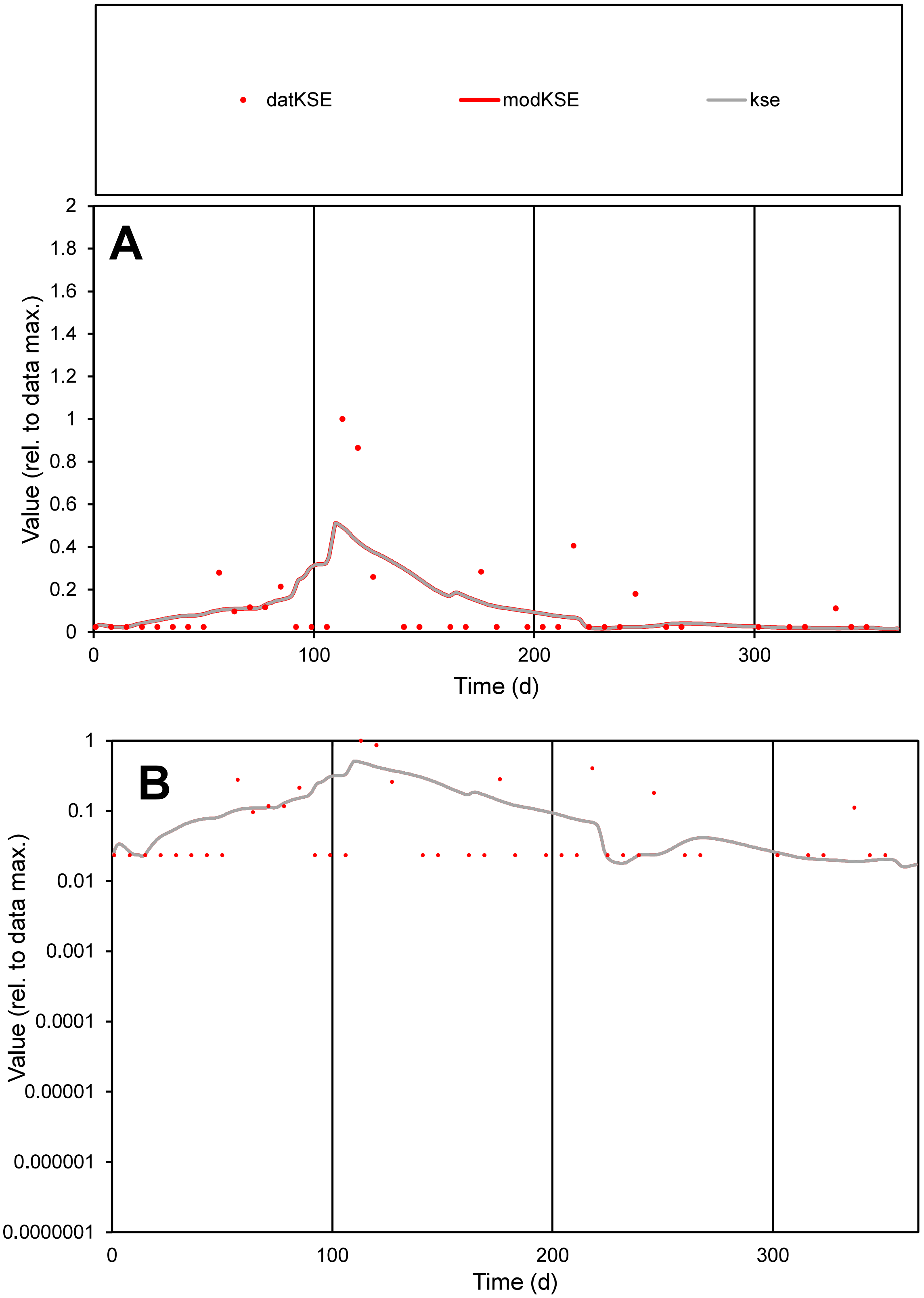

C1

Variables
KSE Provide jtd or Dst
CalibOnly?
2 1 to reproduce of
oNormalize?
2 1=no, 2=yes

ofxn calc*
1.43966
ofxn mod
1.45916

sRun
RunA1
zout
1

Ave
Data
1.8E-05
Model
1.9E-05

data/vMax
0.00016

ofxng
319.391

Min d/m
1.2757

ave/min d
4.78084
ave/min m
6.39953

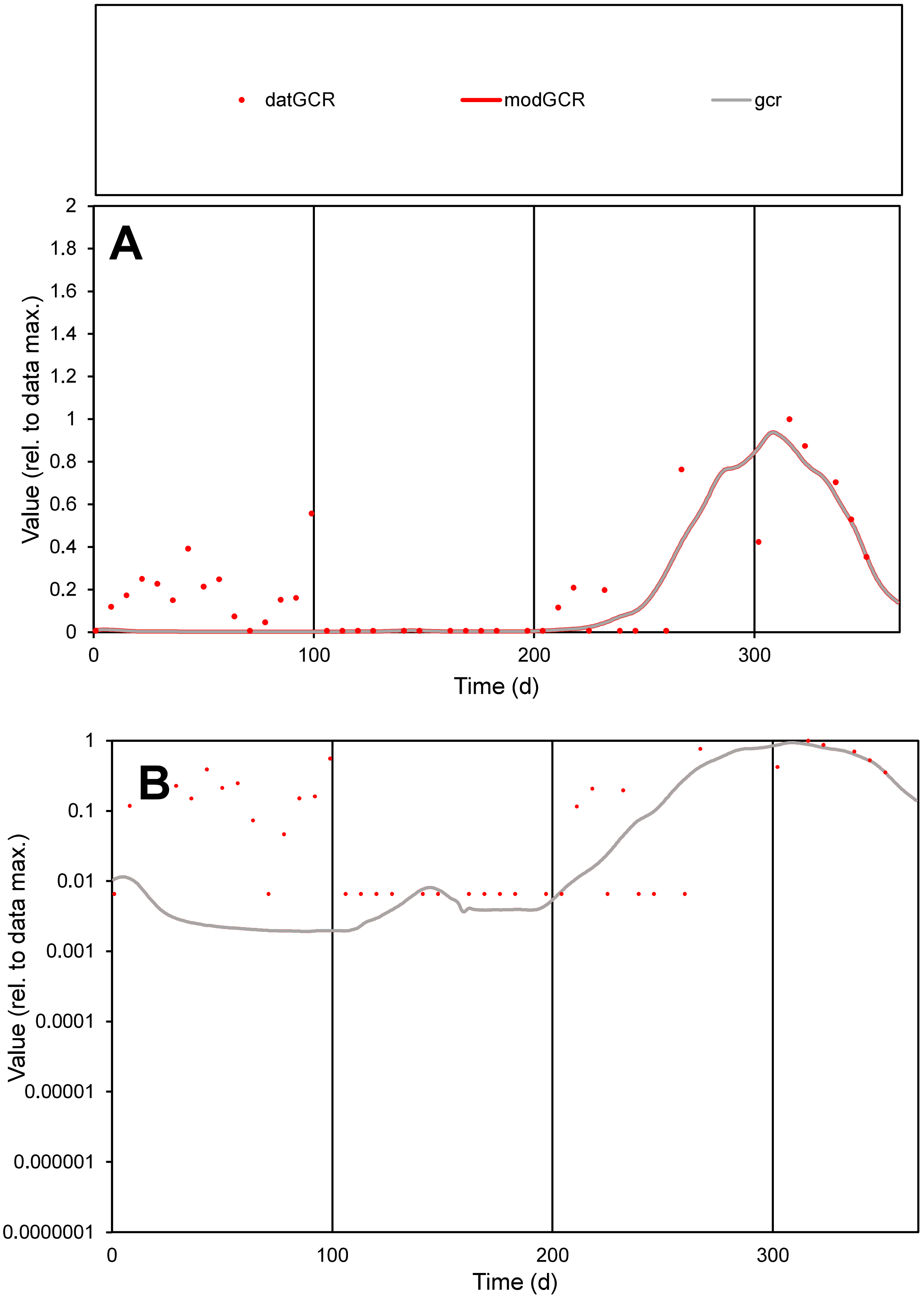

C1

Variables
GCR Provide jtd or Dst
CalibOnly?
2 1 to reproduce of
oNormalize?
2 1=no, 2=yes

ofxn calc*
0.89939
ofxn mod
0.9168

sRun
RunA1
zout
1

Ave
Data
9.4E-05
Model
5.9E-05

data/vMax
0.00048

ofxng
319.391

Min d/m
3.41023

ave/min d
29.914
ave/min m
63.9773

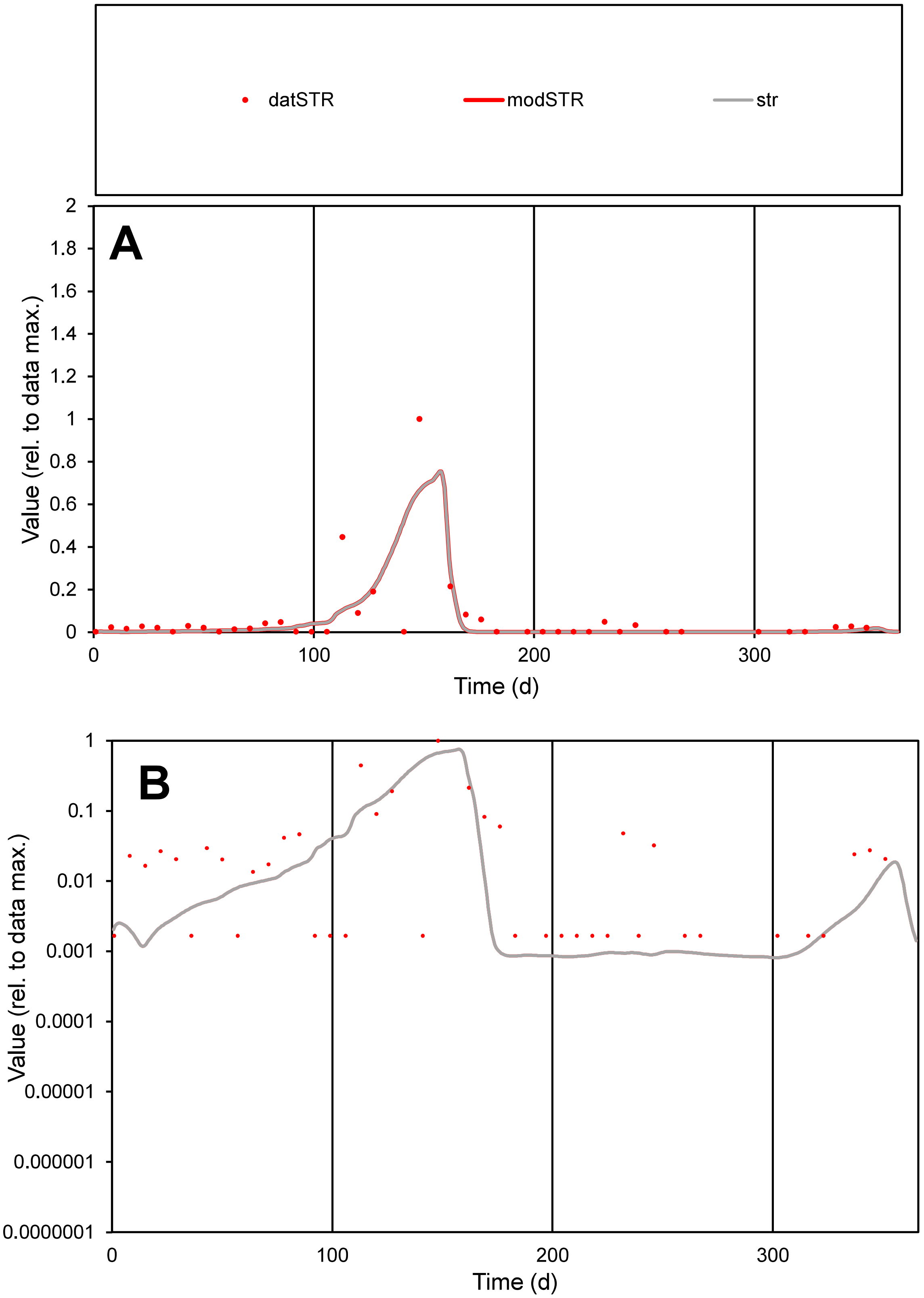

C1

Variables

STR Provide jtd or Dst

CalibOnly?

2 1 to reproduce of

oNormalize?

2 1=no, 2=yes

ofxn calc*

1.78298

ofxn mod

1.82809

sRun

RunA1

zout

1

Ave

Data

6E-05

Model

5.1E-05

data/vMax

0.00098

ofxng

319.391

Min d/m

2.036

ave/min d

37.1043

ave/min m

63.7124

330_Calib_S_A

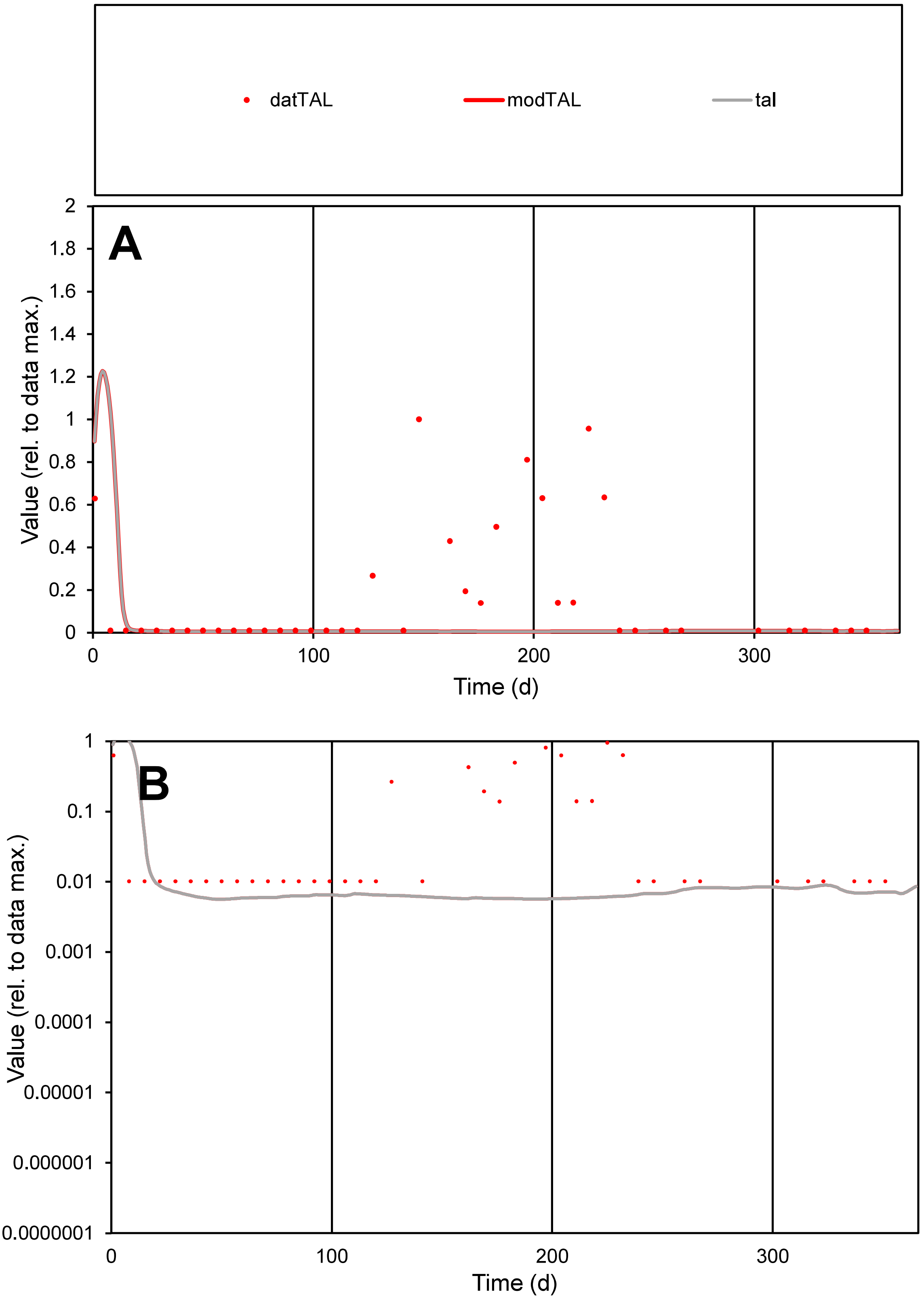

C1

Variables

TAL	Provide jtd or Dst
CalibOnly?	
2	1 to reproduce of
oNormalize?	
2	1=no, 2=yes

ofxn calc*
1.94984
ofxn mod
1.98683

sRun
RunA1
zout
1

Ave
Data
6.7E-05
Model
1.7E-05

data/vMax
0.00041

ofxng
319.391

Min d/m
1.80252

ave/min d
16.1528
ave/min m
7.38497

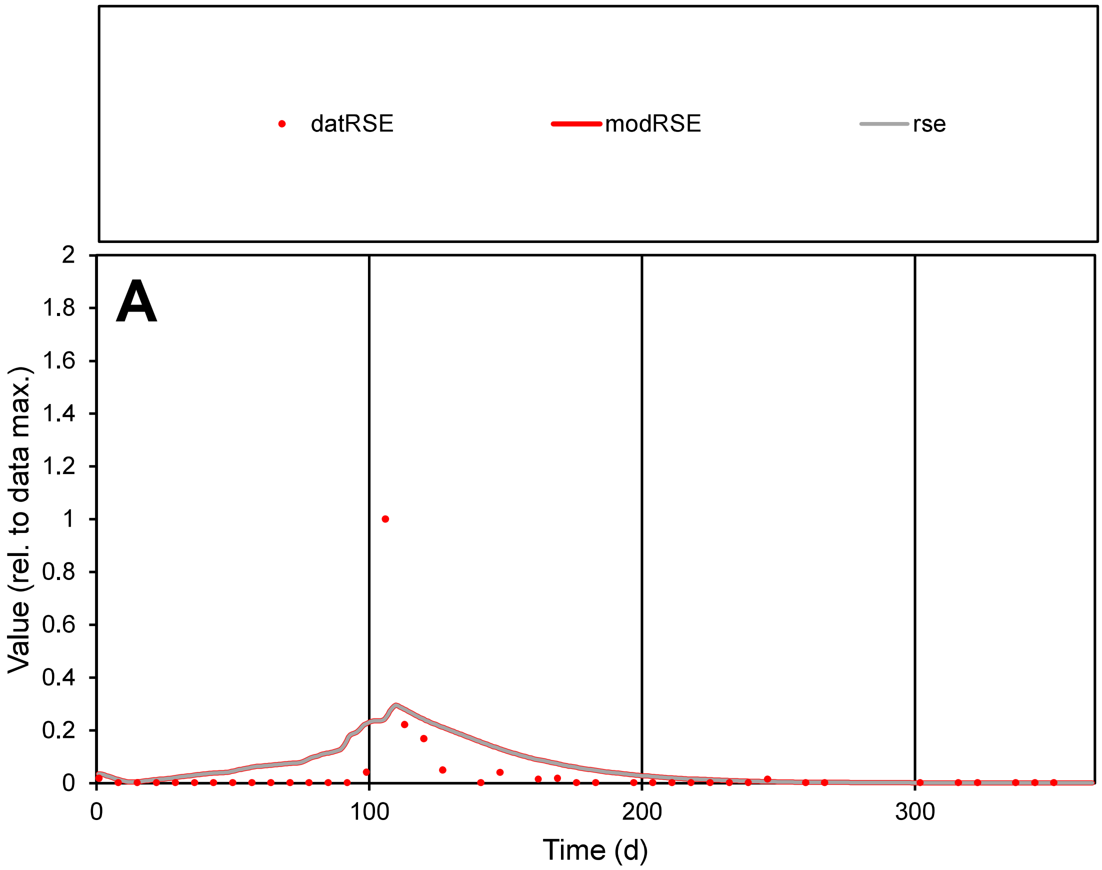

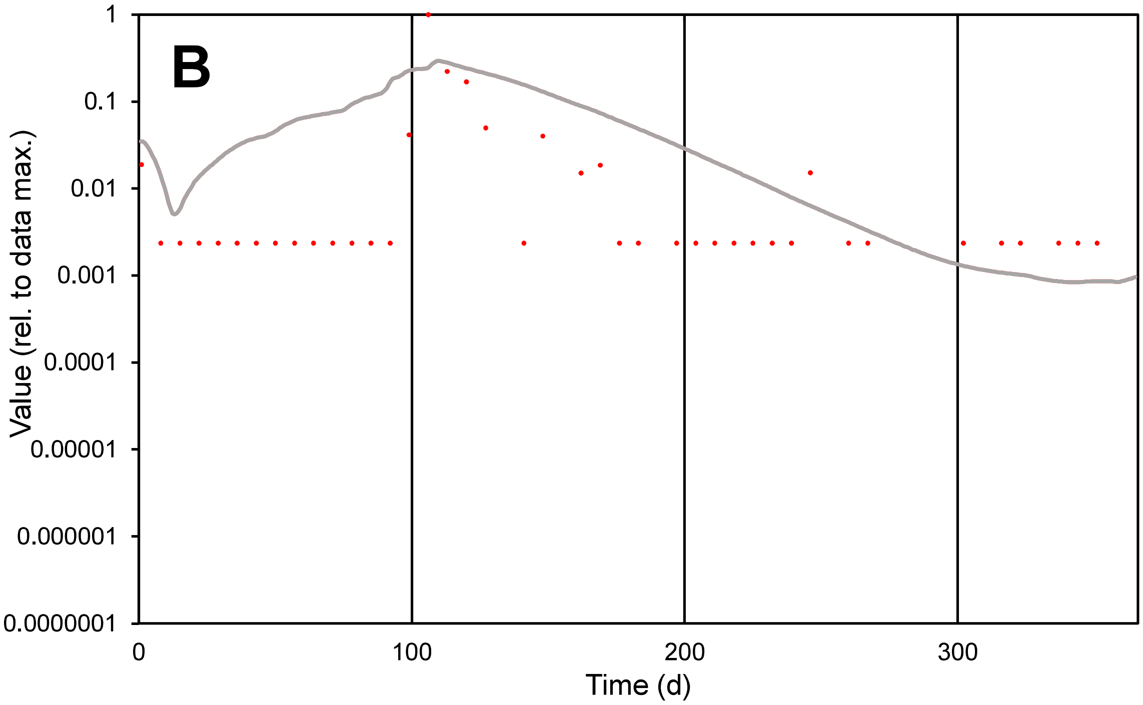

C1

Variables
RSE Provide jtd or Dst
CalibOnly?
2 1 to reproduce of
oNormalize?
2 1=no, 2=yes

ofxn calc*
3.20749
ofxn mod
3.24172

sRun
RunA1
zout
1

Ave
Data
8E-05
Model
0.00013

data/vMax
0.00198

ofxng
319.391

Min d/m
2.79054

ave/min d
17.183
ave/min m
75.7627

330_Calib_S_A

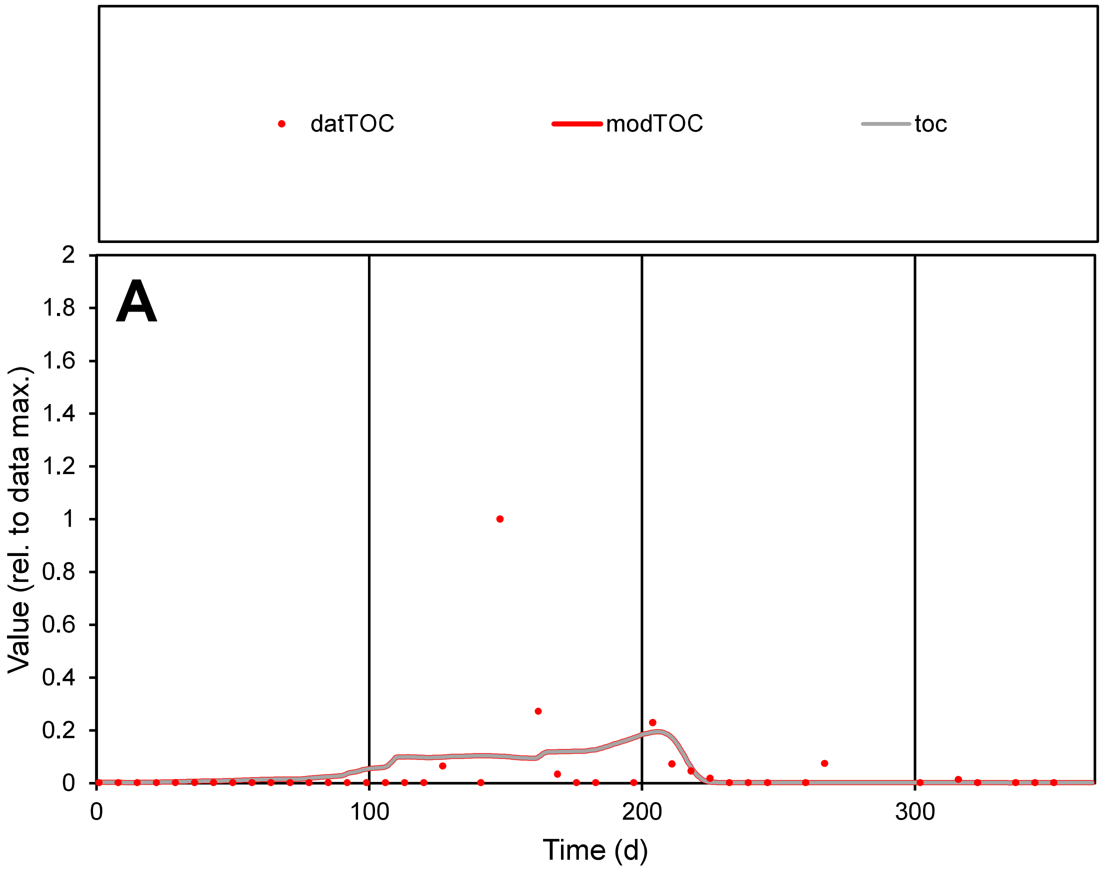

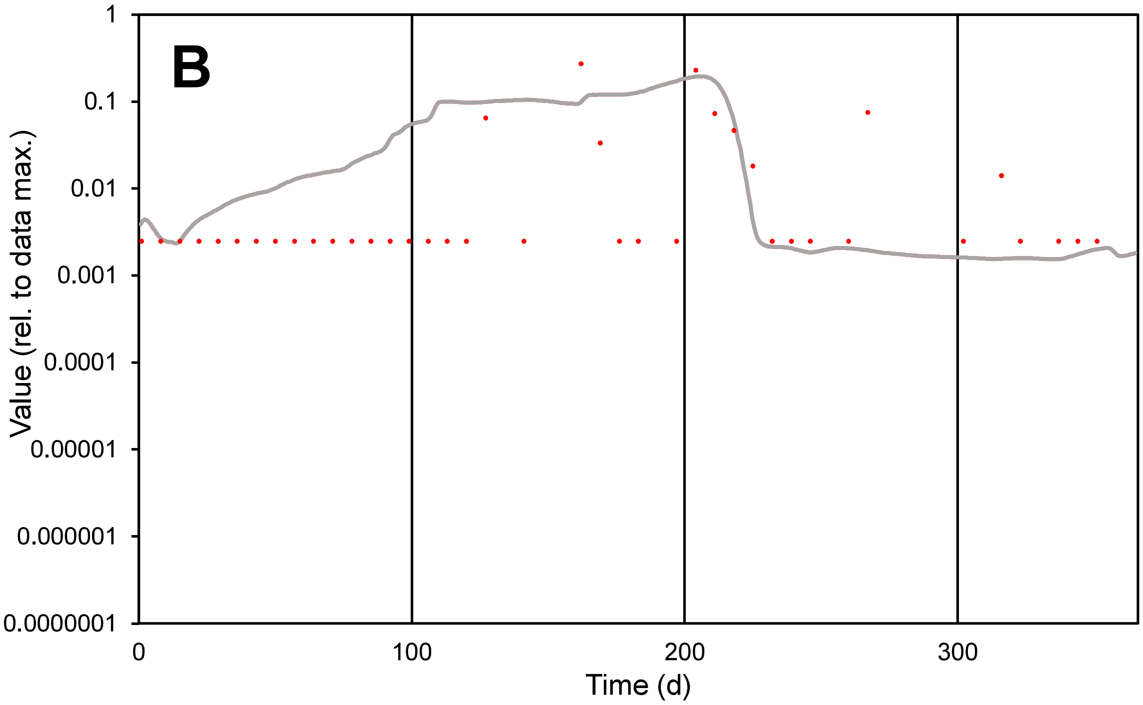

C1

Variables
TOC Provide jtd or Dst
CalibOnly?
2 1 to reproduce of
oNormalize?
2 1=no, 2=yes

ofxn calc*
3.28214
ofxn mod
3.28995

sRun
RunA1
zout
1

Ave
Data
8.1E-05
Model
7.9E-05

data/vMax
0.00174

ofxng
319.391

Min d/m
1.58664

ave/min d
18.7205
ave/min m
28.8237

330_Calib_S_A

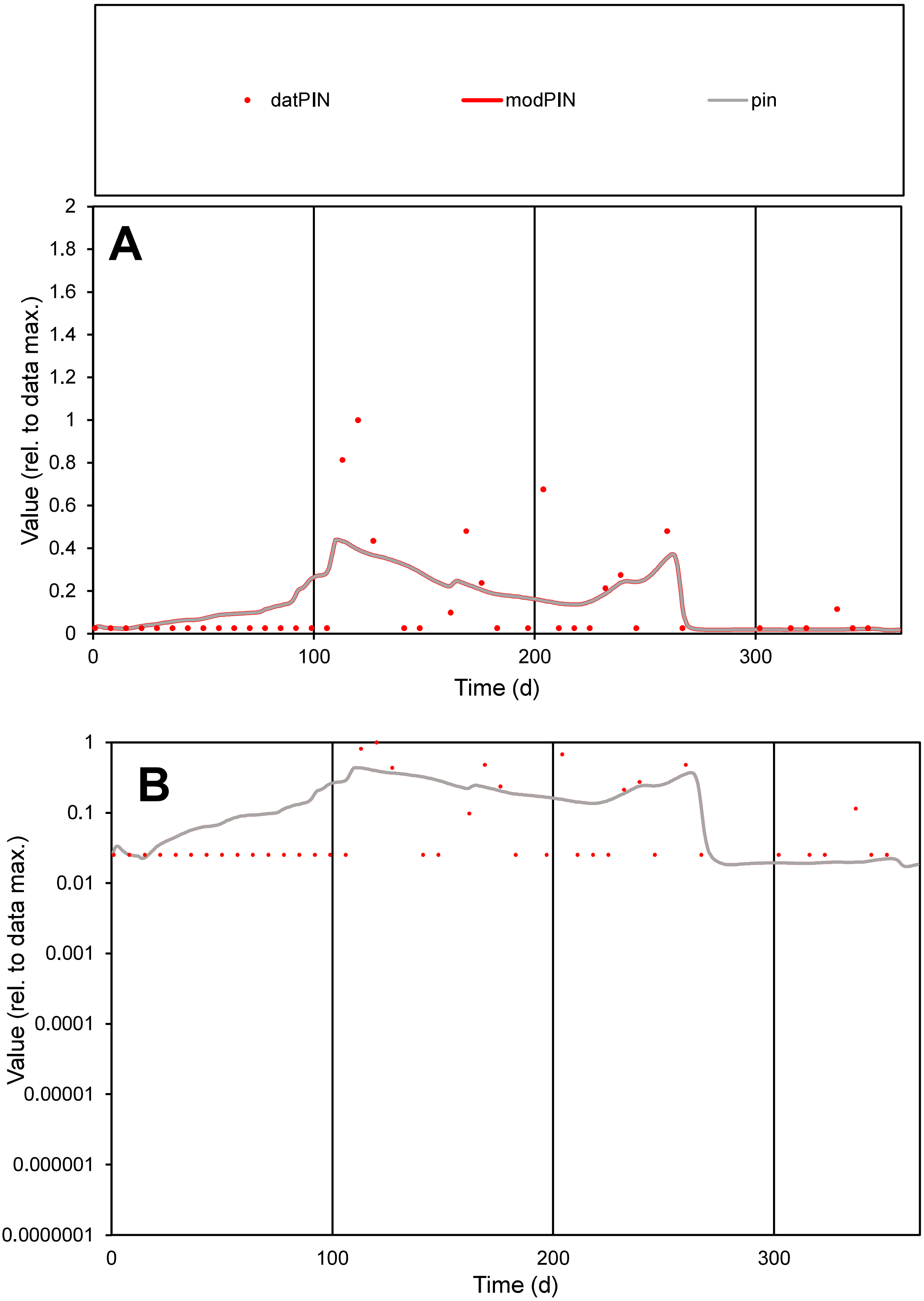

C1

Variables

PIN Provide jtd or Dst

CalibOnly?

2 1 to reproduce of

oNormalize?

2 1=no, 2=yes

ofxn calc*

1.34117

ofxn mod

1.3566

sRun

RunA1

zout

1

Ave

Data

2.2E-05

Model

2.4E-05

data/vMax

0.00016

ofxng

319.391

Min d/m

1.323

ave/min d

5.36798

ave/min m

7.82719

330_Calib_S_A

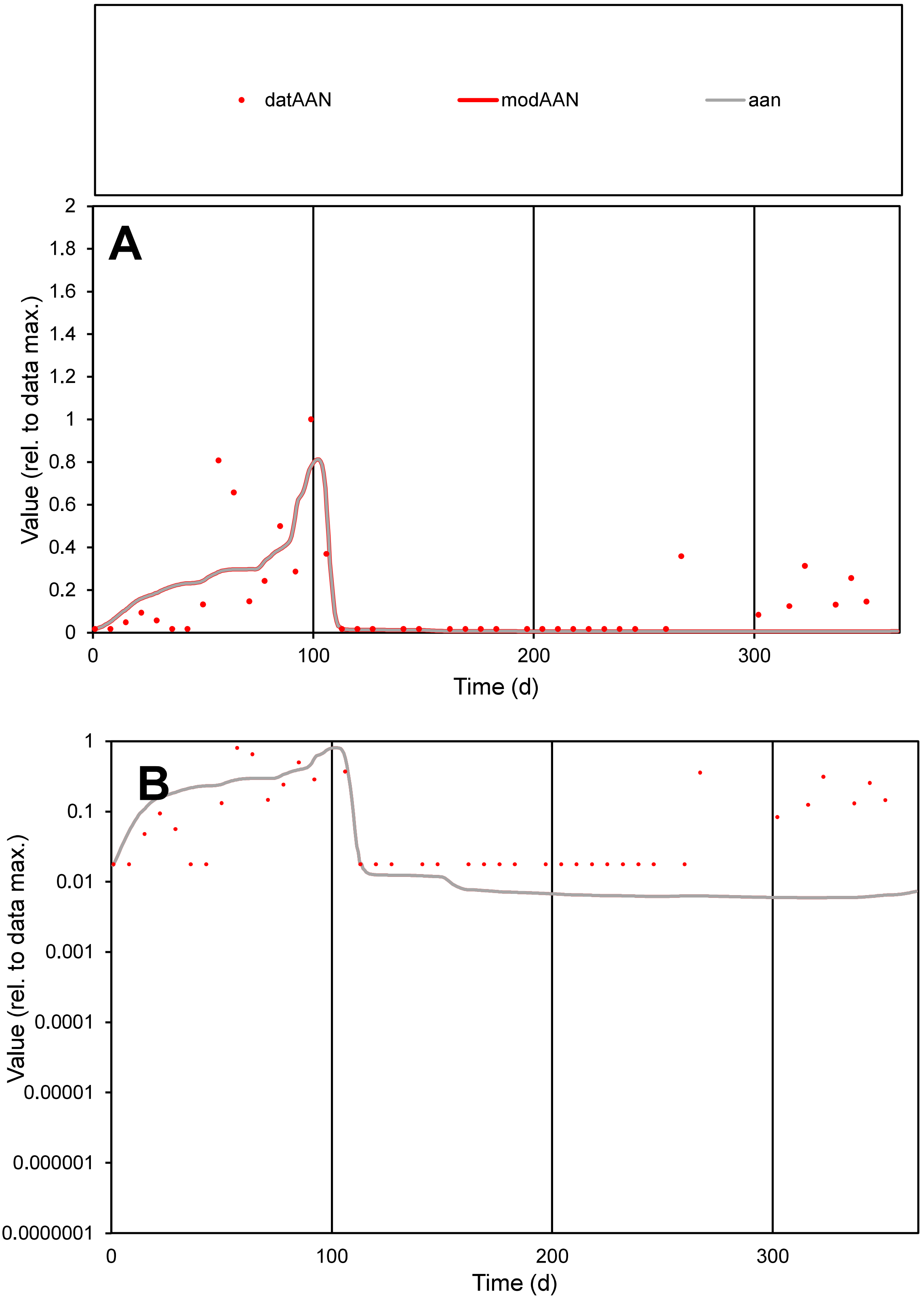

C1

Variables
AAN Provide jtd or Dst
CalibOnly?
2 1 to reproduce of
oNormalize?
2 1=no, 2=yes

ofxn calc*
1.0615
ofxn mod
1.08076

sRun
RunA1
zout
1

Ave
Data
4.7E-05
Model
3.6E-05

data/vMax
0.00031

ofxng
319.391

Min d/m
3.00863
ave/min d
8.42026
ave/min m
19.682

330_Calib_S_A

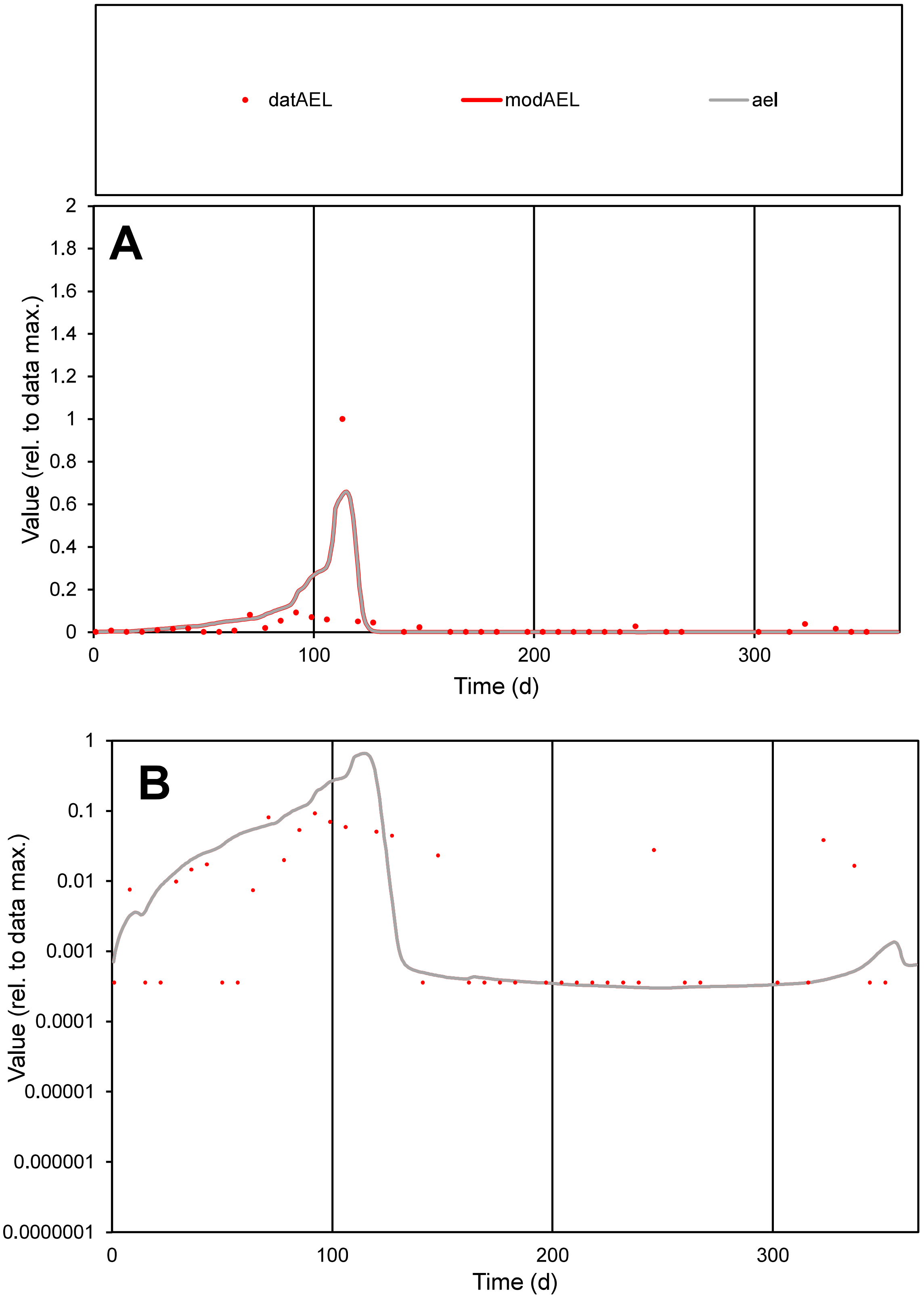

C1

Variables
AEL Provide jtd or Dst
CalibOnly?
2 1 to reproduce of
oNormalize?
2 1=no, 2=yes

ofxn calc*
1.93073
ofxn mod
1.98195

sRun
RunA1
zout
1

Ave
Data
7.8E-05
Model
9.5E-05

data/vMax
0.00194

ofxng
319.391

Min d/m
1.19193

ave/min d
111.497
ave/min m
162.837

330_Calib_S_A

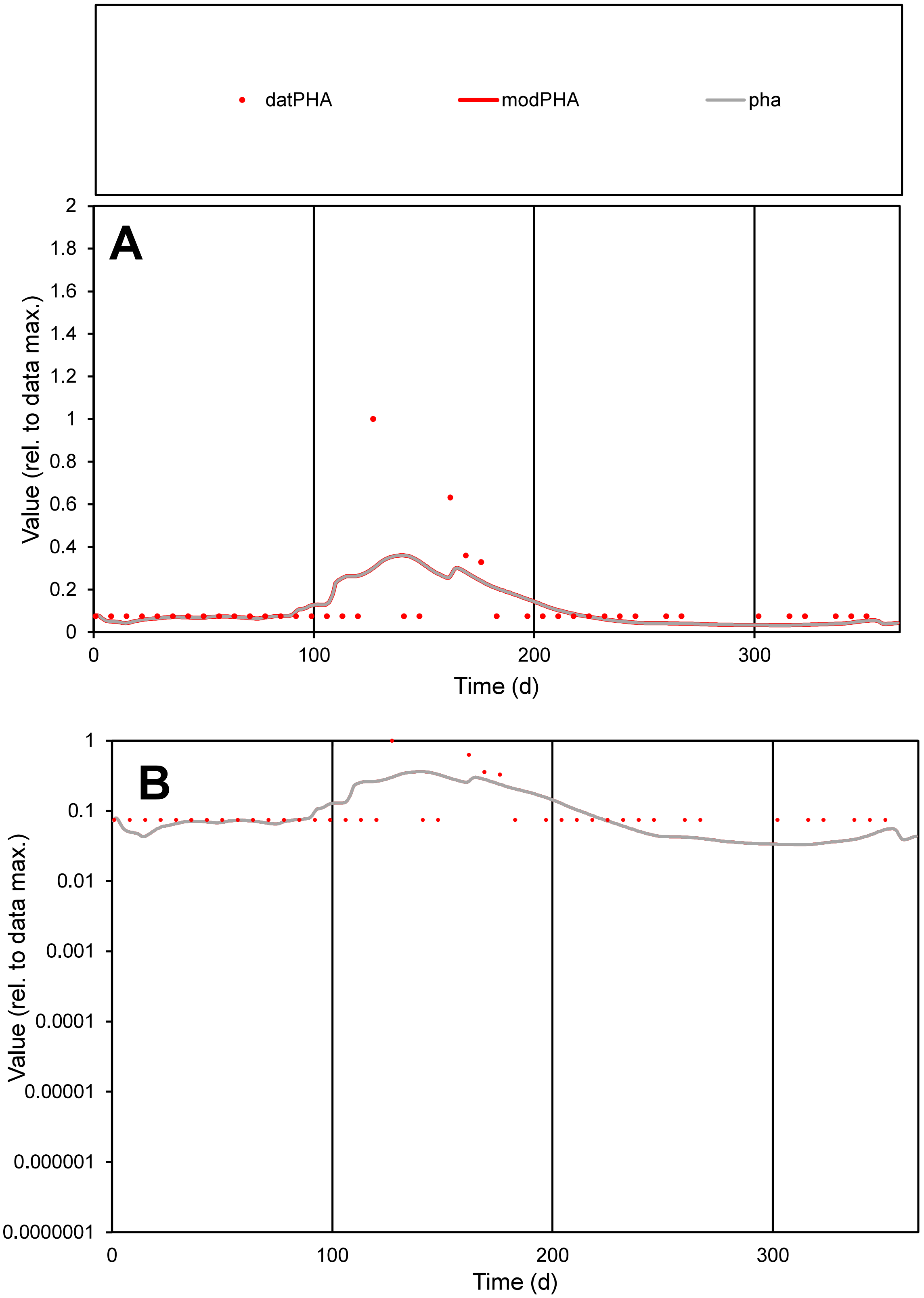

C1

Variables
PHA Provide jtd or Dst
CalibOnly?
2 1 to reproduce of
oNormalize?
2 1=no, 2=yes

ofxn calc*
1.17442
ofxn mod
1.18181

sRun
RunA1
zout
1

Ave
Data
1.7E-05
Model
1.6E-05

data/vMax
0.00014

ofxng
319.391

Min d/m
2.23391

ave/min d
1.66097
ave/min m
3.47313

330_Calib_S_A

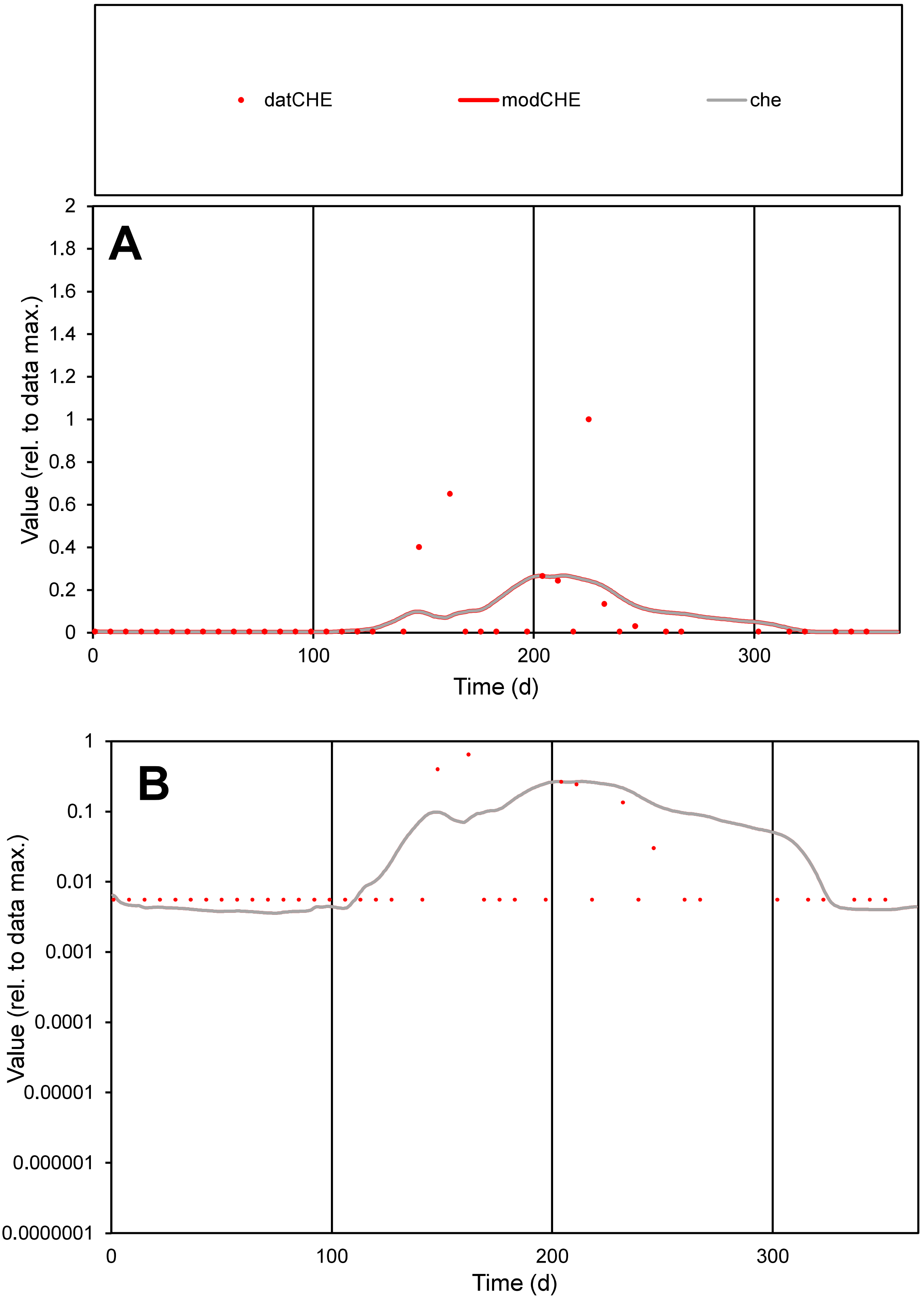

C1

Variables

CHE Provide jtd or Dst

CalibOnly?

2 1 to reproduce of

oNormalize?

2 1=no, 2=yes

ofxn calc*

2.41218

ofxn mod

2.42903

sRun

RunA1

zout

1

Ave

Data

8.9E-05

Model

8.3E-05

data/vMax

0.00125

ofxng

319.391

Min d/m

1.55723

ave/min d

12.7071

ave/min m

18.5212

330_Calib_S_A

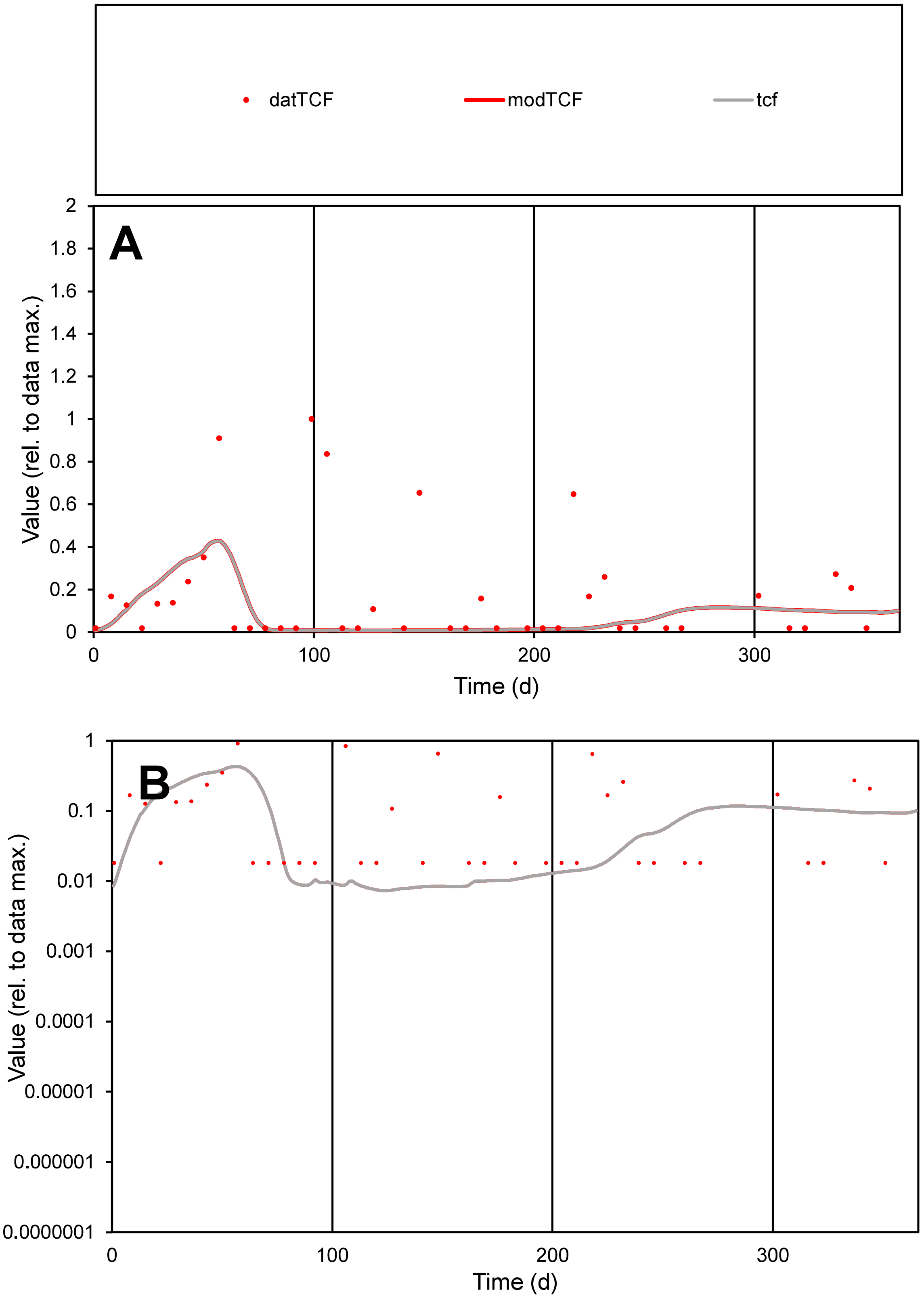

C1

Variables
TCF Provide jtd or Dst
CalibOnly?
2 1 to reproduce of
oNormalize?
2 1=no, 2=yes

ofxn calc*
1.60143
ofxn mod
1.61272

sRun
RunA1
zout
1

Ave
Data
2.9E-05
Model
1.5E-05

data/vMax
0.00017

ofxng
319.391

Min d/m
2.44819

ave/min d
9.35452
ave/min m
11.48

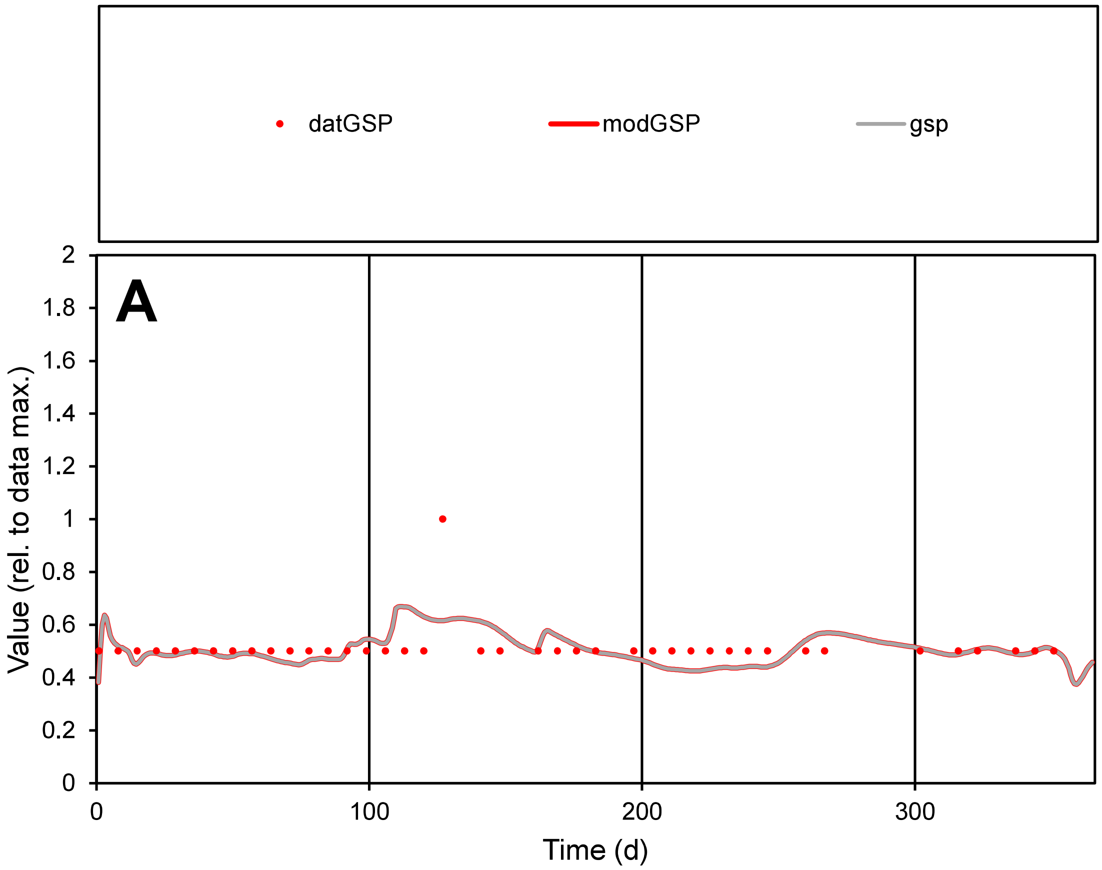

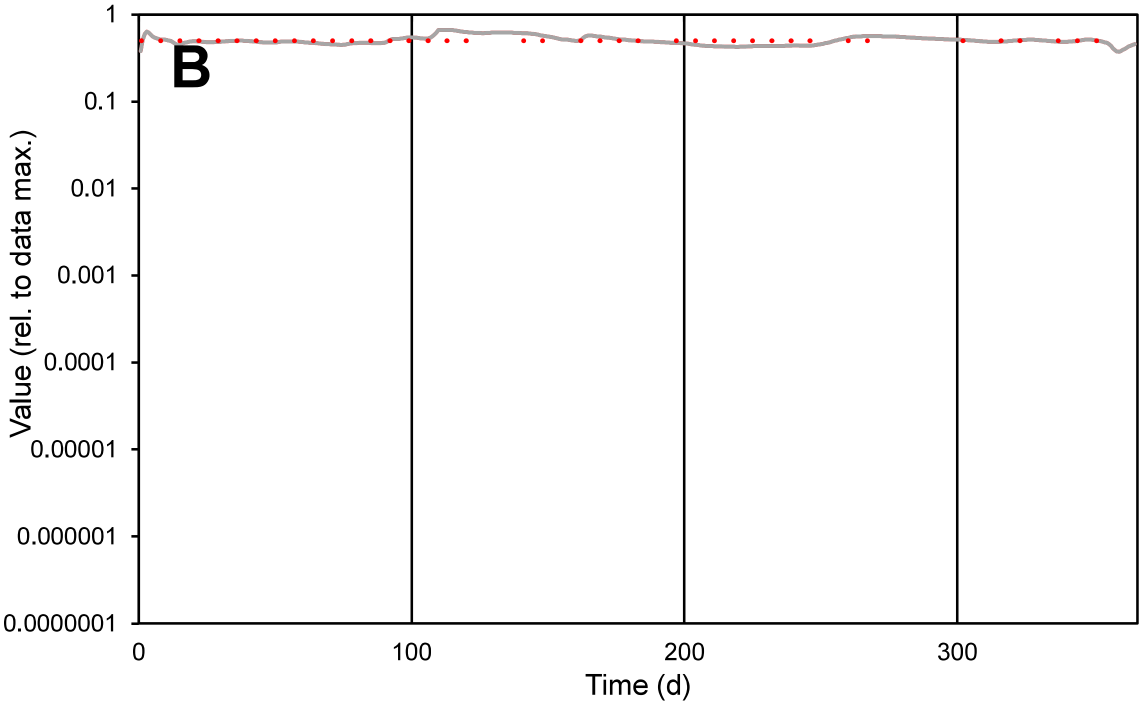

C1

Variables
GSP Provide jtd or Dst
CalibOnly?
2 1 to reproduce of
oNormalize?
2 1=no, 2=yes

ofxn calc*
0.15587
ofxn mod
0.18888

sRun
RunA1
zout
1

Ave
Data
9.2E-06
Model
9.1E-06

data/vMax
1.8E-05

ofxng
319.391

Min d/m
1.17577

ave/min d
1.02438
ave/min m
1.19144

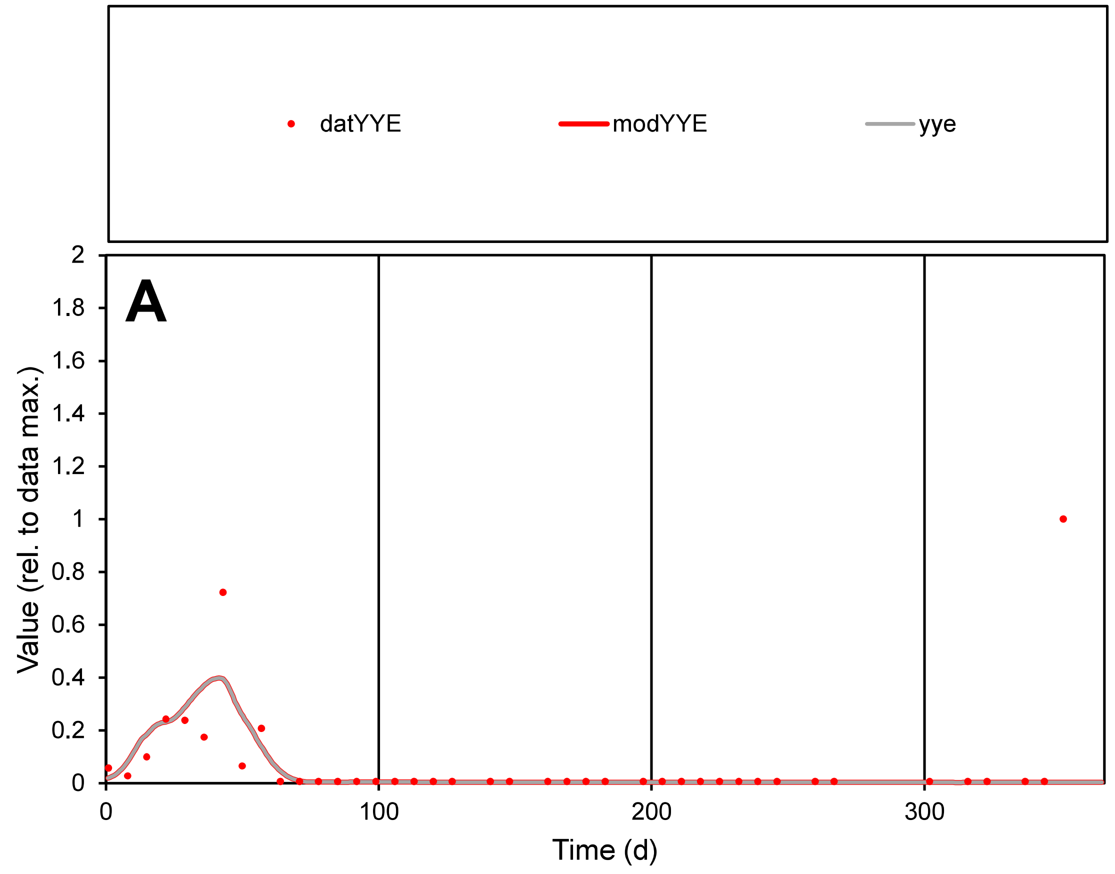

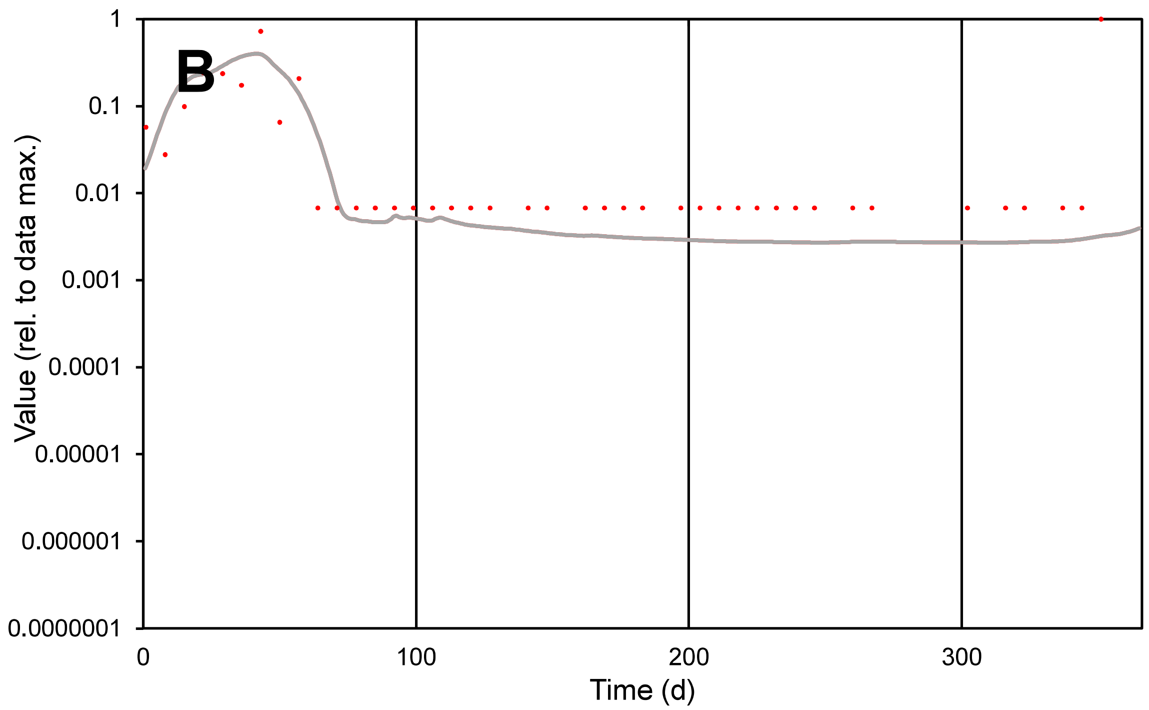

C1

Variables

YYE Provide jtd or Dst

CalibOnly?

2 1 to reproduce of

oNormalize?

2 1=no, 2=yes

ofxn calc*

2.29917

ofxn mod

2.31377

sRun

RunA1

zout

1

Ave

Data

4.1E-05

Model

2.8E-05

data/vMax

0.00056

ofxng

319.391

Min d/m

2.51034

ave/min d

10.9517

ave/min m

18.9267

330_Calib_S_A

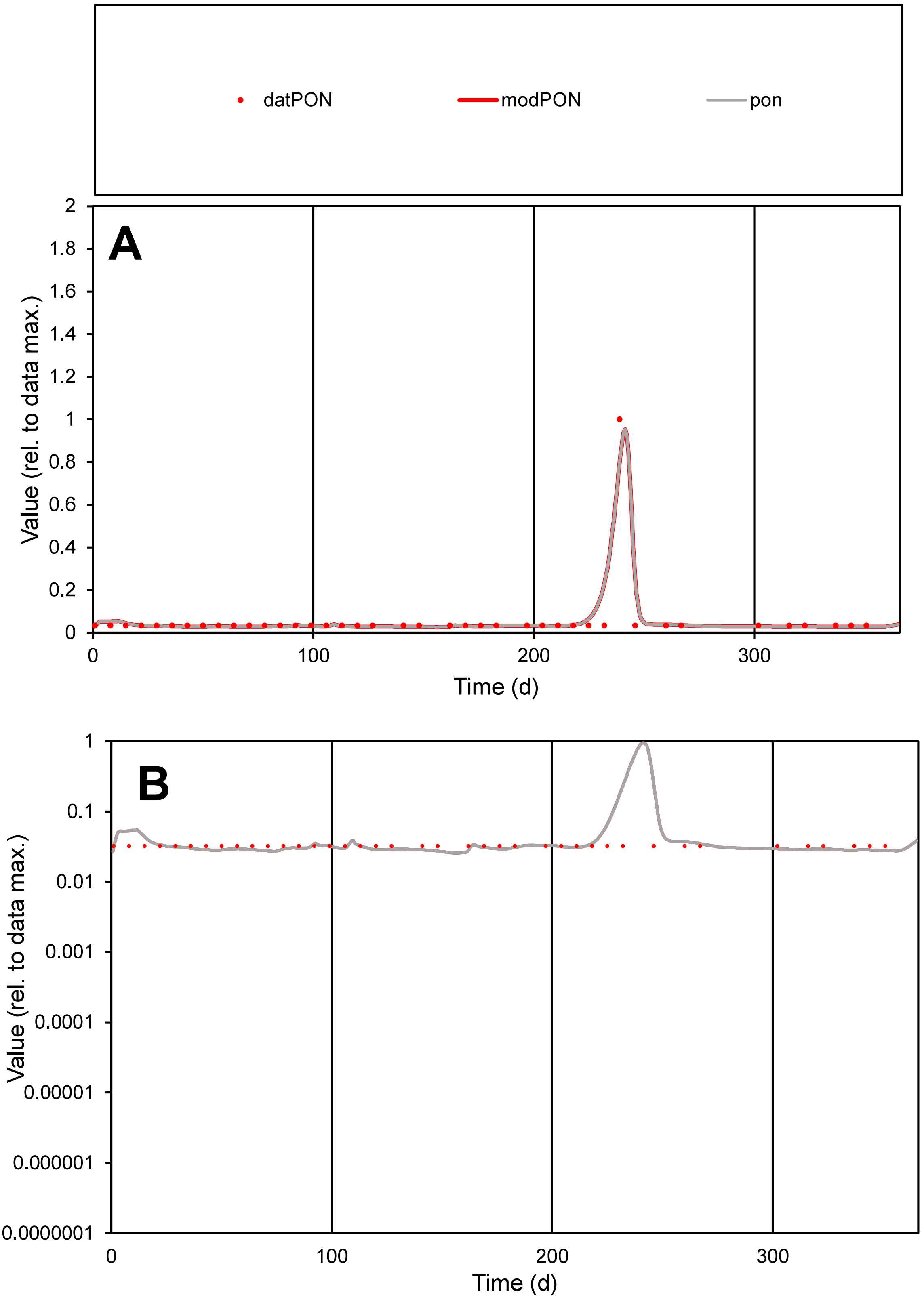

C1

Variables
PON Provide jtd or Dst
CalibOnly?
2 1 to reproduce of
oNormalize?
2 1=no, 2=yes

ofxn calc*
0.45509
ofxn mod
0.71149

sRun
RunA1
zout
1

Ave
Data
5.1E-06
Model
5.3E-06

data/vMax
9.1E-05

ofxng
319.391

Min d/m
1.1956

ave/min d
1.72885
ave/min m
2.16982

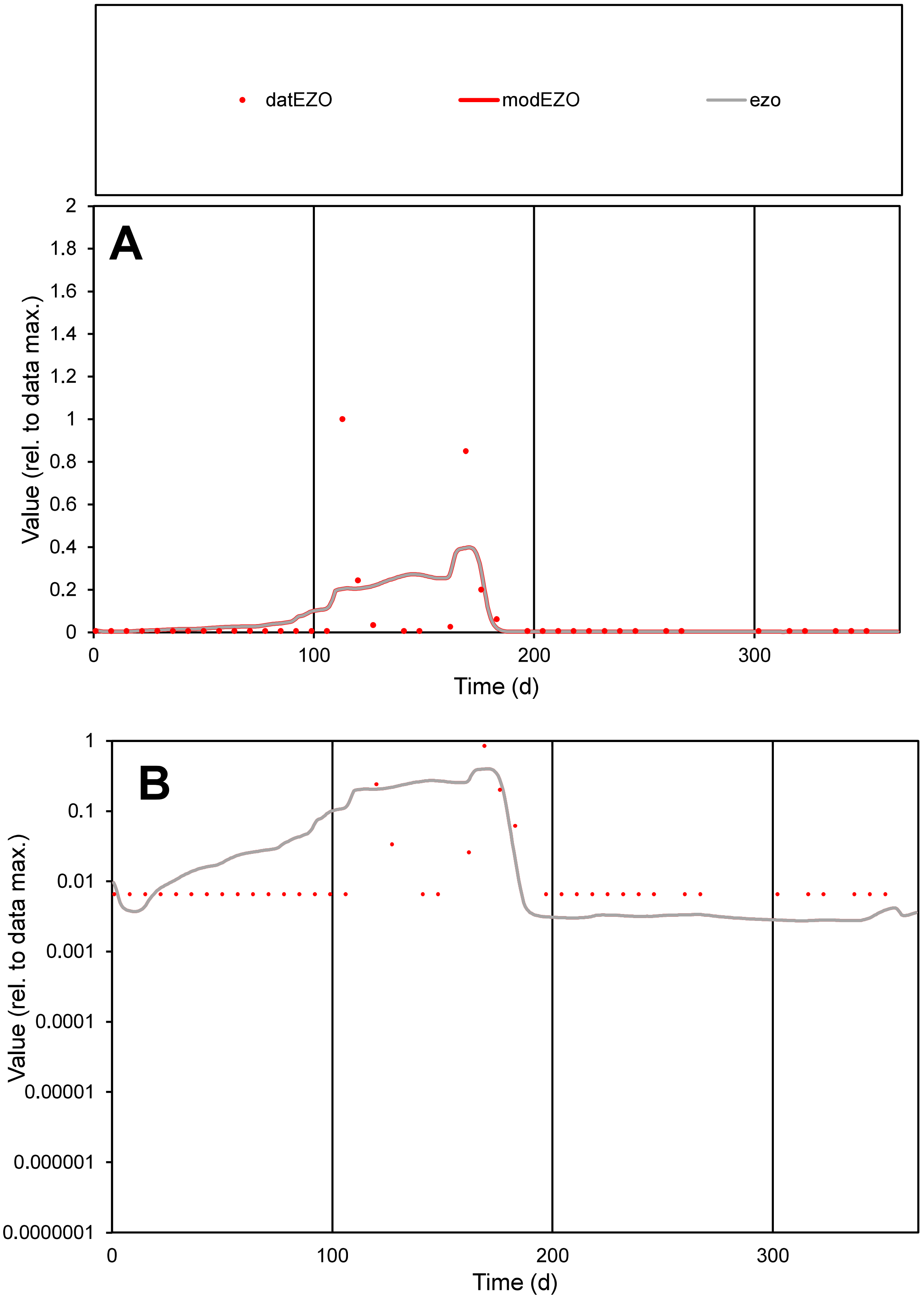

C1

Variables

EZO Provide jtd or Dst

CalibOnly?

2 1 to reproduce of

oNormalize?

2 1=no, 2=yes

ofxn calc*

2.52927

ofxn mod

2.549

sRun

RunA1

zout

1

Ave

Data

3.9E-05

Model

3.8E-05

data/vMax

0.0006

ofxng

319.391

Min d/m

2.38104

ave/min d

9.78456

ave/min m

23.1944

330_Calib_S_A

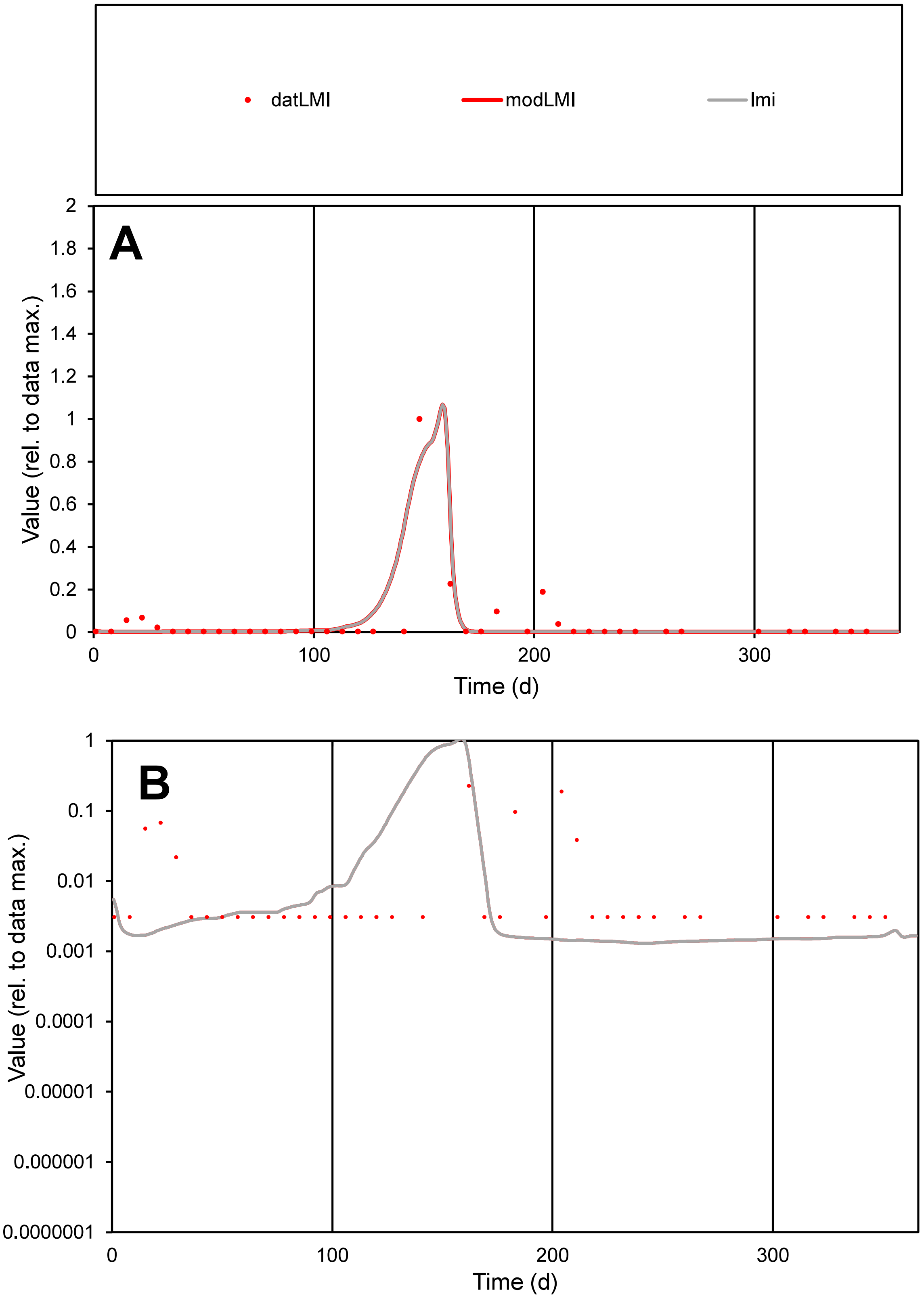

C1

Variables

LMI Provide jtd or Dst

CalibOnly?

2 1 to reproduce of

oNormalize?

2 1=no, 2=yes

ofxn calc*

1.66945

ofxn mod

1.97973

sRun

RunA1

zout

1

Ave

Data

4.3E-05

Model

3.9E-05

data/vMax

0.00098

ofxng

319.391

Min d/m

2.36871

ave/min d

14.2257

ave/min m

30.964

330_Calib_S_A

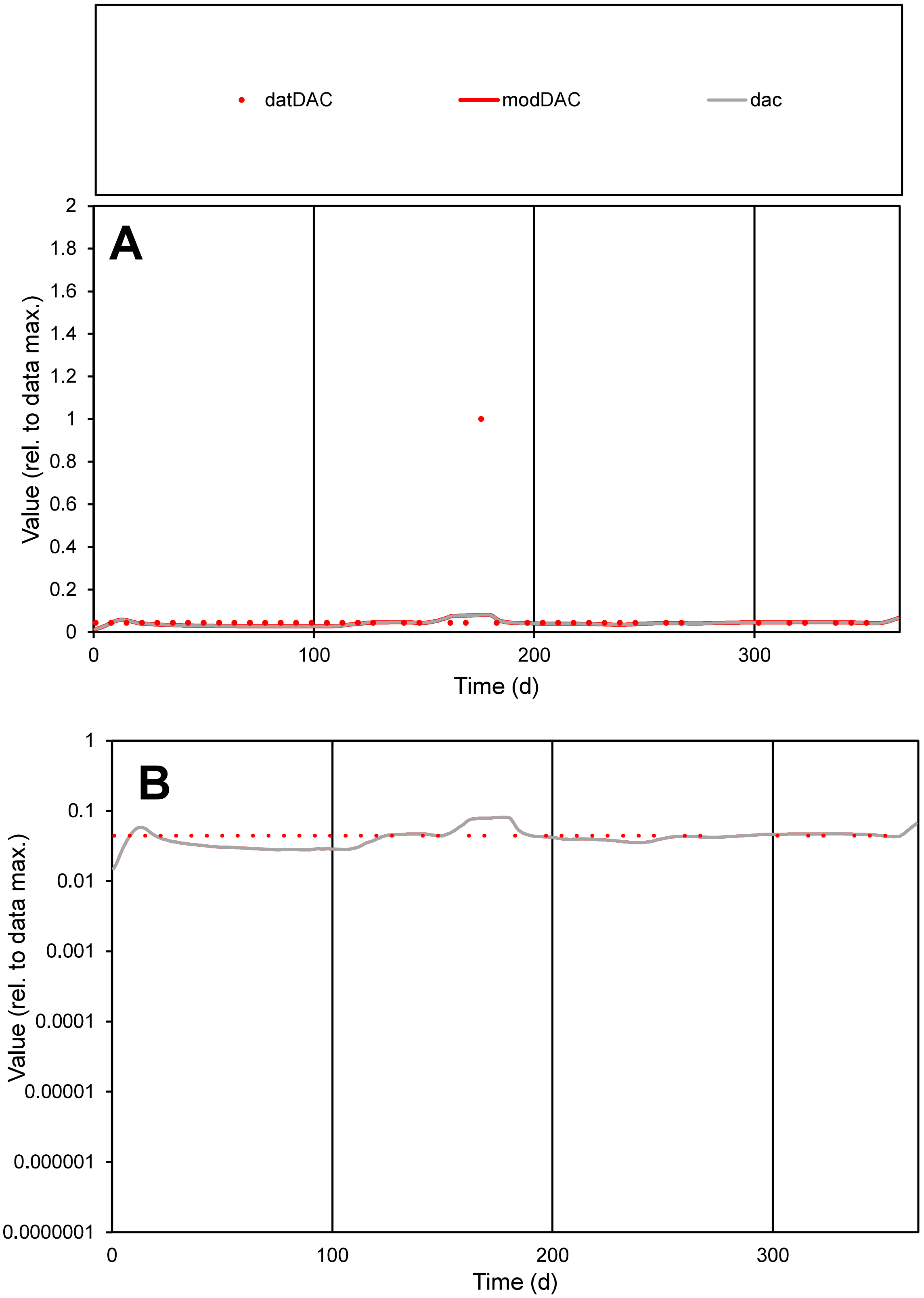

C1

Variables

DAC Provide jtd or Dst

CalibOnly?

2 1 to reproduce of

oNormalize?

2 1=no, 2=yes

ofxn calc*

2.12648

ofxn mod

2.1314

sRun

RunA1

zout

1

Ave

Data

1.2E-05

Model

7.2E-06

data/vMax

0.00017

ofxng

319.391

Min d/m

1.88273

ave/min d

1.52507

ave/min m

1.75259

330_Calib_S_A

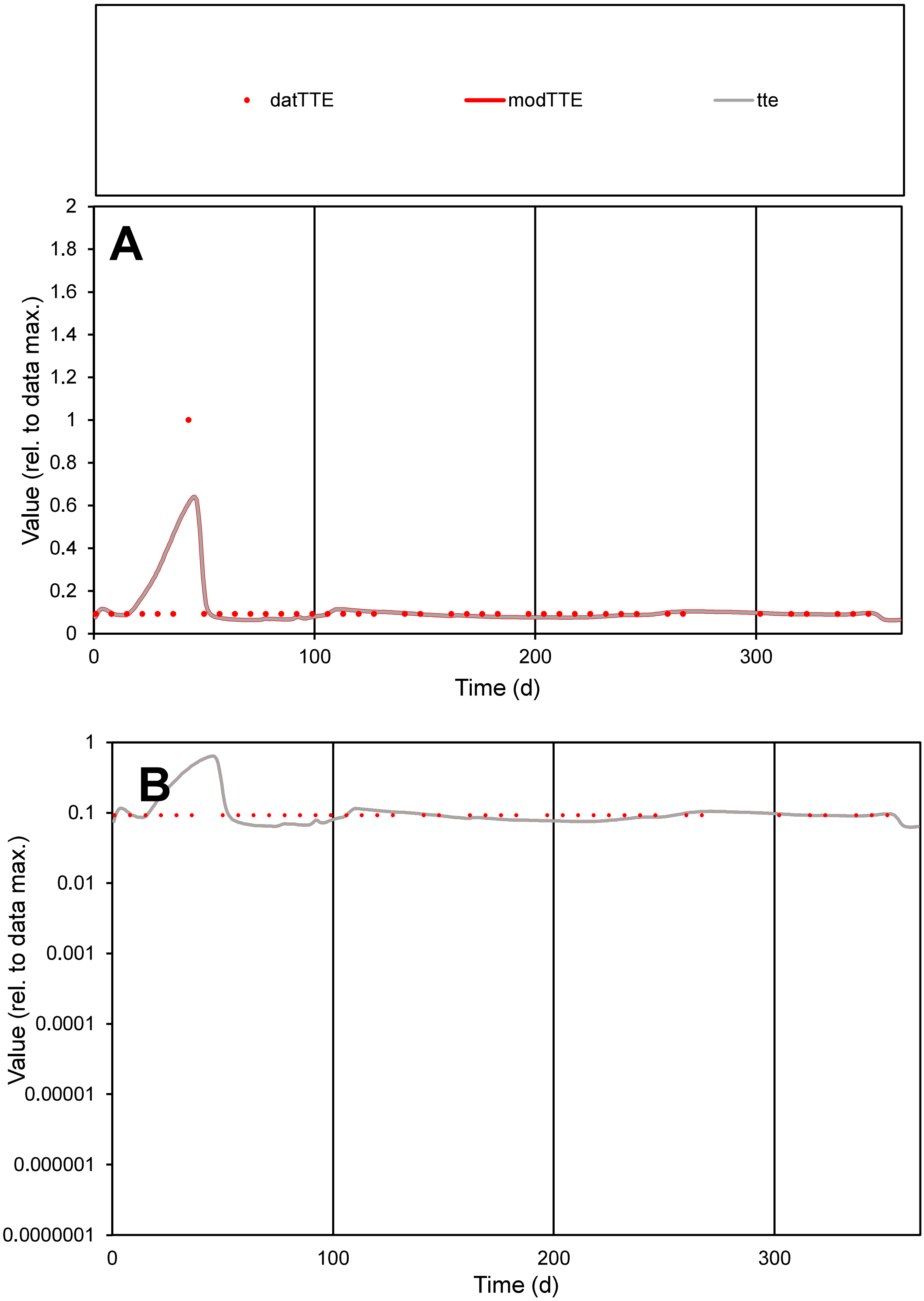

C1

Variables

TTE Provide jtd or Dst

CalibOnly?

2 1 to reproduce of

oNormalize?

2 1=no, 2=yes

ofxn calc*

0.78732

ofxn mod

0.80982

sRun

RunA1

zout

1

Ave

Data

4.7E-06

Model

4.8E-06

data/vMax

4.1E-05

ofxng

319.391

Min d/m

1.43934

ave/min d

1.23851

ave/min m

1.82217

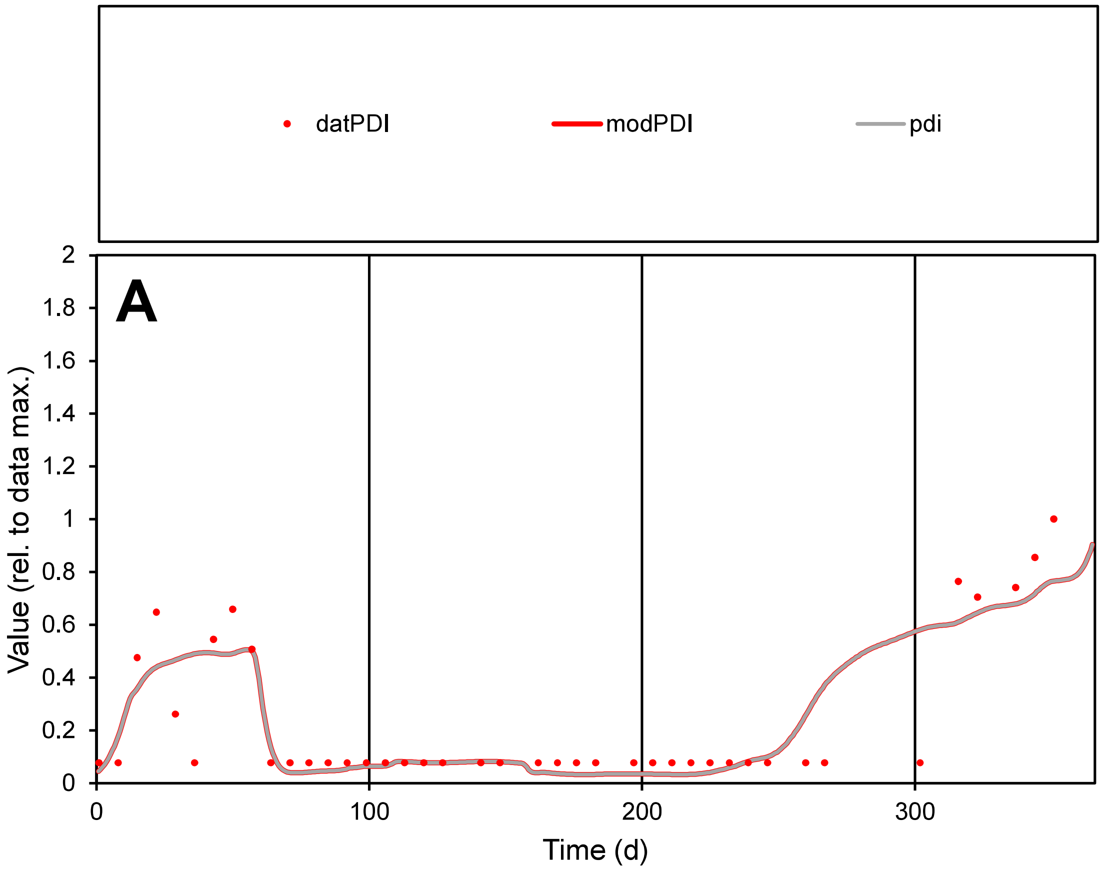

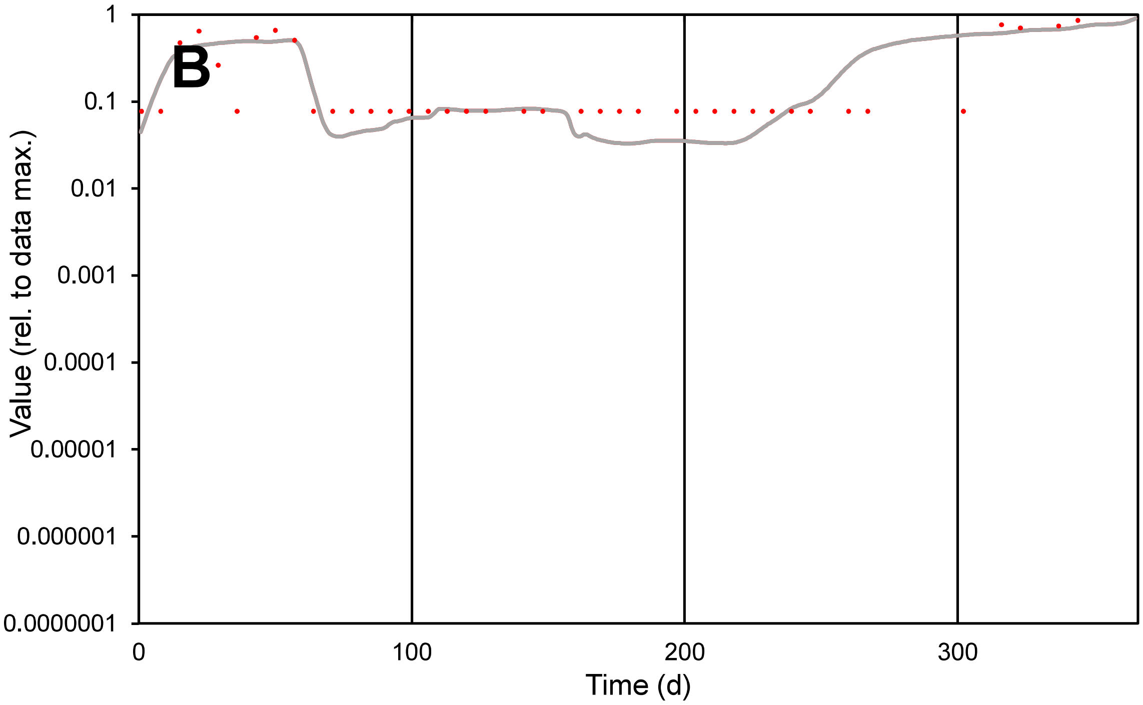

C1

Variables
PDI Provide jtd or Dst
CalibOnly?
2 1 to reproduce of
oNormalize?
2 1=no, 2=yes

ofxn calc*
0.60658
ofxn mod
0.62588

sRun
RunA1
zout
1

Ave
Data
1.3E-05
Model
1.3E-05

data/vMax
5.7E-05

ofxng
319.391

Min d/m
2.34704

ave/min d
2.99496
ave/min m
7.0951

330_Calib_S_A

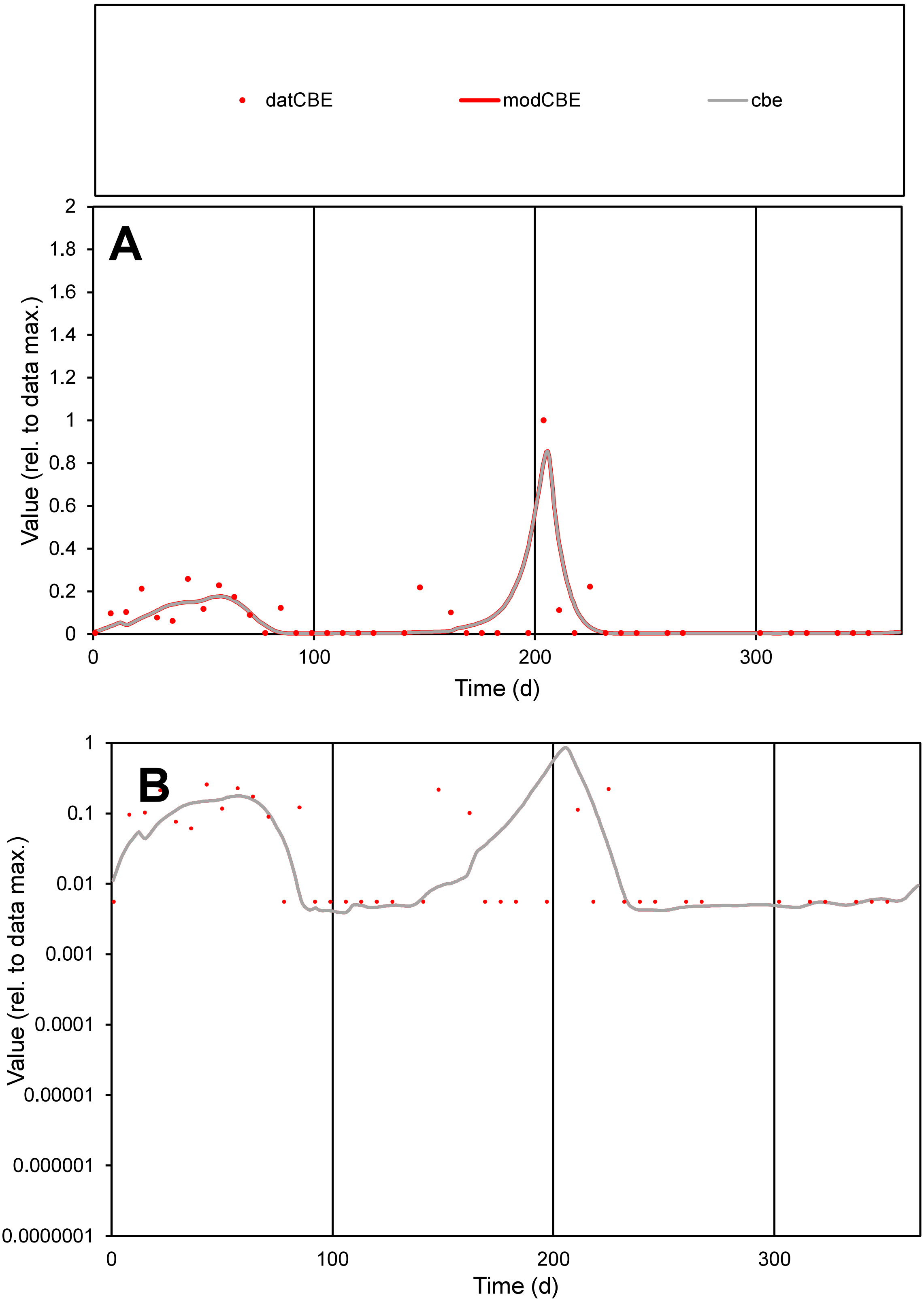

C1

Variables
CBE Provide jtd or Dst
CalibOnly?
2 1 to reproduce of
oNormalize?
2 1=no, 2=yes

ofxn calc*
1.06958
ofxn mod
1.19274

sRun
RunA1
zout
1

Ave
Data
2.4E-05
Model
2.2E-05

data/vMax
0.0003

ofxng
319.391

Min d/m
1.36609

ave/min d
14.5773
ave/min m
18.2663

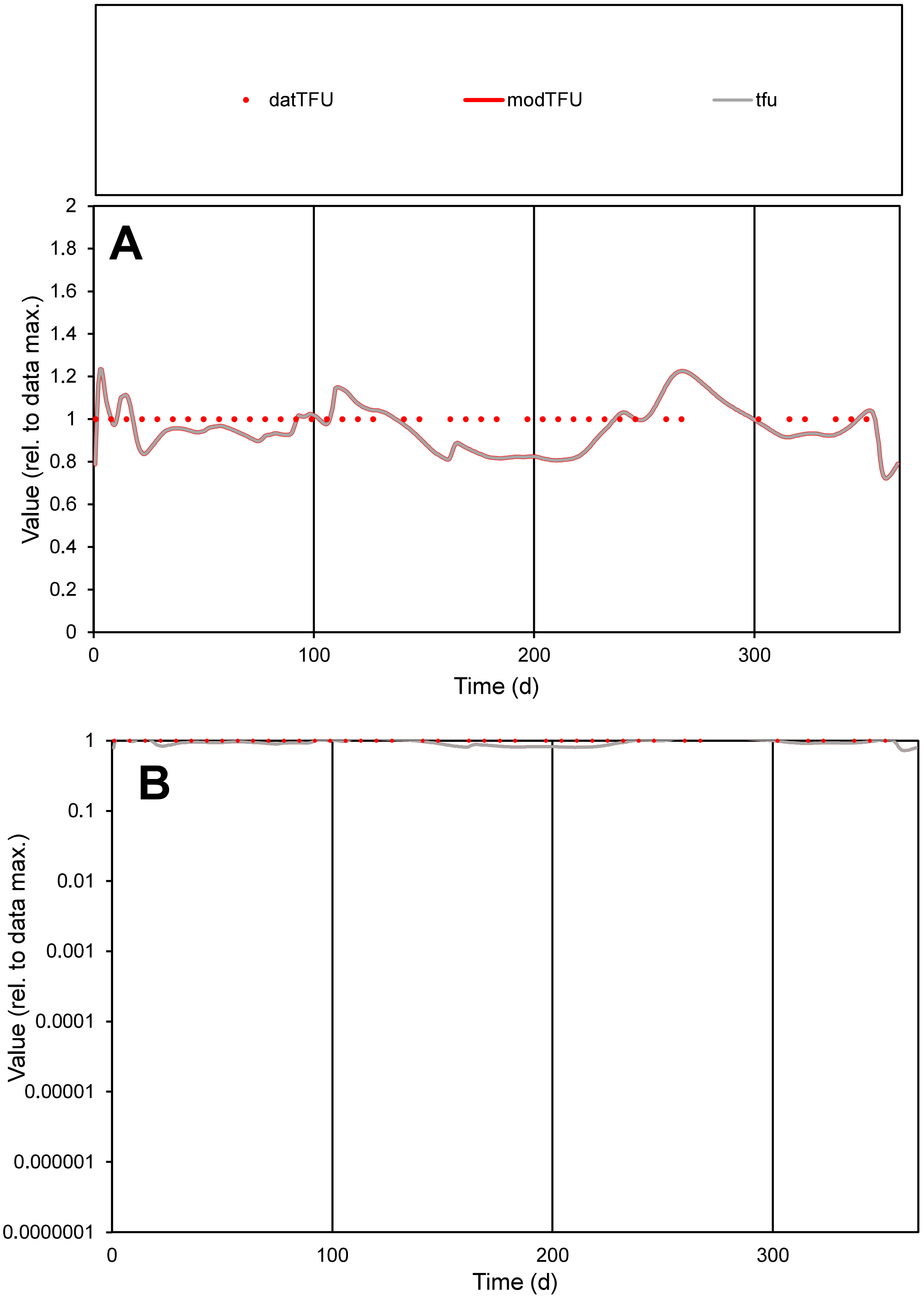

C1

Variables

TFU Provide jtd or Dst

CalibOnly?

2 1 to reproduce of

oNormalize?

2 1=no, 2=yes

ofxn calc*

0.10523

ofxn mod

0.14979

sRun

RunA1

zout

1

Ave

Data

1.3E-05

Model

1.2E-05

data/vMax

1.3E-05

ofxng

319.391

Min d/m

1.23786

ave/min d

1

ave/min m

1.18212

330_Calib_S_A

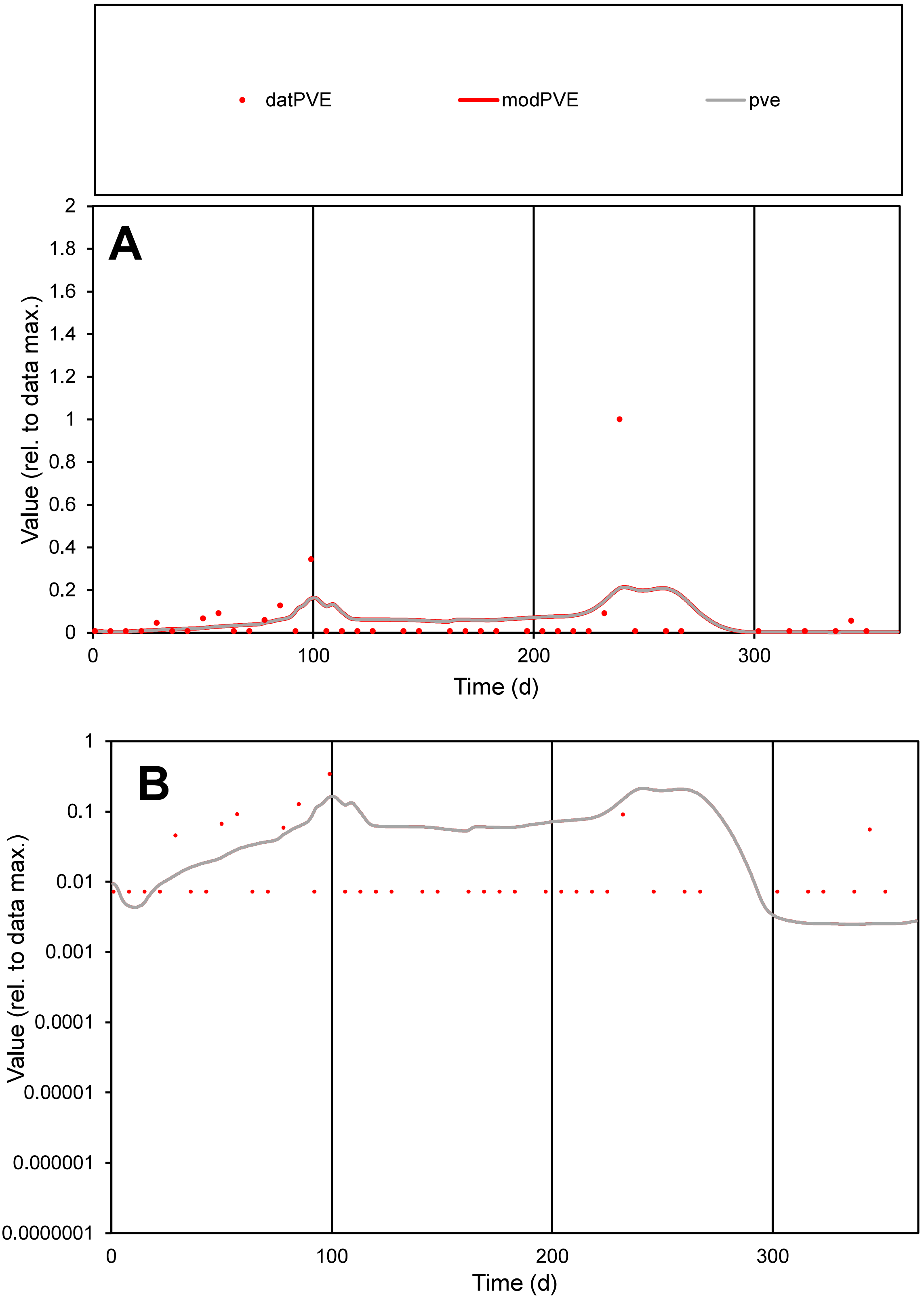

C1

Variables
PVE Provide jtd or Dst
CalibOnly?
2 1 to reproduce of
oNormalize?
2 1=no, 2=yes

ofxn calc*
2.7692
ofxn mod
2.80033

sRun
RunA1
zout
1

Ave
Data
1.5E-05
Model
1.8E-05

data/vMax
0.00029

ofxng
319.391

Min d/m
2.91771

ave/min d
7.12079
ave/min m
25.1509

330_Calib_S_A

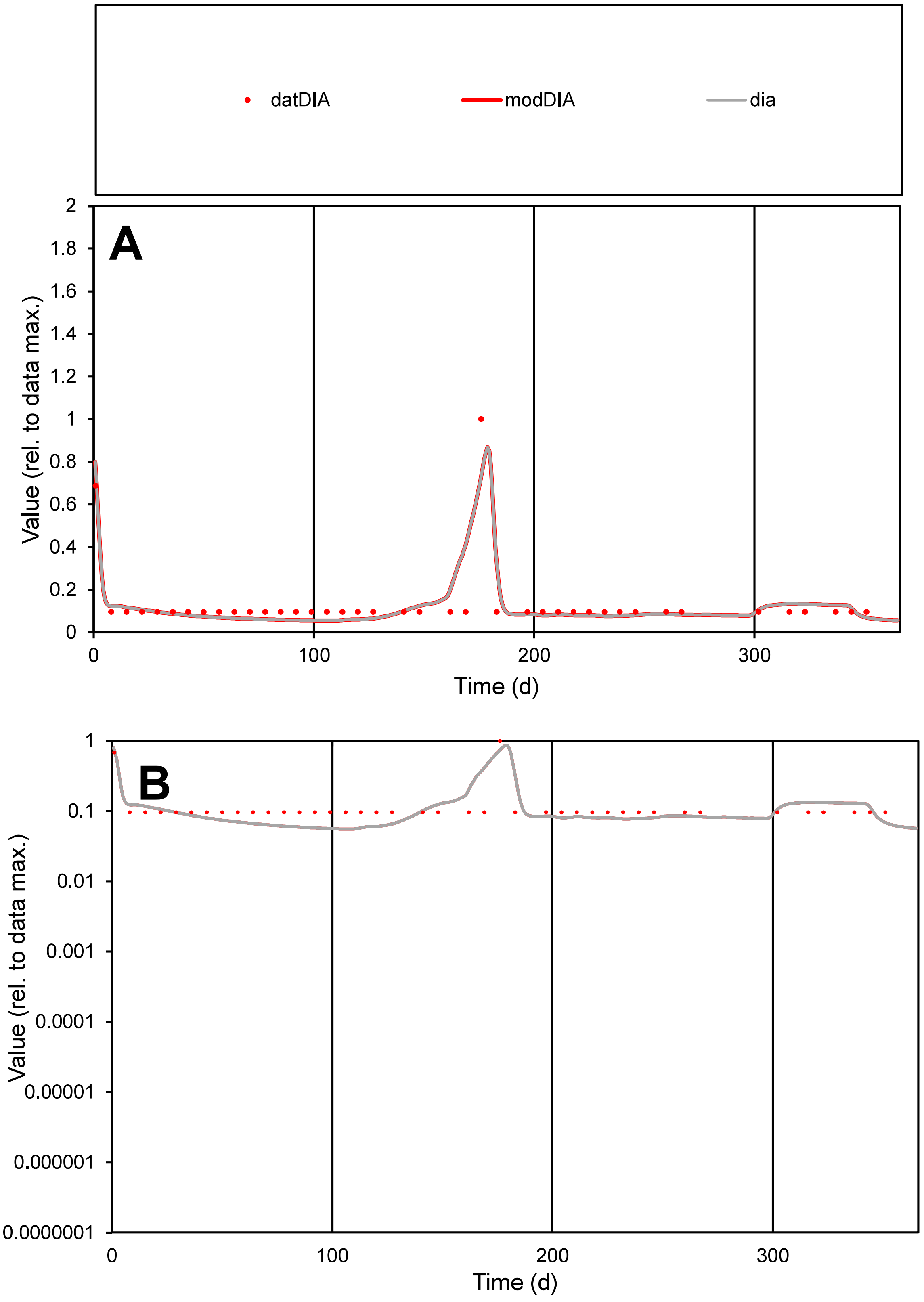

C1

Variables

DIA Provide jtd or Dst

CalibOnly?

2 1 to reproduce of

oNormalize?

2 1=no, 2=yes

ofxn calc*

0.46685

ofxn mod

0.51819

sRun

RunA1

zout

1

Ave

Data

8.6E-06

Model

8.4E-06

data/vMax

6.5E-05

ofxng

319.391

Min d/m

1.72684

ave/min d

1.3809

ave/min m

2.34331

330_Calib_S_A

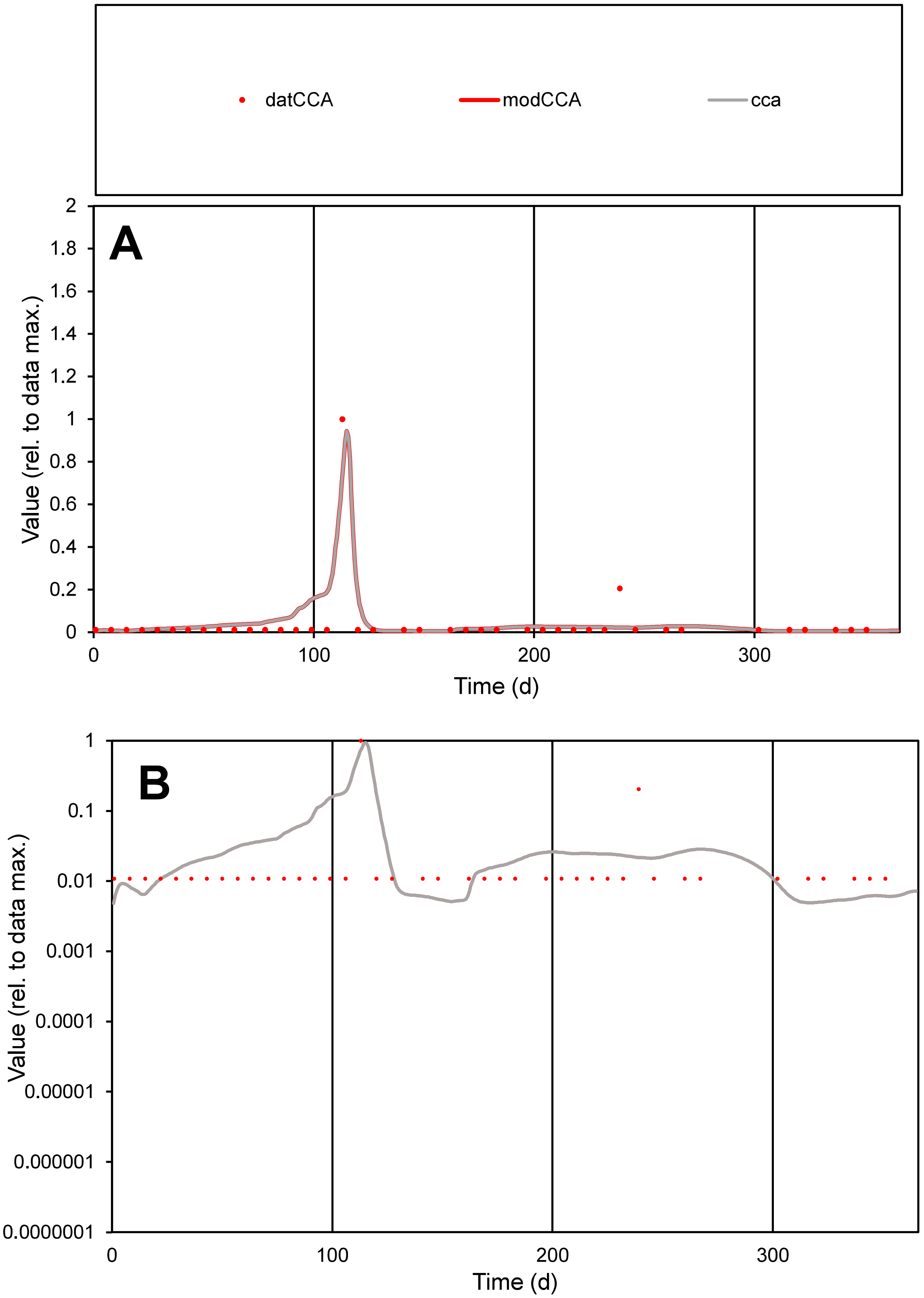

C1

Variables

CCA Provide jtd or Dst

CalibOnly?

2 1 to reproduce of

oNormalize?

2 1=no, 2=yes

ofxn calc*

1.30384

ofxn mod

1.38027

sRun

RunA1

zout

1

Ave

Data

6.7E-06

Model

8.5E-06

data/vMax

0.00017

ofxng

319.391

Min d/m

2.20135

ave/min d

3.67071

ave/min m

10.2644

330_Calib_S_A

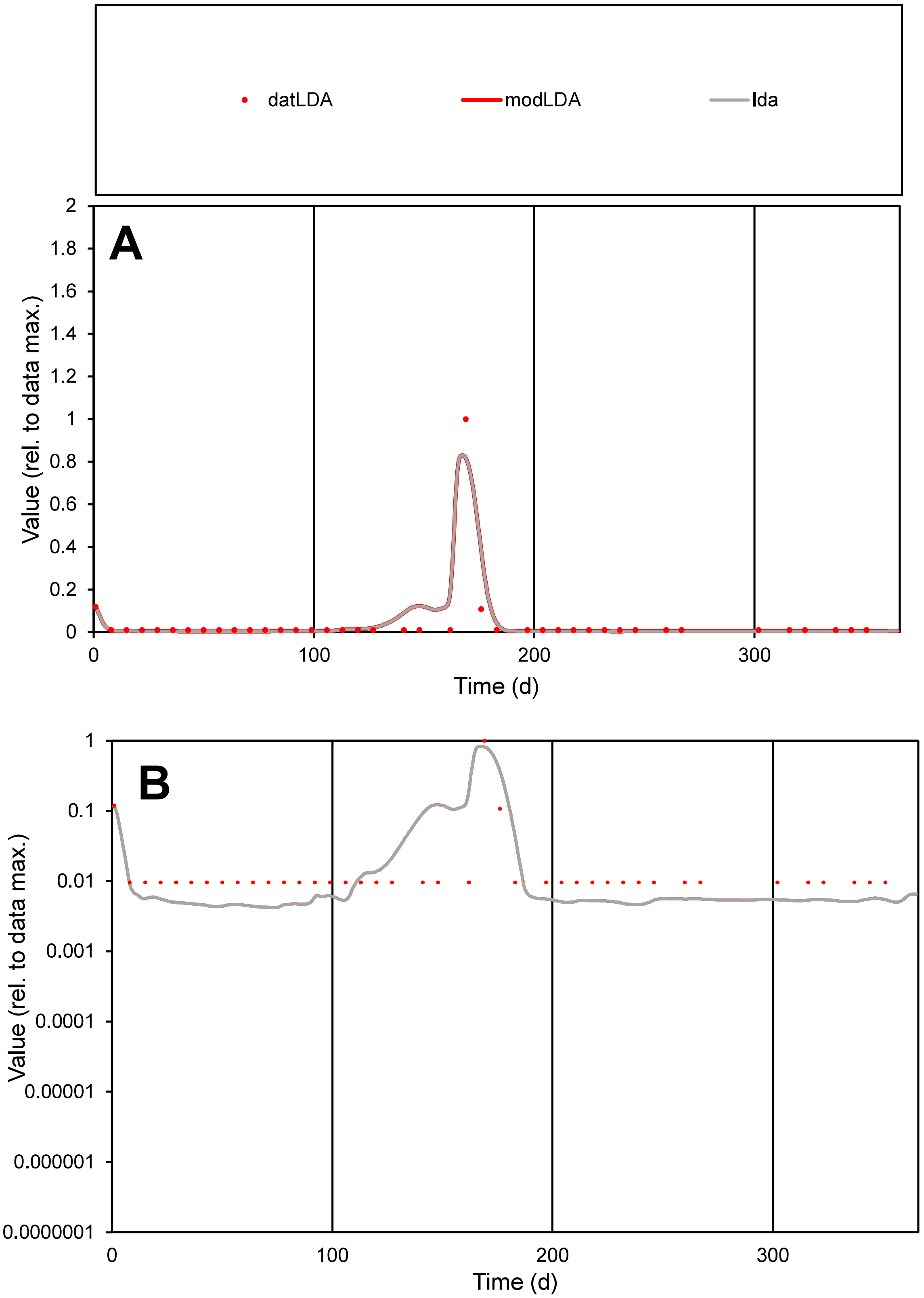

C1

Variables	
LDA	Provide jtd or Dst
CalibOnly?	
2	1 to reproduce of
oNormalize?	
2	1=no, 2=yes
ofxn calc*	
1.20894	
ofxn mod	
1.35209	
sRun	
RunA1	
zout	
1	
Ave	
Data	
2.8E-05	
Model	
3E-05	
data/vMax	
0.00072	
ofxng	
319.391	
Min d/m	
2.26739	
ave/min d	
4.05839	
ave/min m	
9.70536	

330_Calib_S_A

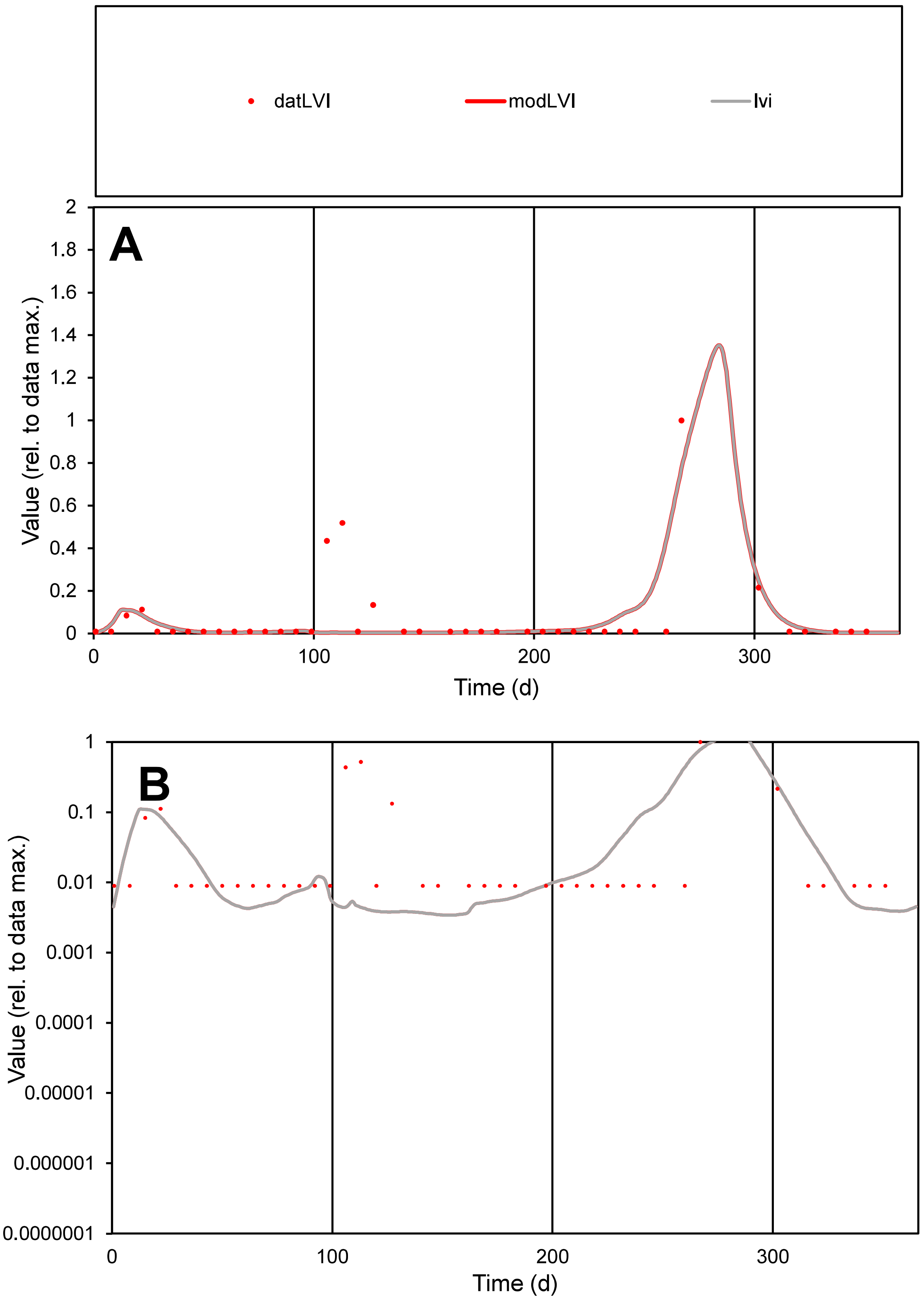

C1

Variables

LVI Provide jtd or Dst

CalibOnly?

2 1 to reproduce of

oNormalize?

2 1=no, 2=yes

ofxn calc*

1.82124

ofxn mod

1.8961

sRun

RunA1

zout

1

Ave

Data

1.1E-05

Model

8.4E-06

data/vMax

0.00016

ofxng

319.391

Min d/m

2.58123

ave/min d

7.65356

ave/min m

15.3367

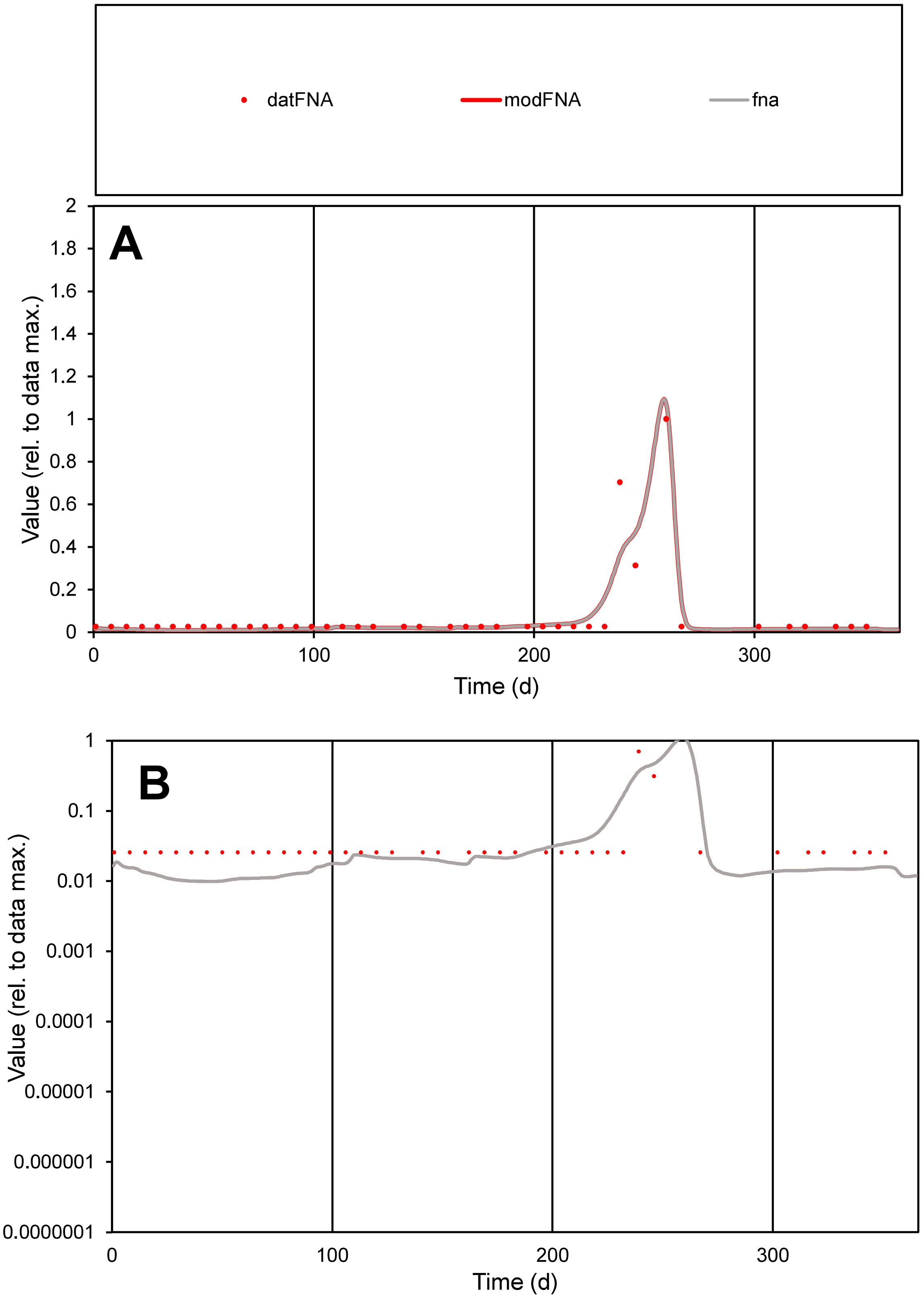

C1

Variables
FNA Provide jtd or Dst
CalibOnly?
2 1 to reproduce of
oNormalize?
2 1=no, 2=yes

ofxn calc*
0.80406
ofxn mod
0.86611

sRun
RunA1
zout
1

Ave
Data
8.9E-06
Model
8.2E-06

data/vMax
0.00012

ofxng
319.391

Min d/m
2.60921

ave/min d
2.8434
ave/min m
6.87582

330_Calib_S_A

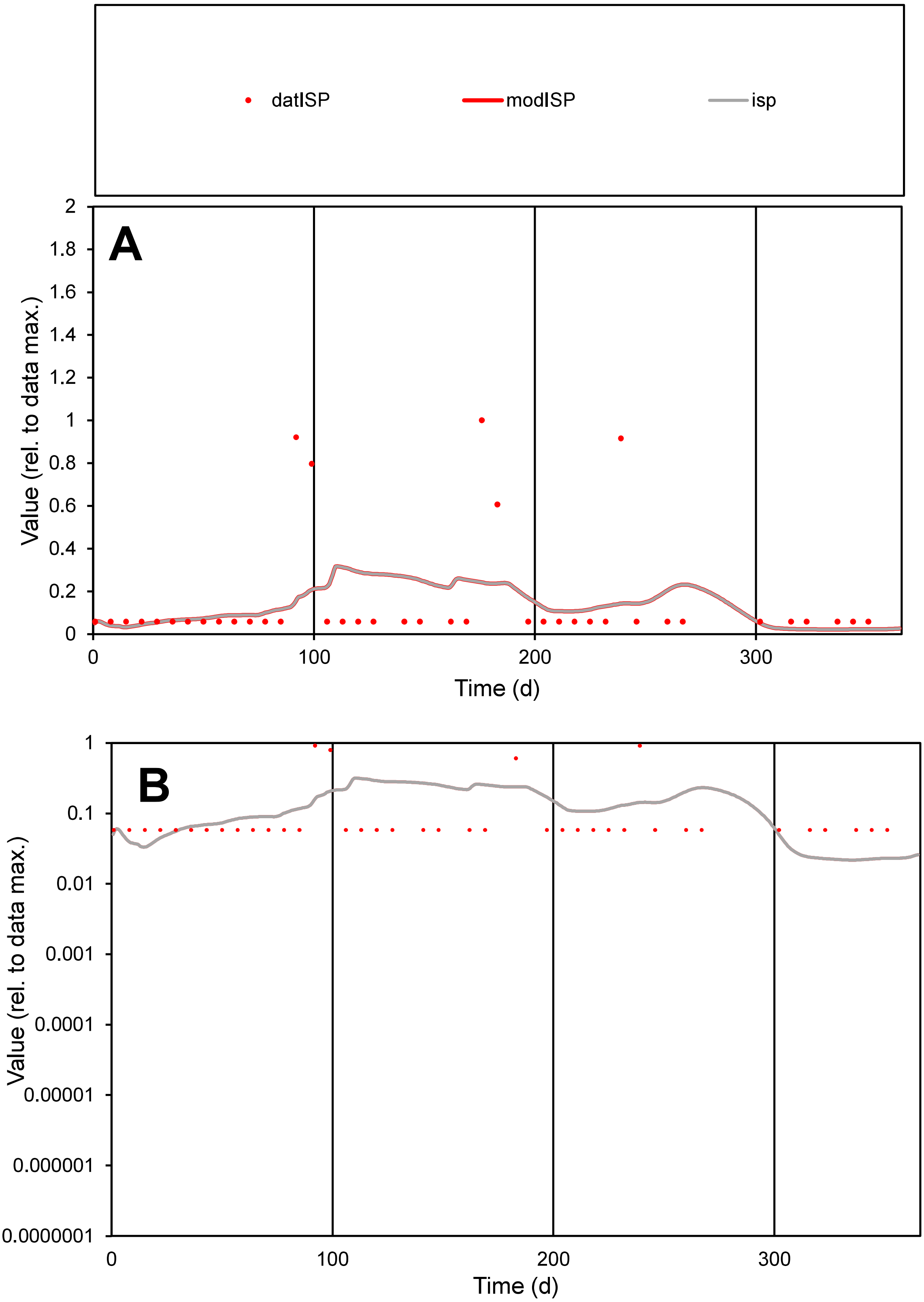

C1

Variables
ISP Provide jtd or Dst
CalibOnly?
2 1 to reproduce of
oNormalize?
2 1=no, 2=yes

ofxn calc*
1.64784
ofxn mod
1.65616

sRun
RunA1
zout
1

Ave
Data
1.3E-05
Model
1.2E-05

data/vMax
8.7E-05

ofxng
319.391

Min d/m
2.64394

ave/min d
2.66035
ave/min m
6.14027

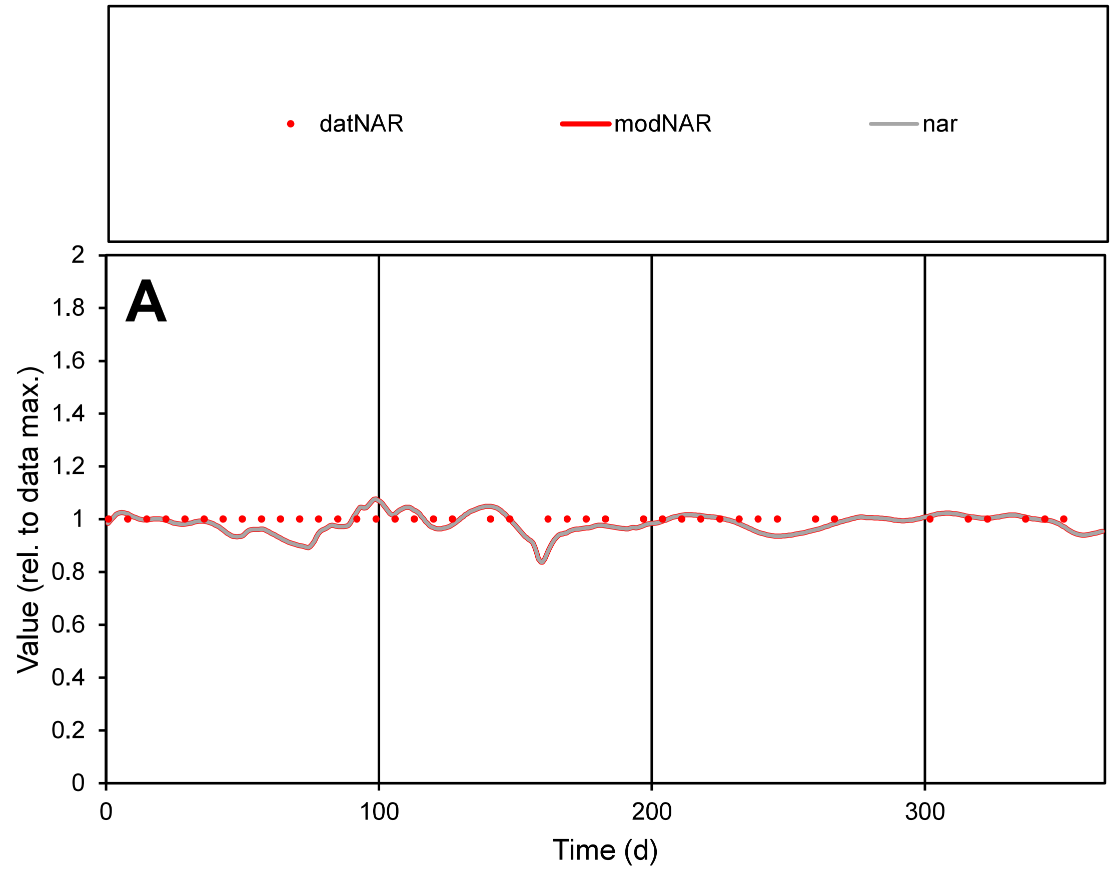

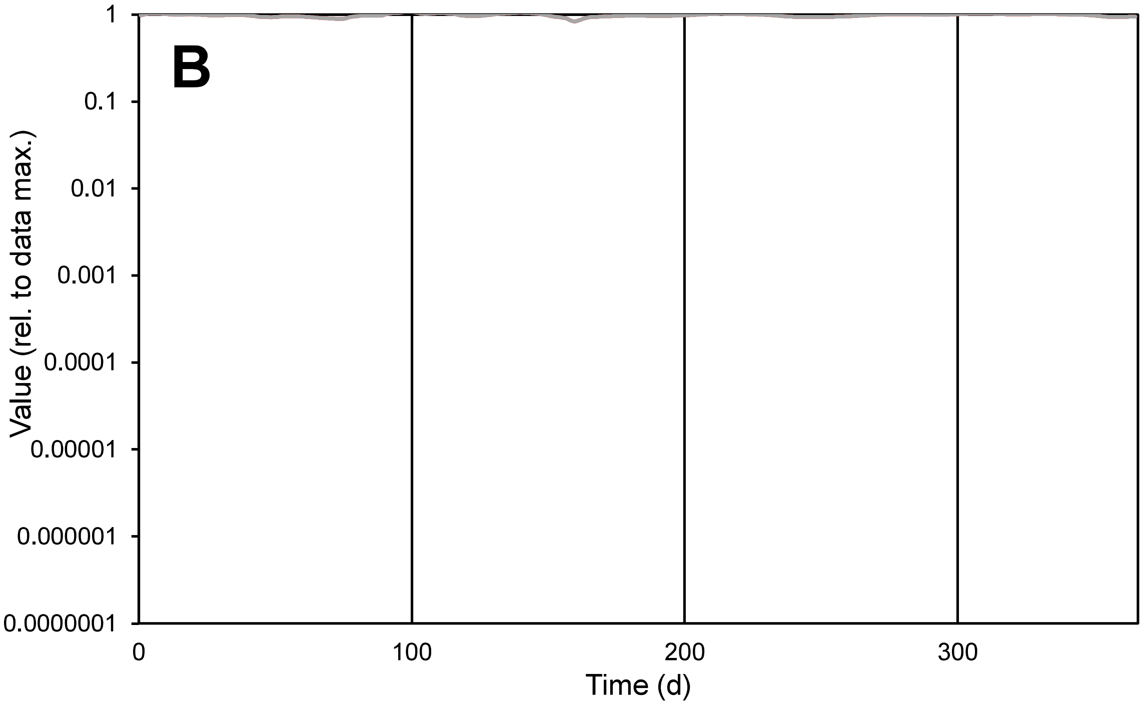

C1

Variables
NAR Provide jtd or Dst
CalibOnly?
2 1 to reproduce of
oNormalize?
2 1=no, 2=yes

ofxn calc*
0.0386
ofxn mod
0.115

sRun
RunA1
zout
1

Ave
Data
2.8E-06
Model
2.8E-06

data/vMax
2.8E-06

ofxng
319.391

Min d/m
1.11396

ave/min d
1
ave/min m
1.09684

330_Calib_S_A

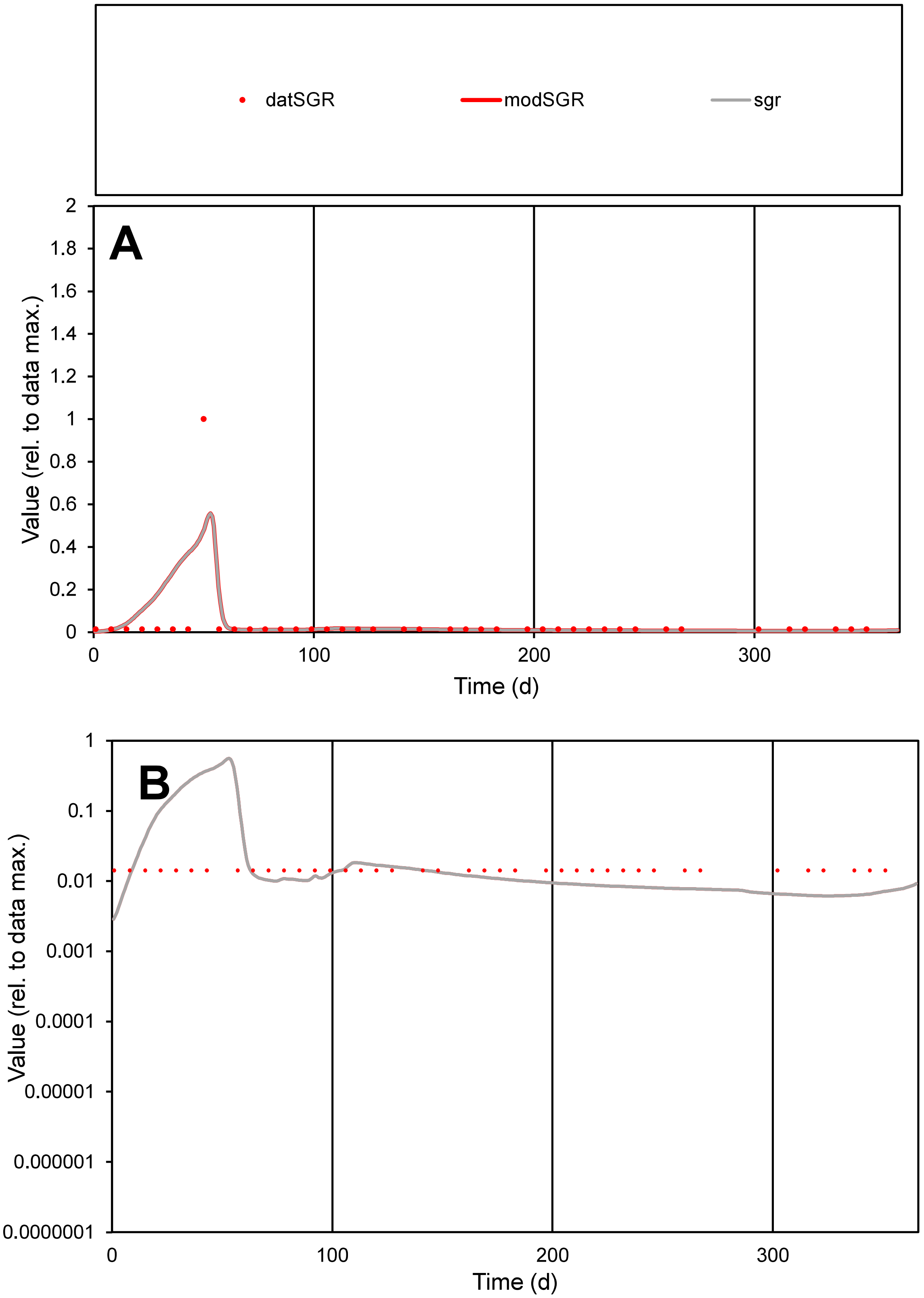

C1

Variables
SGR Provide jtd or Dst
CalibOnly?
2 1 to reproduce of
oNormalize?
2 1=no, 2=yes

ofxn calc*
2.69175
ofxn mod
2.81405

sRun
RunA1
zout
1

Ave
Data
1.2E-05
Model
1.4E-05

data/vMax
0.00032

ofxng
319.391

Min d/m
2.91828

ave/min d
2.69933
ave/min m
9.26919

330_Calib_S_A

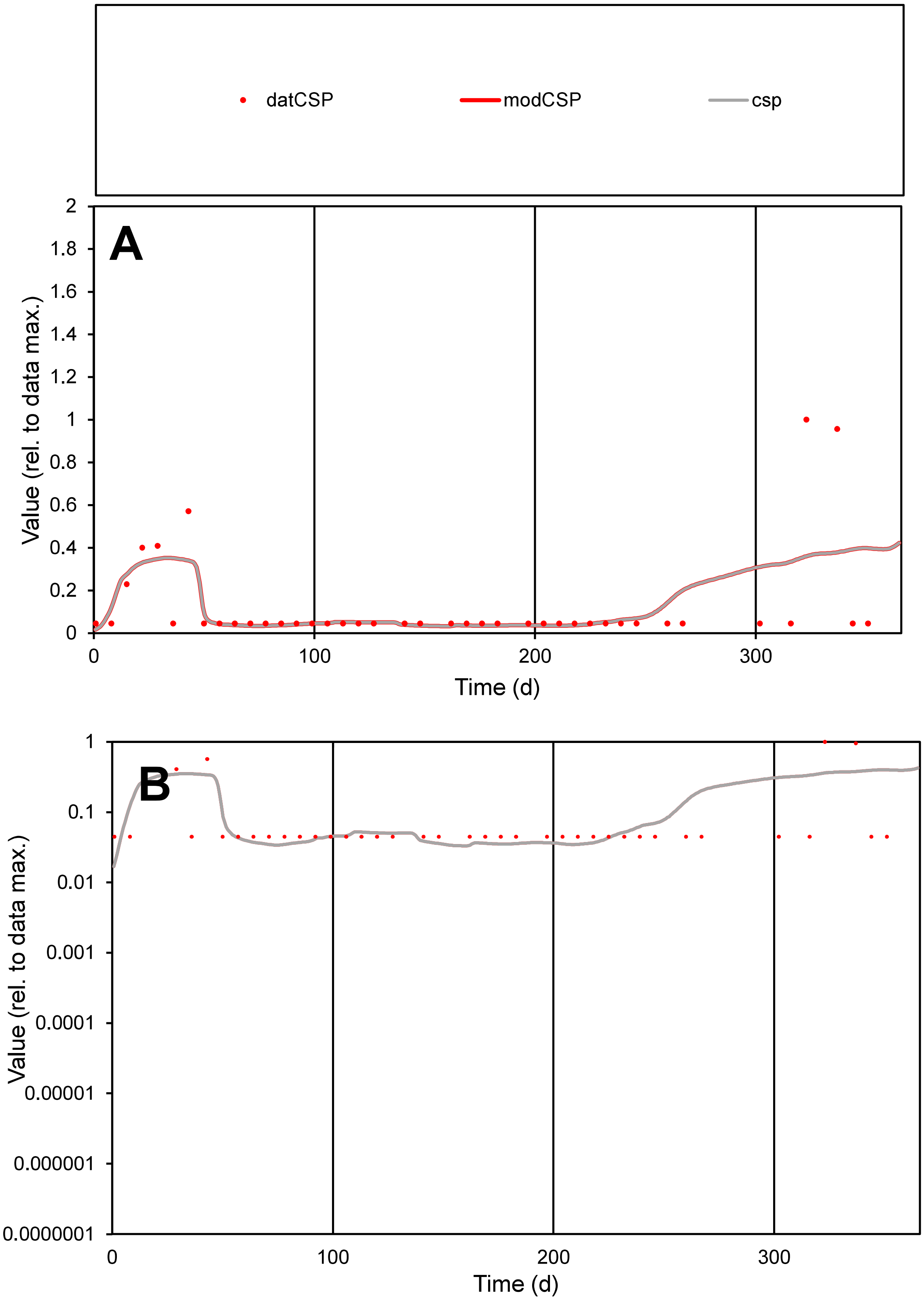

C1

Variables

CSP Provide jtd or Dst

CalibOnly?

2 1 to reproduce of

oNormalize?

2 1=no, 2=yes

ofxn calc*

1.44234

ofxn mod

1.45568

sRun

RunA1

zout

1

Ave

Data

6.7E-06

Model

7.1E-06

data/vMax

5.3E-05

ofxng

319.391

Min d/m

1.30295

ave/min d

2.79006

ave/min m

3.86608

330_Calib_S_A

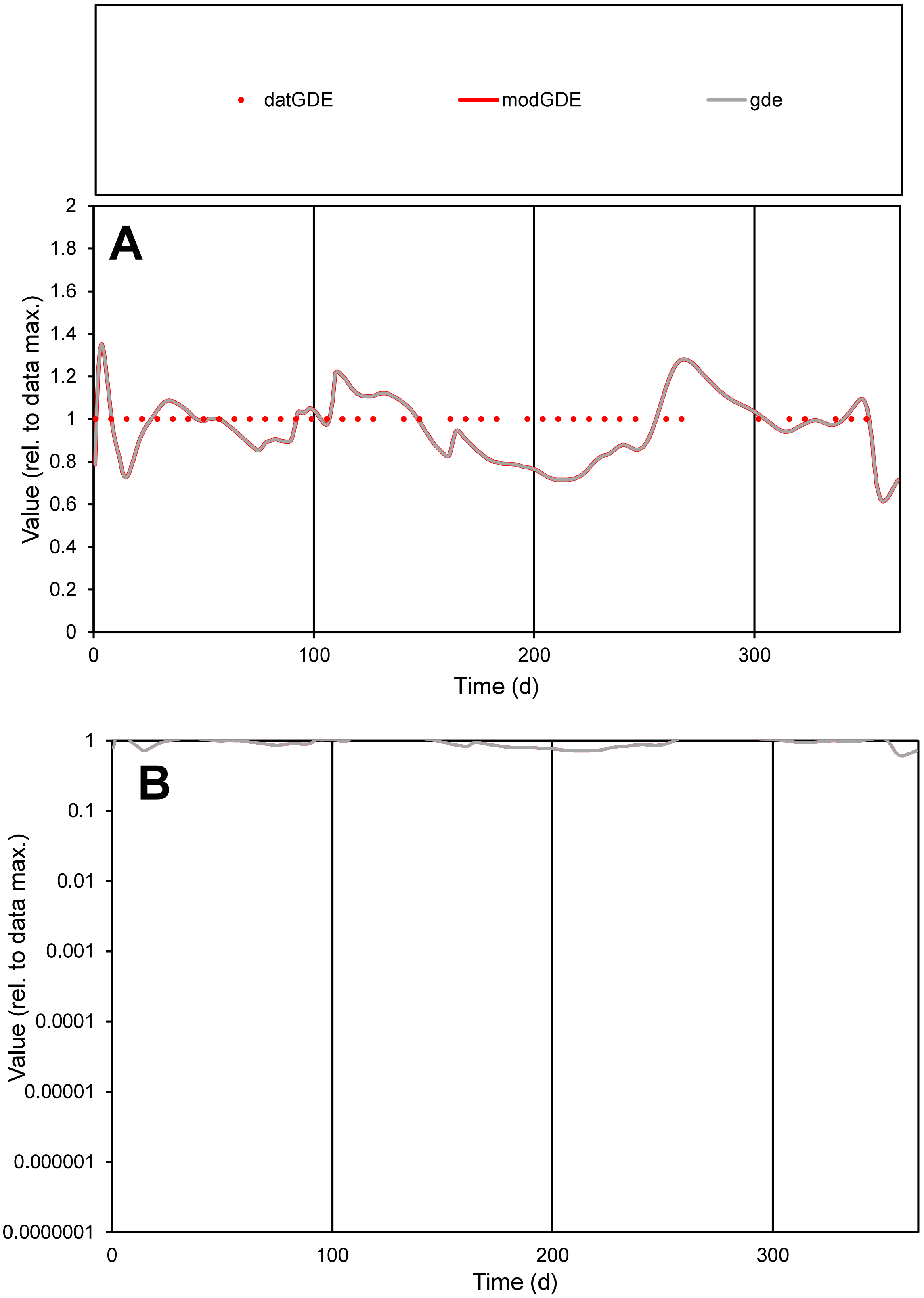

C1

Variables
GDE Provide jtd or Dst
CalibOnly?
2 1 to reproduce of
oNormalize?
2 1=no, 2=yes

ofxn calc*
0.13863
ofxn mod
0.17424

sRun
RunA1
zout
1

Ave
Data
2.7E-05
Model
2.6E-05

data/vMax
2.7E-05

ofxng
319.391

Min d/m
1.39776

ave/min d
1
ave/min m
1.33548

330_Calib_S_A

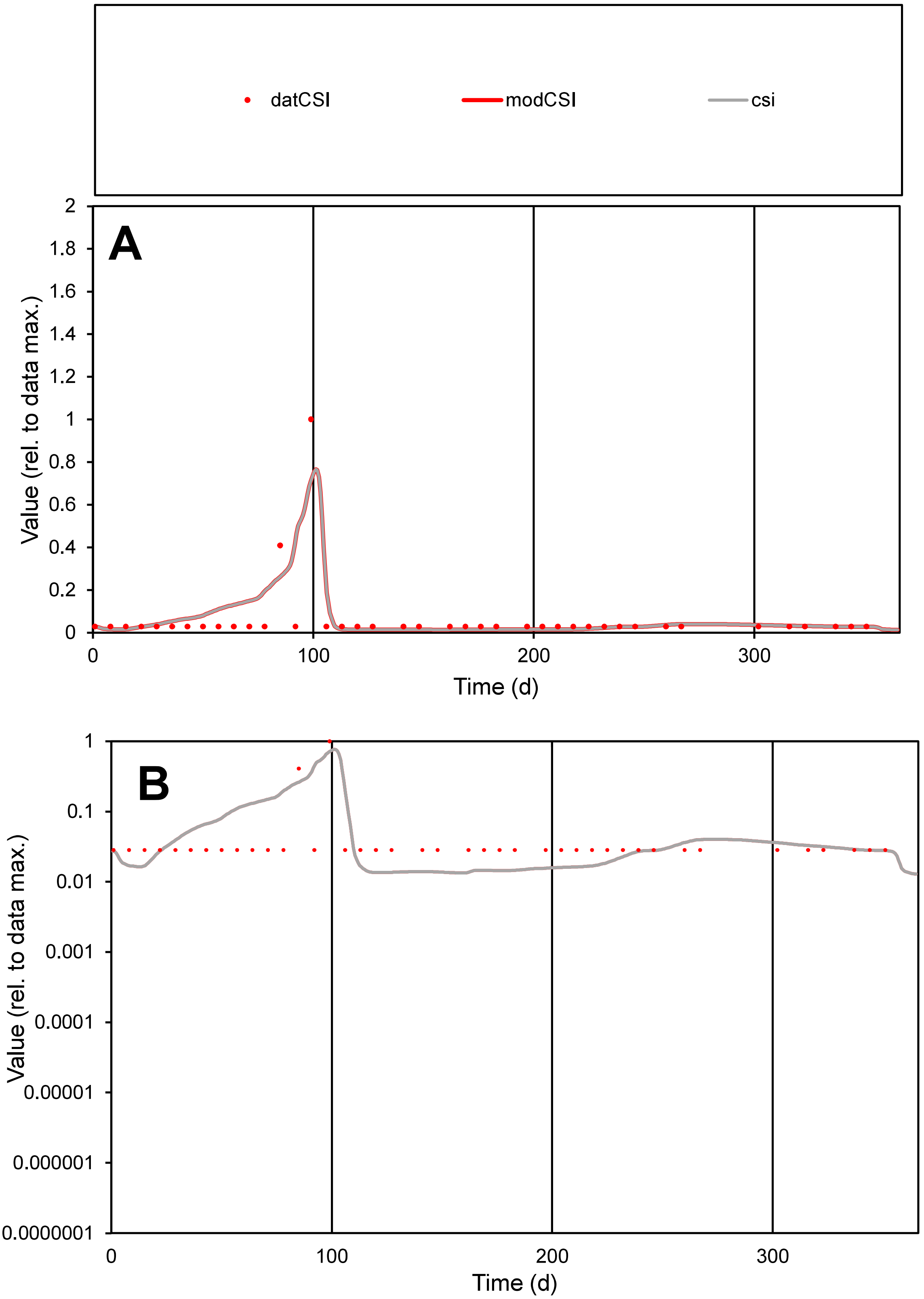

C1

Variables
CSI Provide jtd or Dst
CalibOnly?
2 1 to reproduce of
oNormalize?
2 1=no, 2=yes

ofxn calc*
1.24474
ofxn mod
1.39401

sRun
RunA1
zout
1

Ave
Data
5E-06
Model
5.8E-06

data/vMax
8.1E-05

ofxng
319.391

Min d/m
2.09239

ave/min d
2.16271
ave/min m
5.21841

330_Calib_S_A

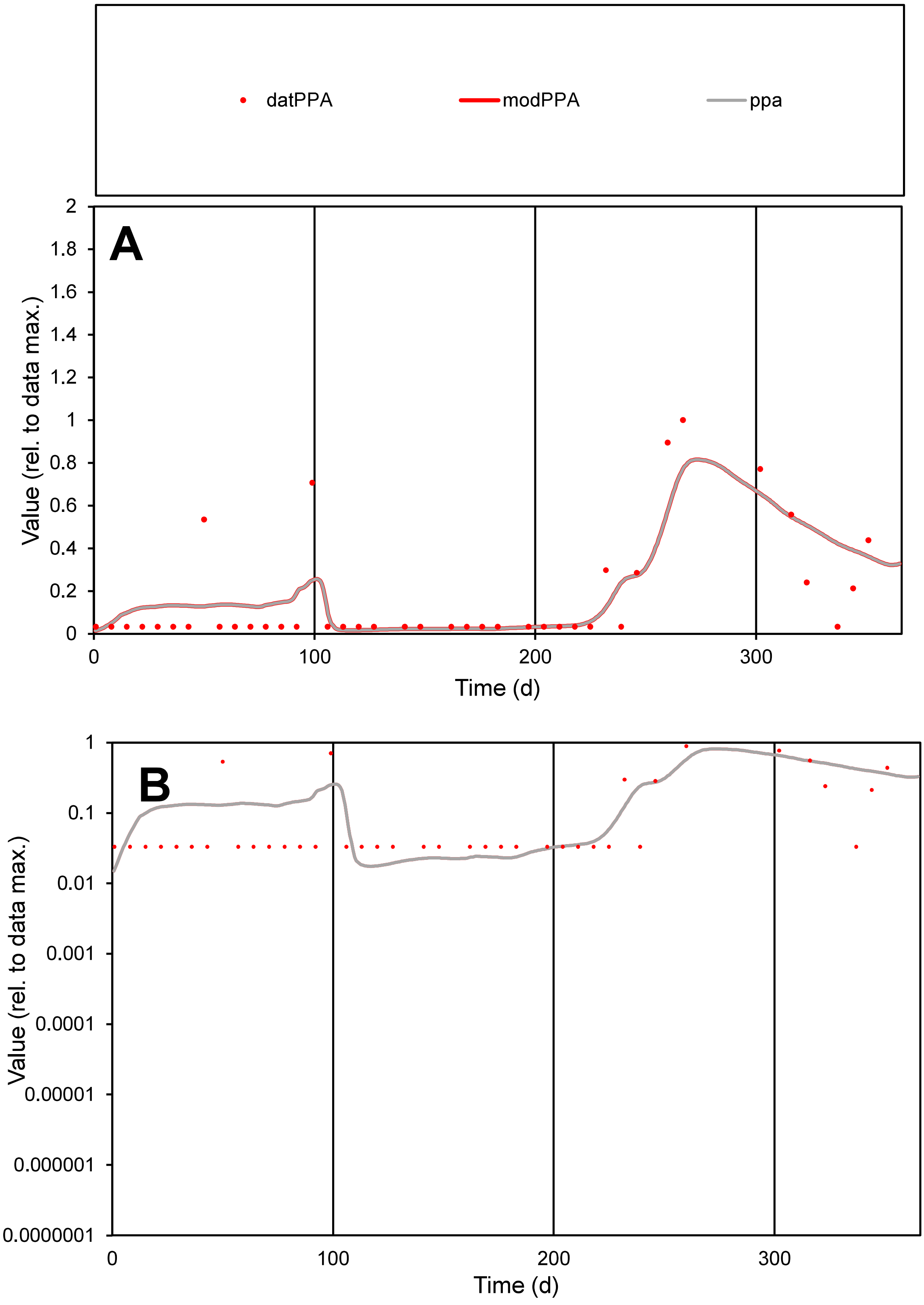

C1

Variables
PPA Provide jtd or Dst
CalibOnly?
2 1 to reproduce of
oNormalize?
2 1=no, 2=yes

ofxn calc*
0.88874
ofxn mod
0.91635

sRun
RunA1
zout
1

Ave
Data
1.6E-05
Model
1.7E-05

data/vMax
9.7E-05

ofxng
319.391

Min d/m
1.855

ave/min d
5.10843
ave/min m
9.92541

330_Calib_S_A

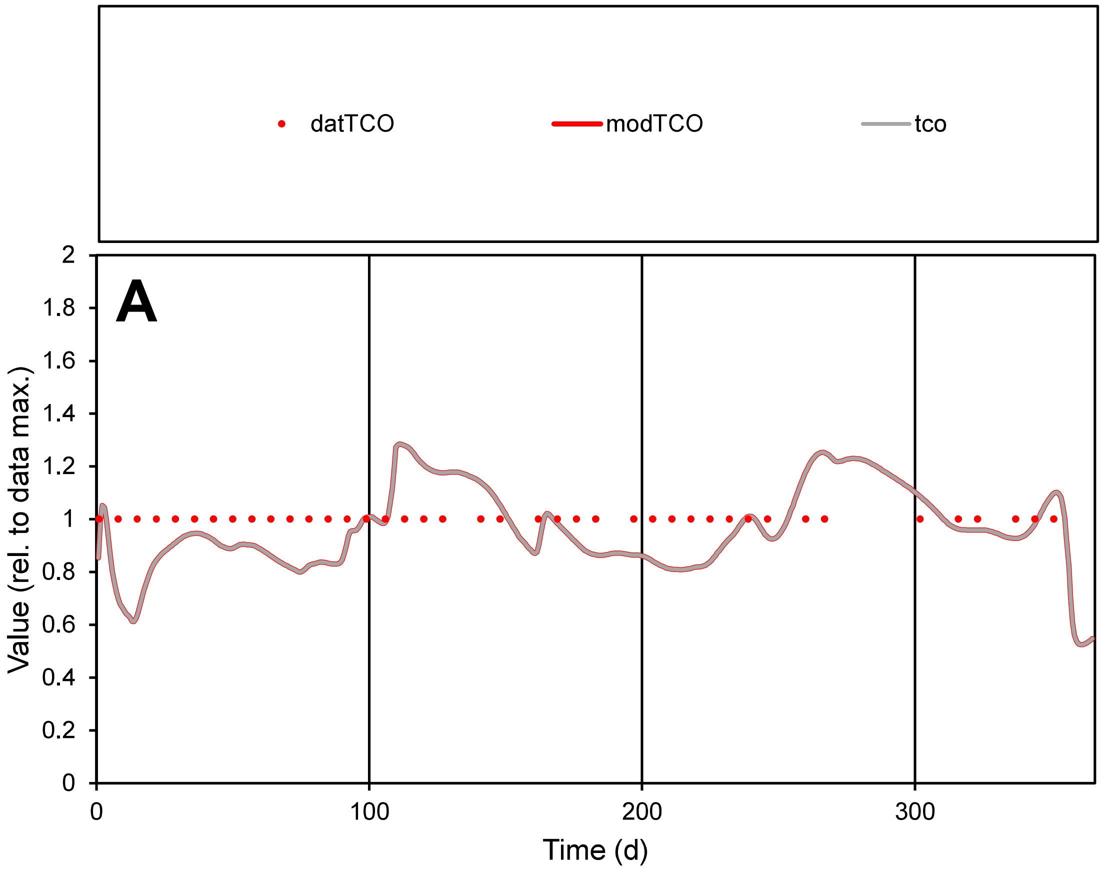

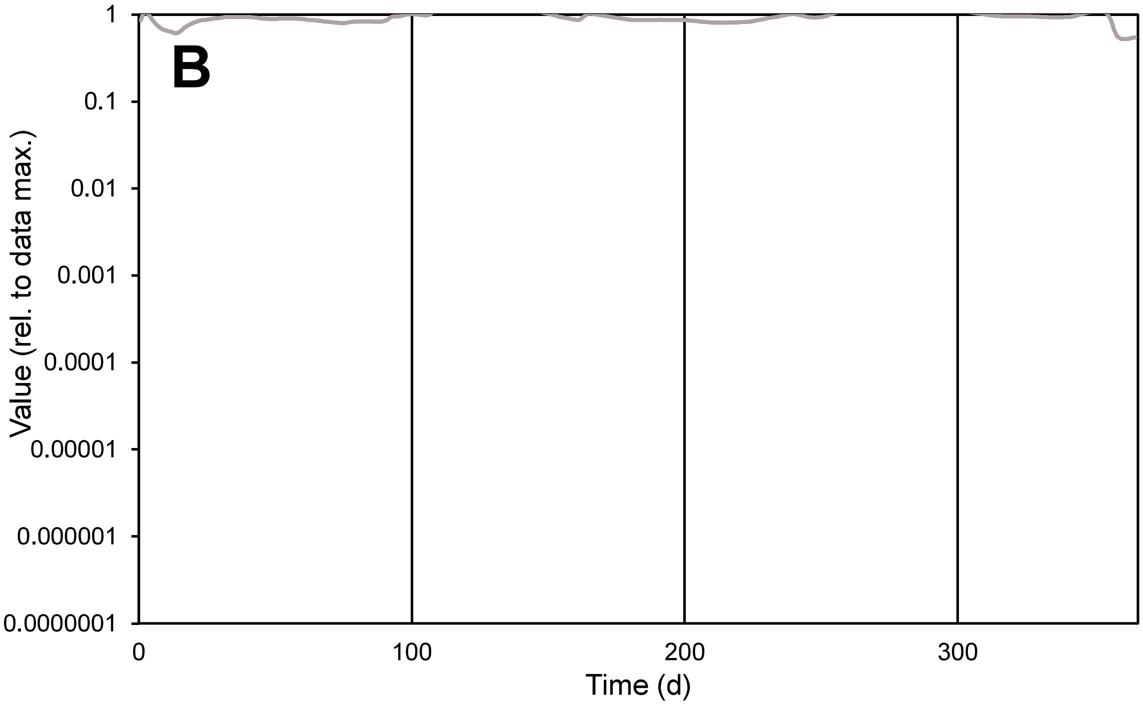

C1

Variables
TCO Provide jtd or Dst
CalibOnly?
2 1 to reproduce of
oNormalize?
2 1=no, 2=yes

ofxn calc*
0.14275
ofxn mod
0.17753

sRun
RunA1
zout
1

Ave
Data
8.6E-06
Model
8.2E-06

data/vMax
8.6E-06

ofxng
319.391

Min d/m
1.49835

ave/min d
1
ave/min m
1.43233

330_Calib_S_A

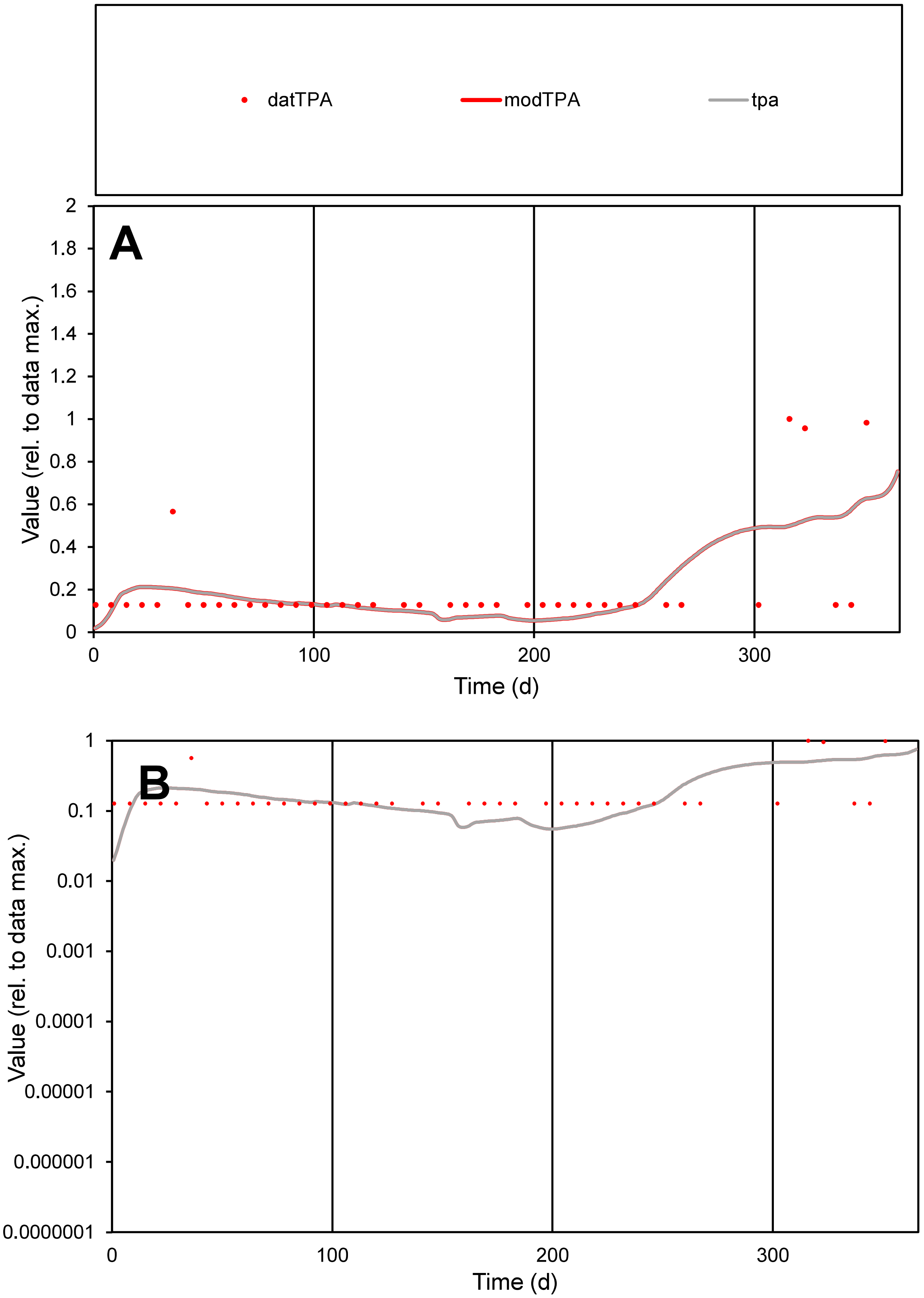

C1

Variables
TPA Provide jtd or Dst
CalibOnly?
2 1 to reproduce of
oNormalize?
2 1=no, 2=yes

ofxn calc*
0.89186
ofxn mod
0.90314

sRun
RunA1
zout
1

Ave
Data
7.4E-06
Model
7.1E-06

data/vMax
3.7E-05

ofxng
319.391

Min d/m
3.27828

ave/min d
1.57307
ave/min m
4.91424

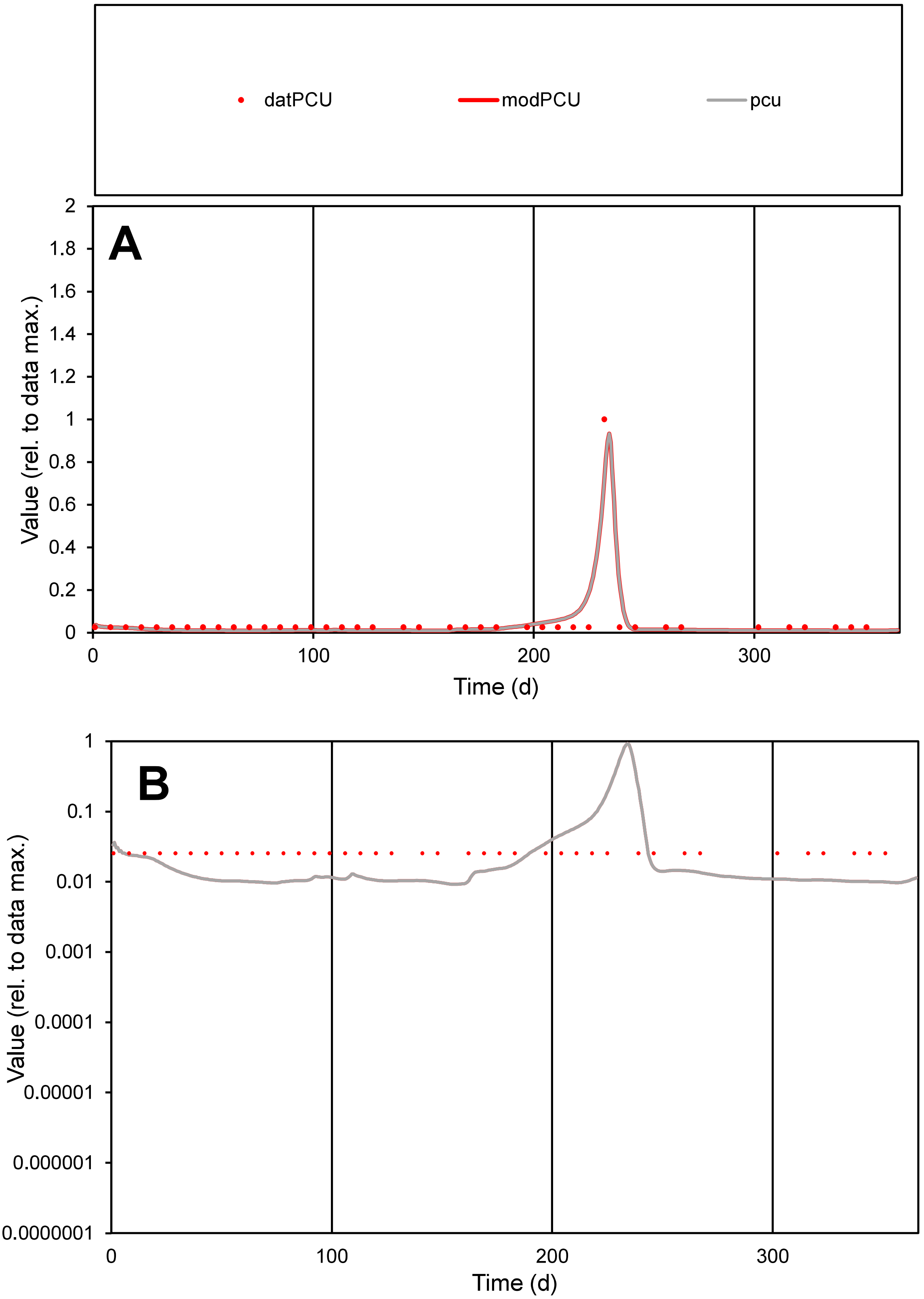

C1

Variables
PCU Provide jtd or Dst
CalibOnly?
2 1 to reproduce of
oNormalize?
2 1=no, 2=yes

ofxn calc*
0.6202
ofxn mod
0.82545

sRun
RunA1
zout
1

Ave
Data
1.2E-05
Model
1.1E-05

data/vMax
0.00024

ofxng
319.391

Min d/m
2.63687

ave/min d
1.93326
ave/min m
4.51848

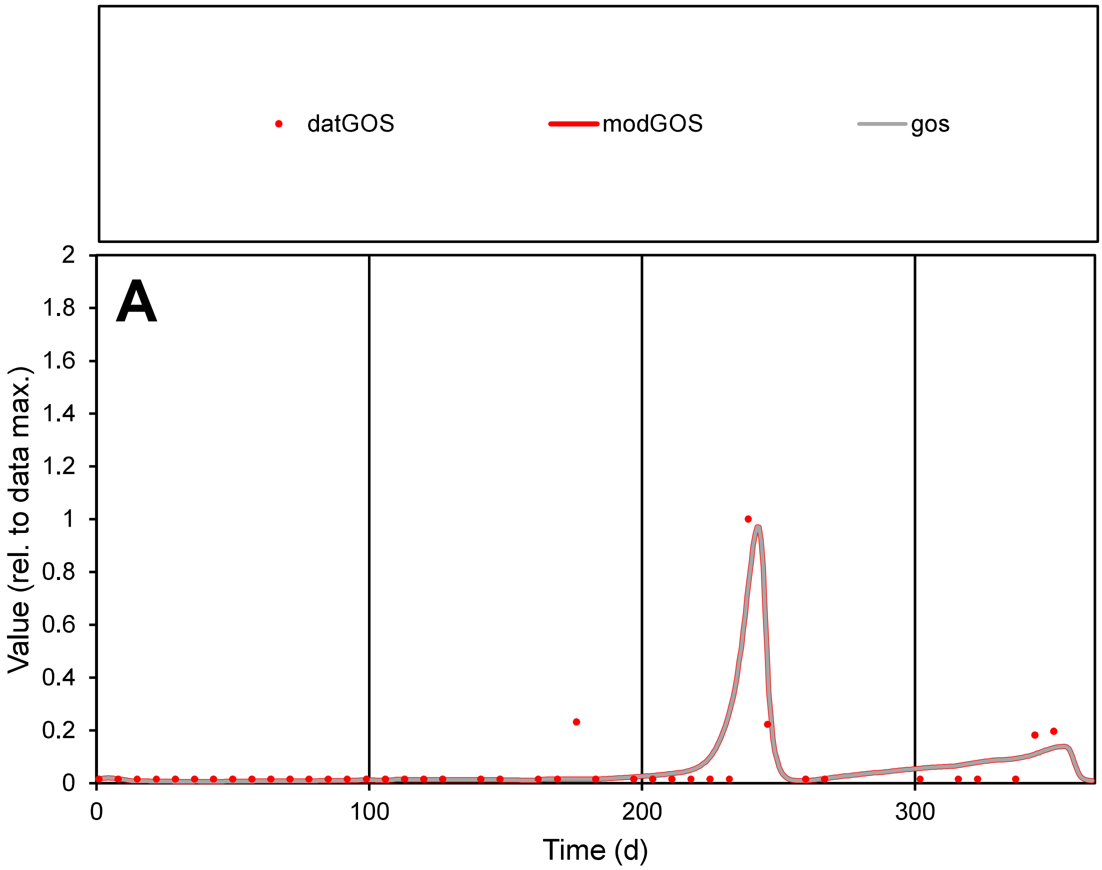

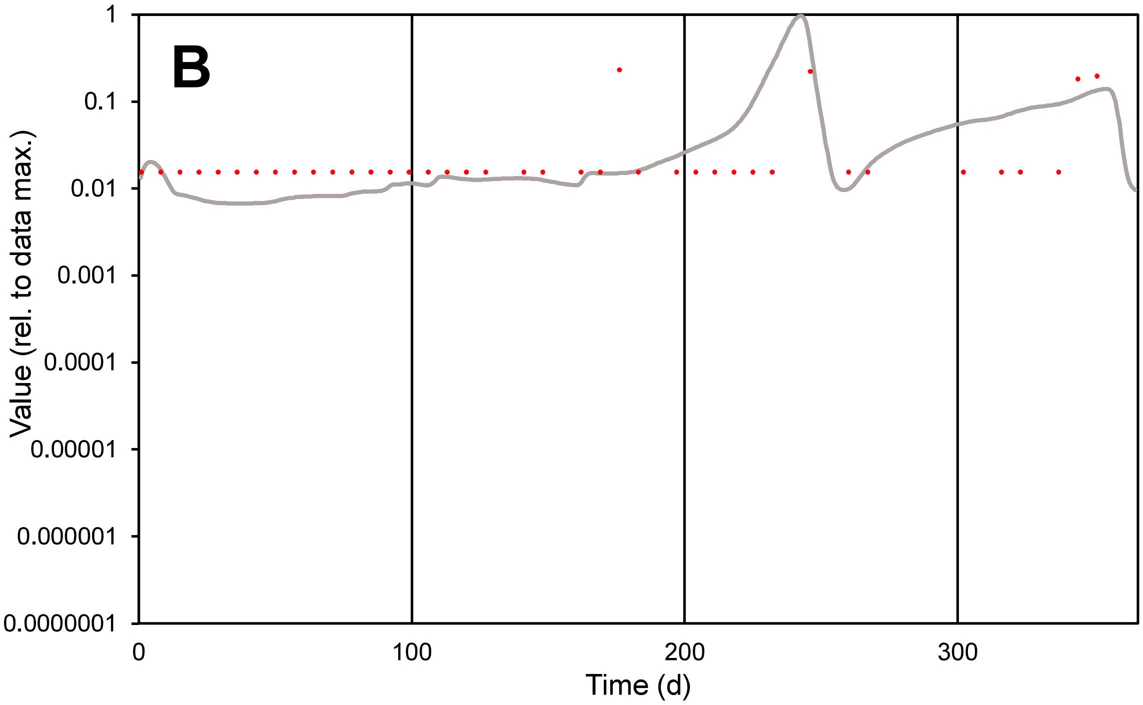

C1

Variables
GOS Provide jtd or Dst
CalibOnly?
2 1 to reproduce of
oNormalize?
2 1=no, 2=yes

ofxn calc*
0.959
ofxn mod
1.09533

sRun
RunA1
zout
1

Ave
Data
6.8E-06
Model
7E-06

data/vMax
0.00012

ofxng
319.391

Min d/m
2.30154

ave/min d
3.76477
ave/min m
8.83473

330_Calib_S_A

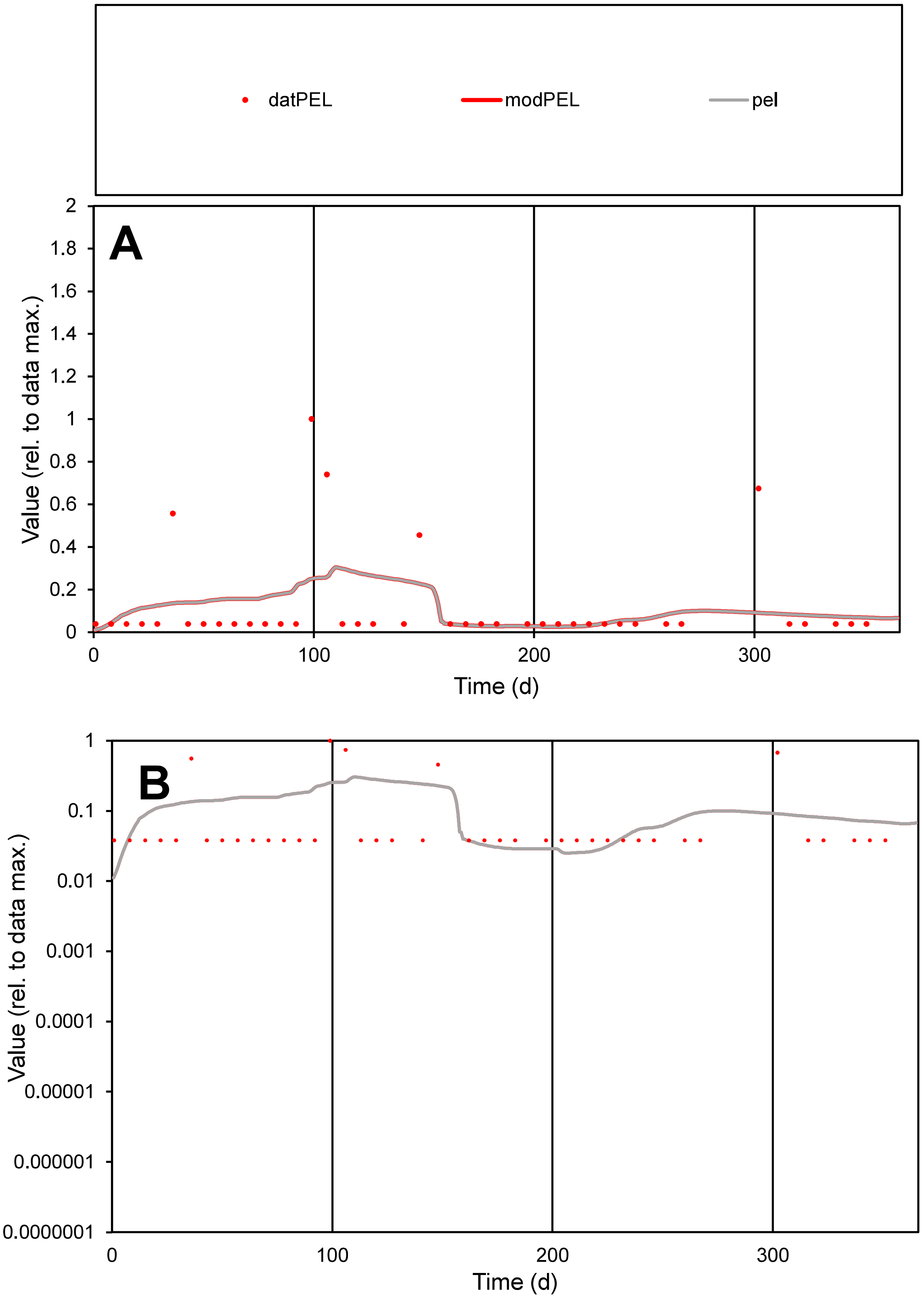

C1

Variables

PEL Provide jtd or Dst

CalibOnly?

2 1 to reproduce of

oNormalize?

2 1=no, 2=yes

ofxn calc*

1.74879

ofxn mod

1.75934

sRun

RunA1

zout

1

Ave

Data

1E-05

Model

1E-05

data/vMax

8.6E-05

ofxng

319.391

Min d/m

1.93059

ave/min d

3.08016

ave/min m

5.87301

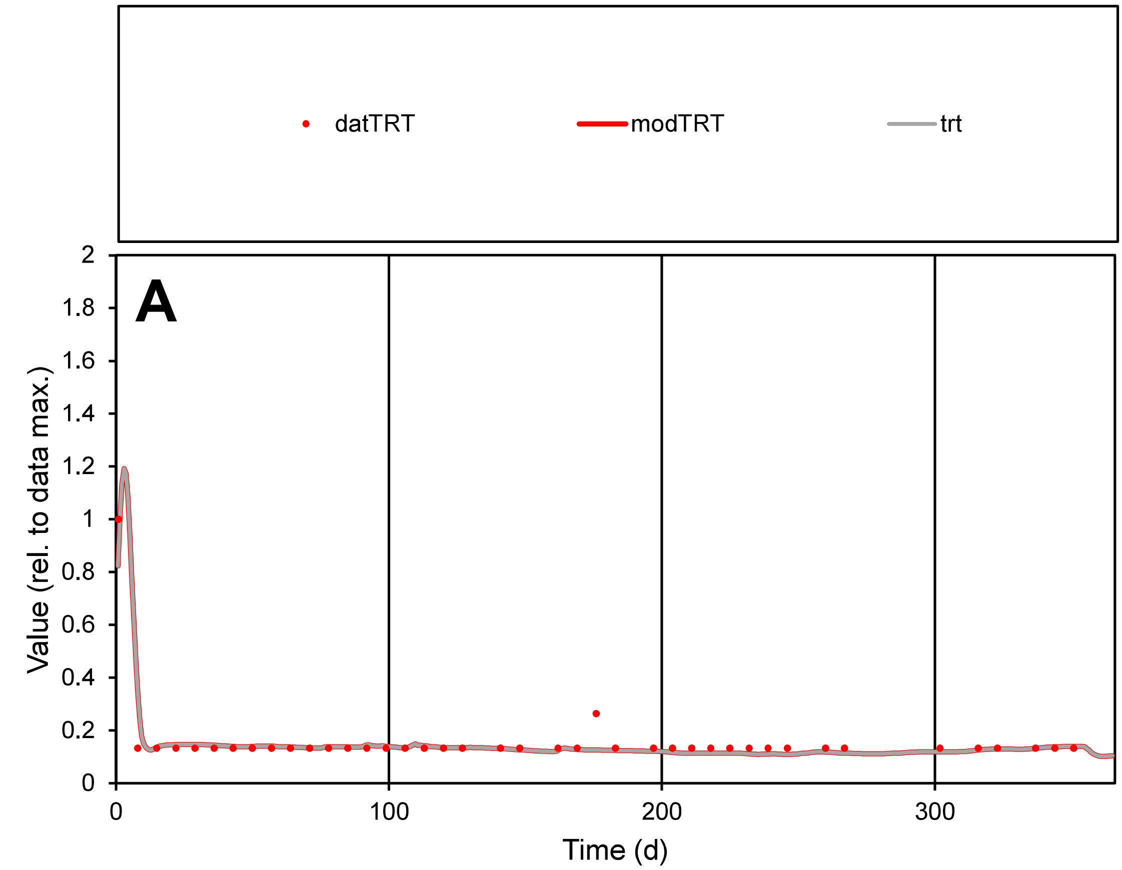

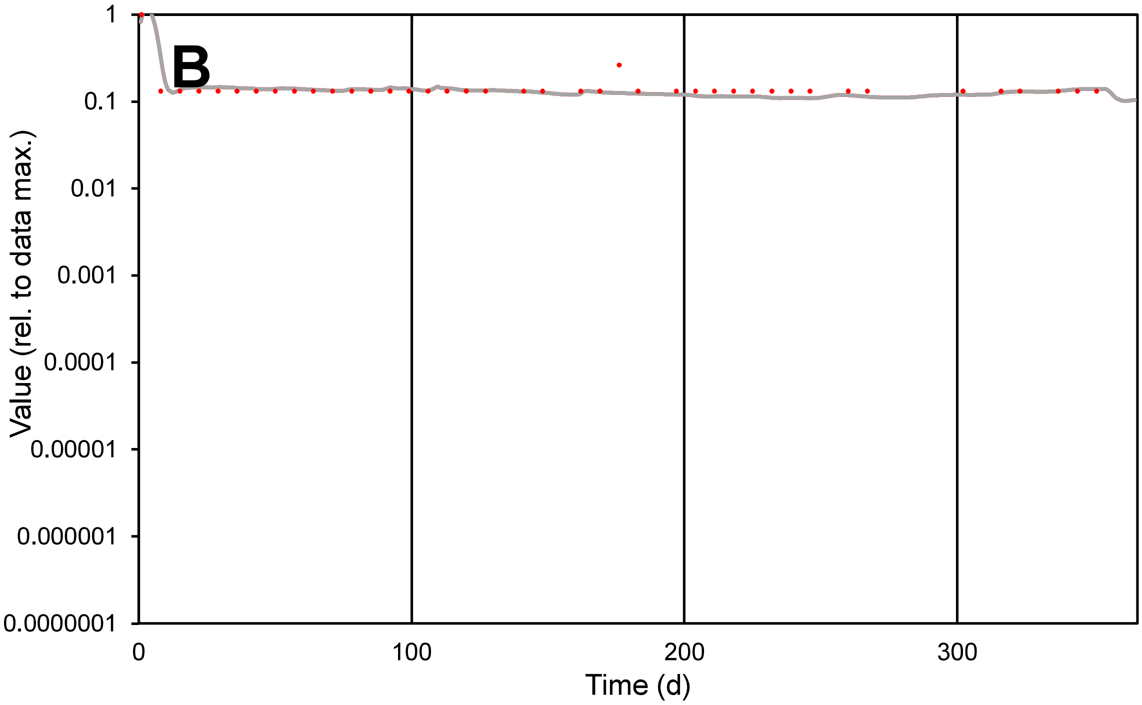

C1

Variables
TRT Provide jtd or Dst
CalibOnly?
2 1 to reproduce of
oNormalize?
2 1=no, 2=yes

ofxn calc*
0.16211
ofxn mod
0.20418

sRun
RunA1
zout
1

Ave
Data
2.7E-05
Model
2.7E-05

data/vMax
0.00018

ofxng
319.391

Min d/m
1.20489

ave/min d
1.18506
ave/min m
1.39336

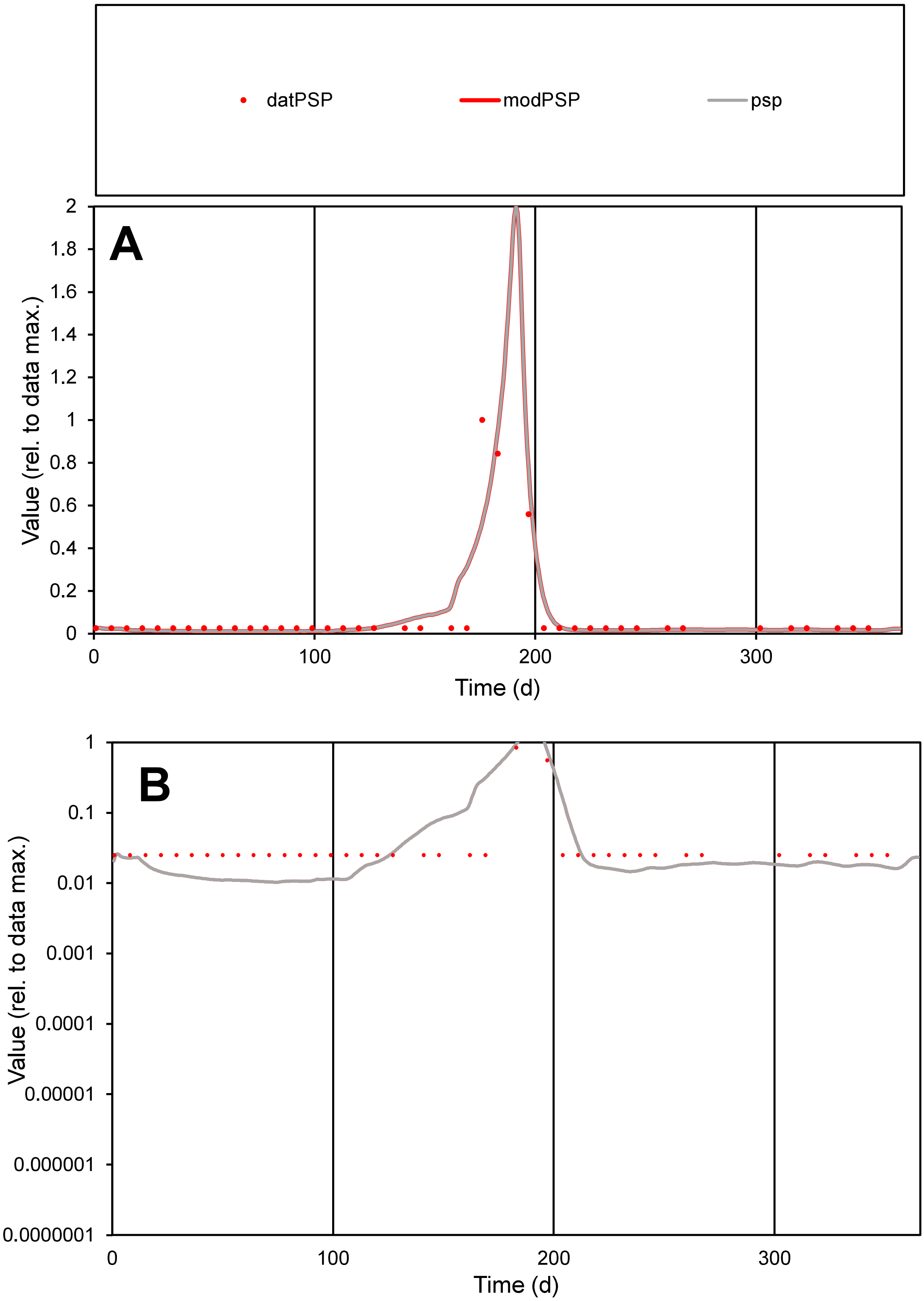

C1

Variables
PSP Provide jtd or Dst
CalibOnly?
2 1 to reproduce of
oNormalize?
2 1=no, 2=yes

ofxn calc*
1.01105
ofxn mod
1.10863

sRun
RunA1
zout
1

Ave
Data
1.4E-05
Model
1.4E-05

data/vMax
0.00017

ofxng
319.391

Min d/m
2.41825

ave/min d
3.25703
ave/min m
7.73063

330_Calib_S_A

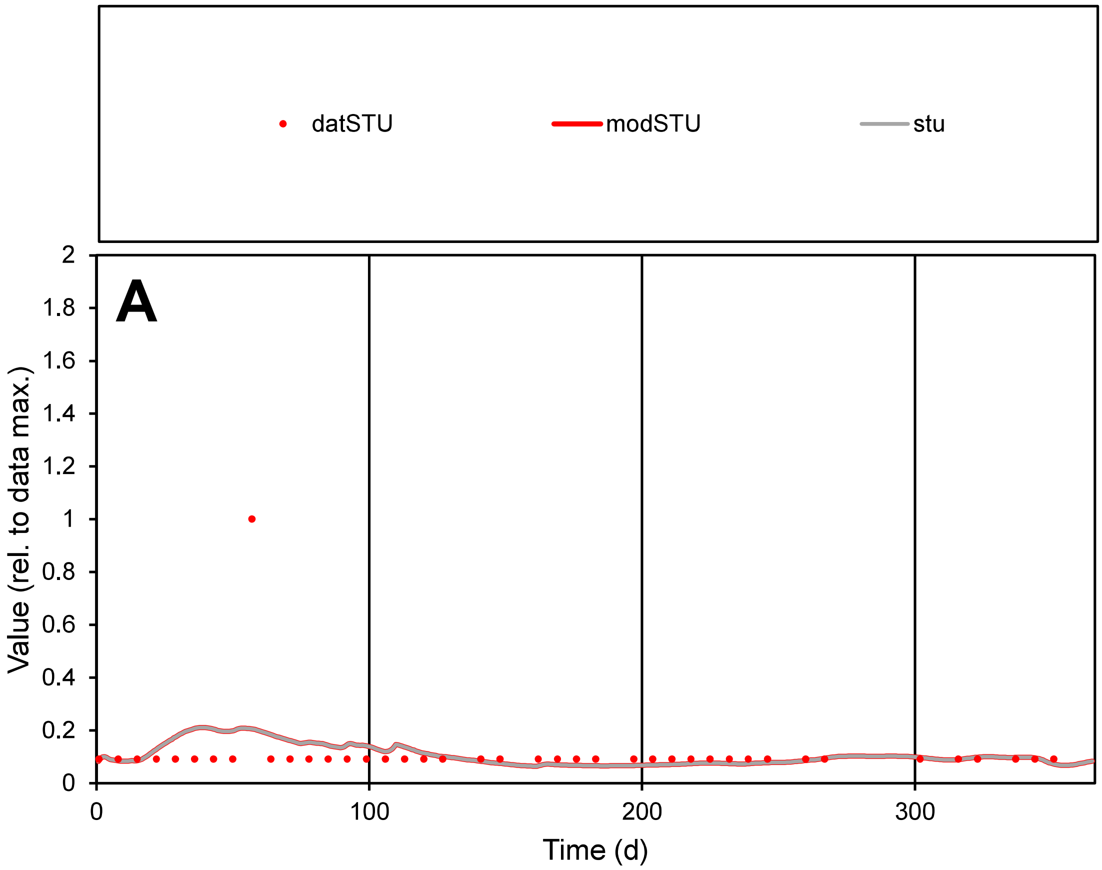

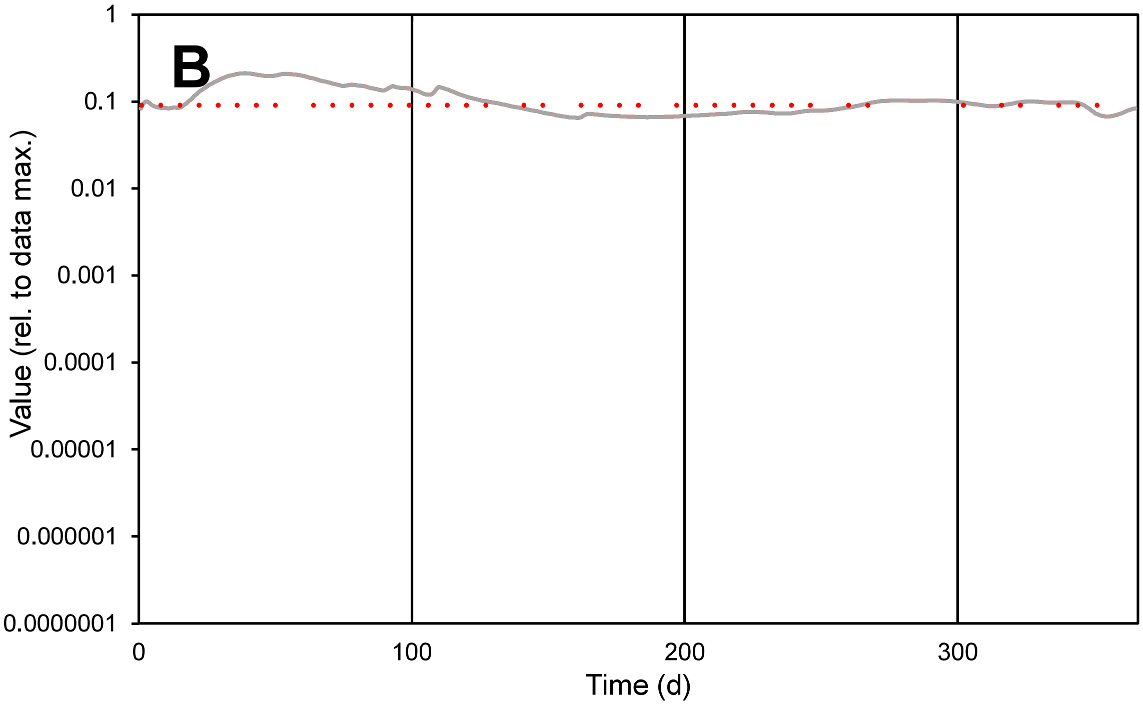

C1

Variables
STU Provide jtd or Dst
CalibOnly?
2 1 to reproduce of
oNormalize?
2 1=no, 2=yes

ofxn calc*
1.16839
ofxn mod
1.17657

sRun
RunA1
zout
1

Ave
Data
3.1E-06
Model
3E-06

data/vMax
2.7E-05

ofxng
319.391

Min d/m
1.36678

ave/min d
1.24417
ave/min m
1.66069

330_Calib_S_A

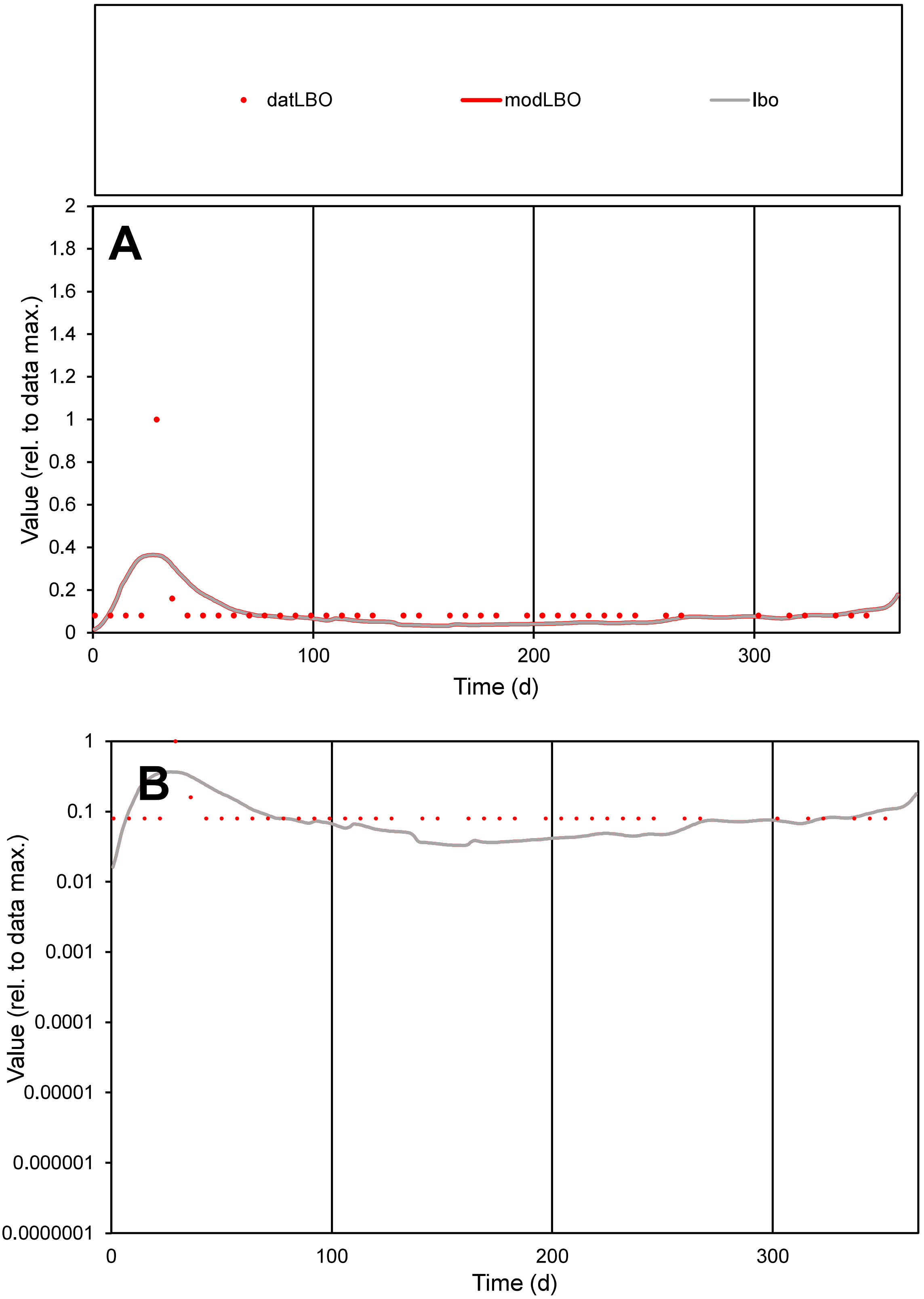

C1

Variables
LBO Provide jtd or Dst
CalibOnly?
2 1 to reproduce of
oNormalize?
2 1=no, 2=yes

ofxn calc*
1.15823
ofxn mod
1.17106

sRun
RunA1
zout
1

Ave
Data
1.8E-06
Model
1.6E-06

data/vMax
1.7E-05

ofxng
319.391

Min d/m
2.3163

ave/min d
1.30563
ave/min m
2.75346

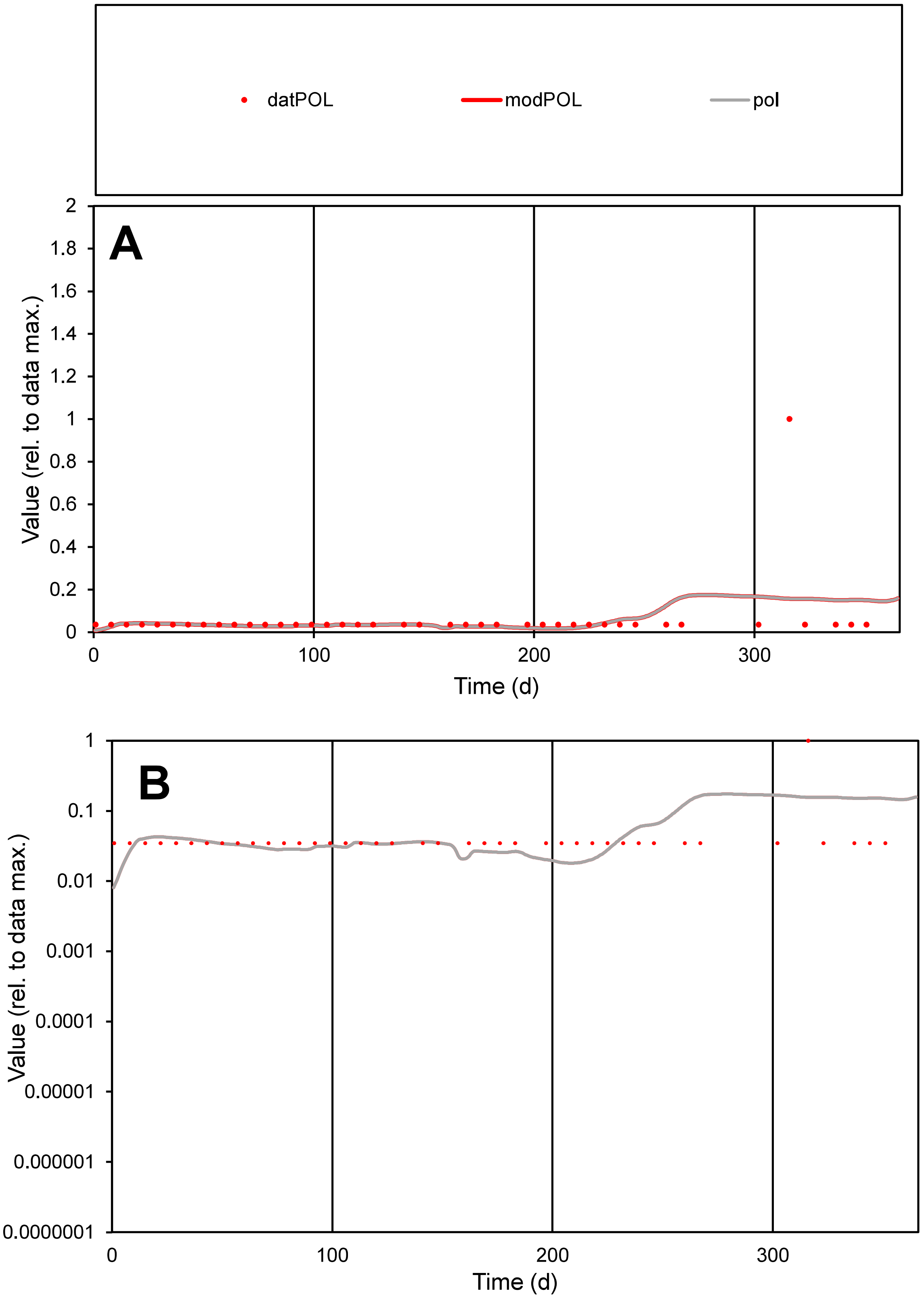

C1

Variables
POL Provide jtd or Dst
CalibOnly?
2 1 to reproduce of
oNormalize?
2 1=no, 2=yes

ofxn calc*
2.41897
ofxn mod
2.42371

sRun
RunA1
zout
1

Ave
Data
2.7E-06
Model
2.6E-06

data/vMax
4.6E-05

ofxng
319.391

Min d/m
2.57752

ave/min d
1.6792
ave/min m
4.17449

330_Calib_S_A

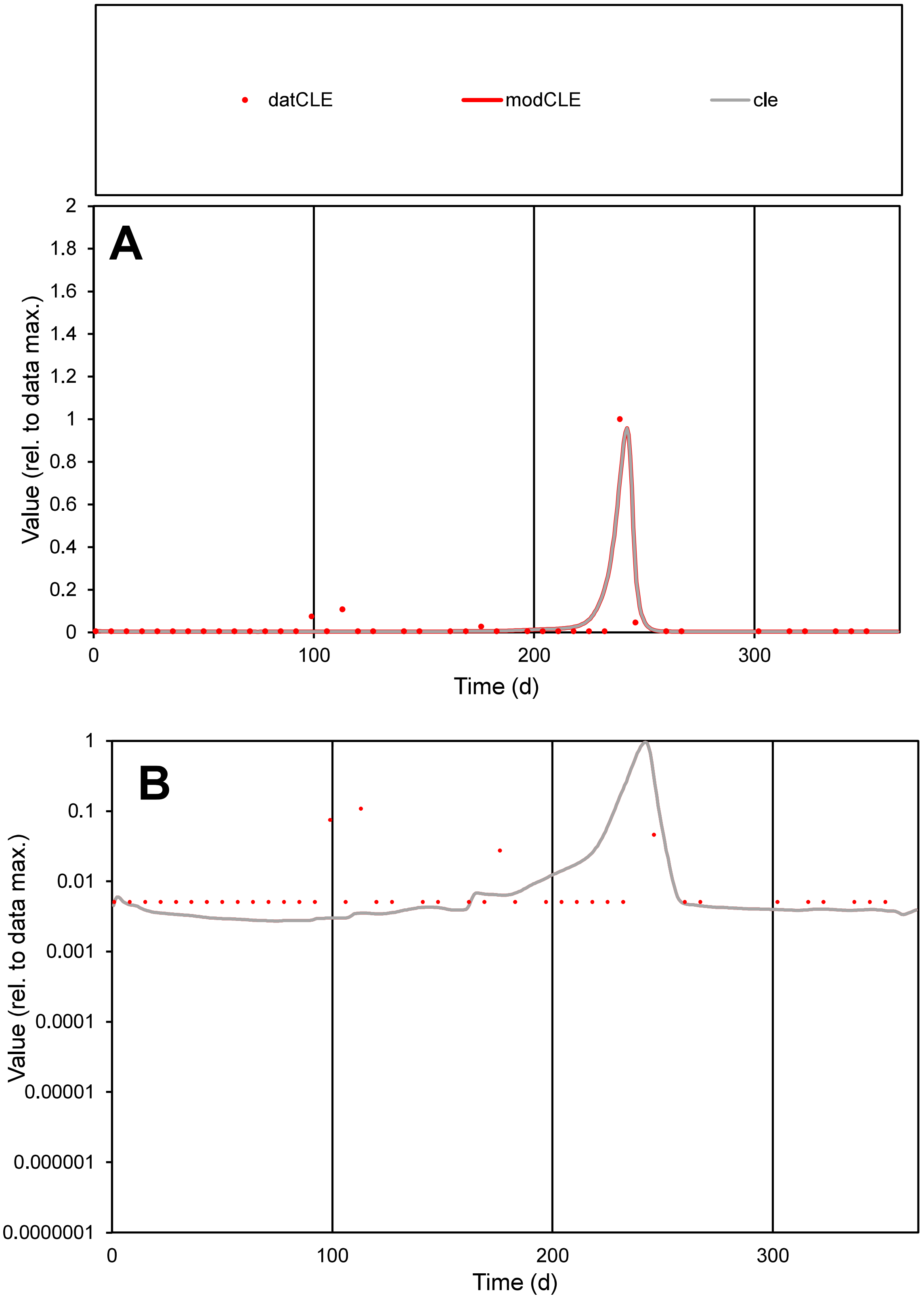

C1

Variables

CLE Provide jtd or Dst

CalibOnly?

2 1 to reproduce of

oNormalize?

2 1=no, 2=yes

ofxn calc*

0.96343

ofxn mod

1.32801

sRun

RunA1

zout

1

Ave

Data

3.2E-05

Model

3E-05

data/vMax

0.00092

ofxng

319.391

Min d/m

1.86349

ave/min d

6.90305

ave/min m

12.0053

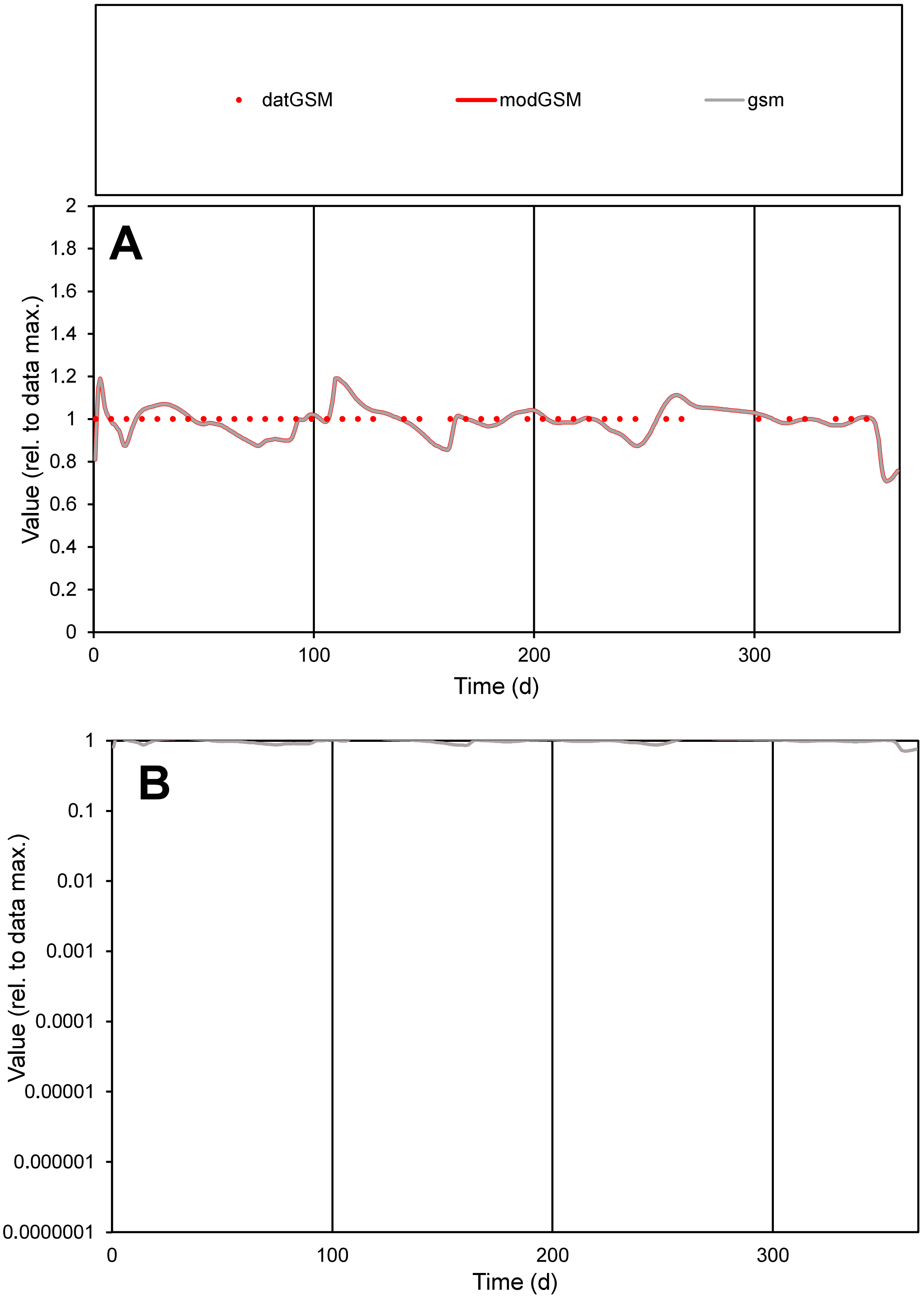

**C1**	
Variables	
GSM	Provide jtd or Dst
CalibOnly?	
2	1 to reproduce of
oNormalize?	
2	1=no, 2=yes
ofxn calc*	
0.05861	
ofxn mod	
0.1232	
sRun	
RunA1	
zout	
1	
Ave	
Data	
1.6E-06	
Model	
1.6E-06	
data/vMax	
1.6E-06	
ofxng	
319.391	
Min d/m	
1.14255	
ave/min d	
1	
ave/min m	
1.13623	

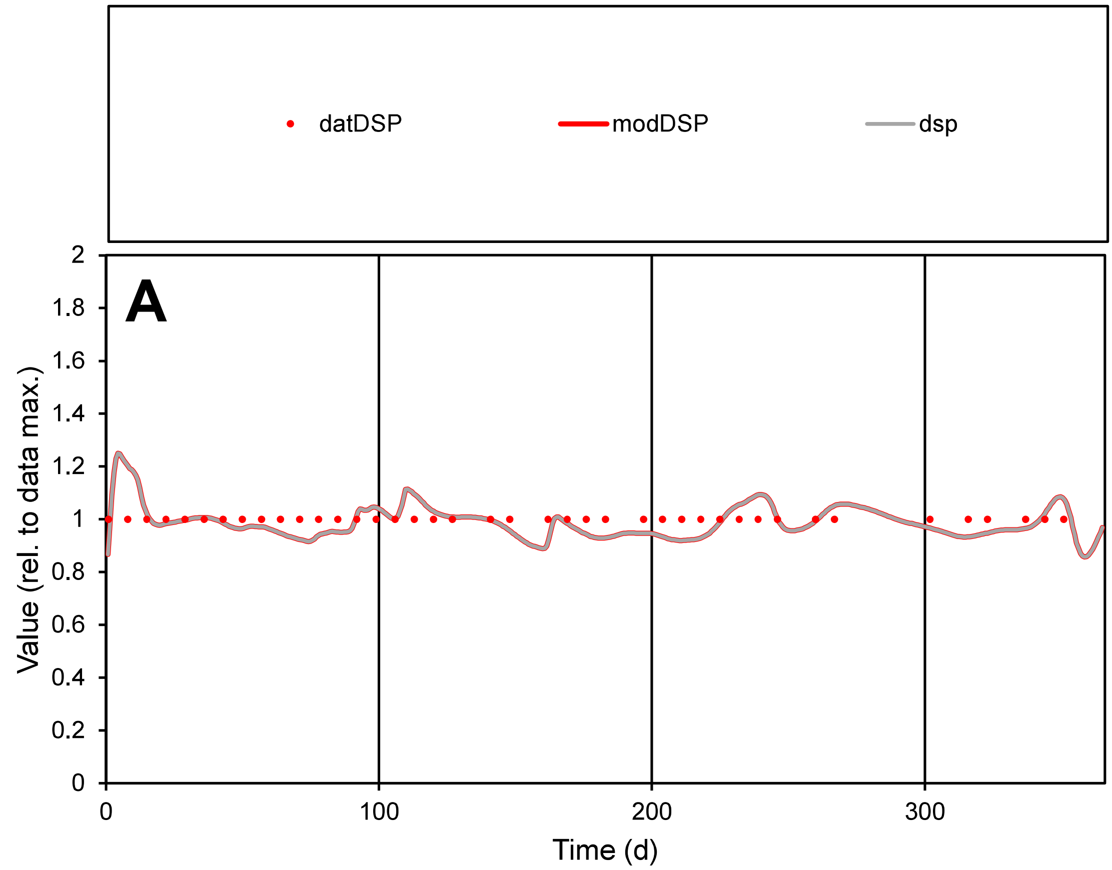

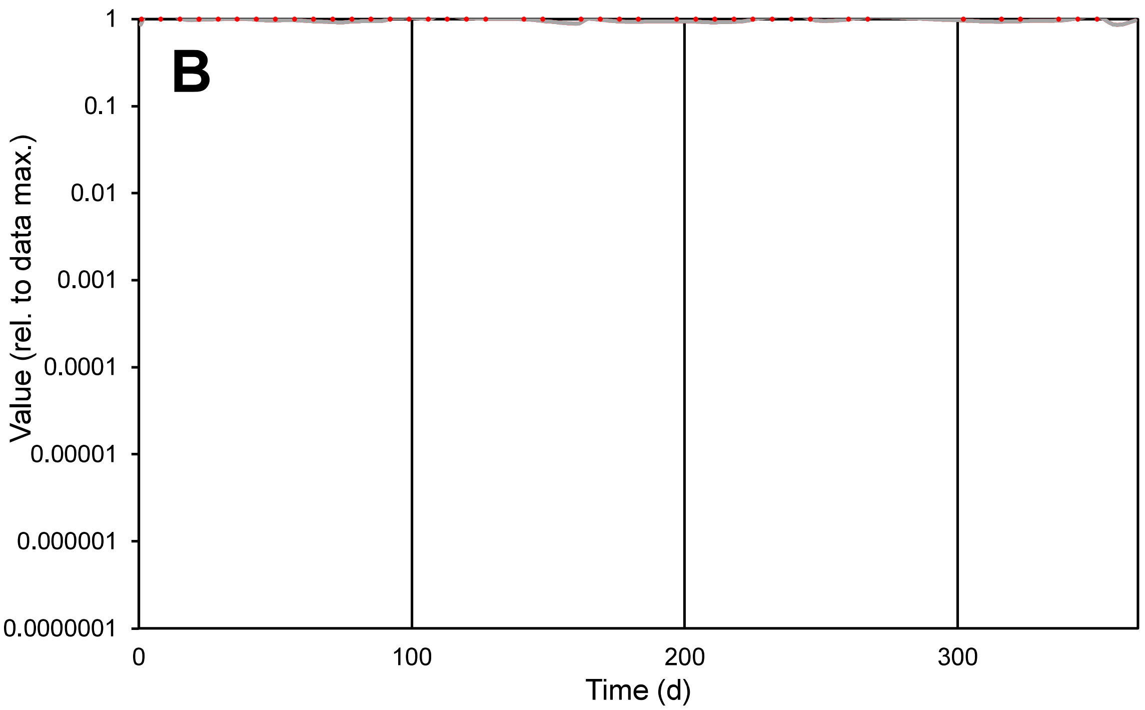

C1

Variables
DSP Provide jtd or Dst
CalibOnly?
2 1 to reproduce of
oNormalize?
2 1=no, 2=yes

ofxn calc*
0.05523
ofxn mod
0.12194

sRun
RunA1
zout
1

Ave
Data
5E-06
Model
5E-06

data/vMax
5E-06

ofxng
319.391

Min d/m
1.08663

ave/min d
1
ave/min m
1.07762

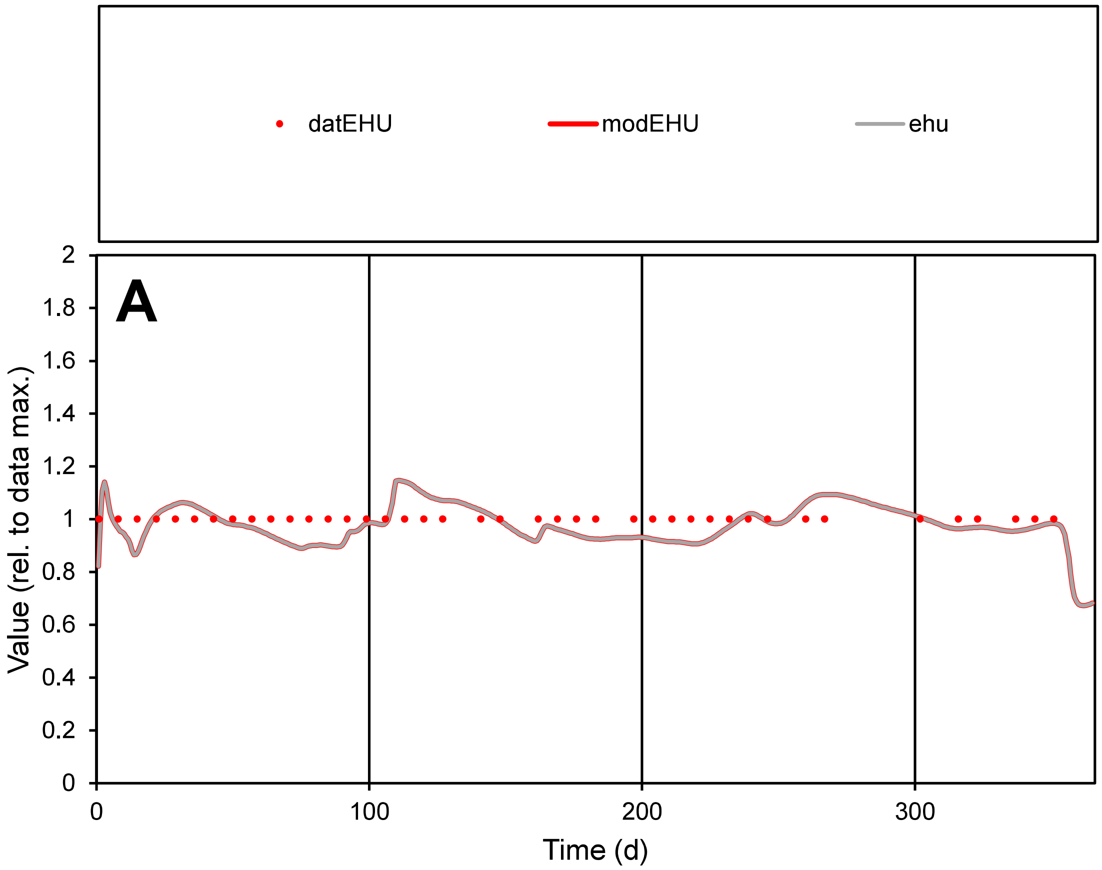

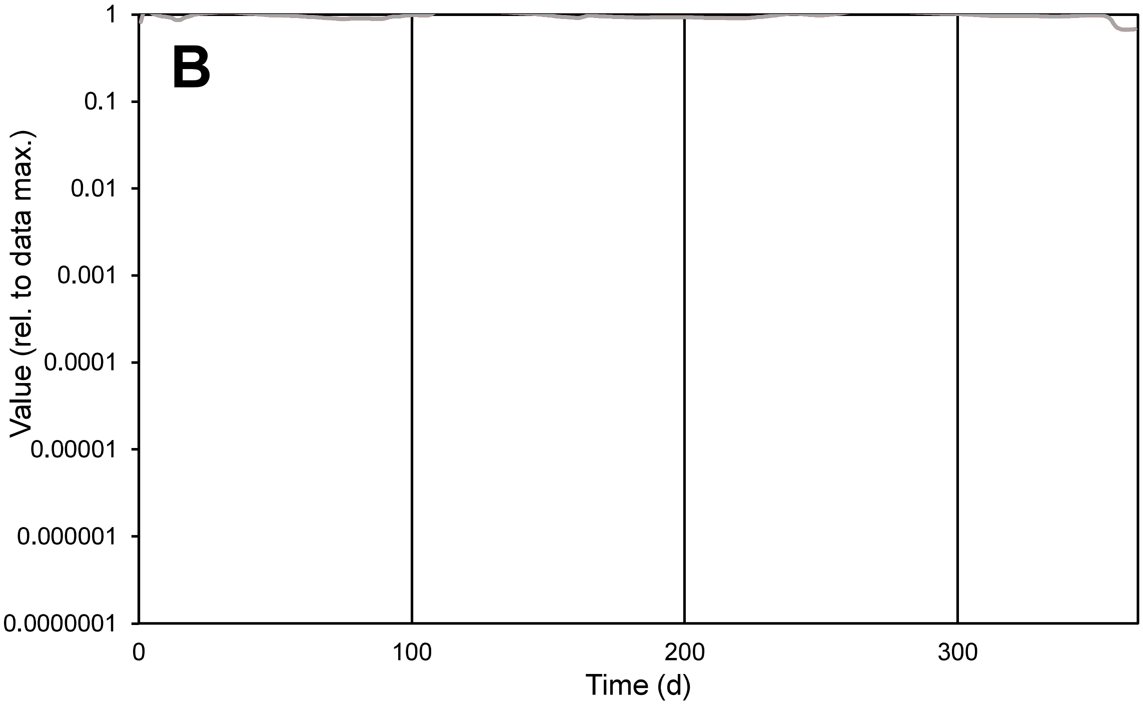

C1

Variables
EHU Provide jtd or Dst
CalibOnly?
2 1 to reproduce of
oNormalize?
2 1=no, 2=yes

ofxn calc*
0.06129
ofxn mod
0.12398

sRun
RunA1
zout
1

Ave
Data
5.8E-06
Model
5.6E-06

data/vMax
5.8E-06

ofxng
319.391

Min d/m
1.11596

ave/min d
1
ave/min m
1.09502

330_Calib_S_A

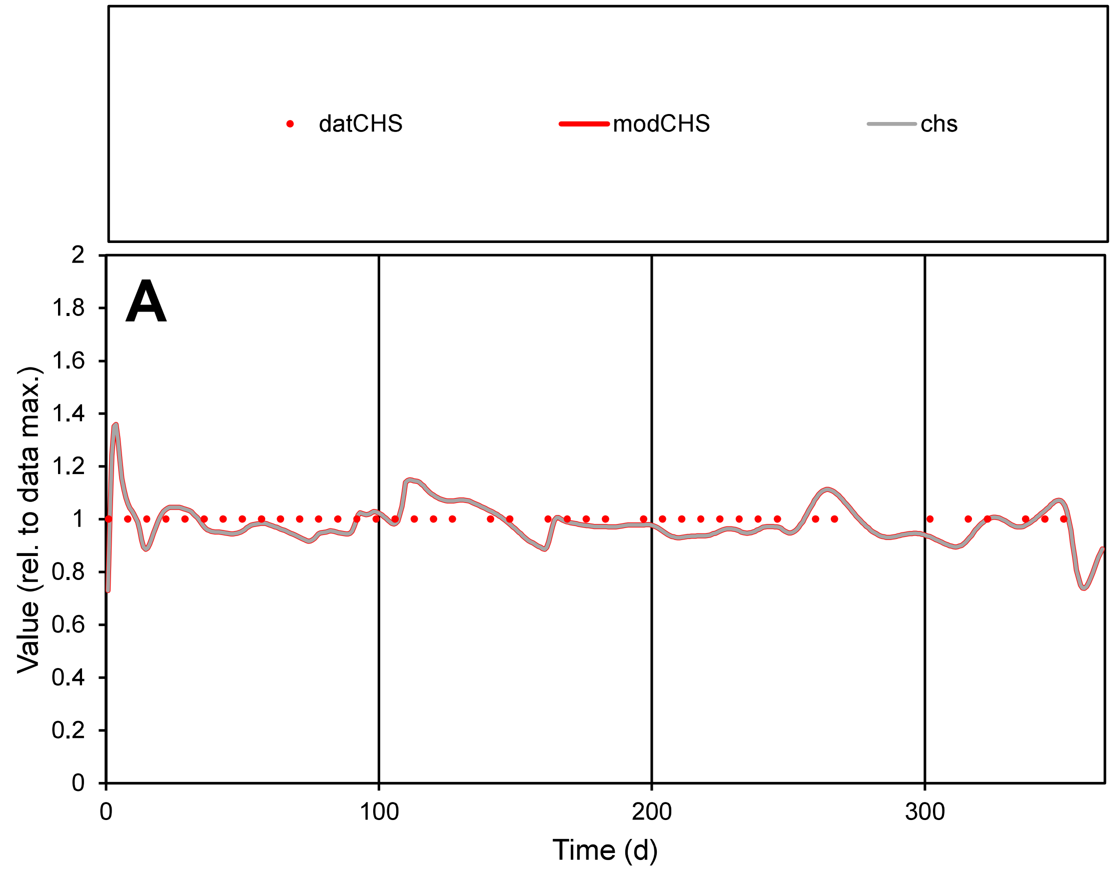

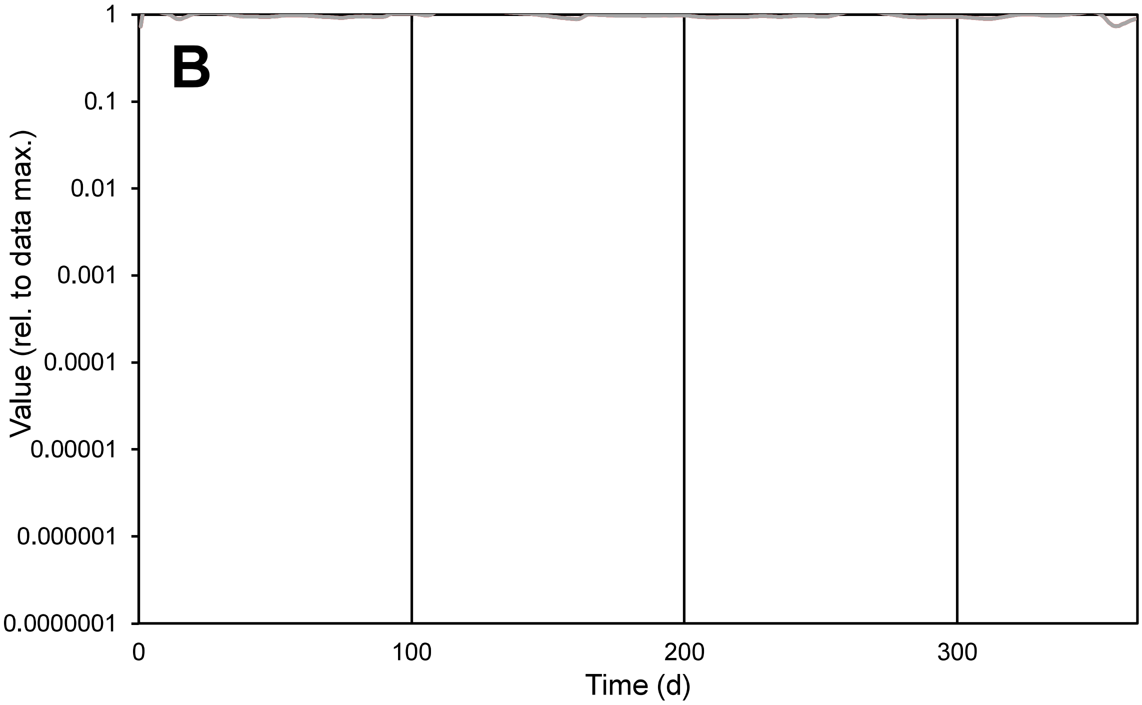

C1

Variables

CHS Provide jtd or Dst

CalibOnly?

2 1 to reproduce of

oNormalize?

2 1=no, 2=yes

ofxn calc*

0.05223

ofxn mod

0.12015

sRun

RunA1

zout

1

Ave

Data

2.1E-05

Model

2.1E-05

data/vMax

2.1E-05

ofxng

319.391

Min d/m

1.08085

ave/min d

1

ave/min m

1.07315

330_Calib_S_A

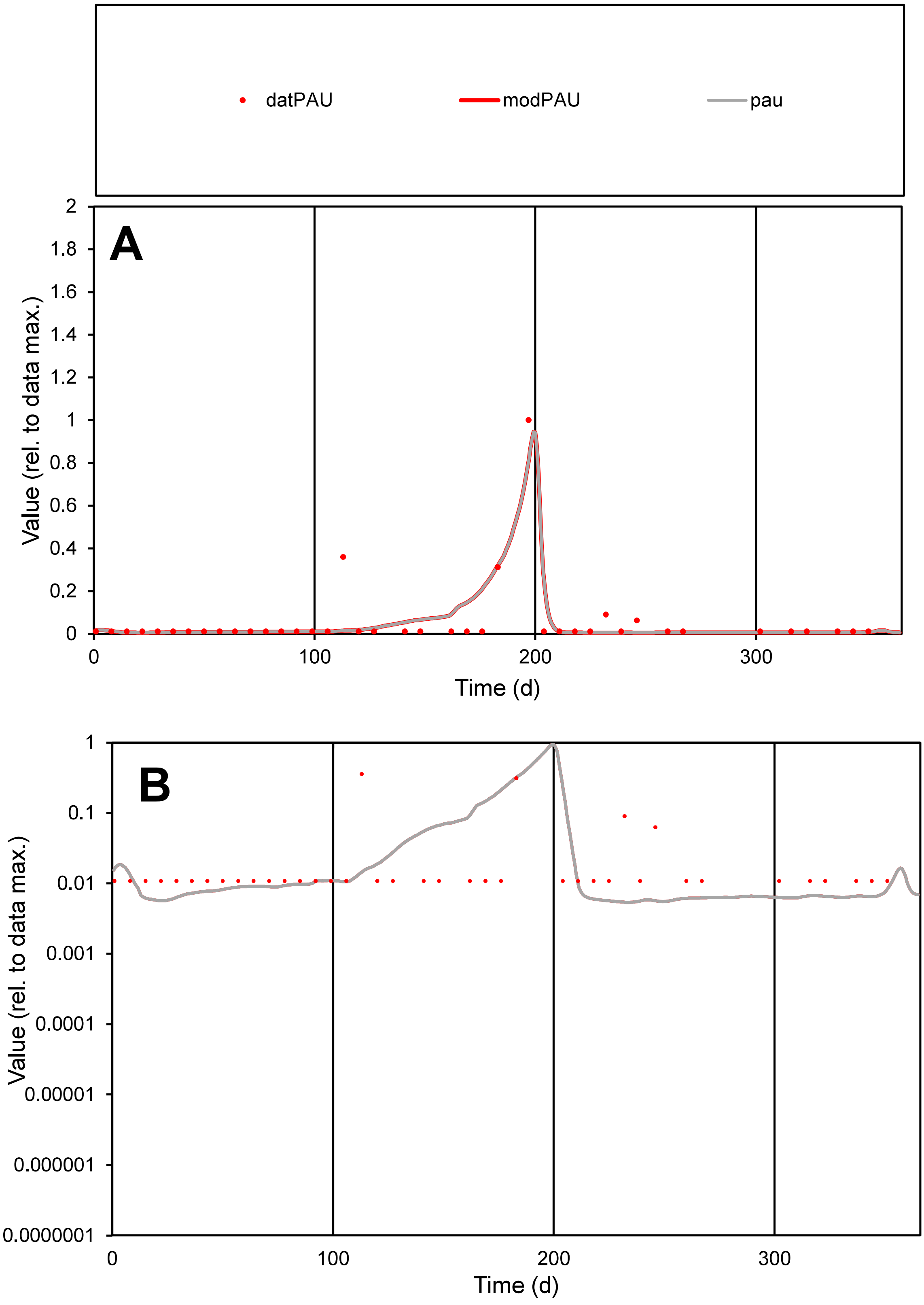

C1

Variables

PAU	Provide jtd or Dst
CalibOnly?	
2	1 to reproduce of
oNormalize?	
2	1=no, 2=yes

ofxn calc*
1.31348
ofxn mod
1.38391

sRun
RunA1
zout
1

Ave
Data
2E-05
Model
1.9E-05

data/vMax
0.00036

ofxng
319.391

Min d/m
2.01688

ave/min d
4.98969
ave/min m
9.95065

330_Calib_S_A

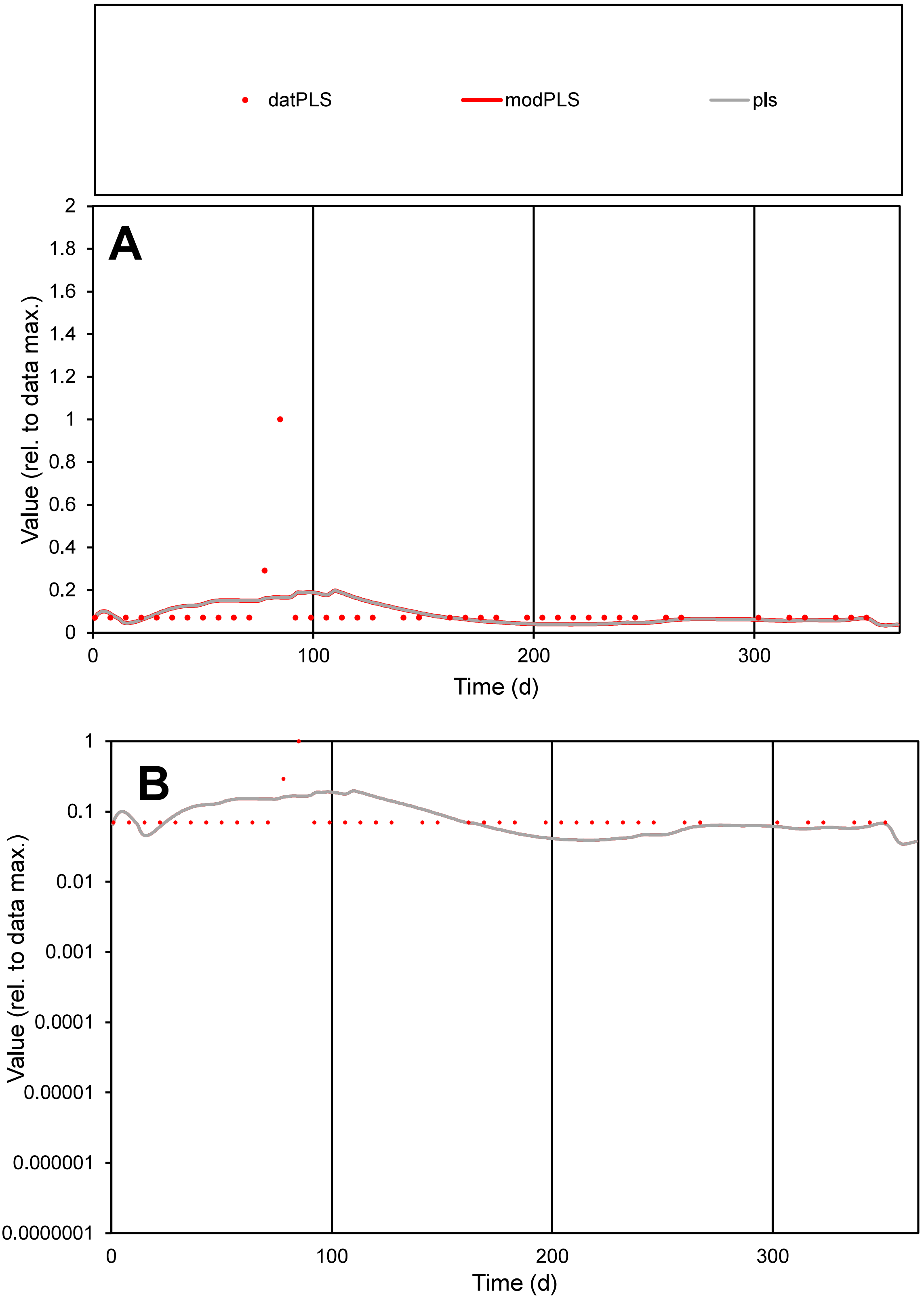

C1

Variables

PLS	Provide jtd or Dst
CalibOnly?	
2	1 to reproduce of
oNormalize?	
2	1=no, 2=yes
ofxn calc*	
1.4403	
ofxn mod	
1.44895	
sRun	
RunA1	
zout	
1	
Ave	
Data	
5.4E-06	
Model	
5.1E-06	
data/vMax	
5.5E-05	
ofxng	
319.391	
Min d/m	
1.79151	
ave/min d	
1.40162	
ave/min m	
2.37052	

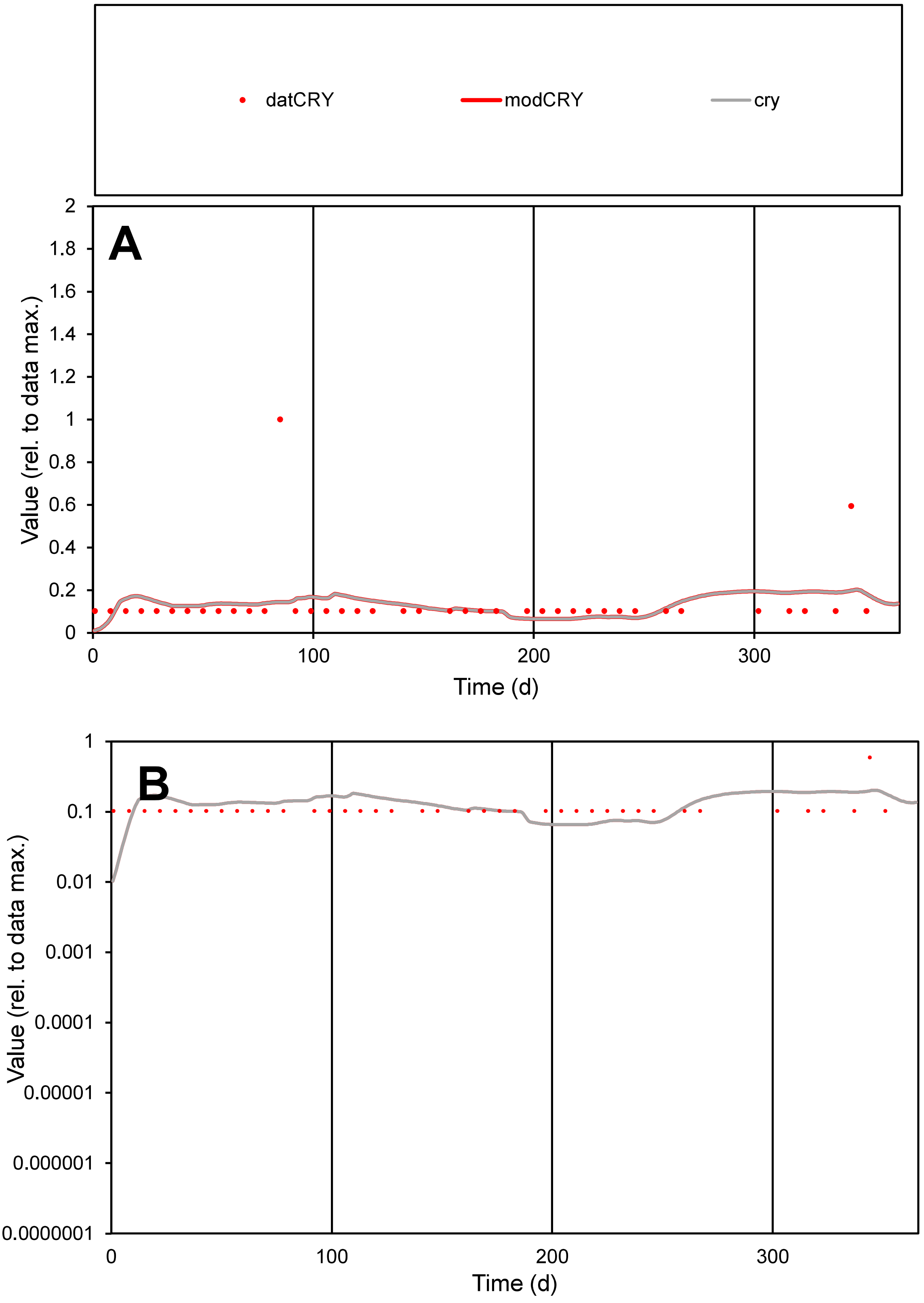

C1

Variables
CRY Provide jtd or Dst
CalibOnly?
2 1 to reproduce of
oNormalize?
2 1=no, 2=yes

ofxn calc*
1.13068
ofxn mod
1.13884

sRun
RunA1
zout
1

Ave
Data
4E-06
Model
3.7E-06

data/vMax
2.9E-05

ofxng
319.391

Min d/m
4.66635

ave/min d
1.32908
ave/min m
5.84134

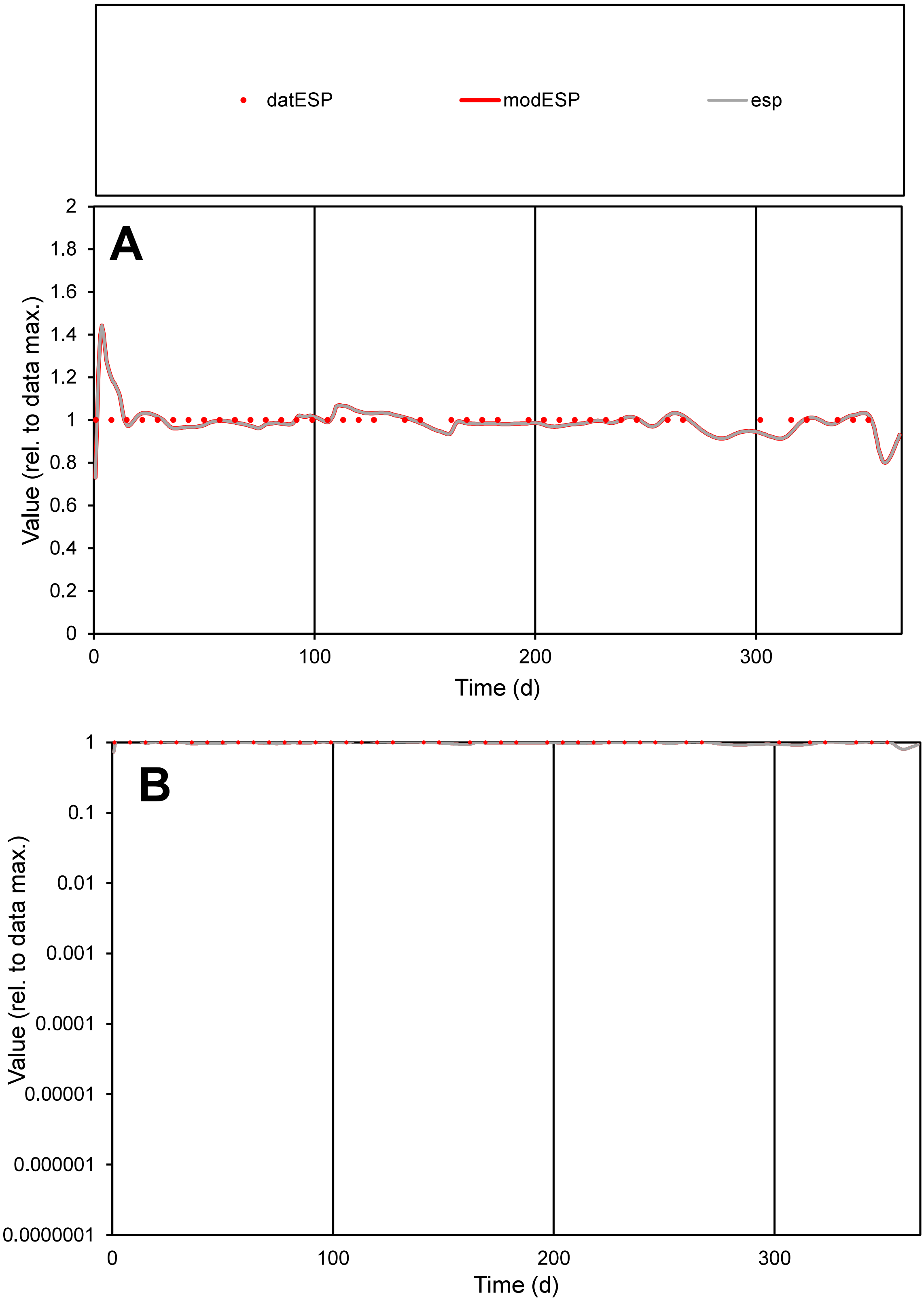

C1

Variables	
ESP	Provide jtd or Dst
CalibOnly?	
2	1 to reproduce of
oNormalize?	
2	1=no, 2=yes
ofxn calc*	
0.03662	
ofxn mod	
0.11502	
sRun	
RunA1	
zout	
1	
Ave	
Data	
8E-06	
Model	
8E-06	
data/vMax	
8E-06	
ofxng	
319.391	
Min d/m	
1.06875	
ave/min d	
1	
ave/min m	
1.06823	

330_Calib_S_A

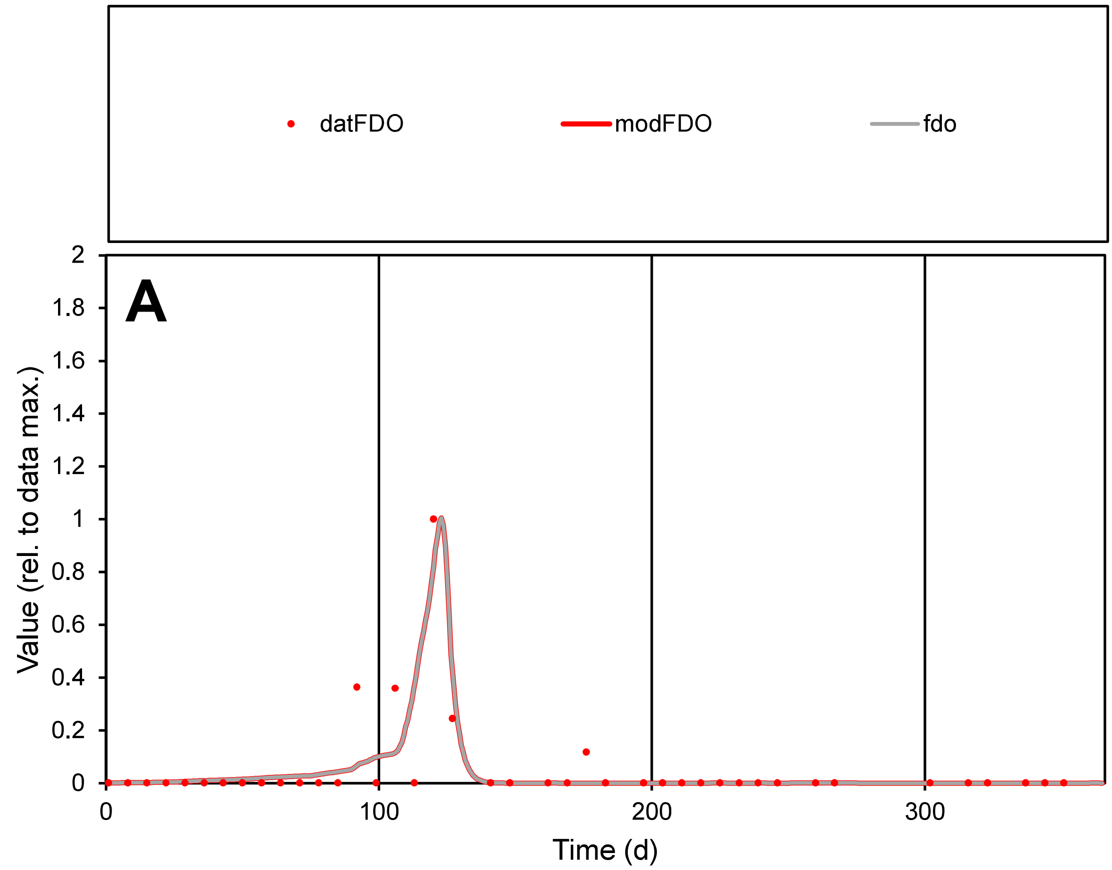

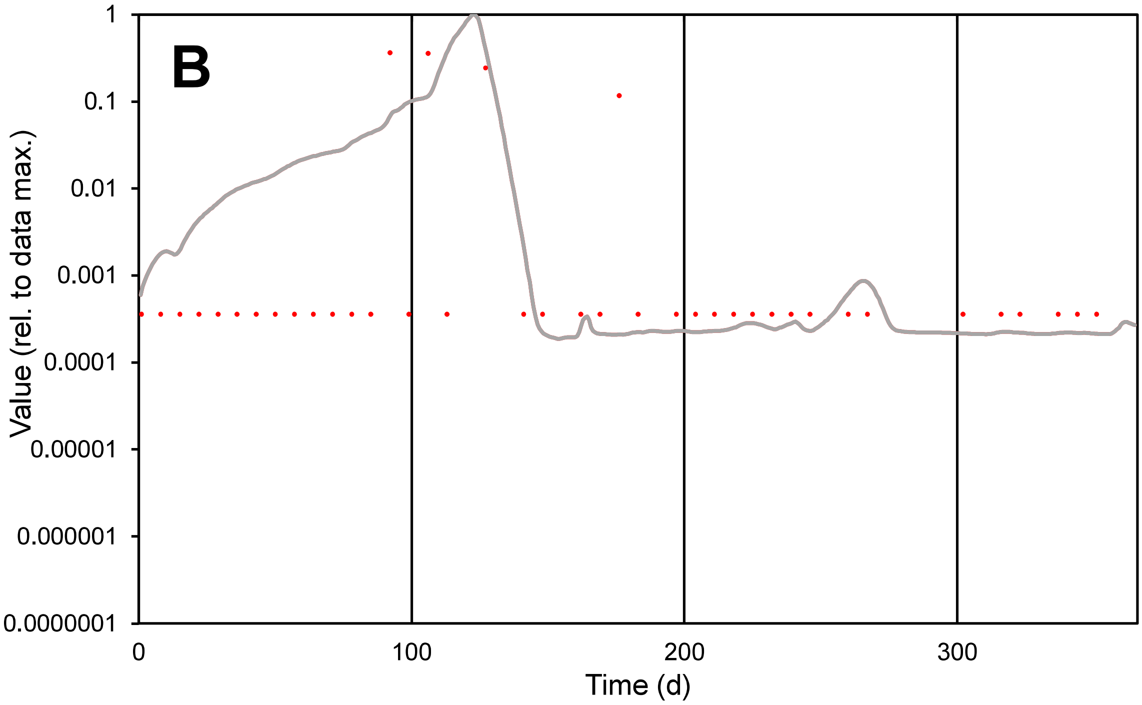

C1

Variables
FDO Provide jtd or Dst
CalibOnly?
2 1 to reproduce of
oNormalize?
2 1=no, 2=yes

ofxn calc*
1.50668
ofxn mod
1.61125

sRun
RunA1
zout
1

Ave
Data
1.4E-05
Model
1.4E-05

data/vMax
0.00028

ofxng
319.391

Min d/m
1.71421

ave/min d
143.114
ave/min m
237.136

330_Calib_S_A

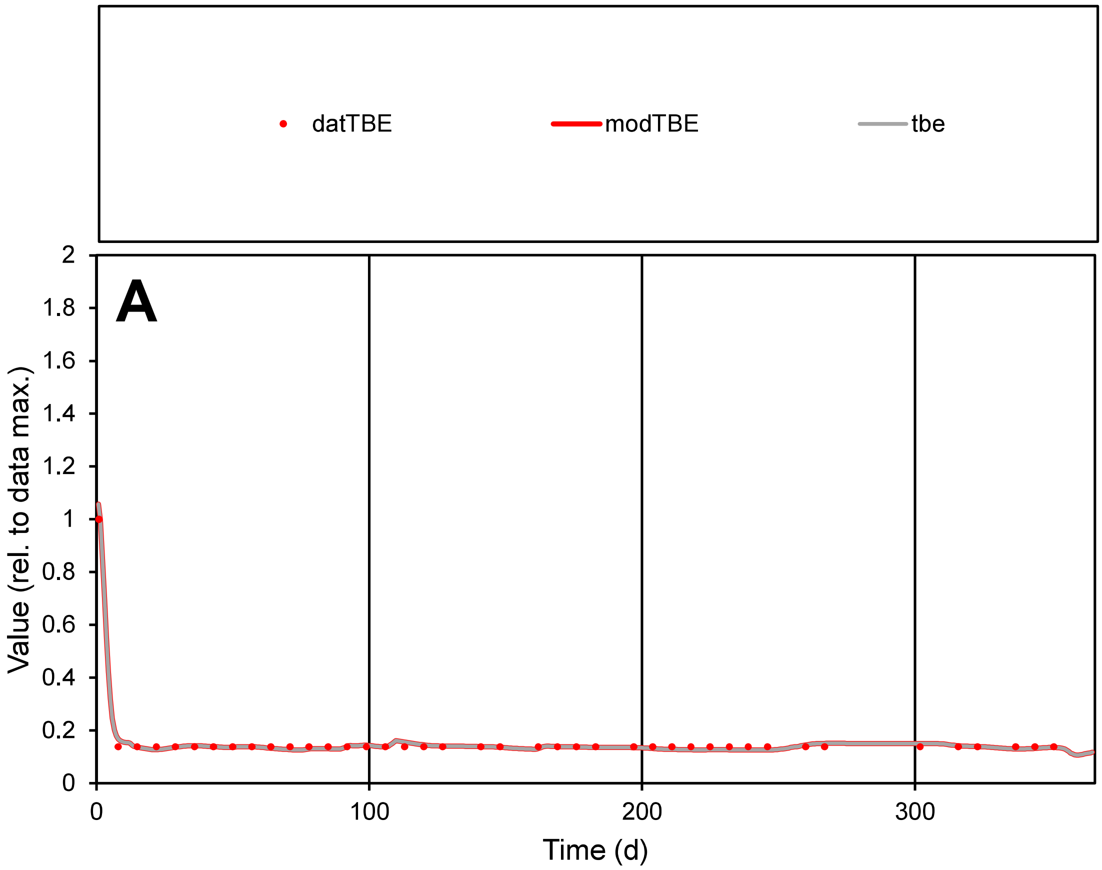

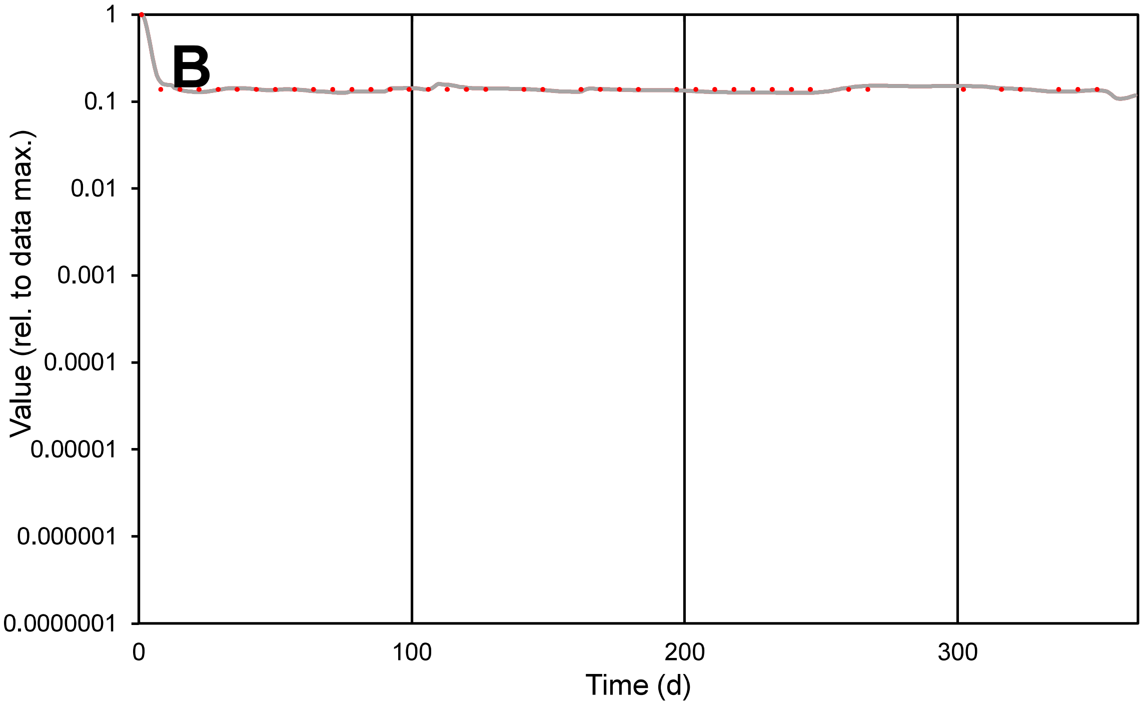

C1

Variables
TBE Provide jtd or Dst
CalibOnly?
2 1 to reproduce of
oNormalize?
2 1=no, 2=yes

ofxn calc*
0.0487
ofxn mod
0.13267

sRun
RunA1
zout
1

Ave
Data
7.7E-06
Model
7.7E-06

data/vMax
4.8E-05

ofxng
319.391

Min d/m
1.0882

ave/min d
1.15279
ave/min m
1.24857

330_Calib_S_A

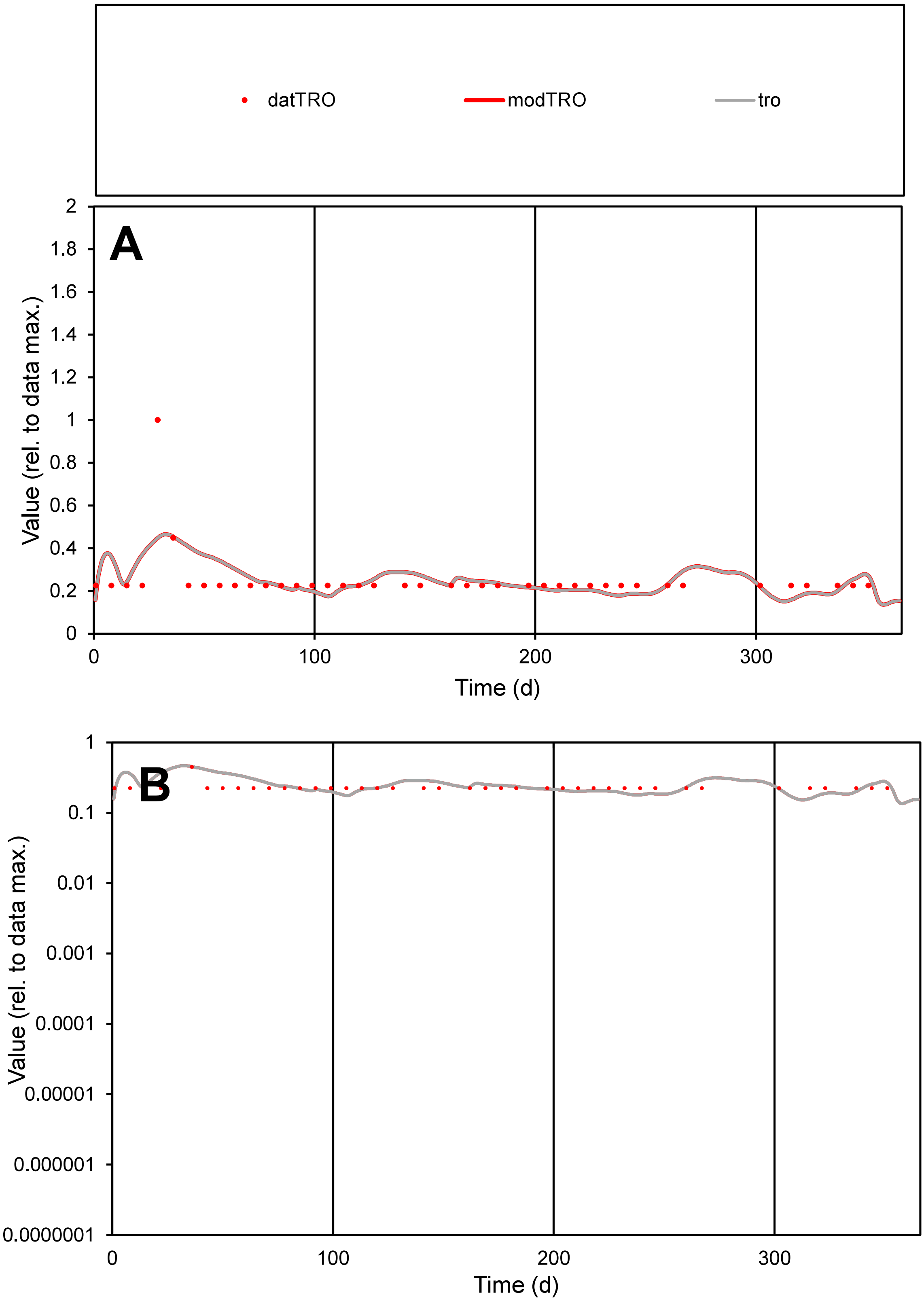

C1

Variables

TRO	Provide jtd or Dst
CalibOnly?	
2	1 to reproduce of
oNormalize?	
2	1=no, 2=yes

ofxn calc*
0.40986
ofxn mod
0.42823

sRun
RunA1
zout
1

Ave
Data
5.6E-06
Model
5.7E-06

data/vMax
2.2E-05

ofxng
319.391

Min d/m
1.37377

ave/min d
1.10863
ave/min m
1.55608

330_Calib_S_A

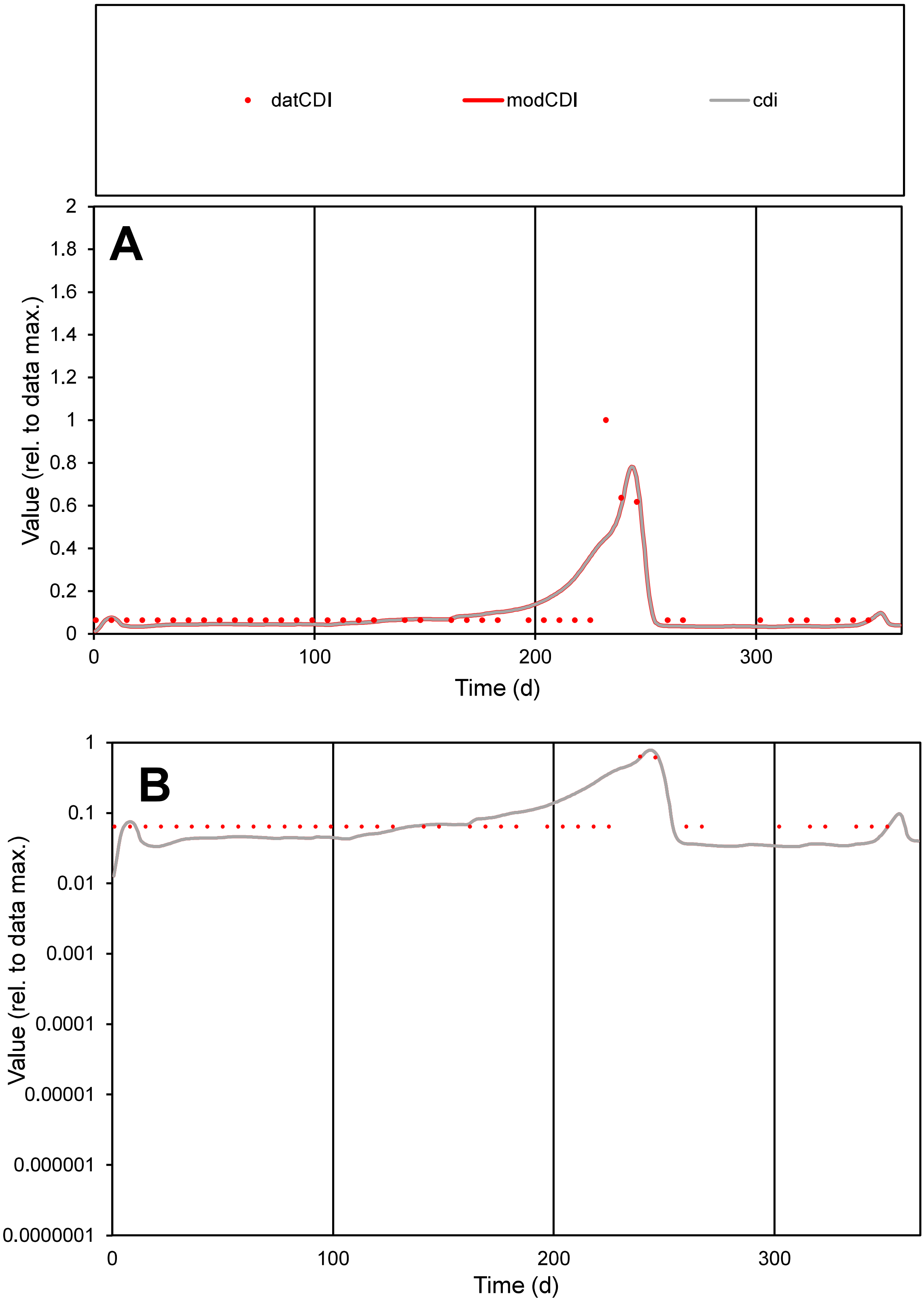

C1

Variables

CDI Provide jtd or Dst

CalibOnly?

2 1 to reproduce of

oNormalize?

2 1=no, 2=yes

ofxn calc*

0.93726

ofxn mod

0.9675

sRun

RunA1

zout

1

Ave

Data

3.1E-07

Model

3E-07

data/vMax

2.7E-06

ofxng

319.391

Min d/m

1.90288

ave/min d

1.78443

ave/min m

3.32716

330_Calib_S_A

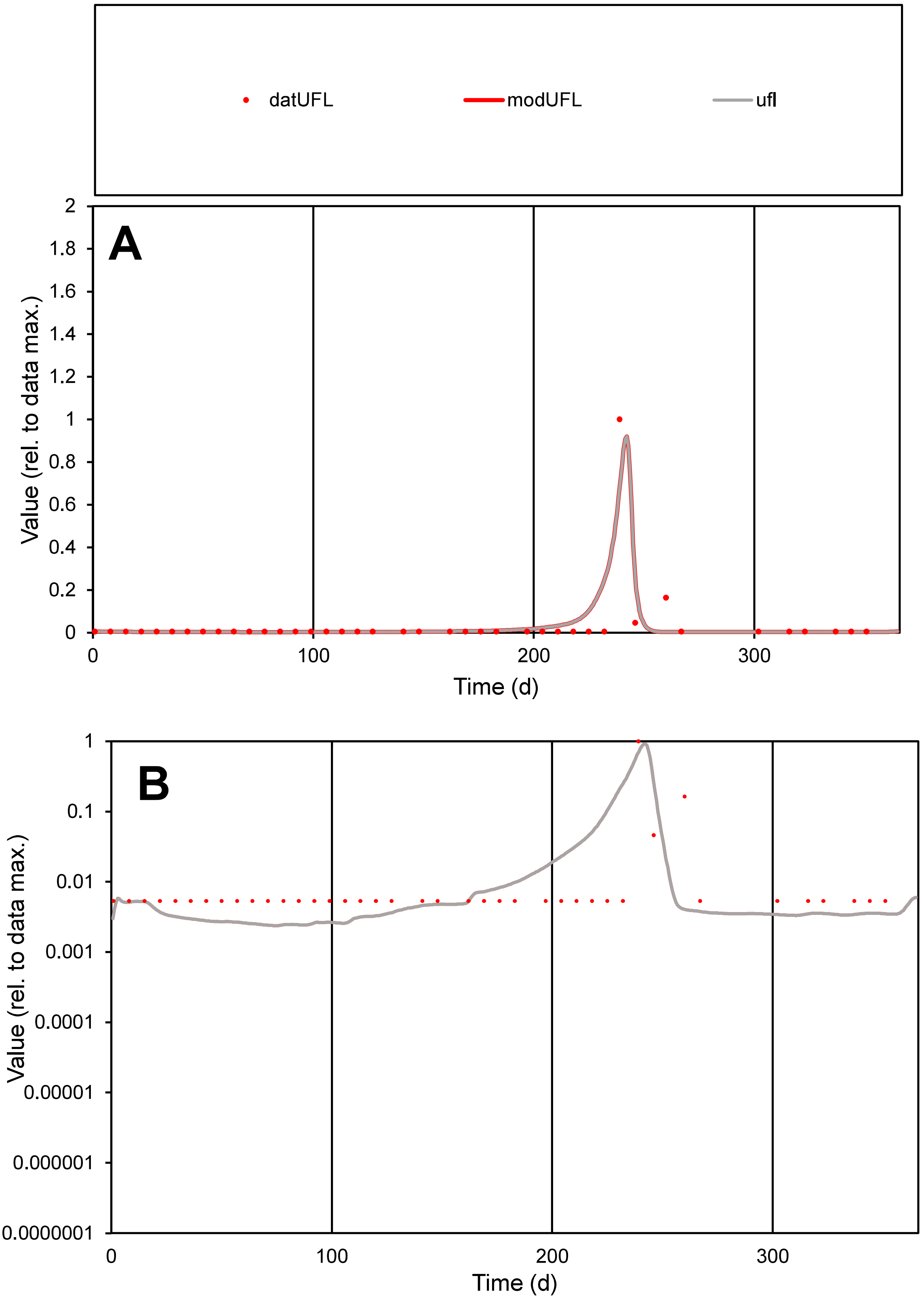

C1

Variables
UFL Provide jtd or Dst
CalibOnly?
2 1 to reproduce of
oNormalize?
2 1=no, 2=yes

ofxn calc*
1.31414
ofxn mod
1.64734

sRun
RunA1
zout
1

Ave
Data
2E-05
Model
2E-05

data/vMax
0.00057

ofxng
319.391

Min d/m
2.22045

ave/min d
6.45852
ave/min m
14.6012

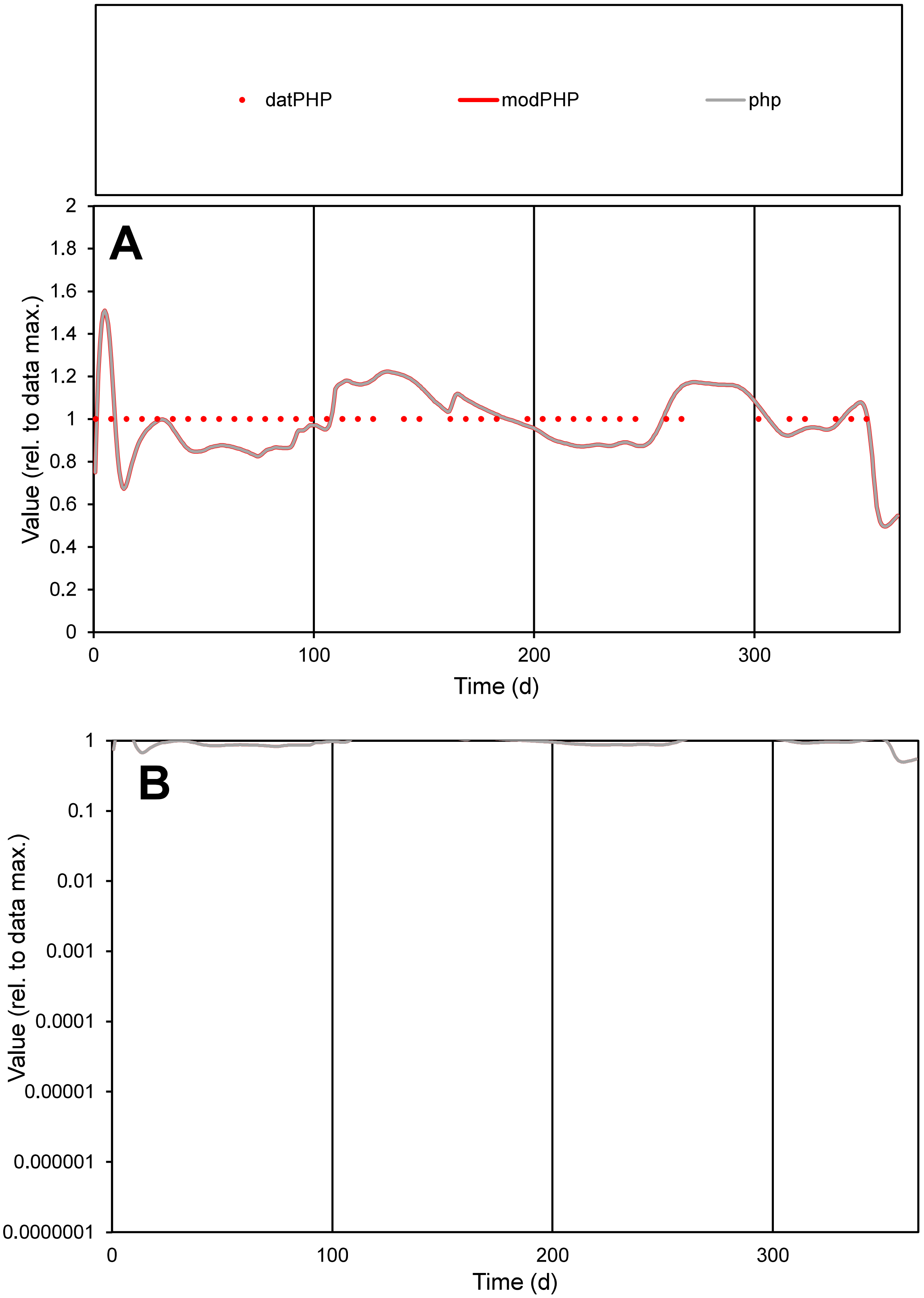

C1

Variables
PHP Provide jtd or Dst
CalibOnly?
2 1 to reproduce of
oNormalize?
2 1=no, 2=yes

ofxn calc*
0.1131
ofxn mod
0.15571

sRun
RunA1
zout
1

Ave
Data
6.9E-06
Model
6.8E-06

data/vMax
6.9E-06

ofxng
319.391

Min d/m
1.31036

ave/min d
1
ave/min m
1.2808

330_Calib_S_A

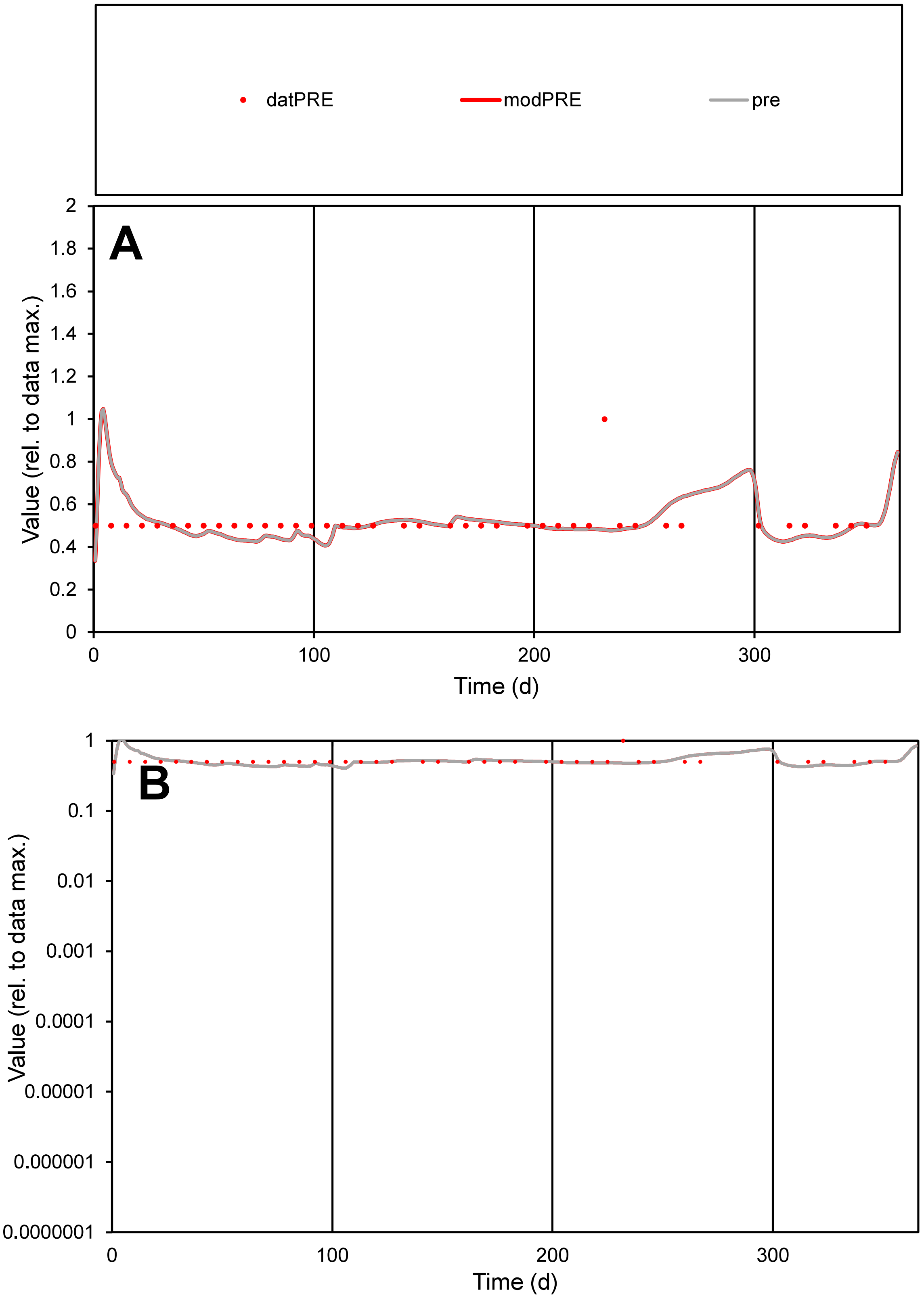

C1

Variables

PRE	Provide jtd or Dst
CalibOnly?	
2	1 to reproduce of
oNormalize?	
2	1=no, 2=yes

ofxn calc*
0.19794
ofxn mod
0.22632

sRun
RunA1
zout
1

Ave
Data
1.4E-05
Model
1.3E-05

data/vMax
2.7E-05

ofxng
319.391

Min d/m
1.16884

ave/min d
1.02439
ave/min m
1.16999

330_Calib_S_A

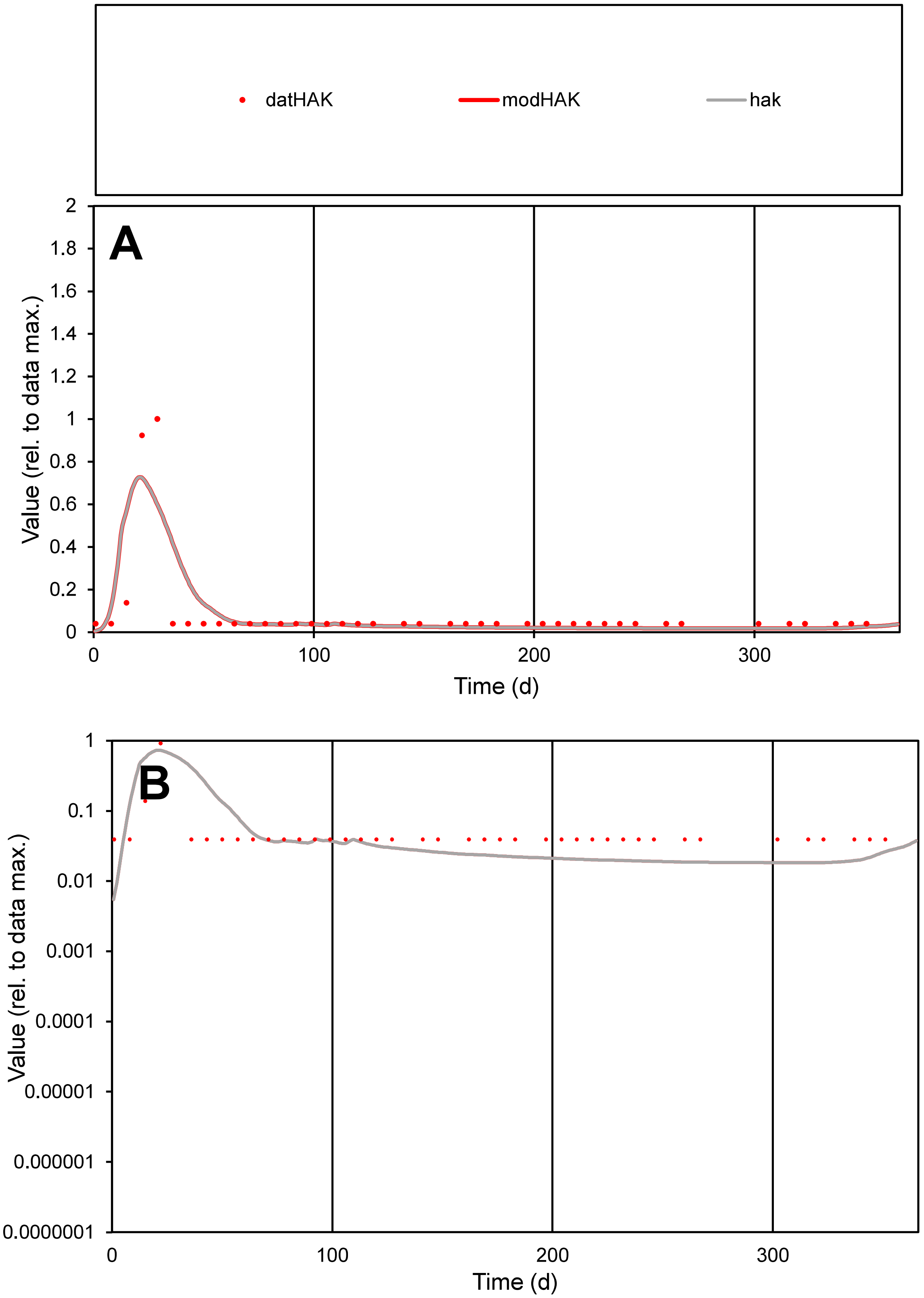

C1

Variables
HAK Provide jtd or Dst
CalibOnly?
2 1 to reproduce of
oNormalize?
2 1=no, 2=yes

ofxn calc*
1.32246
ofxn mod
1.39881

sRun
RunA1
zout
1

Ave
Data
1.1E-05
Model
1.1E-05

data/vMax
0.00013

ofxng
319.391

Min d/m
2.1587

ave/min d
2.20592
ave/min m
4.7648

330_Calib_S_A

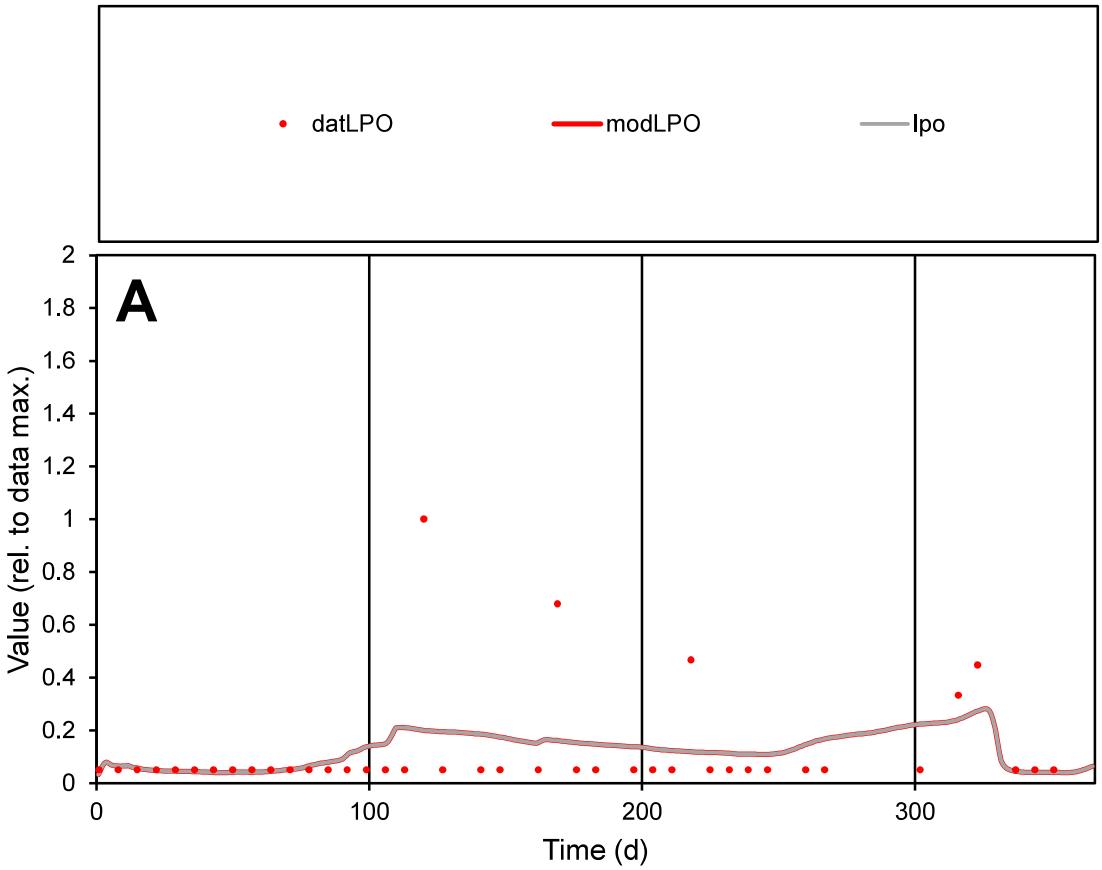

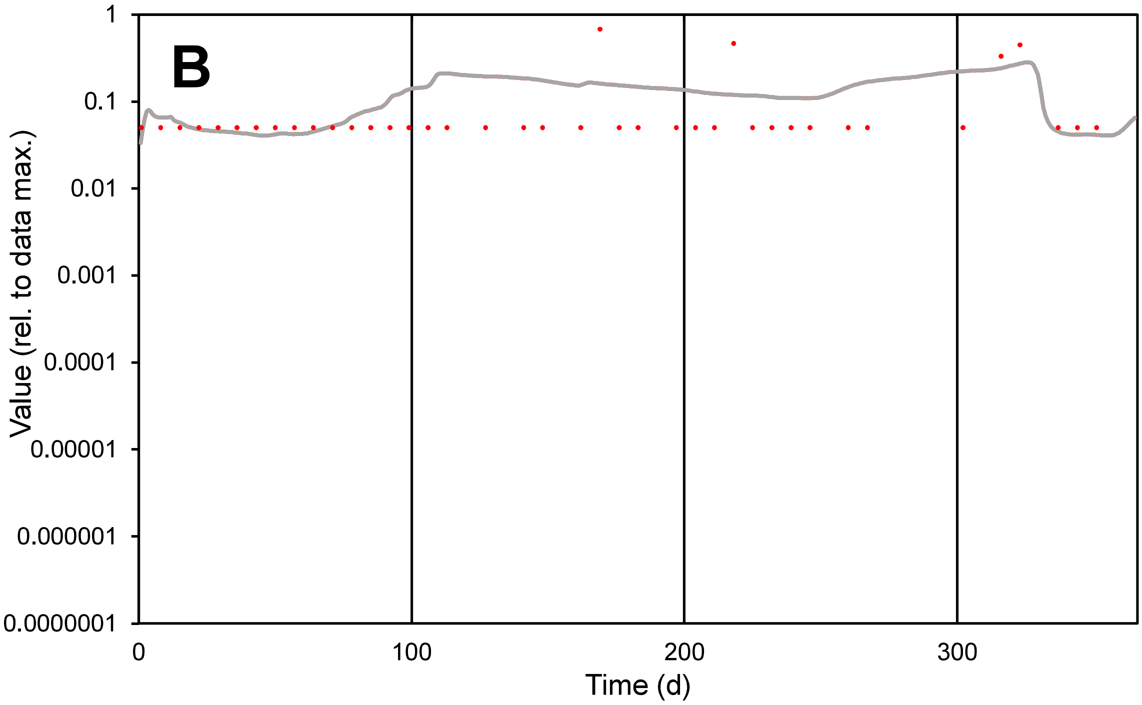

C1

Variables
LPO Provide jtd or Dst
CalibOnly?
2 1 to reproduce of
oNormalize?
2 1=no, 2=yes

ofxn calc*
1.53732
ofxn mod
1.54473

sRun
RunA1
zout
1

Ave
Data
1.3E-05
Model
1.4E-05

data/vMax
0.00012

ofxng
319.391

Min d/m
1.22999

ave/min d
2.30085
ave/min m
2.87259

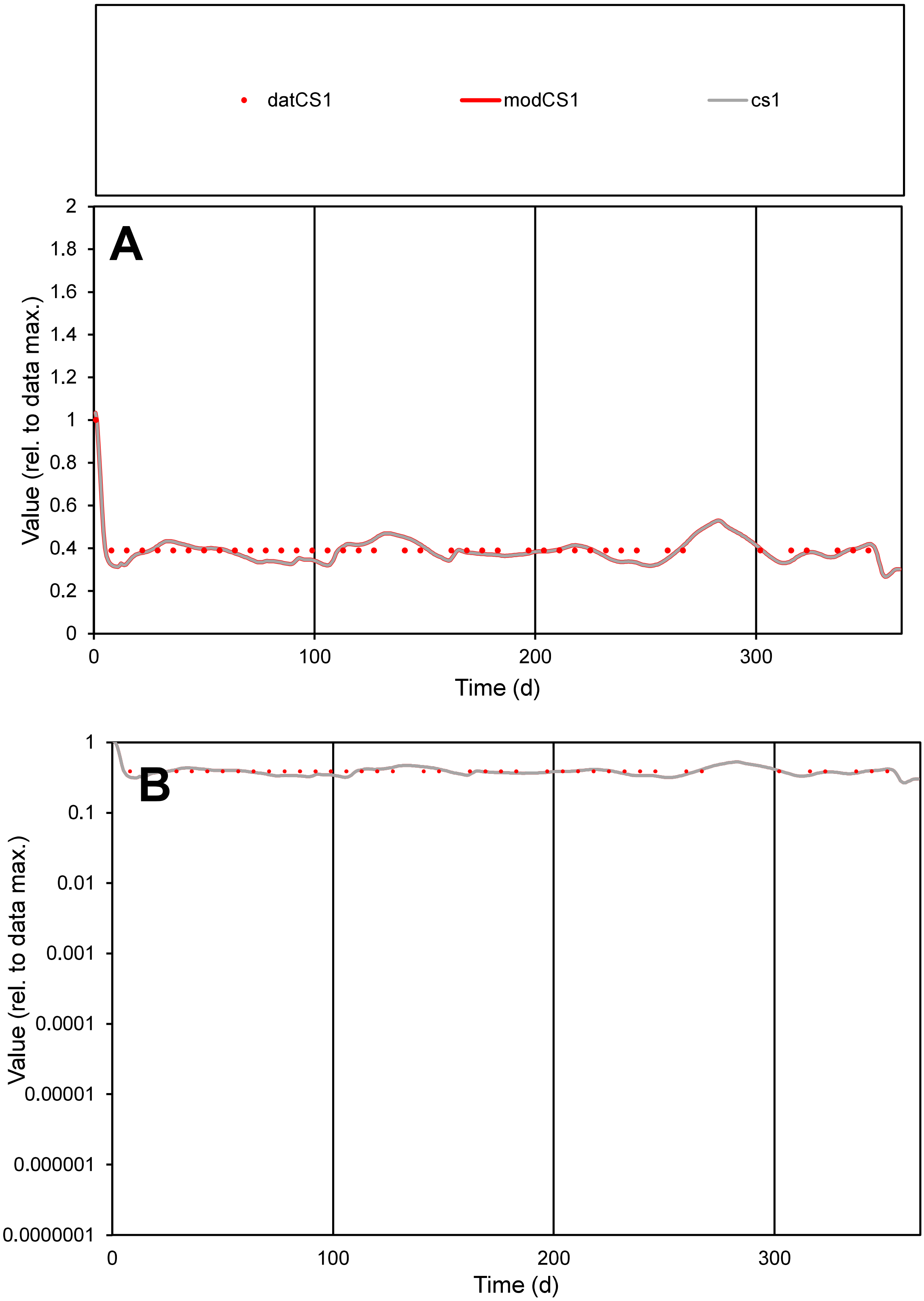

C1

Variables
CS1 Provide jtd or Dst
CalibOnly?
2 1 to reproduce of
oNormalize?
2 1=no, 2=yes

ofxn calc*
0.08548
ofxn mod
0.13734

sRun
RunA1
zout
1

Ave
Data
1.8E-05
Model
1.8E-05

data/vMax
4.5E-05

ofxng
319.391

Min d/m
1.23293

ave/min d
1.03828
ave/min m
1.25326

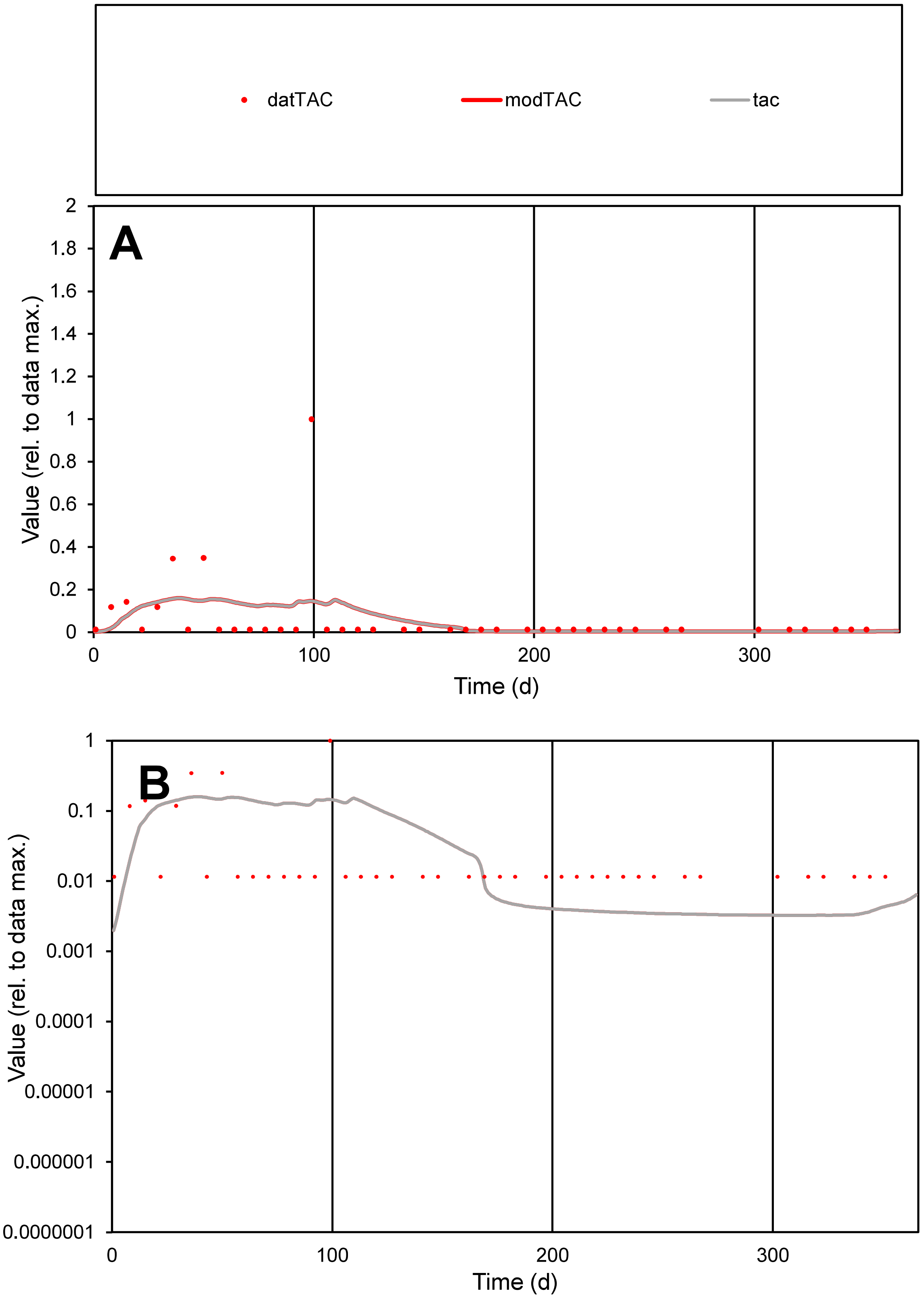

C1

Variables
TAC Provide jtd or Dst
CalibOnly?
2 1 to reproduce of
oNormalize?
2 1=no, 2=yes

ofxn calc*
2.56295
ofxn mod
2.58147

sRun
RunA1
zout
1

Ave
Data
6.8E-06
Model
6.7E-06

data/vMax
0.00011

ofxng
319.391

Min d/m
3.54697

ave/min d
5.24786
ave/min m
18.4565

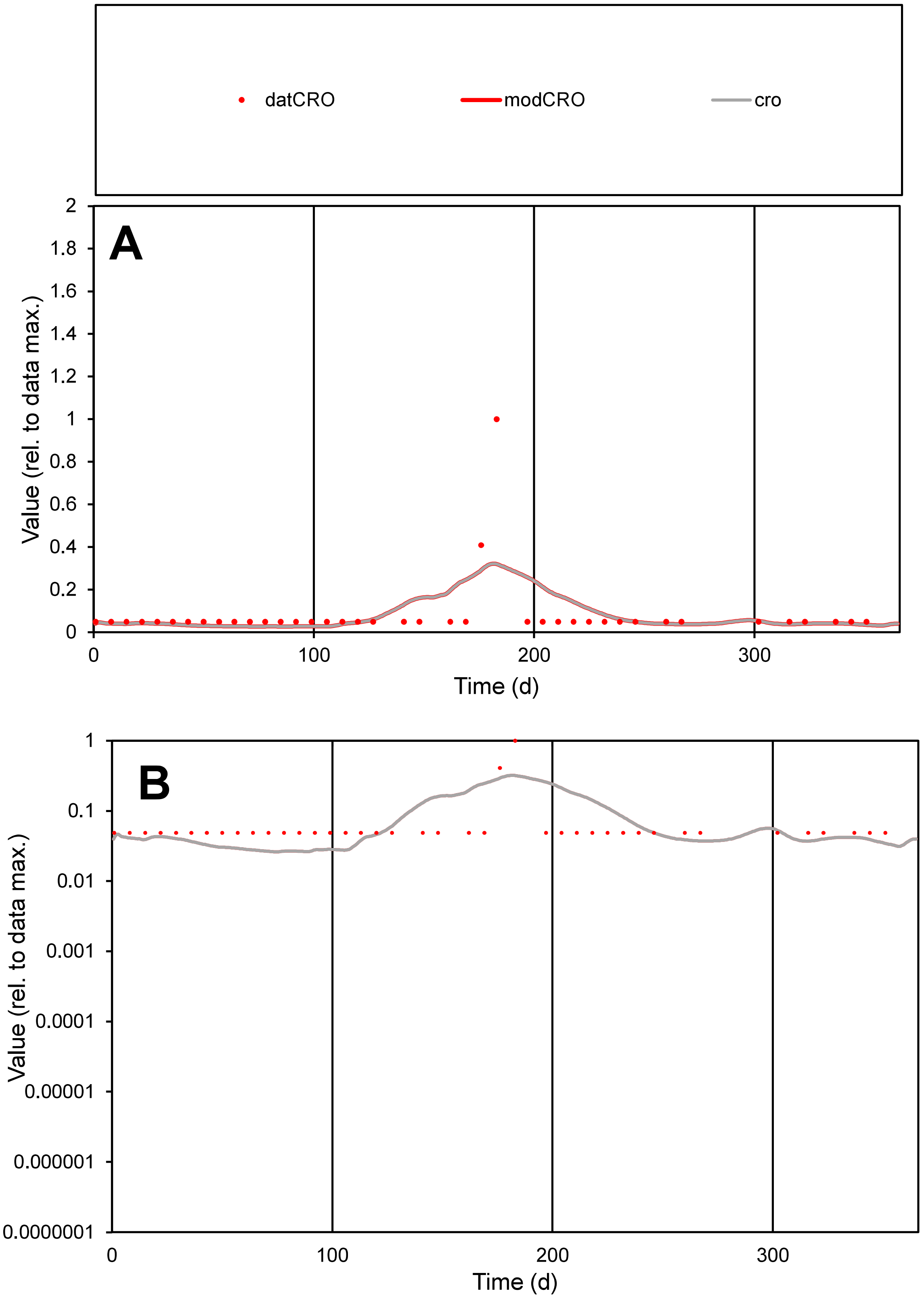

C1

Variables
CRO Provide jtd or Dst
CalibOnly?
2 1 to reproduce of
oNormalize?
2 1=no, 2=yes

ofxn calc*
1.55917
ofxn mod
1.58871

sRun
RunA1
zout
1

Ave
Data
1.7E-05
Model
1.7E-05

data/vMax
0.00021

ofxng
319.391

Min d/m
1.84106

ave/min d
1.65604
ave/min m
3.12507

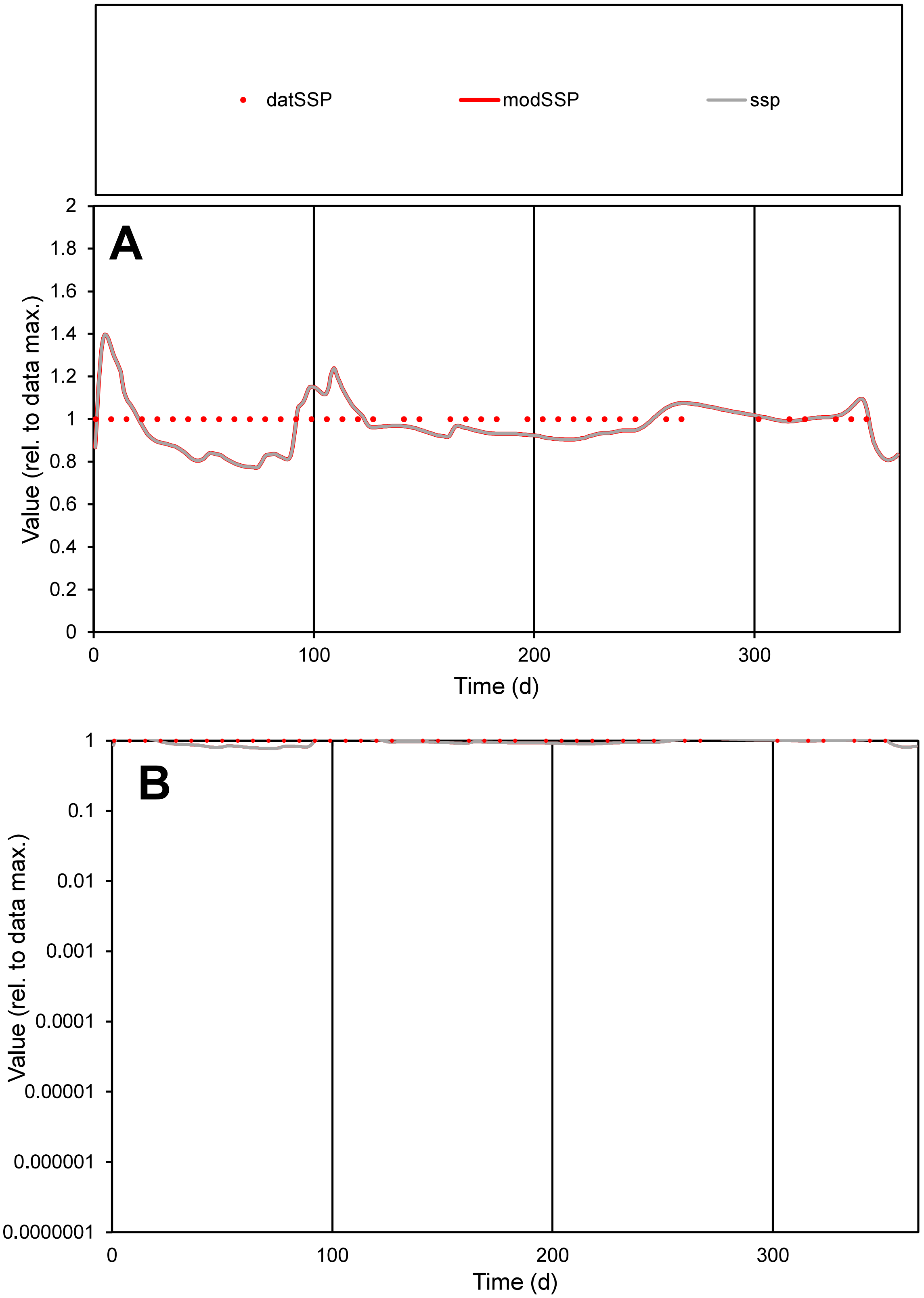

C1

Variables

SSP	Provide jtd or Dst
CalibOnly?	
2	1 to reproduce of
oNormalize?	
2	1=no, 2=yes
ofxn calc*	
0.11354	
ofxn mod	
0.15608	
sRun	
RunA1	
zout	
1	
Ave	
Data	
1.3E-05	
Model	
1.3E-05	
data/vMax	
1.3E-05	
ofxng	
319.391	
Min d/m	
1.29073	
ave/min d	
1	
ave/min m	
1.24657	

330_Calib_S_A

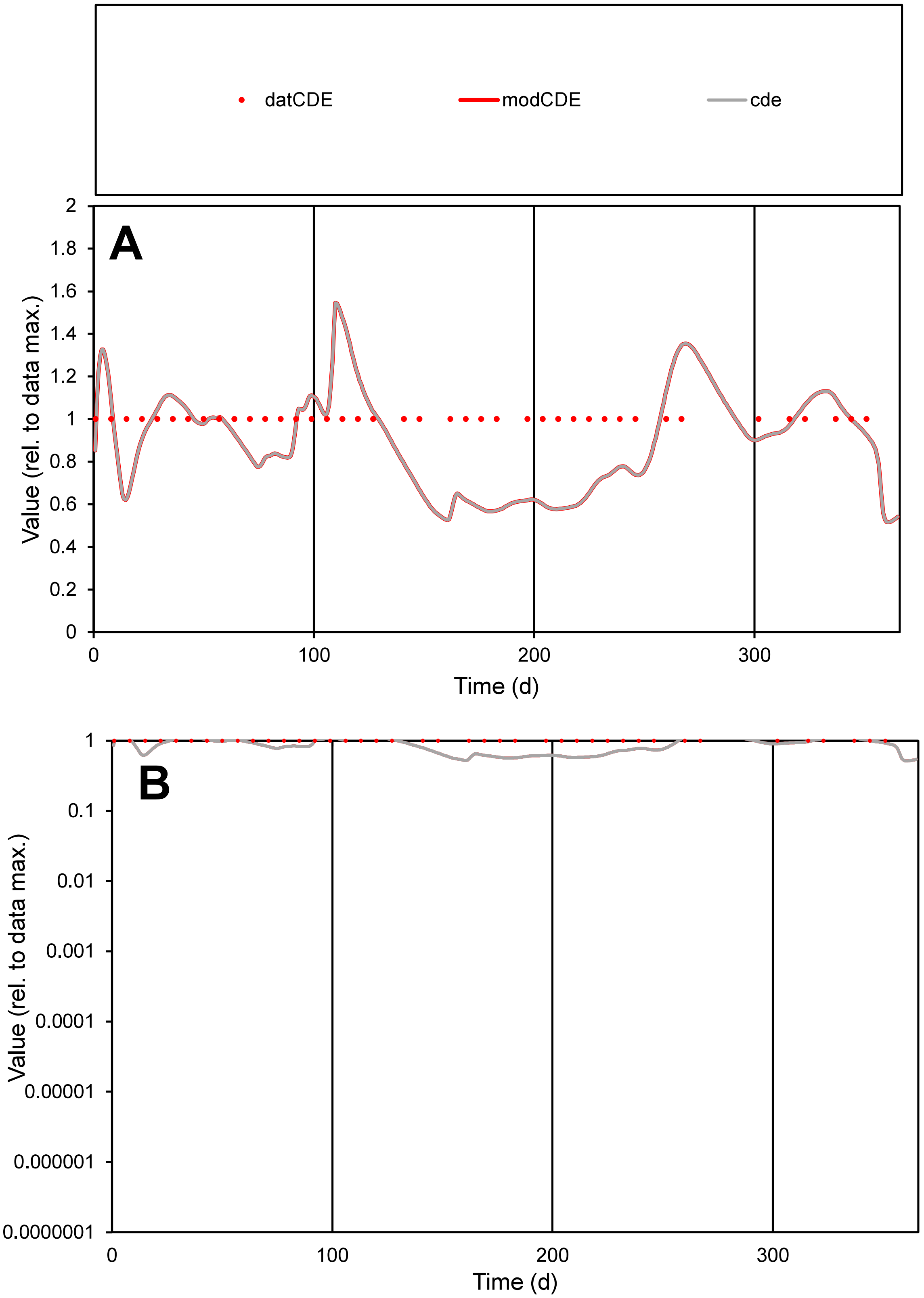

C1

Variables

CDE Provide jtd or Dst

CalibOnly?

2 1 to reproduce of

oNormalize?

2 1=no, 2=yes

ofxn calc*

0.24452

ofxn mod

0.26504

sRun

RunA1

zout

1

Ave

Data

1E-05

Model

8.9E-06

data/vMax

1E-05

ofxng

319.391

Min d/m

1.74598

ave/min d

1

ave/min m

1.56331

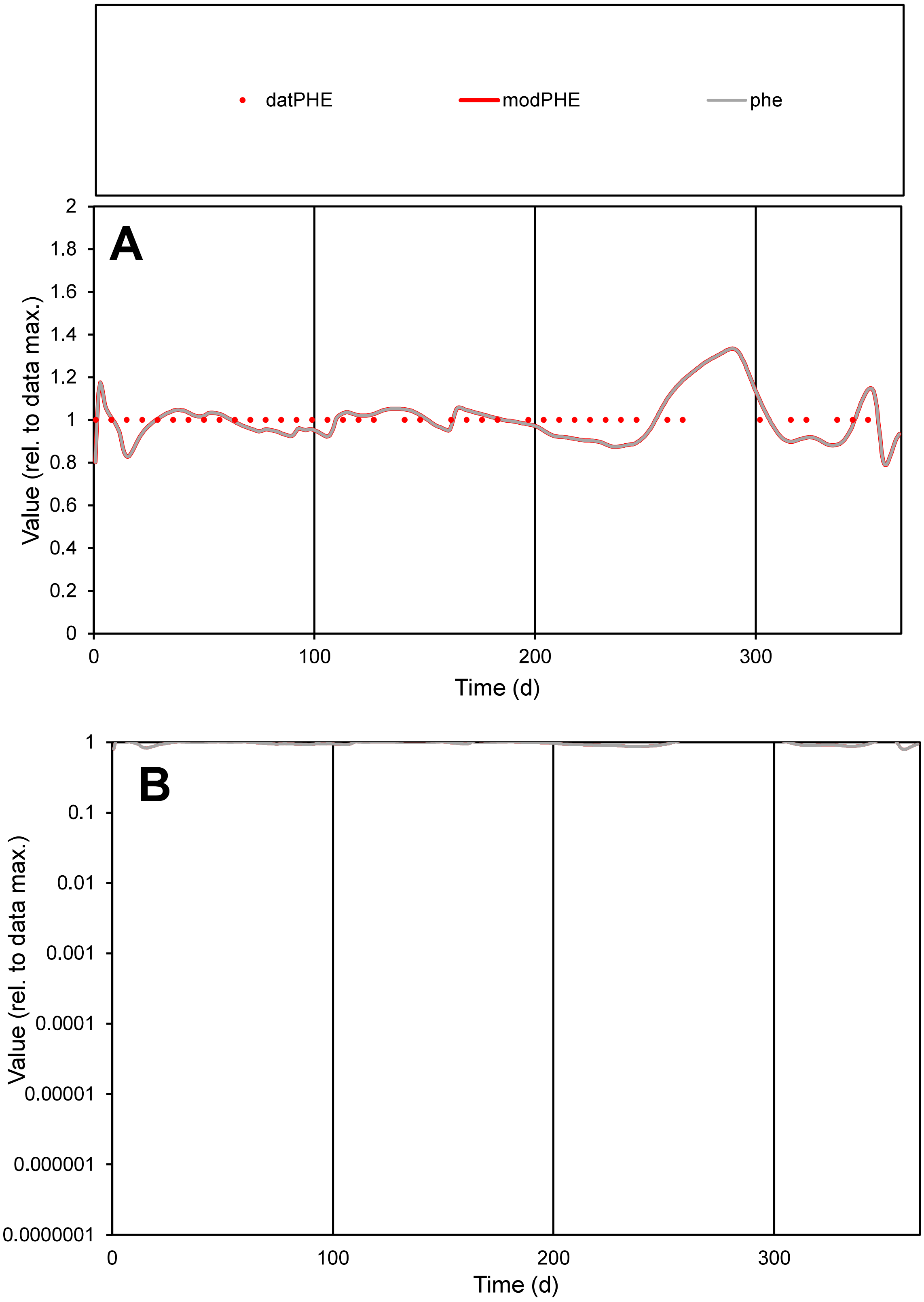

C1

Variables
PHE Provide jtd or Dst
CalibOnly?
2 1 to reproduce of
oNormalize?
2 1=no, 2=yes

ofxn calc*
0.07498
ofxn mod
0.13128

sRun
RunA1
zout
1

Ave
Data
4E-06
Model
3.9E-06

data/vMax
4E-06

ofxng
319.391

Min d/m
1.19022

ave/min d
1
ave/min m
1.17348

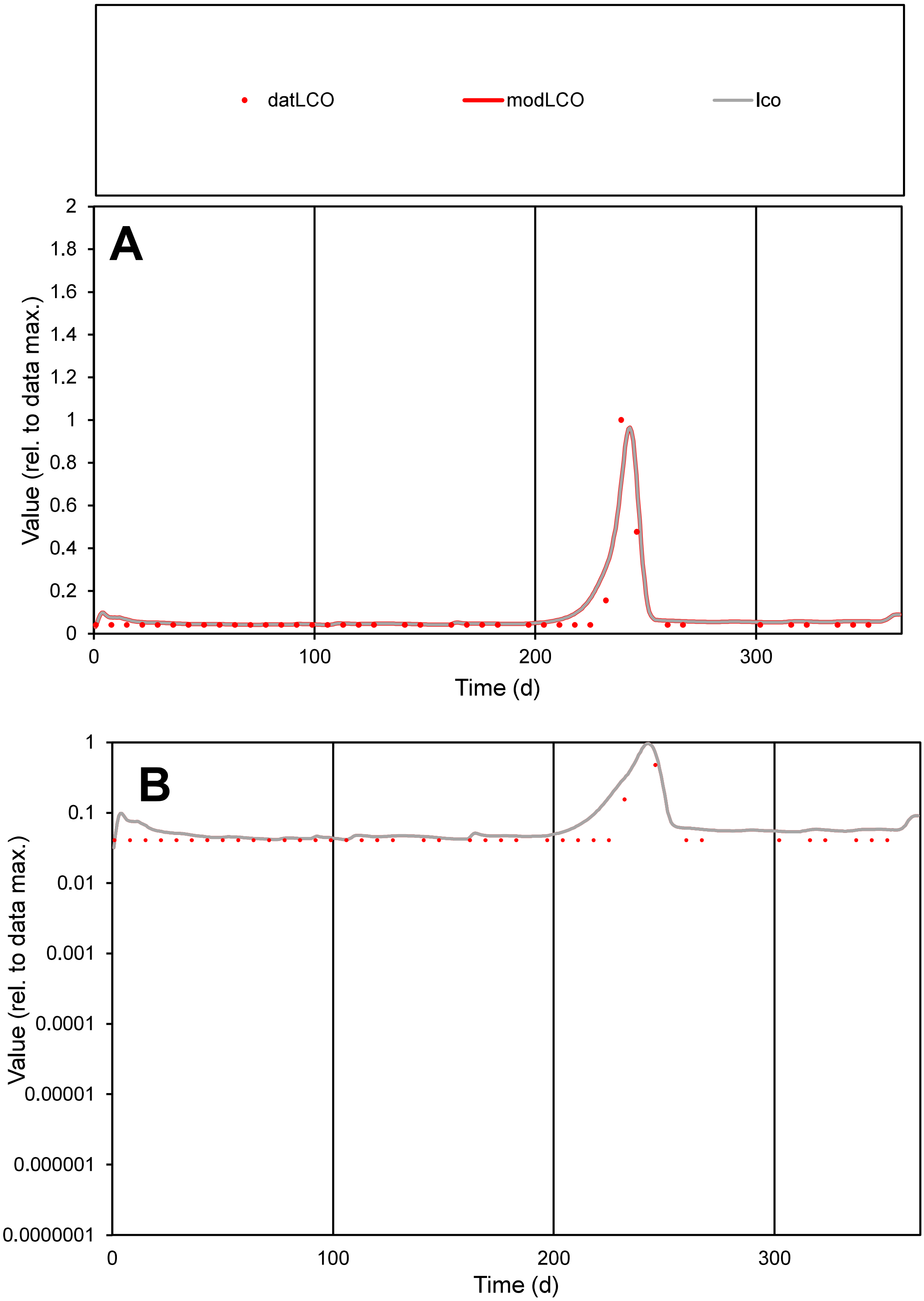

C1

Variables
LCO Provide jtd or Dst
CalibOnly?
2 1 to reproduce of
oNormalize?
2 1=no, 2=yes

ofxn calc*
0.54052
ofxn mod
0.63356

sRun
RunA1
zout
1

Ave
Data
1.4E-05
Model
1.7E-05

data/vMax
0.00018

ofxng
319.391

Min d/m
0.97396

ave/min d
1.90326
ave/min m
2.22383

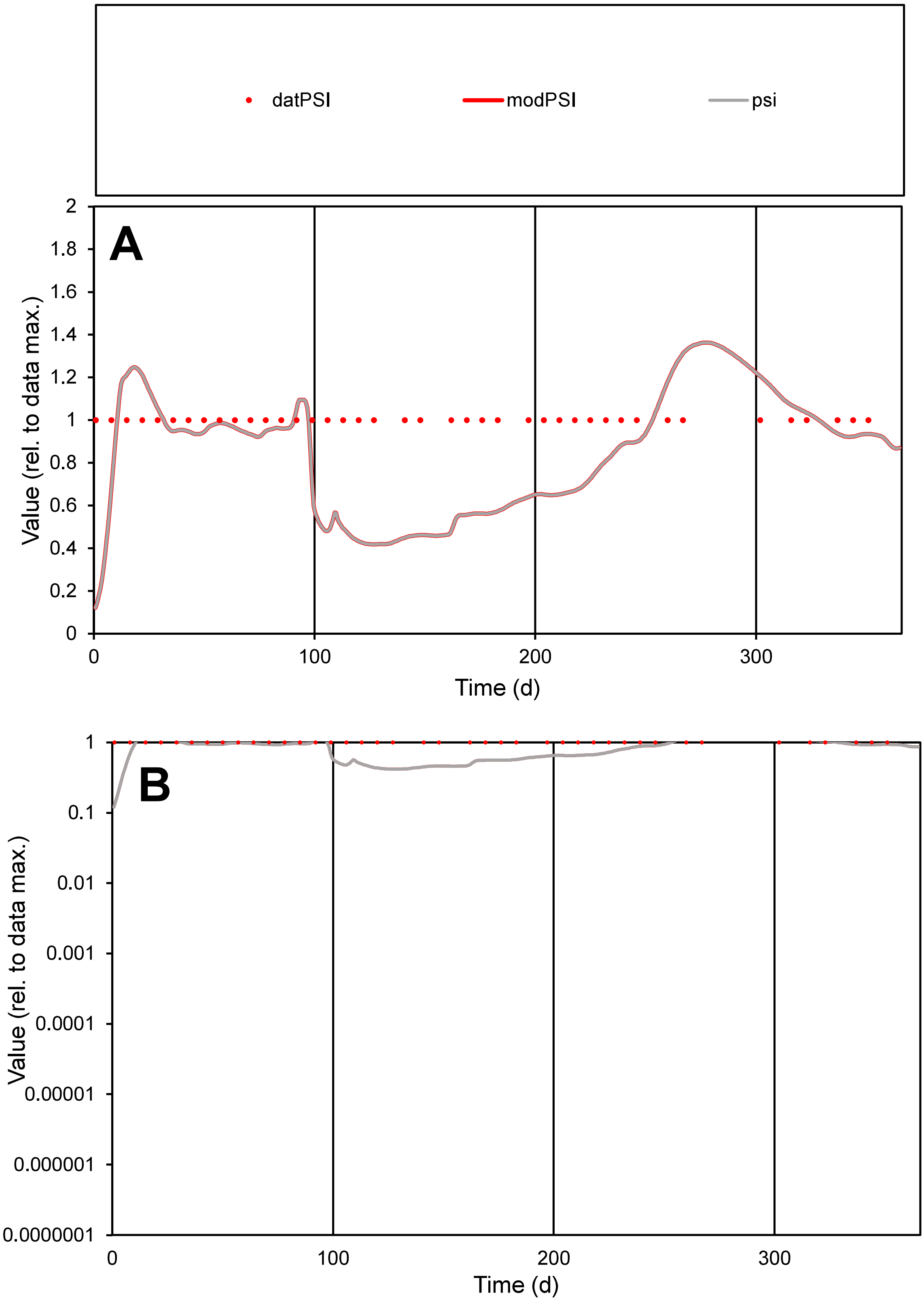

C1

Variables
PSI Provide jtd or Dst
CalibOnly?
2 1 to reproduce of
oNormalize?
2 1=no, 2=yes

ofxn calc*
0.32036
ofxn mod
0.3386

sRun
RunA1
zout
1

Ave
Data
3.6E-06
Model
2.9E-06

data/vMax
3.6E-06

ofxng
319.391

Min d/m
3.97608

ave/min d
1
ave/min m
3.24597

330_Calib_S_A

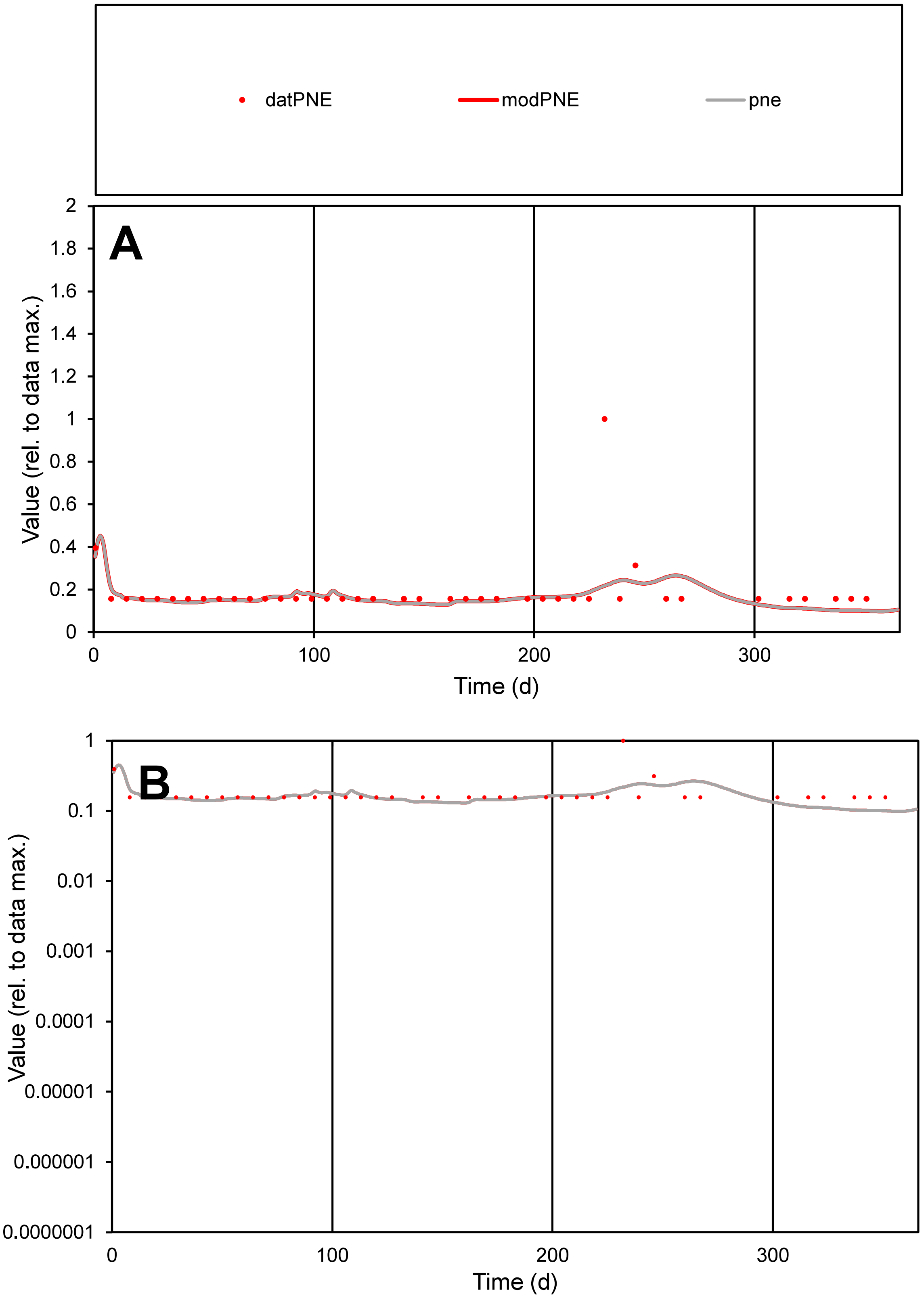

C1

Variables

PNE	Provide jtd or Dst
CalibOnly?	
2	1 to reproduce of
oNormalize?	
2	1=no, 2=yes

ofxn calc*
0.67951
ofxn mod
0.69054

sRun
RunA1
zout
1

Ave
Data
1.7E-05
Model
1.6E-05

data/vMax
9.3E-05

ofxng
319.391

Min d/m
1.57024

ave/min d
1.19294
ave/min m
1.67816

330_Calib_S_A

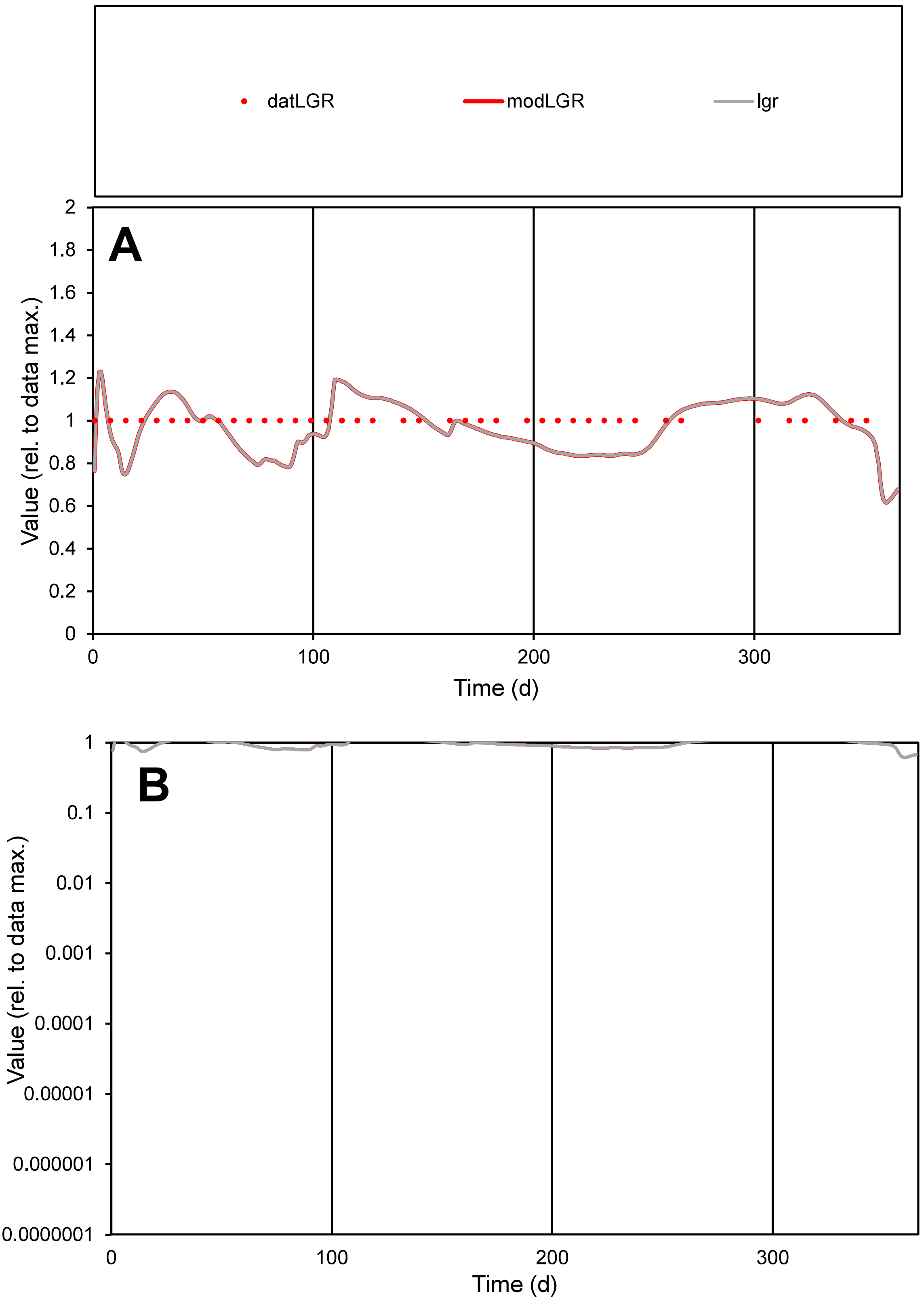

C1

Variables
LGR Provide jtd or Dst
CalibOnly?
2 1 to reproduce of
oNormalize?
2 1=no, 2=yes

ofxn calc*
0.11235
ofxn mod
0.15467

sRun
RunA1
zout
1

Ave
Data
5.2E-06
Model
5E-06

data/vMax
5.2E-06

ofxng
319.391

Min d/m
1.26986

ave/min d
1
ave/min m
1.22527

330_Calib_S_A

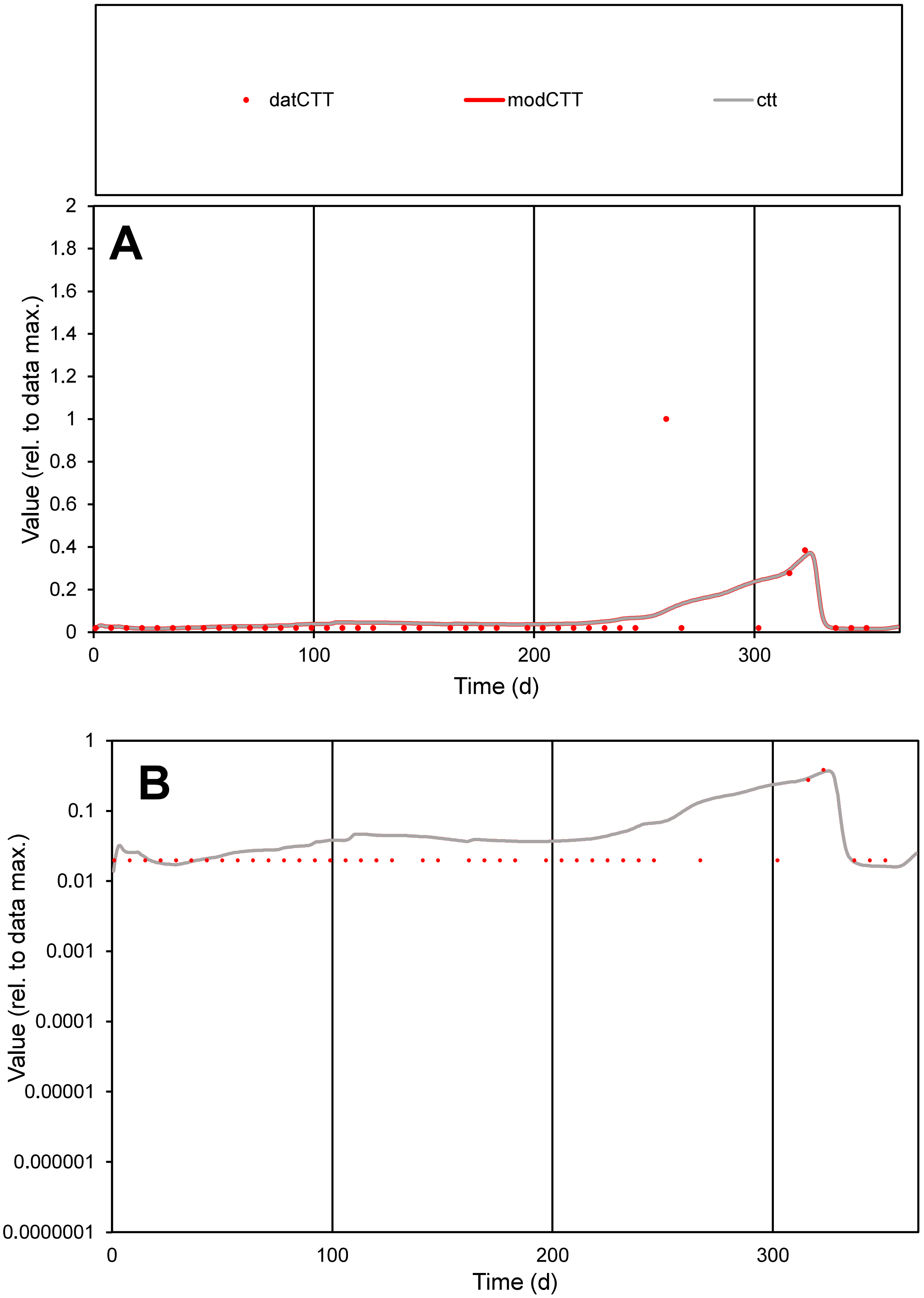

C1

Variables
CTT Provide jtd or Dst
CalibOnly?
2 1 to reproduce of
oNormalize?
2 1=no, 2=yes

ofxn calc*
2.48309
ofxn mod
2.49104

sRun
RunA1
zout
1

Ave
Data
4.4E-06
Model
4.4E-06

data/vMax
7.5E-05

ofxng
319.391

Min d/m
1.22158

ave/min d
2.98117
ave/min m
3.59137

330_Calib_S_A

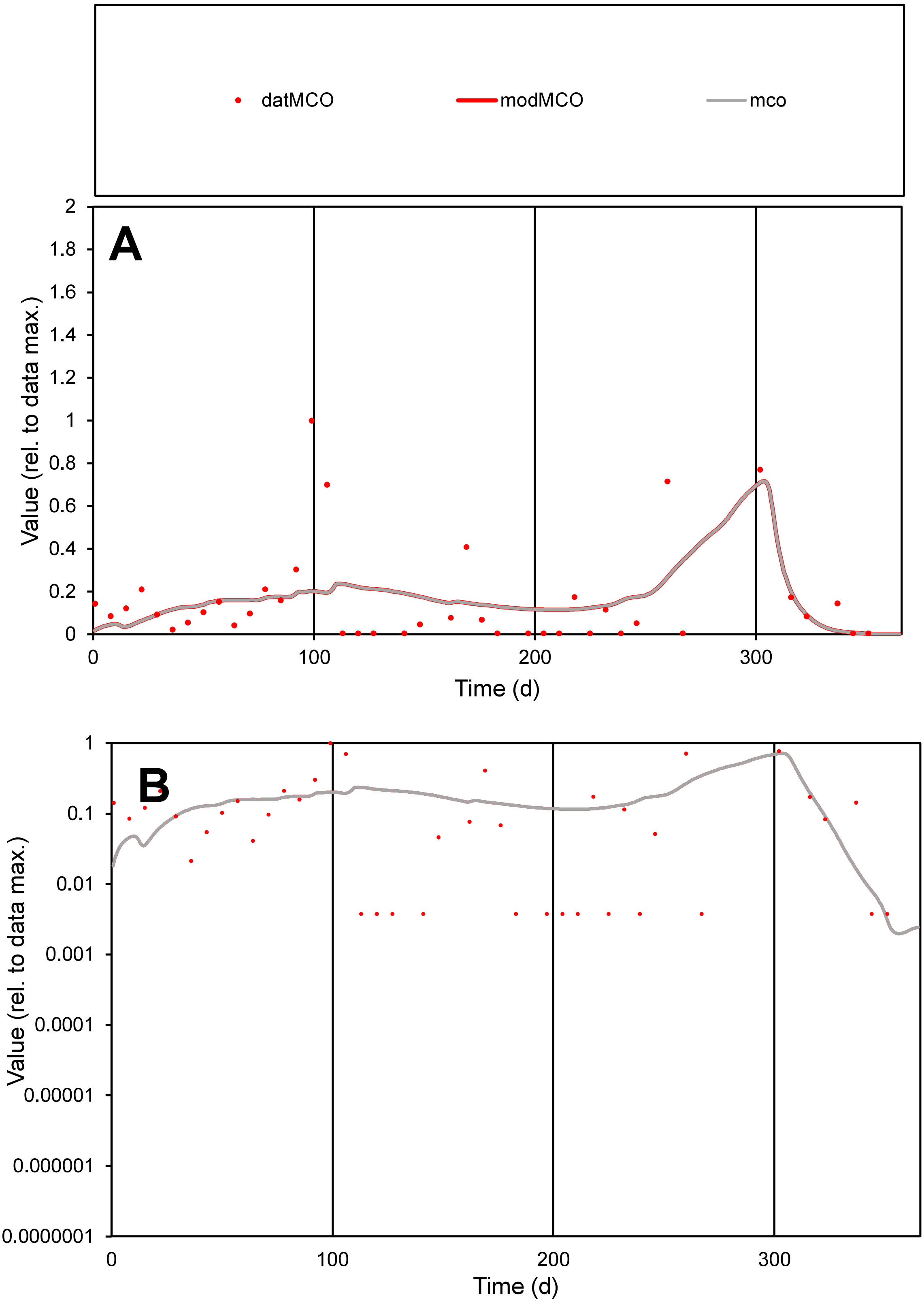

C1

Variables
MCO Provide jtd or Dst
CalibOnly?
2 1 to reproduce of
oNormalize?
2 1=no, 2=yes

ofxn calc*
1.30156
ofxn mod
1.31376

sRun
RunA1
zout
1

Ave
Data
2.5E-06
Model
2.5E-06

data/vMax
1.6E-05

ofxng
319.391

Min d/m
1.25694

ave/min d
41.0663
ave/min m
51.6157

330_Calib_S_A

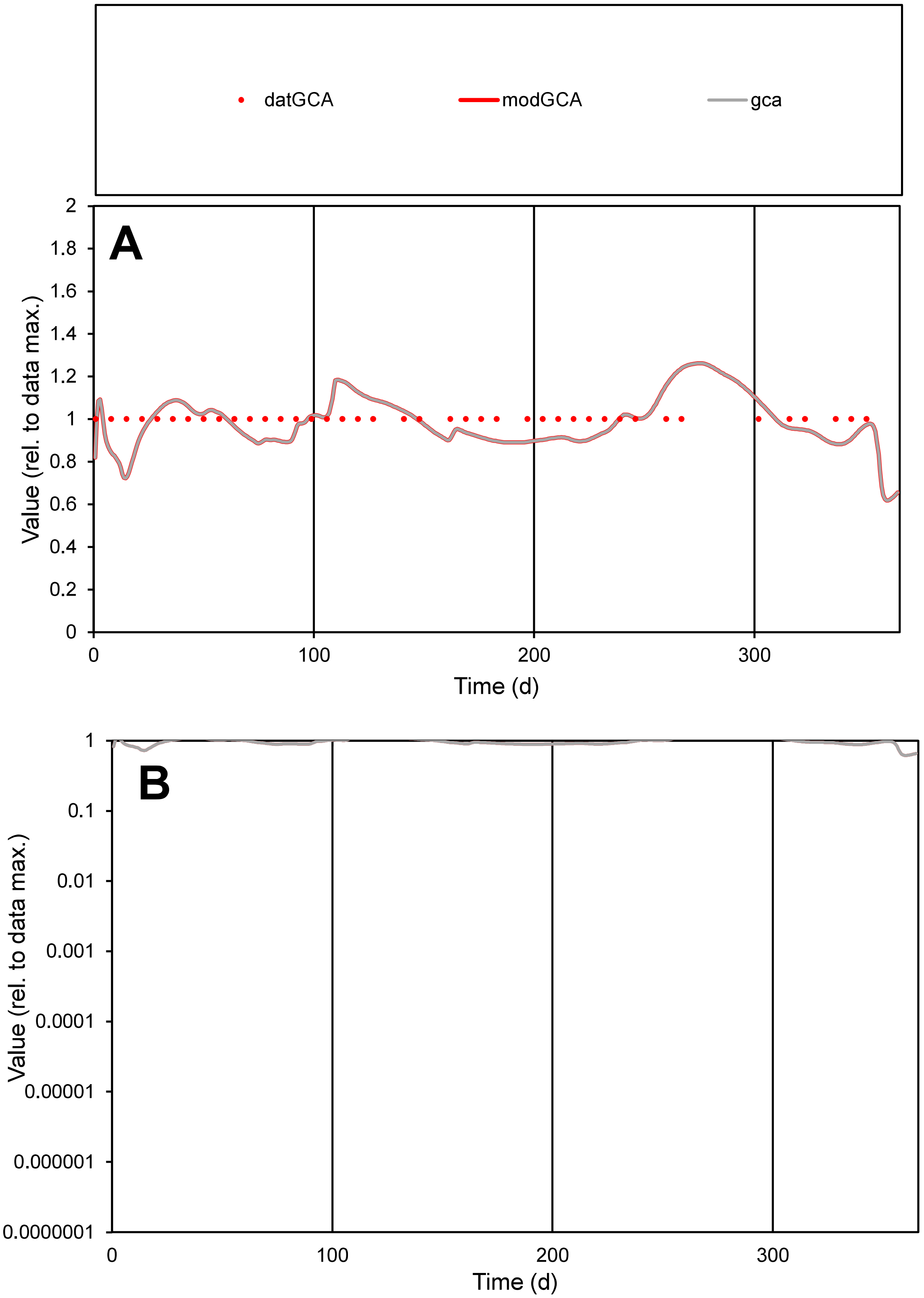

C1

Variables

GCA Provide jtd or Dst

CalibOnly?

2 1 to reproduce of

oNormalize?

2 1=no, 2=yes

ofxn calc*

0.09702

ofxn mod

0.1449

sRun

RunA1

zout

1

Ave

Data

8.4E-06

Model

8.2E-06

data/vMax

8.4E-06

ofxng

319.391

Min d/m

1.29584

ave/min d

1

ave/min m

1.27205

330_Calib_S_A

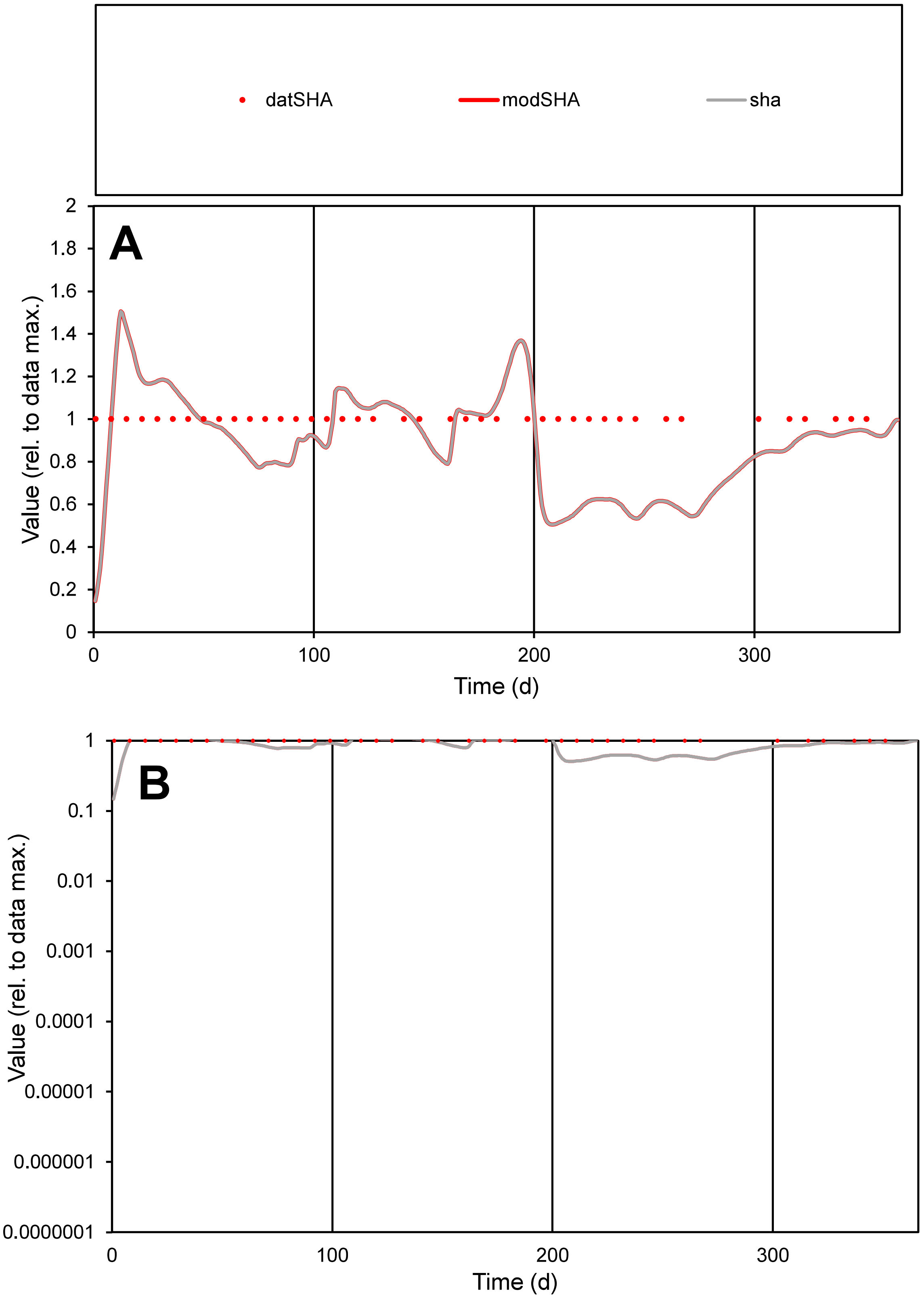

C1

Variables

SHA Provide jtd or Dst

CalibOnly?

2 1 to reproduce of

oNormalize?

2 1=no, 2=yes

ofxn calc*

0.25027

ofxn mod

0.27554

sRun

RunA1

zout

1

Ave

Data

7E-07

Model

6.2E-07

data/vMax

7E-07

ofxng

319.391

Min d/m

2.65171

ave/min d

1

ave/min m

2.37104

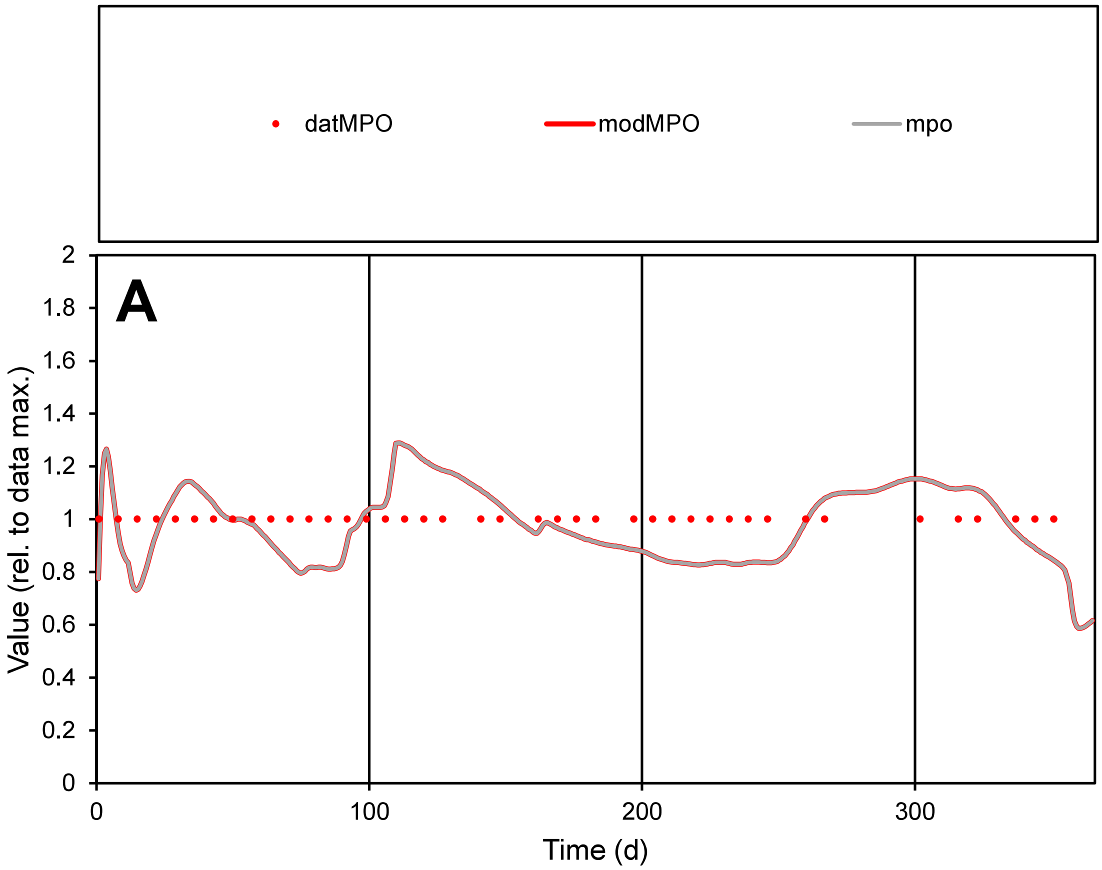

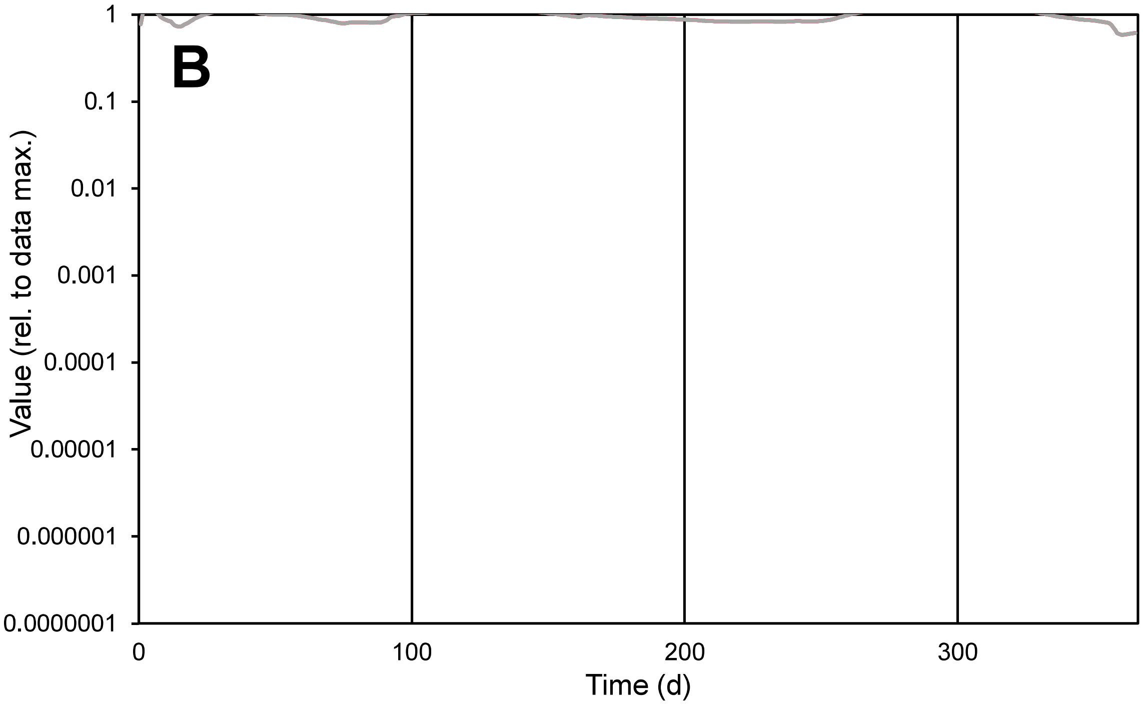

C1

Variables
MPO Provide jtd or Dst
CalibOnly?
2 1 to reproduce of
oNormalize?
2 1=no, 2=yes

ofxn calc*
0.13198
ofxn mod
0.16974

sRun
RunA1
zout
1

Ave
Data
1.2E-05
Model
1.2E-05

data/vMax
1.2E-05

ofxng
319.391

Min d/m
1.30811

ave/min d
1
ave/min m
1.27038

330_Calib_S_A

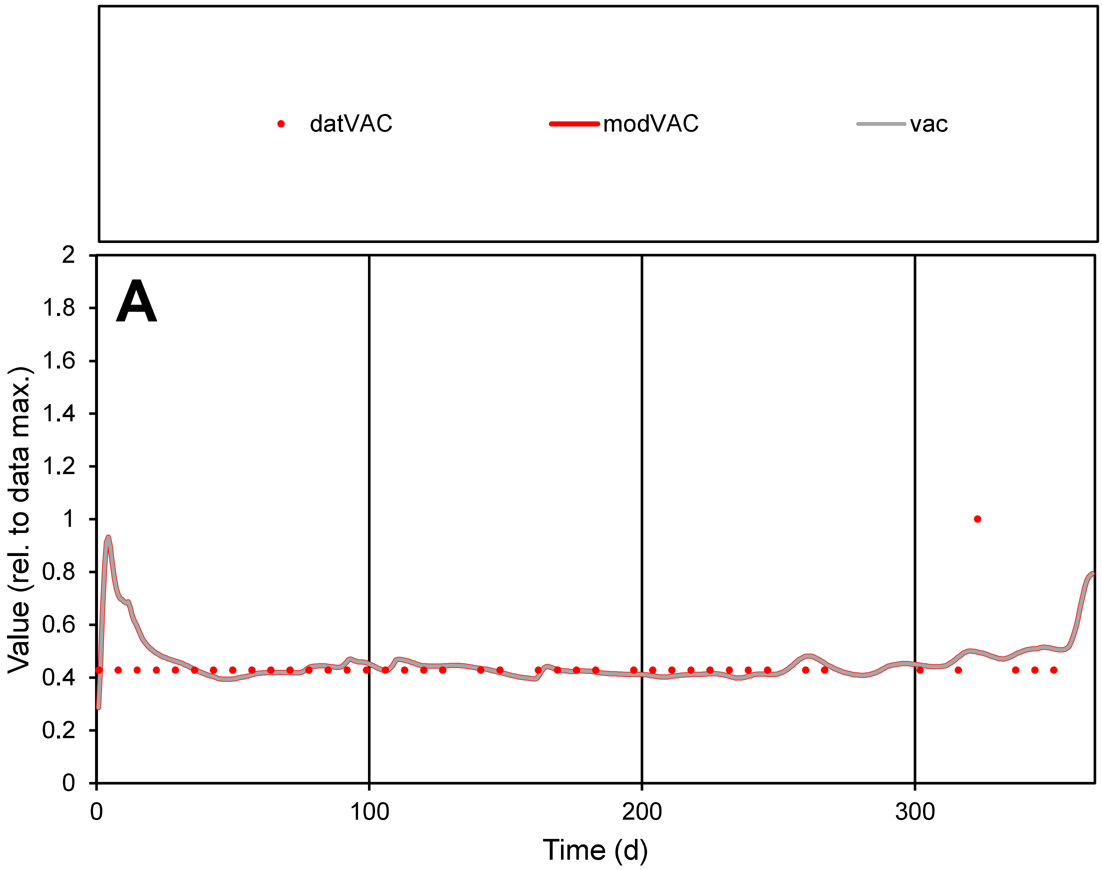

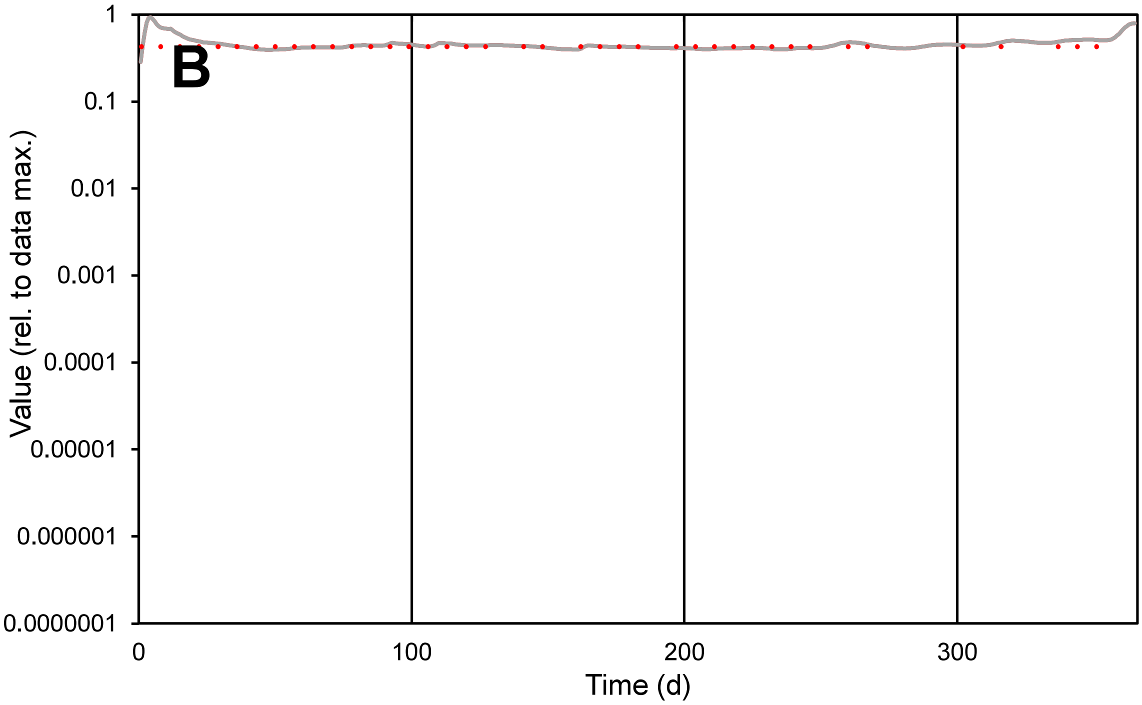

C1

Variables
VAC Provide jtd or Dst
CalibOnly?
2 1 to reproduce of
oNormalize?
2 1=no, 2=yes

ofxn calc*
0.21818
ofxn mod
0.24483

sRun
RunA1
zout
1

Ave
Data
9.8E-06
Model
1E-05

data/vMax
2.2E-05

ofxng
319.391

Min d/m
1.0749

ave/min d
1.03261
ave/min m
1.12598

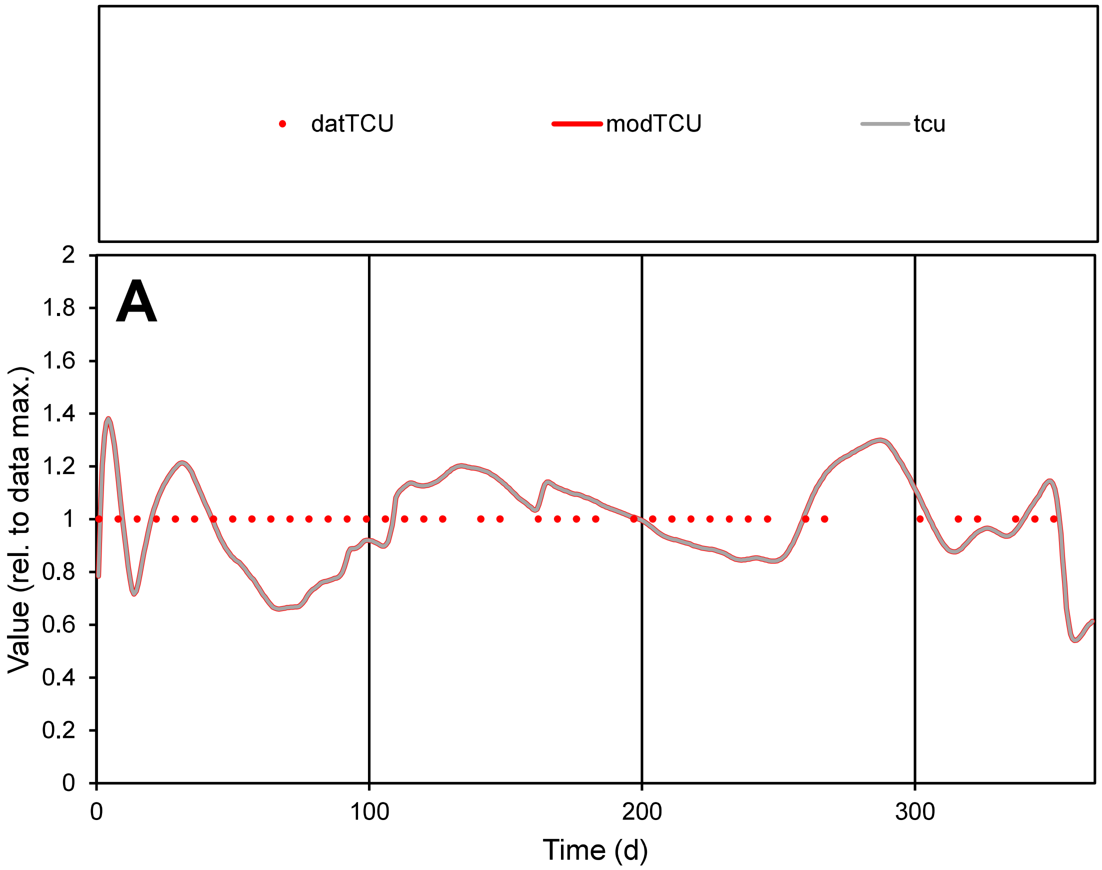

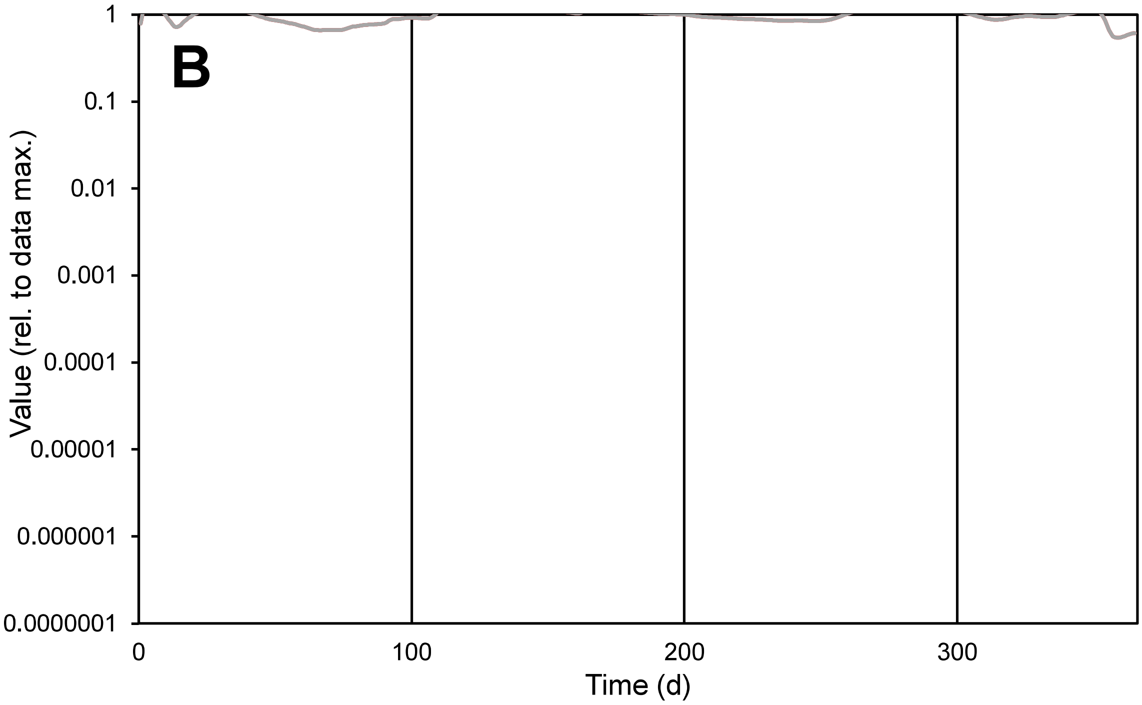

C1

Variables
TCU Provide jtd or Dst
CalibOnly?
2 1 to reproduce of
oNormalize?
2 1=no, 2=yes

ofxn calc*
0.14564
ofxn mod
0.18068

sRun
RunA1
zout
1

Ave
Data
9.8E-06
Model
9.5E-06

data/vMax
9.8E-06

ofxng
319.391

Min d/m
1.49824

ave/min d
1
ave/min m
1.464

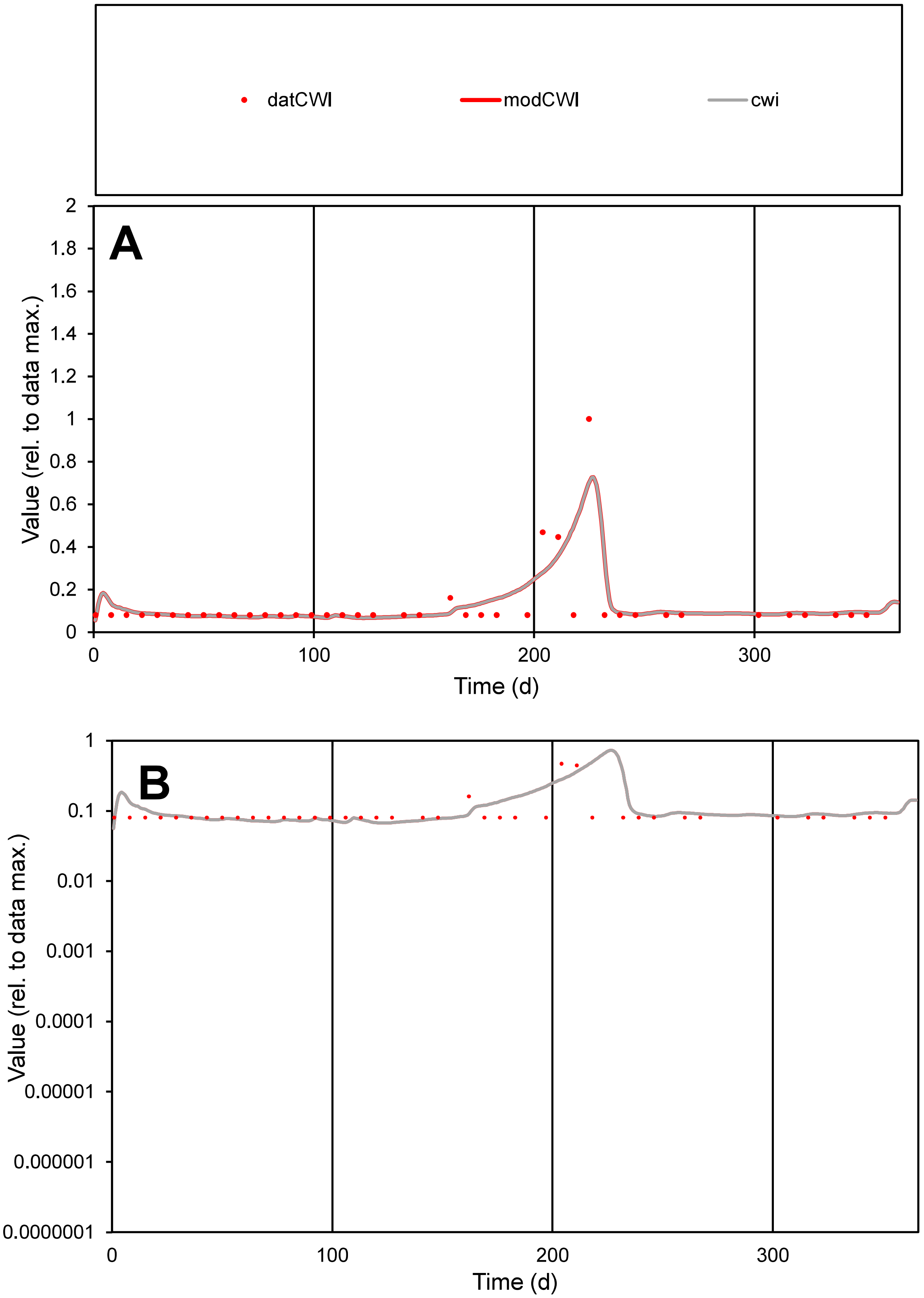

C1

Variables
CWI Provide jtd or Dst
CalibOnly?
2 1 to reproduce of
oNormalize?
2 1=no, 2=yes

ofxn calc*
0.71527
ofxn mod
0.75661

sRun
RunA1
zout
1

Ave
Data
2E-05
Model
2.2E-05

data/vMax
0.00017

ofxng
319.391

Min d/m
1.19086

ave/min d
1.53139
ave/min m
1.92862

330_Calib_S_A

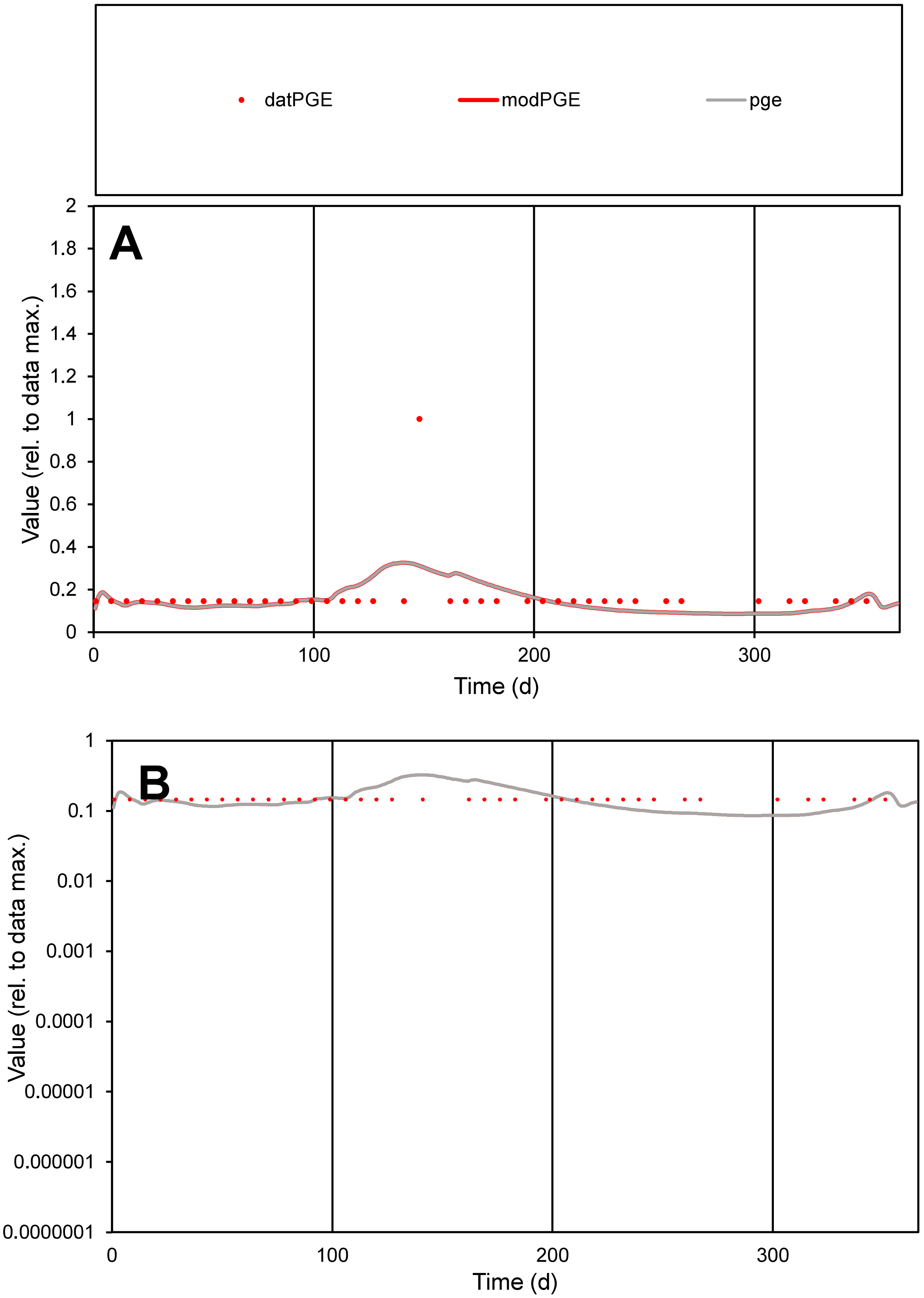

C1

Variables
PGE Provide jtd or Dst
CalibOnly?
2 1 to reproduce of
oNormalize?
2 1=no, 2=yes

ofxn calc*
0.73038
ofxn mod
0.74002

sRun
RunA1
zout
1

Ave
Data
6.7E-06
Model
6.2E-06

data/vMax
4E-05

ofxng
319.391

Min d/m
1.67759

ave/min d
1.1433
ave/min m
1.78135

330_Calib_S_A

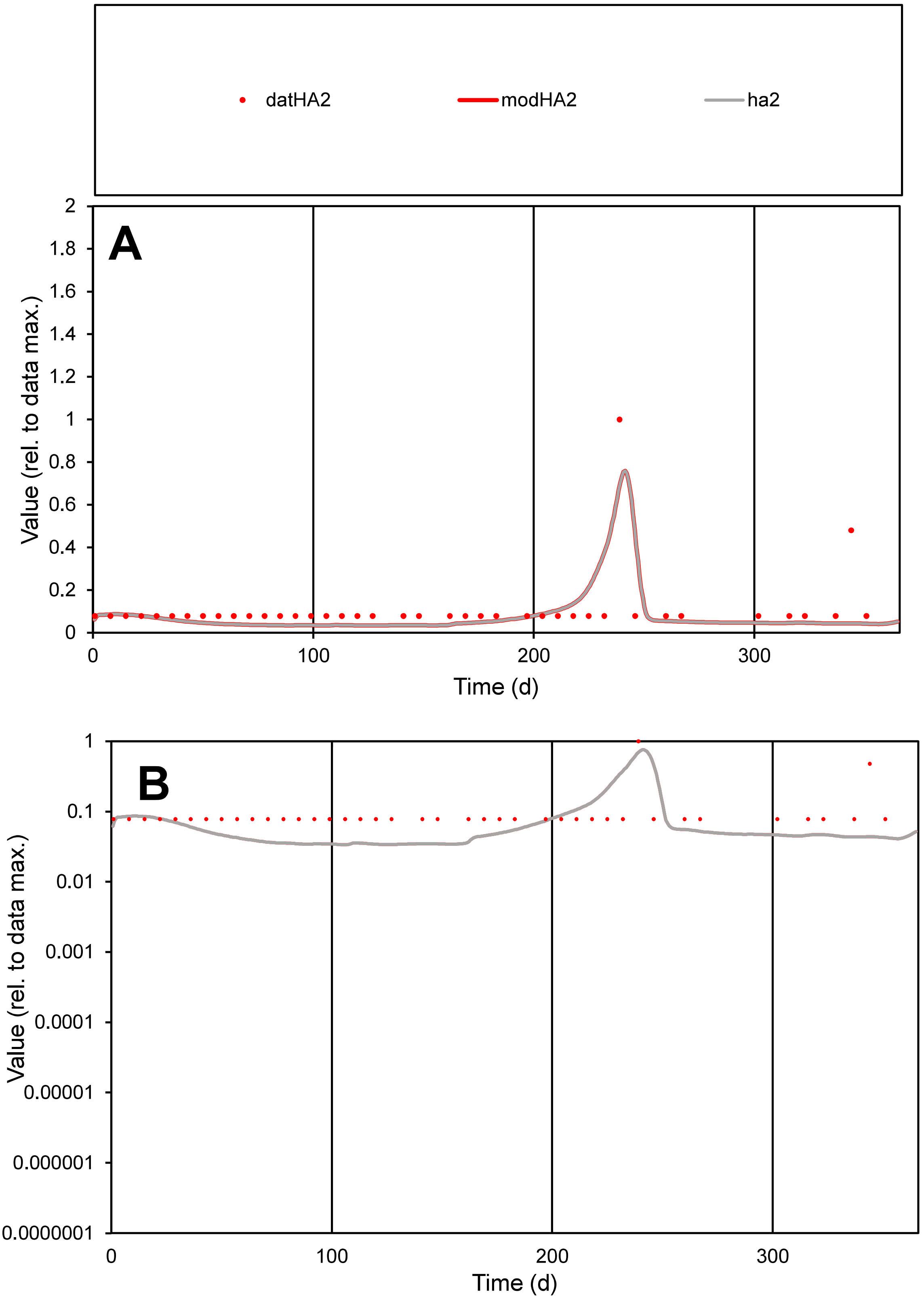

C1

Variables
HA2 Provide jtd or Dst
CalibOnly?
2 1 to reproduce of
oNormalize?
2 1=no, 2=yes

ofxn calc*
0.90256
ofxn mod
0.95517

sRun
RunA1
zout
1

Ave
Data
3.7E-06
Model
2.9E-06

data/vMax
3.4E-05

ofxng
319.391

Min d/m
2.28943

ave/min d
1.41401
ave/min m
2.53056

330_Calib_S_A

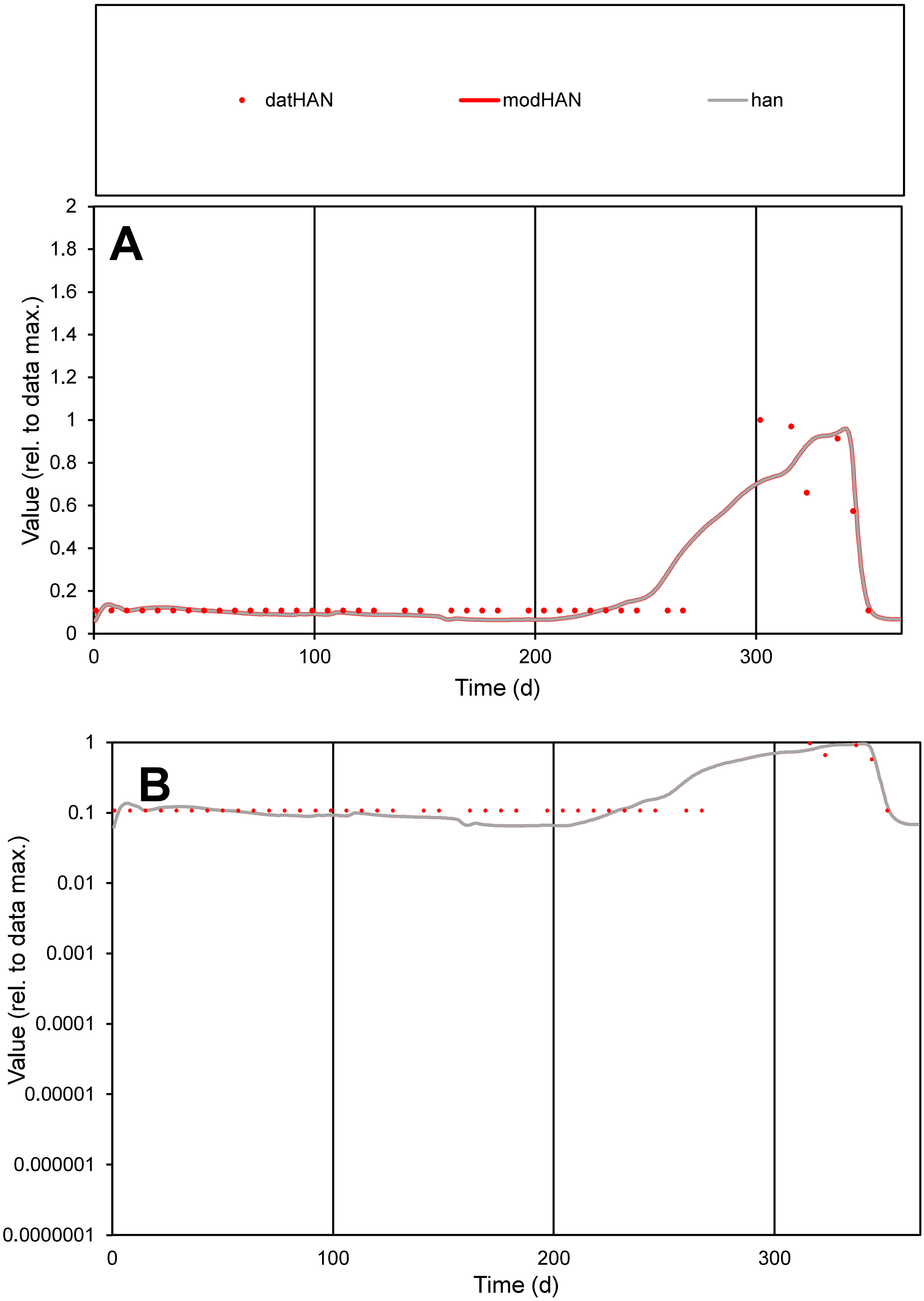

C1

Variables
HAN Provide jtd or Dst
CalibOnly?
2 1 to reproduce of
oNormalize?
2 1=no, 2=yes

ofxn calc*
0.4419
ofxn mod
0.4678

sRun
RunA1
zout
1

Ave
Data
9.3E-06
Model
9.2E-06

data/vMax
4.8E-05

ofxng
319.391

Min d/m
1.65664

ave/min d
1.81096
ave/min m
2.9688

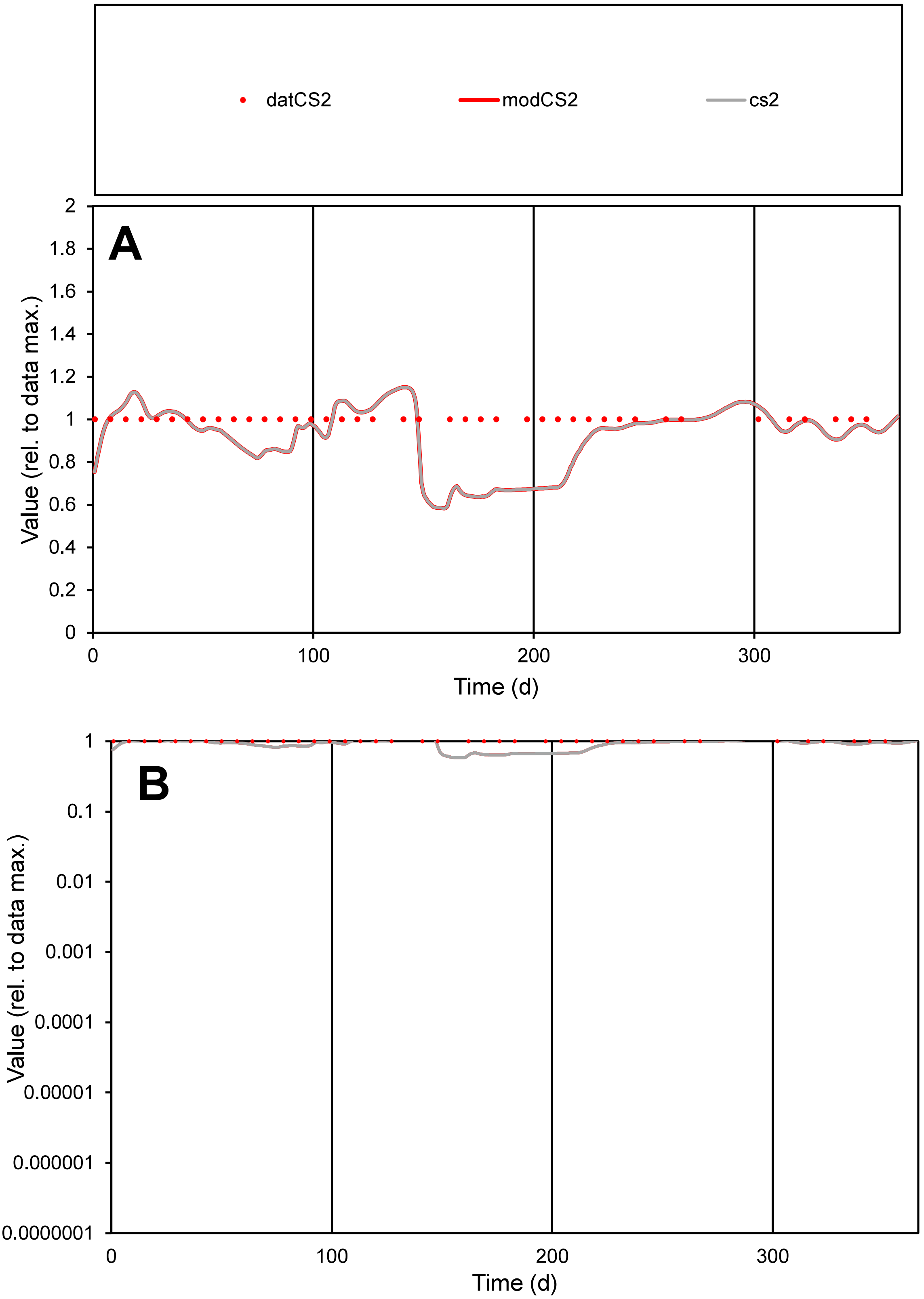

C1

Variables

CS2 Provide jtd or Dst

CalibOnly?

2 1 to reproduce of

oNormalize?

2 1=no, 2=yes

ofxn calc*

0.15969

ofxn mod

0.19283

sRun

RunA1

zout

1

Ave

Data

7.6E-06

Model

7E-06

data/vMax

7.6E-06

ofxng

319.391

Min d/m

1.56765

ave/min d

1

ave/min m

1.43791

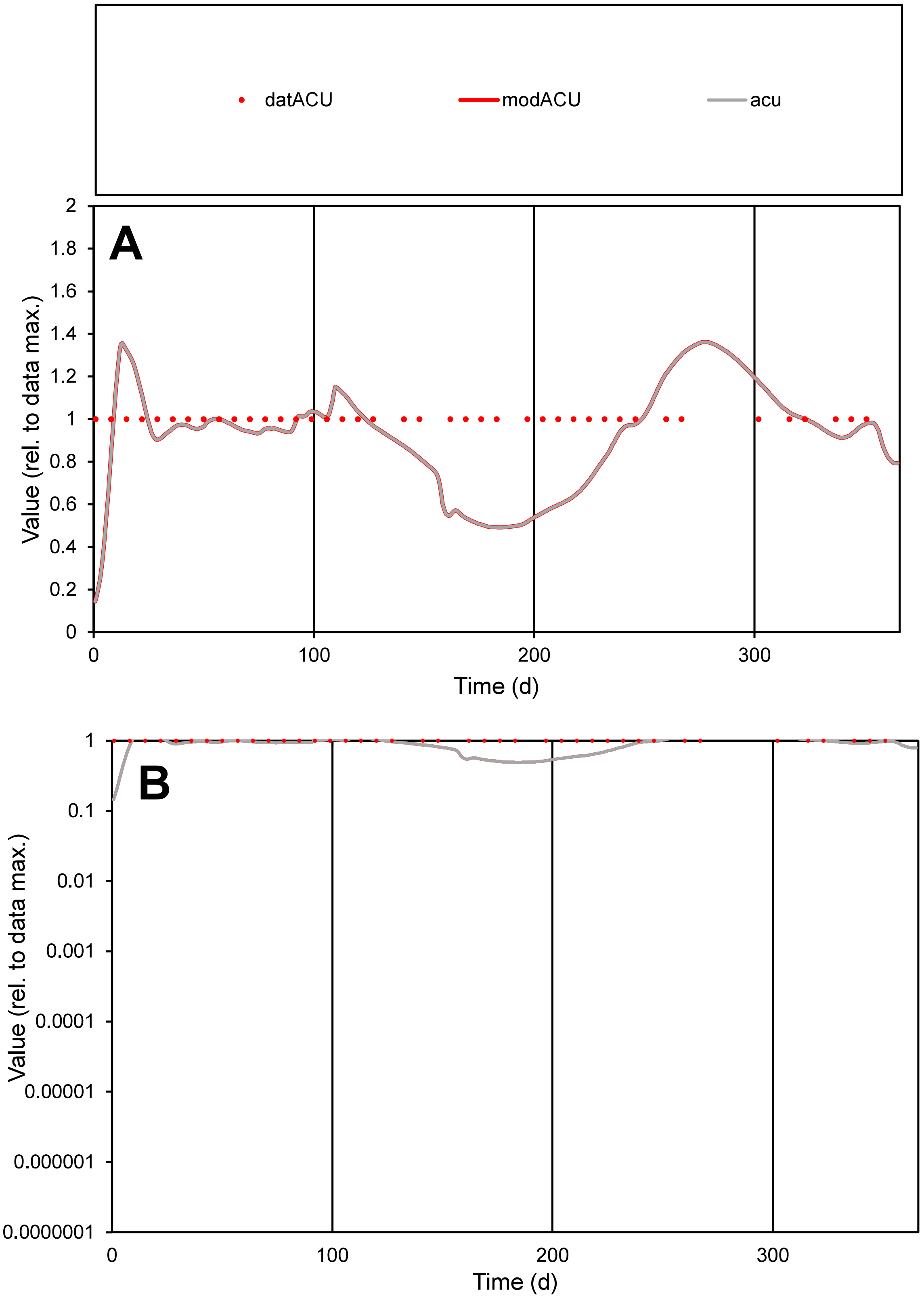

C1

Variables
ACU Provide jtd or Dst
CalibOnly?
2 1 to reproduce of
oNormalize?
2 1=no, 2=yes

ofxn calc*
0.24999
ofxn mod
0.27392

sRun
RunA1
zout
1

Ave
Data
1.1E-05
Model
9.9E-06

data/vMax
1.1E-05

ofxng
319.391

Min d/m
3.27246

ave/min d
1
ave/min m
2.92962

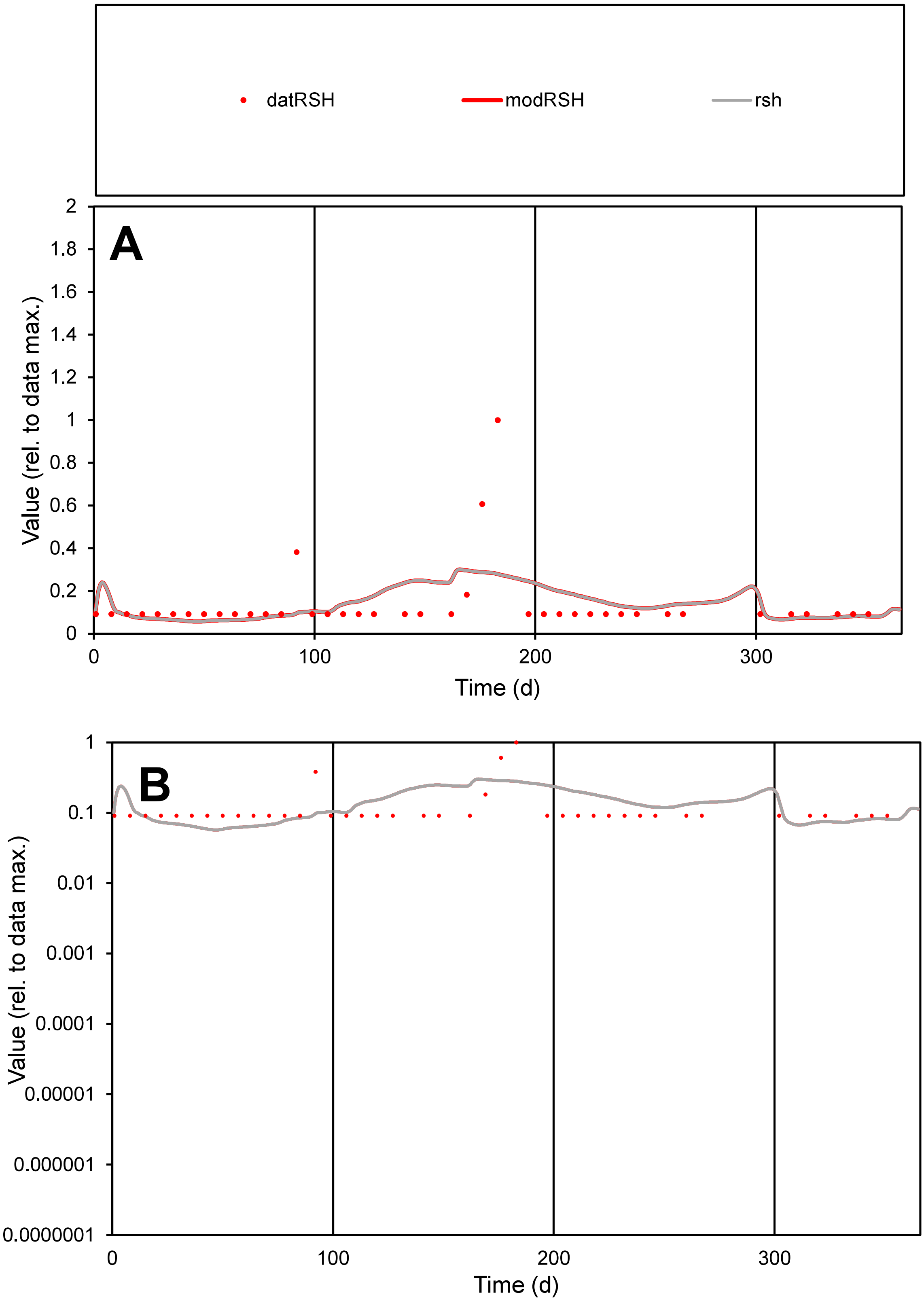

C1

Variables
RSH Provide jtd or Dst
CalibOnly?
2 1 to reproduce of
oNormalize?
2 1=no, 2=yes

ofxn calc*
1.09312
ofxn mod
1.10588

sRun
RunA1
zout
1

Ave
Data
2.7E-05
Model
2.7E-05

data/vMax
0.0002

ofxng
319.391

Min d/m
1.57218

ave/min d
1.48557
ave/min m
2.35486

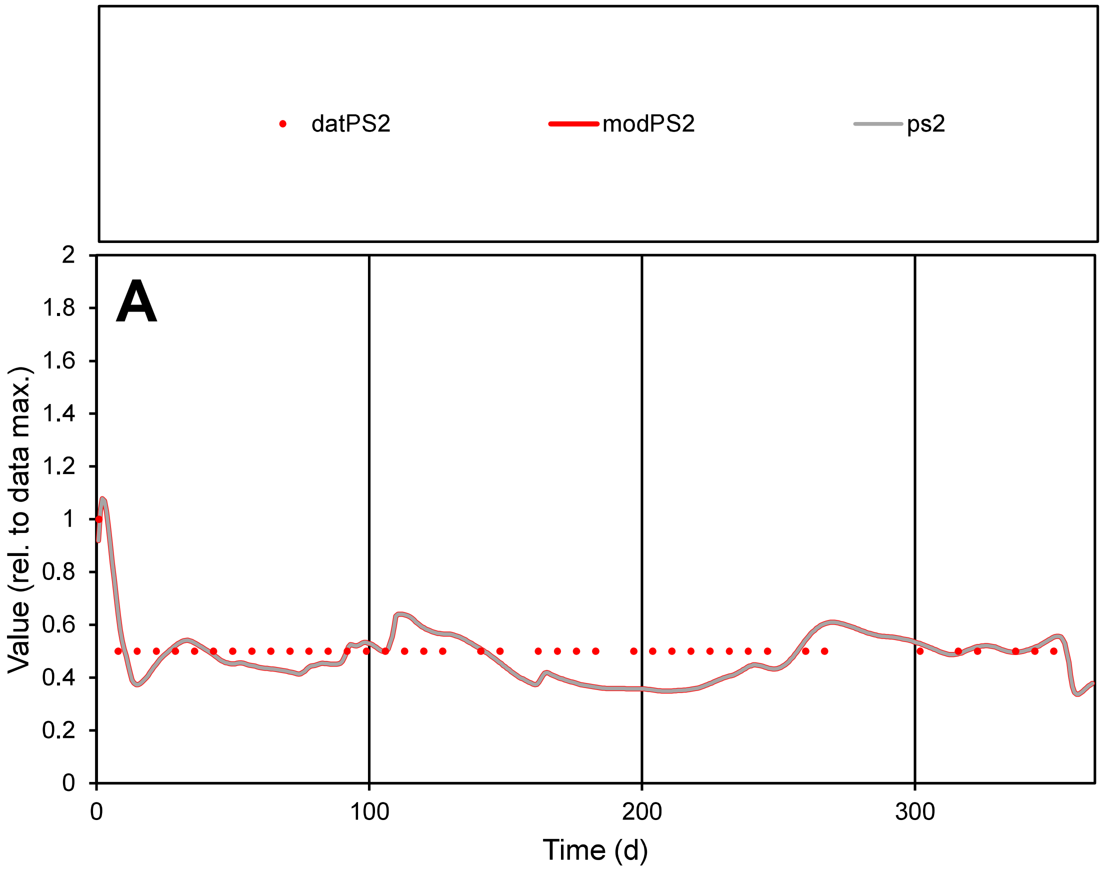

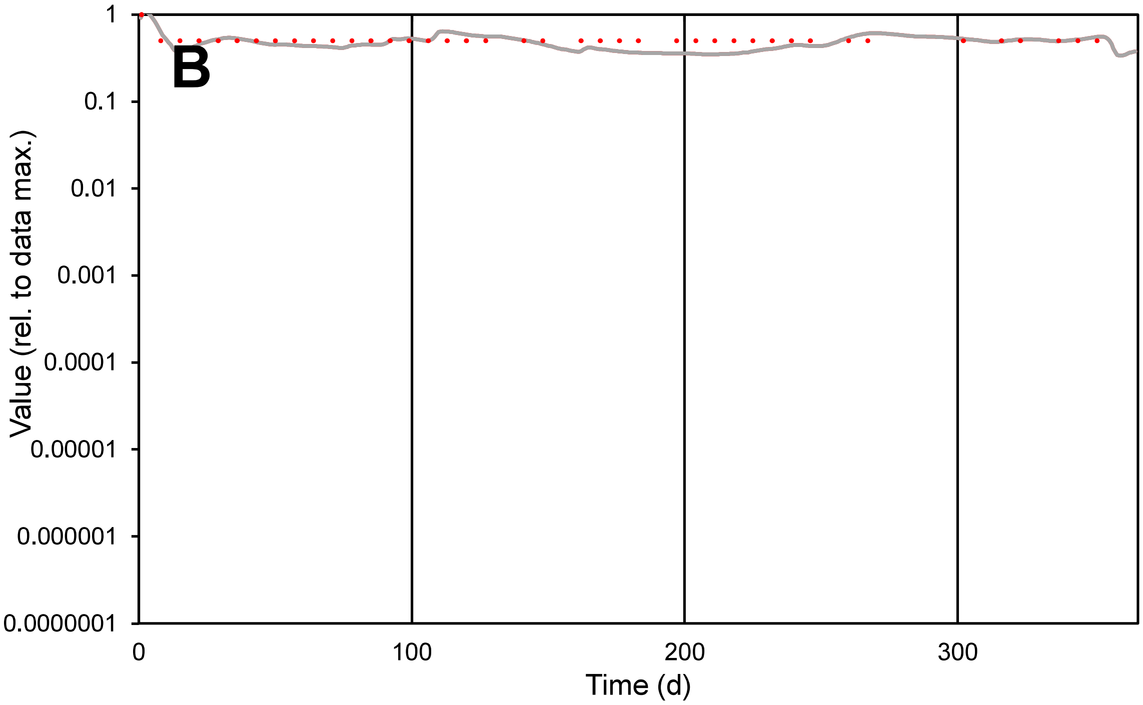

C1

Variables
PS2 Provide jtd or Dst
CalibOnly?
2 1 to reproduce of
oNormalize?
2 1=no, 2=yes

ofxn calc*
0.15631
ofxn mod
0.18797

sRun
RunA1
zout
1

Ave
Data
2E-05
Model
1.8E-05

data/vMax
3.8E-05

ofxng
319.391

Min d/m
1.4259

ave/min d
1.02439
ave/min m
1.3784

330_Calib_S_A

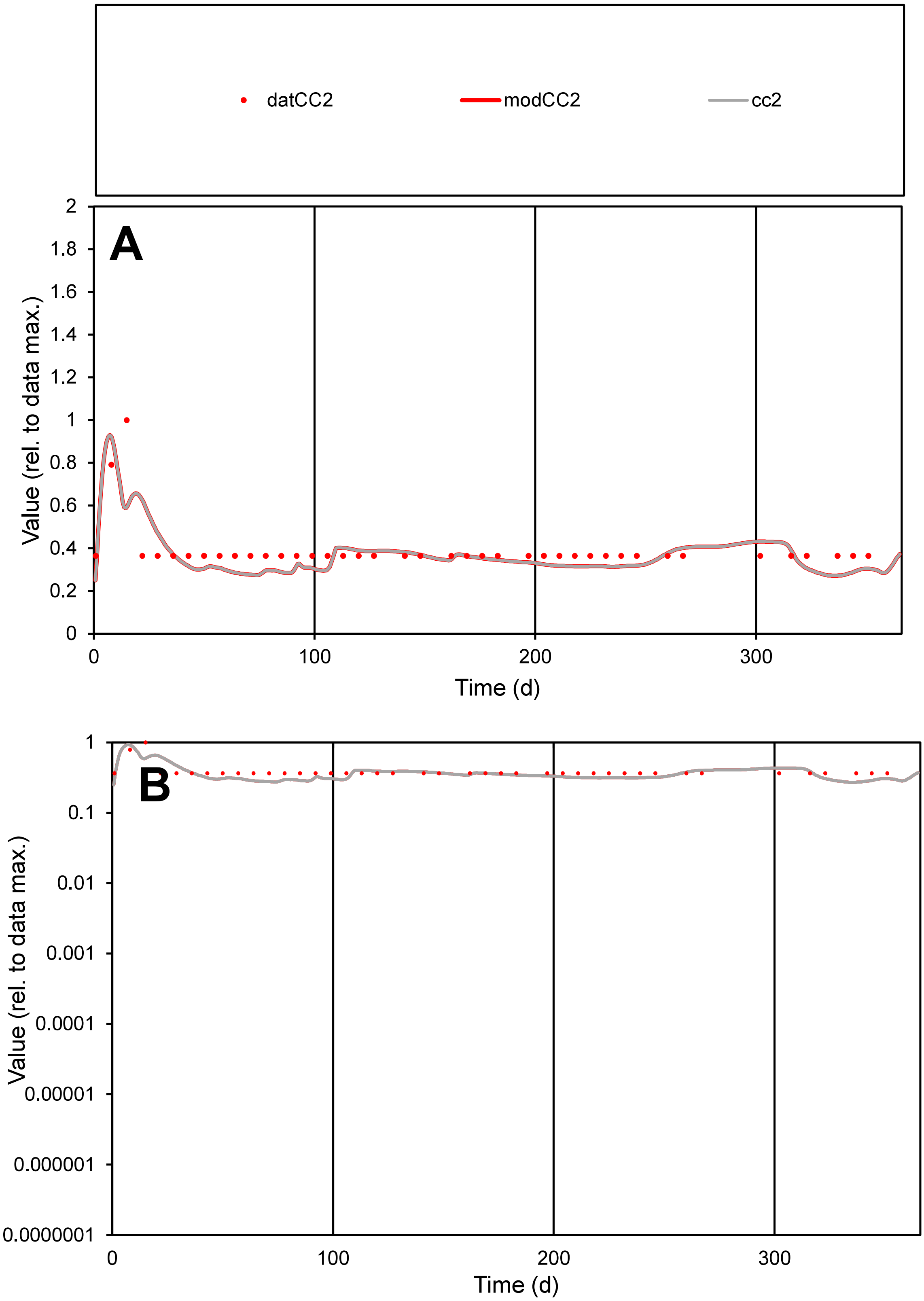

C1

Variables

CC2 Provide jtd or Dst

CalibOnly?

2 1 to reproduce of

oNormalize?

2 1=no, 2=yes

ofxn calc*

0.21532

ofxn mod

0.24311

sRun

RunA1

zout

1

Ave

Data

6.3E-06

Model

5.9E-06

data/vMax

1.6E-05

ofxng

319.391

Min d/m

1.3353

ave/min d

1.07113

ave/min m

1.33558

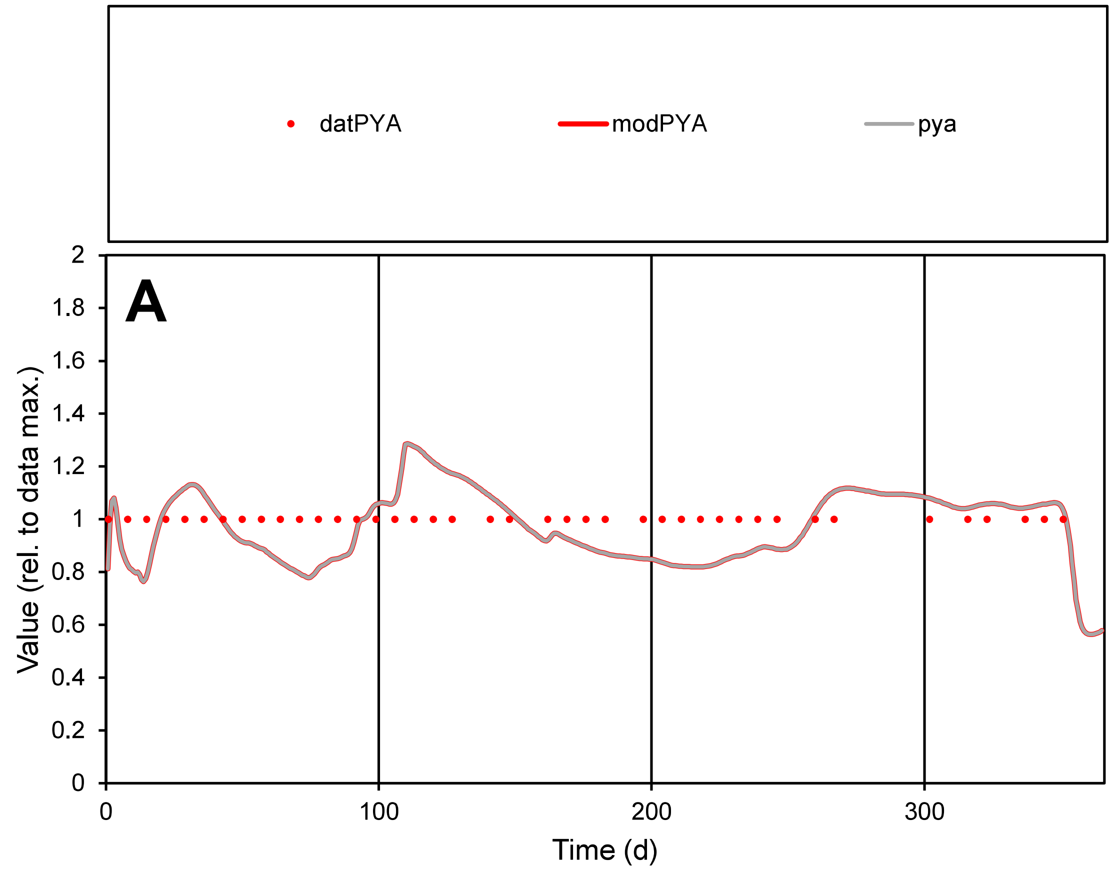

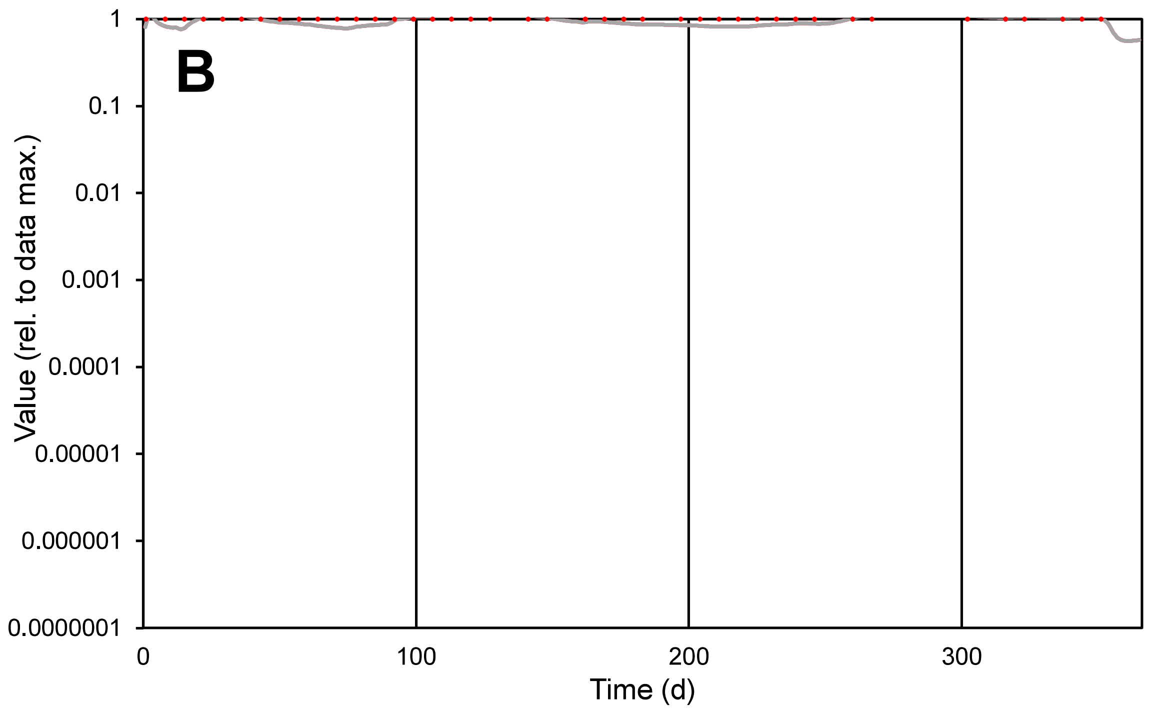

C1

Variables
PYA Provide jtd or Dst
CalibOnly?
2 1 to reproduce of
oNormalize?
2 1=no, 2=yes

ofxn calc*
0.1261
ofxn mod
0.16497

sRun
RunA1
zout
1

Ave
Data
1.8E-06
Model
1.8E-06

data/vMax
1.8E-06

ofxng
319.391

Min d/m
1.26713

ave/min d
1
ave/min m
1.2291

330_Calib_S_A

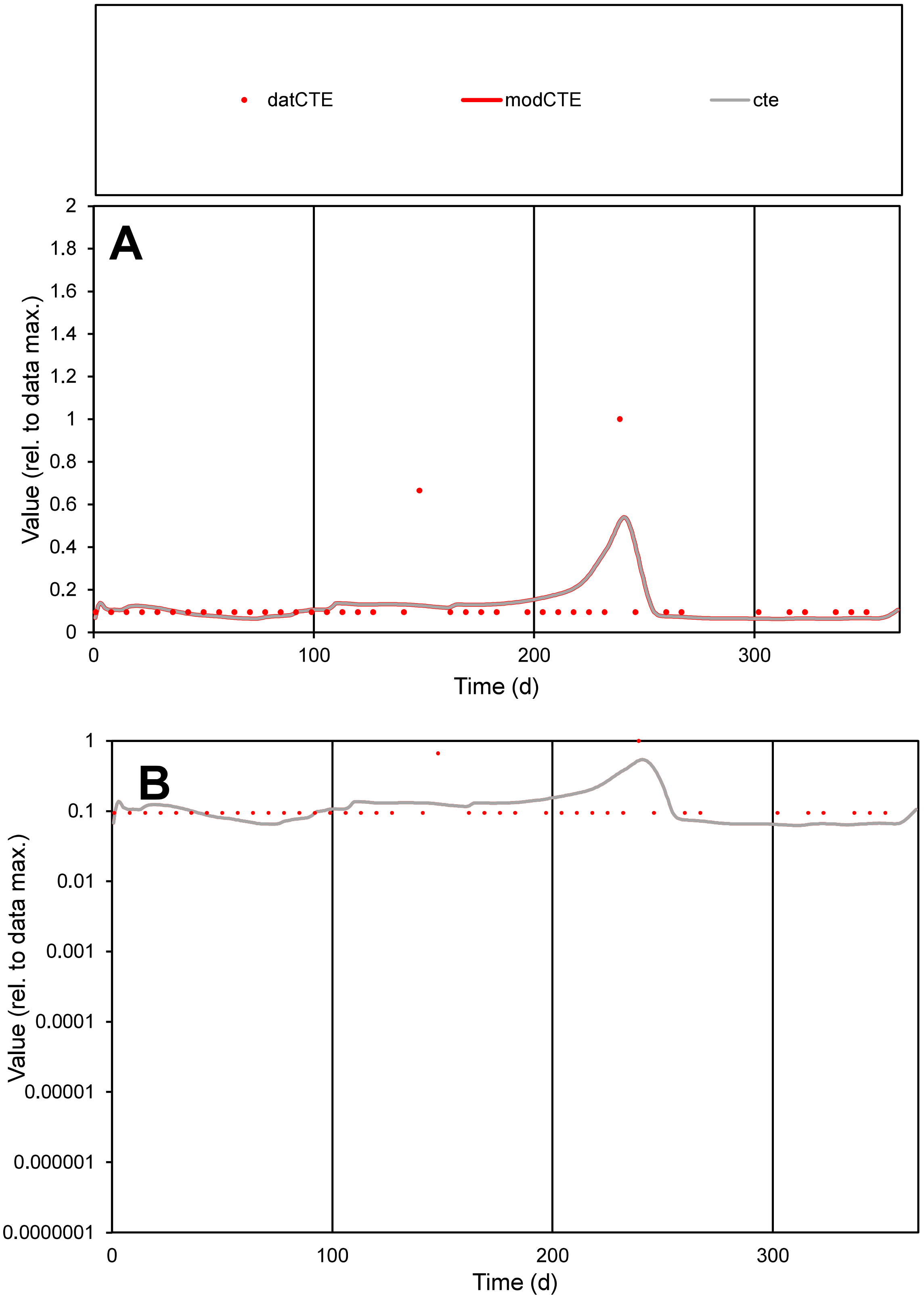

C1

Variables
CTE Provide jtd or Dst
CalibOnly?
2 1 to reproduce of
oNormalize?
2 1=no, 2=yes

ofxn calc*
1.00704
ofxn mod
1.03189

sRun
RunA1
zout
1

Ave
Data
8.5E-06
Model
8.5E-06

data/vMax
6.5E-05

ofxng
319.391

Min d/m
1.4733

ave/min d
1.3809
ave/min m
2.05423

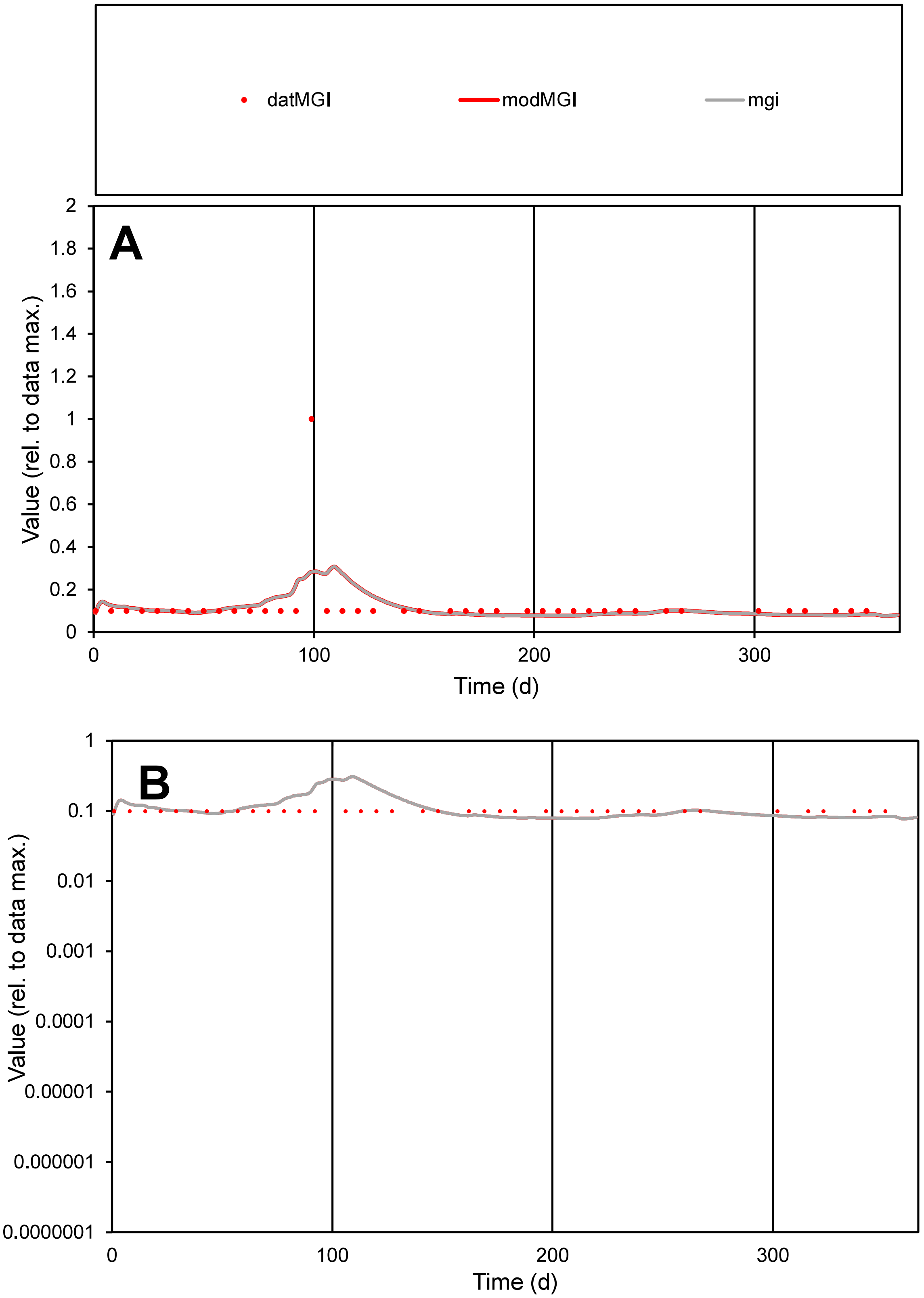

C1

Variables
MGI Provide jtd or Dst
CalibOnly?
2 1 to reproduce of
oNormalize?
2 1=no, 2=yes

ofxn calc*
1.01668
ofxn mod
1.02758

sRun
RunA1
zout
1

Ave
Data
1.3E-05
Model
1.2E-05

data/vMax
0.0001

ofxng
319.391

Min d/m
1.26828

ave/min d
1.22211
ave/min m
1.50969

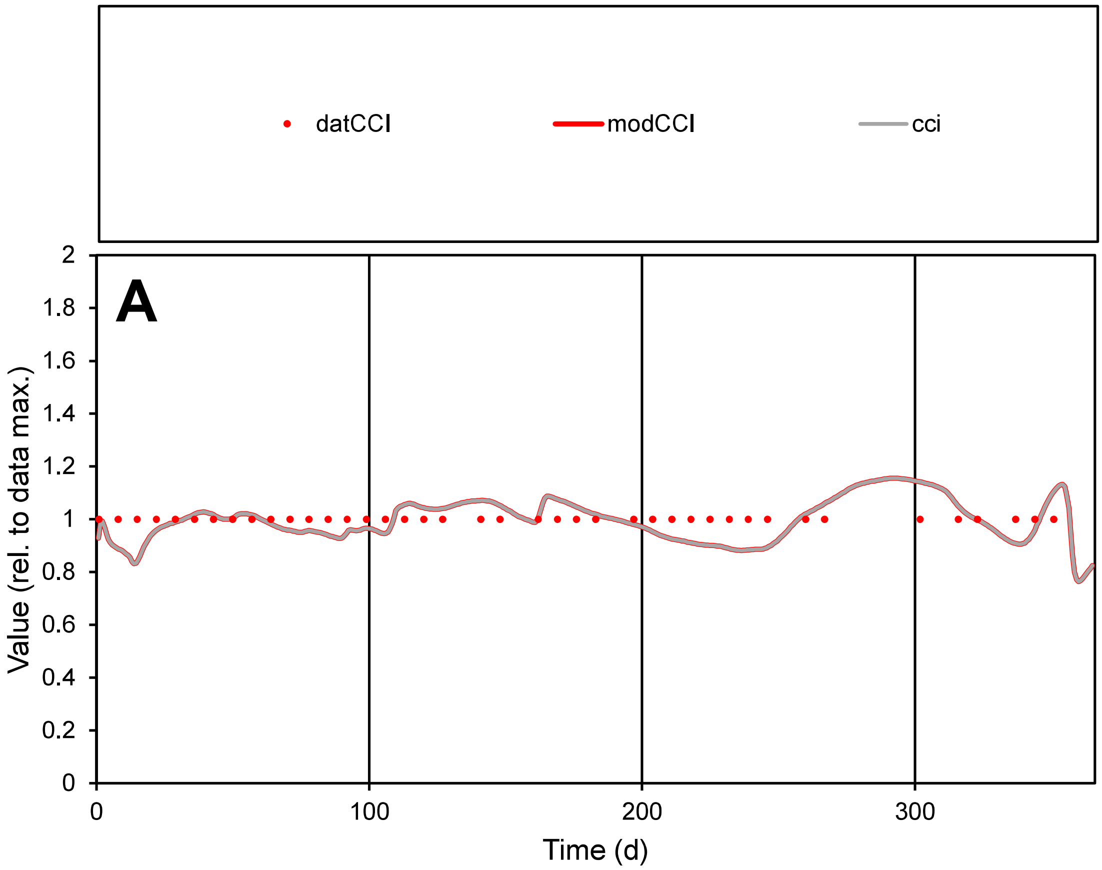

C1

Variables
CCI Provide jtd or Dst
CalibOnly?
2 1 to reproduce of
oNormalize?
2 1=no, 2=yes

ofxn calc*
0.0665
ofxn mod
0.12672

sRun
RunA1
zout
1

Ave
Data
1.1E-05
Model
1.1E-05

data/vMax
1.1E-05

ofxng
319.391

Min d/m
1.13776

ave/min d
1
ave/min m
1.12483

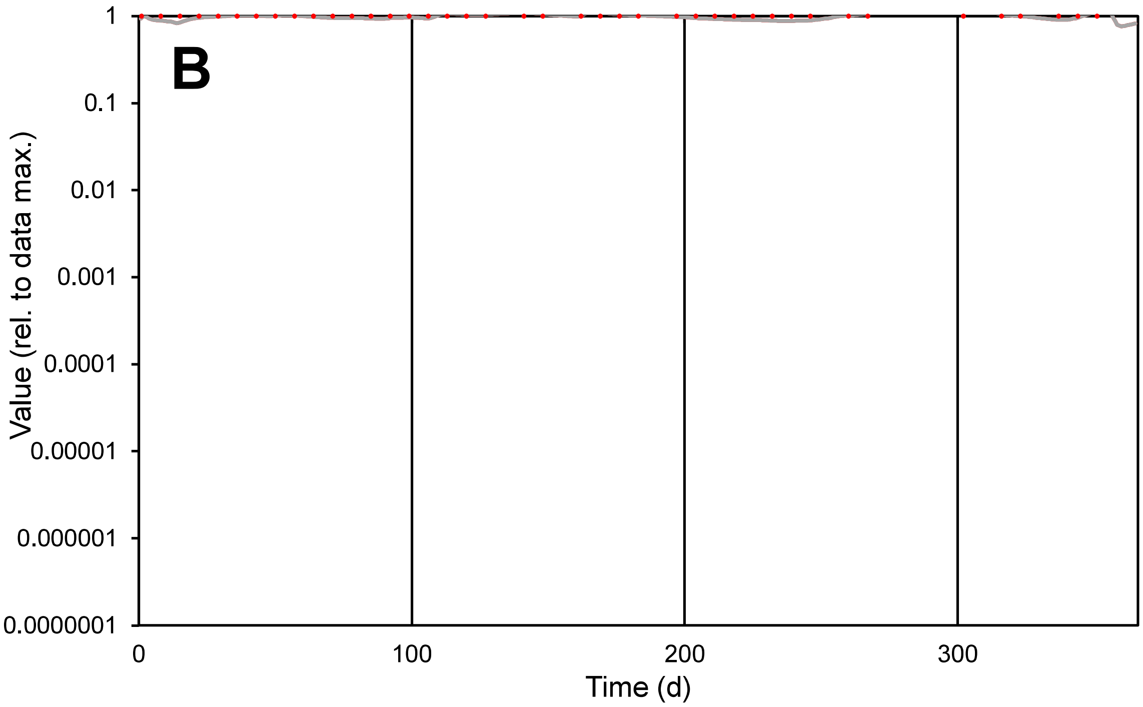

330_Calib_S_A

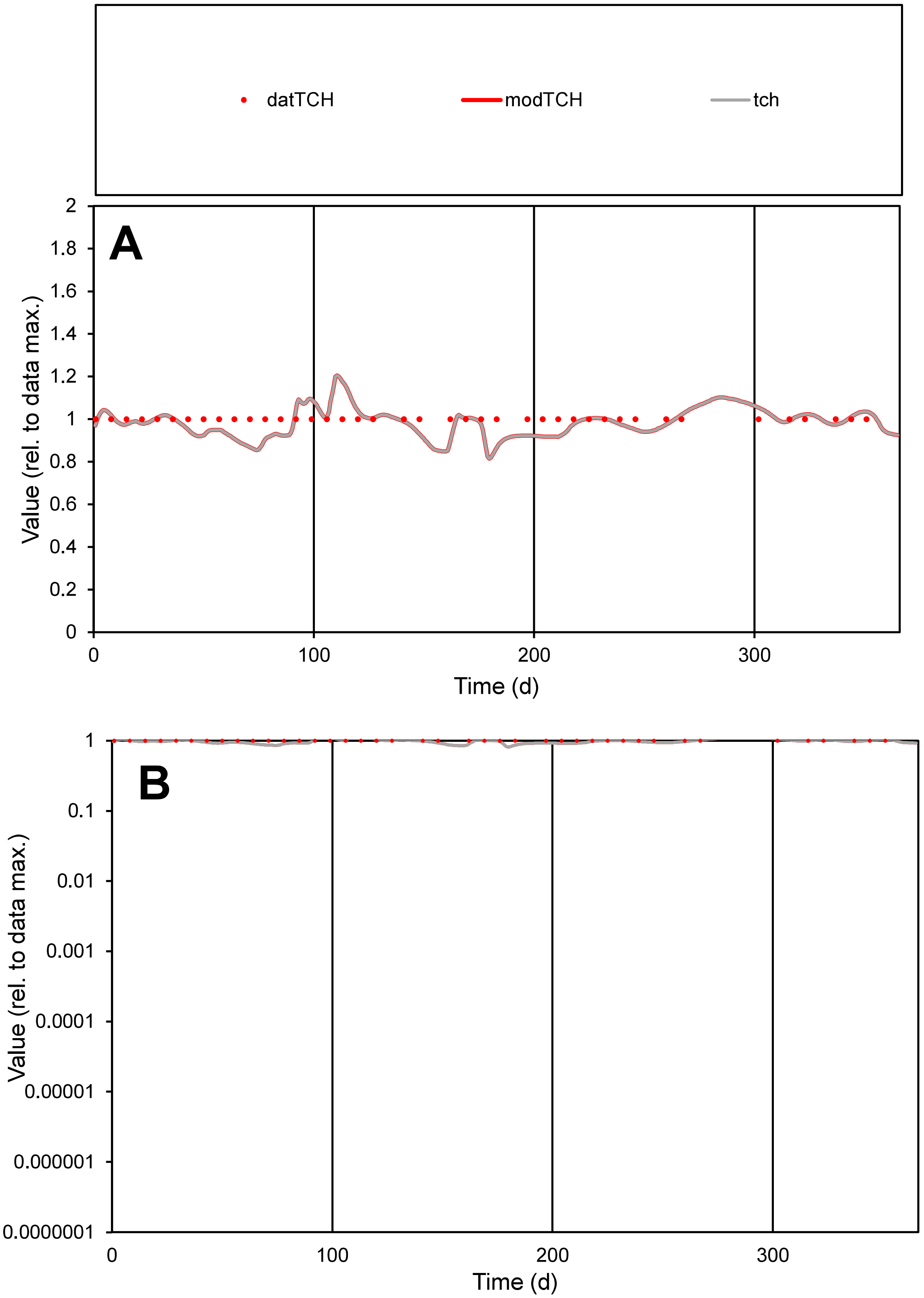

C1

Variables

TCH Provide jtd or Dst

CalibOnly?

2 1 to reproduce of

oNormalize?

2 1=no, 2=yes

ofxn calc*

0.06207

ofxn mod

0.12437

sRun

RunA1

zout

1

Ave

Data

1.7E-06

Model

1.7E-06

data/vMax

1.7E-06

ofxng

319.391

Min d/m

1.1584

ave/min d

1

ave/min m

1.13826

330_Calib_S_A

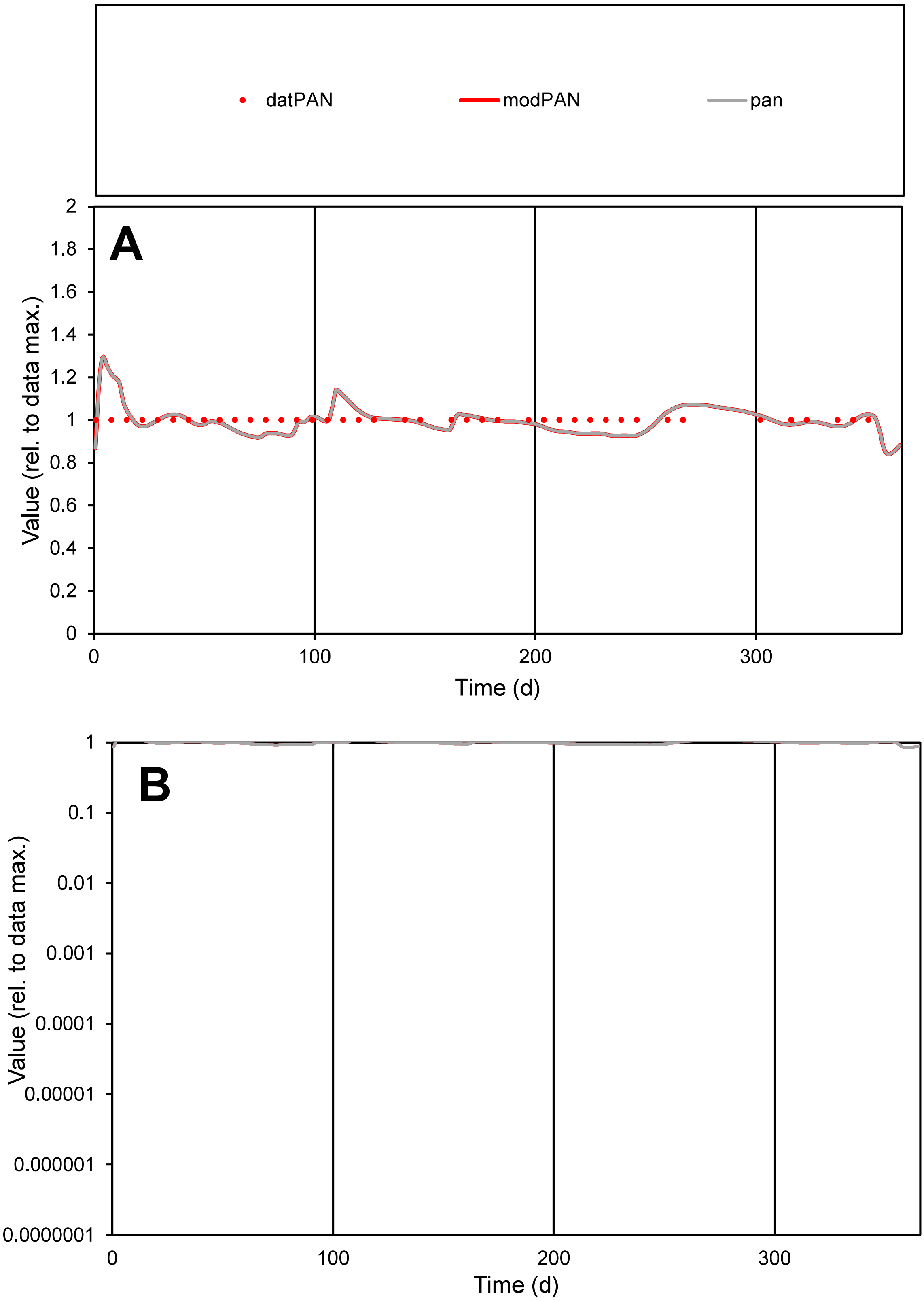

C1

Variables

PAN Provide jtd or Dst

CalibOnly?

2 1 to reproduce of

oNormalize?

2 1=no, 2=yes

ofxn calc*

0.05194

ofxn mod

0.12046

sRun

RunA1

zout

1

Ave

Data

9.1E-06

Model

9.1E-06

data/vMax

9.1E-06

ofxng

319.391

Min d/m

1.08326

ave/min d

1

ave/min m

1.07783

330_Calib_S_A

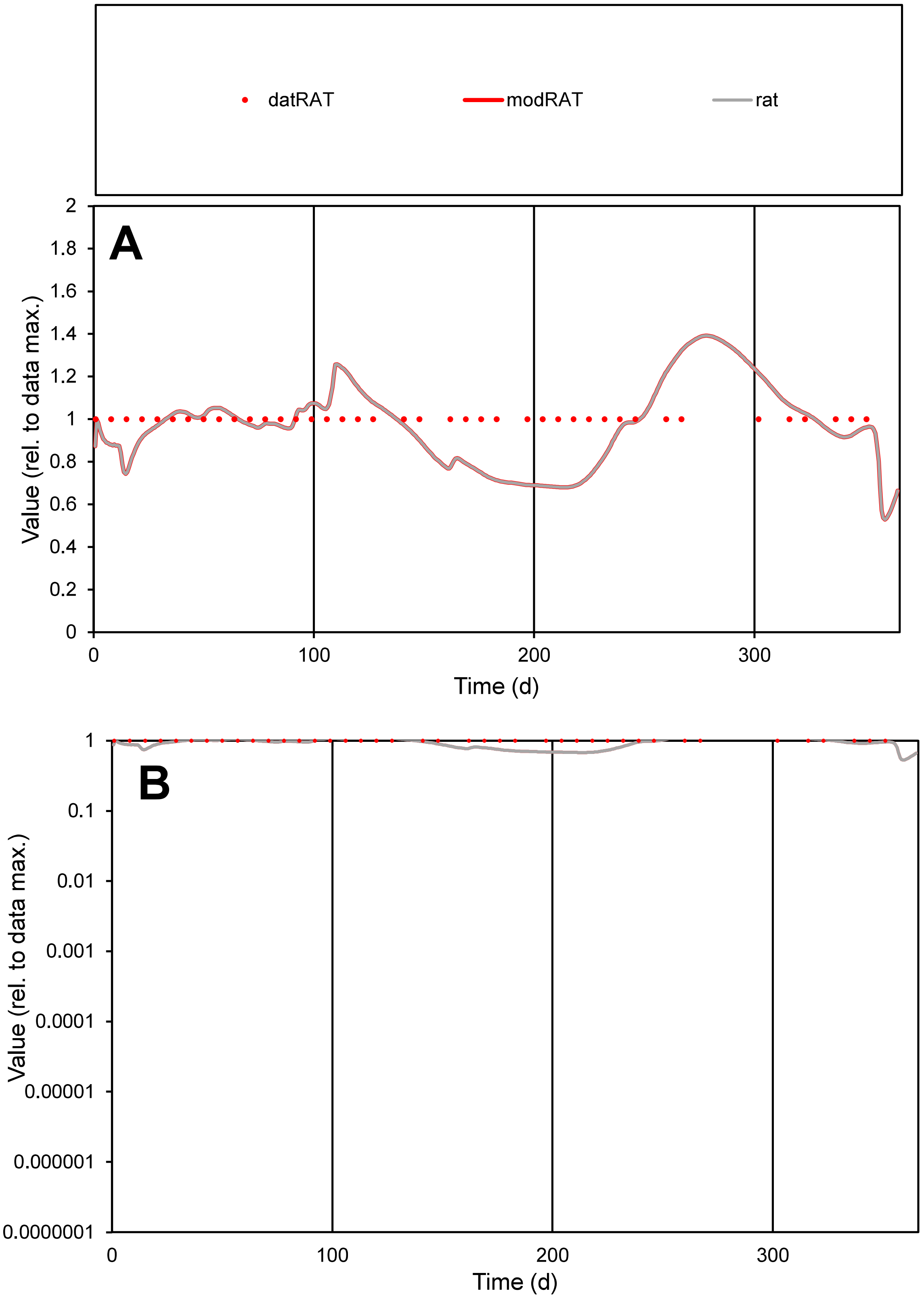

C1

Variables
RAT Provide jtd or Dst
CalibOnly?
2 1 to reproduce of
oNormalize?
2 1=no, 2=yes

ofxn calc*
0.16524
ofxn mod
0.1963

sRun
RunA1
zout
1

Ave
Data
1.1E-05
Model
1.1E-05

data/vMax
1.1E-05

ofxng
319.391

Min d/m
1.46956

ave/min d
1
ave/min m
1.40381

330_Calib_S_A

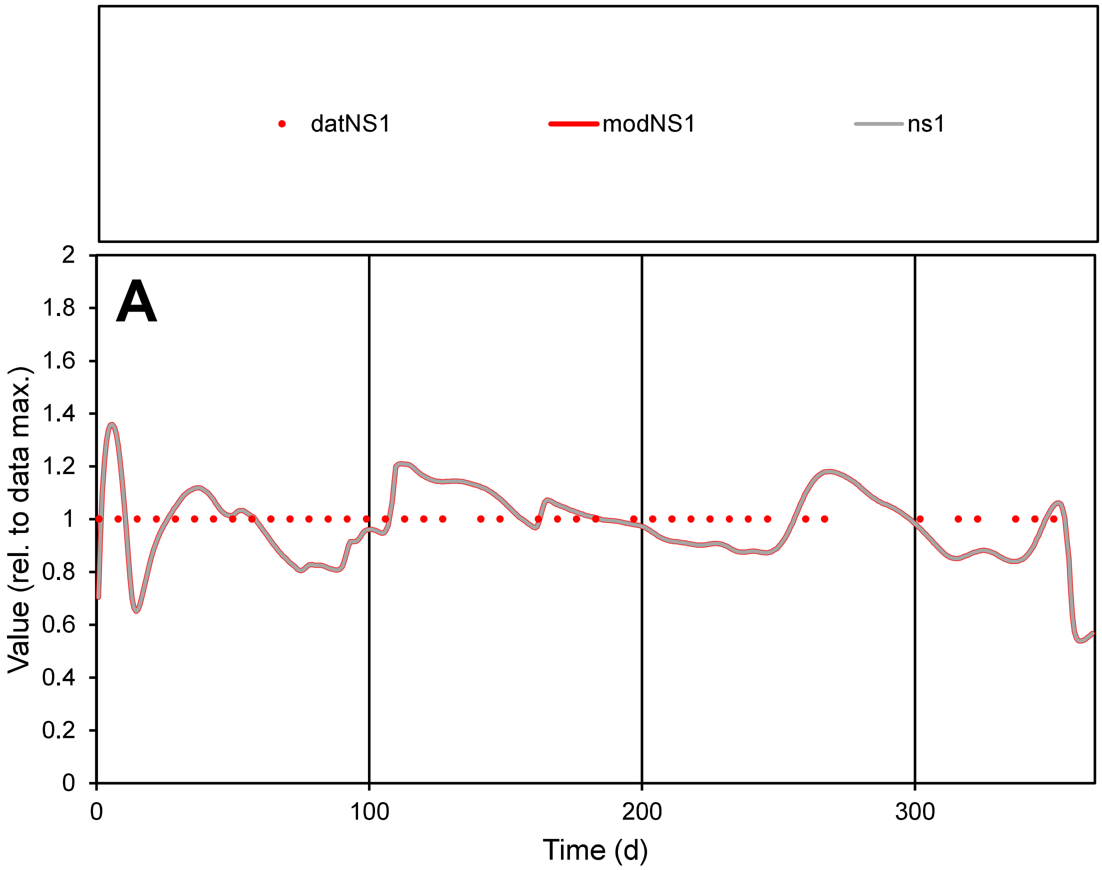

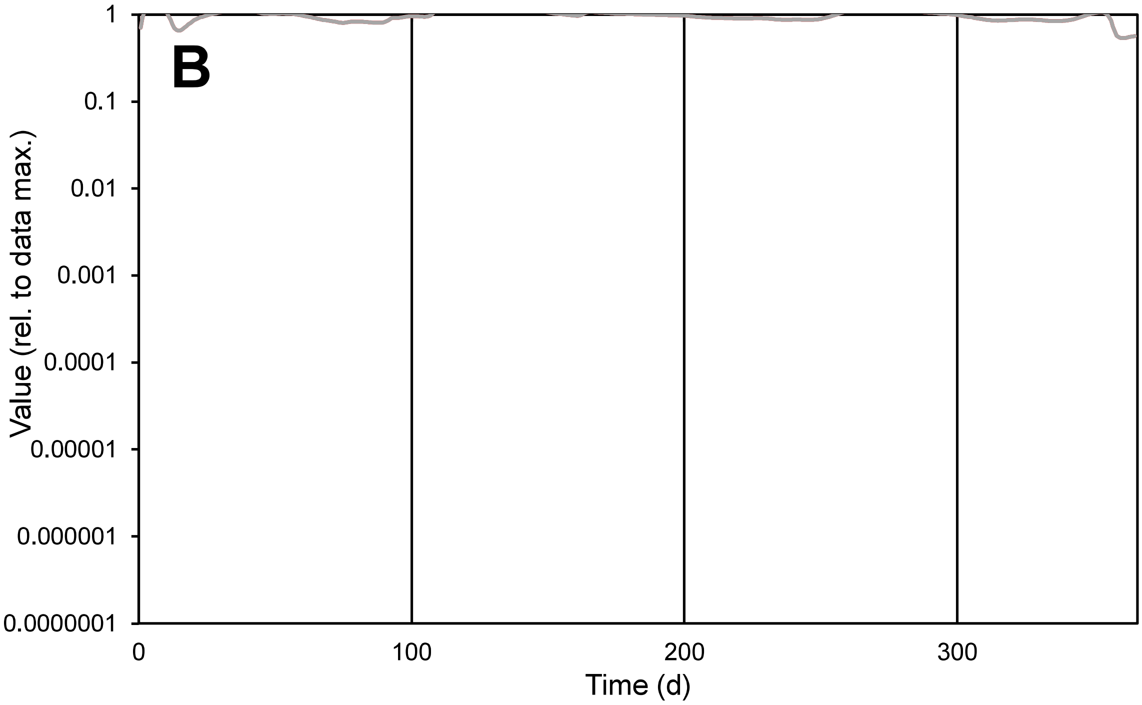

C1

Variables
NS1 Provide jtd or Dst
CalibOnly?
2 1 to reproduce of
oNormalize?
2 1=no, 2=yes

ofxn calc*
0.11571
ofxn mod
0.1578

sRun
RunA1
zout
1

Ave
Data
2.7E-05
Model
2.7E-05

data/vMax
2.7E-05

ofxng
319.391

Min d/m
1.41473

ave/min d
1
ave/min m
1.3866

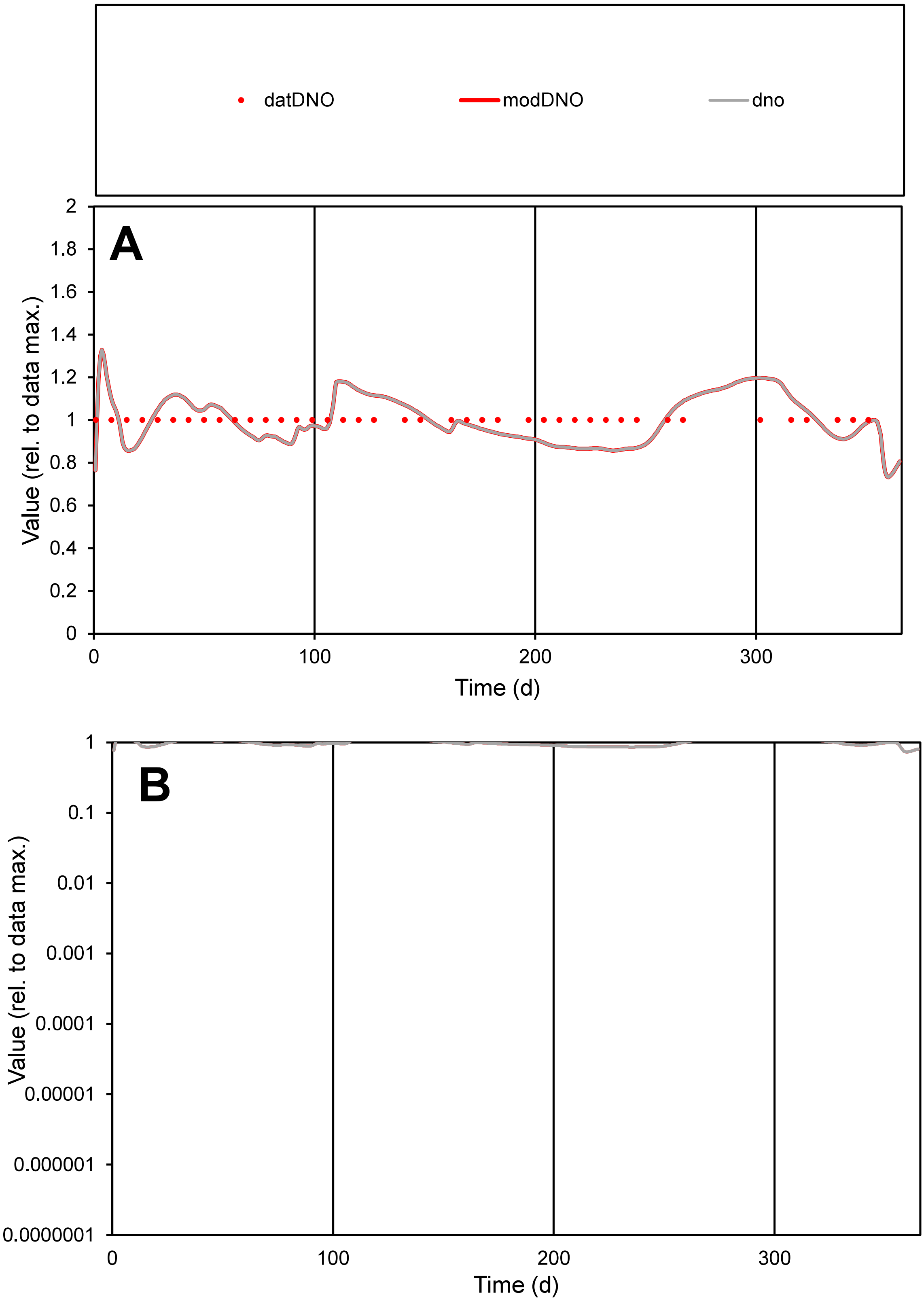

C1

Variables

DNO	Provide jtd or Dst
CalibOnly?	
2	1 to reproduce of
oNormalize?	
2	1=no, 2=yes

ofxn calc*
0.09241
ofxn mod
0.14201

sRun
RunA1
zout
1

Ave
Data
1.8E-05
Model
1.8E-05

data/vMax
1.8E-05

ofxng
319.391

Min d/m
1.16549

ave/min d
1
ave/min m
1.15404

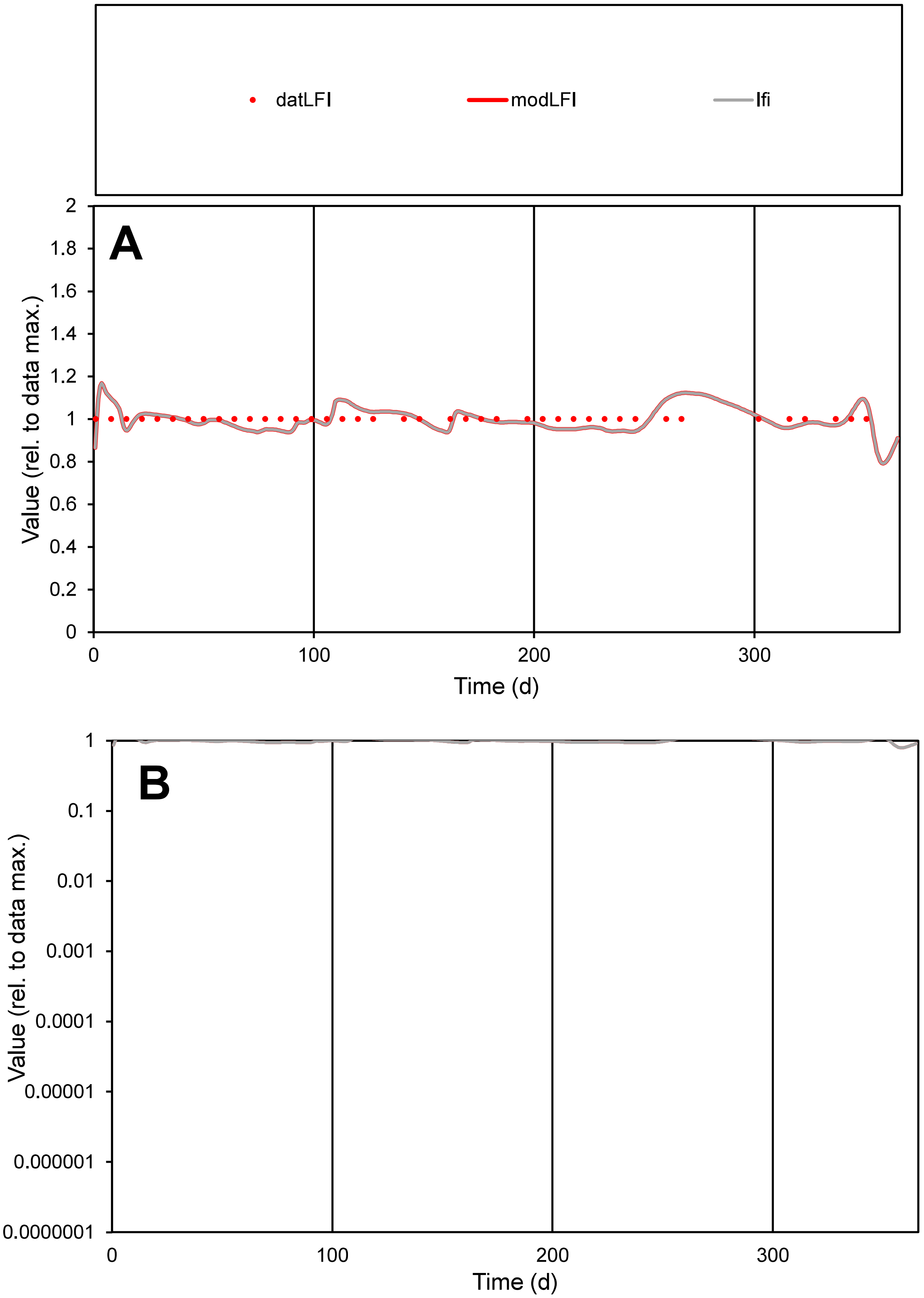

C1

Variables
LFI Provide jtd or Dst
CalibOnly?
2 1 to reproduce of
oNormalize?
2 1=no, 2=yes

ofxn calc*
0.04436
ofxn mod
0.11737

sRun
RunA1
zout
1

Ave
Data
8.3E-06
Model
8.3E-06

data/vMax
8.3E-06

ofxng
319.391

Min d/m
1.06212

ave/min d
1
ave/min m
1.06067

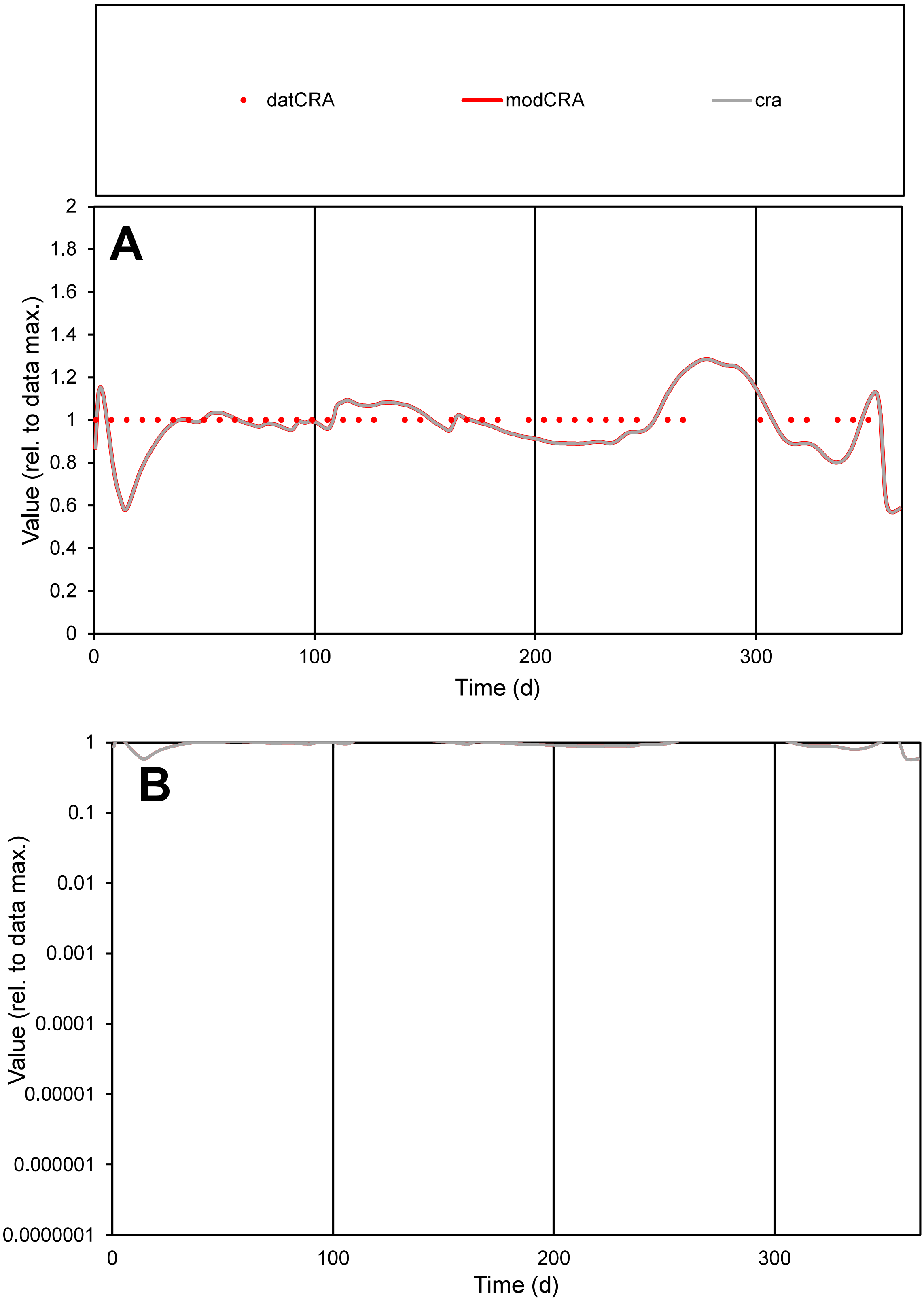

C1

Variables

CRA Provide jtd or Dst

CalibOnly?

2 1 to reproduce of

oNormalize?

2 1=no, 2=yes

ofxn calc*

0.11199

ofxn mod

0.15464

sRun

RunA1

zout

1

Ave

Data

4.9E-06

Model

4.7E-06

data/vMax

4.9E-06

ofxng

319.391

Min d/m

1.59371

ave/min d

1

ave/min m

1.54284

330_Calib_S_A

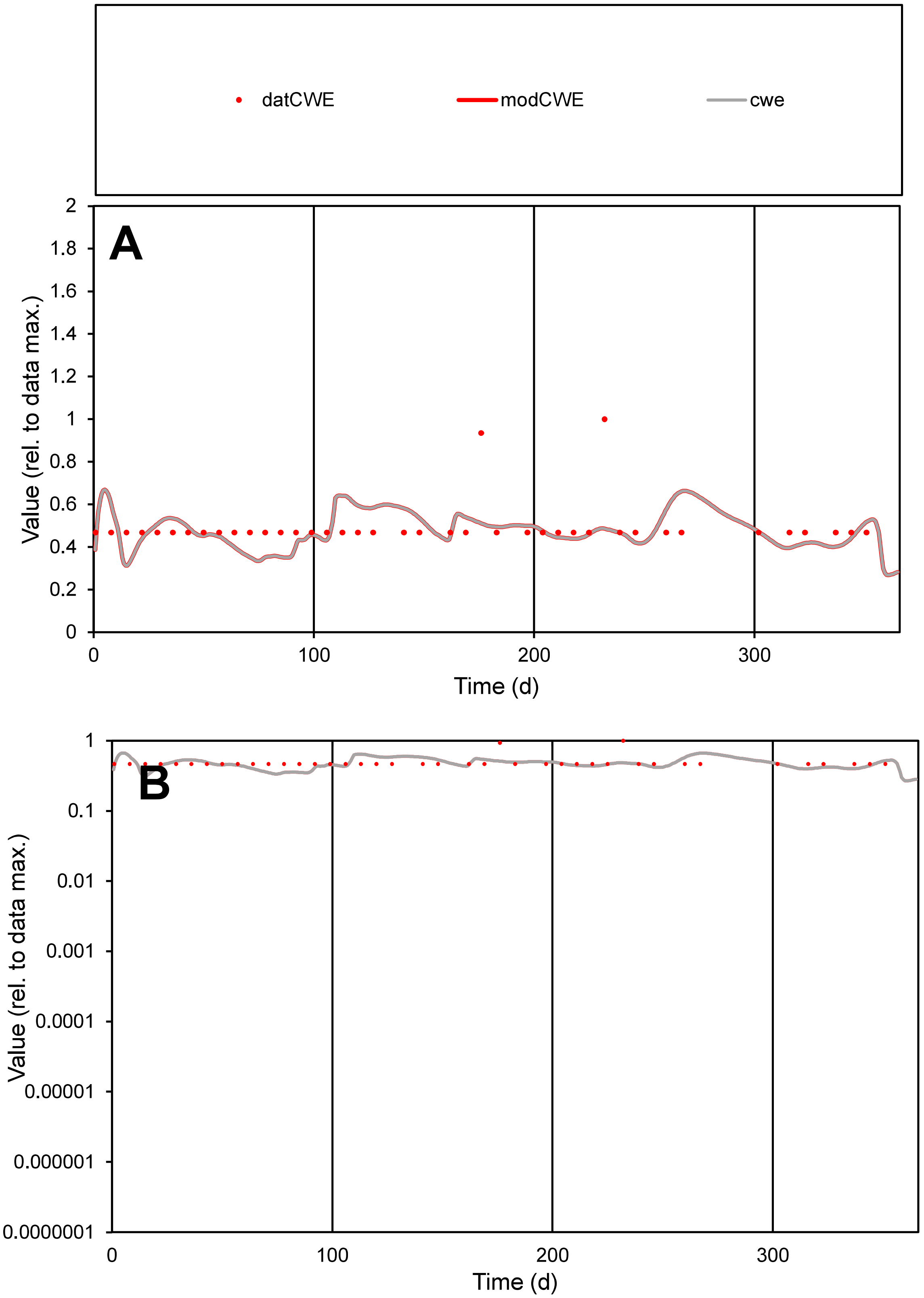

C1

Variables
CWE Provide jtd or Dst
CalibOnly?
2 1 to reproduce of
oNormalize?
2 1=no, 2=yes

ofxn calc*
0.26315
ofxn mod
0.28455

sRun
RunA1
zout
1

Ave
Data
2.7E-05
Model
2.6E-05

data/vMax
5.5E-05

ofxng
319.391

Min d/m
1.40341

ave/min d
1.05219
ave/min m
1.43795

330_Calib_S_A

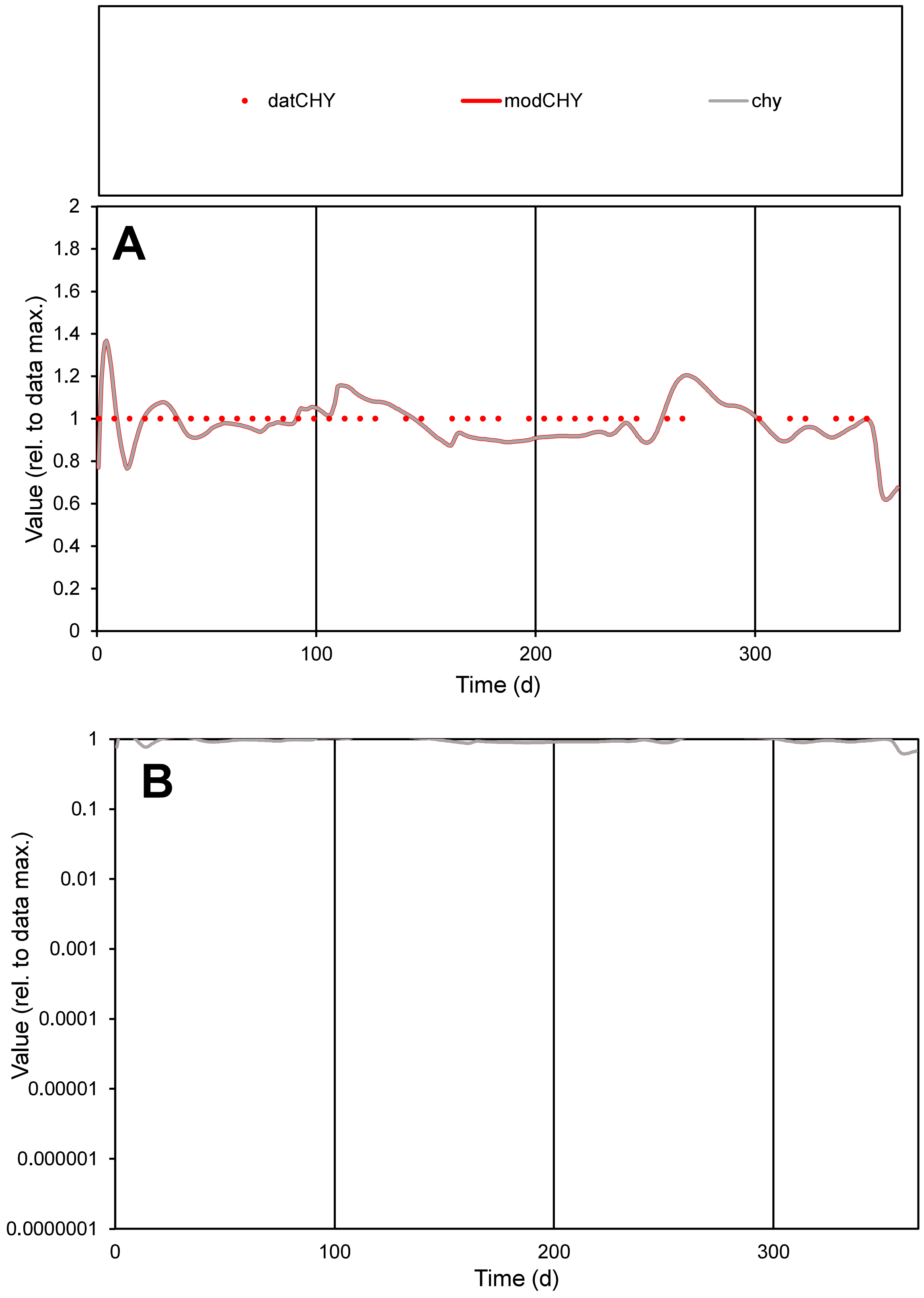

C1

Variables

CHY Provide jtd or Dst

CalibOnly?

2 1 to reproduce of

oNormalize?

2 1=no, 2=yes

ofxn calc*

0.07724

ofxn mod

0.13239

sRun

RunA1

zout

1

Ave

Data

6.7E-06

Model

6.6E-06

data/vMax

6.7E-06

ofxng

319.391

Min d/m

1.17409

ave/min d

1

ave/min m

1.15072

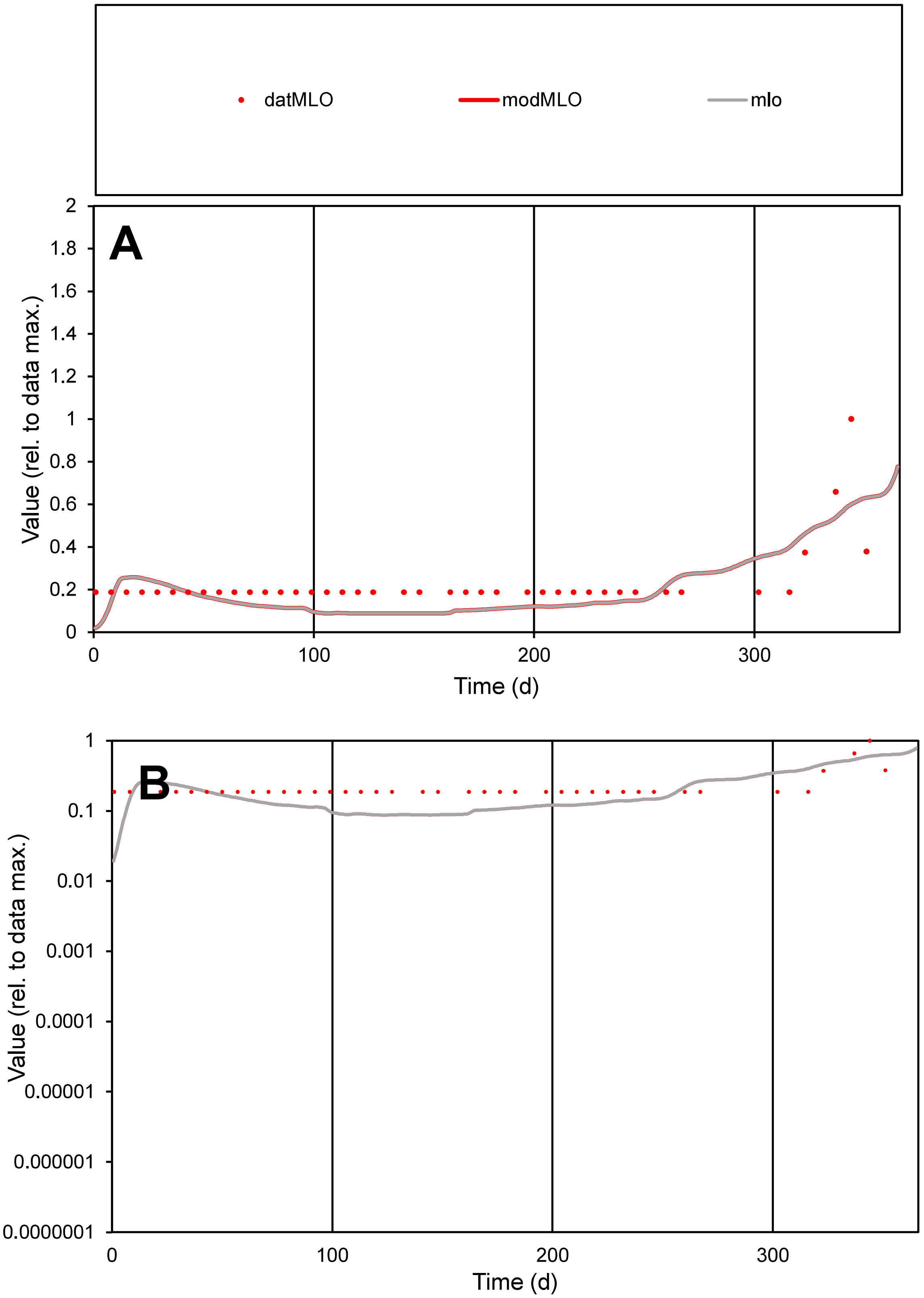

C1

Variables
MLO Provide jtd or Dst
CalibOnly?
2 1 to reproduce of
oNormalize?
2 1=no, 2=yes

ofxn calc*
0.4834
ofxn mod
0.49744

sRun
RunA1
zout
1

Ave
Data
1.3E-05
Model
1.1E-05

data/vMax
5.6E-05

ofxng
319.391

Min d/m
4.09662

ave/min d
1.21671
ave/min m
4.22958

330_Calib_S_A

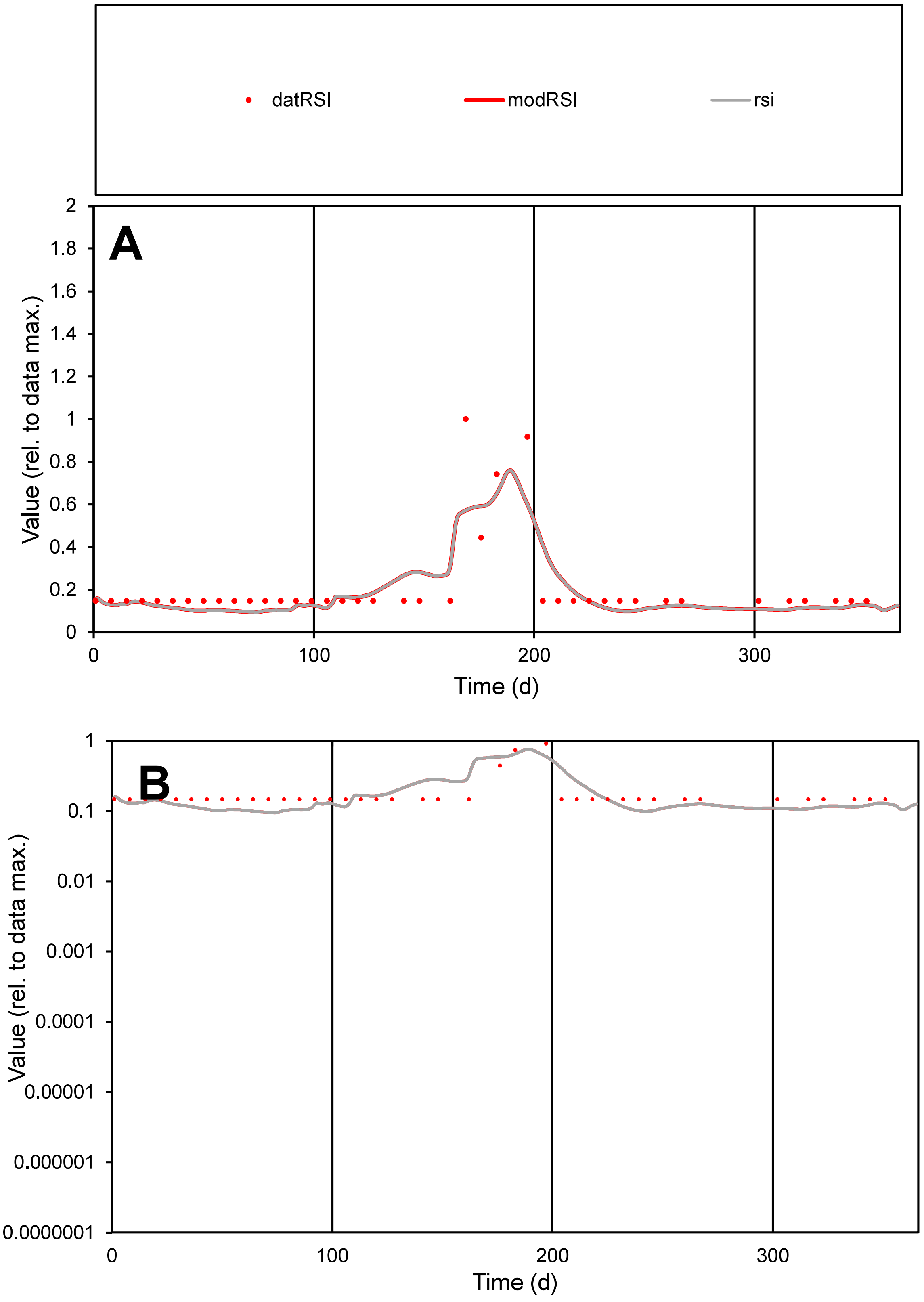

C1

Variables
RSI Provide jtd or Dst
CalibOnly?
2 1 to reproduce of
oNormalize?
2 1=no, 2=yes

ofxn calc*
0.49716
ofxn mod
0.54269

sRun
RunA1
zout
1

Ave
Data
1.5E-05
Model
1.3E-05

data/vMax
7E-05

ofxng
319.391

Min d/m
1.54479

ave/min d
1.41501
ave/min m
2.01608

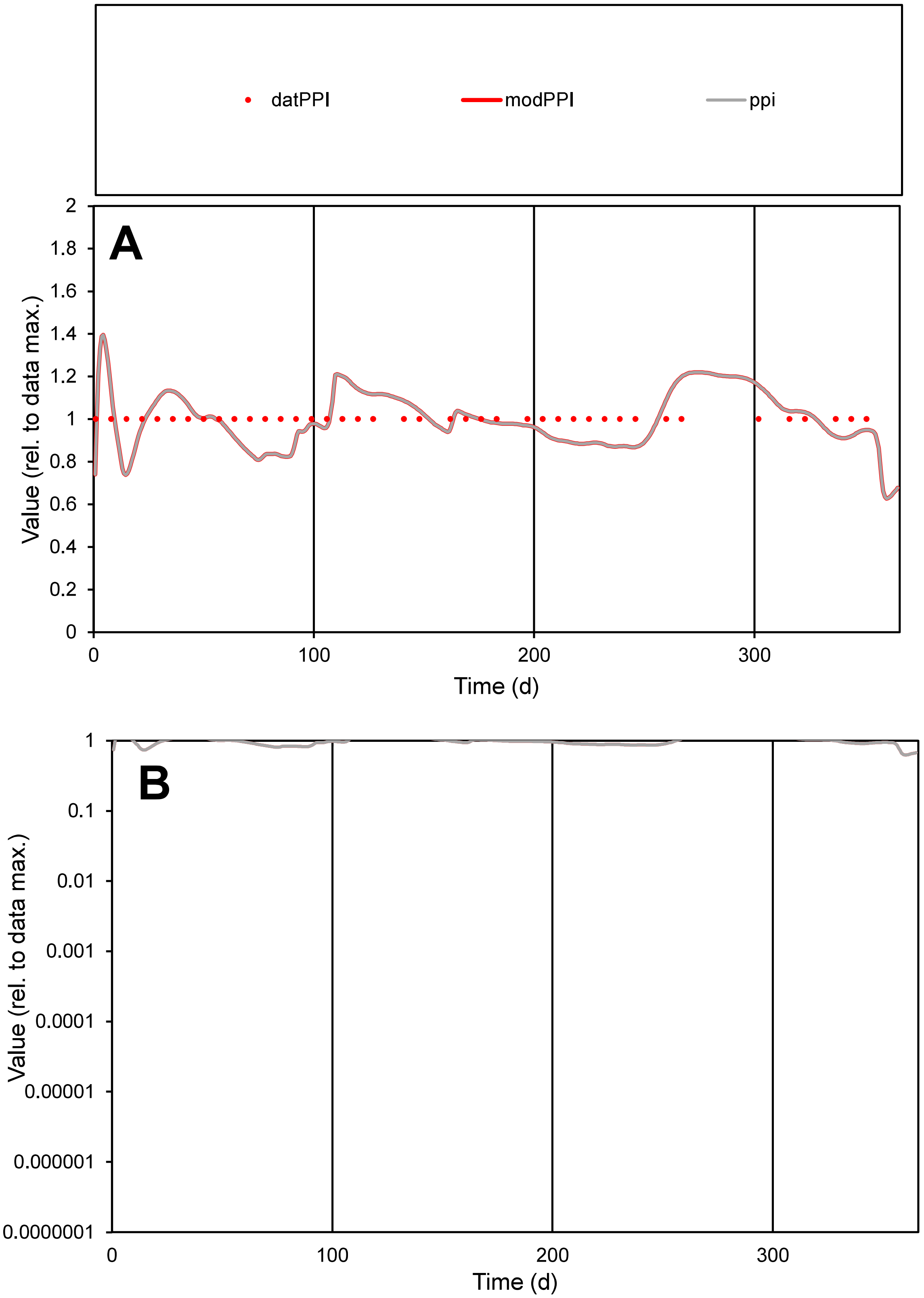

C1

Variables
PPI Provide jtd or Dst
CalibOnly?
2 1 to reproduce of
oNormalize?
2 1=no, 2=yes

ofxn calc*
0.10364
ofxn mod
0.14951

sRun
RunA1
zout
1

Ave
Data
1.4E-06
Model
1.4E-06

data/vMax
1.4E-06

ofxng
319.391

Min d/m
1.27965

ave/min d
1
ave/min m
1.26348

330_Calib_S_A

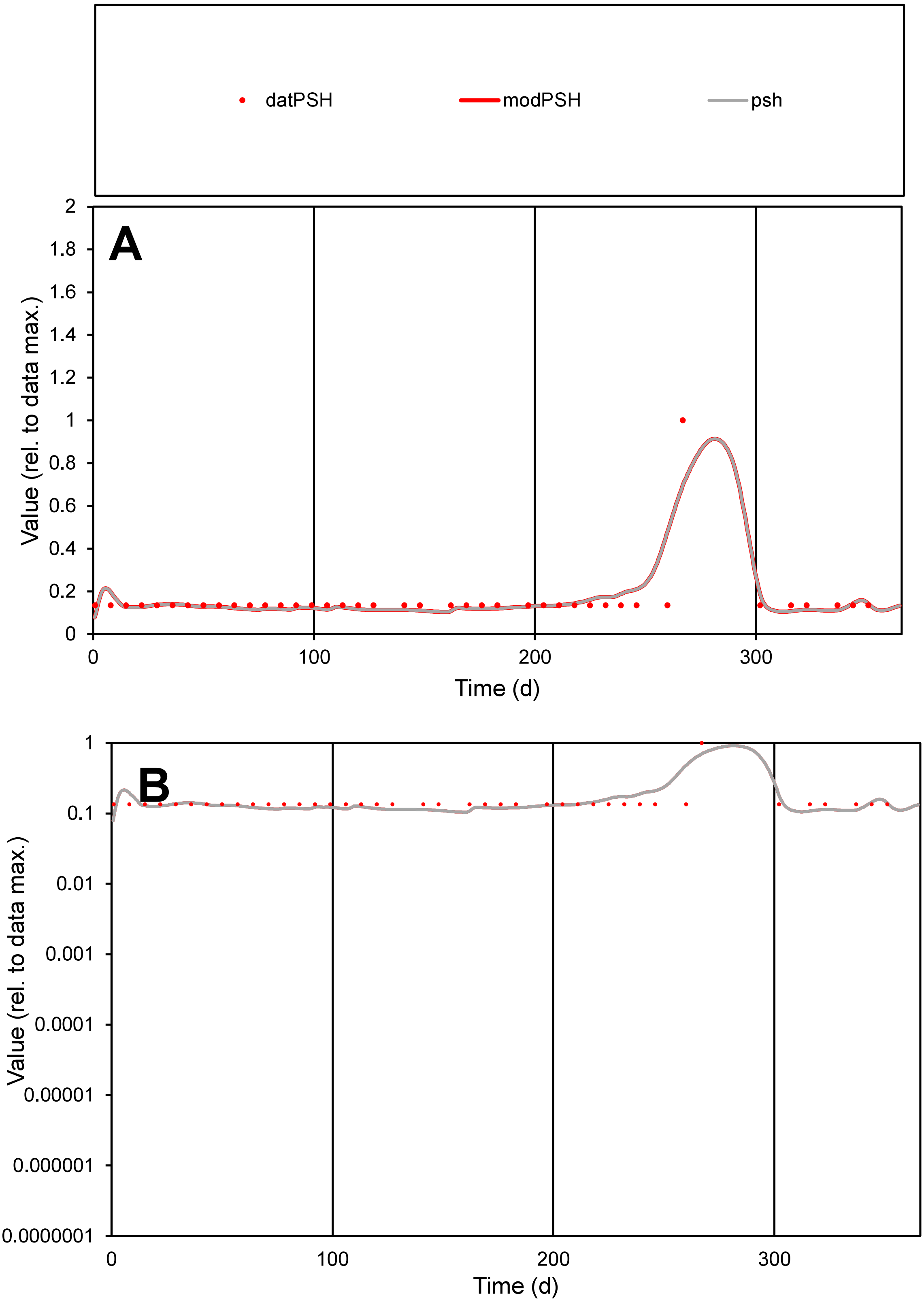

C1

Variables
PSH Provide jtd or Dst
CalibOnly?
2 1 to reproduce of
oNormalize?
2 1=no, 2=yes

ofxn calc*
0.47089
ofxn mod
0.49568

sRun
RunA1
zout
1

Ave
Data
1.5E-05
Model
1.5E-05

data/vMax
9.6E-05

ofxng
319.391

Min d/m
1.23563

ave/min d
1.15716
ave/min m
1.43825

330_Calib_S_A

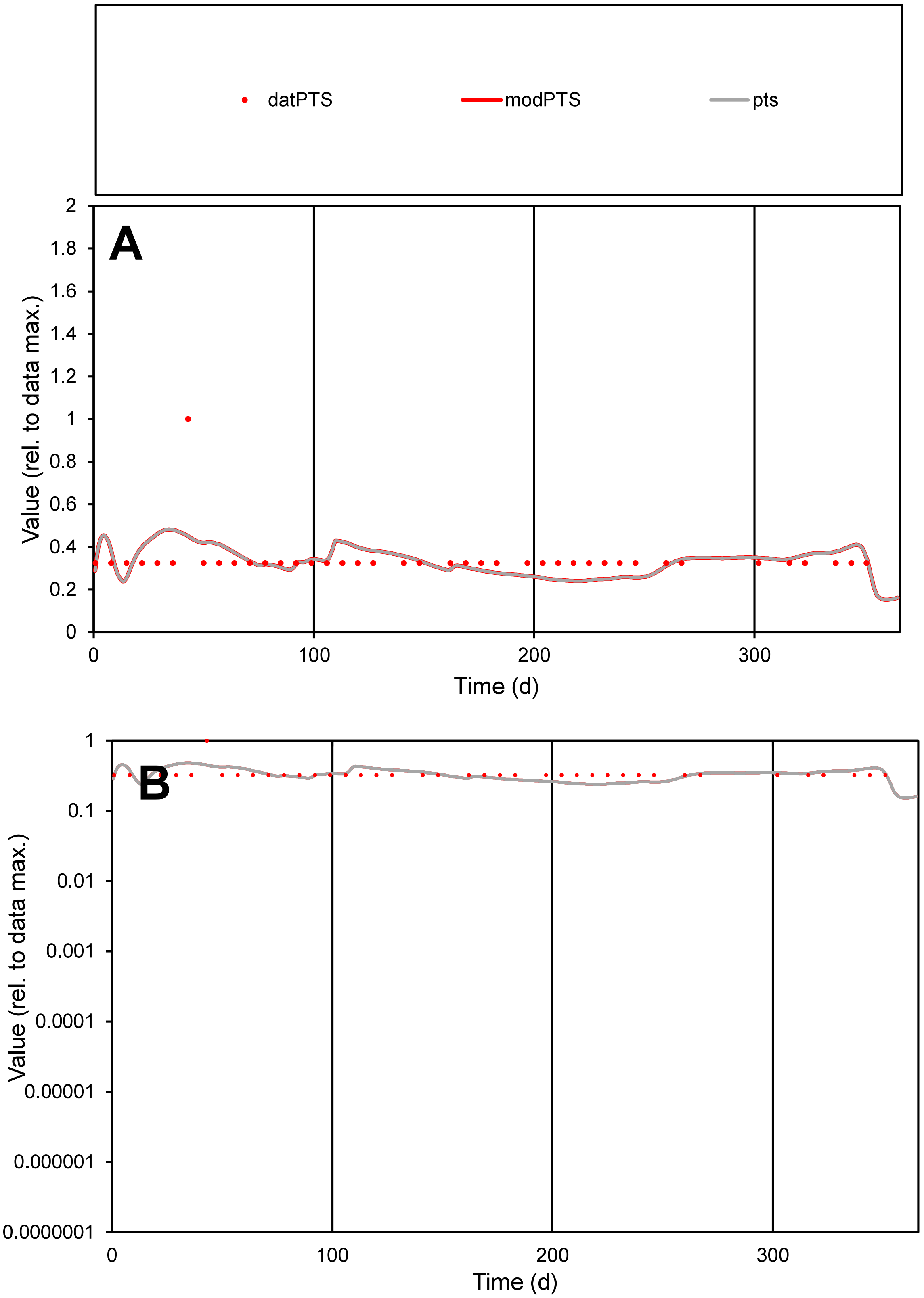

C1

Variables
PTS Provide jtd or Dst
CalibOnly?
2 1 to reproduce of
oNormalize?
2 1=no, 2=yes

ofxn calc*
0.31238
ofxn mod
0.33295

sRun
RunA1
zout
1

Ave
Data
1E-05
Model
1E-05

data/vMax
3E-05

ofxng
319.391

Min d/m
1.34804

ave/min d
1.05095
ave/min m
1.40621

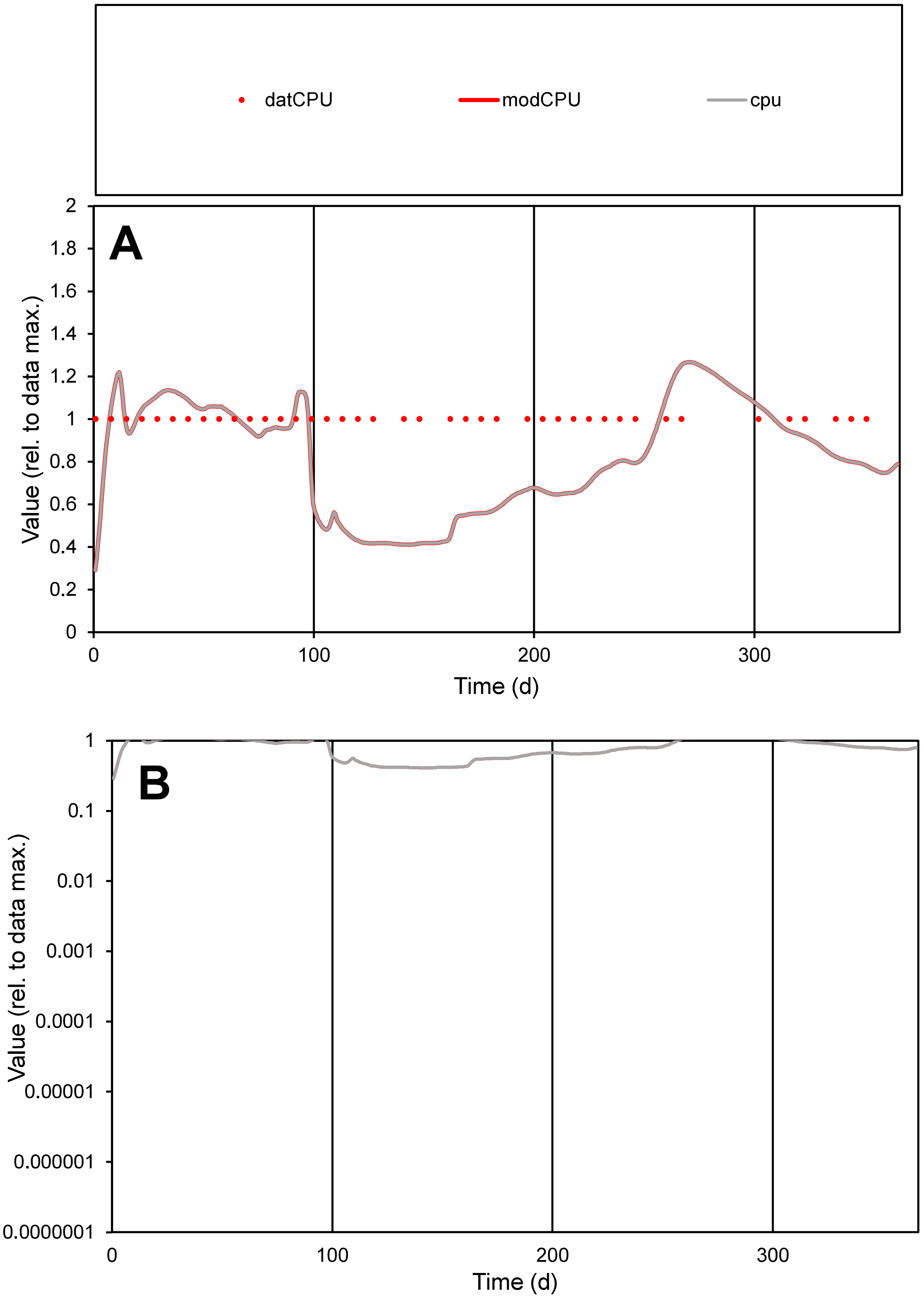

C1

Variables

CPU Provide jtd or Dst

CalibOnly?

2 1 to reproduce of

oNormalize?

2 1=no, 2=yes

ofxn calc*

0.31106

ofxn mod

0.33043

sRun

RunA1

zout

1

Ave

Data

4.4E-07

Model

3.6E-07

data/vMax

4.4E-07

ofxng

319.391

Min d/m

2.43234

ave/min d

1

ave/min m

1.96282

330_Calib_S_A

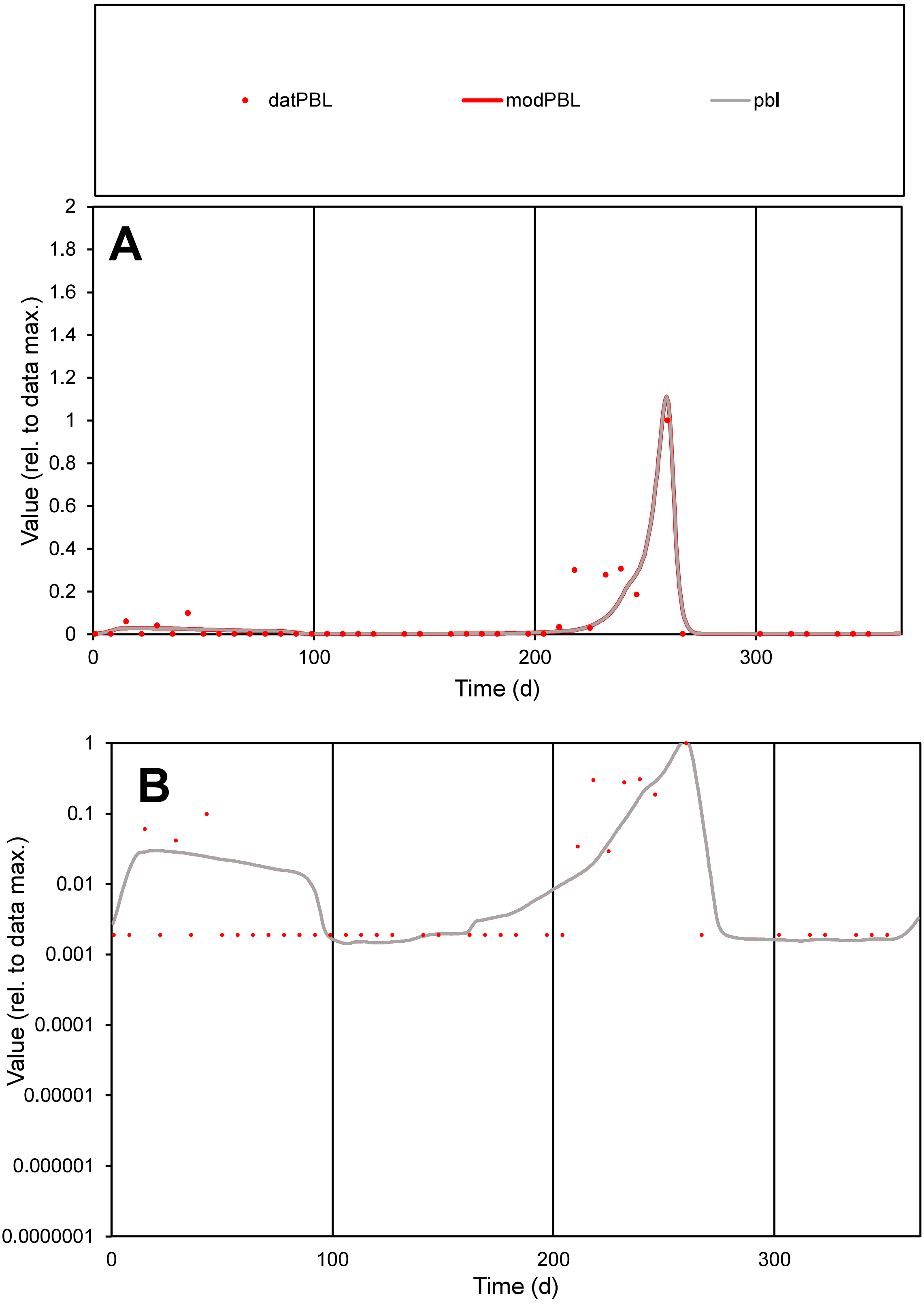

C1

Variables

PBL Provide jtd or Dst

CalibOnly?

2 1 to reproduce of

oNormalize?

2 1=no, 2=yes

ofxn calc*

1.01636

ofxn mod

1.0586

sRun

RunA1

zout

1

Ave

Data

1.3E-06

Model

1.1E-06

data/vMax

2.2E-05

ofxng

319.391

Min d/m

1.32462

ave/min d

30.7758

ave/min m

34.1073

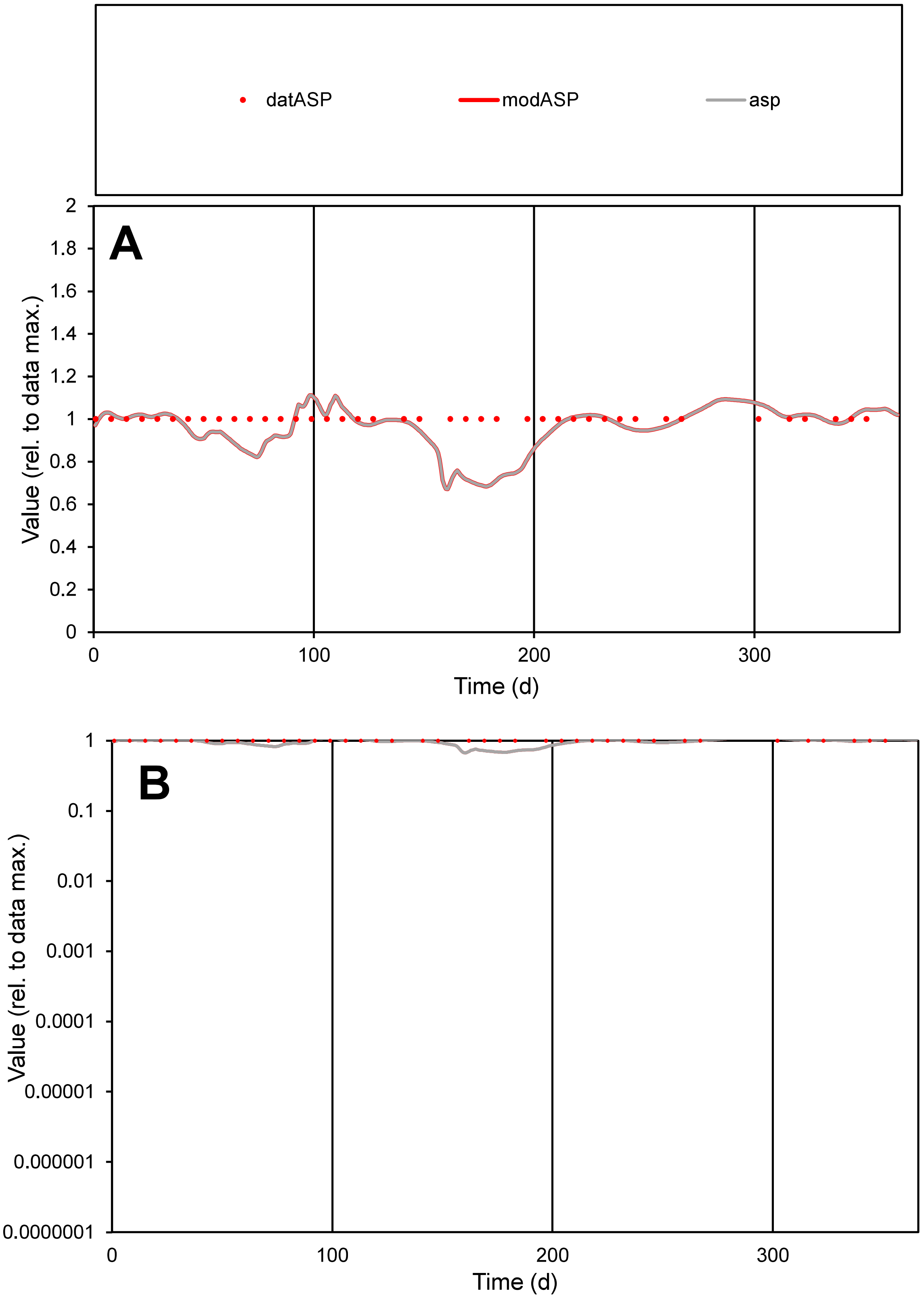

C1

Variables

ASP Provide jtd or Dst

CalibOnly?

2 1 to reproduce of

oNormalize?

2 1=no, 2=yes

ofxn calc*

0.10708

ofxn mod

0.15108

sRun

RunA1

zout

1

Ave

Data

5.8E-06

Model

5.5E-06

data/vMax

5.8E-06

ofxng

319.391

Min d/m

1.46045

ave/min d

1

ave/min m

1.39713

330_Calib_S_A

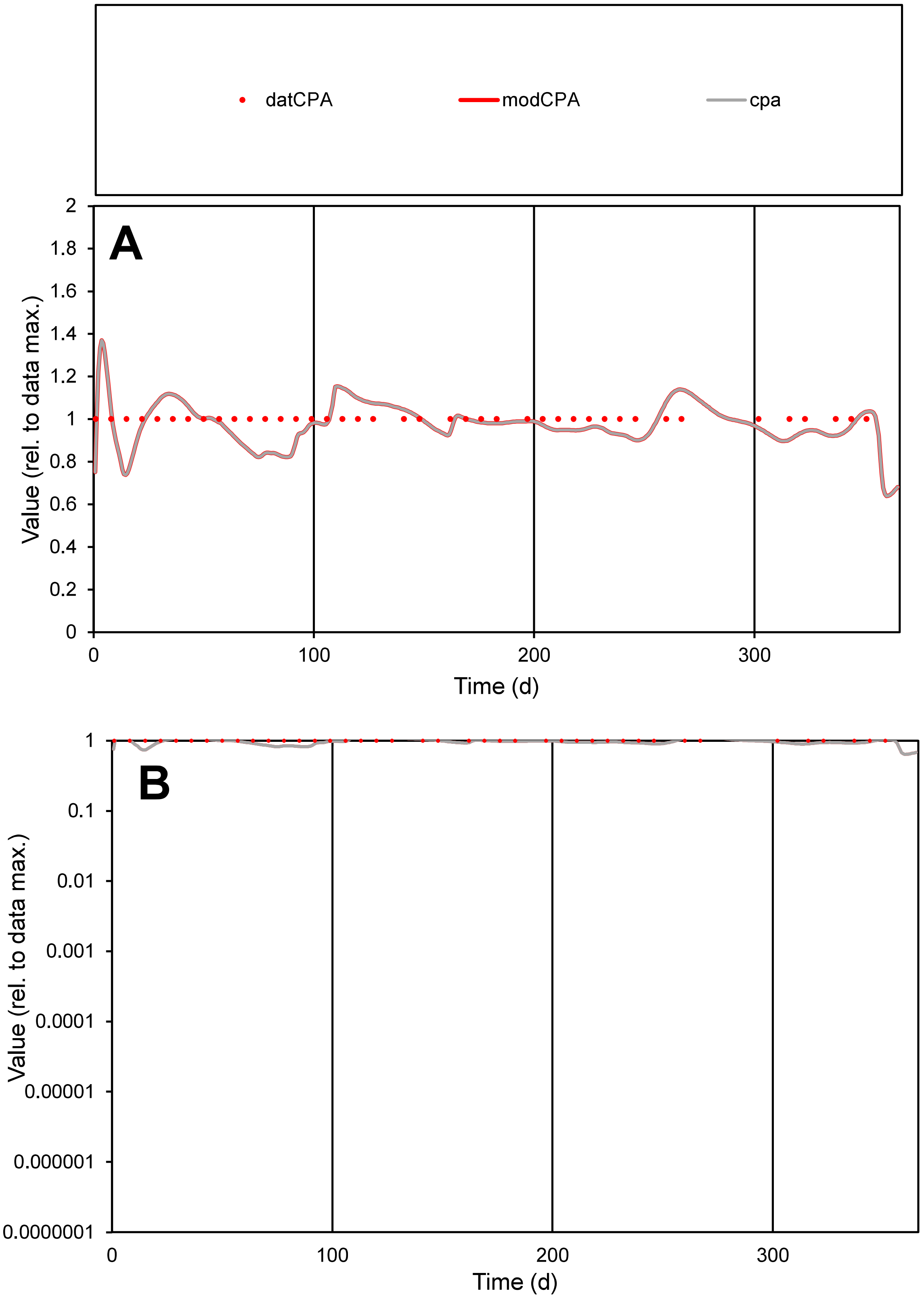

C1

Variables
CPA Provide jtd or Dst
CalibOnly?
2 1 to reproduce of
oNormalize?
2 1=no, 2=yes

ofxn calc*
0.08259
ofxn mod
0.13564

sRun
RunA1
zout
1

Ave
Data
6.6E-08
Model
6.5E-08

data/vMax
6.6E-08

ofxng
319.391

Min d/m
1.2695

ave/min d
1
ave/min m
1.24482

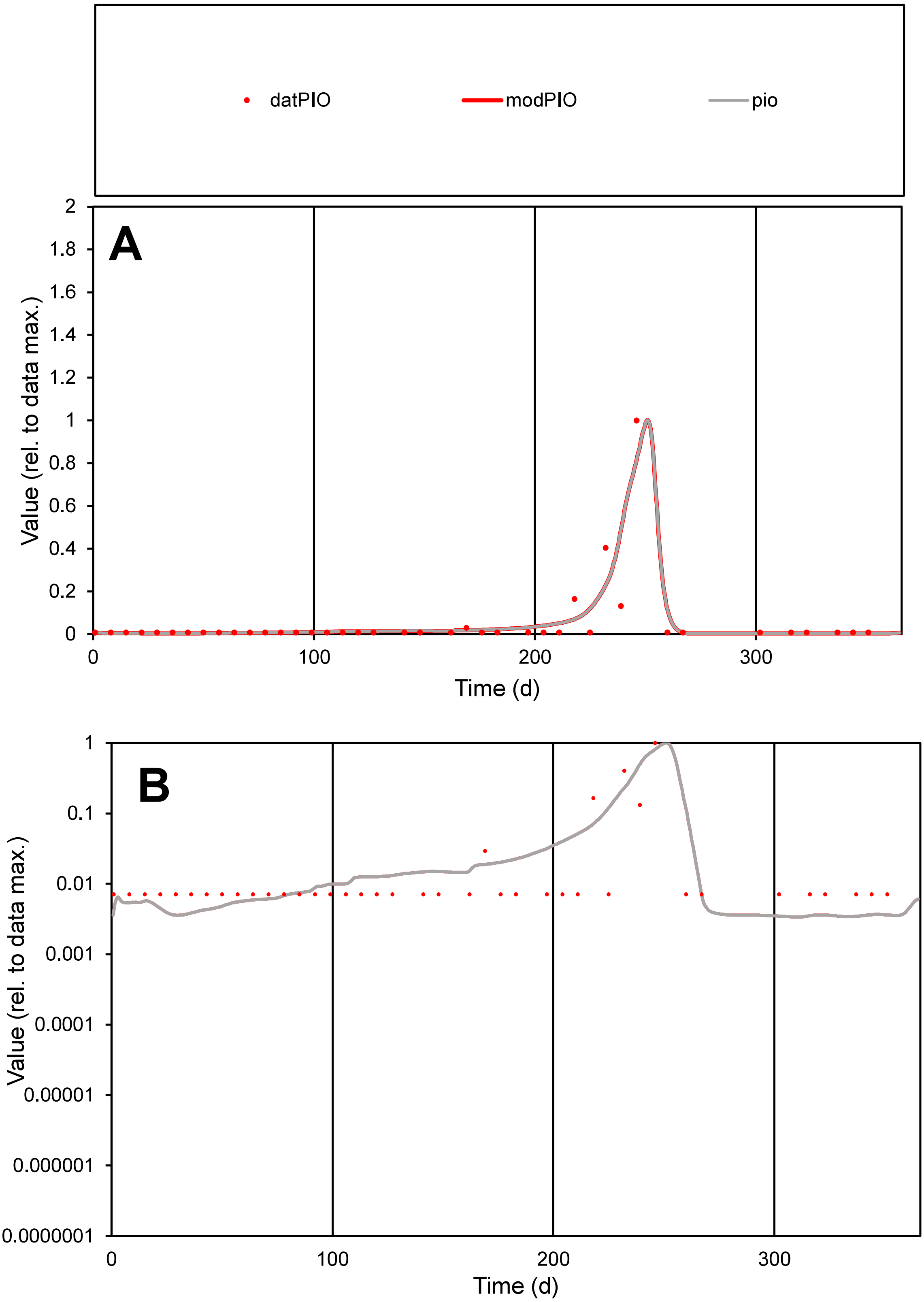

C1

Variables
PIO Provide jtd or Dst
CalibOnly?
2 1 to reproduce of
oNormalize?
2 1=no, 2=yes

ofxn calc*
1.33418
ofxn mod
1.47572

sRun
RunA1
zout
1

Ave
Data
9.9E-07
Model
1.1E-06

data/vMax
2.1E-05

ofxng
319.391

Min d/m
2.04421
ave/min d
6.8376
ave/min m
15.6115

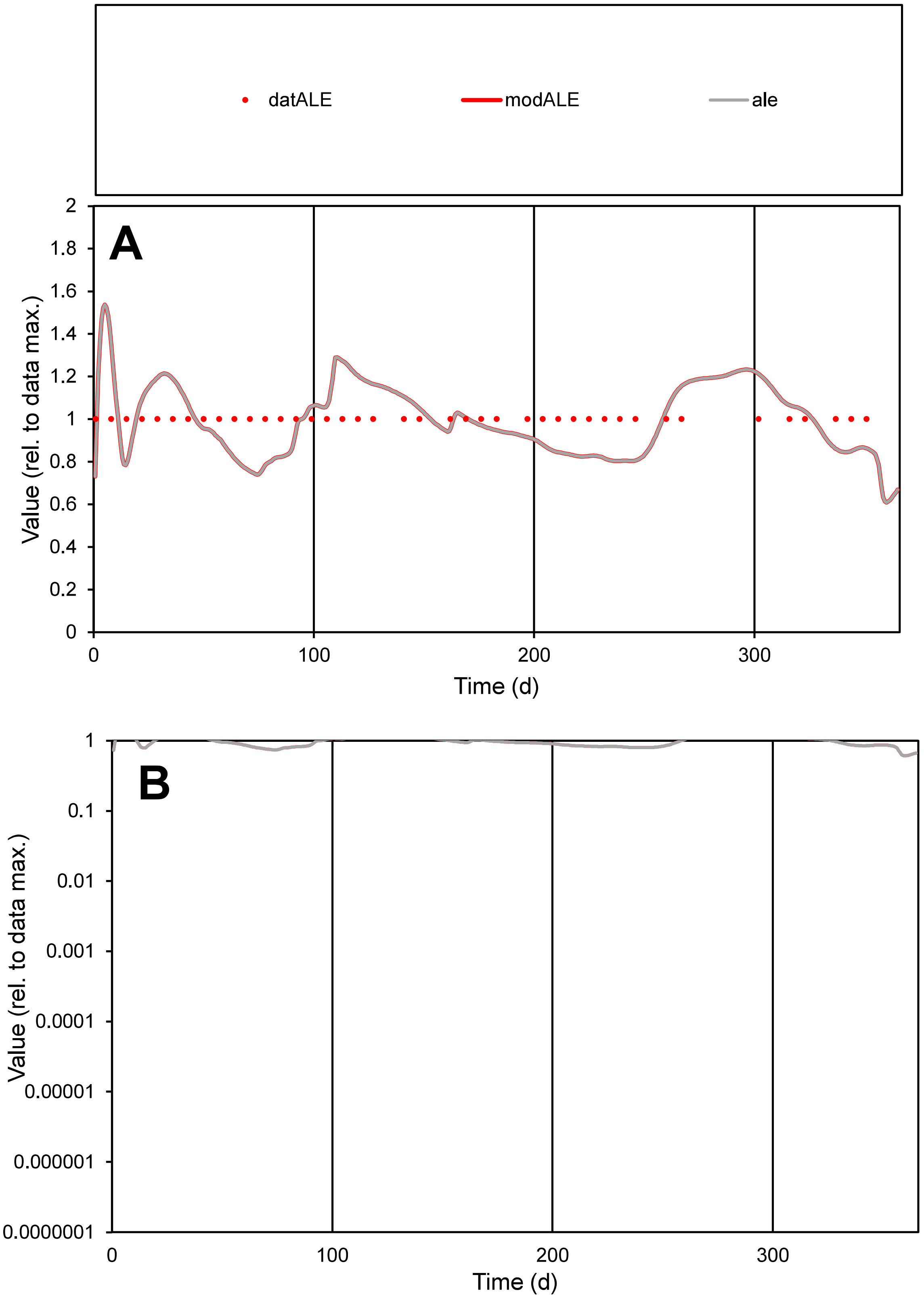

C1

Variables
ALE Provide jtd or Dst
CalibOnly?
2 1 to reproduce of
oNormalize?
2 1=no, 2=yes

ofxn calc*
0.14398
ofxn mod
0.17963

sRun
RunA1
zout
1

Ave
Data
1.1E-05
Model
1.1E-05

data/vMax
1.1E-05

ofxng
319.391

Min d/m
1.33333

ave/min d
1
ave/min m
1.30926

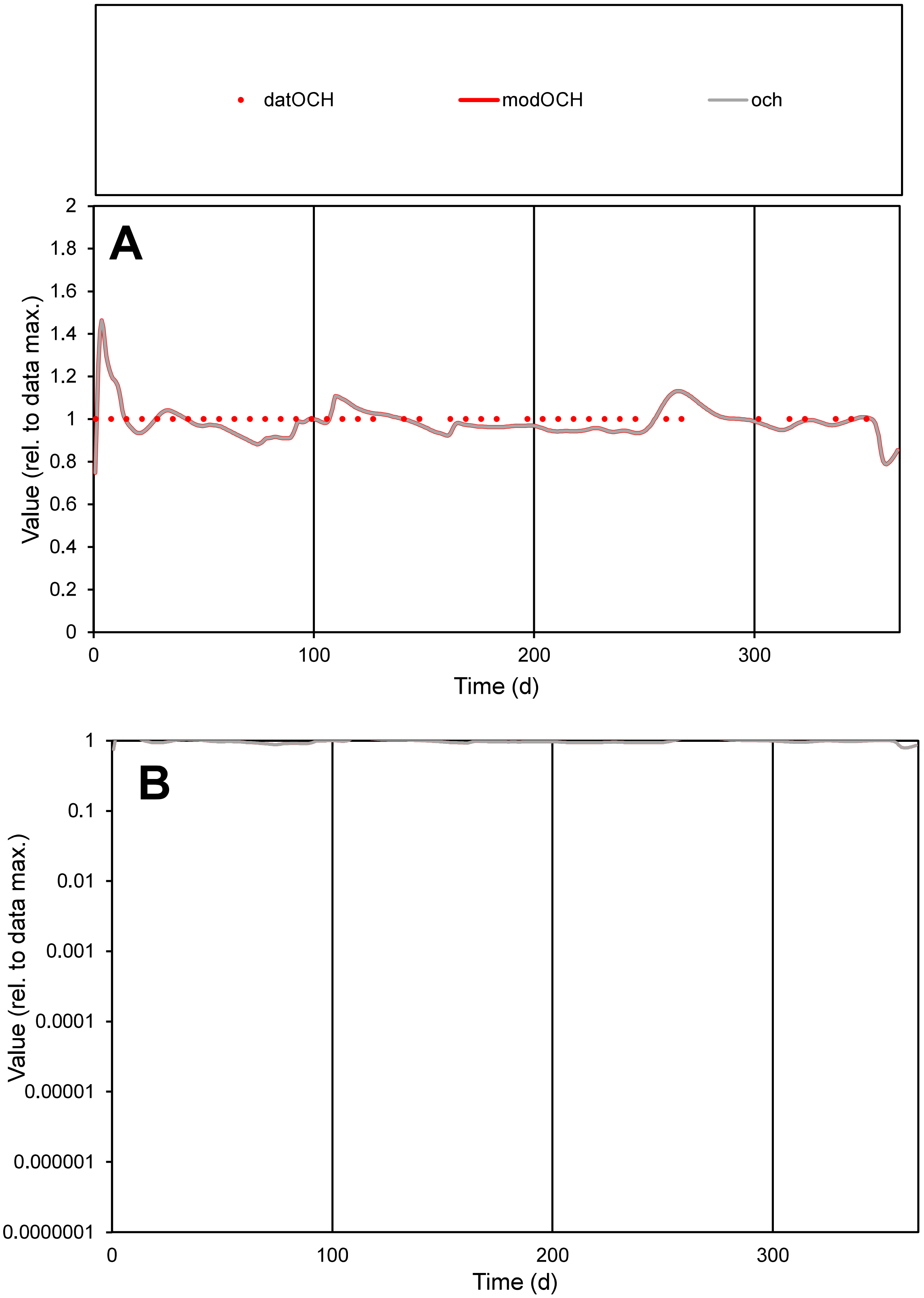

C1

Variables
OCH Provide jtd or Dst
CalibOnly?
2 1 to reproduce of
oNormalize?
2 1=no, 2=yes

ofxn calc*
0.05878
ofxn mod
0.12311

sRun
RunA1
zout
1

Ave
Data
4.4E-06
Model
4.3E-06

data/vMax
4.4E-06

ofxng
319.391

Min d/m
1.1214

ave/min d
1
ave/min m
1.10624

330_Calib_S_A

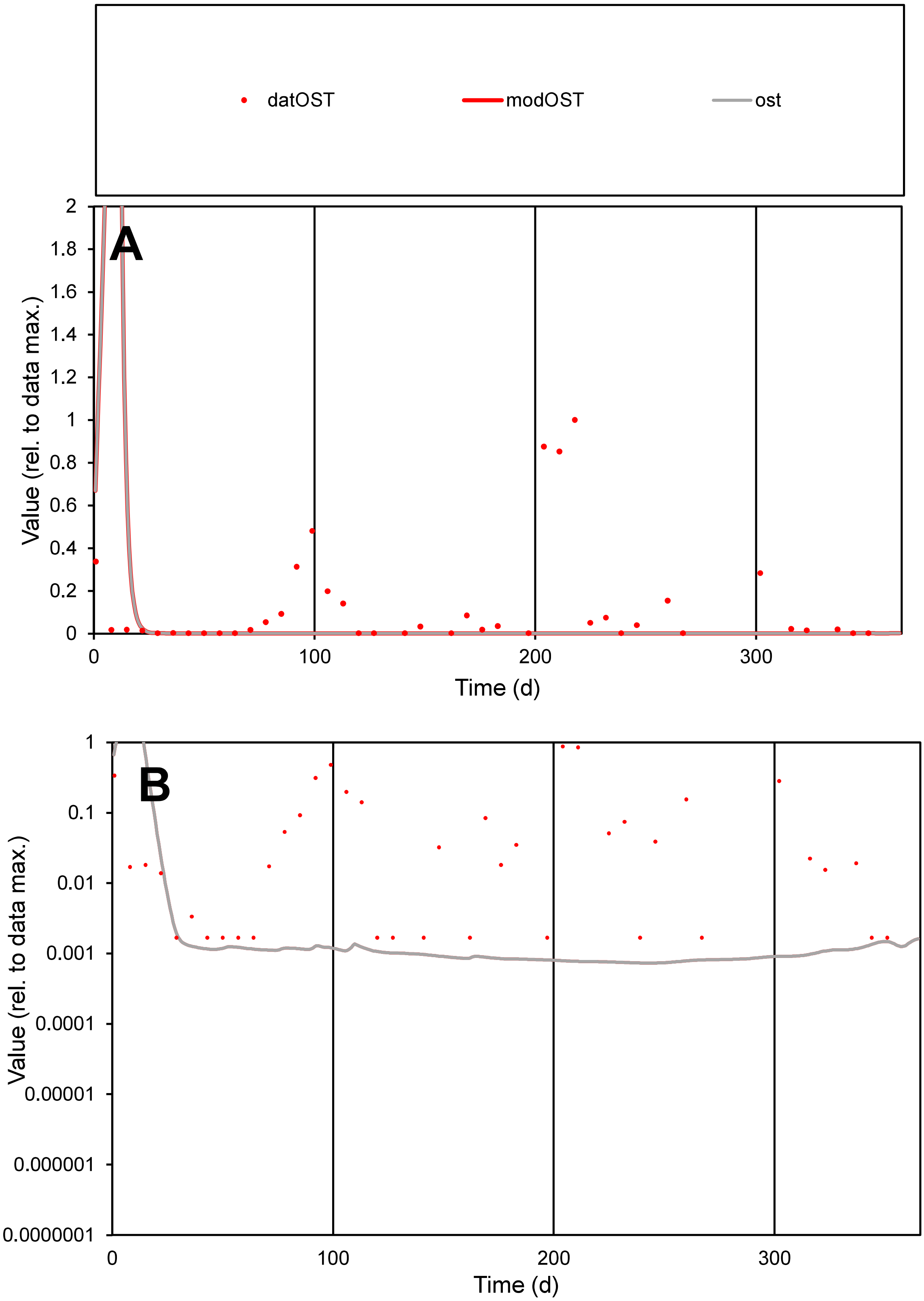

C1

Variables

OST Provide jtd or Dst

CalibOnly?

2 1 to reproduce of

oNormalize?

2 1=no, 2=yes

ofxn calc*

3.05106

ofxn mod

3.83584

sRun

RunA1

zout

1

Ave

Data

0.00017

Model

9.1E-05

data/vMax

0.00129

ofxng

319.391

Min d/m

2.2837

ave/min d

76.6297

ave/min m

95.599

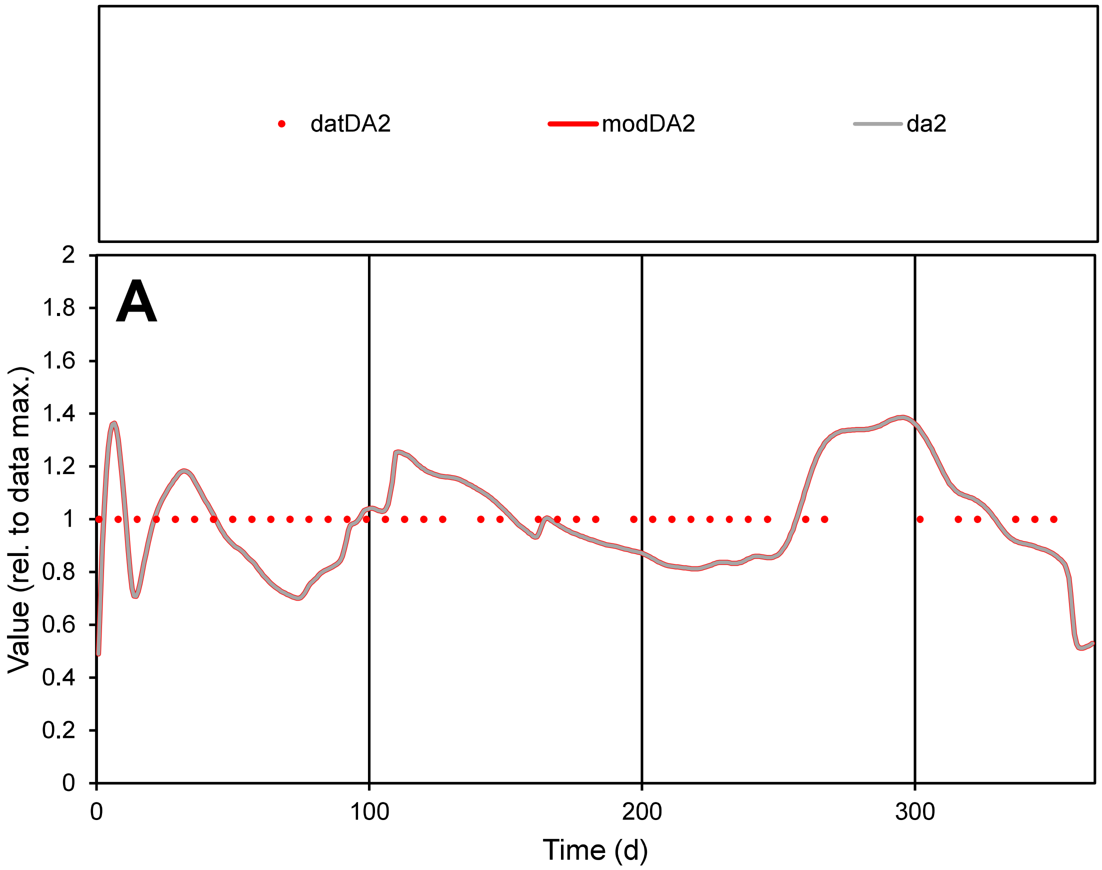

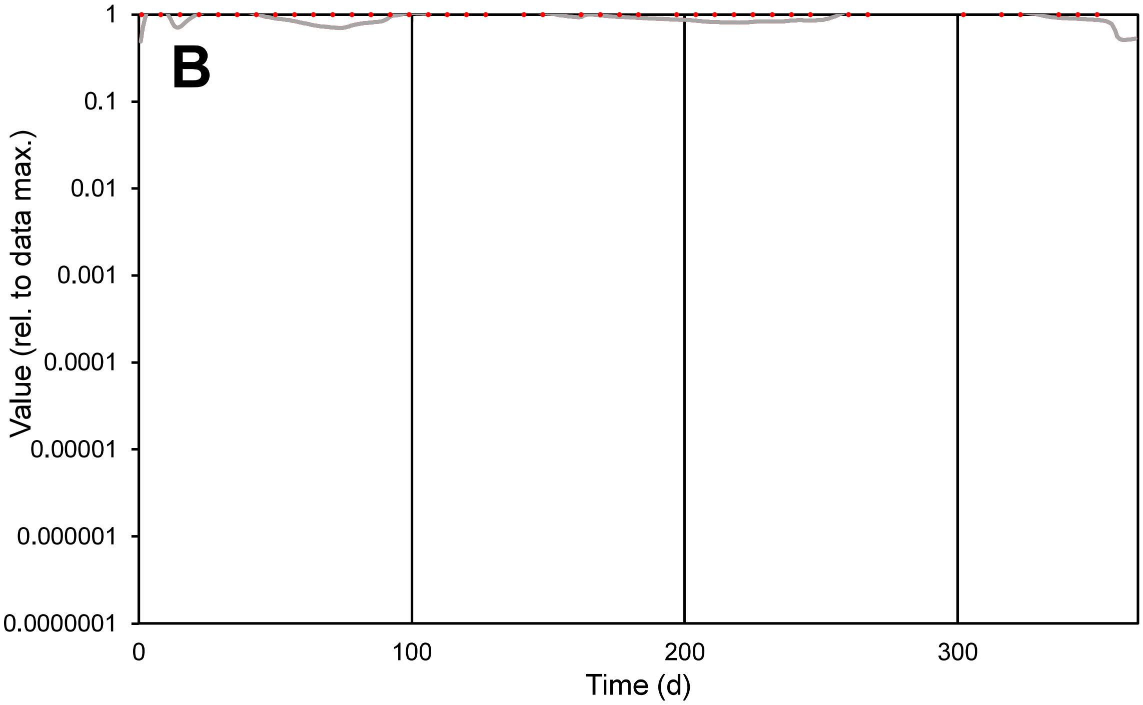

C1

Variables
DA2 Provide jtd or Dst
CalibOnly?
2 1 to reproduce of
oNormalize?
2 1=no, 2=yes

ofxn calc*
0.15858
ofxn mod
0.19169

sRun
RunA1
zout
1

Ave
Data
3.1E-06
Model
3E-06

data/vMax
3.1E-06

ofxng
319.391

Min d/m
1.41689

ave/min d
1
ave/min m
1.37901

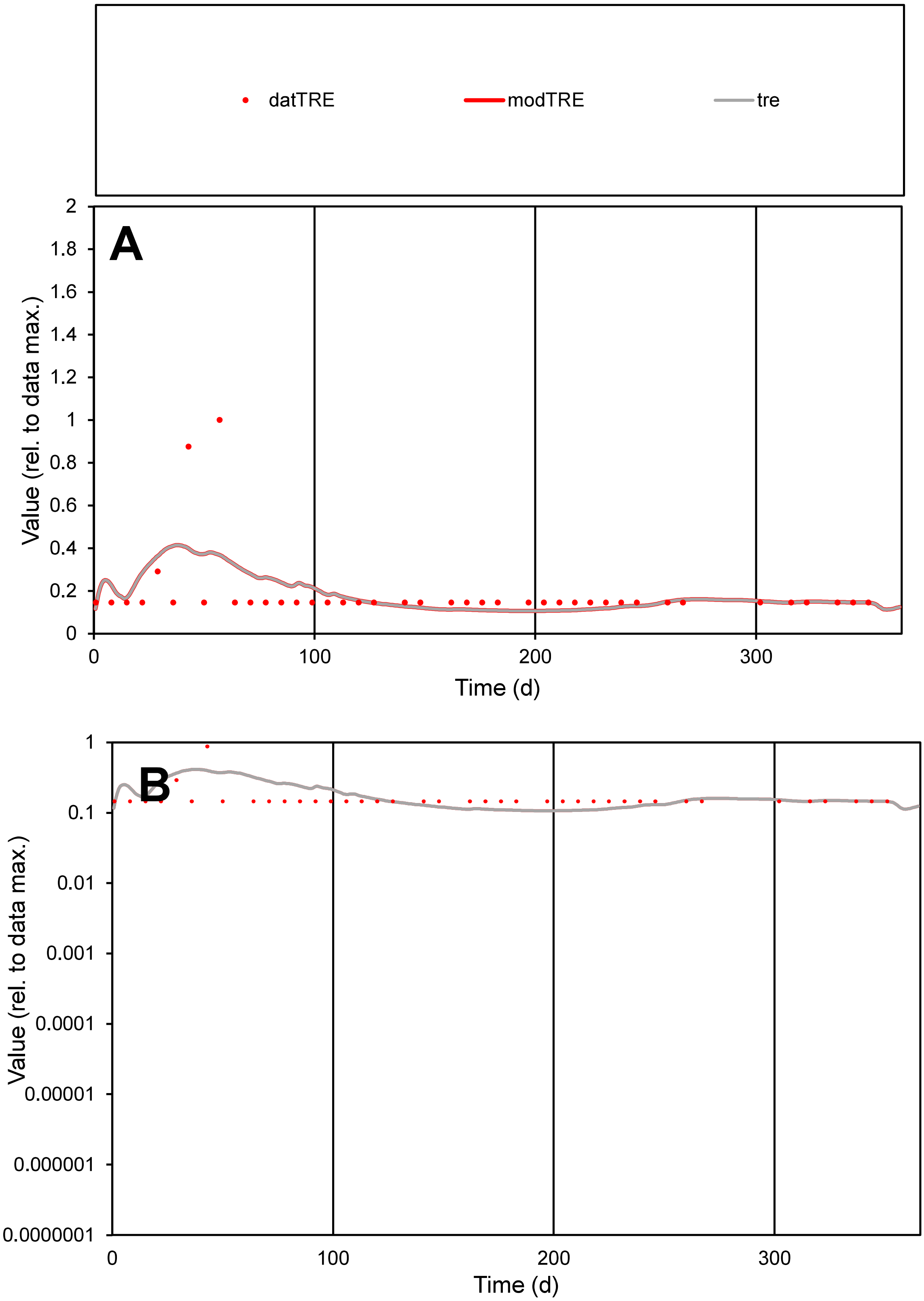

C1

Variables

TRE	Provide jtd or Dst
CalibOnly?	
2	1 to reproduce of
oNormalize?	
2	1=no, 2=yes
ofxn calc*	
0.78473	
ofxn mod	
0.79831	
sRun	
RunA1	
zout	
1	
Ave Data	
8.9E-06	
Model	
9E-06	
data/vMax	
4.8E-05	
ofxng	
319.391	
Min d/m	
1.3636	
ave/min d	
1.28988	
ave/min m	
1.76713	

330_Calib_S_A

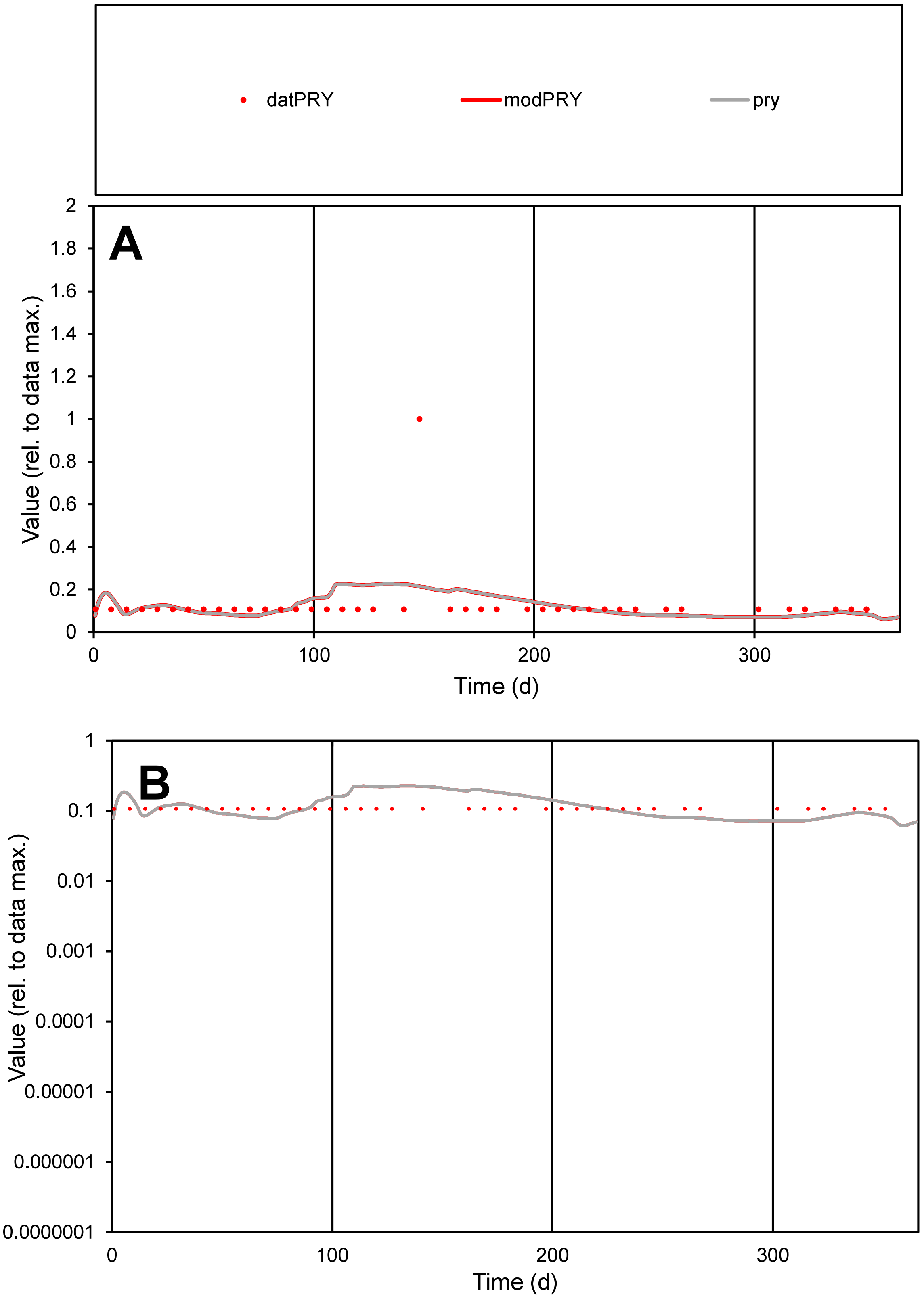

C1

Variables
PRY Provide jtd or Dst
CalibOnly?
2 1 to reproduce of
oNormalize?
2 1=no, 2=yes

ofxn calc*
1.02642
ofxn mod
1.03359

sRun
RunA1
zout
1

Ave
Data
1.1E-05
Model
1E-05

data/vMax
8.2E-05

ofxng
319.391

Min d/m
1.49161

ave/min d
1.20312
ave/min m
1.75527

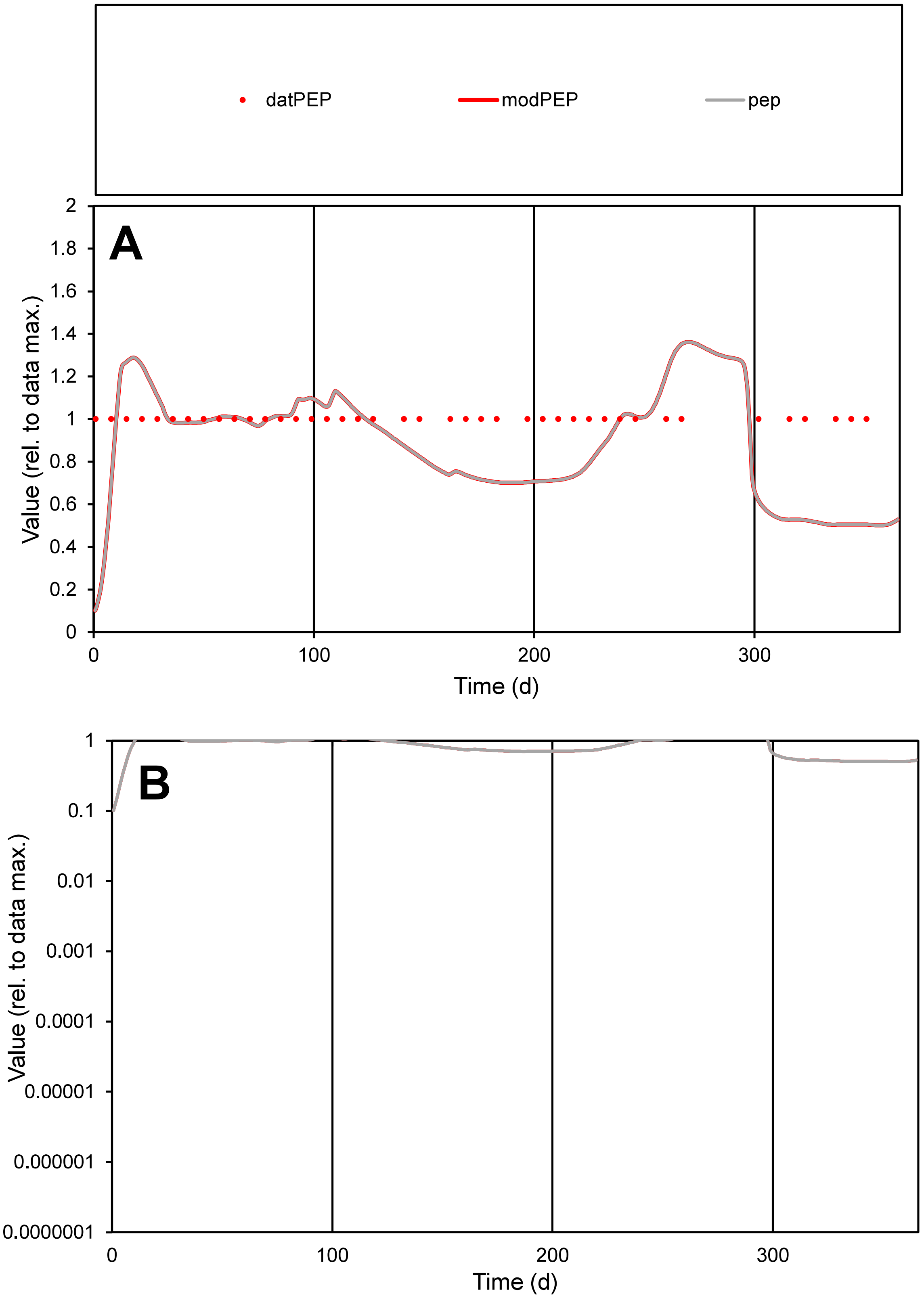

C1

Variables
PEP Provide jtd or Dst
CalibOnly?
2 1 to reproduce of
oNormalize?
2 1=no, 2=yes

ofxn calc*
0.27183
ofxn mod
0.29406

sRun
RunA1
zout
1

Ave
Data
4.9E-06
Model
4.3E-06

data/vMax
4.9E-06

ofxng
319.391

Min d/m
4.26448

ave/min d
1
ave/min m
3.74227

330_Calib_S_A

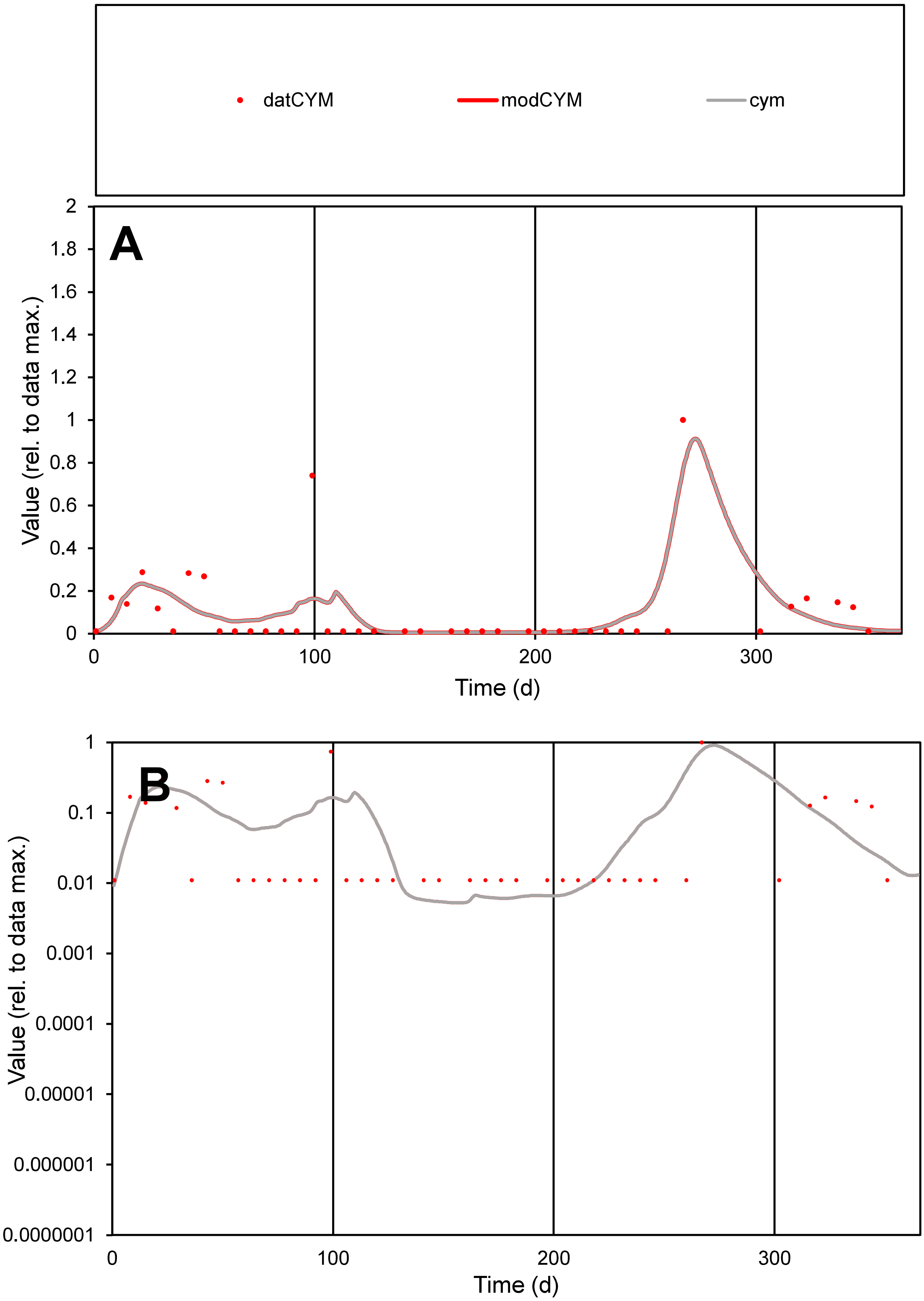

C1

Variables
CYM Provide jtd or Dst
CalibOnly?
2 1 to reproduce of
oNormalize?
2 1=no, 2=yes

ofxn calc*
1.36571
ofxn mod
1.43439

sRun
RunA1
zout
1

Ave
Data
1.2E-05
Model
1.2E-05

data/vMax
0.00012

ofxng
319.391

Min d/m
2.03013

ave/min d
8.60485
ave/min m
17.7562

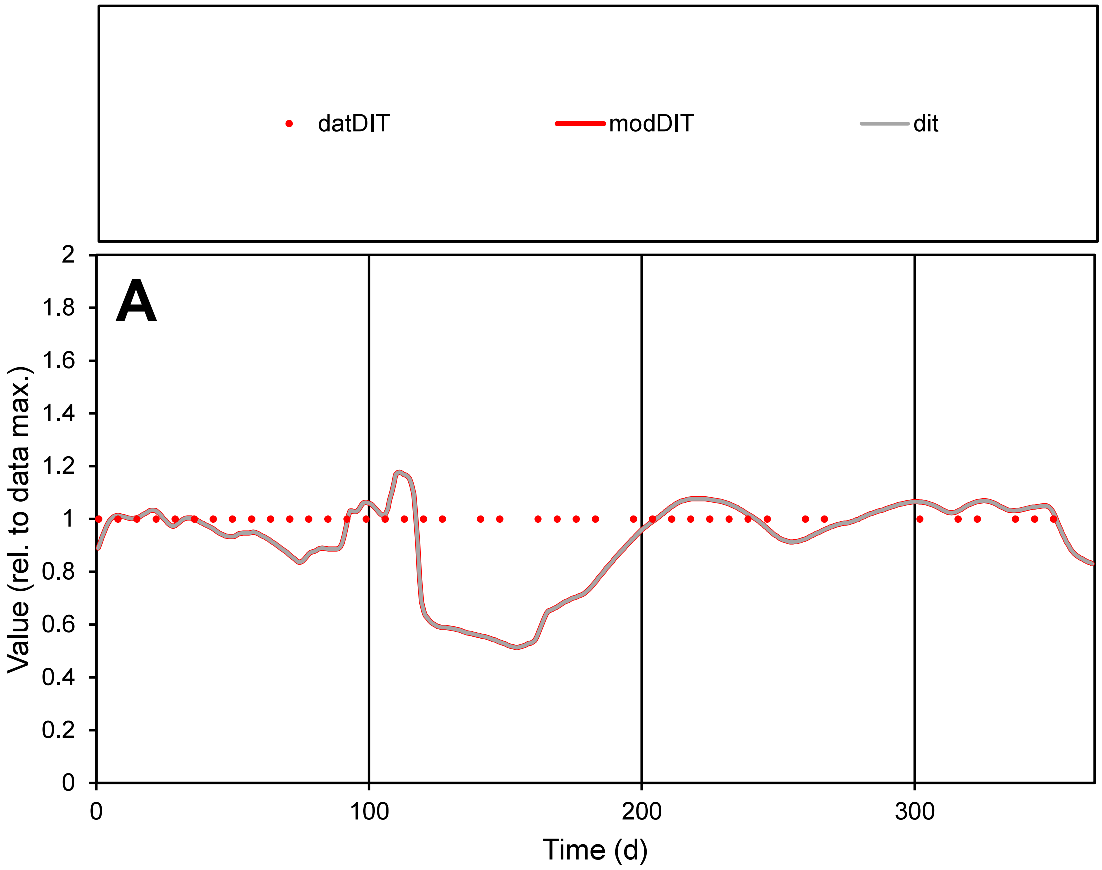

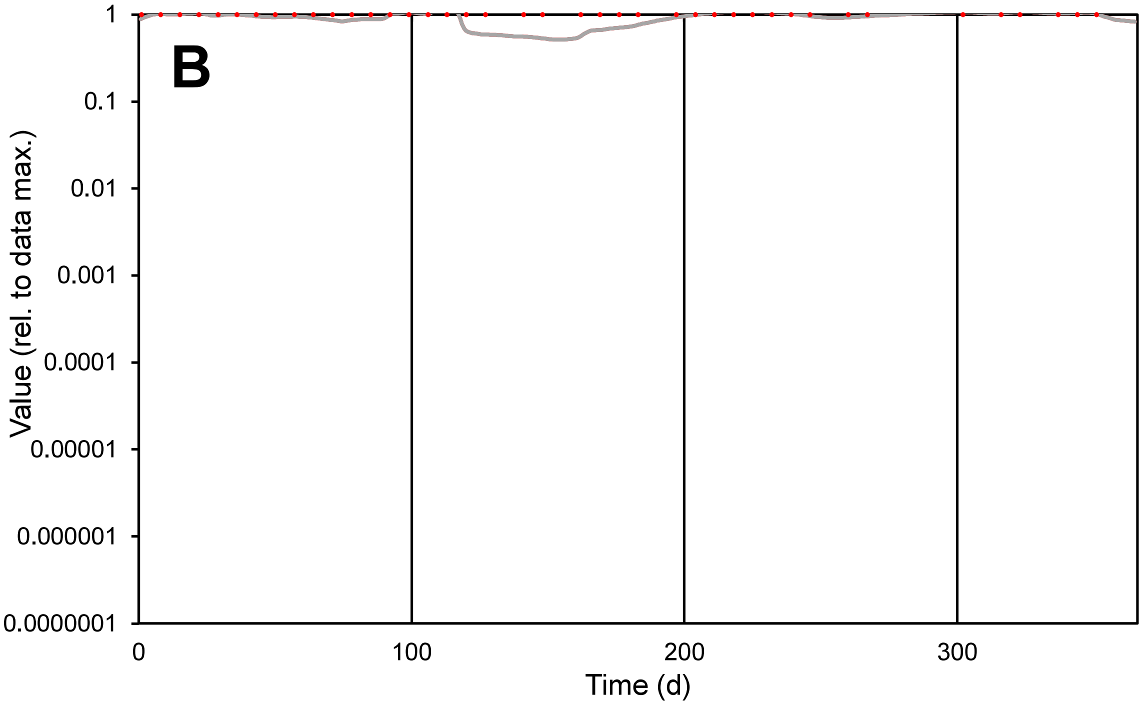

C1

Variables
DIT Provide jtd or Dst
CalibOnly?
2 1 to reproduce of
oNormalize?
2 1=no, 2=yes

ofxn calc*
0.17629
ofxn mod
0.20571

sRun
RunA1
zout
1

Ave
Data
5.1E-06
Model
4.7E-06

data/vMax
5.1E-06

ofxng
319.391

Min d/m
1.88844

ave/min d
1
ave/min m
1.75052

330_Calib_S_A

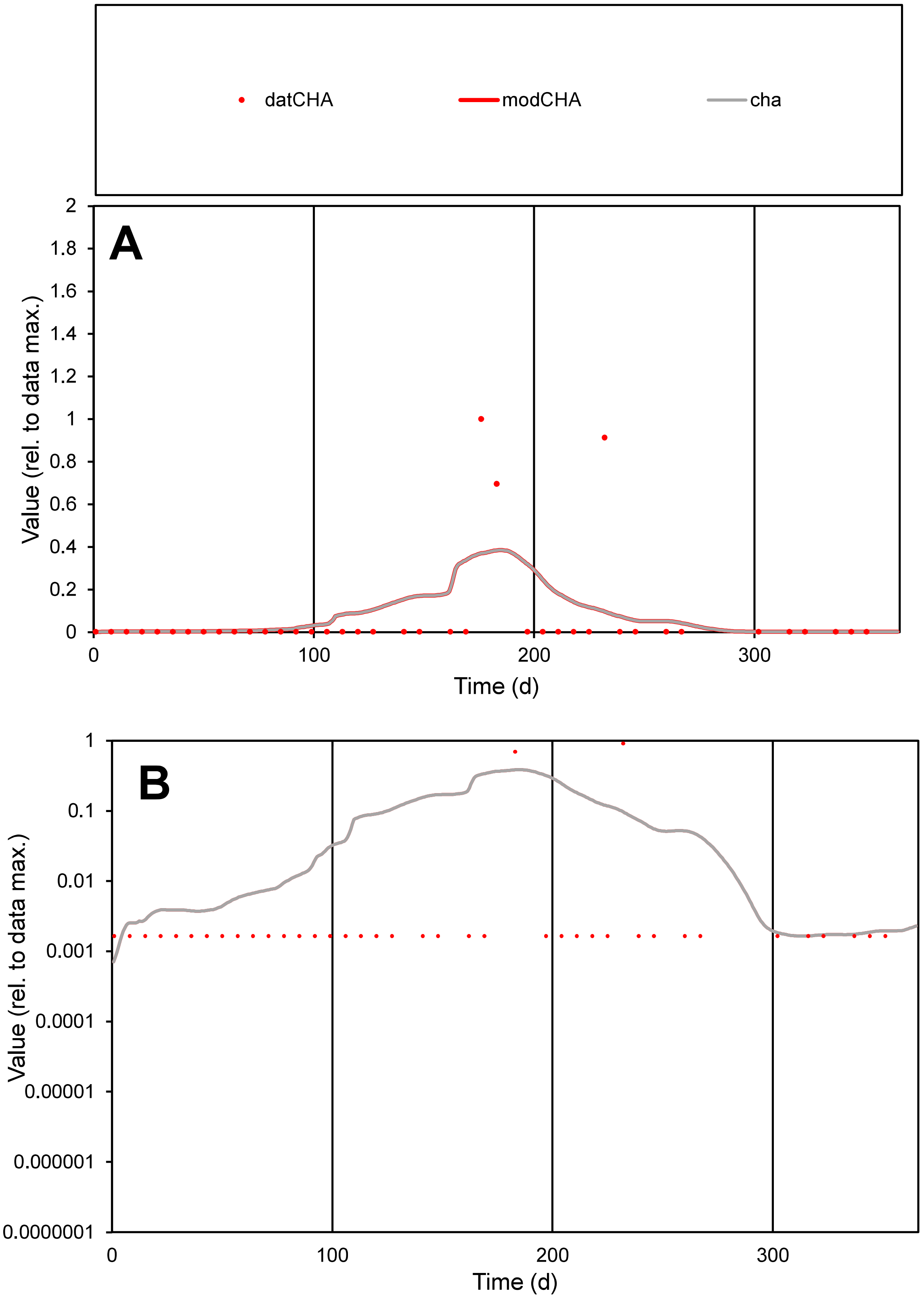

C1

Variables
CHA Provide jtd or Dst
CalibOnly?
2 1 to reproduce of
oNormalize?
2 1=no, 2=yes

ofxn calc*
3.02777
ofxn mod
3.05705

sRun
RunA1
zout
1

Ave
Data
3.4E-06
Model
4.2E-06

data/vMax
5.2E-05

ofxng
319.391

Min d/m
1.19462

ave/min d
39.4017
ave/min m
58.021

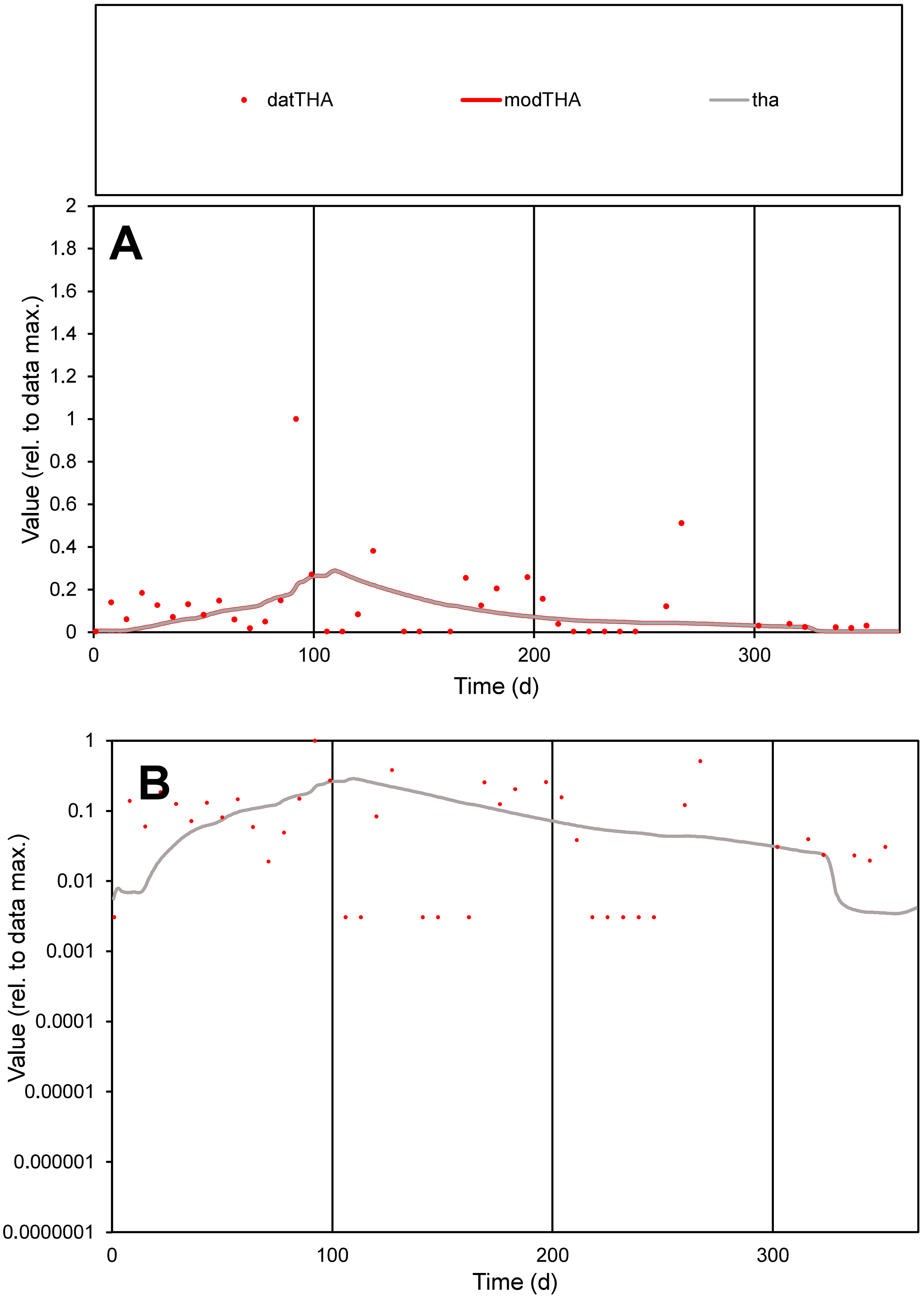

C1

Variables
THA Provide jtd or Dst
CalibOnly?
2 1 to reproduce of
oNormalize?
2 1=no, 2=yes

ofxn calc*
1.48424
ofxn mod
1.49053

sRun
RunA1
zout
1

Ave
Data
0.00011
Model
8.3E-05

data/vMax
0.0009

ofxng
319.391

Min d/m
0.87588

ave/min d
38.5364
ave/min m
26.5574

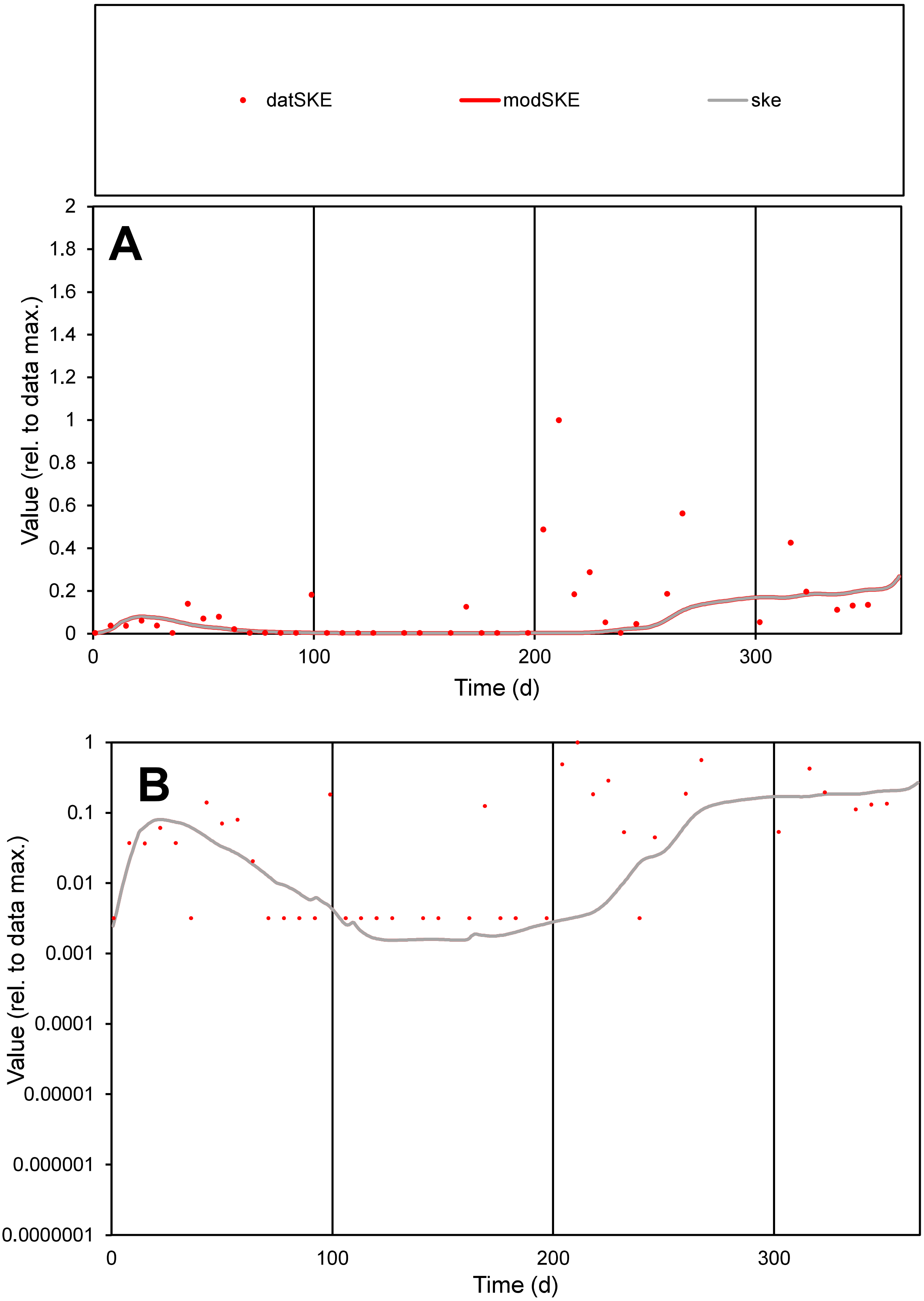

C1

Variables
SKE Provide jtd or Dst
CalibOnly?
2 1 to reproduce of
oNormalize?
2 1=no, 2=yes

ofxn calc*
1.78261
ofxn mod
1.78744

sRun
RunA1
zout
1

Ave
Data
8.3E-05
Model
3.3E-05

data/vMax
0.00072

ofxng
319.391

Min d/m
2.05263

ave/min d
36.1063
ave/min m
29.2578

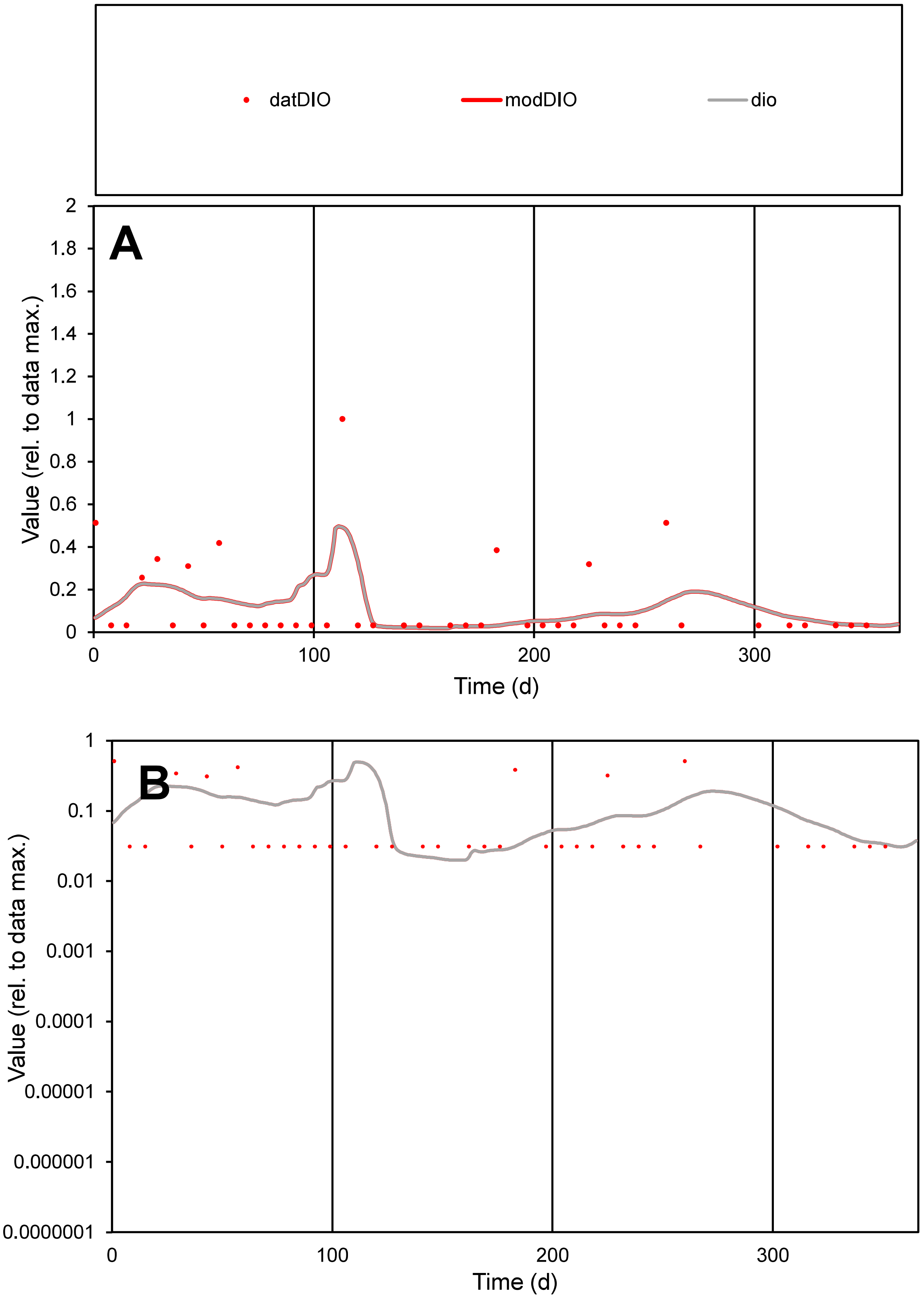

C1

Variables

DIO Provide jtd or Dst

CalibOnly?

2 1 to reproduce of

oNormalize?

2 1=no, 2=yes

ofxn calc*

1.37324

ofxn mod

1.39913

sRun

RunA1

zout

1

Ave

Data

1.8E-05

Model

1.8E-05

data/vMax

0.00015

ofxng

319.391

Min d/m

1.4984

ave/min d

3.94934

ave/min m

5.66211

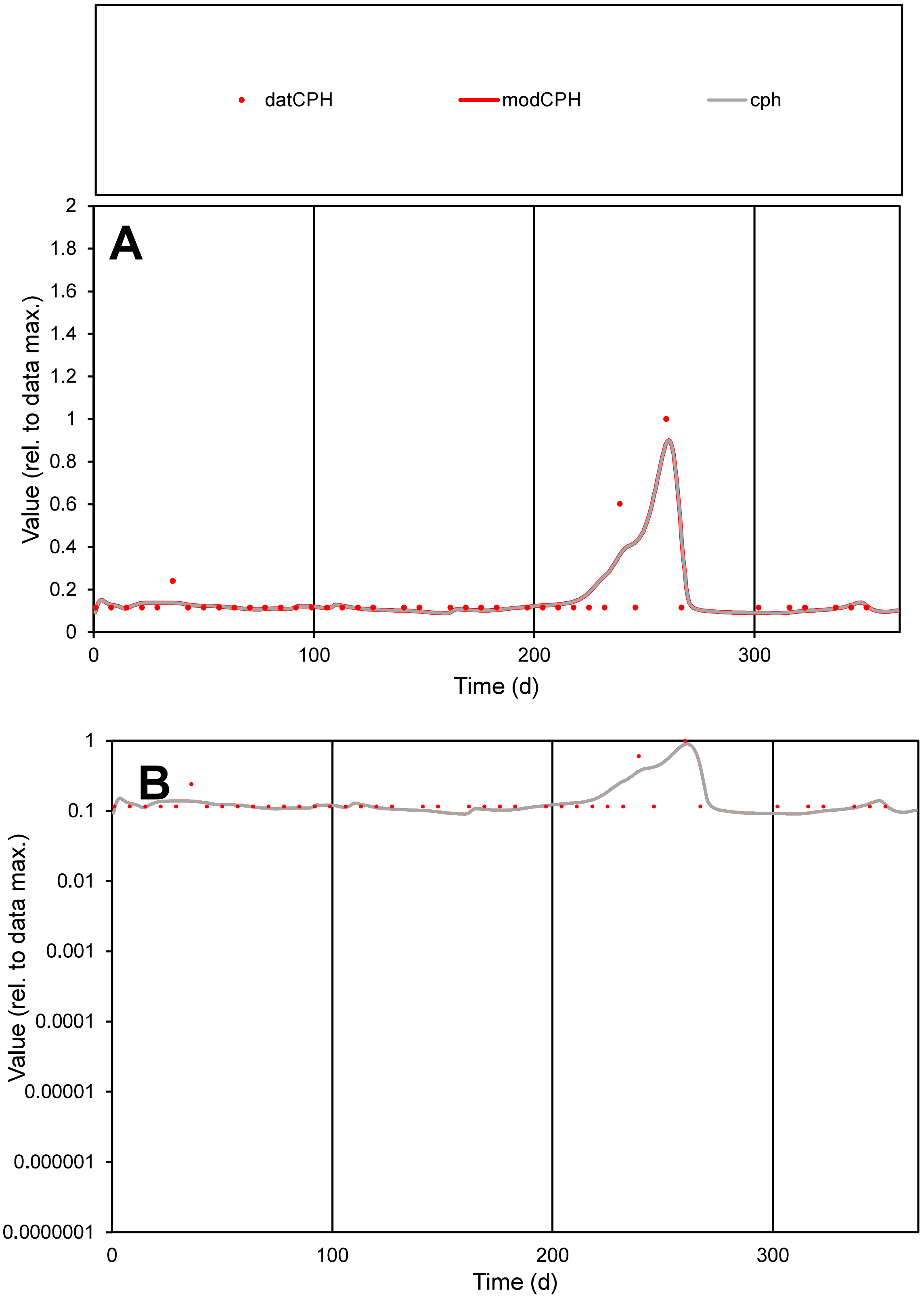

C1

Variables
CPH Provide jtd or Dst
CalibOnly?
2 1 to reproduce of
oNormalize?
2 1=no, 2=yes

ofxn calc*
0.46903
ofxn mod
0.5006

sRun
RunA1
zout
1

Ave
Data
1.3E-05
Model
1.3E-05

data/vMax
8.3E-05

ofxng
319.391

Min d/m
1.26295

ave/min d
1.31681
ave/min m
1.72585

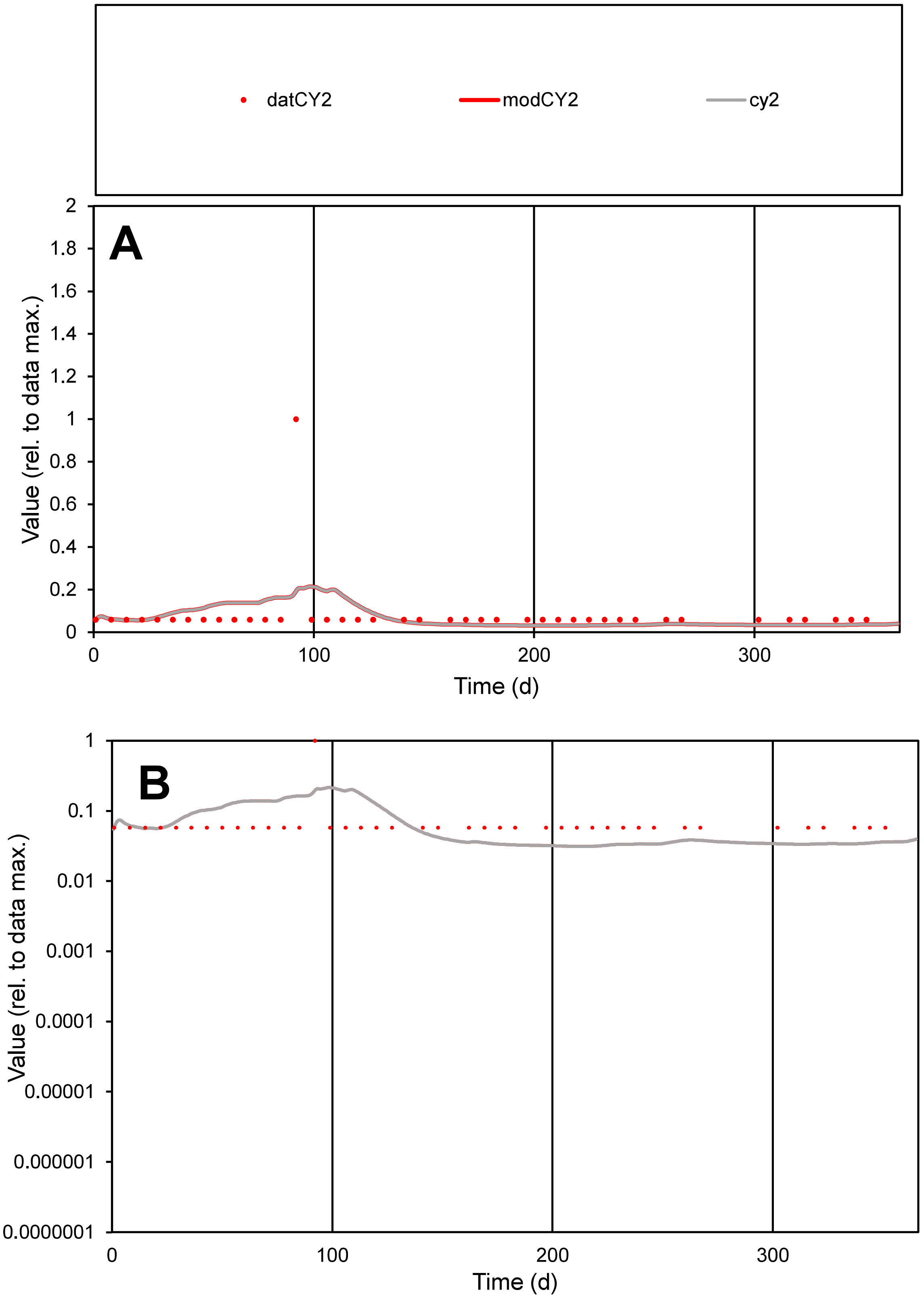

C1

Variables
CY2 Provide jtd or Dst
CalibOnly?
2 1 to reproduce of
oNormalize?
2 1=no, 2=yes

ofxn calc*
1.66992
ofxn mod
1.67605

sRun
RunA1
zout
1

Ave
Data
1.6E-05
Model
1.5E-05

data/vMax
0.00019

ofxng
319.391

Min d/m
1.84774

ave/min d
1.39795
ave/min m
2.41954

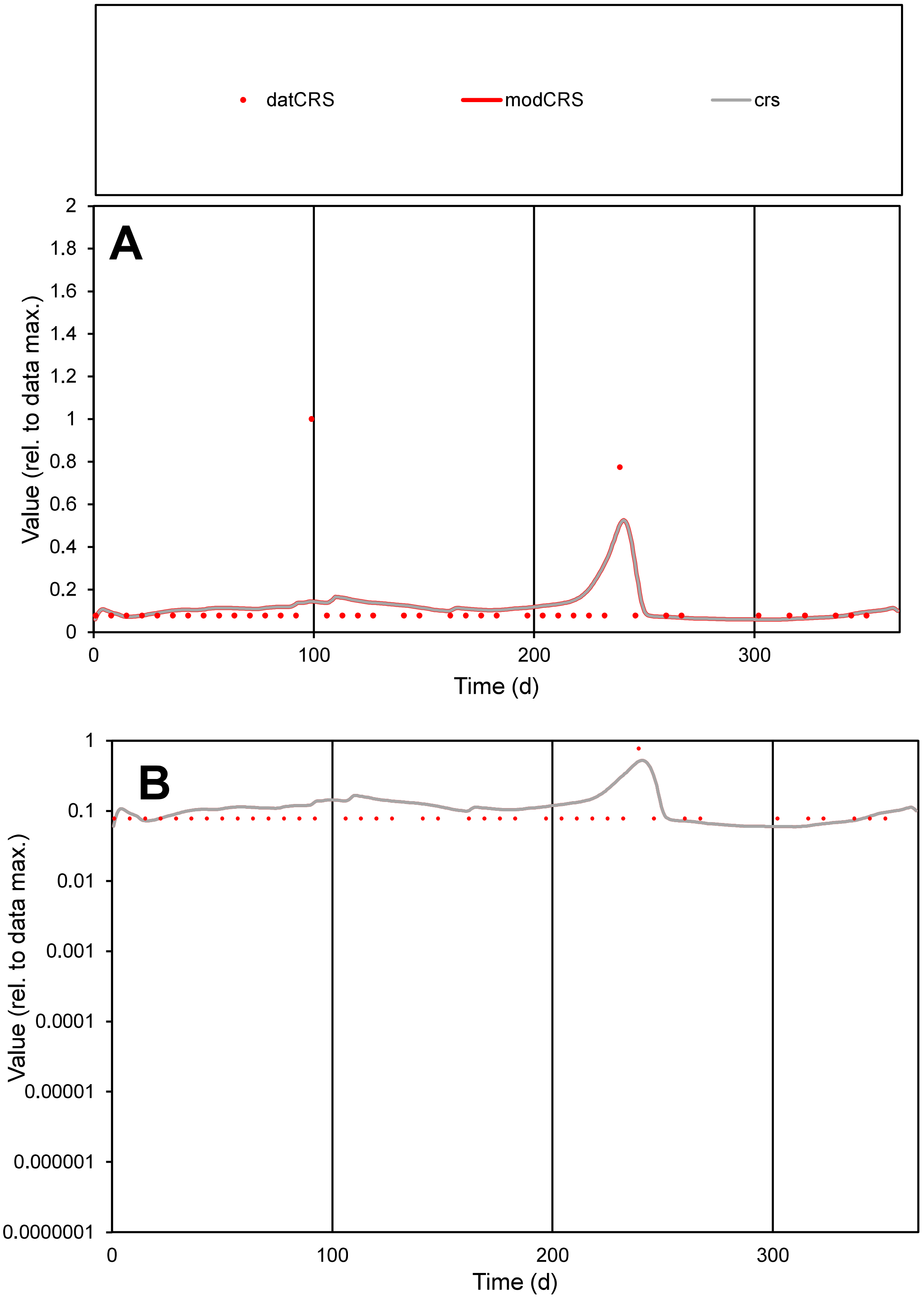

C1

Variables
CRS Provide jtd or Dst
CalibOnly?
2 1 to reproduce of
oNormalize?
2 1=no, 2=yes

ofxn calc*
1.27245
ofxn mod
1.29851

sRun
RunA1
zout
1

Ave
Data
8.1E-06
Model
8.6E-06

data/vMax
6.9E-05

ofxng
319.391

Min d/m
1.30898

ave/min d
1.5044
ave/min m
2.08866

330_Calib_S_A

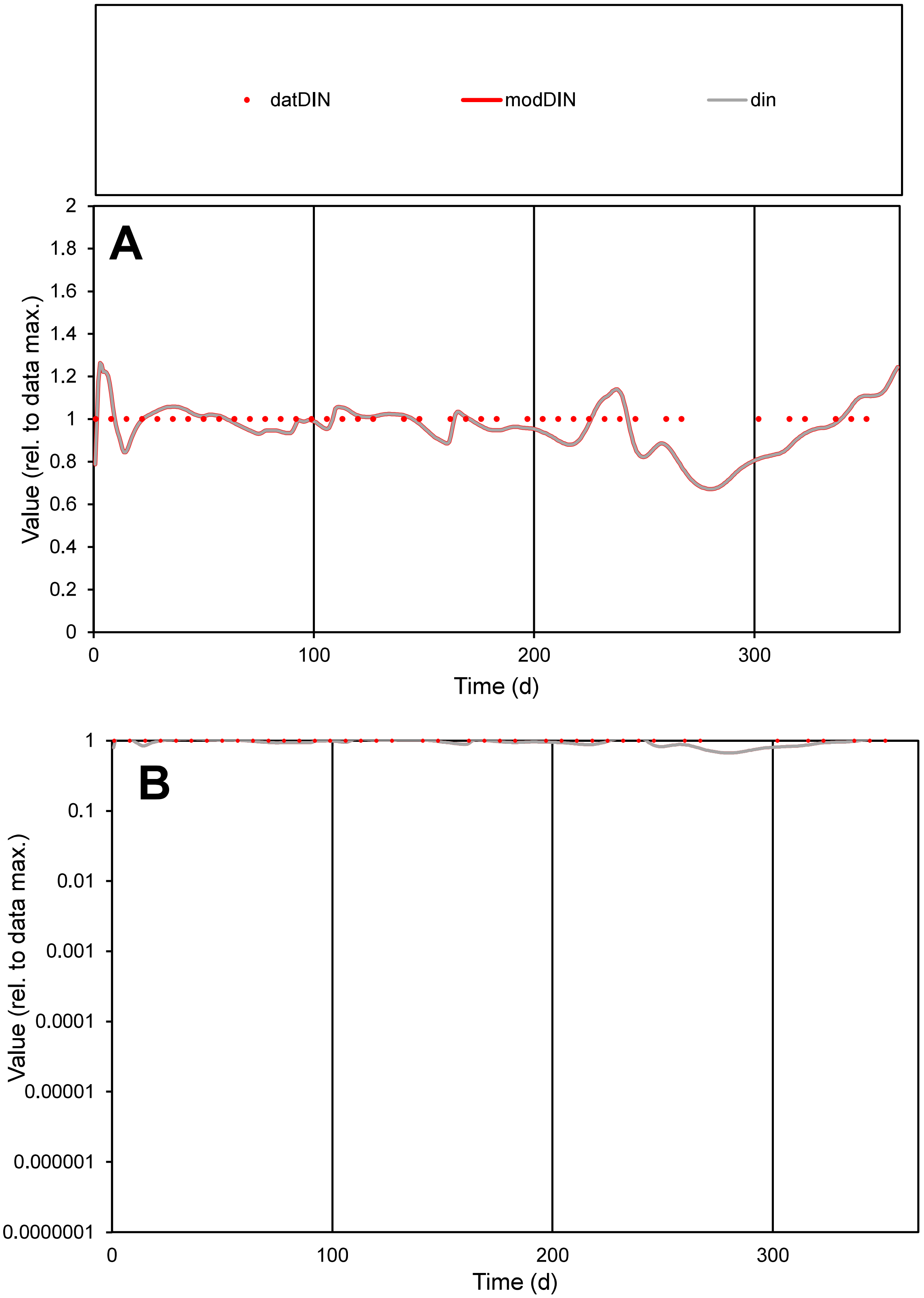

C1

Variables
DIN Provide jtd or Dst
CalibOnly?
2 1 to reproduce of
oNormalize?
2 1=no, 2=yes

ofxn calc*
0.07929
ofxn mod
0.13376

sRun
RunA1
zout
1

Ave
Data
2.9E-06
Model
2.8E-06

data/vMax
2.9E-06

ofxng
319.391

Min d/m
1.32294

ave/min d
1
ave/min m
1.28885

330_Calib_S_A

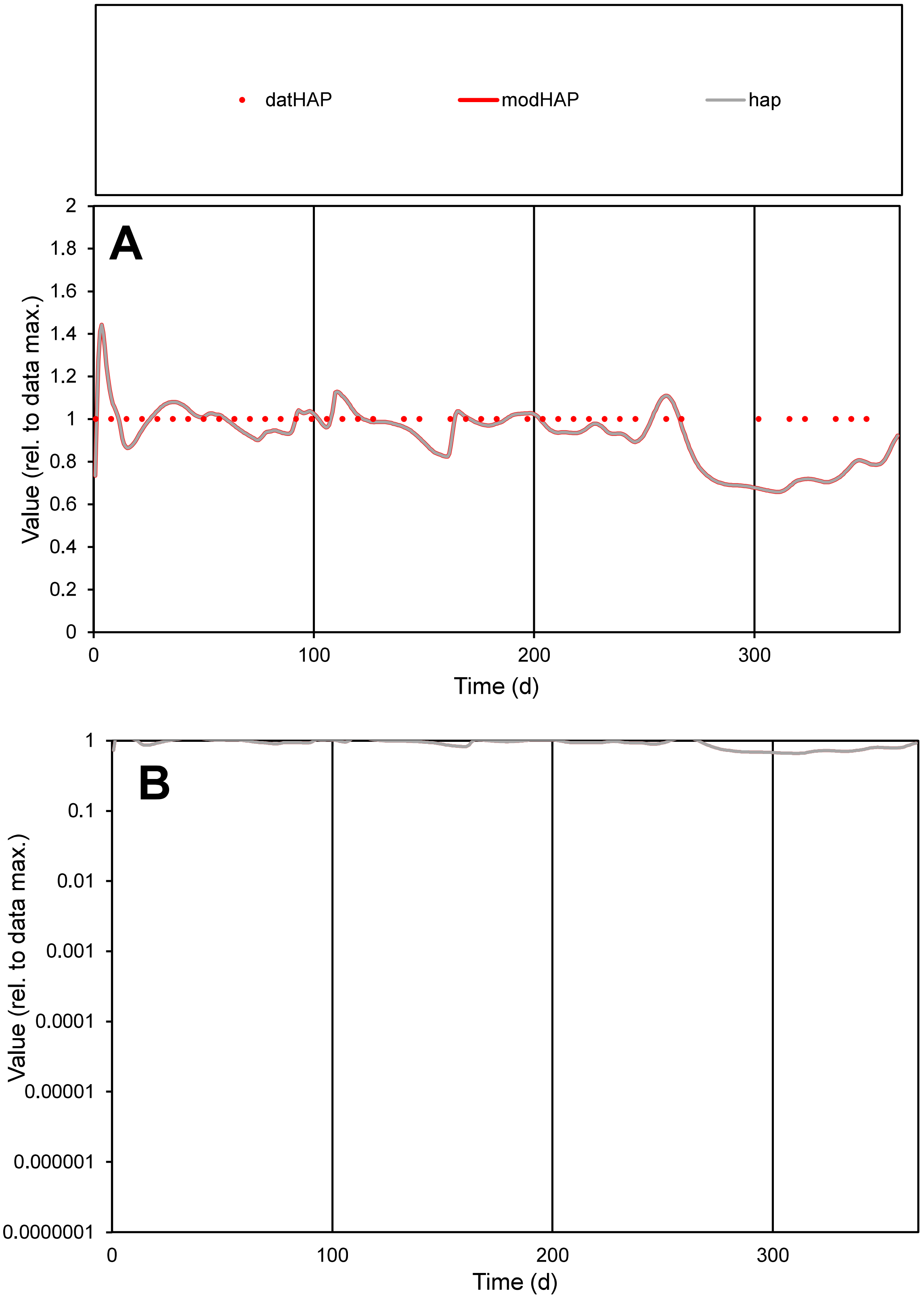

C1

Variables
HAP Provide jtd or Dst
CalibOnly?
2 1 to reproduce of
oNormalize?
2 1=no, 2=yes

ofxn calc*
0.11681
ofxn mod
0.15812

sRun
RunA1
zout
1

Ave
Data
1.1E-05
Model
1.1E-05

data/vMax
1.1E-05

ofxng
319.391

Min d/m
1.48931

ave/min d
1
ave/min m
1.41205

330_Calib_S_A

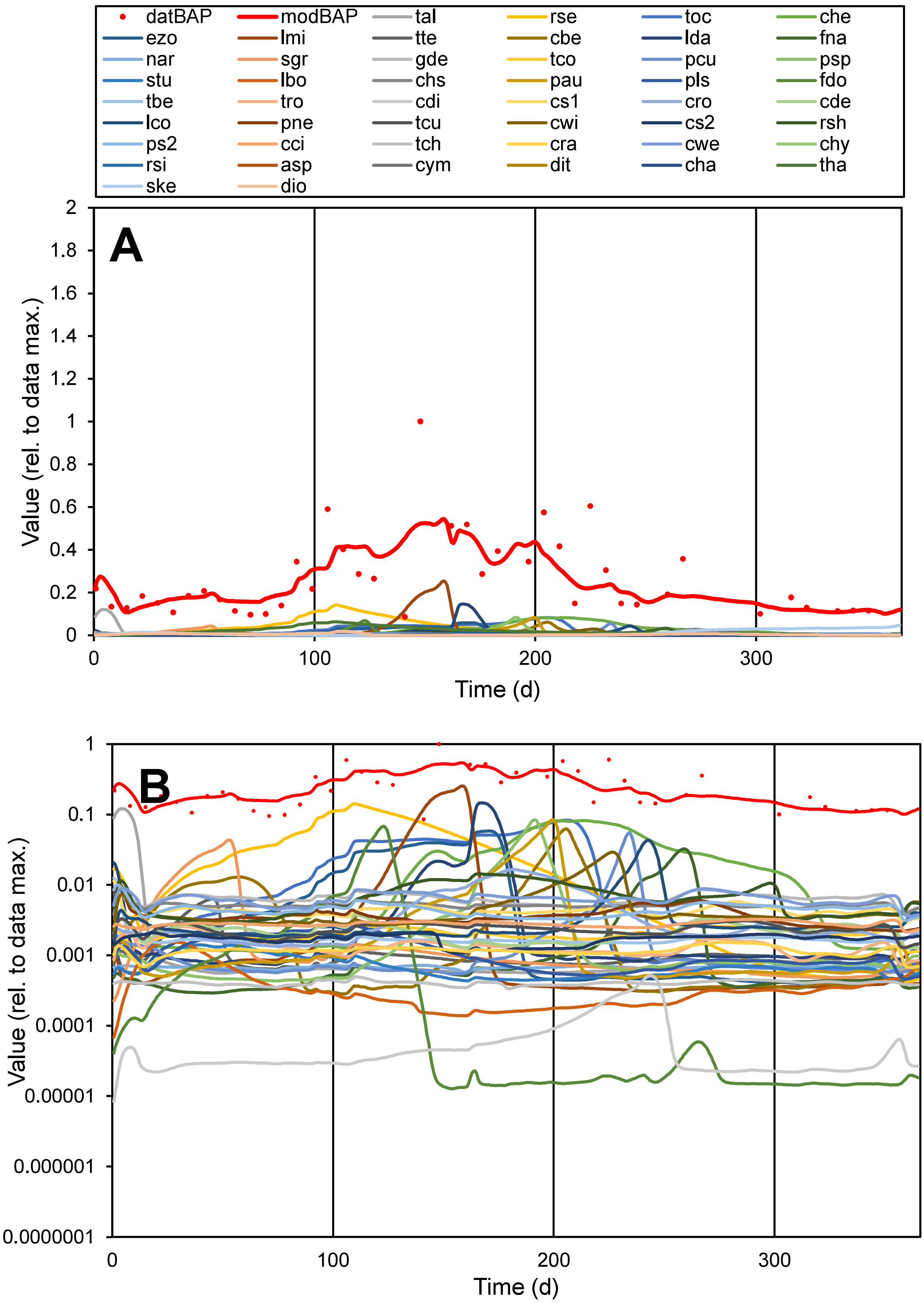

C1

Variables

BAP Provide jtd or Dst

CalibOnly?

2 1 to reproduce of

oNormalize?

2 1=no, 2=yes

ofxn calc*

0.51704

ofxn mod

0.5312

sRun

RunA1

zout

1

Ave

Data

0.00108

Model

0.00102

data/vMax

0.0041

ofxng

319.391

Min d/m

0.78613

ave/min d

3.07631

ave/min m

2.29166

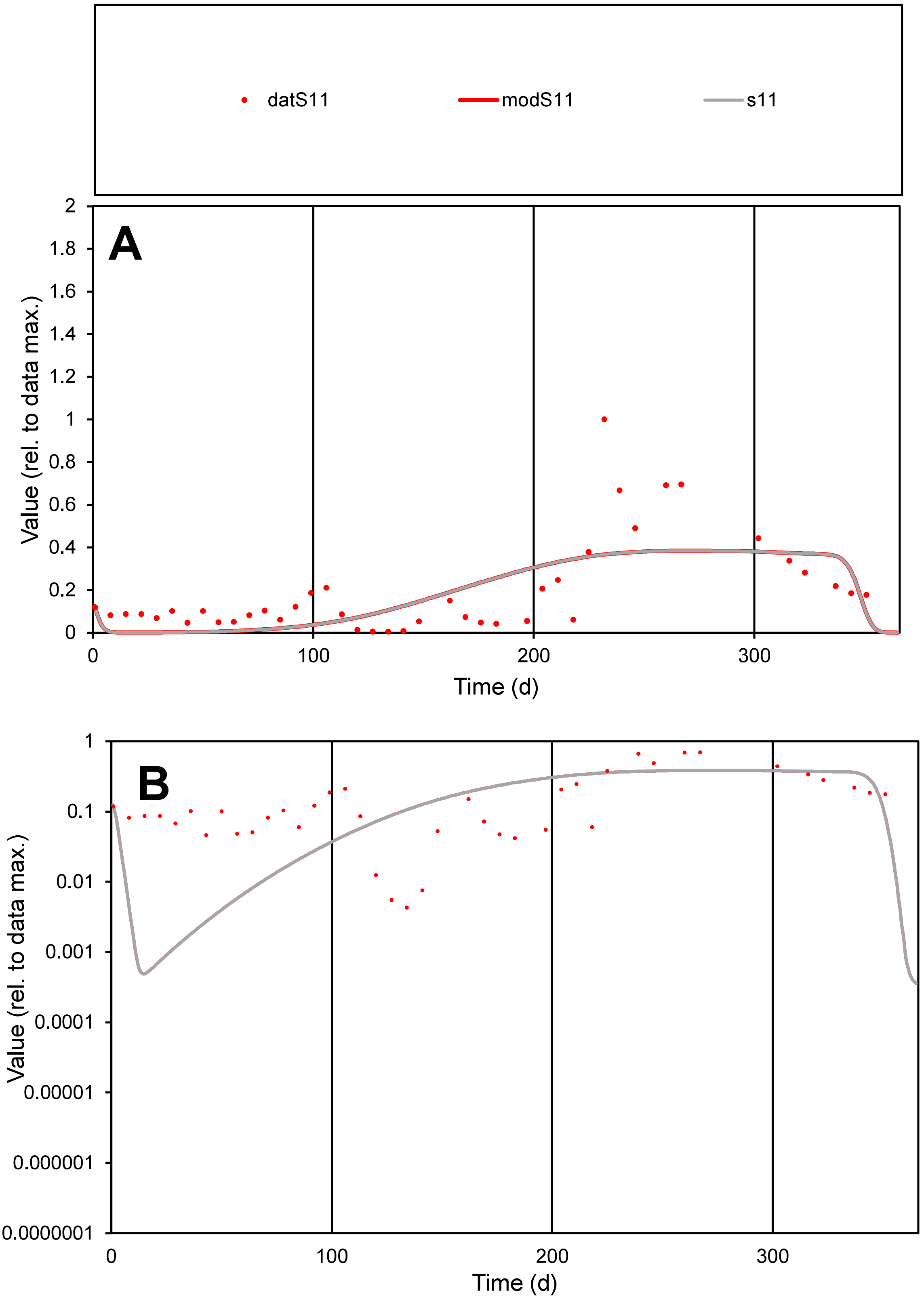

C1

Variables
S11 Provide jtd or Dst
CalibOnly?
2 1 to reproduce of
oNormalize?
2 1=no, 2=yes

ofxn calc*
0.85597
ofxn mod
0.8723

sRun
RunA1
zout
1

Ave
Data
0.08708
Model
0.07663

data/vMax
0.448

ofxng
319.391

Min d/m
8.19344

ave/min d
45.2833
ave/min m
326.484

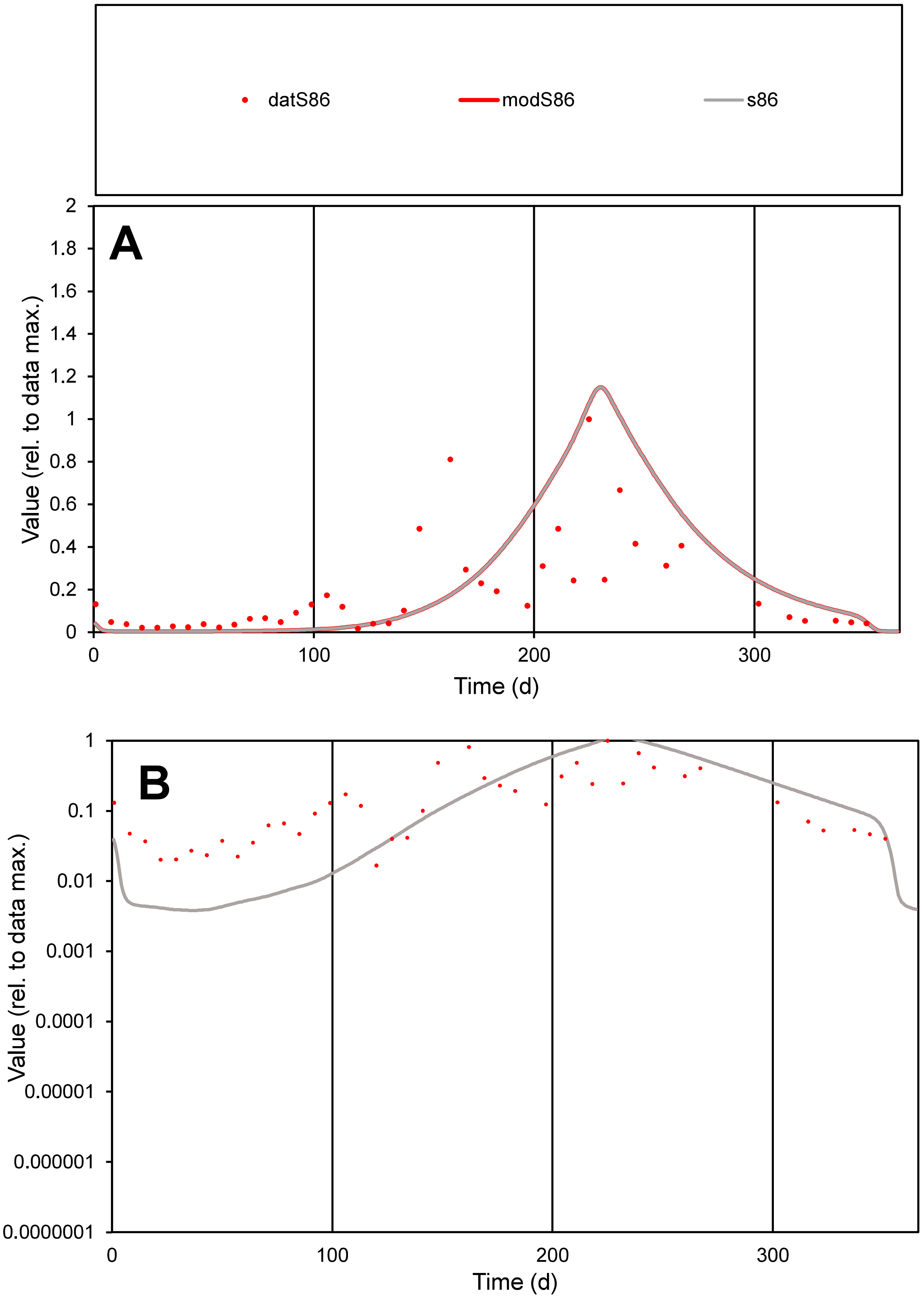

C1

Variables
S86 Provide jtd or Dst
CalibOnly?
2 1 to reproduce of
oNormalize?
2 1=no, 2=yes

ofxn calc*
1.35367
ofxn mod
1.37687

sRun
RunA1
zout
1

Ave
Data
0.04823
Model
0.06393

data/vMax
0.2564

ofxng
319.391

Min d/m
4.36617
ave/min d
11.3103
ave/min m
65.4635

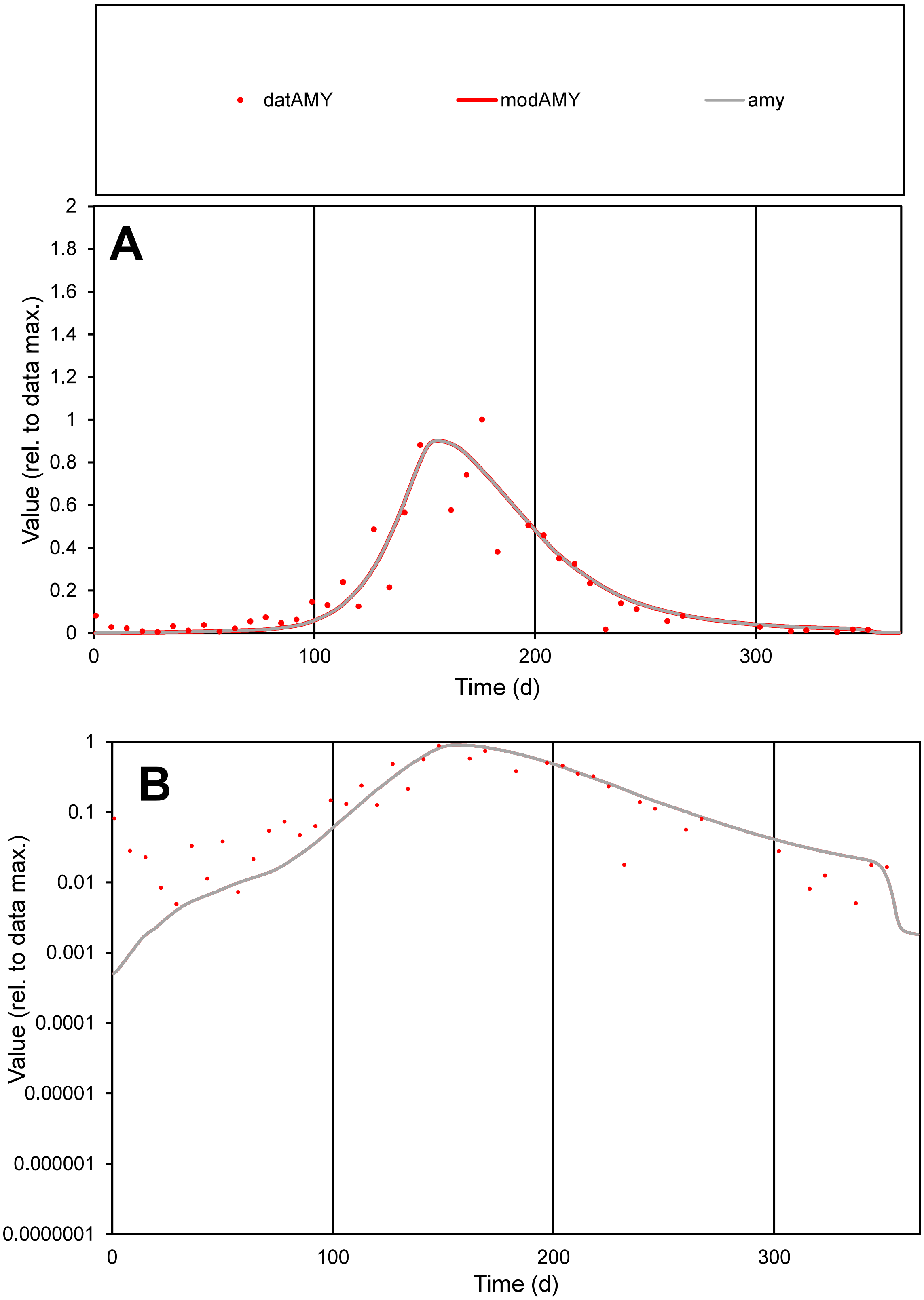

C1

Variables
AMY Provide jtd or Dst
CalibOnly?
2 1 to reproduce of
oNormalize?
2 1=no, 2=yes

ofxn calc*
0.50247
ofxn mod
0.53339

sRun
RunA1
zout
1

Ave
Data
0.03608
Model
0.03768

data/vMax
0.1814

ofxng
319.391

Min d/m
9.31491

ave/min d
40.3848
ave/min m
392.863

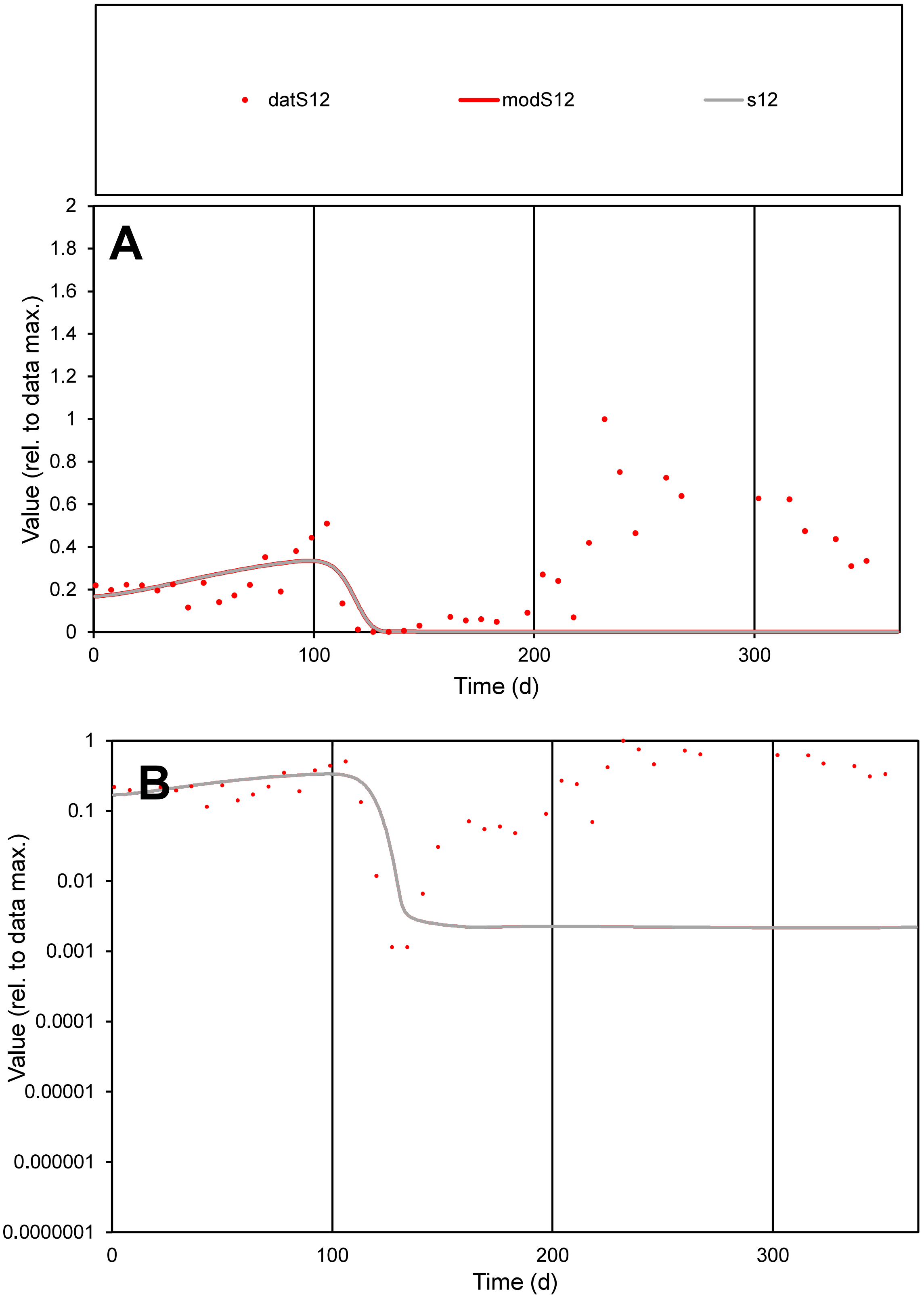

C1

Variables
S12 Provide jtd or Dst
CalibOnly?
2 1 to reproduce of
oNormalize?
2 1=no, 2=yes

ofxn calc*
1.16116
ofxn mod
1.16533

sRun
RunA1
zout
1

Ave
Data
0.03686
Model
0.01408

data/vMax
0.1298

ofxng
319.391

Min d/m
0.52901

ave/min d
248.049
ave/min m
50.1086

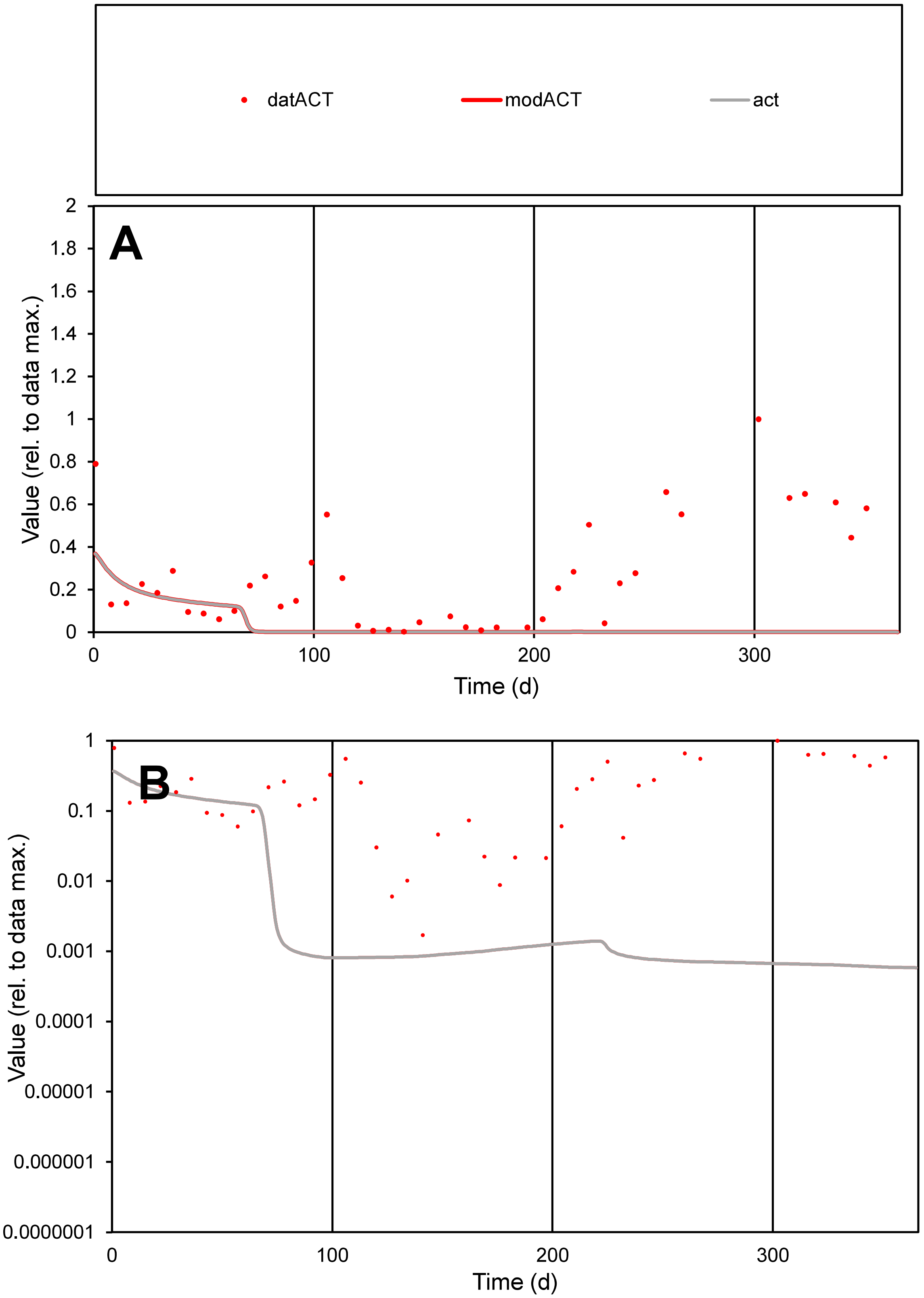

C1

Variables
ACT Provide jtd or Dst
CalibOnly?
2 1 to reproduce of
oNormalize?
2 1=no, 2=yes

ofxn calc*
1.29926
ofxn mod
1.3028

sRun
RunA1
zout
1

Ave
Data
0.01784
Model
0.00317

data/vMax
0.06853

ofxng
319.391

Min d/m
2.85819

ave/min d
152.614
ave/min m
77.464

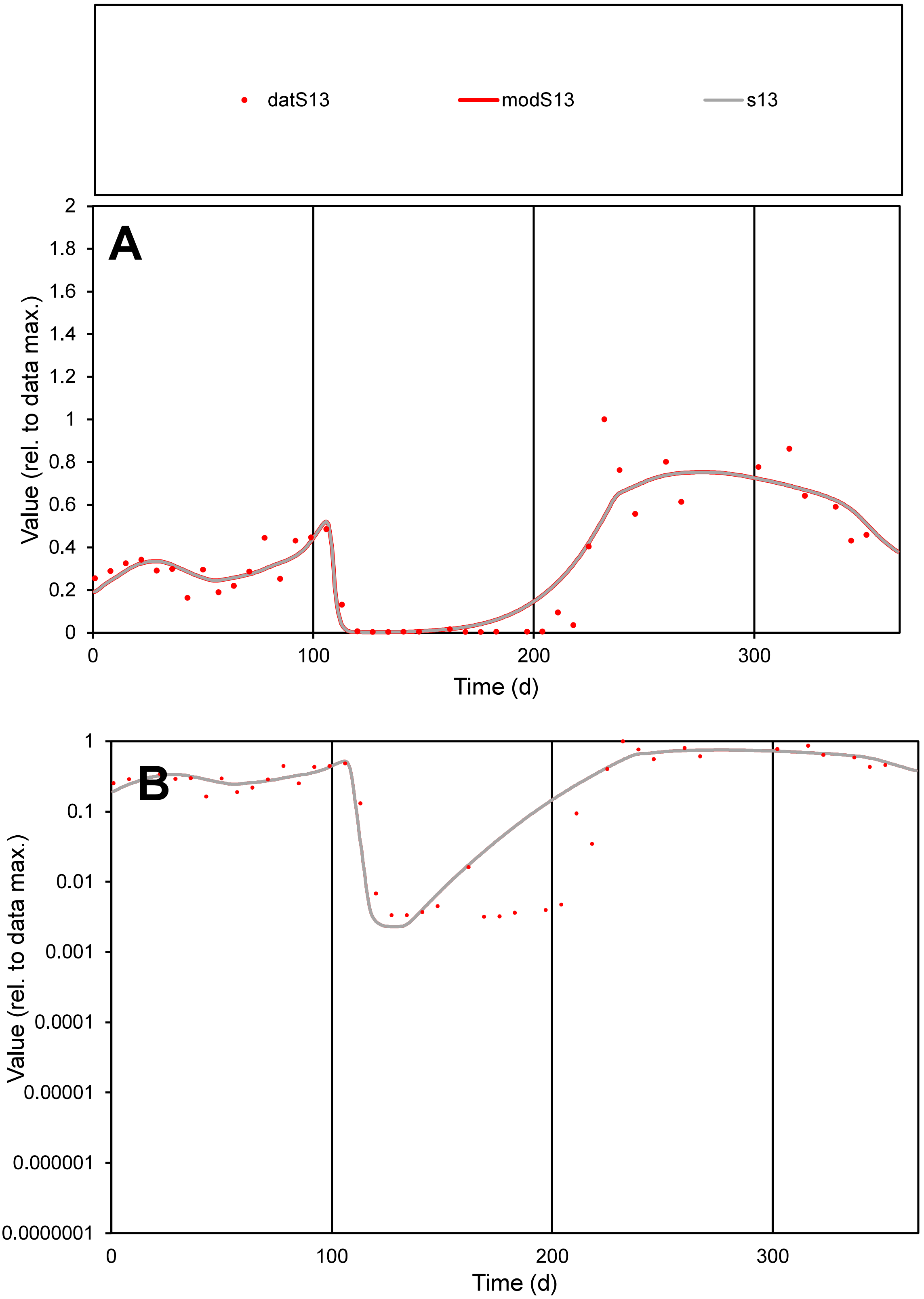

C1

Variables
S13 Provide jtd or Dst
CalibOnly?
2 1 to reproduce of
oNormalize?
2 1=no, 2=yes

ofxn calc*
0.34581
ofxn mod
0.37875

sRun
RunA1
zout
1

Ave
Data
0.02779
Model
0.02876

data/vMax
0.08824

ofxng
319.391

Min d/m
1.39286

ave/min d
98.955
ave/min m
142.657

330_Calib_S_A

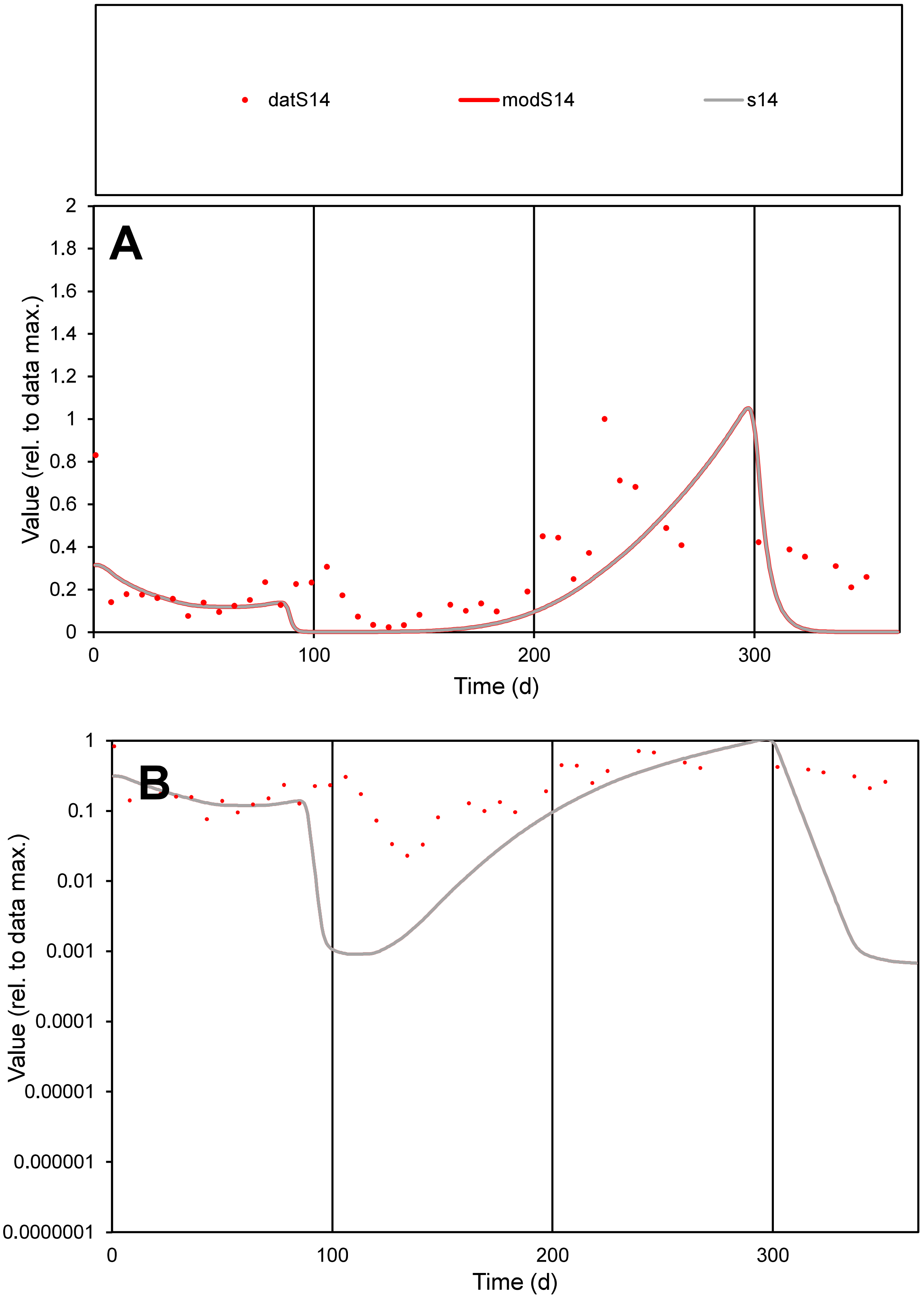

C1

Variables
S14 Provide jtd or Dst
CalibOnly?
2 1 to reproduce of
oNormalize?
2 1=no, 2=yes

ofxn calc*
0.81078
ofxn mod
0.8262

sRun
RunA1
zout
1

Ave
Data
0.02659
Model
0.01483

data/vMax
0.1

ofxng
319.391

Min d/m
31.0401

ave/min d
11.5555
ave/min m
200.097

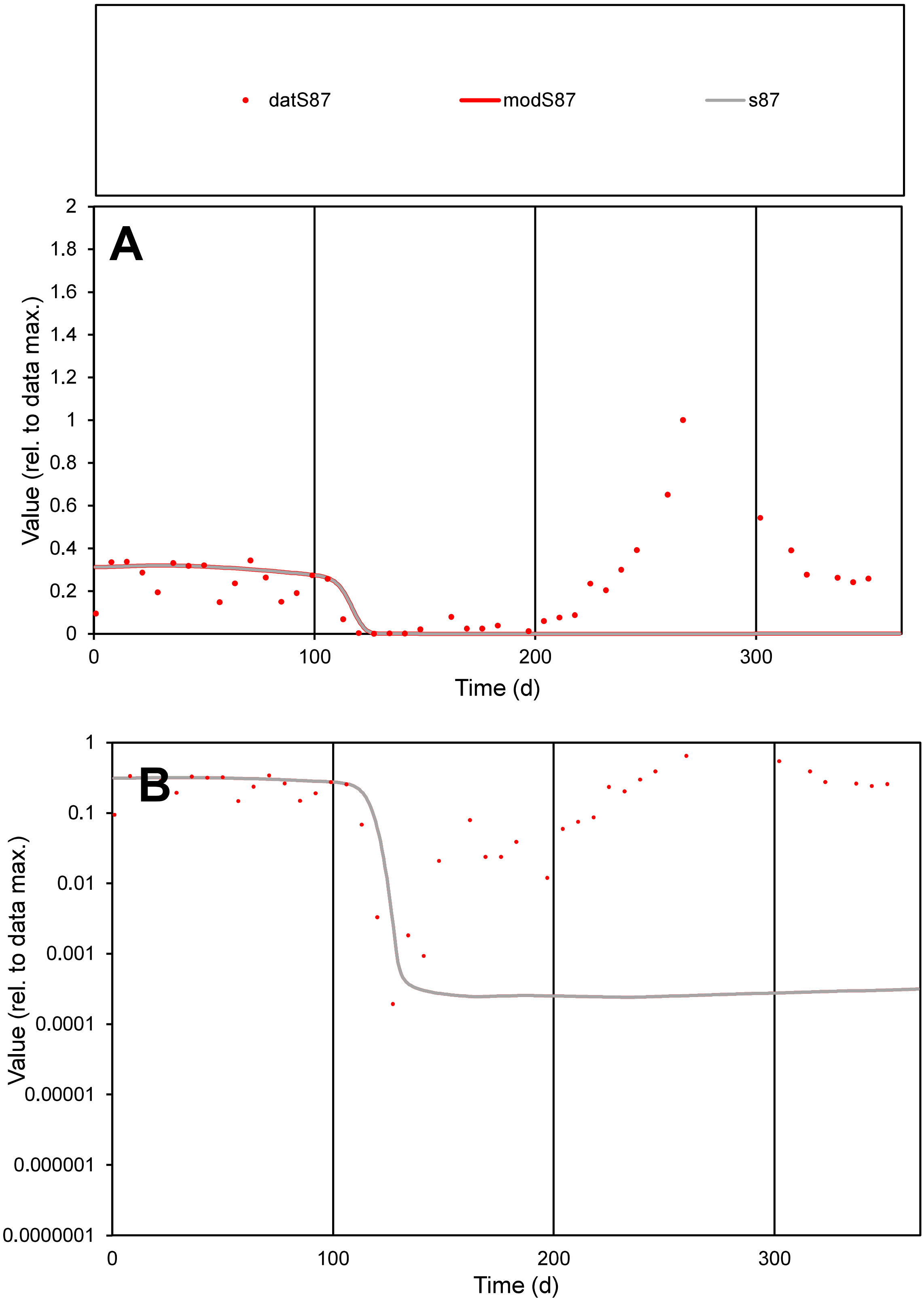

C1

Variables

S87	Provide jtd or Dst
CalibOnly?	
2	1 to reproduce of
oNormalize?	
2	1=no, 2=yes

ofxn calc*
1.12907
ofxn mod
1.13506

sRun
RunA1
zout
1

Ave
Data
0.02158
Model
0.01163

data/vMax
0.09722

ofxng
319.391

Min d/m
0.79957

ave/min d
1148.53
ave/min m
494.803

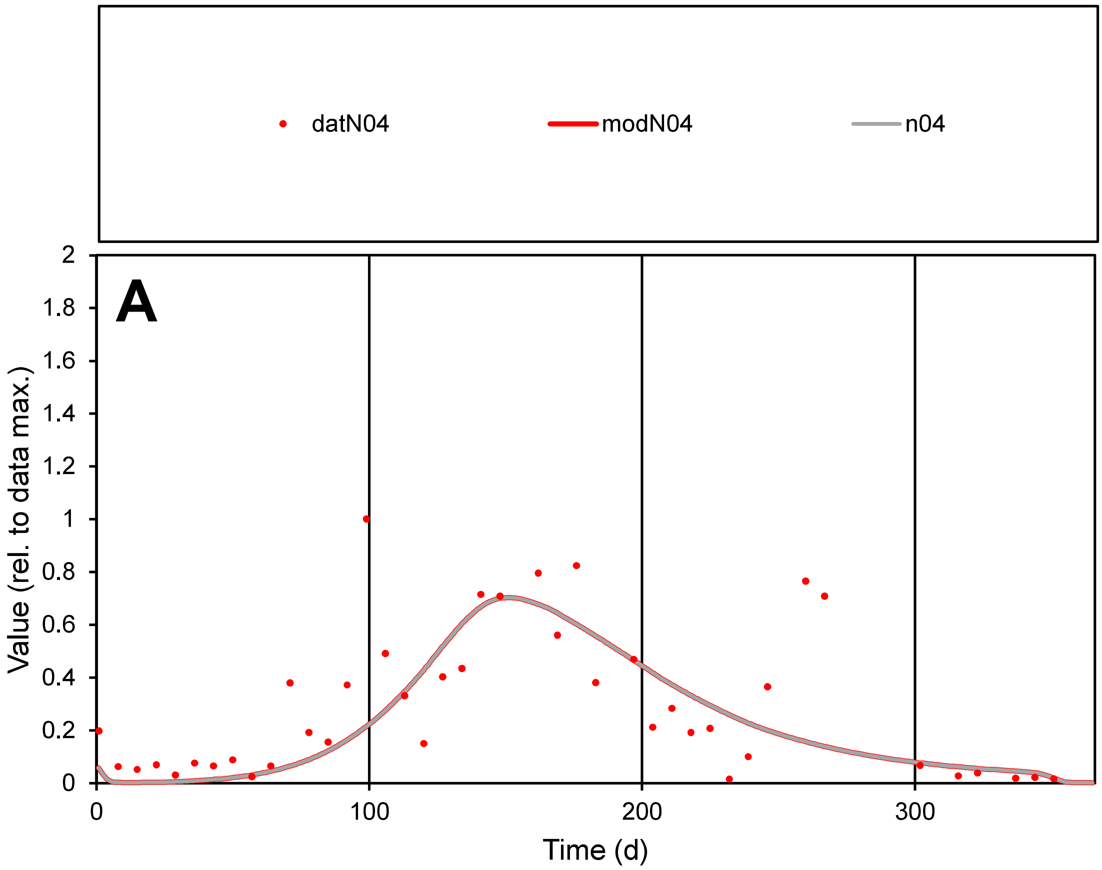

C1

Variables
N04 Provide jtd or Dst
CalibOnly?
2 1 to reproduce of
oNormalize?
2 1=no, 2=yes

ofxn calc*
0.74309
ofxn mod
0.75544

sRun
RunA1
zout
1

Ave
Data
0.02153
Model
0.01784

data/vMax
0.07461

ofxng
319.391

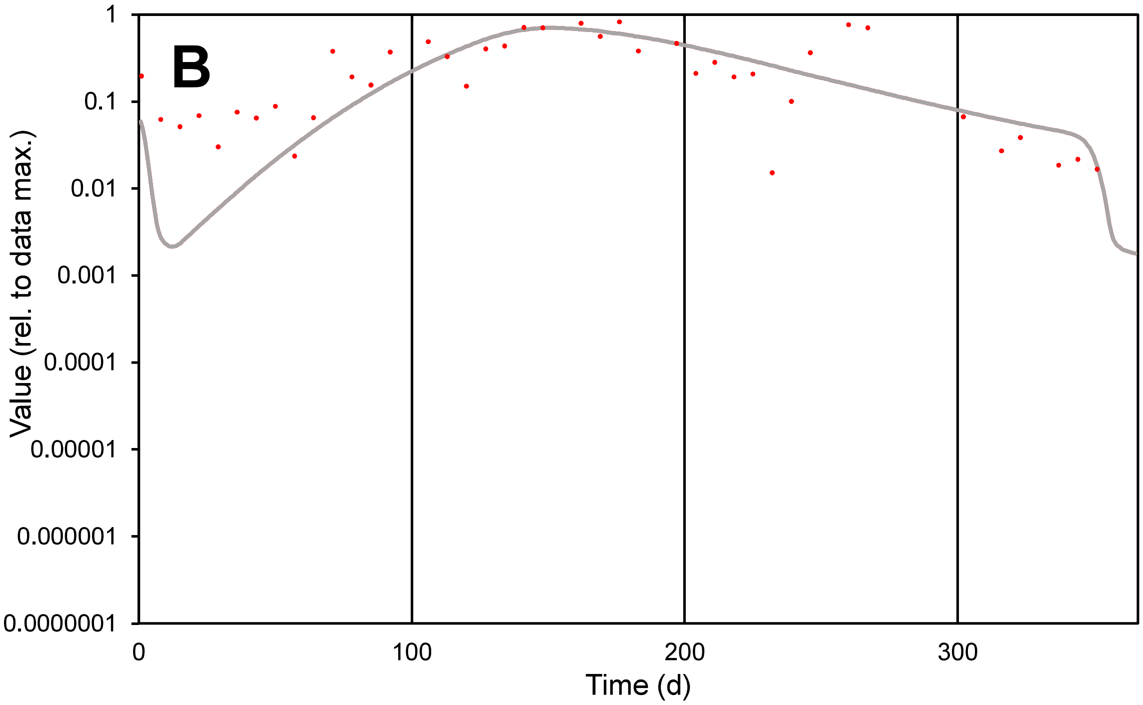

Min d/m
6.41605

ave/min d
18.9687
ave/min m
100.848

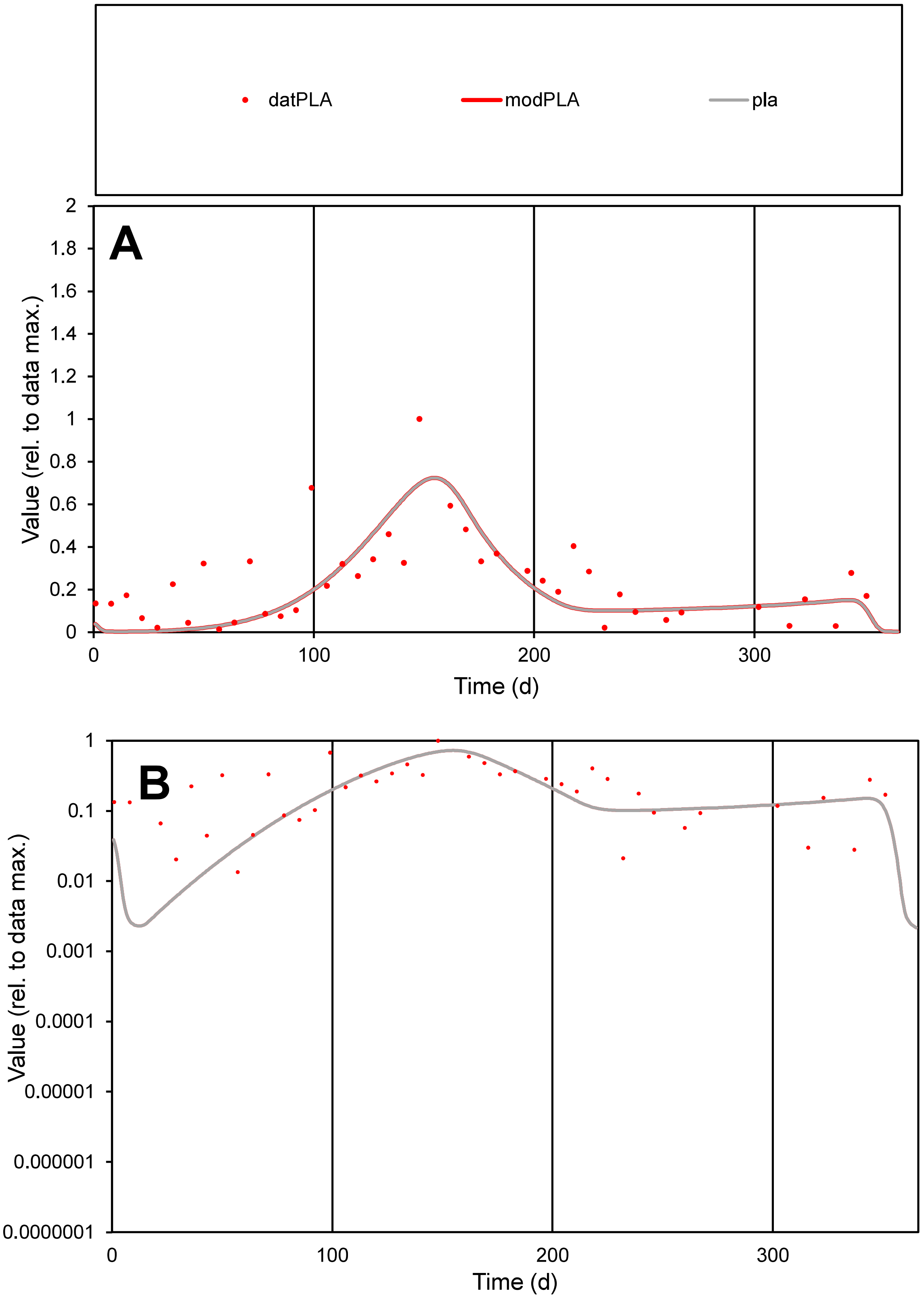

C1

Variables
PLA Provide jtd or Dst
CalibOnly?
2 1 to reproduce of
oNormalize?
2 1=no, 2=yes

ofxn calc*
0.64499
ofxn mod
0.66067

sRun
RunA1
zout
1

Ave
Data
0.01251
Model
0.01056

data/vMax
0.05374

ofxng
319.391

Min d/m
5.48897

ave/min d
17.3362
ave/min m
80.2677

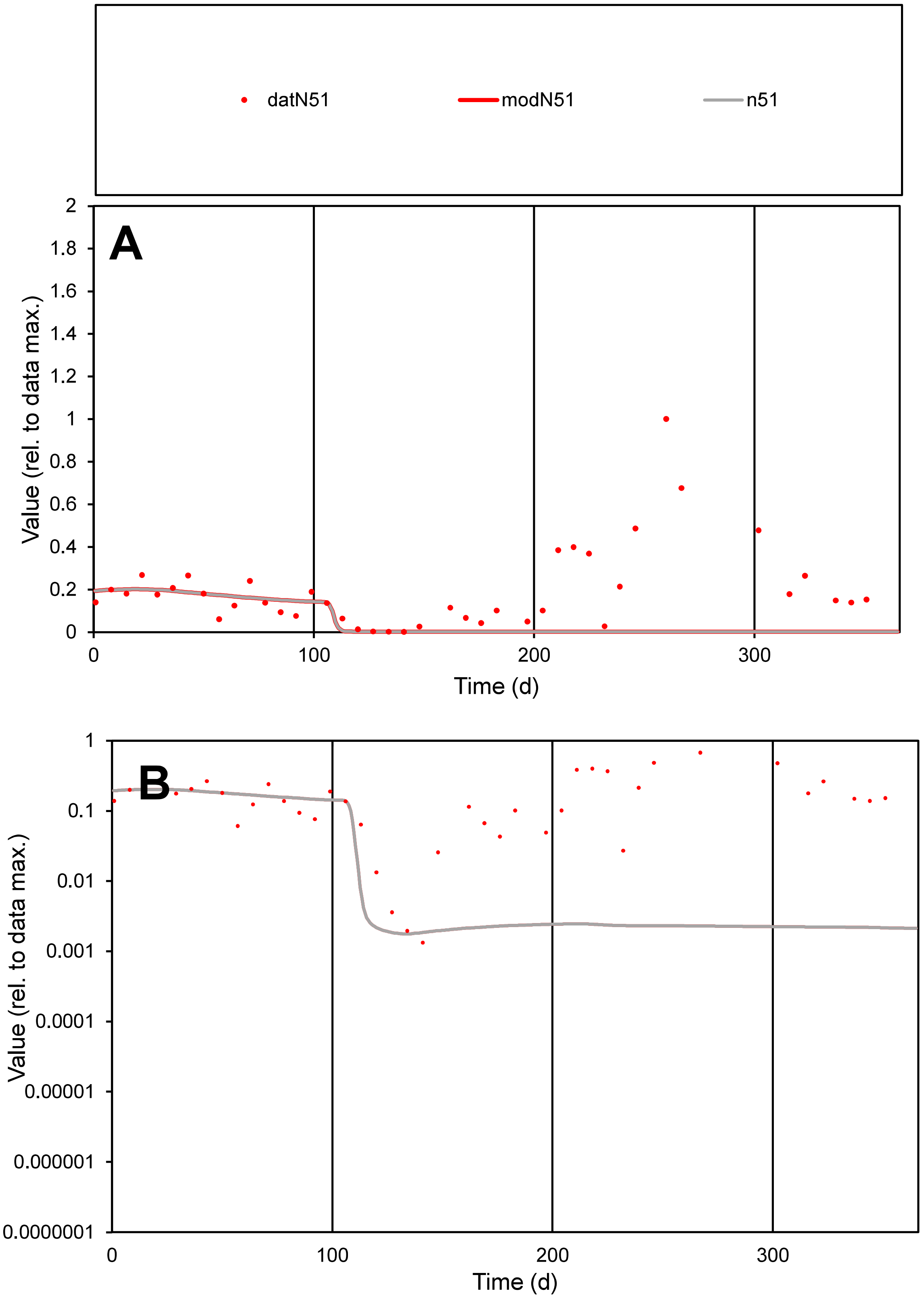

C1

Variables	
N51	Provide jtd or Dst
CalibOnly?	
2	1 to reproduce of
oNormalize?	
2	1=no, 2=yes
ofxn calc*	
1.28857	
ofxn mod	
1.2922	
sRun	
RunA1	
zout	
1	
Ave Data	
0.01328	
Model	
0.00464	
data/vMax	
0.06822	
ofxng	
319.391	
Min d/m	
0.75319	
ave/min d	
146.106	
ave/min m	
38.4474	

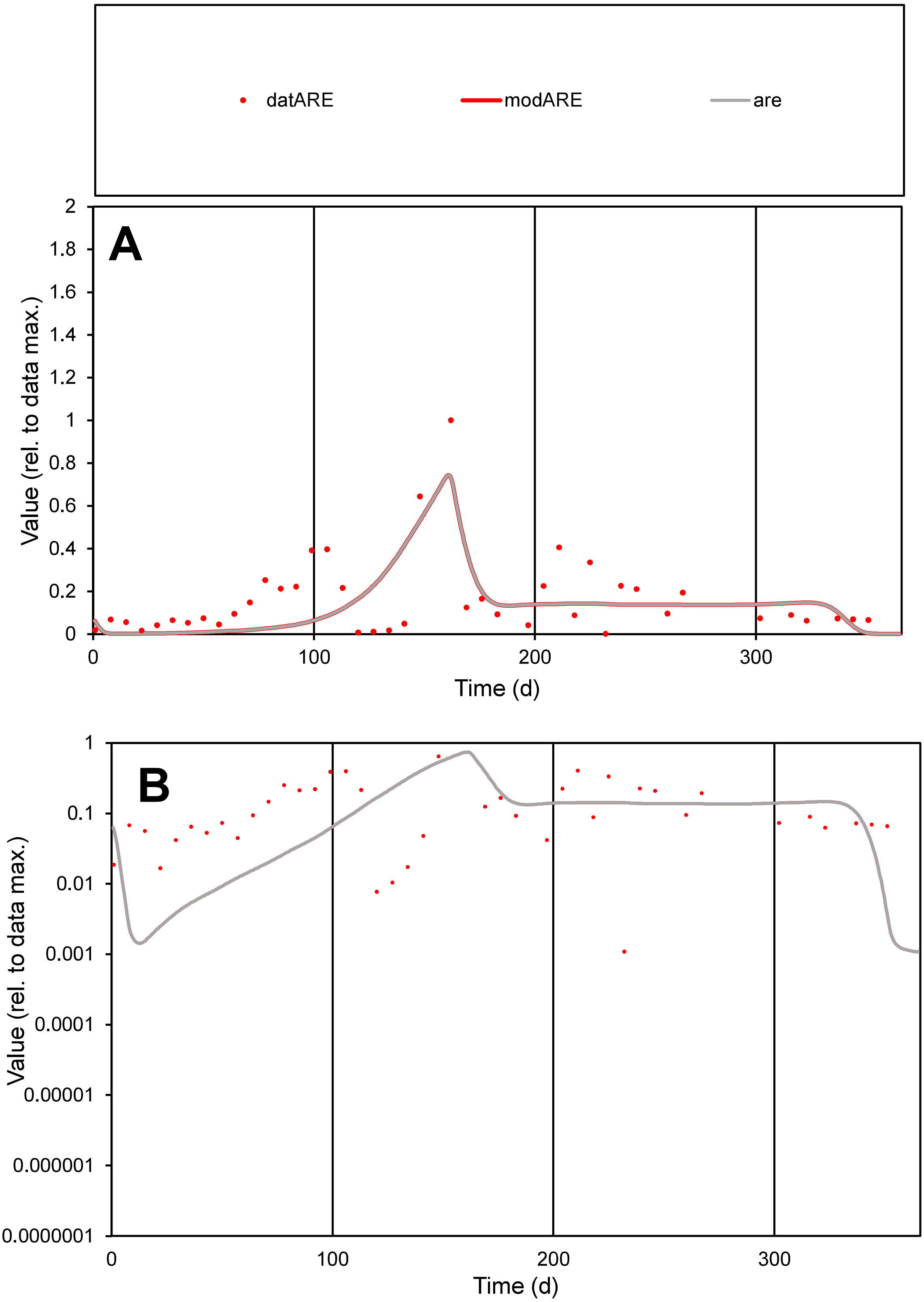

C1

Variables
ARE Provide jtd or Dst
CalibOnly?
2 1 to reproduce of
oNormalize?
2 1=no, 2=yes

ofxn calc*
0.93579
ofxn mod
0.97258

sRun
RunA1
zout
1

Ave
Data
0.00853
Model
0.00691

data/vMax
0.05316

ofxng
319.391

Min d/m
0.63068

ave/min d
147.333
ave/min m
75.2974

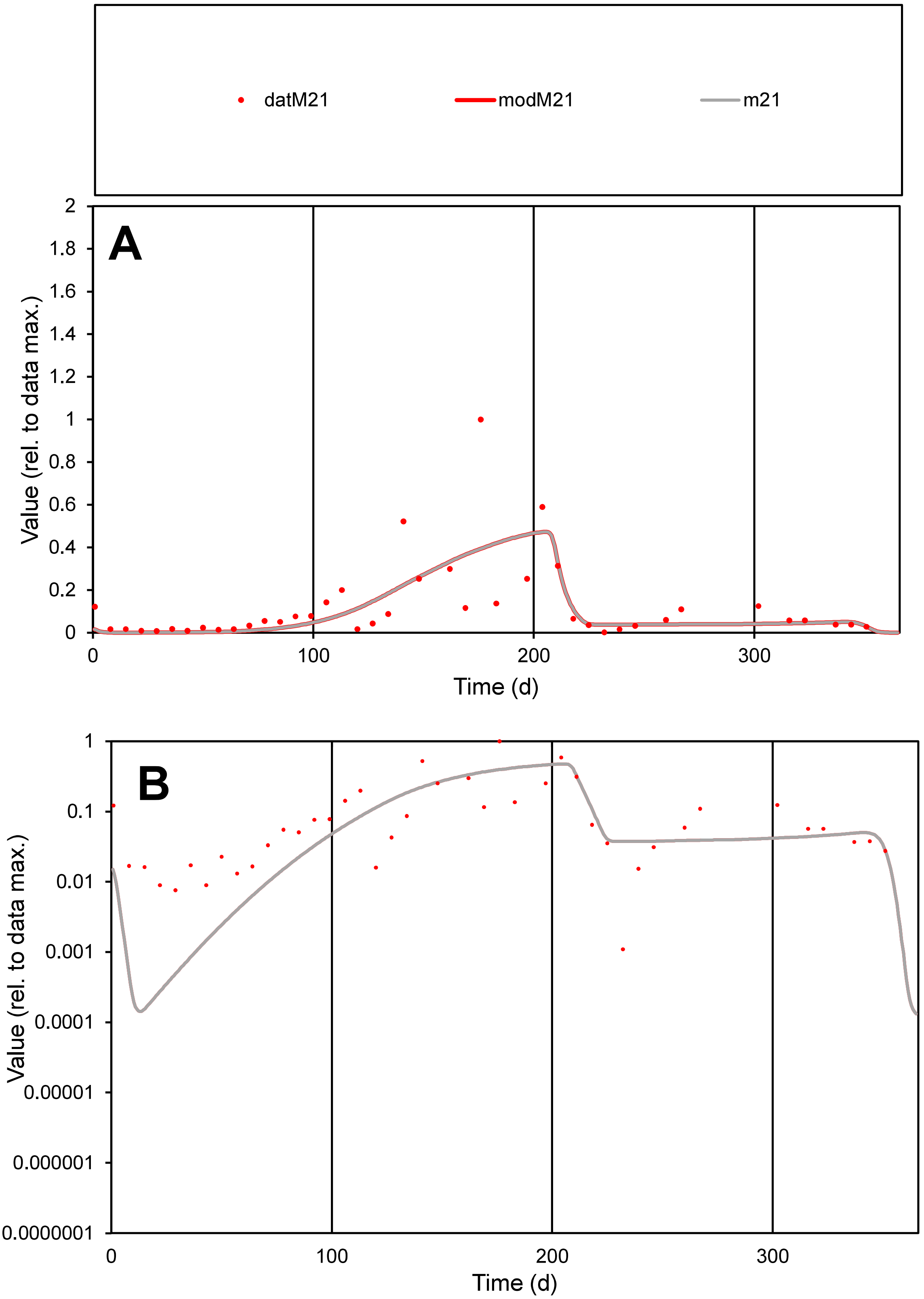

C1

Variables
M21 Provide jtd or Dst
CalibOnly?
2 1 to reproduce of
oNormalize?
2 1=no, 2=yes

ofxn calc*
1.07393
ofxn mod
1.08818

sRun
RunA1
zout
1

Ave
Data
0.01593
Model
0.01419

data/vMax
0.1295

ofxng
319.391

Min d/m
6.21007

ave/min d
112.259
ave/min m
621.221

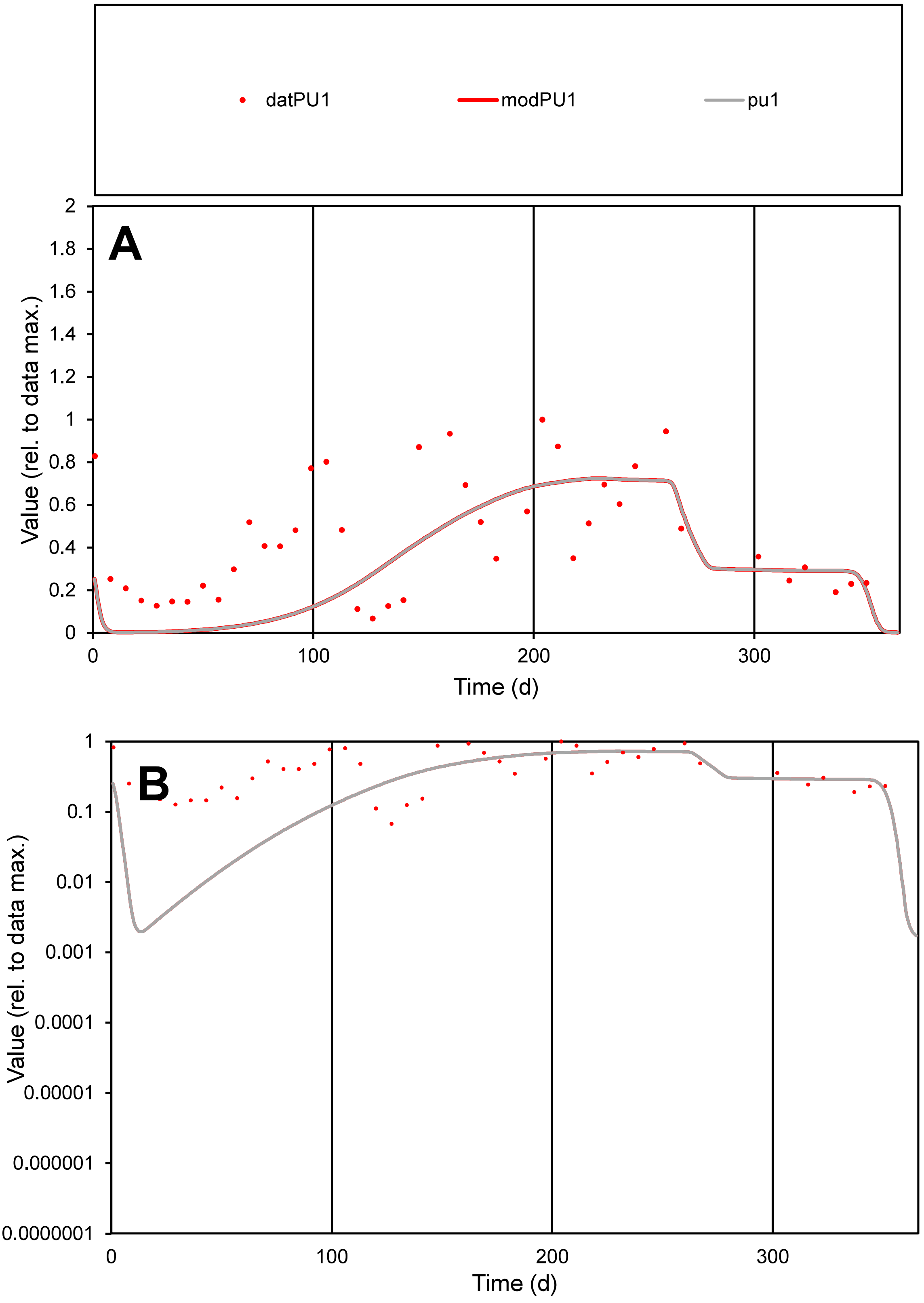

C1

Variables
PU1 Provide jtd or Dst
CalibOnly?
2 1 to reproduce of
oNormalize?
2 1=no, 2=yes

ofxn calc*
0.62593
ofxn mod
0.63743

sRun
RunA1
zout
1

Ave
Data
0.01056
Model
0.00773

data/vMax
0.02386

ofxng
319.391

Min d/m
29.9683

ave/min d
6.57794
ave/min m
144.329

330_Calib_S_A

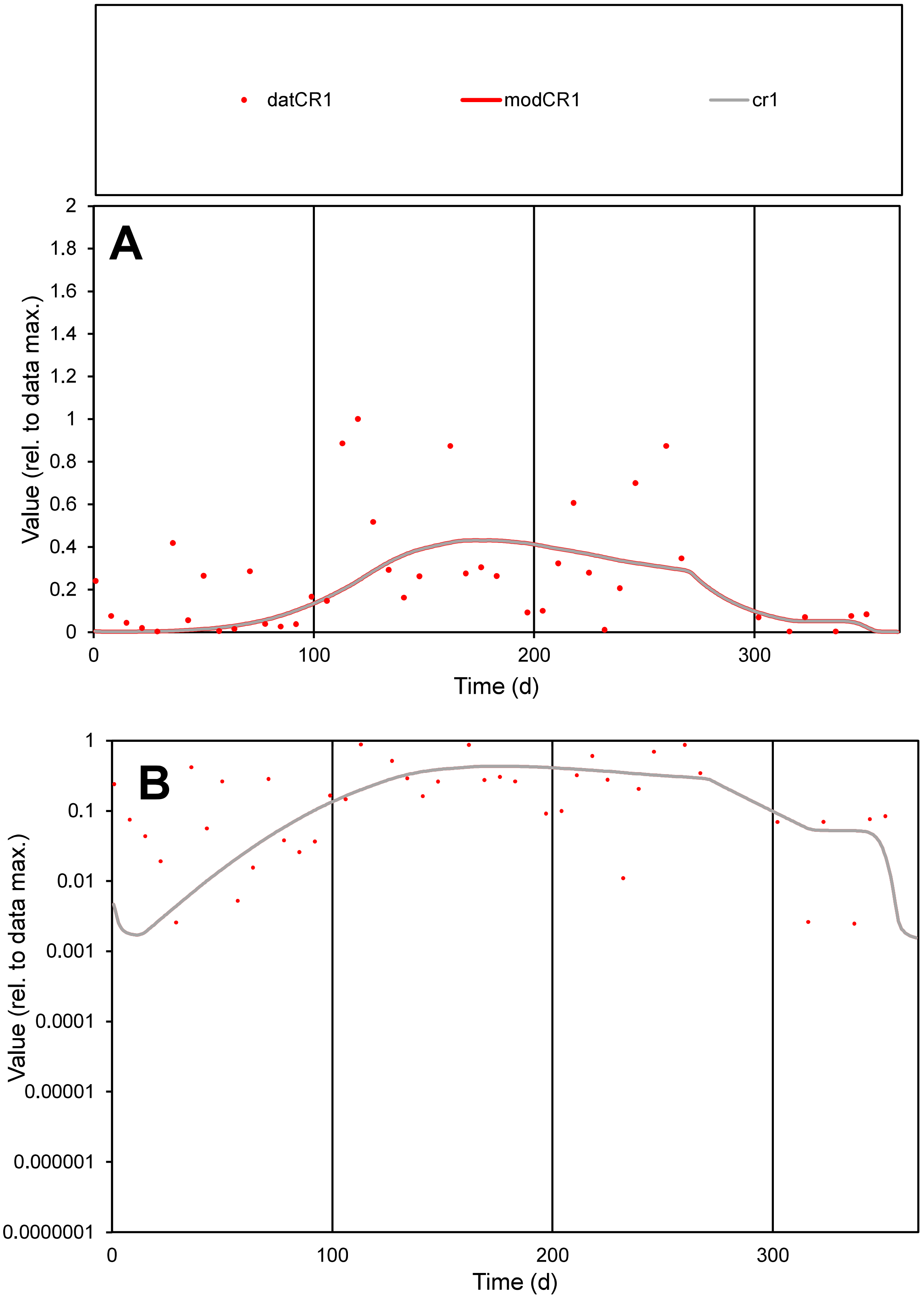

C1

Variables

CR1 Provide jtd or Dst

CalibOnly?

2 1 to reproduce of

oNormalize?

2 1=no, 2=yes

ofxn calc*

1.00102

ofxn mod

1.00883

sRun

RunA1

zout

1

Ave

Data

0.00713

Model

0.0055

data/vMax

0.02847

ofxng

319.391

Min d/m

1.43805

ave/min d

100.541

ave/min m

111.571

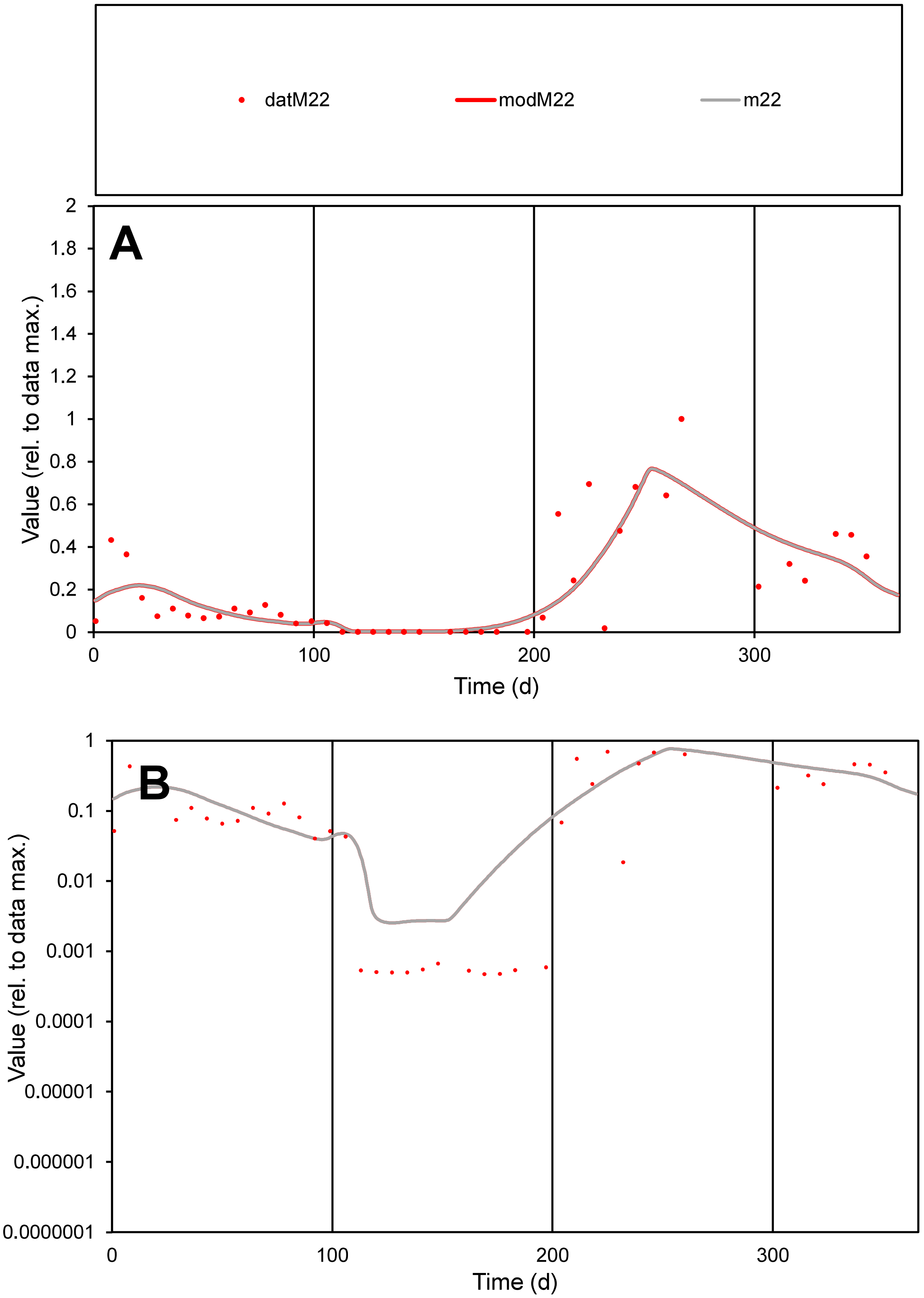

C1

Variables
M22 Provide jtd or Dst
CalibOnly?
2 1 to reproduce of
oNormalize?
2 1=no, 2=yes

ofxn calc*
0.70355
ofxn mod
0.72542

sRun
RunA1
zout
1

Ave
Data
0.00914
Model
0.00865

data/vMax
0.04578

ofxng
319.391

Min d/m
0.18554

ave/min d
421.49
ave/min m
74.0086

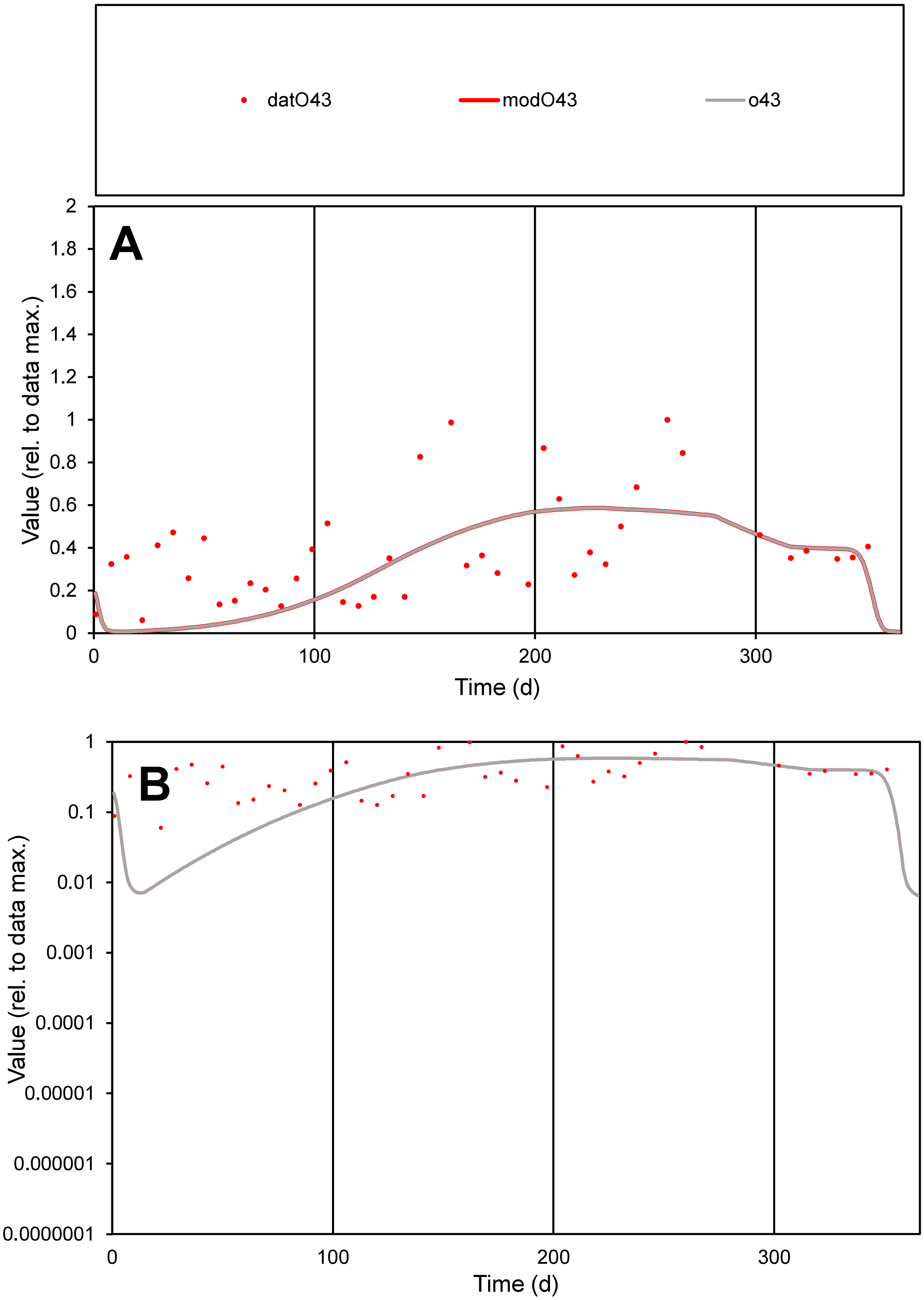

C1

Variables
O43 Provide jtd or Dst
CalibOnly?
2 1 to reproduce of
oNormalize?
2 1=no, 2=yes

ofxn calc*
0.62126
ofxn mod
0.63272

sRun
RunA1
zout
1

Ave
Data
0.01006
Model
0.00808

data/vMax
0.0261

ofxng
319.391

Min d/m
7.65251

ave/min d
6.41738
ave/min m
39.4242

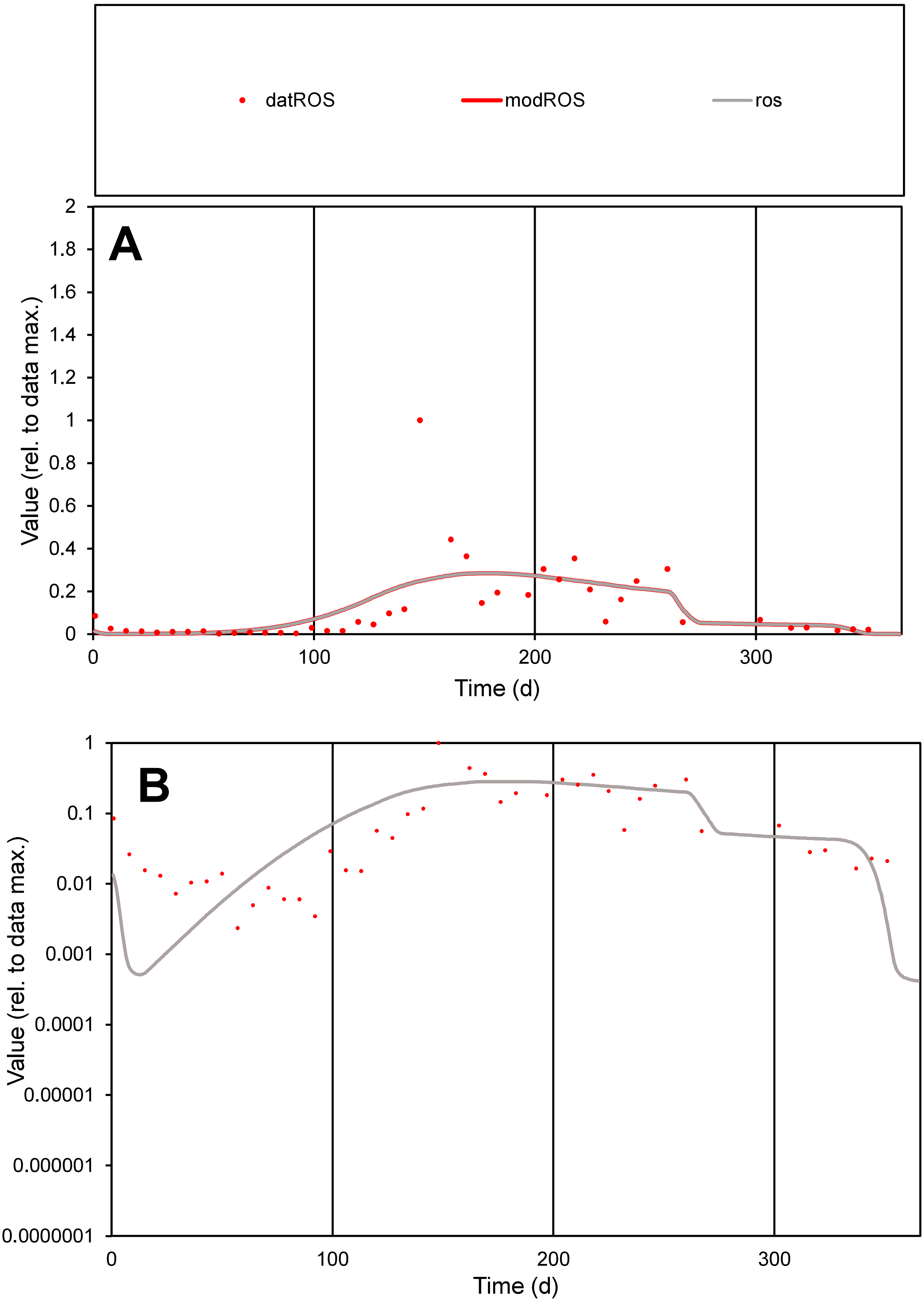

C1

Variables
ROS Provide jtd or Dst
CalibOnly?
2 1 to reproduce of
oNormalize?
2 1=no, 2=yes

ofxn calc*
1.12594
ofxn mod
1.13847

sRun
RunA1
zout
1

Ave
Data
0.0094
Model
0.00937

data/vMax
0.07815

ofxng
319.391

Min d/m
4.15082

ave/min d
51.2901
ave/min m
212.2

330_Calib_S_A

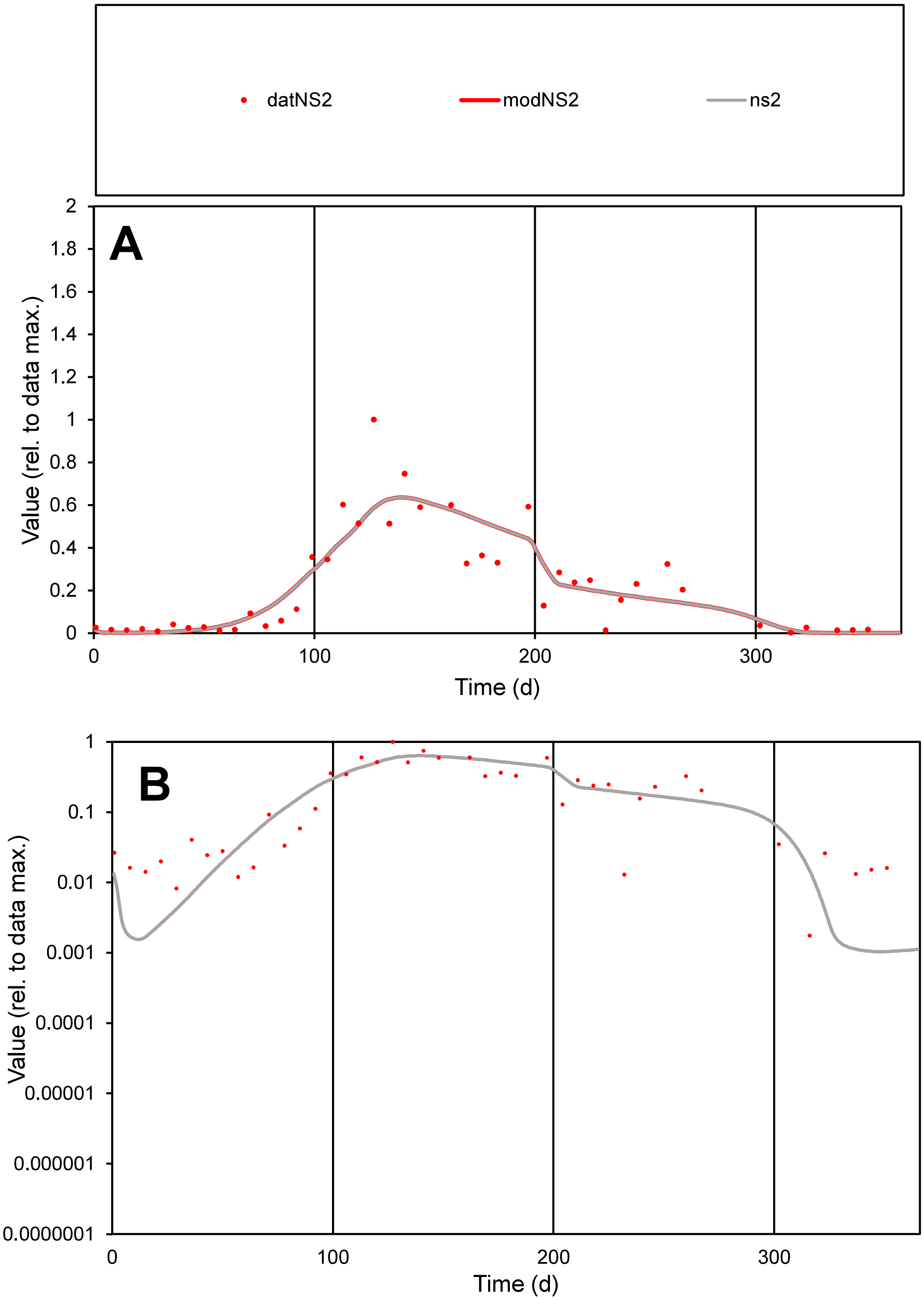

C1

Variables
NS2 Provide jtd or Dst
CalibOnly?
2 1 to reproduce of
oNormalize?
2 1=no, 2=yes

ofxn calc*
0.48563
ofxn mod
0.50787

sRun
RunA1
zout
1

Ave
Data
0.00826
Model
0.00822

data/vMax
0.03725

ofxng
319.391

Min d/m
1.68205

ave/min d
126.635
ave/min m
211.916

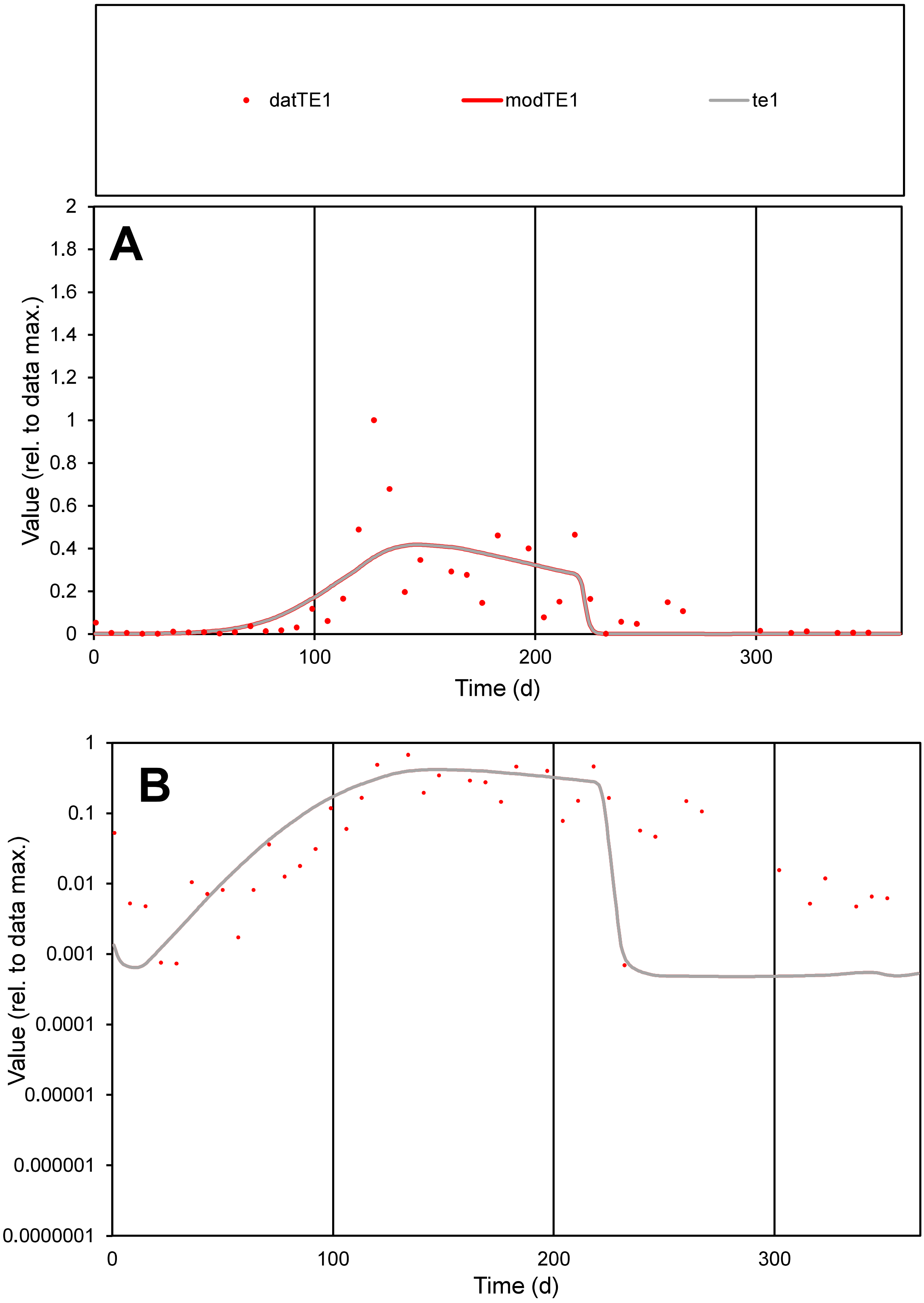

C1

Variables

TE1 Provide jtd or Dst

CalibOnly?

2 1 to reproduce of

oNormalize?

2 1=no, 2=yes

ofxn calc*

0.98824

ofxn mod

1.0066

sRun

RunA1

zout

1

Ave

Data

0.01157

Model

0.01103

data/vMax

0.07975

ofxng

319.391

Min d/m

1.44725

ave/min d

207.719

ave/min m

286.693

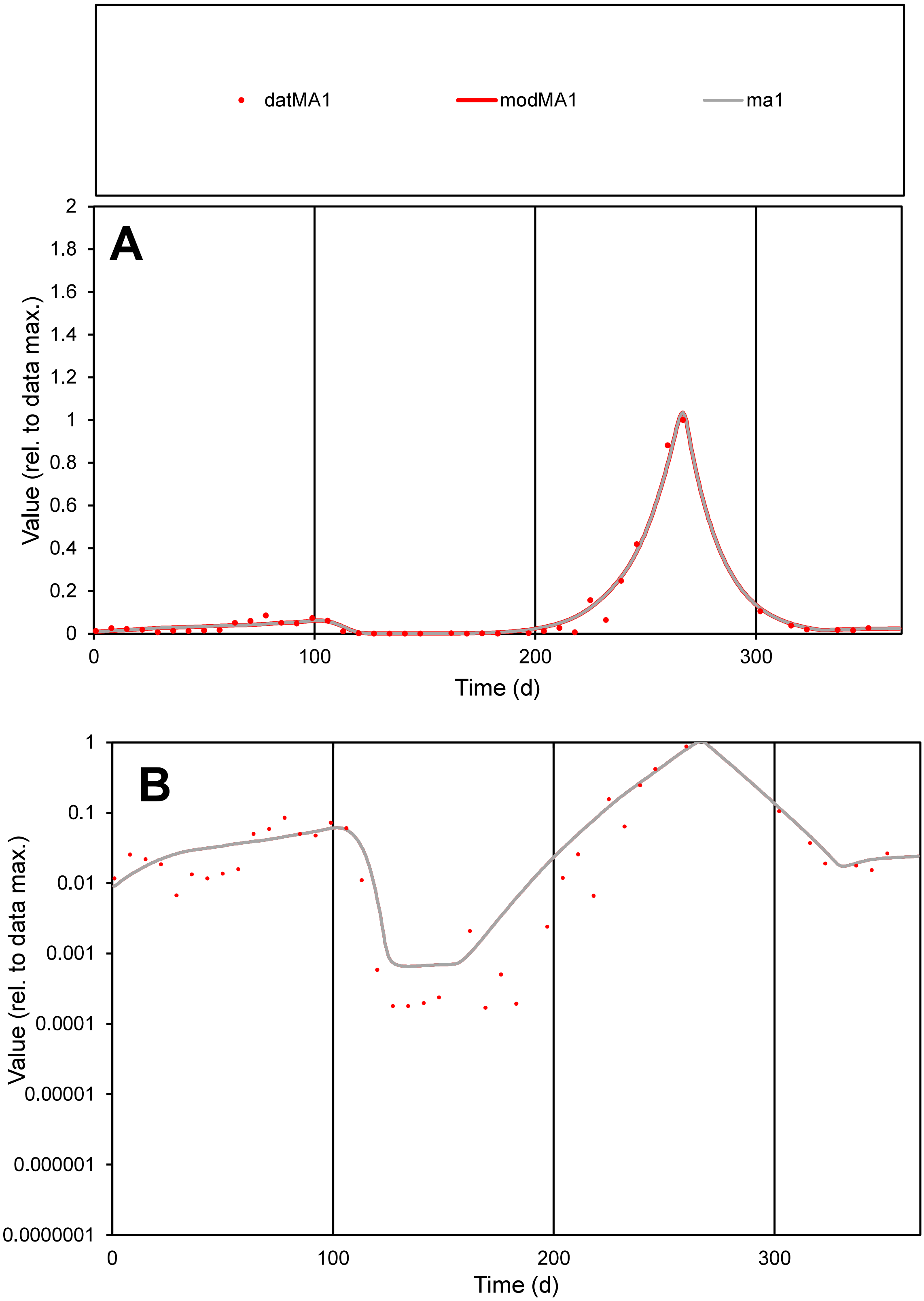

C1

Variables
MA1 Provide jtd or Dst
CalibOnly?
2 1 to reproduce of
oNormalize?
2 1=no, 2=yes

ofxn calc*
0.31028
ofxn mod
0.42731

sRun
RunA1
zout
1

Ave
Data
0.00737
Model
0.00788

data/vMax
0.08566

ofxng
319.391

Min d/m
0.25699

ave/min d
507.315
ave/min m
139.429

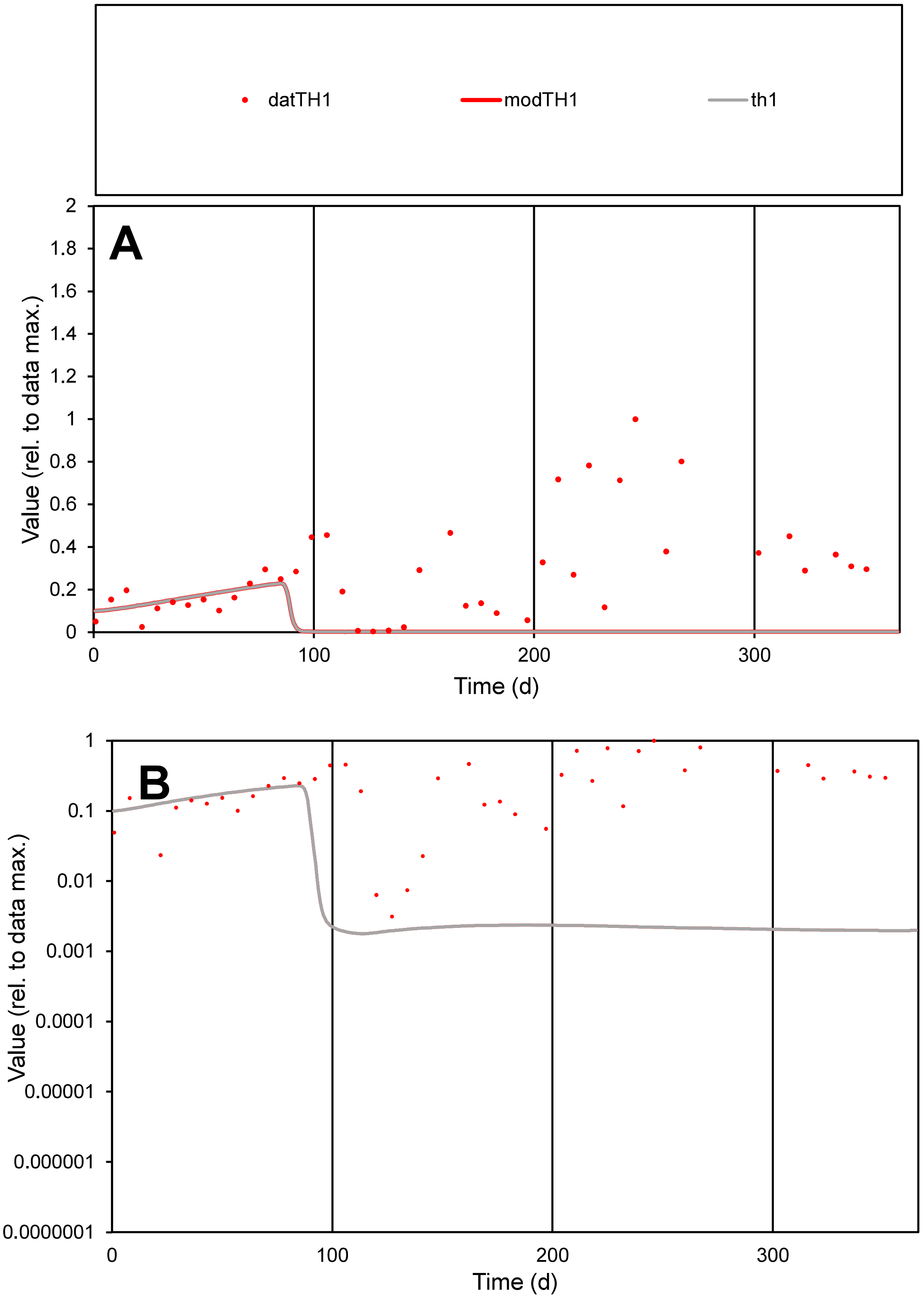

C1

Variables
TH1 Provide jtd or Dst
CalibOnly?
2 1 to reproduce of
oNormalize?
2 1=no, 2=yes

ofxn calc*
1.25196
ofxn mod
1.2604

sRun
RunA1
zout
1

Ave
Data
0.00881
Model
0.00179

data/vMax
0.03151

ofxng
319.391

Min d/m
1.76769

ave/min d
89.3031
ave/min m
32.0442

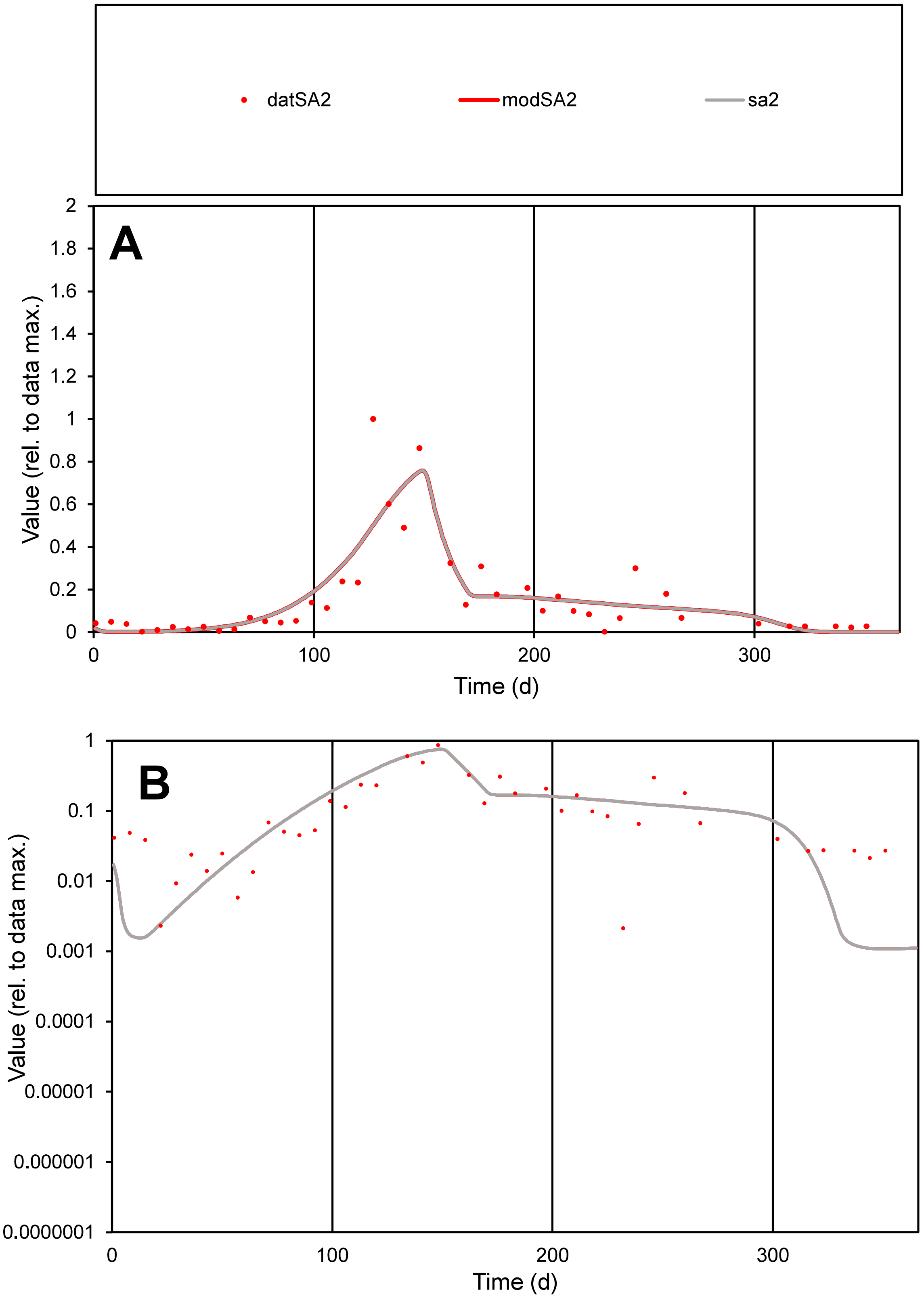

C1

Variables
SA2 Provide jtd or Dst
CalibOnly?
2 1 to reproduce of
oNormalize?
2 1=no, 2=yes

ofxn calc*
0.67623
ofxn mod
0.69752

sRun
RunA1
zout
1

Ave
Data
0.0079
Model
0.00796

data/vMax
0.05107

ofxng
319.391

Min d/m
1.97311

ave/min d
72.7358
ave/min m
144.585

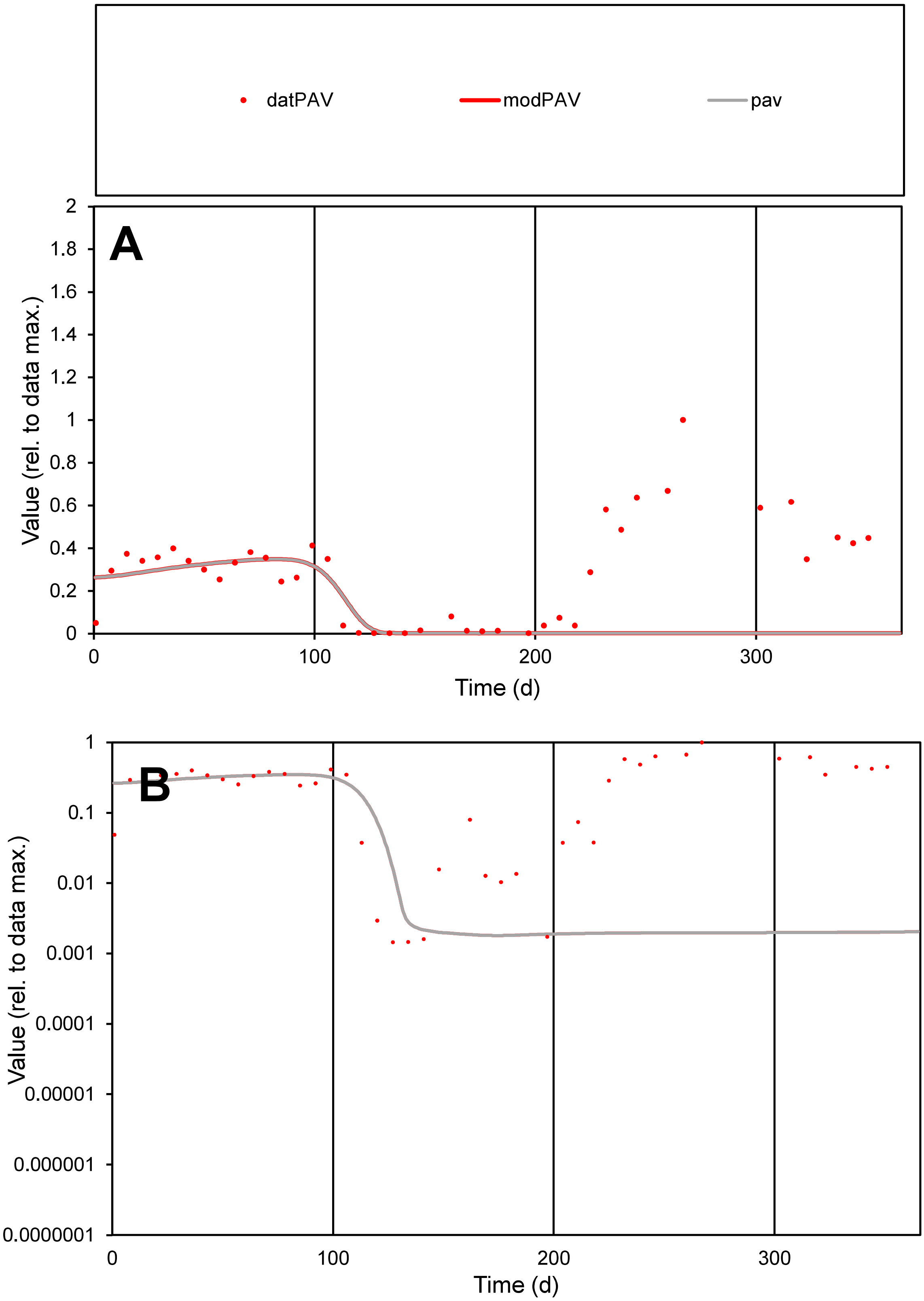

C1

Variables

PAV Provide jtd or Dst

CalibOnly?

2 1 to reproduce of

oNormalize?

2 1=no, 2=yes

ofxn calc*

1.096

ofxn mod

1.10081

sRun

RunA1

zout

1

Ave

Data

0.00772

Model

0.00338

data/vMax

0.02724

ofxng

319.391

Min d/m

0.80506

ave/min d

195.586

ave/min m

68.8347

330_Calib_S_A

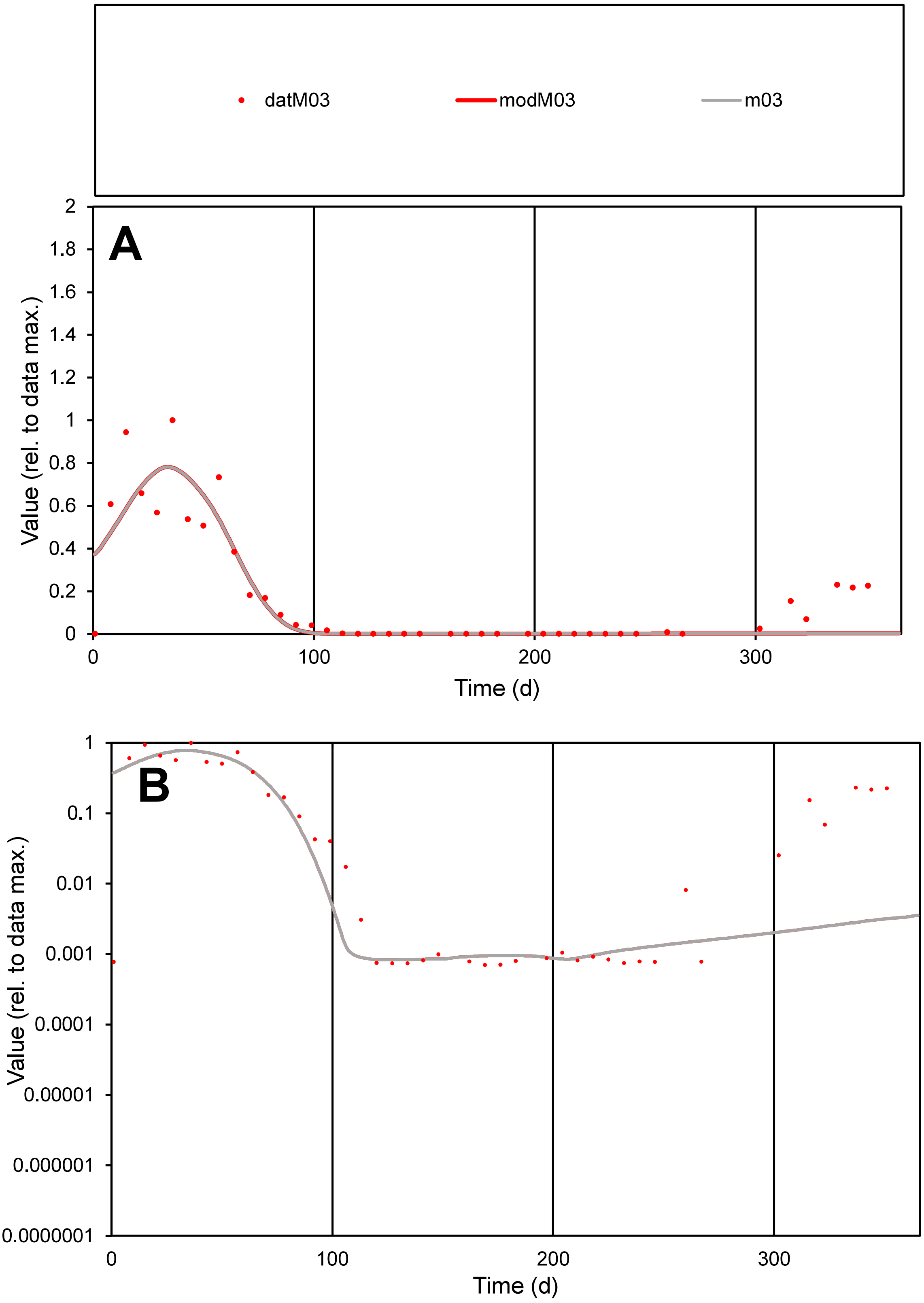

C1

Variables

M03 Provide jtd or Dst

CalibOnly?

2 1 to reproduce of

oNormalize?

2 1=no, 2=yes

ofxn calc*

0.69233

ofxn mod

0.72512

sRun

RunA1

zout

1

Ave

Data

0.00446

Model

0.0039

data/vMax

0.02519

ofxng

319.391

Min d/m

0.84715

ave/min d

252.091

ave/min m

186.839

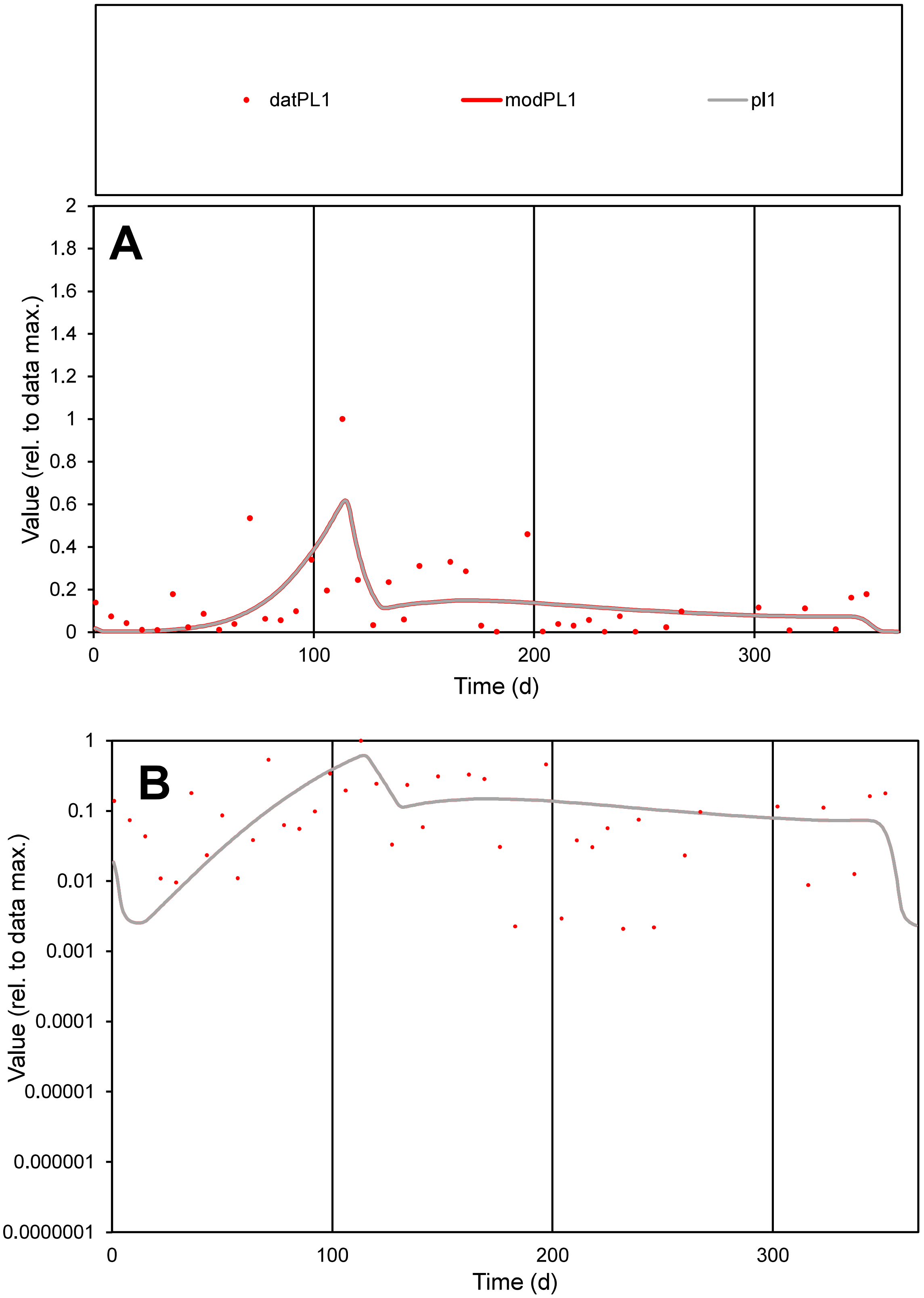

C1

Variables
PL1 Provide jtd or Dst
CalibOnly?
2 1 to reproduce of
oNormalize?
2 1=no, 2=yes

ofxn calc*
1.0554
ofxn mod
1.0684

sRun
RunA1
zout
1

Ave
Data
0.00244
Model
0.0023

data/vMax
0.01766

ofxng
319.391

Min d/m
0.8077

ave/min d
65.7738
ave/min m
50.0284

330_Calib_S_A

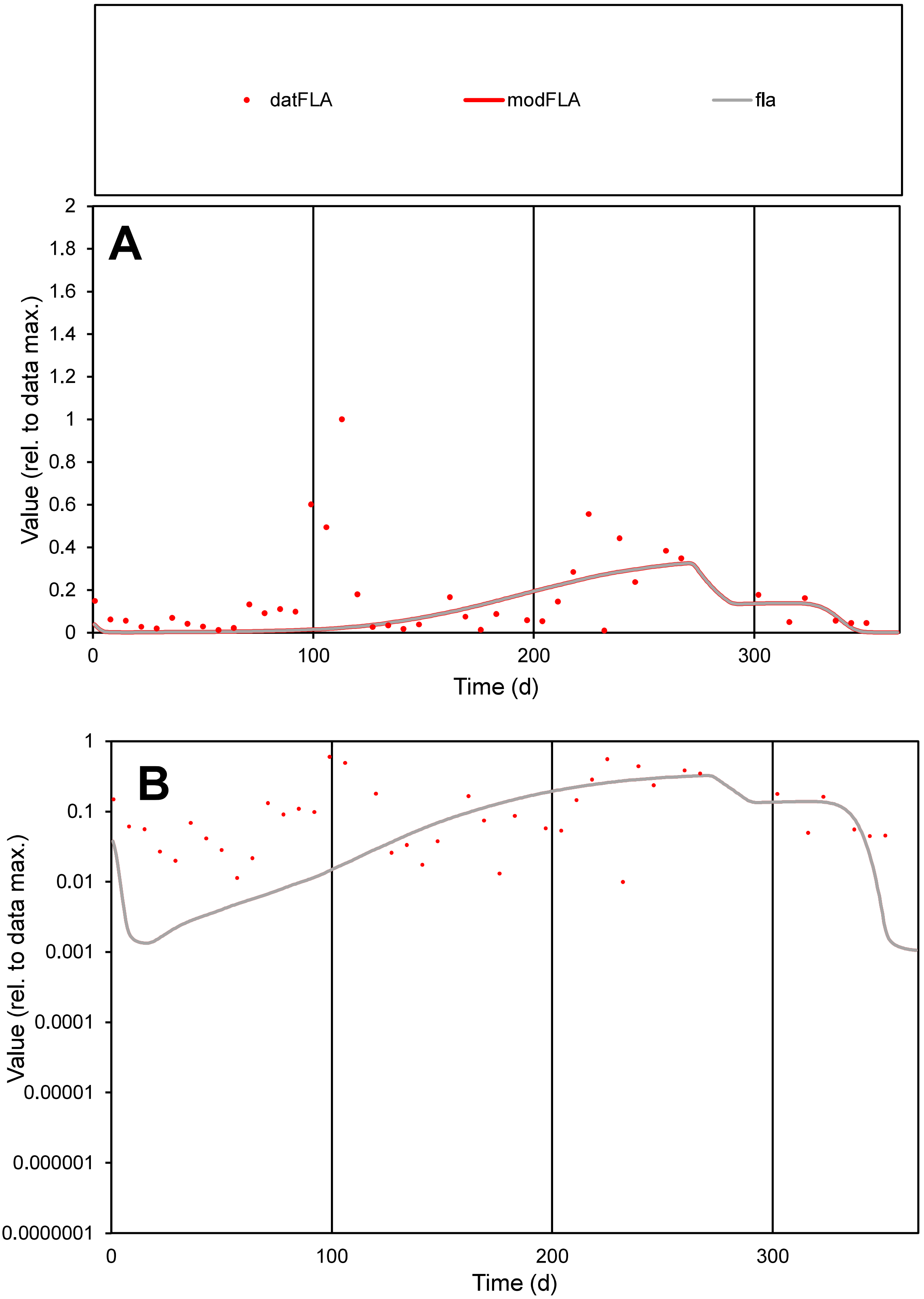

C1

Variables
FLA Provide jtd or Dst
CalibOnly?
2 1 to reproduce of
oNormalize?
2 1=no, 2=yes

ofxn calc*
1.32912
ofxn mod
1.33583

sRun
RunA1
zout
1

Ave
Data
0.00499
Model
0.003

data/vMax
0.03126

ofxng
319.391

Min d/m
7.45581

ave/min d
15.9752
ave/min m
71.6033

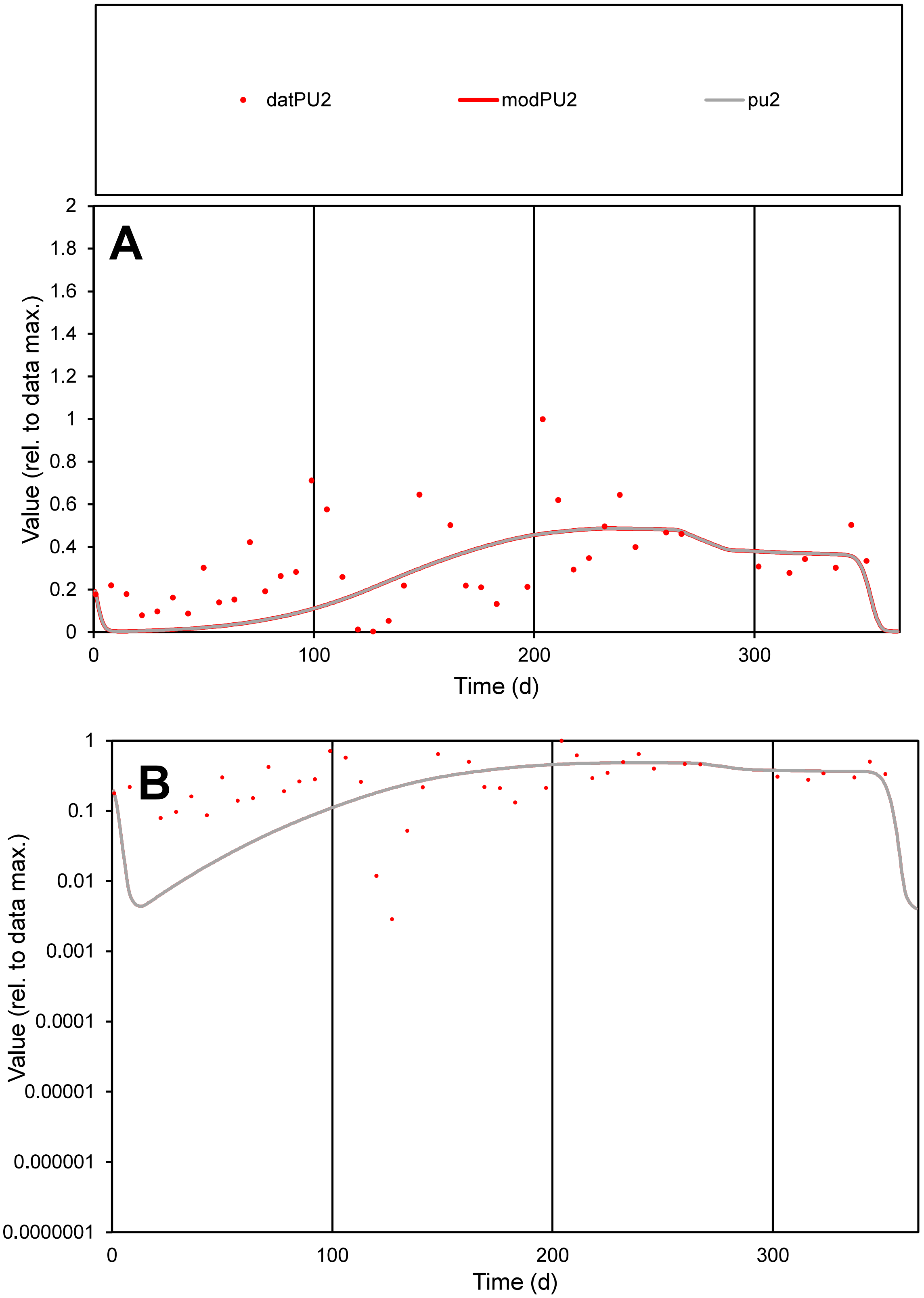

C1

Variables
PU2 Provide jtd or Dst
CalibOnly?
2 1 to reproduce of
oNormalize?
2 1=no, 2=yes

ofxn calc*
0.67386
ofxn mod
0.68463

sRun
RunA1
zout
1

Ave
Data
0.0064
Model
0.0051

data/vMax
0.02022

ofxng
319.391

Min d/m
0.58236

ave/min d
109.975
ave/min m
50.9875

330_Calib_S_A

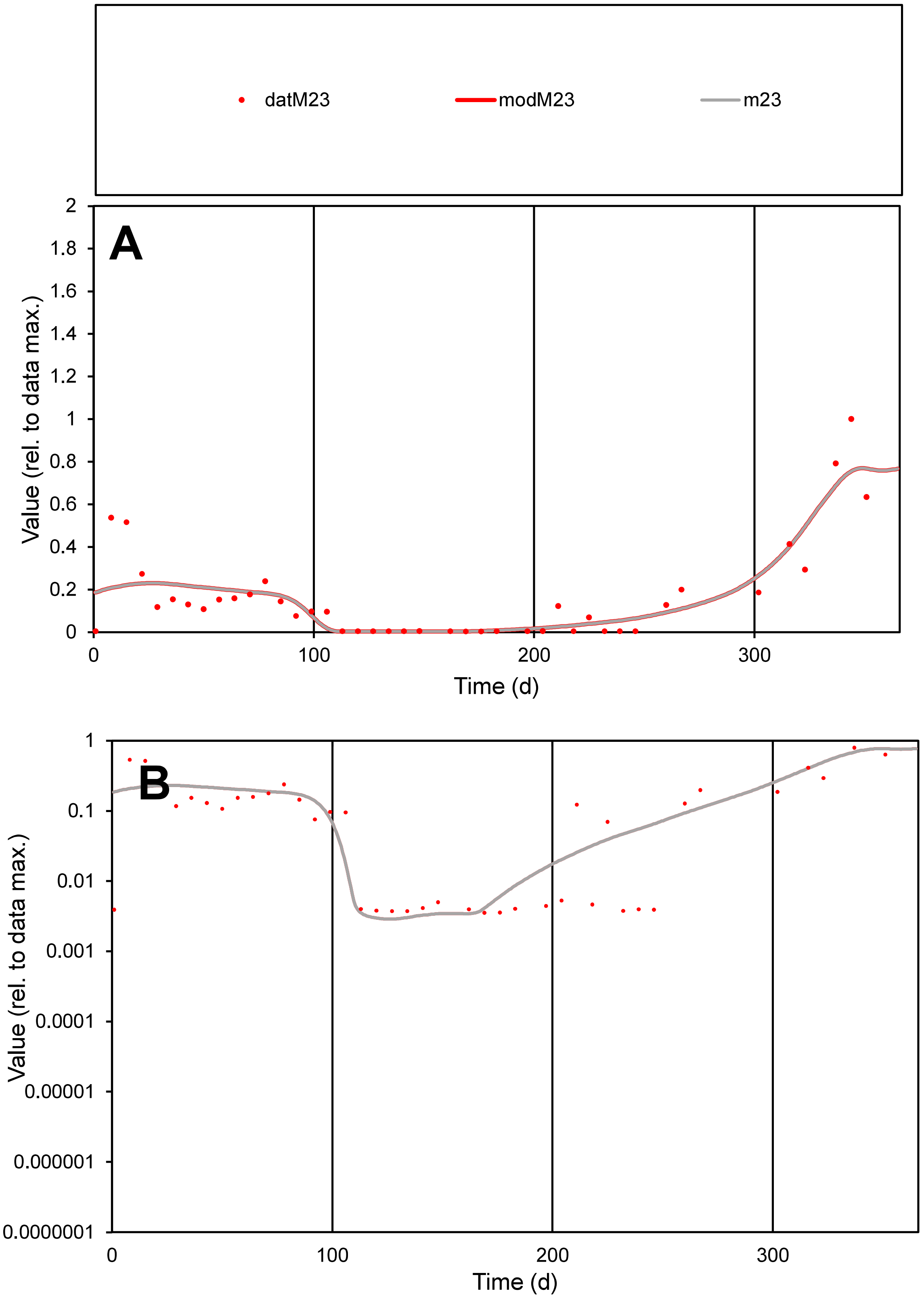

C1

Variables

M23 Provide jtd or Dst

CalibOnly?

2 1 to reproduce of

oNormalize?

2 1=no, 2=yes

ofxn calc*

0.62611

ofxn mod

0.64477

sRun

RunA1

zout

1

Ave

Data

0.005

Model

0.005

data/vMax

0.03054

ofxng

319.391

Min d/m

1.22843

ave/min d

46.2506

ave/min m

56.7131

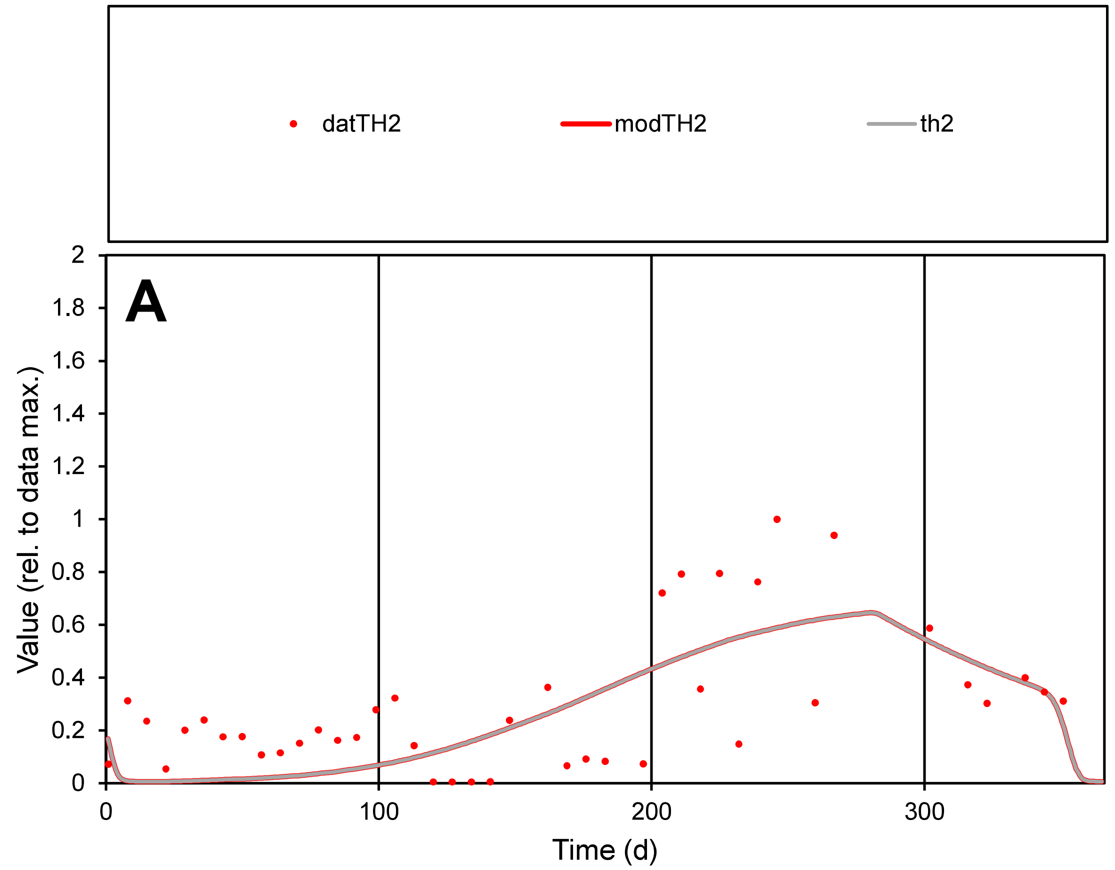

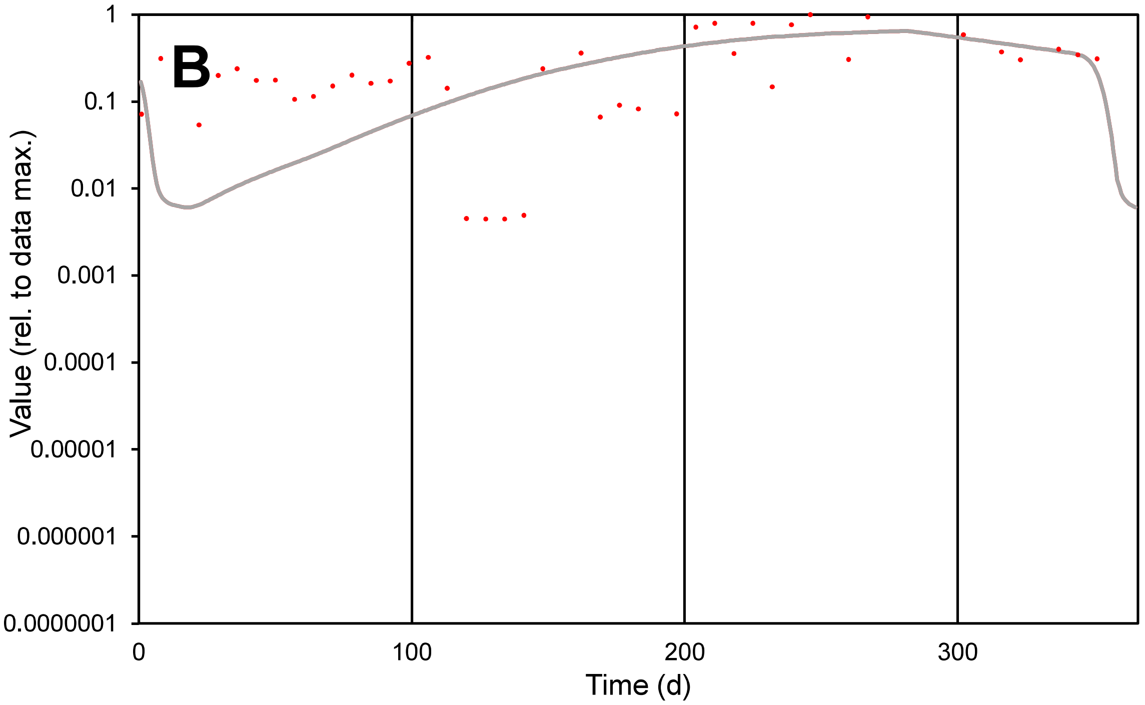

C1

Variables
TH2 Provide jtd or Dst
CalibOnly?
2 1 to reproduce of
oNormalize?
2 1=no, 2=yes

ofxn calc*
0.70034
ofxn mod
0.71521

sRun
RunA1
zout
1

Ave
Data
0.0073
Model
0.00625

data/vMax
0.02519

ofxng
319.391

Min d/m
0.72875

ave/min d
65.0364
ave/min m
40.574

330_Calib_S_A

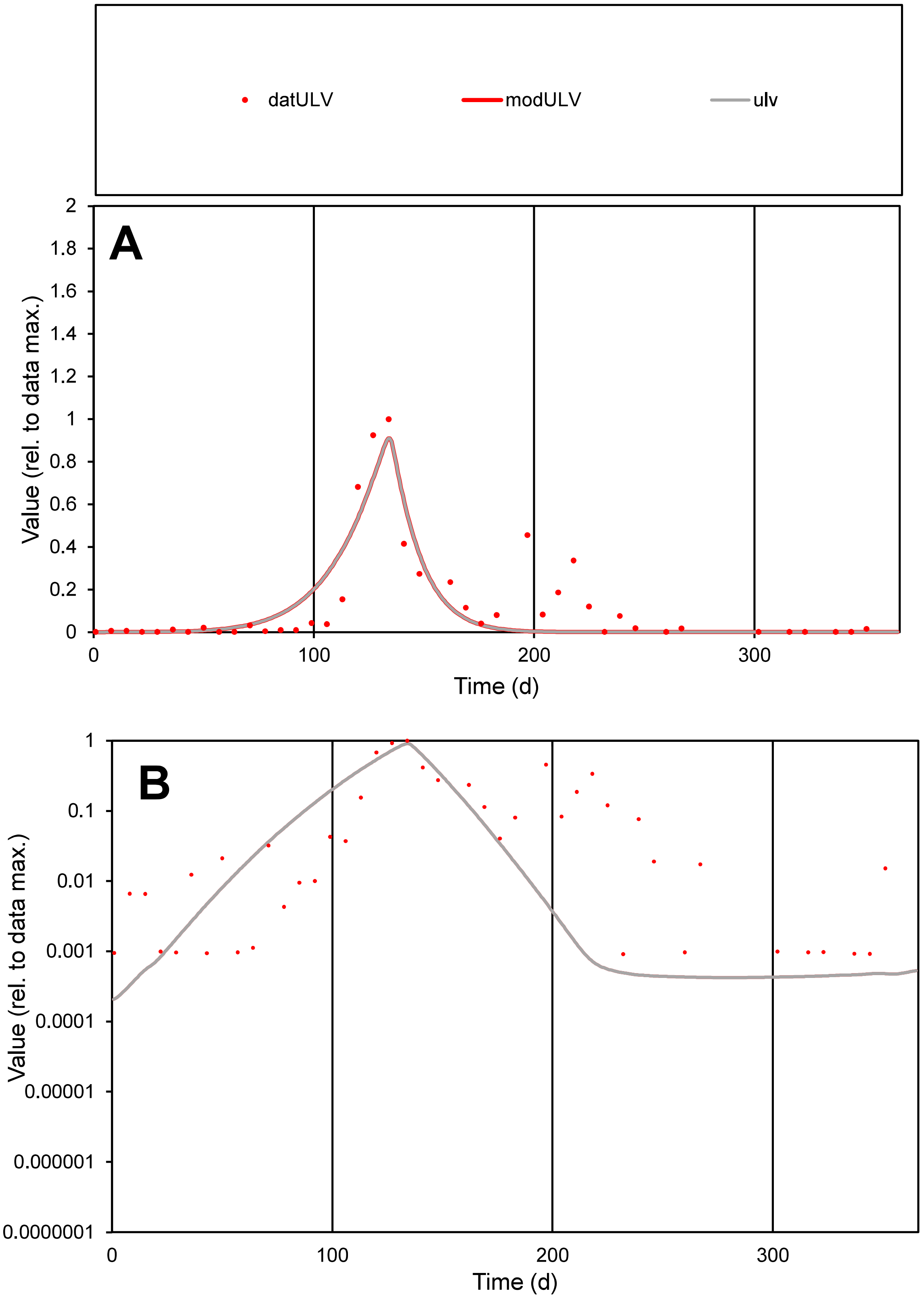

C1

Variables

ULV Provide jtd or Dst

CalibOnly?

2 1 to reproduce of

oNormalize?

2 1=no, 2=yes

ofxn calc*

0.95049

ofxn mod

0.97463

sRun

RunA1

zout

1

Ave

Data

0.00261

Model

0.00219

data/vMax

0.02021

ofxng

319.391

Min d/m

4.14937

ave/min d

141.925

ave/min m

494.699

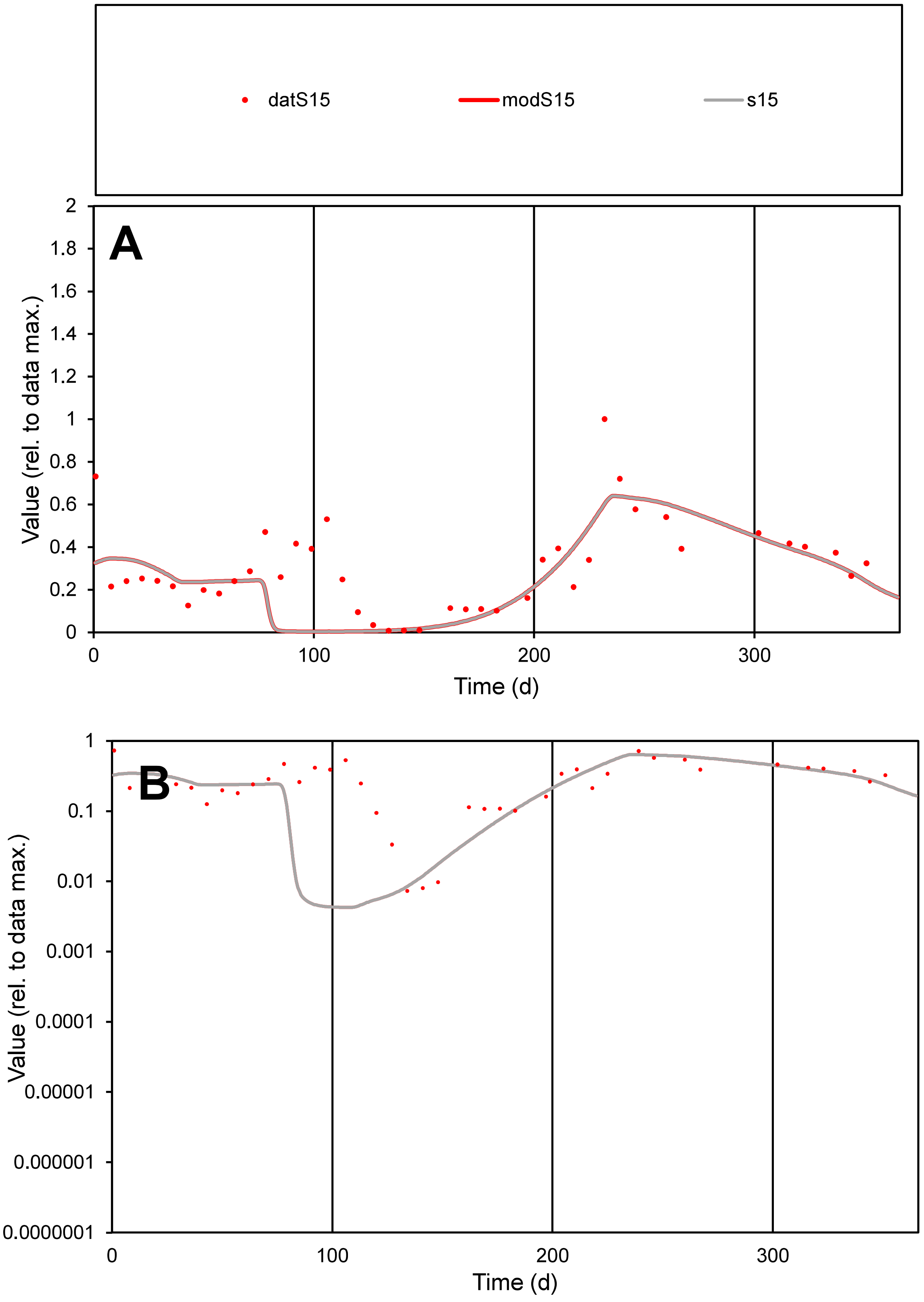

C1

Variables
S15 Provide jtd or Dst
CalibOnly?
2 1 to reproduce of
oNormalize?
2 1=no, 2=yes

ofxn calc*
0.57776
ofxn mod
0.59852

sRun
RunA1
zout
1

Ave
Data
0.0055
Model
0.00451

data/vMax
0.01813

ofxng
319.391

Min d/m
1.71903

ave/min d
41.5623
ave/min m
58.5152

330_Calib_S_A

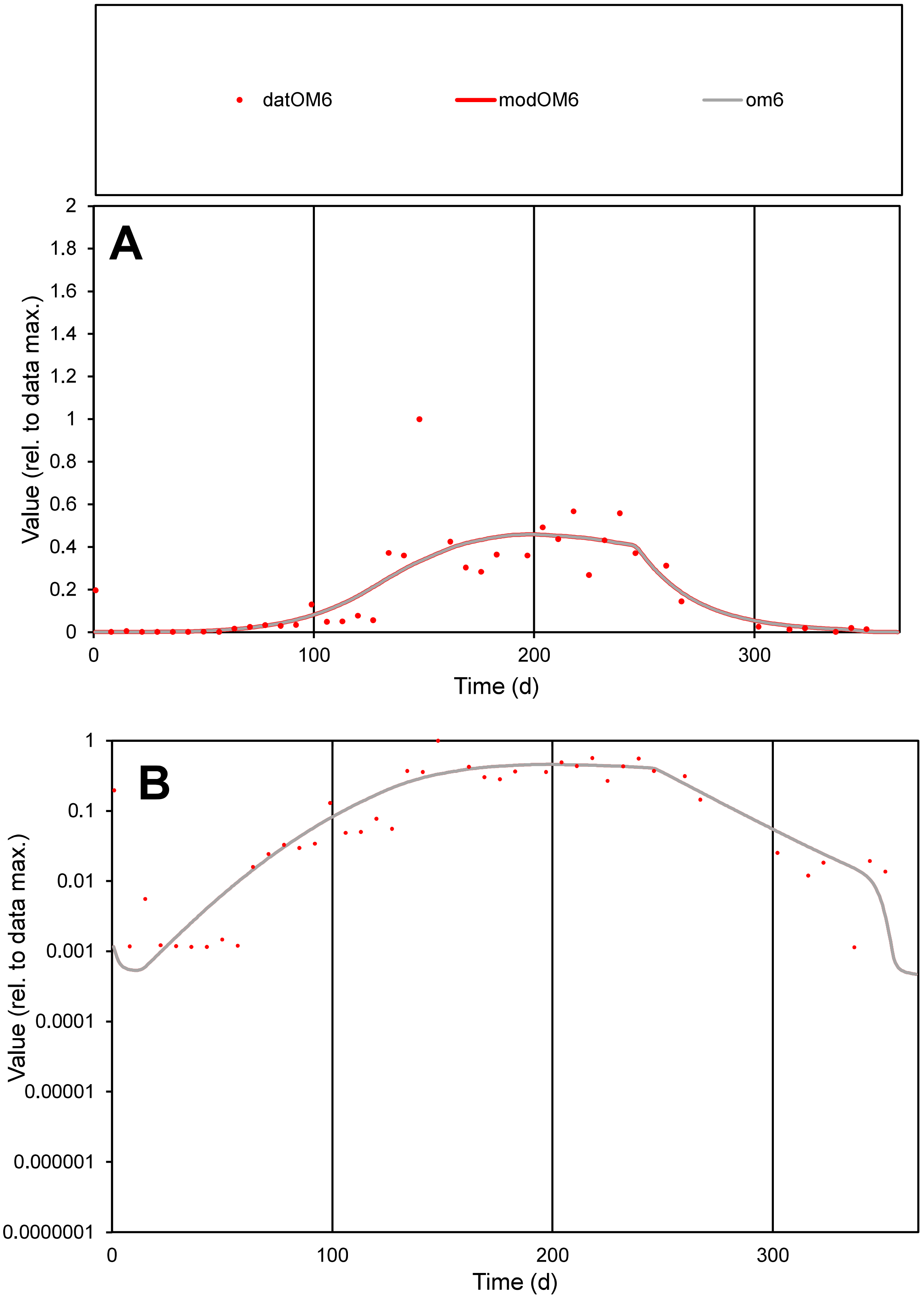

C1

Variables

OM6 Provide jtd or Dst

CalibOnly?

2 1 to reproduce of

oNormalize?

2 1=no, 2=yes

ofxn calc*

0.67584

ofxn mod

0.69701

sRun

RunA1

zout

1

Ave

Data

0.00443

Model

0.00427

data/vMax

0.02375

ofxng

319.391

Min d/m

2.12363

ave/min d

163.321

ave/min m

334.007

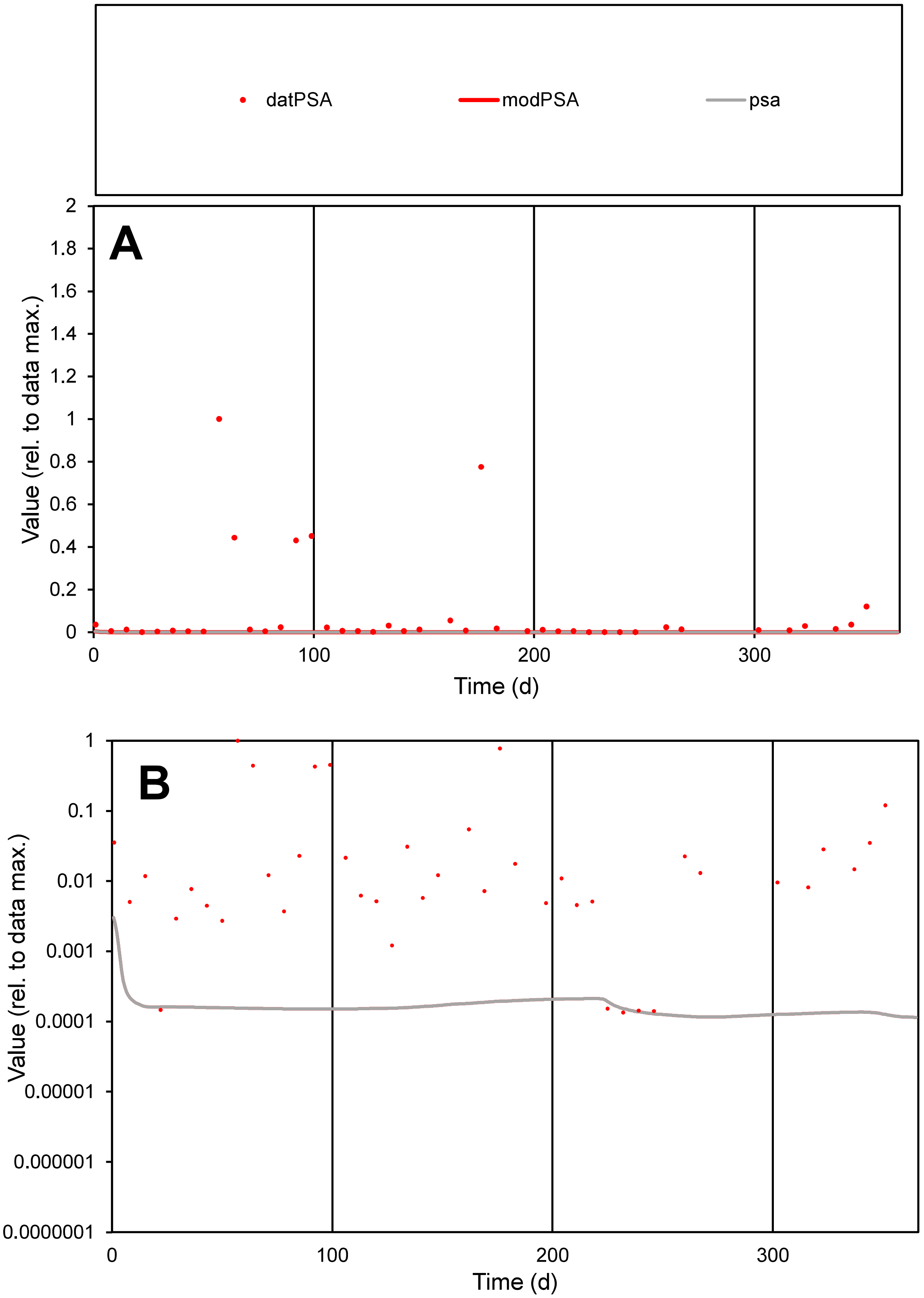

C1

Variables
PSA Provide jtd or Dst
CalibOnly?
2 1 to reproduce of
oNormalize?
2 1=no, 2=yes

ofxn calc*
2.63997
ofxn mod
2.64348

sRun
RunA1
zout
1

Ave
Data
0.00607
Model
1.5E-05

data/vMax
0.06991

ofxng
319.391

Min d/m
1.16431

ave/min d
642.432
ave/min m
1.85595

330_Calib_S_A

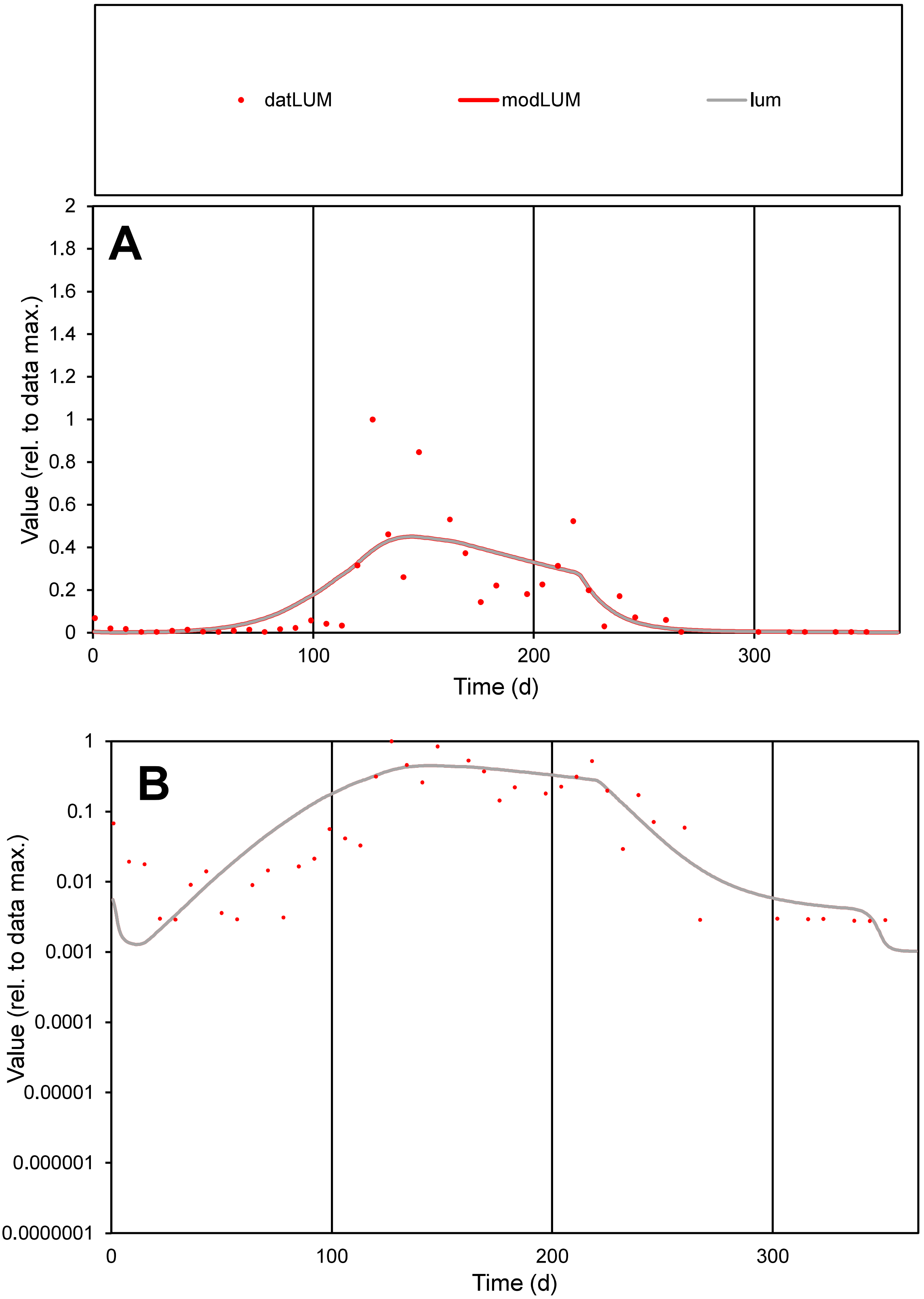

C1	
Variables	
LUM	Provide jtd or Dst
CalibOnly?	
2	1 to reproduce of
oNormalize?	
2	1=no, 2=yes
ofxn calc*	
0.98006	
ofxn mod	
0.99487	
sRun	
RunA1	
zout	
1	
Ave	
Data	
0.0026	
Model	
0.00269	
data/vMax	
0.01739	
ofxng	
319.391	
Min d/m	
2.10722	
ave/min d	
53.9663	
ave/min m	
117.656	

330_Calib_S_A

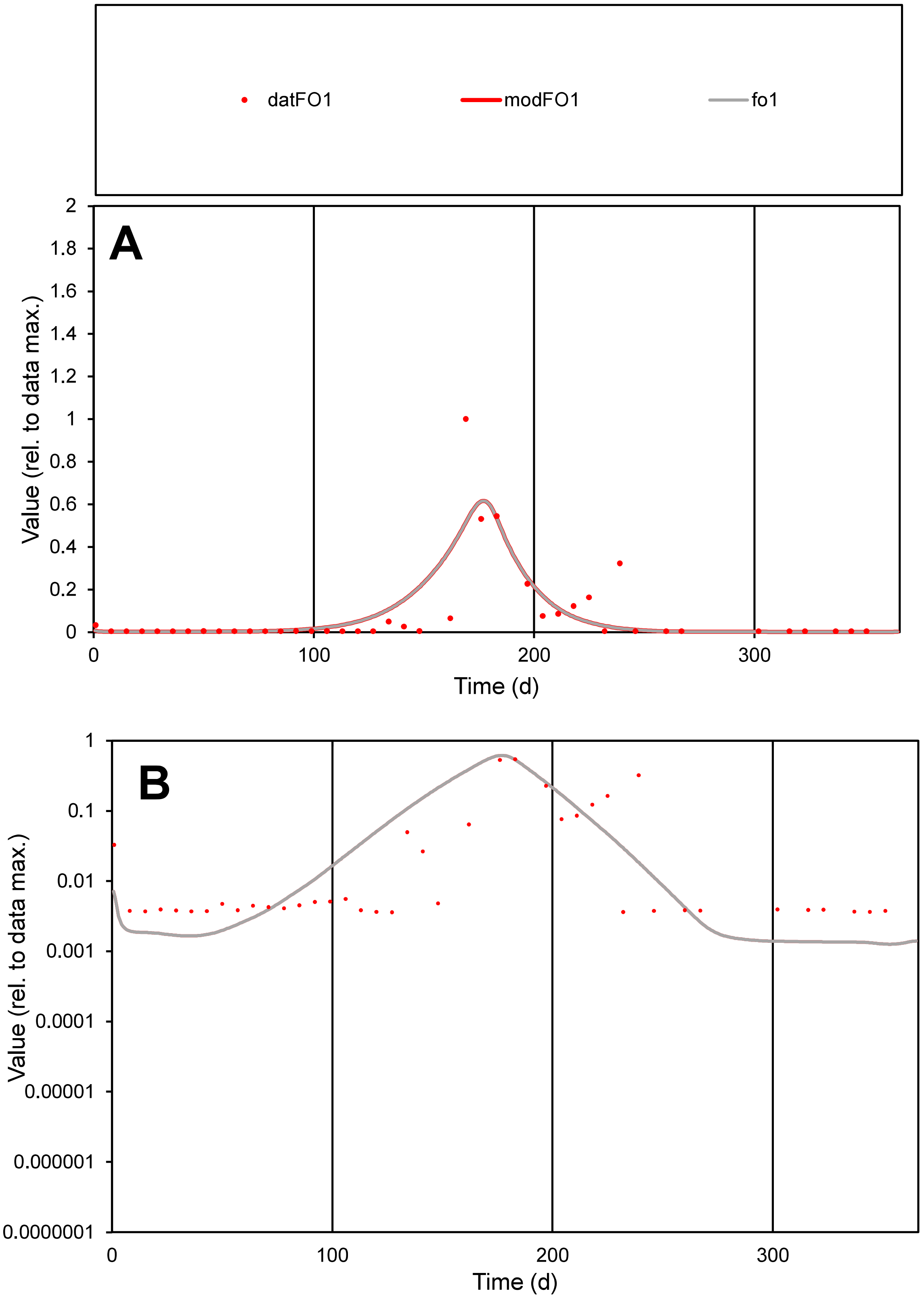

C1

Variables
FO1 Provide jtd or Dst
CalibOnly?
2 1 to reproduce of
oNormalize?
2 1=no, 2=yes

ofxn calc*
1.3866
ofxn mod
1.41787

sRun
RunA1
zout
1

Ave
Data
0.00138
Model
0.0014

data/vMax
0.01716

ofxng
319.391

Min d/m
2.85088

ave/min d
22.2704
ave/min m
64.748

330_Calib_S_A

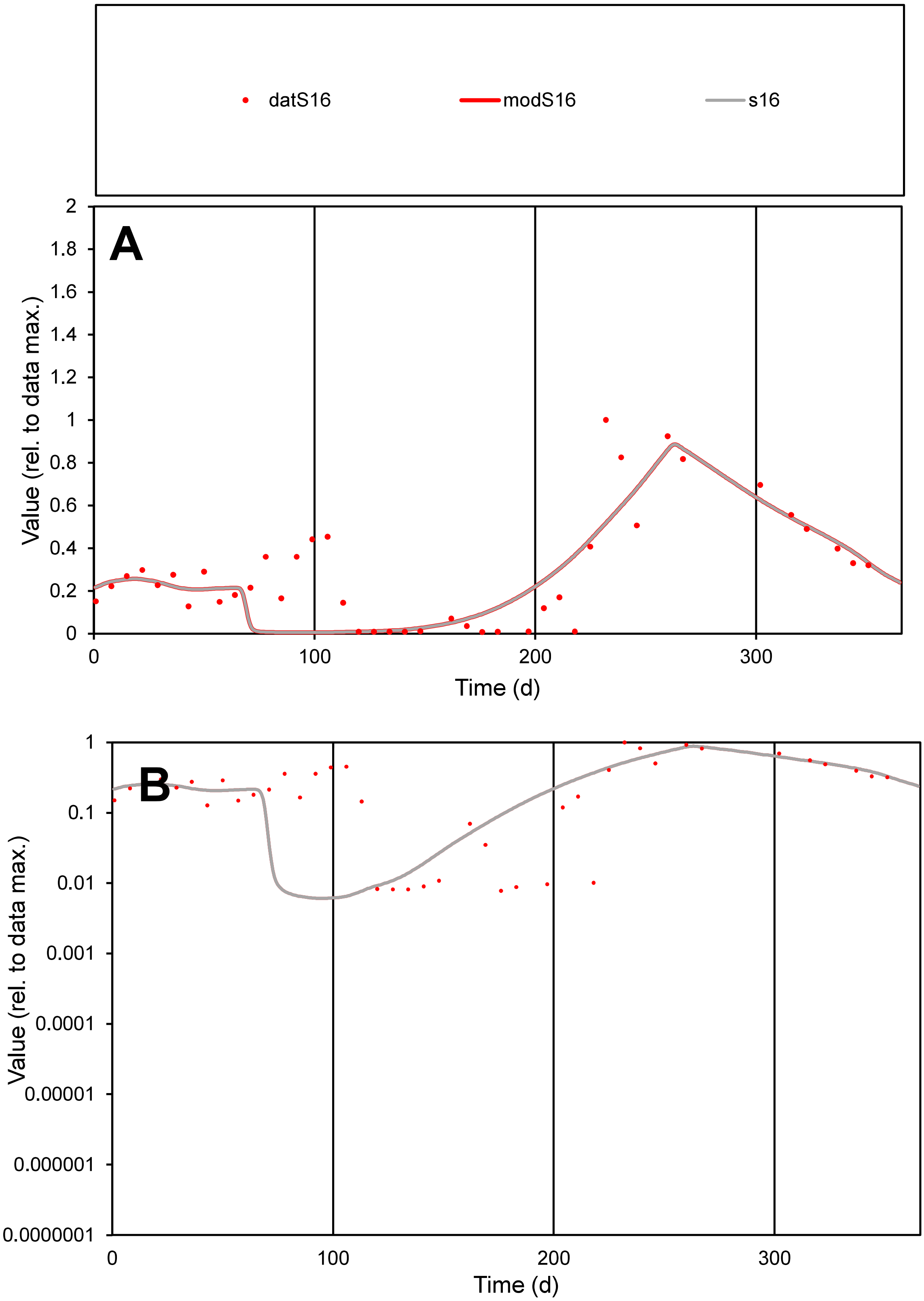

C1

Variables
S16 Provide jtd or Dst
CalibOnly?
2 1 to reproduce of
oNormalize?
2 1=no, 2=yes

ofxn calc*
0.60219
ofxn mod
0.62201

sRun
RunA1
zout
1

Ave
Data
0.00466
Model
0.00417

data/vMax
0.01623

ofxng
319.391

Min d/m
1.27703

ave/min d
36.9037
ave/min m
42.1273

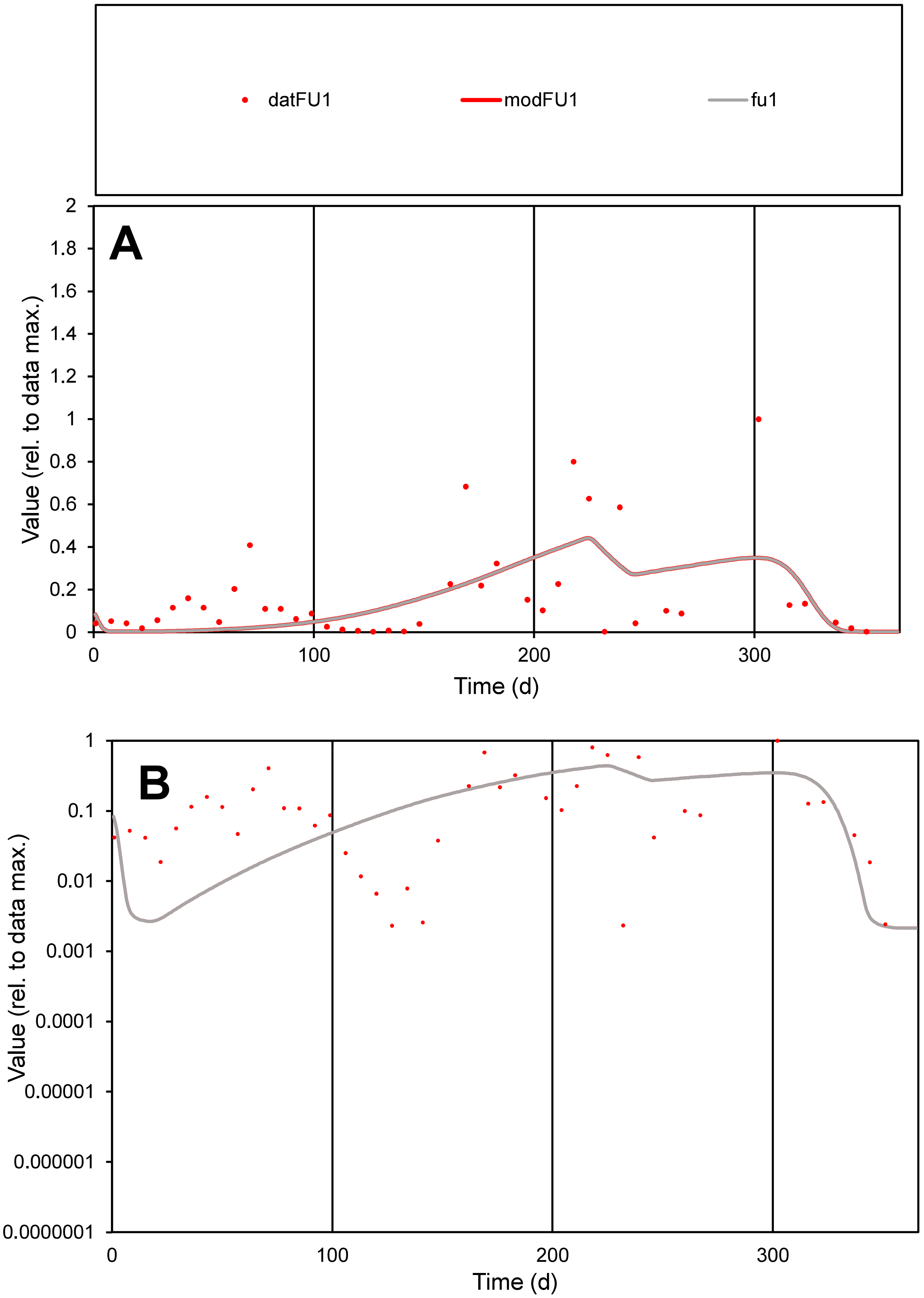

C1

Variables
FU1 Provide jtd or Dst
CalibOnly?
2 1 to reproduce of
oNormalize?
2 1=no, 2=yes

ofxn calc*
1.14489
ofxn mod
1.16062

sRun
RunA1
zout
1

Ave
Data
0.00365
Model
0.00321

data/vMax
0.02124

ofxng
319.391

Min d/m
1.03999

ave/min d
73.8558
ave/min m
67.6692

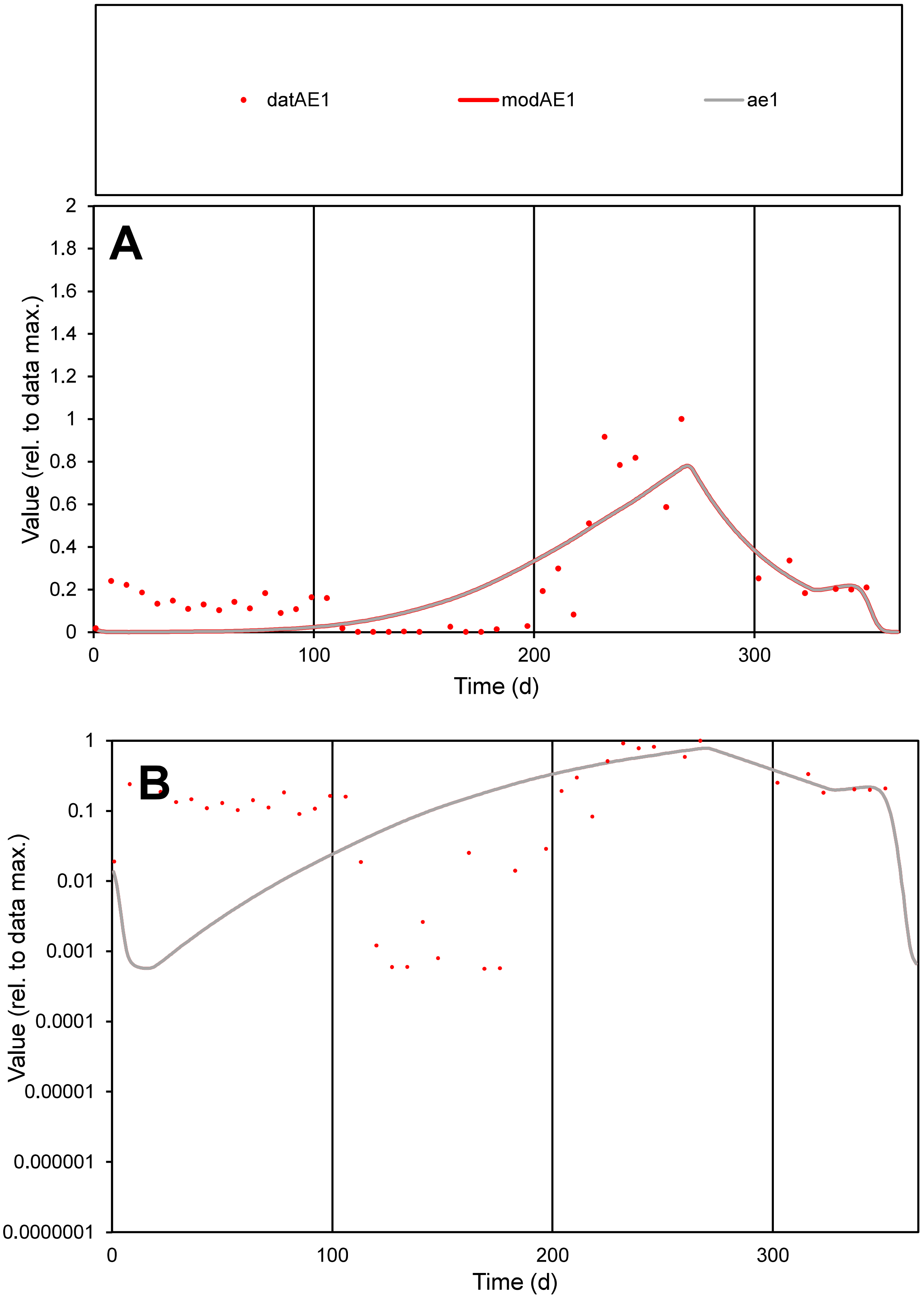

C1

Variables
AE1 Provide jtd or Dst
CalibOnly?
2 1 to reproduce of
oNormalize?
2 1=no, 2=yes

ofxn calc*
0.74985
ofxn mod
0.7711

sRun
RunA1
zout
1

Ave
Data
0.00525
Model
0.00474

data/vMax
0.02476

ofxng
319.391

Min d/m
0.99083

ave/min d
374.268
ave/min m
334.284

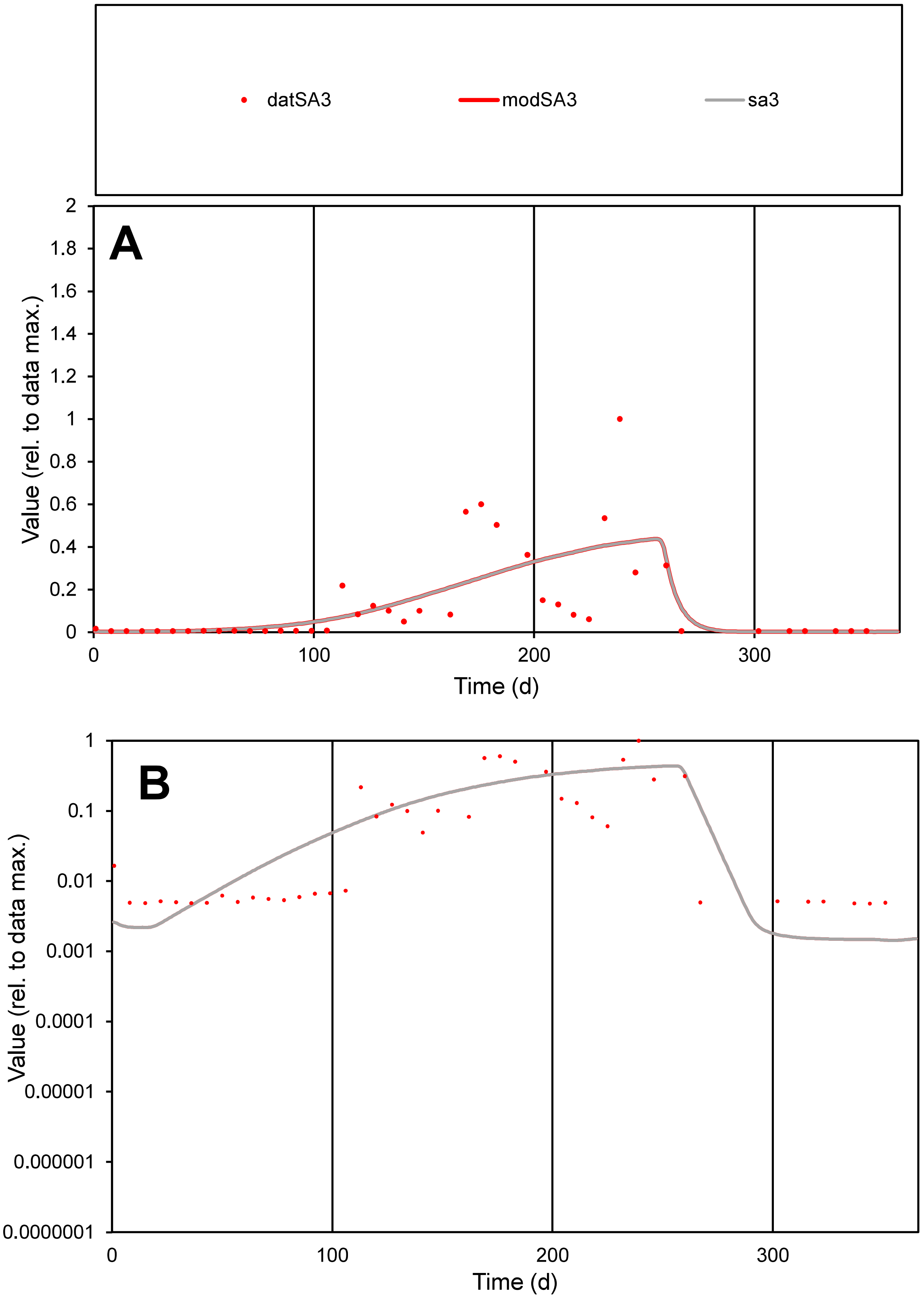

C1

Variables
SA3 Provide jtd or Dst
CalibOnly?
2 1 to reproduce of
oNormalize?
2 1=no, 2=yes

ofxn calc*
1.18316
ofxn mod
1.20417

sRun
RunA1
zout
1

Ave
Data
0.00288
Model
0.00285

data/vMax
0.02213

ofxng
319.391

Min d/m
3.31868

ave/min d
27.2778
ave/min m
89.5072

330_Calib_S_A

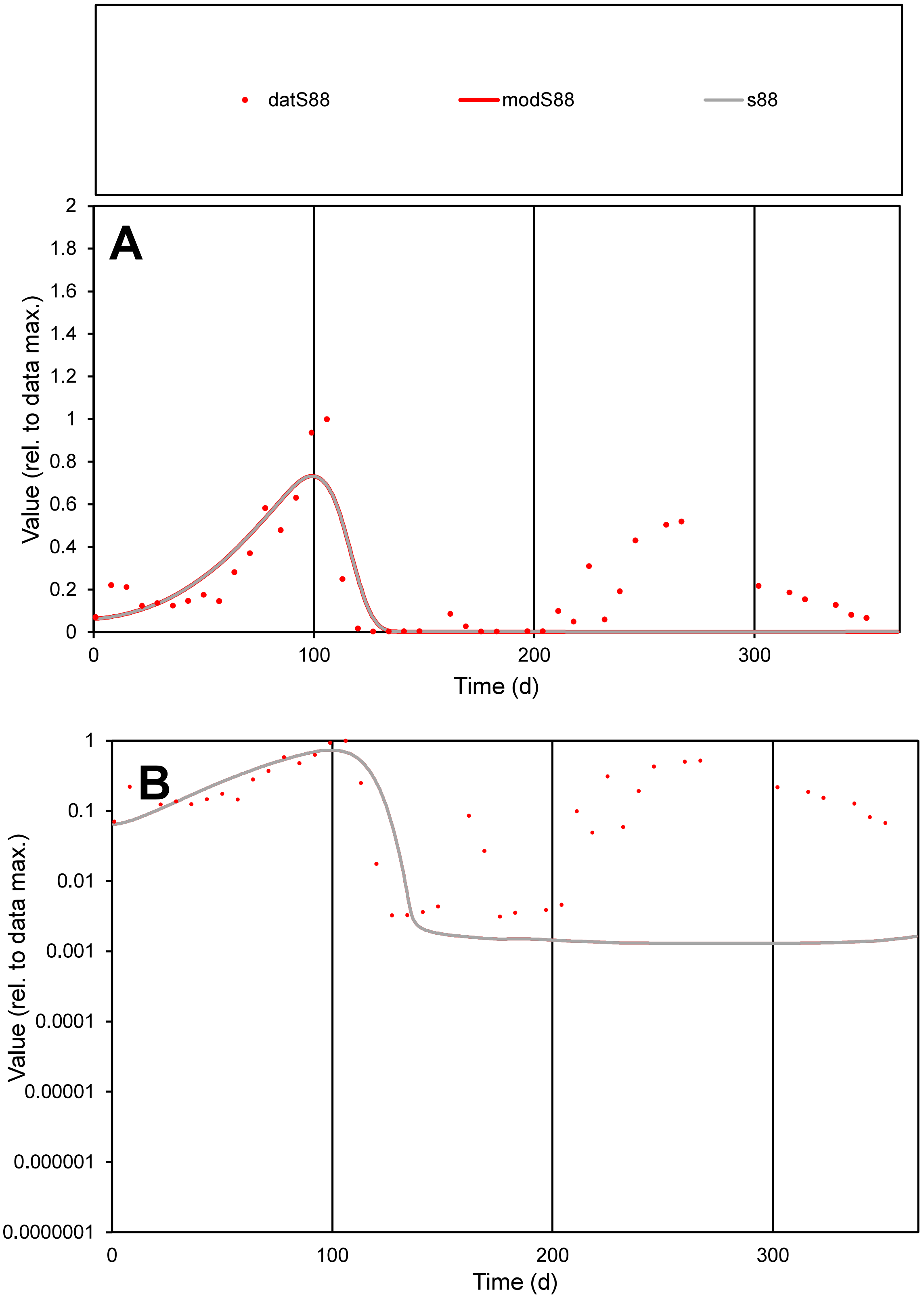

C1

Variables

S88	Provide jtd or Dst
CalibOnly?	
2	1 to reproduce of
oNormalize?	
2	1=no, 2=yes

ofxn calc*
0.83237
ofxn mod
0.84896

sRun
RunA1
zout
1

Ave
Data
0.00404
Model
0.0028

data/vMax
0.01876

ofxng
319.391

Min d/m
2.40188

ave/min d
68.7337
ave/min m
114.667

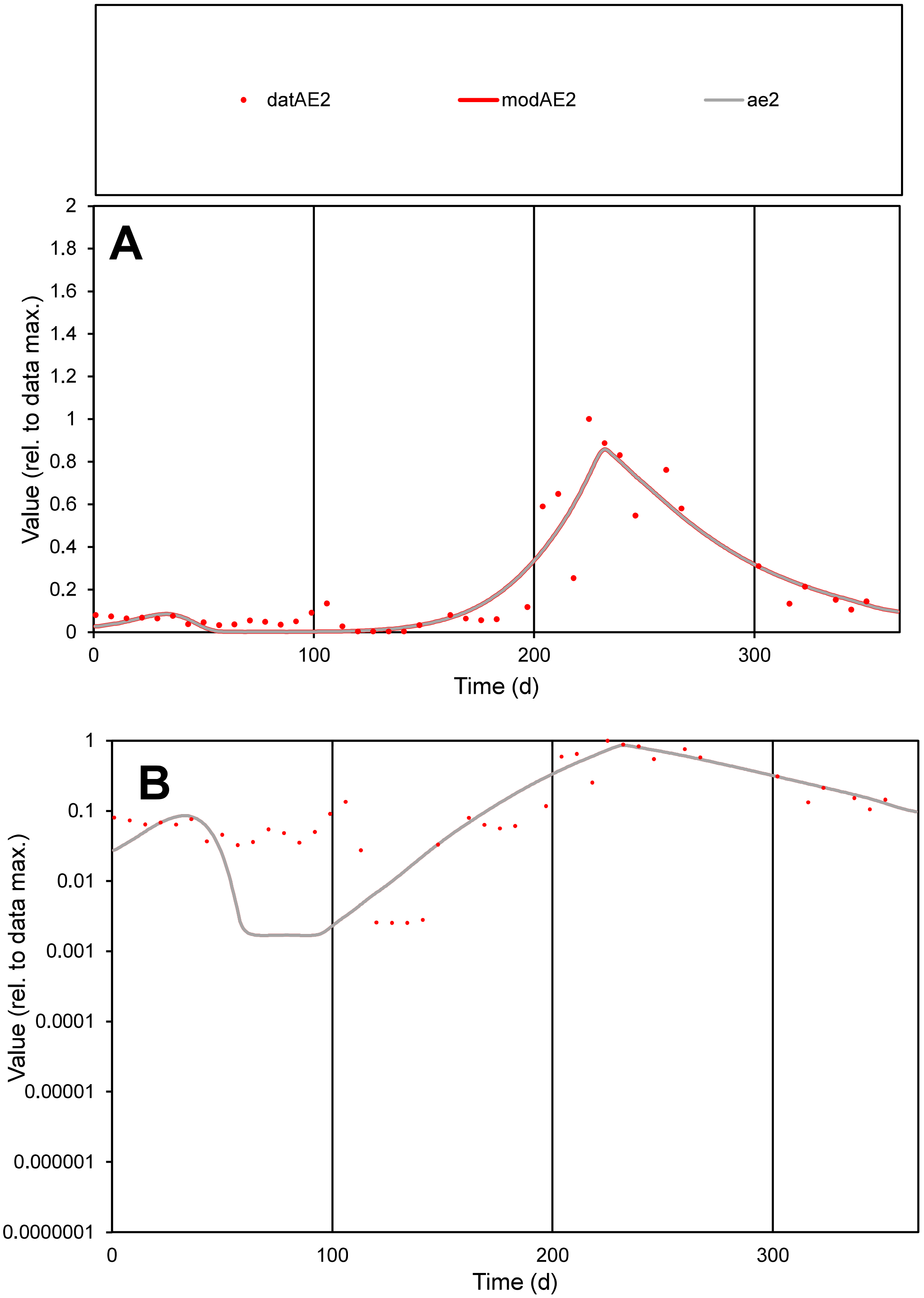

C1

Variables
AE2 Provide jtd or Dst
CalibOnly?
2 1 to reproduce of
oNormalize?
2 1=no, 2=yes

ofxn calc*
0.48109
ofxn mod
0.52186

sRun
RunA1
zout
1

Ave
Data
0.00495
Model
0.00477

data/vMax
0.0242

ofxng
319.391

Min d/m
1.50282

ave/min d
80.7435
ave/min m
116.869

330_Calib_S_A

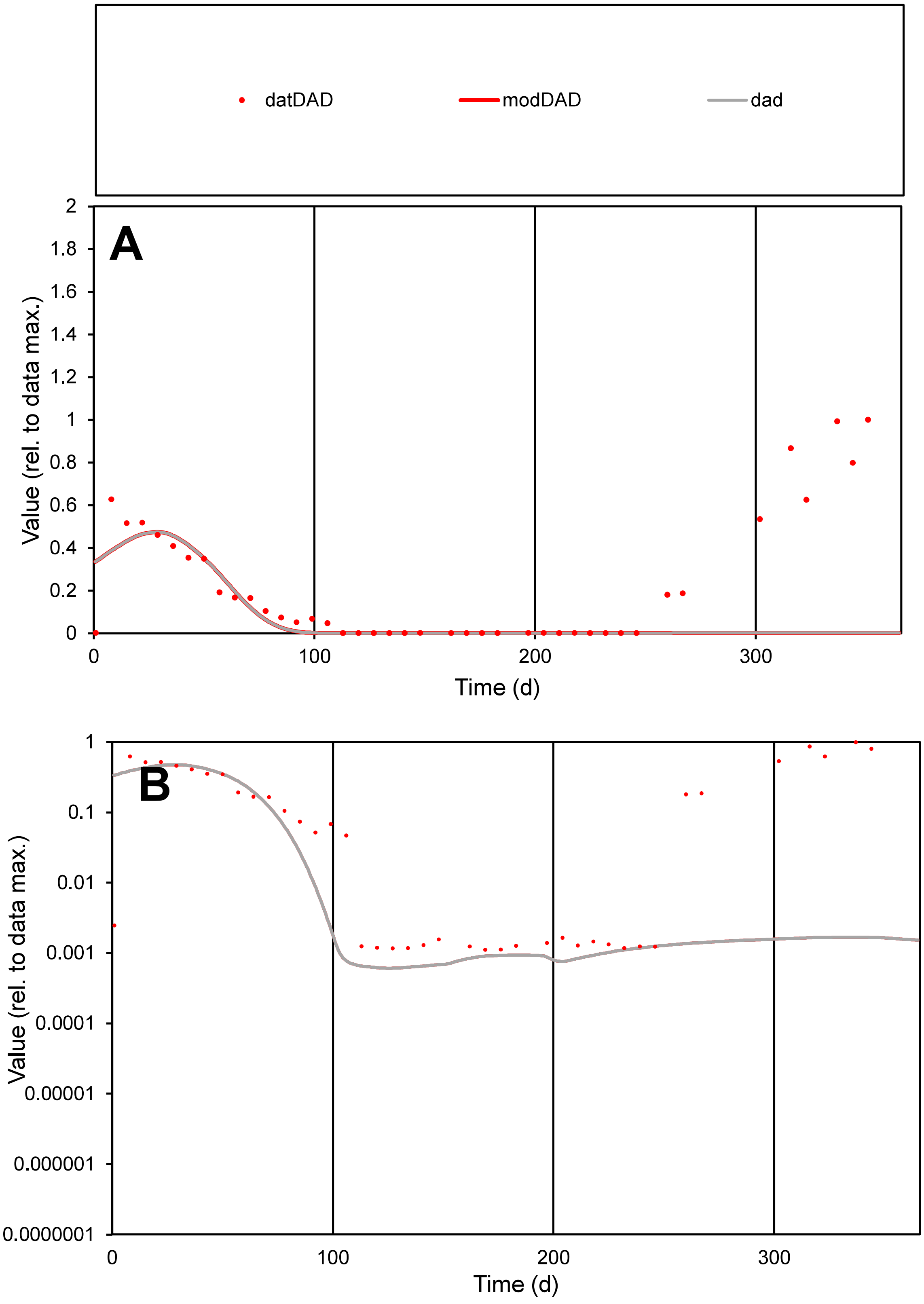

C1

Variables
DAD Provide jtd or Dst
CalibOnly?
2 1 to reproduce of
oNormalize?
2 1=no, 2=yes

ofxn calc*
1.44286
ofxn mod
1.44768

sRun
RunA1
zout
1

Ave
Data
0.00311
Model
0.00133

data/vMax
0.01401

ofxng
319.391

Min d/m
1.81648

ave/min d
199.744
ave/min m
155.418

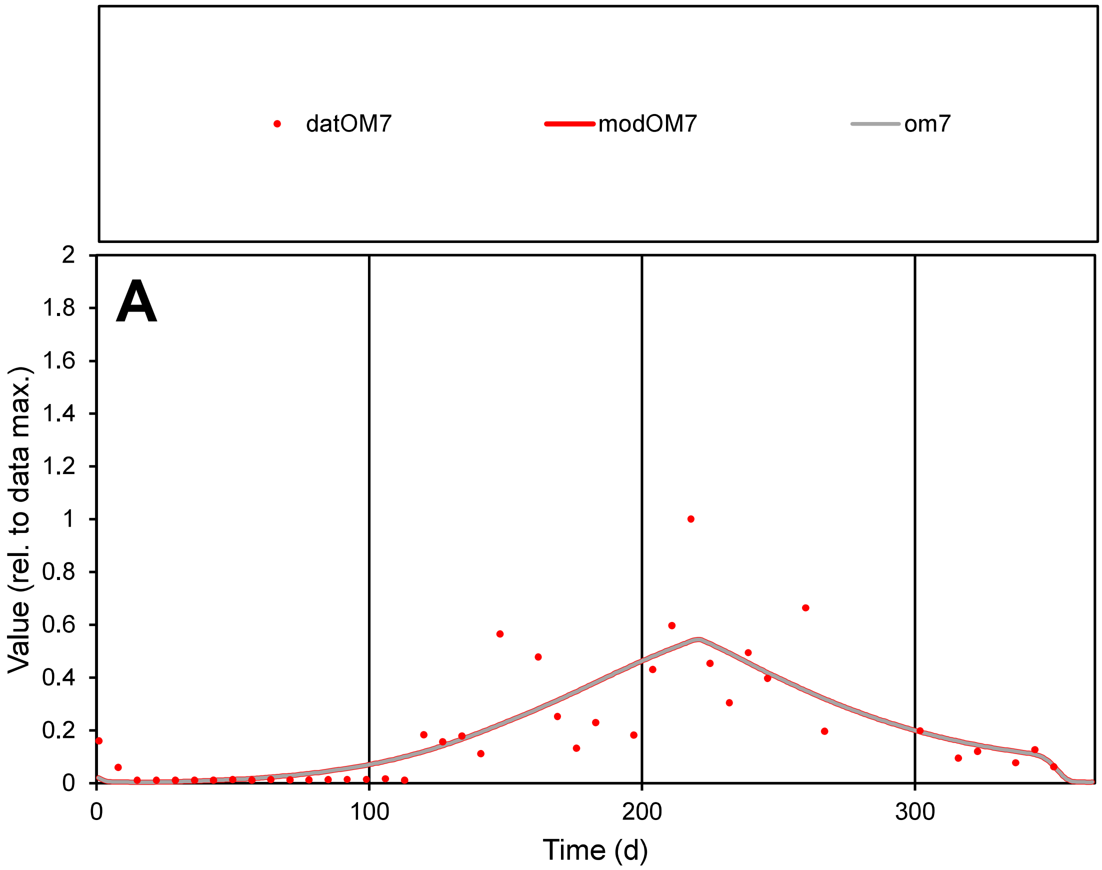

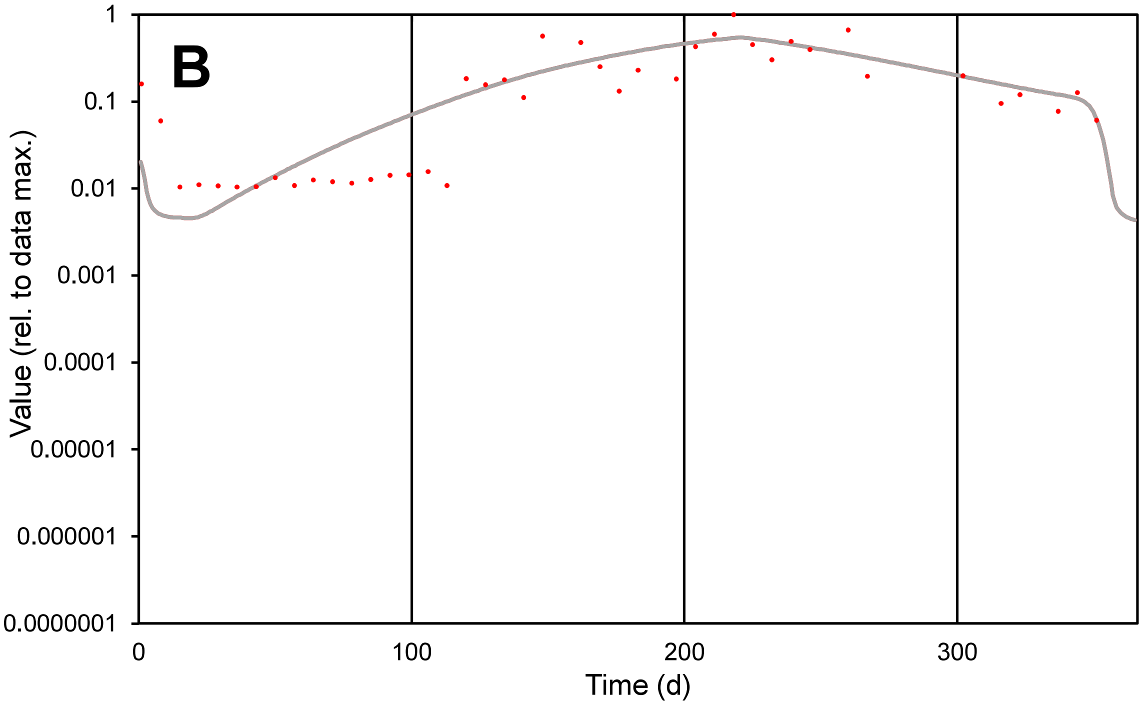

C1

Variables
OM7 Provide jtd or Dst
CalibOnly?
2 1 to reproduce of
oNormalize?
2 1=no, 2=yes

ofxn calc*
0.68436
ofxn mod
0.70934

sRun
RunA1
zout
1

Ave
Data
0.00189
Model
0.00188

data/vMax
0.00981

ofxng
319.391

Min d/m
2.28552

ave/min d
18.4419
ave/min m
41.8903

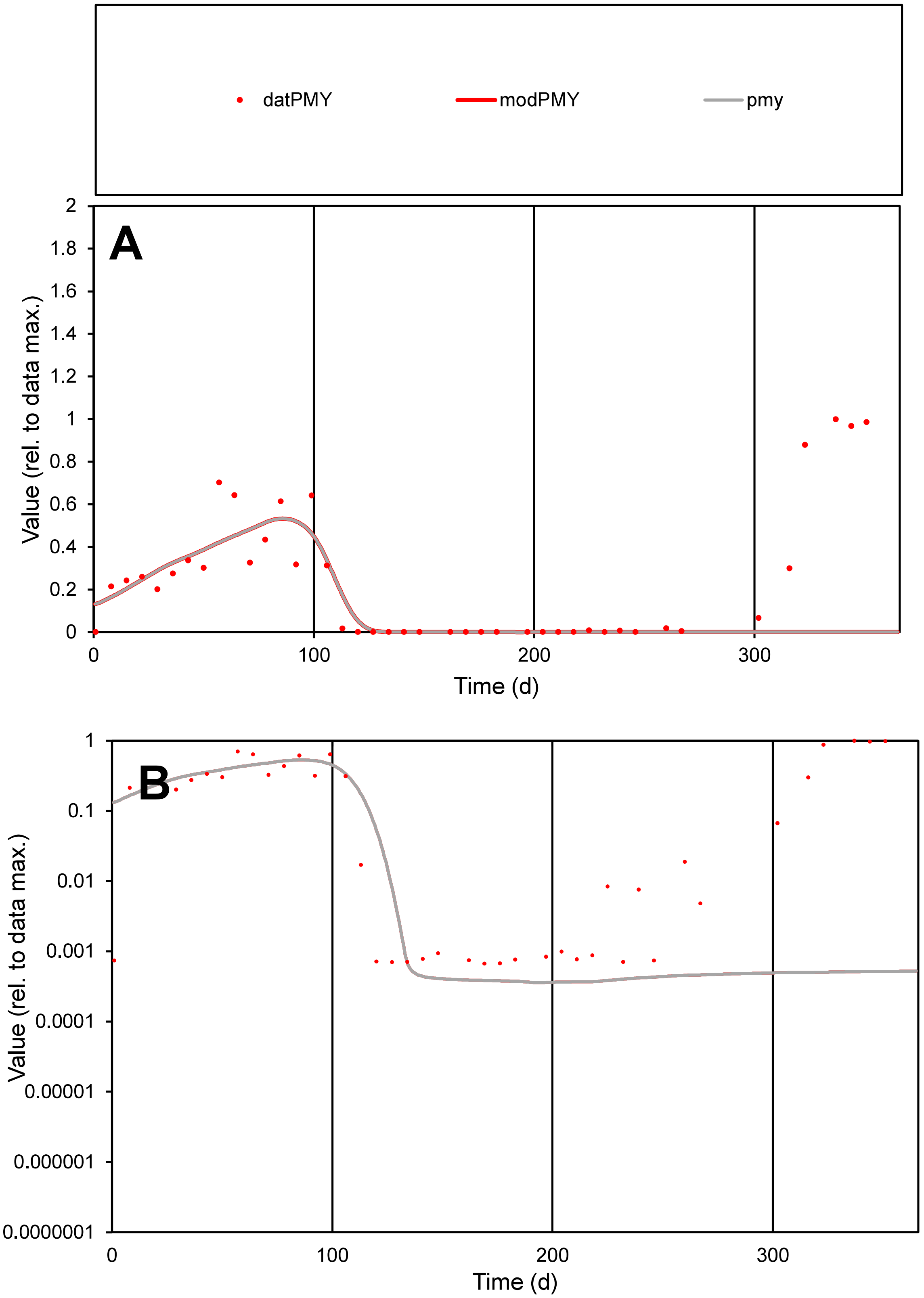

C1

Variables
PMY Provide jtd or Dst
CalibOnly?
2 1 to reproduce of
oNormalize?
2 1=no, 2=yes

ofxn calc*
1.29245
ofxn mod
1.29891

sRun
RunA1
zout
1

Ave
Data
0.00349
Model
0.00207

data/vMax
0.01451

ofxng
319.391

Min d/m
1.84021

ave/min d
360.092
ave/min m
394.168

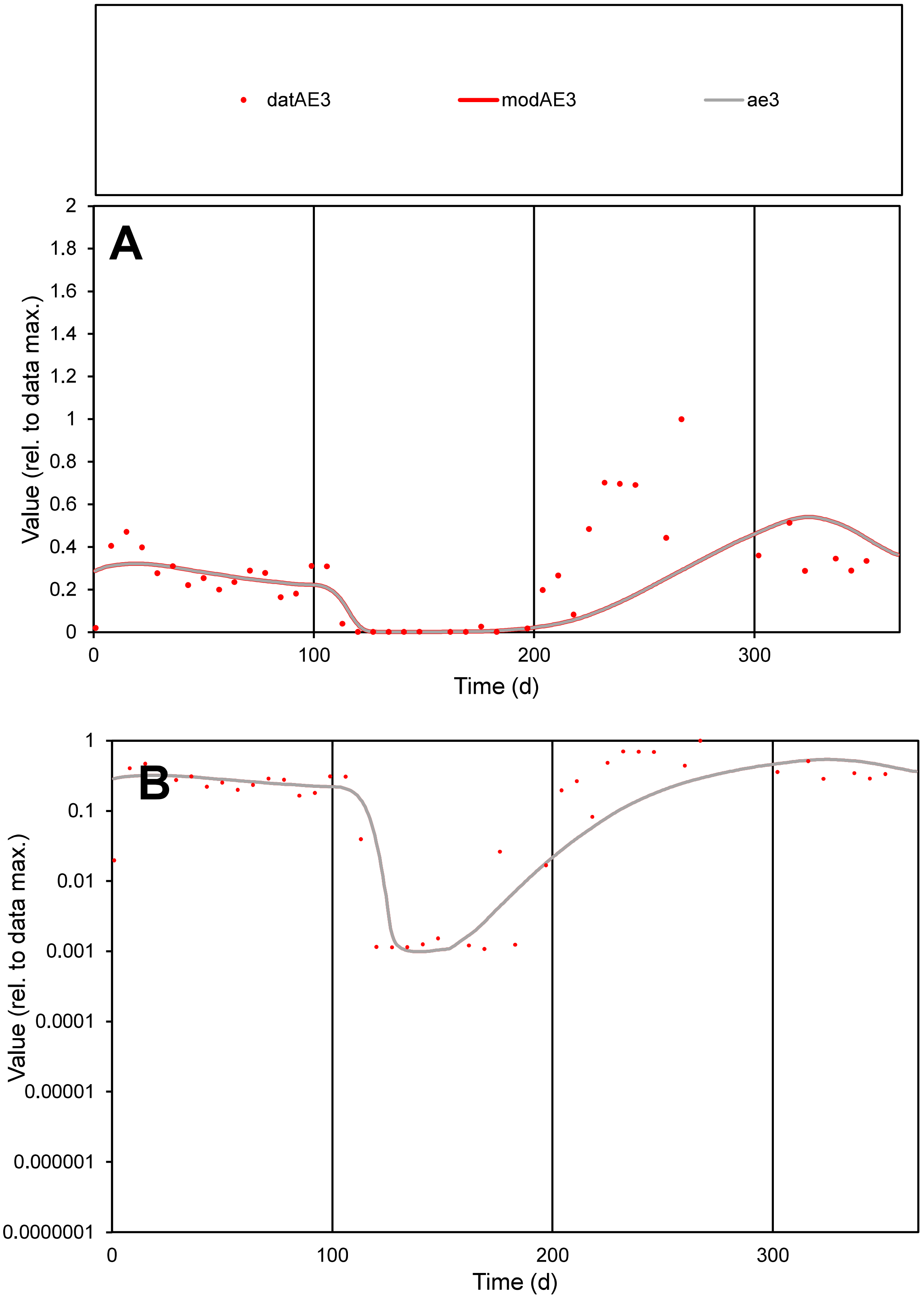

C1

Variables
AE3 Provide jtd or Dst
CalibOnly?
2 1 to reproduce of
oNormalize?
2 1=no, 2=yes

ofxn calc*
0.81937
ofxn mod
0.82872

sRun
RunA1
zout
1

Ave
Data
0.00414
Model
0.00323

data/vMax
0.01565

ofxng
319.391

Min d/m
1.08515

ave/min d
244.034
ave/min m
206.706

330_Calib_S_A

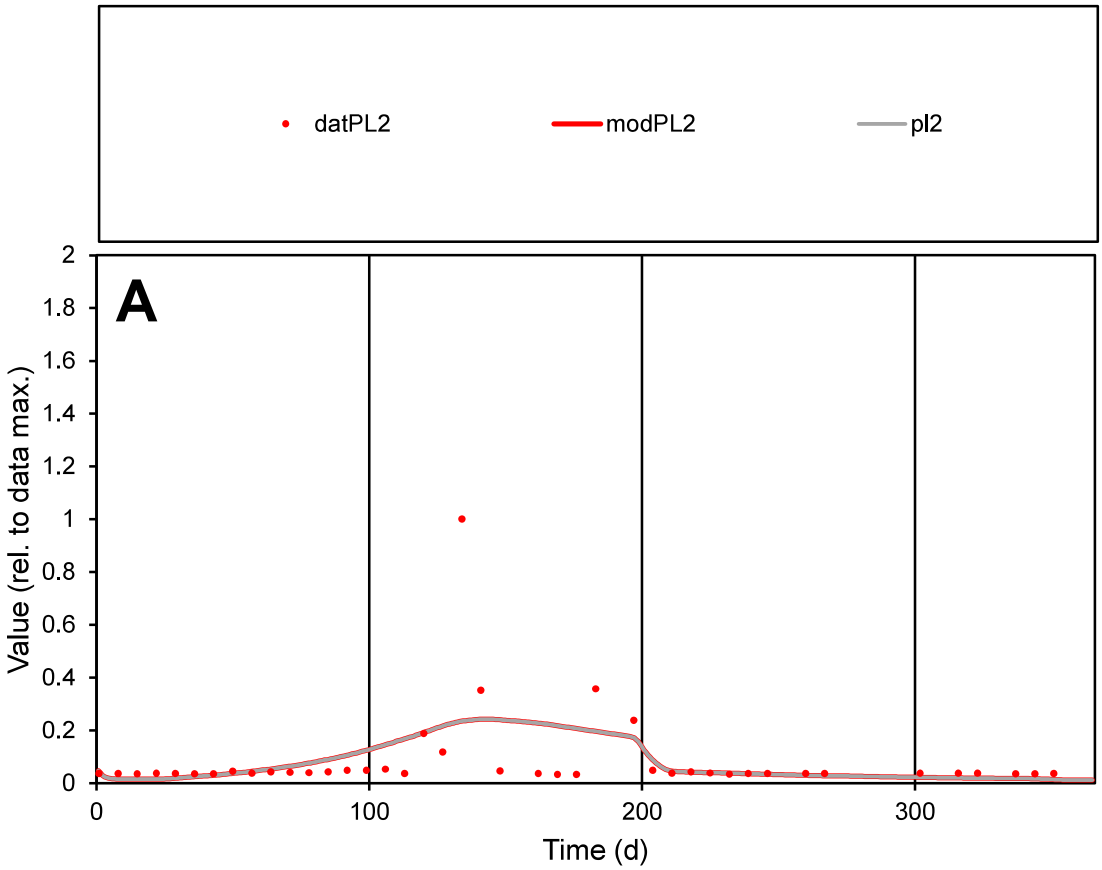

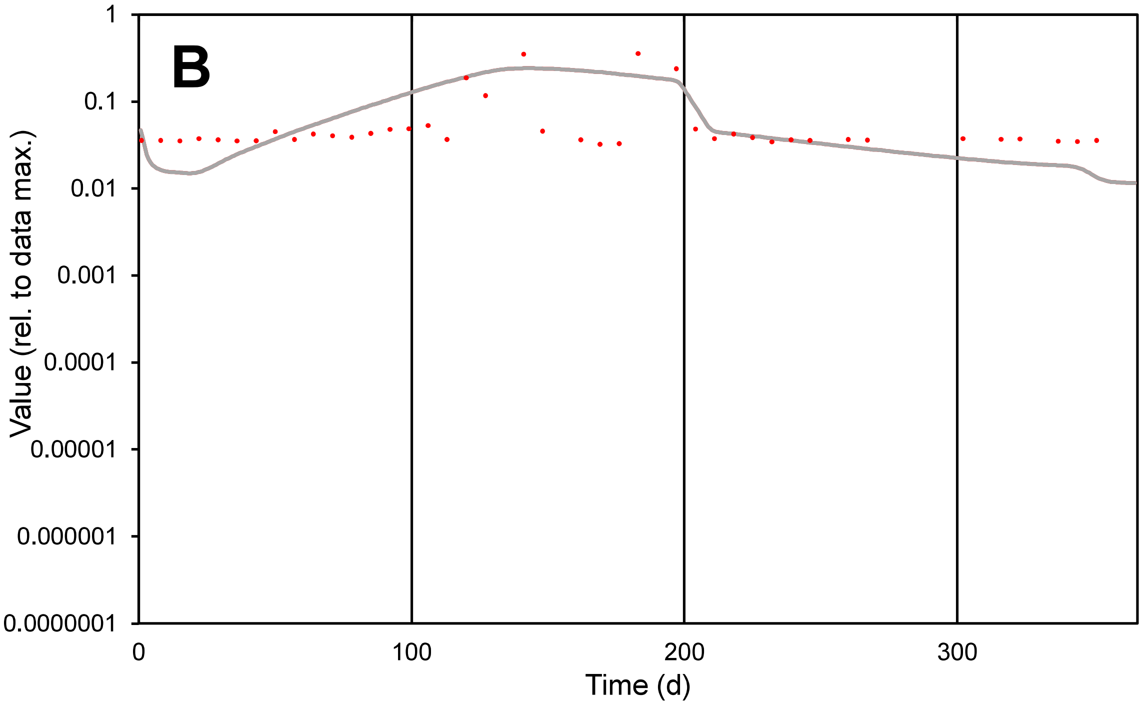

C1

Variables
PL2 Provide jtd or Dst
CalibOnly?
2 1 to reproduce of
oNormalize?
2 1=no, 2=yes

ofxn calc*
1.60625
ofxn mod
1.61836

sRun
RunA1
zout
1

Ave
Data
0.00044
Model
0.00045

data/vMax
0.00506

ofxng
319.391

Min d/m
2.47286

ave/min d
2.67844
ave/min m
6.7423

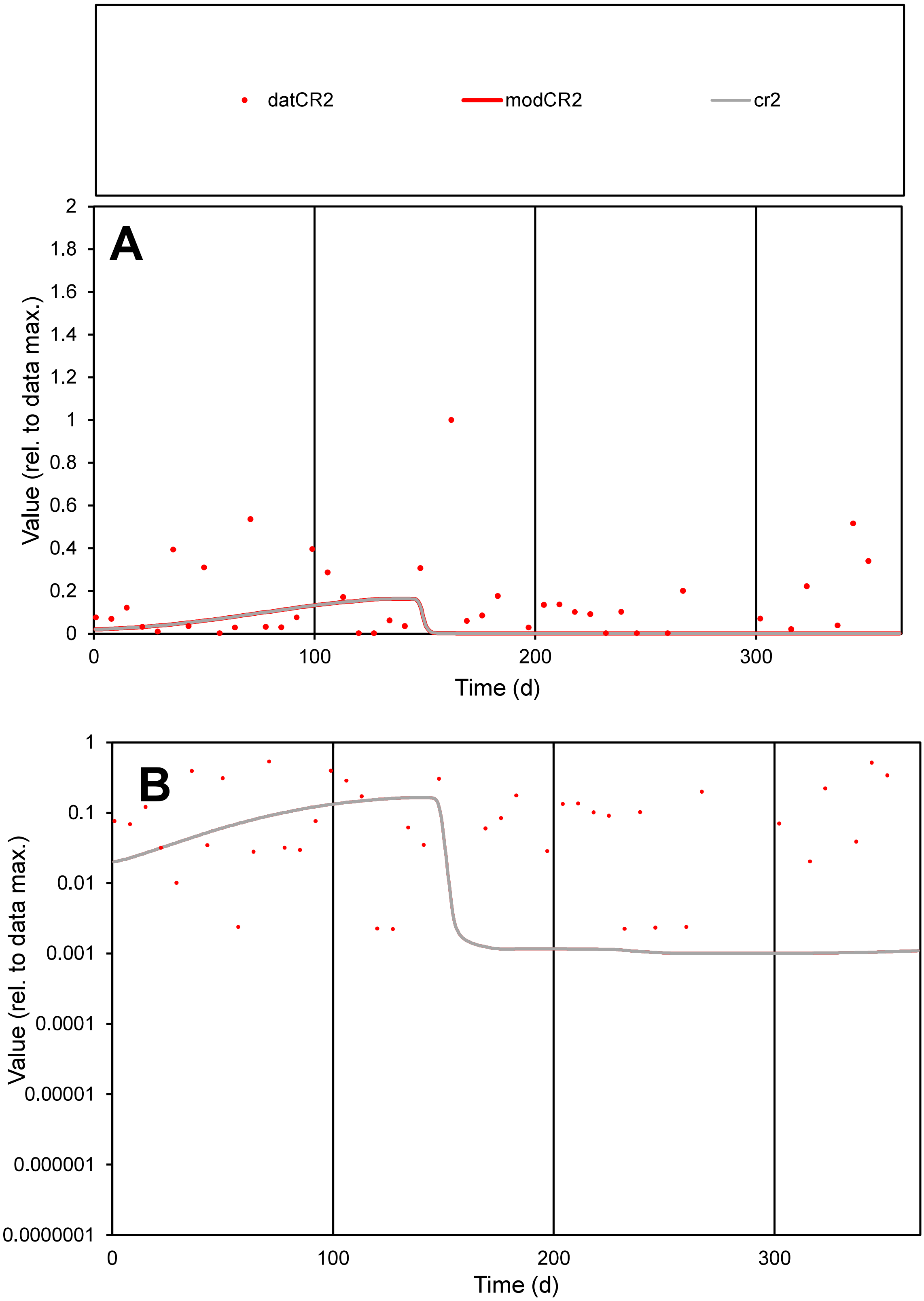

C1

Variables
CR2 Provide jtd or Dst
CalibOnly?
2 1 to reproduce of
oNormalize?
2 1=no, 2=yes

ofxn calc*
1.51205
ofxn mod
1.51912

sRun
RunA1
zout
1

Ave
Data
0.00287
Model
0.00095

data/vMax
0.019

ofxng
319.391

Min d/m
2.20229

ave/min d
67.74
ave/min m
49.6082

330_Calib_S_A

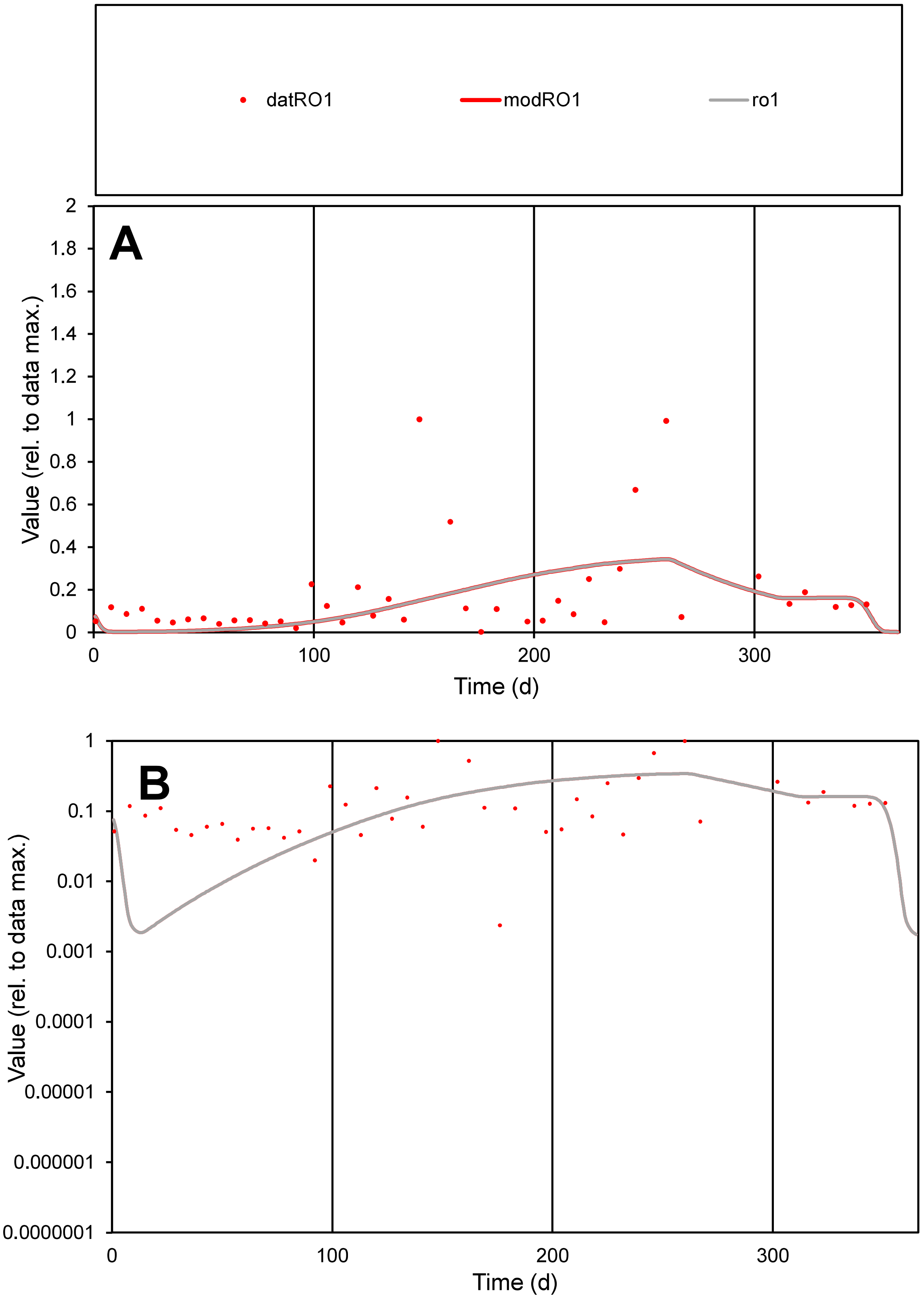

C1

Variables

RO1	Provide jtd or Dst
CalibOnly?	
2	1 to reproduce of
oNormalize?	
2	1=no, 2=yes

ofxn calc*
1.23743
ofxn mod
1.24498

sRun
RunA1
zout
1

Ave
Data
0.00258
Model
0.00213

data/vMax
0.01519

ofxng
319.391

Min d/m
1.12774

ave/min d
71.5522
ave/min m
66.5331

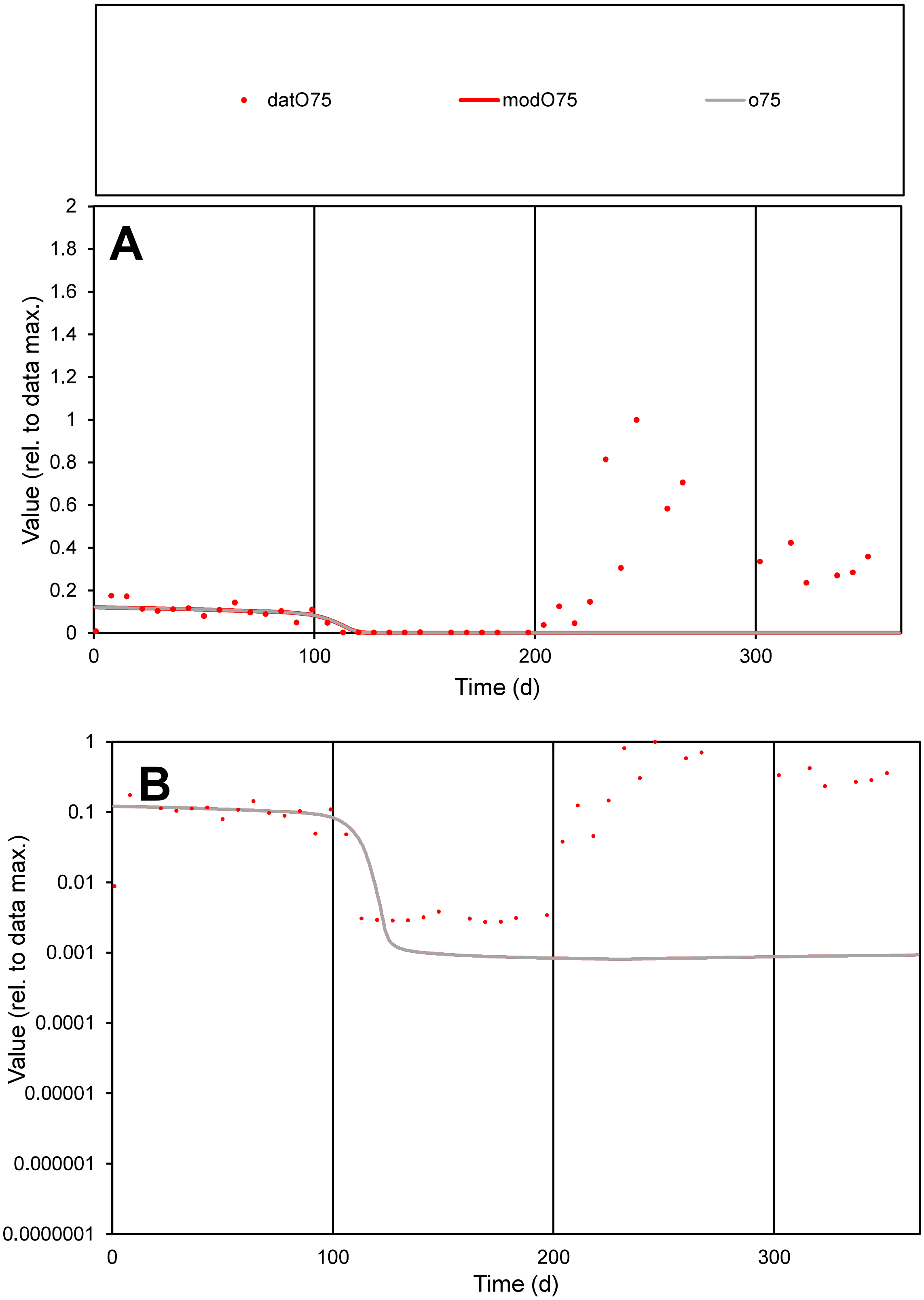

C1

Variables
O75 Provide jtd or Dst
CalibOnly?
2 1 to reproduce of
oNormalize?
2 1=no, 2=yes

ofxn calc*
1.59968
ofxn mod
1.60274

sRun
RunA1
zout
1

Ave
Data
0.00451
Model
0.00106

data/vMax
0.02581

ofxng
319.391

Min d/m
3.38506

ave/min d
63.7963
ave/min m
50.764

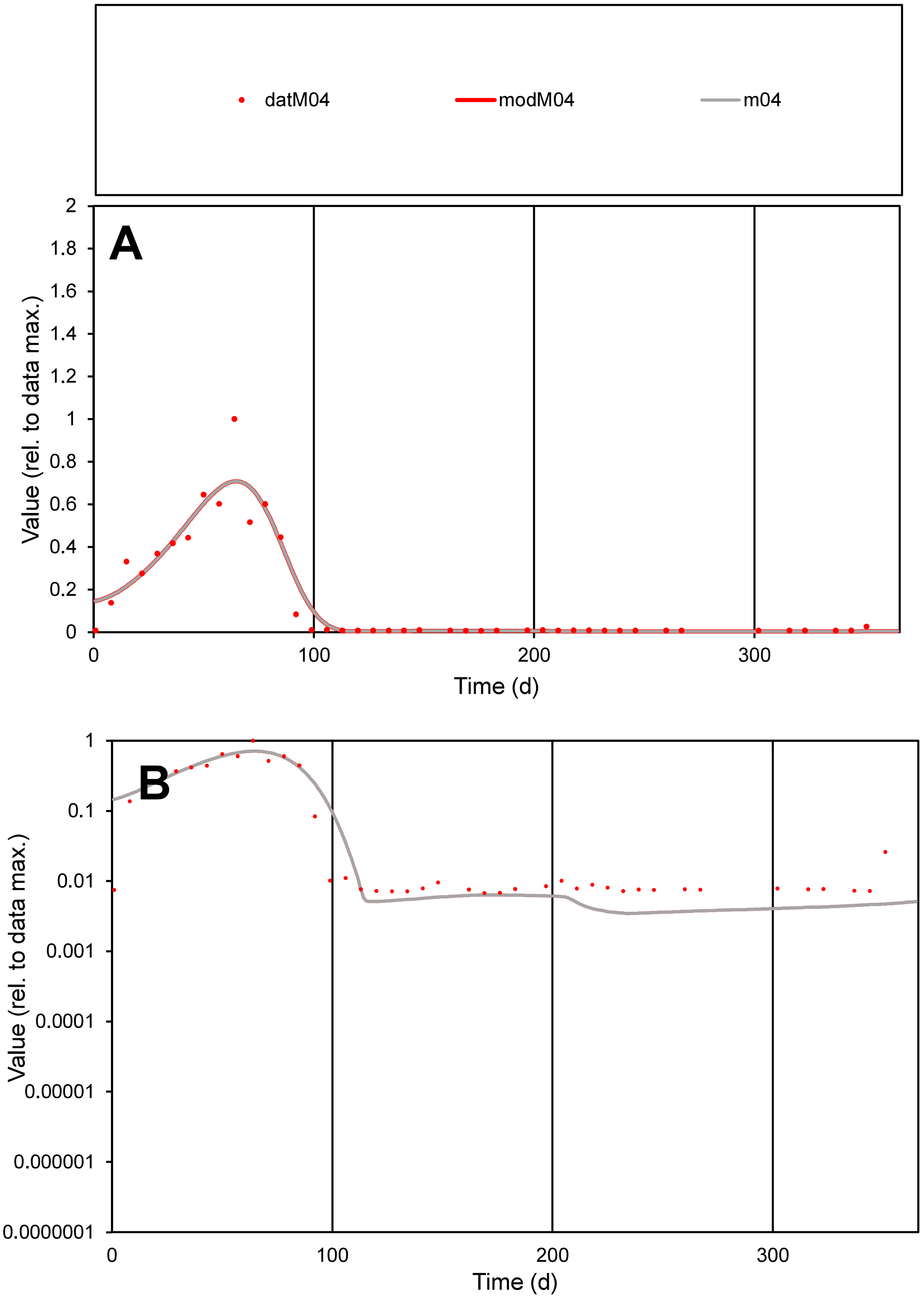

C1

Variables
M04 Provide jtd or Dst
CalibOnly?
2 1 to reproduce of
oNormalize?
2 1=no, 2=yes

ofxn calc*
0.44777
ofxn mod
0.50642

sRun
RunA1
zout
1

Ave
Data
0.00076
Model
0.00078

data/vMax
0.00525

ofxng
319.391

Min d/m
1.94688

ave/min d
21.5139
ave/min m
42.5119

330_Calib_S_A

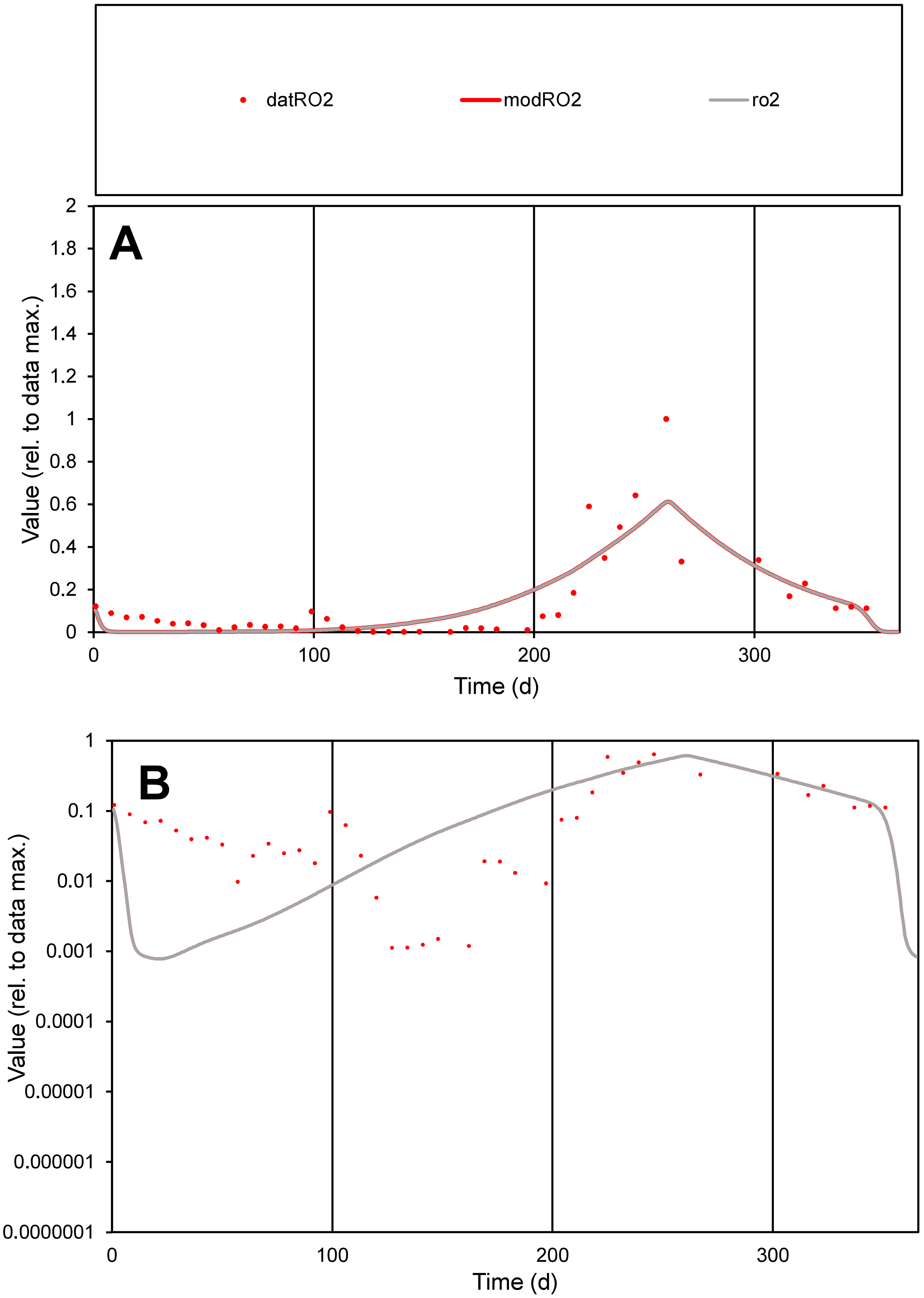

C1

Variables
RO2 Provide jtd or Dst
CalibOnly?
2 1 to reproduce of
oNormalize?
2 1=no, 2=yes

ofxn calc*
0.76861
ofxn mod
0.79199

sRun
RunA1
zout
1

Ave
Data
0.00496
Model
0.00485

data/vMax
0.03637

ofxng
319.391

Min d/m
1.42648

ave/min d
121.563
ave/min m
169.377

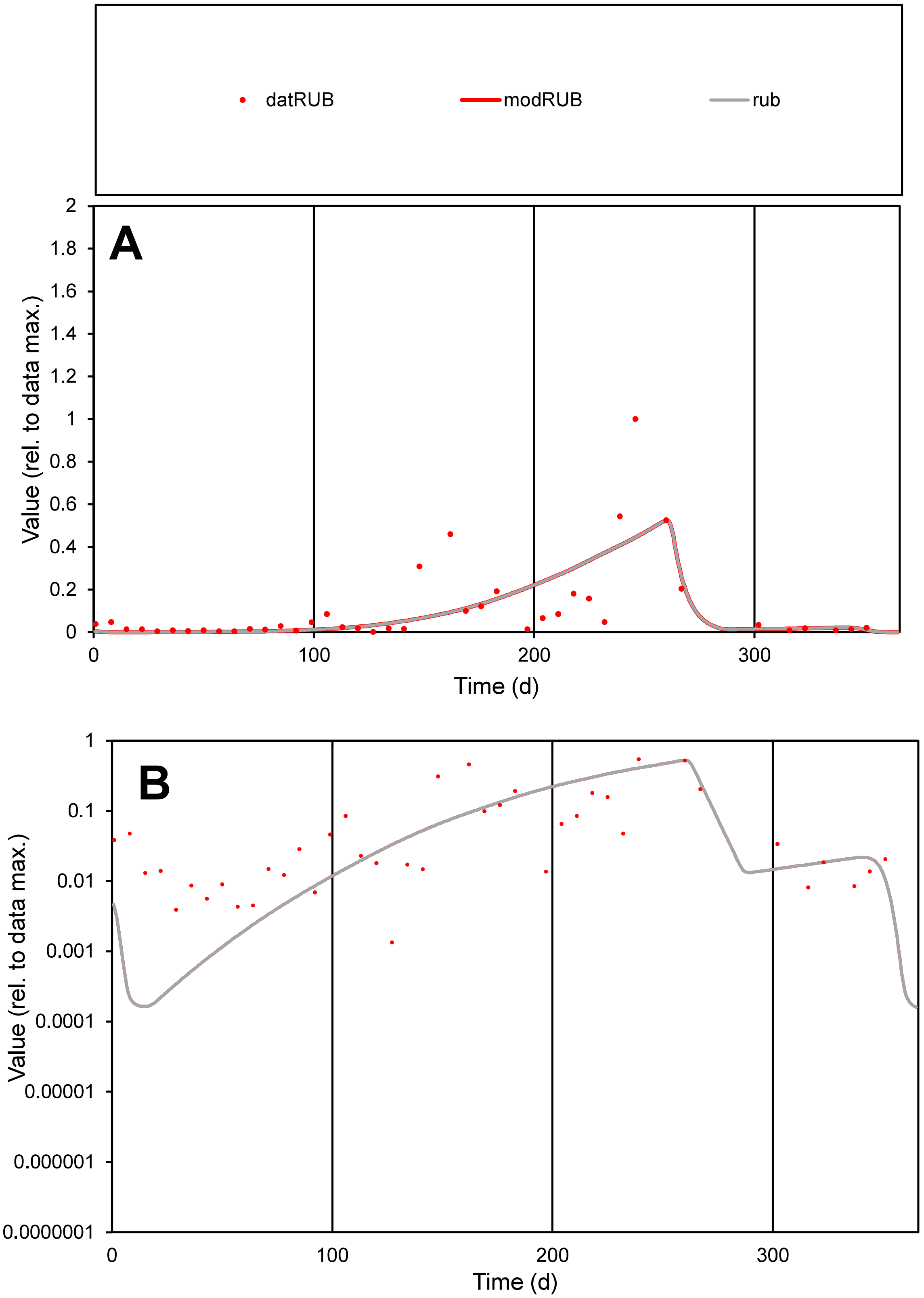

C1

Variables

RUB Provide jtd or Dst

CalibOnly?

2 1 to reproduce of

oNormalize?

2 1=no, 2=yes

ofxn calc*

1.26606

ofxn mod

1.28467

sRun

RunA1

zout

1

Ave

Data

0.00341

Model

0.00321

data/vMax

0.03168

ofxng

319.391

Min d/m

8.04883

ave/min d

80.2349

ave/min m

608.426

330_Calib_S_A

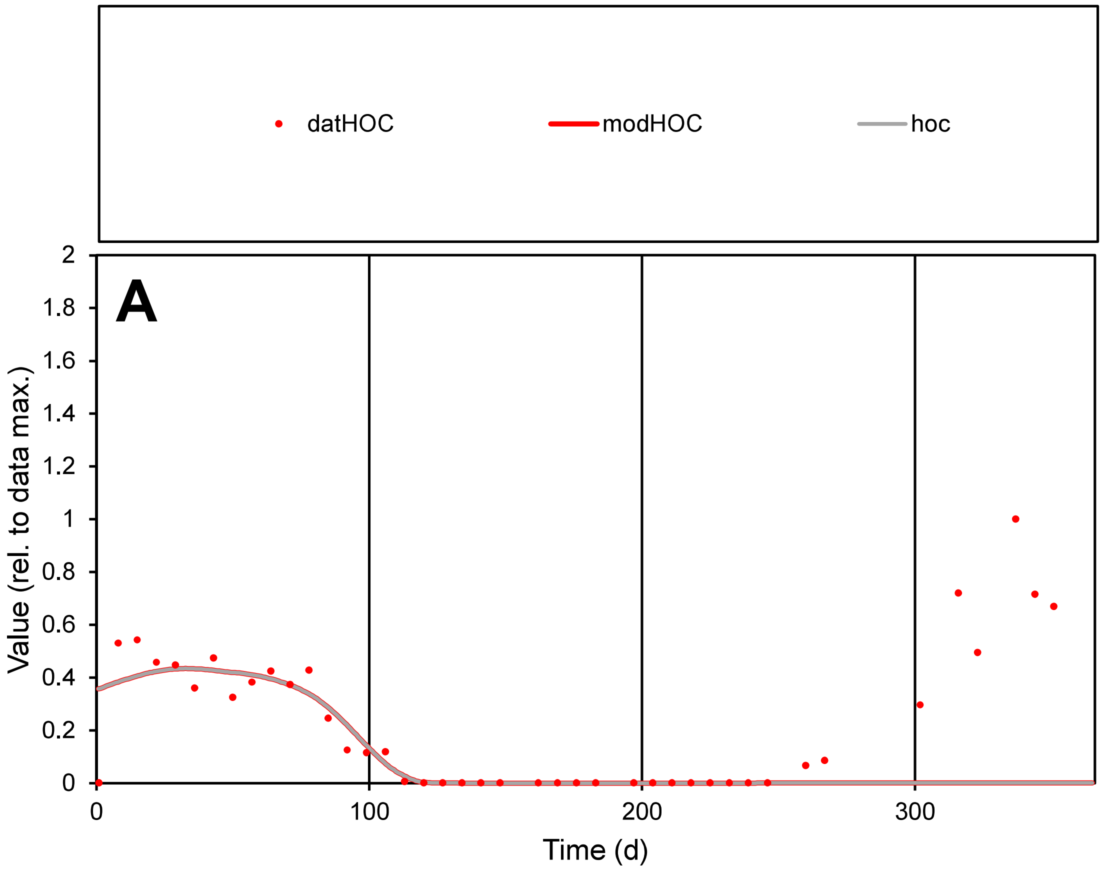

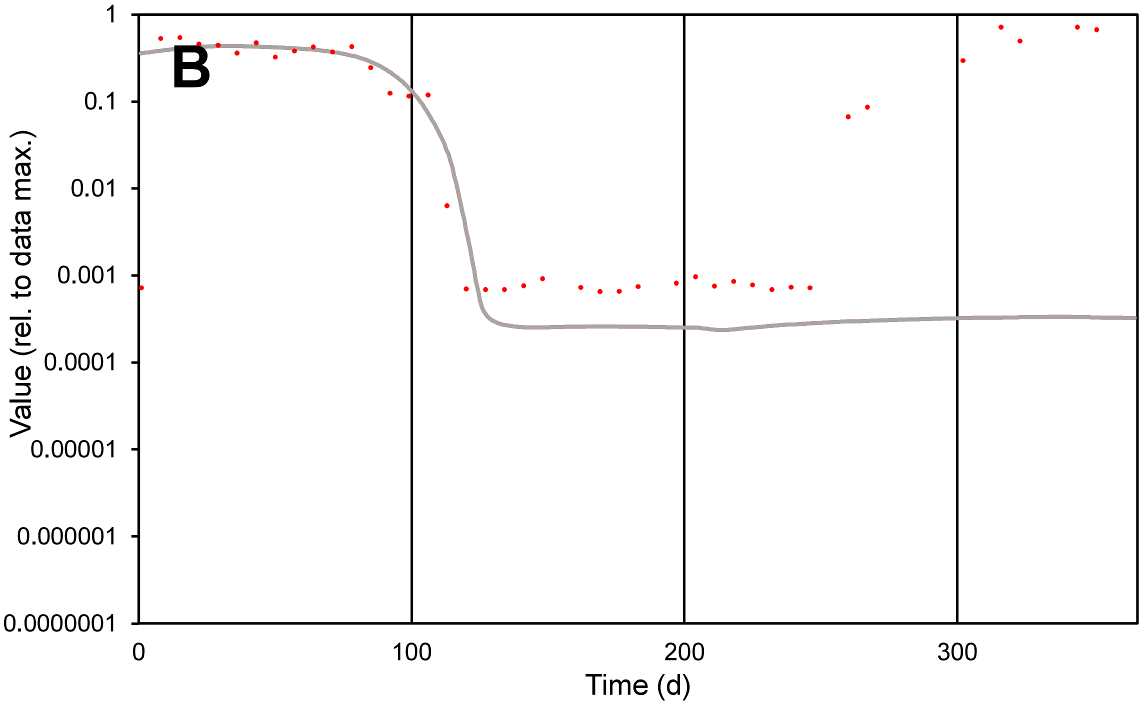

C1

Variables

HOC	Provide jtd or Dst
CalibOnly?	
2	1 to reproduce of
oNormalize?	
2	1=no, 2=yes

ofxn calc*
1.19644
ofxn mod
1.20299

sRun
RunA1
zout
1

Ave
Data
0.00436
Model
0.00254

data/vMax
0.01942

ofxng
319.391

Min d/m
2.75054

ave/min d
343.612
ave/min m
551.74

330_Calib_S_A

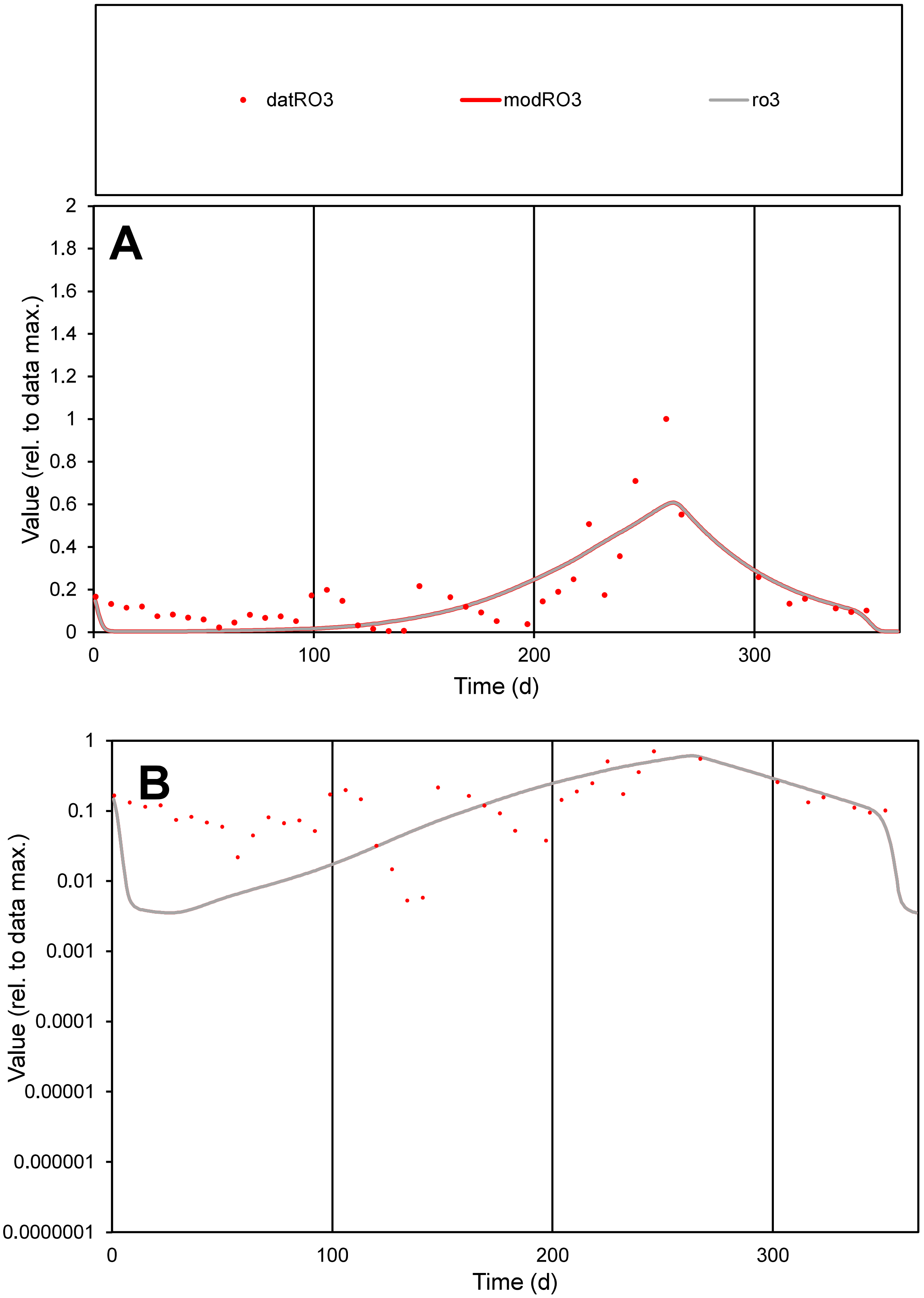

C1

Variables
RO3 Provide jtd or Dst
CalibOnly?
2 1 to reproduce of
oNormalize?
2 1=no, 2=yes

ofxn calc*
0.68176
ofxn mod
0.70021

sRun
RunA1
zout
1

Ave
Data
0.00331
Model
0.00285

data/vMax
0.01945

ofxng
319.391

Min d/m
1.48508

ave/min d
32.2849
ave/min m
41.2852

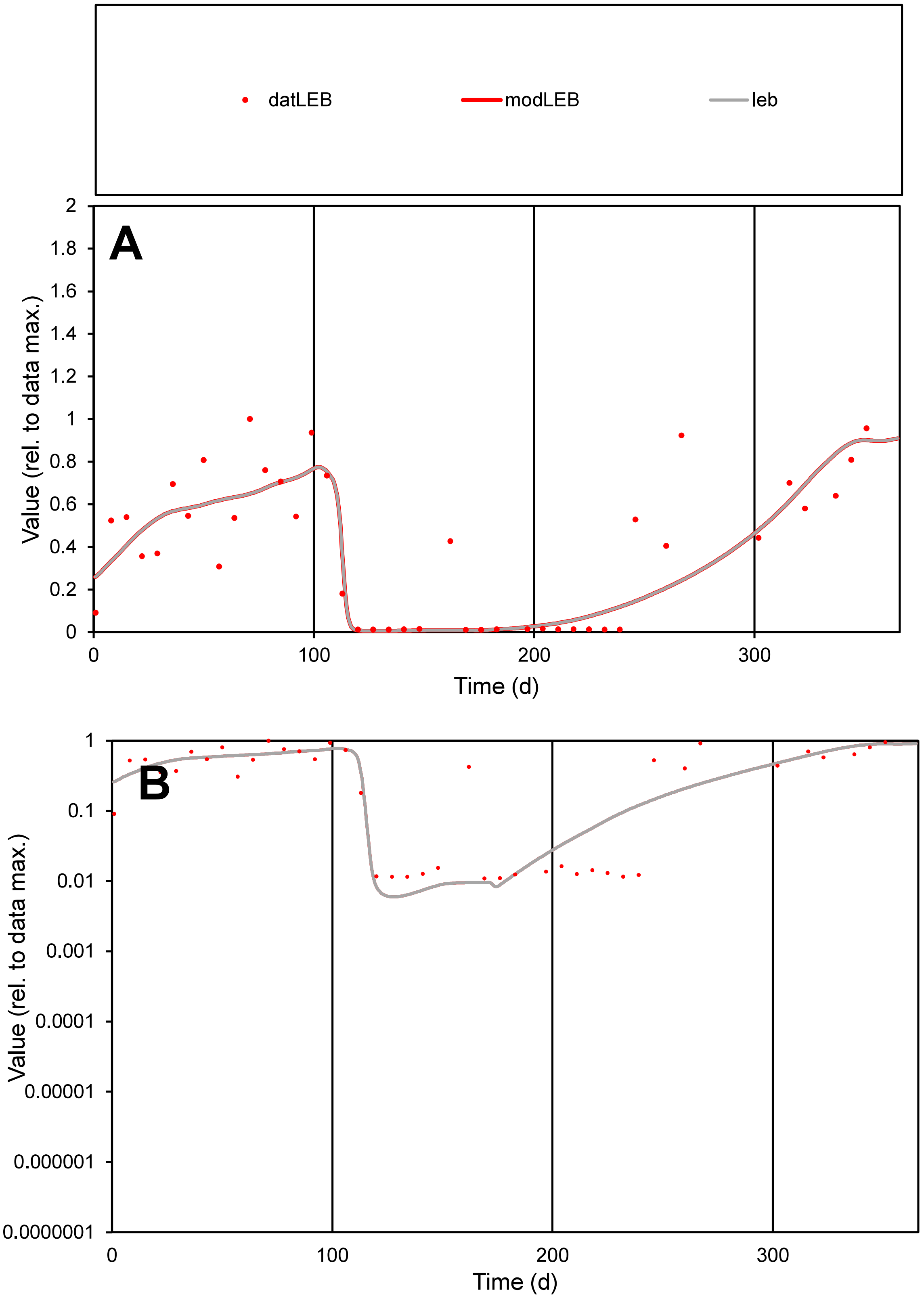

C1

Variables
LEB Provide jtd or Dst
CalibOnly?
2 1 to reproduce of
oNormalize?
2 1=no, 2=yes

ofxn calc*
0.47014
ofxn mod
0.48799

sRun
RunA1
zout
1

Ave
Data
0.00249
Model
0.00232

data/vMax
0.00643

ofxng
319.391

Min d/m
1.82692

ave/min d
35.3733
ave/min m
60.3955

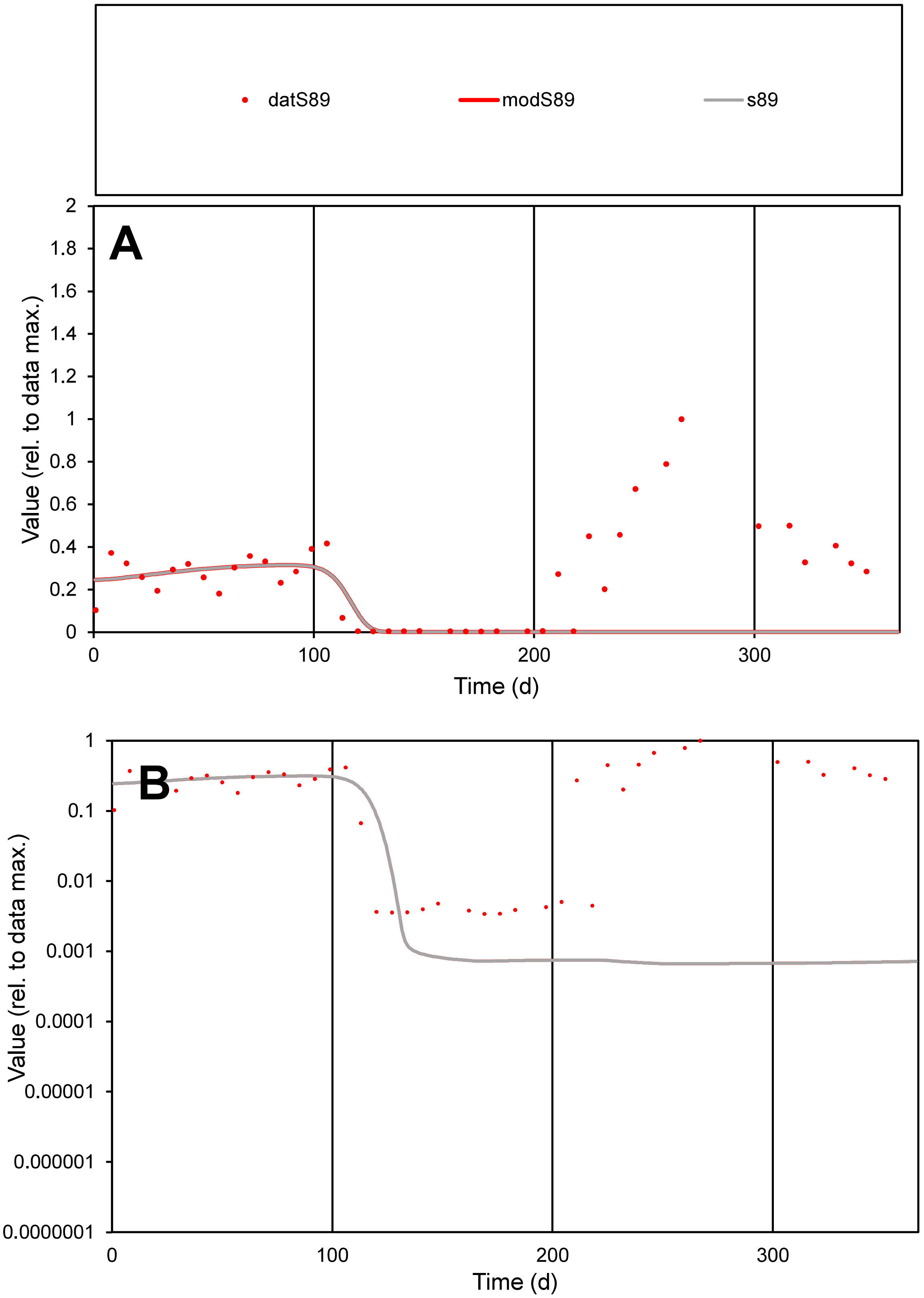

C1

Variables	
S89	Provide jtd or Dst
CalibOnly?	
2	1 to reproduce of
oNormalize?	
2	1=no, 2=yes
ofxn calc*	
1.13629	
ofxn mod	
1.14121	
sRun	
RunA1	
zout	
1	
Ave Data	
0.00278	
Model	
0.00123	
data/vMax	
0.01069	
ofxng	
319.391	
Min d/m	
5.09969	
ave/min d	
76.4223	
ave/min m	
172.937	

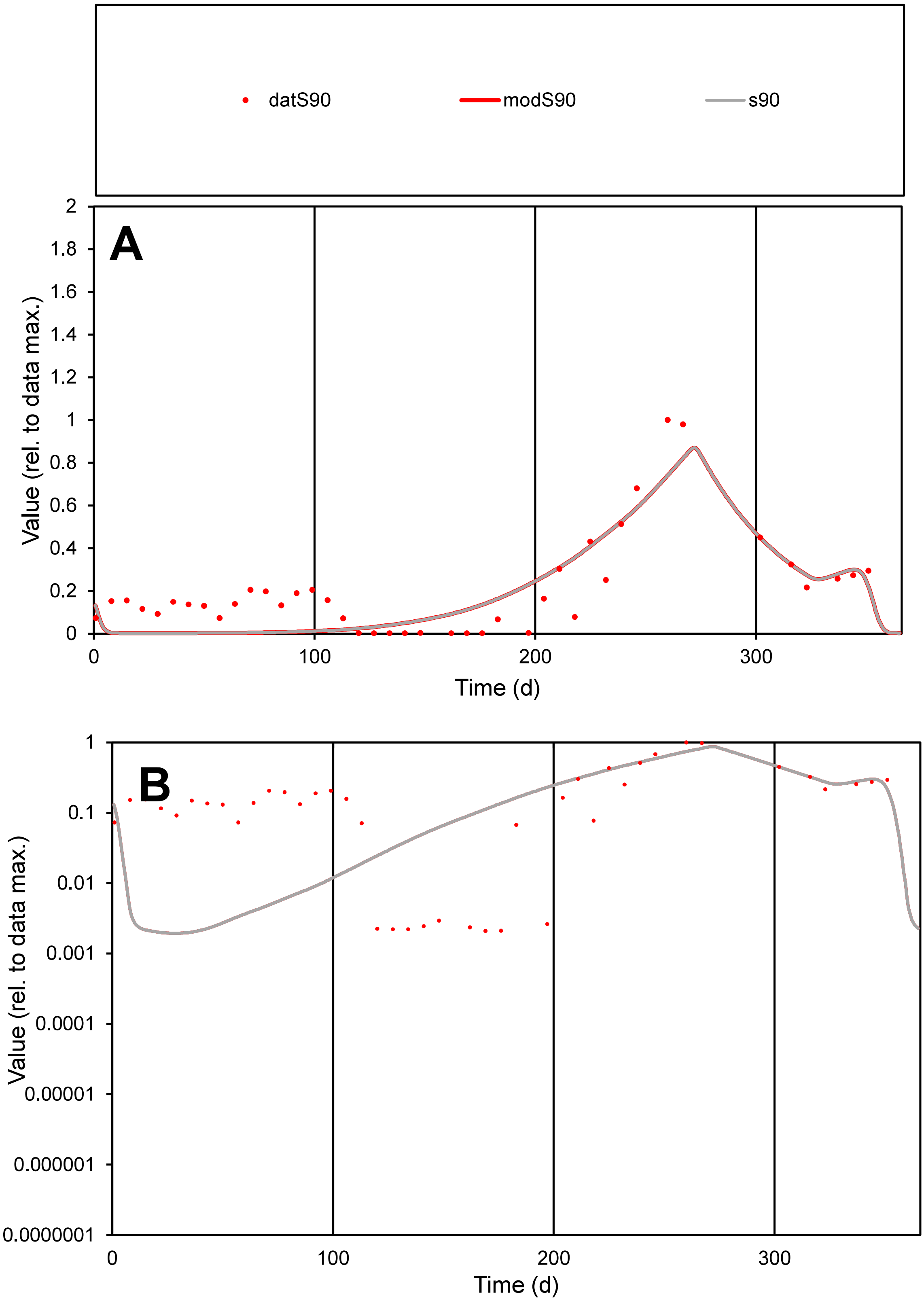

C1

Variables

S90	Provide jtd or Dst
CalibOnly?	
2	1 to reproduce of
oNormalize?	
2	1=no, 2=yes

ofxn calc*
0.6144
ofxn mod
0.63613

sRun
RunA1
zout
1

Ave
Data
0.00198
Model
0.00172

data/vMax
0.00959

ofxng
319.391

Min d/m
1.07953

ave/min d
98.5909
ave/min m
92.1832

330_Calib_S_A

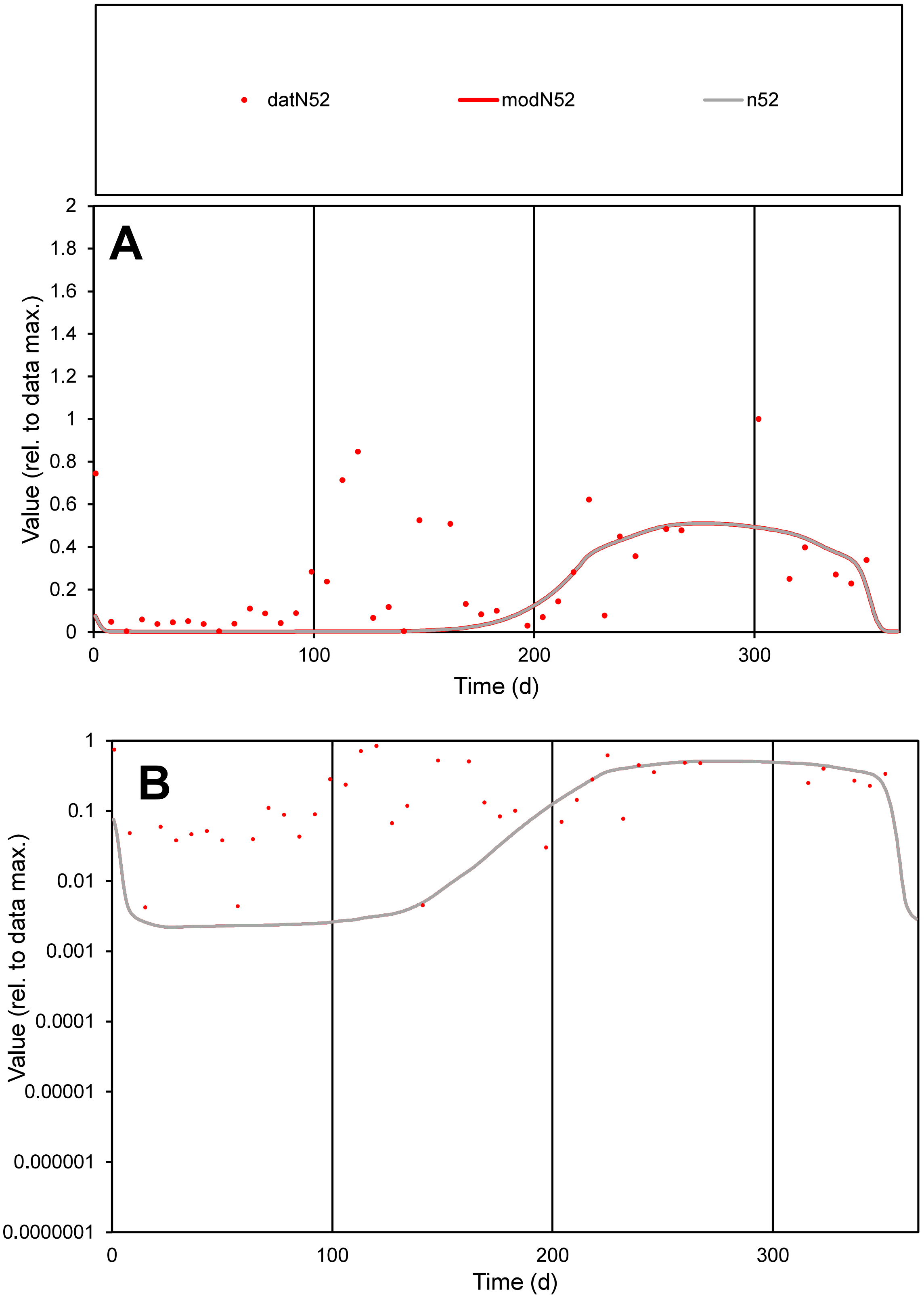

C1

Variables
N52 Provide jtd or Dst
CalibOnly?
2 1 to reproduce of
oNormalize?
2 1=no, 2=yes

ofxn calc*
1.05977
ofxn mod
1.06654

sRun
RunA1
zout
1

Ave
Data
0.00155
Model
0.00089

data/vMax
0.00622

ofxng
319.391

Min d/m
1.91232

ave/min d
58.922
ave/min m
64.3855

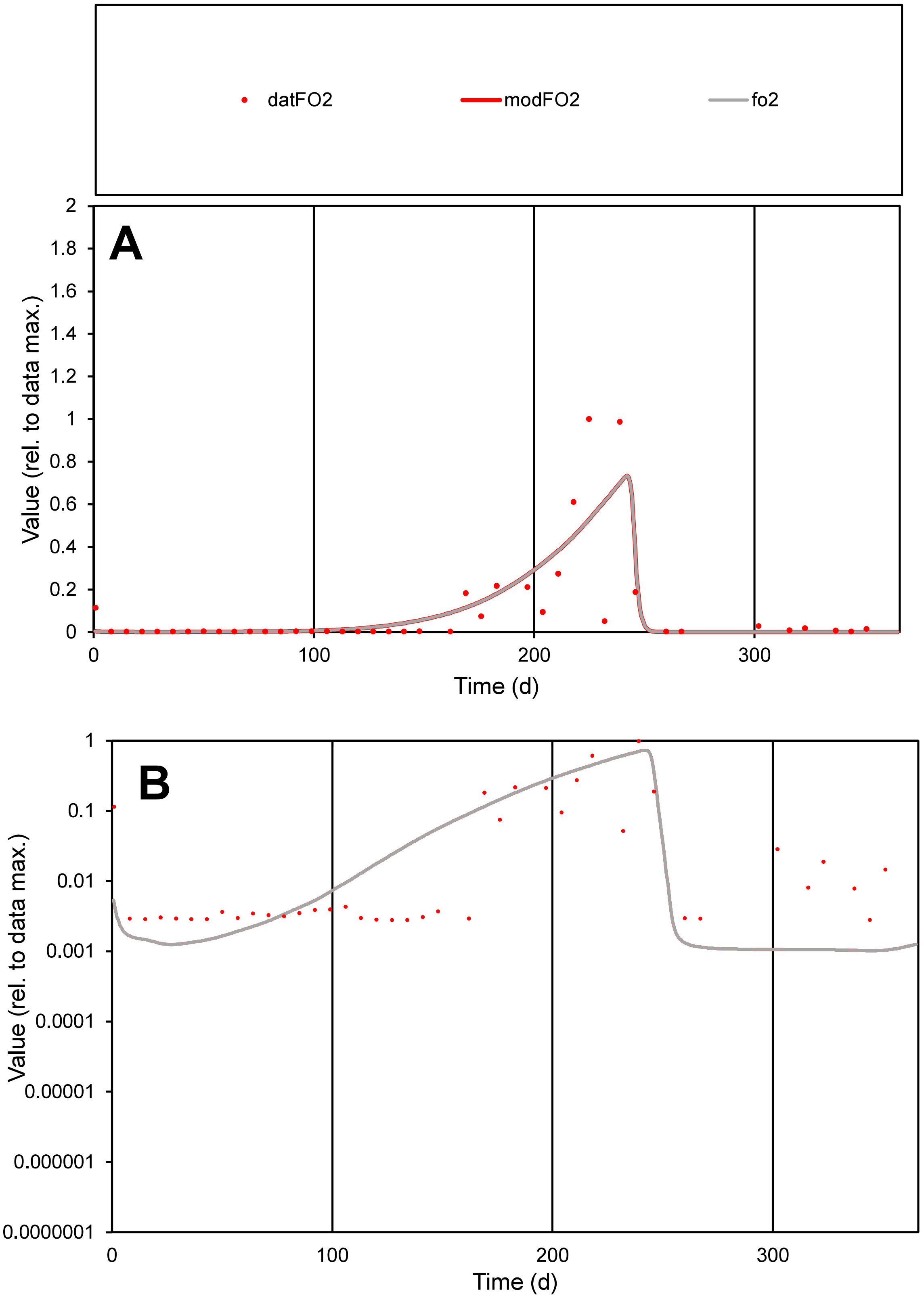

C1

Variables
FO2 Provide jtd or Dst
CalibOnly?
2 1 to reproduce of
oNormalize?
2 1=no, 2=yes

ofxn calc*
1.33176
ofxn mod
1.36789

sRun
RunA1
zout
1

Ave
Data
0.00255
Model
0.00264

data/vMax
0.02566

ofxng
319.391

Min d/m
2.72137

ave/min d
35.7057
ave/min m
100.949

330_Calib_S_A

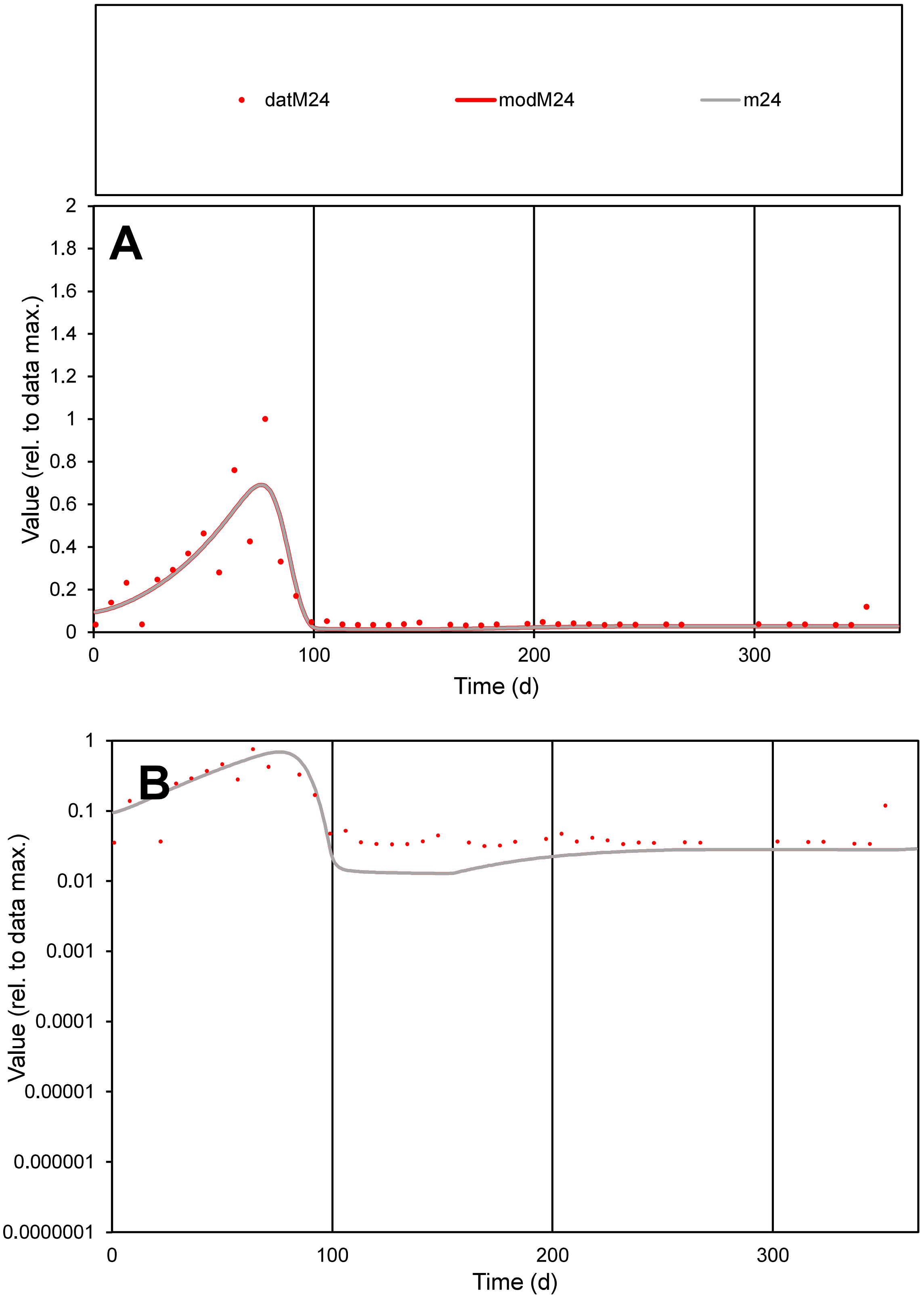

C1

Variables
M24 Provide jtd or Dst
CalibOnly?
2 1 to reproduce of
oNormalize?
2 1=no, 2=yes

ofxn calc*
0.60761
ofxn mod
0.63987

sRun
RunA1
zout
1

Ave
Data
0.00032
Model
0.0003

data/vMax
0.00229

ofxng
319.391

Min d/m
2.47955

ave/min d
4.43257
ave/min m
10.2466

330_Calib_S_A

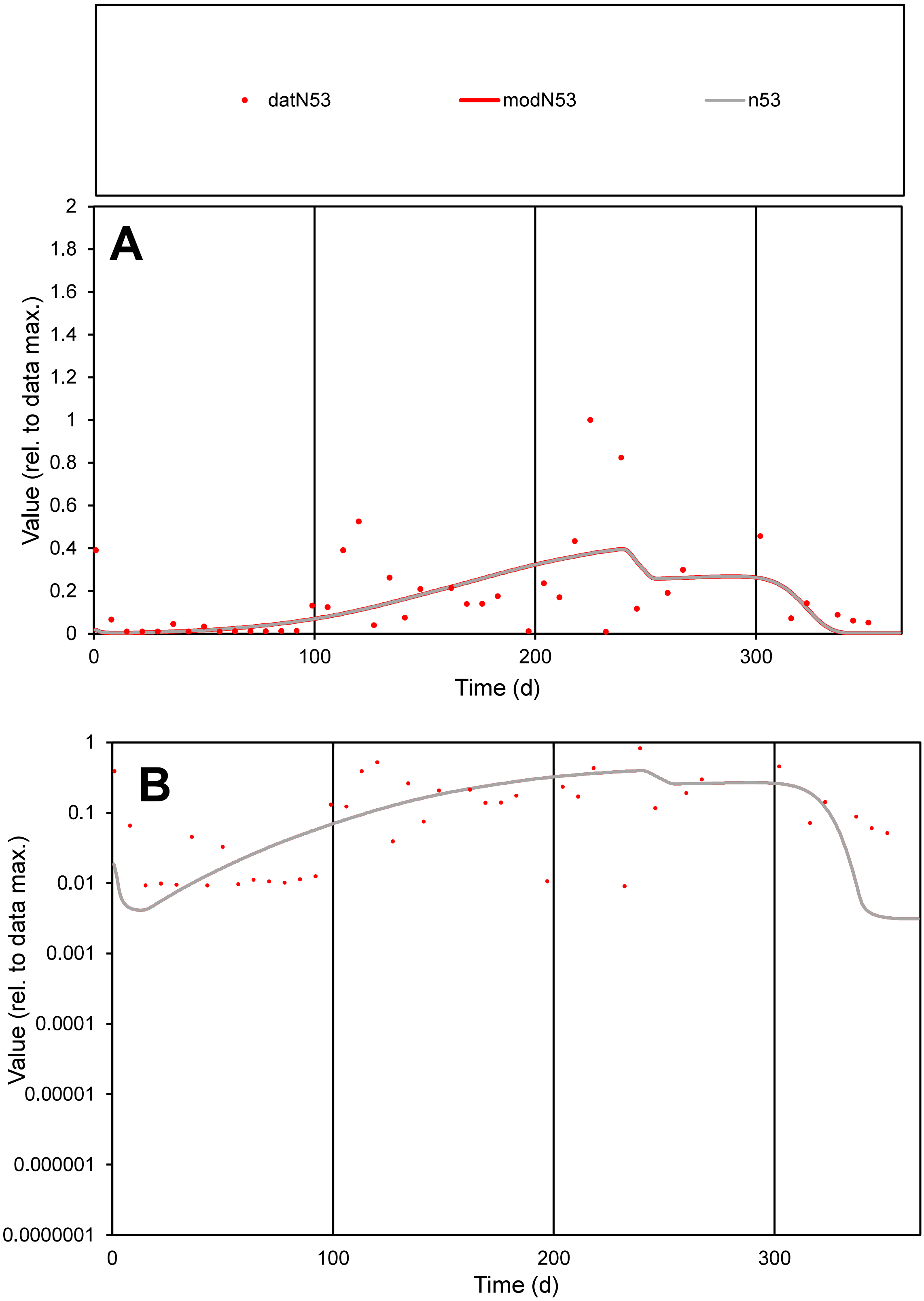

C1

Variables
N53 Provide jtd or Dst
CalibOnly?
2 1 to reproduce of
oNormalize?
2 1=no, 2=yes

ofxn calc*
1.07845
ofxn mod
1.09205

sRun
RunA1
zout
1

Ave
Data
0.00074
Model
0.00064

data/vMax
0.00432

ofxng
319.391

Min d/m
2.81962

ave/min d
19.0072
ave/min m
46.241

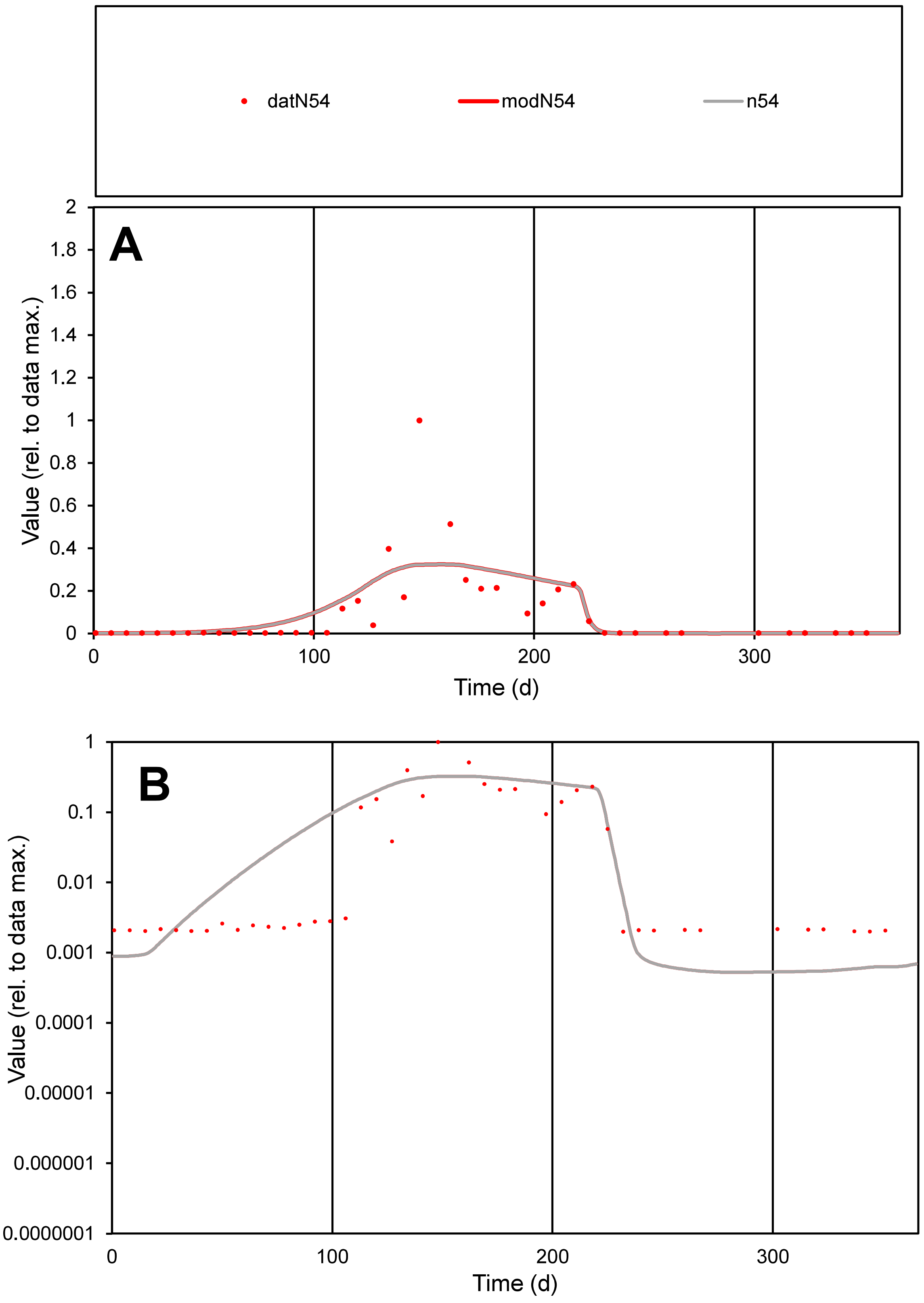

C1

Variables
N54 Provide jtd or Dst
CalibOnly?
2 1 to reproduce of
oNormalize?
2 1=no, 2=yes

ofxn calc*
1.3723
ofxn mod
1.38793

sRun
RunA1
zout
1

Ave
Data
0.00107
Model
0.00118

data/vMax
0.01164

ofxng
319.391

Min d/m
3.73486

ave/min d
46.1564
ave/min m
190.405

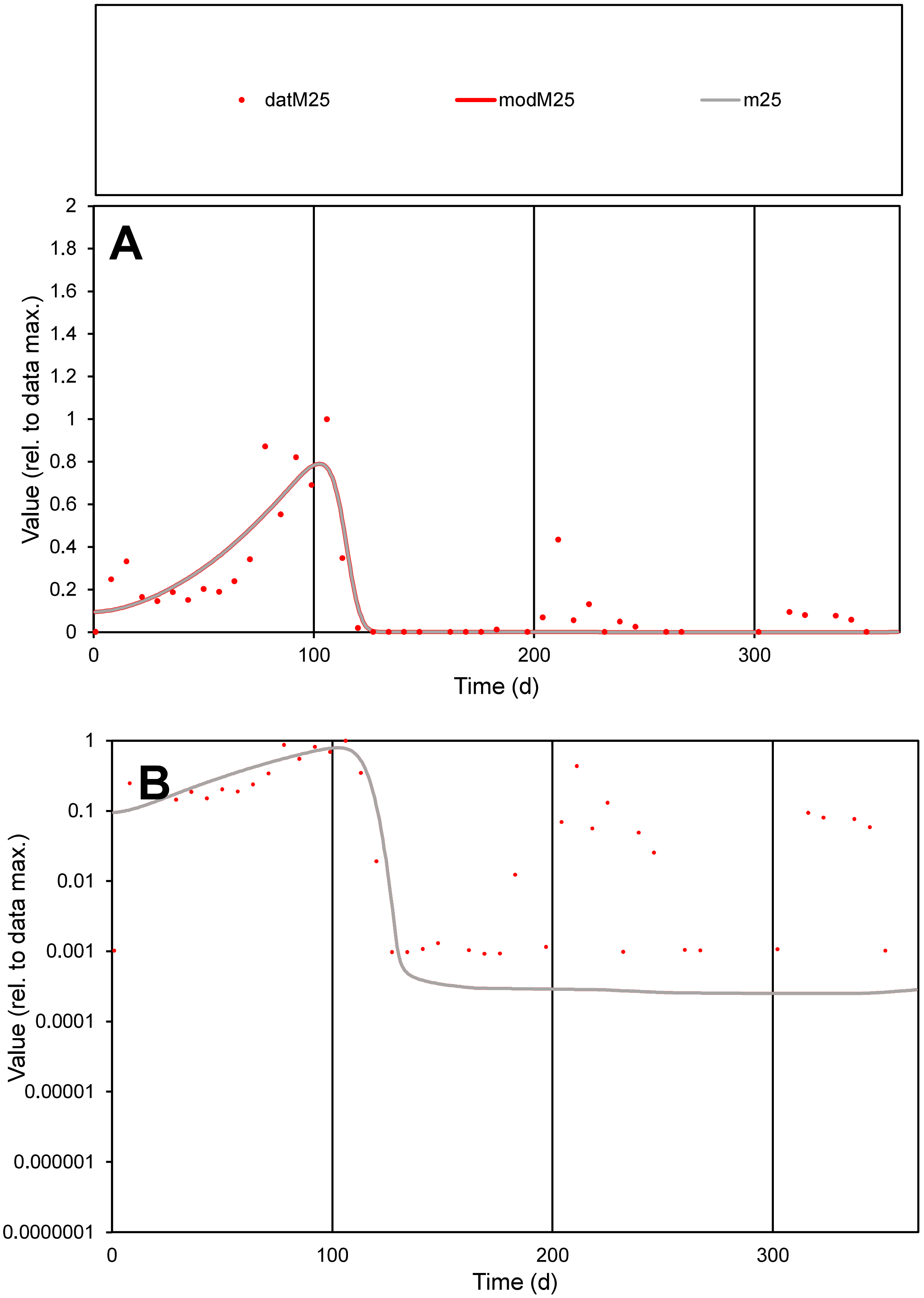

C1

Variables
M25 Provide jtd or Dst
CalibOnly?
2 1 to reproduce of
oNormalize?
2 1=no, 2=yes

ofxn calc*
0.67044
ofxn mod
0.6958

sRun
RunA1
zout
1

Ave
Data
0.00189
Model
0.00167

data/vMax
0.01046

ofxng
319.391

Min d/m
3.69656

ave/min d
195.596
ave/min m
637.24

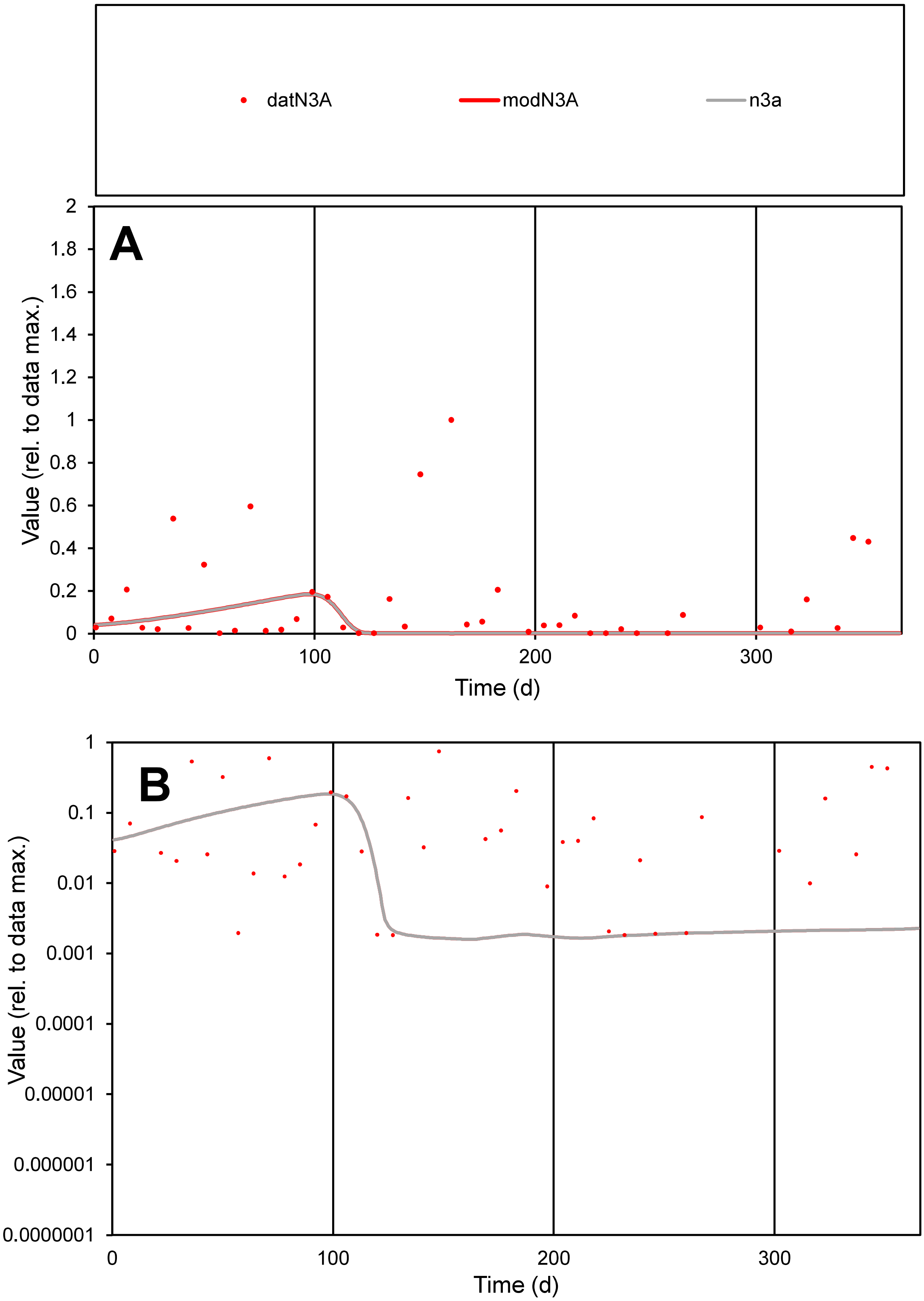

C1

Variables
N3A Provide jtd or Dst
CalibOnly?
2 1 to reproduce of
oNormalize?
2 1=no, 2=yes

ofxn calc*
1.75411
ofxn mod
1.75795

sRun
RunA1
zout
1

Ave
Data
0.00148
Model
0.00047

data/vMax
0.01041

ofxng
319.391

Min d/m
1.14864

ave/min d
77.9781
ave/min m
28.1045

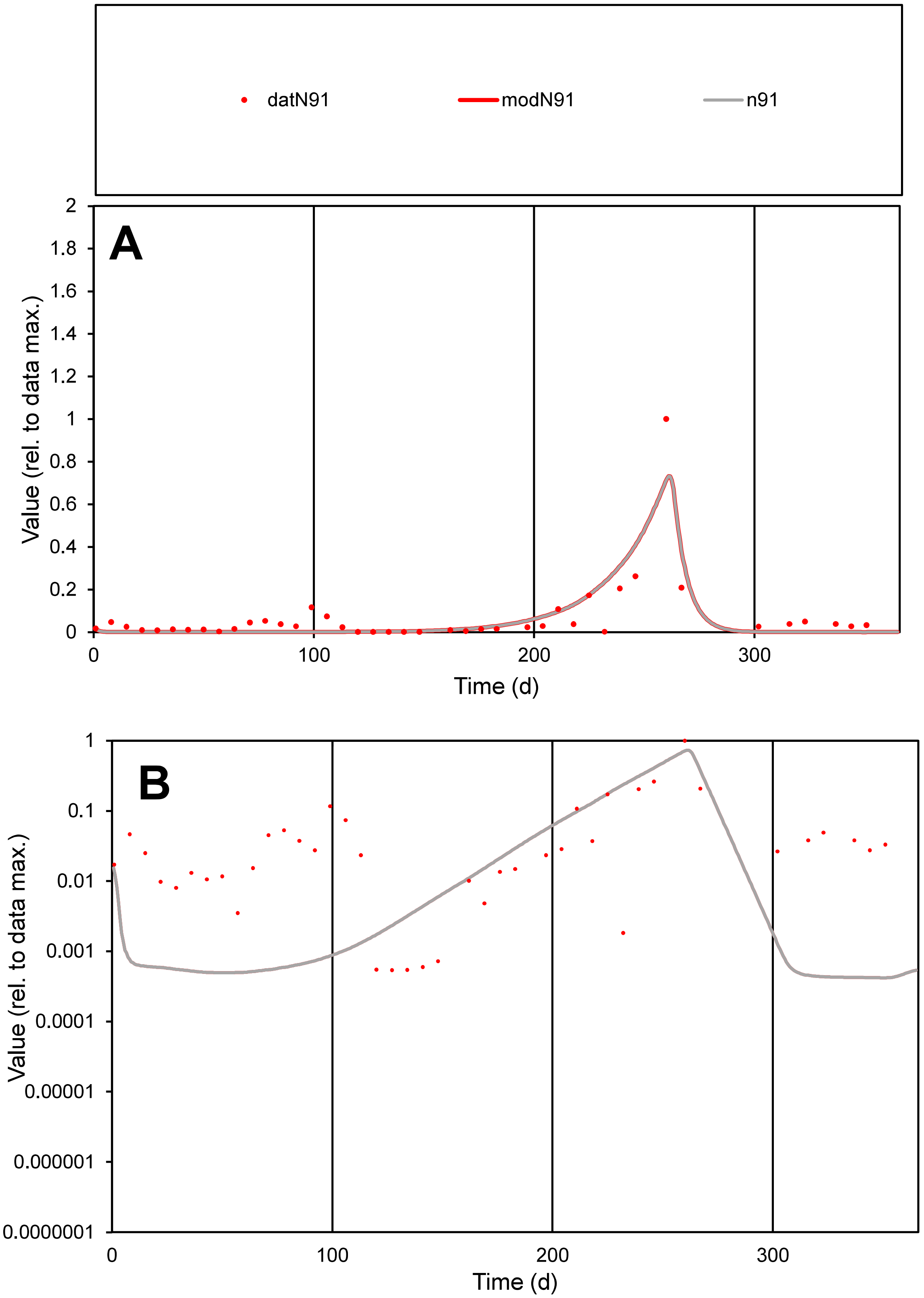

C1

Variables
N91 Provide jtd or Dst
CalibOnly?
2 1 to reproduce of
oNormalize?
2 1=no, 2=yes

ofxn calc*
1.06367
ofxn mod
1.10422

sRun
RunA1
zout
1

Ave
Data
0.0042
Model
0.00394

data/vMax
0.06206

ofxng
319.391

Min d/m
1.28725

ave/min d
125.335
ave/min m
151.35

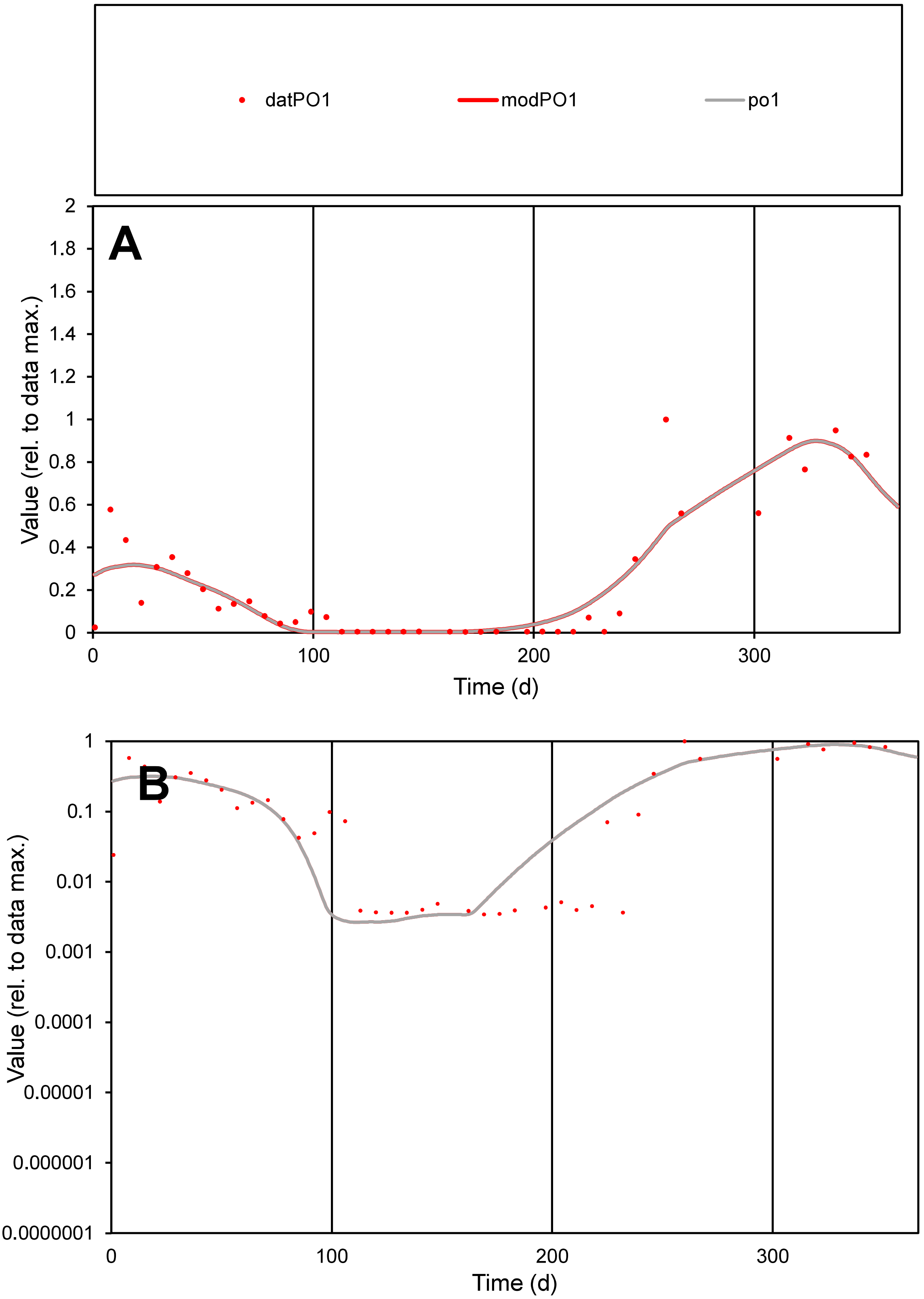

C1

Variables

PO1 Provide jtd or Dst

CalibOnly?

2 1 to reproduce of

oNormalize?

2 1=no, 2=yes

ofxn calc*

0.50908

ofxn mod

0.53147

sRun

RunA1

zout

1

Ave

Data

0.00319

Model

0.00318

data/vMax

0.01336

ofxng

319.391

Min d/m

1.28663

ave/min d

69.4367

ave/min m

89.1613

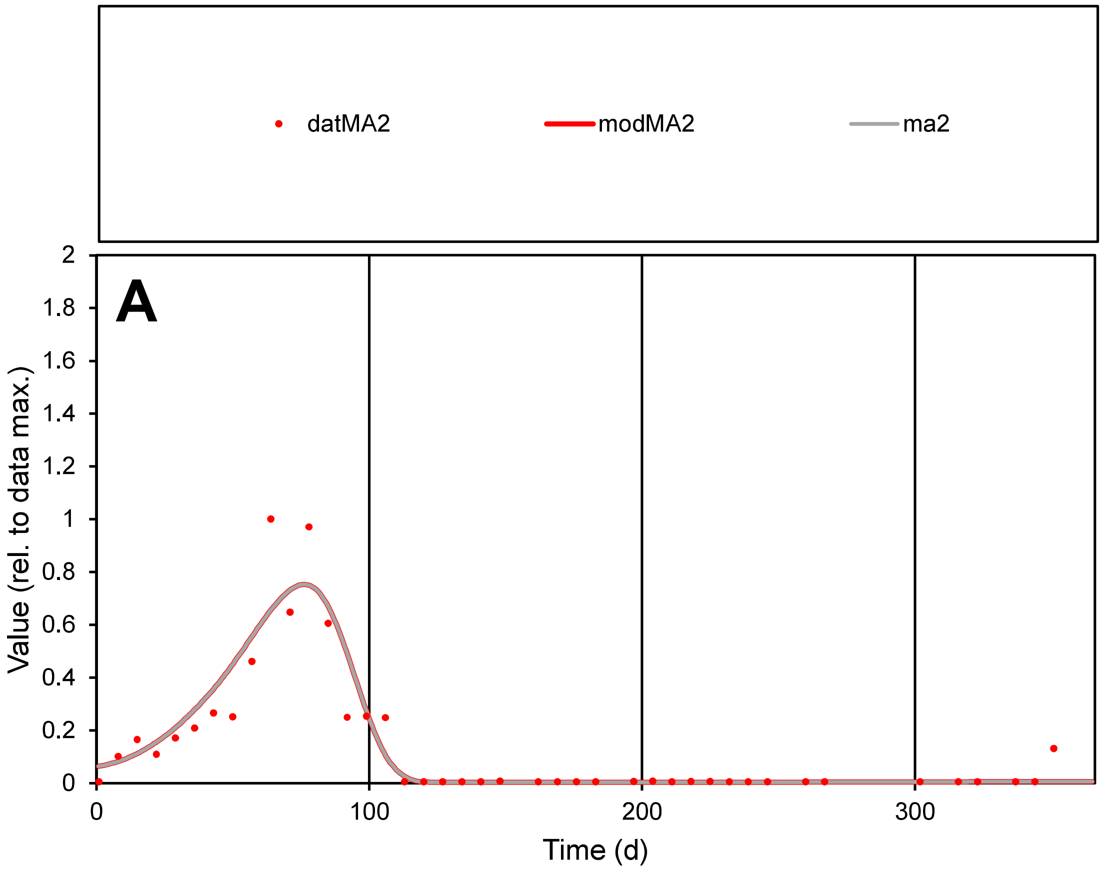

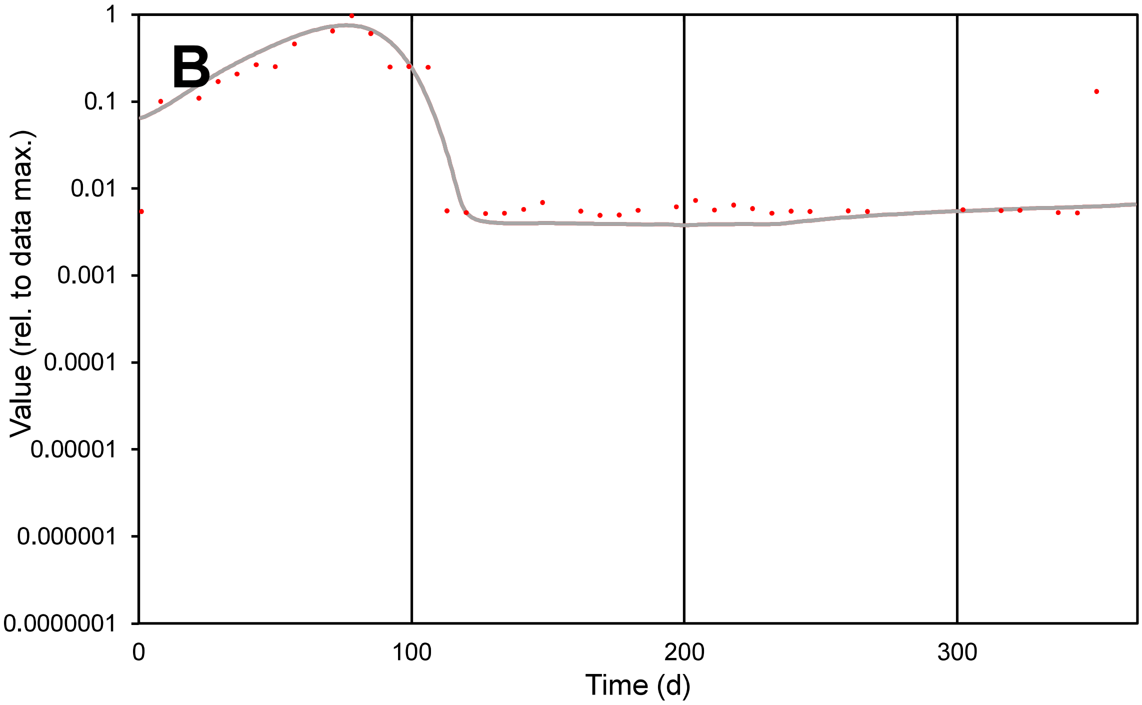

C1

Variables
MA2 Provide jtd or Dst
CalibOnly?
2 1 to reproduce of
oNormalize?
2 1=no, 2=yes

ofxn calc*
0.62013
ofxn mod
0.66125

sRun
RunA1
zout
1

Ave
Data
0.00052
Model
0.00053

data/vMax
0.00365

ofxng
319.391

Min d/m
1.29654

ave/min d
28.9702
ave/min m
38.0192

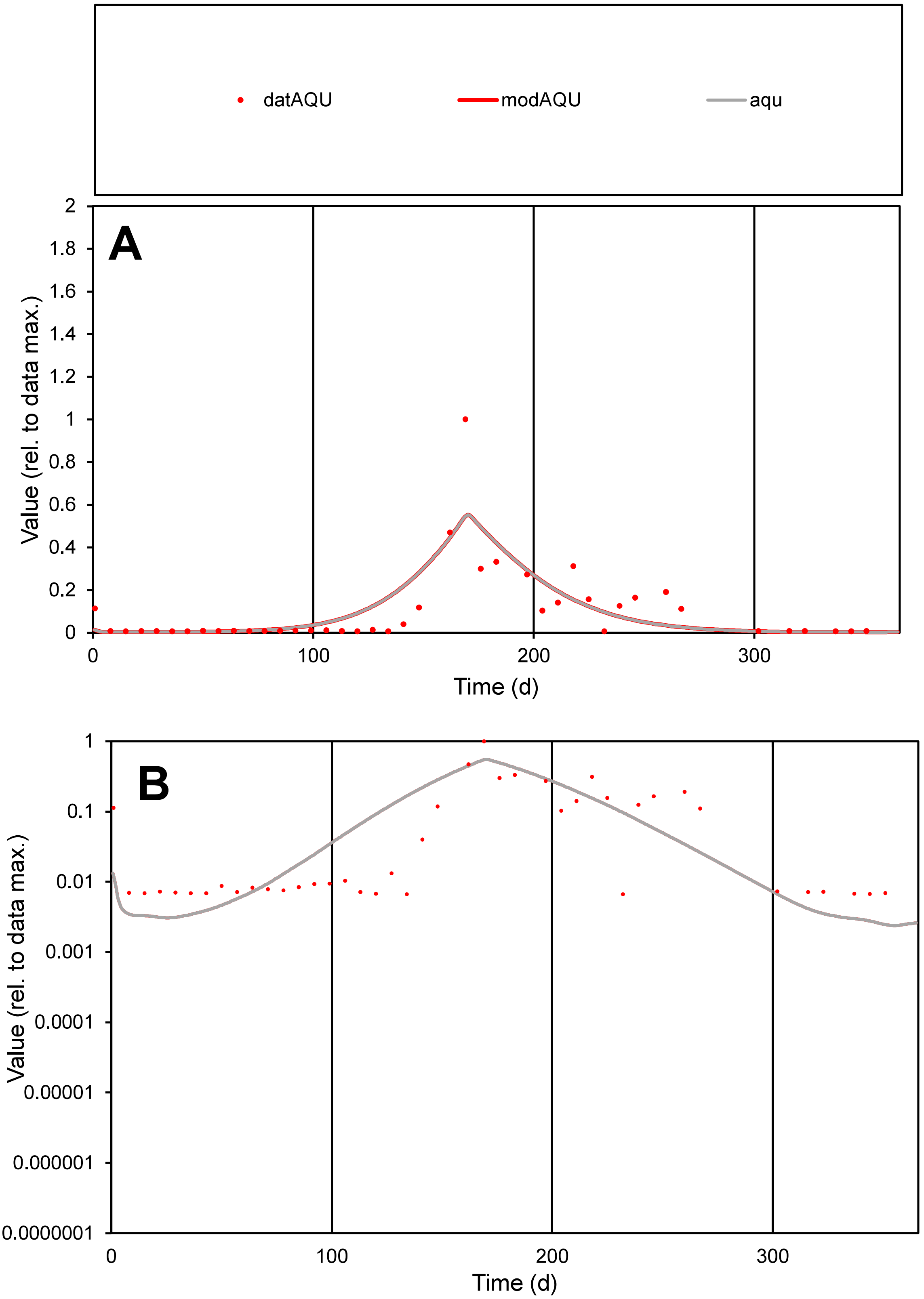

C1

Variables
AQU Provide jtd or Dst
CalibOnly?
2 1 to reproduce of
oNormalize?
2 1=no, 2=yes

ofxn calc*
1.0537
ofxn mod
1.08005

sRun
RunA1
zout
1

Ave
Data
0.00206
Model
0.00214

data/vMax
0.02081

ofxng
319.391

Min d/m
2.74525

ave/min d
14.8205
ave/min m
42.3596

330_Calib_S_A

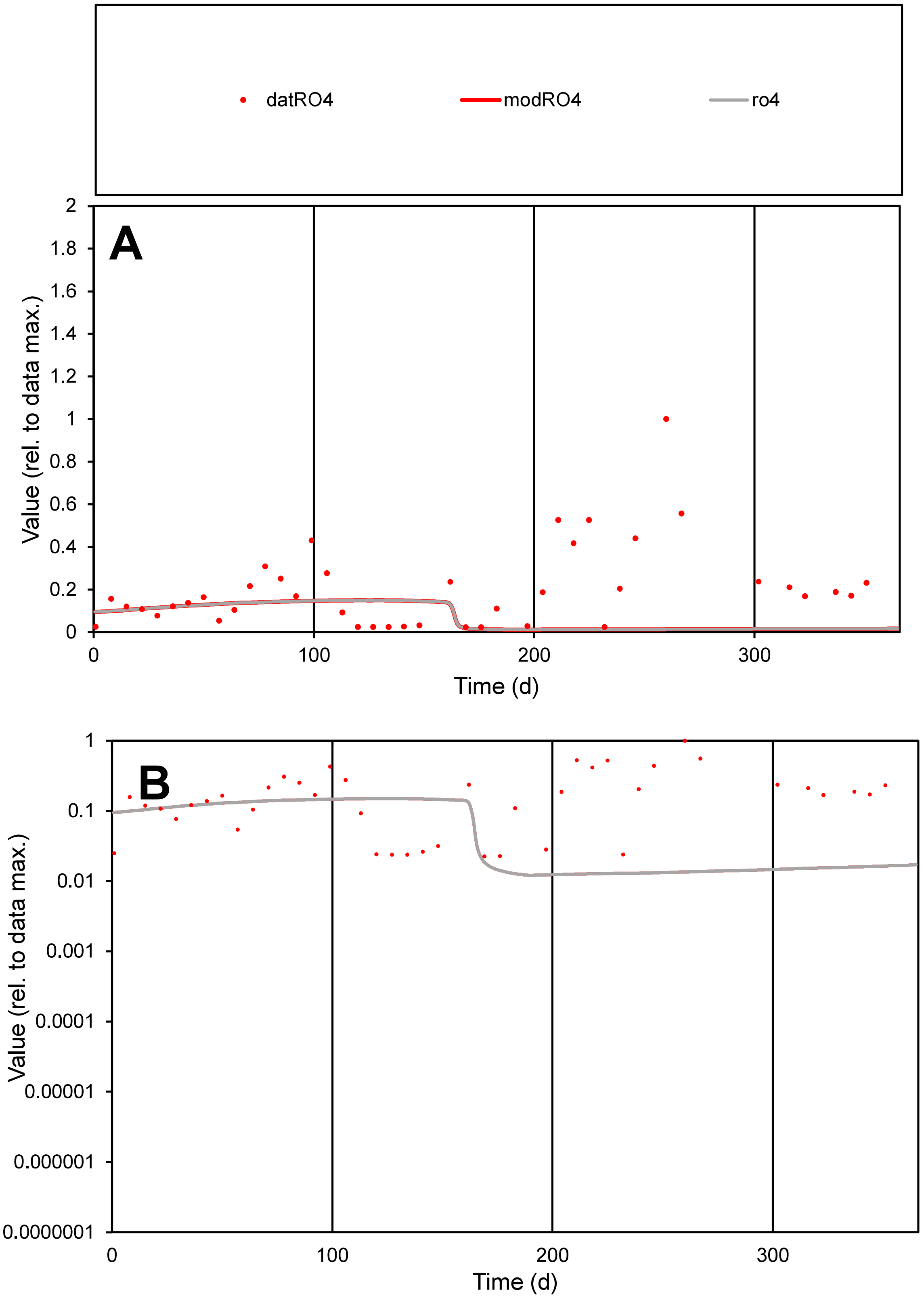

C1

Variables

RO4 Provide jtd or Dst

CalibOnly?

2 1 to reproduce of

oNormalize?

2 1=no, 2=yes

ofxn calc*

1.25577

ofxn mod

1.25919

sRun

RunA1

zout

1

Ave

Data

0.00196

Model

0.00077

data/vMax

0.00973

ofxng

319.391

Min d/m

1.82721

ave/min d

8.93566

ave/min m

6.42742

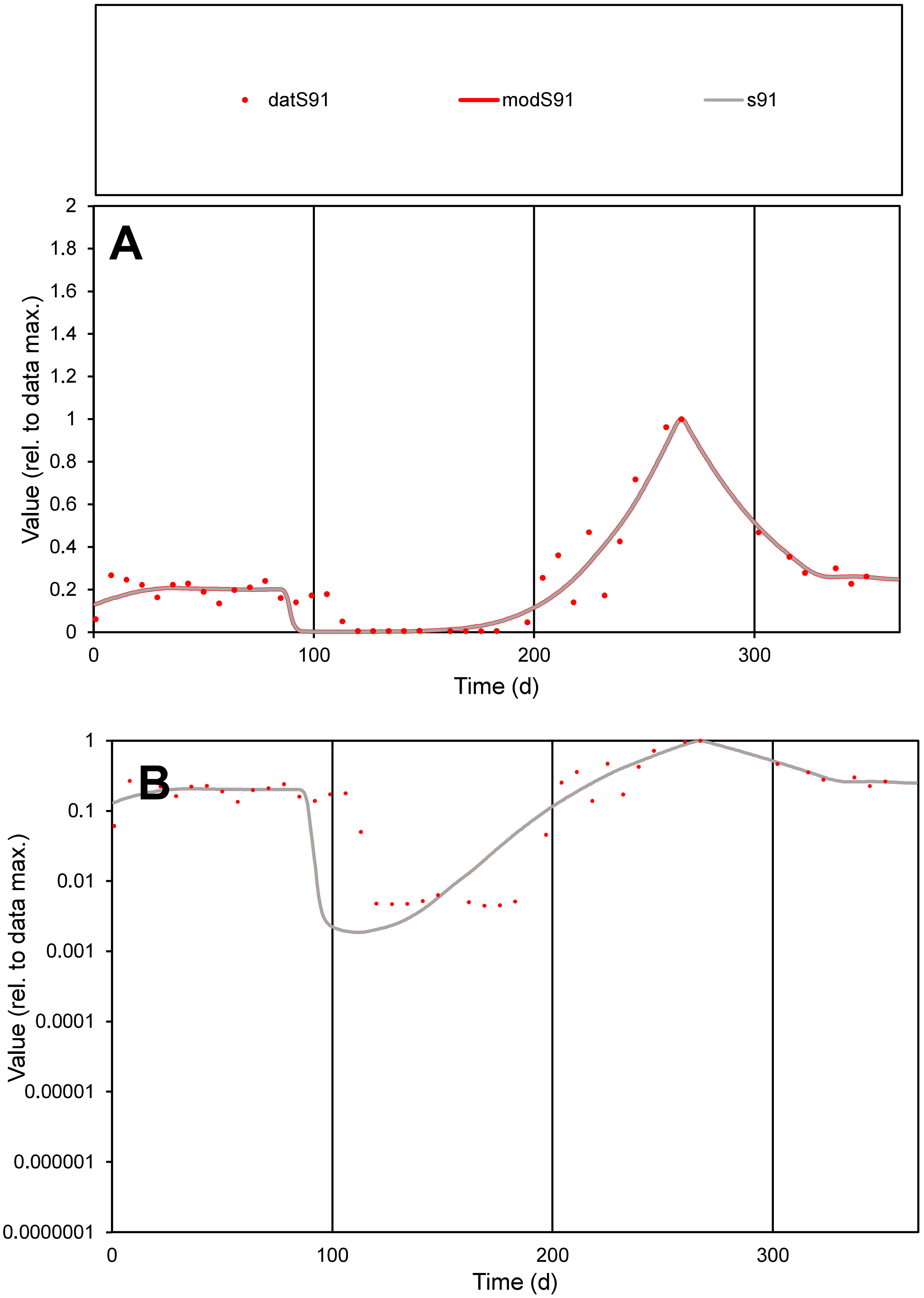

C1

Variables
S91 Provide jtd or Dst
CalibOnly?
2 1 to reproduce of
oNormalize?
2 1=no, 2=yes

ofxn calc*
0.34004
ofxn mod
0.37894

sRun
RunA1
zout
1

Ave
Data
0.00335
Model
0.00319

data/vMax
0.01474

ofxng
319.391

Min d/m
2.37987

ave/min d
50.8406
ave/min m
115.239

330_Calib_S_A

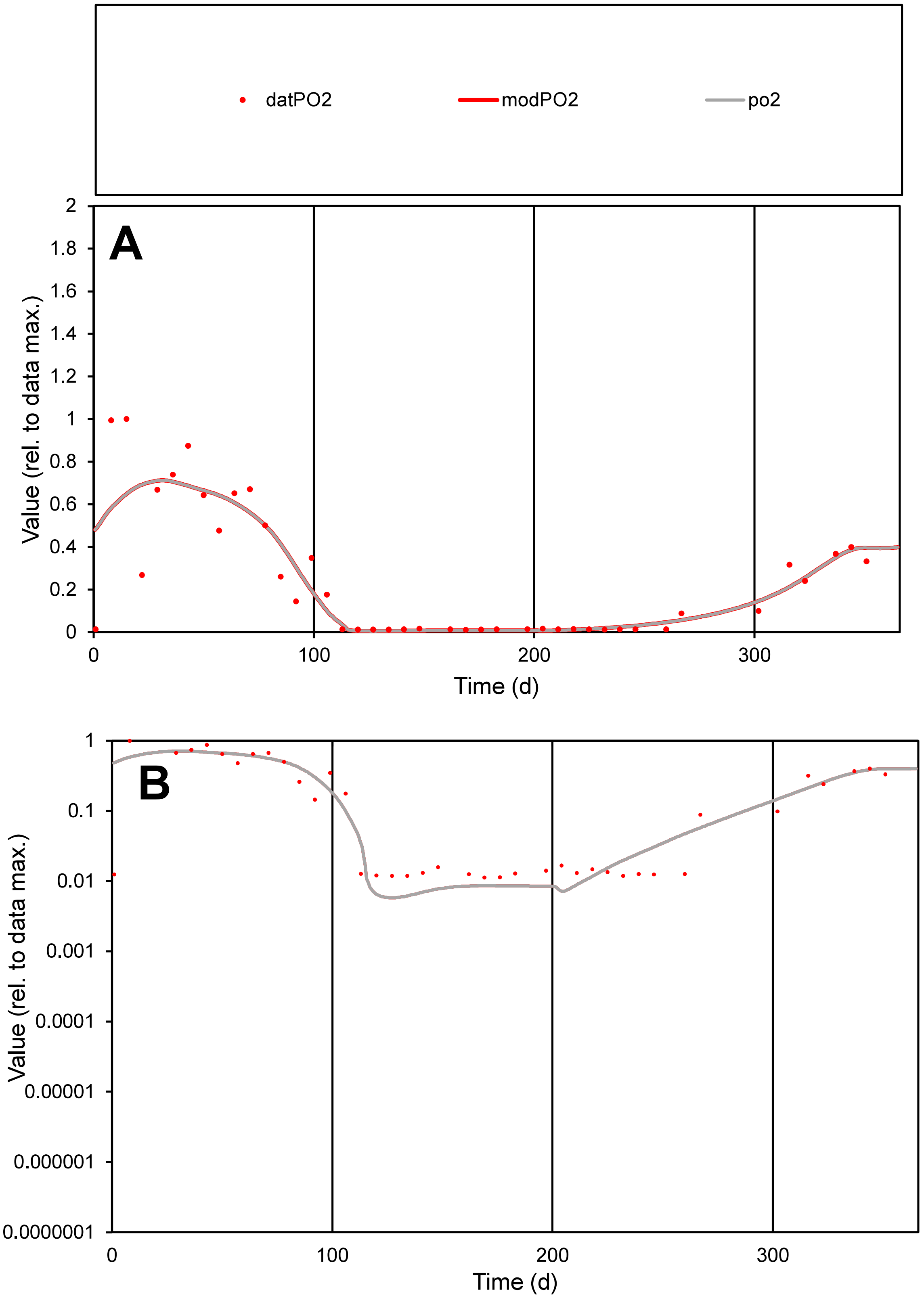

C1

Variables
PO2 Provide jtd or Dst
CalibOnly?
2 1 to reproduce of
oNormalize?
2 1=no, 2=yes

ofxn calc*
0.56963
ofxn mod
0.59503

sRun
RunA1
zout
1

Ave
Data
0.00178
Model
0.00179

data/vMax
0.00712

ofxng
319.391

Min d/m
1.94411

ave/min d
22.2766
ave/min m
43.4347

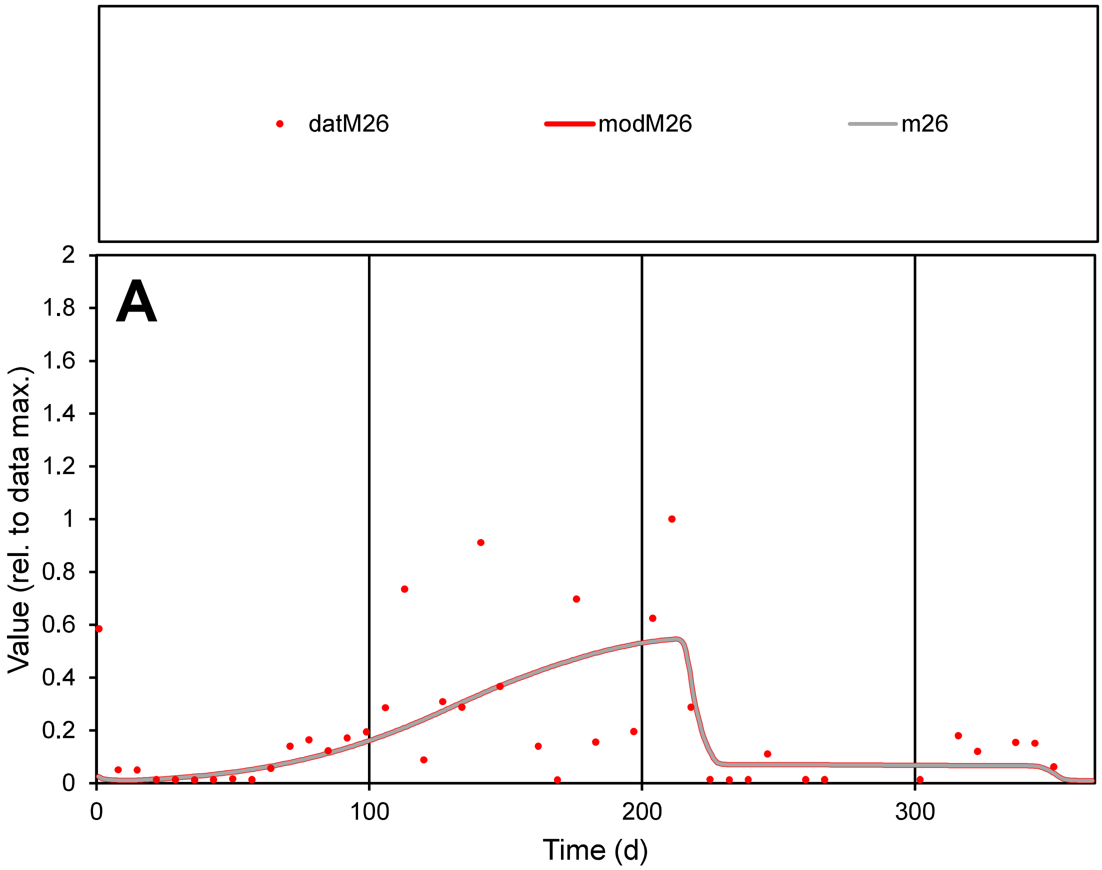

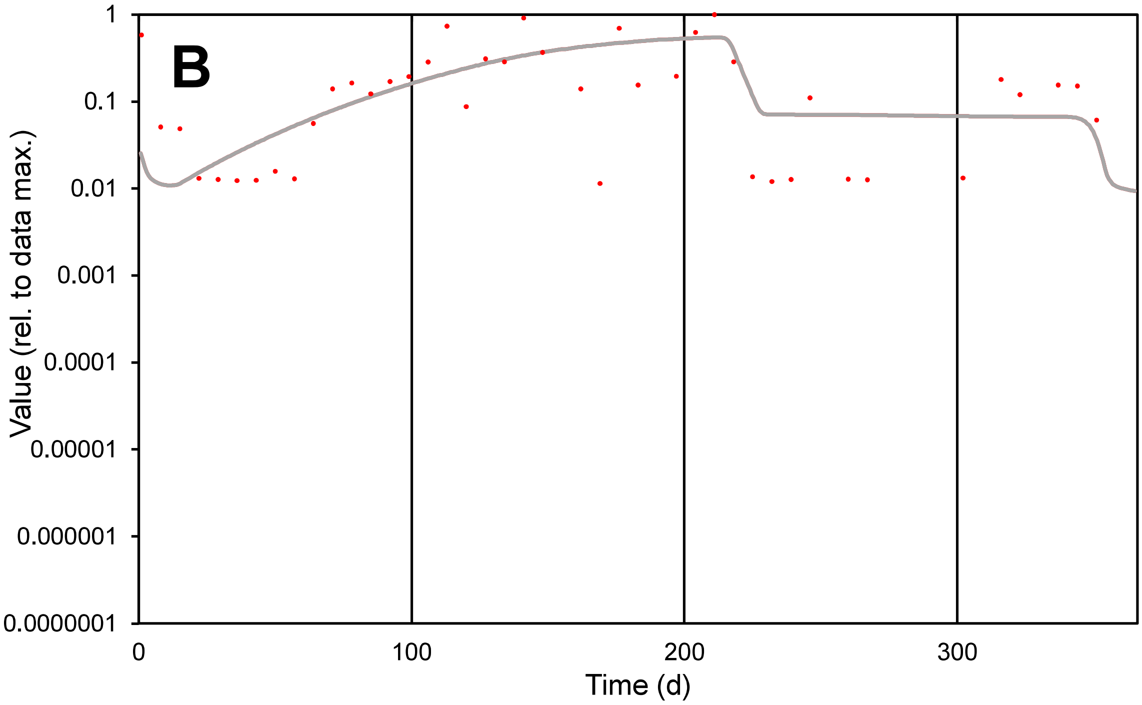

C1

Variables

M26	Provide jtd or Dst
CalibOnly?	
2	1 to reproduce of
oNormalize?	
2	1=no, 2=yes
ofxn calc*	
1.00783	
ofxn mod	
1.02019	
sRun	
RunA1	
zout	
1	
Ave	
Data	
0.00147	
Model	
0.00127	
data/vMax	
0.00723	
ofxng	
319.391	
Min d/m	
1.03495	
ave/min d	
17.8861	
ave/min m	
16.0241	

330_Calib_S_A

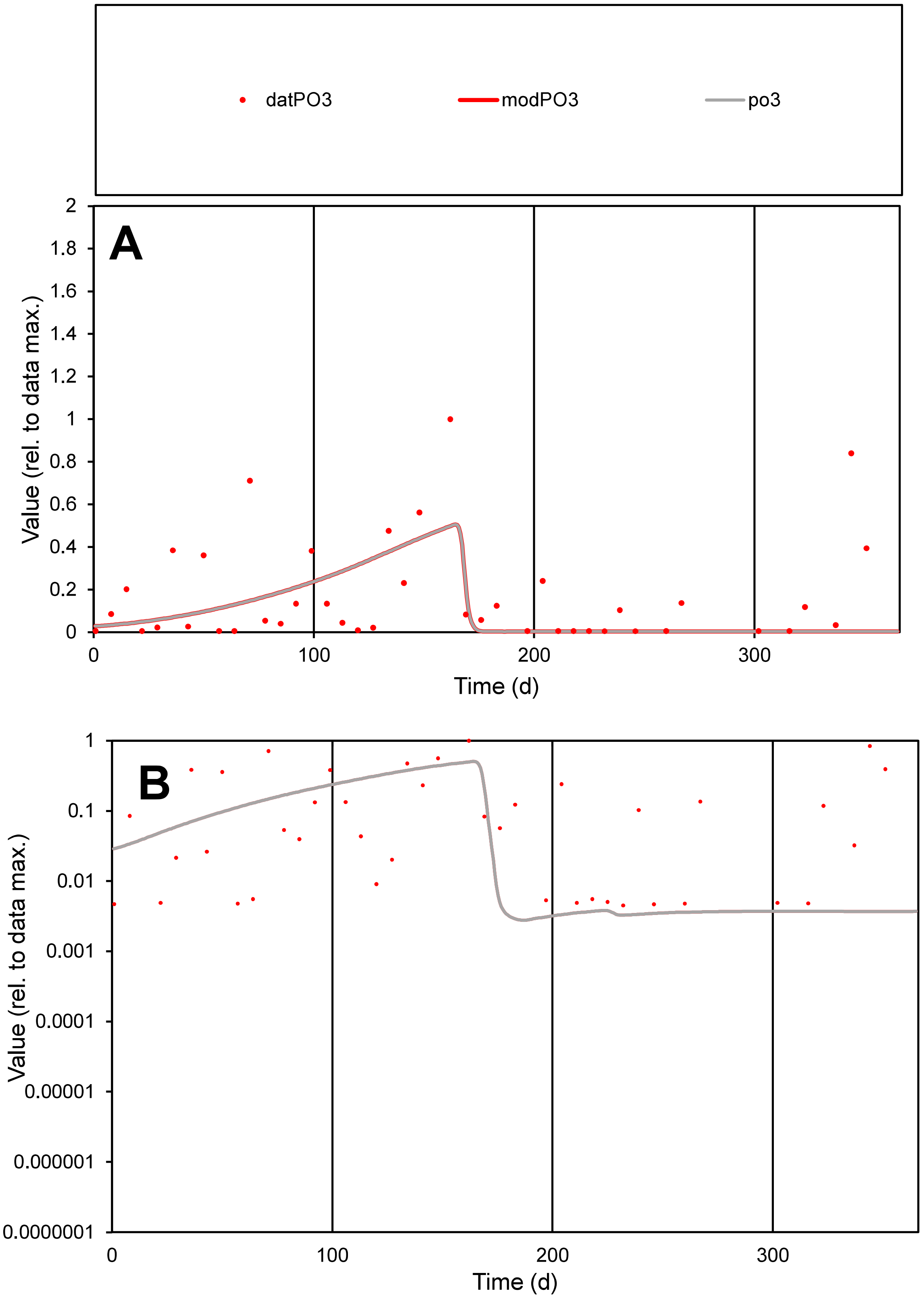

C1

Variables
PO3 Provide jtd or Dst
CalibOnly?
2 1 to reproduce of
oNormalize?
2 1=no, 2=yes

ofxn calc*
1.34526
ofxn mod
1.35458

sRun
RunA1
zout
1

Ave
Data
0.00186
Model
0.00127

data/vMax
0.01104

ofxng
319.391

Min d/m
1.60103

ave/min d
37.3487
ave/min m
41.0162

330_Calib_S_A

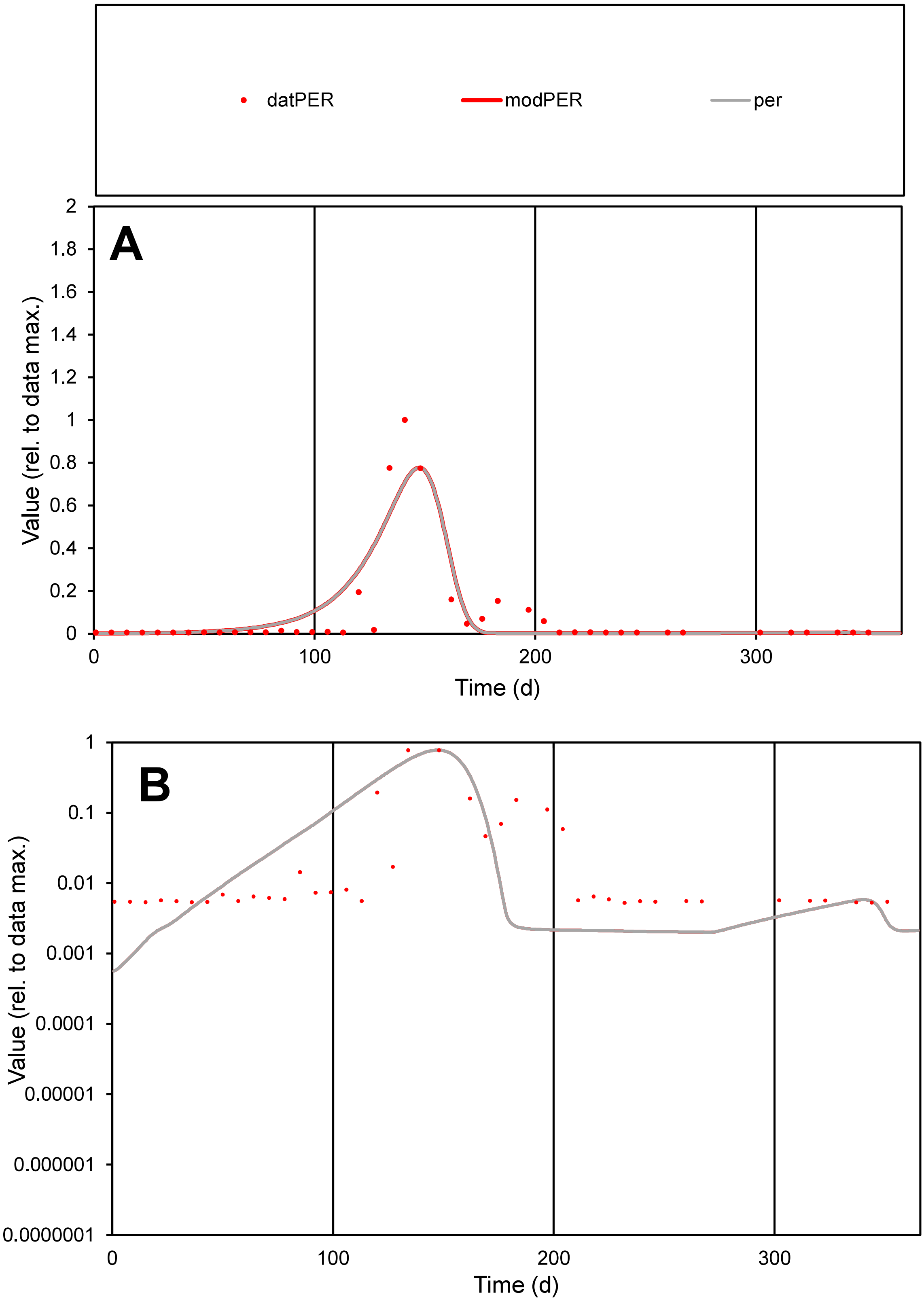

C1

Variables

PER Provide jtd or Dst

CalibOnly?

2 1 to reproduce of

oNormalize?

2 1=no, 2=yes

ofxn calc*

1.13654

ofxn mod

1.19571

sRun

RunA1

zout

1

Ave

Data

0.00032

Model

0.00034

data/vMax

0.00373

ofxng

319.391

Min d/m

8.76682

ave/min d

16.1149

ave/min m

154.013

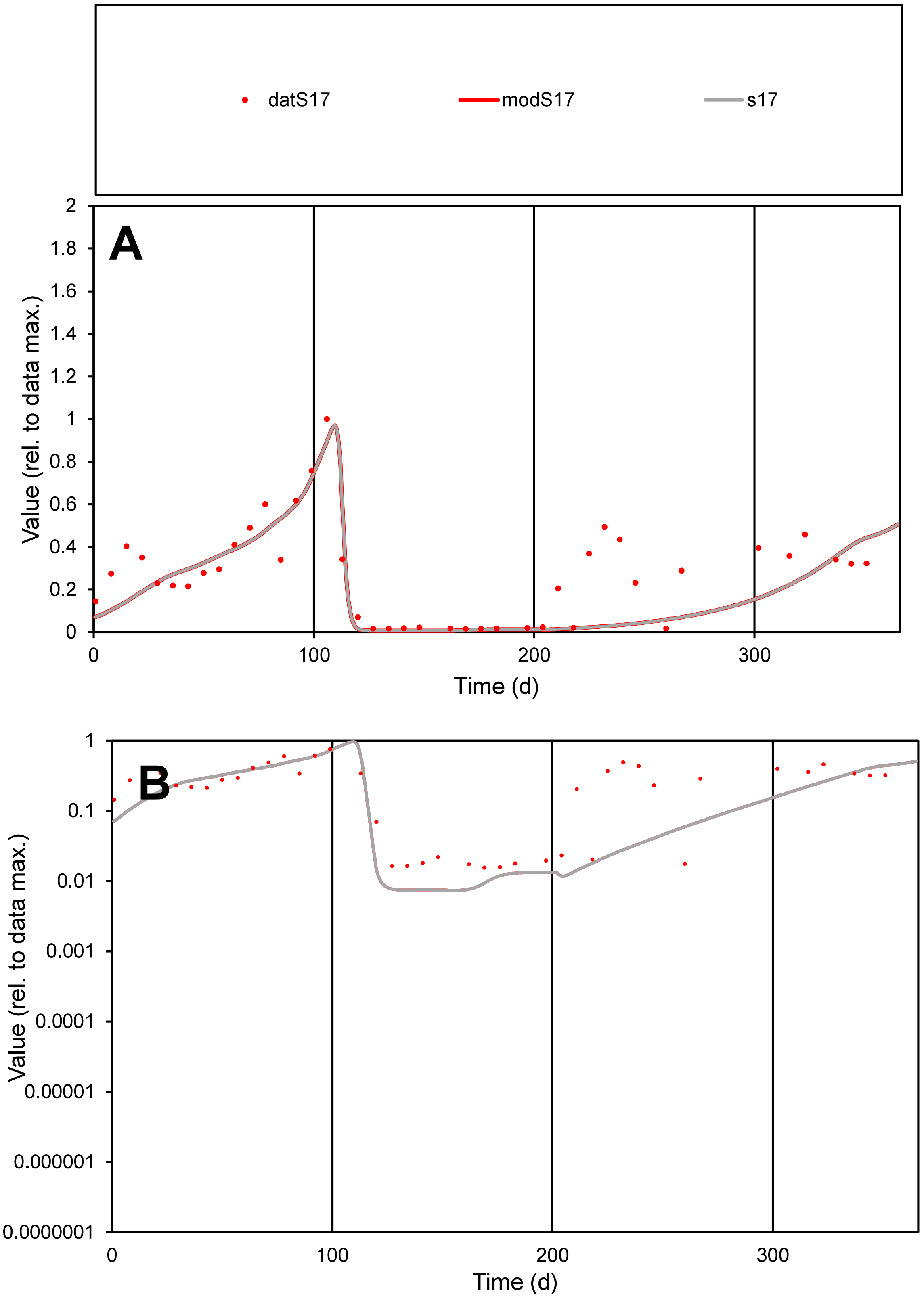

C1

Variables
S17 Provide jtd or Dst
CalibOnly?
2 1 to reproduce of
oNormalize?
2 1=no, 2=yes

ofxn calc*
0.5483
ofxn mod
0.56459

sRun
RunA1
zout
1

Ave
Data
0.00192
Model
0.00149

data/vMax
0.00701

ofxng
319.391

Min d/m
2.09489

ave/min d
17.4988
ave/min m
28.5309

330_Calib_S_A

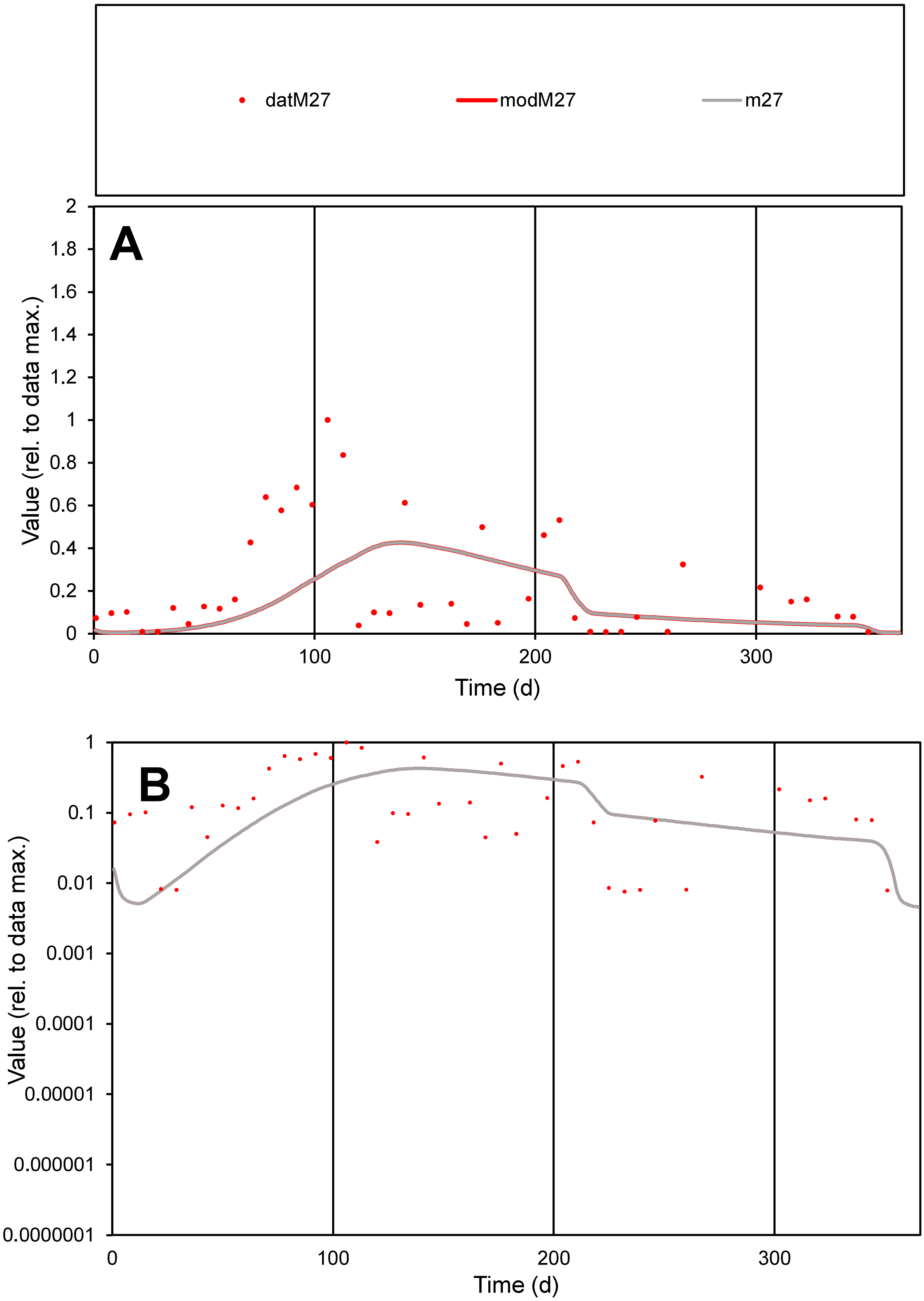

C1

Variables
M27 Provide jtd or Dst
CalibOnly?
2 1 to reproduce of
oNormalize?
2 1=no, 2=yes

ofxn calc*
1.08245
ofxn mod
1.08984

sRun
RunA1
zout
1

Ave
Data
0.00211
Model
0.00152

data/vMax
0.00914

ofxng
319.391

Min d/m
1.4531

ave/min d
30.398
ave/min m
31.8391

330_Calib_S_A

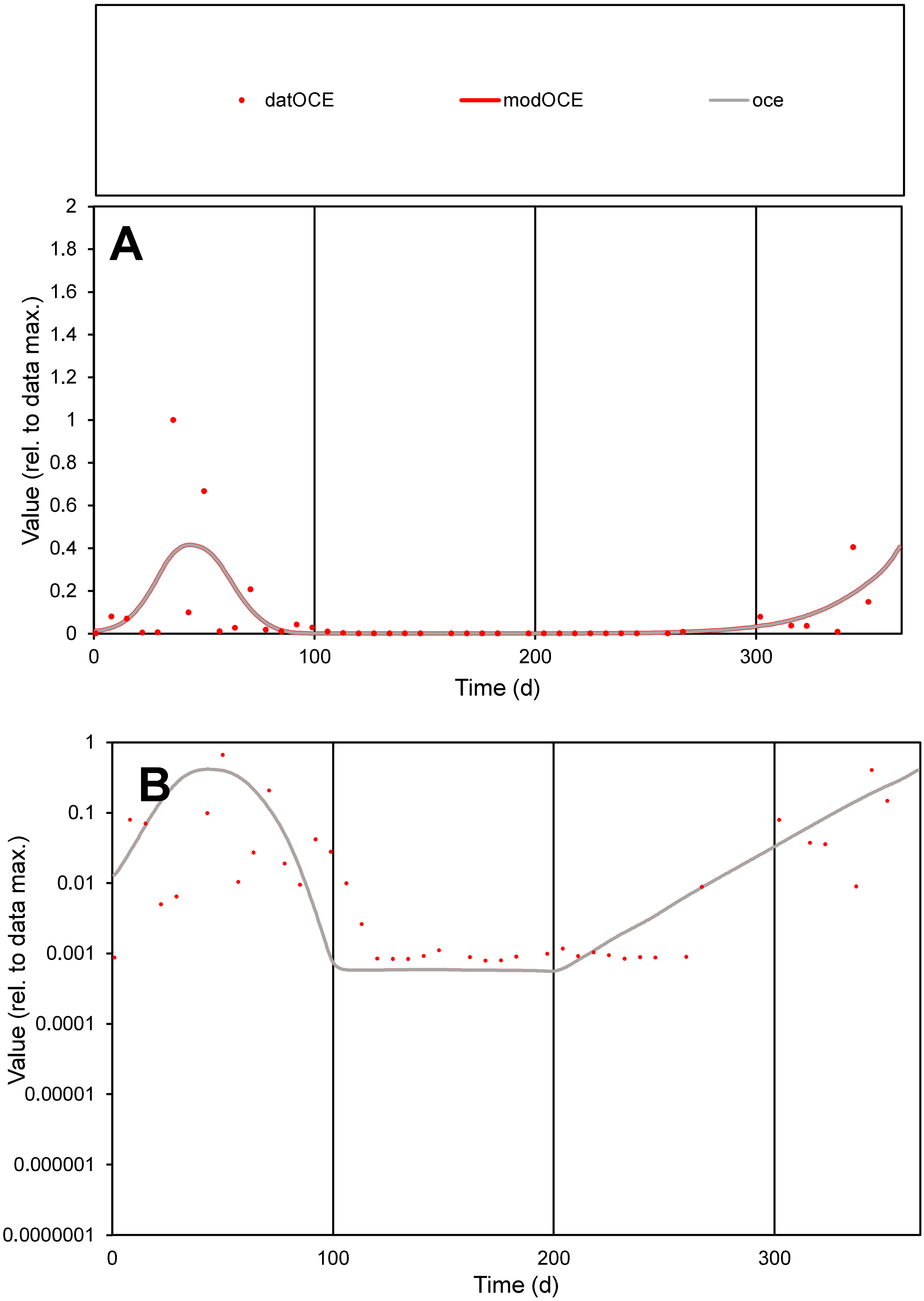

C1

Variables
OCE Provide jtd or Dst
CalibOnly?
2 1 to reproduce of
oNormalize?
2 1=no, 2=yes

ofxn calc*
1.91527
ofxn mod
1.97029

sRun
RunA1
zout
1

Ave
Data
0.0034
Model
0.00348

data/vMax
0.04711

ofxng
319.391

Min d/m
1.41692

ave/min d
90.9111
ave/min m
131.996

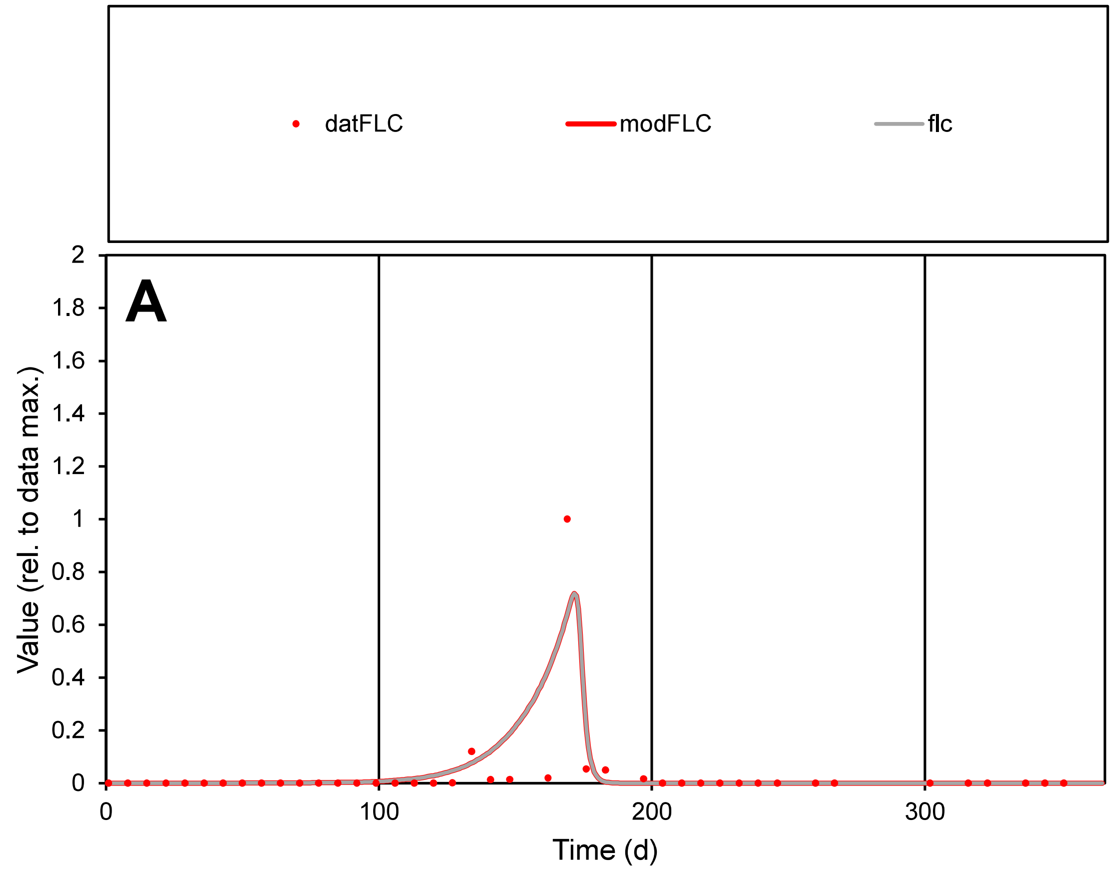

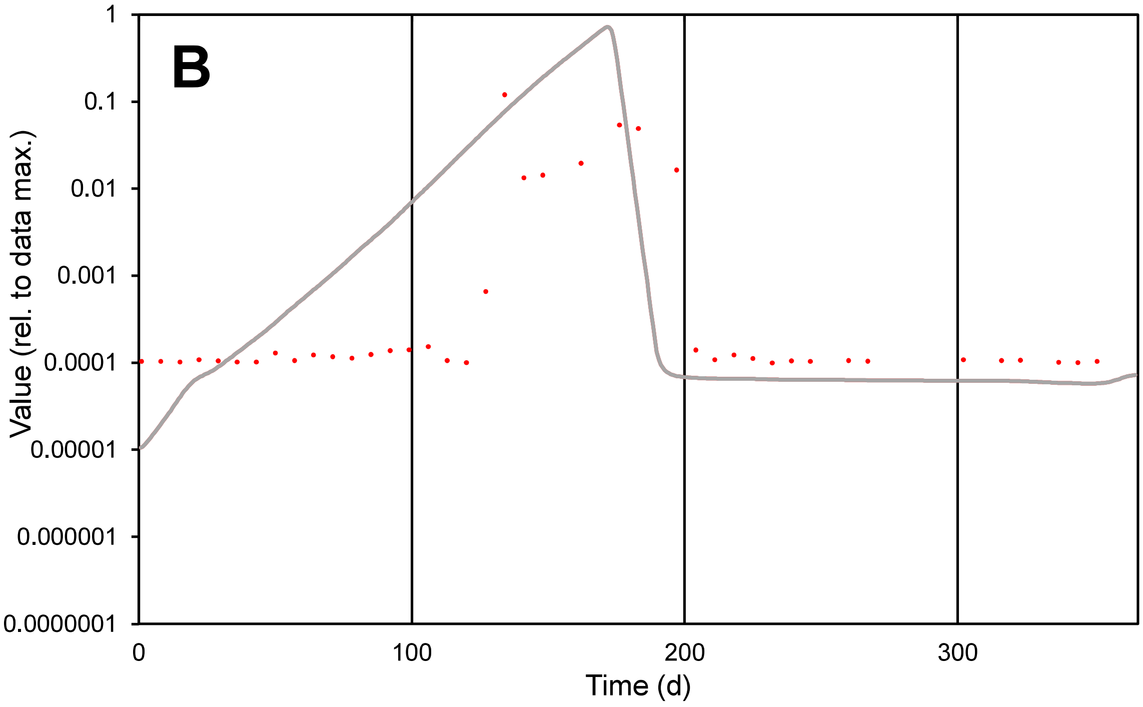

C1

Variables

FLC Provide jtd or Dst

CalibOnly?

2 1 to reproduce of

oNormalize?

2 1=no, 2=yes

ofxn calc*

2.62388

ofxn mod

2.8574

sRun

RunA1

zout

1

Ave

Data

0.00397

Model

0.00511

data/vMax

0.1292

ofxng

319.391

Min d/m

8.66487

ave/min d

309.188

ave/min m

3446.66

330_Calib_S_A

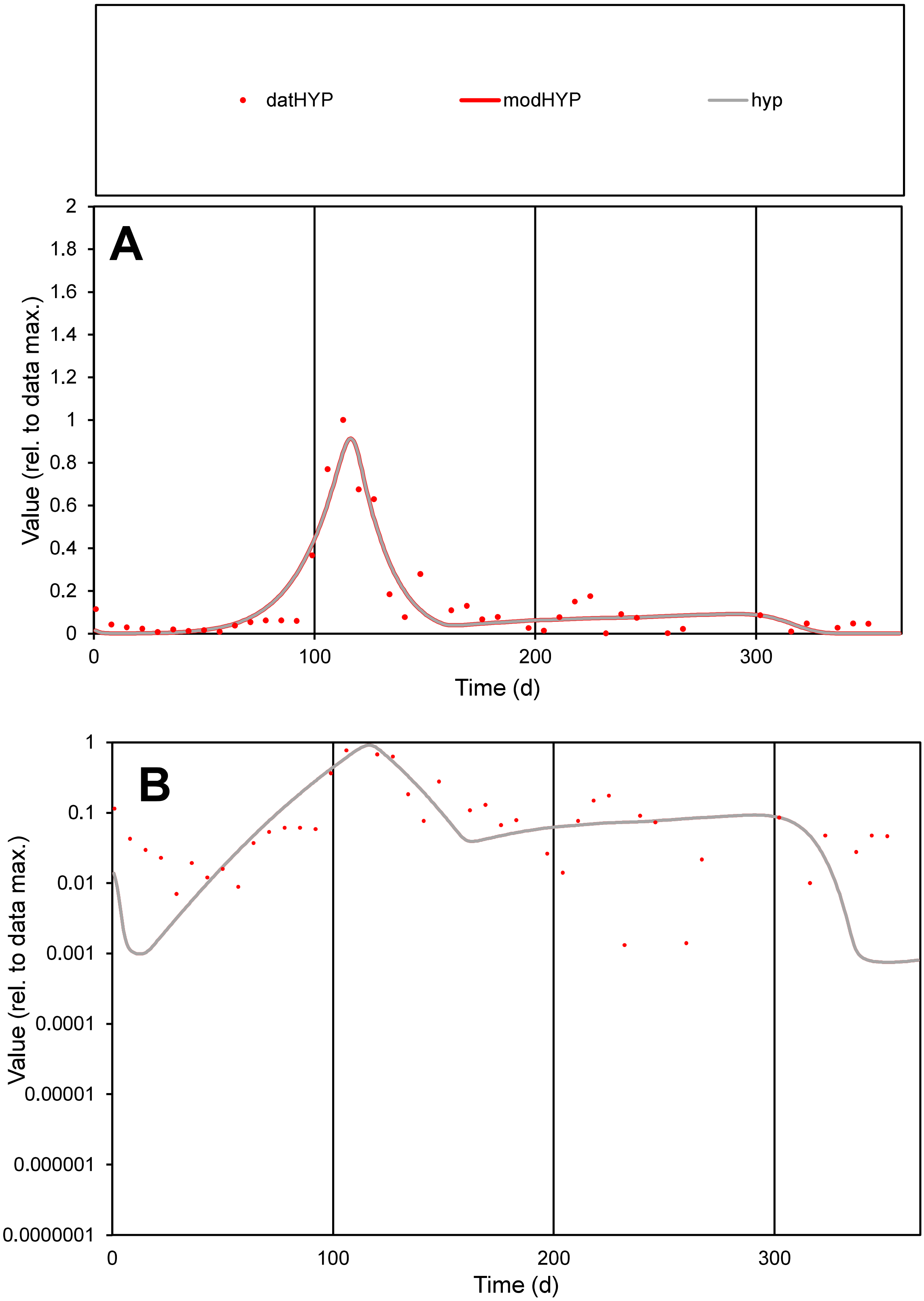

C1

Variables

HYP	Provide jtd or Dst
CalibOnly?	
2	1 to reproduce of
oNormalize?	
2	1=no, 2=yes

ofxn calc*
0.55936
ofxn mod
0.59119

sRun
RunA1
zout
1

Ave
Data
0.00178
Model
0.00175

data/vMax
0.01289

ofxng
319.391

Min d/m
1.75916

ave/min d
104.857
ave/min m
181.314

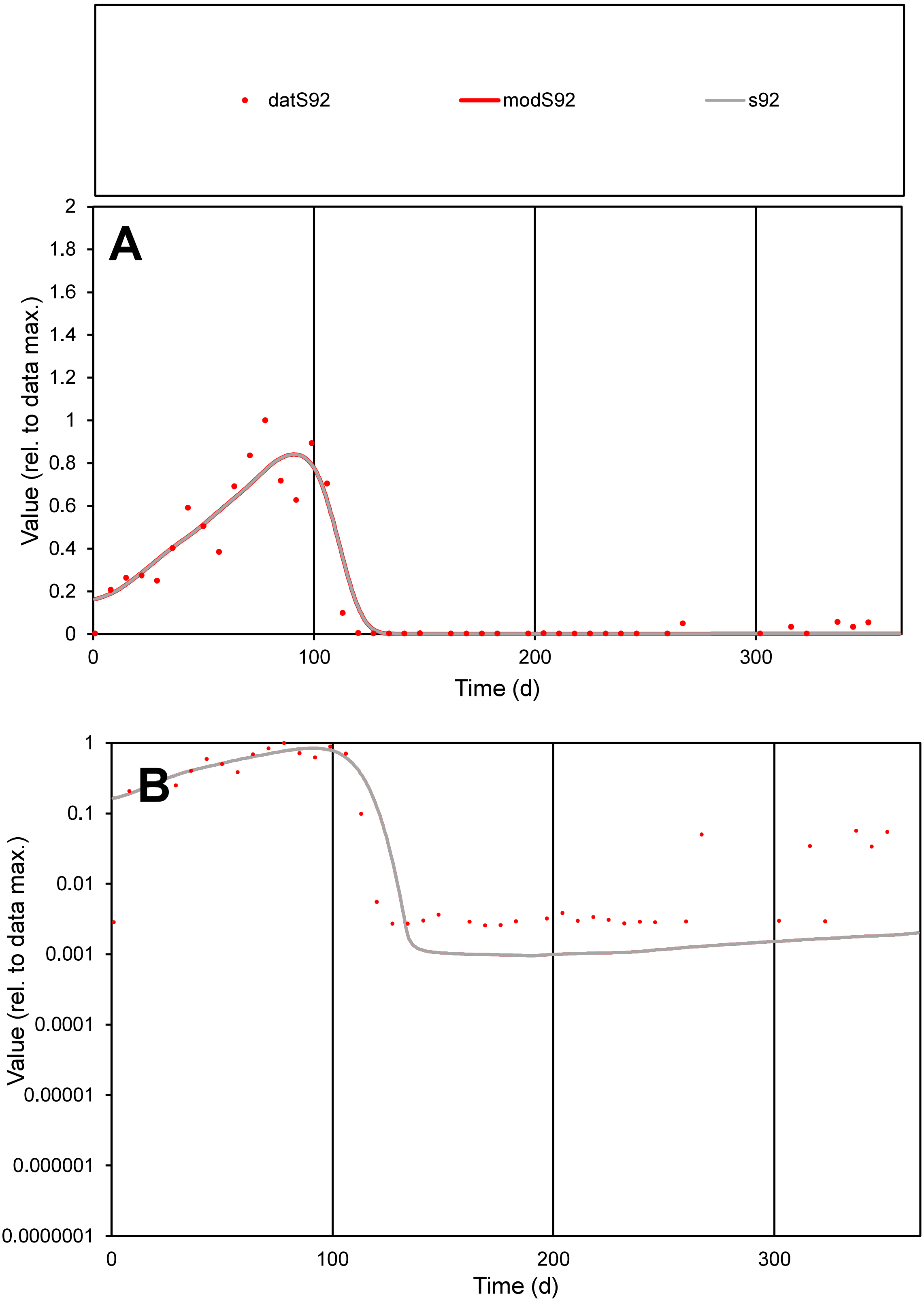

C1

Variables
S92 Provide jtd or Dst
CalibOnly?
2 1 to reproduce of
oNormalize?
2 1=no, 2=yes

ofxn calc*
0.40066
ofxn mod
0.45001

sRun
RunA1
zout
1

Ave
Data
0.00189
Model
0.00189

data/vMax
0.00906

ofxng
319.391

Min d/m
2.67673

ave/min d
80.5844
ave/min m
216.178

330_Calib_S_A

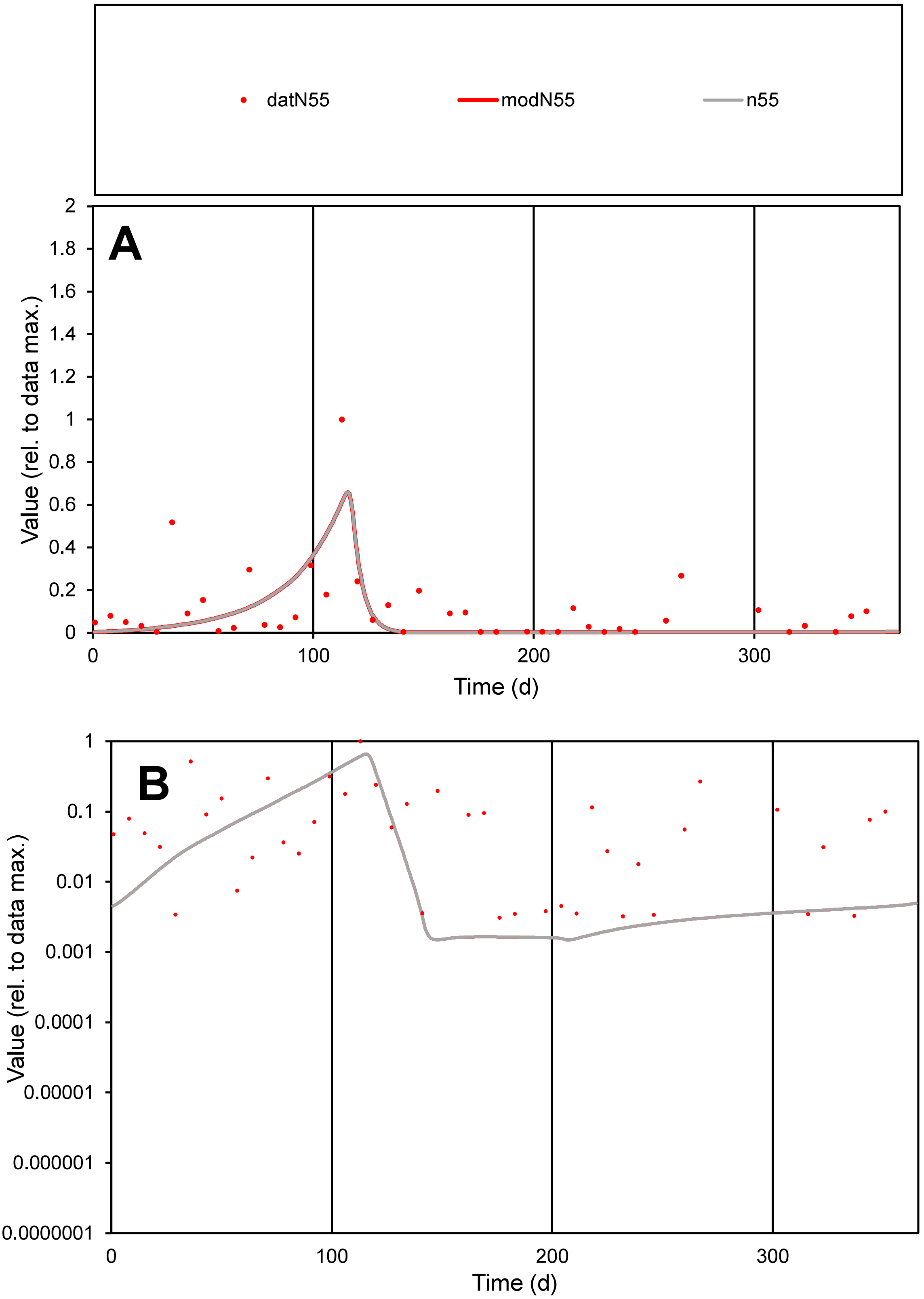

C1

Variables

N55 Provide jtd or Dst

CalibOnly?

2 1 to reproduce of

oNormalize?

2 1=no, 2=yes

ofxn calc*

1.24951

ofxn mod

1.26253

sRun

RunA1

zout

1

Ave

Data

0.00134

Model

0.00088

data/vMax

0.01229

ofxng

319.391

Min d/m

2.05643

ave/min d

35.2529

ave/min m

47.7351

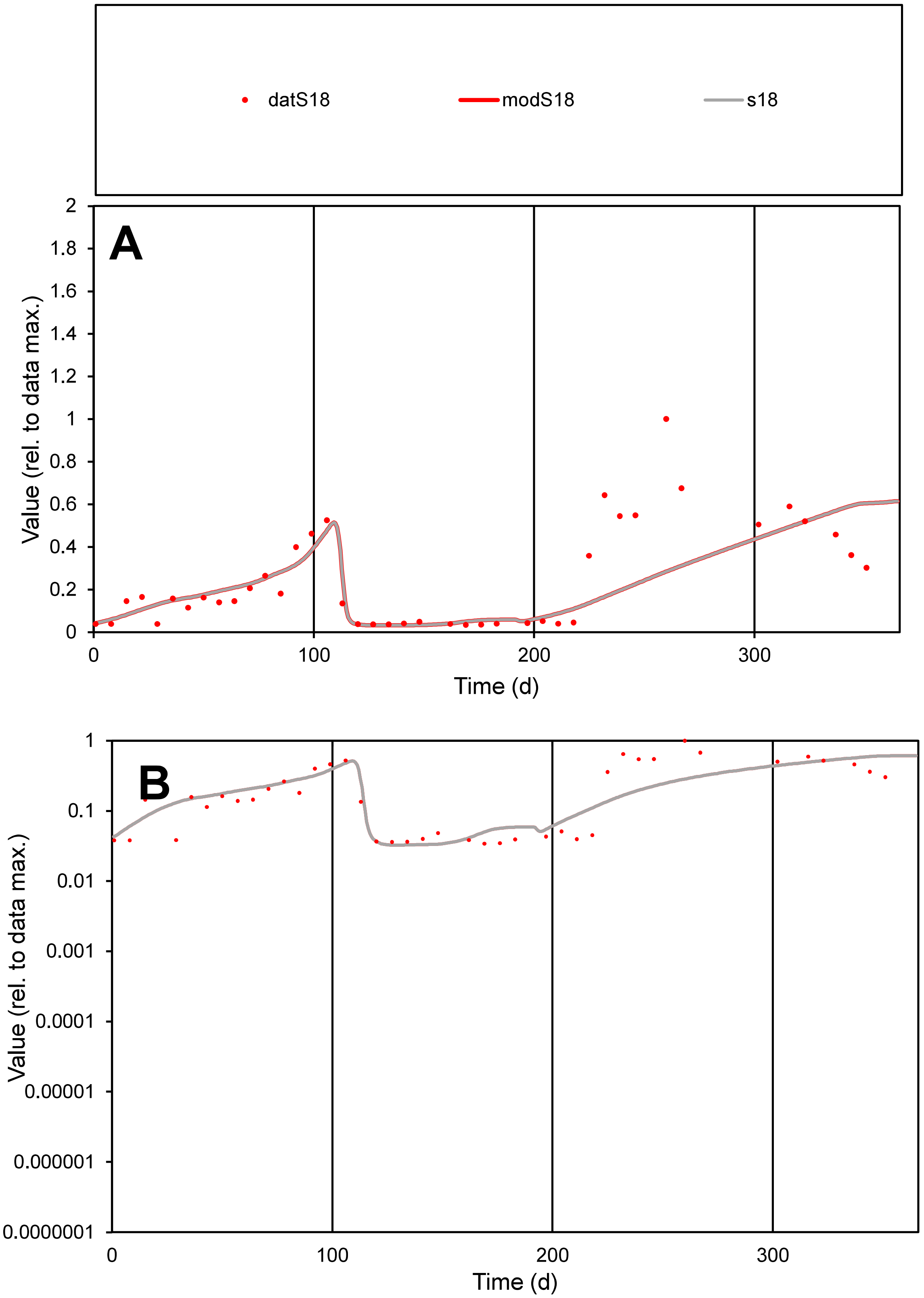

C1

Variables
S18 Provide jtd or Dst
CalibOnly?
2 1 to reproduce of
oNormalize?
2 1=no, 2=yes

ofxn calc*
0.72775
ofxn mod
0.73863

sRun
RunA1
zout
1

Ave
Data
0.00192
Model
0.00164

data/vMax
0.00781

ofxng
319.391

Min d/m
1.05722

ave/min d
7.18014
ave/min m
6.46156

330_Calib_S_A

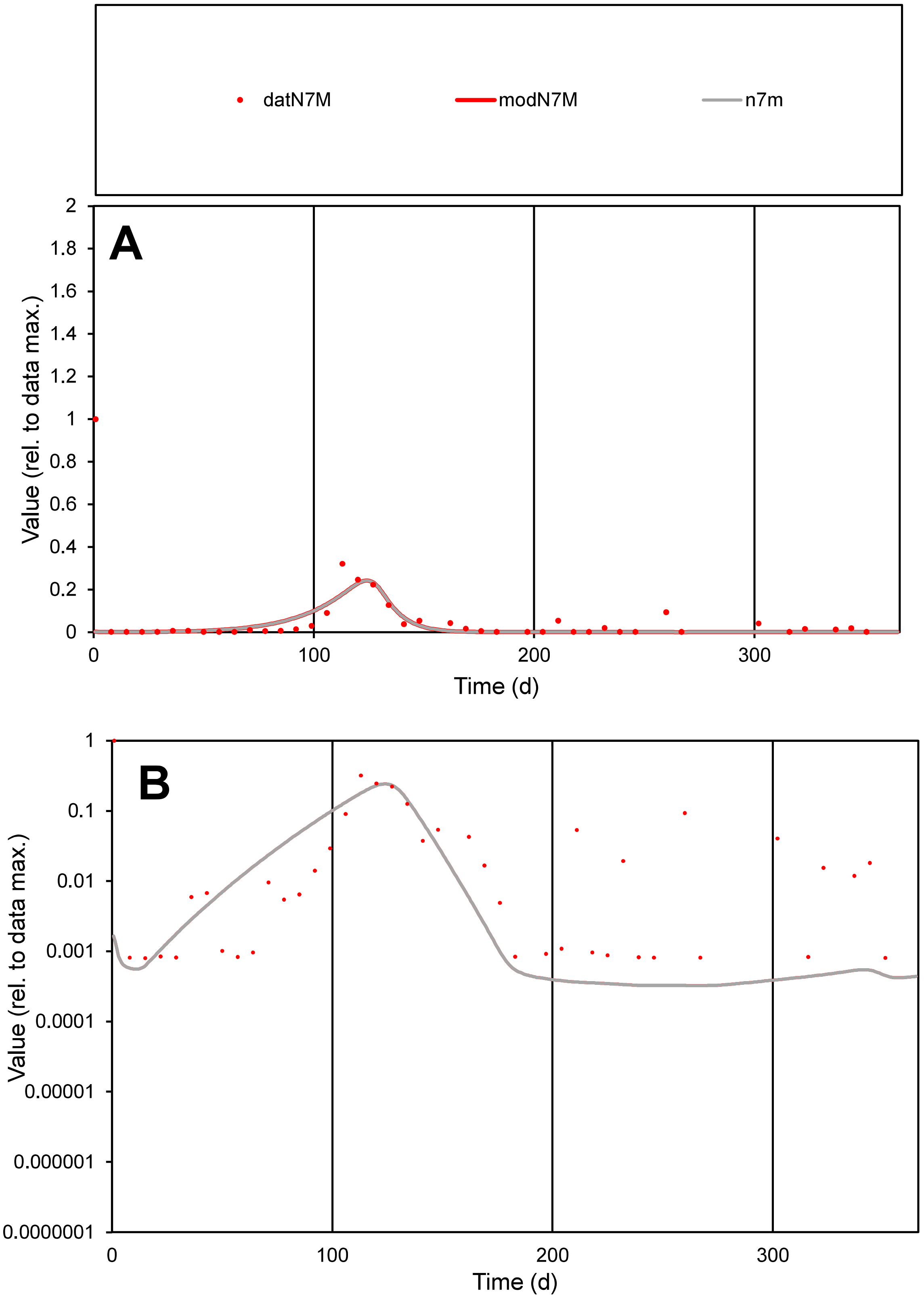

C1

Variables

N7M	Provide jtd or Dst
CalibOnly?	
2	1 to reproduce of
oNormalize?	
2	1=no, 2=yes
ofxn calc*	
2.6435	
ofxn mod	
2.64846	
sRun	
RunA1	
zout	
1	
Ave	
Data	
0.00079	
Model	
0.00042	
data/vMax	
0.01321	
ofxng	
319.391	
Min d/m	
2.4696	
ave/min d	
74.6303	
ave/min m	
97.9569	

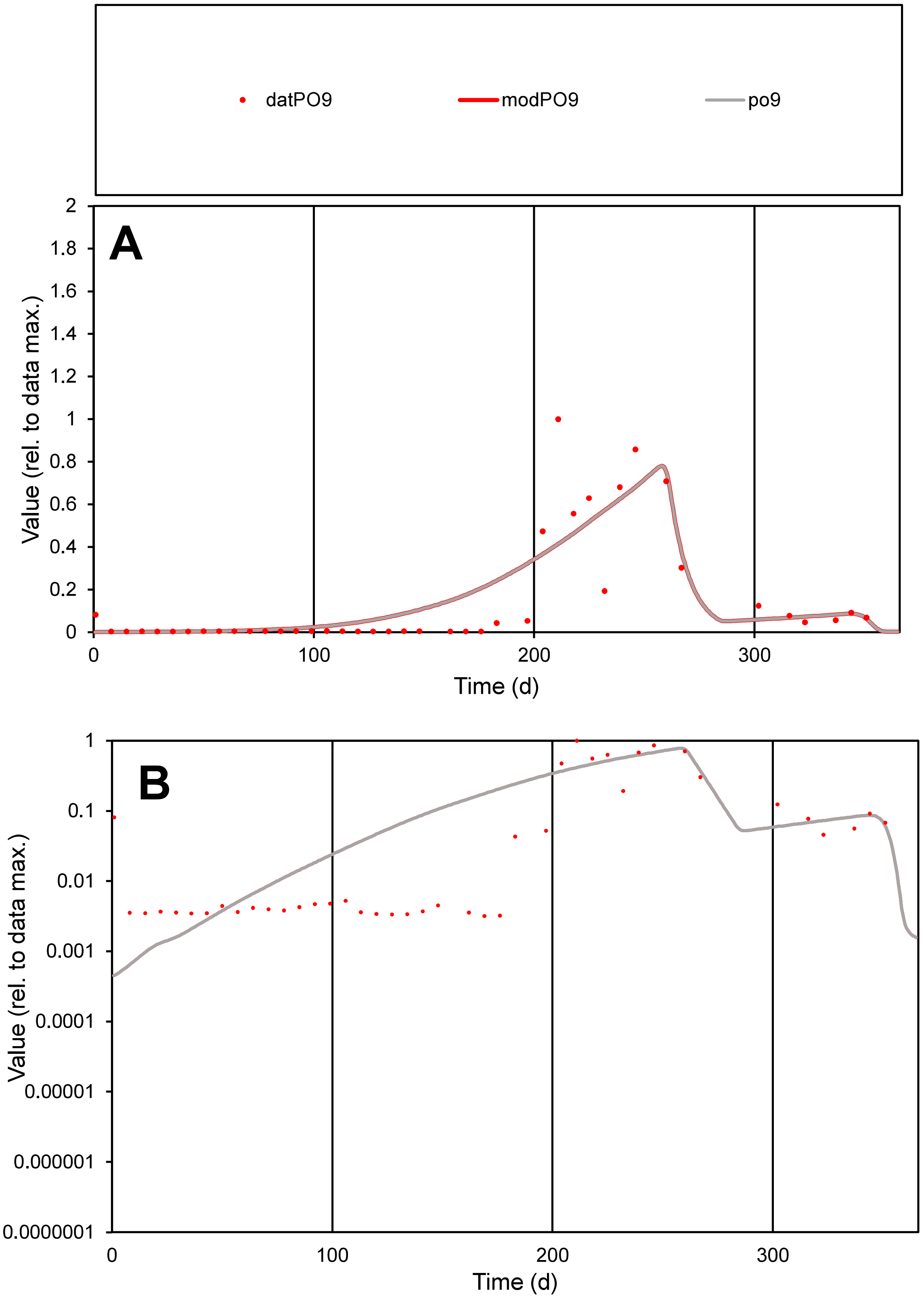

C1

Variables
PO9 Provide jtd or Dst
CalibOnly?
2 1 to reproduce of
oNormalize?
2 1=no, 2=yes

ofxn calc*
0.93631
ofxn mod
0.96465

sRun
RunA1
zout
1

Ave
Data
0.00268
Model
0.00298

data/vMax
0.01839

ofxng
319.391

Min d/m
6.92753

ave/min d
45.7217
ave/min m
352.327

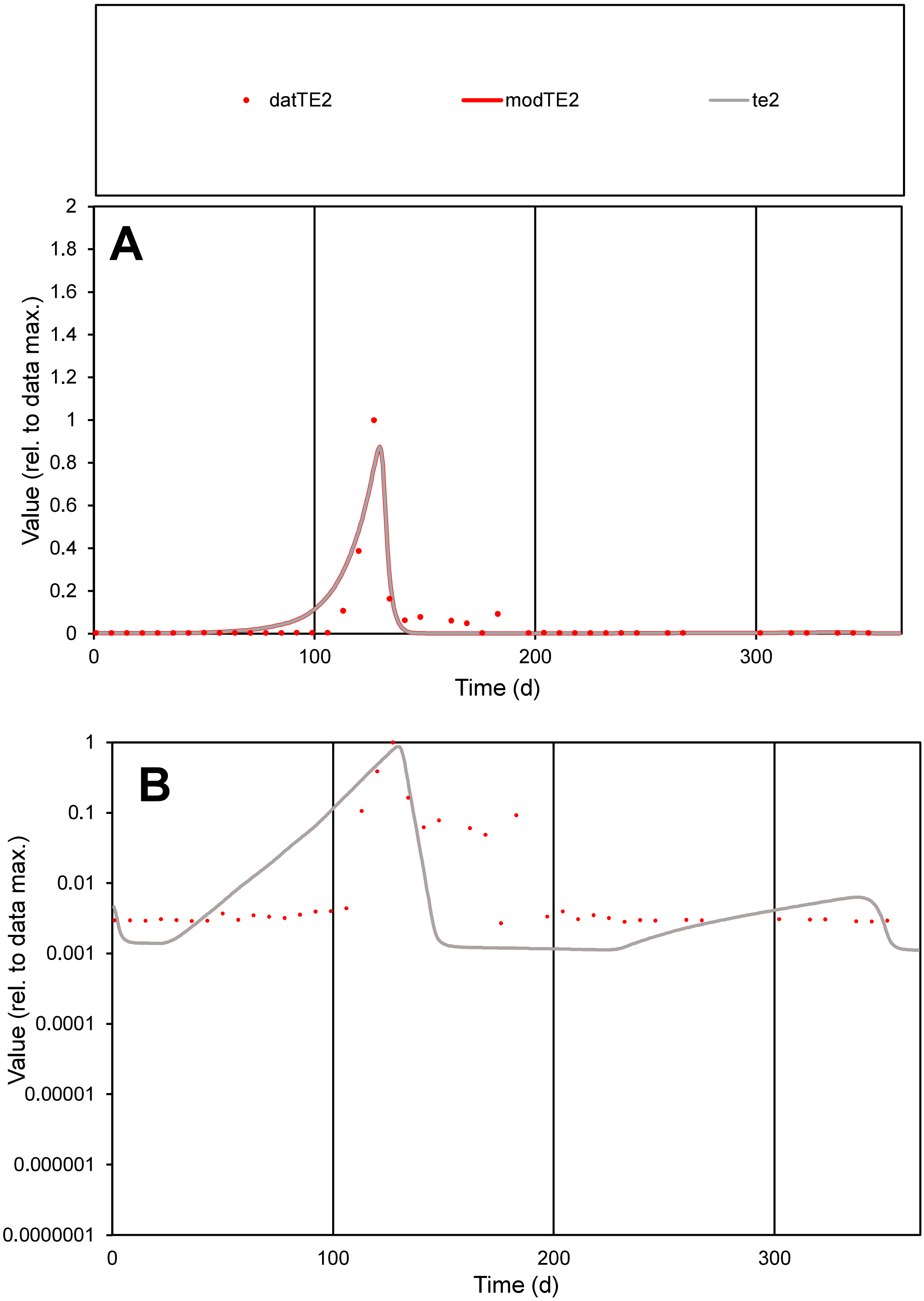

C1

Variables
TE2 Provide jtd or Dst
CalibOnly?
2 1 to reproduce of
oNormalize?
2 1=no, 2=yes

ofxn calc*
1.07691
ofxn mod
1.2283

sRun
RunA1
zout
1

Ave
Data
0.00161
Model
0.00175

data/vMax
0.03218

ofxng
319.391

Min d/m
2.38709

ave/min d
18.5528
ave/min m
48.0965

330_Calib_S_A

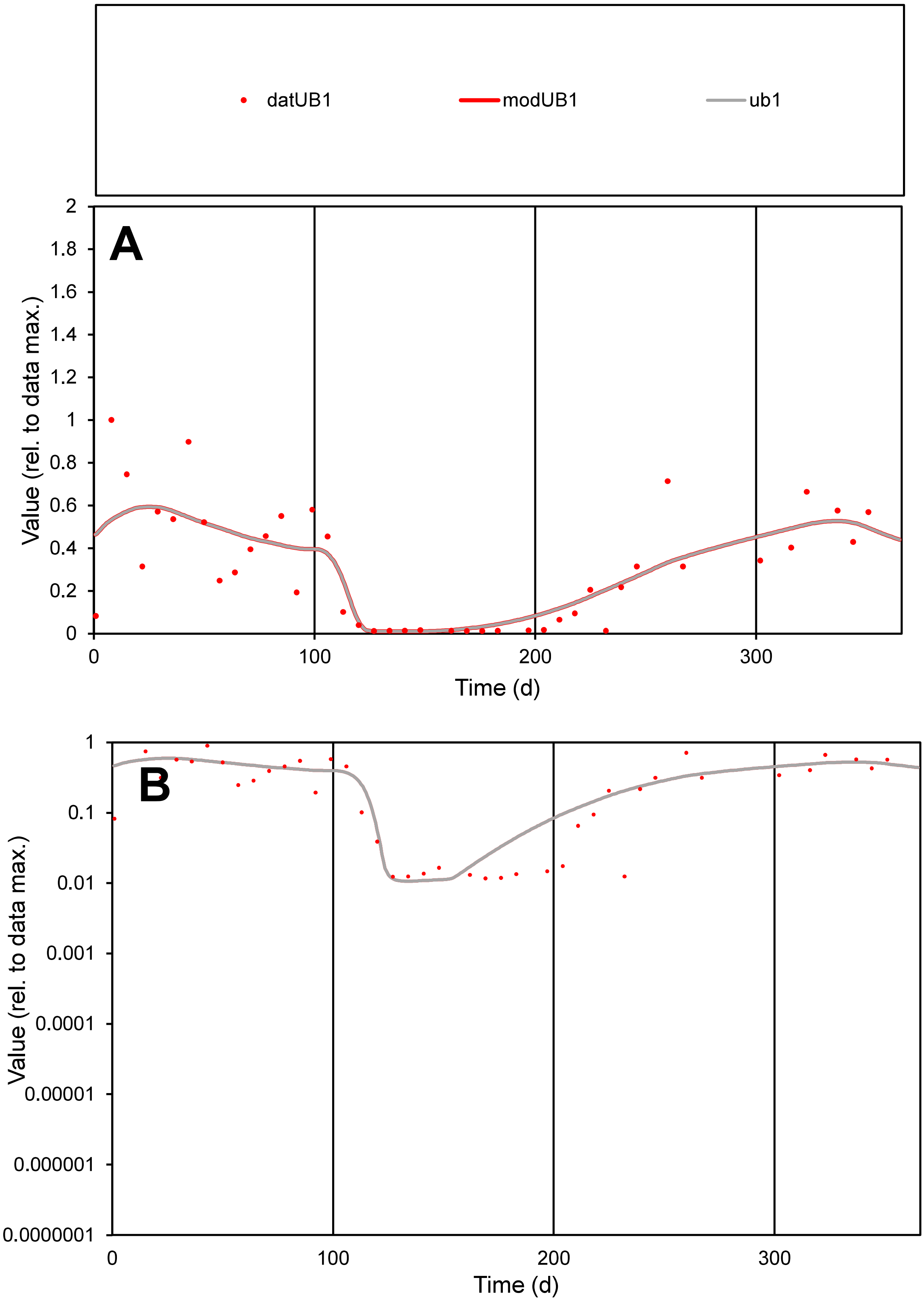

C1

Variables

UB1 Provide jtd or Dst

CalibOnly?

2 1 to reproduce of

oNormalize?

2 1=no, 2=yes

ofxn calc*

0.51295

ofxn mod

0.53231

sRun

RunA1

zout

1

Ave

Data

0.00152

Model

0.00154

data/vMax

0.00489

ofxng

319.391

Min d/m

1.10398

ave/min d

26.4227

ave/min m

29.5695

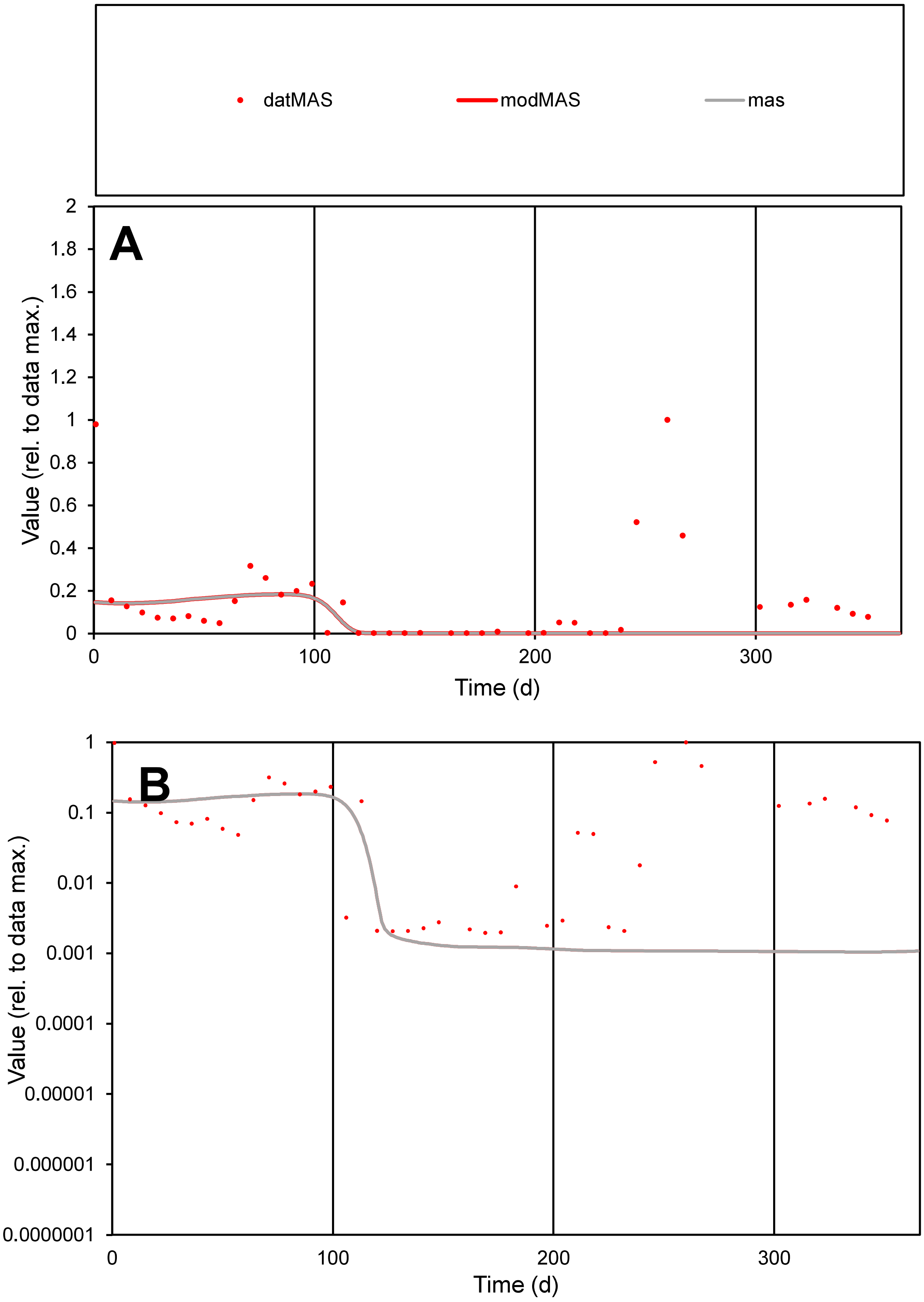

C1

Variables
MAS Provide jtd or Dst
CalibOnly?
2 1 to reproduce of
oNormalize?
2 1=no, 2=yes

ofxn calc*
1.65029
ofxn mod
1.6562

sRun
RunA1
zout
1

Ave
Data
0.00088
Model
0.00038

data/vMax
0.00617

ofxng
319.391

Min d/m
1.87781

ave/min d
73.0427
ave/min m
59.4665

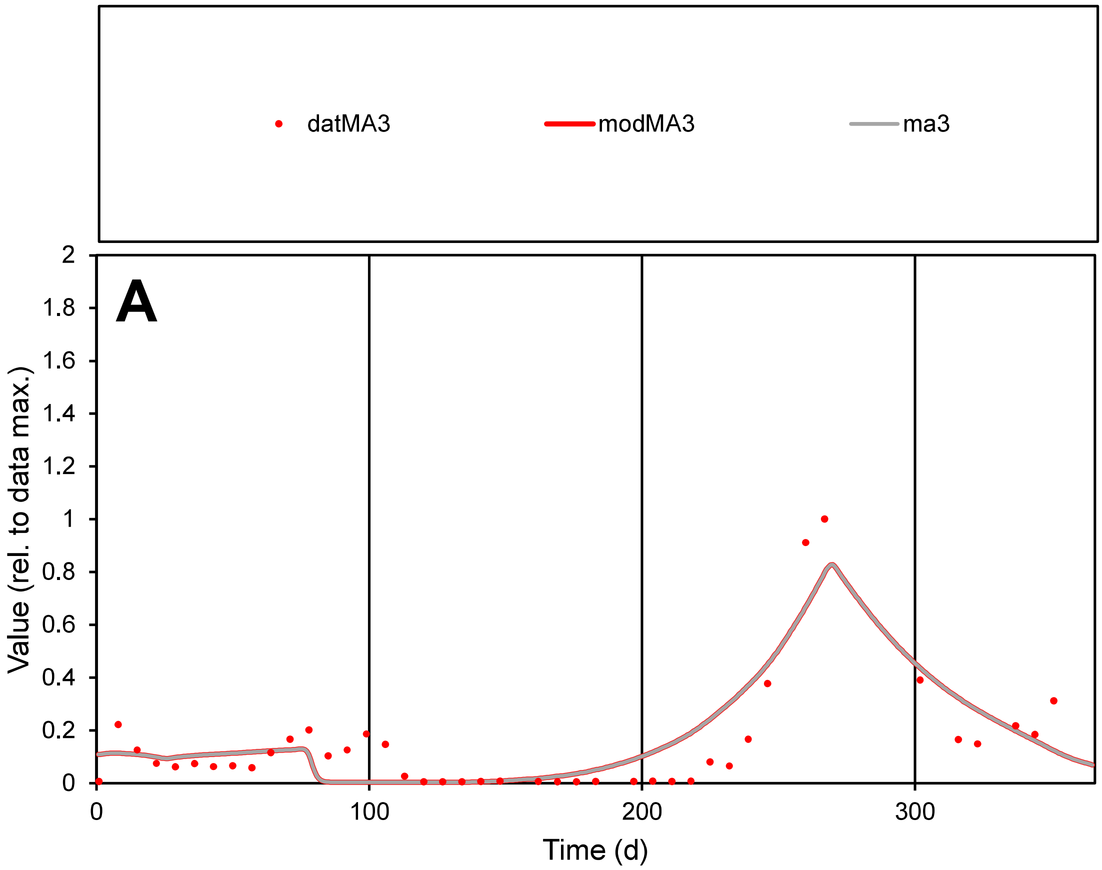

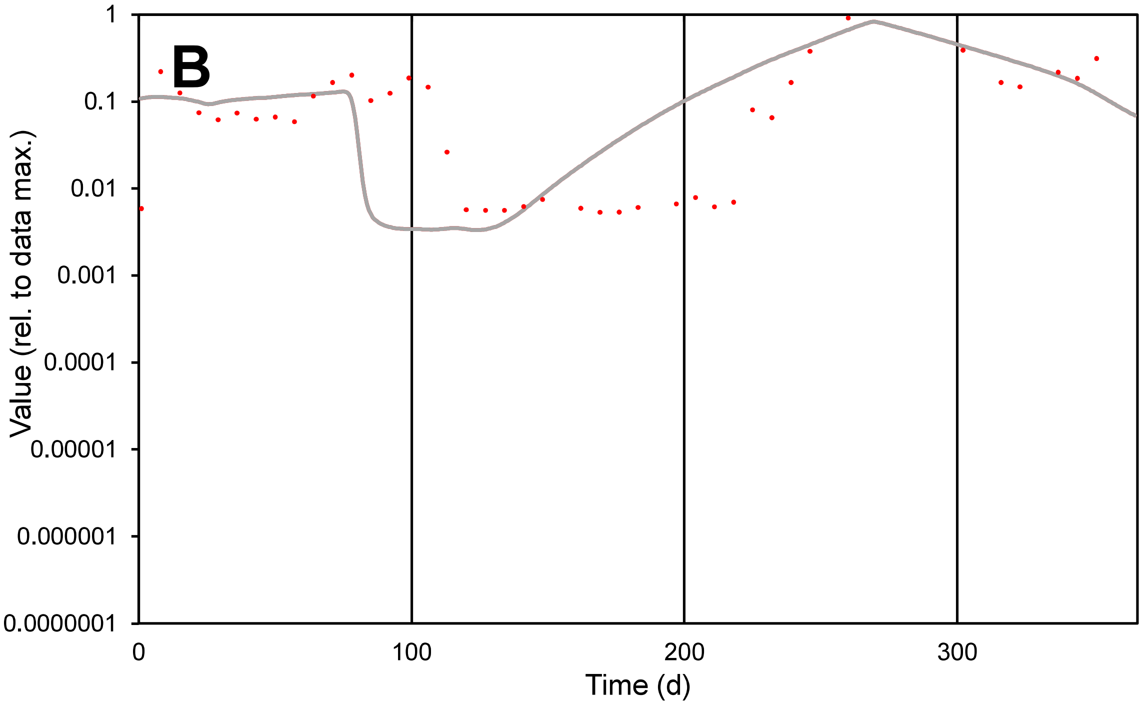

**C1**	
Variables	
MA3	Provide jtd or Dst
CalibOnly?	
2	1 to reproduce of
oNormalize?	
2	1=no, 2=yes
ofxn calc*	
0.76919	
ofxn mod	
0.7987	
sRun	
RunA1	
zout	
1	
Ave	
Data	
0.00165	
Model	
0.0018	
data/vMax	
0.01173	
ofxng	
319.391	
Min d/m	
1.57794	
ave/min d	
26.5125	
ave/min m	
45.5198	

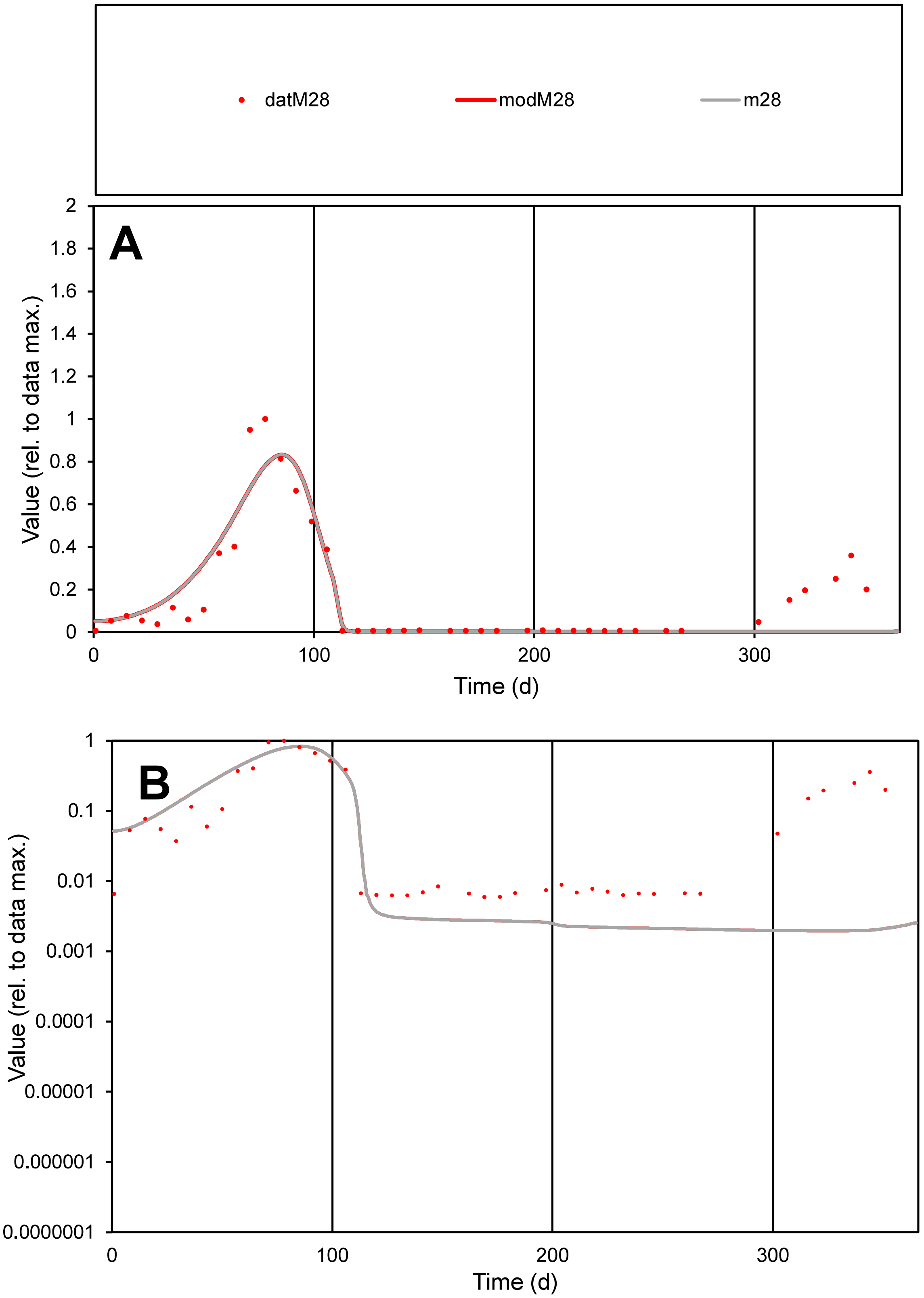

C1

Variables
M28 Provide jtd or Dst
CalibOnly?
2 1 to reproduce of
oNormalize?
2 1=no, 2=yes

ofxn calc*
0.68146
ofxn mod
0.71199

sRun
RunA1
zout
1

Ave
Data
0.00151
Model
0.00134

data/vMax
0.00913

ofxng
319.391

Min d/m
3.05177

ave/min d
27.887
ave/min m
75.6492

330_Calib_S_A

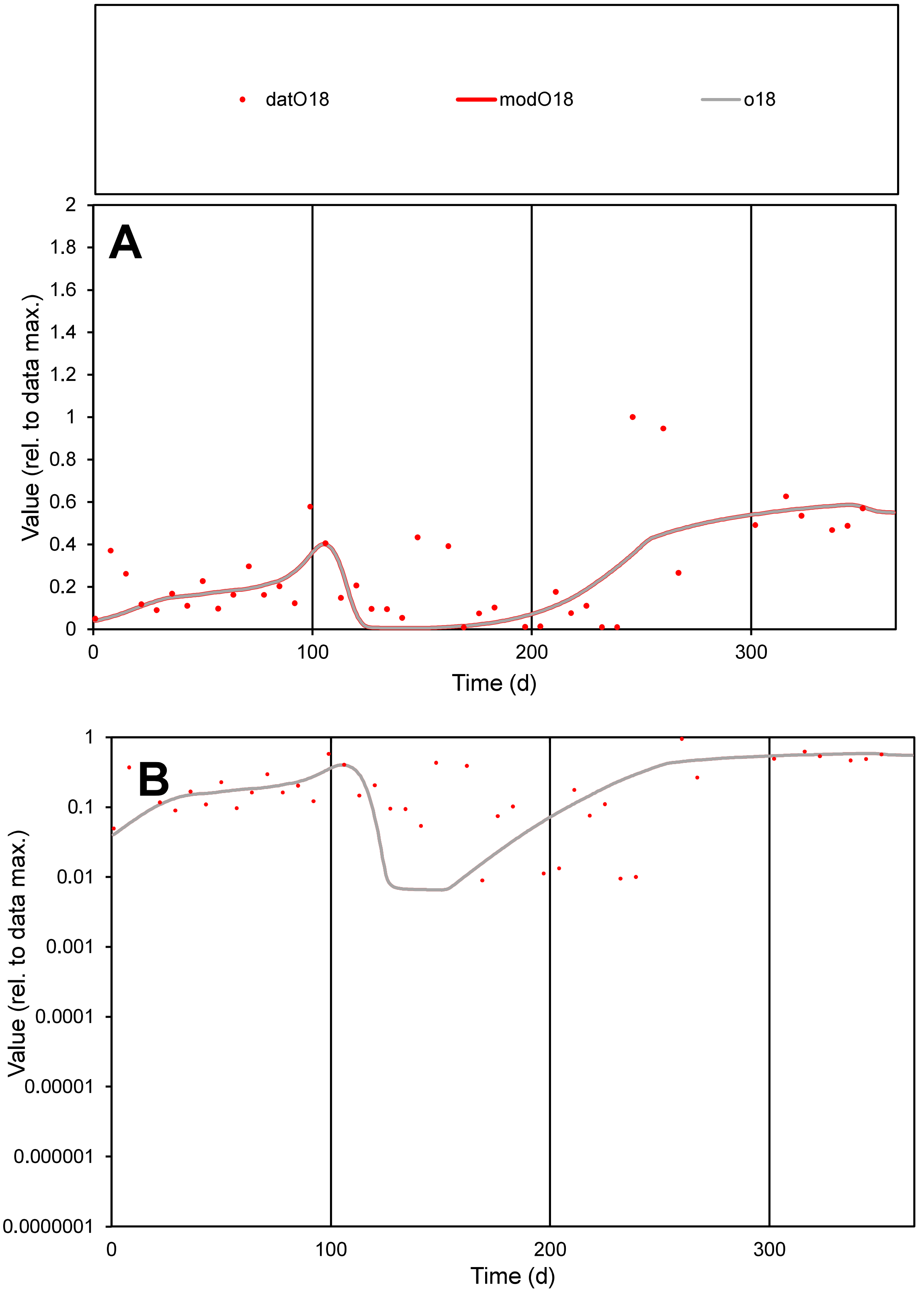

C1

Variables
O18 Provide jtd or Dst
CalibOnly?
2 1 to reproduce of
oNormalize?
2 1=no, 2=yes

ofxn calc*
0.72544
ofxn mod
0.73932

sRun
RunA1
zout
1

Ave
Data
0.00149
Model
0.00127

data/vMax
0.00579

ofxng
319.391

Min d/m
1.36481

ave/min d
28.742
ave/min m
33.4478

330_Calib_S_A

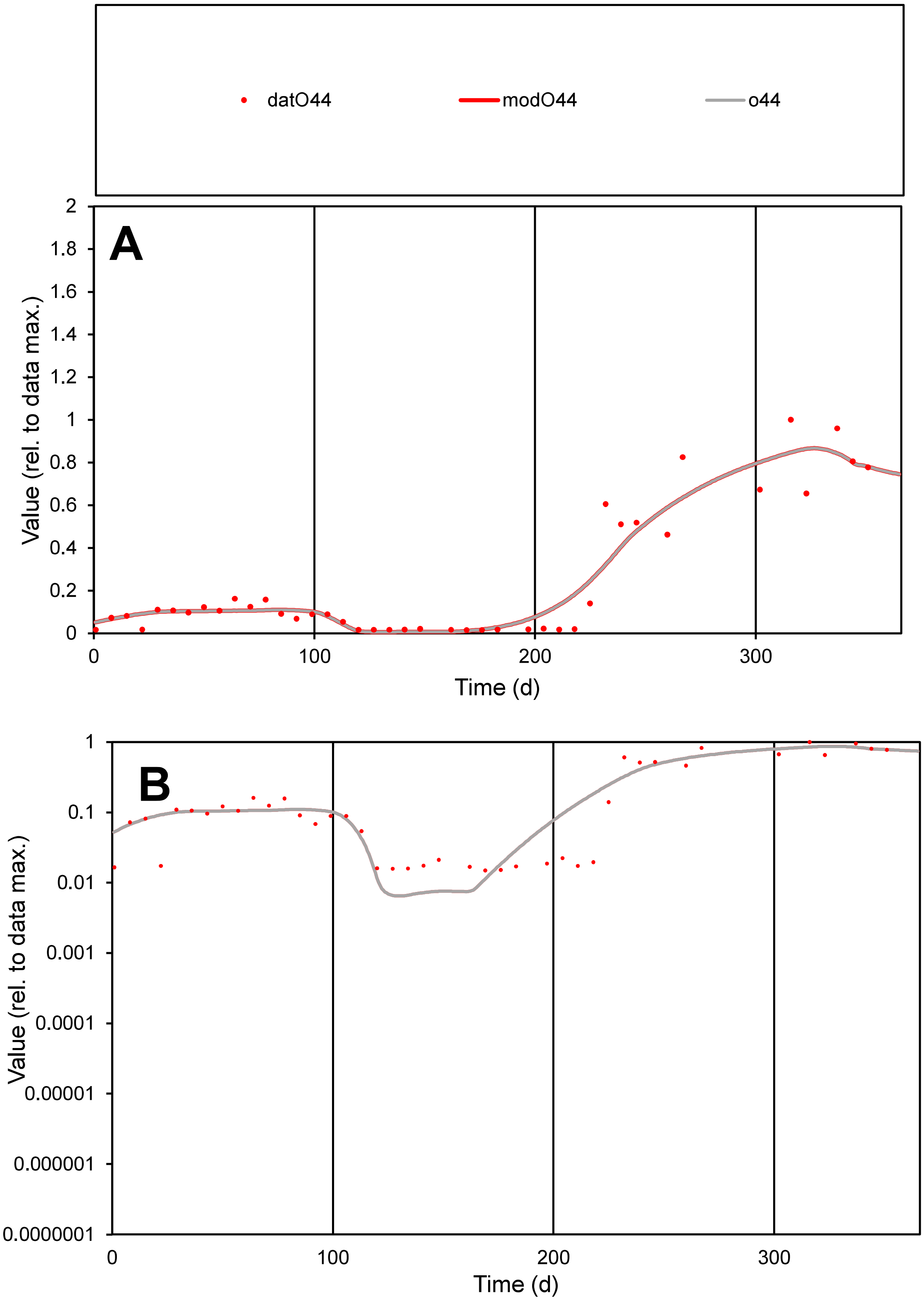

C1

Variables
O44 Provide jtd or Dst
CalibOnly?
2 1 to reproduce of
oNormalize?
2 1=no, 2=yes

ofxn calc*
0.36712
ofxn mod
0.40195

sRun
RunA1
zout
1

Ave
Data
0.00171
Model
0.00173

data/vMax
0.00741

ofxng
319.391

Min d/m
2.29261

ave/min d
15.4338
ave/min m
35.7807

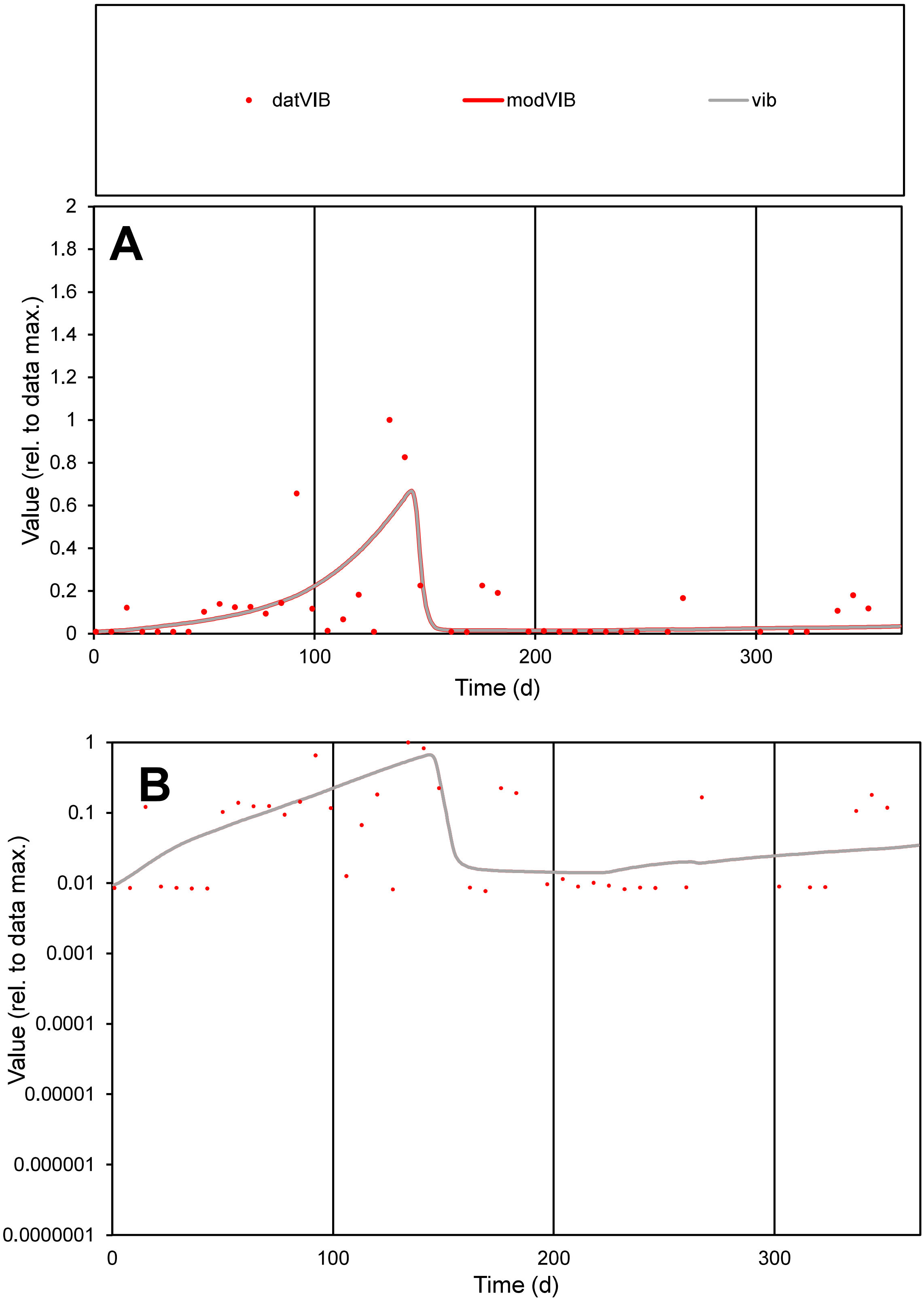

C1

Variables
VIB Provide jtd or Dst
CalibOnly?
2 1 to reproduce of
oNormalize?
2 1=no, 2=yes

ofxn calc*
1.26027
ofxn mod
1.27751

sRun
RunA1
zout
1

Ave
Data
0.00028
Model
0.00025

data/vMax
0.00231

ofxng
319.391

Min d/m
0.80415

ave/min d
15.7703
ave/min m
11.1645

330_Calib_S_A

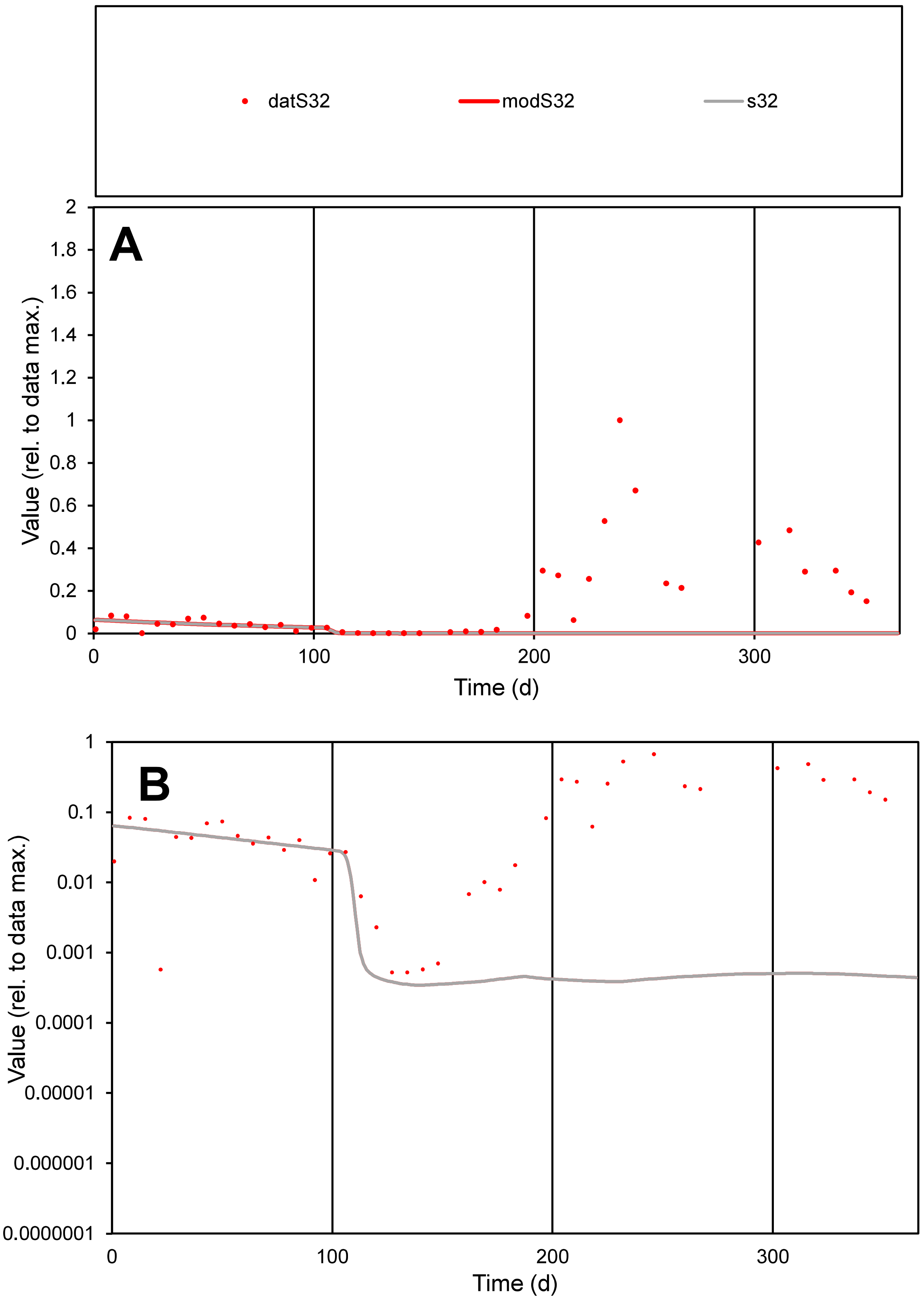

C1

Variables
S32 Provide jtd or Dst
CalibOnly?
2 1 to reproduce of
oNormalize?
2 1=no, 2=yes

ofxn calc*
1.72824
ofxn mod
1.73202

sRun
RunA1
zout
1

Ave
Data
0.00322
Model
0.00036

data/vMax
0.02187

ofxng
319.391

Min d/m
1.51363

ave/min d
281.297
ave/min m
48.1304

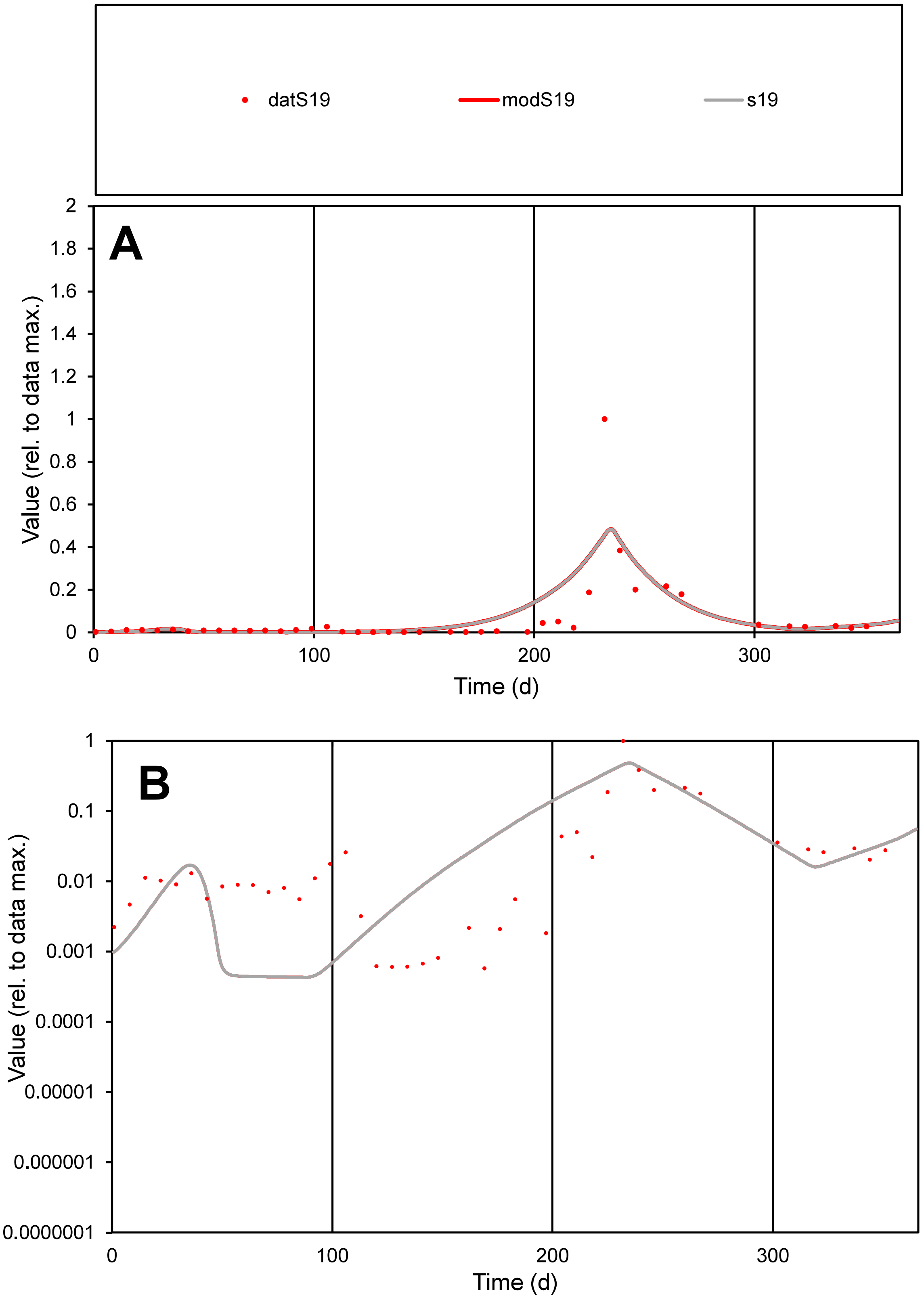

C1

Variables
S19 Provide jtd or Dst
CalibOnly?
2 1 to reproduce of
oNormalize?
2 1=no, 2=yes

ofxn calc*
1.65637
ofxn mod
1.70385

sRun
RunA1
zout
1

Ave
Data
0.00515
Model
0.00602

data/vMax
0.08234

ofxng
319.391

Min d/m
1.33568

ave/min d
108.425
ave/min m
169.367

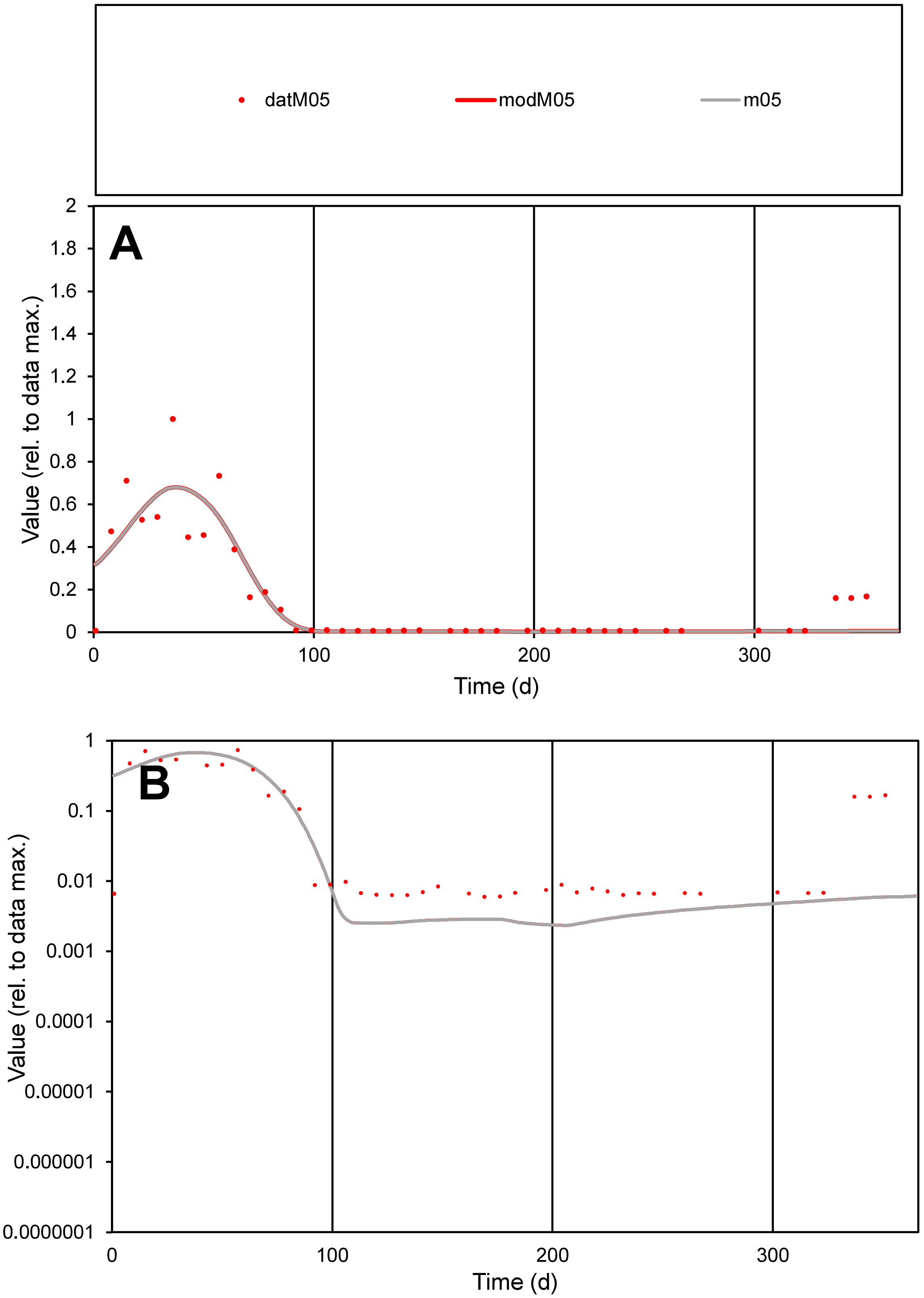

C1

Variables
M05 Provide jtd or Dst
CalibOnly?
2 1 to reproduce of
oNormalize?
2 1=no, 2=yes

ofxn calc*
0.69022
ofxn mod
0.72175

sRun
RunA1
zout
1

Ave
Data
0.00111
Model
0.00104

data/vMax
0.00726

ofxng
319.391

Min d/m
2.54157

ave/min d
25.5457
ave/min m
60.6275

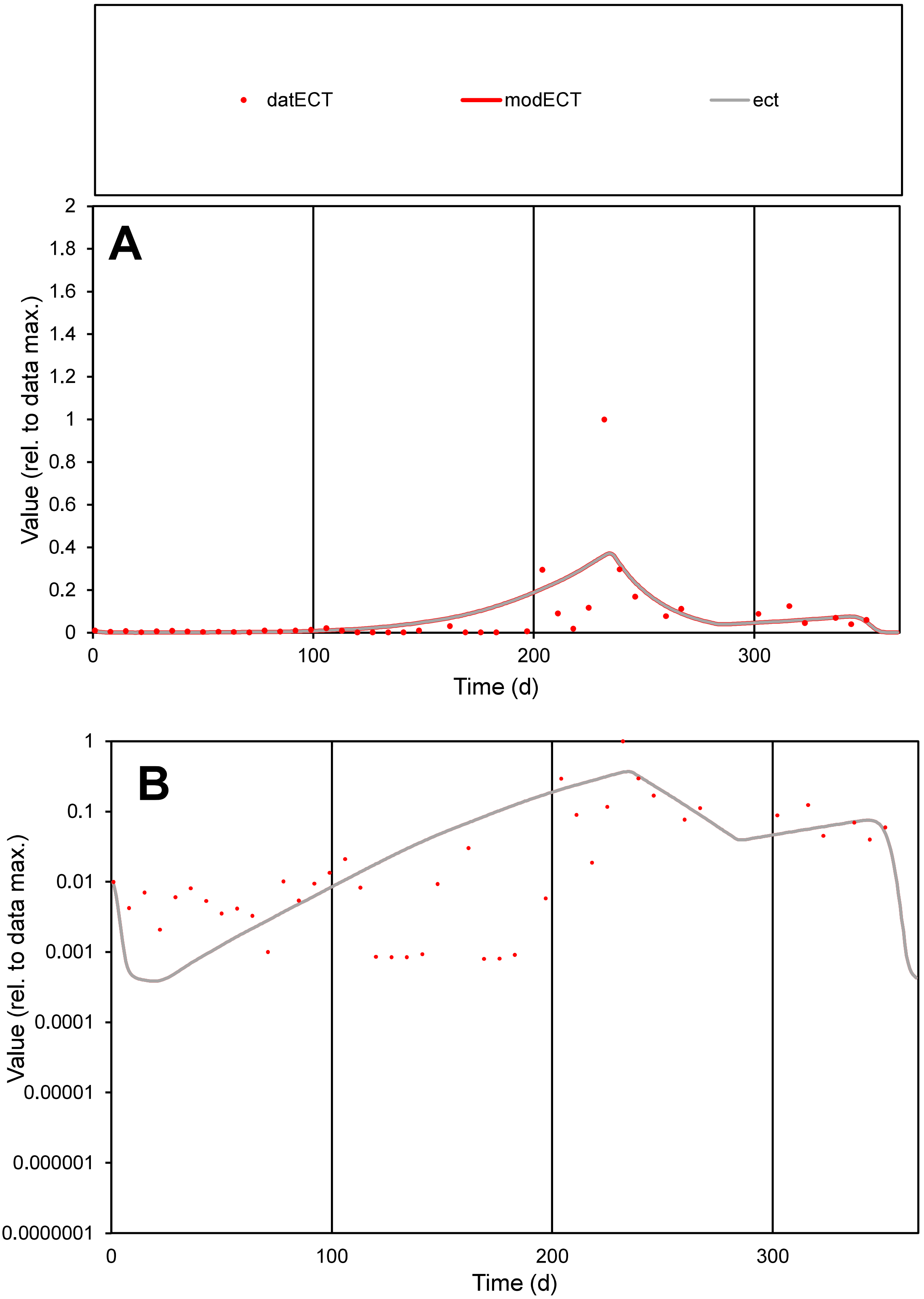

C1

Variables
ECT Provide jtd or Dst
CalibOnly?
2 1 to reproduce of
oNormalize?
2 1=no, 2=yes

ofxn calc*
1.83522
ofxn mod
1.86169

sRun
RunA1
zout
1

Ave
Data
0.00235
Model
0.00279

data/vMax
0.03564

ofxng
319.391

Min d/m
2.03574

ave/min d
82.5938
ave/min m
199.43

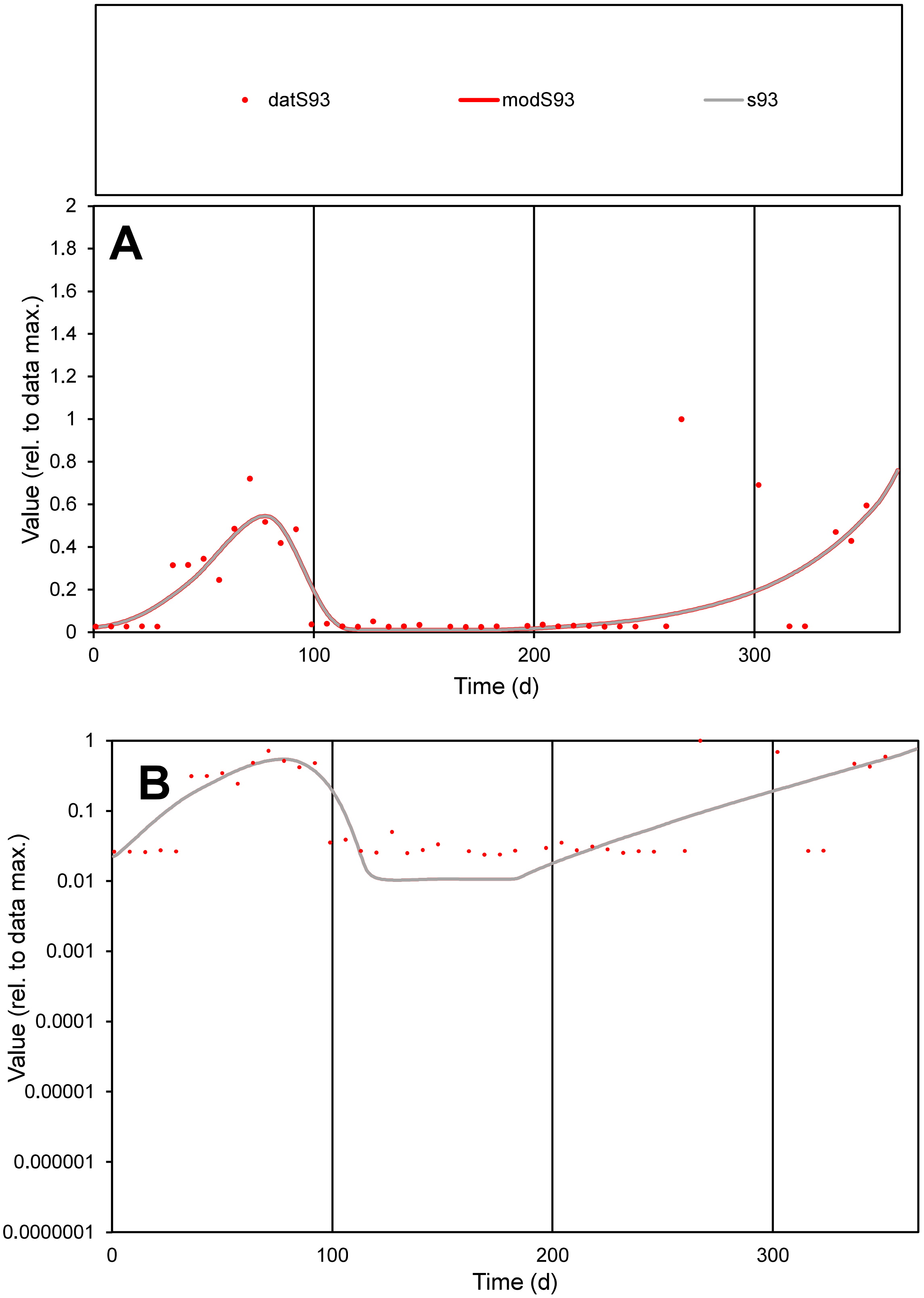

C1

Variables
S93 Provide jtd or Dst
CalibOnly?
2 1 to reproduce of
oNormalize?
2 1=no, 2=yes

ofxn calc*
0.95691
ofxn mod
0.97093

sRun
RunA1
zout
1

Ave
Data
0.00051
Model
0.00045

data/vMax
0.00276

ofxng
319.391

Min d/m
2.29713

ave/min d
7.85082
ave/min m
15.734

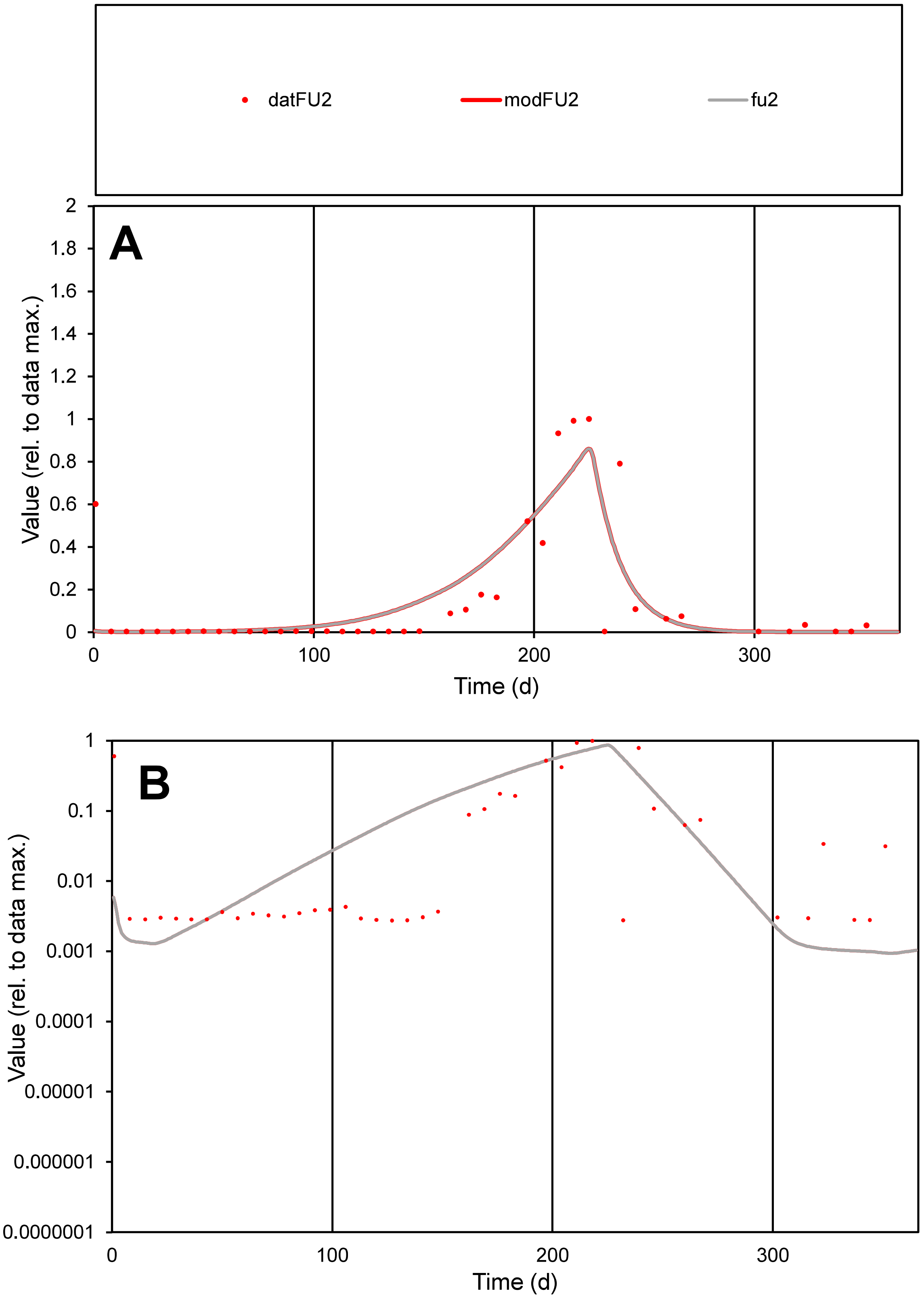

C1

Variables
FU2 Provide jtd or Dst
CalibOnly?
2 1 to reproduce of
oNormalize?
2 1=no, 2=yes

ofxn calc*
1.08758
ofxn mod
1.13953

sRun
RunA1
zout
1

Ave
Data
0.00145
Model
0.00148

data/vMax
0.00983

ofxng
319.391

Min d/m
2.92472

ave/min d
53.438
ave/min m
160.302

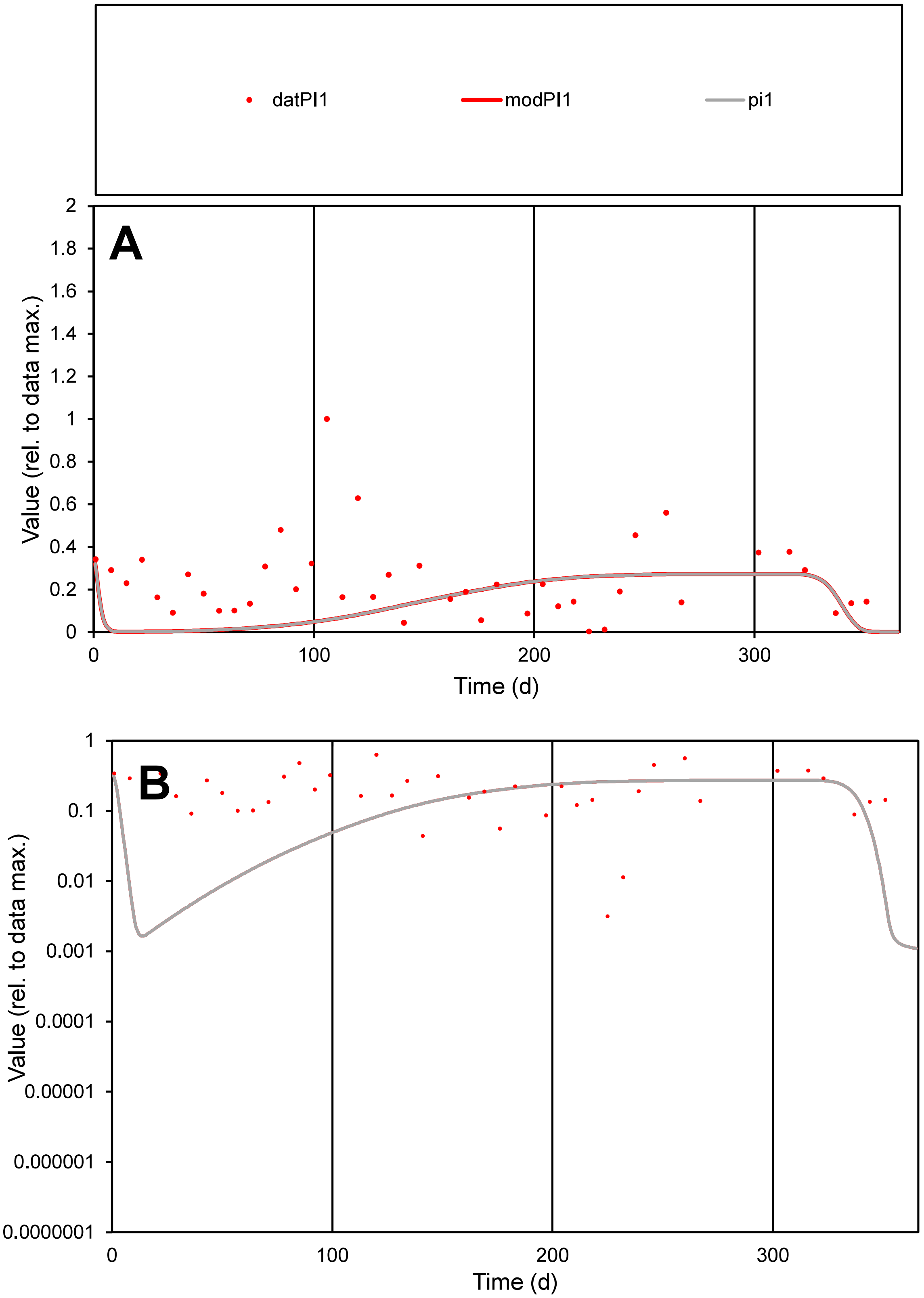

C1

Variables
PI1 Provide jtd or Dst
CalibOnly?
2 1 to reproduce of
oNormalize?
2 1=no, 2=yes

ofxn calc*
1.00127
ofxn mod
1.00648

sRun
RunA1
zout
1

Ave
Data
0.00121
Model
0.00067

data/vMax
0.00501

ofxng
319.391

Min d/m
1.72844

ave/min d
76.2386
ave/min m
73.2524

330_Calib_S_A

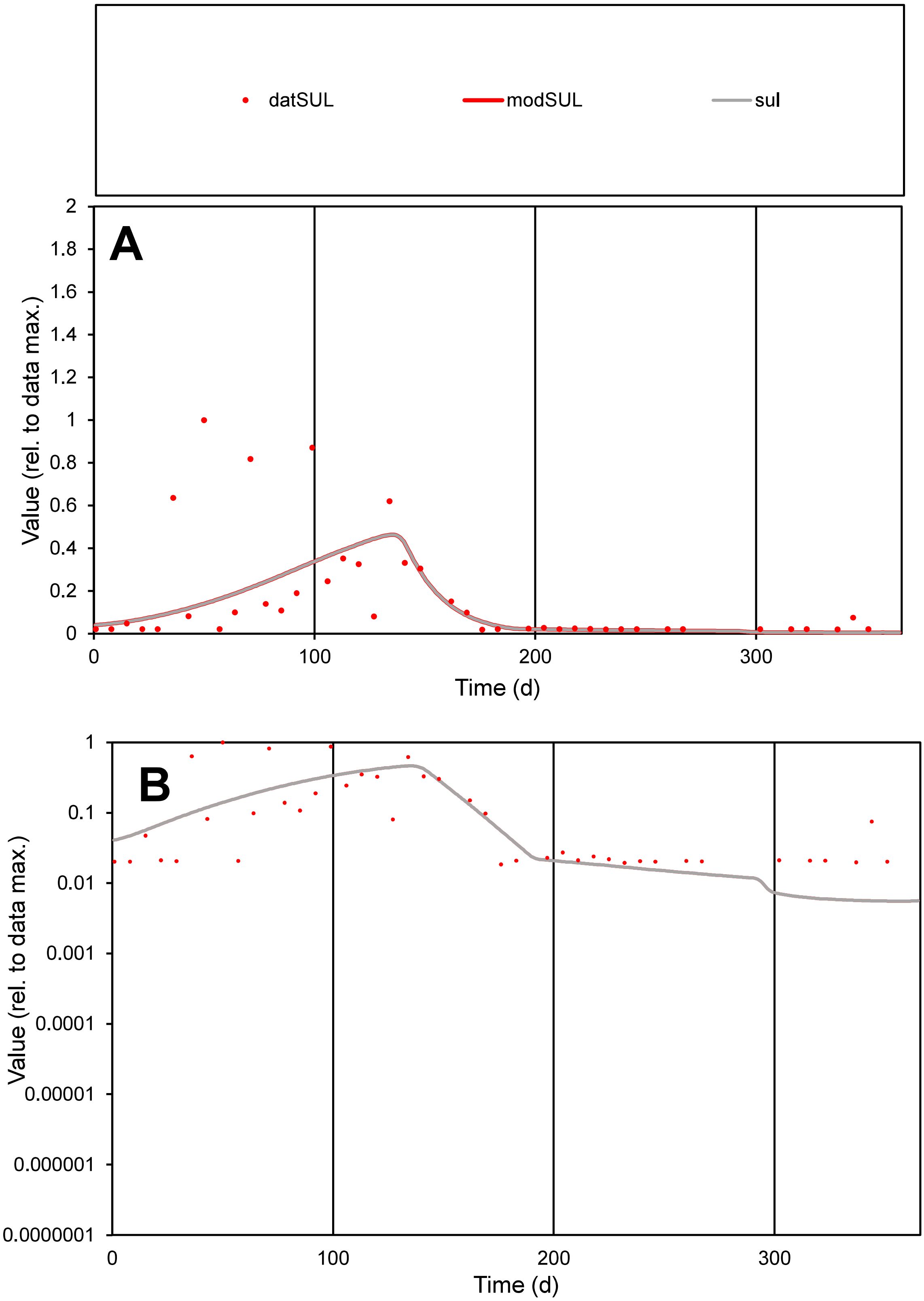

C1

Variables

SUL Provide jtd or Dst

CalibOnly?

2 1 to reproduce of

oNormalize?

2 1=no, 2=yes

ofxn calc*

1.2857

ofxn mod

1.29469

sRun

RunA1

zout

1

Ave

Data

0.00073

Model

0.00059

data/vMax

0.00436

ofxng

319.391

Min d/m

3.3177

ave/min d

9.04543

ave/min m

24.2034

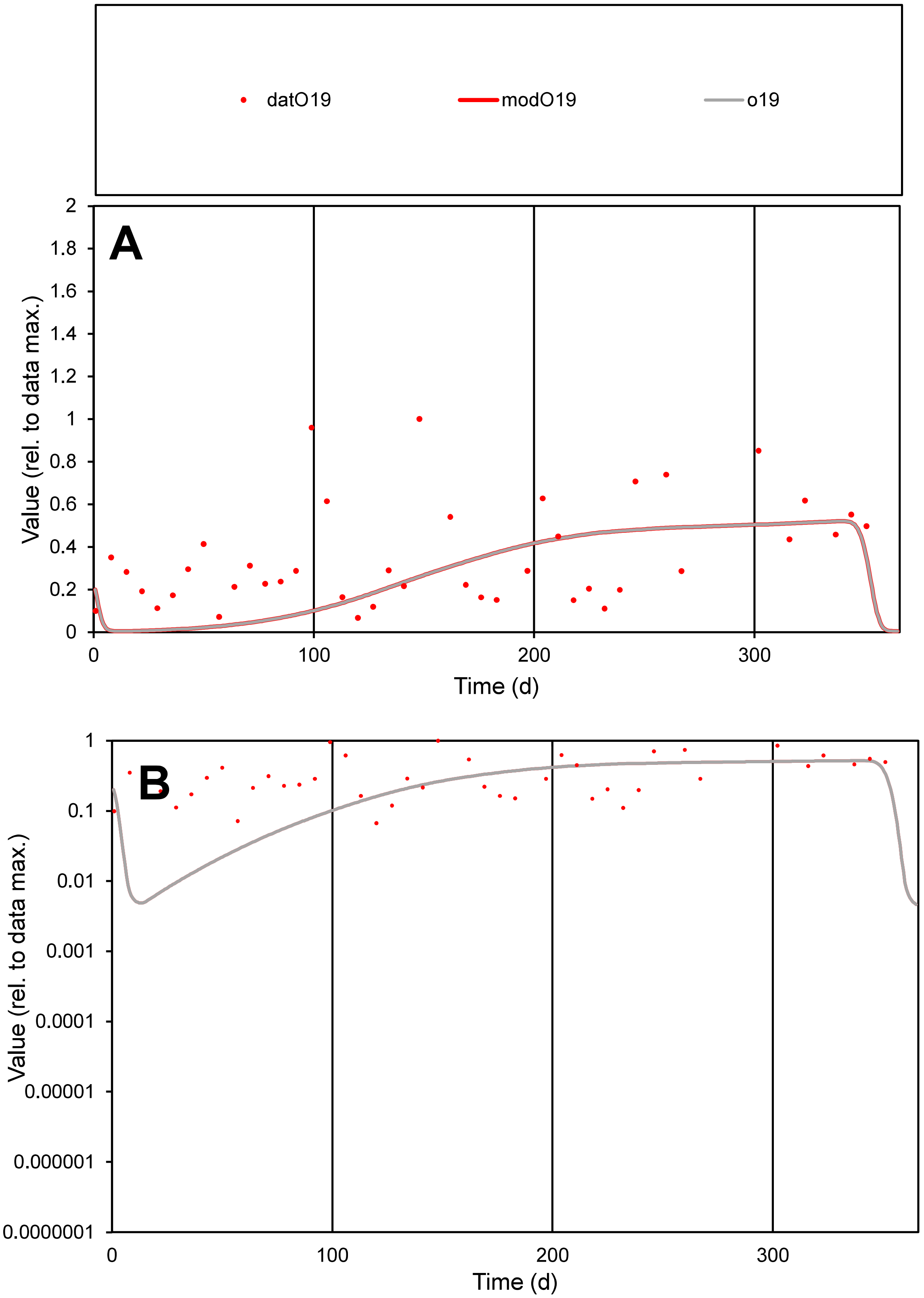

C1

Variables

O19 Provide jtd or Dst

CalibOnly?

2 1 to reproduce of

oNormalize?

2 1=no, 2=yes

ofxn calc*

0.76784

ofxn mod

0.77581

sRun

RunA1

zout

1

Ave

Data

0.00165

Model

0.0012

data/vMax

0.00464

ofxng

319.391

Min d/m

12.3168

ave/min d

5.30262

ave/min m

47.4252

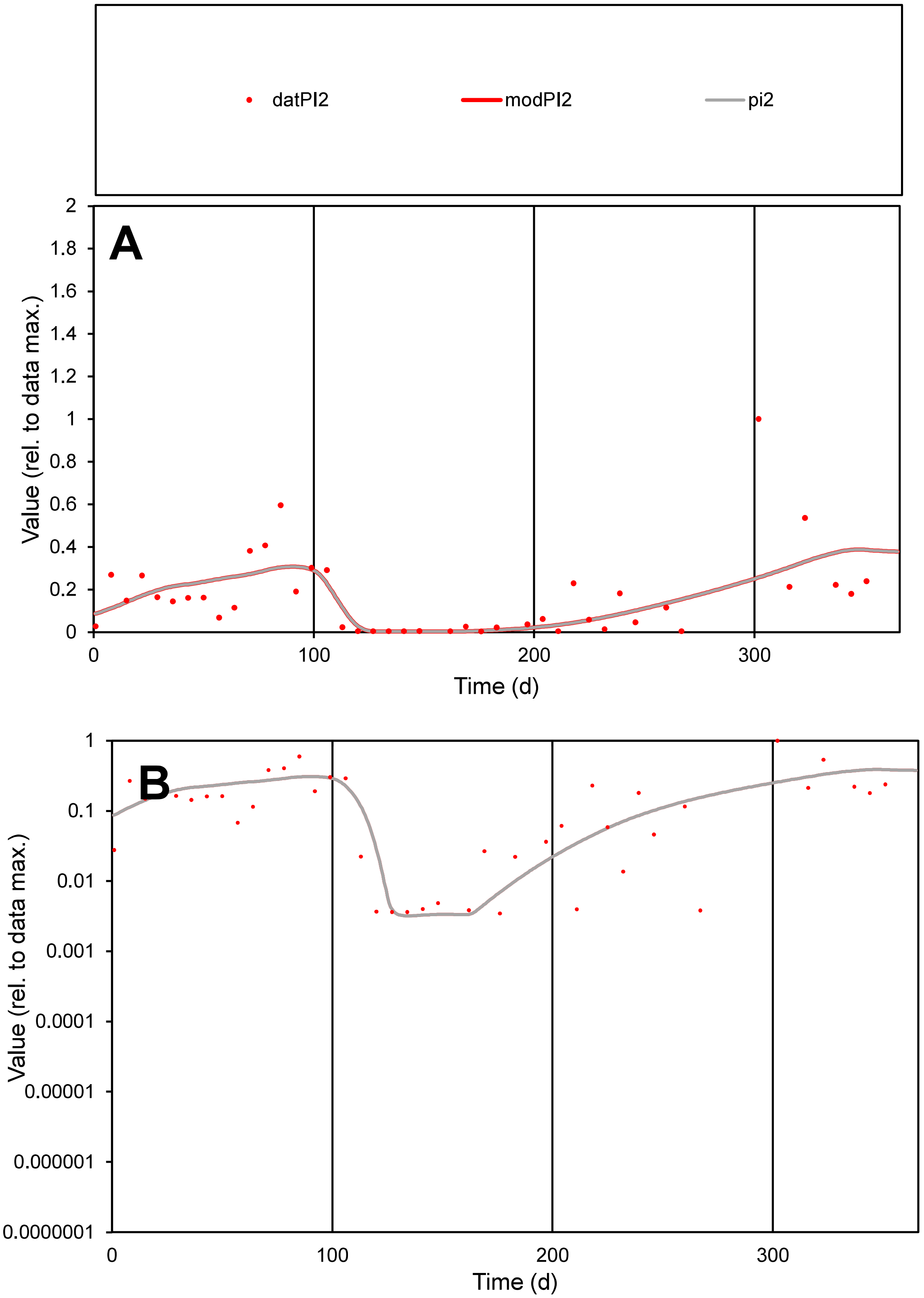

C1

Variables
PI2 Provide jtd or Dst
CalibOnly?
2 1 to reproduce of
oNormalize?
2 1=no, 2=yes

ofxn calc*
0.93312
ofxn mod
0.9448

sRun
RunA1
zout
1

Ave
Data
0.00137
Model
0.0013

data/vMax
0.00829

ofxng
319.391

Min d/m
1.08727

ave/min d
47.538
ave/min m
48.9618

330_Calib_S_A

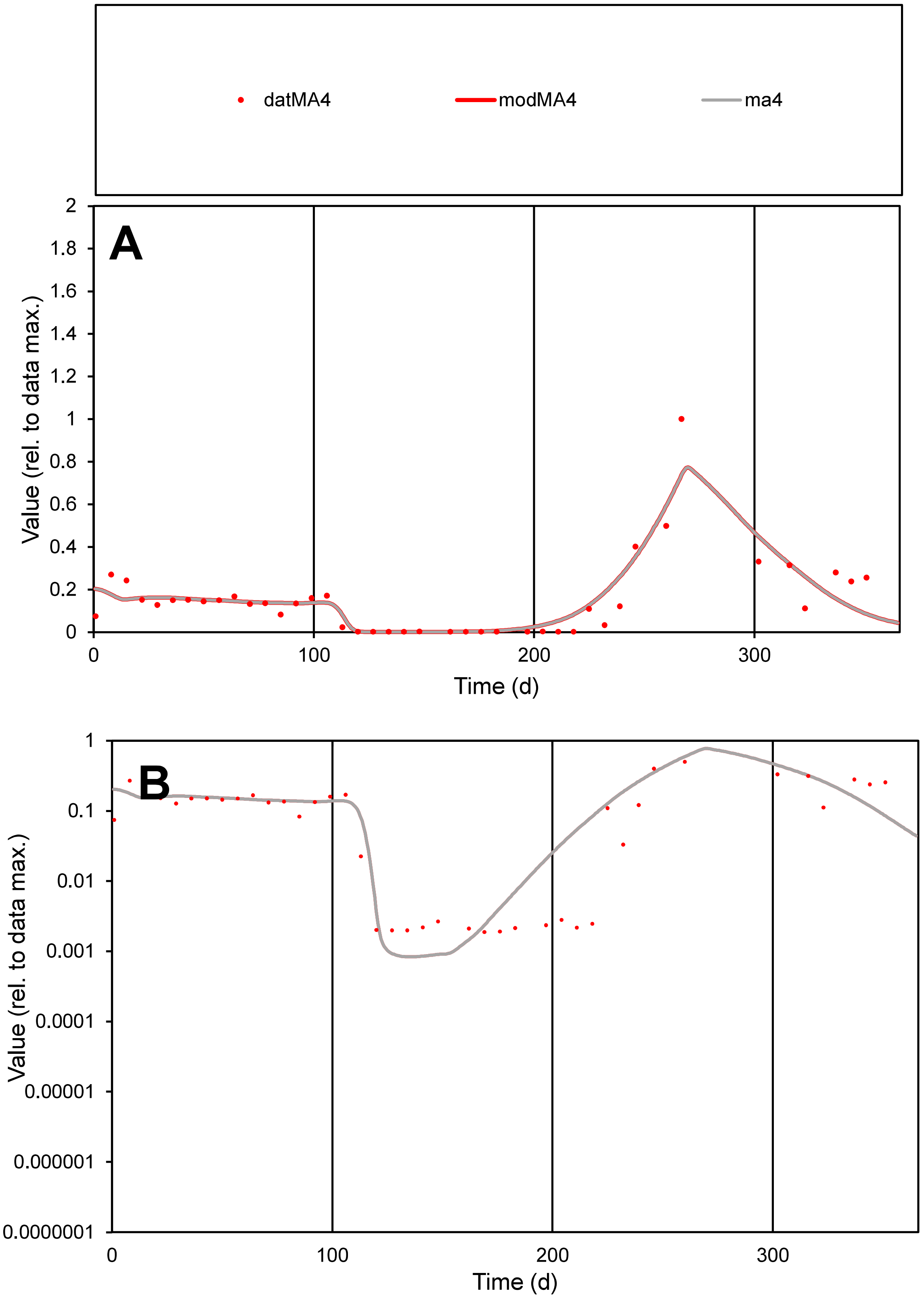

C1

Variables

MA4 Provide jtd or Dst

CalibOnly?

2 1 to reproduce of

oNormalize?

2 1=no, 2=yes

ofxn calc*

0.52891

ofxn mod

0.5614

sRun

RunA1

zout

1

Ave

Data

0.00152

Model

0.00156

data/vMax

0.0103

ofxng

319.391

Min d/m

2.24954

ave/min d

78.2604

ave/min m

180.632

330_Calib_S_A

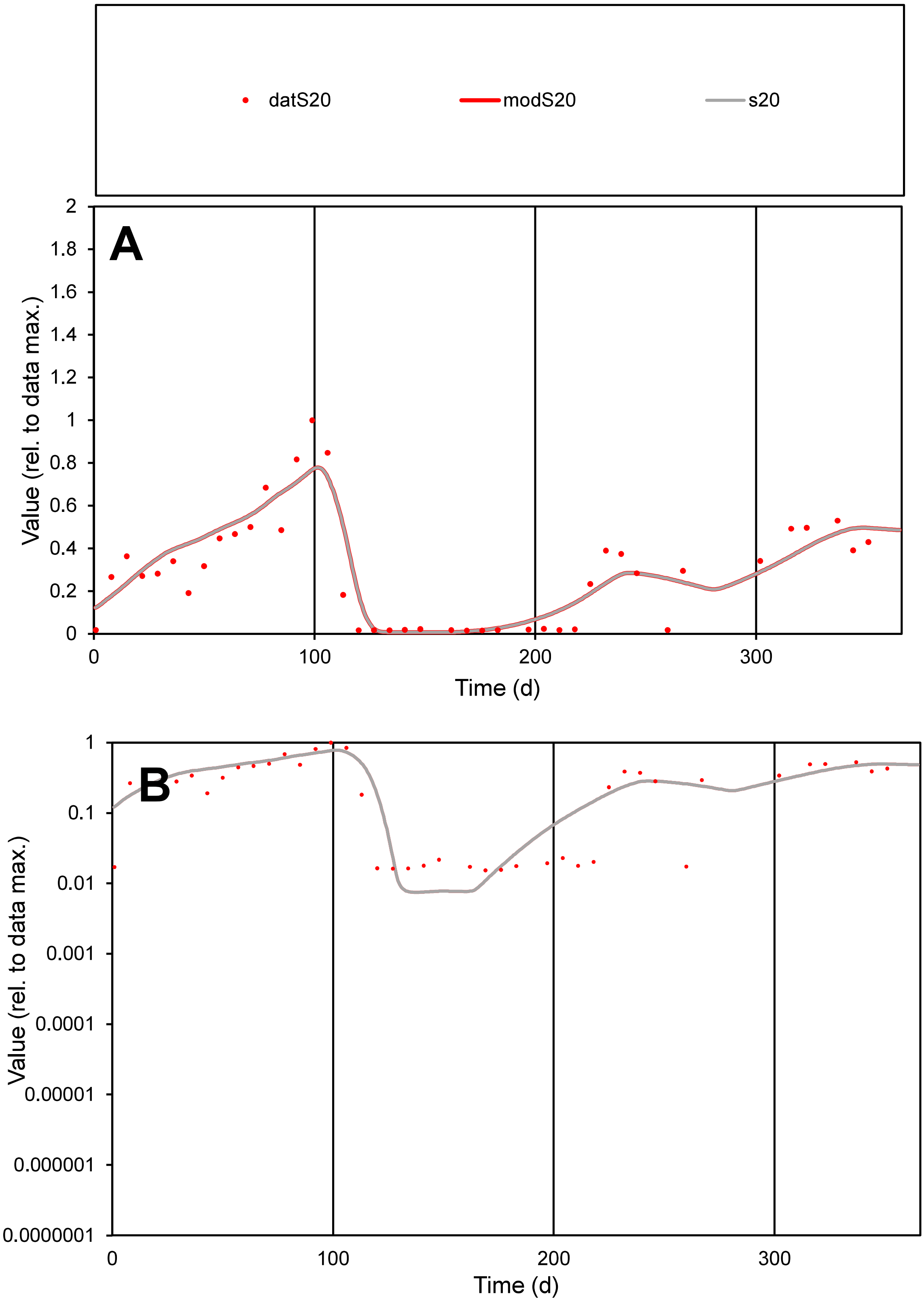

C1

Variables
S20 Provide jtd or Dst
CalibOnly?
2 1 to reproduce of
oNormalize?
2 1=no, 2=yes

ofxn calc*
0.3653
ofxn mod
0.40251

sRun
RunA1
zout
1

Ave
Data
0.00145
Model
0.00153

data/vMax
0.0051

ofxng
319.391

Min d/m
2.04186

ave/min d
18.5248
ave/min m
39.8249

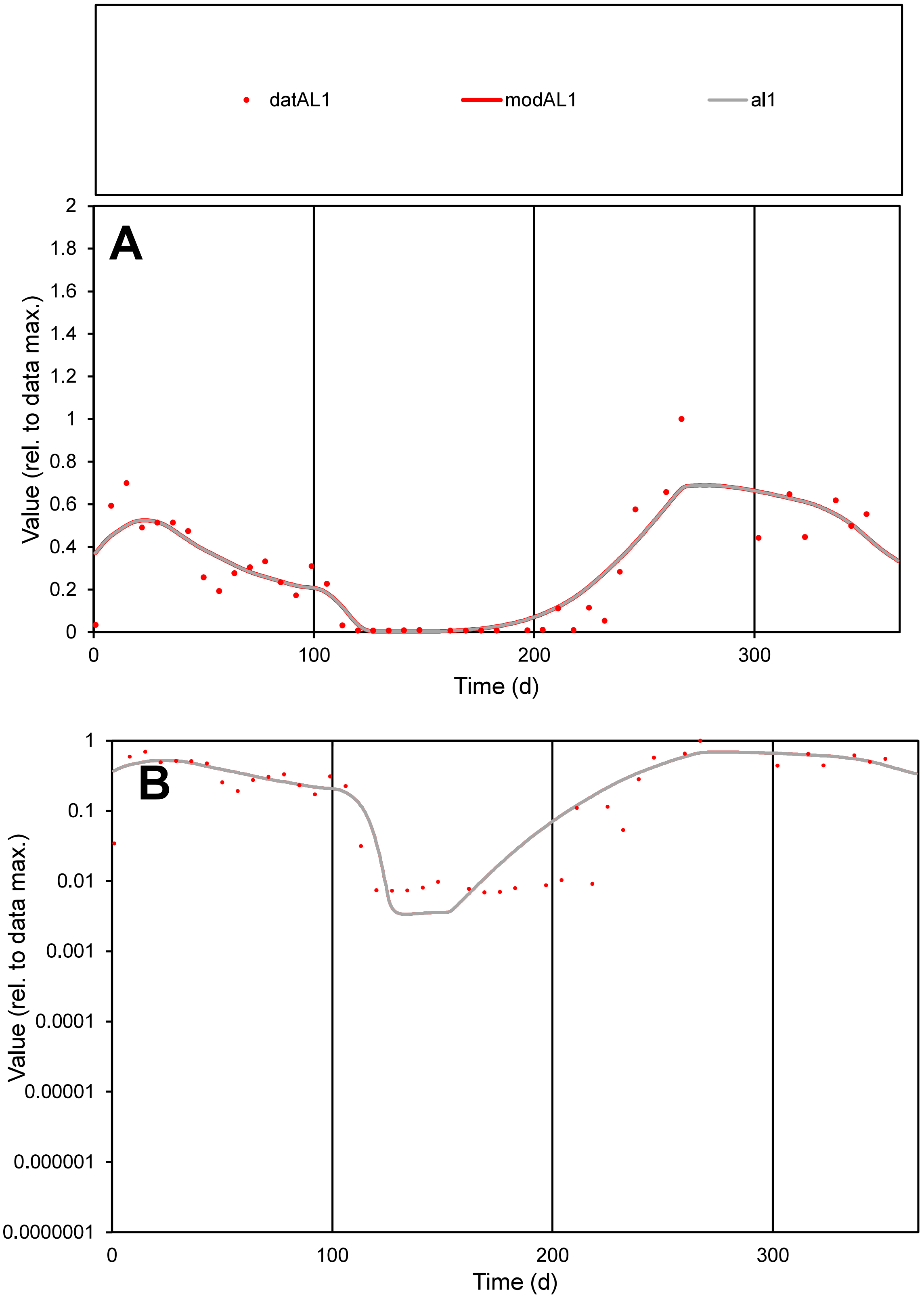

C1

Variables
AL1 Provide jtd or Dst
CalibOnly?
2 1 to reproduce of
oNormalize?
2 1=no, 2=yes

ofxn calc*
0.41089
ofxn mod
0.43634

sRun
RunA1
zout
1

Ave
Data
0.00178
Model
0.00185

data/vMax
0.00636

ofxng
319.391

Min d/m
2.05393

ave/min d
40.2678
ave/min m
86.1117

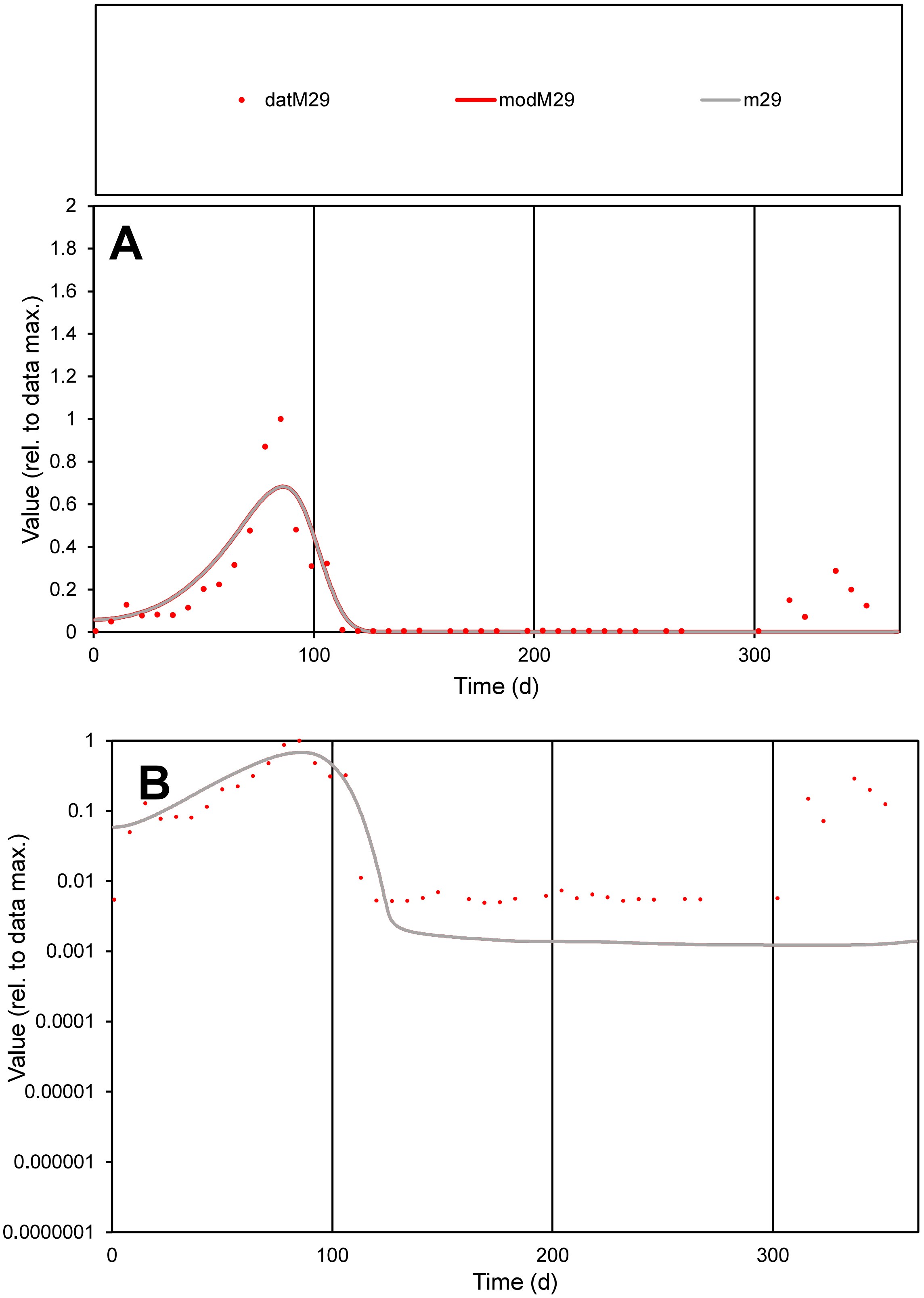

C1

Variables
M29 Provide jtd or Dst
CalibOnly?
2 1 to reproduce of
oNormalize?
2 1=no, 2=yes

ofxn calc*
0.75308
ofxn mod
0.7822

sRun
RunA1
zout
1

Ave
Data
0.0015
Model
0.00138

data/vMax
0.01109

ofxng
319.391

Min d/m
4.03461

ave/min d
27.4578
ave/min m
101.826

330_Calib_S_A

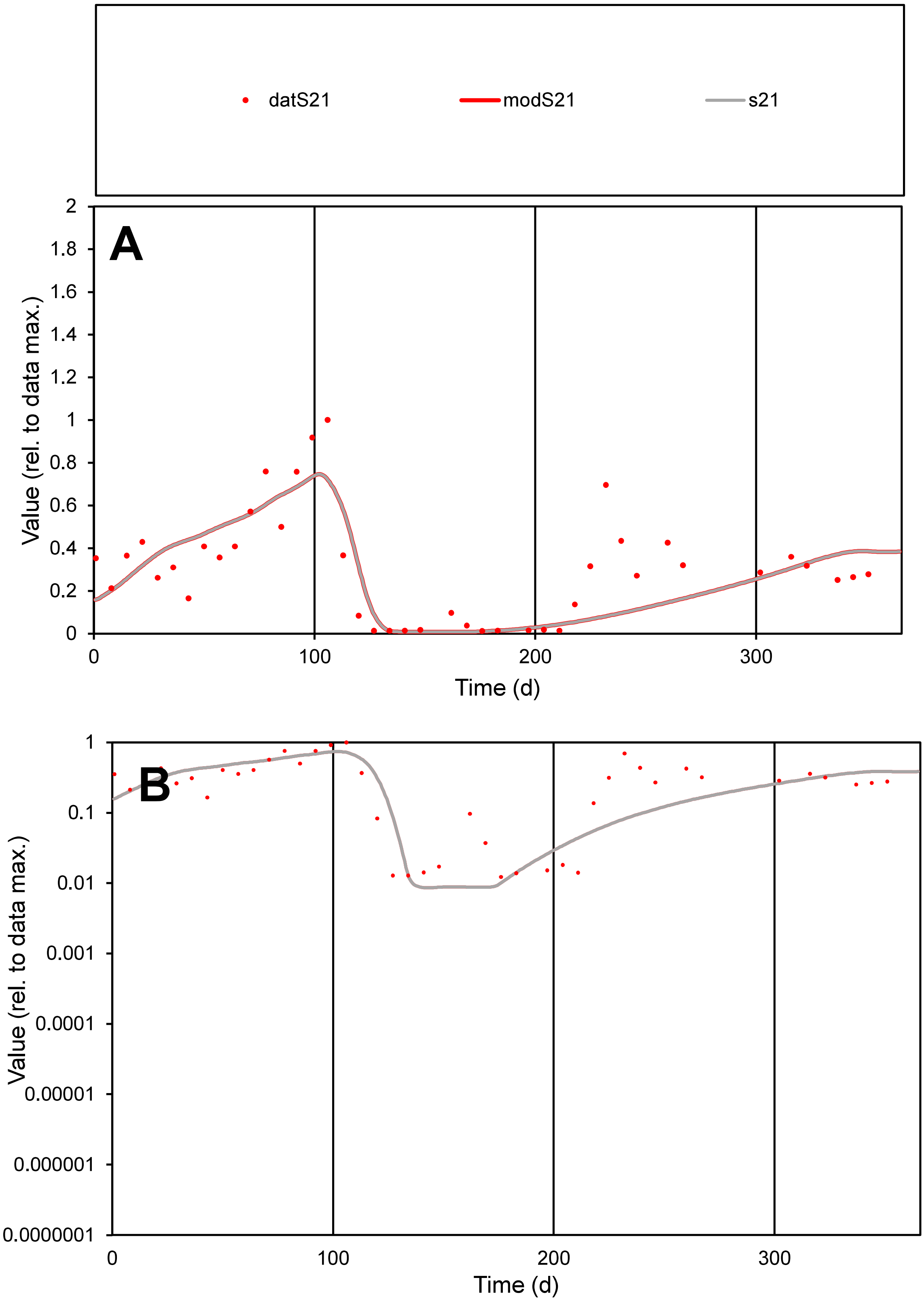

C1

Variables
S21 Provide jtd or Dst
CalibOnly?
2 1 to reproduce of
oNormalize?
2 1=no, 2=yes

ofxn calc*
0.54272
ofxn mod
0.56014

sRun
RunA1
zout
1

Ave
Data
0.00103
Model
0.00093

data/vMax
0.00338

ofxng
319.391

Min d/m
1.42695

ave/min d
24.8524
ave/min m
31.8087

330_Calib_S_A

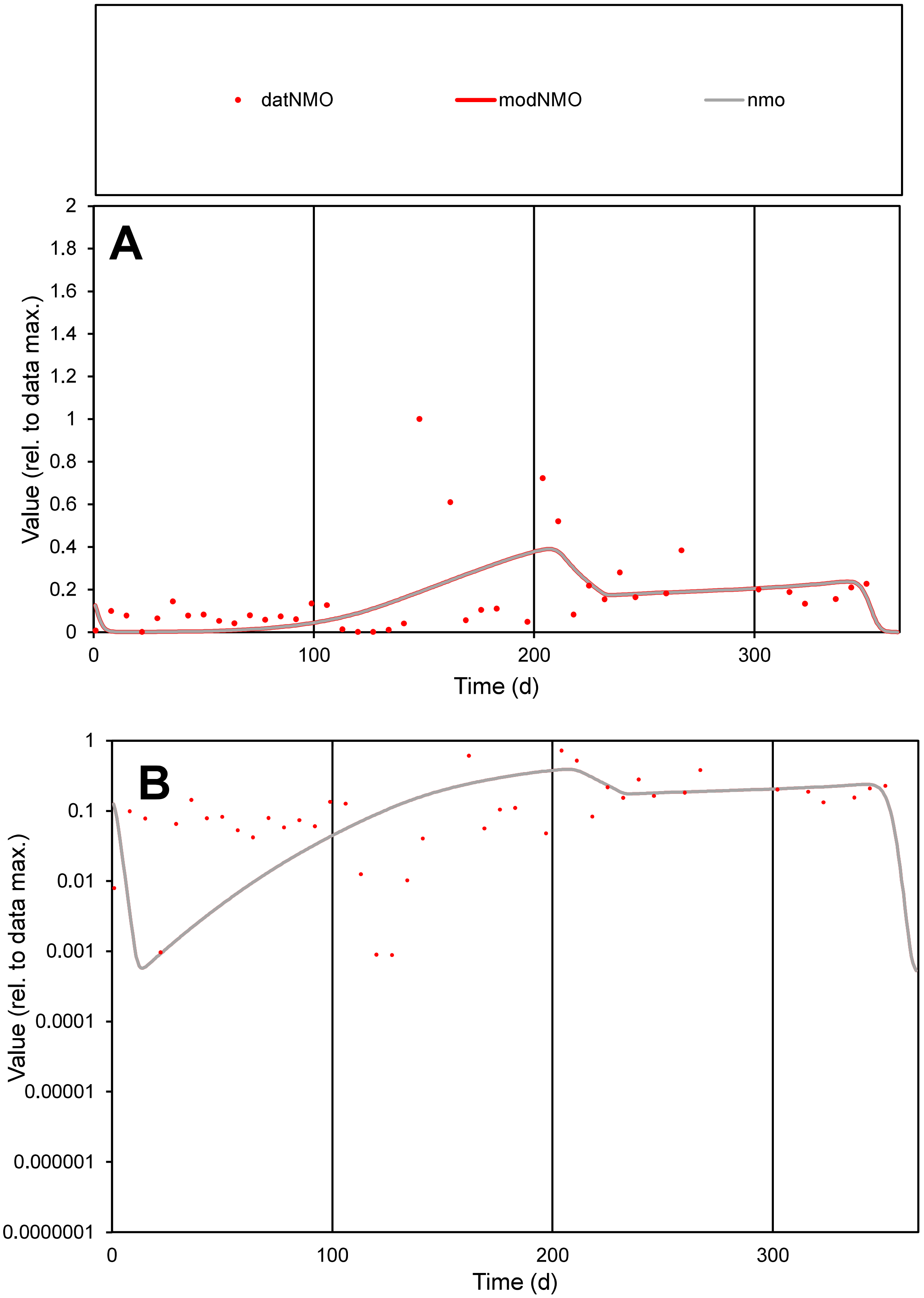

C1

Variables
NMO Provide jtd or Dst
CalibOnly?
2 1 to reproduce of
oNormalize?
2 1=no, 2=yes

ofxn calc*
1.09096
ofxn mod
1.10186

sRun
RunA1
zout
1

Ave
Data
0.00144
Model
0.00124

data/vMax
0.00866

ofxng
319.391

Min d/m
1.34233

ave/min d
188.404
ave/min m
216.943

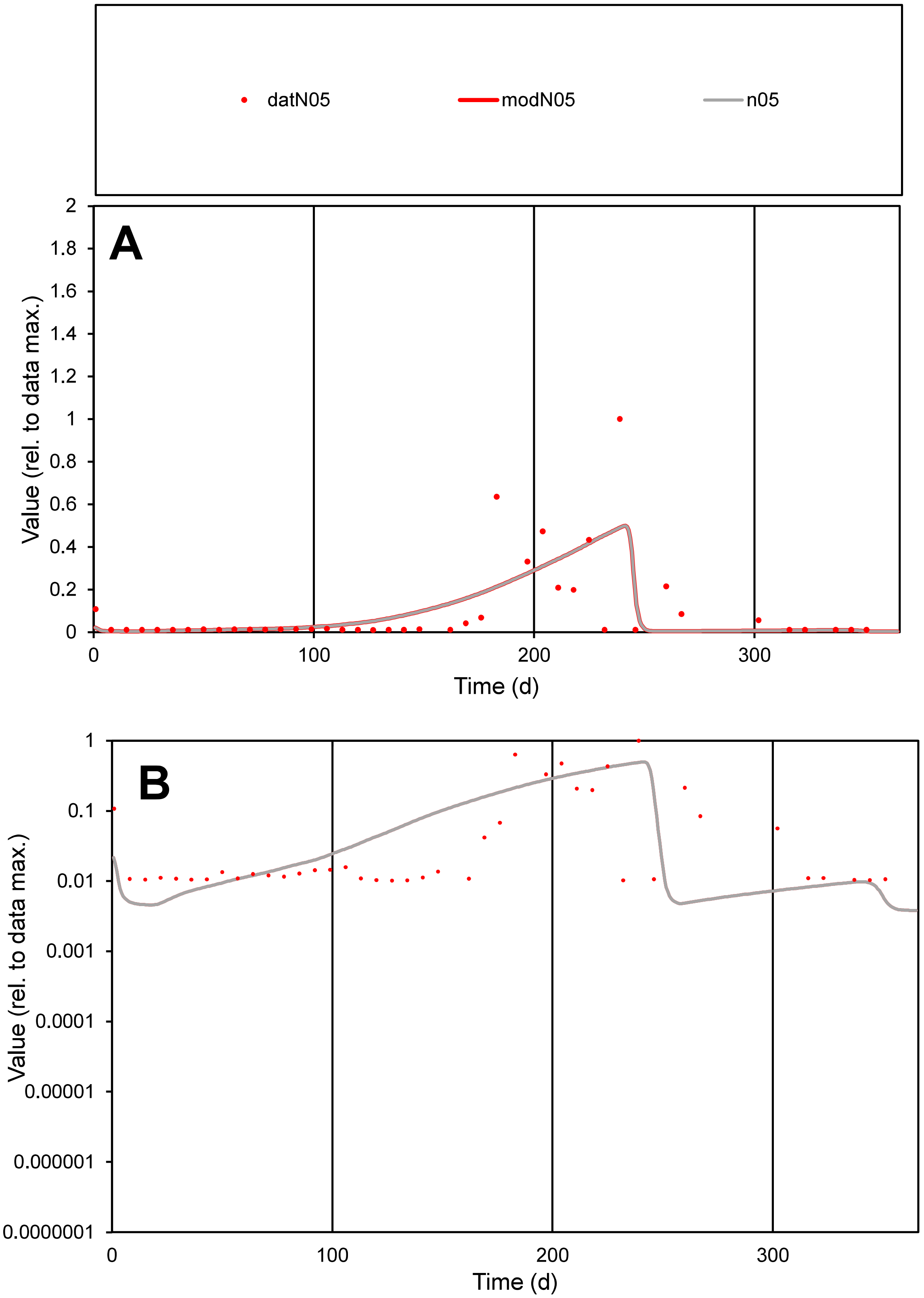

C1

Variables
N05 Provide jtd or Dst
CalibOnly?
2 1 to reproduce of
oNormalize?
2 1=no, 2=yes

ofxn calc*
1.40863
ofxn mod
1.4359

sRun
RunA1
zout
1

Ave
Data
0.00041
Model
0.0004

data/vMax
0.00415

ofxng
319.391

Min d/m
2.23835

ave/min d
9.78169
ave/min m
21.3893

330_Calib_S_A

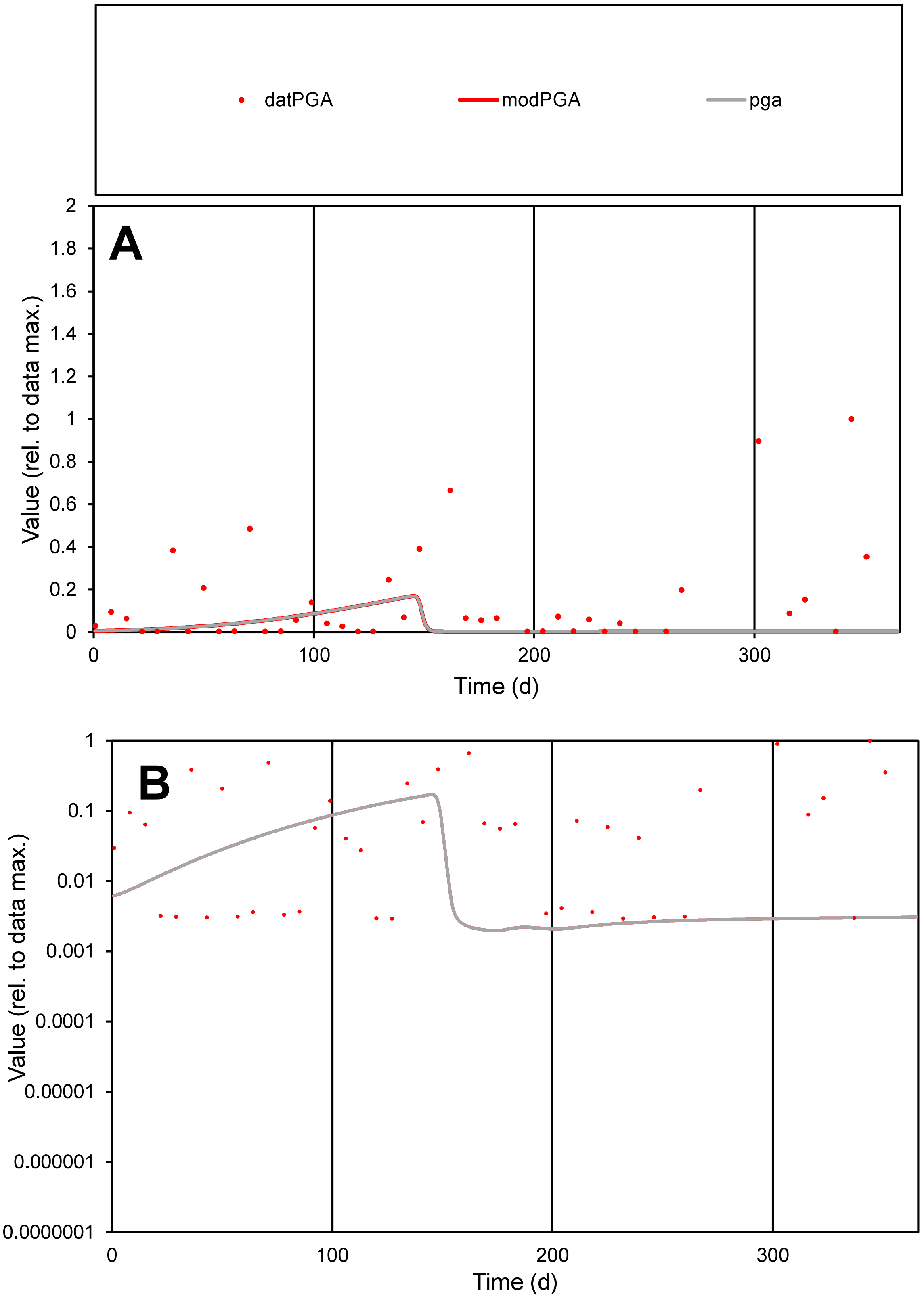

C1

Variables
PGA Provide jtd or Dst
CalibOnly?
2 1 to reproduce of
oNormalize?
2 1=no, 2=yes

ofxn calc*
1.85553
ofxn mod
1.85922

sRun
RunA1
zout
1

Ave
Data
0.00088
Model
0.00022

data/vMax
0.00619

ofxng
319.391

Min d/m
1.46272

ave/min d
48.9886
ave/min m
17.7917

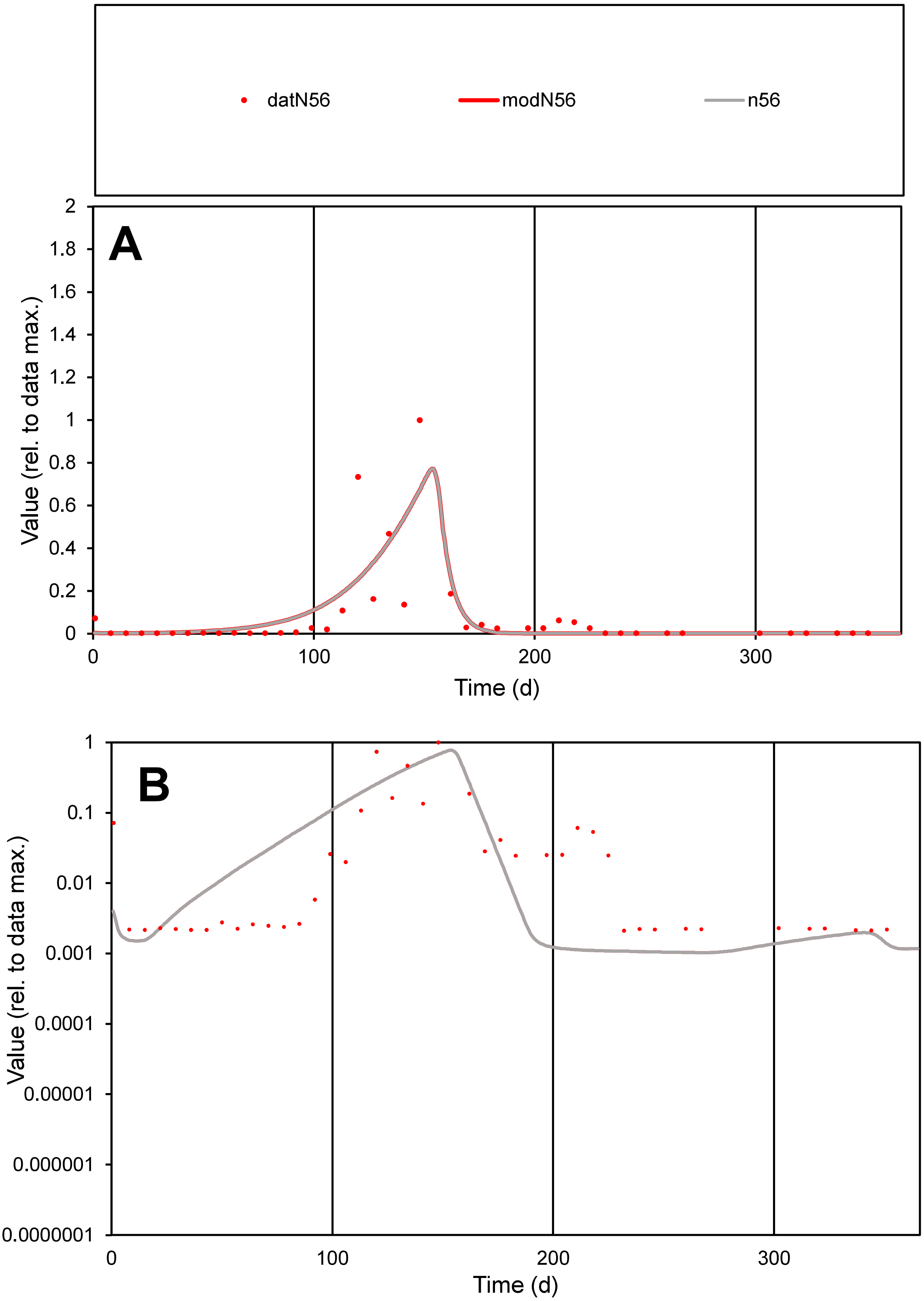

C1

Variables
N56 Provide jtd or Dst
CalibOnly?
2 1 to reproduce of
oNormalize?
2 1=no, 2=yes

ofxn calc*
1.4915
ofxn mod
1.51808

sRun
RunA1
zout
1

Ave
Data
0.00134
Model
0.00137

data/vMax
0.01727

ofxng
319.391

Min d/m
2.04724

ave/min d
36.7313
ave/min m
76.808

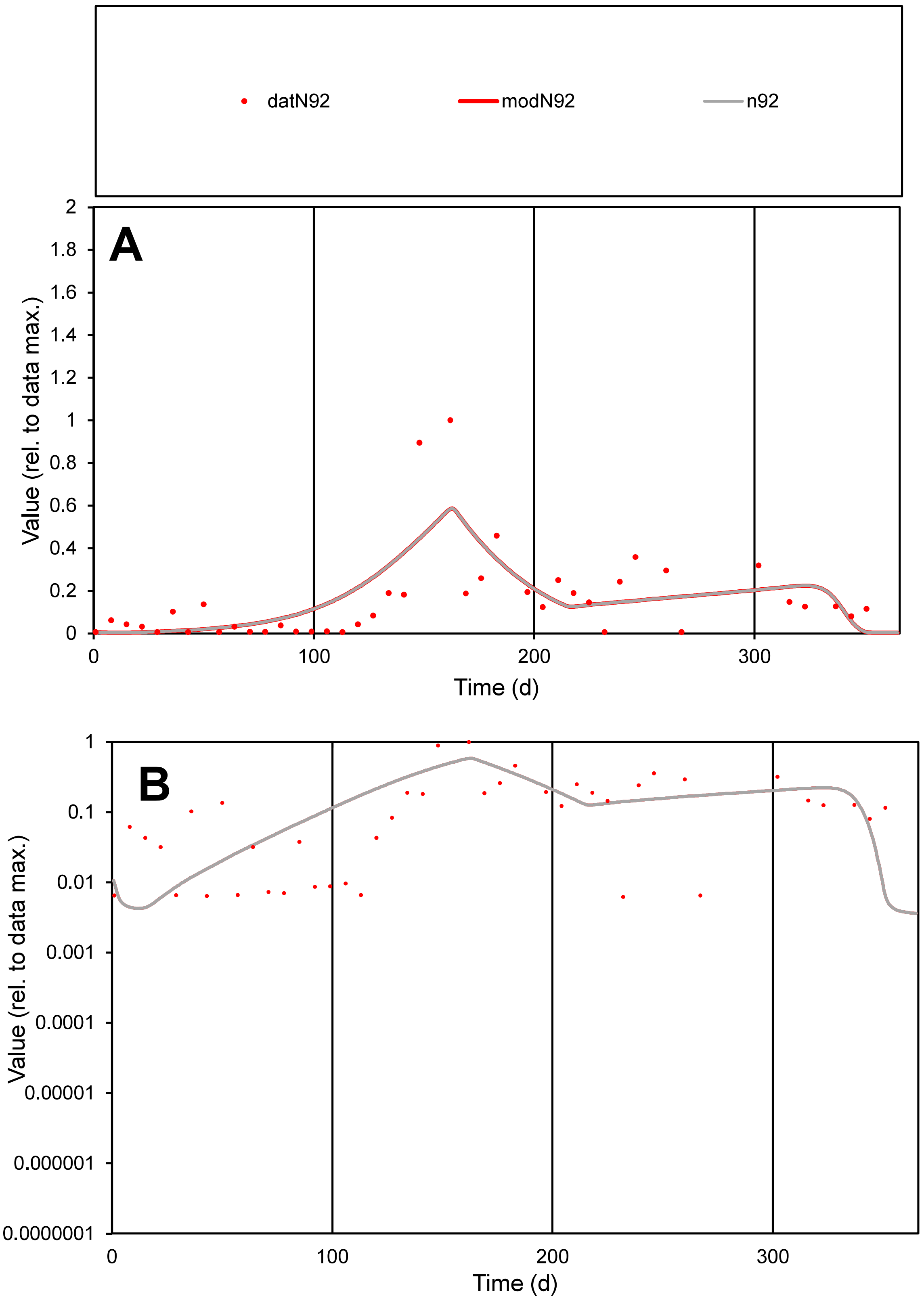

C1

Variables
N92 Provide jtd or Dst
CalibOnly?
2 1 to reproduce of
oNormalize?
2 1=no, 2=yes

ofxn calc*
0.92932
ofxn mod
0.9509

sRun
RunA1
zout
1

Ave
Data
0.00101
Model
0.00105

data/vMax
0.0065

ofxng
319.391

Min d/m
1.43094

ave/min d
25.014
ave/min m
36.926

330_Calib_S_A

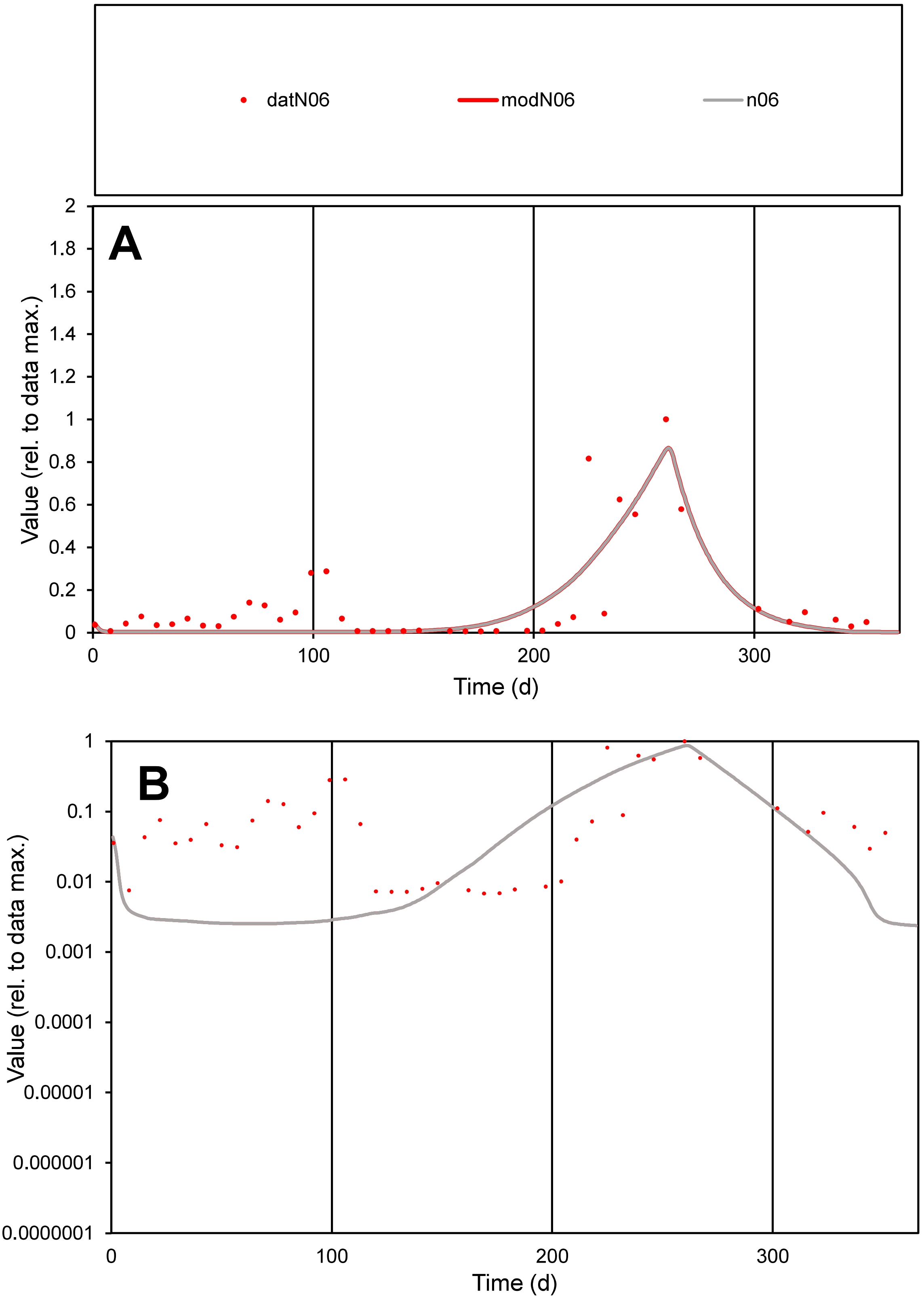

C1

Variables
N06 Provide jtd or Dst
CalibOnly?
2 1 to reproduce of
oNormalize?
2 1=no, 2=yes

ofxn calc*
0.9384
ofxn mod
0.96234

sRun
RunA1
zout
1

Ave
Data
0.00132
Model
0.00104

data/vMax
0.00959

ofxng
319.391

Min d/m
2.67939

ave/min d
20.1487
ave/min m
42.6206

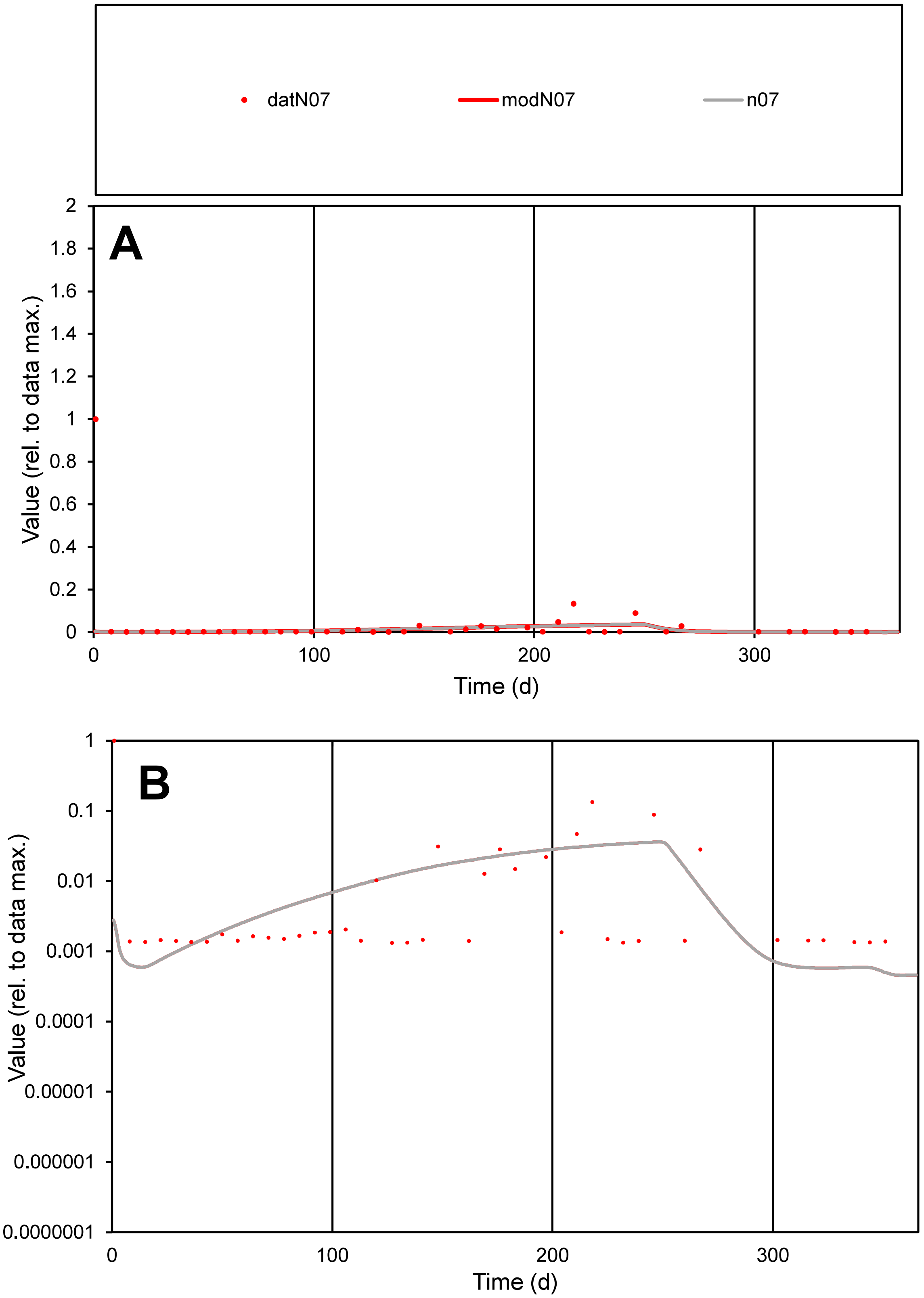

C1

Variables
N07 Provide jtd or Dst
CalibOnly?
2 1 to reproduce of
oNormalize?
2 1=no, 2=yes

ofxn calc*
4.46271
ofxn mod
4.46691

sRun
RunA1
zout
1

Ave
Data
0.00048
Model
0.00016

data/vMax
0.01367

ofxng
319.391

Min d/m
2.70088

ave/min d
26.3756
ave/min m
24.2147

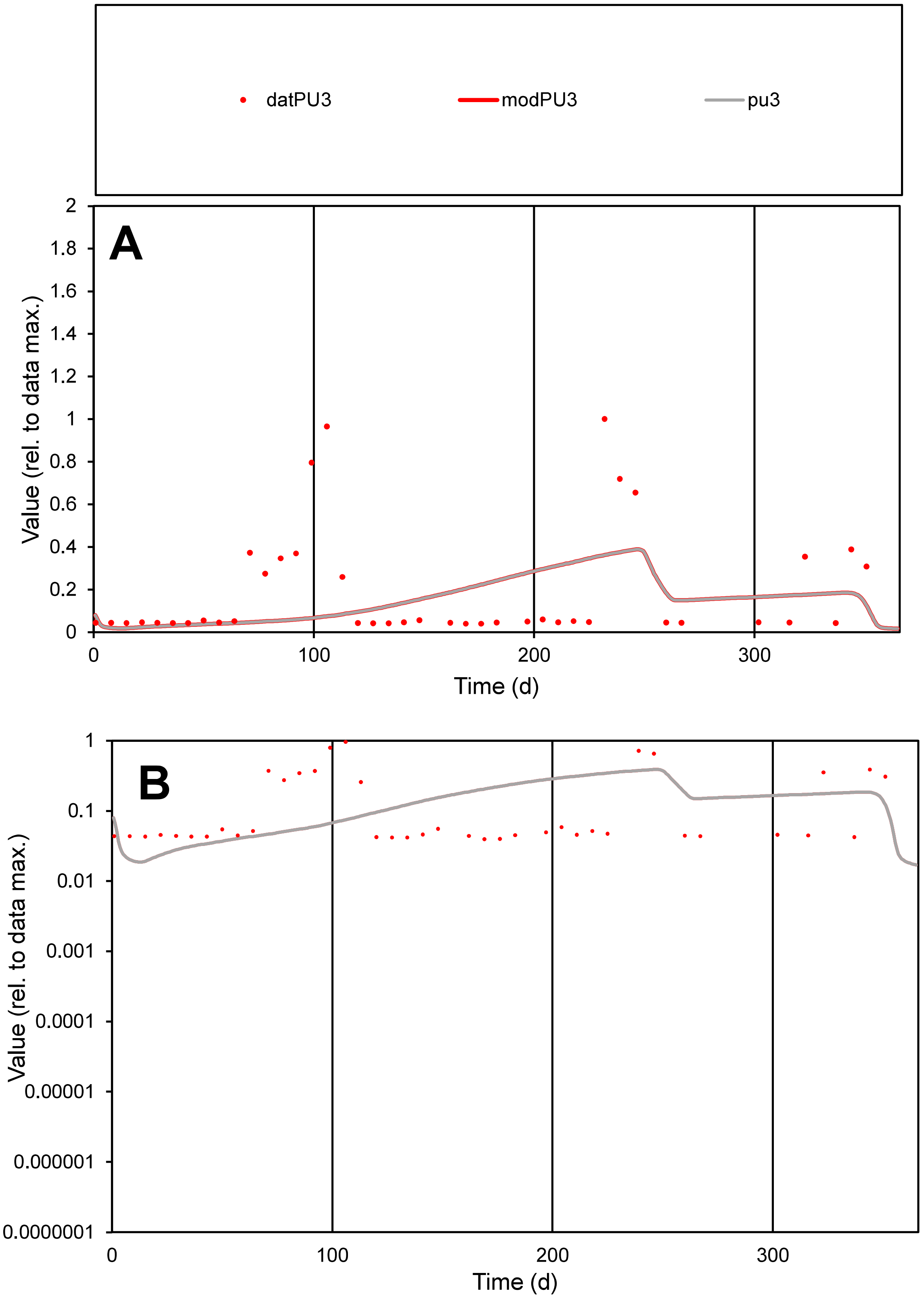

C1

Variables
PU3 Provide jtd or Dst
CalibOnly?
2 1 to reproduce of
oNormalize?
2 1=no, 2=yes

ofxn calc*
1.37131
ofxn mod
1.38187

sRun
RunA1
zout
1

Ave
Data
0.00074
Model
0.00057

data/vMax
0.00383

ofxng
319.391

Min d/m
2.03169

ave/min d
4.90243
ave/min m
7.68228

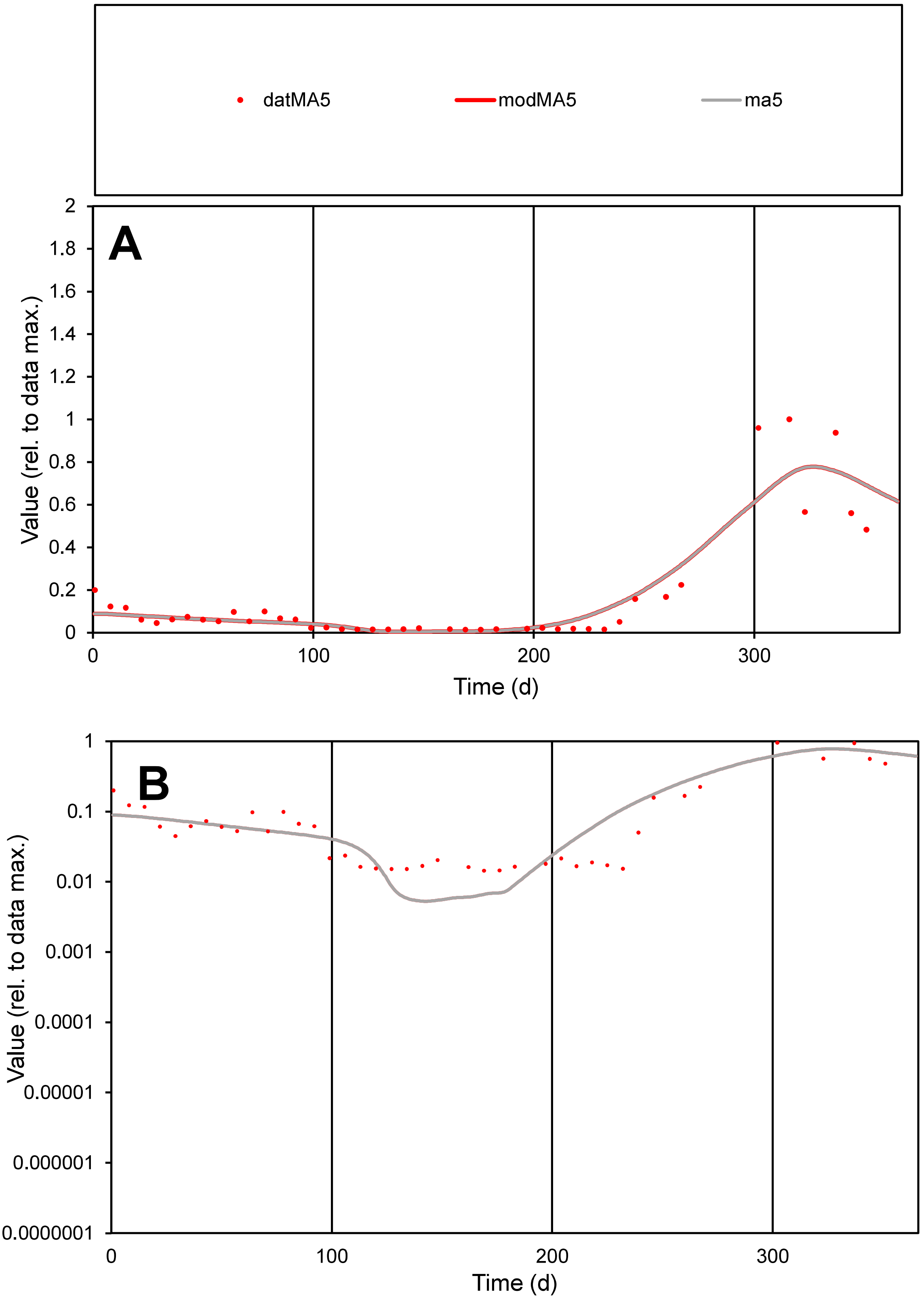

C1

Variables
MA5 Provide jtd or Dst
CalibOnly?
2 1 to reproduce of
oNormalize?
2 1=no, 2=yes

ofxn calc*
0.60347
ofxn mod
0.62745

sRun
RunA1
zout
1

Ave
Data
0.00103
Model
0.00104

data/vMax
0.00655

ofxng
319.391

Min d/m
2.72977

ave/min d
10.8851
ave/min m
30.131

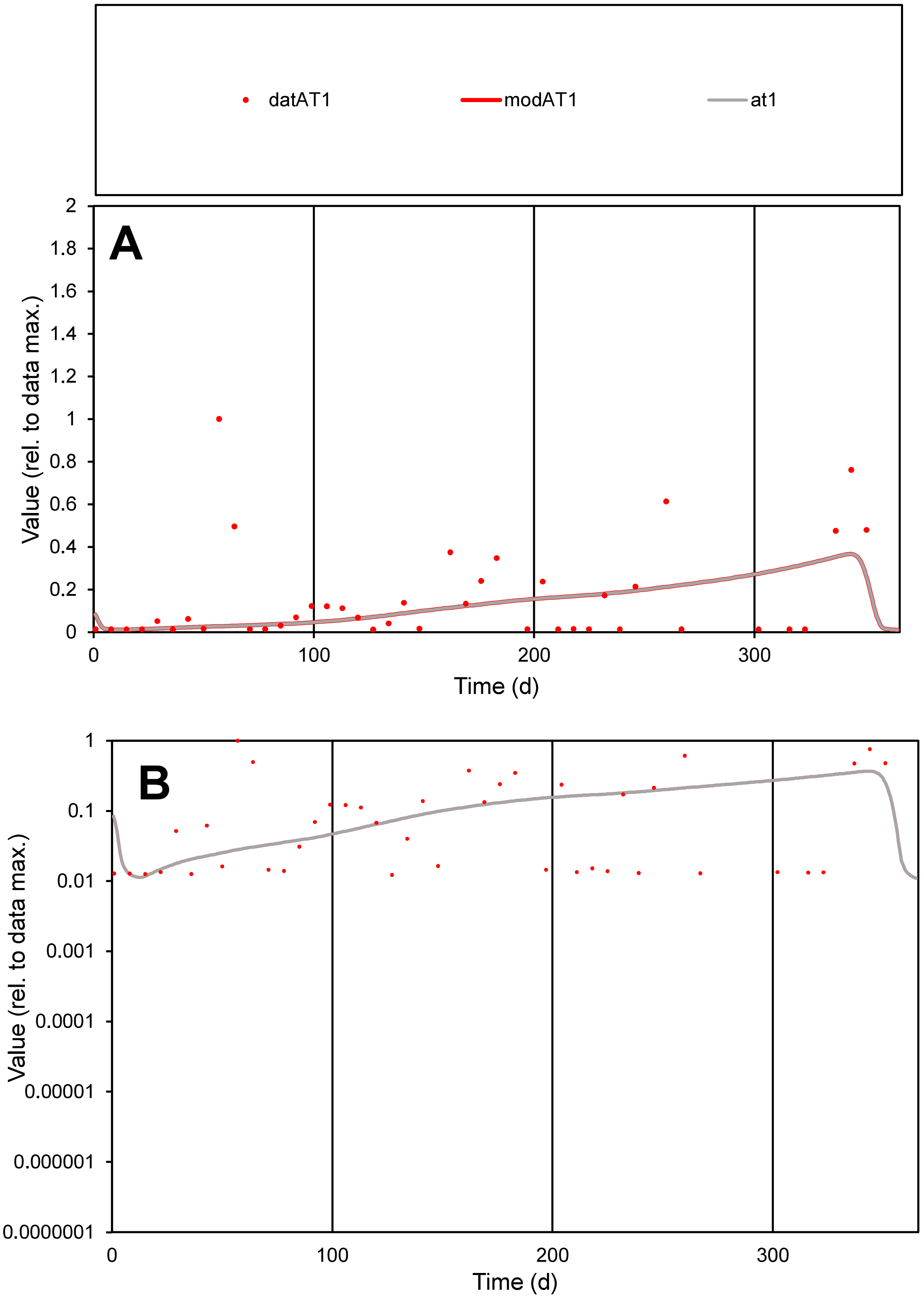

C1

Variables
AT1 Provide jtd or Dst
CalibOnly?
2 1 to reproduce of
oNormalize?
2 1=no, 2=yes

ofxn calc*
1.42469
ofxn mod
1.43262

sRun
RunA1
zout
1

Ave
Data
0.00065
Model
0.00051

data/vMax
0.00414

ofxng
319.391

Min d/m
1.03195

ave/min d
12.8605
ave/min m
10.3008

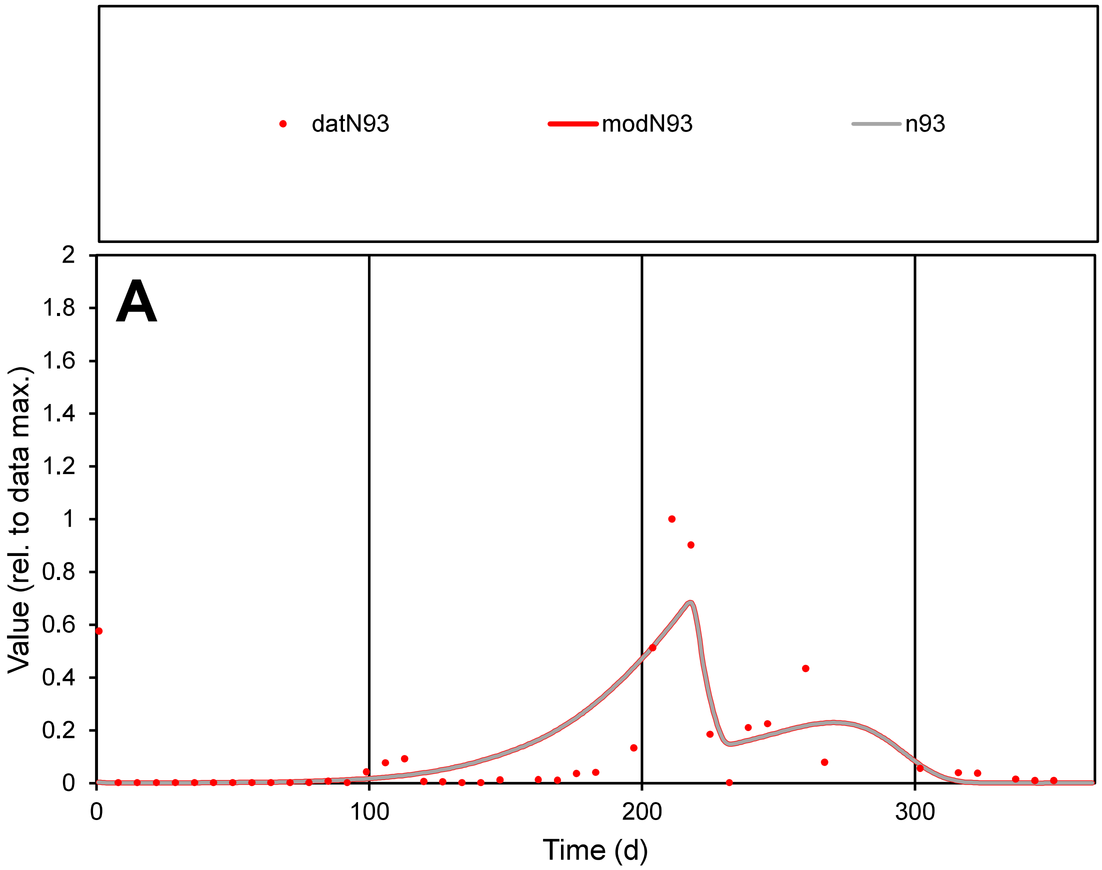

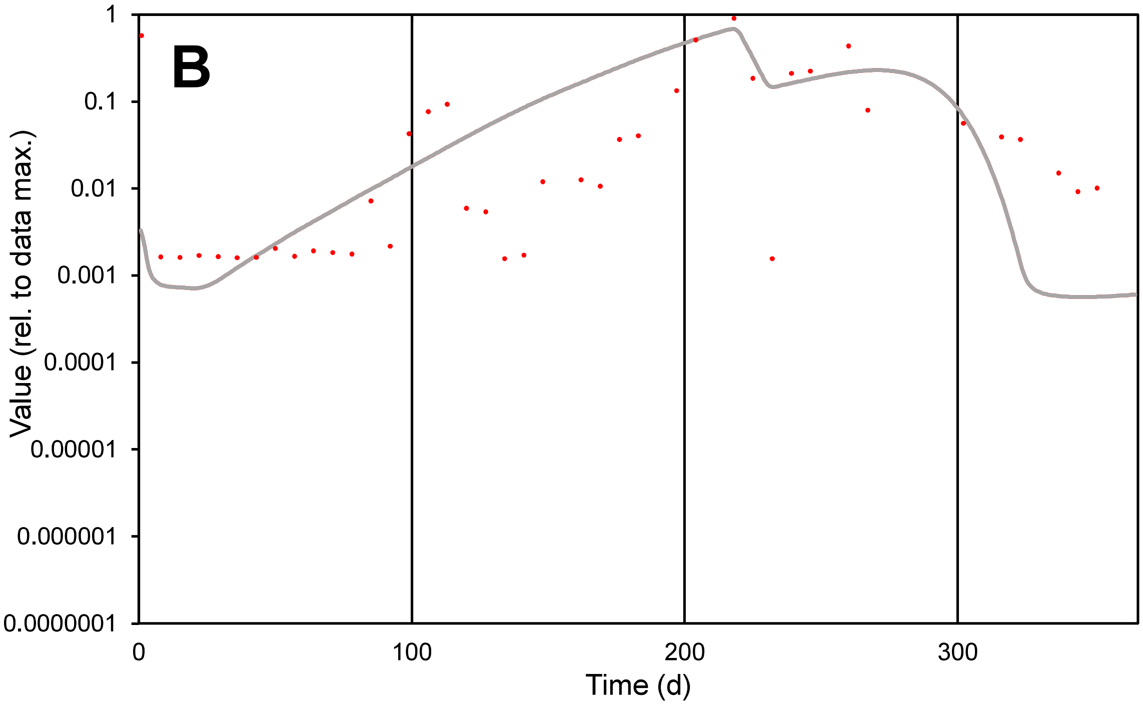

C1

Variables
N93 Provide jtd or Dst
CalibOnly?
2 1 to reproduce of
oNormalize?
2 1=no, 2=yes

ofxn calc*
1.29562
ofxn mod
1.33196

sRun
RunA1
zout
1

Ave
Data
0.00135
Model
0.0014

data/vMax
0.01183

ofxng
319.391

Min d/m
2.75713

ave/min d
73.1509
ave/min m
208.526

330_Calib_S_A

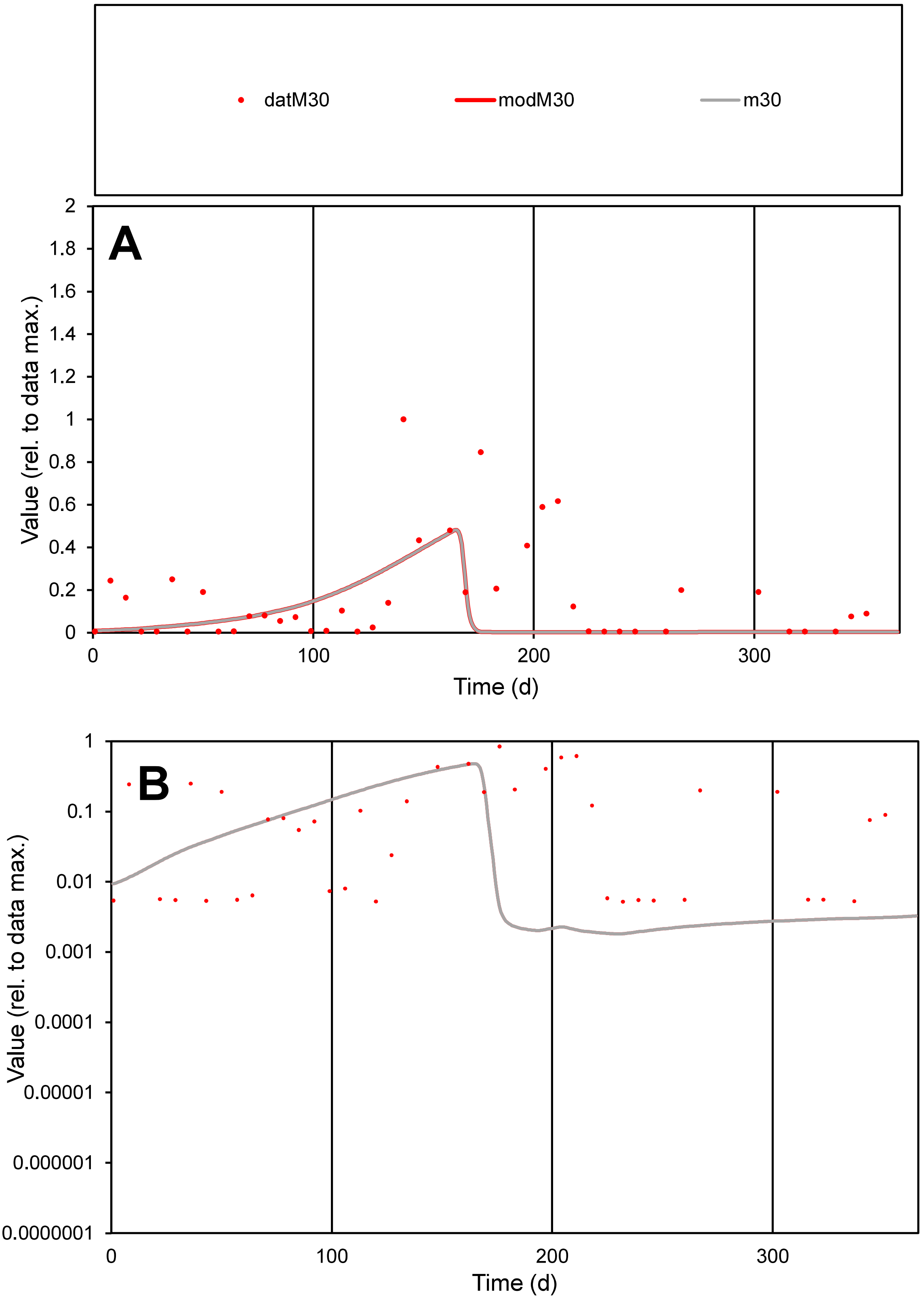

C1

Variables

M30 Provide jtd or Dst

CalibOnly?

2 1 to reproduce of

oNormalize?

2 1=no, 2=yes

ofxn calc*

1.47831

ofxn mod

1.4851

sRun

RunA1

zout

1

Ave

Data

0.00139

Model

0.0007

data/vMax

0.0084

ofxng

319.391

Min d/m

2.82783

ave/min d

31.7965

ave/min m

45.4326

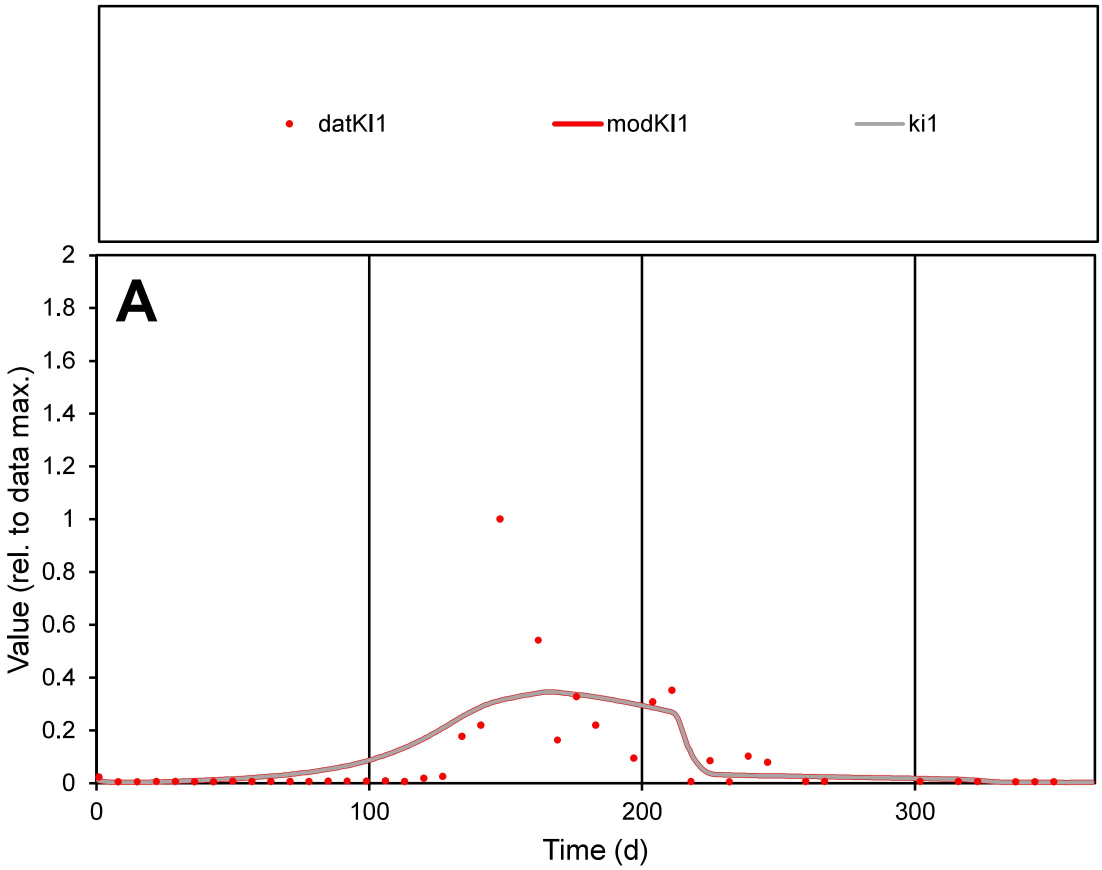

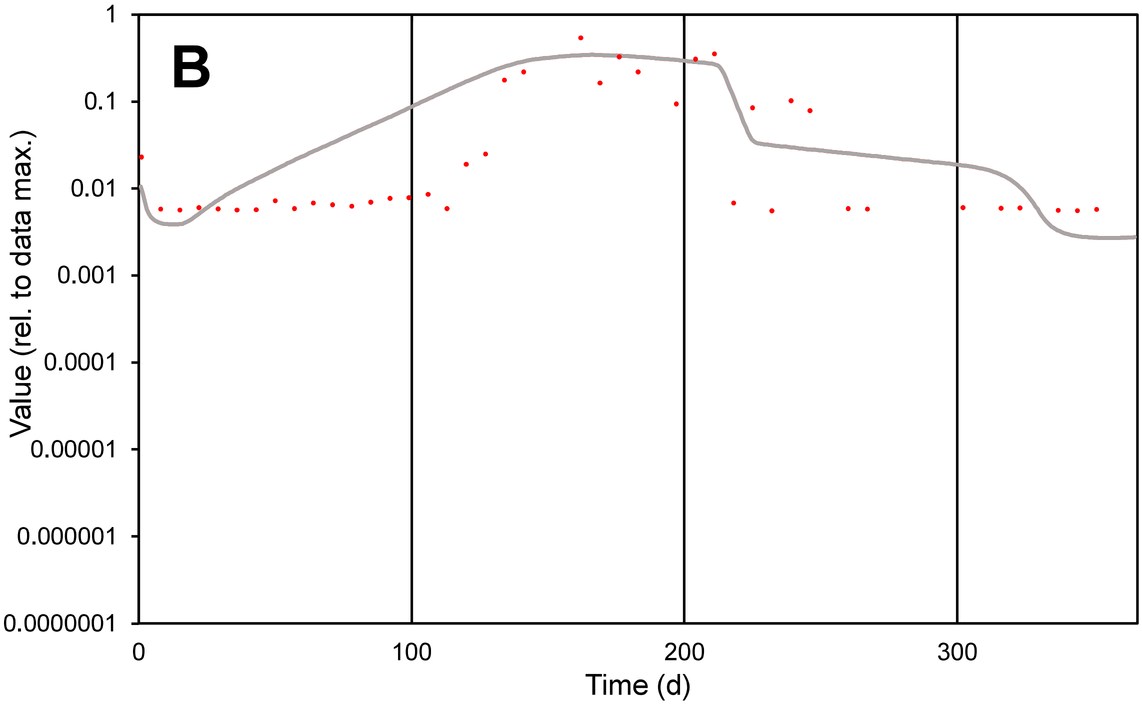

C1

Variables
KI1 Provide jtd or Dst
CalibOnly?
2 1 to reproduce of
oNormalize?
2 1=no, 2=yes

ofxn calc*
1.41411
ofxn mod
1.43285

sRun
RunA1
zout
1

Ave
Data
0.00083
Model
0.00093

data/vMax
0.00899

ofxng
319.391

Min d/m
2.04979

ave/min d
16.7421
ave/min m
38.4361

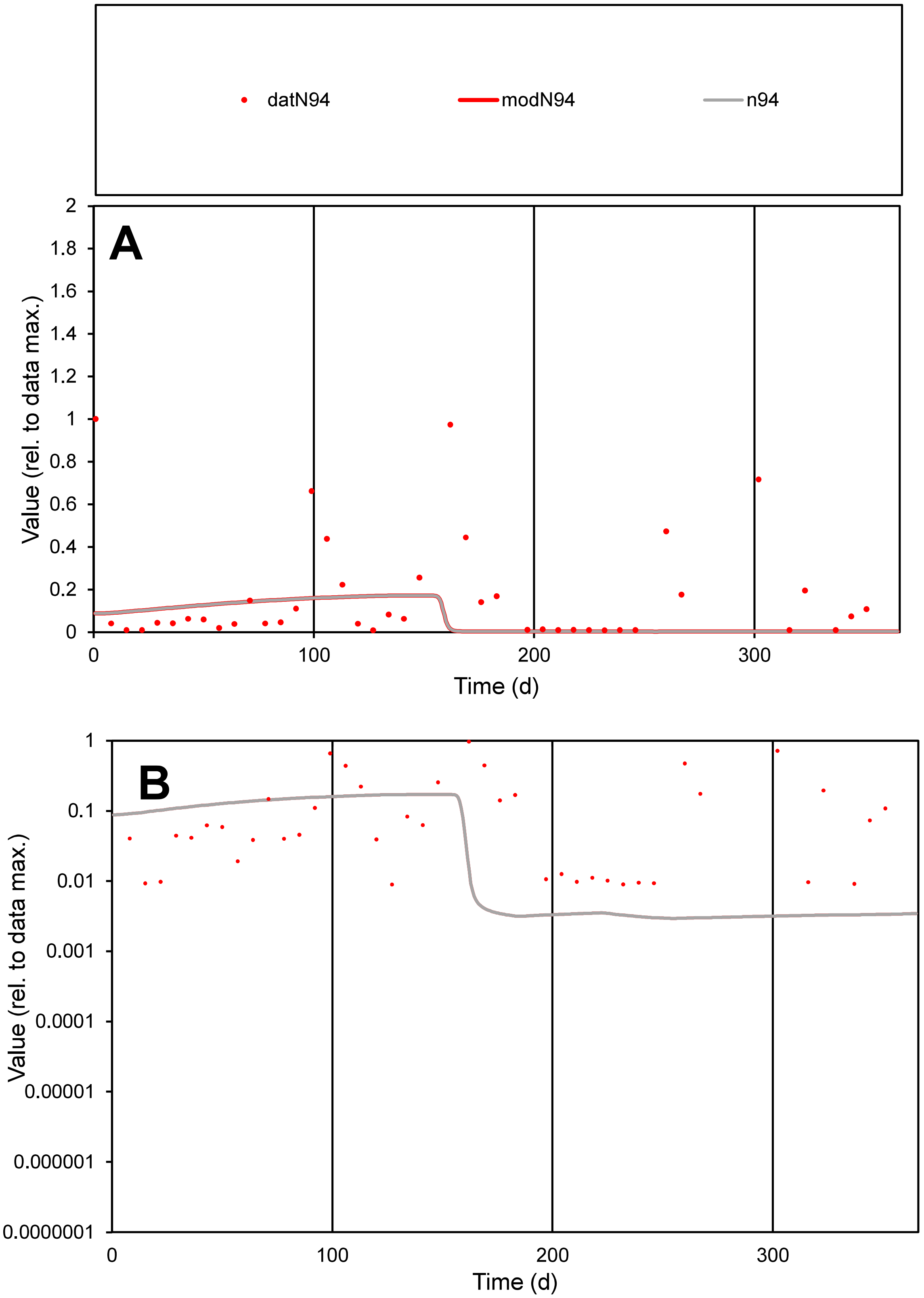

C1

Variables
N94 Provide jtd or Dst
CalibOnly?
2 1 to reproduce of
oNormalize?
2 1=no, 2=yes

ofxn calc*
1.67682
ofxn mod
1.68282

sRun
RunA1
zout
1

Ave
Data
0.0009
Model
0.0004

data/vMax
0.00536

ofxng
319.391

Min d/m
3.01759

ave/min d
18.6407
ave/min m
25.2755

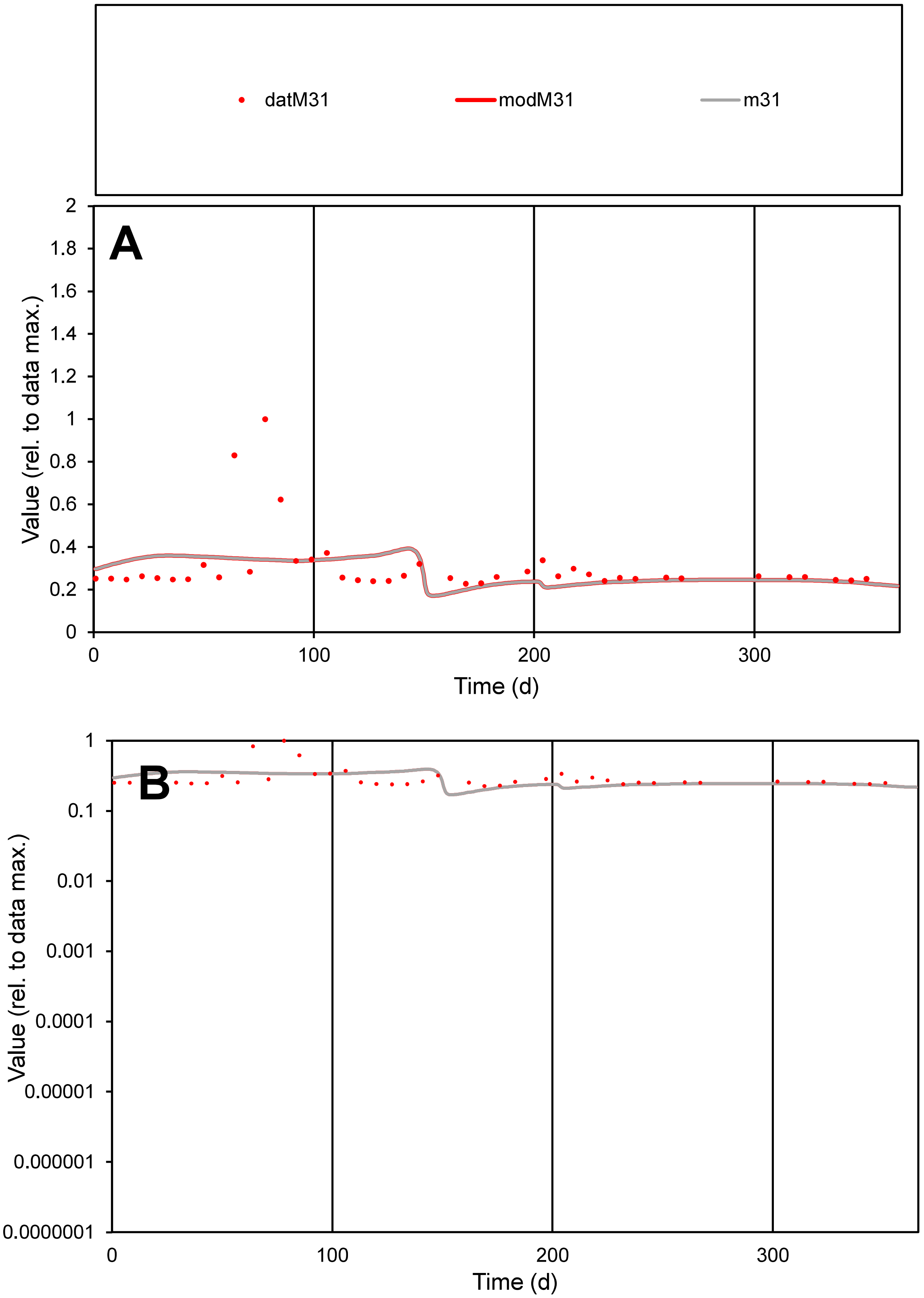

C1

Variables
M31 Provide jtd or Dst
CalibOnly?
2 1 to reproduce of
oNormalize?
2 1=no, 2=yes

ofxn calc*
0.48576
ofxn mod
0.49864

sRun
RunA1
zout
1

Ave
Data
0.00011
Model
0.0001

data/vMax
0.00036

ofxng
319.391

Min d/m
1.20962

ave/min d
1.35107
ave/min m
1.54944

330_Calib_S_A

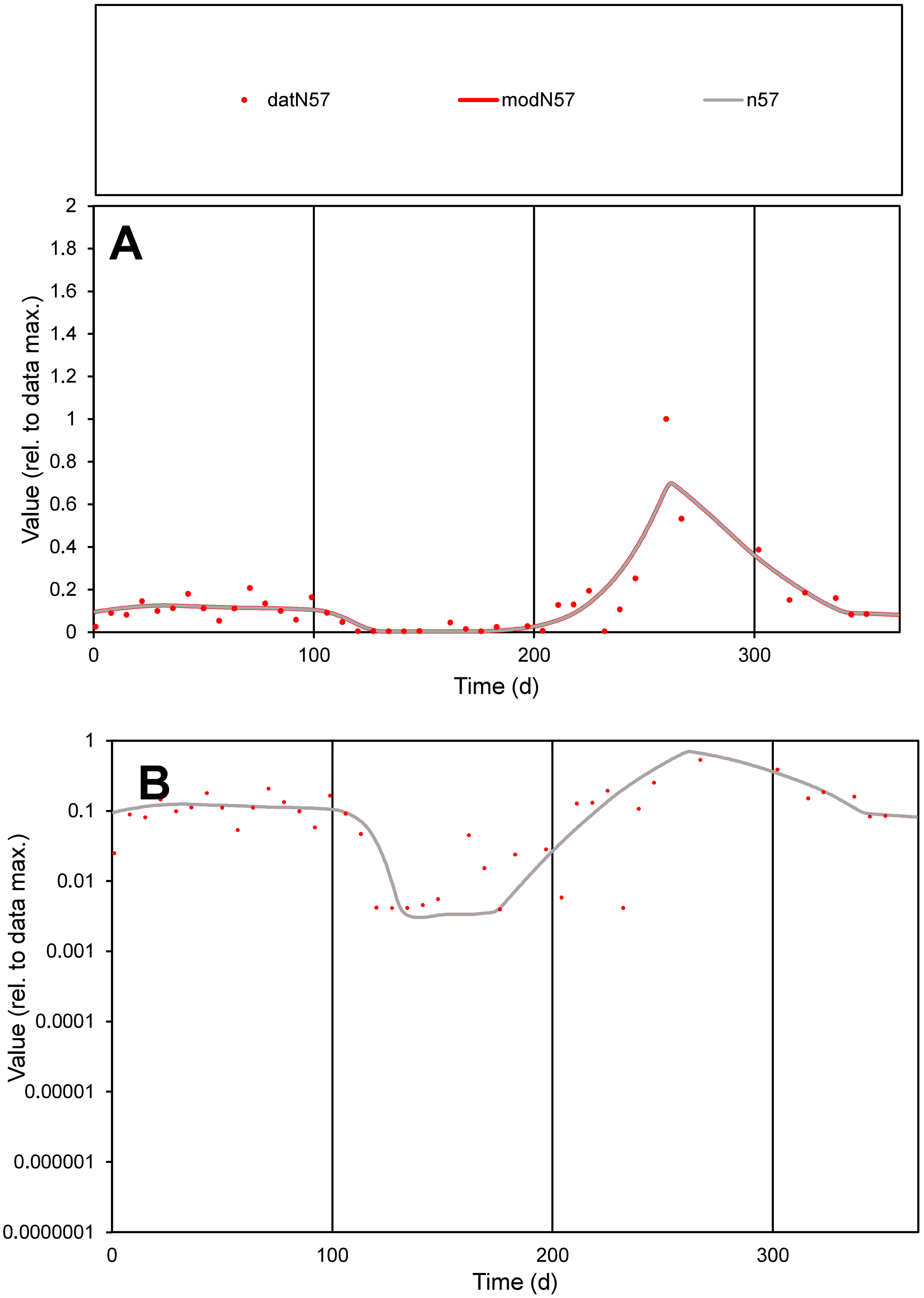

C1

Variables
N57 Provide jtd or Dst
CalibOnly?
2 1 to reproduce of
oNormalize?
2 1=no, 2=yes

ofxn calc*
0.61034
ofxn mod
0.63823

sRun
RunA1
zout
1

Ave
Data
0.00118
Model
0.00122

data/vMax
0.00925

ofxng
319.391

Min d/m
1.28887

ave/min d
32.0985
ave/min m
42.8756

330_Calib_S_A

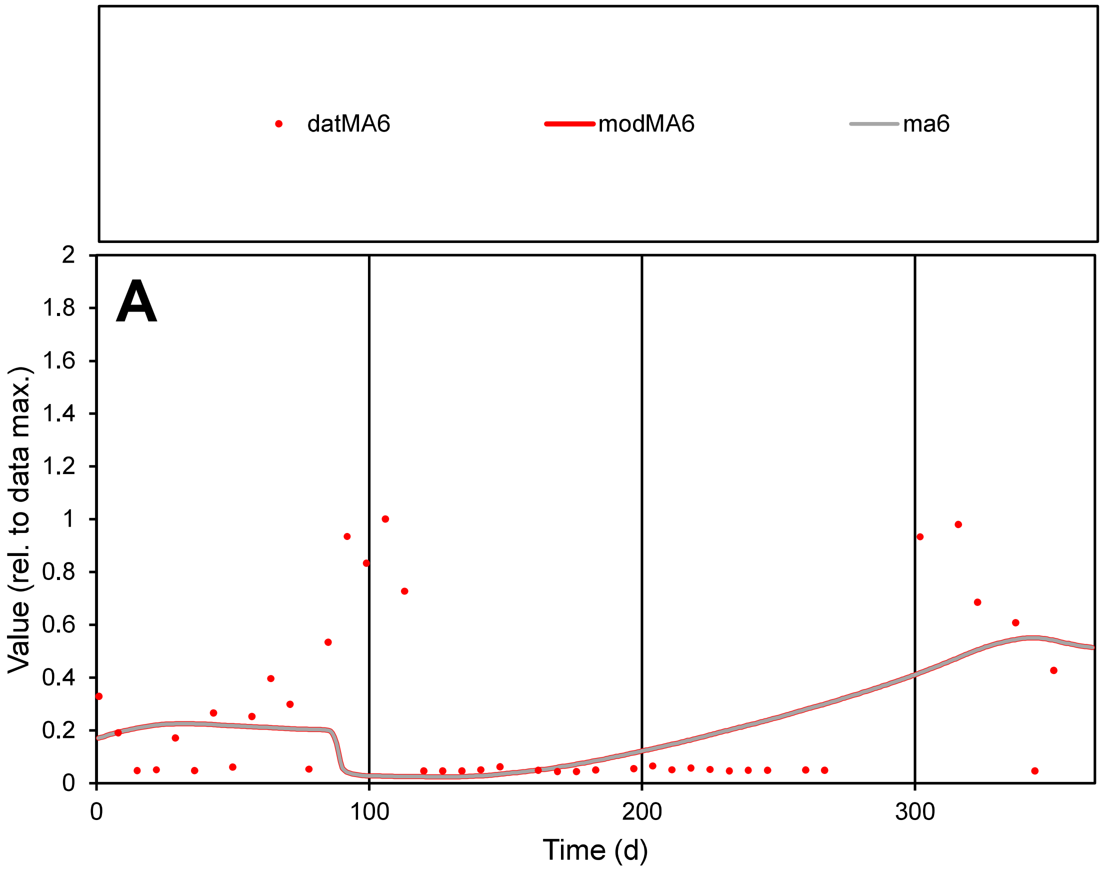

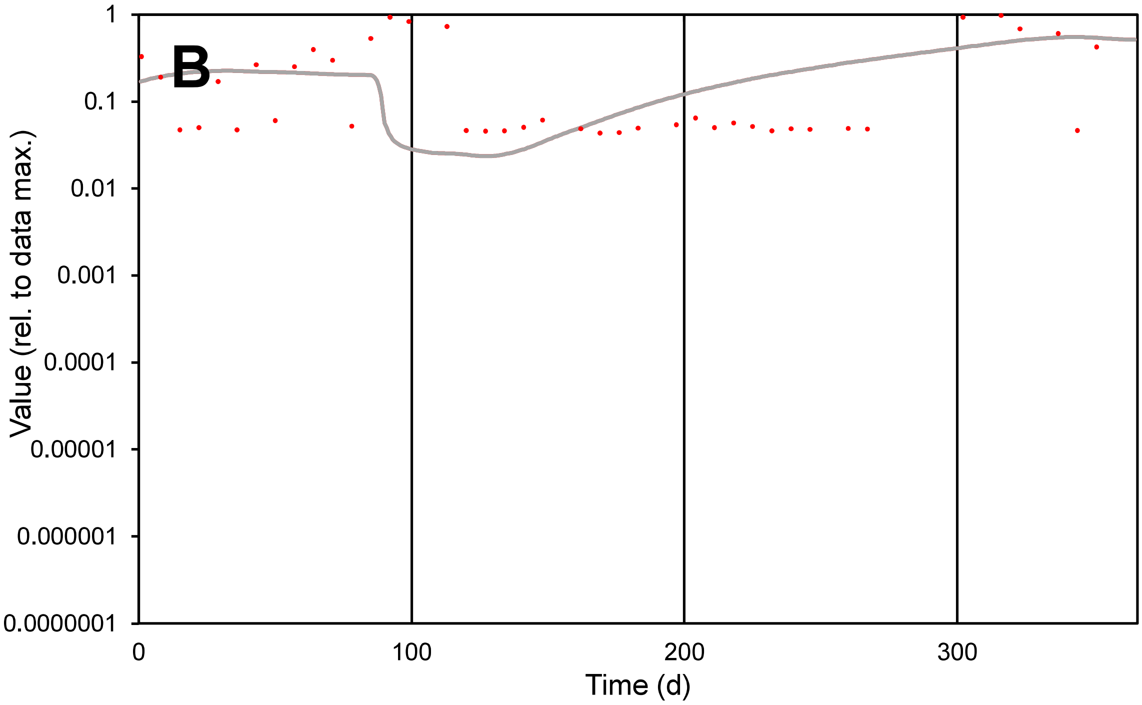

C1

Variables

MA6 Provide jtd or Dst

CalibOnly?

2 1 to reproduce of

oNormalize?

2 1=no, 2=yes

ofxn calc*

1.23982

ofxn mod

1.24616

sRun

RunA1

zout

1

Ave

Data

0.0007

Model

0.00054

data/vMax

0.00272

ofxng

319.391

Min d/m

1.83566

ave/min d

5.92749

ave/min m

8.34277

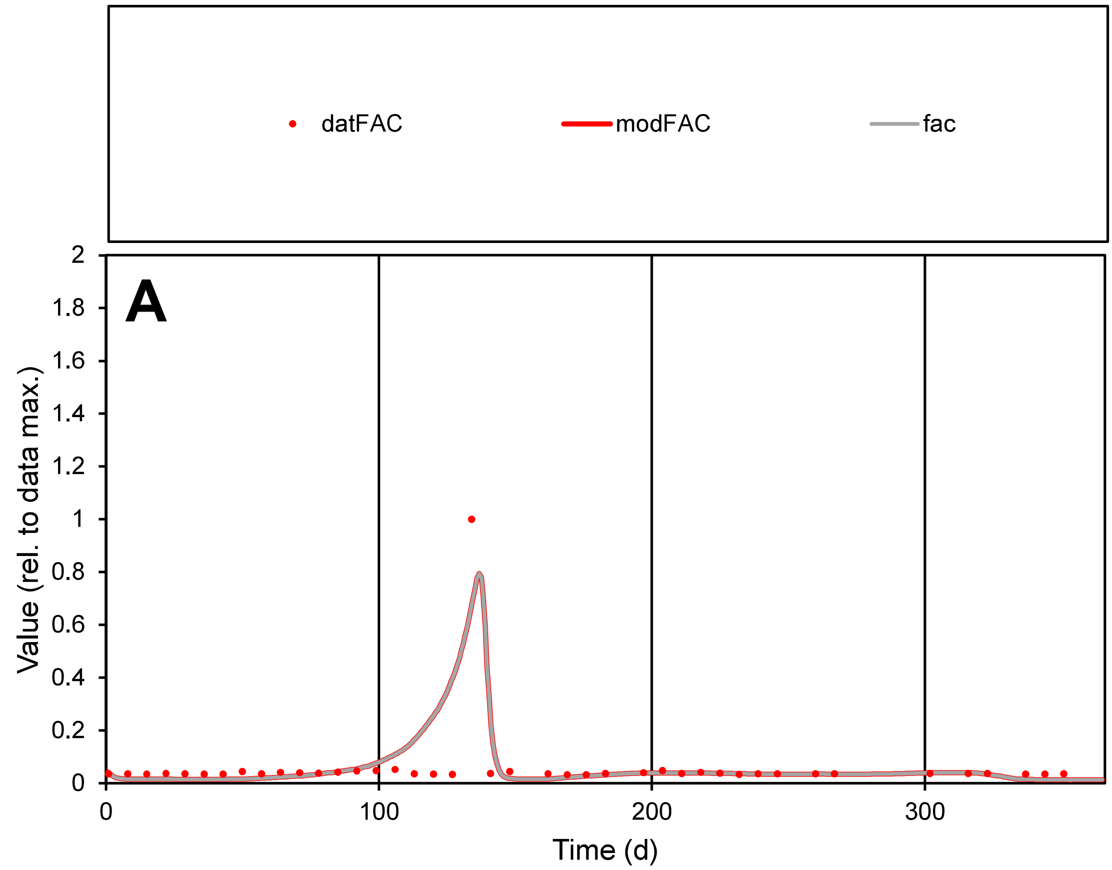

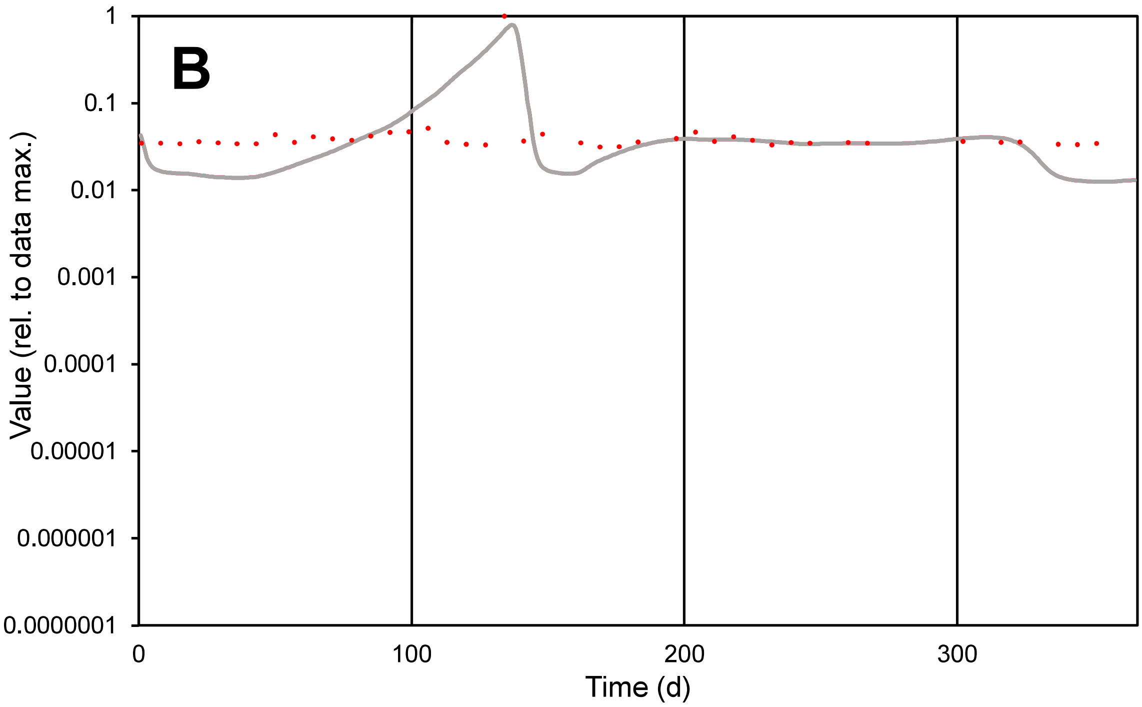

C1

Variables

FAC	Provide jtd or Dst
CalibOnly?	
2	1 to reproduce of
oNormalize?	
2	1=no, 2=yes

ofxn calc*
1.25203
ofxn mod
1.38176

sRun
RunA1
zout
1

Ave
Data
0.00017
Model
0.00018

data/vMax
0.00277

ofxng
319.391

Min d/m
2.51405

ave/min d
1.91946
ave/min m
5.23017

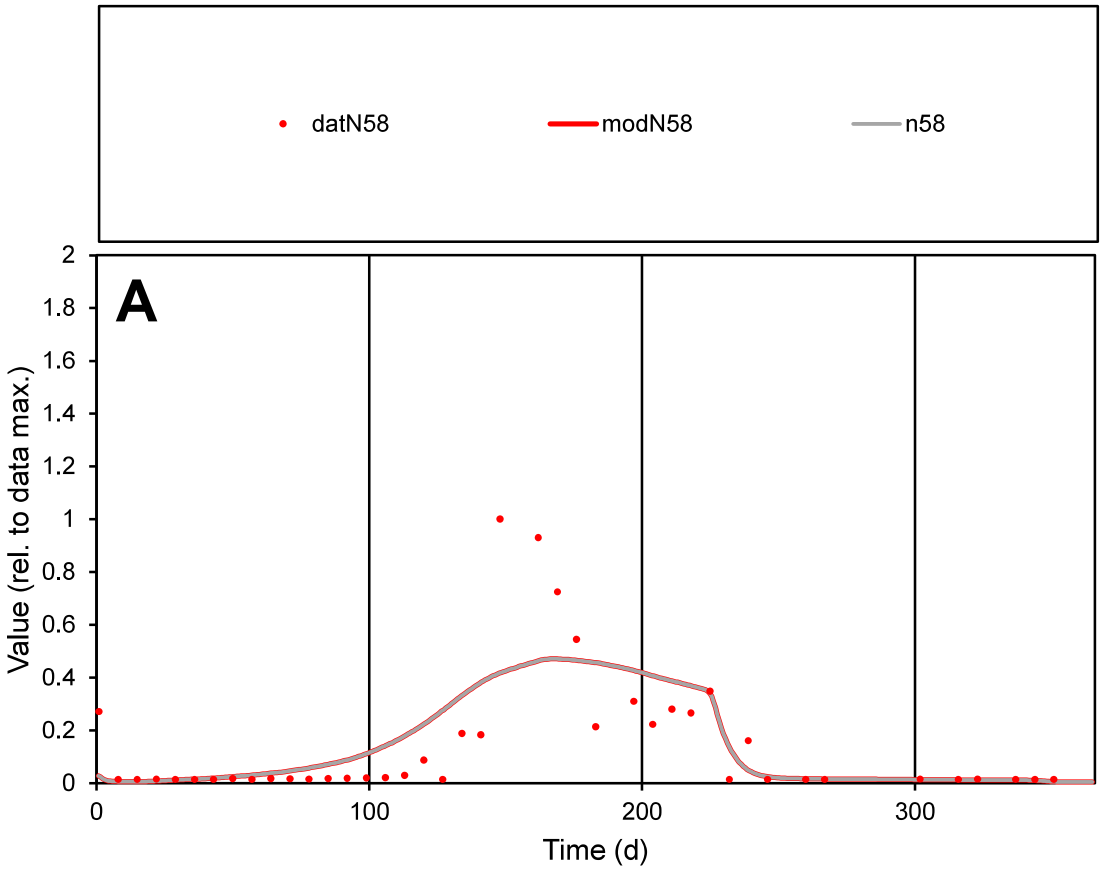

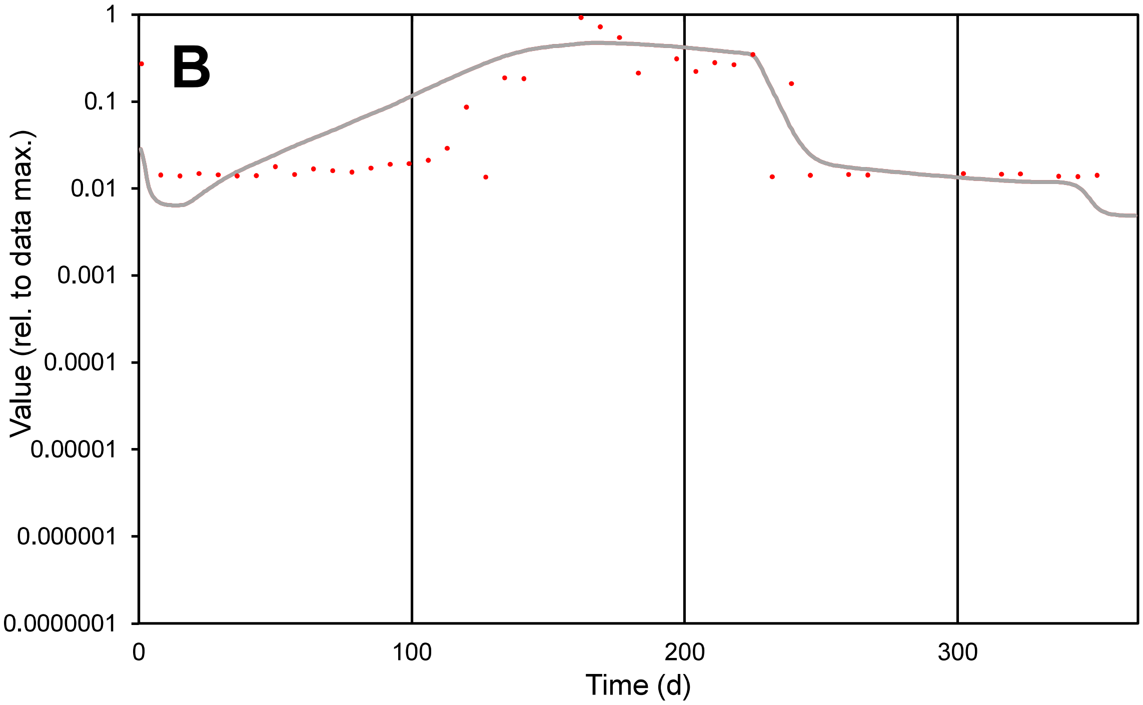

C1

Variables
N58 Provide jtd or Dst
CalibOnly?
2 1 to reproduce of
oNormalize?
2 1=no, 2=yes

ofxn calc*
1.06984
ofxn mod
1.0899

sRun
RunA1
zout
1

Ave
Data
0.00094
Model
0.00099

data/vMax
0.00639

ofxng
319.391

Min d/m
2.26129

ave/min d
10.8038
ave/min m
25.9146

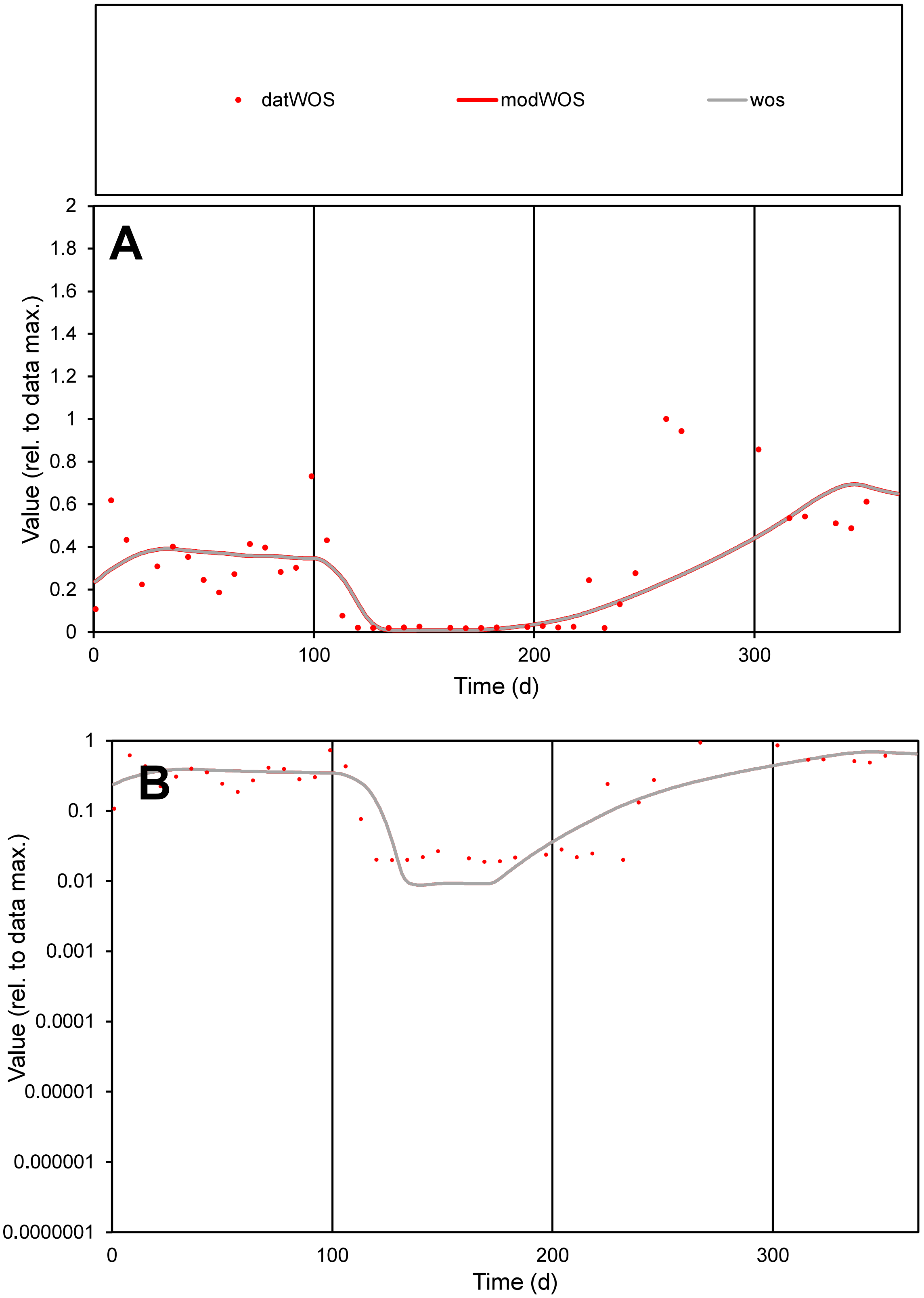

C1

Variables
WOS Provide jtd or Dst
CalibOnly?
2 1 to reproduce of
oNormalize?
2 1=no, 2=yes

ofxn calc*
0.69318
ofxn mod
0.70562

sRun
RunA1
zout
1

Ave
Data
0.00106
Model
0.00095

data/vMax
0.00363

ofxng
319.391

Min d/m
2.14063

ave/min d
15.3988
ave/min m
29.5303

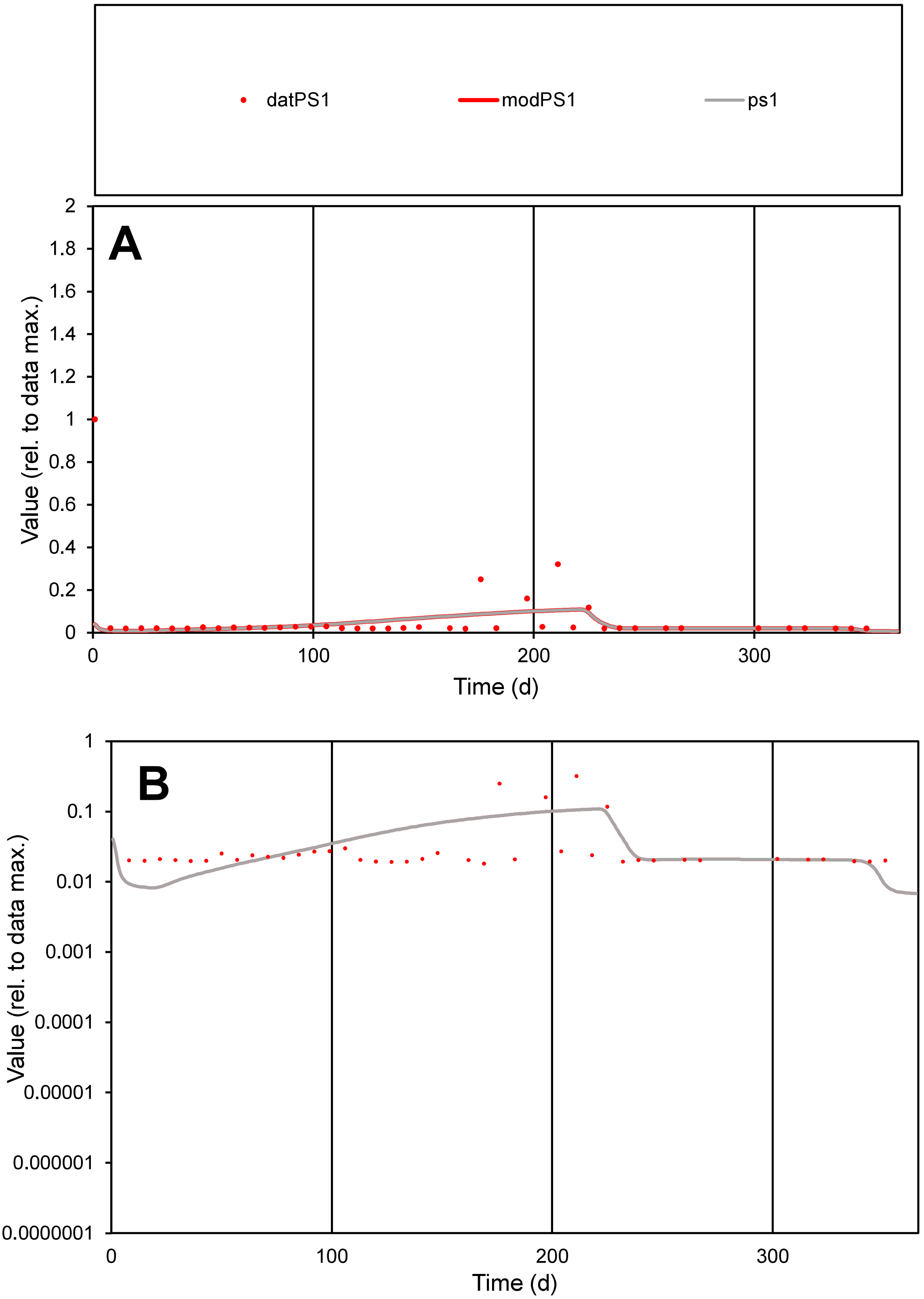

C1

Variables
PS1 Provide jtd or Dst
CalibOnly?
2 1 to reproduce of
oNormalize?
2 1=no, 2=yes

ofxn calc*
2.49966
ofxn mod
2.50475

sRun
RunA1
zout
1

Ave
Data
0.00016
Model
0.00011

data/vMax
0.0026

ofxng
319.391

Min d/m
2.21292

ave/min d
3.46202
ave/min m
5.04151

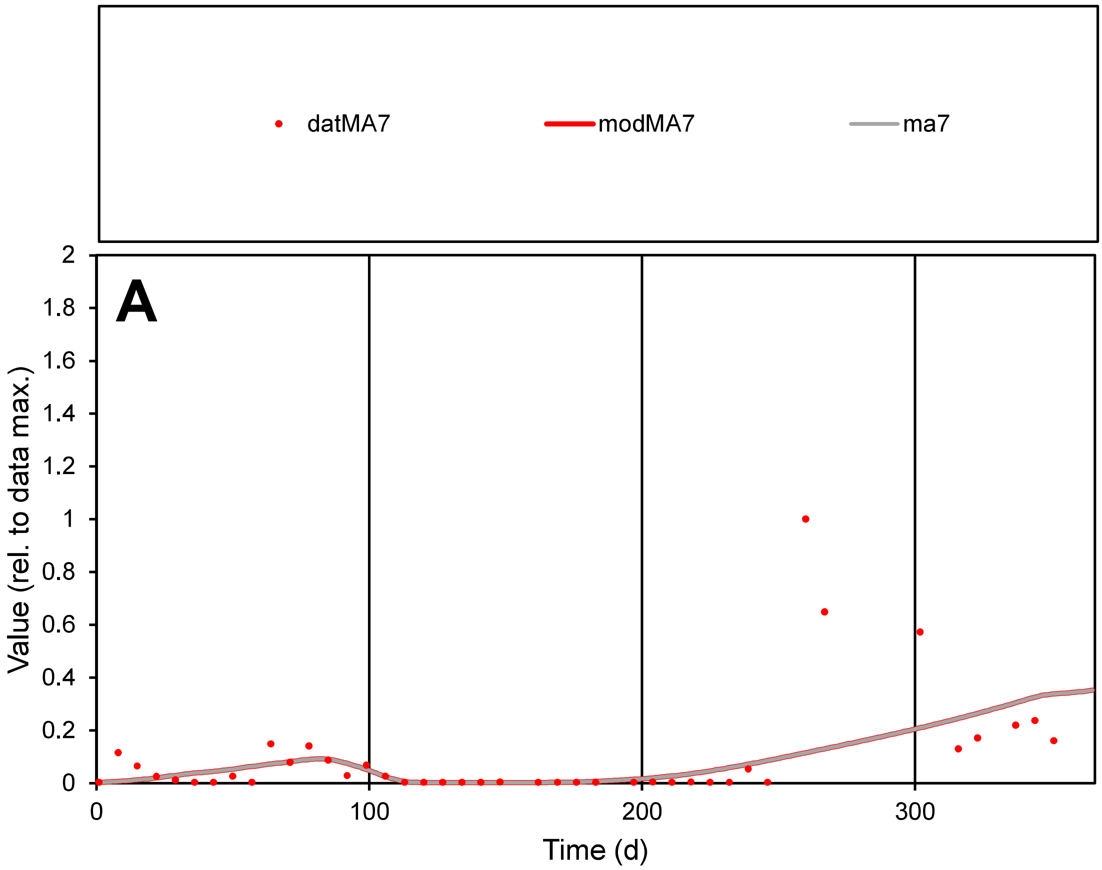

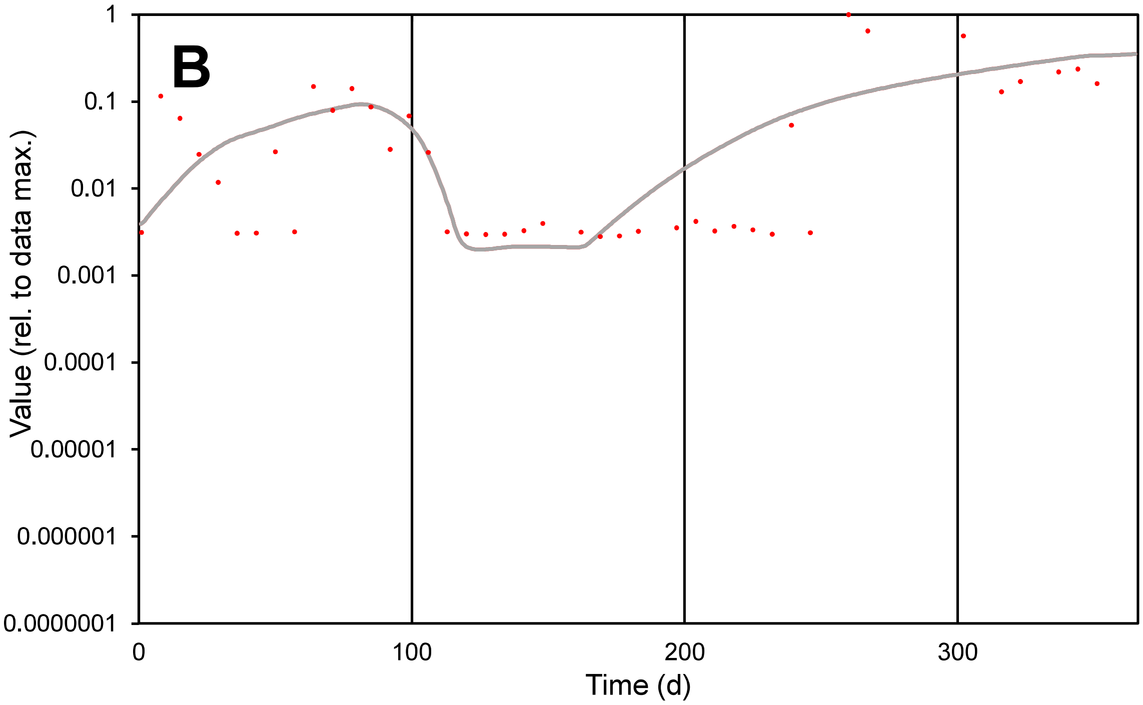

C1

Variables

MA7 Provide jtd or Dst

CalibOnly?

2 1 to reproduce of

oNormalize?

2 1=no, 2=yes

ofxn calc*

1.80412

ofxn mod

1.81101

sRun

RunA1

zout

1

Ave

Data

0.00047

Model

0.00036

data/vMax

0.0048

ofxng

319.391

Min d/m

1.4

ave/min d

34.4805

ave/min m

36.8535

330_Calib_S_A

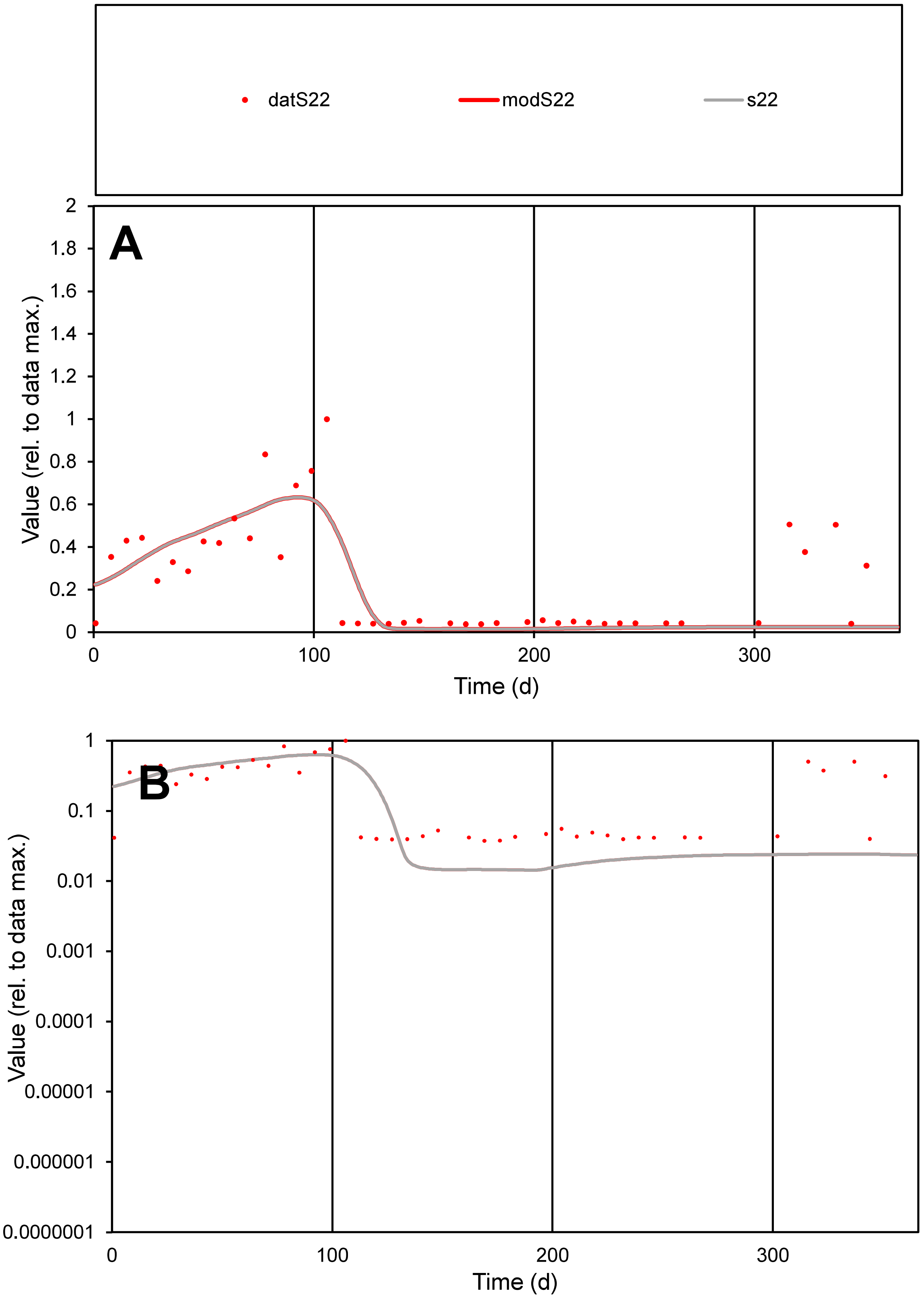

C1

Variables

S22	Provide jtd or Dst
CalibOnly?	
2	1 to reproduce of
oNormalize?	
2	1=no, 2=yes

ofxn calc*
0.72083
ofxn mod
0.74636

sRun
RunA1
zout
1

Ave
Data
0.00096
Model
0.0008

data/vMax
0.00395

ofxng
319.391

Min d/m
2.5846

ave/min d
6.5078
ave/min m
14.0961

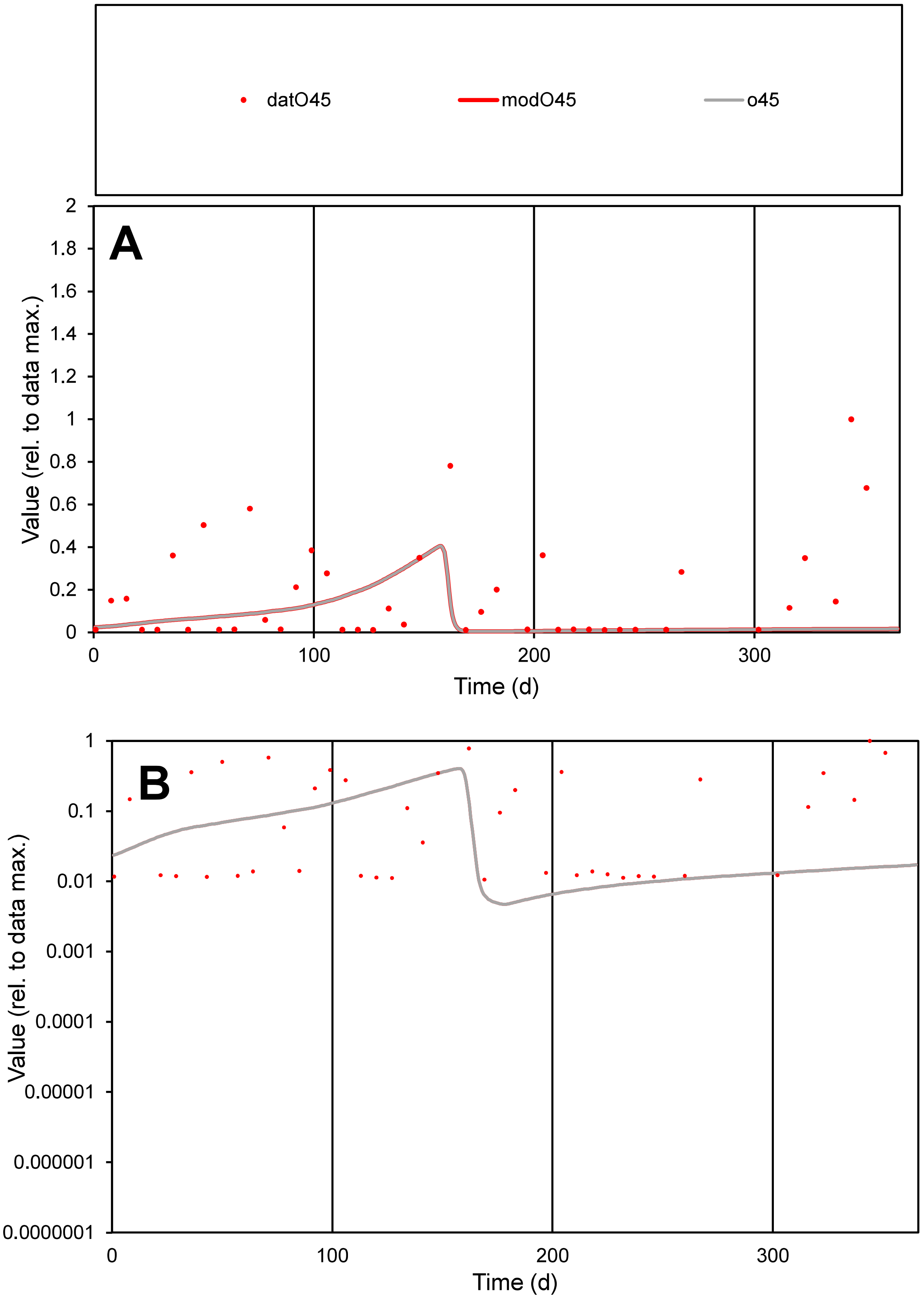

C1

Variables
O45 Provide jtd or Dst
CalibOnly?
2 1 to reproduce of
oNormalize?
2 1=no, 2=yes

ofxn calc*
1.47342
ofxn mod
1.51464

sRun
RunA1
zout
1

Ave
Data
0.00096
Model
0.00042

data/vMax
0.00542

ofxng
319.391

Min d/m
2.23241

ave/min d
16.6956
ave/min m
16.3337

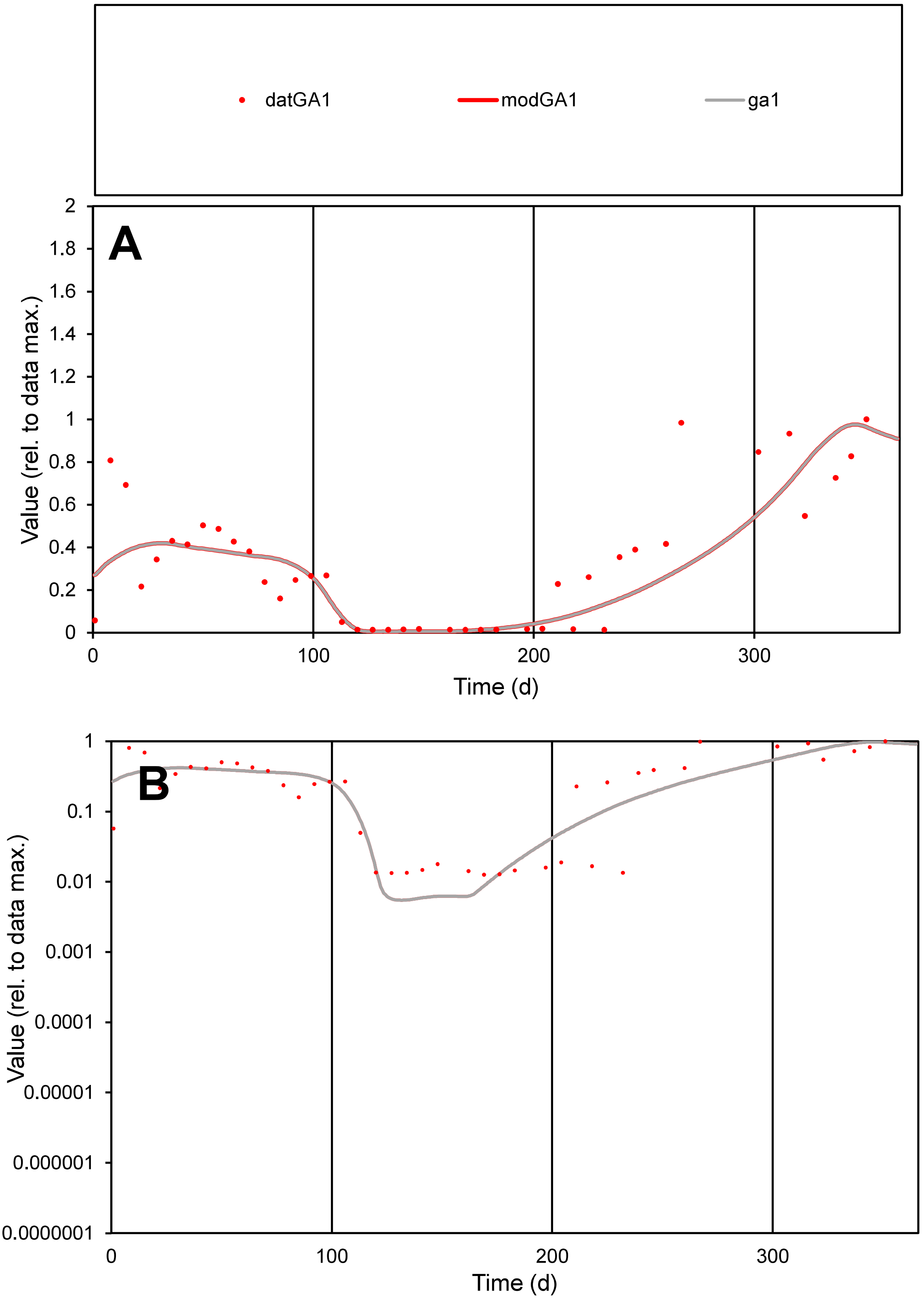

C1

Variables
GA1 Provide jtd or Dst
CalibOnly?
2 1 to reproduce of
oNormalize?
2 1=no, 2=yes

ofxn calc*
0.55229
ofxn mod
0.56696

sRun
RunA1
zout
1

Ave
Data
0.00091
Model
0.00081

data/vMax
0.0028

ofxng
319.391

Min d/m
2.27447

ave/min d
25.6945
ave/min m
51.8199

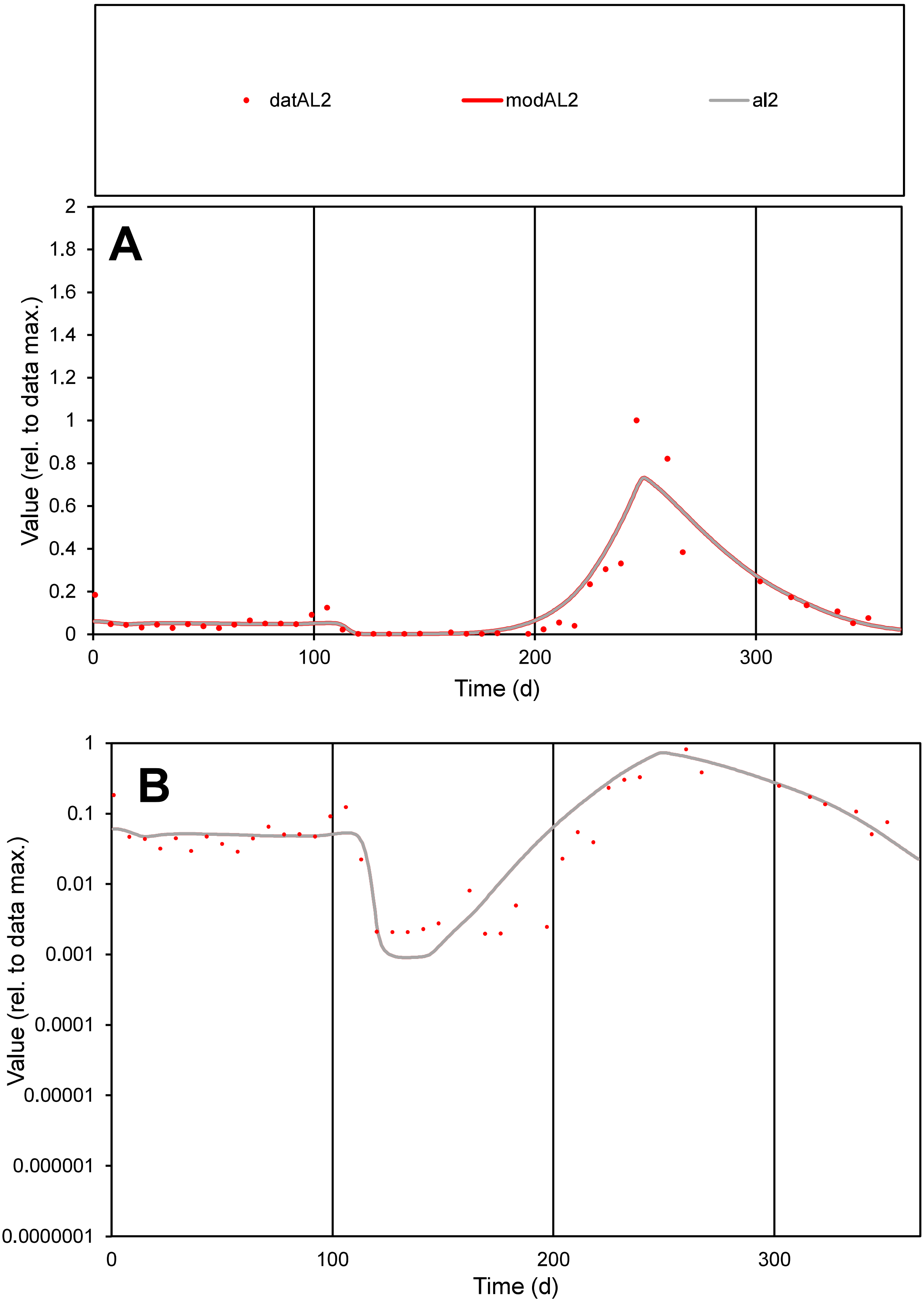

C1

Variables
AL2 Provide jtd or Dst
CalibOnly?
2 1 to reproduce of
oNormalize?
2 1=no, 2=yes

ofxn calc*
0.66334
ofxn mod
0.70118

sRun
RunA1
zout
1

Ave
Data
0.00132
Model
0.00138

data/vMax
0.0111

ofxng
319.391

Min d/m
2.17729

ave/min d
60.4456
ave/min m
137.269

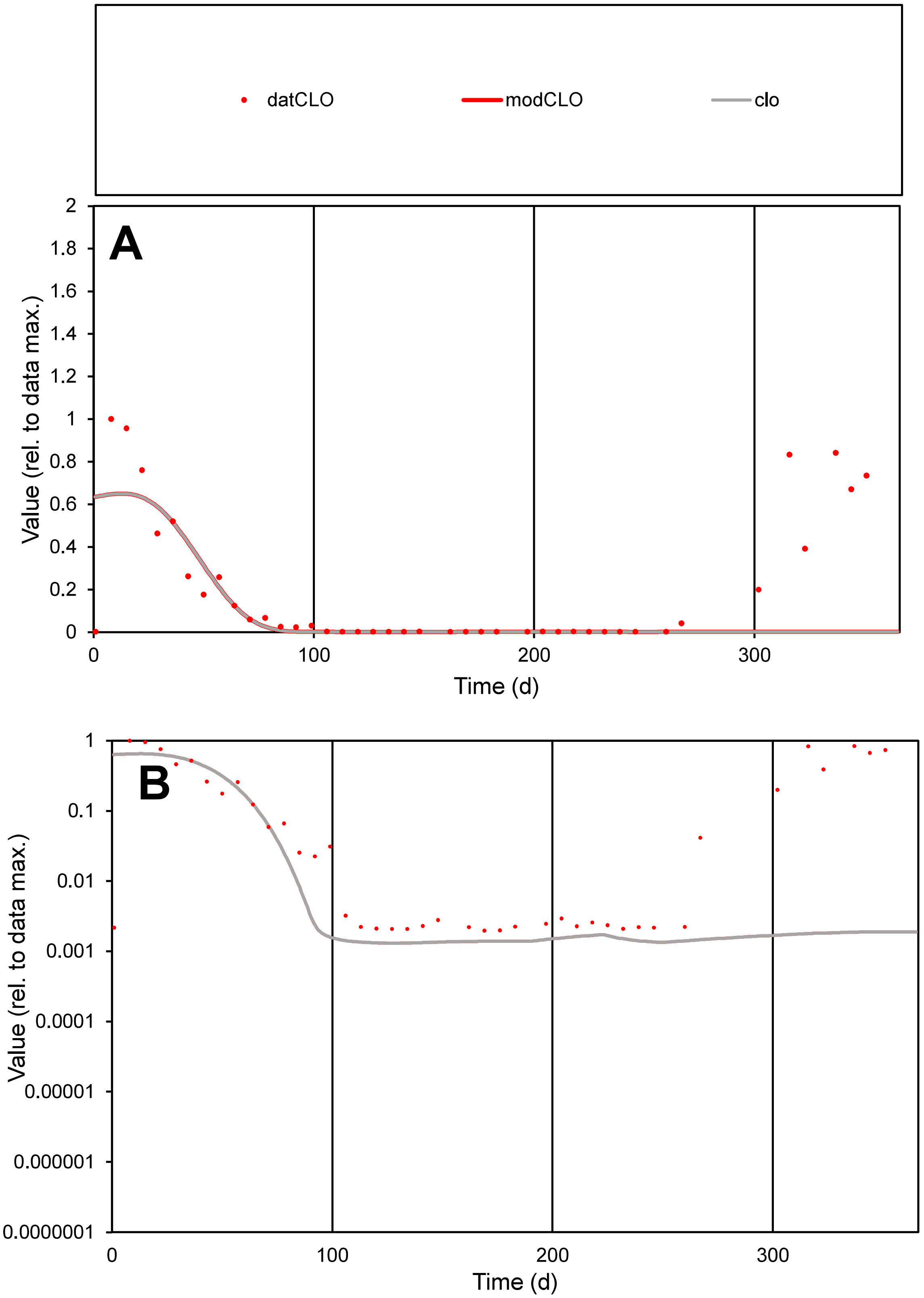

C1

Variables
CLO Provide jtd or Dst
CalibOnly?
2 1 to reproduce of
oNormalize?
2 1=no, 2=yes

ofxn calc*
1.37907
ofxn mod
1.38797

sRun
RunA1
zout
1

Ave
Data
0.00136
Model
0.00076

data/vMax
0.00672

ofxng
319.391

Min d/m
1.50722

ave/min d
102.458
ave/min m
86.9552

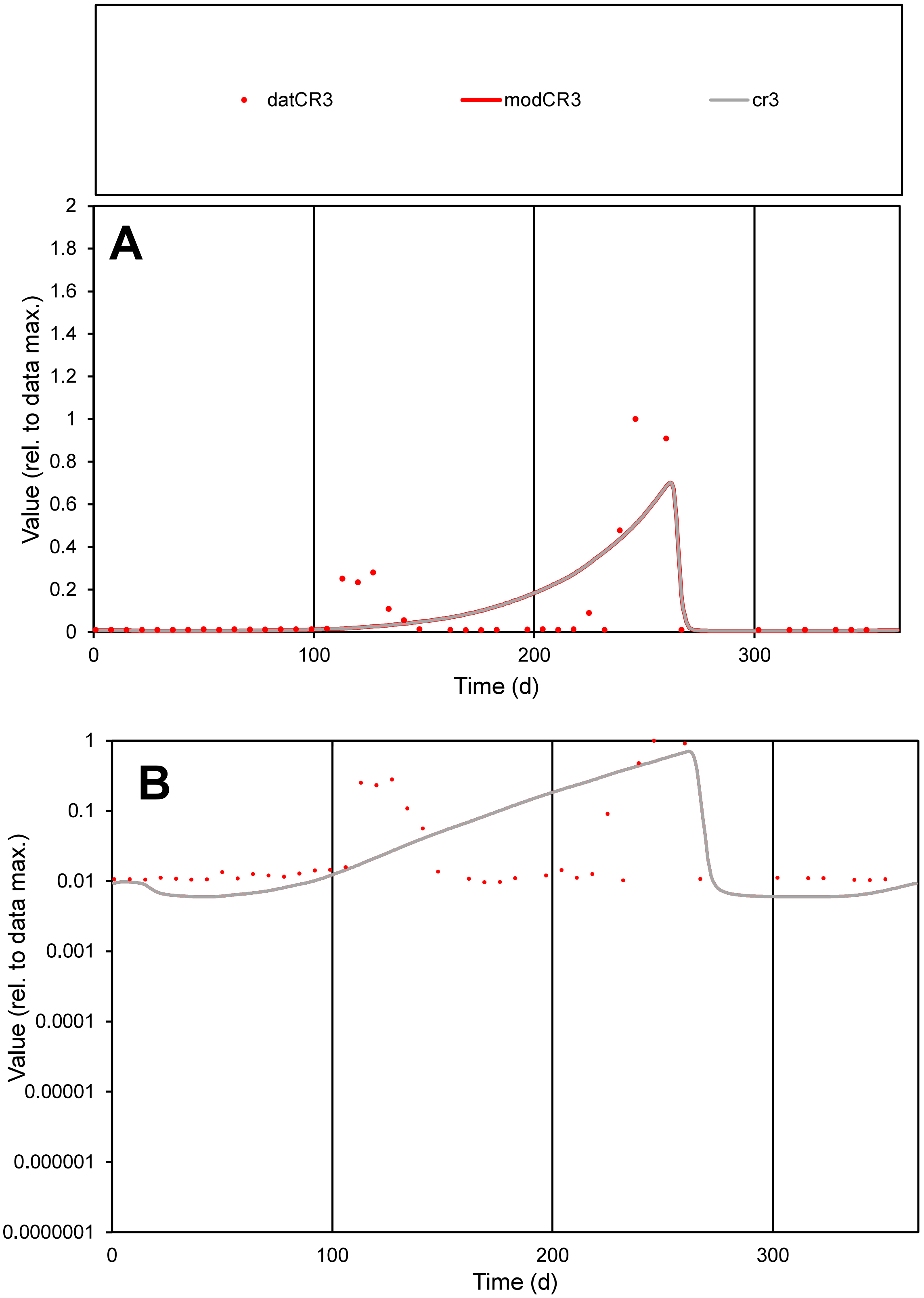

C1

Variables
CR3 Provide jtd or Dst
CalibOnly?
2 1 to reproduce of
oNormalize?
2 1=no, 2=yes

ofxn calc*
1.57971
ofxn mod
1.59817

sRun
RunA1
zout
1

Ave
Data
0.00033
Model
0.00036

data/vMax
0.00369

ofxng
319.391

Min d/m
1.61368

ave/min d
9.34884
ave/min m
16.1664

330_Calib_S_A

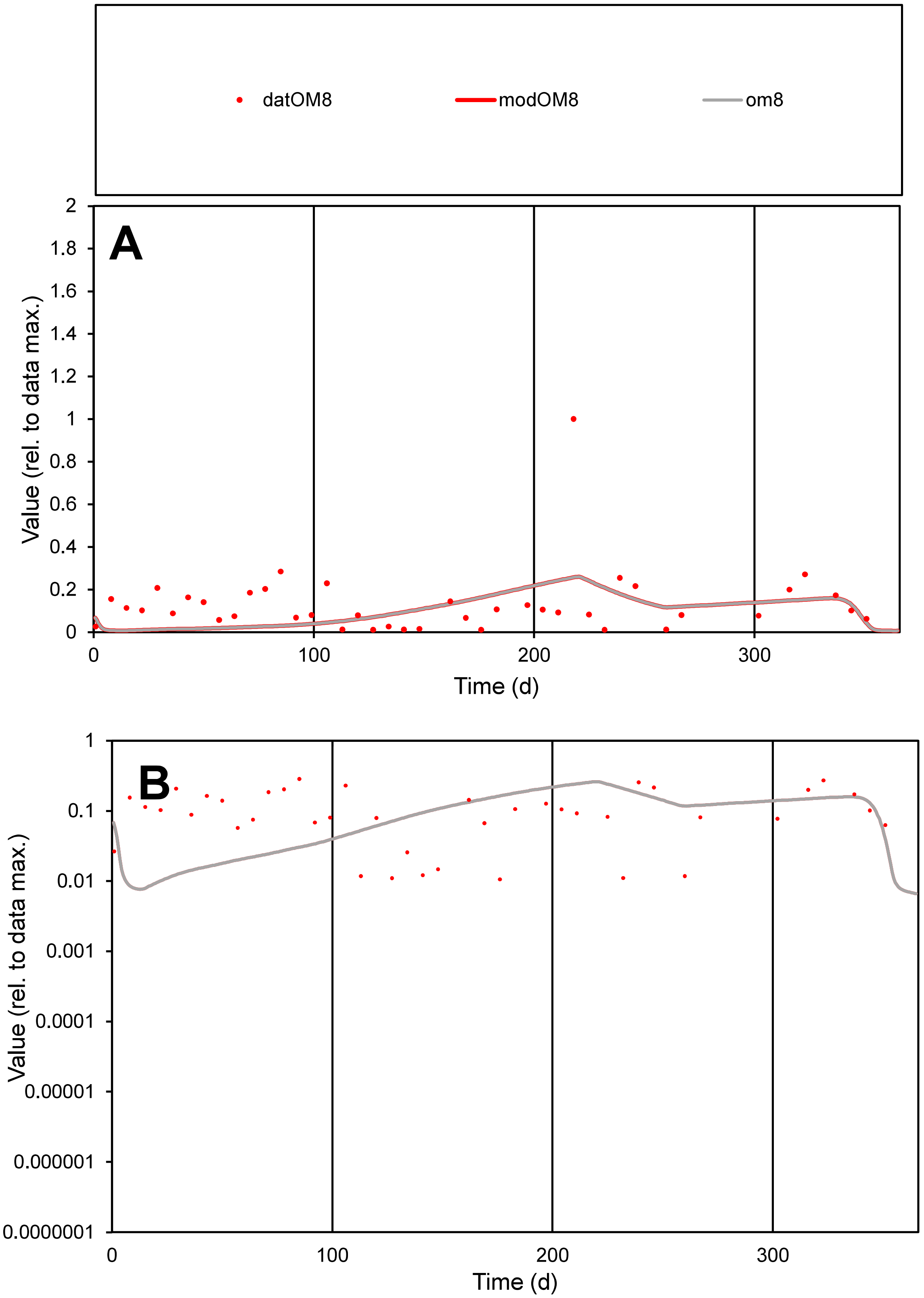

C1

Variables
OM8 Provide jtd or Dst
CalibOnly?
2 1 to reproduce of
oNormalize?
2 1=no, 2=yes

ofxn calc*
1.1955
ofxn mod
1.2058

sRun
RunA1
zout
1

Ave
Data
0.00068
Model
0.00052

data/vMax
0.00519

ofxng
319.391

Min d/m
1.30835

ave/min d
12.453
ave/min m
12.3591

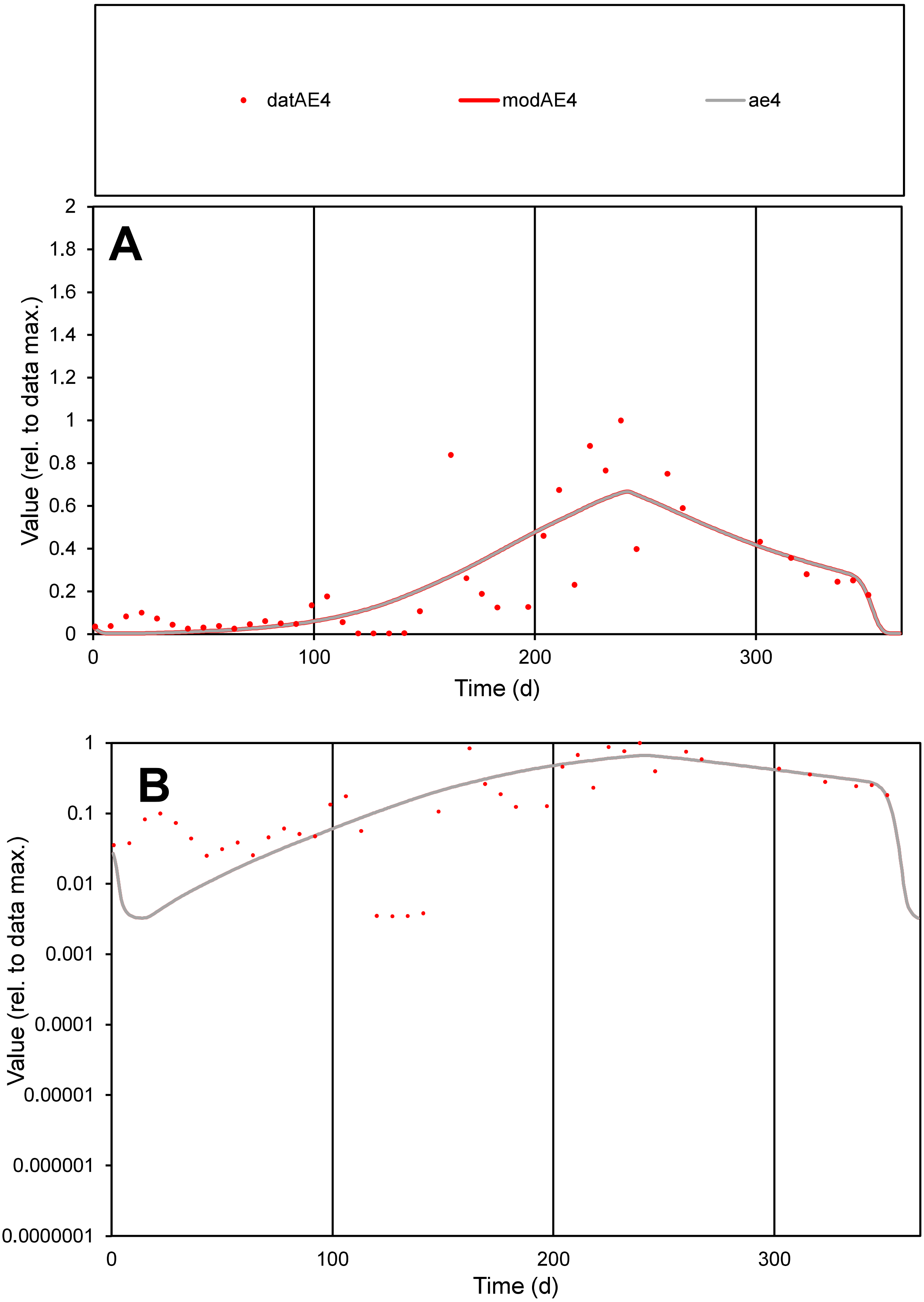

C1

Variables
AE4 Provide jtd or Dst
CalibOnly?
2 1 to reproduce of
oNormalize?
2 1=no, 2=yes

ofxn calc*
0.65965
ofxn mod
0.6838

sRun
RunA1
zout
1

Ave
Data
0.00087
Model
0.00086

data/vMax
0.00356

ofxng
319.391

Min d/m
1.02235

ave/min d
70.128
ave/min m
71.3802

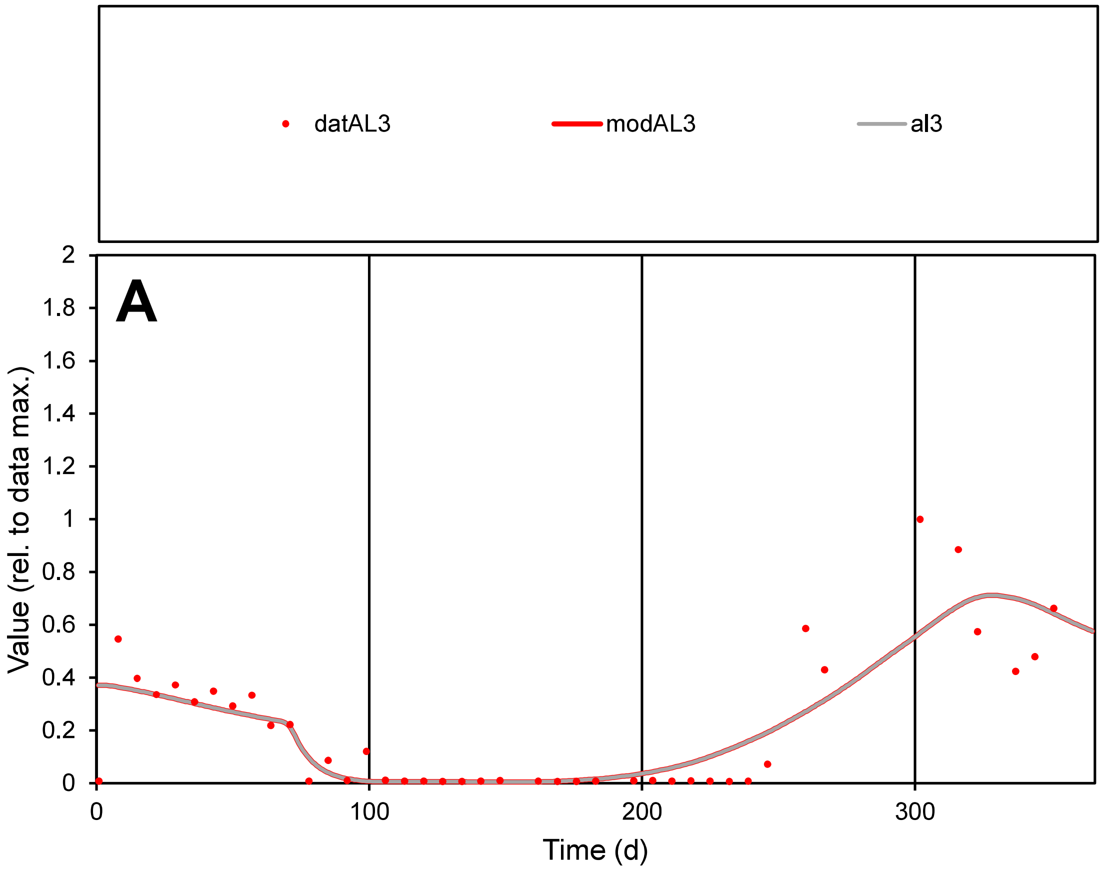

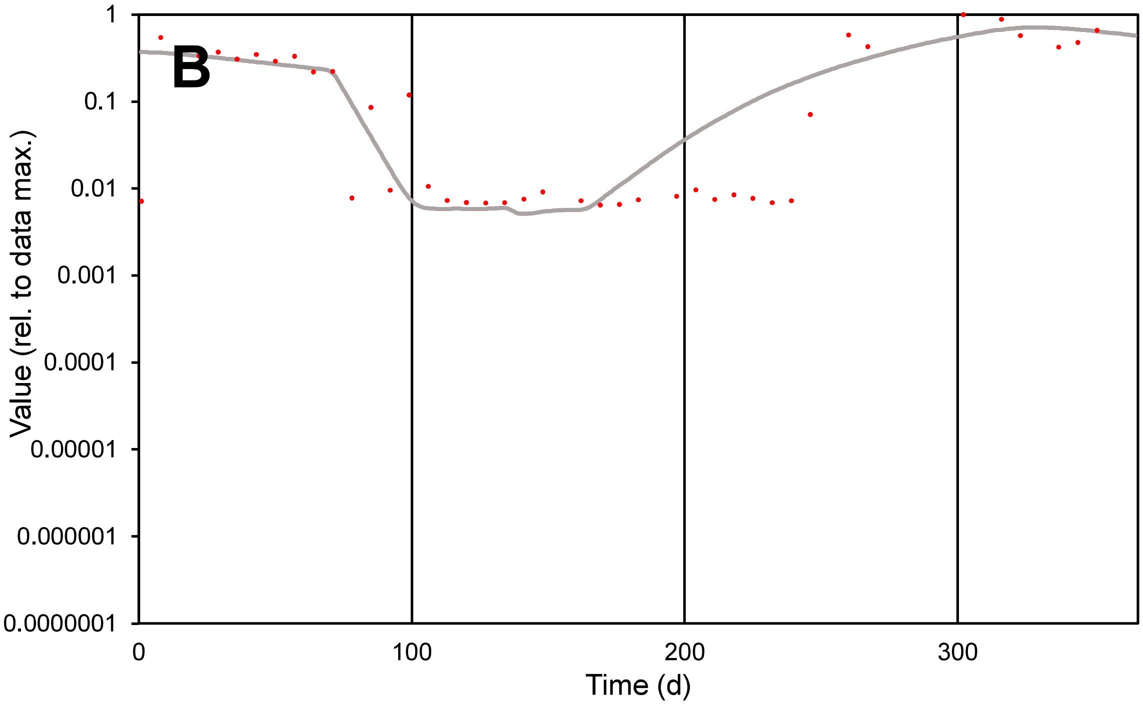

C1

Variables
AL3 Provide jtd or Dst
CalibOnly?
2 1 to reproduce of
oNormalize?
2 1=no, 2=yes

ofxn calc*
0.62479
ofxn mod
0.64297

sRun
RunA1
zout
1

Ave
Data
0.00079
Model
0.00079

data/vMax
0.00375

ofxng
319.391

Min d/m
1.25323

ave/min d
32.5352
ave/min m
40.7278

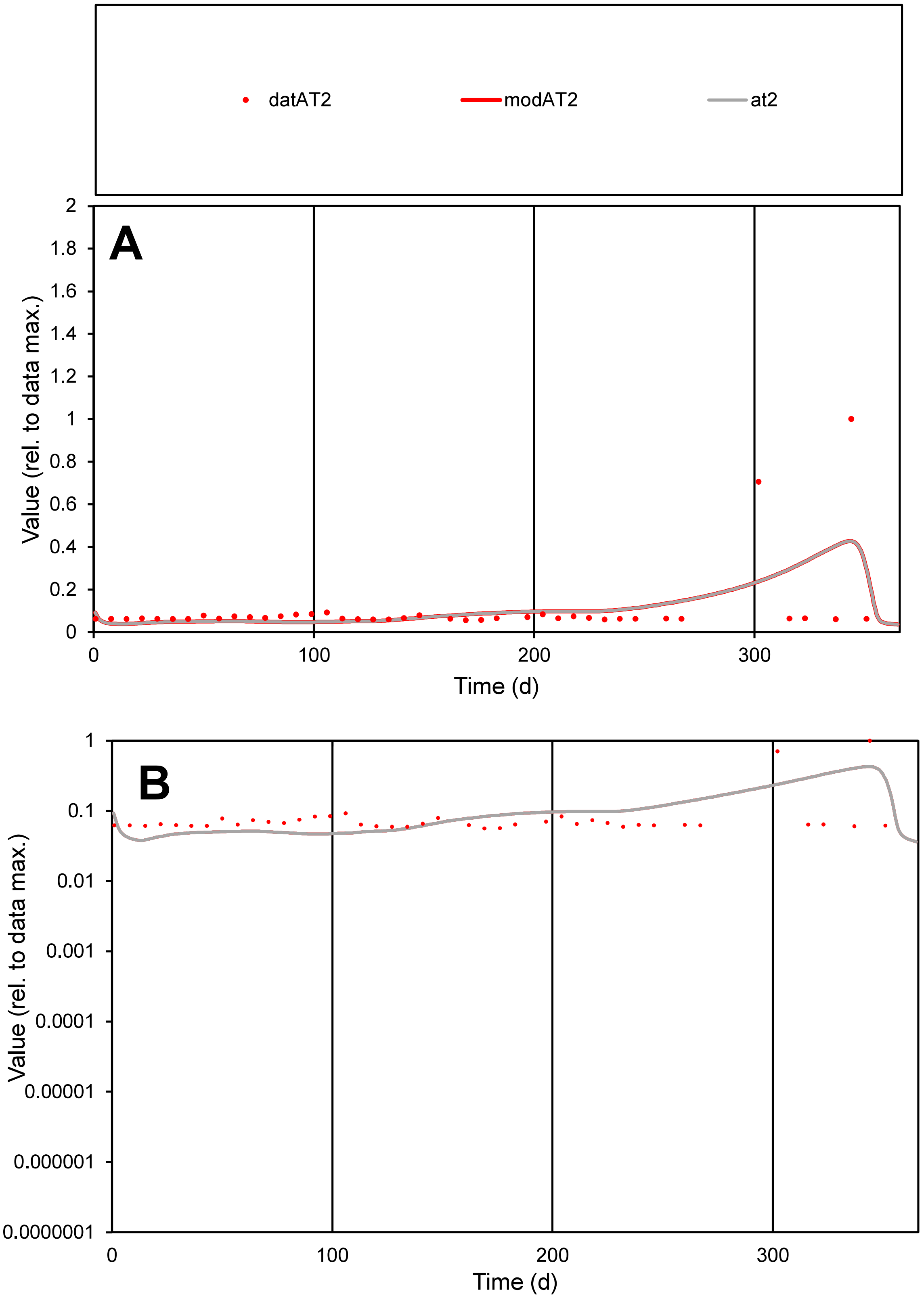

C1

Variables
AT2 Provide jtd or Dst
CalibOnly?
2 1 to reproduce of
oNormalize?
2 1=no, 2=yes

ofxn calc*
1.35825
ofxn mod
1.37305

sRun
RunA1
zout
1

Ave
Data
0.00015
Model
0.00015

data/vMax
0.00142

ofxng
319.391

Min d/m
1.42197

ave/min d
1.85343
ave/min m
2.66969

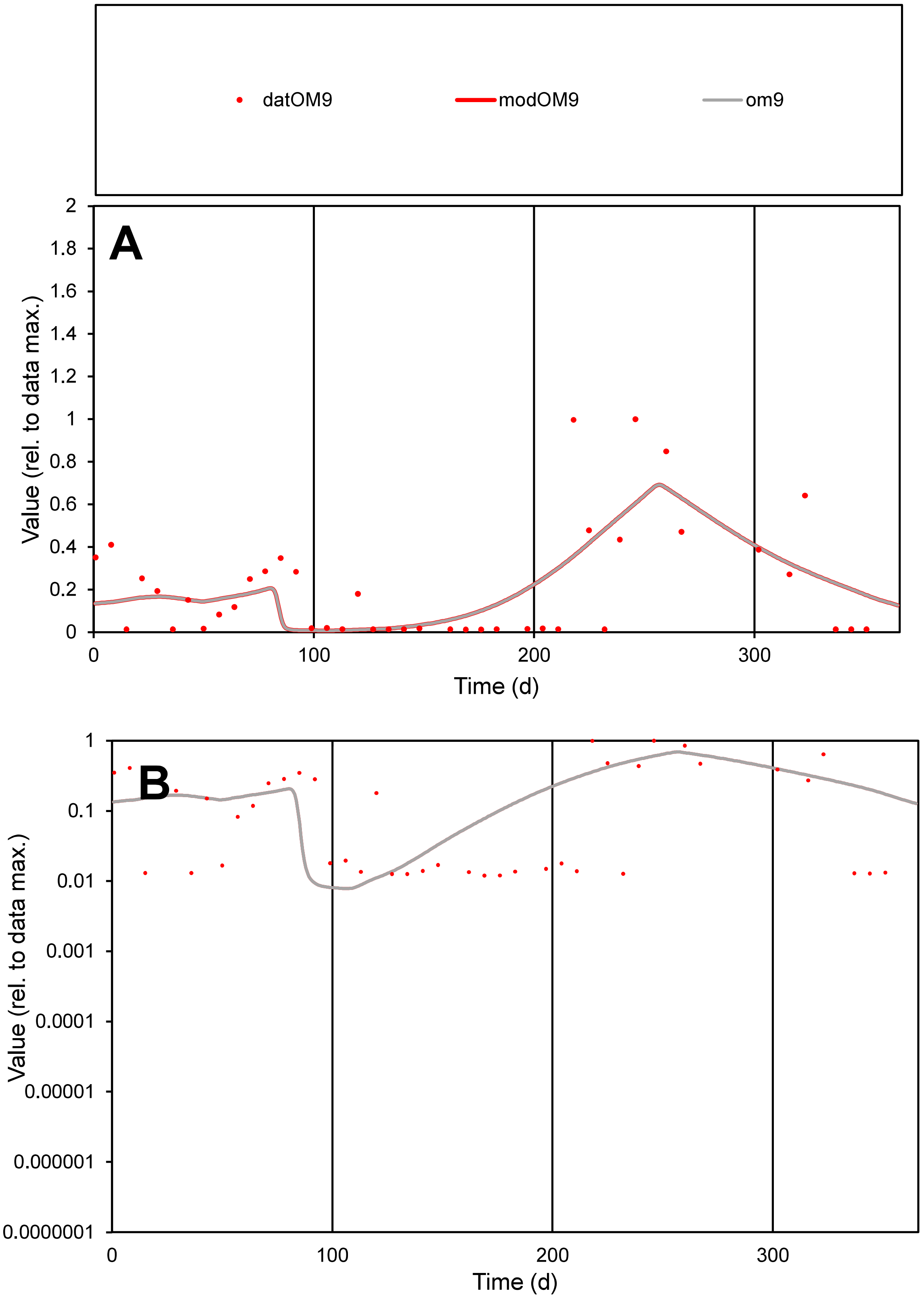

C1

Variables
OM9 Provide jtd or Dst
CalibOnly?
2 1 to reproduce of
oNormalize?
2 1=no, 2=yes

ofxn calc*
0.94361
ofxn mod
0.96658

sRun
RunA1
zout
1

Ave
Data
0.00075
Model
0.00075

data/vMax
0.00363

ofxng
319.391

Min d/m
1.52221

ave/min d
17.344
ave/min m
26.1985

330_Calib_S_A

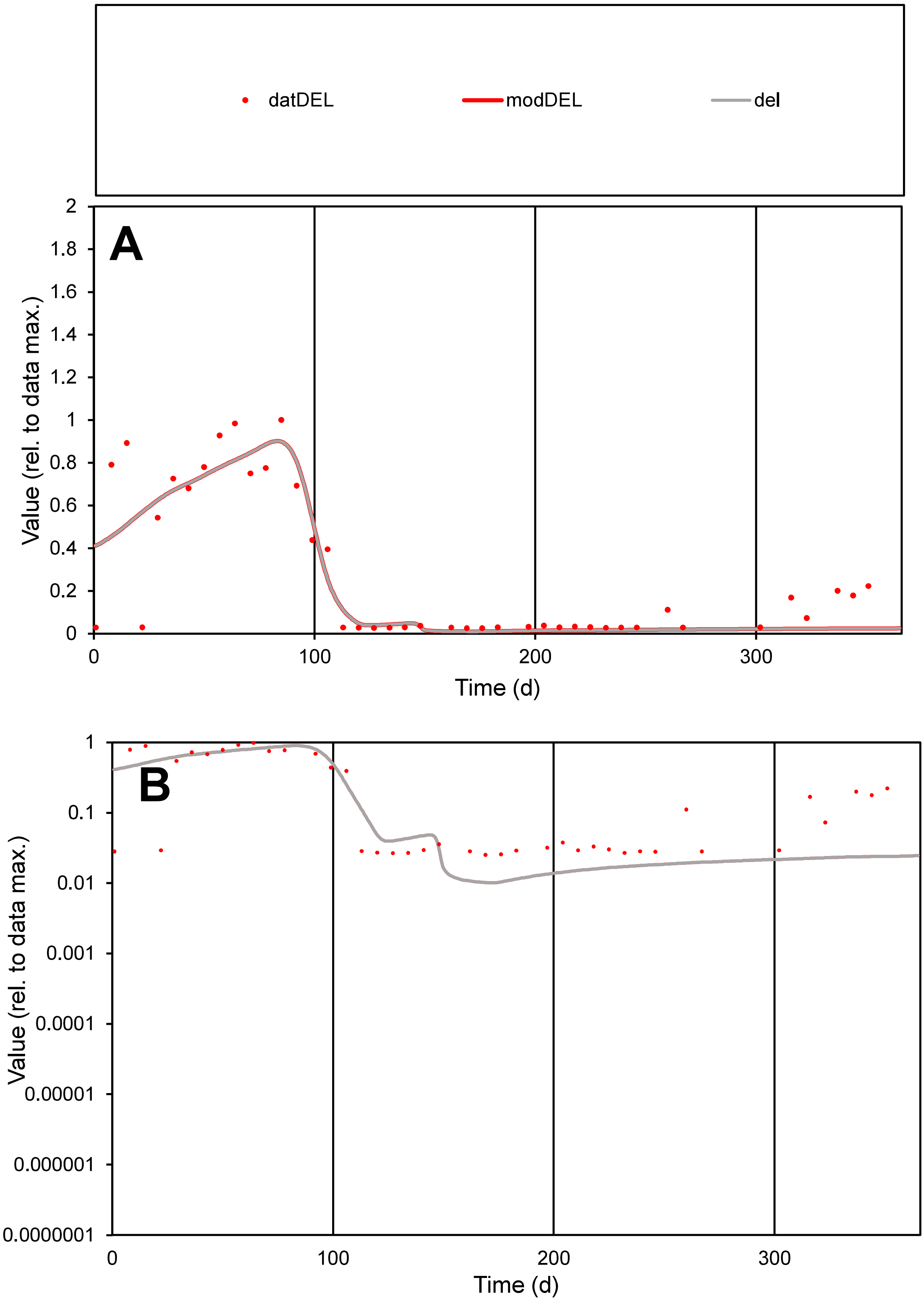

C1

Variables

DEL	Provide jtd or Dst
CalibOnly?	
2	1 to reproduce of
oNormalize?	
2	1=no, 2=yes

ofxn calc*
0.52702
ofxn mod
0.55535

sRun
RunA1
zout
1

Ave
Data
0.0007
Model
0.00065

data/vMax
0.00246

ofxng
319.391

Min d/m
2.4938

ave/min d
11.2393
ave/min m
26.0478

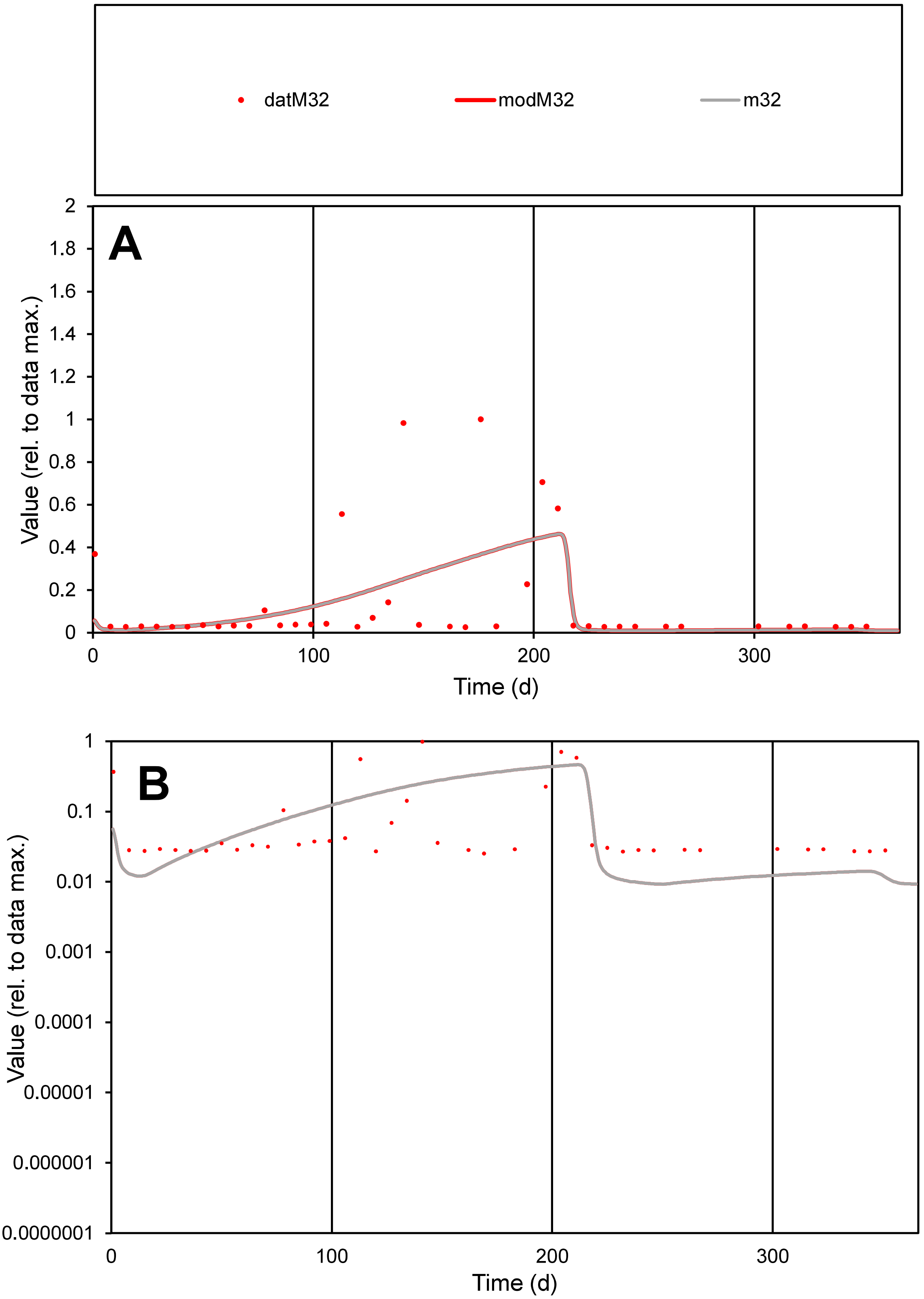

C1		
Variables		
M32	Provide jtd or Dst	
CalibOnly?		
2	1 to reproduce of	
oNormalize?		
2	1=no, 2=yes	
ofxn calc*		
1.50804		
ofxn mod		
1.51933		
sRun		
RunA1		
zout		
1		
Ave		
Data		
0.00113		
Model		
0.00102		
data/vMax		
0.0083		
ofxng		
319.391		
Min d/m		
2.71894		
ave/min d		
5.35359		
ave/min m		
13.2264		

330_Calib_S_A

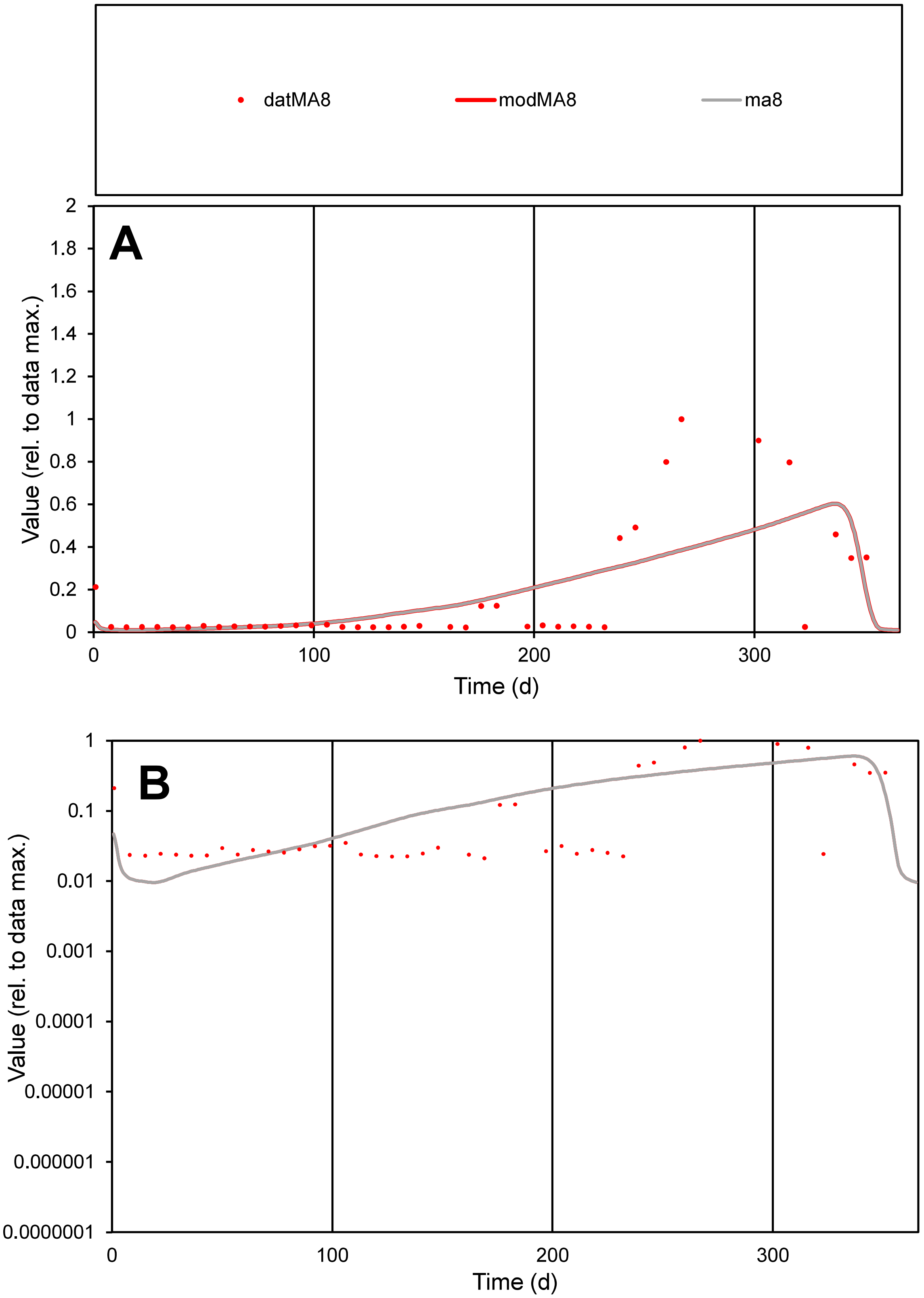

C1

Variables
MA8 Provide jtd or Dst
CalibOnly?
2 1 to reproduce of
oNormalize?
2 1=no, 2=yes

ofxn calc*
1.18989
ofxn mod
1.20357

sRun
RunA1
zout
1

Ave
Data
0.00046
Model
0.00048

data/vMax
0.00283

ofxng
319.391

Min d/m
2.20147

ave/min d
7.63703
ave/min m
17.611

330_Calib_S_A

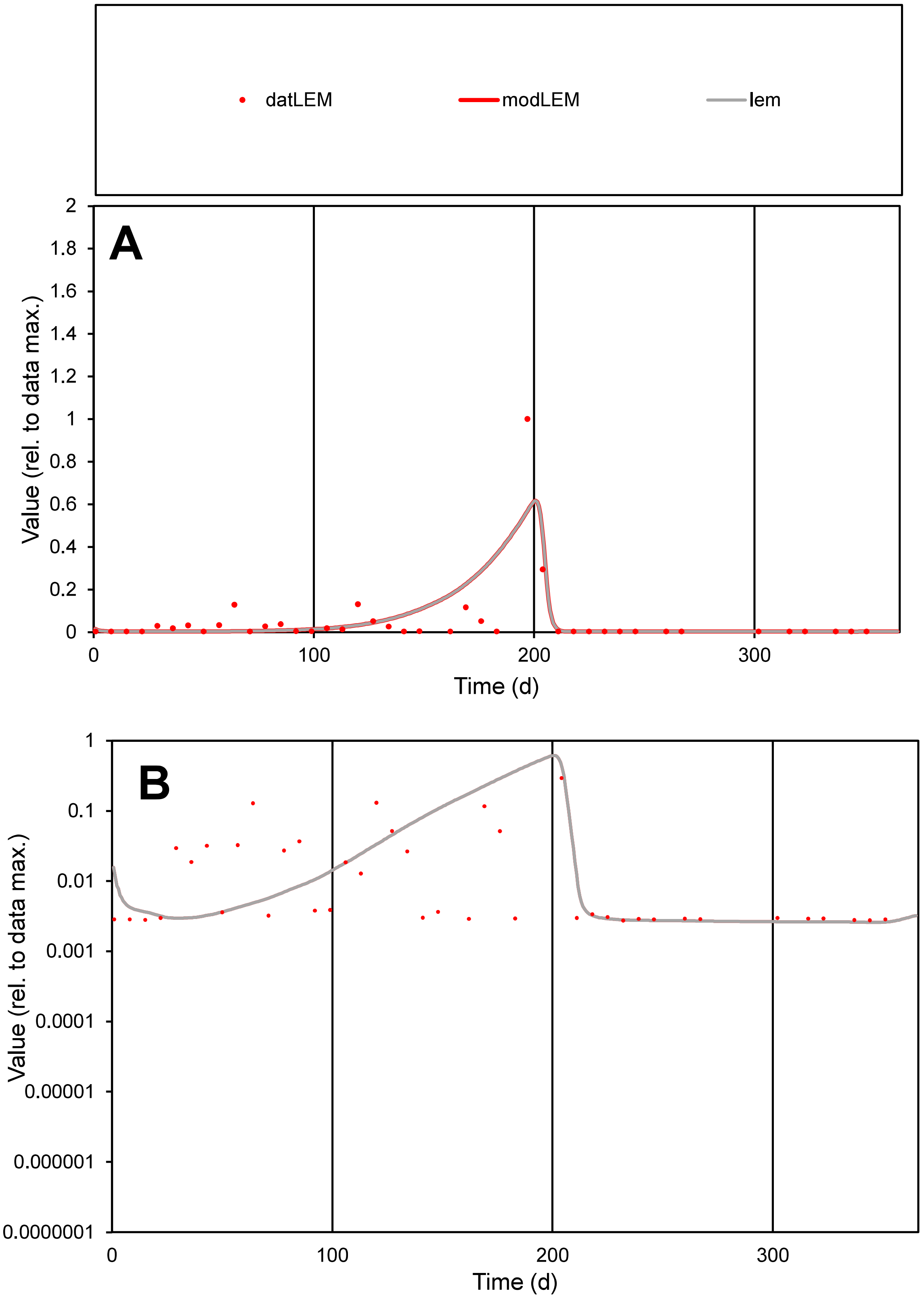

C1

Variables
LEM Provide jtd or Dst
CalibOnly?
2 1 to reproduce of
oNormalize?
2 1=no, 2=yes

ofxn calc*
2.06865
ofxn mod
2.10559

sRun
RunA1
zout
1

Ave
Data
0.00042
Model
0.0005

data/vMax
0.00841

ofxng
319.391

Min d/m
1.05782

ave/min d
18.1379
ave/min m
22.7646

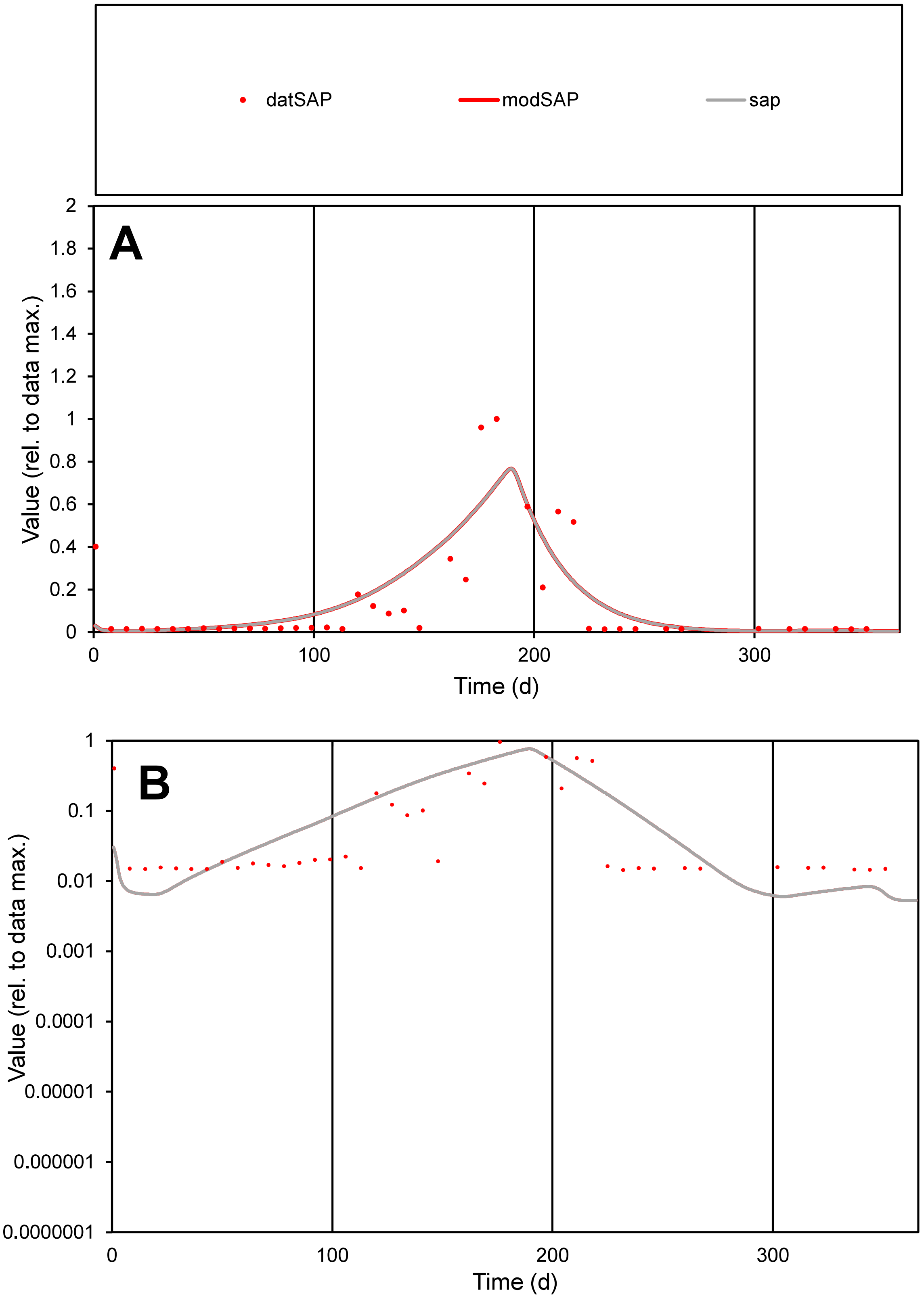

C1

Variables
SAP Provide jtd or Dst
CalibOnly?
2 1 to reproduce of
oNormalize?
2 1=no, 2=yes

ofxn calc*
1.02638
ofxn mod
1.05702

sRun
RunA1
zout
1

Ave
Data
0.00034
Model
0.00036

data/vMax
0.00243

ofxng
319.391

Min d/m
2.38425

ave/min d
9.55036
ave/min m
24.4893

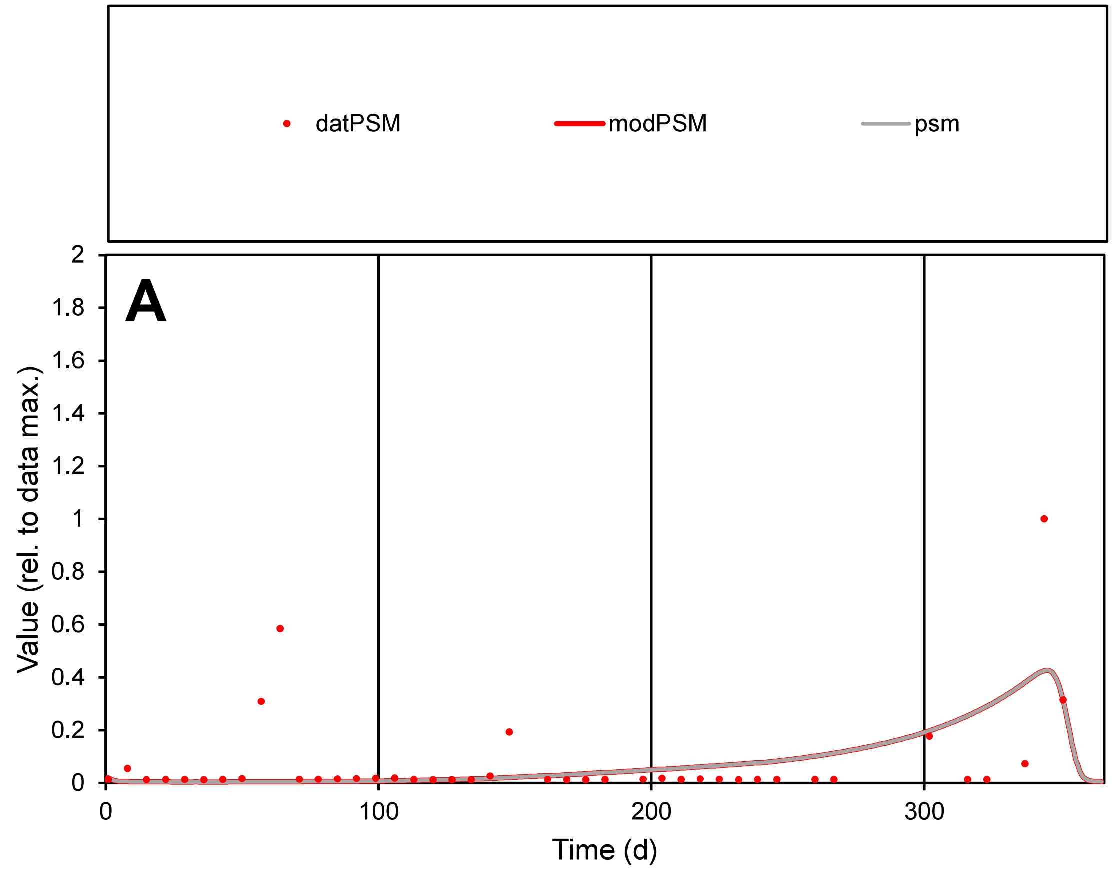

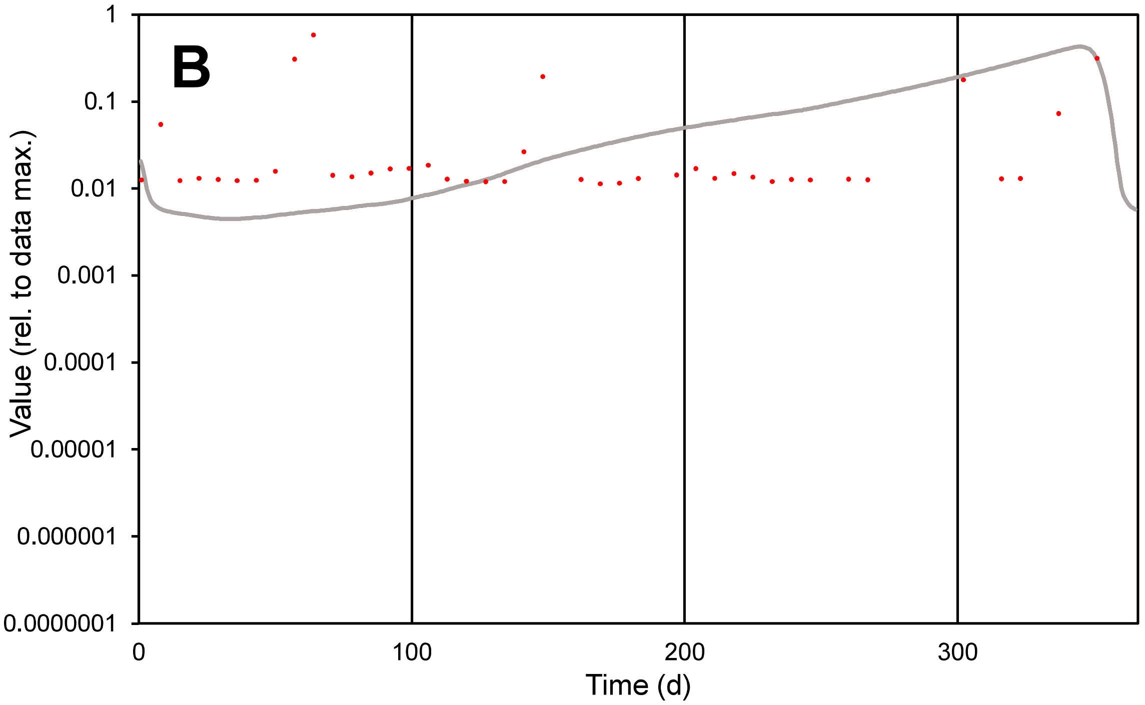

C1

Variables

PSM	Provide jtd or Dst
CalibOnly?	
2	1 to reproduce of
oNormalize?	
2	1=no, 2=yes

ofxn calc*
2.10849
ofxn mod
2.11968

sRun
RunA1
zout
1

Ave
Data
0.00019
Model
0.00017

data/vMax
0.00245

ofxng
319.391

Min d/m
2.53461

ave/min d
6.65686
ave/min m
15.3193

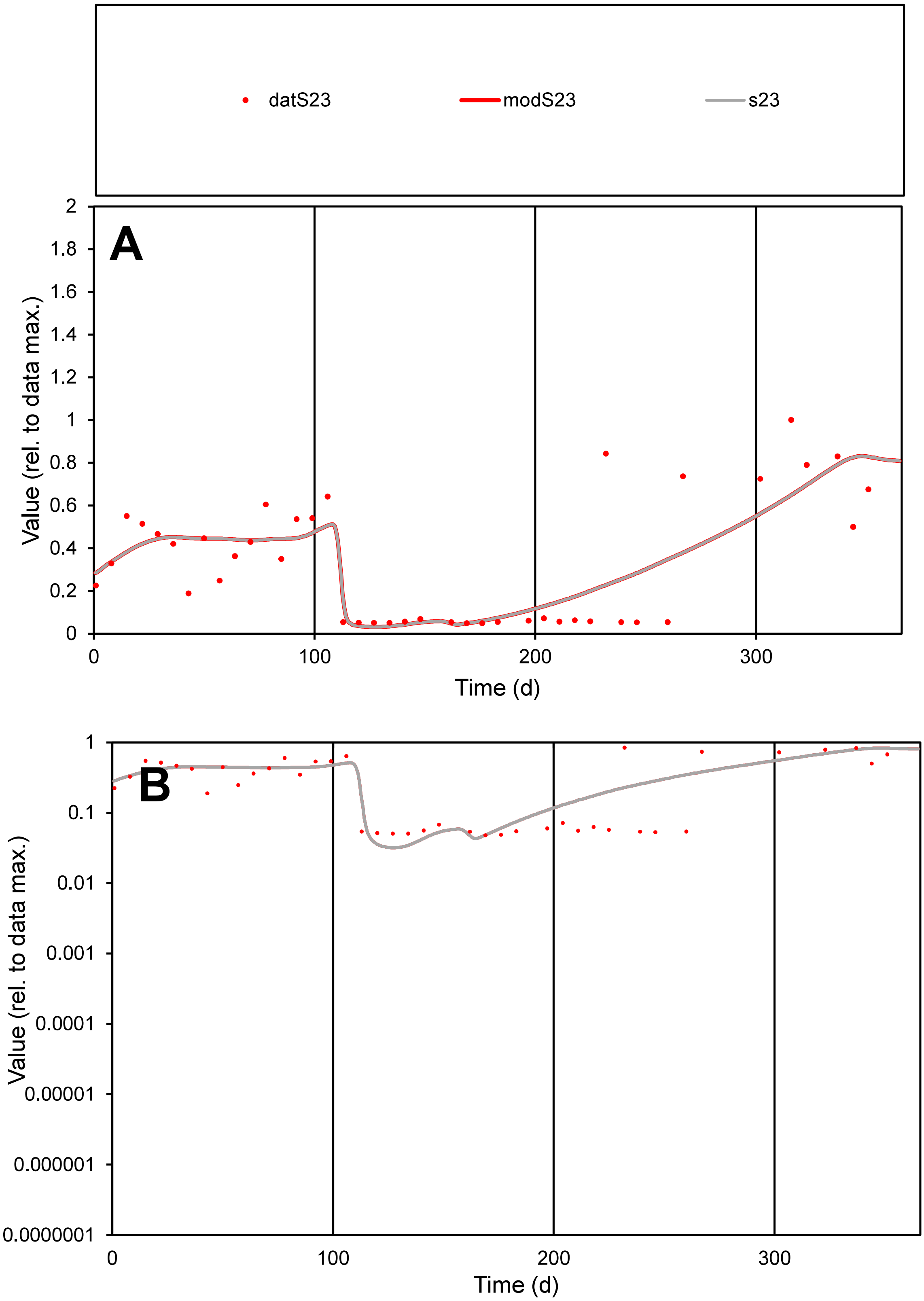

C1

Variables
S23 Provide jtd or Dst
CalibOnly?
2 1 to reproduce of
oNormalize?
2 1=no, 2=yes

ofxn calc*
0.513
ofxn mod
0.5299

sRun
RunA1
zout
1

Ave
Data
0.00112
Model
0.00113

data/vMax
0.00337

ofxng
319.391

Min d/m
1.5098

ave/min d
6.91938
ave/min m
10.5383

330_Calib_S_A

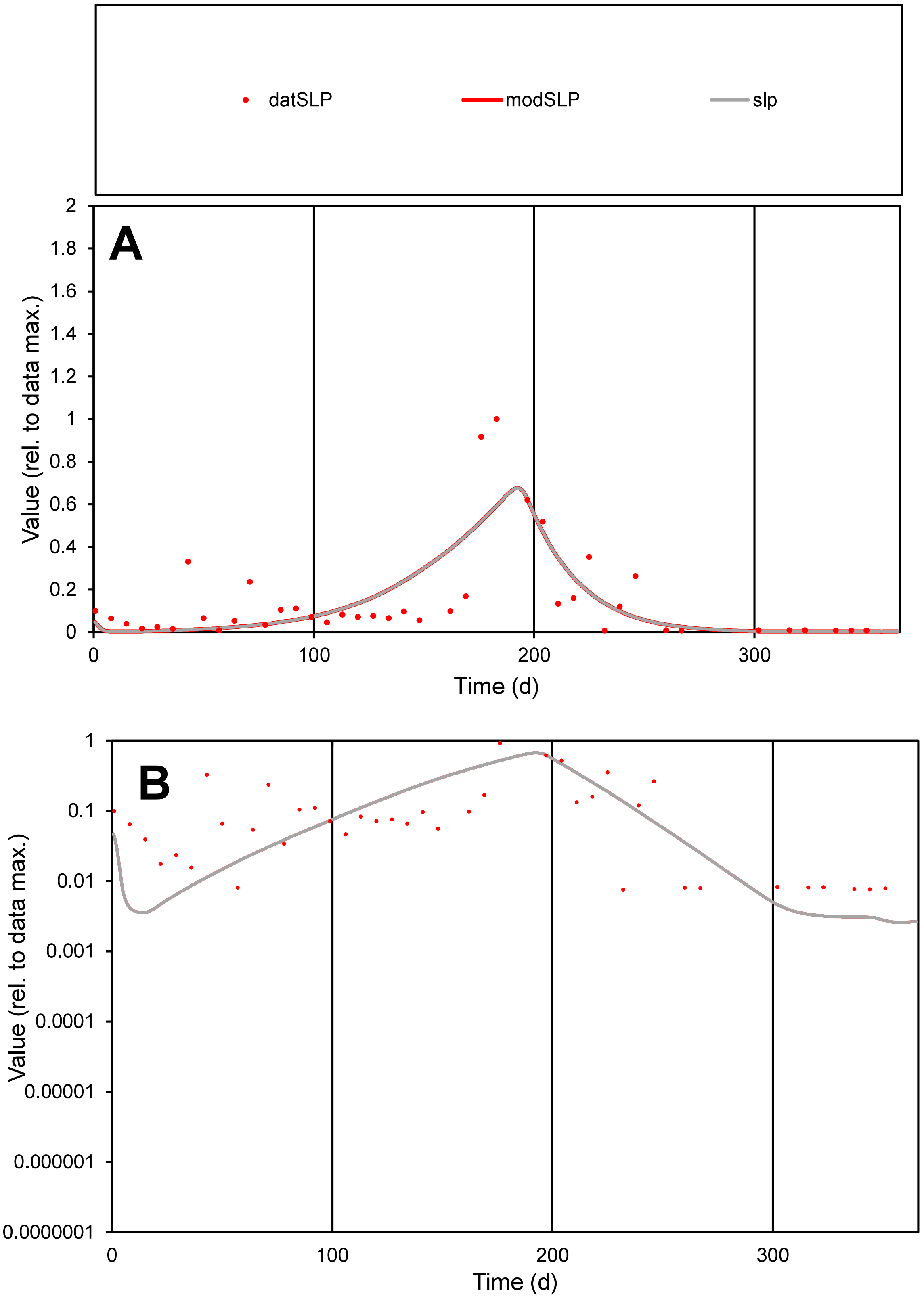

C1

Variables
SLP Provide jtd or Dst
CalibOnly?
2 1 to reproduce of
oNormalize?
2 1=no, 2=yes

ofxn calc*
0.97582
ofxn mod
1.00157

sRun
RunA1
zout
1

Ave
Data
0.0005
Model
0.00046

data/vMax
0.00337

ofxng
319.391

Min d/m
2.81284

ave/min d
19.4107
ave/min m
51.0426

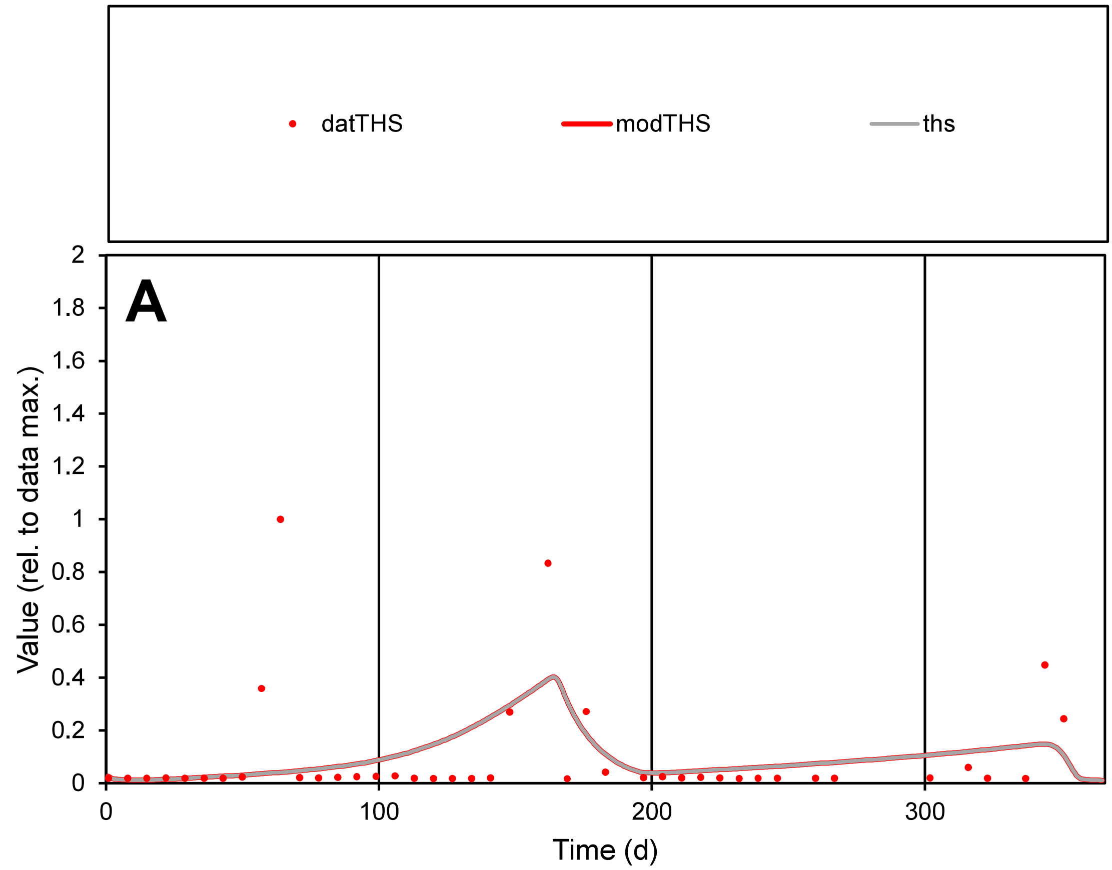

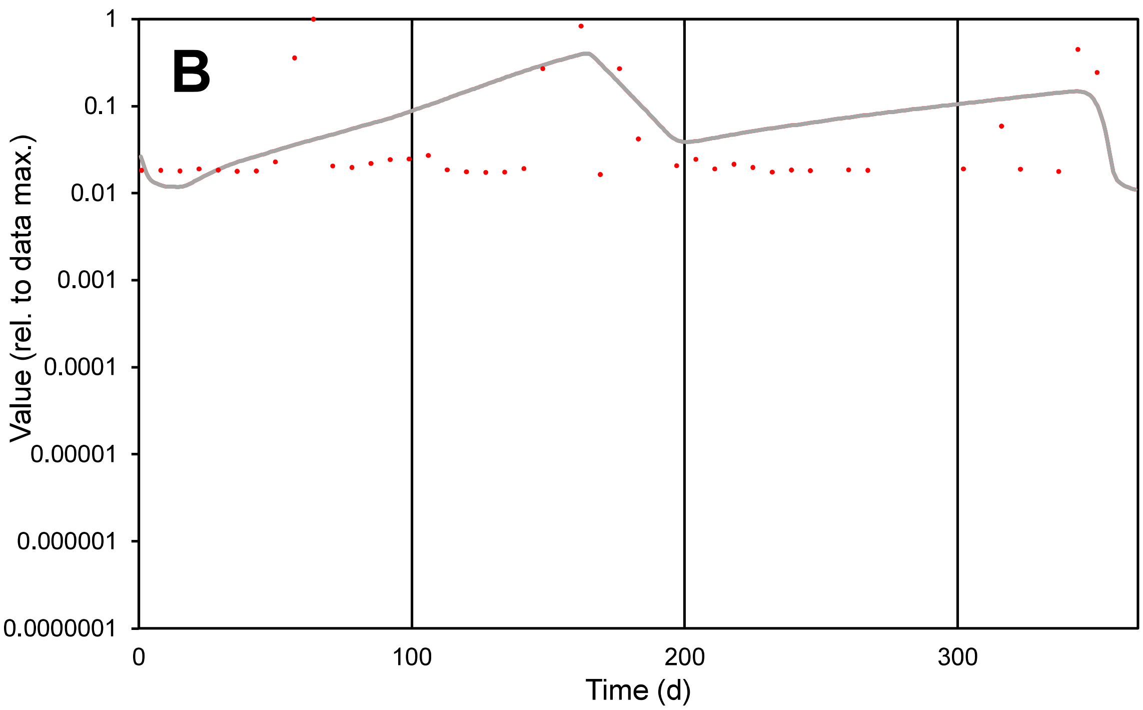

C1

Variables

THS Provide jtd or Dst

CalibOnly?

2 1 to reproduce of

oNormalize?

2 1=no, 2=yes

ofxn calc*

1.96694

ofxn mod

1.98339

sRun

RunA1

zout

1

Ave

Data

0.00017

Model

0.00017

data/vMax

0.00176

ofxng

319.391

Min d/m

1.36527

ave/min d

6.02957

ave/min m

8.15305

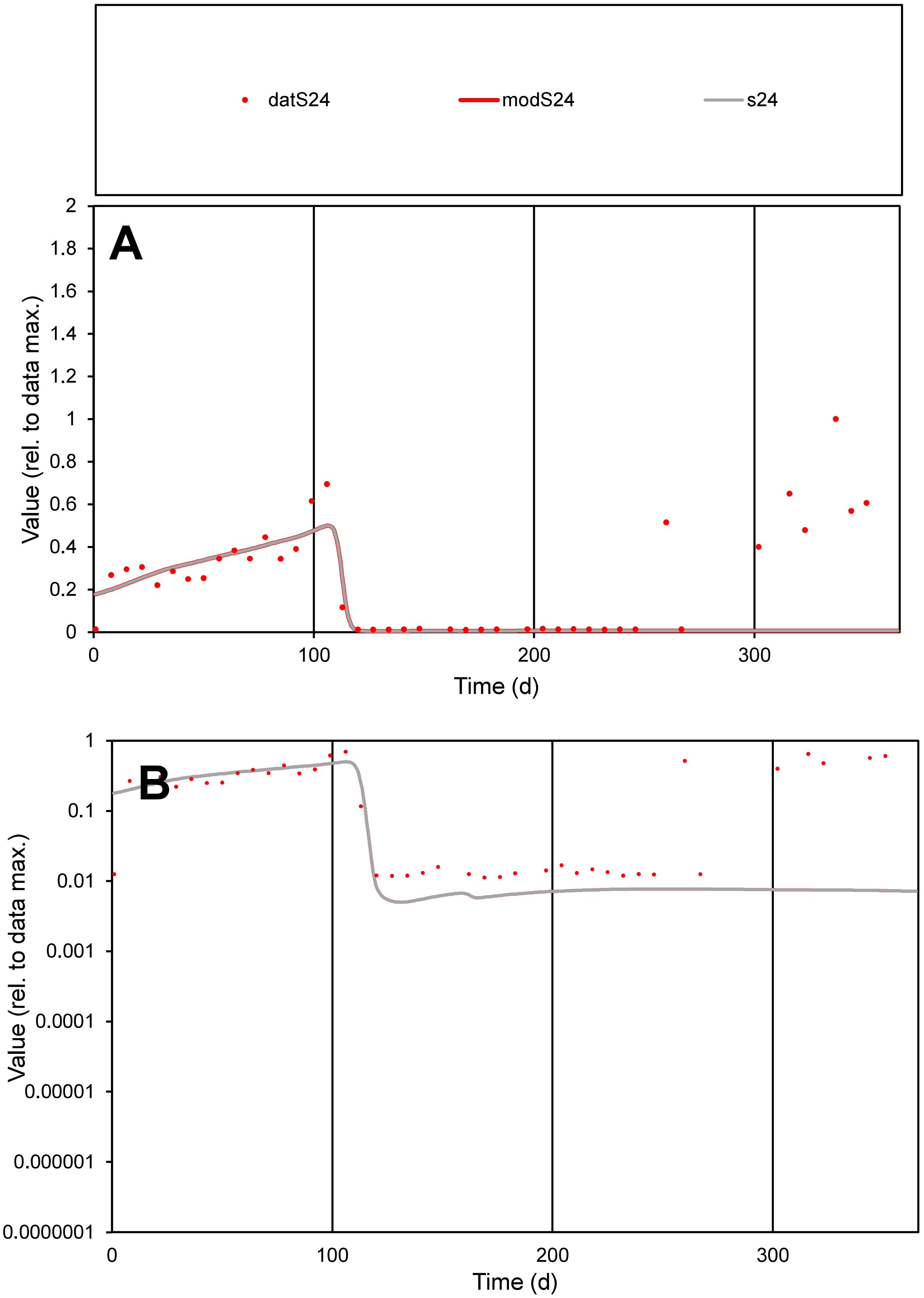

C1

Variables
S24 Provide jtd or Dst
CalibOnly?
2 1 to reproduce of
oNormalize?
2 1=no, 2=yes

ofxn calc*
1.08867
ofxn mod
1.09497

sRun
RunA1
zout
1

Ave
Data
0.00124
Model
0.00072

data/vMax
0.00519

ofxng
319.391

Min d/m
2.20618

ave/min d
21.1414
ave/min m
27.3066

330_Calib_S_A

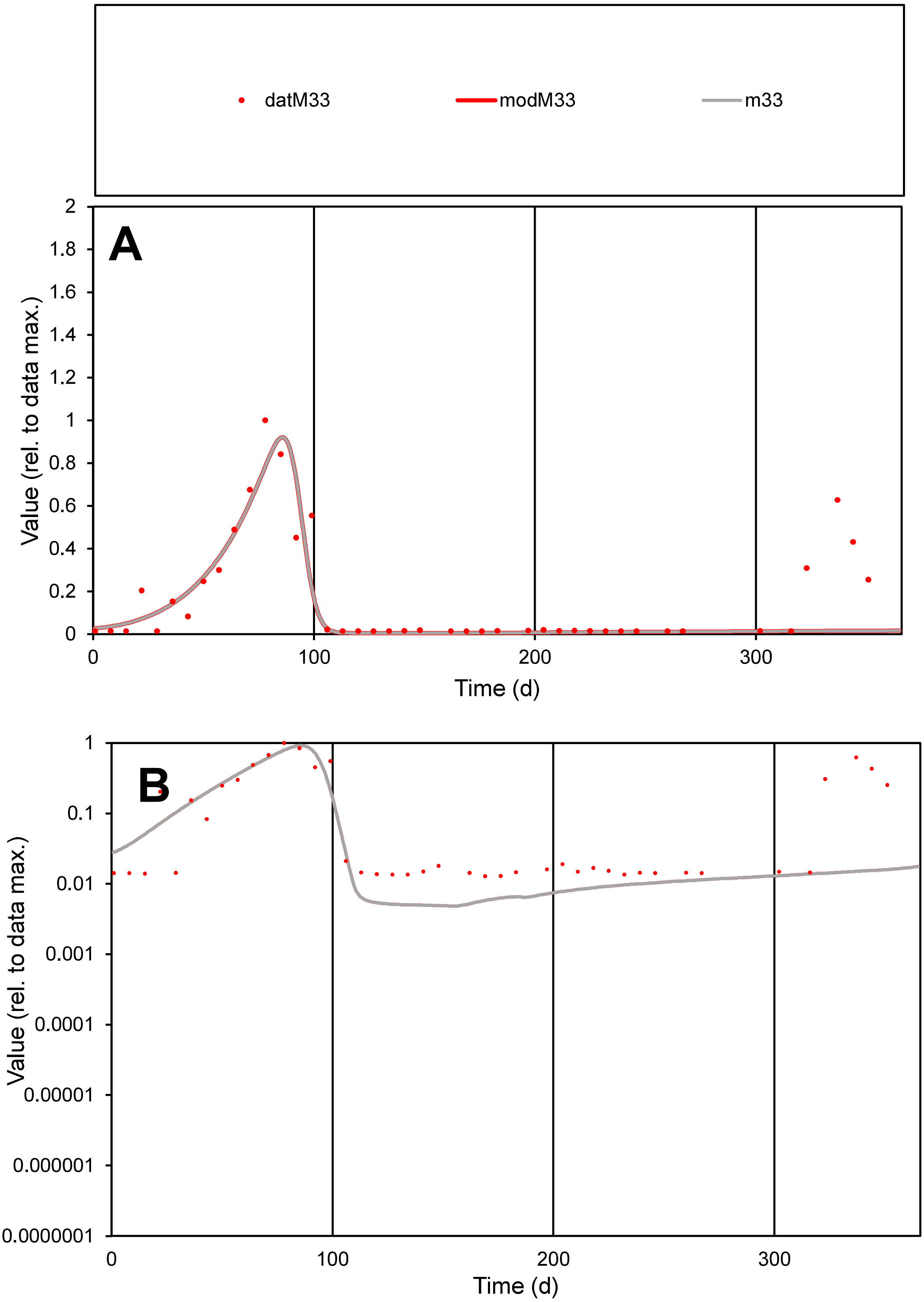

C1

Variables
M33 Provide jtd or Dst
CalibOnly?
2 1 to reproduce of
oNormalize?
2 1=no, 2=yes

ofxn calc*
0.87506
ofxn mod
0.90922

sRun
RunA1
zout
1

Ave
Data
0.0009
Model
0.00068

data/vMax
0.00538

ofxng
319.391

Min d/m
2.6174

ave/min d
13.0582
ave/min m
25.9279

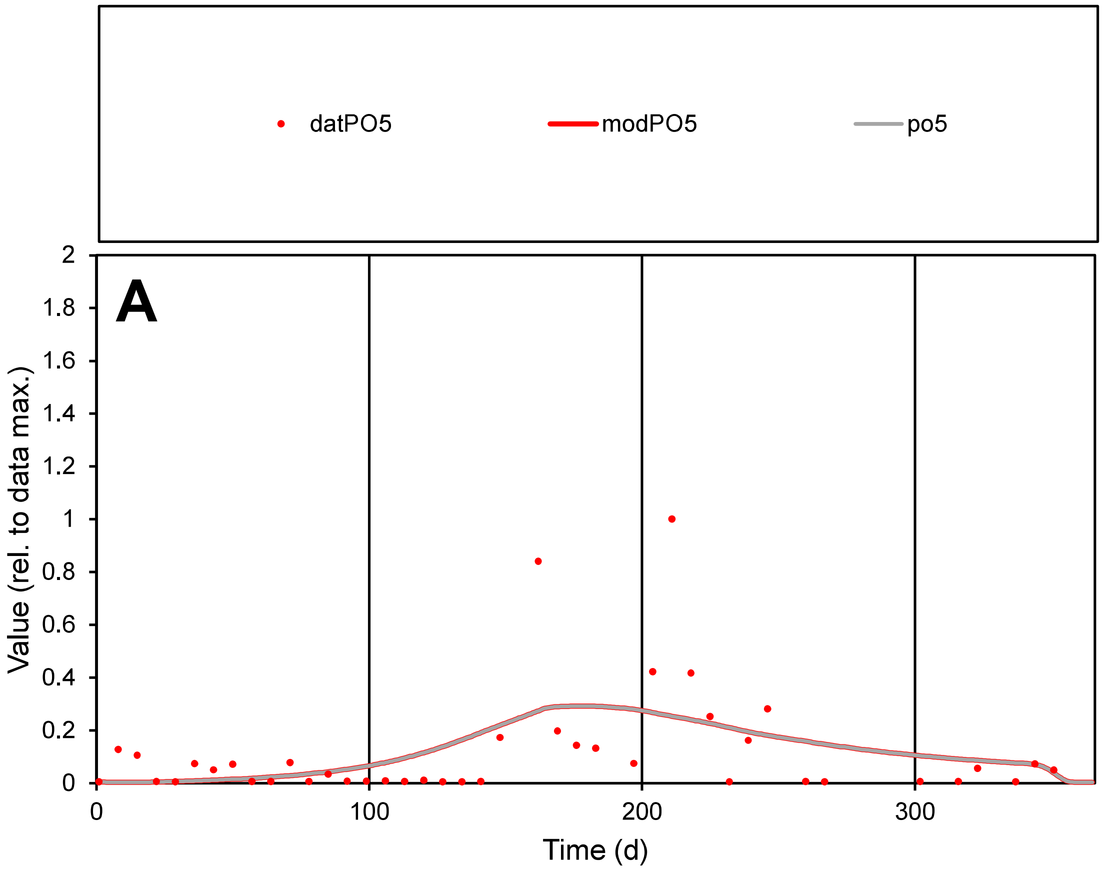

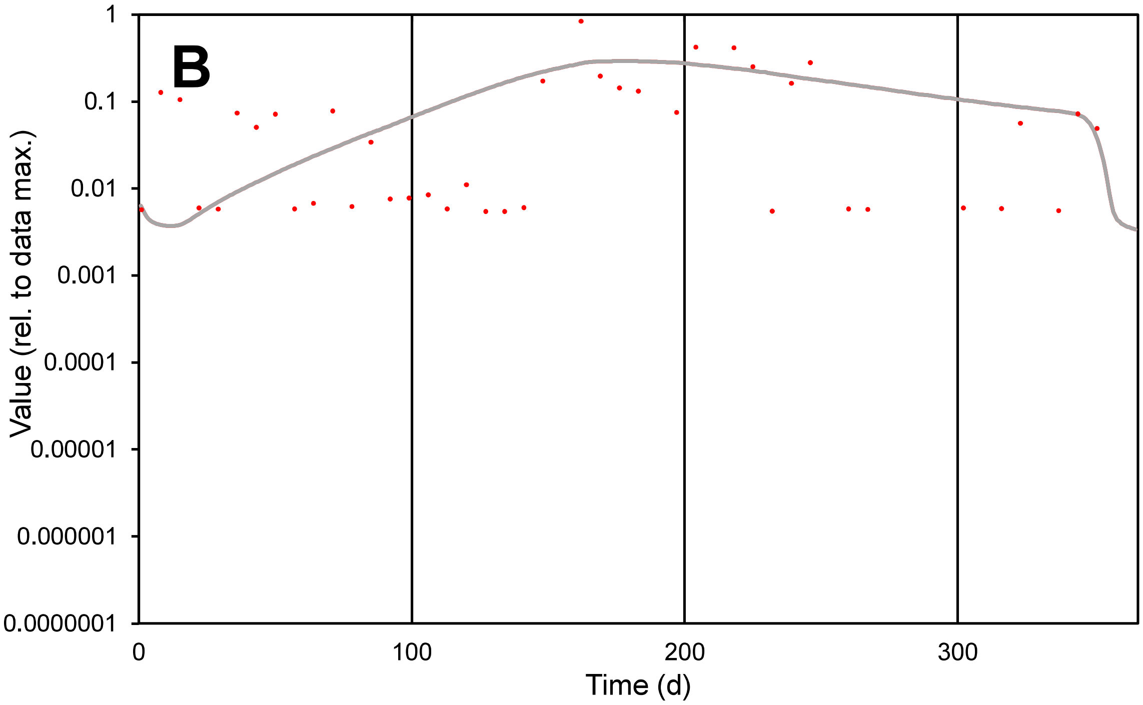

C1

Variables
PO5 Provide jtd or Dst
CalibOnly?
2 1 to reproduce of
oNormalize?
2 1=no, 2=yes

ofxn calc*
1.49167
ofxn mod
1.50454

sRun
RunA1
zout
1

Ave
Data
0.00107
Model
0.0011

data/vMax
0.00905

ofxng
319.391

Min d/m
1.45075

ave/min d
21.5851
ave/min m
32.2949

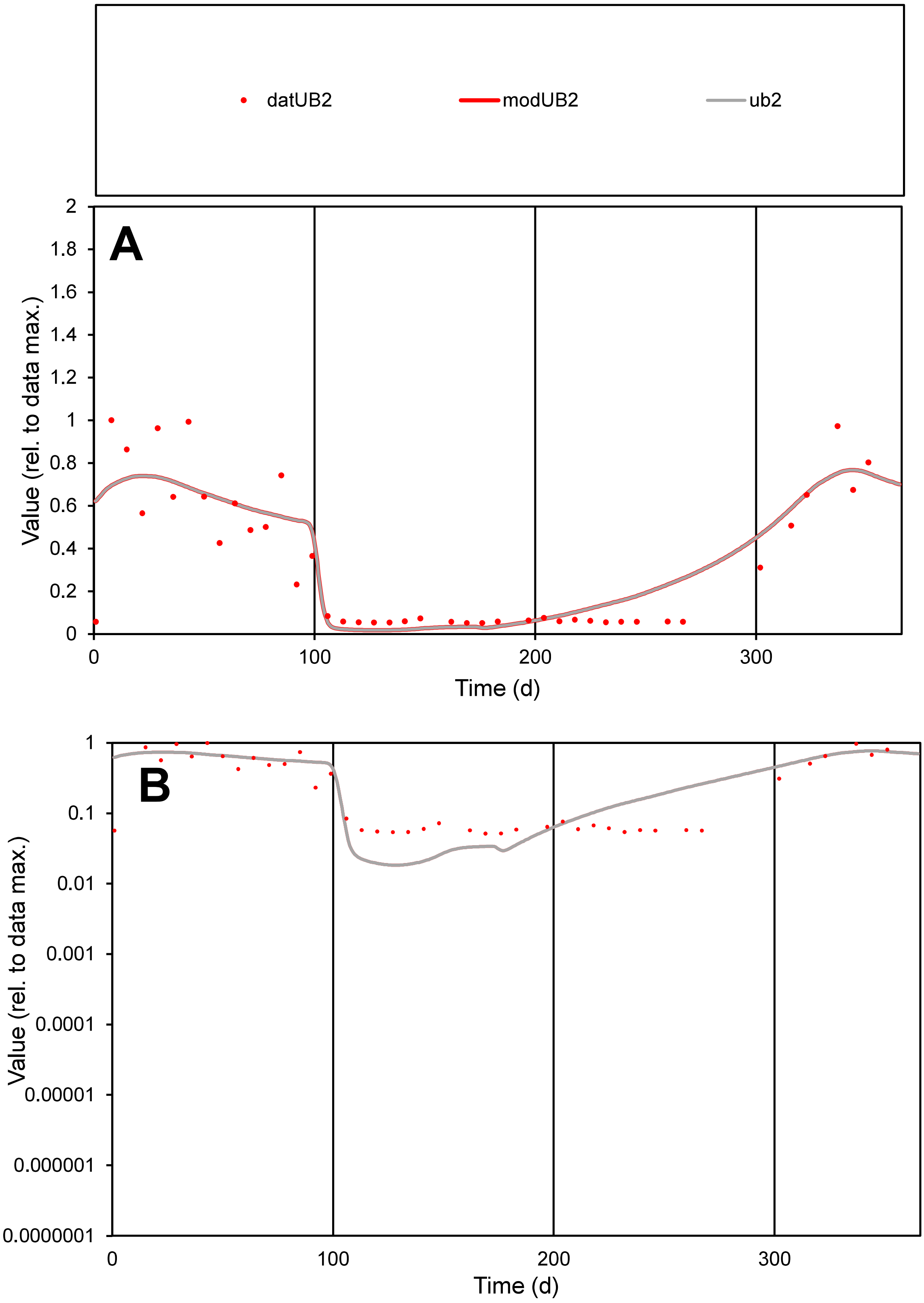

C1

Variables
UB2 Provide jtd or Dst
CalibOnly?
2 1 to reproduce of
oNormalize?
2 1=no, 2=yes

ofxn calc*
0.4591
ofxn mod
0.48251

sRun
RunA1
zout
1

Ave
Data
0.00069
Model
0.00074

data/vMax
0.00204

ofxng
319.391

Min d/m
2.78946

ave/min d
6.62836
ave/min m
19.6831

330_Calib_S_A

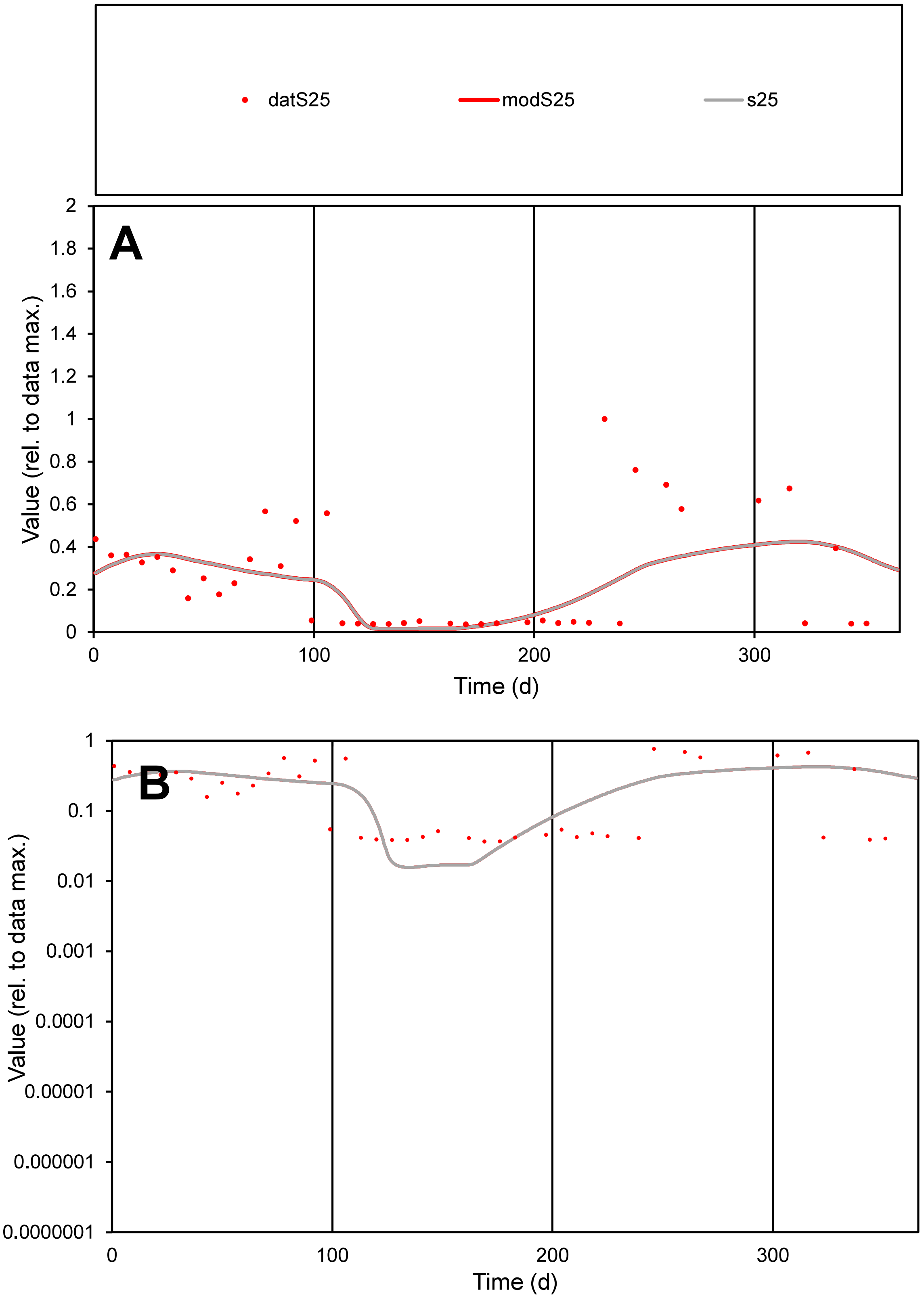

datS25
modS25
s25
A
Value (rel. to data max.)
Time (d)
B

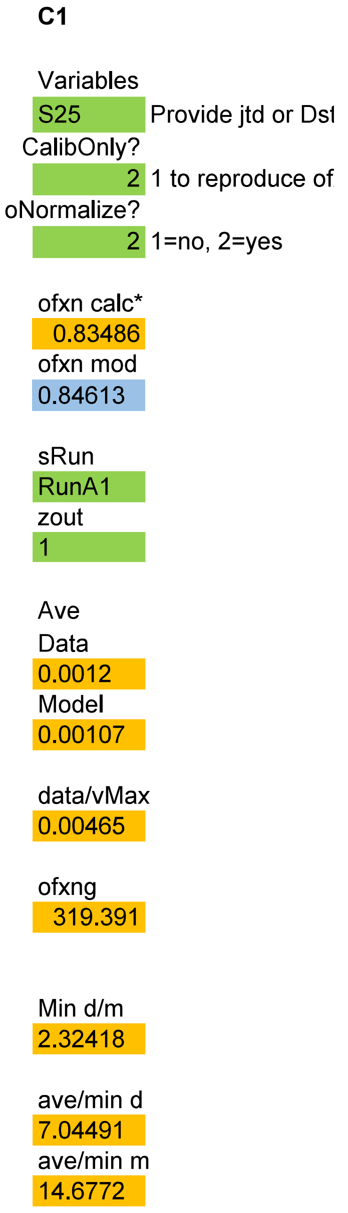

C1
Variables
S25 Provide jtd or Dst
CalibOnly?
2 1 to reproduce of
oNormalize?
2 1=no, 2=yes
ofxn calc*
0.83486
ofxn mod
0.84613
sRun
RunA1
zout
1
Ave
Data
0.0012
Model
0.00107
data/vMax
0.00465
ofxng
319.391
Min d/m
2.32418
ave/min d
7.04491
ave/min m
14.6772

330_Calib_S_A

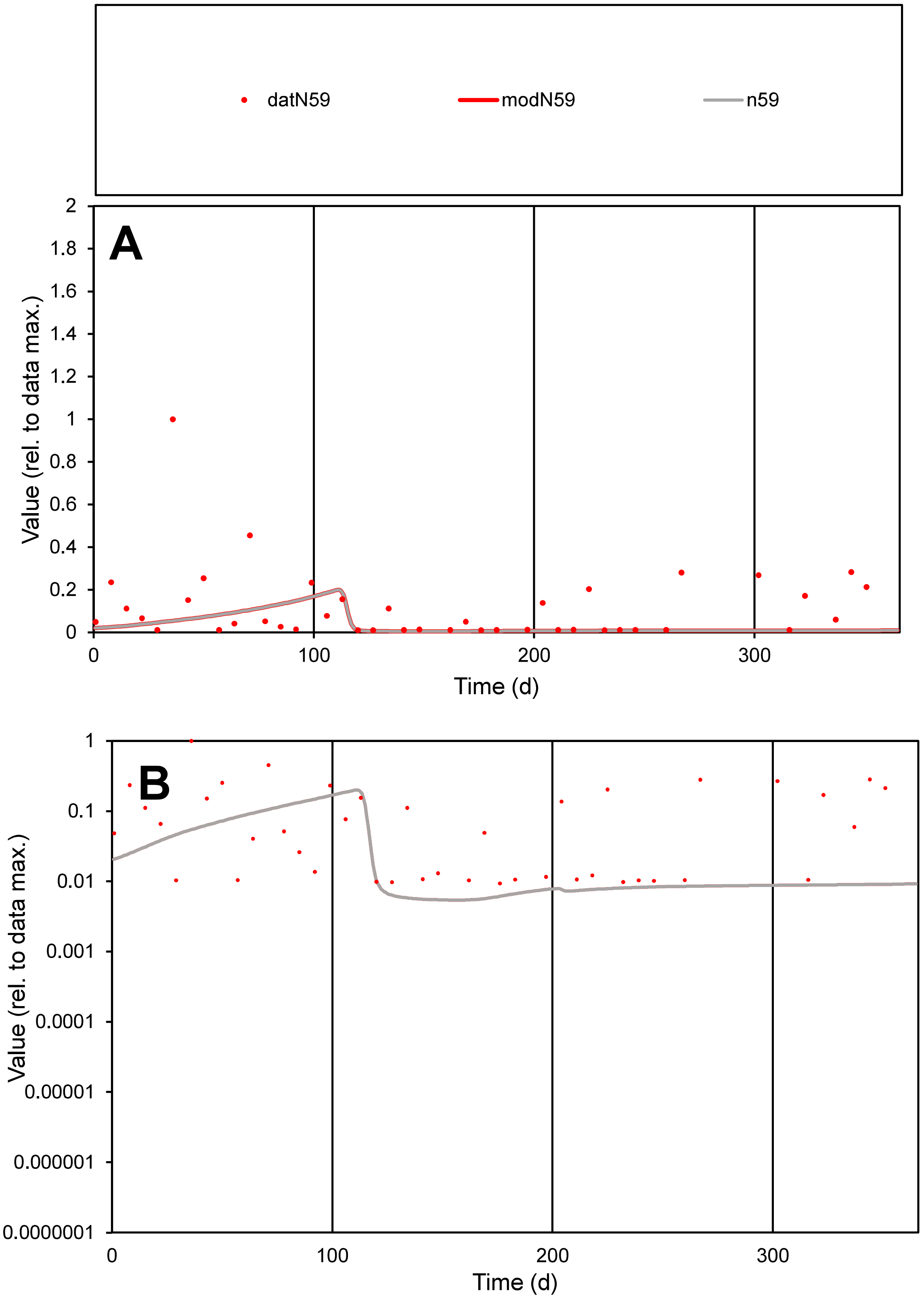

C1

Variables
N59 Provide jtd or Dst
CalibOnly?
2 1 to reproduce of
oNormalize?
2 1=no, 2=yes

ofxn calc*
1.63908
ofxn mod
1.64261

sRun
RunA1
zout
1

Ave
Data
0.00052
Model
0.00019

data/vMax
0.0045

ofxng
319.391

Min d/m
1.71621

ave/min d
12.4018
ave/min m
7.64306

330_Calib_S_A

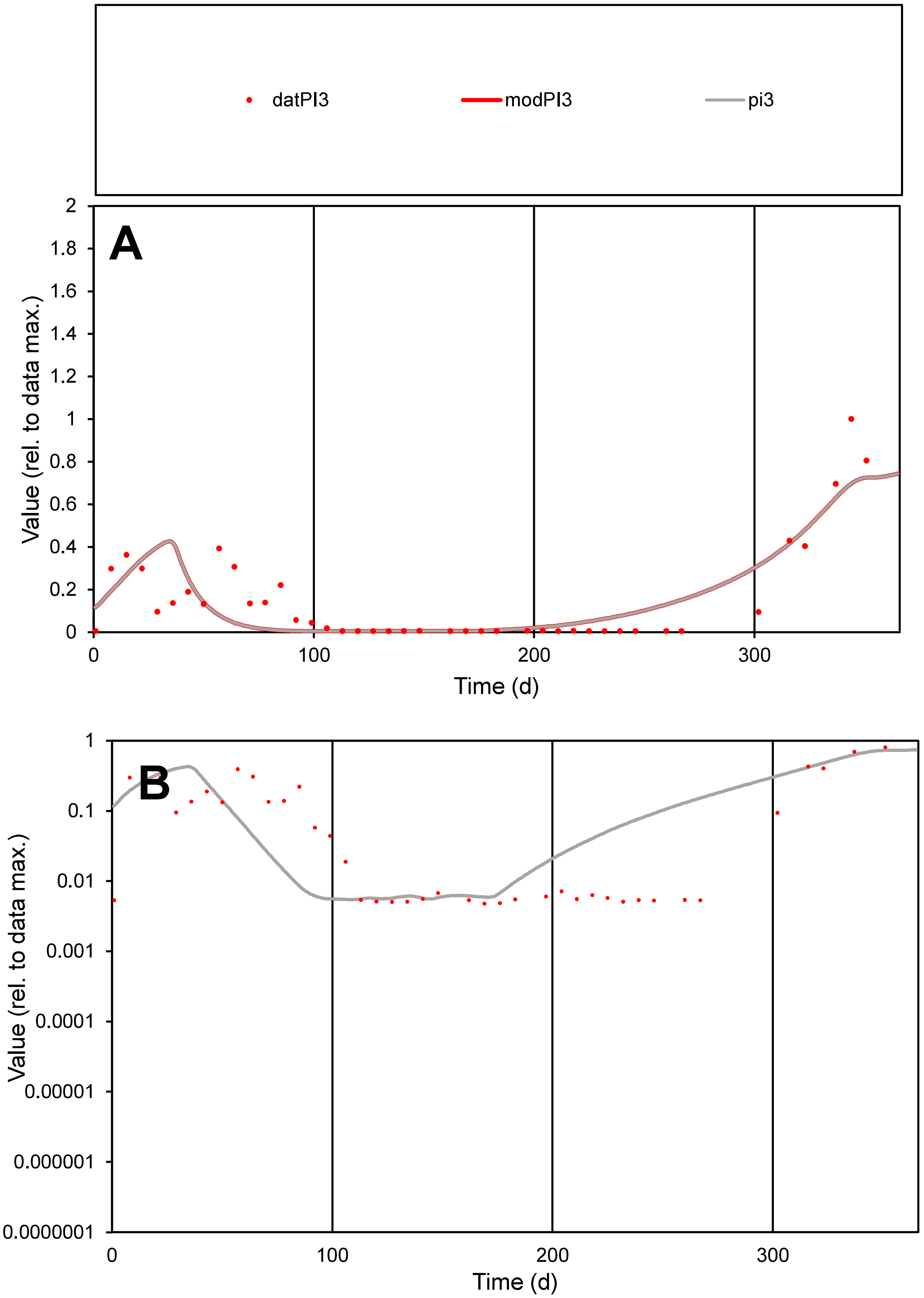

C1

Variables

PI3	Provide jtd or Dst
CalibOnly?	
2	1 to reproduce of
oNormalize?	
2	1=no, 2=yes

ofxn calc*
0.8109
ofxn mod
0.83689

sRun
RunA1
zout
1

Ave
Data
0.00226
Model
0.00225

data/vMax
0.01493

ofxng
319.391

Min d/m
0.88469

ave/min d
31.5937
ave/min m
27.7731

330_Calib_S_A

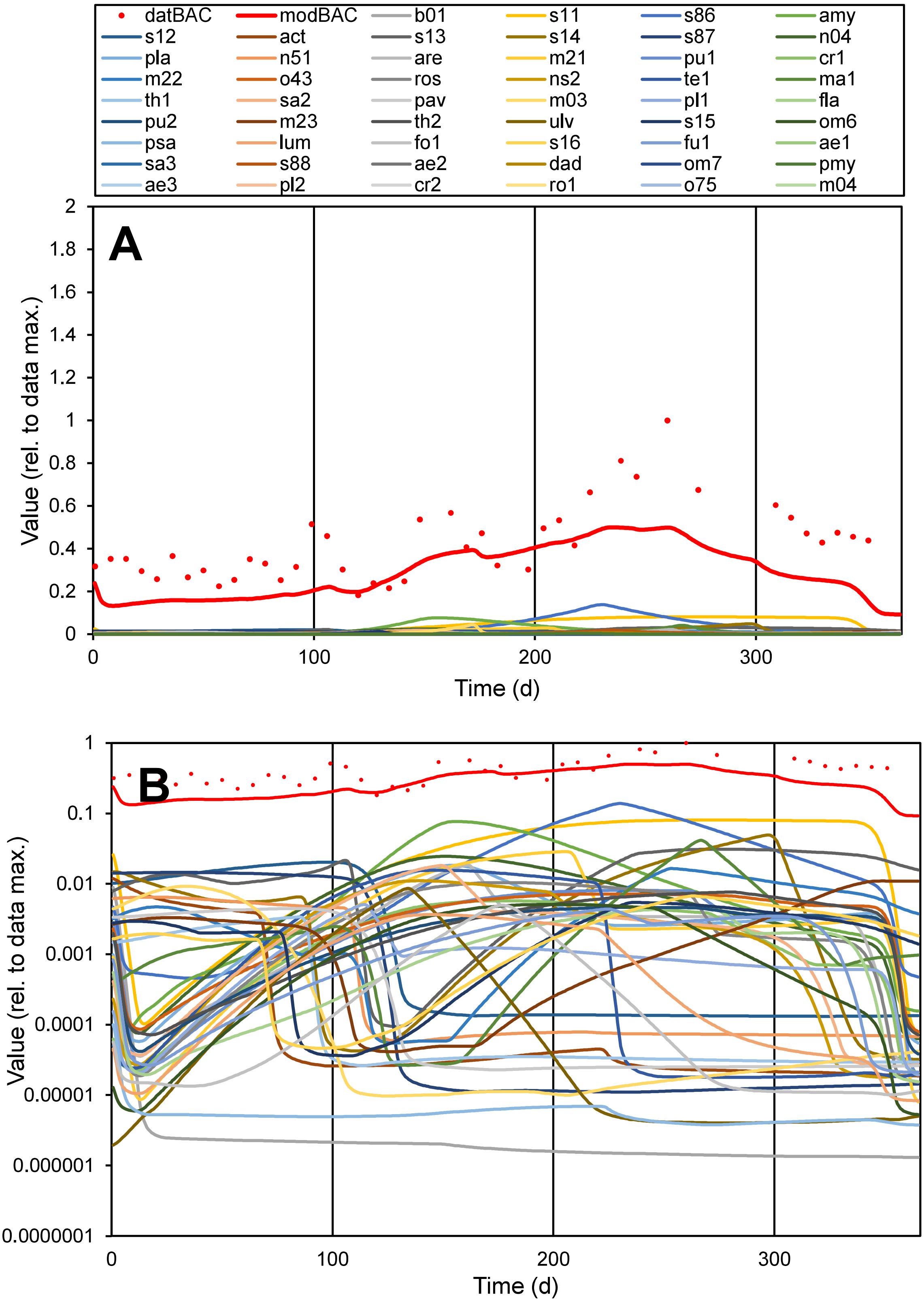

C1

Variables

BAC Provide jtd or Dst

CalibOnly?

2 1 to reproduce of

oNormalize?

2 1=no, 2=yes

ofxn calc*

0.45462

ofxn mod

0.46585

sRun

RunA1

zout

1

Ave

Data

0.90039

Model

0.58465

data/vMax

2.134

ofxng

319.391

Min d/m

1.36456

ave/min d

2.32418

ave/min m

2.05937

331_Calib_S_A

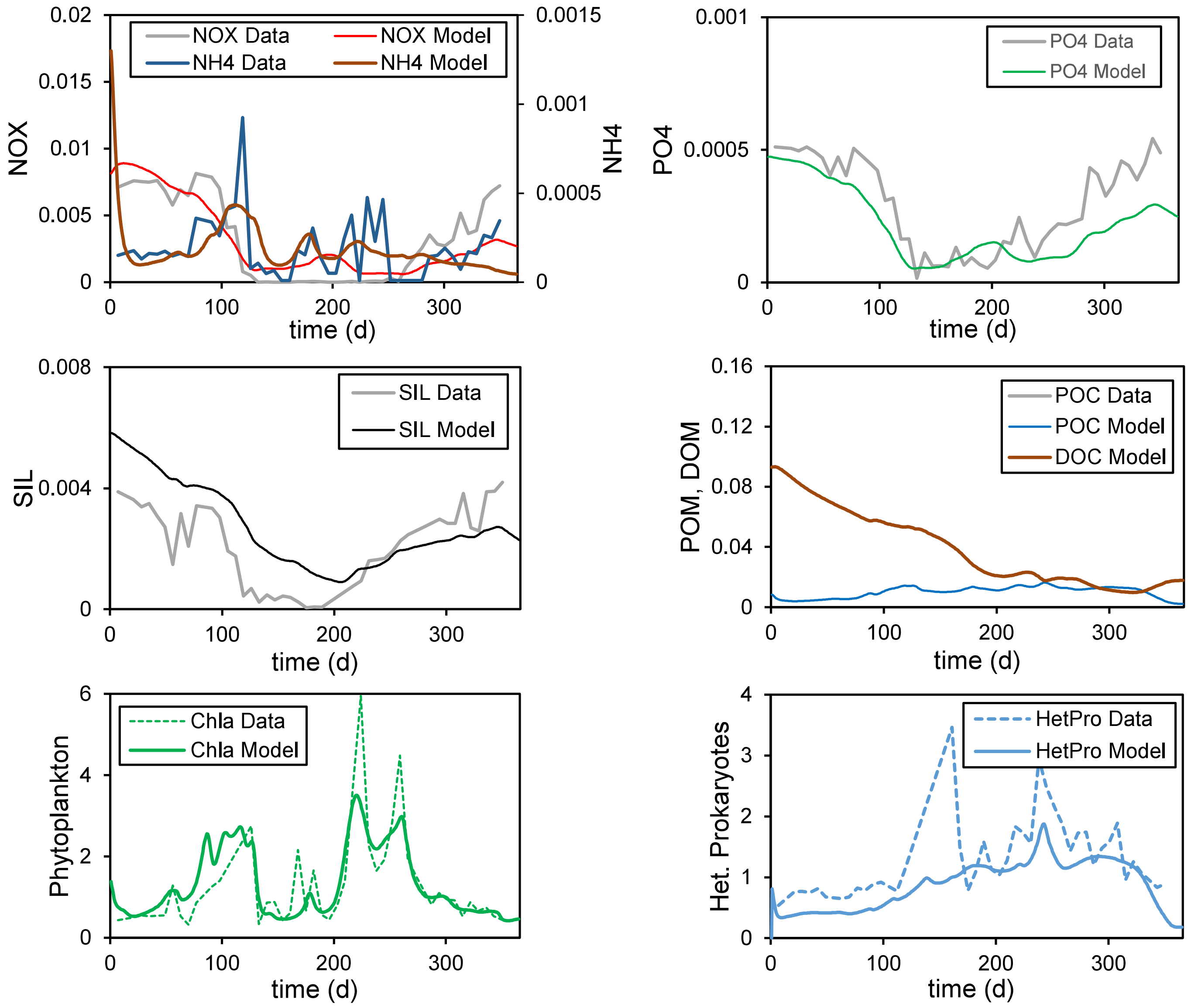
NOX Data
NOX Model
NH4 Data
NH4 Model
NOX
NH4
0.02
0.015
0.01
0.005
0
0.0015
0.001
0.0005
0
0
100
200
300
time (d)
PO4 Data
PO4 Model
PO4
0.001
0.0005
0
0
100
200
300
time (d)
SIL Data
SIL Model
SIL
0.008
0.004
0
0
100
200
300
time (d)
POC Data
POC Model
DOC Model
POM, DOM
0.16
0.12
0.08
0.04
0
0
100
200
300
time (d)
Chla Data
Chla Model
Phytoplankton
6
4
2
0
0
100
200
300
time (d)
HetPro Data
HetPro Model
Het. Prokaryotes
4
3
2
1
0
0
100
200
300
time (d)

331_Calib_S_A

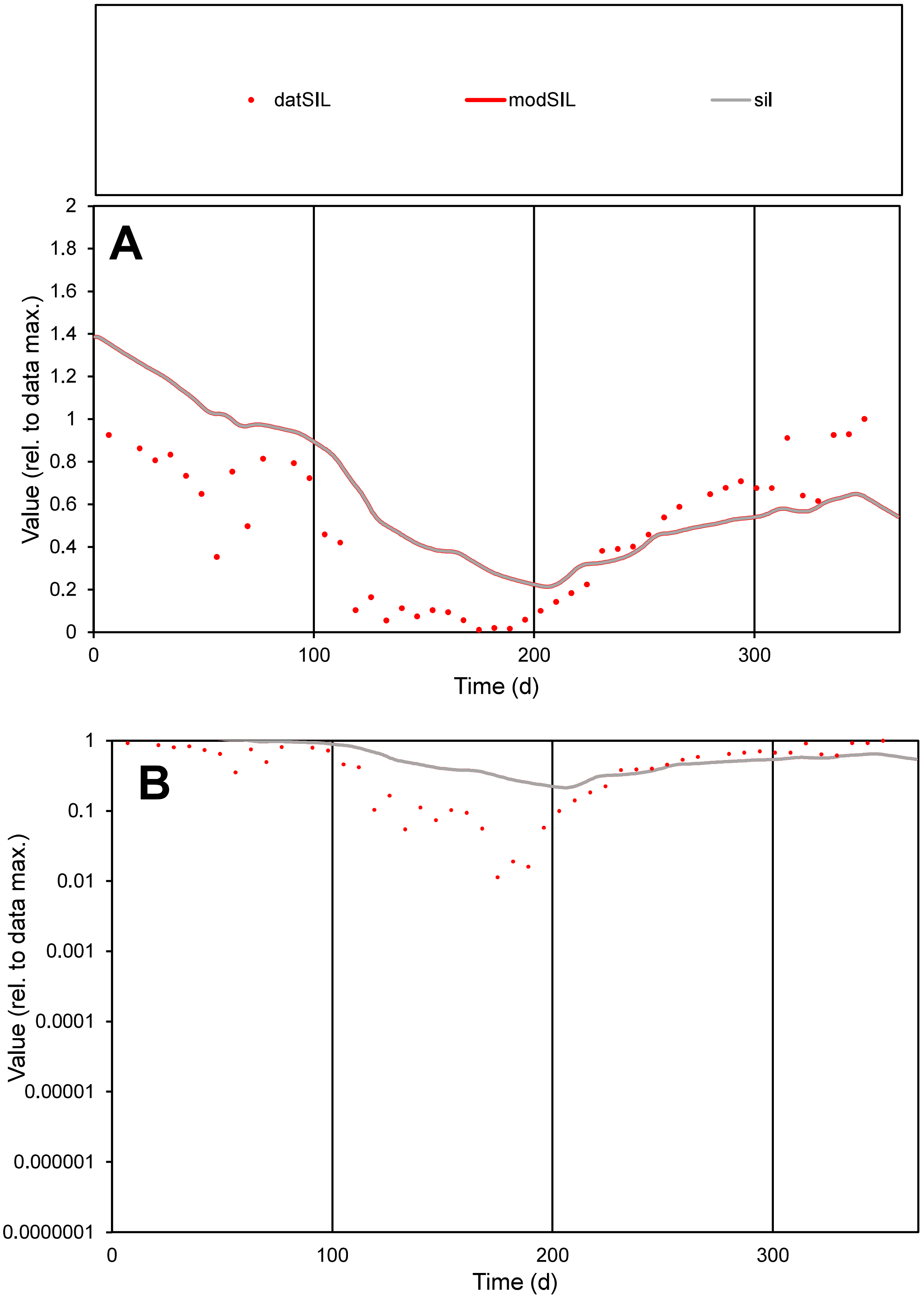

C1

Variables
SIL Provide jtd or Dst
CalibOnly?
2 1 to reproduce of
oNormalize?
2 1=no, 2=yes

ofxn calc*
1.8352
ofxn mod
1.88636

sRun
RunA1
zout
1

Ave
Data
0.00199
Model
0.0026

data/vMax
0.0042

ofxng
311.734

Min d/m
0.05269

ave/min d
41.9455
ave/min m
2.88296

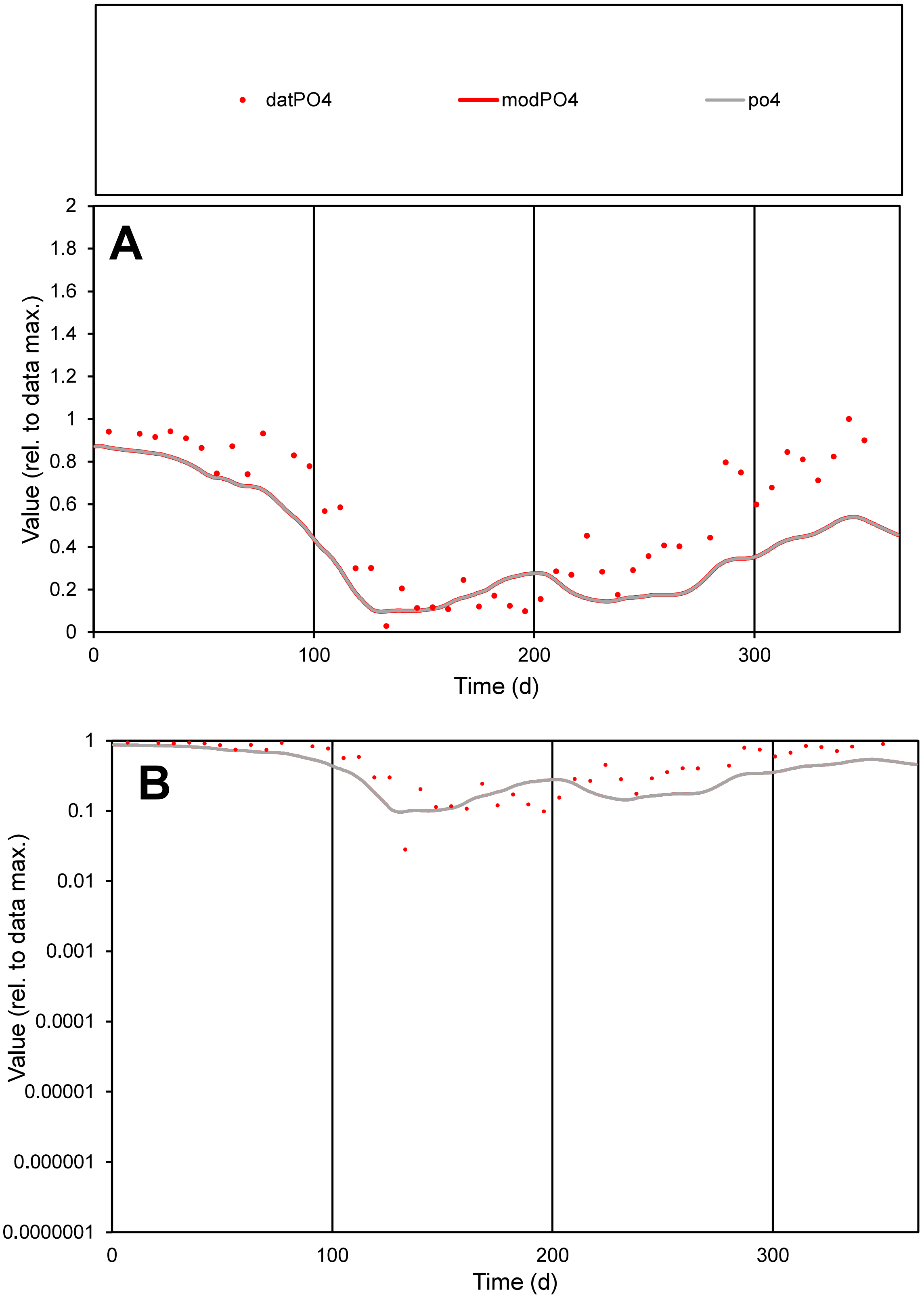

C1

Variables
PO4 Provide jtd or Dst
CalibOnly?
2 1 to reproduce of
oNormalize?
2 1=no, 2=yes

ofxn calc*
1.23266
ofxn mod
1.28349

sRun
RunA1
zout
1

Ave
Data
0.00029
Model
0.00021

data/vMax
0.00054

ofxng
311.734

Min d/m
0.28762

ave/min d
18.851
ave/min m
3.87598

331_Calib_S_A

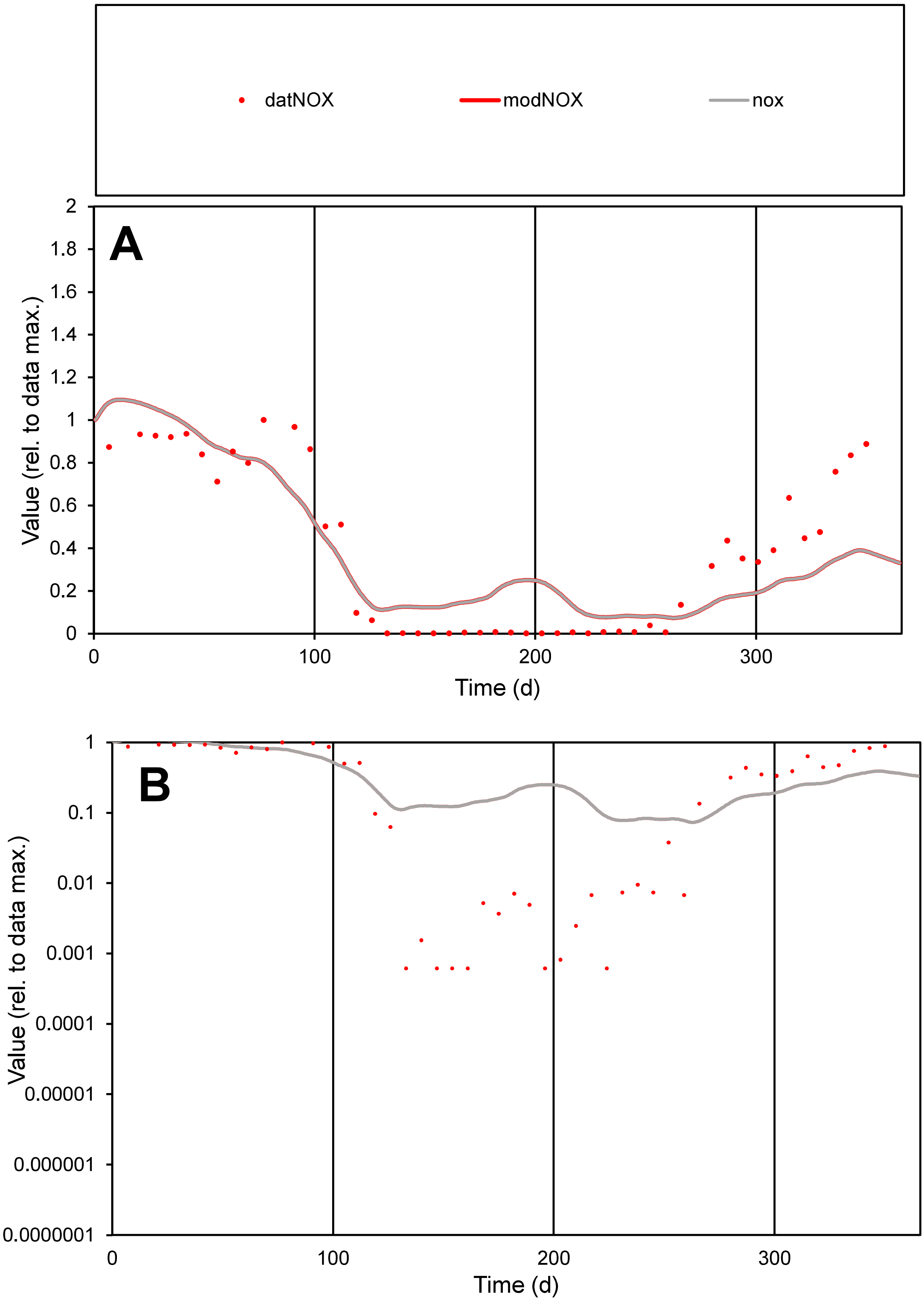

C1

Variables
NOX Provide jtd or Dst
CalibOnly?
2 1 to reproduce of
oNormalize?
2 1=no, 2=yes

ofxn calc*
1.55154
ofxn mod
1.62701

sRun
RunA1
zout
1

Ave
Data
0.0031
Model
0.00298

data/vMax
0.00814

ofxng
311.734

Min d/m
0.00818

ave/min d
619.62
ave/min m
4.87733

331_Calib_S_A

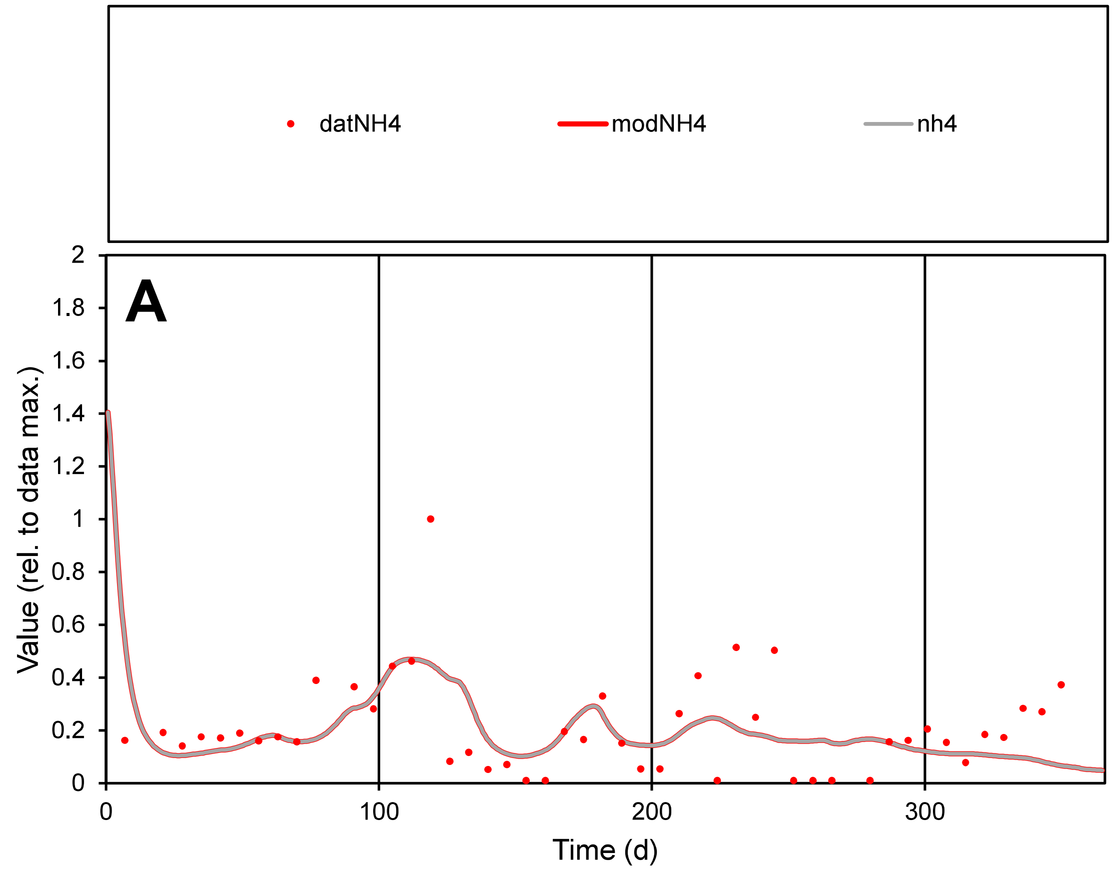

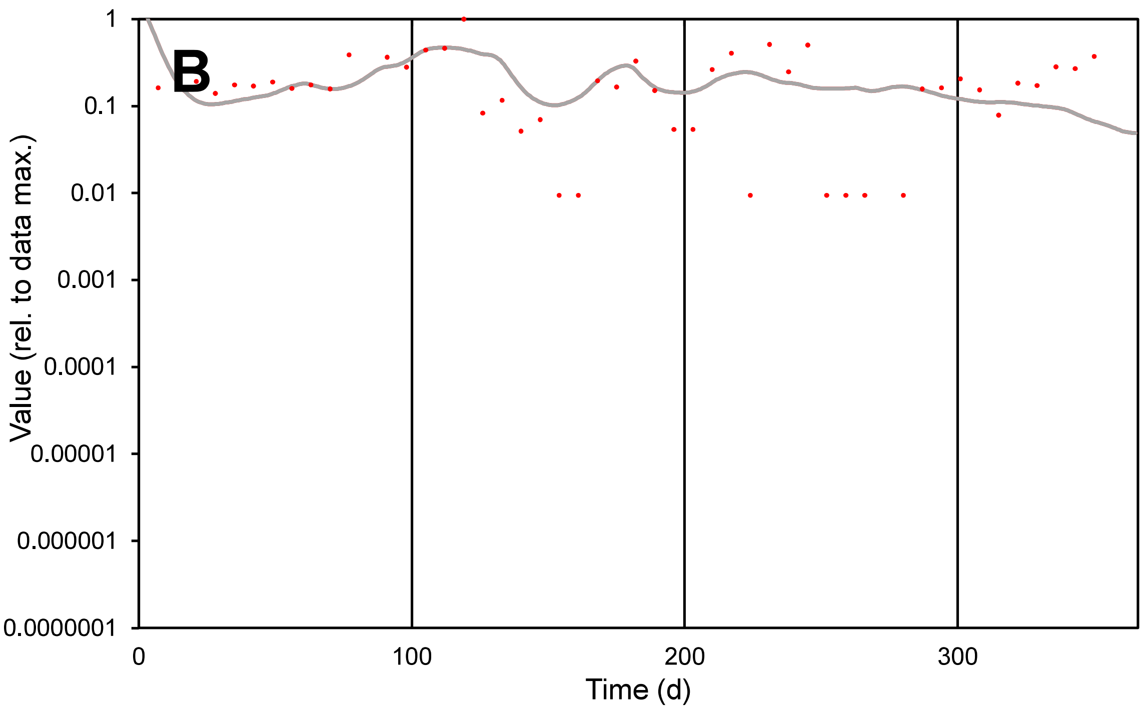

C1

Variables
NH4 Provide jtd or Dst
CalibOnly?
2 1 to reproduce of
oNormalize?
2 1=no, 2=yes

ofxn calc*
2.29179
ofxn mod
2.34057

sRun
RunA1
zout
1

Ave
Data
0.00019
Model
0.00018

data/vMax
0.00093

ofxng
311.734

Min d/m
0.1453

ave/min d
21.9847
ave/min m
2.91055

331_Calib_S_A

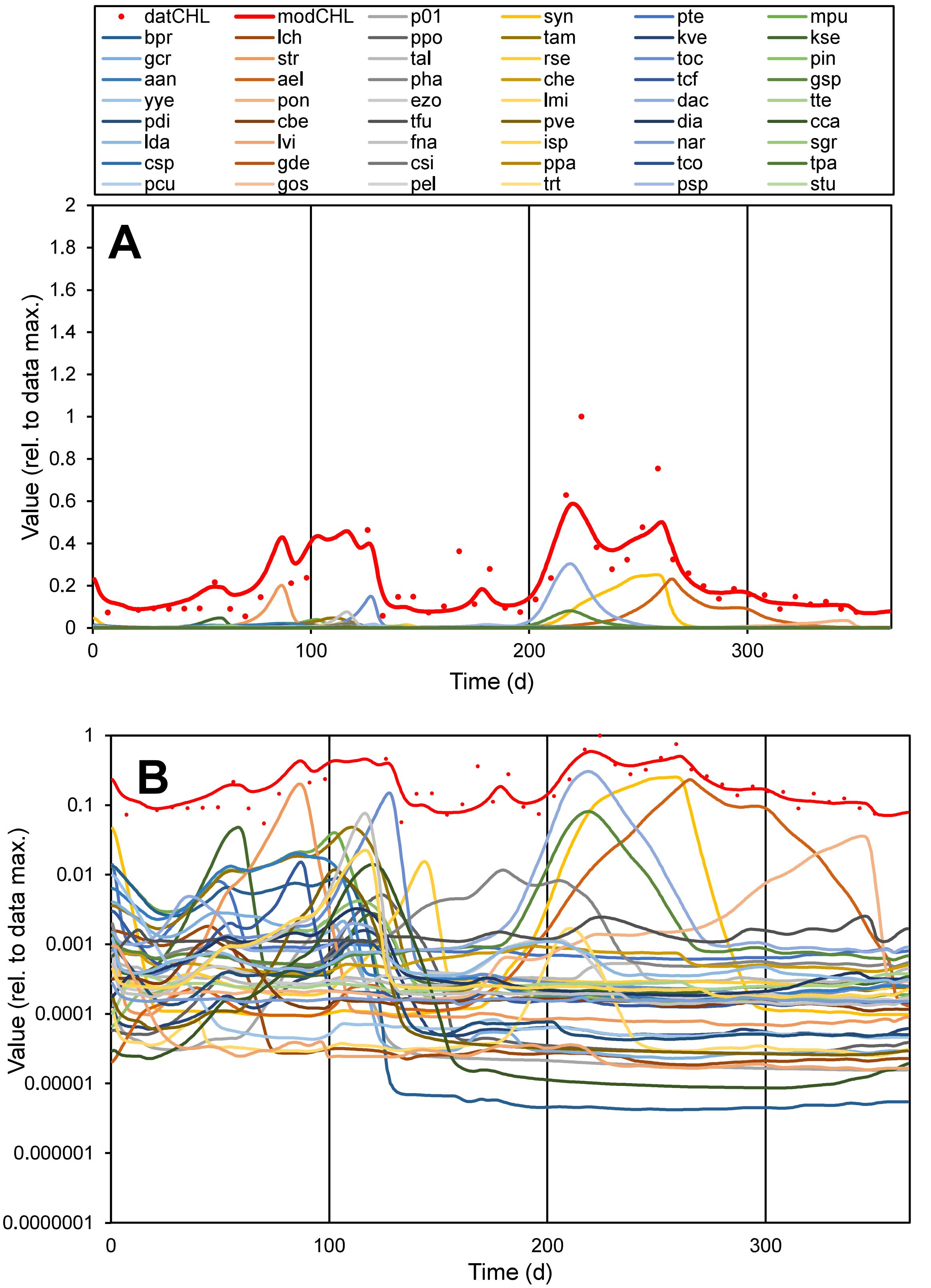

C1

Variables

CHL Provide jtd or Dst

CalibOnly?

2 1 to reproduce of

oNormalize?

2 1=no, 2=yes

ofxn calc*

4.74745

ofxn mod

5.00005

sRun

RunA1

zout

1

Ave

Data

1.27698

Model

1.19777

data/vMax

5.949

ofxng

311.734

Min d/m

0.74674

ave/min d

3.91232

ave/min m

2.74027

331_Calib_S_A

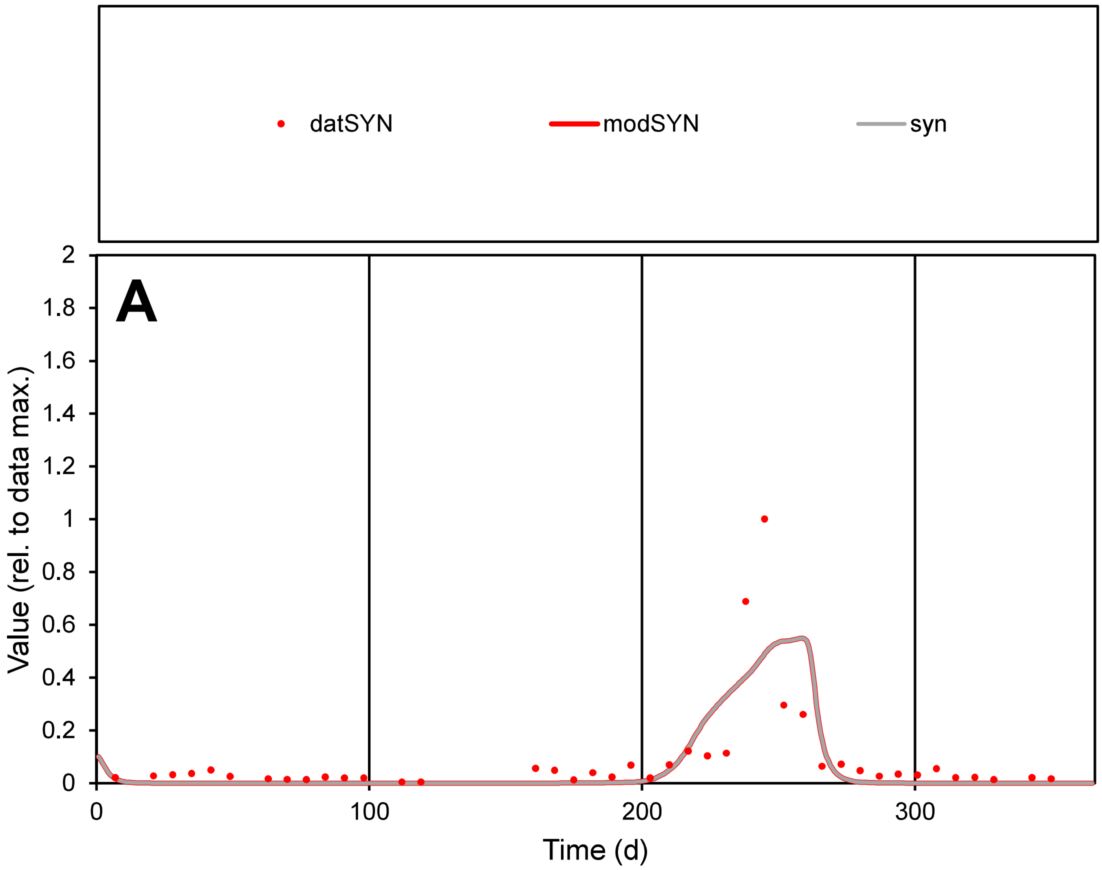

C1

Variables

SYN Provide jtd or Dst

CalibOnly?

2 1 to reproduce of

oNormalize?

2 1=no, 2=yes

ofxn calc*

1.31368

ofxn mod

1.33653

sRun

RunA1

zout

1

Ave

Data

28.8899

Model

23.7637

data/vMax

325.2

ofxng

311.734

Min d/m

21.1427

ave/min d

20.3307

ave/min m

353.573

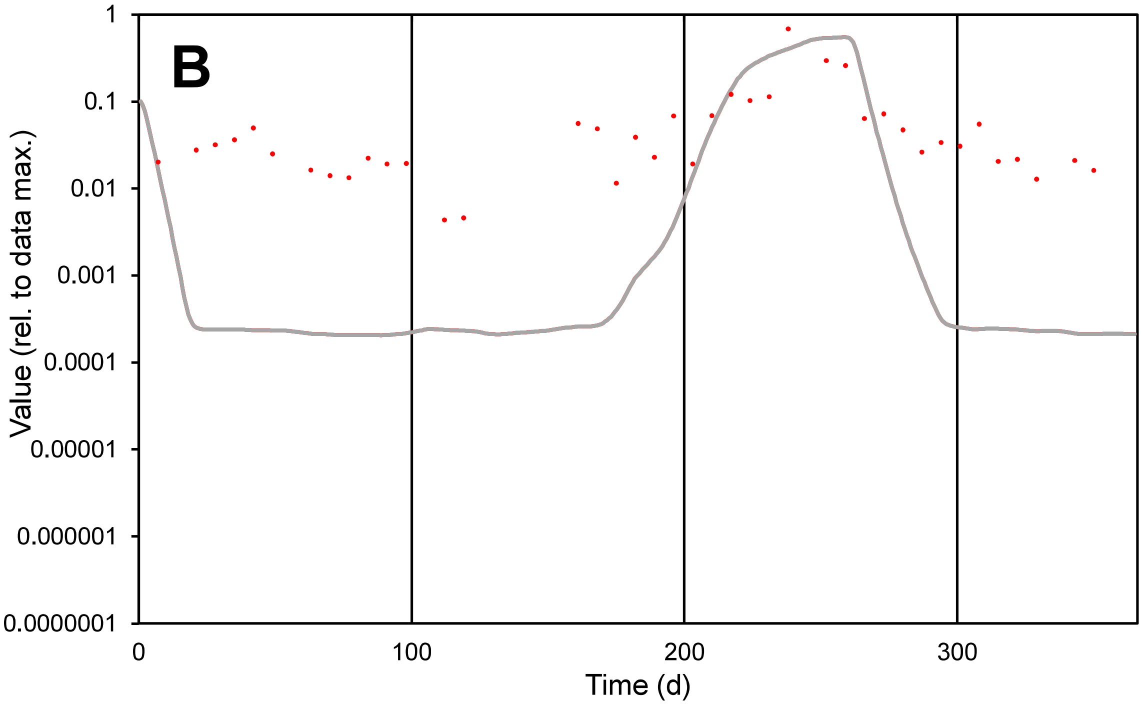

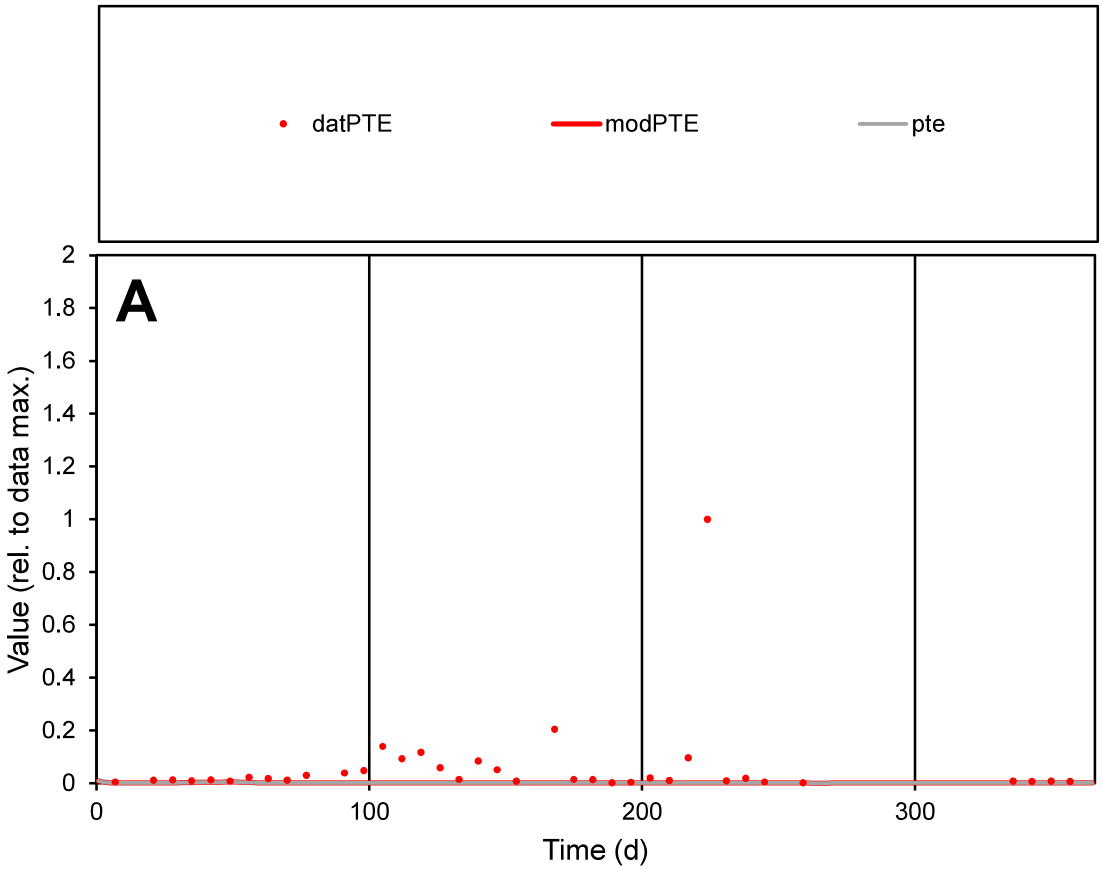

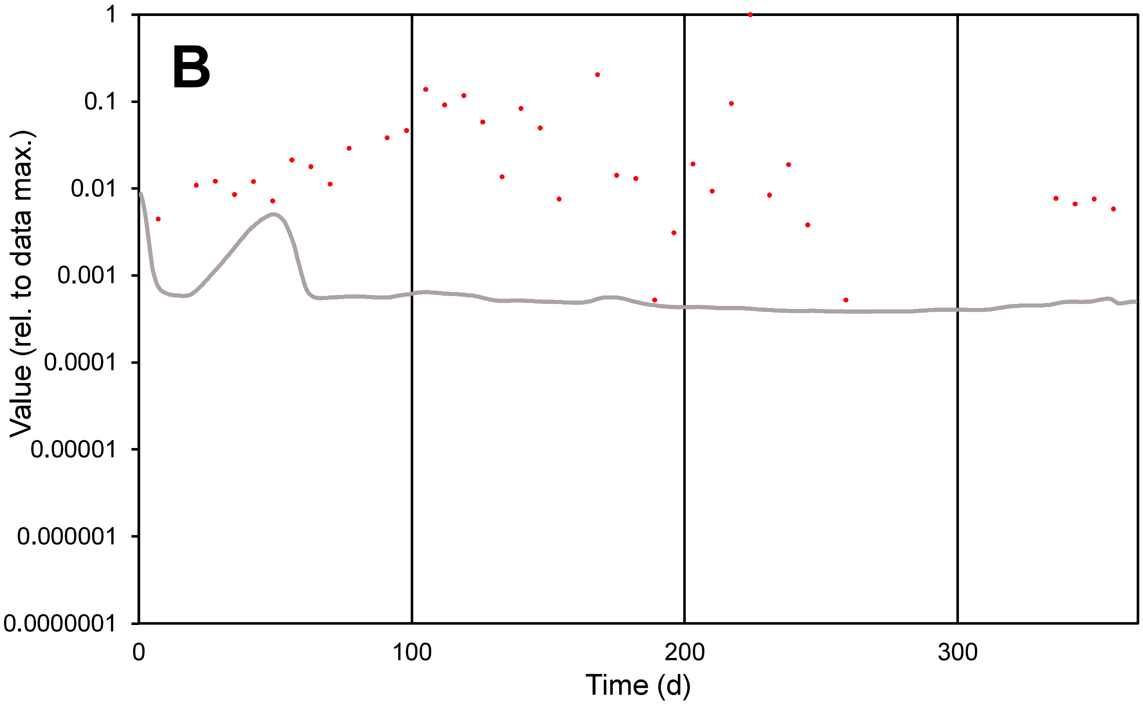

C1

Variables

PTE Provide jtd or Dst

CalibOnly?

2 1 to reproduce of

oNormalize?

2 1=no, 2=yes

ofxn calc*

2.91538

ofxn mod

2.92202

sRun

RunA1

zout

1

Ave

Data

0.00095

Model

1.4E-05

data/vMax

0.01592

ofxng

311.734

Min d/m

1.36646

ave/min d

113.15

ave/min m

2.21159

331_Calib_S_A

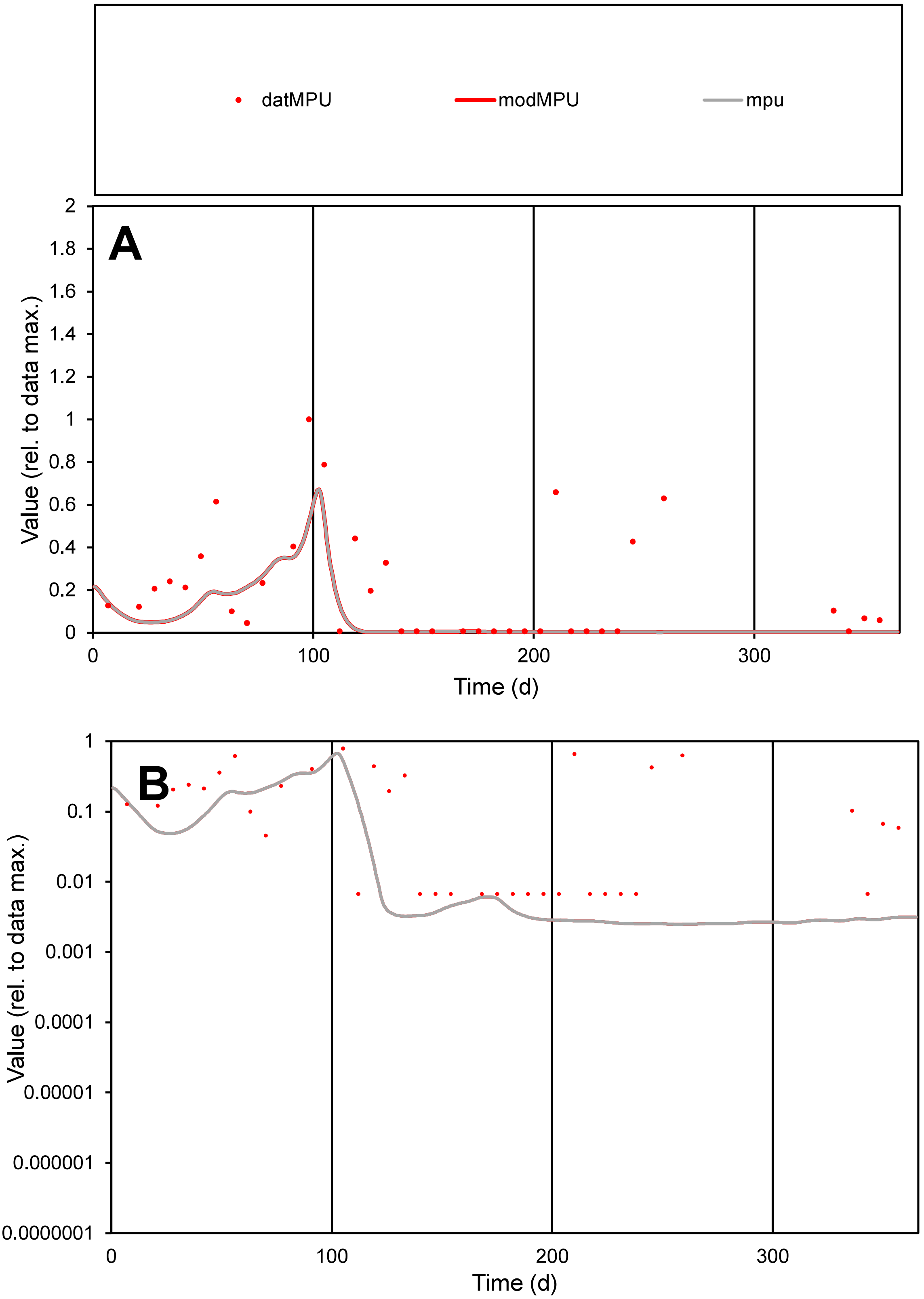

C1

Variables

MPU Provide jtd or Dst

CalibOnly?

2 1 to reproduce of

oNormalize?

2 1=no, 2=yes

ofxn calc*

1.13391

ofxn mod

1.1476

sRun

RunA1

zout

1

Ave

Data

0.00012

Model

5E-05

data/vMax

0.00061

ofxng

311.734

Min d/m

2.70596

ave/min d

29.9103

ave/min m

32.8051

331_Calib_S_A

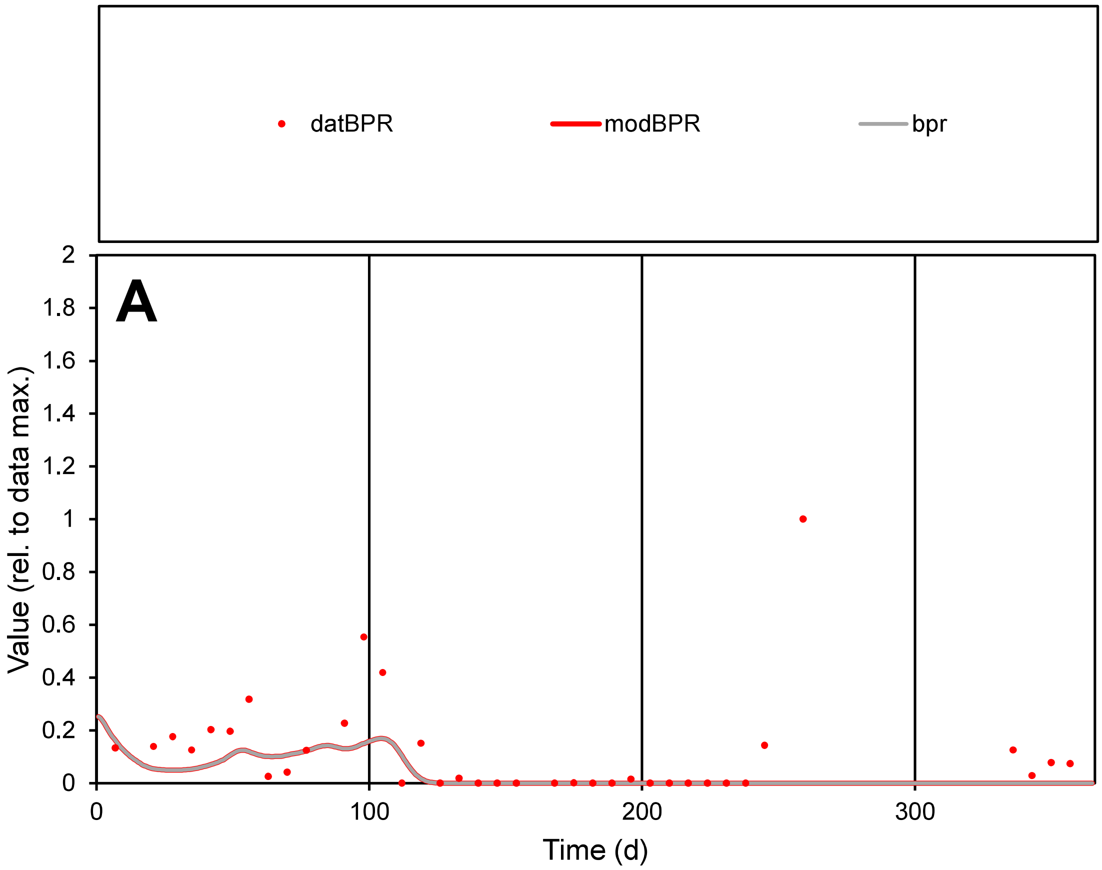

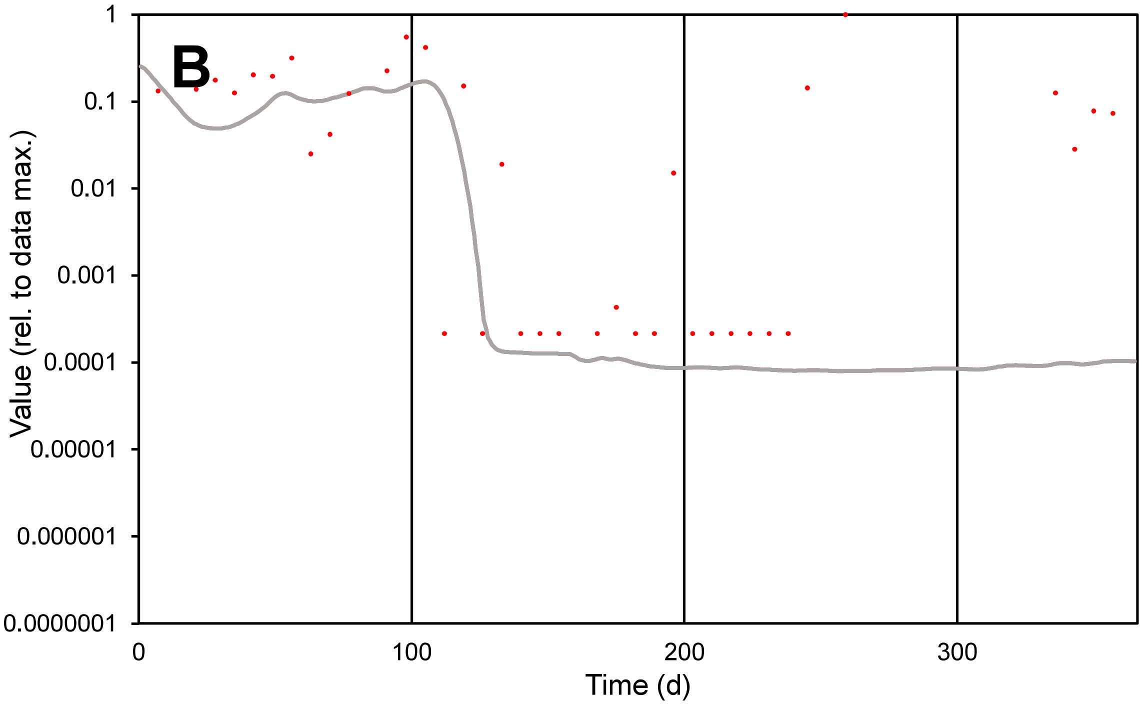

C1

Variables

BPR Provide jtd or Dst

CalibOnly?

2 1 to reproduce of

oNormalize?

2 1=no, 2=yes

ofxn calc*

1.66792

ofxn mod

1.67492

sRun

RunA1

zout

1

Ave

Data

0.0001

Model

3.4E-05

data/vMax

0.00086

ofxng

311.734

Min d/m

2.70038

ave/min d

540.902

ave/min m

498.264

331_Calib_S_A

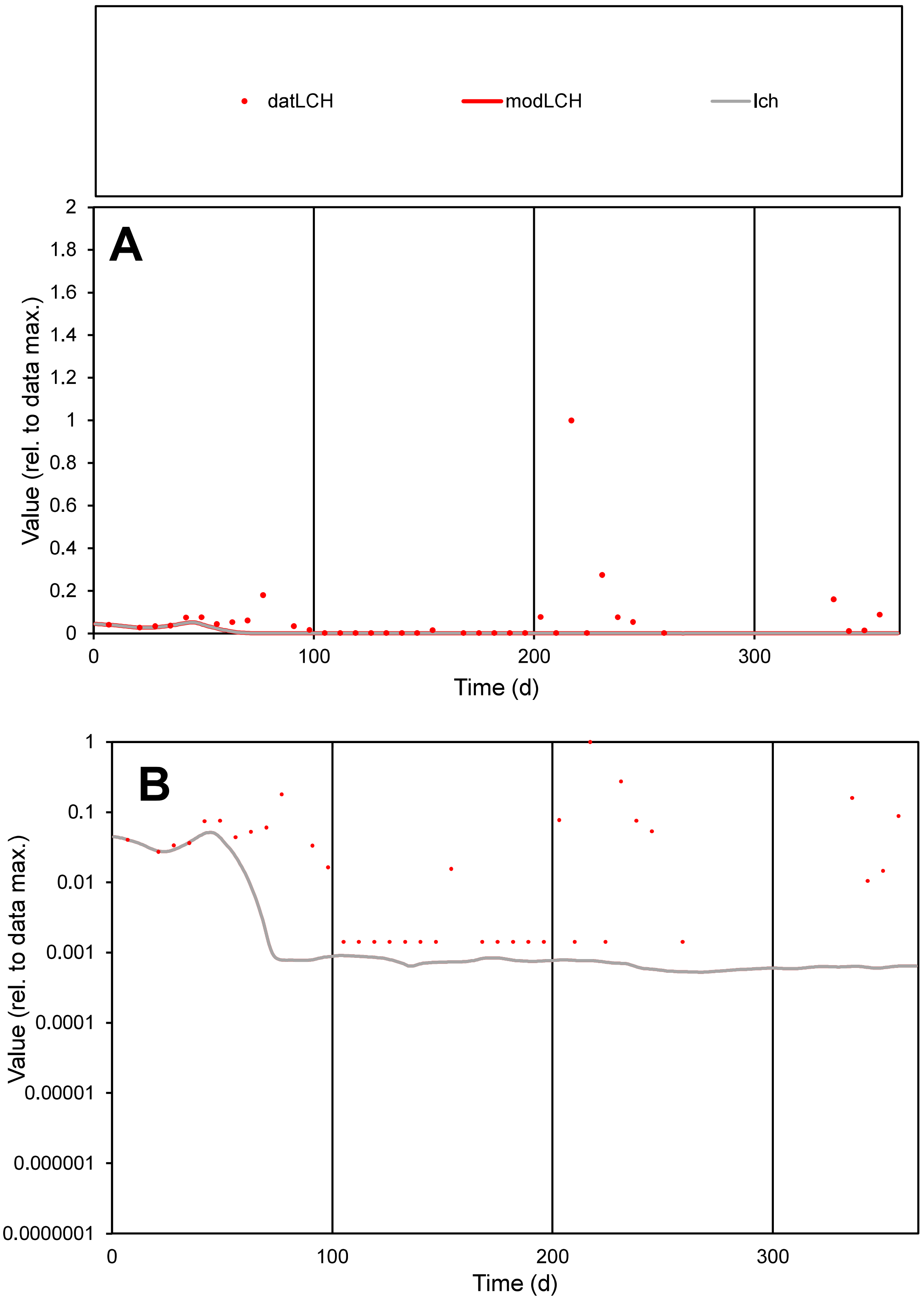

C1

Variables
LCH Provide jtd or Dst
CalibOnly?
2 1 to reproduce of
oNormalize?
2 1=no, 2=yes

ofxn calc*
2.65949
ofxn mod
2.66341

sRun
RunA1
zout
1

Ave
Data
0.00011
Model
1.3E-05

data/vMax
0.00171

ofxng
311.734

Min d/m
2.68821

ave/min d
46.585
ave/min m
14.3698

331_Calib_S_A

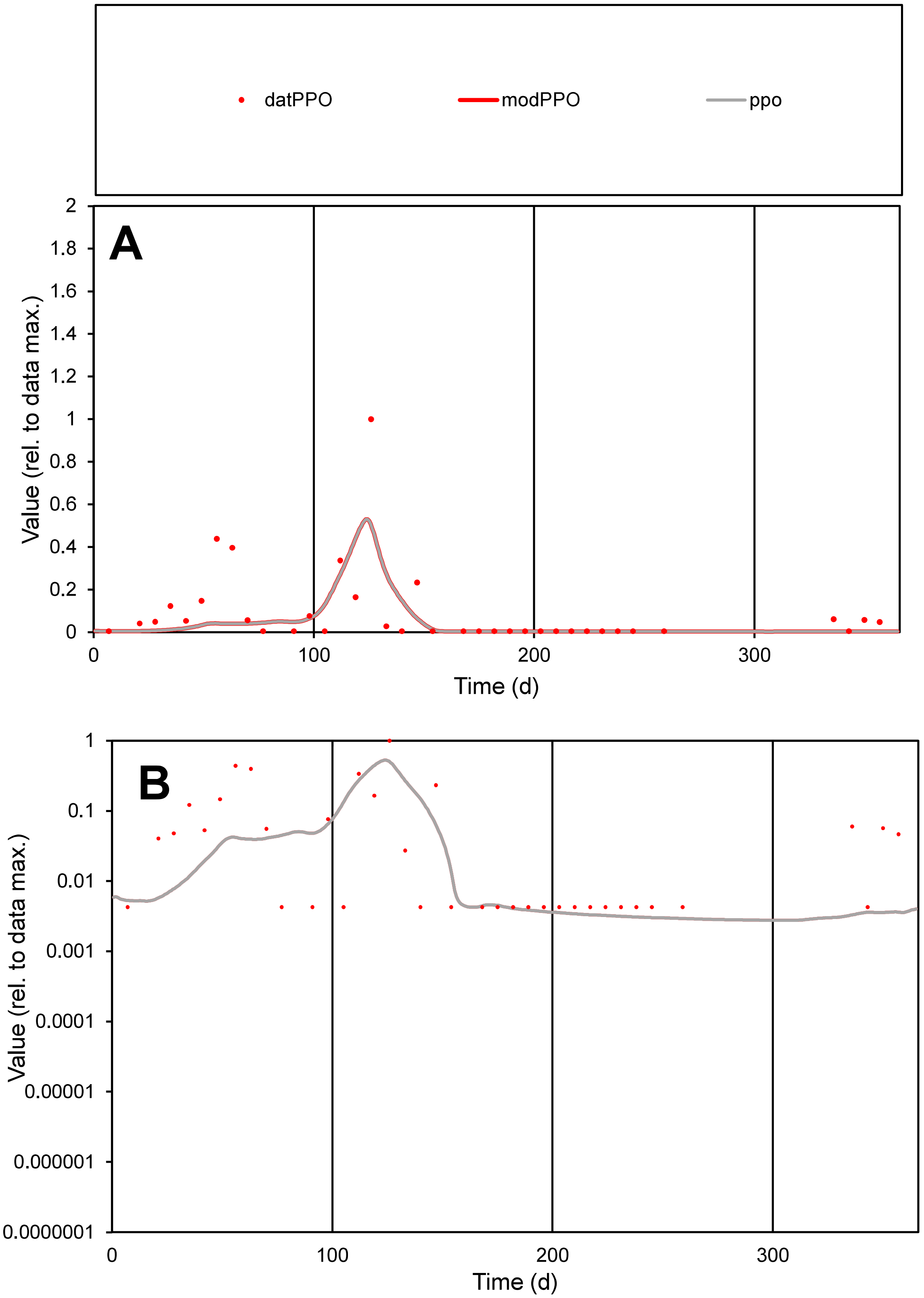

C1

Variables
PPO Provide jtd or Dst
CalibOnly?
2 1 to reproduce of
oNormalize?
2 1=no, 2=yes

ofxn calc*
1.56249
ofxn mod
1.59871

sRun
RunA1
zout
1

Ave
Data
4.1E-05
Model
2.6E-05

data/vMax
0.00045

ofxng
311.734

Min d/m
1.45808

ave/min d
21.4693
ave/min m
20.1478

331_Calib_S_A

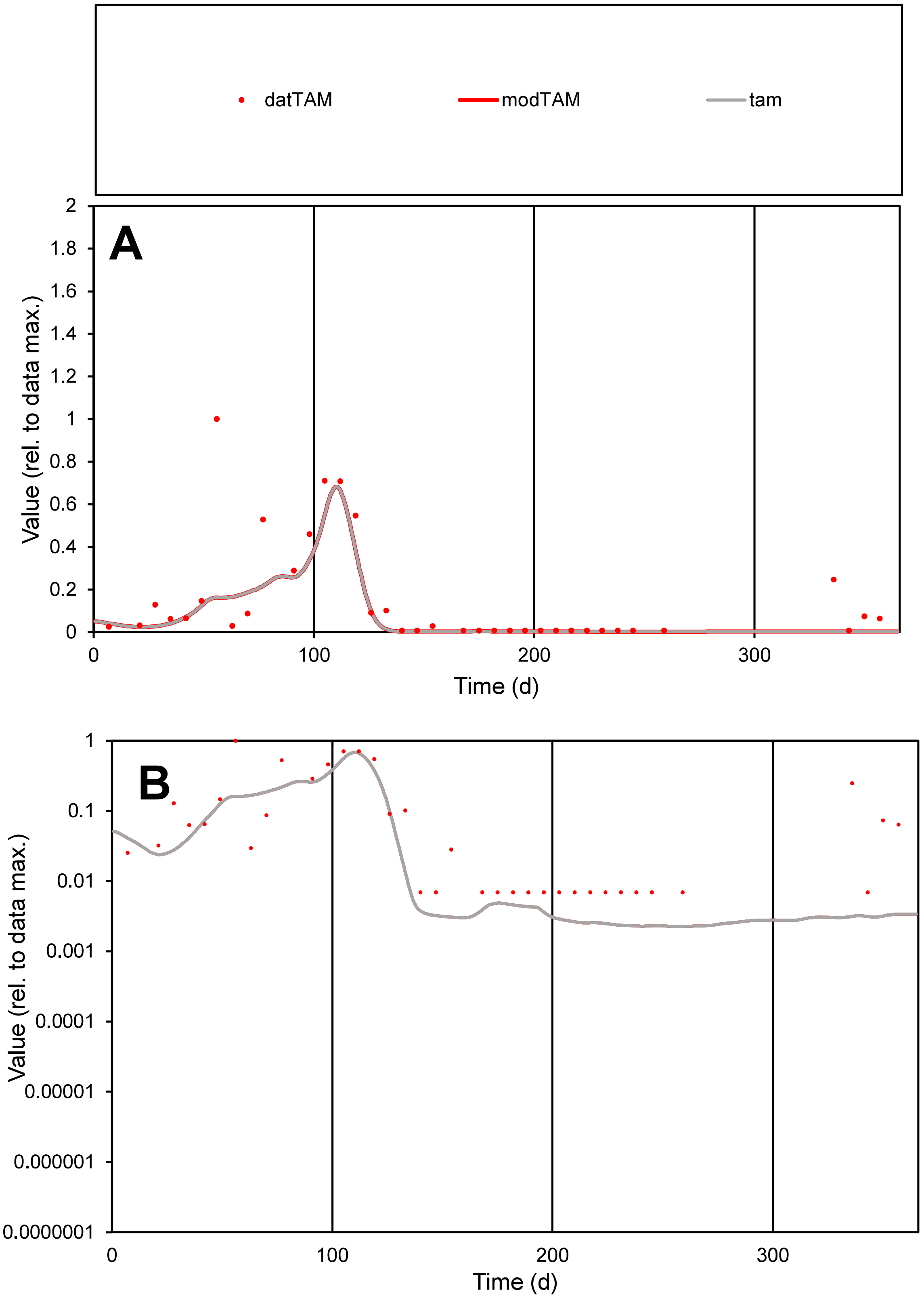

C1

Variables

TAM Provide jtd or Dst

CalibOnly?

2 1 to reproduce of

oNormalize?

2 1=no, 2=yes

ofxn calc*

1.08213

ofxn mod

1.09837

sRun

RunA1

zout

1

Ave

Data

0.00012

Model

7.9E-05

data/vMax

0.00083

ofxng

311.734

Min d/m

3.04226

ave/min d

21.6657

ave/min m

41.5068

331_Calib_S_A

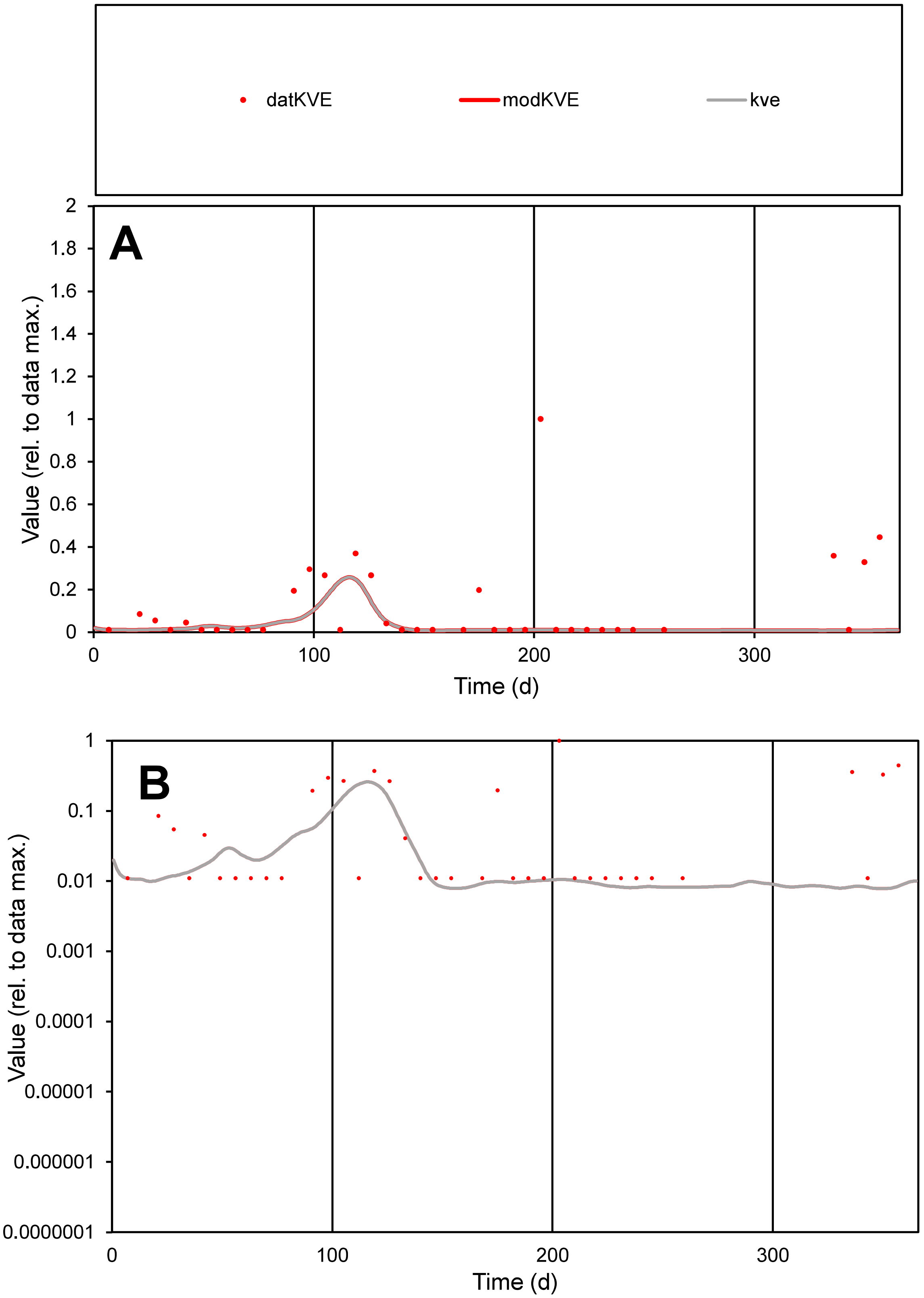

C1

Variables
KVE Provide jtd or Dst
CalibOnly?
2 1 to reproduce of
oNormalize?
2 1=no, 2=yes

ofxn calc*
1.82603
ofxn mod
1.83133

sRun
RunA1
zout
1

Ave
Data
3.2E-05
Model
1.1E-05

data/vMax
0.00029

ofxng
311.734

Min d/m
1.39528

ave/min d
10.3372
ave/min m
4.69994

331_Calib_S_A

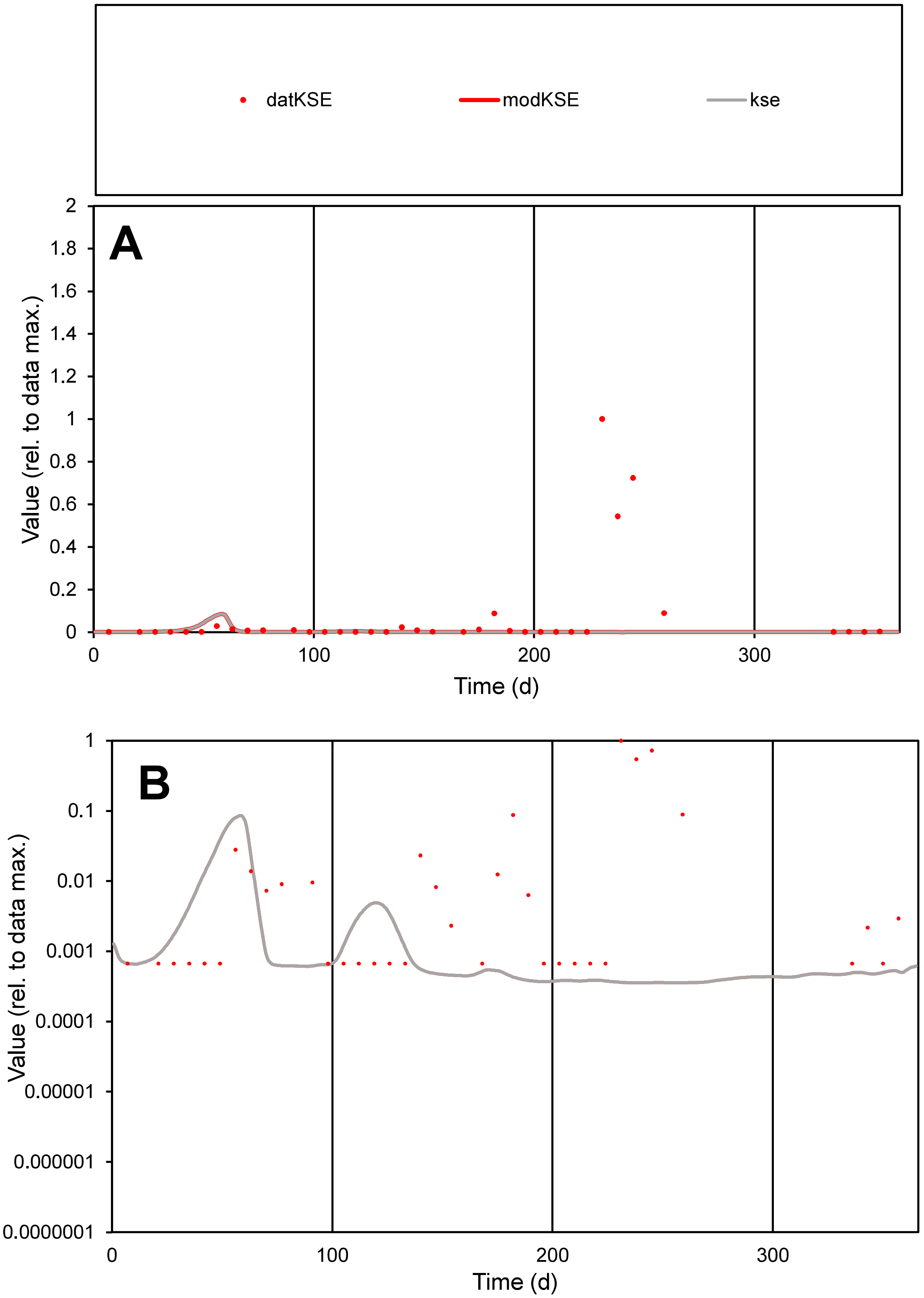

C1

Variables
KSE Provide jtd or Dst
CalibOnly?
2 1 to reproduce of
oNormalize?
2 1=no, 2=yes

ofxn calc*
3.19259
ofxn mod
3.20087

sRun
RunA1
zout
1

Ave
Data
0.00039
Model
2.5E-05

data/vMax
0.00559

ofxng
311.734

Min d/m
1.89965

ave/min d
104.061
ave/min m
12.5844

331_Calib_S_A

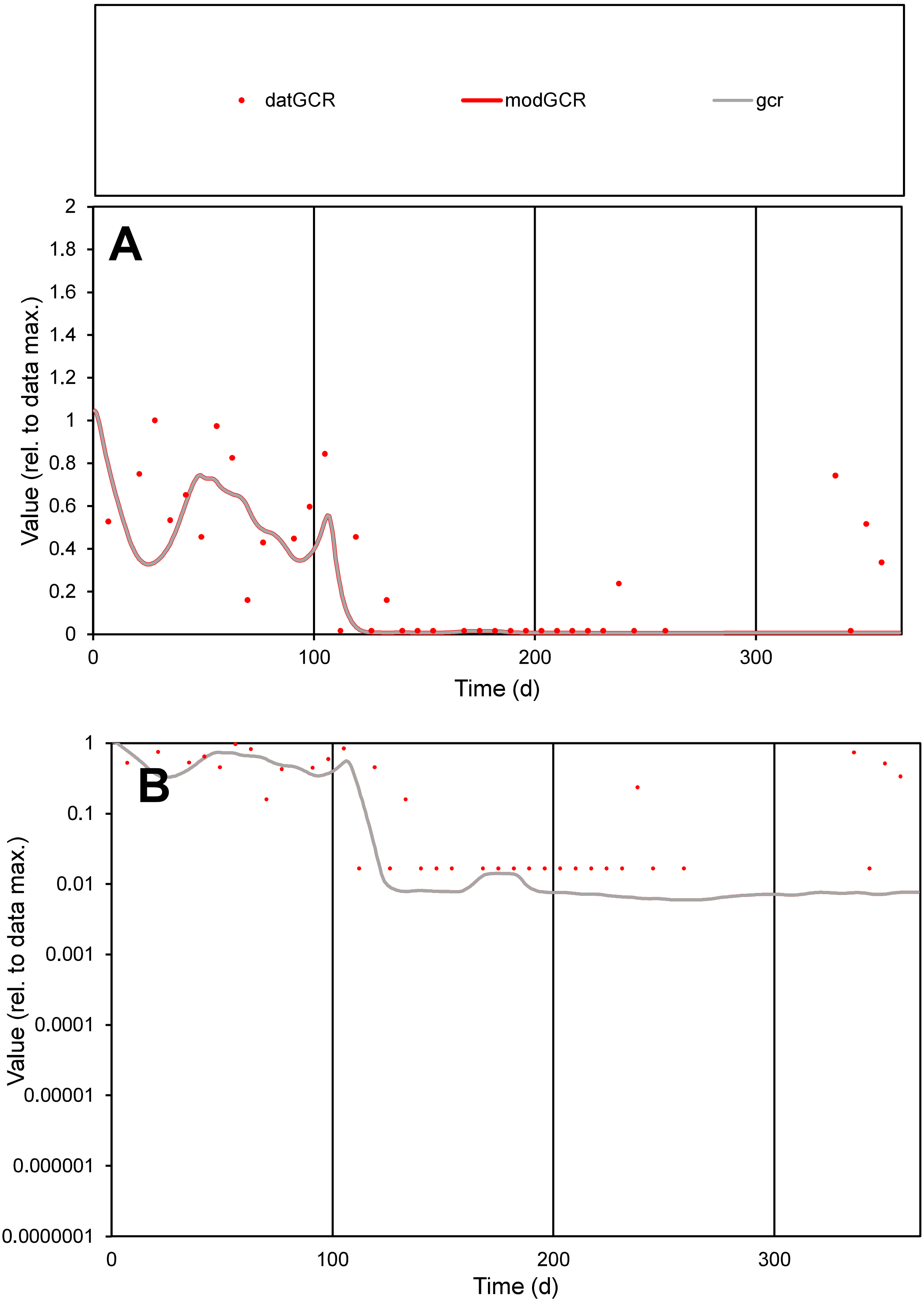

C1

Variables
GCR Provide jtd or Dst
CalibOnly?
2 1 to reproduce of
oNormalize?
2 1=no, 2=yes

ofxn calc*
0.8414
ofxn mod
0.85759

sRun
RunA1
zout
1

Ave
Data
5.6E-05
Model
3.7E-05

data/vMax
0.00019

ofxng
311.734

Min d/m
2.78578

ave/min d
17.7323
ave/min m
32.8892

331_Calib_S_A

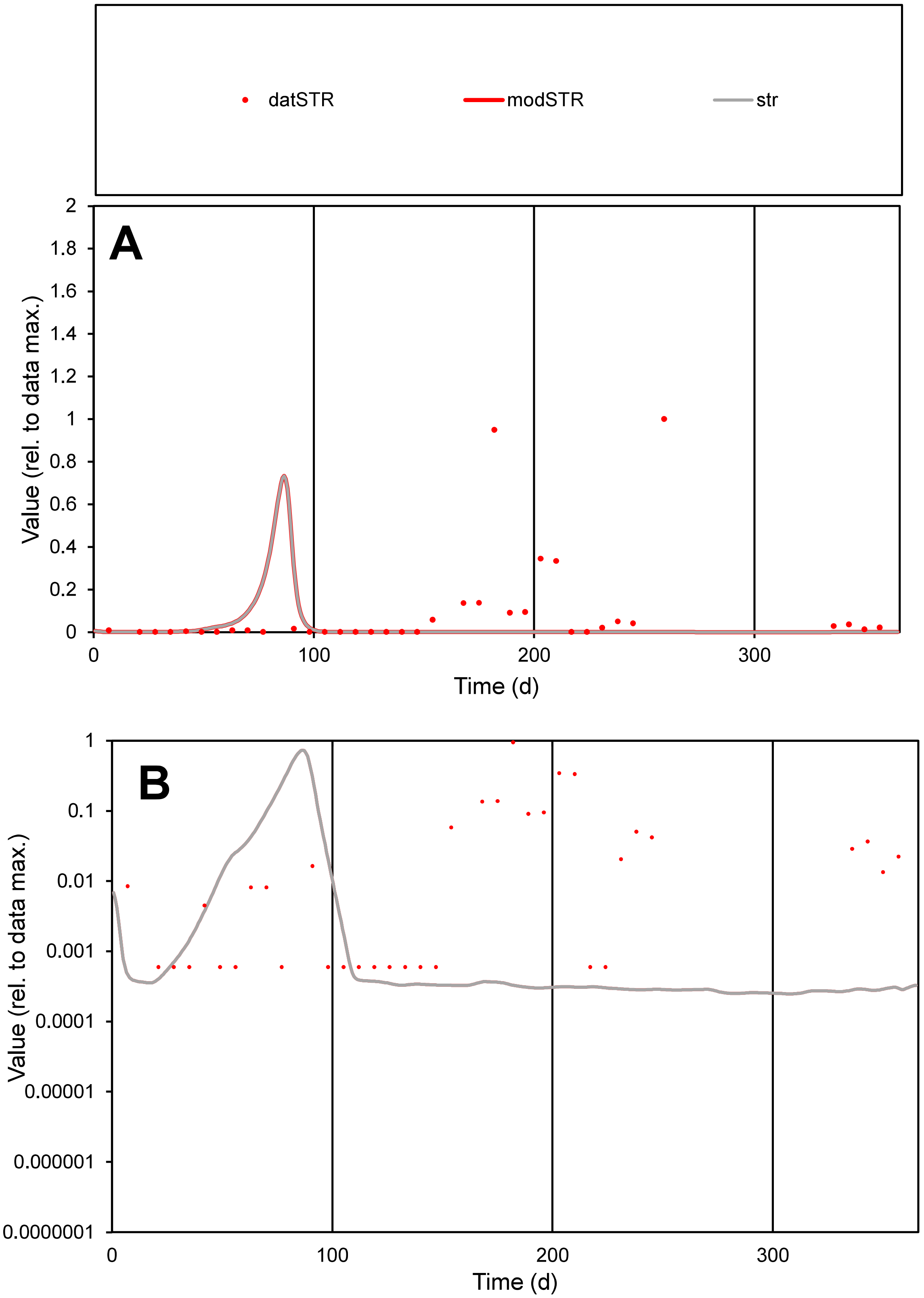

C1

Variables

STR Provide jtd or Dst

CalibOnly?

2 1 to reproduce of

oNormalize?

2 1=no, 2=yes

ofxn calc*

2.66084

ofxn mod

2.68302

sRun

RunA1

zout

1

Ave

Data

0.00025

Model

3E-05

data/vMax

0.00273

ofxng

311.734

Min d/m

2.12054

ave/min d

154.411

ave/min m

39.2669

331_Calib_S_A

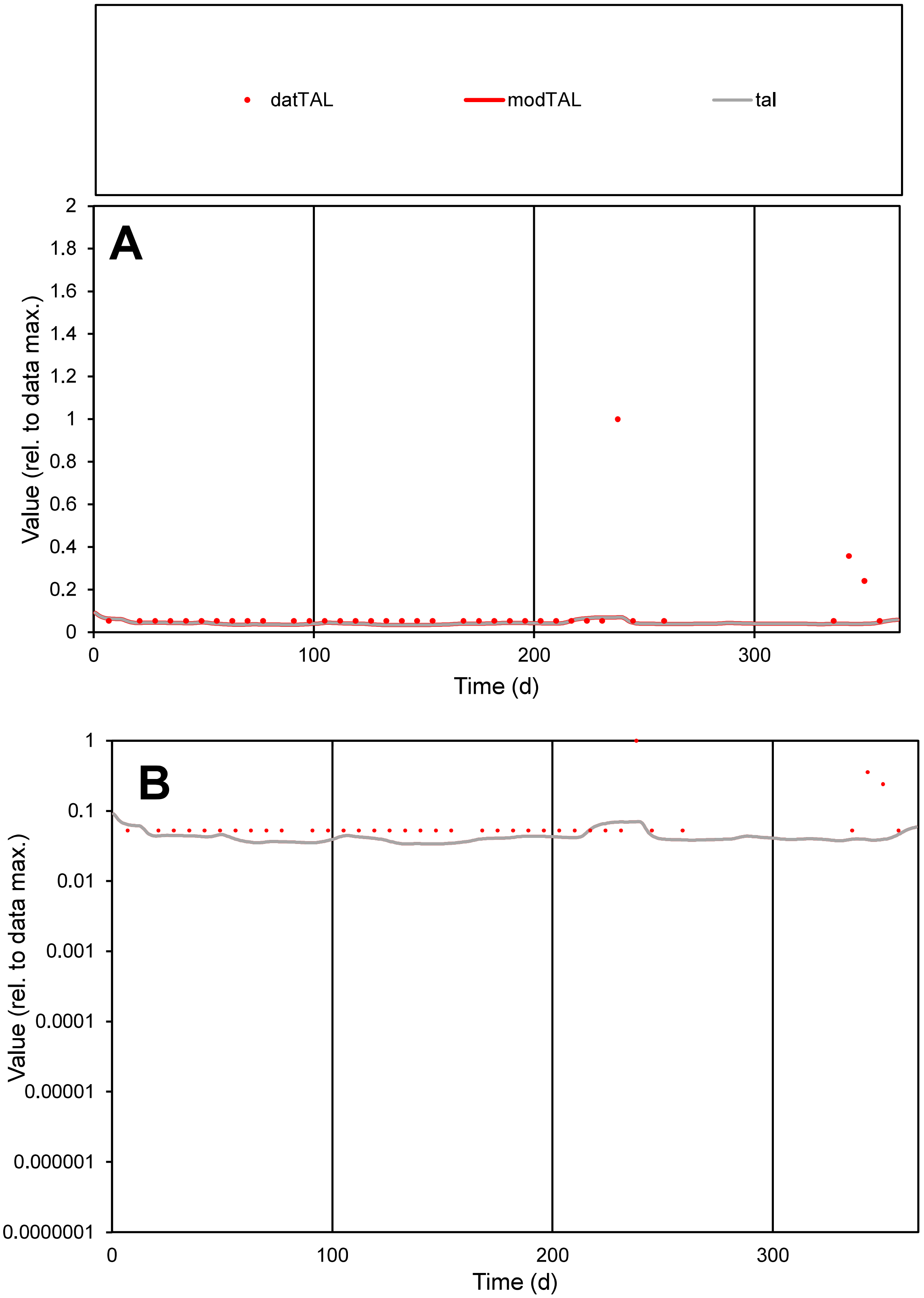

C1

Variables
TAL Provide jtd or Dst
CalibOnly?
2 1 to reproduce of
oNormalize?
2 1=no, 2=yes

ofxn calc*
1.80467
ofxn mod
1.80847

sRun
RunA1
zout
1

Ave
Data
7.2E-06
Model
3.4E-06

data/vMax
7.9E-05

ofxng
311.734

Min d/m
1.55148

ave/min d
1.73749
ave/min m
1.28281

331_Calib_S_A

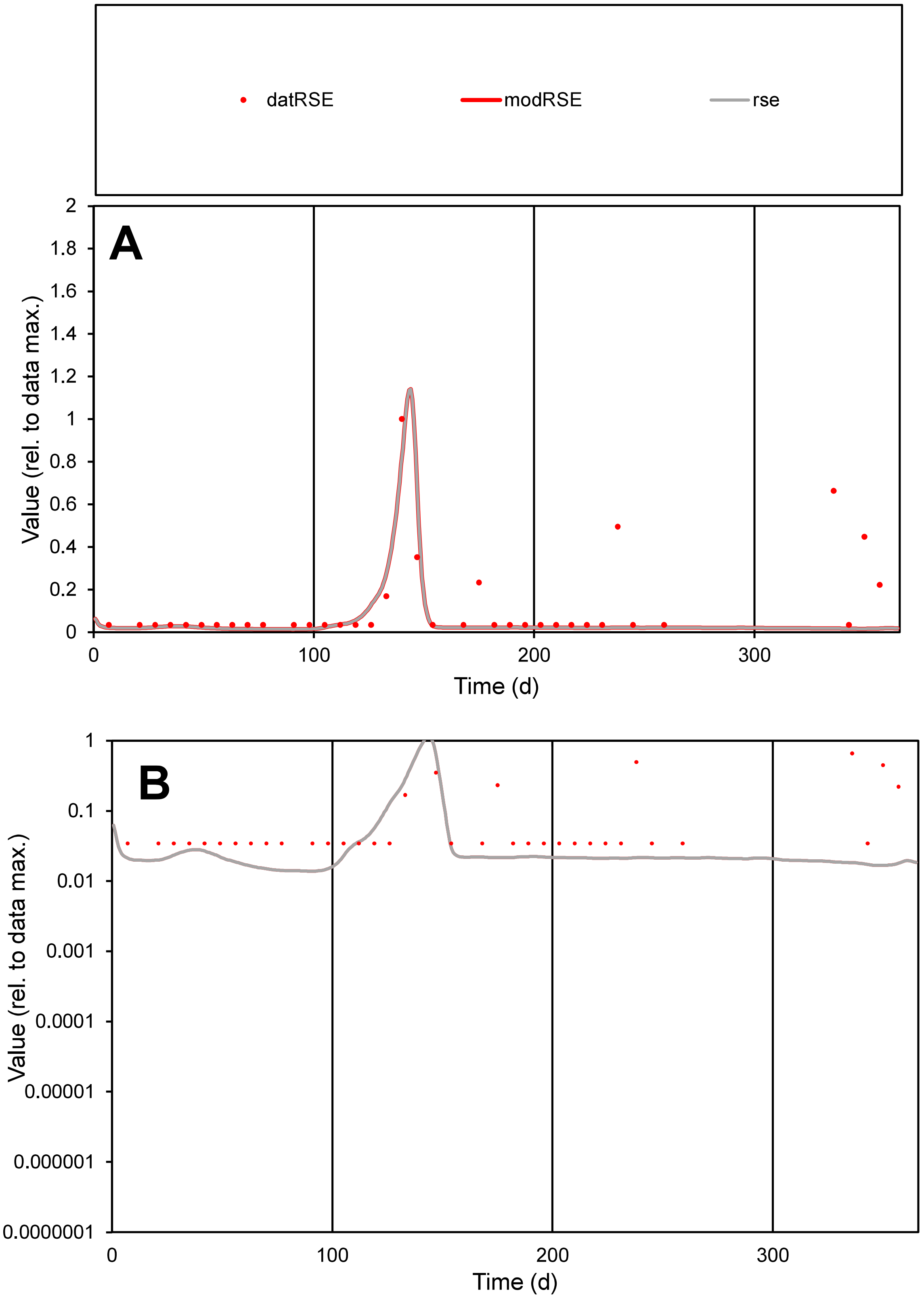

C1

Variables
RSE Provide jtd or Dst
CalibOnly?
2 1 to reproduce of
oNormalize?
2 1=no, 2=yes

ofxn calc*
1.28668
ofxn mod
1.29683

sRun
RunA1
zout
1

Ave
Data
1.7E-05
Model
8.9E-06

data/vMax
0.00014

ofxng
311.734

Min d/m
2.48959

ave/min d
3.59087
ave/min m
4.77811

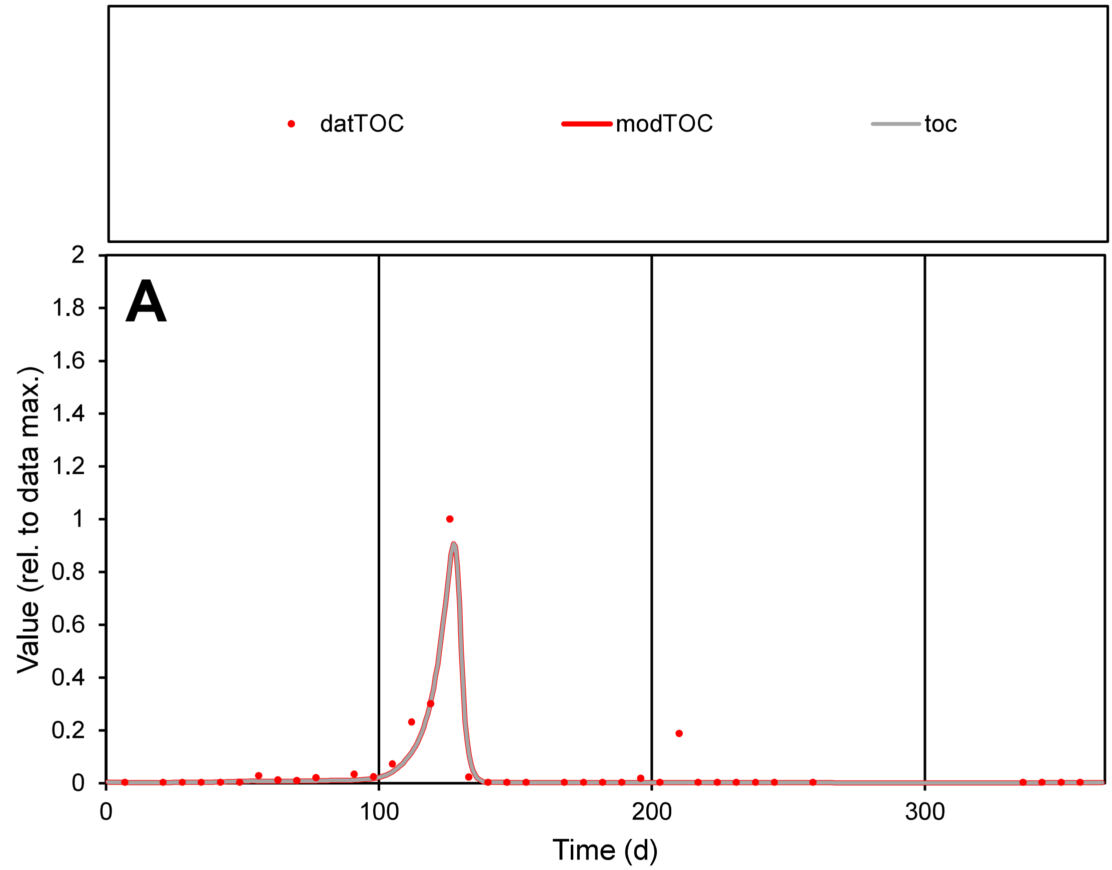

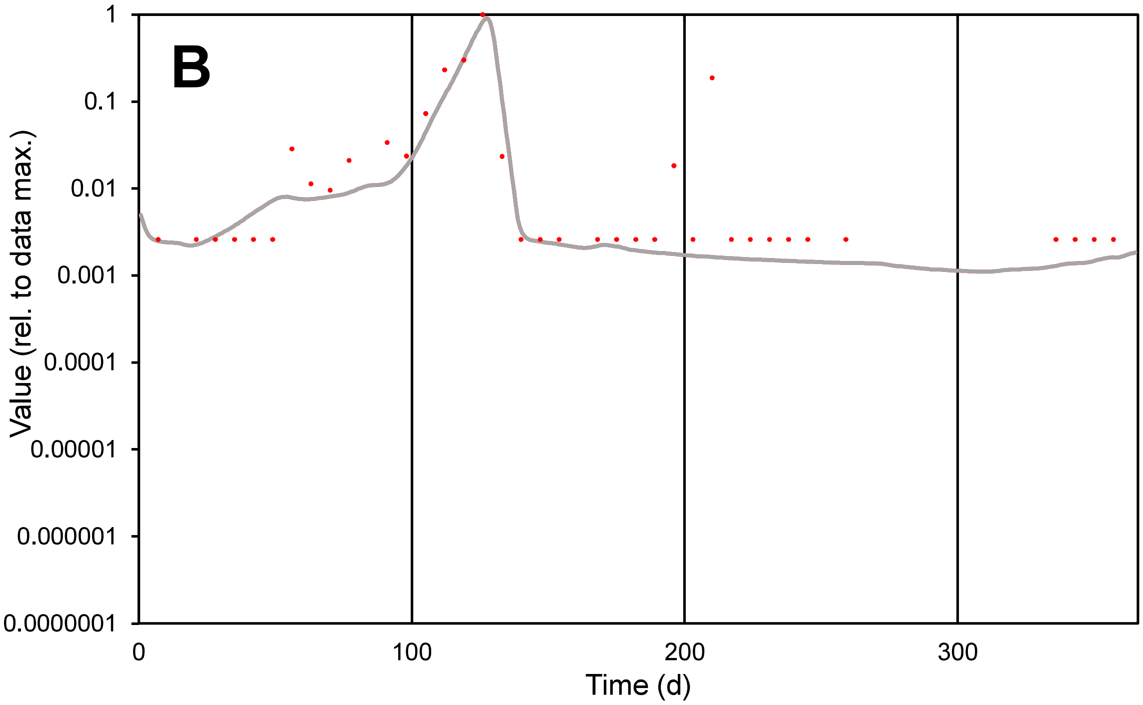

C1

Variables
TOC Provide jtd or Dst
CalibOnly?
2 1 to reproduce of
oNormalize?
2 1=no, 2=yes

ofxn calc*
0.71896
ofxn mod
0.77695

sRun
RunA1
zout
1

Ave
Data
9.1E-05
Model
7.3E-05

data/vMax
0.00166

ofxng
311.734

Min d/m
1.96544

ave/min d
21.0813
ave/min m
33.4184

331_Calib_S_A

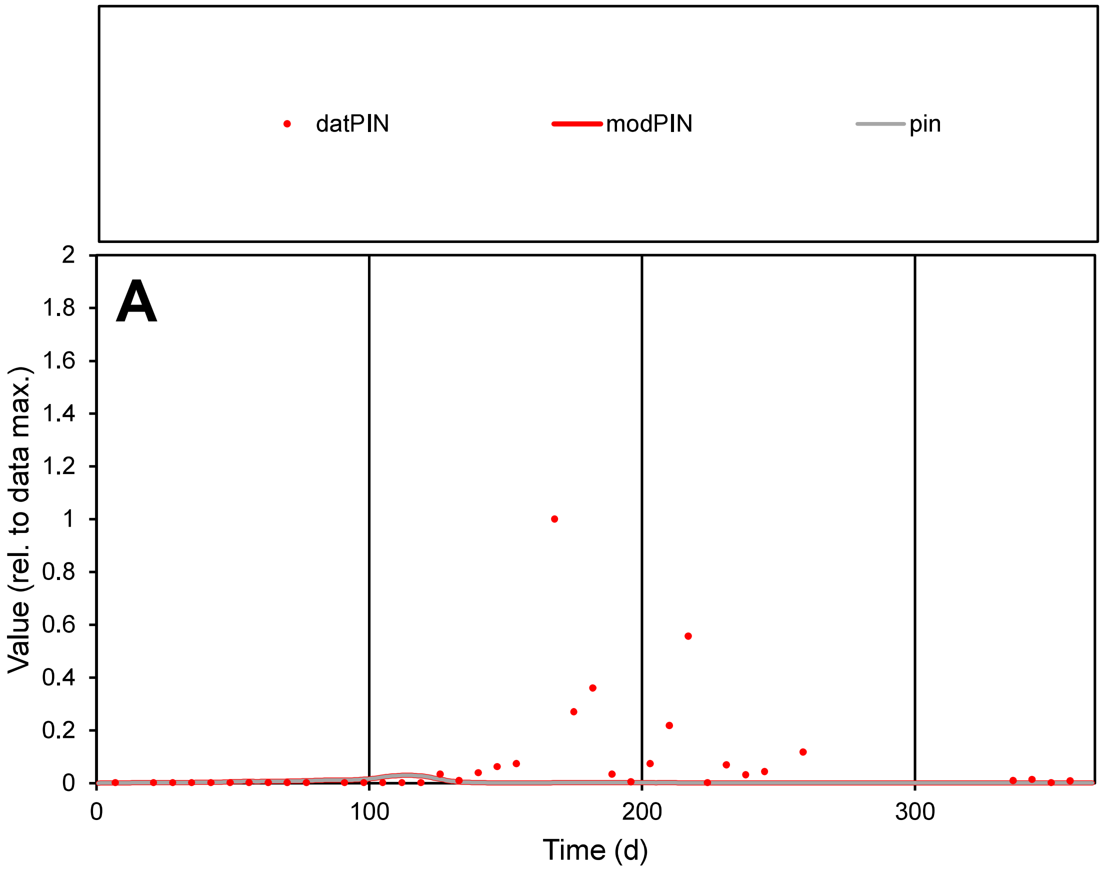

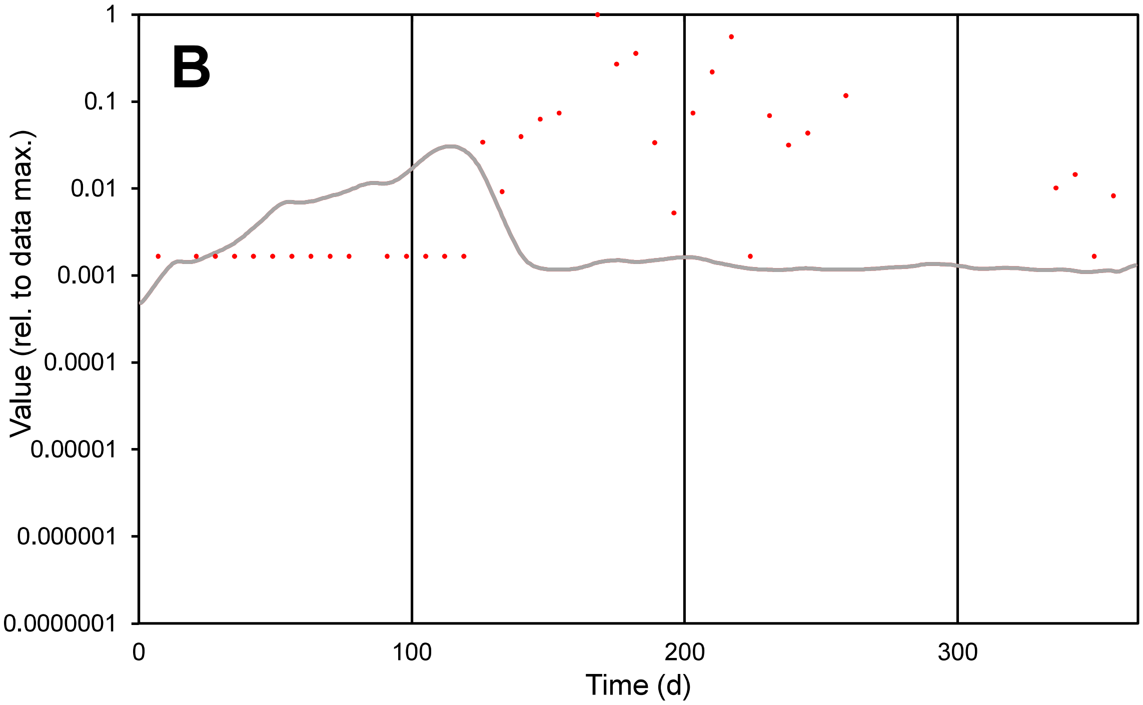

C1

Variables
PIN Provide jtd or Dst
CalibOnly?
2 1 to reproduce of
oNormalize?
2 1=no, 2=yes

ofxn calc*
2.50911
ofxn mod
2.51217

sRun
RunA1
zout
1

Ave
Data
0.0002
Model
1.3E-05

data/vMax
0.00241

ofxng
311.734

Min d/m
1.74576

ave/min d
49.6997
ave/min m
5.53046

331_Calib_S_A

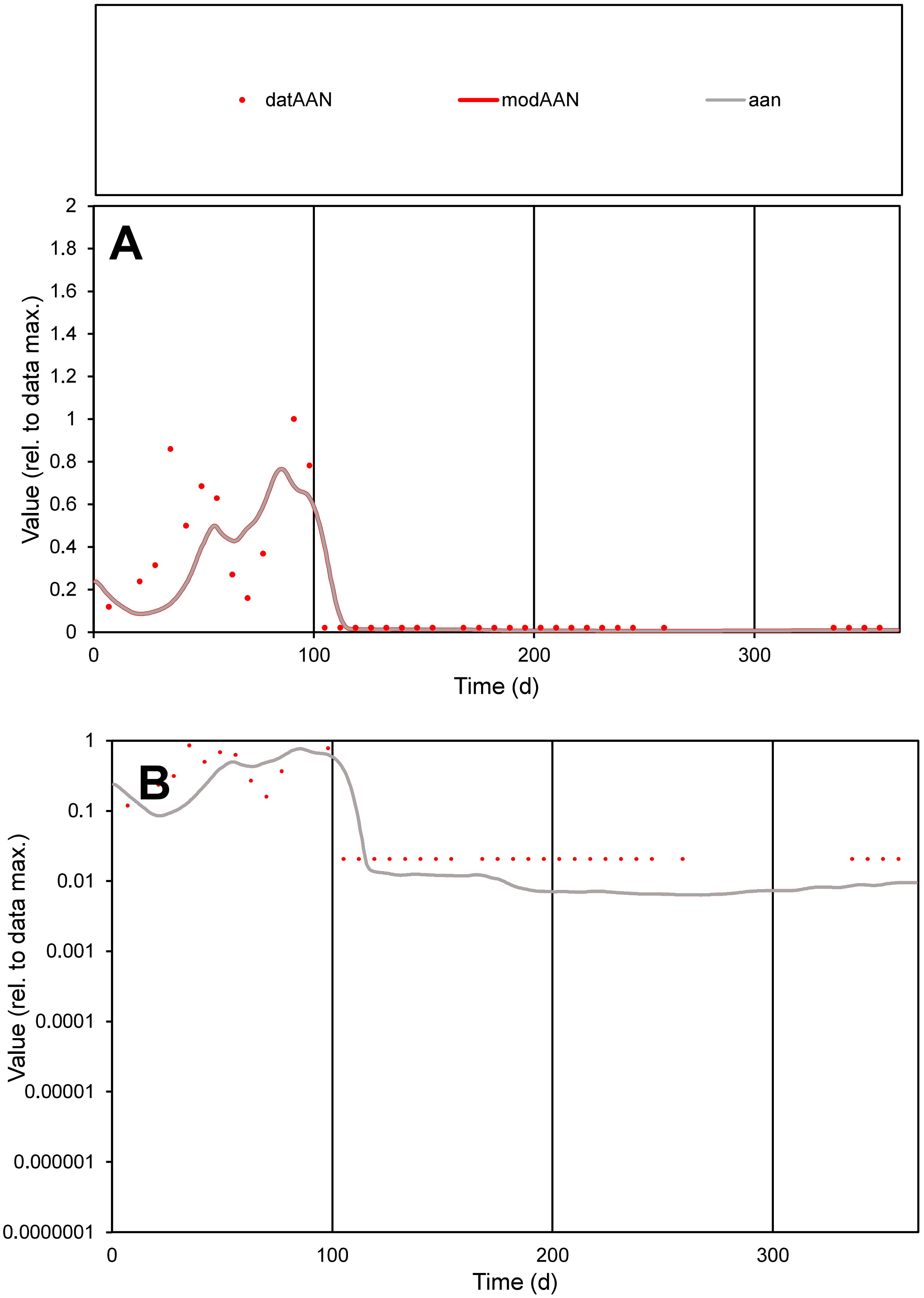

C1

Variables

AAN Provide jtd or Dst

CalibOnly?

2 1 to reproduce of

oNormalize?

2 1=no, 2=yes

ofxn calc*

0.98936

ofxn mod

1.02252

sRun

RunA1

zout

1

Ave

Data

4.7E-05

Model

3.6E-05

data/vMax

0.00027

ofxng

311.734

Min d/m

3.23362

ave/min d

8.4217

ave/min m

20.7524

331_Calib_S_A

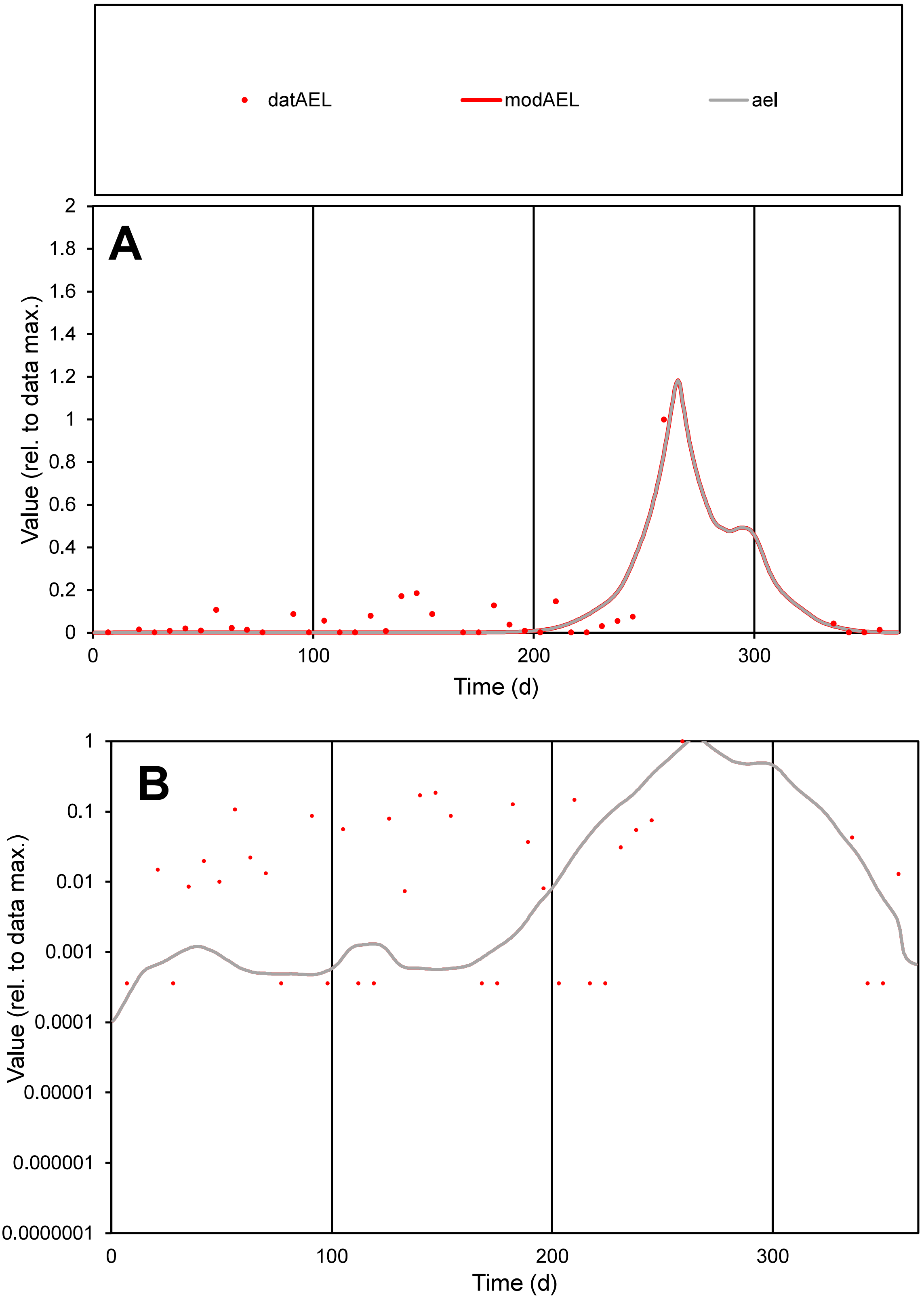

C1

Variables
AEL Provide jtd or Dst
CalibOnly?
2 1 to reproduce of
oNormalize?
2 1=no, 2=yes

ofxn calc*
1.20989
ofxn mod
1.28609

sRun
RunA1
zout
1

Ave
Data
0.00013
Model
9.3E-05

data/vMax
0.00195

ofxng
311.734

Min d/m
1.43115

ave/min d
181.702
ave/min m
191.546

331_Calib_S_A

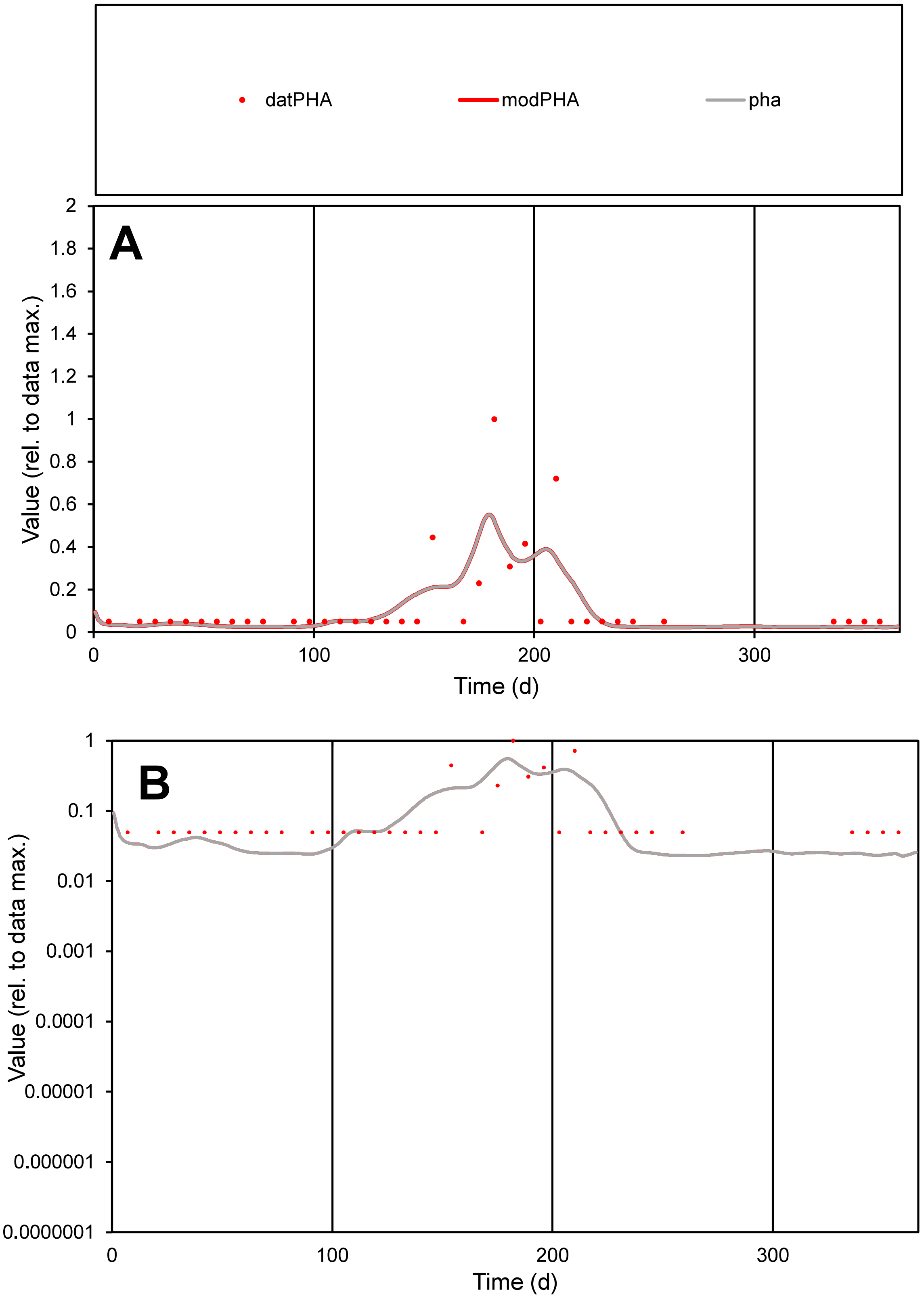

C1

Variables
PHA Provide jtd or Dst
CalibOnly?
2 1 to reproduce of
oNormalize?
2 1=no, 2=yes

ofxn calc*
1.11121
ofxn mod
1.15124

sRun
RunA1
zout
1

Ave
Data
2.6E-05
Model
2.5E-05

data/vMax
0.00021

ofxng
311.734

Min d/m
2.14109

ave/min d
2.54168
ave/min m
5.1175

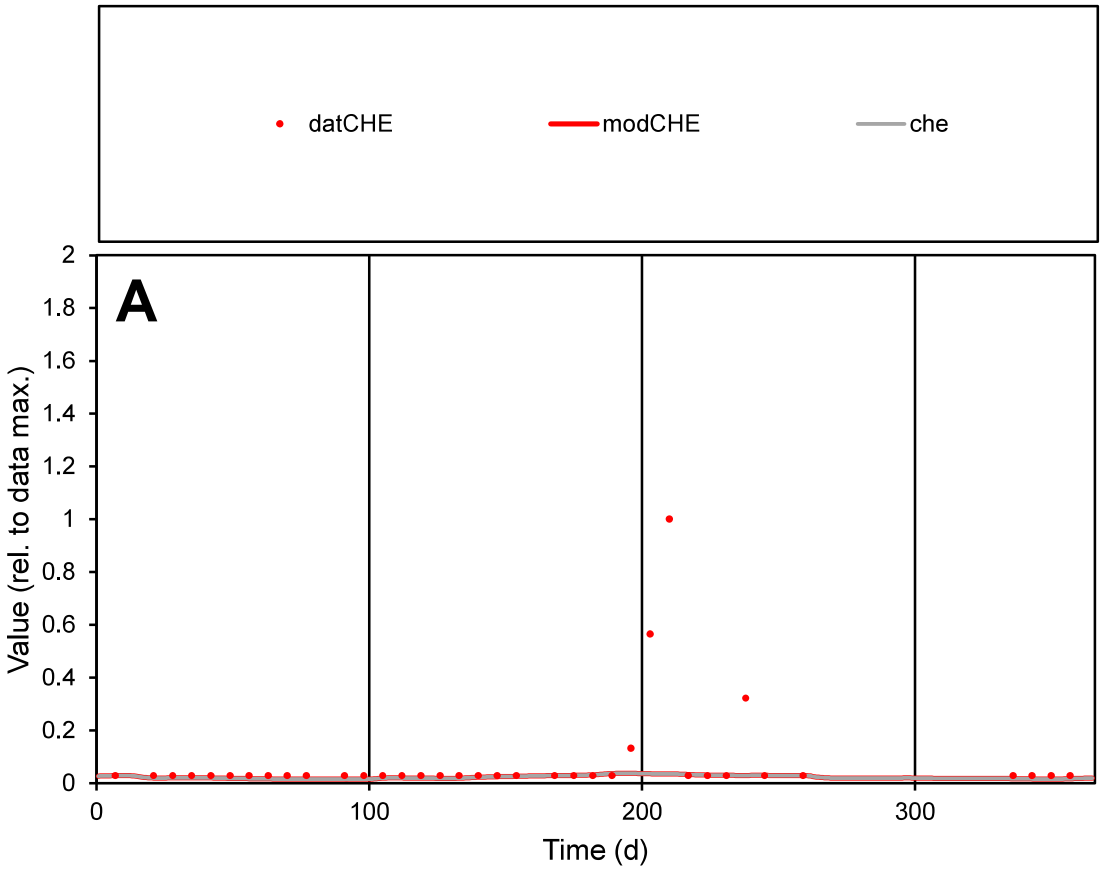

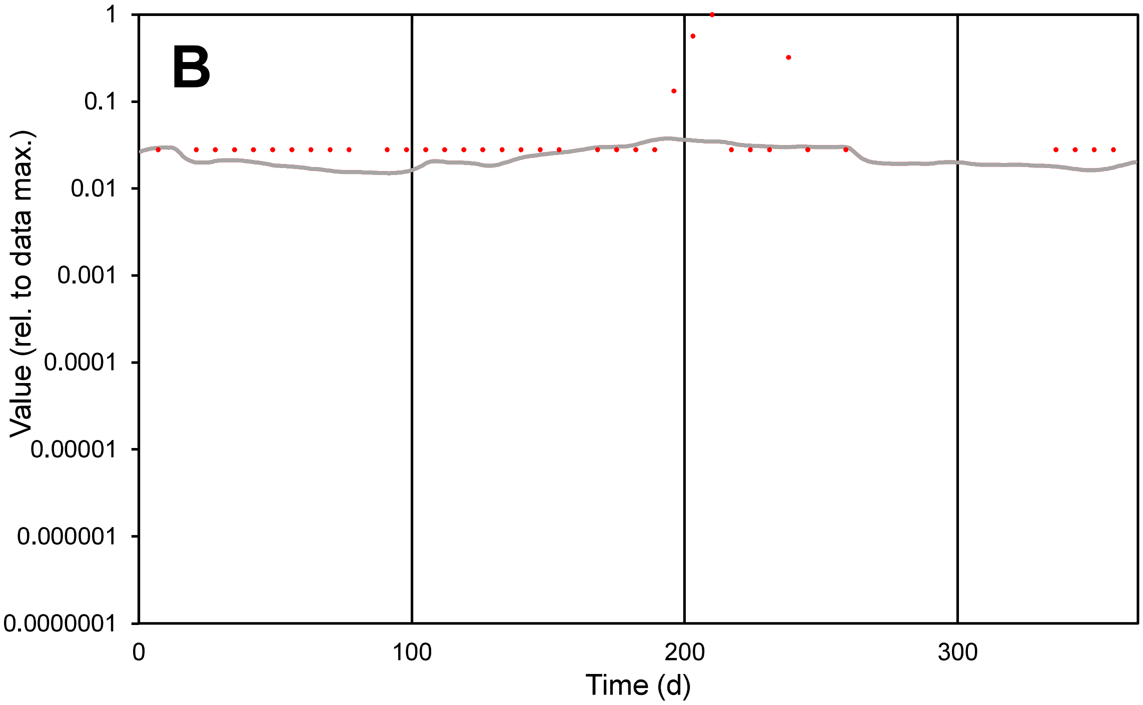

C1

Variables

CHE Provide jtd or Dst

CalibOnly?

2 1 to reproduce of

oNormalize?

2 1=no, 2=yes

ofxn calc*

2.36616

ofxn mod

2.37322

sRun

RunA1

zout

1

Ave

Data

2E-05

Model

6E-06

data/vMax

0.00025

ofxng

311.734

Min d/m

1.86256

ave/min d

2.84692

ave/min m

1.59936

331_Calib_S_A

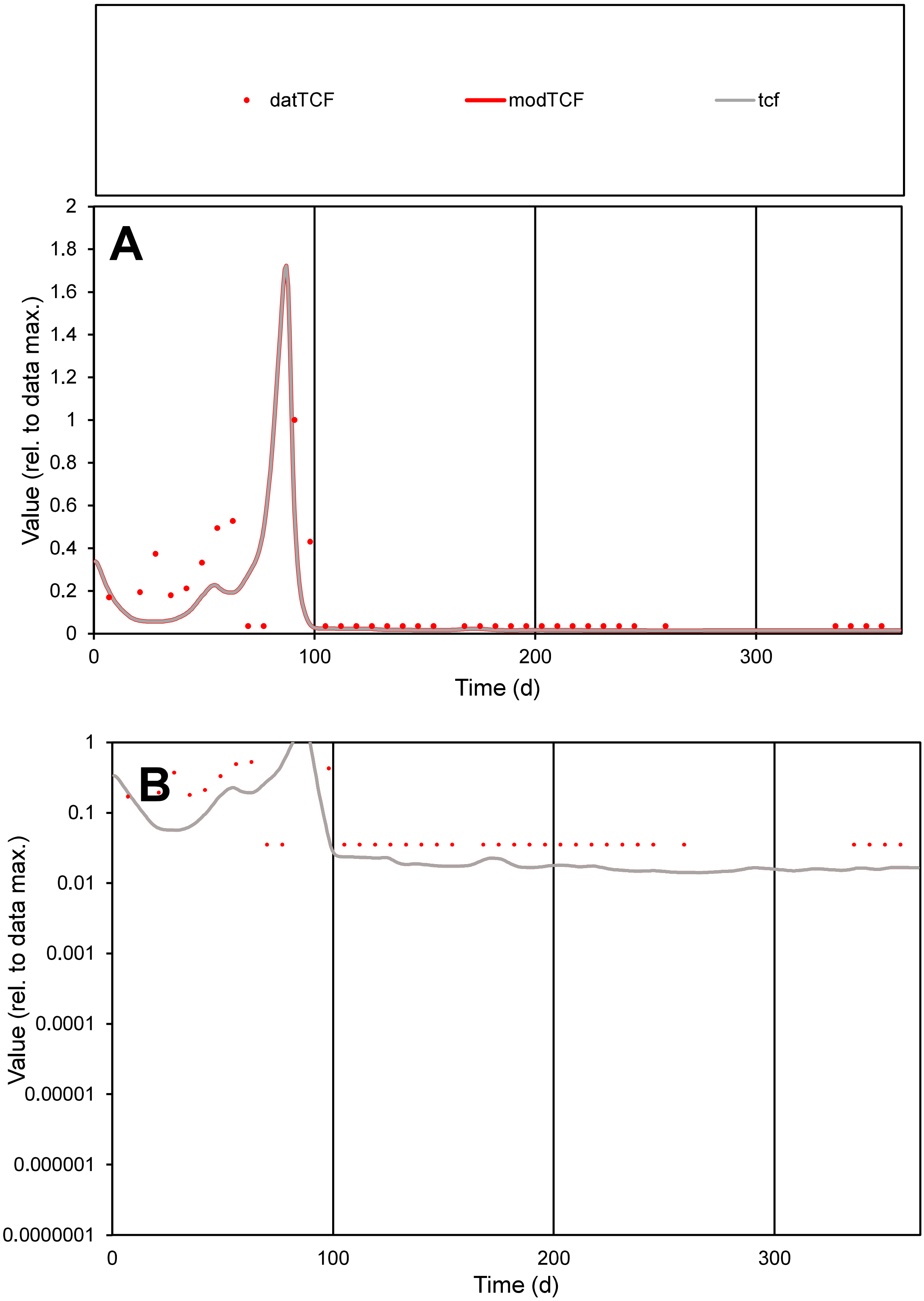

C1

Variables

TCF	Provide jtd or Dst
CalibOnly?	
2	1 to reproduce of
oNormalize?	
2	1=no, 2=yes

ofxn calc*
1.0772
ofxn mod
1.21483

sRun
RunA1
zout
1

Ave
Data
1.2E-05
Model
7.3E-06

data/vMax
8.8E-05

ofxng
311.734

Min d/m
2.47933

ave/min d
3.72454
ave/min m
5.78726

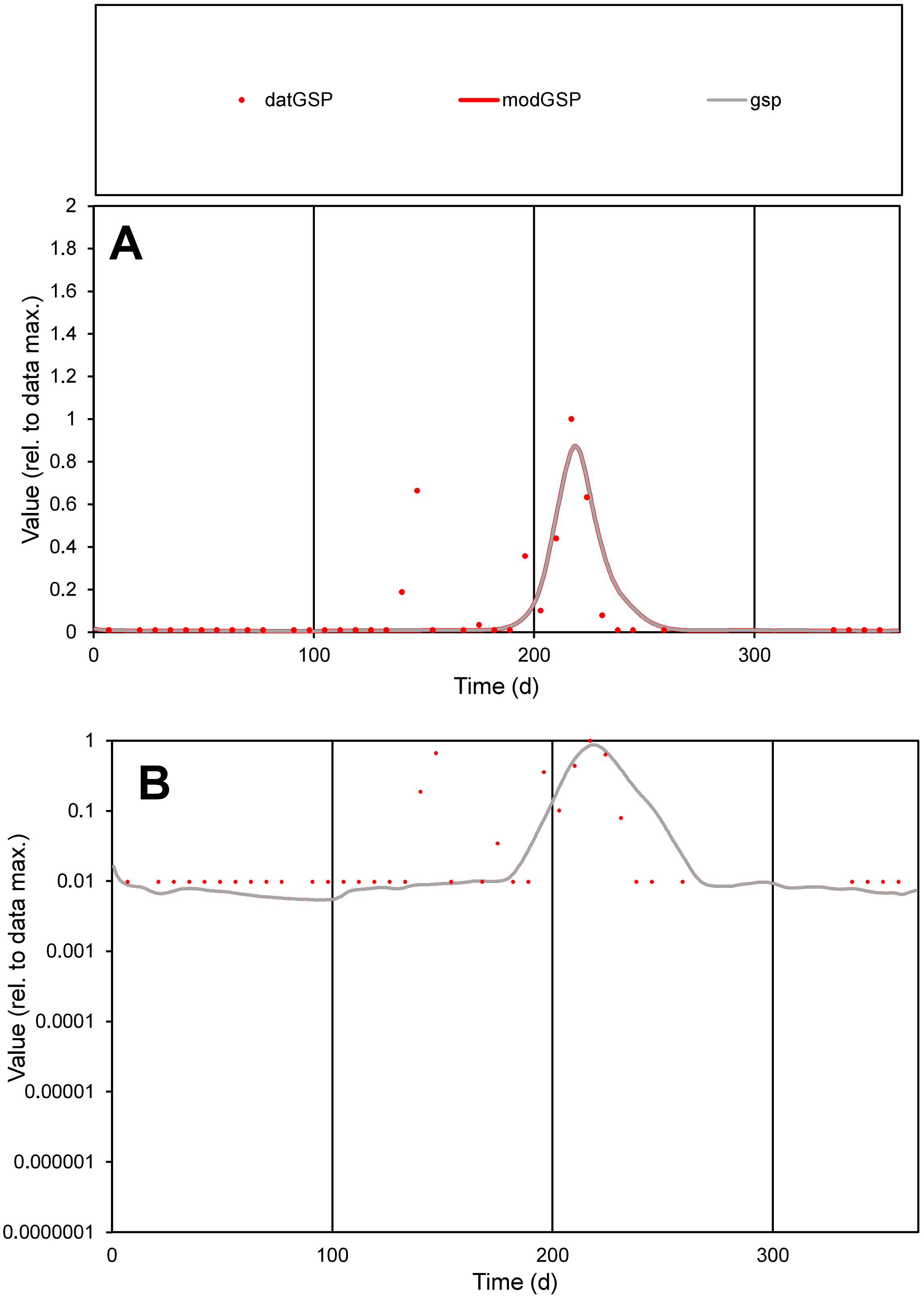

C1

Variables
GSP Provide jtd or Dst
CalibOnly?
2 1 to reproduce of
oNormalize?
2 1=no, 2=yes

ofxn calc*
1.2919
ofxn mod
1.3514

sRun
RunA1
zout
1

Ave
Data
9.4E-05
Model
7.9E-05

data/vMax
0.00092

ofxng
311.734

Min d/m
1.8099

ave/min d
10.4277
ave/min m
15.849

331_Calib_S_A

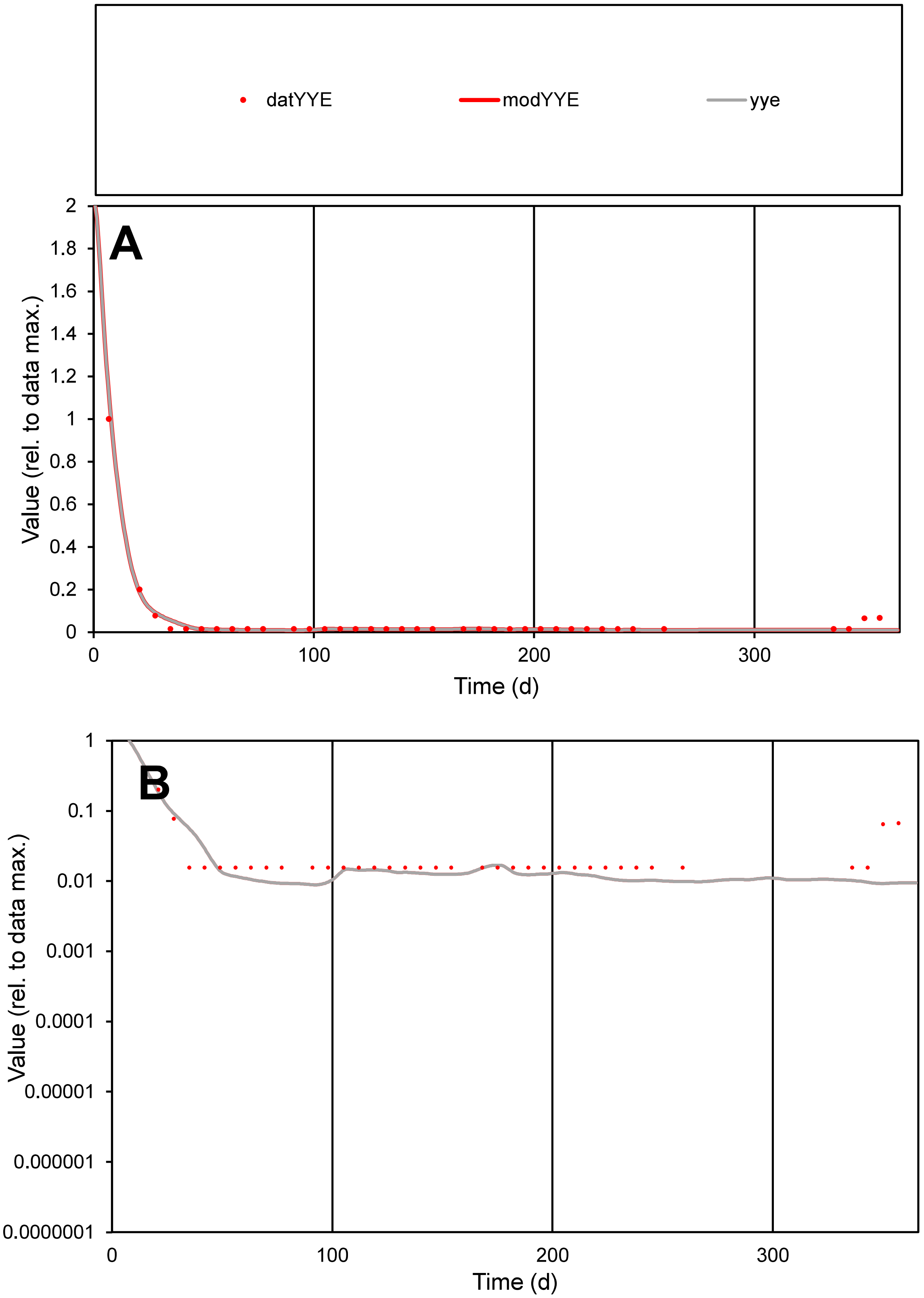

C1

Variables
YYE Provide jtd or Dst
CalibOnly?
2 1 to reproduce of
oNormalize?
2 1=no, 2=yes

ofxn calc*
0.2924
ofxn mod
0.36869

sRun
RunA1
zout
1

Ave
Data
1.2E-05
Model
1.1E-05

data/vMax
0.00024

ofxng
311.734

Min d/m
1.76501

ave/min d
3.30534
ave/min m
5.22855

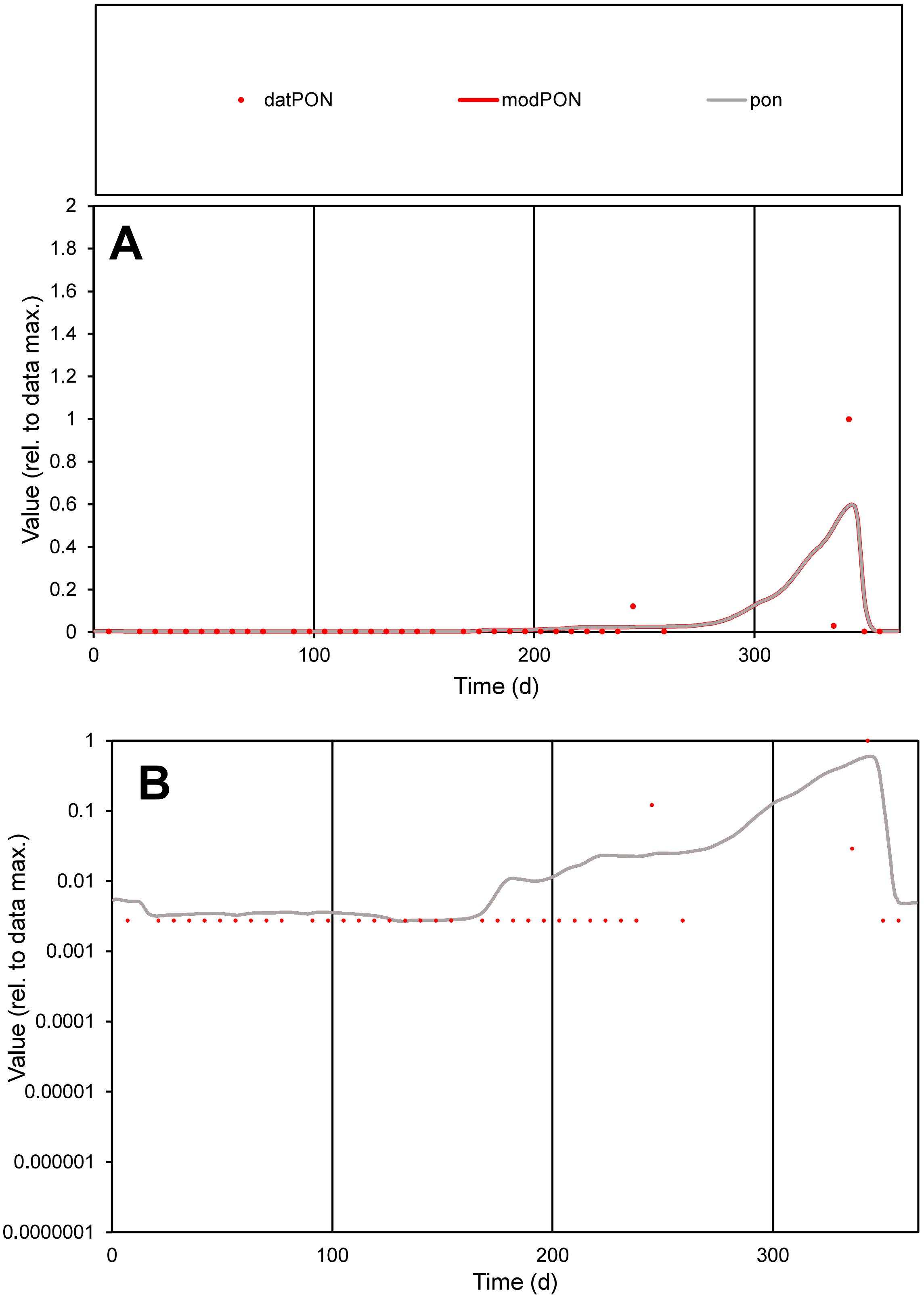

C1

Variables
PON Provide jtd or Dst
CalibOnly?
2 1 to reproduce of
oNormalize?
2 1=no, 2=yes

ofxn calc*
3.04595
ofxn mod
3.07578

sRun
RunA1
zout
1

Ave
Data
3.6E-05
Model
4E-05

data/vMax
0.00107

ofxng
311.734

Min d/m
1.00894

ave/min d
12.2562
ave/min m
13.8227

331_Calib_S_A

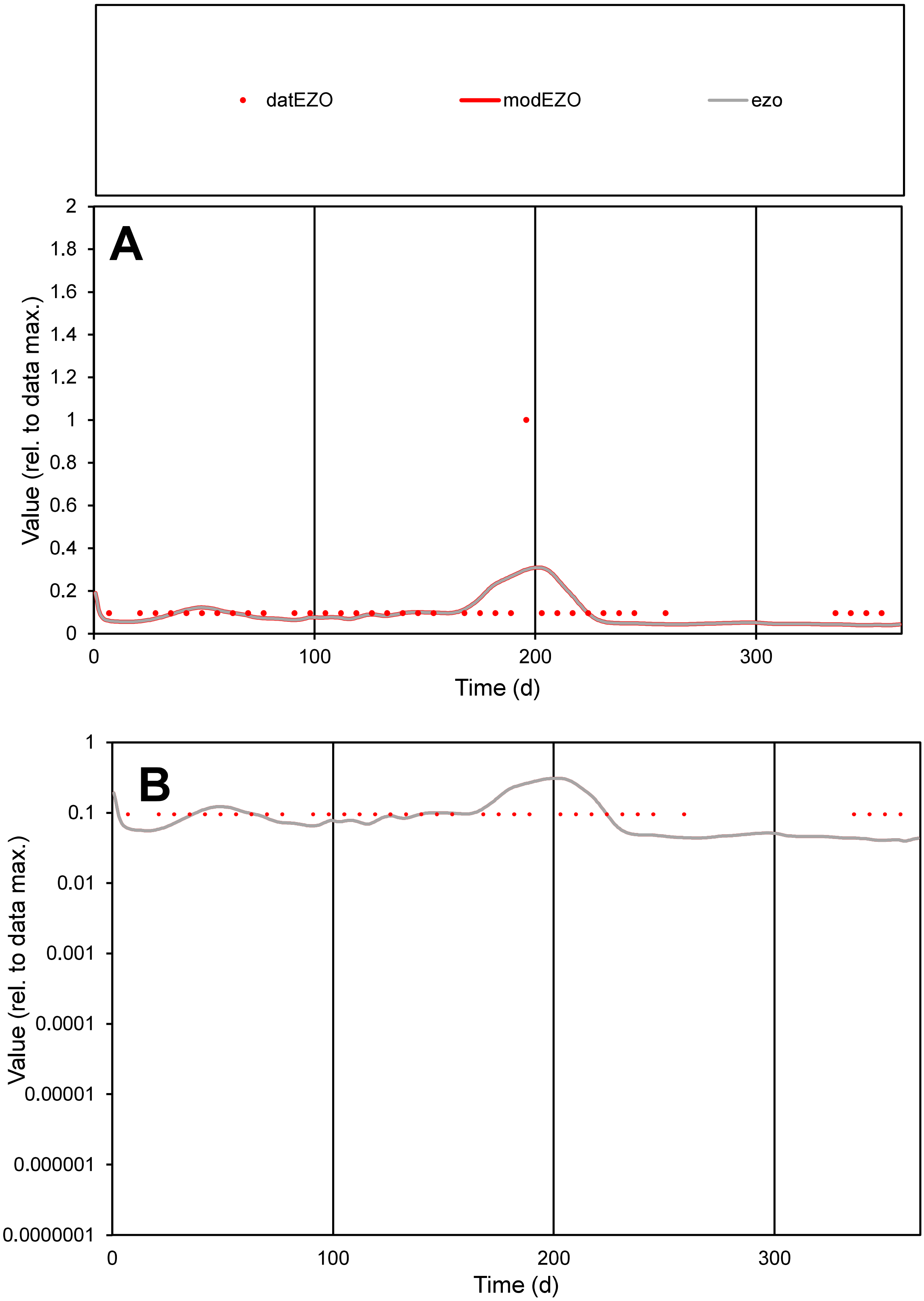

C1

Variables
EZO Provide jtd or Dst
CalibOnly?
2 1 to reproduce of
oNormalize?
2 1=no, 2=yes

ofxn calc*
1.09433
ofxn mod
1.10322

sRun
RunA1
zout
1

Ave
Data
5E-06
Model
4.4E-06

data/vMax
4.1E-05

ofxng
311.734

Min d/m
2.40134

ave/min d
1.25606
ave/min m
2.70214

331_Calib_S_A

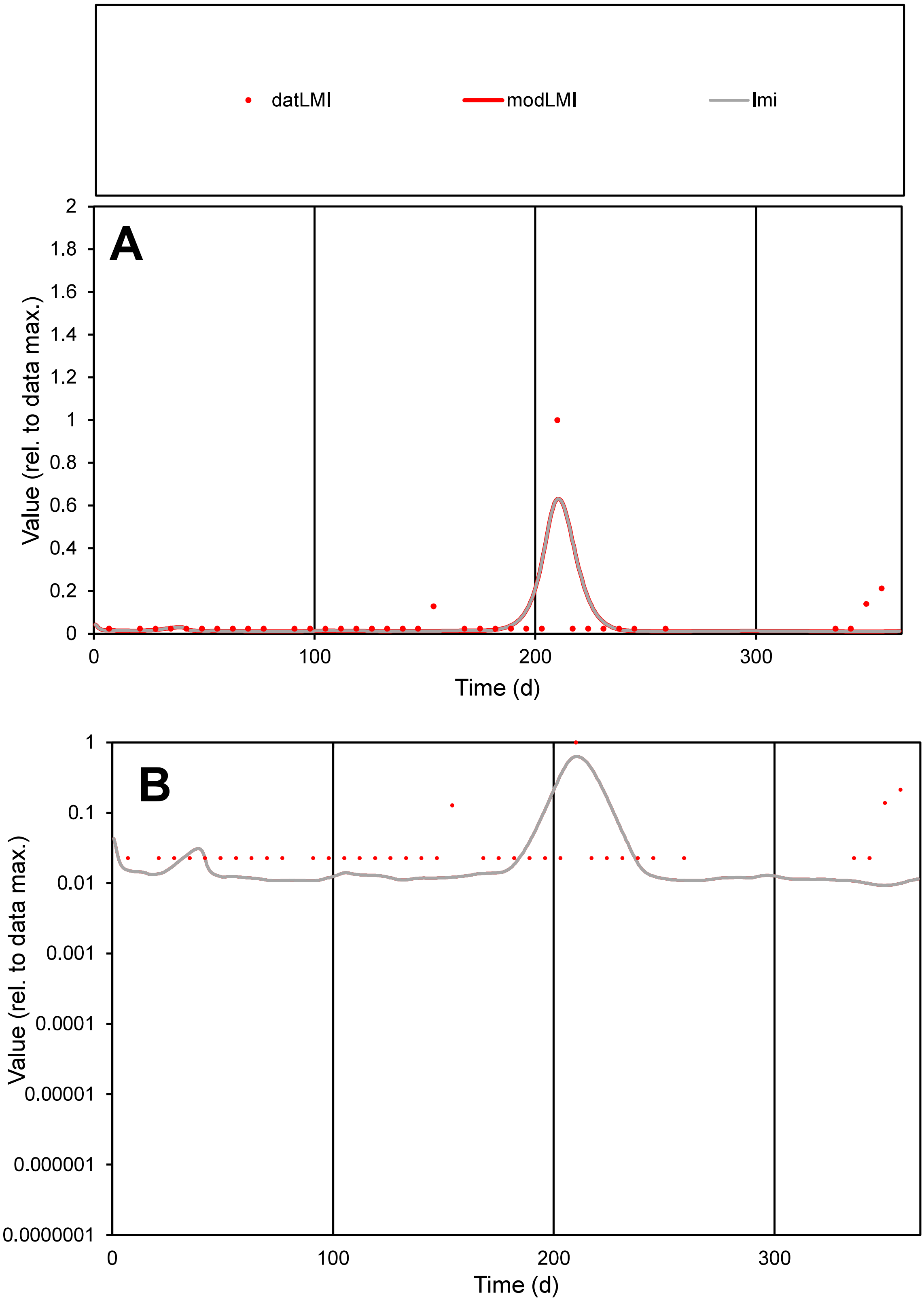

C1

Variables
LMI Provide jtd or Dst
CalibOnly?
2 1 to reproduce of
oNormalize?
2 1=no, 2=yes

ofxn calc*
1.61849
ofxn mod
1.76893

sRun
RunA1
zout
1

Ave
Data
8E-06
Model
6.6E-06

data/vMax
0.00013

ofxng
311.734

Min d/m
2.42203

ave/min d
2.65417
ave/min m
5.29819

331_Calib_S_A

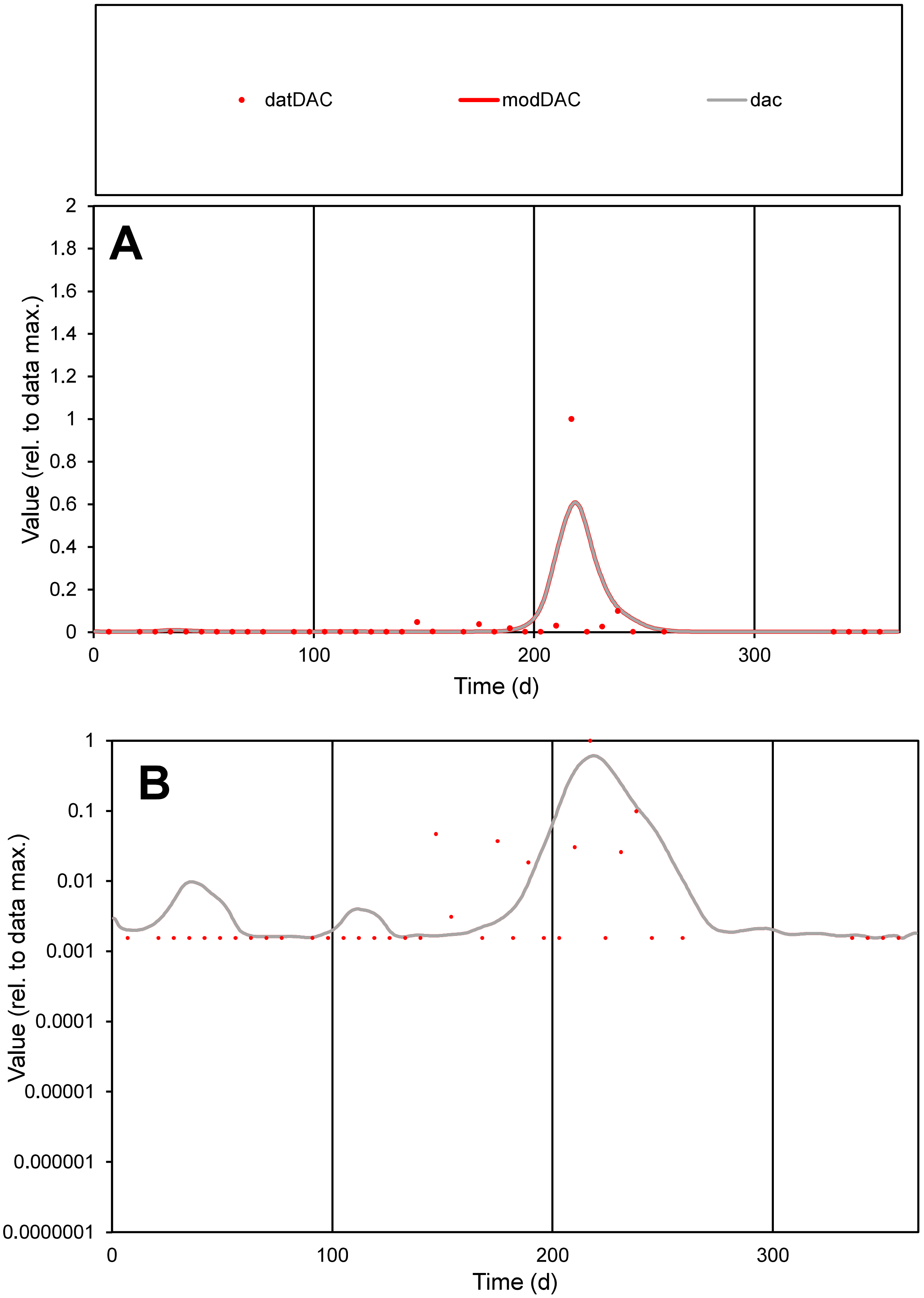

C1

Variables
DAC Provide jtd or Dst
CalibOnly?
2 1 to reproduce of
oNormalize?
2 1=no, 2=yes

ofxn calc*
2.80708
ofxn mod
3.14319

sRun
RunA1
zout
1

Ave
Data
0.00017
Model
0.00023

data/vMax
0.00496

ofxng
311.734

Min d/m
1.00903

ave/min d
22.6938
ave/min m
30.6719

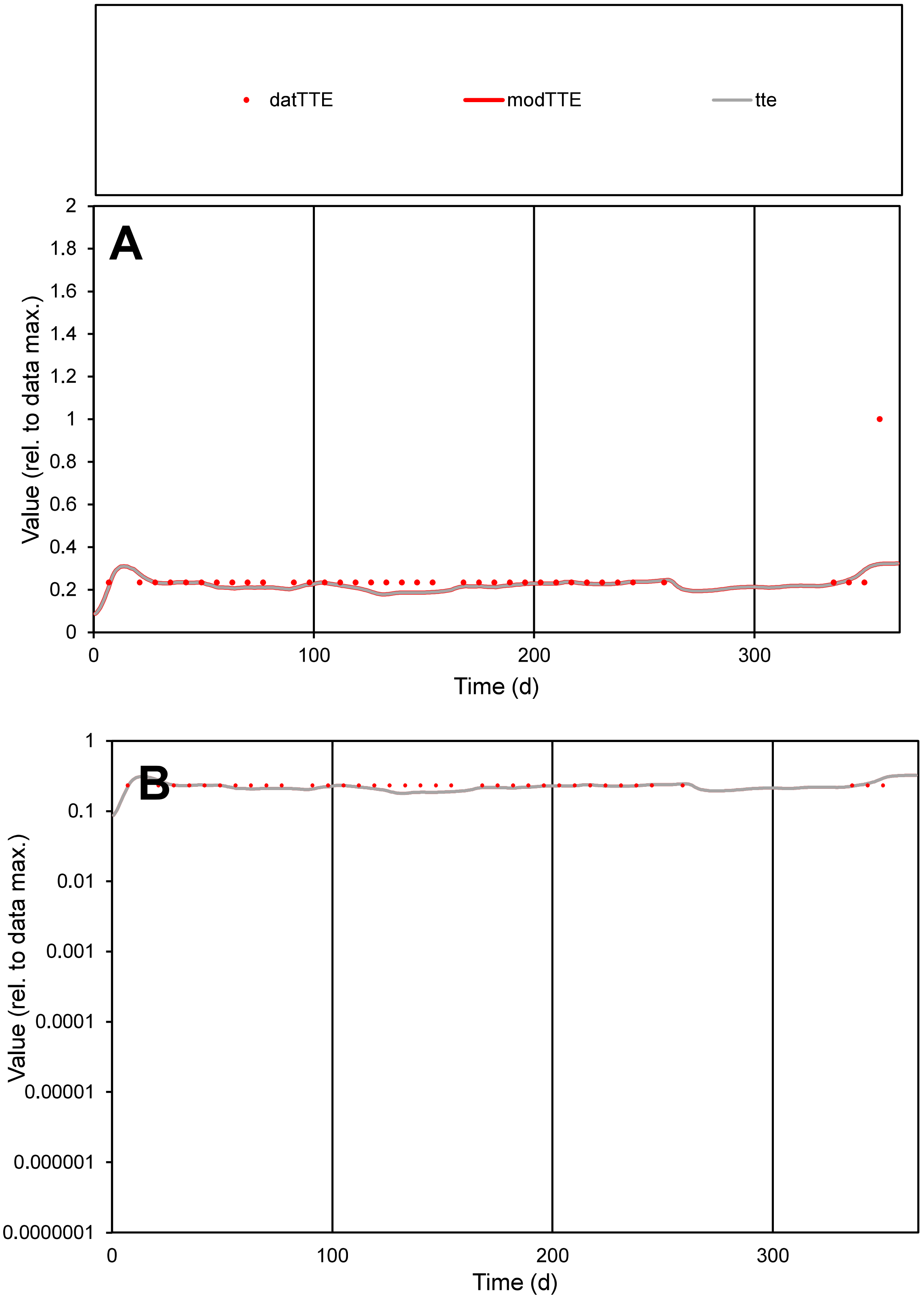

C1

Variables
TTE Provide jtd or Dst
CalibOnly?
2 1 to reproduce of
oNormalize?
2 1=no, 2=yes

ofxn calc*
0.4485
ofxn mod
0.46215

sRun
RunA1
zout
1

Ave
Data
4.1E-06
Model
3.6E-06

data/vMax
1.6E-05

ofxng
311.734

Min d/m
1.29511

ave/min d
1.08845
ave/min m
1.25101

331_Calib_S_A

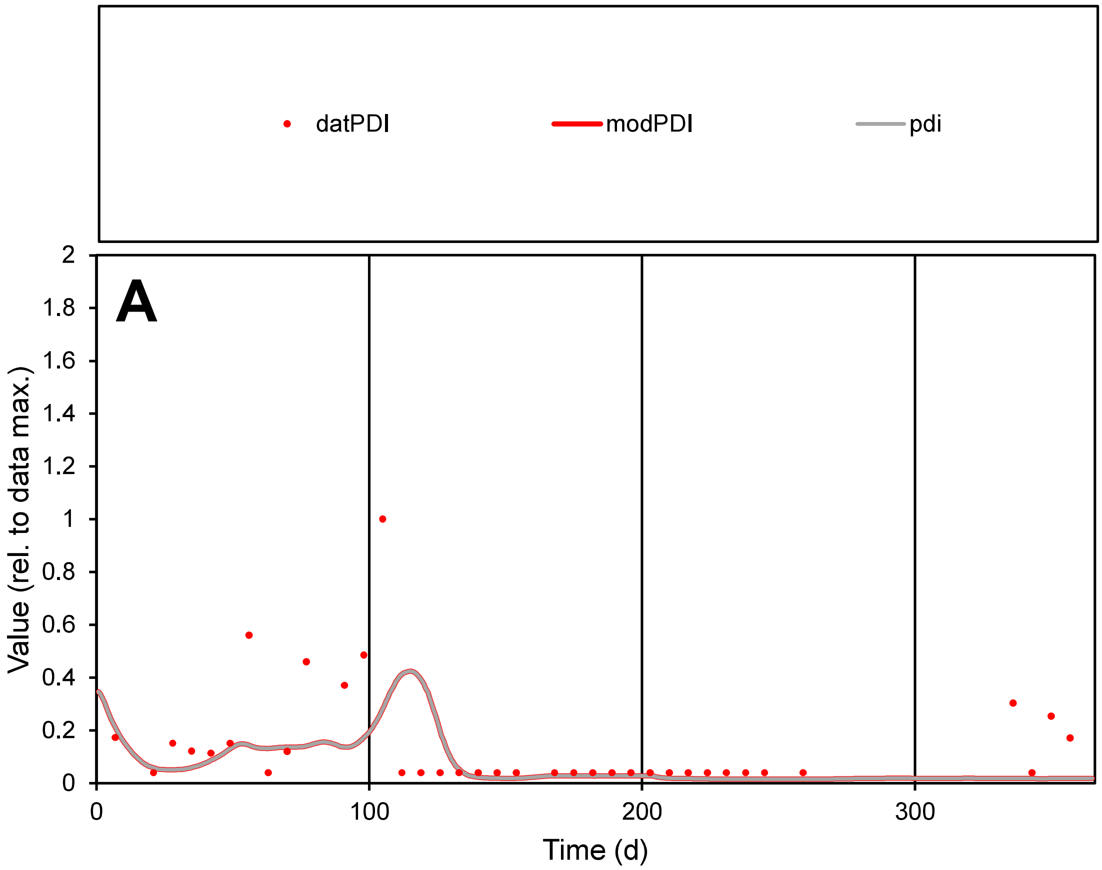

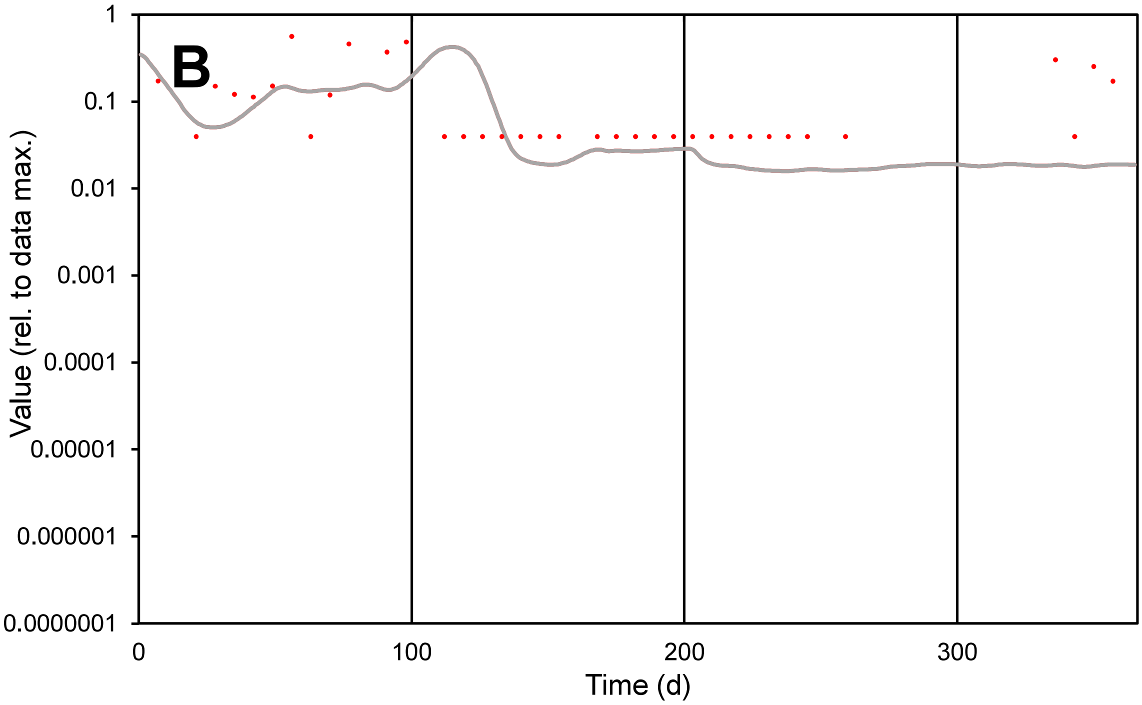

C1

Variables

PDI Provide jtd or Dst

CalibOnly?

2 1 to reproduce of

oNormalize?

2 1=no, 2=yes

ofxn calc*

1.30596

ofxn mod

1.31946

sRun

RunA1

zout

1

Ave

Data

1.6E-05

Model

9.3E-06

data/vMax

0.00011

ofxng

311.734

Min d/m

2.48924

ave/min d

3.63769

ave/min m

5.25073

331_Calib_S_A

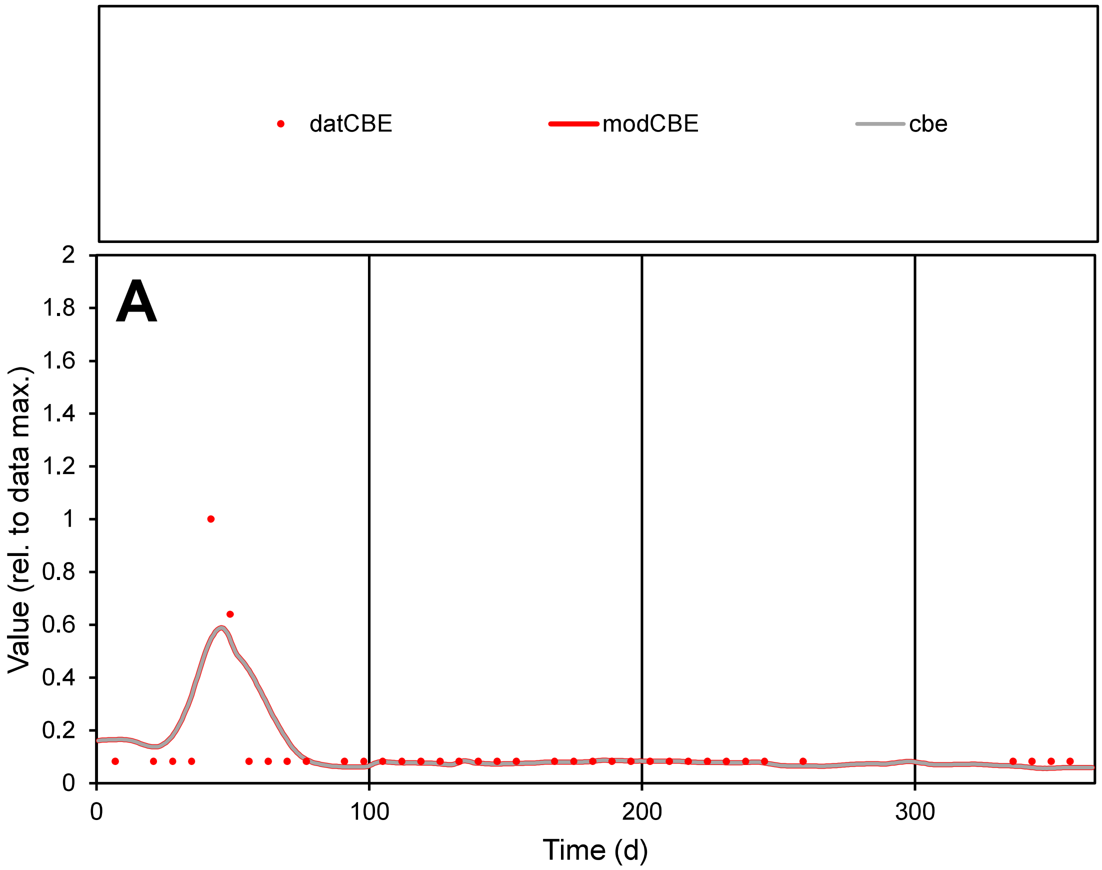

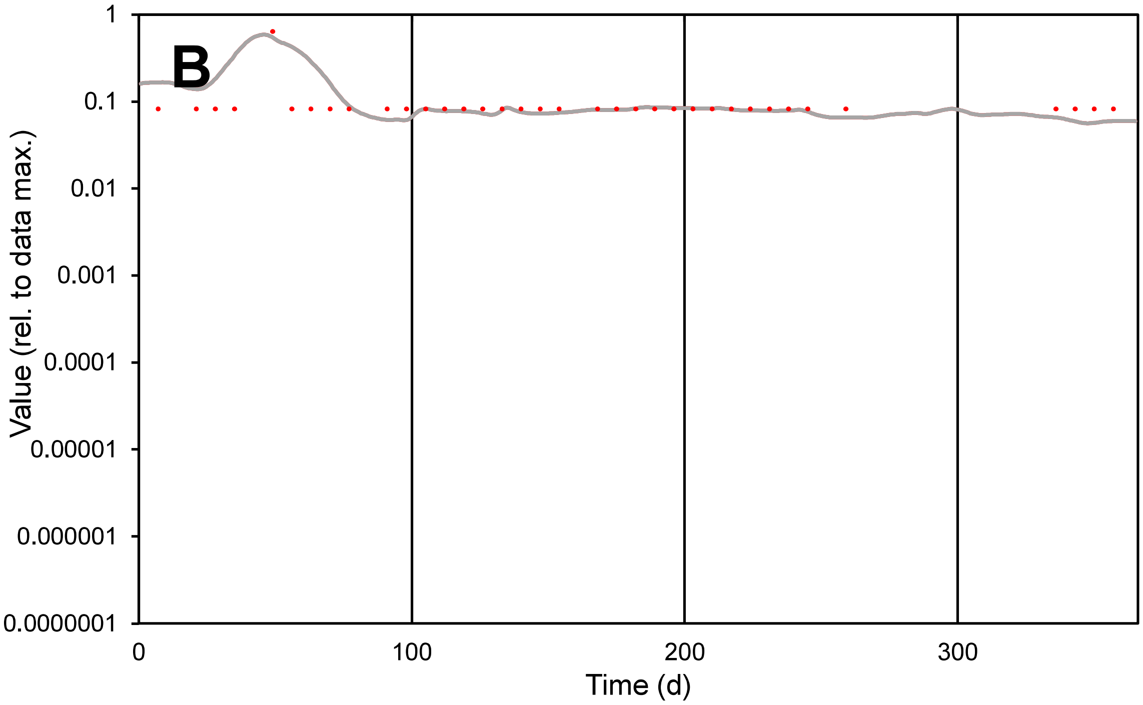

C1

Variables

CBE Provide jtd or Dst

CalibOnly?

2 1 to reproduce of

oNormalize?

2 1=no, 2=yes

ofxn calc*

0.86211

ofxn mod

0.8967

sRun

RunA1

zout

1

Ave

Data

2.5E-06

Model

2.6E-06

data/vMax

2E-05

ofxng

311.734

Min d/m

1.43053

ave/min d

1.48438

ave/min m

2.25251

331_Calib_S_A

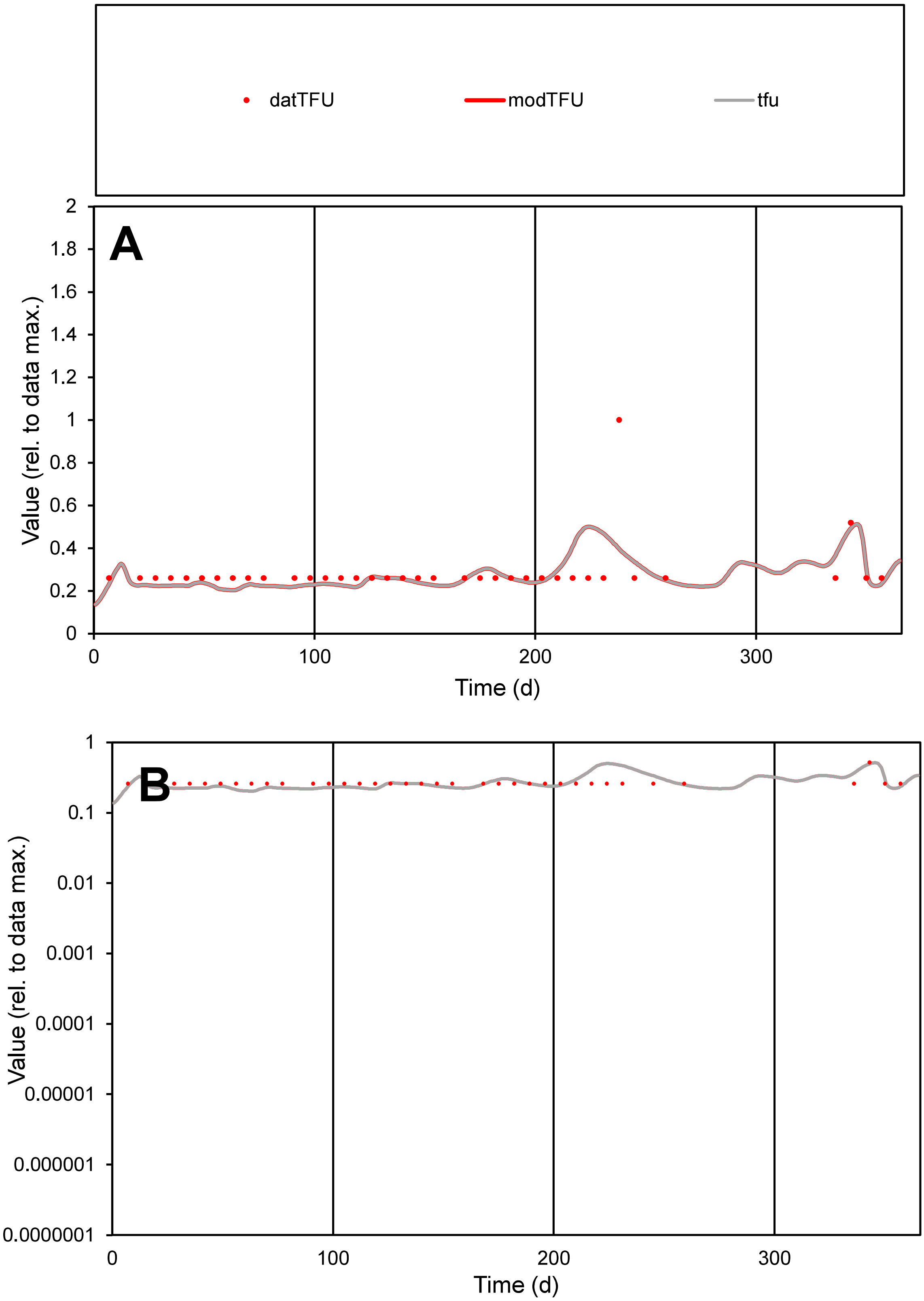

C1

Variables
TFU Provide jtd or Dst
CalibOnly?
2 1 to reproduce of
oNormalize?
2 1=no, 2=yes

ofxn calc*
0.42116
ofxn mod
0.4392

sRun
RunA1
zout
1

Ave
Data
1.4E-05
Model
1.4E-05

data/vMax
4.9E-05

ofxng
311.734

Min d/m
1.26238

ave/min d
1.10397
ave/min m
1.3426

331_Calib_S_A

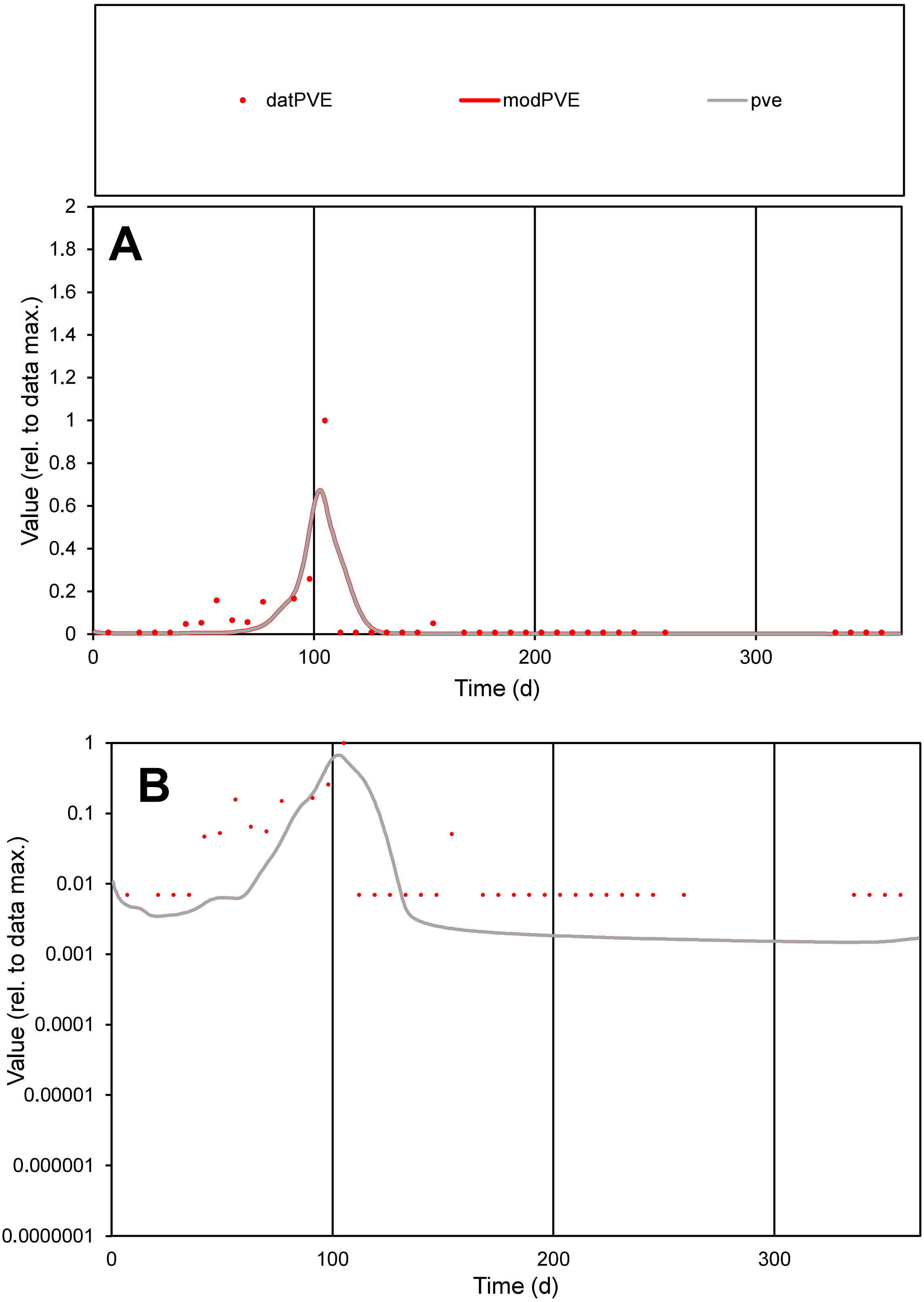

C1

Variables
PVE Provide jtd or Dst
CalibOnly?
2 1 to reproduce of
oNormalize?
2 1=no, 2=yes

ofxn calc*
1.58302
ofxn mod
1.71532

sRun
RunA1
zout
1

Ave
Data
1.8E-05
Model
1.5E-05

data/vMax
0.0003

ofxng
311.734

Min d/m
4.72235

ave/min d
8.47416
ave/min m
33.0211

331_Calib_S_A

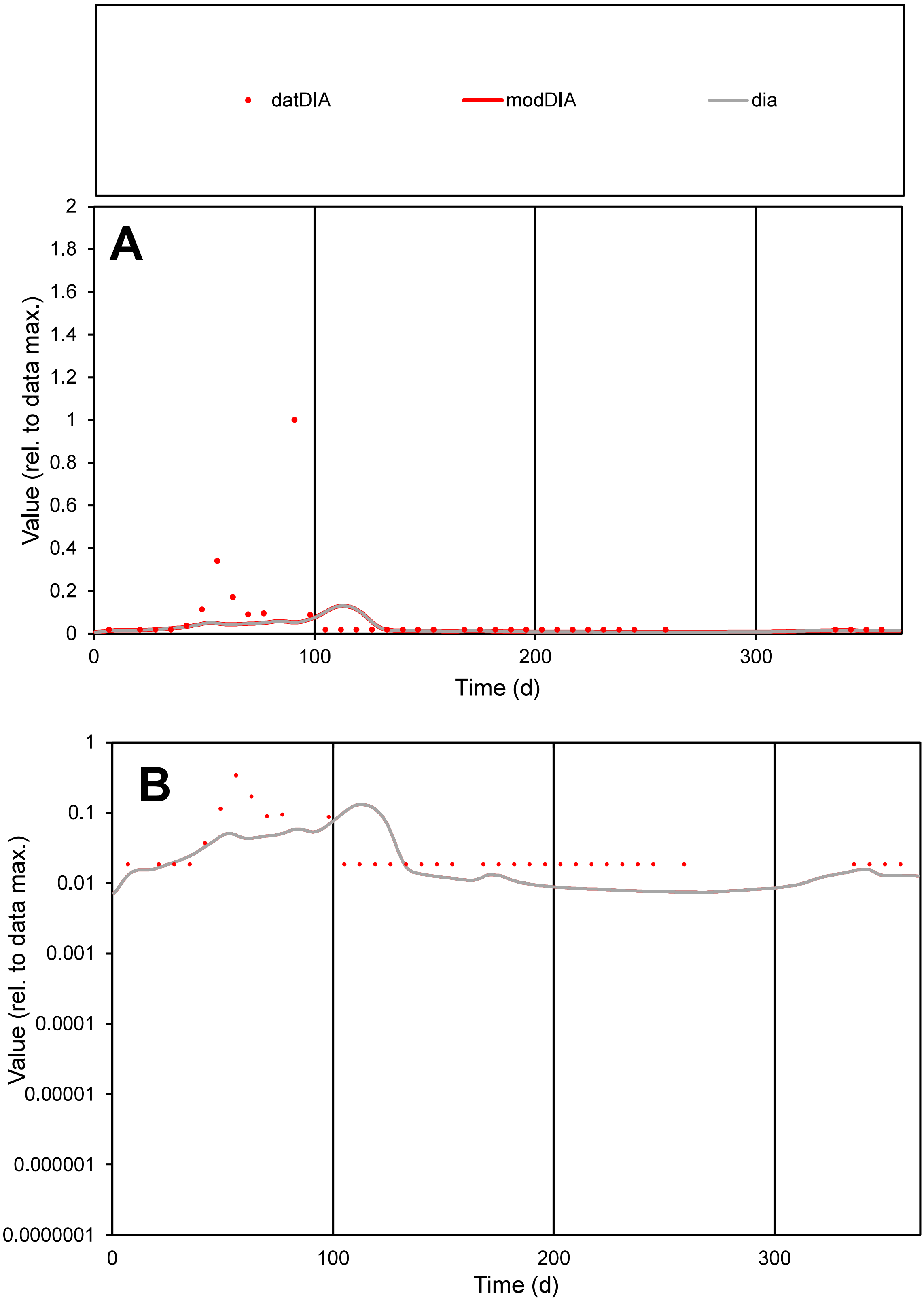

C1

Variables
DIA Provide jtd or Dst
CalibOnly?
2 1 to reproduce of
oNormalize?
2 1=no, 2=yes

ofxn calc*
2.49909
ofxn mod
2.50817

sRun
RunA1
zout
1

Ave
Data
2.2E-05
Model
9.7E-06

data/vMax
0.00034

ofxng
311.734

Min d/m
2.4918

ave/min d
3.6032
ave/min m
3.87637

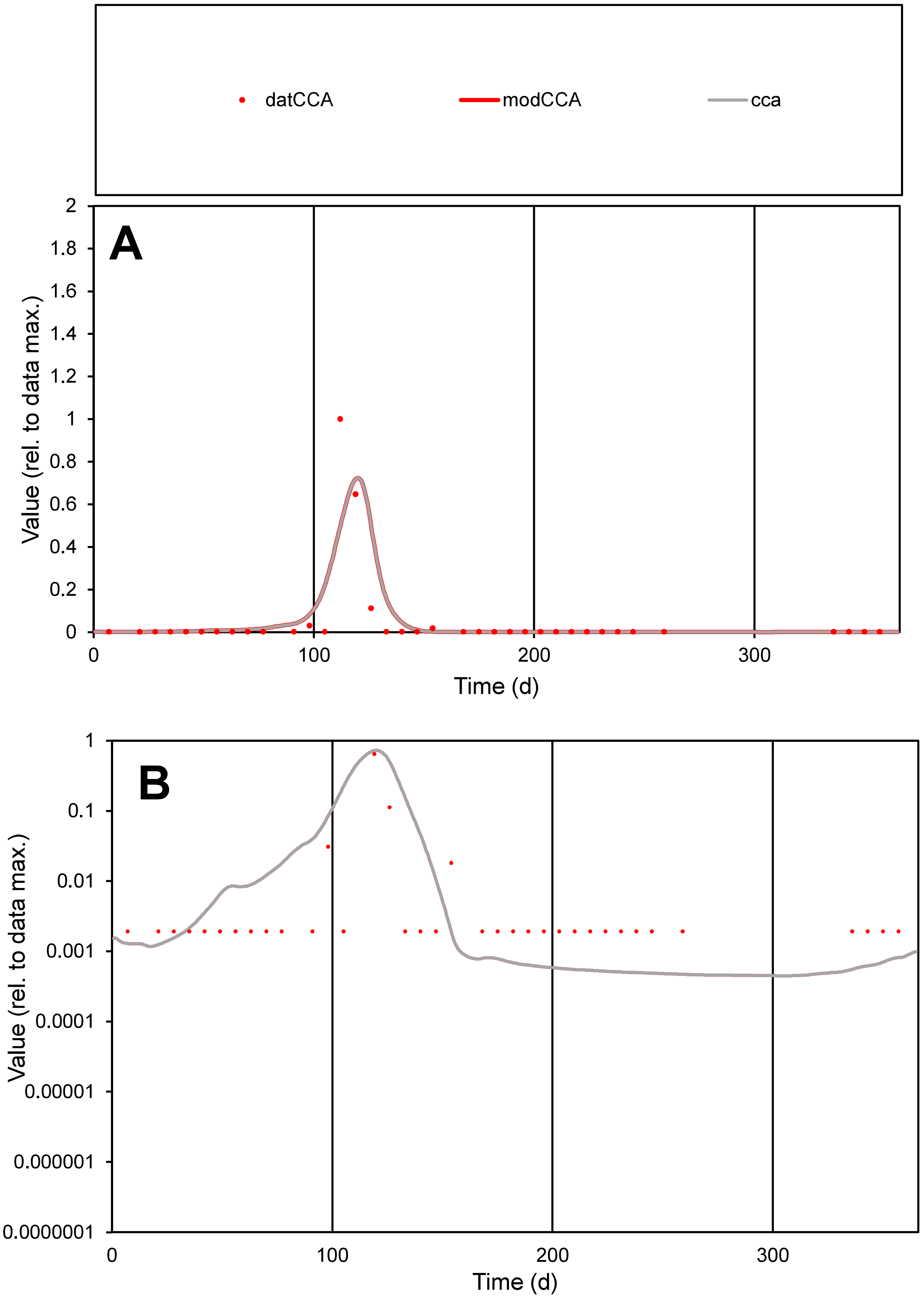

C1

Variables

CCA	Provide jtd or Dst

CalibOnly?

2	1 to reproduce of

oNormalize?

2	1=no, 2=yes

ofxn calc*
1.70983
ofxn mod
1.88427

sRun
RunA1
zout
1

Ave
Data
4.8E-05
Model
5.3E-05

data/vMax
0.00095

ofxng
311.734

Min d/m
4.09682

ave/min d
26.2346
ave/min m
118.146

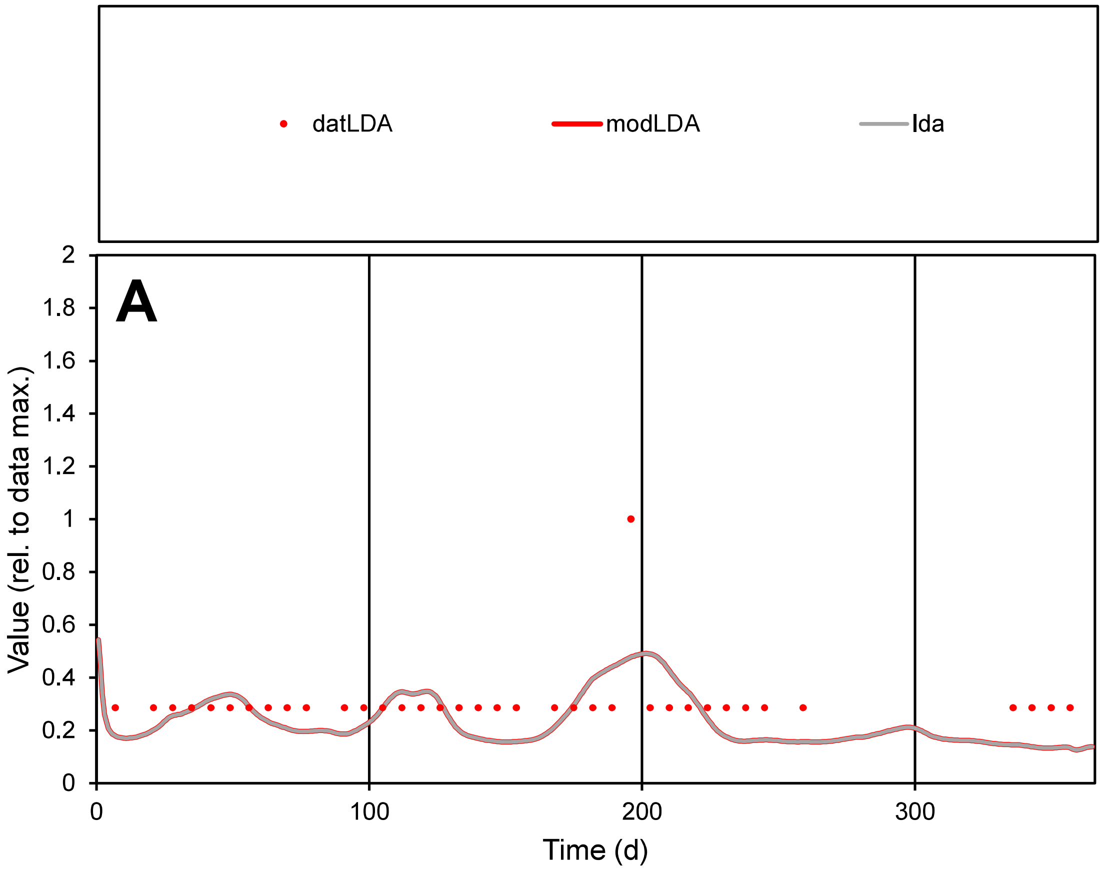

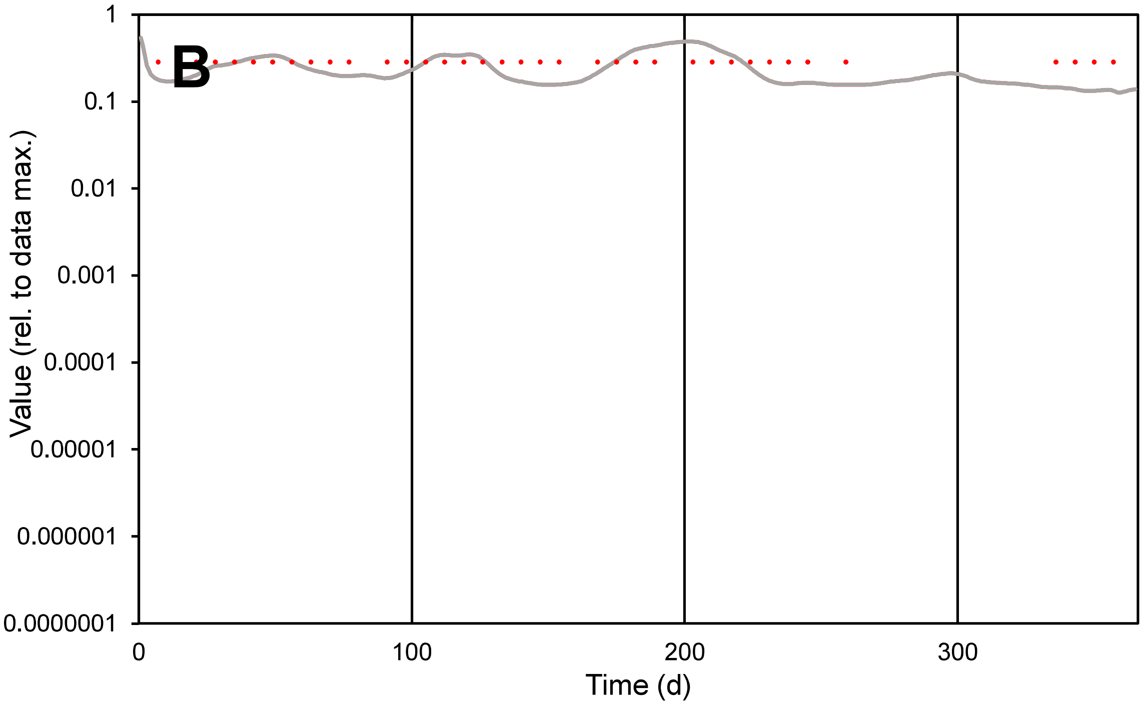

C1

Variables
LDA Provide jtd or Dst
CalibOnly?
2 1 to reproduce of
oNormalize?
2 1=no, 2=yes

ofxn calc*
0.43015
ofxn mod
0.44332

sRun
RunA1
zout
1

Ave
Data
7.4E-06
Model
6.2E-06

data/vMax
2.4E-05

ofxng
311.734

Min d/m
2.14029

ave/min d
1.06777
ave/min m
1.90704

331_Calib_S_A

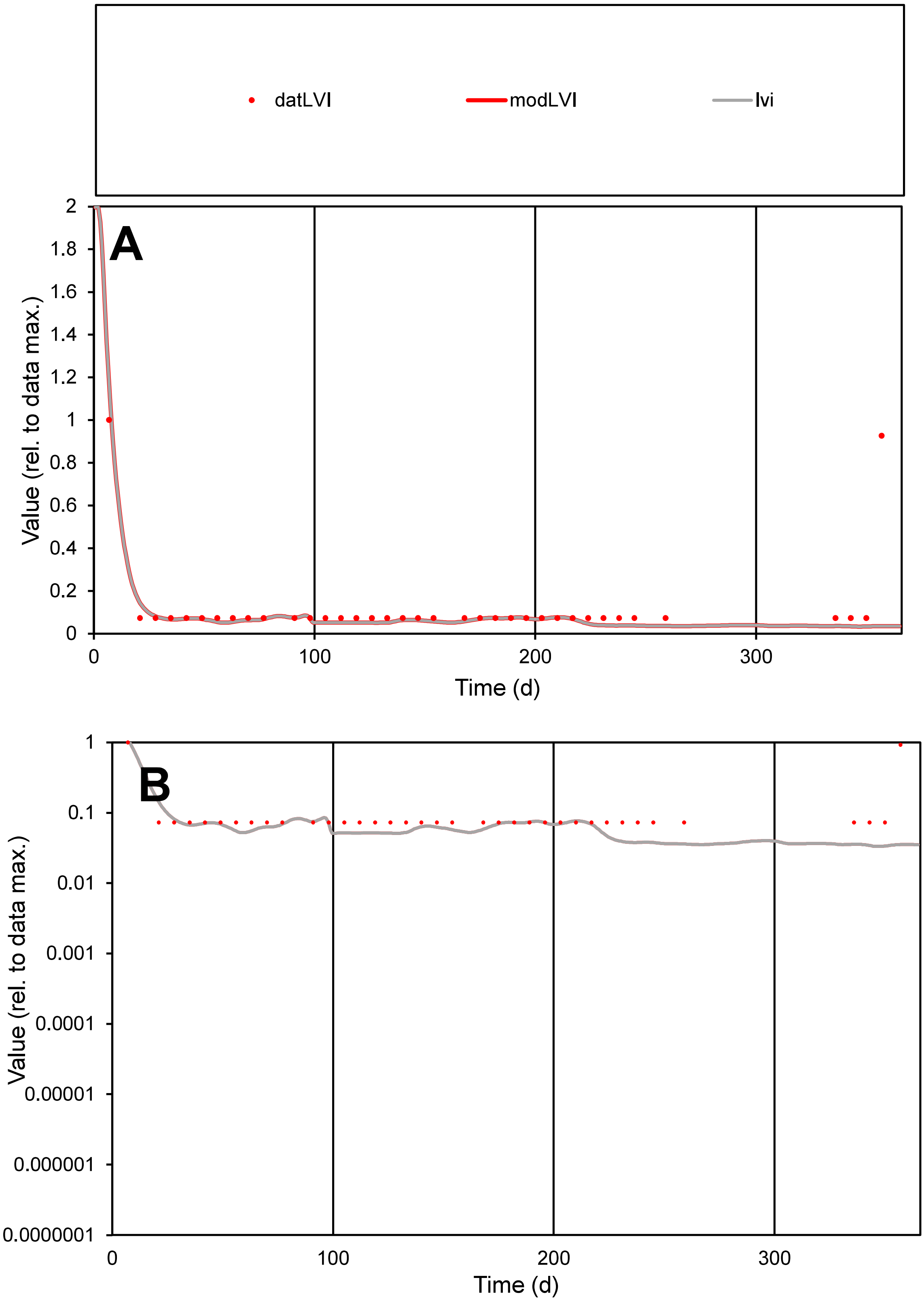

C1

Variables

LVI Provide jtd or Dst

CalibOnly?

2 1 to reproduce of

oNormalize?

2 1=no, 2=yes

ofxn calc*

1.22262

ofxn mod

1.22905

sRun

RunA1

zout

1

Ave

Data

2.4E-06

Model

1.6E-06

data/vMax

1.9E-05

ofxng

311.734

Min d/m

2.15161

ave/min d

1.65944

ave/min m

2.49447

331_Calib_S_A

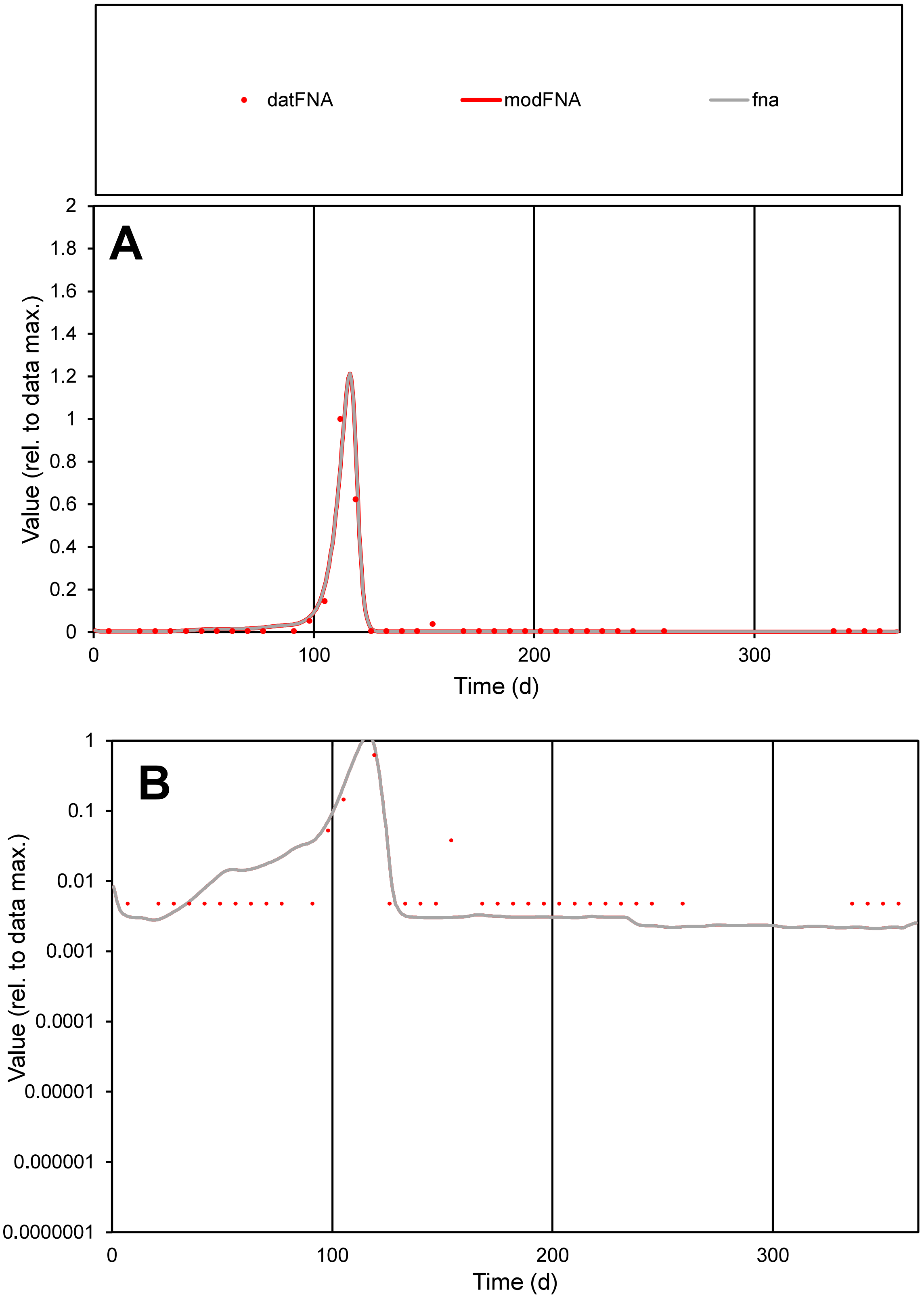

C1

Variables

FNA Provide jtd or Dst

CalibOnly?

2 1 to reproduce of

oNormalize?

2 1=no, 2=yes

ofxn calc*

0.35974

ofxn mod

0.47051

sRun

RunA1

zout

1

Ave

Data

3.5E-05

Model

3.7E-05

data/vMax

0.00065

ofxng

311.734

Min d/m

2.24478

ave/min d

11.3841

ave/min m

26.6431

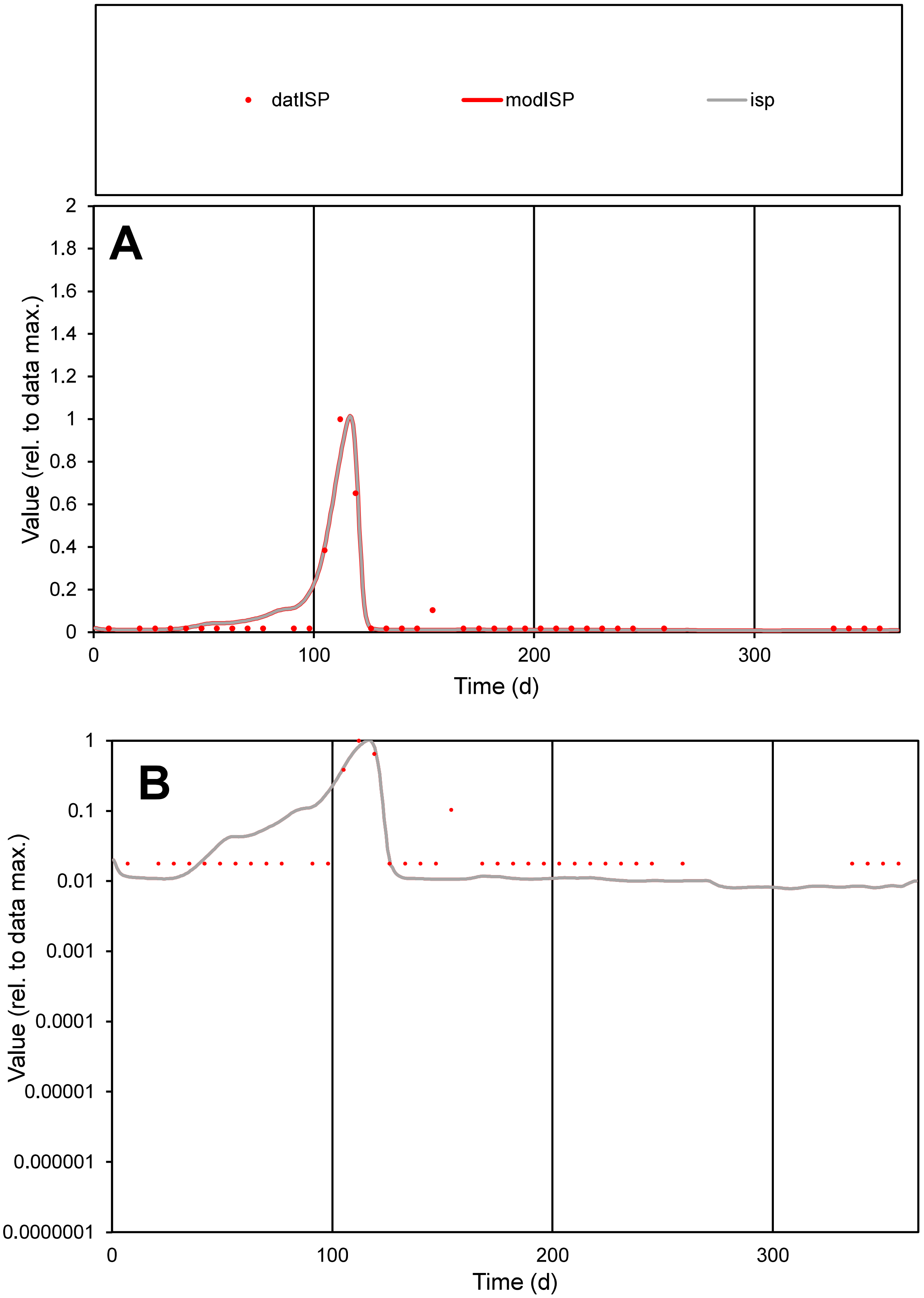

C1

Variables
ISP Provide jtd or Dst
CalibOnly?
2 1 to reproduce of
oNormalize?
2 1=no, 2=yes

ofxn calc*
0.55335
ofxn mod
0.62732

sRun
RunA1
zout
1

Ave
Data
2.1E-05
Model
2.2E-05

data/vMax
0.00028

ofxng
311.734

Min d/m
2.1808

ave/min d
4.13733
ave/min m
9.4118

331_Calib_S_A

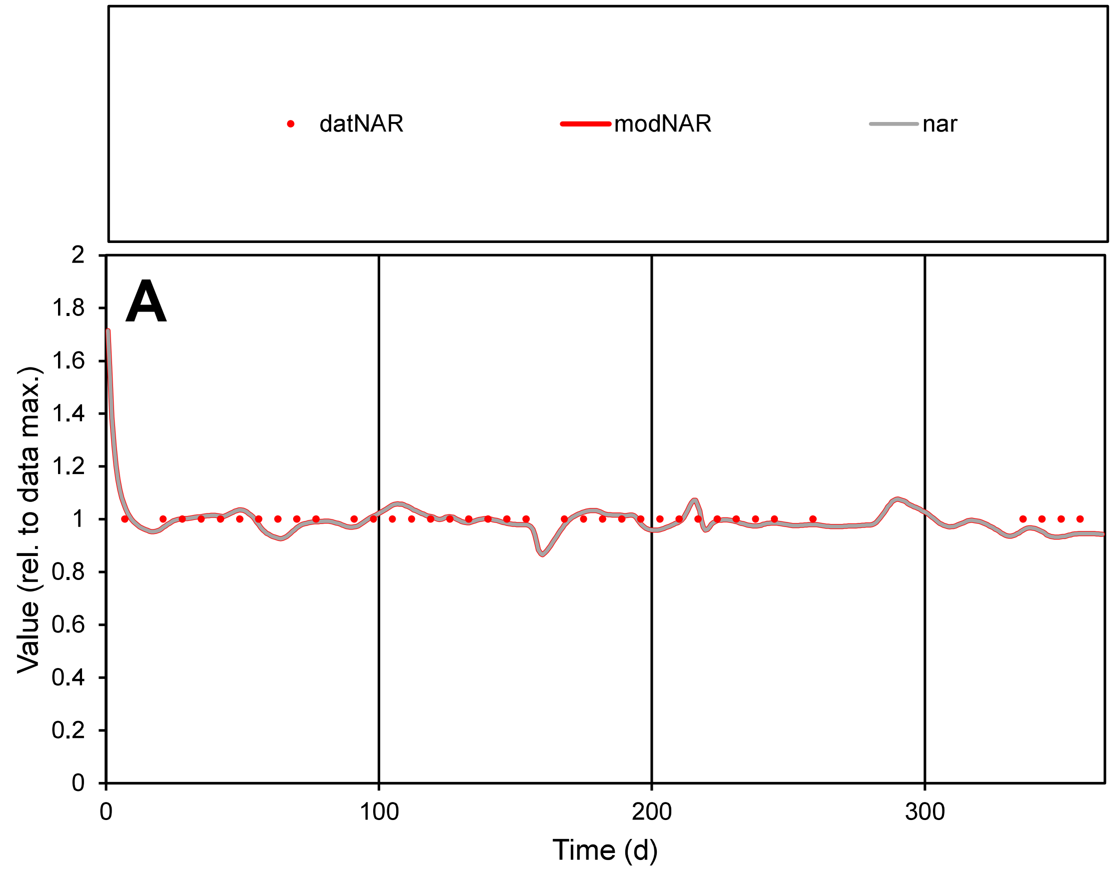

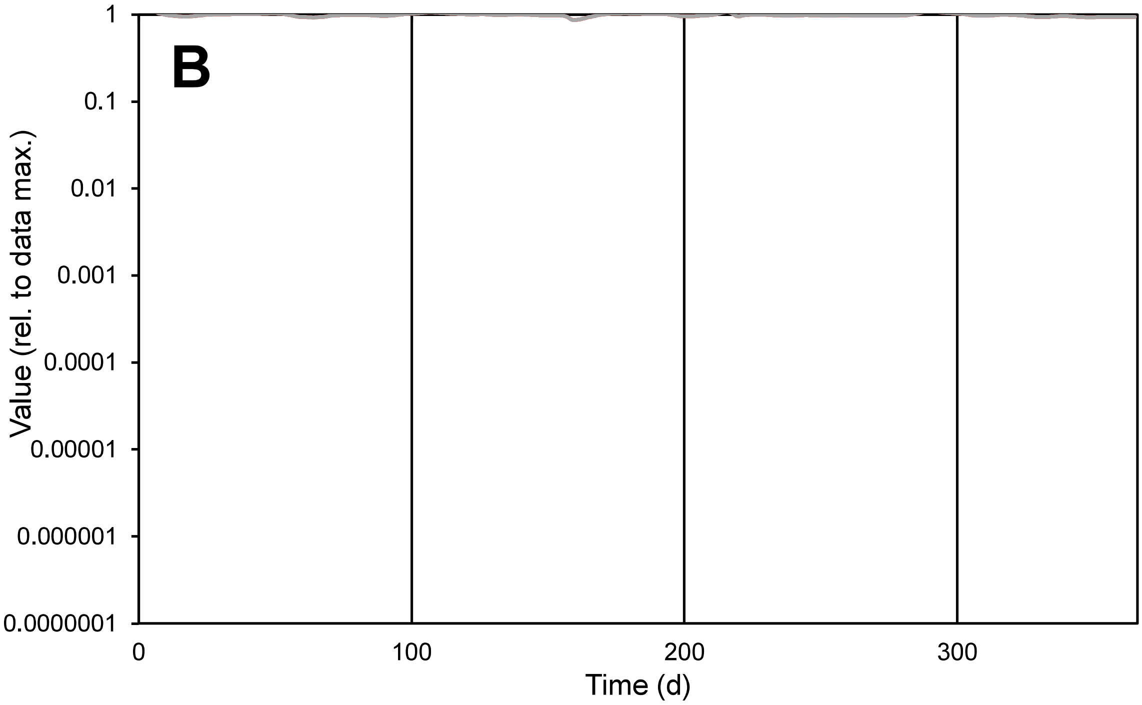

C1

Variables

NAR Provide jtd or Dst

CalibOnly?

2 1 to reproduce of

oNormalize?

2 1=no, 2=yes

ofxn calc*

0.02942

ofxn mod

0.11493

sRun

RunA1

zout

1

Ave

Data

2.8E-06

Model

2.8E-06

data/vMax

2.8E-06

ofxng

311.734

Min d/m

1.07742

ave/min d

1

ave/min m

1.06837

331_Calib_S_A

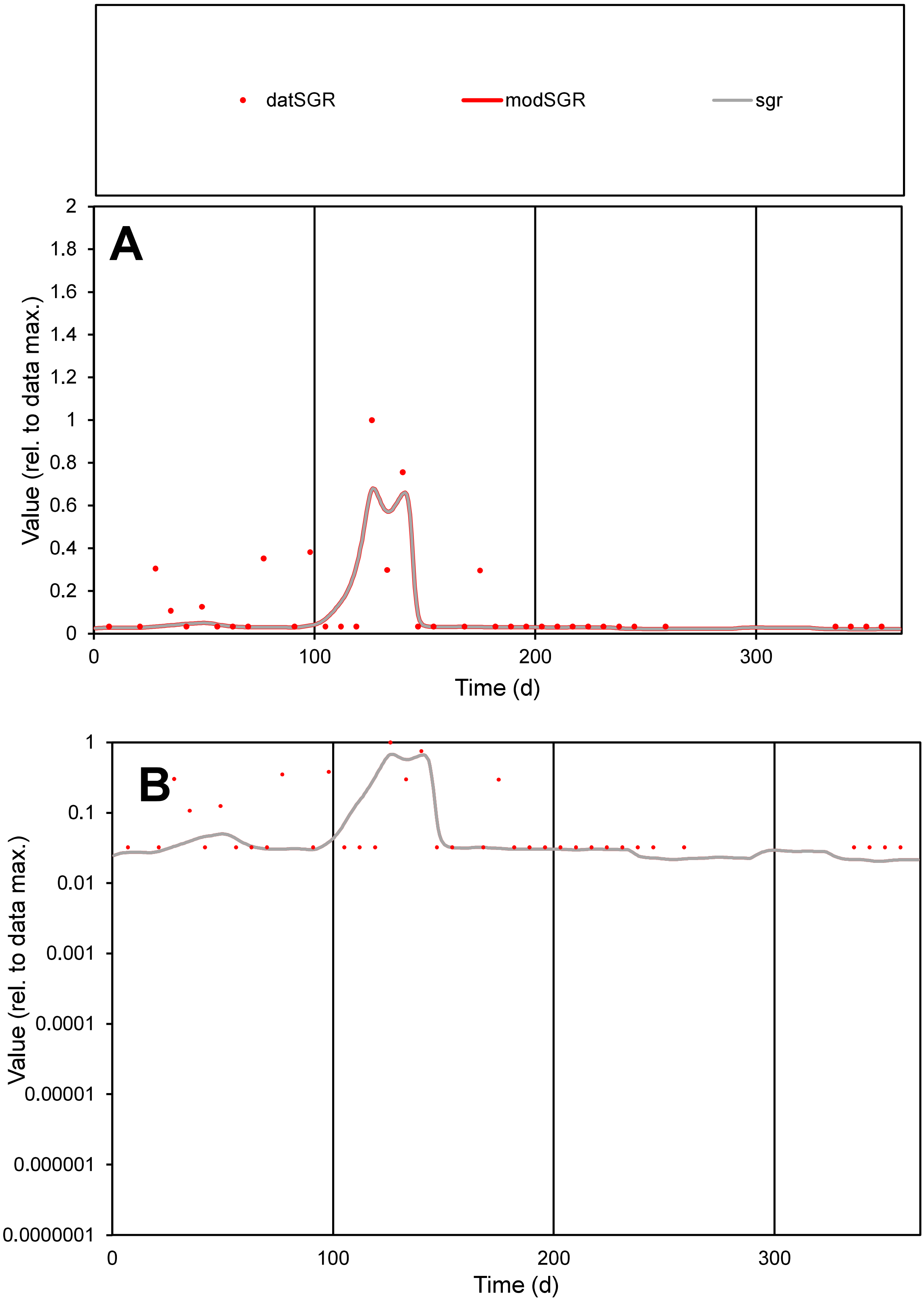

C1

Variables

SGR Provide jtd or Dst

CalibOnly?

2 1 to reproduce of

oNormalize?

2 1=no, 2=yes

ofxn calc*

1.06183

ofxn mod

1.0951

sRun

RunA1

zout

1

Ave

Data

1.7E-05

Model

1.3E-05

data/vMax

0.00014

ofxng

311.734

Min d/m

1.55735

ave/min d

3.78319

ave/min m

4.35028

331_Calib_S_A

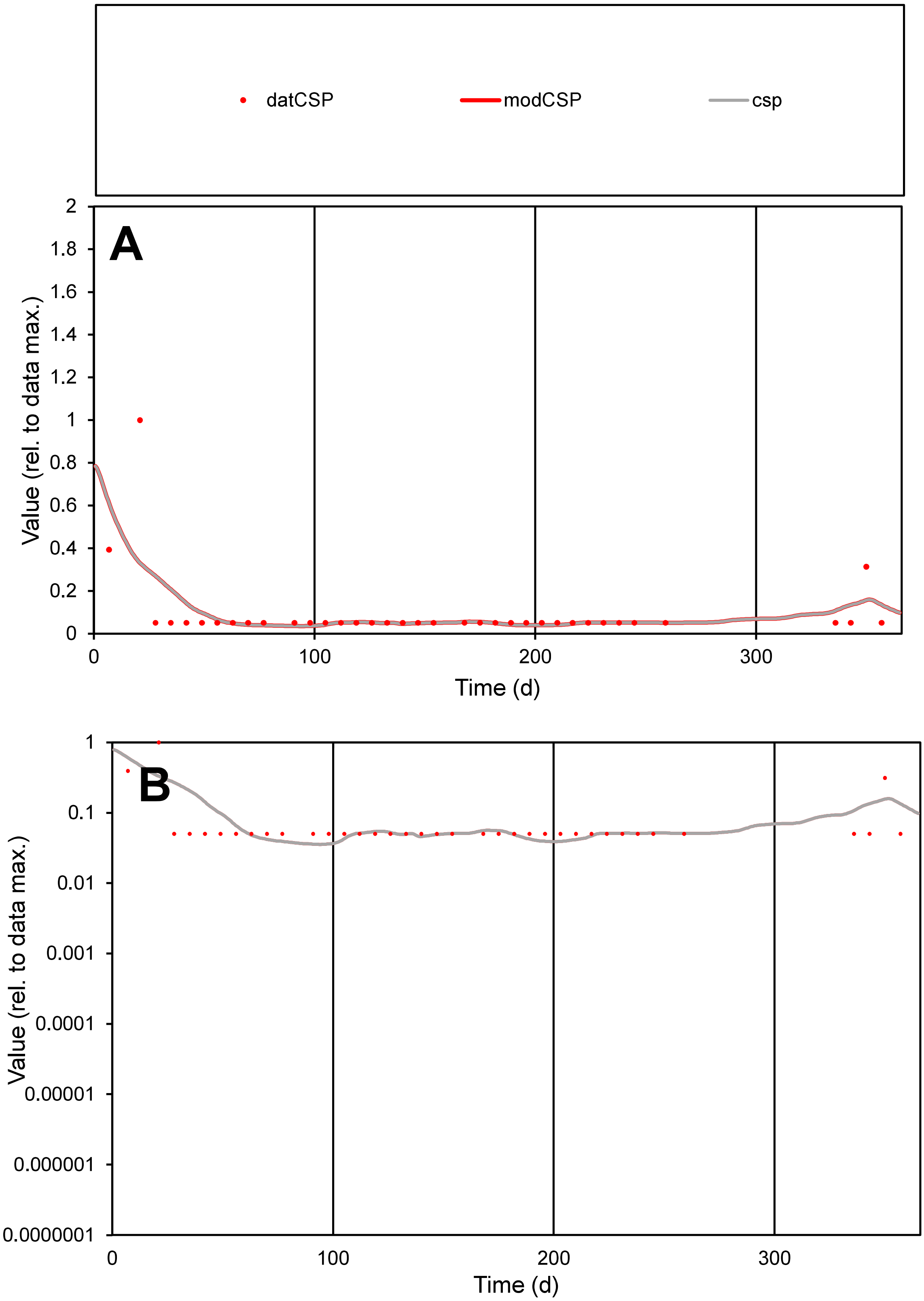

C1

Variables
CSP Provide jtd or Dst
CalibOnly?
2 1 to reproduce of
oNormalize?
2 1=no, 2=yes

ofxn calc*
1.36991
ofxn mod
1.4037

sRun
RunA1
zout
1

Ave
Data
4.4E-06
Model
4.3E-06

data/vMax
4.7E-05

ofxng
311.734

Min d/m
1.41494

ave/min d
1.83469
ave/min m
2.57311

331_Calib_S_A

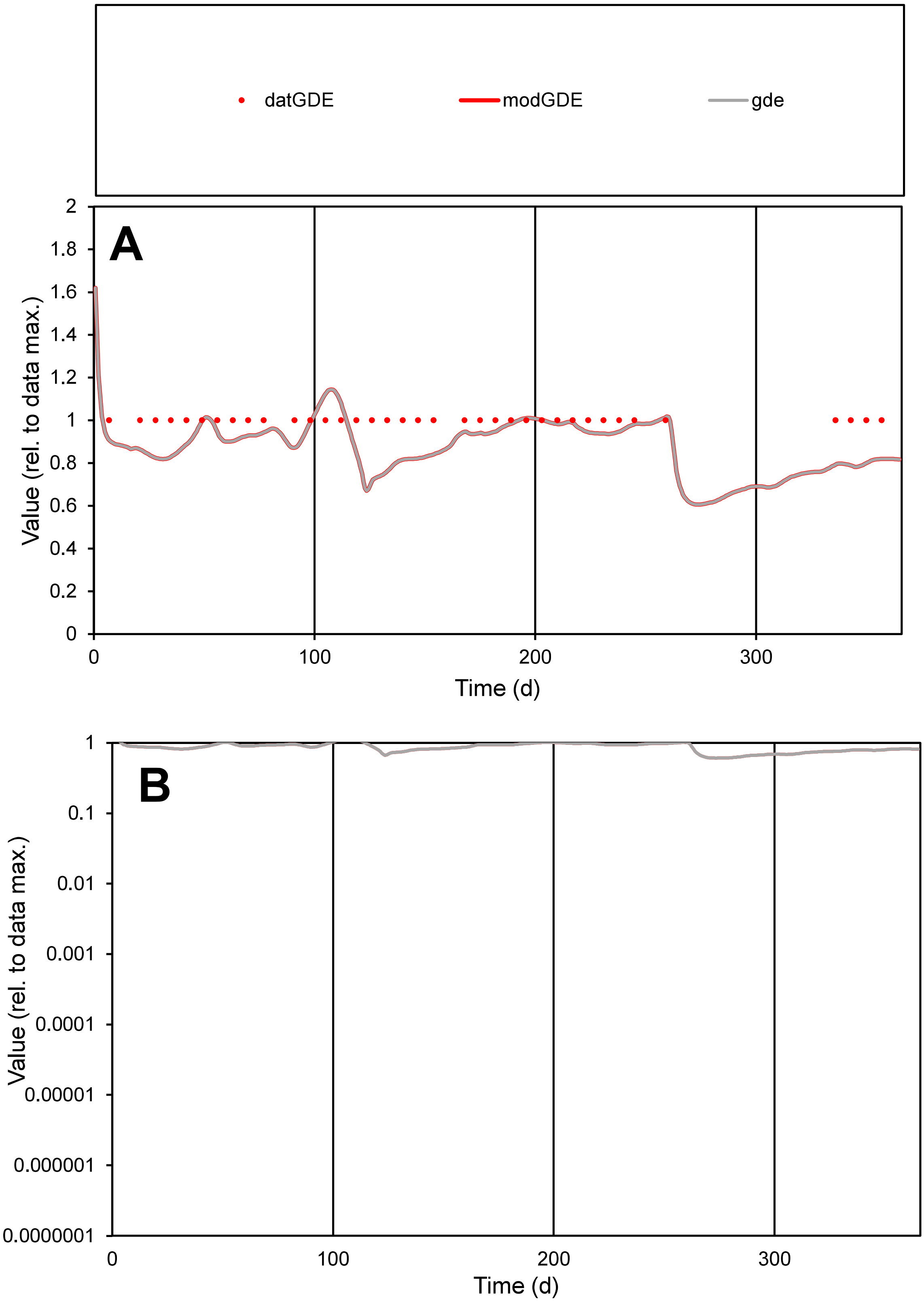

C1

Variables
GDE Provide jtd or Dst
CalibOnly?
2 1 to reproduce of
oNormalize?
2 1=no, 2=yes

ofxn calc*
0.12482
ofxn mod
0.16452

sRun
RunA1
zout
1

Ave
Data
2.7E-05
Model
2.5E-05

data/vMax
2.7E-05

ofxng
311.734

Min d/m
1.37194

ave/min d
1
ave/min m
1.2522

331_Calib_S_A

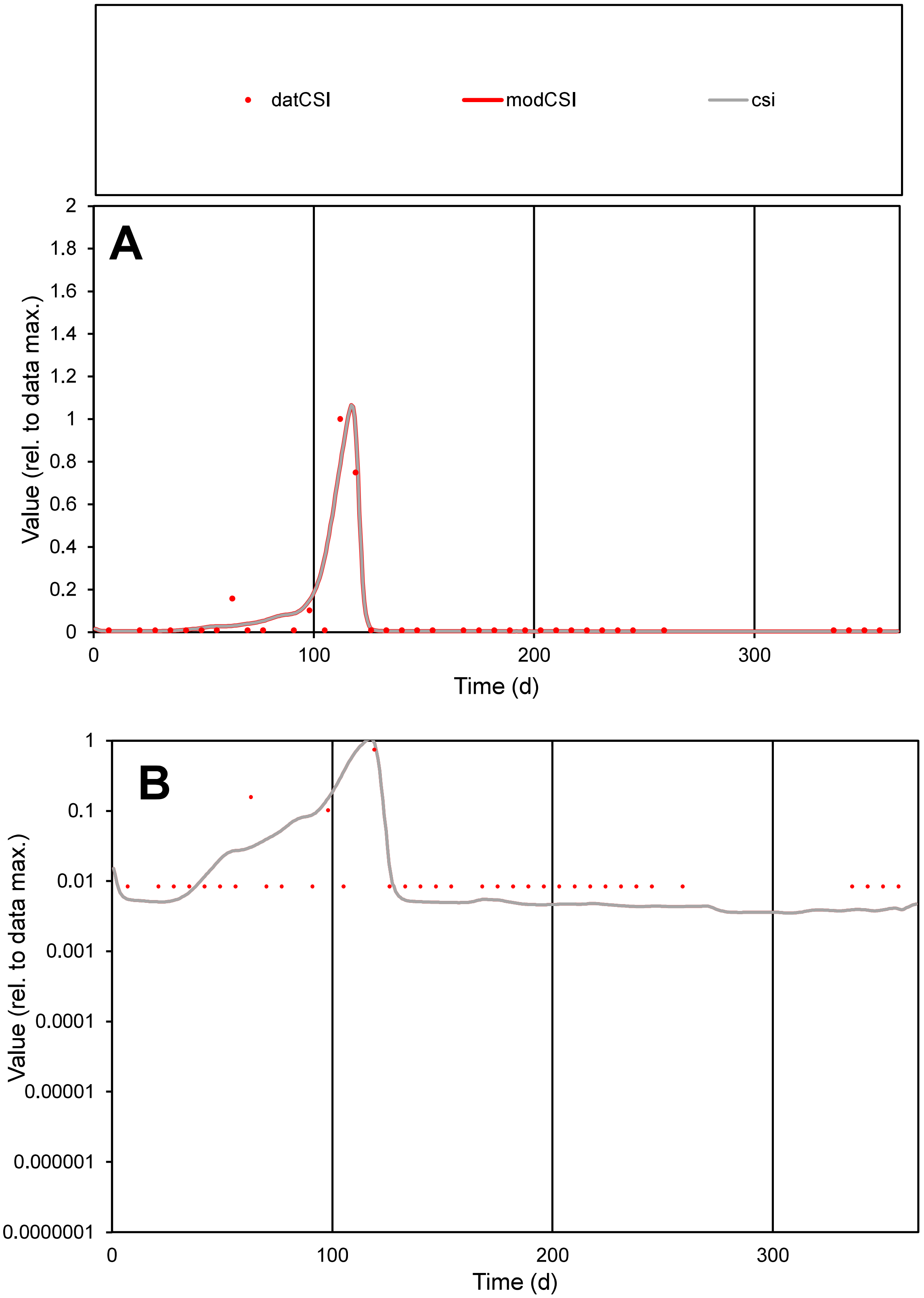

C1

Variables
CSI Provide jtd or Dst
CalibOnly?
2 1 to reproduce of
oNormalize?
2 1=no, 2=yes

ofxn calc*
0.90567
ofxn mod
1.03124

sRun
RunA1
zout
1

Ave
Data
1.7E-05
Model
1.8E-05

data/vMax
0.00028

ofxng
311.734

Min d/m
2.19791

ave/min d
7.36843
ave/min m
17.4231

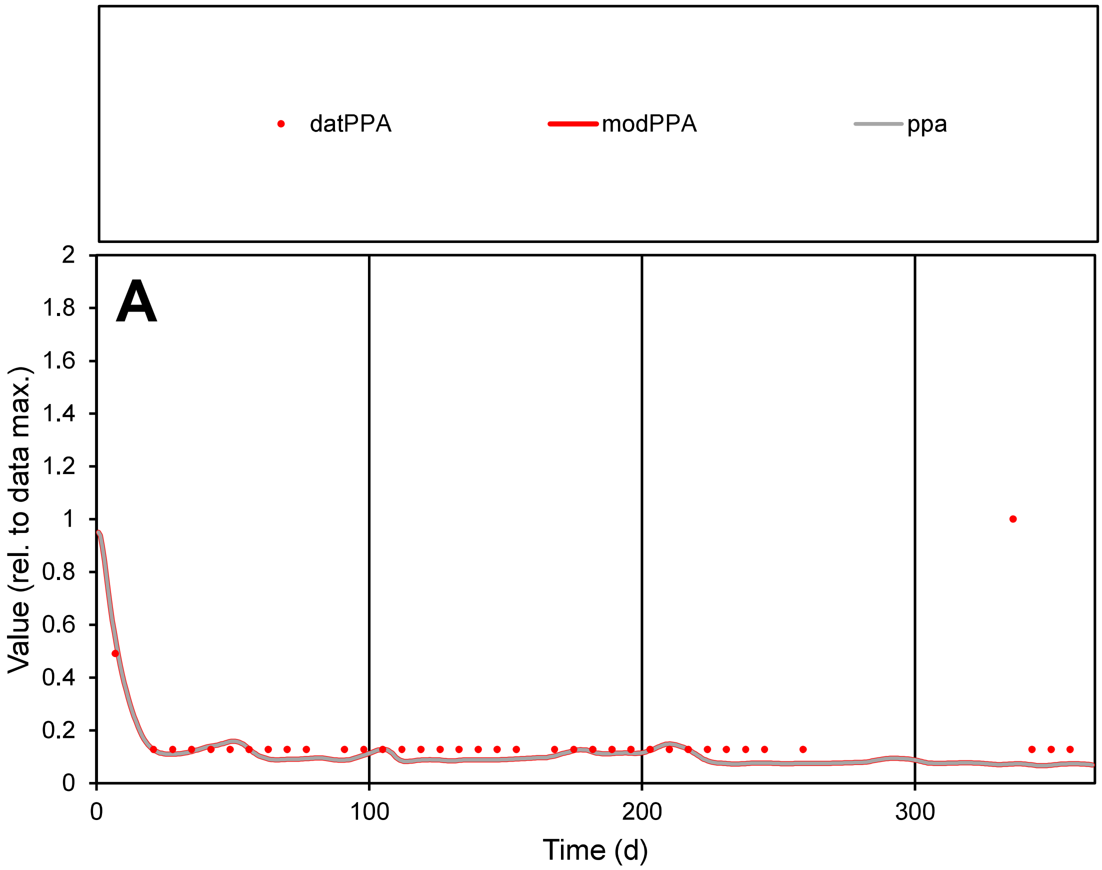

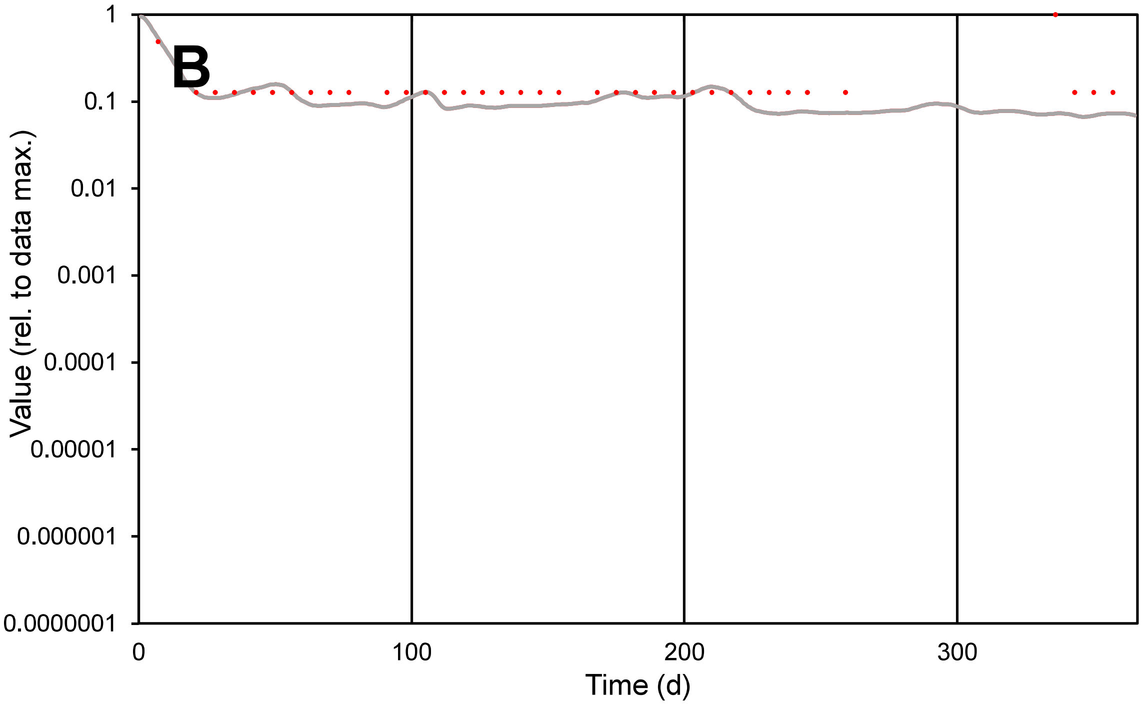

C1

Variables
PPA Provide jtd or Dst
CalibOnly?
2 1 to reproduce of
oNormalize?
2 1=no, 2=yes

ofxn calc*
0.96654
ofxn mod
0.9714

sRun
RunA1
zout
1

Ave
Data
4E-06
Model
2.8E-06

data/vMax
2.5E-05

ofxng
311.734

Min d/m
1.87778

ave/min d
1.26015
ave/min m
1.63134

331_Calib_S_A

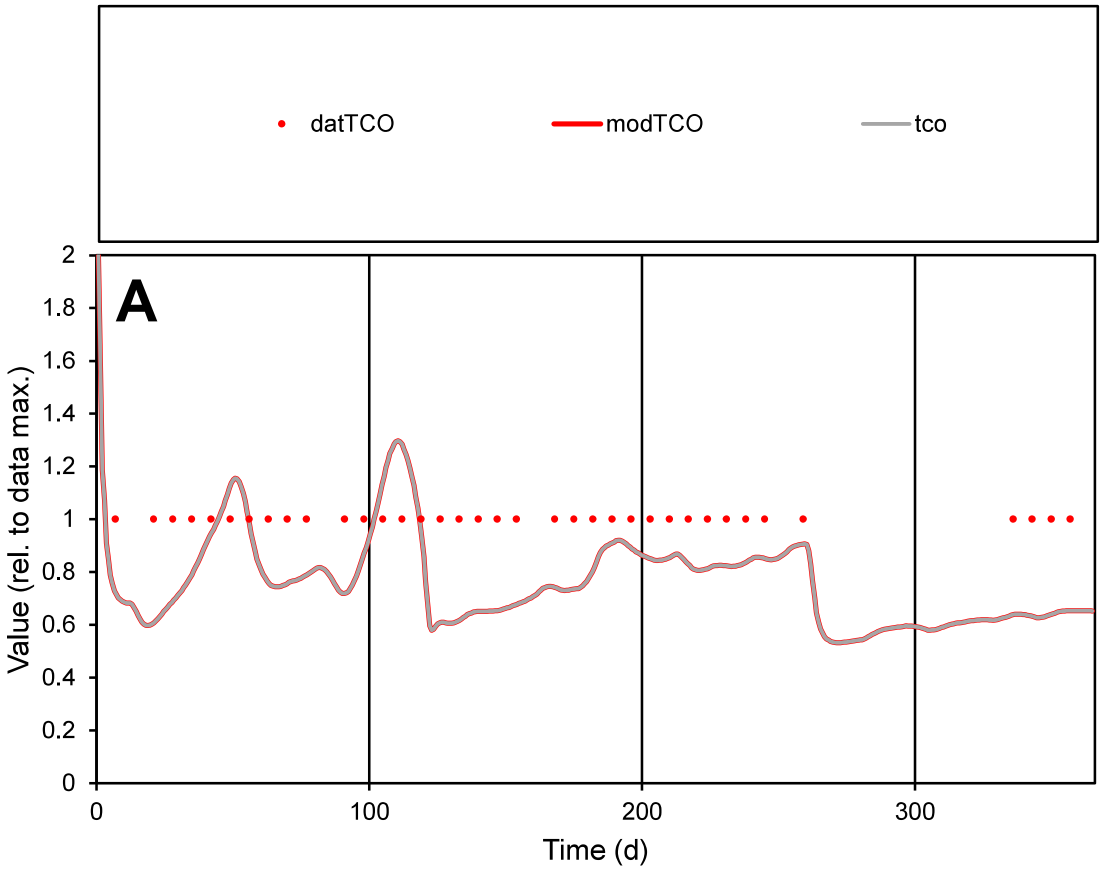

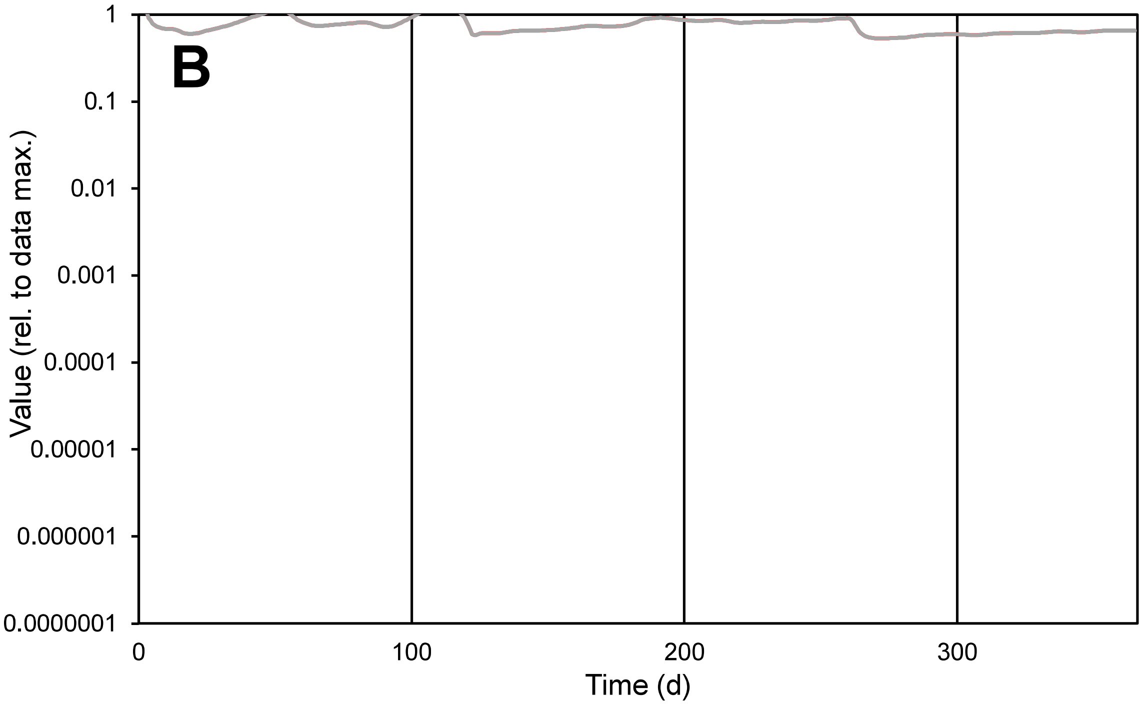

C1

Variables
TCO Provide jtd or Dst
CalibOnly?
2 1 to reproduce of
oNormalize?
2 1=no, 2=yes

ofxn calc*
0.24751
ofxn mod
0.26748

sRun
RunA1
zout
1

Ave
Data
8.6E-06
Model
7E-06

data/vMax
8.6E-06

ofxng
311.734

Min d/m
1.64091

ave/min d
1
ave/min m
1.32323

331_Calib_S_A

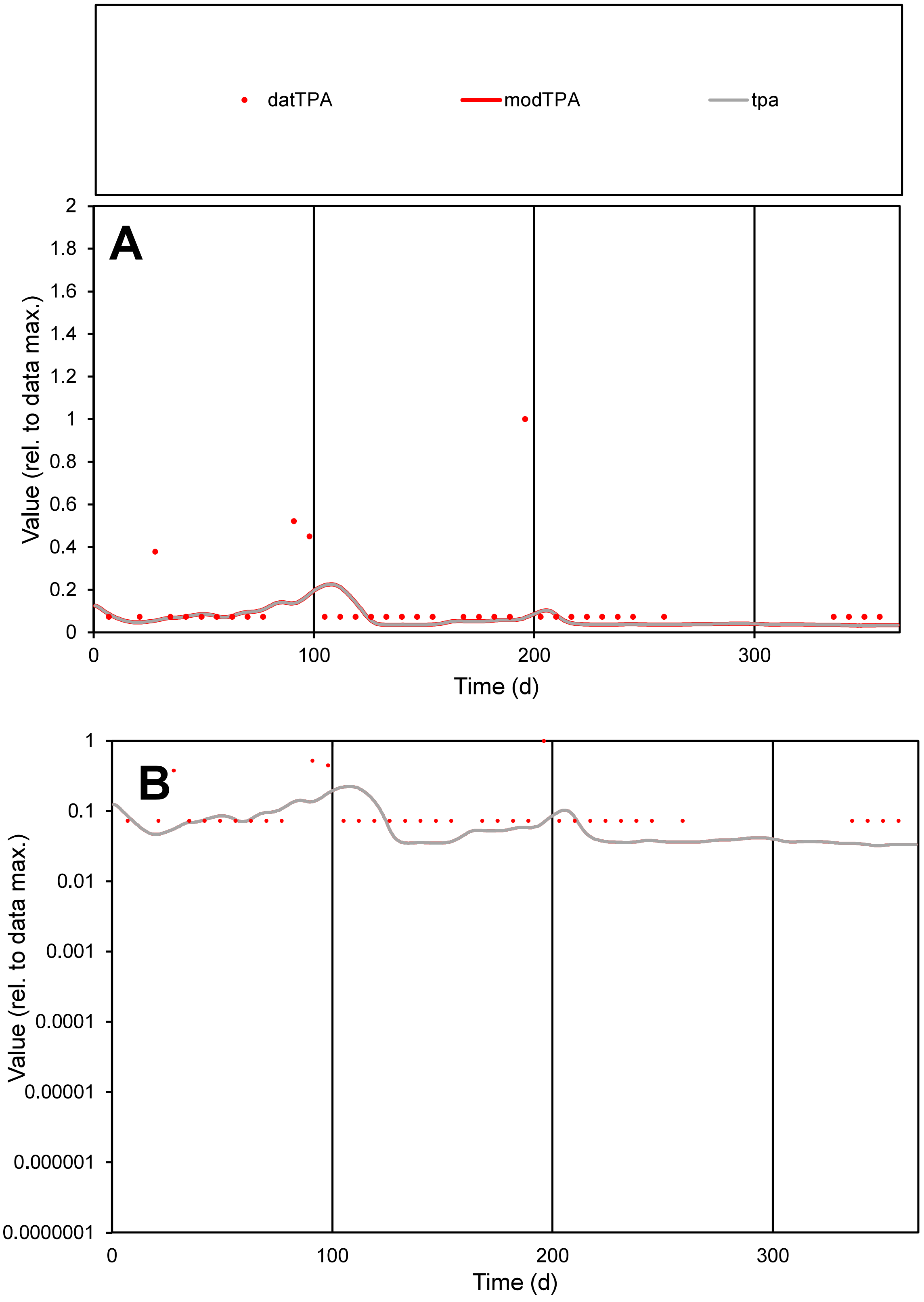

C1

Variables
TPA Provide jtd or Dst
CalibOnly?
2 1 to reproduce of
oNormalize?
2 1=no, 2=yes

ofxn calc*
1.42414
ofxn mod
1.42973

sRun
RunA1
zout
1

Ave
Data
8.3E-06
Model
4.7E-06

data/vMax
6.5E-05

ofxng
311.734

Min d/m
2.22515

ave/min d
1.76529
ave/min m
2.19984

331_Calib_S_A

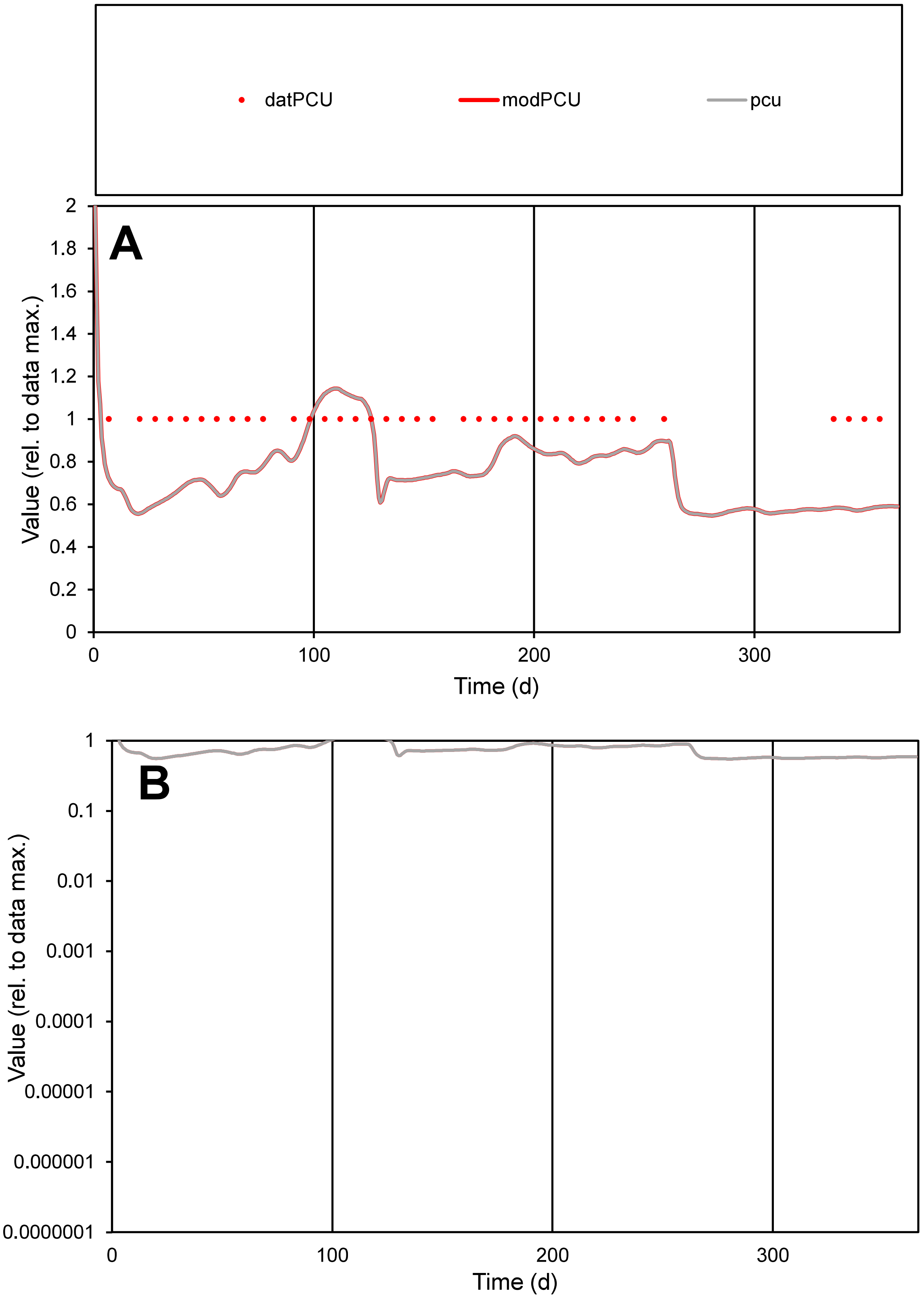

C1

Variables

PCU Provide jtd or Dst

CalibOnly?

2 1 to reproduce of

oNormalize?

2 1=no, 2=yes

ofxn calc*

0.26078

ofxn mod

0.27926

sRun

RunA1

zout

1

Ave

Data

6.2E-06

Model

4.9E-06

data/vMax

6.2E-06

ofxng

311.734

Min d/m

1.77275

ave/min d

1

ave/min m

1.39131

331_Calib_S_A

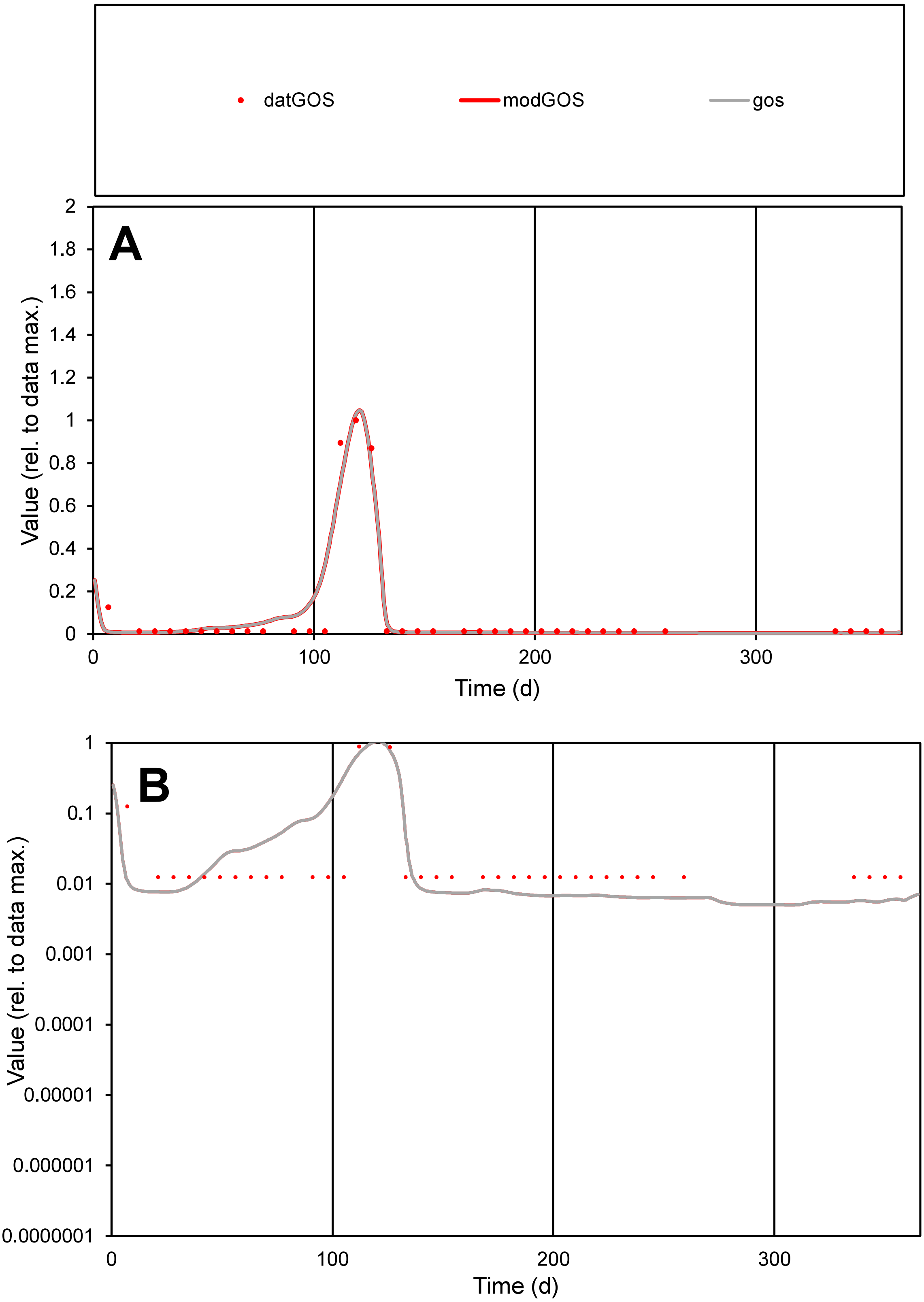

C1

Variables

GOS Provide jtd or Dst

CalibOnly?

2 1 to reproduce of

oNormalize?

2 1=no, 2=yes

ofxn calc*

0.62507

ofxn mod

0.77767

sRun

RunA1

zout

1

Ave

Data

1.3E-05

Model

1.4E-05

data/vMax

0.00015

ofxng

311.734

Min d/m

2.21868

ave/min d

7.17851

ave/min m

16.7653

331_Calib_S_A

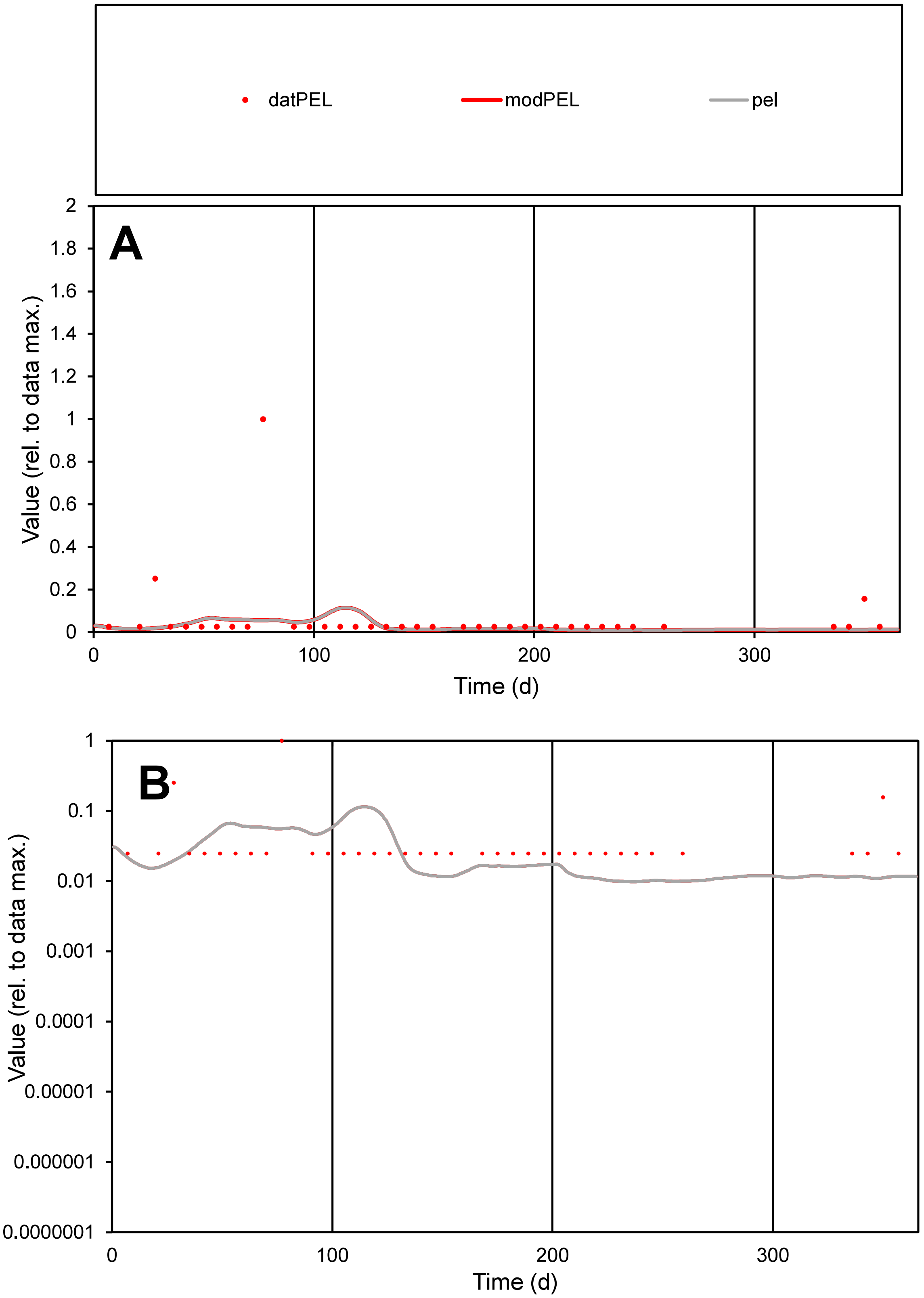

C1

Variables

PEL Provide jtd or Dst

CalibOnly?

2 1 to reproduce of

oNormalize?

2 1=no, 2=yes

ofxn calc*

2.68922

ofxn mod

2.69799

sRun

RunA1

zout

1

Ave

Data

8.1E-06

Model

4E-06

data/vMax

0.00013

ofxng

311.734

Min d/m

2.52267

ave/min d

2.45366

ave/min m

3.07905

331_Calib_S_A

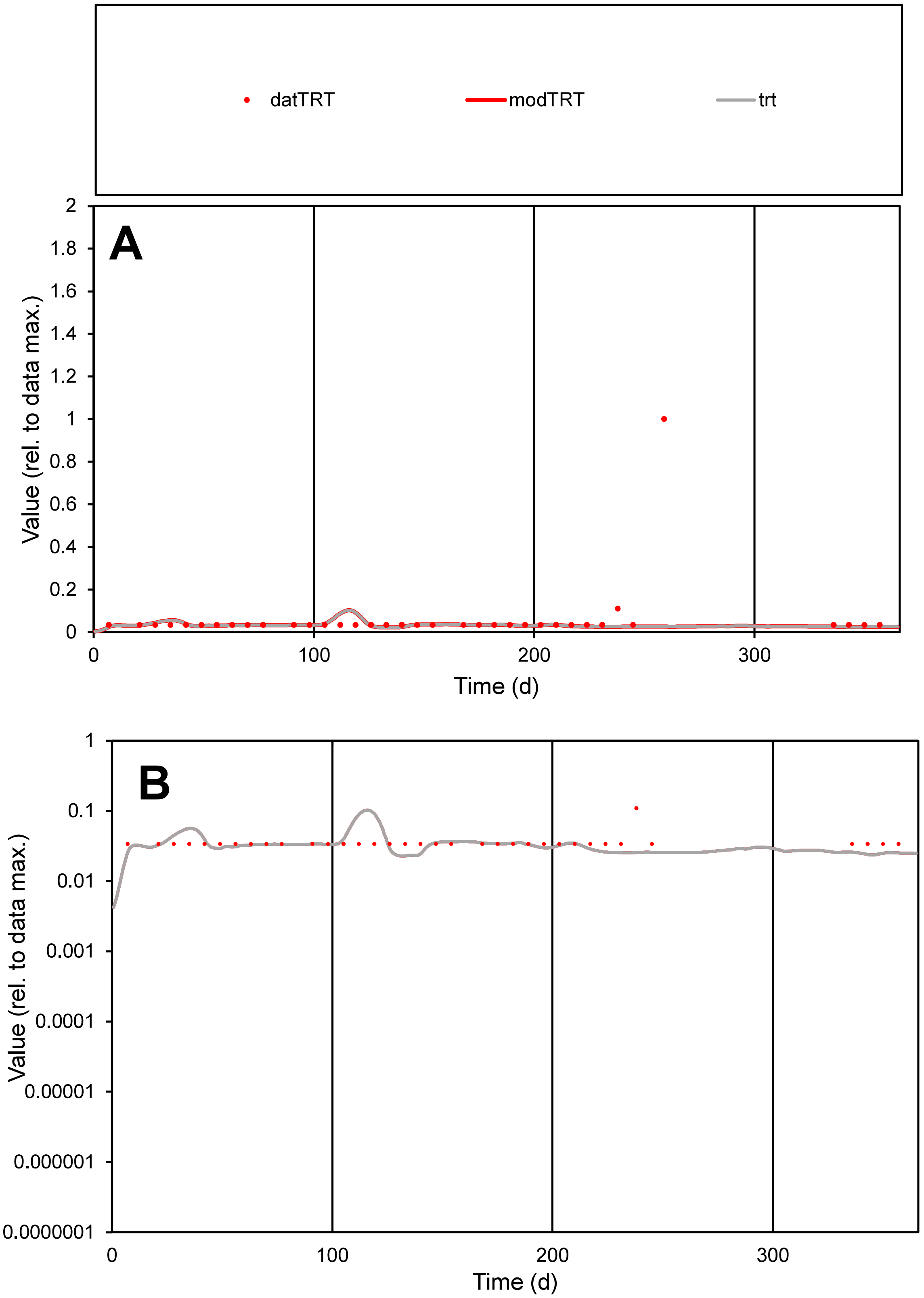

C1

Variables

TRT Provide jtd or Dst

CalibOnly?

2 1 to reproduce of

oNormalize?

2 1=no, 2=yes

ofxn calc*

2.59523

ofxn mod

2.6029

sRun

RunA1

zout

1

Ave

Data

4.2E-05

Model

2.3E-05

data/vMax

0.00068

ofxng

311.734

Min d/m

1.47486

ave/min d

1.82866

ave/min m

1.48174

331_Calib_S_A

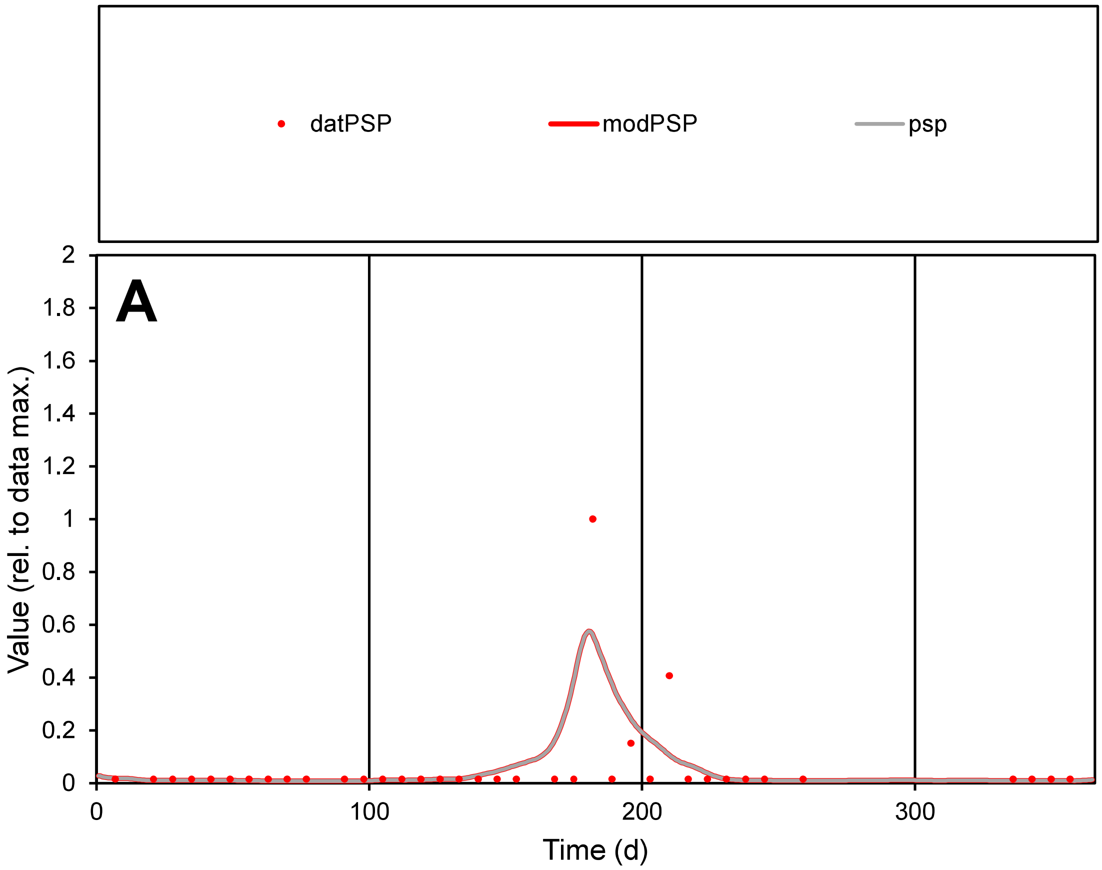

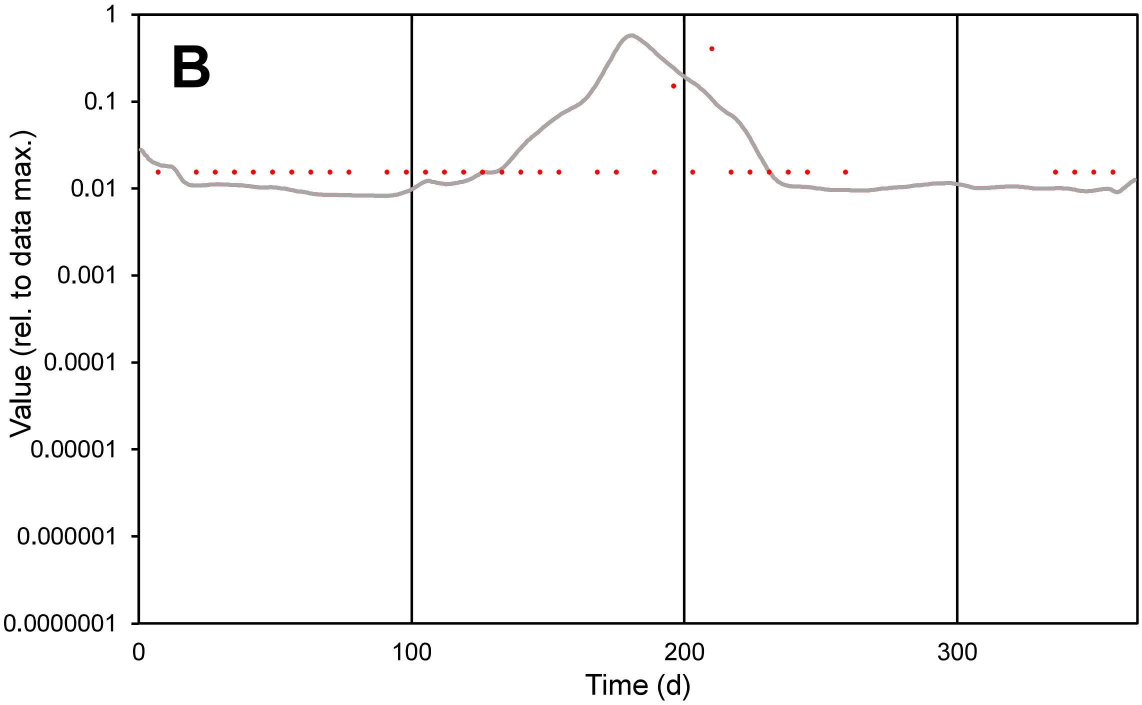

C1

Variables

PSP Provide jtd or Dst

CalibOnly?

2 1 to reproduce of

oNormalize?

2 1=no, 2=yes

ofxn calc*

2.06767

ofxn mod

2.23455

sRun

RunA1

zout

1

Ave

Data

1.6E-05

Model

1.7E-05

data/vMax

0.00028

ofxng

311.734

Min d/m

1.8697

ave/min d

3.63946

ave/min m

7.43861

331_Calib_S_A

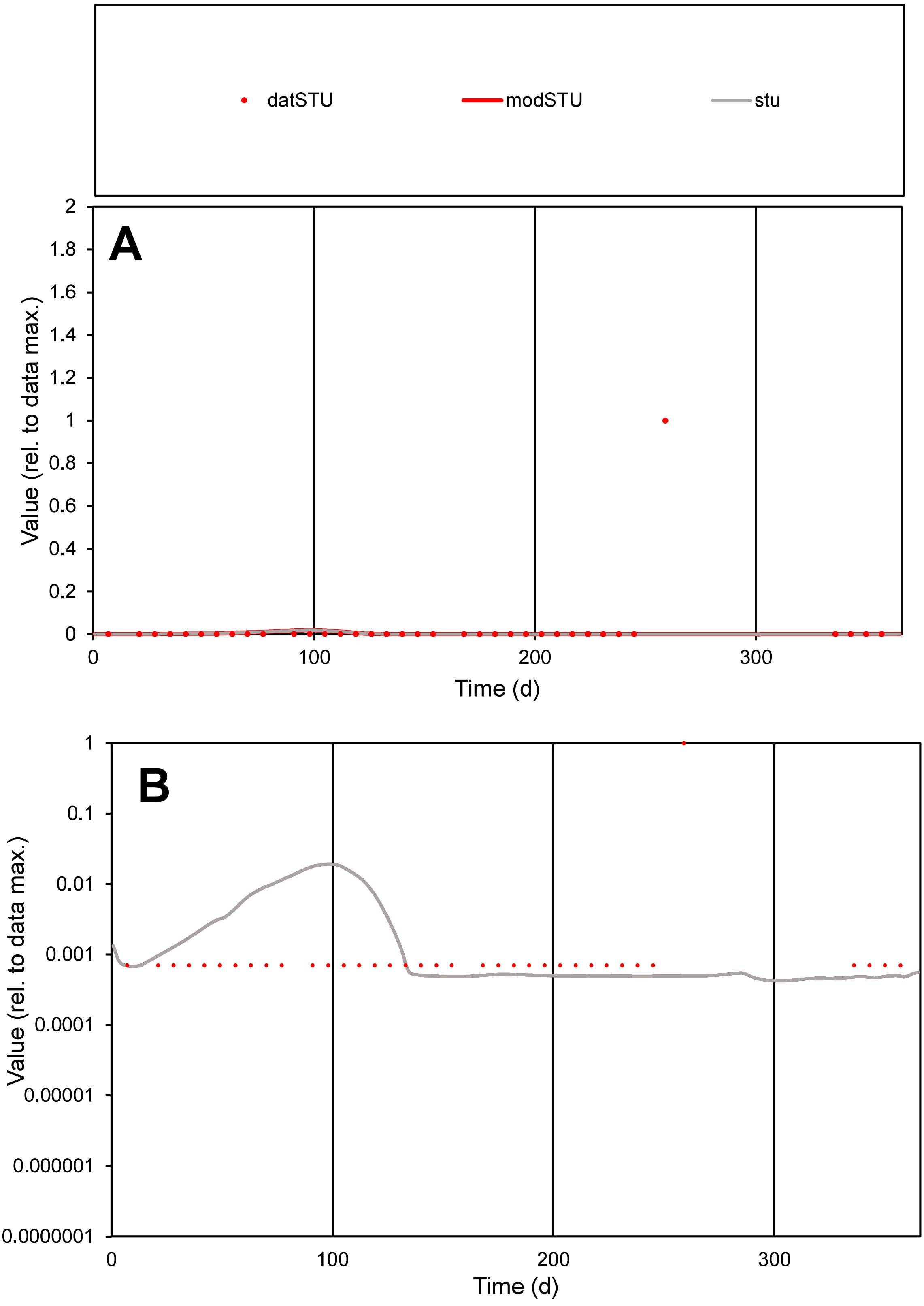

C1

Variables

STU Provide jtd or Dst

CalibOnly?

2 1 to reproduce of

oNormalize?

2 1=no, 2=yes

ofxn calc*

5.93337

ofxn mod

5.9456

sRun

RunA1

zout

1

Ave

Data

9.8E-05

Model

1.2E-05

data/vMax

0.00355

ofxng

311.734

Min d/m

1.47865

ave/min d

39.4483

ave/min m

6.93763

331_Calib_S_A

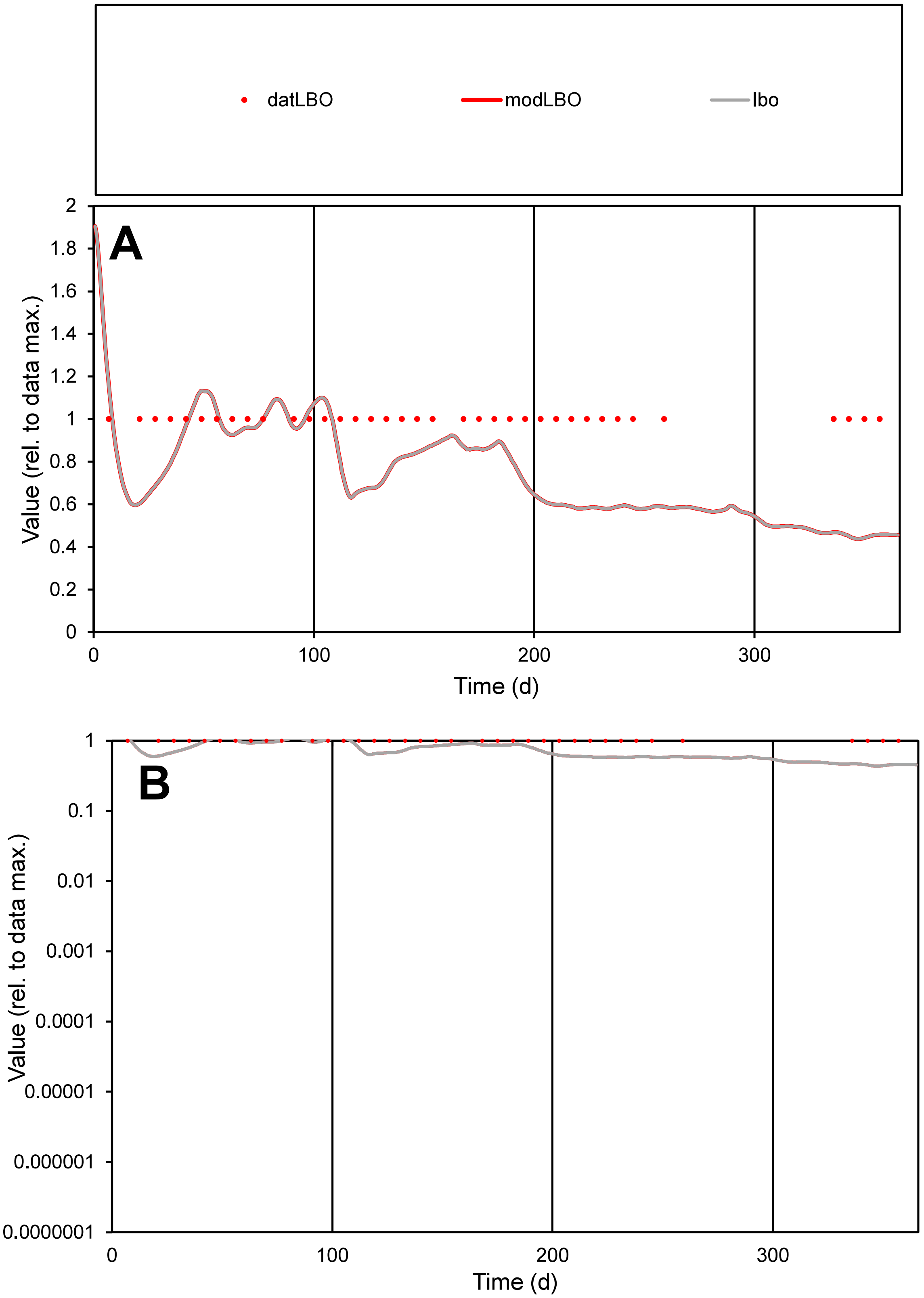

C1

Variables

LBO Provide jtd or Dst

CalibOnly?

2 1 to reproduce of

oNormalize?

2 1=no, 2=yes

ofxn calc*

0.30541

ofxn mod

0.32116

sRun

RunA1

zout

1

Ave

Data

1.4E-06

Model

1.1E-06

data/vMax

1.4E-06

ofxng

311.734

Min d/m

2.24434

ave/min d

1

ave/min m

1.72107

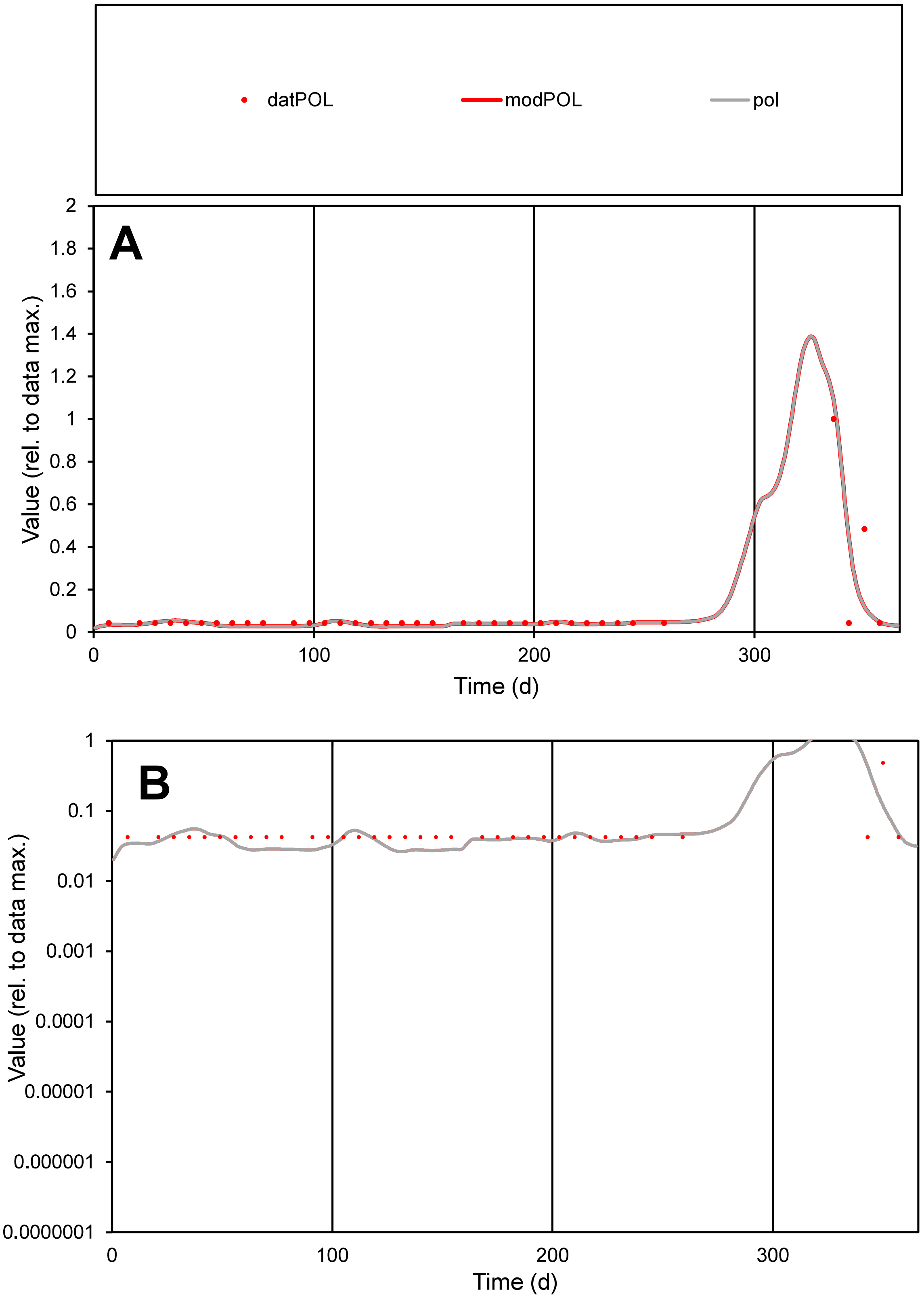

C1

Variables
POL Provide jtd or Dst
CalibOnly?
2 1 to reproduce of
oNormalize?
2 1=no, 2=yes

ofxn calc*
0.89616
ofxn mod
0.94443

sRun
RunA1
zout
1

Ave
Data
3E-06
Model
2.7E-06

data/vMax
3.8E-05

ofxng
311.734

Min d/m
1.55859

ave/min d
1.89443
ave/min m
2.64493

331_Calib_S_A

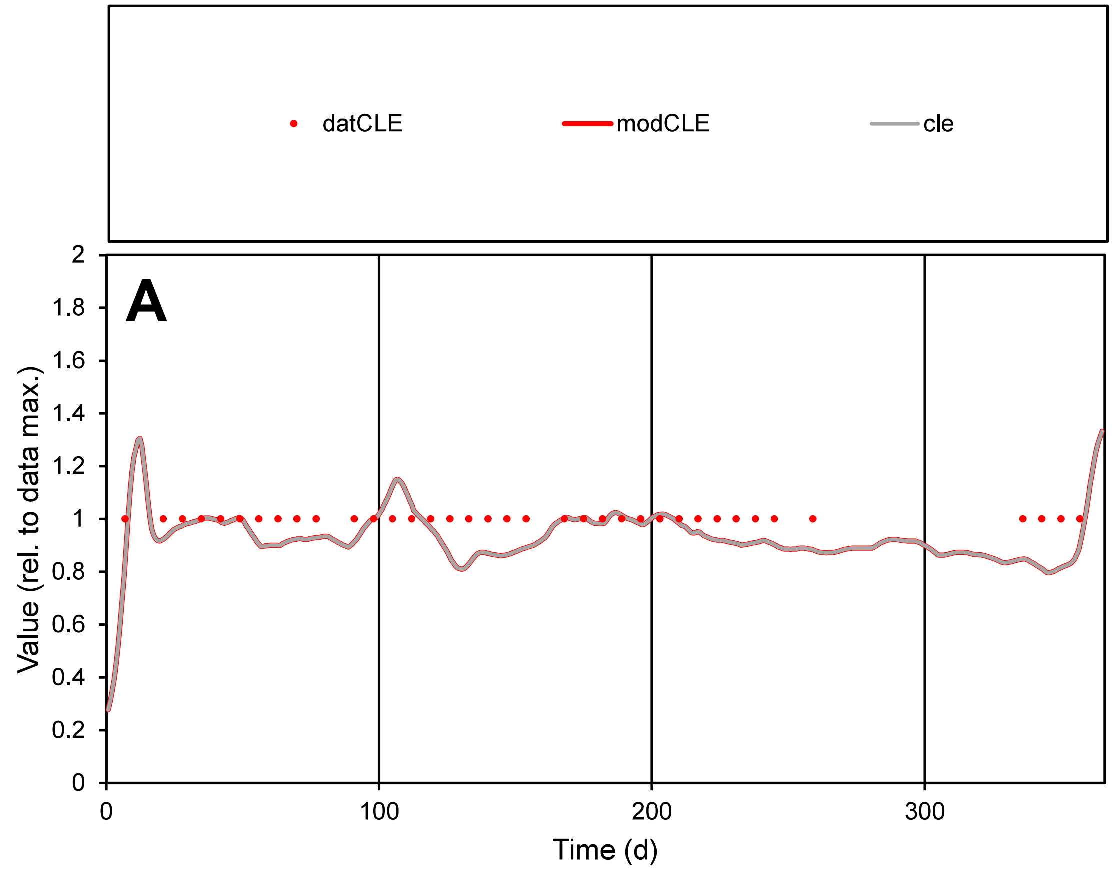

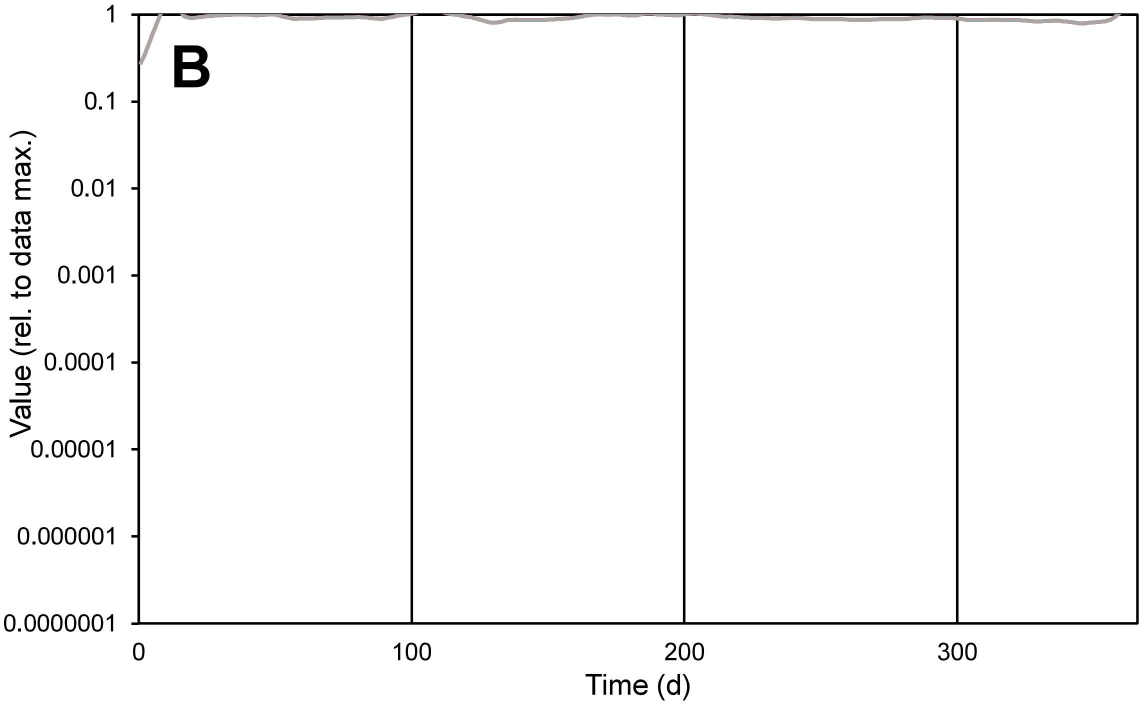

C1

Variables
CLE Provide jtd or Dst
CalibOnly?
2 1 to reproduce of
oNormalize?
2 1=no, 2=yes

ofxn calc*
0.0912
ofxn mod
0.1415

sRun
RunA1
zout
1

Ave
Data
4.7E-06
Model
4.4E-06

data/vMax
4.7E-06

ofxng
311.734

Min d/m
1.24463

ave/min d
1
ave/min m
1.17189

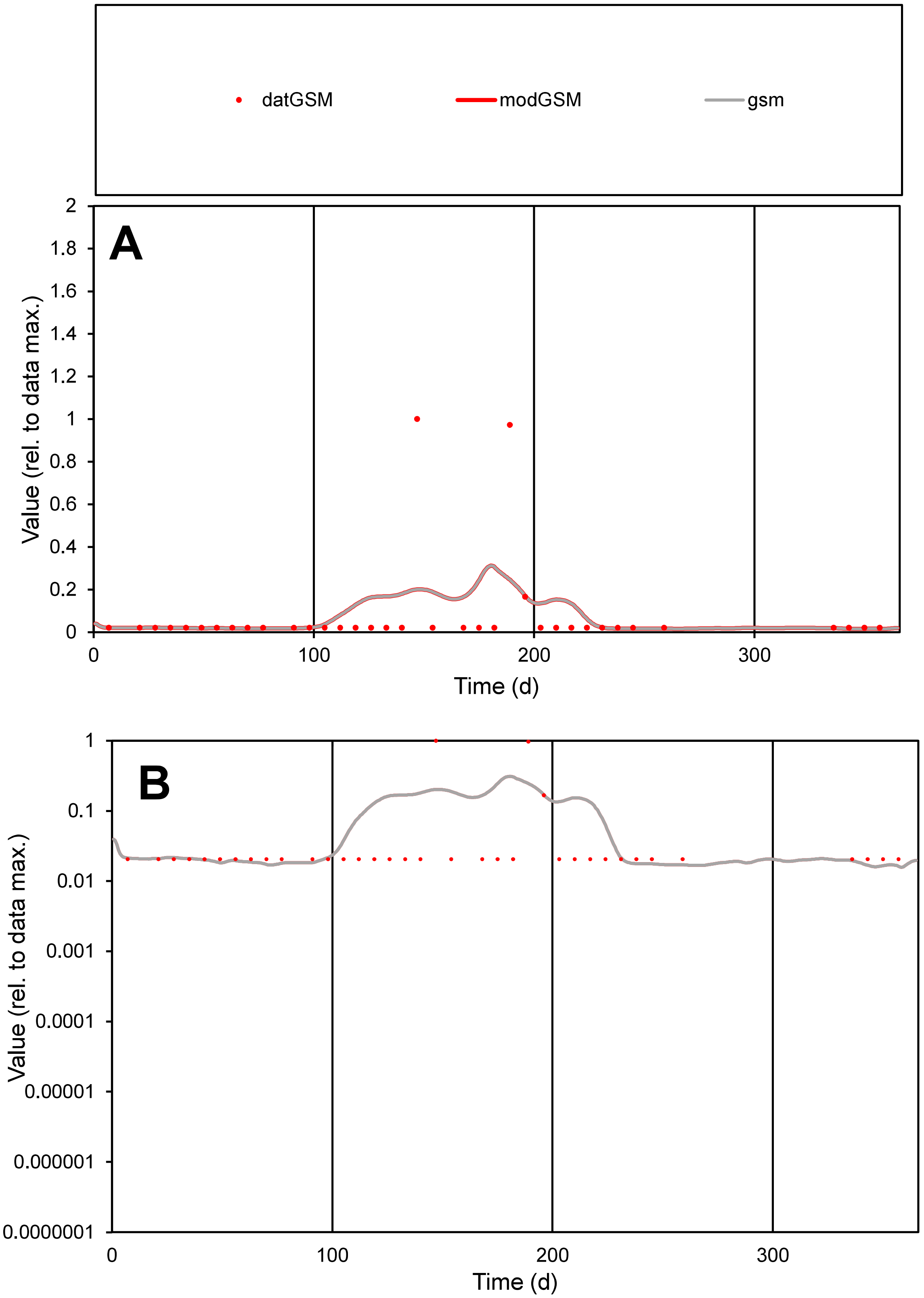

C1

Variables
GSM Provide jtd or Dst
CalibOnly?
2 1 to reproduce of
oNormalize?
2 1=no, 2=yes

ofxn calc*
2.57115
ofxn mod
2.61182

sRun
RunA1
zout
1

Ave
Data
5.9E-06
Model
6.3E-06

data/vMax
7.7E-05

ofxng
311.734

Min d/m
1.3082

ave/min d
3.72278
ave/min m
5.19203

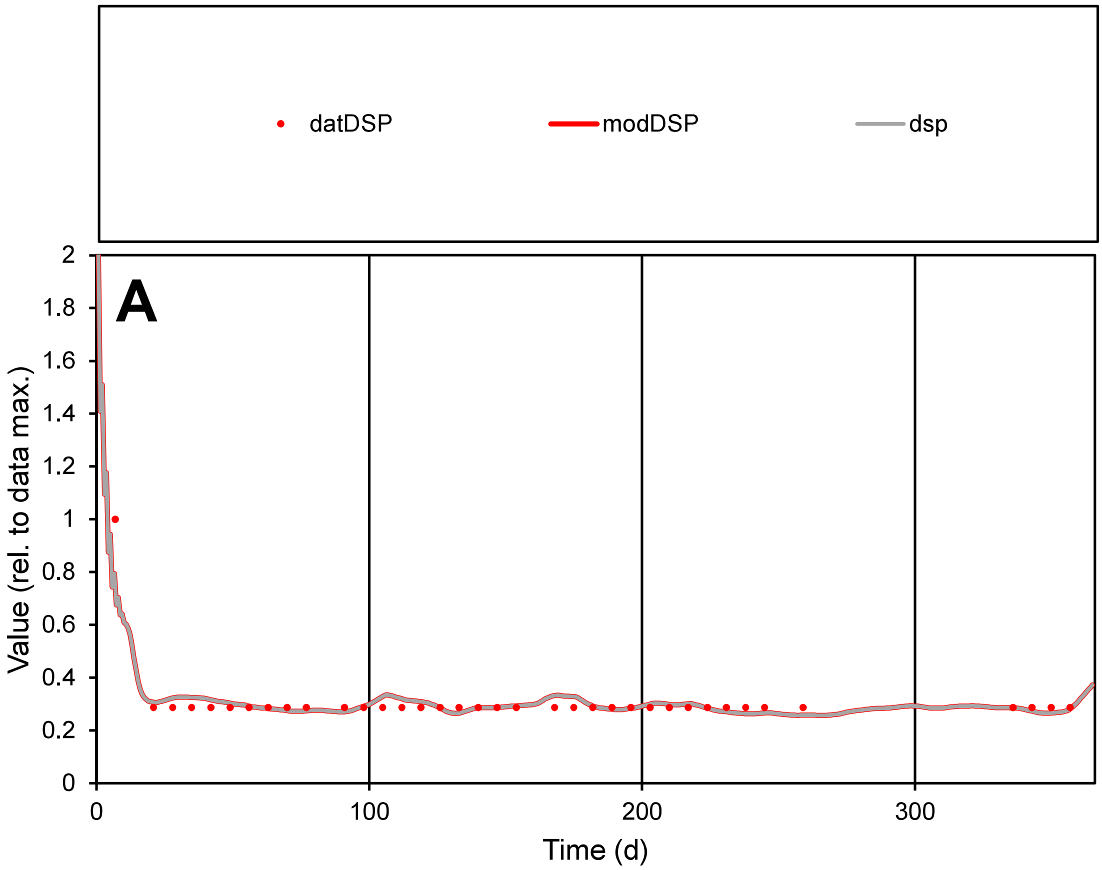

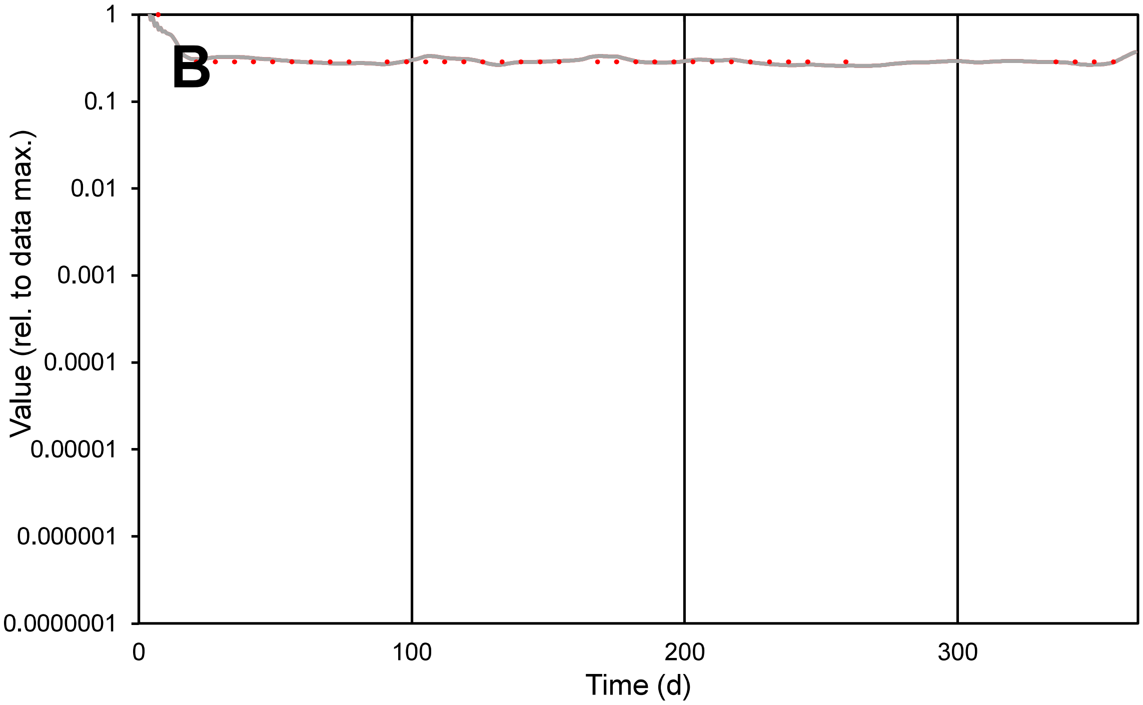

C1

Variables
DSP Provide jtd or Dst
CalibOnly?
2 1 to reproduce of
oNormalize?
2 1=no, 2=yes

ofxn calc*
0.17329
ofxn mod
0.20375

sRun
RunA1
zout
1

Ave
Data
5.3E-06
Model
5.3E-06

data/vMax
1.7E-05

ofxng
311.734

Min d/m
1.11198

ave/min d
1.0672
ave/min m
1.17408

331_Calib_S_A

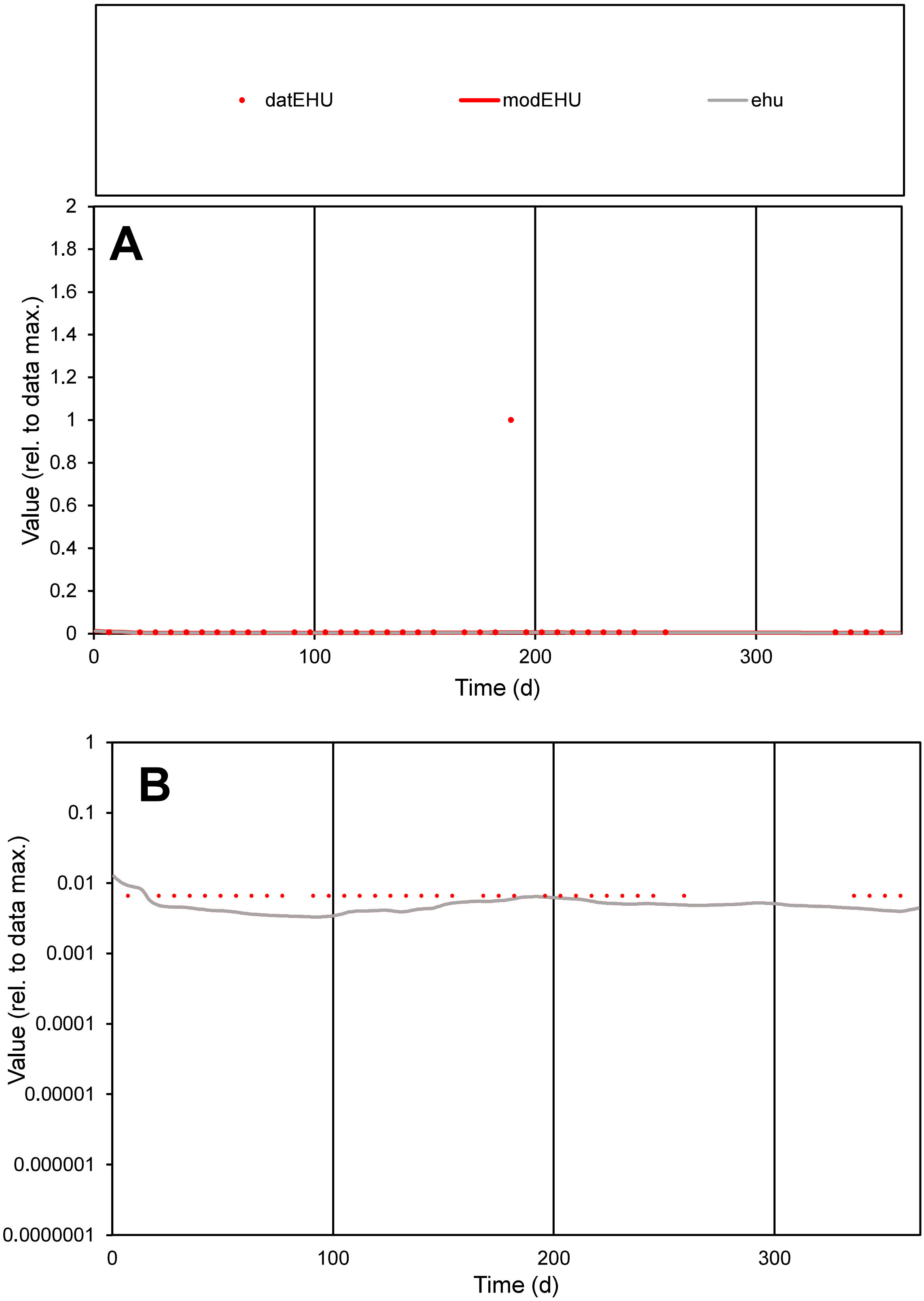

C1

Variables
EHU Provide jtd or Dst
CalibOnly?
2 1 to reproduce of
oNormalize?
2 1=no, 2=yes

ofxn calc*
4.88354
ofxn mod
4.89269

sRun
RunA1
zout
1

Ave
Data
2.9E-05
Model
4.1E-06

data/vMax
0.00087

ofxng
311.734

Min d/m
1.98997

ave/min d
5.06624
ave/min m
1.43227

331_Calib_S_A

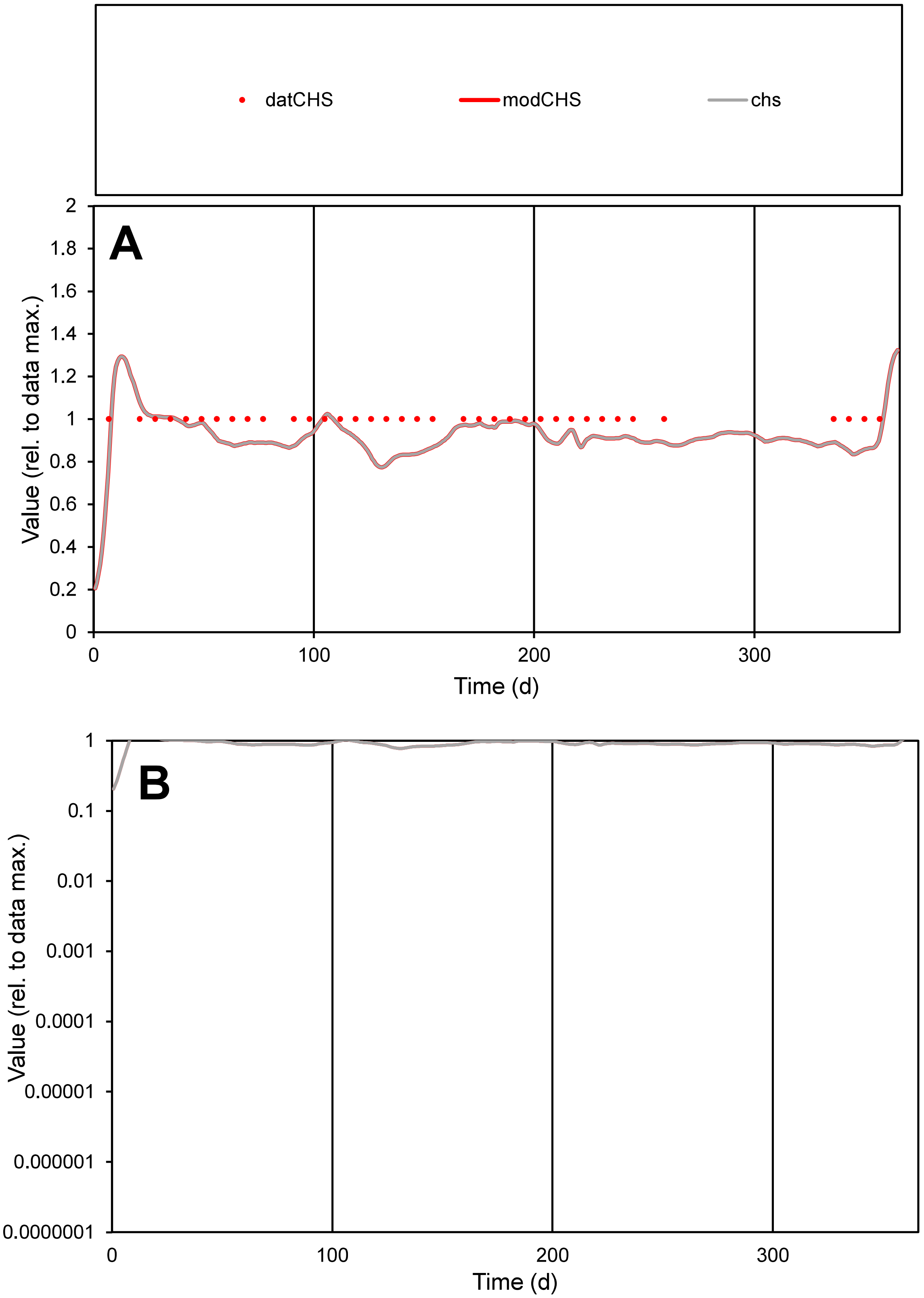

C1

Variables

CHS Provide jtd or Dst

CalibOnly?

2 1 to reproduce of

oNormalize?

2 1=no, 2=yes

ofxn calc*

0.09828

ofxn mod

0.14549

sRun

RunA1

zout

1

Ave

Data

2.1E-05

Model

2E-05

data/vMax

2.1E-05

ofxng

311.734

Min d/m

1.24796

ave/min d

1

ave/min m

1.15275

331_Calib_S_A

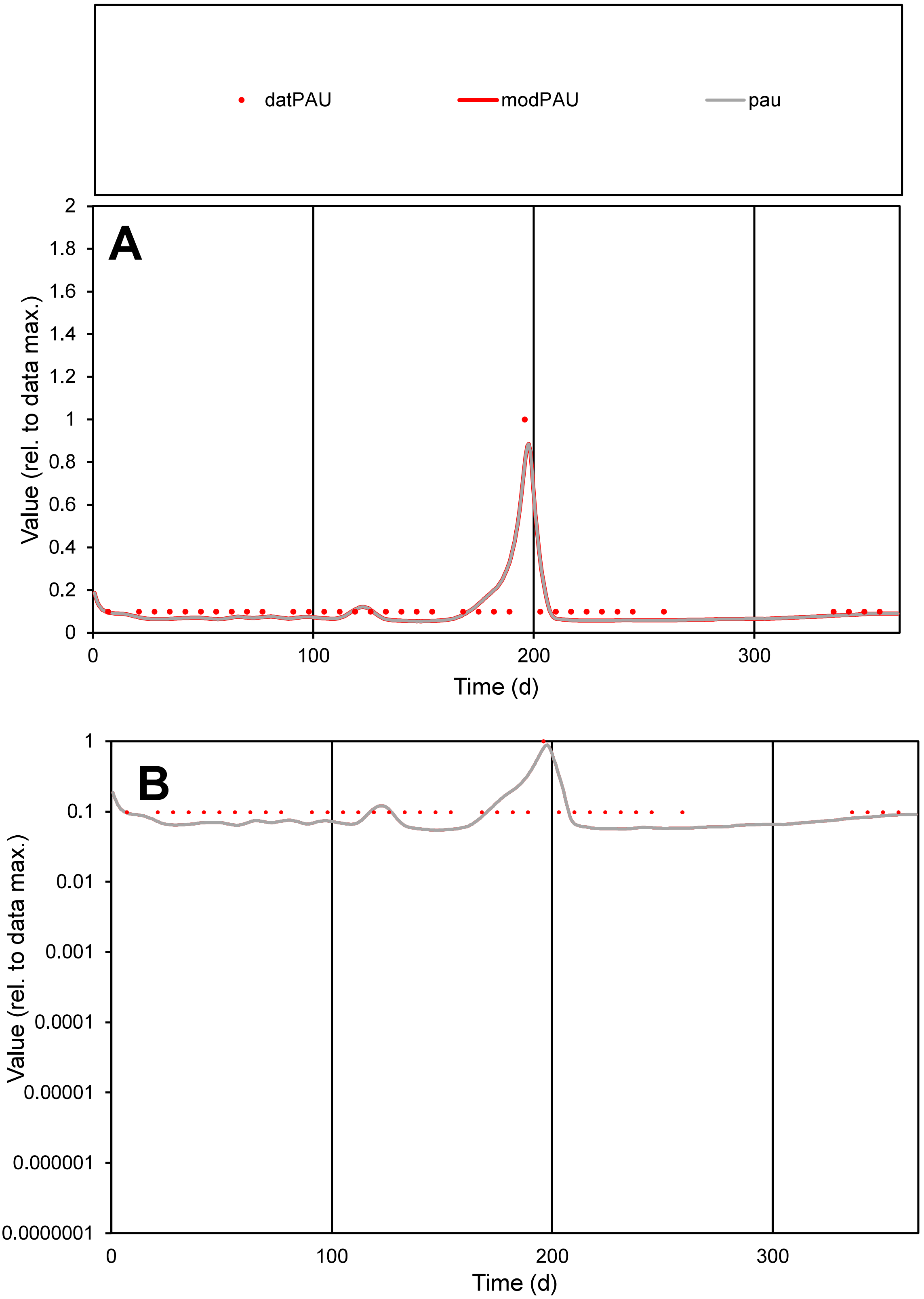

C1

Variables	
PAU	Provide jtd or Dst
CalibOnly?	
2	1 to reproduce of
oNormalize?	
2	1=no, 2=yes
ofxn calc*	
0.45132	
ofxn mod	
0.4989	
sRun	
RunA1	
zout	
1	
Ave	
Data	
4.9E-06	
Model	
4.4E-06	
data/vMax	
4E-05	
ofxng	
311.734	
Min d/m	
1.80375	
ave/min d	
1.24914	
ave/min m	
1.99812	

331_Calib_S_A

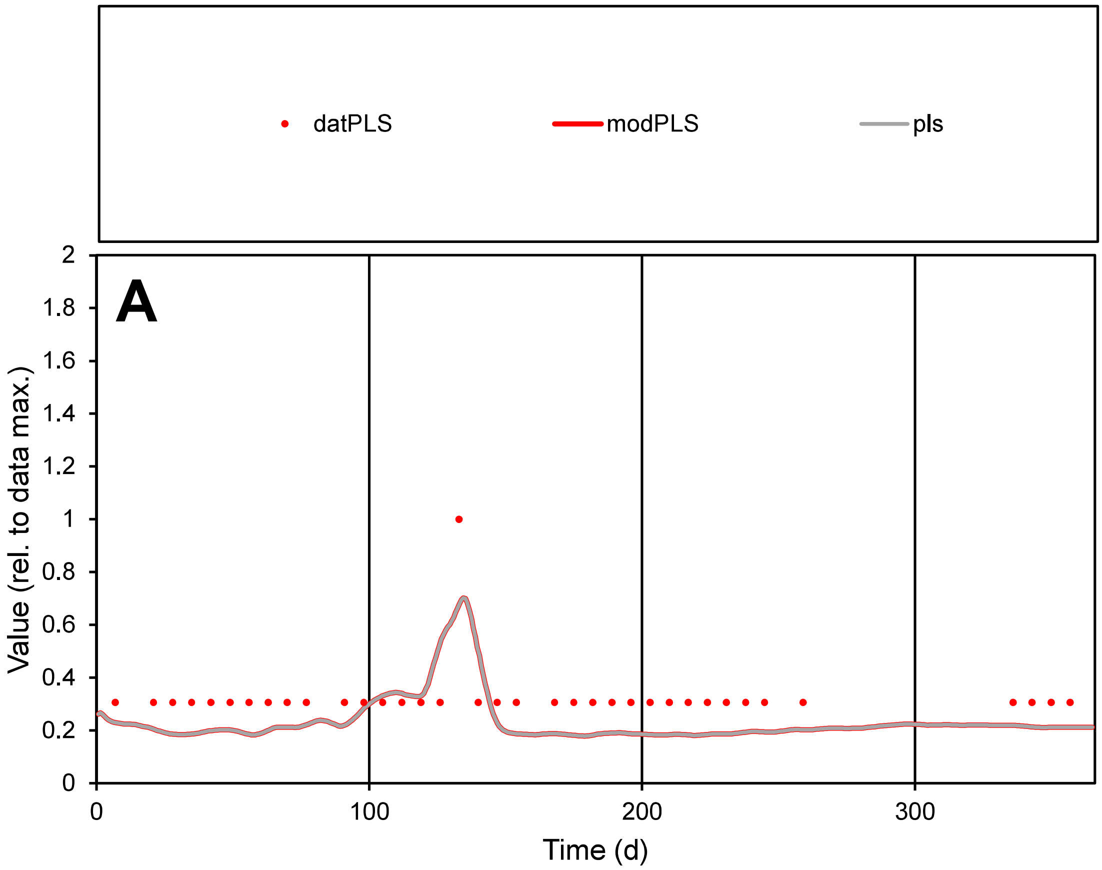

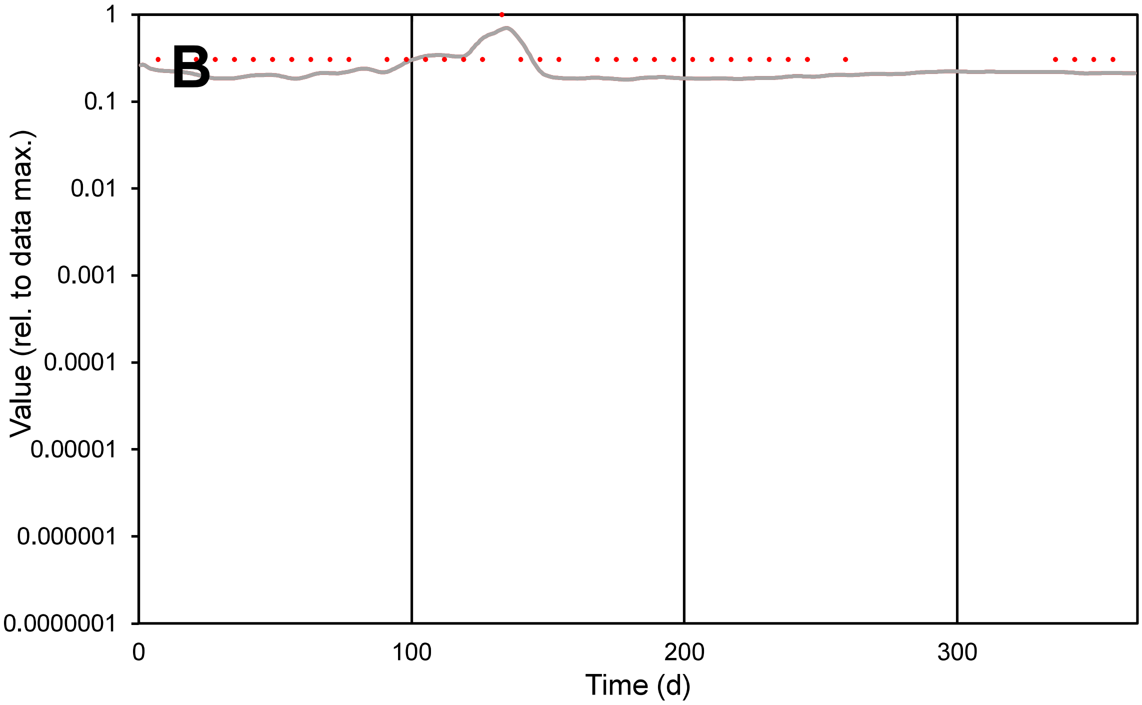

C1

Variables

PLS Provide jtd or Dst

CalibOnly?

2 1 to reproduce of

oNormalize?

2 1=no, 2=yes

ofxn calc*

0.36493

ofxn mod

0.38079

sRun

RunA1

zout

1

Ave

Data

4.1E-06

Model

3E-06

data/vMax

1.3E-05

ofxng

311.734

Min d/m

1.69207

ave/min d

1.06142

ave/min m

1.34178

331_Calib_S_A

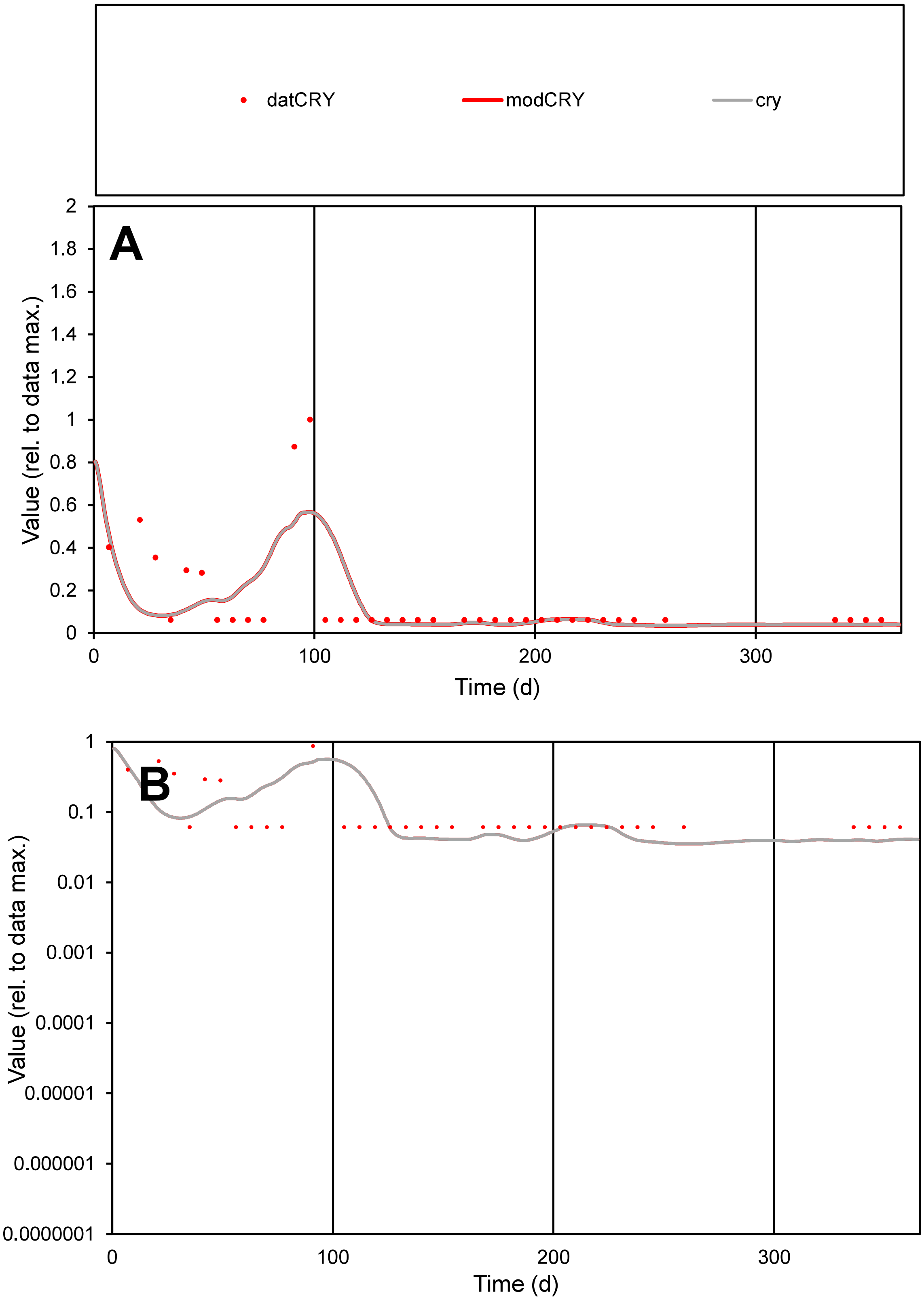

C1

Variables
CRY Provide jtd or Dst
CalibOnly?
2 1 to reproduce of
oNormalize?
2 1=no, 2=yes

ofxn calc*
1.06147
ofxn mod
1.09056

sRun
RunA1
zout
1

Ave
Data
7.3E-06
Model
6.4E-06

data/vMax
4.9E-05

ofxng
311.734

Min d/m
1.73706

ave/min d
2.4556
ave/min m
3.71454

331_Calib_S_A

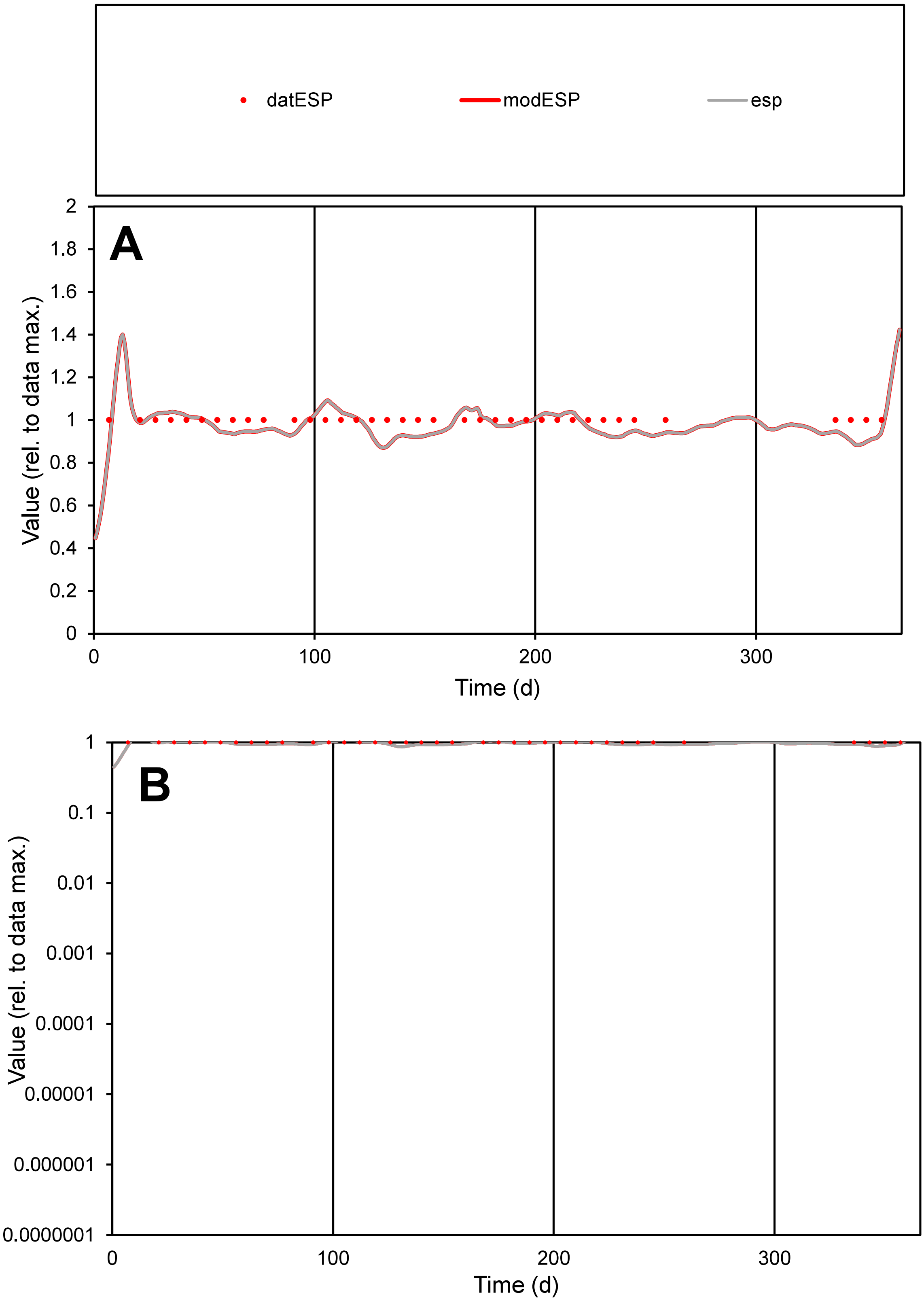

C1

Variables
ESP Provide jtd or Dst
CalibOnly?
2 1 to reproduce of
oNormalize?
2 1=no, 2=yes

ofxn calc*
0.05473
ofxn mod
0.12297

sRun
RunA1
zout
1

Ave
Data
8E-06
Model
7.8E-06

data/vMax
8E-06

ofxng
311.734

Min d/m
1.11941

ave/min d
1
ave/min m
1.09201

331_Calib_S_A

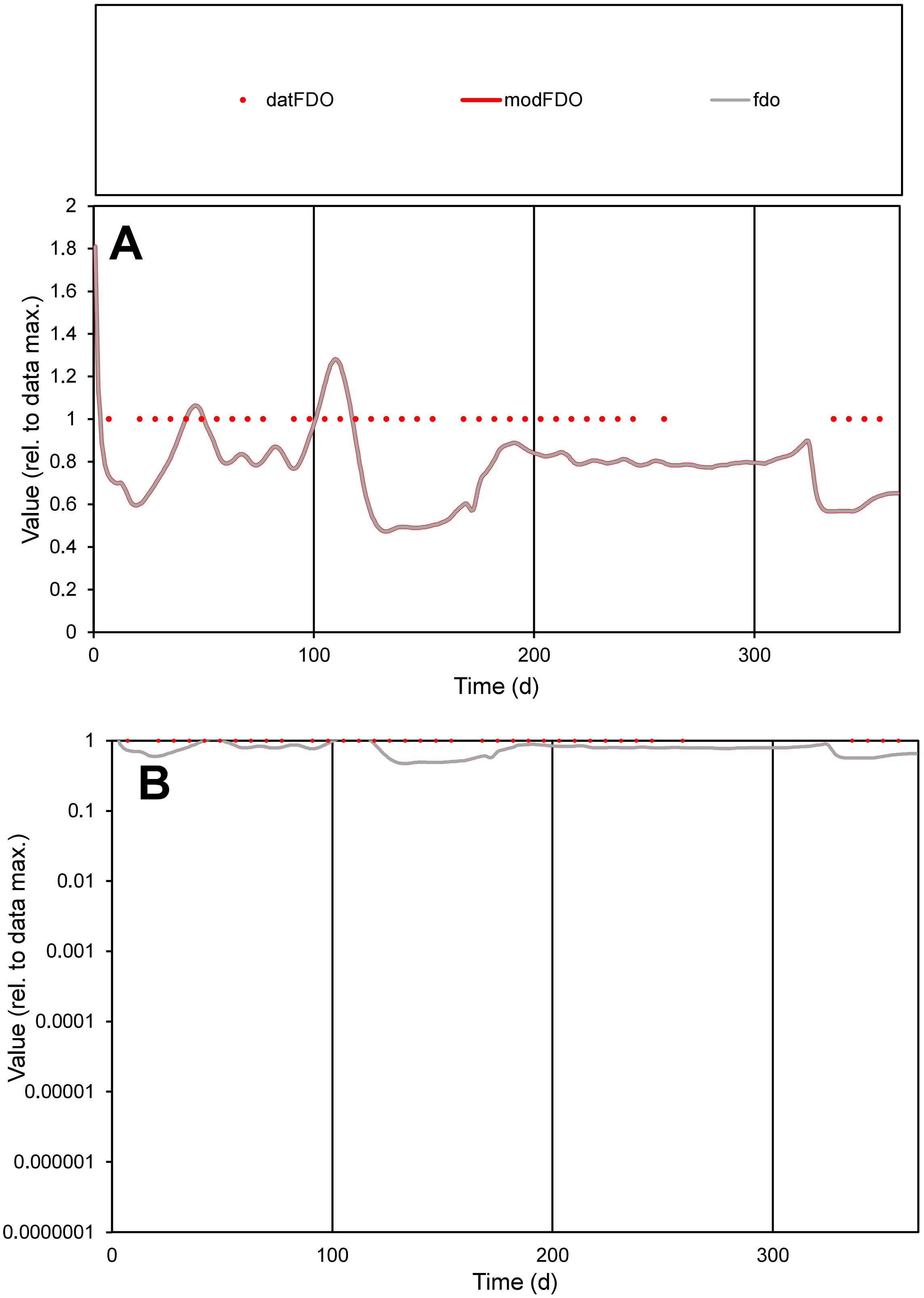

C1

Variables
FDO Provide jtd or Dst
CalibOnly?
2 1 to reproduce of
oNormalize?
2 1=no, 2=yes

ofxn calc*
0.29057
ofxn mod
0.30774

sRun
RunA1
zout
1

Ave
Data
1E-07
Model
7.7E-08

data/vMax
1E-07

ofxng
311.734

Min d/m
2.09924

ave/min d
1
ave/min m
1.61414

331_Calib_S_A

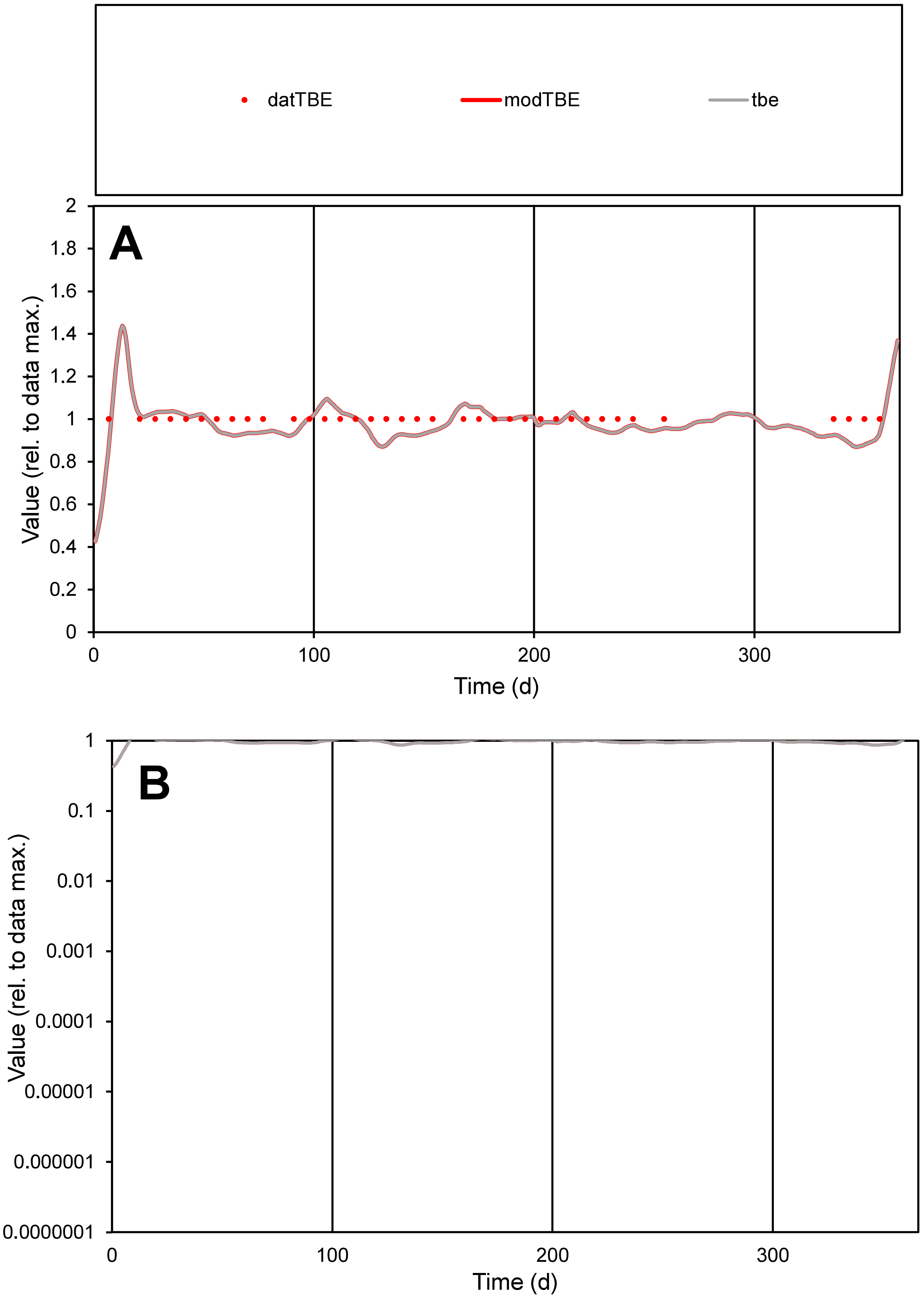

C1

Variables
TBE Provide jtd or Dst
CalibOnly?
2 1 to reproduce of
oNormalize?
2 1=no, 2=yes

ofxn calc*
0.05671
ofxn mod
0.12384

sRun
RunA1
zout
1

Ave
Data
6.7E-06
Model
6.5E-06

data/vMax
6.7E-06

ofxng
311.734

Min d/m
1.13598

ave/min d
1
ave/min m
1.10937

331_Calib_S_A

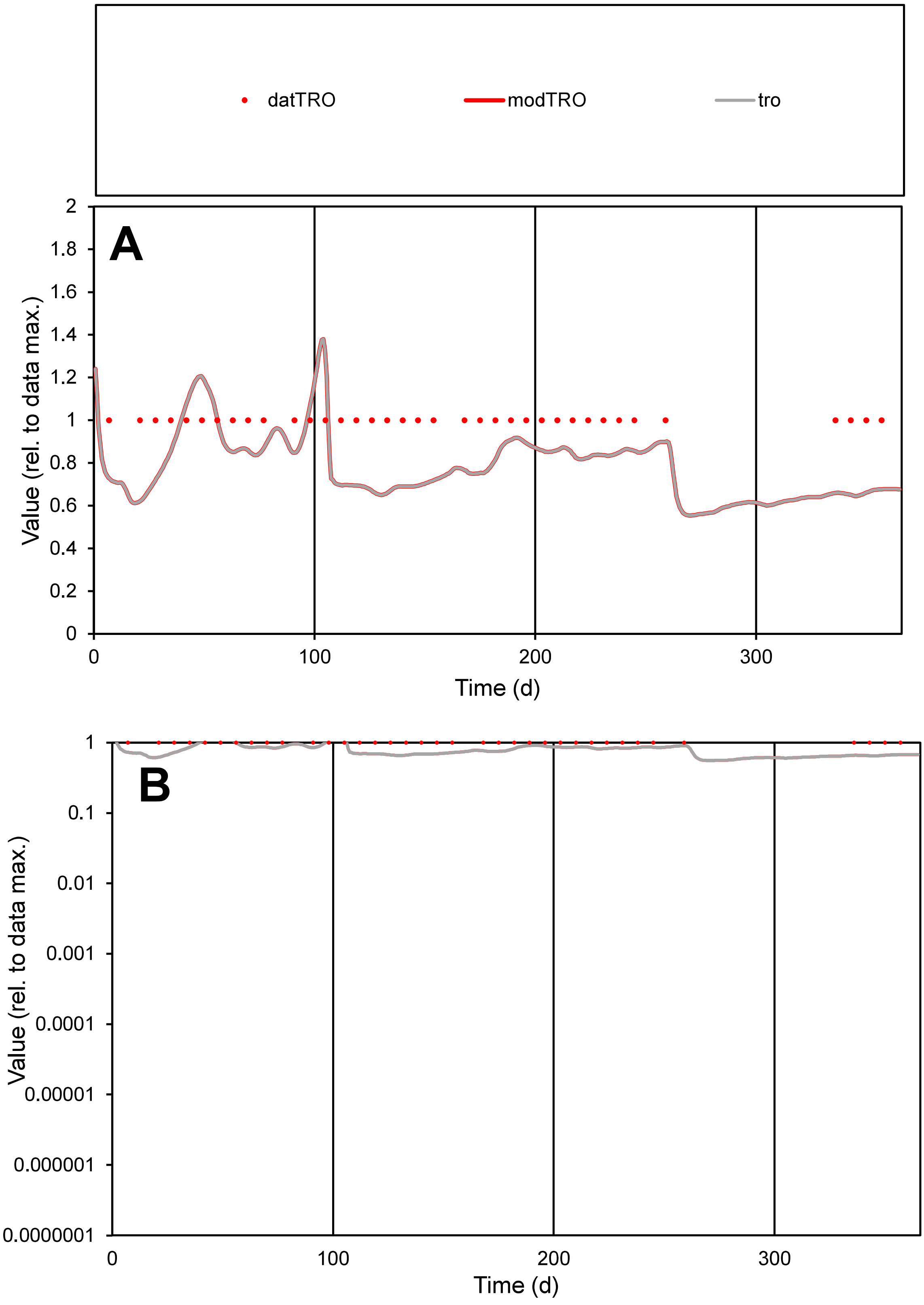

C1

Variables

TRO Provide jtd or Dst

CalibOnly?

2 1 to reproduce of

oNormalize?

2 1=no, 2=yes

ofxn calc*

0.22716

ofxn mod

0.24944

sRun

RunA1

zout

1

Ave

Data

5E-06

Model

4.1E-06

data/vMax

5E-06

ofxng

311.734

Min d/m

1.57125

ave/min d

1

ave/min m

1.28938

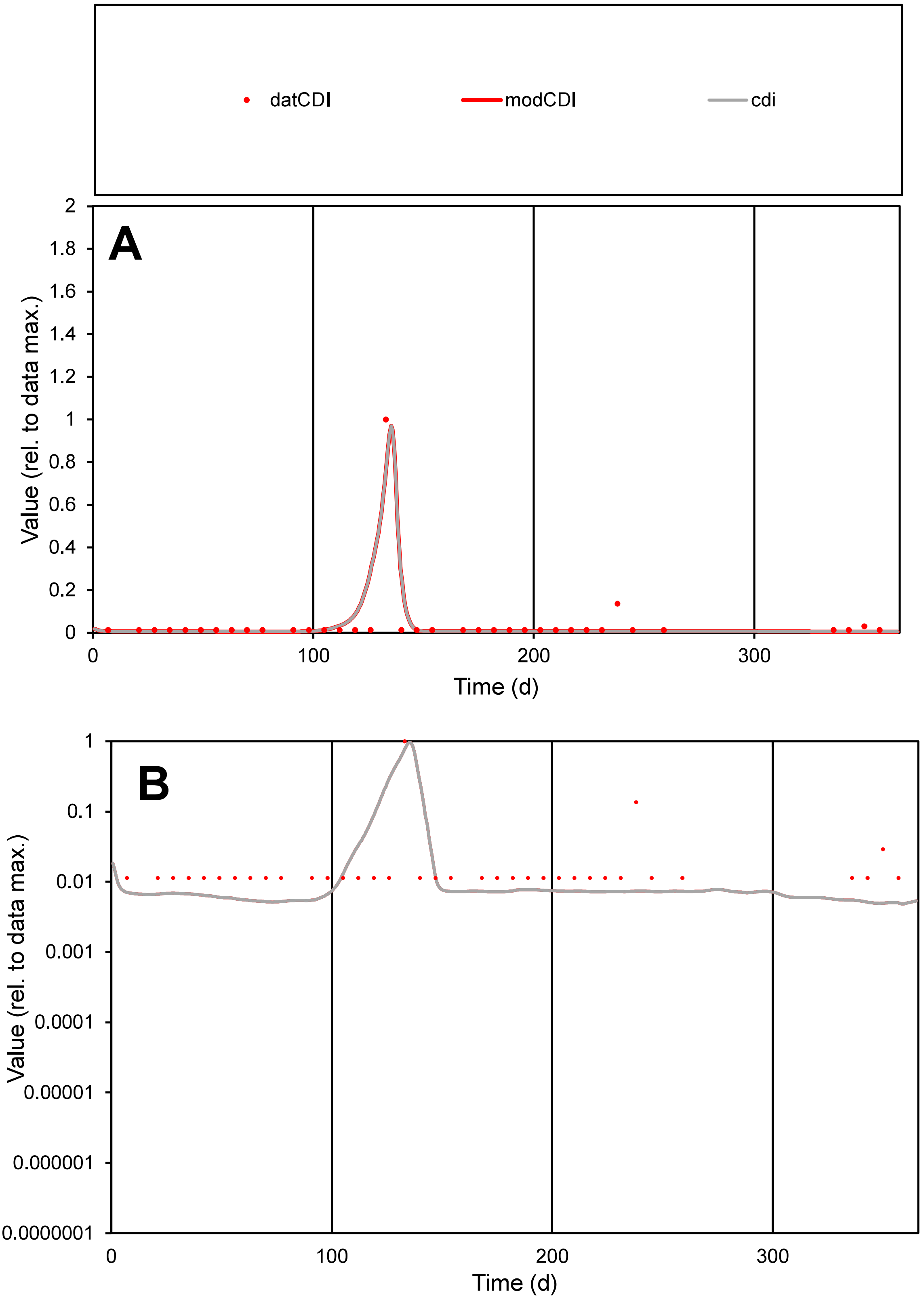

C1

Variables
CDI Provide jtd or Dst
CalibOnly?
2 1 to reproduce of
oNormalize?
2 1=no, 2=yes

ofxn calc*
1.08836
ofxn mod
1.29371

sRun
RunA1
zout
1

Ave
Data
6.4E-07
Model
6.4E-07

data/vMax
1.5E-05

ofxng
311.734

Min d/m
2.33549

ave/min d
3.70033
ave/min m
8.65951

331_Calib_S_A

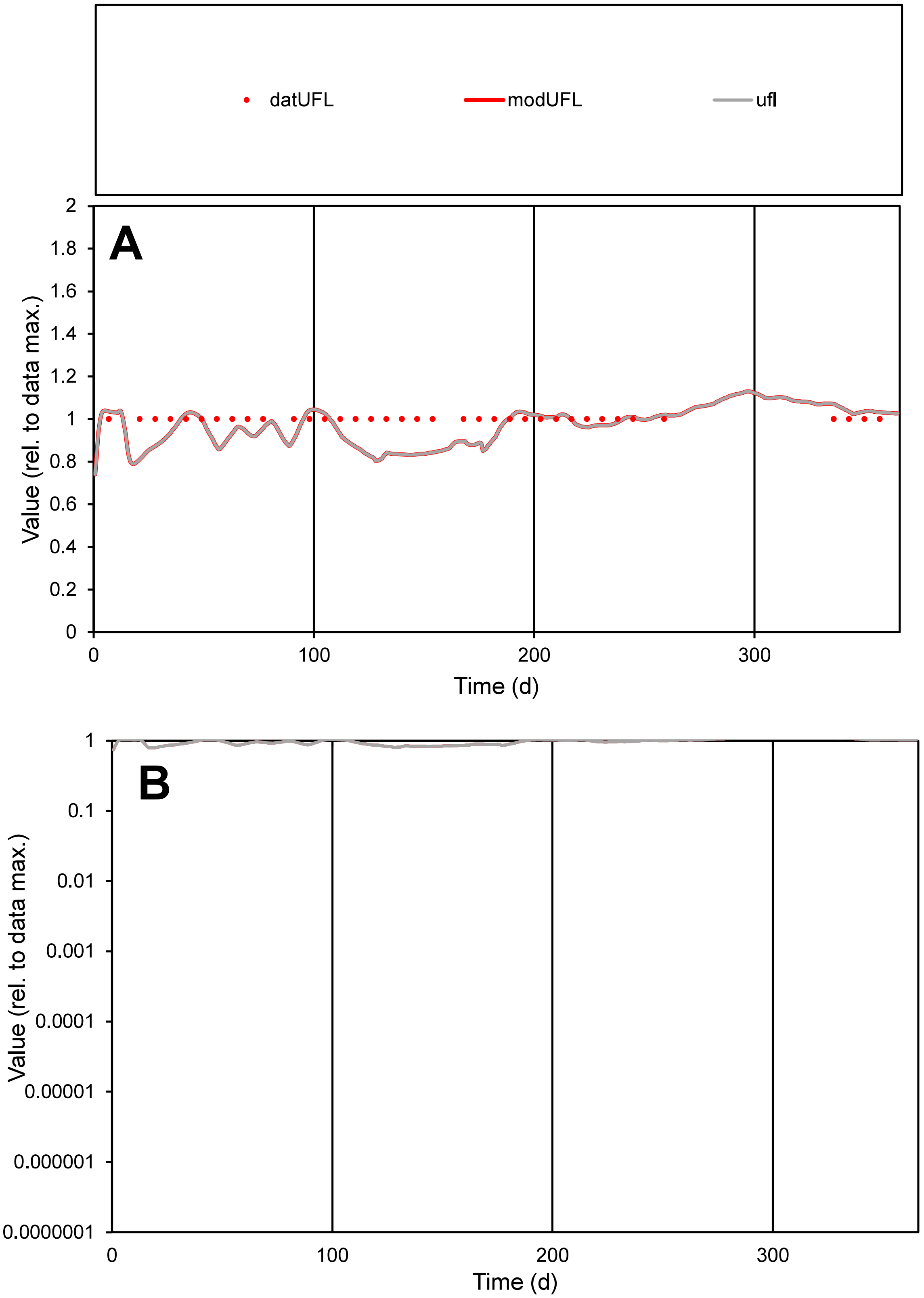

C1

Variables

UFL Provide jtd or Dst

CalibOnly?

2 1 to reproduce of

oNormalize?

2 1=no, 2=yes

ofxn calc*

0.08826

ofxn mod

0.14047

sRun

RunA1

zout

1

Ave

Data

3.1E-06

Model

2.9E-06

data/vMax

3.1E-06

ofxng

311.734

Min d/m

1.22333

ave/min d

1

ave/min m

1.16527

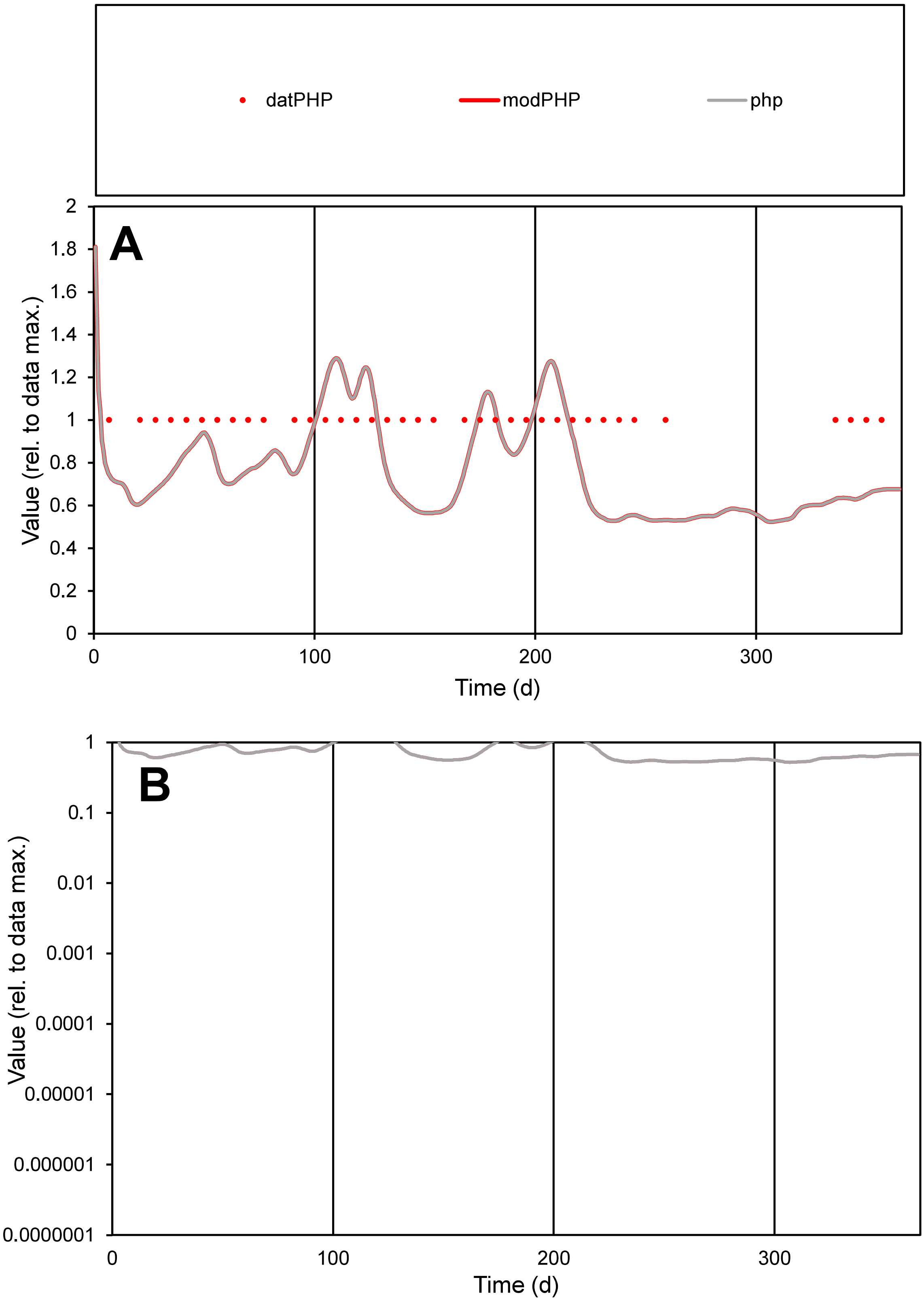

C1

Variables
PHP Provide jtd or Dst
CalibOnly?
2 1 to reproduce of
oNormalize?
2 1=no, 2=yes

ofxn calc*
0.2874
ofxn mod
0.30481

sRun
RunA1
zout
1

Ave
Data
6.9E-06
Model
5.6E-06

data/vMax
6.9E-06

ofxng
311.734

Min d/m
1.88052

ave/min d
1
ave/min m
1.51586

331_Calib_S_A

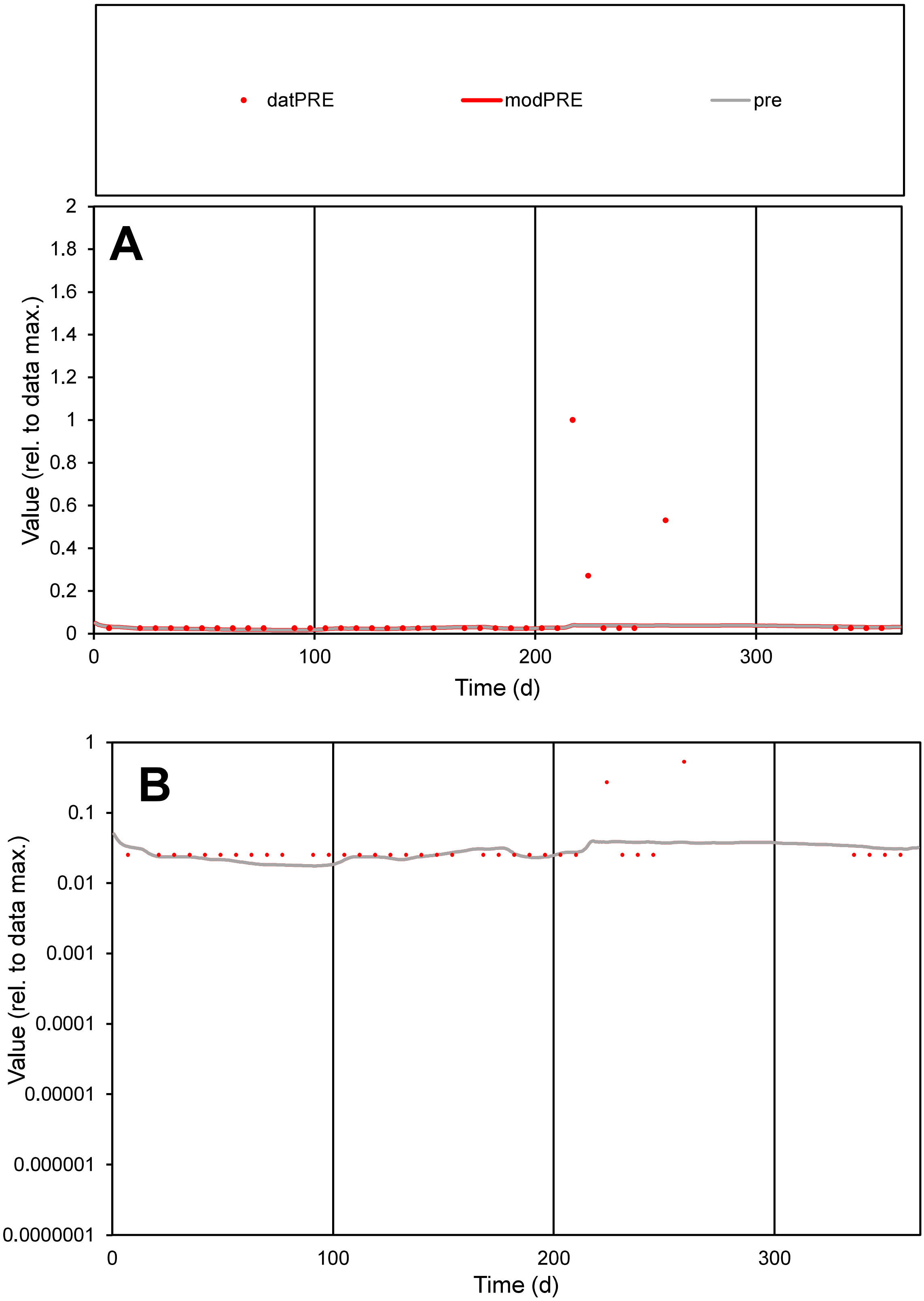

C1

Variables
PRE Provide jtd or Dst
CalibOnly?
2 1 to reproduce of
oNormalize?
2 1=no, 2=yes

ofxn calc*
2.52263
ofxn mod
2.52782

sRun
RunA1
zout
1

Ave
Data
3.8E-05
Model
1.4E-05

data/vMax
0.00053

ofxng
311.734

Min d/m
1.44161

ave/min d
2.83798
ave/min m
1.52811

331_Calib_S_A

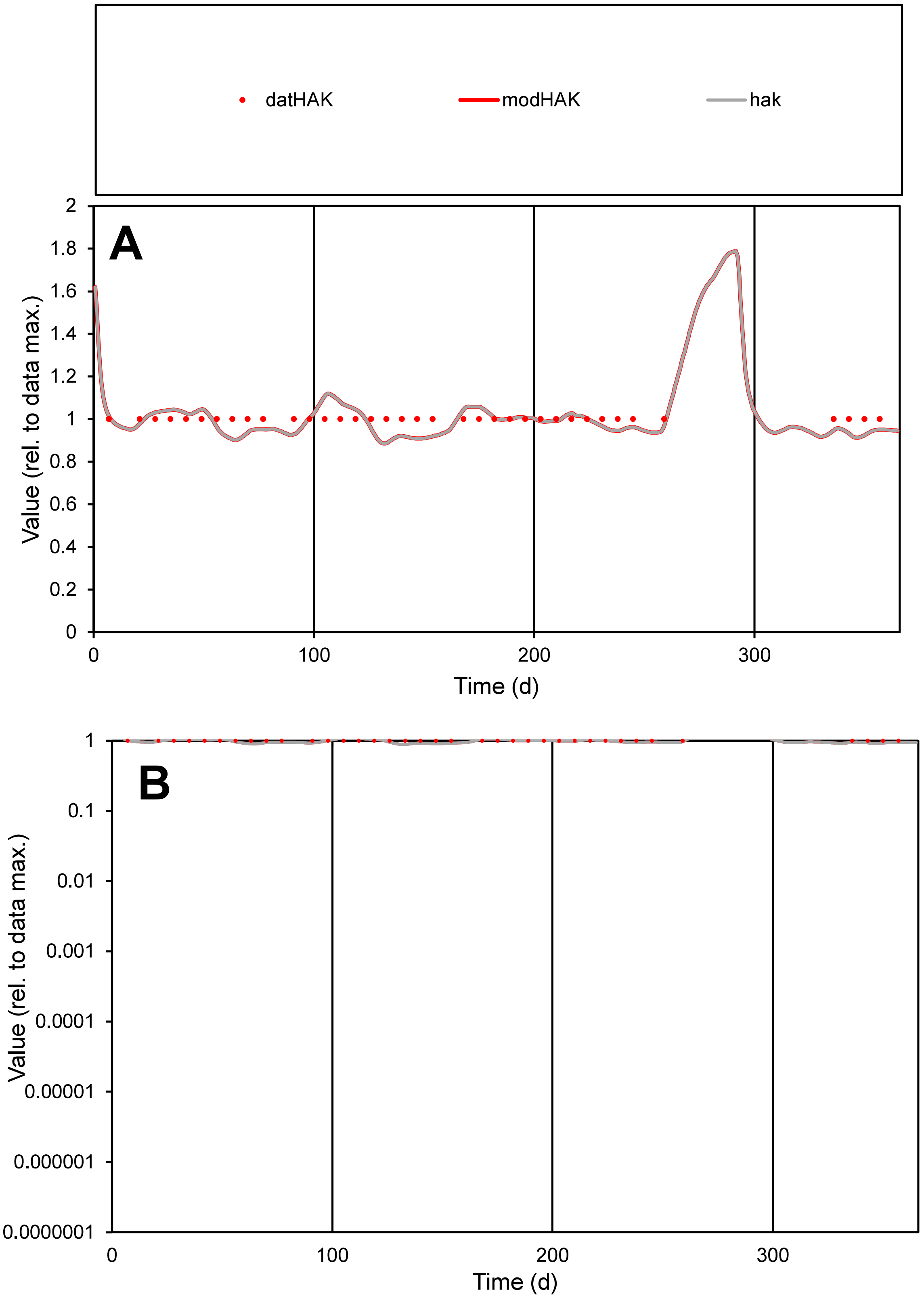

C1

Variables
HAK Provide jtd or Dst
CalibOnly?
2 1 to reproduce of
oNormalize?
2 1=no, 2=yes

ofxn calc*
0.055
ofxn mod
0.12338

sRun
RunA1
zout
1

Ave
Data
5.1E-06
Model
5E-06

data/vMax
5.1E-06

ofxng
311.734

Min d/m
1.11546

ave/min d
1
ave/min m
1.09803

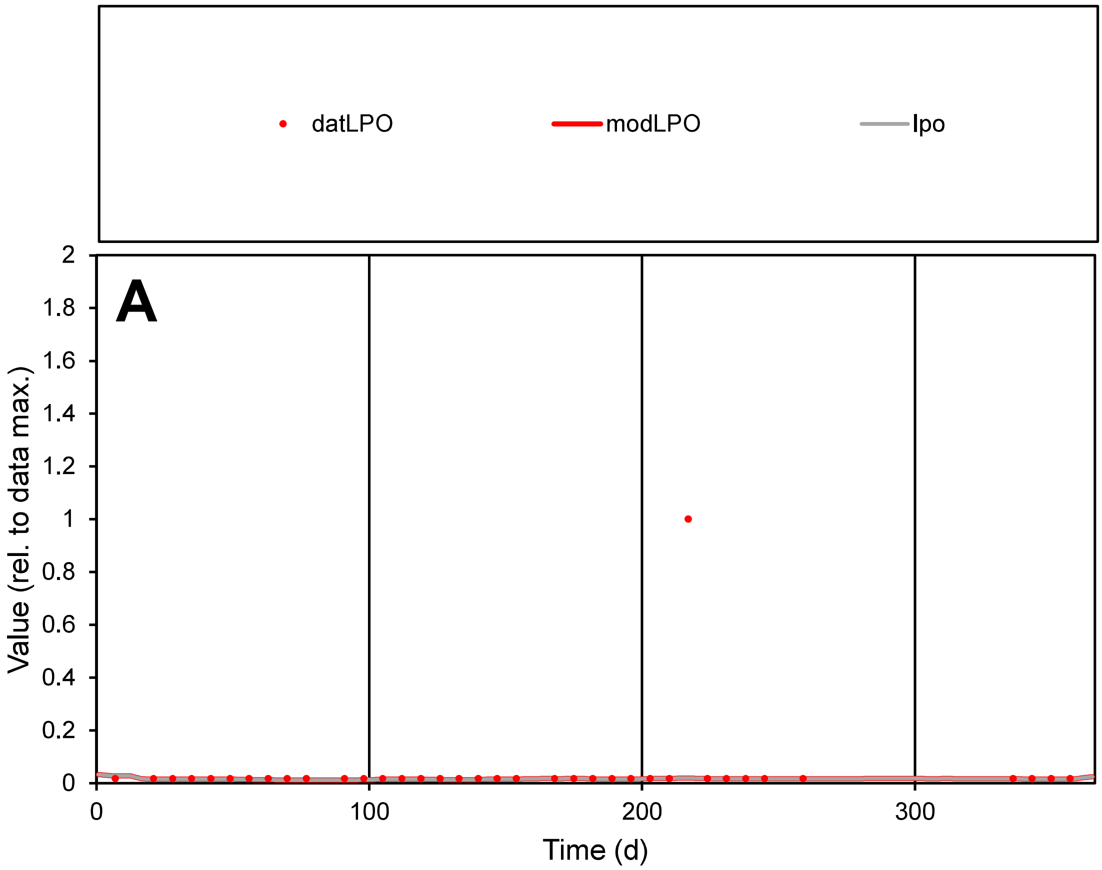

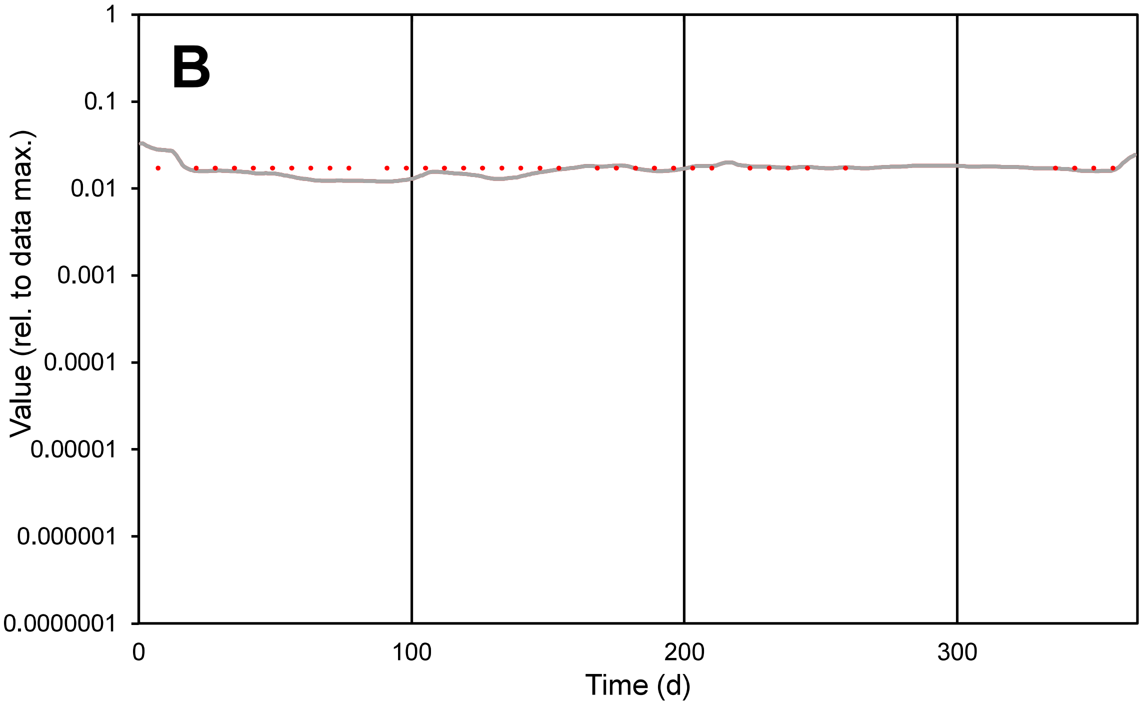

C1

Variables
LPO Provide jtd or Dst
CalibOnly?
2 1 to reproduce of
oNormalize?
2 1=no, 2=yes

ofxn calc*
3.68022
ofxn mod
3.68548

sRun
RunA1
zout
1

Ave
Data
1.5E-05
Model
5.5E-06

data/vMax
0.00034

ofxng
311.734

Min d/m
1.42412

ave/min d
2.54032
ave/min m
1.3264

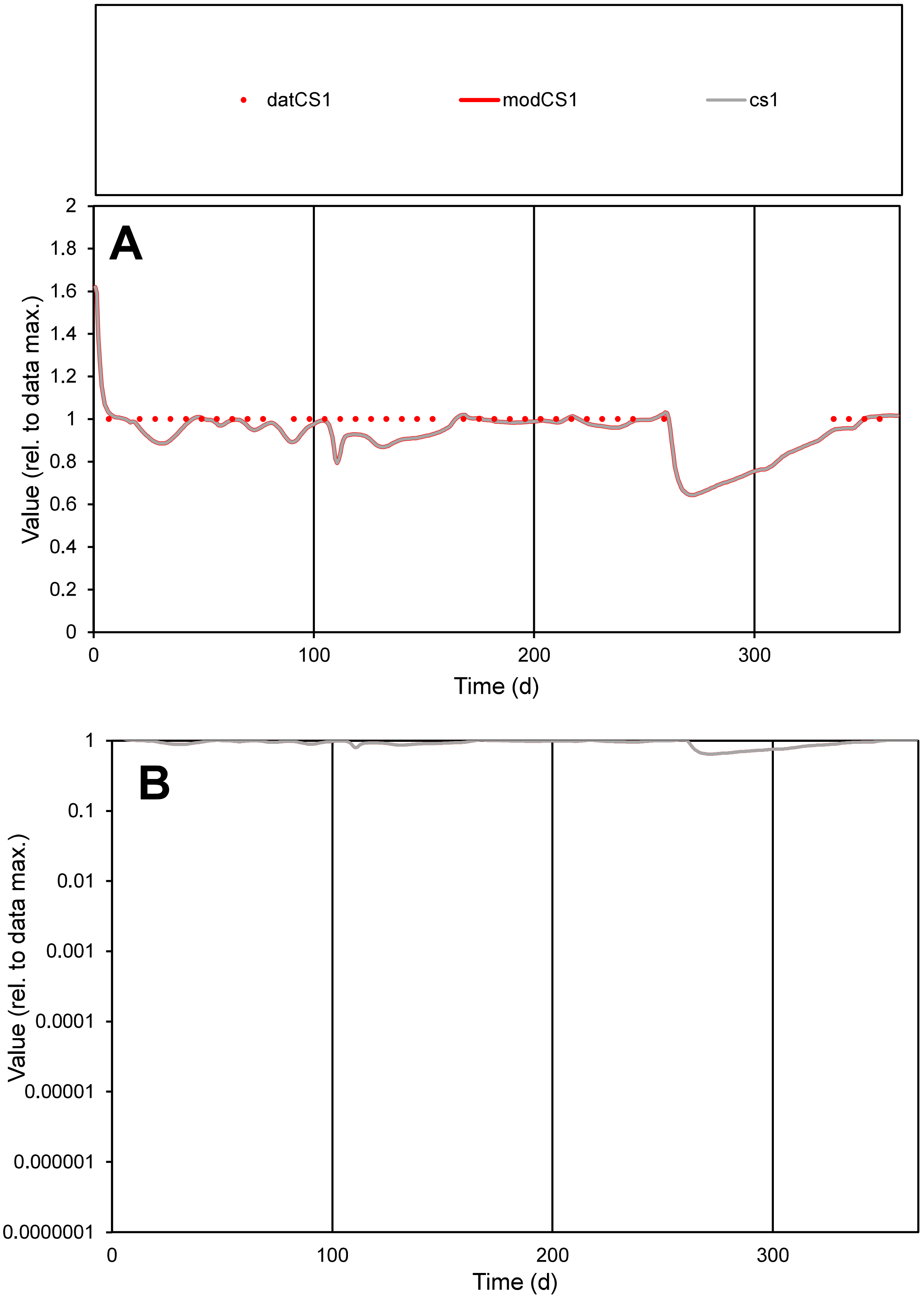

C1

Variables
CS1 Provide jtd or Dst
CalibOnly?
2 1 to reproduce of
oNormalize?
2 1=no, 2=yes

ofxn calc*
0.0541
ofxn mod
0.12249

sRun
RunA1
zout
1

Ave
Data
1.8E-05
Model
1.7E-05

data/vMax
1.8E-05

ofxng
311.734

Min d/m
1.13692

ave/min d
1
ave/min m
1.09694

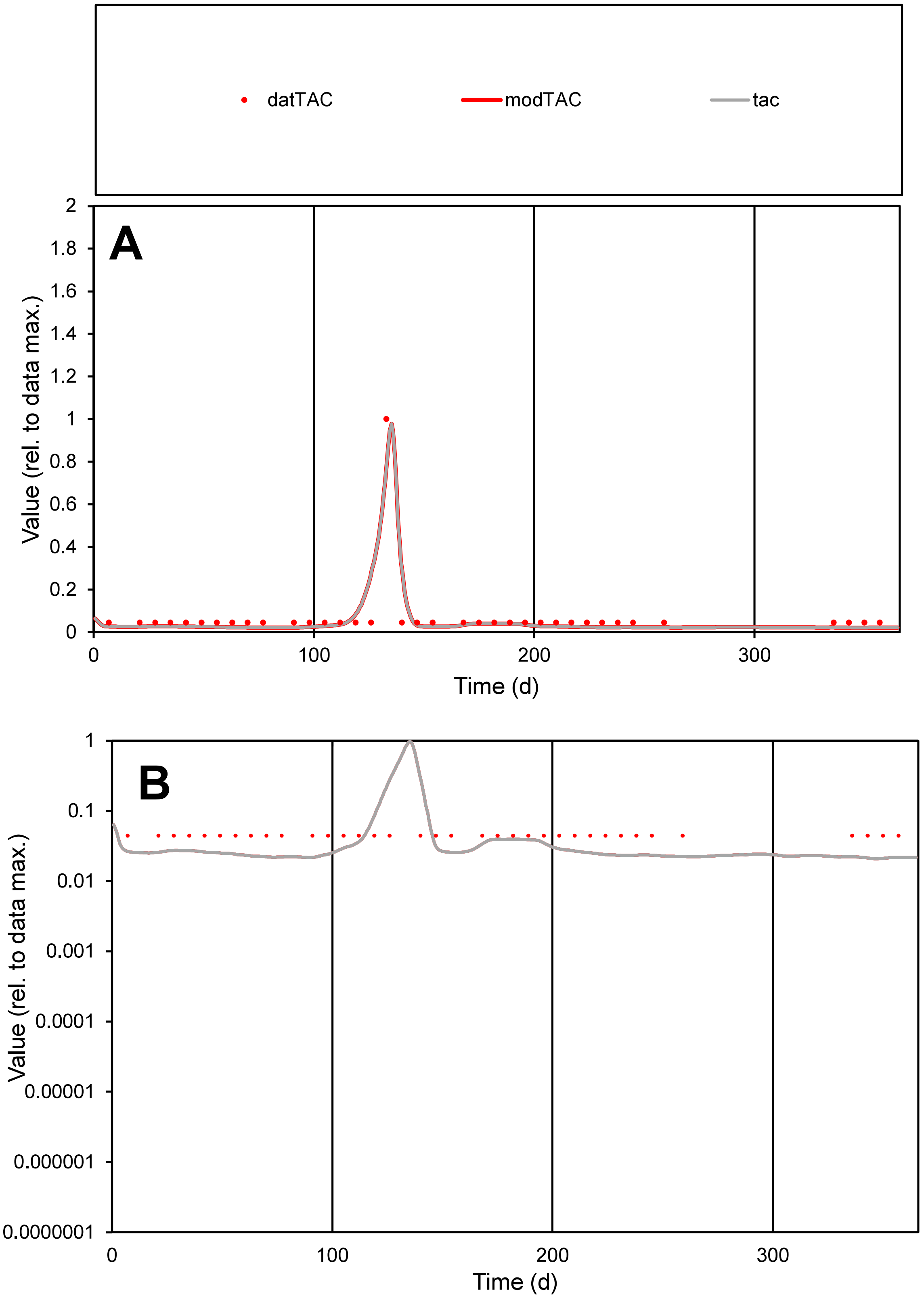

C1

Variables
TAC Provide jtd or Dst
CalibOnly?
2 1 to reproduce of
oNormalize?
2 1=no, 2=yes

ofxn calc*
0.5678
ofxn mod
0.67379

sRun
RunA1
zout
1

Ave
Data
2E-06
Model
1.8E-06

data/vMax
2.9E-05

ofxng
311.734

Min d/m
2.10638

ave/min d
1.5803
ave/min m
2.91817

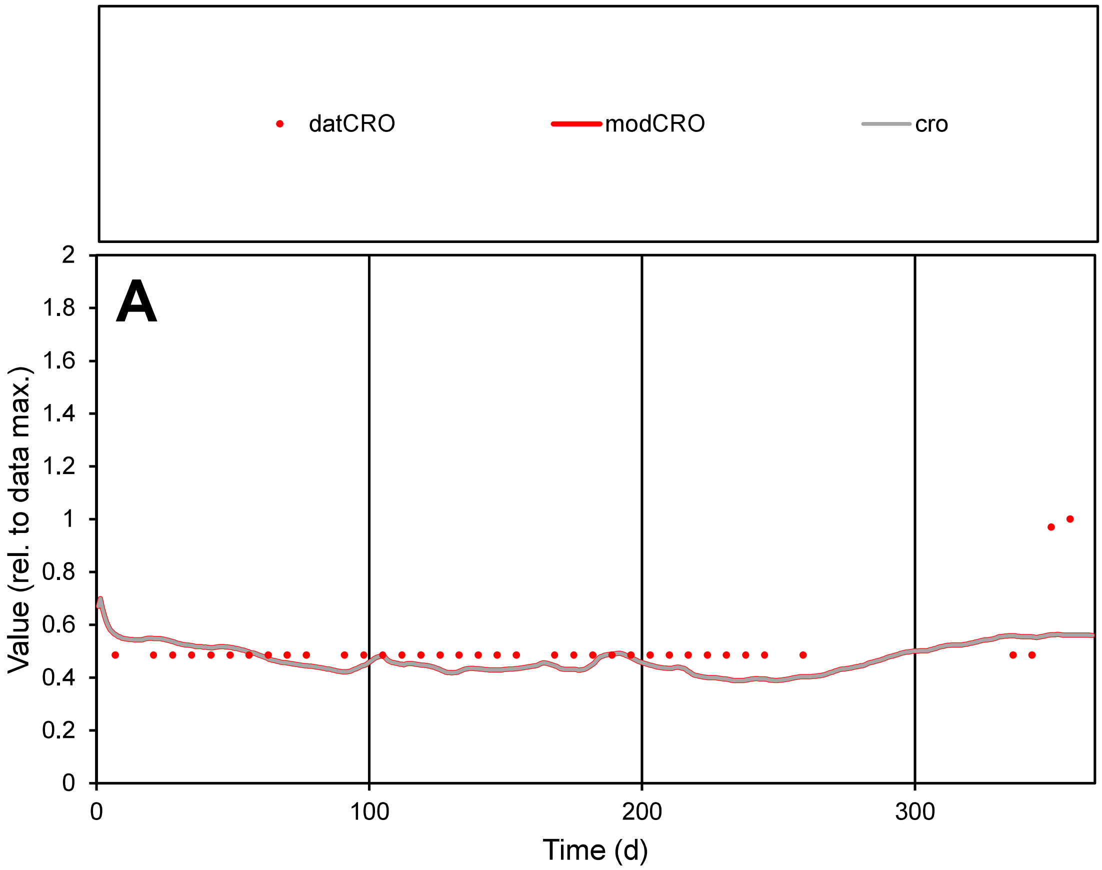

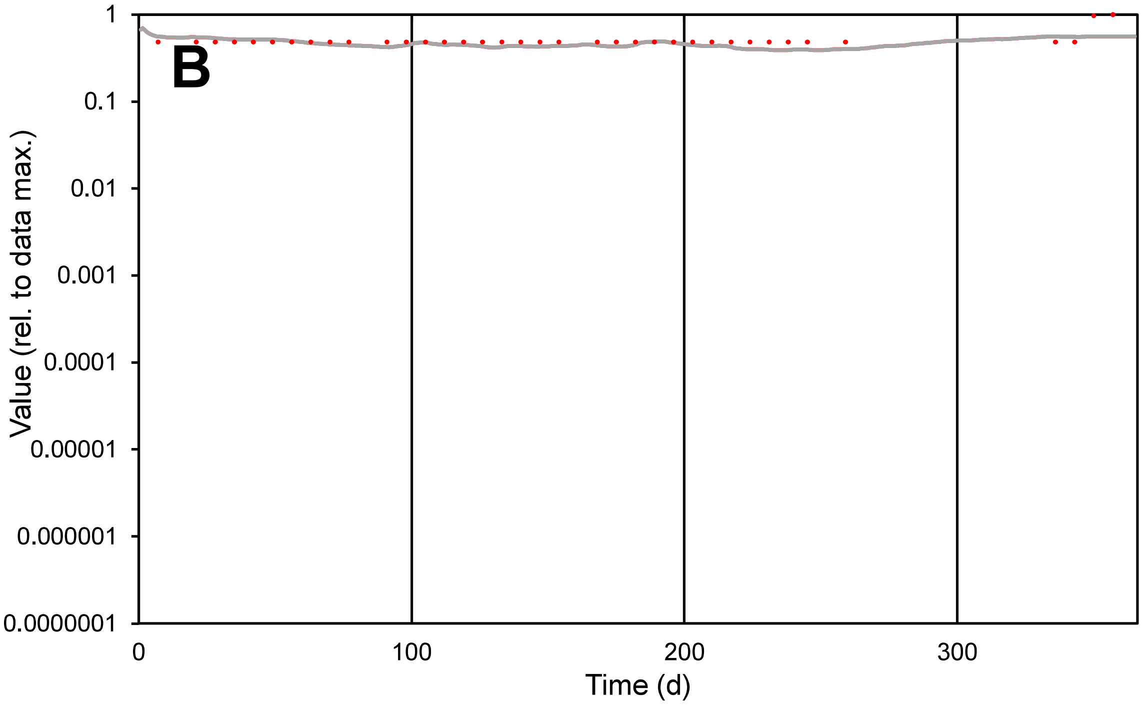

C1

Variables
CRO Provide jtd or Dst
CalibOnly?
2 1 to reproduce of
oNormalize?
2 1=no, 2=yes

ofxn calc*
0.21846
ofxn mod
0.24325

sRun
RunA1
zout
1

Ave
Data
1.1E-05
Model
9.8E-06

data/vMax
2.1E-05

ofxng
311.734

Min d/m
1.24121

ave/min d
1.0558
ave/min m
1.19358

331_Calib_S_A

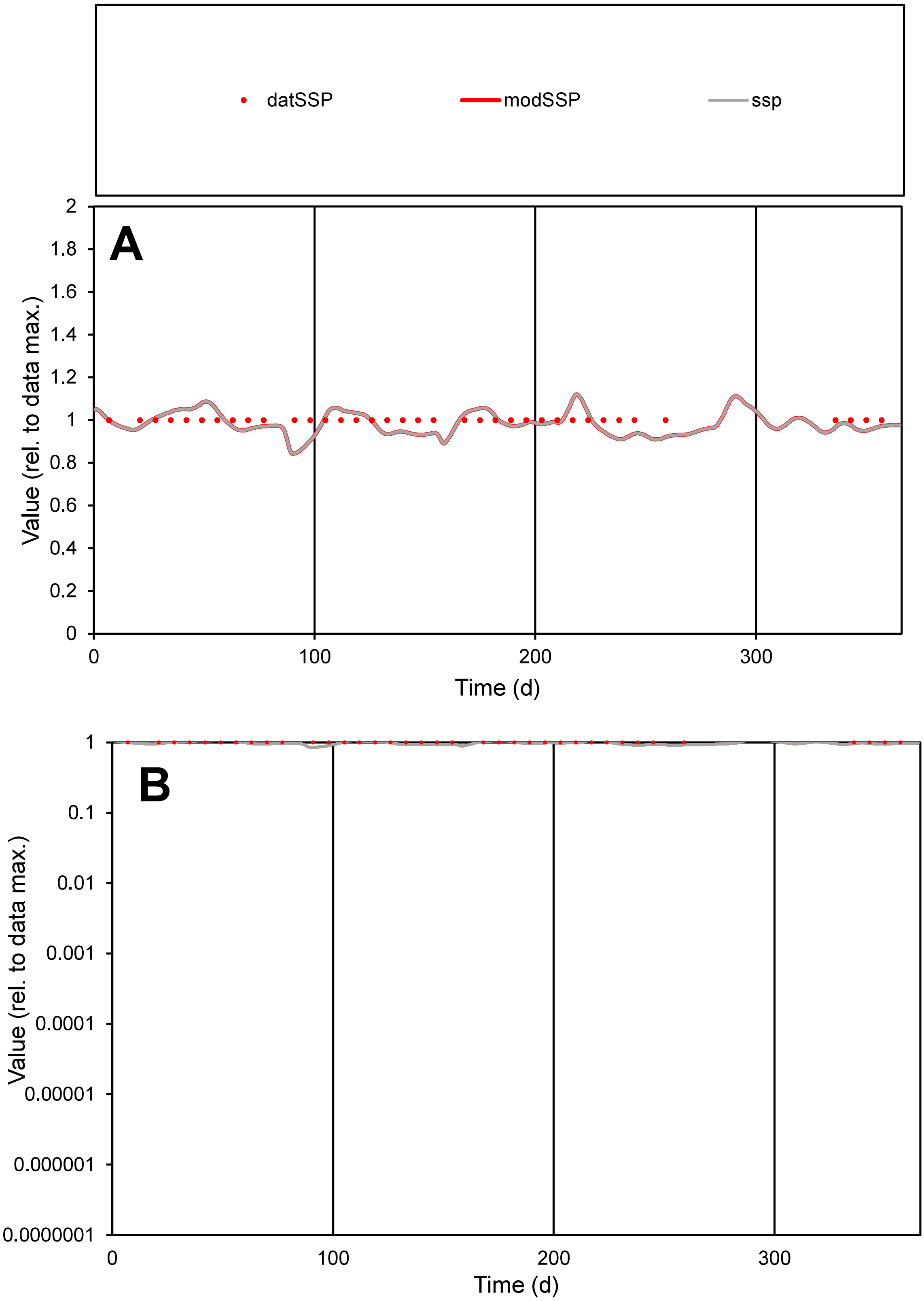

C1

Variables
SSP Provide jtd or Dst
CalibOnly?
2 1 to reproduce of
oNormalize?
2 1=no, 2=yes

ofxn calc*
0.0555
ofxn mod
0.12371

sRun
RunA1
zout
1

Ave
Data
1.3E-05
Model
1.3E-05

data/vMax
1.3E-05

ofxng
311.734

Min d/m
1.176

ave/min d
1
ave/min m
1.15863

331_Calib_S_A

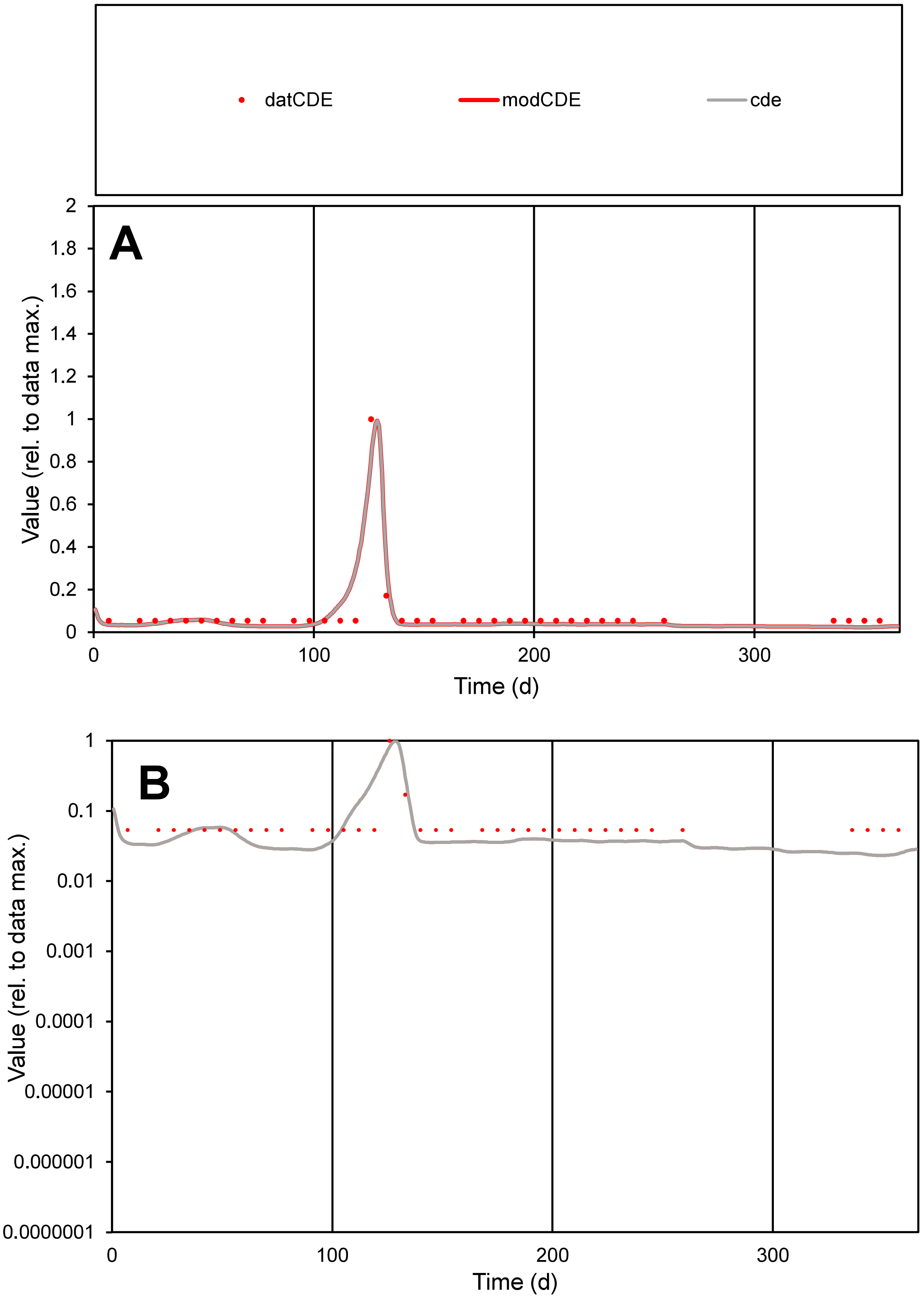

C1

Variables
CDE Provide jtd or Dst
CalibOnly?
2 1 to reproduce of
oNormalize?
2 1=no, 2=yes

ofxn calc*
0.45873
ofxn mod
0.58619

sRun
RunA1
zout
1

Ave
Data
1.5E-05
Model
1.4E-05

data/vMax
0.00019

ofxng
311.734

Min d/m
2.30327

ave/min d
1.53815
ave/min m
3.14414

331_Calib_S_A

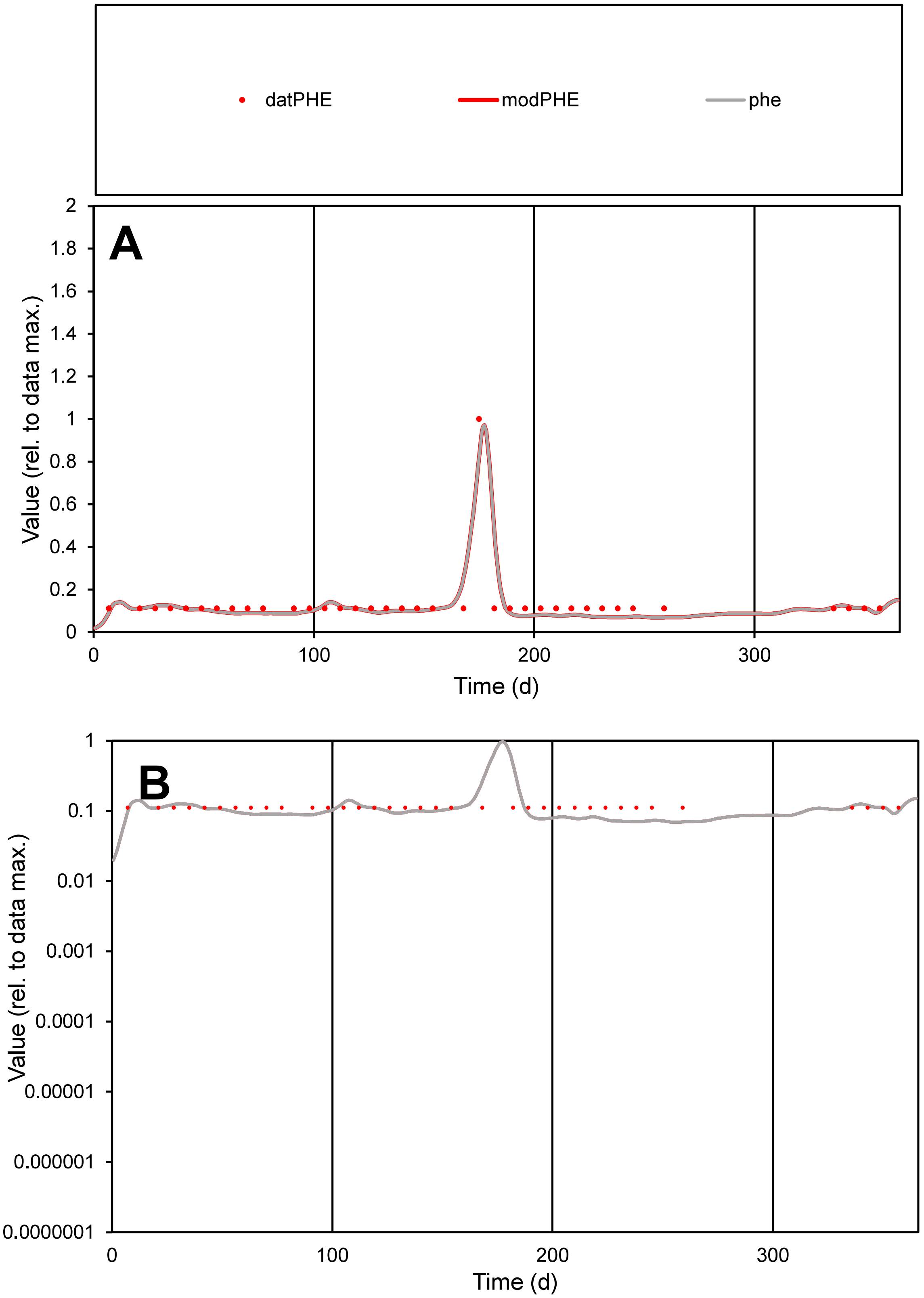

C1

Variables

PHE Provide jtd or Dst

CalibOnly?

2 1 to reproduce of

oNormalize?

2 1=no, 2=yes

ofxn calc*

0.25739

ofxn mod

0.33031

sRun

RunA1

zout

1

Ave

Data

4.8E-06

Model

4.5E-06

data/vMax

3.6E-05

ofxng

311.734

Min d/m

1.59189

ave/min d

1.21538

ave/min m

1.80783

331_Calib_S_A

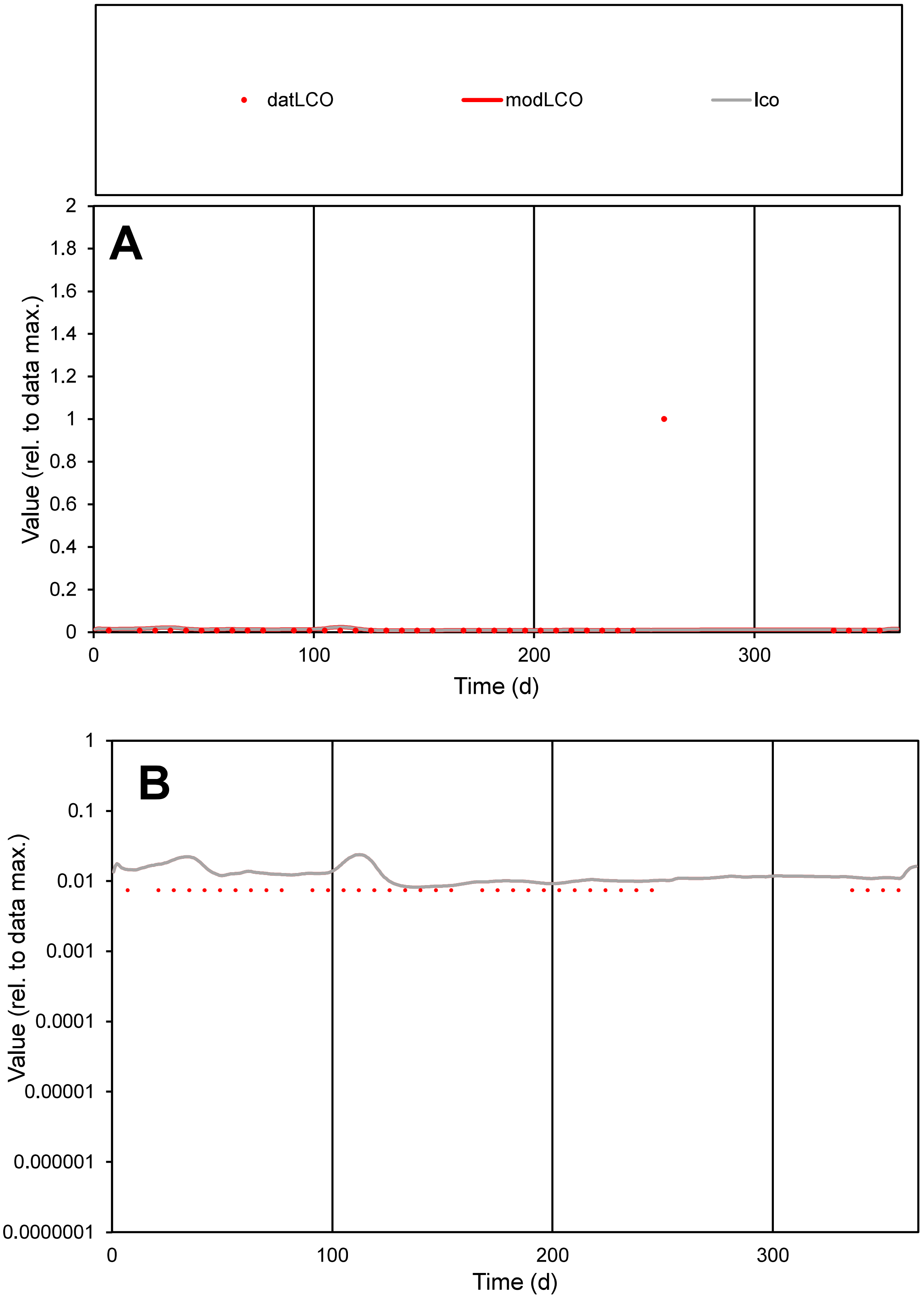

C1

Variables
LCO Provide jtd or Dst
CalibOnly?
2 1 to reproduce of
oNormalize?
2 1=no, 2=yes

ofxn calc*
4.74818
ofxn mod
4.75855

sRun
RunA1
zout
1

Ave
Data
3.4E-05
Model
1.2E-05

data/vMax
0.001

ofxng
311.734

Min d/m
0.90307

ave/min d
4.60613
ave/min m
1.48459

331_Calib_S_A

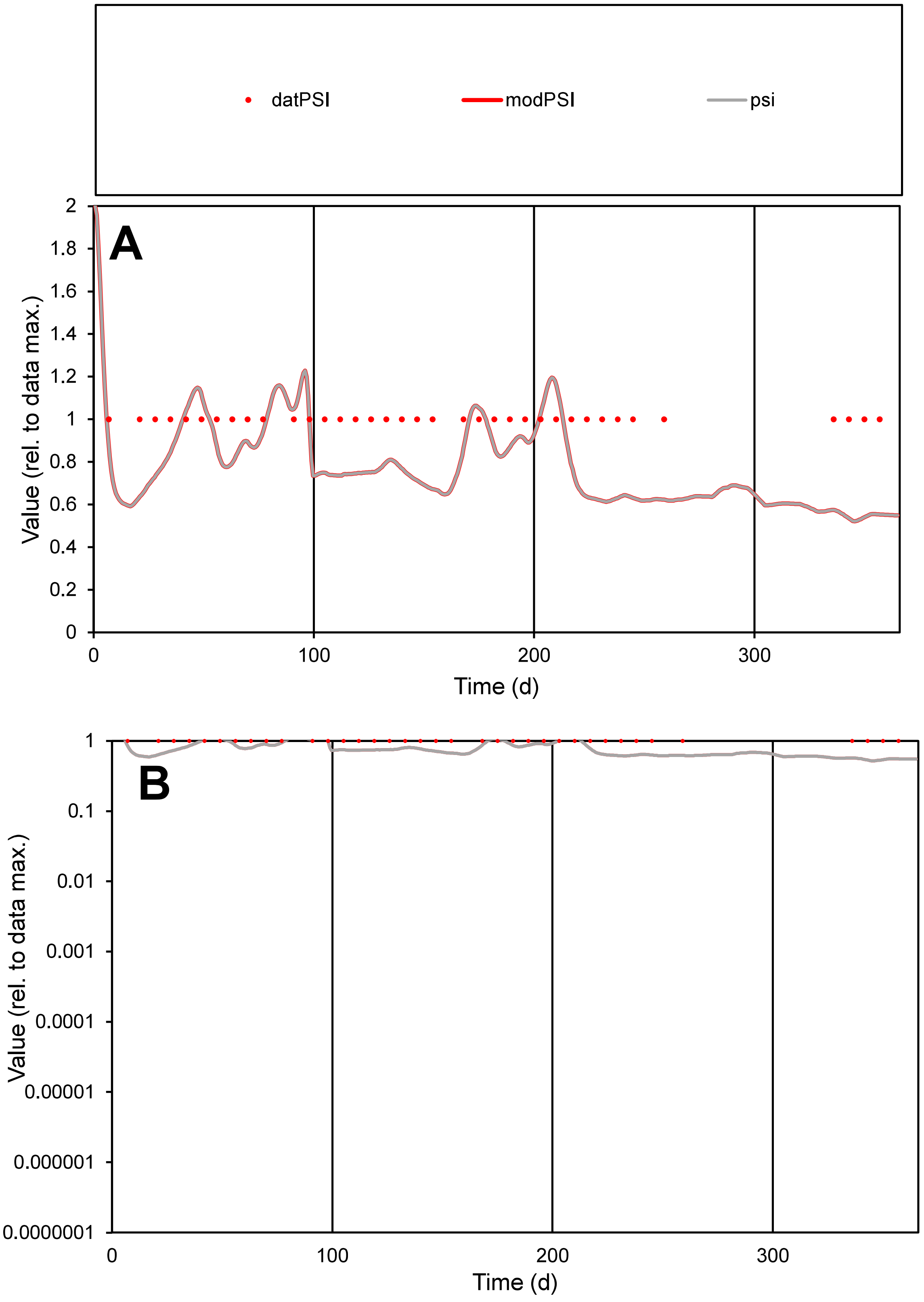

C1

Variables

PSI Provide jtd or Dst

CalibOnly?

2 1 to reproduce of

oNormalize?

2 1=no, 2=yes

ofxn calc*

0.26174

ofxn mod

0.2827

sRun

RunA1

zout

1

Ave

Data

3.6E-06

Model

2.8E-06

data/vMax

3.6E-06

ofxng

311.734

Min d/m

1.89505

ave/min d

1

ave/min m

1.51449

331_Calib_S_A

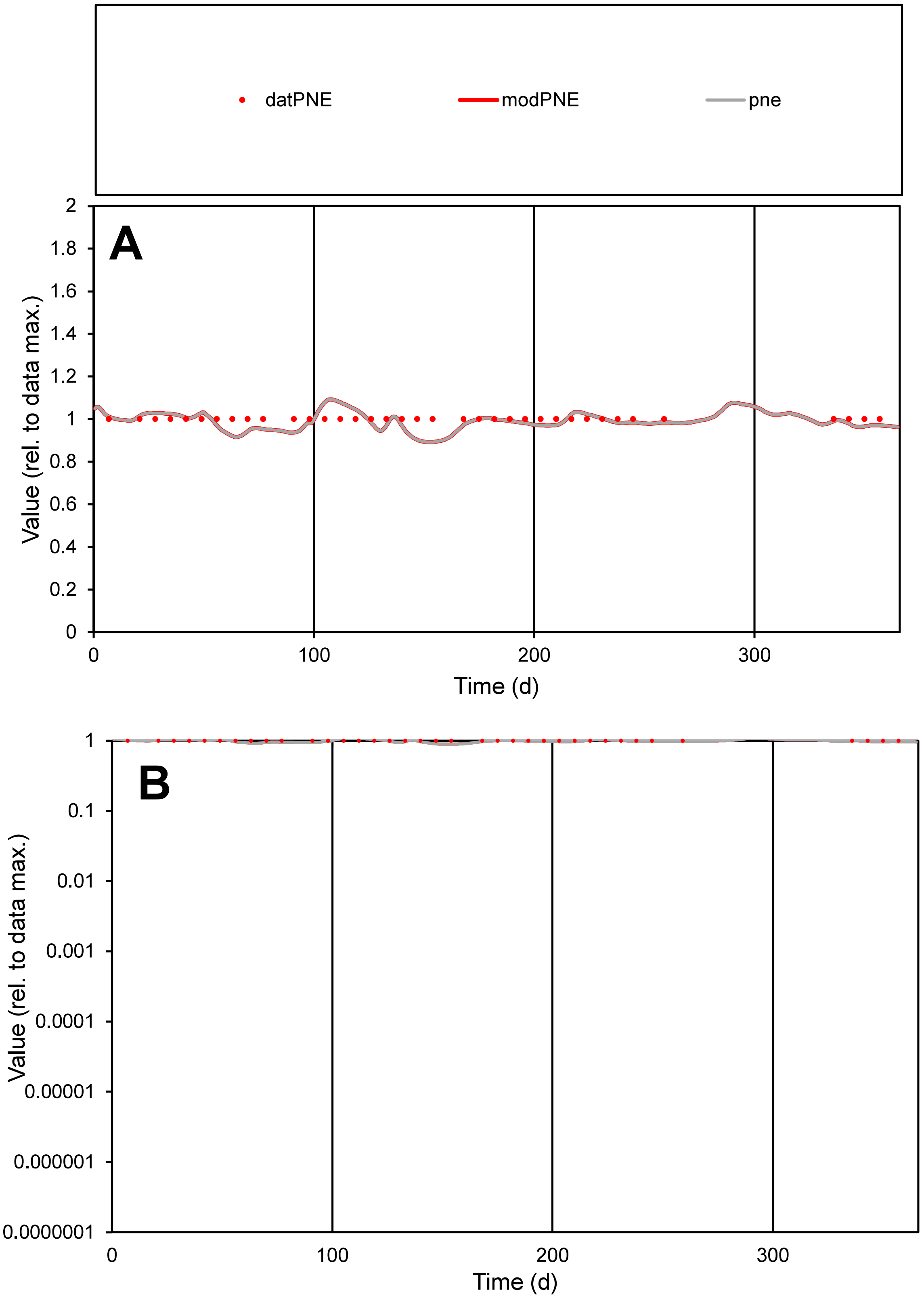

C1

Variables

PNE Provide jtd or Dst

CalibOnly?

2 1 to reproduce of

oNormalize?

2 1=no, 2=yes

ofxn calc*

0.04231

ofxn mod

0.11852

sRun

RunA1

zout

1

Ave

Data

1.5E-05

Model

1.4E-05

data/vMax

1.5E-05

ofxng

311.734

Min d/m

1.11778

ave/min d

1

ave/min m

1.10405

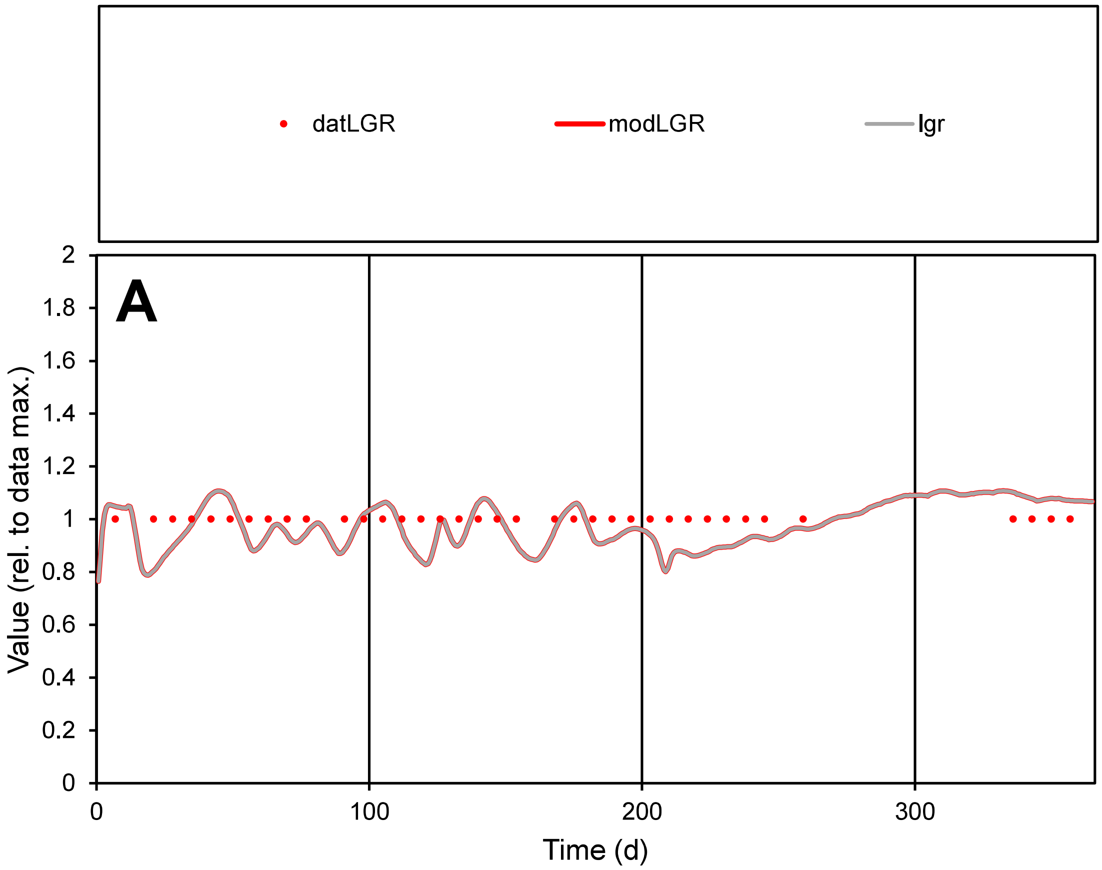

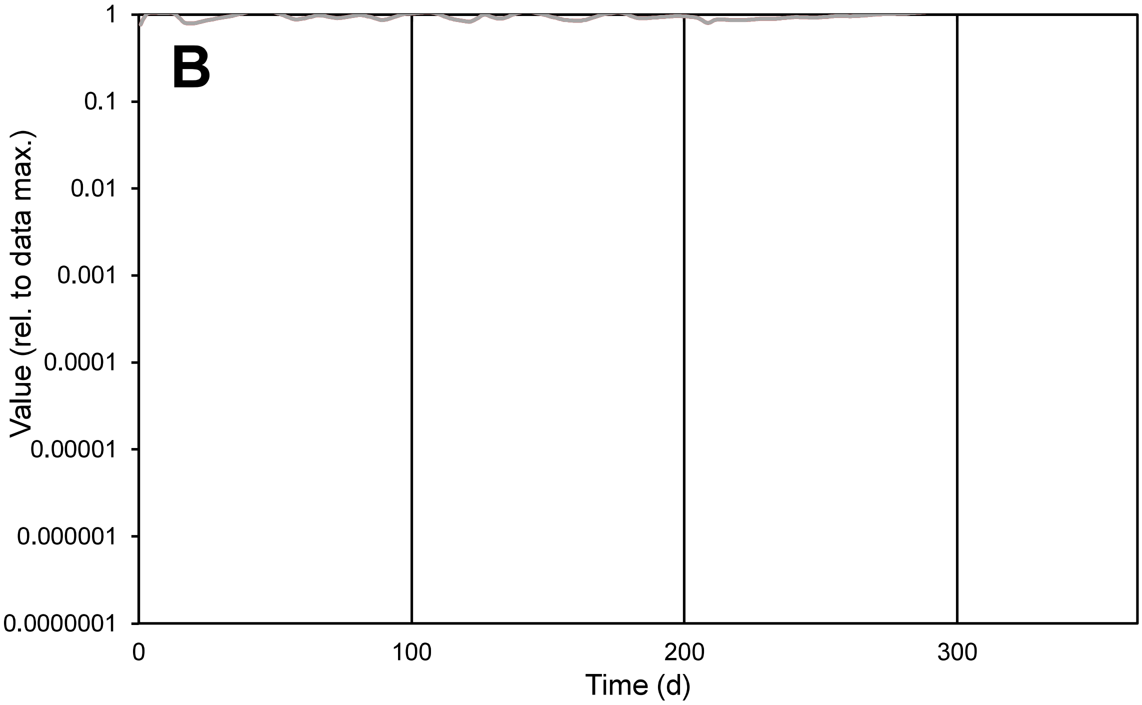

C1

Variables
LGR Provide jtd or Dst
CalibOnly?
2 1 to reproduce of
oNormalize?
2 1=no, 2=yes

ofxn calc*
0.08577
ofxn mod
0.13881

sRun
RunA1
zout
1

Ave
Data
5.2E-06
Model
5E-06

data/vMax
5.2E-06

ofxng
311.734

Min d/m
1.2143

ave/min d
1
ave/min m
1.17306

331_Calib_S_A

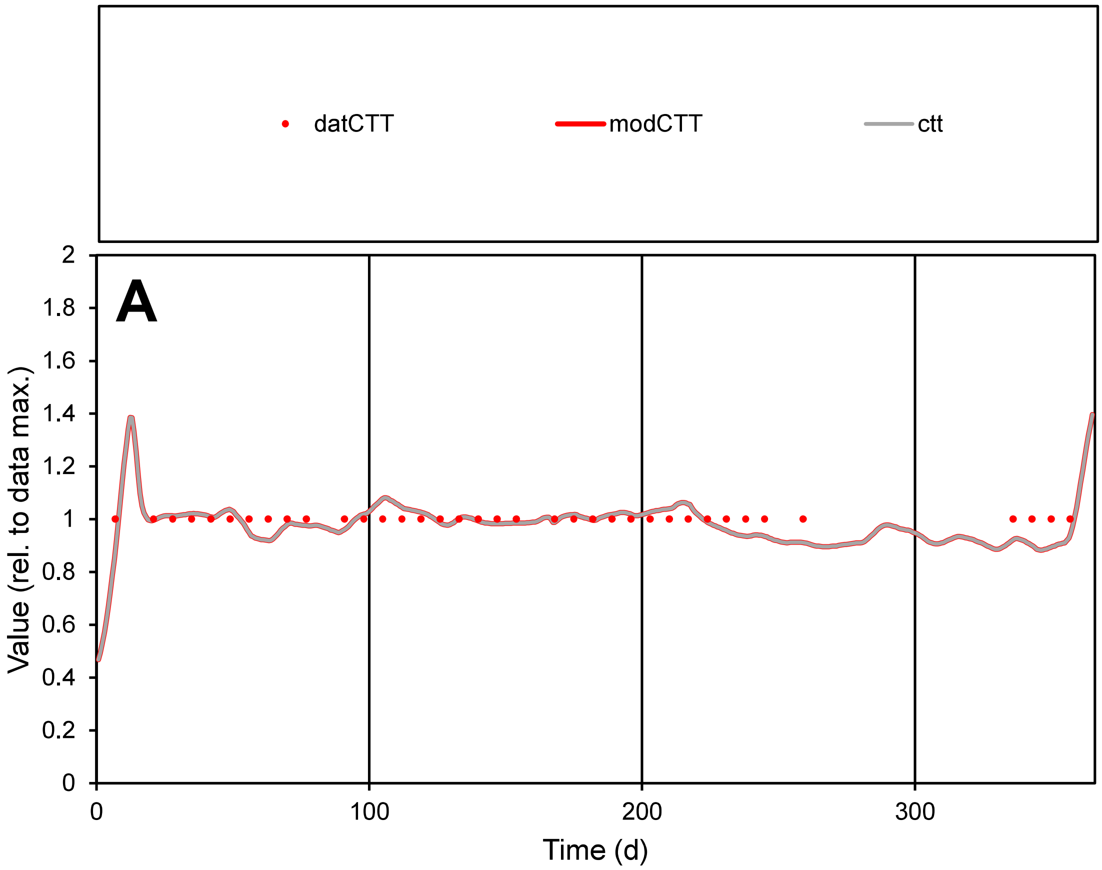

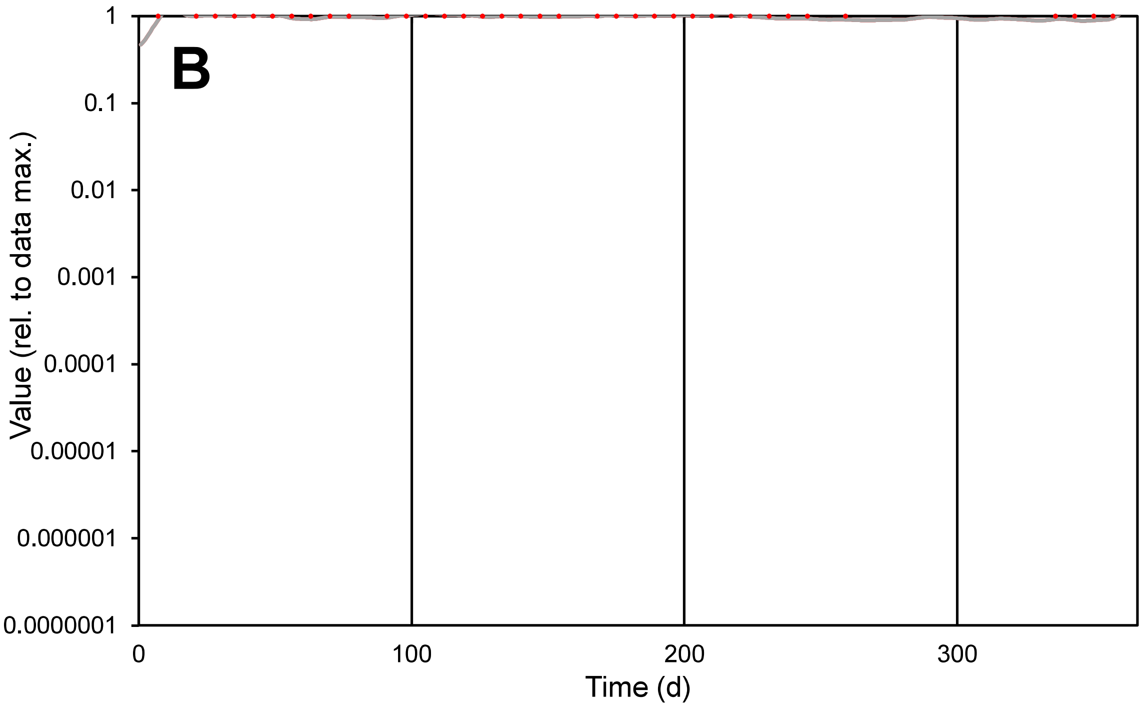

C1

Variables
CTT Provide jtd or Dst
CalibOnly?
2 1 to reproduce of
oNormalize?
2 1=no, 2=yes

ofxn calc*
0.04553
ofxn mod
0.11984

sRun
RunA1
zout
1

Ave
Data
1.5E-06
Model
1.5E-06

data/vMax
1.5E-06

ofxng
311.734

Min d/m
1.12094

ave/min d
1
ave/min m
1.1085

331_Calib_S_A

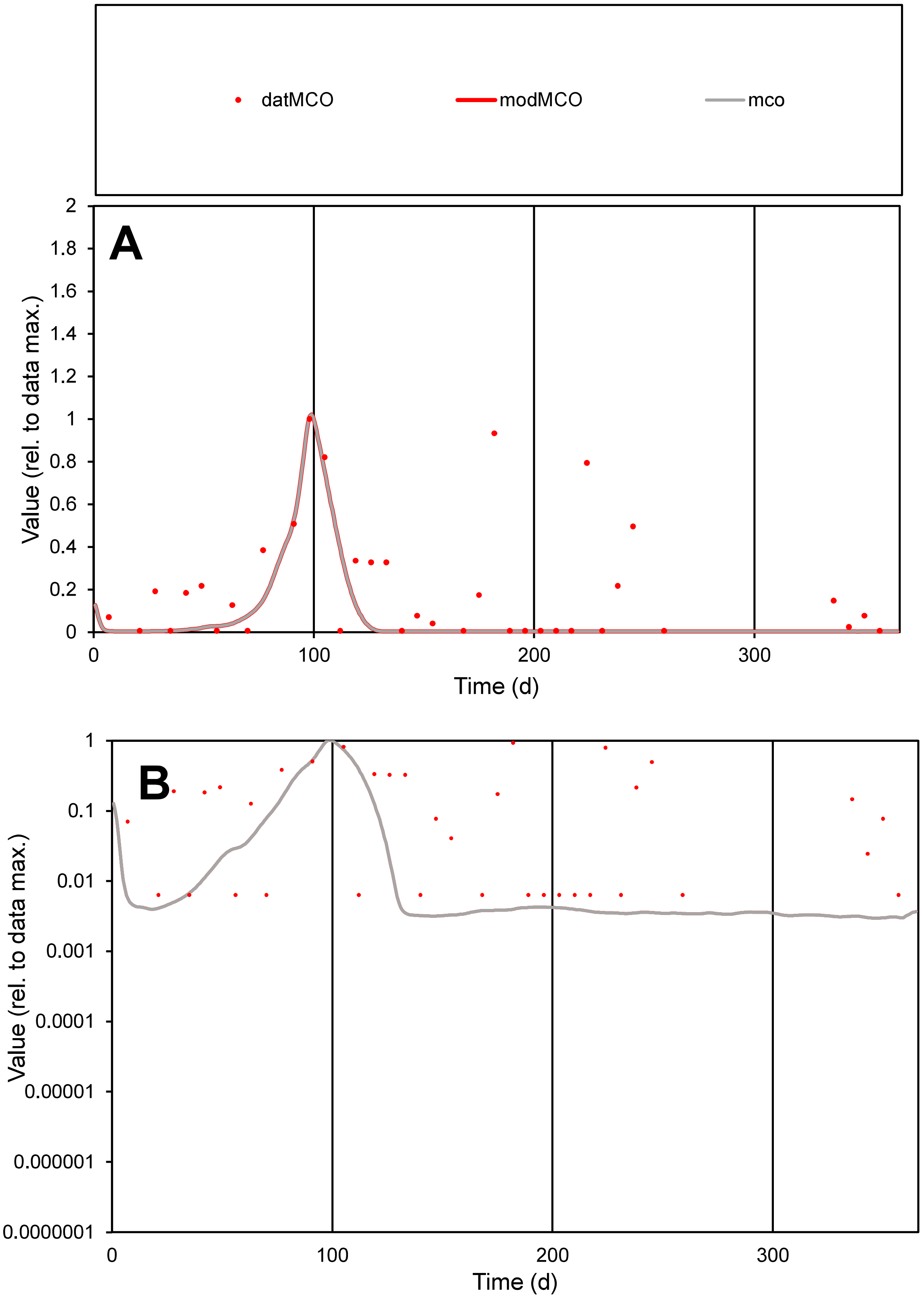

C1

Variables
MCO Provide jtd or Dst
CalibOnly?
2 1 to reproduce of
oNormalize?
2 1=no, 2=yes

ofxn calc*
1.22179
ofxn mod
1.23916

sRun
RunA1
zout
1

Ave
Data
2E-06
Model
8.2E-07

data/vMax
9.6E-06

ofxng
311.734

Min d/m
2.12474
ave/min d
32.1084
ave/min m
28.4844

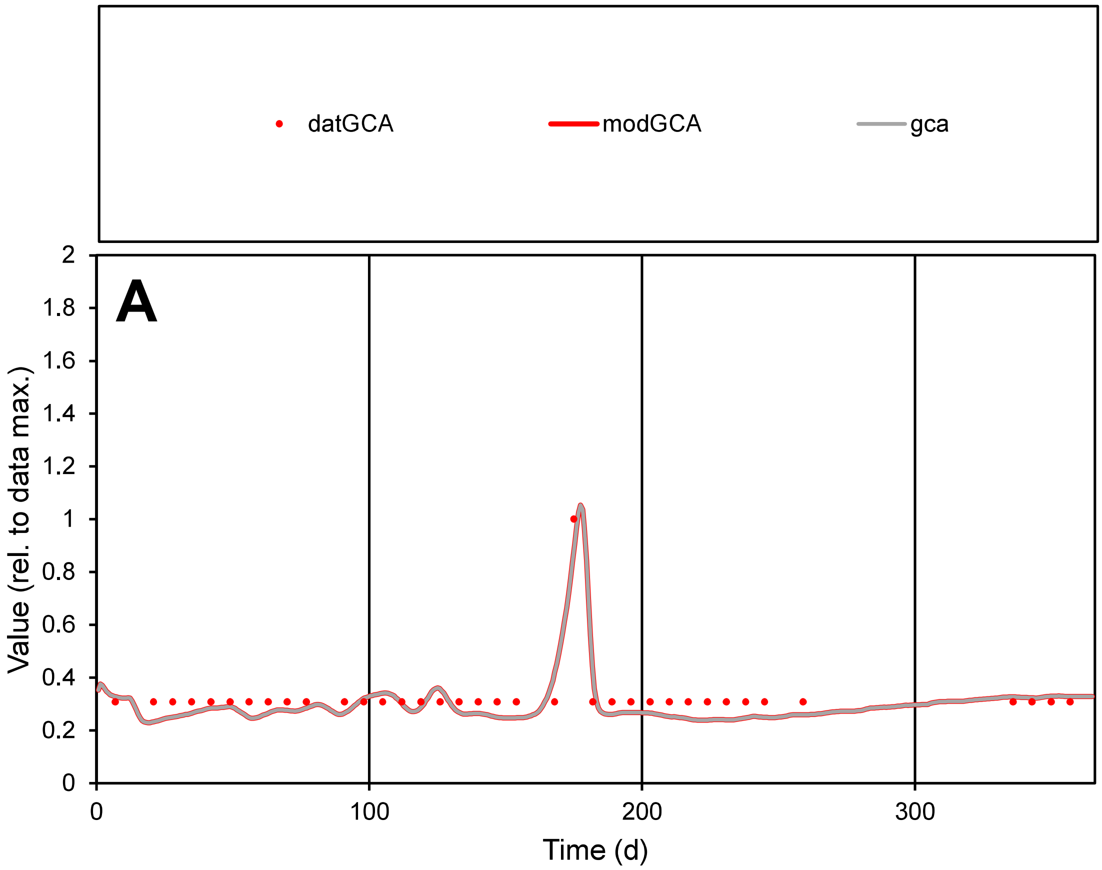

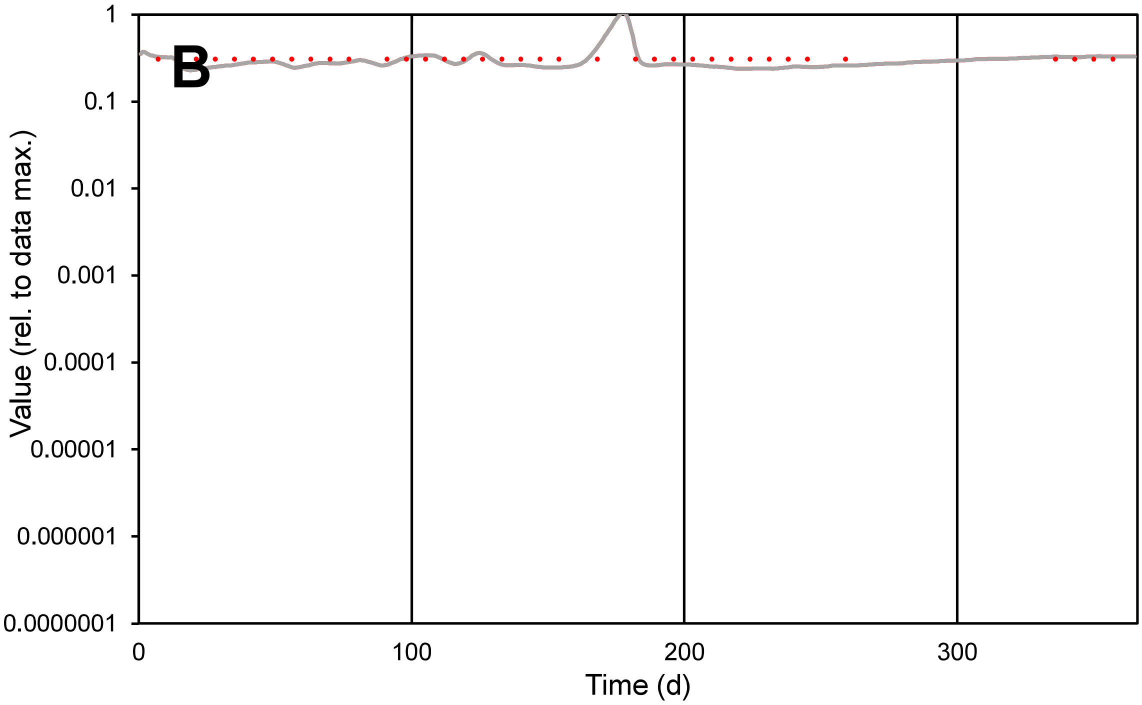

C1

Variables
GCA Provide jtd or Dst
CalibOnly?
2 1 to reproduce of
oNormalize?
2 1=no, 2=yes

ofxn calc*
0.14066
ofxn mod
0.17932

sRun
RunA1
zout
1

Ave
Data
8.9E-06
Model
8.2E-06

data/vMax
2.7E-05

ofxng
311.734

Min d/m
1.29624

ave/min d
1.06088
ave/min m
1.2731

331_Calib_S_A

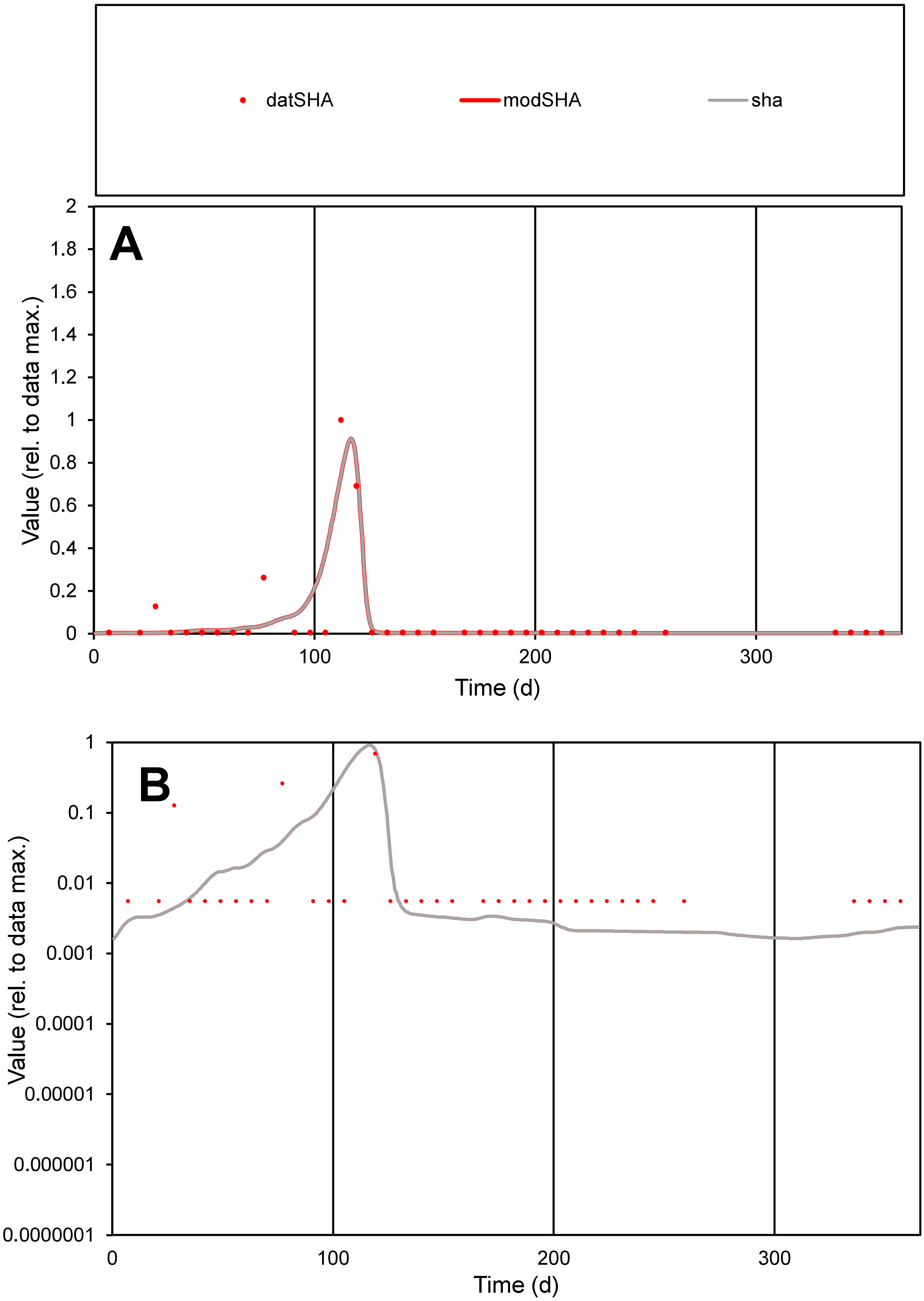

C1

Variables

SHA	Provide jtd or Dst
CalibOnly?	
2	1 to reproduce of
oNormalize?	
2	1=no, 2=yes

ofxn calc*
1.24309
ofxn mod
1.35724

sRun
RunA1
zout
1

Ave
Data
7.6E-06
Model
7.6E-06

data/vMax
0.00012

ofxng
311.734

Min d/m
2.87706

ave/min d
10.9412
ave/min m
31.2424

331_Calib_S_A

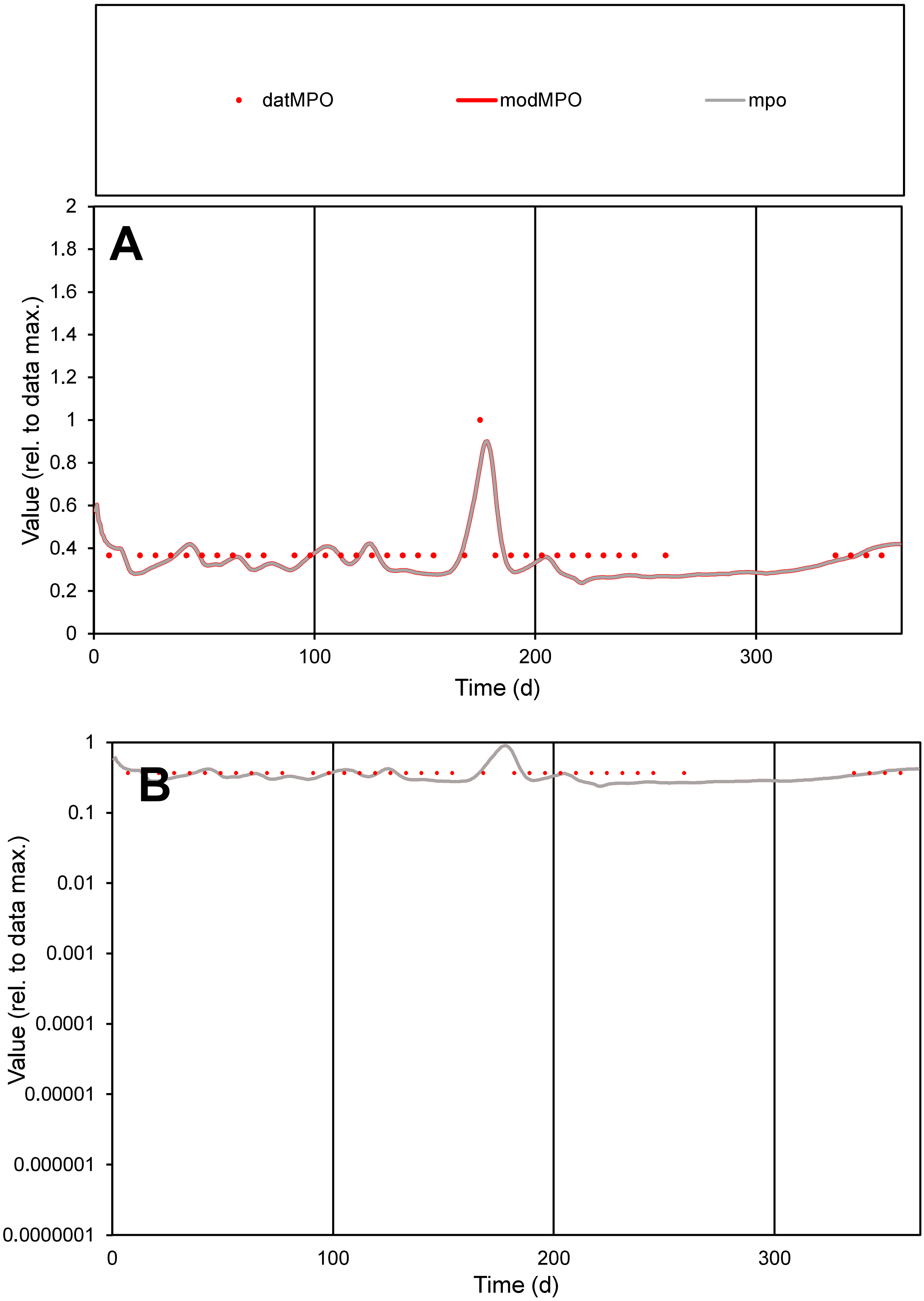

C1	
Variables	
MPO	Provide jtd or Dst
CalibOnly?	
2	1 to reproduce of
oNormalize?	
2	1=no, 2=yes
ofxn calc*	
0.18586	
ofxn mod	
0.21741	
sRun	
RunA1	
zout	
1	
Ave	
Data	
1.3E-05	
Model	
1.2E-05	
data/vMax	
3.4E-05	
ofxng	
311.734	
Min d/m	
1.43154	
ave/min d	
1.04682	
ave/min m	
1.36939	

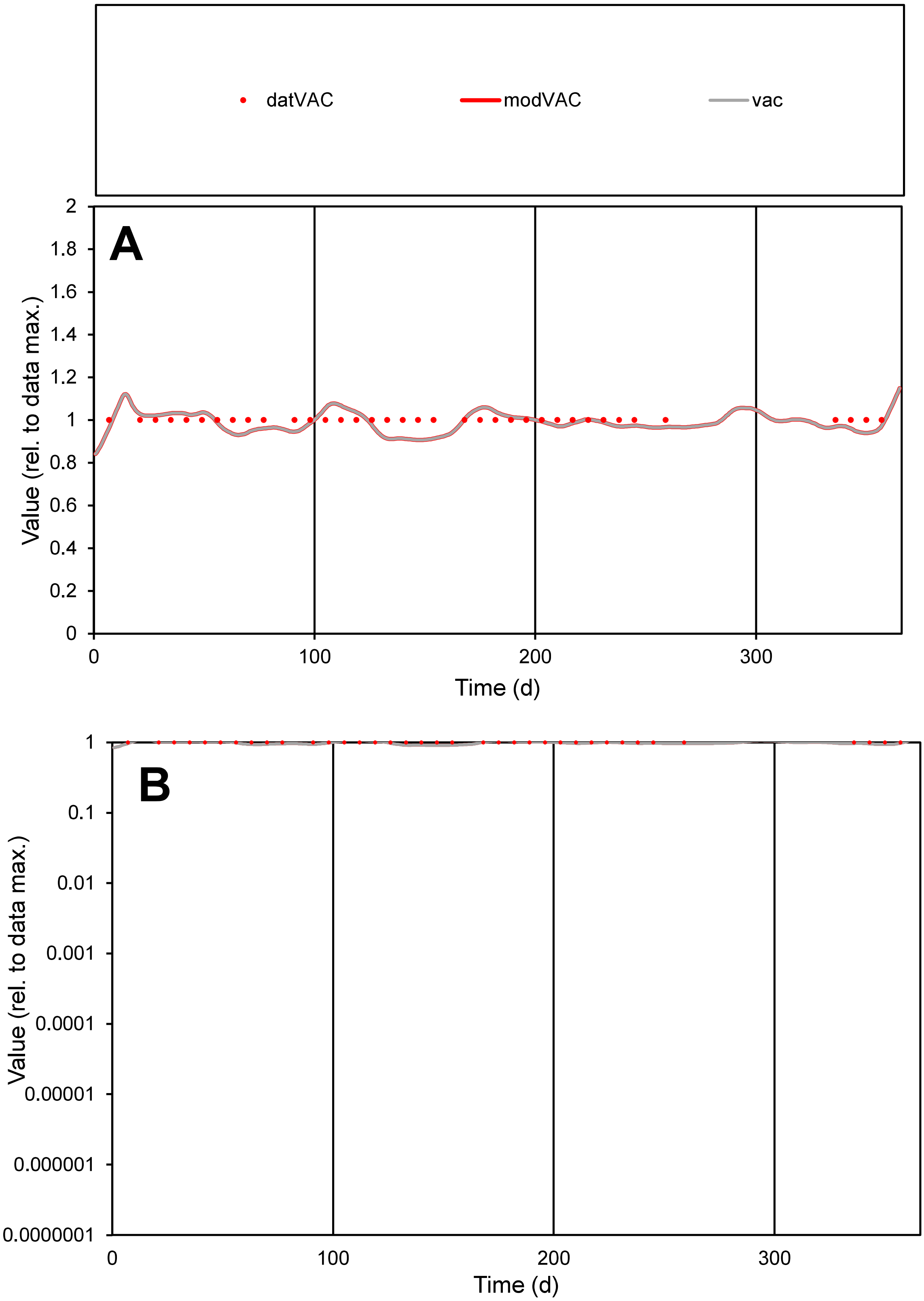

C1

Variables
VAC Provide jtd or Dst
CalibOnly?
2 1 to reproduce of
oNormalize?
2 1=no, 2=yes

ofxn calc*
0.0446
ofxn mod
0.11937

sRun
RunA1
zout
1

Ave
Data
9.5E-06
Model
9.4E-06

data/vMax
9.5E-06

ofxng
311.734

Min d/m
1.10311

ave/min d
1
ave/min m
1.08874

331_Calib_S_A

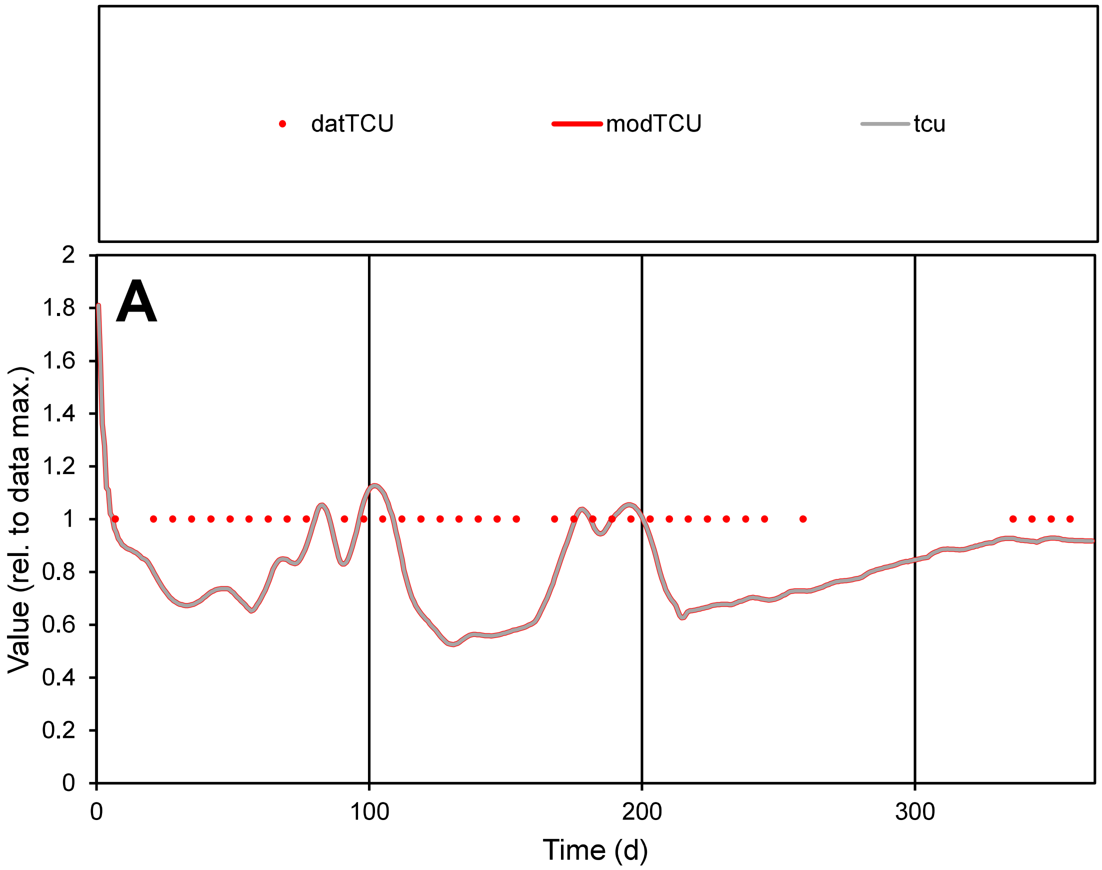

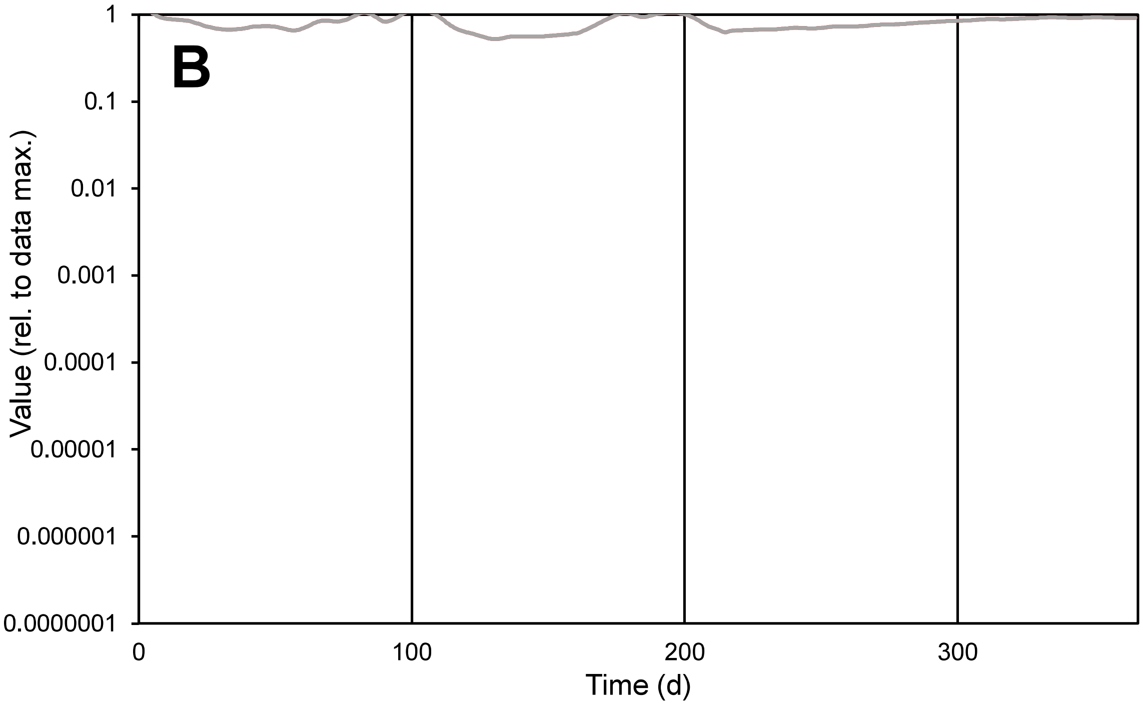

C1

Variables

TCU Provide jtd or Dst

CalibOnly?

2 1 to reproduce of

oNormalize?

2 1=no, 2=yes

ofxn calc*

0.26235

ofxn mod

0.28062

sRun

RunA1

zout

1

Ave

Data

9.8E-06

Model

7.7E-06

data/vMax

9.8E-06

ofxng

311.734

Min d/m

1.83983

ave/min d

1

ave/min m

1.45499

331_Calib_S_A

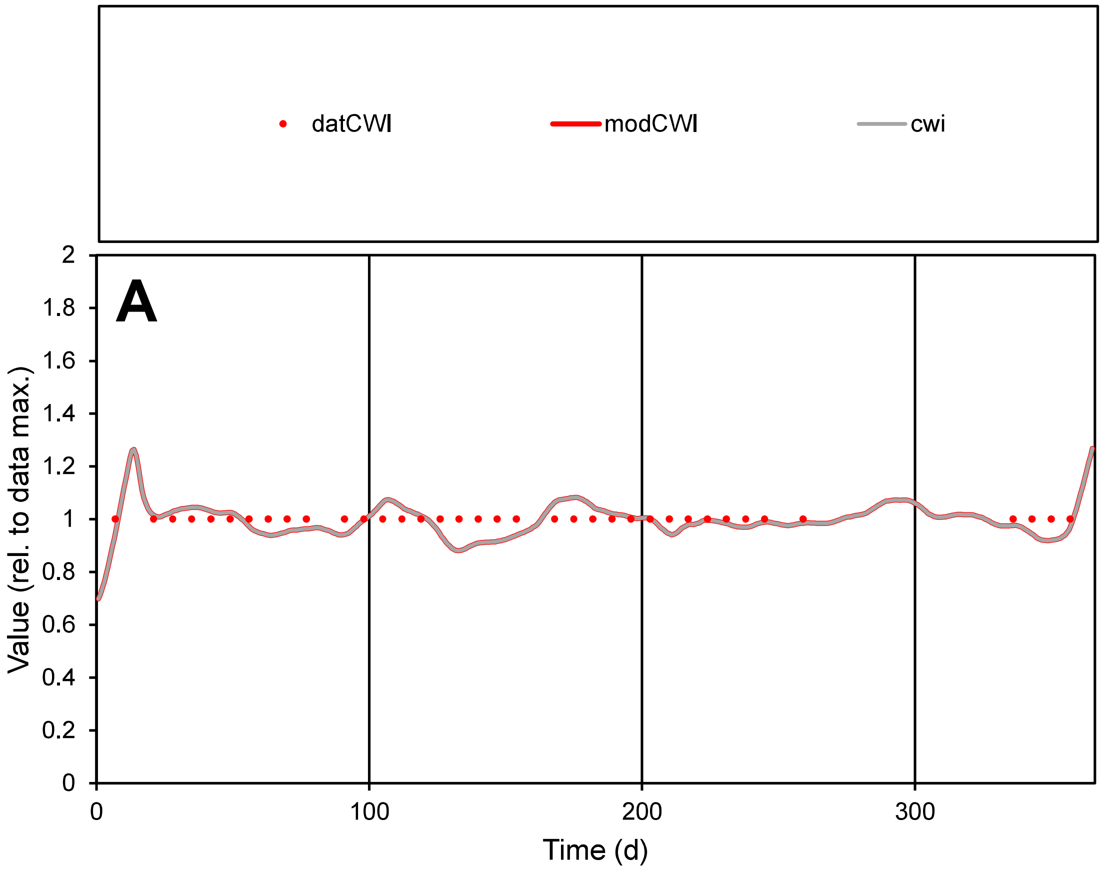

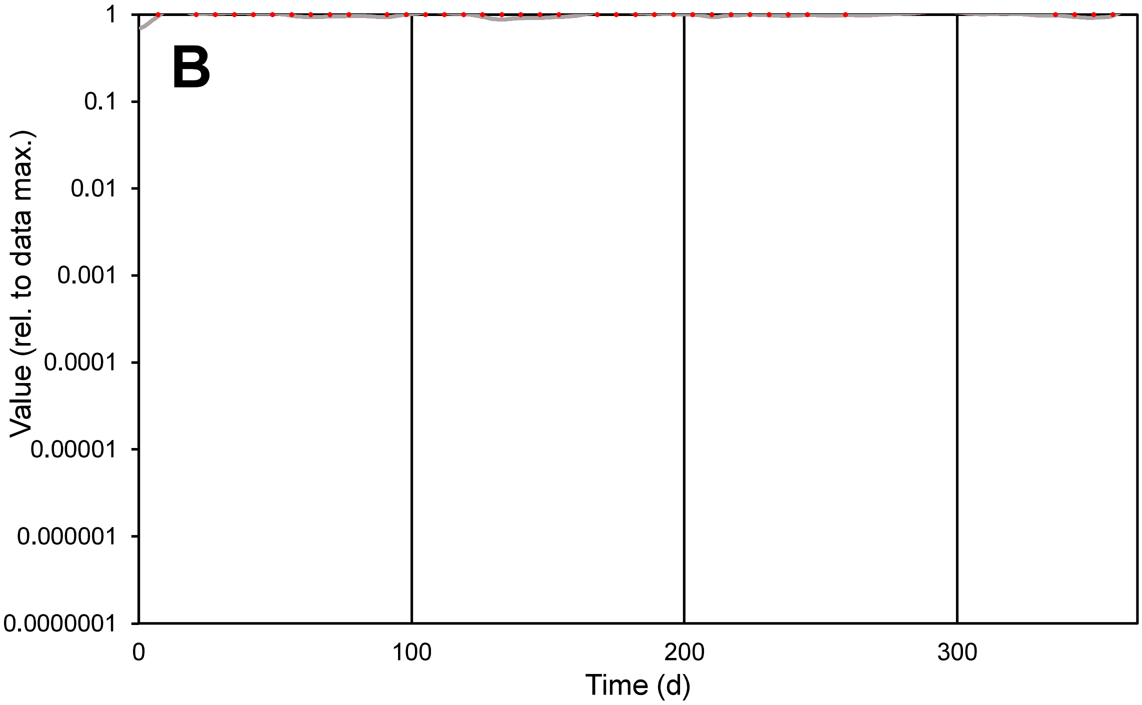

C1

Variables

CWI Provide jtd or Dst

CalibOnly?

2 1 to reproduce of

oNormalize?

2 1=no, 2=yes

ofxn calc*

0.04886

ofxn mod

0.12104

sRun

RunA1

zout

1

Ave

Data

1.3E-05

Model

1.3E-05

data/vMax

1.3E-05

ofxng

311.734

Min d/m

1.1301

ave/min d

1

ave/min m

1.11597

331_Calib_S_A

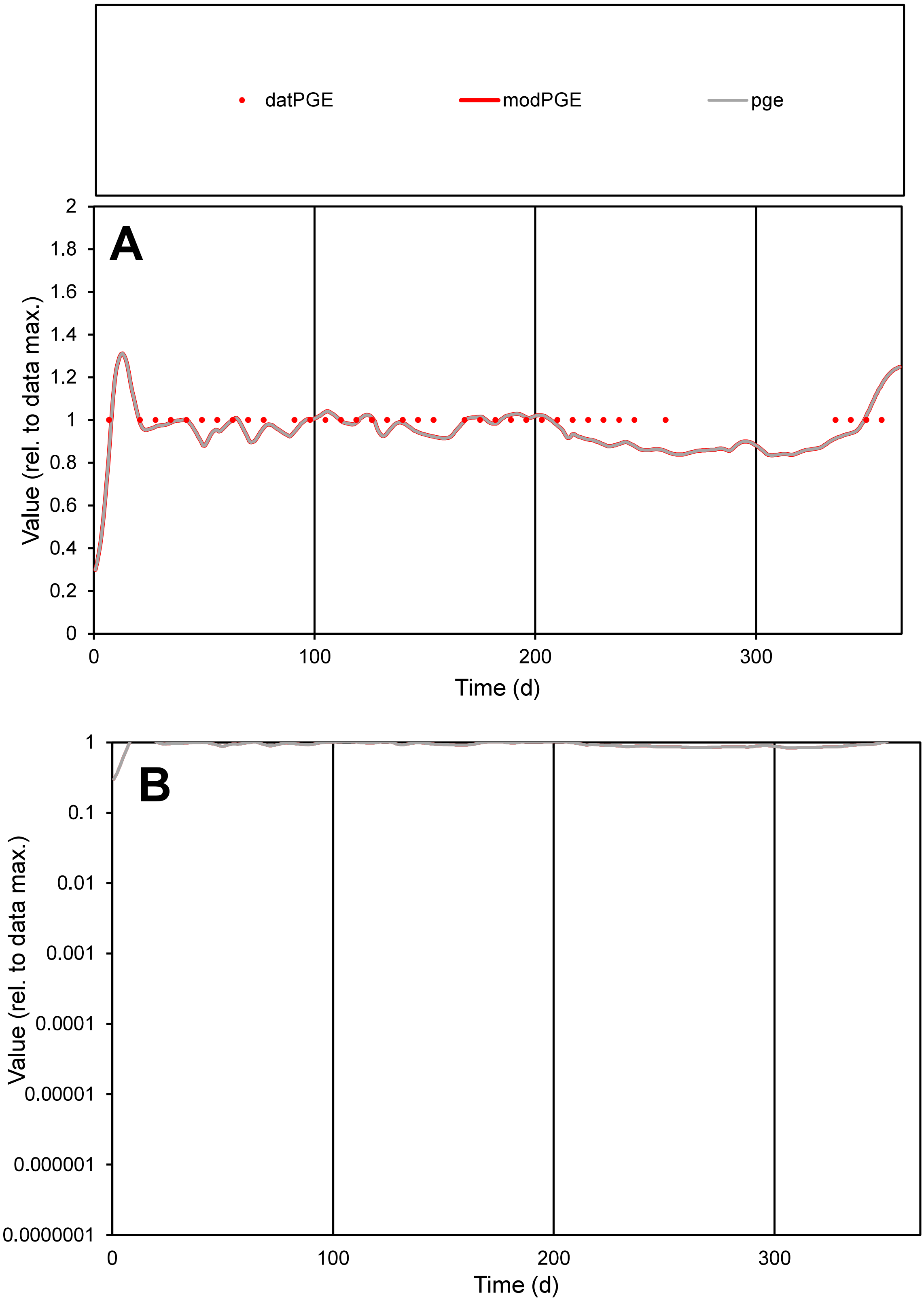

C1

Variables

PGE Provide jtd or Dst

CalibOnly?

2 1 to reproduce of

oNormalize?

2 1=no, 2=yes

ofxn calc*

0.06951

ofxn mod

0.12998

sRun

RunA1

zout

1

Ave

Data

5.9E-06

Model

5.7E-06

data/vMax

5.9E-06

ofxng

311.734

Min d/m

1.17818

ave/min d

1

ave/min m

1.14258

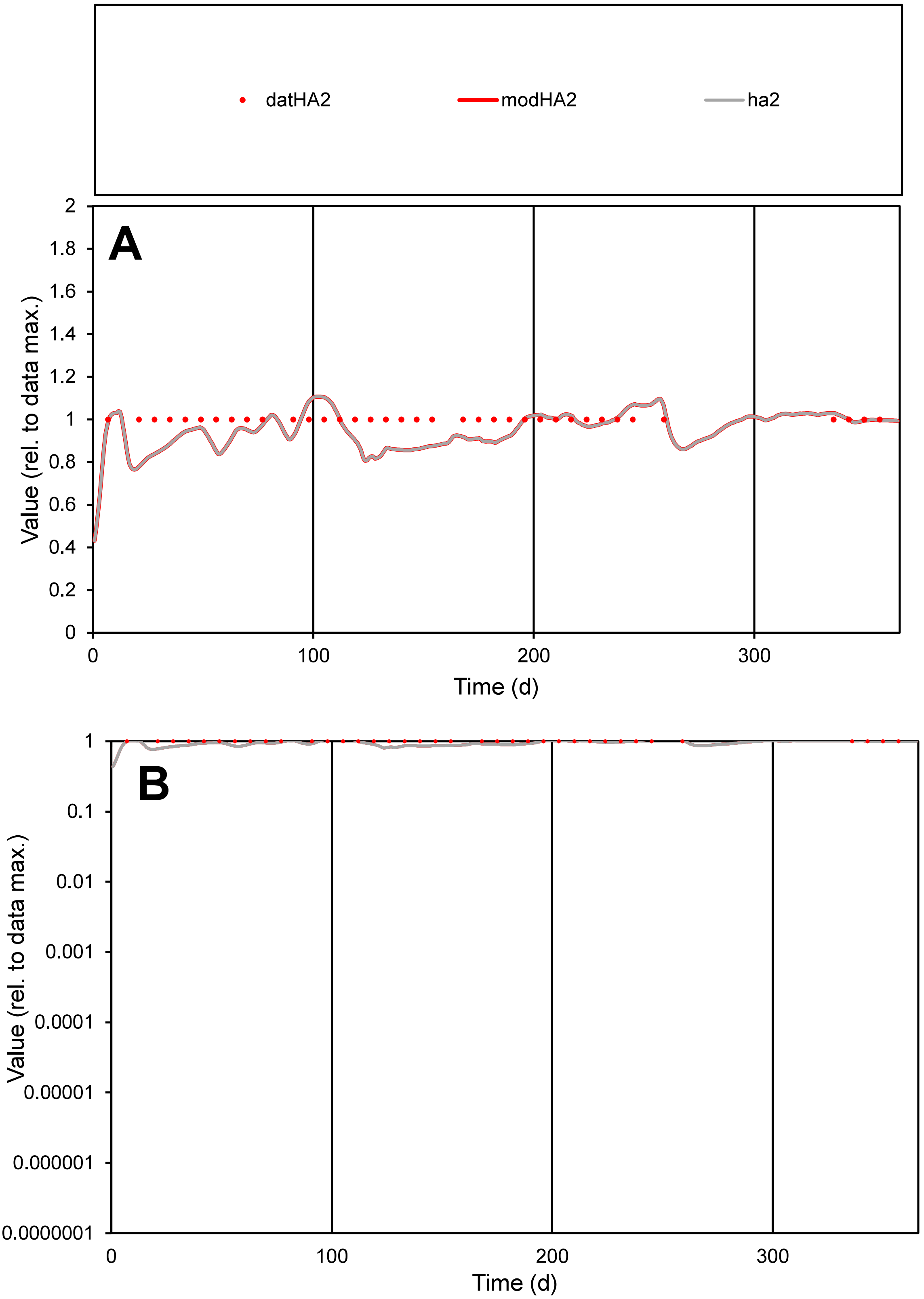

C1

Variables

HA2	Provide jtd or Dst
CalibOnly?	
2	1 to reproduce of
oNormalize?	
2	1=no, 2=yes

ofxn calc*
0.08867
ofxn mod
0.14053

sRun
RunA1
zout
1

Ave
Data
2.6E-06
Model
2.5E-06

data/vMax
2.6E-06

ofxng
311.734

Min d/m
1.2569

ave/min d
1
ave/min m
1.19611

331_Calib_S_A

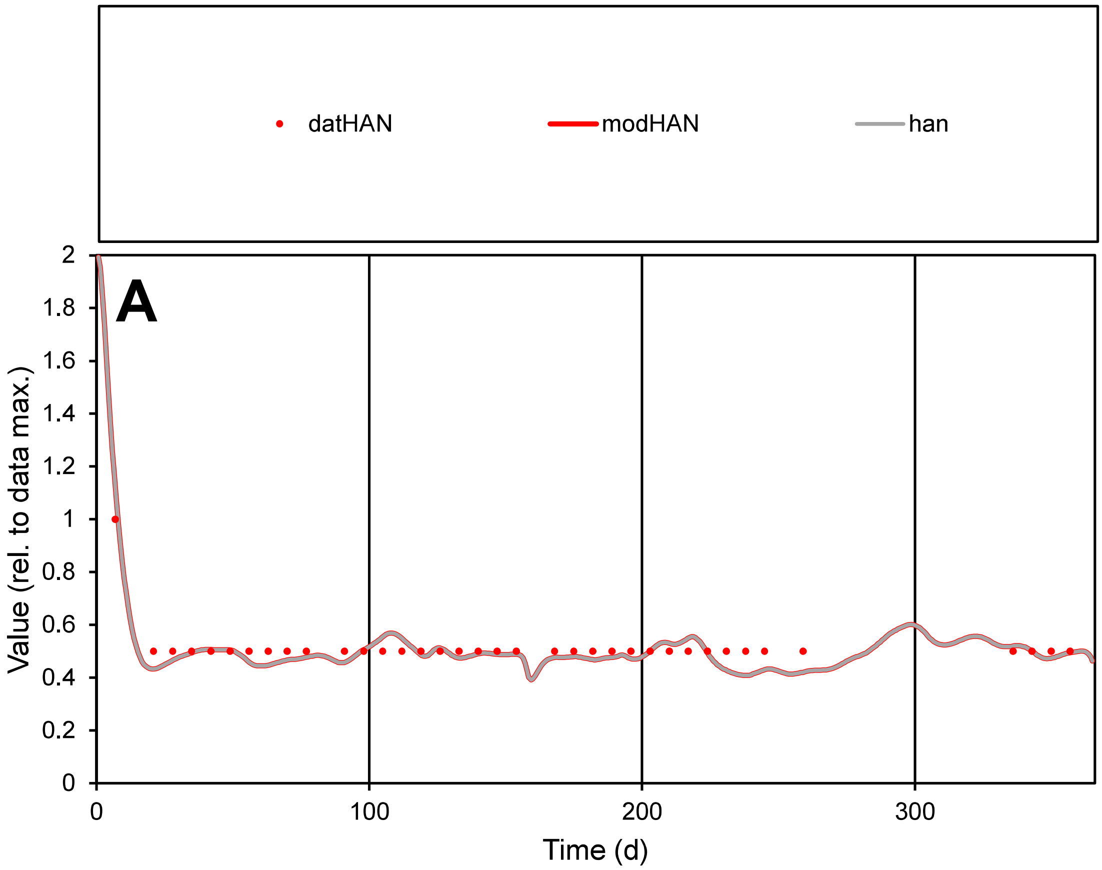

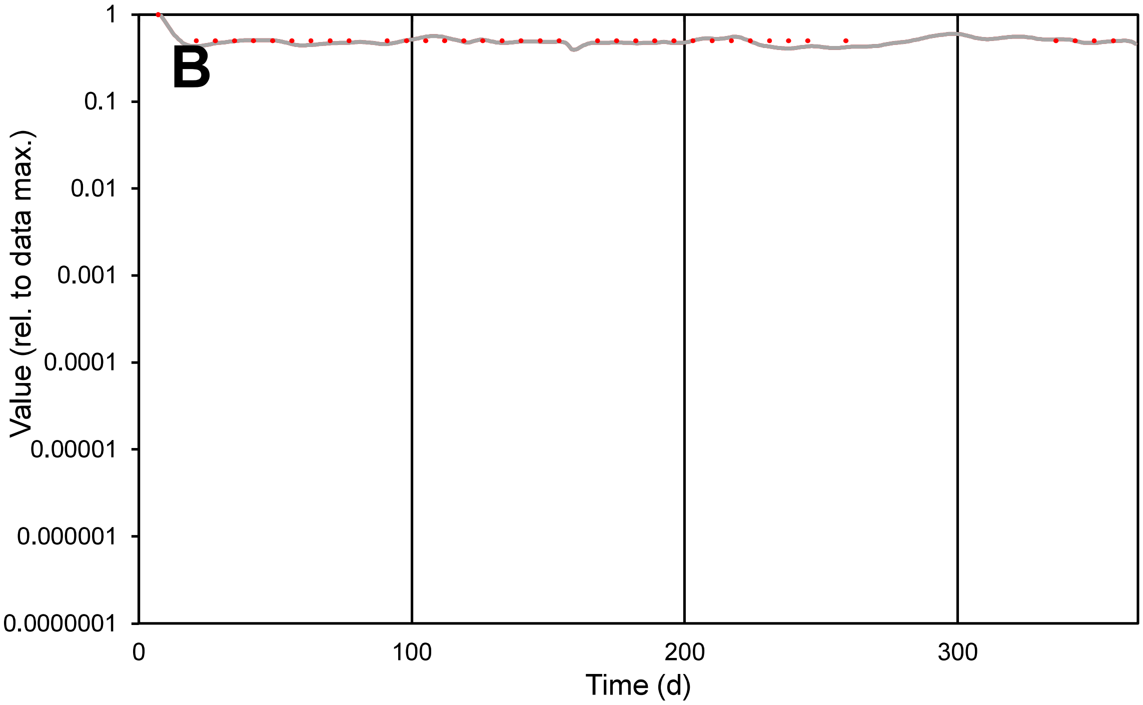

C1

Variables
HAN Provide jtd or Dst
CalibOnly?
2 1 to reproduce of
oNormalize?
2 1=no, 2=yes

ofxn calc*
0.07349
ofxn mod
0.13035

sRun
RunA1
zout
1

Ave
Data
5.3E-06
Model
5.1E-06

data/vMax
1E-05

ofxng
311.734

Min d/m
1.22087

ave/min d
1.02704
ave/min m
1.21371

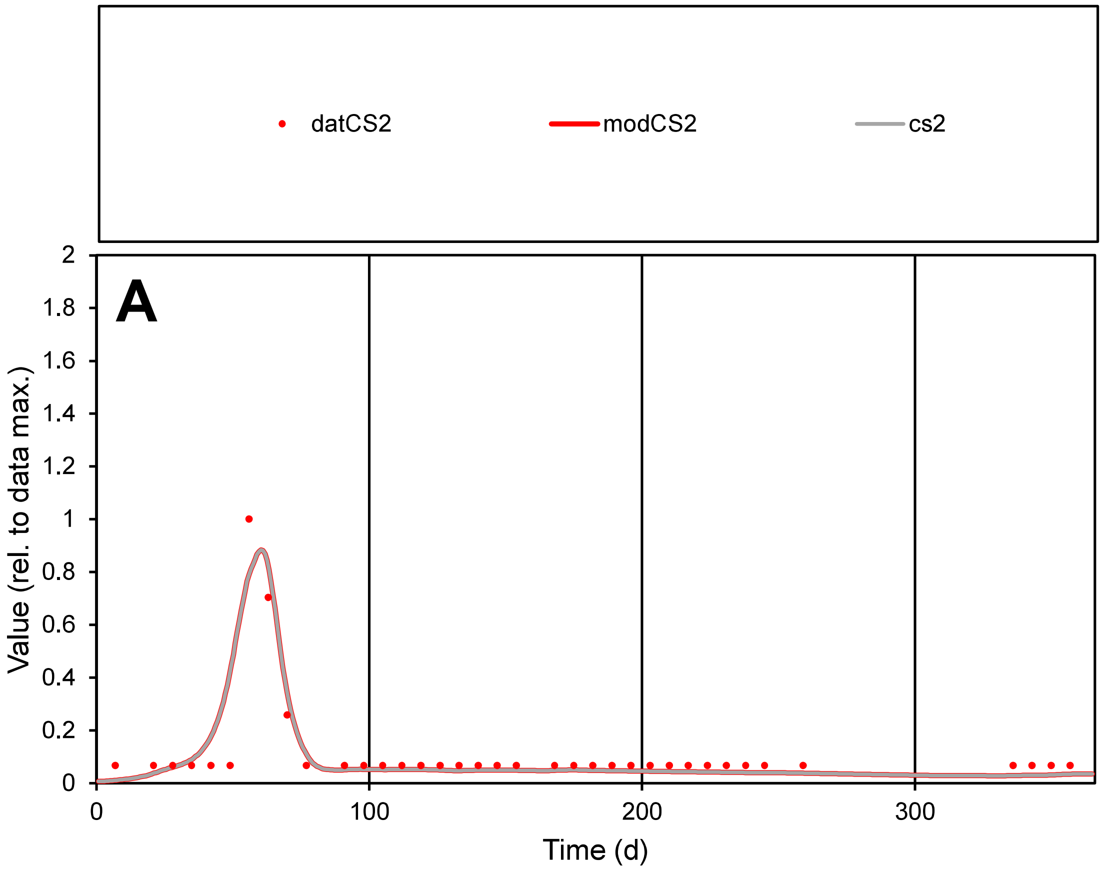

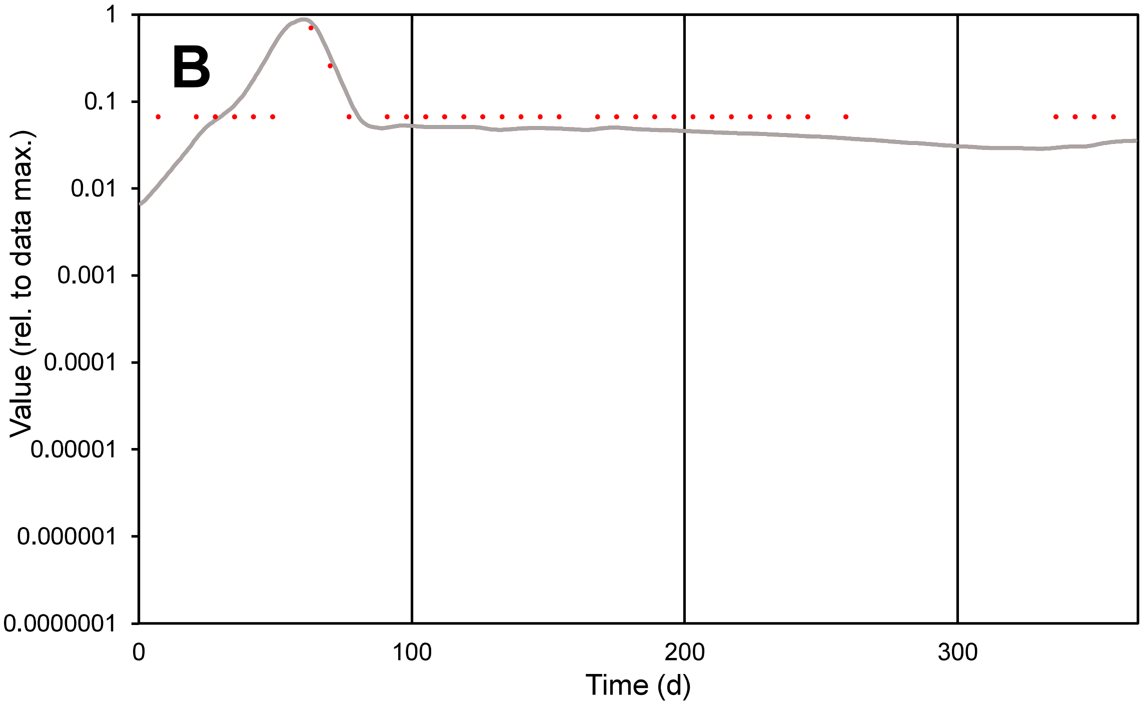

C1

Variables
CS2 Provide jtd or Dst
CalibOnly?
2 1 to reproduce of
oNormalize?
2 1=no, 2=yes

ofxn calc*
0.52237
ofxn mod
0.66208

sRun
RunA1
zout
1

Ave
Data
1.3E-05
Model
1.2E-05

data/vMax
0.00011

ofxng
311.734

Min d/m
5.68066

ave/min d
1.71297
ave/min m
8.81523

331_Calib_S_A

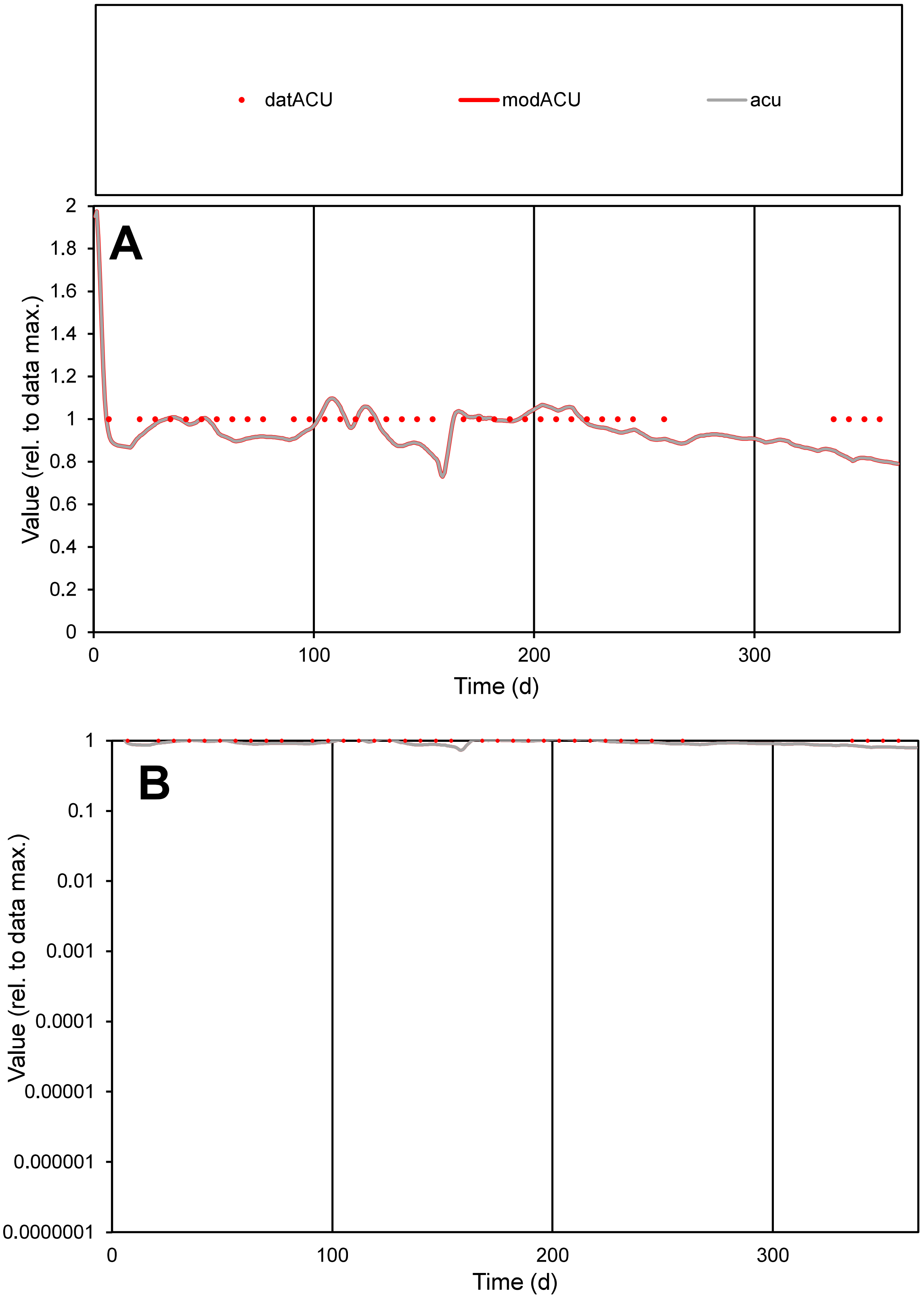

C1

Variables
ACU Provide jtd or Dst
CalibOnly?
2 1 to reproduce of
oNormalize?
2 1=no, 2=yes

ofxn calc*
0.08937
ofxn mod
0.1409

sRun
RunA1
zout
1

Ave
Data
1.1E-05
Model
1.1E-05

data/vMax
1.1E-05

ofxng
311.734

Min d/m
1.24705

ave/min d
1
ave/min m
1.18462

331_Calib_S_A

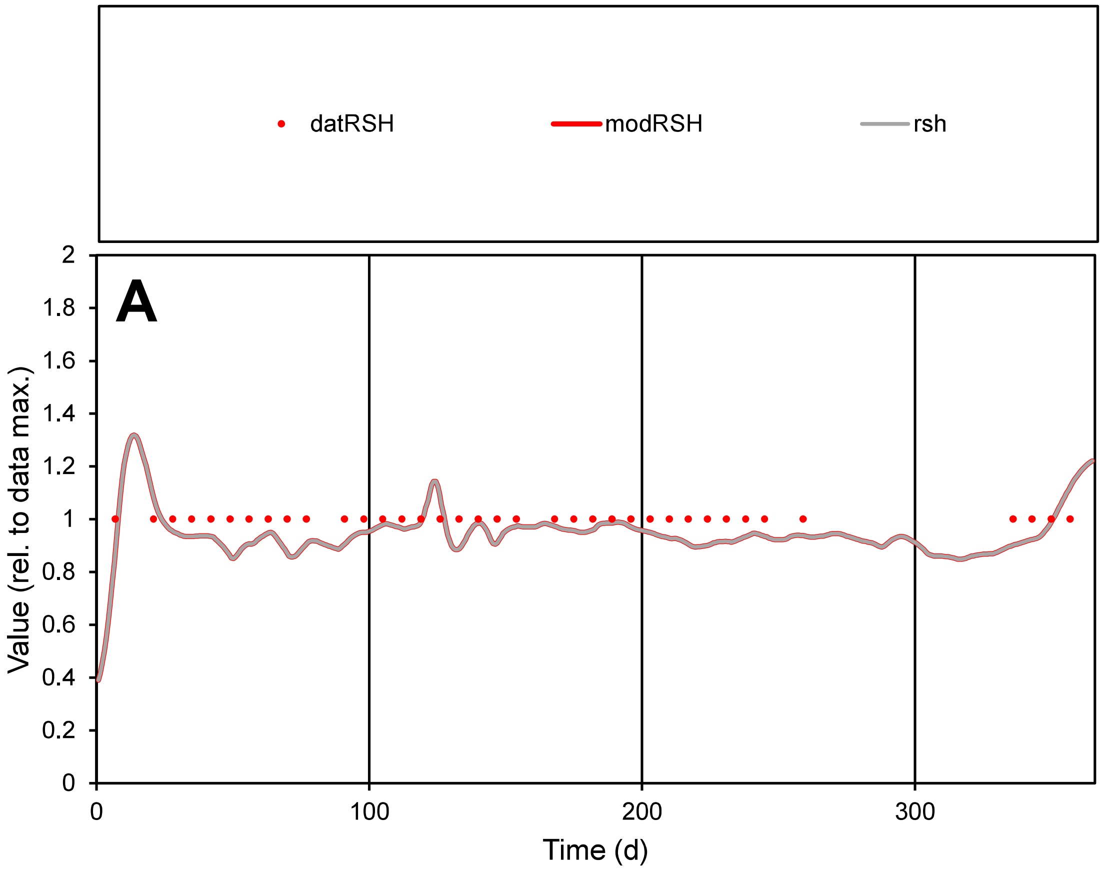

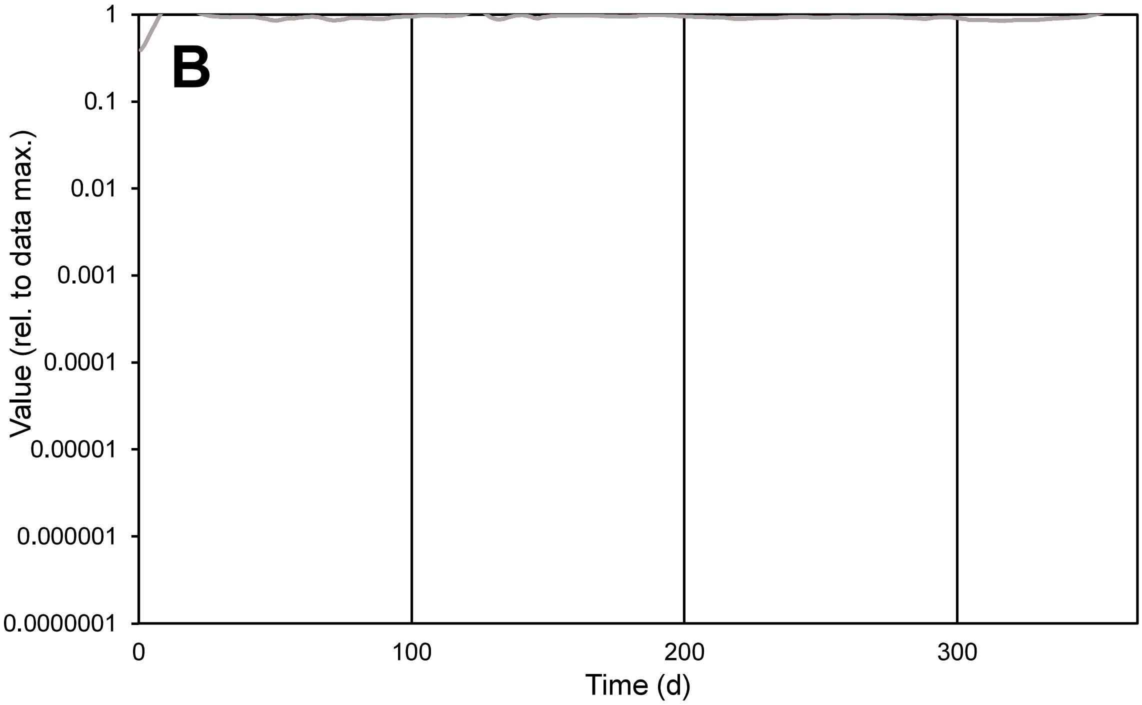

C1

Variables
RSH Provide jtd or Dst
CalibOnly?
2 1 to reproduce of
oNormalize?
2 1=no, 2=yes

ofxn calc*
0.0715
ofxn mod
0.13025

sRun
RunA1
zout
1

Ave
Data
1.8E-05
Model
1.7E-05

data/vMax
1.8E-05

ofxng
311.734

Min d/m
1.17423

ave/min d
1
ave/min m
1.11735

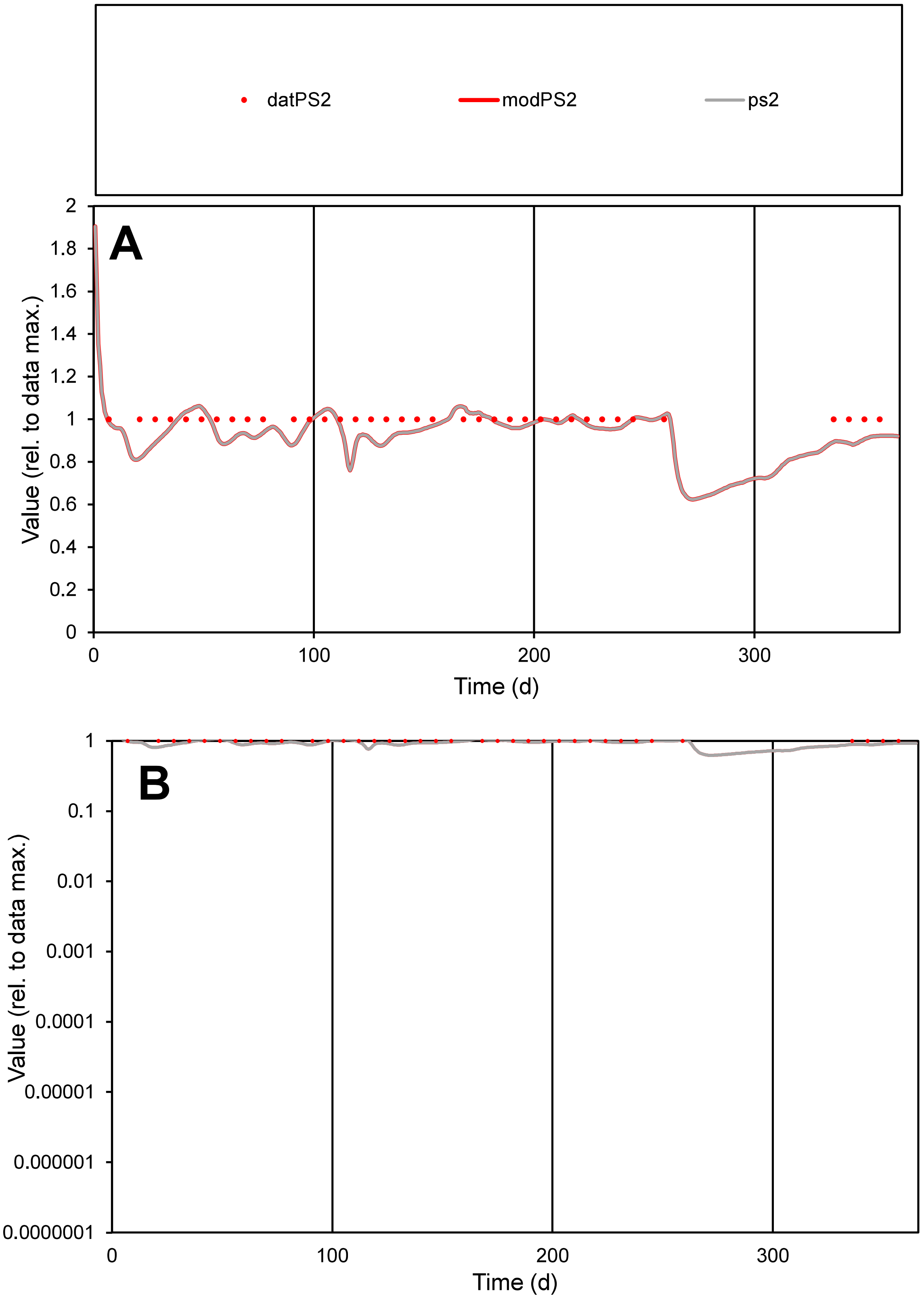

C1

Variables
PS2 Provide jtd or Dst
CalibOnly?
2 1 to reproduce of
oNormalize?
2 1=no, 2=yes

ofxn calc*
0.0682
ofxn mod
0.12889

sRun
RunA1
zout
1

Ave
Data
1.9E-05
Model
1.8E-05

data/vMax
1.9E-05

ofxng
311.734

Min d/m
1.20493

ave/min d
1
ave/min m
1.15327

331_Calib_S_A

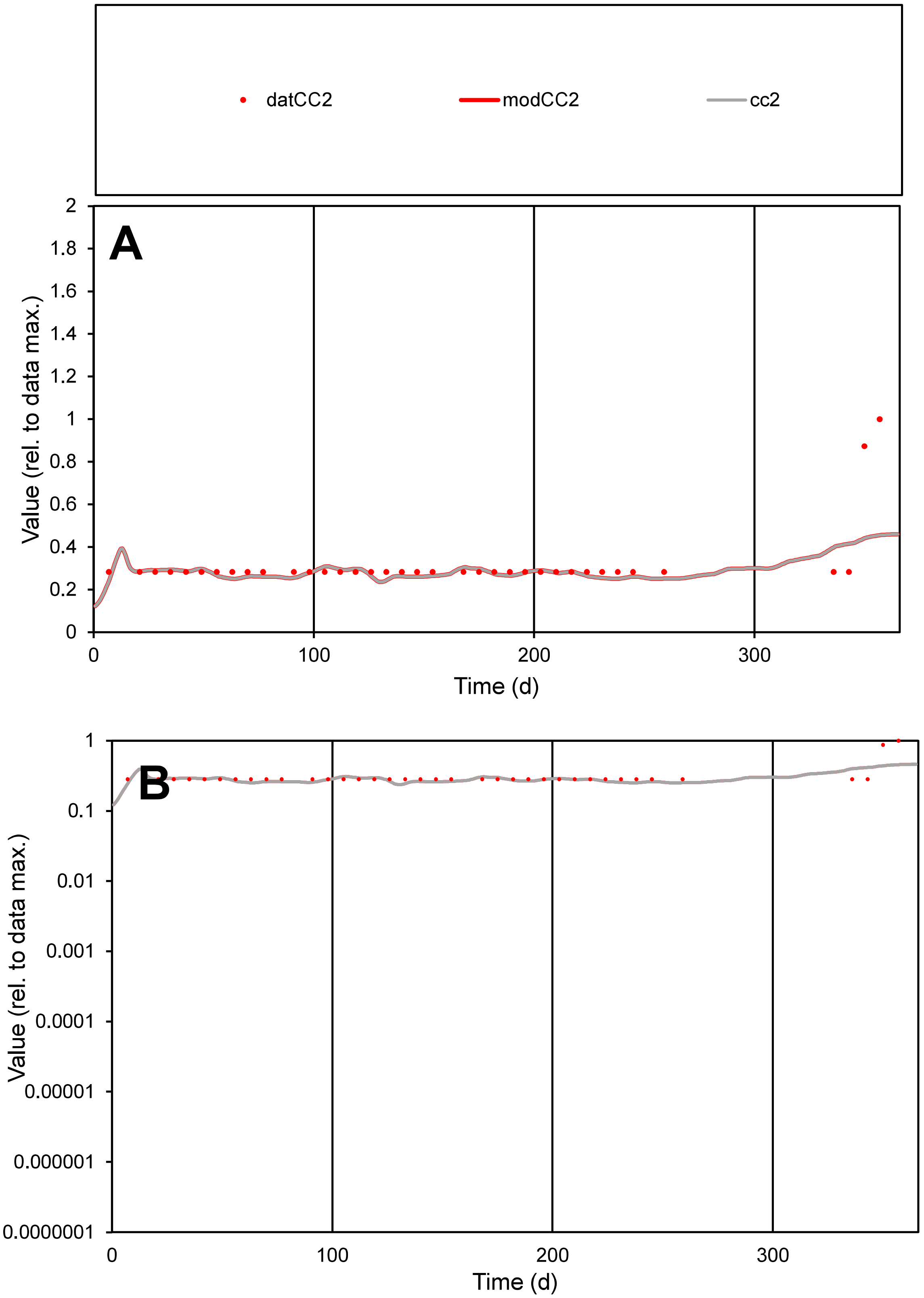

C1

Variables
CC2 Provide jtd or Dst
CalibOnly?
2 1 to reproduce of
oNormalize?
2 1=no, 2=yes

ofxn calc*
0.37398
ofxn mod
0.39002

sRun
RunA1
zout
1

Ave
Data
6.6E-06
Model
6E-06

data/vMax
2.1E-05

ofxng
311.734

Min d/m
1.12625

ave/min d
1.12496
ave/min m
1.16059

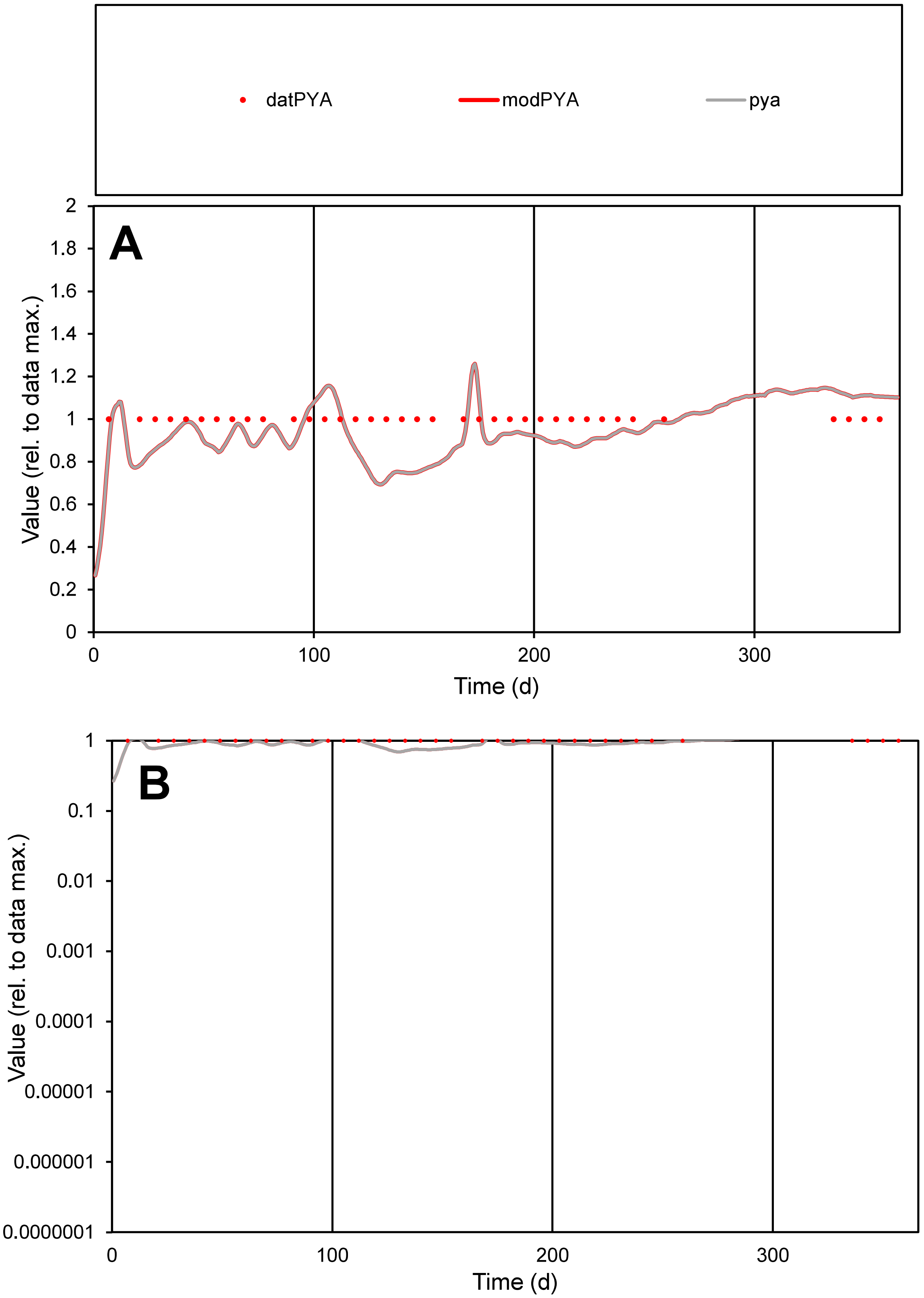

C1

Variables
PYA Provide jtd or Dst
CalibOnly?
2 1 to reproduce of
oNormalize?
2 1=no, 2=yes

ofxn calc*
0.13059
ofxn mod
0.16897

sRun
RunA1
zout
1

Ave
Data
1.8E-06
Model
1.7E-06

data/vMax
1.8E-06

ofxng
311.734

Min d/m
1.40031

ave/min d
1
ave/min m
1.30079

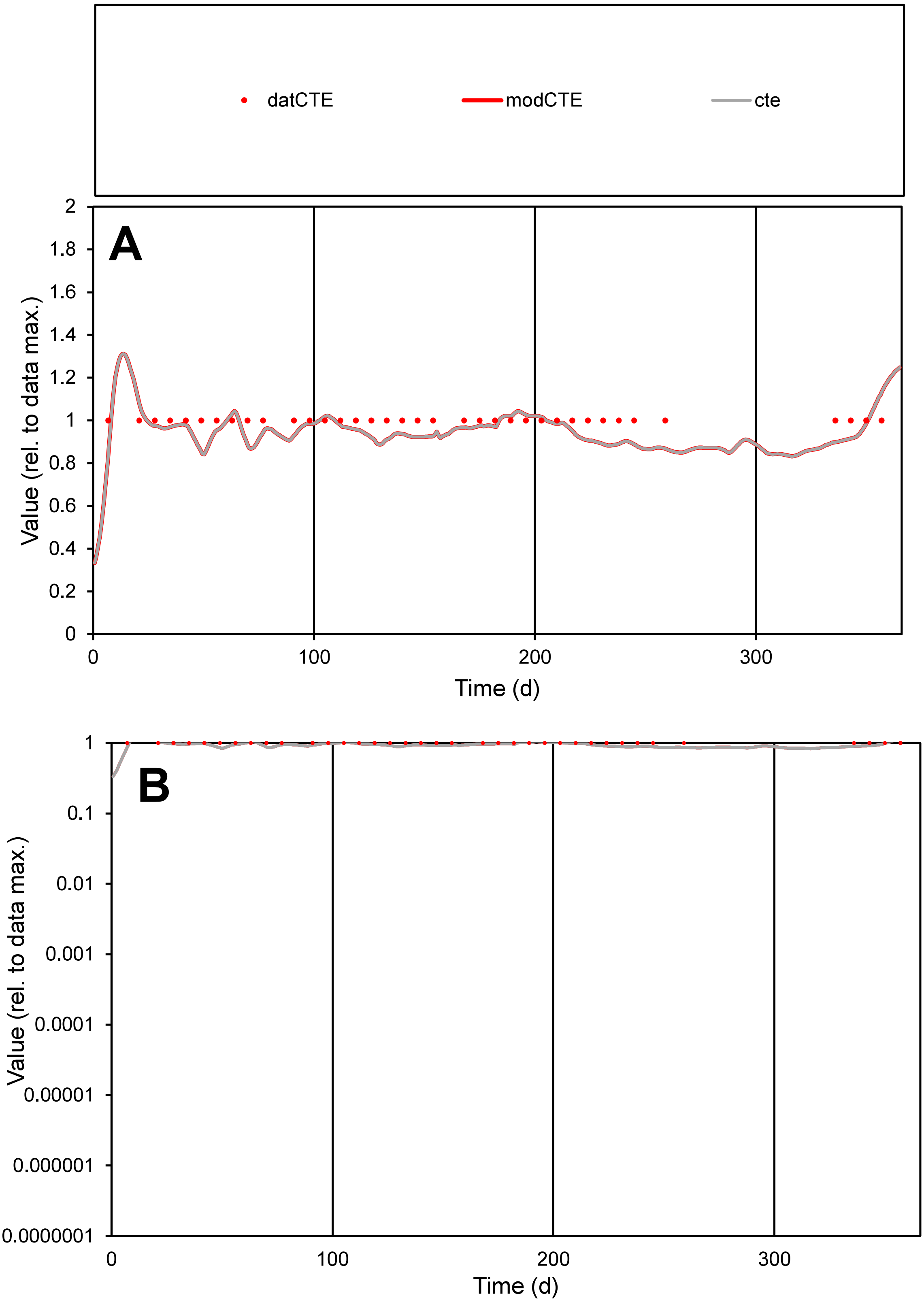

C1

Variables
CTE Provide jtd or Dst
CalibOnly?
2 1 to reproduce of
oNormalize?
2 1=no, 2=yes

ofxn calc*
0.07414
ofxn mod
0.1322

sRun
RunA1
zout
1

Ave
Data
6.1E-06
Model
5.9E-06

data/vMax
6.1E-06

ofxng
311.734

Min d/m
1.18744

ave/min d
1
ave/min m
1.1372

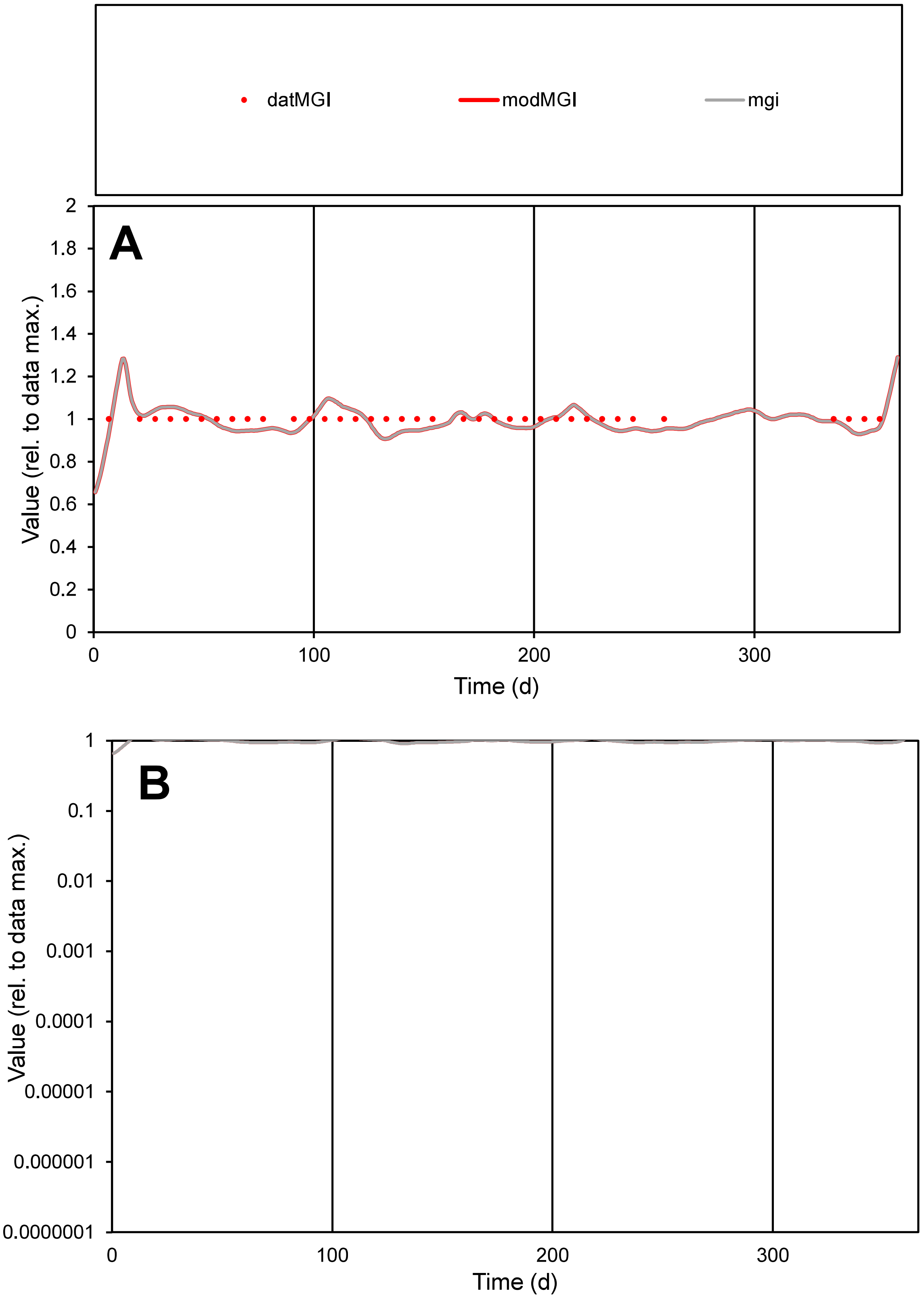

C1

Variables
MGI Provide jtd or Dst
CalibOnly?
2 1 to reproduce of
oNormalize?
2 1=no, 2=yes

ofxn calc*
0.04487
ofxn mod
0.11965

sRun
RunA1
zout
1

Ave
Data
1E-05
Model
1E-05

data/vMax
1E-05

ofxng
311.734

Min d/m
1.09589

ave/min d
1
ave/min m
1.08285

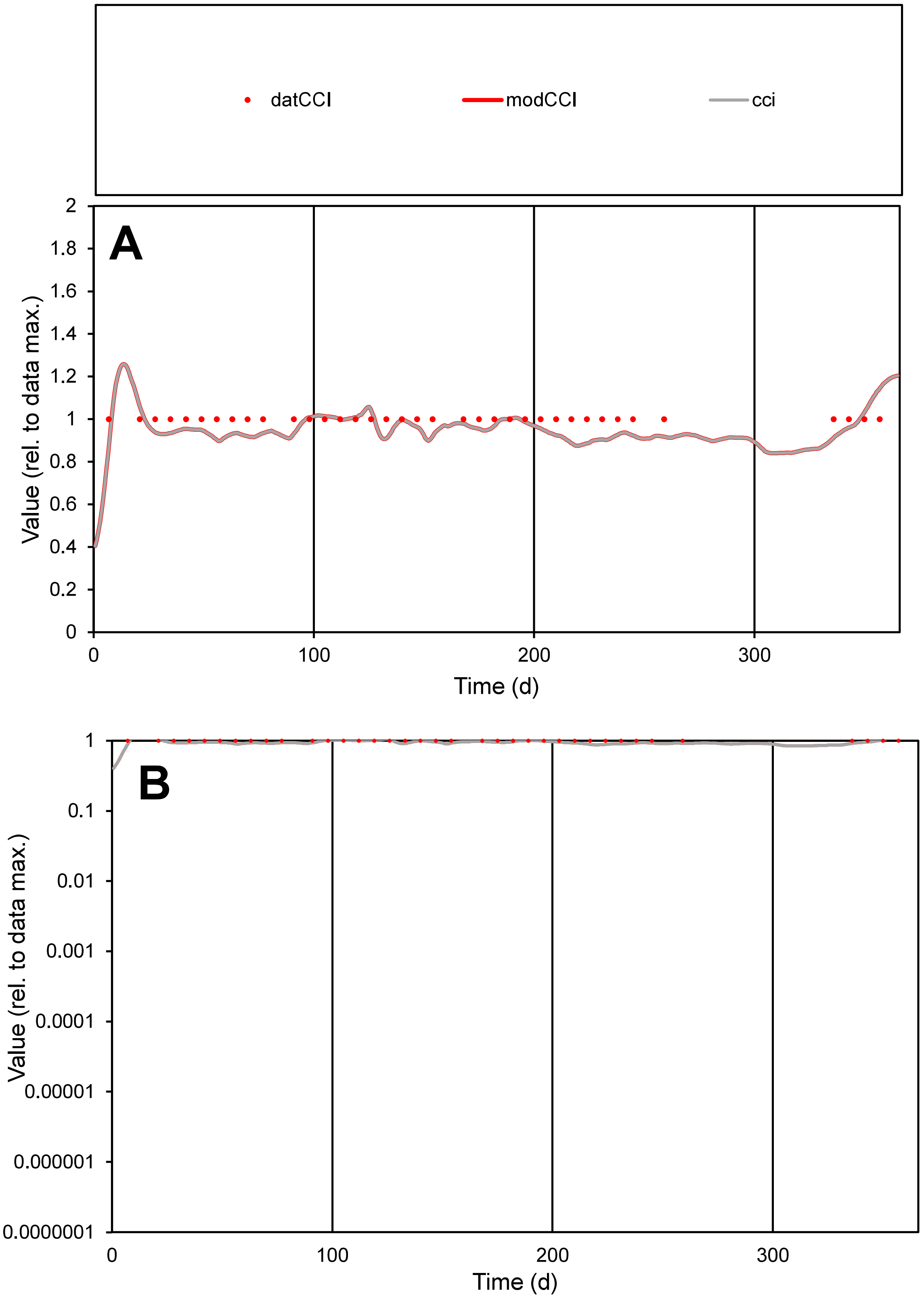

C1

Variables
CCI Provide jtd or Dst
CalibOnly?
2 1 to reproduce of
oNormalize?
2 1=no, 2=yes

ofxn calc*
0.06537
ofxn mod
0.12721

sRun
RunA1
zout
1

Ave
Data
1.1E-05
Model
1.1E-05

data/vMax
1.1E-05

ofxng
311.734

Min d/m
1.13776

ave/min d
1
ave/min m
1.09405

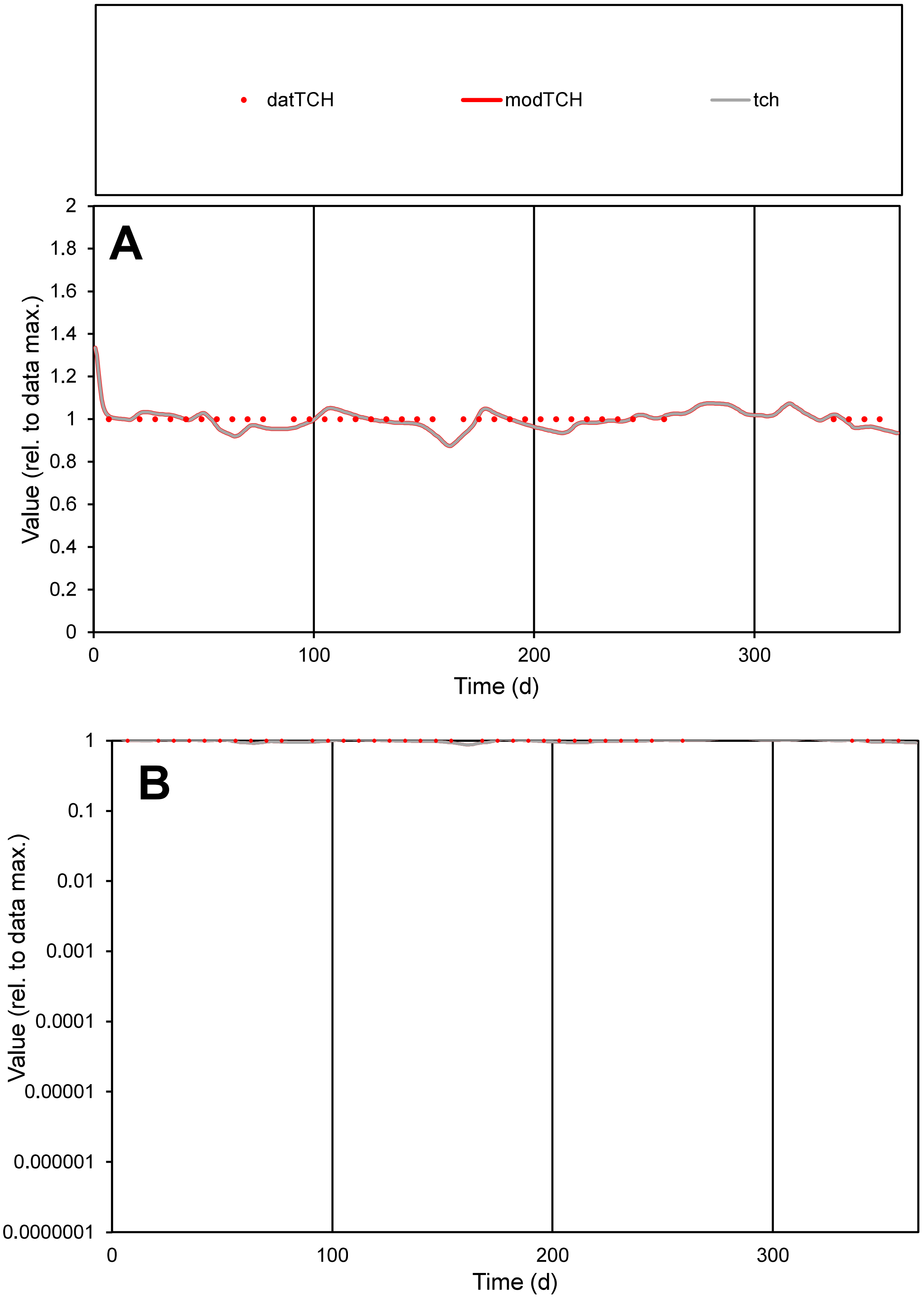

C1

Variables
TCH Provide jtd or Dst
CalibOnly?
2 1 to reproduce of
oNormalize?
2 1=no, 2=yes

ofxn calc*
0.03543
ofxn mod
0.11628

sRun
RunA1
zout
1

Ave
Data
1.7E-06
Model
1.7E-06

data/vMax
1.7E-06

ofxng
311.734

Min d/m
1.08727

ave/min d
1
ave/min m
1.07434

331_Calib_S_A

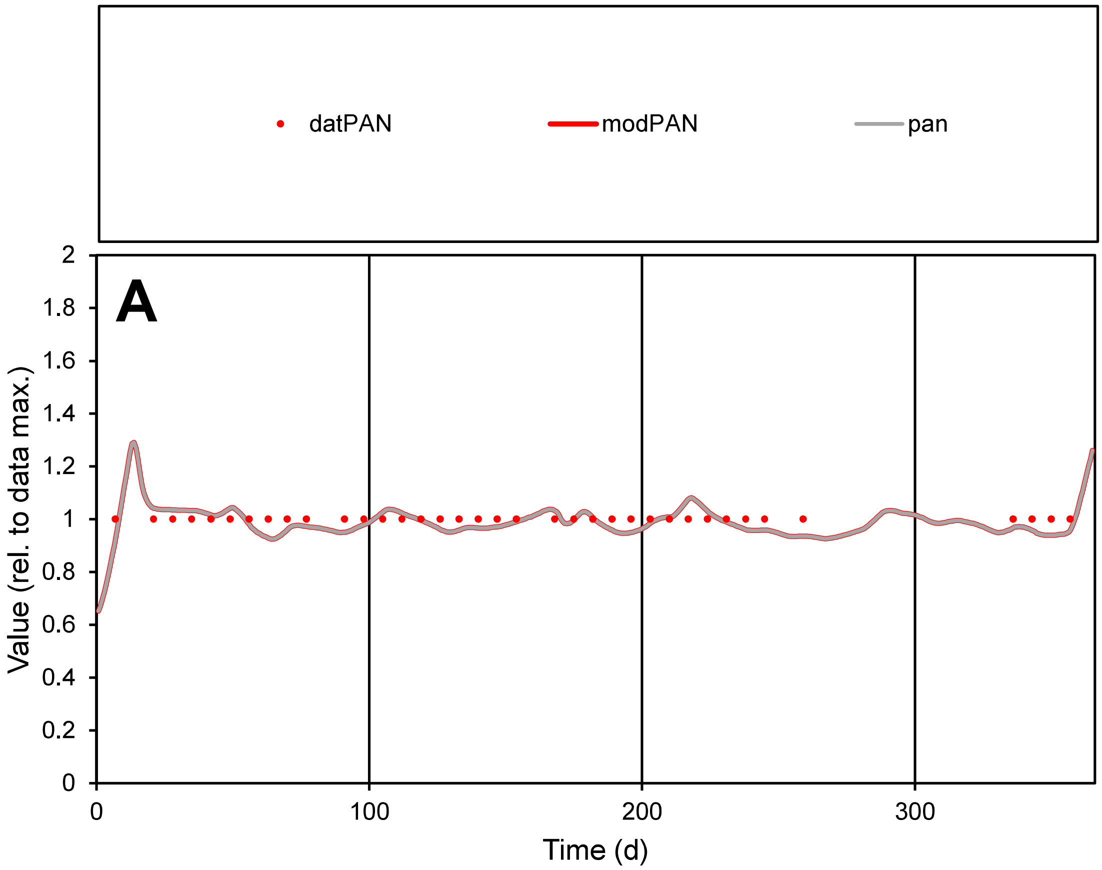

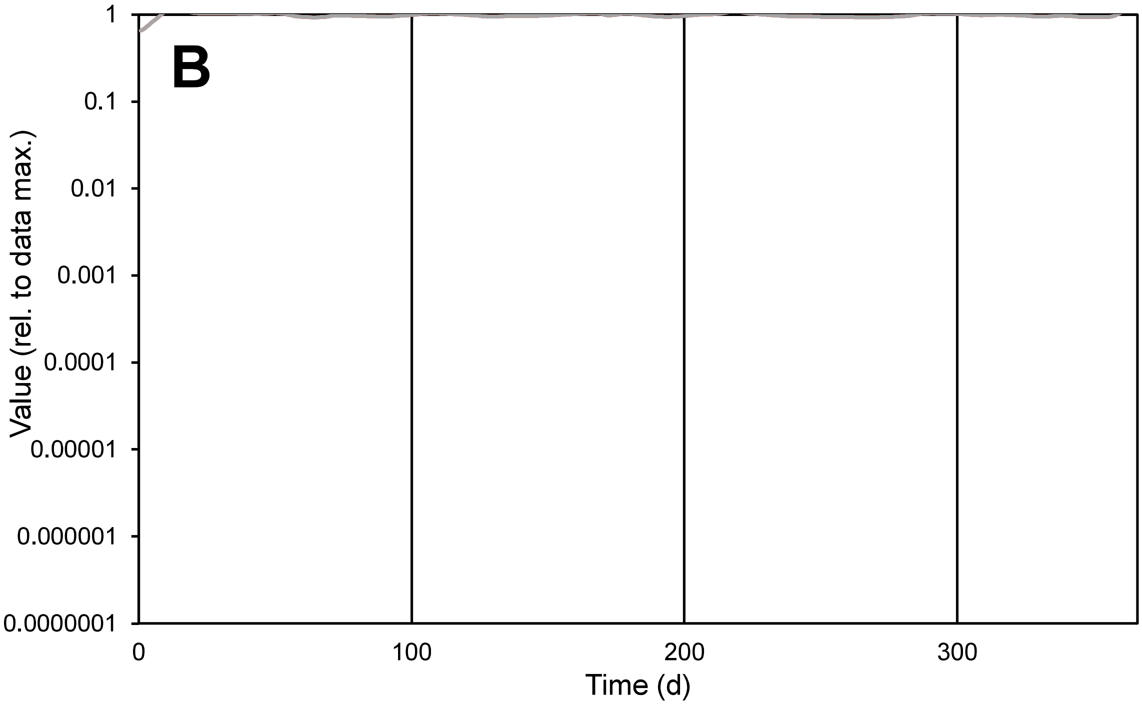

C1

Variables
PAN Provide jtd or Dst
CalibOnly?
2 1 to reproduce of
oNormalize?
2 1=no, 2=yes

ofxn calc*
0.03677
ofxn mod
0.11693

sRun
RunA1
zout
1

Ave
Data
9.1E-06
Model
9E-06

data/vMax
9.1E-06

ofxng
311.734

Min d/m
1.08044

ave/min d
1
ave/min m
1.06765

331_Calib_S_A

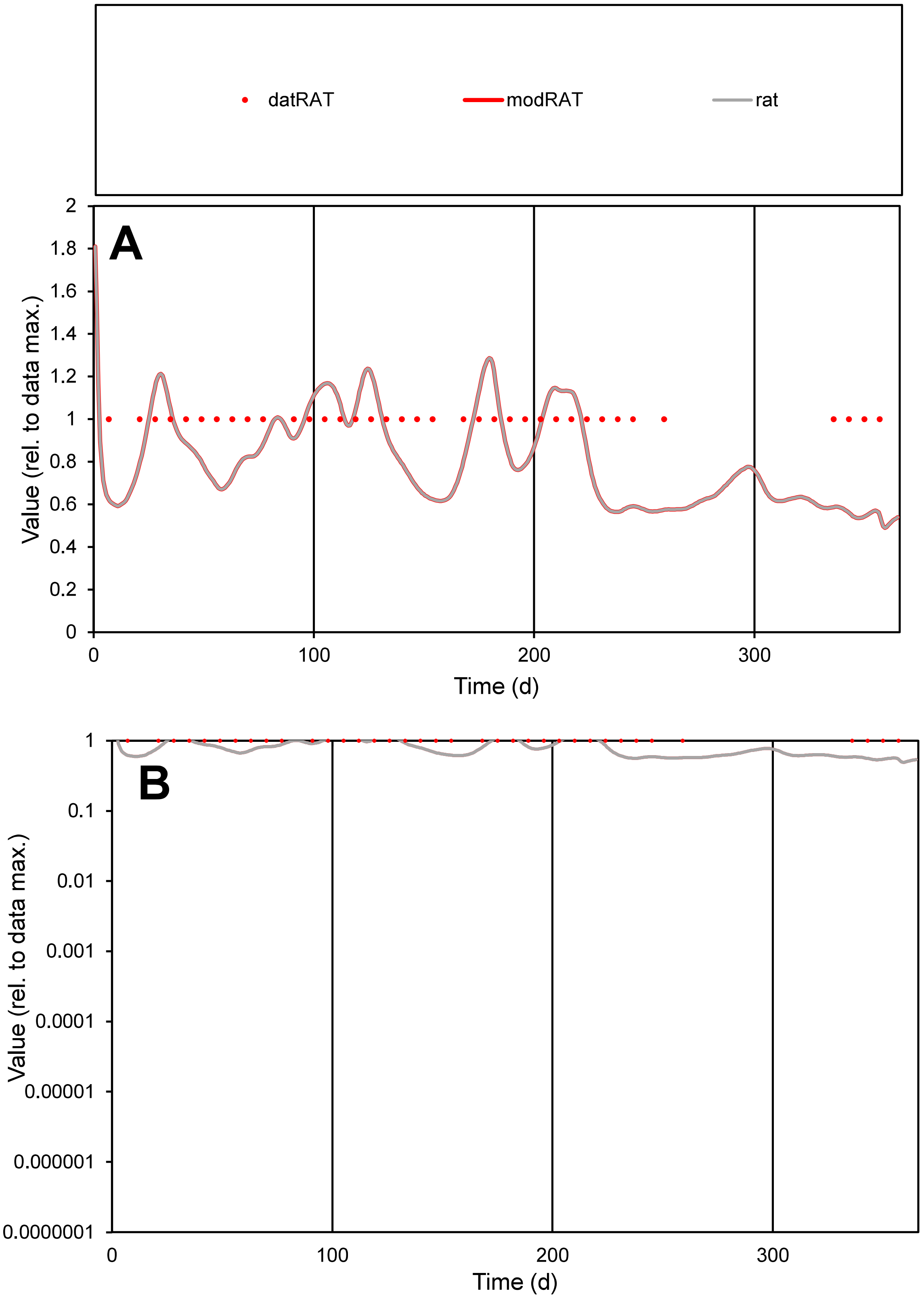

C1

Variables

RAT Provide jtd or Dst

CalibOnly?

2 1 to reproduce of

oNormalize?

2 1=no, 2=yes

ofxn calc*

0.26391

ofxn mod

0.28459

sRun

RunA1

zout

1

Ave

Data

1.1E-05

Model

9.6E-06

data/vMax

1.1E-05

ofxng

311.734

Min d/m

1.83594

ave/min d

1

ave/min m

1.55351

331_Calib_S_A

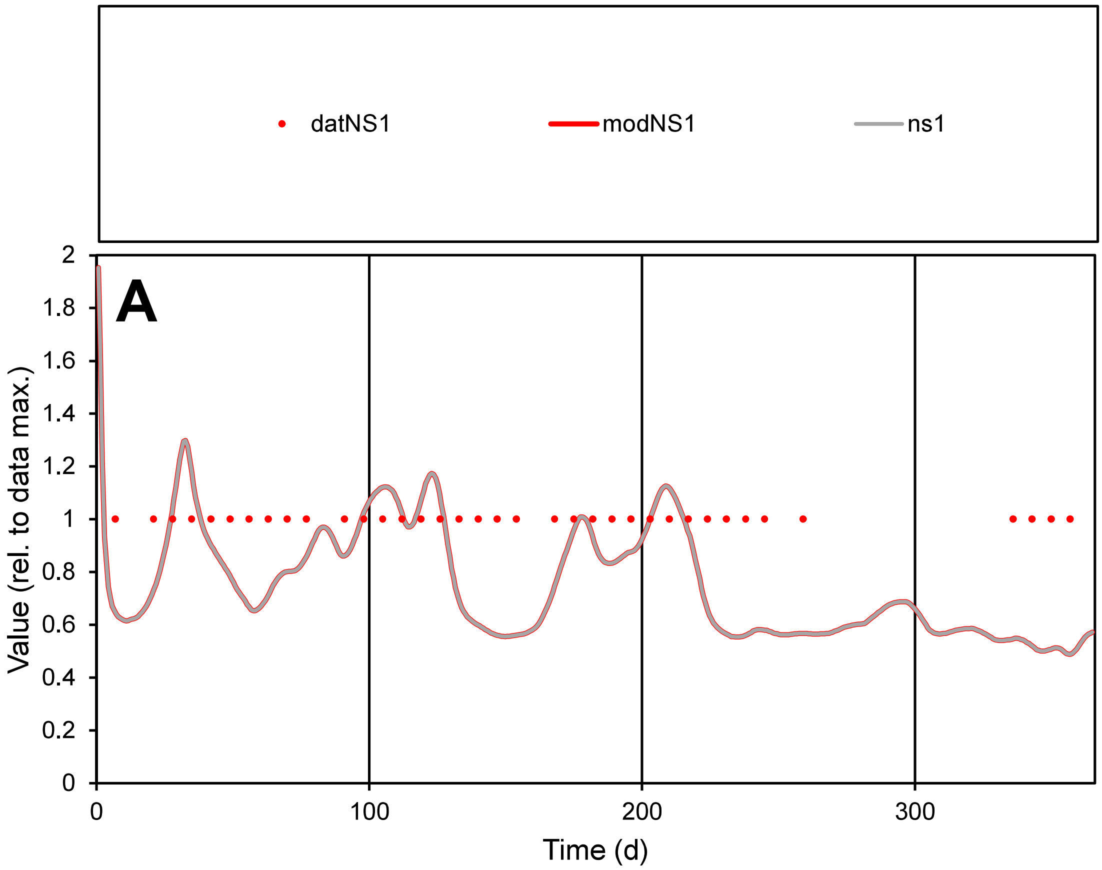

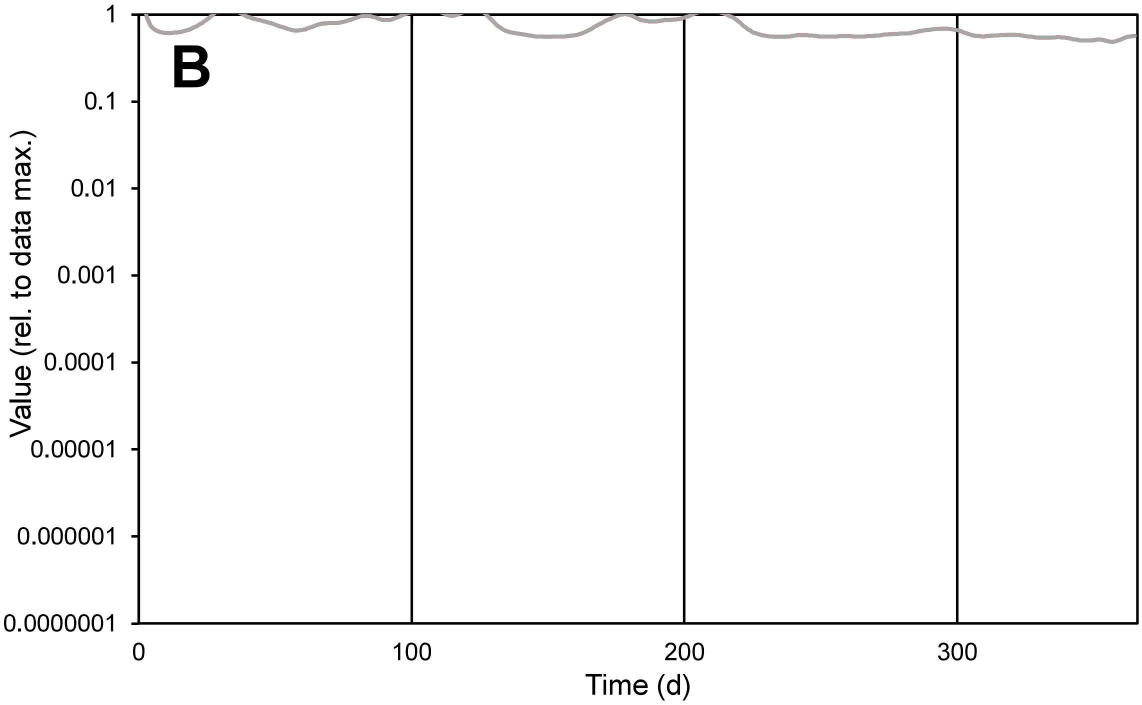

C1

Variables

NS1 Provide jtd or Dst

CalibOnly?

2 1 to reproduce of

oNormalize?

2 1=no, 2=yes

ofxn calc*

0.29226

ofxn mod

0.30895

sRun

RunA1

zout

1

Ave

Data

2.7E-05

Model

2.2E-05

data/vMax

2.7E-05

ofxng

311.734

Min d/m

2.00292

ave/min d

1

ave/min m

1.58527

331_Calib_S_A

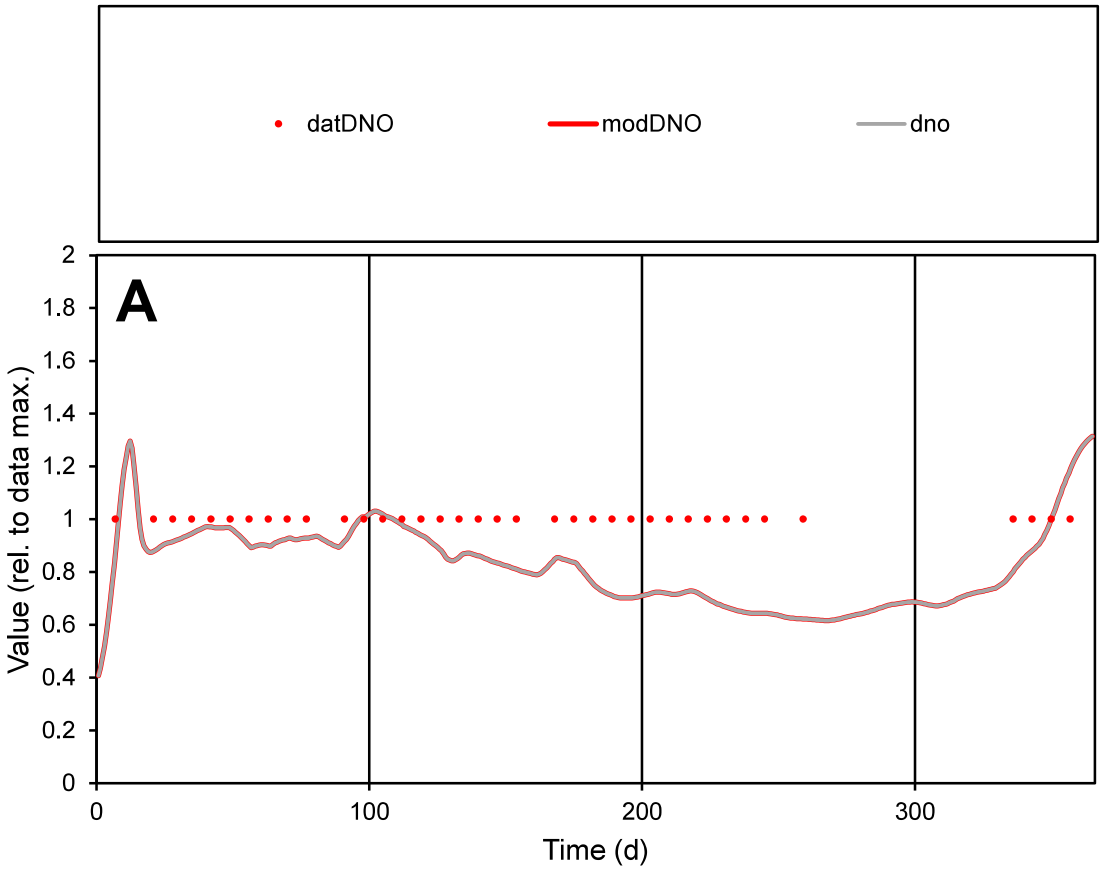

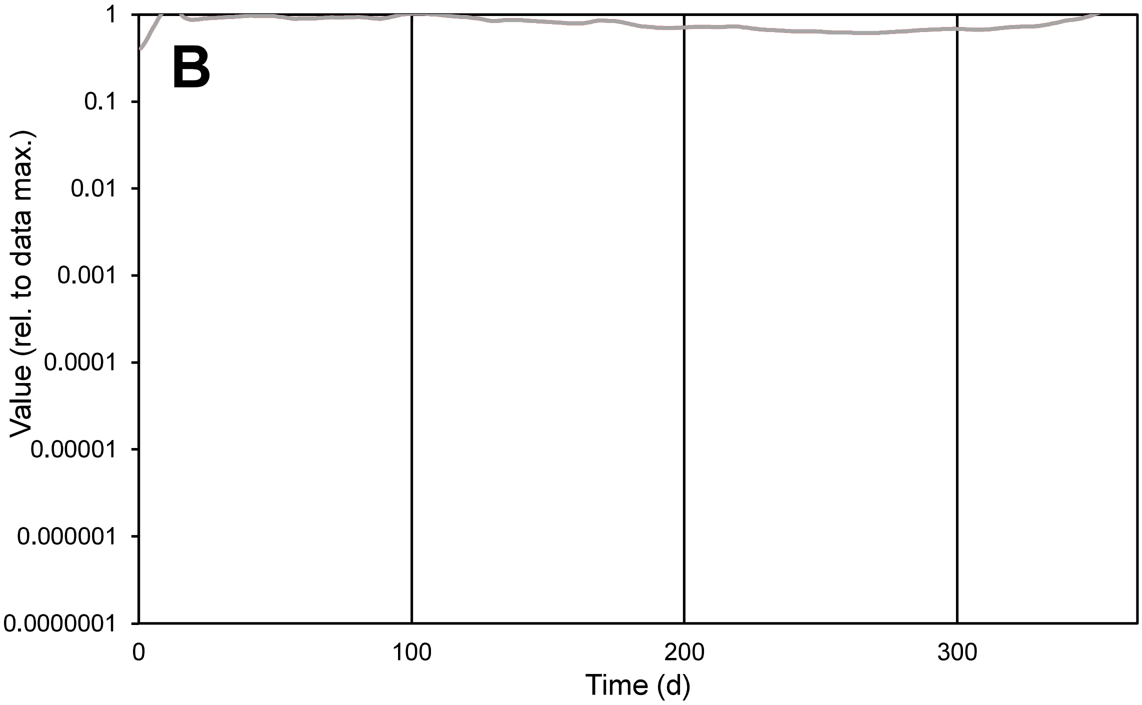

C1

Variables

DNO Provide jtd or Dst

CalibOnly?

2 1 to reproduce of

oNormalize?

2 1=no, 2=yes

ofxn calc*

0.19505

ofxn mod

0.22067

sRun

RunA1

zout

1

Ave

Data

1.8E-05

Model

1.5E-05

data/vMax

1.8E-05

ofxng

311.734

Min d/m

1.60889

ave/min d

1

ave/min m

1.37715

331_Calib_S_A

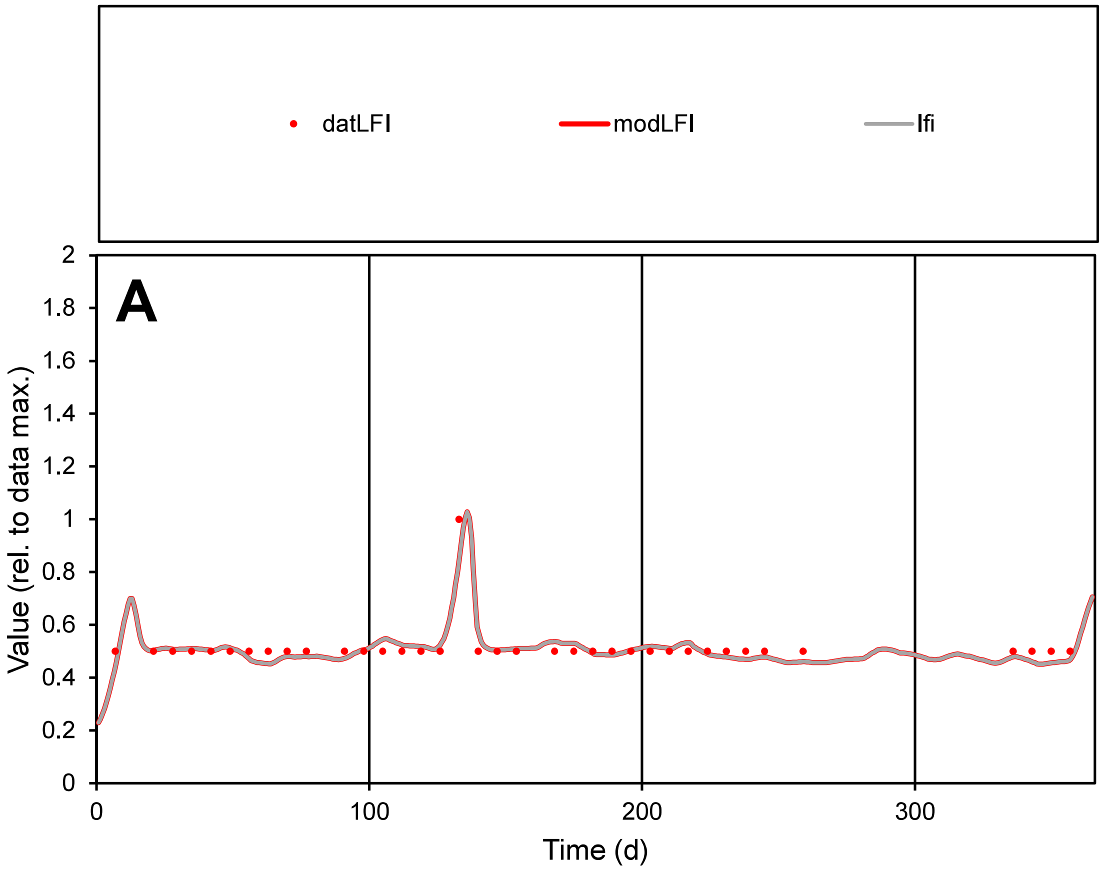

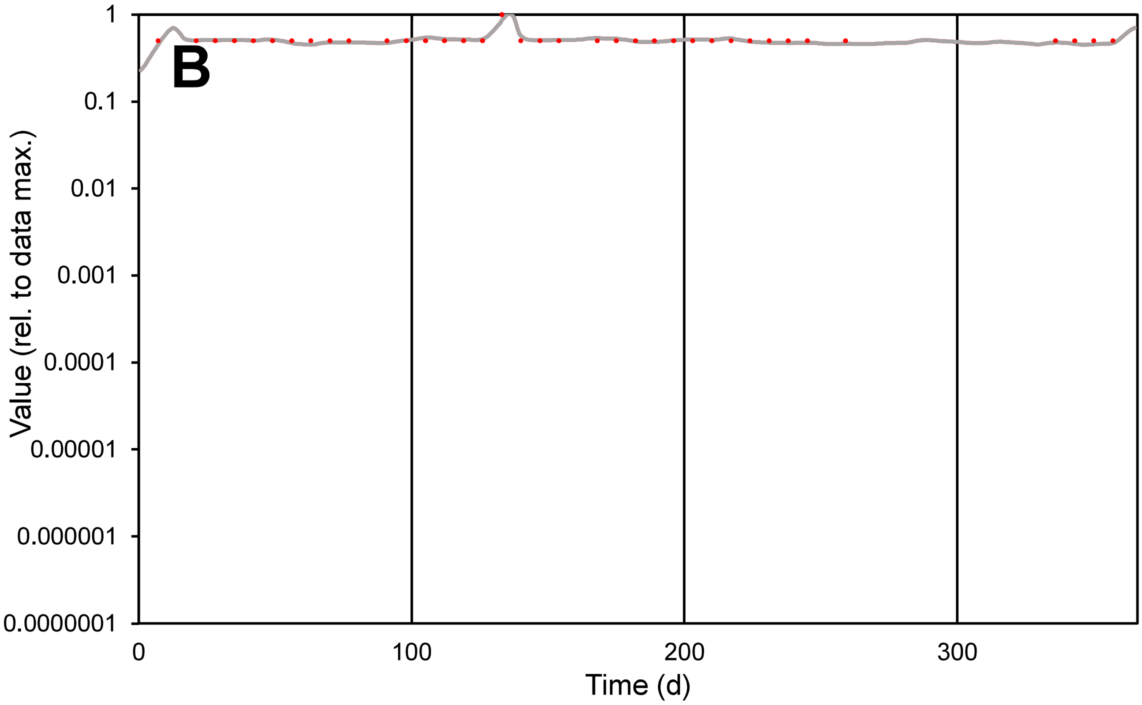

C1

Variables
LFI Provide jtd or Dst
CalibOnly?
2 1 to reproduce of
oNormalize?
2 1=no, 2=yes

ofxn calc*
0.04978
ofxn mod
0.12267

sRun
RunA1
zout
1

Ave
Data
8.5E-06
Model
8.5E-06

data/vMax
1.7E-05

ofxng
311.734

Min d/m
1.09661

ave/min d
1.02703
ave/min m
1.12097

331_Calib_S_A

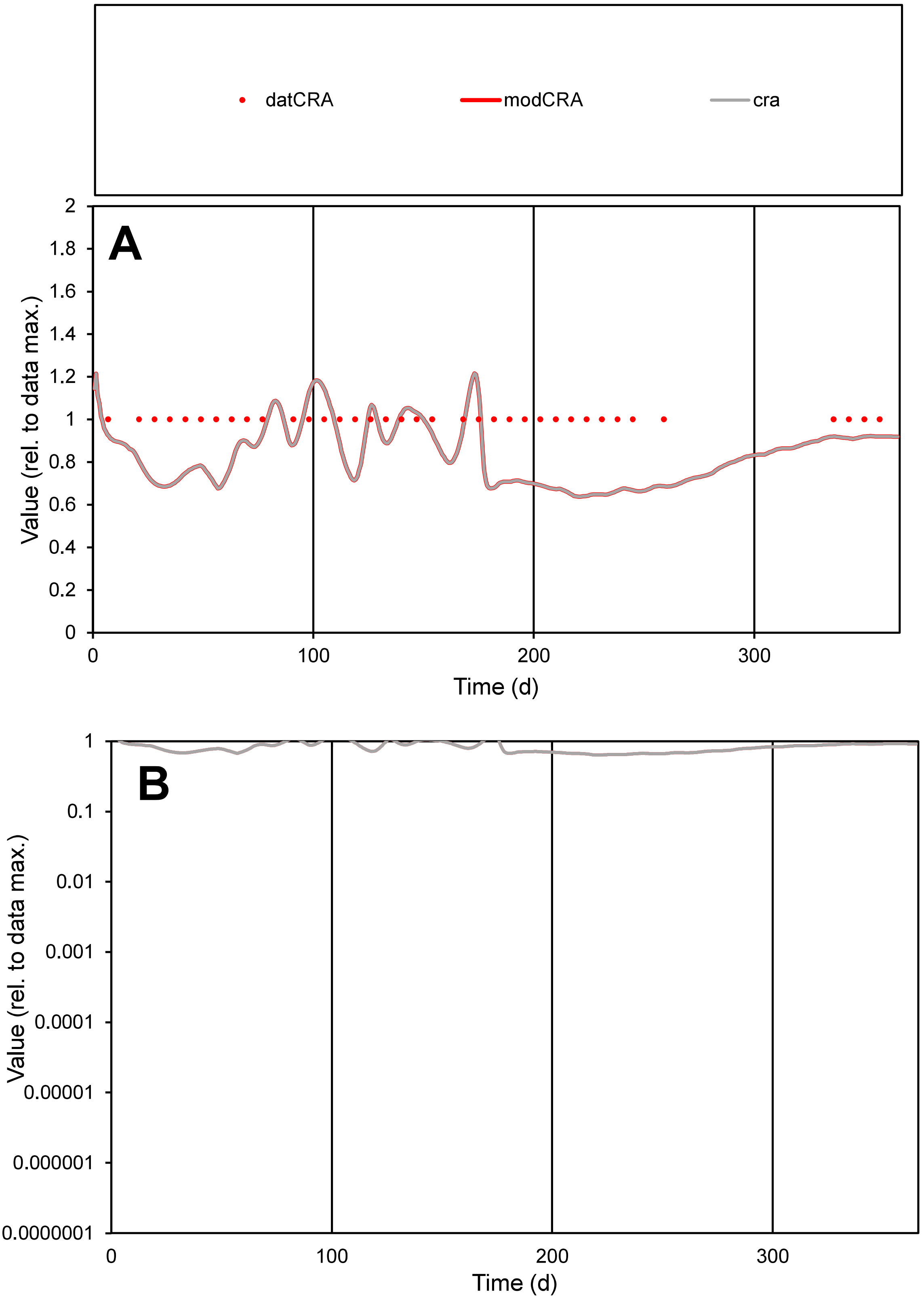

C1

Variables
CRA Provide jtd or Dst
CalibOnly?
2 1 to reproduce of
oNormalize?
2 1=no, 2=yes

ofxn calc*
0.22474
ofxn mod
0.24661

sRun
RunA1
zout
1

Ave
Data
4.9E-06
Model
4E-06

data/vMax
4.9E-06

ofxng
311.734

Min d/m
1.55698

ave/min d
1
ave/min m
1.29586

331_Calib_S_A

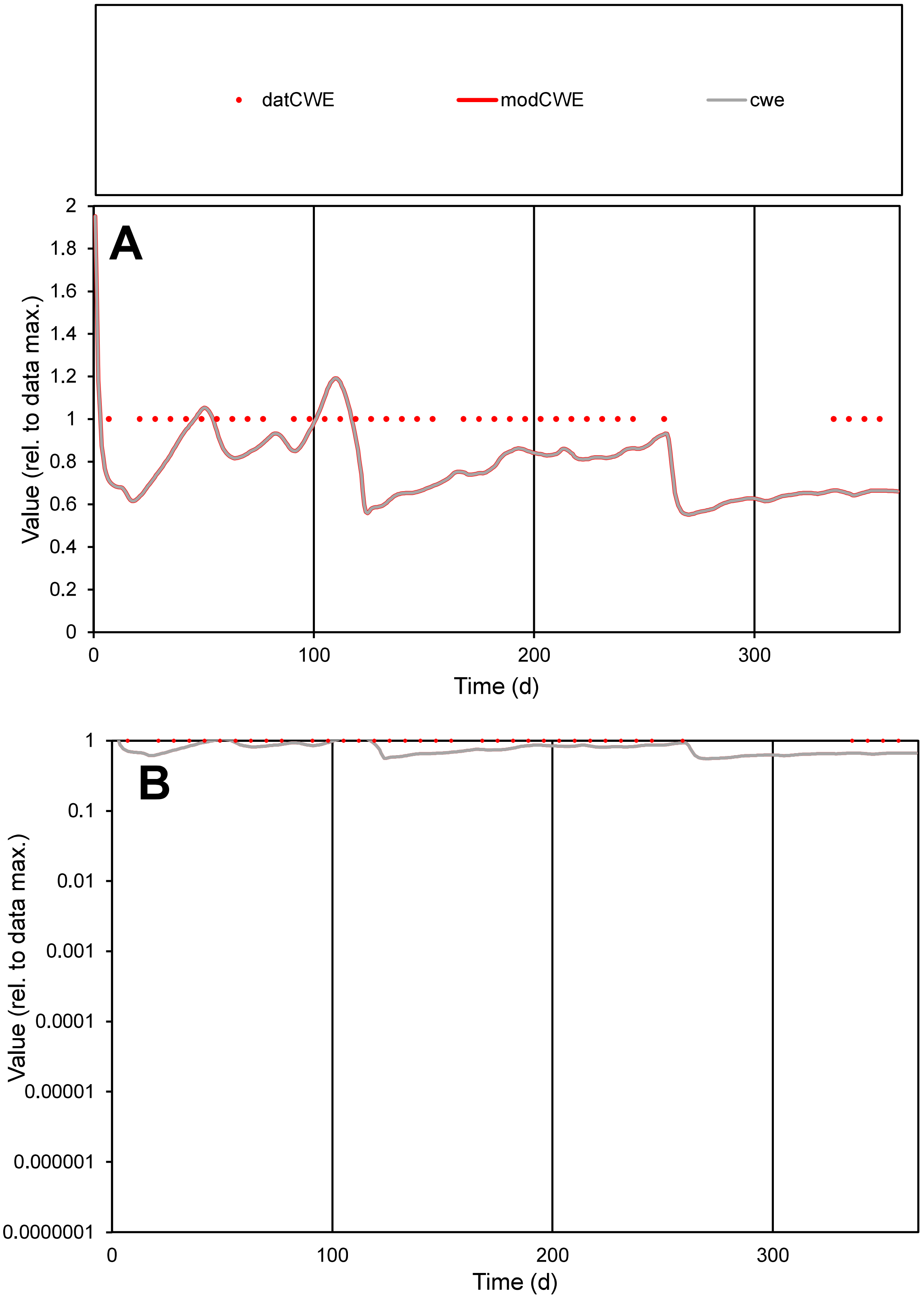

C1

Variables

CWE Provide jtd or Dst

CalibOnly?

2 1 to reproduce of

oNormalize?

2 1=no, 2=yes

ofxn calc*

0.23122

ofxn mod

0.25263

sRun

RunA1

zout

1

Ave

Data

2.6E-05

Model

2.1E-05

data/vMax

2.6E-05

ofxng

311.734

Min d/m

1.70912

ave/min d

1

ave/min m

1.38736

331_Calib_S_A

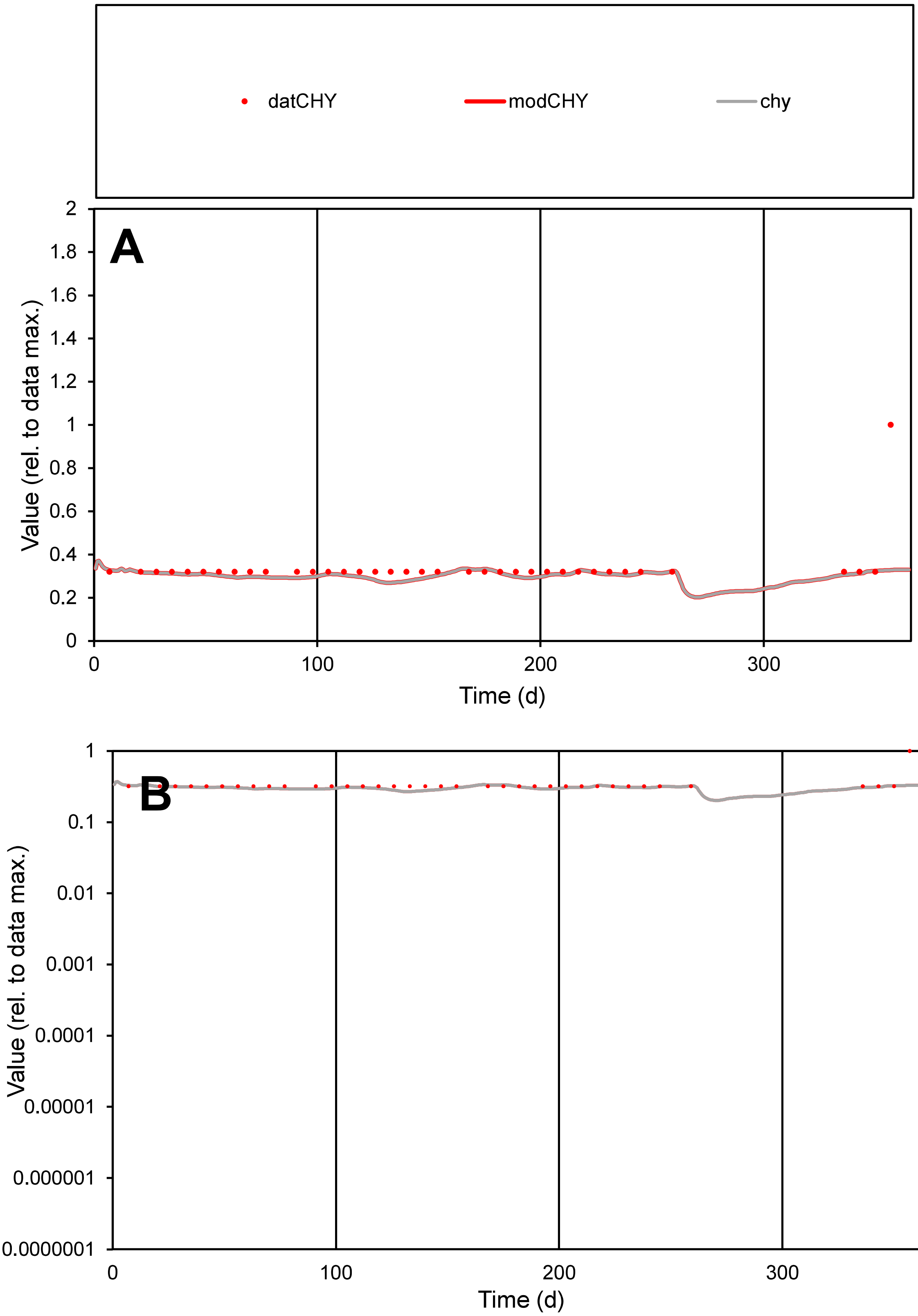

C1

Variables
CHY Provide jtd or Dst
CalibOnly?
2 1 to reproduce of
oNormalize?
2 1=no, 2=yes

ofxn calc*
0.3313
ofxn mod
0.34866

sRun
RunA1
zout
1

Ave
Data
7.1E-06
Model
6.4E-06

data/vMax
2.1E-05

ofxng
311.734

Min d/m
1.18217

ave/min d
1.05752
ave/min m
1.13484

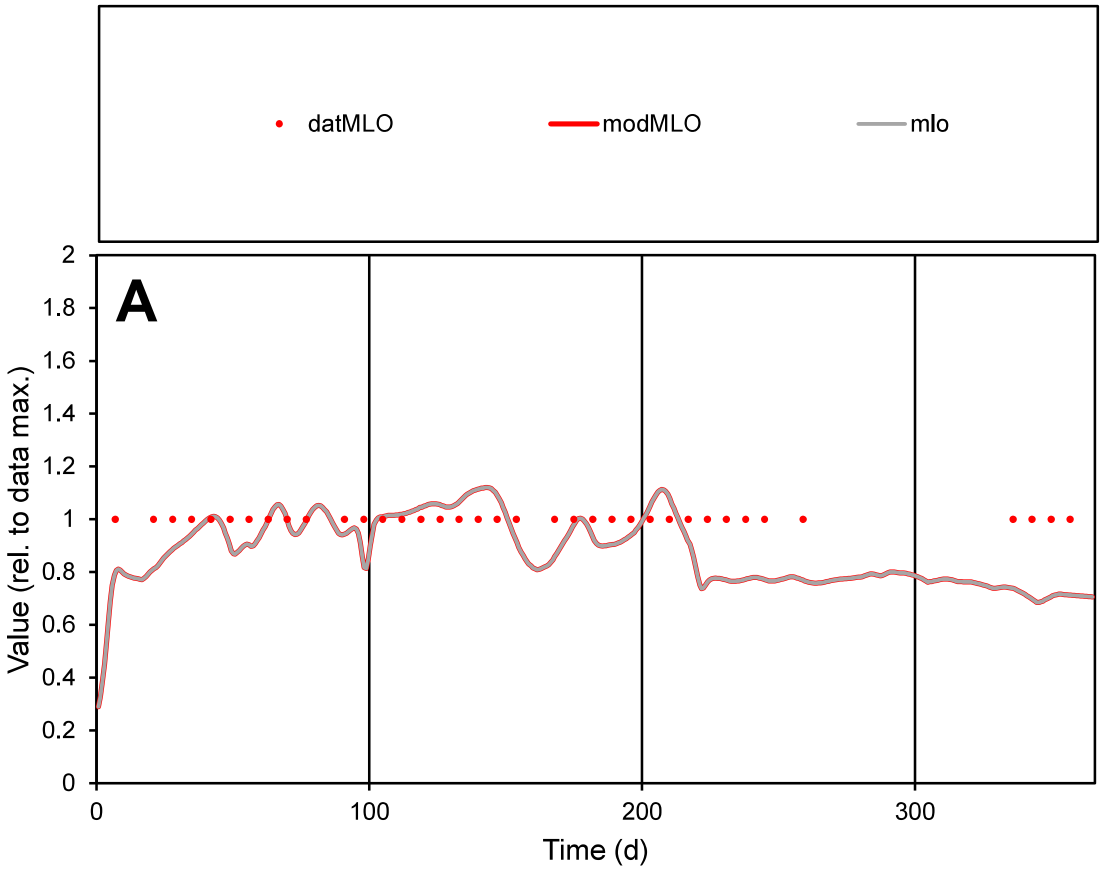

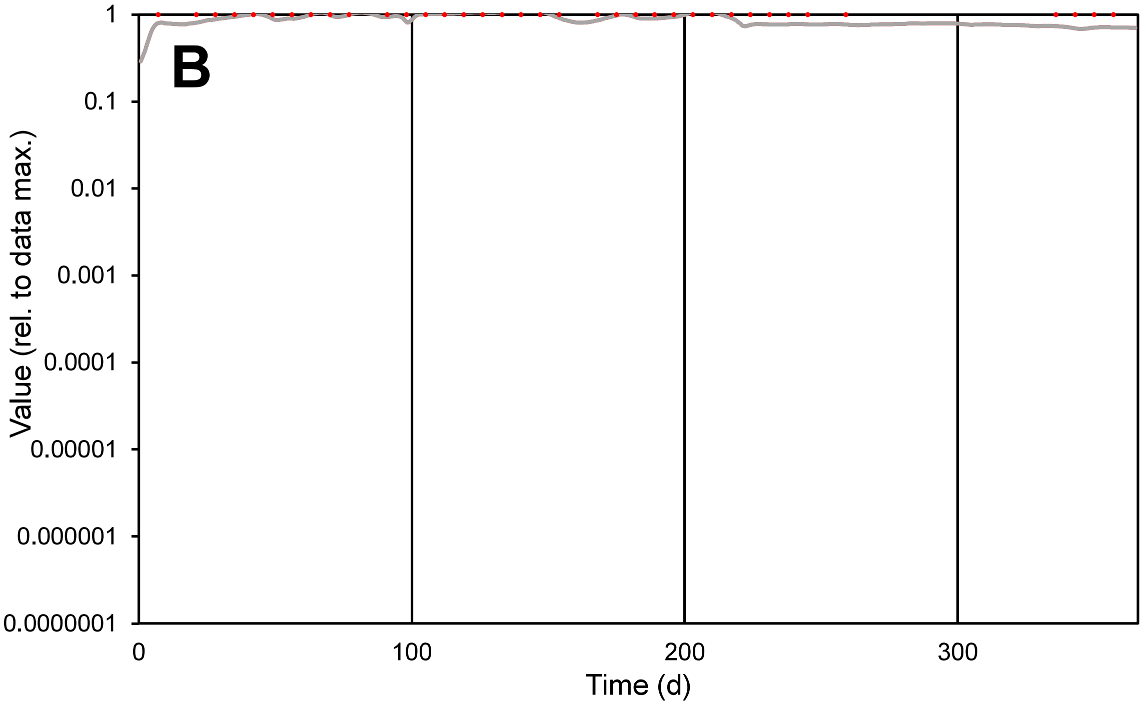

C1

Variables
MLO Provide jtd or Dst
CalibOnly?
2 1 to reproduce of
oNormalize?
2 1=no, 2=yes

ofxn calc*
0.15036
ofxn mod
0.18457

sRun
RunA1
zout
1

Ave
Data
1E-05
Model
9.5E-06

data/vMax
1E-05

ofxng
311.734

Min d/m
1.44921

ave/min d
1
ave/min m
1.32

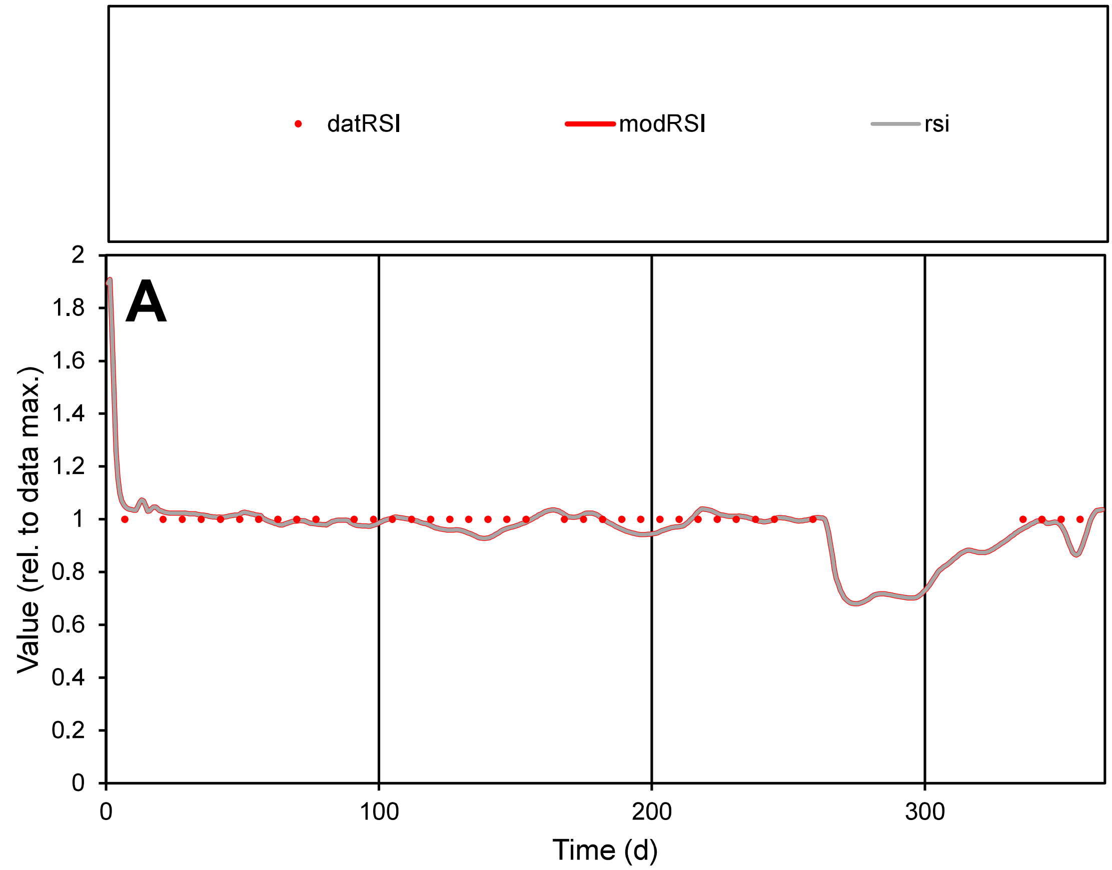

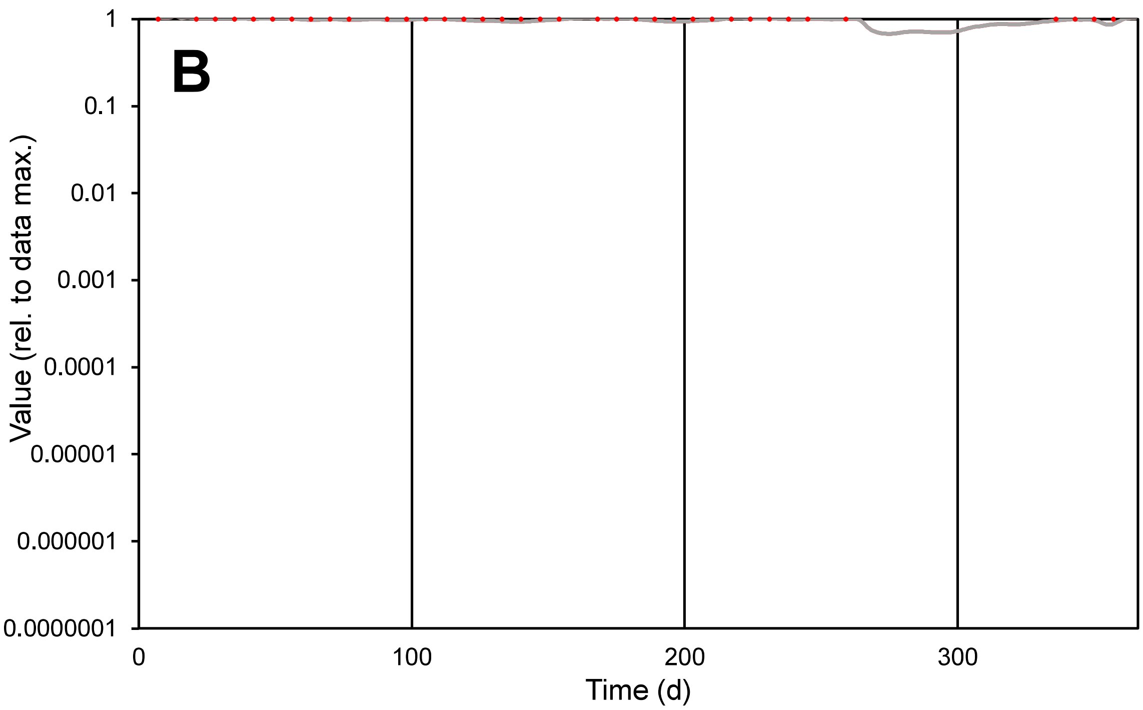

C1

Variables
RSI Provide jtd or Dst
CalibOnly?
2 1 to reproduce of
oNormalize?
2 1=no, 2=yes

ofxn calc*
0.03137
ofxn mod
0.11549

sRun
RunA1
zout
1

Ave
Data
1E-05
Model
1E-05

data/vMax
1E-05

ofxng
311.734

Min d/m
1.08213

ave/min d
1
ave/min m
1.07085

331_Calib_S_A

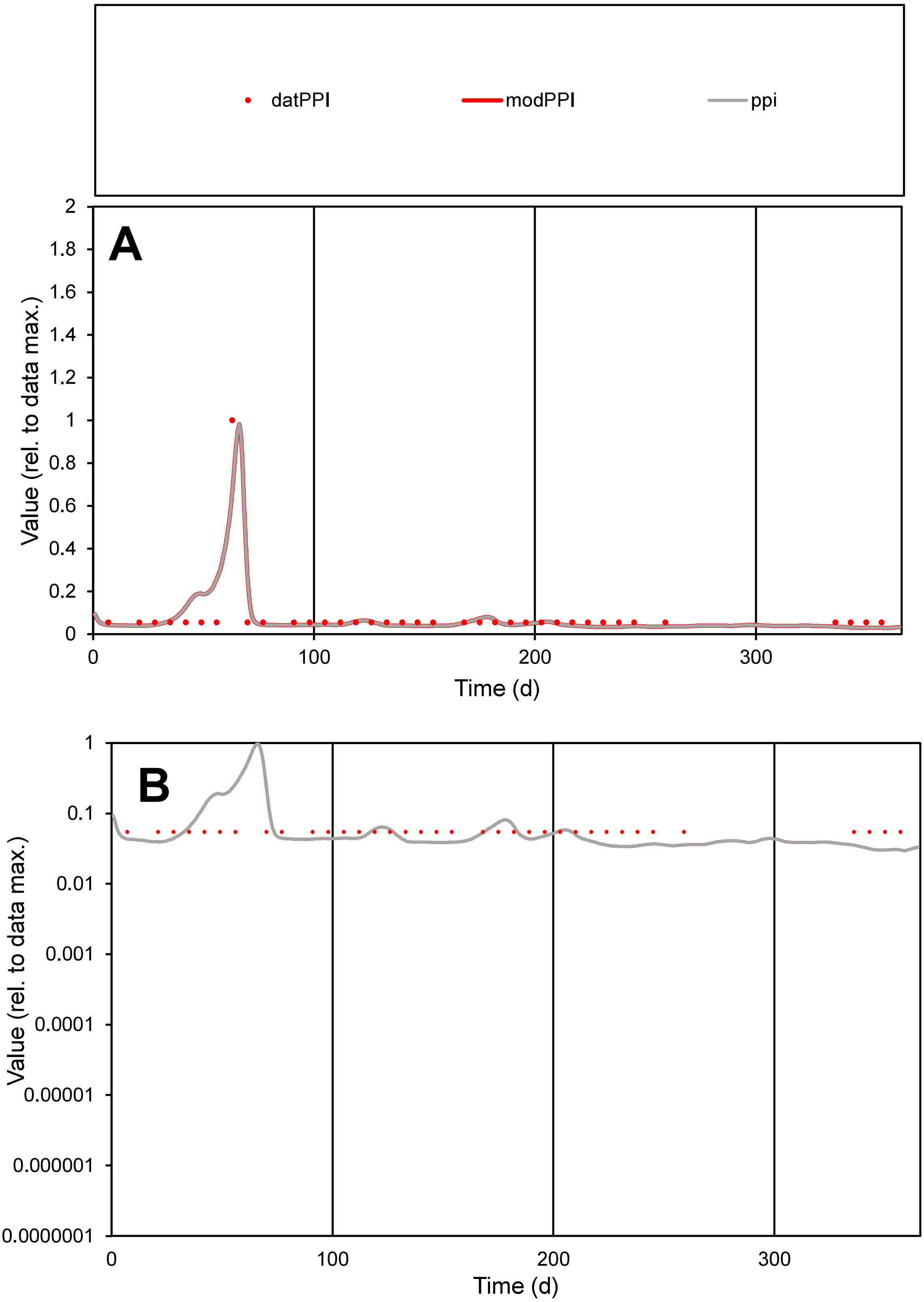

C1

Variables

PPI	Provide jtd or Dst
CalibOnly?	
2	1 to reproduce of
oNormalize?	
2	1=no, 2=yes

ofxn calc*
0.57798
ofxn mod
0.67815

sRun
RunA1
zout
1

Ave
Data
2.1E-06
Model
2.1E-06

data/vMax
2.7E-05

ofxng
311.734

Min d/m
1.84533

ave/min d
1.46866
ave/min m
2.68293

331_Calib_S_A

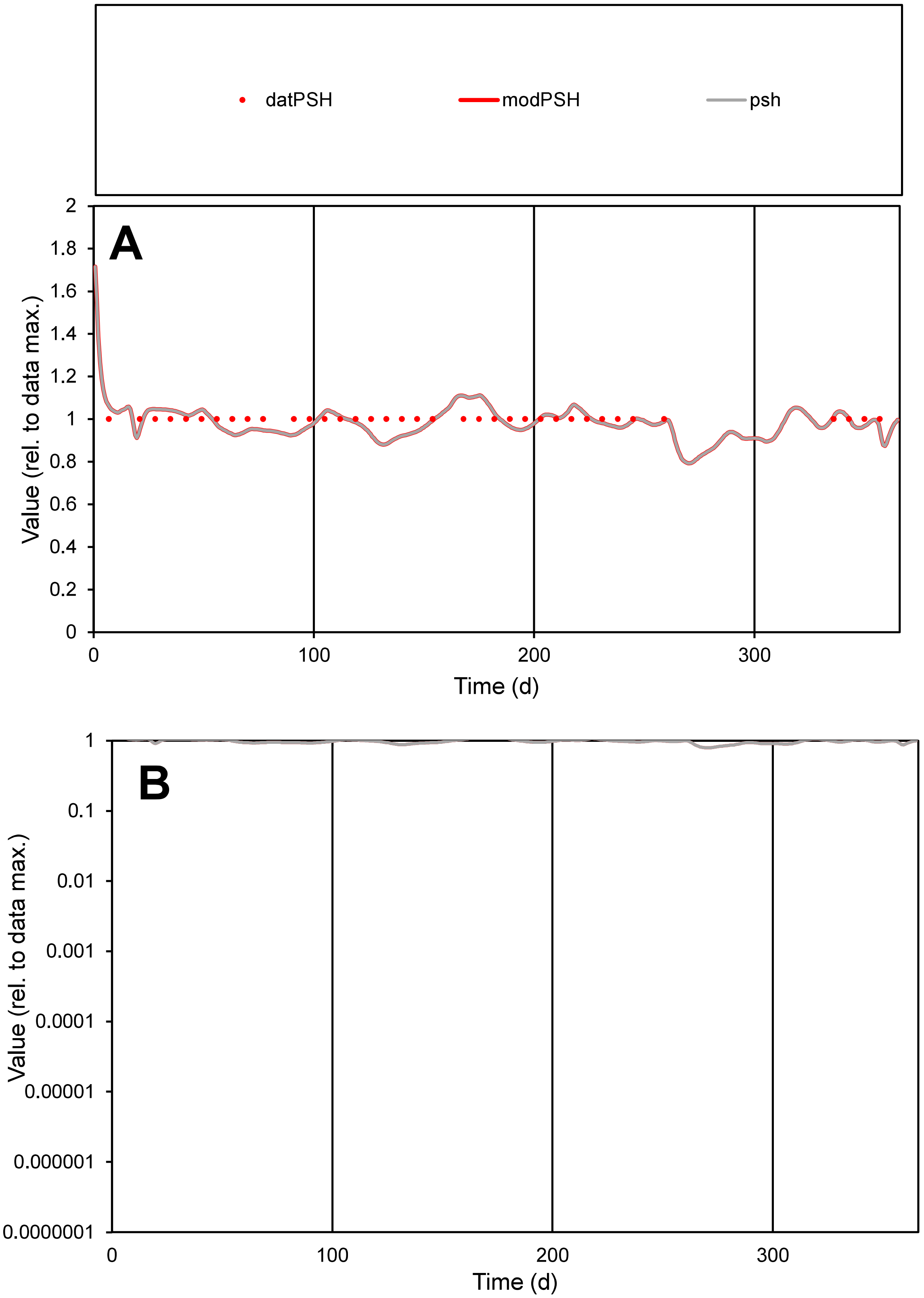

C1

Variables
PSH Provide jtd or Dst
CalibOnly?
2 1 to reproduce of
oNormalize?
2 1=no, 2=yes

ofxn calc*
0.05329
ofxn mod
0.12302

sRun
RunA1
zout
1

Ave
Data
1.3E-05
Model
1.3E-05

data/vMax
1.3E-05

ofxng
311.734

Min d/m
1.13757

ave/min d
1
ave/min m
1.12691

331_Calib_S_A

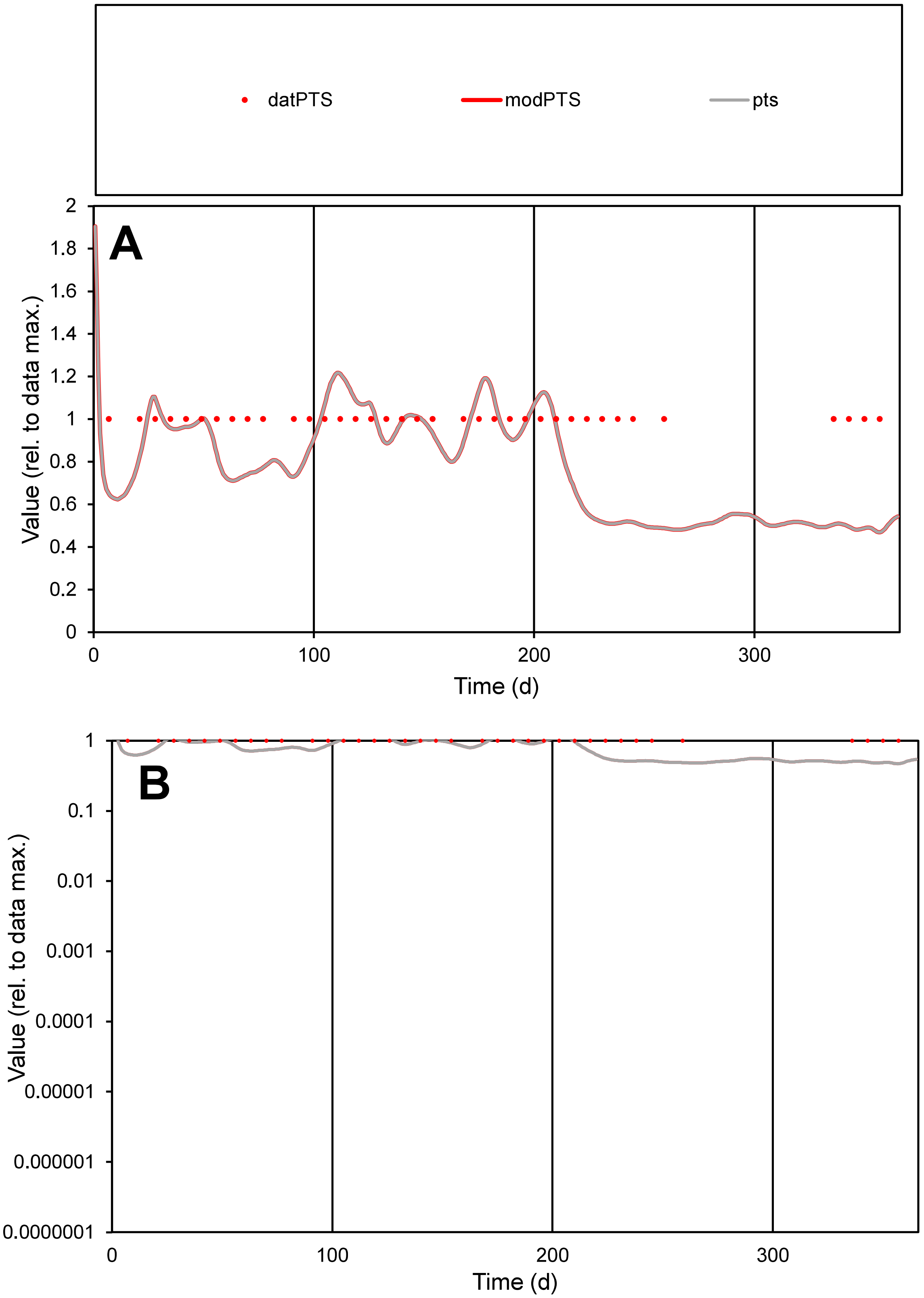

C1

Variables
PTS Provide jtd or Dst
CalibOnly?
2 1 to reproduce of
oNormalize?
2 1=no, 2=yes

ofxn calc*
0.28041
ofxn mod
0.29866

sRun
RunA1
zout
1

Ave
Data
9.7E-06
Model
8E-06

data/vMax
9.7E-06

ofxng
311.734

Min d/m
2.09272

ave/min d
1
ave/min m
1.73917

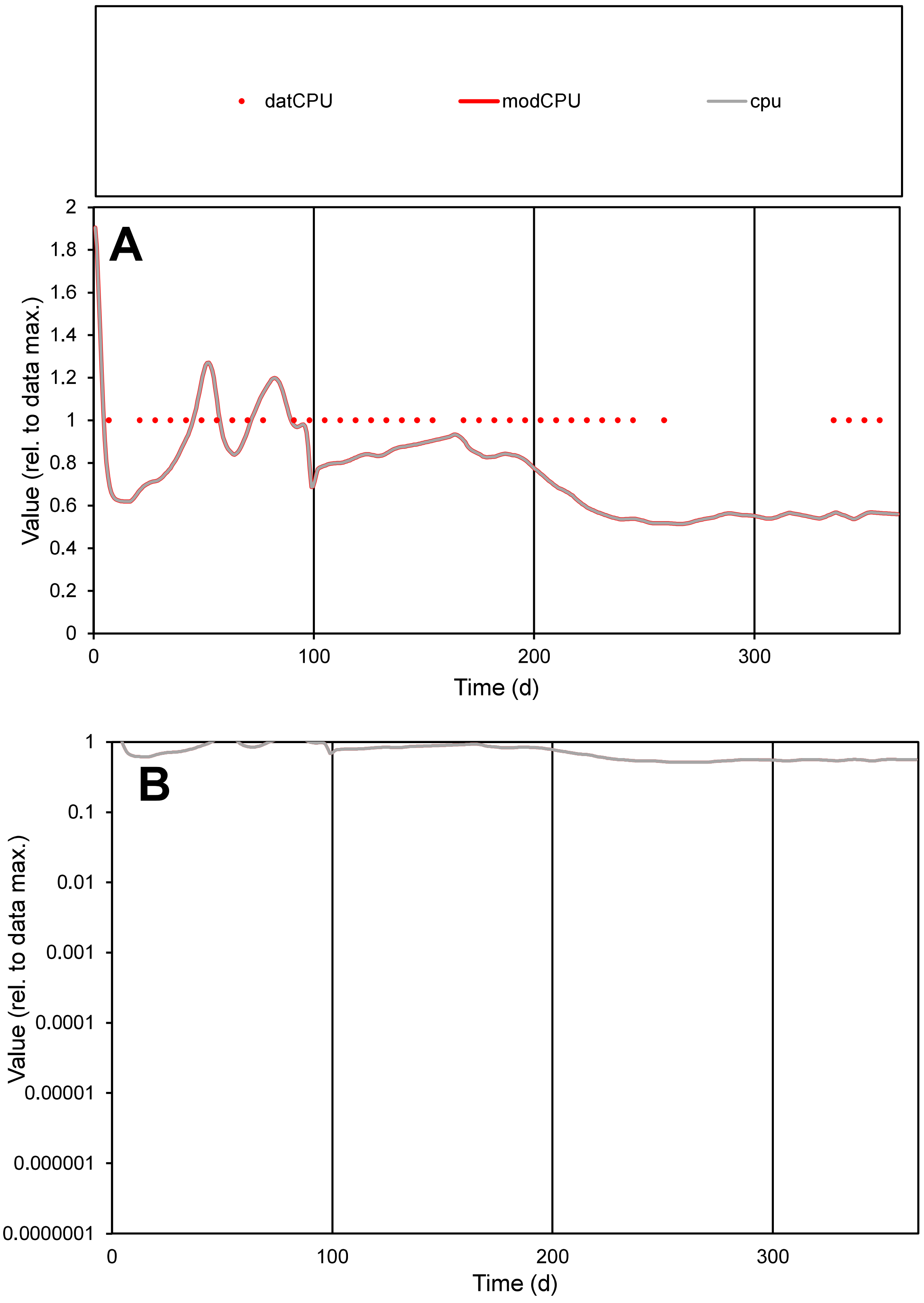

C1

Variables
CPU Provide jtd or Dst
CalibOnly?
2 1 to reproduce of
oNormalize?
2 1=no, 2=yes

ofxn calc*
0.28112
ofxn mod
0.29956

sRun
RunA1
zout
1

Ave
Data
4.4E-07
Model
3.4E-07

data/vMax
4.4E-07

ofxng
311.734

Min d/m
1.931

ave/min d
1
ave/min m
1.50577

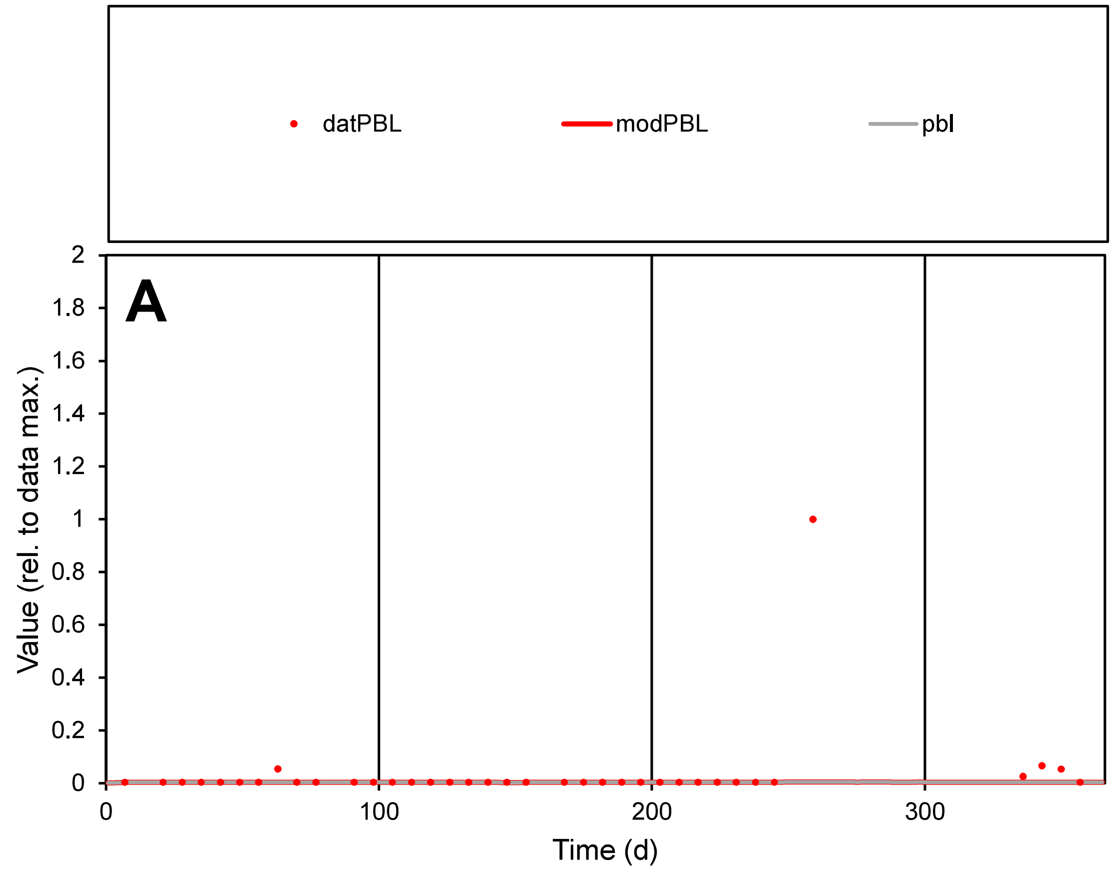

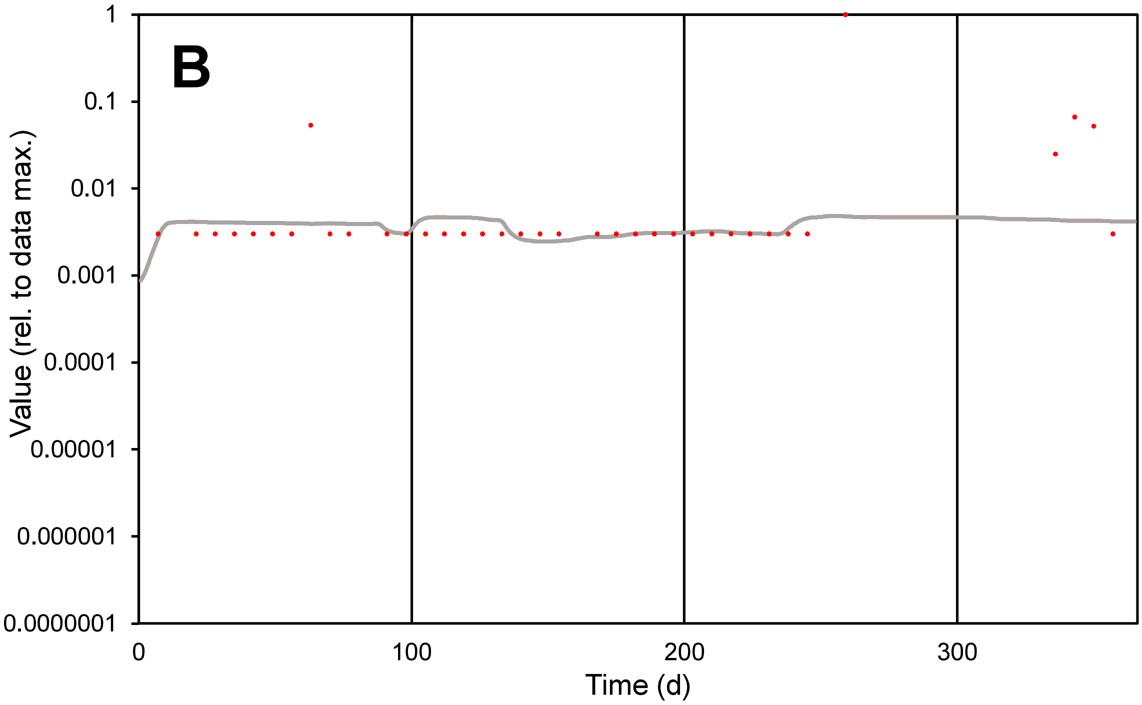

C1

Variables
PBL Provide jtd or Dst
CalibOnly?
2 1 to reproduce of
oNormalize?
2 1=no, 2=yes

ofxn calc*
4.70228
ofxn mod
4.71182

sRun
RunA1
zout
1

Ave
Data
4.9E-07
Model
5.2E-08

data/vMax
1.4E-05

ofxng
311.734

Min d/m
1.22687

ave/min d
11.6294
ave/min m
1.49955

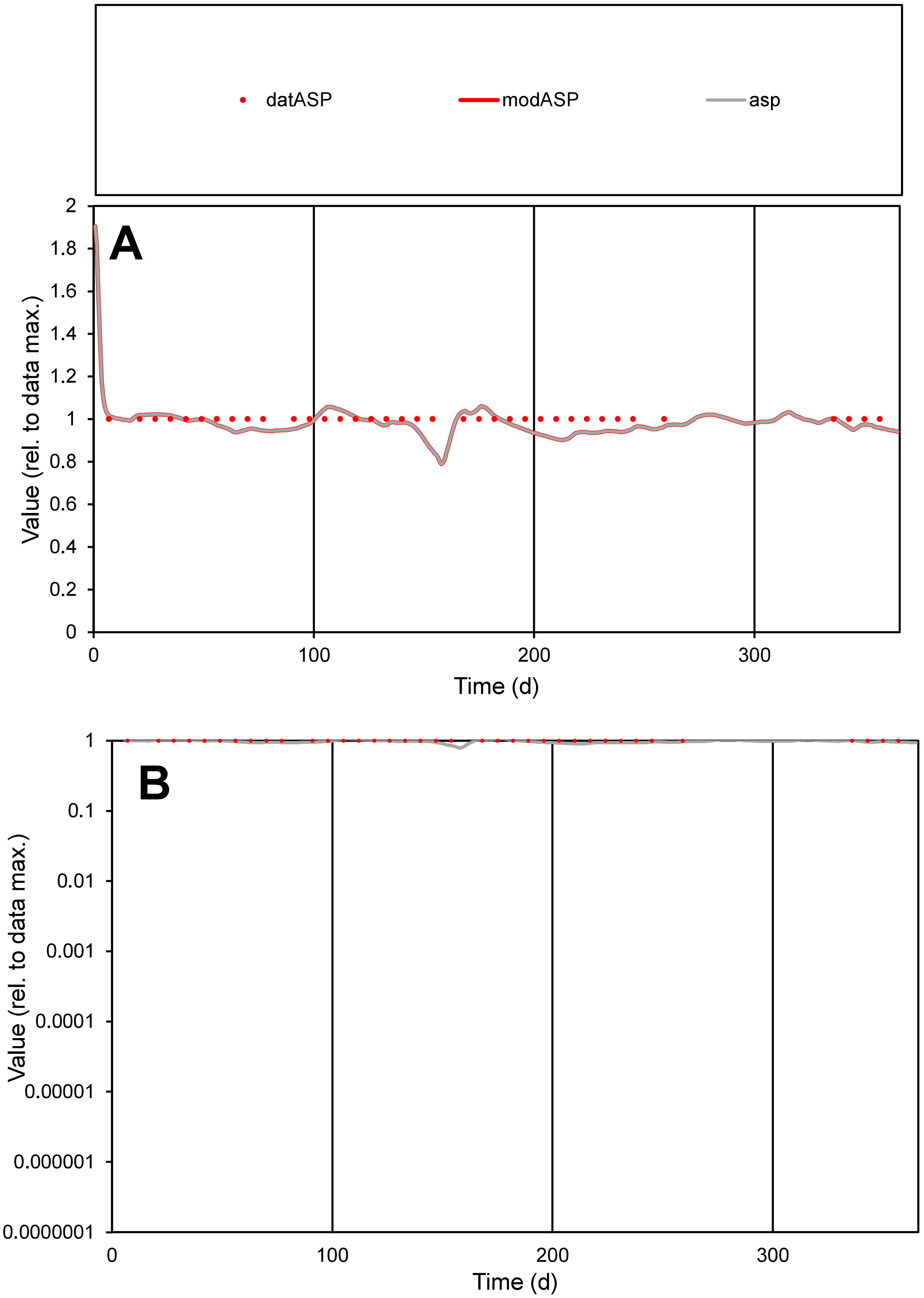

C1

Variables
ASP Provide jtd or Dst
CalibOnly?
2 1 to reproduce of
oNormalize?
2 1=no, 2=yes

ofxn calc*
0.05138
ofxn mod
0.12126

sRun
RunA1
zout
1

Ave
Data
5.8E-06
Model
5.6E-06

data/vMax
5.8E-06

ofxng
311.734

Min d/m
1.20241

ave/min d
1
ave/min m
1.17161

331_Calib_S_A

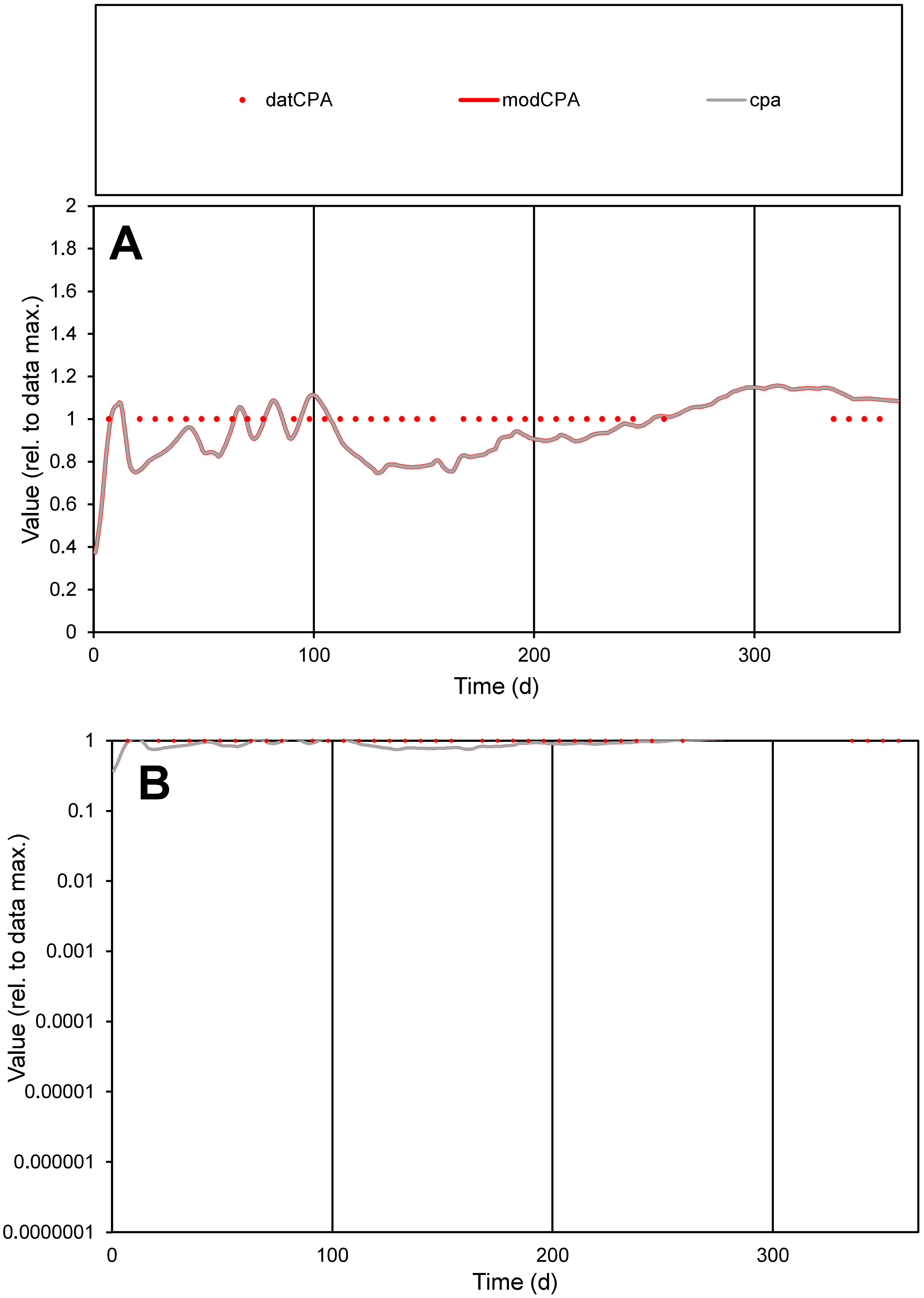

C1

Variables

CPA Provide jtd or Dst

CalibOnly?

2 1 to reproduce of

oNormalize?

2 1=no, 2=yes

ofxn calc*

0.12969

ofxn mod

0.16857

sRun

RunA1

zout

1

Ave

Data

6.6E-08

Model

6.1E-08

data/vMax

6.6E-08

ofxng

311.734

Min d/m

1.30851

ave/min d

1

ave/min m

1.2081

331_Calib_S_A

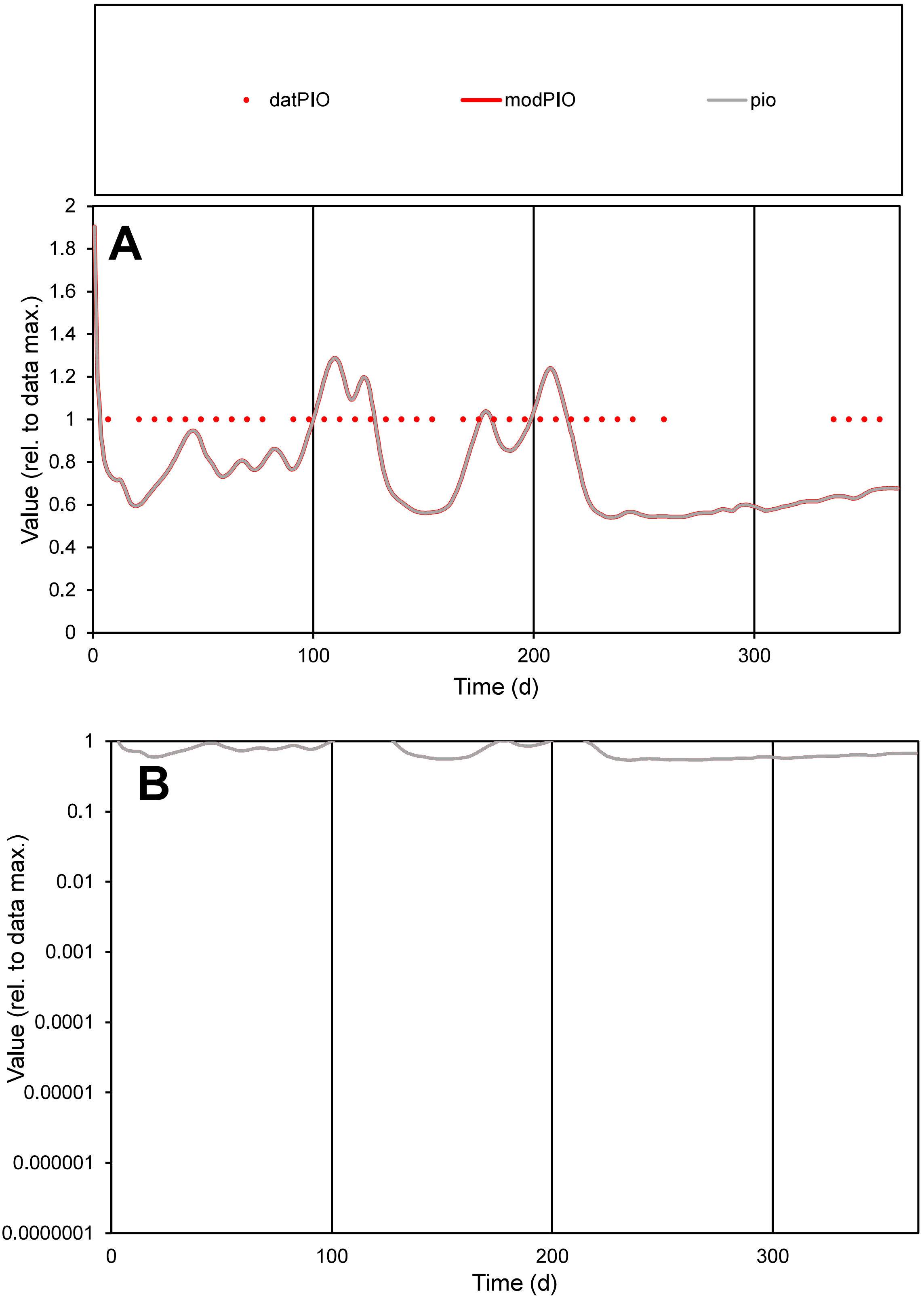

C1

Variables

PIO Provide jtd or Dst

CalibOnly?

2 1 to reproduce of

oNormalize?

2 1=no, 2=yes

ofxn calc*

0.28258

ofxn mod

0.3

sRun

RunA1

zout

1

Ave

Data

1.5E-07

Model

1.2E-07

data/vMax

1.5E-07

ofxng

311.734

Min d/m

1.84054

ave/min d

1

ave/min m

1.4801

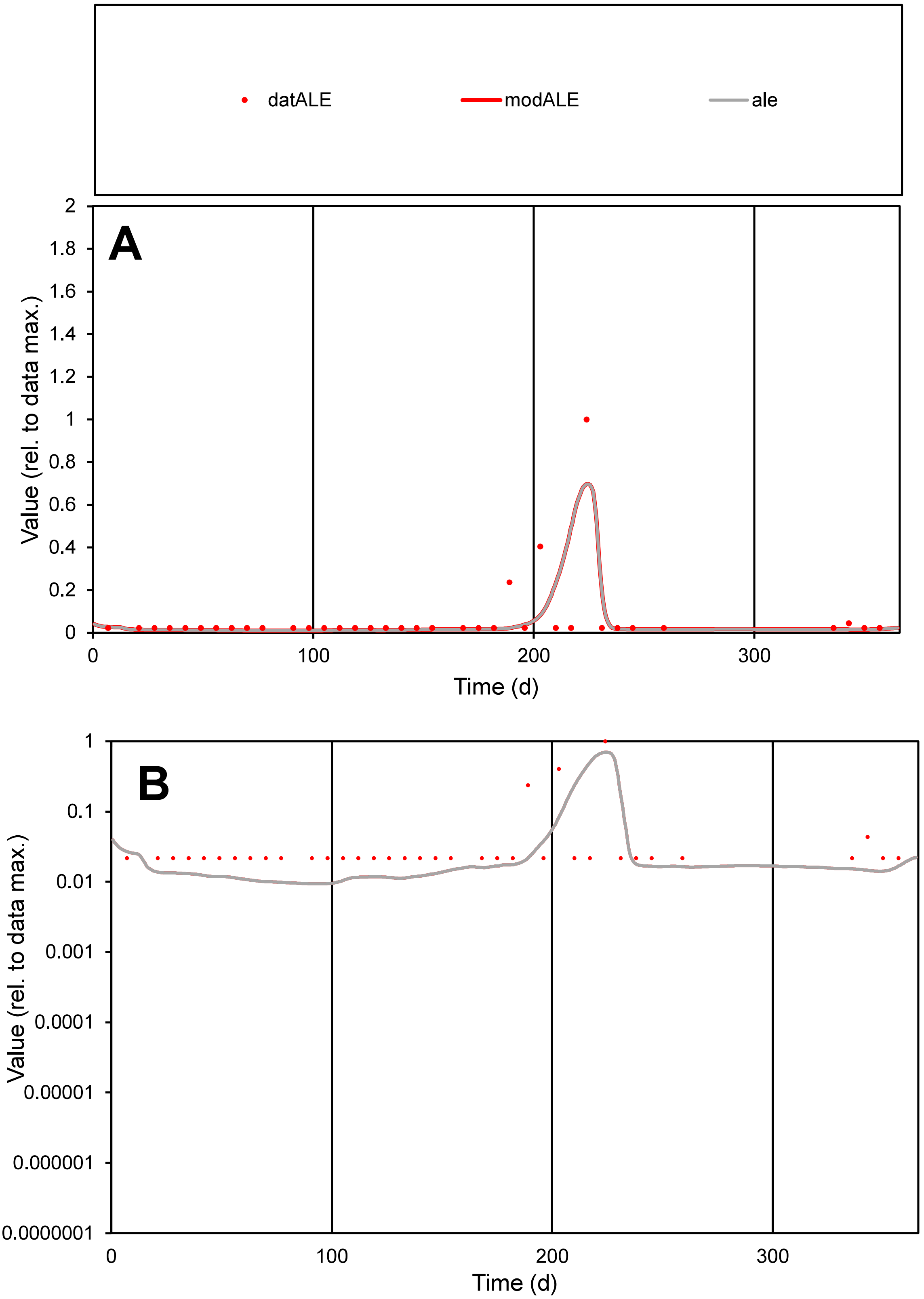

**C1**	
Variables	
ALE	Provide jtd or Dst
CalibOnly?	
2	1 to reproduce of
oNormalize?	
2	1=no, 2=yes
ofxn calc*	
1.62138	
ofxn mod	
1.77942	
sRun	
RunA1	
zout	
1	
Ave	
Data	
3.3E-05	
Model	
2.6E-05	
data/vMax	
0.00051	
ofxng	
311.734	
Min d/m	
2.3172	
ave/min d	
2.98668	
ave/min m	
5.49134	

331_Calib_S_A

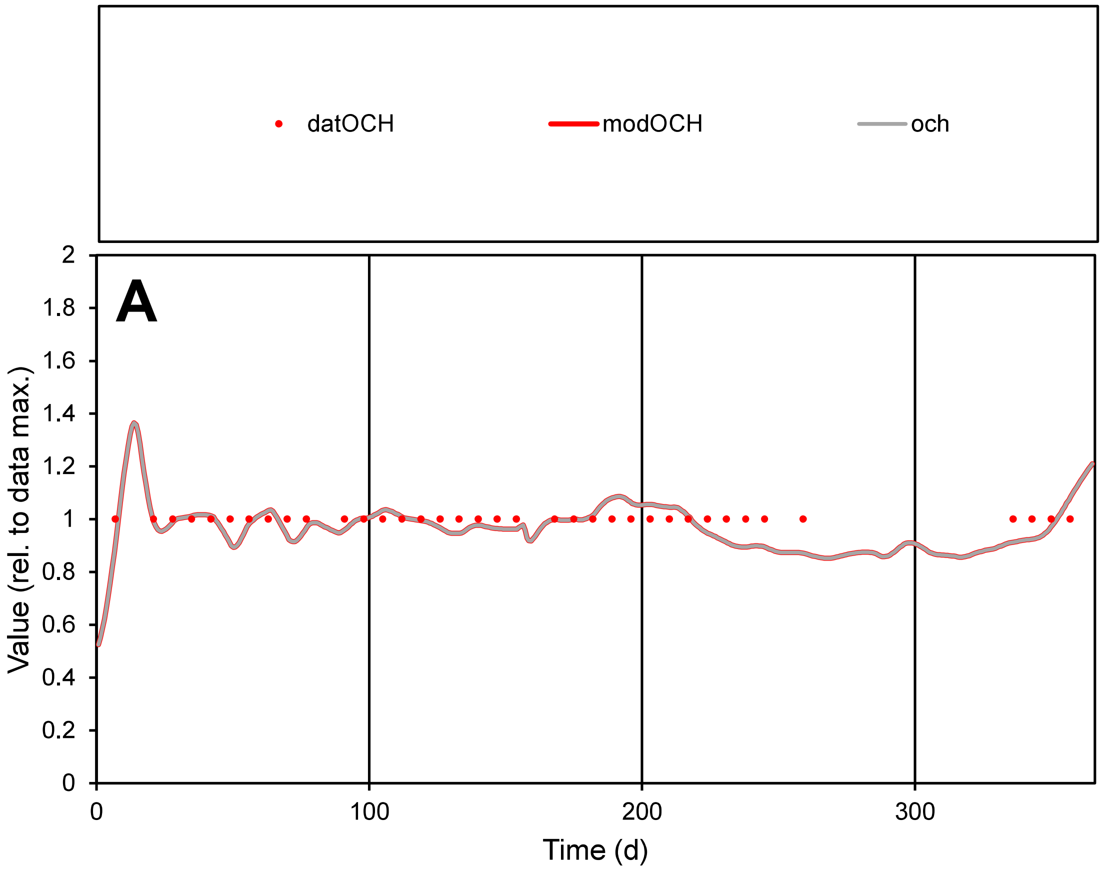

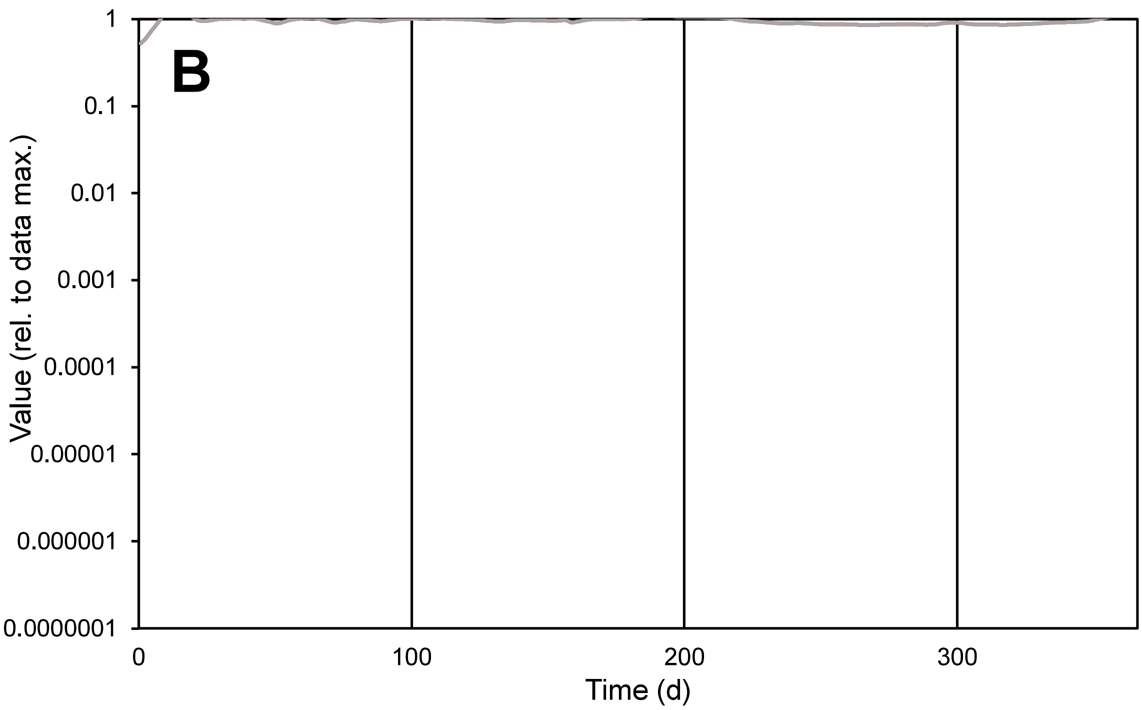

C1

Variables

OCH Provide jtd or Dst

CalibOnly?

2 1 to reproduce of

oNormalize?

2 1=no, 2=yes

ofxn calc*

0.05792

ofxn mod

0.12506

sRun

RunA1

zout

1

Ave

Data

4.4E-06

Model

4.3E-06

data/vMax

4.4E-06

ofxng

311.734

Min d/m

1.15135

ave/min d

1

ave/min m

1.13059

331_Calib_S_A

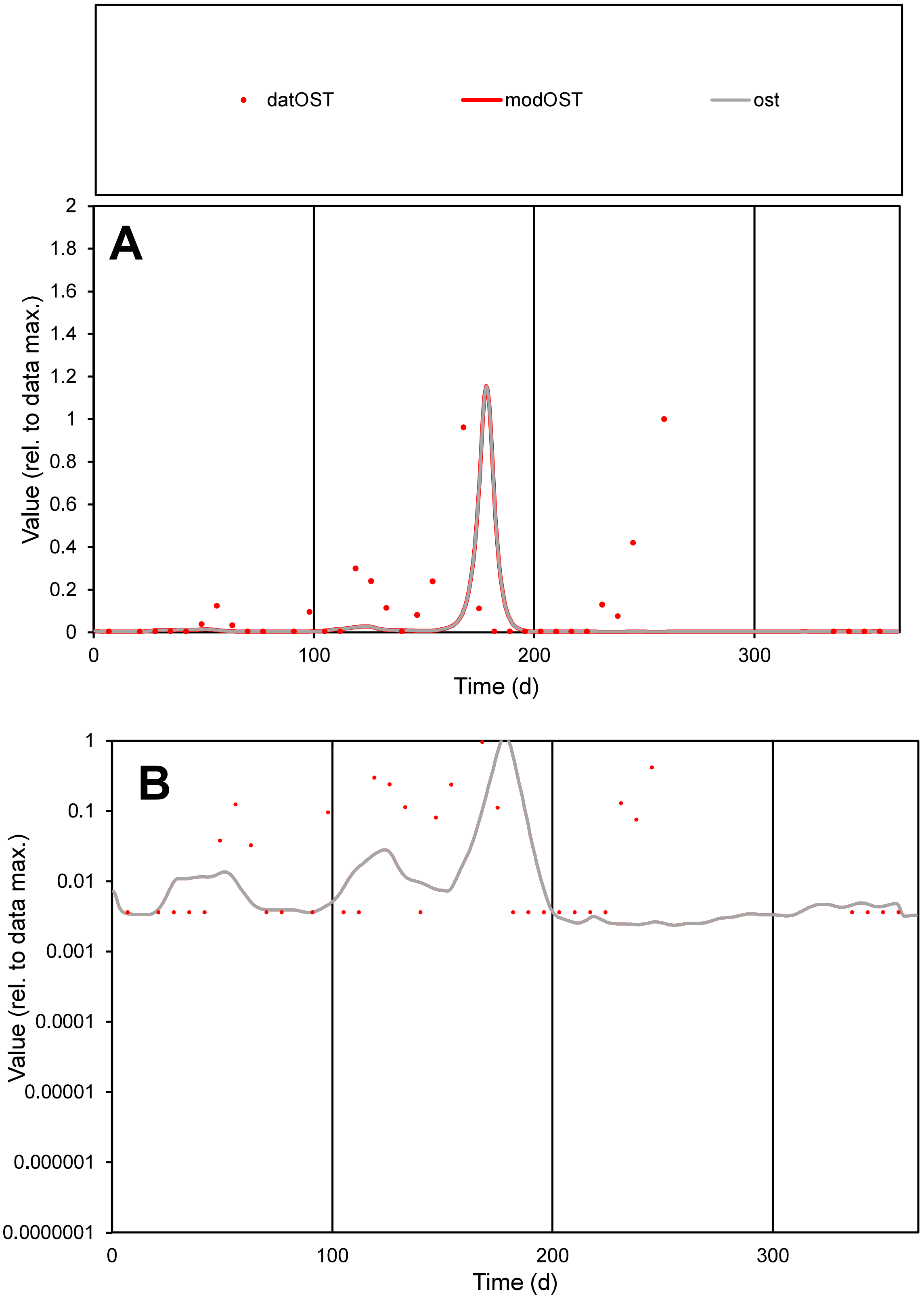

C1

Variables
OST Provide jtd or Dst
CalibOnly?
2 1 to reproduce of
oNormalize?
2 1=no, 2=yes

ofxn calc*
2.12948
ofxn mod
2.29212

sRun
RunA1
zout
1

Ave
Data
6.6E-05
Model
1.8E-05

data/vMax
0.0006

ofxng
311.734

Min d/m
1.49345
ave/min d
30.2443
ave/min m
12.7262

331_Calib_S_A

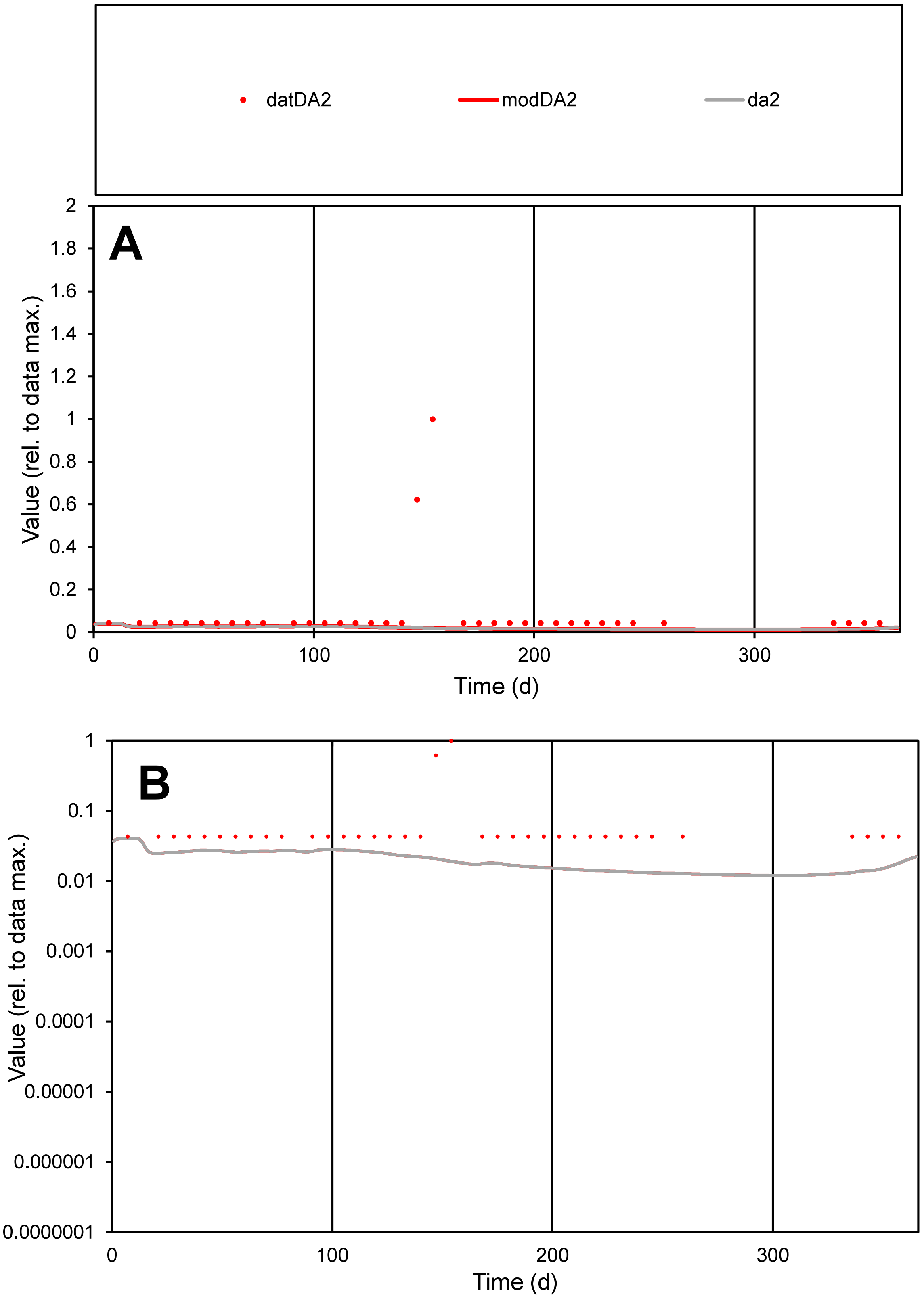

C1

Variables
DA2 Provide jtd or Dst
CalibOnly?
2 1 to reproduce of
oNormalize?
2 1=no, 2=yes

ofxn calc*
2.25243
ofxn mod
2.25792

sRun
RunA1
zout
1

Ave
Data
6.1E-06
Model
1.5E-06

data/vMax
7.2E-05

ofxng
311.734

Min d/m
3.38762

ave/min d
1.9631
ave/min m
1.65331

331_Calib_S_A

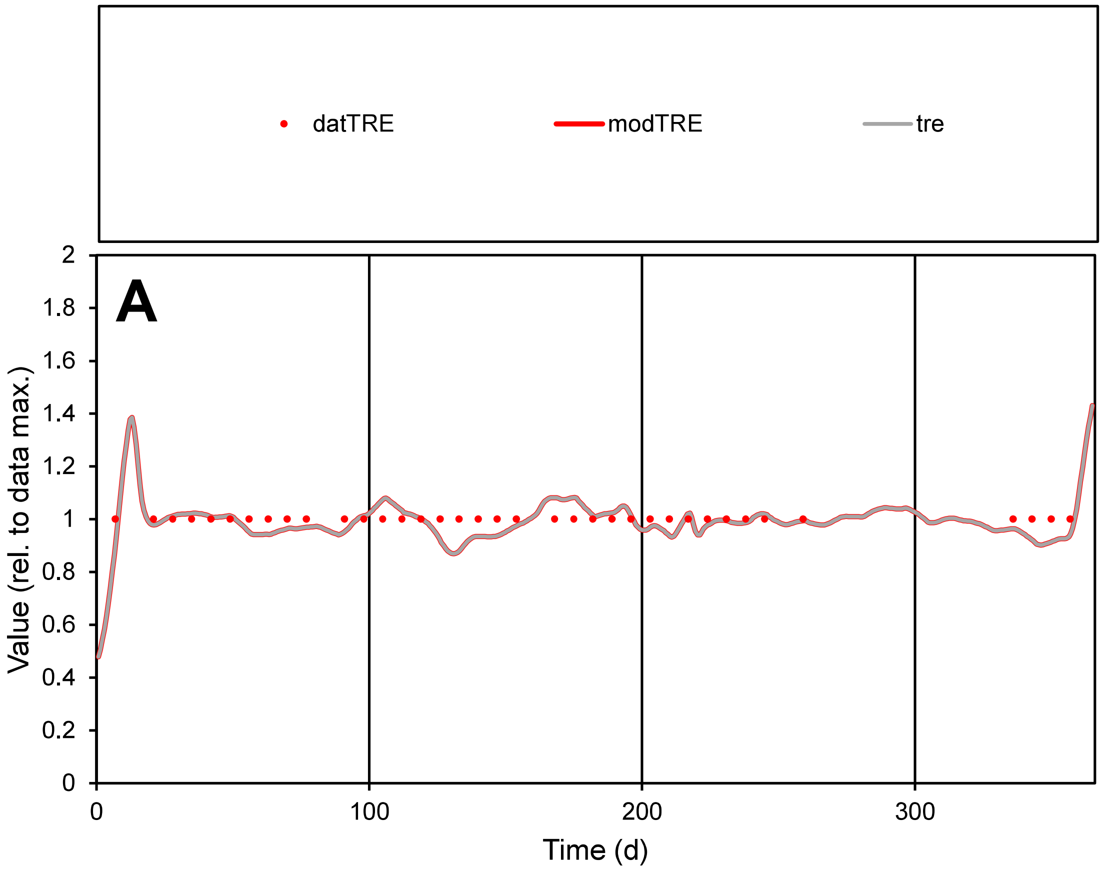

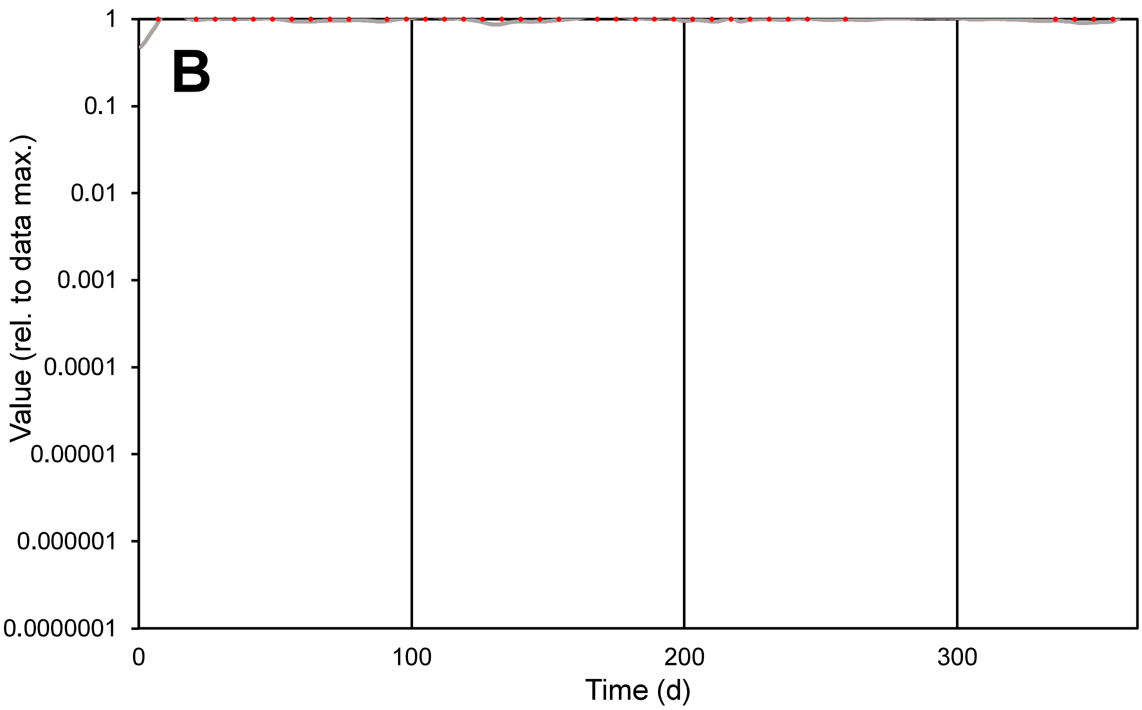

C1

Variables
TRE Provide jtd or Dst
CalibOnly?
2 1 to reproduce of
oNormalize?
2 1=no, 2=yes

ofxn calc*
0.04664
ofxn mod
0.12008

sRun
RunA1
zout
1

Ave
Data
6.9E-06
Model
6.8E-06

data/vMax
6.9E-06

ofxng
311.734

Min d/m
1.11338

ave/min d
1
ave/min m
1.09773

331_Calib_S_A

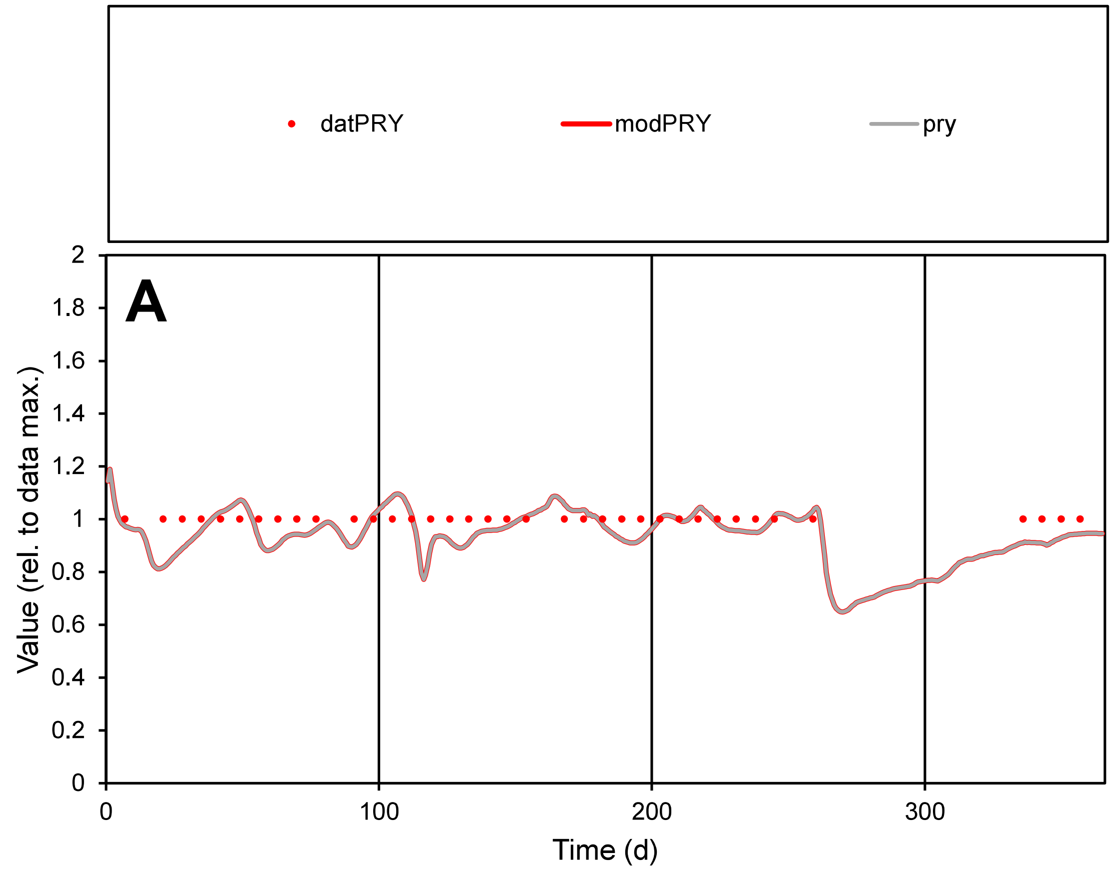

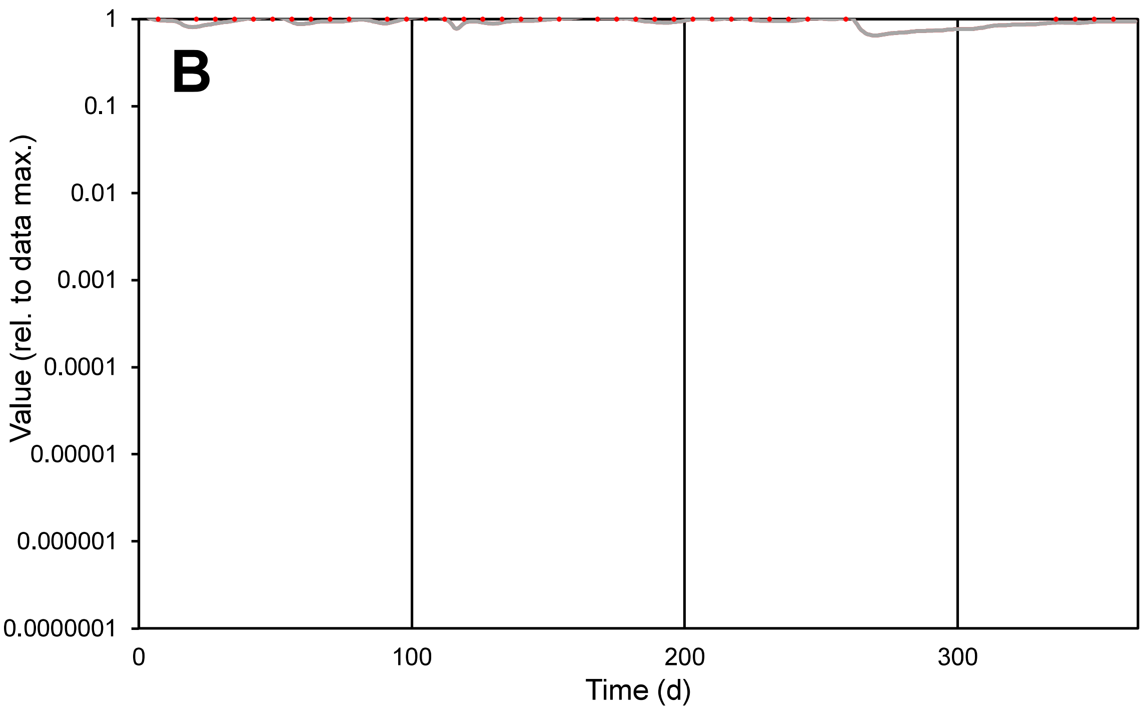

C1

Variables

PRY Provide jtd or Dst

CalibOnly?

2 1 to reproduce of

oNormalize?

2 1=no, 2=yes

ofxn calc*

0.06498

ofxn mod

0.12771

sRun

RunA1

zout

1

Ave

Data

8.8E-06

Model

8.5E-06

data/vMax

8.8E-06

ofxng

311.734

Min d/m

1.20295

ave/min d

1

ave/min m

1.16437

331_Calib_S_A

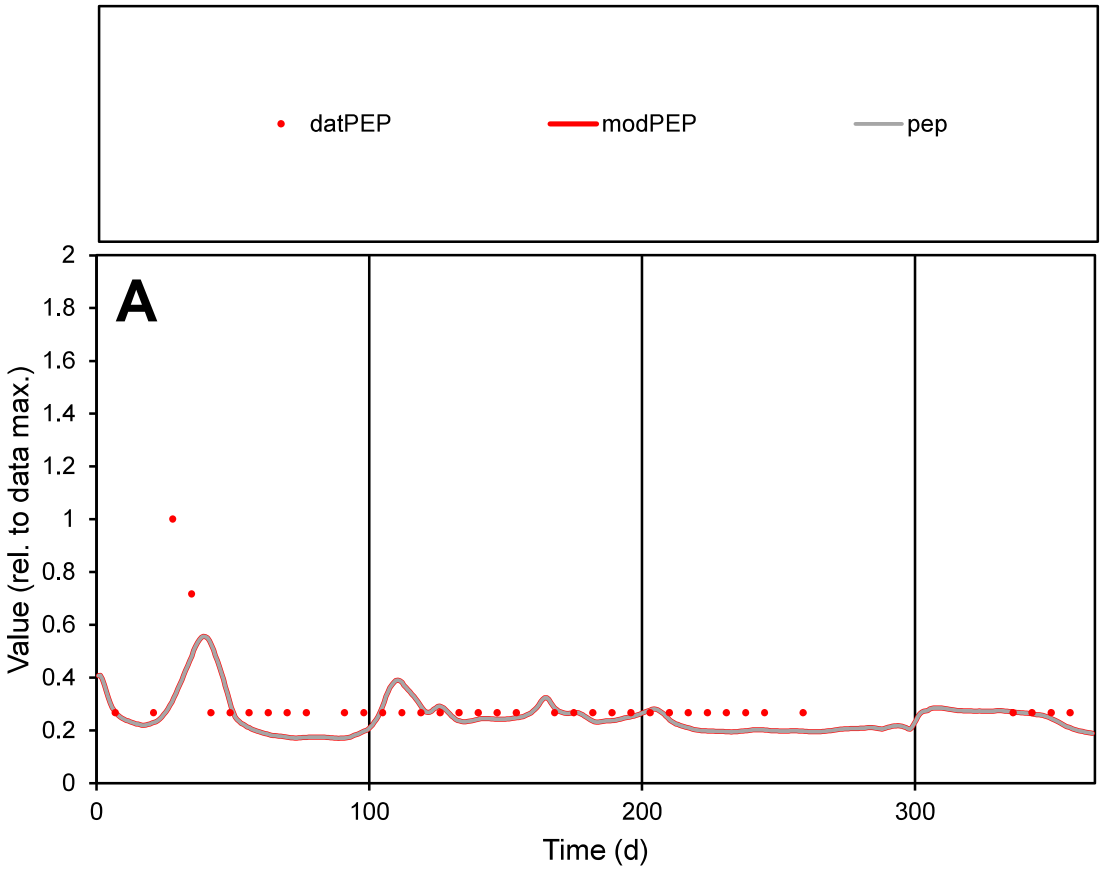

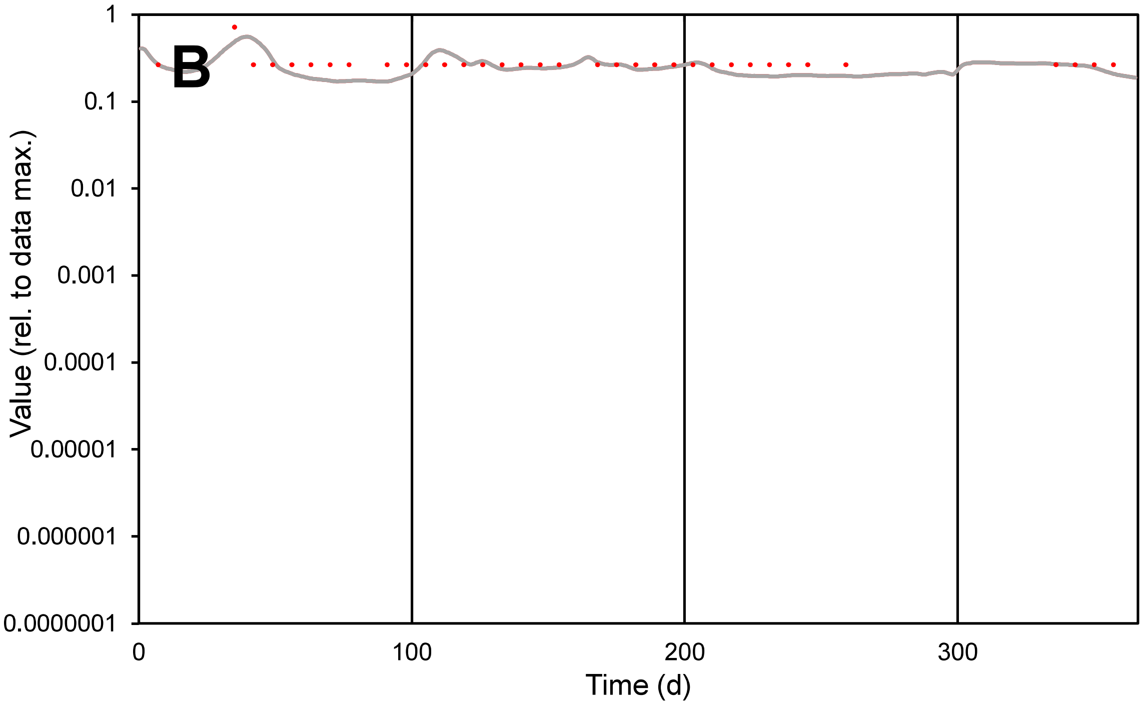

C1

Variables

PEP Provide jtd or Dst

CalibOnly?

2 1 to reproduce of

oNormalize?

2 1=no, 2=yes

ofxn calc*

0.43217

ofxn mod

0.44522

sRun

RunA1

zout

1

Ave

Data

5.5E-06

Model

4.7E-06

data/vMax

1.9E-05

ofxng

311.734

Min d/m

1.5506

ave/min d

1.11997

ave/min m

1.47404

331_Calib_S_A

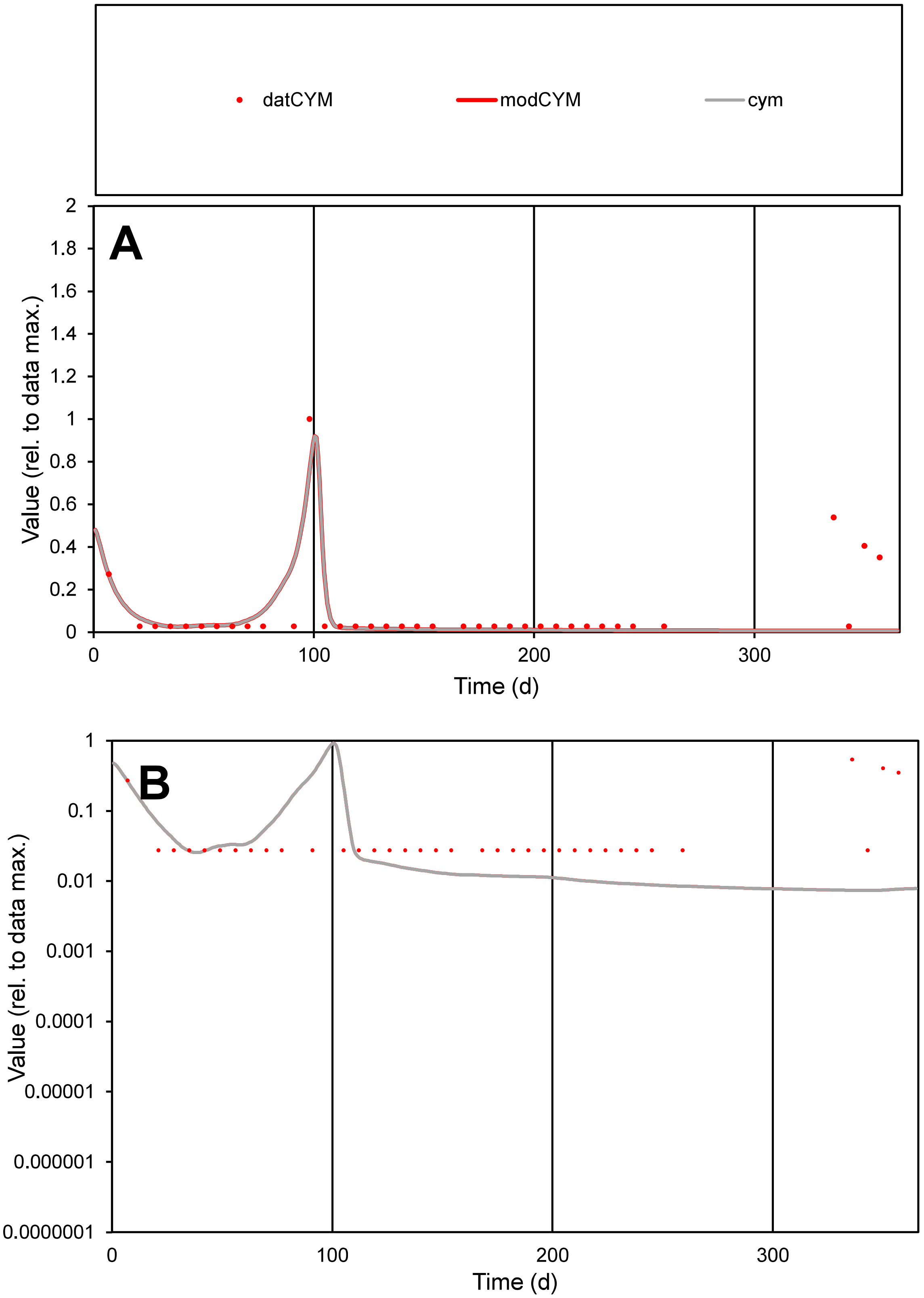

C1

Variables
CYM Provide jtd or Dst
CalibOnly?
2 1 to reproduce of
oNormalize?
2 1=no, 2=yes

ofxn calc*
1.4412
ofxn mod
1.47257

sRun
RunA1
zout
1

Ave
Data
4.6E-06
Model
2.9E-06

data/vMax
4.9E-05

ofxng
311.734

Min d/m
3.71756

ave/min d
3.38626
ave/min m
7.9671

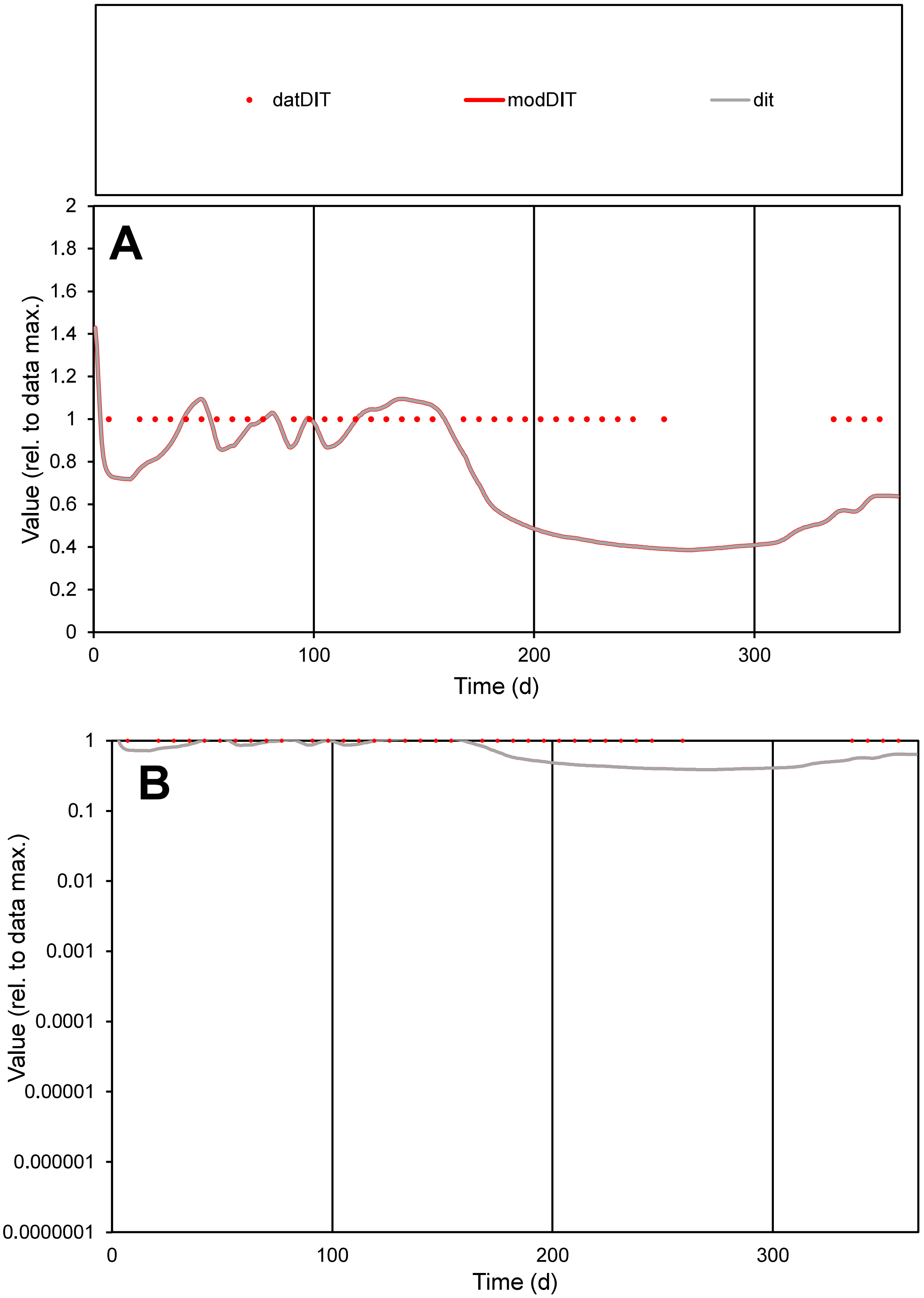

C1

Variables
DIT Provide jtd or Dst
CalibOnly?
2 1 to reproduce of
oNormalize?
2 1=no, 2=yes

ofxn calc*
0.34314
ofxn mod
0.35702

sRun
RunA1
zout
1

Ave
Data
5.1E-06
Model
3.9E-06

data/vMax
5.1E-06

ofxng
311.734

Min d/m
2.56241

ave/min d
1
ave/min m
1.93944

331_Calib_S_A

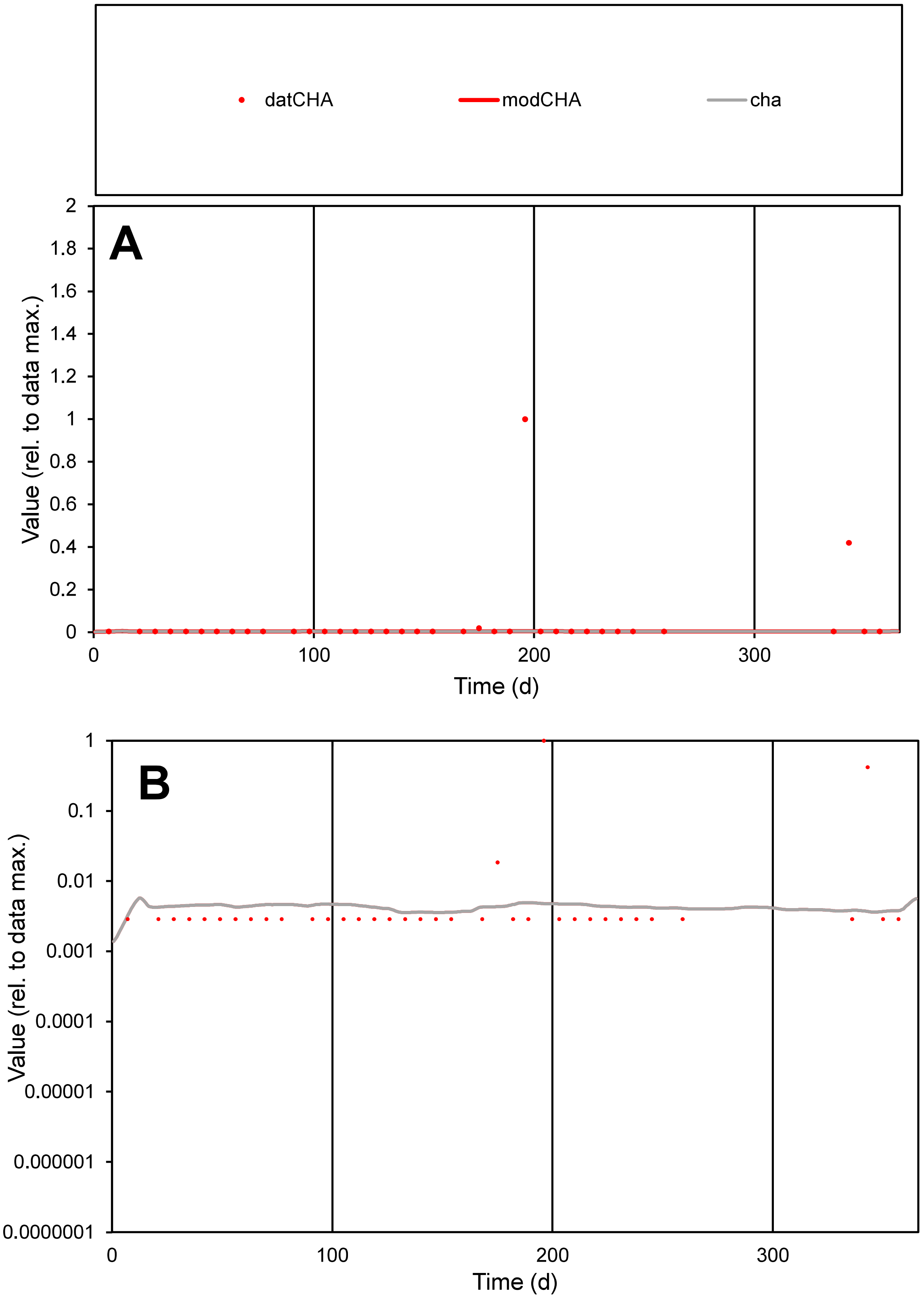

C1

Variables

CHA Provide jtd or Dst

CalibOnly?

2 1 to reproduce of

oNormalize?

2 1=no, 2=yes

ofxn calc*

4.27373

ofxn mod

4.27673

sRun

RunA1

zout

1

Ave

Data

1.3E-06

Model

1.3E-07

data/vMax

3E-05

ofxng

311.734

Min d/m

0.87327

ave/min d

14.4796

ave/min m

1.29946

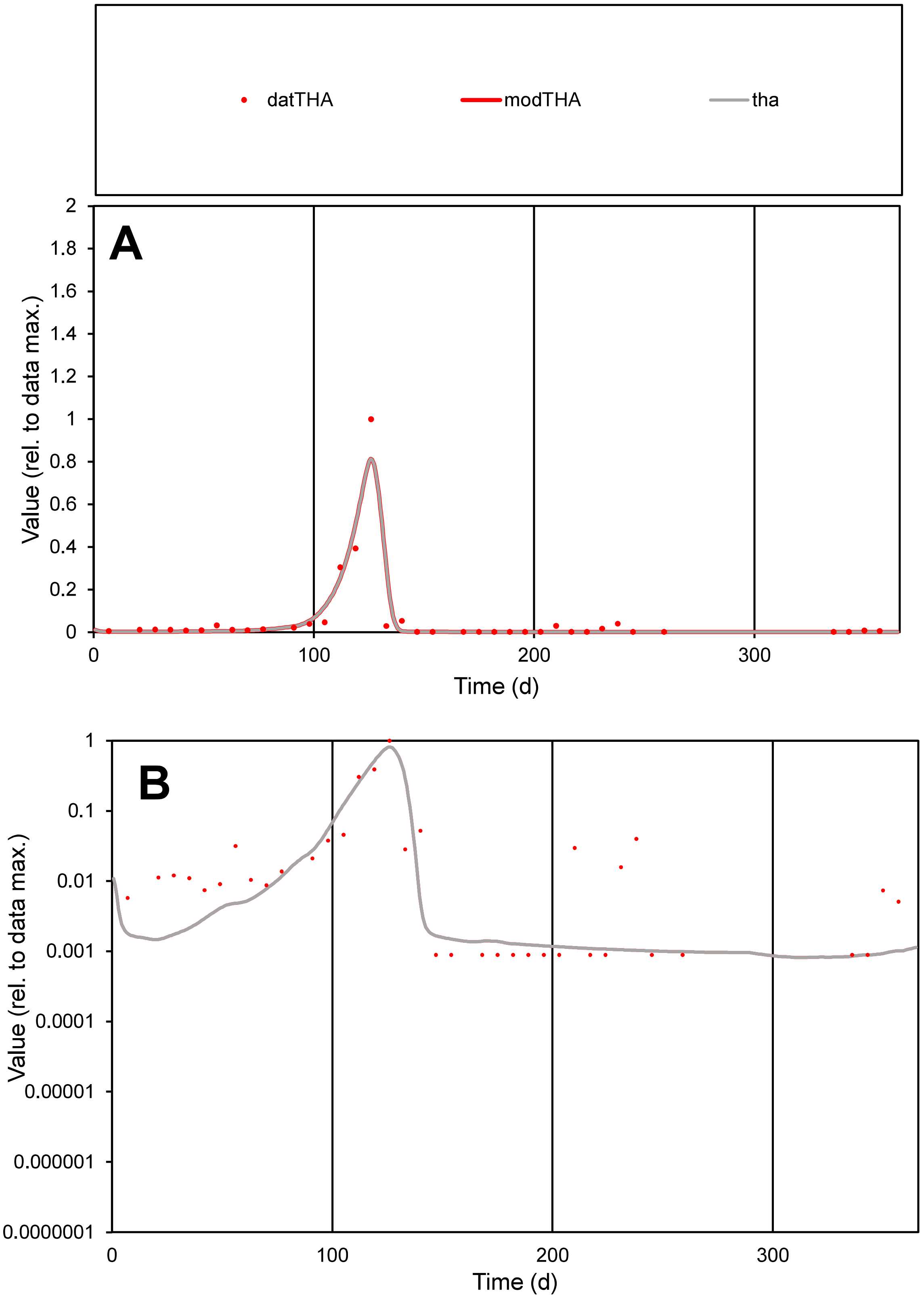

C1

Variables
THA Provide jtd or Dst
CalibOnly?
2 1 to reproduce of
oNormalize?
2 1=no, 2=yes

ofxn calc*
0.80221
ofxn mod
0.92106

sRun
RunA1
zout
1

Ave
Data
0.00018
Model
0.00017

data/vMax
0.0031

ofxng
311.734

Min d/m
1.02687
ave/min d
64.3153
ave/min m
62.862

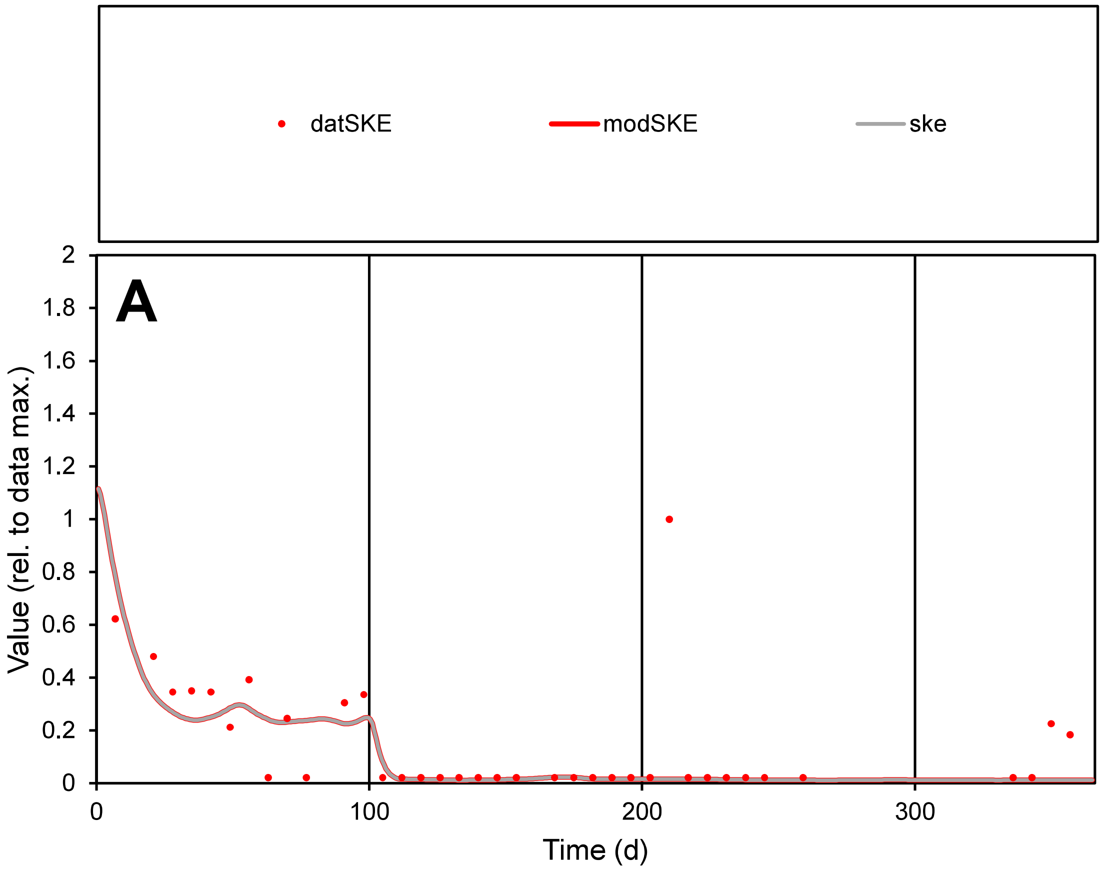

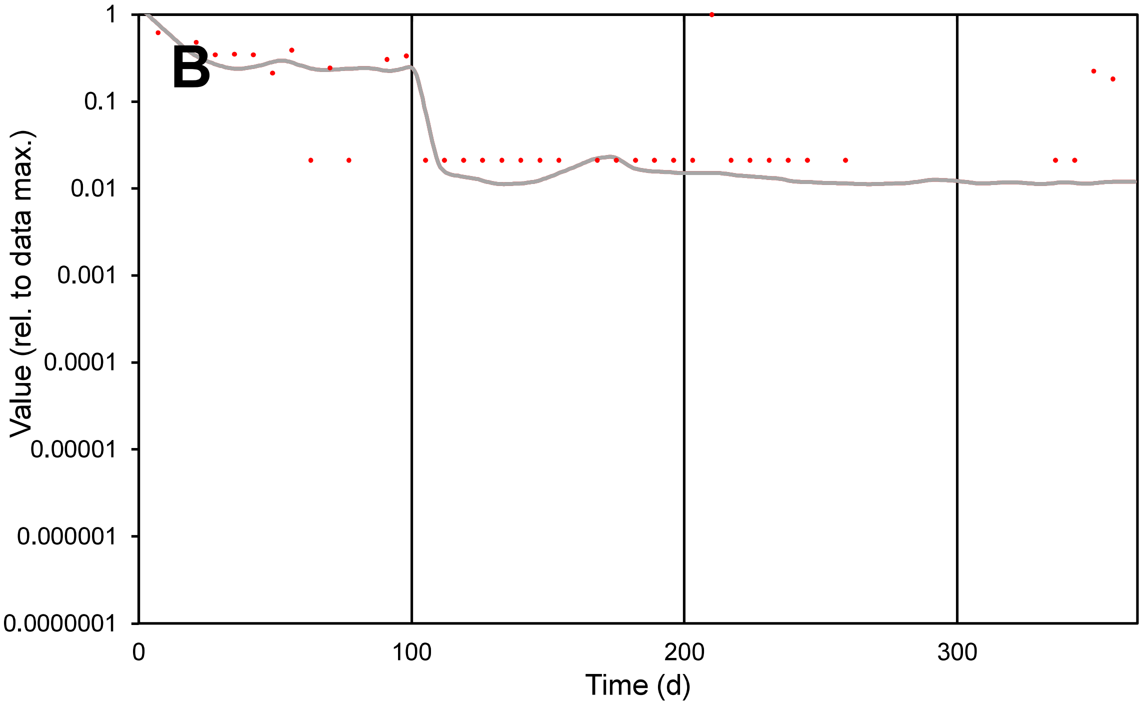

C1

Variables
SKE Provide jtd or Dst
CalibOnly?
2 1 to reproduce of
oNormalize?
2 1=no, 2=yes

ofxn calc*
1.22065
ofxn mod
1.23091

sRun
RunA1
zout
1

Ave
Data
1.6E-05
Model
1.2E-05

data/vMax
0.00011

ofxng
311.734

Min d/m
1.87531

ave/min d
7.0905
ave/min m
9.40759

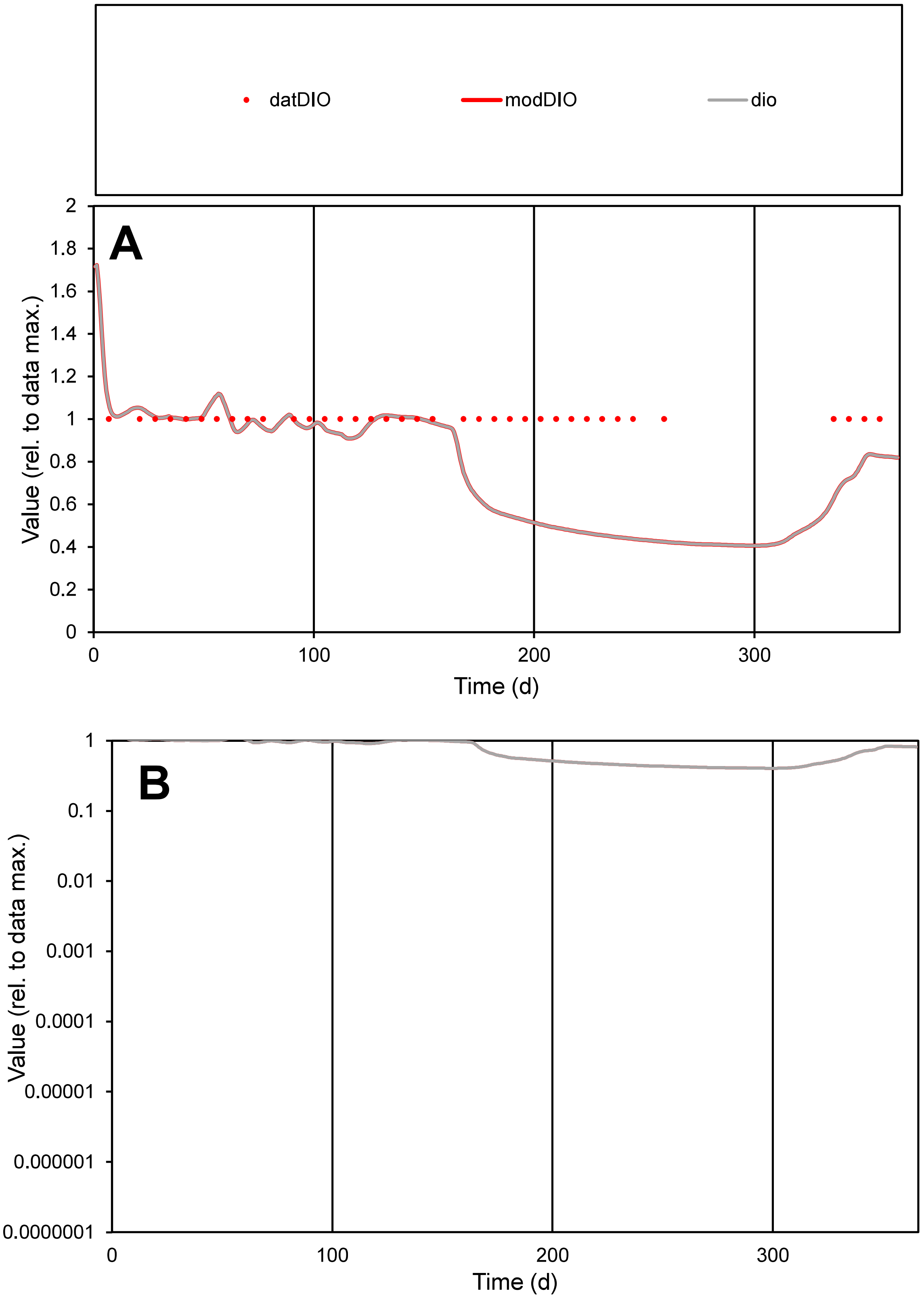

C1

Variables
DIO Provide jtd or Dst
CalibOnly?
2 1 to reproduce of
oNormalize?
2 1=no, 2=yes

ofxn calc*
0.3069
ofxn mod
0.32312

sRun
RunA1
zout
1

Ave
Data
4.7E-06
Model
3.7E-06

data/vMax
4.7E-06

ofxng
311.734

Min d/m
2.36166

ave/min d
1
ave/min m
1.8854

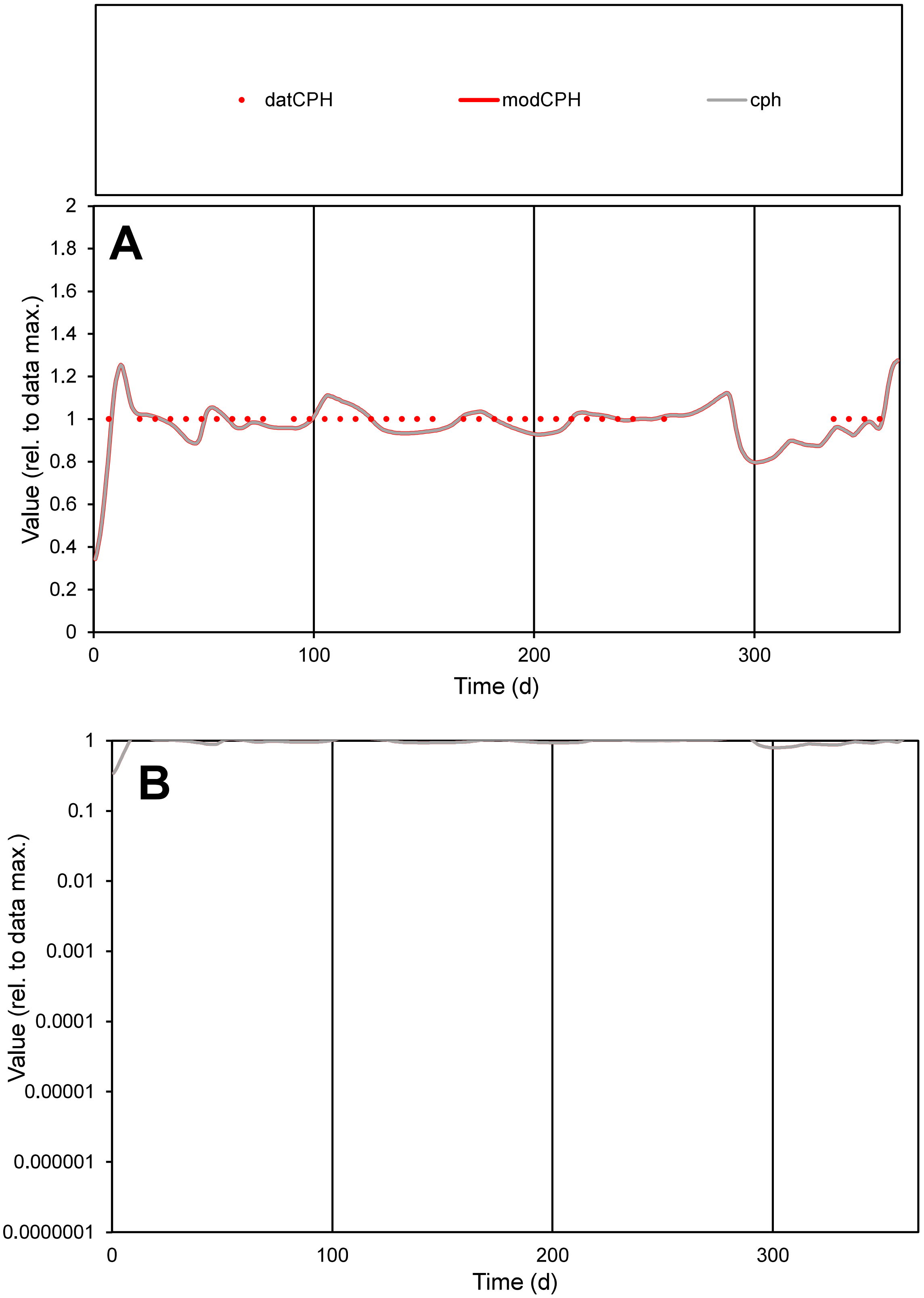

C1

Variables
CPH Provide jtd or Dst
CalibOnly?
2 1 to reproduce of
oNormalize?
2 1=no, 2=yes

ofxn calc*
0.04645
ofxn mod
0.1201

sRun
RunA1
zout
1

Ave
Data
9.6E-06
Model
9.5E-06

data/vMax
9.6E-06

ofxng
311.734

Min d/m
1.11822

ave/min d
1
ave/min m
1.10446

331_Calib_S_A

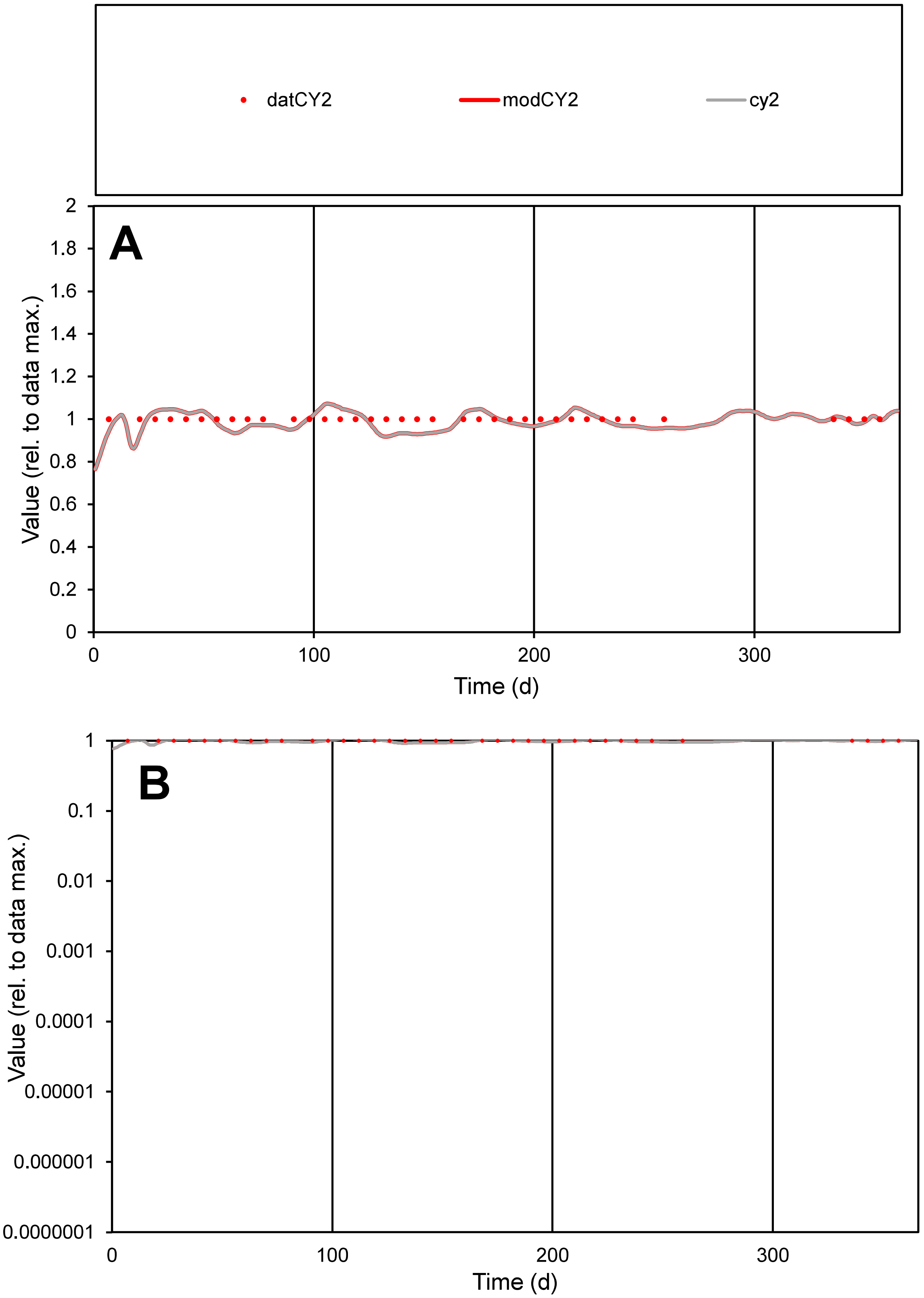

C1

Variables
CY2 Provide jtd or Dst
CalibOnly?
2 1 to reproduce of
oNormalize?
2 1=no, 2=yes

ofxn calc*
0.04022
ofxn mod
0.11805

sRun
RunA1
zout
1

Ave
Data
1.1E-05
Model
1.1E-05

data/vMax
1.1E-05

ofxng
311.734

Min d/m
1.08683

ave/min d
1
ave/min m
1.07808

331_Calib_S_A

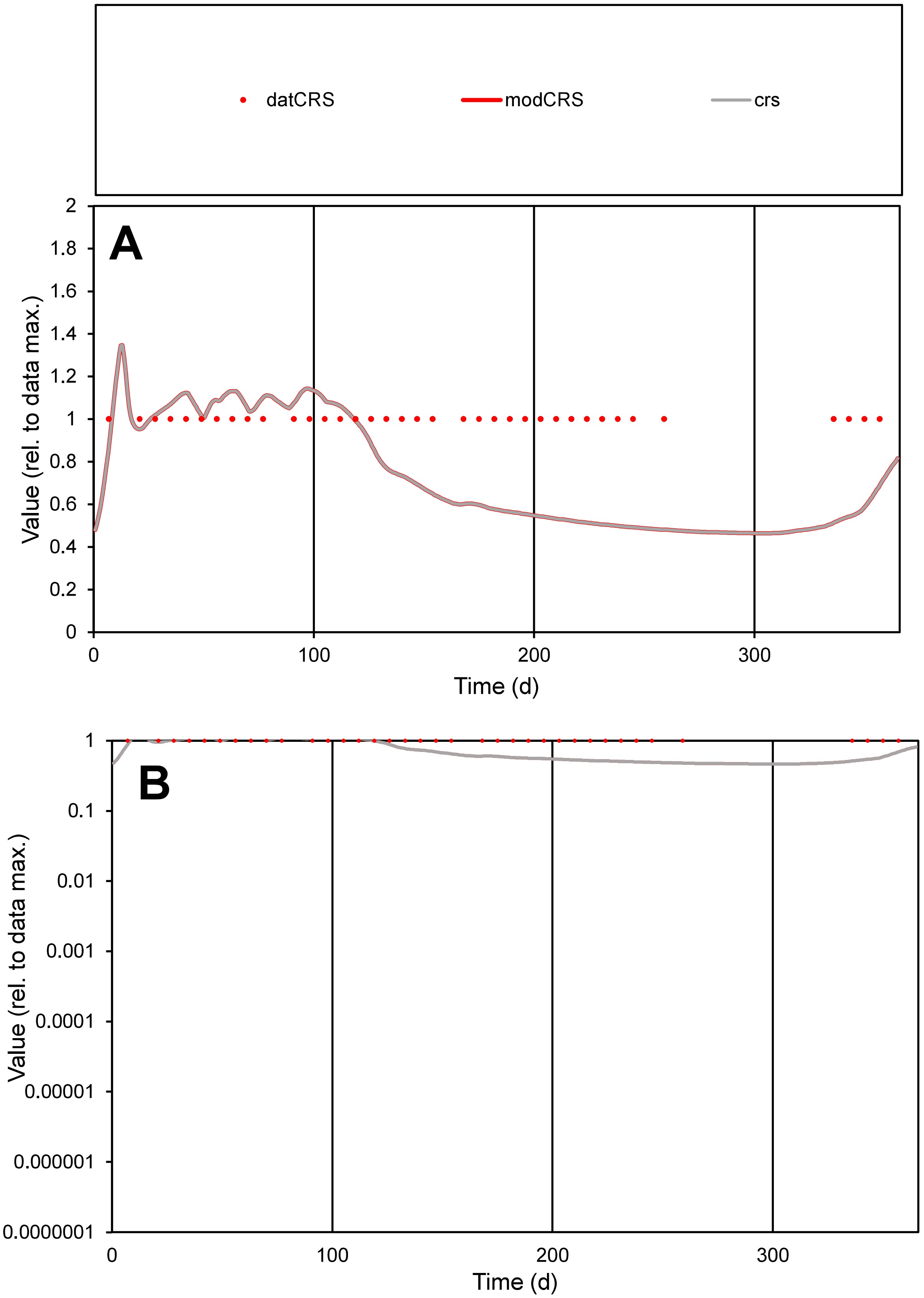

C1

Variables
CRS Provide jtd or Dst
CalibOnly?
2 1 to reproduce of
oNormalize?
2 1=no, 2=yes

ofxn calc*
0.32753
ofxn mod
0.3427

sRun
RunA1
zout
1

Ave
Data
5.4E-06
Model
4.2E-06

data/vMax
5.4E-06

ofxng
311.734

Min d/m
2.08157

ave/min d
1
ave/min m
1.62509

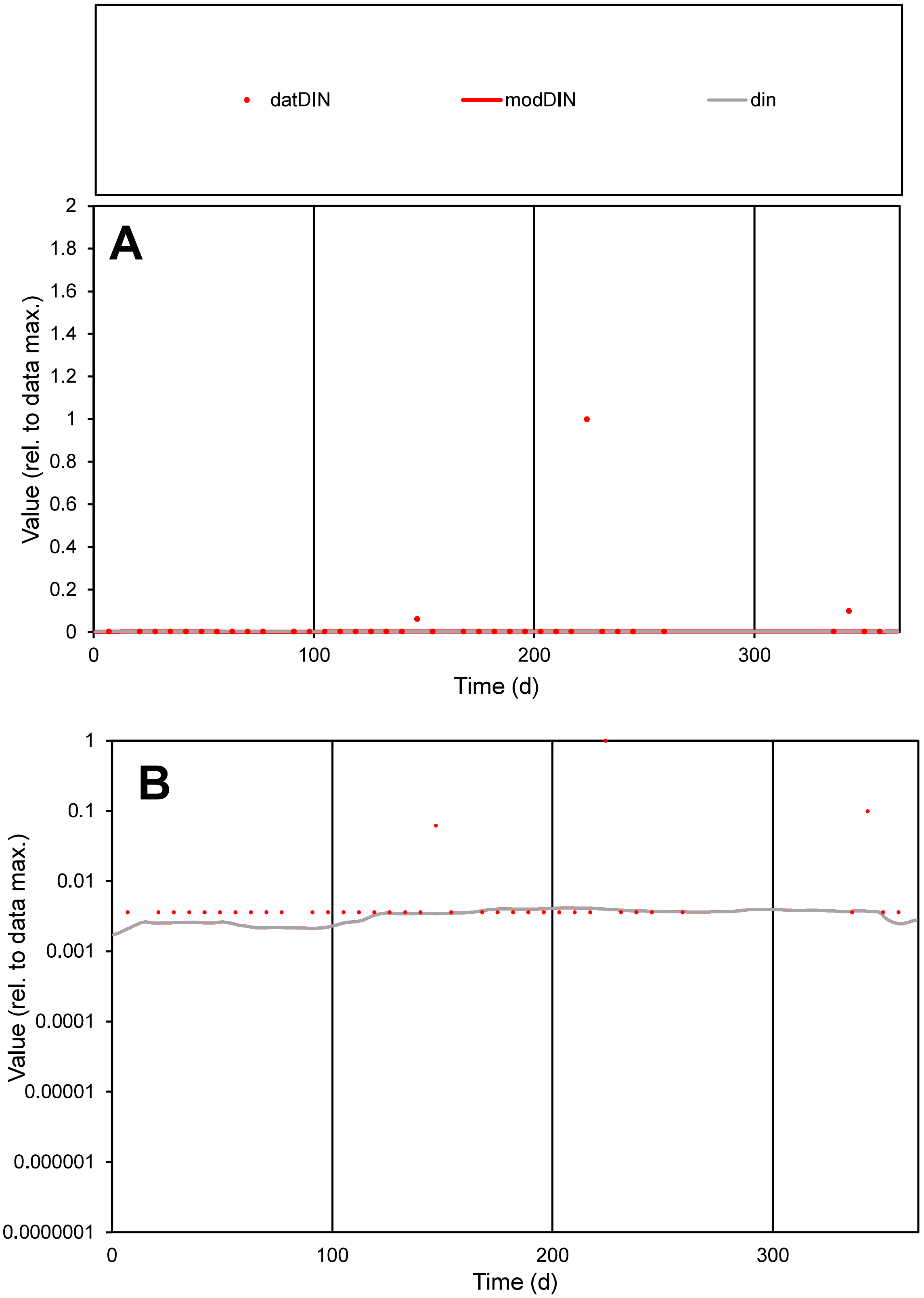

C1

Variables
DIN Provide jtd or Dst
CalibOnly?
2 1 to reproduce of
oNormalize?
2 1=no, 2=yes

ofxn calc*
4.75139
ofxn mod
4.76316

sRun
RunA1
zout
1

Ave
Data
2.8E-05
Model
2.6E-06

data/vMax
0.0008

ofxng
311.734

Min d/m
1.6905

ave/min d
9.66943
ave/min m
1.50821

331_Calib_S_A

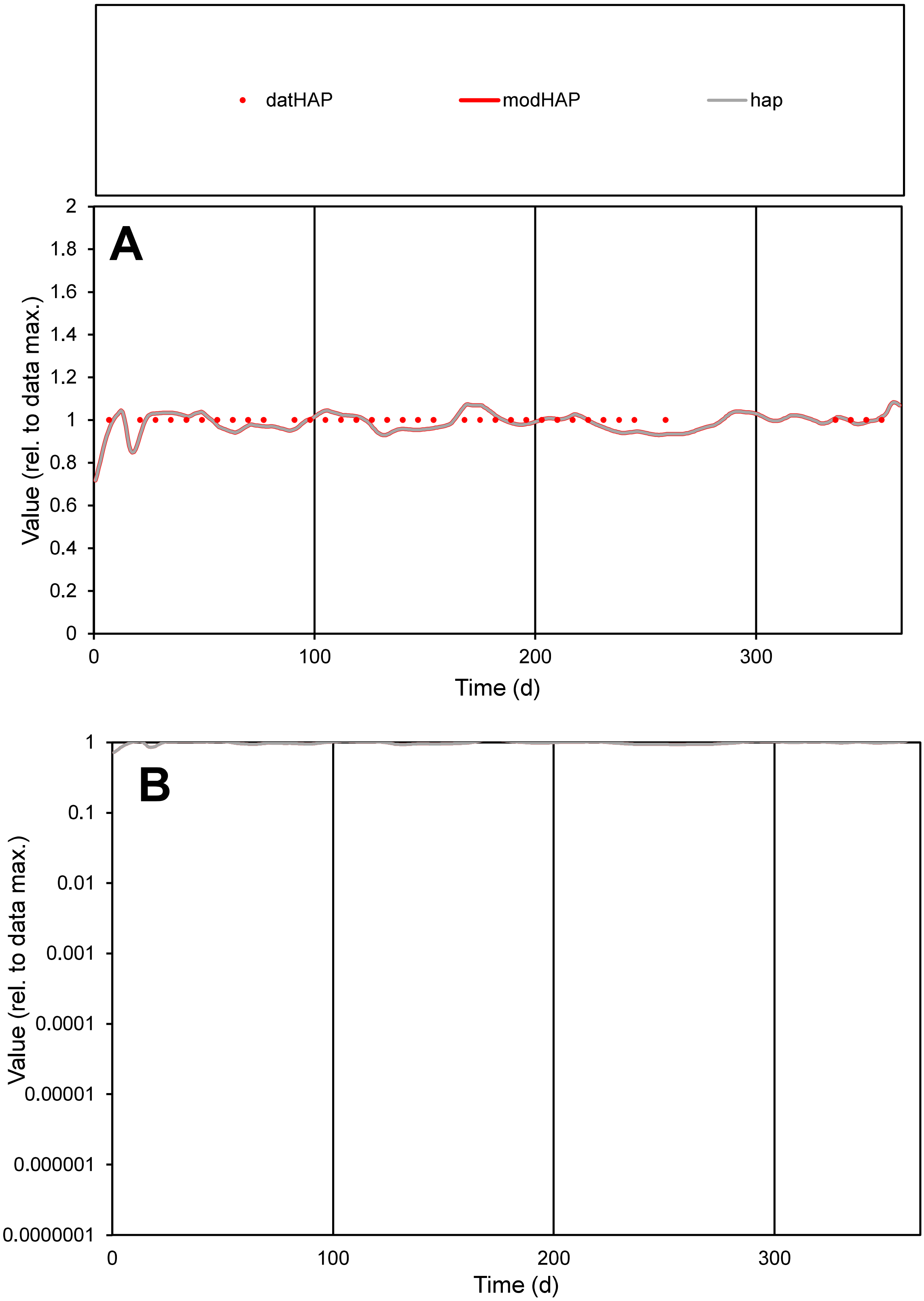

C1

Variables
HAP Provide jtd or Dst
CalibOnly?
2 1 to reproduce of
oNormalize?
2 1=no, 2=yes

ofxn calc*
0.03644
ofxn mod
0.11675

sRun
RunA1
zout
1

Ave
Data
1.1E-05
Model
1.1E-05

data/vMax
1.1E-05

ofxng
311.734

Min d/m
1.06975

ave/min d
1
ave/min m
1.06185

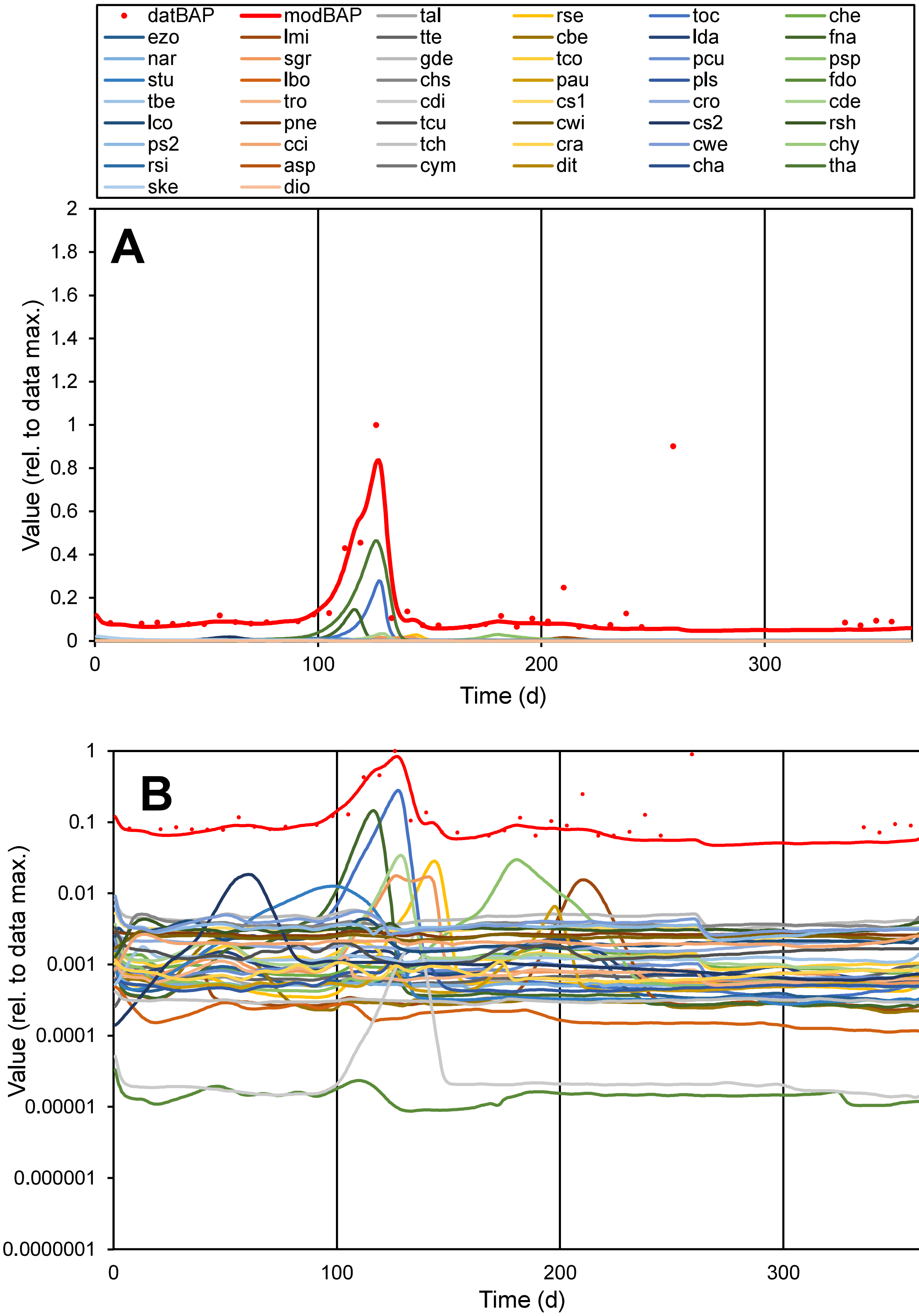

C1

Variables

BAP Provide jtd or Dst

CalibOnly?

2 1 to reproduce of

oNormalize?

2 1=no, 2=yes

ofxn calc*

0.93567

ofxn mod

0.94652

sRun

RunA1

zout

1

Ave

Data

0.00086

Model

0.00067

data/vMax

0.00542

ofxng

311.734

Min d/m

1.24654

ave/min d

2.44435

ave/min m

2.38654

331_Calib_S_A

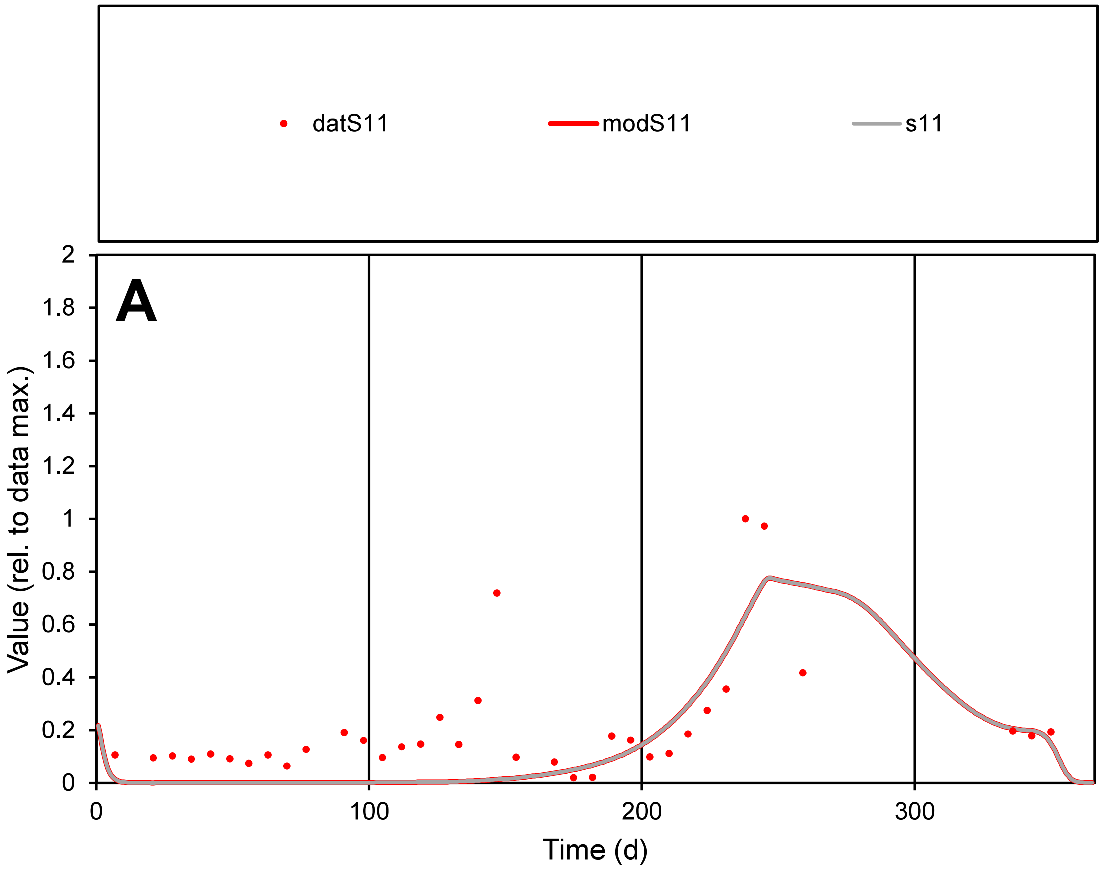

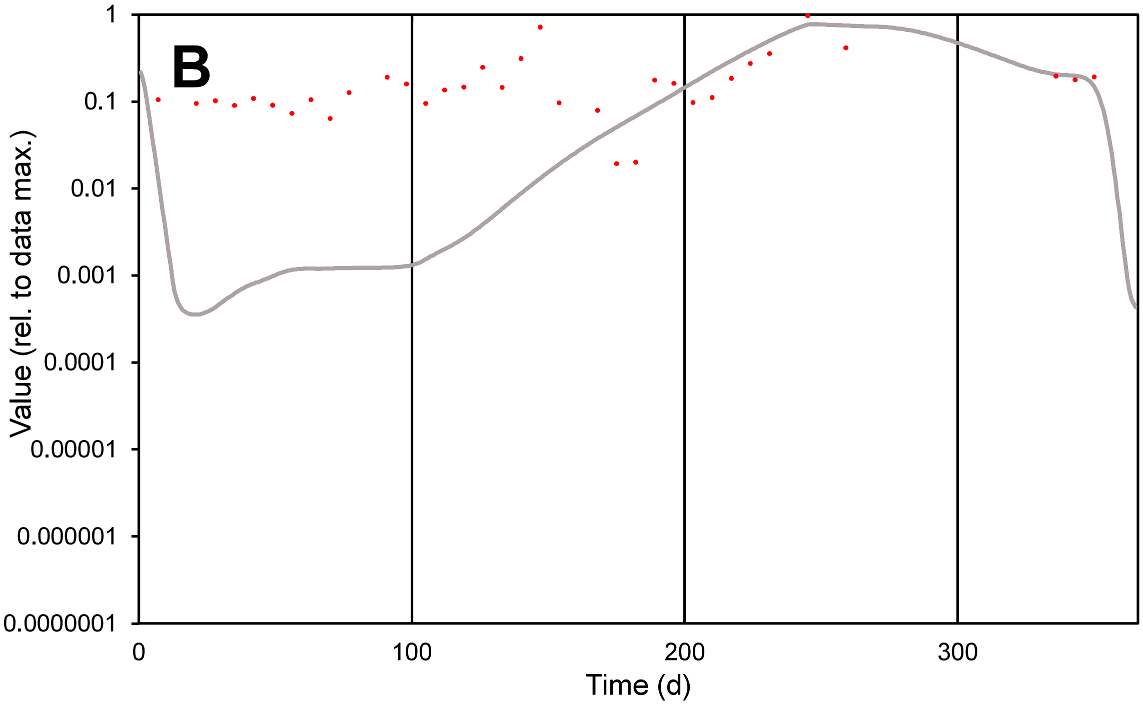

C1

Variables
S11 Provide jtd or Dst
CalibOnly?
2 1 to reproduce of
oNormalize?
2 1=no, 2=yes

ofxn calc*
0.78379
ofxn mod
0.80298

sRun
RunA1
zout
1

Ave
Data
0.08793
Model
0.05898

data/vMax
0.4403

ofxng
311.734

Min d/m
53.6008

ave/min d
10.3631
ave/min m
372.57

331_Calib_S_A

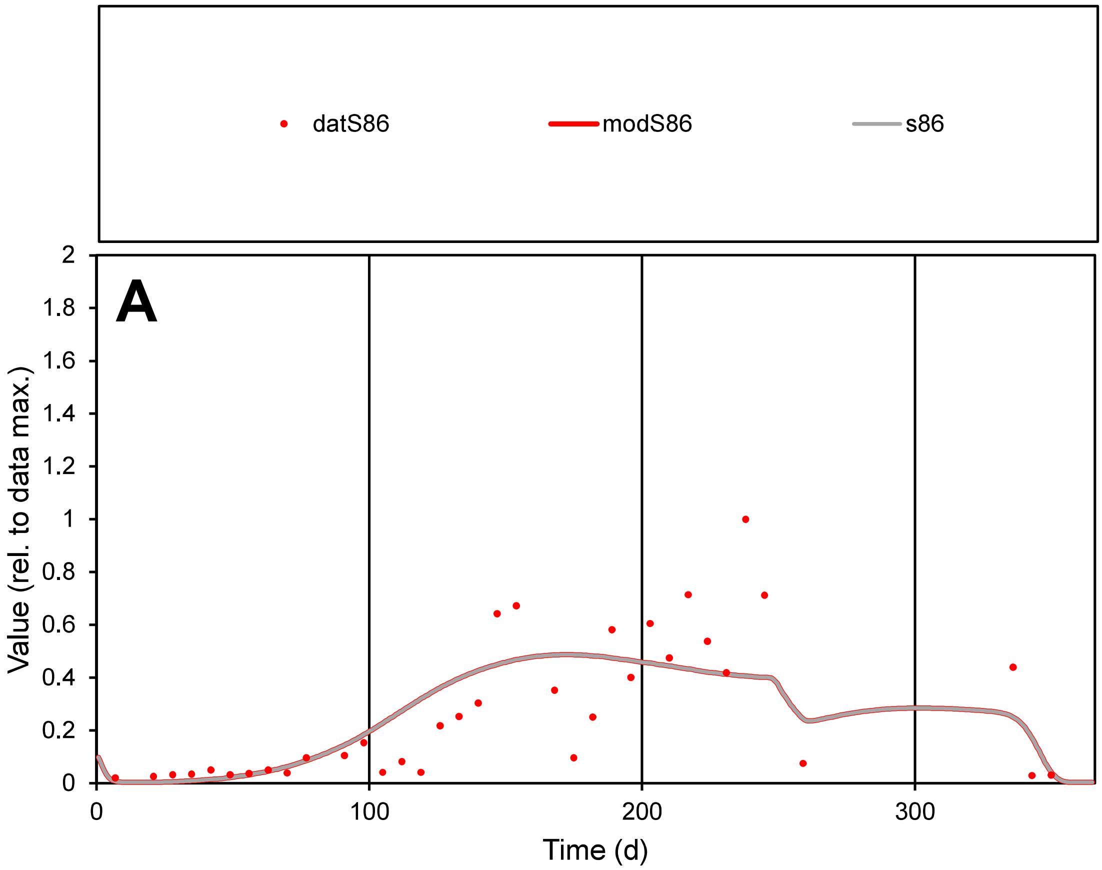

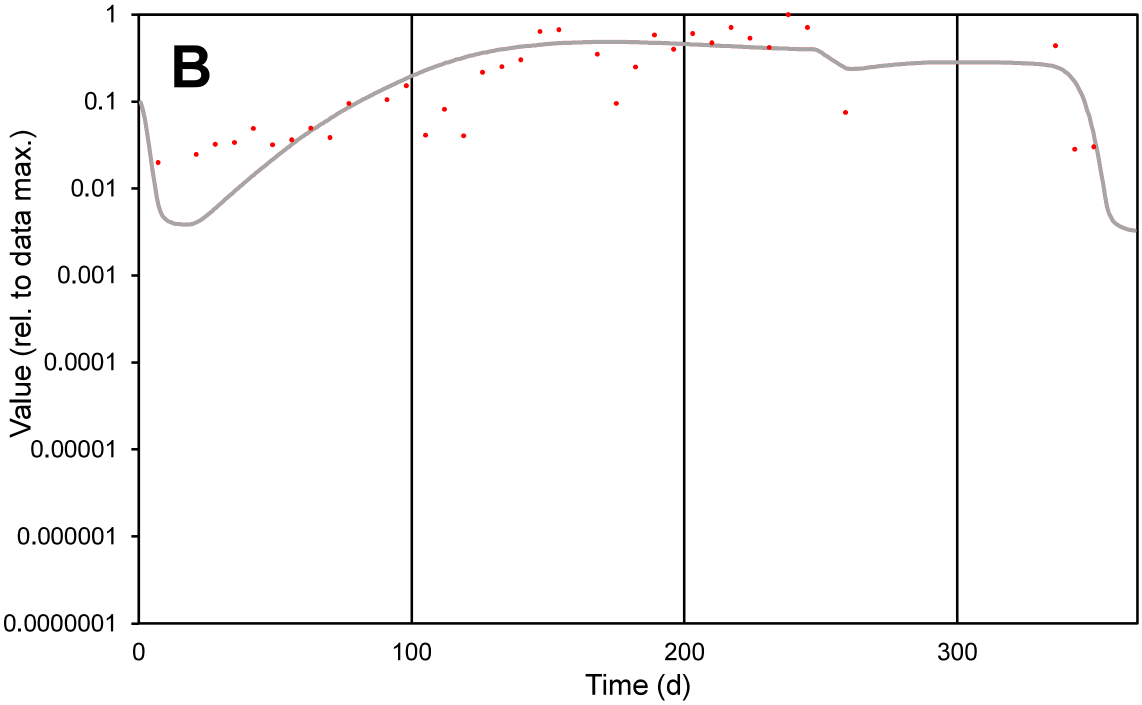

C1

Variables

S86 Provide jtd or Dst

CalibOnly?

2 1 to reproduce of

oNormalize?

2 1=no, 2=yes

ofxn calc*

0.73051

ofxn mod

0.7481

sRun

RunA1

zout

1

Ave

Data

0.07221

Model

0.0768

data/vMax

0.2882

ofxng

311.734

Min d/m

4.63636

ave/min d

12.5304

ave/min m

61.7884

331_Calib_S_A

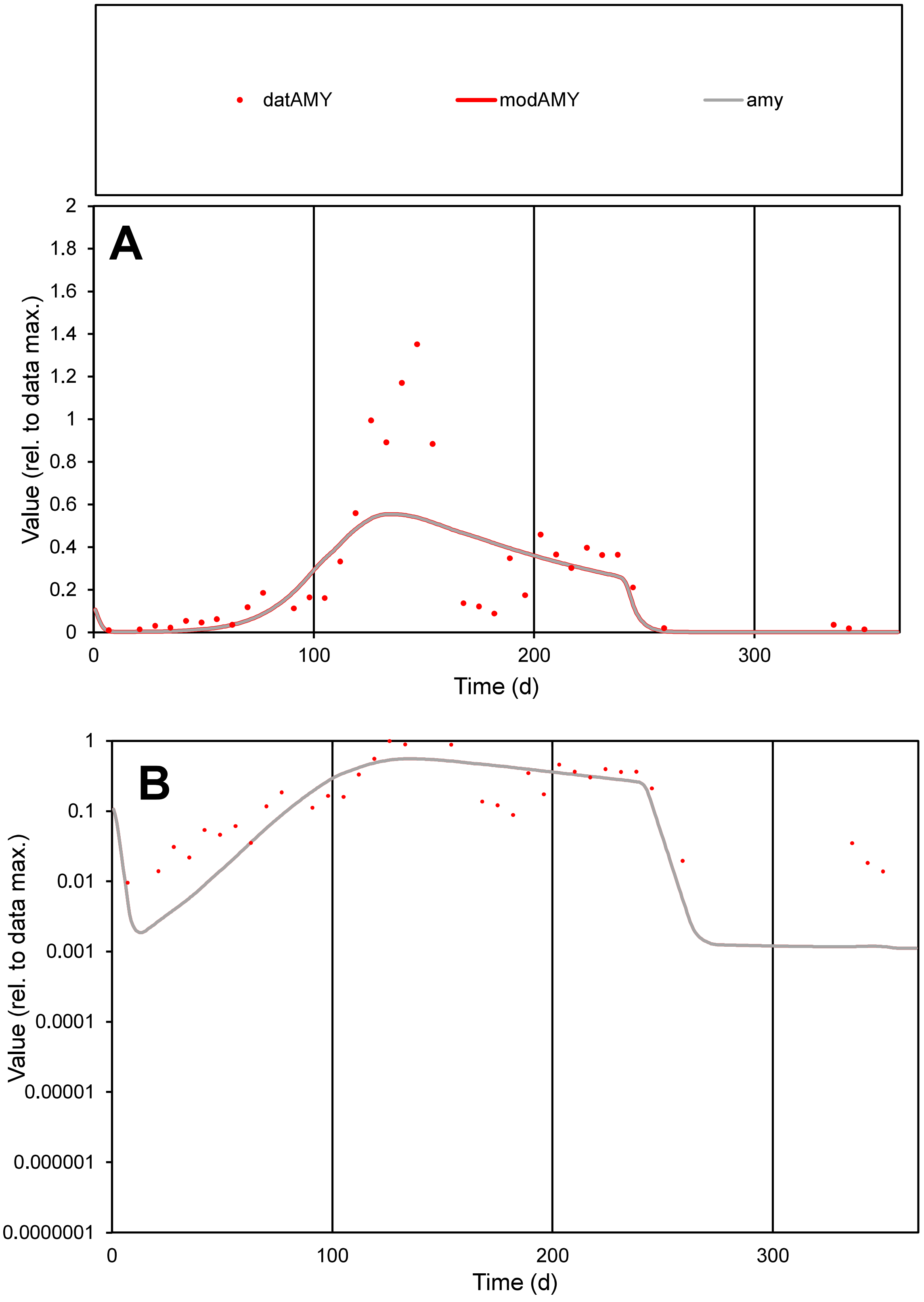

C1

Variables

AMY Provide jtd or Dst

CalibOnly?

2 1 to reproduce of

oNormalize?

2 1=no, 2=yes

ofxn calc*

0.58753

ofxn mod

0.60676

sRun

RunA1

zout

1

Ave

Data

0.05253

Model

0.051

data/vMax

0.2106

ofxng

311.734

Min d/m

8.17628

ave/min d

26.0955

ave/min m

207.142

331_Calib_S_A

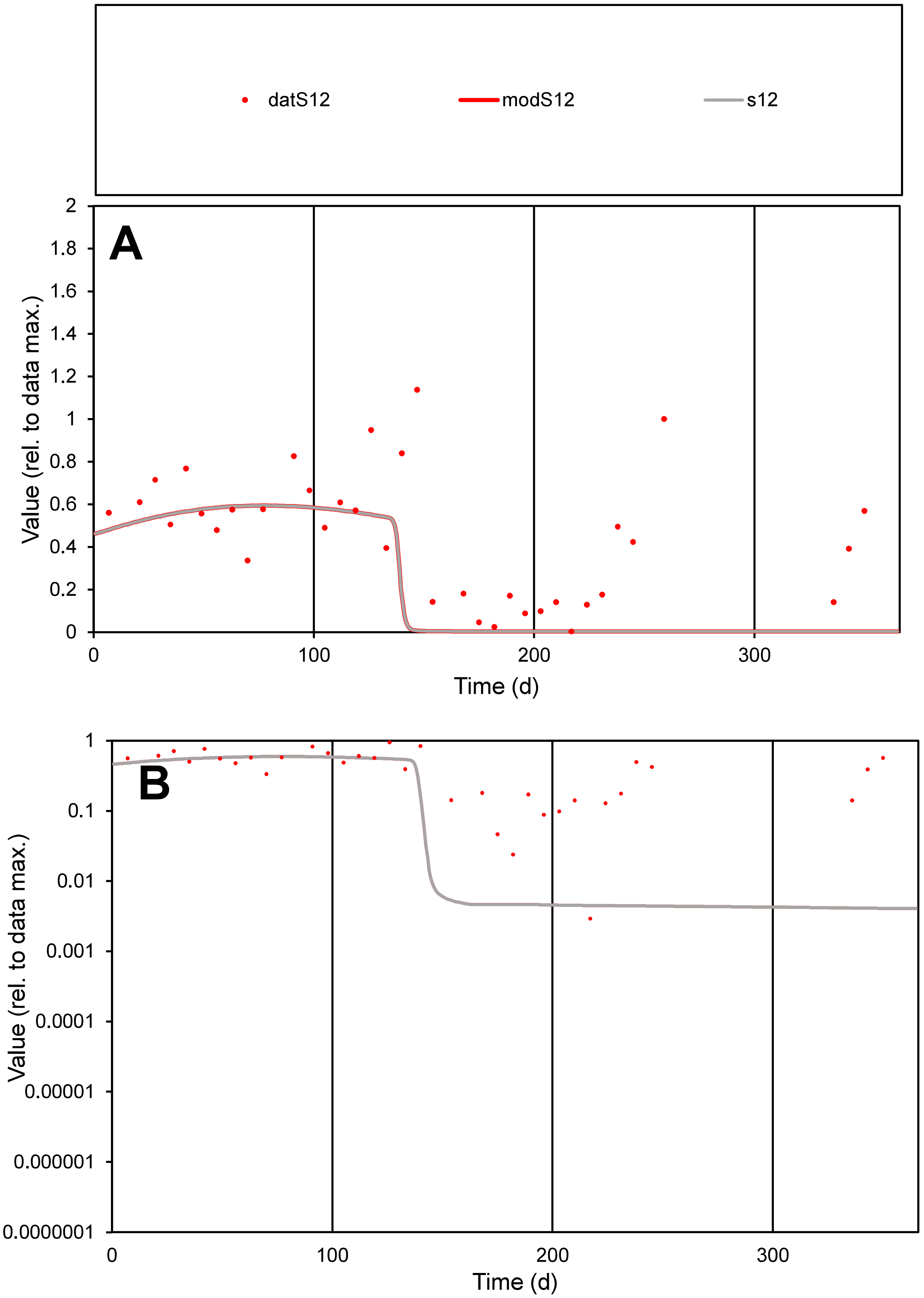

C1

Variables
S12 Provide jtd or Dst
CalibOnly?
2 1 to reproduce of
oNormalize?
2 1=no, 2=yes

ofxn calc*
0.68693
ofxn mod
0.70297

sRun
RunA1
zout
1

Ave
Data
0.02883
Model
0.01898

data/vMax
0.06785

ofxng
311.734

Min d/m
0.71552

ave/min d
145.086
ave/min m
68.3348

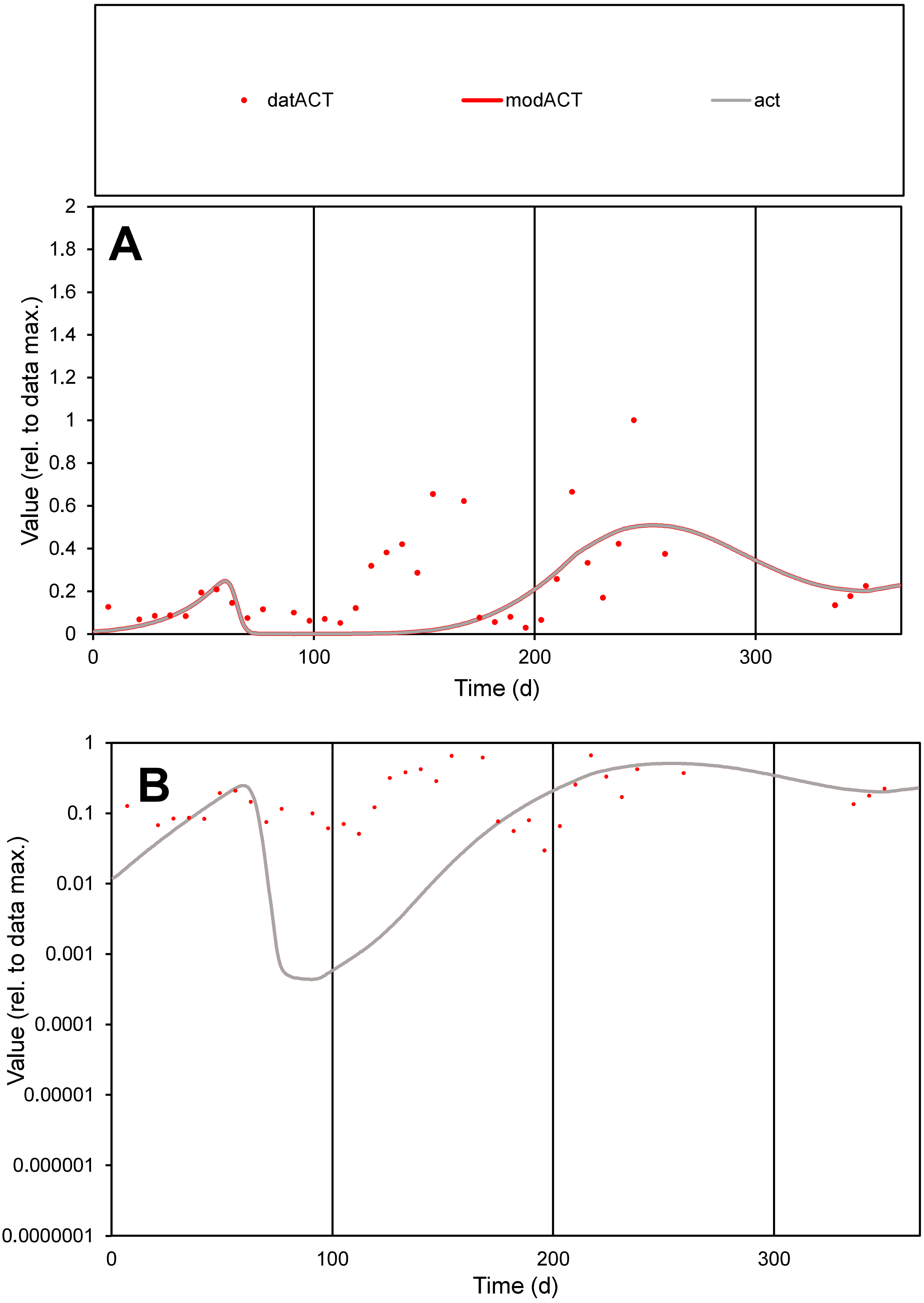

C1

Variables

ACT Provide jtd or Dst

CalibOnly?

2 1 to reproduce of

oNormalize?

2 1=no, 2=yes

ofxn calc*

0.89586

ofxn mod

0.90721

sRun

RunA1

zout

1

Ave Data

0.03595

Model

0.02531

data/vMax

0.1678

ofxng

311.734

Min d/m

67.2428

ave/min d

7.21885

ave/min m

341.745

331_Calib_S_A

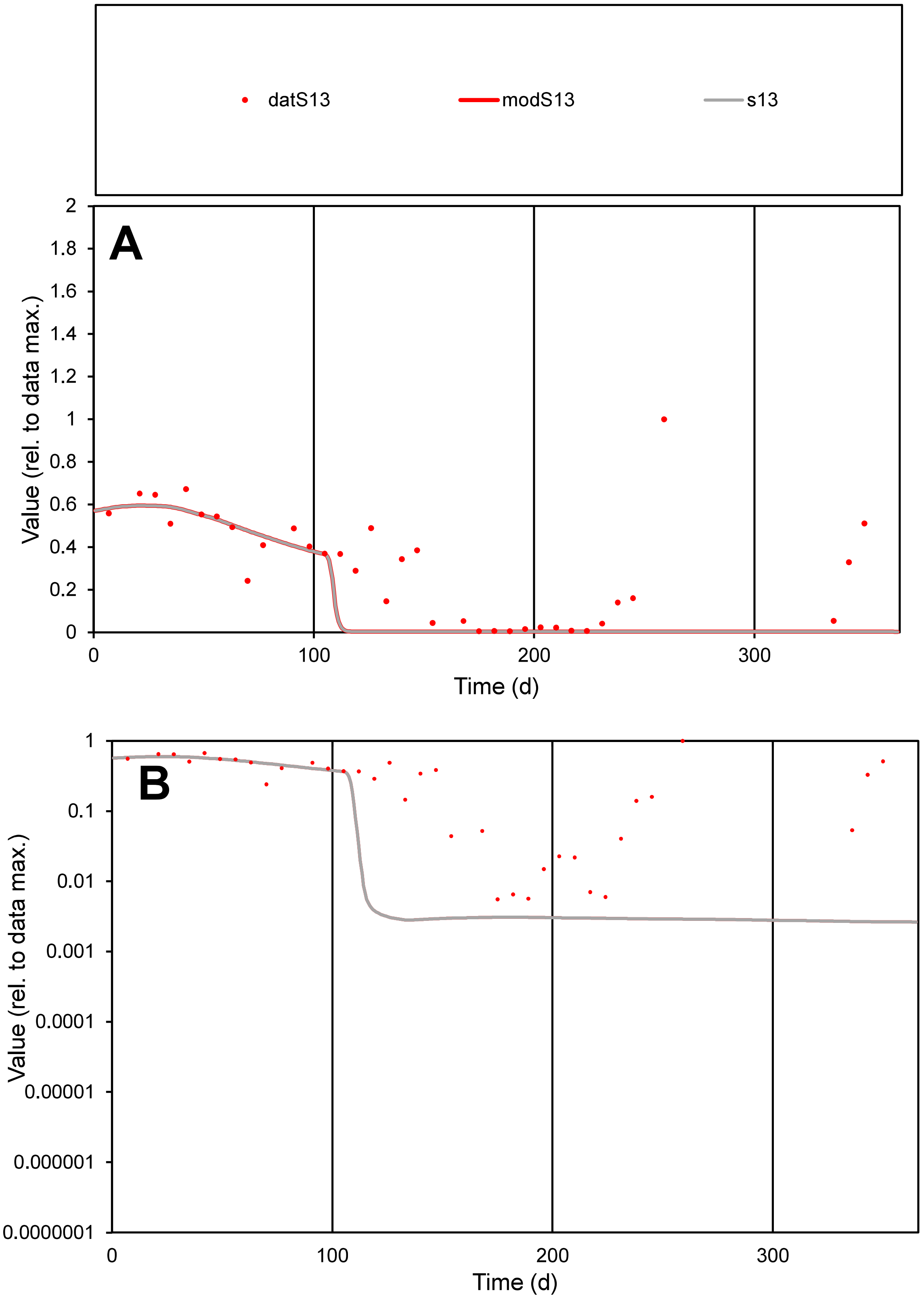

C1

Variables

S13 Provide jtd or Dst

CalibOnly?

2 1 to reproduce of

oNormalize?

2 1=no, 2=yes

ofxn calc*

0.77634

ofxn mod

0.79767

sRun

RunA1

zout

1

Ave

Data

0.01647

Model

0.01065

data/vMax

0.05632

ofxng

311.734

Min d/m

2.08166

ave/min d

52.9709

ave/min m

71.2855

331_Calib_S_A

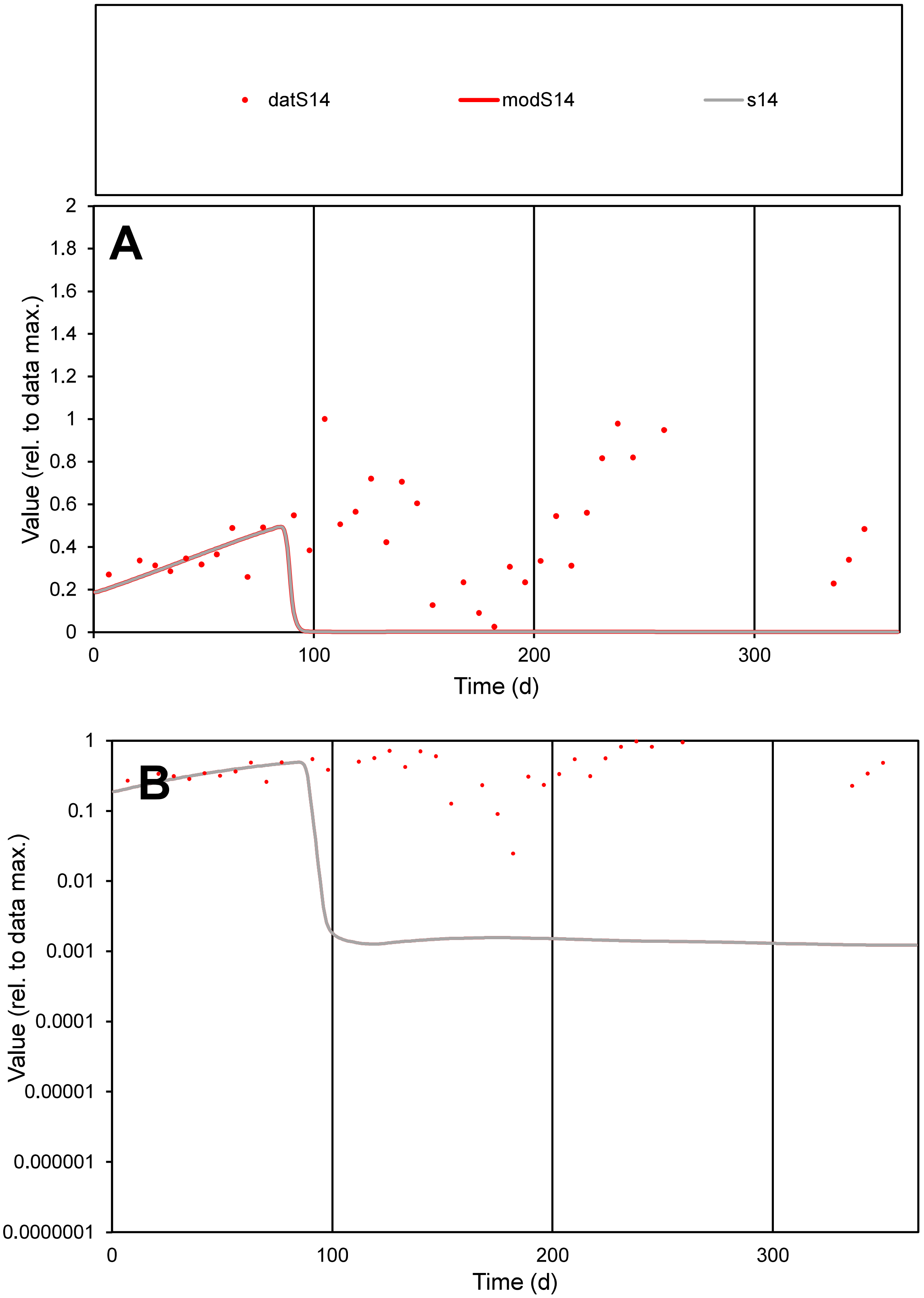

C1

Variables

S14 Provide jtd or Dst

CalibOnly?

2 1 to reproduce of

oNormalize?

2 1=no, 2=yes

ofxn calc*

1.03369

ofxn mod

1.04558

sRun

RunA1

zout

1

Ave

Data

0.02647

Model

0.00681

data/vMax

0.06152

ofxng

311.734

Min d/m

20.1058

ave/min d

17.4125

ave/min m

90.0623

331_Calib_S_A

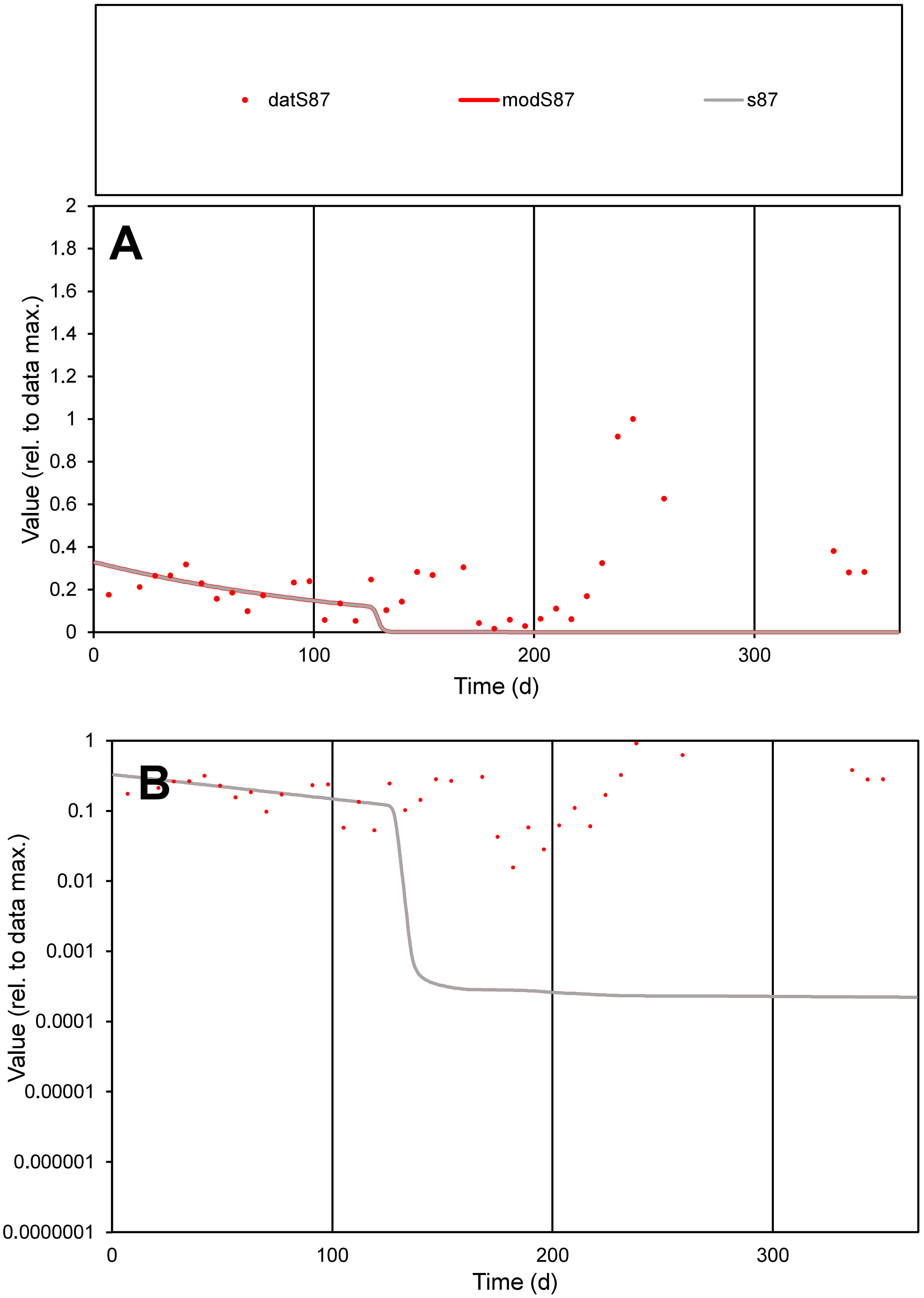

C1

Variables
S87 Provide jtd or Dst
CalibOnly?
2 1 to reproduce of
oNormalize?
2 1=no, 2=yes

ofxn calc*
1.25747
ofxn mod
1.26209

sRun
RunA1
zout
1

Ave
Data
0.01942
Model
0.00757

data/vMax
0.08536

ofxng
311.734

Min d/m
70.5728

ave/min d
14.4609
ave/min m
397.851

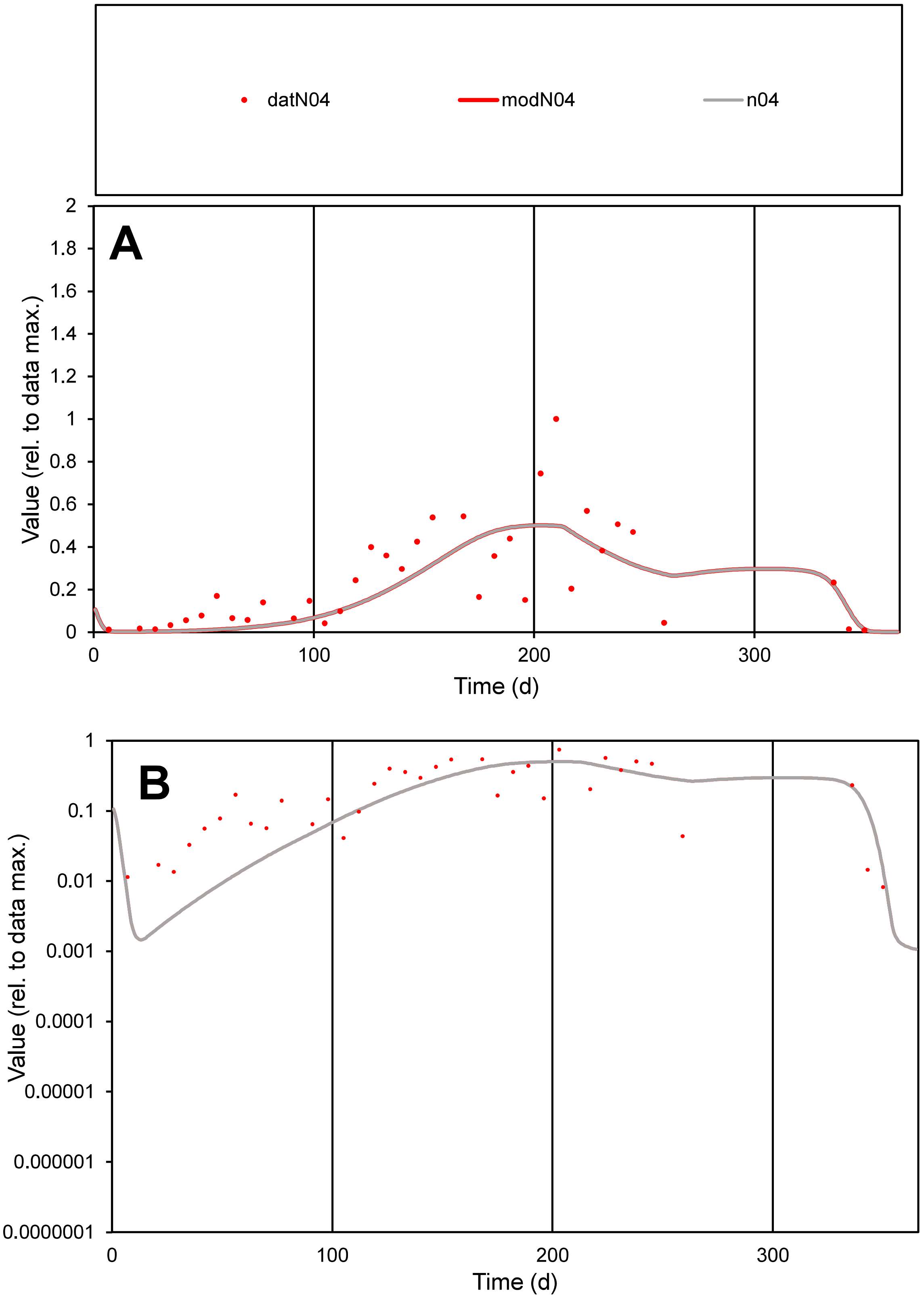

C1

Variables
N04 Provide jtd or Dst
CalibOnly?
2 1 to reproduce of
oNormalize?
2 1=no, 2=yes

ofxn calc*
0.64728
ofxn mod
0.66527

sRun
RunA1
zout
1

Ave
Data
0.02782
Model
0.02484

data/vMax
0.118

ofxng
311.734

Min d/m
3.4982

ave/min d
28.673
ave/min m
89.5546

331_Calib_S_A

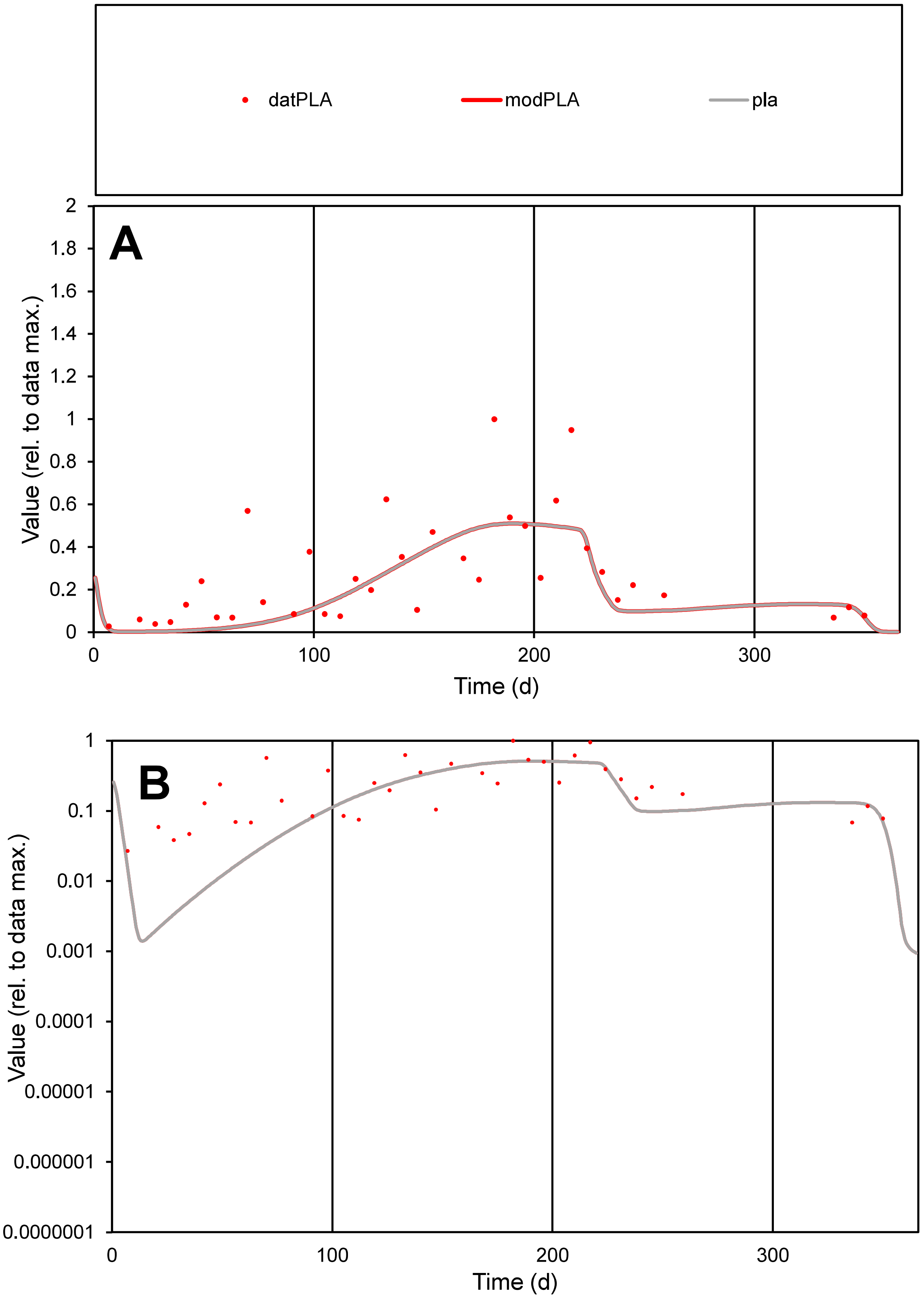

C1

Variables
PLA Provide jtd or Dst
CalibOnly?
2 1 to reproduce of
oNormalize?
2 1=no, 2=yes

ofxn calc*
0.70988
ofxn mod
0.72252

sRun
RunA1
zout
1

Ave
Data
0.03125
Model
0.02559

data/vMax
0.1197

ofxng
311.734

Min d/m
11.421

ave/min d
9.67243
ave/min m
90.4426

331_Calib_S_A

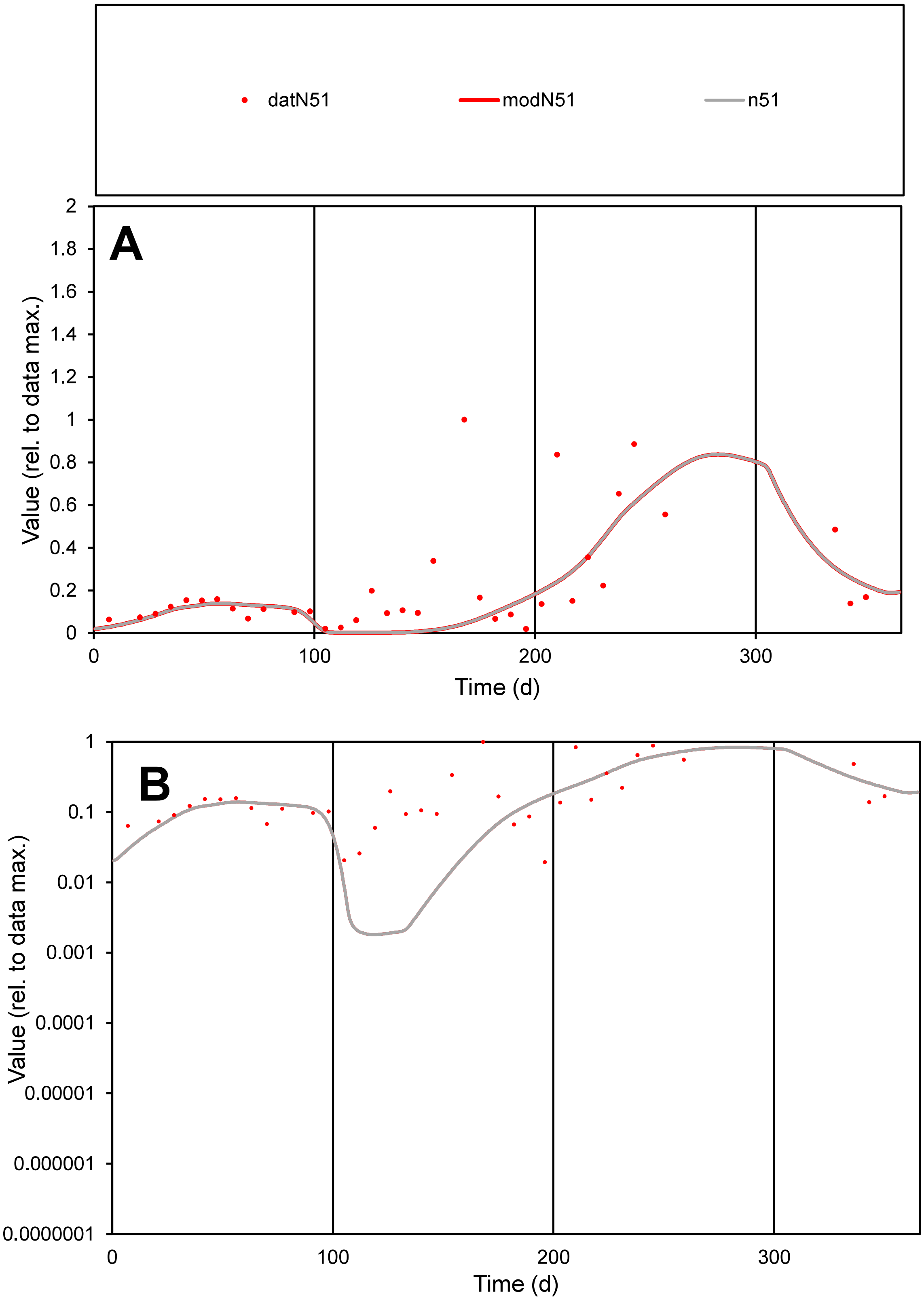

C1

Variables

N51	Provide jtd or Dst
CalibOnly?	
2	1 to reproduce of
oNormalize?	
2	1=no, 2=yes

ofxn calc*
0.96185
ofxn mod
0.97435

sRun
RunA1
zout
1

Ave
Data
0.01746
Model
0.01341

data/vMax
0.07922

ofxng
311.734

Min d/m
10.693

ave/min d
11.3176
ave/min m
92.9395

331_Calib_S_A

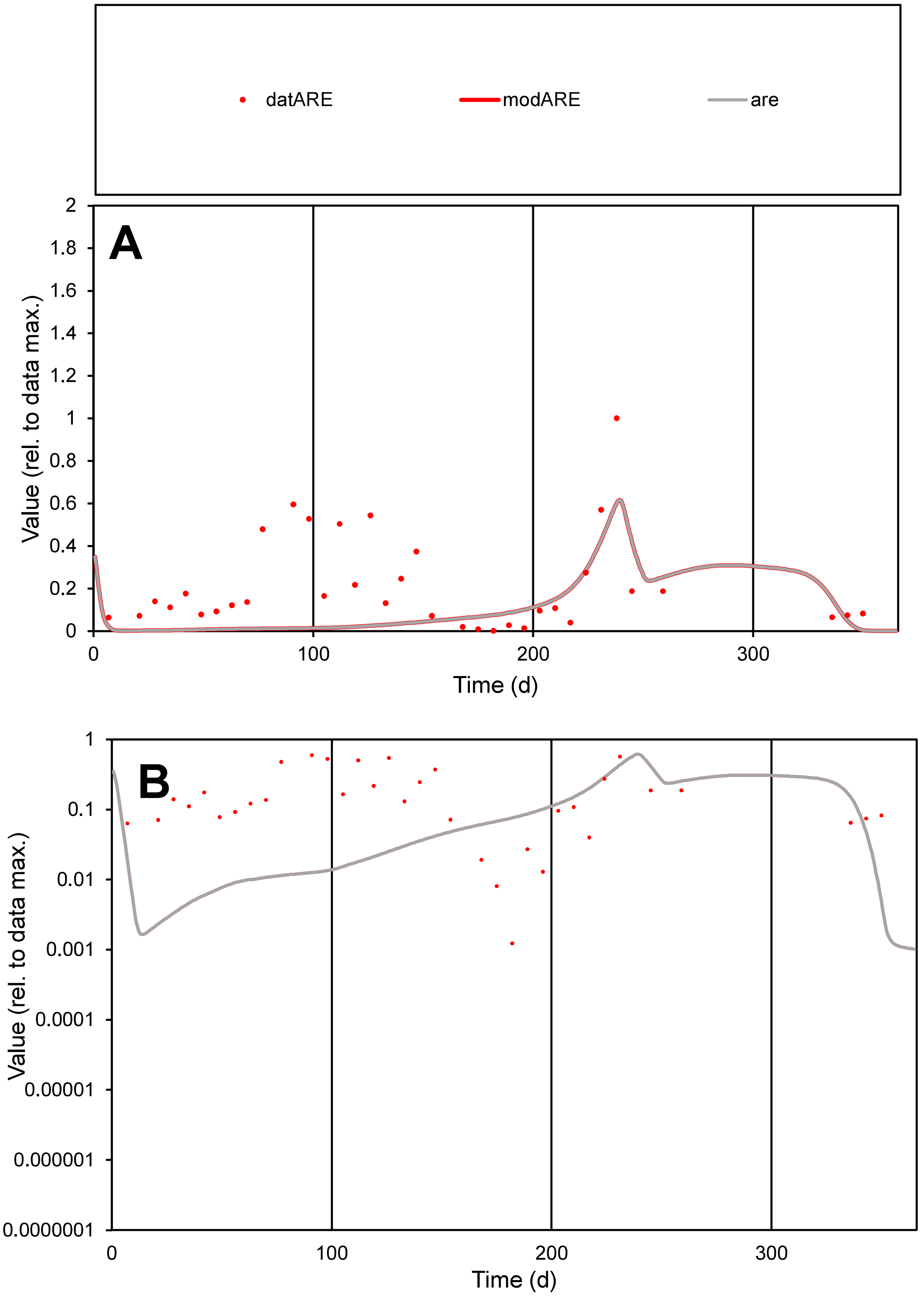

C1

Variables
ARE Provide jtd or Dst
CalibOnly?
2 1 to reproduce of
oNormalize?
2 1=no, 2=yes

ofxn calc*
1.08167
ofxn mod
1.0901

sRun
RunA1
zout
1

Ave
Data
0.01153
Model
0.00555

data/vMax
0.05779

ofxng
311.734

Min d/m
0.49179

ave/min d
161.664
ave/min m
38.2608

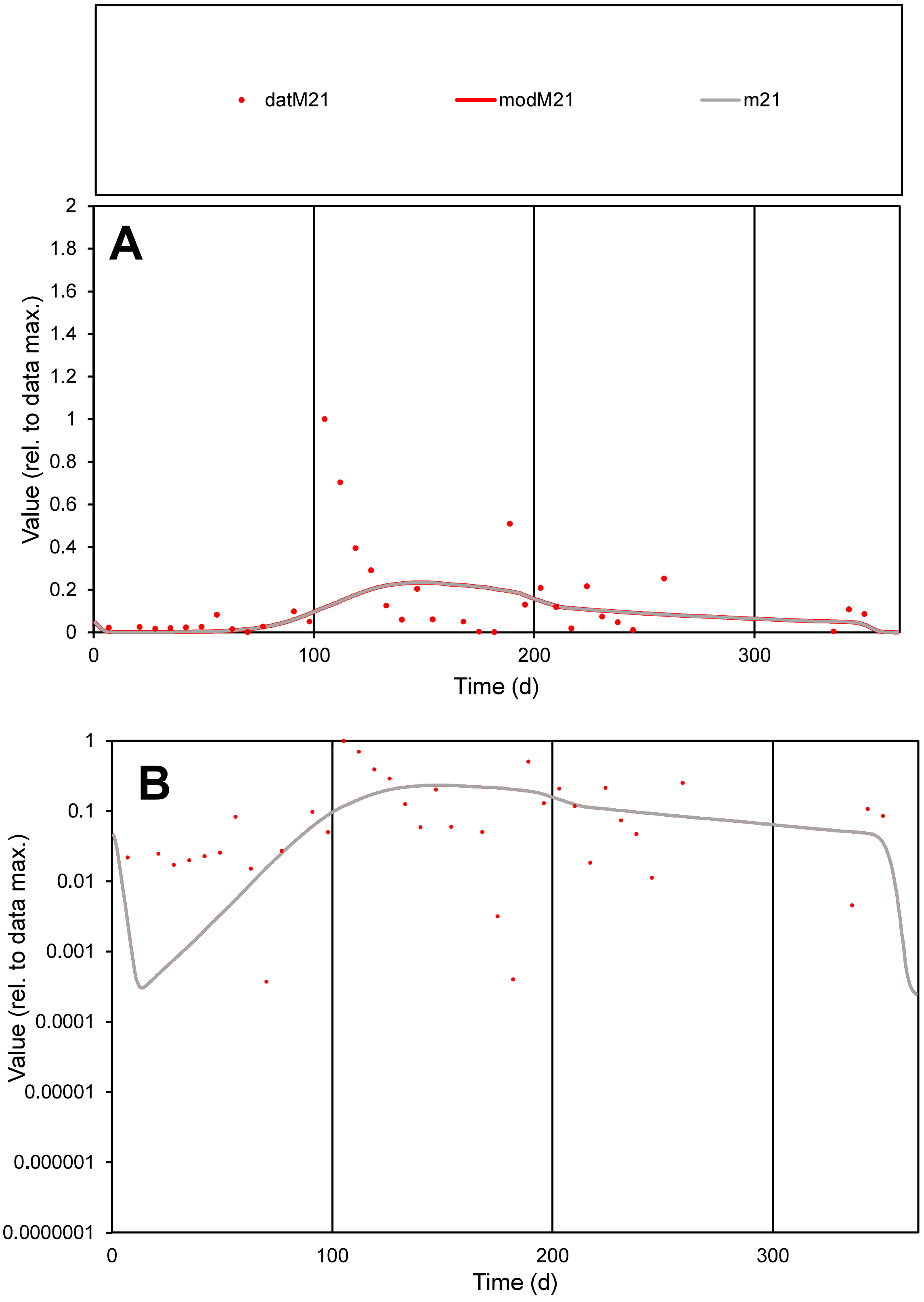

C1

Variables

M21 Provide jtd or Dst

CalibOnly?

2 1 to reproduce of

oNormalize?

2 1=no, 2=yes

ofxn calc*

1.51922

ofxn mod

1.52448

sRun

RunA1

zout

1

Ave

Data

0.00881

Model

0.00686

data/vMax

0.06514

ofxng

311.734

Min d/m

0.70345

ave/min d

362.51

ave/min m

198.72

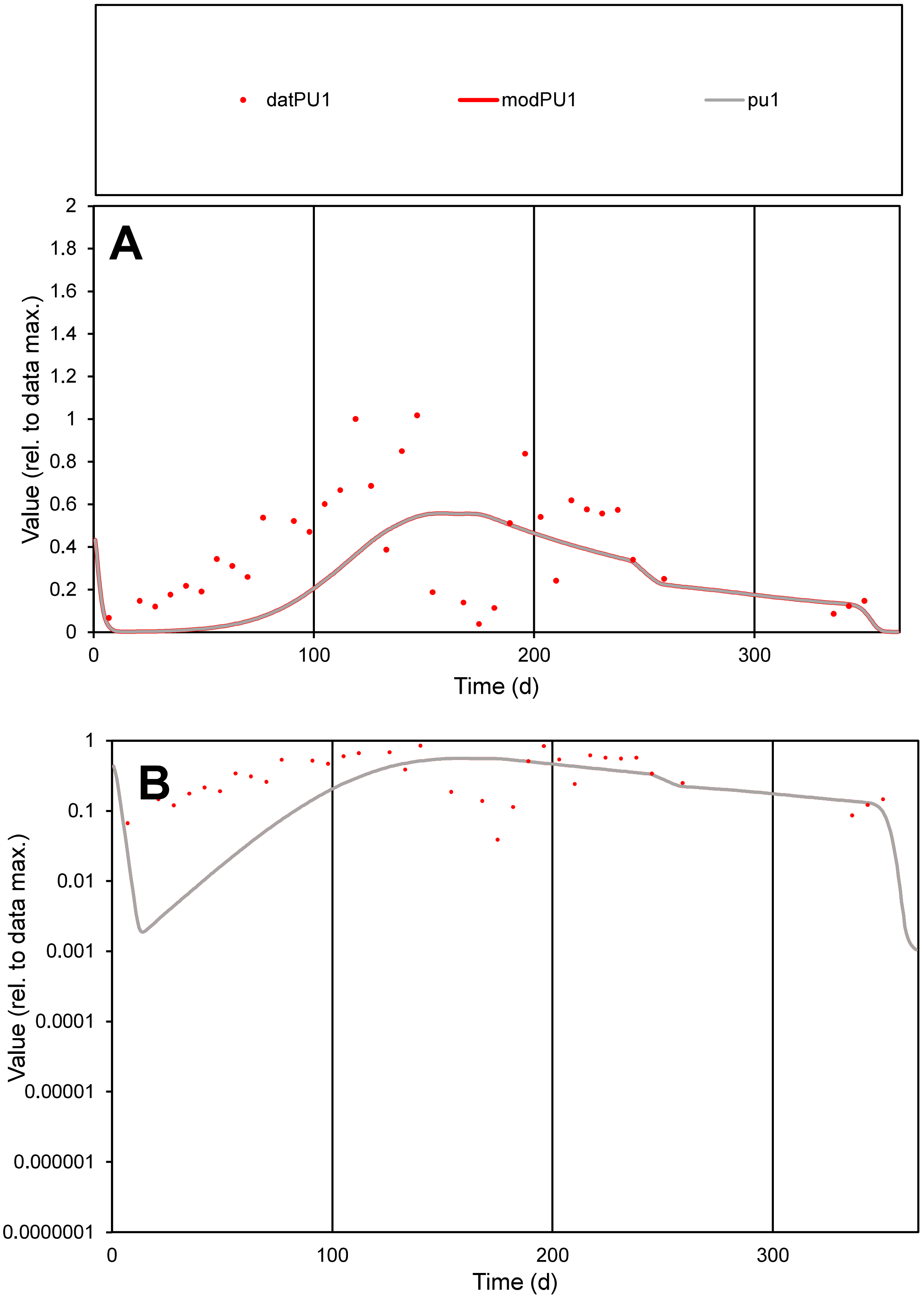

C1

Variables
PU1 Provide jtd or Dst
CalibOnly?
2 1 to reproduce of
oNormalize?
2 1=no, 2=yes

ofxn calc*
0.72274
ofxn mod
0.7309

sRun
RunA1
zout
1

Ave
Data
0.014
Model
0.01044

data/vMax
0.03745

ofxng
311.734

Min d/m
12.383

ave/min d
9.61949
ave/min m
88.8743

331_Calib_S_A

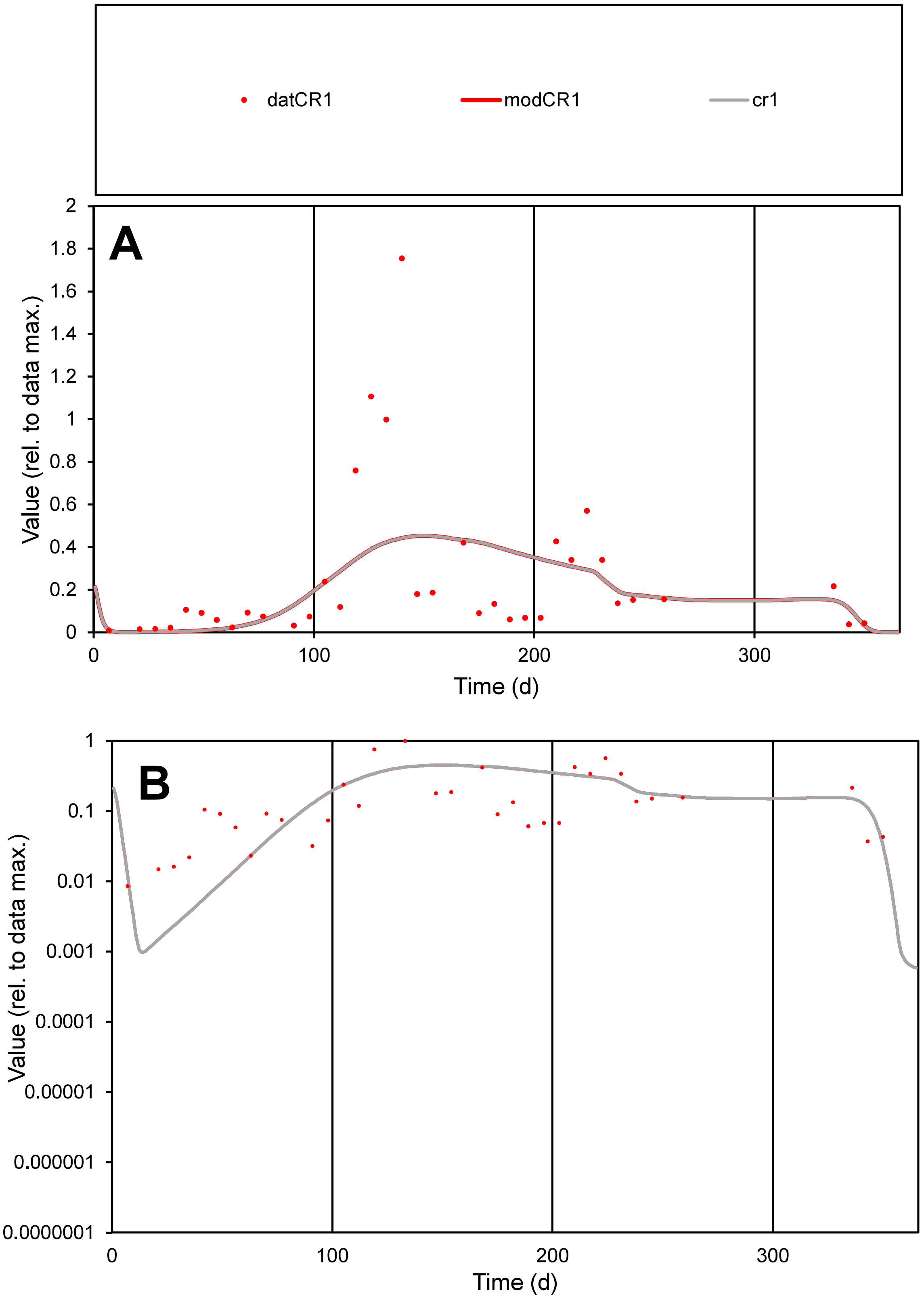

C1

Variables

CR1 Provide jtd or Dst

CalibOnly?

2 1 to reproduce of

oNormalize?

2 1=no, 2=yes

ofxn calc*

0.95544

ofxn mod

0.96719

sRun

RunA1

zout

1

Ave

Data

0.01686

Model

0.01701

data/vMax

0.07827

ofxng

311.734

Min d/m

5.19189

ave/min d

25.3234

ave/min m

132.691

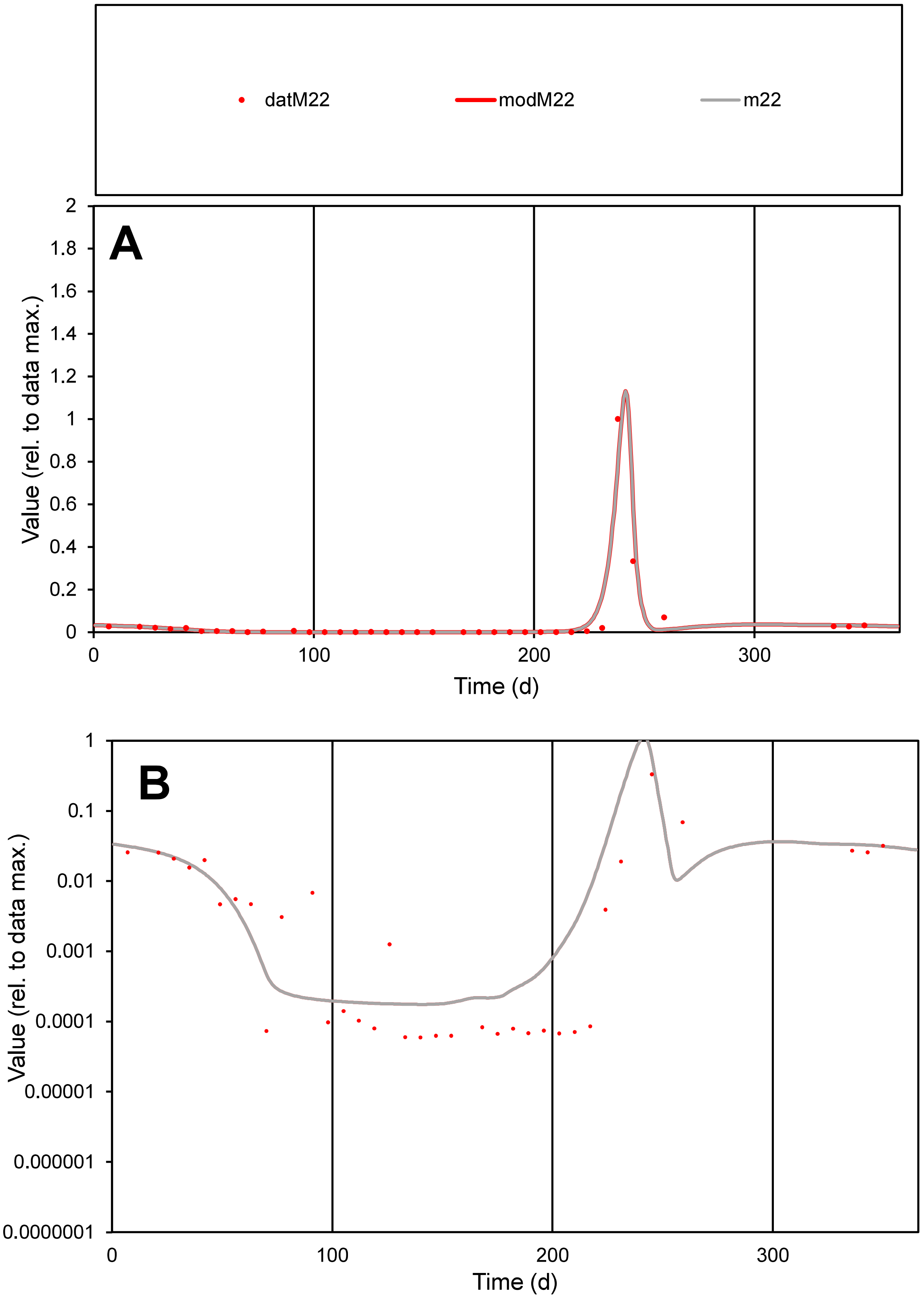

C1

Variables
M22 Provide jtd or Dst
CalibOnly?
2 1 to reproduce of
oNormalize?
2 1=no, 2=yes

ofxn calc*
0.46024
ofxn mod
0.68581

sRun
RunA1
zout
1

Ave
Data
0.01637
Model
0.01639

data/vMax
0.3584

ofxng
311.734

Min d/m
0.34014

ave/min d
767.291
ave/min m
261.351

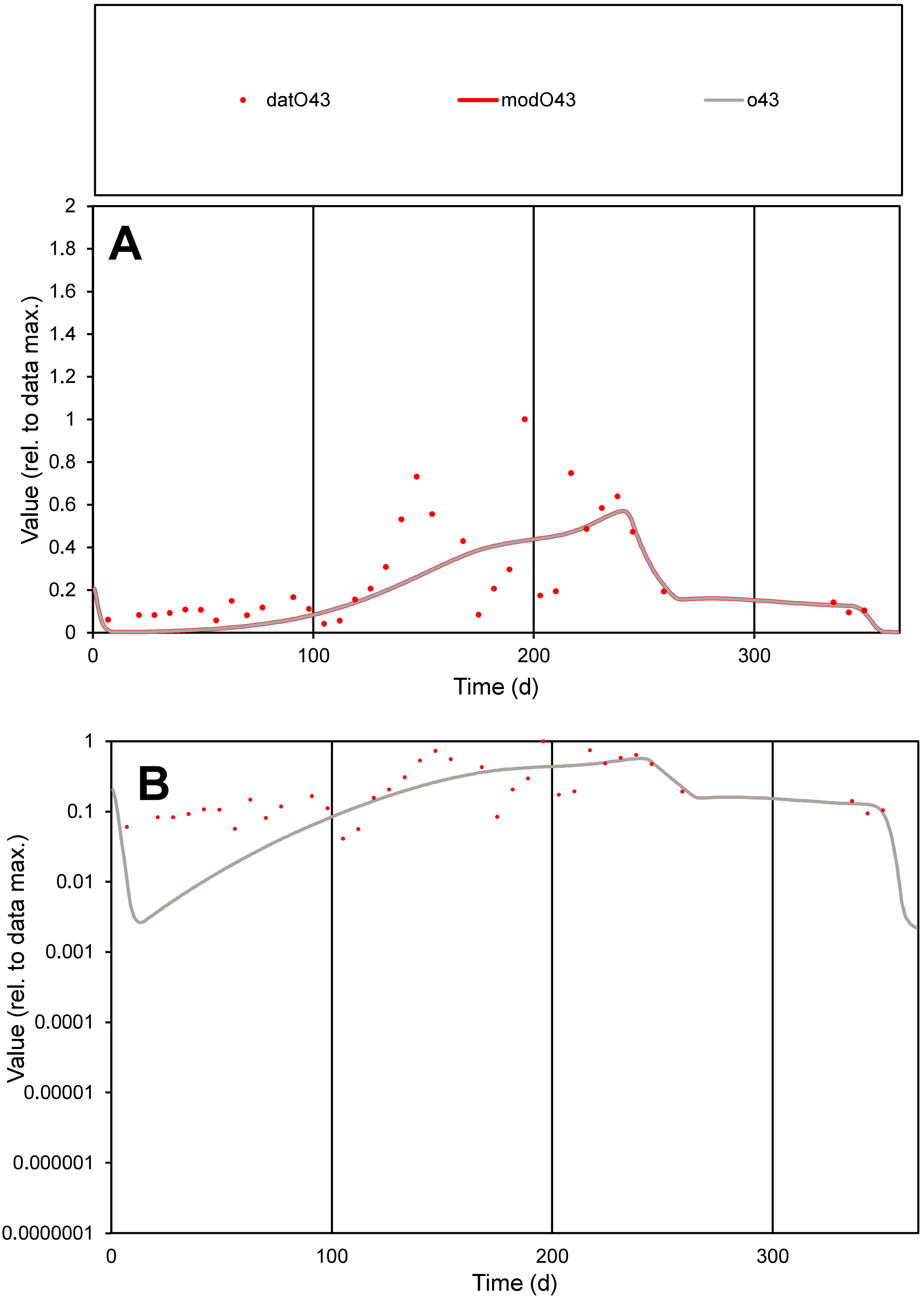

C1

Variables
O43 Provide jtd or Dst
CalibOnly?
2 1 to reproduce of
oNormalize?
2 1=no, 2=yes

ofxn calc*
0.62719
ofxn mod
0.64318

sRun
RunA1
zout
1

Ave
Data
0.019
Model
0.01686

data/vMax
0.07654

ofxng
311.734

Min d/m
10.1028

ave/min d
6.03897
ave/min m
54.1374

331_Calib_S_A

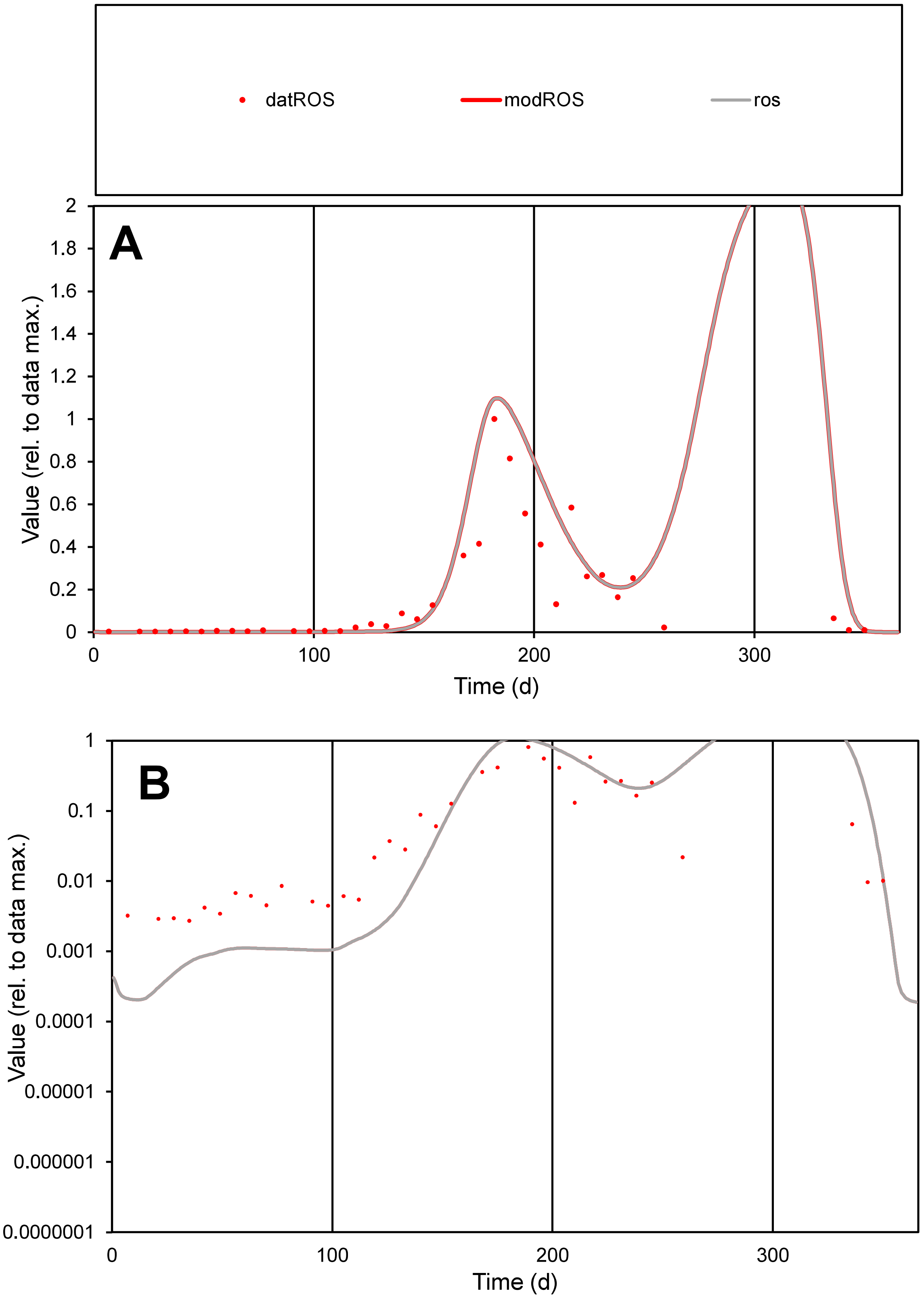

C1

Variables

ROS Provide jtd or Dst

CalibOnly?

2 1 to reproduce of

oNormalize?

2 1=no, 2=yes

ofxn calc*

1.03903

ofxn mod

1.12623

sRun

RunA1

zout

1

Ave

Data

0.03007

Model

0.04315

data/vMax

0.1916

ofxng

311.734

Min d/m

13.0702

ave/min d

57.4754

ave/min m

1077.85

331_Calib_S_A

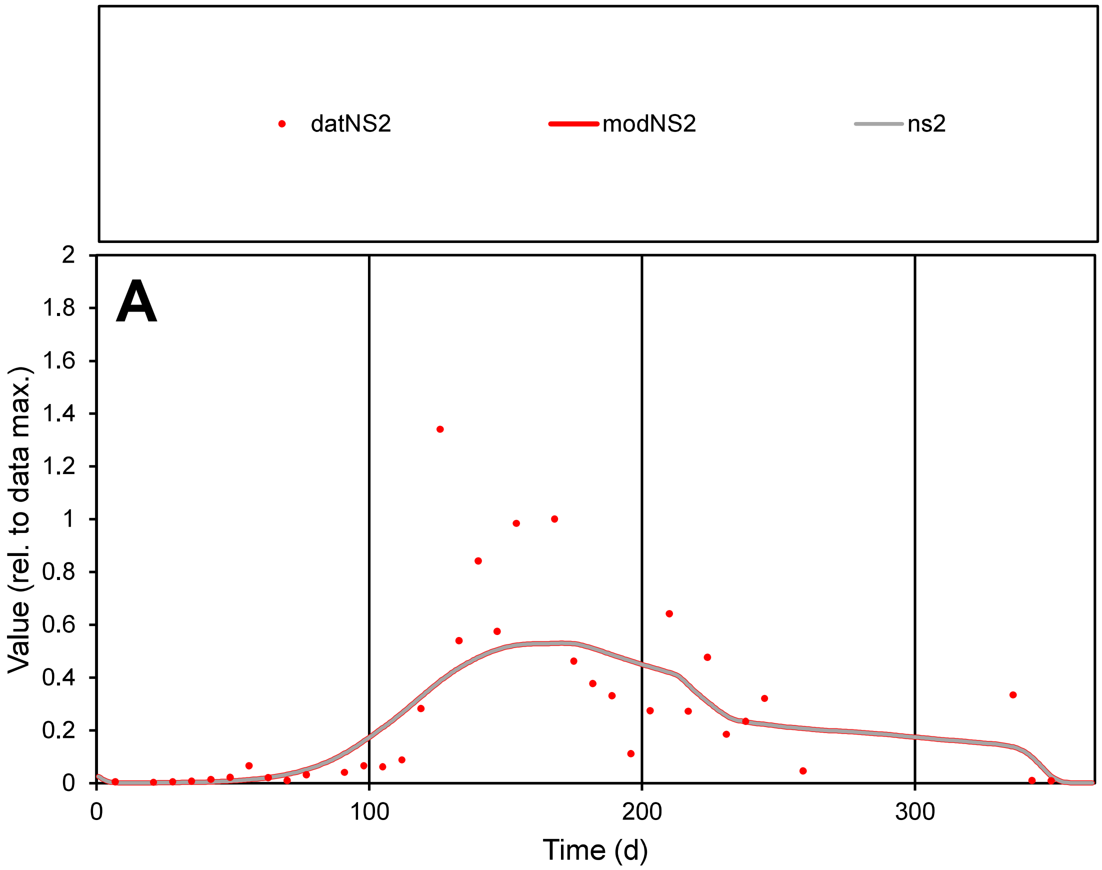

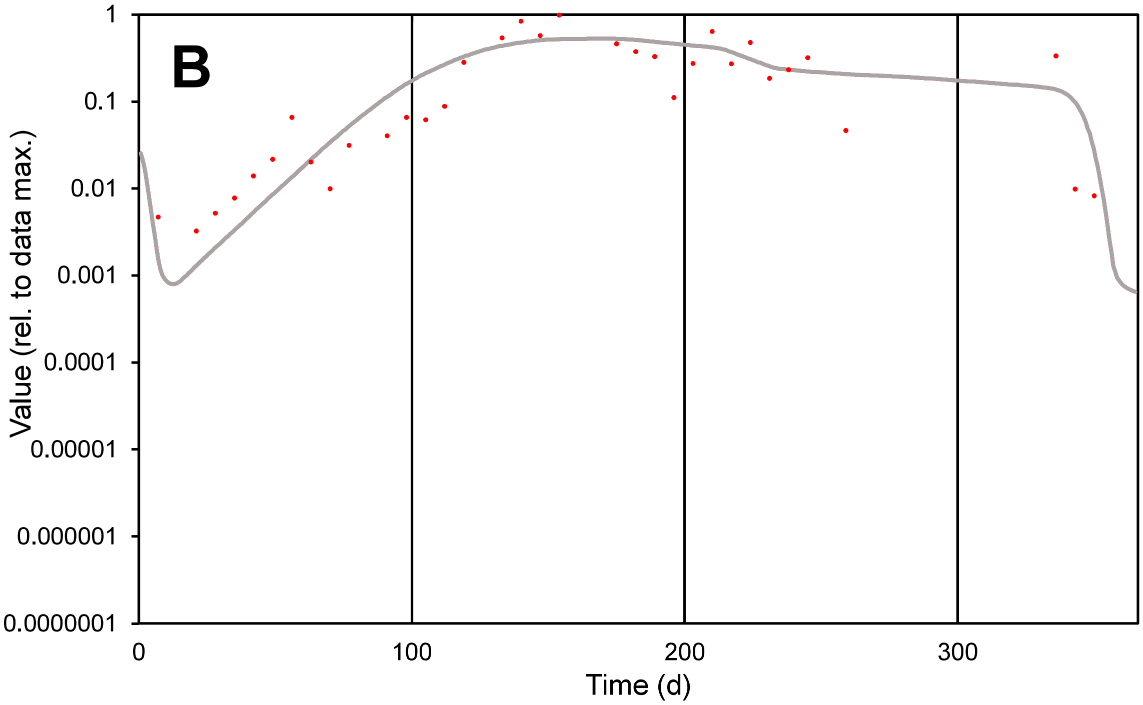

C1

Variables
NS2 Provide jtd or Dst
CalibOnly?
2 1 to reproduce of
oNormalize?
2 1=no, 2=yes

ofxn calc*
0.69128
ofxn mod
0.70793

sRun
RunA1
zout
1

Ave
Data
0.0193
Model
0.01922

data/vMax
0.07918

ofxng
311.734

Min d/m
2.26643

ave/min d
74.6426
ave/min m
168.432

331_Calib_S_A

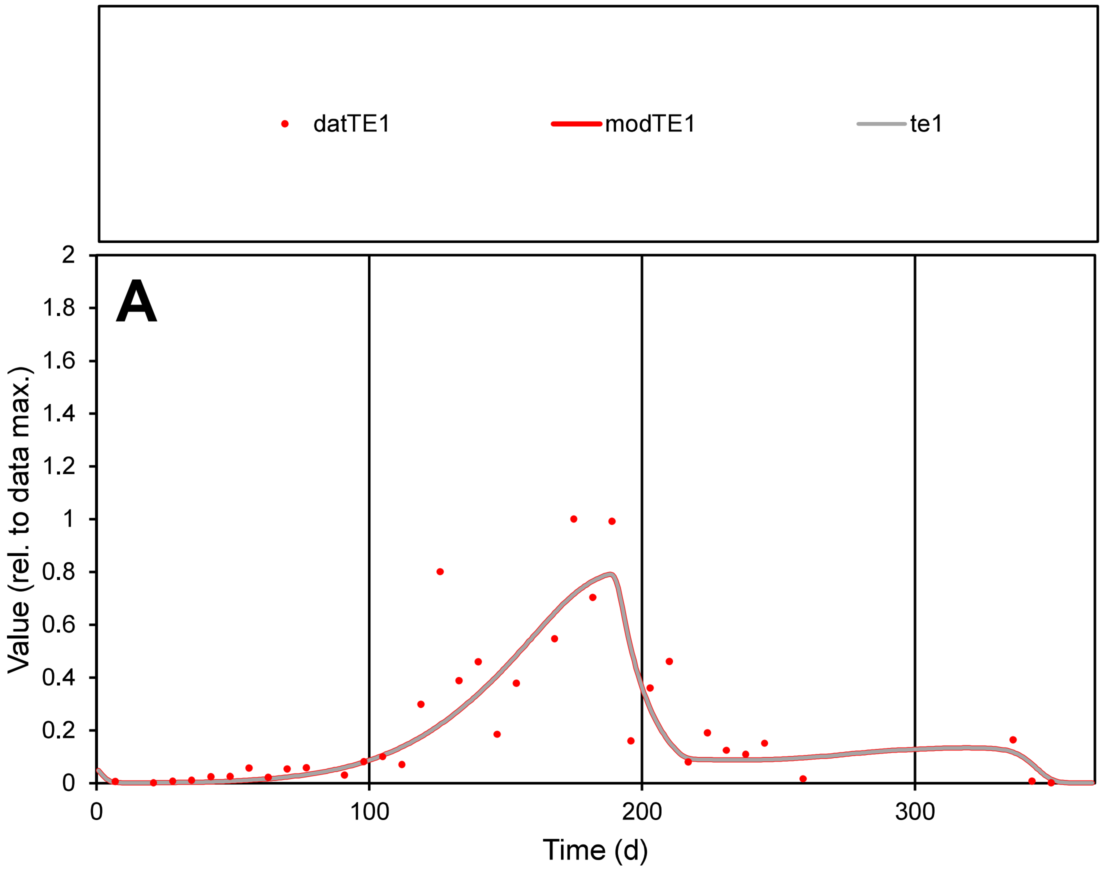

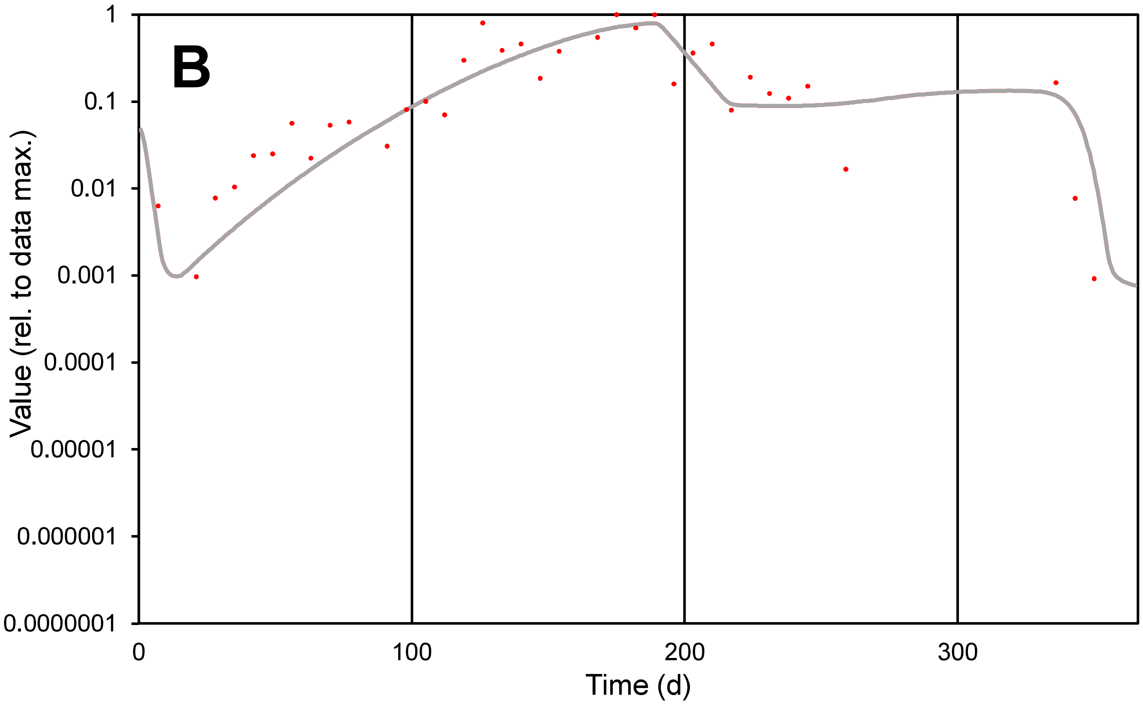

C1

Variables

TE1 Provide jtd or Dst

CalibOnly?

2 1 to reproduce of

oNormalize?

2 1=no, 2=yes

ofxn calc*

0.62588

ofxn mod

0.65778

sRun

RunA1

zout

1

Ave

Data

0.01219

Model

0.01136

data/vMax

0.05898

ofxng

311.734

Min d/m

0.63847

ave/min d

225.203

ave/min m

133.992

331_Calib_S_A

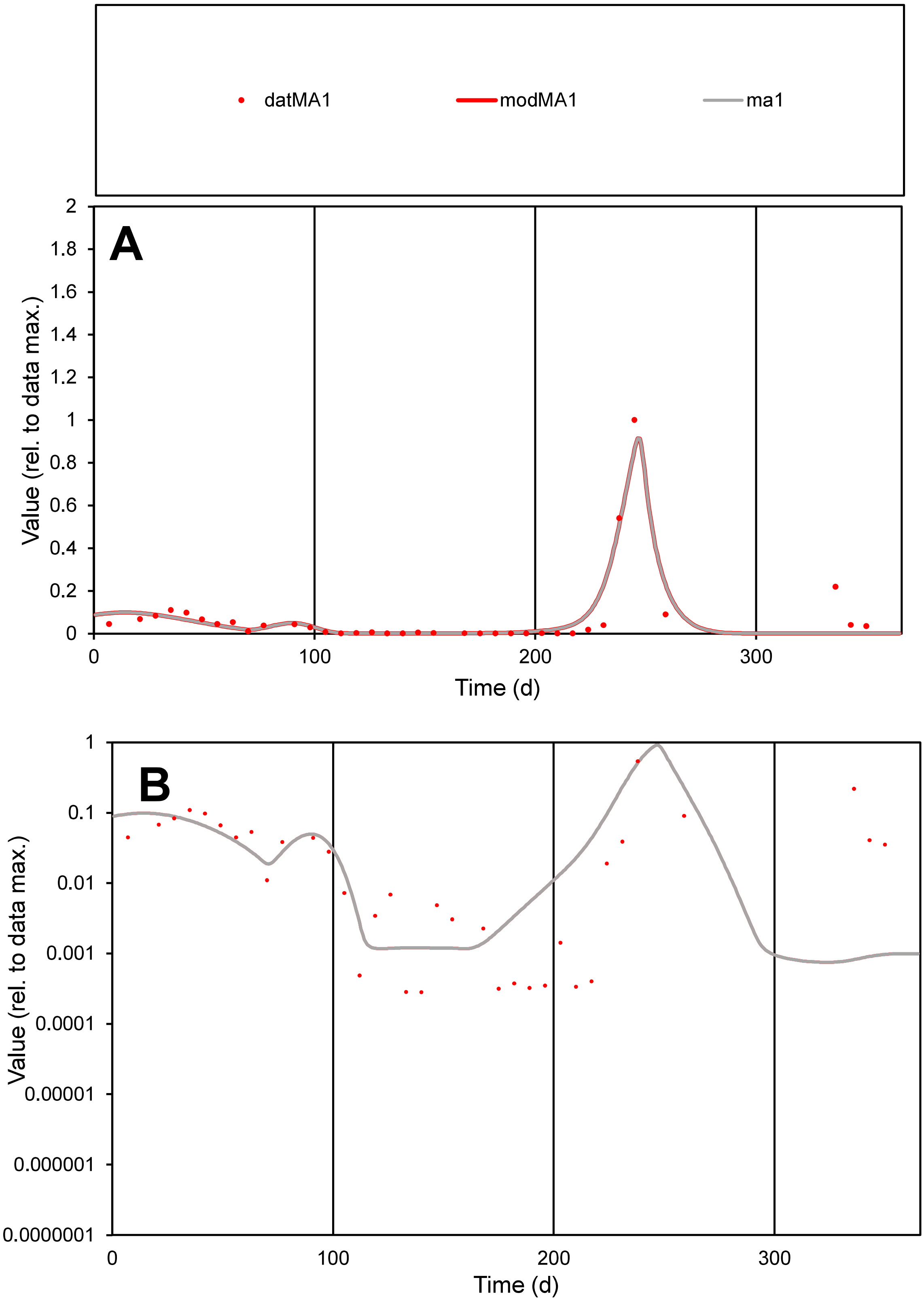

C1

Variables
MA1 Provide jtd or Dst
CalibOnly?
2 1 to reproduce of
oNormalize?
2 1=no, 2=yes

ofxn calc*
0.70103
ofxn mod
0.81201

sRun
RunA1
zout
1

Ave
Data
0.00381
Model
0.00381

data/vMax
0.0507

ofxng
311.734

Min d/m
0.33695

ave/min d
266.437
ave/min m
89.8314

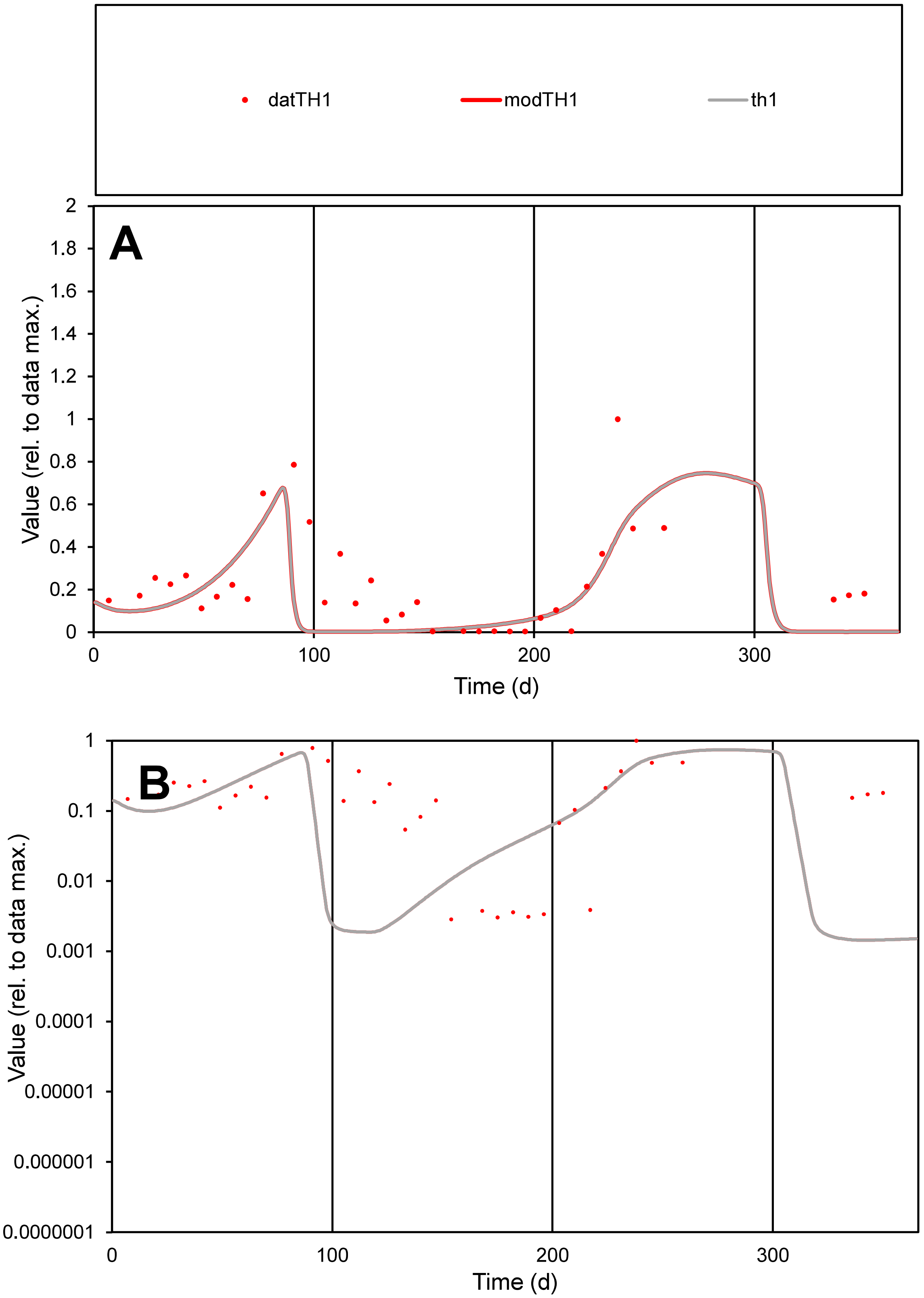

C1

Variables
TH1 Provide jtd or Dst
CalibOnly?
2 1 to reproduce of
oNormalize?
2 1=no, 2=yes

ofxn calc*
0.79428
ofxn mod
0.8951

sRun
RunA1
zout
1

Ave
Data
0.0075
Model
0.0055

data/vMax
0.03403

ofxng
311.734

Min d/m
1.97252

ave/min d
77.3733
ave/min m
111.979

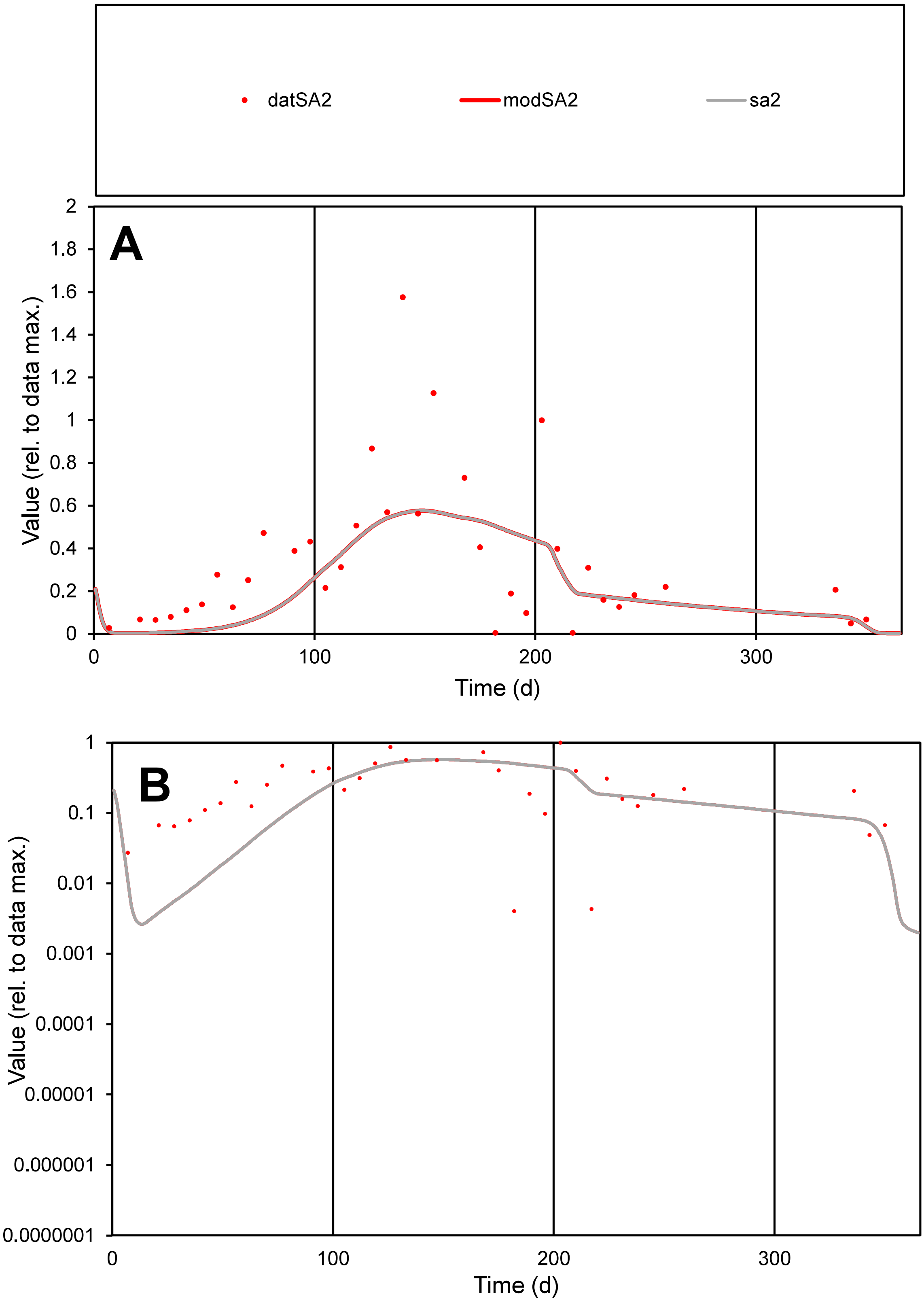

C1

Variables
SA2 Provide jtd or Dst
CalibOnly?
2 1 to reproduce of
oNormalize?
2 1=no, 2=yes

ofxn calc*
0.68563
ofxn mod
0.69799

sRun
RunA1
zout
1

Ave
Data
0.00993
Model
0.00841

data/vMax
0.03318

ofxng
311.734

Min d/m
0.97236

ave/min d
74.263
ave/min m
61.1615

331_Calib_S_A

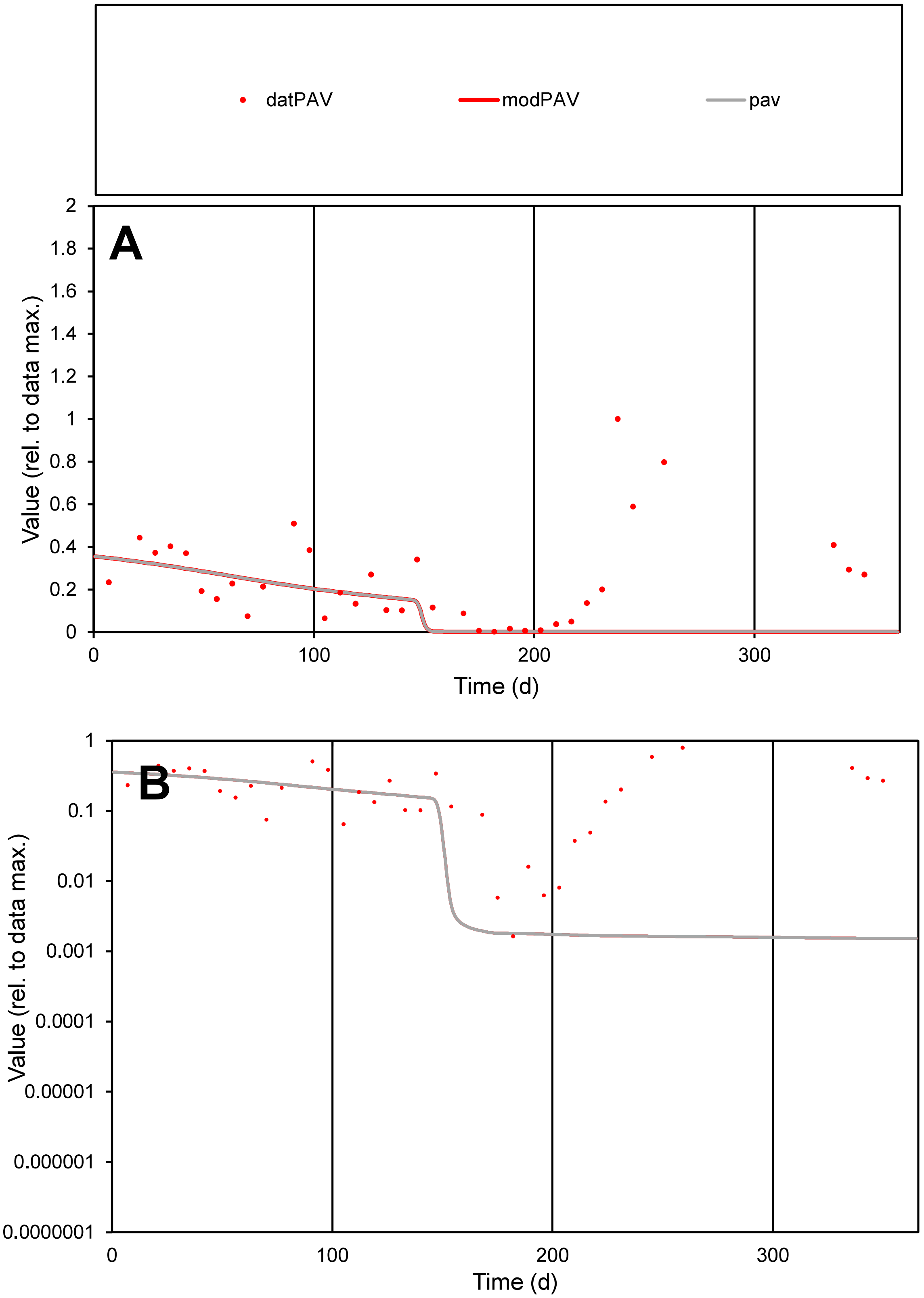

C1

Variables
PAV Provide jtd or Dst
CalibOnly?
2 1 to reproduce of
oNormalize?
2 1=no, 2=yes

ofxn calc*
1.14168
ofxn mod
1.14836

sRun
RunA1
zout
1

Ave
Data
0.00708
Model
0.00381

data/vMax
0.02986

ofxng
311.734

Min d/m
1.07273

ave/min d
144.537
ave/min m
83.4051

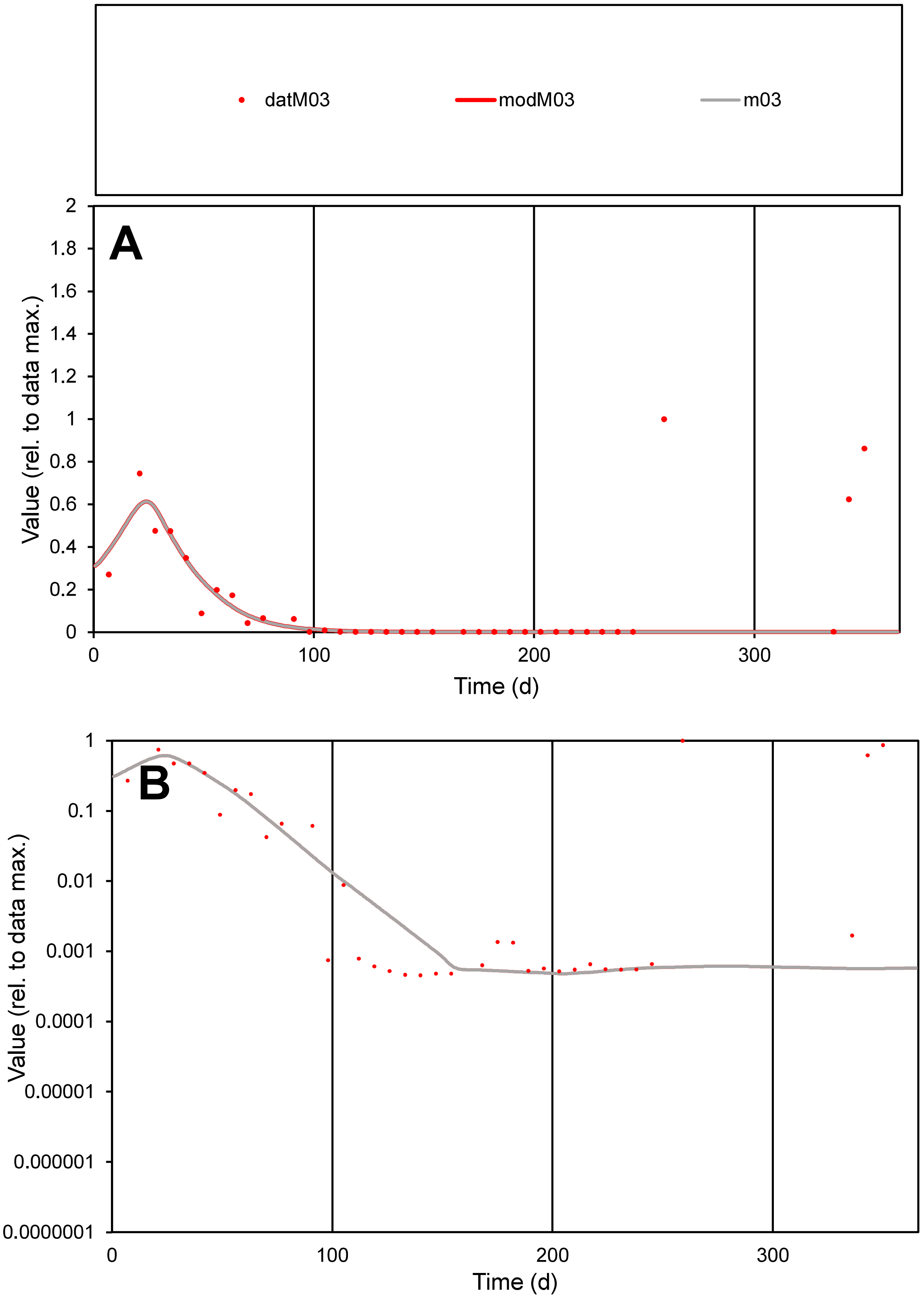

C1

Variables
M03 Provide jtd or Dst
CalibOnly?
2 1 to reproduce of
oNormalize?
2 1=no, 2=yes

ofxn calc*
1.63362
ofxn mod
1.64201

sRun
RunA1
zout
1

Ave
Data
0.00577
Model
0.00322

data/vMax
0.03809

ofxng
311.734

Min d/m
0.95392

ave/min d
331.519
ave/min m
176.494

331_Calib_S_A

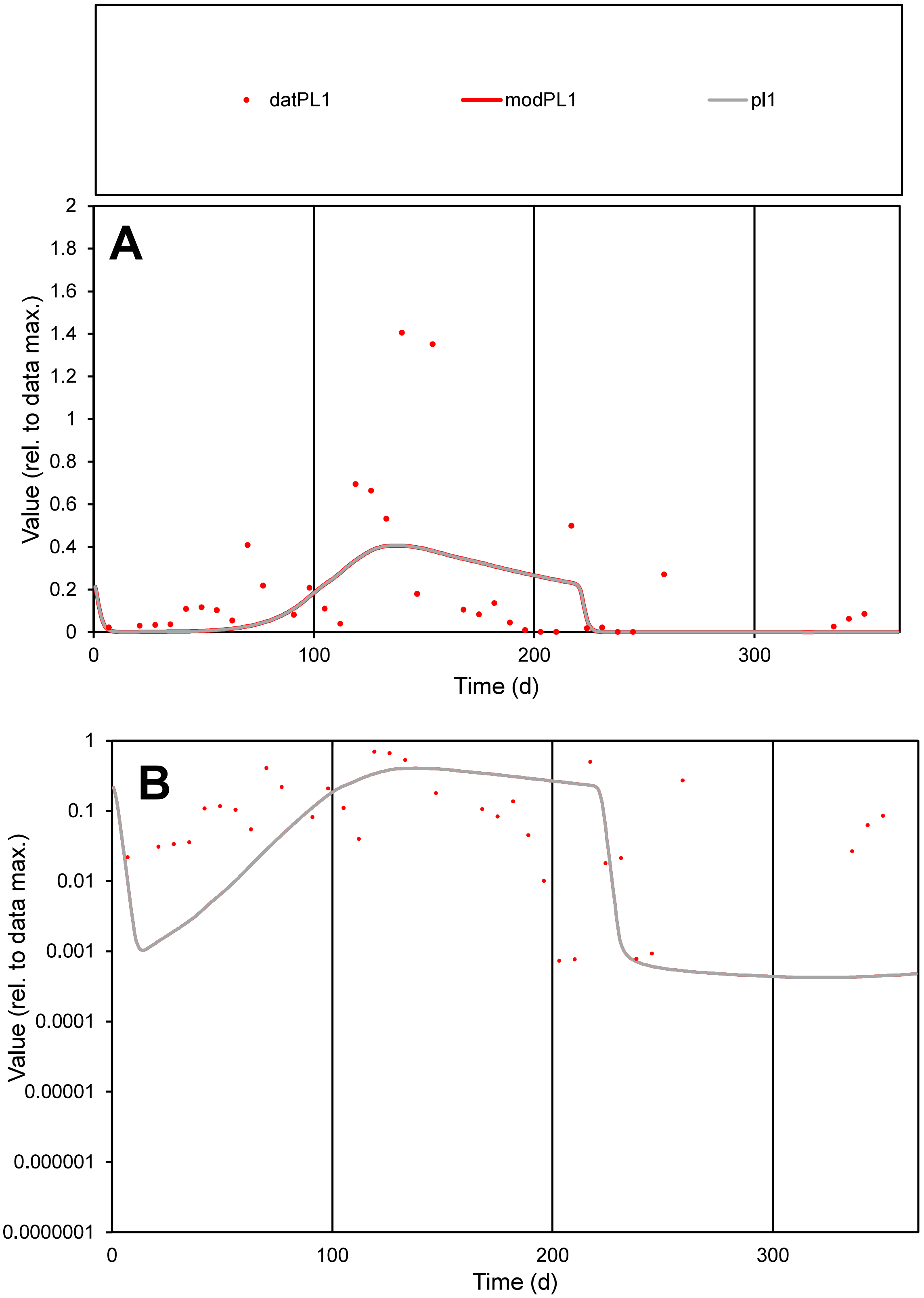

C1

Variables

PL1 Provide jtd or Dst

CalibOnly?

2 1 to reproduce of

oNormalize?

2 1=no, 2=yes

ofxn calc*

1.17292

ofxn mod

1.1822

sRun

RunA1

zout

1

Ave

Data

0.00952

Model

0.00826

data/vMax

0.05336

ofxng

311.734

Min d/m

1.70335

ave/min d

243.136

ave/min m

359.223

331_Calib_S_A

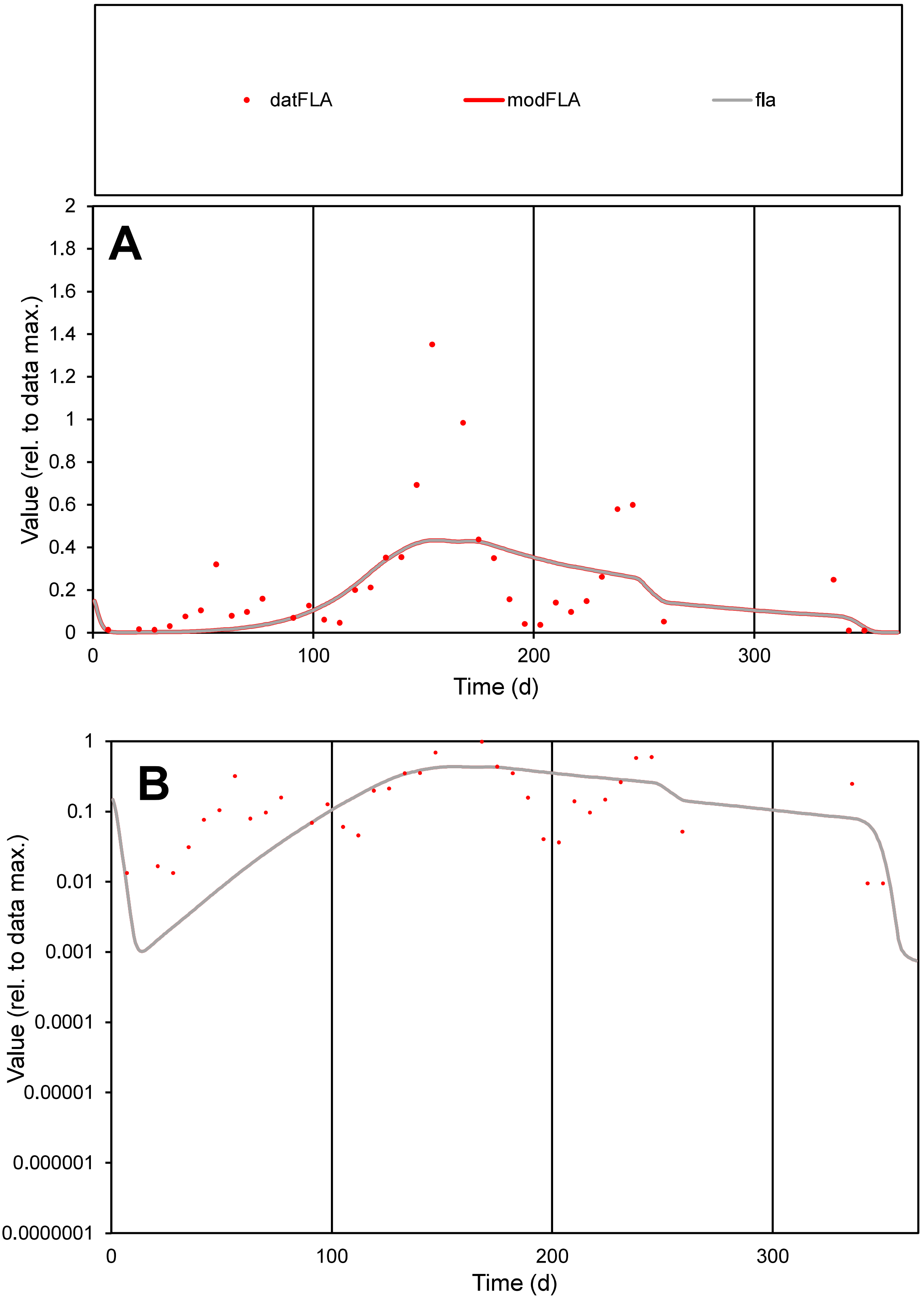

C1

Variables

FLA Provide jtd or Dst

CalibOnly?

2 1 to reproduce of

oNormalize?

2 1=no, 2=yes

ofxn calc*

0.94493

ofxn mod

0.9564

sRun

RunA1

zout

1

Ave

Data

0.01061

Model

0.00983

data/vMax

0.04981

ofxng

311.734

Min d/m

5.80118

ave/min d

22.4807

ave/min m

120.766

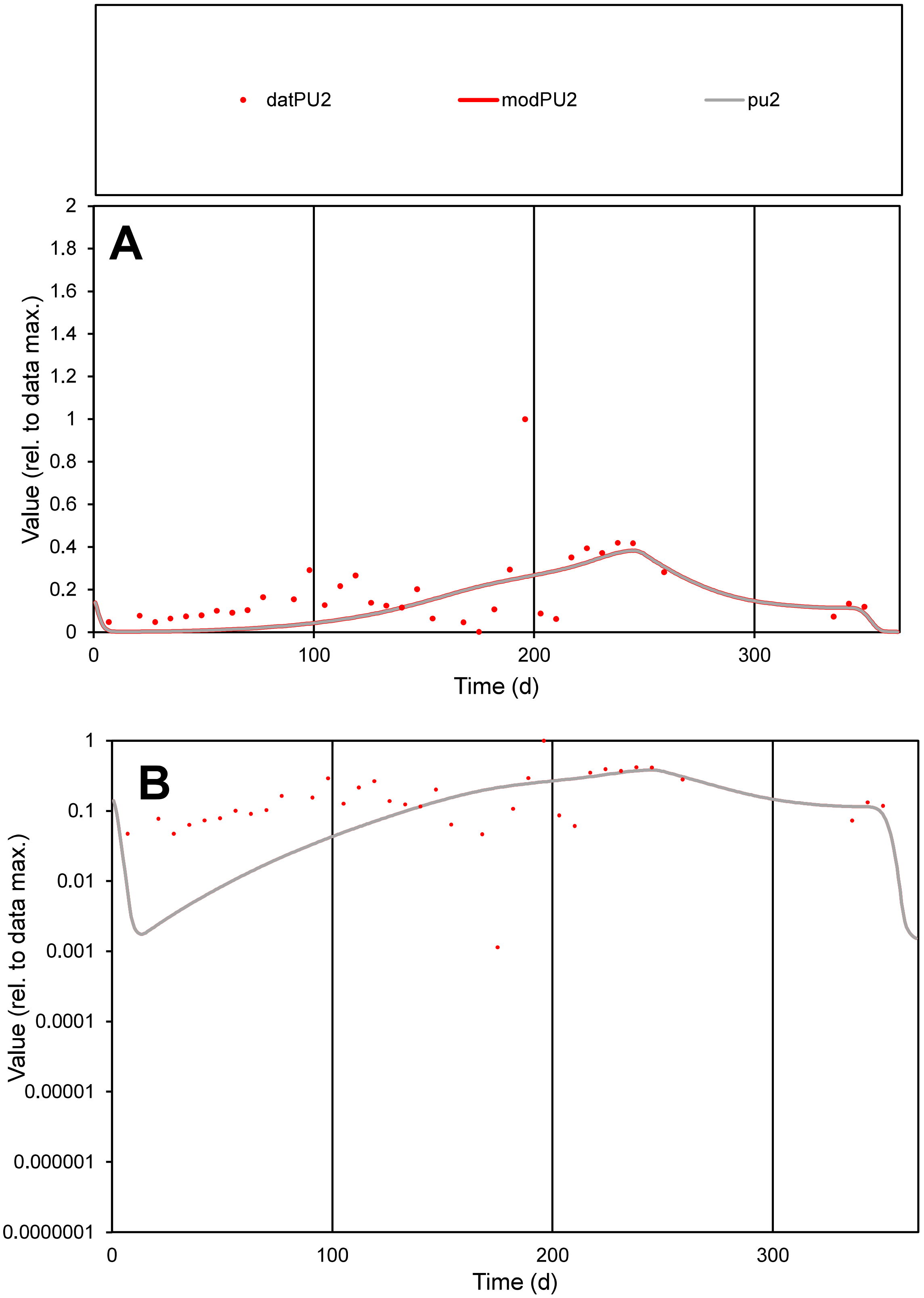

C1

Variables
PU2 Provide jtd or Dst
CalibOnly?
2 1 to reproduce of
oNormalize?
2 1=no, 2=yes

ofxn calc*
0.8998
ofxn mod
0.91073

sRun
RunA1
zout
1

Ave
Data
0.00966
Model
0.00753

data/vMax
0.05347

ofxng
311.734

Min d/m
0.43655

ave/min d
157.885
ave/min m
53.7616

331_Calib_S_A

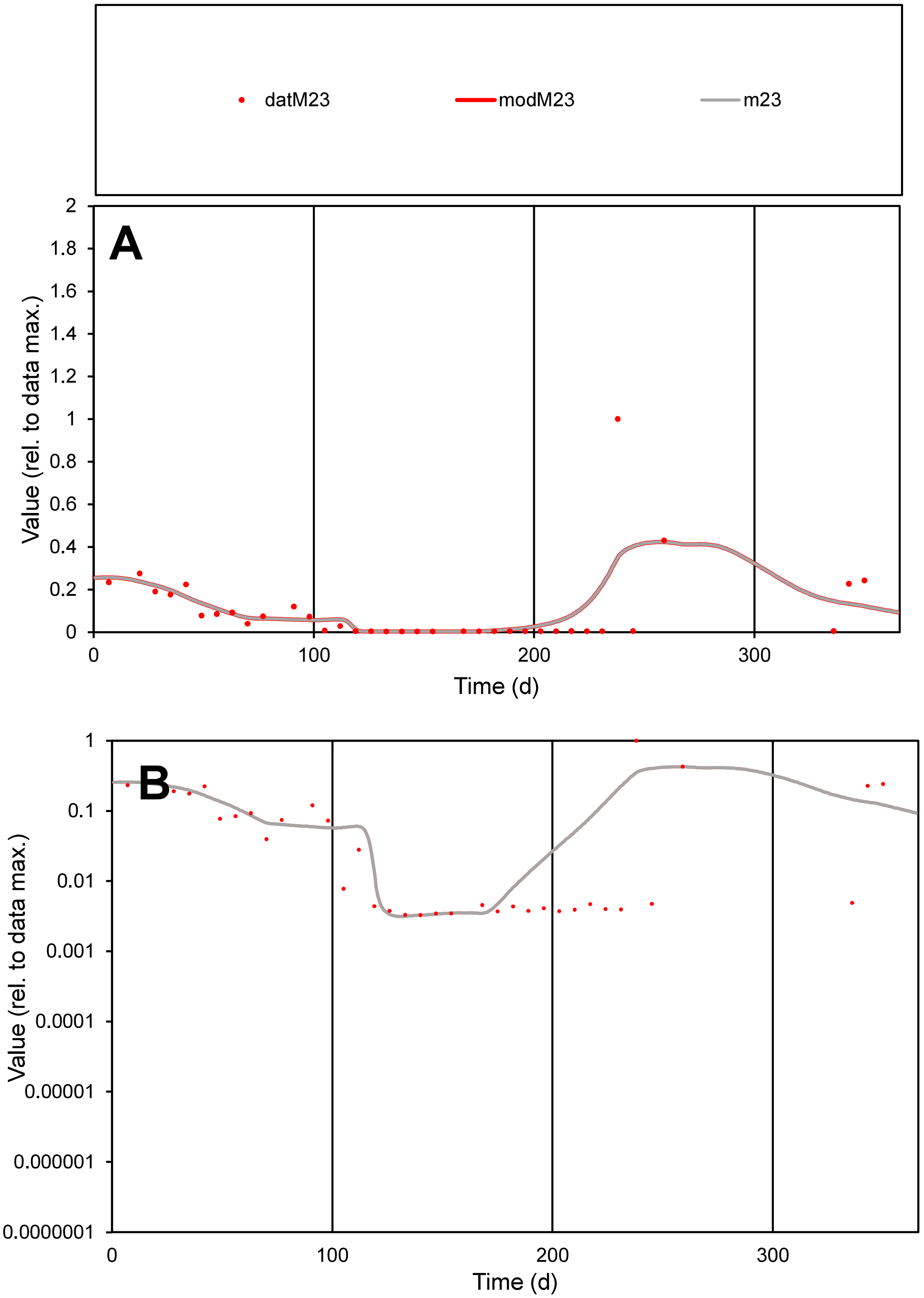

C1

Variables

M23 Provide jtd or Dst

CalibOnly?

2 1 to reproduce of

oNormalize?

2 1=no, 2=yes

ofxn calc*

1.34741

ofxn mod

1.37321

sRun

RunA1

zout

1

Ave

Data

0.00331

Model

0.00349

data/vMax

0.03243

ofxng

311.734

Min d/m

1.02899

ave/min d

31.0388

ave/min m

33.7358

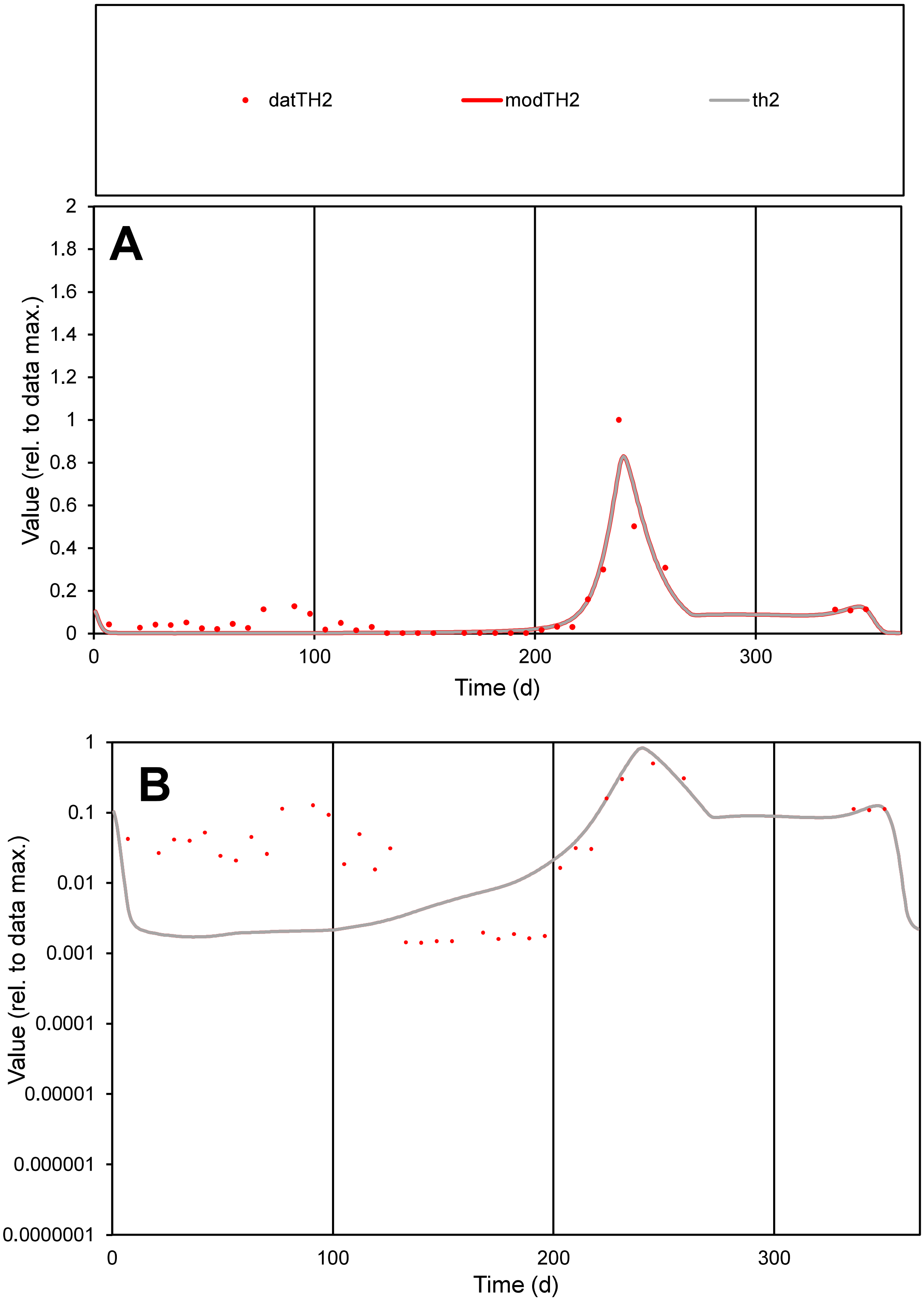

C1

Variables

TH2 Provide jtd or Dst

CalibOnly?

2 1 to reproduce of

oNormalize?

2 1=no, 2=yes

ofxn calc*

0.57977

ofxn mod

0.63295

sRun

RunA1

zout

1

Ave

Data

0.00709

Model

0.00577

data/vMax

0.07386

ofxng

311.734

Min d/m

0.82715

ave/min d

67.6315

ave/min m

45.5324

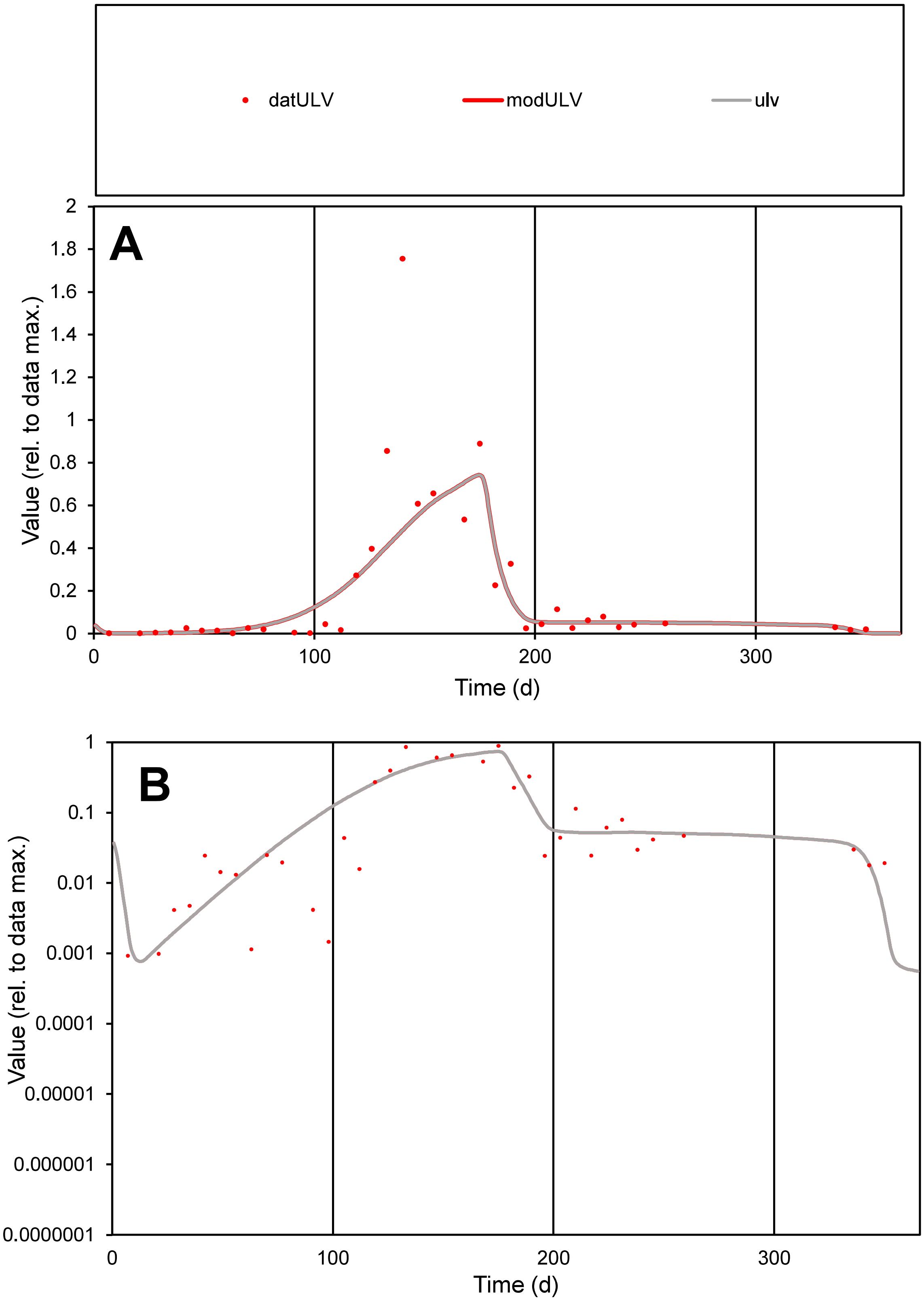

C1

Variables

ULV Provide jtd or Dst

CalibOnly?

2 1 to reproduce of

oNormalize?

2 1=no, 2=yes

ofxn calc*

0.73239

ofxn mod

0.7548

sRun

RunA1

zout

1

Ave

Data

0.00308

Model

0.00313

data/vMax

0.01916

ofxng

311.734

Min d/m

0.73915

ave/min d

173.653

ave/min m

130.762

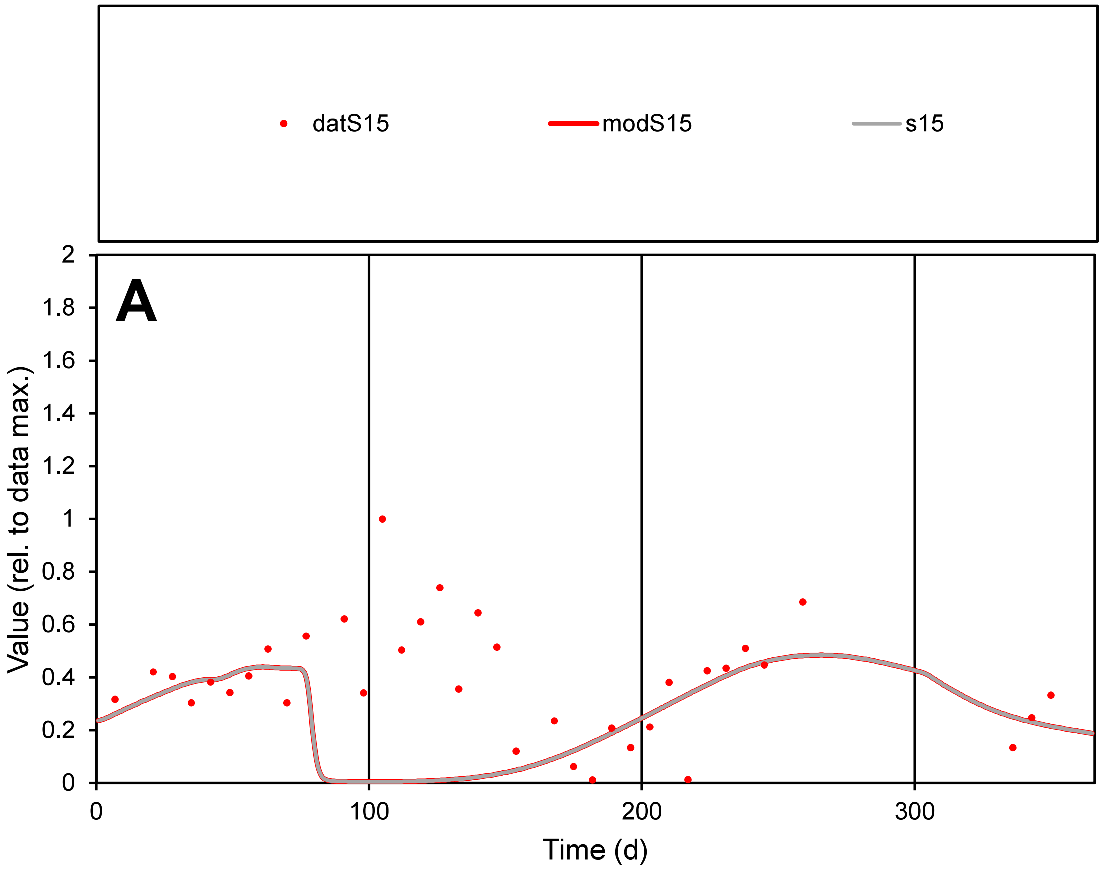

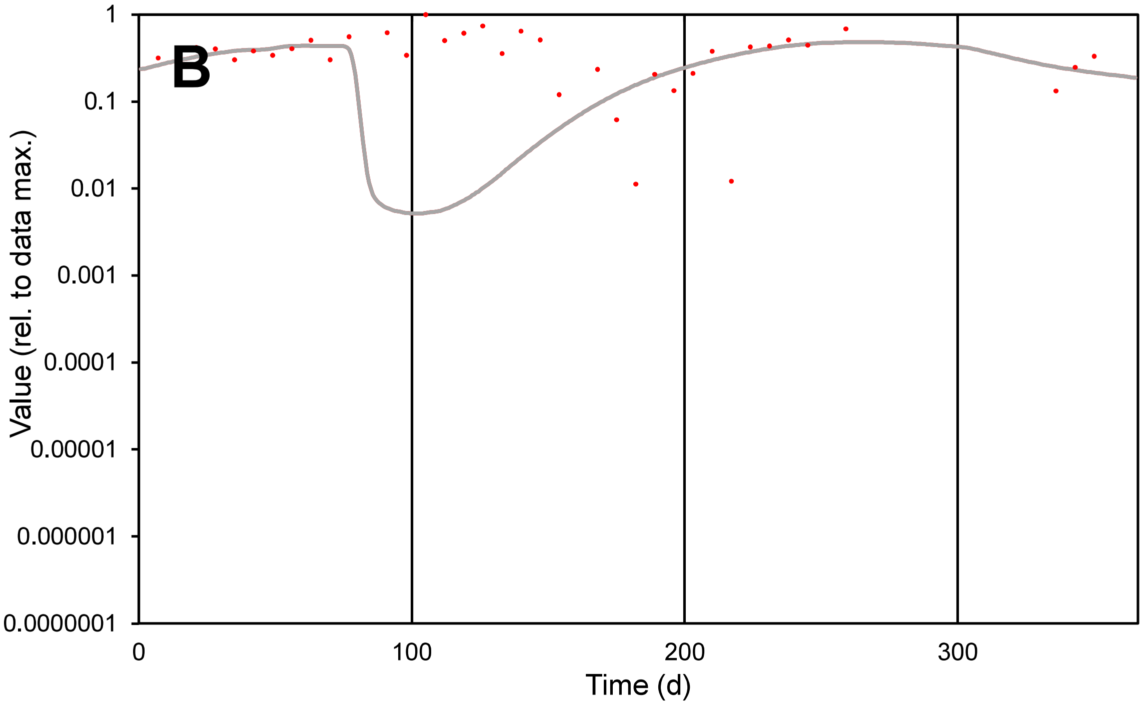

C1

Variables
S15 Provide jtd or Dst
CalibOnly?
2 1 to reproduce of
oNormalize?
2 1=no, 2=yes

ofxn calc*
0.78907
ofxn mod
0.79768

sRun
RunA1
zout
1

Ave
Data
0.00528
Model
0.00347

data/vMax
0.01453

ofxng
311.734

Min d/m
2.16861

ave/min d
32.2859
ave/min m
46.0329

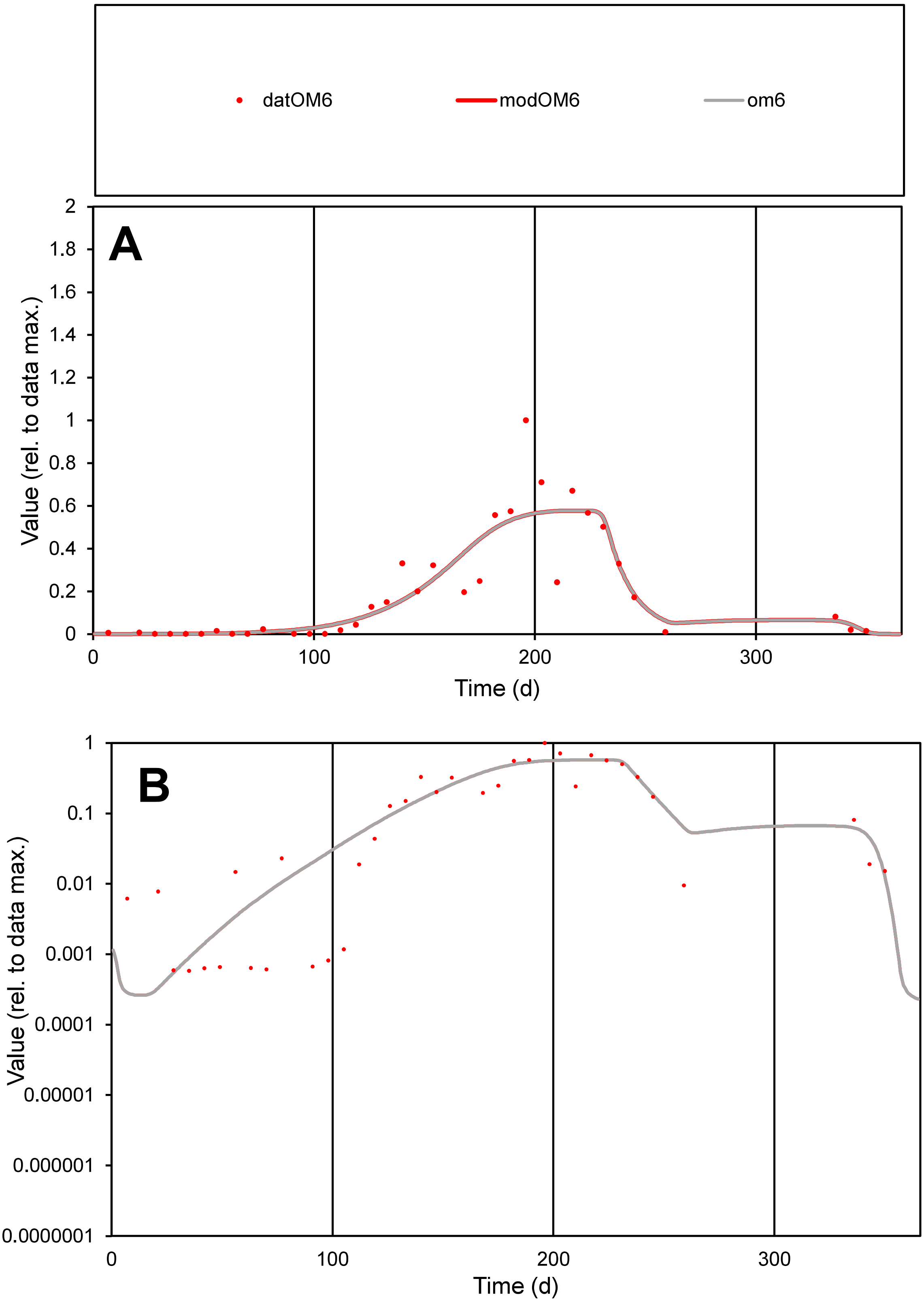

C1

Variables
OM6 Provide jtd or Dst
CalibOnly?
2 1 to reproduce of
oNormalize?
2 1=no, 2=yes

ofxn calc*
0.57935
ofxn mod
0.61125

sRun
RunA1
zout
1

Ave
Data
0.00939
Model
0.00964

data/vMax
0.04982

ofxng
311.734

Min d/m
2.10618

ave/min d
324.361
ave/min m
701.44

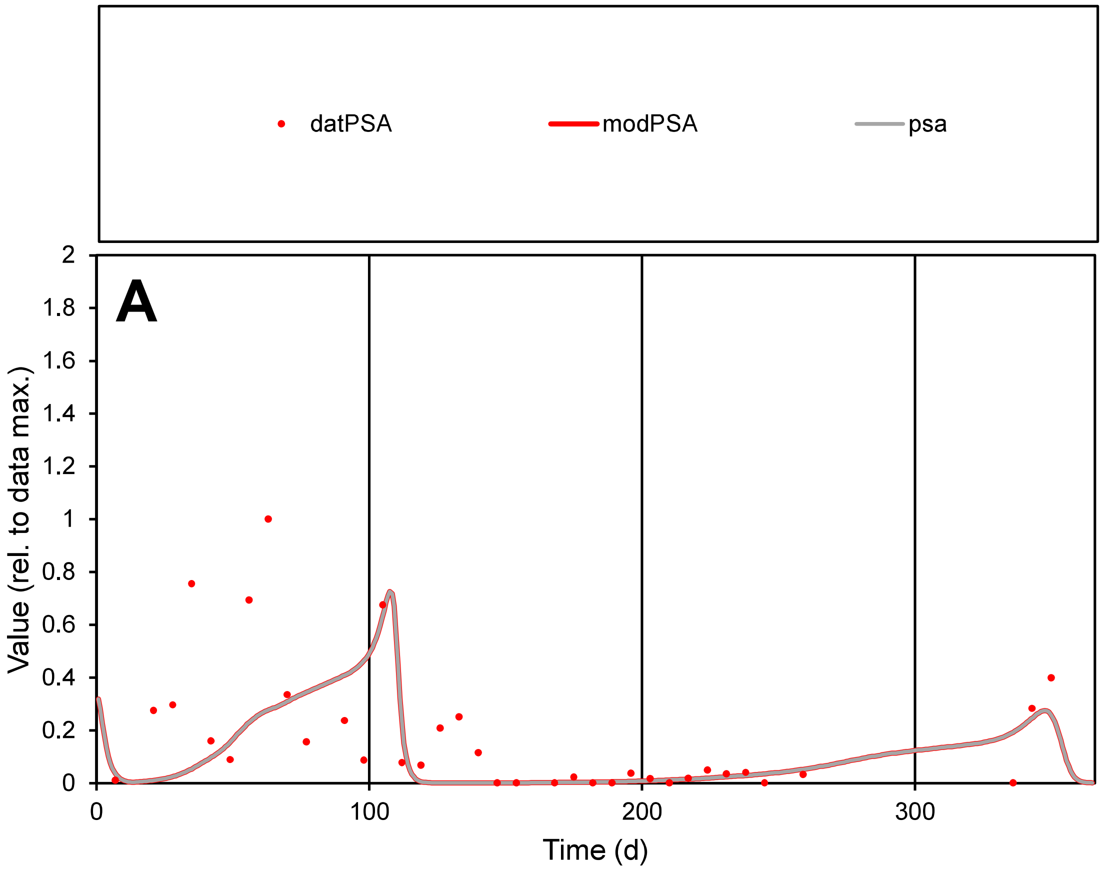

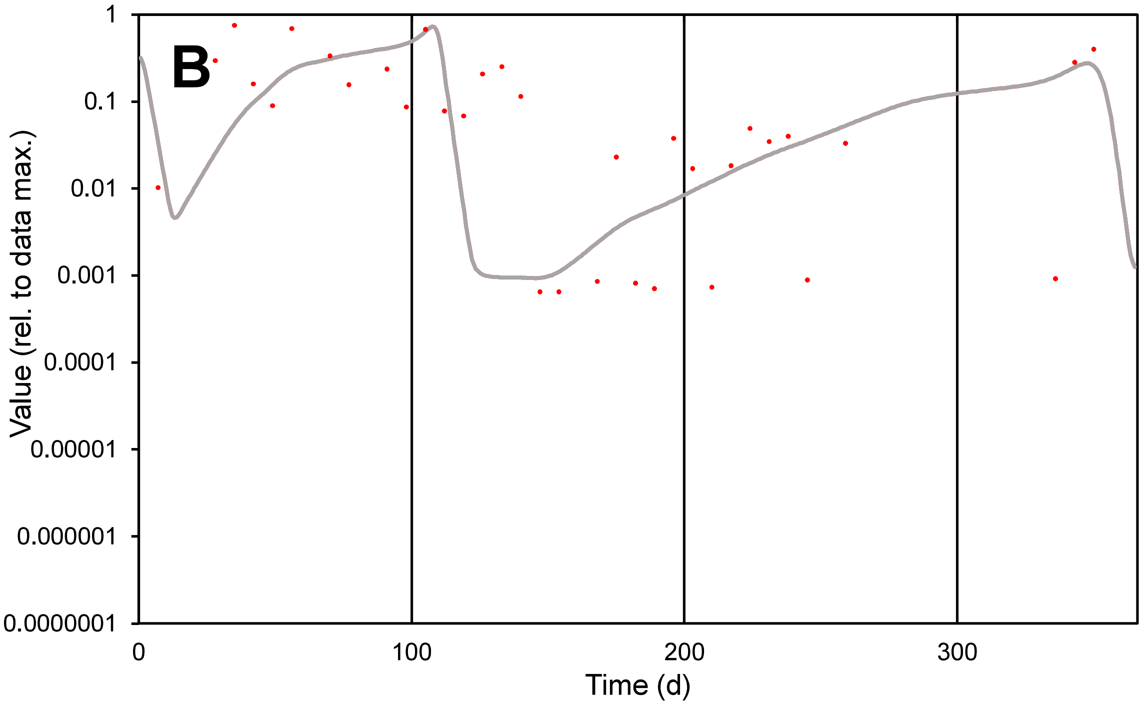

C1

Variables
PSA Provide jtd or Dst
CalibOnly?
2 1 to reproduce of
oNormalize?
2 1=no, 2=yes

ofxn calc*
1.24436
ofxn mod
1.25823

sRun
RunA1
zout
1

Ave
Data
0.00247
Model
0.00166

data/vMax
0.01424

ofxng
311.734

Min d/m
0.68484

ave/min d
268.224
ave/min m
123.447

331_Calib_S_A

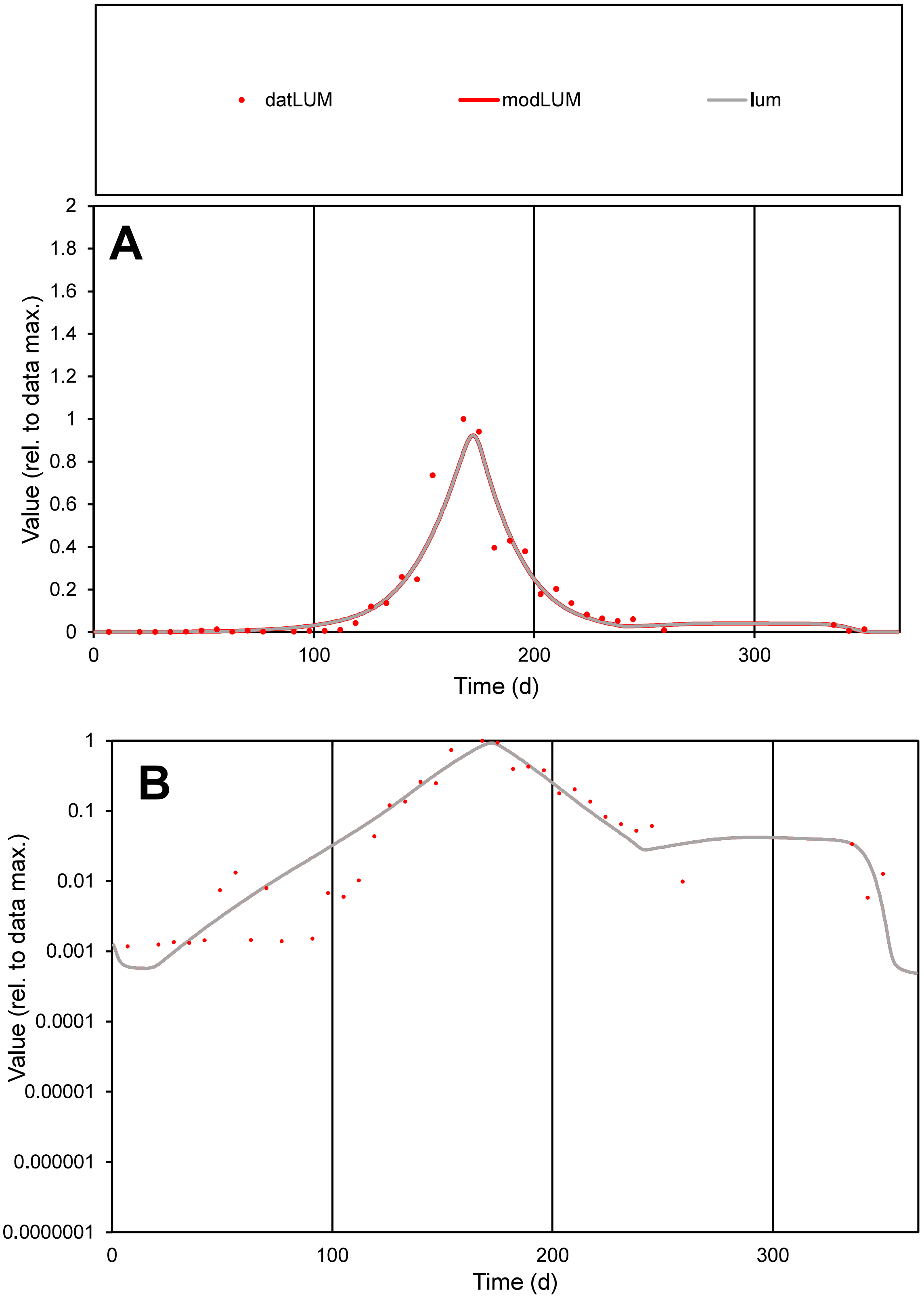

C1

Variables

LUM Provide jtd or Dst

CalibOnly?

2 1 to reproduce of

oNormalize?

2 1=no, 2=yes

ofxn calc*

0.41287

ofxn mod

0.45572

sRun

RunA1

zout

1

Ave

Data

0.00561

Model

0.00583

data/vMax

0.03921

ofxng

311.734

Min d/m

1.97258

ave/min d

121.783

ave/min m

249.996

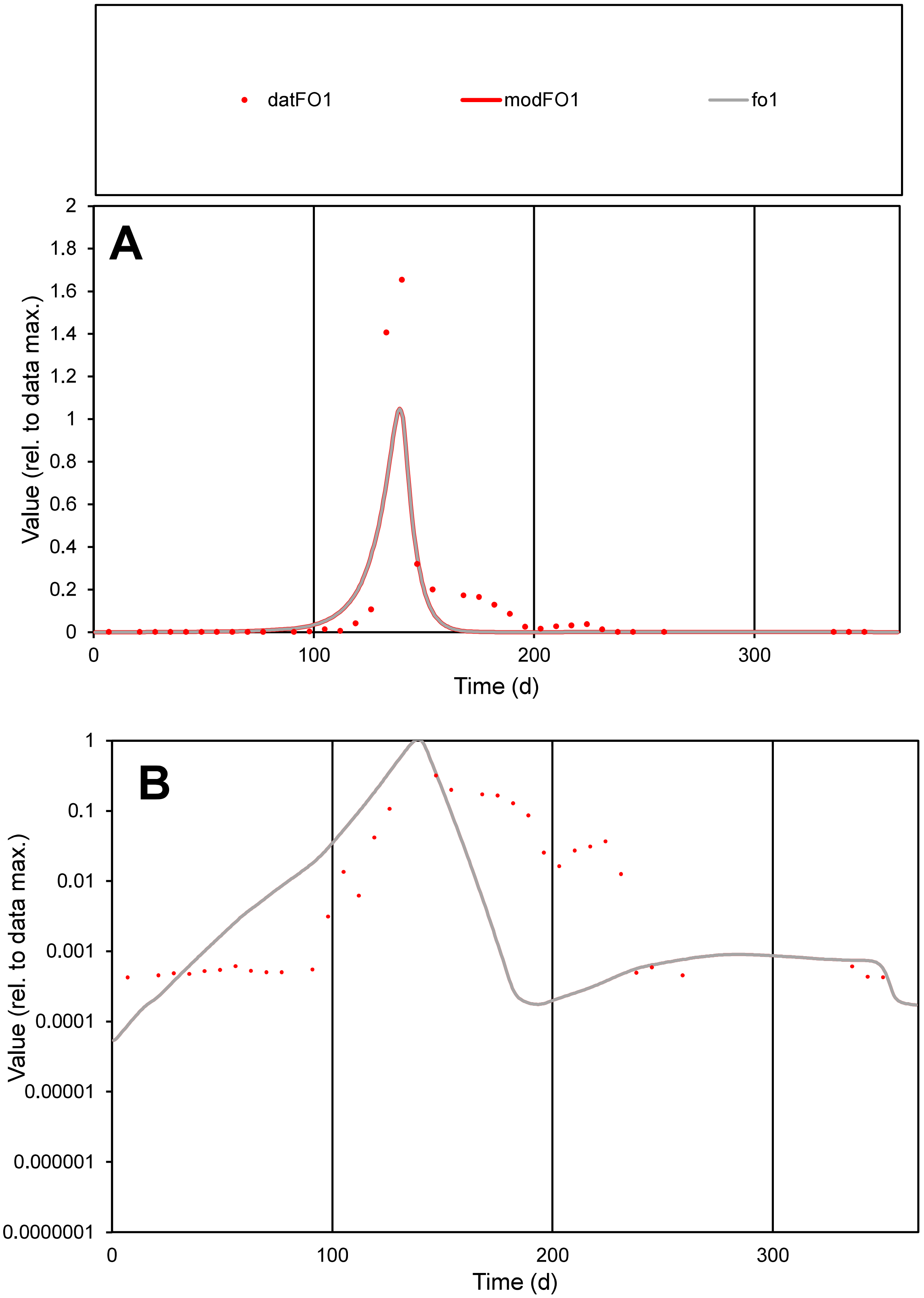

C1

Variables
FO1 Provide jtd or Dst
CalibOnly?
2 1 to reproduce of
oNormalize?
2 1=no, 2=yes

ofxn calc*
0.87486
ofxn mod
0.94627

sRun
RunA1
zout
1

Ave
Data
0.01244
Model
0.01111

data/vMax
0.1408

ofxng
311.734

Min d/m
4.4694

ave/min d
207.651
ave/min m
829.396

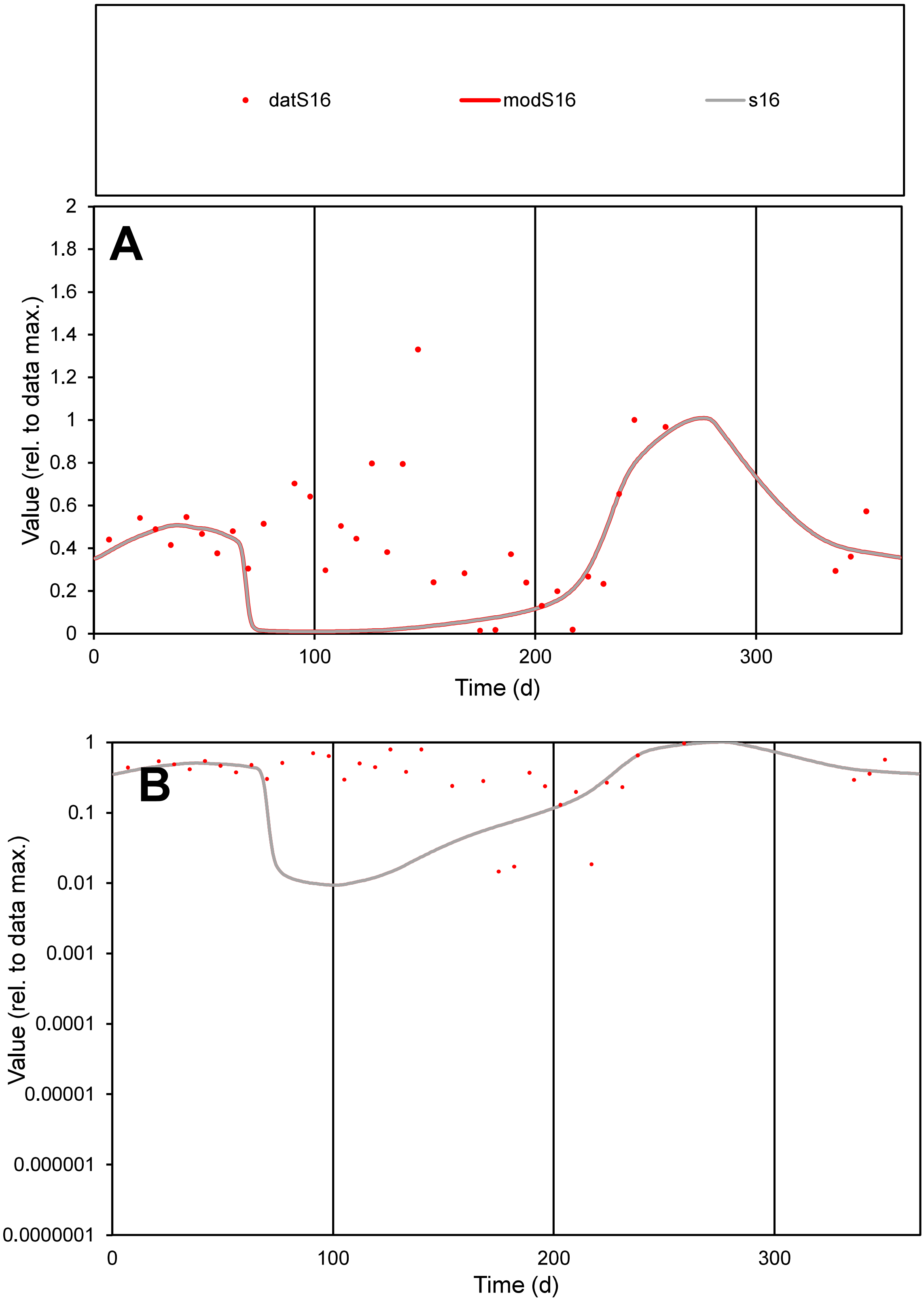

C1

Variables
S16 Provide jtd or Dst
CalibOnly?
2 1 to reproduce of
oNormalize?
2 1=no, 2=yes

ofxn calc*
0.73617
ofxn mod
0.74604

sRun
RunA1
zout
1

Ave
Data
0.00401
Model
0.0025

data/vMax
0.00949

ofxng
311.734

Min d/m
1.55374

ave/min d
28.9872
ave/min m
28.035

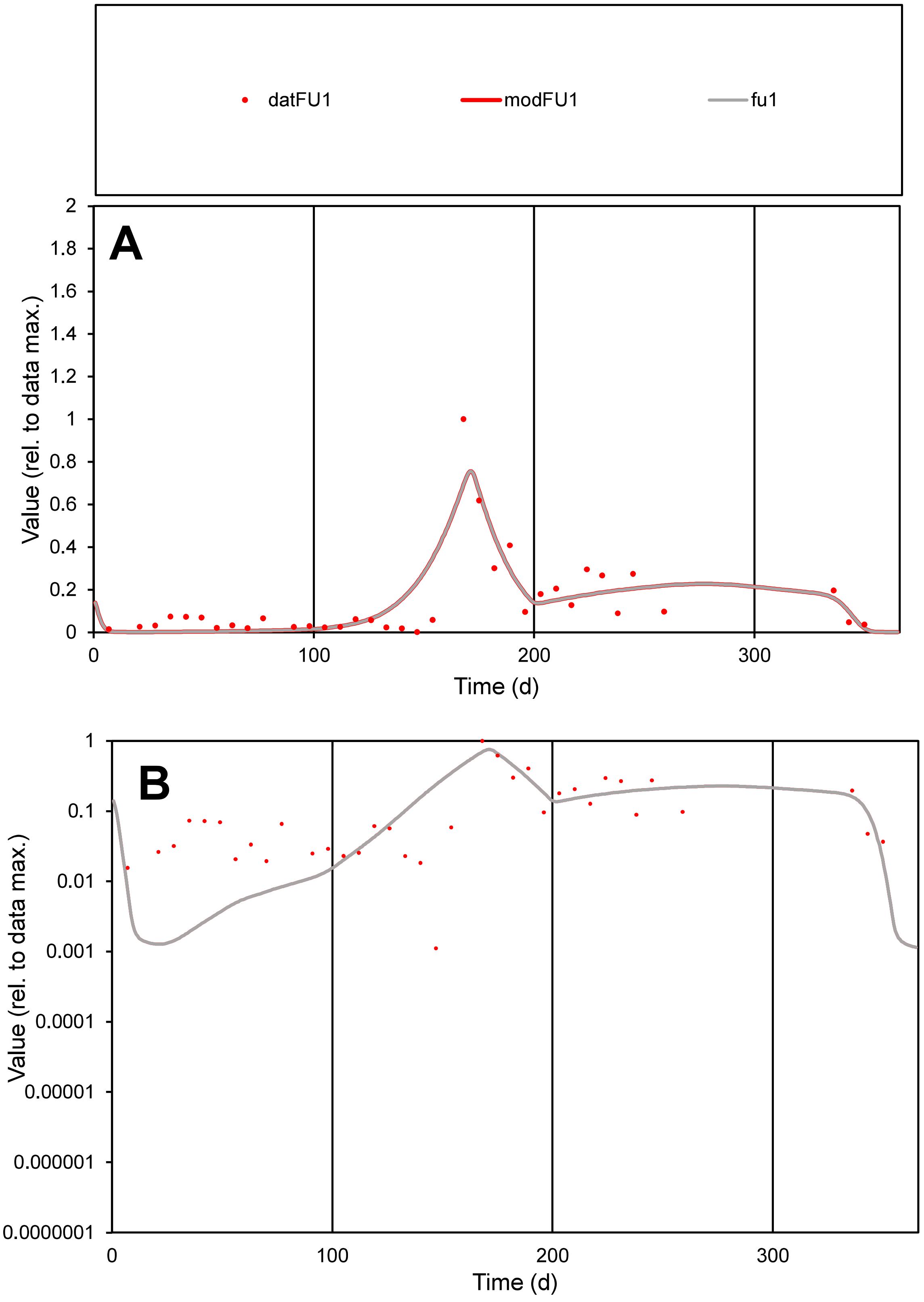

C1

Variables
FU1 Provide jtd or Dst
CalibOnly?
2 1 to reproduce of
oNormalize?
2 1=no, 2=yes

ofxn calc*
0.71498
ofxn mod
0.75651

sRun
RunA1
zout
1

Ave
Data
0.00599
Model
0.00604

data/vMax
0.04365

ofxng
311.734

Min d/m
0.86932

ave/min d
123.264
ave/min m
107.997

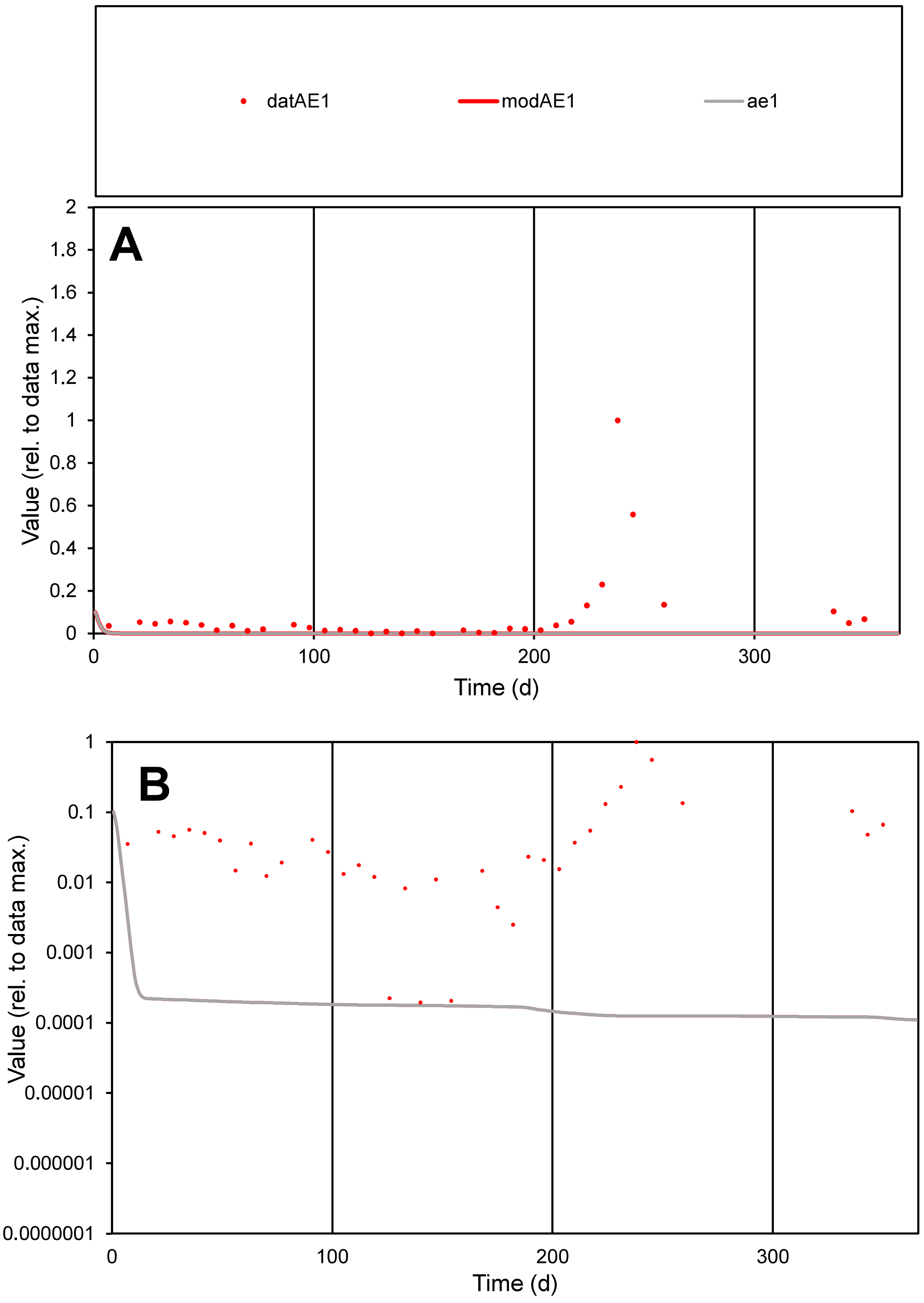

C1

Variables

AE1	Provide jtd or Dst
CalibOnly?	
2	1 to reproduce of
oNormalize?	
2	1=no, 2=yes

ofxn calc*
2.4557
ofxn mod
2.45986

sRun
RunA1
zout
1

Ave
Data
0.00575
Model
6.4E-05

data/vMax
0.07062

ofxng
311.734

Min d/m
1.66446

ave/min d
416.27
ave/min m
7.65969

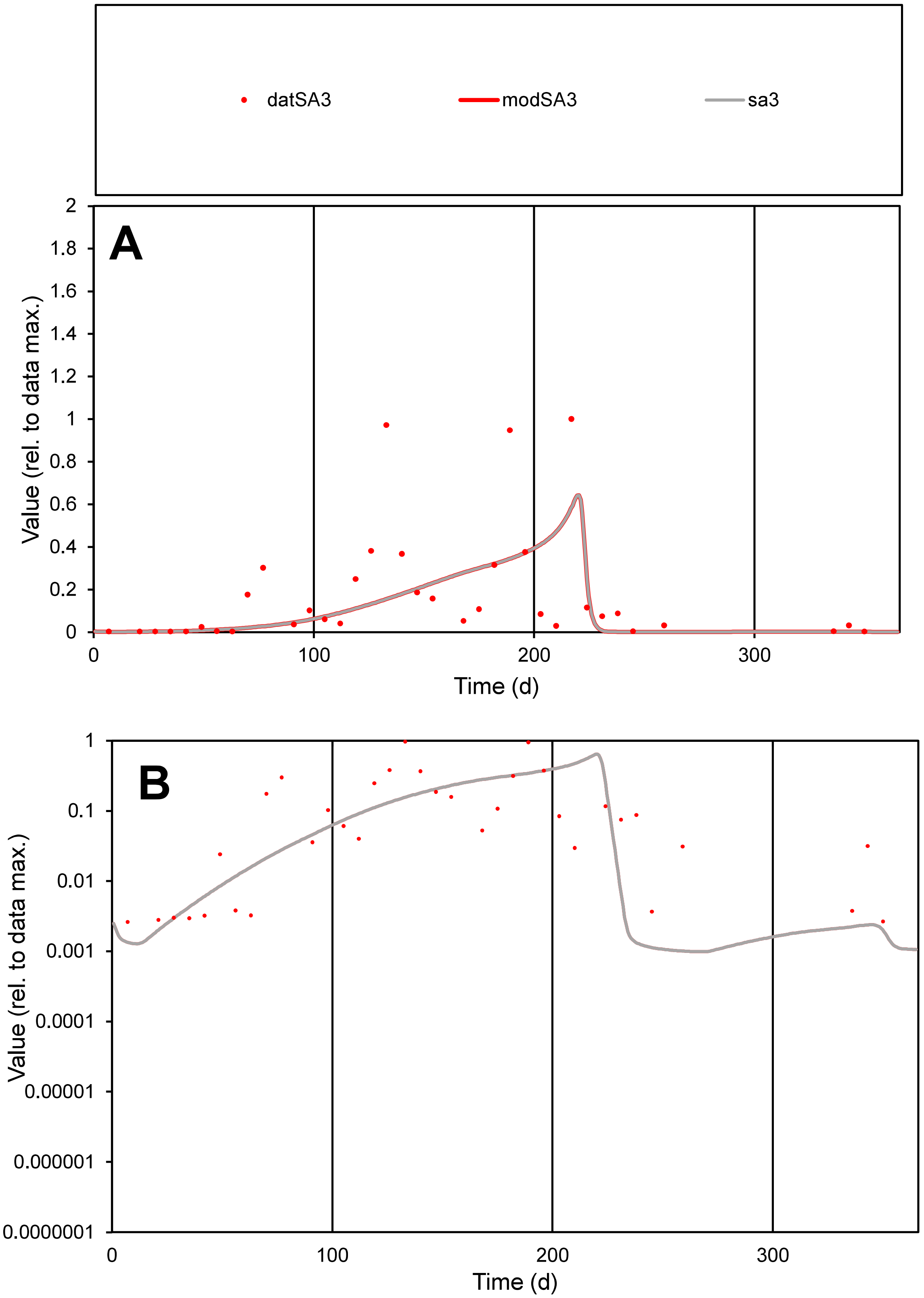

C1

Variables
SA3 Provide jtd or Dst
CalibOnly?
2 1 to reproduce of
oNormalize?
2 1=no, 2=yes

ofxn calc*
1.22371
ofxn mod
1.23663

sRun
RunA1
zout
1

Ave
Data
0.00609
Model
0.00504

data/vMax
0.03844

ofxng
311.734

Min d/m
2.61375
ave/min d
60.234
ave/min m
130.277

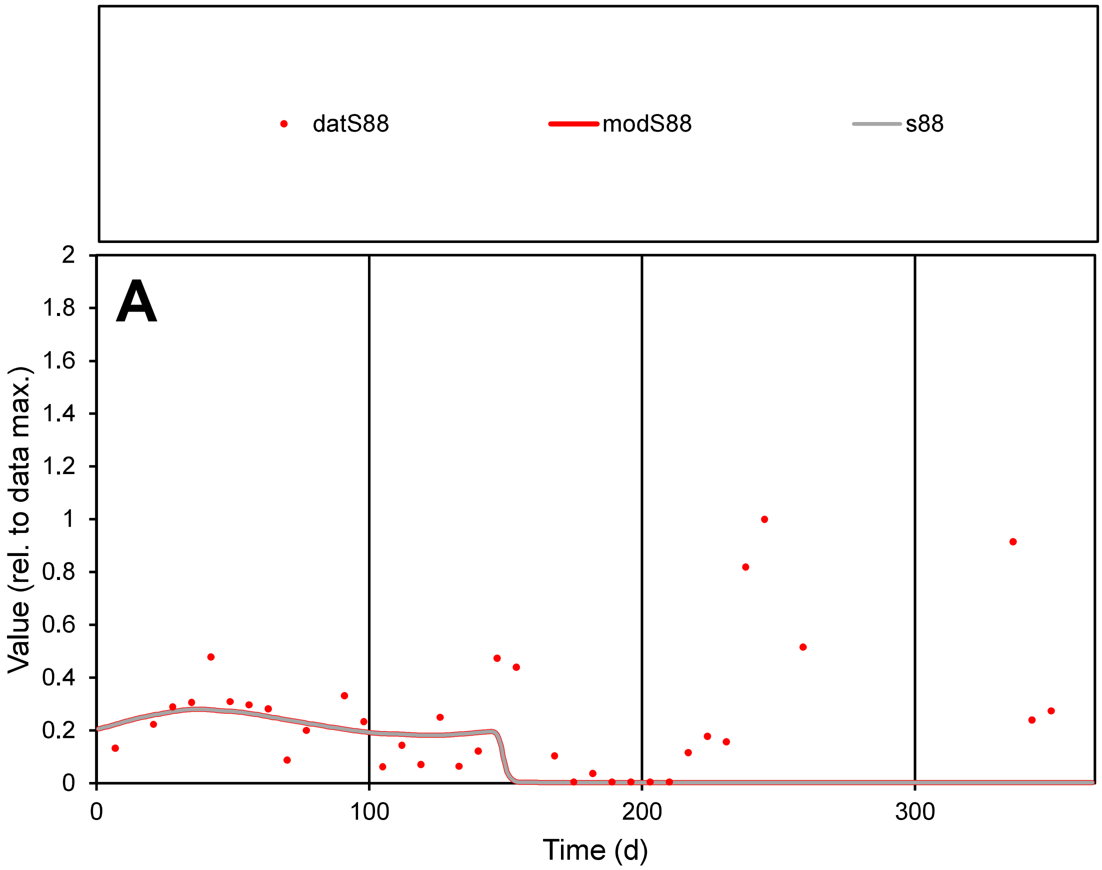

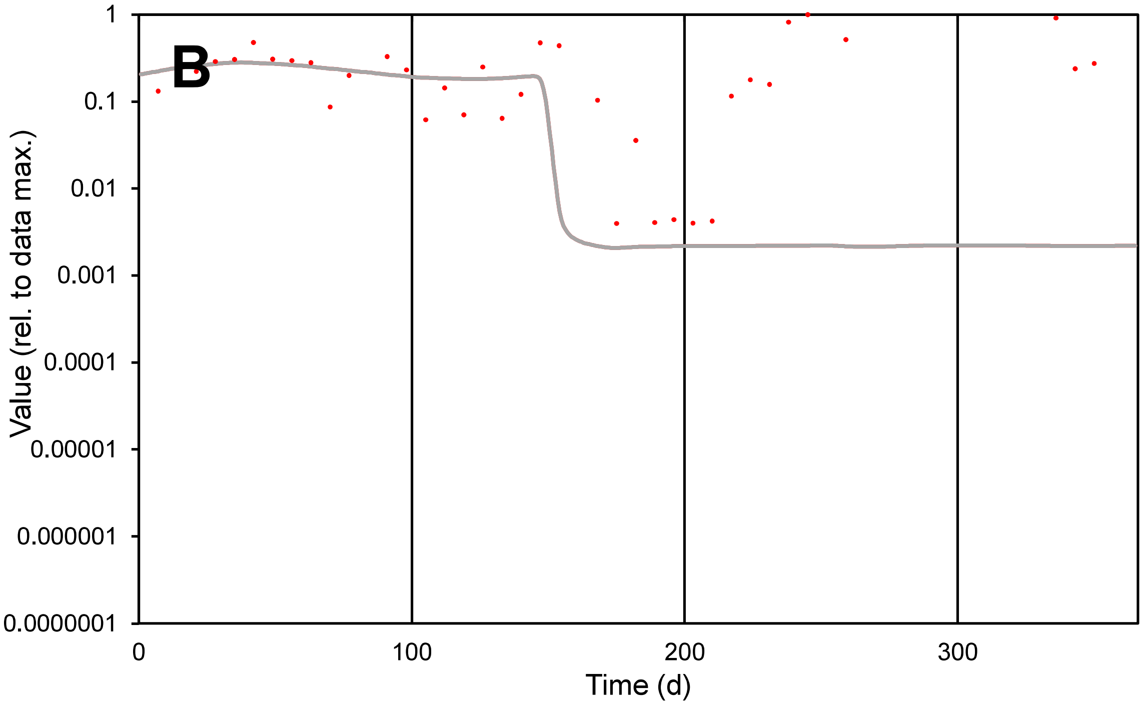

C1

Variables

S88 Provide jtd or Dst

CalibOnly?

2 1 to reproduce of

oNormalize?

2 1=no, 2=yes

ofxn calc*

1.23518

ofxn mod

1.23999

sRun

RunA1

zout

1

Ave

Data

0.00396

Model

0.00193

data/vMax

0.01623

ofxng

311.734

Min d/m

1.9095

ave/min d

61.4742

ave/min m

57.3013

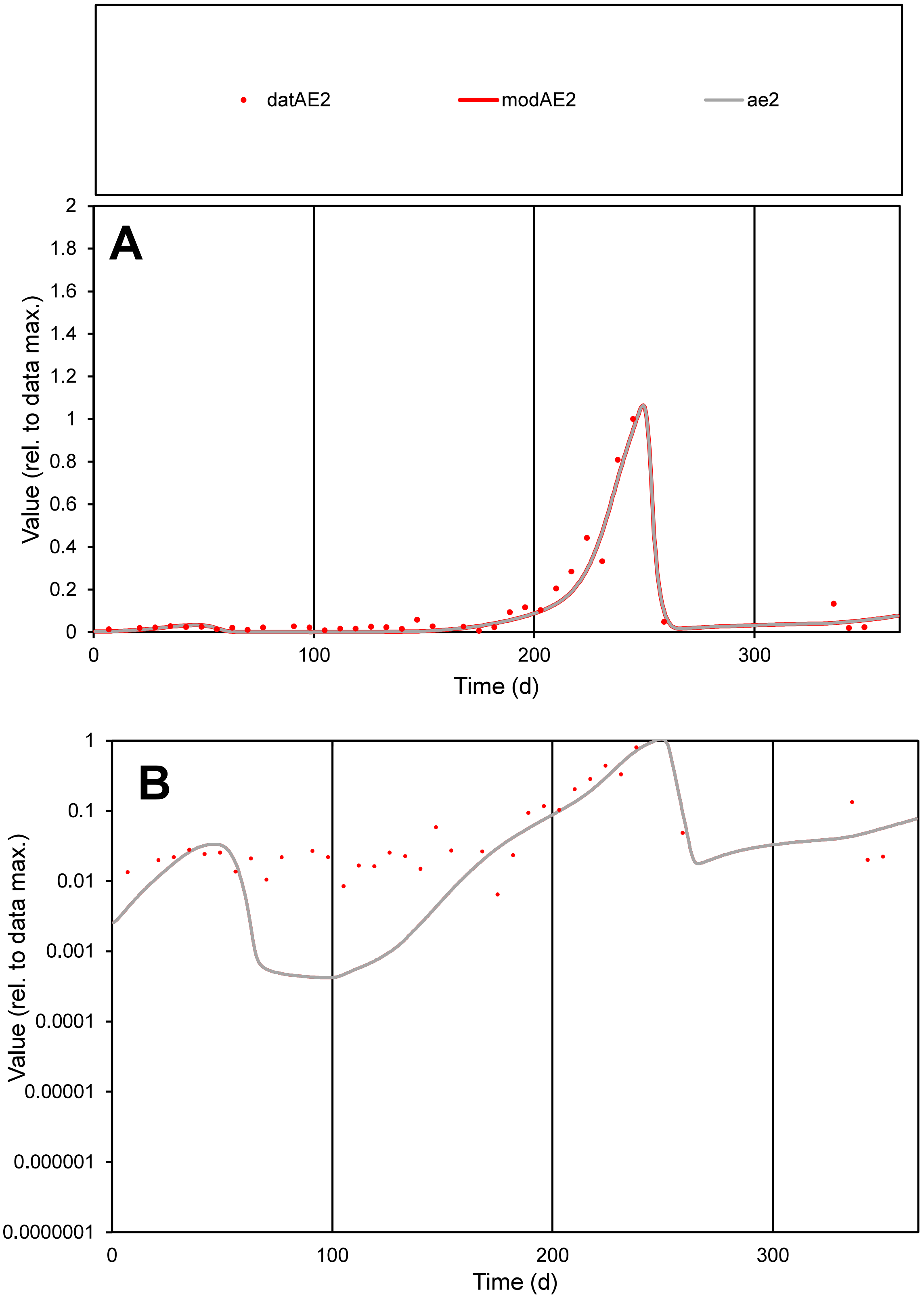

C1

Variables
AE2 Provide jtd or Dst
CalibOnly?
2 1 to reproduce of
oNormalize?
2 1=no, 2=yes

ofxn calc*
0.3813
ofxn mod
0.46273

sRun
RunA1
zout
1

Ave
Data
0.00846
Model
0.00757

data/vMax
0.07489

ofxng
311.734

Min d/m
15.3352

ave/min d
17.4387
ave/min m
239.268

331_Calib_S_A

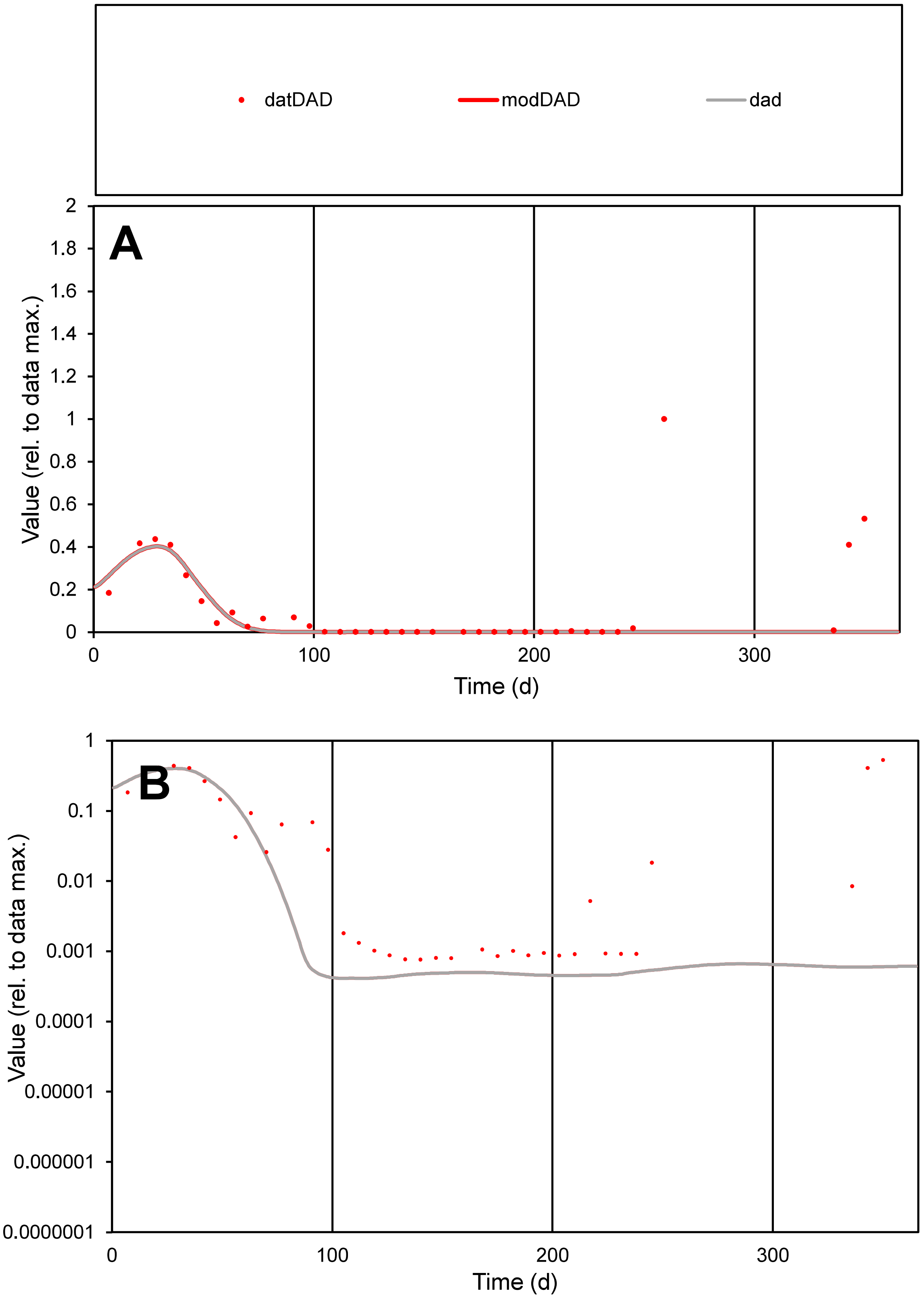

C1

Variables

DAD	Provide jtd or Dst
CalibOnly?	
2	1 to reproduce of
oNormalize?	
2	1=no, 2=yes

ofxn calc*
1.74787
ofxn mod
1.75644

sRun
RunA1
zout
1

Ave
Data
0.00233
Model
0.00118

data/vMax
0.02007

ofxng
311.734

Min d/m
1.8559

ave/min d
152.096
ave/min m
142.889

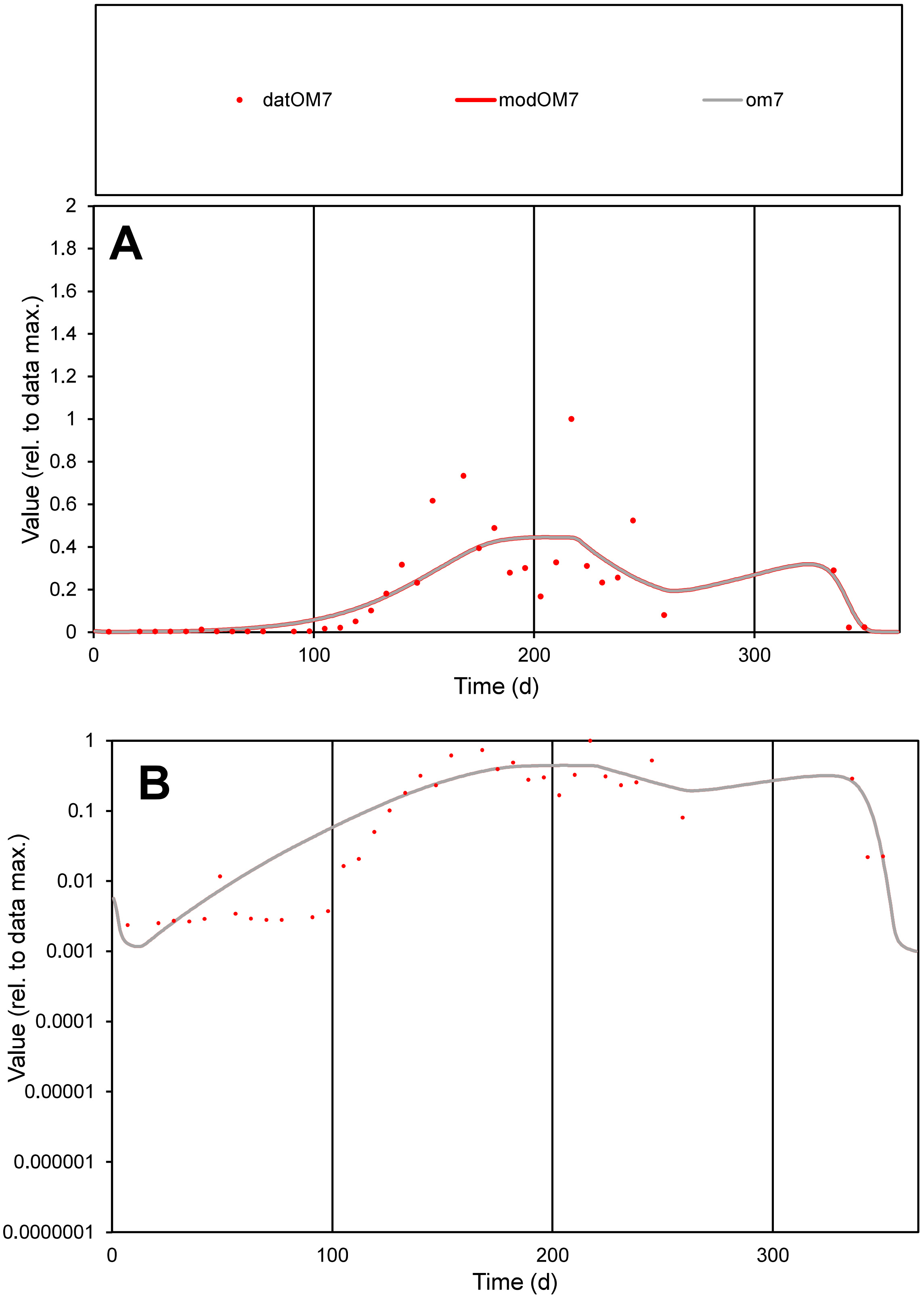

C1

Variables
OM7 Provide jtd or Dst
CalibOnly?
2 1 to reproduce of
oNormalize?
2 1=no, 2=yes

ofxn calc*
0.79056
ofxn mod
0.80783

sRun
RunA1
zout
1

Ave
Data
0.00742
Model
0.00762

data/vMax
0.04064

ofxng
311.734

Min d/m
1.89939

ave/min d
77.1919
ave/min m
150.537

331_Calib_S_A

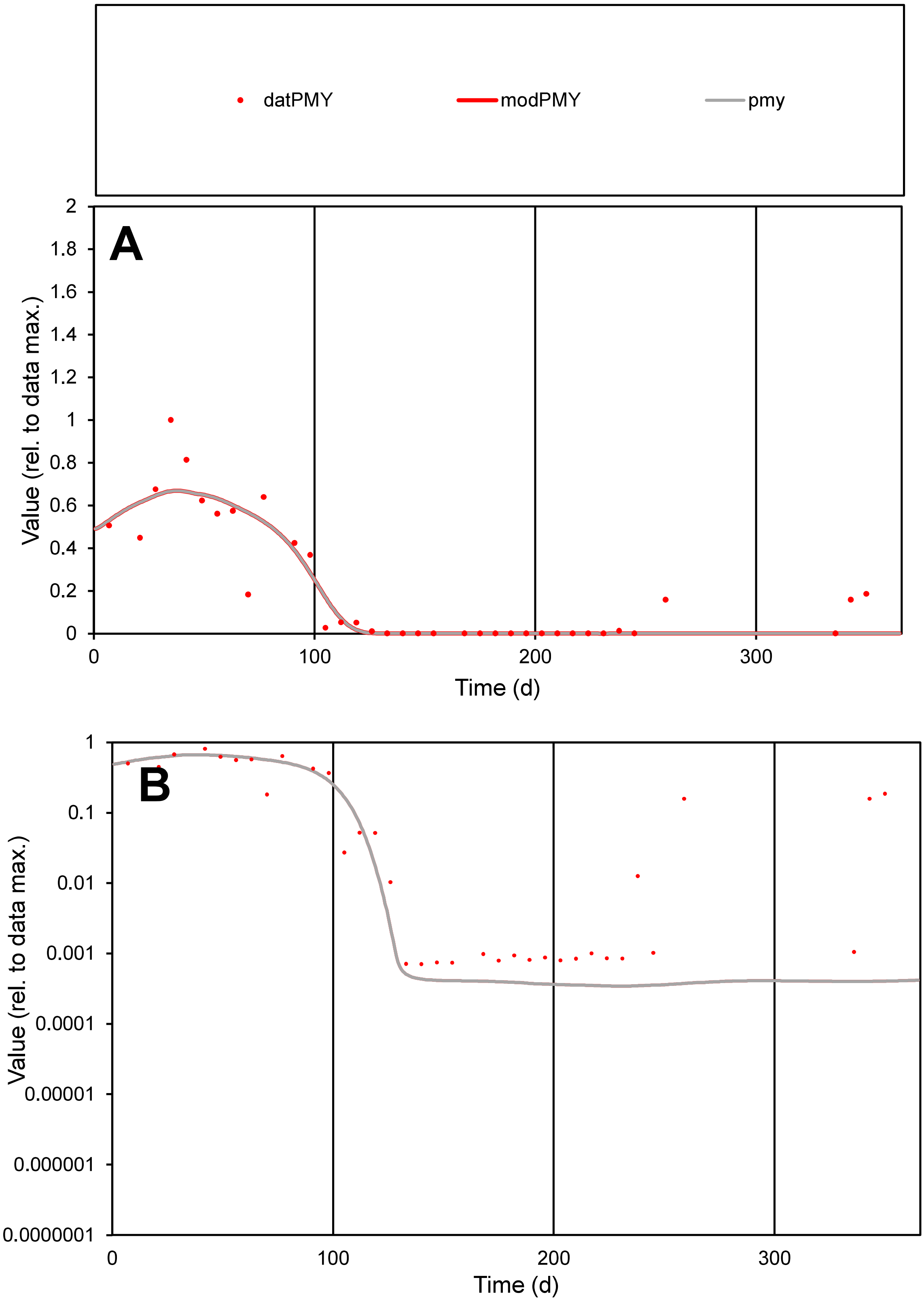

C1

Variables
PMY Provide jtd or Dst
CalibOnly?
2 1 to reproduce of
oNormalize?
2 1=no, 2=yes

ofxn calc*
0.52814
ofxn mod
0.56708

sRun
RunA1
zout
1

Ave
Data
0.00281
Model
0.00261

data/vMax
0.01351

ofxng
311.734

Min d/m
2.05279

ave/min d
294.956
ave/min m
562.21

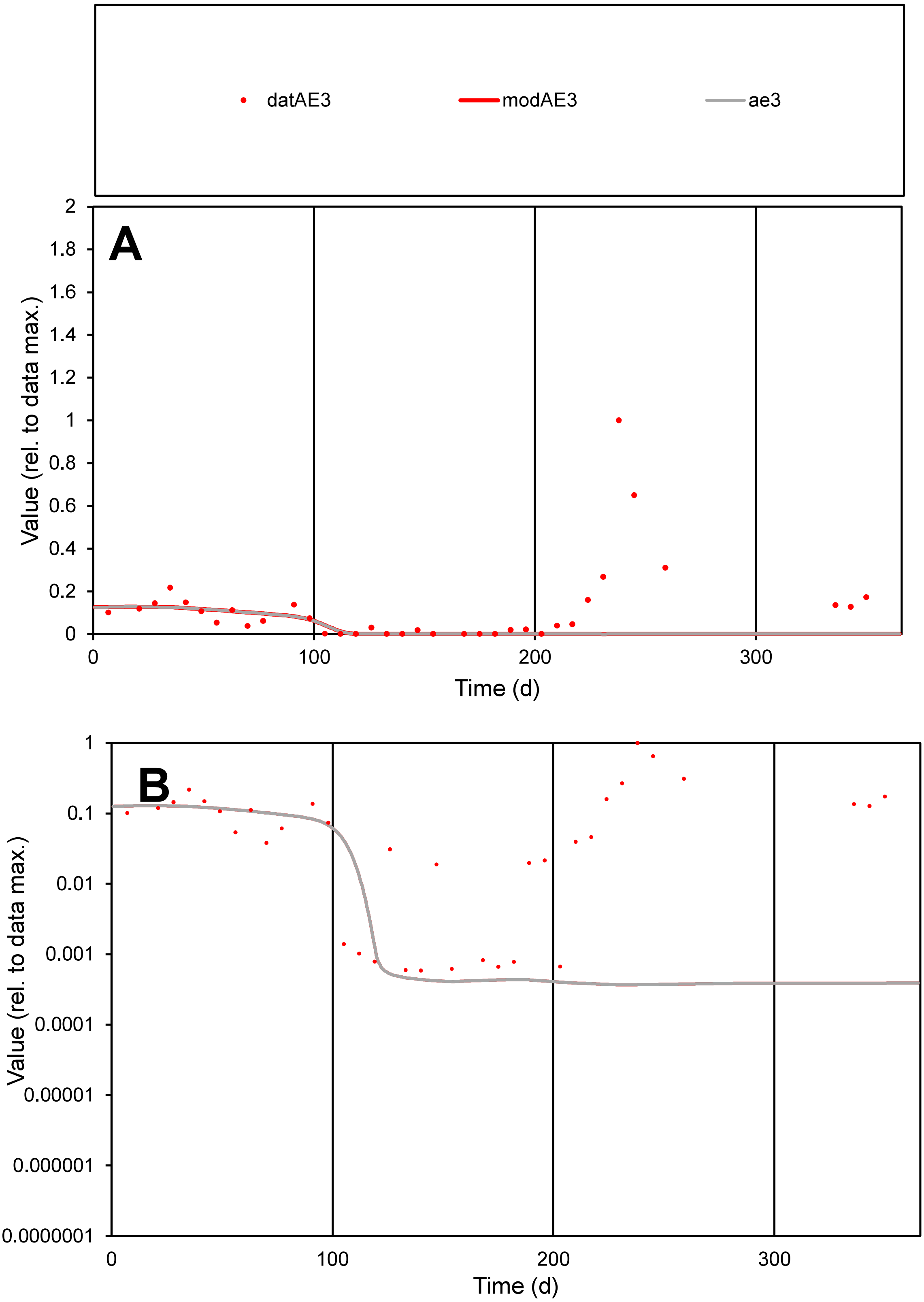

C1

Variables
AE3 Provide jtd or Dst
CalibOnly?
2 1 to reproduce of
oNormalize?
2 1=no, 2=yes

ofxn calc*
1.81879
ofxn mod
1.82283

sRun
RunA1
zout
1

Ave
Data
0.00338
Model
0.00106

data/vMax
0.02827

ofxng
311.734

Min d/m
1.60134

ave/min d
203.013
ave/min m
101.447

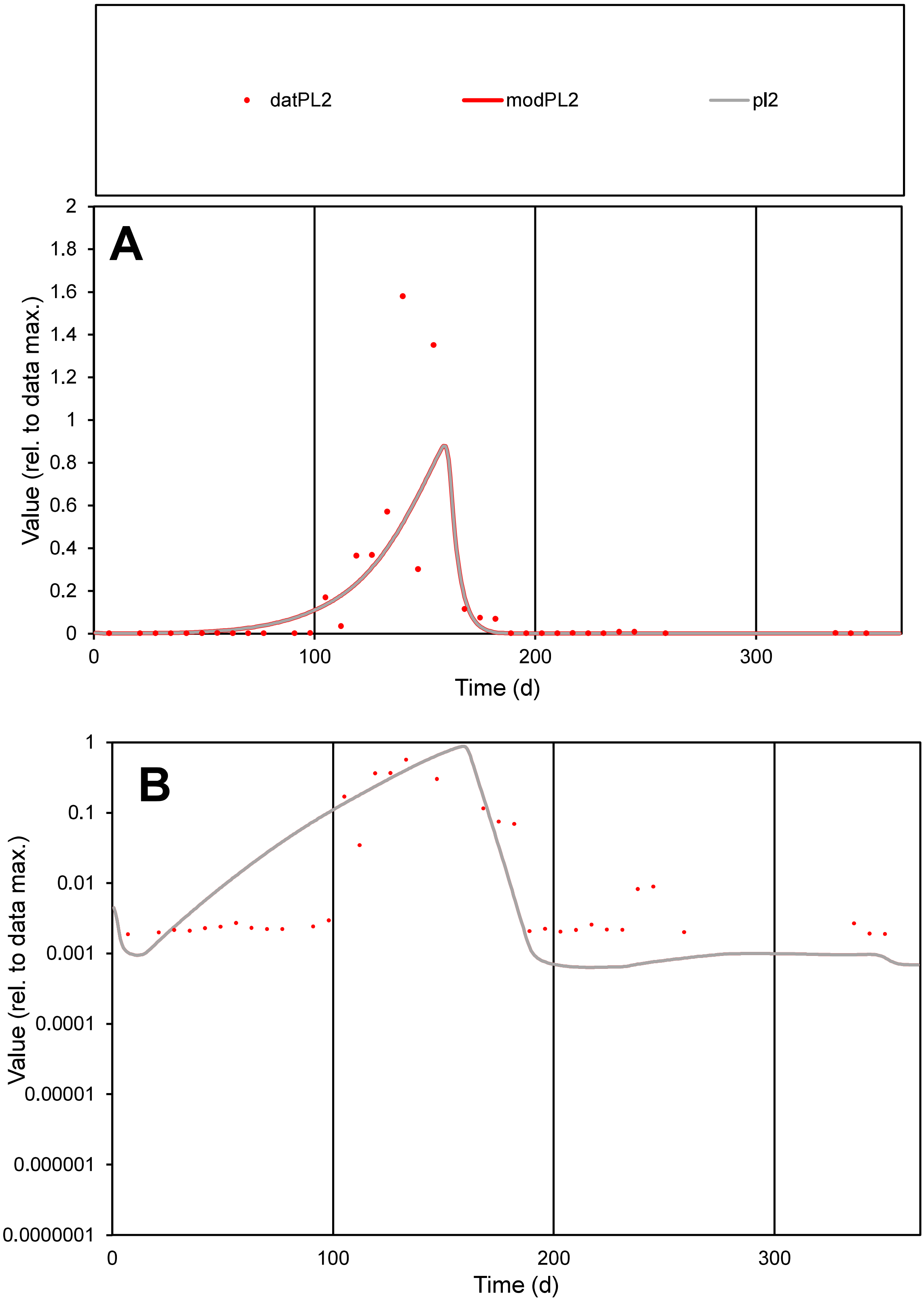

C1

Variables
PL2 Provide jtd or Dst
CalibOnly?
2 1 to reproduce of
oNormalize?
2 1=no, 2=yes

ofxn calc*
1.04995
ofxn mod
1.08502

sRun
RunA1
zout
1

Ave
Data
0.00916
Model
0.00944

data/vMax
0.08937

ofxng
311.734

Min d/m
2.95715

ave/min d
54.6326
ave/min m
166.54

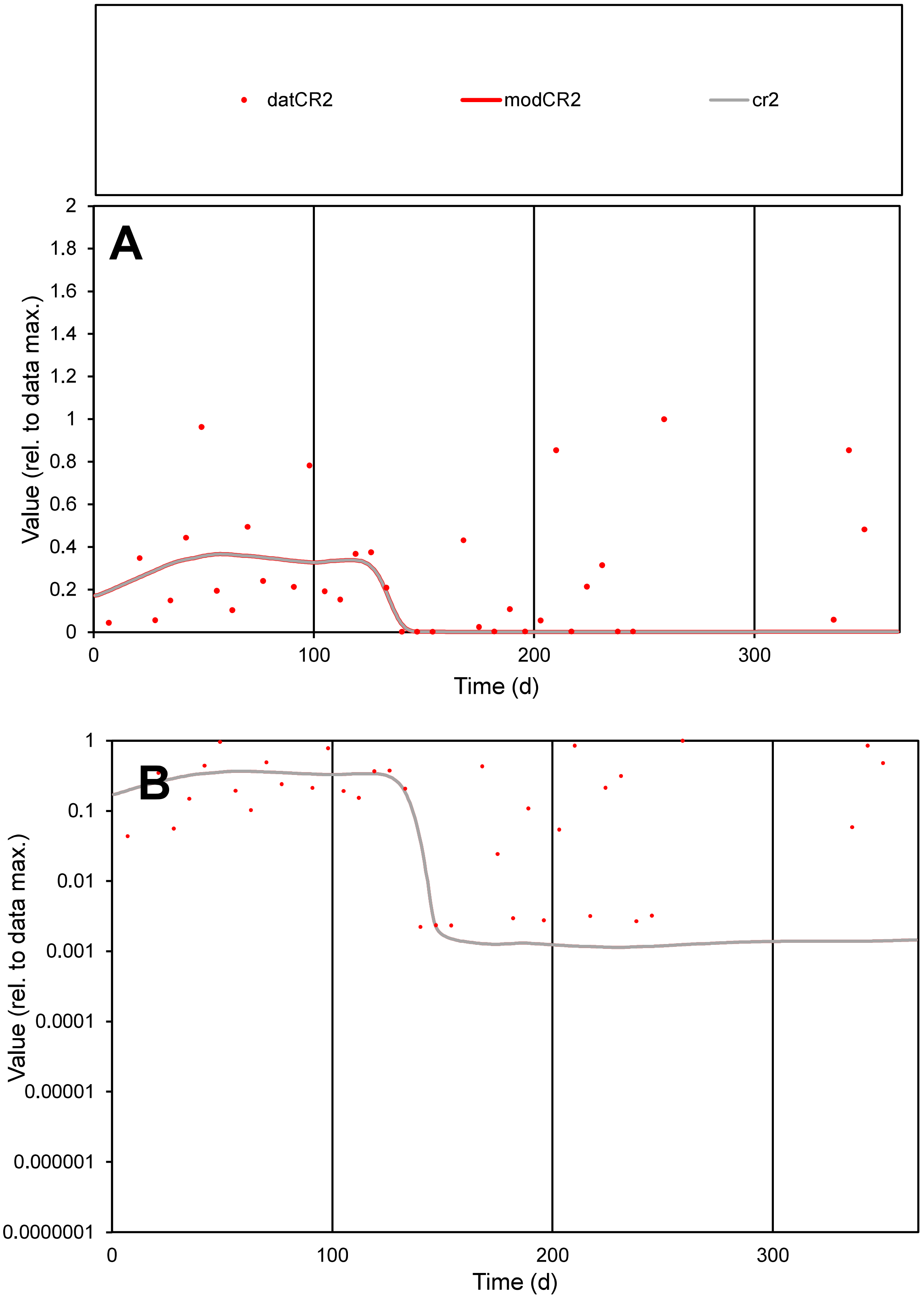

C1

Variables
CR2 Provide jtd or Dst
CalibOnly?
2 1 to reproduce of
oNormalize?
2 1=no, 2=yes

ofxn calc*
1.24199
ofxn mod
1.24786

sRun
RunA1
zout
1

Ave
Data
0.00472
Model
0.00265

data/vMax
0.01776

ofxng
311.734

Min d/m
1.95353

ave/min d
119.462
ave/min m
130.771

331_Calib_S_A

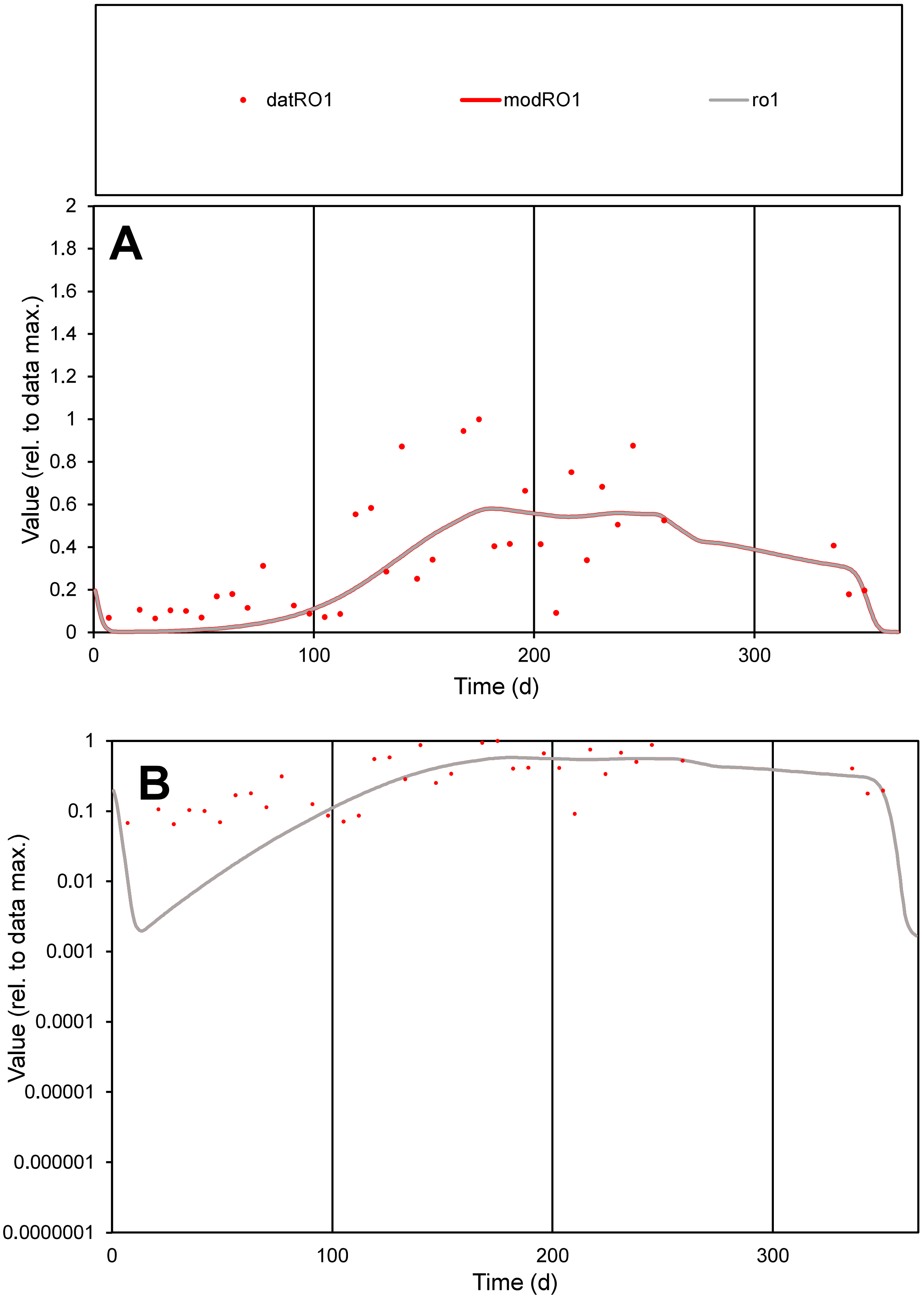

C1

Variables
RO1 Provide jtd or Dst
CalibOnly?
2 1 to reproduce of
oNormalize?
2 1=no, 2=yes

ofxn calc*
0.56187
ofxn mod
0.57657

sRun
RunA1
zout
1

Ave
Data
0.00541
Model
0.00479

data/vMax
0.01602

ofxng
311.734

Min d/m
20.4301

ave/min d
5.17334
ave/min m
93.5531

331_Calib_S_A

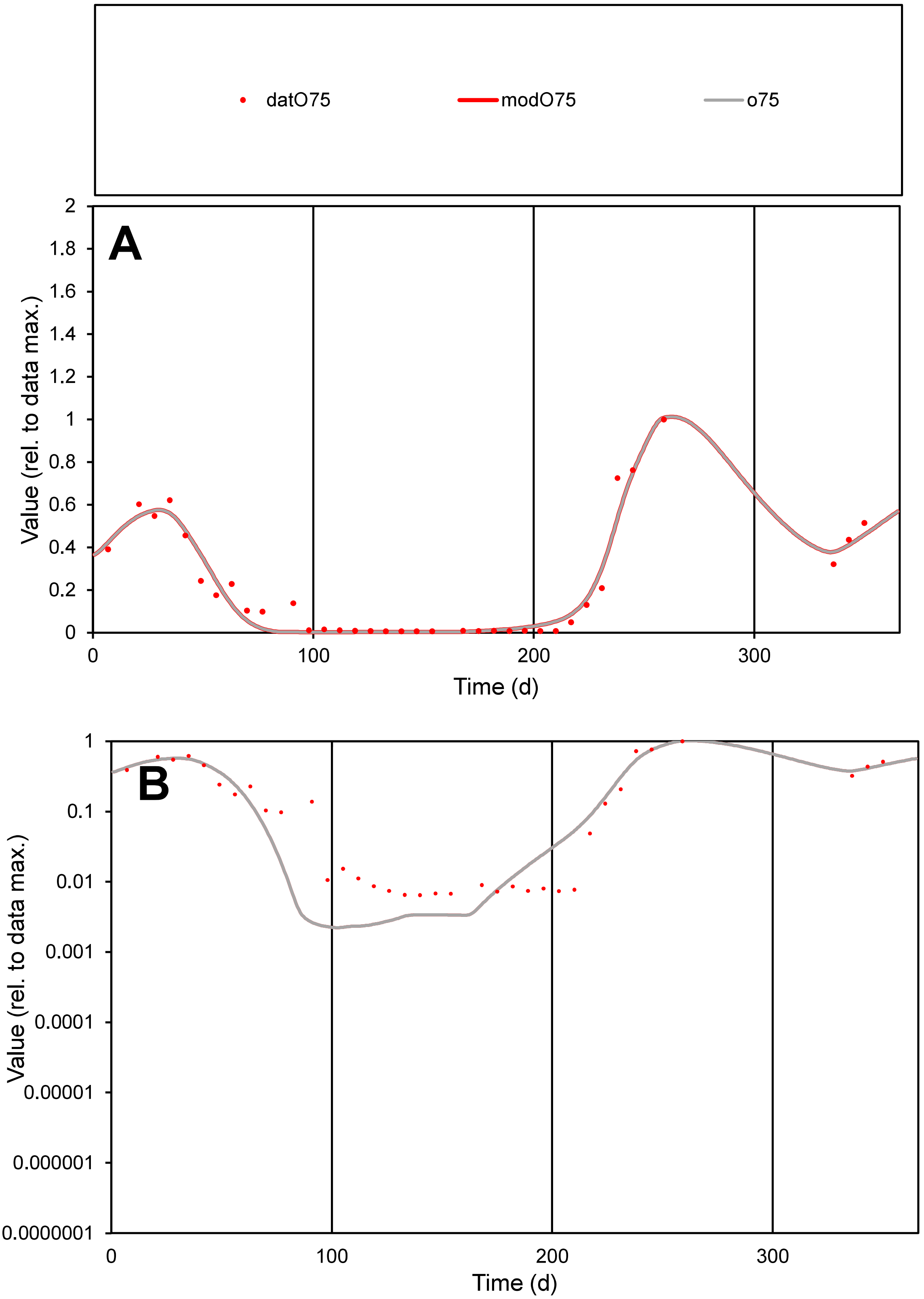

C1

Variables

O75 Provide jtd or Dst

CalibOnly?

2 1 to reproduce of

oNormalize?

2 1=no, 2=yes

ofxn calc*

0.24757

ofxn mod

0.30756

sRun

RunA1

zout

1

Ave

Data

0.00236

Model

0.00231

data/vMax

0.01077

ofxng

311.734

Min d/m

2.86249

ave/min d

33.9107

ave/min m

95.2747

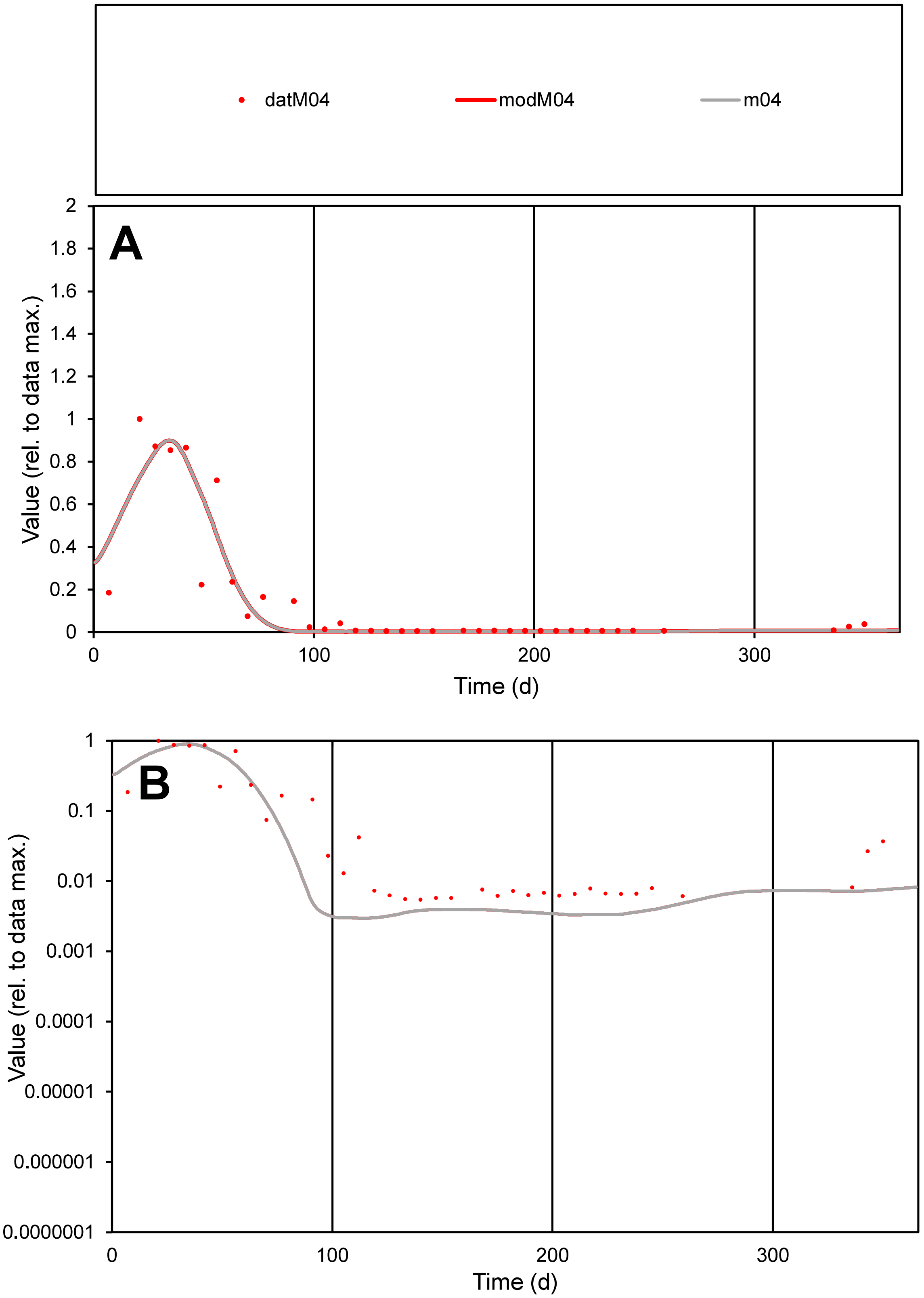

C1

Variables

M04	Provide jtd or Dst
CalibOnly?	
2	1 to reproduce of
oNormalize?	
2	1=no, 2=yes

ofxn calc*
0.68025
ofxn mod
0.72686

sRun
RunA1
zout
1

Ave
Data
0.001
Model
0.00094

data/vMax
0.00639

ofxng
311.734

Min d/m
1.85321

ave/min d
28.4735
ave/min m
49.6544

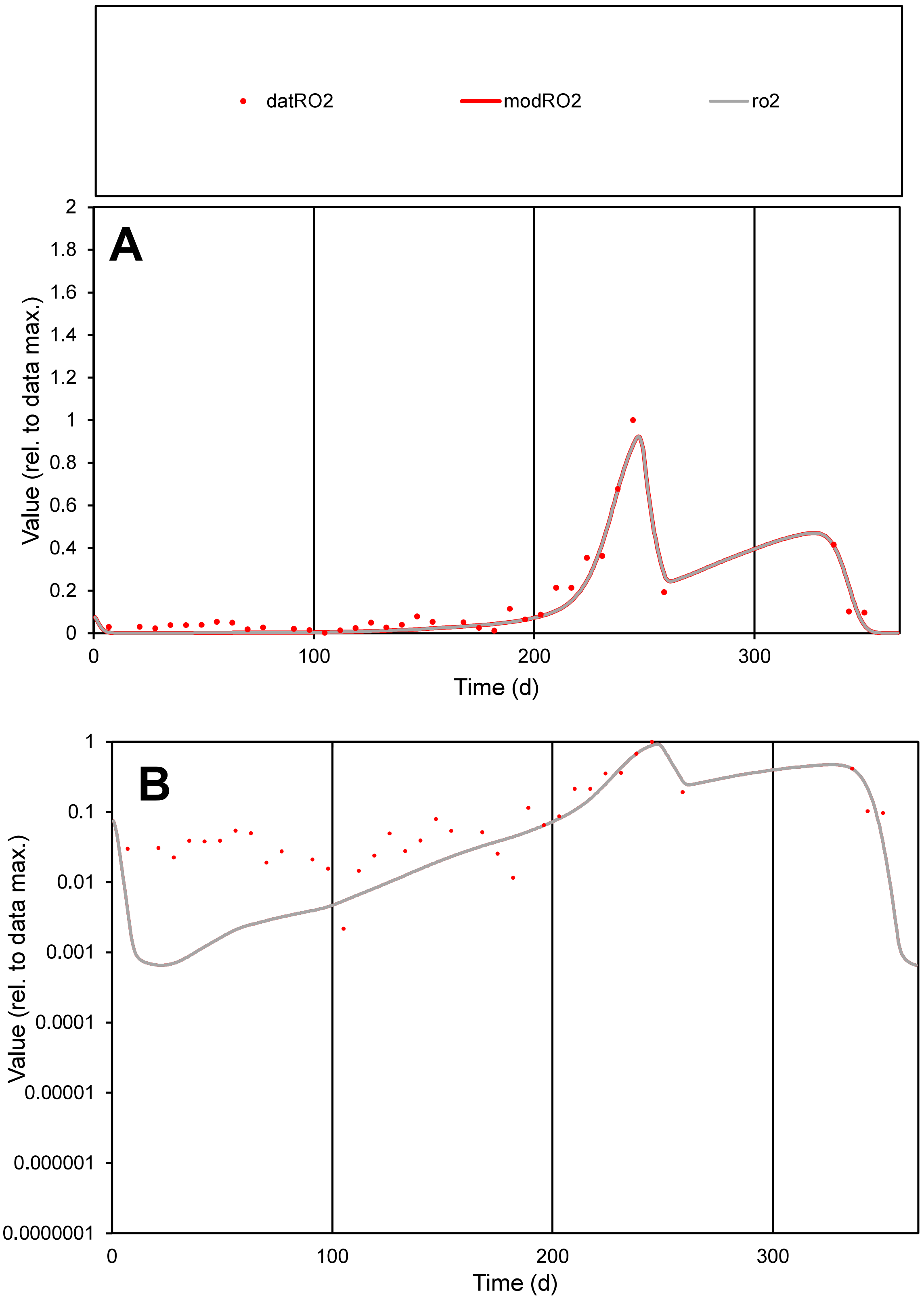

C1

Variables
RO2 Provide jtd or Dst
CalibOnly?
2 1 to reproduce of
oNormalize?
2 1=no, 2=yes

ofxn calc*
0.31944
ofxn mod
0.4021

sRun
RunA1
zout
1

Ave
Data
0.0053
Model
0.00452

data/vMax
0.04158

ofxng
311.734

Min d/m
3.30721

ave/min d
58.6695
ave/min m
165.539

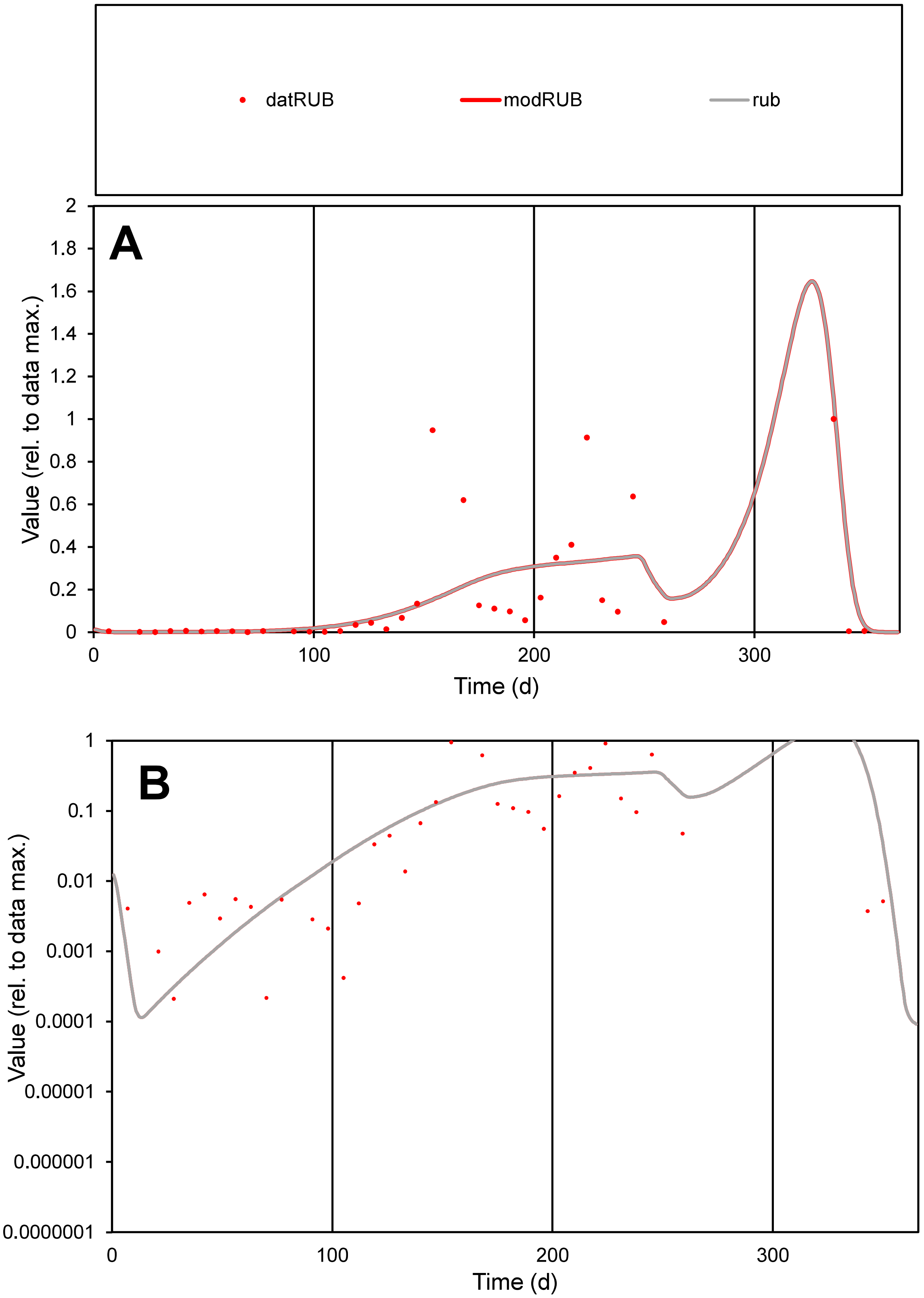

C1

Variables
RUB Provide jtd or Dst
CalibOnly?
2 1 to reproduce of
oNormalize?
2 1=no, 2=yes

ofxn calc*
1.13994
ofxn mod
1.16192

sRun
RunA1
zout
1

Ave
Data
0.00891
Model
0.00872

data/vMax
0.05588

ofxng
311.734

Min d/m
1.0034

ave/min d
755.805
ave/min m
742.43

331_Calib_S_A

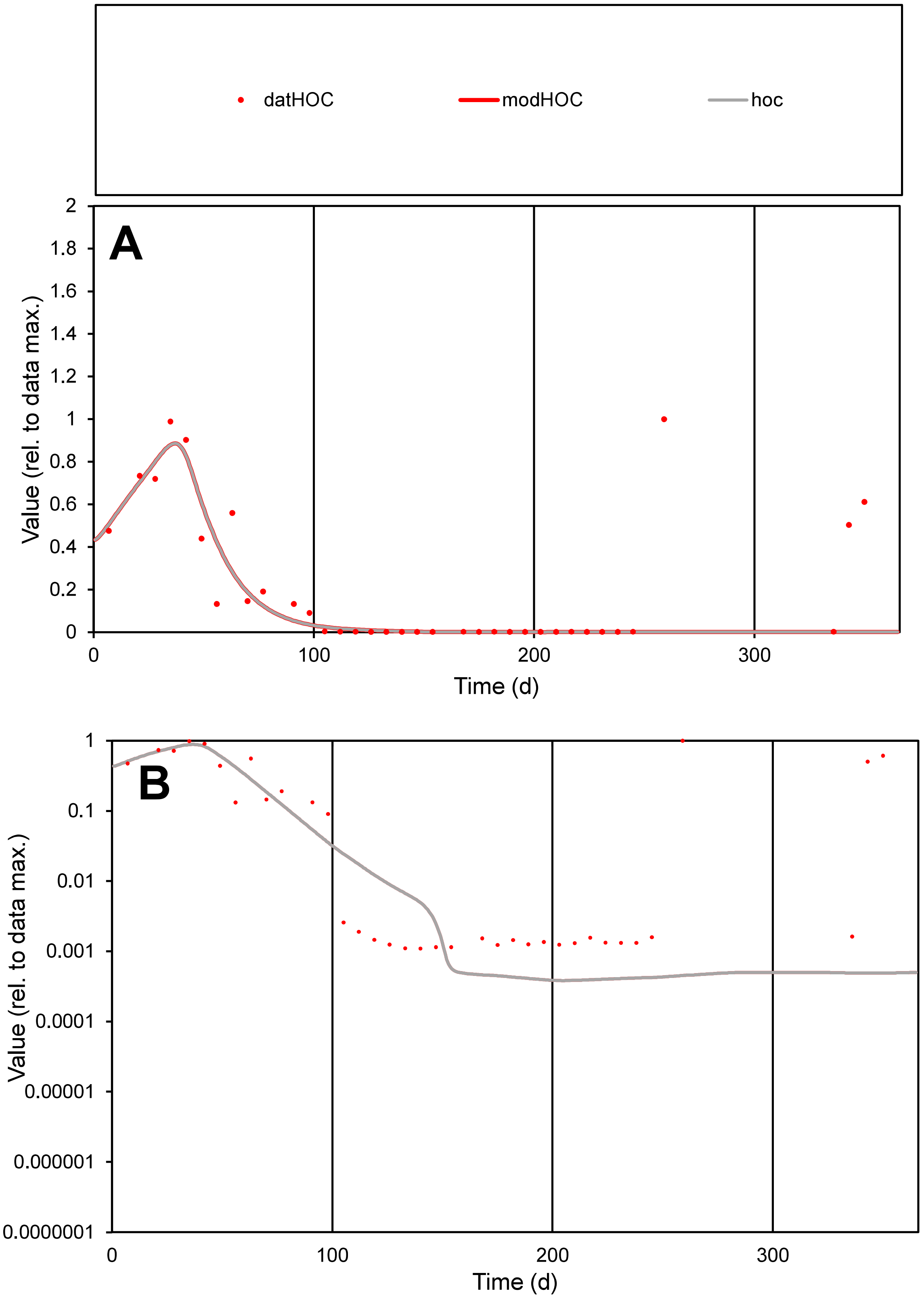

C1

Variables

HOC	Provide jtd or Dst
CalibOnly?	
2	1 to reproduce of
oNormalize?	
2	1=no, 2=yes

ofxn calc*
1.06381
ofxn mod
1.08101

sRun
RunA1
zout
1

Ave
Data
0.00243
Model
0.00172

data/vMax
0.01141

ofxng
311.734

Min d/m
2.86535

ave/min d
194.512
ave/min m
395.793

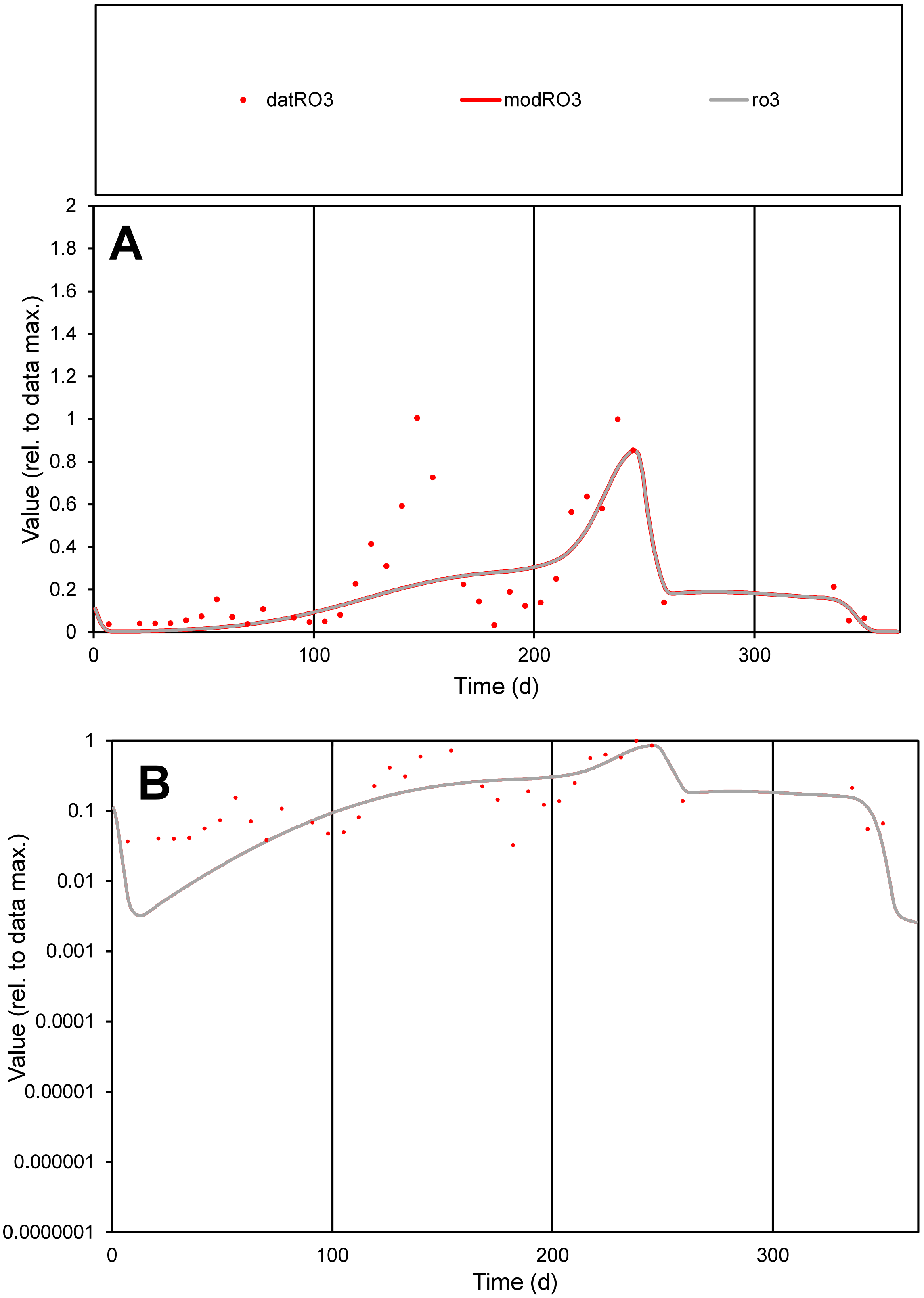

C1

Variables
RO3 Provide jtd or Dst
CalibOnly?
2 1 to reproduce of
oNormalize?
2 1=no, 2=yes

ofxn calc*
0.5898
ofxn mod
0.6122

sRun
RunA1
zout
1

Ave
Data
0.00568
Model
0.00507

data/vMax
0.02411

ofxng
311.734

Min d/m
6.57789

ave/min d
7.22844
ave/min m
42.426

331_Calib_S_A

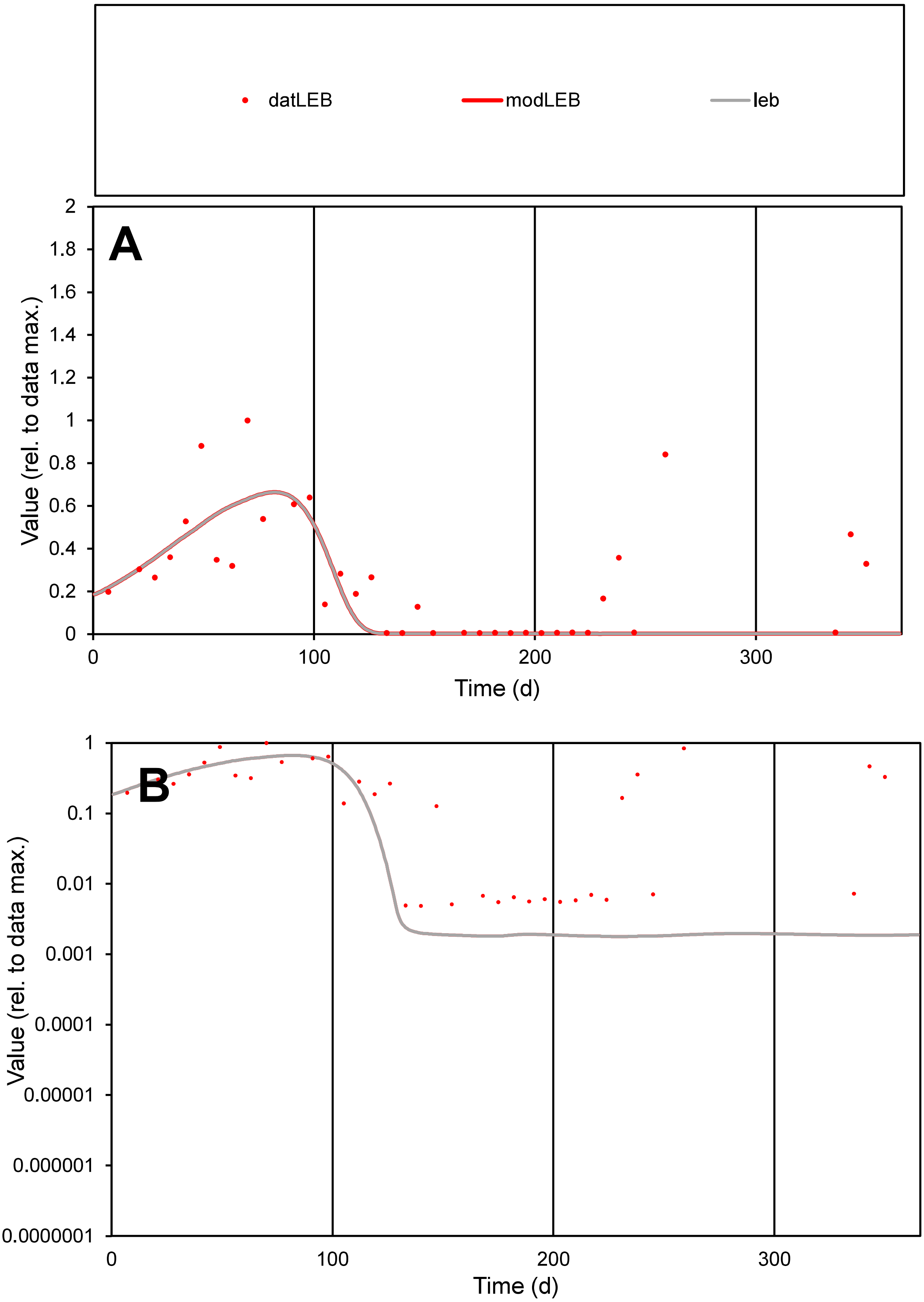

C1

Variables

LEB	Provide jtd or Dst
CalibOnly?	
2	1 to reproduce of
oNormalize?	
2	1=no, 2=yes

ofxn calc*
0.8612
ofxn mod
0.87924

sRun
RunA1
zout
1

Ave
Data
0.00359
Model
0.0026

data/vMax
0.01419

ofxng
311.734

Min d/m
2.73753

ave/min d
51.9599
ave/min m
102.783

331_Calib_S_A

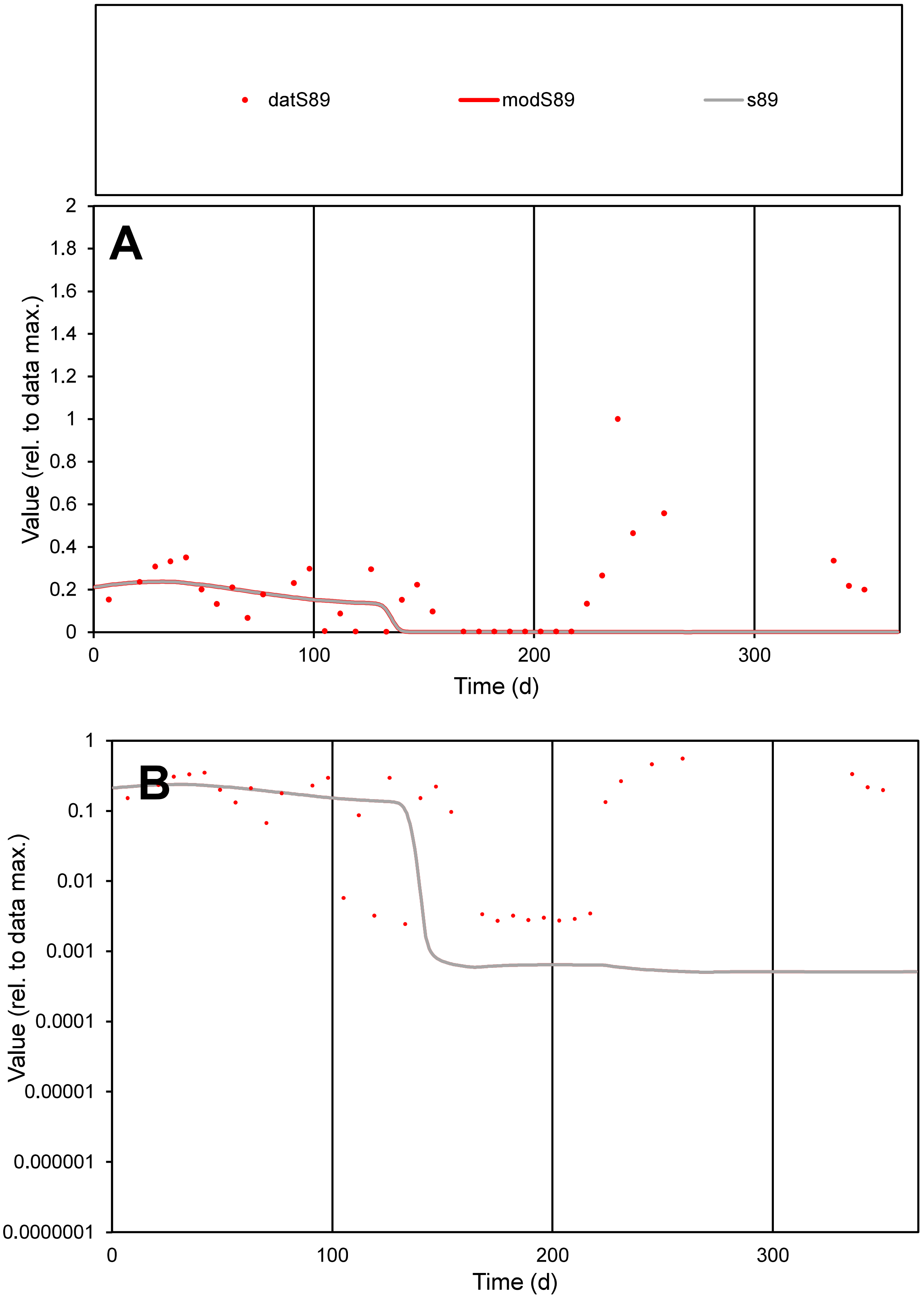

C1

Variables
S89 Provide jtd or Dst
CalibOnly?
2 1 to reproduce of
oNormalize?
2 1=no, 2=yes

ofxn calc*
1.29242
ofxn mod
1.29968

sRun
RunA1
zout
1

Ave
Data
0.00267
Model
0.00128

data/vMax
0.01476

ofxng
311.734

Min d/m
4.81912

ave/min d
74.0681
ave/min m
171.433

331_Calib_S_A

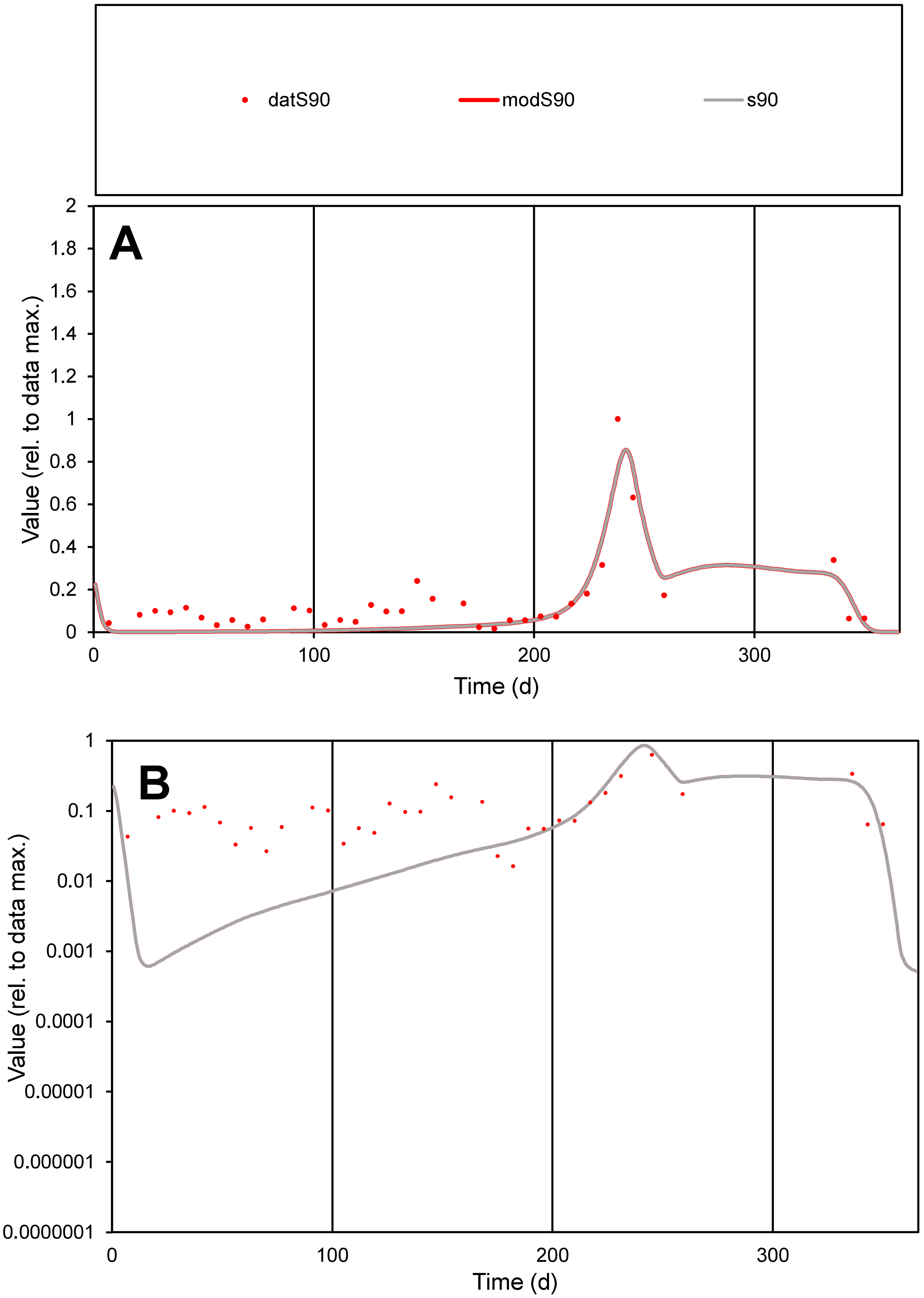

C1

Variables
S90 Provide jtd or Dst
CalibOnly?
2 1 to reproduce of
oNormalize?
2 1=no, 2=yes

ofxn calc*
0.56311
ofxn mod
0.59964

sRun
RunA1
zout
1

Ave
Data
0.00505
Model
0.00369

data/vMax
0.03733

ofxng
311.734

Min d/m
21.6869

ave/min d
8.30812
ave/min m
131.447

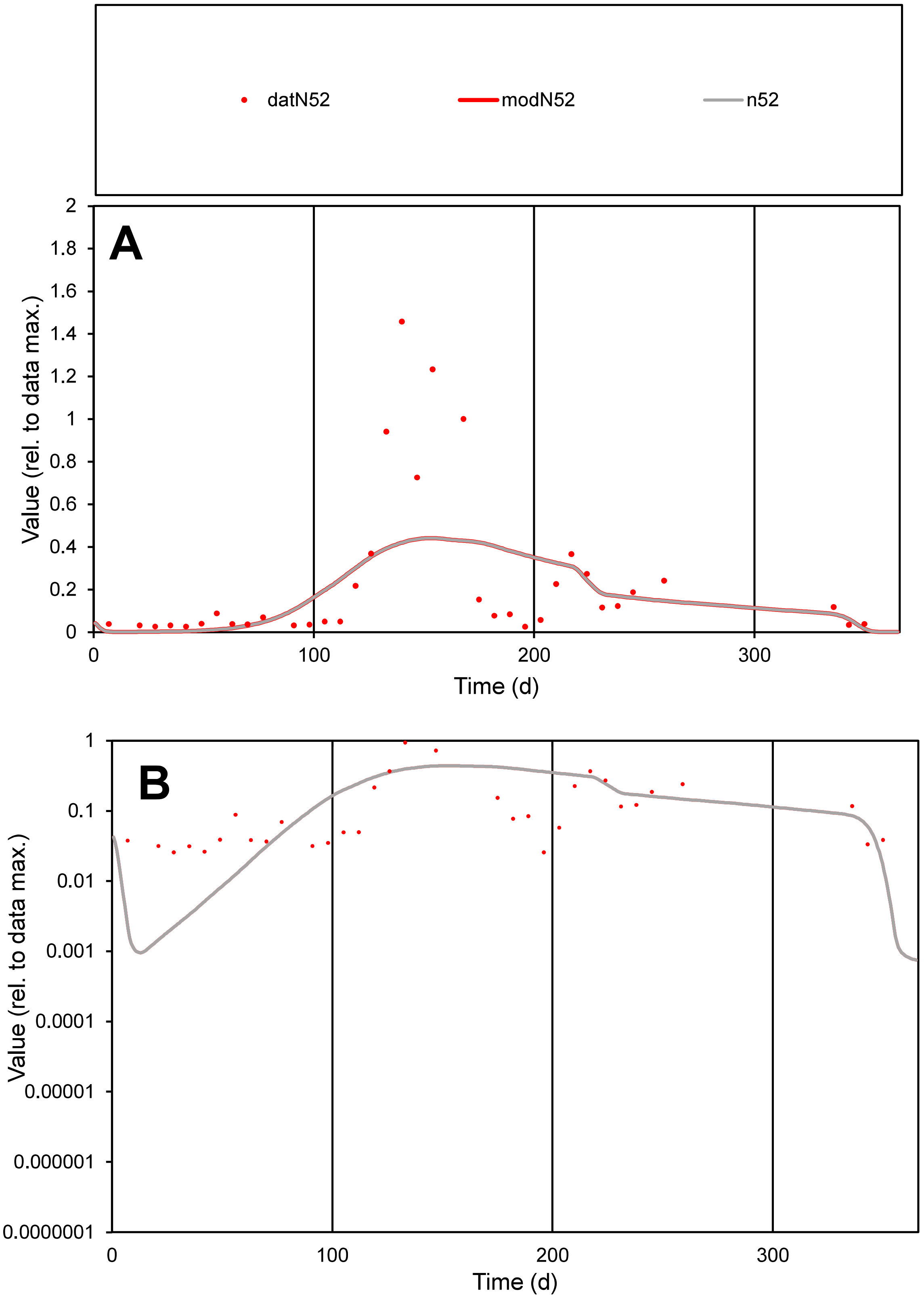

C1

Variables
N52 Provide jtd or Dst
CalibOnly?
2 1 to reproduce of
oNormalize?
2 1=no, 2=yes

ofxn calc*
0.99153
ofxn mod
1.00421

sRun
RunA1
zout
1

Ave
Data
0.00416
Model
0.00423

data/vMax
0.02098

ofxng
311.734

Min d/m
16.0173

ave/min d
7.73497
ave/min m
125.882

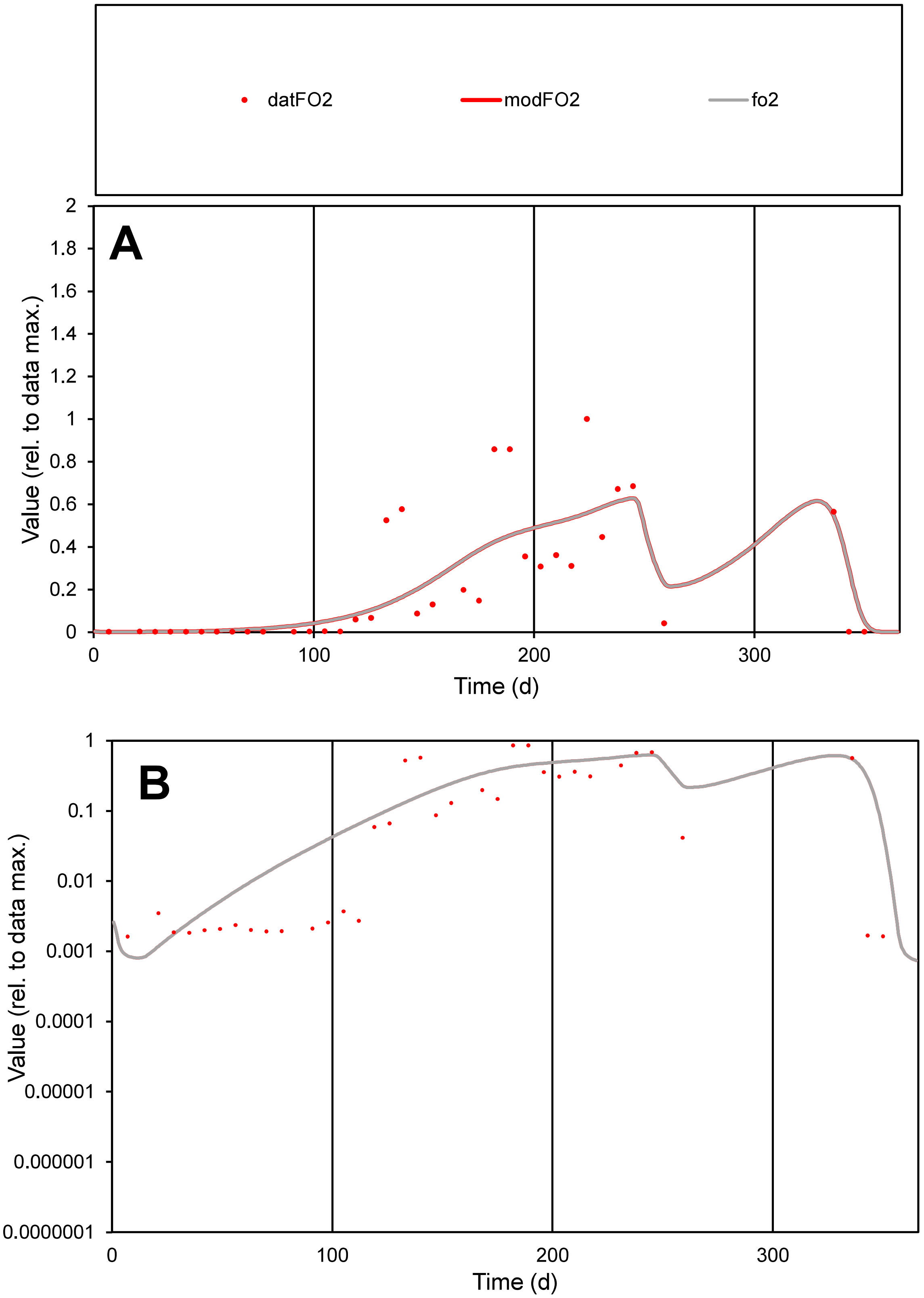

C1

Variables
FO2 Provide jtd or Dst
CalibOnly?
2 1 to reproduce of
oNormalize?
2 1=no, 2=yes

ofxn calc*
0.75042
ofxn mod
0.78147

sRun
RunA1
zout
1

Ave
Data
0.0092
Model
0.00957

data/vMax
0.04241

ofxng
311.734

Min d/m
1.94268

ave/min d
133.095
ave/min m
268.865

331_Calib_S_A

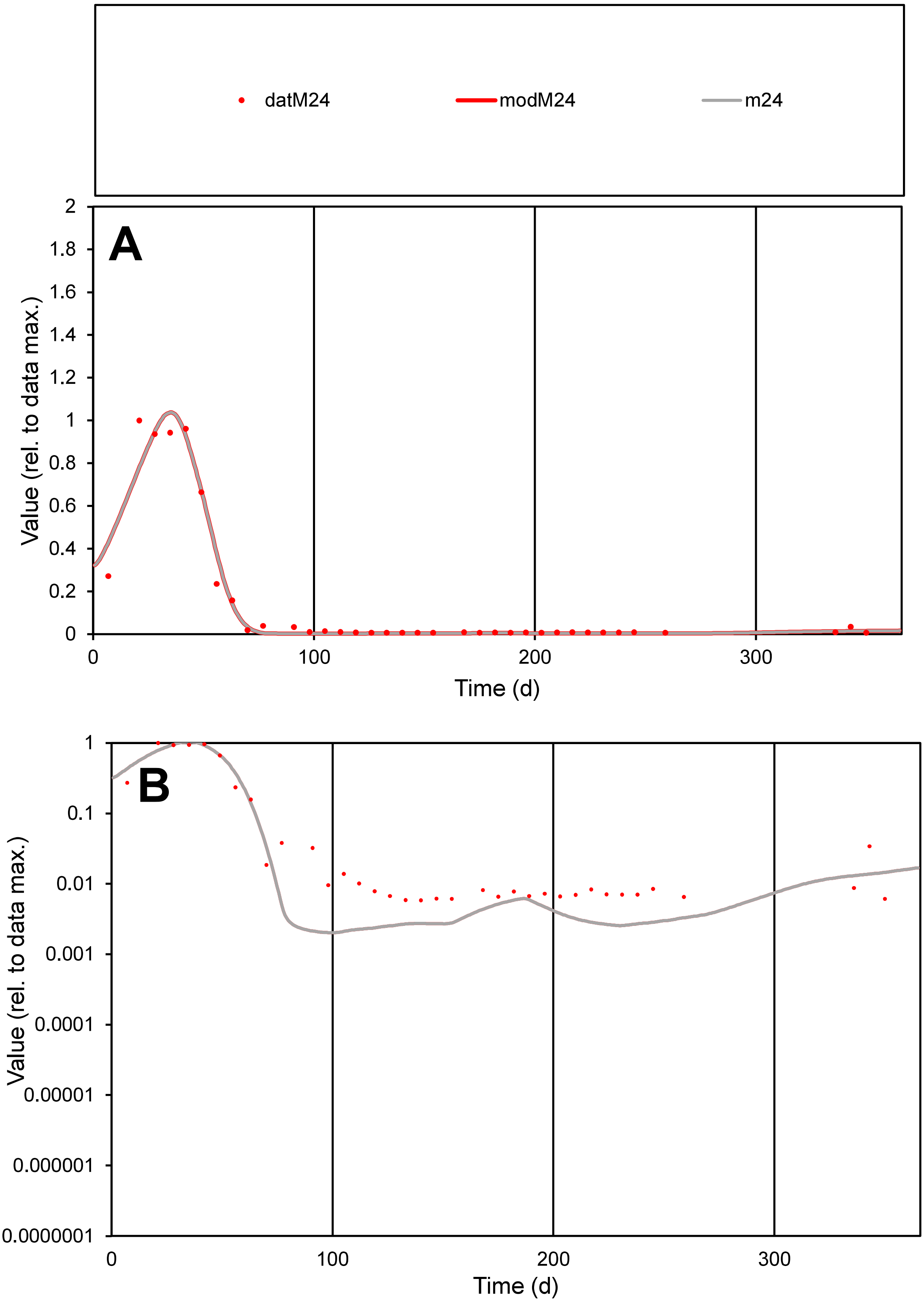

C1

Variables	
M24	Provide jtd or Dst
CalibOnly?	
2	1 to reproduce of
oNormalize?	
2	1=no, 2=yes
ofxn calc*	
0.32875	
ofxn mod	
0.43035	
sRun	
RunA1	
zout	
1	
Ave	
Data	
0.00186	
Model	
0.00183	
data/vMax	
0.01227	
ofxng	
311.734	
Min d/m	
2.88084	
ave/min d	
26.0553	
ave/min m	
73.5862	

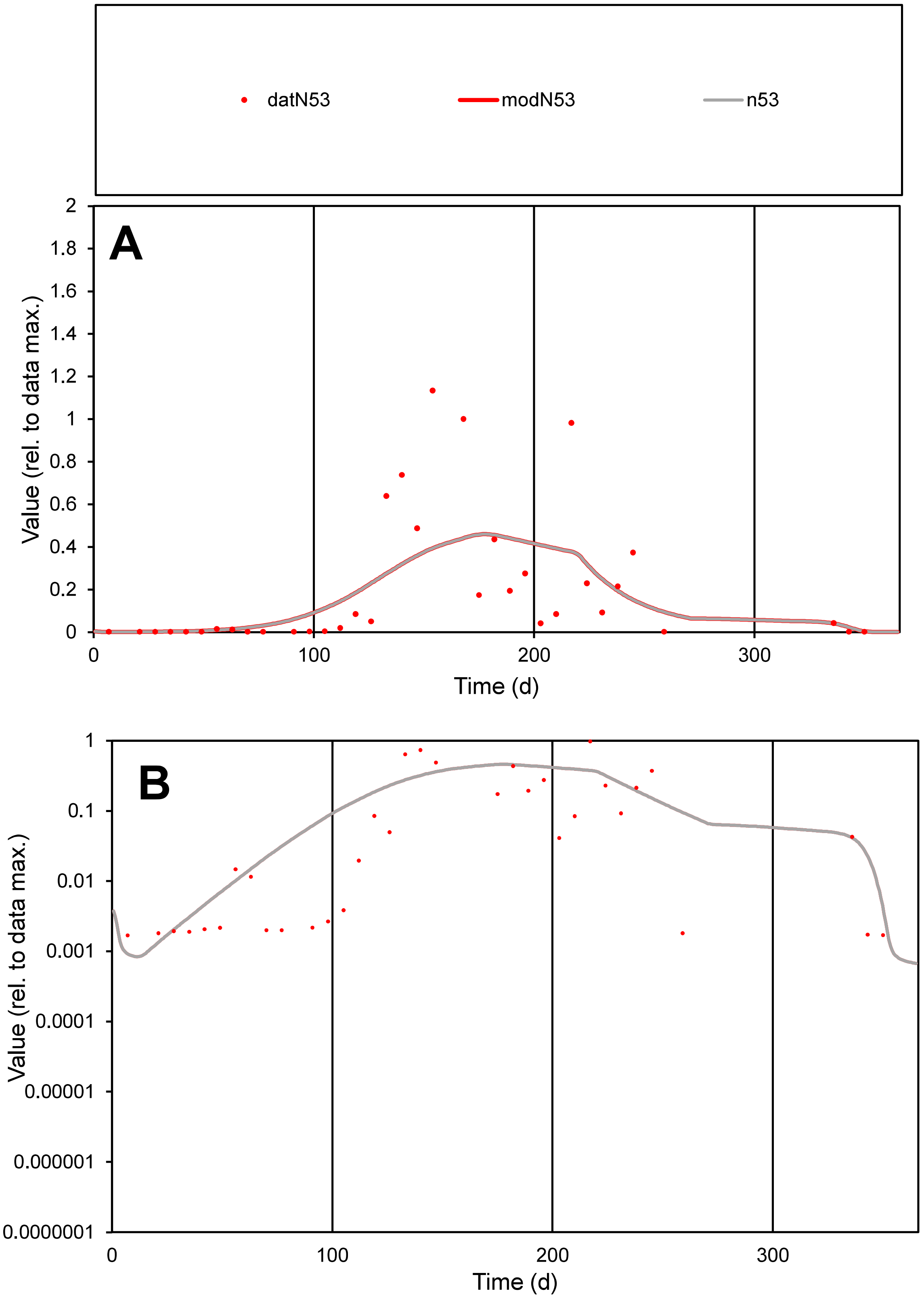

C1

Variables

N53 Provide jtd or Dst

CalibOnly?

2 1 to reproduce of

oNormalize?

2 1=no, 2=yes

ofxn calc*

1.13196

ofxn mod

1.14643

sRun

RunA1

zout

1

Ave

Data

0.00397

Model

0.00415

data/vMax

0.02228

ofxng

311.734

Min d/m

1.90293

ave/min d

105.388

ave/min m

209.619

331_Calib_S_A

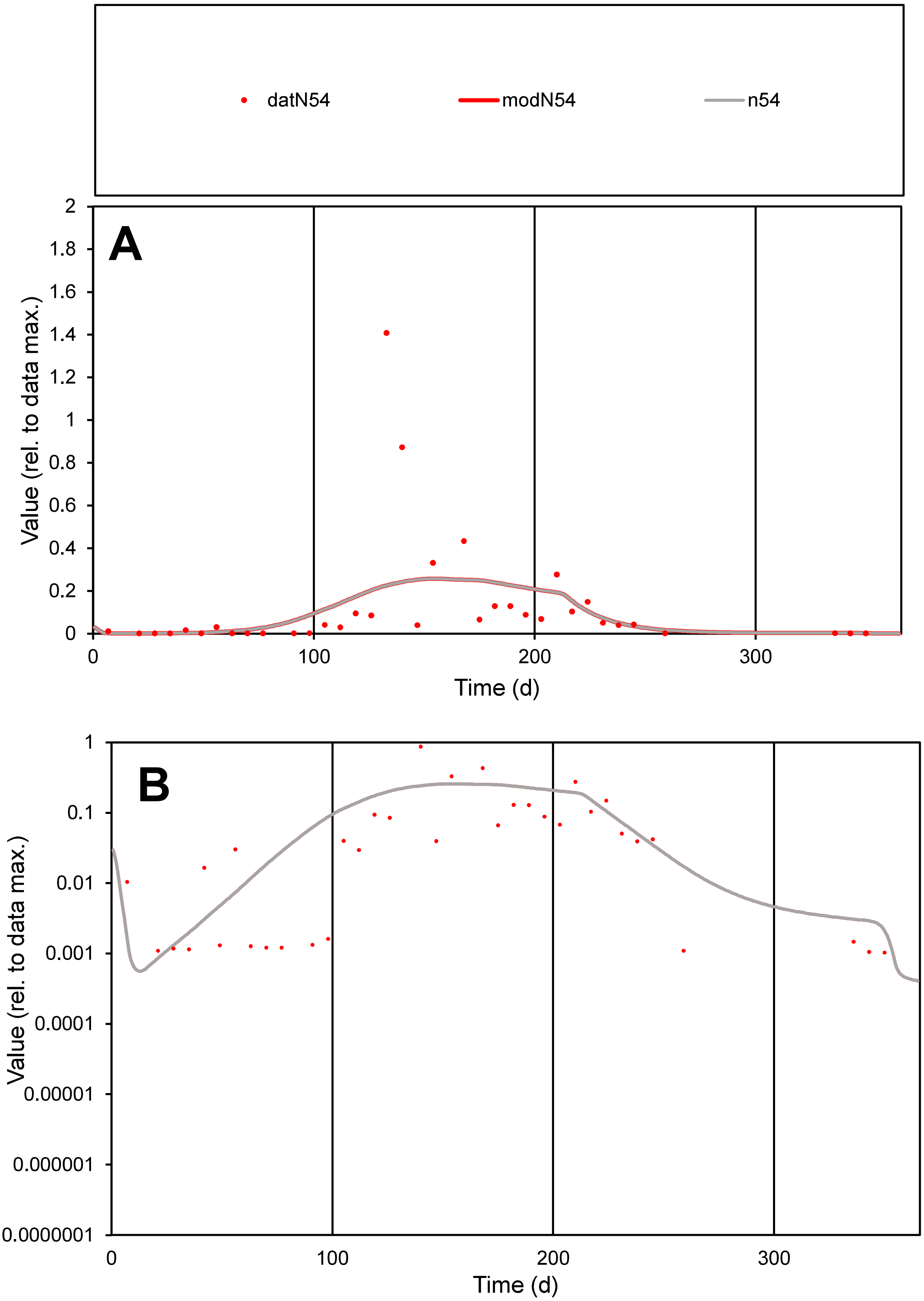

C1

Variables
N54 Provide jtd or Dst
CalibOnly?
2 1 to reproduce of
oNormalize?
2 1=no, 2=yes

ofxn calc*
1.56086
ofxn mod
1.57322

sRun
RunA1
zout
1

Ave
Data
0.0022
Model
0.00232

data/vMax
0.02174

ofxng
311.734

Min d/m
1.0977

ave/min d
97.9383
ave/min m
113.21

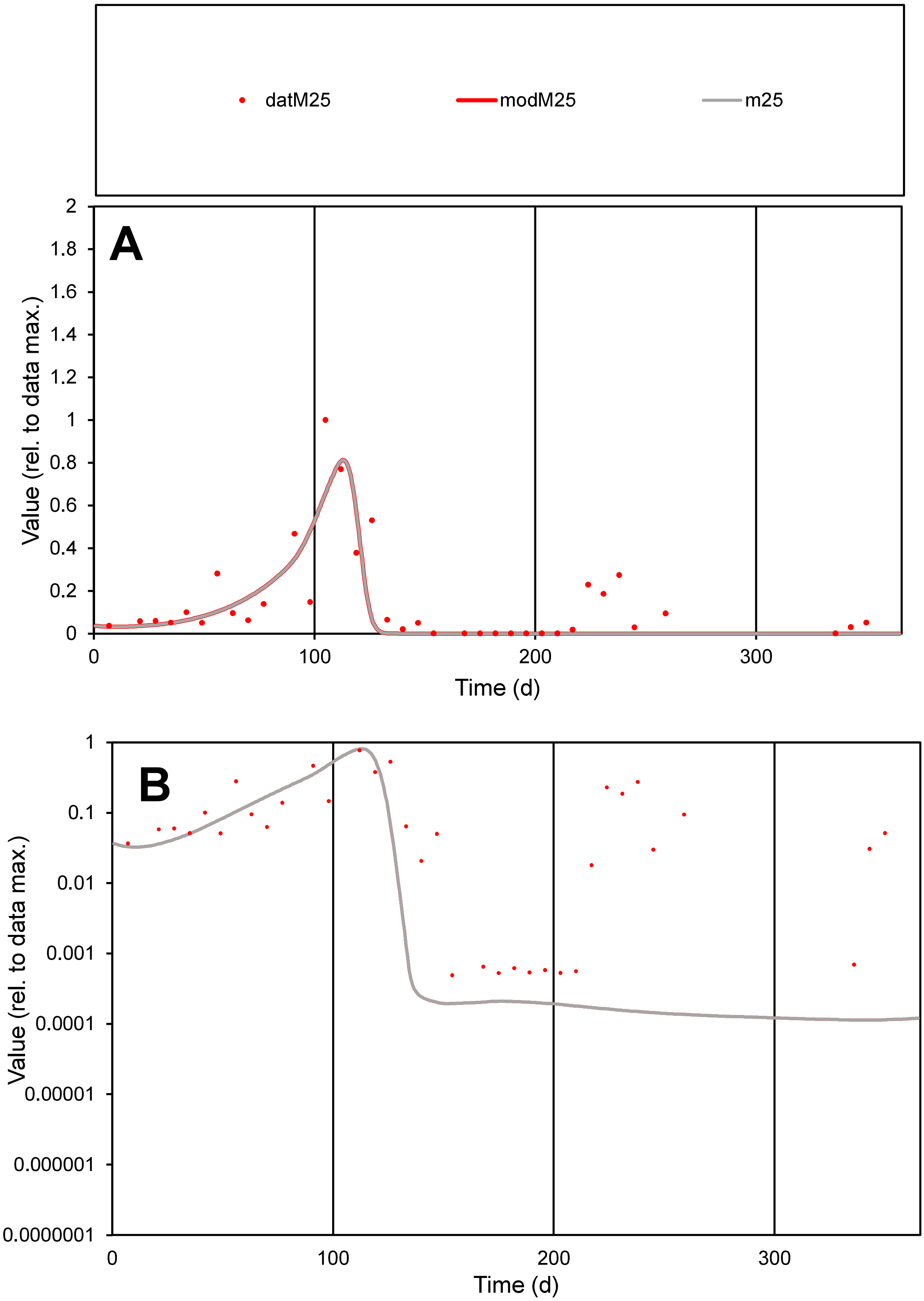

C1

Variables
M25 Provide jtd or Dst
CalibOnly?
2 1 to reproduce of
oNormalize?
2 1=no, 2=yes

ofxn calc*
0.85077
ofxn mod
0.89079

sRun
RunA1
zout
1

Ave
Data
0.00288
Model
0.00223

data/vMax
0.02038

ofxng
311.734

Min d/m
4.34952

ave/min d
287.339
ave/min m
965.463

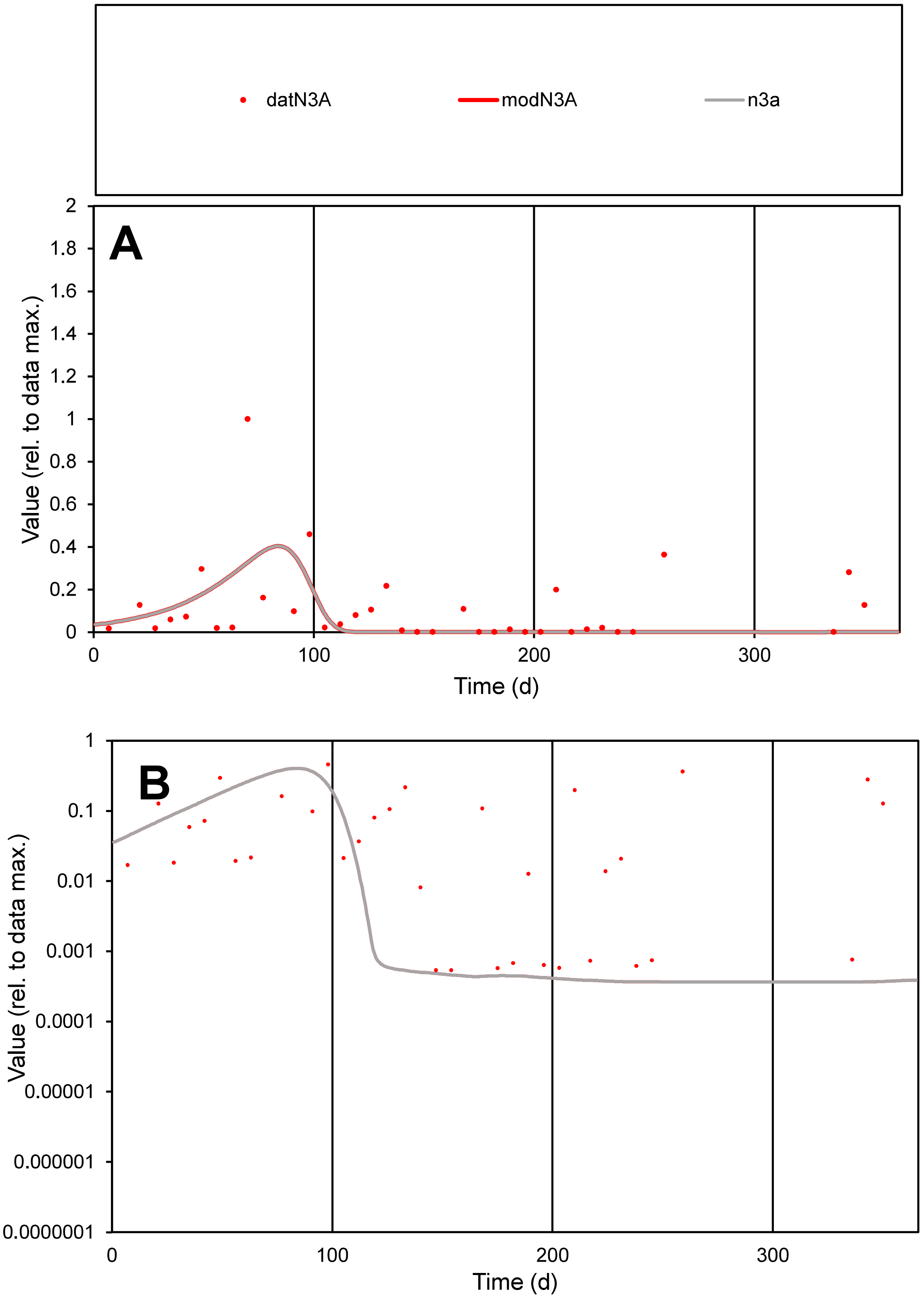

C1

Variables
N3A Provide jtd or Dst
CalibOnly?
2 1 to reproduce of
oNormalize?
2 1=no, 2=yes

ofxn calc*
1.5906
ofxn mod
1.60789

sRun
RunA1
zout
1

Ave
Data
0.00371
Model
0.00241

data/vMax
0.03457

ofxng
311.734

Min d/m
1.47393

ave/min d
198.577
ave/min m
190.243

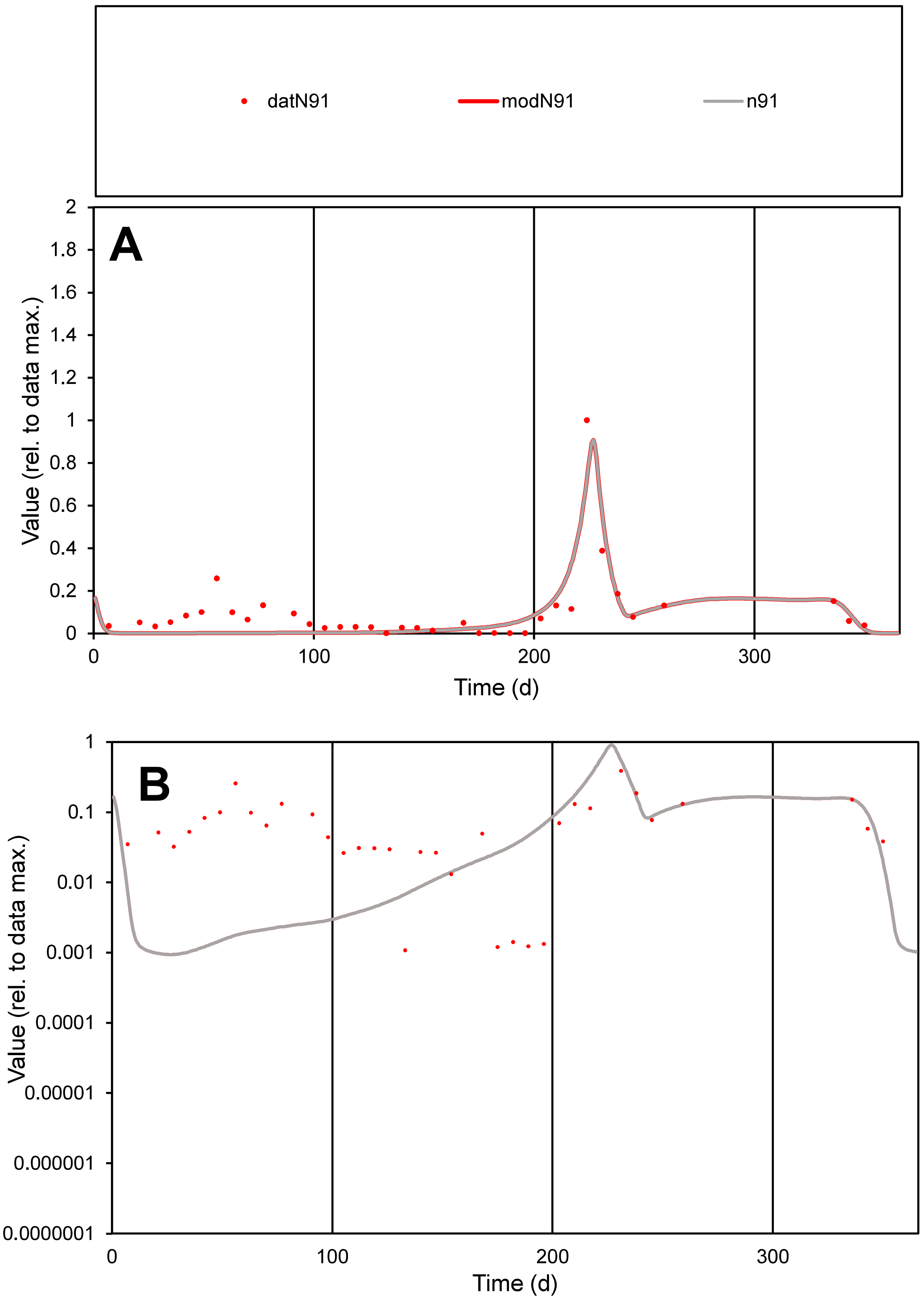

C1

Variables
N91 Provide jtd or Dst
CalibOnly?
2 1 to reproduce of
oNormalize?
2 1=no, 2=yes

ofxn calc*
0.76526
ofxn mod
0.83134

sRun
RunA1
zout
1

Ave
Data
0.00294
Model
0.00228

data/vMax
0.02934

ofxng
311.734

Min d/m
1.13669

ave/min d
92.9145
ave/min m
82.0823

331_Calib_S_A

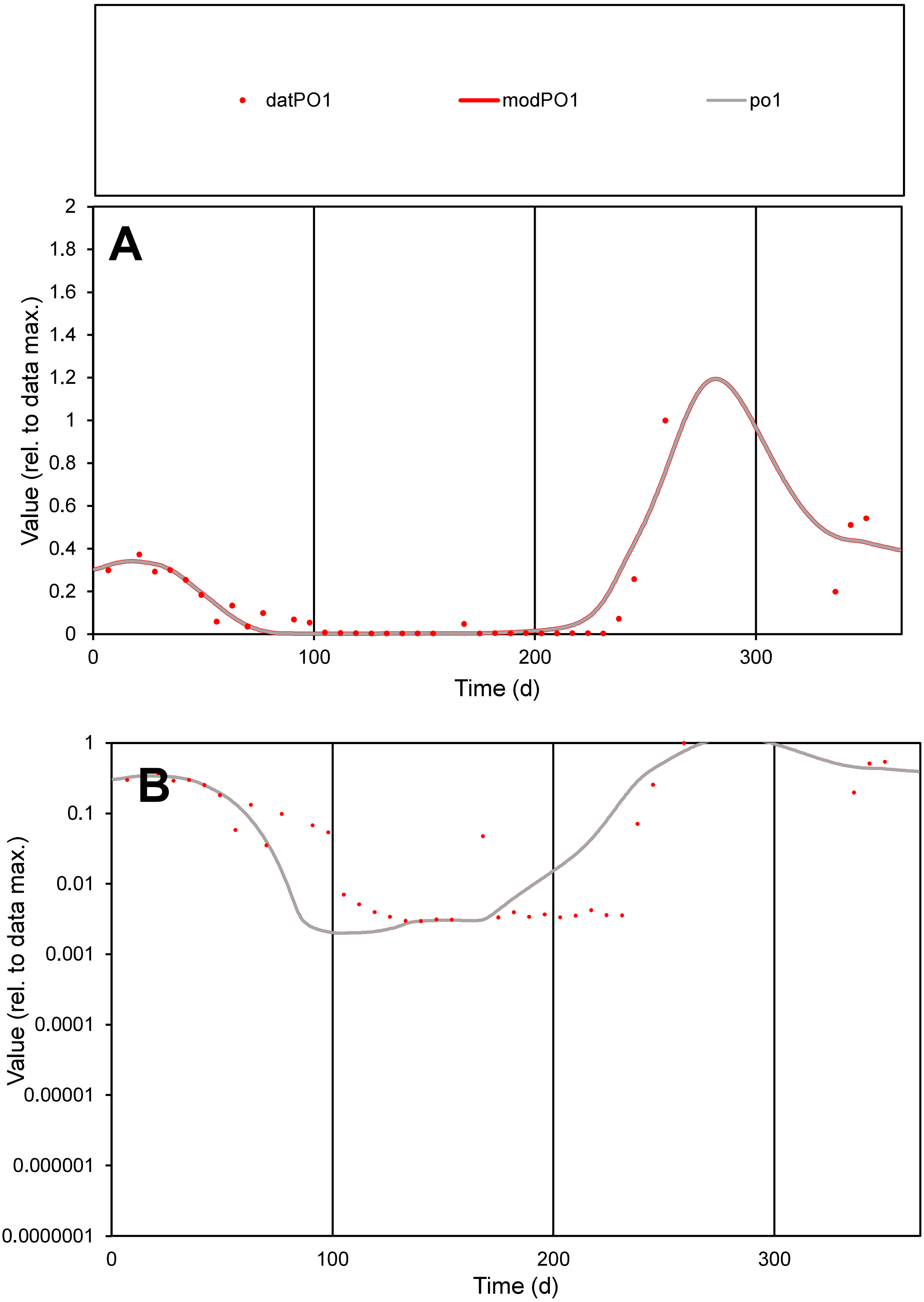

C1

Variables

PO1 Provide jtd or Dst

CalibOnly?

2 1 to reproduce of

oNormalize?

2 1=no, 2=yes

ofxn calc*

0.60738

ofxn mod

0.66067

sRun

RunA1

zout

1

Ave

Data

0.00205

Model

0.00217

data/vMax

0.01527

ofxng

311.734

Min d/m

1.4747

ave/min d

45.413

ave/min m

70.807

331_Calib_S_A

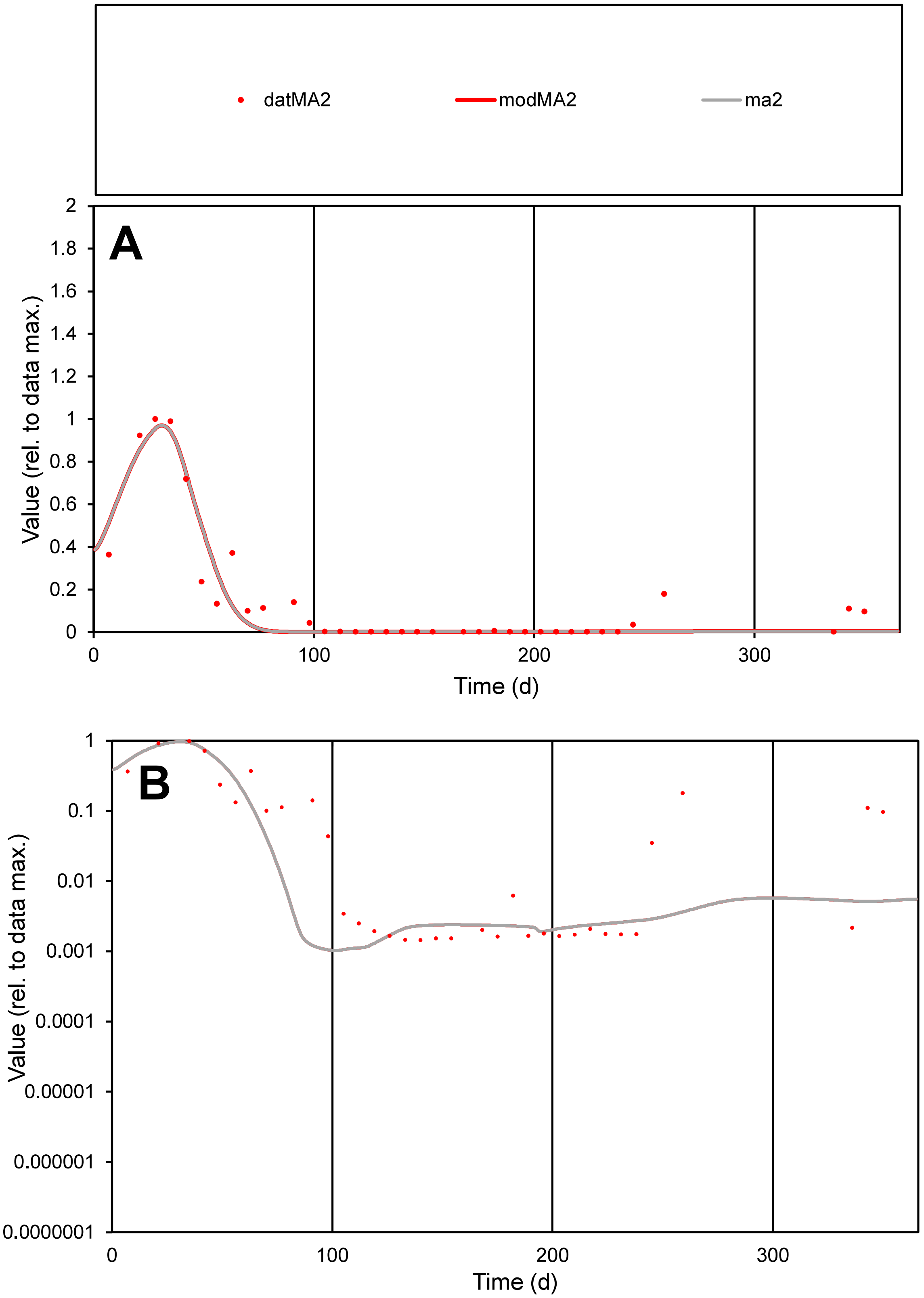

C1

Variables

MA2 Provide jtd or Dst

CalibOnly?

2 1 to reproduce of

oNormalize?

2 1=no, 2=yes

ofxn calc*

0.53951

ofxn mod

0.59084

sRun

RunA1

zout

1

Ave

Data

0.0019

Model

0.00168

data/vMax

0.01221

ofxng

311.734

Min d/m

1.39905

ave/min d

107.451

ave/min m

132.803

331_Calib_S_A

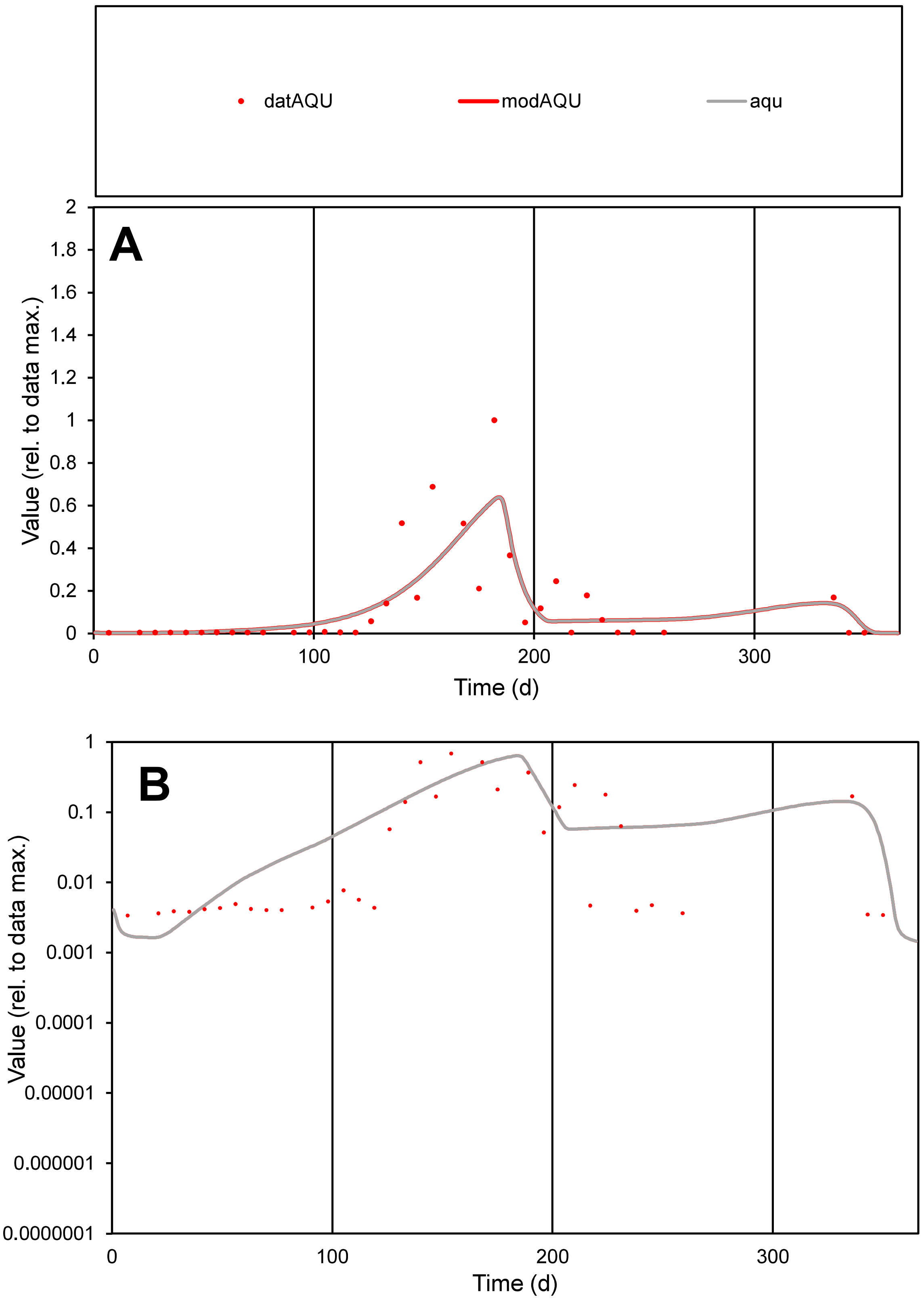

C1

Variables
AQU Provide jtd or Dst
CalibOnly?
2 1 to reproduce of
oNormalize?
2 1=no, 2=yes

ofxn calc*
0.95273
ofxn mod
0.98022

sRun
RunA1
zout
1

Ave
Data
0.00447
Model
0.00481

data/vMax
0.0395

ofxng
311.734

Min d/m
1.97117

ave/min d
33.391
ave/min m
70.8263

331_Calib_S_A

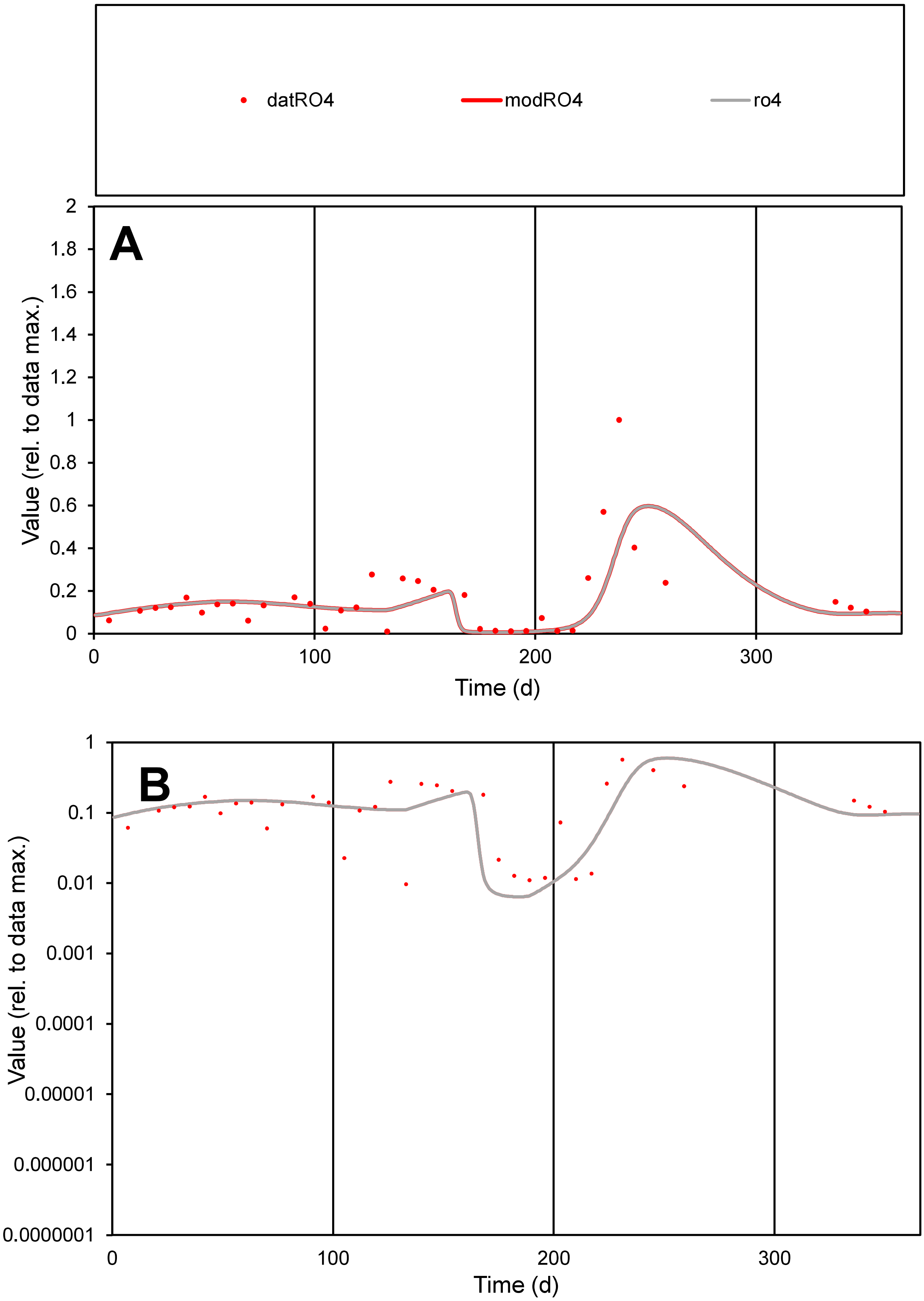

C1

Variables
RO4 Provide jtd or Dst
CalibOnly?
2 1 to reproduce of
oNormalize?
2 1=no, 2=yes

ofxn calc*
0.84285
ofxn mod
0.86945

sRun
RunA1
zout
1

Ave
Data
0.00349
Model
0.00319

data/vMax
0.02251

ofxng
311.734

Min d/m
1.51321

ave/min d
16.0267
ave/min m
22.1821

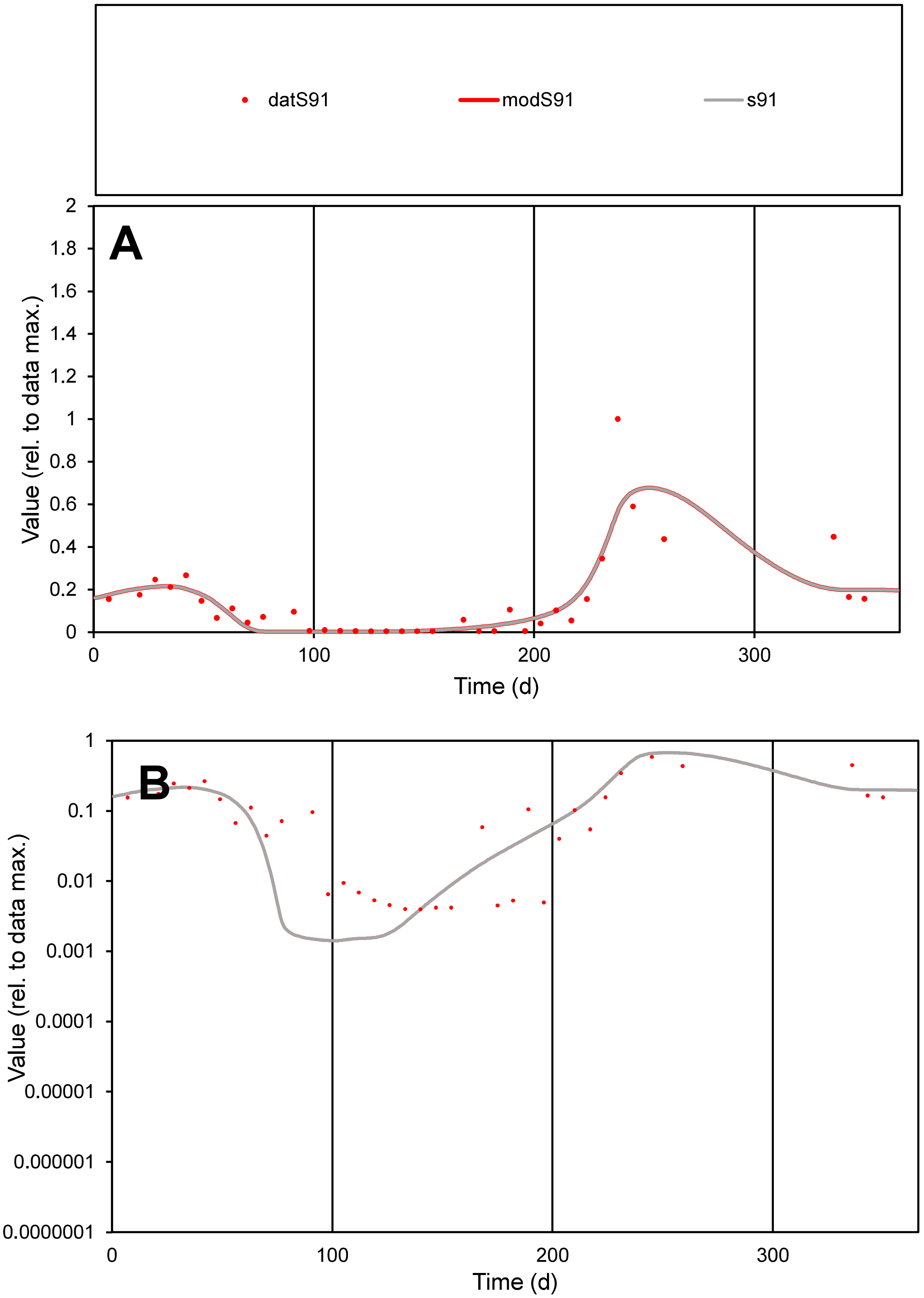

C1

Variables
S91 Provide jtd or Dst
CalibOnly?
2 1 to reproduce of
oNormalize?
2 1=no, 2=yes

ofxn calc*
0.65722
ofxn mod
0.68494

sRun
RunA1
zout
1

Ave
Data
0.00241
Model
0.00231

data/vMax
0.0163

ofxng
311.734

Min d/m
2.80302

ave/min d
37.0984
ave/min m
99.7178

331_Calib_S_A

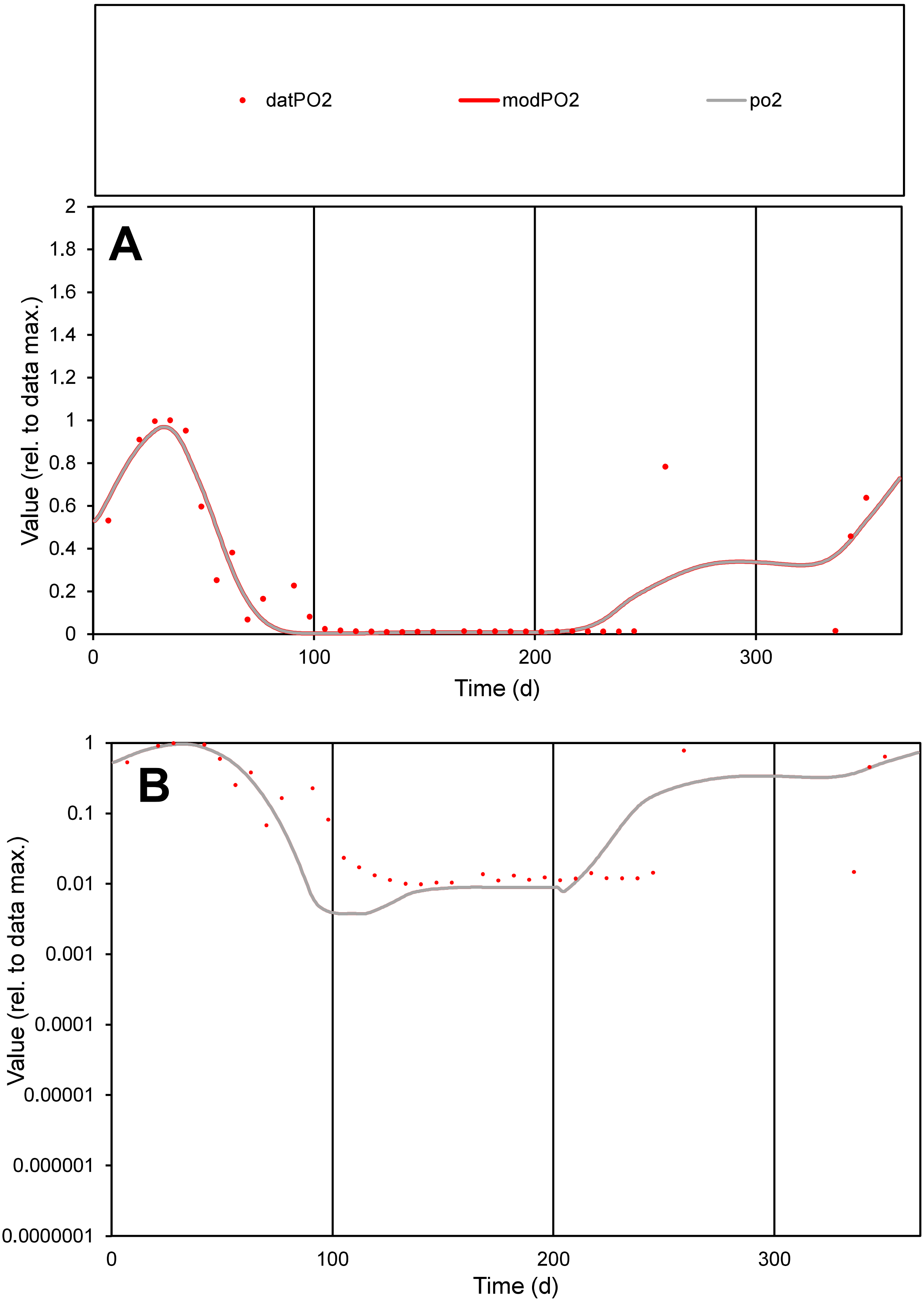

C1

Variables
PO2 Provide jtd or Dst
CalibOnly?
2 1 to reproduce of
oNormalize?
2 1=no, 2=yes

ofxn calc*
0.5618
ofxn mod
0.59733

sRun
RunA1
zout
1

Ave
Data
0.00183
Model
0.00177

data/vMax
0.00794

ofxng
311.734

Min d/m
2.6486

ave/min d
23.2917
ave/min m
59.582

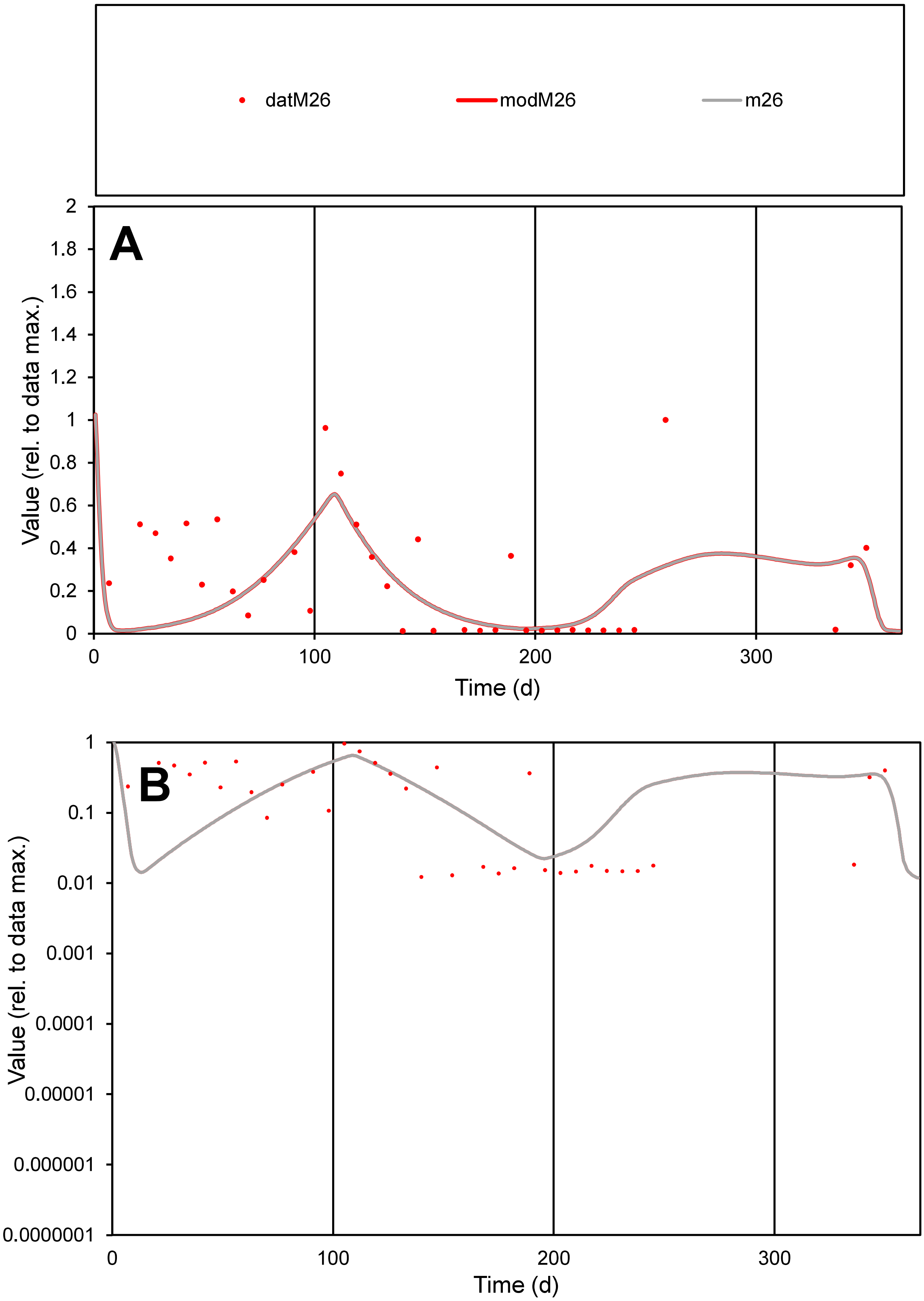

C1

Variables
M26 Provide jtd or Dst
CalibOnly?
2 1 to reproduce of
oNormalize?
2 1=no, 2=yes

ofxn calc*
0.9602
ofxn mod
0.97489

sRun
RunA1
zout
1

Ave
Data
0.00168
Model
0.00131

data/vMax
0.00661

ofxng
311.734

Min d/m
0.54138

ave/min d
20.6839
ave/min m
8.75943

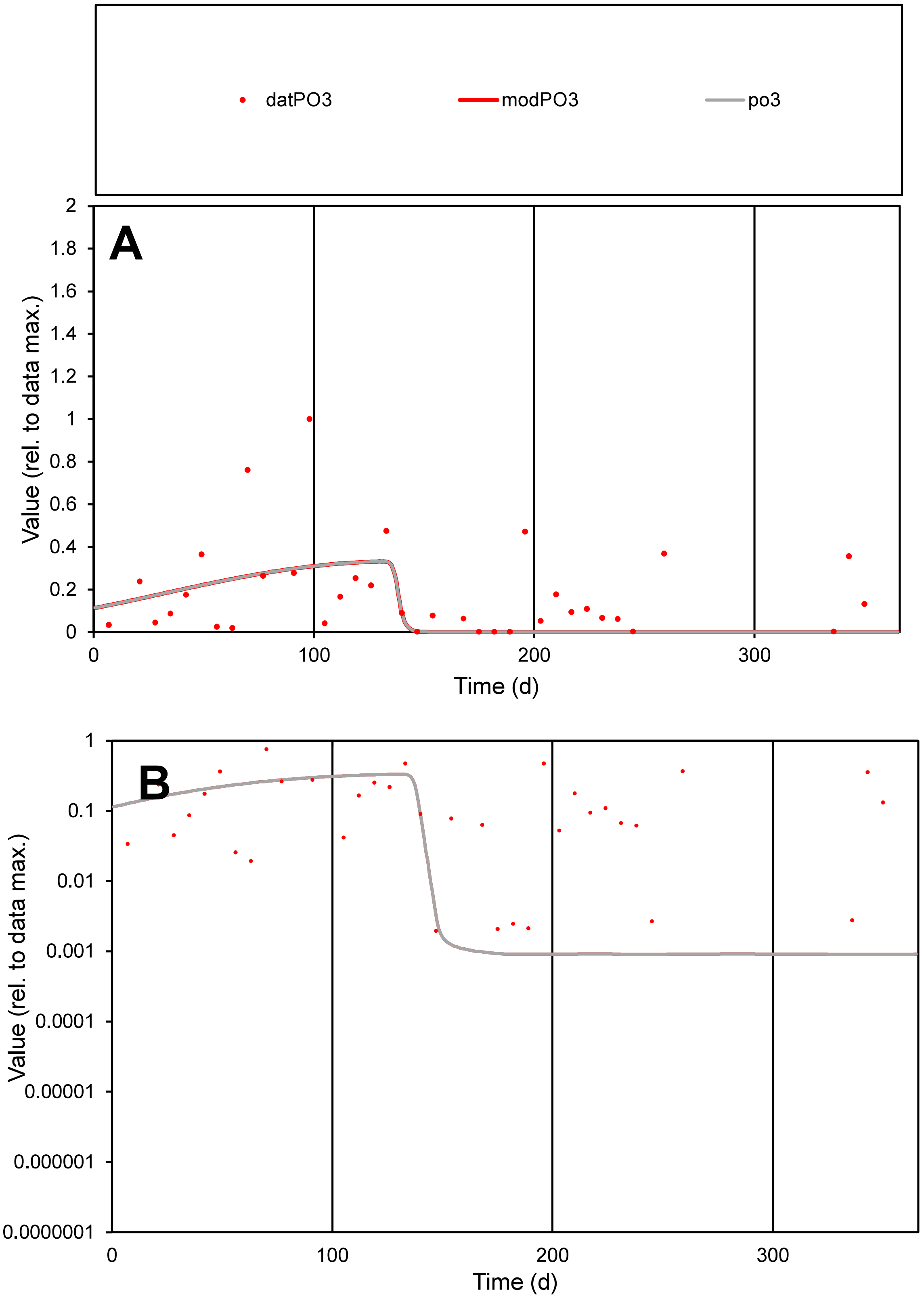

C1

Variables
PO3 Provide jtd or Dst
CalibOnly?
2 1 to reproduce of
oNormalize?
2 1=no, 2=yes

ofxn calc*
1.19607
ofxn mod
1.20413

sRun
RunA1
zout
1

Ave
Data
0.00436
Model
0.00305

data/vMax
0.02484

ofxng
311.734

Min d/m
2.17608

ave/min d
89.849
ave/min m
136.727

331_Calib_S_A

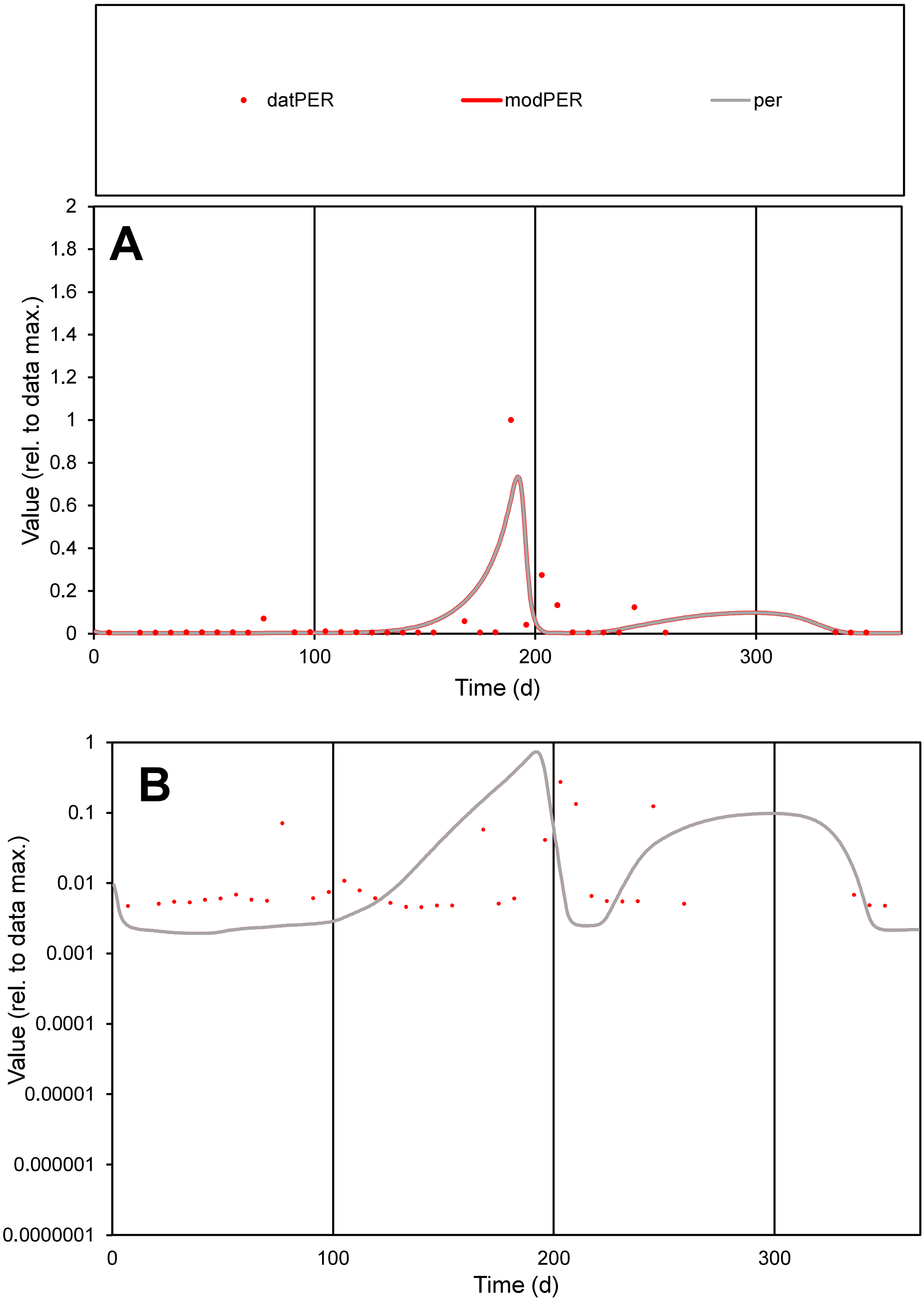

C1

Variables
PER Provide jtd or Dst
CalibOnly?
2 1 to reproduce of
oNormalize?
2 1=no, 2=yes

ofxn calc*
1.94703
ofxn mod
2.06039

sRun
RunA1
zout
1

Ave
Data
0.00021
Model
0.00021

data/vMax
0.00397

ofxng
311.734

Min d/m
2.36546
ave/min d
11.3797
ave/min m
26.824

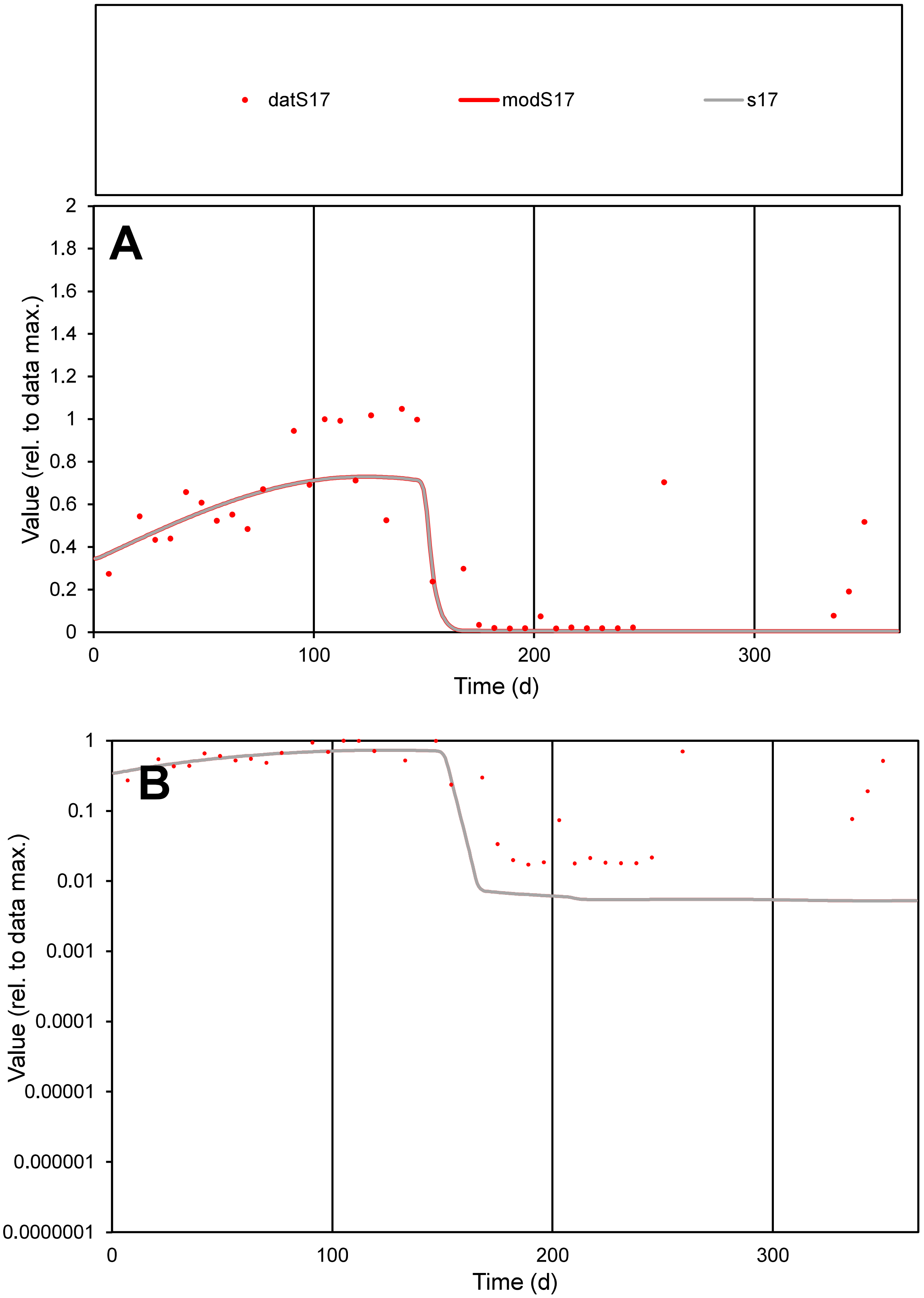

C1

Variables
S17 Provide jtd or Dst
CalibOnly?
2 1 to reproduce of
oNormalize?
2 1=no, 2=yes

ofxn calc*
0.48369
ofxn mod
0.50144

sRun
RunA1
zout
1

Ave
Data
0.00284
Model
0.00244

data/vMax
0.0072

ofxng
311.734

Min d/m
3.28738

ave/min d
22.8805
ave/min m
64.5712

331_Calib_S_A

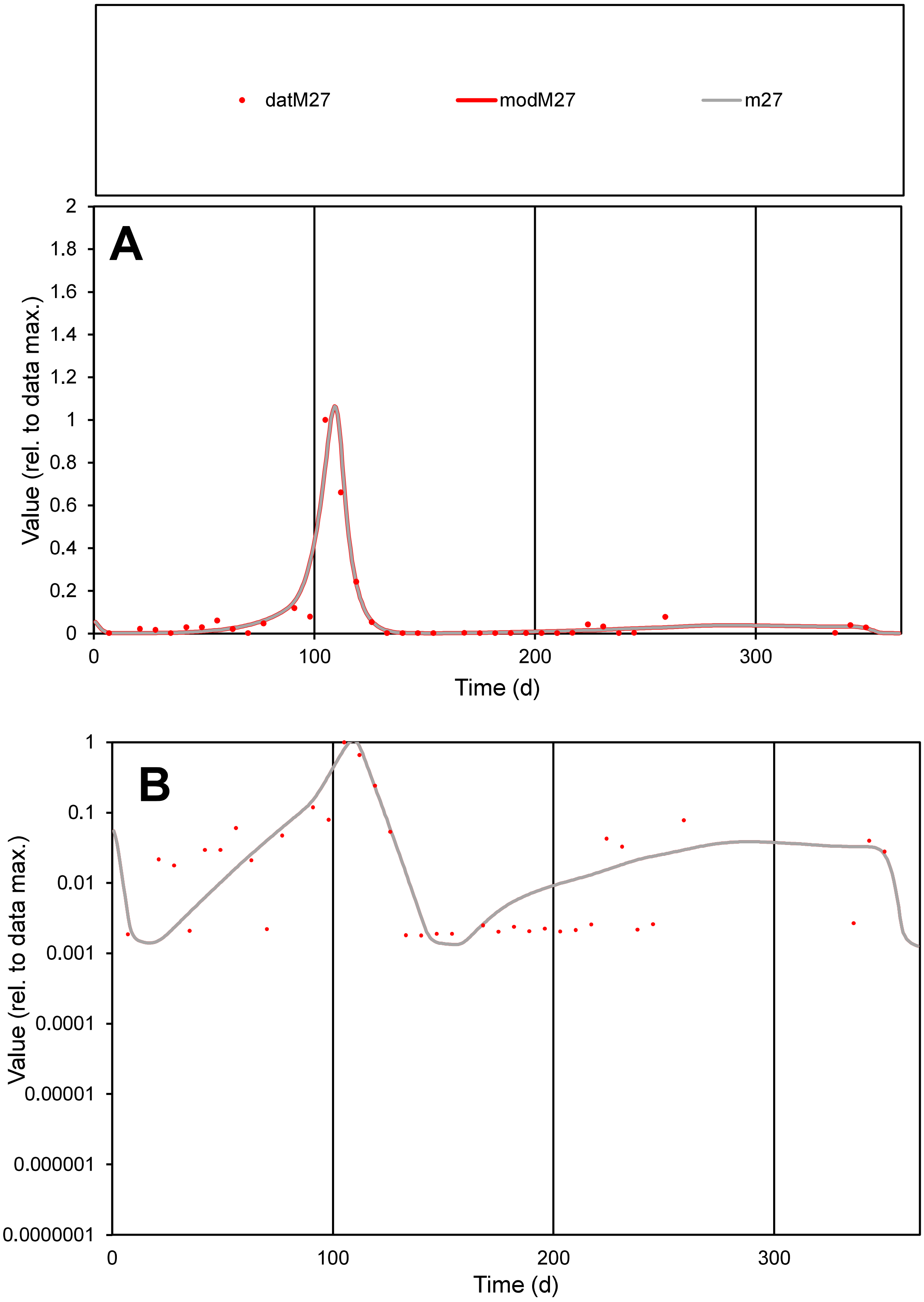

C1

Variables

M27 Provide jtd or Dst

CalibOnly?

2 1 to reproduce of

oNormalize?

2 1=no, 2=yes

ofxn calc*

0.56209

ofxn mod

0.7349

sRun

RunA1

zout

1

Ave

Data

0.00261

Model

0.00267

data/vMax

0.03575

ofxng

311.734

Min d/m

1.34956

ave/min d

40.5536

ave/min m

56.0829

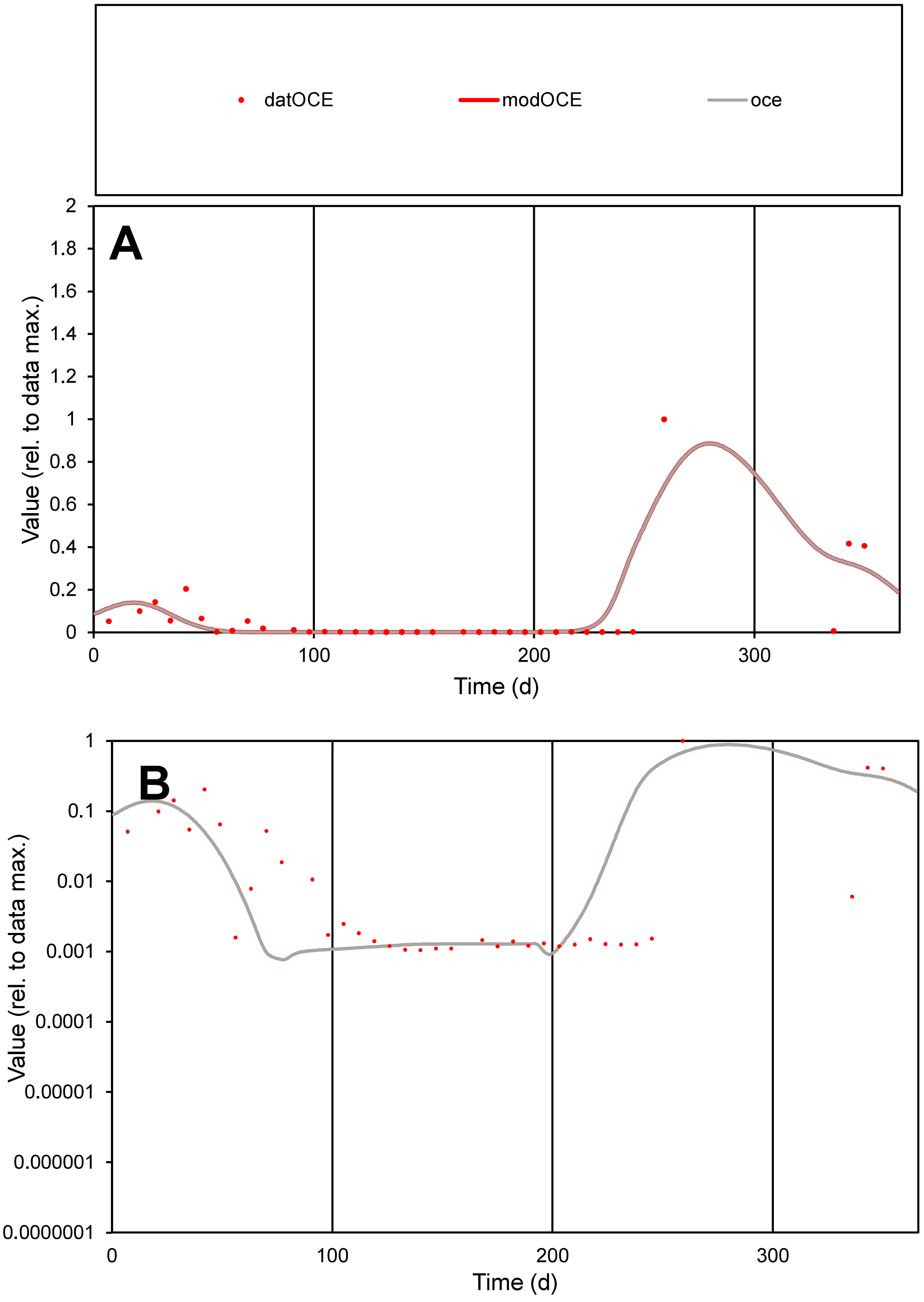

C1

Variables
OCE Provide jtd or Dst
CalibOnly?
2 1 to reproduce of
oNormalize?
2 1=no, 2=yes

ofxn calc*
1.44165
ofxn mod
1.56623

sRun
RunA1
zout
1

Ave
Data
0.00249
Model
0.00267

data/vMax
0.03496

ofxng
311.734

Min d/m
1.36024

ave/min d
67.7569
ave/min m
98.7077

331_Calib_S_A

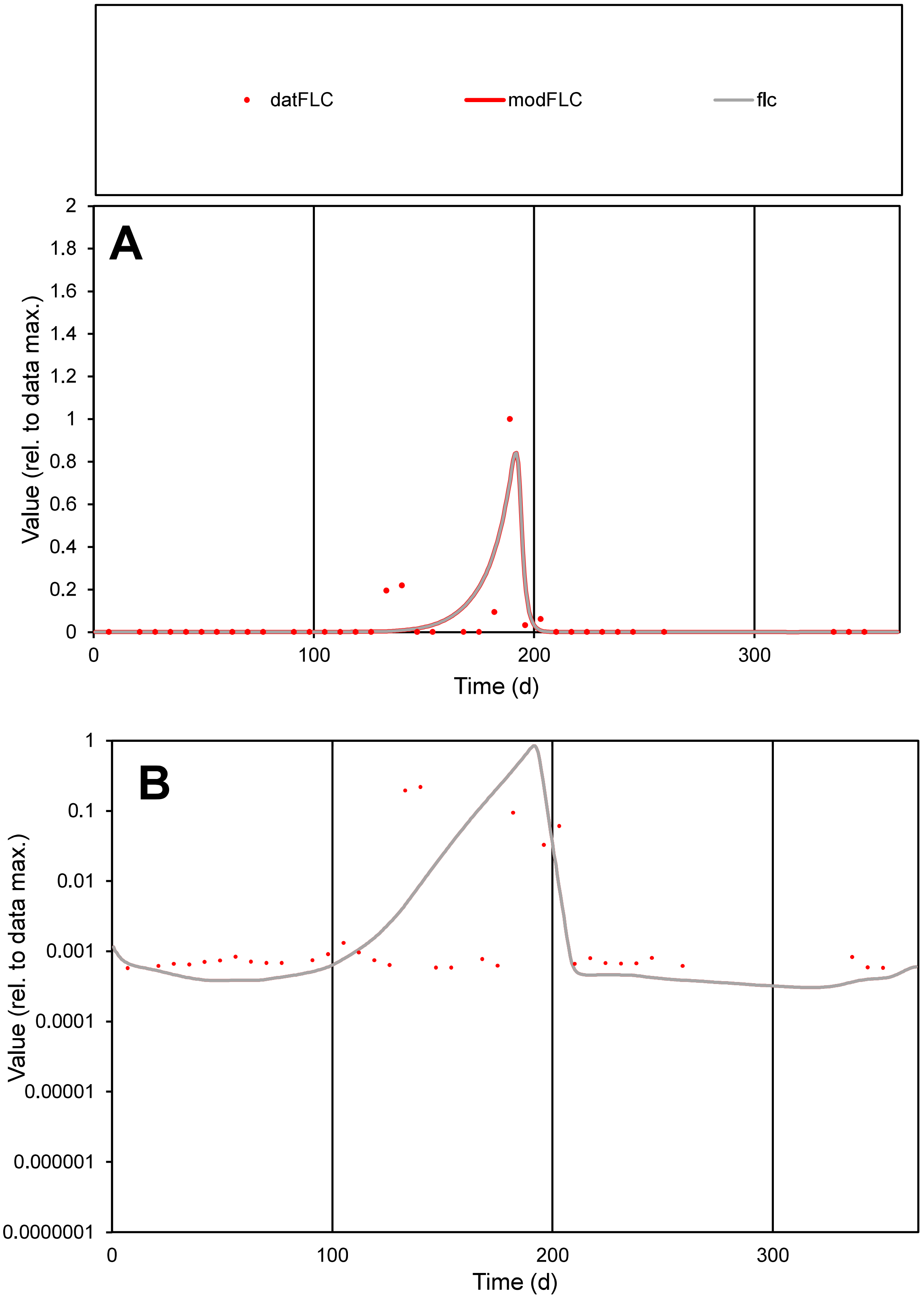

C1

Variables

FLC Provide jtd or Dst

CalibOnly?

2 1 to reproduce of

oNormalize?

2 1=no, 2=yes

ofxn calc*

1.71927

ofxn mod

1.9236

sRun

RunA1

zout

1

Ave

Data

0.00088

Model

0.00097

data/vMax

0.02139

ofxng

311.734

Min d/m

1.55002

ave/min d

70.7119

ave/min m

120.847

331_Calib_S_A

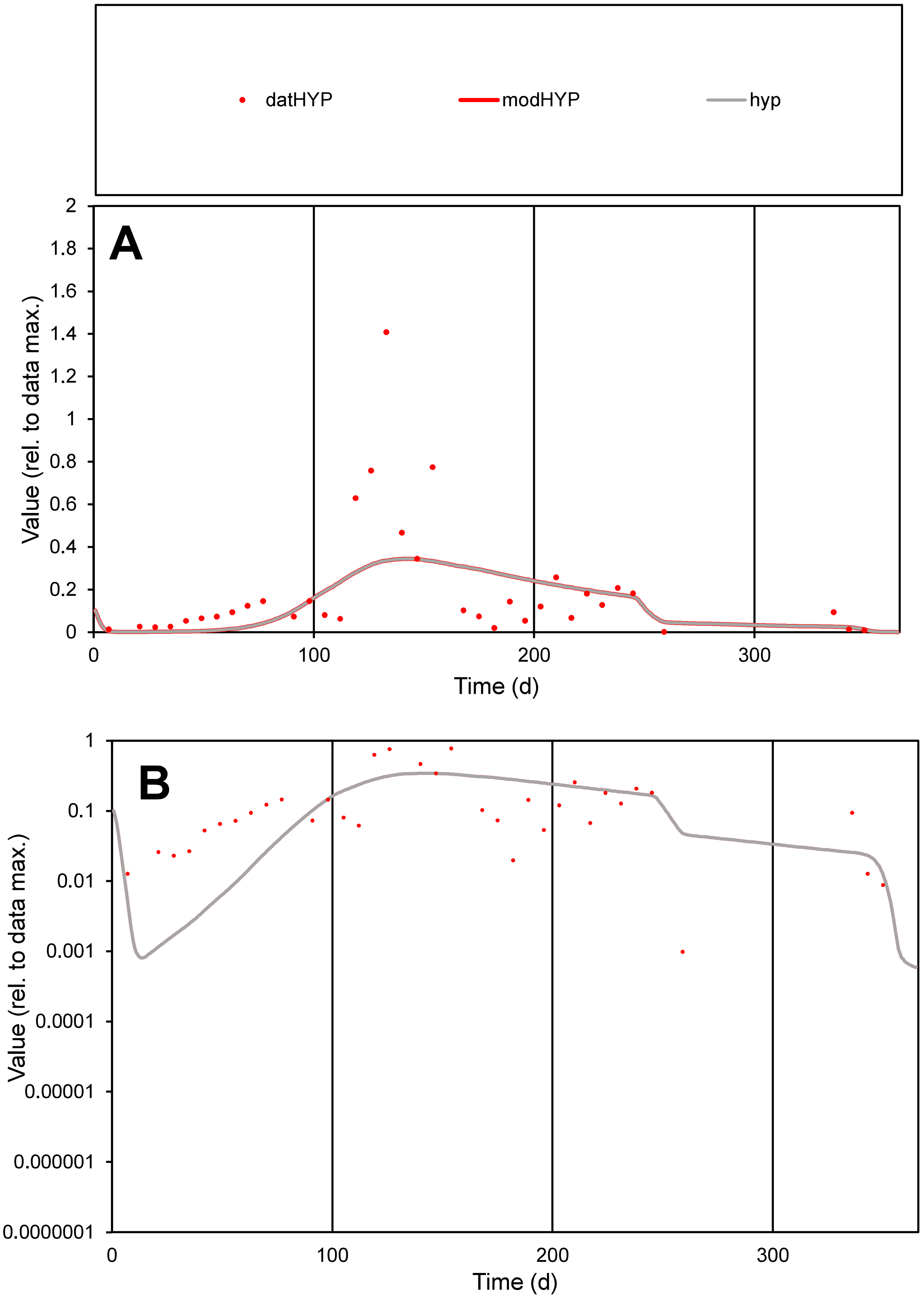

C1

Variables
HYP Provide jtd or Dst
CalibOnly?
2 1 to reproduce of
oNormalize?
2 1=no, 2=yes

ofxn calc*
1.01785
ofxn mod
1.02928

sRun
RunA1
zout
1

Ave
Data
0.00294
Model
0.0028

data/vMax
0.01788

ofxng
311.734

Min d/m
0.78183

ave/min d
167.23
ave/min m
124.83

331_Calib_S_A

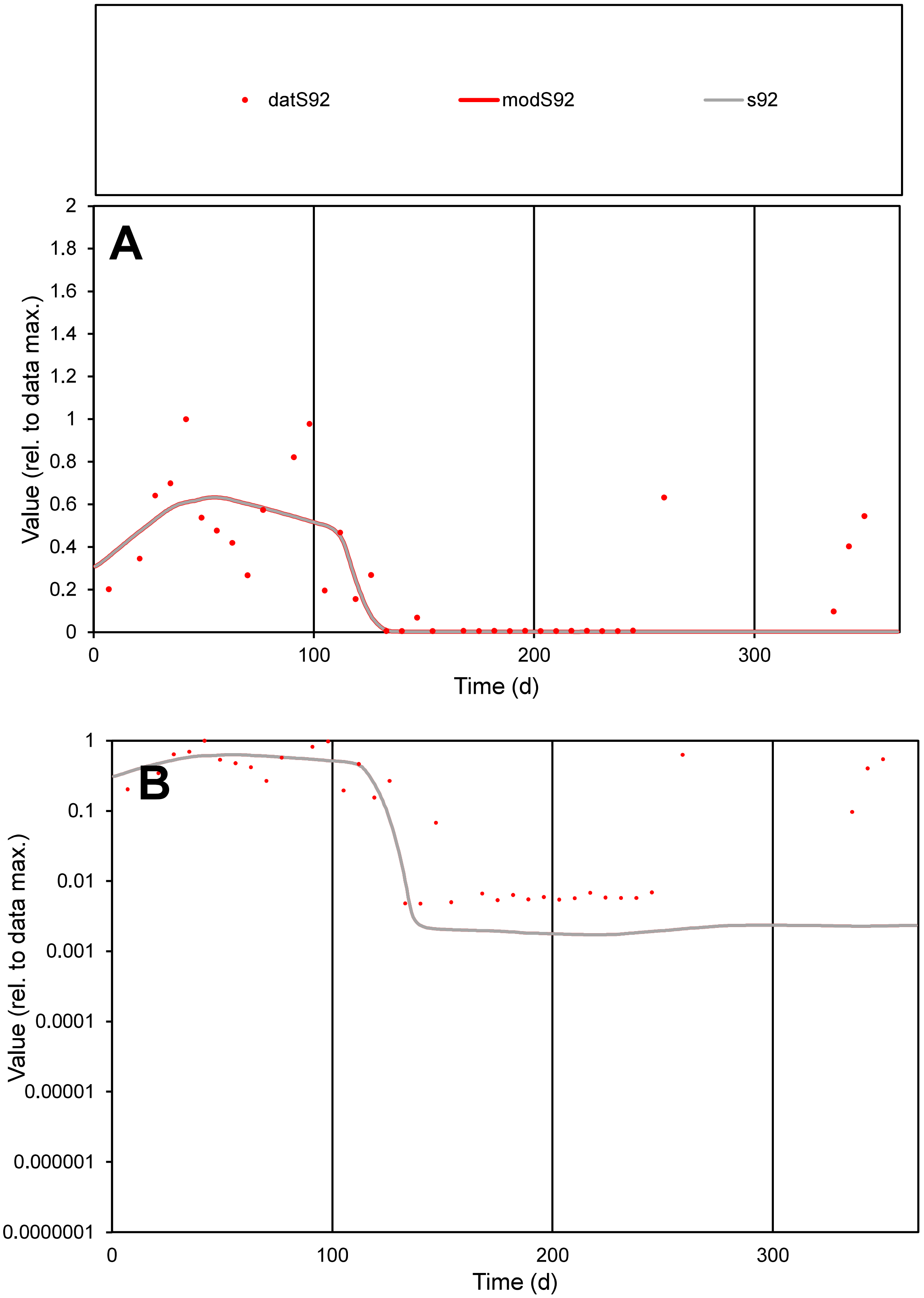

C1

Variables
S92 Provide jtd or Dst
CalibOnly?
2 1 to reproduce of
oNormalize?
2 1=no, 2=yes

ofxn calc*
0.79072
ofxn mod
0.80831

sRun
RunA1
zout
1

Ave
Data
0.00131
Model
0.00107

data/vMax
0.00482

ofxng
311.734

Min d/m
2.76882

ave/min d
56.8588
ave/min m
128.358

331_Calib_S_A

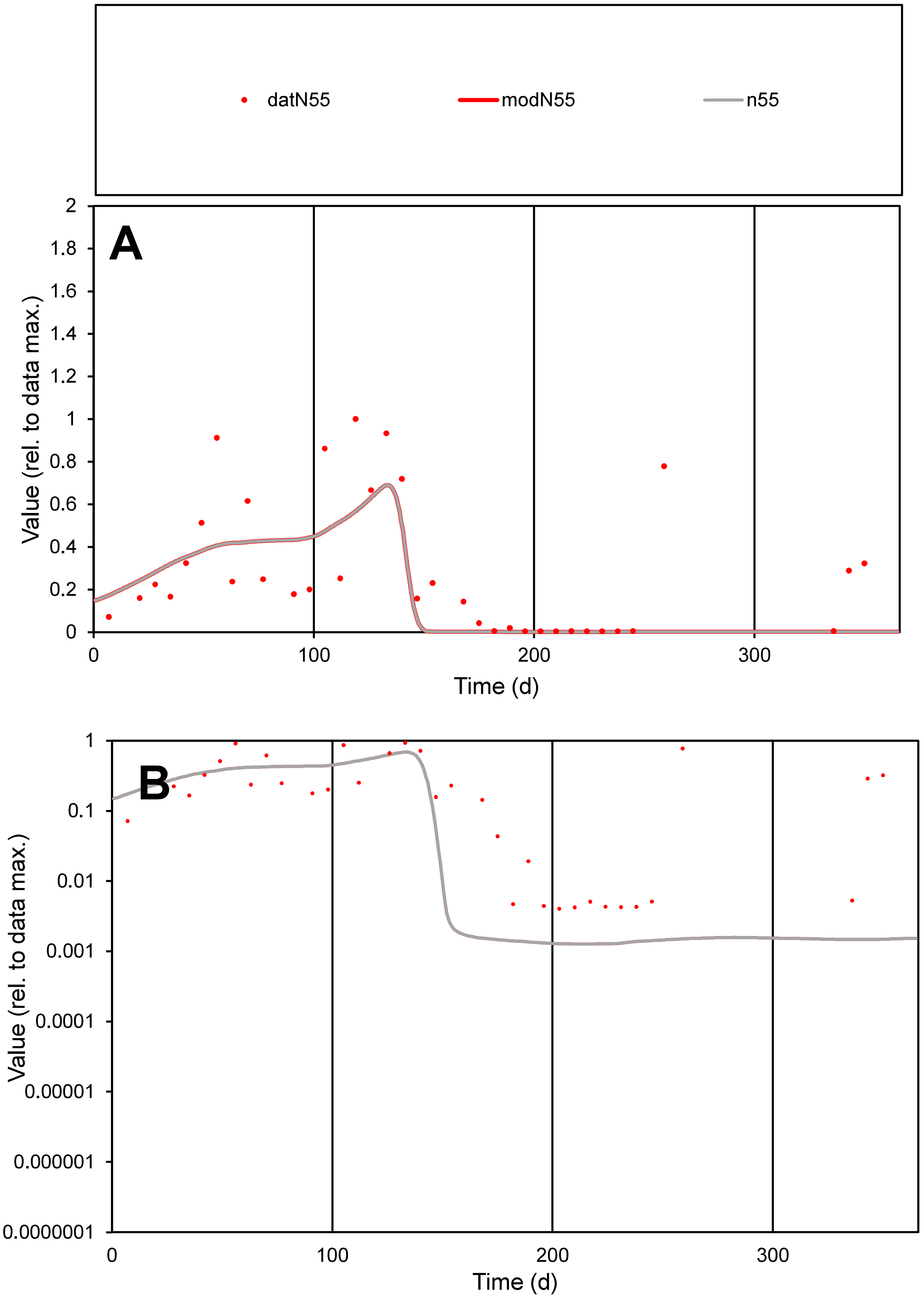

C1

Variables

N55 Provide jtd or Dst

CalibOnly?

2 1 to reproduce of

oNormalize?

2 1=no, 2=yes

ofxn calc*

0.85703

ofxn mod

0.8683

sRun

RunA1

zout

1

Ave

Data

0.00273

Model

0.00226

data/vMax

0.01041

ofxng

311.734

Min d/m

3.18196

ave/min d

65.0297

ave/min m

171.061

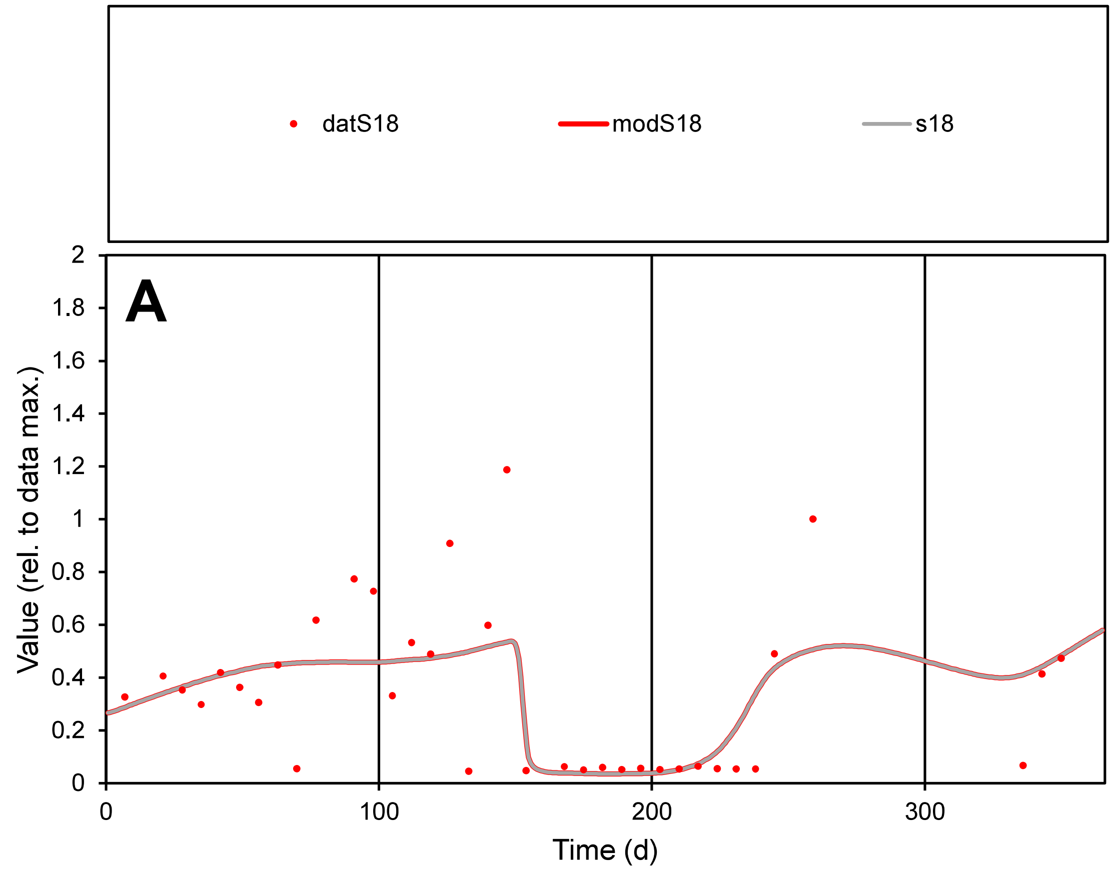

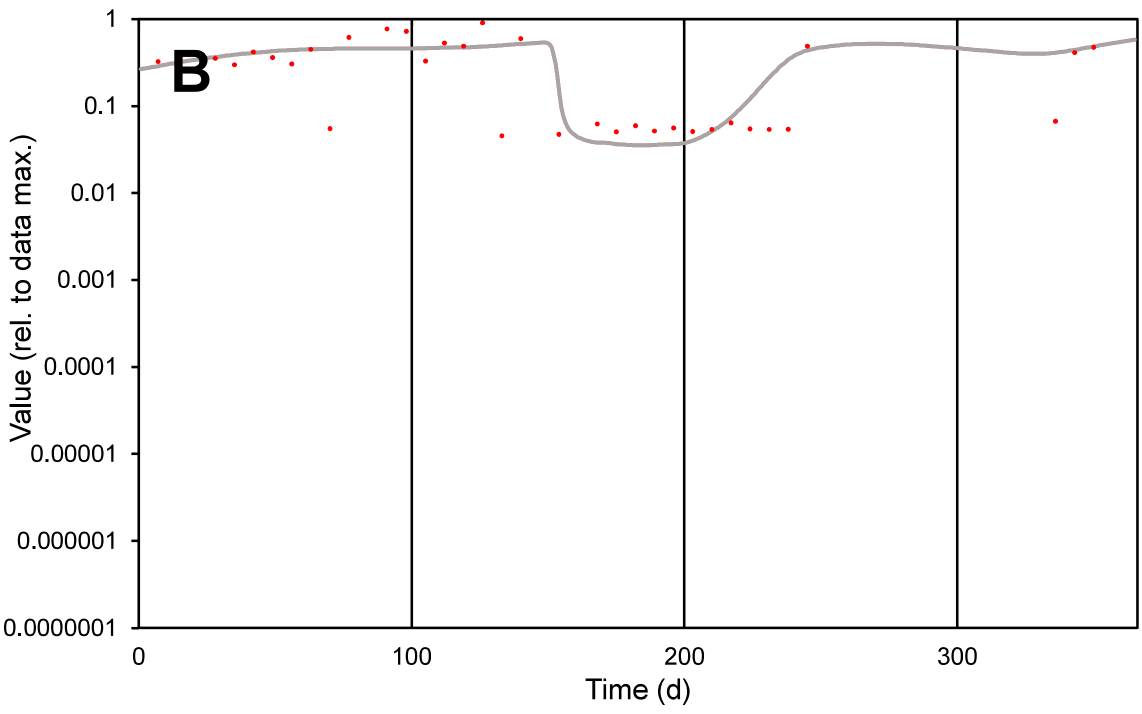

C1

Variables
S18 Provide jtd or Dst
CalibOnly?
2 1 to reproduce of
oNormalize?
2 1=no, 2=yes

ofxn calc*
0.58778
ofxn mod
0.60802

sRun
RunA1
zout
1

Ave
Data
0.00187
Model
0.00193

data/vMax
0.00588

ofxng
311.734

Min d/m
1.28214

ave/min d
7.02044
ave/min m
9.2867

331_Calib_S_A

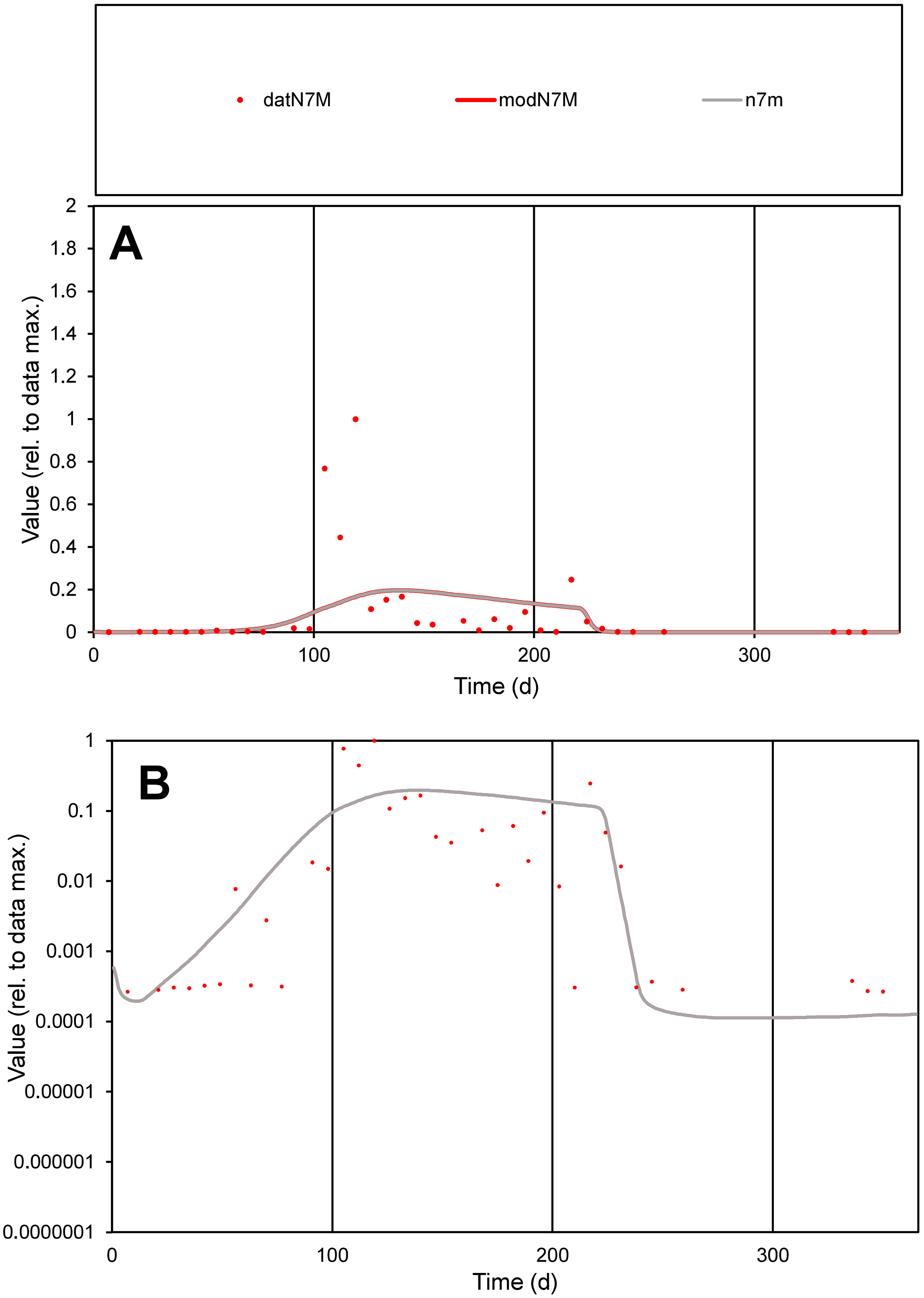

C1

Variables

N7M Provide jtd or Dst

CalibOnly?

2 1 to reproduce of

oNormalize?

2 1=no, 2=yes

ofxn calc*

2.24978

ofxn mod

2.25399

sRun

RunA1

zout

1

Ave

Data

0.00328

Model

0.00288

data/vMax

0.03743

ofxng

311.734

Min d/m

2.21301

ave/min d

331.296

ave/min m

644.091

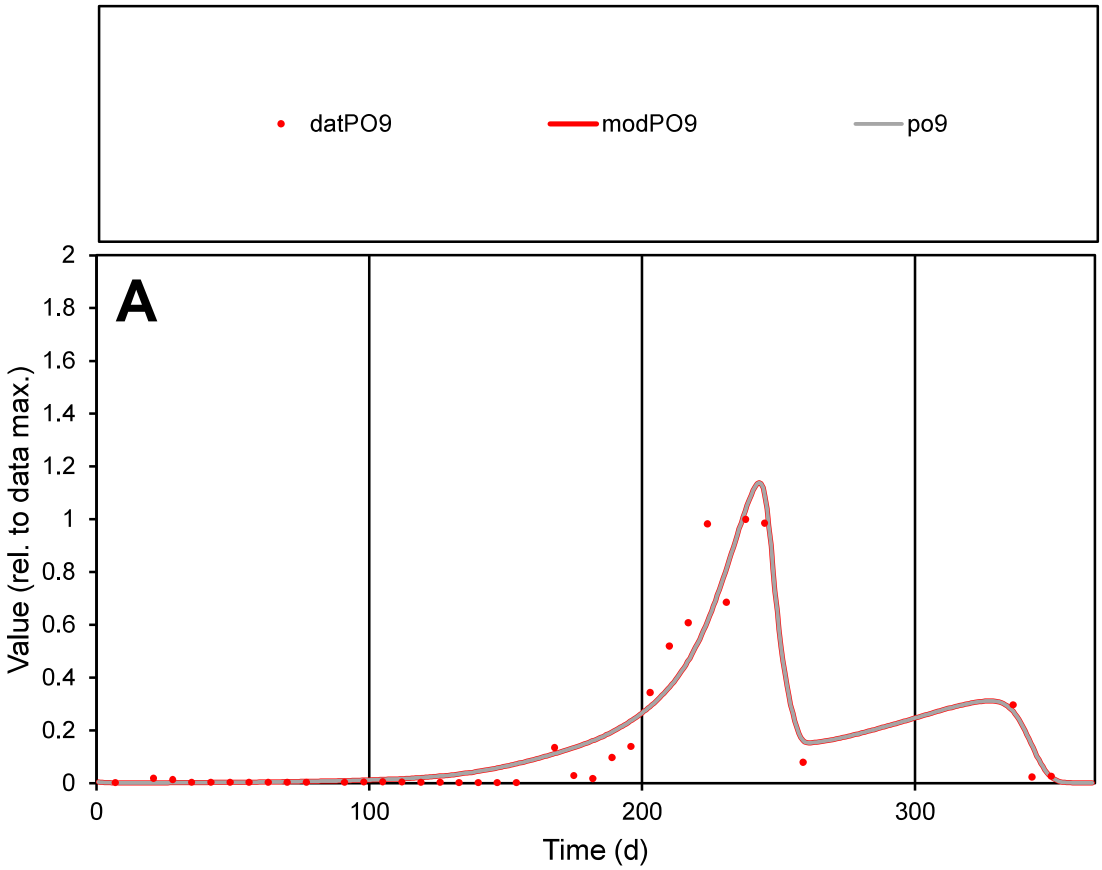

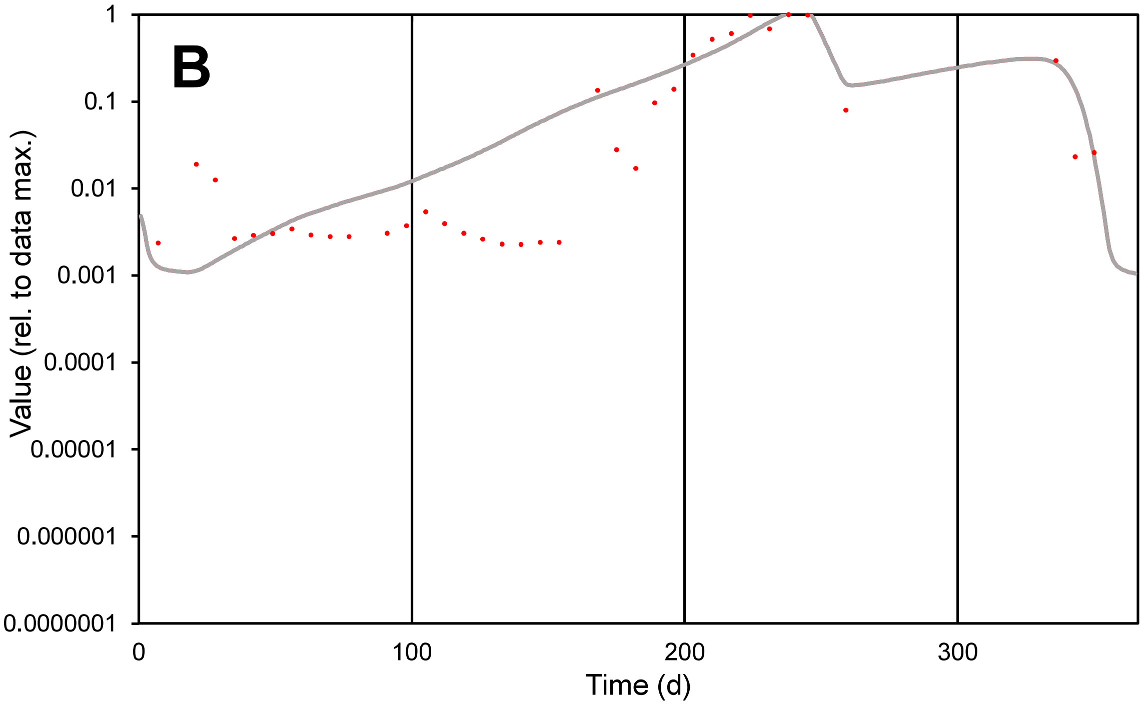

C1

Variables
PO9 Provide jtd or Dst
CalibOnly?
2 1 to reproduce of
oNormalize?
2 1=no, 2=yes

ofxn calc*
0.49194
ofxn mod
0.56381

sRun
RunA1
zout
1

Ave
Data
0.00425
Model
0.0045

data/vMax
0.02531

ofxng
311.734

Min d/m
1.93077

ave/min d
73.6246
ave/min m
150.668

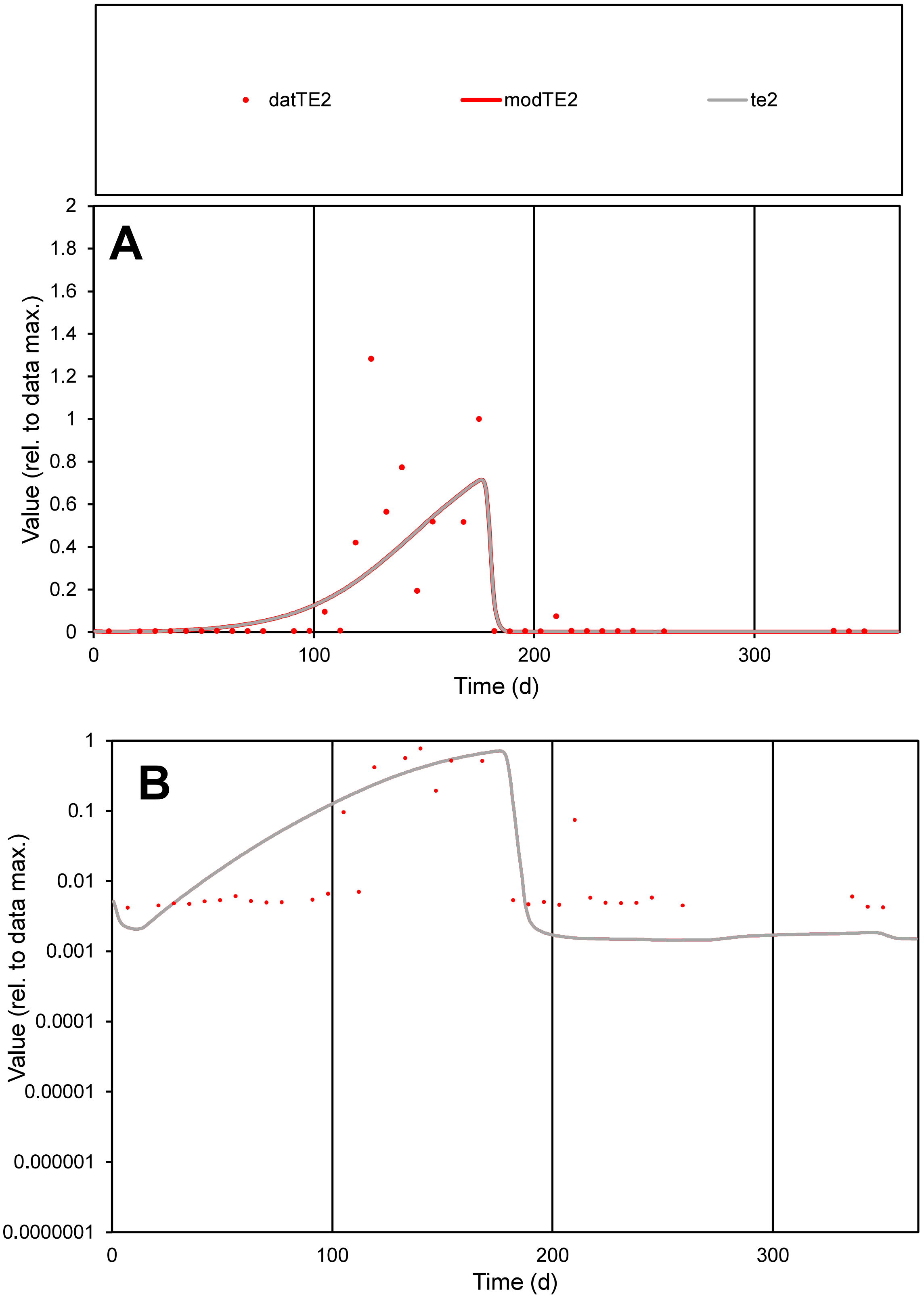

C1

Variables
TE2 Provide jtd or Dst
CalibOnly?
2 1 to reproduce of
oNormalize?
2 1=no, 2=yes

ofxn calc*
1.12687
ofxn mod
1.14306

sRun
RunA1
zout
1

Ave
Data
0.00262
Model
0.00263

data/vMax
0.02088

ofxng
311.734

Min d/m
2.90479

ave/min d
29.8619
ave/min m
87.092

331_Calib_S_A

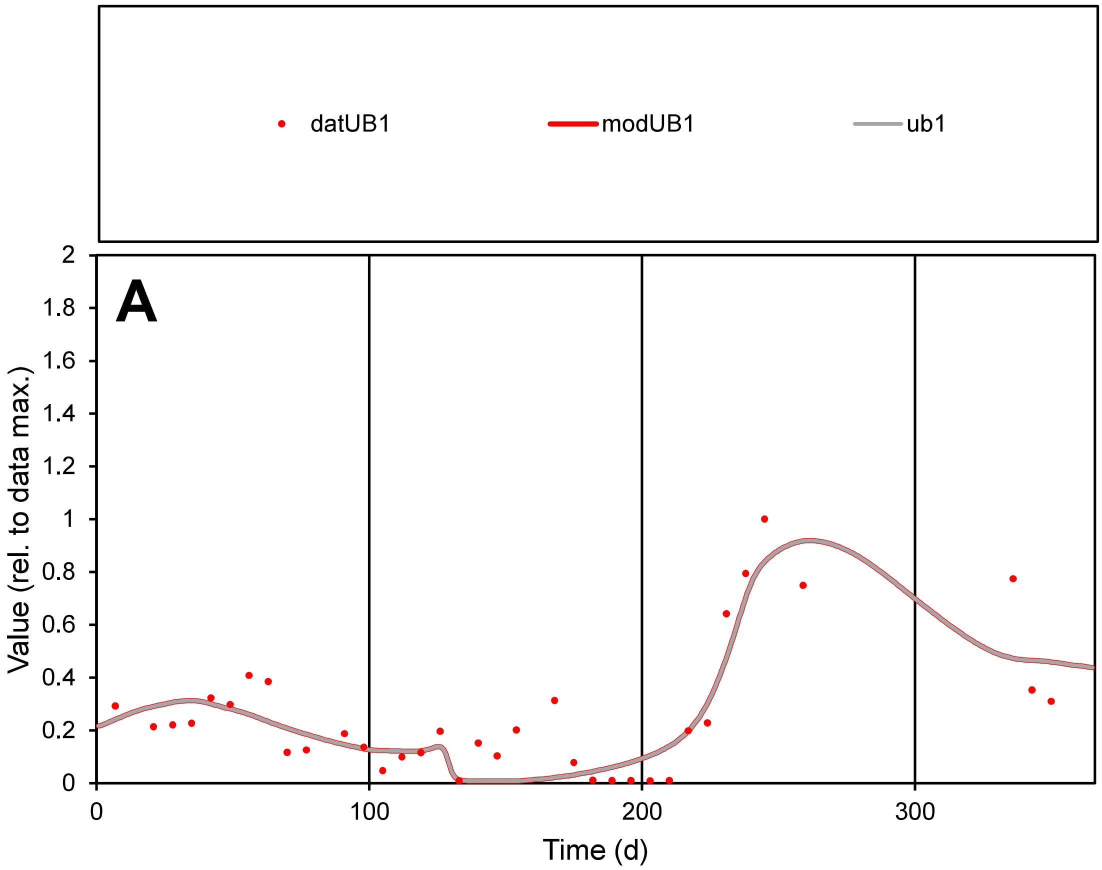

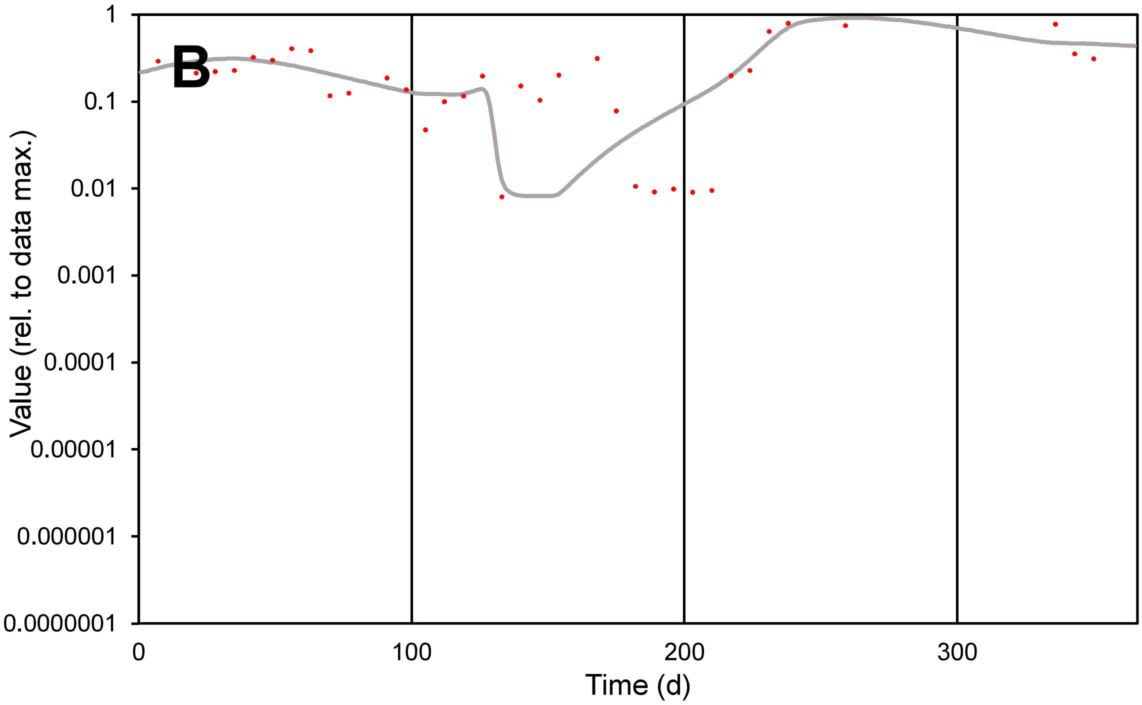

C1

Variables
UB1 Provide jtd or Dst
CalibOnly?
2 1 to reproduce of
oNormalize?
2 1=no, 2=yes

ofxn calc*
0.43471
ofxn mod
0.46385

sRun
RunA1
zout
1

Ave
Data
0.0018
Model
0.00175

data/vMax
0.0071

ofxng
311.734

Min d/m
0.98211

ave/min d
31.6009
ave/min m
30.0889

331_Calib_S_A

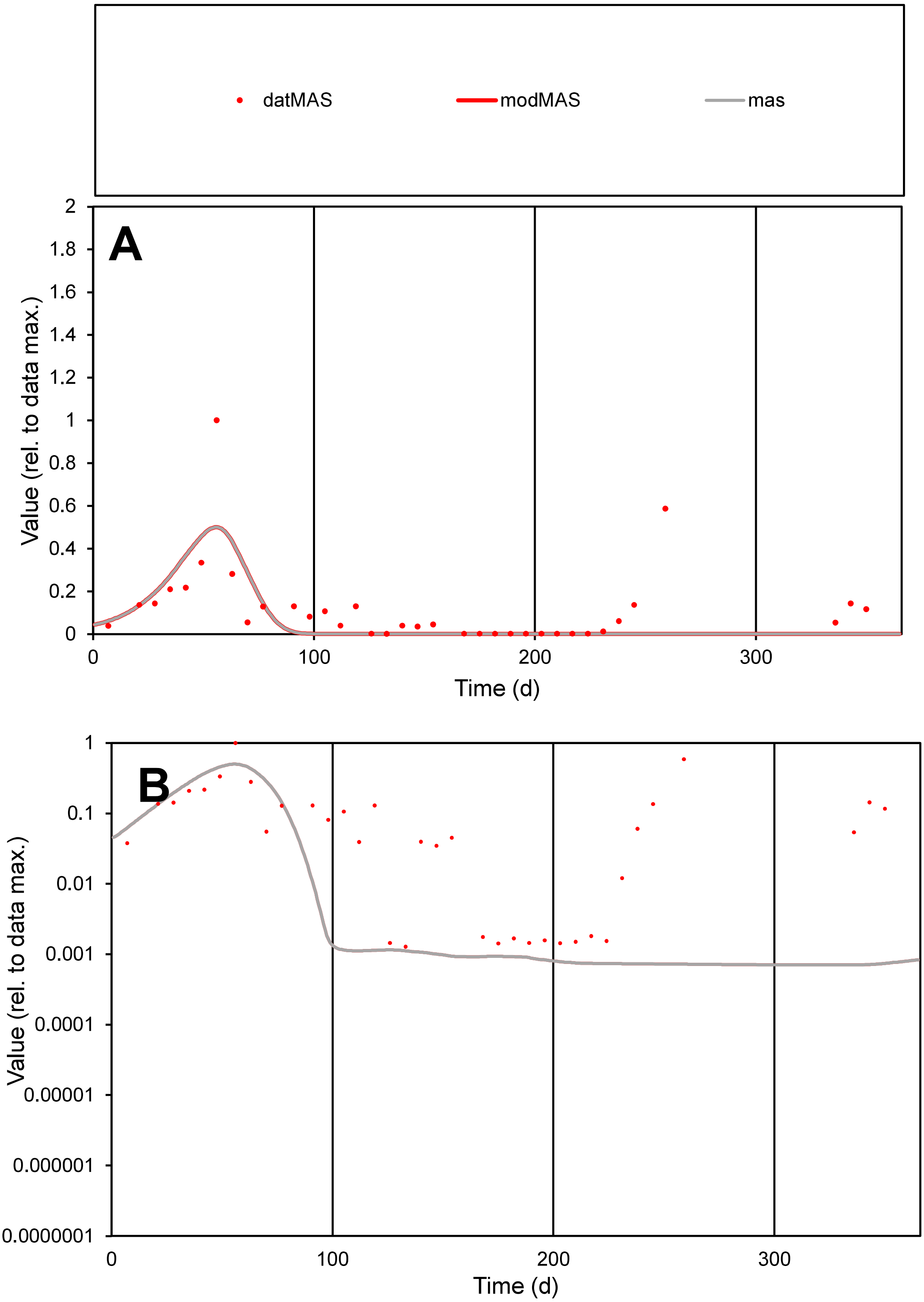

C1

Variables
MAS Provide jtd or Dst
CalibOnly?
2 1 to reproduce of
oNormalize?
2 1=no, 2=yes

ofxn calc*
1.26247
ofxn mod
1.29161

sRun
RunA1
zout
1

Ave
Data
0.00111
Model
0.00073

data/vMax
0.00944

ofxng
311.734

Min d/m
1.8009

ave/min d
92.3111
ave/min m
109.575

331_Calib_S_A

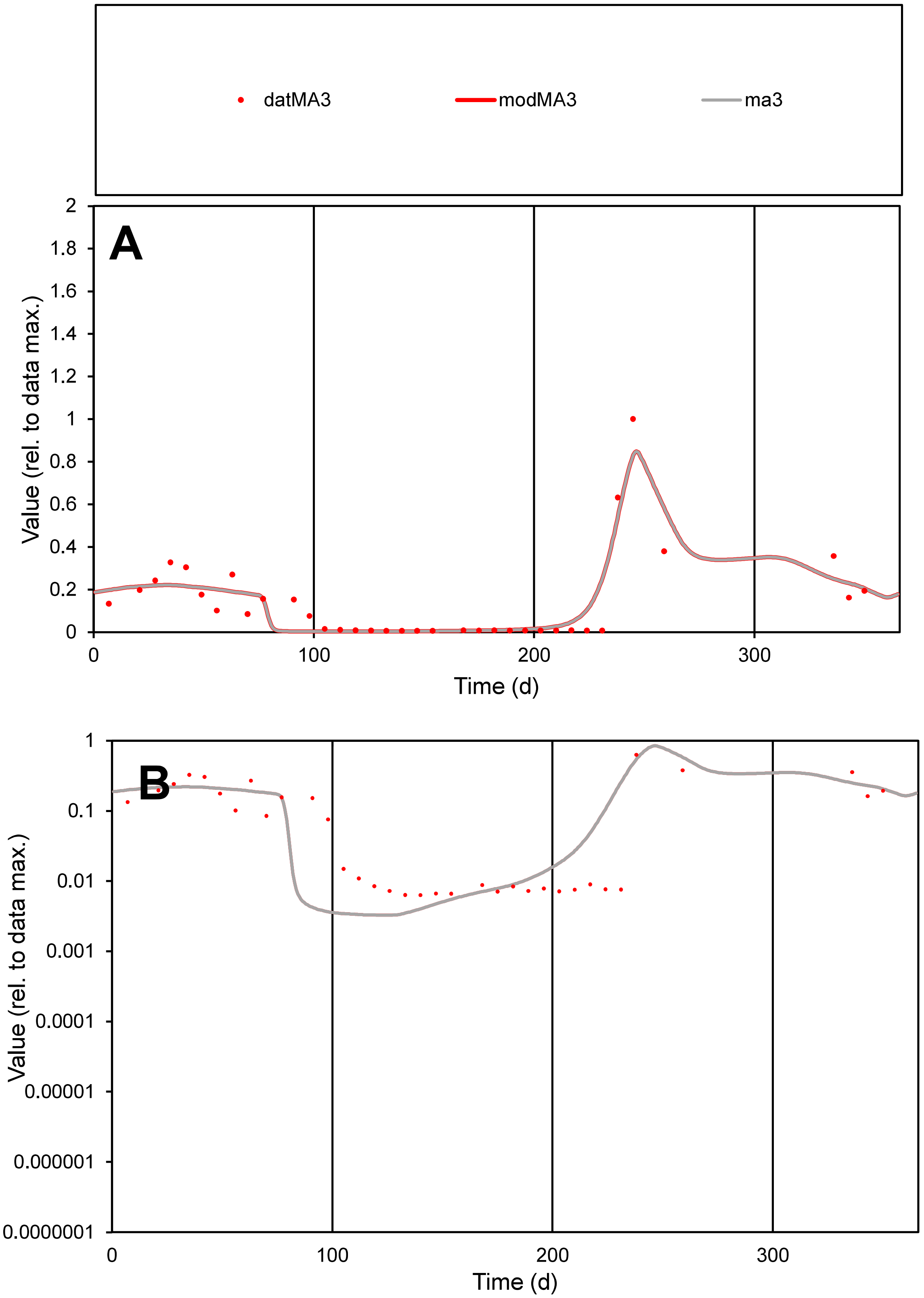

C1

Variables
MA3 Provide jtd or Dst
CalibOnly?
2 1 to reproduce of
oNormalize?
2 1=no, 2=yes

ofxn calc*
0.52045
ofxn mod
0.57543

sRun
RunA1
zout
1

Ave
Data
0.00138
Model
0.00139

data/vMax
0.00974

ofxng
311.734

Min d/m
1.92409

ave/min d
22.452
ave/min m
43.5997

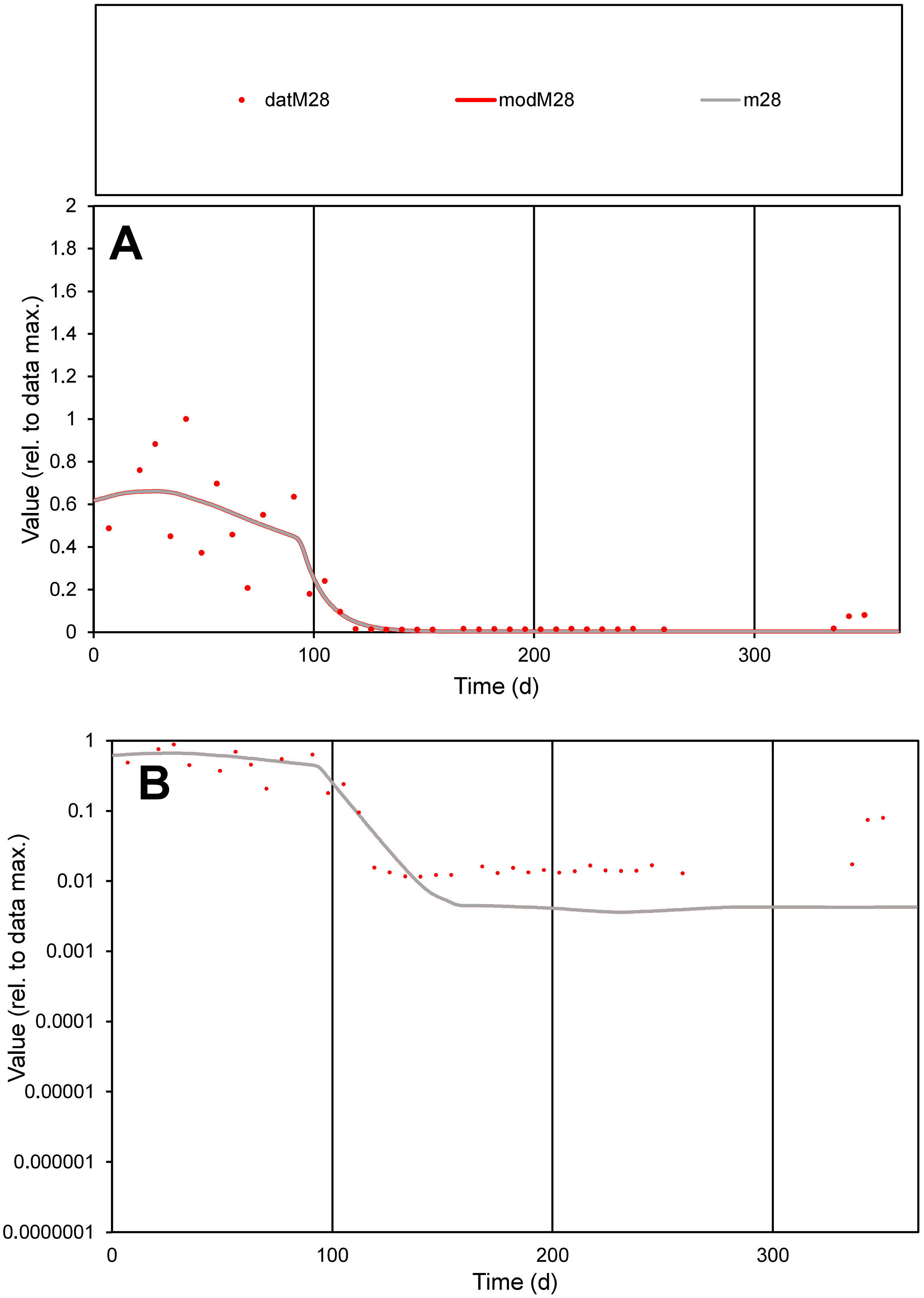

C1

Variables
M28 Provide jtd or Dst
CalibOnly?
2 1 to reproduce of
oNormalize?
2 1=no, 2=yes

ofxn calc*
0.57203
ofxn mod
0.60538

sRun
RunA1
zout
1

Ave
Data
0.00095
Model
0.00091

data/vMax
0.0046

ofxng
311.734

Min d/m
3.20806

ave/min d
17.8333
ave/min m
54.6021

331_Calib_S_A

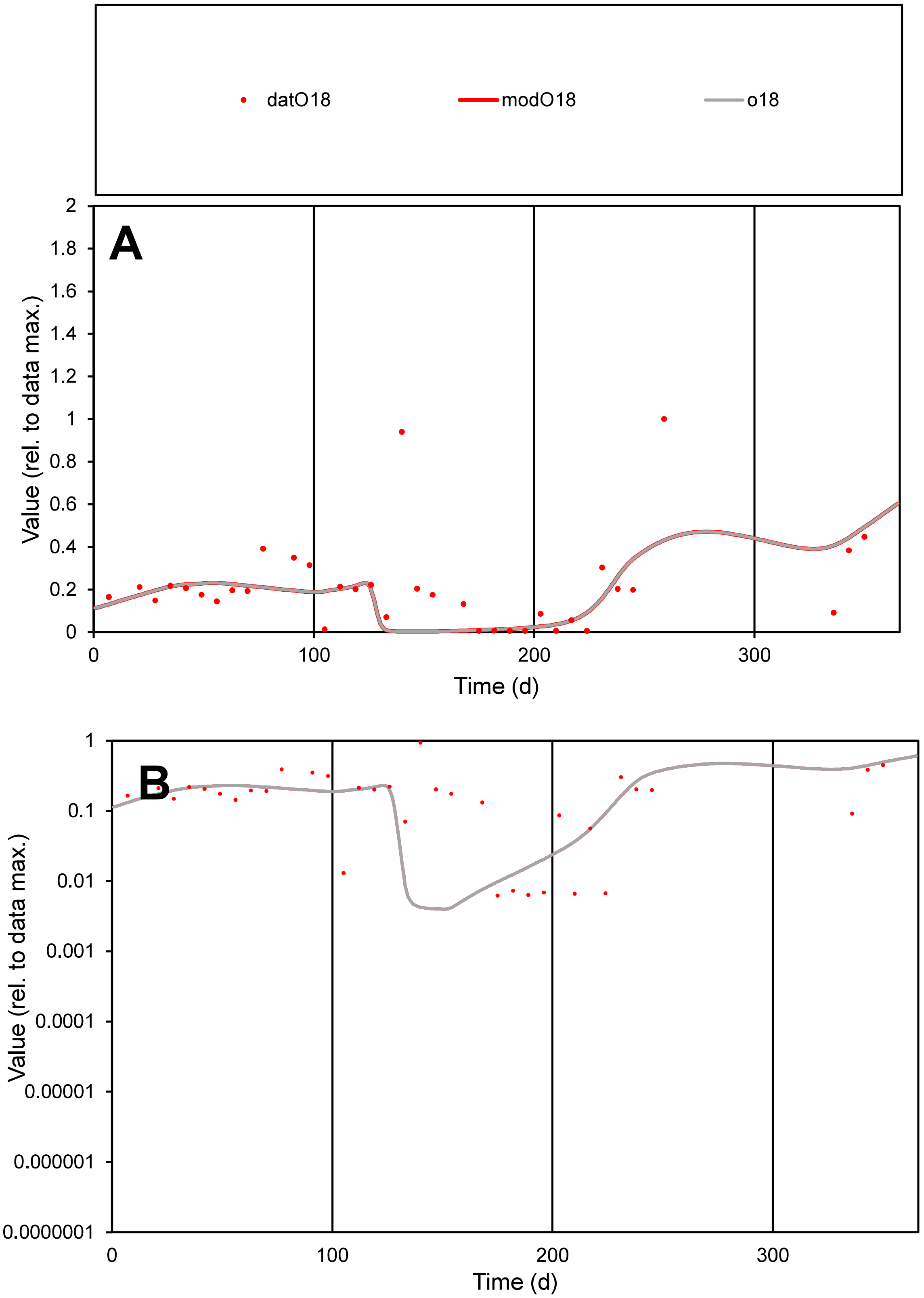

C1

Variables
O18 Provide jtd or Dst
CalibOnly?
2 1 to reproduce of
oNormalize?
2 1=no, 2=yes

ofxn calc*
0.81556
ofxn mod
0.83037

sRun
RunA1
zout
1

Ave
Data
0.00183
Model
0.00158

data/vMax
0.00926

ofxng
311.734

Min d/m
1.54462

ave/min d
31.8354
ave/min m
42.5587

331_Calib_S_A

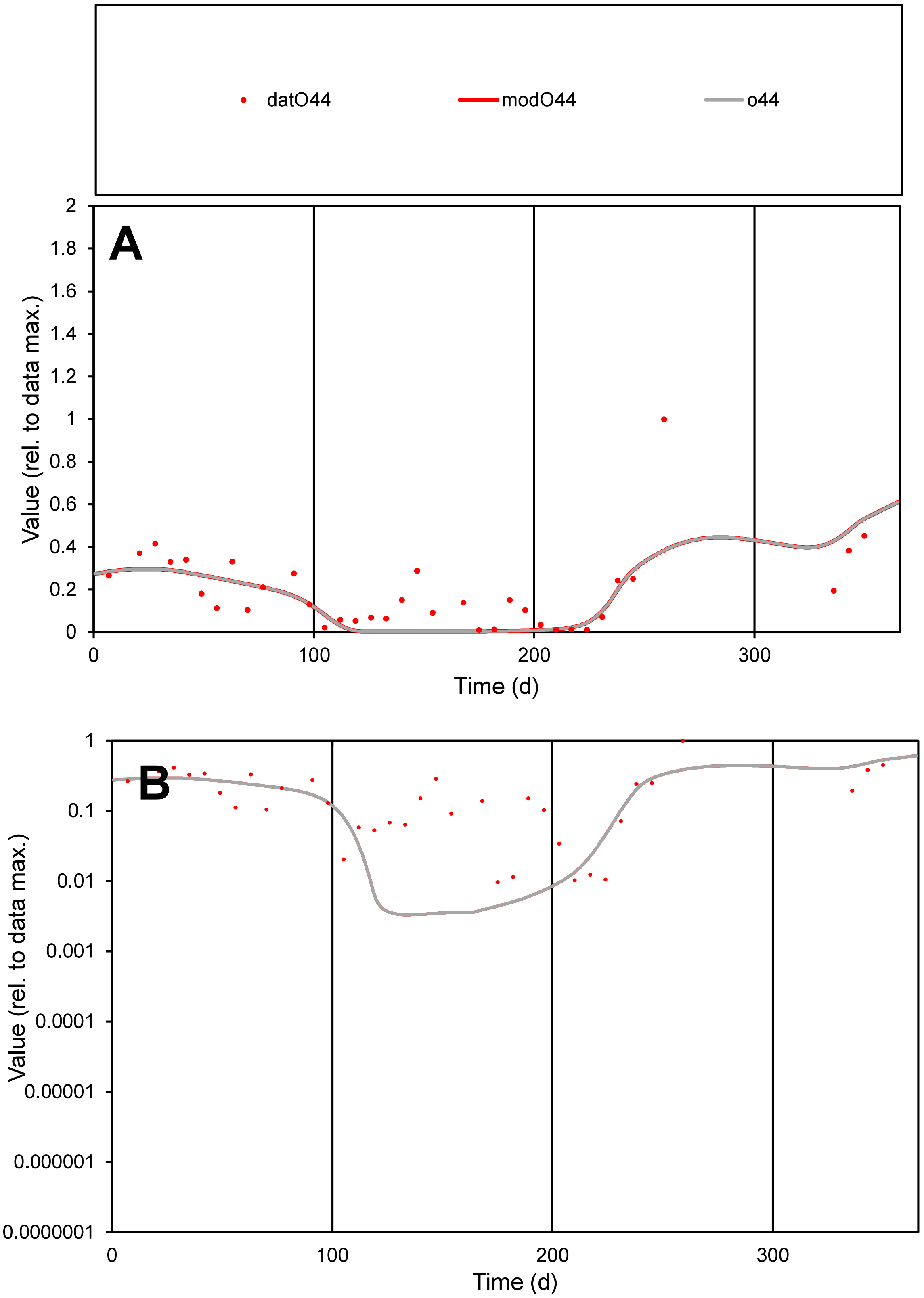

C1

Variables
O44 Provide jtd or Dst
CalibOnly?
2 1 to reproduce of
oNormalize?
2 1=no, 2=yes

ofxn calc*
0.72004
ofxn mod
0.73448

sRun
RunA1
zout
1

Ave
Data
0.00238
Model
0.00199

data/vMax
0.0127

ofxng
311.734

Min d/m
2.91331

ave/min d
19.3126
ave/min m
47.0492

331_Calib_S_A

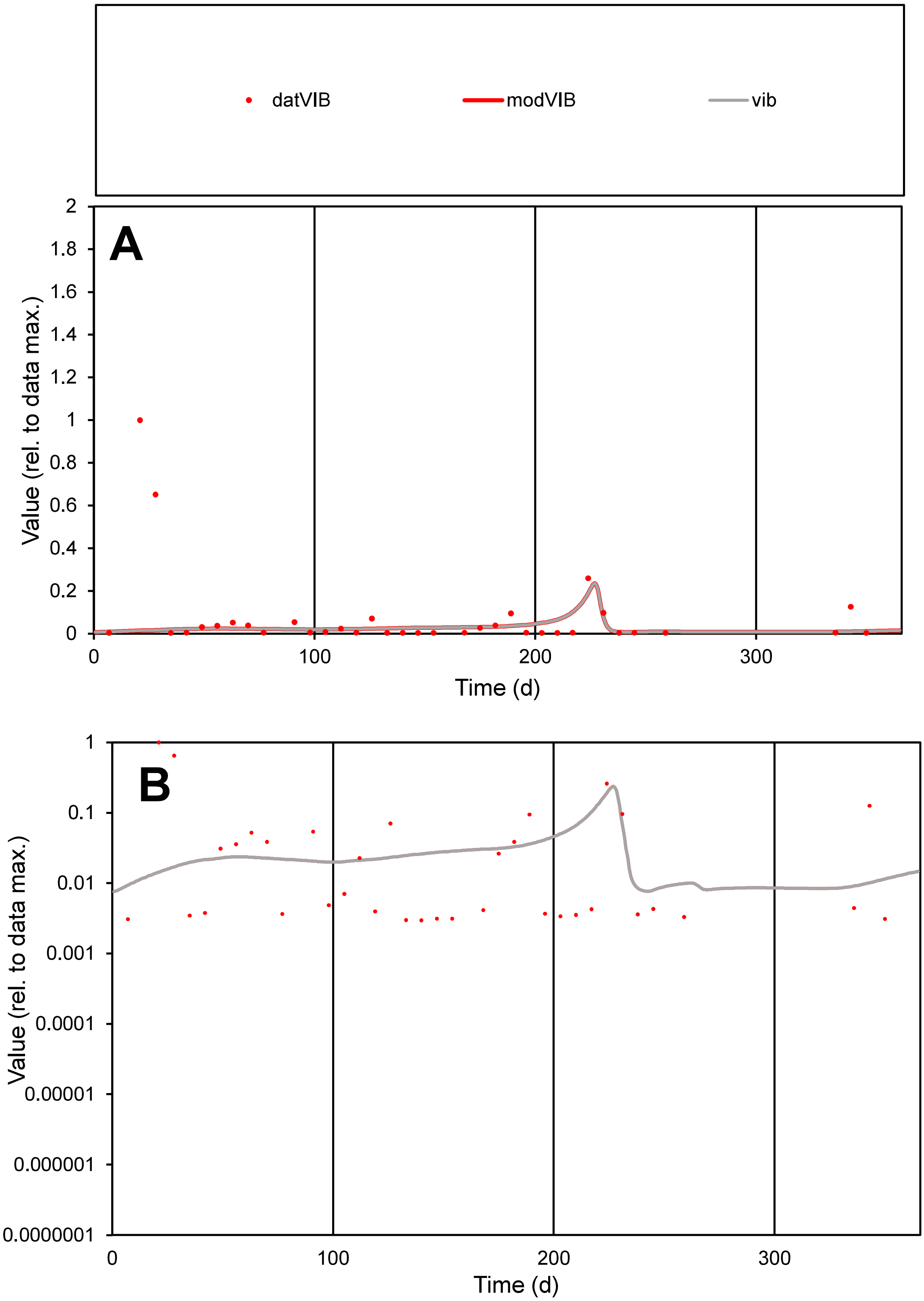

C1

Variables
VIB Provide jtd or Dst
CalibOnly?
2 1 to reproduce of
oNormalize?
2 1=no, 2=yes

ofxn calc*
2.6865
ofxn mod
2.6944

sRun
RunA1
zout
1

Ave
Data
0.00044
Model
0.00019

data/vMax
0.00591

ofxng
311.734

Min d/m
0.37234

ave/min d
24.8766
ave/min m
4.00192

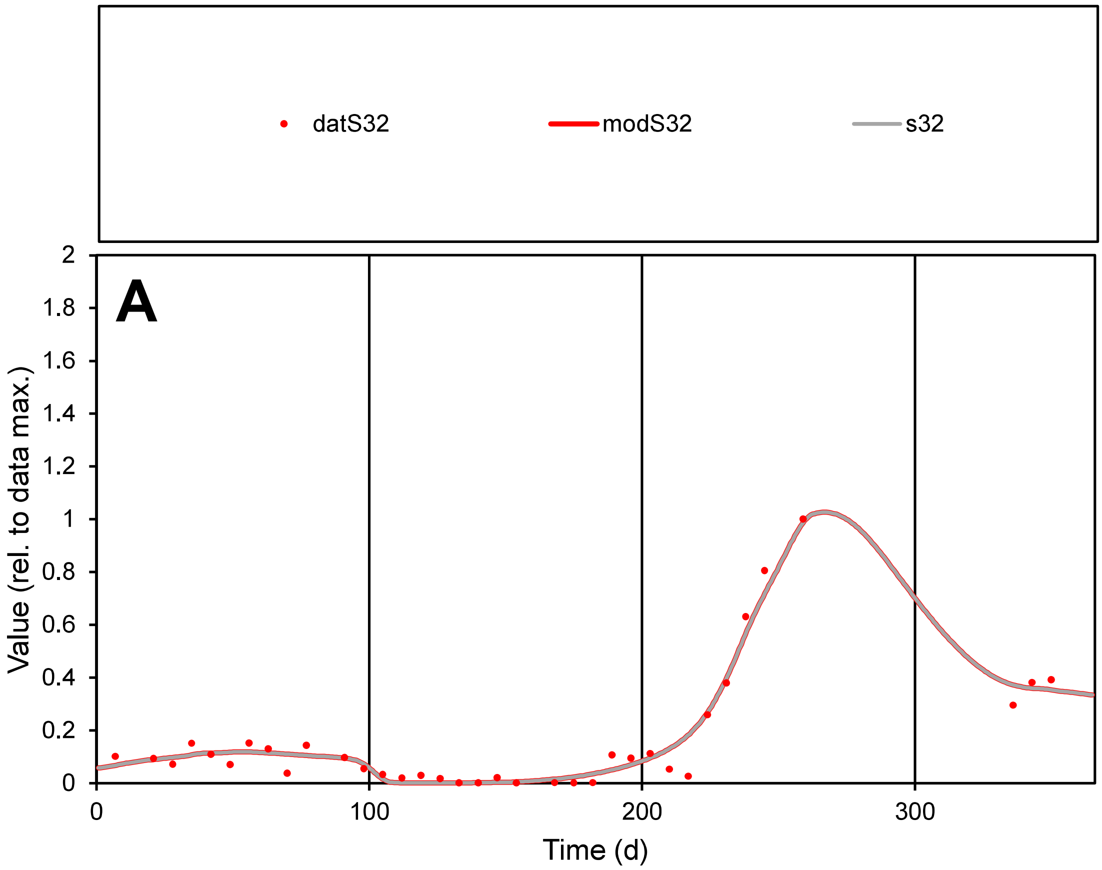

C1

Variables
S32 Provide jtd or Dst
CalibOnly?
2 1 to reproduce of
oNormalize?
2 1=no, 2=yes

ofxn calc*
0.25766
ofxn mod
0.33526

sRun
RunA1
zout
1

Ave
Data
0.00165
Model
0.00168

data/vMax
0.01012

ofxng
311.734

Min d/m
1.09774

ave/min d
154.469
ave/min m
172.566

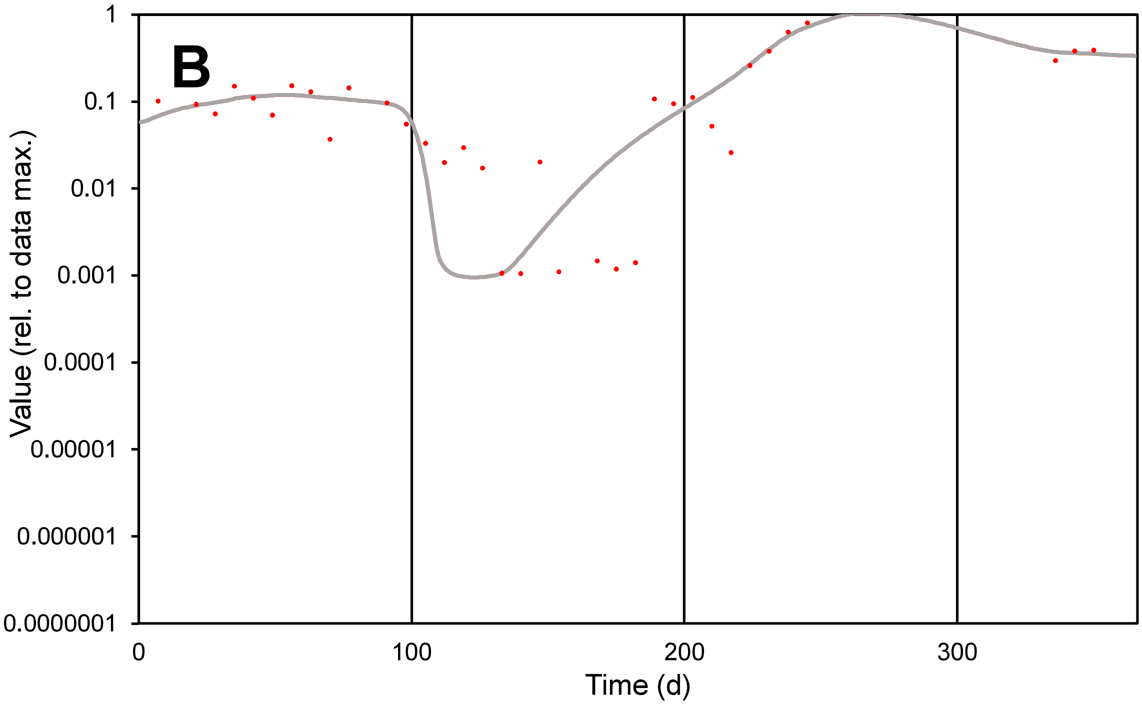

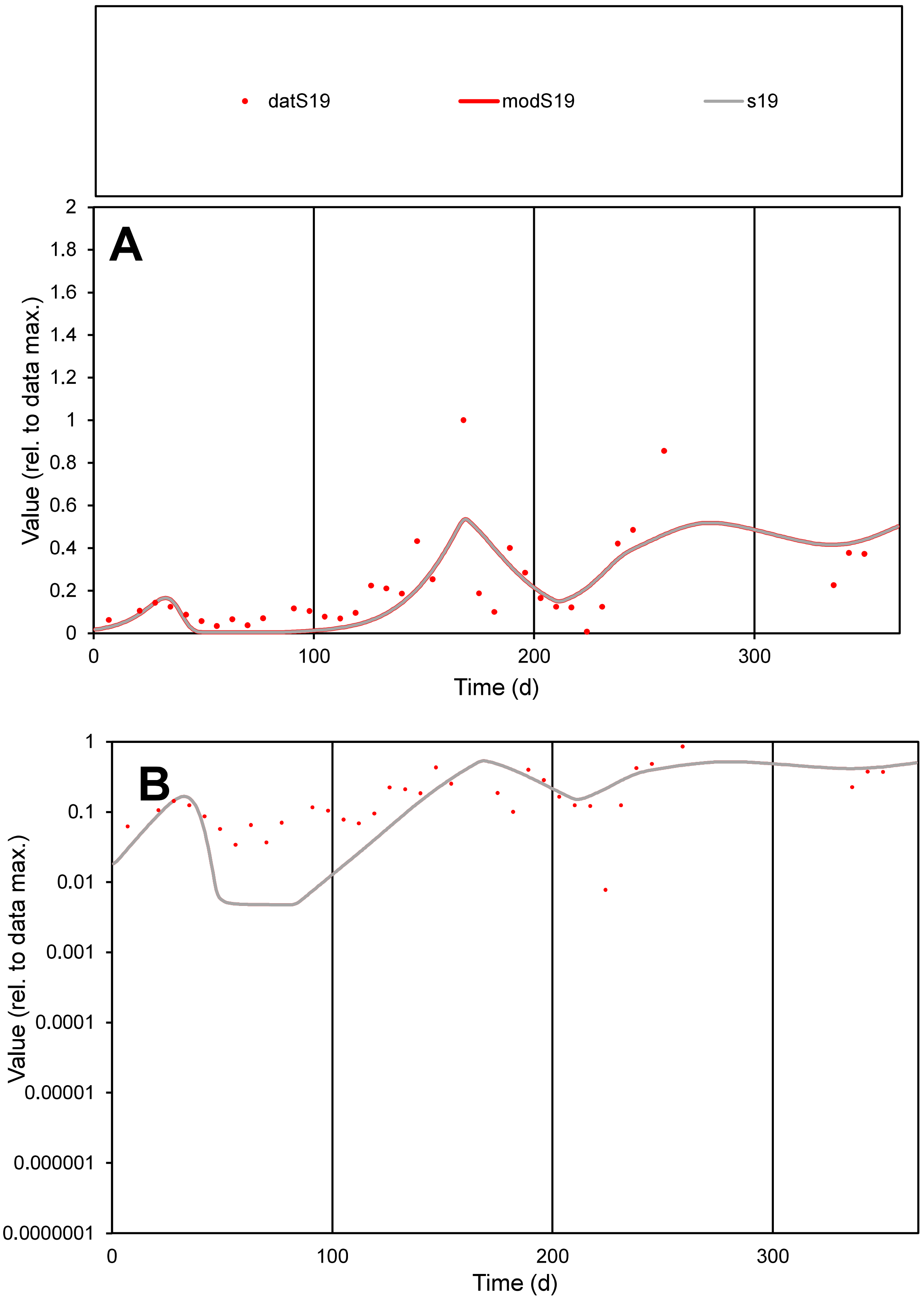

C1

Variables
S19 Provide jtd or Dst
CalibOnly?
2 1 to reproduce of
oNormalize?
2 1=no, 2=yes

ofxn calc*
0.6934
ofxn mod
0.70966

sRun
RunA1
zout
1

Ave
Data
0.00151
Model
0.0014

data/vMax
0.00731

ofxng
311.734

Min d/m
1.63537

ave/min d
26.4897
ave/min m
40.2399

331_Calib_S_A

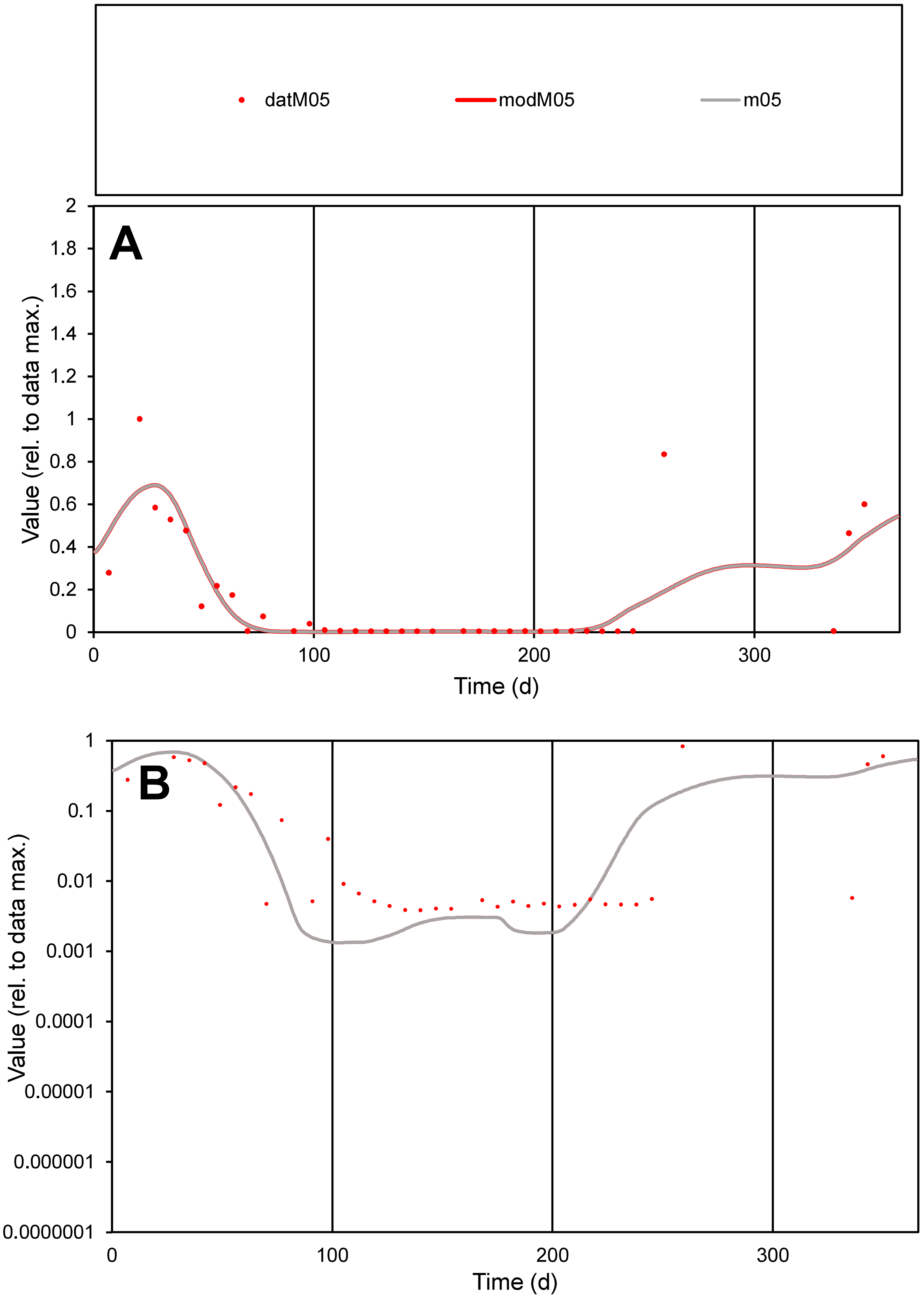

C1

Variables
M05 Provide jtd or Dst
CalibOnly?
2 1 to reproduce of
oNormalize?
2 1=no, 2=yes

ofxn calc*
0.94849
ofxn mod
0.98028

sRun
RunA1
zout
1

Ave
Data
0.0017
Model
0.00158

data/vMax
0.0111

ofxng
311.734

Min d/m
2.88319

ave/min d
39.7852
ave/min m
106.7

331_Calib_S_A

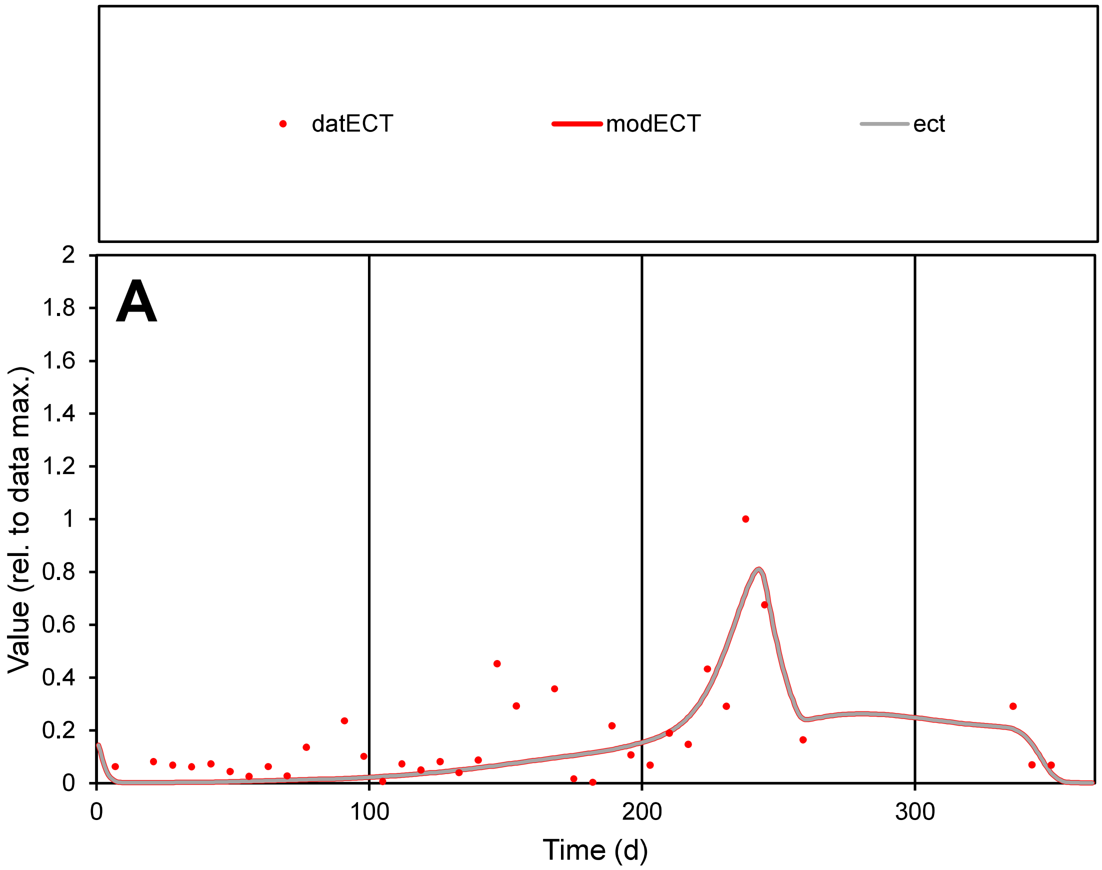

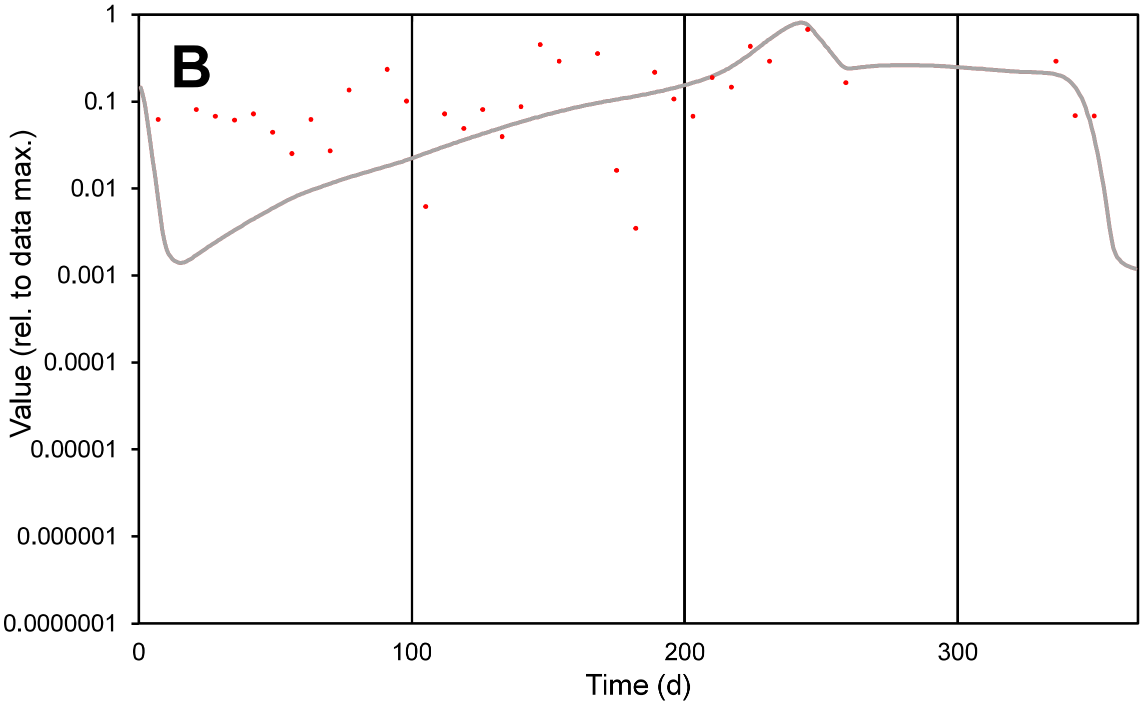

C1

Variables
ECT Provide jtd or Dst
CalibOnly?
2 1 to reproduce of
oNormalize?
2 1=no, 2=yes

ofxn calc*
0.67439
ofxn mod
0.70109

sRun
RunA1
zout
1

Ave
Data
0.00175
Model
0.0014

data/vMax
0.01071

ofxng
311.734

Min d/m
1.88322

ave/min d
47.0199
ave/min m
70.7415

331_Calib_S_A

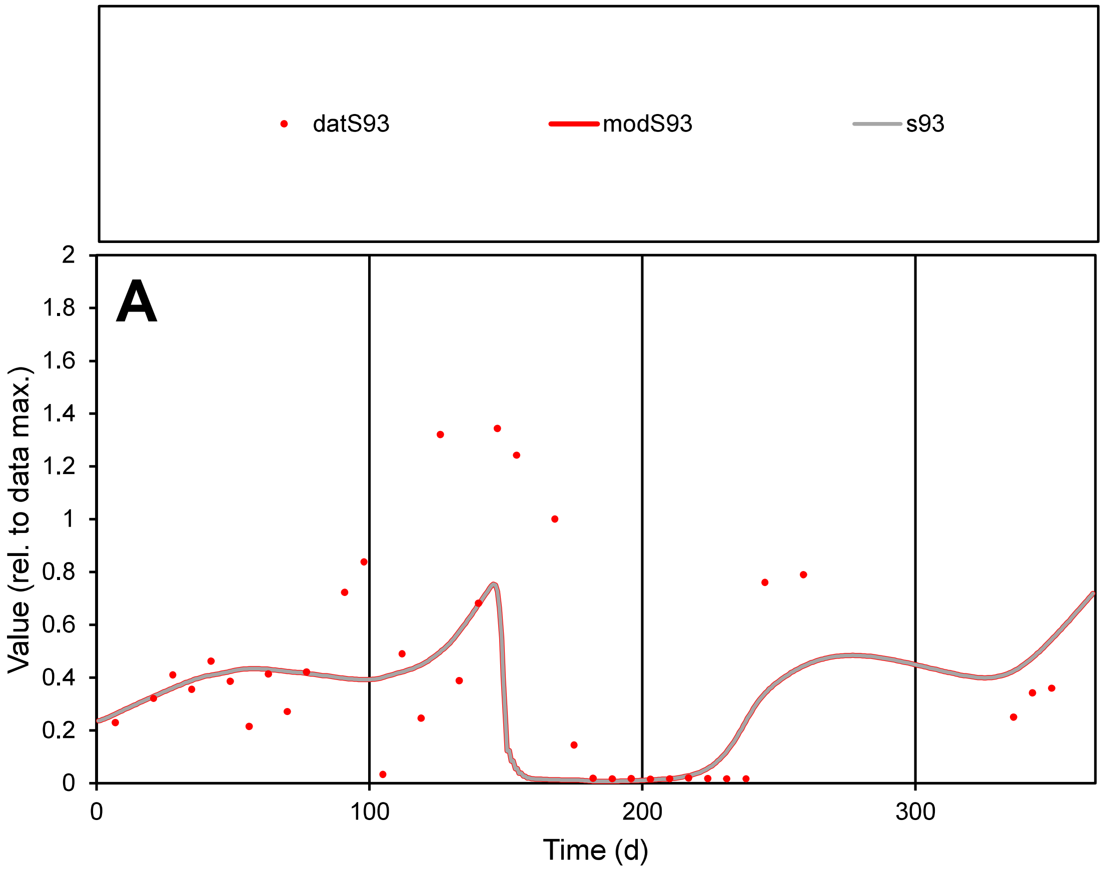

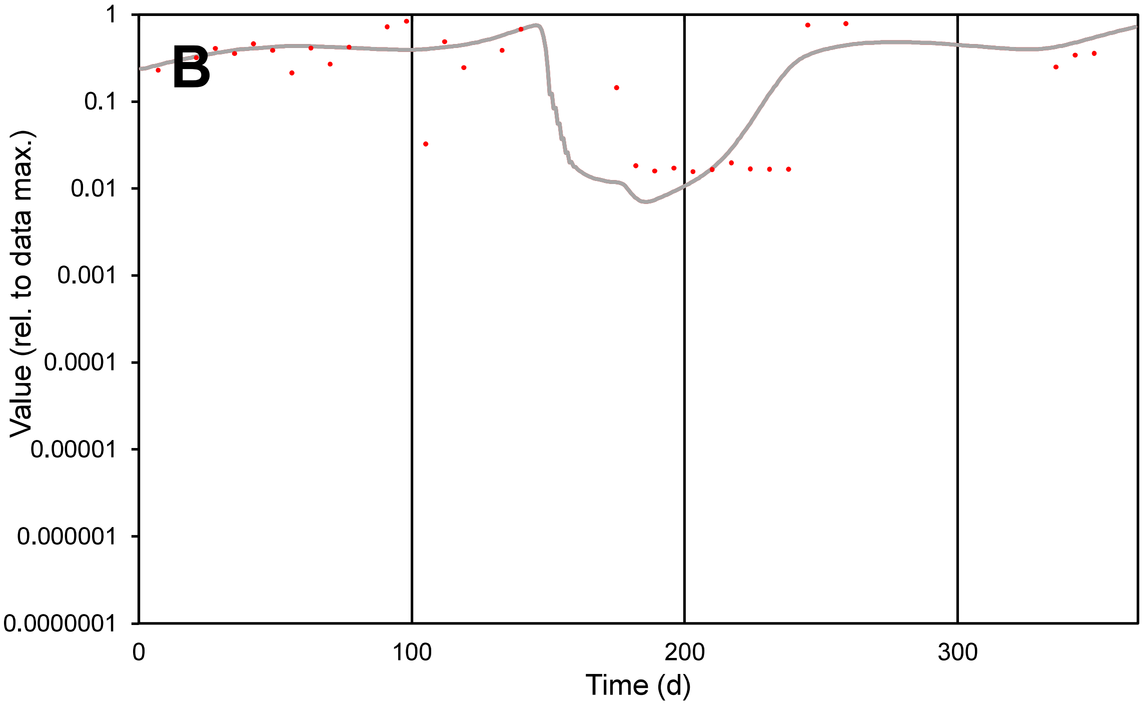

C1

Variables
S93 Provide jtd or Dst
CalibOnly?
2 1 to reproduce of
oNormalize?
2 1=no, 2=yes

ofxn calc*
0.81855
ofxn mod
0.83205

sRun
RunA1
zout
1

Ave
Data
0.0017
Model
0.00146

data/vMax
0.00467

ofxng
311.734

Min d/m
2.06528

ave/min d
23.2068
ave/min m
41.0422

331_Calib_S_A

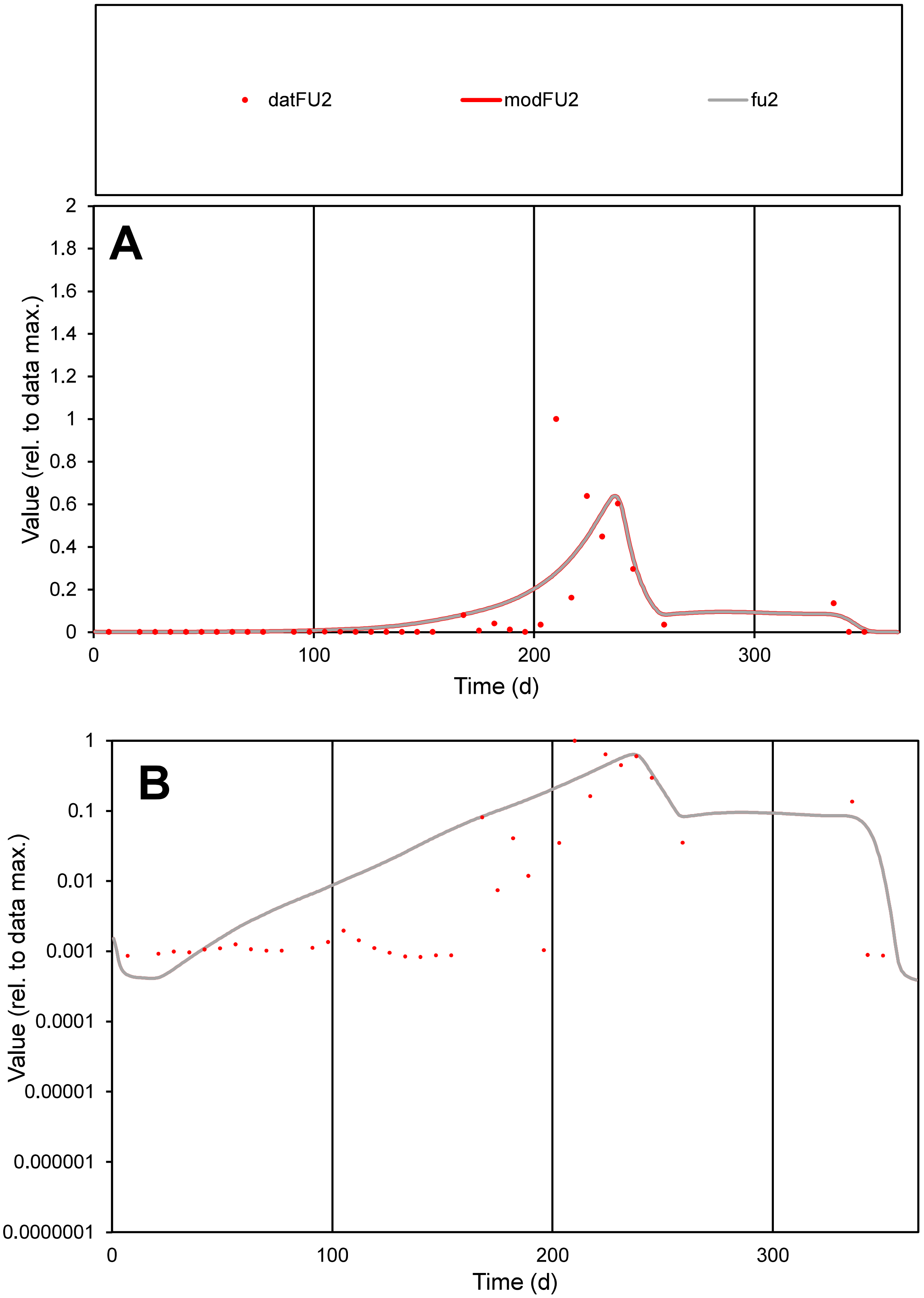

C1

Variables

FU2 Provide jtd or Dst

CalibOnly?

2 1 to reproduce of

oNormalize?

2 1=no, 2=yes

ofxn calc*

1.42166

ofxn mod

1.45711

sRun

RunA1

zout

1

Ave

Data

0.00297

Model

0.00328

data/vMax

0.03034

ofxng

311.734

Min d/m

1.89288

ave/min d

117.404

ave/min m

245.428

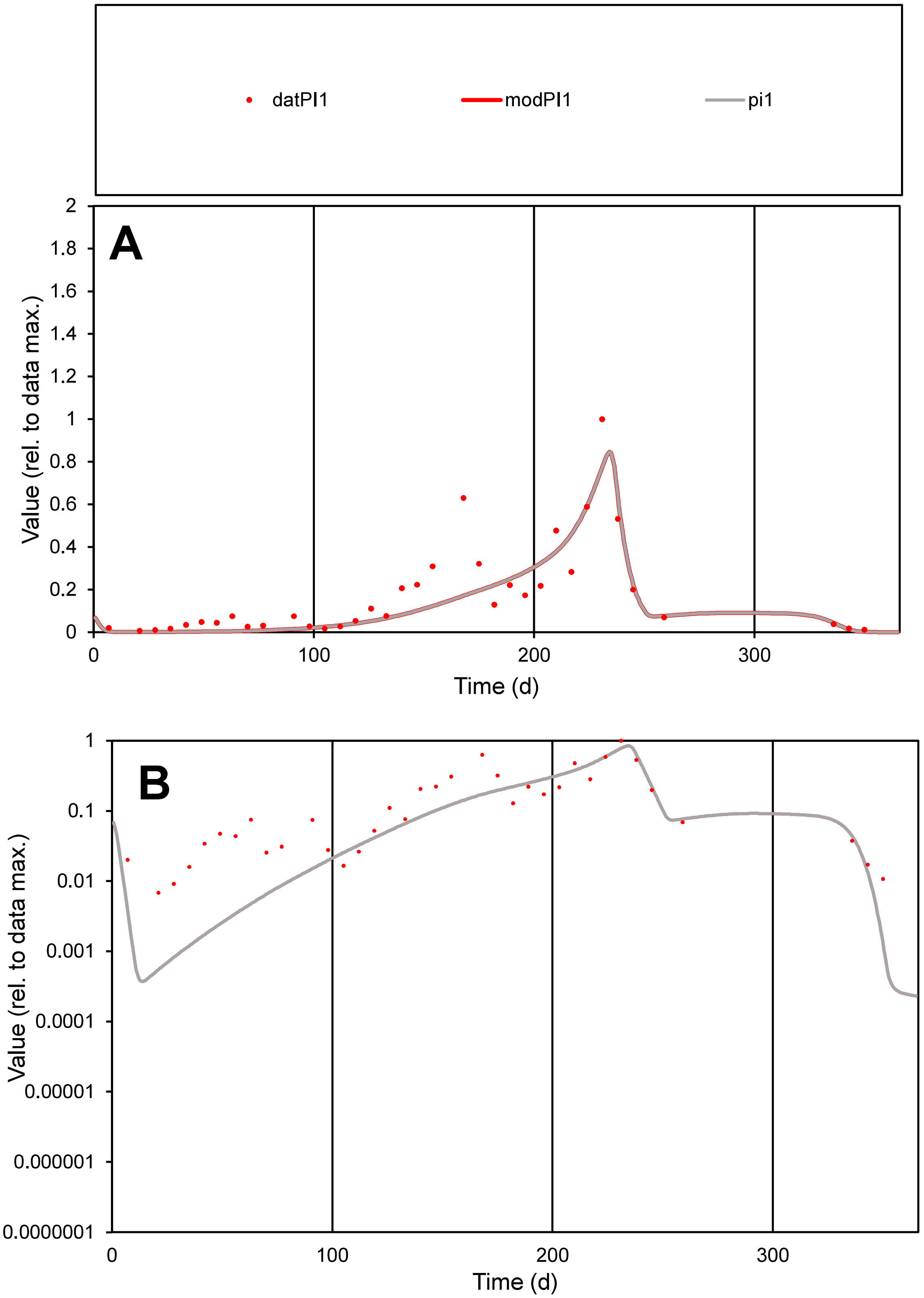

C1

Variables
PI1 Provide jtd or Dst
CalibOnly?
2 1 to reproduce of
oNormalize?
2 1=no, 2=yes

ofxn calc*
0.59562
ofxn mod
0.6311

sRun
RunA1
zout
1

Ave
Data
0.00409
Model
0.00354

data/vMax
0.02432

ofxng
311.734

Min d/m
11.4139

ave/min d
24.5763
ave/min m
243.027

331_Calib_S_A

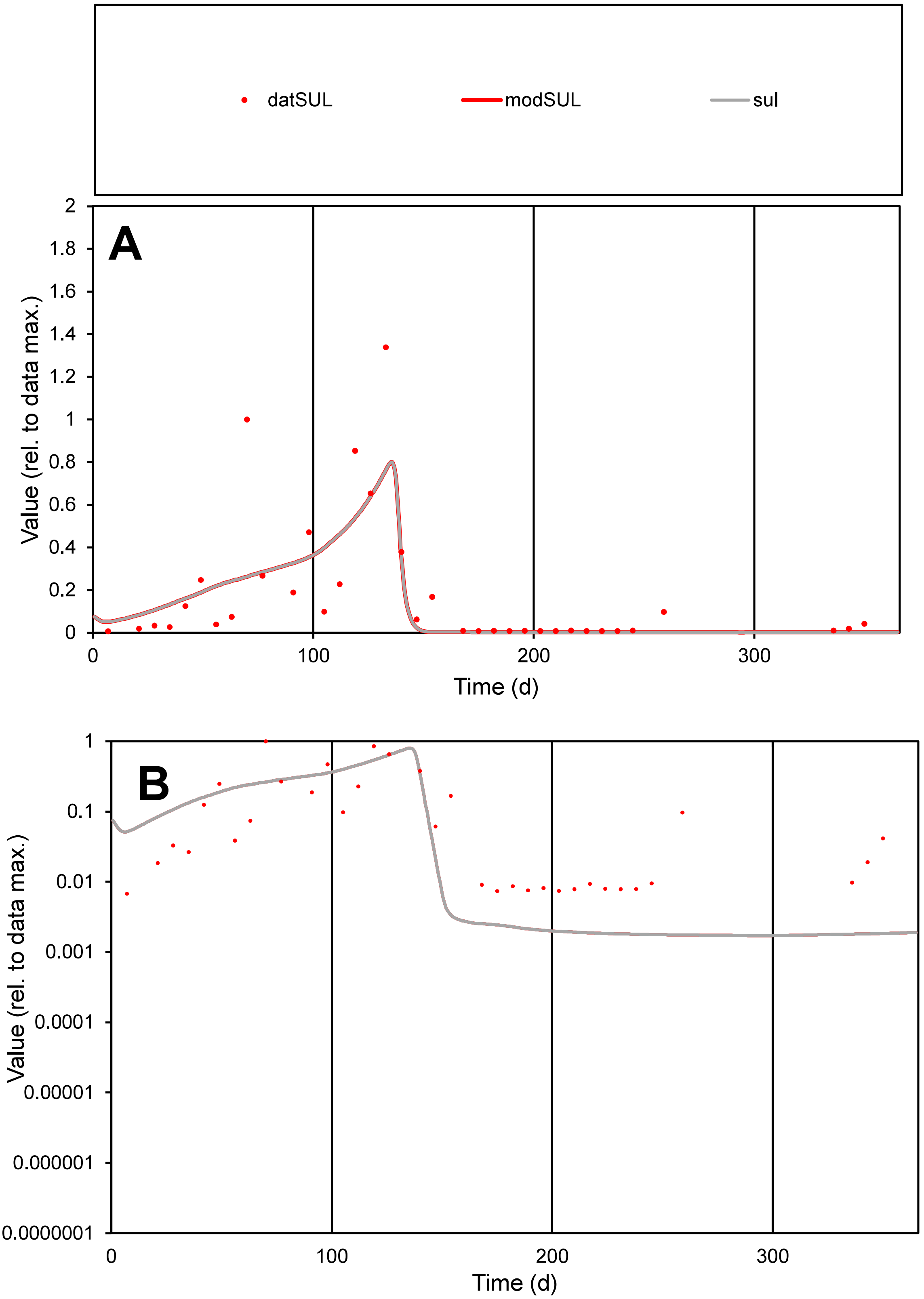

C1

Variables
SUL Provide jtd or Dst
CalibOnly?
2 1 to reproduce of
oNormalize?
2 1=no, 2=yes

ofxn calc*
1.0402
ofxn mod
1.05978

sRun
RunA1
zout
1

Ave
Data
0.00191
Model
0.00187

data/vMax
0.01201

ofxng
311.734

Min d/m
3.88608

ave/min d
23.4749
ave/min m
89.1797

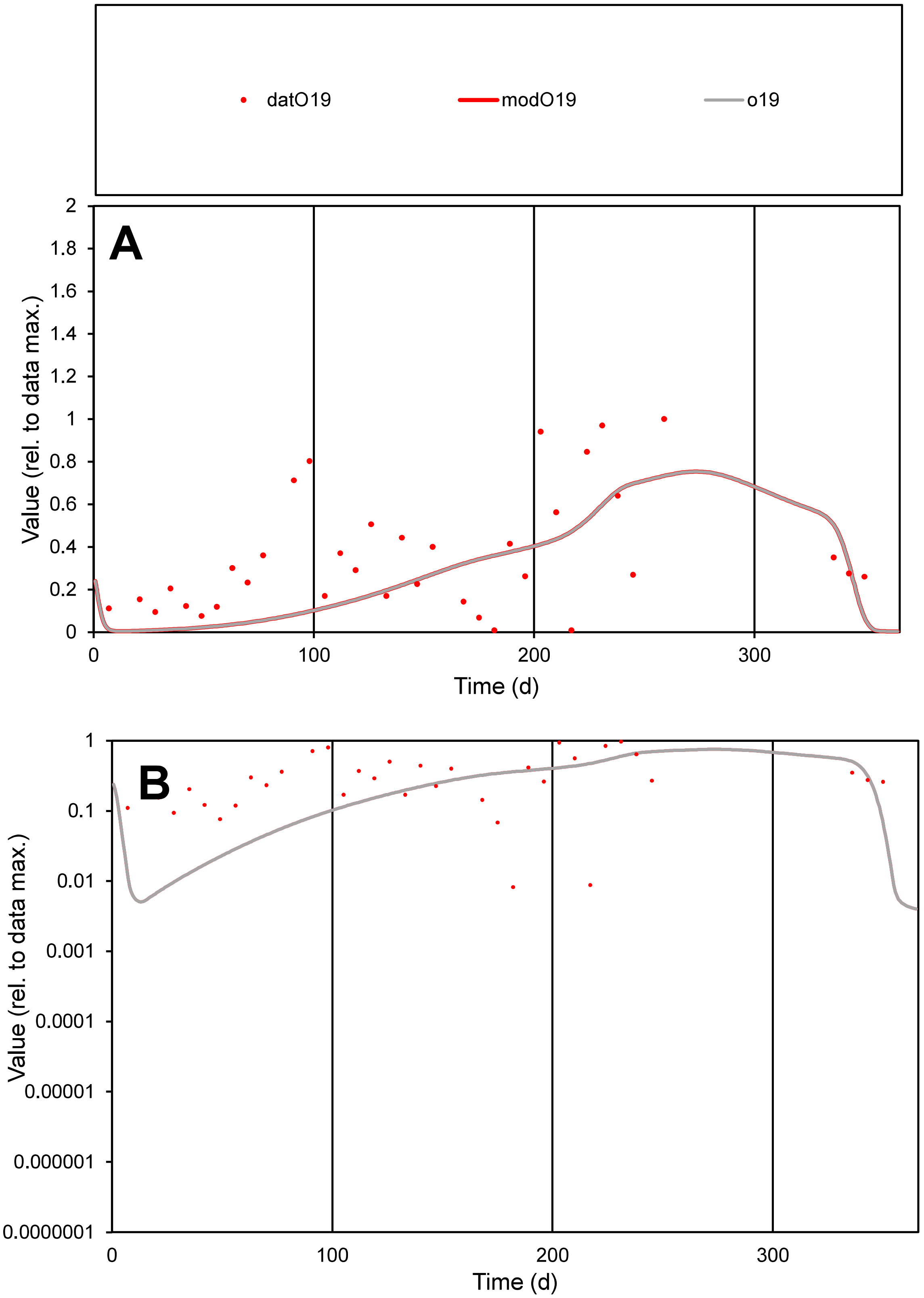

C1

Variables
O19 Provide jtd or Dst
CalibOnly?
2 1 to reproduce of
oNormalize?
2 1=no, 2=yes

ofxn calc*
0.78054
ofxn mod
0.79411

sRun
RunA1
zout
1

Ave
Data
0.00156
Model
0.00117

data/vMax
0.00455

ofxng
311.734

Min d/m
1.08137

ave/min d
41.8653
ave/min m
34.1202

331_Calib_S_A

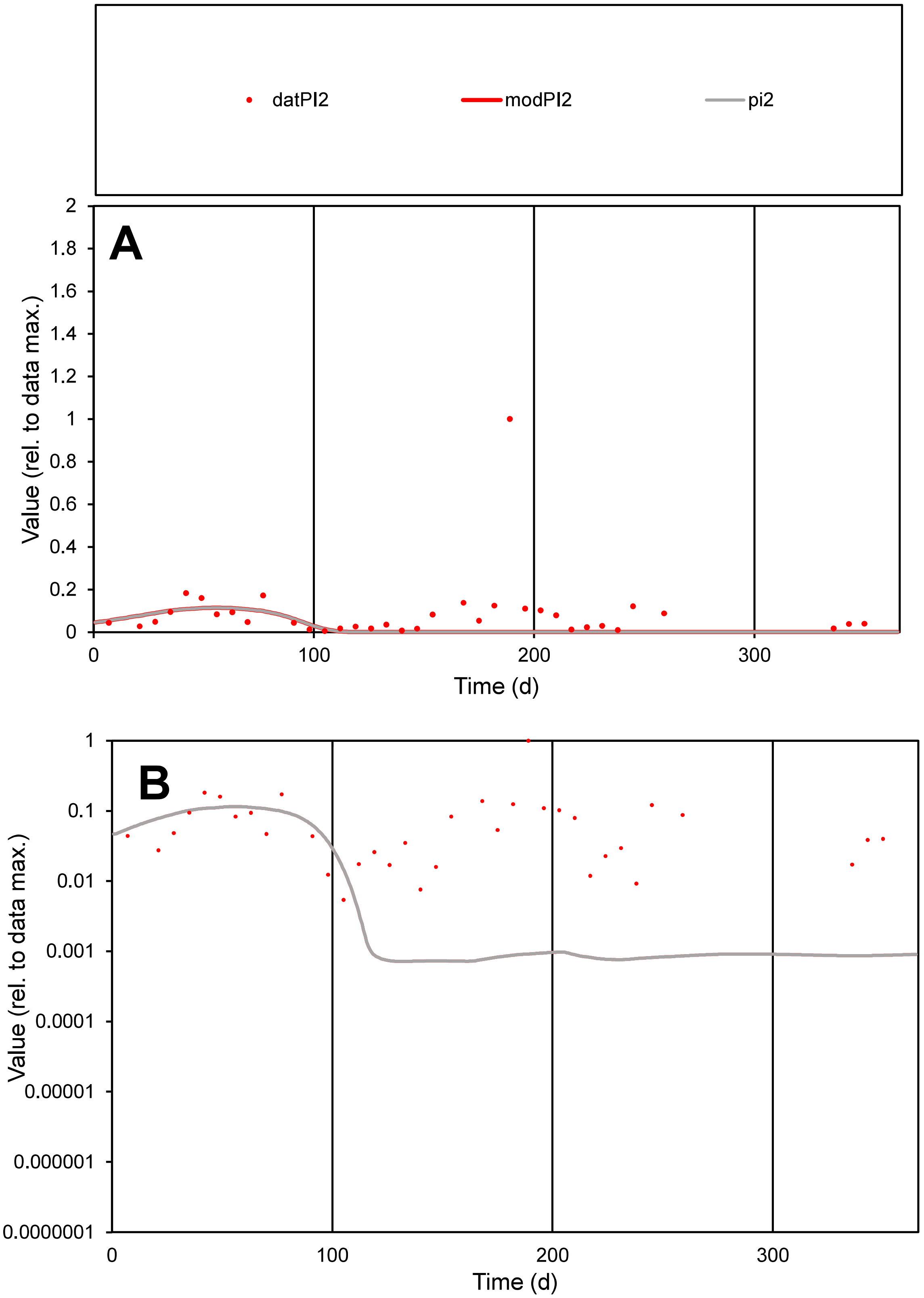

C1

Variables
PI2 Provide jtd or Dst
CalibOnly?
2 1 to reproduce of
oNormalize?
2 1=no, 2=yes

ofxn calc*
2.01109
ofxn mod
2.01621

sRun
RunA1
zout
1

Ave
Data
0.00271
Model
0.00095

data/vMax
0.03089

ofxng
311.734

Min d/m
5.99012

ave/min d
20.2961
ave/min m
42.4358

331_Calib_S_A

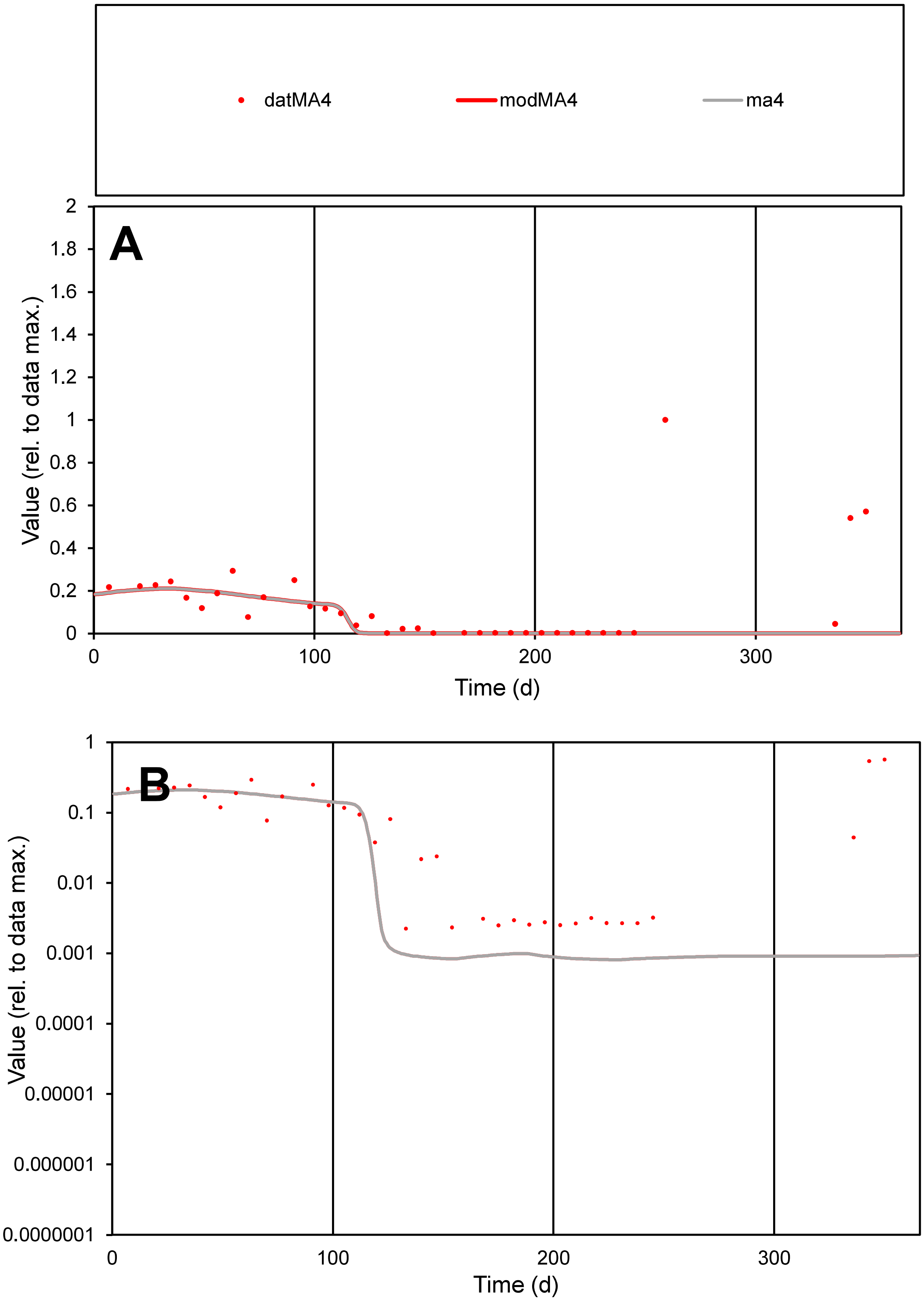

C1

Variables
MA4 Provide jtd or Dst
CalibOnly?
2 1 to reproduce of
oNormalize?
2 1=no, 2=yes

ofxn calc*
1.60039
ofxn mod
1.6063

sRun
RunA1
zout
1

Ave
Data
0.00115
Model
0.0006

data/vMax
0.00858

ofxng
311.734

Min d/m
2.76654

ave/min d
59.7654
ave/min m
86.1309

331_Calib_S_A

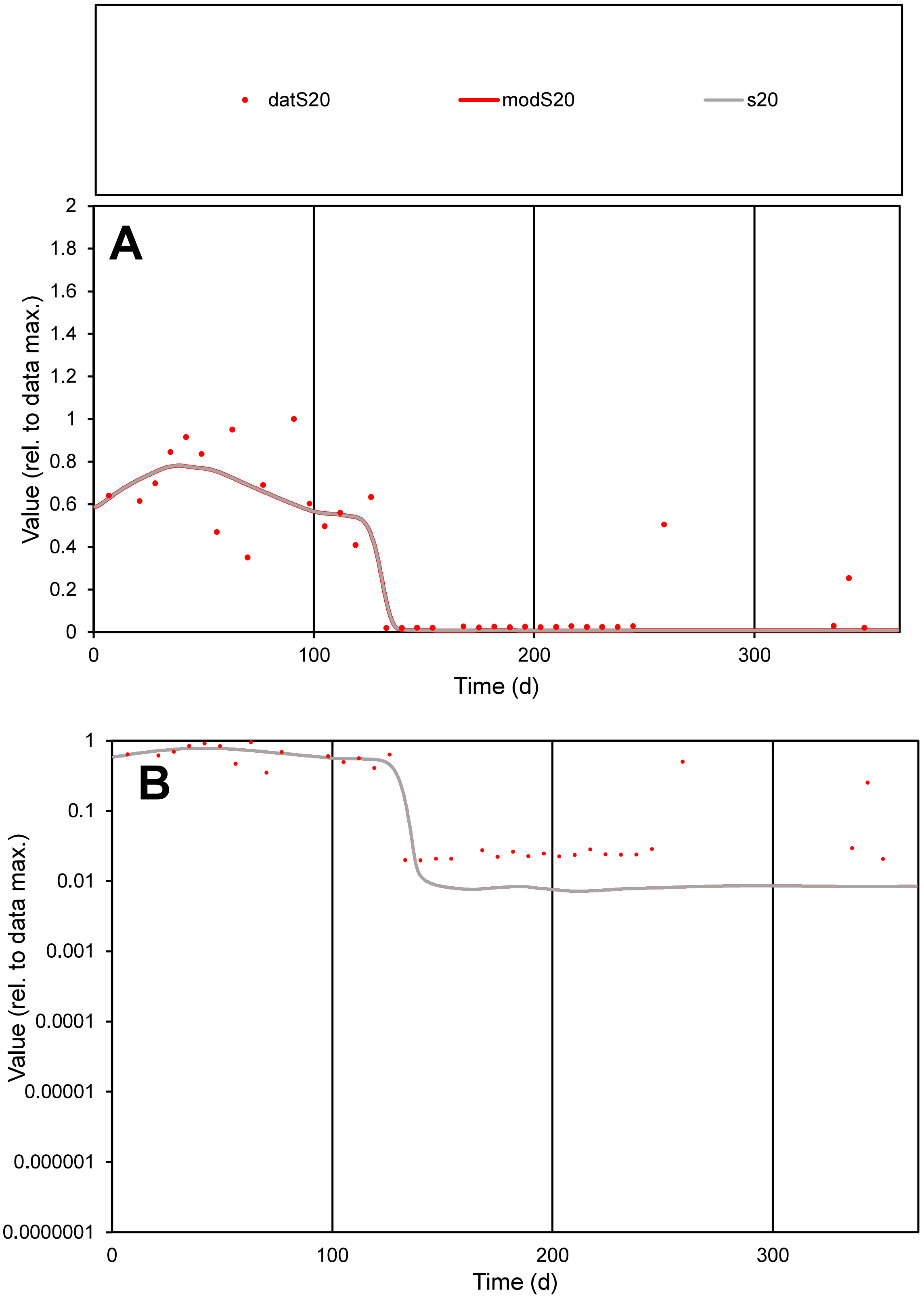

C1

Variables
S20 Provide jtd or Dst
CalibOnly?
2 1 to reproduce of
oNormalize?
2 1=no, 2=yes

ofxn calc*
0.45533
ofxn mod
0.48399

sRun
RunA1
zout
1

Ave
Data
0.00127
Model
0.00116

data/vMax
0.0039

ofxng
311.734

Min d/m
2.76189

ave/min d
16.4507
ave/min m
41.4258

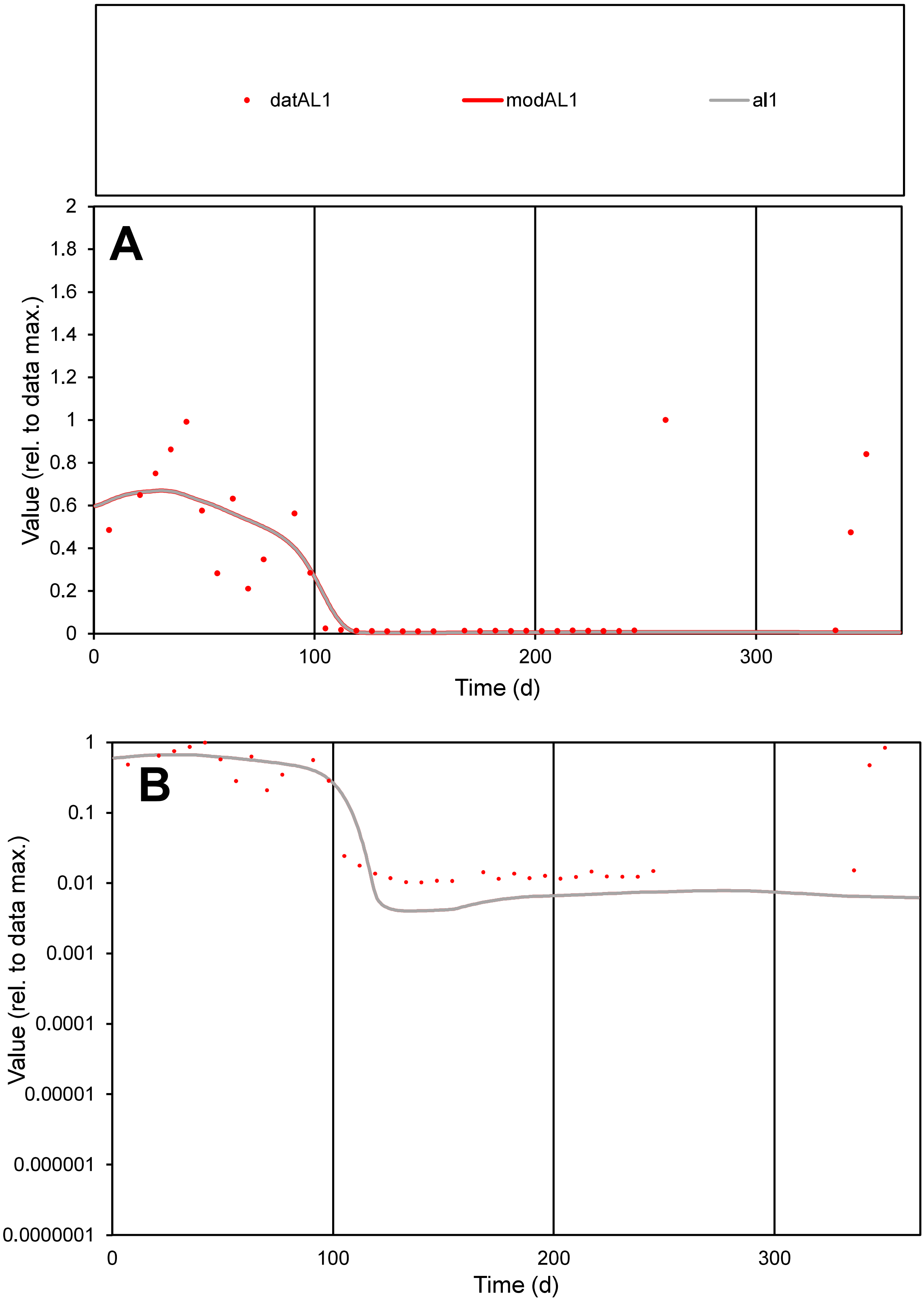

C1

Variables
AL1 Provide jtd or Dst
CalibOnly?
2 1 to reproduce of
oNormalize?
2 1=no, 2=yes

ofxn calc*
0.99717
ofxn mod
1.01145

sRun
RunA1
zout
1

Ave
Data
0.00109
Model
0.00082

data/vMax
0.00425

ofxng
311.734

Min d/m
2.53945

ave/min d
25.0421
ave/min m
48.1426

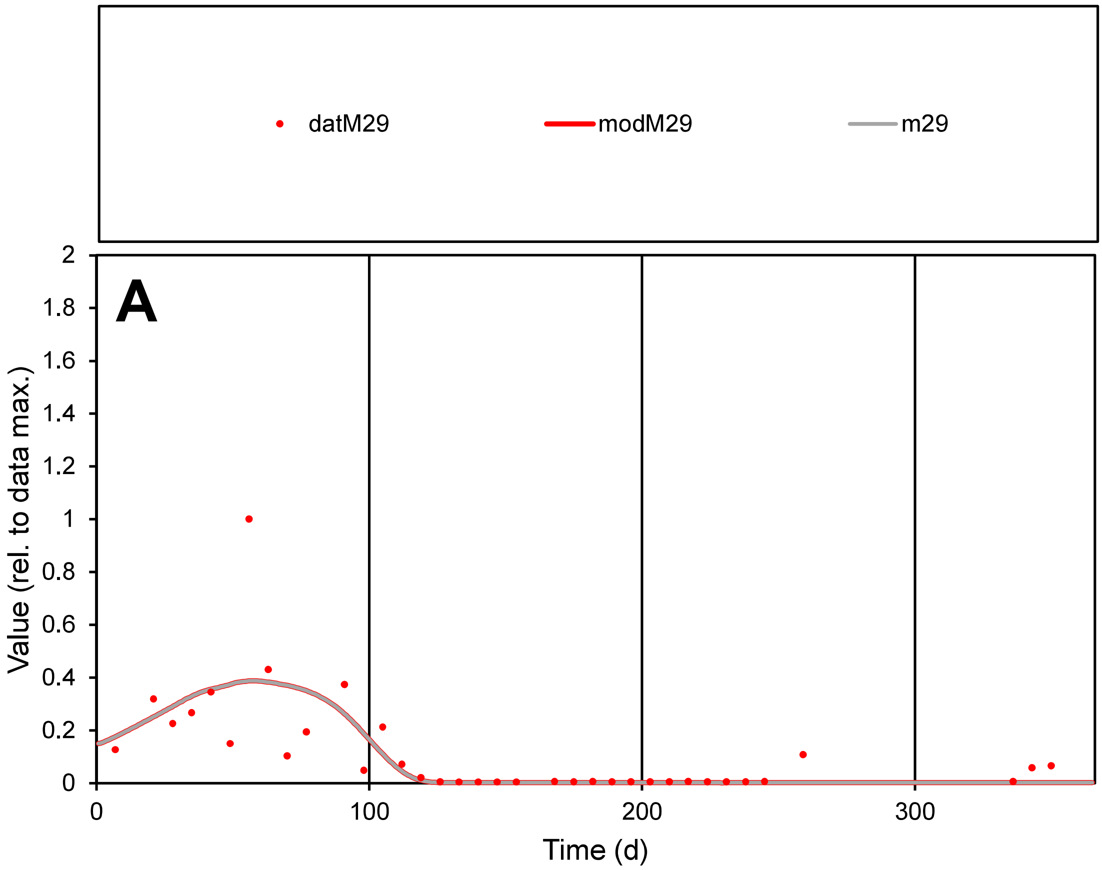

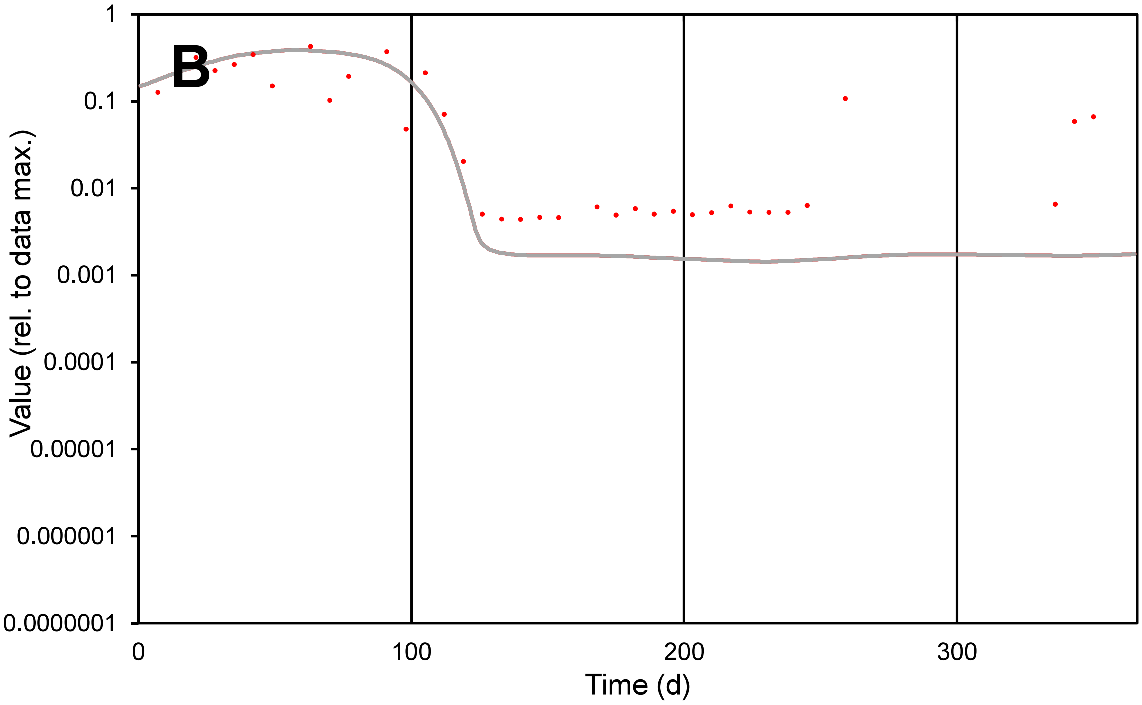

C1

Variables
M29 Provide jtd or Dst
CalibOnly?
2 1 to reproduce of
oNormalize?
2 1=no, 2=yes

ofxn calc*
1.10072
ofxn mod
1.12539

sRun
RunA1
zout
1

Ave
Data
0.00144
Model
0.00134

data/vMax
0.01229

ofxng
311.734

Min d/m
3.04291

ave/min d
26.6939
ave/min m
75.4503

331_Calib_S_A

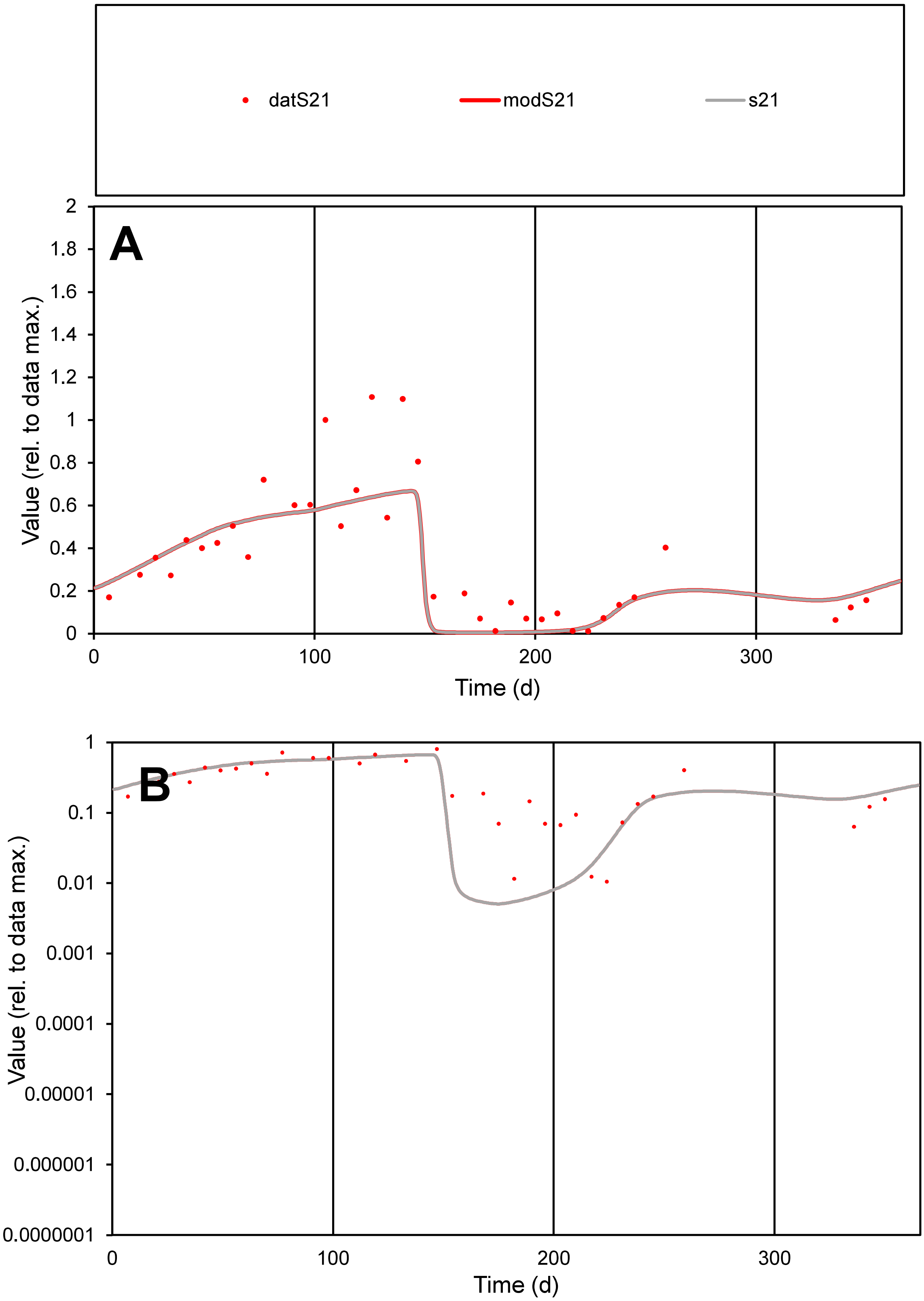

C1

Variables
S21 Provide jtd or Dst
CalibOnly?
2 1 to reproduce of
oNormalize?
2 1=no, 2=yes

ofxn calc*
0.36589
ofxn mod
0.39319

sRun
RunA1
zout
1

Ave
Data
0.00151
Model
0.00145

data/vMax
0.00469

ofxng
311.734

Min d/m
2.0648

ave/min d
30.5919
ave/min m
60.5826

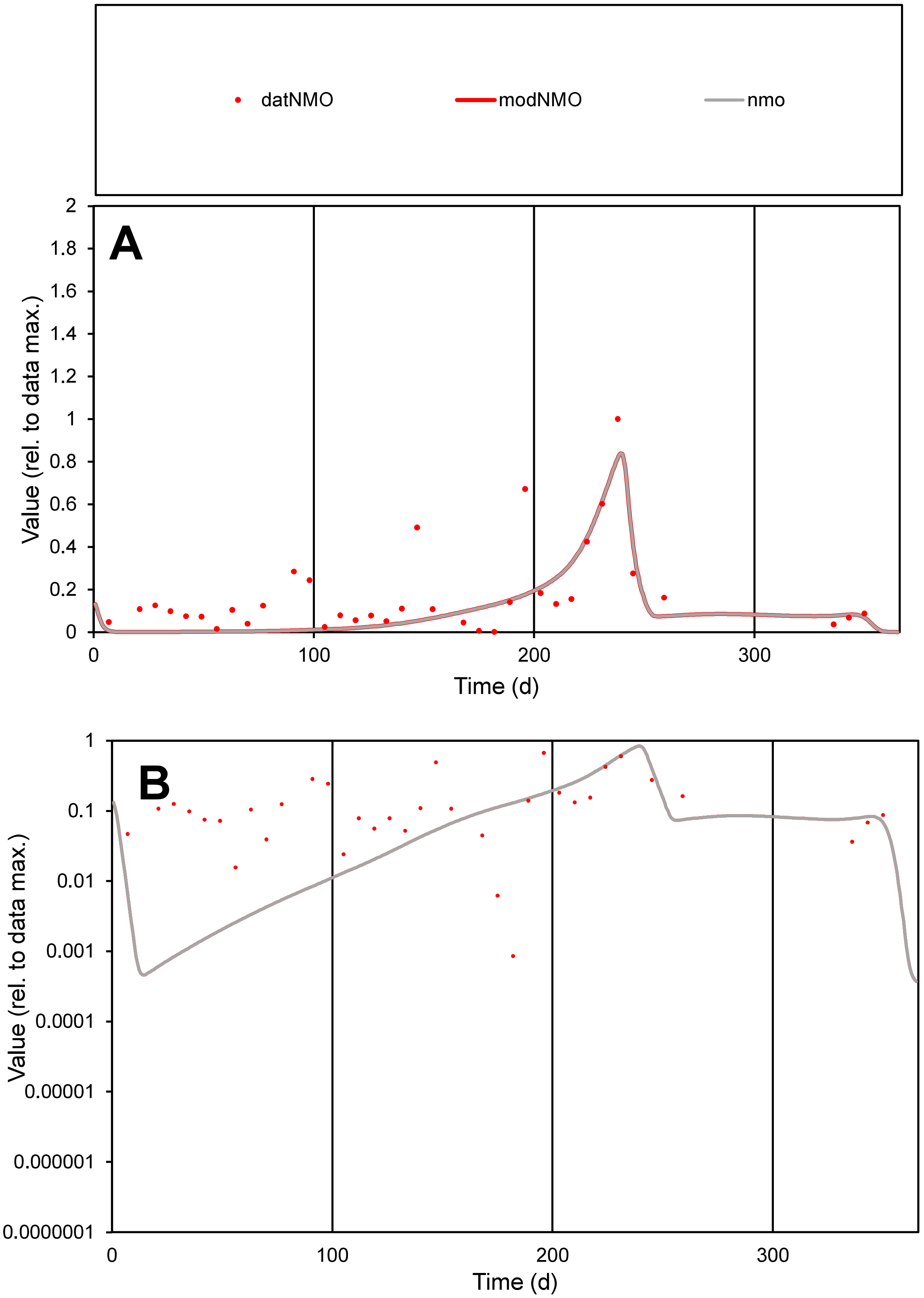

C1

Variables

NMO Provide jtd or Dst

CalibOnly?

2 1 to reproduce of

oNormalize?

2 1=no, 2=yes

ofxn calc*

0.8044

ofxn mod

0.8236

sRun

RunA1

zout

1

Ave

Data

0.00188

Model

0.00136

data/vMax

0.01111

ofxng

311.734

Min d/m

1.30862

ave/min d

197.745

ave/min m

187.772

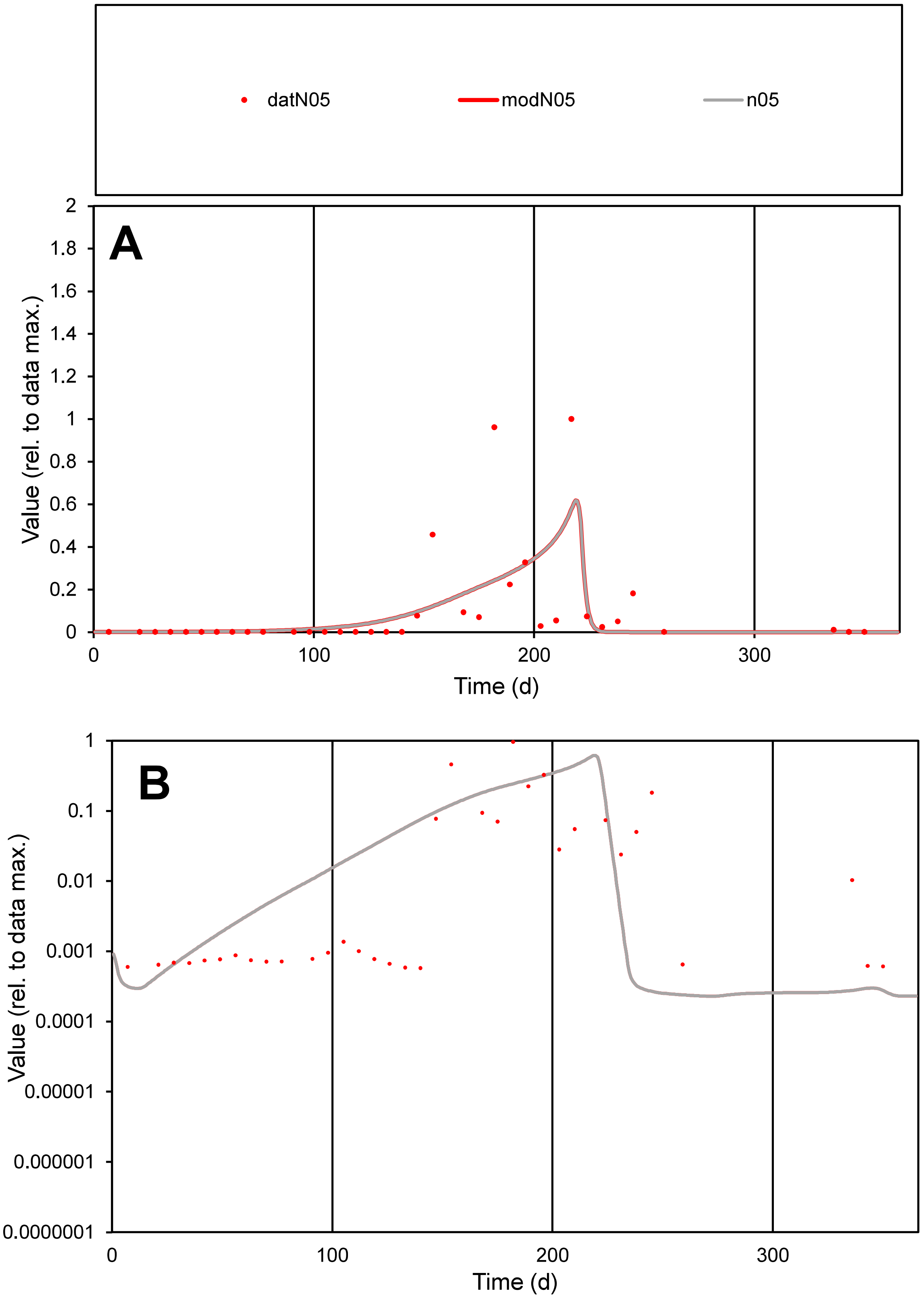

C1

Variables
N05 Provide jtd or Dst
CalibOnly?
2 1 to reproduce of
oNormalize?
2 1=no, 2=yes

ofxn calc*
1.76002
ofxn mod
1.77613

sRun
RunA1
zout
1

Ave
Data
0.00663
Model
0.0062

data/vMax
0.06794

ofxng
311.734

Min d/m
2.43457

ave/min d
168.11
ave/min m
382.415

331_Calib_S_A

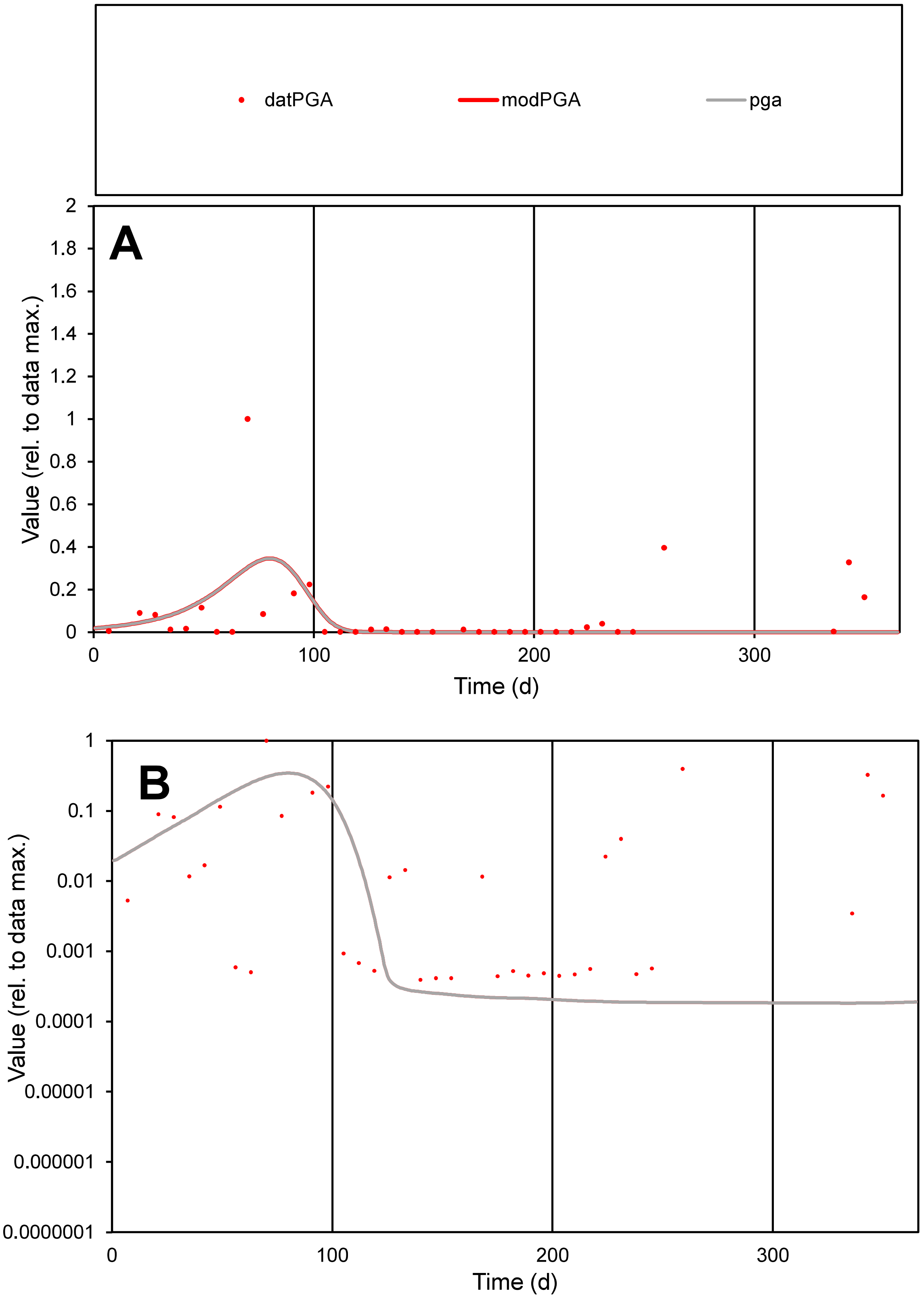

C1

Variables
PGA Provide jtd or Dst
CalibOnly?
2 1 to reproduce of
oNormalize?
2 1=no, 2=yes

ofxn calc*
2.09592
ofxn mod
2.11071

sRun
RunA1
zout
1

Ave
Data
0.00334
Model
0.00245

data/vMax
0.04285

ofxng
311.734

Min d/m
2.14431

ave/min d
197.954
ave/min m
312.022

331_Calib_S_A

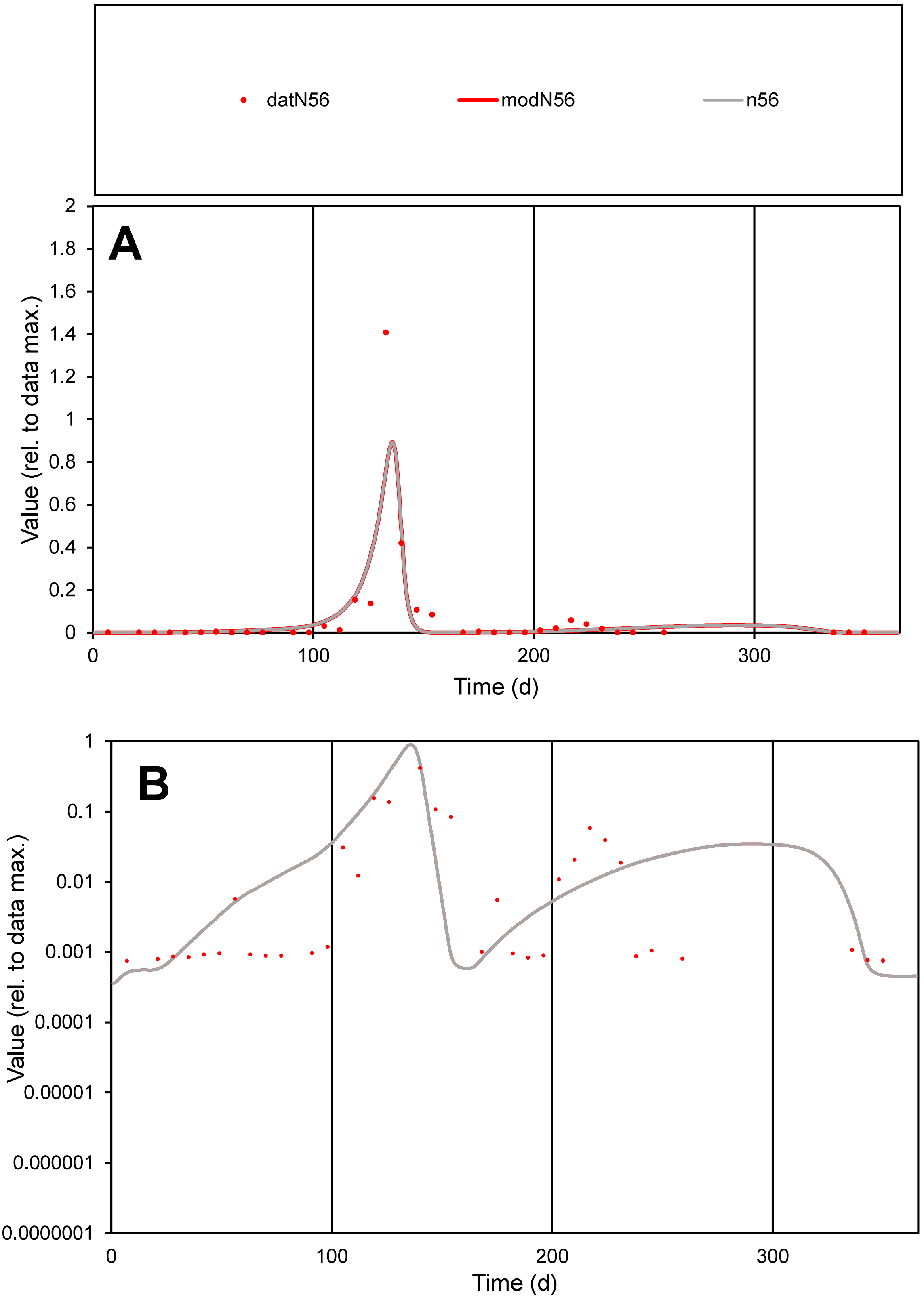

C1

Variables

N56 Provide jtd or Dst

CalibOnly?

2 1 to reproduce of

oNormalize?

2 1=no, 2=yes

ofxn calc*

1.01532

ofxn mod

1.16146

sRun

RunA1

zout

1

Ave

Data

0.00241

Model

0.00275

data/vMax

0.0468

ofxng

311.734

Min d/m

1.62843

ave/min d

68.7216

ave/min m

127.636

331_Calib_S_A

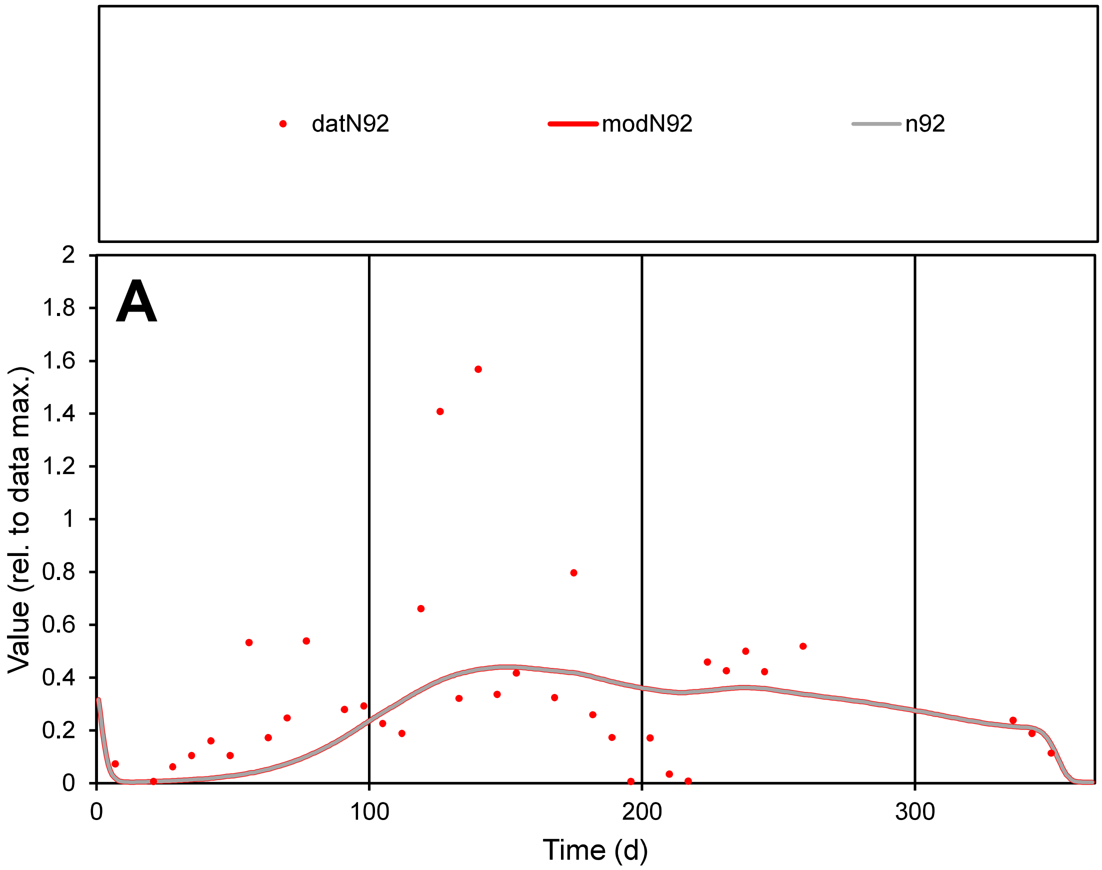

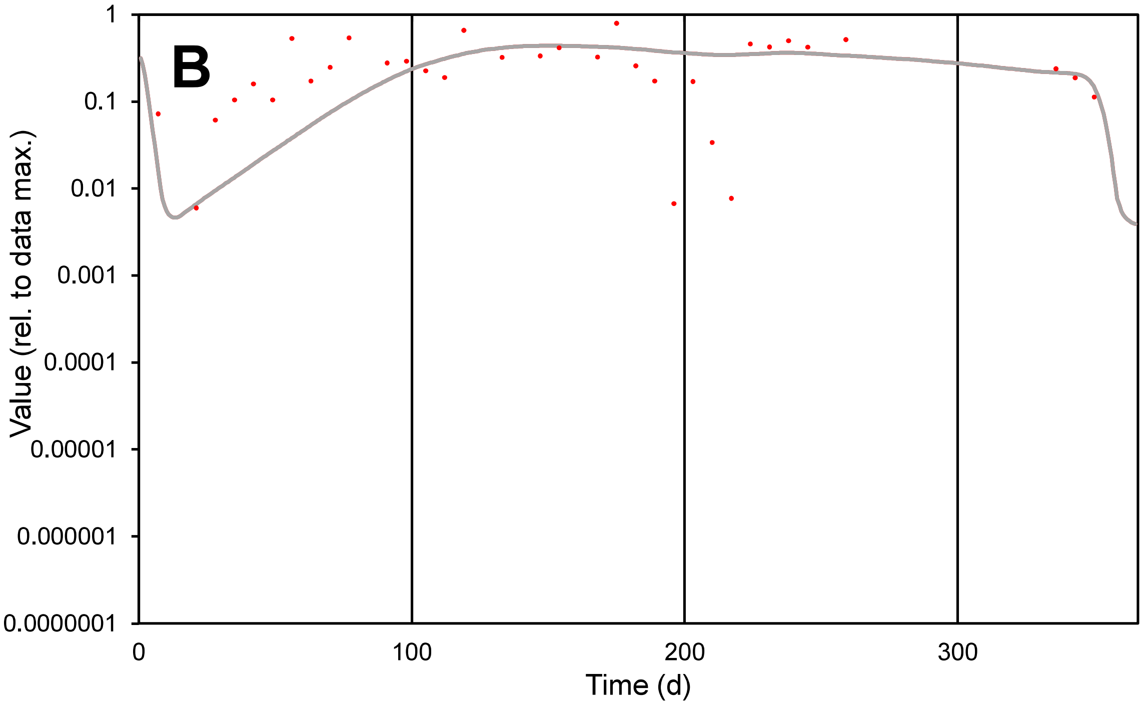

C1

Variables
N92 Provide jtd or Dst
CalibOnly?
2 1 to reproduce of
oNormalize?
2 1=no, 2=yes

ofxn calc*
0.76656
ofxn mod
0.77507

sRun
RunA1
zout
1

Ave
Data
0.00212
Model
0.00179

data/vMax
0.00696

ofxng
311.734

Min d/m
0.8357

ave/min d
50.778
ave/min m
35.8213

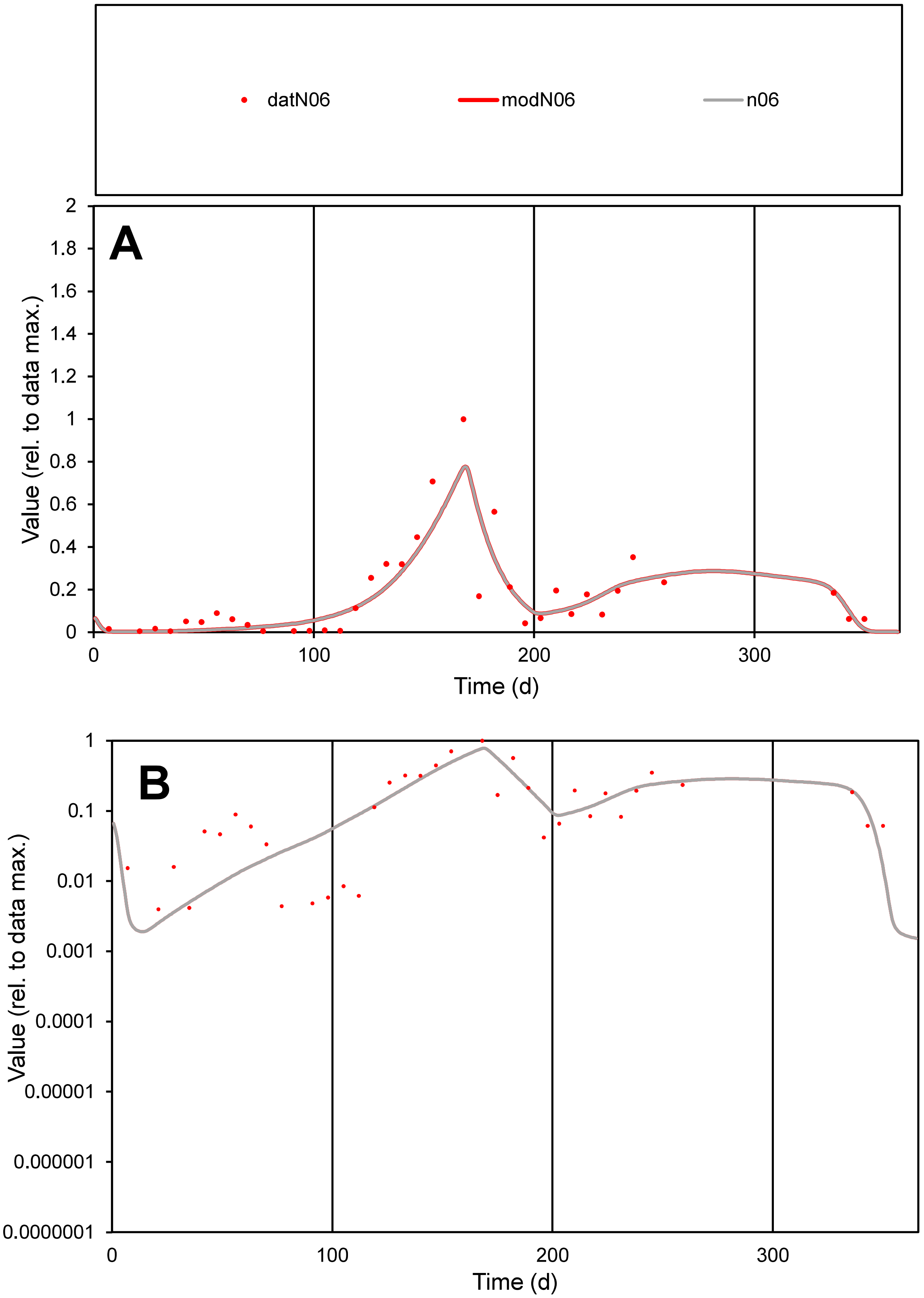

C1

Variables
N06 Provide jtd or Dst
CalibOnly?
2 1 to reproduce of
oNormalize?
2 1=no, 2=yes

ofxn calc*
0.57013
ofxn mod
0.6195

sRun
RunA1
zout
1

Ave
Data
0.00278
Model
0.0028

data/vMax
0.01796

ofxng
311.734

Min d/m
1.44753

ave/min d
39.0394
ave/min m
56.794

331_Calib_S_A

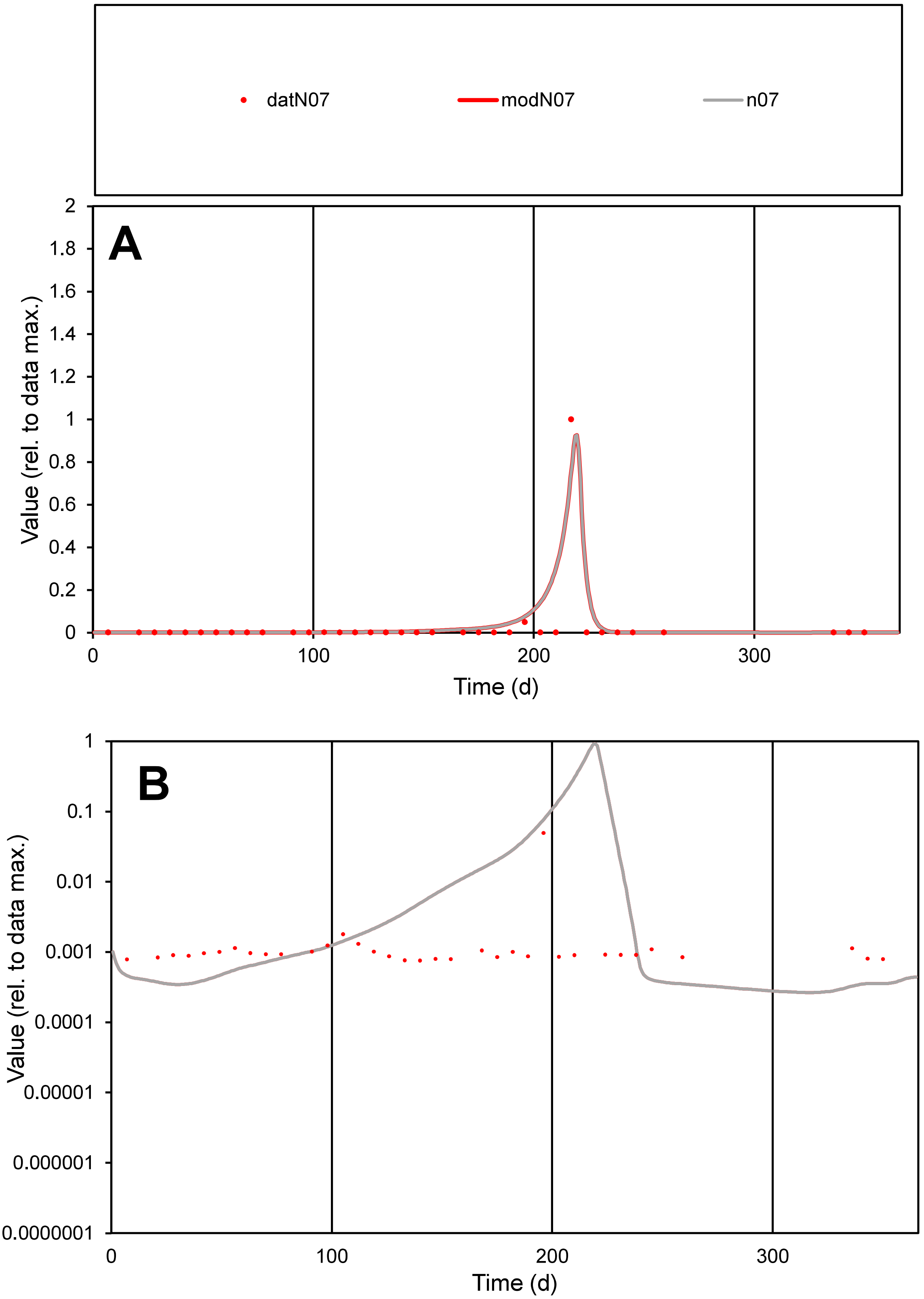

C1

Variables
N07 Provide jtd or Dst
CalibOnly?
2 1 to reproduce of
oNormalize?
2 1=no, 2=yes

ofxn calc*
1.64793
ofxn mod
2.02995

sRun
RunA1
zout
1

Ave
Data
0.00067
Model
0.00094

data/vMax
0.02228

ofxng
311.734

Min d/m
2.25902

ave/min d
39.7409
ave/min m
126.003

331_Calib_S_A

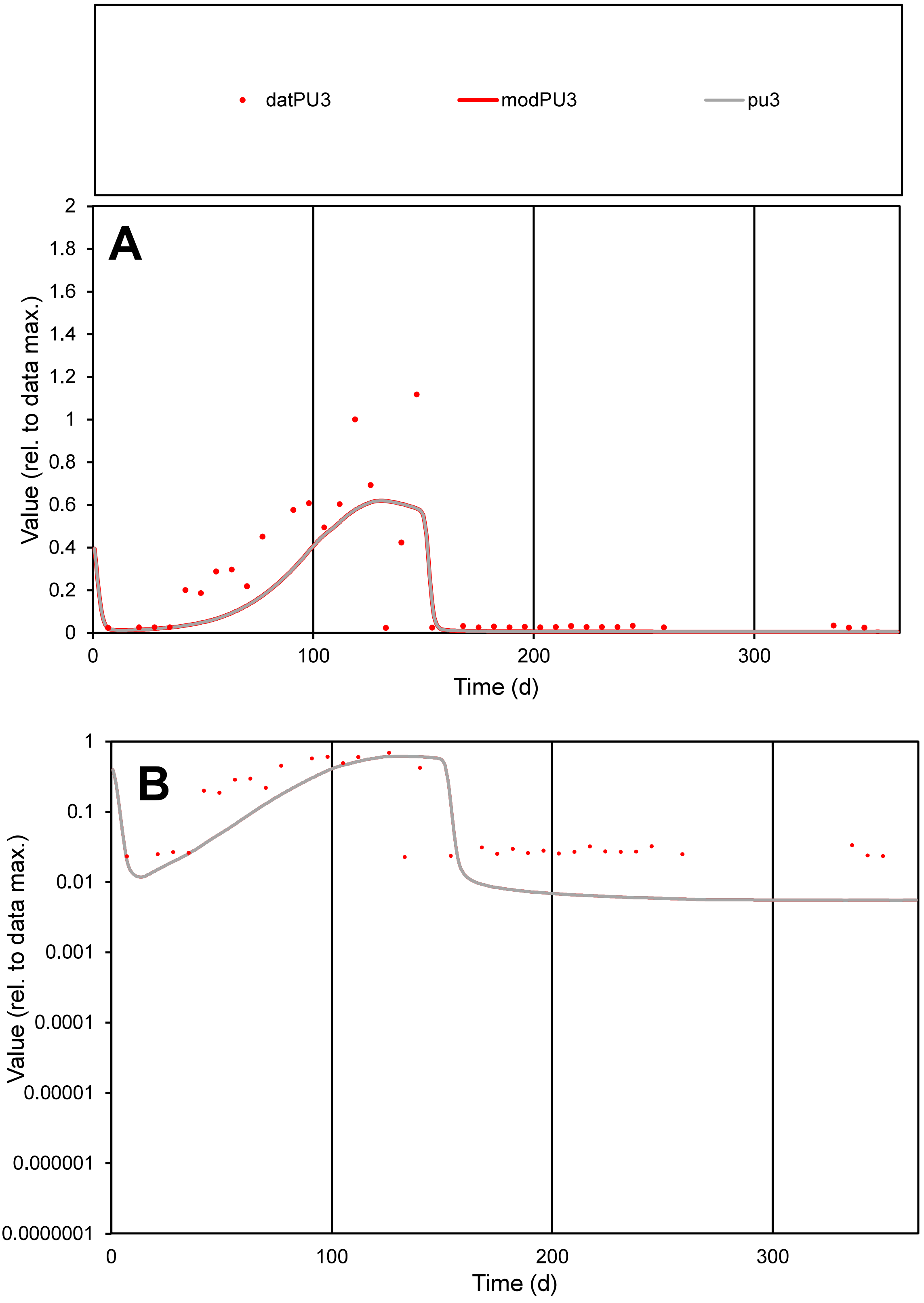

C1

Variables
PU3 Provide jtd or Dst
CalibOnly?
2 1 to reproduce of
oNormalize?
2 1=no, 2=yes

ofxn calc*
0.87484
ofxn mod
0.88681

sRun
RunA1
zout
1

Ave
Data
0.0013
Model
0.00102

data/vMax
0.00664

ofxng
311.734

Min d/m
4.08424

ave/min d
8.68023
ave/min m
27.7435

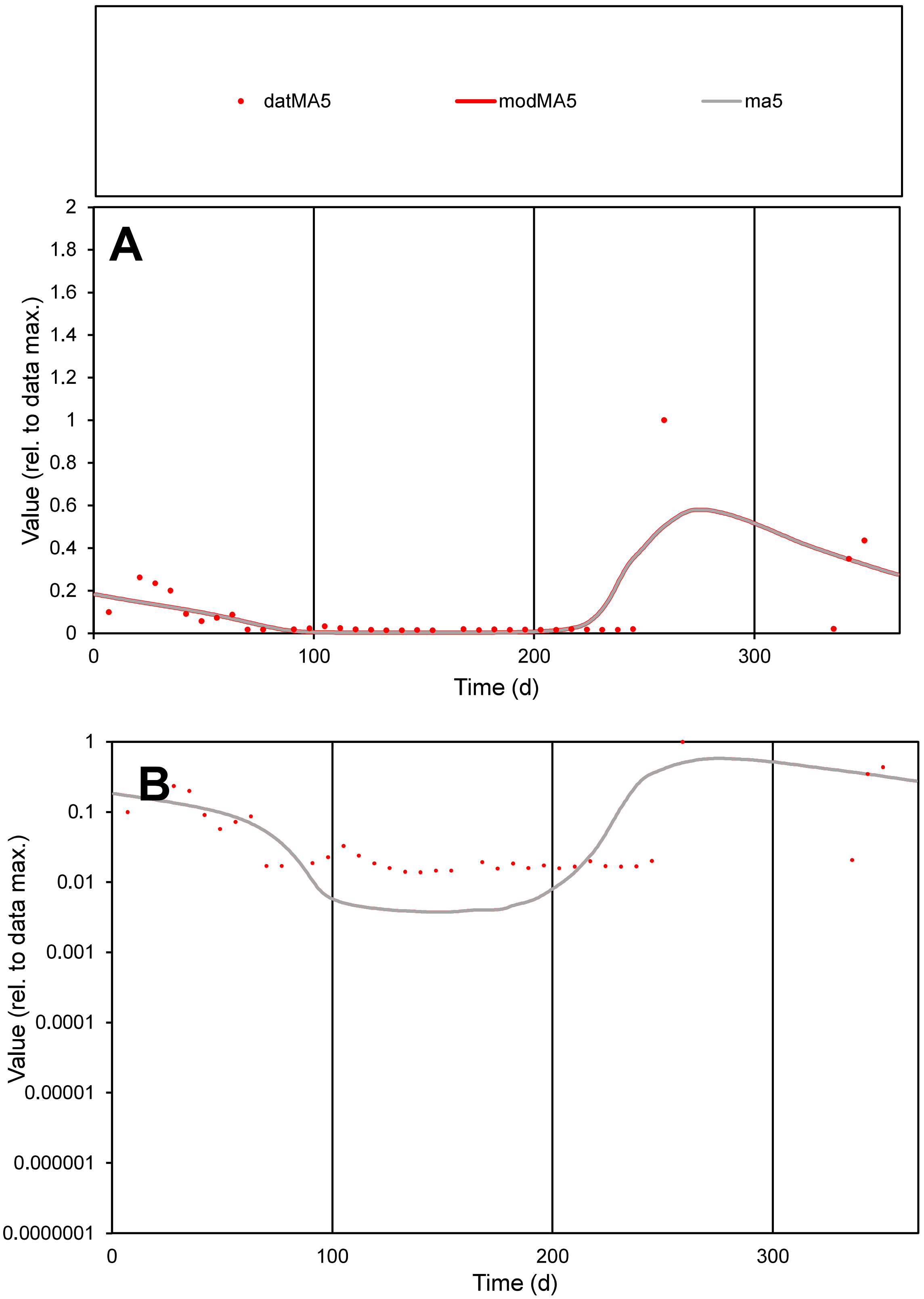

C1

Variables
MA5 Provide jtd or Dst
CalibOnly?
2 1 to reproduce of
oNormalize?
2 1=no, 2=yes

ofxn calc*
1.35283
ofxn mod
1.38036

sRun
RunA1
zout
1

Ave
Data
0.00062
Model
0.00063

data/vMax
0.00669

ofxng
311.734

Min d/m
3.71195

ave/min d
6.68737
ave/min m
25.0699

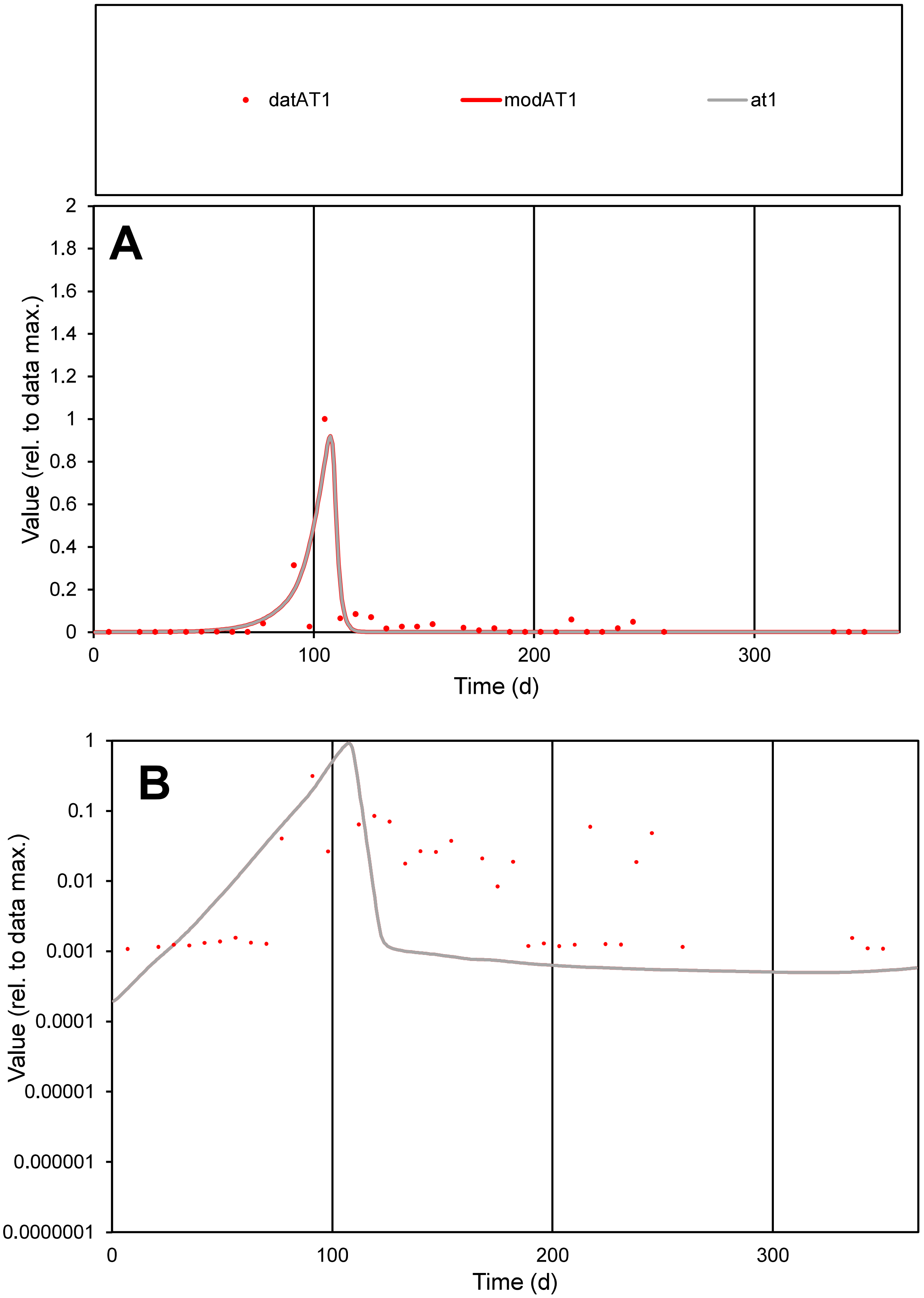

C1

Variables
AT1 Provide jtd or Dst
CalibOnly?
2 1 to reproduce of
oNormalize?
2 1=no, 2=yes

ofxn calc*
1.18767
ofxn mod
1.36358

sRun
RunA1
zout
1

Ave
Data
0.00235
Model
0.00216

data/vMax
0.04552

ofxng
311.734

Min d/m
3.41765

ave/min d
47.6938
ave/min m
149.874

331_Calib_S_A

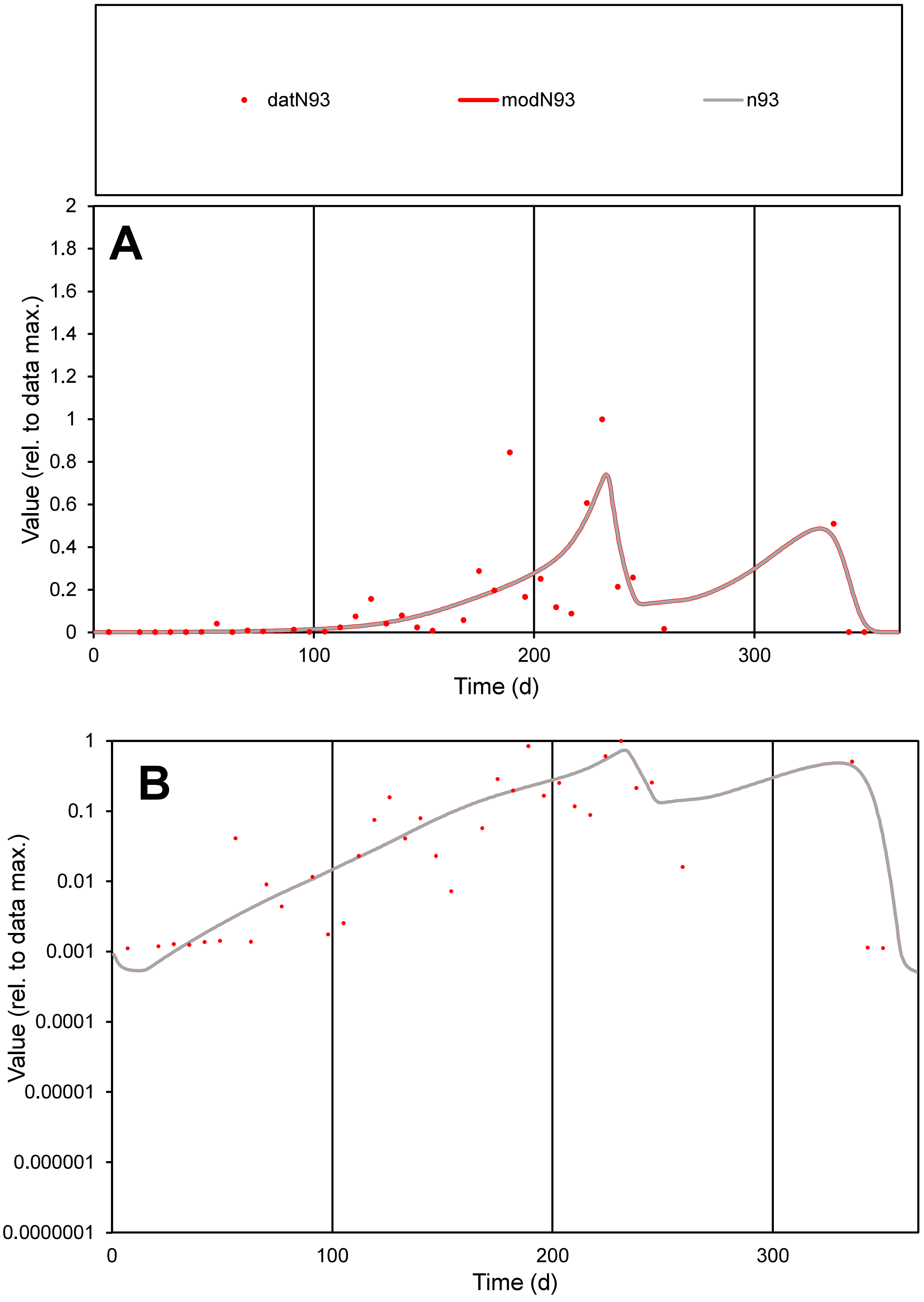

C1

Variables

N93 Provide jtd or Dst

CalibOnly?

2 1 to reproduce of

oNormalize?

2 1=no, 2=yes

ofxn calc*

1.03072

ofxn mod

1.07037

sRun

RunA1

zout

1

Ave

Data

0.00222

Model

0.00225

data/vMax

0.016

ofxng

311.734

Min d/m

2.03351

ave/min d

124.532

ave/min m

256.904

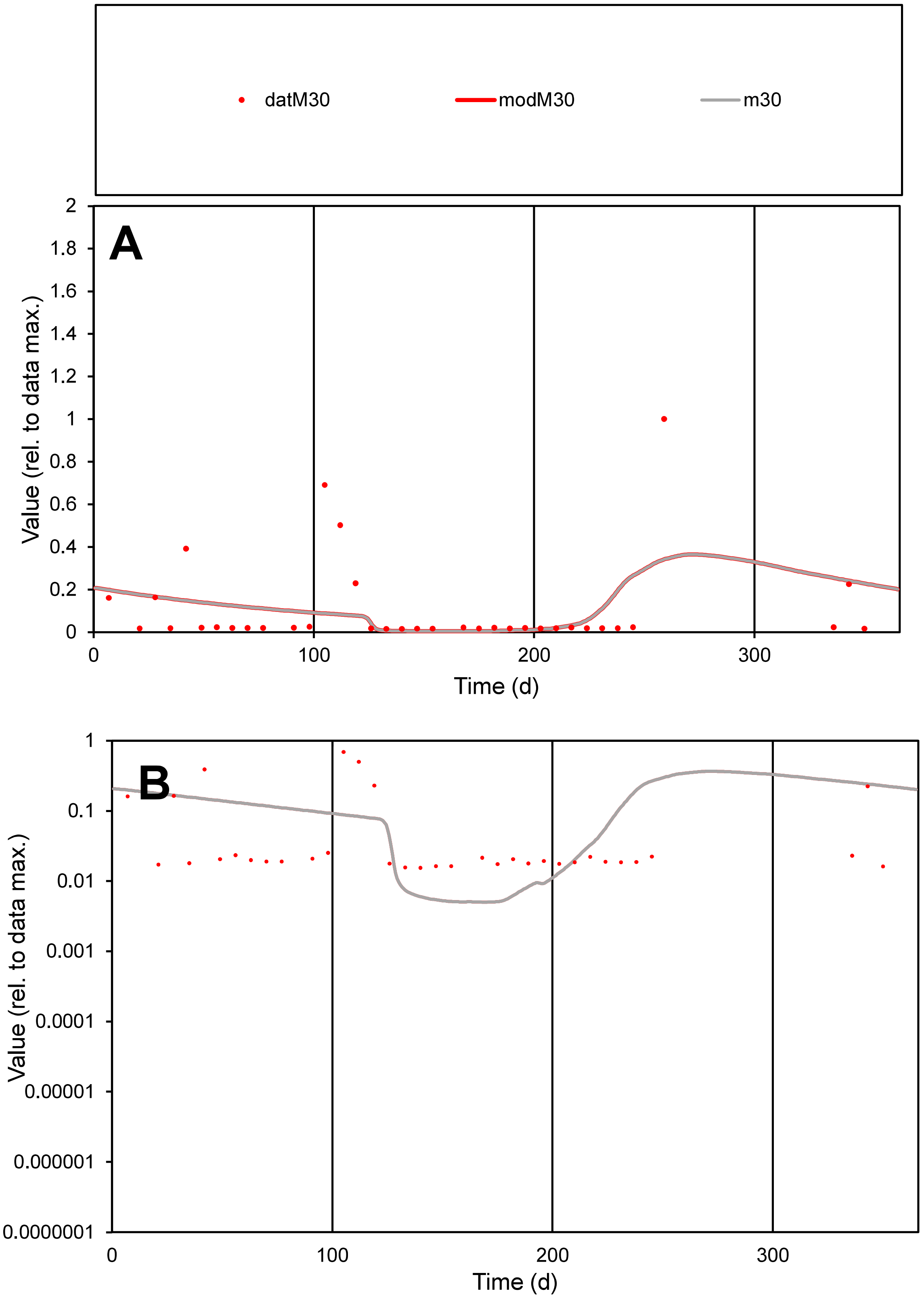

C1

Variables
M30 Provide jtd or Dst
CalibOnly?
2 1 to reproduce of
oNormalize?
2 1=no, 2=yes

ofxn calc*
1.78883
ofxn mod
1.80193

sRun
RunA1
zout
1

Ave
Data
0.00028
Model
0.00027

data/vMax
0.00262

ofxng
311.734

Min d/m
3.09793

ave/min d
7.00123
ave/min m
20.6144

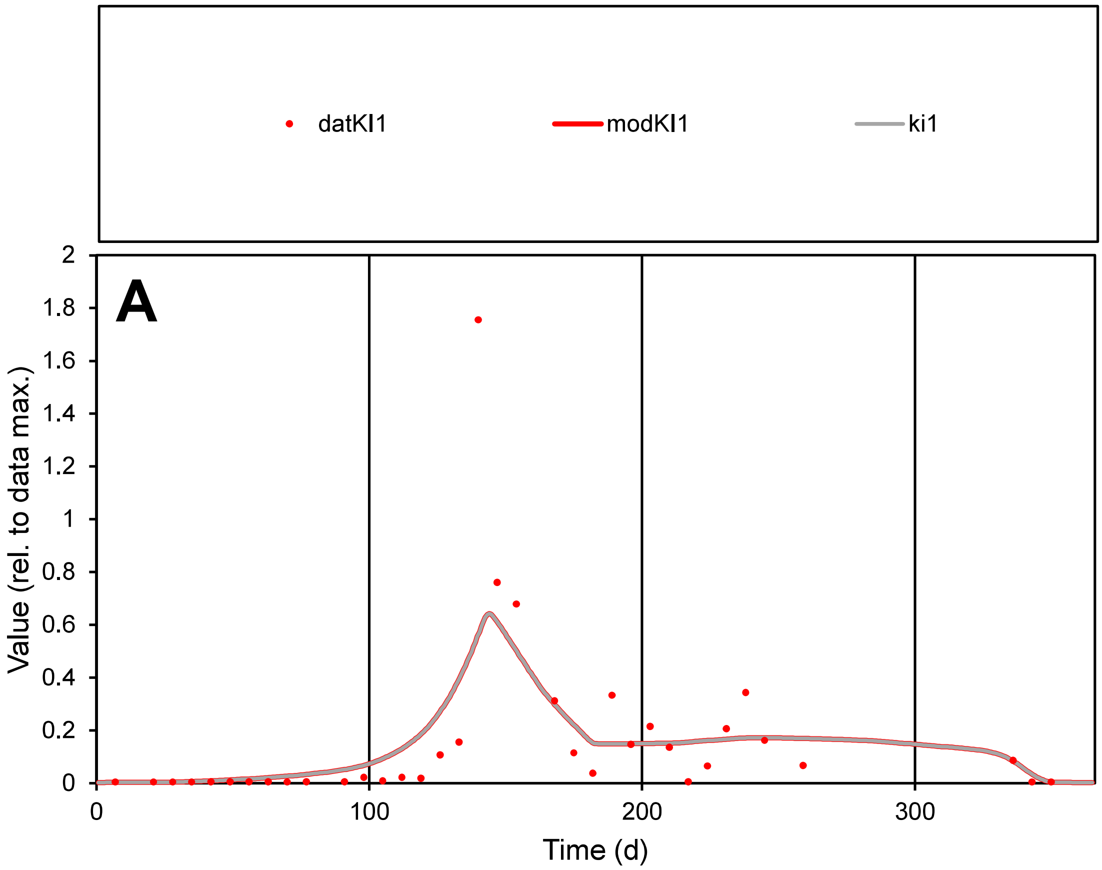

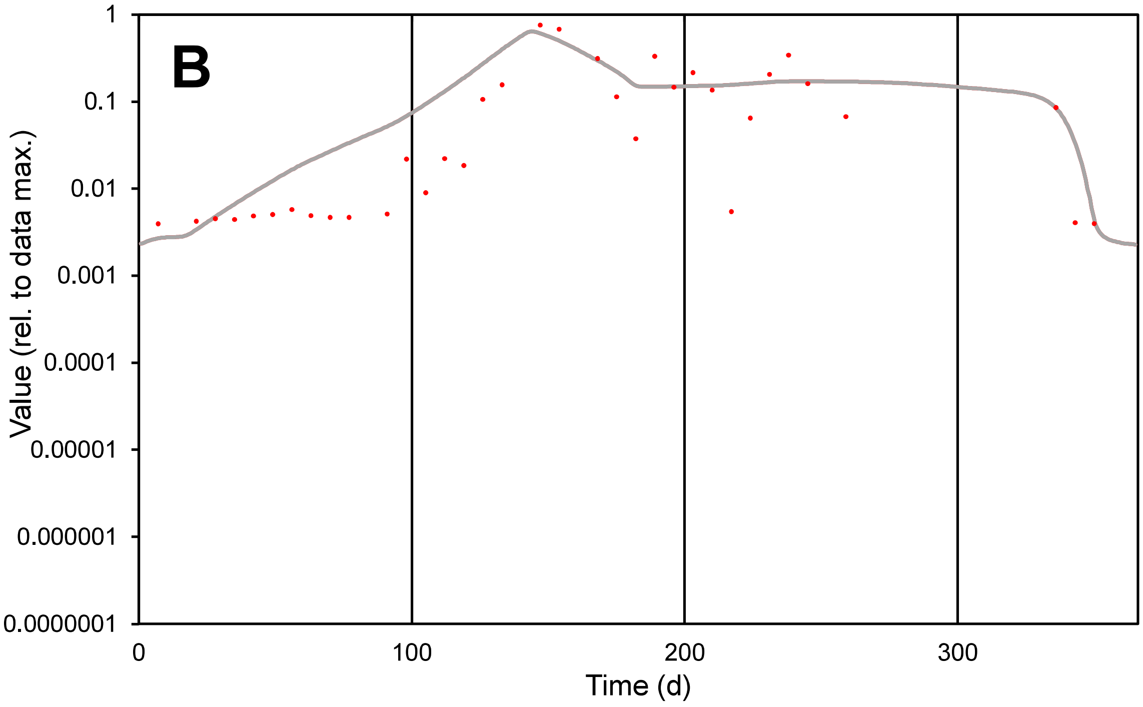

C1

Variables
KI1 Provide jtd or Dst
CalibOnly?
2 1 to reproduce of
oNormalize?
2 1=no, 2=yes

ofxn calc*
0.89144
ofxn mod
0.92374

sRun
RunA1
zout
1

Ave
Data
0.00155
Model
0.00179

data/vMax
0.01214

ofxng
311.734

Min d/m
1.45659

ave/min d
32.4057
ave/min m
54.4064

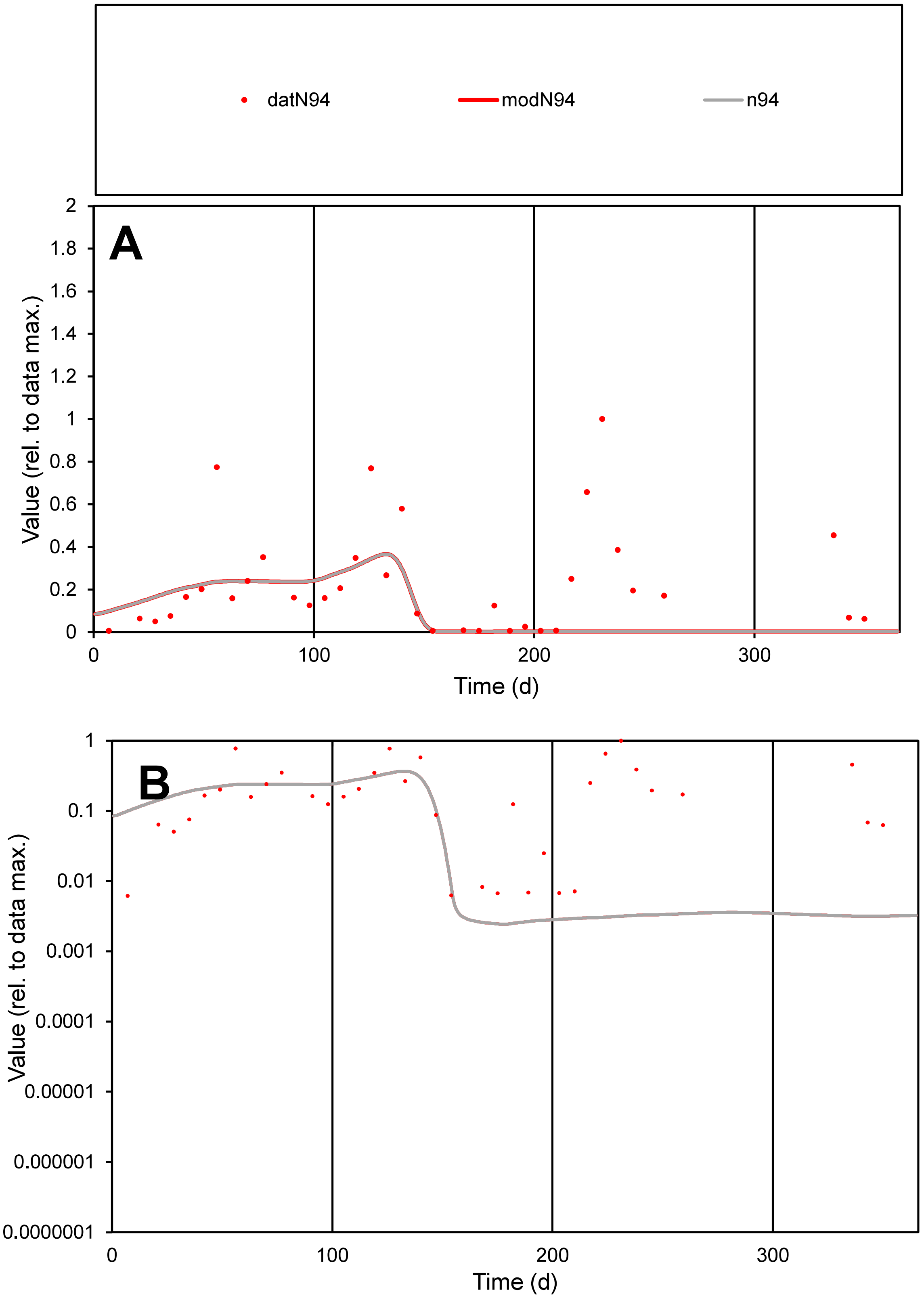

C1

Variables
N94 Provide jtd or Dst
CalibOnly?
2 1 to reproduce of
oNormalize?
2 1=no, 2=yes

ofxn calc*
1.20936
ofxn mod
1.21615

sRun
RunA1
zout
1

Ave
Data
0.0016
Model
0.00093

data/vMax
0.00752

ofxng
311.734

Min d/m
2.53428

ave/min d
34.3515
ave/min m
50.585

331_Calib_S_A

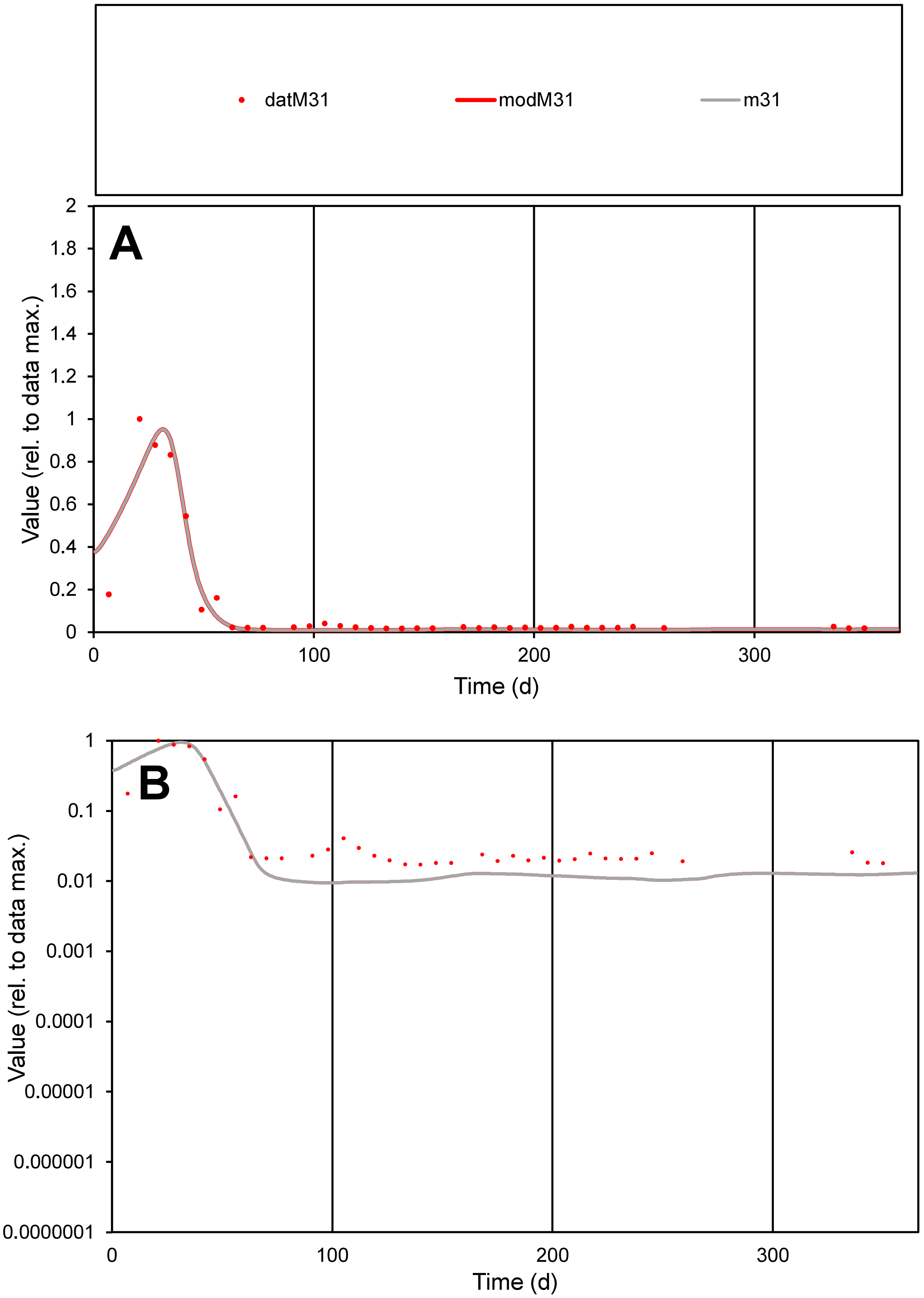

C1

Variables
M31 Provide jtd or Dst
CalibOnly?
2 1 to reproduce of
oNormalize?
2 1=no, 2=yes

ofxn calc*
0.53577
ofxn mod
0.6085

sRun
RunA1
zout
1

Ave
Data
0.00056
Model
0.00053

data/vMax
0.00467

ofxng
311.734

Min d/m
1.82362

ave/min d
7.004
ave/min m
12.1235

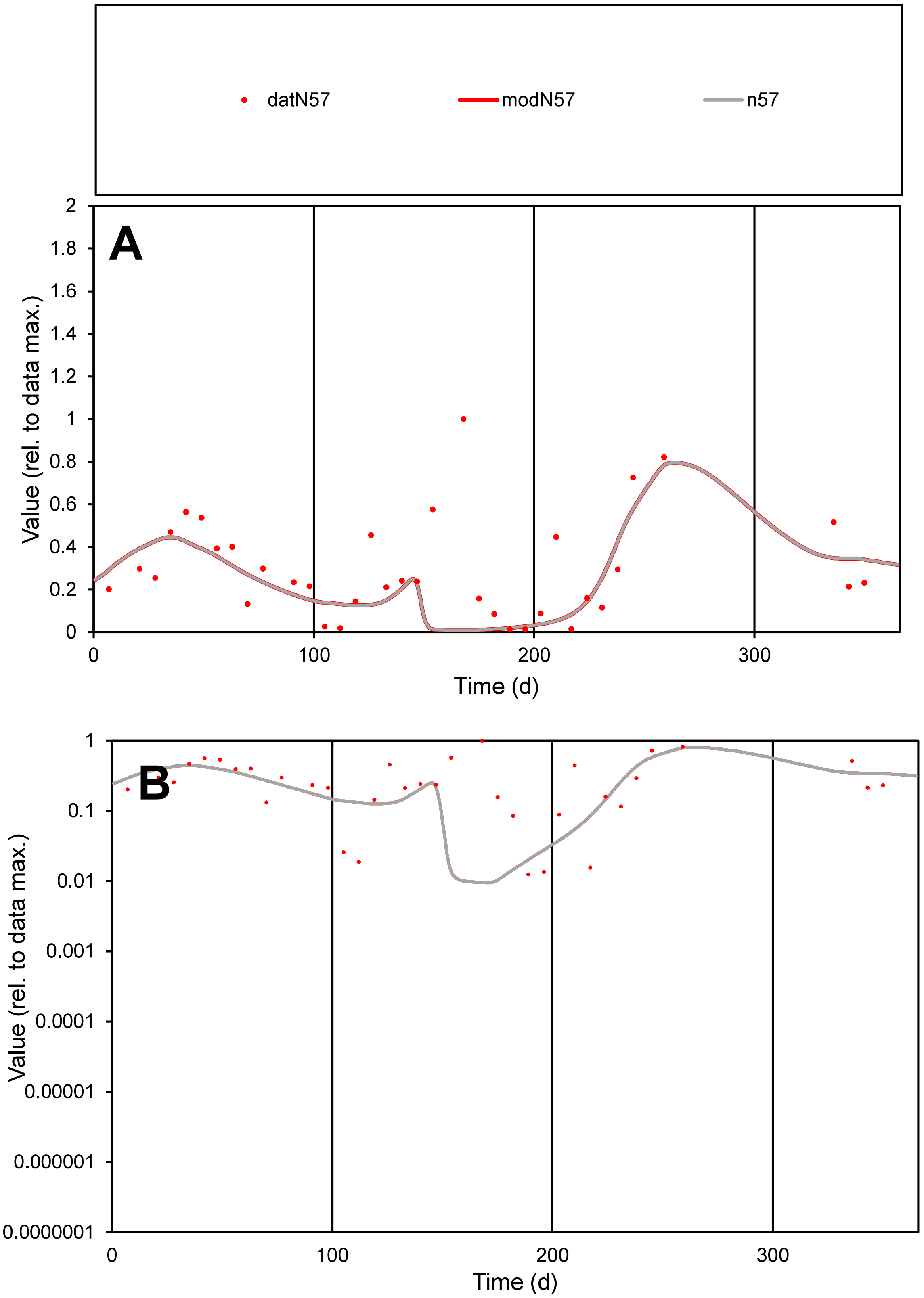

C1

Variables
N57 Provide jtd or Dst
CalibOnly?
2 1 to reproduce of
oNormalize?
2 1=no, 2=yes

ofxn calc*
0.75114
ofxn mod
0.76347

sRun
RunA1
zout
1

Ave
Data
0.00094
Model
0.00077

data/vMax
0.00329

ofxng
311.734

Min d/m
1.31386

ave/min d
22.8739
ave/min m
24.7198

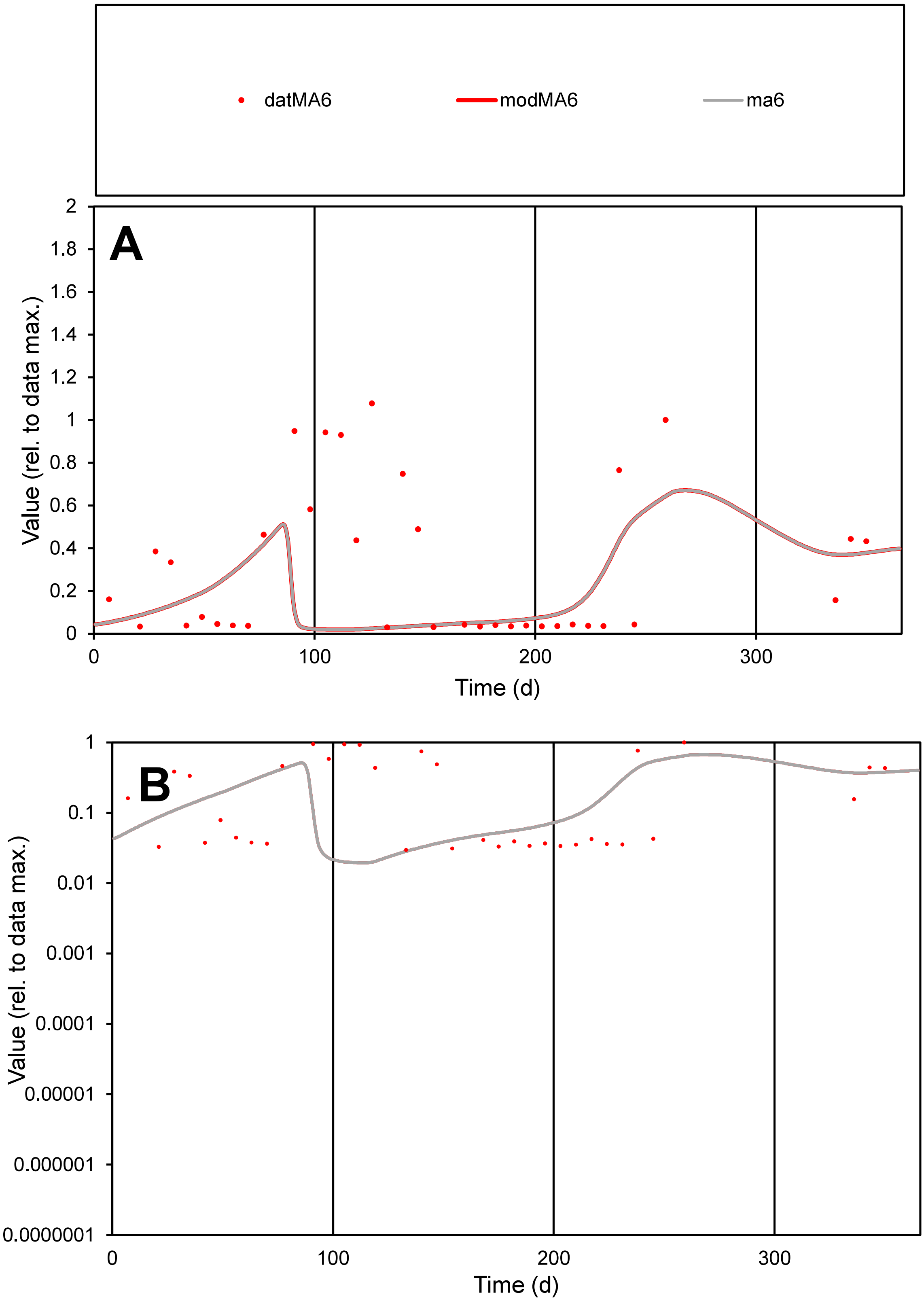

C1

Variables
MA6 Provide jtd or Dst
CalibOnly?
2 1 to reproduce of
oNormalize?
2 1=no, 2=yes

ofxn calc*
1.1986
ofxn mod
1.25316

sRun
RunA1
zout
1

Ave
Data
0.00113
Model
0.00072

data/vMax
0.00395

ofxng
311.734

Min d/m
1.53655

ave/min d
9.56384
ave/min m
9.38444

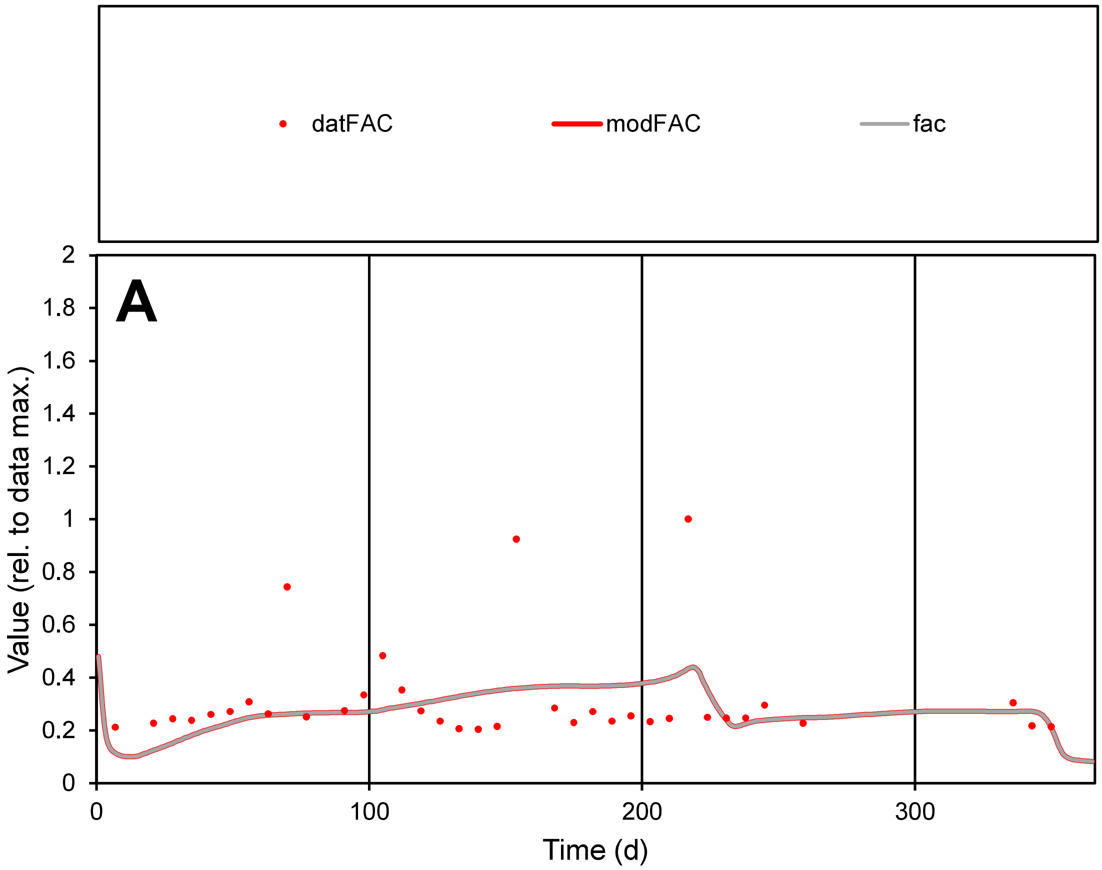

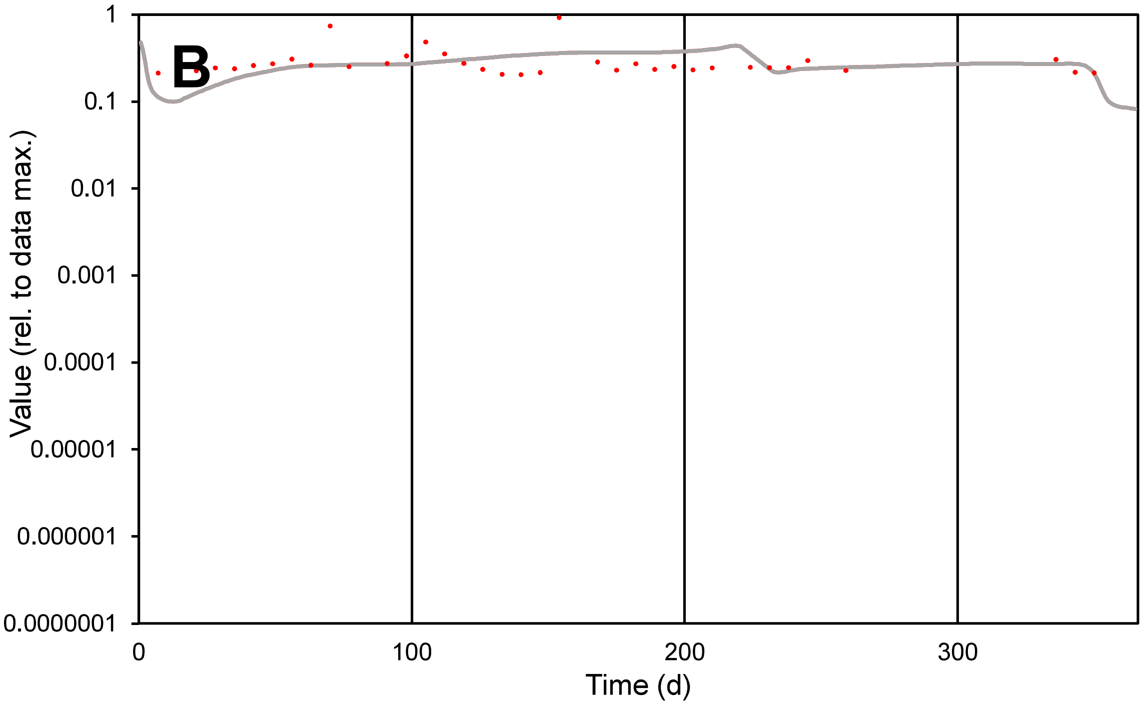

C1

Variables
FAC Provide jtd or Dst
CalibOnly?
2 1 to reproduce of
oNormalize?
2 1=no, 2=yes

ofxn calc*
0.52499
ofxn mod
0.53698

sRun
RunA1
zout
1

Ave
Data
0.00013
Model
0.00012

data/vMax
0.00042

ofxng
311.734

Min d/m
1.8843

ave/min d
1.50204
ave/min m
2.63005

331_Calib_S_A

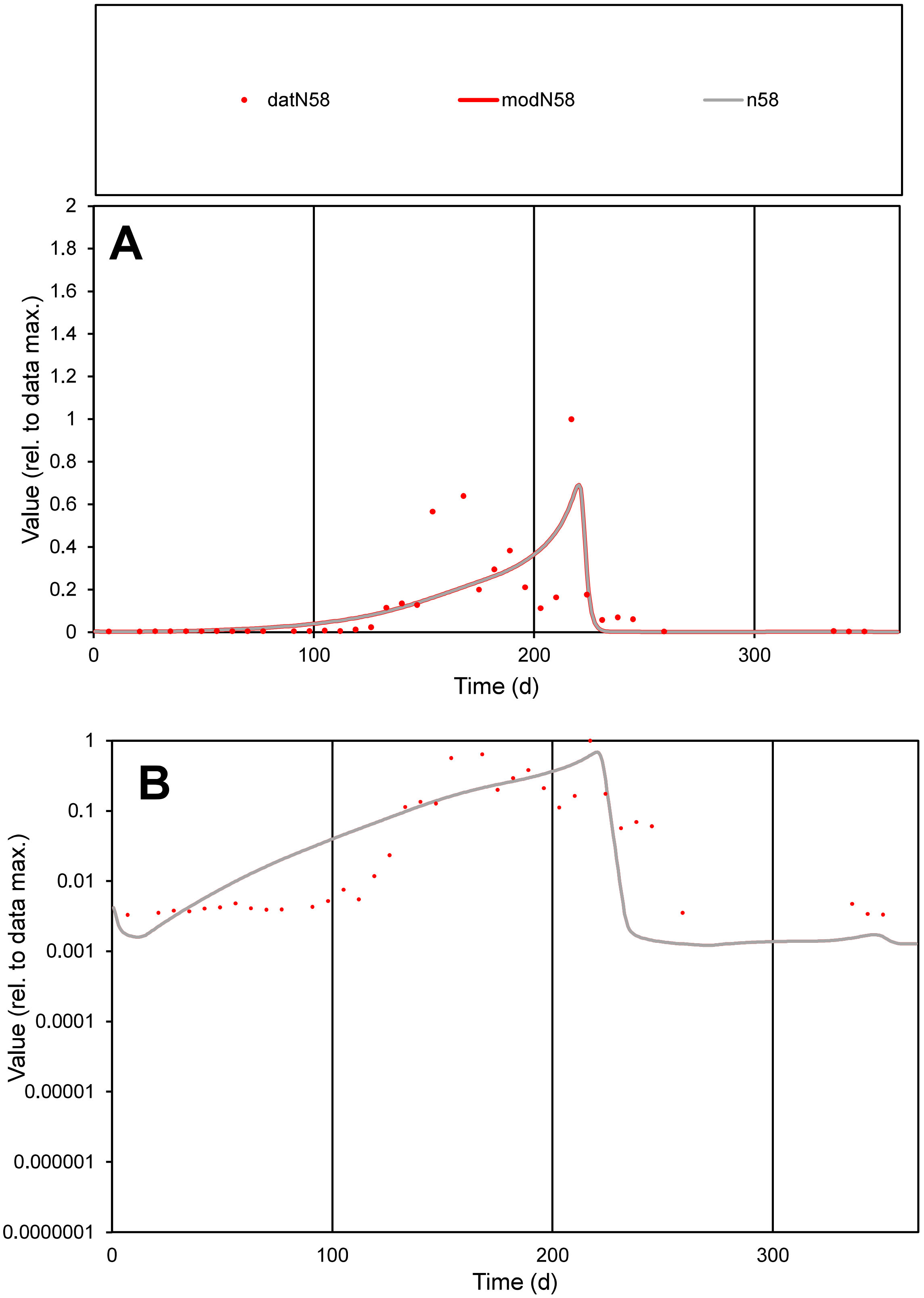

C1

Variables
N58 Provide jtd or Dst
CalibOnly?
2 1 to reproduce of
oNormalize?
2 1=no, 2=yes

ofxn calc*
1.11694
ofxn mod
1.13648

sRun
RunA1
zout
1

Ave
Data
0.00292
Model
0.00286

data/vMax
0.02537

ofxng
311.734

Min d/m
2.6155

ave/min d
34.7308
ave/min m
89.0931

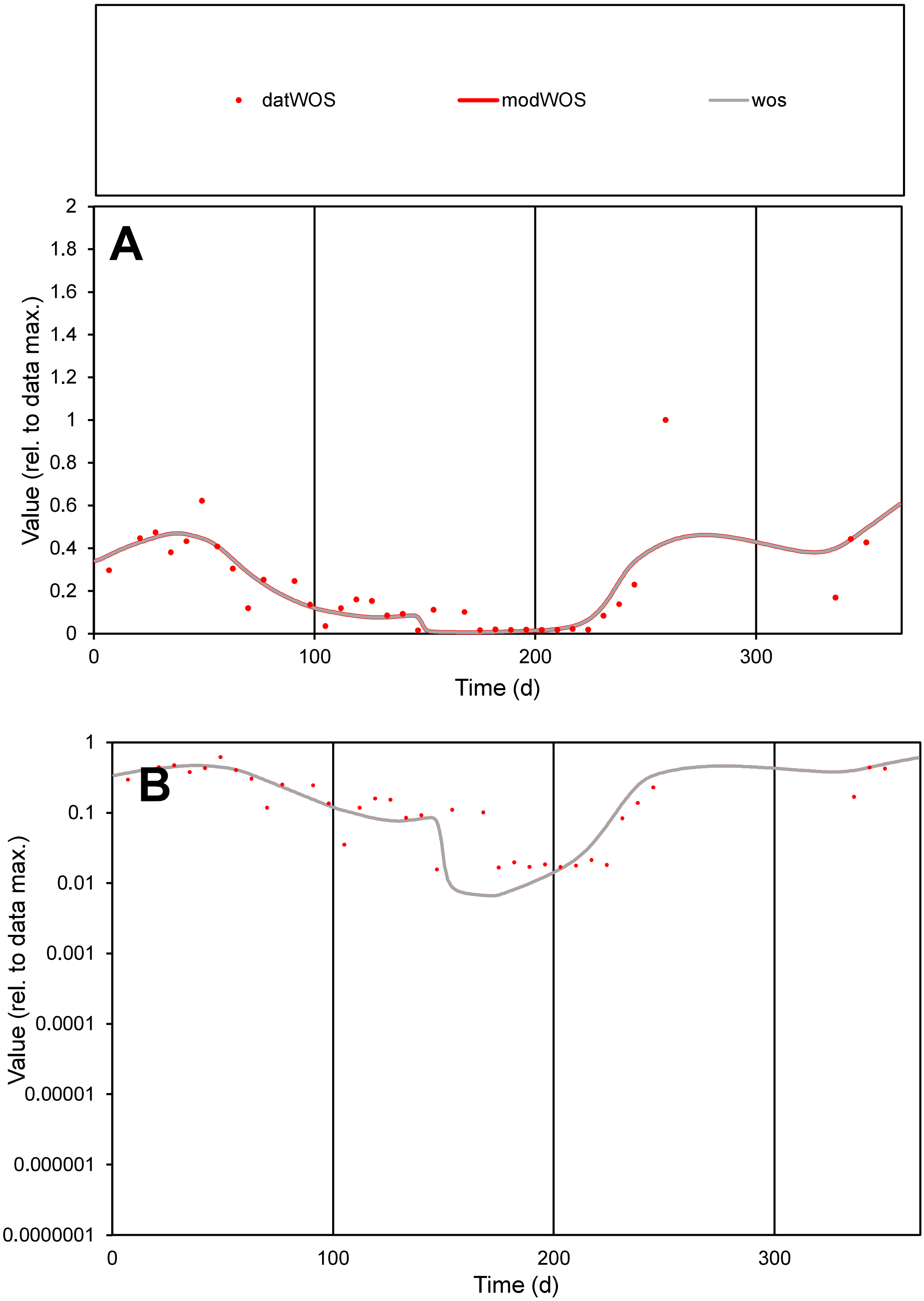

C1

Variables
WOS Provide jtd or Dst
CalibOnly?
2 1 to reproduce of
oNormalize?
2 1=no, 2=yes

ofxn calc*
0.5819
ofxn mod
0.60458

sRun
RunA1
zout
1

Ave
Data
0.00095
Model
0.00093

data/vMax
0.00455

ofxng
311.734

Min d/m
2.34618

ave/min d
13.2681
ave/min m
30.7879

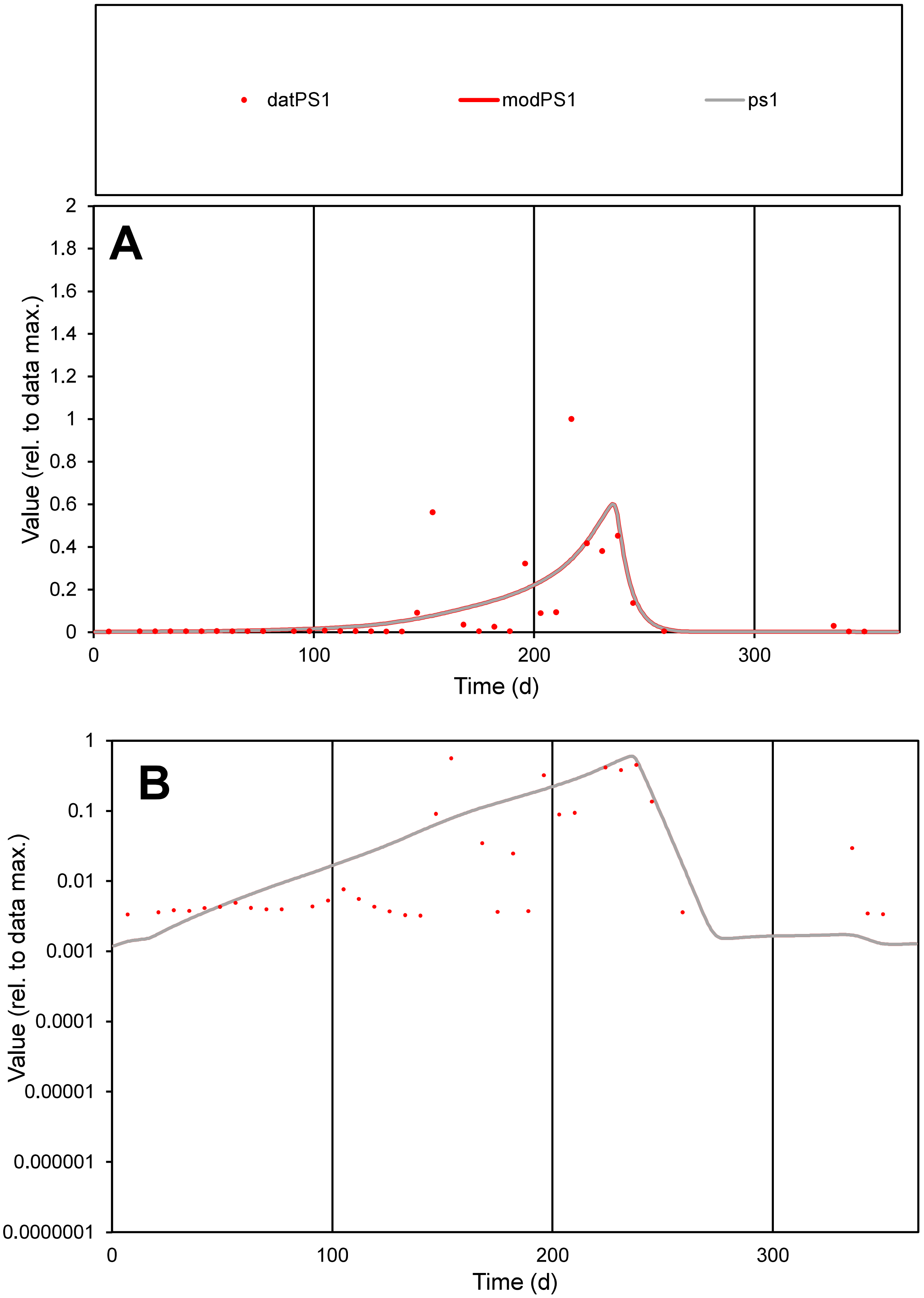

C1

Variables
PS1 Provide jtd or Dst
CalibOnly?
2 1 to reproduce of
oNormalize?
2 1=no, 2=yes

ofxn calc*
1.41025
ofxn mod
1.43473

sRun
RunA1
zout
1

Ave
Data
0.00142
Model
0.00146

data/vMax
0.01438

ofxng
311.734

Min d/m
2.55044

ave/min d
30.5509
ave/min m
80.2113

331_Calib_S_A

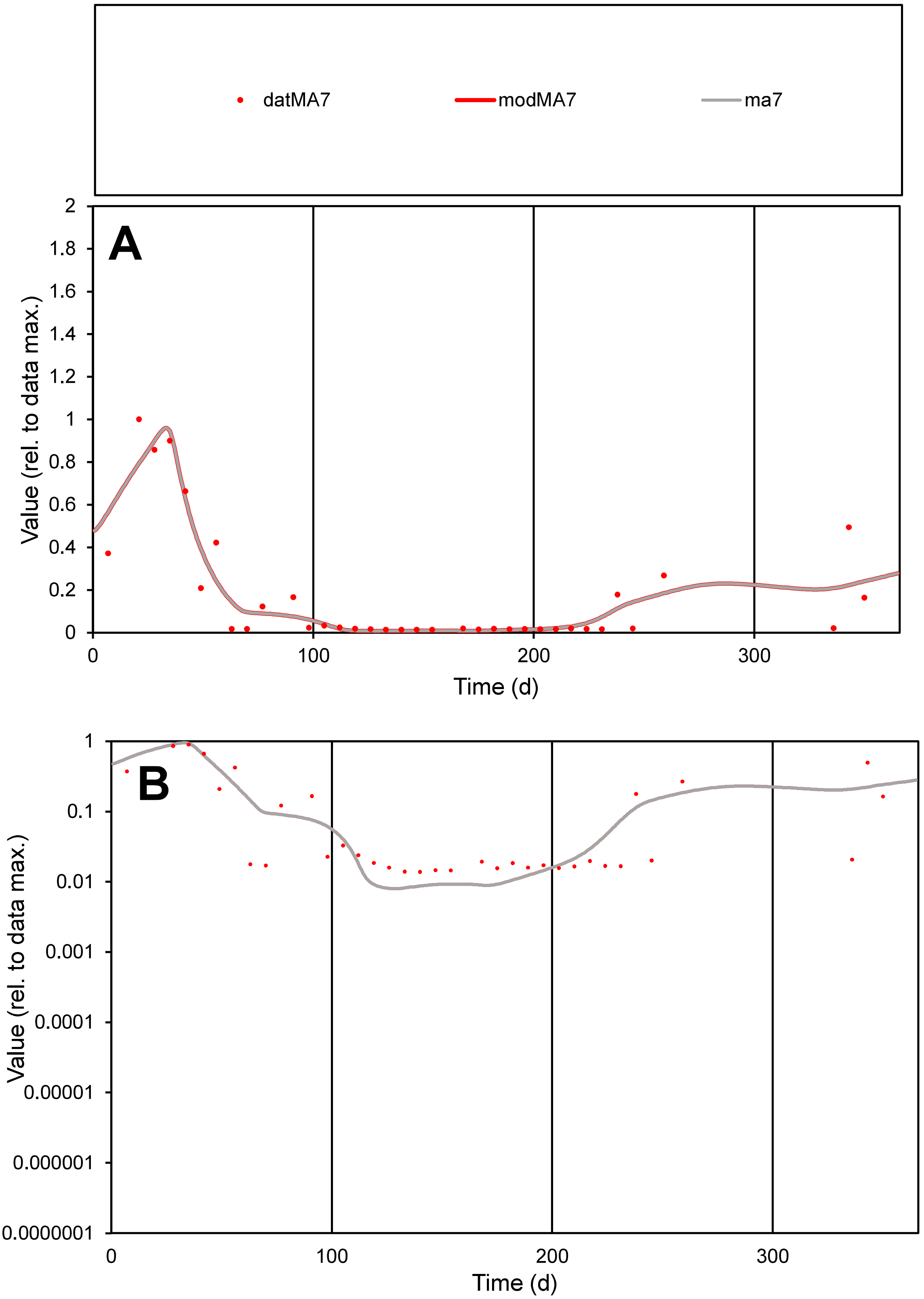

C1

Variables
MA7 Provide jtd or Dst
CalibOnly?
2 1 to reproduce of
oNormalize?
2 1=no, 2=yes

ofxn calc*
0.54048
ofxn mod
0.58609

sRun
RunA1
zout
1

Ave
Data
0.00017
Model
0.00017

data/vMax
0.00096

ofxng
311.734

Min d/m
1.72912

ave/min d
12.5141
ave/min m
22.053

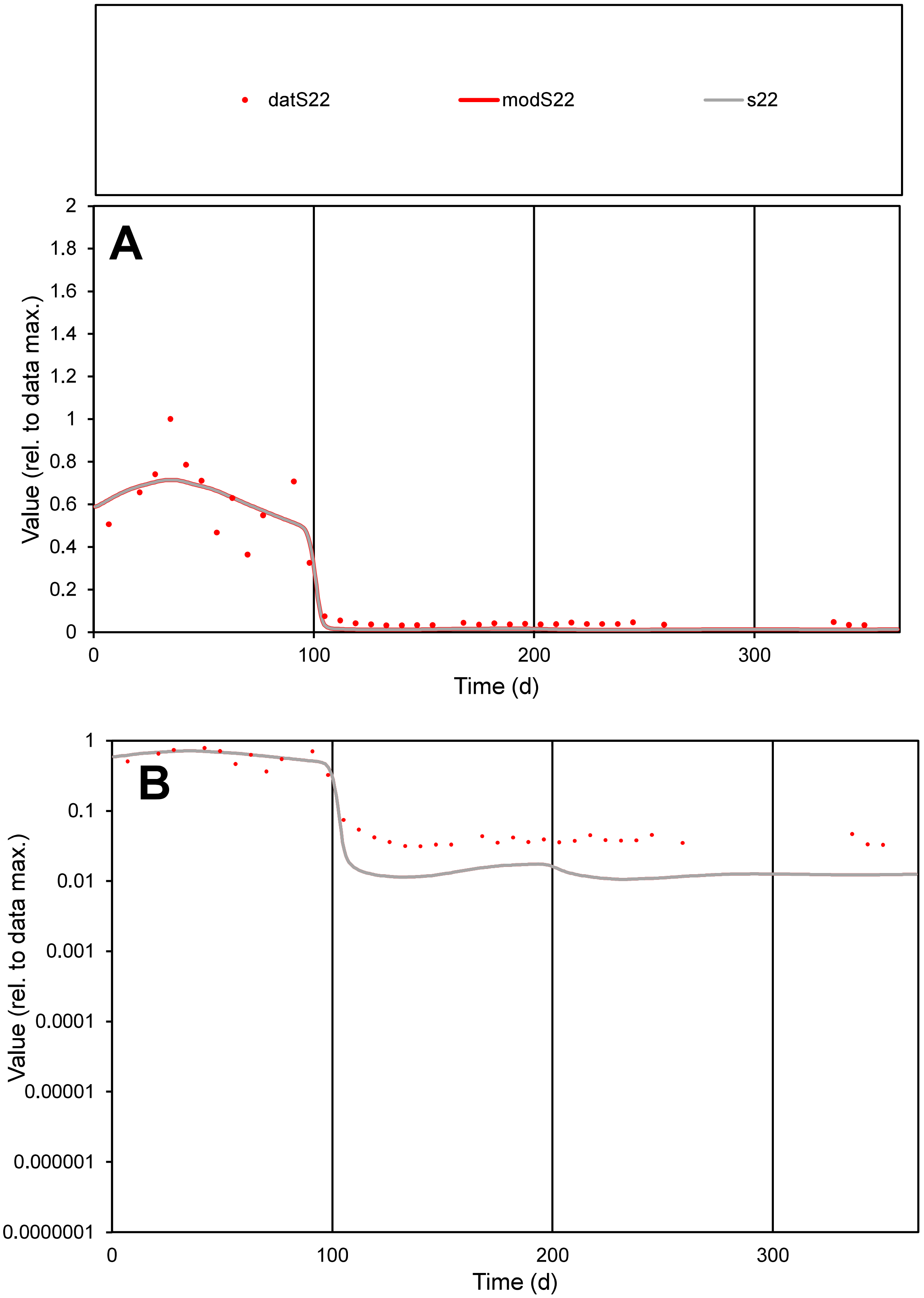

C1

Variables
S22 Provide jtd or Dst
CalibOnly?
2 1 to reproduce of
oNormalize?
2 1=no, 2=yes

ofxn calc*
0.3588
ofxn mod
0.40682

sRun
RunA1
zout
1

Ave
Data
0.00108
Model
0.00099

data/vMax
0.00462

ofxng
311.734

Min d/m
2.97192

ave/min d
7.43006
ave/min m
20.3895

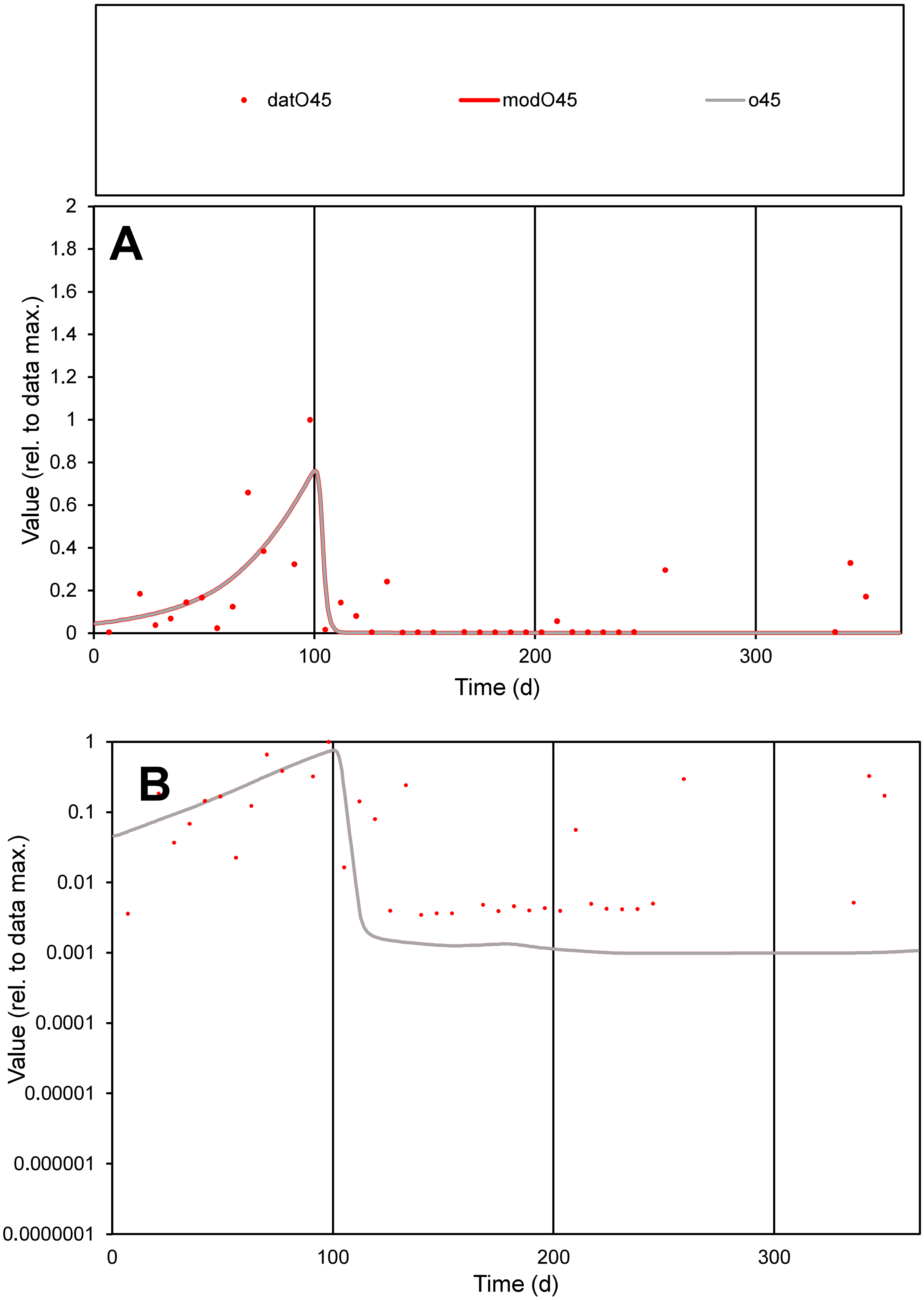

C1

Variables
O45 Provide jtd or Dst
CalibOnly?
2 1 to reproduce of
oNormalize?
2 1=no, 2=yes

ofxn calc*
1.04724
ofxn mod
1.07556

sRun
RunA1
zout
1

Ave
Data
0.00201
Model
0.00151

data/vMax
0.01628

ofxng
311.734

Min d/m
3.54899

ave/min d
35.567
ave/min m
95.0833

331_Calib_S_A

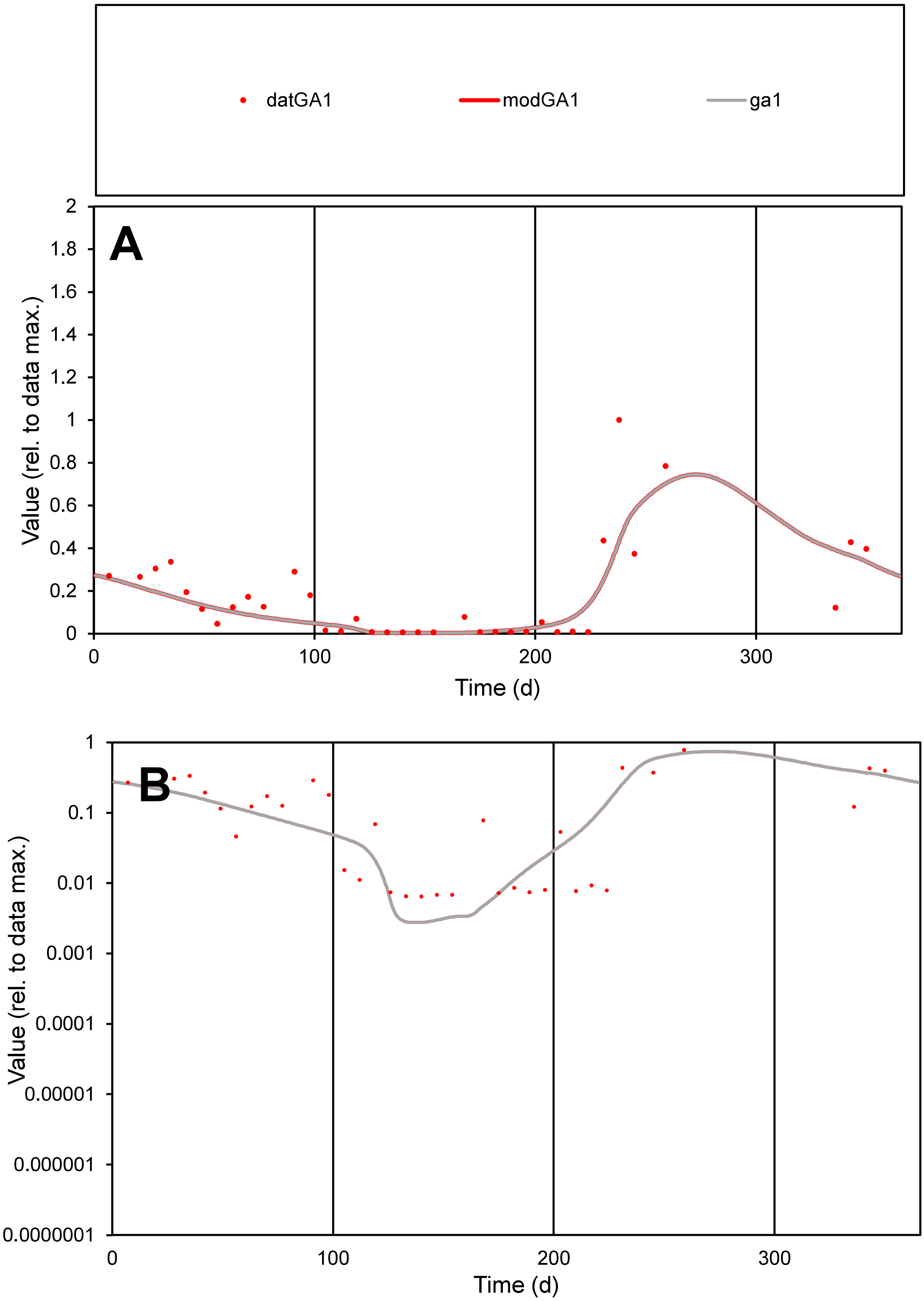

C1

Variables
GA1 Provide jtd or Dst
CalibOnly?
2 1 to reproduce of
oNormalize?
2 1=no, 2=yes

ofxn calc*
0.73939
ofxn mod
0.75855

sRun
RunA1
zout
1

Ave
Data
0.00094
Model
0.00078

data/vMax
0.00541

ofxng
311.734

Min d/m
2.32205

ave/min d
27.0003
ave/min m
51.9279

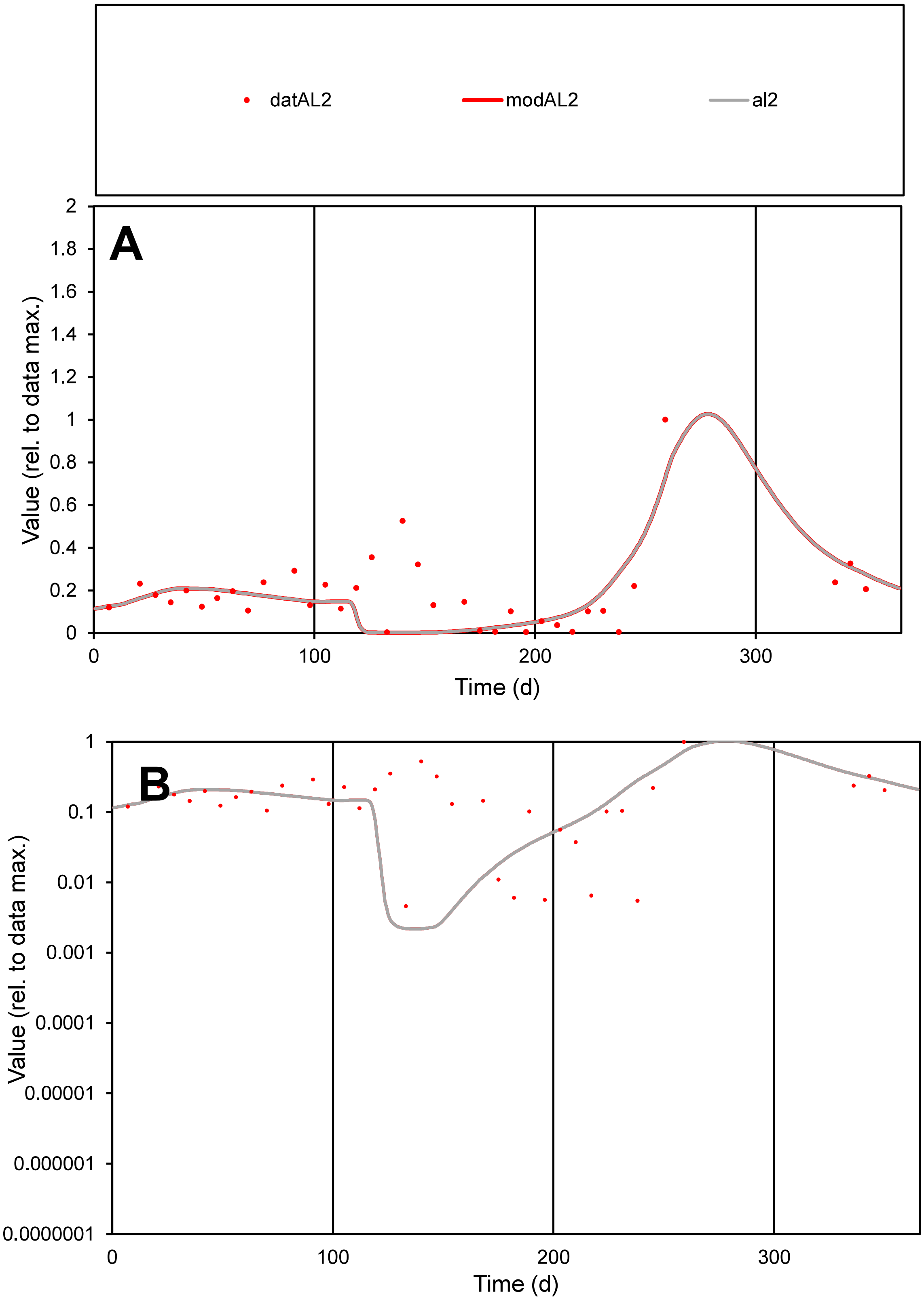

C1

Variables
AL2 Provide jtd or Dst
CalibOnly?
2 1 to reproduce of
oNormalize?
2 1=no, 2=yes

ofxn calc*
0.68113
ofxn mod
0.70733

sRun
RunA1
zout
1

Ave
Data
0.0008
Model
0.00074

data/vMax
0.00472

ofxng
311.734

Min d/m
2.09547

ave/min d
37.0452
ave/min m
70.99

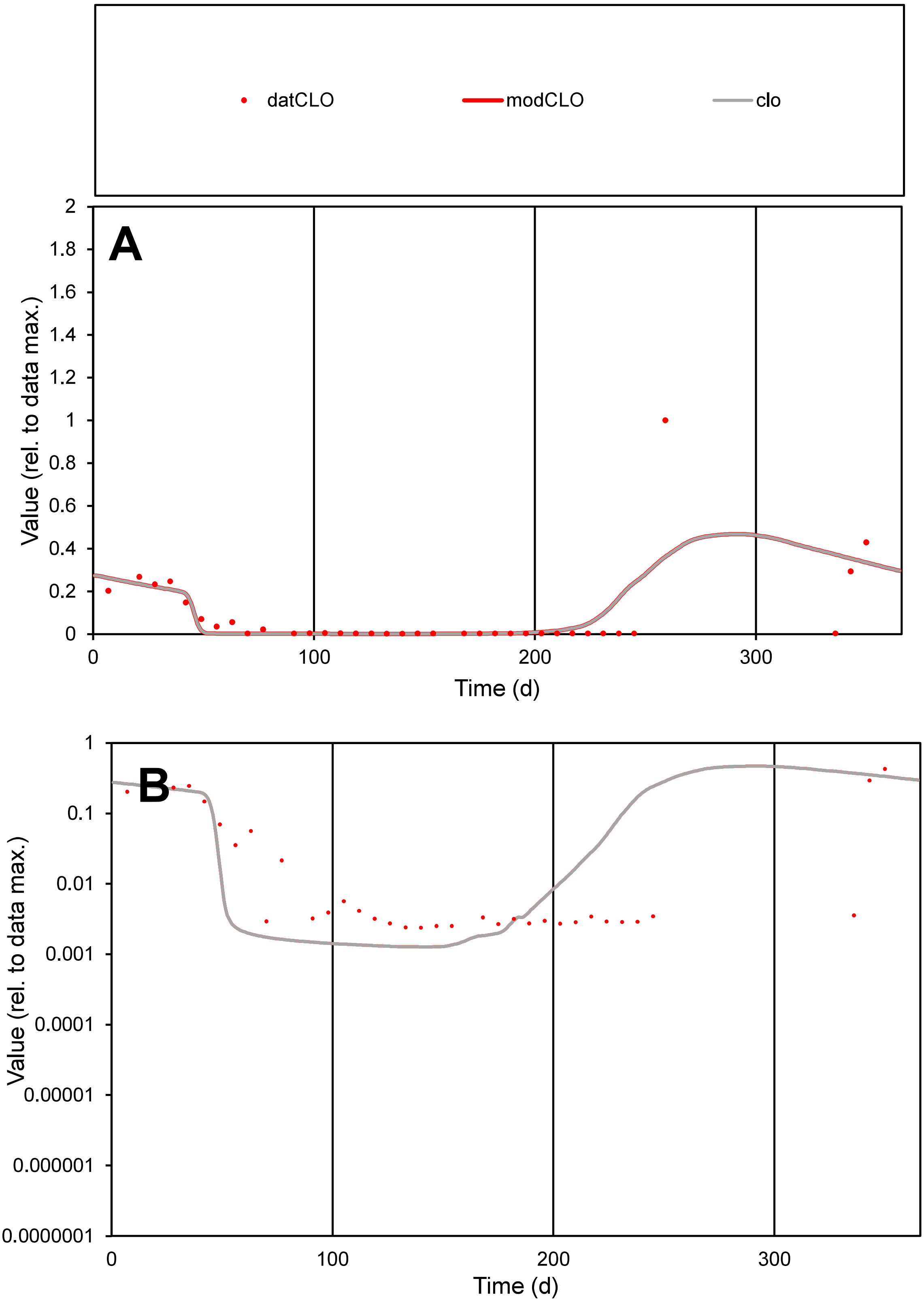

C1

Variables
CLO Provide jtd or Dst
CalibOnly?
2 1 to reproduce of
oNormalize?
2 1=no, 2=yes

ofxn calc*
1.55875
ofxn mod
1.58425

sRun
RunA1
zout
1

Ave
Data
0.00047
Model
0.00048

data/vMax
0.00545

ofxng
311.734

Min d/m
1.88595

ave/min d
35.7365
ave/min m
69.3354

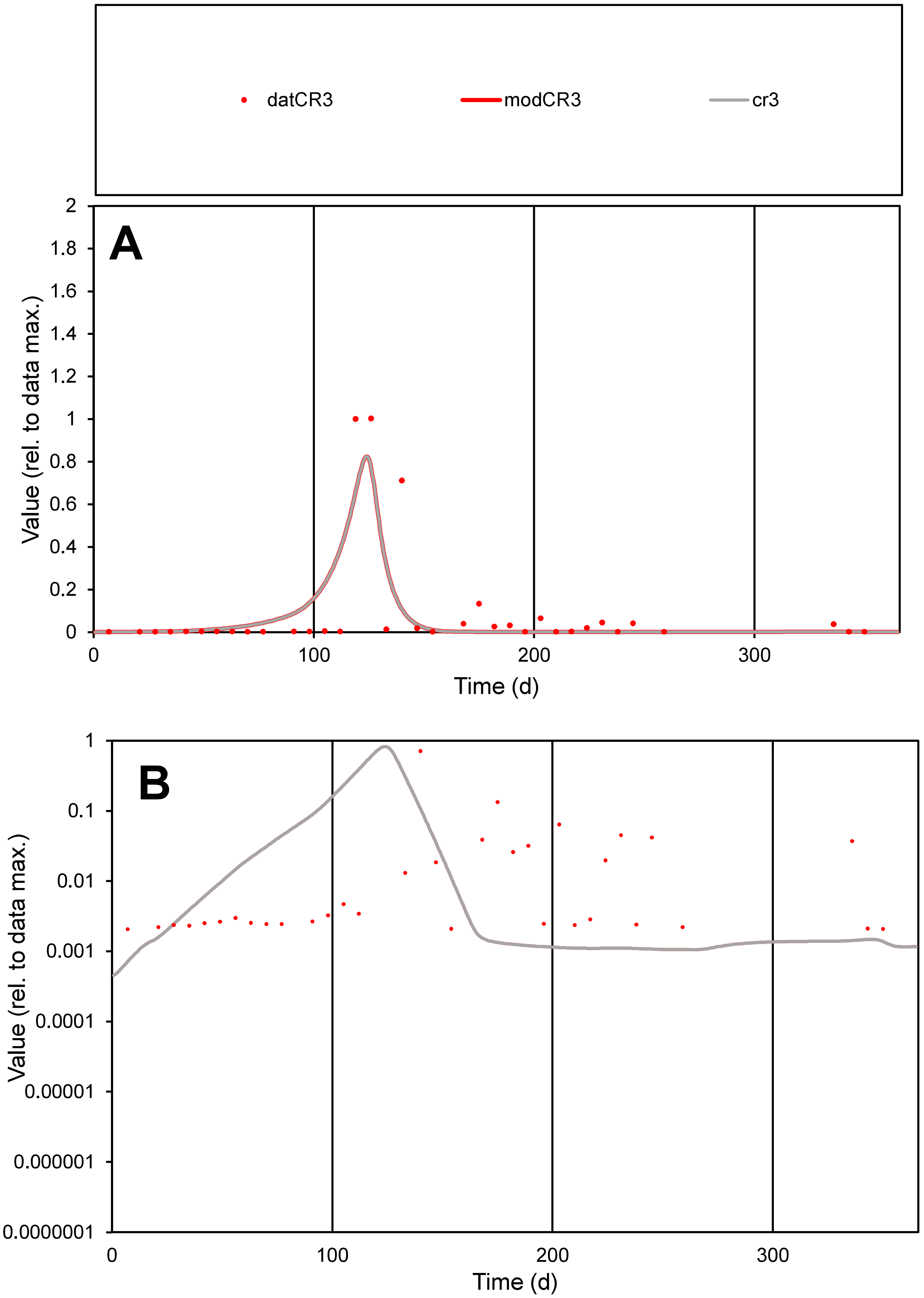

C1

Variables
CR3 Provide jtd or Dst
CalibOnly?
2 1 to reproduce of
oNormalize?
2 1=no, 2=yes

ofxn calc*
1.49449
ofxn mod
1.58319

sRun
RunA1
zout
1

Ave
Data
0.00129
Model
0.0013

data/vMax
0.01765

ofxng
311.734

Min d/m
2.69453

ave/min d
35.4846
ave/min m
96.333

331_Calib_S_A

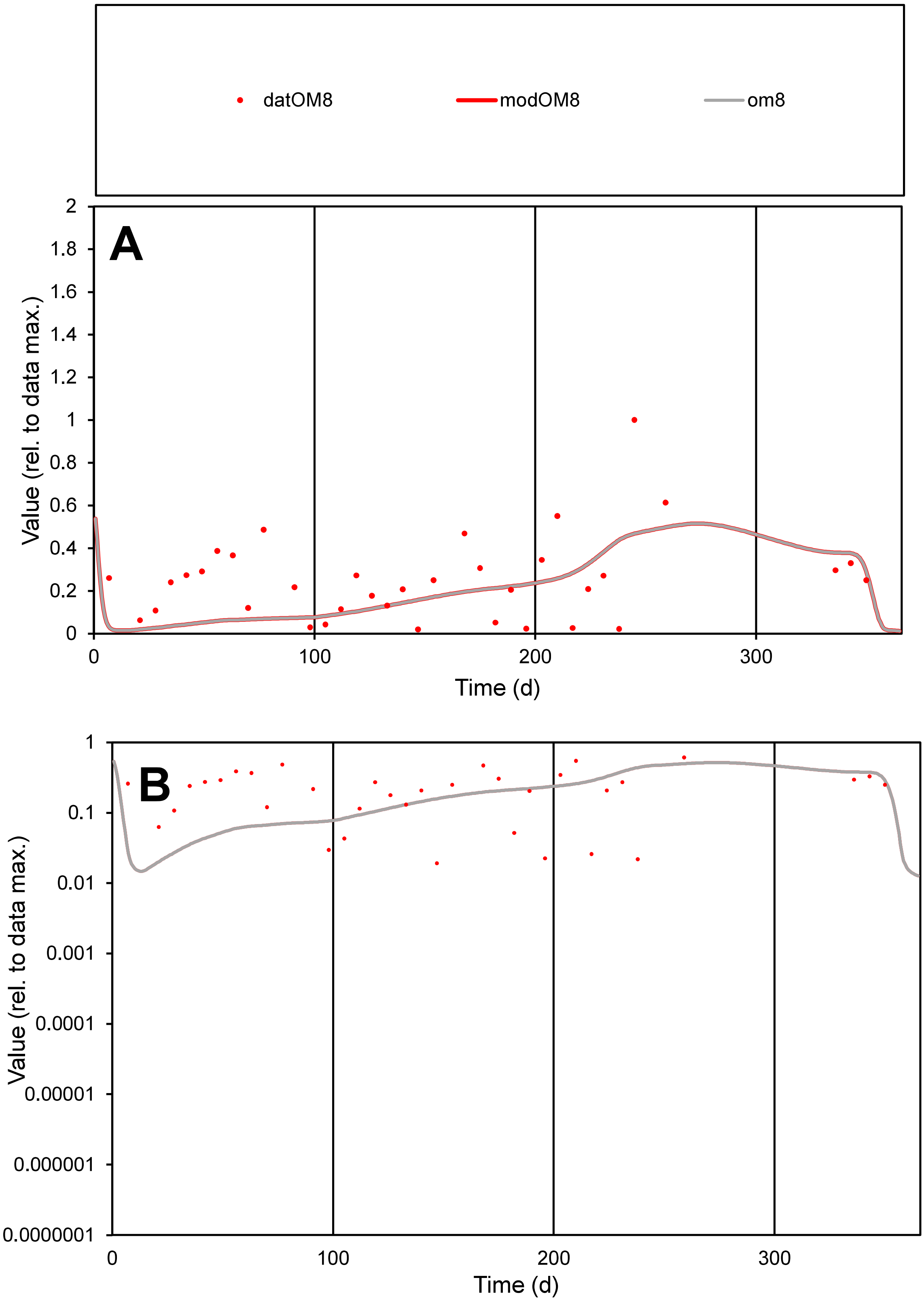

C1

Variables
OM8 Provide jtd or Dst
CalibOnly?
2 1 to reproduce of
oNormalize?
2 1=no, 2=yes

ofxn calc*
0.82153
ofxn mod
0.83846

sRun
RunA1
zout
1

Ave
Data
0.00072
Model
0.00055

data/vMax
0.00294

ofxng
311.734

Min d/m
0.88922

ave/min d
12.7363
ave/min m
8.69708

331_Calib_S_A

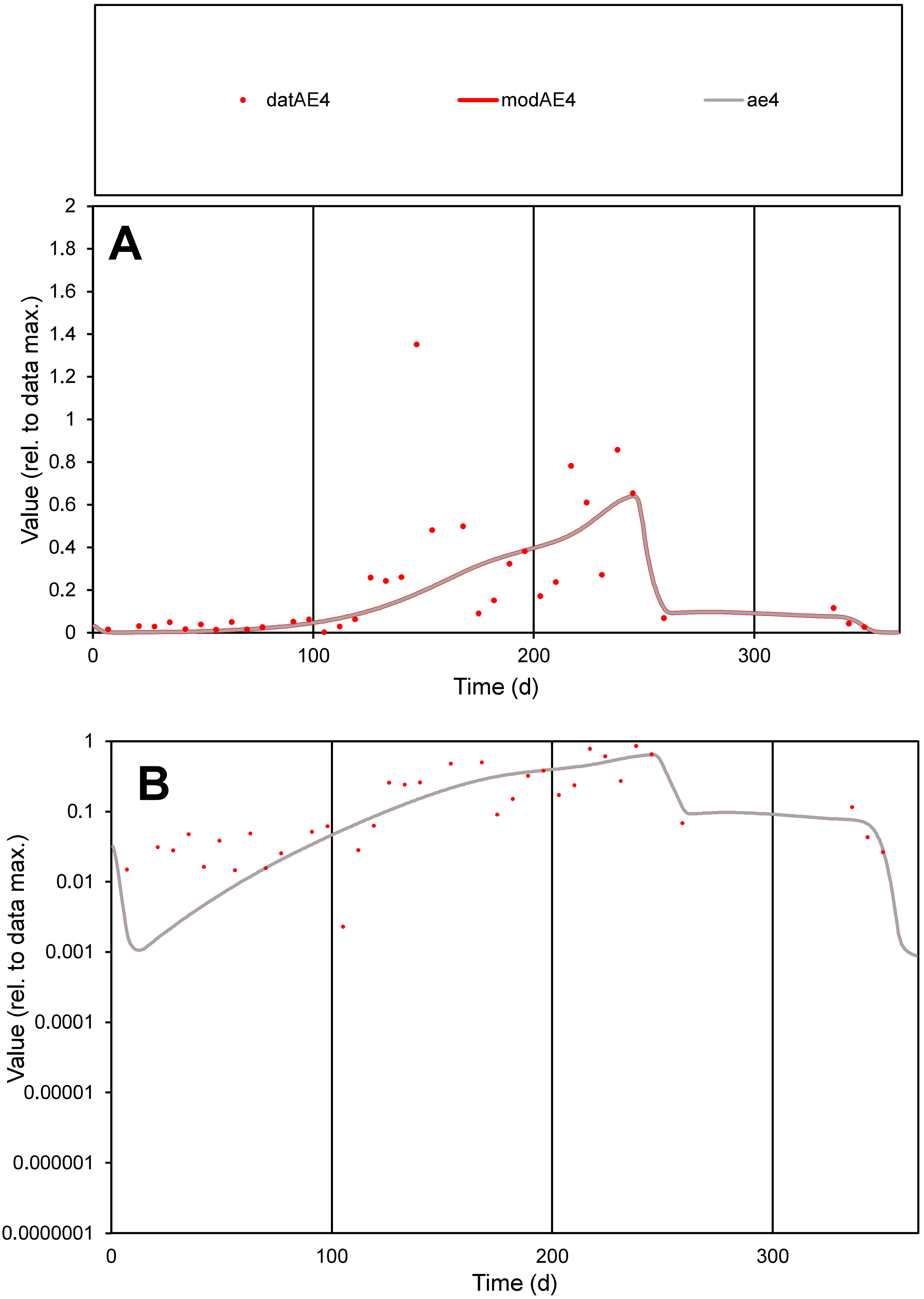

C1

Variables
AE4 Provide jtd or Dst
CalibOnly?
2 1 to reproduce of
oNormalize?
2 1=no, 2=yes

ofxn calc*
0.85312
ofxn mod
0.87041

sRun
RunA1
zout
1

Ave
Data
0.00252
Model
0.00225

data/vMax
0.0119

ofxng
311.734

Min d/m
1.33301

ave/min d
92.364
ave/min m
109.803

331_Calib_S_A

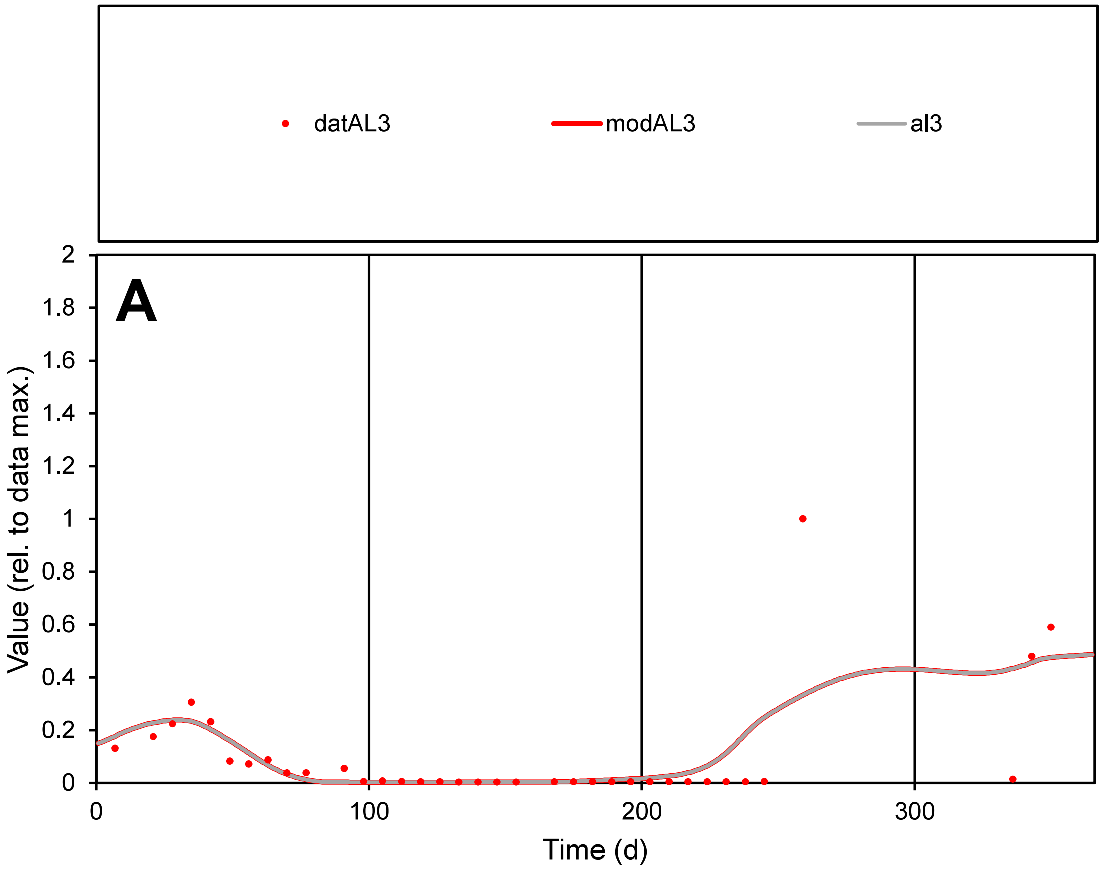

C1

Variables

AL3	Provide jtd or Dst
CalibOnly?	
2	1 to reproduce of
oNormalize?	
2	1=no, 2=yes

ofxn calc*
1.44018
ofxn mod
1.46302

sRun
RunA1
zout
1

Ave
Data
0.00073
Model
0.00079

data/vMax
0.00729

ofxng
311.734

Min d/m
1.57804

ave/min d
30.6591
ave/min m
52.1071

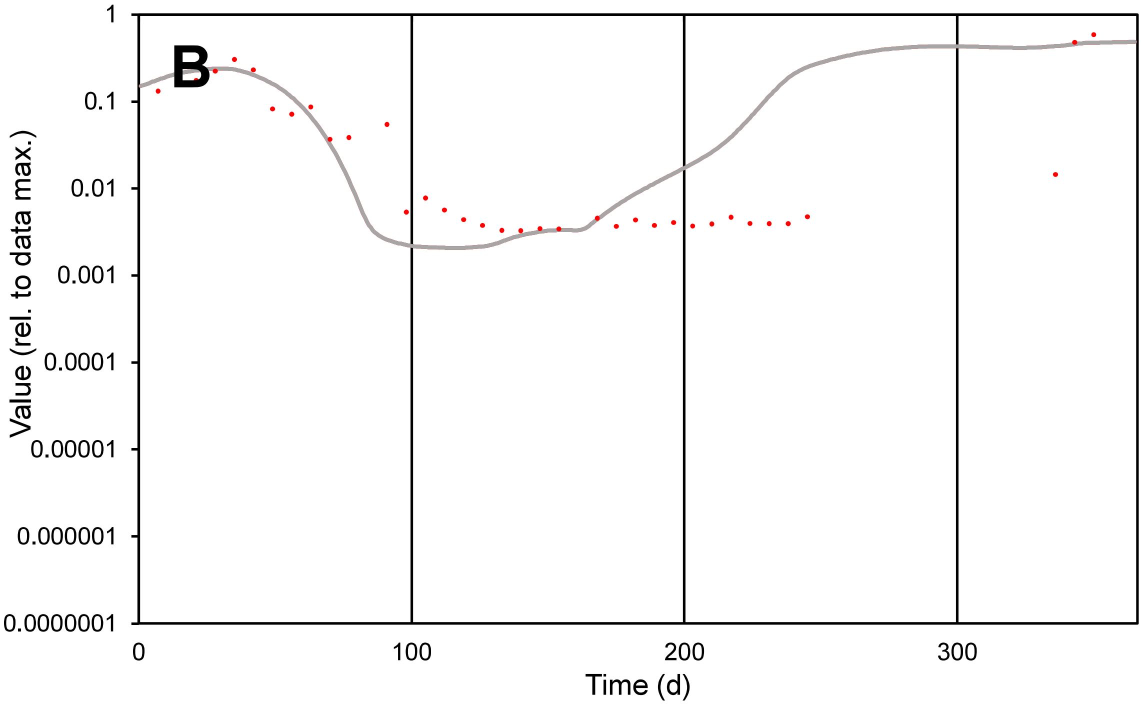

331_Calib_S_A

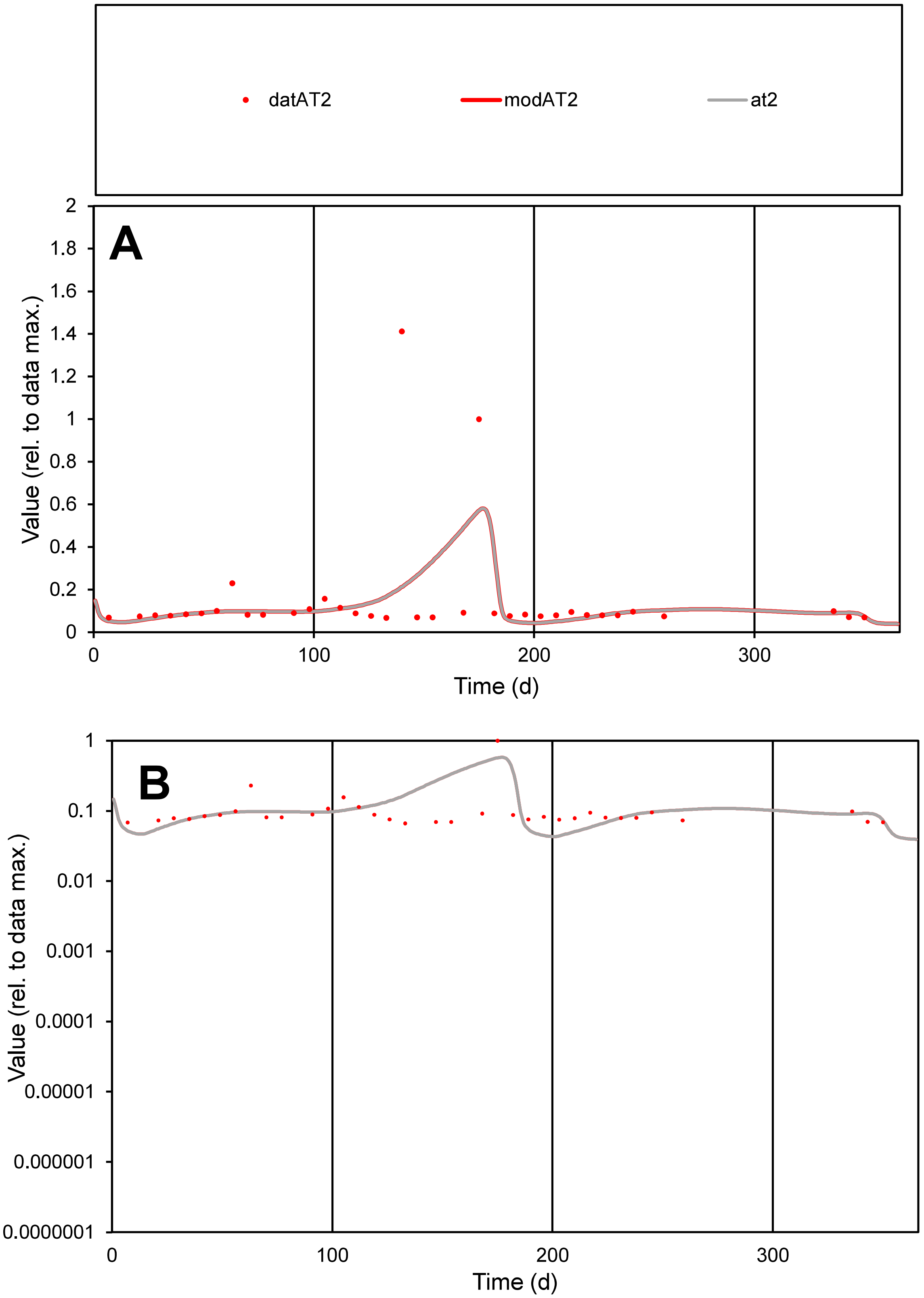

C1

Variables
AT2 Provide jtd or Dst
CalibOnly?
2 1 to reproduce of
oNormalize?
2 1=no, 2=yes

ofxn calc*
1.13584
ofxn mod
1.15229

sRun
RunA1
zout
1

Ave
Data
0.00016
Model
0.00015

data/vMax
0.0012

ofxng
311.734

Min d/m
1.50959

ave/min d
2.01642
ave/min m
2.92914

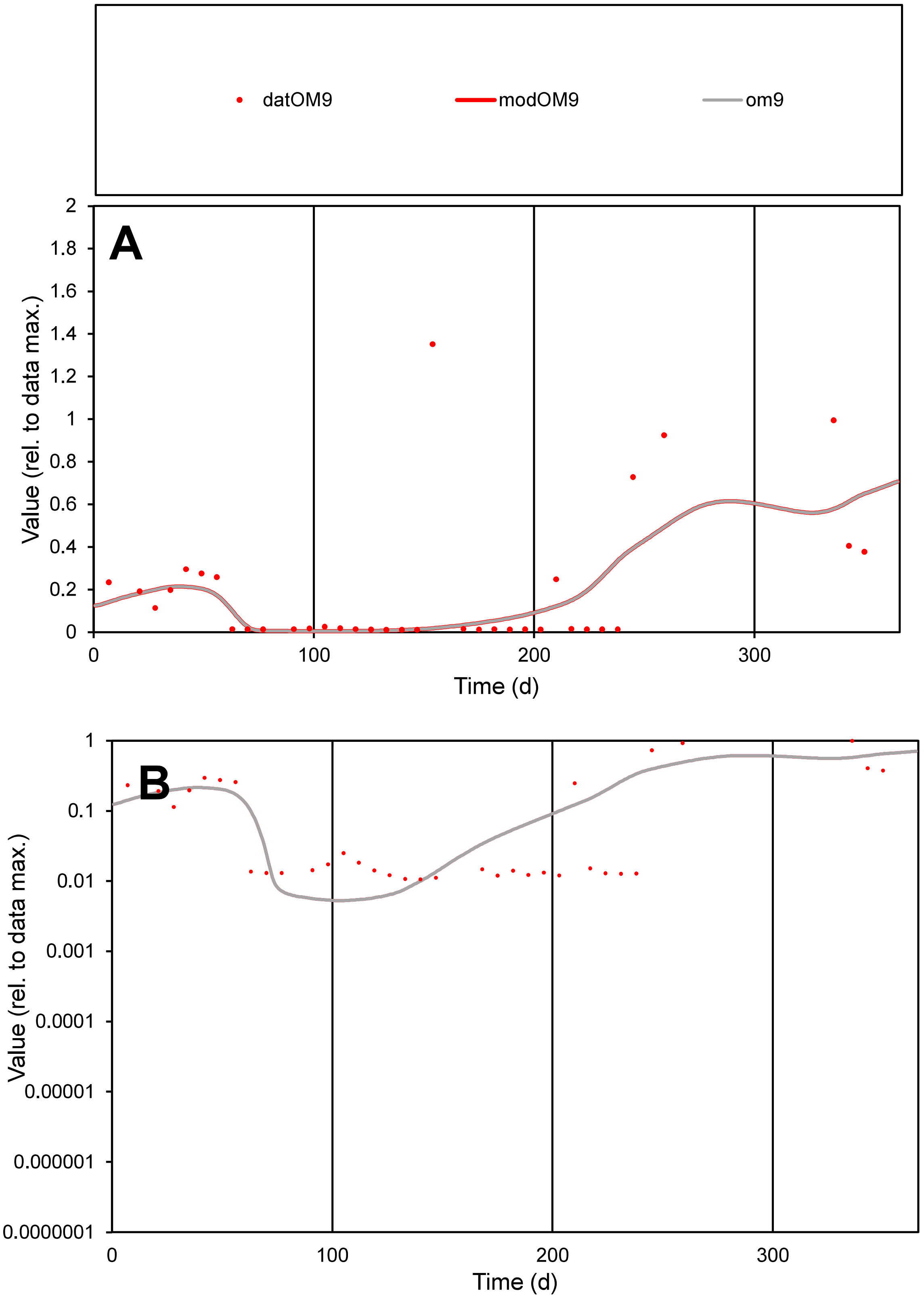

C1

Variables
OM9 Provide jtd or Dst
CalibOnly?
2 1 to reproduce of
oNormalize?
2 1=no, 2=yes

ofxn calc*
1.23434
ofxn mod
1.25086

sRun
RunA1
zout
1

Ave
Data
0.00073
Model
0.00064

data/vMax
0.00404

ofxng
311.734

Min d/m
2.00234

ave/min d
17.1455
ave/min m
29.9307

331_Calib_S_A

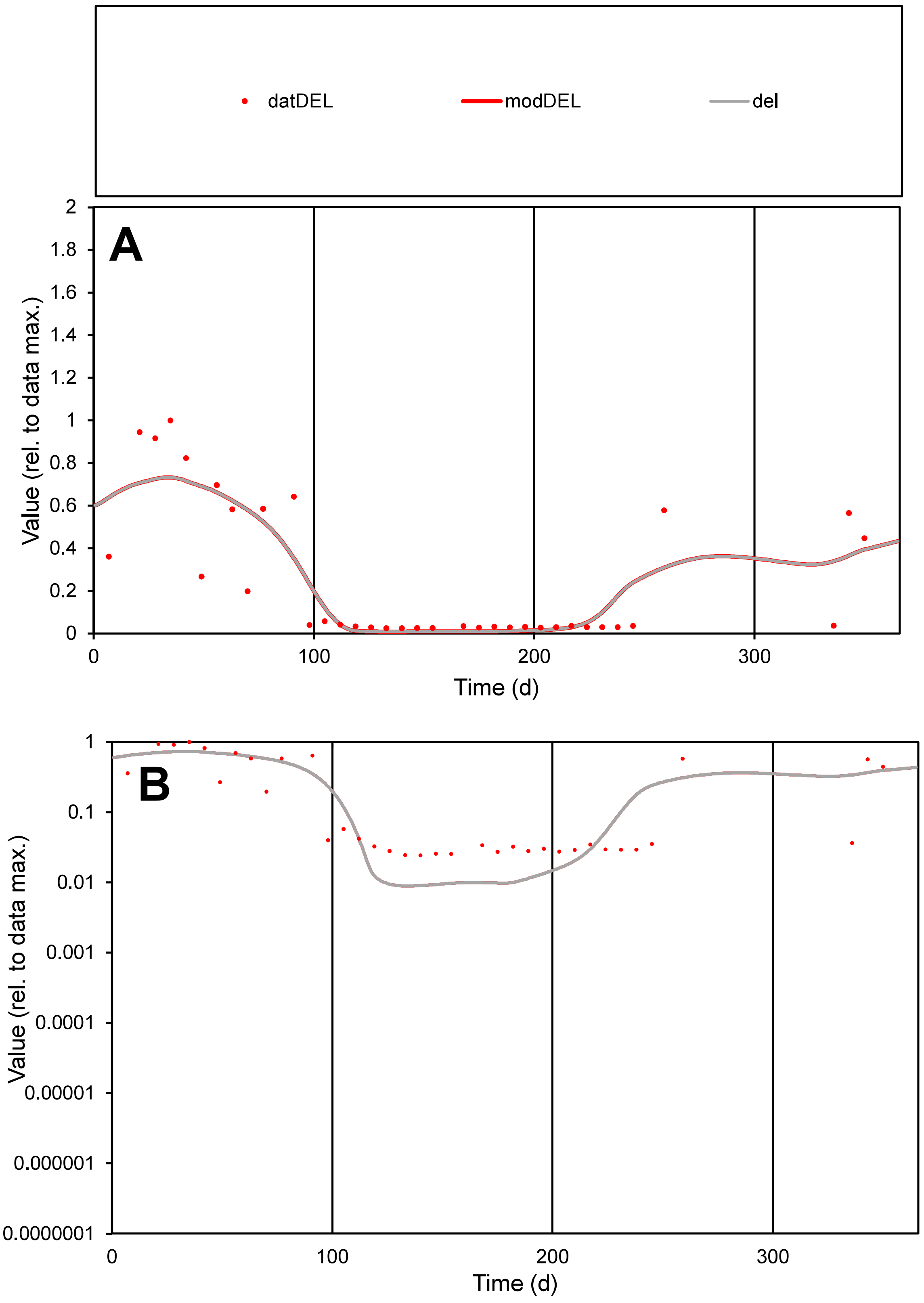

C1

Variables
DEL Provide jtd or Dst
CalibOnly?
2 1 to reproduce of
oNormalize?
2 1=no, 2=yes

ofxn calc*
0.63343
ofxn mod
0.66238

sRun
RunA1
zout
1

Ave
Data
0.00065
Model
0.00066

data/vMax
0.00253

ofxng
311.734

Min d/m
2.72444

ave/min d
10.6426
ave/min m
29.2532

331_Calib_S_A

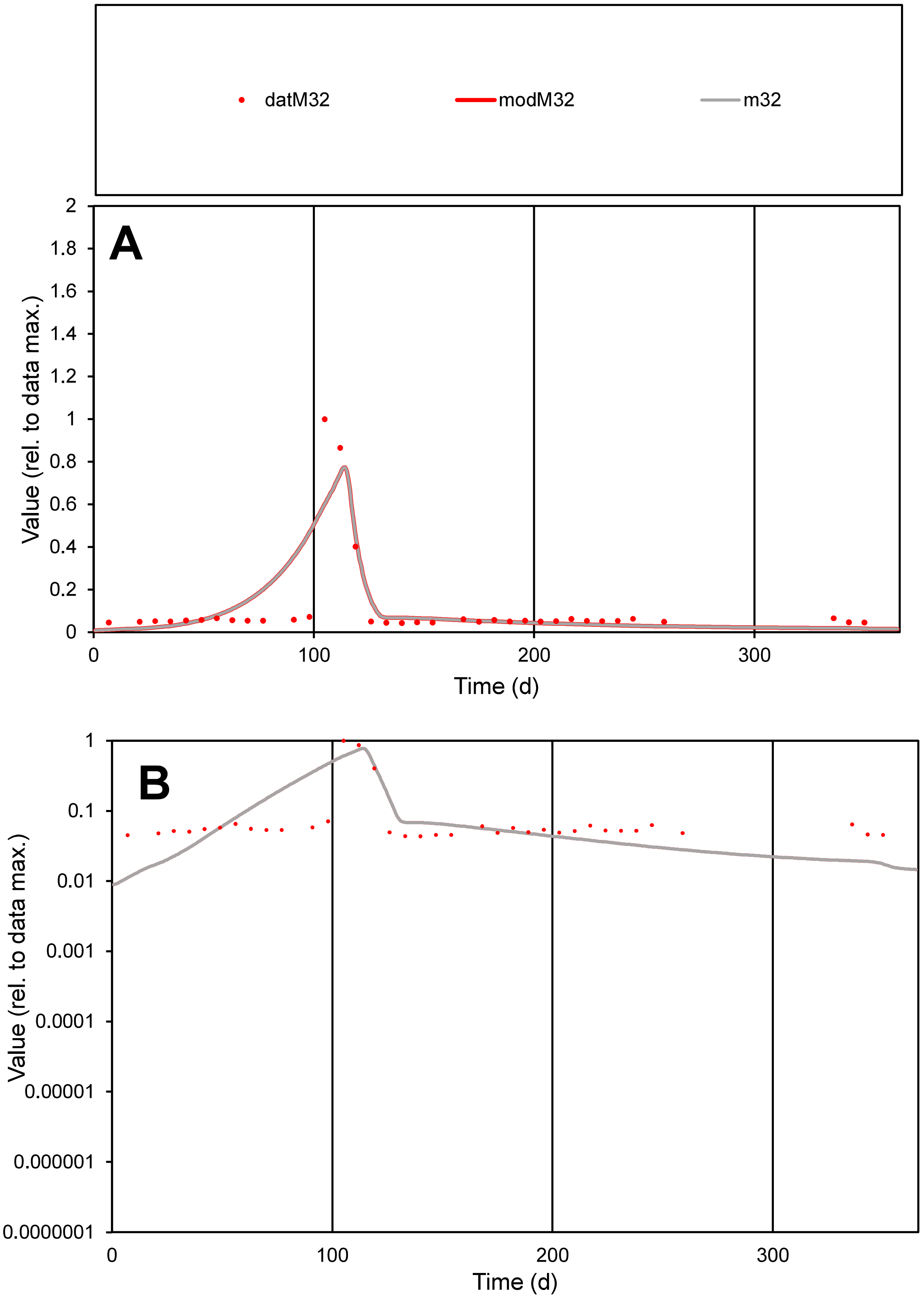

C1

Variables

M32 Provide jtd or Dst

CalibOnly?

2 1 to reproduce of

oNormalize?

2 1=no, 2=yes

ofxn calc*

0.99534

ofxn mod

1.02725

sRun

RunA1

zout

1

Ave

Data

0.00053

Model

0.00057

data/vMax

0.00479

ofxng

311.734

Min d/m

3.73175

ave/min d

2.5794

ave/min m

10.2926

331_Calib_S_A

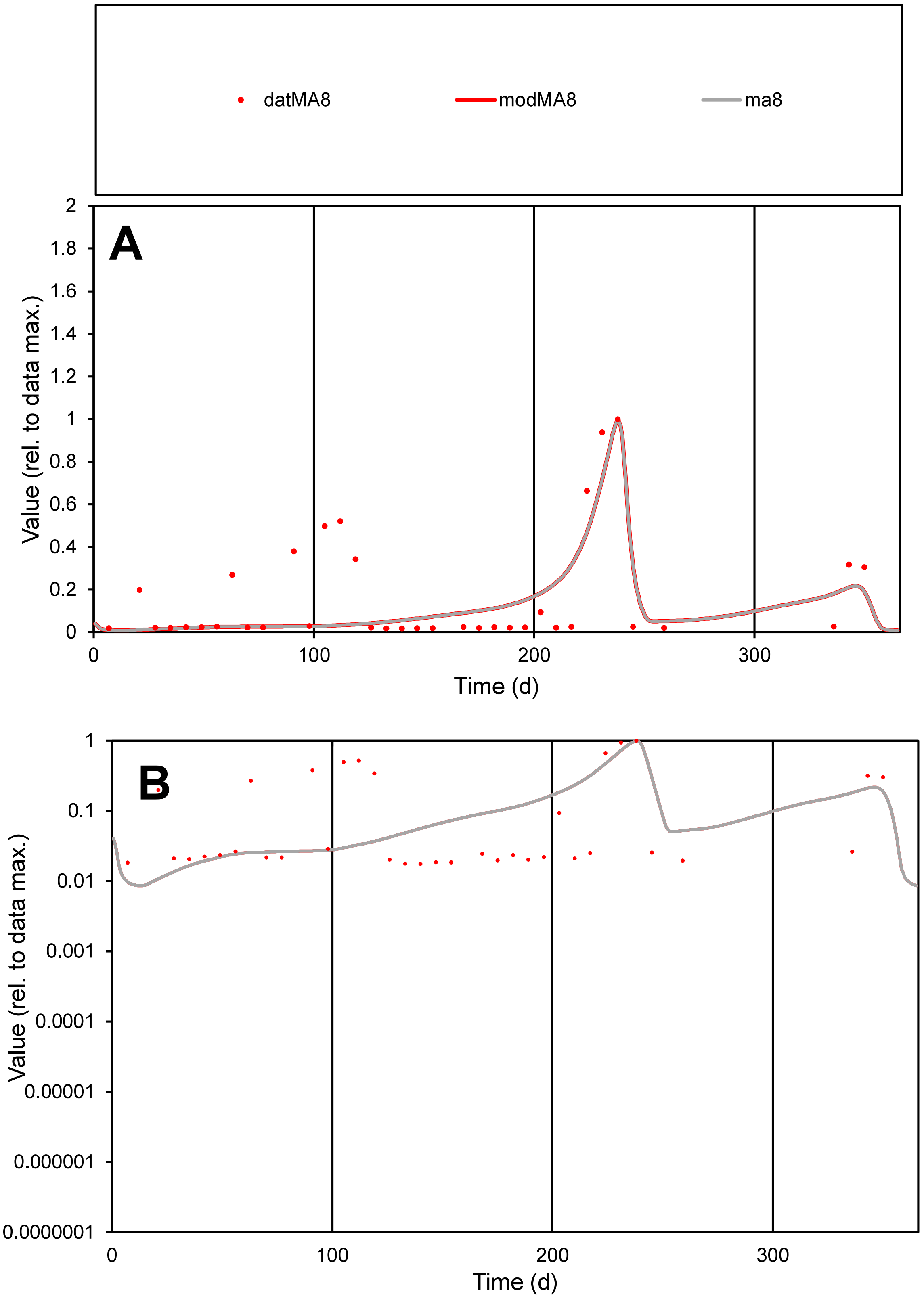

C1

Variables
MA8 Provide jtd or Dst
CalibOnly?
2 1 to reproduce of
oNormalize?
2 1=no, 2=yes

ofxn calc*
1.02265
ofxn mod
1.05307

sRun
RunA1
zout
1

Ave
Data
0.00056
Model
0.00047

data/vMax
0.00336

ofxng
311.734

Min d/m
1.87949

ave/min d
9.55304
ave/min m
14.8038

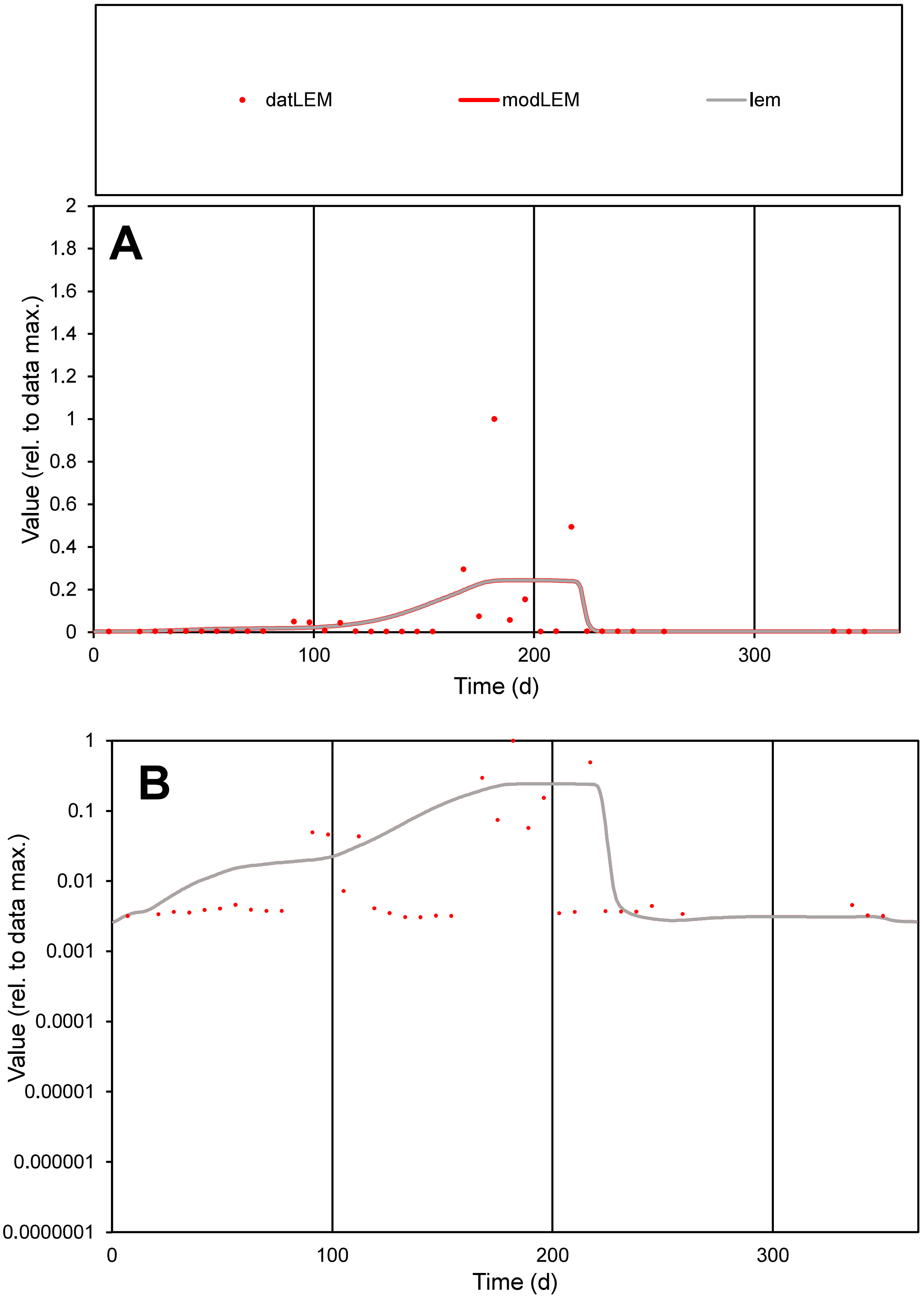

C1

Variables
LEM Provide jtd or Dst
CalibOnly?
2 1 to reproduce of
oNormalize?
2 1=no, 2=yes

ofxn calc*
2.42546
ofxn mod
2.43837

sRun
RunA1
zout
1

Ave
Data
0.00045
Model
0.00051

data/vMax
0.00699

ofxng
311.734

Min d/m
1.09147

ave/min d
21.0616
ave/min m
26.1885

331_Calib_S_A

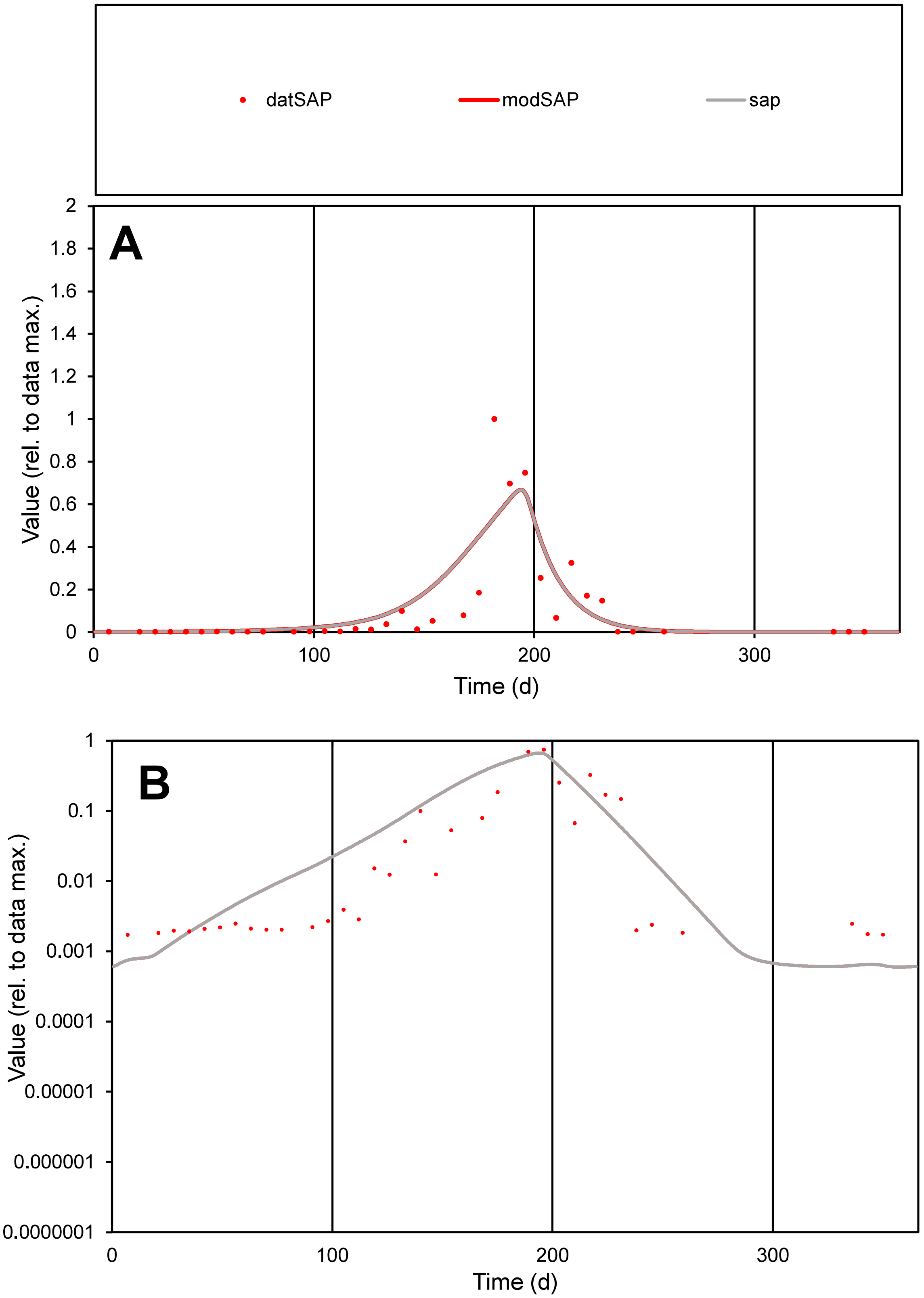

C1

Variables
SAP Provide jtd or Dst
CalibOnly?
2 1 to reproduce of
oNormalize?
2 1=no, 2=yes

ofxn calc*
1.12443
ofxn mod
1.15717

sRun
RunA1
zout
1

Ave
Data
0.00213
Model
0.00245

data/vMax
0.01976

ofxng
311.734

Min d/m
2.76163

ave/min d
62.8583
ave/min m
199.95

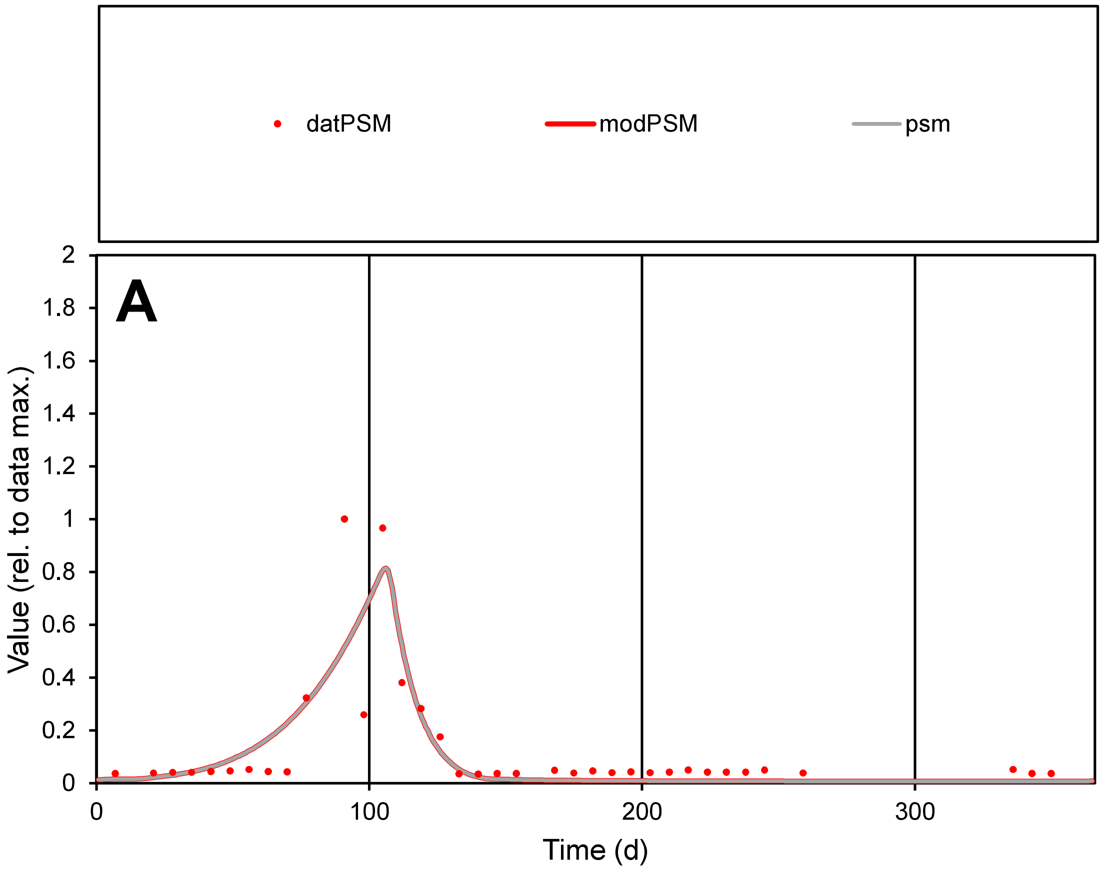

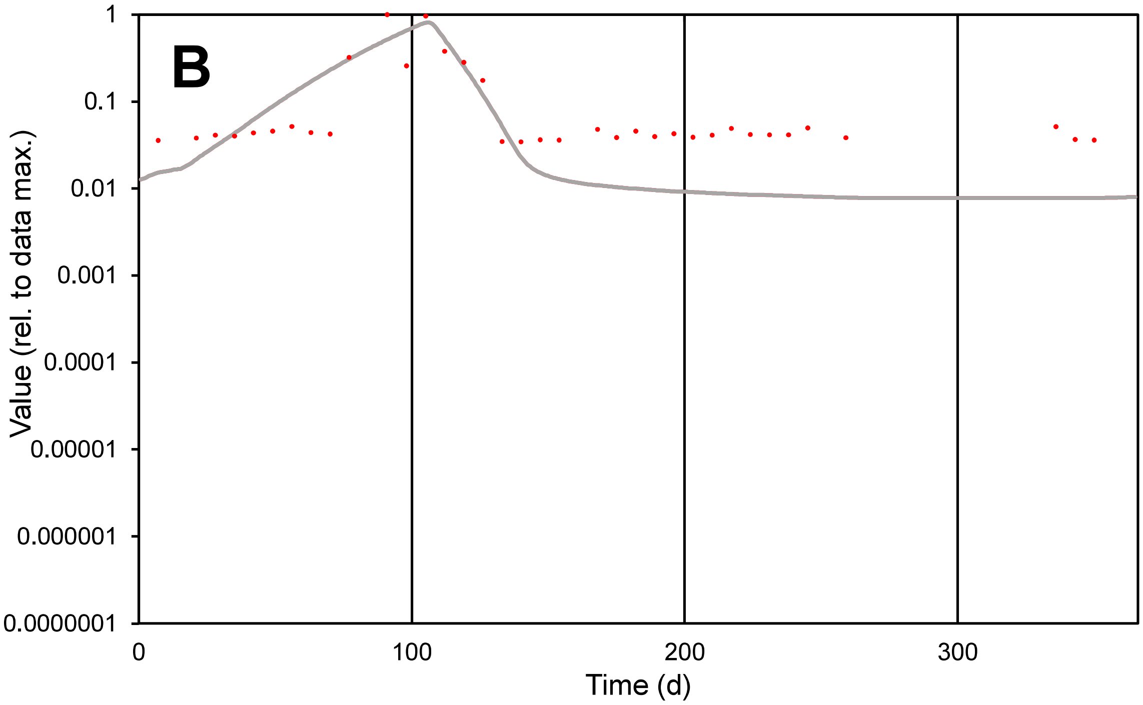

C1

Variables

PSM	Provide jtd or Dst
CalibOnly?	
2	1 to reproduce of
oNormalize?	
2	1=no, 2=yes

ofxn calc*
0.91281
ofxn mod
0.94422

sRun
RunA1
zout
1

Ave
Data
0.0001
Model
9.2E-05

data/vMax
0.0008

ofxng
311.734

Min d/m
4.4403

ave/min d
3.67103
ave/min m
14.8843

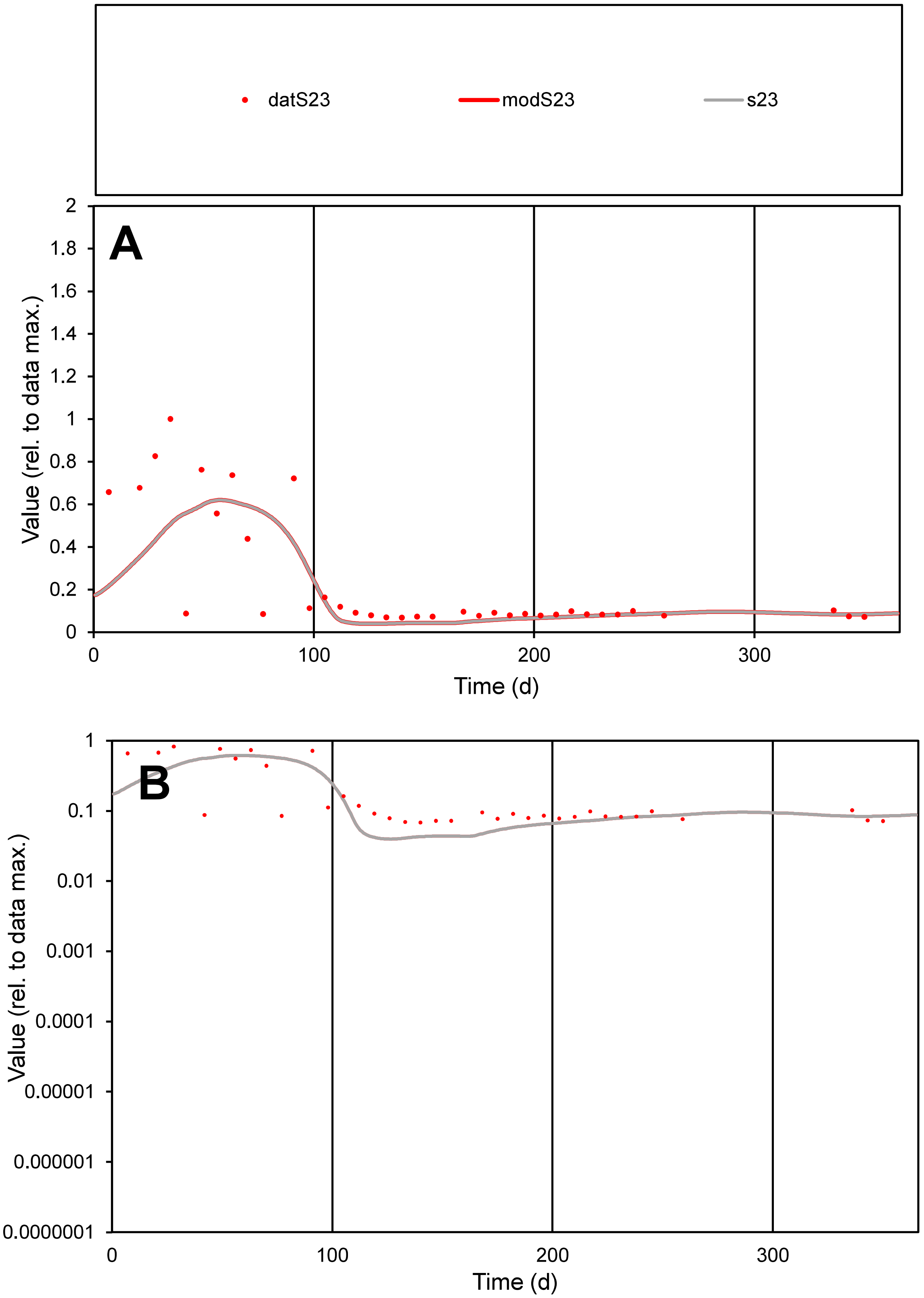

C1

Variables
S23 Provide jtd or Dst
CalibOnly?
2 1 to reproduce of
oNormalize?
2 1=no, 2=yes

ofxn calc*
0.78229
ofxn mod
0.79998

sRun
RunA1
zout
1

Ave
Data
0.00056
Model
0.00047

data/vMax
0.00232

ofxng
311.734

Min d/m
1.71907

ave/min d
3.54576
ave/min m
5.1222

331_Calib_S_A

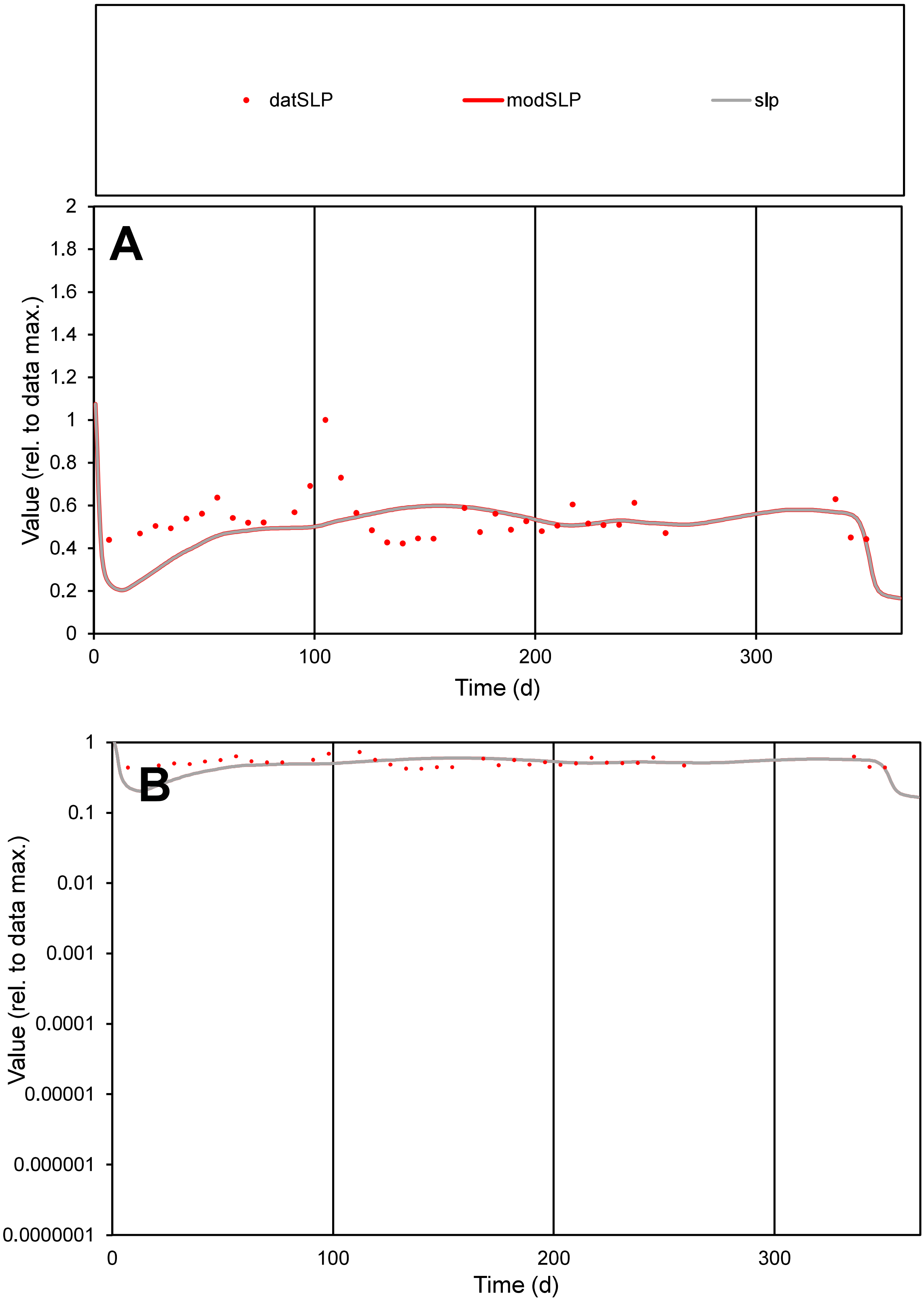

C1

Variables

SLP Provide jtd or Dst

CalibOnly?

2 1 to reproduce of

oNormalize?

2 1=no, 2=yes

ofxn calc*

0.25861

ofxn mod

0.28086

sRun

RunA1

zout

1

Ave

Data

3E-05

Model

2.8E-05

data/vMax

5.6E-05

ofxng

311.734

Min d/m

1.88474

ave/min d

1.27437

ave/min m

2.22231

331_Calib_S_A

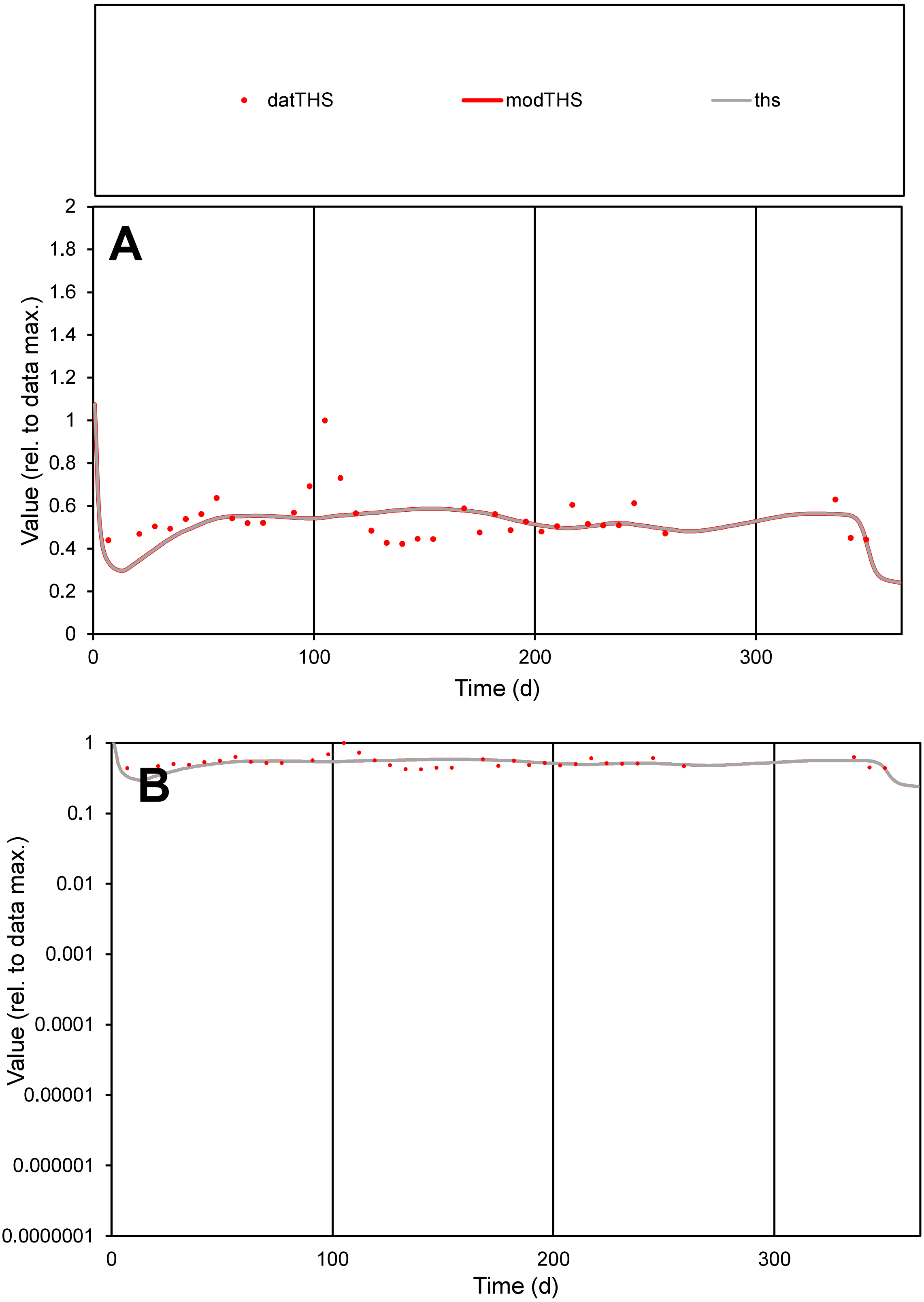

C1

Variables
THS Provide jtd or Dst
CalibOnly?
2 1 to reproduce of
oNormalize?
2 1=no, 2=yes

ofxn calc*
0.20951
ofxn mod
0.23659

sRun
RunA1
zout
1

Ave
Data
3.6E-05
Model
3.5E-05

data/vMax
6.7E-05

ofxng
311.734

Min d/m
1.3067

ave/min d
1.27456
ave/min m
1.60827

331_Calib_S_A

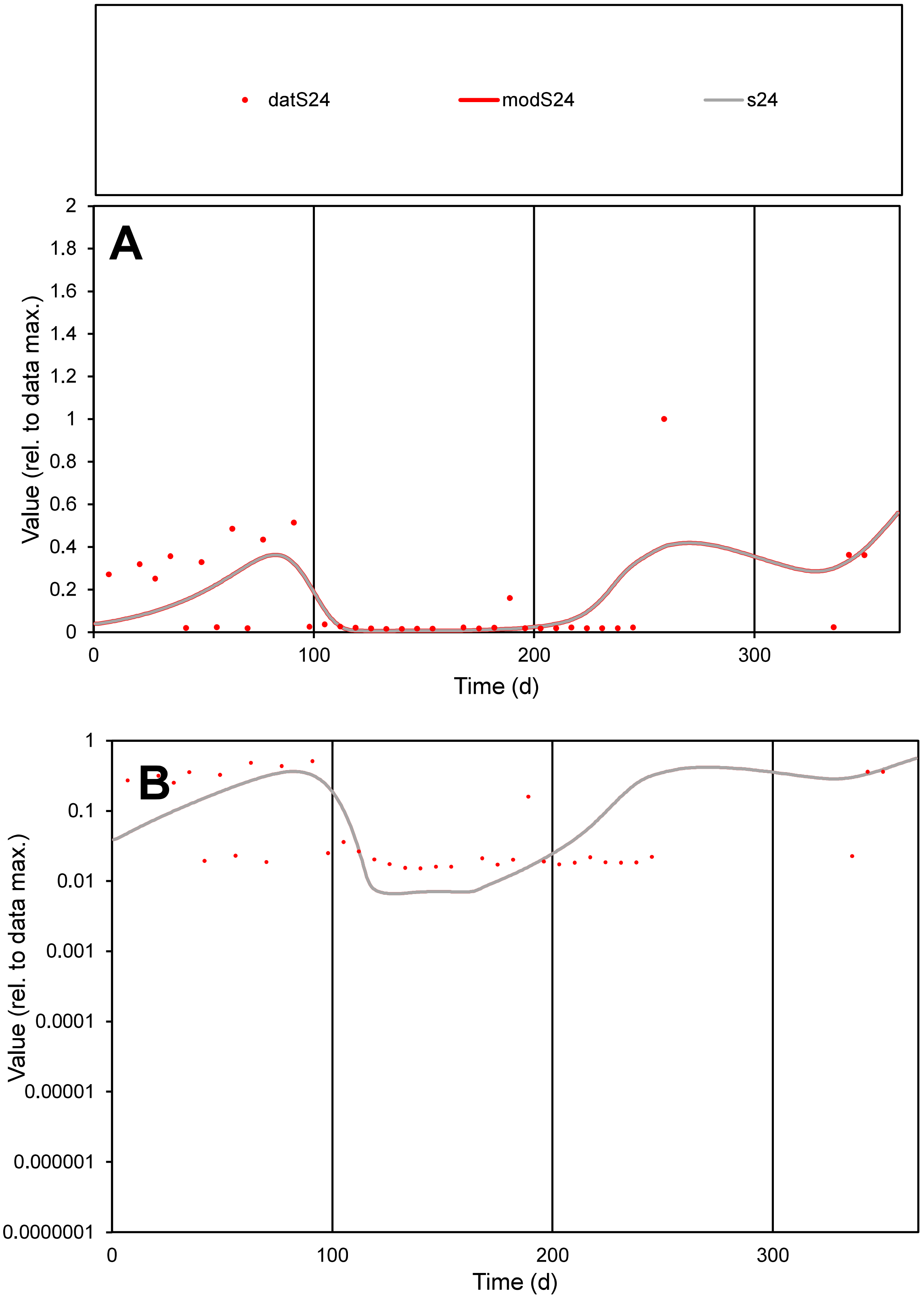

C1

Variables
S24 Provide jtd or Dst
CalibOnly?
2 1 to reproduce of
oNormalize?
2 1=no, 2=yes

ofxn calc*
1.16333
ofxn mod
1.18631

sRun
RunA1
zout
1

Ave
Data
0.00056
Model
0.00052

data/vMax
0.00377

ofxng
311.734

Min d/m
2.30188

ave/min d
9.69919
ave/min m
20.7987

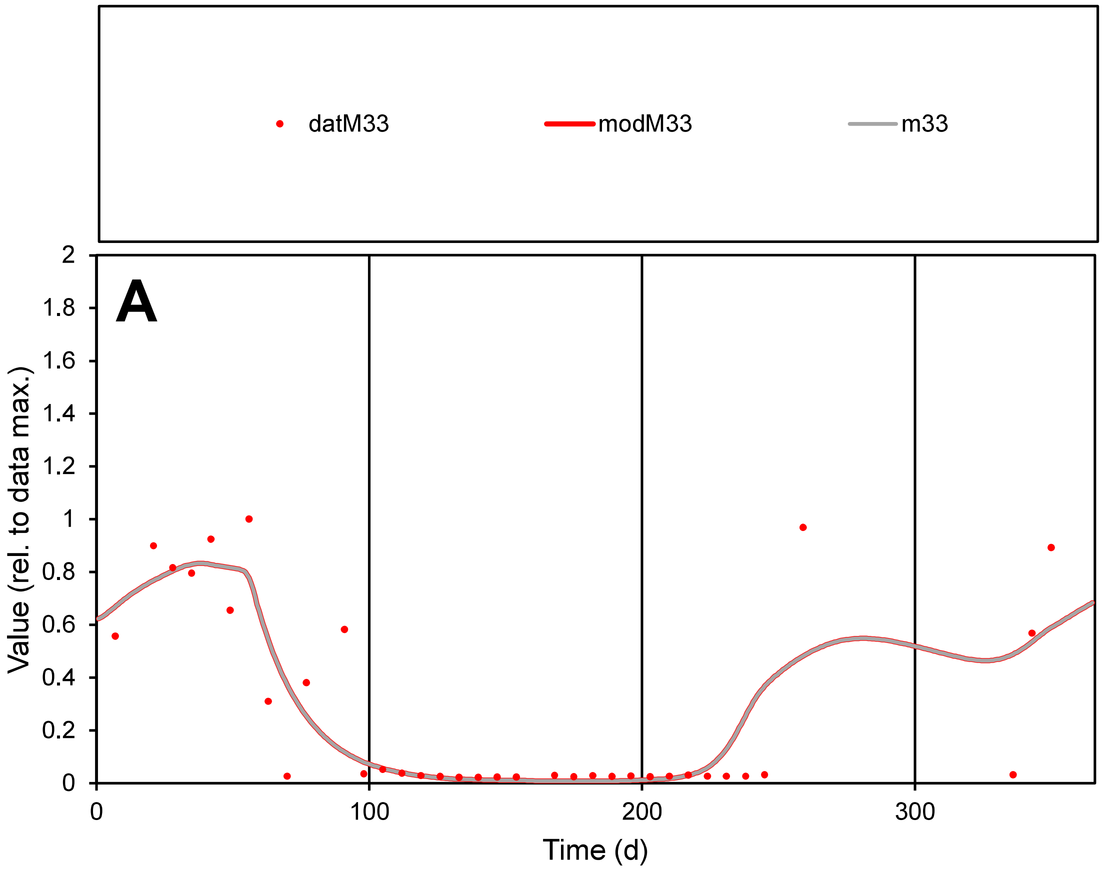

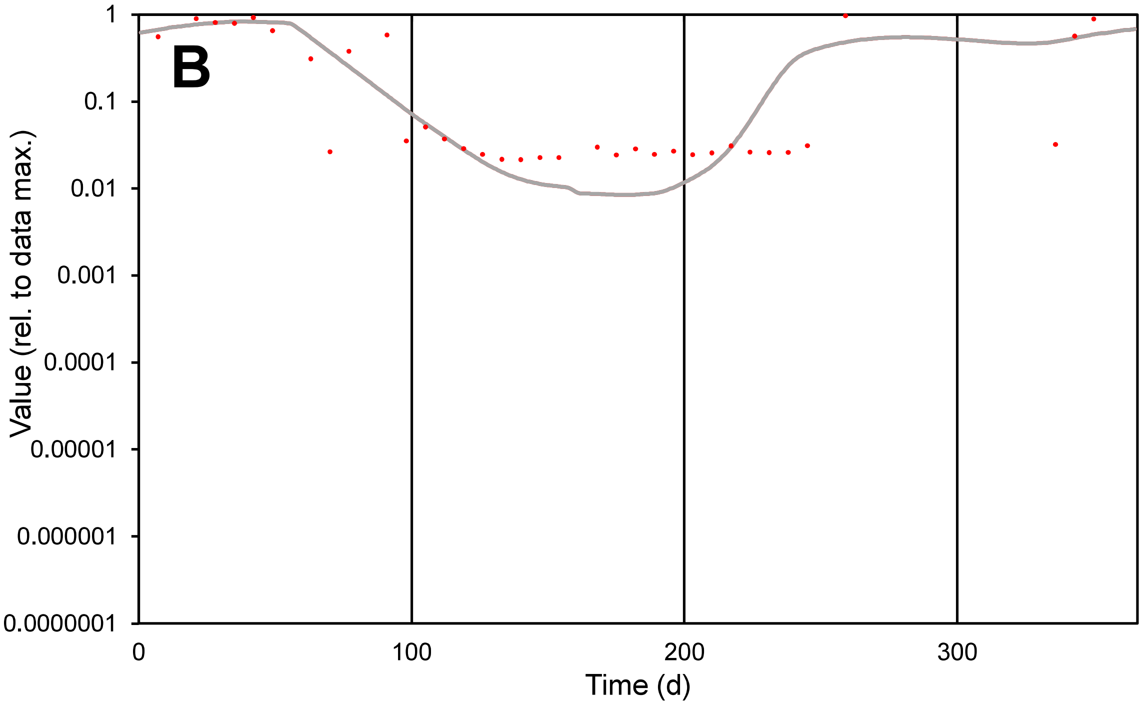

C1

Variables
M33 Provide jtd or Dst
CalibOnly?
2 1 to reproduce of
oNormalize?
2 1=no, 2=yes

ofxn calc*
0.64907
ofxn mod
0.68315

sRun
RunA1
zout
1

Ave
Data
0.00087
Model
0.00087

data/vMax
0.00315

ofxng
311.734

Min d/m
2.53403

ave/min d
12.8898
ave/min m
32.4215

331_Calib_S_A

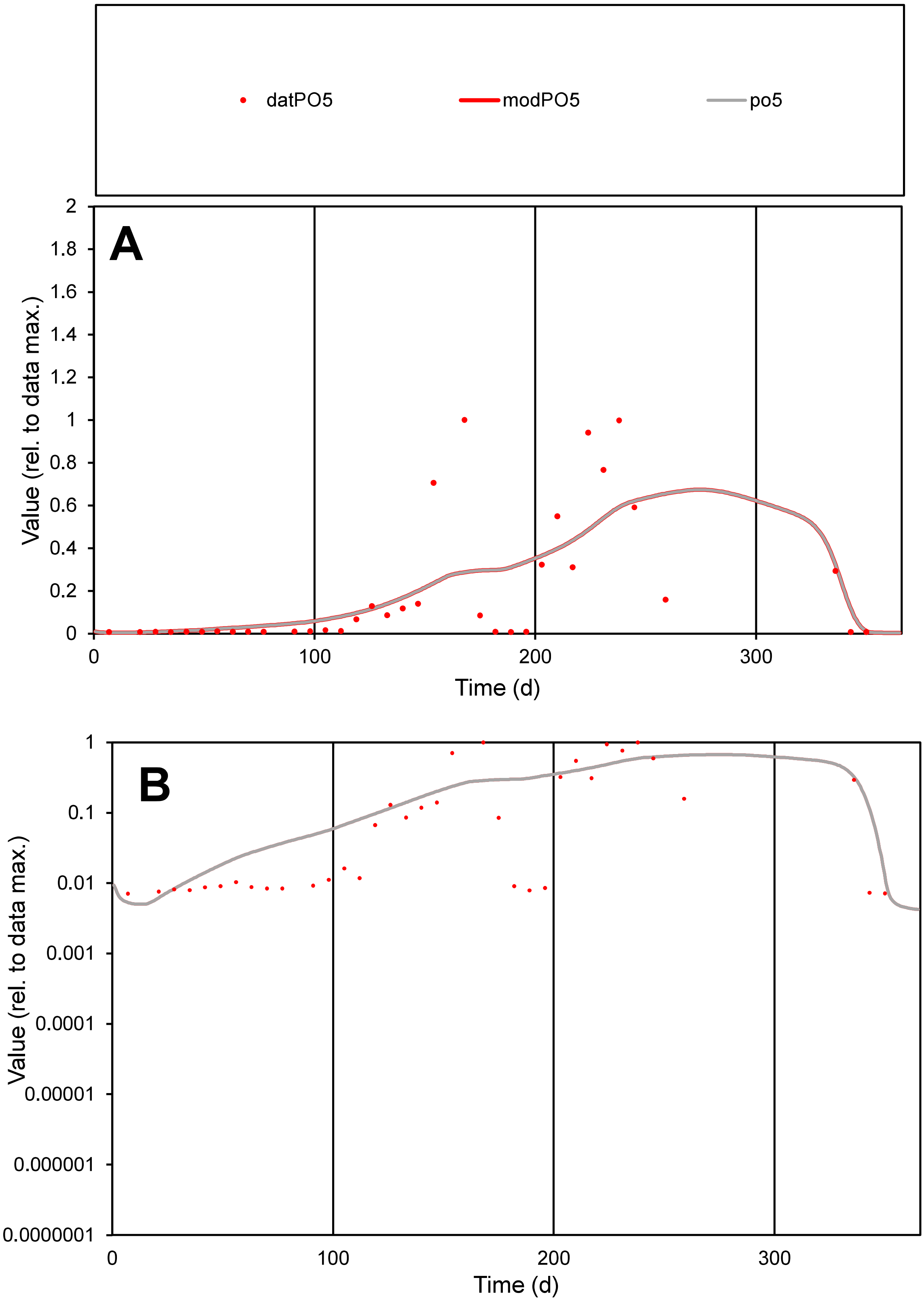

C1

Variables

PO5	Provide jtd or Dst
CalibOnly?	
2	1 to reproduce of
oNormalize?	
2	1=no, 2=yes

ofxn calc*
1.08351
ofxn mod
1.10284

sRun
RunA1
zout
1

Ave
Data
0.00133
Model
0.00139

data/vMax
0.00674

ofxng
311.734

Min d/m
1.36208

ave/min d
27.7821
ave/min m
39.7039

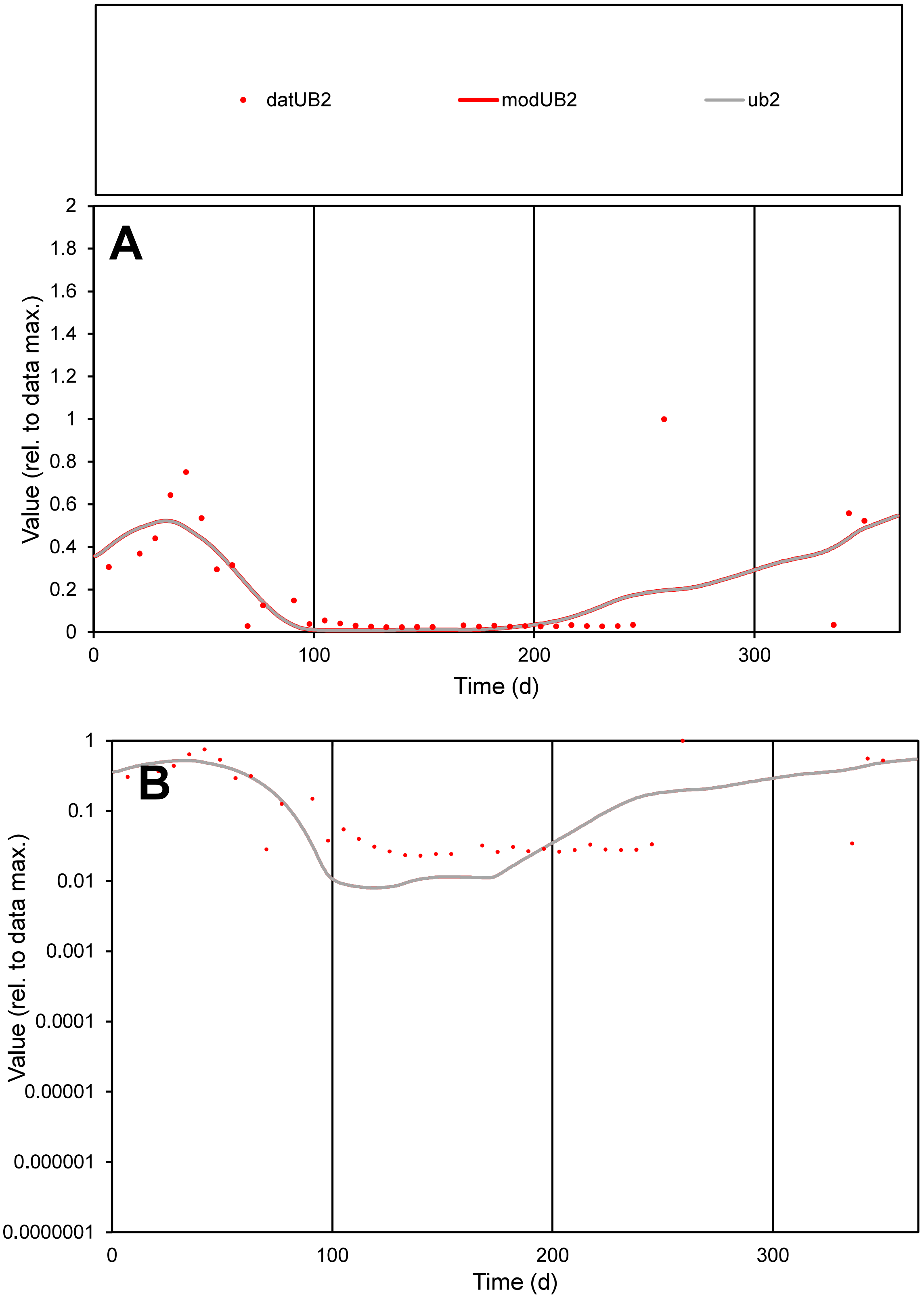

C1

Variables
UB2 Provide jtd or Dst
CalibOnly?
2 1 to reproduce of
oNormalize?
2 1=no, 2=yes

ofxn calc*
0.90522
ofxn mod
0.92535

sRun
RunA1
zout
1

Ave
Data
0.00083
Model
0.00078

data/vMax
0.00446

ofxng
311.734

Min d/m
2.88311

ave/min d
8.05857
ave/min m
21.7498

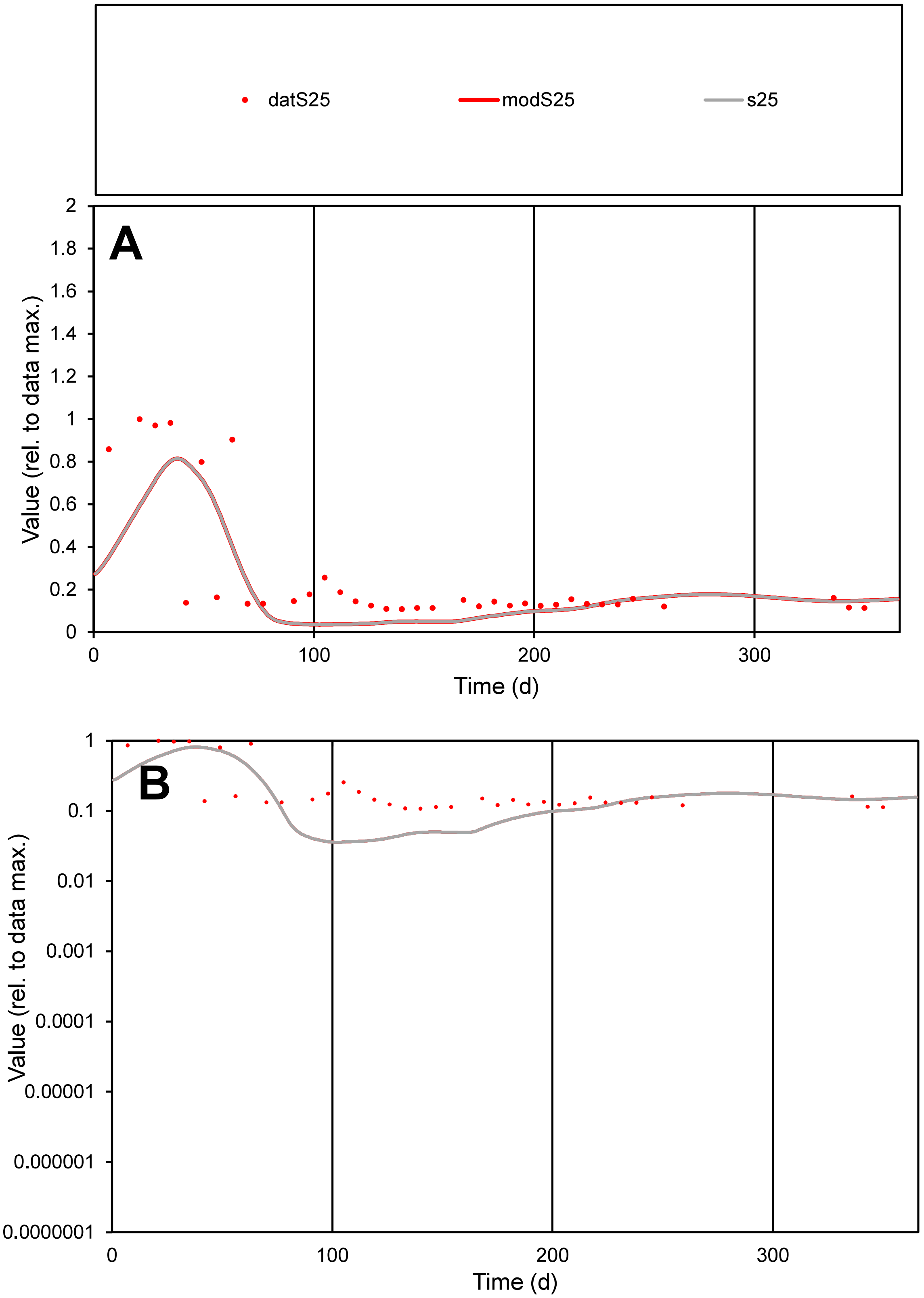

C1

Variables
S25 Provide jtd or Dst
CalibOnly?
2 1 to reproduce of
oNormalize?
2 1=no, 2=yes

ofxn calc*
0.74914
ofxn mod
0.76503

sRun
RunA1
zout
1

Ave
Data
0.00042
Model
0.00033

data/vMax
0.00155

ofxng
311.734

Min d/m
2.99428

ave/min d
2.49498
ave/min m
5.83956

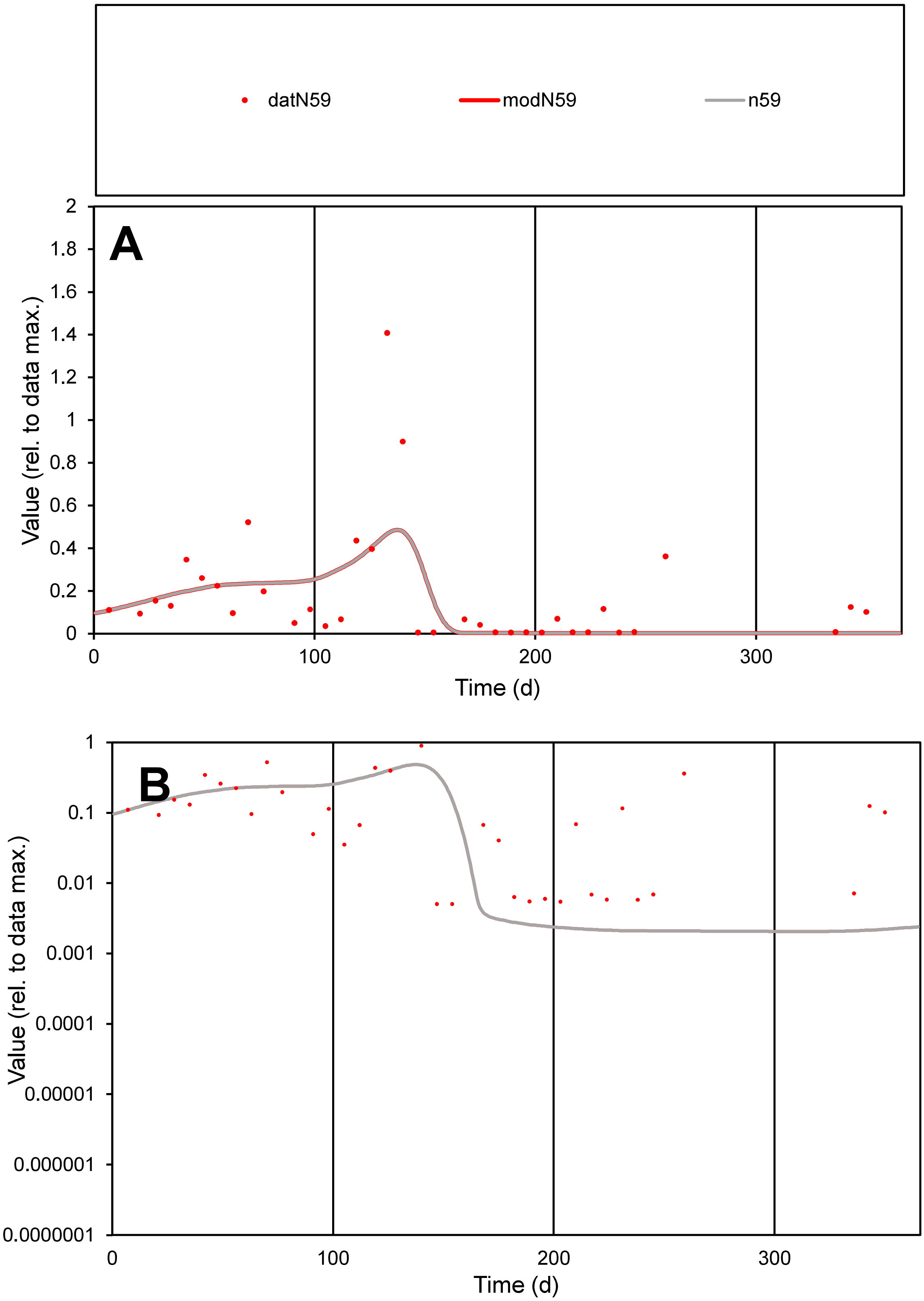

C1

Variables
N59 Provide jtd or Dst
CalibOnly?
2 1 to reproduce of
oNormalize?
2 1=no, 2=yes

ofxn calc*
0.99187
ofxn mod
1.01537

sRun
RunA1
zout
1

Ave
Data
0.00132
Model
0.00122

data/vMax
0.00854

ofxng
311.734

Min d/m
2.41662

ave/min d
30.7328
ave/min m
68.4321

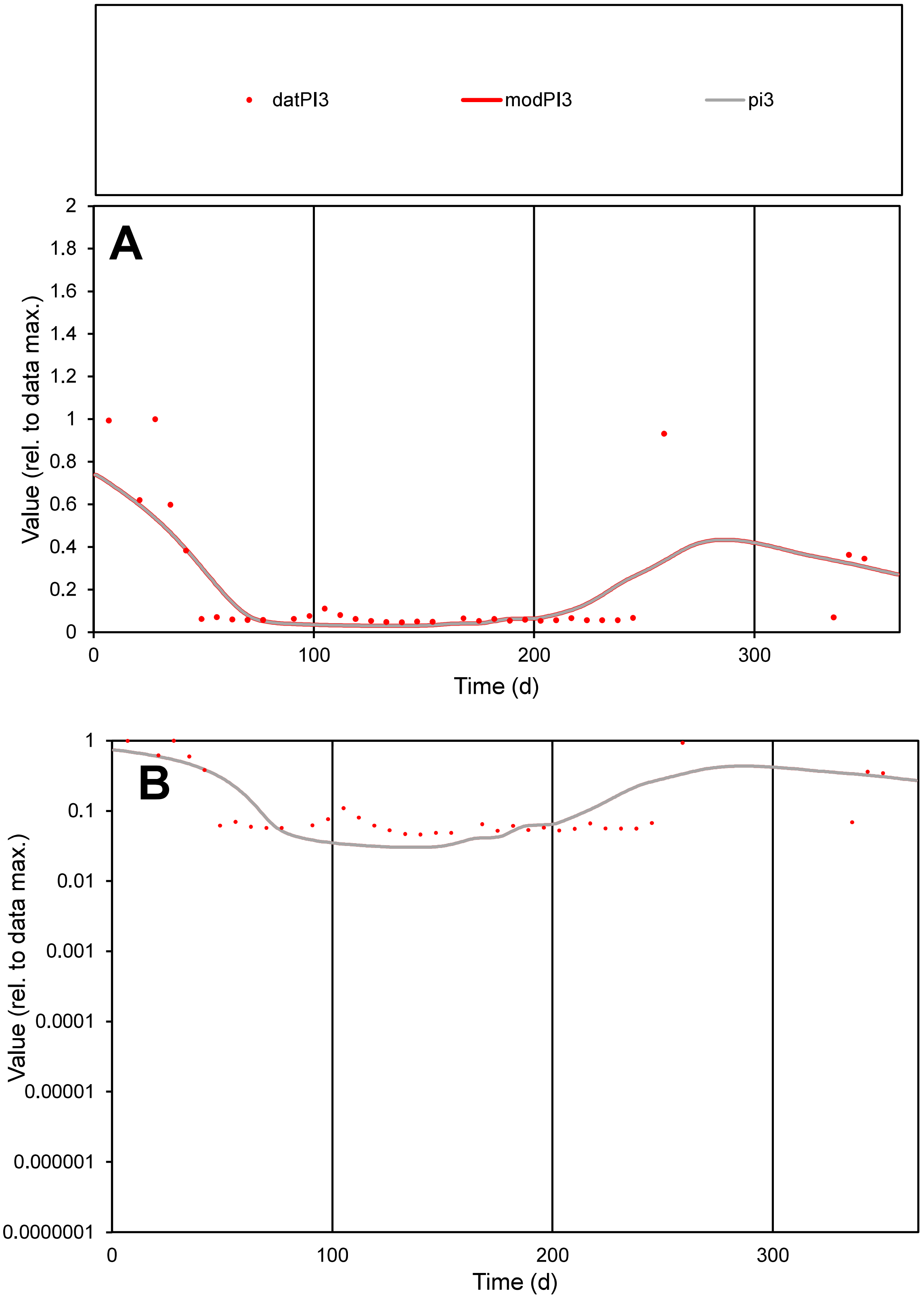

C1

Variables
PI3 Provide jtd or Dst
CalibOnly?
2 1 to reproduce of
oNormalize?
2 1=no, 2=yes

ofxn calc*
0.83089
ofxn mod
0.84853

sRun
RunA1
zout
1

Ave
Data
0.00029
Model
0.00027

data/vMax
0.00152

ofxng
311.734

Min d/m
1.5304

ave/min d
4.15905
ave/min m
5.78103

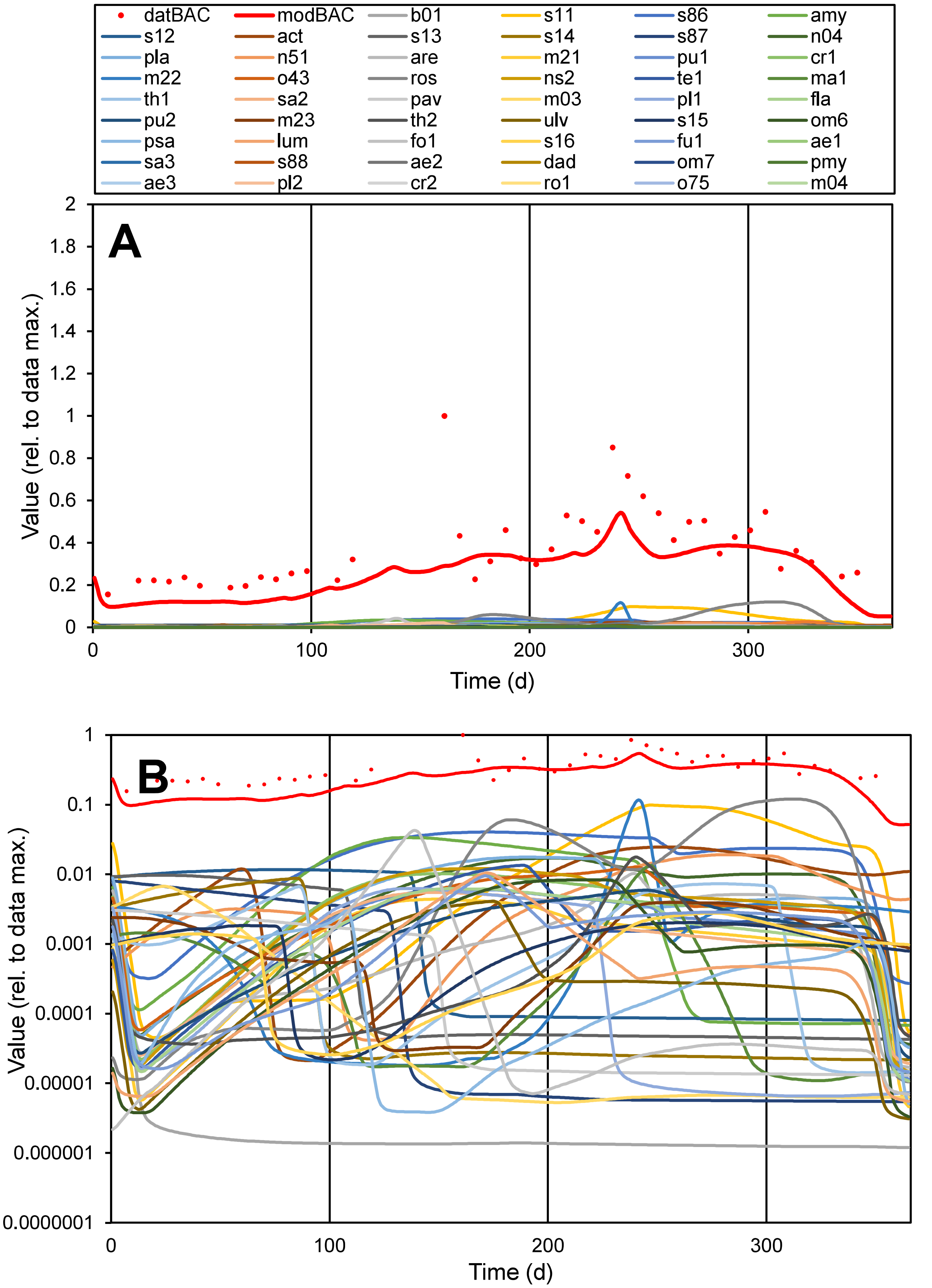

C1

Variables
BAC Provide jtd or Dst
CalibOnly?
2 1 to reproduce of
oNormalize?
2 1=no, 2=yes

ofxn calc*
0.44162
ofxn mod
0.45584

sRun
RunA1
zout
1

Ave
Data
1.30276
Model
0.93351

data/vMax
3.463

ofxng
311.734

Min d/m
1.72425

ave/min d
2.41699
ave/min m
2.98628
